# Integrated Glycosylation Analysis of Immunoglobulin Isotypes Reveals Expanded Humoral Remodeling in Elderly Tuberculosis Infection

**DOI:** 10.1016/j.mcpro.2025.101438

**Published:** 2025-10-30

**Authors:** Yun-Jung Yang, Chih-Hsin Lee, San-Yuan Wang, Yung-Kun Chuang, Michael X. Chen, Hsi-Chang Shih, I-Lin Tsai

**Affiliations:** 1 Department of Biochemistry and Molecular Cell Biology, School of Medicine, College of Medicine, Taipei Medical University, Taipei, Taiwan; 2 Graduate Institute of Medical Sciences, College of Medicine, Taipei Medical University, Taipei, Taiwan; 3 Pulmonary Research Center, Wan Fang Hospital, Taipei Medical University, Taipei, Taiwan; 4 Department of Internal Medicine, Wan Fang Hospital, Taipei Medical University, Taipei, Taiwan; 5 Division of Pulmonary Medicine, Department of Internal Medicine, School of Medicine, College of Medicine, Taipei Medical University, Taipei, Taiwan; 6 Master Program in Clinical Genomics and Proteomics, College of Pharmacy, Taipei Medical University, Taipei, Taiwan; 7 Master Program in Food Safety, College of Nutrition, Taipei Medical University, Taipei, Taiwan; 8 Department of Pathology and Laboratory Medicine, The University of British Columbia, Victoria, British Columbia, Canada; 9 Division of Medical Sciences, University of Victoria, Victoria, British Columbia, Canada; 10 Department of Physiology, Pharmacology & Therapeutics, Johns Hopkins University School of Medicine, Baltimore, Maryland, USA

**Keywords:** immunoglobulin, Fc-glycosylation, tuberculosis, active TB, latent TB, LC–MS

## Abstract

Antibody fragment crystallizable region (Fc) glycosylation critically modulates immune signaling, yet characterization of glycosylation beyond the immunoglobulin G (IgG) isotype remains limited. Here, we present the first site-specific glycoprofiling of immunoglobulin A (IgA) and immunoglobulin M (IgM) in elderly individuals with tuberculosis (TB), a population particularly susceptible to disease reactivation. Using dual-enzyme digestion and targeted LC–MS/MS analysis, we quantified Fc glycosylation of IgG, IgA, and IgM in plasma from 20 patients with active TB (ATB), 18 with latent TB infection (LTBI), and 20 controls. Consistent with previous studies, IgG1 and IgG2 in ATB displayed reduced galactosylation and elevated fucosylation compared with LTBI. Extending the analysis to other isotypes, we identified analogous alterations in IgA and IgM. ATB samples showed reduced digalactosylation and increased monogalactosylation at IgA1/2-N144/131, indicating a shift toward agalactosylation. In IgM, decreased galactosylation at N171, N332, and N395, increased agalactosylation at N563, and increased fucosylation and sialylation at N71 were observed in ATB relative to LTBI and controls. Integrating 18 significantly altered glycosylation traits across all three Ig isotypes revealed coordinated humoral remodeling associated with active disease. Collectively, these findings indicate that IgA and IgM, like IgG, undergo infection-associated proinflammatory glycan remodeling, underscoring their overlooked roles in antibody-mediated immune modulation and providing a broader framework for understanding humoral responses in aging and chronic infection.

## Highlights

• First glycoprofiling of IgA and IgM in elderly tuberculosis (TB) patients.
• Site-specific Fc glycan shifts observed in IgG, IgA, and IgM during TB.
• IgA-N144/131 and IgM-N171/N332/N395 show decreased galactosylation in active TB.
• Multi-isotype analysis reveals coordinated humoral remodeling in aging TB immunity.

## Graphical Abstract

**Figure:**
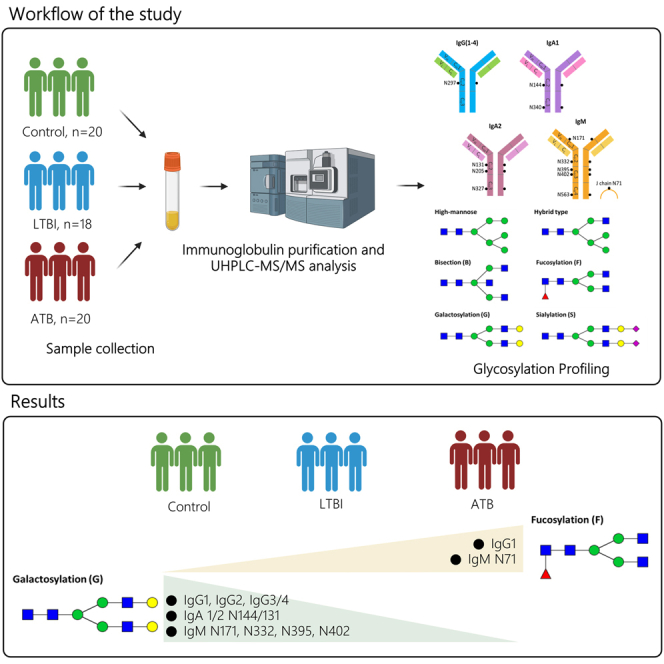

## In Brief

Antibody Fc glycosylation regulates immune effector functions, yet its broader remodeling across antibody isotypes during tuberculosis infection remains underexplored. By performing sensitive and site-specific LC–MS/MS profiling of IgG, IgA, and IgM glycosylation from elderly adults with tuberculosis, this study reveals coordinated proinflammatory glycan alterations across antibody isotypes. IgA and IgM exhibit complementary patterns of humoral remodeling, providing integrated insight into antibody diversification and immune modulation during *Mycobacterium tuberculosis* infection.

Tuberculosis (TB), caused by *Mycobacterium tuberculosis* (*Mtb*) infection, remains a significant global health threat, responsible for 1.3 million deaths in 2022 (1, 2). Patients are classified as having active tuberculosis (ATB) or latent tuberculosis infection (LTBI) based on clinical symptoms and bacterial activity; in LTBI, bacteria remain dormant without active replication (3). Patients with ATB exhibit symptoms, such as cough, fever, fatigue, and weight loss, and are potentially contagious (4). In contrast, patients with LTBI show no clinical symptoms, imaging findings, or microbiological evidence (3). However, approximately 5% to 15% of patients will develop TB reactivation and progress to ATB (5).

In terms of disease control, accurate diagnosis, treatment, and management of TB infections are of the utmost importance, particularly when providing distinct medical treatments for patients with LTBI and ATB. Currently, diagnostic tools for TB infection primarily rely on tuberculin skin tests and interferon-gamma release assays (IGRAs) (6). However, these tests cannot effectively differentiate between LTBI and ATB states. While chest radiography, sputum acid–fast *bacillus* smears, sputum culture for *Mtb*, and molecular diagnostics have been utilized for ATB diagnosis, challenges arise because of the limited availability of sputum samples in some patients, the lengthy time required for bacterial culturing (7), and the inability to identify extrapulmonary TB using these diagnostic methods. Predicting the reactivation and progression of untreated LTBI in the absence of clinical symptoms poses a significant challenge. The shift from LTBI to a reactivated state can turn patients into inadvertent carriers of the disease, thereby increasing public health concerns.

Aging is an important risk factor for an increased incidence of ATB. During aging, we face the challenges of lower lung function, immunosenescence or chronic inflammation, low tolerance to anti-TB drugs, a higher possibility of adverse drug reactions, and more comorbidities (8, 9). A special focus on the elderly population, including a better understanding of the molecular and cellular mechanisms of lung aging and infection, and incorporating more predictive factors, is recommended for disease control (8, 10). To address this issue and enhance disease control measures, the development of supplementary biomarkers capable of predicting disease progression holds immense promise.

**Fig. 1:**
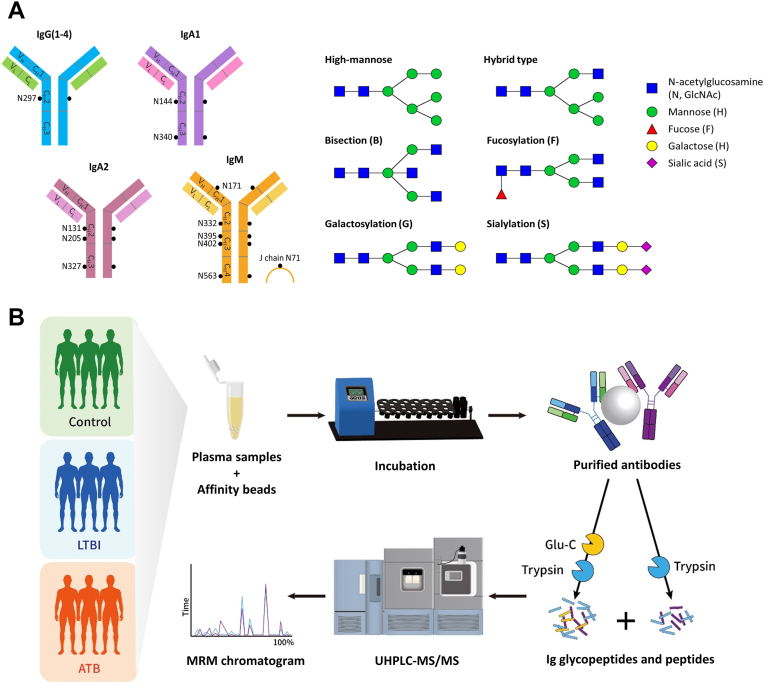
**Demonstration of *N*-glycan types and *N*-glycosylation sites on human immunoglobulin (Ig) and the experimental design.***A*, *black spots* indicate the *N*-glycosylation sites on the IgG, IgA, and IgM isotypes with the sequence number of asparagine. Six major *N*-glycan types including high-mannose, hybrid type, bisection, fucosylation, galactosylation, and sialylation. *B*, three groups of clinical samples from the controls, patients with latent tuberculosis (TB) infection (LTBI), and patients with active TB (ATB) infection. Plasma samples were incubated with Ig affinity beads followed by on-bead digestion. Trypsin or Glu-C/trypsin were used for Ig digestion, and the supernatants were collected for UHPLC–MS/MS analysis. Glycosylation traits were calculated and used for between-group comparison. IgA, immunoglobulin A; IgG, immunoglobulin G; IgM, immunoglobulin M.

Antibodies, also known as immunoglobulins (Igs), consist of two regions: the fragment crystallizable region (Fc) and the fragment antigen-binding region (11). Figure 1*A* shows the glycosylation sites of immunoglobulin G (IgG), immunoglobulin A (IgA), and immunoglobulin M (IgM), as well as the major glycoforms, including high-mannose, hybrid type, fucosylation (F), bisection (B), galactosylation (Gal)/agalactosylation (G0), and sialylation (S) (12, 13, 14). Antibody glycosylation affects the conformation, stability, and affinity to corresponding targets (15, 16, 17). Glycosylation of the Fc region triggers immune responses, such as inflammation, antibody-dependent cell-mediated cytotoxicity (ADCC), and antibody-dependent cellular phagocytosis by interacting with receptors on effector cells (18, 19). IgG from patients with LTBI contained less fucose but more galactose and sialic acid than IgG from patients with ATB, which presented fewer inflammatory features in the LTBI group (20). Glycosylation of the Fc domain also showed the potential to discriminate between LTBI and ATB infections, and galactosylation profiles were evaluated as auxiliary diagnostic biomarkers (21, 22). Distinct IgG glycosylation and antigen-specific antibodies have also been associated with TB drug treatment (23). The average age of the participants in these studies was approximately 25 to 35 years, and we found limited reports regarding antibody molecular profiles in older TB patients. In this study, we focused on elderly adults with TB (mean age, 54–60 years), representing an older cohort than those previously analyzed and providing an opportunity to investigate age-associated differences in antibody glycosylation.

Despite extensive efforts characterizing IgG Fc glycosylation in infection and inflammation, glycosylation profiles of other Ig isotypes remain largely unexplored. A recent murine study by Kumagai *et al.* (24) demonstrated that IgM glycosylation is dynamically regulated during TB infection. However, whether such glycan remodeling occurs in human IgA and IgM during TB infection remains unknown. To address this gap, we investigated the levels and Fc glycosylation patterns of IgG, IgA, and IgM across elderly individuals with ATB, LTBI, and controls. While our analysis focused on total plasma antibodies because of the limited availability of antigen-specific fractions, this study represents the first comprehensive human glycoprofiling of all three major antibody isotypes in the context of TB. By extending glycosylation analysis beyond IgG, we provide new insight into isotype-specific humoral modulation during infection and highlight previously unrecognized proinflammatory glycan signatures in IgA and IgM, particularly relevant to aging-associated immune responses.

## Experimental Procedures

### Reagents and Materials

Affinity beads, CaptureSelect KappaXL Affinity Matrix, and CaptureSelect LC-lambda (Hu) Affinity Matrix were purchased from Thermo Fisher Scientific. Ammonium bicarbonate, DTT, iodoacetamide, and formic acid were purchased from Sigma–Aldrich. Acetonitrile was purchased from J. T. Baker (Phillips). PBS was obtained from VWR International, LLC. Trypsin and Glu-C were purchased from Promega. Stable isotope–labeled peptides, used as internal standards (ISs), were synthesized by Genomics. Blood samples (10 ml each) were collected in BD Vacutainer tubes containing sodium heparin as an anticoagulant (BD). Following collection, samples were centrifuged at 2000 rpm for 10 min at 4 °C (HITACHI Himac CR21G, Rotor-R3S) to separate the plasma. The resulting plasma was divided into 1.5 ml microtubes (Axygen) and stored at −80 °C. All plasma specimens used in this study underwent no more than three freeze–thaw cycles.

### Experimental Design and Statistical Rationale

This study aimed to discover whether IgA and IgM glycosylation profiles provide complementary information to IgG glycosylation in TB infection. The study represents a discovery-phase clinical investigation. A total of 58 individuals were recruited from Taipei Municipal Wan Fang Hospital, including 20 patients with ATB, 18 with LTBI, and 20 healthy controls (Table 1 and Supplemental Table S1). Given the challenges of enrolling clinically confirmed TB patients, the sample size was determined by clinical feasibility, and efforts were made to balance age and sex across groups to minimize biological bias (25, 26). The mean ages were 54.1, 55.8, and 60.2 years in the control, LTBI, and ATB groups, respectively. ATB diagnosis was based on a positive *Mtb* sputum culture, whereas LTBI was defined by a positive IGRA in the absence of clinical or radiographic evidence of active disease. Controls were confirmed to be IGRA negative. The study protocol was approved by the Research Ethics Committee of Taipei Medical University (Institutional Review Board no:. N201903025), following the Declaration of Helsinki, and written informed consent was obtained from each participant. Each plasma sample was analyzed in duplicate using the validated multiple reaction monitoring method to minimize missing injections and ensure analytical reproducibility. All 58 samples passed quality control filtering and were included in downstream analysis.

**Table 1:** Basic characteristics of controls, LTBI, and ATB patients

Demographic variables	Controls	LTBI	ATB	*p*
	(n = 20)	(n = 18)	(n = 20)	
Age (mean ± SD)	54.1 ± 8.8	55.8 ± 13.3	60.2 ± 18.8	0.3942
Gender (male/female)	13/7	12/6	14/6	0.9430

Statistics of age distribution was calculated using one-way ANOVA. Statistics of gender distribution was calculated using Chi-squared test.

Comparisons between each combination of ATB, LTBI, and controls were performed using the nonparametric unpaired Mann–Whitney test. This nonparametric method was selected because of the modest group sizes and inherent variability in clinical samples, where normality cannot be assumed.

We constructed supervised classification models to distinguish LTBI from ATB using percentages of glycosylation traits calculated for each sample. To mitigate overfitting and prevent information leakage, we implemented stratified 10-fold crossvalidation: all preprocessing, univariate screening, and model fitting/tuning were performed within each training fold, predictions were generated for the held-out fold, and fold-level predictions were aggregated for evaluation. Glycosylation traits across IgG, IgA, and IgM were first screened using nonparametric, unpaired Mann–Whitney *U* tests; features with *p* < 0.05 were retained. A liberal screening threshold was used to avoid prematurely discarding potentially informative features, with final feature selection and evaluation conducted strictly within the cross-validation framework to prevent information leakage.

Partial least squares discriminant analysis (PLS-DA) models were fit *via* the R package *pls* (interfaced through *caret*) and trained separately on four prespecified feature sets: IgG-only, IgA-only, IgM-only, and all retained features combined. PLS-DA was chosen as an initial multivariate approach suited for high-dimensional data with correlated predictors, whereas logistic regression was applied to a reduced set of nominated features to improve interpretability and robustness in a low-dimensional setting.

To reduce model complexity, decision trees were used to nominate a small set of representative predictors; within each training fold, trees were trained with the R package *rpart* on a randomly selected 80% subsample (maxdepth = 2, minsplit = 5, and minbucket = 3), and the nominated features were then used to specify logistic regression models fit with R’s *glm* function (family = binomial). Cross-validation workflows, including stratified fold creation and resampling, were implemented in R using *caret*, and model performance was summarized from cross-validated predictions using accuracy, sensitivity, specificity, precision, recall, and F1 score.

### Ig Purification and Protein Digestion

An overview of the workflow is presented in Figure 1*B*. To purify all classes of Igs from human plasma, affinity purification beads, CaptureSelect KappaXL Affinity Matrix, and CaptureSelect LC-lambda (Hu) Affinity Matrix were used to capture the constant region of Ig light chains (27). The two types of bead slurry were mixed in a 1:1 ratio. Next, 40 μl of the mixed slurry was conditioned with 150 μl PBS twice, and 16 μl of plasma was added to beads in 184 μl of PBS. Two Ig purification samples were prepared from each plasma sample for further dual-enzyme digestion. The samples were incubated at 4 °C overnight on a mixer. After incubation, the supernatants were removed, and the beads were washed with 150 μl PBS twice to prevent nonspecific binding.

After the Igs were purified from human plasma, on-bead enzymatic protein digestion was performed. Fifty microliters of 50 mM ammonium bicarbonate were added, containing four stable isotope–labeled peptides as ISs: 240 ng of IS 1, 200 ng of IS 2, 400 ng of IS 3, and 1 ng of IS 4. One microliter of 550 mM DTT was added to the solution as a reducing reagent and incubated for 45 min at 56 °C to disrupt the disulfide bonds in the protein. Then, 2 μl of 450 mM iodoacetamide was added as the alkylating reagent and incubated for 45 min in the dark at room temperature. Duplexed Ig purification samples prepared from each plasma sample were treated with trypsin and Glu-C/trypsin in parallel. Specifically, one Ig purification sample was treated sequentially with 5 μl of Glu-C (0.1 μg/μl) and incubated for 1 h at 37 °C on a desktop shaker (300 rpm model CB-1703; CLUBIO). After the Glu-C digestion, 5 μl of trypsin (0.2 μg/μl) was added, and the sample was incubated overnight. The other Ig purification sample was treated with trypsin alone, without prior Glu-C treatment, and also incubated overnight. At the end of the on-bead digestion, 6 μl of 10% formic acid was added to stop the enzymatic digestion reaction. For LC–MS/MS analysis, the samples were centrifuged at 12,000 rpm (approximately 13,800*g*) for 10 min, and equal volumes of supernatants from each paired sample with and without Glu-C treatment were pooled together. Because of their relatively high concentration in human plasma, the pooled samples were diluted fivefold with 0.1% formic acid as an additional step for IgG peptide and glycopeptide analysis.

### LC–MS/MS Glycopeptide Analysis

A Xevo TQ-XS Triple Quadrupole Mass Spectrometry system (Waters Corporation) was used to analyze the targets. For chromatographic separation, a Core-Shell C18 Kinetex column with 50 mm of length, 2.1 mm of internal diameter, and 2.6 μm of particle size was used. The mobile phase comprised solvent A (0.1% formic acid in water) and solvent B (0.1% formic acid in acetonitrile). The flow rate was set to 0.3 ml/min.

For IgG peptide and glycopeptide analyses, the following elution gradient was used: 2% solvent B at 0 min and for the first 0.5 min, then organic solvent B was increased to 50% at 8 min and lasted for 0.5 min, and then increased to 100% at 9 min. After maintaining the mobile phase at 100% solvent B for 2 min, the gradient was restored to 2% solvent B at 11.5 min and maintained for 1.5 min to achieve system equilibration.

For IgA, IgM peptide, and glycopeptide analyses, the elution gradient was set as follows: 2% solvent B at 0 min and for the first 0.5 min, then solvent B was increased to 30% at 9 min and 35% at 9.5 min. Solvent B was increased to 100% after 10 min and maintained at 100% for 1 min. The gradient was then restored to 2% solvent B at 11.5 min and maintained for 1.5 min to achieve system equilibration.

The samples were stored in an autosampler set at 4 °C, and the column oven was set at 40 °C. The injection volume was 1 μl for IgG analytes and 5 μl for IgA and IgM analytes, and Waters partial loop injection mode was used. The parameters of electrospray ionization positive mode were set as follows: capillary voltage was 2.5 kV, source offset was 30 V, source temperature was 150 °C, desolvation temperature was 450 °C, collision gas flow was 0.15 ml/min, and the nebulizer gas flow was 7 bar. The optimal multiple reaction monitoring transitions, retention times, cone voltages, and collision energies for IgG, IgA, and IgM peptides and glycopeptides can be found in Supplemental Table S2 and our previously published study (27). Quality control samples were prepared by pooling aliquots from all clinical plasma samples and were injected at the beginning, at the end, and after every 12 analytical injections. The relative standard deviation of the response for each analyte is provided in Supplemental Table S3. The relative standard deviations of analytes were below 30%, ranging from 4.93% to 19.81% for IgG-associated analytes, 6.44% to 26.43% for IgA-associated analytes, and 2.53% to 29.45% for IgM-associated analytes.

### Data Analysis and Software

UHPLC–MS/MS tier 2 data processing and glycosylation trait calculations were performed using the TargetLynx software (MassLynx V4.2; Waters Corporation) and Microsoft Excel (2016 MSO 16.0.4266.1001). Integration values and peak area ratios (target/IS response) can be manually exported from TargetLynx software to Excel. In the previous software, an annotation of the primary FLAG indicated the condition of the integration baseline. The glycosylation traits are summarized in Supplemental Table S4. Score plot of the sPLS-DA analysis, heatmap of the glycosylation profile, correlation heatmaps, and the results of correlation analysis for IgG, IgA, IgM glycosylation traits were performed using MetaboAnalyst 6.0 (https://www.metaboanalyst.ca/). Boxplots and statistical analysis for glycosylation traits or glycopeptide comparisons were conducted in GraphPad Prism, version 8.0, for Windows (GraphPad Software). The classification model and decision tree were conducted in R (version 4.5.1, https://www.r-project.org/). Cross-validation and preprocessing pipelines were implemented with caret (version 7.0-1, https://cran.r-project.org/package=caret); PLS-DA models were fit with pls (version 2.8-5, https://cran.r-project.org/package=pls); decision trees with rpart (version 4.1.24, https://cran.r-project.org/package=rpart); and logistic regression with the base stats::glm function.

## Results

### Distinct IgG Glycosylation of ATB, LTBI, and Control Groups

**Fig. 2:**
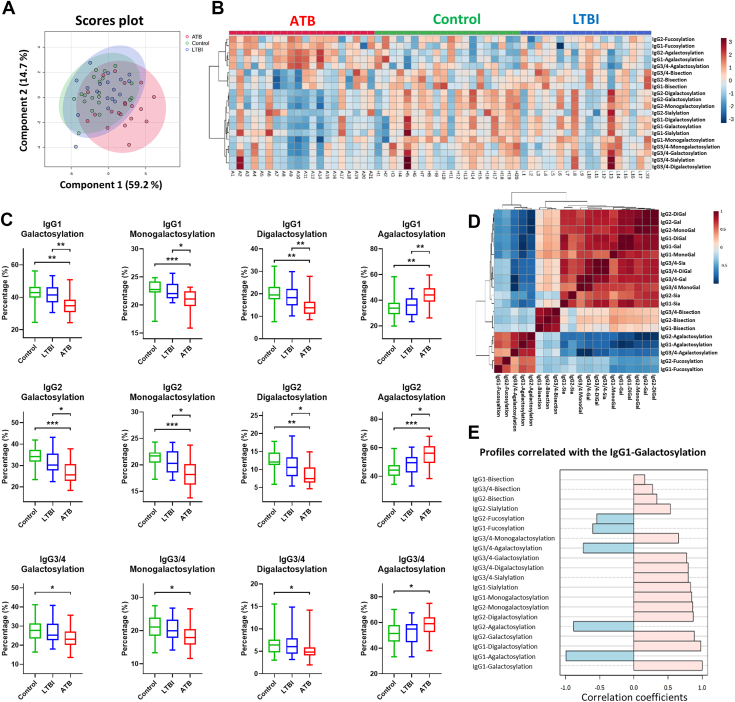
**Differential IgG glycosylation profiles among the control, latent tuberculosis (TB) infection (LTBI), and active TB (ATB) groups.***A*, score plot of the sPLS-DA analysis. *Red*: ATB group; *green*: control group; *blue:* LTBI group. *B*, heatmap of the glycosylation profile differences among the three groups. *C*, boxplots and the statistical results of IgG galactosylation–related traits among the three groups. ∗*p* < 0.05; ∗∗*p* < 0.01; and ∗∗∗*p* < 0.001. *D*, correlation heatmaps of the 20 IgG glycosylation traits. *E*, correlation coefficients between each IgG glycosylation trait and IgG1 galactosylation. IgG, immunoglobulin G; PLS-DA, partial least squares discriminant analysis.

Plasma levels of total IgG and IgG subclasses were not very different among the controls, patients with LTBI, and patients with ATB (Supplemental Fig. S1*A*). The only IgG subclass that showed a marginally significant difference was IgG2 (*p* = 0.051), with higher levels observed in the control group than in the LTBI group. When we focused on the 20 IgG glycosylation traits, we observed similar glycosylation profiles in the control and LTBI groups, whereas the ATB group showed distinct glycosylation patterns. On the score plot of sPLS-DA, sample clusters of the control and LTBI groups overlapped, and the ATB sample cluster was slightly shifted to the right side of the plot (Fig. 2*A*). The heatmap (Fig. 2*B*) shows that the ATB group showed higher fucosylation, lower galactosylation, and sialylation of IgG1 and IgG2 compared with LTBI and controls. These results were supported and validated by statistical analysis; the boxplots are shown in Figure 2*C* and Supplemental Fig. S1. Regarding the monogalactosylation and digalactosylation of IgG subclasses, we also observed a decreasing trend in the ATB group (Fig. 2*C*). The fucosylation levels were significantly higher in the ATB group in IgG1 but not IgG2 (Supplemental Fig. S1*B*). IgG1 sialylation was not significantly different among the three groups; however, the LTBI group showed lower IgG2 sialylation compared with controls. IgG3/4 sialylation levels were reduced in the ATB group compared with the controls (Supplemental Fig. S1*C*). Bisection was only found to be lower in the ATB group with IgG3/4 (Supplemental Fig. S1*D*). As shown in Figure 2*D*, three groups of glycosylation traits were identified based on their correlations: (first group) fucosylation and agalactosylation; (second group) bisection; and (third group) galactosylation and sialylation. The first group of glycosylation traits was higher in the ATB group, which was the opposite of the third group of glycosylation traits. The latter was higher in the control and LTBI groups (Fig. 2*E*).

Although it is more complicated to investigate the biological functions of individual glycopeptides, we summarized 11 of 26 IgG glycopeptides that were statistically different among the three groups (Supplemental Fig. S2). Similar to our investigation of glycosylation traits, glycopeptides carrying more galactose, such as IgG1 H5N4, IgG1 H5N5F1, and IgG2 H5N4F1, were downregulated in the ATB and LTBI groups. Glycopeptides carrying fucose but less galactose, such as IgG1 H3N4F1 and IgG2 H3N4F1, were higher in the ATB group. The response of the glycopeptides was normalized to the response of their respective IgG subclasses; therefore, the comparison was not affected by the protein level in each sample.

### Significant Differences in the Level of Iga Subclasses and Glycosylation Profiles

**Fig. 3:**
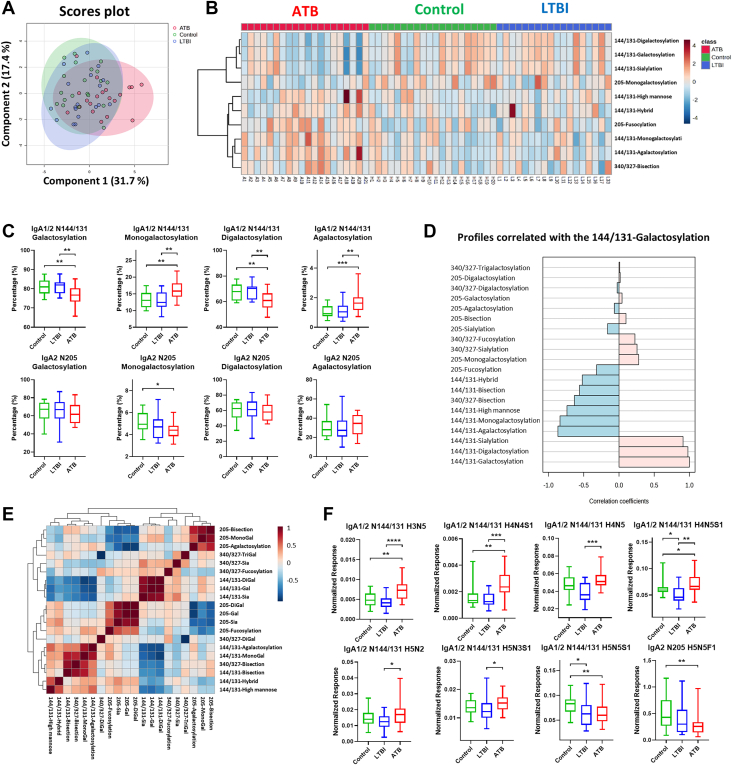
**Distinct IgA glycosylation traits among the control, latent tuberculosis (TB) infection (LTBI), and active TB (ATB) groups.***A*, score plot of the sPLS-DA analysis. *Red*: ATB group; *green*: control group; *blue*: LTBI group. *B*, heatmap of top 10 IgA glycosylation traits demonstrating the glycosylation profile differences among the three groups. *C*, boxplots and the statistical results of IgA1/2-N144/131 and IgA2-N205 galactosylation–related traits among the three groups. ∗*p* < 0.05; ∗∗*p* < 0.01; and ∗∗∗*p* < 0.001. *D*, correlation coefficients between each IgA glycosylation trait and IgA1/2 N144/131 galactosylation. *E*, correlation heatmaps of the 20 IgA glycosylation traits. *F*, IgA glycopeptides, which showed statistical differences among the three groups. ∗*p* < 0.05; ∗∗*p* < 0.01; ∗∗∗*p* < 0.001; and ∗∗∗∗*p* < 0.0001. IgA, immunoglobulin A; PLS-DA, partial least squares discriminant analysis.

Neither IgA1 nor IgA2 levels were significantly different between the control, LTBI, and ATB groups (Supplemental Fig. S3*A*). Regarding the IgA glycosylation traits, the glycosylation patterns were similar between the LTBI and control groups, whereas the ATB group had relatively different glycosylation profiles, which resulted in the sample cluster shifting to the right side of the sPLS-DA score plot (Fig. 3*A*). Notably, distinct galactosylation-related profiles were found only in the IgA1/2-N144/131 position, rather than in IgA2-N205 or IgA1/2-N340/327 (Fig. 3, *B* and *C*, Supplemental Fig. S3*C*). As shown in Figure 3*C*, on IgA1/2-N144/131, patients with ATB showed lower total galactosylation, which resulted from a combination of lower digalactosylation and higher monogalactosylation. We assumed that the galactosylation profile at this site shifted from digalactosylation to monogalactosylation or nongalactosylation (agalactosylation) in the ATB group. The lower galactosylation trend in the ATB group was similar to that observed for the IgG isotype. When we investigated the correlations among different glycosylation traits, the sialylation and digalactosylation of N144/131 had higher positive correlation coefficients with galactosylation (Fig. 3*D*), which were clustered together in Figure 3*E*. In contrast, the monogalactosylation and agalactosylation of N144/131 were negatively correlated with galactosylation and clustered independently in Figure 3*E*. Other glycosylation traits of IgA, such as fucosylation, sialylation, and bisection, were similar among the three groups (Supplemental Fig. S3, *B*–*D*).

We have also reported distinct glycopeptides for the IgA isotype. Six IgA glycopeptides with glycosylation sites, IgA1/2-N144/131, were found to be more abundant in ATB than in LTBI (Fig. 3*F*): H3N5, H4N4S1, H4N5, H4N5S1, H5N2, and H5N3S1. Compared with controls, patients with ATB showed higher IgA1/2-N144/131 H3N5, H4N4S1, and H4N5S1 and lower IgA1/2-N144/131 H5N5S1 and IgA2-N205 H5N5F1 levels. To discriminate between LTBI and controls, significantly decreased IgA1/2-N144/131 H4N5S1 and H5N5S1 levels were found in LTBI groups.

### Lower Galactosylation of IgM Was Identified in the ATB Group

**Fig. 4:**
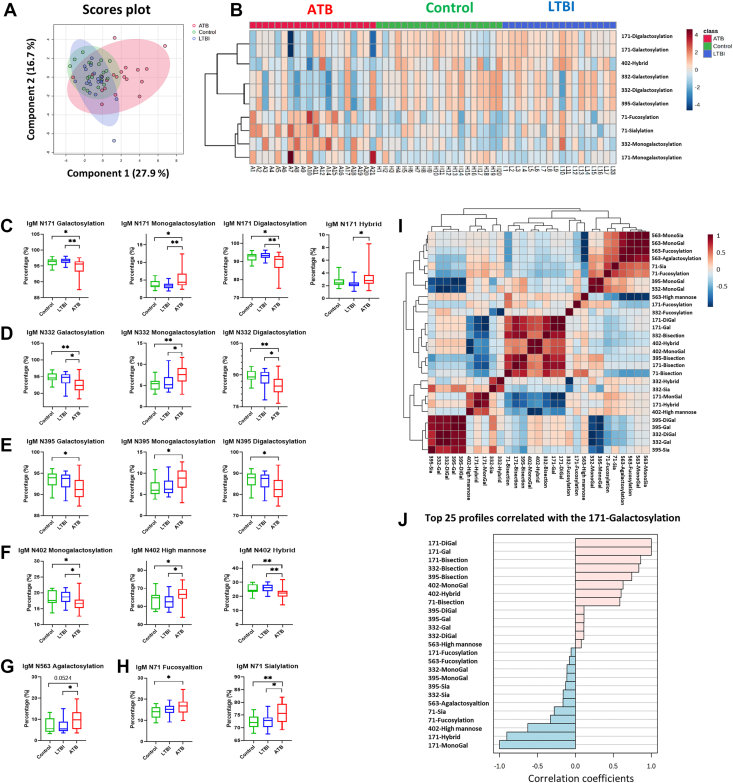
**Divergent IgM glycosylation traits among the control, latent tuberculosis (TB) infection (LTBI), and active TB (ATB) groups.***A*, score plot of the sPLS-DA analysis. *Red*, ATB group; *green*, control group; *blue*, LTBI group. *B*, heatmap of top 10 IgM glycosylation traits demonstrating the glycosylation profile differences among the three groups. *C*, boxplots and the statistical results of IgM-N171 glycosylation traits among the three groups. *D*, boxplots and the statistical results of IgM-N332 galactosylation–related traits among the three groups. *E*, boxplots and the statistical results of IgM-N395 galactosylation–related traits among the three groups. *F*, boxplots and the statistical results of IgM-N402 glycosylation traits among the three groups. *G*, boxplot and the statistical results of IgM-N563 agalactosylation among the three groups. *H*, boxplots and the statistical results of IgM-N71 fucosylation and sialylation among the three groups. ∗*p* < 0.05; ∗∗*p* < 0.01. *I*, correlation heatmaps of the 29 IgM glycosylation traits. *J*, correlation coefficients between each IgM glycosylation trait and IgM N171 galactosylation. IgM, immunoglobulin M; PLS-DA, partial least squares discriminant analysis.

No significant differences were observed in plasma levels of IgM among the control, LTBI, and ATB groups (data not shown). However, when 29 IgM glycosylation traits were input into the sPLS-DA analysis, a significant cluster shift was observed in the ATB group (Fig. 4*A*, *red cluster*). In Figure 4*B*, we constructed a heatmap of the top 10 features that showed significant differences among the three groups. Galactosylation of IgM-N171, N332, and N395 was lower, whereas N71 fucosylation and sialylation were higher in the ATB group. These findings were supported by the results of the statistical analysis, and the box plots are summarized in Figure 4, *C*–*E*, and *H*. Furthermore, we observed lower monogalactosylation, higher mannosylation, and lower hybrid-type glycosylation on IgM N402 in the ATB group (Fig. 4*F*). For N563, only agalactosylation was significantly higher in ATB, which also represents lower galactosylation at this site (Fig. 4*G*). Looking into the details, we observed special patterns of monogalactosylation and digalactosylation for N171, N332, and N395 (Fig. 4, *C*–*E*). Increased monogalactosylation was accompanied by decreased digalactosylation in the ATB group. This trend highlighted the shift from digalactosylation to monogalactosylation or agalactosylation, which resulted in reduced galactosylation in the ATB group. The inversely proportional relationships between (di)galactosylation and monogalactosylation at the three N-glycosylation sites can also be found in Figure 4, *I* and *J*.

Seven IgM glycopeptides showed the potential ability to discriminate ATB from LTBI, as they increased significantly in ATB (Supplemental Fig. S4): N171 H4N3F1S1, N171 H5N3F1S1, N171 H6N3F1S1, N402 H9N2, N563 H3N5F1, N71 H5N4S2, and N71 H5N4F1S2. Among them, N171 H4N3F1S1, N402 H9N2, and N71 H5N4F1S2 also significantly increased in patients with ATB when compared with controls.

Multi-isotype glycosylation features reveal complementary humoral remodeling patterns. After comparing the differences in Ig glycosylation profiles among the control, LTBI, and ATB groups, we evaluated whether incorporating IgA and IgM glycosylation traits into IgG glycosylation could provide complementary information on humoral remodeling. We first imported all Ig glycosylation traits into the Statistical Analysis tool in MetaboAnalyst 6.0 and filtered out 18 glycosylation traits that showed statistical differences between the ATB and LTBI groups. Among the 18 glycosylation traits, seven belonged to IgG, four belonged to IgA, and six belonged to IgM.

When we evaluated the univariate performance of individual glycosylation traits in GraphPad Prism, all 18 traits showed area under the curves (AUCs) higher than 0.736, with IgG1-fucosylation providing the highest AUC of 0.799 (Supplemental Fig. S5). These findings indicated that individual traits carry discriminatory information but may be limited in differentiating LTBI from ATB when considered alone.

**Fig. 5:**
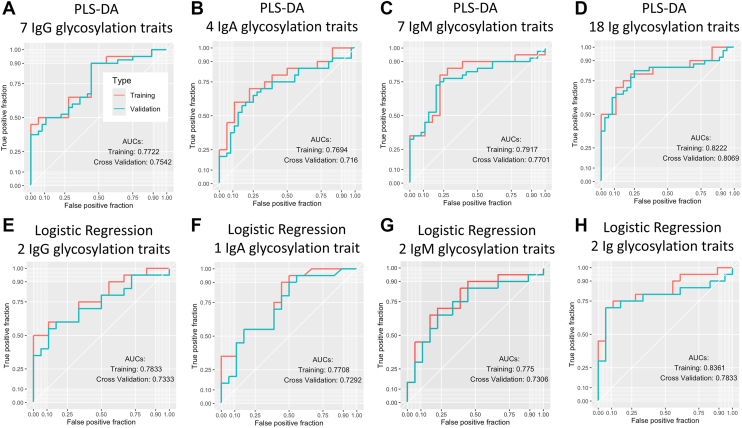
**Receiver operating characteristic (ROC) curves for models distinguishing latent tuberculosis (TB) infection (LTBI) from active TB (ATB) using serum immunoglobulin features.***A*–*D* show partial least squares discriminant analysis (PLS-DA) models built with (*A*) 7 IgG features, (*B*) 4 IgA features, (*C*) 7 IgM features, and (*D*) 18 combined Ig features. *E*–*H* show logistic regression models built with (*E*) 2 IgG features, (*F*) 1 IgA feature, (*G*) 2 IgM features, and (*H*) 2 combined Ig features. In each panel, the training ROC curve is shown in *red* and the cross-validation ROC curve (10-fold) in *teal*; the corresponding area under the curves (AUCs) for training and crossvalidation are reported within each subplot. *Axes* indicate true-positive fraction *versus* false-positive fraction. IgA, immunoglobulin A; IgG, immunoglobulin G; IgM, immunoglobulin M.

To assess whether multifeature integration could enhance classification, we constructed predictive models using three approaches: PLS-DA, logistic regression, and decision tree classifiers. For each model, we applied a stratified 10-fold cross-validation strategy to evaluate generalizability and to minimize overfitting. Predictive performance metrics are summarized in Supplemental Table S5, with the corresponding receiver operating characteristic curves shown in Figure 5.

Models built using features from a single Ig isotype showed modest classification ability. For example, the seven-feature IgG PLS-DA model (Fig. 5*A*) achieved an AUC of 0.7458, an accuracy of 0.6053, and an F1 score of 0.6341. In contrast, models incorporating multiple isotypes demonstrated higher performance: the combined 18-feature PLS-DA model (Fig. 5*D*) reached an AUC of 0.8246 with an accuracy of 0.7632. Similarly, in logistic regression, the integration of IgG1-monogalactosylation and IgM 402-high-mannose (Fig. 5*H*) produced the best performance, with an AUC of 0.7833, accuracy of 0.8158, and F1 score of 0.8108.

To further illustrate the discriminative contribution of individual features, we also constructed decision tree classifiers using minimal subsets of traits. The resulting classification rules and feature distributions are presented in Supplemental Fig. S6.

## Discussion

The addition of *N*-glycans, such as galactosylation, sialylation, fucosylation, and bisection, to the Fc domain of antibodies is an important post-translational modification that can greatly affect immune functions. For TB infection, specifically, Lu *et al.* (20) have demonstrated that the *in vitro* protective functions of IgG, including antibody-dependent cellular phagocytosis and ADCC, were associated with distinct glycosylation profiles in the IgG Fc region.

IgG galactosylation structures can change quickly in one’s inflammatory status (15). Galactose-lacking IgG glycoforms possess proinflammatory activity by binding to mannose-binding lectin and subsequently activating complement *via* alternative and lectin pathways (28). Galactosylation levels are decreased in various infectious diseases, including TB, which may provide another means for TB diagnosis (15). Liu *et al.* (22) demonstrated the diagnostic value of a high IgG G0/(G1+G2 × 2) ratio for ATB infections. Furthermore, IgG galactosylation levels varied significantly between patients with LTBI and those with ATB. Lu *et al.* (20) reported significantly more digalactosylated IgG glycoforms in individuals with LTBI than in those with ATB. These may reflect that controlled LTBI does not induce as much inflammatory activity as ATB (29, 30). Consistent with previous studies, our study revealed a significant decrease in IgG galactosylation levels in the ATB group (Fig. 2), indicating a proinflammatory status in patients with active infection. In line with this, Burel *et al.* (31) recently demonstrated that specific IgG1 glycosylation traits, including galactosylation and the H3N4F1 motif, could robustly discriminate ATB from LTBI and even identify LTBI individuals at higher risk of progression. Their IgG1-based “AbGlyc score” highlights the diagnostic and prognostic potential of antibody glycosylation, which our work extends by integrating IgA and IgM features. Furthermore, we found that galactosylation changes were not limited to IgG but were also observed in IgA and IgM glycans. These novel findings suggest that IgA and IgM may also contribute to inflammation through glycosylation and have regulatory effects similar to those of IgG. Notably, we found distinct galactosylation patterns for IgA1/2-N144/131, IgM-N171, IgM-N332, and IgM-N395 in ATB: increased monogalactosylation and decreased digalactosylation (Figs. 3*C* and 4, *C*–*E*). This may reflect a shift from digalactosylation to monogalactosylation or agalactosylation, resulting in an overall decrease in the galactosylation levels.

The sialylation of IgG is responsible for its anti-inflammatory activity. Two known mechanisms mediate the anti-inflammatory effects: (1) activation of the inhibitory FcγRIIB *via* dendritic cell–specific intercellular adhesion molecule grabbing nonintegrin (DC-SIGN) and (2) decreased affinity for activating FcγRIIIA on NK cells, resulting in reduced ADCC (15, 28). Decreased IgG sialylation has been observed in several proinflammatory diseases, such as rheumatoid arthritis, HIV infection, and hepatitis B (15, 30). Regarding TB infection, significantly decreased IgG sialylation has been reported in patients with ATB compared with LTBI (20). This reduced sialylation, together with the above-mentioned galactosylation profiles, may indicate an active inflammatory response in ATB infection compared with the controlled LTBI state (29, 30). Although we did not observe a significant decrease in IgG1 sialylation in the ATB group, we observed a decrease not only in IgG3/4 of ATB individuals but also in IgG2 of LTBI individuals when compared with controls (Supplemental Fig. S1*C*). The activated immune response indicated by the decrease in IgG2 sialylation might be associated with a better defense mechanism against bacteria in patients with LTBI, as IgG2, a poor complement activator, is responsible for the bacterial capsular polysaccharide antigen response (32, 33).

Core fucose is present in over 90% of serum IgG. The lack of core fucose on IgG glycans results in a significant increase in ADCC because of up to 100-fold enhanced affinity for the activating FcγRIIIA and FcγRIIIB (15). Lu *et al.* (20) reported that IgG isolated from individuals with LTBI contained less fucose. Meanwhile, they did find higher binding of IgG to FcγRIIIA along with enhanced PPD-specific ADCC in LTBI. These unique features may be responsible for the enhanced killing of intracellular *Mtb* by infected macrophages, indicating that distinct Fc glycosylation patterns in LTBI are associated with enhanced *Mtb* control (30). In contrast, Liu *et al.* (22) found that IgG afucosylated glycans did not differ significantly between patients with ATB and healthy donors. In addition to the relatively well-studied IgG glycoprofiles, Kumagai *et al.* (24) used a mouse infection model to characterize the changes in IgM glycosylation. They observed a >5-fold increase in IgM core fucosylation after *Mtb* infection in BCG-naïve mice. Notably, BCG vaccination attenuated this increase. Consistent with the literature, our study found that patients with ATB had higher levels of IgG1 fucosylation than those with LTBI and controls (Supplemental Fig. S1*B*). However, we did not observe significant differences in IgA and IgM fucosylation at most *N*-glycosylation sites, except for IgM N71 (Supplemental Fig. S3, *C* and *D* and Fig. 4*H*), which is inconsistent with a previous study in mice (24).

Bisecting GlcNAc indirectly affects the antibody effector function by inhibiting the addition of fucose at the glycan synthesis level (30). As a result, although to a lower degree, the presence of bisecting GlcNAc had similar effects as the lack of fucose. That is, a bisecting GlcNAc on IgG is associated with greater affinity for FcγRIII and consequently enhances ADCC activity (28). Decreased levels of IgG-bisecting glycans in patients with ATB compared with healthy donors have been reported by Liu *et al.* (22). Reasonably, in patients with ATB, IgG bisection and fucosylation change in opposite directions, thus having the same ADCC-modulating effect. However, Lu *et al.* (20) did not observe a significant difference in bisecting GlcNAc between patients with LTBI and ATB. Consistent with the findings of Liu *et al.*, our study observed significantly lower levels of IgG3/4 bisecting glycans in the ATB group than in the LTBI and control groups (Supplemental Fig. S1*D*). Similar to fucosylation, the levels of bisecting GlcNAc in IgA and IgM appeared to have little association with TB infection (Supplemental Fig. S3, *B*–*D*; data not shown for IgM).

In our study, individual glycosylation traits already demonstrated appreciable discriminatory power, with all 18 traits achieving AUCs above 0.73 and IgG1-fucosylation reaching an AUC of 0.799 (Supplemental Fig. S5). These findings suggest that single glycosylation markers can carry clinically relevant information for distinguishing LTBI from ATB. However, the performance of single features was variable, and classification based on one trait may be vulnerable to interindividual heterogeneity.

By comparison, models integrating multiple isotypes offered more balanced and, in some cases, improved performance. For example, the combined 18-feature PLS-DA model achieved an AUC of 0.825, surpassing the best-performing single trait (Fig. 5*D*). Logistic regression models using a small subset of features, such as IgG1-monogalactosylation and IgM 402-high-mannose, provided slightly lower AUCs than the top univariate trait but yielded higher overall accuracy and F1 scores, reflecting more stable classification. These results indicate that while individual traits may be informative, incorporating complementary features across IgA, IgG, and IgM can enhance robustness and mitigate the limitations of single-marker variability.

This study has several limitations that warrant consideration. First, the relatively modest sample size may constrain the statistical power and generalizability of our findings across broader populations. To mitigate this, we applied rigorous statistical analyses, including multivariate receiver operating characteristic modeling and feature selection, and observed consistent patterns across all three Ig isotypes. Second, although we initially attempted to enrich for antigen-specific Igs, we found that their abundance in elderly individuals was insufficient for robust glycosylation analysis. Therefore, our glycoprofiling was conducted on total Ig pools, which—despite lower antigen specificity—still reflect systemic immune remodeling and offer translational potential for clinical biomarker development. Third, certain *N*-glycosylation sites were represented by a limited number of glycopeptides, potentially underrepresenting site-specific heterogeneity. Future studies with improved antigen-specific enrichment and expanded peptide coverage will help refine these findings. Despite these limitations, our integrated multi-isotype approach provides novel insights into humoral glycosylation in TB and establishes a strong foundation for future mechanistic and translational studies.

This study provides the first comprehensive glycoprofiling of all three major Ig isotypes—IgG, IgA, and IgM—in elderly individuals with TB, unveiling distinct and coordinated proinflammatory Fc glycosylation signatures in active disease. Beyond replicating known IgG alterations, we demonstrate that IgA and IgM also undergo site-specific glycan remodeling during infection, particularly in galactosylation and fucosylation patterns. These findings expand the understanding of humoral glycosylation beyond the well-studied IgG isotype and suggest that IgA and IgM glycosylation changes may modulate immune effector balance and inflammatory tone during infection and aging. Overall, our data indicate that IgA and IgM glycosylation, though less explored, participate in coordinated inflammatory remodeling during TB infection, warranting further mechanistic and physiological investigation into their roles in host–pathogen interaction and immune regulation.

## Data Availability

Chromatograms of the UHPLC–QqQ–MS/MS analysis are provided as supplemental data (Supplemental Data—Chromatograms and Integration Results).

## Supplemental Data

This article contains supplemental data.

## Conflict Of Interest

The authors declare no competing interests.
