# Supplementary material for: Integrated Glycosylation Analysis of Immunoglobulin Isotypes Reveals Expanded Humoral Remodeling in Elderly Tuberculosis Infection
Source: Mol Cell Proteomics. 2025 Oct 30;24(12):101438. doi: 10.1016/j.mcpro.2025.101438 (PMC12718469; doi:10.1016/j.mcpro.2025.101438)
Supplement: Supplementary Table 1 [file mmc1.docx]

**Supplementary Table 1. Demographic and Immunological Characteristics of Study Participants**

| Sample ID | Age (years) | Sex | Height (cm) | Weight (kg) | BMI (kg/m²) | IGRA antigen (IU/mL) | IGRA nil (IU/mL) | IGRA response  antigen – nil (IU/mL) |
| --- | --- | --- | --- | --- | --- | --- | --- | --- |
| Control-01 | 69 | female | 153 | 62 | 26.5 | 0.29 | 0.11 | 0.18 |
| Control-02 | 52 | female | 162 | 70 | 26.7 | 0.09 | 0.07 | 0.02 |
| Control-03 | 45 | male | 174 | 96 | 31.7 | 0.44 | 0.29 | 0.15 |
| Control-04 | 52 | female | 159.5 | 60.5 | 23.8 | 0.09 | 0.08 | 0.01 |
| Control-05 | 56 | female | 161 | 56 | 21.6 | 0.05 | 0.04 | 0.01 |
| Control-06 | 58 | male | 166 | 64 | 23.2 | 0.02 | 0.03 | -0.01 |
| Control-07 | 66 | male | 166 | 70 | 25.4 | 0.19 | 0.04 | 0.15 |
| Control-08 | 58 | male | 165 | 72 | 26.4 | 0.02 | 0.03 | -0.01 |
| Control-09 | 50 | male | 172 | 83 | 28.1 | 0.11 | 0.14 | -0.03 |
| Control-10 | 68 | male | 173.3 | 90.1 | 30 | 0.29 | 0.03 | 0.26 |
| Control-11 | 52 | female | 155 | 64 | 26.6 | 0.10 | 0.10 | 0.00 |
| Control-12 | 52 | male | 172 | 74 | 25 | 0.03 | 0.04 | -0.01 |
| Control-13 | 53 | male | 163 | 68.4 | 25.7 | 0.08 | 0.16 | -0.08 |
| Control-14 | 45 | female | 165 | 72 | 26.4 | 0.03 | 0.03 | 0.00 |
| Control-15 | 62 | male | 167 | 62 | 22.2 | 0.17 | 0.10 | 0.07 |
| Control-16 | 62 | male | 163 | 49 | 18.4 | 0.04 | 0.03 | 0.01 |
| Control-17 | 52 | female | 163 | 51 | 19.38 | 0.13 | 0.1 | 0.03 |
| Control-18 | 55 | male | 172 | 75 | 25.4 | 0.08 | 0.11 | -0.03 |
| Control-19 | 37 | male | 173 | 62 | 20.7 | 0.3 | 0.08 | 0.22 |
| Control-20 | 38 | male | 178 | 62 | 19.57 | 0.04 | 0.04 | 0 |
| LTBI-01 | 52 | female | 160 | 55 | 21.5 | >10 | 0.20 | 9.80 |
| LTBI-02 | 81 | male | 156 | 58 | 23.8 | >10 | 0.69 | 9.31 |
| LTBI-03 | 59 | female | 154 | 56.5 | 23.8 | 4.41 | 0.49 | 3.92 |
| LTBI-04 | 57 | male | 171 | 84.6 | 28.9 | 4.80 | 0.02 | 4.78 |
| LTBI-05 | 51 | male | 174 | 84 | 27.7 | 4.64 | 0.07 | 4.57 |
| LTBI-06 | 54 | male | 168 | 67 | 23.7 | 7.79 | 0.30 | 7.49 |
| LTBI-07 | 58 | male | 180 | 67 | 20.7 | 8.56 | 0.28 | 8.28 |
| LTBI-08 | 38 | male | 168 | 105 | 37.2 | 1.03 | 0.02 | 1.01 |
| LTBI-09 | 63 | male | 173 | 73 | 24.4 | 0.50 | 0.02 | 0.48 |
| LTBI-10 | 82 | male | 168 | 72 | 25.5 | 0.53 | 0.09 | 0.44 |
| LTBI-11 | 63 | male | 173 | 85 | 28.4 | 4.03 | 0.37 | 3.66 |
| LTBI-12 | 51 | female | 162 | 70 | 26.7 | 3.34 | 0.05 | 3.29 |
| LTBI-13 | 25 | male | 178 | 60 | 18.9 | 0.44 | 0.03 | 0.41 |
| LTBI-14 | 61 | female | 159 | 61 | 24.1 | >10 | 0.22 | 9.78 |
| LTBI-15 | 44 | female | 165 | 69 | 25.3 | 9.27 | 0.17 | 9.10 |
| LTBI-16 | 48 | male | 168 | 75 | 26.6 | 3.45 | 0.08 | 3.37 |
| LTBI-17 | 58 | female | 161 | 59 | 22.8 | 5.64 | 0.45 | 5.19 |
| LTBI-18 | 60 | male | 170 | 75 | 26 | 8.99 | 0.08 | 8.91 |
| ATB-01 | 74 | male | 173 | 83 | 27.7 | >10 | 0.78 | 9.22 |
| ATB-02 | 50 | female | 164 | 63 | 23.4 | 1.10 | 0.10 | 1.00 |
| ATB-03 | 92 | male | 165 | 64 | 23.5 | >10 | 0.10 | 9.90 |
| ATB-04 | 43 | male | 166 | 60.5 | 22 | 0.83 | 0.04 | 0.79 |
| ATB-05 | 58 | male | 168 | 81 | 28.7 | 5.63 | 0.37 | 5.26 |
| ATB-06 | 34 | male | 171 | 68 | 23.3 | 0.87 | 0.04 | 0.83 |
| ATB-07 | 50 | male | 181 | 69 | 21.1 | 1.88 | 0.47 | 1.41 |
| ATB-08 | 44 | male | 172 | 50 | 16.9 | 3.25 | 0.30 | 2.95 |
| ATB-09 | 77 | female | 148.3 | 47.8 | 21.7 | 8.24 | 0.06 | 8.18 |
| ATB-10 | 74 | male | 153 | 49 | 20.9 | 1.63 | 0.14 | 1.49 |
| ATB-11 | 71 | male | 167 | 65.5 | 23.5 | 0.06 | 0.07 | -0.01 |
| ATB-12 | 80 | male | 173 | 70 | 23.4 | 0.94 | 0.39 | 0.55 |
| ATB-13 | 64 | male | 167.6 | 56.3 | 20 | 6.08 | 0.17 | 5.91 |
| ATB-14 | 45 | female | 148 | 38.5 | 17.6 | 0.49 | 0.09 | 0.40 |
| ATB-15 | 87 | male | 159 | 47 | 18.6 | 1.21 | 0.04 | 1.17 |
| ATB-16 | 39 | female | 153.1 | 44.7 | 19.1 | 3.69 | 0.18 | 3.51 |
| ATB-17 | 79 | male | 152 | 50 | 21.6 | 1.87 | 0.12 | 1.75 |
| ATB-18 | 56 | female | 154 | 47 | 19.8 | >10 | >10 | 0.00 |
| ATB-19 | 62 | male | 177 | 63.5 | 20.3 | >10 | 1.17 | 8.83 |
| ATB-20 | 24 | female | 159 | 43 | 17 | >10 | 0.37 | 9.63 |
