# Supplementary material for: Integrated Glycosylation Analysis of Immunoglobulin Isotypes Reveals Expanded Humoral Remodeling in Elderly Tuberculosis Infection
Source: Mol Cell Proteomics. 2025 Oct 30;24(12):101438. doi: 10.1016/j.mcpro.2025.101438 (PMC12718469; doi:10.1016/j.mcpro.2025.101438)
Supplement: Supplementary Table 2 [file mmc2.docx]

**Supplementary Table 2. Parameters of multiple reaction monitoring acquisition mode for analytes.**

| **Analytes** | **RT (min)** | **Cone**  **(V)** | **CE**  **(eV)** | **Precursor ions**  **(m / z)** | **Product ions**  **(m / z)** |
| --- | --- | --- | --- | --- | --- |
| **IgG glycopeptides^a, b^** | | | | | |
| (1)  IgG1 H3N4F1 | 2.0 | 35 | 15 | 878.8 (3+) | 204.1 |
| (2)   IgG1 H3N5F1 | 2.0 | 35 | 30 | 946.5 (3+) | 204.1 |
| (3)   IgG1 H4N4 | 2.0 | 35 | 10 | 884.1 (3+) | 204.1 |
| (4)   IgG1 H4N4F1 | 2.0 | 35 | 25 | 932.8 (3+) | 204.1 |
| (5)   IgG1 H4N4F1S1 | 2.1 | 35 | 40 | 1029.8 (3+) | 204.1 |
| (6)   IgG1 H4N5 | 2.0 | 35 | 25 | 951.7 (3+) | 204.1 |
| (7)   IgG1 H4N5F1 | 2.0 | 35 | 20 | 1000.5 (3+) | 204.1 |
| (8)   IgG1 H5N4 | 2.0 | 35 | 10 | 938.1 (3+) | 366.1 |
| (9)   IgG1 H5N4F1 | 2.0 | 35 | 20 | 986.8 (3+) | 366.1 |
| (10)   IgG1 H5N4F1S1 | 2.1 | 35 | 25 | 1083.8 (3+) | 366.1 |
| (11)   IgG1 H5N5F1 | 2.0 | 35 | 30 | 1054.5 (3+) | 366.1 |
| (12)   IgG2 H3N4F1 | 2.7 | 35 | 25 | 868.1 (3+) | 204.1 |
| (13)   IgG2 H3N5F1 | 2.7 | 35 | 15 | 935.8 (3+) | 204.1 |
| (14)   IgG2 H4N4 | 2.7 | 35 | 15 | 873.4 (3+) | 204.1 |
| (15)   IgG2 H4N4F1 | 2.7 | 35 | 30 | 922.1 (3+) | 204.1 |
| (16)   IgG2 H4N4F1S1 | 2.8 | 35 | 40 | 1019.1 (3+) | 204.1 |
| (17)   IgG2 H4N5 | 2.7 | 35 | 30 | 941.1 (3+) | 204.1 |
| (18)   IgG2 H4N5F1 | 2.7 | 35 | 15 | 989.9 (3+) | 204.1 |
| (19)   IgG2 H5N4F1 | 2.7 | 35 | 25 | 976.1 (3+) | 366.1 |
| (20)   IgG2 H5N4F1S1 | 2.8 | 35 | 35 | 1073.1 (3+) | 366.1 |
| (21)   IgG2 H5N5F1 | 2.7 | 35 | 20 | 1043.8 (3+) | 366.1 |
| (22)  IgG3/4 H3N4F1 | 2.3 | 35 | 25 | 873.4 (3+) | 204.1 |
| (23)  IgG3/4 H3N5F1 | 2.4 | 35 | 10 | 941.4 (3+) | 204.1 |
| (24)  IgG3/4 H4N4F1 | 2.3 | 35 | 30 | 927.4 (3+) | 204.1 |
| (25)  IgG3/4 H4N5F1 | 2.4 | 35 | 20 | 995.1 (3+) | 204.1 |
| (26)  IgG3/4 H5N4F1S1 | 2.4 | 35 | 25 | 1078.4 (3+) | 366.1 |
| **IgA glycopeptides^a,c^** | | | | | |
| (27) IgA1/2 N144/131 H3N5 | 8.8 | 35 | 35 | 1117.1 (4+) | 366.1 |
| (28) IgA1/2 N144/131 H4N4S1 | 9.0 | 35 | 35 | 1179.6 (4+) | 204.1 |
|  |  |  |  | 943.9 (5+) | 366.1 |
| (29) IgA1/2 N144/131 H4N5 | 8.7 | 35 | 35 | 1157.6 (4+) | 204.1 |
| (30) IgA1/2 N144/131 H4N5S1 | 9.0 | 35 | 35 | 984.5 (5+) | 204.1 |
|  |  |  |  | 1230.4 (4+) | 366.1 |
| (31) IgA1/2 N144/131 H5N2 | 8.8 | 35 | 35 | 1045.8 (4+) | 204.1 |
| (32) IgA1/2 N144/131 H5N3S1 | 9.0 | 35 | 35 | 1169.3 (4+) | 204.1 |
| (33) IgA1/2 N144/131 H5N4S1 | 9.0 | 35 | 35 | 976.3 (5+) | 204.1 / 366.1 |
|  |  |  |  | 1220.1 (4+) | 366.1 |
| (34) IgA1 / 2 N144 / 131 H5N4S2 | 9.3 | 35 | 25 | 1034.5 (5+) | 204.1 |
|  |  |  |  | 1292.9 (4+) | 366.1 |
| (35) IgA1/2 N144/131 H5N5 | 8.7 | 35 | 25 | 1198.1 (4+) | 366.1 |
| (36) IgA1/2 N144/131 H5N5S1 | 9.0 | 35 | 25 | 1016.9 (4+) | 366.1 |
| (37) IgA1/2 N144/131 H5N5S2 | 9.3 | 35 | 25 | 1075.1 (4+) | 366.1 |
| (38) IgA1/2 N144/131 H6N2 | 8.8 | 35 | 25 | 1086.2 (4+) | 204.1 |
| (39) IgA1/2 N144/131 H6N3S1 | 9.0 | 35 | 25 | 968.1 (5+) | 366.1 |
| (40) IgA1/2 N144/131 H7N2 | 8.8 | 35 | 25 | 1126.7 (4+) | 204.1 |
| (41) IgA1/2 N144/131 H8N2 | 8.7 | 35 | 25 | 1167.3 (4+) | 204.1 |
| (42) IgA1/2 N340/327 H5N4F1S1 | 6.7 | 35 | 25 | 1102.8 (4+) | 204.1 |
| (43) IgA1/2 N340/327 H5N4F1S2 | 6.9 | 35 | 25 | 1175.2 (4+) | 366.1 |
| (44) IgA1/2 N340/327 H5N4S1 | 6.8 | 35 | 25 | 1066.3 (4+) | 204.1 |
| (45) IgA1/2 N340/327 H5N5F1S2 | 6.9 | 35 | 25 | 1226 (4+) | 366.1 |
| (46) IgA1/2 N340/327 H6N5F1S1 | 6.8 | 35 | 25 | 1193.7 (4+) | 366.1 |
| (47) IgA1/2 N340/327tr H5N5F1S1 | 6.3 | 35 | 25 | 1112.5 (4+) | 366.1 |
| (48) IgA1/2 N340/327tr H5N5F1S2 | 6.6 | 35 | 35 | 1580.0 (3+) | 366.1 |
|  |  |  |  | 948.4 (5+) | 366.1 |
| (49) IgA1/2 N340/327tr H5N4F1S1 | 6.4 | 35 | 25 | 1061.7 (4+) | 366.1 |
| (50) IgA1/2 N340/327tr H5N4F1S2 | 6.5 | 35 | 25 | 1134.5 (4+) | 204.1 |
| (51)  IgA2 N205 H4N5F1 | 3.7 | 35 | 25 | 923.5 (3+) | 366.1 |
| (52)  IgA2 N205 H3N4F1 | 3.8 | 35 | 25 | 801.7 (3+) | 204.1 |
| (53)  IgA2 N205 H3N5F1 | 3.7 | 35 | 25 | 869.3 (3+) | 204.1 |
| (54)  IgA2 N205 H4N4F1S1 | 3.8 | 35 | 25 | 952.7 (3+) | 204.1 |
| (55)  IgA2 N205 H5N4F1S1 | 3.9 | 35 | 25 | 1006.8 (3+) | 366.1 |
| (56)  IgA2 N205 H5N4F1S2 | 4.0 | 35 | 25 | 828.1 (4+) | 204.1 |
| (57)IgA2 N205 H5N4S1 | 3.8 | 35 | 25 | 958.0 (3+) | 366.1 |
| (58) IgA2 N205 H5N5F1 | 3.7 | 35 | 25 | 977.4 (3+) | 366.1 |
| (59) IgA2 N205 H5N5F1S1 | 3.8 | 35 | 25 | 1074.5 (3+) | 366.1 |
| **IgM glycopeptides^a,d^** | | | | | |
| (60)  IgM N171 H4N3F1S1 | 2.1 | 35 | 25 | 993.7 (3+) | 204.1 |
| (61)   IgM N171 H5N3F1S1 | 2.1 | 35 | 25 | 1047.7 (3+) | 366.1 |
| (62)   IgM N171 H5N4S1 | 2.0 | 35 | 25 | 800.3 (4+) | 204.1 / 274.1 |
| (63)   IgM N171 H5N4F1S1 | 2.1 | 35 | 25 | 1115.4 (3+) | 366.1 |
| (64)   IgM N171 H5N5S1 | 2.1 | 35 | 25 | 851.0 (4+) | 204.1 |
| (65)   IgM N171 H5N5F1S1 | 2.2 | 35 | 25 | 887.6 (4+) | 204.1 |
| (66)   IgM N171 H6N3F1S1 | 2.1 | 35 | 25 | 826.5 (4+) | 204.1 / 274.1 |
| (67)   IgM N332 H4N5F1S1 | 5.9 | 35 | 25 | 1110.7 (4+) | 204.1 |
| (68)   IgM N332 H5N4F1S1 | 5.8 | 35 | 25 | 1100.4 (4+) | 366.1 |
| (69)   IgM N332 H5N4F1S2 | 6.0 | 35 | 25 | 1173.2 (4+) | 366.1 |
| (70)   IgM N332 H5N5F1S1 | 5.8 | 35 | 25 | 1151.2 (4+) | 366.1 |
| (71)   IgM N332 H5N5F1S2 | 6.0 | 35 | 25 | 1224.0 (4+) | 366.1 |
| (72)   IgM N332 H6N4S1 | 6.0 | 35 | 25 | 1104.4 (4+) | 204.1 |
| (73)   IgM N395 H4N5F1 | 2.2 | 35 | 25 | 871.0 (3+) | 204.1 / 274.1 |
| (74)   IgM N395 H5N4F1S2 | 2.3 | 35 | 25 | 1051.4 (3+) | 366.1 |
| (75)   IgM N395 H5N5F1S1 | 2.3 | 35 | 25 | 1022.0 (3+) | 366.1 |
| (76)   IgM N395 H5N5F1S2 | 2.4 | 35 | 25 | 839.5 (4+) | 204.1 |
| (77)   IgM N395 H5N4F1S1 | 2.2 | 35 | 25 | 954.3 (3+) | 204.1 |
| (78)   IgM N395 H5N5F1 | 2.2 | 35 | 25 | 925.0 (3+) | 366.1 |
| (79)   IgM N402 H4N2 | 3.8 | 35 | 25 | 757.6 (3+) | 204.1 |
| (80)   IgM N402 H4N3 | 3.8 | 35 | 25 | 825.3 (3+) | 204.1 |
| (81)   IgM N402 H5N2 | 3.7 | 35 | 25 | 811.6 (3+) | 204.1 |
| (82)   IgM N402 H5N3 | 3.7 | 35 | 25 | 879.3 (3+) | 204.1 |
| (83)   IgM N402 H9N2 | 3.6 | 35 | 25 | 1027.7 (3+) | 204.1 / 274.1 / 366.1 |
| (84)   IgM N563 H3N5F1 | 6.5 | 35 | 25 | 1003.9 (4+) | 204.1 |
| (85)   IgM N563 H4N5F1 | 6.5 | 35 | 25 | 1044.4 (4+) | 204.1 |
| (86)   IgM N563 H4N5F1S1 | 6.8 | 35 | 25 | 1117.2 (4+) | 204.1 |
| (87)   IgM N563 H5N2 | 6.4 | 35 | 25 | 1194.5 (3+) | 204.1 |
| (88)   IgM N563 H6N2 | 6.8 | 35 | 25 | 1248.5 (3+) | 204.1 |
| (89)   IgM N563 H7N2 | 6.4 | 35 | 25 | 1302.5 (3+) | 204.1 |
| (90)   IgM N563 H8N2 | 6.7 | 35 | 25 | 1356.5 (3+) | 204.1 |
| (91)   IgM J chain N71 H5N4S1 | 4.3 | 35 | 25 | 1048.1 (3+) | 366.1 |
| (92)   IgM J chain N71 H5N4S2 | 4.4 | 35 | 25 | 859.1 (4+) | 204.1 |
| (93)   IgM J chain N71 H5N4F1S1 | 4.3 | 35 | 25 | 1096.7 (3+) | 366.1 |
| (94)   IgM J chain N71 H5N4F1S2 | 4.4 | 35 | 25 | 895.6 (4+) | 204.1 / 274.1 / 366.1 |
| (95)   IgM J chain N71 H5N5S1 | 4.3 | 35 | 25 | 1115.7 (3+) | 366.1 |
| **Ig surrogate peptides and internal standard (IS)** | | | | | |
| IgG - DTLMISR | 3.0 | 35 | 14 | 418.2 (2+) | 506.3 / 619.4 / 310.2 |
| IgG1 - GPSVFPLAPSSK | 3.8 | 35 | 21 | 593.8 (2+) | 699.4 / 846.5 / 418.2 |
| IgG2 - TTPPMLDSDGSFFLYSK | 4.7 | 35 | 34 | 953.5 (2+) | 852.4 / 804.4 / 1150.5 |
| IgG3 - WYVDGVEVHNAK | 3.2 | 35 | 16 | 472.9 (3+) | 534.2 / 484.7 / 697.3 |
| IgG4 - TTPPVLDSDGSFFLYSR | 4.7 | 35 | 34 | 951.5 (2+) | 850.4 / 1293.6 |
| IgA1 - TPLTATLSK | 4.4 | 35 | 16 | 466.3 (2+) | 415.8 / 620.4 / 733.4 |
| IgA2 - DASGATFTWTPSSGK | 5.4 | 35 | 27 | 756.9 (2+) | 475.3 / 111.5 / 863.4 |
| IgM - FTCTVTHTDLPSPLK | 5.5 | 35 | 25 | 572.9 (3+) | 734.8 / 654.8 / 785.4 / 870.5 |
| IS 1^e^ | 1.8 | 35 | 25 | 500.7 | 570.3 / 772.4 / 685.4 |
| IS 2 | 6.0 | 35 | 25 | 782.3 | 458.2 / 818.4 / 915.5 |
| IS 3 | 7.3 | 35 | 25 | 791.1 | 374.2 / 848.5 / 962.5 |
| IS 4: | 4.3 | 35 | 25 | 469.9 | 419.2 / 627.3 / 740.5 |

^a^ H, hexose; N, *N*-acetylglucosamine; F, fucose; S, *N*-acetylneuraminic acid.

^b^ Peptide sequence of IgG1 glycopeptides: EEQY**N**STYR.

Peptide sequence of IgG2 glycopeptides: EEQF**N**STFR.

Peptide sequence of IgG3 glycopeptides: EEQY**N**STFR.

Peptide sequence of IgG4 glycopeptides: EEQF**N**STYR.

^c^ Peptide sequence of IgA1/2 N144/131 glycopeptides: LSLHRPALEDLLLGSEA**N**LTCTLTGLR.

Peptide sequence of IgA1/2 N340/327 glycopeptides: LAGKPTHV**N**VSVVMAEVDGTCY.

Peptide sequence of IgA1/2 N340/327 truncated glycopeptides: LAGKPTHV**N**VSVVMAEVDGTC.

Peptide sequence of IgA2 N205 glycopeptides: TPLTA**N**ITK.

^d^ Peptide sequence of IgM N171 glycopeptides: YK**N**NSDISSTR.

Peptide sequence of IgM N332 glycopeptides: GLTFQQ**N**ASSMCVPDQDTAIR.

Peptide sequence of IgM N395 glycopeptides: THT**N**ISE.

Peptide sequence of IgM N402 glycopeptides: SHP**N**ATFSAVGE.

Peptide sequence of IgM N563 glycopeptides: STGKPTLY**N**VSLVMSDTAGTCY.

Peptide sequence of IgM J chain N71 glycopeptides: E**N**ISDPTSPLR.

^e^ IS1: ITFSWK **YKNNSDI{^13^C_6_, ^15^N}SSTR** GFPSVLR was used for IgM N171, N395 and N402 glycopeptides.

IS2: VDHR **GLTFQQNASSMCV{^13^C_5_, ^15^N}PDQDTAIR** VFAIPPSF was used for IgM N332, J chain N71, IgG glycopeptides, IgGs and IgM surrogate peptides.

IS3: TVDK **STGKPTLYNVSLV{^13^C_5_, ^15^N}MSDTAGTCY** was used for IgM N563 glycopeptides.

IS4: AYPESK **TPLTATL{^13^C_6_, ^15^N}SK** SGNTFR was used for IgA1, IgA2 and IgA glycopeptides.
