# Supplementary material for: Integrated Glycosylation Analysis of Immunoglobulin Isotypes Reveals Expanded Humoral Remodeling in Elderly Tuberculosis Infection
Source: Mol Cell Proteomics. 2025 Oct 30;24(12):101438. doi: 10.1016/j.mcpro.2025.101438 (PMC12718469; doi:10.1016/j.mcpro.2025.101438)
Supplement: Supplementary Table 4 [file mmc4.docx]

**Supplementary Table 4.** Calculation of derived glycosylation traits. Each glycopeptide's response is normalized to a percentage by dividing its individual response by the cumulative response of all glycopeptides at the respective N-glycosylation sites.

| **IgG glycosylation traits** | |
| --- | --- |
| **IgG1** | |
| **Glycosylation trait** | **Formula** |
| **Agalactosylation** | H3N4F1 + H3N5F1 |
| **Galactosylation** | (H4N4 + H4N4F1 + H4N4F1S1 + H4N5 + H4N5F1) *0.5 + H5N4 + H5N4F1 + H5N4F1S1 + H5N5F1 |
| **Monogalactosylation** | (H4N4 + H4N4F1 + H4N4F1S1 + H4N5 + H4N5F1) *0.5 |
| **Digalactosylation** | H5N4 + H5N4F1 + H5N4F1S1 + H5N5F1 |
| **Sialylation** | (H4N4F1S1 + H5N4F1S1) *0.5 |
| **Fucosylation** | H3N4F1 + H3N5F1 + H4N4F1 + H4N4F1S1 + H4N5F1 + H5N4F1 + H5N4F1S1 + H5N5F1 |
| **Bisection** | H3N5F1 + H4N5 + H4N5F1 + H5N5F1 |
| **IgG2** | |
| **Agalactosylation** | H3N4F1 + H3N5F1 |
| **Galactosylation** | (H4N4 + H4N4F1 + H4N4F1S1 + H4N5 + H4N5F1) *0.5 + H5N4F1 + H5N4F1S1 + H5N5F1 |
| **Monogalactosylation** | (H4N4 + H4N4F1 + H4N4F1S1 + H4N5 + H4N5F1) *0.5 |
| **Digalactosylation** | H5N4F1 + H5N4F1S1 + H5N5F1 |
| **Sialylation** | (H4N4F1S1 + H5N4F1S1) *0.5 |
| **Fucosylation** | H3N4F1 + H3N5F1 + H4N4F1 + H4N4F1S1 + H4N5F1 + H5N4F1 + H5N4F1S1 + H5N5F1 |
| **Bisection** | H3N5F1 + H4N5 + H4N5F1 + H5N5F1 |
| **IgG3/4** | |
| **Agalactosylation** | H3N4F1 + H3N5F1 |
| **Galactosylation** | (H4N4F1 + H4N5F1) *0.5 + H5N4F1S1 |
| **Monogalactosylation** | (H4N4F1 + H4N5F1) *0.5 |
| **Digalactosylation** | H5N4F1S1 |
| **Sialylation** | H5N4F1S1 *0.5 |
| **Fucosylation** | H3N4F1 + H3N5F1+ H4N4F1 + H4N5F1+ H5N4F1S1 |
| **Bisection** | H3N5F1 + H4N5F1 |
|  |  |
| **IgA glycosylation traits** | |
| **IgA1/2 N144/131^a^** | |
| **Agalactosylation** | H3N5 |
| **Galactosylation** | (H4N4S1 + H4N5 + H4N5S1 + H5N3S1 + H6N3S1) *0.5 + H5N4S1 + H5N4S2 + H5N5 + H5N5S1 + H5N5S2 |
| **Monogalactosylation** | (H4N4S1 + H4N5 + H4N5S1 + H5N3S1 + H6N3S1) *0.5 |
| **Digalactosylation** | H5N4S1 + H5N4S2 + H5N5 + H5N5S1 + H5N5S2 |
| **Trigalactosylation** | NA |
| **Sialylation** | (H4N4S1 + H4N5S1 + H5N3S1 + H5N4S1 + H5N5S1 + H6N3S1) *0.5 + H5N4S2 + H5N5S2 |
| **Fucosylation** | NA |
| **Bisection** | H3N5 + H4N5 + H4N5S1 + H5N5 + H5N5S1 + H5N5S2 |
| **Oligo mannose** | H5N2 + H6N2 + H7N2 + H8N2 |
| **Hybrid type** | H5N3S1 + H6N3S1 |
| **IgA1/2 N340/327 ^b^** | |
| **Agalactosylation** | NA |
| **Galactosylation** | H5N4F1S1 + H5N4F1S2 + H5N4S1 + H5N5F1S2 + tr-H5N5F1S1 + tr-H5N5F1S2 + tr-H5N4F1S1 + tr-H5N4F1S2+ H6N5F1S1 |
| **Monogalactosylation** | NA |
| **Digalactosylation** | H5N4F1S1 + H5N4F1S2 + H5N4S1 + H5N5F1S2 + tr-H5N5F1S1 + tr-H5N5F1S2 + tr-H5N4F1S1 + tr-H5N4F1S2 |
| **Trigalactosylation** | H6N5F1S1 |
| **Sialylation** | (H5N4F1S1 + H5N4S1 + H6N5F1S1 + tr-H5N5F1S1 + tr-H5N4F1S1) *0.5 + H5N4F1S2 + H5N5F1S2 + tr-H5N5F1S2 + tr-H5N4F1S2 |
| **Fucosylation** | H5N4F1S1 + H5N4F1S2 + H5N5F1S2 + H6N5F1S1 + tr-H5N5F1S1 + tr-H5N5F1S2 + tr-H5N4F1S1 + tr-H5N4F1S2 |
| **Bisection** | H5N5F1S2 + tr-H5N5F1S1 + tr-H5N5F1S2 |
| **Oligo mannose** | NA |
| **Hybrid type** | NA |
| **IgA2 N205** | |
| **Agalactosylation** | H3N4F1 + H3N5F1 |
| **Galactosylation** | (H4N5F1 + H4N4F1S1) *0.5 + H5N4F1S1 + H5N4F1S2 + H5N4S1 + H5N5F1 + H5N5F1S1 |
| **Monogalactosylation** | (H4N5F1 + H4N4F1S1) *0.5 |
| **Digalactosylation** | H5N4F1S1 + H5N4F1S2 + H5N4S1 + H5N5F1 + H5N5F1S1 |
| **Trigalactosylation** | NA |
| **Sialylation** | (H4N4F1S1 + H5N4F1S1 + H5N4S1 + H5N5F1S1) *0.5 + H5N4F1S2 |
| **Fucosylation** | H4N5F1 + H3N4F1 + H3N5F1 + H4N4F1S1 + H5N4F1S1 + H5N4F1S2 + H5N5F1 + H5N5F1S1 |
| **Bisection** | H4N5F1 + H3N5F1 + H5N5F1 + H5N5F1S1 |
| **Oligo mannose** | NA |
| **Hybrid type** | NA |
| ^a^ Glycopeptides from site IgA1 N144 and IgA2 N131 are isobaric ions which were analyzed concurrently in our study.  ^b^ Glycopeptides from site IgA1 N340 and IgA2 N327 are isobaric ions which were analyzed concurrently in our study. | |
| **IgM glycosylation traits** | |
| **IgM N171** | |
| **Agalactosylation** | NA |
| **Galactosylation** | (H4N3F1S1+H5N3F1S1+H6N3F1S1)*0.5+ H5N4S1+ H5N4F1S1+ H5N5S1+ H5N5F1S1 |
| **Monogalactosylation** | (H4N3F1S1+H5N3F1S1+H6N3F1S1)*0.5 |
| **Digalactosylation** | H5N4S1+ H5N4F1S1+ H5N5S1+ H5N5F1S1 |
| **Sialylation** | (H4N3F1S1+ H5N3F1S1+ H5N4S1+ H5N4F1S1+ H5N5S1+ H5N5F1S1+ H6N3F1S1)*0.5 |
| **Fucosylation** | H4N3F1S1+ H5N3F1S1+ H5N4F1S1+ H5N5F1S1+ H6N3F1S1 |
| **Bisection** | H5N5S1+ H5N5F1S1 |
| **Oligo mannose** | NA |
| **Hybrid type** | H5N3F1S1+ H6N3F1S1 |
| **IgM N323** | |
| **Agalactosylation** | NA |
| **Galactosylation** | (H4N5F1S1+ H6N4S1)*0.5+ H5N4F1S1+ H5N4F1S2+ H5N5F1S1+ H5N5F1S2 |
| **Monogalactosylation** | (H4N5F1S1+ H6N4S1)*0.5 |
| **Digalactosylation** | H5N4F1S1+ H5N4F1S2+ H5N5F1S1+ H5N5F1S2 |
| **Sialylation** | (H4N5F1S1+H5N4F1S1+H5N5F1S1+ H6N4S1)*0.5+(H5N4F1S2+H5N5F1S2) |
| **Fucosylation** | H4N5F1S1+ H5N4F1S1+ H5N4F1S2+ H5N5F1S1+ H5N5F1S2 |
| **Bisection** | H5N5F1S1+ H5N5F1S2+ H6N4S1 |
| **Oligo mannose** | NA |
| **Hybrid type** | H6N4S1 |
| **IgM N395** | |
| **Agalactosylation** | NA |
| **Galactosylation** | H4N5F1*0.5+ H5N4F1S2+H5N5F1S1+H5N5F1S2+H5N4F1S1+  H5N5F1 |
| **Monogalactosylation** | H4N5F1*0.5 |
| **Digalactosylation** | H5N4F1S2+H5N5F1S1+H5N5F1S2+H5N4F1S1+H5N5F1 |
| **Sialylation** | (H5N5F1S1+ H5N4F1S1)*0.5+ H5N4F1S2+ H5N5F1S2 |
| **Fucosylation** | H4N5F1+H5N4F1S2+H5N5F1S1+H5N5F1S2+H5N4F1S1+  H5N5F1 |
| **Bisection** | H4N5F1+H5N5F1S1+H5N5F1S2+H5N5F1 |
| **Oligo mannose** | NA |
| **Hybrid type** | NA |
| **IgM N402** | |
| **Agalactosylation** | NA |
| **Galactosylation** | (H4N3+ H5N3)*0.5 |
| **Monogalactosylation** | (H4N3+ H5N3)*0.5 |
| **Digalactosylation** | NA |
| **Sialylation** | NA |
| **Fucosylation** | NA |
| **Bisection** | NA |
| **Oligo mannose** | H4N2+H5N2+H9N2 |
| **Hybrid type** | H5N3 |
| **IgM N563** | |
| **Agalactosylation** | H3N5F1 |
| **Galactosylation** | (H4N5F1+H4N5F1S1)*0.5 |
| **Monogalactosylation** | (H4N5F1+H4N5F1S1)*0.5 |
| **Digalactosylation** | NA |
| **Sialylation** | H4N5F1S1*0.5 |
| **Fucosylation** | H3N5F1+H4N5F1+H4N5F1S1 |
| **Bisection** | H3N5F1+H4N5F1+H4N5F1S1 |
| **Oligo mannose** | H5N2+H6N2+H7N2+H8N2 |
| **Hybrid type** | NA |
| **IgM J chain N71** | |
| **Agalactosylation** | NA |
| **Galactosylation** | H5N4S1+H5N4S2+H5N4F1S1+H5N4F1S2+H5N5S1 |
| **Monogalactosylation** | NA |
| **Digalactosylation** | H5N4S1+H5N4S2+H5N4F1S1+H5N4F1S2+H5N5S1 |
| **Sialylation** | (H5N4S1+H5N4F1S1+H5N5S1)*0.5+(H5N4S2+H5N4F1S2) |
| **Fucosylation** | H5N4F1S1+H5N4F1S2 |
| **Bisection** | H5N5S1 |
| **Oligo mannose** | NA |
| **Hybrid type** | NA |

H: hexose, N: N-acetylglucosamine, F: fucose, S: sialic acid
