# Supplementary material for: Integrated Glycosylation Analysis of Immunoglobulin Isotypes Reveals Expanded Humoral Remodeling in Elderly Tuberculosis Infection
Source: Mol Cell Proteomics. 2025 Oct 30;24(12):101438. doi: 10.1016/j.mcpro.2025.101438 (PMC12718469; doi:10.1016/j.mcpro.2025.101438)
Supplement: Supplementary Table 5 [file mmc5.docx]

| **Supplementary Table 5.** Model_Performance — Overview of classification performance of models built using immunoglobulin features to distinguish ATB from LTBI. | | | | | | |
| --- | --- | --- | --- | --- | --- | --- |
| Feature Set | Model | Accuracy | Sensitivity | Specificity | Precision | F1 Score |
| 4 IgA Features | PLSDA | 0.6842 | 0.75 | 0.6111 | 0.6818 | 0.7143 |
| 7 IgG Features | PLSDA | 0.6053 | 0.65 | 0.5556 | 0.619 | 0.6341 |
| 7 IgM Features | PLSDA | 0.7632 | 0.75 | 0.7778 | 0.7895 | 0.7692 |
| 18 Ig Features | PLSDA | 0.7632 | 0.8 | 0.7222 | 0.7619 | 0.7805 |
| IgA N144/131-Monogalactosylation | Decision Tree | 0.7368 | 0.95 | 0.5 | 0.6786 | 0.7917 |
| IgG1-Monogalactosylation and IgG1-Fucosylation | Decision Tree | 0.8158 | 0.7 | 0.9444 | 0.9333 | 0.8 |
| IgM 171-Hybrid and IgM 402-High-mannose | Decision Tree | 0.7895 | 0.65 | 0.9444 | 0.9286 | 0.7647 |
| IgG1-Monogalactosylation and IgM 402-High-mannose | Decision Tree | 0.9211 | 0.9 | 0.9444 | 0.9474 | 0.9231 |
| IgA N144/131-Monogalactosylation | Logistic Regression | 0.6842 | 0.75 | 0.6111 | 0.6818 | 0.7143 |
| IgG1-Monogalactosylation and IgG1-Fucosylation | Logistic Regression | 0.7105 | 0.75 | 0.6667 | 0.7143 | 0.7317 |
| IgM 171-Hybrid and IgM 402-High-mannose | Logistic Regression | 0.7105 | 0.7 | 0.7222 | 0.7368 | 0.7179 |
| IgG1-Monogalactosylation and IgM 402-High-mannose | Logistic Regression | 0.8158 | 0.75 | 0.8889 | 0.8824 | 0.8108 |
