# Supplementary material for: Integrated Glycosylation Analysis of Immunoglobulin Isotypes Reveals Expanded Humoral Remodeling in Elderly Tuberculosis Infection
Source: Mol Cell Proteomics. 2025 Oct 30;24(12):101438. doi: 10.1016/j.mcpro.2025.101438 (PMC12718469; doi:10.1016/j.mcpro.2025.101438)
Supplement: Supplementary Figure [file mmc6.pdf]

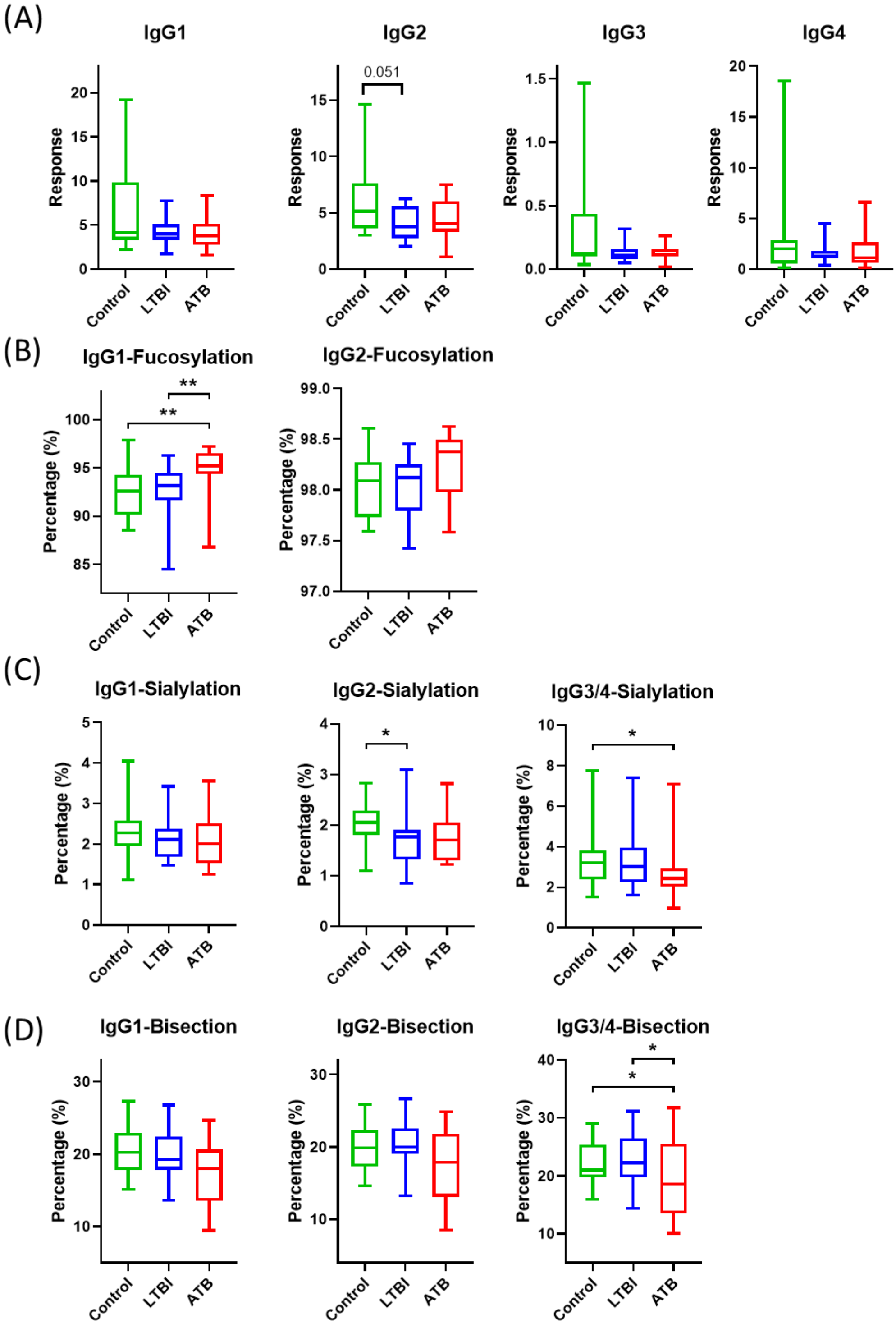

Supplementary Figure 1. Differences in IgG and IgG glycosylation profiles among the control, latent tuberculosis infection (LTBI), and active tuberculosis (ATB) groups. Boxplots showing the statistical comparisons of (A) the four IgG subclasses (IgG1, IgG2, IgG3, and IgG4), (B) IgG subclass fucosylation, (C) IgG subclass sialylation, and (D) IgG subclass bisection among the three groups. *, $p < 0.05$; **, $p < 0.01$; ***, $p < 0.001$.

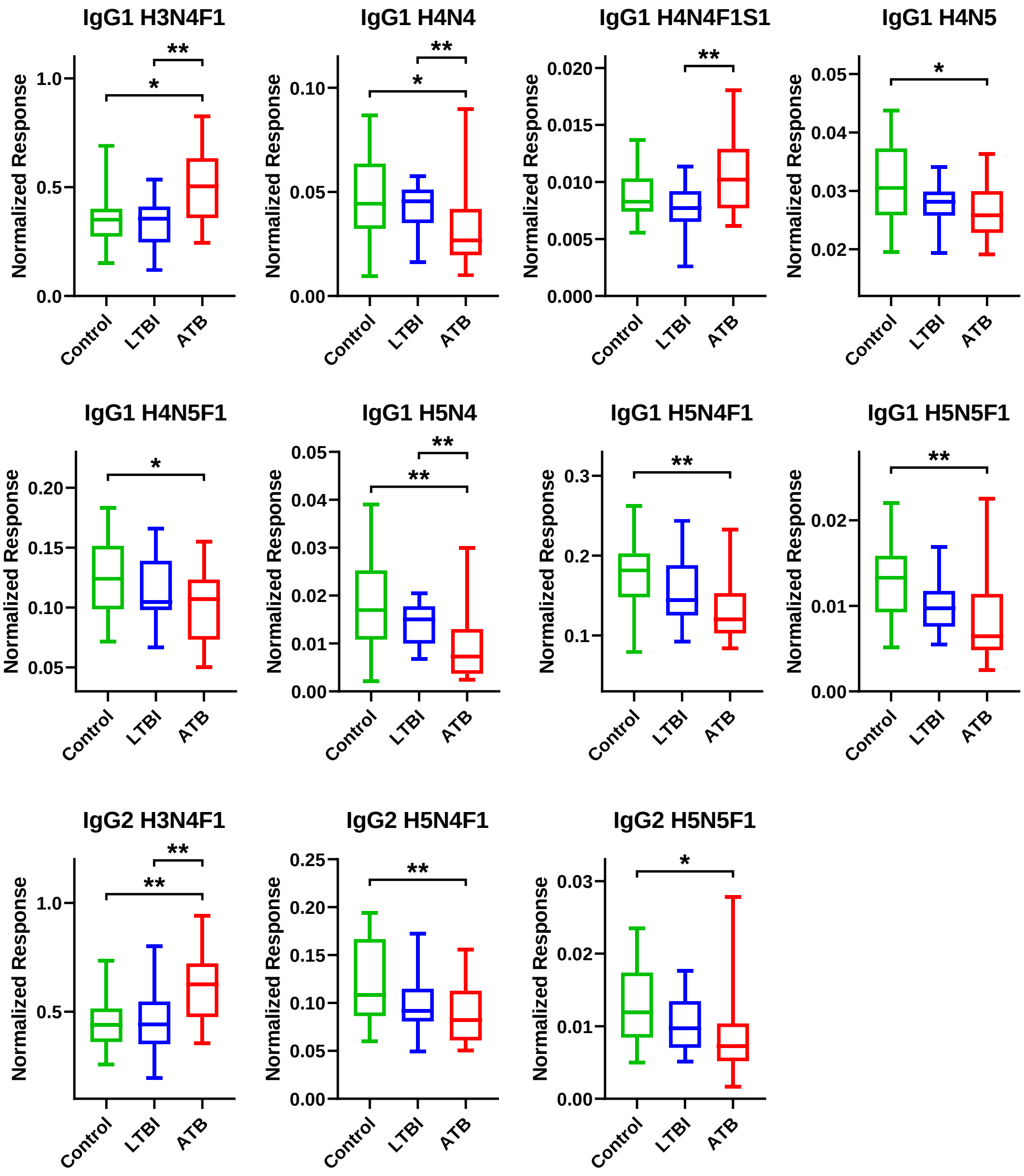

Supplementary Figure 2. Differences in specific IgG glycopeptides among the control, latent tuberculosis infection (LTBI), and active tuberculosis (ATB) groups. Boxplots show statistical comparisons performed using the Mann-Whitney test. *, $p < 0.05$; **, $p < 0.01$; ***, $p < 0.001$.

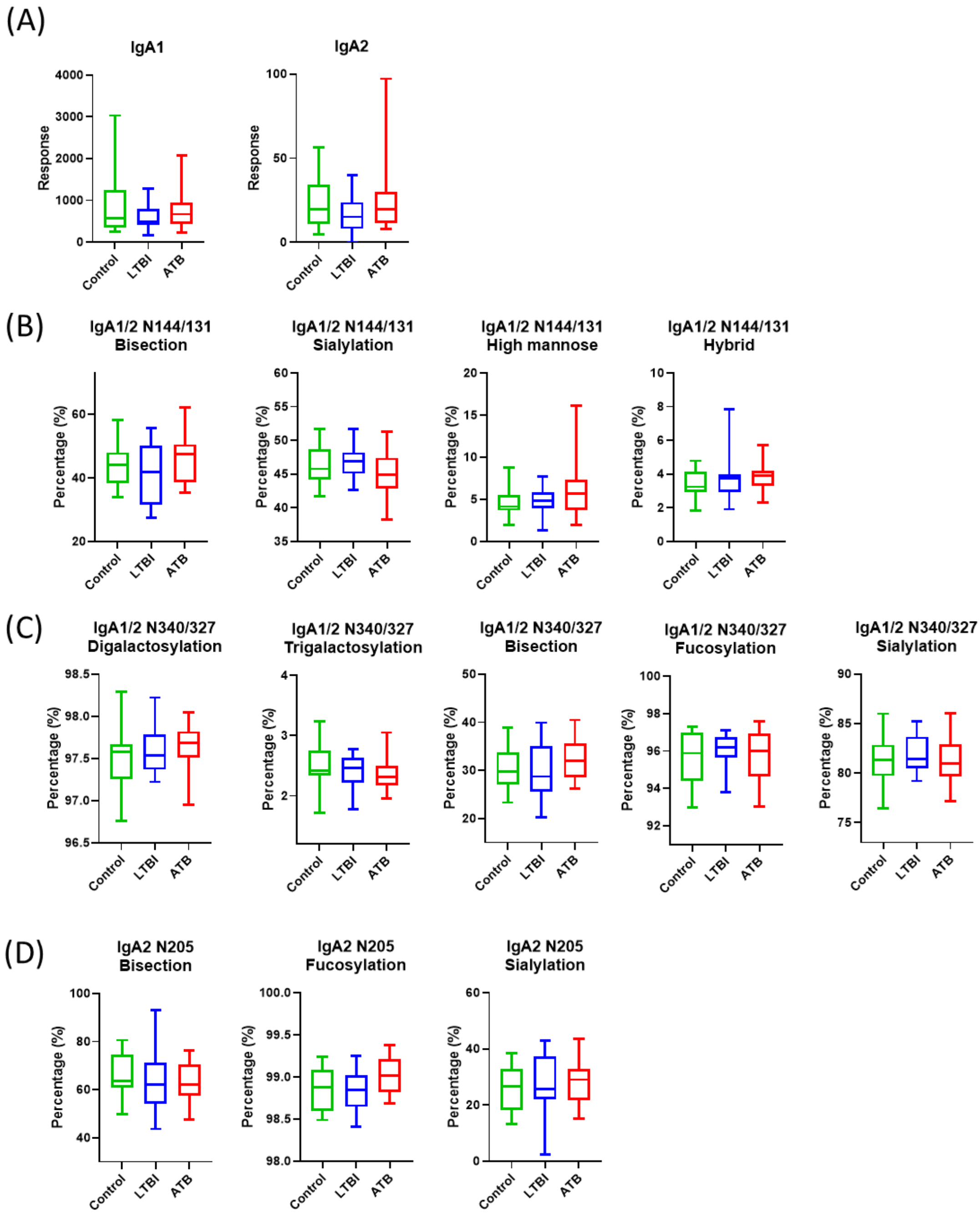

Supplementary Figure 3. Comparison of IgA1, IgA2, and IgA glycosylation profiles among the control, latent tuberculosis infection (LTBI), and active tuberculosis (ATB) groups. Boxplots show the statistical results of the Mann-Whitney test among the three groups, with no significant differences observed in these profiles.

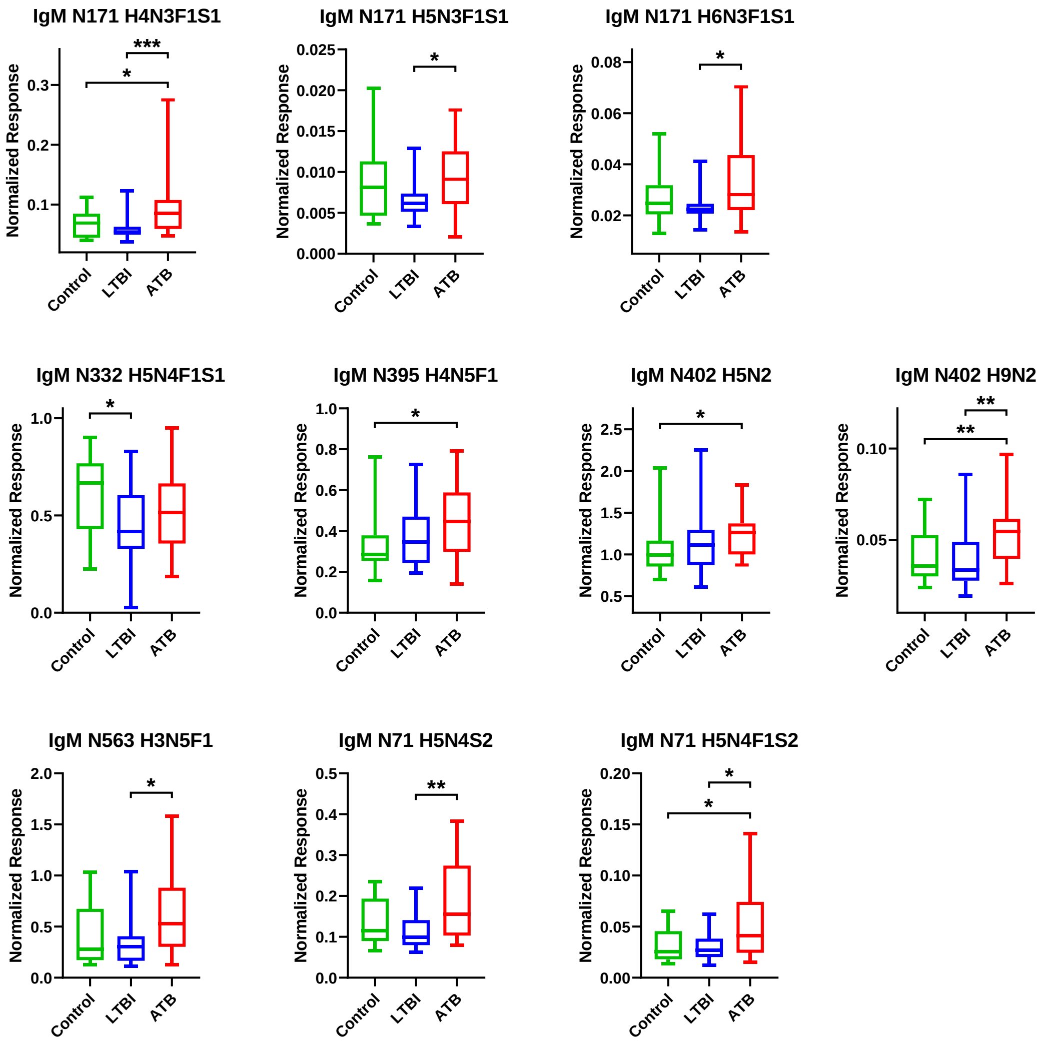

Supplementary Figure 4. Differences in specific IgM glycopeptides among the control, latent tuberculosis infection (LTBI), and active tuberculosis (ATB) groups. Boxplots show statistical comparisons performed using the Mann-Whitney test. *, $p < 0.05$; **, $p < 0.01$; ***, $p < 0.001$.

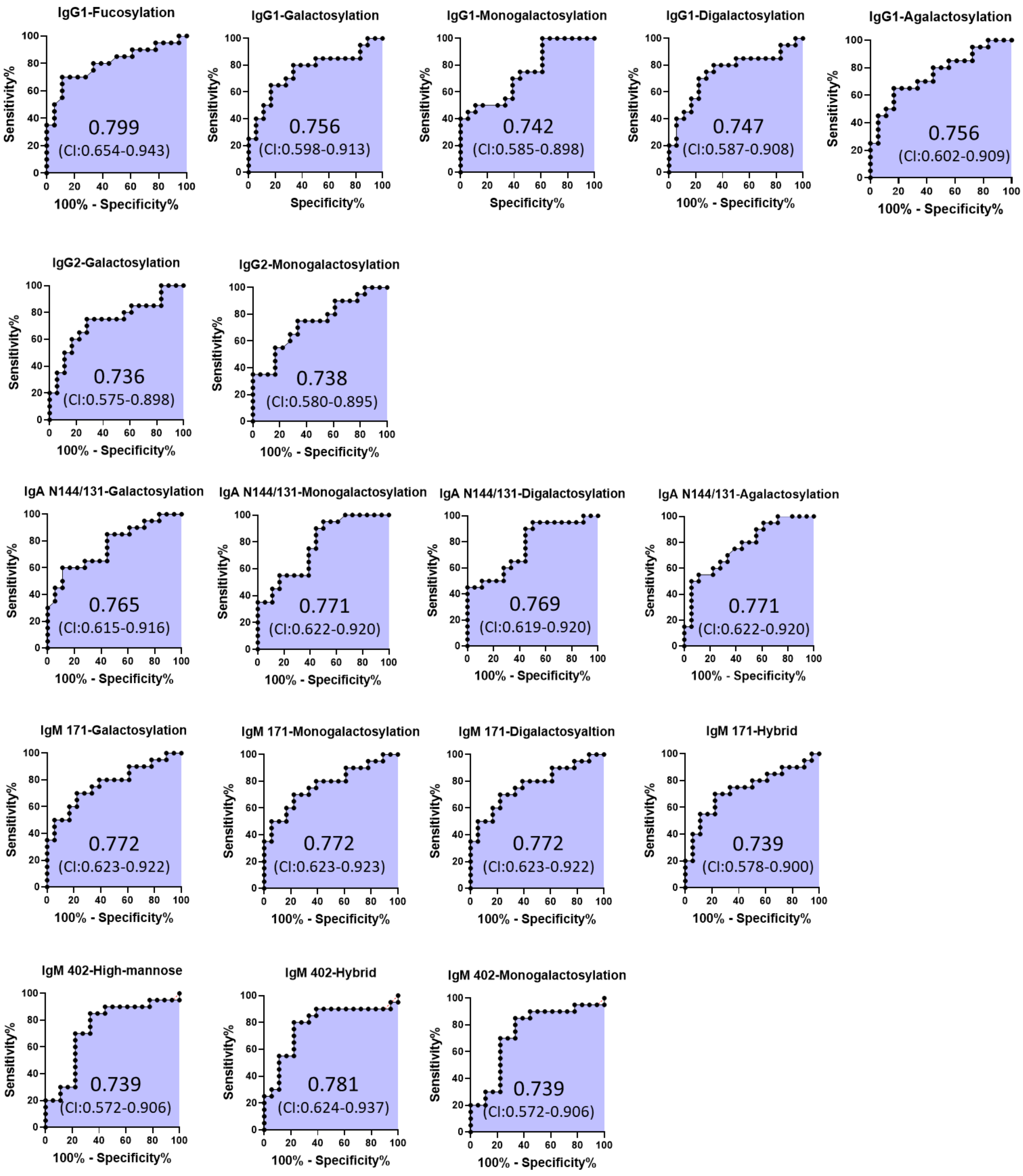

Supplementary Figure 5. Receiver Operating Characteristic (ROC) curves illustrating the performance of IgG, IgA, and IgM glycosylation profiles in distinguishing active tuberculosis (ATB) from latent tuberculosis infection (LTBI). Each subplot represents an individual glycosylation feature, with the area under the curve (AUC) value displayed at the top and the corresponding confidence interval (CI) below. The shaded region indicates the distribution of the ROC curve across different samples. Higher AUC values suggest better discriminatory power between ATB and LTBI.

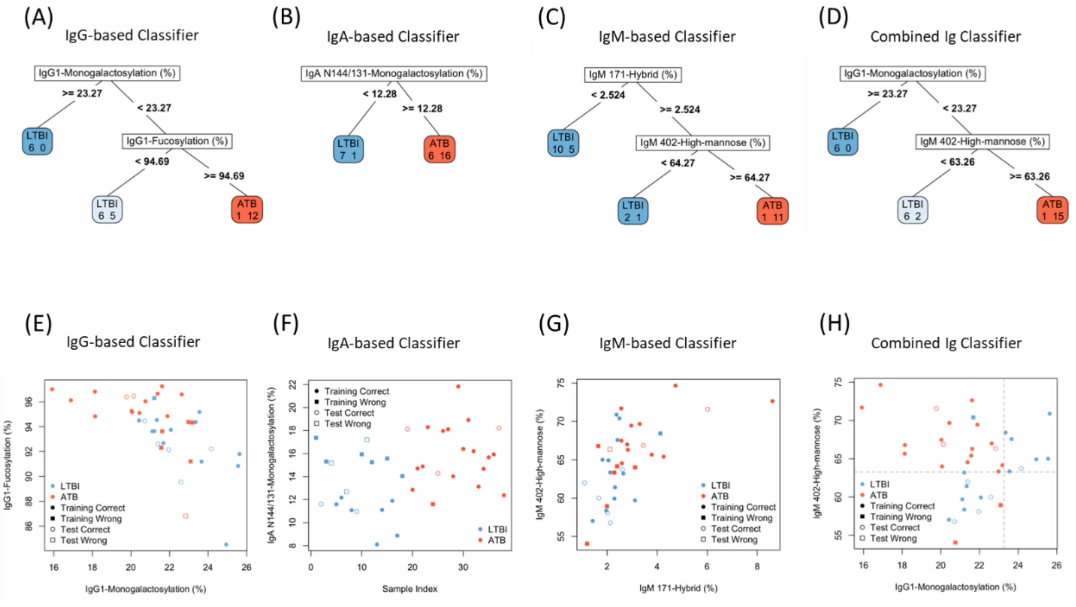

Supplementary Figure 6. Decision tree models for classifying LTBI (blue) and ATB (red) based on immunoglobulin glycosylation profiles, and corresponding feature distributions. (A–D) Decision tree classifiers built using: (A) 7 IgG features, (B) 4 IgA features, (C) 7 IgM features, and (D) combined 4 IgA, 7 IgG, and 7 IgM features. Each node indicates the decision rule (feature and threshold), the number of LTBI/ATB samples classified at that node. Node background color intensity corresponds to the proportion of samples of the predicted class. (E–H) Scatter plots of the key immunoglobulin glycosylation features used in the corresponding decision trees (A–D). Dashed lines represent the threshold values applied in each decision node. Circles indicate correctly predicted samples, squares indicate misclassified samples; solid symbols correspond to training set samples, and hollow symbols correspond to test set samples.
