# Supplementary material for: Integrated Glycosylation Analysis of Immunoglobulin Isotypes Reveals Expanded Humoral Remodeling in Elderly Tuberculosis Infection
Source: Mol Cell Proteomics. 2025 Oct 30;24(12):101438. doi: 10.1016/j.mcpro.2025.101438 (PMC12718469; doi:10.1016/j.mcpro.2025.101438)
Supplement: Supplementary Data [file mmc7.pdf]

Supplementary Data-Chromatograms and Integration Results

Table of Contents

(1) IgG1 H3N4F1 1–19

(2) IgG1 H3N5F1 20–38

(3) IgG1 H4N4 39–57

(4) IgG1 H4N4F1 58–76

(5) IgG1 H4N4F1S1 77–95

(6) IgG1 H4N5 96–114

(7) IgG1 H4N5F1 115–133

(8) IgG1 H5N4 134–152

(9) IgG1 H5N4F1 153–171

(10) IgG1 H5N4F1S1 172–190

(11) IgG1 H5N5F1 191–209

(12) IgG2 H3N4F1 210–228

(13) IgG2 H3N5F1 229–261

(14) IgG2 H4N4 262–280

(15) IgG2 H4N4F1 281–299

(16) IgG2 H4N4F1S1 300–318

(17) IgG2 H4N5 319–337

(18) IgG2 H4N5F1 338–356

(19) IgG2 H5N4F1 357–375

(20) IgG2 H5N4F1S1 376–394

(21) IgG2 H5N5F1 395–413

(22) IgG3/4 H3N4F1 414–432

(23) IgG3/4 H3N5F1 433–451

(24) IgG3/4 H4N4F1 452–470

(25) IgG3/4 H4N5F1 471–489

(26) IgG3/4 H5N4F1S1 490–508

(27) IgA1/2 N144/131 H3N5 509–527

(28) IgA1/2 N144/131 H4N4S1 528–560

(29) IgA1/2 N144/131 H4N5 561–579

(30) IgA1/2 N144/131 H4N5S1 580–612

(31) IgA1/2 N144/131 H5N2 613–631

(32) IgA1/2 N144/131 H5N3S1 632–650

(33) IgA1/2 N144/131 H5N4S1 651–697

(34) IgA1 / 2 N144 / 131 H5N4S2 698–730

(35) IgA1/2 N144/131 H5N5 731–749

(36) IgA1/2 N144/131 H5N5S1 750–768

(37) IgA1/2 N144/131 H5N5S2 769–787

(38) IgA1/2 N144/131 H6N2 788–806

(39) IgA1/2 N144/131 H6N3S1 807–825

(40) IgA1/2 N144/131 H7N2 826–844

(41) IgA1/2 N144/131 H8N2 845–863

(42) IgA1/2 N340/327 H5N4F1S1 864–882

(43) IgA1/2 N340/327 H5N4F1S2 883–901

(44) IgA1/2 N340/327 H5N4S1 902–920

(45) IgA1/2 N340/327 H5N5F1S2 921–939

(46) IgA1/2 N340/327 H6N5F1S1 940–958

(47) IgA1/2 N340/327tr H5N5F1S1 959–977

(48) IgA1/2 N340/327tr H5N5F1S2 978–1010

(49) IgA1/2 N340/327tr H5N4F1S1 1011–1029

(50) IgA1/2 N340/327tr H5N4F1S2 1030–1048

(51) IgA2 N205 H4N5F1 1049–1067

(52) IgA2 N205 H3N4F1 1068–1086

(53) IgA2 N205 H3N5F1 1087–1119

(54) IgA2 N205 H4N4F1S1 1120–1152

(55) IgA2 N205 H5N4F1S1 1153–1171

(56) IgA2 N205 H5N4F1S2 1172–1190

(57)IgA2 N205 H5N4S1 1191–1237

(58) IgA2 N205 H5N5F1 1238–1284

(59) IgA2 N205 H5N5F1S1 1285–1303

(60) IgM N171 H4N3F1S1 1304–1322

(61) IgM N171 H5N3F1S1 1323–1341

(62) IgM N171 H5N4S1 1342–1374

(63) IgM N171 H5N4F1S1 1375–1393

(64) IgM N171 H5N5S1 1394–1412

(65) IgM N171 H5N5F1S1 1413–1431

(66) IgM N171 H6N3F1S1 1432–1464

(67) IgM N332 H4N5F1S1 1465–1483

(68) IgM N332 H5N4F1S1 1484–1502

(69) IgM N332 H5N4F1S2 1503–1521

(70) IgM N332 H5N5F1S1 1522–1540

(71) IgM N332 H5N5F1S2 1541–1559

(72) IgM N332 H6N4S1 1560–1578

(73) IgM N395 H4N5F1 1579–1611

(74) IgM N395 H5N4F1S2 1612–1630

(75) IgM N395 H5N5F1S1 1631–1649

(76) IgM N395 H5N5F1S2 1650–1668

(77) IgM N395 H5N4F1S1 1669–1687

(78) IgM N395 H5N5F1 1688–1706

(79) IgM N402 H4N2 1707–1725

(80) IgM N402 H4N3 1726–1744

(81) IgM N402 H5N2 1745–1763

(82) IgM N402 H5N3....1764–1782

(83) IgM N402 H9N2....1783–1829

(84) IgM N563 H3N5F1....1830–1848

(85) IgM N563 H4N5F1....1849–1867

(86) IgM N563 H4N5F1S1....1868–1886

(87) IgM N563 H5N2....1887–1905

(88) IgM N563 H6N2....1906–1924

(89) IgM N563 H7N2....1925–1943

(90) IgM N563 H8N2....1944–1962

(91) IgM J chain N71 H5N4S1....1963–1981

(92) IgM J chain N71 H5N4S2....1982–2000

(93) IgM J chain N71 H5N4F1S1....2001–2019

(94) IgM J chain N71 H5N4F1S2....2020–2066

(95) IgM J chain N71 H5N5S1....2067–2085

IgG - DTLMISR....2086–2131

IgG1 - GPSVFPLAPSSK....2132–2177

IgG2 - TTPPMLDSDGSFFLYSK....2178–2223

IgG3 - WYVDGVEVHNAK....2224–2269

IgG4 - TTPPVLDSDGSFFLYSR....2270–2302

IgA1 - TPLTATLSK....2303–2349

IgA2 - DASGATFTWTPSSGK....2350–2396

IgM - FTCTVTHTDLPSPLK....2397–2457

IS 1e....2458–2503

IS 2....2504–2550

IS 3....2551–2583

IS 4....2584–2644

Dataset: C:\MassLynx\10.pro\20211115_clinicalsamples_IgG.qld

Last Altered: Sunday, October 02, 2022 16:02:30 台北標準時間
Printed: Friday, August 01, 2025 16:26:04 台北標準時間

Method: C:\MassLynx\10_Pro_IgA.PRO\MethDB\20210926_IgG_DMRM_IgM_IS.mdb 16 Feb 2022 11:58:21
Calibration: 18 Nov 2021 13:56:22

Sample Name: 20211111_QC_R1_IgG

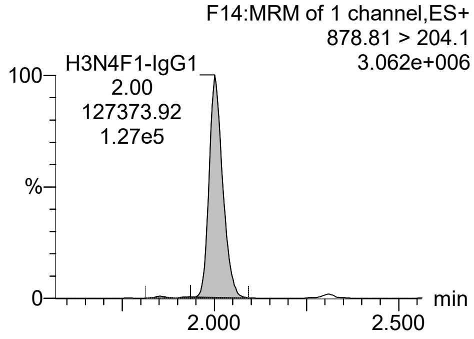

Sample Name: 20211111_A1_R1_IgG

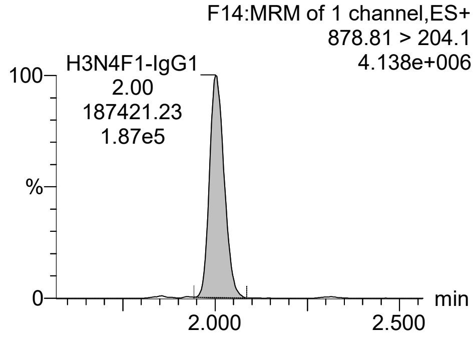

Sample Name: 20211111_A2_R1_IgG

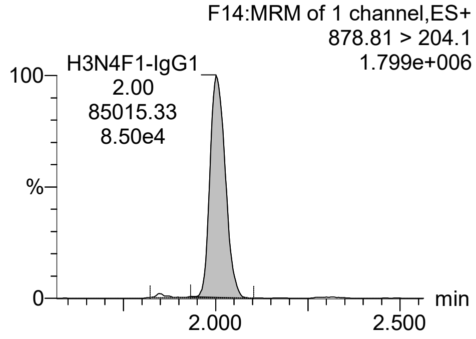

Sample Name: 20211111_L1_R1_IgG

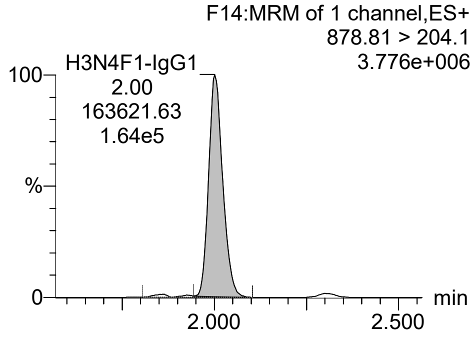

Sample Name: 20211111_L2_R1_IgG

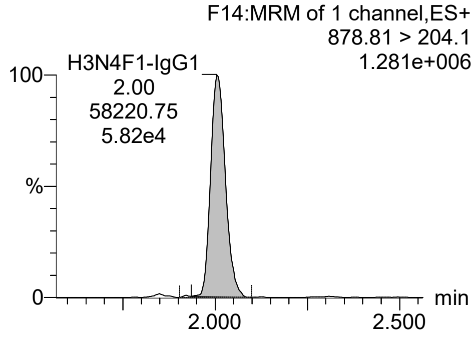

Sample Name: 20211111_H1_R1_IgG

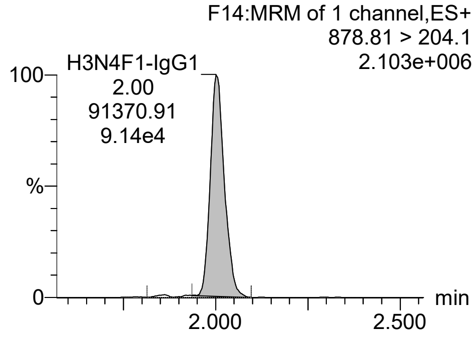

Sample Name: 20211111_A3_R1_IgG

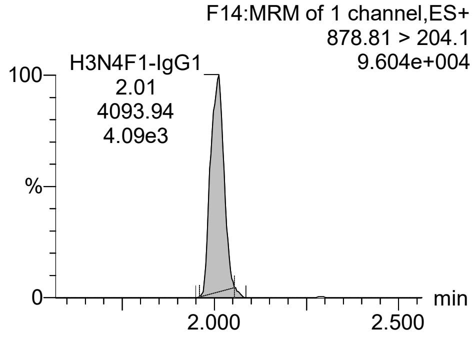

Sample Name: 20211111_A4_R1_IgG

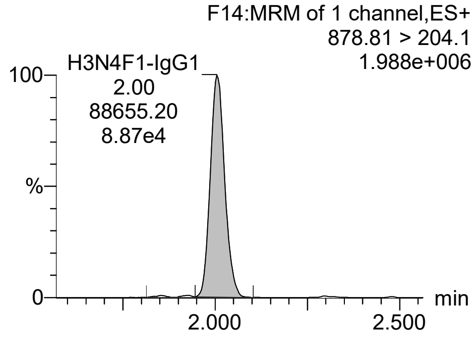

Sample Name: 20211111_L3_R1_IgG

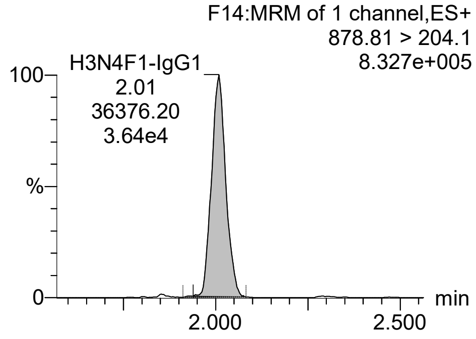

Sample Name: 20211111_L4_R1_IgG

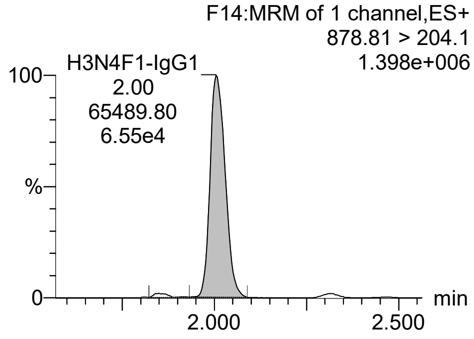

Sample Name: 20211111_H2_R1_IgG

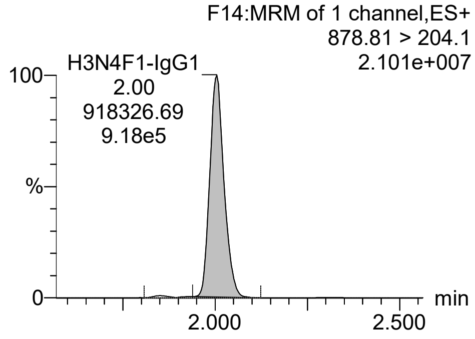

Sample Name: 20211111_A5_R1_IgG

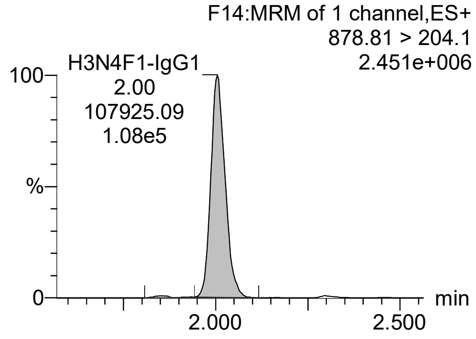

Dataset: C:\MassLynx\10.pro\20211115_clinicalsamples_IgG.qld

Last Altered: Sunday, October 02, 2022 16:02:30 台北標準時間
Printed: Friday, August 01, 2025 16:26:04 台北標準時間

Sample Name: 20211111_A6_R1_IgG

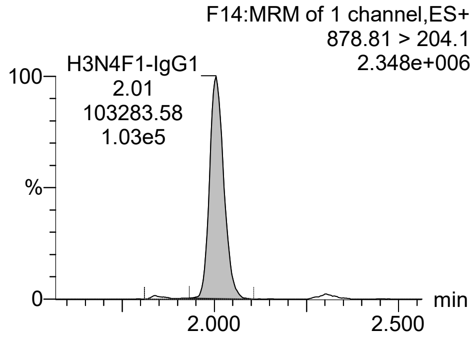

Sample Name: 20211111_QC_R2_IgG

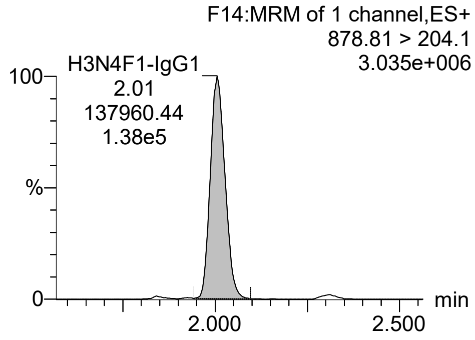

Sample Name: 20211111_L5_R1_IgG

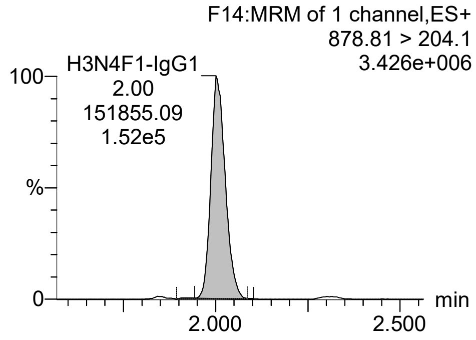

Sample Name: 20211111_L6_R1_IgG

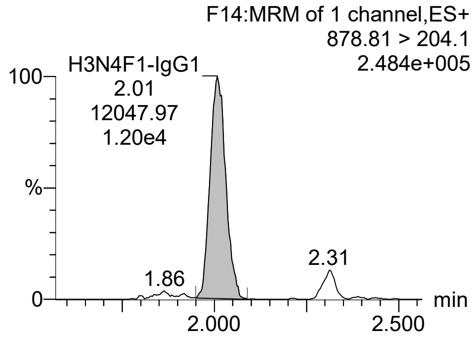

Sample Name: 20211111_H3_R1_IgG

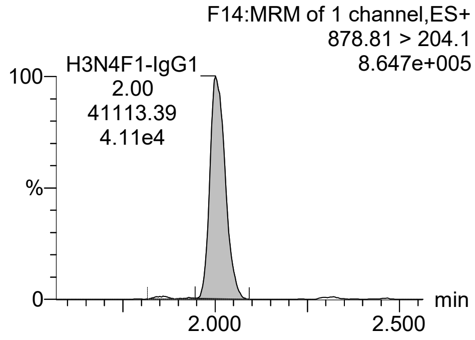

Sample Name: 20211111_A7_R1_IgG

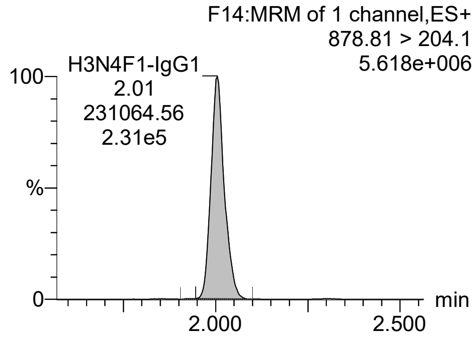

Sample Name: 20211111_A8_R1_IgG

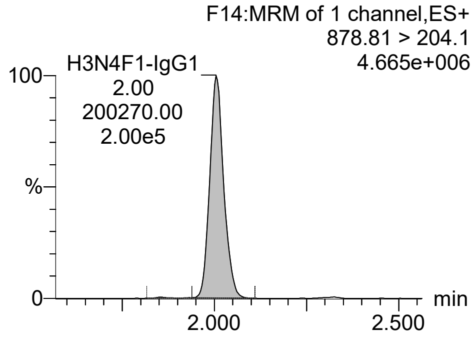

Sample Name: 20211111_L7_R1_IgG

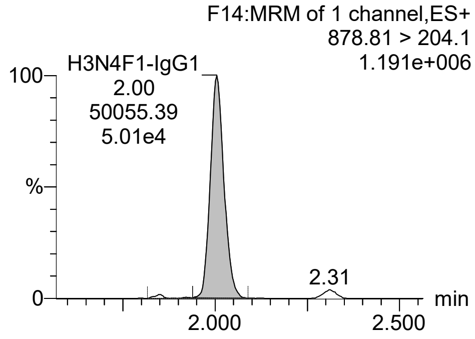

Sample Name: 20211111_L8_R1_IgG

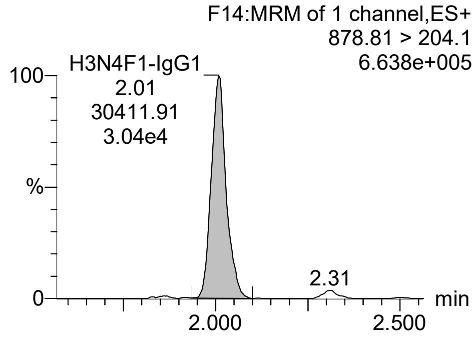

Sample Name: 20211111_H4_R1_IgG

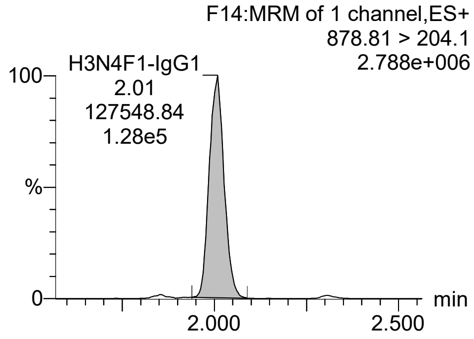

Sample Name: 20211111_A9_R1_IgG

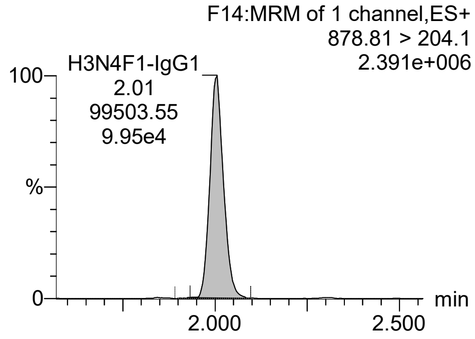

Sample Name: 20211111_A10_R1_IgG

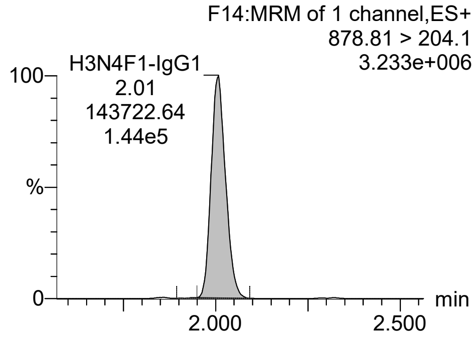

Sample Name: 20211111_L9_R1_IgG

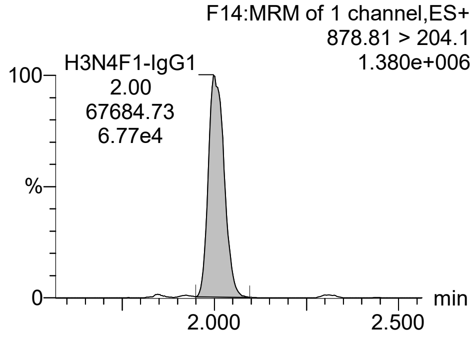

Sample Name: 20211111_L10_R1_IgG

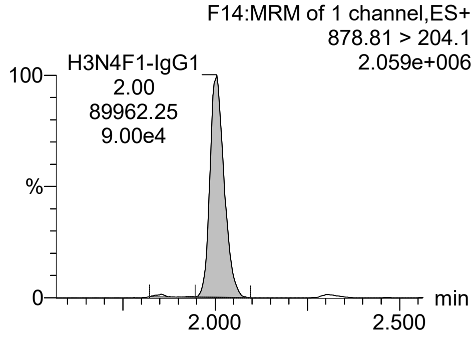

Sample Name: 20211111_QC_R3_IgG

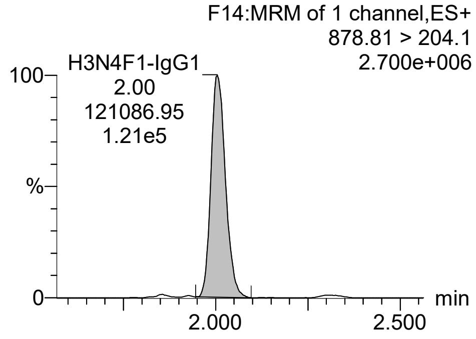

Sample Name: 20211111_H5_R1_IgG

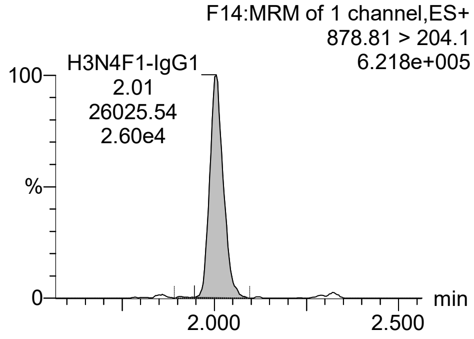

Sample Name: 20211111_A11_R1_IgG

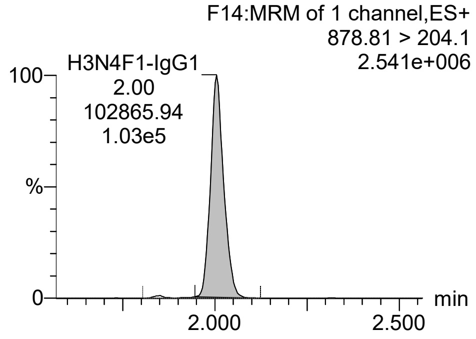

Sample Name: 20211111_A12_R1_IgG

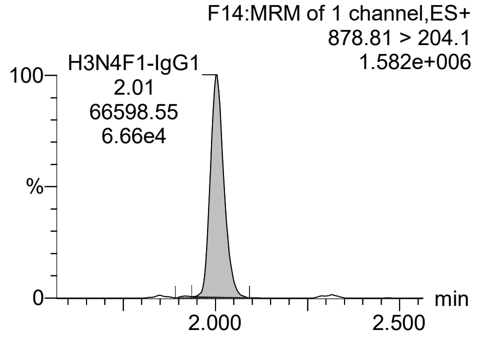

Sample Name: 20211111_L11_R1_IgG

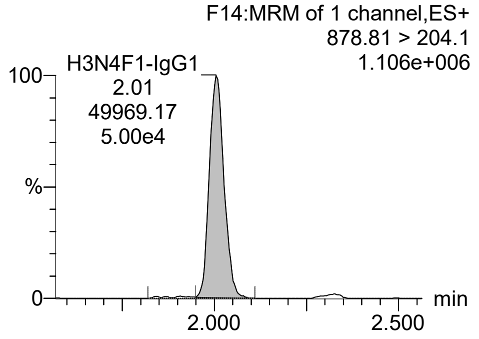

Sample Name: 20211111_L12_R1_IgG

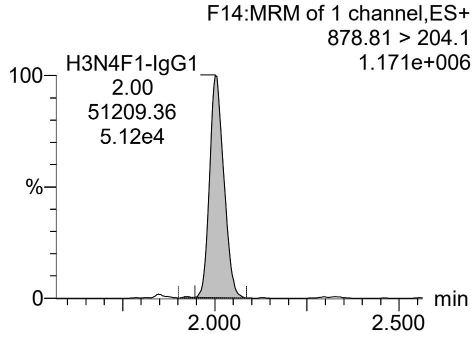

Sample Name: 20211111_H6_R1_IgG

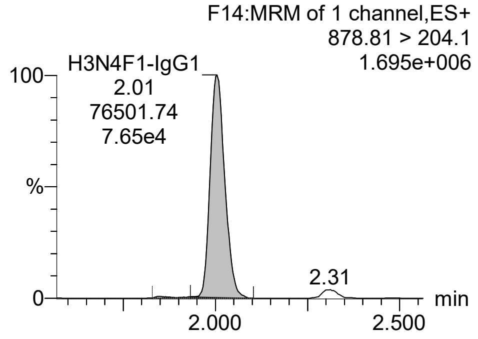

Sample Name: 20211111_A13_R1_IgG

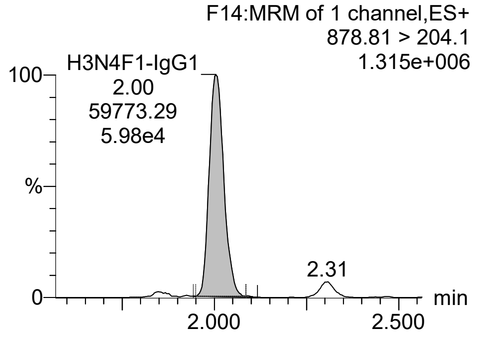

Sample Name: 20211111_A14_R1_IgG

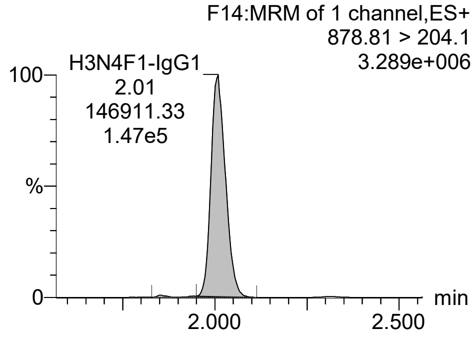

Sample Name: 20211111_L13_R1_IgG

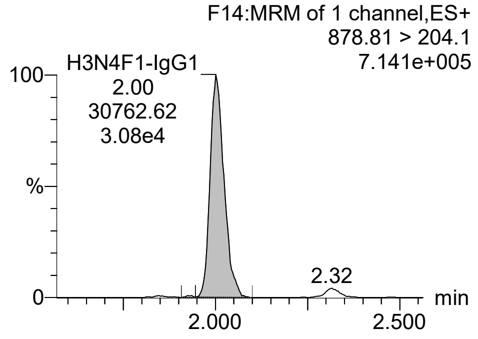

Sample Name: 20211111_L14_R1_IgG

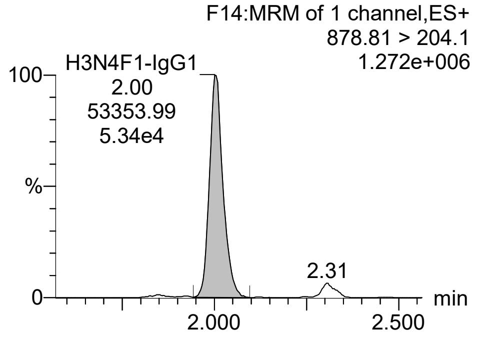

Sample Name: 20211111_H7_R1_IgG

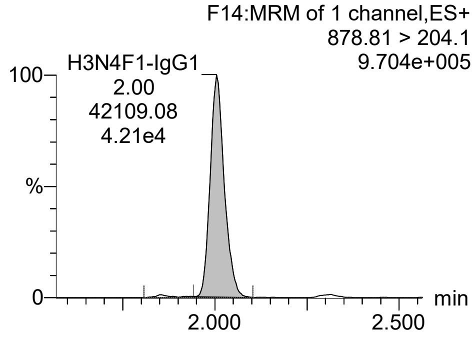

Sample Name: 20211111_A15_R1_IgG

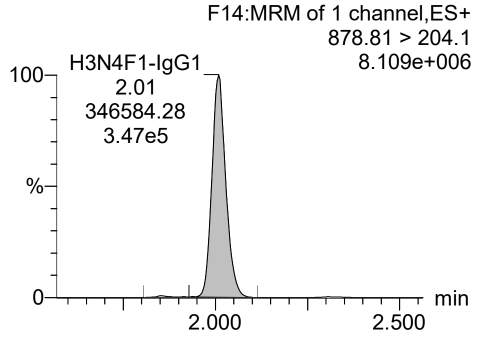

Sample Name: 20211111_QC_R4_IgG

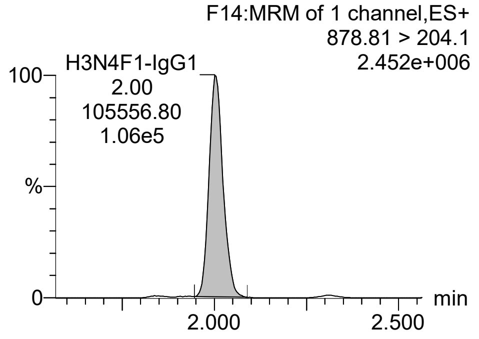

Sample Name: 20211111_A16_R1_IgG

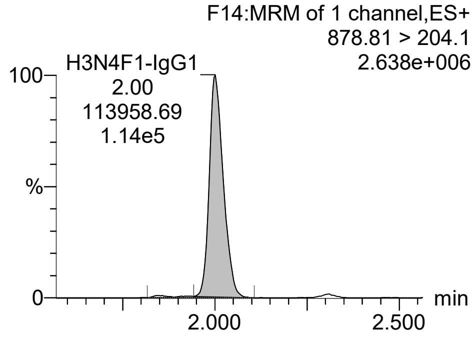

Sample Name: 20211111_L15_R1_IgG

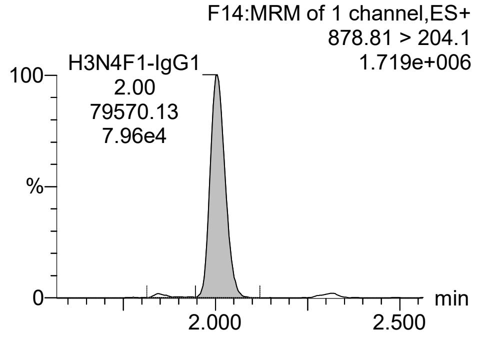

Dataset: C:\MassLynx\10.pro\20211115_clinicalsamples_IgG.qld

Last Altered: Sunday, October 02, 2022 16:02:30 台北標準時間
Printed: Friday, August 01, 2025 16:26:04 台北標準時間

Sample Name: 20211111_L16_R1_IgG

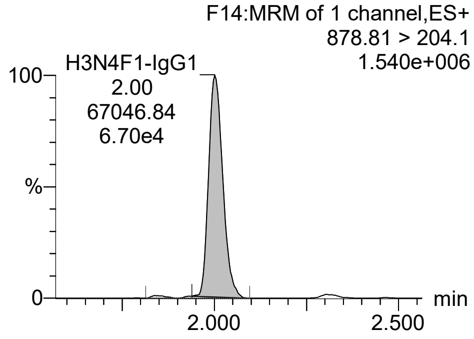

Sample Name: 20211111_H8_R1_IgG

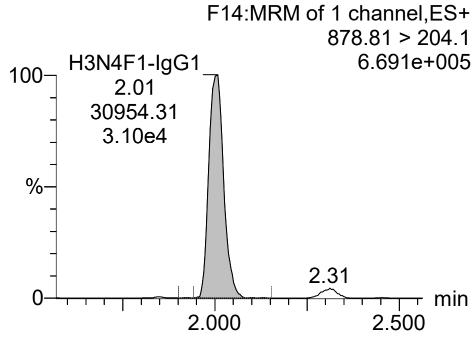

Sample Name: 20211111_A17_R1_IgG

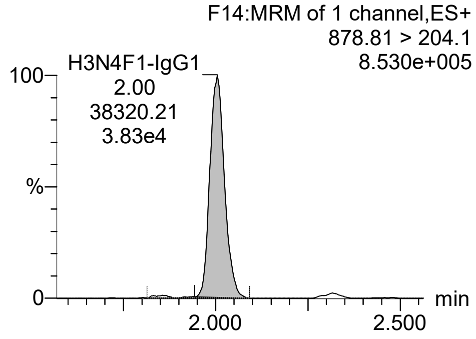

Sample Name: 20211111_A18_R1_IgG

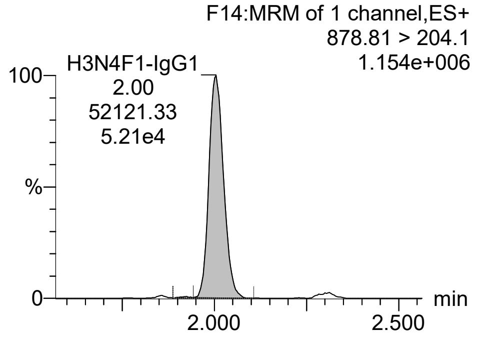

Sample Name: 20211111_L17_R1_IgG

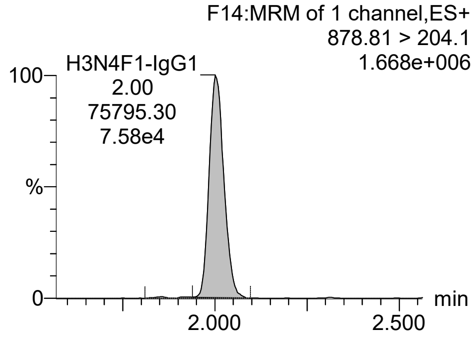

Sample Name: 20211111_L18_R1_IgG

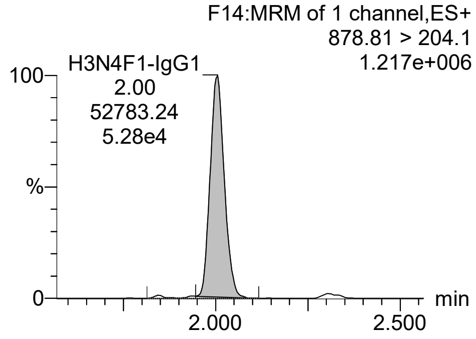

Sample Name: 20211111_H9_R1_IgG

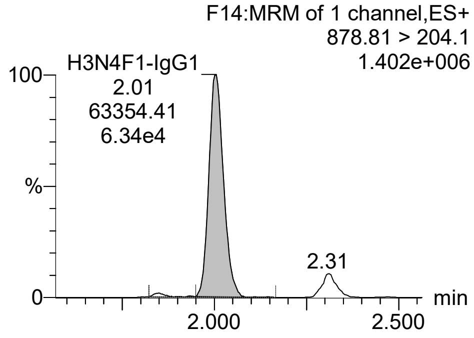

Sample Name: 20211111_A19_R1_IgG

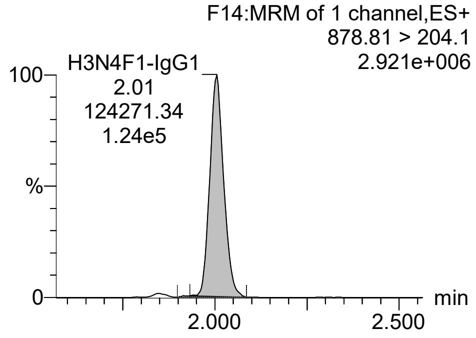

Sample Name: 20211111_A20_R1_IgG

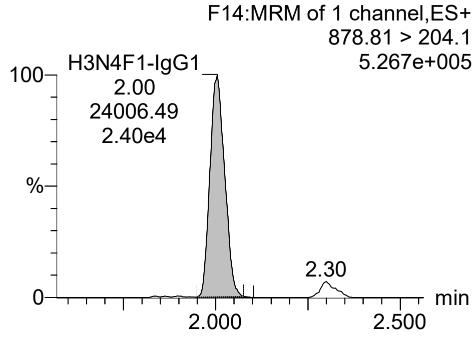

Sample Name: 20211111_L19_R1_IgG

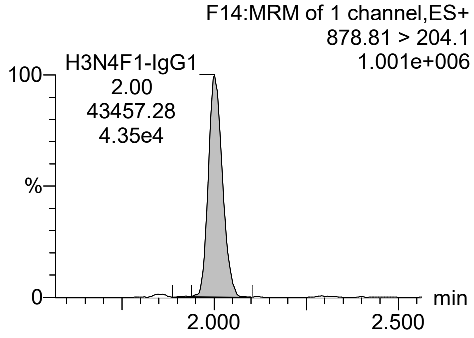

Sample Name: 20211111_QC_R5_IgG

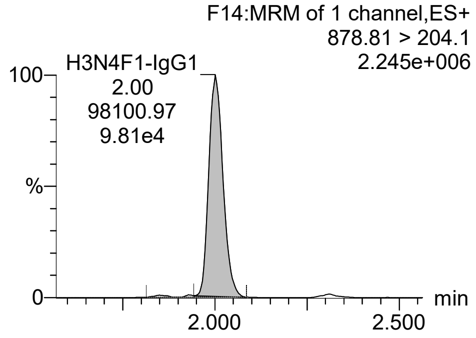

Sample Name: 20211111_L20_R1_IgG

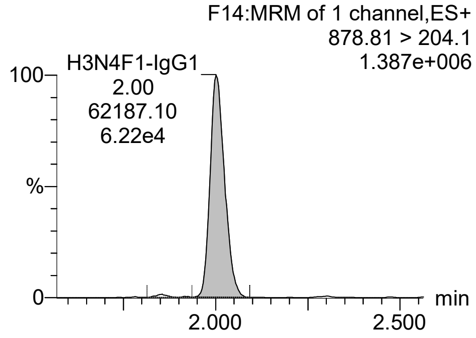

Sample Name: 20211111_H10_R1_IgG

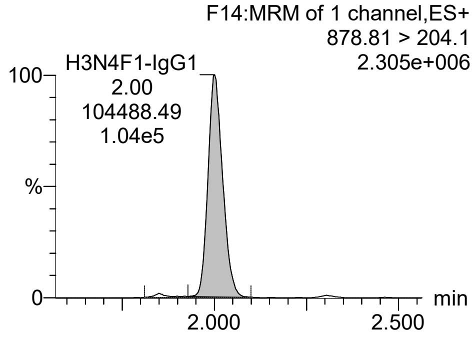

Sample Name: 20211111_A21_R1_IgG

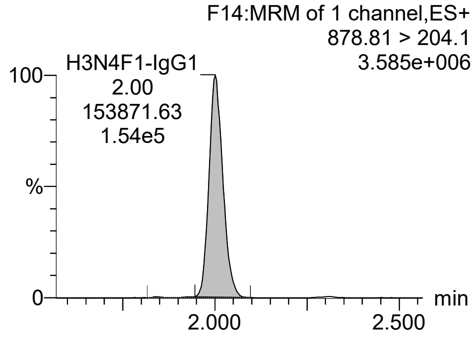

Sample Name: 20211111_A22_R1_IgG

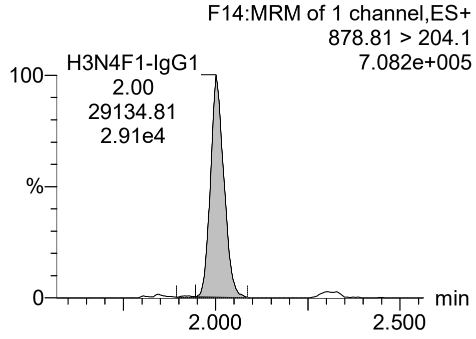

Sample Name: 20211111_L21_R1_IgG

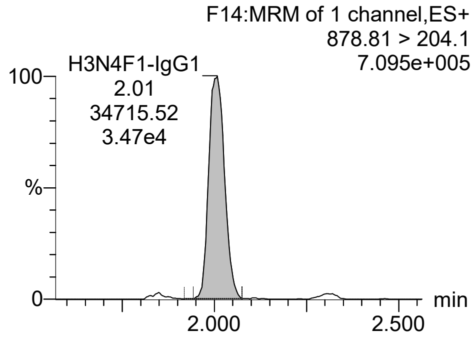

Sample Name: 20211111_L22_R1_IgG

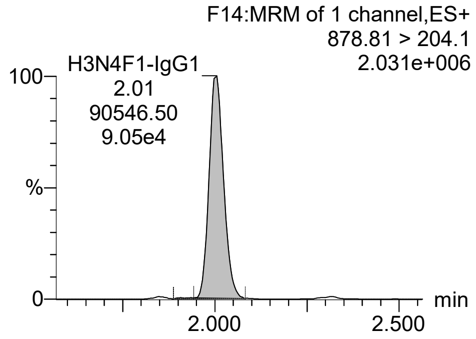

Sample Name: 20211111_H11_R1_IgG

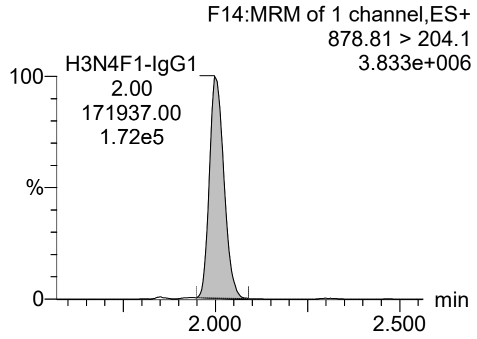

Sample Name: 20211111_A23_R1_IgG

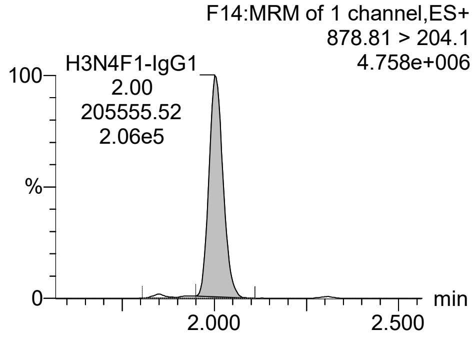

Sample Name: 20211111_A24_R1_IgG

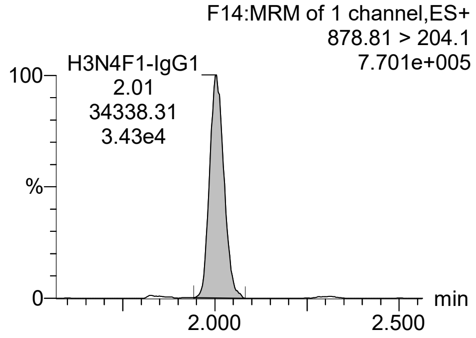

Sample Name: 20211111_L23_R1_IgG

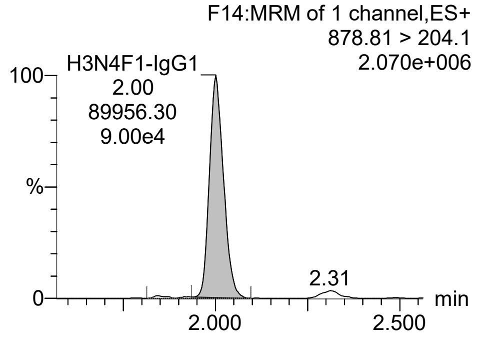

Sample Name: 20211111_L24_R1_IgG

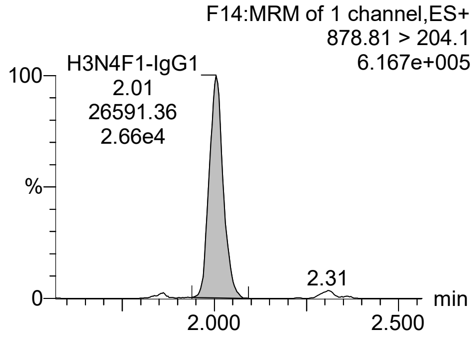

Sample Name: 20211111_H12_R1_IgG

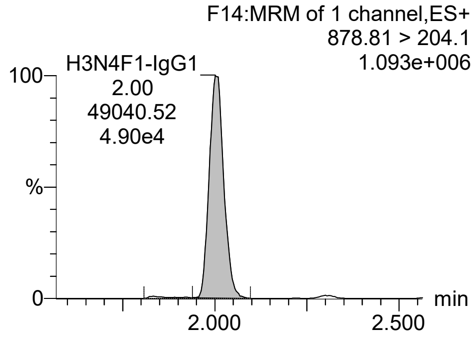

Sample Name: 20211111_QC_R6_IgG

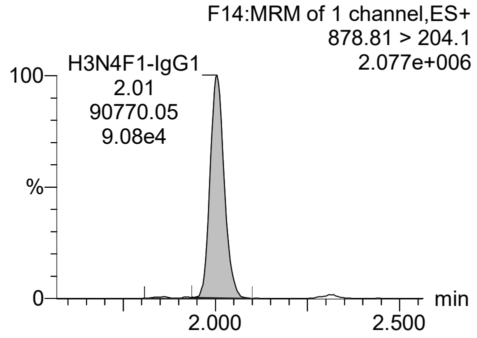

Sample Name: 20211111_A25_R1_IgG

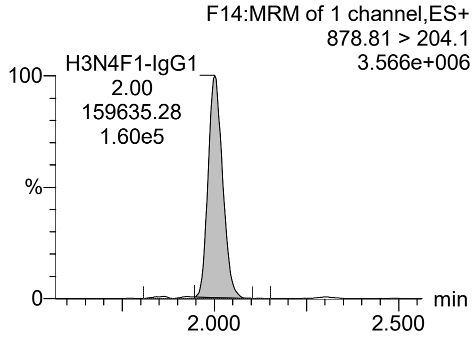

Sample Name: 20211111_A26_R1_IgG

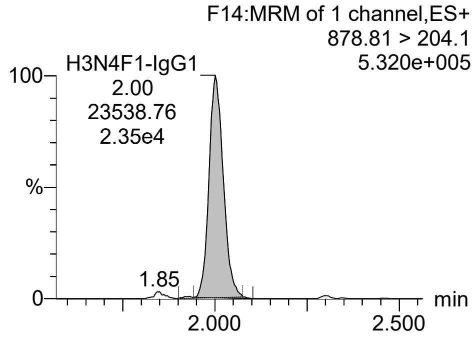

Sample Name: 20211111_L25_R1_IgG

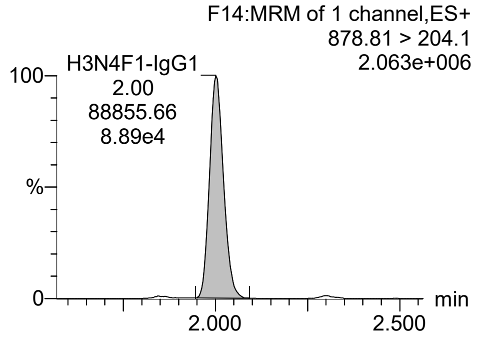

Sample Name: 20211111_L26_R1_IgG

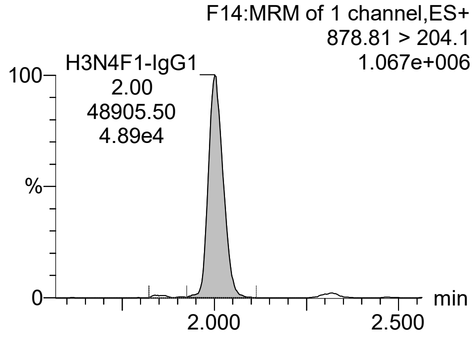

Sample Name: 20211111_H13_R1_IgG

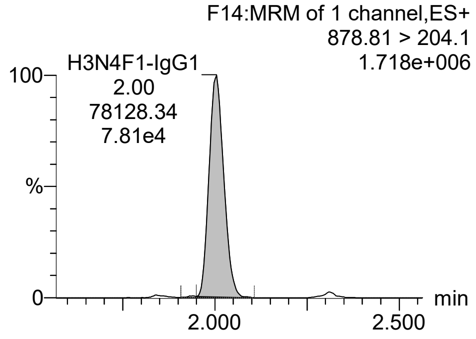

Sample Name: 20211111_A27_R1_IgG

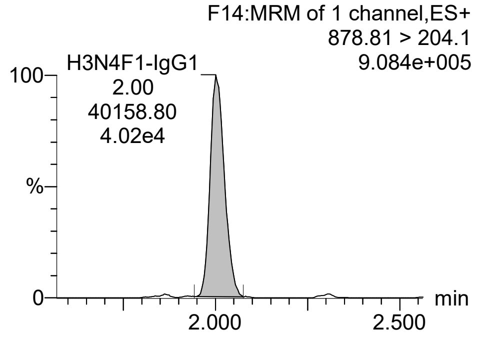

Dataset: C:\MassLynx\10.pro\20211115_clinicalsamples_IgG.qld

Last Altered: Sunday, October 02, 2022 16:02:30 台北標準時間
Printed: Friday, August 01, 2025 16:26:04 台北標準時間

Sample Name: 20211111_A28_R1_IgG

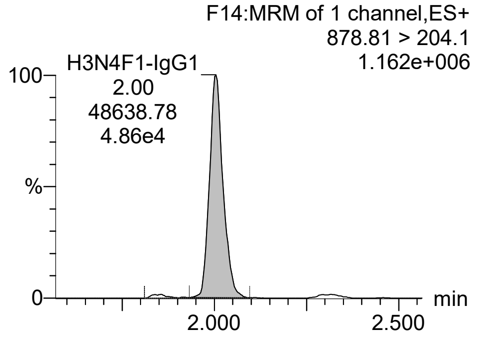

Sample Name: 20211111_L27_R1_IgG

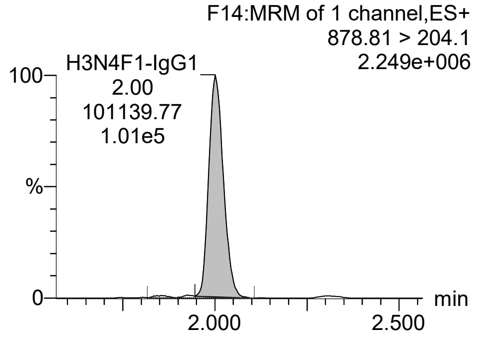

Sample Name: 20211111_L28_R1_IgG

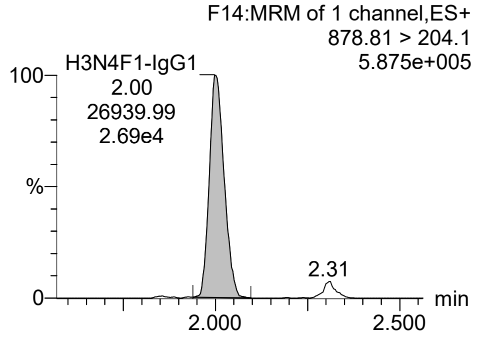

Sample Name: 20211111_H14_R1_IgG

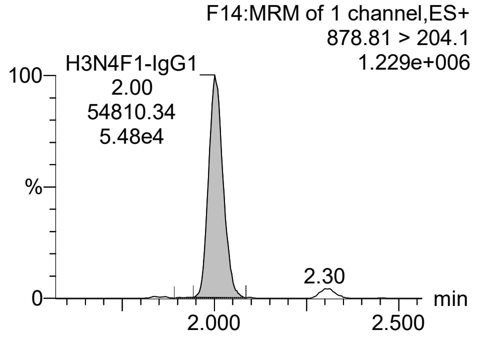

Sample Name: 20211111_A29_R1_IgG

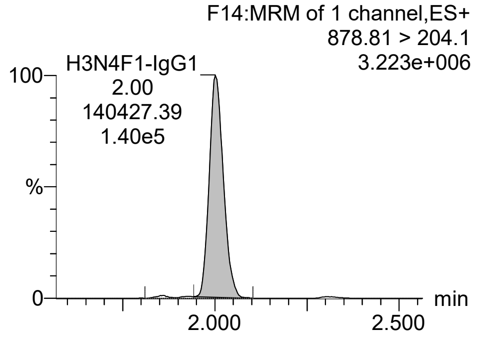

Sample Name: 20211111_A30_R1_IgG

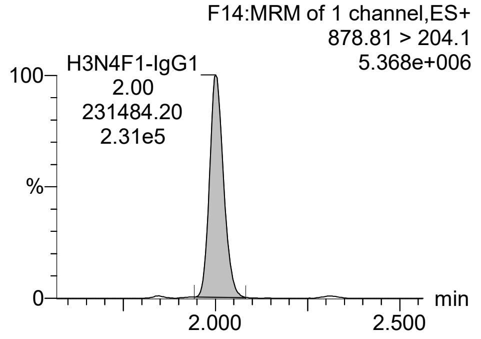

Sample Name: 20211111_QC_R7_IgG

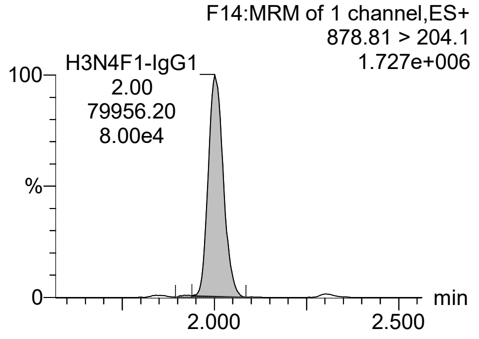

Sample Name: 20211111_L29_R1_IgG

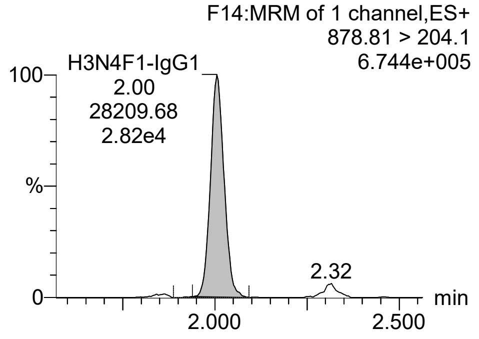

Sample Name: 20211111_L30_R1_IgG

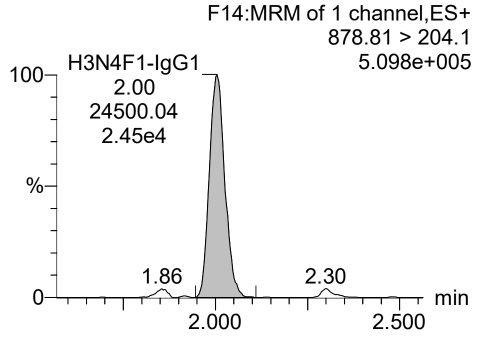

Sample Name: 20211111_H15_R1_IgG

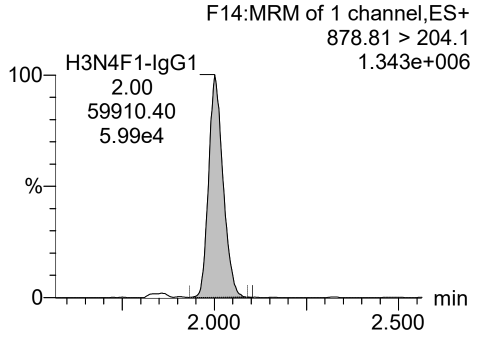

Sample Name: 20211111_A31_R1_IgG

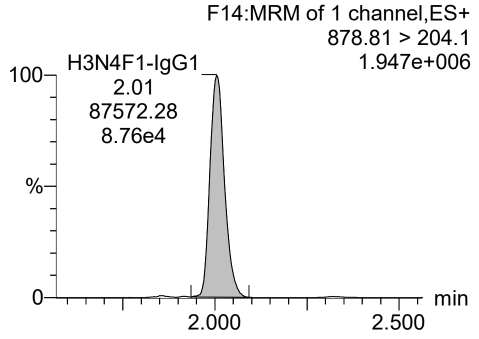

Sample Name: 20211111_A32_R1_IgG

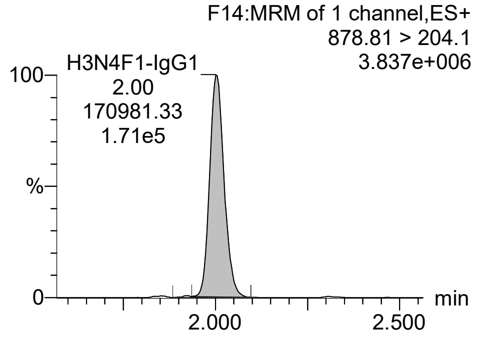

Sample Name: 20211111_L31_R1_IgG

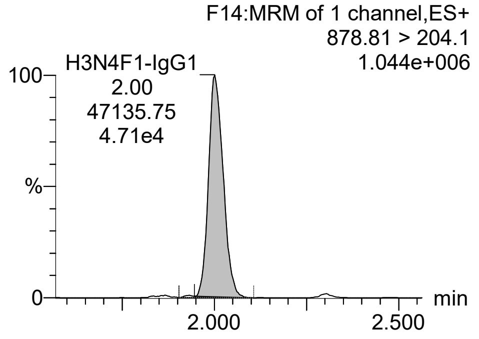

Sample Name: 20211111_L32_R1_IgG

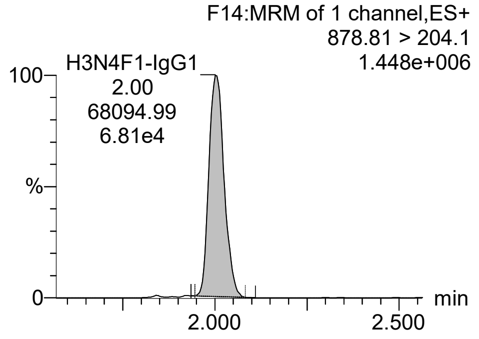

Sample Name: 20211111_H16_R1_IgG

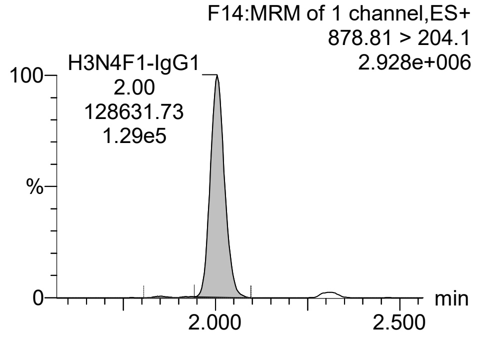

Dataset: C:\MassLynx\10.pro\20211115_clinicalsamples_IgG.qld

Last Altered: Sunday, October 02, 2022 16:02:30 台北標準時間
Printed: Friday, August 01, 2025 16:26:04 台北標準時間

Sample Name: 20211111_A33_R1_IgG

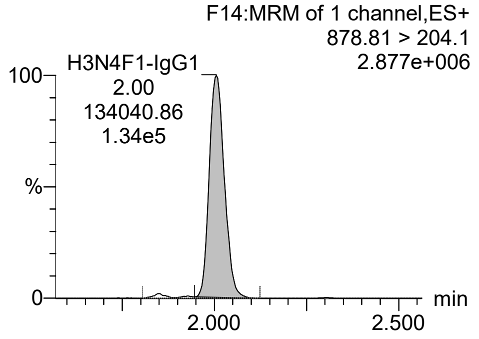

Sample Name: 20211111_A34_R1_IgG

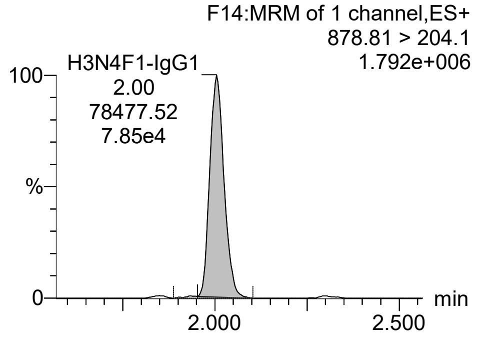

Sample Name: 20211111_H17_R1_IgG

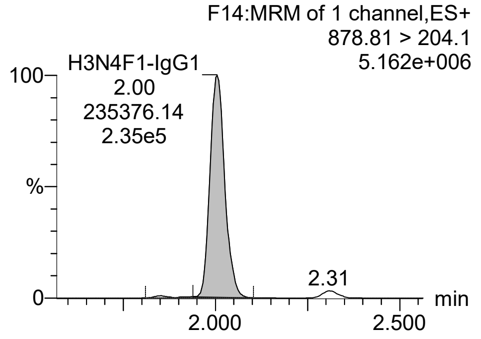

Sample Name: 20211111_A35_R1_IgG

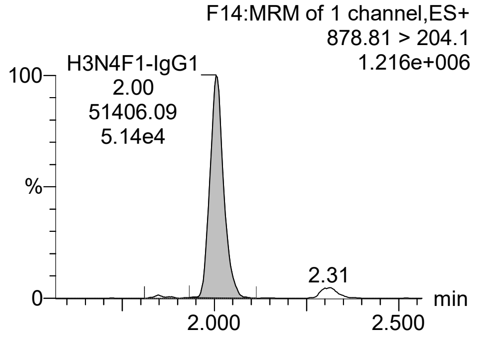

Sample Name: 20211111_QC_R8_IgG

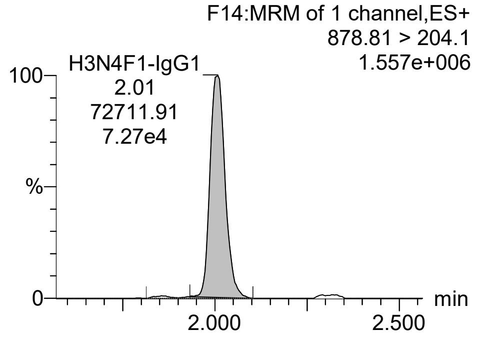

Sample Name: 20211111_A36_R1_IgG

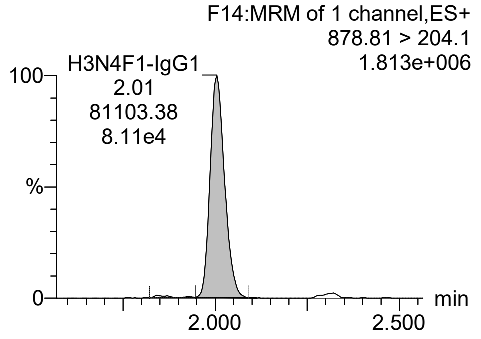

Sample Name: 20211111_H18_R1_IgG

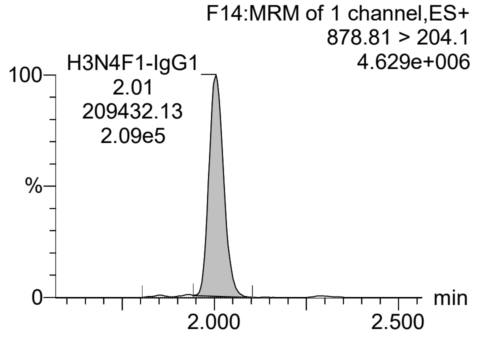

Sample Name: 20211111_A37_R1_IgG

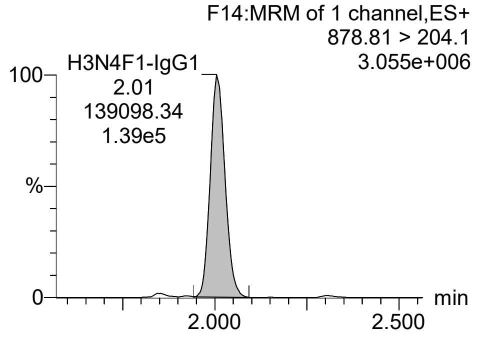

Sample Name: 20211111_A38_R1_IgG

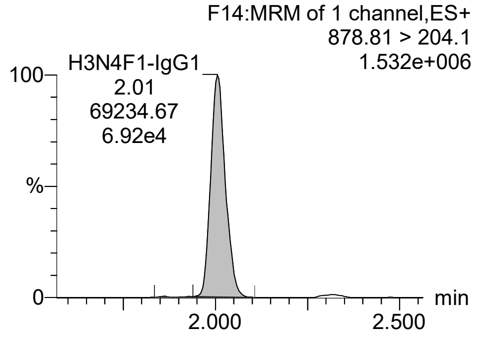

Sample Name: 20211111_H19_R1_IgG

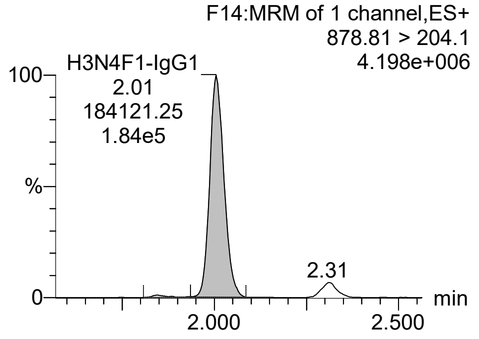

Sample Name: 20211111_A39_R1_IgG

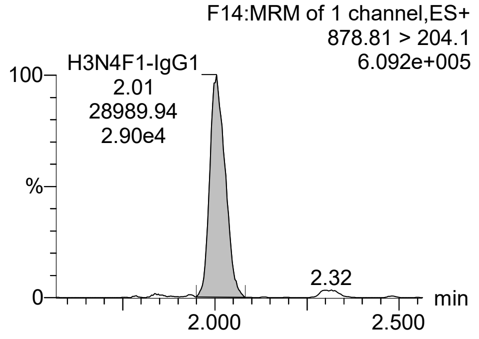

Sample Name: 20211111_A40_R1_IgG

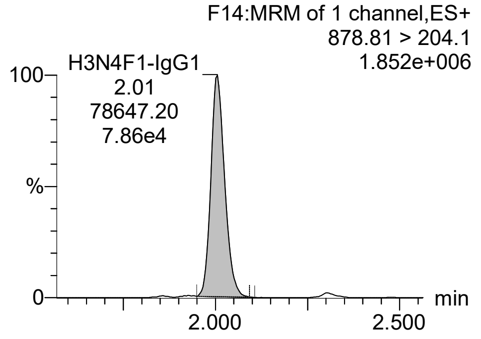

Sample Name: 20211111_H20_R1_IgG

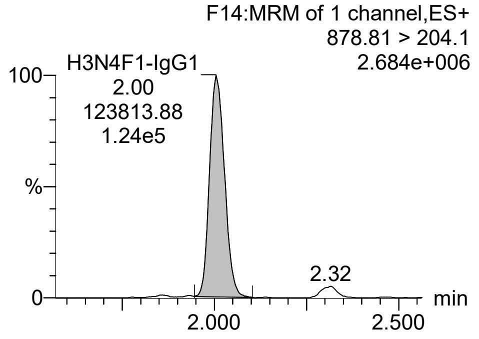

Sample Name: 20211111_A41_R1_IgG

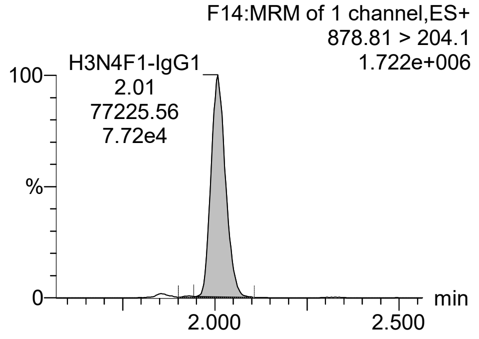

Sample Name: 20211111_A42_R1_IgG

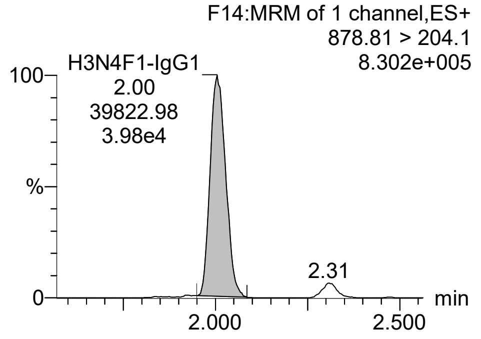

Dataset: C:\MassLynx\10.pro\20211115_clinicalsamples_IgG.qld

Last Altered: Sunday, October 02, 2022 16:02:30 台北標準時間
Printed: Friday, August 01, 2025 16:26:04 台北標準時間

Sample Name: 20211111_A43_R1_IgG

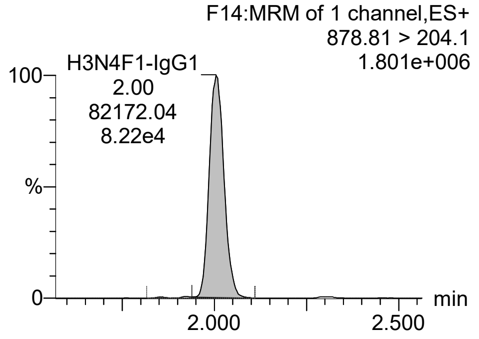

Sample Name: 20211111_A44_R1_IgG

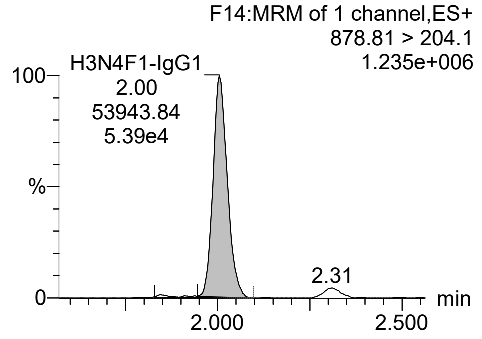

Sample Name: 20211111_QC_R9_IgG

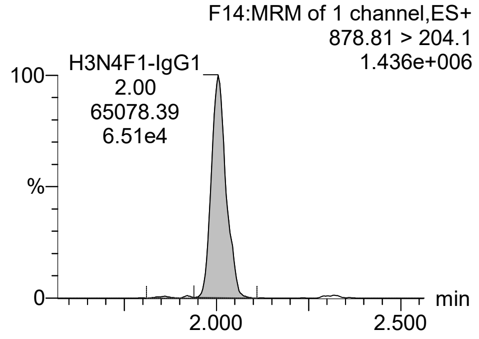

Sample Name: 20211111_A1_R2_IgG

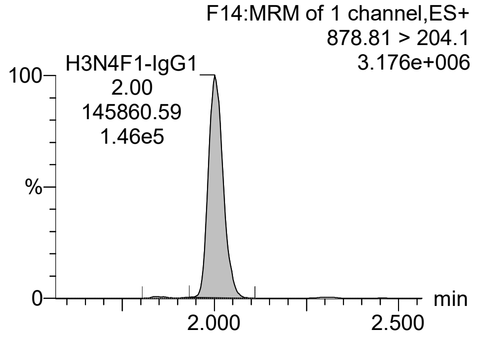

Sample Name: 20211111_A2_R2_IgG

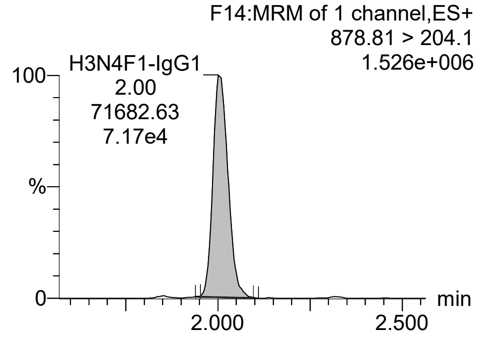

Sample Name: 20211111_L1_R2_IgG

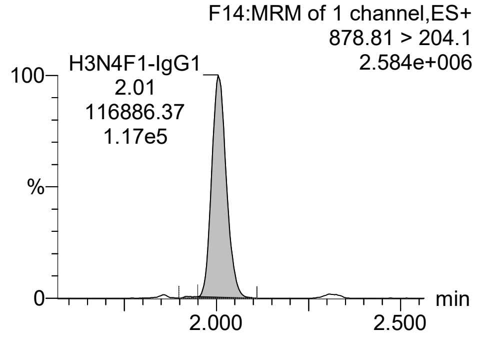

Sample Name: 20211111_L2_R2_IgG

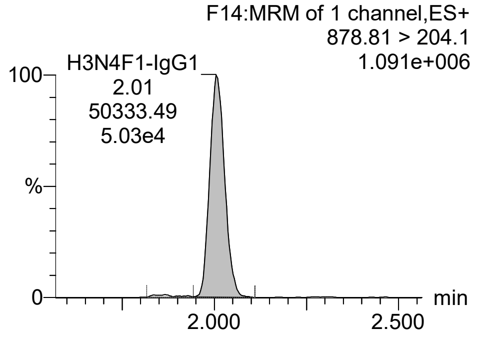

Sample Name: 20211111_H1_R2_IgG

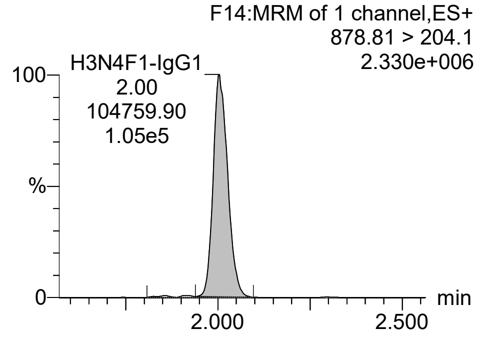

Sample Name: 20211111_A3_R2_IgG

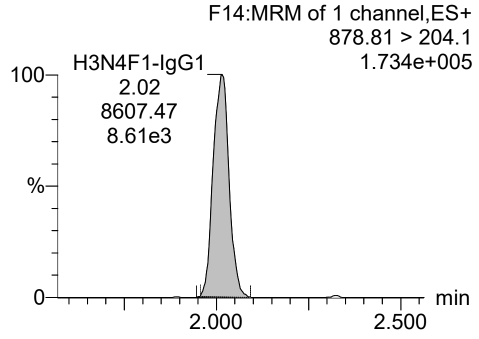

Sample Name: 20211111_A4_R2_IgG

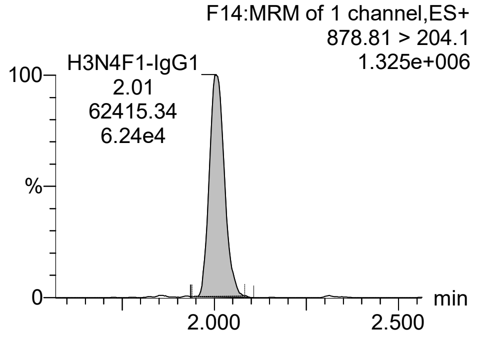

Sample Name: 20211111_L3_R2_IgG

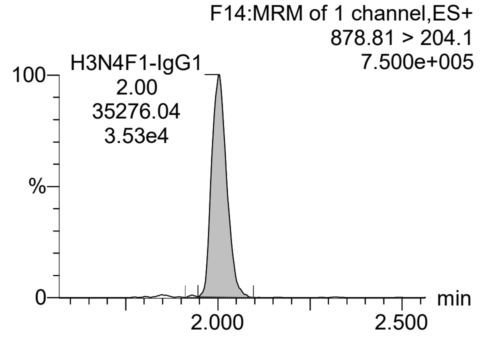

Sample Name: 20211111_L4_R2_IgG

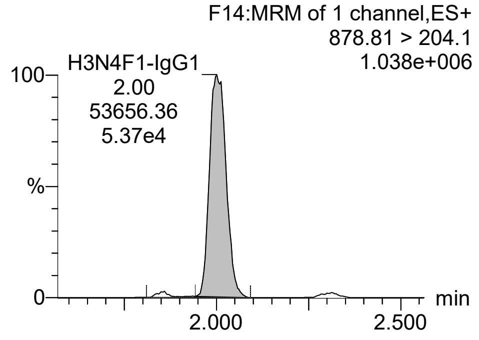

Sample Name: 20211111_H2_R2_IgG

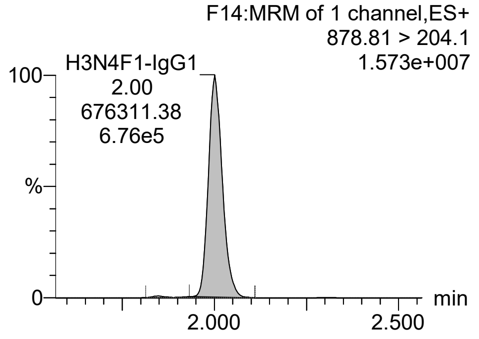

Sample Name: 20211111_A5_R2_IgG

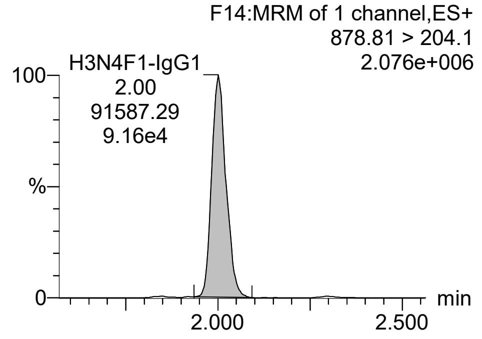

Sample Name: 20211111_A6_R2_IgG

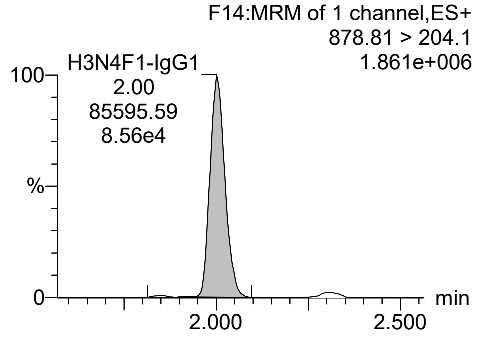

Dataset: C:\MassLynx\10.pro\20211115_clinicalsamples_IgG.qld

Last Altered: Sunday, October 02, 2022 16:02:30 台北標準時間
Printed: Friday, August 01, 2025 16:26:04 台北標準時間

Sample Name: 20211111_QC_R10_IgG
F14:MRM of 1 channel,ES+
878.81 > 204.1
1.402e+006

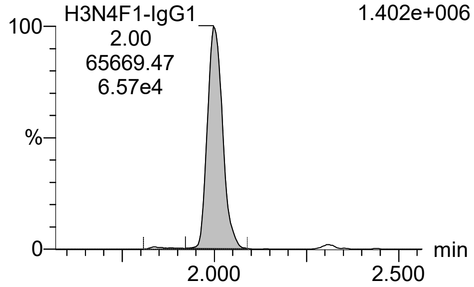

Sample Name: 20211111_L5_R2_IgG
F14:MRM of 1 channel,ES+
878.81 > 204.1
2.990e+006

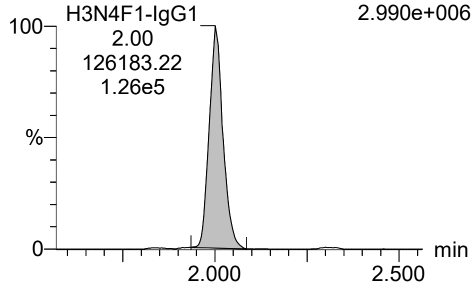

Sample Name: 20211111_L6_R2_IgG
F14:MRM of 1 channel,ES+
878.81 > 204.1
2.089e+005

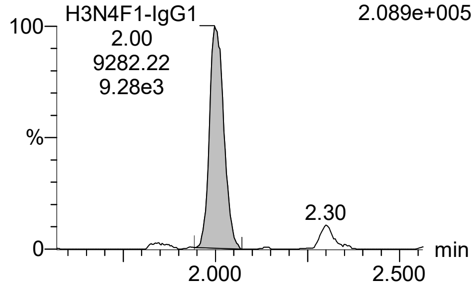

Sample Name: 20211111_H3_R2_IgG
F14:MRM of 1 channel,ES+
878.81 > 204.1
7.042e+005

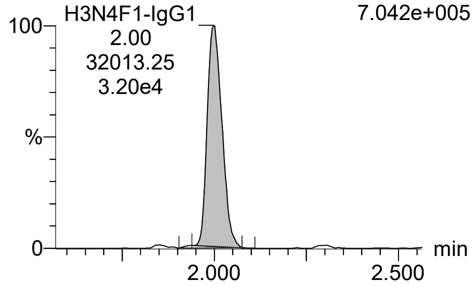

Sample Name: 20211111_A7_R2_IgG
F14:MRM of 1 channel,ES+
878.81 > 204.1
3.470e+006

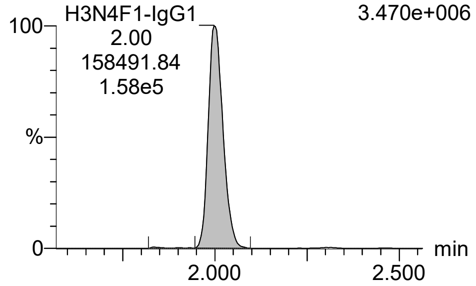

Sample Name: 20211111_A8_R2_IgG
F14:MRM of 1 channel,ES+
878.81 > 204.1
2.992e+006

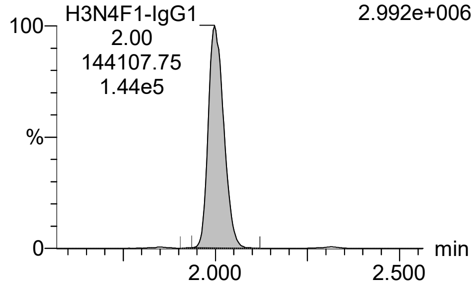

Sample Name: 20211111_L7_R2_IgG
F14:MRM of 1 channel,ES+
878.81 > 204.1
9.662e+005

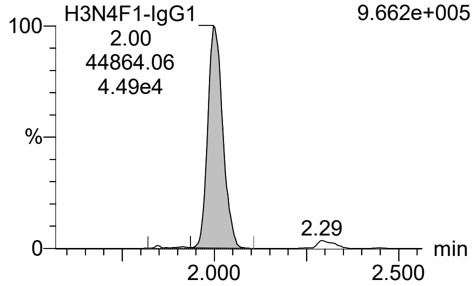

Sample Name: 20211111_L8_R2_IgG
F14:MRM of 1 channel,ES+
878.81 > 204.1
5.358e+005

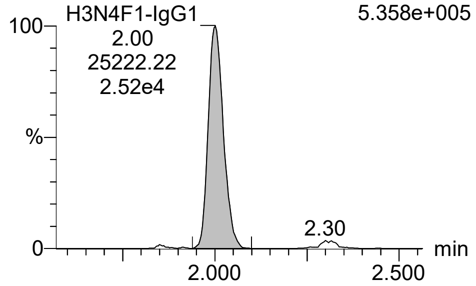

Sample Name: 20211111_H4_R2_IgG
F14:MRM of 1 channel,ES+
878.81 > 204.1
2.245e+006

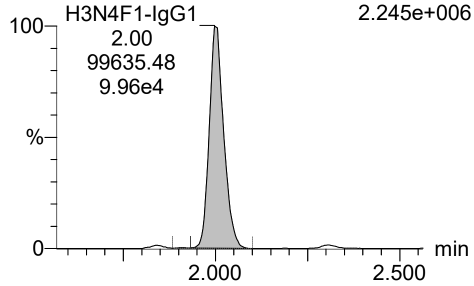

Sample Name: 20211111_A9_R2_IgG
F14:MRM of 1 channel,ES+
878.81 > 204.1
1.654e+006

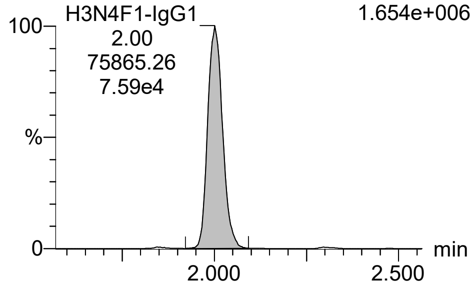

Sample Name: 20211111_A10_R2_IgG
F14:MRM of 1 channel,ES+
878.81 > 204.1
2.562e+006

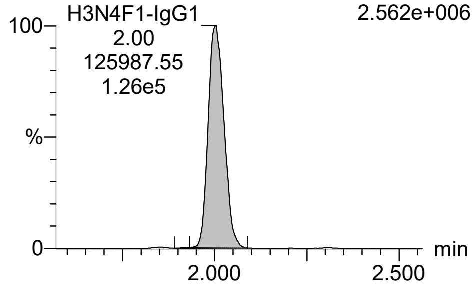

Sample Name: 20211111_L9_R2_IgG
F14:MRM of 1 channel,ES+
878.81 > 204.1
1.370e+006

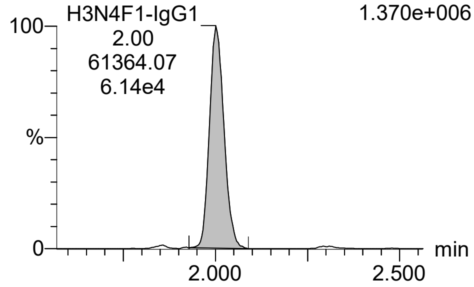

Sample Name: 20211111_L10_R2_IgG
F14:MRM of 1 channel,ES+
878.81 > 204.1
1.784e+006

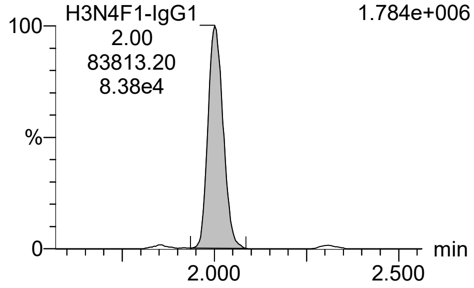

Sample Name: 20211111_QC_R11_IgG
F14:MRM of 1 channel,ES+
878.81 > 204.1
1.470e+006

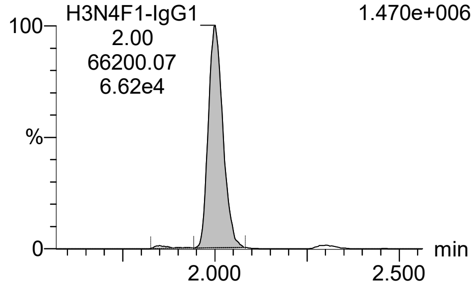

Sample Name: 20211111_H5_R2_IgG
F14:MRM of 1 channel,ES+
878.81 > 204.1
4.861e+005

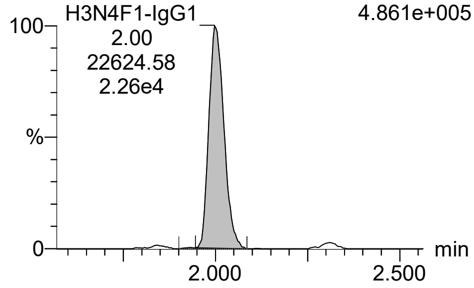

Dataset: C:\MassLynx\10.pro\20211115_clinicalsamples_IgG.qld

Last Altered: Sunday, October 02, 2022 16:02:30 台北標準時間
Printed: Friday, August 01, 2025 16:26:04 台北標準時間

Sample Name: 20211111_A11_R2_IgG

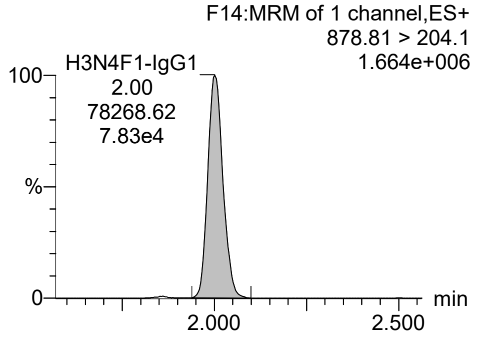

Sample Name: 20211111_A12_R2_IgG

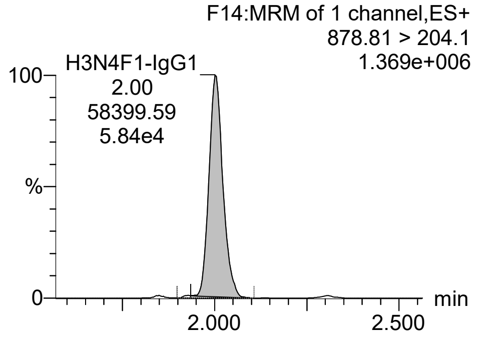

Sample Name: 20211111_L11_R2_IgG

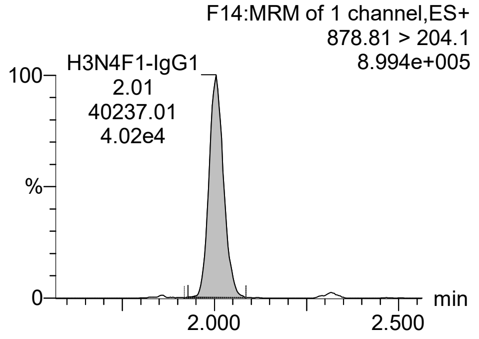

Sample Name: 20211111_L12_R2_IgG

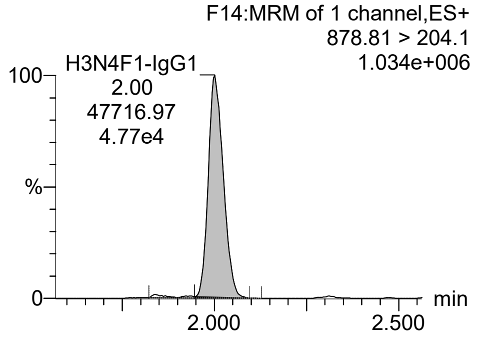

Sample Name: 20211111_H6_R2_IgG

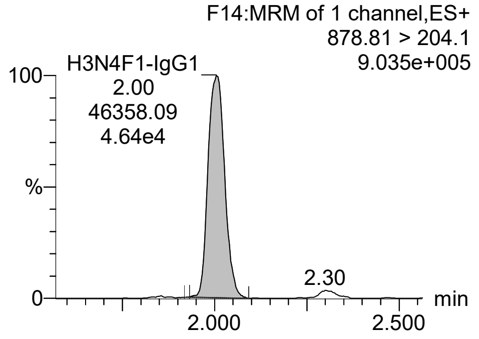

Sample Name: 20211111_A13_R2_IgG

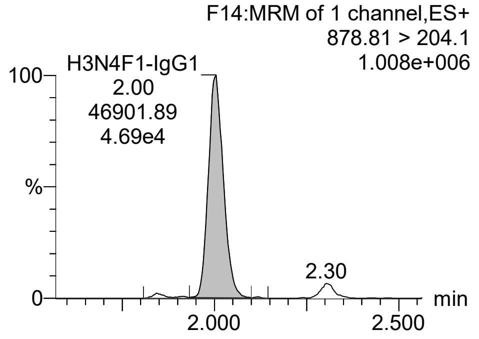

Sample Name: 20211111_A14_R2_IgG

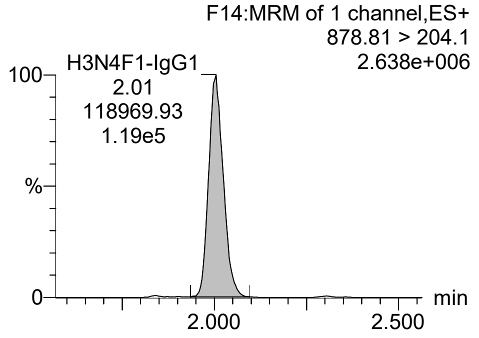

Sample Name: 20211111_L13_R2_IgG

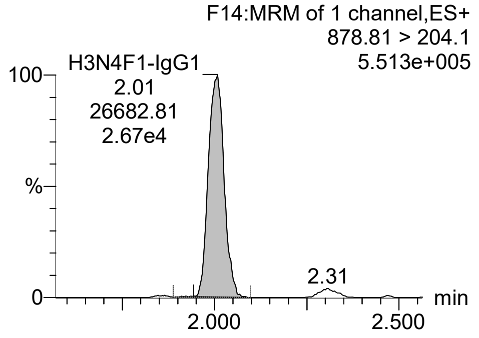

Sample Name: 20211111_L14_R2_IgG

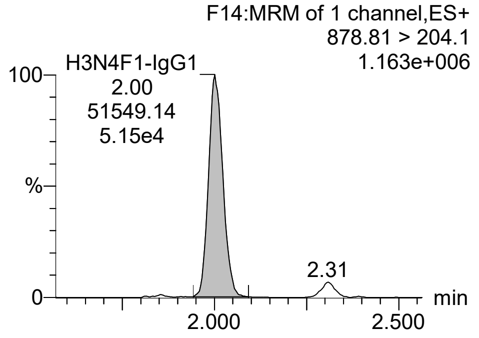

Sample Name: 20211111_H7_R2_IgG

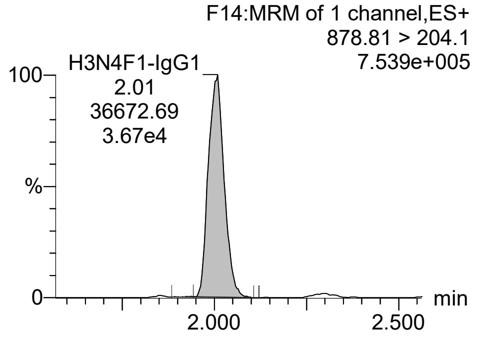

Sample Name: 20211111_A15_R2_IgG

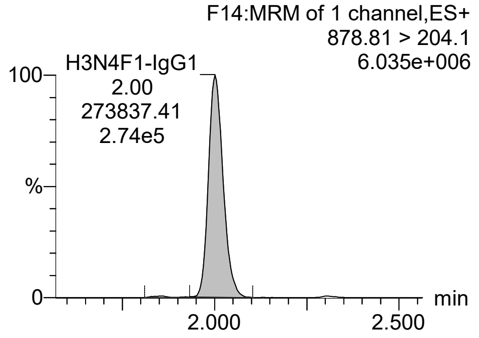

Sample Name: 20211111_QC_R12_IgG

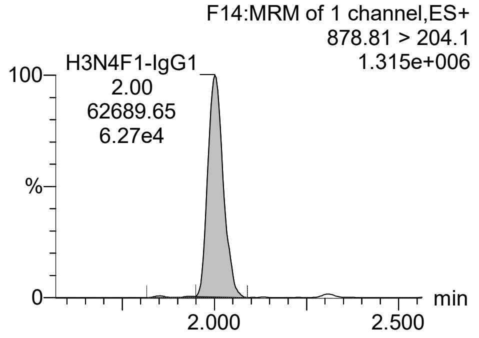

Sample Name: 20211111_A16_R2_IgG

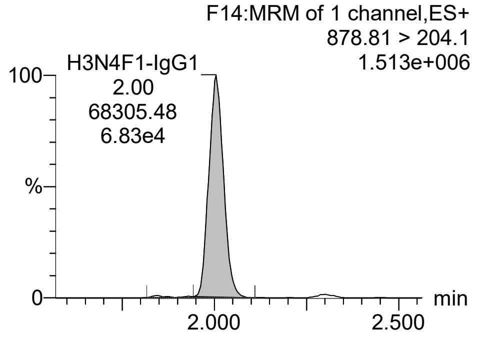

Sample Name: 20211111_L15_R2_IgG

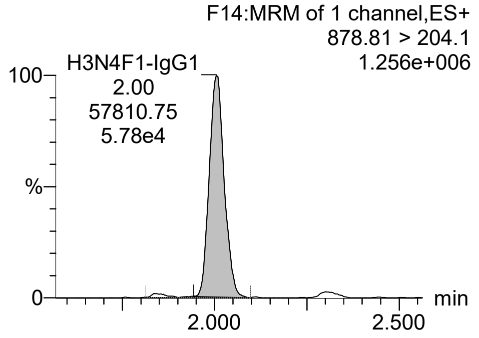

Sample Name: 20211111_L16_R2_IgG

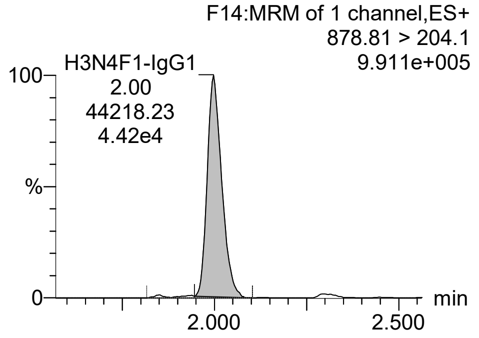

Sample Name: 20211111_H8_R2_IgG

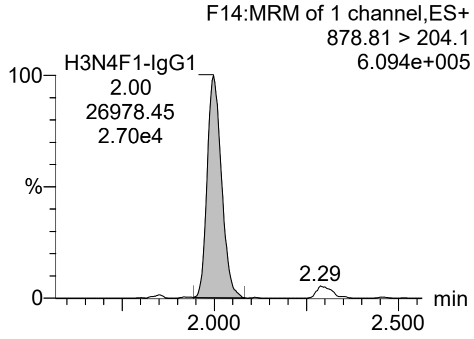

Sample Name: 20211111_A17_R2_IgG

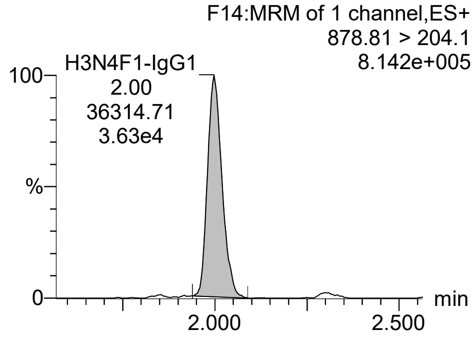

Sample Name: 20211111_A18_R2_IgG

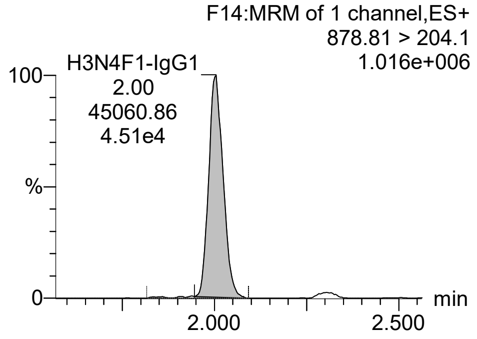

Sample Name: 20211111_L17_R2_IgG

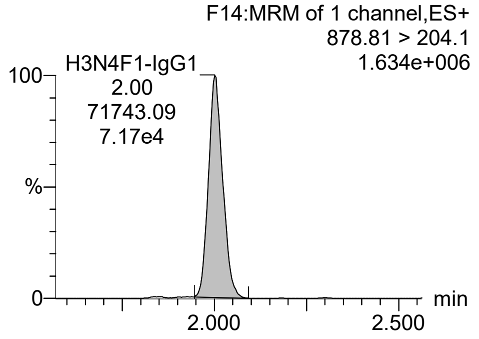

Sample Name: 20211111_L18_R2_IgG

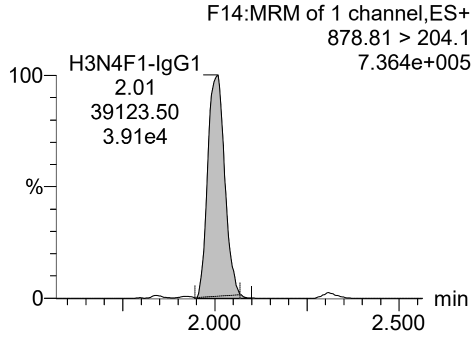

Sample Name: 20211111_H9_R2_IgG

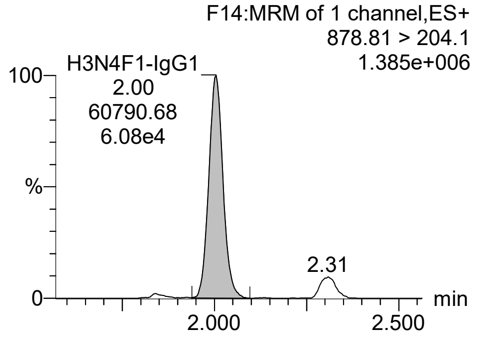

Sample Name: 20211111_A19_R2_IgG

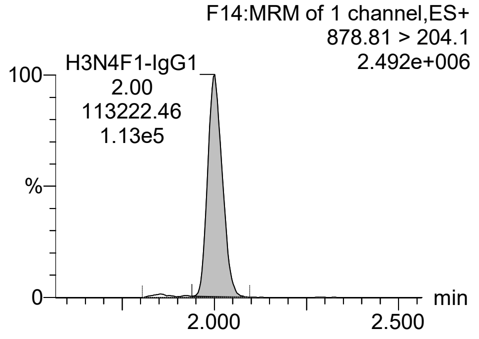

Sample Name: 20211111_A20_R2_IgG

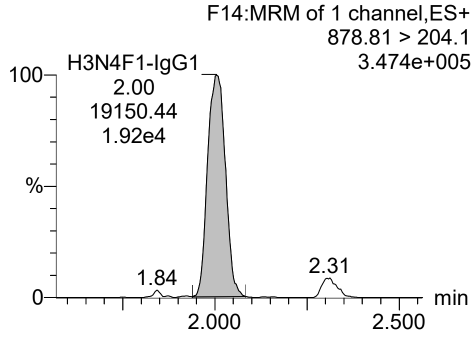

Sample Name: 20211111_L19_R2_IgG

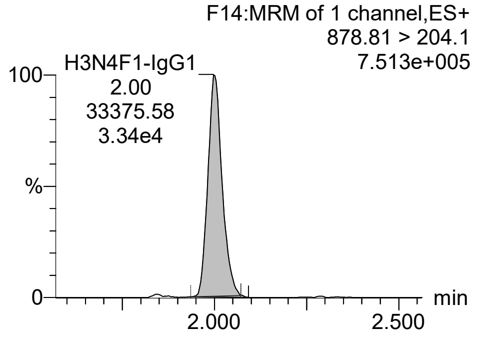

Sample Name: 20211111_QC_R13_IgG

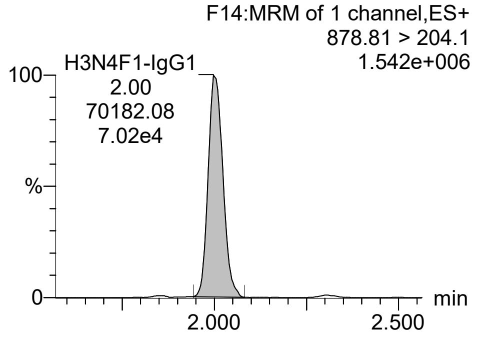

Sample Name: 20211111_L20_R2_IgG

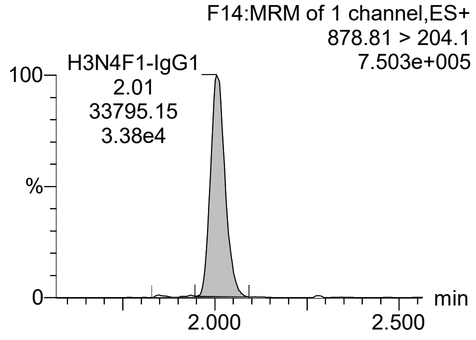

Sample Name: 20211111_H10_R2_IgG

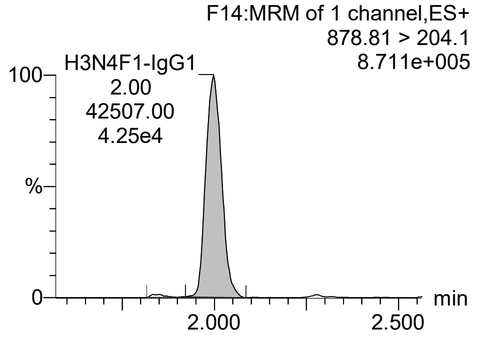

Sample Name: 20211111_A21_R2_IgG

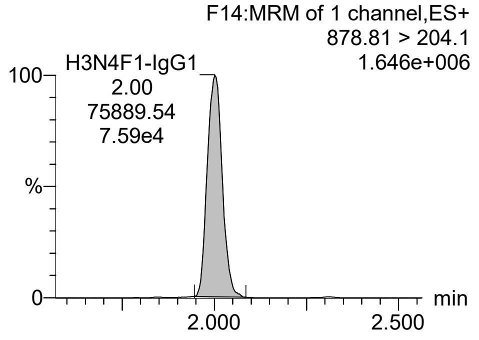

Sample Name: 20211111_A22_R2_IgG

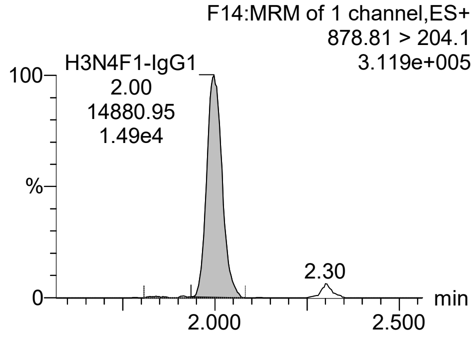

Sample Name: 20211111_L21_R2_IgG

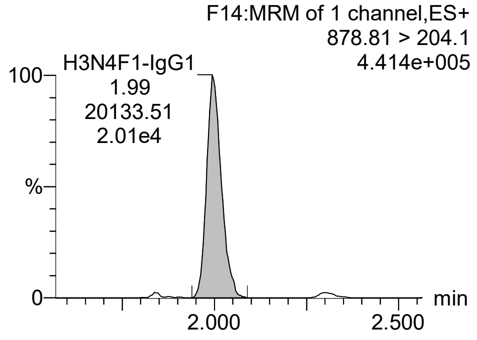

Sample Name: 20211111_L22_R2_IgG

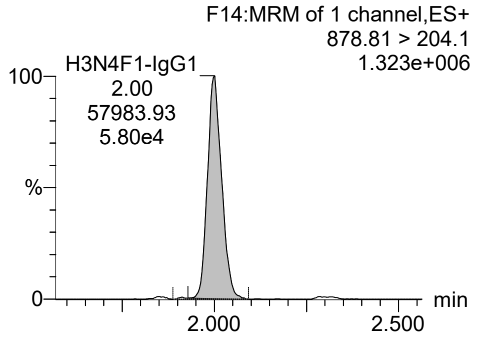

Sample Name: 20211111_H11_R2_IgG

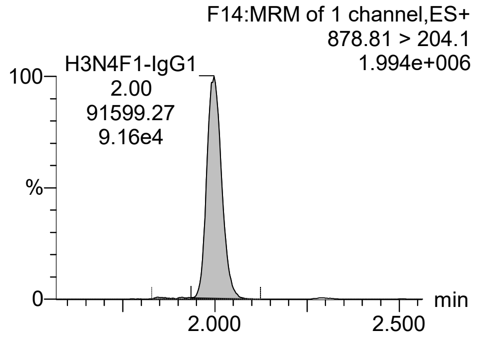

Sample Name: 20211111_A23_R2_IgG

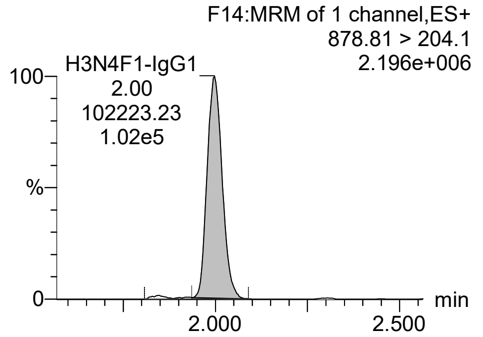

Sample Name: 20211111_A24_R2_IgG

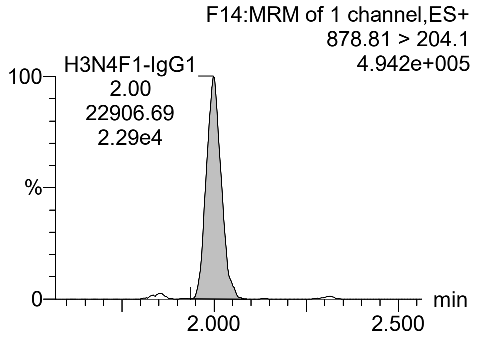

Sample Name: 20211111_L23_R2_IgG

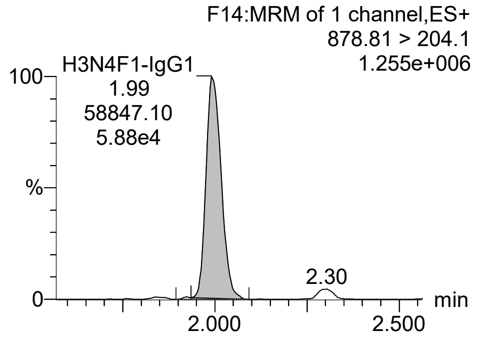

Sample Name: 20211111_L24_R2_IgG

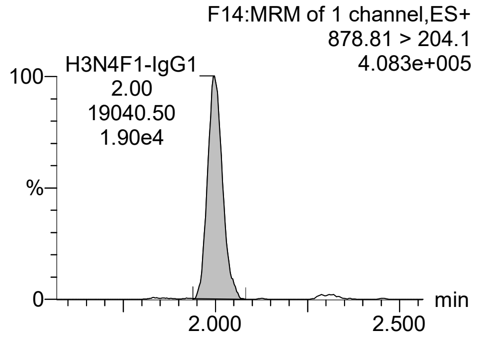

Sample Name: 20211111_H12_R2_IgG

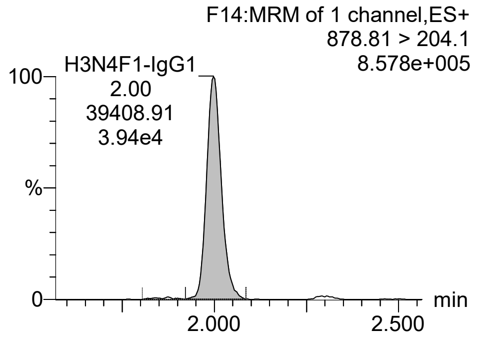

Sample Name: 20211111_QC_R14_IgG

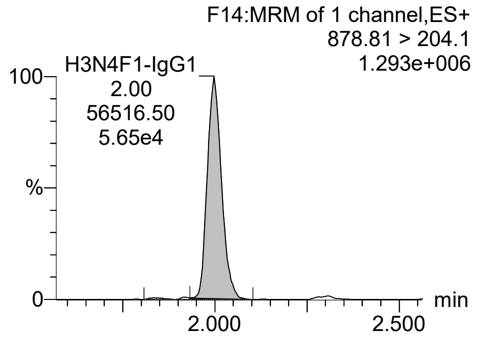

Sample Name: 20211111_A25_R2_IgG

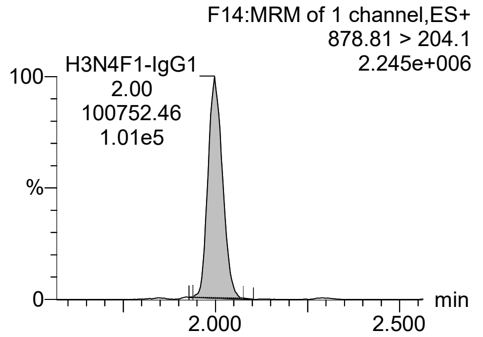

Sample Name: 20211111_A26_R2_IgG

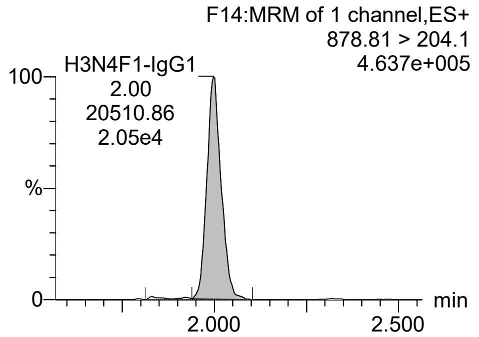

Sample Name: 20211111_L25_R2_IgG

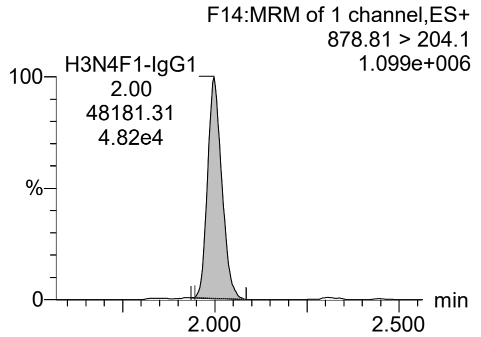

Sample Name: 20211111_L26_R2_IgG

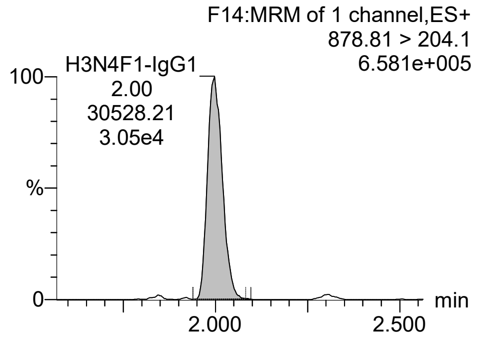

Sample Name: 20211111_H13_R2_IgG

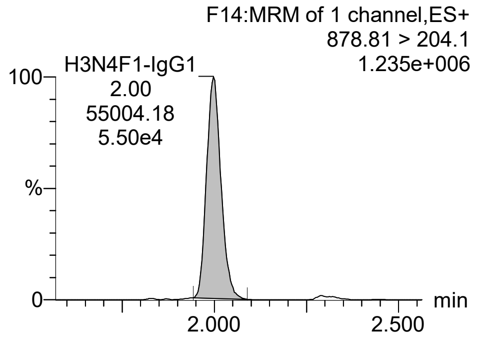

Sample Name: 20211111_A27_R2_IgG

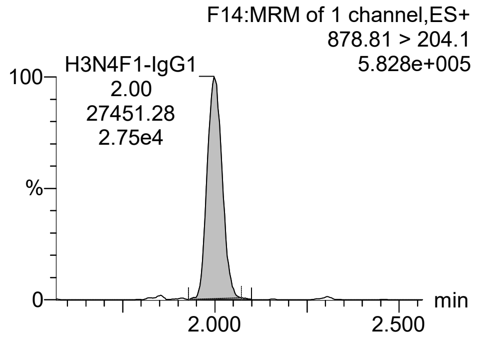

Sample Name: 20211111_A28_R2_IgG

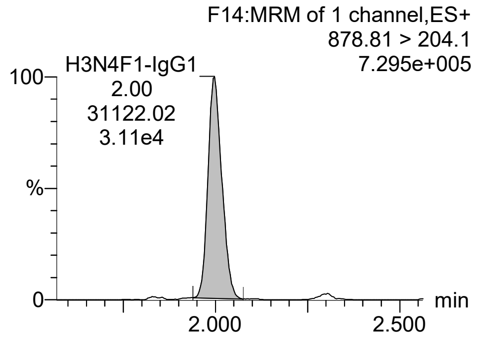

Sample Name: 20211111_L27_R2_IgG

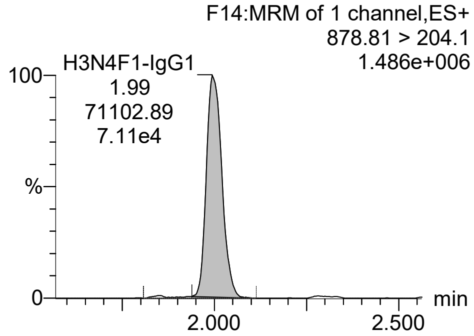

Sample Name: 20211111_L28_R2_IgG

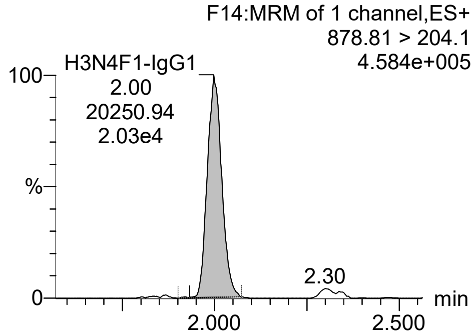

Sample Name: 20211111_H14_R2_IgG

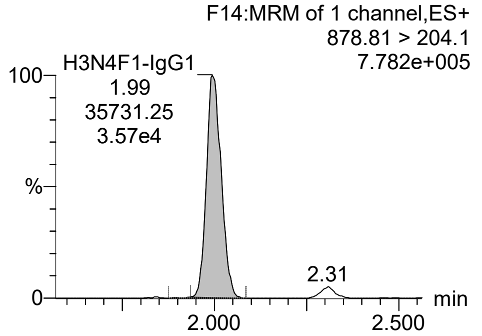

Sample Name: 20211111_A29_R2_IgG

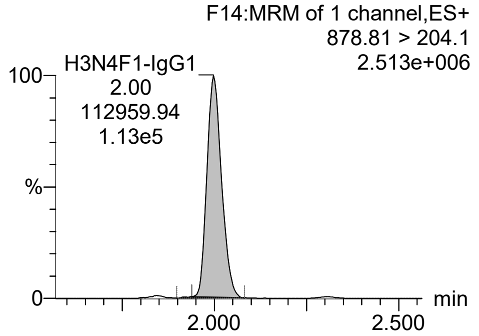

Sample Name: 20211111_A30_R2_IgG

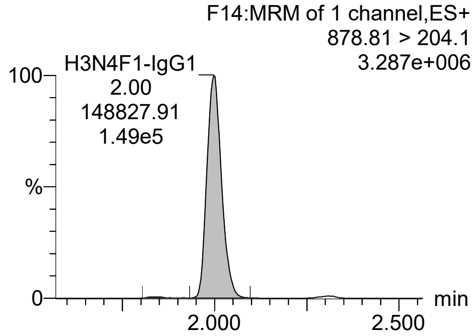

Sample Name: 20211111_QC_R15_IgG

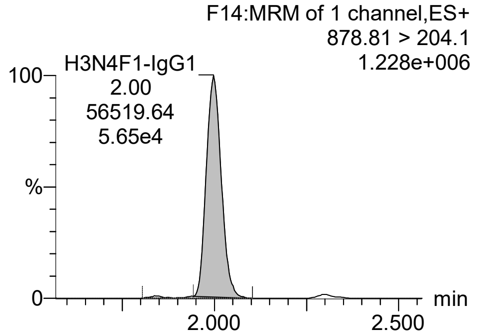

Sample Name: 20211111_L29_R2_IgG

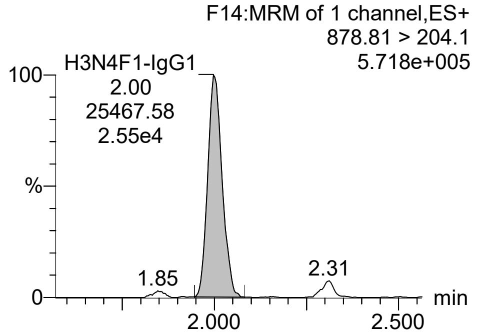

Sample Name: 20211111_L30_R2_IgG

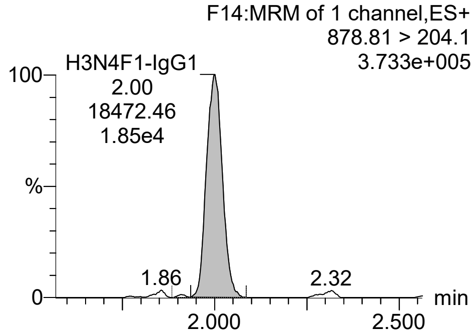

Sample Name: 20211111_H15_R2_IgG

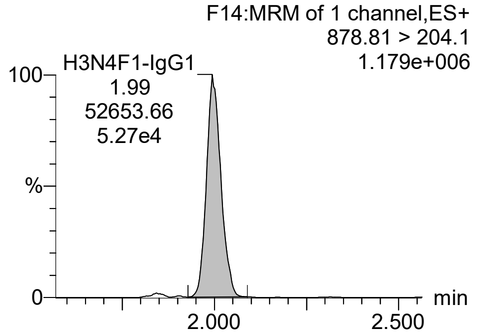

Sample Name: 20211111_A31_R2_IgG

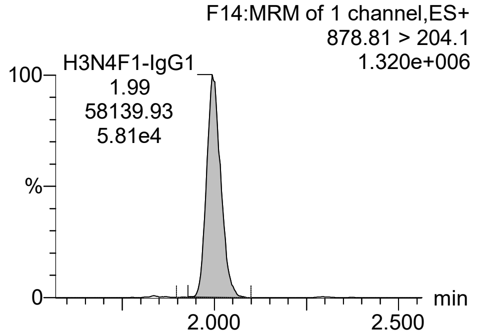

Sample Name: 20211111_A32_R2_IgG

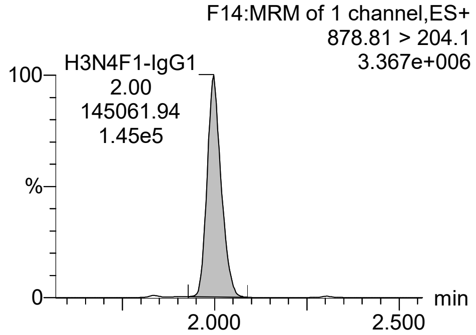

Sample Name: 20211111_L31_R2_IgG

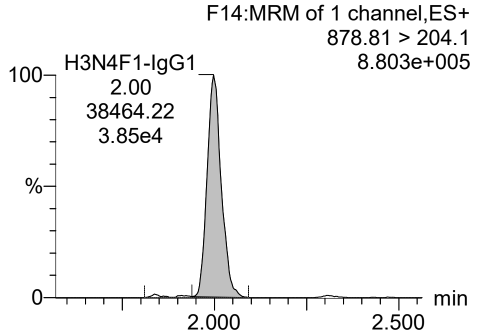

Sample Name: 20211111_L32_R2_IgG

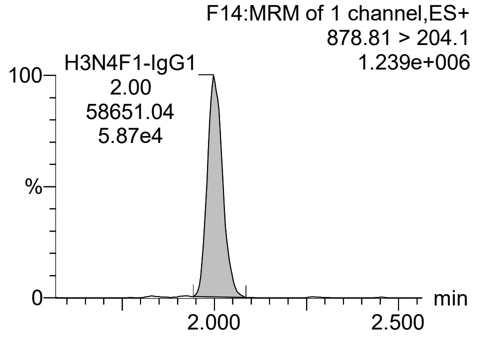

Sample Name: 20211111_H16_R2_IgG

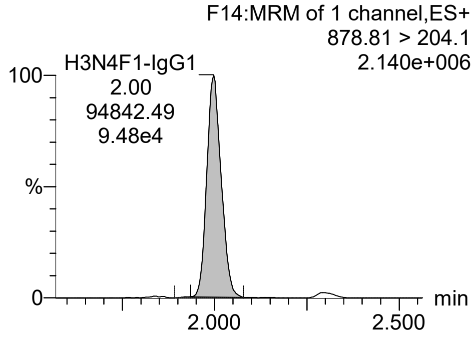

Sample Name: 20211111_A33_R2_IgG

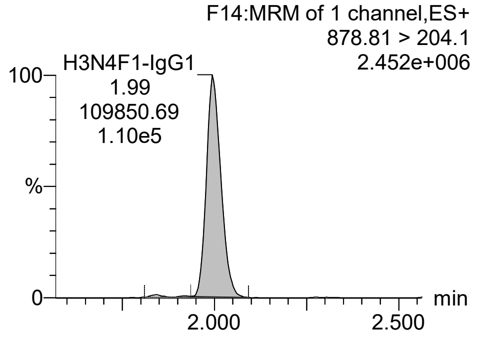

Dataset: C:\MassLynx\10.pro\20211115_clinicalsamples_IgG.qld

Last Altered: Sunday, October 02, 2022 16:02:30 台北標準時間
Printed: Friday, August 01, 2025 16:26:04 台北標準時間

Sample Name: 20211111_A34_R2_IgG

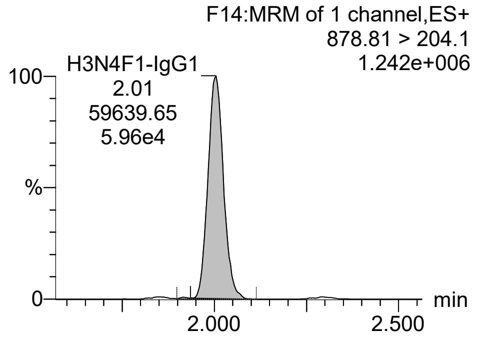

Sample Name: 20211111_H17_R2_IgG

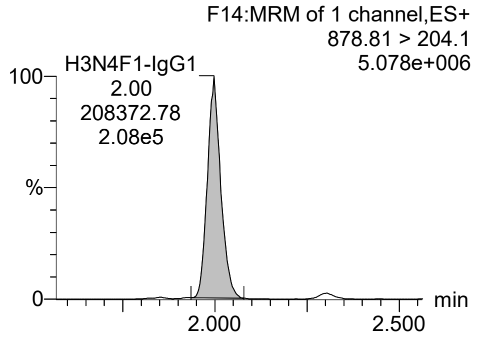

Sample Name: 20211111_A35_R2_IgG

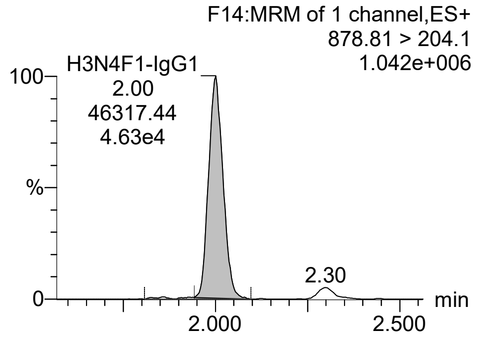

Sample Name: 20211111_QC_R16_IgG

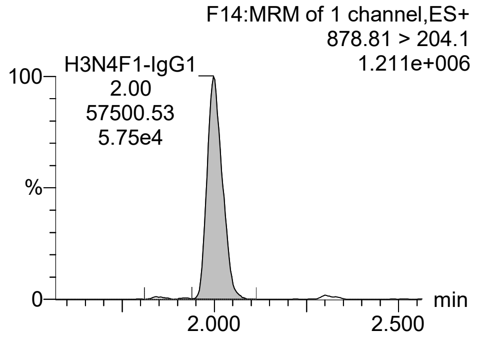

Sample Name: 20211111_A36_R2_IgG

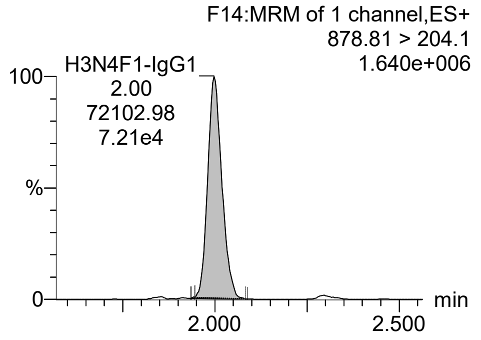

Sample Name: 20211111_H18_R2_IgG

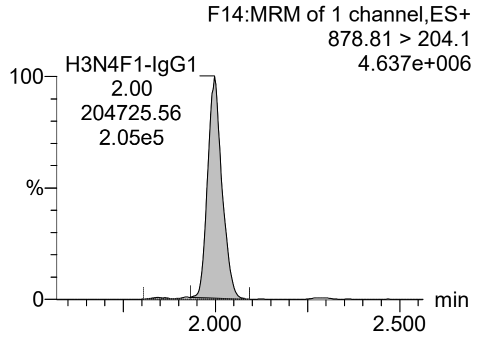

Sample Name: 20211111_A37_R2_IgG

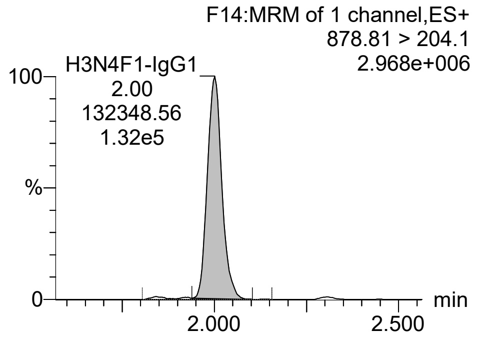

Sample Name: 20211111_A38_R2_IgG

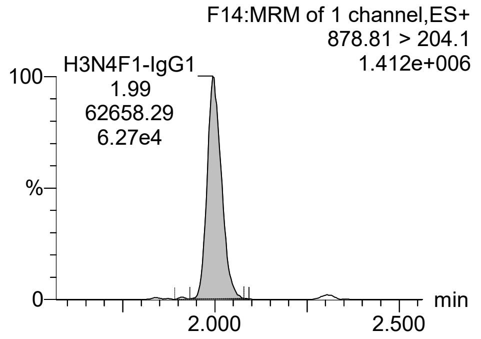

Sample Name: 20211111_H19_R2_IgG

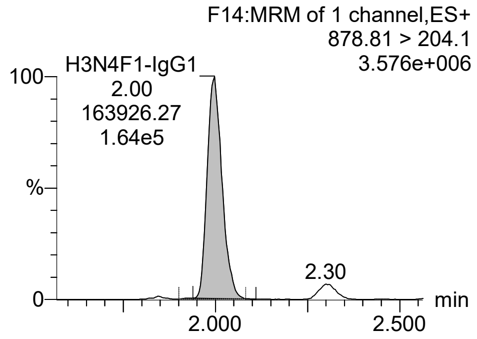

Sample Name: 20211111_A39_R2_IgG

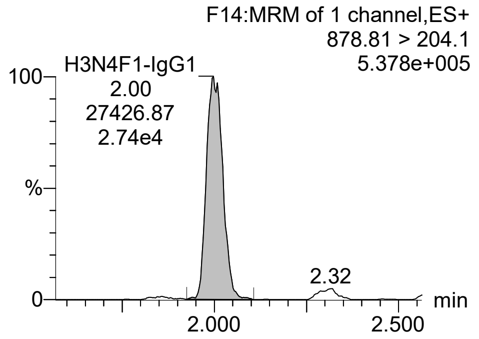

Sample Name: 20211111_A40_R2_IgG

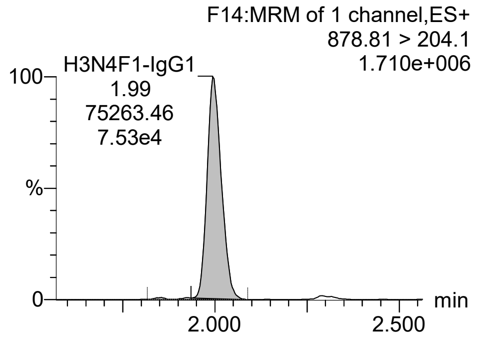

Sample Name: 20211111_H20_R2_IgG

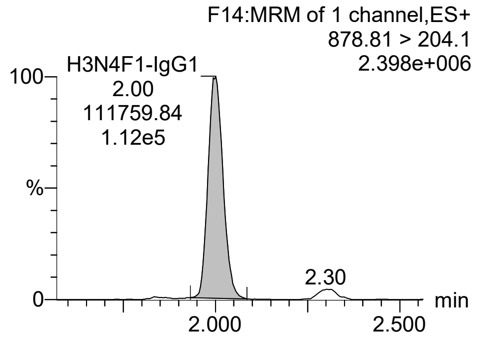

Sample Name: 20211111_A41_R2_IgG

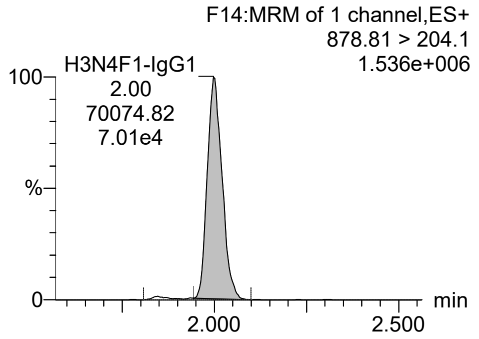

Sample Name: 20211111_A42_R2_IgG

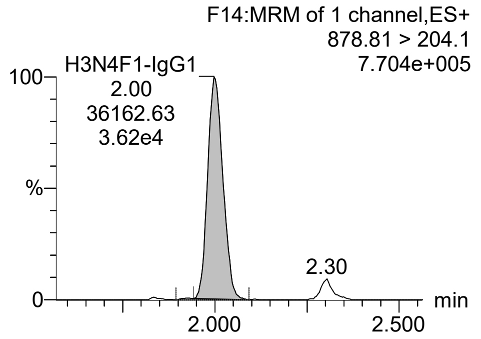

Sample Name: 20211111_A43_R2_IgG

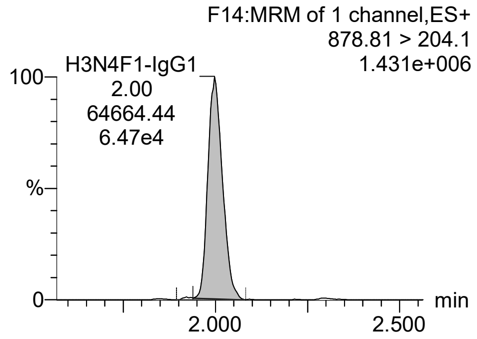

Dataset: C:\MassLynx\10.pro\20211115_clinicalsamples_IgG.qld

Last Altered: Sunday, October 02, 2022 16:02:30 台北標準時間
Printed: Friday, August 01, 2025 16:26:04 台北標準時間

Sample Name: 20211111_A44_R2_IgG

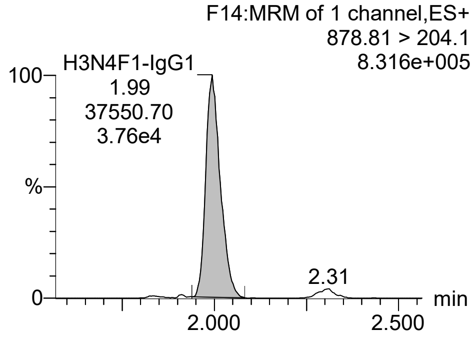

Sample Name: 20211111_QC_R17_IgG

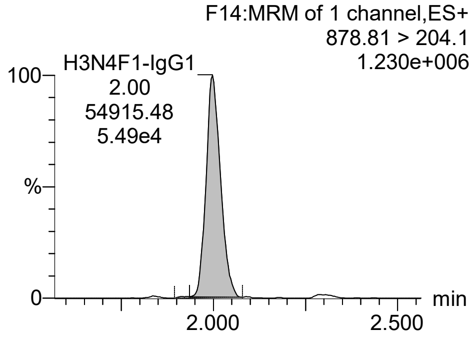

Dataset: C:\MassLynx\10.pro\20211115_clinicalsamples_IgG.qld

Last Altered: Sunday, October 02, 2022 16:02:30 台北標準時間
Printed: Friday, August 01, 2025 16:26:04 台北標準時間

	#	Name	RT	Area	IS Area	Response	Prima...
1	1	20211111_QC_R1_IgG	2.00	127373.9	64968.0	1.961	MM
2	2	20211111_A1_R1_IgG	2.00	187421.2	59059.2	3.173	MM
3	3	20211111_A2_R1_IgG	2.00	85015.3	66621.2	1.276	MM
4	4	20211111_L1_R1_IgG	2.00	163621.6	64201.0	2.549	MM
5	5	20211111_L2_R1_IgG	2.00	58220.8	46950.2	1.240	MM
6	6	20211111_H1_R1_IgG	2.00	91370.9	38648.2	2.364	MM
7	7	20211111_A3_R1_IgG	2.01	4093.9	2603.7	1.572	MM
8	8	20211111_A4_R1_IgG	2.00	88655.2	69960.4	1.267	MM
9	9	20211111_L3_R1_IgG	2.01	36376.2	38864.7	0.936	MM
10	10	20211111_L4_R1_IgG	2.00	65489.8	43380.5	1.510	MM
11	11	20211111_H2_R1_IgG	2.00	918326.7	71349.2	12.871	MM
12	12	20211111_A5_R1_IgG	2.00	107925.1	65569.7	1.646	MM
13	13	20211111_A6_R1_IgG	2.01	103283.6	53120.7	1.944	MM
14	14	20211111_QC_R2_IgG	2.01	137960.4	81034.1	1.702	MM
15	15	20211111_L5_R1_IgG	2.00	151855.1	50994.6	2.978	MM
16	16	20211111_L6_R1_IgG	2.01	12048.0	58058.0	0.208	bb
17	17	20211111_H3_R1_IgG	2.00	41113.4	64028.6	0.642	MM
18	18	20211111_A7_R1_IgG	2.01	231064.6	82996.1	2.784	MM
19	19	20211111_A8_R1_IgG	2.00	200270.0	77731.7	2.576	MM
20	20	20211111_L7_R1_IgG	2.00	50055.4	38827.3	1.289	MM
21	21	20211111_L8_R1_IgG	2.01	30411.9	44220.1	0.688	bb
22	22	20211111_H4_R1_IgG	2.01	127548.8	65826.3	1.938	bb
23	23	20211111_A9_R1_IgG	2.01	99503.6	60771.1	1.637	MM
24	24	20211111_A10_R1_IgG	2.01	143722.6	48578.7	2.959	MM
25	25	20211111_L9_R1_IgG	2.00	67684.7	50830.7	1.332	bb
26	26	20211111_L10_R1_IgG	2.00	89962.3	38034.2	2.365	MM
27	27	20211111_QC_R3_IgG	2.00	121086.9	67025.5	1.807	bb
28	28	20211111_H5_R1_IgG	2.01	26025.5	44544.9	0.584	MM
29	29	20211111_A11_R1_IgG	2.00	102865.9	66408.5	1.549	MM
30	30	20211111_A12_R1_IgG	2.01	66598.5	46624.5	1.428	MM
31	31	20211111_L11_R1_IgG	2.01	49969.2	33425.1	1.495	MM
32	32	20211111_L12_R1_IgG	2.00	51209.4	41178.0	1.244	MM
33	33	20211111_H6_R1_IgG	2.01	76501.7	58593.4	1.306	MM
34	34	20211111_A13_R1_IgG	2.00	59773.3	54133.0	1.104	MM
35	35	20211111_A14_R1_IgG	2.01	146911.3	56467.3	2.602	MM
36	36	20211111_L13_R1_IgG	2.00	30762.6	36024.4	0.854	MM
37	37	20211111_L14_R1_IgG	2.00	53354.0	37181.6	1.435	bb
38	38	20211111_H7_R1_IgG	2.00	42109.1	42850.6	0.983	MM
39	39	20211111_A15_R1_IgG	2.01	346584.3	62015.1	5.589	MM
40	40	20211111_QC_R4_IgG	2.00	105556.8	58572.5	1.802	bb
41	41	20211111_A16_R1_IgG	2.00	113958.7	78283.8	1.456	MM
42	42	20211111_L15_R1_IgG	2.00	79570.1	44158.2	1.802	MM
43	43	20211111_L16_R1_IgG	2.00	67046.8	55150.8	1.216	MM
44	44	20211111_H8_R1_IgG	2.01	30954.3	42965.2	0.720	MM
45	45	20211111_A17_R1_IgG	2.00	38320.2	39717.7	0.965	MM
46	46	20211111_A18_R1_IgG	2.00	52121.3	44675.4	1.167	MM
47	47	20211111_L17_R1_IgG	2.00	75795.3	37854.1	2.002	MM
48	48	20211111_L18_R1_IgG	2.00	52783.2	50716.9	1.041	MM
49	49	20211111_H9_R1_IgG	2.01	63354.4	36276.0	1.746	MM
50	50	20211111_A19_R1_IgG	2.01	124271.3	42862.5	2.899	MM
51	51	20211111_A20_R1_IgG	2.00	24006.5	36542.7	0.657	MM
52	52	20211111_L19_R1_IgG	2.00	43457.3	53892.8	0.806	MM
53	53	20211111_QC_R5_IgG	2.00	98101.0	56513.2	1.736	MM
54	54	20211111_L20_R1_IgG	2.00	62187.1	56499.9	1.101	MM
55	55	20211111_H10_R1_IgG	2.00	104488.5	68120.5	1.534	MM
56	56	20211111_A21_R1_IgG	2.00	153871.6	63699.8	2.416	MM

Dataset: C:\MassLynx\10.pro\20211115_clinicalsamples_IgG.qld

Last Altered: Sunday, October 02, 2022 16:02:30 台北標準時間
Printed: Friday, August 01, 2025 16:26:04 台北標準時間

	# Name	RT	Area	IS Area	Response	Prima...
57	57 20211111_A22_R1_IgG	2.00	29134.8	52222.1	0.558	MM
58	58 20211111_L21_R1_IgG	2.01	34715.5	54372.5	0.638	MM
59	59 20211111_L22_R1_IgG	2.01	90546.5	41353.1	2.190	MM
60	60 20211111_H11_R1_IgG	2.00	171937.0	65579.1	2.622	MM
61	61 20211111_A23_R1_IgG	2.00	205555.5	64131.1	3.205	MM
62	62 20211111_A24_R1_IgG	2.01	34338.3	38724.8	0.887	bb
63	63 20211111_L23_R1_IgG	2.00	89956.3	64245.2	1.400	MM
64	64 20211111_L24_R1_IgG	2.01	26591.4	45298.0	0.587	bb
65	65 20211111_H12_R1_IgG	2.00	49040.5	44679.3	1.098	MM
66	66 20211111_QC_R6_IgG	2.01	90770.0	48165.0	1.885	MM
67	67 20211111_A25_R1_IgG	2.00	159635.3	57169.9	2.792	MM
68	68 20211111_A26_R1_IgG	2.00	23538.8	34254.0	0.687	MM
69	69 20211111_L25_R1_IgG	2.00	88855.7	67491.5	1.317	bb
70	70 20211111_L26_R1_IgG	2.00	48905.5	55934.1	0.874	MM
71	71 20211111_H13_R1_IgG	2.00	78128.3	48767.3	1.602	MM
72	72 20211111_A27_R1_IgG	2.00	40158.8	57269.7	0.701	bb
73	73 20211111_A28_R1_IgG	2.00	48638.8	49231.1	0.988	MM
74	74 20211111_L27_R1_IgG	2.00	101139.8	60014.5	1.685	MM
75	75 20211111_L28_R1_IgG	2.00	26940.0	35062.6	0.768	bb
76	76 20211111_H14_R1_IgG	2.00	54810.3	62371.9	0.879	MM
77	77 20211111_A29_R1_IgG	2.00	140427.4	43858.2	3.202	MM
78	78 20211111_A30_R1_IgG	2.00	231484.2	62500.4	3.704	bb
79	79 20211111_QC_R7_IgG	2.00	79956.2	43946.9	1.819	MM
80	80 20211111_L29_R1_IgG	2.00	28209.7	38728.9	0.728	MM
81	81 20211111_L30_R1_IgG	2.00	24500.0	42613.2	0.575	bb
82	82 20211111_H15_R1_IgG	2.00	59910.4	39874.6	1.502	MM
83	83 20211111_A31_R1_IgG	2.01	87572.3	53414.3	1.639	bb
84	84 20211111_A32_R1_IgG	2.00	170981.3	41844.6	4.086	MM
85	85 20211111_L31_R1_IgG	2.00	47135.7	40553.7	1.162	MM
86	86 20211111_L32_R1_IgG	2.00	68095.0	49618.7	1.372	MM
87	87 20211111_H16_R1_IgG	2.00	128631.7	60170.9	2.138	MM
88	88 20211111_A33_R1_IgG	2.00	134040.9	48588.2	2.759	MM
89	89 20211111_A34_R1_IgG	2.00	78477.5	43288.0	1.813	MM
90	90 20211111_H17_R1_IgG	2.00	235376.1	47104.5	4.997	MM
91	91 20211111_A35_R1_IgG	2.00	51406.1	41025.9	1.253	MM
92	92 20211111_QC_R8_IgG	2.01	72711.9	40546.3	1.793	MM
93	93 20211111_A36_R1_IgG	2.01	81103.4	44504.4	1.822	MM
94	94 20211111_H18_R1_IgG	2.01	209432.1	34162.2	6.131	MM
95	95 20211111_A37_R1_IgG	2.01	139098.3	39760.0	3.498	bb
96	96 20211111_A38_R1_IgG	2.01	69234.7	43982.6	1.574	MM
97	97 20211111_H19_R1_IgG	2.01	184121.3	45314.8	4.063	MM
98	98 20211111_A39_R1_IgG	2.01	28989.9	39388.0	0.736	bb
99	99 20211111_A40_R1_IgG	2.01	78647.2	38726.4	2.031	MM
100	1... 20211111_H20_R1_IgG	2.00	123813.9	37814.4	3.274	bb
101	1... 20211111_A41_R1_IgG	2.01	77225.6	48930.3	1.578	MM
102	1... 20211111_A42_R1_IgG	2.00	39823.0	40406.8	0.986	bb
103	1... 20211111_A43_R1_IgG	2.00	82172.0	44421.5	1.850	MM
104	1... 20211111_A44_R1_IgG	2.00	53943.8	53128.2	1.015	MM
105	1... 20211111_QC_R9_IgG	2.00	65078.4	37049.0	1.757	MM
106	1... 20211111_A1_R2_IgG	2.00	145860.6	46499.4	3.137	MM
107	1... 20211111_A2_R2_IgG	2.00	71682.6	57260.7	1.252	MM
108	1... 20211111_L1_R2_IgG	2.01	116886.4	48062.9	2.432	MM
109	1... 20211111_L2_R2_IgG	2.01	50333.5	38745.2	1.299	MM
110	1... 20211111_H1_R2_IgG	2.00	104759.9	44273.5	2.366	MM
111	1... 20211111_A3_R2_IgG	2.02	8607.5	4755.5	1.810	MM
112	1... 20211111_A4_R2_IgG	2.01	62415.3	50546.7	1.235	MM

Dataset: C:\MassLynx\10.pro\20211115_clinicalsamples_IgG.qld

Last Altered: Sunday, October 02, 2022 16:02:30 台北標準時間
Printed: Friday, August 01, 2025 16:26:04 台北標準時間

	# Name	RT	Area	IS Area	Response	Prima...
113	1... 20211111_L3_R2_IgG	2.00	35276.0	34825.8	1.013	MM
114	1... 20211111_L4_R2_IgG	2.00	53656.4	39107.7	1.372	MM
115	1... 20211111_H2_R2_IgG	2.00	676311.4	49750.0	13.594	MM
116	1... 20211111_A5_R2_IgG	2.00	91587.3	52536.2	1.743	bb
117	1... 20211111_A6_R2_IgG	2.00	85595.6	42050.0	2.036	MM
118	1... 20211111_QC_R10_IgG	2.00	65669.5	35350.8	1.858	MM
119	1... 20211111_L5_R2_IgG	2.00	126183.2	38039.3	3.317	bb
120	1... 20211111_L6_R2_IgG	2.00	9282.2	44907.4	0.207	bb
121	1... 20211111_H3_R2_IgG	2.00	32013.3	47729.1	0.671	MM
122	1... 20211111_A7_R2_IgG	2.00	158491.8	57347.1	2.764	MM
123	1... 20211111_A8_R2_IgG	2.00	144107.8	52397.8	2.750	MM
124	1... 20211111_L7_R2_IgG	2.00	44864.1	36318.4	1.235	MM
125	1... 20211111_L8_R2_IgG	2.00	25222.2	38131.9	0.661	bb
126	1... 20211111_H4_R2_IgG	2.00	99635.5	46849.6	2.127	MM
127	1... 20211111_A9_R2_IgG	2.00	75865.3	47759.6	1.588	bb
128	1... 20211111_A10_R2_IgG	2.00	125987.5	41366.2	3.046	MM
129	1... 20211111_L9_R2_IgG	2.00	61364.1	43052.1	1.425	bb
130	1... 20211111_L10_R2_IgG	2.00	83813.2	31487.4	2.662	bb
131	1... 20211111_QC_R11_IgG	2.00	66200.1	37804.6	1.751	MM
132	1... 20211111_H5_R2_IgG	2.00	22624.6	40380.0	0.560	MM
133	1... 20211111_A11_R2_IgG	2.00	78268.6	49533.3	1.580	bb
134	1... 20211111_A12_R2_IgG	2.00	58399.6	40226.3	1.452	MM
135	1... 20211111_L11_R2_IgG	2.01	40237.0	28884.0	1.393	MM
136	1... 20211111_L12_R2_IgG	2.00	47717.0	34351.3	1.389	MM
137	1... 20211111_H6_R2_IgG	2.00	46358.1	40466.6	1.146	MM
138	1... 20211111_A13_R2_IgG	2.00	46901.9	42306.7	1.109	MM
139	1... 20211111_A14_R2_IgG	2.01	118969.9	44904.0	2.649	bb
140	1... 20211111_L13_R2_IgG	2.01	26682.8	31913.0	0.836	MM
141	1... 20211111_L14_R2_IgG	2.00	51549.1	31527.4	1.635	bb
142	1... 20211111_H7_R2_IgG	2.01	36672.7	40838.2	0.898	MM
143	1... 20211111_A15_R2_IgG	2.00	273837.4	49389.7	5.544	MM
144	1... 20211111_QC_R12_IgG	2.00	62689.6	35168.7	1.783	MM
145	1... 20211111_A16_R2_IgG	2.00	68305.5	47440.5	1.440	MM
146	1... 20211111_L15_R2_IgG	2.00	57810.8	33145.9	1.744	MM
147	1... 20211111_L16_R2_IgG	2.00	44218.2	37596.6	1.176	MM
148	1... 20211111_H8_R2_IgG	2.00	26978.4	43933.3	0.614	bb
149	1... 20211111_A17_R2_IgG	2.00	36314.7	43605.5	0.833	bb
150	1... 20211111_A18_R2_IgG	2.00	45060.9	37581.8	1.199	MM
151	1... 20211111_L17_R2_IgG	2.00	71743.1	33942.5	2.114	bb
152	1... 20211111_L18_R2_IgG	2.01	39123.5	37831.5	1.034	MM
153	1... 20211111_H9_R2_IgG	2.00	60790.7	30898.4	1.967	bb
154	1... 20211111_A19_R2_IgG	2.00	113222.5	39352.1	2.877	MM
155	1... 20211111_A20_R2_IgG	2.00	19150.4	34661.2	0.553	bb
156	1... 20211111_L19_R2_IgG	2.00	33375.6	39918.3	0.836	MM
157	1... 20211111_QC_R13_IgG	2.00	70182.1	36239.1	1.937	bb
158	1... 20211111_L20_R2_IgG	2.01	33795.1	32897.7	1.027	MM
159	1... 20211111_H10_R2_IgG	2.00	42507.0	31394.5	1.354	MM
160	1... 20211111_A21_R2_IgG	2.00	75889.5	34863.7	2.177	bb
161	1... 20211111_A22_R2_IgG	2.00	14880.9	30342.1	0.490	MM
162	1... 20211111_L21_R2_IgG	1.99	20133.5	33706.2	0.597	bb
163	1... 20211111_L22_R2_IgG	2.00	57983.9	31298.6	1.853	MM
164	1... 20211111_H11_R2_IgG	2.00	91599.3	38904.1	2.354	MM
165	1... 20211111_A23_R2_IgG	2.00	102223.2	32382.3	3.157	MM
166	1... 20211111_A24_R2_IgG	2.00	22906.7	30261.9	0.757	bb
167	1... 20211111_L23_R2_IgG	1.99	58847.1	43037.5	1.367	MM
168	1... 20211111_L24_R2_IgG	2.00	19040.5	37023.3	0.514	bb

Dataset: C:\MassLynx\10.pro\20211115_clinicalsamples_IgG.qld

Last Altered: Sunday, October 02, 2022 16:02:30 台北標準時間
Printed: Friday, August 01, 2025 16:26:04 台北標準時間

	# Name	RT	Area	IS Area	Response	Prima...
169	1... 20211111_H12_R2_IgG	2.00	39408.9	39478.2	0.998	MM
170	1... 20211111_QC_R14_IgG	2.00	56516.5	34463.2	1.640	MM
171	1... 20211111_A25_R2_IgG	2.00	100752.5	39945.3	2.522	MM
172	1... 20211111_A26_R2_IgG	2.00	20510.9	29663.2	0.691	MM
173	1... 20211111_L25_R2_IgG	2.00	48181.3	38589.3	1.249	MM
174	1... 20211111_L26_R2_IgG	2.00	30528.2	38405.2	0.795	MM
175	1... 20211111_H13_R2_IgG	2.00	55004.2	37771.2	1.456	bb
176	1... 20211111_A27_R2_IgG	2.00	27451.3	42634.1	0.644	MM
177	1... 20211111_A28_R2_IgG	2.00	31122.0	33231.1	0.937	bb
178	1... 20211111_L27_R2_IgG	1.99	71102.9	41761.1	1.703	MM
179	1... 20211111_L28_R2_IgG	2.00	20250.9	26968.6	0.751	MM
180	1... 20211111_H14_R2_IgG	1.99	35731.3	49250.2	0.726	MM
181	1... 20211111_A29_R2_IgG	2.00	112959.9	37601.7	3.004	MM
182	1... 20211111_A30_R2_IgG	2.00	148827.9	40366.8	3.687	MM
183	1... 20211111_QC_R15_IgG	2.00	56519.6	35156.1	1.608	MM
184	1... 20211111_L29_R2_IgG	2.00	25467.6	33813.1	0.753	bb
185	1... 20211111_L30_R2_IgG	2.00	18472.5	33488.6	0.552	MM
186	1... 20211111_H15_R2_IgG	1.99	52653.7	38501.0	1.368	bb
187	1... 20211111_A31_R2_IgG	1.99	58139.9	37525.5	1.549	MM
188	1... 20211111_A32_R2_IgG	2.00	145061.9	35770.8	4.055	bb
189	1... 20211111_L31_R2_IgG	2.00	38464.2	33282.4	1.156	MM
190	1... 20211111_L32_R2_IgG	2.00	58651.0	42049.6	1.395	bb
191	1... 20211111_H16_R2_IgG	2.00	94842.5	45947.6	2.064	MM
192	1... 20211111_A33_R2_IgG	1.99	109850.7	40202.3	2.732	MM
193	1... 20211111_A34_R2_IgG	2.01	59639.6	36747.0	1.623	MM
194	1... 20211111_H17_R2_IgG	2.00	208372.8	40920.9	5.092	bb
195	1... 20211111_A35_R2_IgG	2.00	46317.4	33689.2	1.375	MM
196	1... 20211111_QC_R16_IgG	2.00	57500.5	32783.6	1.754	MM
197	1... 20211111_A36_R2_IgG	2.00	72103.0	36822.4	1.958	MM
198	1... 20211111_H18_R2_IgG	2.00	204725.6	31769.3	6.444	MM
199	1... 20211111_A37_R2_IgG	2.00	132348.6	37581.8	3.522	MM
200	2... 20211111_A38_R2_IgG	1.99	62658.3	38390.4	1.632	MM
201	2... 20211111_H19_R2_IgG	2.00	163926.3	41217.0	3.977	MM
202	2... 20211111_A39_R2_IgG	2.00	27426.9	37392.7	0.733	bb
203	2... 20211111_A40_R2_IgG	1.99	75263.5	38295.1	1.965	MM
204	2... 20211111_H20_R2_IgG	2.00	111759.8	35941.8	3.109	bb
205	2... 20211111_A41_R2_IgG	2.00	70074.8	45304.9	1.547	MM
206	2... 20211111_A42_R2_IgG	2.00	36162.6	37803.8	0.957	MM
207	2... 20211111_A43_R2_IgG	2.00	64664.4	38161.8	1.694	MM
208	2... 20211111_A44_R2_IgG	1.99	37550.7	37819.8	0.993	bb
209	2... 20211111_QC_R17_IgG	2.00	54915.5	31731.8	1.731	MM

Dataset: C:\MassLynx\10.pro\20211115_clinicalsamples_IgG.qld

Last Altered: Sunday, October 02, 2022 16:02:30 台北標準時間
Printed: Friday, August 01, 2025 16:42:42 台北標準時間

Method: C:\MassLynx\10_Pro_IgA.PRO\MethDB\20210926_IgG_DMRM_IgM_IS.mdb 16 Feb 2022 11:58:21
Calibration: 18 Nov 2021 13:56:22

Sample Name: 20211111_QC_R1_IgG

F23:MRM of 1 channel,ES+
946.54 > 204.1

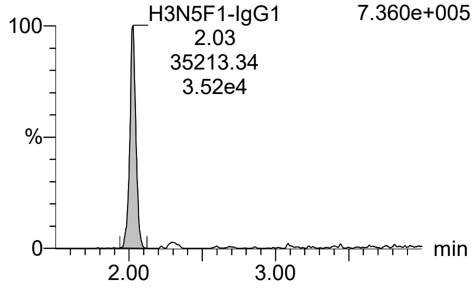

Sample Name: 20211111_A1_R1_IgG

F23:MRM of 1 channel,ES+
946.54 > 204.1

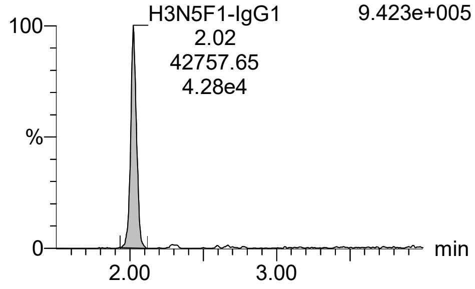

Sample Name: 20211111_A2_R1_IgG

F23:MRM of 1 channel,ES+
946.54 > 204.1

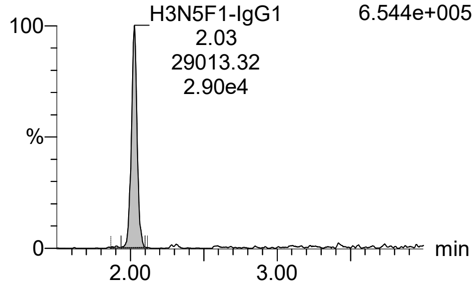

Sample Name: 20211111_L1_R1_IgG

F23:MRM of 1 channel,ES+
946.54 > 204.1

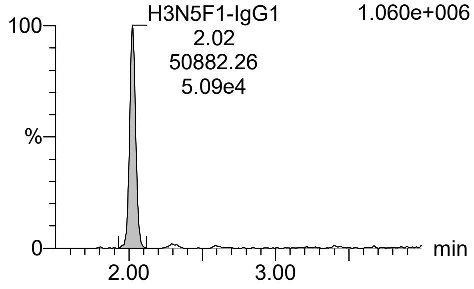

Sample Name: 20211111_L2_R1_IgG

F23:MRM of 1 channel,ES+
946.54 > 204.1

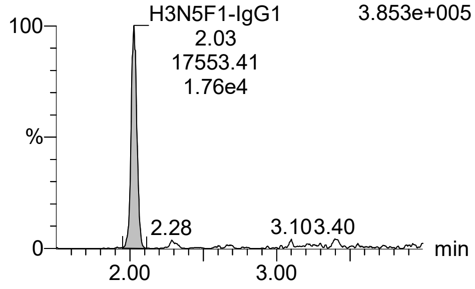

Sample Name: 20211111_H1_R1_IgG

F23:MRM of 1 channel,ES+
946.54 > 204.1

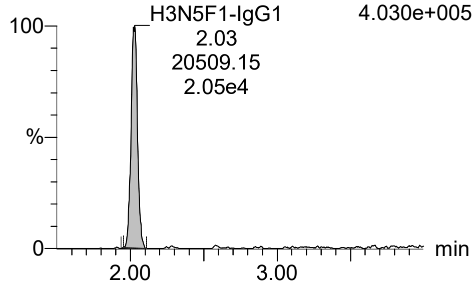

Sample Name: 20211111_A3_R1_IgG

F23:MRM of 1 channel,ES+
946.54 > 204.1

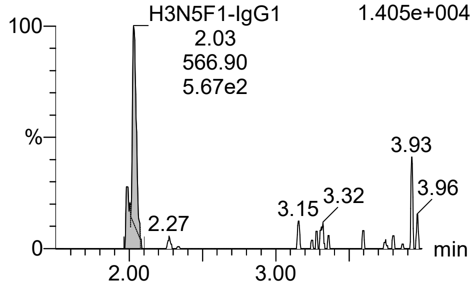

Sample Name: 20211111_A4_R1_IgG

F23:MRM of 1 channel,ES+
946.54 > 204.1

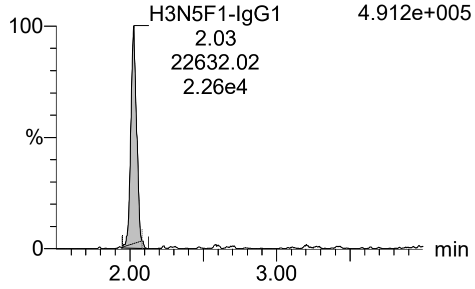

Sample Name: 20211111_L3_R1_IgG

F23:MRM of 1 channel,ES+
946.54 > 204.1

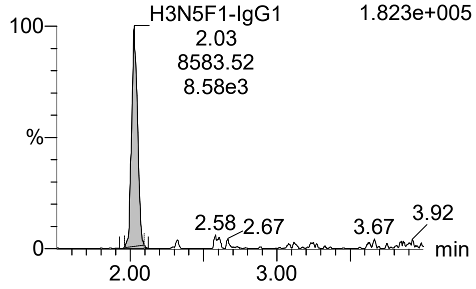

Sample Name: 20211111_L4_R1_IgG

F23:MRM of 1 channel,ES+
946.54 > 204.1

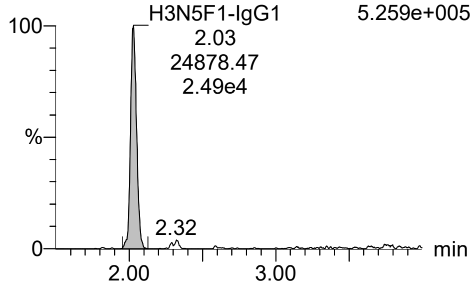

Sample Name: 20211111_H2_R1_IgG

F23:MRM of 1 channel,ES+
946.54 > 204.1

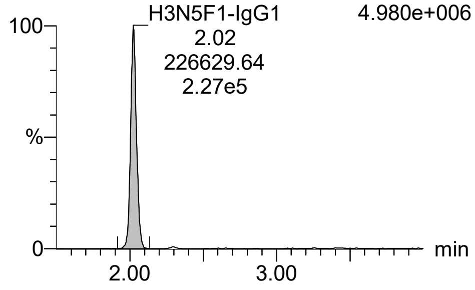

Sample Name: 20211111_A5_R1_IgG

F23:MRM of 1 channel,ES+
946.54 > 204.1

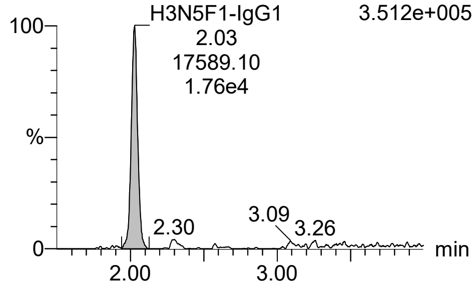

Dataset: C:\MassLynx\10.pro\20211115_clinicalsamples_IgG.qld

Last Altered: Sunday, October 02, 2022 16:02:30 台北標準時間
Printed: Friday, August 01, 2025 16:42:42 台北標準時間

Sample Name: 20211111_A6_R1_IgG

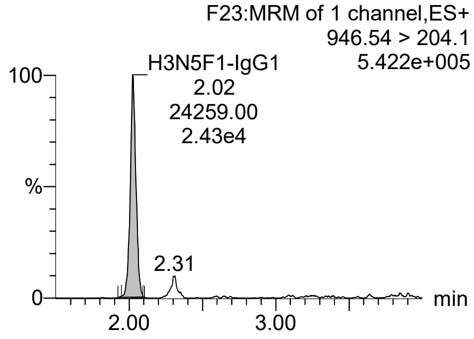

Sample Name: 20211111_QC_R2_IgG

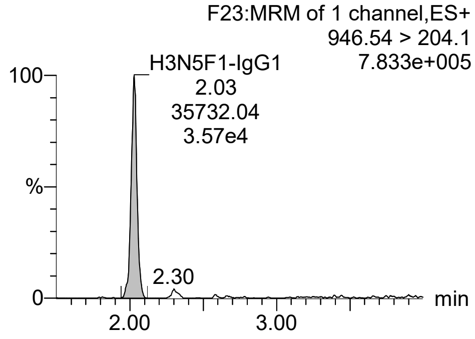

Sample Name: 20211111_L5_R1_IgG

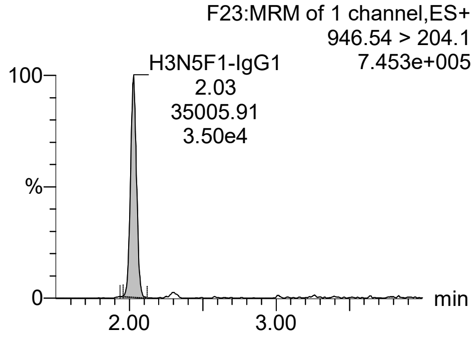

Sample Name: 20211111_L6_R1_IgG

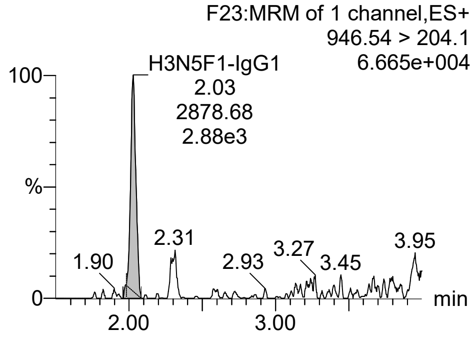

Sample Name: 20211111_H3_R1_IgG

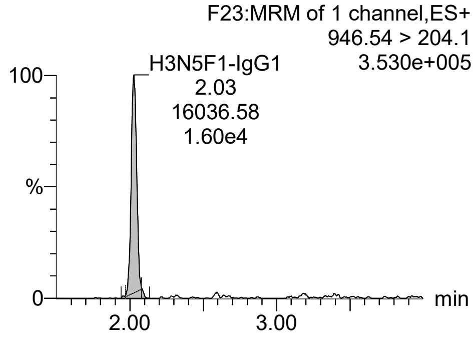

Sample Name: 20211111_A7_R1_IgG

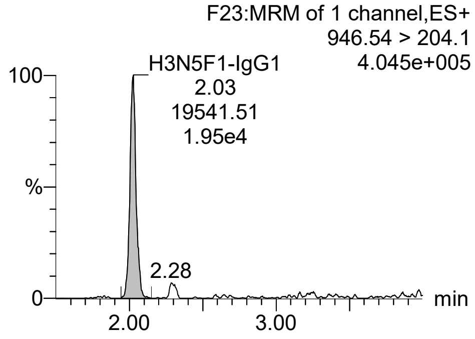

Sample Name: 20211111_A8_R1_IgG

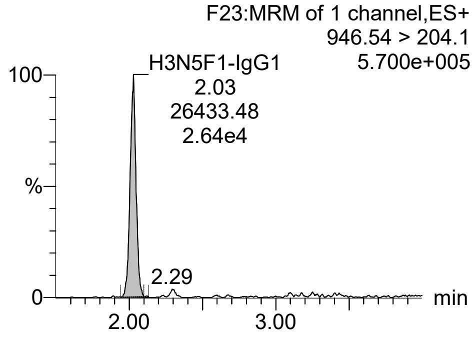

Sample Name: 20211111_L7_R1_IgG

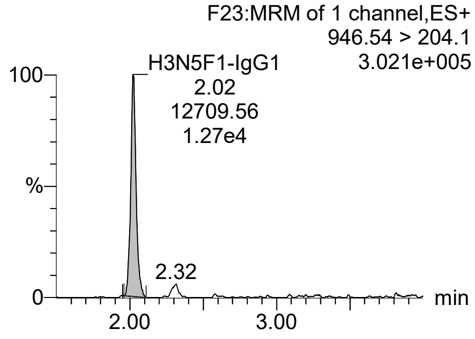

Sample Name: 20211111_L8_R1_IgG

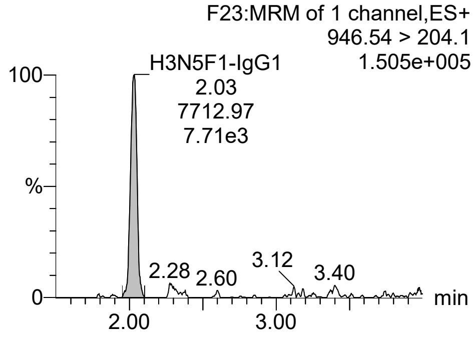

Sample Name: 20211111_H4_R1_IgG

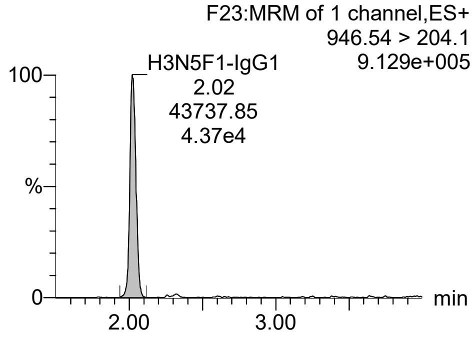

Sample Name: 20211111_A9_R1_IgG

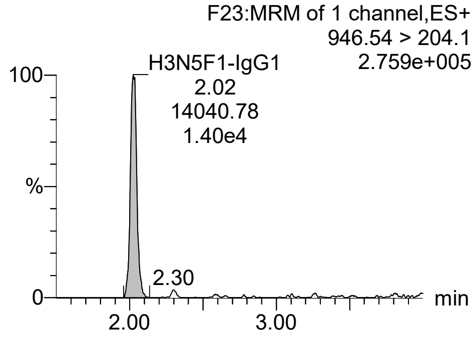

Sample Name: 20211111_A10_R1_IgG

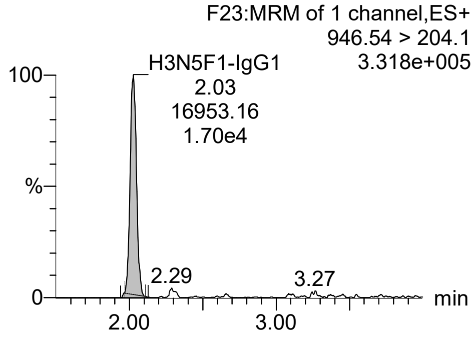

Sample Name: 20211111_L9_R1_IgG

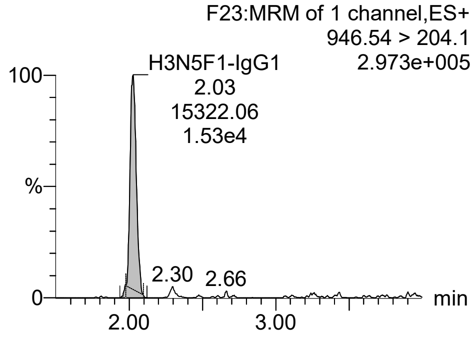

Sample Name: 20211111_L10_R1_IgG

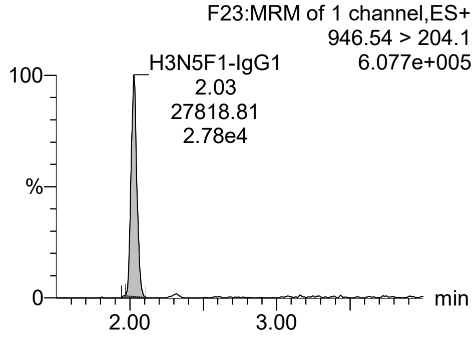

Sample Name: 20211111_QC_R3_IgG

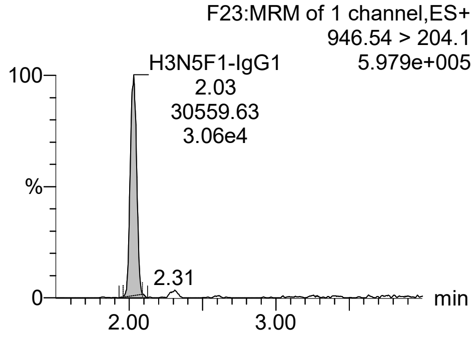

Sample Name: 20211111_H5_R1_IgG

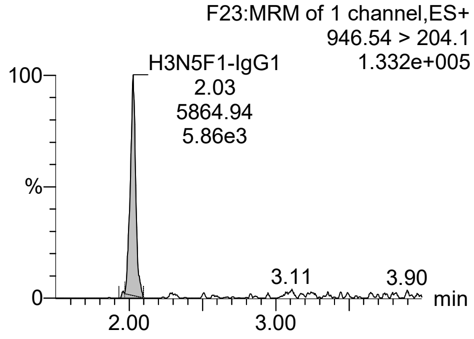

Sample Name: 20211111_A11_R1_IgG

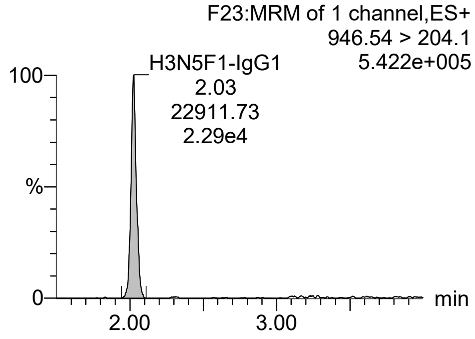

Sample Name: 20211111_A12_R1_IgG

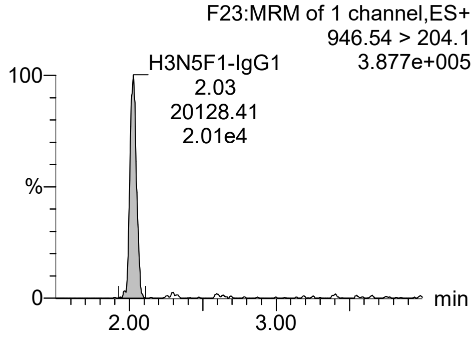

Sample Name: 20211111_L11_R1_IgG

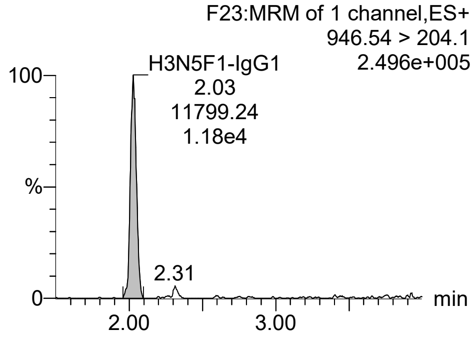

Sample Name: 20211111_L12_R1_IgG

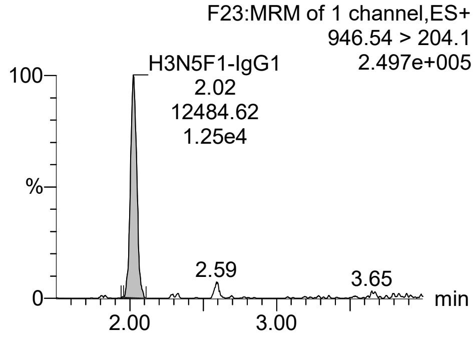

Sample Name: 20211111_H6_R1_IgG

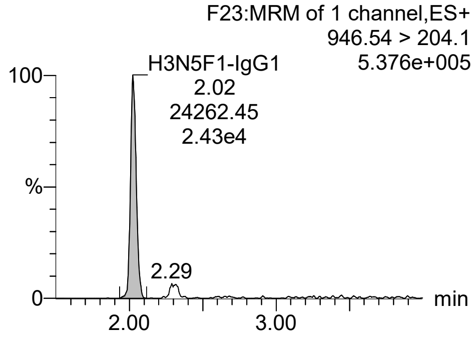

Sample Name: 20211111_A13_R1_IgG

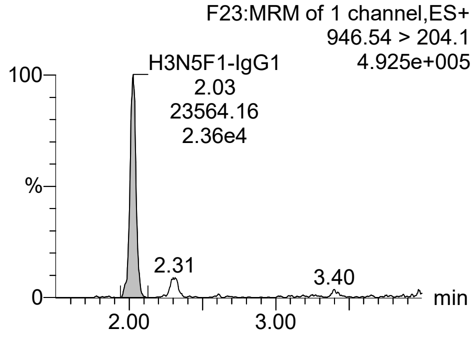

Sample Name: 20211111_A14_R1_IgG

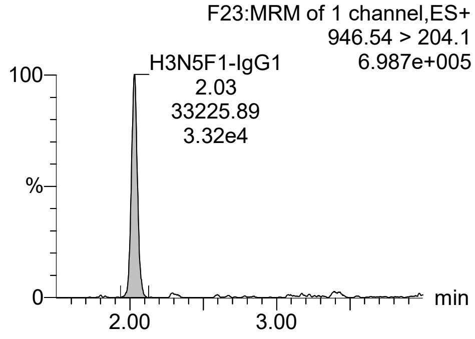

Sample Name: 20211111_L13_R1_IgG

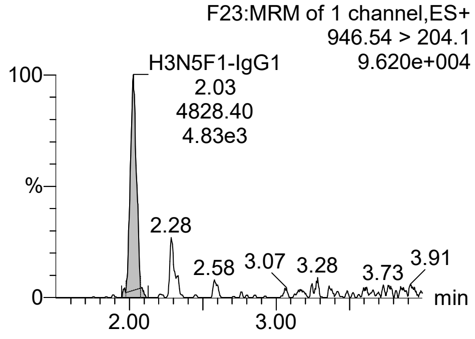

Sample Name: 20211111_L14_R1_IgG

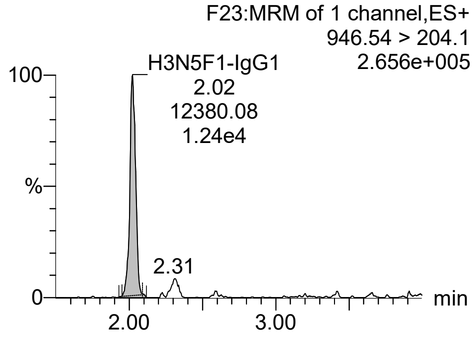

Sample Name: 20211111_H7_R1_IgG

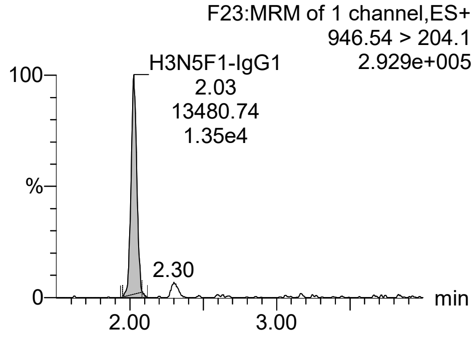

Sample Name: 20211111_A15_R1_IgG

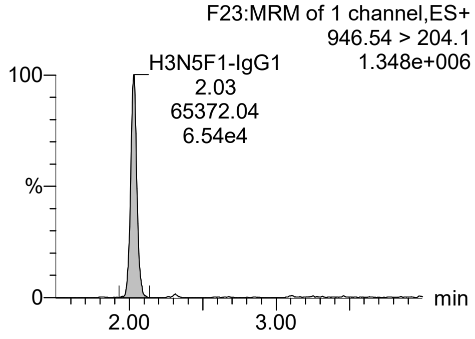

Sample Name: 20211111_QC_R4_IgG

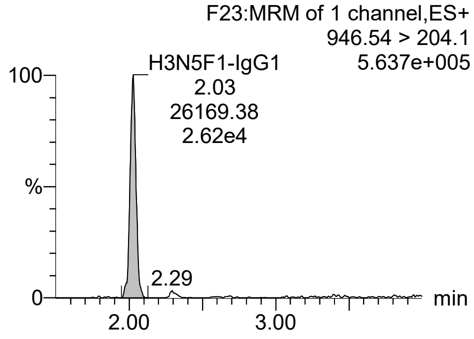

Sample Name: 20211111_A16_R1_IgG

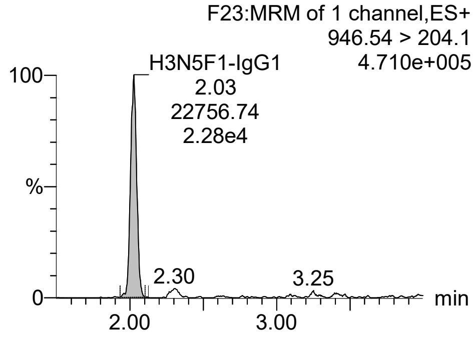

Sample Name: 20211111_L15_R1_IgG

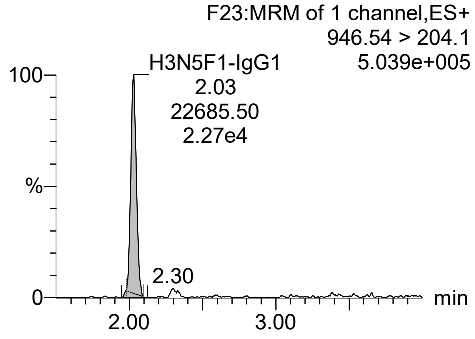

Sample Name: 20211111_L16_R1_IgG
F23:MRM of 1 channel,ES+
946.54 > 204.1

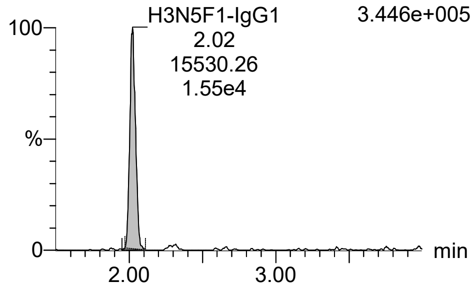

Sample Name: 20211111_H8_R1_IgG
F23:MRM of 1 channel,ES+
946.54 > 204.1

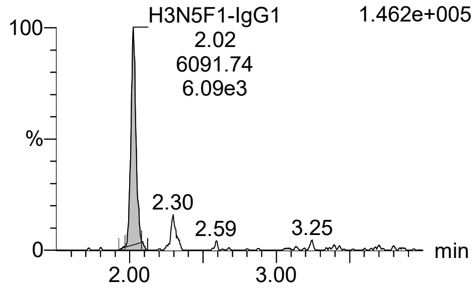

Sample Name: 20211111_A17_R1_IgG
F23:MRM of 1 channel,ES+
946.54 > 204.1

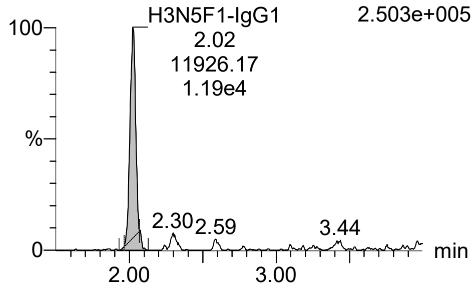

Sample Name: 20211111_A18_R1_IgG
F23:MRM of 1 channel,ES+
946.54 > 204.1

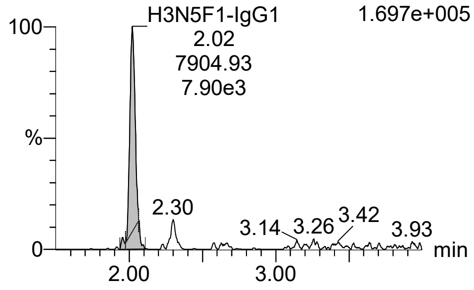

Sample Name: 20211111_L17_R1_IgG
F23:MRM of 1 channel,ES+
946.54 > 204.1

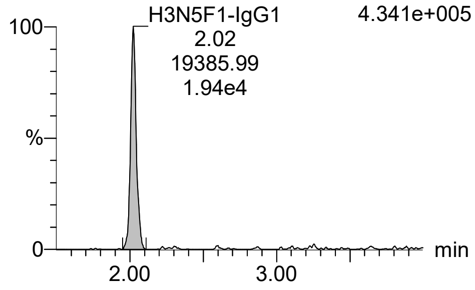

Sample Name: 20211111_L18_R1_IgG
F23:MRM of 1 channel,ES+
946.54 > 204.1

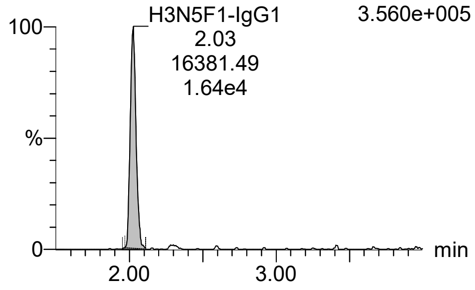

Sample Name: 20211111_H9_R1_IgG
F23:MRM of 1 channel,ES+
946.54 > 204.1

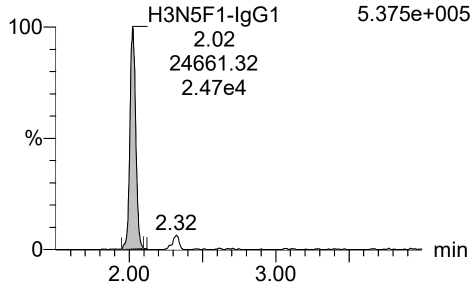

Sample Name: 20211111_A19_R1_IgG
F23:MRM of 1 channel,ES+
946.54 > 204.1

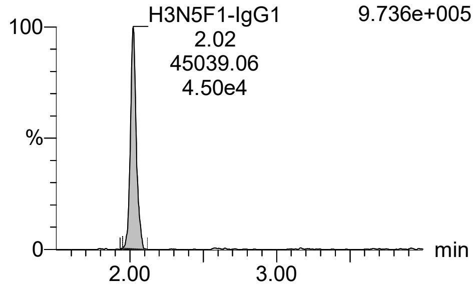

Sample Name: 20211111_A20_R1_IgG
F23:MRM of 1 channel,ES+
946.54 > 204.1

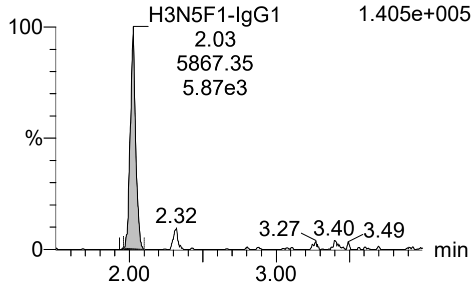

Sample Name: 20211111_L19_R1_IgG
F23:MRM of 1 channel,ES+
946.54 > 204.1

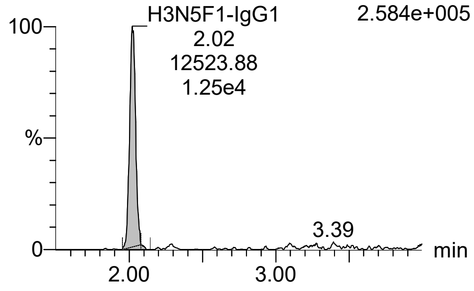

Sample Name: 20211111_QC_R5_IgG
F23:MRM of 1 channel,ES+
946.54 > 204.1

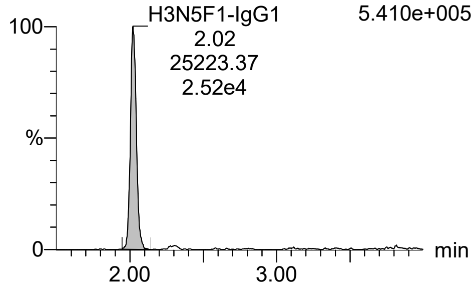

Sample Name: 20211111_L20_R1_IgG
F23:MRM of 1 channel,ES+
946.54 > 204.1

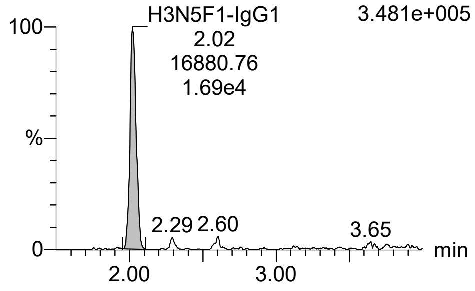

Sample Name: 20211111_H10_R1_IgG
F23:MRM of 1 channel,ES+
946.54 > 204.1

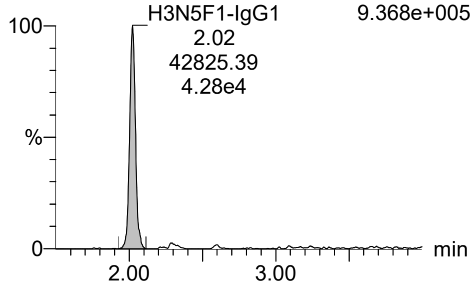

Sample Name: 20211111_A21_R1_IgG
F23:MRM of 1 channel,ES+
946.54 > 204.1

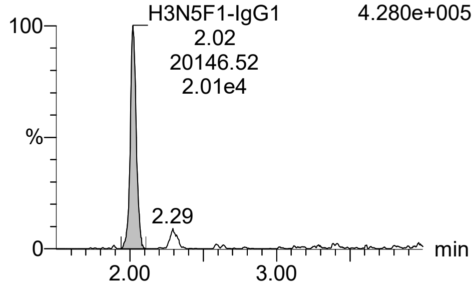

Sample Name: 20211111_A22_R1_IgG
F23:MRM of 1 channel,ES+
946.54 > 204.1

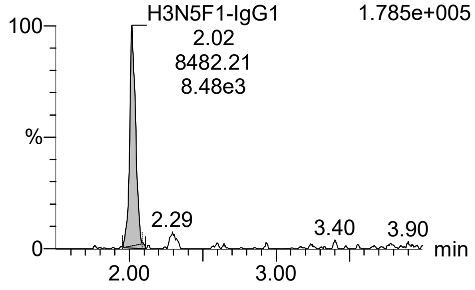

Sample Name: 20211111_L21_R1_IgG

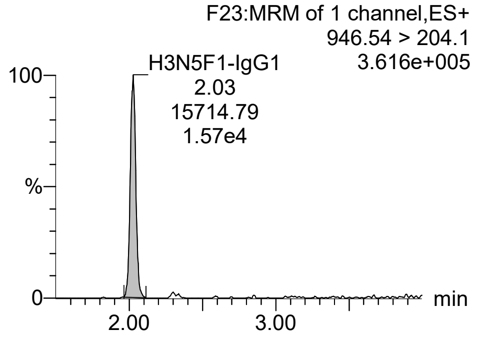

Sample Name: 20211111_L22_R1_IgG

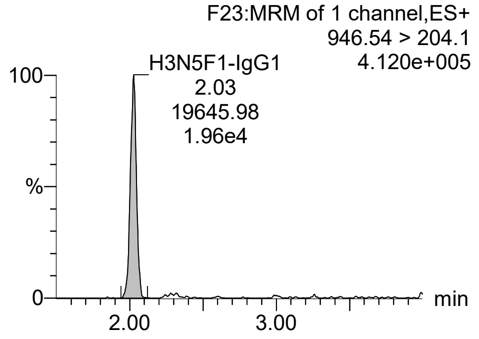

Sample Name: 20211111_H11_R1_IgG

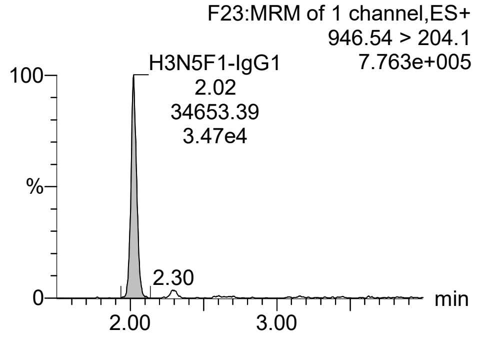

Sample Name: 20211111_A23_R1_IgG

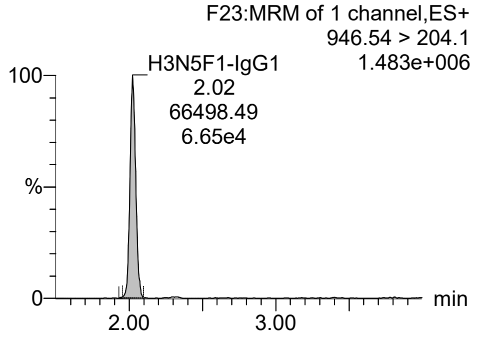

Sample Name: 20211111_A24_R1_IgG

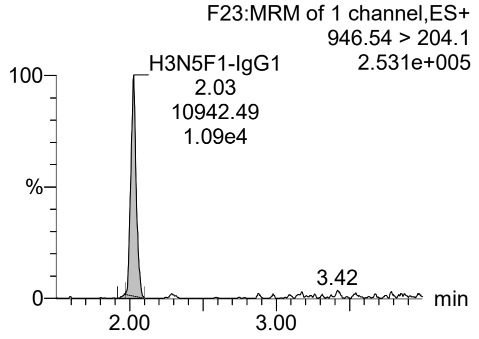

Sample Name: 20211111_L23_R1_IgG

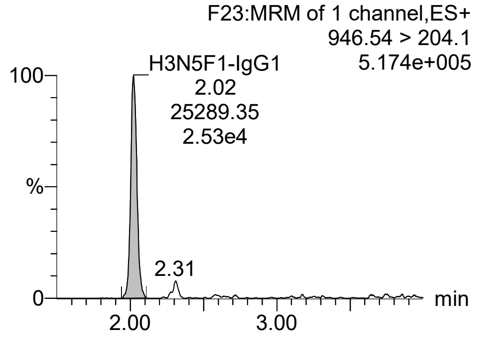

Sample Name: 20211111_L24_R1_IgG

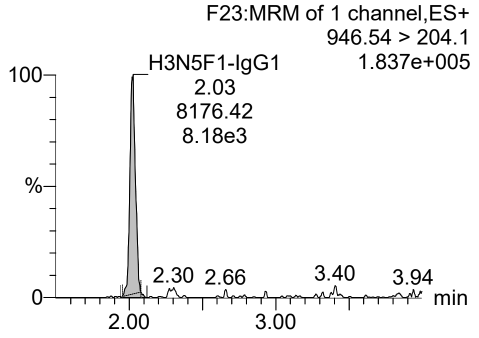

Sample Name: 20211111_H12_R1_IgG

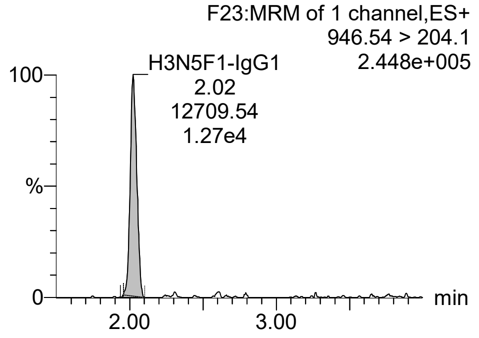

Sample Name: 20211111_QC_R6_IgG

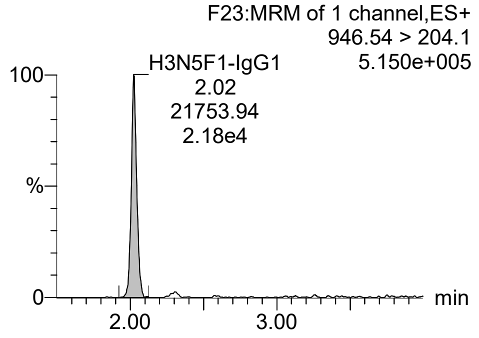

Sample Name: 20211111_A25_R1_IgG

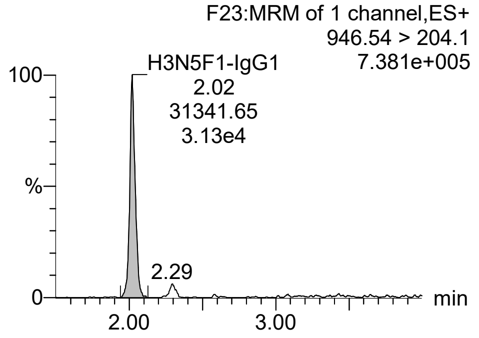

Sample Name: 20211111_A26_R1_IgG

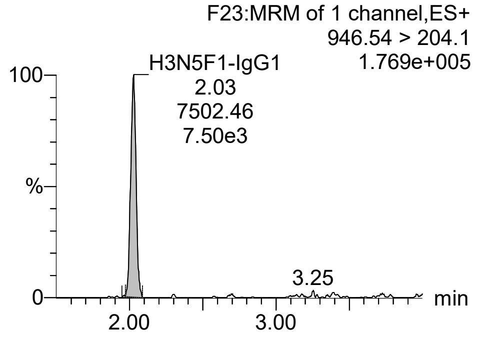

Sample Name: 20211111_L25_R1_IgG

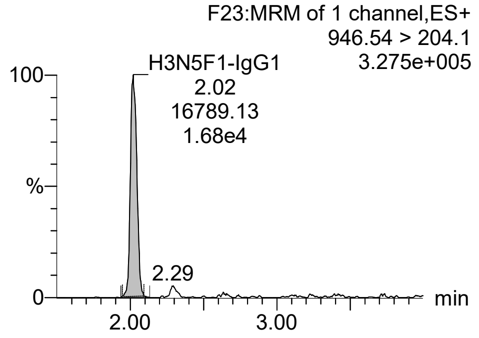

Sample Name: 20211111_L26_R1_IgG

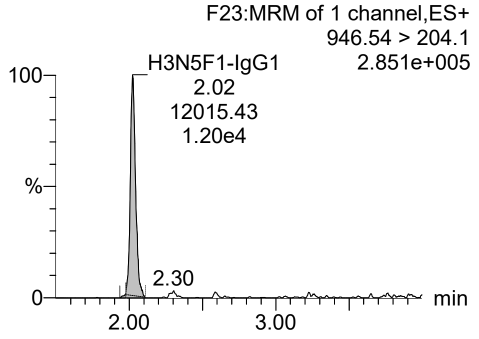

Sample Name: 20211111_H13_R1_IgG

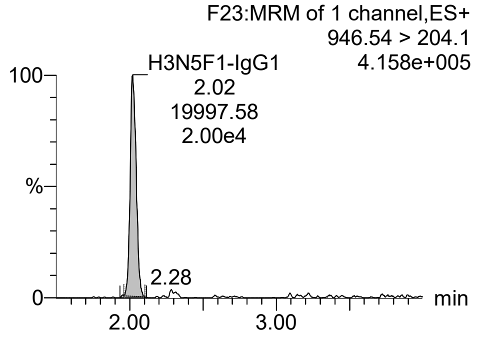

Sample Name: 20211111_A27_R1_IgG

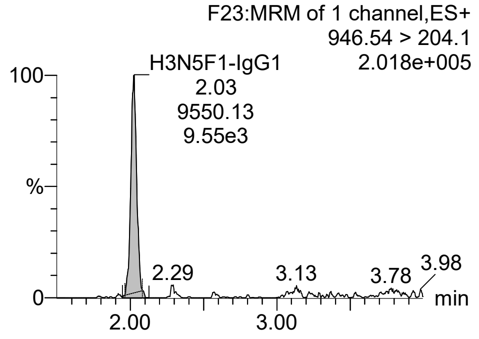

Dataset: C:\MassLynx\10.pro\20211115_clinicalsamples_IgG.qld

Last Altered: Sunday, October 02, 2022 16:02:30 台北標準時間
Printed: Friday, August 01, 2025 16:42:42 台北標準時間

Sample Name: 20211111_A28_R1_IgG

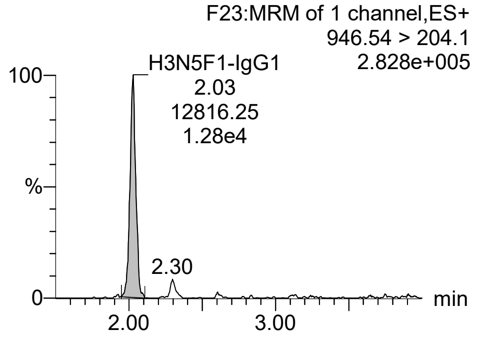

Sample Name: 20211111_L27_R1_IgG

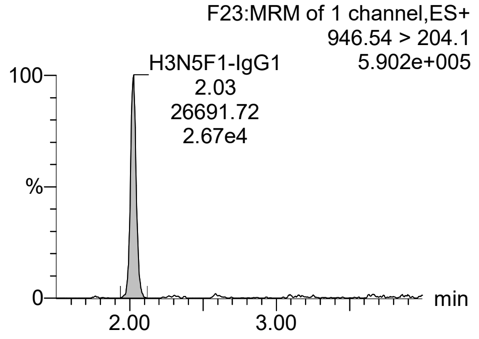

Sample Name: 20211111_L28_R1_IgG

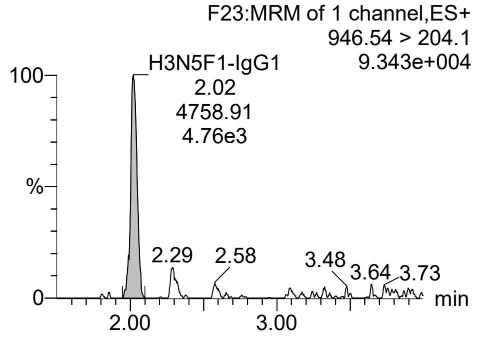

Sample Name: 20211111_H14_R1_IgG

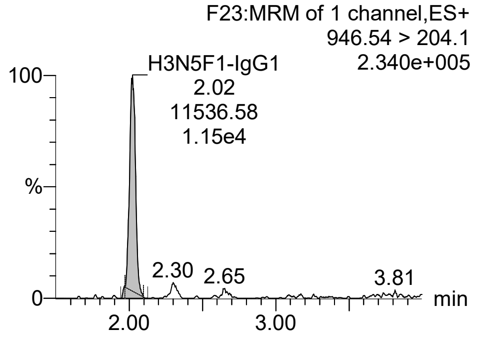

Sample Name: 20211111_A29_R1_IgG

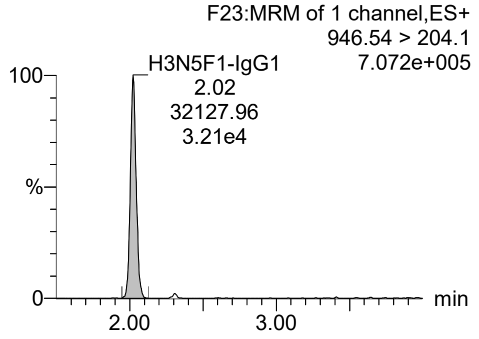

Sample Name: 20211111_A30_R1_IgG

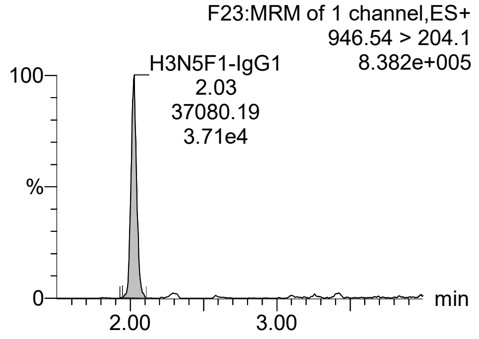

Sample Name: 20211111_QC_R7_IgG

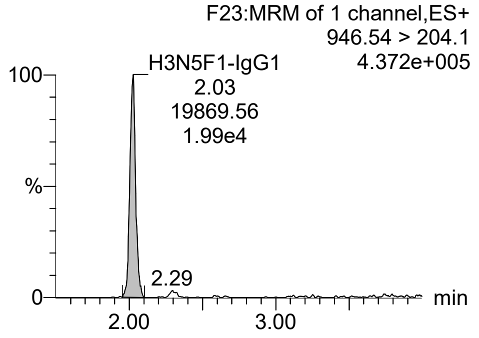

Sample Name: 20211111_L29_R1_IgG

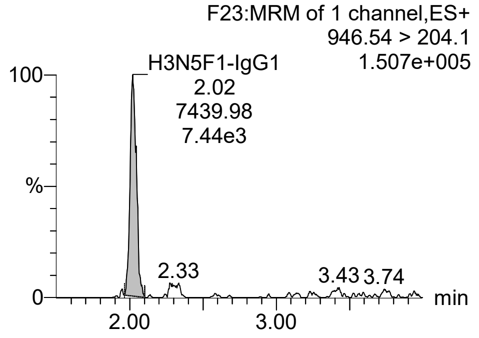

Sample Name: 20211111_L30_R1_IgG

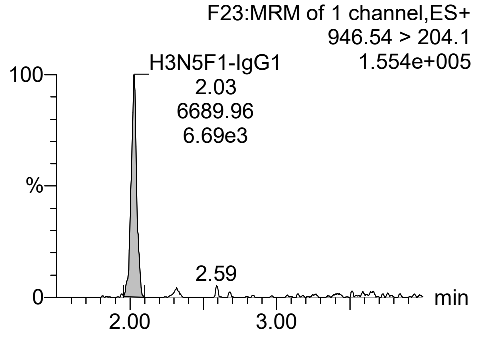

Sample Name: 20211111_H15_R1_IgG

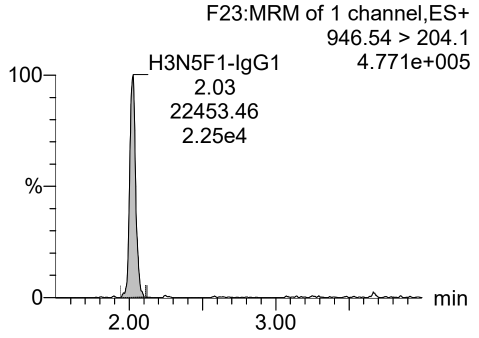

Sample Name: 20211111_A31_R1_IgG

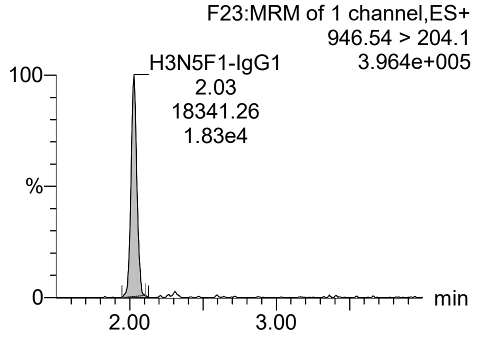

Sample Name: 20211111_A32_R1_IgG

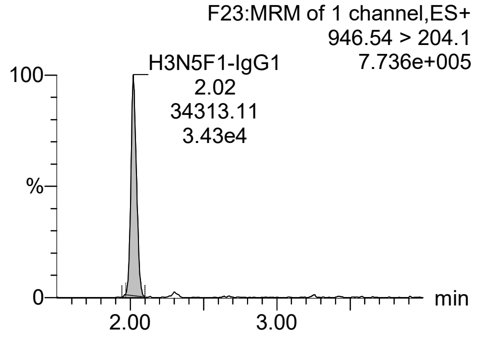

Sample Name: 20211111_L31_R1_IgG

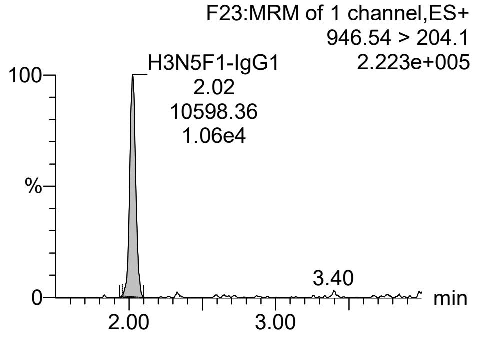

Sample Name: 20211111_L32_R1_IgG

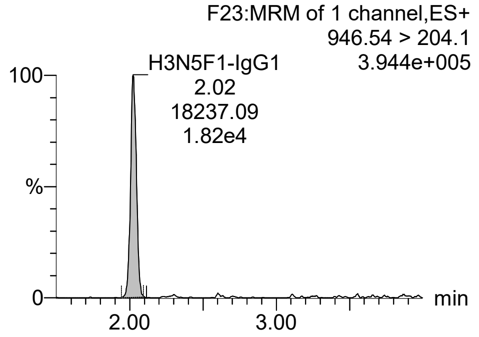

Sample Name: 20211111_H16_R1_IgG

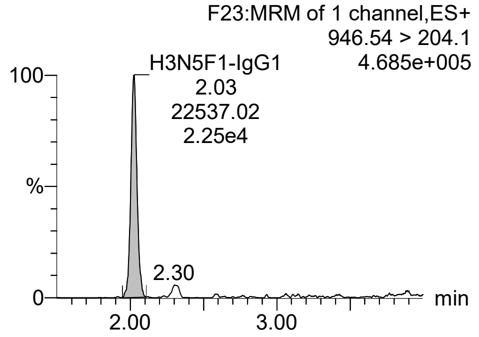

Sample Name: 20211111_A33_R1_IgG

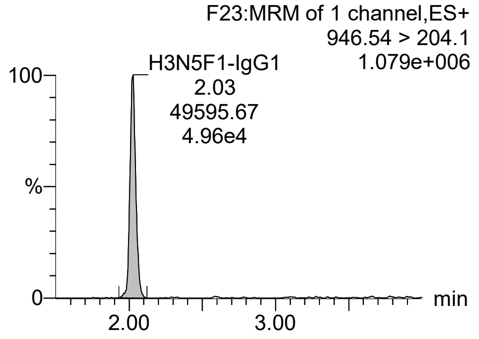

Sample Name: 20211111_A34_R1_IgG

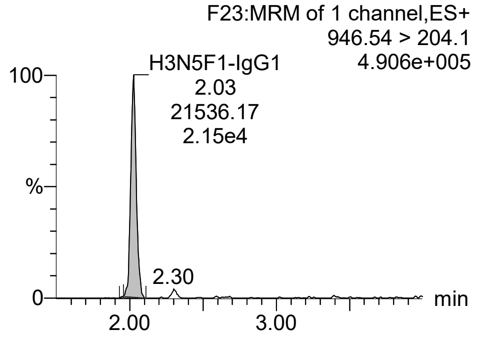

Sample Name: 20211111_H17_R1_IgG

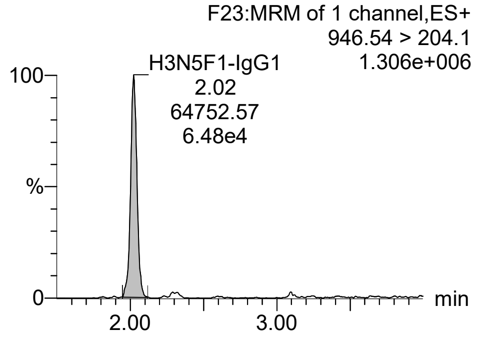

Sample Name: 20211111_A35_R1_IgG

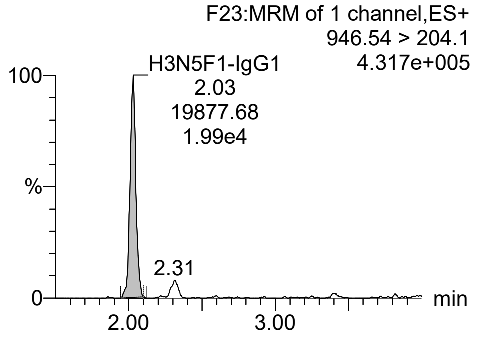

Sample Name: 20211111_QC_R8_IgG

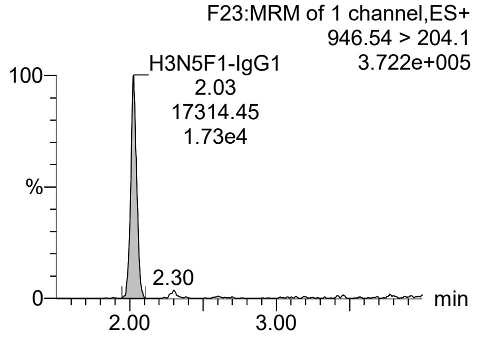

Sample Name: 20211111_A36_R1_IgG

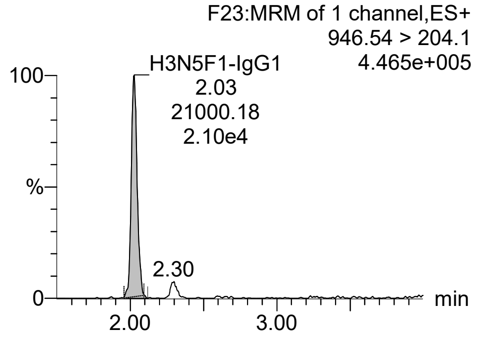

Sample Name: 20211111_H18_R1_IgG

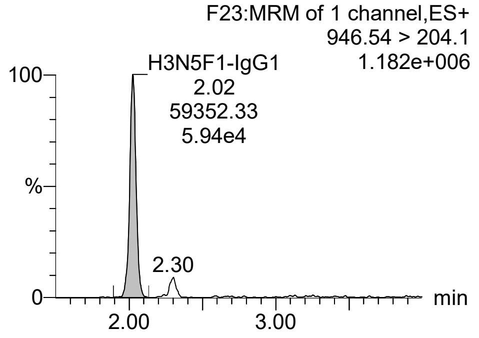

Sample Name: 20211111_A37_R1_IgG

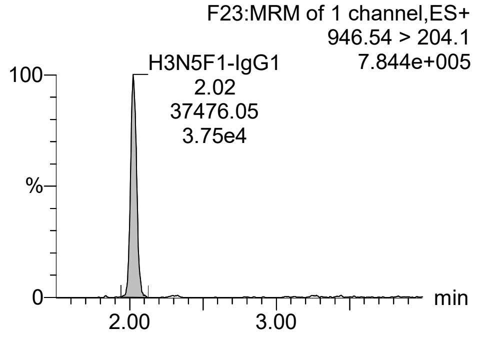

Sample Name: 20211111_A38_R1_IgG

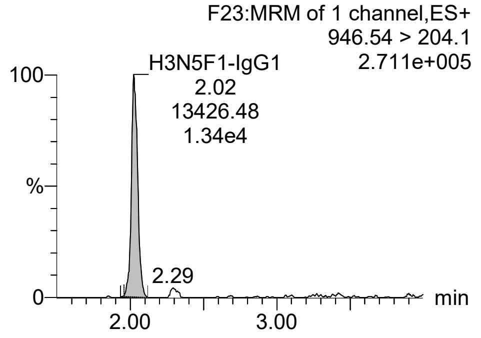

Sample Name: 20211111_H19_R1_IgG

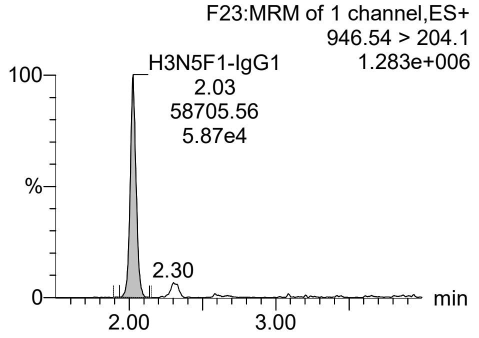

Sample Name: 20211111_A39_R1_IgG

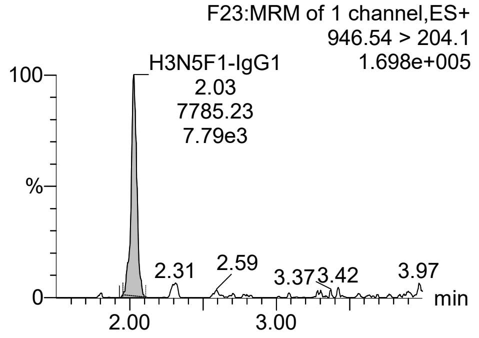

Sample Name: 20211111_A40_R1_IgG

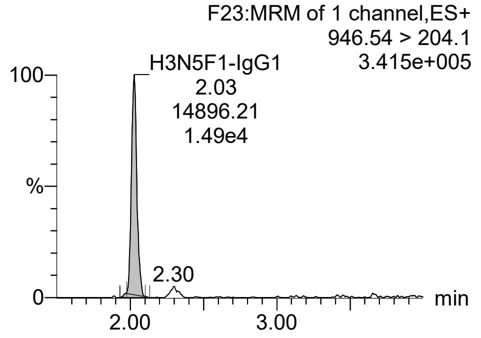

Sample Name: 20211111_H20_R1_IgG

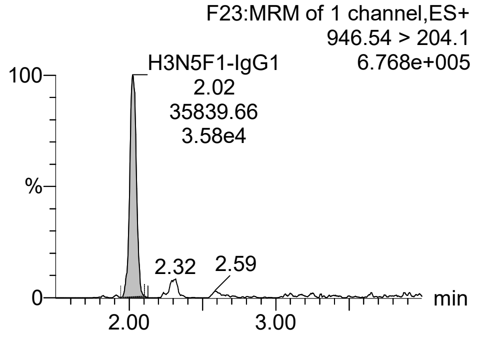

Sample Name: 20211111_A41_R1_IgG

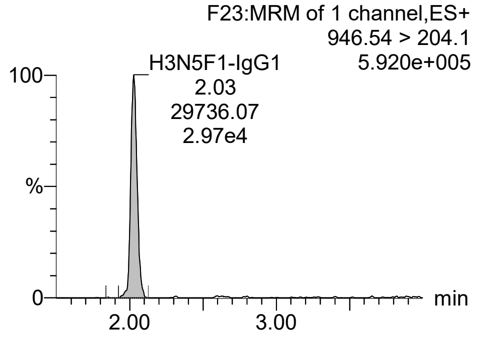

Sample Name: 20211111_A42_R1_IgG

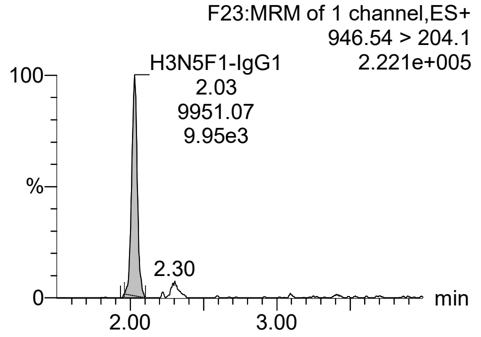

Dataset: C:\MassLynx\10.pro\20211115_clinicalsamples_IgG.qld

Last Altered: Sunday, October 02, 2022 16:02:30 台北標準時間
Printed: Friday, August 01, 2025 16:42:42 台北標準時間

Sample Name: 20211111_A43_R1_IgG

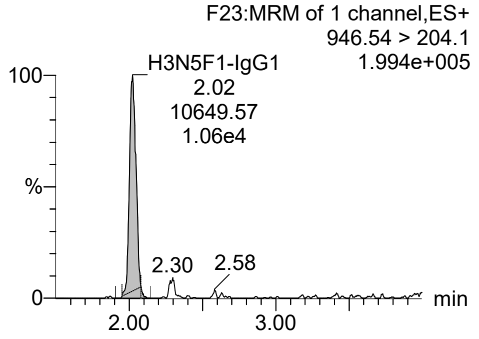

Sample Name: 20211111_A44_R1_IgG

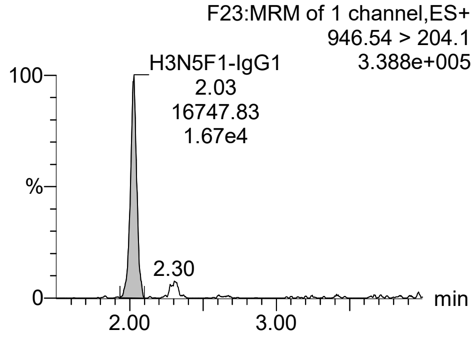

Sample Name: 20211111_QC_R9_IgG

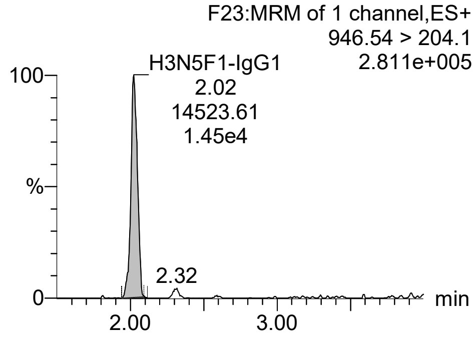

Sample Name: 20211111_A1_R2_IgG

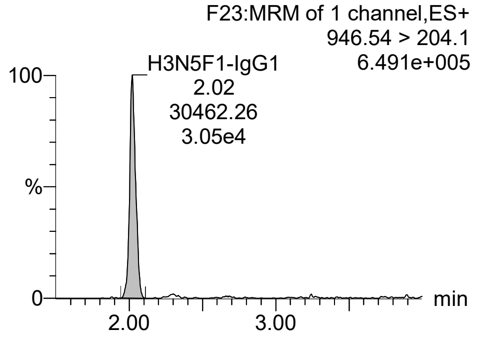

Sample Name: 20211111_A2_R2_IgG

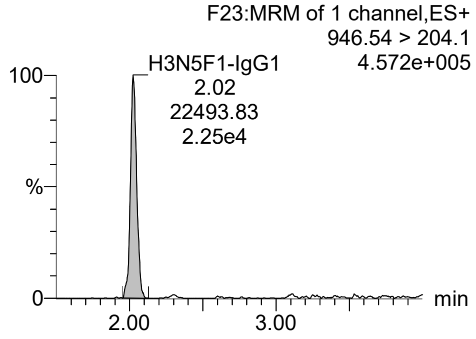

Sample Name: 20211111_L1_R2_IgG

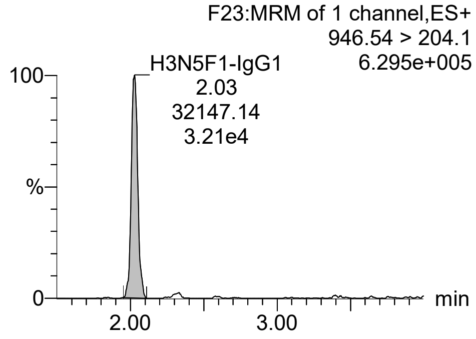

Sample Name: 20211111_L2_R2_IgG

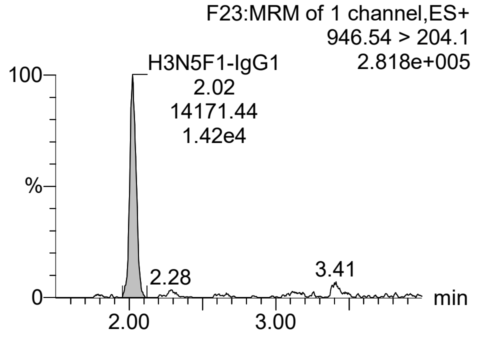

Sample Name: 20211111_H1_R2_IgG

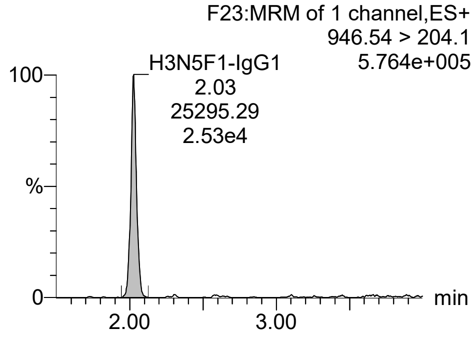

Sample Name: 20211111_A3_R2_IgG

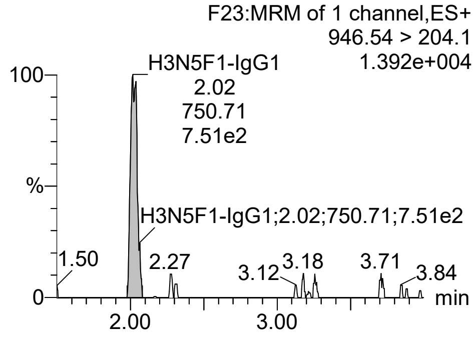

Sample Name: 20211111_A4_R2_IgG

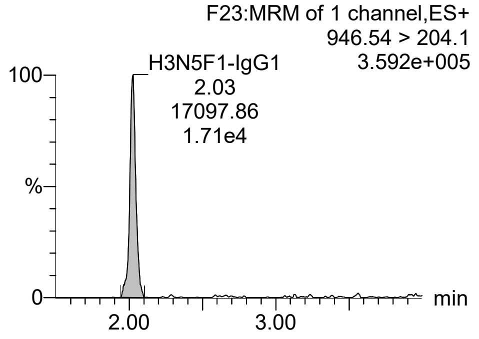

Sample Name: 20211111_L3_R2_IgG

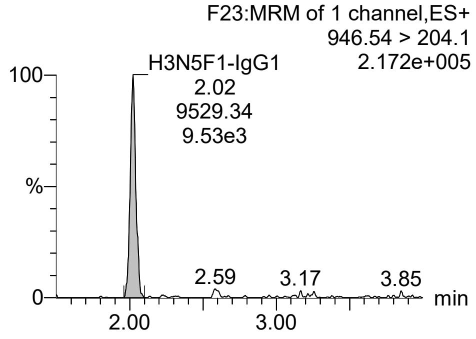

Sample Name: 20211111_L4_R2_IgG

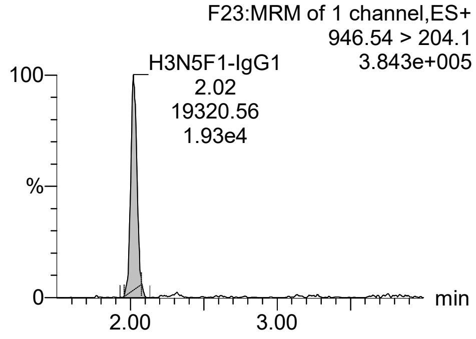

Sample Name: 20211111_H2_R2_IgG

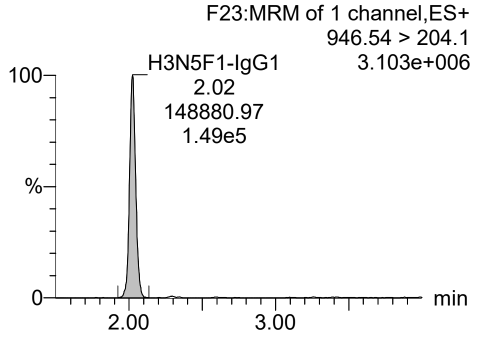

Sample Name: 20211111_A5_R2_IgG

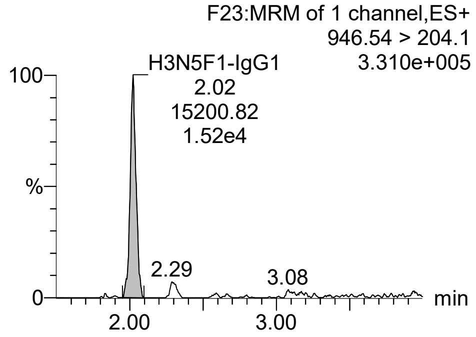

Sample Name: 20211111_A6_R2_IgG

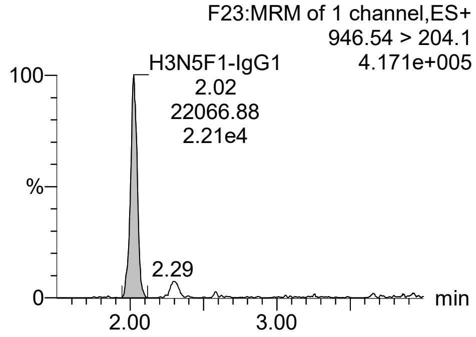

Dataset: C:\MassLynx\10.pro\20211115_clinicalsamples_IgG.qld

Last Altered: Sunday, October 02, 2022 16:02:30 台北標準時間
Printed: Friday, August 01, 2025 16:42:42 台北標準時間

Sample Name: 20211111_QC_R10_IgG

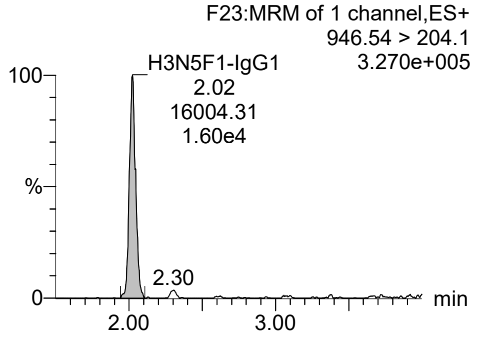

Sample Name: 20211111_L5_R2_IgG

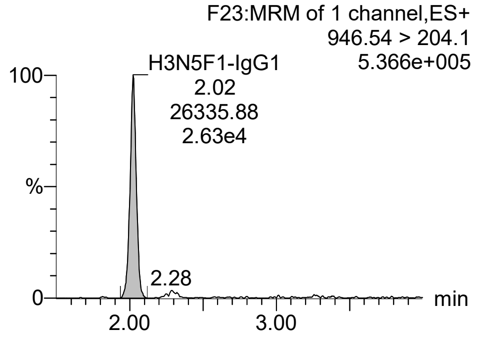

Sample Name: 20211111_L6_R2_IgG

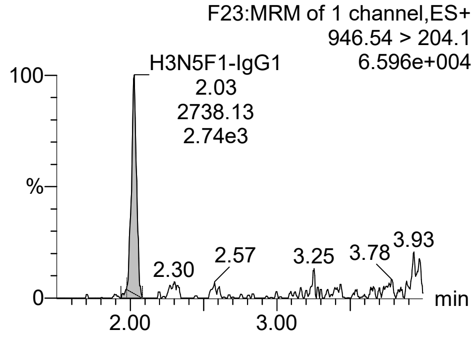

Sample Name: 20211111_H3_R2_IgG

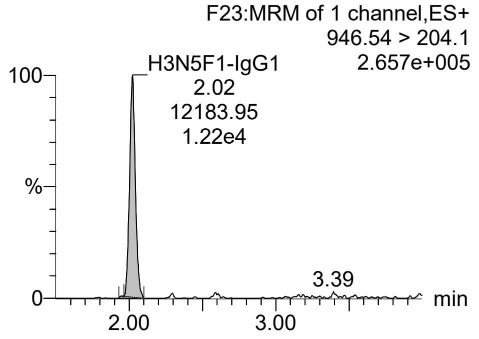

Sample Name: 20211111_A7_R2_IgG

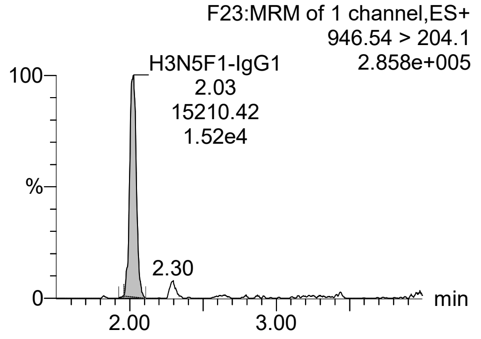

Sample Name: 20211111_A8_R2_IgG

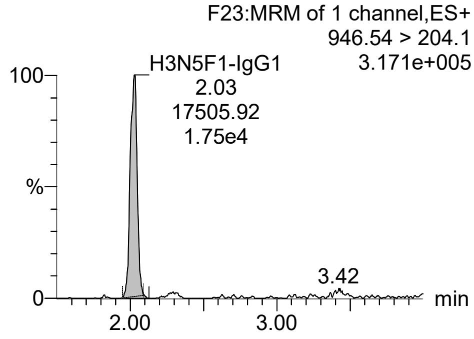

Sample Name: 20211111_L7_R2_IgG

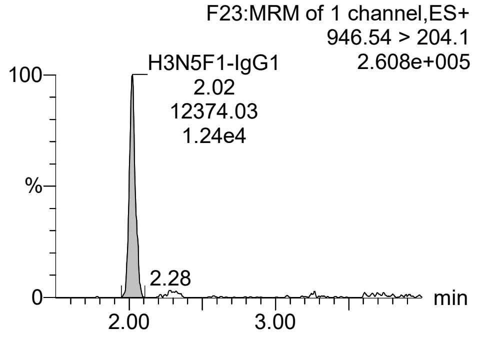

Sample Name: 20211111_L8_R2_IgG

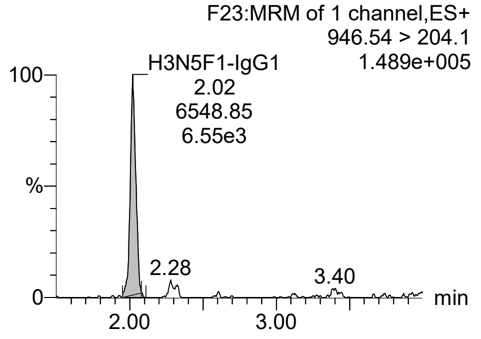

Sample Name: 20211111_H4_R2_IgG

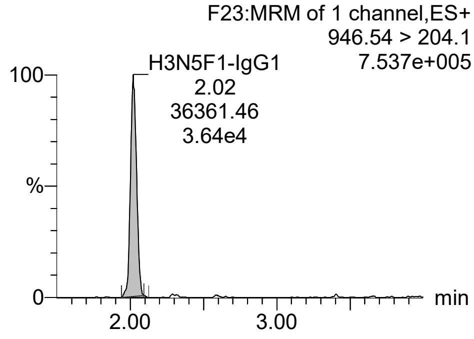

Sample Name: 20211111_A9_R2_IgG

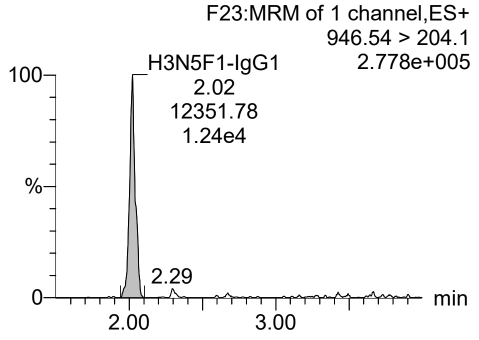

Sample Name: 20211111_A10_R2_IgG

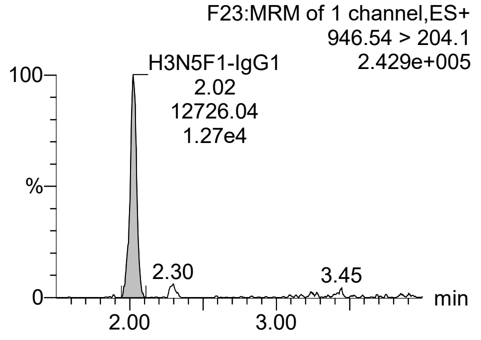

Sample Name: 20211111_L9_R2_IgG

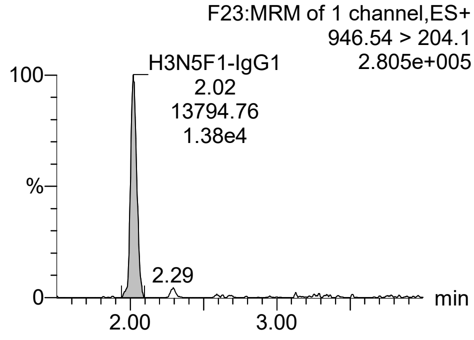

Sample Name: 20211111_L10_R2_IgG

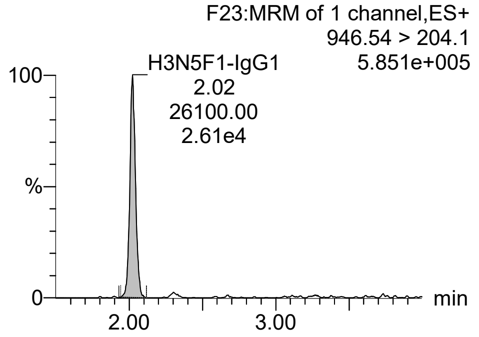

Sample Name: 20211111_QC_R11_IgG

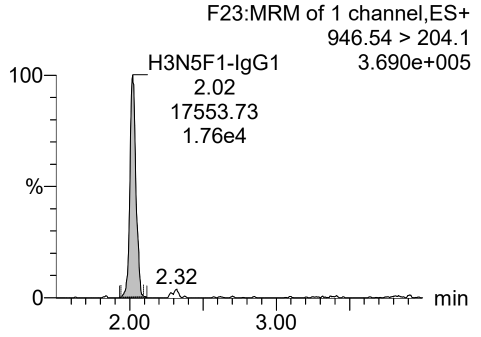

Sample Name: 20211111_H5_R2_IgG

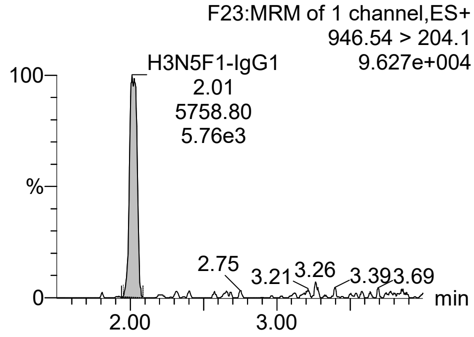

Sample Name: 20211111_A11_R2_IgG

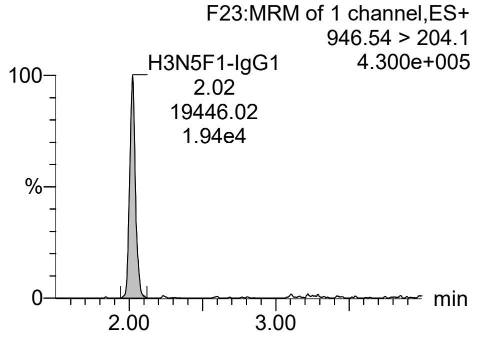

Sample Name: 20211111_A12_R2_IgG

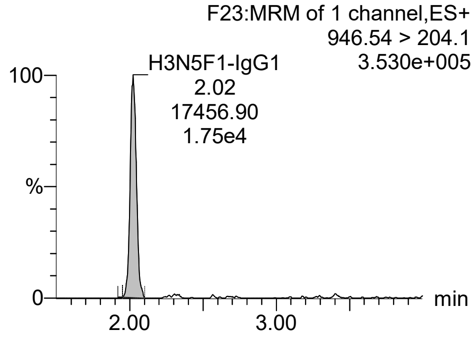

Sample Name: 20211111_L11_R2_IgG

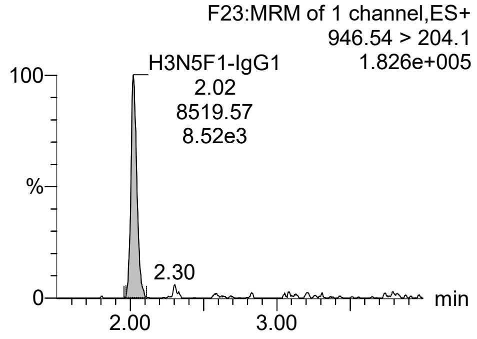

Sample Name: 20211111_L12_R2_IgG

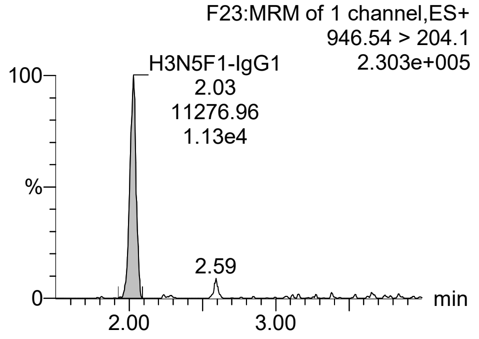

Sample Name: 20211111_H6_R2_IgG

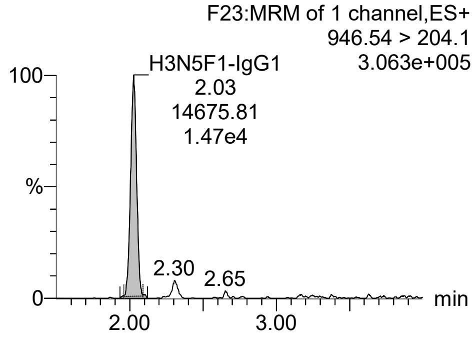

Sample Name: 20211111_A13_R2_IgG

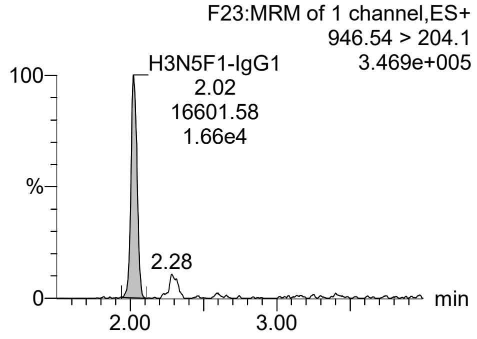

Sample Name: 20211111_A14_R2_IgG

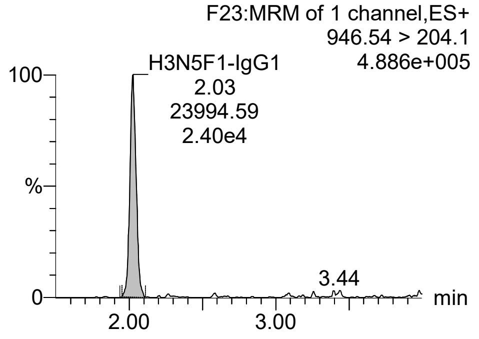

Sample Name: 20211111_L13_R2_IgG

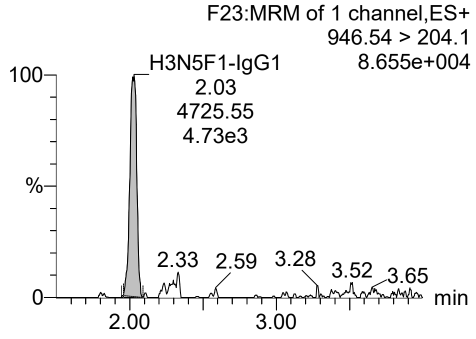

Sample Name: 20211111_L14_R2_IgG

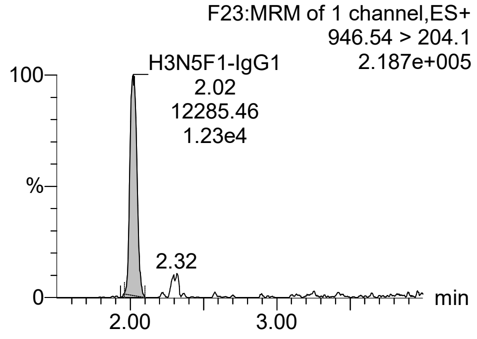

Sample Name: 20211111_H7_R2_IgG

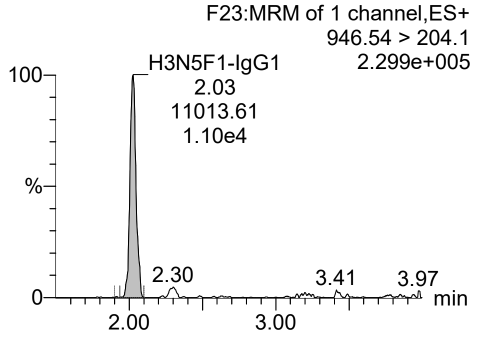

Sample Name: 20211111_A15_R2_IgG

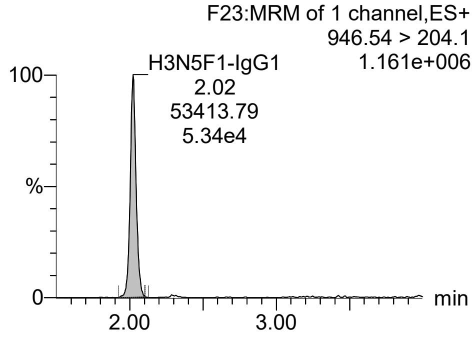

Sample Name: 20211111_QC_R12_IgG

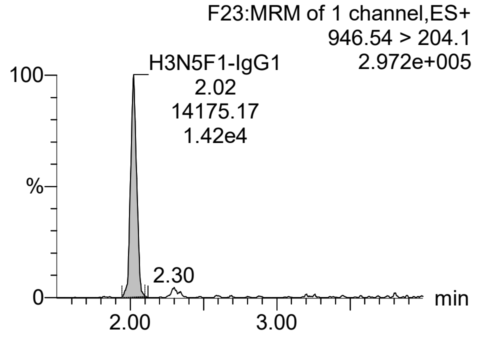

Sample Name: 20211111_A16_R2_IgG

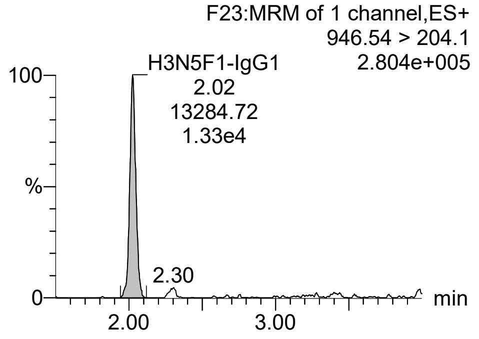

Sample Name: 20211111_L15_R2_IgG

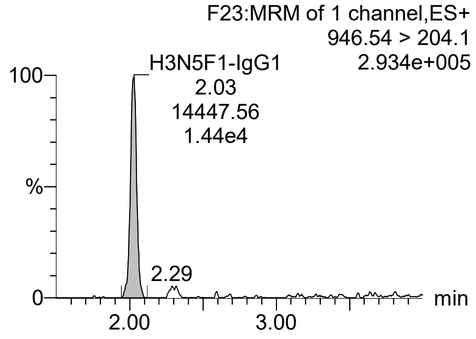

Sample Name: 20211111_L16_R2_IgG

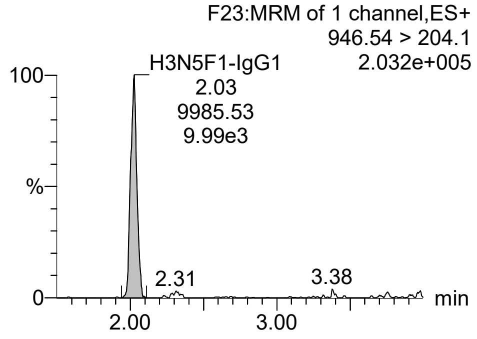

Sample Name: 20211111_H8_R2_IgG

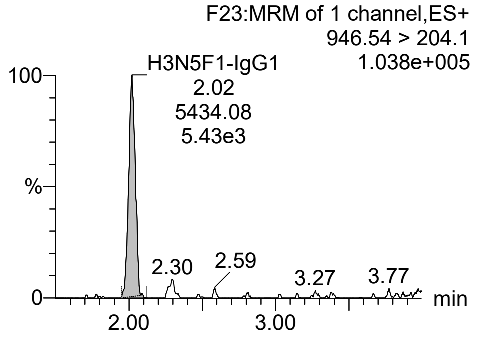

Sample Name: 20211111_A17_R2_IgG

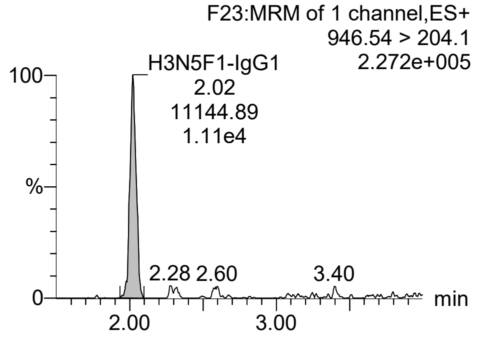

Sample Name: 20211111_A18_R2_IgG

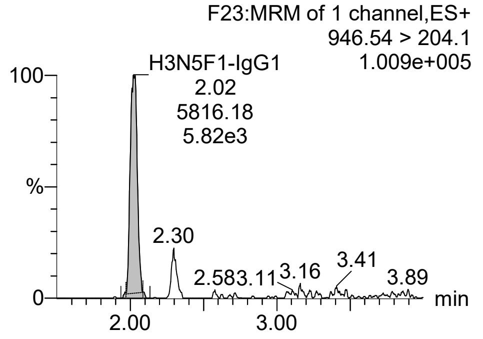

Sample Name: 20211111_L17_R2_IgG

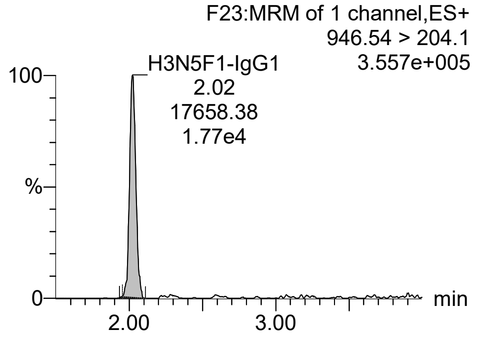

Sample Name: 20211111_L18_R2_IgG

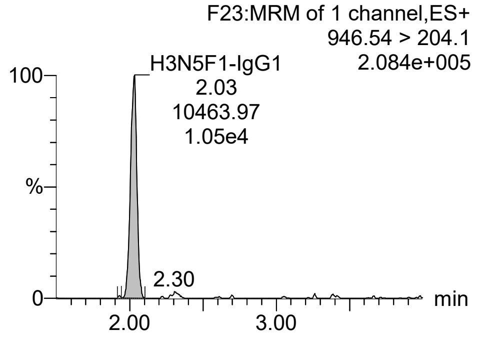

Sample Name: 20211111_H9_R2_IgG

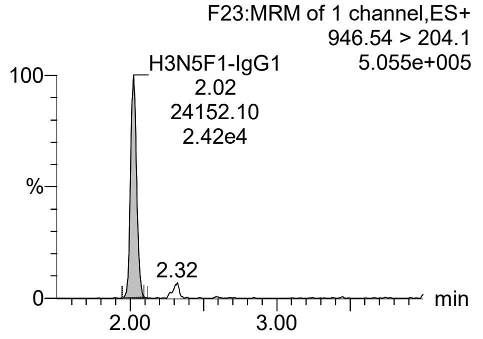

Sample Name: 20211111_A19_R2_IgG

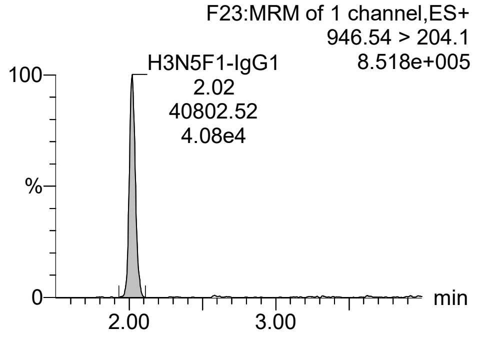

Sample Name: 20211111_A20_R2_IgG

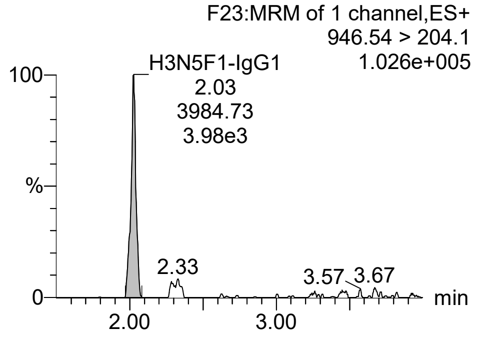

Sample Name: 20211111_L19_R2_IgG

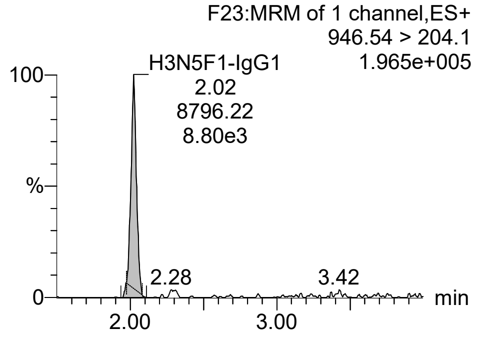

Sample Name: 20211111_QC_R13_IgG

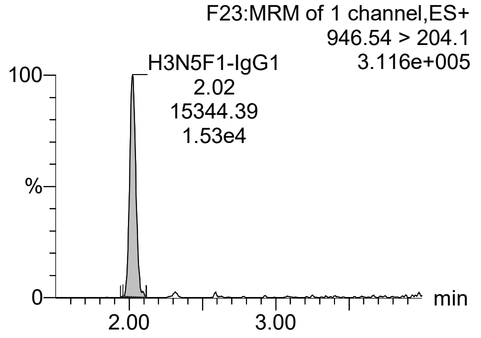

Sample Name: 20211111_L20_R2_IgG

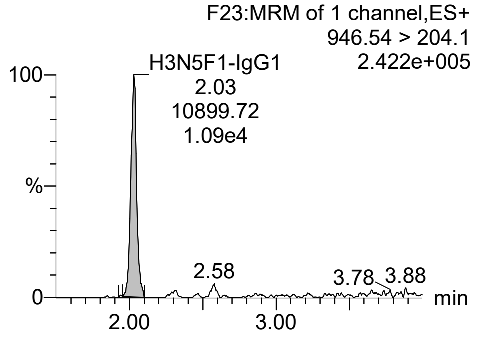

Sample Name: 20211111_H10_R2_IgG

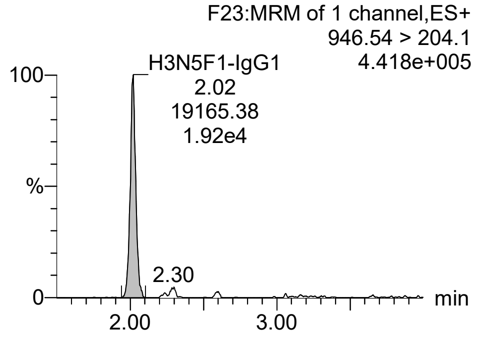

Sample Name: 20211111_A21_R2_IgG

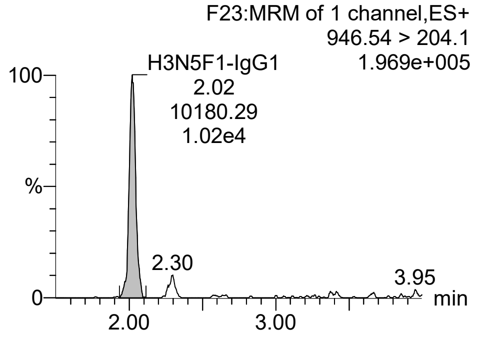

Sample Name: 20211111_A22_R2_IgG

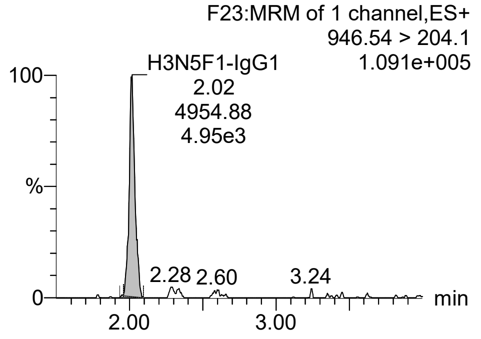

Sample Name: 20211111_L21_R2_IgG

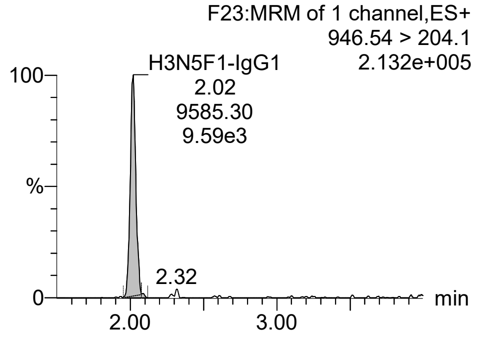

Sample Name: 20211111_L22_R2_IgG

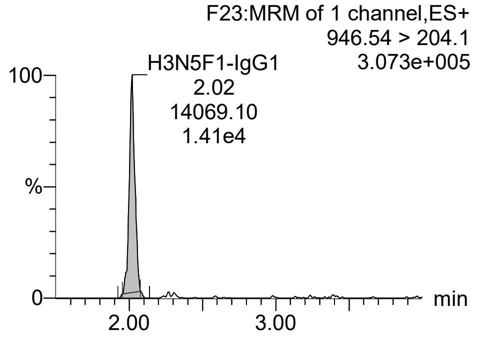

Sample Name: 20211111_H11_R2_IgG

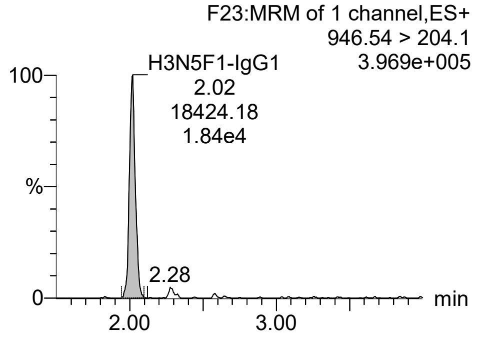

Sample Name: 20211111_A23_R2_IgG

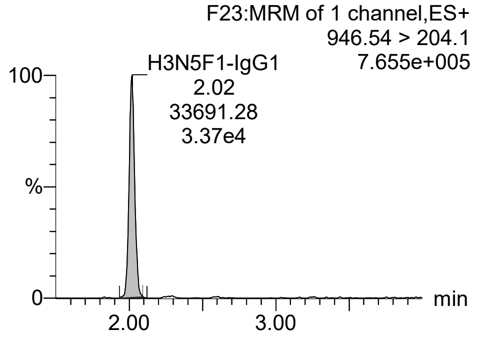

Sample Name: 20211111_A24_R2_IgG

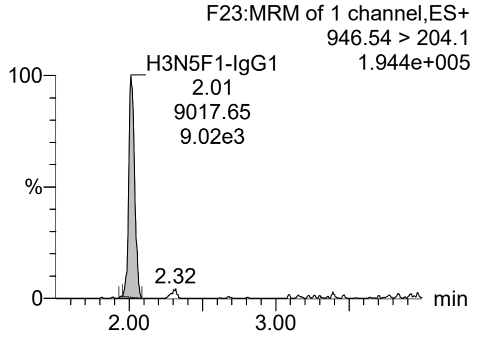

Sample Name: 20211111_L23_R2_IgG

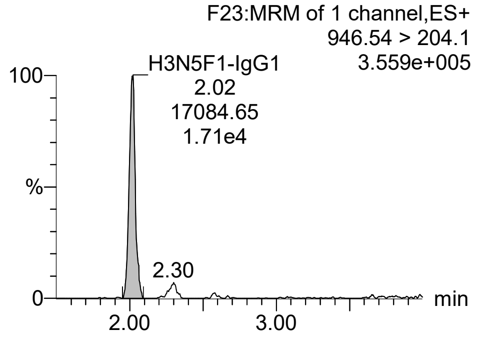

Sample Name: 20211111_L24_R2_IgG

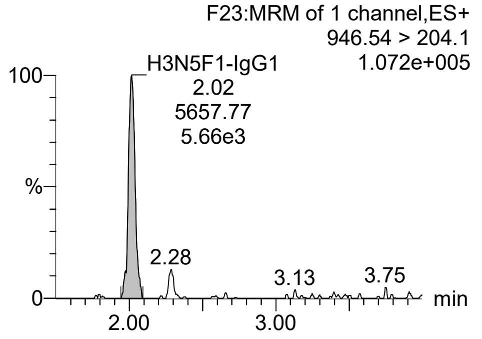

Sample Name: 20211111_H12_R2_IgG

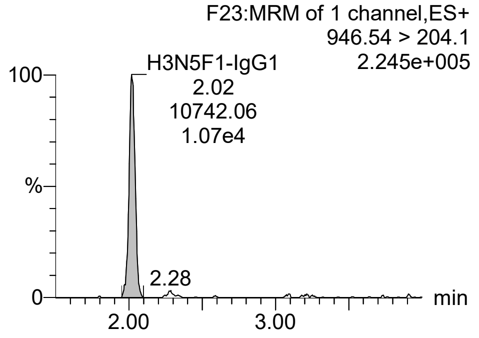

Sample Name: 20211111_QC_R14_IgG

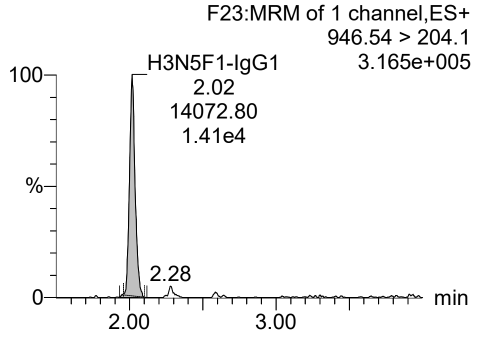

Sample Name: 20211111_A25_R2_IgG

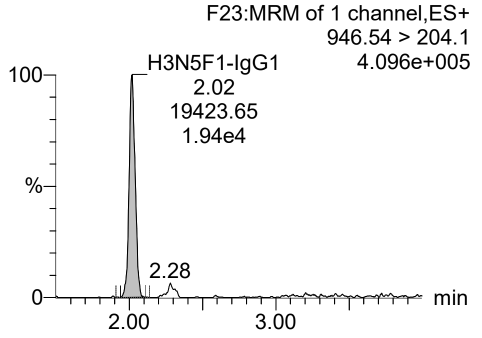

Sample Name: 20211111_A26_R2_IgG

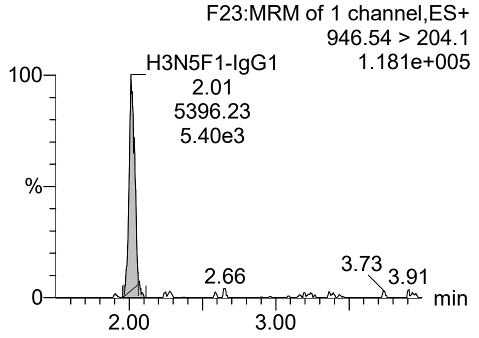

Sample Name: 20211111_L25_R2_IgG

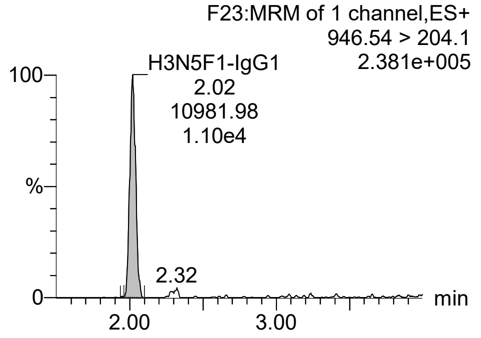

Sample Name: 20211111_L26_R2_IgG

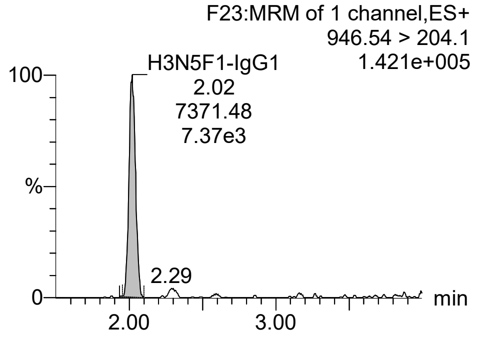

Sample Name: 20211111_H13_R2_IgG

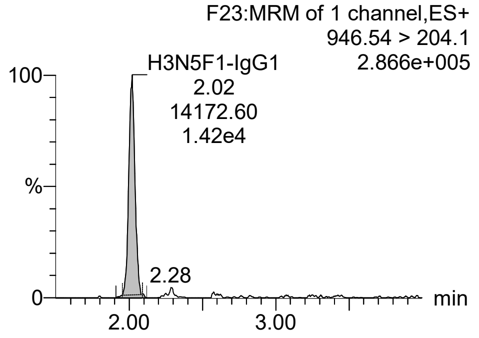

Sample Name: 20211111_A27_R2_IgG

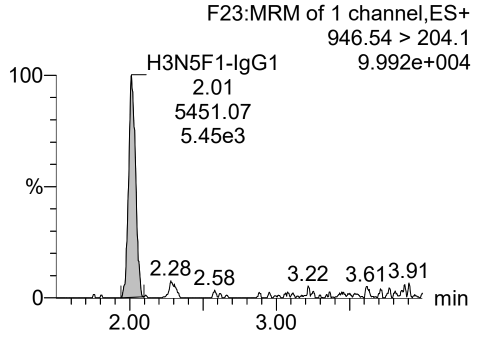

Sample Name: 20211111_A28_R2_IgG

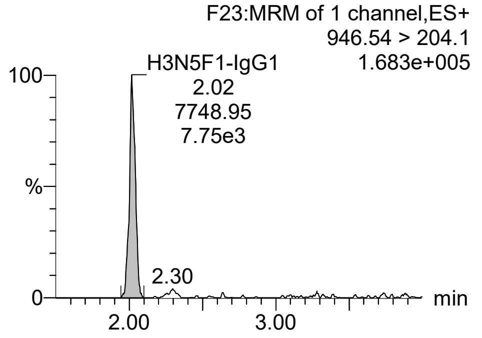

Sample Name: 20211111_L27_R2_IgG

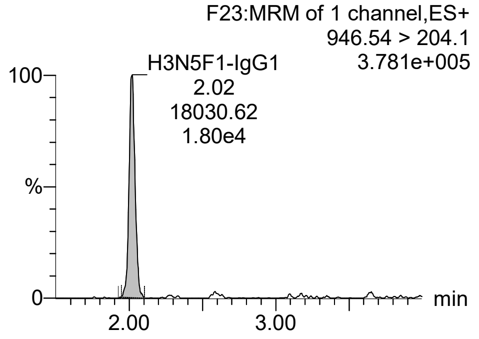

Sample Name: 20211111_L28_R2_IgG

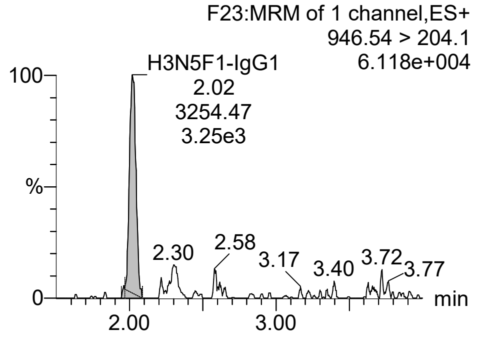

Sample Name: 20211111_H14_R2_IgG

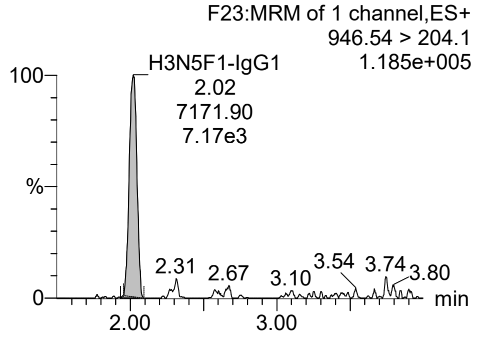

Sample Name: 20211111_A29_R2_IgG

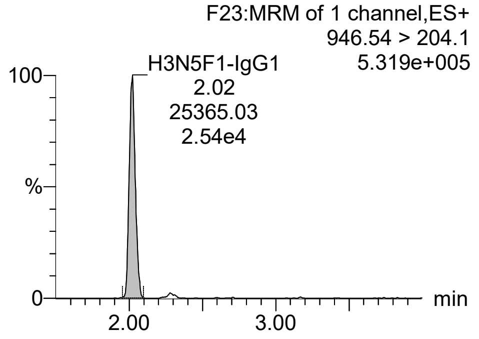

Sample Name: 20211111_A30_R2_IgG

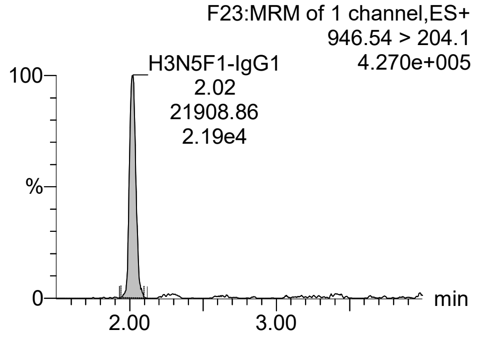

Sample Name: 20211111_QC_R15_IgG

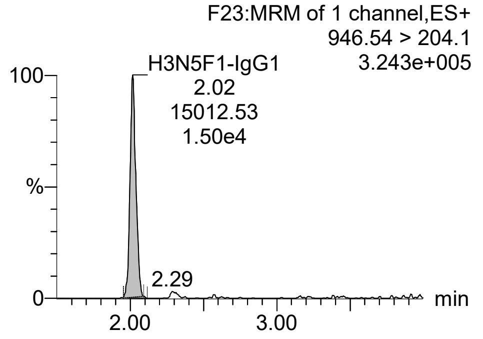

Sample Name: 20211111_L29_R2_IgG

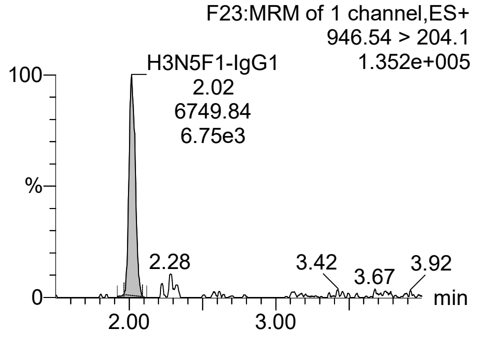

Sample Name: 20211111_L30_R2_IgG

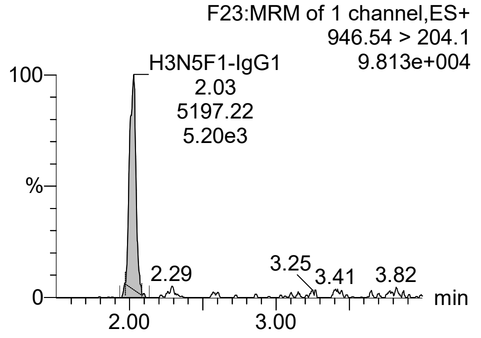

Sample Name: 20211111_H15_R2_IgG

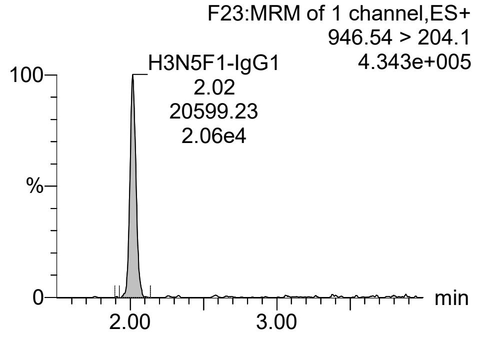

Sample Name: 20211111_A31_R2_IgG

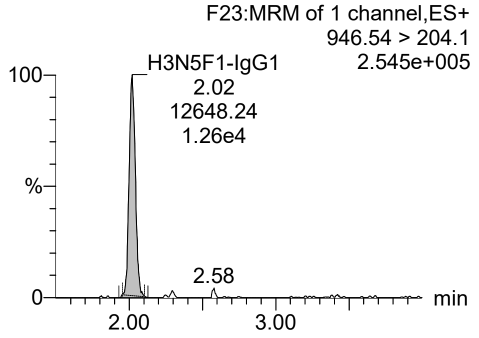

Sample Name: 20211111_A32_R2_IgG

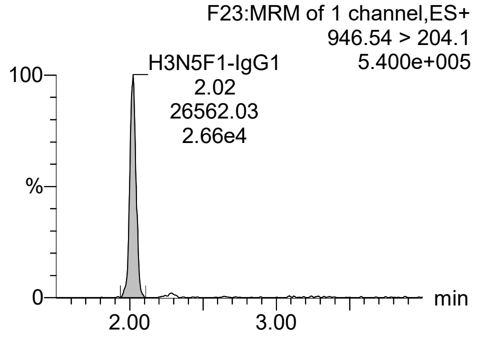

Sample Name: 20211111_L31_R2_IgG

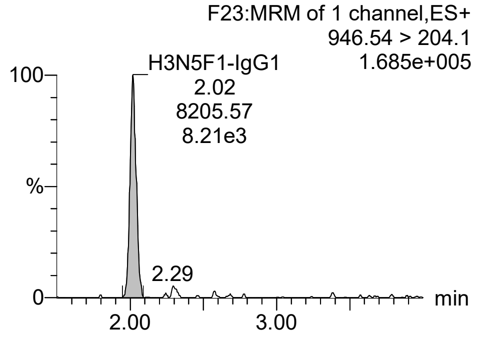

Sample Name: 20211111_L32_R2_IgG

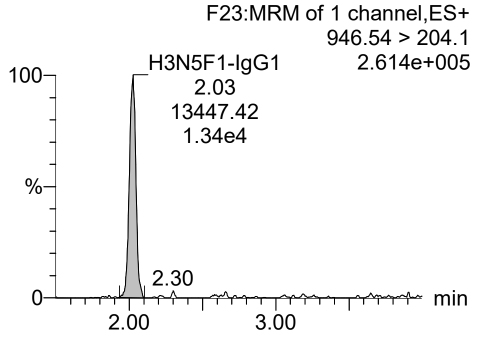

Sample Name: 20211111_H16_R2_IgG

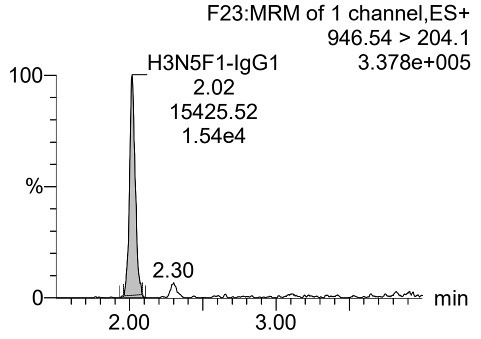

Sample Name: 20211111_A33_R2_IgG

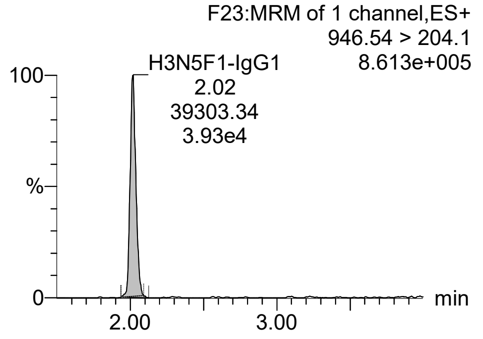

Sample Name: 20211111_A34_R2_IgG
F23:MRM of 1 channel,ES+
946.54 > 204.1

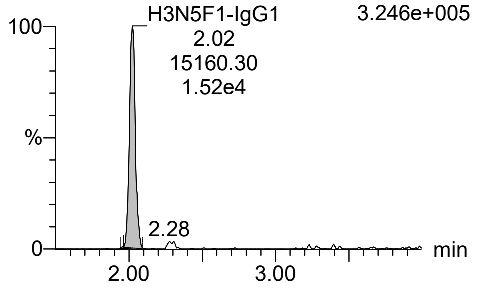

Sample Name: 20211111_H17_R2_IgG
F23:MRM of 1 channel,ES+
946.54 > 204.1

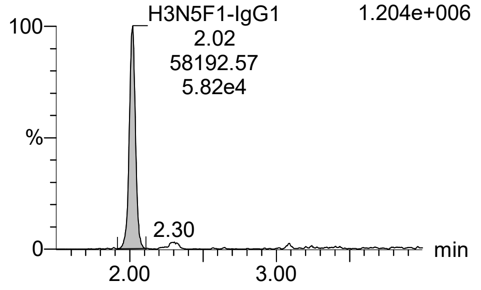

Sample Name: 20211111_A35_R2_IgG
F23:MRM of 1 channel,ES+
946.54 > 204.1

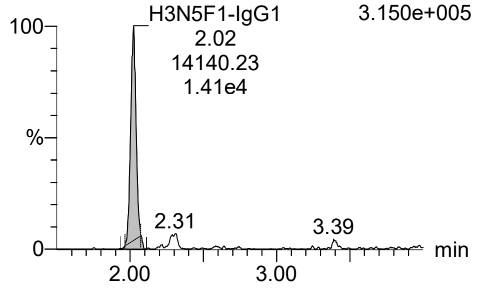

Sample Name: 20211111_QC_R16_IgG
F23:MRM of 1 channel,ES+
946.54 > 204.1

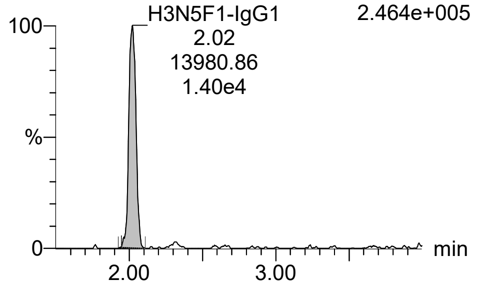

Sample Name: 20211111_A36_R2_IgG
F23:MRM of 1 channel,ES+
946.54 > 204.1

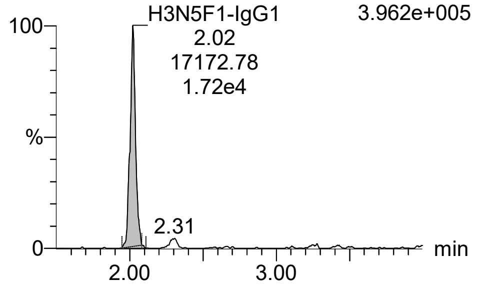

Sample Name: 20211111_H18_R2_IgG
F23:MRM of 1 channel,ES+
946.54 > 204.1

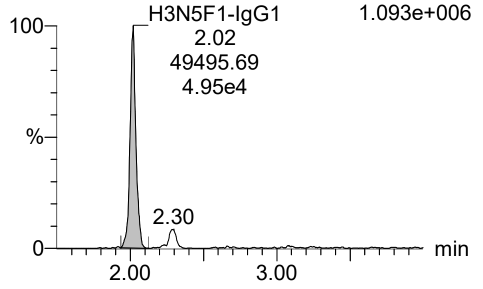

Sample Name: 20211111_A37_R2_IgG
F23:MRM of 1 channel,ES+
946.54 > 204.1

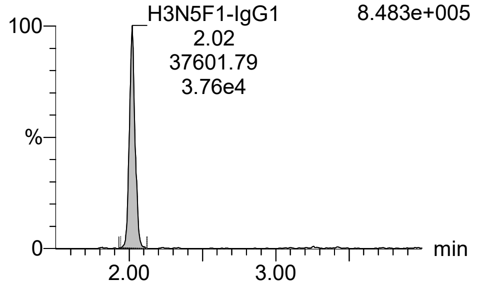

Sample Name: 20211111_A38_R2_IgG
F23:MRM of 1 channel,ES+
946.54 > 204.1

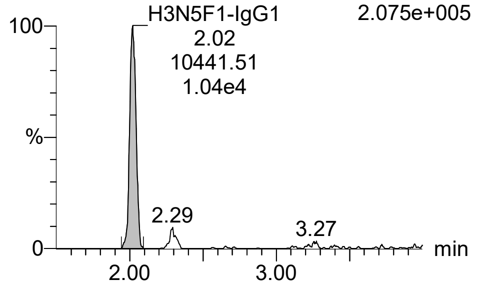

Sample Name: 20211111_H19_R2_IgG
F23:MRM of 1 channel,ES+
946.54 > 204.1

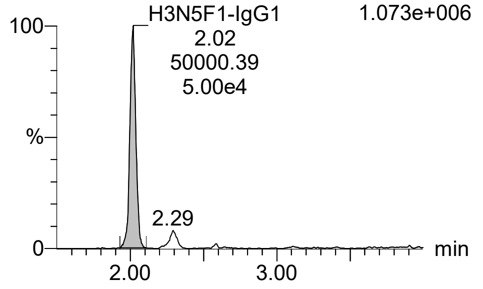

Sample Name: 20211111_A39_R2_IgG
F23:MRM of 1 channel,ES+
946.54 > 204.1

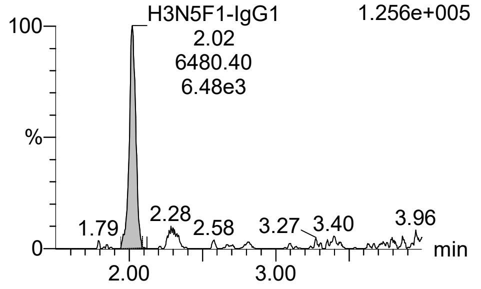

Sample Name: 20211111_A40_R2_IgG
F23:MRM of 1 channel,ES+
946.54 > 204.1

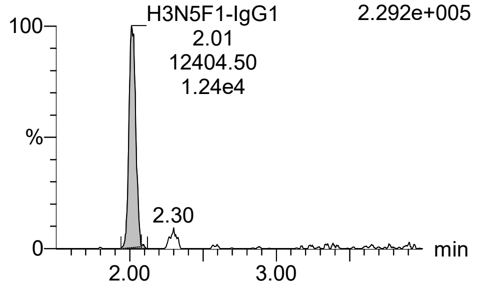

Sample Name: 20211111_H20_R2_IgG
F23:MRM of 1 channel,ES+
946.54 > 204.1

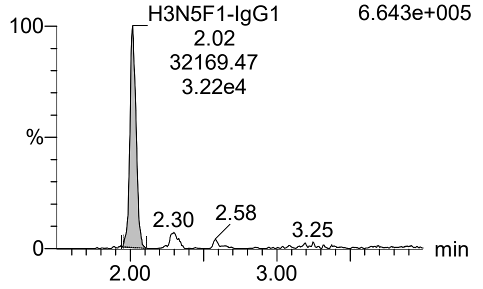

Sample Name: 20211111_A41_R2_IgG
F23:MRM of 1 channel,ES+
946.54 > 204.1

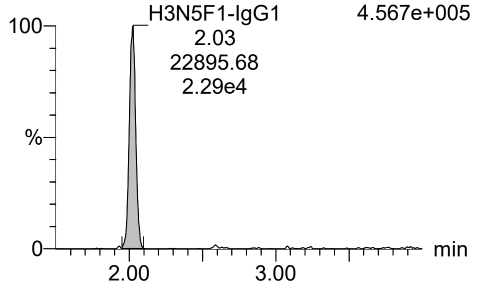

Sample Name: 20211111_A42_R2_IgG
F23:MRM of 1 channel,ES+
946.54 > 204.1

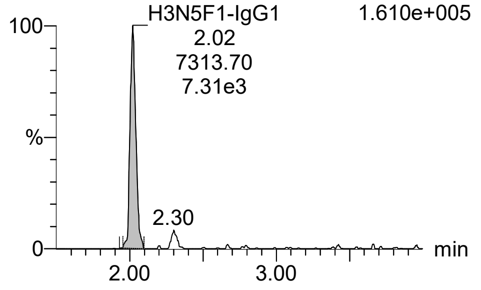

Sample Name: 20211111_A43_R2_IgG
F23:MRM of 1 channel,ES+
946.54 > 204.1

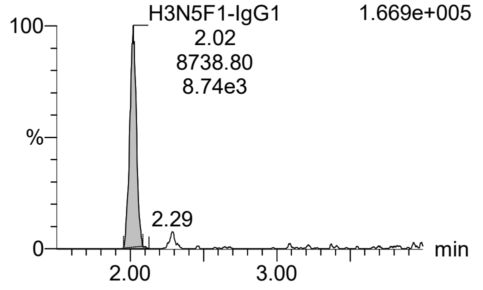

Dataset: C:\MassLynx\10.pro\20211115_clinicalsamples_IgG.qld

Last Altered: Sunday, October 02, 2022 16:02:30 台北標準時間
Printed: Friday, August 01, 2025 16:42:42 台北標準時間

Sample Name: 20211111_A44_R2_IgG

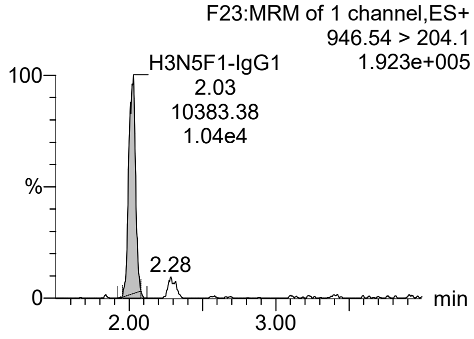

Sample Name: 20211111_QC_R17_IgG

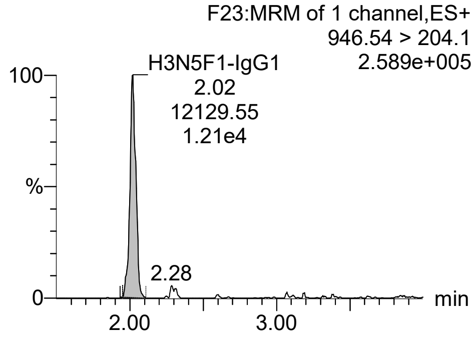

	# Name	RT	Area	IS Area	Response	Prima...
1	1 20211111_QC_R1_IgG	2.03	35213.3	64968.0	0.542	bb
2	2 20211111_A1_R1_IgG	2.02	42757.7	59059.2	0.724	bb
3	3 20211111_A2_R1_IgG	2.03	29013.3	66621.2	0.435	MM
4	4 20211111_L1_R1_IgG	2.02	50882.3	64201.0	0.793	bb
5	5 20211111_L2_R1_IgG	2.03	17553.4	46950.2	0.374	bb
6	6 20211111_H1_R1_IgG	2.03	20509.2	38648.2	0.531	MM
7	7 20211111_A3_R1_IgG	2.03	566.9	2603.7	0.218	MM
8	8 20211111_A4_R1_IgG	2.03	22632.0	69960.4	0.323	MM
9	9 20211111_L3_R1_IgG	2.03	8583.5	38864.7	0.221	MM
10	10 20211111_L4_R1_IgG	2.03	24878.5	43380.5	0.573	bb
11	11 20211111_H2_R1_IgG	2.02	226629.6	71349.2	3.176	bb
12	12 20211111_A5_R1_IgG	2.03	17589.1	65569.7	0.268	bb
13	13 20211111_A6_R1_IgG	2.02	24259.0	53120.7	0.457	MM
14	14 20211111_QC_R2_IgG	2.03	35732.0	81034.1	0.441	bb
15	15 20211111_L5_R1_IgG	2.03	35005.9	50994.6	0.686	MM
16	16 20211111_L6_R1_IgG	2.03	2878.7	58058.0	0.050	MM
17	17 20211111_H3_R1_IgG	2.03	16036.6	64028.6	0.250	MM
18	18 20211111_A7_R1_IgG	2.03	19541.5	82996.1	0.235	bb
19	19 20211111_A8_R1_IgG	2.03	26433.5	77731.7	0.340	MM
20	20 20211111_L7_R1_IgG	2.02	12709.6	38827.3	0.327	MM
21	21 20211111_L8_R1_IgG	2.03	7713.0	44220.1	0.174	bb
22	22 20211111_H4_R1_IgG	2.02	43737.8	65826.3	0.664	bb
23	23 20211111_A9_R1_IgG	2.02	14040.8	60771.1	0.231	bb
24	24 20211111_A10_R1_IgG	2.03	16953.2	48578.7	0.349	MM
25	25 20211111_L9_R1_IgG	2.03	15322.1	50830.7	0.301	MM
26	26 20211111_L10_R1_IgG	2.03	27818.8	38034.2	0.731	MM
27	27 20211111_QC_R3_IgG	2.03	30559.6	67025.5	0.456	MM
28	28 20211111_H5_R1_IgG	2.03	5864.9	44544.9	0.132	MM
29	29 20211111_A11_R1_IgG	2.03	22911.7	66408.5	0.345	bb
30	30 20211111_A12_R1_IgG	2.03	20128.4	46624.5	0.432	bb
31	31 20211111_L11_R1_IgG	2.03	11799.2	33425.1	0.353	bb
32	32 20211111_L12_R1_IgG	2.02	12484.6	41178.0	0.303	MM
33	33 20211111_H6_R1_IgG	2.02	24262.4	58593.4	0.414	bb
34	34 20211111_A13_R1_IgG	2.03	23564.2	54133.0	0.435	bb
35	35 20211111_A14_R1_IgG	2.03	33225.9	56467.3	0.588	bb
36	36 20211111_L13_R1_IgG	2.03	4828.4	36024.4	0.134	MM
37	37 20211111_L14_R1_IgG	2.02	12380.1	37181.6	0.333	MM
38	38 20211111_H7_R1_IgG	2.03	13480.7	42850.6	0.315	MM
39	39 20211111_A15_R1_IgG	2.03	65372.0	62015.1	1.054	bb
40	40 20211111_QC_R4_IgG	2.03	26169.4	58572.5	0.447	bb
41	41 20211111_A16_R1_IgG	2.03	22756.7	78283.8	0.291	MM
42	42 20211111_L15_R1_IgG	2.03	22685.5	44158.2	0.514	MM
43	43 20211111_L16_R1_IgG	2.02	15530.3	55150.8	0.282	MM
44	44 20211111_H8_R1_IgG	2.02	6091.7	42965.2	0.142	MM
45	45 20211111_A17_R1_IgG	2.02	11926.2	39717.7	0.300	MM
46	46 20211111_A18_R1_IgG	2.02	7904.9	44675.4	0.177	MM
47	47 20211111_L17_R1_IgG	2.02	19386.0	37854.1	0.512	bb
48	48 20211111_L18_R1_IgG	2.03	16381.5	50716.9	0.323	MM
49	49 20211111_H9_R1_IgG	2.02	24661.3	36276.0	0.680	MM
50	50 20211111_A19_R1_IgG	2.02	45039.1	42862.5	1.051	MM
51	51 20211111_A20_R1_IgG	2.03	5867.3	36542.7	0.161	MM
52	52 20211111_L19_R1_IgG	2.02	12523.9	53892.8	0.232	MM
53	53 20211111_QC_R5_IgG	2.02	25223.4	56513.2	0.446	bb
54	54 20211111_L20_R1_IgG	2.02	16880.8	56499.9	0.299	bb
55	55 20211111_H10_R1_IgG	2.02	42825.4	68120.5	0.629	bb
56	56 20211111_A21_R1_IgG	2.02	20146.5	63699.8	0.316	bb

Dataset: C:\MassLynx\10.pro\20211115_clinicalsamples_IgG.qld

Last Altered: Sunday, October 02, 2022 16:02:30 台北標準時間
Printed: Friday, August 01, 2025 16:42:42 台北標準時間

	# Name	RT	Area	IS Area	Response	Prima...
57	57 20211111_A22_R1_IgG	2.02	8482.2	52222.1	0.162	MM
58	58 20211111_L21_R1_IgG	2.03	15714.8	54372.5	0.289	bb
59	59 20211111_L22_R1_IgG	2.03	19646.0	41353.1	0.475	bb
60	60 20211111_H11_R1_IgG	2.02	34653.4	65579.1	0.528	bb
61	61 20211111_A23_R1_IgG	2.02	66498.5	64131.1	1.037	MM
62	62 20211111_A24_R1_IgG	2.03	10942.5	38724.8	0.283	MM
63	63 20211111_L23_R1_IgG	2.02	25289.4	64245.2	0.394	bb
64	64 20211111_L24_R1_IgG	2.03	8176.4	45298.0	0.181	MM
65	65 20211111_H12_R1_IgG	2.02	12709.5	44679.3	0.284	MM
66	66 20211111_QC_R6_IgG	2.02	21753.9	48165.0	0.452	bb
67	67 20211111_A25_R1_IgG	2.02	31341.7	57169.9	0.548	bb
68	68 20211111_A26_R1_IgG	2.03	7502.5	34254.0	0.219	MM
69	69 20211111_L25_R1_IgG	2.02	16789.1	67491.5	0.249	MM
70	70 20211111_L26_R1_IgG	2.02	12015.4	55934.1	0.215	MM
71	71 20211111_H13_R1_IgG	2.02	19997.6	48767.3	0.410	MM
72	72 20211111_A27_R1_IgG	2.03	9550.1	57269.7	0.167	MM
73	73 20211111_A28_R1_IgG	2.03	12816.3	49231.1	0.260	bb
74	74 20211111_L27_R1_IgG	2.03	26691.7	60014.5	0.445	bb
75	75 20211111_L28_R1_IgG	2.02	4758.9	35062.6	0.136	bb
76	76 20211111_H14_R1_IgG	2.02	11536.6	62371.9	0.185	MM
77	77 20211111_A29_R1_IgG	2.02	32128.0	43858.2	0.733	bb
78	78 20211111_A30_R1_IgG	2.03	37080.2	62500.4	0.593	MM
79	79 20211111_QC_R7_IgG	2.03	19869.6	43946.9	0.452	bb
80	80 20211111_L29_R1_IgG	2.02	7440.0	38728.9	0.192	MM
81	81 20211111_L30_R1_IgG	2.03	6690.0	42613.2	0.157	bb
82	82 20211111_H15_R1_IgG	2.03	22453.5	39874.6	0.563	MM
83	83 20211111_A31_R1_IgG	2.03	18341.3	53414.3	0.343	MM
84	84 20211111_A32_R1_IgG	2.02	34313.1	41844.6	0.820	MM
85	85 20211111_L31_R1_IgG	2.02	10598.4	40553.7	0.261	MM
86	86 20211111_L32_R1_IgG	2.02	18237.1	49618.7	0.368	MM
87	87 20211111_H16_R1_IgG	2.03	22537.0	60170.9	0.375	bb
88	88 20211111_A33_R1_IgG	2.03	49595.7	48588.2	1.021	bb
89	89 20211111_A34_R1_IgG	2.03	21536.2	43288.0	0.498	MM
90	90 20211111_H17_R1_IgG	2.02	64752.6	47104.5	1.375	bb
91	91 20211111_A35_R1_IgG	2.03	19877.7	41025.9	0.485	MM
92	92 20211111_QC_R8_IgG	2.03	17314.4	40546.3	0.427	bb
93	93 20211111_A36_R1_IgG	2.03	21000.2	44504.4	0.472	MM
94	94 20211111_H18_R1_IgG	2.02	59352.3	34162.2	1.737	bb
95	95 20211111_A37_R1_IgG	2.02	37476.0	39760.0	0.943	bb
96	96 20211111_A38_R1_IgG	2.02	13426.5	43982.6	0.305	MM
97	97 20211111_H19_R1_IgG	2.03	58705.6	45314.8	1.296	MM
98	98 20211111_A39_R1_IgG	2.03	7785.2	39388.0	0.198	MM
99	99 20211111_A40_R1_IgG	2.03	14896.2	38726.4	0.385	MM
100	1... 20211111_H20_R1_IgG	2.02	35839.7	37814.4	0.948	MM
101	1... 20211111_A41_R1_IgG	2.03	29736.1	48930.3	0.608	MM
102	1... 20211111_A42_R1_IgG	2.03	9951.1	40406.8	0.246	MM
103	1... 20211111_A43_R1_IgG	2.02	10649.6	44421.5	0.240	MM
104	1... 20211111_A44_R1_IgG	2.03	16747.8	53128.2	0.315	bb
105	1... 20211111_QC_R9_IgG	2.02	14523.6	37049.0	0.392	MM
106	1... 20211111_A1_R2_IgG	2.02	30462.3	46499.4	0.655	bb
107	1... 20211111_A2_R2_IgG	2.02	22493.8	57260.7	0.393	bb
108	1... 20211111_L1_R2_IgG	2.03	32147.1	48062.9	0.669	bb
109	1... 20211111_L2_R2_IgG	2.02	14171.4	38745.2	0.366	bb
110	1... 20211111_H1_R2_IgG	2.03	25295.3	44273.5	0.571	bb
111	1... 20211111_A3_R2_IgG	2.02	750.7	4755.5	0.158	bb
112	1... 20211111_A4_R2_IgG	2.03	17097.9	50546.7	0.338	bb

Dataset: C:\MassLynx\10.pro\20211115_clinicalsamples_IgG.qld

Last Altered: Sunday, October 02, 2022 16:02:30 台北標準時間
Printed: Friday, August 01, 2025 16:42:42 台北標準時間

	# Name	RT	Area	IS Area	Response	Prima...
113	1... 20211111_L3_R2_IgG	2.02	9529.3	34825.8	0.274	bb
114	1... 20211111_L4_R2_IgG	2.02	19320.6	39107.7	0.494	MM
115	1... 20211111_H2_R2_IgG	2.02	148881.0	49750.0	2.993	bb
116	1... 20211111_A5_R2_IgG	2.02	15200.8	52536.2	0.289	bb
117	1... 20211111_A6_R2_IgG	2.02	22066.9	42050.0	0.525	bb
118	1... 20211111_QC_R10_IgG	2.02	16004.3	35350.8	0.453	bb
119	1... 20211111_L5_R2_IgG	2.02	26335.9	38039.3	0.692	bb
120	1... 20211111_L6_R2_IgG	2.03	2738.1	44907.4	0.061	MM
121	1... 20211111_H3_R2_IgG	2.02	12183.9	47729.1	0.255	MM
122	1... 20211111_A7_R2_IgG	2.03	15210.4	57347.1	0.265	MM
123	1... 20211111_A8_R2_IgG	2.03	17505.9	52397.8	0.334	MM
124	1... 20211111_L7_R2_IgG	2.02	12374.0	36318.4	0.341	bb
125	1... 20211111_L8_R2_IgG	2.02	6548.8	38131.9	0.172	MM
126	1... 20211111_H4_R2_IgG	2.02	36361.5	46849.6	0.776	MM
127	1... 20211111_A9_R2_IgG	2.02	12351.8	47759.6	0.259	bb
128	1... 20211111_A10_R2_IgG	2.02	12726.0	41366.2	0.308	bb
129	1... 20211111_L9_R2_IgG	2.02	13794.8	43052.1	0.320	bb
130	1... 20211111_L10_R2_IgG	2.02	26100.0	31487.4	0.829	MM
131	1... 20211111_QC_R11_IgG	2.02	17553.7	37804.6	0.464	MM
132	1... 20211111_H5_R2_IgG	2.01	5758.8	40380.0	0.143	MM
133	1... 20211111_A11_R2_IgG	2.02	19446.0	49533.3	0.393	bb
134	1... 20211111_A12_R2_IgG	2.02	17456.9	40226.3	0.434	MM
135	1... 20211111_L11_R2_IgG	2.02	8519.6	28884.0	0.295	MM
136	1... 20211111_L12_R2_IgG	2.03	11277.0	34351.3	0.328	bb
137	1... 20211111_H6_R2_IgG	2.03	14675.8	40466.6	0.363	MM
138	1... 20211111_A13_R2_IgG	2.02	16601.6	42306.7	0.392	bb
139	1... 20211111_A14_R2_IgG	2.03	23994.6	44904.0	0.534	MM
140	1... 20211111_L13_R2_IgG	2.03	4725.5	31913.0	0.148	MM
141	1... 20211111_L14_R2_IgG	2.02	12285.5	31527.4	0.390	MM
142	1... 20211111_H7_R2_IgG	2.03	11013.6	40838.2	0.270	MM
143	1... 20211111_A15_R2_IgG	2.02	53413.8	49389.7	1.081	MM
144	1... 20211111_QC_R12_IgG	2.02	14175.2	35168.7	0.403	MM
145	1... 20211111_A16_R2_IgG	2.02	13284.7	47440.5	0.280	bb
146	1... 20211111_L15_R2_IgG	2.03	14447.6	33145.9	0.436	bb
147	1... 20211111_L16_R2_IgG	2.03	9985.5	37596.6	0.266	bb
148	1... 20211111_H8_R2_IgG	2.02	5434.1	43933.3	0.124	MM
149	1... 20211111_A17_R2_IgG	2.02	11144.9	43605.5	0.256	bb
150	1... 20211111_A18_R2_IgG	2.02	5816.2	37581.8	0.155	MM
151	1... 20211111_L17_R2_IgG	2.02	17658.4	33942.5	0.520	MM
152	1... 20211111_L18_R2_IgG	2.03	10464.0	37831.5	0.277	MM
153	1... 20211111_H9_R2_IgG	2.02	24152.1	30898.4	0.782	MM
154	1... 20211111_A19_R2_IgG	2.02	40802.5	39352.1	1.037	bb
155	1... 20211111_A20_R2_IgG	2.03	3984.7	34661.2	0.115	bb
156	1... 20211111_L19_R2_IgG	2.02	8796.2	39918.3	0.220	MM
157	1... 20211111_QC_R13_IgG	2.02	15344.4	36239.1	0.423	MM
158	1... 20211111_L20_R2_IgG	2.03	10899.7	32897.7	0.331	MM
159	1... 20211111_H10_R2_IgG	2.02	19165.4	31394.5	0.610	bb
160	1... 20211111_A21_R2_IgG	2.02	10180.3	34863.7	0.292	bb
161	1... 20211111_A22_R2_IgG	2.02	4954.9	30342.1	0.163	MM
162	1... 20211111_L21_R2_IgG	2.02	9585.3	33706.2	0.284	MM
163	1... 20211111_L22_R2_IgG	2.02	14069.1	31298.6	0.450	MM
164	1... 20211111_H11_R2_IgG	2.02	18424.2	38904.1	0.474	MM
165	1... 20211111_A23_R2_IgG	2.02	33691.3	32382.3	1.040	MM
166	1... 20211111_A24_R2_IgG	2.01	9017.6	30261.9	0.298	MM
167	1... 20211111_L23_R2_IgG	2.02	17084.7	43037.5	0.397	bb
168	1... 20211111_L24_R2_IgG	2.02	5657.8	37023.3	0.153	bb

Dataset: C:\MassLynx\10.pro\20211115_clinicalsamples_IgG.qld

Last Altered: Sunday, October 02, 2022 16:02:30 台北標準時間
Printed: Friday, August 01, 2025 16:42:42 台北標準時間

	# Name	RT	Area	IS Area	Response	Prima...
169	1... 20211111_H12_R2_IgG	2.02	10742.1	39478.2	0.272	bb
170	1... 20211111_QC_R14_IgG	2.02	14072.8	34463.2	0.408	MM
171	1... 20211111_A25_R2_IgG	2.02	19423.6	39945.3	0.486	MM
172	1... 20211111_A26_R2_IgG	2.01	5396.2	29663.2	0.182	MM
173	1... 20211111_L25_R2_IgG	2.02	10982.0	38589.3	0.285	MM
174	1... 20211111_L26_R2_IgG	2.02	7371.5	38405.2	0.192	MM
175	1... 20211111_H13_R2_IgG	2.02	14172.6	37771.2	0.375	MM
176	1... 20211111_A27_R2_IgG	2.01	5451.1	42634.1	0.128	bb
177	1... 20211111_A28_R2_IgG	2.02	7748.9	33231.1	0.233	bb
178	1... 20211111_L27_R2_IgG	2.02	18030.6	41761.1	0.432	MM
179	1... 20211111_L28_R2_IgG	2.02	3254.5	26968.6	0.121	MM
180	1... 20211111_H14_R2_IgG	2.02	7171.9	49250.2	0.146	MM
181	1... 20211111_A29_R2_IgG	2.02	25365.0	37601.7	0.675	MM
182	1... 20211111_A30_R2_IgG	2.02	21908.9	40366.8	0.543	MM
183	1... 20211111_QC_R15_IgG	2.02	15012.5	35156.1	0.427	MM
184	1... 20211111_L29_R2_IgG	2.02	6749.8	33813.1	0.200	MM
185	1... 20211111_L30_R2_IgG	2.03	5197.2	33488.6	0.155	MM
186	1... 20211111_H15_R2_IgG	2.02	20599.2	38501.0	0.535	MM
187	1... 20211111_A31_R2_IgG	2.02	12648.2	37525.5	0.337	MM
188	1... 20211111_A32_R2_IgG	2.02	26562.0	35770.8	0.743	bb
189	1... 20211111_L31_R2_IgG	2.02	8205.6	33282.4	0.247	bb
190	1... 20211111_L32_R2_IgG	2.03	13447.4	42049.6	0.320	bb
191	1... 20211111_H16_R2_IgG	2.02	15425.5	45947.6	0.336	MM
192	1... 20211111_A33_R2_IgG	2.02	39303.3	40202.3	0.978	MM
193	1... 20211111_A34_R2_IgG	2.02	15160.3	36747.0	0.413	MM
194	1... 20211111_H17_R2_IgG	2.02	58192.6	40920.9	1.422	bb
195	1... 20211111_A35_R2_IgG	2.02	14140.2	33689.2	0.420	MM
196	1... 20211111_QC_R16_IgG	2.02	13980.9	32783.6	0.426	MM
197	1... 20211111_A36_R2_IgG	2.02	17172.8	36822.4	0.466	MM
198	1... 20211111_H18_R2_IgG	2.02	49495.7	31769.3	1.558	bb
199	1... 20211111_A37_R2_IgG	2.02	37601.8	37581.8	1.001	MM
200	2... 20211111_A38_R2_IgG	2.02	10441.5	38390.4	0.272	bb
201	2... 20211111_H19_R2_IgG	2.02	50000.4	41217.0	1.213	bb
202	2... 20211111_A39_R2_IgG	2.02	6480.4	37392.7	0.173	MM
203	2... 20211111_A40_R2_IgG	2.01	12404.5	38295.1	0.324	MM
204	2... 20211111_H20_R2_IgG	2.02	32169.5	35941.8	0.895	MM
205	2... 20211111_A41_R2_IgG	2.03	22895.7	45304.9	0.505	bb
206	2... 20211111_A42_R2_IgG	2.02	7313.7	37803.8	0.193	MM
207	2... 20211111_A43_R2_IgG	2.02	8738.8	38161.8	0.229	MM
208	2... 20211111_A44_R2_IgG	2.03	10383.4	37819.8	0.275	MM
209	2... 20211111_QC_R17_IgG	2.02	12129.5	31731.8	0.382	MM

Dataset: C:\MassLynx\10.pro\20211115_clinicalsamples_IgG.qld

Last Altered: Sunday, October 02, 2022 16:02:30 台北標準時間
Printed: Friday, August 01, 2025 16:43:22 台北標準時間

Method: C:\MassLynx\10_Pro_IgA.PRO\MethDB\20210926_IgG_DMRM_IgM_IS.mdb 16 Feb 2022 11:58:21
Calibration: 18 Nov 2021 13:56:22

Sample Name: 20211111_QC_R1_IgG

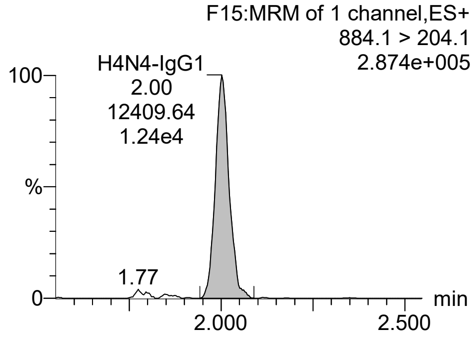

Sample Name: 20211111_A1_R1_IgG

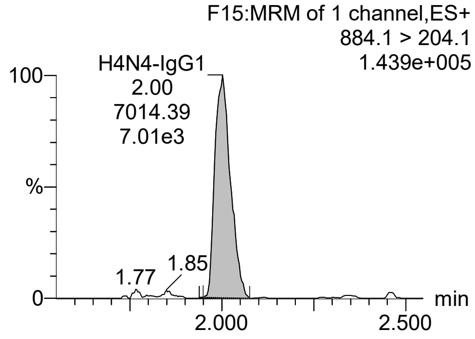

Sample Name: 20211111_A2_R1_IgG

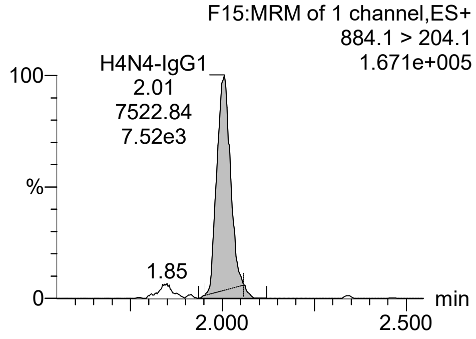

Sample Name: 20211111_L1_R1_IgG

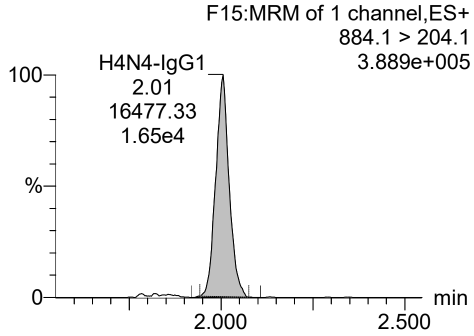

Sample Name: 20211111_L2_R1_IgG

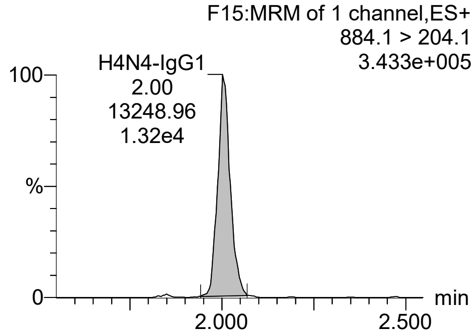

Sample Name: 20211111_H1_R1_IgG

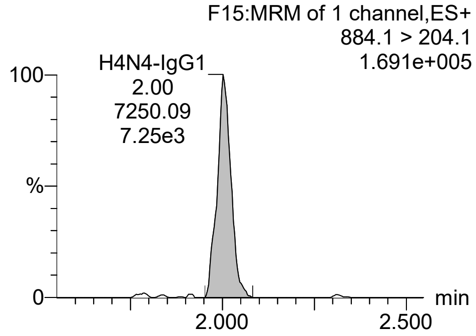

Sample Name: 20211111_A3_R1_IgG

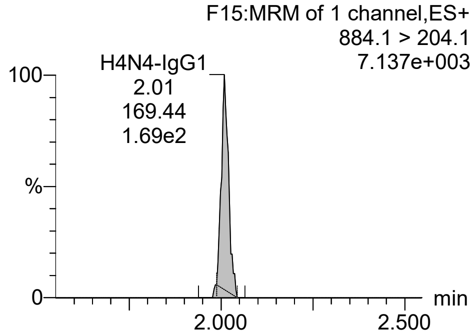

Sample Name: 20211111_A4_R1_IgG

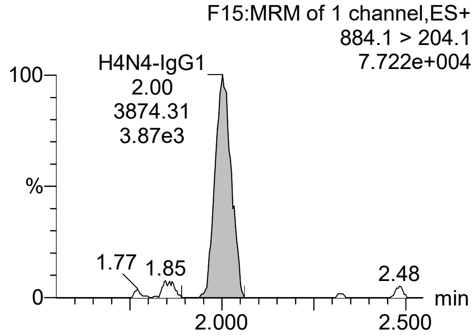

Sample Name: 20211111_L3_R1_IgG

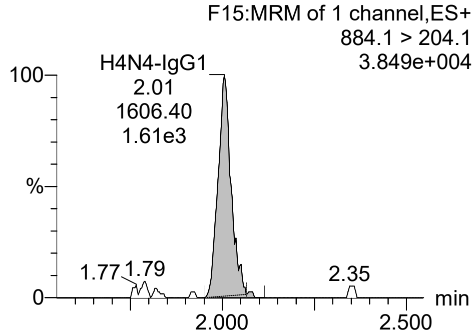

Sample Name: 20211111_L4_R1_IgG

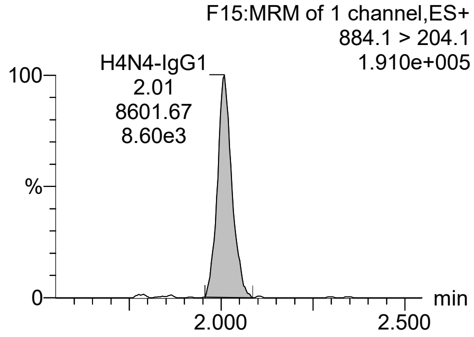

Sample Name: 20211111_H2_R1_IgG

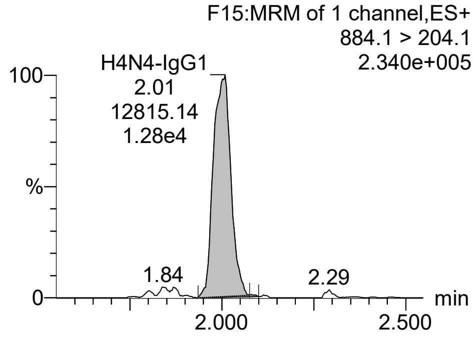

Sample Name: 20211111_A5_R1_IgG

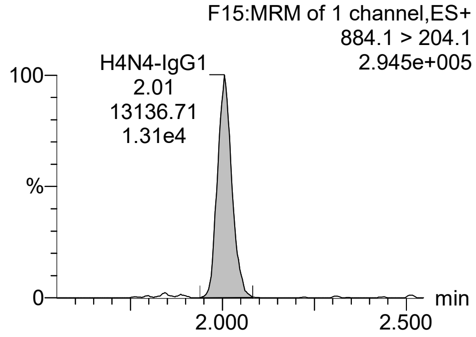

Dataset: C:\MassLynx\10.pro\20211115_clinicalsamples_IgG.qld

Last Altered: Sunday, October 02, 2022 16:02:30 台北標準時間
Printed: Friday, August 01, 2025 16:43:22 台北標準時間

Sample Name: 20211111_A6_R1_IgG

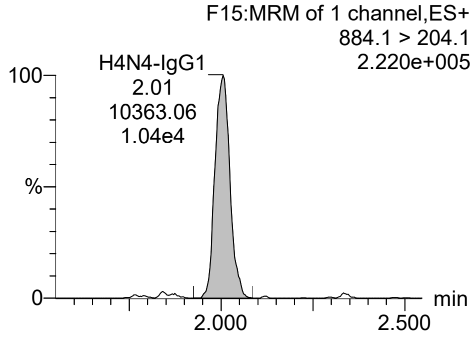

Sample Name: 20211111_QC_R2_IgG

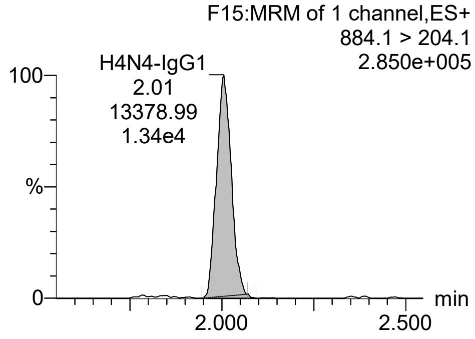

Sample Name: 20211111_L5_R1_IgG

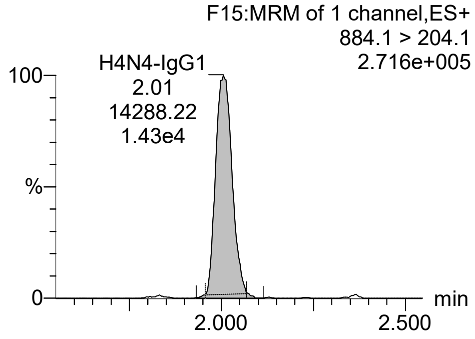

Sample Name: 20211111_L6_R1_IgG

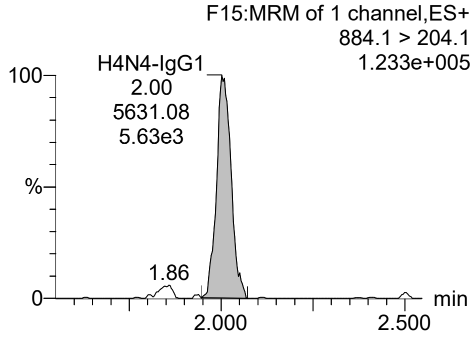

Sample Name: 20211111_H3_R1_IgG

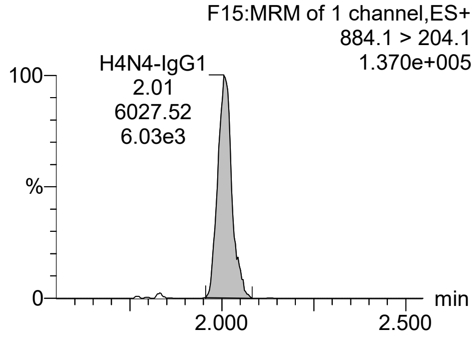

Sample Name: 20211111_A7_R1_IgG

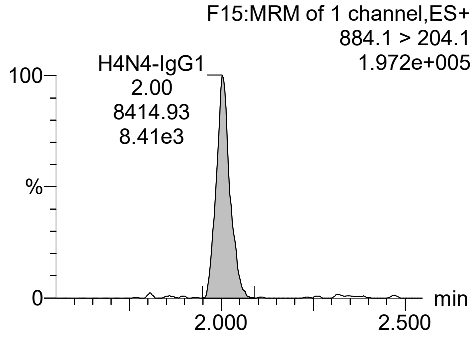

Sample Name: 20211111_A8_R1_IgG

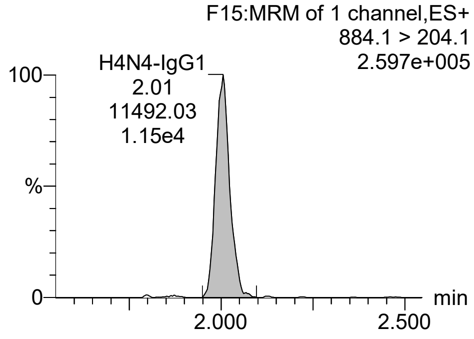

Sample Name: 20211111_L7_R1_IgG

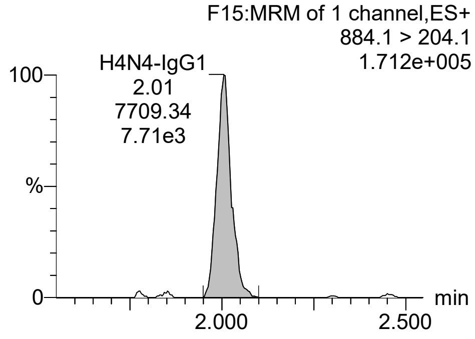

Sample Name: 20211111_L8_R1_IgG

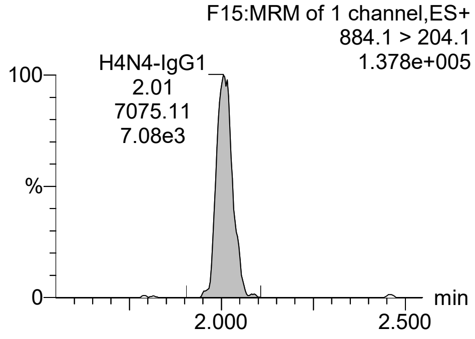

Sample Name: 20211111_H4_R1_IgG

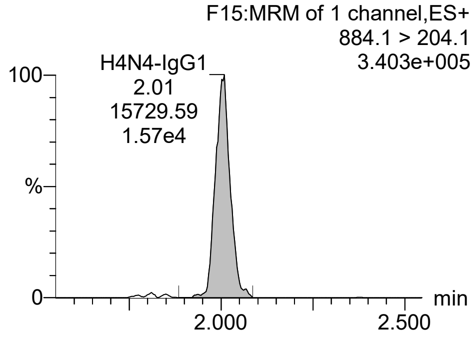

Sample Name: 20211111_A9_R1_IgG

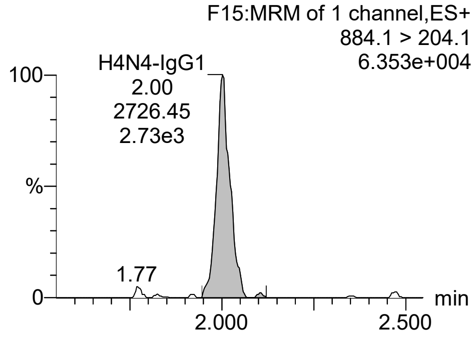

Sample Name: 20211111_A10_R1_IgG

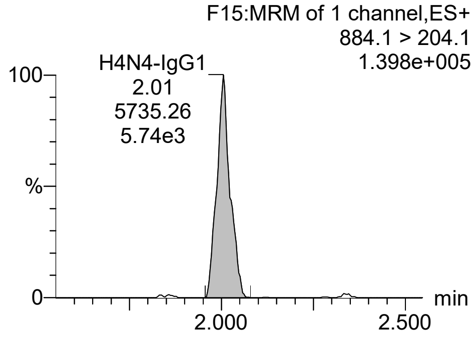

Sample Name: 20211111_L9_R1_IgG

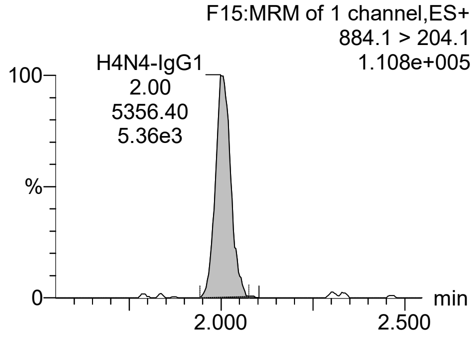

Sample Name: 20211111_L10_R1_IgG

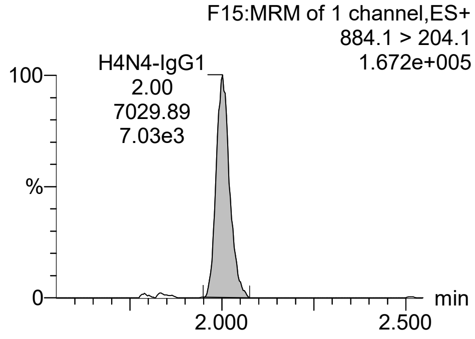

Sample Name: 20211111_QC_R3_IgG

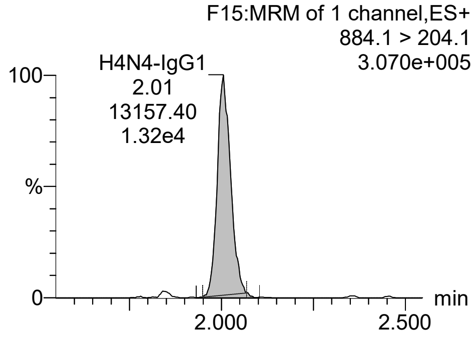

Dataset: C:\MassLynx\10.pro\20211115_clinicalsamples_IgG.qld

Last Altered: Sunday, October 02, 2022 16:02:30 台北標準時間
Printed: Friday, August 01, 2025 16:43:22 台北標準時間

Sample Name: 20211111_H5_R1_IgG

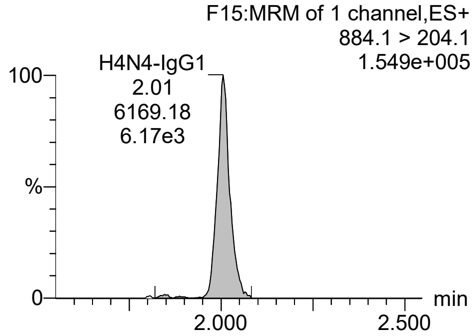

Sample Name: 20211111_A11_R1_IgG

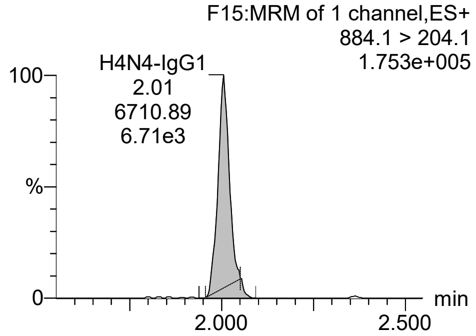

Sample Name: 20211111_A12_R1_IgG

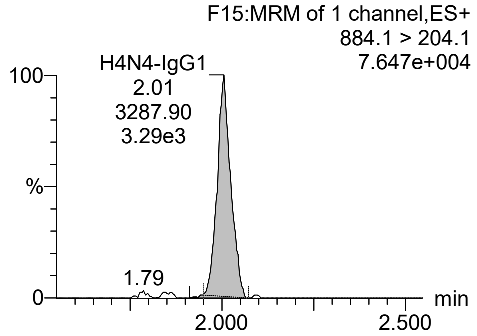

Sample Name: 20211111_L11_R1_IgG

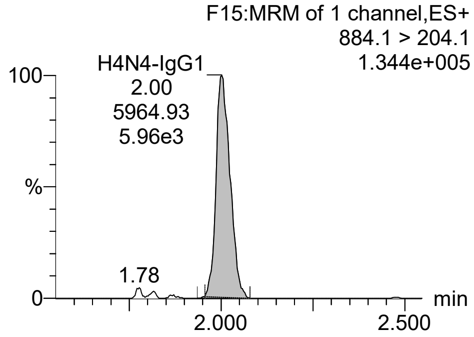

Sample Name: 20211111_L12_R1_IgG

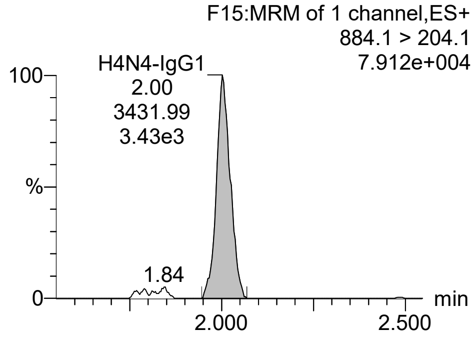

Sample Name: 20211111_H6_R1_IgG

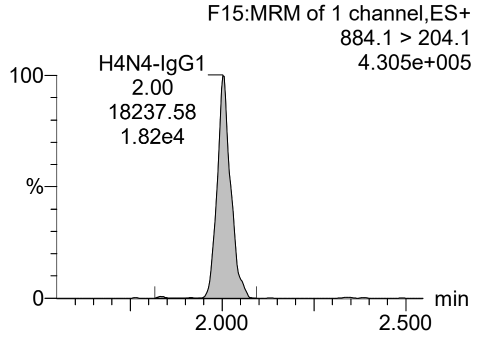

Sample Name: 20211111_A13_R1_IgG

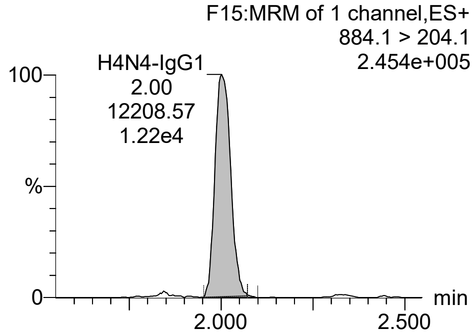

Sample Name: 20211111_A14_R1_IgG

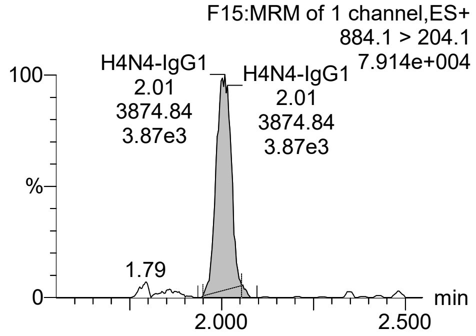

Sample Name: 20211111_L13_R1_IgG

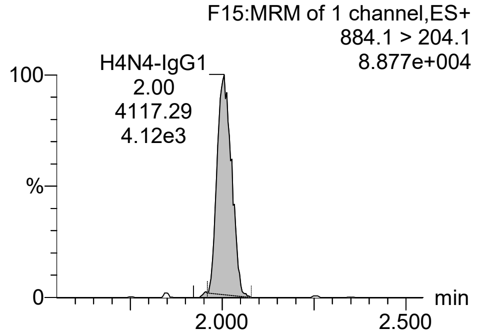

Sample Name: 20211111_L14_R1_IgG

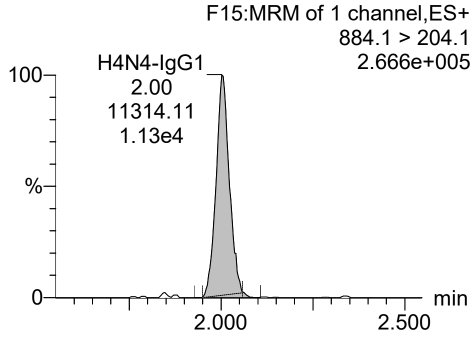

Sample Name: 20211111_H7_R1_IgG

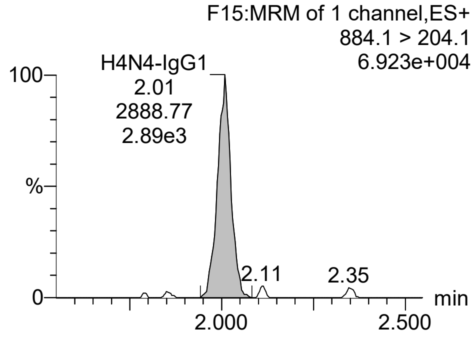

Sample Name: 20211111_A15_R1_IgG

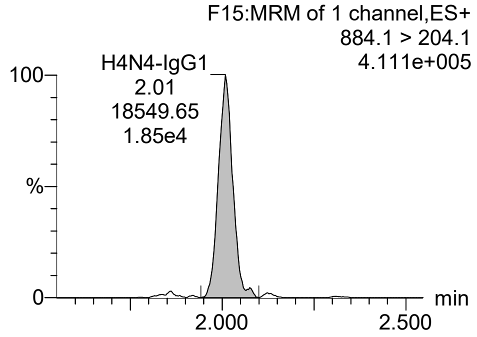

Sample Name: 20211111_QC_R4_IgG

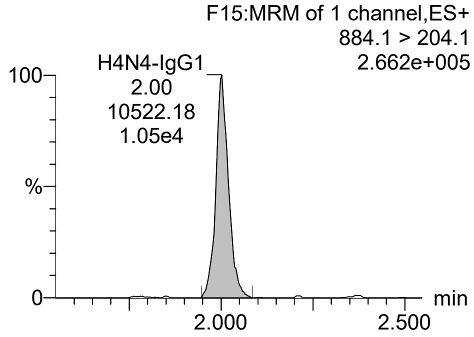

Sample Name: 20211111_A16_R1_IgG

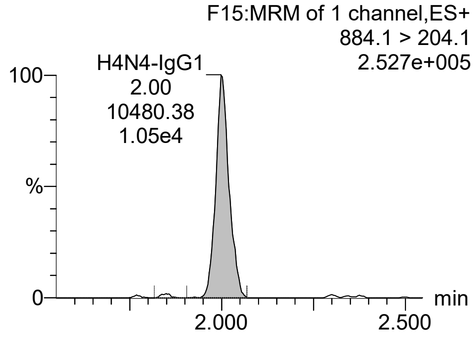

Sample Name: 20211111_L15_R1_IgG

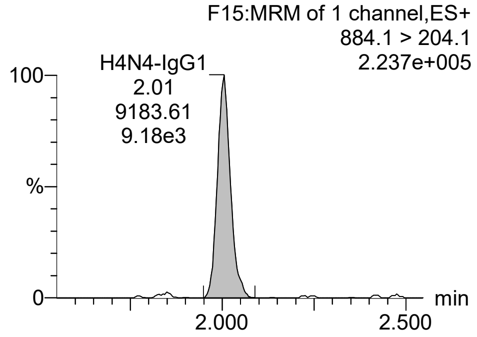

Sample Name: 20211111_L16_R1_IgG

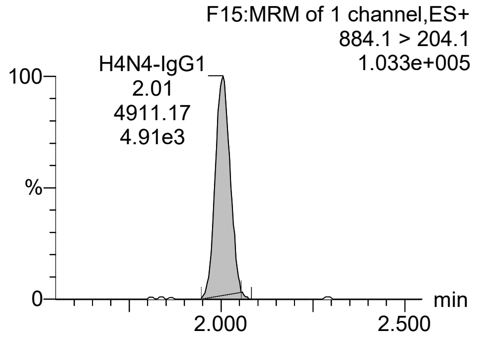

Sample Name: 20211111_H8_R1_IgG

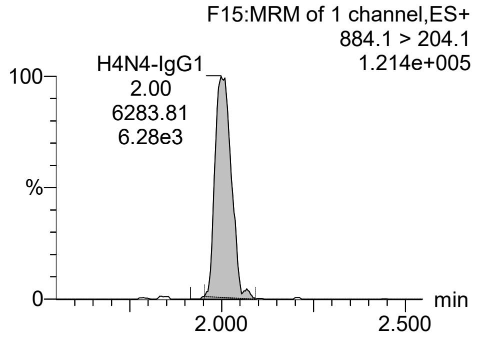

Sample Name: 20211111_A17_R1_IgG

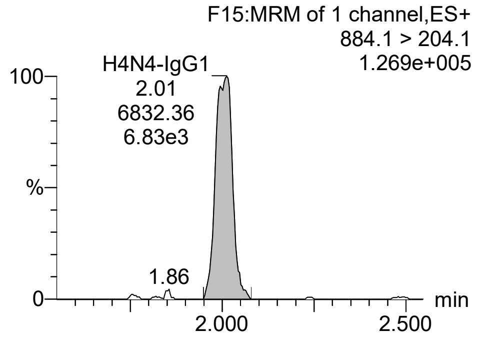

Sample Name: 20211111_A18_R1_IgG

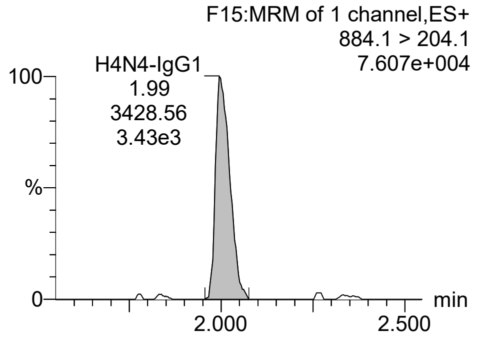

Sample Name: 20211111_L17_R1_IgG

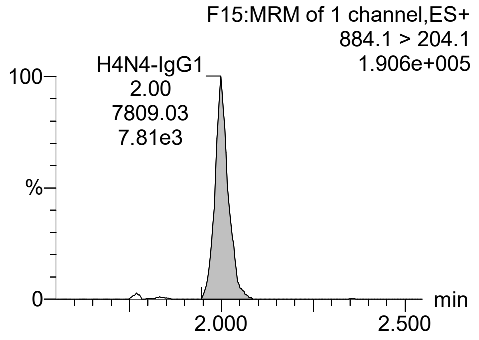

Sample Name: 20211111_L18_R1_IgG

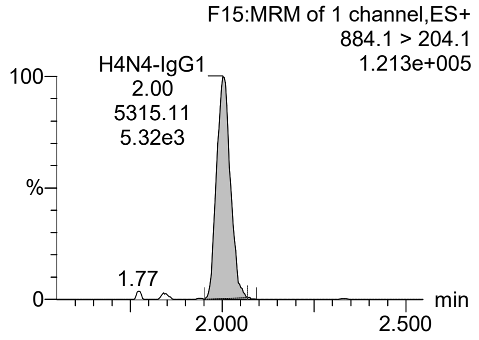

Sample Name: 20211111_H9_R1_IgG

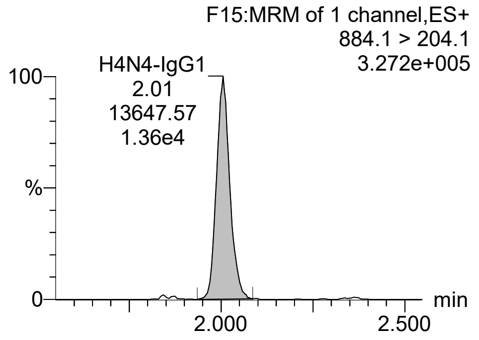

Sample Name: 20211111_A19_R1_IgG

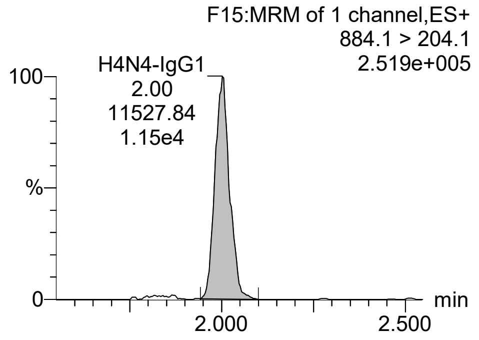

Sample Name: 20211111_A20_R1_IgG

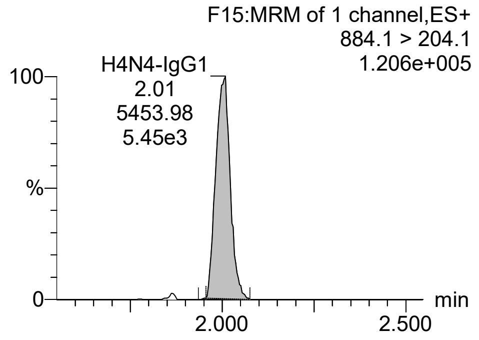

Sample Name: 20211111_L19_R1_IgG

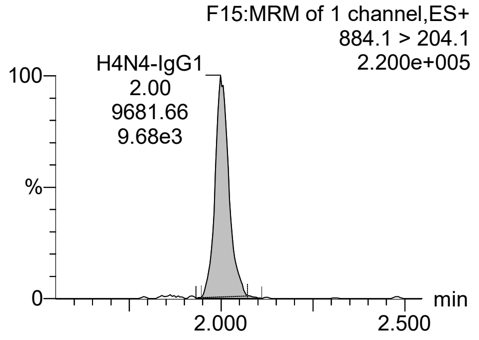

Sample Name: 20211111_QC_R5_IgG

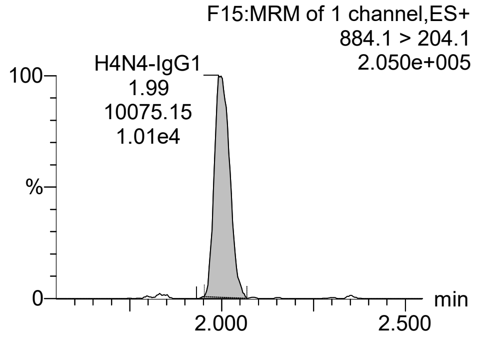

Sample Name: 20211111_L20_R1_IgG

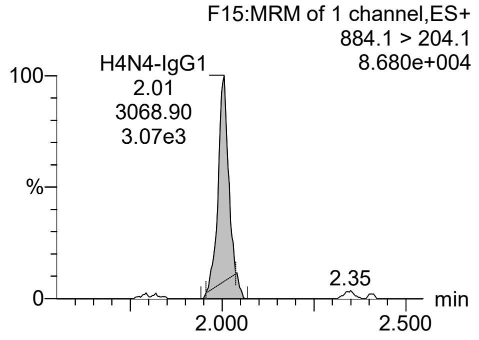

Sample Name: 20211111_H10_R1_IgG

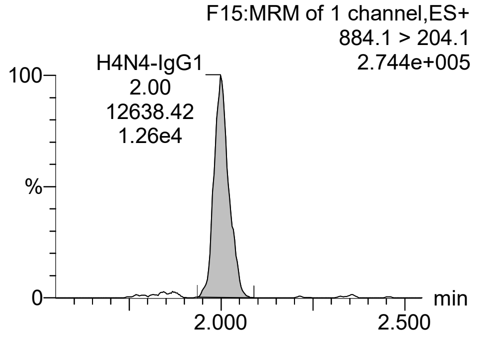

Sample Name: 20211111_A21_R1_IgG

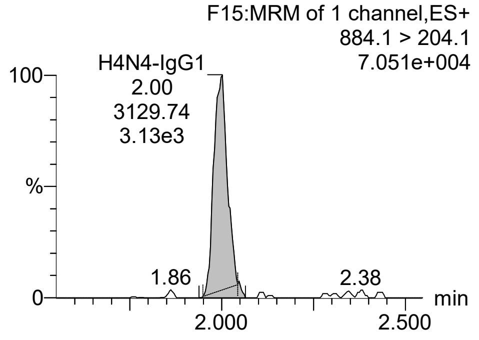

Sample Name: 20211111_A22_R1_IgG

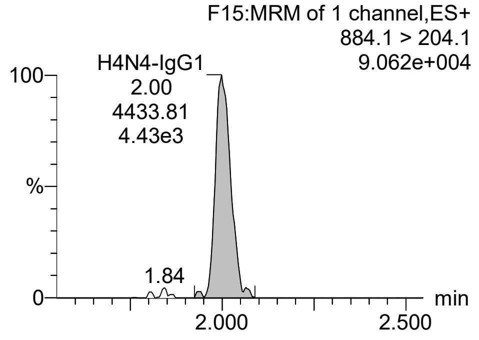

Sample Name: 20211111_L21_R1_IgG

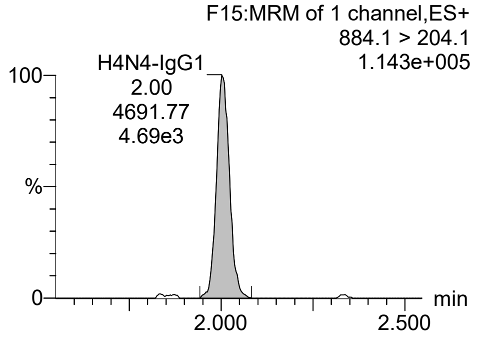

Sample Name: 20211111_L22_R1_IgG

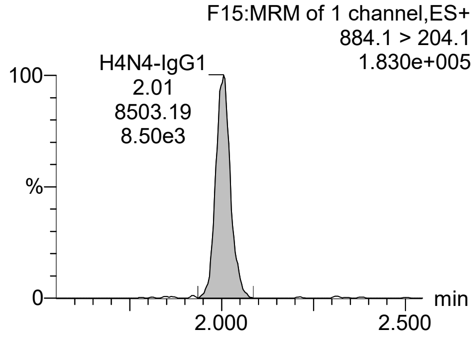

Sample Name: 20211111_H11_R1_IgG

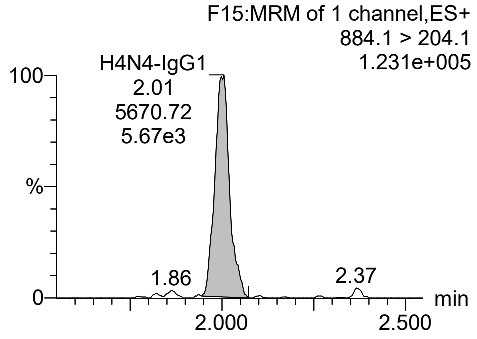

Sample Name: 20211111_A23_R1_IgG

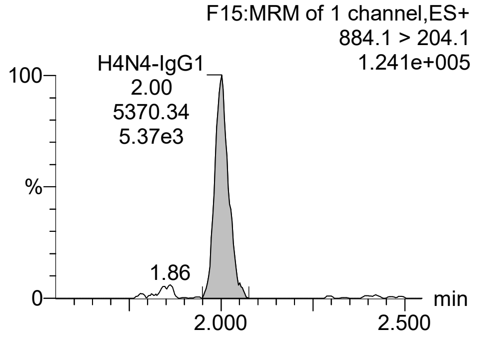

Sample Name: 20211111_A24_R1_IgG

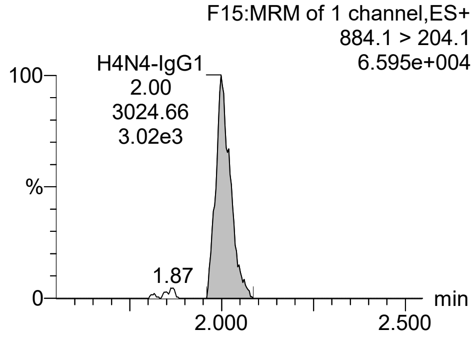

Sample Name: 20211111_L23_R1_IgG

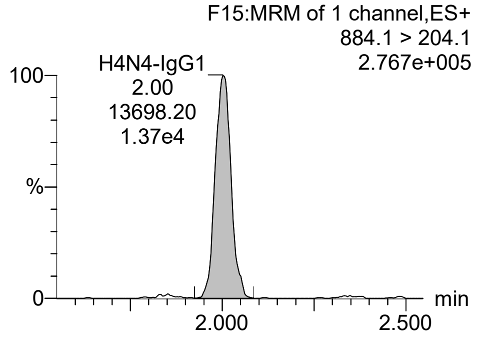

Sample Name: 20211111_L24_R1_IgG

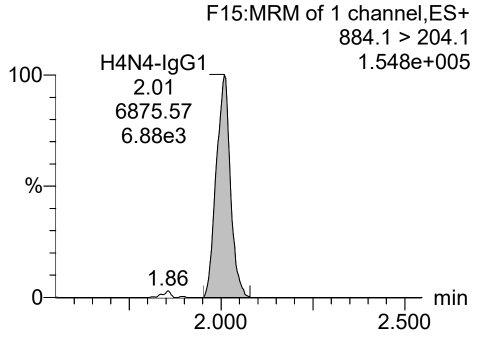

Sample Name: 20211111_H12_R1_IgG

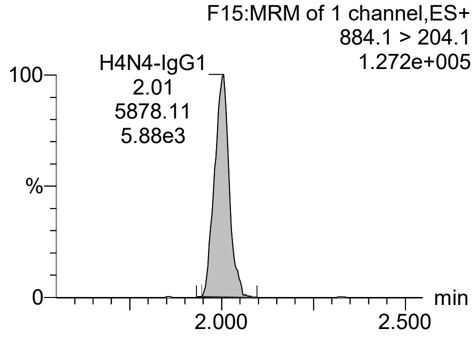

Sample Name: 20211111_QC_R6_IgG

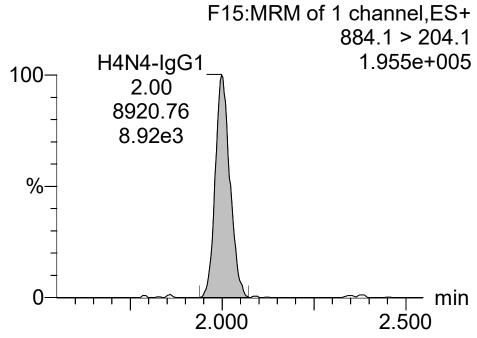

Sample Name: 20211111_A25_R1_IgG

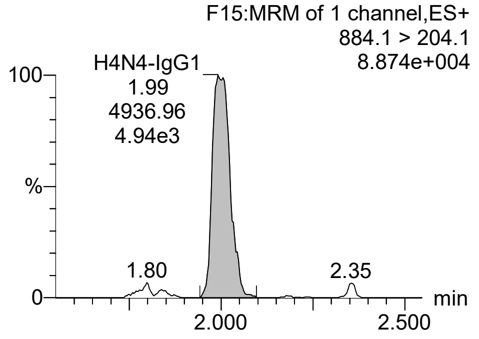

Sample Name: 20211111_A26_R1_IgG

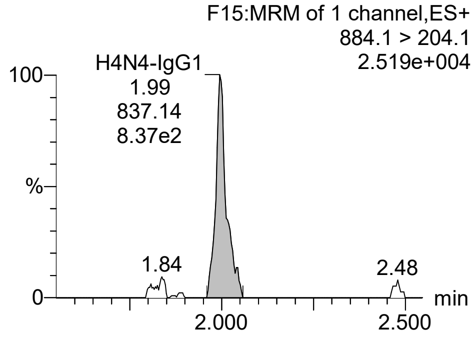

Sample Name: 20211111_L25_R1_IgG

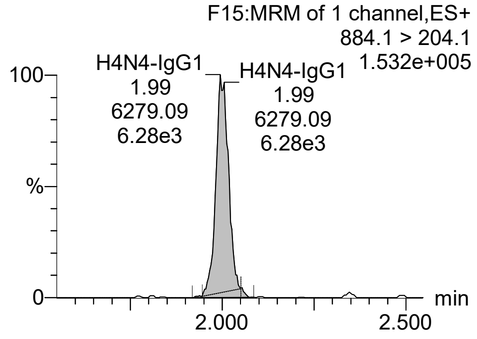

Sample Name: 20211111_L26_R1_IgG

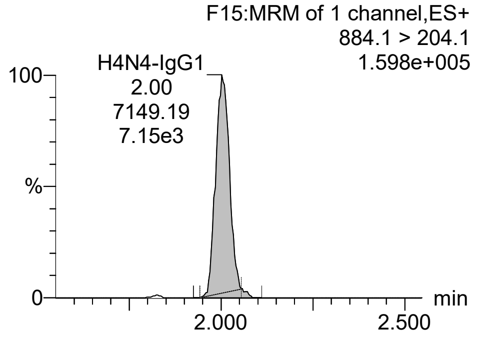

Sample Name: 20211111_H13_R1_IgG

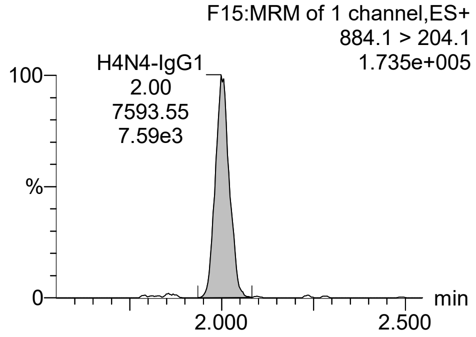

Sample Name: 20211111_A27_R1_IgG

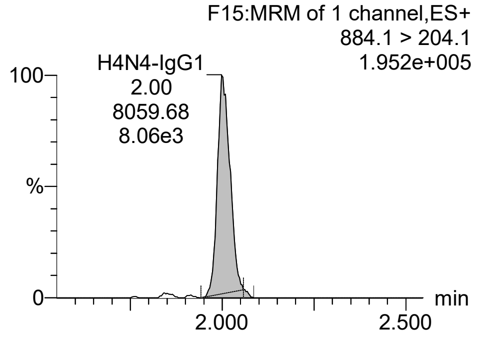

Dataset: C:\MassLynx\10.pro\20211115_clinicalsamples_IgG.qld

Last Altered: Sunday, October 02, 2022 16:02:30 台北標準時間
Printed: Friday, August 01, 2025 16:43:22 台北標準時間

Sample Name: 20211111_A28_R1_IgG

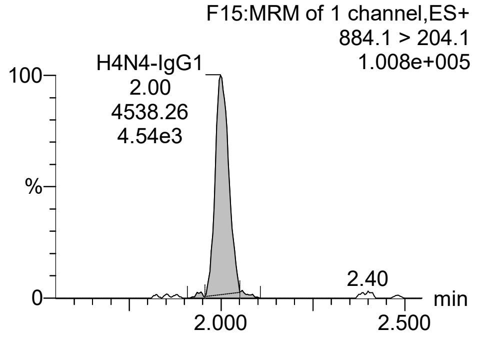

Sample Name: 20211111_L27_R1_IgG

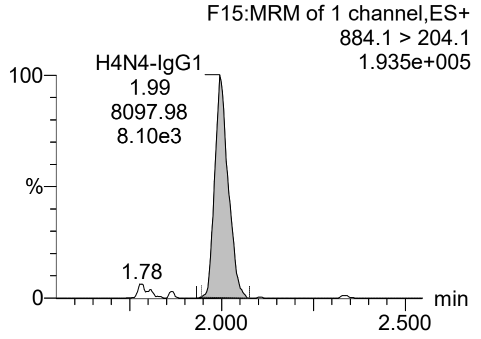

Sample Name: 20211111_L28_R1_IgG

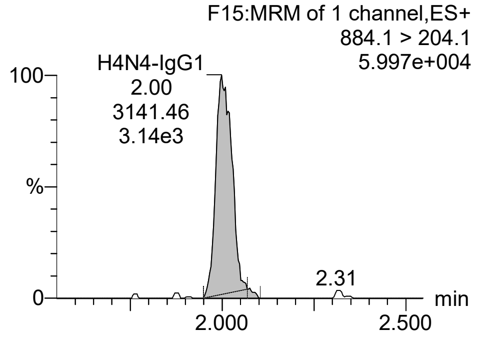

Sample Name: 20211111_H14_R1_IgG

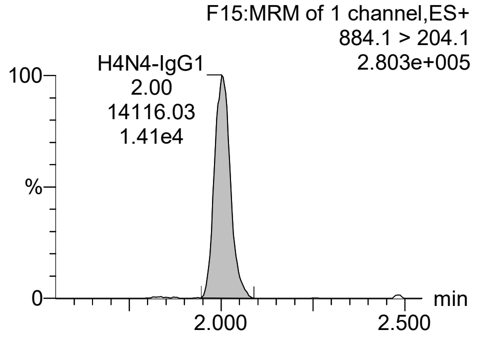

Sample Name: 20211111_A29_R1_IgG

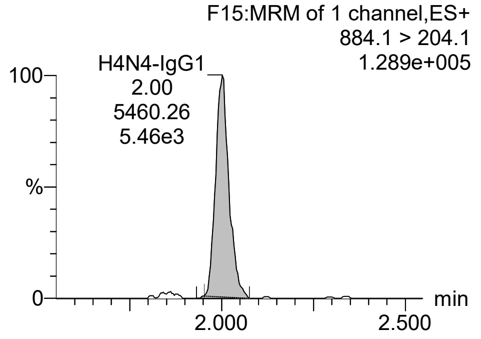

Sample Name: 20211111_A30_R1_IgG

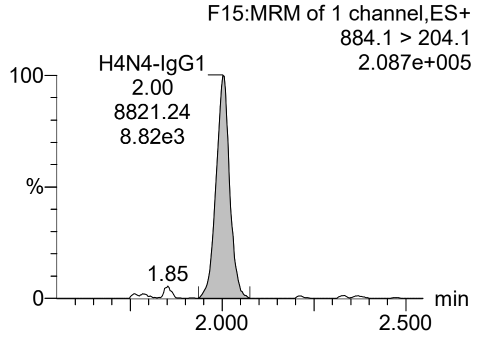

Sample Name: 20211111_QC_R7_IgG

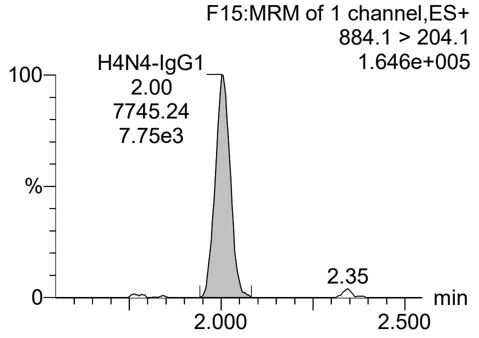

Sample Name: 20211111_L29_R1_IgG

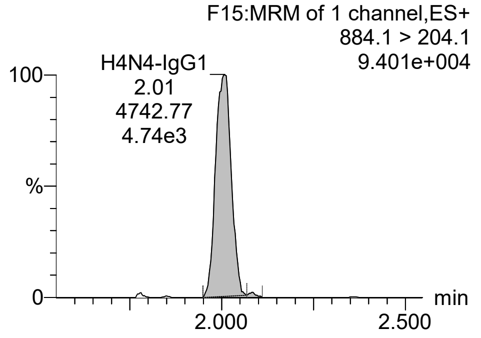

Sample Name: 20211111_L30_R1_IgG

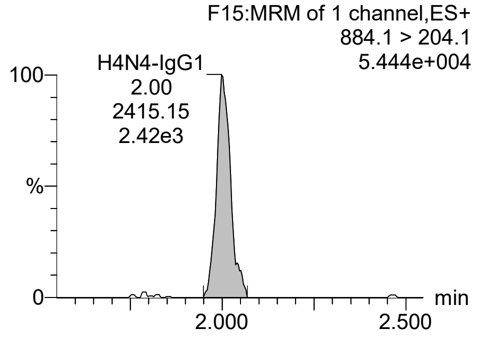

Sample Name: 20211111_H15_R1_IgG

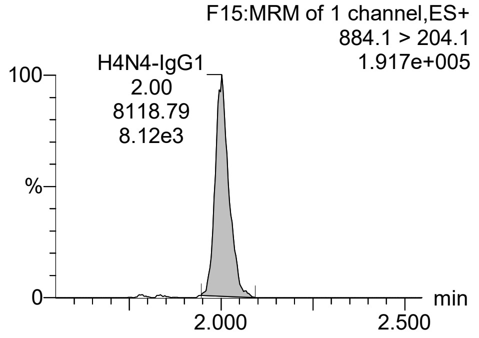

Sample Name: 20211111_A31_R1_IgG

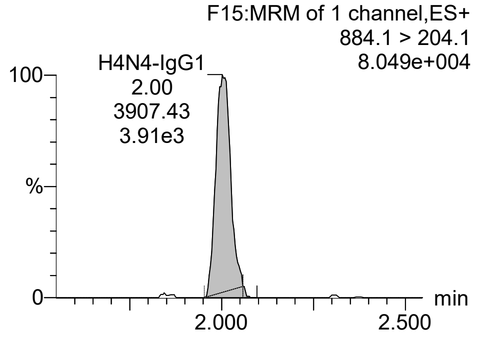

Sample Name: 20211111_A32_R1_IgG

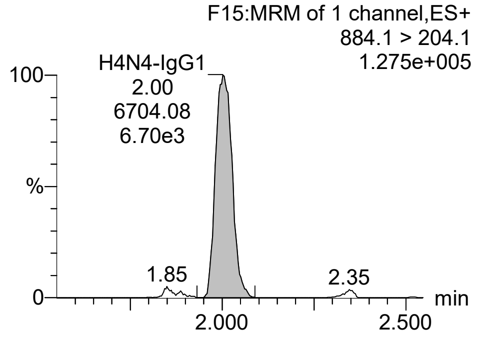

Sample Name: 20211111_L31_R1_IgG

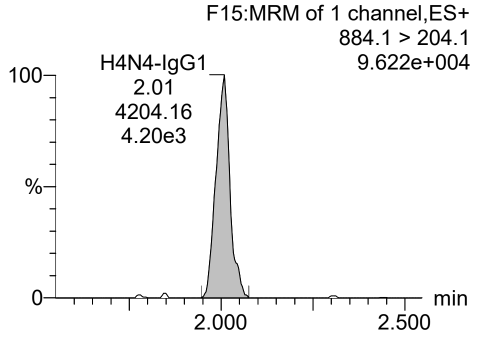

Sample Name: 20211111_L32_R1_IgG

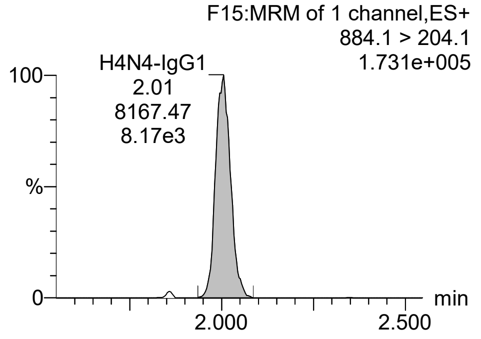

Sample Name: 20211111_H16_R1_IgG

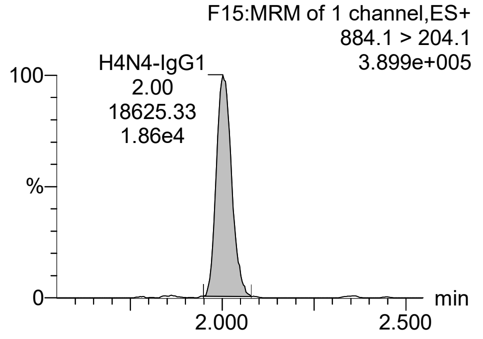

Dataset: C:\MassLynx\10.pro\20211115_clinicalsamples_IgG.qld

Last Altered: Sunday, October 02, 2022 16:02:30 台北標準時間
Printed: Friday, August 01, 2025 16:43:22 台北標準時間

Sample Name: 20211111_A33_R1_IgG

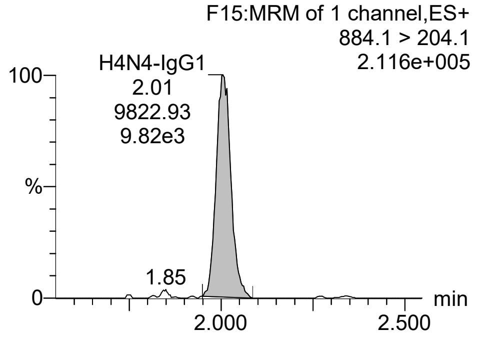

Sample Name: 20211111_A34_R1_IgG

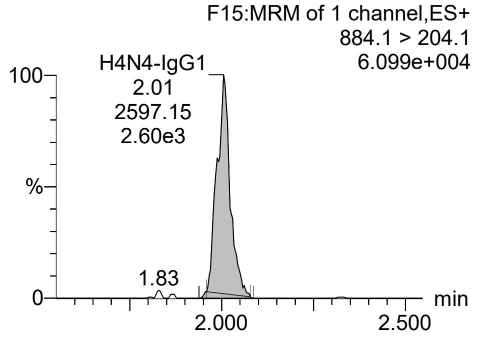

Sample Name: 20211111_H17_R1_IgG

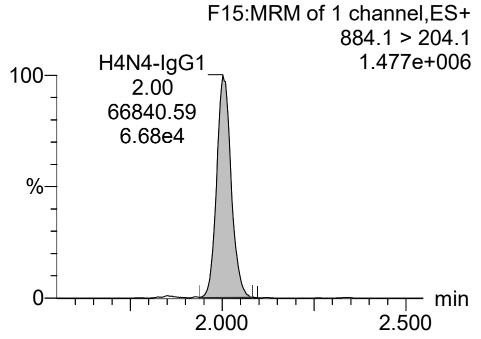

Sample Name: 20211111_A35_R1_IgG

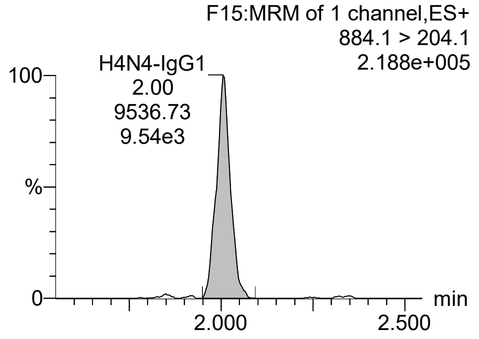

Sample Name: 20211111_QC_R8_IgG

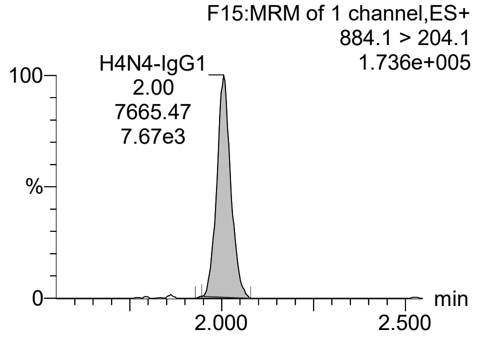

Sample Name: 20211111_A36_R1_IgG

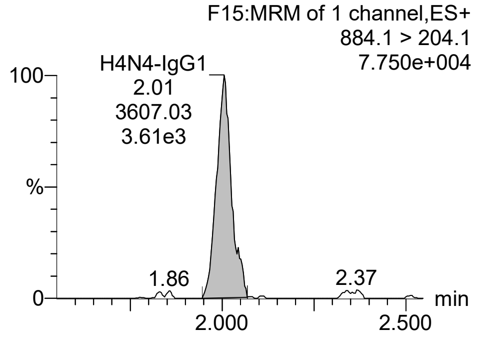

Sample Name: 20211111_H18_R1_IgG

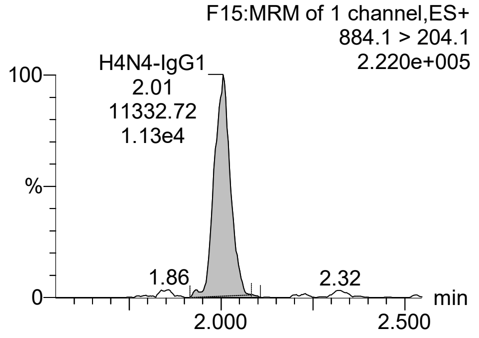

Sample Name: 20211111_A37_R1_IgG

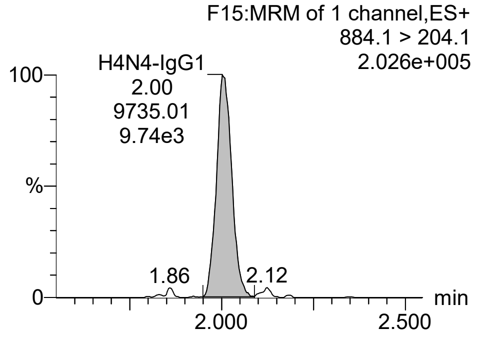

Sample Name: 20211111_A38_R1_IgG

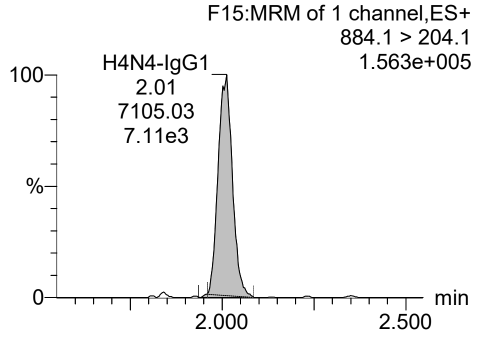

Sample Name: 20211111_H19_R1_IgG

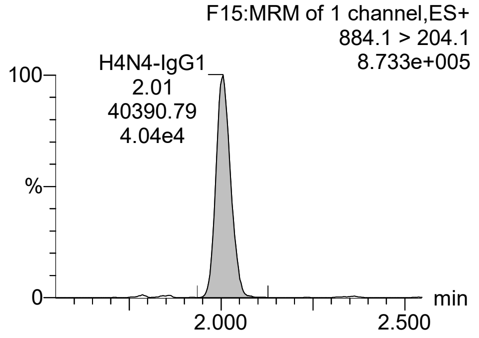

Sample Name: 20211111_A39_R1_IgG

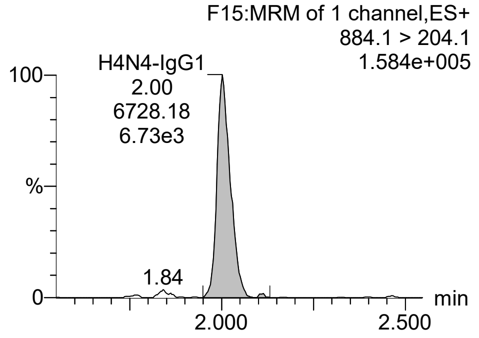

Sample Name: 20211111_A40_R1_IgG

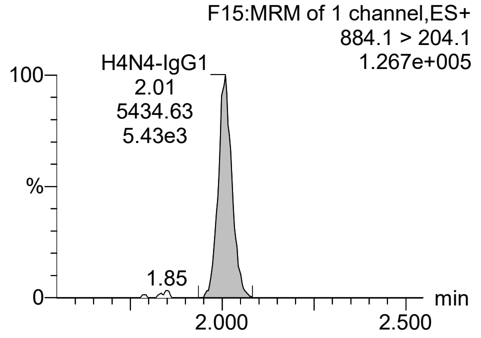

Sample Name: 20211111_H20_R1_IgG

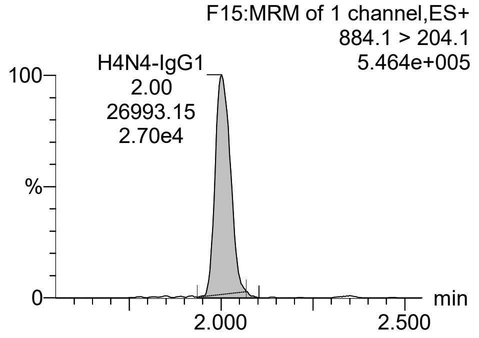

Sample Name: 20211111_A41_R1_IgG

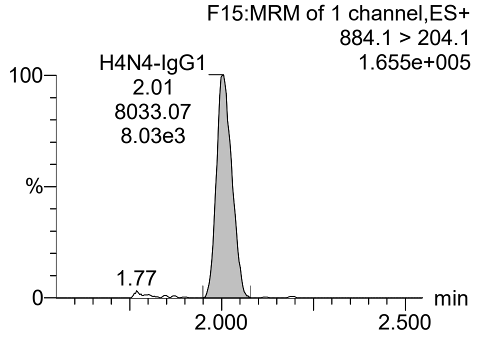

Sample Name: 20211111_A42_R1_IgG

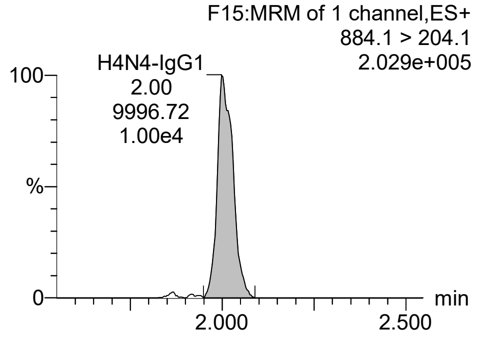

Dataset: C:\MassLynx\10.pro\20211115_clinicalsamples_IgG.qld

Last Altered: Sunday, October 02, 2022 16:02:30 台北標準時間
Printed: Friday, August 01, 2025 16:43:22 台北標準時間

Sample Name: 20211111_A43_R1_IgG

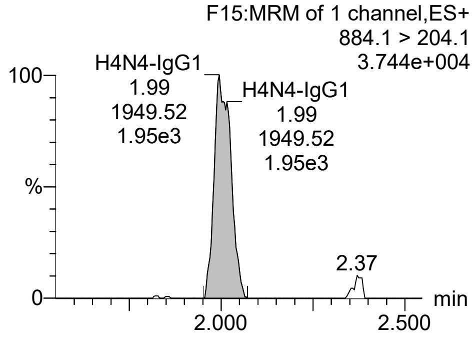

Sample Name: 20211111_A44_R1_IgG

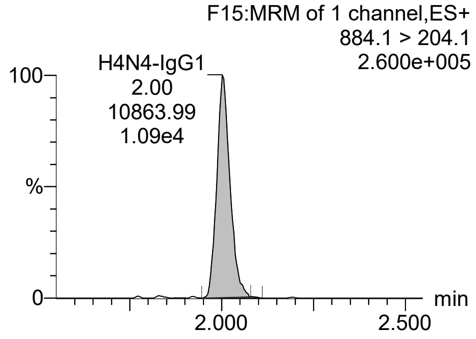

Sample Name: 20211111_QC_R9_IgG

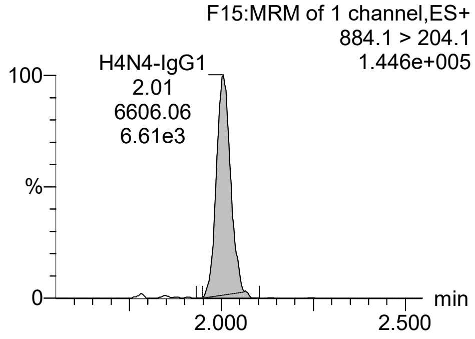

Sample Name: 20211111_A1_R2_IgG

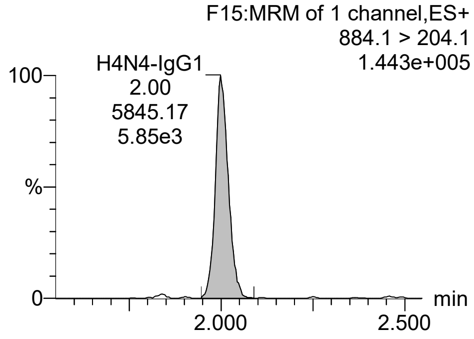

Sample Name: 20211111_A2_R2_IgG

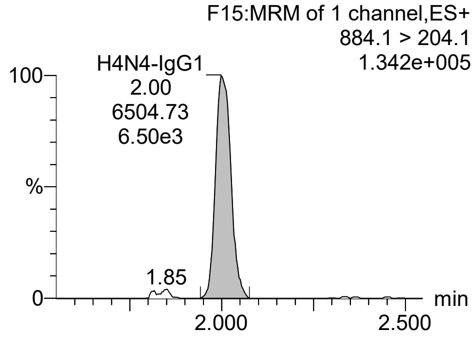

Sample Name: 20211111_L1_R2_IgG

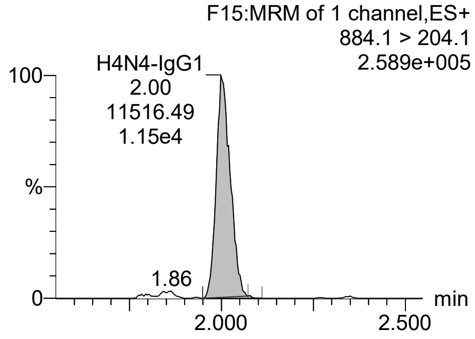

Sample Name: 20211111_L2_R2_IgG

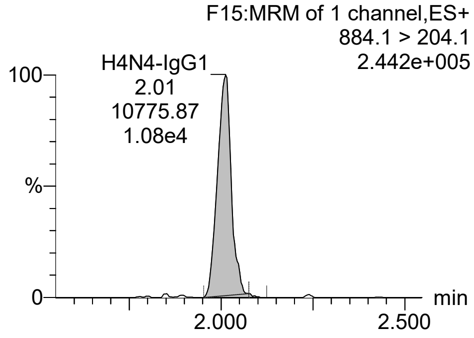

Sample Name: 20211111_H1_R2_IgG

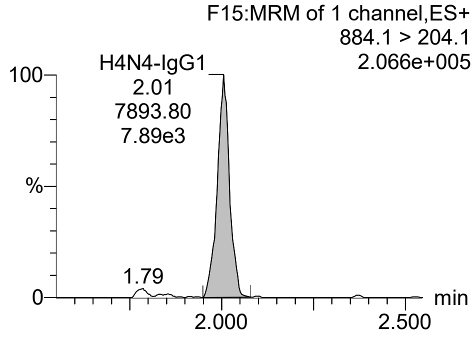

Sample Name: 20211111_A3_R2_IgG

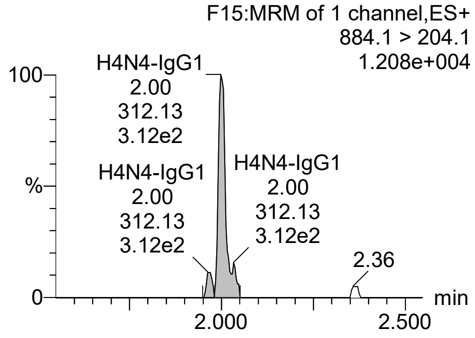

Sample Name: 20211111_A4_R2_IgG

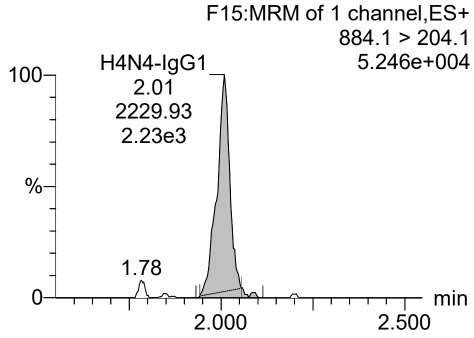

Sample Name: 20211111_L3_R2_IgG

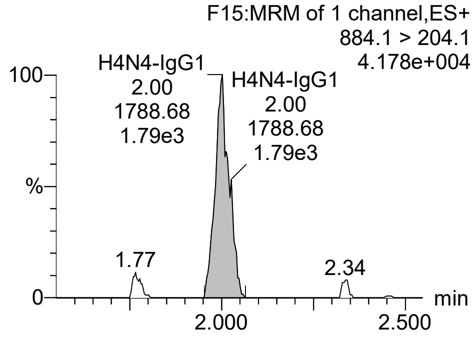

Sample Name: 20211111_L4_R2_IgG

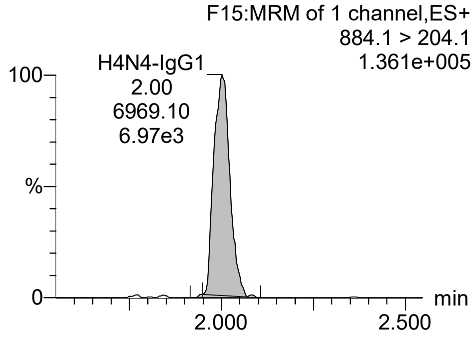

Sample Name: 20211111_H2_R2_IgG

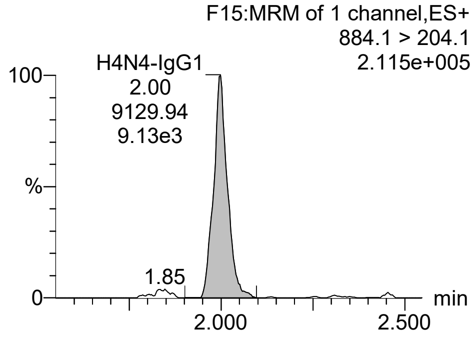

Sample Name: 20211111_A5_R2_IgG

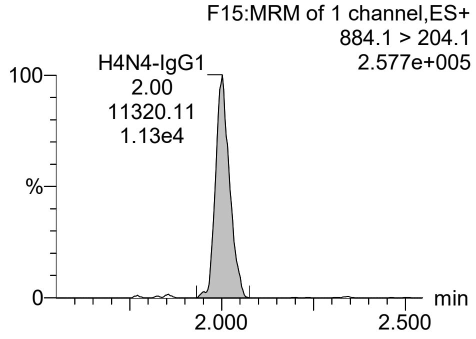

Sample Name: 20211111_A6_R2_IgG

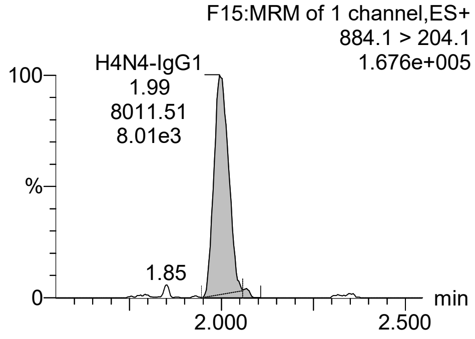

Sample Name: 20211111_QC_R10_IgG

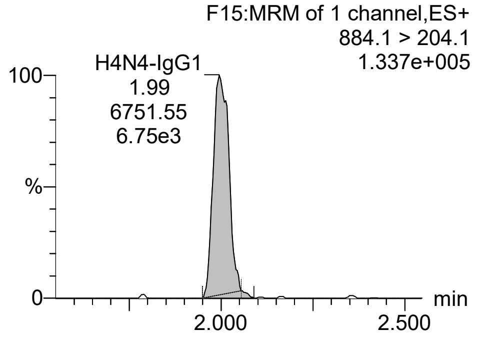

Sample Name: 20211111_L5_R2_IgG

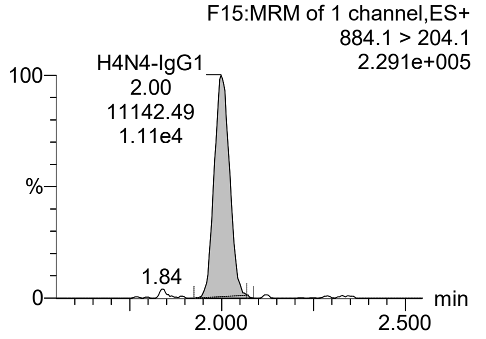

Sample Name: 20211111_L6_R2_IgG

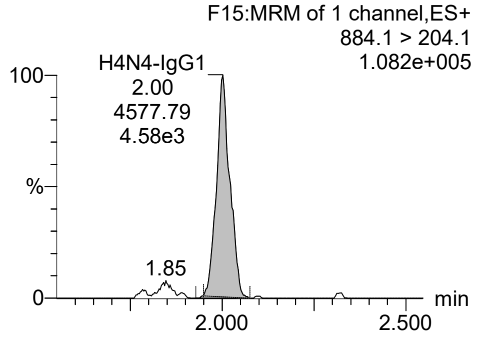

Sample Name: 20211111_H3_R2_IgG

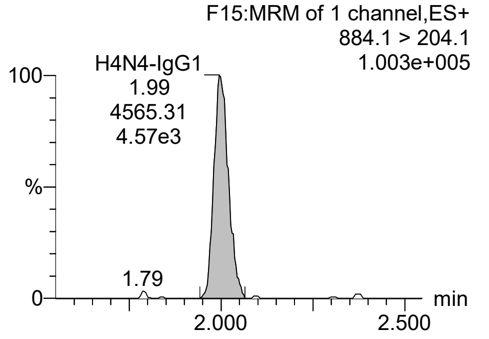

Sample Name: 20211111_A7_R2_IgG

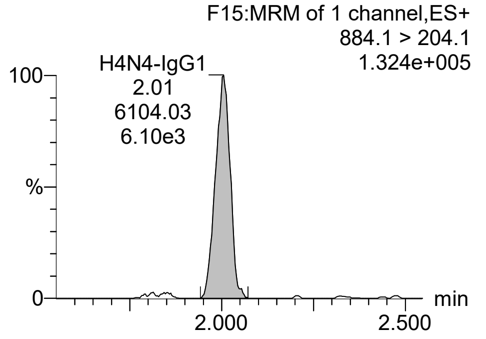

Sample Name: 20211111_A8_R2_IgG

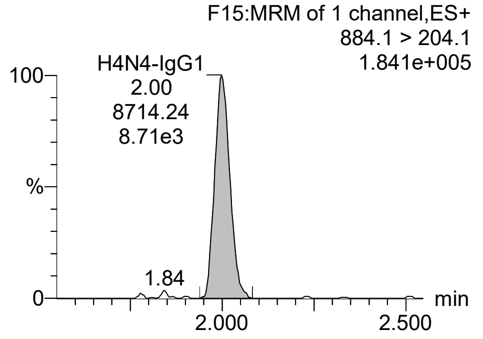

Sample Name: 20211111_L7_R2_IgG

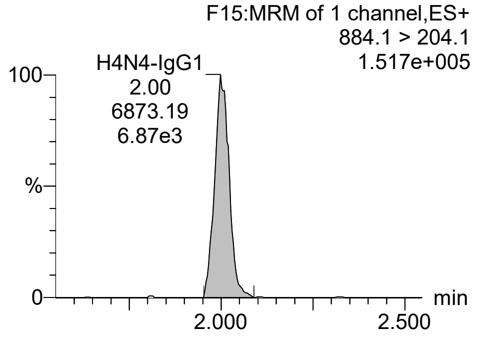

Sample Name: 20211111_L8_R2_IgG

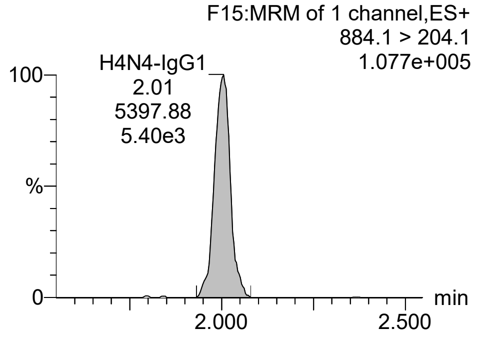

Sample Name: 20211111_H4_R2_IgG

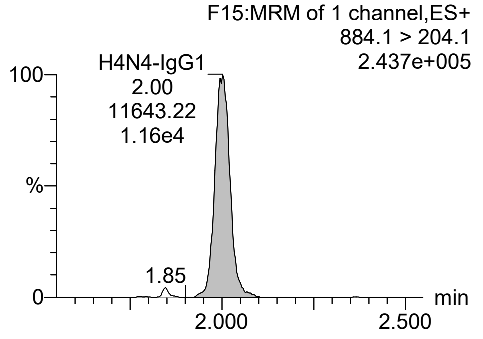

Sample Name: 20211111_A9_R2_IgG

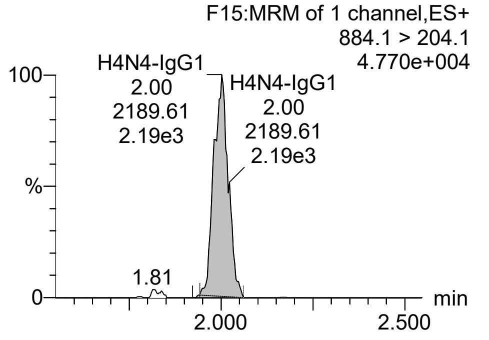

Sample Name: 20211111_A10_R2_IgG

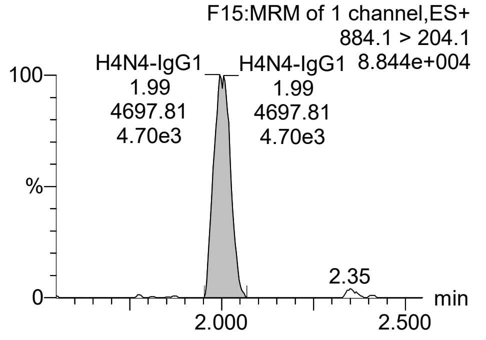

Sample Name: 20211111_L9_R2_IgG

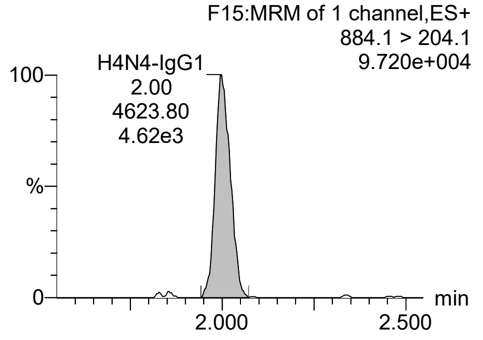

Sample Name: 20211111_L10_R2_IgG

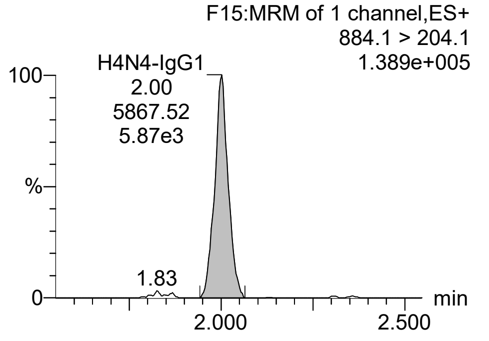

Sample Name: 20211111_QC_R11_IgG

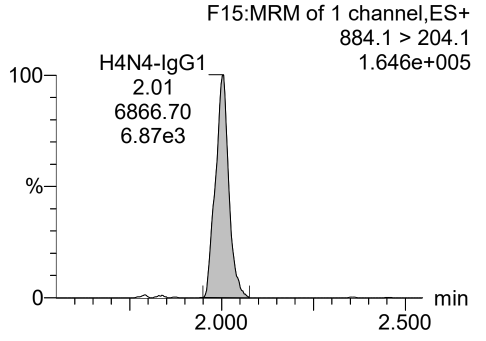

Sample Name: 20211111_H5_R2_IgG

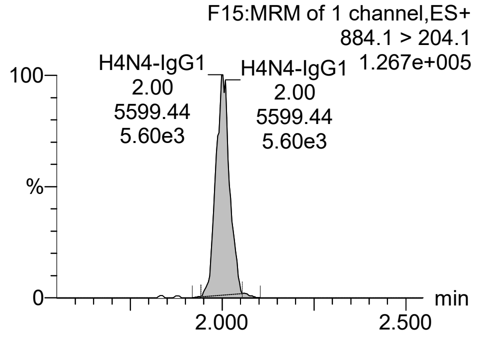

Dataset: C:\MassLynx\10.pro\20211115_clinicalsamples_IgG.qld

Last Altered: Sunday, October 02, 2022 16:02:30 台北標準時間
Printed: Friday, August 01, 2025 16:43:22 台北標準時間

Sample Name: 20211111_A11_R2_IgG

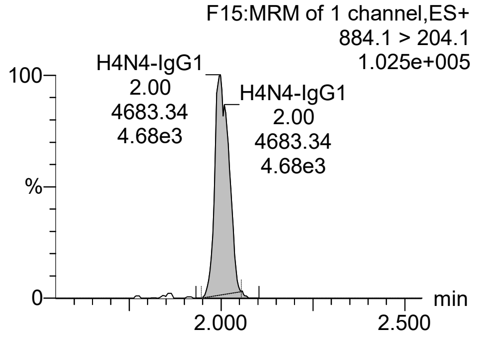

Sample Name: 20211111_A12_R2_IgG

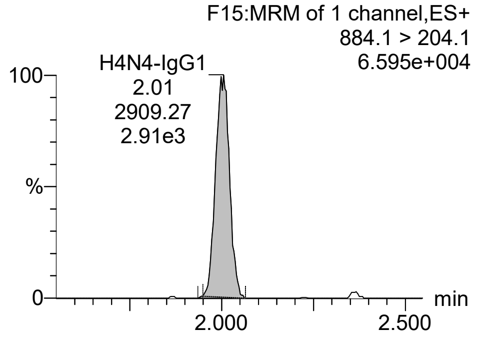

Sample Name: 20211111_L11_R2_IgG

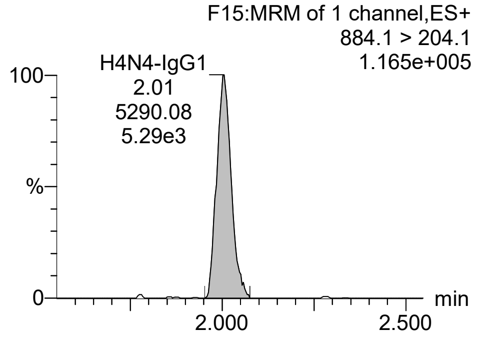

Sample Name: 20211111_L12_R2_IgG

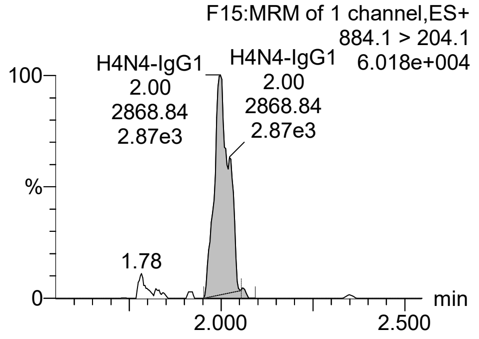

Sample Name: 20211111_H6_R2_IgG

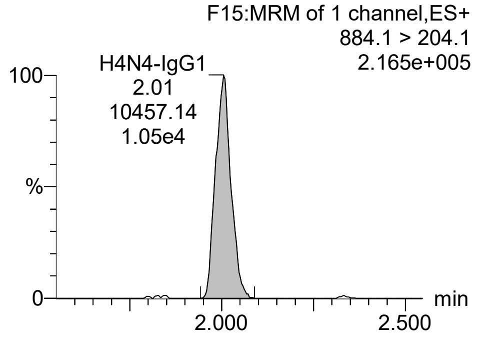

Sample Name: 20211111_A13_R2_IgG

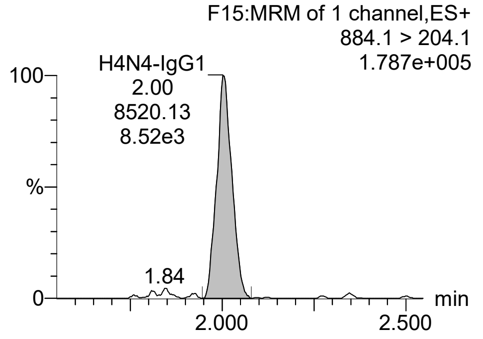

Sample Name: 20211111_A14_R2_IgG

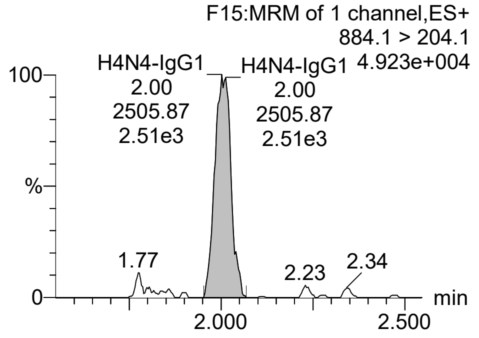

Sample Name: 20211111_L13_R2_IgG

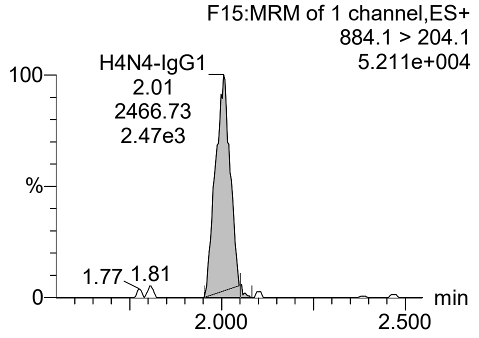

Sample Name: 20211111_L14_R2_IgG

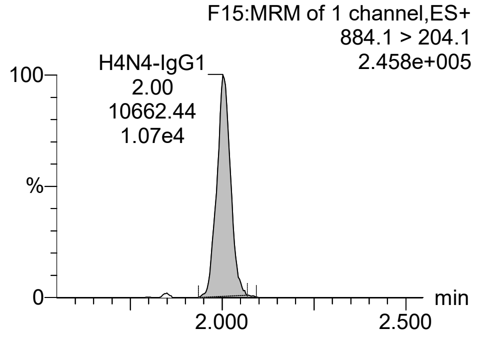

Sample Name: 20211111_H7_R2_IgG

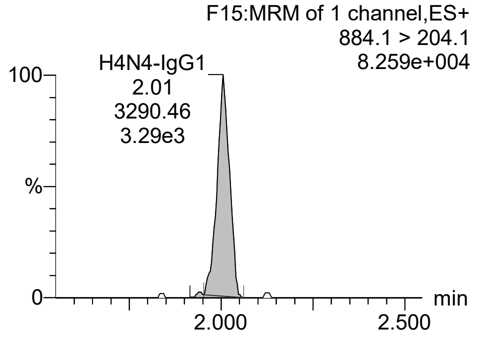

Sample Name: 20211111_A15_R2_IgG

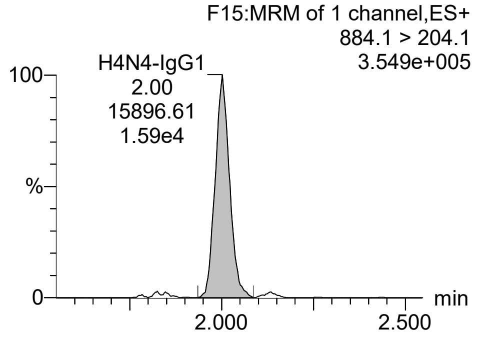

Sample Name: 20211111_QC_R12_IgG

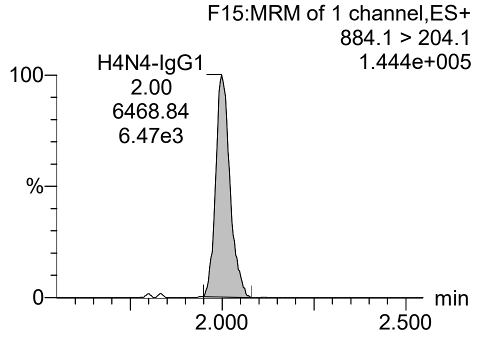

Sample Name: 20211111_A16_R2_IgG

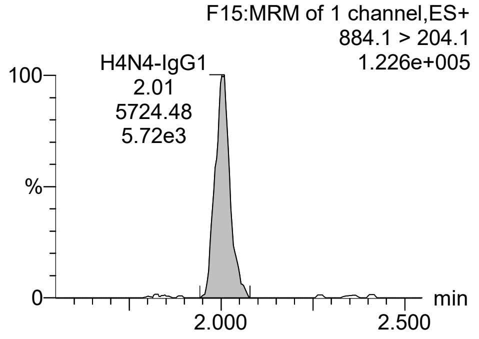

Sample Name: 20211111_L15_R2_IgG

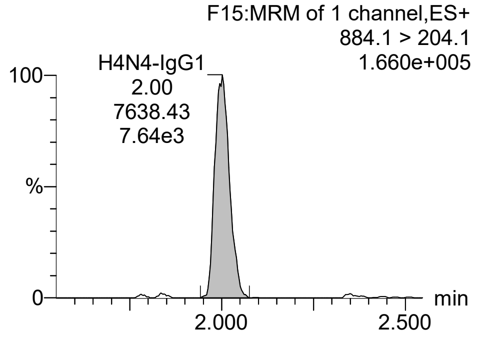

Sample Name: 20211111_L16_R2_IgG

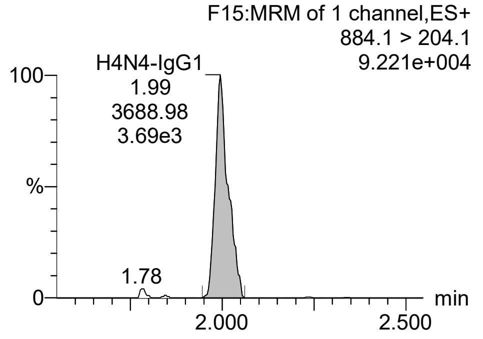

Sample Name: 20211111_H8_R2_IgG

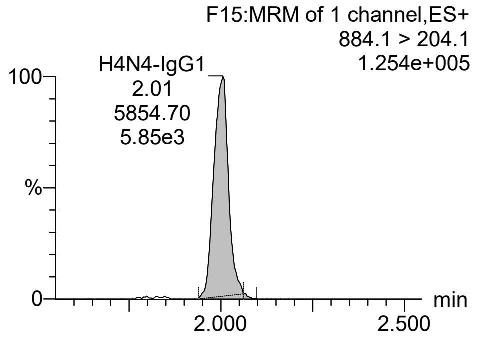

Sample Name: 20211111_A17_R2_IgG

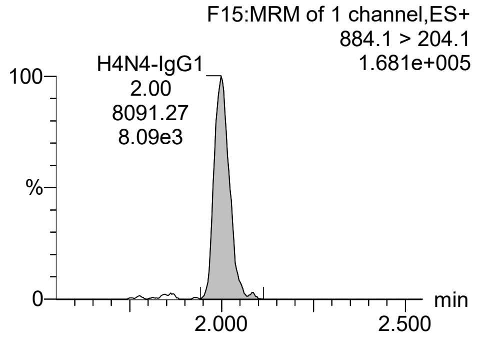

Sample Name: 20211111_A18_R2_IgG

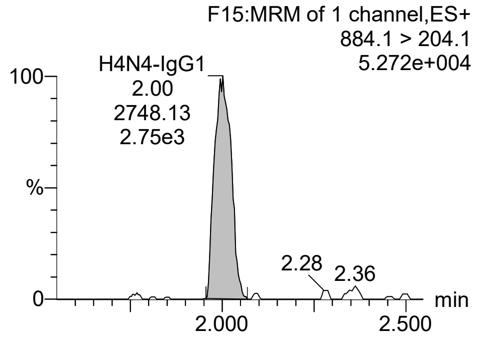

Sample Name: 20211111_L17_R2_IgG

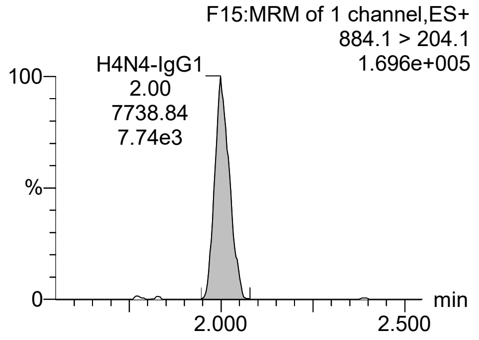

Sample Name: 20211111_L18_R2_IgG

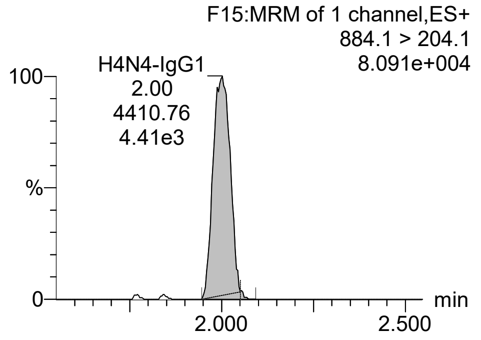

Sample Name: 20211111_H9_R2_IgG

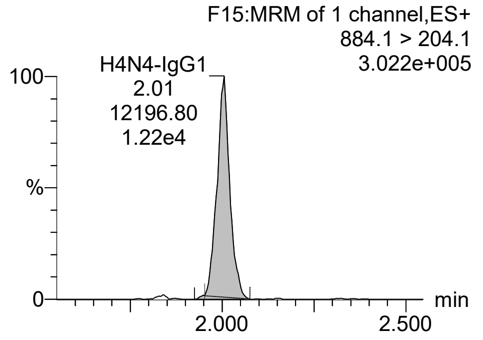

Sample Name: 20211111_A19_R2_IgG

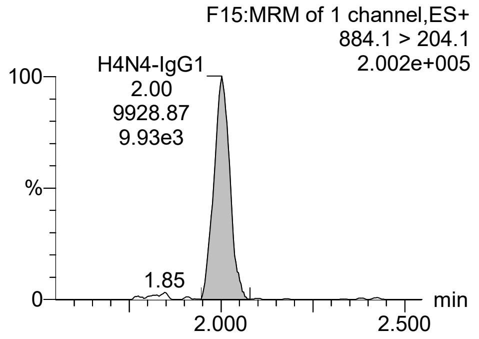

Sample Name: 20211111_A20_R2_IgG

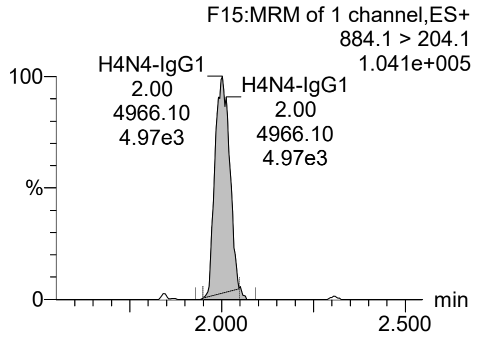

Sample Name: 20211111_L19_R2_IgG

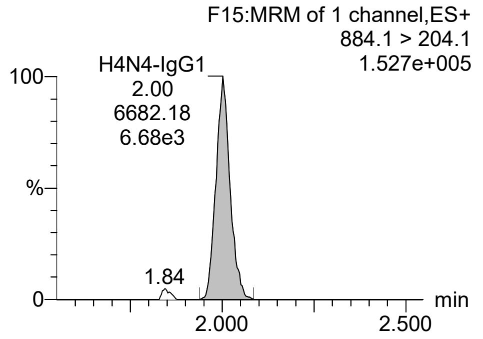

Sample Name: 20211111_QC_R13_IgG

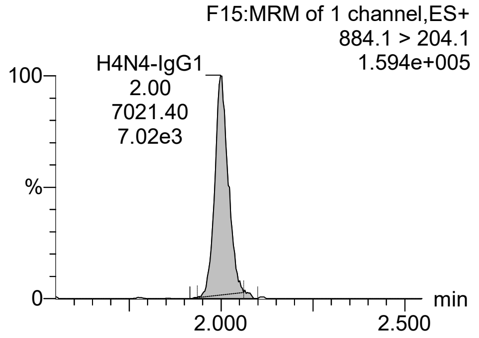

Sample Name: 20211111_L20_R2_IgG

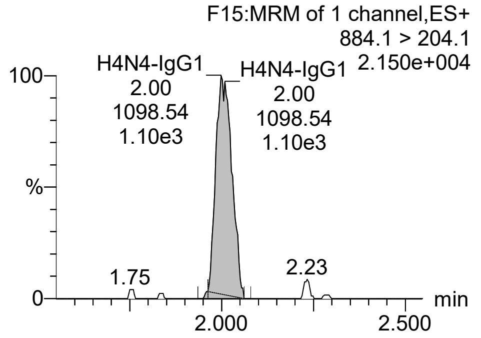

Sample Name: 20211111_H10_R2_IgG

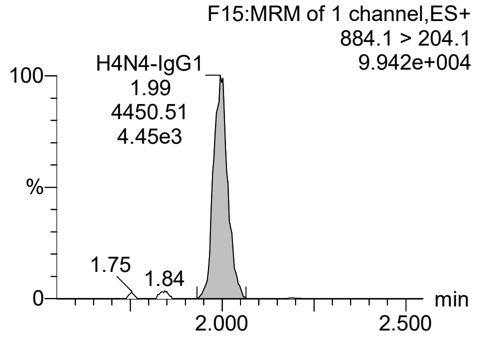

Sample Name: 20211111_A21_R2_IgG

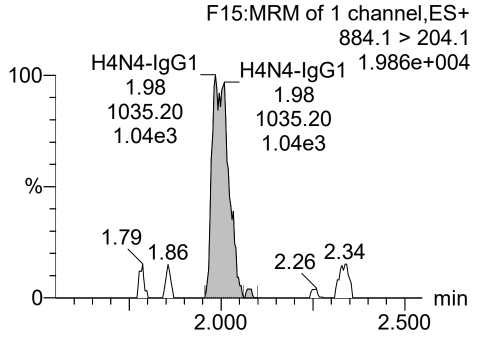

Sample Name: 20211111_A22_R2_IgG

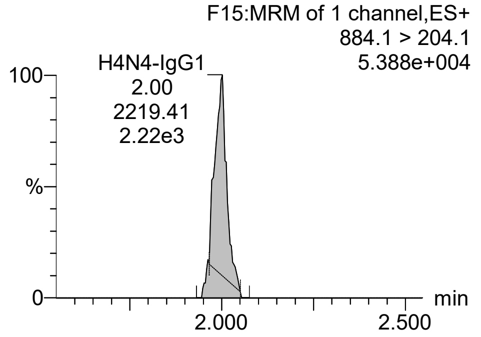

Sample Name: 20211111_L21_R2_IgG

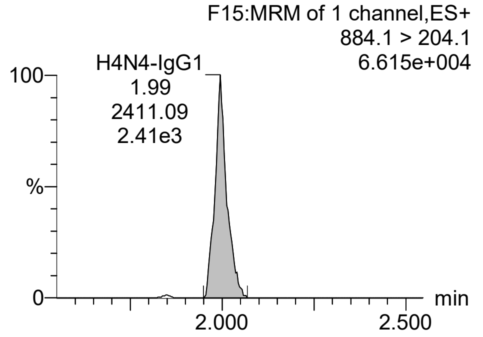

Sample Name: 20211111_L22_R2_IgG

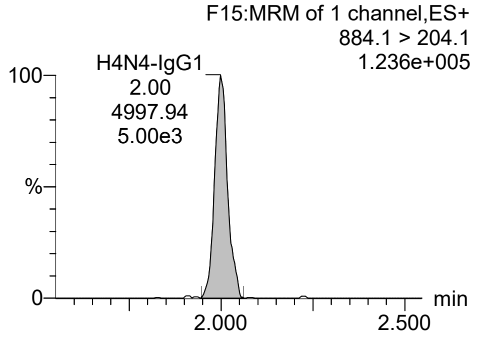

Sample Name: 20211111_H11_R2_IgG

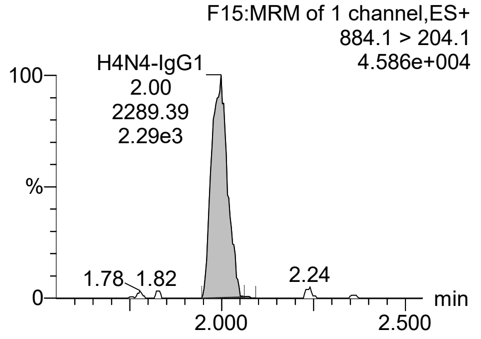

Sample Name: 20211111_A23_R2_IgG

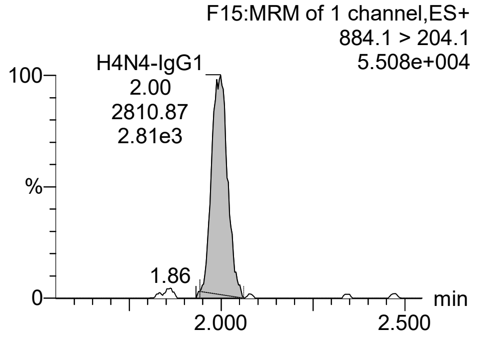

Sample Name: 20211111_A24_R2_IgG

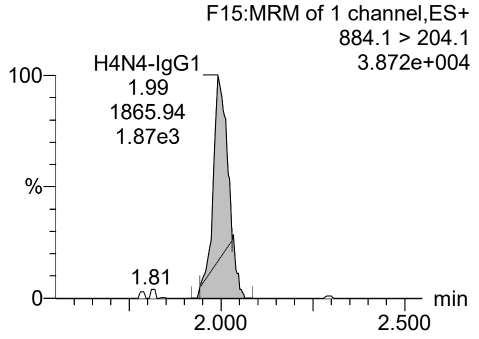

Sample Name: 20211111_L23_R2_IgG

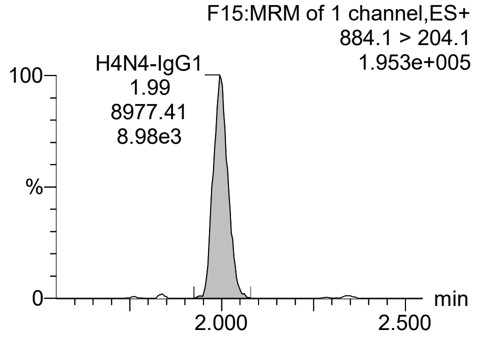

Sample Name: 20211111_L24_R2_IgG

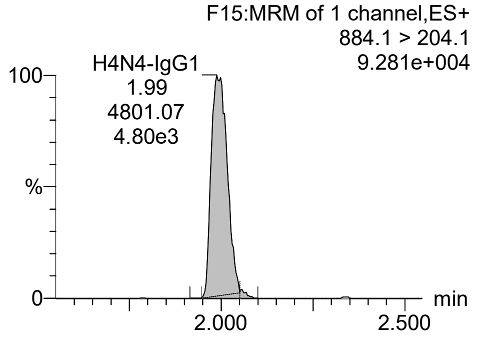

Sample Name: 20211111_H12_R2_IgG

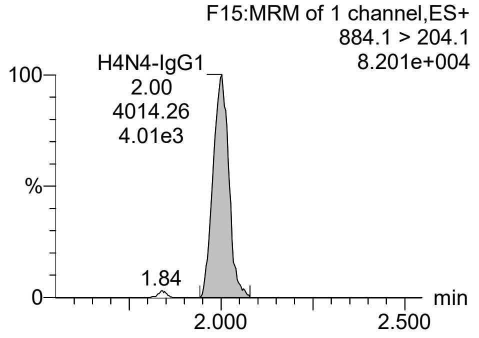

Sample Name: 20211111_QC_R14_IgG

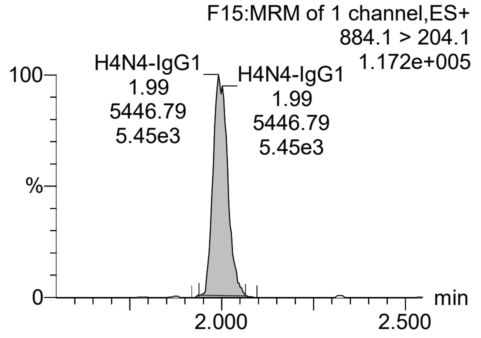

Sample Name: 20211111_A25_R2_IgG

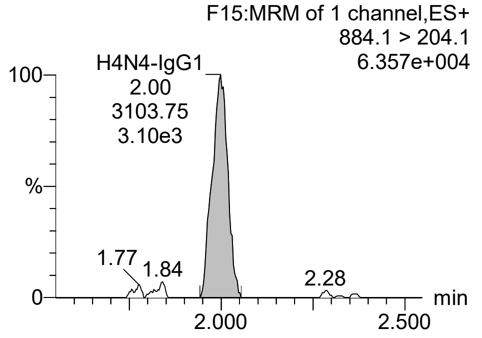

Sample Name: 20211111_A26_R2_IgG

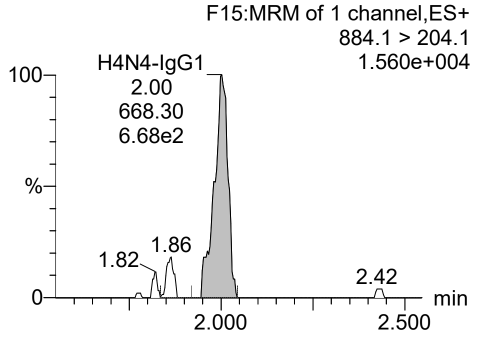

Sample Name: 20211111_L25_R2_IgG

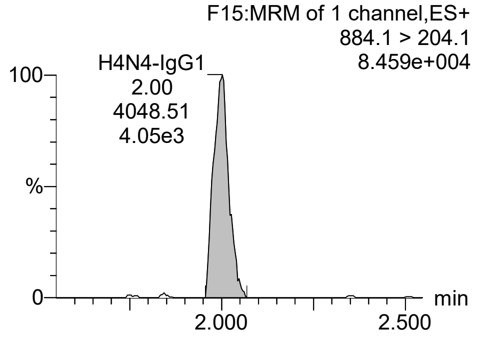

Sample Name: 20211111_L26_R2_IgG

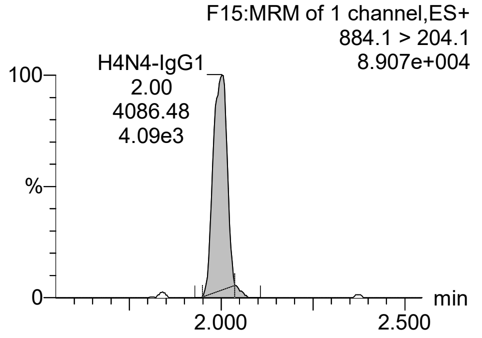

Sample Name: 20211111_H13_R2_IgG

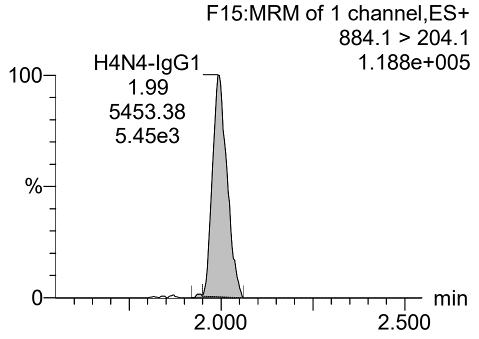

Sample Name: 20211111_A27_R2_IgG

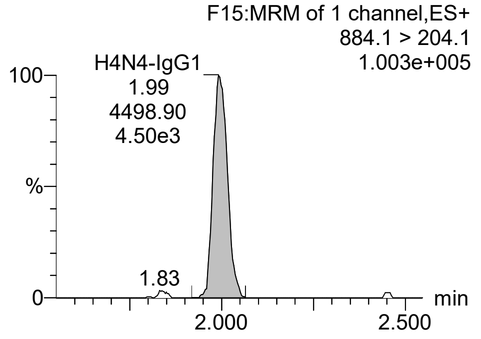

Sample Name: 20211111_A28_R2_IgG

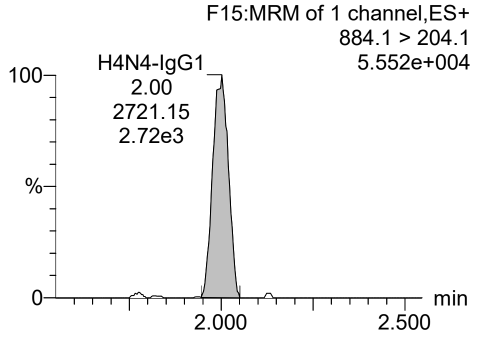

Dataset: C:\MassLynx\10.pro\20211115_clinicalsamples_IgG.qld

Last Altered: Sunday, October 02, 2022 16:02:30 台北標準時間
Printed: Friday, August 01, 2025 16:43:22 台北標準時間

Sample Name: 20211111_L27_R2_IgG

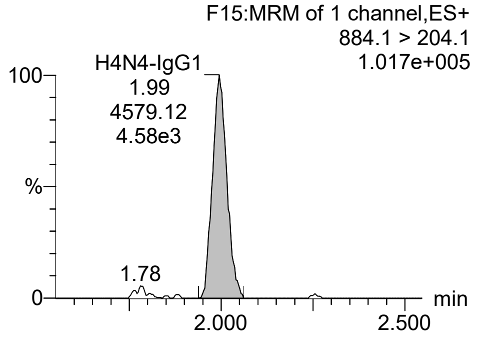

Sample Name: 20211111_L28_R2_IgG

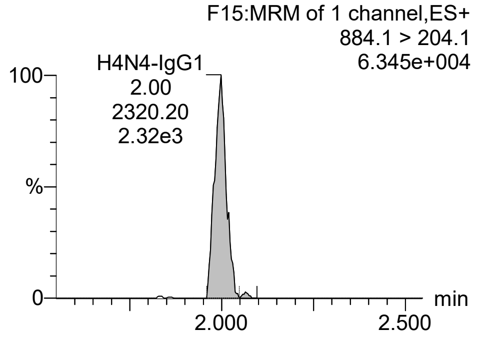

Sample Name: 20211111_H14_R2_IgG

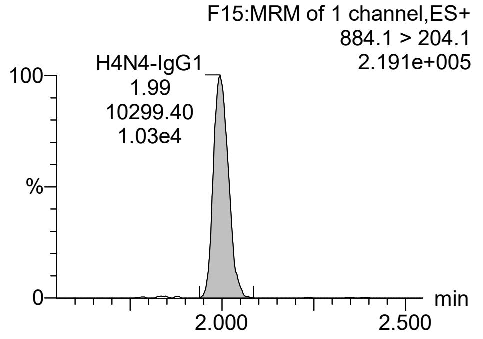

Sample Name: 20211111_A29_R2_IgG

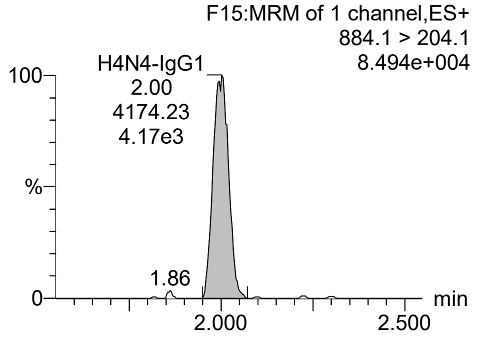

Sample Name: 20211111_A30_R2_IgG

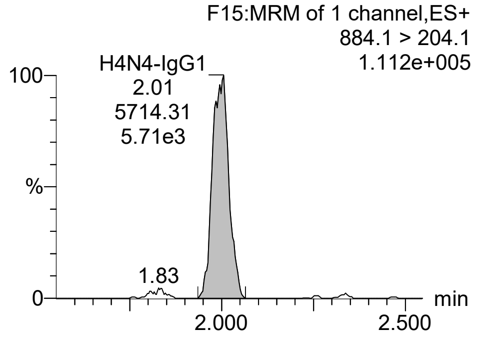

Sample Name: 20211111_QC_R15_IgG

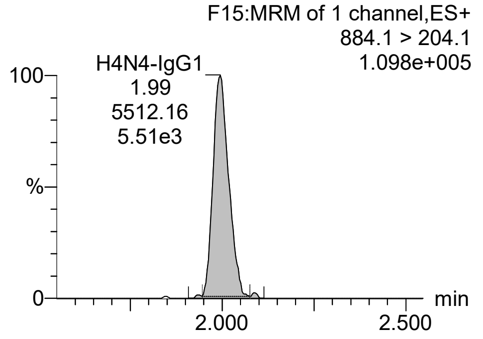

Sample Name: 20211111_L29_R2_IgG

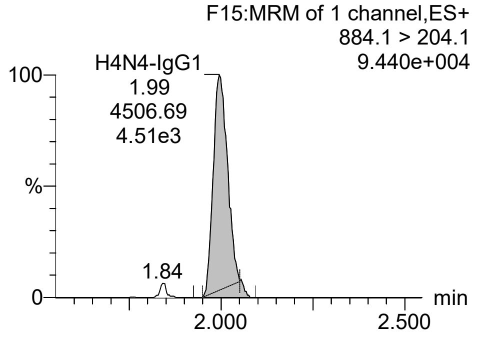

Sample Name: 20211111_L30_R2_IgG

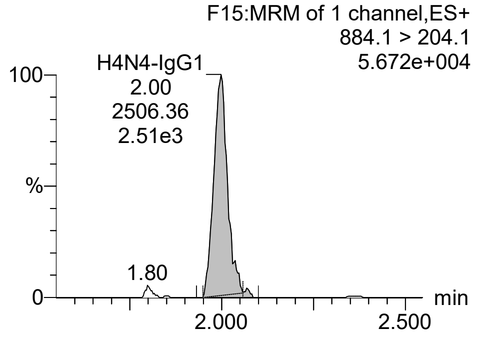

Sample Name: 20211111_H15_R2_IgG

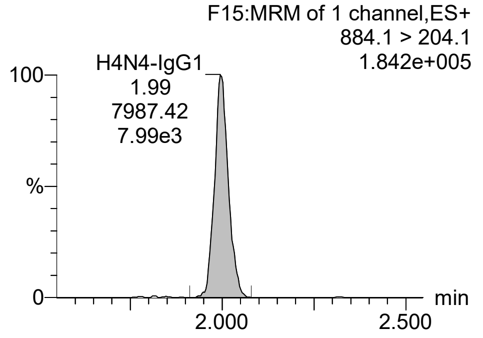

Sample Name: 20211111_A31_R2_IgG

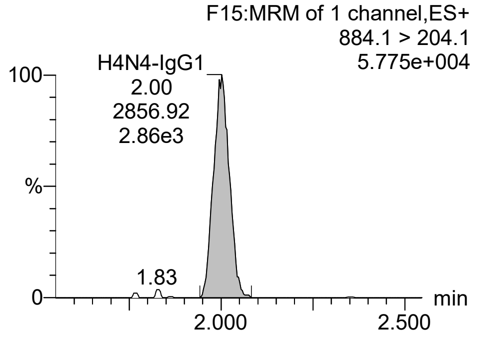

Sample Name: 20211111_A32_R2_IgG

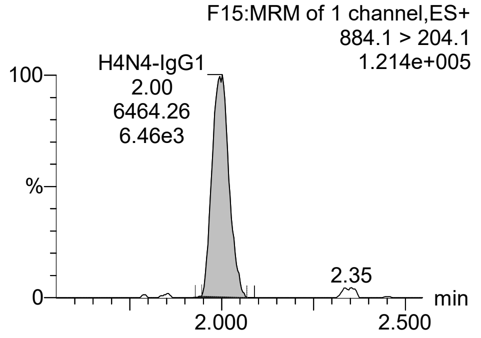

Sample Name: 20211111_L31_R2_IgG

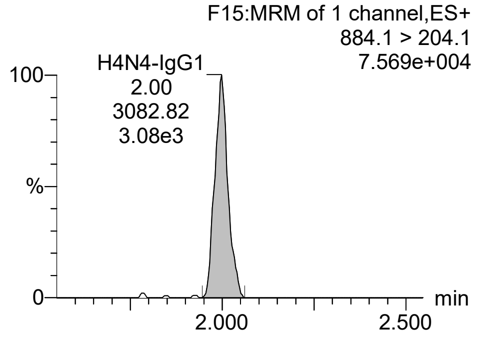

Sample Name: 20211111_L32_R2_IgG

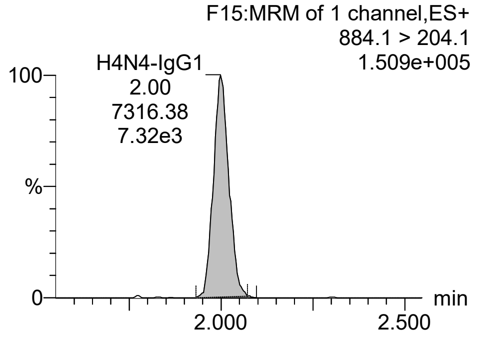

Sample Name: 20211111_H16_R2_IgG

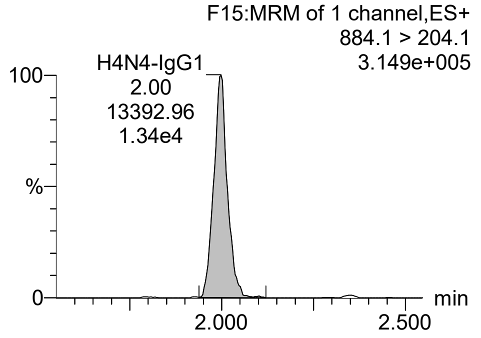

Sample Name: 20211111_A33_R2_IgG

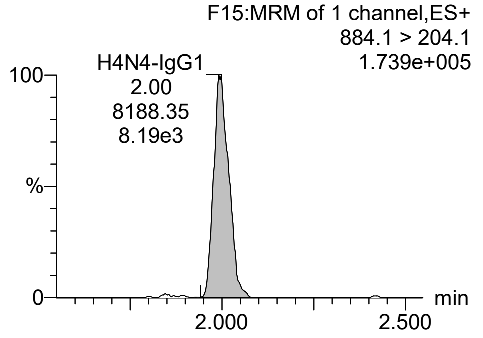

Dataset: C:\MassLynx\10.pro\20211115_clinicalsamples_IgG.qld

Last Altered: Sunday, October 02, 2022 16:02:30 台北標準時間
Printed: Friday, August 01, 2025 16:43:22 台北標準時間

Sample Name: 20211111_A34_R2_IgG

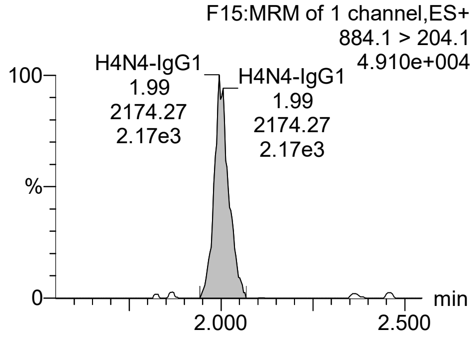

Sample Name: 20211111_H17_R2_IgG

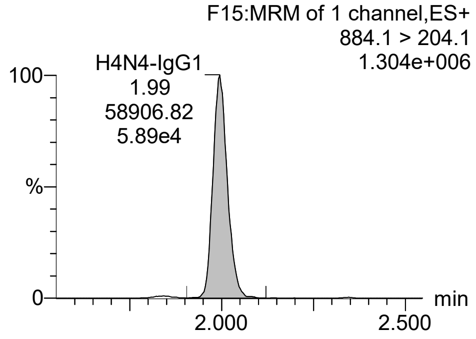

Sample Name: 20211111_A35_R2_IgG

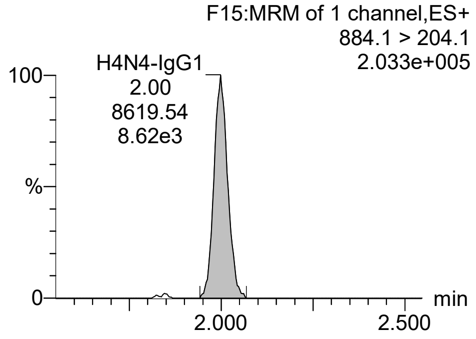

Sample Name: 20211111_QC_R16_IgG

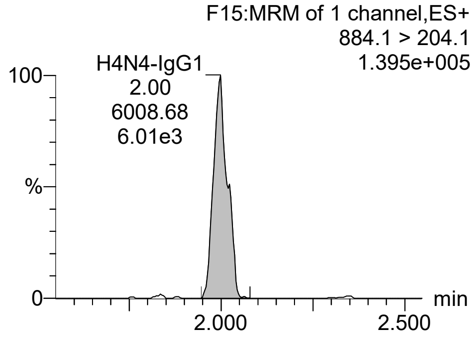

Sample Name: 20211111_A36_R2_IgG

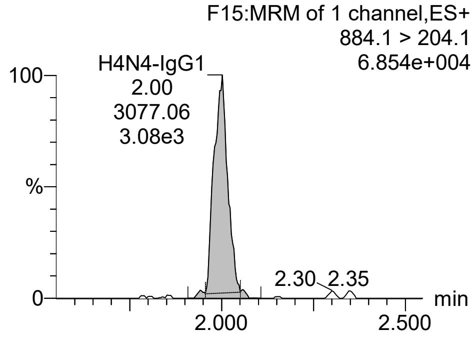

Sample Name: 20211111_H18_R2_IgG

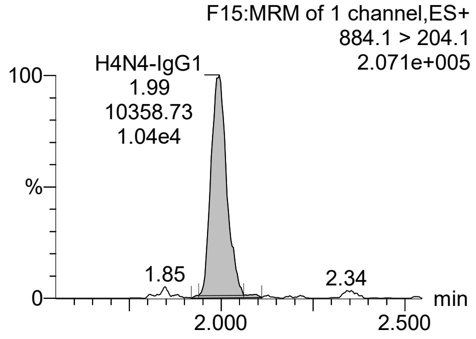

Sample Name: 20211111_A37_R2_IgG

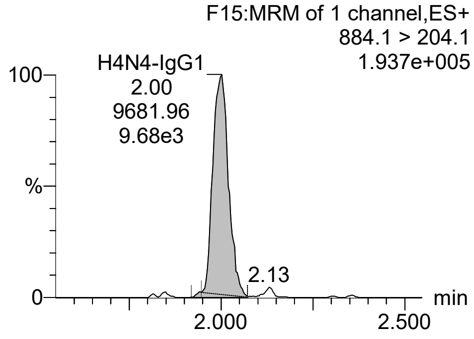

Sample Name: 20211111_A38_R2_IgG

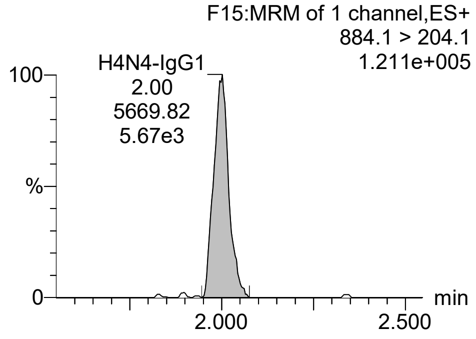

Sample Name: 20211111_H19_R2_IgG

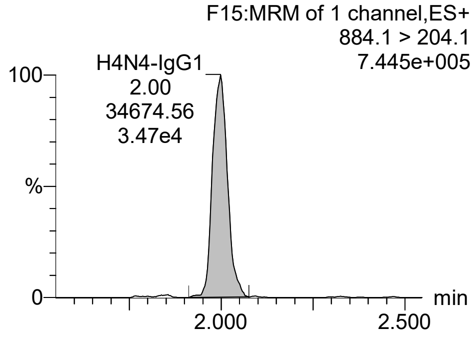

Sample Name: 20211111_A39_R2_IgG

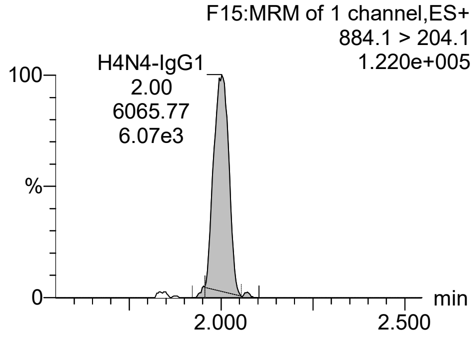

Sample Name: 20211111_A40_R2_IgG

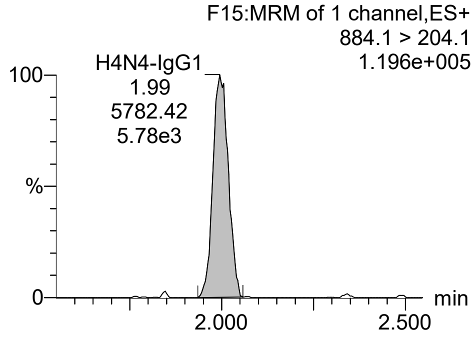

Sample Name: 20211111_H20_R2_IgG

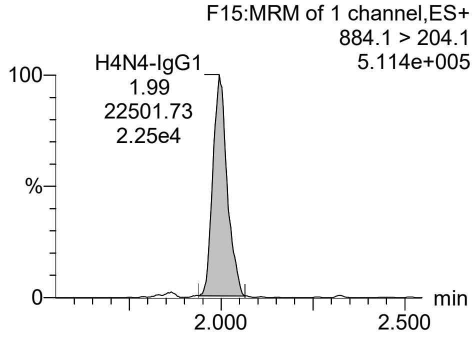

Sample Name: 20211111_A41_R2_IgG

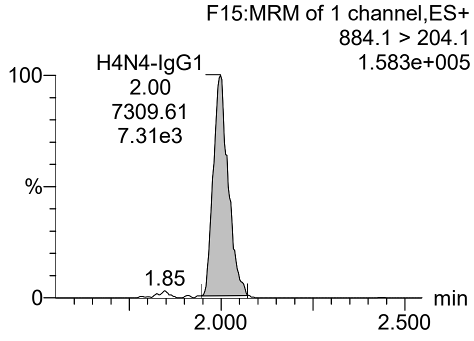

Sample Name: 20211111_A42_R2_IgG

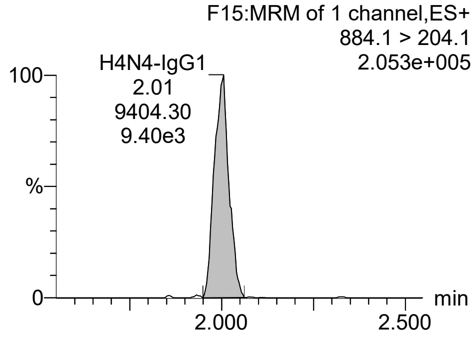

Sample Name: 20211111_A43_R2_IgG

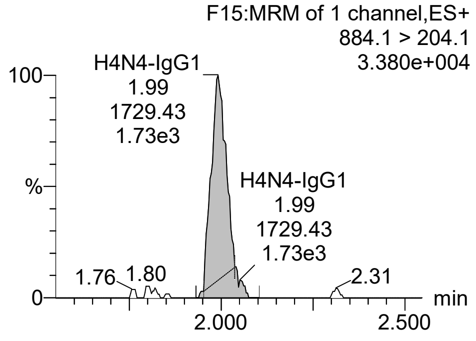

Dataset: C:\MassLynx\10.pro\20211115_clinicalsamples_IgG.qld

Last Altered: Sunday, October 02, 2022 16:02:30 台北標準時間
Printed: Friday, August 01, 2025 16:43:22 台北標準時間

Sample Name: 20211111_A44_R2_IgG

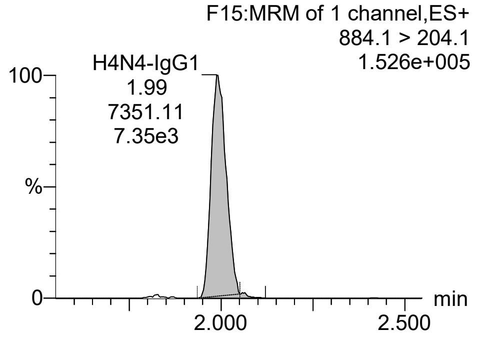

Sample Name: 20211111_QC_R17_IgG

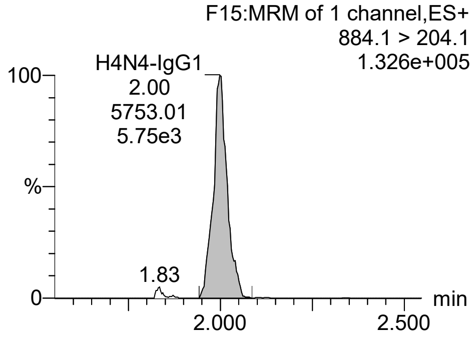

Dataset: C:\MassLynx\10.pro\20211115_clinicalsamples_IgG.qld

Last Altered: Sunday, October 02, 2022 16:02:30 台北標準時間
Printed: Friday, August 01, 2025 16:43:22 台北標準時間

	#	Name	RT	Area	IS Area	Response	Prima...
1	1	20211111_QC_R1_IgG	2.00	12409.6	64968.0	0.191	bb
2	2	20211111_A1_R1_IgG	2.00	7014.4	59059.2	0.119	MM
3	3	20211111_A2_R1_IgG	2.01	7522.8	66621.2	0.113	MM
4	4	20211111_L1_R1_IgG	2.01	16477.3	64201.0	0.257	MM
5	5	20211111_L2_R1_IgG	2.00	13249.0	46950.2	0.282	bb
6	6	20211111_H1_R1_IgG	2.00	7250.1	38648.2	0.188	bb
7	7	20211111_A3_R1_IgG	2.01	169.4	2603.7	0.065	MM
8	8	20211111_A4_R1_IgG	2.00	3874.3	69960.4	0.055	bb
9	9	20211111_L3_R1_IgG	2.01	1606.4	38864.7	0.041	MM
10	10	20211111_L4_R1_IgG	2.01	8601.7	43380.5	0.198	bb
11	11	20211111_H2_R1_IgG	2.01	12815.1	71349.2	0.180	MM
12	12	20211111_A5_R1_IgG	2.01	13136.7	65569.7	0.200	bb
13	13	20211111_A6_R1_IgG	2.01	10363.1	53120.7	0.195	bb
14	14	20211111_QC_R2_IgG	2.01	13379.0	81034.1	0.165	MM
15	15	20211111_L5_R1_IgG	2.01	14288.2	50994.6	0.280	MM
16	16	20211111_L6_R1_IgG	2.00	5631.1	58058.0	0.097	bb
17	17	20211111_H3_R1_IgG	2.01	6027.5	64028.6	0.094	bb
18	18	20211111_A7_R1_IgG	2.00	8414.9	82996.1	0.101	bb
19	19	20211111_A8_R1_IgG	2.01	11492.0	77731.7	0.148	bb
20	20	20211111_L7_R1_IgG	2.01	7709.3	38827.3	0.199	bb
21	21	20211111_L8_R1_IgG	2.01	7075.1	44220.1	0.160	bb
22	22	20211111_H4_R1_IgG	2.01	15729.6	65826.3	0.239	bb
23	23	20211111_A9_R1_IgG	2.00	2726.4	60771.1	0.045	bb
24	24	20211111_A10_R1_IgG	2.01	5735.3	48578.7	0.118	bb
25	25	20211111_L9_R1_IgG	2.00	5356.4	50830.7	0.105	MM
26	26	20211111_L10_R1_IgG	2.00	7029.9	38034.2	0.185	bb
27	27	20211111_QC_R3_IgG	2.01	13157.4	67025.5	0.196	MM
28	28	20211111_H5_R1_IgG	2.01	6169.2	44544.9	0.138	bb
29	29	20211111_A11_R1_IgG	2.01	6710.9	66408.5	0.101	MM
30	30	20211111_A12_R1_IgG	2.01	3287.9	46624.5	0.071	MM
31	31	20211111_L11_R1_IgG	2.00	5964.9	33425.1	0.178	MM
32	32	20211111_L12_R1_IgG	2.00	3432.0	41178.0	0.083	bb
33	33	20211111_H6_R1_IgG	2.00	18237.6	58593.4	0.311	bb
34	34	20211111_A13_R1_IgG	2.00	12208.6	54133.0	0.226	MM
35	35	20211111_A14_R1_IgG	2.01	3874.8	56467.3	0.069	MM
36	36	20211111_L13_R1_IgG	2.00	4117.3	36024.4	0.114	MM
37	37	20211111_L14_R1_IgG	2.00	11314.1	37181.6	0.304	MM
38	38	20211111_H7_R1_IgG	2.01	2888.8	42850.6	0.067	bb
39	39	20211111_A15_R1_IgG	2.01	18549.7	62015.1	0.299	bb
40	40	20211111_QC_R4_IgG	2.00	10522.2	58572.5	0.180	bb
41	41	20211111_A16_R1_IgG	2.00	10480.4	78283.8	0.134	MM
42	42	20211111_L15_R1_IgG	2.01	9183.6	44158.2	0.208	bb
43	43	20211111_L16_R1_IgG	2.01	4911.2	55150.8	0.089	MM
44	44	20211111_H8_R1_IgG	2.00	6283.8	42965.2	0.146	MM
45	45	20211111_A17_R1_IgG	2.01	6832.4	39717.7	0.172	bb
46	46	20211111_A18_R1_IgG	1.99	3428.6	44675.4	0.077	bb
47	47	20211111_L17_R1_IgG	2.00	7809.0	37854.1	0.206	bb
48	48	20211111_L18_R1_IgG	2.00	5315.1	50716.9	0.105	MM
49	49	20211111_H9_R1_IgG	2.01	13647.6	36276.0	0.376	bb
50	50	20211111_A19_R1_IgG	2.00	11527.8	42862.5	0.269	bb
51	51	20211111_A20_R1_IgG	2.01	5454.0	36542.7	0.149	MM
52	52	20211111_L19_R1_IgG	2.00	9681.7	53892.8	0.180	MM
53	53	20211111_QC_R5_IgG	1.99	10075.2	56513.2	0.178	MM
54	54	20211111_L20_R1_IgG	2.01	3068.9	56499.9	0.054	MM
55	55	20211111_H10_R1_IgG	2.00	12638.4	68120.5	0.186	bb
56	56	20211111_A21_R1_IgG	2.00	3129.7	63699.8	0.049	MM

Dataset: C:\MassLynx\10.pro\20211115_clinicalsamples_IgG.qld

Last Altered: Sunday, October 02, 2022 16:02:30 台北標準時間
Printed: Friday, August 01, 2025 16:43:22 台北標準時間

	# Name	RT	Area	IS Area	Response	Prima...
57	57 20211111_A22_R1_IgG	2.00	4433.8	52222.1	0.085	bb
58	58 20211111_L21_R1_IgG	2.00	4691.8	54372.5	0.086	bb
59	59 20211111_L22_R1_IgG	2.01	8503.2	41353.1	0.206	bb
60	60 20211111_H11_R1_IgG	2.01	5670.7	65579.1	0.086	bb
61	61 20211111_A23_R1_IgG	2.00	5370.3	64131.1	0.084	bb
62	62 20211111_A24_R1_IgG	2.00	3024.7	38724.8	0.078	bb
63	63 20211111_L23_R1_IgG	2.00	13698.2	64245.2	0.213	bb
64	64 20211111_L24_R1_IgG	2.01	6875.6	45298.0	0.152	bb
65	65 20211111_H12_R1_IgG	2.01	5878.1	44679.3	0.132	MM
66	66 20211111_QC_R6_IgG	2.00	8920.8	48165.0	0.185	bb
67	67 20211111_A25_R1_IgG	1.99	4937.0	57169.9	0.086	bb
68	68 20211111_A26_R1_IgG	1.99	837.1	34254.0	0.024	bb
69	69 20211111_L25_R1_IgG	1.99	6279.1	67491.5	0.093	MM
70	70 20211111_L26_R1_IgG	2.00	7149.2	55934.1	0.128	MM
71	71 20211111_H13_R1_IgG	2.00	7593.5	48767.3	0.156	bb
72	72 20211111_A27_R1_IgG	2.00	8059.7	57269.7	0.141	MM
73	73 20211111_A28_R1_IgG	2.00	4538.3	49231.1	0.092	MM
74	74 20211111_L27_R1_IgG	1.99	8098.0	60014.5	0.135	MM
75	75 20211111_L28_R1_IgG	2.00	3141.5	35062.6	0.090	MM
76	76 20211111_H14_R1_IgG	2.00	14116.0	62371.9	0.226	bb
77	77 20211111_A29_R1_IgG	2.00	5460.3	43858.2	0.124	MM
78	78 20211111_A30_R1_IgG	2.00	8821.2	62500.4	0.141	bb
79	79 20211111_QC_R7_IgG	2.00	7745.2	43946.9	0.176	bb
80	80 20211111_L29_R1_IgG	2.01	4742.8	38728.9	0.122	MM
81	81 20211111_L30_R1_IgG	2.00	2415.1	42613.2	0.057	bb
82	82 20211111_H15_R1_IgG	2.00	8118.8	39874.6	0.204	bb
83	83 20211111_A31_R1_IgG	2.00	3907.4	53414.3	0.073	MM
84	84 20211111_A32_R1_IgG	2.00	6704.1	41844.6	0.160	bb
85	85 20211111_L31_R1_IgG	2.01	4204.2	40553.7	0.104	bb
86	86 20211111_L32_R1_IgG	2.01	8167.5	49618.7	0.165	bb
87	87 20211111_H16_R1_IgG	2.00	18625.3	60170.9	0.310	bb
88	88 20211111_A33_R1_IgG	2.01	9822.9	48588.2	0.202	bb
89	89 20211111_A34_R1_IgG	2.01	2597.1	43288.0	0.060	MM
90	90 20211111_H17_R1_IgG	2.00	66840.6	47104.5	1.419	MM
91	91 20211111_A35_R1_IgG	2.00	9536.7	41025.9	0.232	bb
92	92 20211111_QC_R8_IgG	2.00	7665.5	40546.3	0.189	MM
93	93 20211111_A36_R1_IgG	2.01	3607.0	44504.4	0.081	bb
94	94 20211111_H18_R1_IgG	2.01	11332.7	34162.2	0.332	MM
95	95 20211111_A37_R1_IgG	2.00	9735.0	39760.0	0.245	bb
96	96 20211111_A38_R1_IgG	2.01	7105.0	43982.6	0.162	MM
97	97 20211111_H19_R1_IgG	2.01	40390.8	45314.8	0.891	bb
98	98 20211111_A39_R1_IgG	2.00	6728.2	39388.0	0.171	bb
99	99 20211111_A40_R1_IgG	2.01	5434.6	38726.4	0.140	bb
100	1... 20211111_H20_R1_IgG	2.00	26993.2	37814.4	0.714	MM
101	1... 20211111_A41_R1_IgG	2.01	8033.1	48930.3	0.164	bb
102	1... 20211111_A42_R1_IgG	2.00	9996.7	40406.8	0.247	bb
103	1... 20211111_A43_R1_IgG	1.99	1949.5	44421.5	0.044	bb
104	1... 20211111_A44_R1_IgG	2.00	10864.0	53128.2	0.204	MM
105	1... 20211111_QC_R9_IgG	2.01	6606.1	37049.0	0.178	MM
106	1... 20211111_A1_R2_IgG	2.00	5845.2	46499.4	0.126	bb
107	1... 20211111_A2_R2_IgG	2.00	6504.7	57260.7	0.114	bb
108	1... 20211111_L1_R2_IgG	2.00	11516.5	48062.9	0.240	MM
109	1... 20211111_L2_R2_IgG	2.01	10775.9	38745.2	0.278	MM
110	1... 20211111_H1_R2_IgG	2.01	7893.8	44273.5	0.178	bb
111	1... 20211111_A3_R2_IgG	2.00	312.1	4755.5	0.066	MM
112	1... 20211111_A4_R2_IgG	2.01	2229.9	50546.7	0.044	MM

Dataset: C:\MassLynx\10.pro\20211115_clinicalsamples_IgG.qld

Last Altered: Sunday, October 02, 2022 16:02:30 台北標準時間
Printed: Friday, August 01, 2025 16:43:22 台北標準時間

	# Name	RT	Area	IS Area	Response	Prima...
113	1... 20211111_L3_R2_IgG	2.00	1788.7	34825.8	0.051	bb
114	1... 20211111_L4_R2_IgG	2.00	6969.1	39107.7	0.178	MM
115	1... 20211111_H2_R2_IgG	2.00	9129.9	49750.0	0.184	bb
116	1... 20211111_A5_R2_IgG	2.00	11320.1	52536.2	0.215	bb
117	1... 20211111_A6_R2_IgG	1.99	8011.5	42050.0	0.191	MM
118	1... 20211111_QC_R10_IgG	1.99	6751.5	35350.8	0.191	MM
119	1... 20211111_L5_R2_IgG	2.00	11142.5	38039.3	0.293	MM
120	1... 20211111_L6_R2_IgG	2.00	4577.8	44907.4	0.102	MM
121	1... 20211111_H3_R2_IgG	1.99	4565.3	47729.1	0.096	bb
122	1... 20211111_A7_R2_IgG	2.01	6104.0	57347.1	0.106	bb
123	1... 20211111_A8_R2_IgG	2.00	8714.2	52397.8	0.166	bb
124	1... 20211111_L7_R2_IgG	2.00	6873.2	36318.4	0.189	bb
125	1... 20211111_L8_R2_IgG	2.01	5397.9	38131.9	0.142	bb
126	1... 20211111_H4_R2_IgG	2.00	11643.2	46849.6	0.249	bb
127	1... 20211111_A9_R2_IgG	2.00	2189.6	47759.6	0.046	MM
128	1... 20211111_A10_R2_IgG	1.99	4697.8	41366.2	0.114	bb
129	1... 20211111_L9_R2_IgG	2.00	4623.8	43052.1	0.107	bb
130	1... 20211111_L10_R2_IgG	2.00	5867.5	31487.4	0.186	bb
131	1... 20211111_QC_R11_IgG	2.01	6866.7	37804.6	0.182	bb
132	1... 20211111_H5_R2_IgG	2.00	5599.4	40380.0	0.139	MM
133	1... 20211111_A11_R2_IgG	2.00	4683.3	49533.3	0.095	MM
134	1... 20211111_A12_R2_IgG	2.01	2909.3	40226.3	0.072	MM
135	1... 20211111_L11_R2_IgG	2.01	5290.1	28884.0	0.183	bb
136	1... 20211111_L12_R2_IgG	2.00	2868.8	34351.3	0.084	MM
137	1... 20211111_H6_R2_IgG	2.01	10457.1	40466.6	0.258	bb
138	1... 20211111_A13_R2_IgG	2.00	8520.1	42306.7	0.201	bb
139	1... 20211111_A14_R2_IgG	2.00	2505.9	44904.0	0.056	bb
140	1... 20211111_L13_R2_IgG	2.01	2466.7	31913.0	0.077	MM
141	1... 20211111_L14_R2_IgG	2.00	10662.4	31527.4	0.338	MM
142	1... 20211111_H7_R2_IgG	2.01	3290.5	40838.2	0.081	MM
143	1... 20211111_A15_R2_IgG	2.00	15896.6	49389.7	0.322	bb
144	1... 20211111_QC_R12_IgG	2.00	6468.8	35168.7	0.184	bb
145	1... 20211111_A16_R2_IgG	2.01	5724.5	47440.5	0.121	bb
146	1... 20211111_L15_R2_IgG	2.00	7638.4	33145.9	0.230	bb
147	1... 20211111_L16_R2_IgG	1.99	3689.0	37596.6	0.098	bb
148	1... 20211111_H8_R2_IgG	2.01	5854.7	43933.3	0.133	MM
149	1... 20211111_A17_R2_IgG	2.00	8091.3	43605.5	0.186	bb
150	1... 20211111_A18_R2_IgG	2.00	2748.1	37581.8	0.073	bb
151	1... 20211111_L17_R2_IgG	2.00	7738.8	33942.5	0.228	bb
152	1... 20211111_L18_R2_IgG	2.00	4410.8	37831.5	0.117	MM
153	1... 20211111_H9_R2_IgG	2.01	12196.8	30898.4	0.395	MM
154	1... 20211111_A19_R2_IgG	2.00	9928.9	39352.1	0.252	bb
155	1... 20211111_A20_R2_IgG	2.00	4966.1	34661.2	0.143	MM
156	1... 20211111_L19_R2_IgG	2.00	6682.2	39918.3	0.167	bb
157	1... 20211111_QC_R13_IgG	2.00	7021.4	36239.1	0.194	MM
158	1... 20211111_L20_R2_IgG	2.00	1098.5	32897.7	0.033	MM
159	1... 20211111_H10_R2_IgG	1.99	4450.5	31394.5	0.142	bb
160	1... 20211111_A21_R2_IgG	1.98	1035.2	34863.7	0.030	MM
161	1... 20211111_A22_R2_IgG	2.00	2219.4	30342.1	0.073	MM
162	1... 20211111_L21_R2_IgG	1.99	2411.1	33706.2	0.072	bb
163	1... 20211111_L22_R2_IgG	2.00	4997.9	31298.6	0.160	bb
164	1... 20211111_H11_R2_IgG	2.00	2289.4	38904.1	0.059	MM
165	1... 20211111_A23_R2_IgG	2.00	2810.9	32382.3	0.087	MM
166	1... 20211111_A24_R2_IgG	1.99	1865.9	30261.9	0.062	MM
167	1... 20211111_L23_R2_IgG	1.99	8977.4	43037.5	0.209	bb
168	1... 20211111_L24_R2_IgG	1.99	4801.1	37023.3	0.130	MM

Dataset: C:\MassLynx\10.pro\20211115_clinicalsamples_IgG.qld

Last Altered: Sunday, October 02, 2022 16:02:30 台北標準時間
Printed: Friday, August 01, 2025 16:43:22 台北標準時間

	# Name	RT	Area	IS Area	Response	Prima...
169	1... 20211111_H12_R2_IgG	2.00	4014.3	39478.2	0.102	bb
170	1... 20211111_QC_R14_IgG	1.99	5446.8	34463.2	0.158	MM
171	1... 20211111_A25_R2_IgG	2.00	3103.8	39945.3	0.078	bb
172	1... 20211111_A26_R2_IgG	2.00	668.3	29663.2	0.023	MM
173	1... 20211111_L25_R2_IgG	2.00	4048.5	38589.3	0.105	bb
174	1... 20211111_L26_R2_IgG	2.00	4086.5	38405.2	0.106	MM
175	1... 20211111_H13_R2_IgG	1.99	5453.4	37771.2	0.144	MM
176	1... 20211111_A27_R2_IgG	1.99	4498.9	42634.1	0.106	bb
177	1... 20211111_A28_R2_IgG	2.00	2721.1	33231.1	0.082	bb
178	1... 20211111_L27_R2_IgG	1.99	4579.1	41761.1	0.110	bb
179	1... 20211111_L28_R2_IgG	2.00	2320.2	26968.6	0.086	MM
180	1... 20211111_H14_R2_IgG	1.99	10299.4	49250.2	0.209	bb
181	1... 20211111_A29_R2_IgG	2.00	4174.2	37601.7	0.111	bb
182	1... 20211111_A30_R2_IgG	2.01	5714.3	40366.8	0.142	bb
183	1... 20211111_QC_R15_IgG	1.99	5512.2	35156.1	0.157	MM
184	1... 20211111_L29_R2_IgG	1.99	4506.7	33813.1	0.133	MM
185	1... 20211111_L30_R2_IgG	2.00	2506.4	33488.6	0.075	MM
186	1... 20211111_H15_R2_IgG	1.99	7987.4	38501.0	0.207	bb
187	1... 20211111_A31_R2_IgG	2.00	2856.9	37525.5	0.076	bb
188	1... 20211111_A32_R2_IgG	2.00	6464.3	35770.8	0.181	MM
189	1... 20211111_L31_R2_IgG	2.00	3082.8	33282.4	0.093	bb
190	1... 20211111_L32_R2_IgG	2.00	7316.4	42049.6	0.174	MM
191	1... 20211111_H16_R2_IgG	2.00	13393.0	45947.6	0.291	bb
192	1... 20211111_A33_R2_IgG	2.00	8188.4	40202.3	0.204	bb
193	1... 20211111_A34_R2_IgG	1.99	2174.3	36747.0	0.059	bb
194	1... 20211111_H17_R2_IgG	1.99	58906.8	40920.9	1.440	bb
195	1... 20211111_A35_R2_IgG	2.00	8619.5	33689.2	0.256	bb
196	1... 20211111_QC_R16_IgG	2.00	6008.7	32783.6	0.183	bb
197	1... 20211111_A36_R2_IgG	2.00	3077.1	36822.4	0.084	MM
198	1... 20211111_H18_R2_IgG	1.99	10358.7	31769.3	0.326	MM
199	1... 20211111_A37_R2_IgG	2.00	9682.0	37581.8	0.258	MM
200	2... 20211111_A38_R2_IgG	2.00	5669.8	38390.4	0.148	bb
201	2... 20211111_H19_R2_IgG	2.00	34674.6	41217.0	0.841	bb
202	2... 20211111_A39_R2_IgG	2.00	6065.8	37392.7	0.162	MM
203	2... 20211111_A40_R2_IgG	1.99	5782.4	38295.1	0.151	bb
204	2... 20211111_H20_R2_IgG	1.99	22501.7	35941.8	0.626	bb
205	2... 20211111_A41_R2_IgG	2.00	7309.6	45304.9	0.161	bb
206	2... 20211111_A42_R2_IgG	2.01	9404.3	37803.8	0.249	bb
207	2... 20211111_A43_R2_IgG	1.99	1729.4	38161.8	0.045	MM
208	2... 20211111_A44_R2_IgG	1.99	7351.1	37819.8	0.194	MM
209	2... 20211111_QC_R17_IgG	2.00	5753.0	31731.8	0.181	bb

Dataset: C:\MassLynx\10.pro\20211115_clinicalsamples_IgG.qld

Last Altered: Sunday, October 02, 2022 16:02:30 台北標準時間
Printed: Friday, August 01, 2025 16:43:53 台北標準時間

Method: C:\MassLynx\10_Pro_IgA.PRO\MethDB\20210926_IgG_DMRM_IgM_IS.mdb 16 Feb 2022 11:58:21
Calibration: 18 Nov 2021 13:56:22

Sample Name: 20211111_QC_R1_IgG
F17:MRM of 1 channel,ES+
932.83 > 204.1

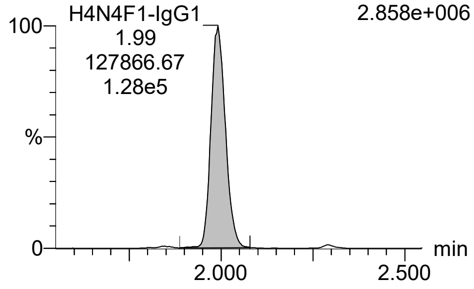

Sample Name: 20211111_A1_R1_IgG
F17:MRM of 1 channel,ES+
932.83 > 204.1

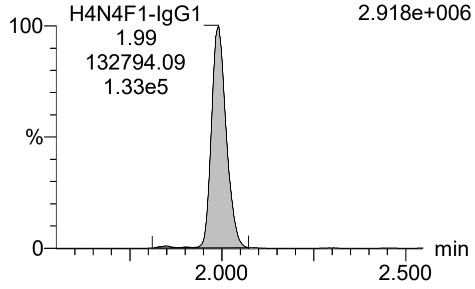

Sample Name: 20211111_A2_R1_IgG
F17:MRM of 1 channel,ES+
932.83 > 204.1

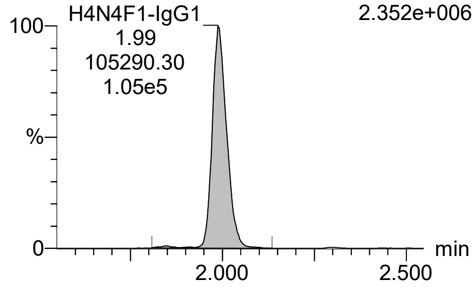

Sample Name: 20211111_L1_R1_IgG
F17:MRM of 1 channel,ES+
932.83 > 204.1

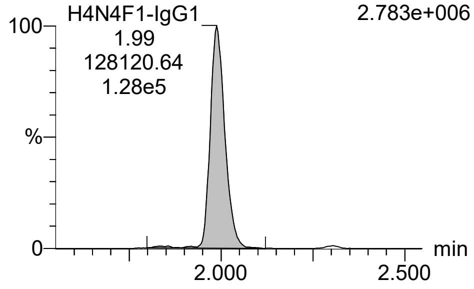

Sample Name: 20211111_L2_R1_IgG
F17:MRM of 1 channel,ES+
932.83 > 204.1

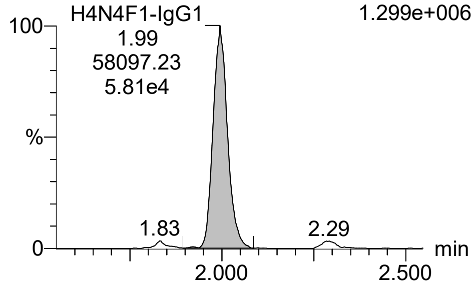

Sample Name: 20211111_H1_R1_IgG
F17:MRM of 1 channel,ES+
932.83 > 204.1

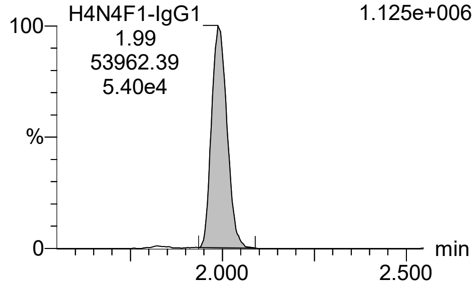

Sample Name: 20211111_A3_R1_IgG
F17:MRM of 1 channel,ES+
932.83 > 204.1

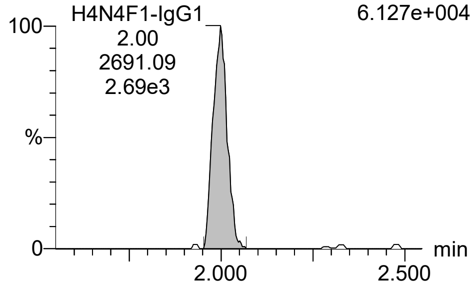

Sample Name: 20211111_A4_R1_IgG
F17:MRM of 1 channel,ES+
932.83 > 204.1

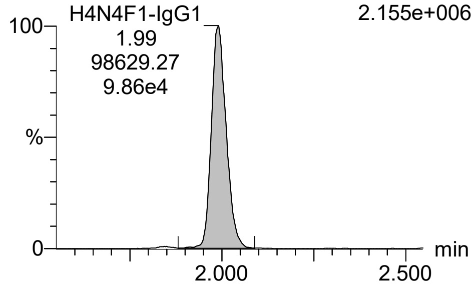

Sample Name: 20211111_L3_R1_IgG
F17:MRM of 1 channel,ES+
932.83 > 204.1

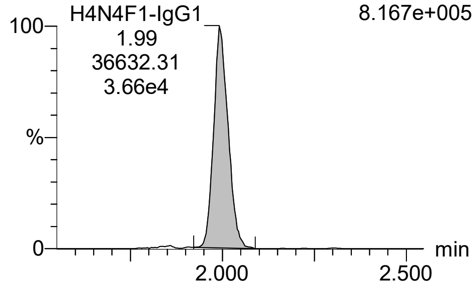

Sample Name: 20211111_L4_R1_IgG
F17:MRM of 1 channel,ES+
932.83 > 204.1

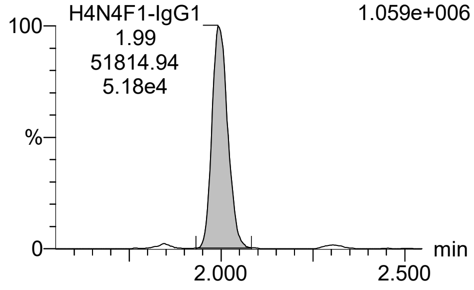

Sample Name: 20211111_H2_R1_IgG
F17:MRM of 1 channel,ES+
932.83 > 204.1

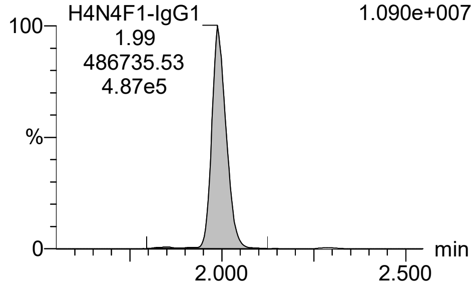

Sample Name: 20211111_A5_R1_IgG
F17:MRM of 1 channel,ES+
932.83 > 204.1

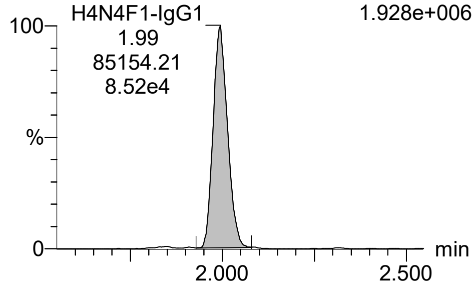

Sample Name: 20211111_A6_R1_IgG

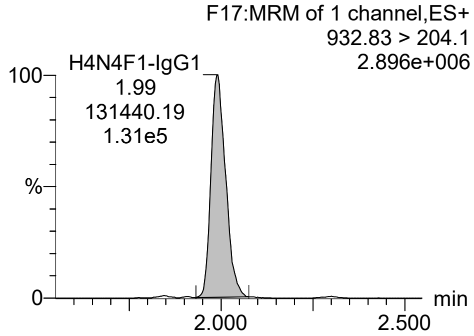

Sample Name: 20211111_QC_R2_IgG

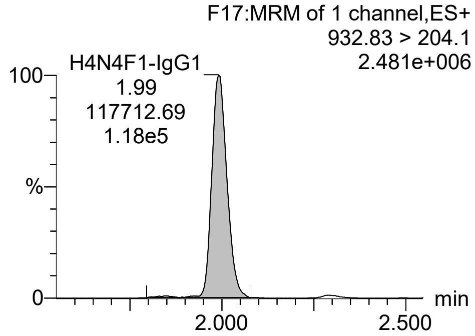

Sample Name: 20211111_L5_R1_IgG

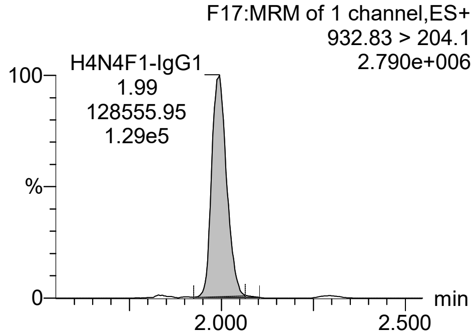

Sample Name: 20211111_L6_R1_IgG

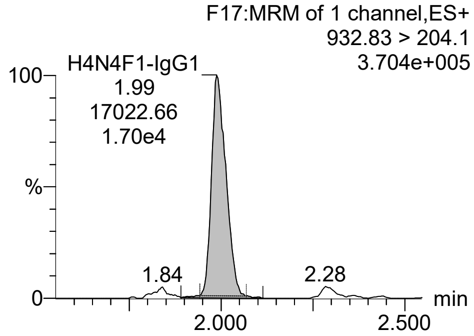

Sample Name: 20211111_H3_R1_IgG

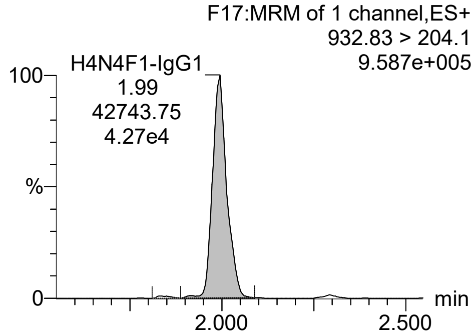

Sample Name: 20211111_A7_R1_IgG

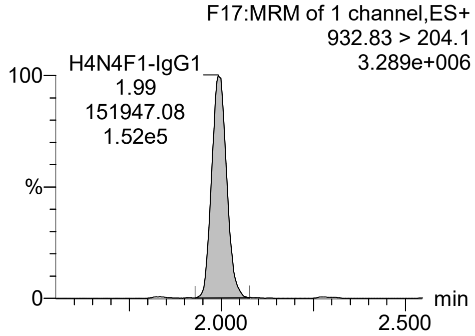

Sample Name: 20211111_A8_R1_IgG

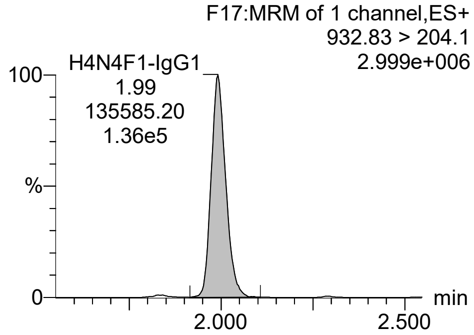

Sample Name: 20211111_L7_R1_IgG

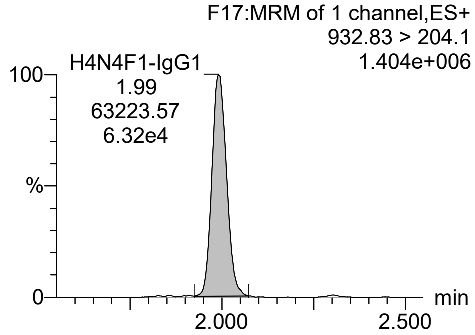

Sample Name: 20211111_L8_R1_IgG

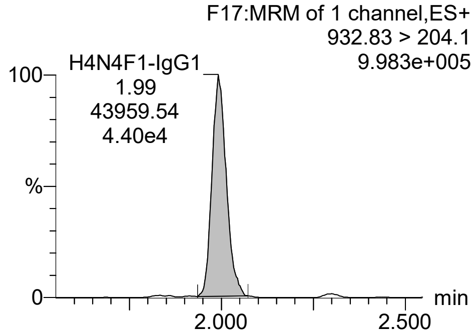

Sample Name: 20211111_H4_R1_IgG

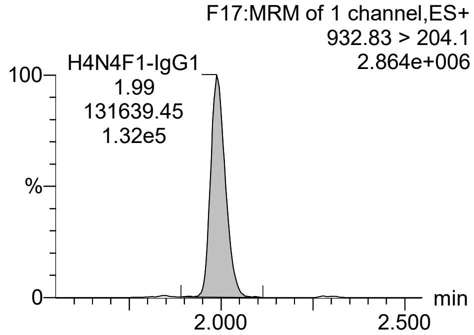

Sample Name: 20211111_A9_R1_IgG

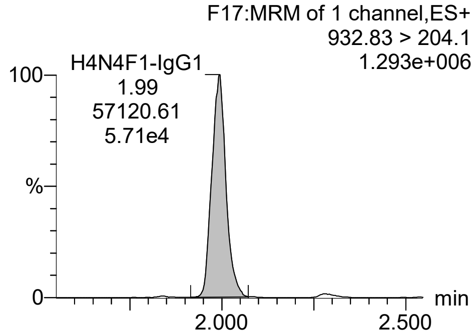

Sample Name: 20211111_A10_R1_IgG

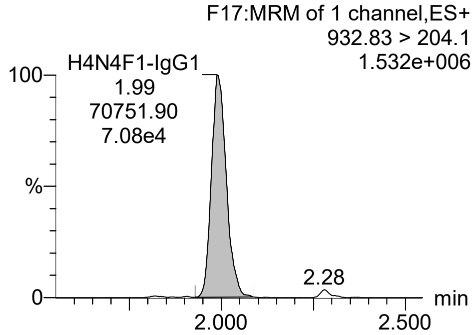

Sample Name: 20211111_L9_R1_IgG

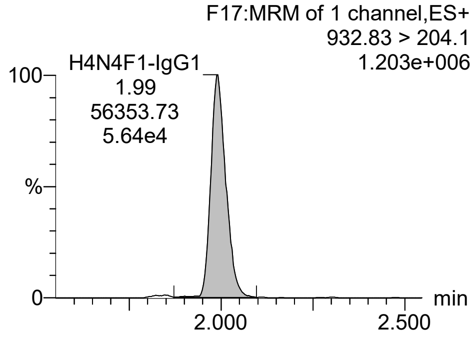

Sample Name: 20211111_L10_R1_IgG

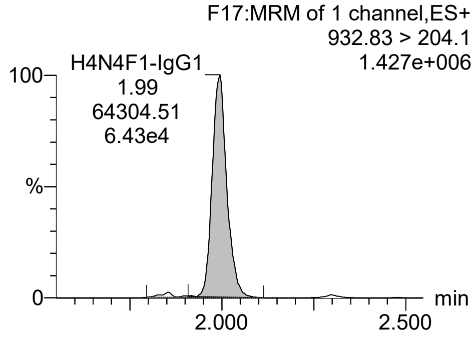

Sample Name: 20211111_QC_R3_IgG

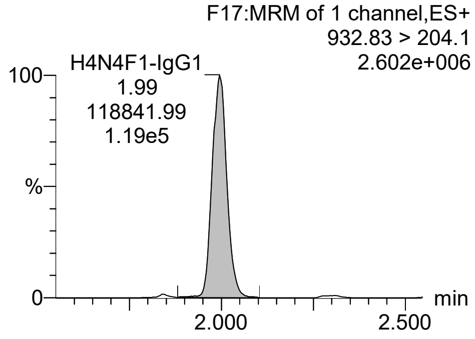

Dataset: C:\MassLynx\10.pro\20211115_clinicalsamples_IgG.qld

Last Altered: Sunday, October 02, 2022 16:02:30 台北標準時間
Printed: Friday, August 01, 2025 16:43:53 台北標準時間

Sample Name: 20211111_H5_R1_IgG

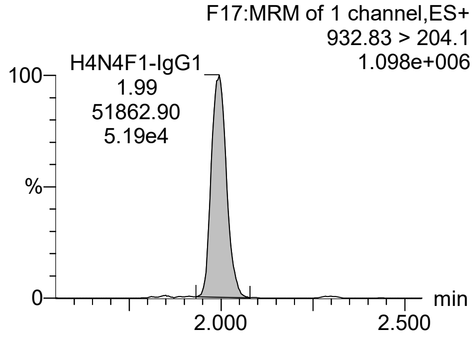

Sample Name: 20211111_A11_R1_IgG

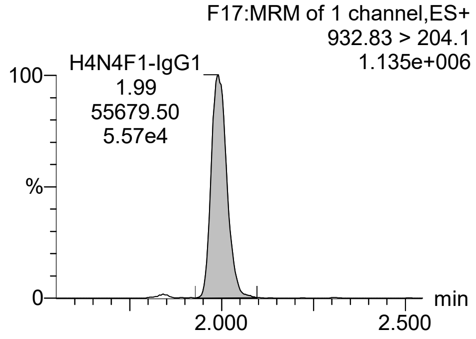

Sample Name: 20211111_A12_R1_IgG

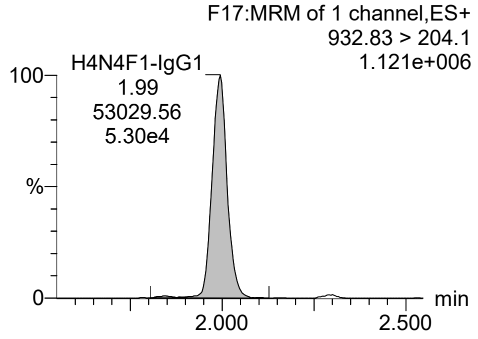

Sample Name: 20211111_L11_R1_IgG

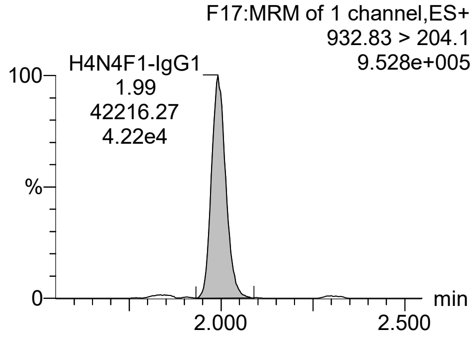

Sample Name: 20211111_L12_R1_IgG

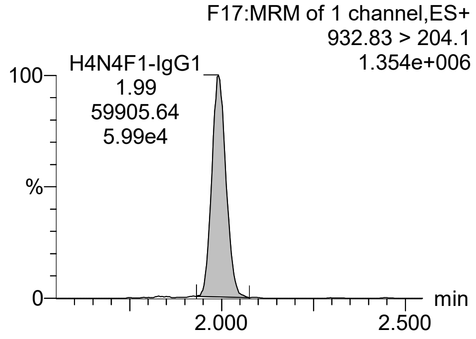

Sample Name: 20211111_H6_R1_IgG

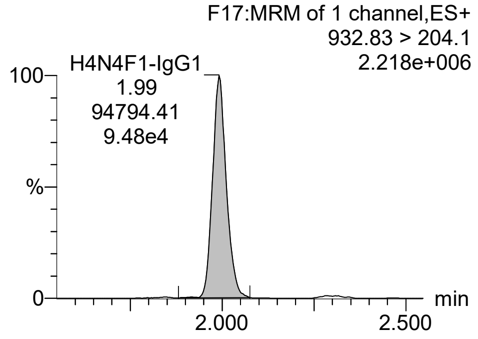

Sample Name: 20211111_A13_R1_IgG

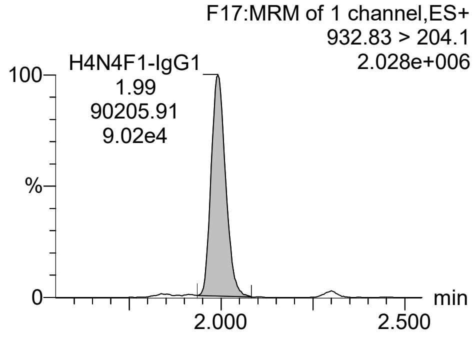

Sample Name: 20211111_A14_R1_IgG

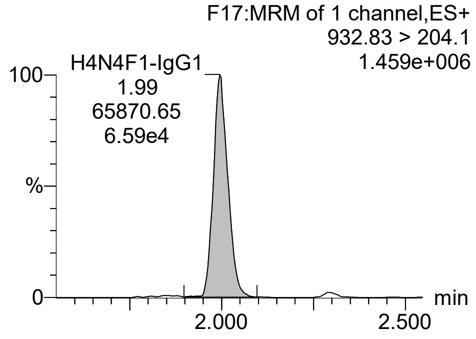

Sample Name: 20211111_L13_R1_IgG

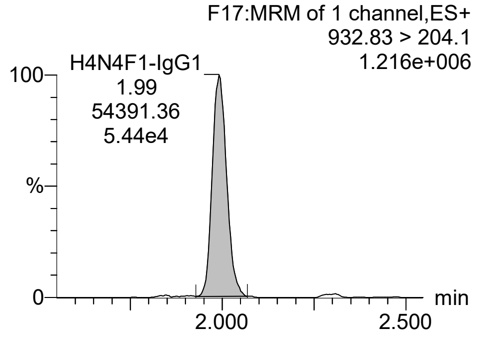

Sample Name: 20211111_L14_R1_IgG

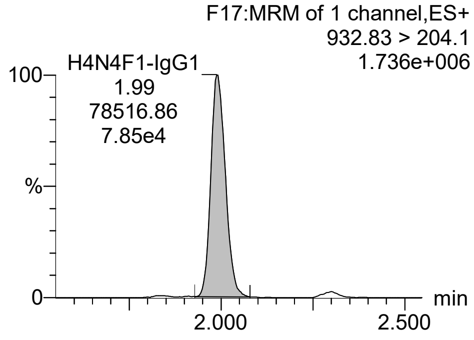

Sample Name: 20211111_H7_R1_IgG

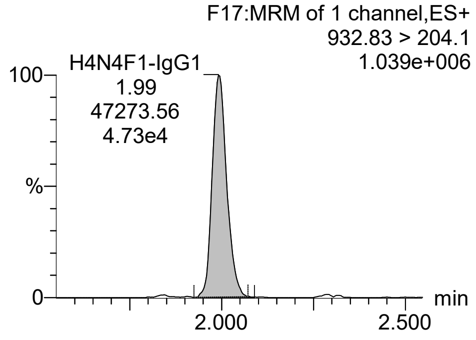

Sample Name: 20211111_A15_R1_IgG

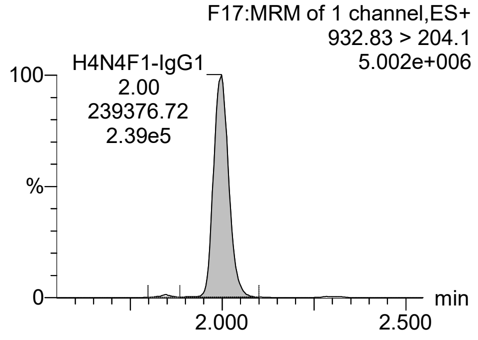

Sample Name: 20211111_QC_R4_IgG

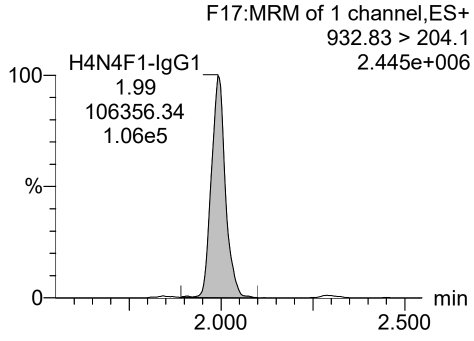

Sample Name: 20211111_A16_R1_IgG

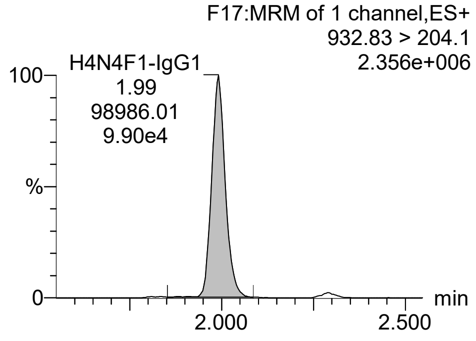

Sample Name: 20211111_L15_R1_IgG

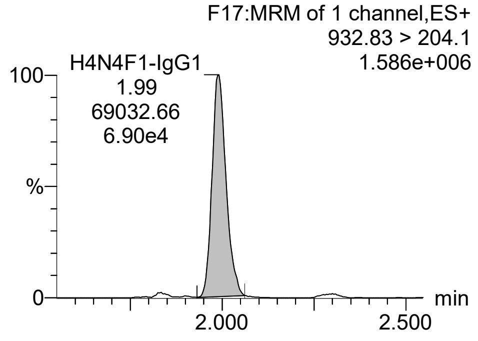

Sample Name: 20211111_L16_R1_IgG

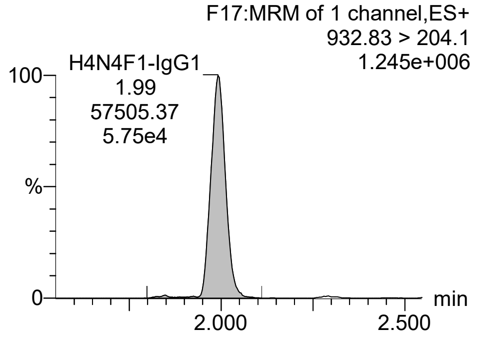

Sample Name: 20211111_H8_R1_IgG

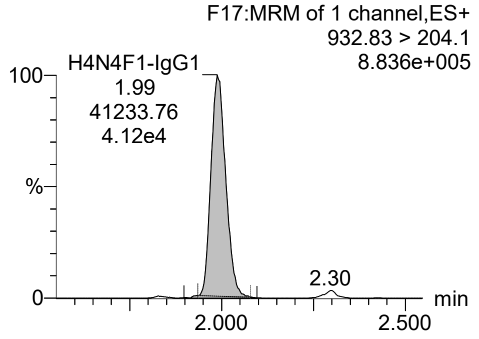

Sample Name: 20211111_A17_R1_IgG

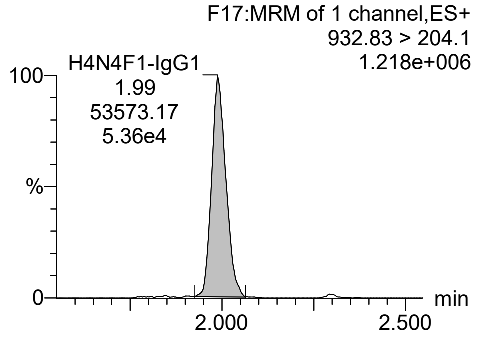

Sample Name: 20211111_A18_R1_IgG

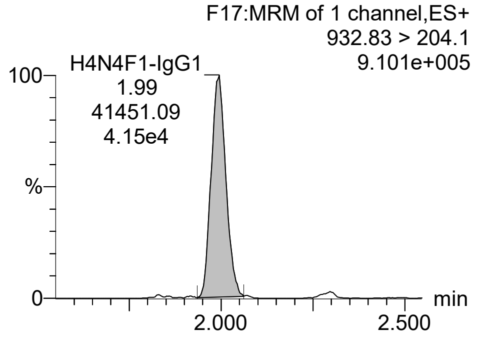

Sample Name: 20211111_L17_R1_IgG

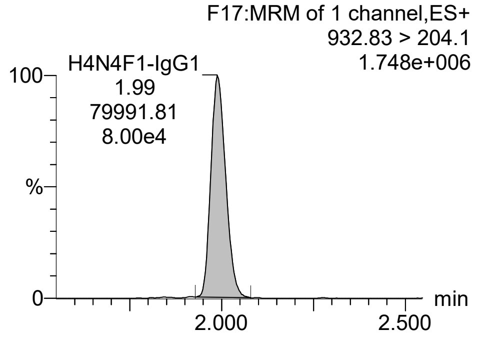

Sample Name: 20211111_L18_R1_IgG

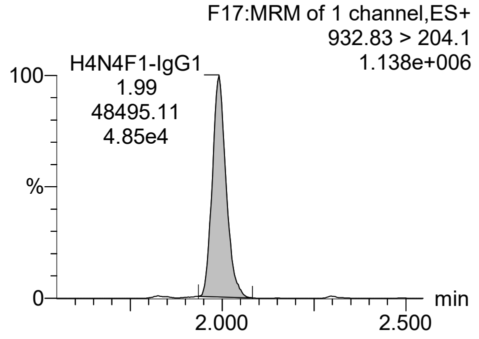

Sample Name: 20211111_H9_R1_IgG

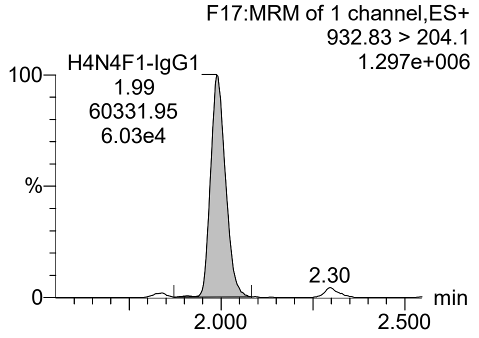

Sample Name: 20211111_A19_R1_IgG

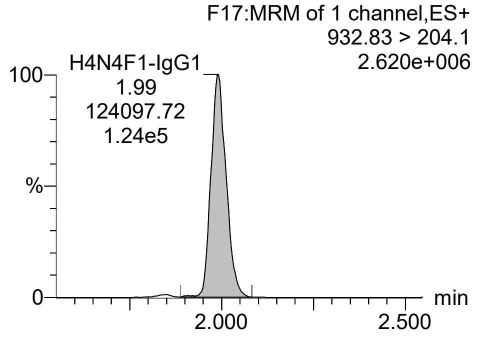

Sample Name: 20211111_A20_R1_IgG

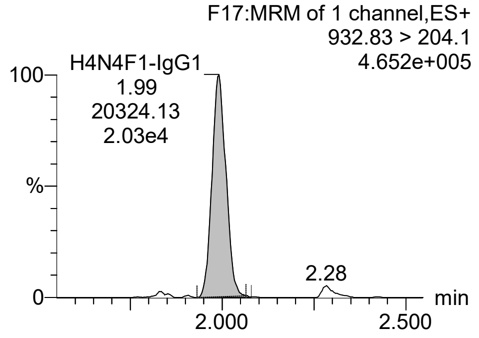

Sample Name: 20211111_L19_R1_IgG

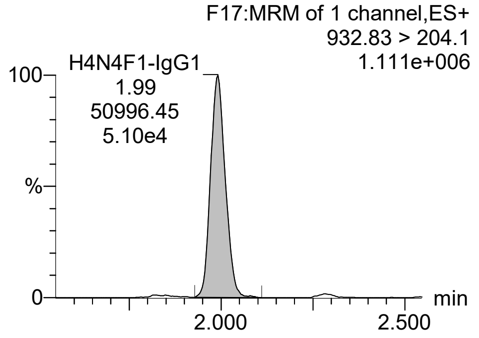

Sample Name: 20211111_QC_R5_IgG

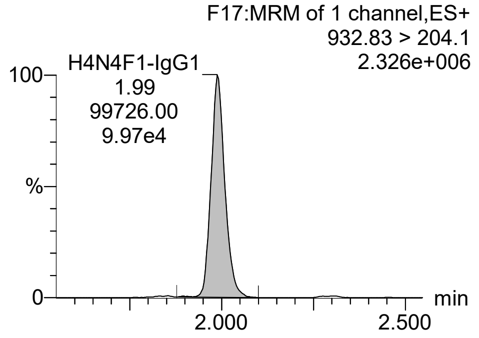

Sample Name: 20211111_L20_R1_IgG

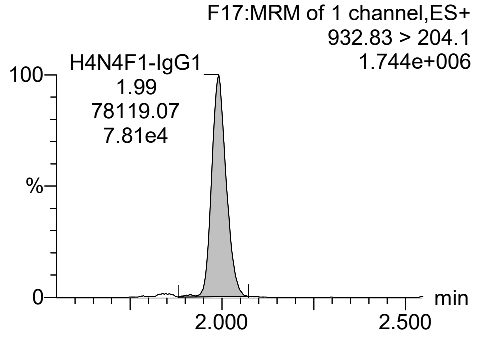

Sample Name: 20211111_H10_R1_IgG

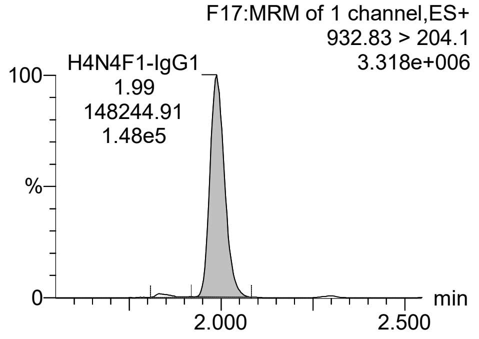

Sample Name: 20211111_A21_R1_IgG

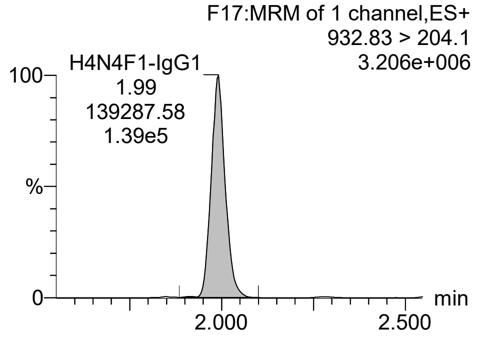

Sample Name: 20211111_A22_R1_IgG

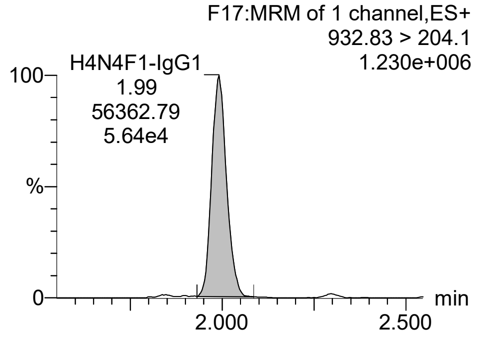

Sample Name: 20211111_L21_R1_IgG

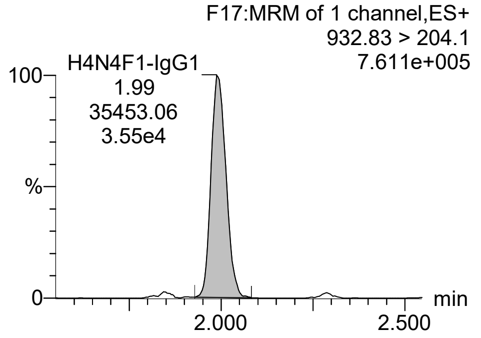

Sample Name: 20211111_L22_R1_IgG

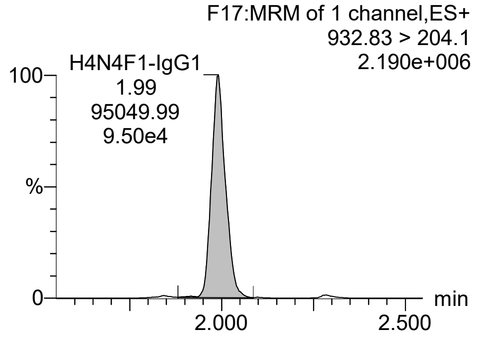

Sample Name: 20211111_H11_R1_IgG

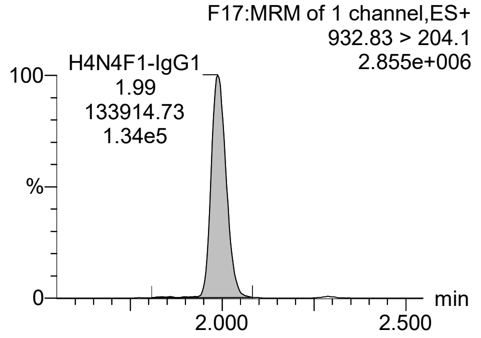

Sample Name: 20211111_A23_R1_IgG

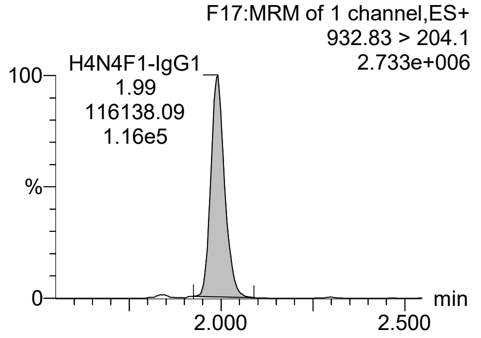

Sample Name: 20211111_A24_R1_IgG

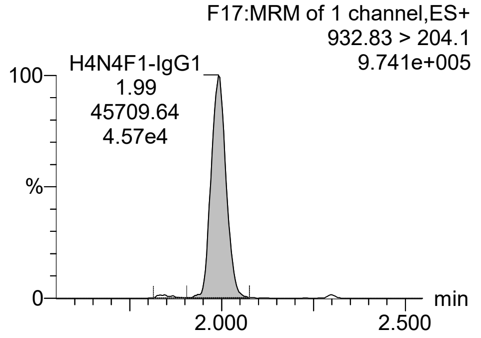

Sample Name: 20211111_L23_R1_IgG

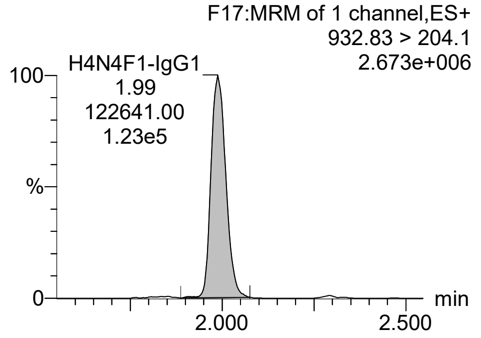

Sample Name: 20211111_L24_R1_IgG

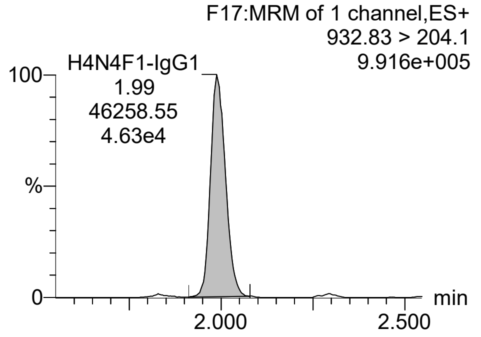

Sample Name: 20211111_H12_R1_IgG

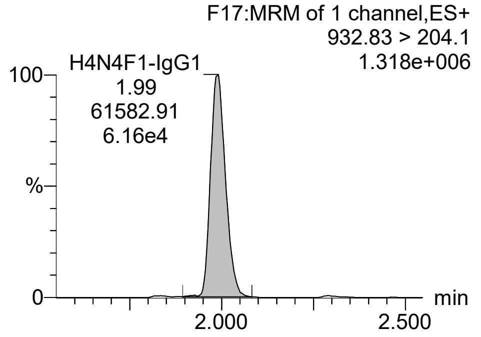

Sample Name: 20211111_QC_R6_IgG

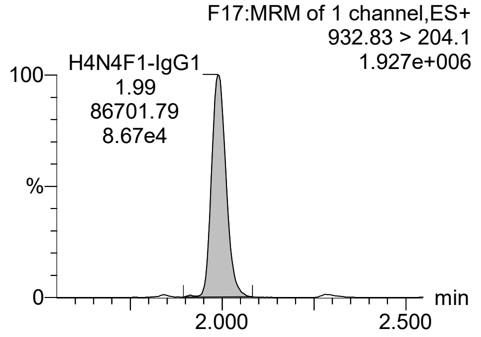

Sample Name: 20211111_A25_R1_IgG

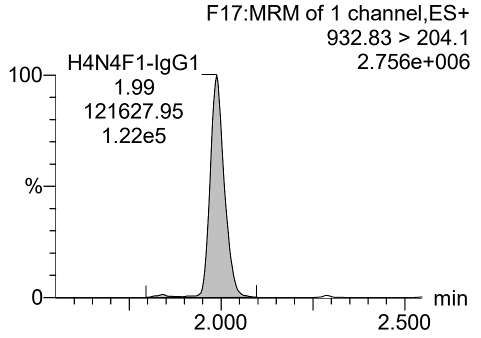

Sample Name: 20211111_A26_R1_IgG

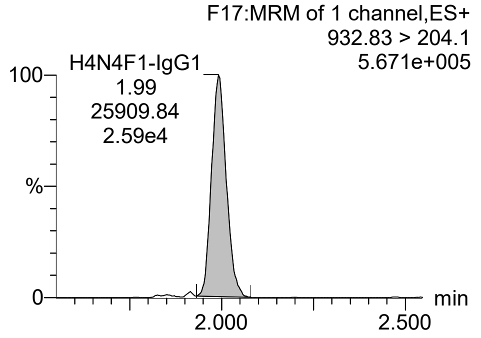

Sample Name: 20211111_L25_R1_IgG

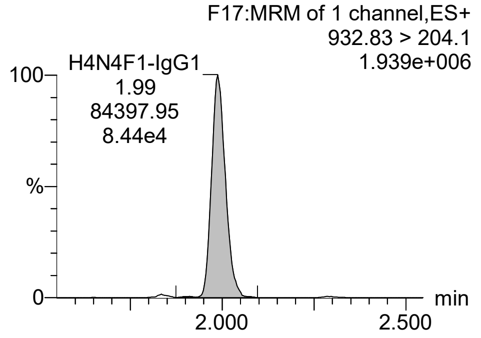

Sample Name: 20211111_L26_R1_IgG

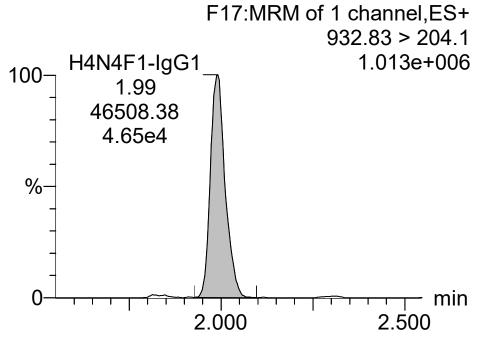

Sample Name: 20211111_H13_R1_IgG

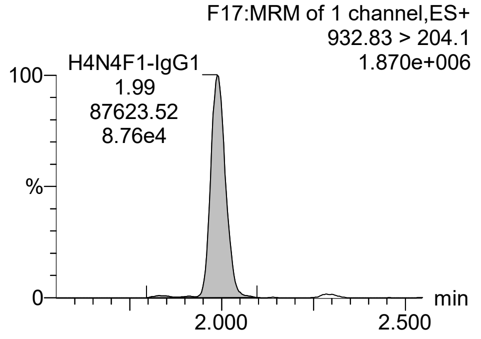

Sample Name: 20211111_A27_R1_IgG

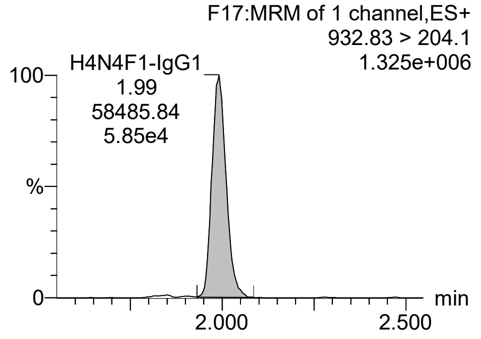

Sample Name: 20211111_A28_R1_IgG

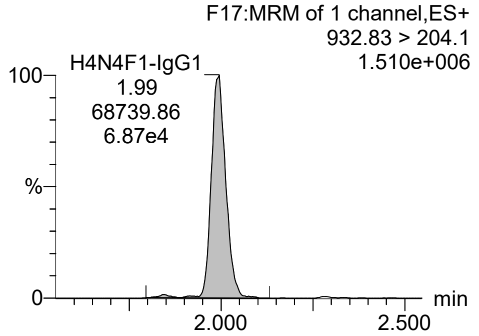

Sample Name: 20211111_L27_R1_IgG

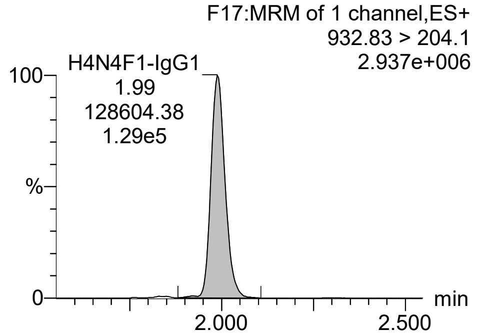

Sample Name: 20211111_L28_R1_IgG

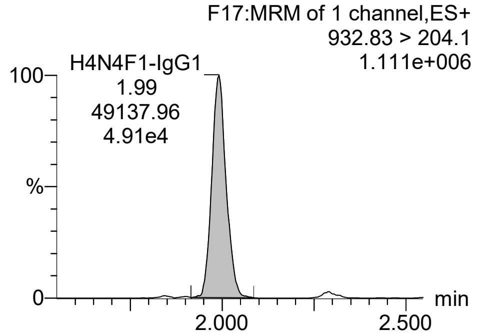

Sample Name: 20211111_H14_R1_IgG

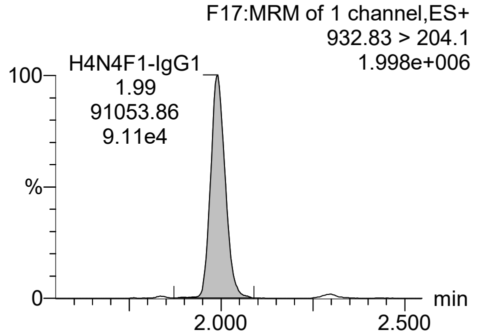

Sample Name: 20211111_A29_R1_IgG

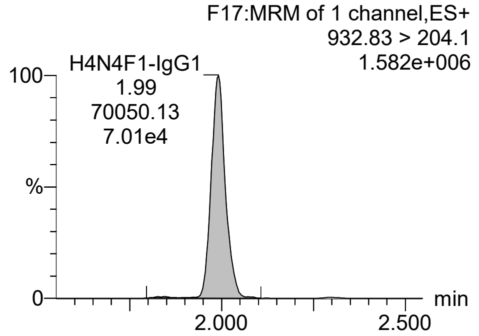

Sample Name: 20211111_A30_R1_IgG

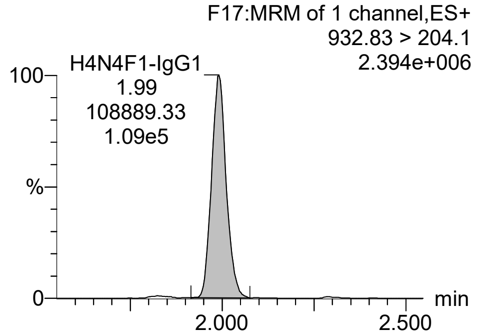

Sample Name: 20211111_QC_R7_IgG

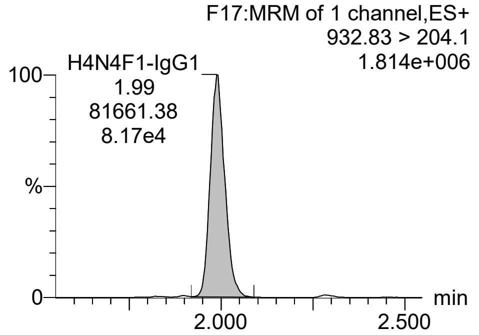

Sample Name: 20211111_L29_R1_IgG

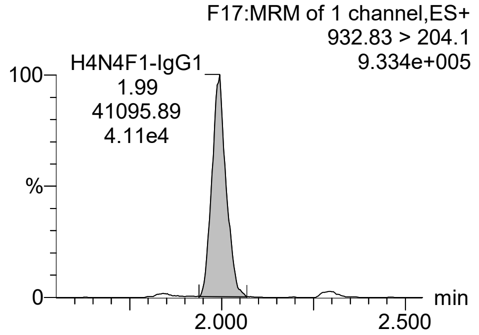

Sample Name: 20211111_L30_R1_IgG

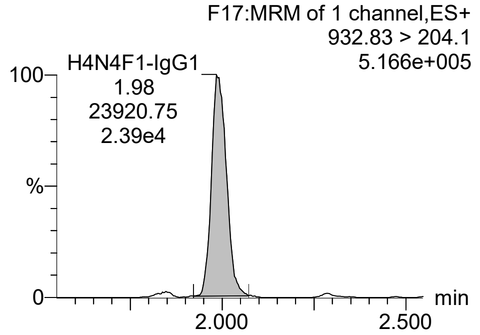

Sample Name: 20211111_H15_R1_IgG

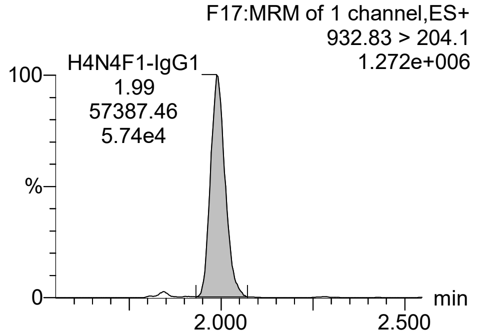

Sample Name: 20211111_A31_R1_IgG

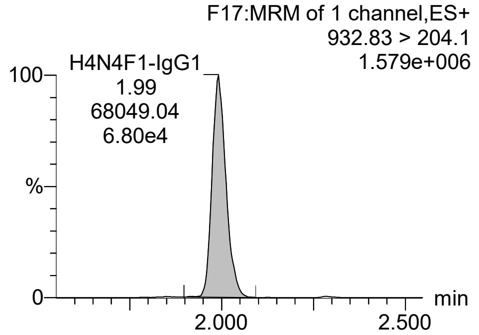

Sample Name: 20211111_A32_R1_IgG

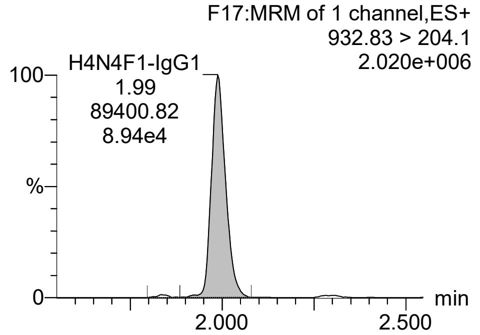

Sample Name: 20211111_L31_R1_IgG

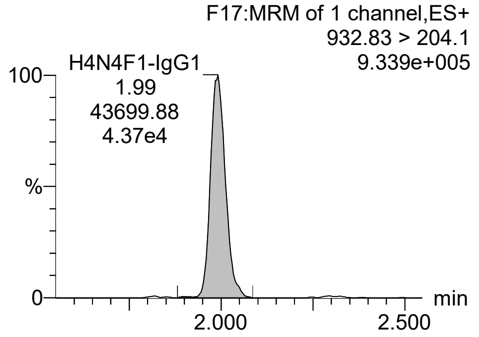

Sample Name: 20211111_L32_R1_IgG

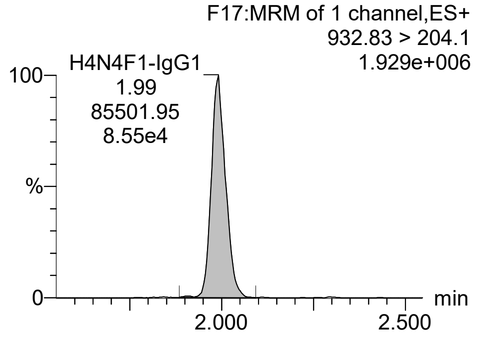

Sample Name: 20211111_H16_R1_IgG

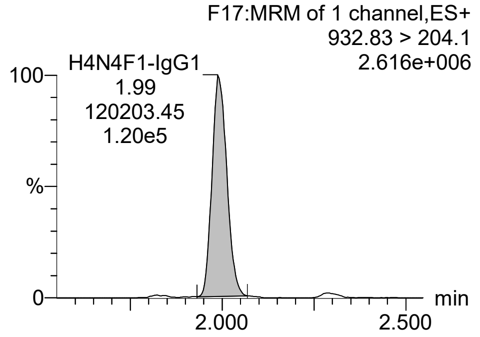

Dataset: C:\MassLynx\10.pro\20211115_clinicalsamples_IgG.qld

Last Altered: Sunday, October 02, 2022 16:02:30 台北標準時間
Printed: Friday, August 01, 2025 16:43:53 台北標準時間

Sample Name: 20211111_A33_R1_IgG

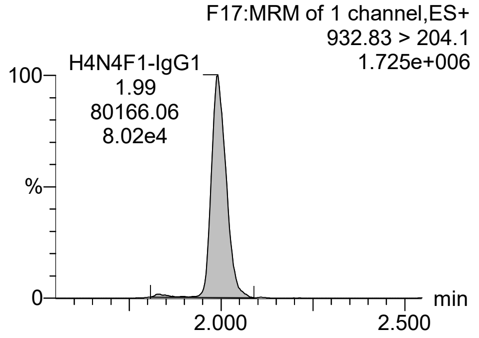

Sample Name: 20211111_A34_R1_IgG

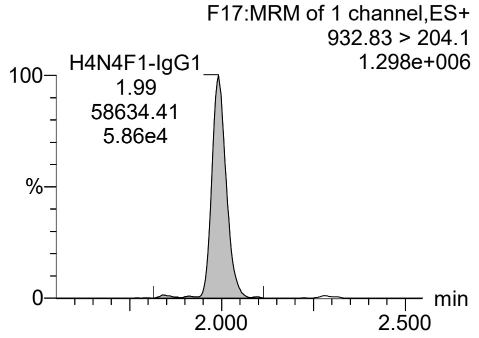

Sample Name: 20211111_H17_R1_IgG

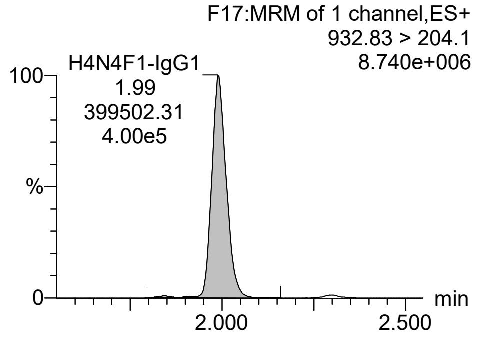

Sample Name: 20211111_A35_R1_IgG

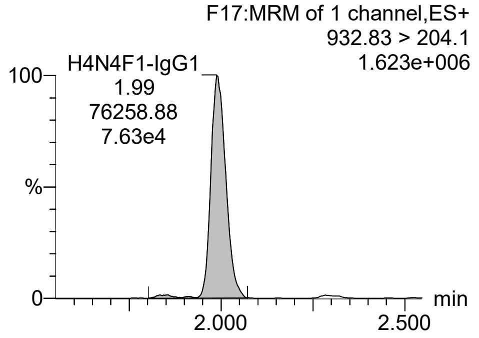

Sample Name: 20211111_QC_R8_IgG

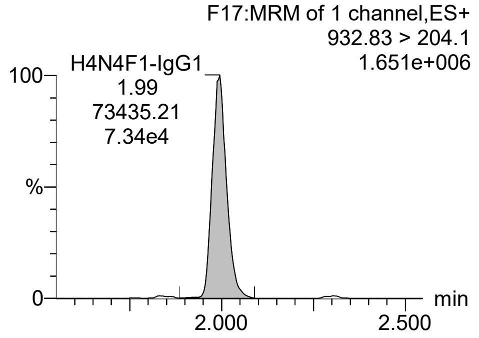

Sample Name: 20211111_A36_R1_IgG

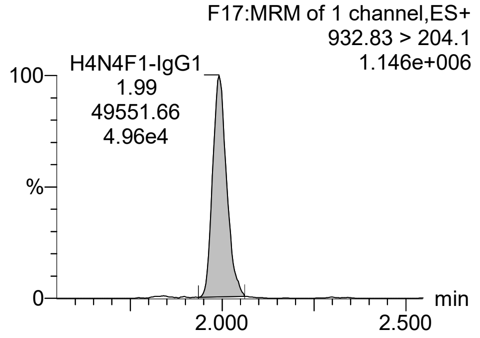

Sample Name: 20211111_H18_R1_IgG

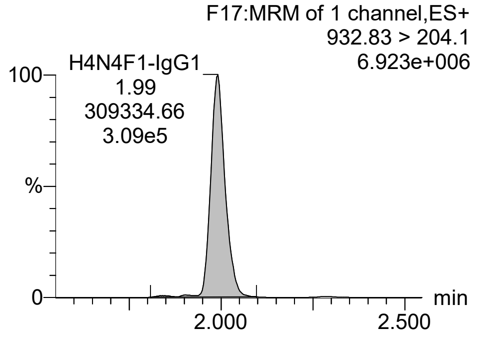

Sample Name: 20211111_A37_R1_IgG

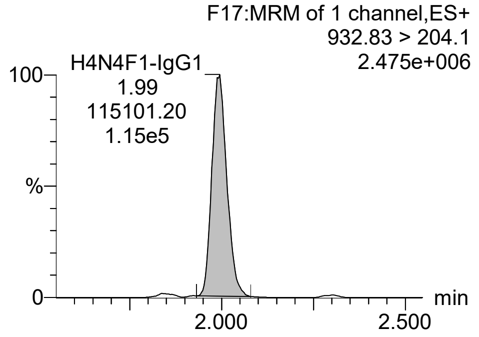

Sample Name: 20211111_A38_R1_IgG

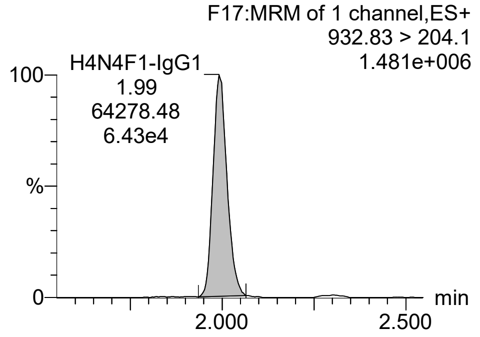

Sample Name: 20211111_H19_R1_IgG

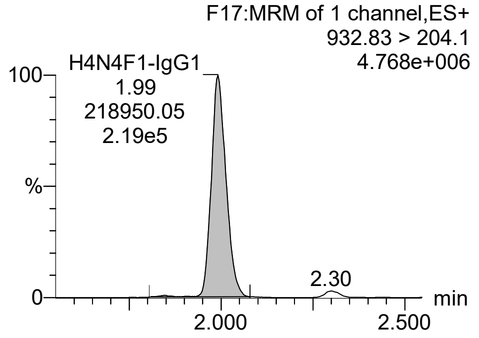

Sample Name: 20211111_A39_R1_IgG

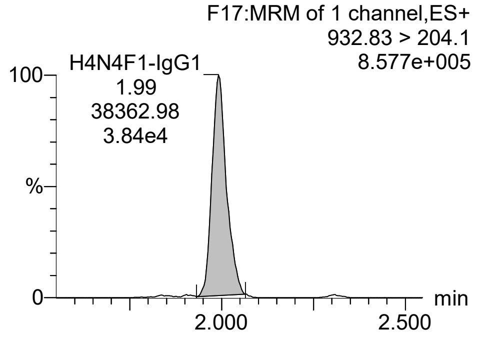

Sample Name: 20211111_A40_R1_IgG

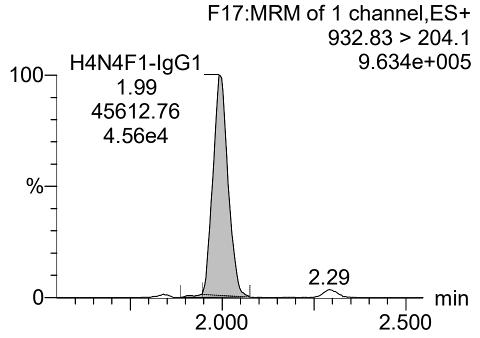

Sample Name: 20211111_H20_R1_IgG

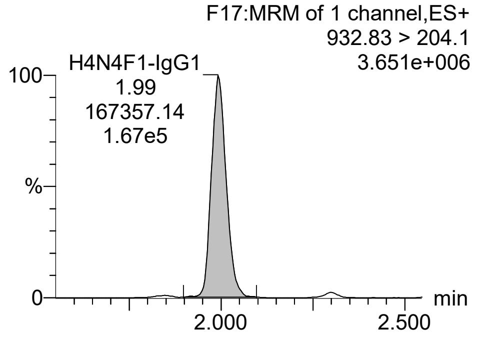

Sample Name: 20211111_A41_R1_IgG

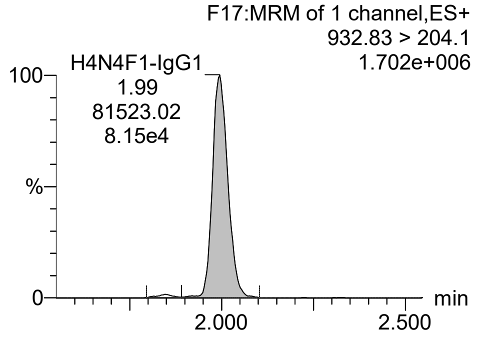

Sample Name: 20211111_A42_R1_IgG

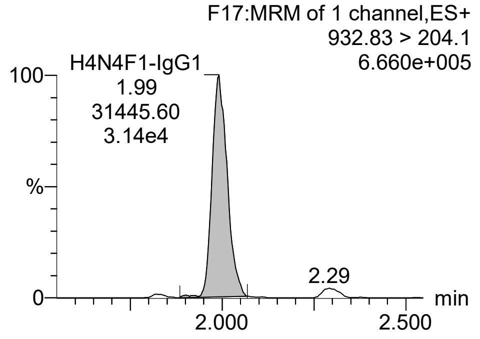

Sample Name: 20211111_A43_R1_IgG

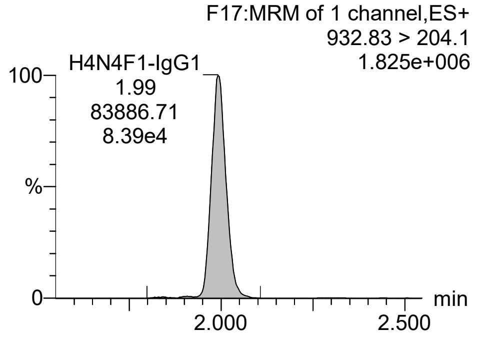

Sample Name: 20211111_A44_R1_IgG

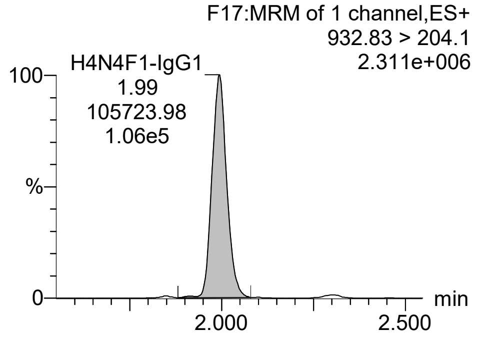

Sample Name: 20211111_QC_R9_IgG

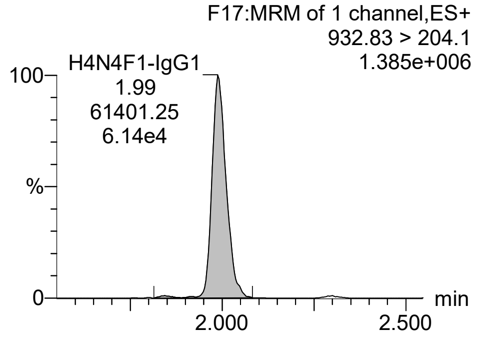

Sample Name: 20211111_A1_R2_IgG

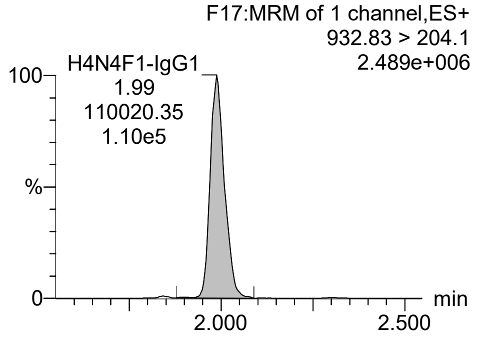

Sample Name: 20211111_A2_R2_IgG

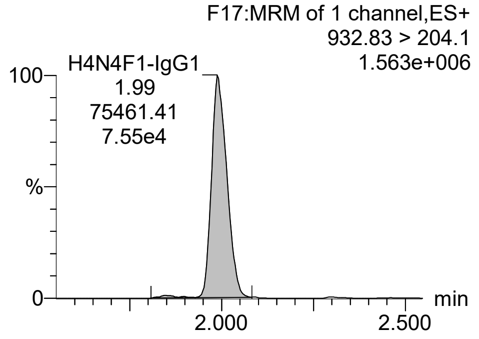

Sample Name: 20211111_L1_R2_IgG

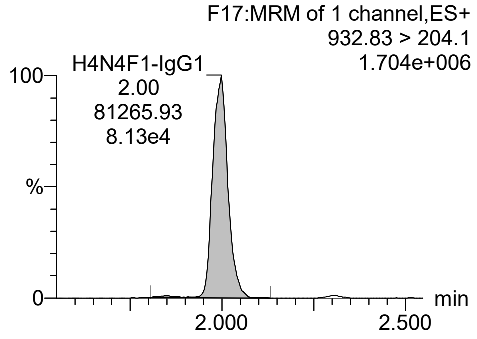

Sample Name: 20211111_L2_R2_IgG

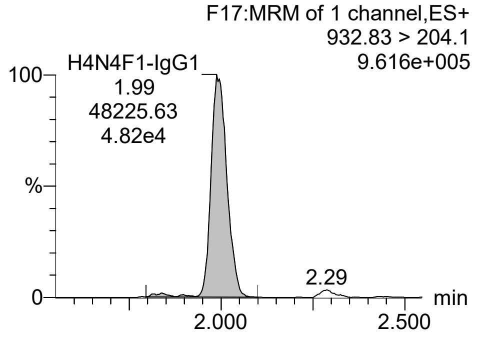

Sample Name: 20211111_H1_R2_IgG

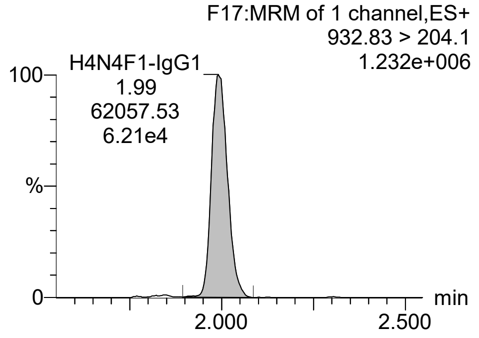

Sample Name: 20211111_A3_R2_IgG

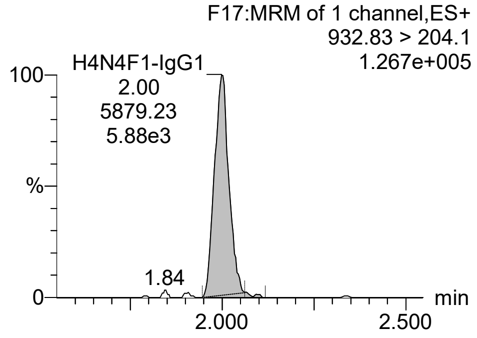

Sample Name: 20211111_A4_R2_IgG

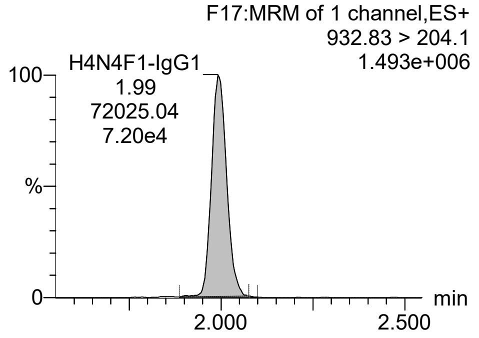

Sample Name: 20211111_L3_R2_IgG

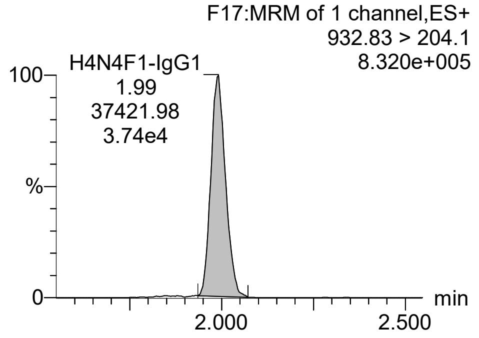

Sample Name: 20211111_L4_R2_IgG

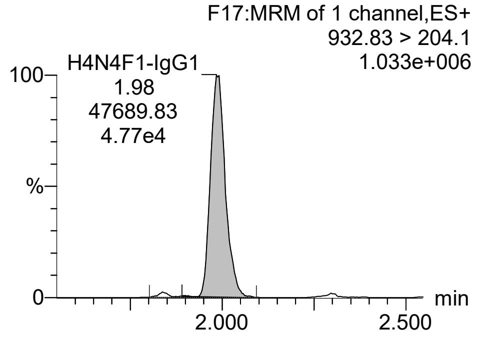

Sample Name: 20211111_H2_R2_IgG

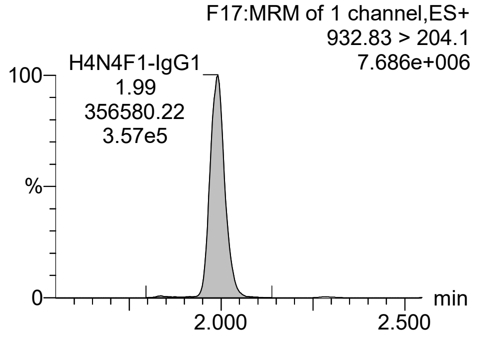

Sample Name: 20211111_A5_R2_IgG

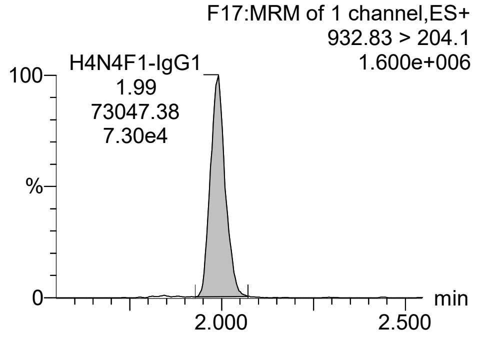

Sample Name: 20211111_A6_R2_IgG

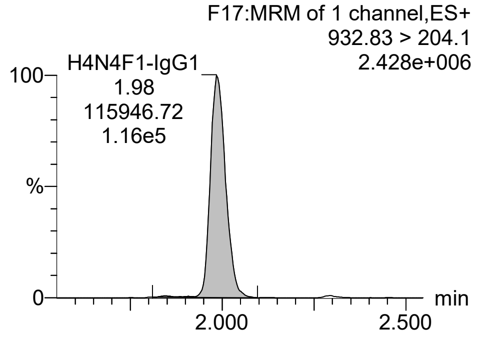

Sample Name: 20211111_QC_R10_IgG

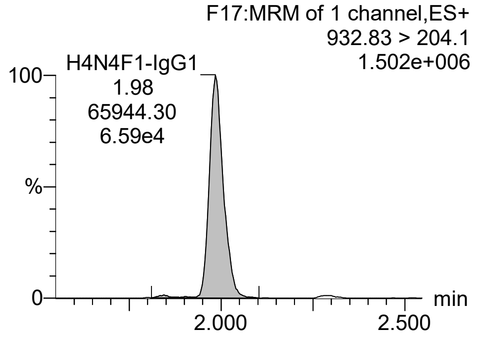

Sample Name: 20211111_L5_R2_IgG

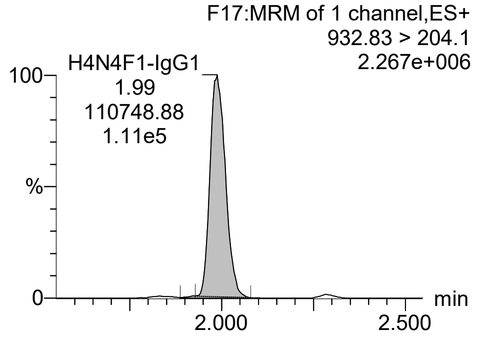

Sample Name: 20211111_L6_R2_IgG

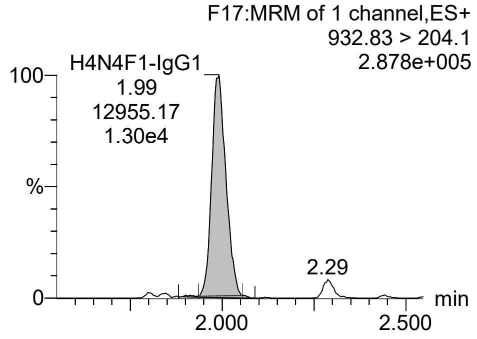

Sample Name: 20211111_H3_R2_IgG

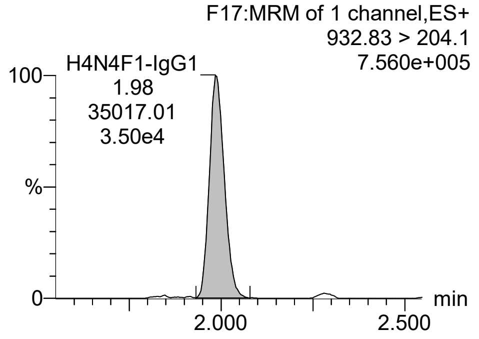

Sample Name: 20211111_A7_R2_IgG

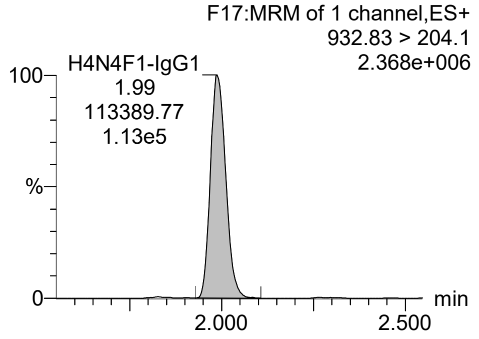

Sample Name: 20211111_A8_R2_IgG

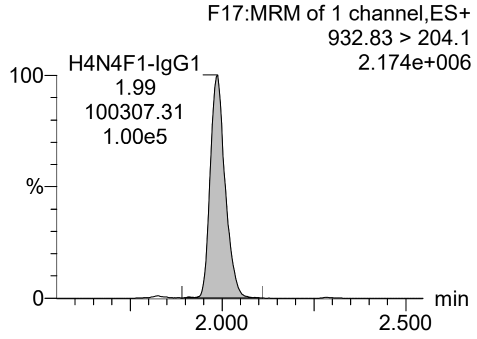

Sample Name: 20211111_L7_R2_IgG

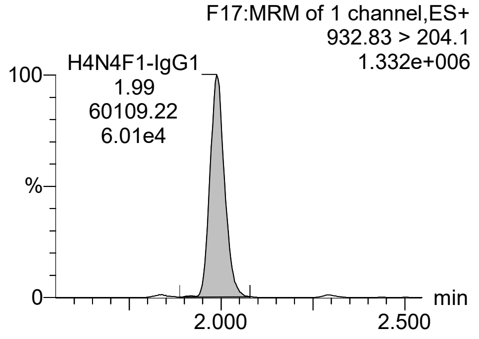

Sample Name: 20211111_L8_R2_IgG

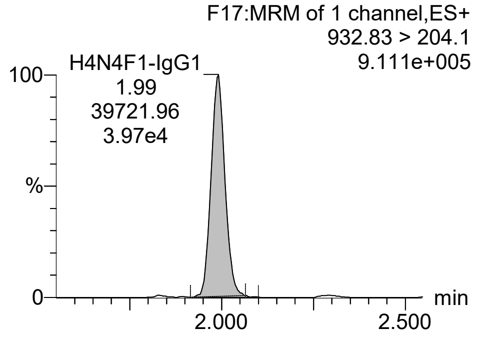

Sample Name: 20211111_H4_R2_IgG

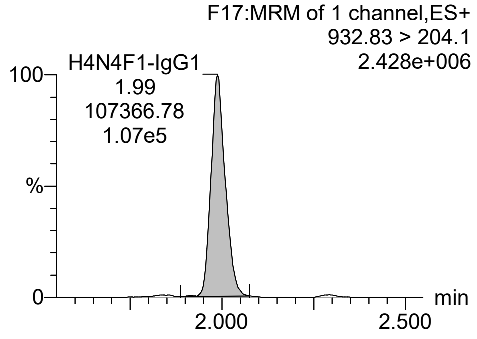

Sample Name: 20211111_A9_R2_IgG

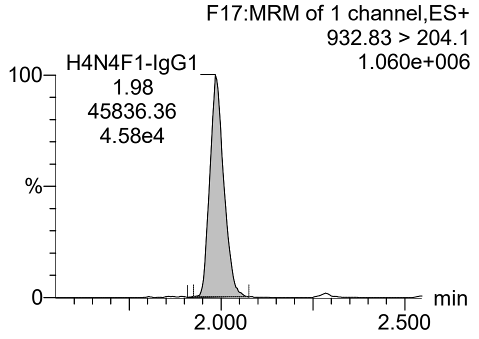

Sample Name: 20211111_A10_R2_IgG

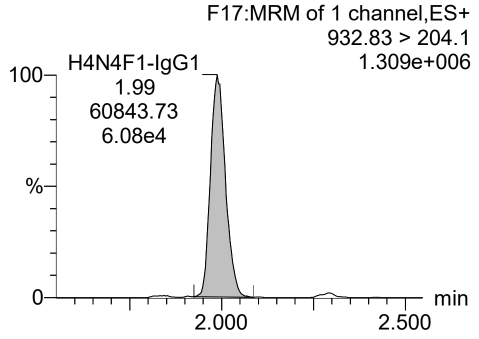

Sample Name: 20211111_L9_R2_IgG

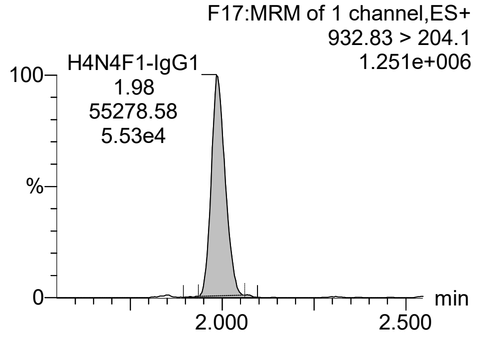

Sample Name: 20211111_L10_R2_IgG

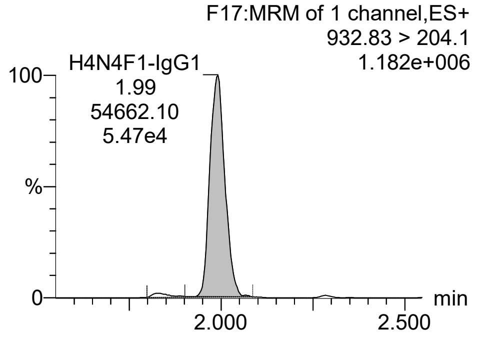

Sample Name: 20211111_QC_R11_IgG

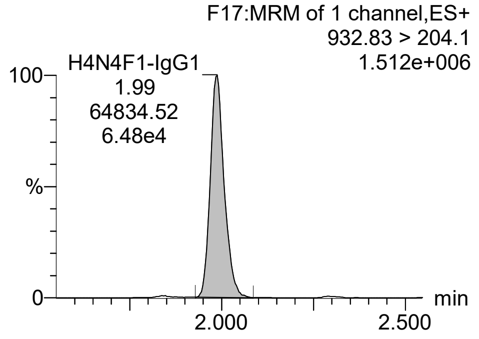

Sample Name: 20211111_H5_R2_IgG

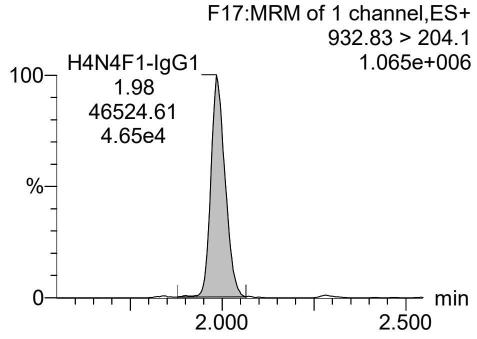

Dataset: C:\MassLynx\10.pro\20211115_clinicalsamples_IgG.qld

Last Altered: Sunday, October 02, 2022 16:02:30 台北標準時間
Printed: Friday, August 01, 2025 16:43:53 台北標準時間

Sample Name: 20211111_A11_R2_IgG

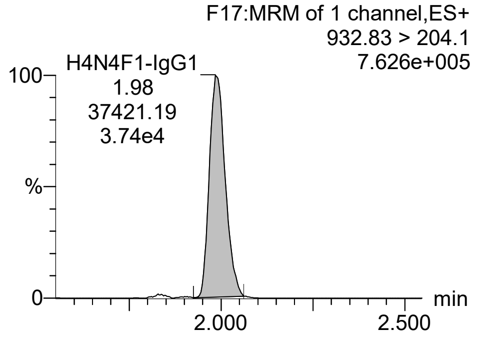

Sample Name: 20211111_A12_R2_IgG

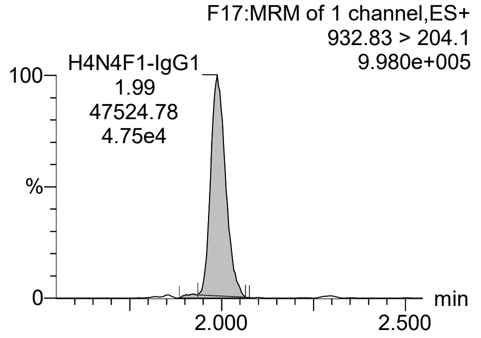

Sample Name: 20211111_L11_R2_IgG

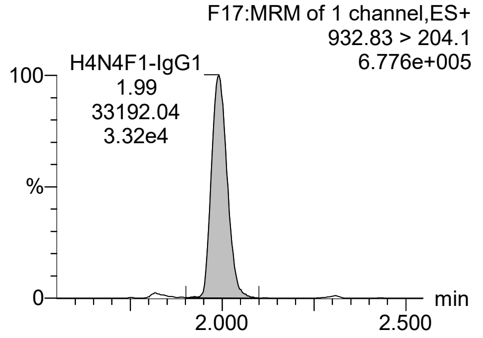

Sample Name: 20211111_L12_R2_IgG

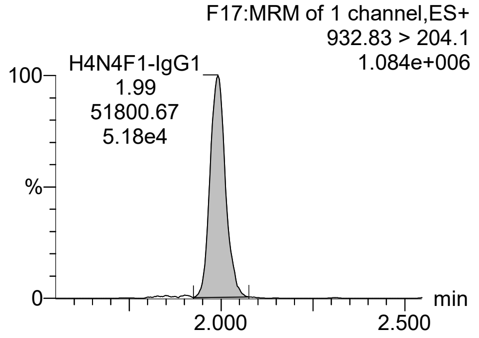

Sample Name: 20211111_H6_R2_IgG

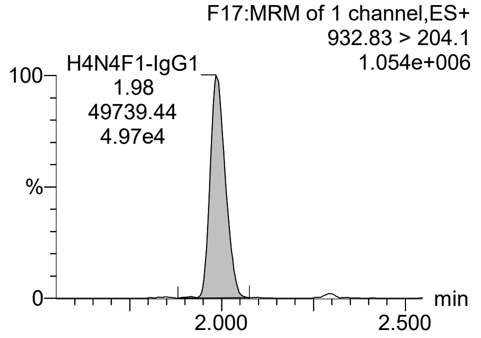

Sample Name: 20211111_A13_R2_IgG

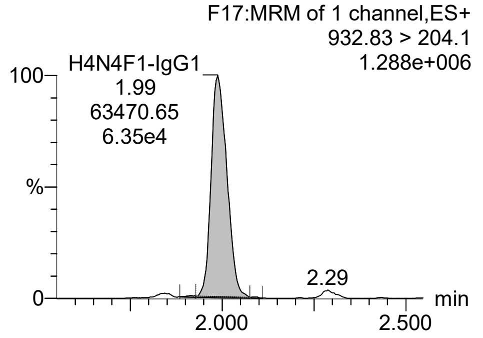

Sample Name: 20211111_A14_R2_IgG

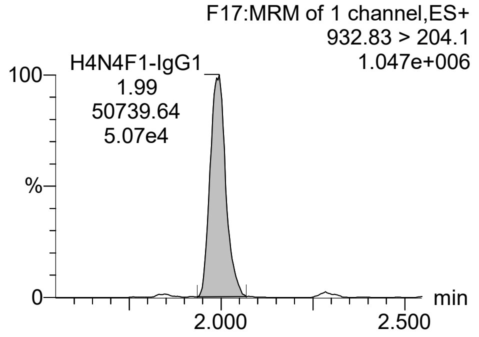

Sample Name: 20211111_L13_R2_IgG

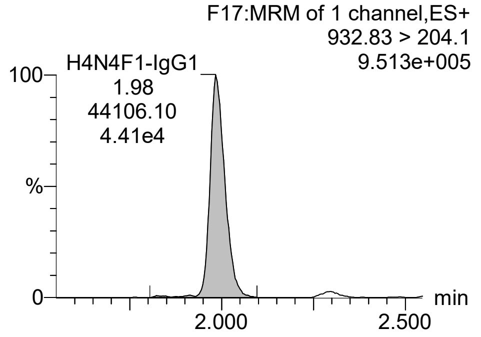

Sample Name: 20211111_L14_R2_IgG

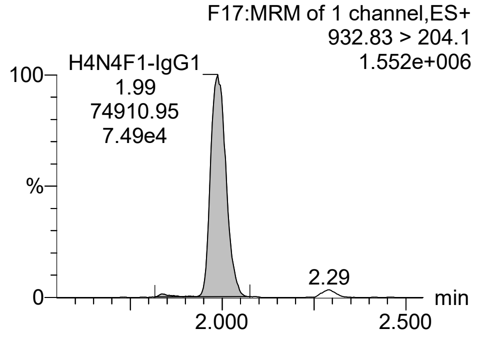

Sample Name: 20211111_H7_R2_IgG

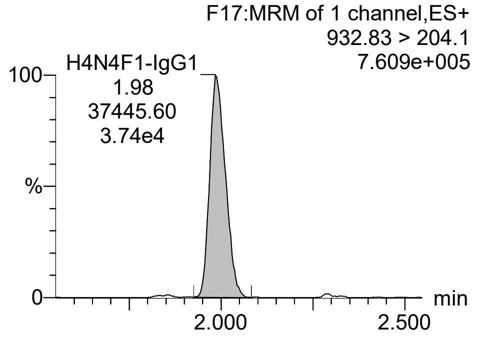

Sample Name: 20211111_A15_R2_IgG

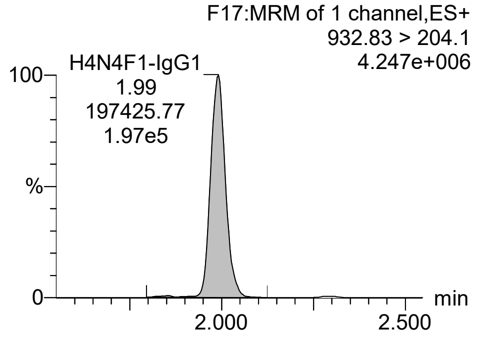

Sample Name: 20211111_QC_R12_IgG

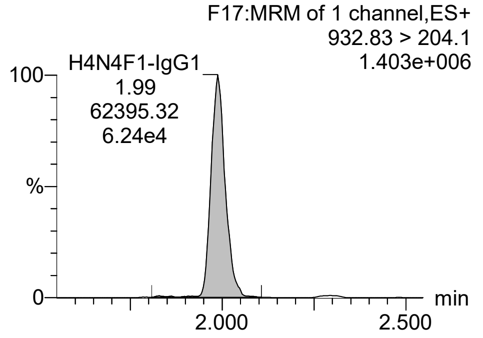

Sample Name: 20211111_A16_R2_IgG

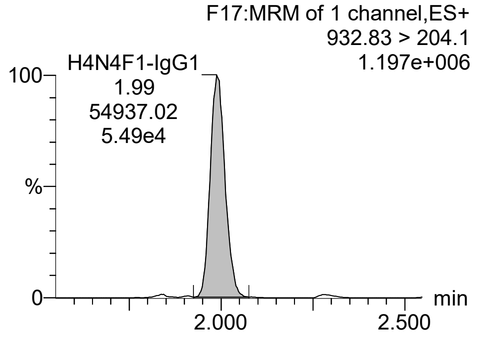

Sample Name: 20211111_L15_R2_IgG

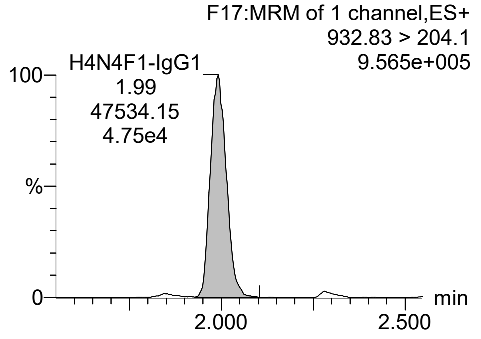

Sample Name: 20211111_L16_R2_IgG

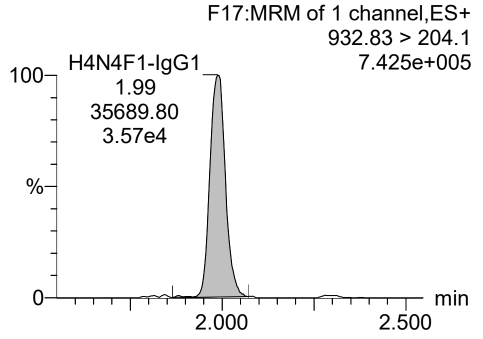

Sample Name: 20211111_H8_R2_IgG

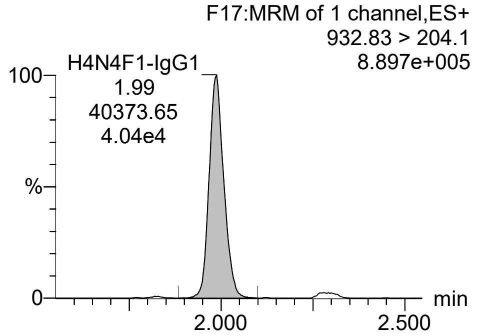

Sample Name: 20211111_A17_R2_IgG

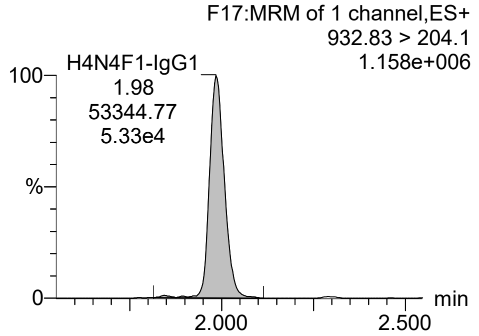

Sample Name: 20211111_A18_R2_IgG

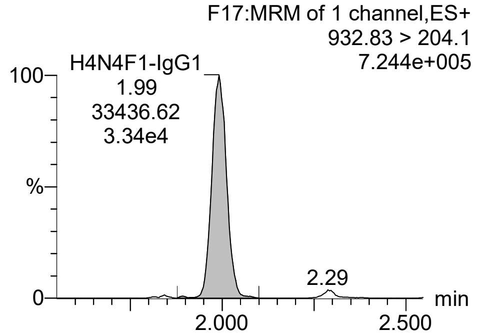

Sample Name: 20211111_L17_R2_IgG

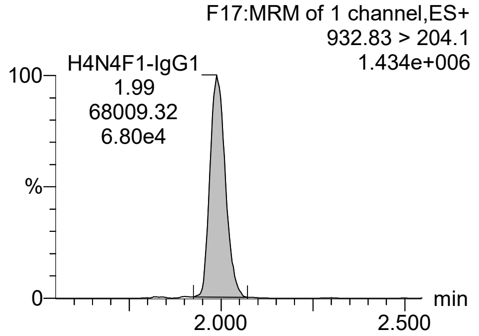

Sample Name: 20211111_L18_R2_IgG

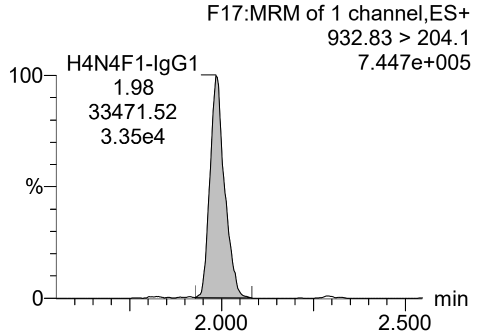

Sample Name: 20211111_H9_R2_IgG

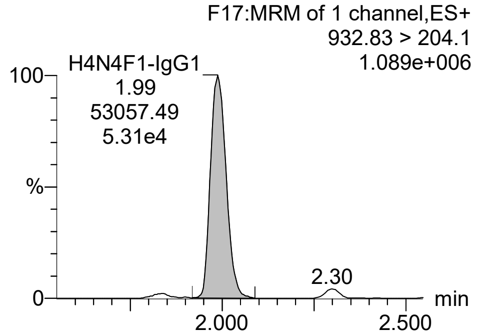

Sample Name: 20211111_A19_R2_IgG

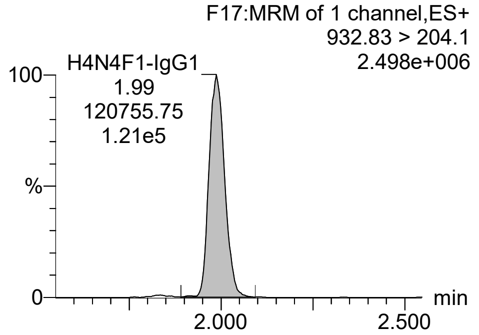

Sample Name: 20211111_A20_R2_IgG

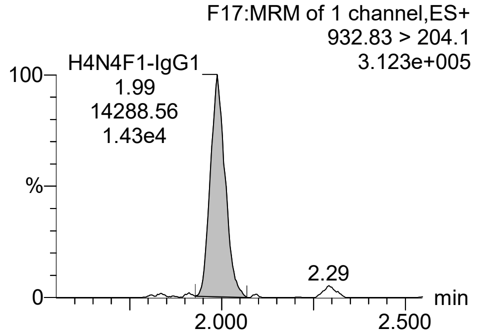

Sample Name: 20211111_L19_R2_IgG

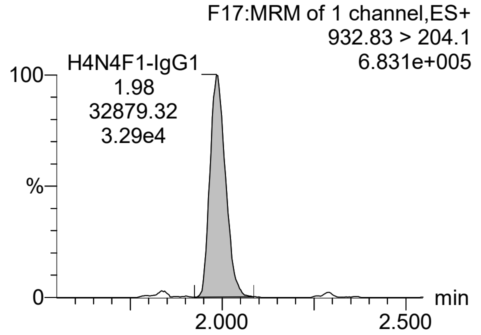

Sample Name: 20211111_QC_R13_IgG

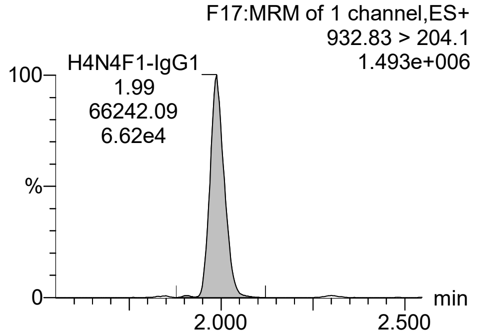

Sample Name: 20211111_L20_R2_IgG

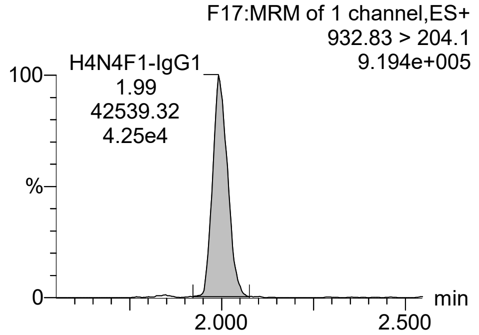

Sample Name: 20211111_H10_R2_IgG

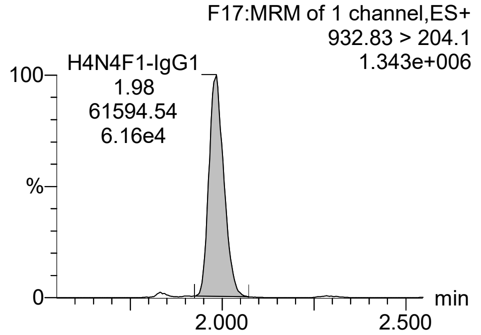

Sample Name: 20211111_A21_R2_IgG

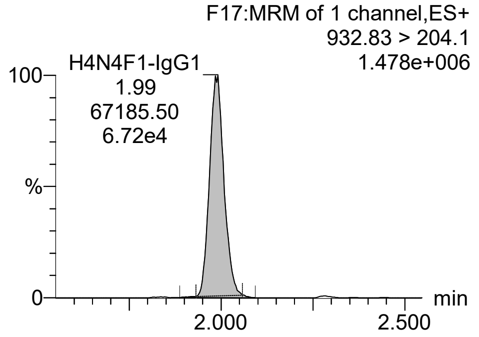

Sample Name: 20211111_A22_R2_IgG

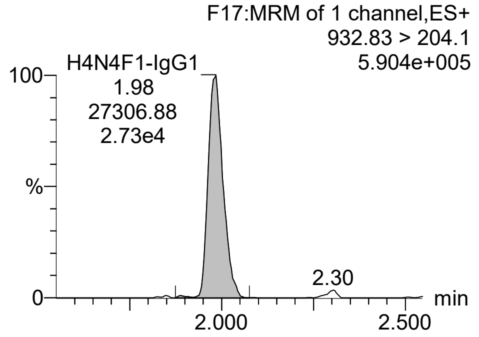

Sample Name: 20211111_L21_R2_IgG

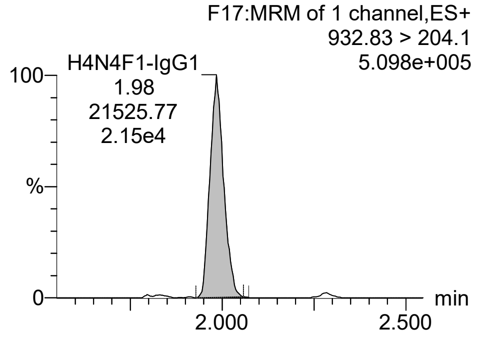

Sample Name: 20211111_L22_R2_IgG

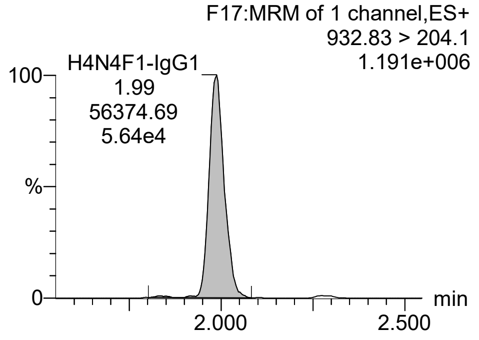

Sample Name: 20211111_H11_R2_IgG

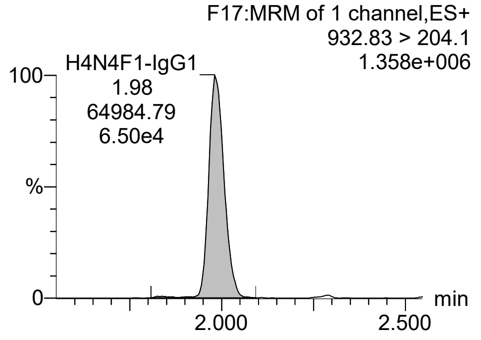

Sample Name: 20211111_A23_R2_IgG

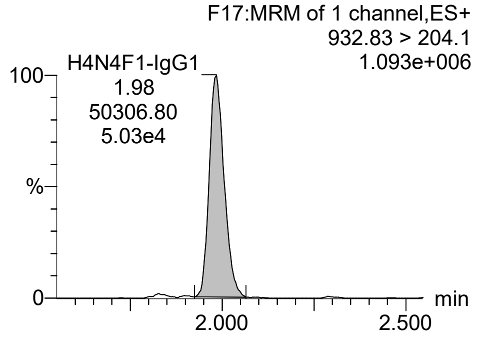

Sample Name: 20211111_A24_R2_IgG

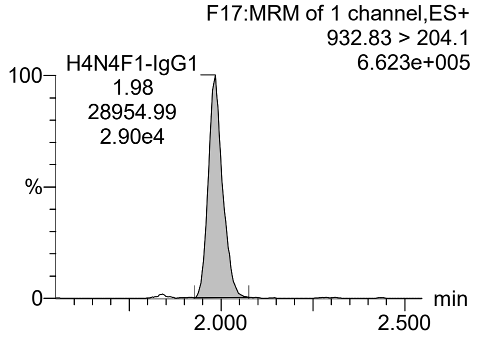

Sample Name: 20211111_L23_R2_IgG

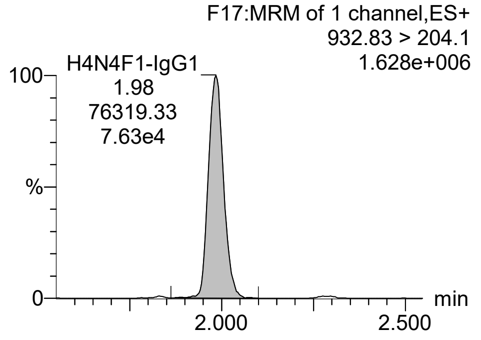

Sample Name: 20211111_L24_R2_IgG

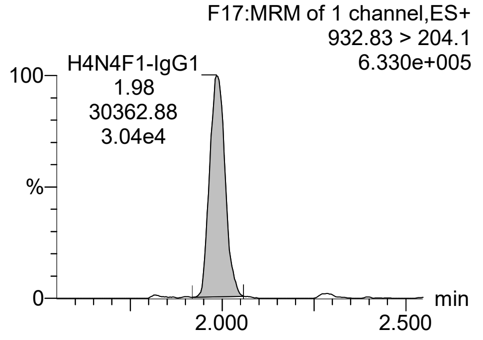

Sample Name: 20211111_H12_R2_IgG

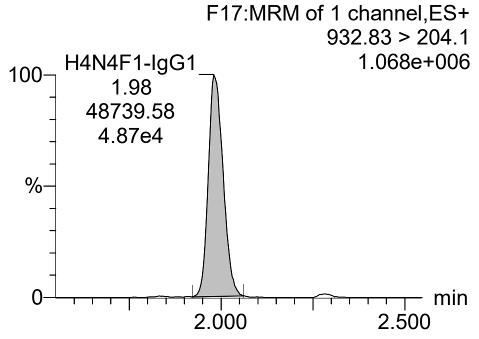

Sample Name: 20211111_QC_R14_IgG

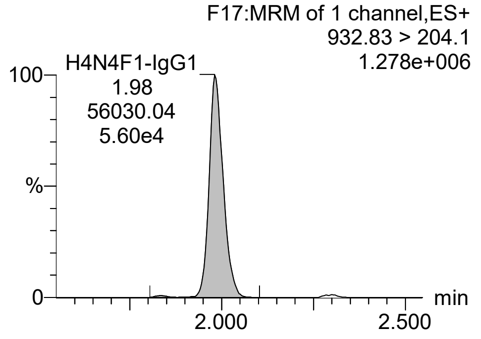

Sample Name: 20211111_A25_R2_IgG

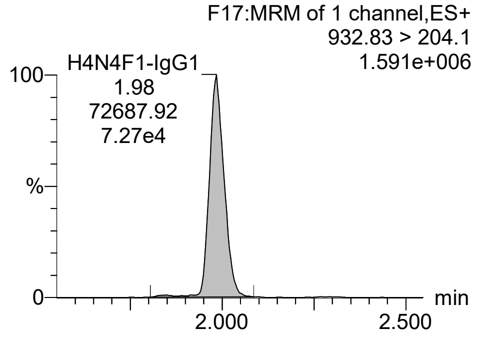

Sample Name: 20211111_A26_R2_IgG

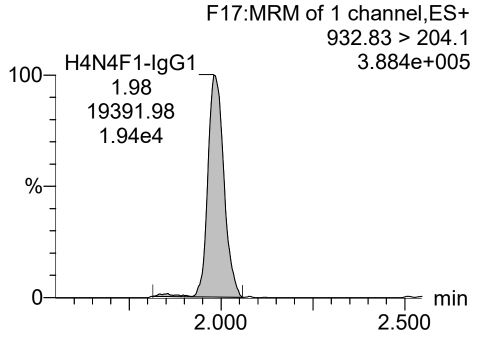

Sample Name: 20211111_L25_R2_IgG

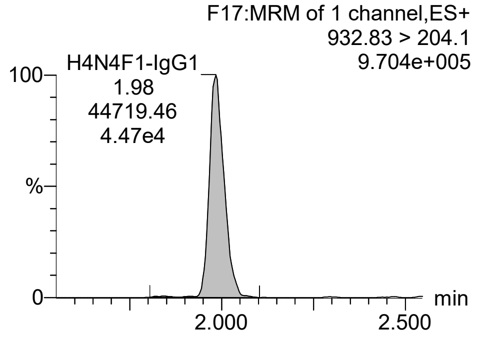

Sample Name: 20211111_L26_R2_IgG

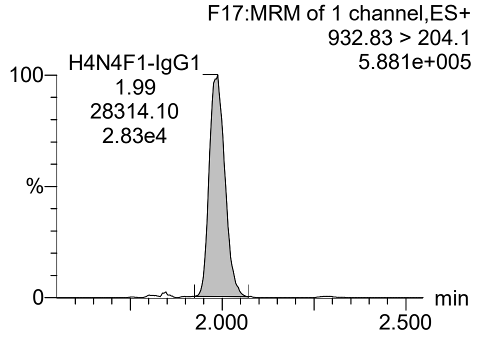

Sample Name: 20211111_H13_R2_IgG

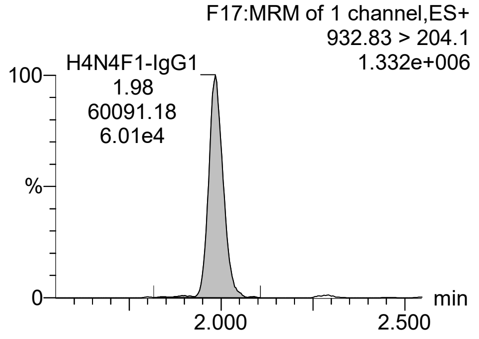

Sample Name: 20211111_A27_R2_IgG

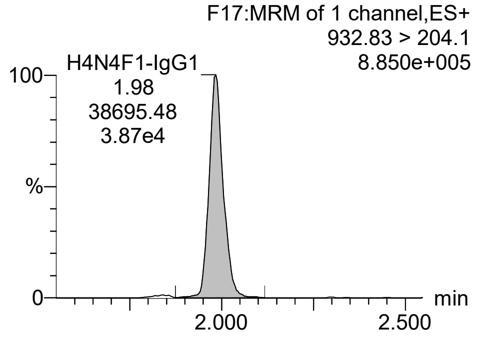

Sample Name: 20211111_A28_R2_IgG

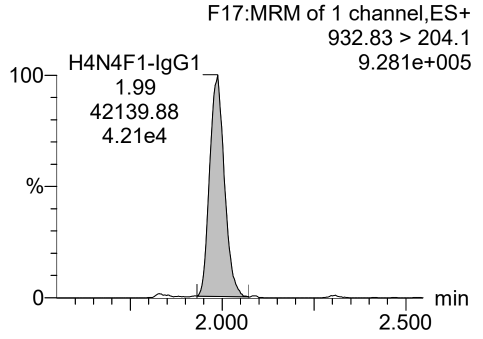

Dataset: C:\MassLynx\10.pro\20211115_clinicalsamples_IgG.qld

Last Altered: Sunday, October 02, 2022 16:02:30 台北標準時間
Printed: Friday, August 01, 2025 16:43:53 台北標準時間

Sample Name: 20211111_L27_R2_IgG

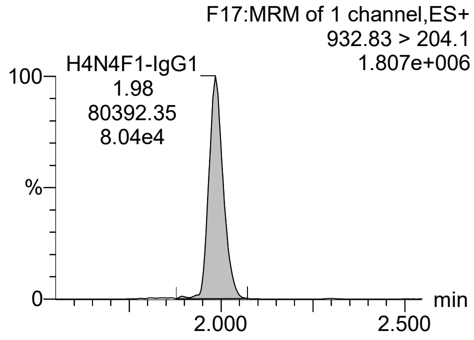

Sample Name: 20211111_L28_R2_IgG

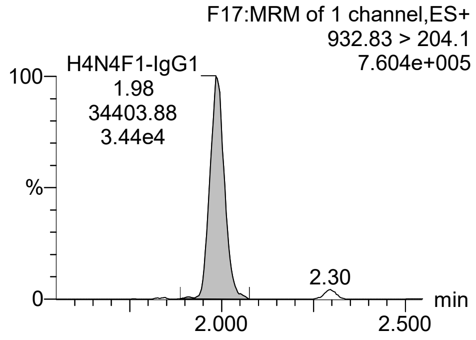

Sample Name: 20211111_H14_R2_IgG

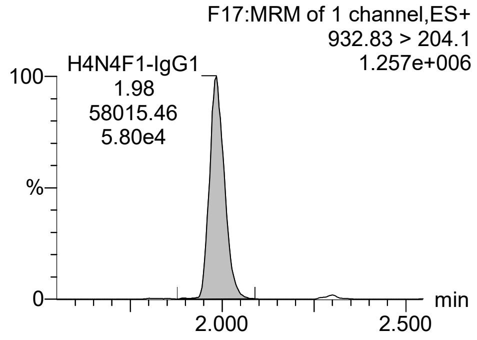

Sample Name: 20211111_A29_R2_IgG

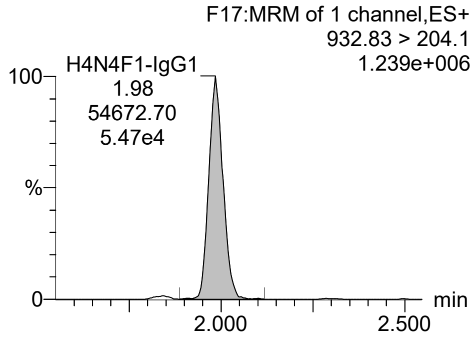

Sample Name: 20211111_A30_R2_IgG

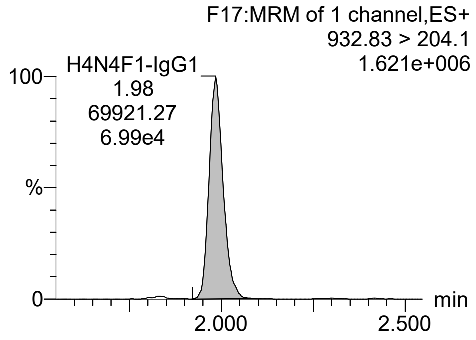

Sample Name: 20211111_QC_R15_IgG

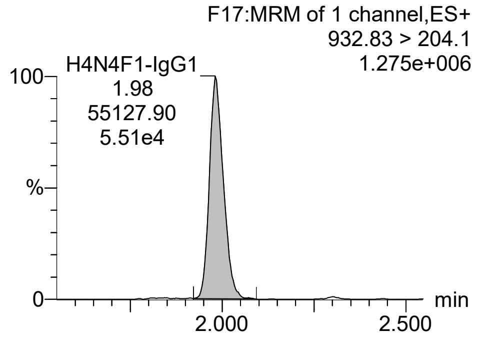

Sample Name: 20211111_L29_R2_IgG

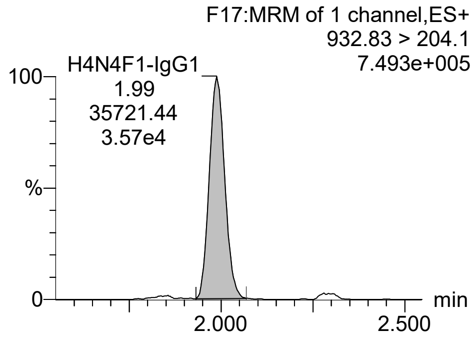

Sample Name: 20211111_L30_R2_IgG

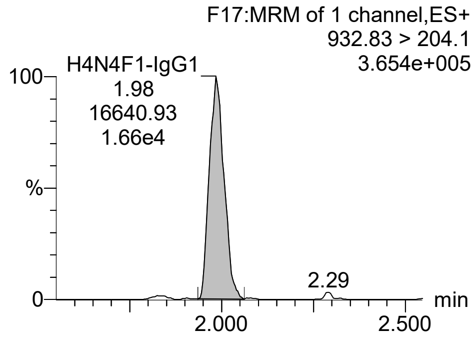

Sample Name: 20211111_H15_R2_IgG

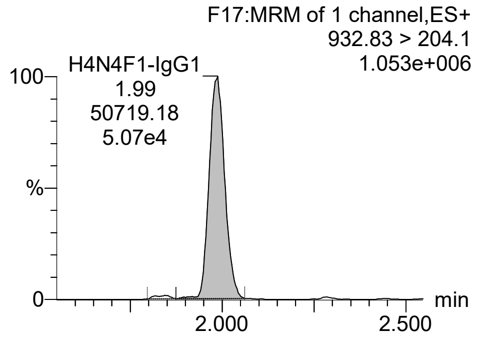

Sample Name: 20211111_A31_R2_IgG

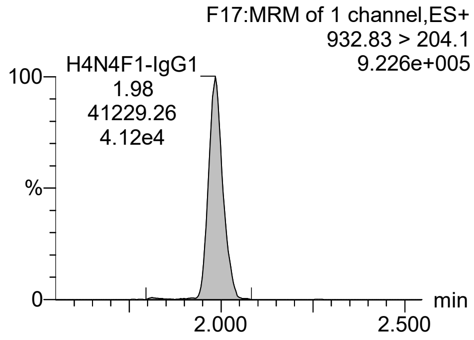

Sample Name: 20211111_A32_R2_IgG

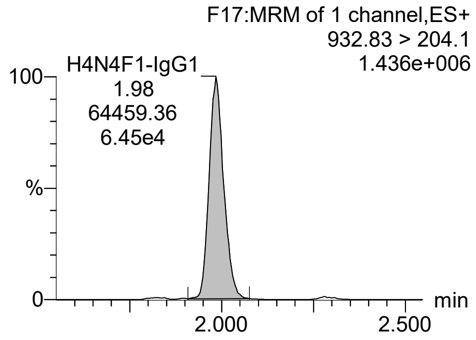

Sample Name: 20211111_L31_R2_IgG

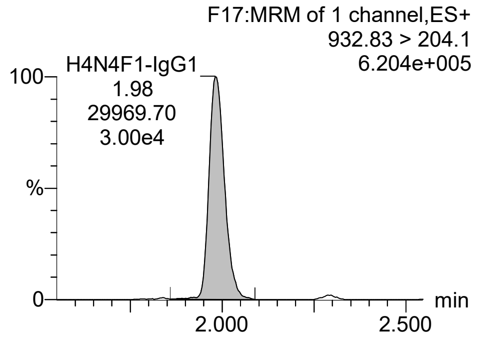

Sample Name: 20211111_L32_R2_IgG

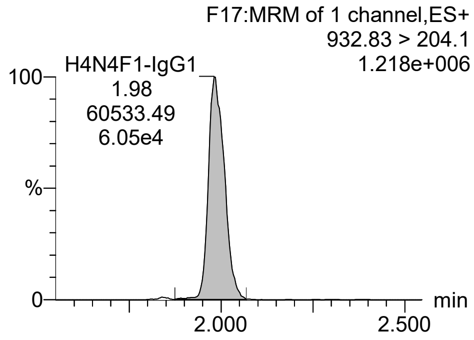

Sample Name: 20211111_H16_R2_IgG

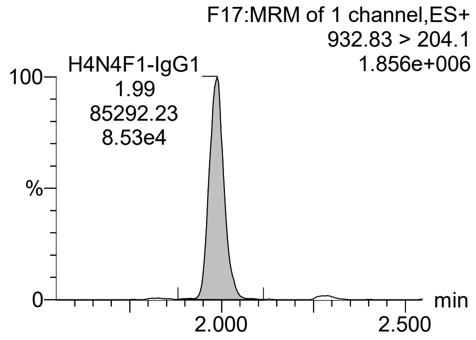

Sample Name: 20211111_A33_R2_IgG

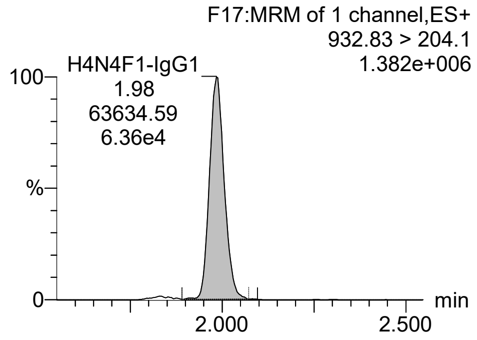

Dataset: C:\MassLynx\10.pro\20211115_clinicalsamples_IgG.qld

Last Altered: Sunday, October 02, 2022 16:02:30 台北標準時間
Printed: Friday, August 01, 2025 16:43:53 台北標準時間

Sample Name: 20211111_A34_R2_IgG

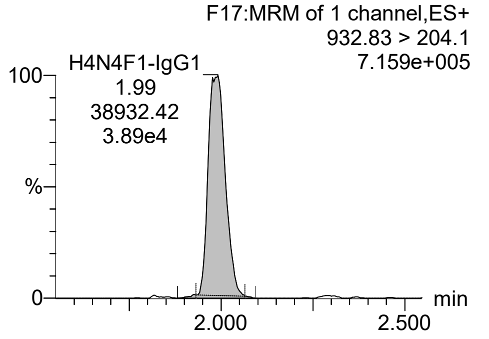

Sample Name: 20211111_H17_R2_IgG

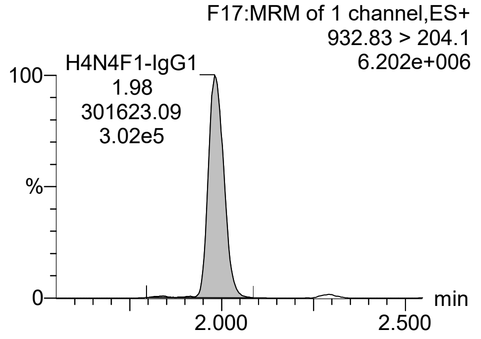

Sample Name: 20211111_A35_R2_IgG

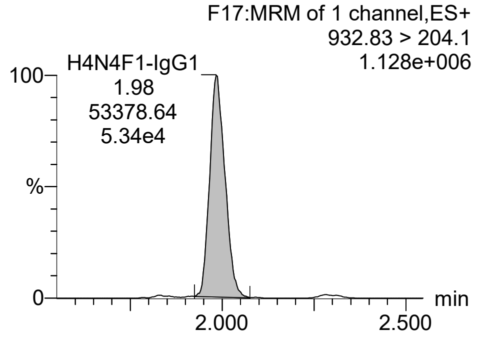

Sample Name: 20211111_QC_R16_IgG

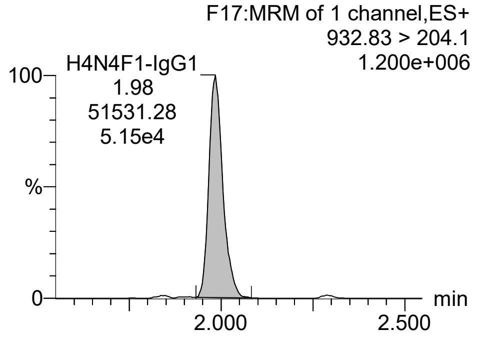

Sample Name: 20211111_A36_R2_IgG

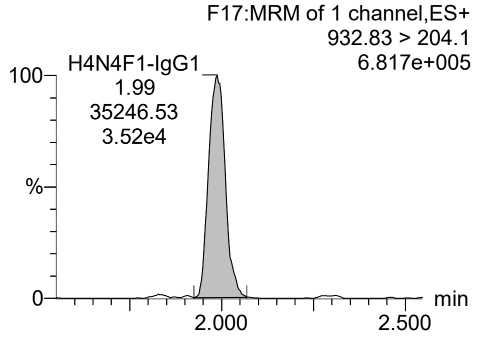

Sample Name: 20211111_H18_R2_IgG

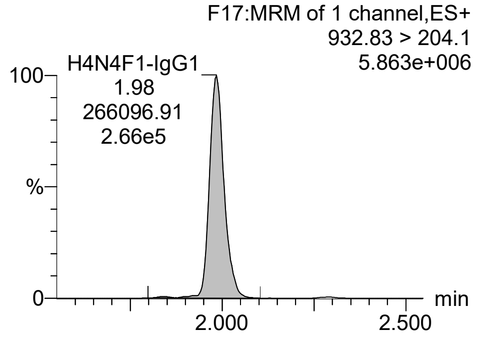

Sample Name: 20211111_A37_R2_IgG

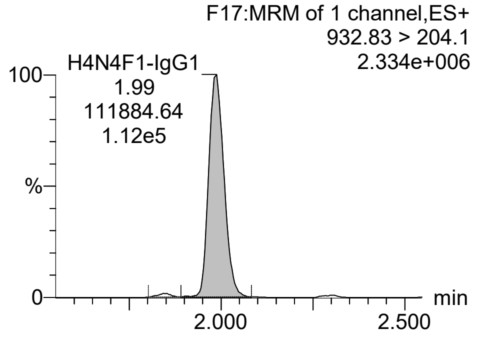

Sample Name: 20211111_A38_R2_IgG

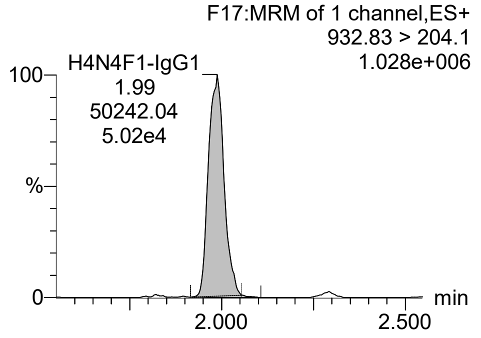

Sample Name: 20211111_H19_R2_IgG

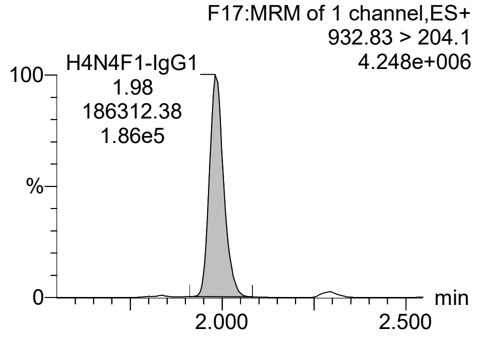

Sample Name: 20211111_A39_R2_IgG

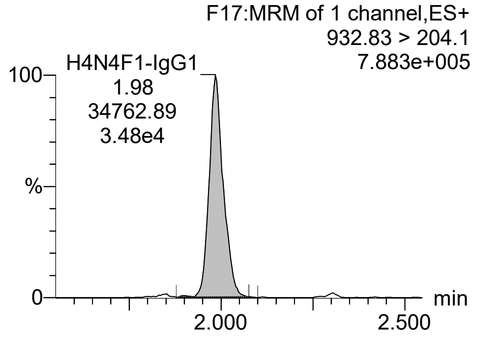

Sample Name: 20211111_A40_R2_IgG

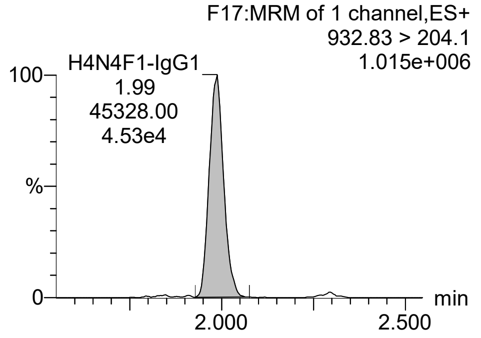

Sample Name: 20211111_H20_R2_IgG

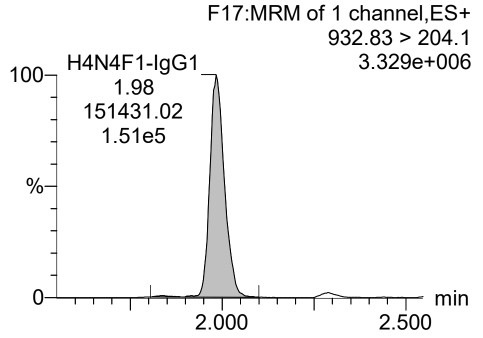

Sample Name: 20211111_A41_R2_IgG

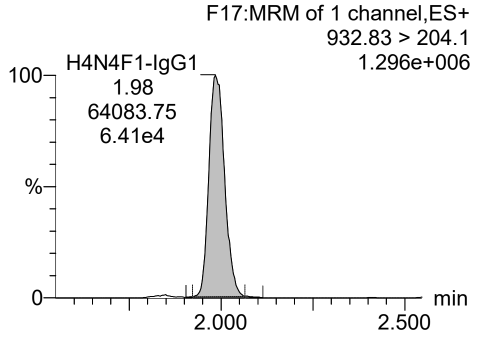

Sample Name: 20211111_A42_R2_IgG

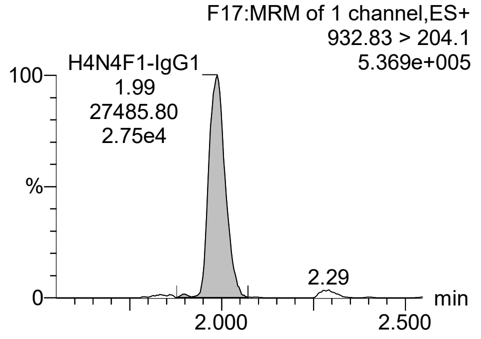

Sample Name: 20211111_A43_R2_IgG

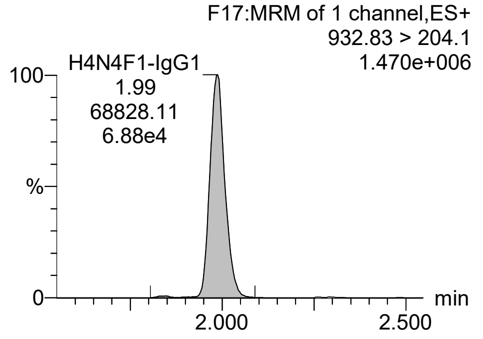

Dataset: C:\MassLynx\10.pro\20211115_clinicalsamples_IgG.qld

Last Altered: Sunday, October 02, 2022 16:02:30 台北標準時間
Printed: Friday, August 01, 2025 16:43:53 台北標準時間

Sample Name: 20211111_A44_R2_IgG

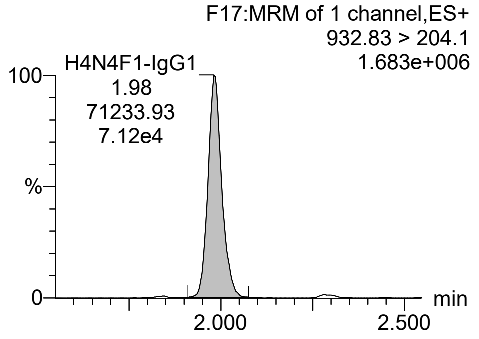

Sample Name: 20211111_QC_R17_IgG

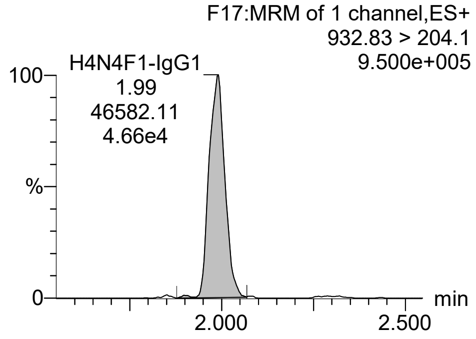

Dataset: C:\MassLynx\10.pro\20211115_clinicalsamples_IgG.qld

Last Altered: Sunday, October 02, 2022 16:02:30 台北標準時間
Printed: Friday, August 01, 2025 16:43:53 台北標準時間

	#	Name	RT	Area	IS Area	Response	Prima...
1	1	20211111_QC_R1_IgG	1.99	127866.7	64968.0	1.968	bb
2	2	20211111_A1_R1_IgG	1.99	132794.1	59059.2	2.248	bb
3	3	20211111_A2_R1_IgG	1.99	105290.3	66621.2	1.580	bb
4	4	20211111_L1_R1_IgG	1.99	128120.6	64201.0	1.996	bb
5	5	20211111_L2_R1_IgG	1.99	58097.2	46950.2	1.237	bb
6	6	20211111_H1_R1_IgG	1.99	53962.4	38648.2	1.396	bb
7	7	20211111_A3_R1_IgG	2.00	2691.1	2603.7	1.034	bb
8	8	20211111_A4_R1_IgG	1.99	98629.3	69960.4	1.410	bb
9	9	20211111_L3_R1_IgG	1.99	36632.3	38864.7	0.943	bb
10	10	20211111_L4_R1_IgG	1.99	51814.9	43380.5	1.194	bb
11	11	20211111_H2_R1_IgG	1.99	486735.5	71349.2	6.822	bb
12	12	20211111_A5_R1_IgG	1.99	85154.2	65569.7	1.299	bb
13	13	20211111_A6_R1_IgG	1.99	131440.2	53120.7	2.474	bb
14	14	20211111_QC_R2_IgG	1.99	117712.7	81034.1	1.453	bb
15	15	20211111_L5_R1_IgG	1.99	128556.0	50994.6	2.521	MM
16	16	20211111_L6_R1_IgG	1.99	17022.7	58058.0	0.293	MM
17	17	20211111_H3_R1_IgG	1.99	42743.8	64028.6	0.668	MM
18	18	20211111_A7_R1_IgG	1.99	151947.1	82996.1	1.831	bb
19	19	20211111_A8_R1_IgG	1.99	135585.2	77731.7	1.744	bb
20	20	20211111_L7_R1_IgG	1.99	63223.6	38827.3	1.628	bb
21	21	20211111_L8_R1_IgG	1.99	43959.5	44220.1	0.994	bb
22	22	20211111_H4_R1_IgG	1.99	131639.5	65826.3	2.000	bb
23	23	20211111_A9_R1_IgG	1.99	57120.6	60771.1	0.940	bb
24	24	20211111_A10_R1_IgG	1.99	70751.9	48578.7	1.456	bb
25	25	20211111_L9_R1_IgG	1.99	56353.7	50830.7	1.109	bb
26	26	20211111_L10_R1_IgG	1.99	64304.5	38034.2	1.691	MM
27	27	20211111_QC_R3_IgG	1.99	118842.0	67025.5	1.773	bb
28	28	20211111_H5_R1_IgG	1.99	51862.9	44544.9	1.164	bb
29	29	20211111_A11_R1_IgG	1.99	55679.5	66408.5	0.838	bb
30	30	20211111_A12_R1_IgG	1.99	53029.6	46624.5	1.137	bb
31	31	20211111_L11_R1_IgG	1.99	42216.3	33425.1	1.263	bb
32	32	20211111_L12_R1_IgG	1.99	59905.6	41178.0	1.455	bb
33	33	20211111_H6_R1_IgG	1.99	94794.4	58593.4	1.618	bb
34	34	20211111_A13_R1_IgG	1.99	90205.9	54133.0	1.666	bb
35	35	20211111_A14_R1_IgG	1.99	65870.6	56467.3	1.167	bb
36	36	20211111_L13_R1_IgG	1.99	54391.4	36024.4	1.510	bb
37	37	20211111_L14_R1_IgG	1.99	78516.9	37181.6	2.112	bb
38	38	20211111_H7_R1_IgG	1.99	47273.6	42850.6	1.103	MM
39	39	20211111_A15_R1_IgG	2.00	239376.7	62015.1	3.860	MM
40	40	20211111_QC_R4_IgG	1.99	106356.3	58572.5	1.816	bb
41	41	20211111_A16_R1_IgG	1.99	98986.0	78283.8	1.264	bb
42	42	20211111_L15_R1_IgG	1.99	69032.7	44158.2	1.563	bb
43	43	20211111_L16_R1_IgG	1.99	57505.4	55150.8	1.043	bb
44	44	20211111_H8_R1_IgG	1.99	41233.8	42965.2	0.960	MM
45	45	20211111_A17_R1_IgG	1.99	53573.2	39717.7	1.349	bb
46	46	20211111_A18_R1_IgG	1.99	41451.1	44675.4	0.928	bb
47	47	20211111_L17_R1_IgG	1.99	79991.8	37854.1	2.113	bb
48	48	20211111_L18_R1_IgG	1.99	48495.1	50716.9	0.956	bb
49	49	20211111_H9_R1_IgG	1.99	60331.9	36276.0	1.663	bb
50	50	20211111_A19_R1_IgG	1.99	124097.7	42862.5	2.895	bb
51	51	20211111_A20_R1_IgG	1.99	20324.1	36542.7	0.556	MM
52	52	20211111_L19_R1_IgG	1.99	50996.5	53892.8	0.946	bb
53	53	20211111_QC_R5_IgG	1.99	99726.0	56513.2	1.765	bb
54	54	20211111_L20_R1_IgG	1.99	78119.1	56499.9	1.383	bb
55	55	20211111_H10_R1_IgG	1.99	148244.9	68120.5	2.176	MM
56	56	20211111_A21_R1_IgG	1.99	139287.6	63699.8	2.187	bb

Dataset: C:\MassLynx\10.pro\20211115_clinicalsamples_IgG.qld

Last Altered: Sunday, October 02, 2022 16:02:30 台北標準時間
Printed: Friday, August 01, 2025 16:43:53 台北標準時間

	# Name	RT	Area	IS Area	Response	Prima...
57	57 20211111_A22_R1_IgG	1.99	56362.8	52222.1	1.079	bb
58	58 20211111_L21_R1_IgG	1.99	35453.1	54372.5	0.652	bb
59	59 20211111_L22_R1_IgG	1.99	95050.0	41353.1	2.298	bb
60	60 20211111_H11_R1_IgG	1.99	133914.7	65579.1	2.042	bb
61	61 20211111_A23_R1_IgG	1.99	116138.1	64131.1	1.811	bb
62	62 20211111_A24_R1_IgG	1.99	45709.6	38724.8	1.180	MM
63	63 20211111_L23_R1_IgG	1.99	122641.0	64245.2	1.909	bb
64	64 20211111_L24_R1_IgG	1.99	46258.6	45298.0	1.021	bb
65	65 20211111_H12_R1_IgG	1.99	61582.9	44679.3	1.378	bb
66	66 20211111_QC_R6_IgG	1.99	86701.8	48165.0	1.800	bb
67	67 20211111_A25_R1_IgG	1.99	121627.9	57169.9	2.127	bb
68	68 20211111_A26_R1_IgG	1.99	25909.8	34254.0	0.756	bb
69	69 20211111_L25_R1_IgG	1.99	84397.9	67491.5	1.250	bb
70	70 20211111_L26_R1_IgG	1.99	46508.4	55934.1	0.831	bb
71	71 20211111_H13_R1_IgG	1.99	87623.5	48767.3	1.797	bb
72	72 20211111_A27_R1_IgG	1.99	58485.8	57269.7	1.021	bb
73	73 20211111_A28_R1_IgG	1.99	68739.9	49231.1	1.396	bb
74	74 20211111_L27_R1_IgG	1.99	128604.4	60014.5	2.143	bb
75	75 20211111_L28_R1_IgG	1.99	49138.0	35062.6	1.401	bb
76	76 20211111_H14_R1_IgG	1.99	91053.9	62371.9	1.460	bb
77	77 20211111_A29_R1_IgG	1.99	70050.1	43858.2	1.597	bb
78	78 20211111_A30_R1_IgG	1.99	108889.3	62500.4	1.742	bb
79	79 20211111_QC_R7_IgG	1.99	81661.4	43946.9	1.858	bb
80	80 20211111_L29_R1_IgG	1.99	41095.9	38728.9	1.061	bb
81	81 20211111_L30_R1_IgG	1.98	23920.8	42613.2	0.561	bb
82	82 20211111_H15_R1_IgG	1.99	57387.5	39874.6	1.439	bb
83	83 20211111_A31_R1_IgG	1.99	68049.0	53414.3	1.274	bb
84	84 20211111_A32_R1_IgG	1.99	89400.8	41844.6	2.136	MM
85	85 20211111_L31_R1_IgG	1.99	43699.9	40553.7	1.078	bb
86	86 20211111_L32_R1_IgG	1.99	85501.9	49618.7	1.723	bb
87	87 20211111_H16_R1_IgG	1.99	120203.4	60170.9	1.998	bb
88	88 20211111_A33_R1_IgG	1.99	80166.1	48588.2	1.650	bb
89	89 20211111_A34_R1_IgG	1.99	58634.4	43288.0	1.355	bb
90	90 20211111_H17_R1_IgG	1.99	399502.3	47104.5	8.481	bb
91	91 20211111_A35_R1_IgG	1.99	76258.9	41025.9	1.859	bb
92	92 20211111_QC_R8_IgG	1.99	73435.2	40546.3	1.811	bb
93	93 20211111_A36_R1_IgG	1.99	49551.7	44504.4	1.113	bb
94	94 20211111_H18_R1_IgG	1.99	309334.7	34162.2	9.055	bb
95	95 20211111_A37_R1_IgG	1.99	115101.2	39760.0	2.895	bb
96	96 20211111_A38_R1_IgG	1.99	64278.5	43982.6	1.461	bb
97	97 20211111_H19_R1_IgG	1.99	218950.0	45314.8	4.832	bb
98	98 20211111_A39_R1_IgG	1.99	38363.0	39388.0	0.974	bb
99	99 20211111_A40_R1_IgG	1.99	45612.8	38726.4	1.178	MM
100	1... 20211111_H20_R1_IgG	1.99	167357.1	37814.4	4.426	bb
101	1... 20211111_A41_R1_IgG	1.99	81523.0	48930.3	1.666	MM
102	1... 20211111_A42_R1_IgG	1.99	31445.6	40406.8	0.778	bb
103	1... 20211111_A43_R1_IgG	1.99	83886.7	44421.5	1.888	bb
104	1... 20211111_A44_R1_IgG	1.99	105724.0	53128.2	1.990	bb
105	1... 20211111_QC_R9_IgG	1.99	61401.2	37049.0	1.657	bb
106	1... 20211111_A1_R2_IgG	1.99	110020.4	46499.4	2.366	bb
107	1... 20211111_A2_R2_IgG	1.99	75461.4	57260.7	1.318	bb
108	1... 20211111_L1_R2_IgG	2.00	81265.9	48062.9	1.691	bb
109	1... 20211111_L2_R2_IgG	1.99	48225.6	38745.2	1.245	bb
110	1... 20211111_H1_R2_IgG	1.99	62057.5	44273.5	1.402	bb
111	1... 20211111_A3_R2_IgG	2.00	5879.2	4755.5	1.236	MM
112	1... 20211111_A4_R2_IgG	1.99	72025.0	50546.7	1.425	MM

Dataset: C:\MassLynx\10.pro\20211115_clinicalsamples_IgG.qld

Last Altered: Sunday, October 02, 2022 16:02:30 台北標準時間
Printed: Friday, August 01, 2025 16:43:53 台北標準時間

	# Name	RT	Area	IS Area	Response	Prima...
113	1... 20211111_L3_R2_IgG	1.99	37422.0	34825.8	1.075	bb
114	1... 20211111_L4_R2_IgG	1.98	47689.8	39107.7	1.219	MM
115	1... 20211111_H2_R2_IgG	1.99	356580.2	49750.0	7.167	bb
116	1... 20211111_A5_R2_IgG	1.99	73047.4	52536.2	1.390	bb
117	1... 20211111_A6_R2_IgG	1.98	115946.7	42050.0	2.757	bb
118	1... 20211111_QC_R10_IgG	1.98	65944.3	35350.8	1.865	bb
119	1... 20211111_L5_R2_IgG	1.99	110748.9	38039.3	2.911	MM
120	1... 20211111_L6_R2_IgG	1.99	12955.2	44907.4	0.288	MM
121	1... 20211111_H3_R2_IgG	1.98	35017.0	47729.1	0.734	bb
122	1... 20211111_A7_R2_IgG	1.99	113389.8	57347.1	1.977	bb
123	1... 20211111_A8_R2_IgG	1.99	100307.3	52397.8	1.914	bb
124	1... 20211111_L7_R2_IgG	1.99	60109.2	36318.4	1.655	bb
125	1... 20211111_L8_R2_IgG	1.99	39722.0	38131.9	1.042	MM
126	1... 20211111_H4_R2_IgG	1.99	107366.8	46849.6	2.292	bb
127	1... 20211111_A9_R2_IgG	1.98	45836.4	47759.6	0.960	MM
128	1... 20211111_A10_R2_IgG	1.99	60843.7	41366.2	1.471	bb
129	1... 20211111_L9_R2_IgG	1.98	55278.6	43052.1	1.284	MM
130	1... 20211111_L10_R2_IgG	1.99	54662.1	31487.4	1.736	MM
131	1... 20211111_QC_R11_IgG	1.99	64834.5	37804.6	1.715	bb
132	1... 20211111_H5_R2_IgG	1.98	46524.6	40380.0	1.152	bb
133	1... 20211111_A11_R2_IgG	1.98	37421.2	49533.3	0.755	bb
134	1... 20211111_A12_R2_IgG	1.99	47524.8	40226.3	1.181	MM
135	1... 20211111_L11_R2_IgG	1.99	33192.0	28884.0	1.149	bb
136	1... 20211111_L12_R2_IgG	1.99	51800.7	34351.3	1.508	bb
137	1... 20211111_H6_R2_IgG	1.98	49739.4	40466.6	1.229	bb
138	1... 20211111_A13_R2_IgG	1.99	63470.7	42306.7	1.500	MM
139	1... 20211111_A14_R2_IgG	1.99	50739.6	44904.0	1.130	bb
140	1... 20211111_L13_R2_IgG	1.98	44106.1	31913.0	1.382	bb
141	1... 20211111_L14_R2_IgG	1.99	74910.9	31527.4	2.376	bb
142	1... 20211111_H7_R2_IgG	1.98	37445.6	40838.2	0.917	bb
143	1... 20211111_A15_R2_IgG	1.99	197425.8	49389.7	3.997	bb
144	1... 20211111_QC_R12_IgG	1.99	62395.3	35168.7	1.774	bb
145	1... 20211111_A16_R2_IgG	1.99	54937.0	47440.5	1.158	bb
146	1... 20211111_L15_R2_IgG	1.99	47534.2	33145.9	1.434	bb
147	1... 20211111_L16_R2_IgG	1.99	35689.8	37596.6	0.949	bb
148	1... 20211111_H8_R2_IgG	1.99	40373.7	43933.3	0.919	bb
149	1... 20211111_A17_R2_IgG	1.98	53344.8	43605.5	1.223	bb
150	1... 20211111_A18_R2_IgG	1.99	33436.6	37581.8	0.890	bb
151	1... 20211111_L17_R2_IgG	1.99	68009.3	33942.5	2.004	bb
152	1... 20211111_L18_R2_IgG	1.98	33471.5	37831.5	0.885	bb
153	1... 20211111_H9_R2_IgG	1.99	53057.5	30898.4	1.717	bb
154	1... 20211111_A19_R2_IgG	1.99	120755.8	39352.1	3.069	bb
155	1... 20211111_A20_R2_IgG	1.99	14288.6	34661.2	0.412	bb
156	1... 20211111_L19_R2_IgG	1.98	32879.3	39918.3	0.824	bb
157	1... 20211111_QC_R13_IgG	1.99	66242.1	36239.1	1.828	bb
158	1... 20211111_L20_R2_IgG	1.99	42539.3	32897.7	1.293	bb
159	1... 20211111_H10_R2_IgG	1.98	61594.5	31394.5	1.962	bb
160	1... 20211111_A21_R2_IgG	1.99	67185.5	34863.7	1.927	MM
161	1... 20211111_A22_R2_IgG	1.98	27306.9	30342.1	0.900	bb
162	1... 20211111_L21_R2_IgG	1.98	21525.8	33706.2	0.639	MM
163	1... 20211111_L22_R2_IgG	1.99	56374.7	31298.6	1.801	bb
164	1... 20211111_H11_R2_IgG	1.98	64984.8	38904.1	1.670	bb
165	1... 20211111_A23_R2_IgG	1.98	50306.8	32382.3	1.554	bb
166	1... 20211111_A24_R2_IgG	1.98	28955.0	30261.9	0.957	bb
167	1... 20211111_L23_R2_IgG	1.98	76319.3	43037.5	1.773	bb
168	1... 20211111_L24_R2_IgG	1.98	30362.9	37023.3	0.820	bb

Dataset: C:\MassLynx\10.pro\20211115_clinicalsamples_IgG.qld

Last Altered: Sunday, October 02, 2022 16:02:30 台北標準時間
Printed: Friday, August 01, 2025 16:43:53 台北標準時間

	# Name	RT	Area	IS Area	Response	Prima...
169	1... 20211111_H12_R2_IgG	1.98	48739.6	39478.2	1.235	bb
170	1... 20211111_QC_R14_IgG	1.98	56030.0	34463.2	1.626	bb
171	1... 20211111_A25_R2_IgG	1.98	72687.9	39945.3	1.820	bb
172	1... 20211111_A26_R2_IgG	1.98	19392.0	29663.2	0.654	bb
173	1... 20211111_L25_R2_IgG	1.98	44719.5	38589.3	1.159	bb
174	1... 20211111_L26_R2_IgG	1.99	28314.1	38405.2	0.737	bb
175	1... 20211111_H13_R2_IgG	1.98	60091.2	37771.2	1.591	bb
176	1... 20211111_A27_R2_IgG	1.98	38695.5	42634.1	0.908	bb
177	1... 20211111_A28_R2_IgG	1.99	42139.9	33231.1	1.268	bb
178	1... 20211111_L27_R2_IgG	1.98	80392.4	41761.1	1.925	bb
179	1... 20211111_L28_R2_IgG	1.98	34403.9	26968.6	1.276	bb
180	1... 20211111_H14_R2_IgG	1.98	58015.5	49250.2	1.178	bb
181	1... 20211111_A29_R2_IgG	1.98	54672.7	37601.7	1.454	bb
182	1... 20211111_A30_R2_IgG	1.98	69921.3	40366.8	1.732	bb
183	1... 20211111_QC_R15_IgG	1.98	55127.9	35156.1	1.568	bb
184	1... 20211111_L29_R2_IgG	1.99	35721.4	33813.1	1.056	bb
185	1... 20211111_L30_R2_IgG	1.98	16640.9	33488.6	0.497	bb
186	1... 20211111_H15_R2_IgG	1.99	50719.2	38501.0	1.317	MM
187	1... 20211111_A31_R2_IgG	1.98	41229.3	37525.5	1.099	bb
188	1... 20211111_A32_R2_IgG	1.98	64459.4	35770.8	1.802	bb
189	1... 20211111_L31_R2_IgG	1.98	29969.7	33282.4	0.900	bb
190	1... 20211111_L32_R2_IgG	1.98	60533.5	42049.6	1.440	bb
191	1... 20211111_H16_R2_IgG	1.99	85292.2	45947.6	1.856	bb
192	1... 20211111_A33_R2_IgG	1.98	63634.6	40202.3	1.583	MM
193	1... 20211111_A34_R2_IgG	1.99	38932.4	36747.0	1.059	MM
194	1... 20211111_H17_R2_IgG	1.98	301623.1	40920.9	7.371	bb
195	1... 20211111_A35_R2_IgG	1.98	53378.6	33689.2	1.584	bb
196	1... 20211111_QC_R16_IgG	1.98	51531.3	32783.6	1.572	bb
197	1... 20211111_A36_R2_IgG	1.99	35246.5	36822.4	0.957	bb
198	1... 20211111_H18_R2_IgG	1.98	266096.9	31769.3	8.376	bb
199	1... 20211111_A37_R2_IgG	1.99	111884.6	37581.8	2.977	MM
200	2... 20211111_A38_R2_IgG	1.99	50242.0	38390.4	1.309	MM
201	2... 20211111_H19_R2_IgG	1.98	186312.4	41217.0	4.520	bb
202	2... 20211111_A39_R2_IgG	1.98	34762.9	37392.7	0.930	MM
203	2... 20211111_A40_R2_IgG	1.99	45328.0	38295.1	1.184	bb
204	2... 20211111_H20_R2_IgG	1.98	151431.0	35941.8	4.213	bb
205	2... 20211111_A41_R2_IgG	1.98	64083.8	45304.9	1.414	MM
206	2... 20211111_A42_R2_IgG	1.99	27485.8	37803.8	0.727	bb
207	2... 20211111_A43_R2_IgG	1.99	68828.1	38161.8	1.804	bb
208	2... 20211111_A44_R2_IgG	1.98	71233.9	37819.8	1.884	bb
209	2... 20211111_QC_R17_IgG	1.99	46582.1	31731.8	1.468	bb

Dataset: C:\MassLynx\10.pro\20211115_clinicalsamples_IgG.qld

Last Altered: Sunday, October 02, 2022 16:02:30 台北標準時間
Printed: Friday, August 01, 2025 16:44:55 台北標準時間

Method: C:\MassLynx\10_Pro_IgA.PRO\MethDB\20210926_IgG_DMRM_IgM_IS.mdb 16 Feb 2022 11:58:21
Calibration: 18 Nov 2021 13:56:22

Sample Name: 20211111_QC_R1_IgG

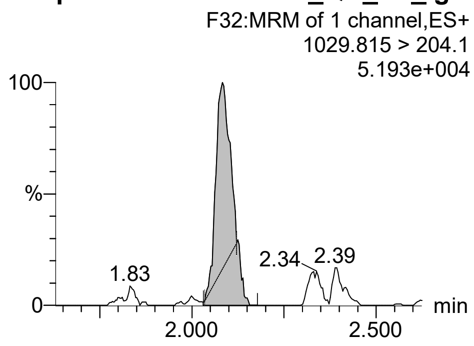

Sample Name: 20211111_A1_R1_IgG

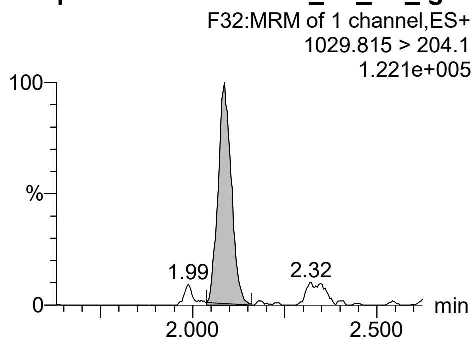

Sample Name: 20211111_A2_R1_IgG

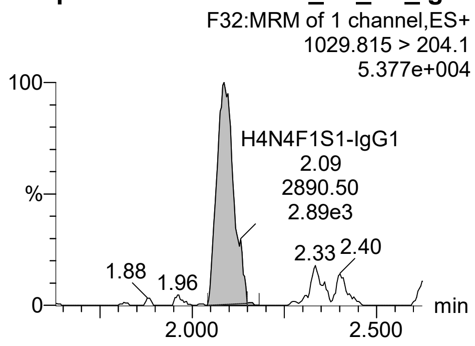

Sample Name: 20211111_L1_R1_IgG

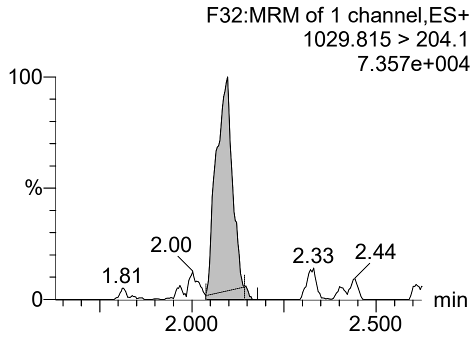

Sample Name: 20211111_L2_R1_IgG

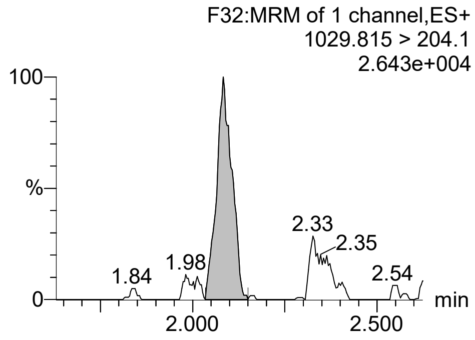

Sample Name: 20211111_H1_R1_IgG

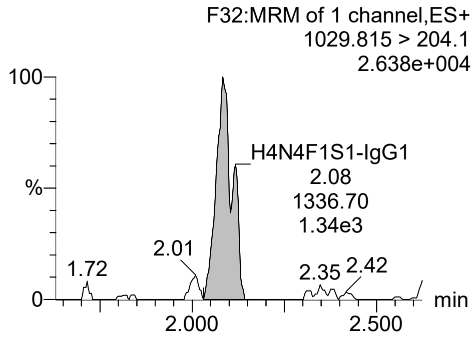

Sample Name: 20211111_A3_R1_IgG

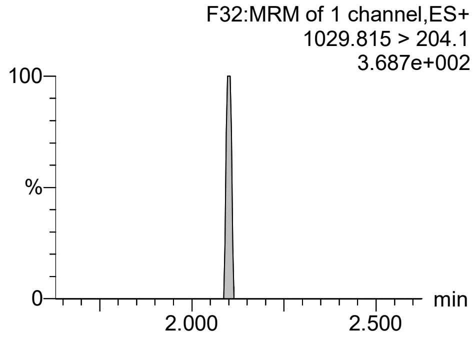

Sample Name: 20211111_A4_R1_IgG

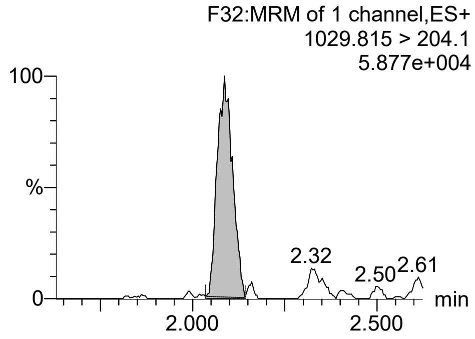

Sample Name: 20211111_L3_R1_IgG

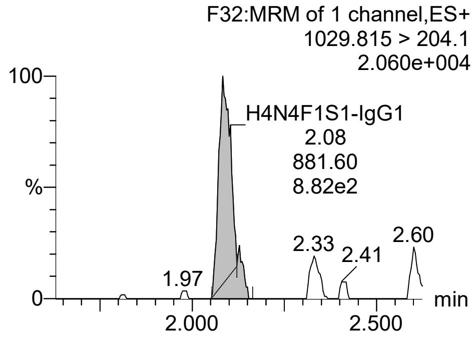

Sample Name: 20211111_L4_R1_IgG

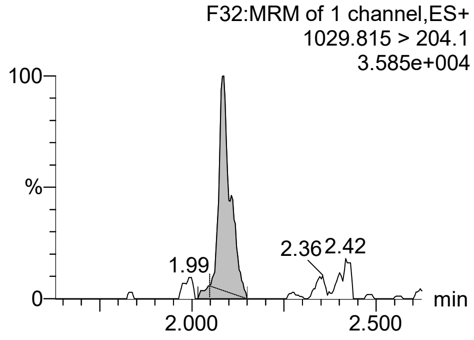

Sample Name: 20211111_H2_R1_IgG

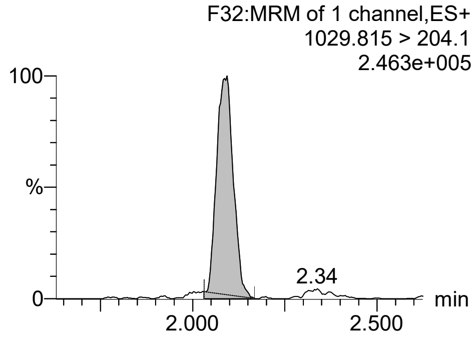

Sample Name: 20211111_A5_R1_IgG

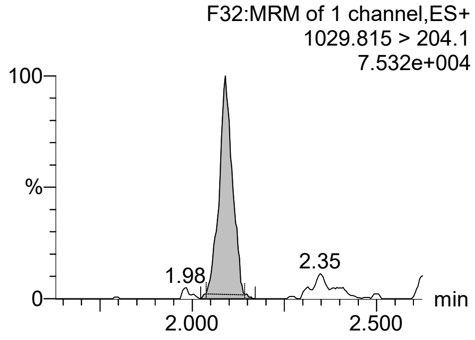

Dataset: C:\MassLynx\10.pro\20211115_clinicalsamples_IgG.qld

Last Altered: Sunday, October 02, 2022 16:02:30 台北標準時間
Printed: Friday, August 01, 2025 16:44:55 台北標準時間

Sample Name: 20211111_A6_R1_IgG

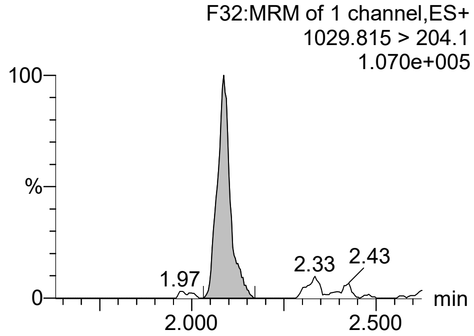

Sample Name: 20211111_QC_R2_IgG

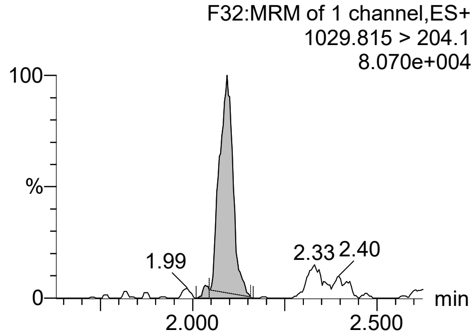

Sample Name: 20211111_L5_R1_IgG

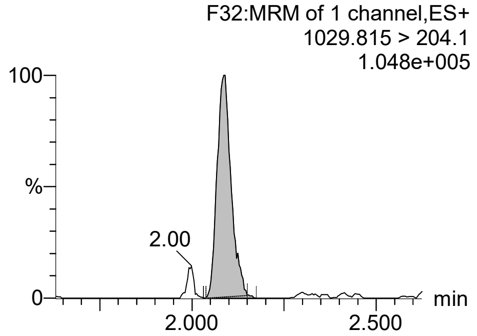

Sample Name: 20211111_L6_R1_IgG

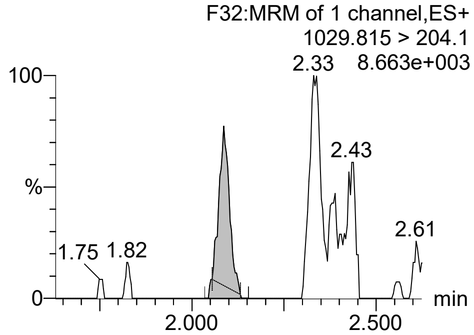

Sample Name: 20211111_H3_R1_IgG

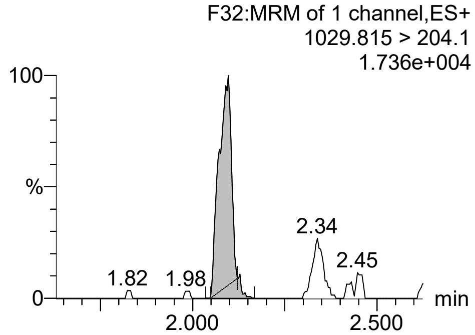

Sample Name: 20211111_A7_R1_IgG

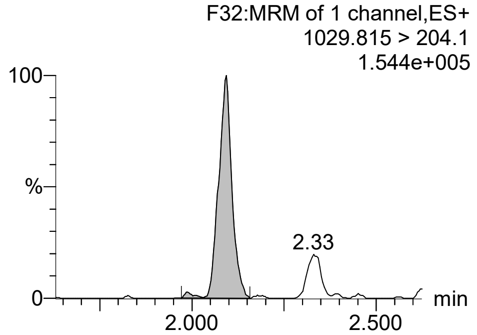

Sample Name: 20211111_A8_R1_IgG

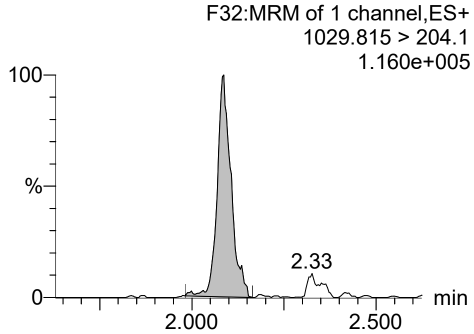

Sample Name: 20211111_L7_R1_IgG

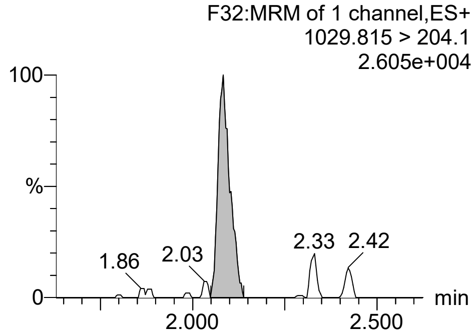

Sample Name: 20211111_L8_R1_IgG

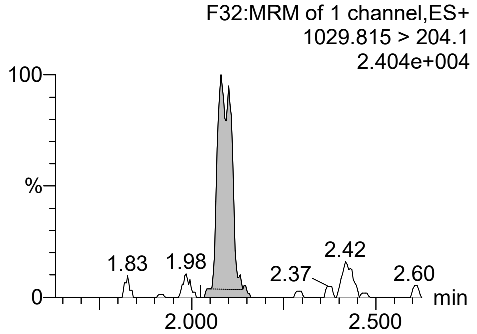

Sample Name: 20211111_H4_R1_IgG

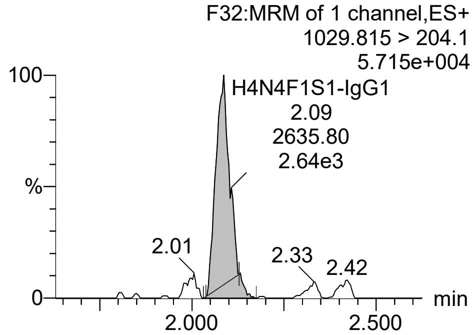

Sample Name: 20211111_A9_R1_IgG

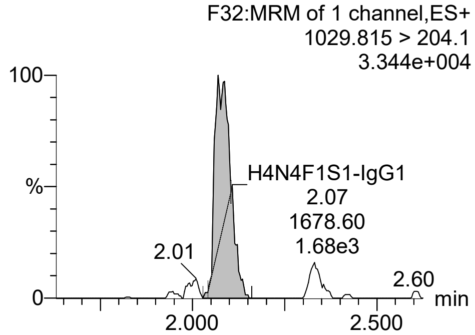

Sample Name: 20211111_A10_R1_IgG

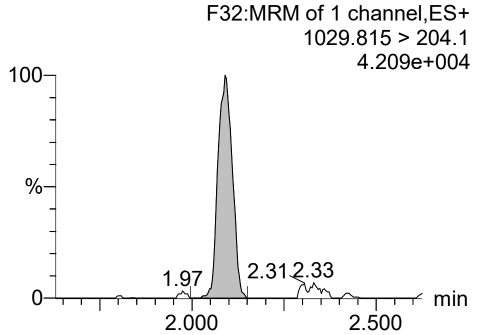

Sample Name: 20211111_L9_R1_IgG

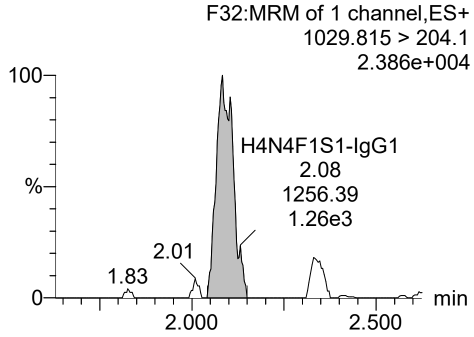

Sample Name: 20211111_L10_R1_IgG

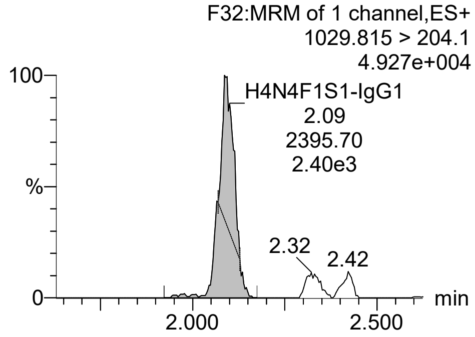

Sample Name: 20211111_QC_R3_IgG

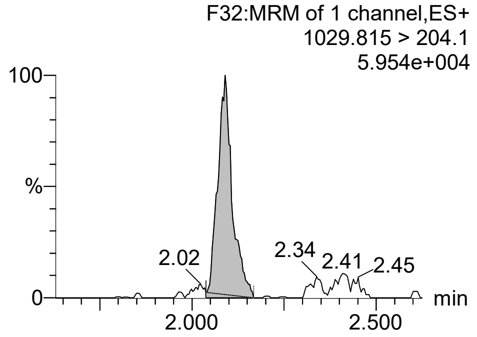

Dataset: C:\MassLynx\10.pro\20211115_clinicalsamples_IgG.qld

Last Altered: Sunday, October 02, 2022 16:02:30 台北標準時間
Printed: Friday, August 01, 2025 16:44:55 台北標準時間

Sample Name: 20211111_H5_R1_IgG
F32:MRM of 1 channel,ES+
1029.815 > 204.1
2.129e+004

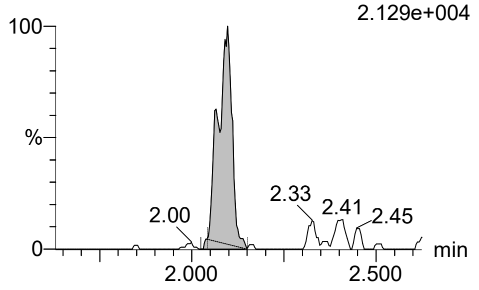

Sample Name: 20211111_A11_R1_IgG
F32:MRM of 1 channel,ES+
1029.815 > 204.1
4.736e+004

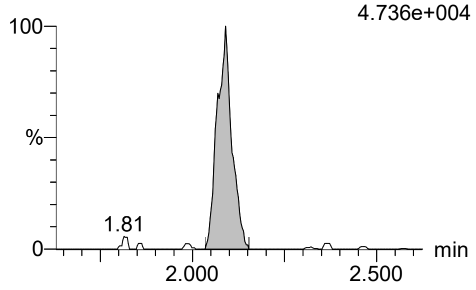

Sample Name: 20211111_A12_R1_IgG
F32:MRM of 1 channel,ES+
1029.815 > 204.1
2.293e+004

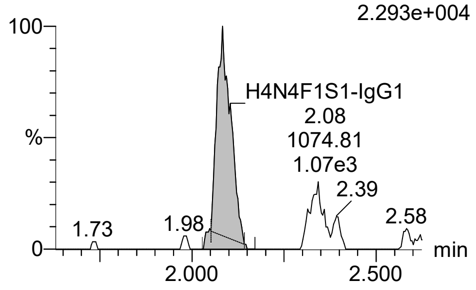

Sample Name: 20211111_L11_R1_IgG
F32:MRM of 1 channel,ES+
1029.815 > 204.1
2.006e+004

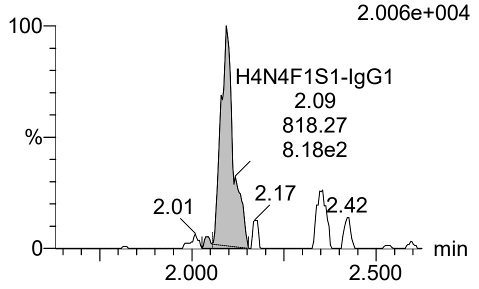

Sample Name: 20211111_L12_R1_IgG
F32:MRM of 1 channel,ES+
1029.815 > 204.1
2.095e+004

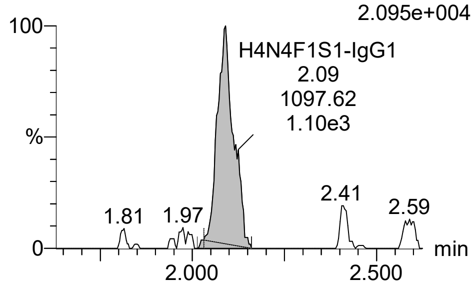

Sample Name: 20211111_H6_R1_IgG
F32:MRM of 1 channel,ES+
1029.815 > 204.1
3.794e+004

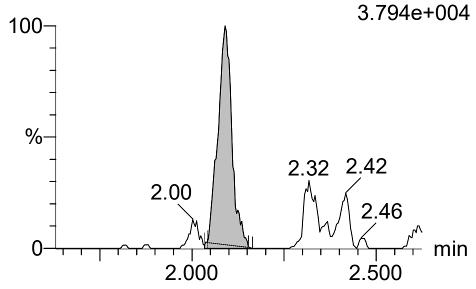

Sample Name: 20211111_A13_R1_IgG
F32:MRM of 1 channel,ES+
1029.815 > 204.1
4.188e+004

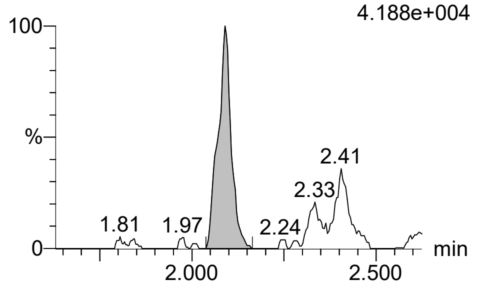

Sample Name: 20211111_A14_R1_IgG
F32:MRM of 1 channel,ES+
1029.815 > 204.1
3.794e+004

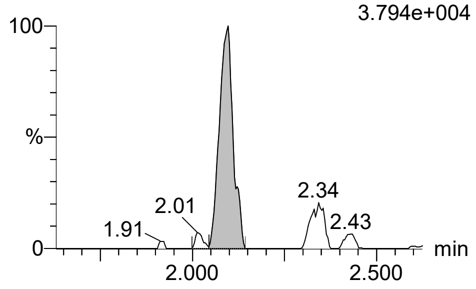

Sample Name: 20211111_L13_R1_IgG
F32:MRM of 1 channel,ES+
1029.815 > 204.1
2.589e+004

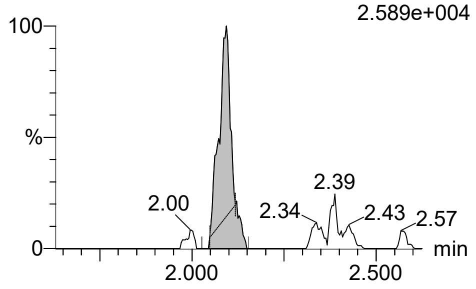

Sample Name: 20211111_L14_R1_IgG
F32:MRM of 1 channel,ES+
1029.815 > 204.1
3.736e+004

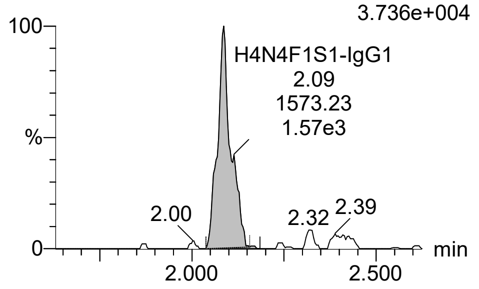

Sample Name: 20211111_H7_R1_IgG
F32:MRM of 1 channel,ES+
1029.815 > 204.1
2.121e+004

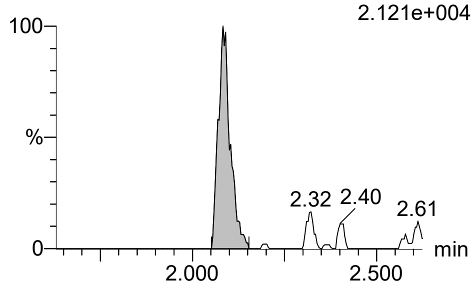

Sample Name: 20211111_A15_R1_IgG
F32:MRM of 1 channel,ES+
1029.815 > 204.1
1.074e+005

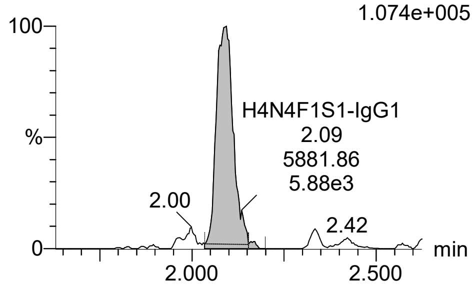

Sample Name: 20211111_QC_R4_IgG
F32:MRM of 1 channel,ES+
1029.815 > 204.1
4.150e+004

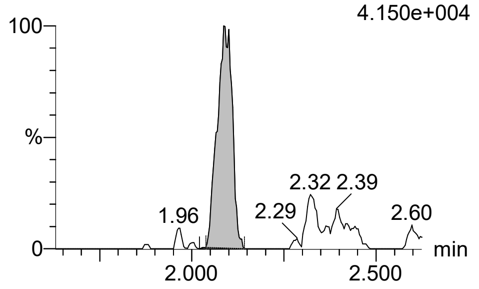

Sample Name: 20211111_A16_R1_IgG
F32:MRM of 1 channel,ES+
1029.815 > 204.1
5.588e+004

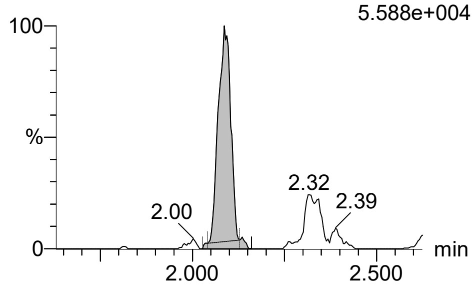

Sample Name: 20211111_L15_R1_IgG
F32:MRM of 1 channel,ES+
1029.815 > 204.1
3.351e+004

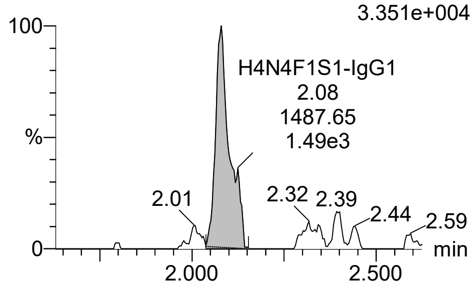

Dataset: C:\MassLynx\10.pro\20211115_clinicalsamples_IgG.qld

Last Altered: Sunday, October 02, 2022 16:02:30 台北標準時間
Printed: Friday, August 01, 2025 16:44:55 台北標準時間

Sample Name: 20211111_L16_R1_IgG
F32:MRM of 1 channel,ES+
1029.815 > 204.1
2.713e+004

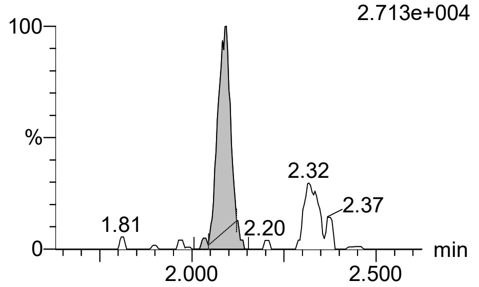

Sample Name: 20211111_H8_R1_IgG
F32:MRM of 1 channel,ES+
1029.815 > 204.1
2.503e+004

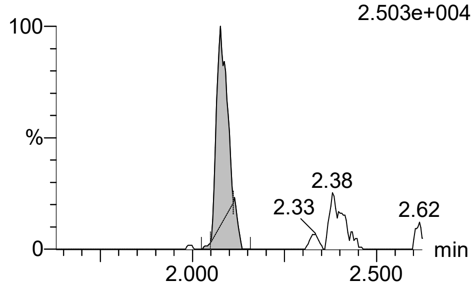

Sample Name: 20211111_A17_R1_IgG
F32:MRM of 1 channel,ES+
1029.815 > 204.1
2.329e+004

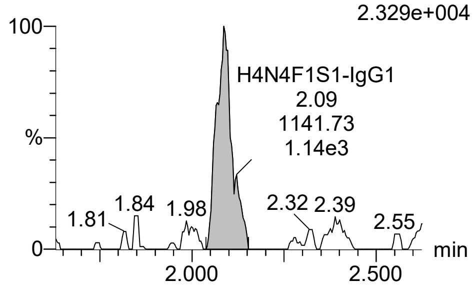

Sample Name: 20211111_A18_R1_IgG
F32:MRM of 1 channel,ES+
1029.815 > 204.1
2.110e+004

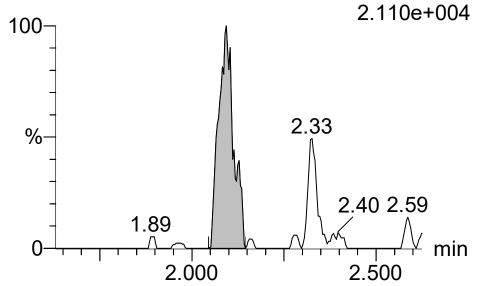

Sample Name: 20211111_L17_R1_IgG
F32:MRM of 1 channel,ES+
1029.815 > 204.1
2.837e+004

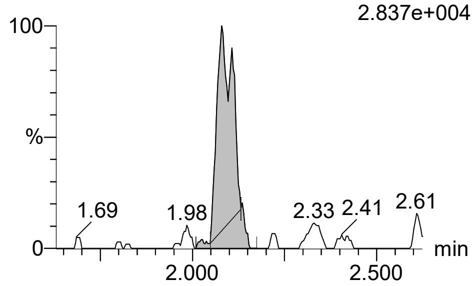

Sample Name: 20211111_L18_R1_IgG
F32:MRM of 1 channel,ES+
1029.815 > 204.1
1.887e+004

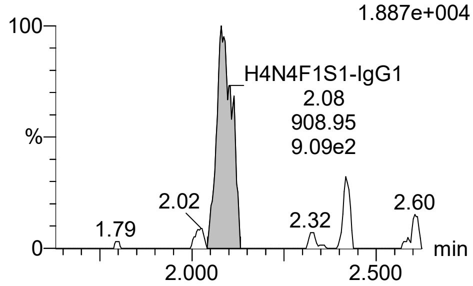

Sample Name: 20211111_H9_R1_IgG
F32:MRM of 1 channel,ES+
1029.815 > 204.1
2.165e+004

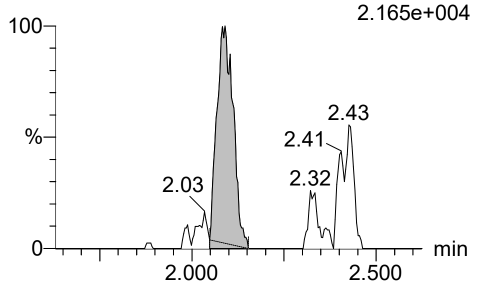

Sample Name: 20211111_A19_R1_IgG
F32:MRM of 1 channel,ES+
1029.815 > 204.1
8.353e+004

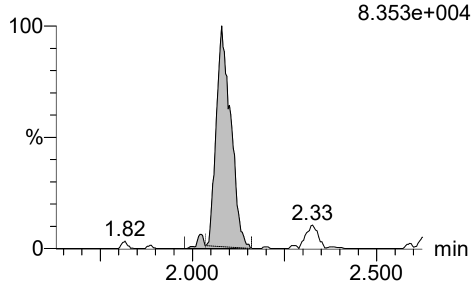

Sample Name: 20211111_A20_R1_IgG
F32:MRM of 1 channel,ES+
1029.815 > 204.1
7.375e+003

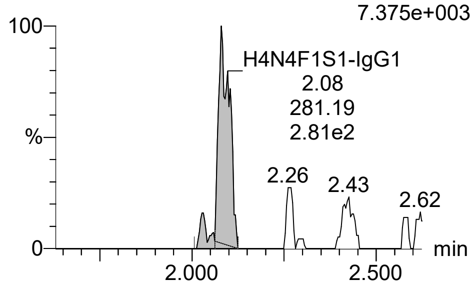

Sample Name: 20211111_L19_R1_IgG
F32:MRM of 1 channel,ES+
1029.815 > 204.1
1.937e+004

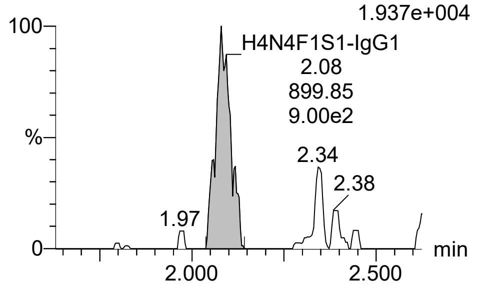

Sample Name: 20211111_QC_R5_IgG
F32:MRM of 1 channel,ES+
1029.815 > 204.1
5.876e+004

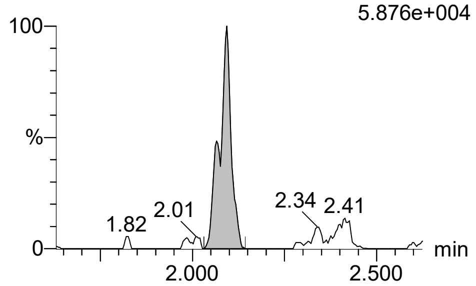

Sample Name: 20211111_L20_R1_IgG
F32:MRM of 1 channel,ES+
1029.815 > 204.1
4.693e+004

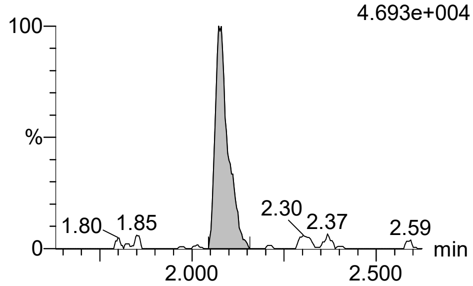

Sample Name: 20211111_H10_R1_IgG
F32:MRM of 1 channel,ES+
1029.815 > 204.1
8.218e+004

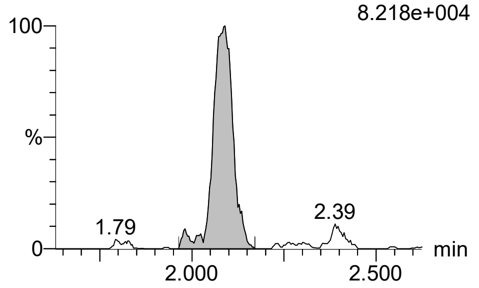

Sample Name: 20211111_A21_R1_IgG
F32:MRM of 1 channel,ES+
1029.815 > 204.1
8.263e+004

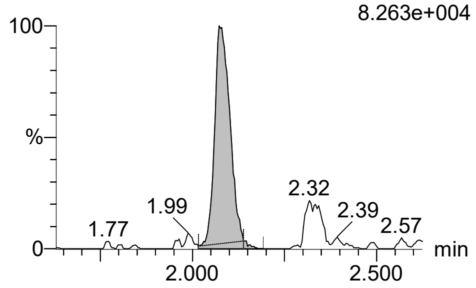

Sample Name: 20211111_A22_R1_IgG
F32:MRM of 1 channel,ES+
1029.815 > 204.1
2.959e+004

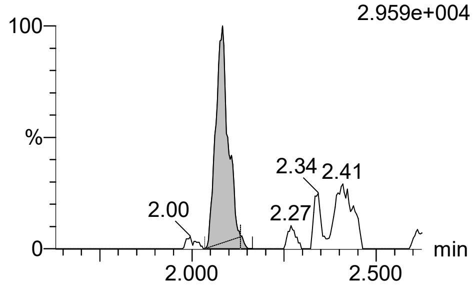

Dataset: C:\MassLynx\10.pro\20211115_clinicalsamples_IgG.qld

Last Altered: Sunday, October 02, 2022 16:02:30 台北標準時間
Printed: Friday, August 01, 2025 16:44:55 台北標準時間

Sample Name: 20211111_L21_R1_IgG

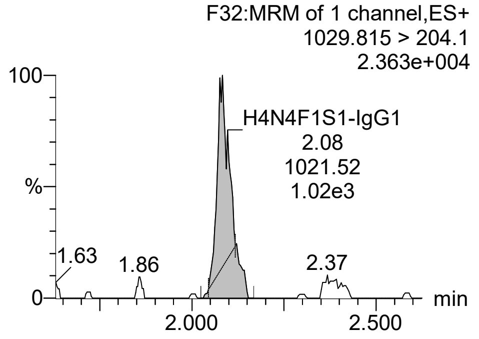

Sample Name: 20211111_L22_R1_IgG

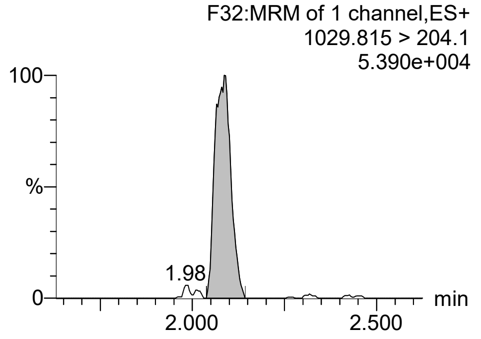

Sample Name: 20211111_H11_R1_IgG

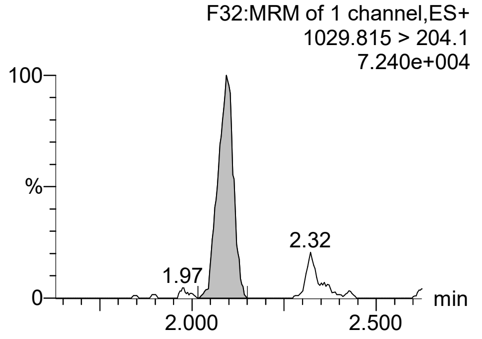

Sample Name: 20211111_A23_R1_IgG

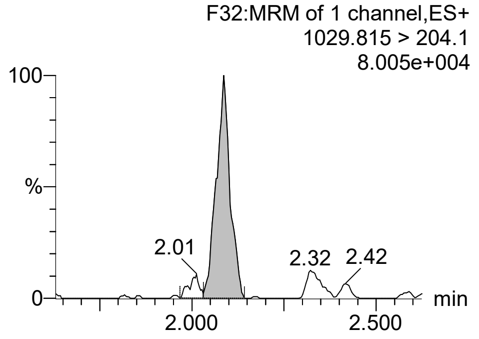

Sample Name: 20211111_A24_R1_IgG

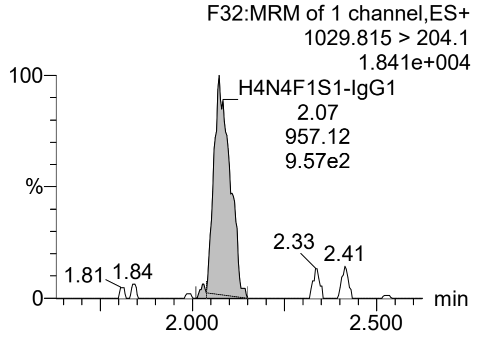

Sample Name: 20211111_L23_R1_IgG

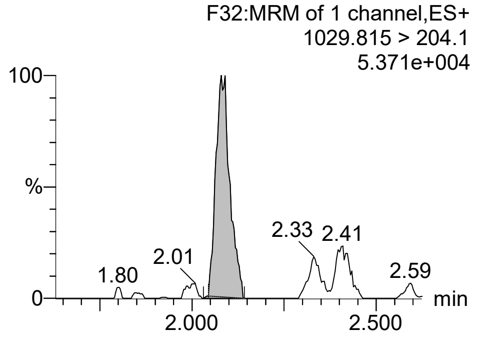

Sample Name: 20211111_L24_R1_IgG

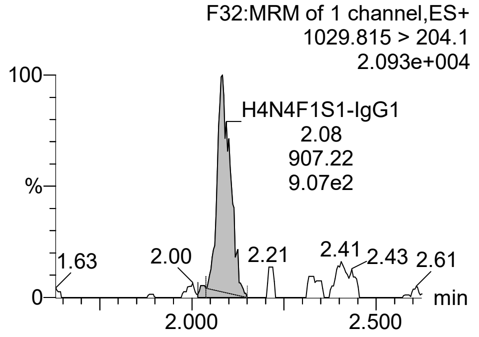

Sample Name: 20211111_H12_R1_IgG

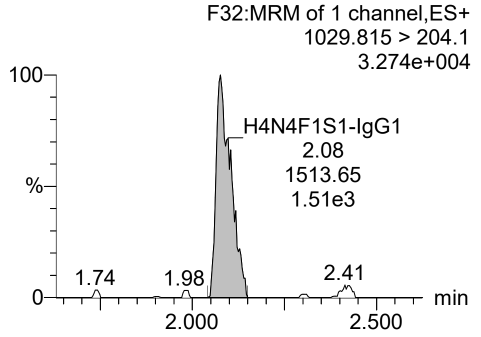

Sample Name: 20211111_QC_R6_IgG

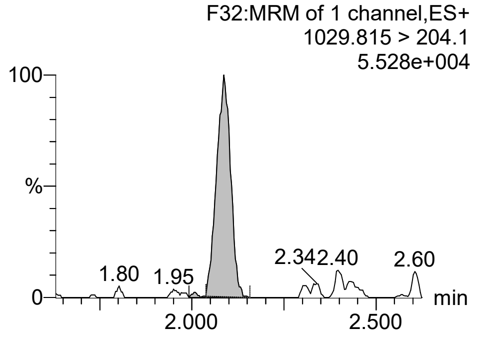

Sample Name: 20211111_A25_R1_IgG

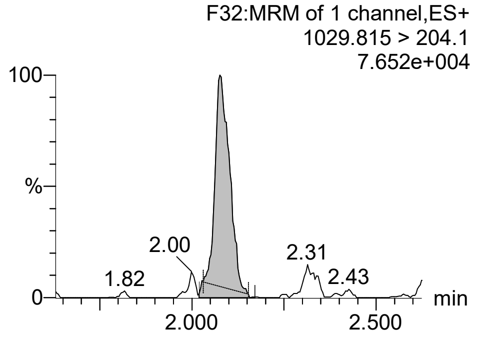

Sample Name: 20211111_A26_R1_IgG

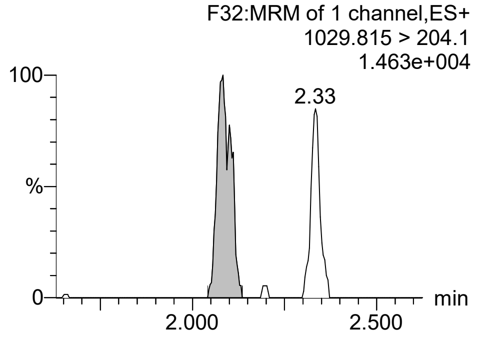

Sample Name: 20211111_L25_R1_IgG

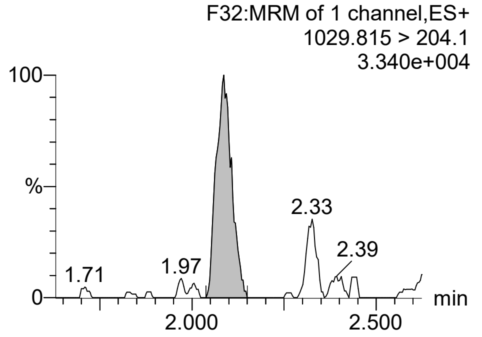

Sample Name: 20211111_L26_R1_IgG

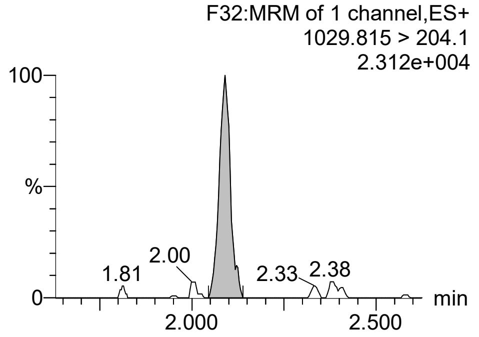

Sample Name: 20211111_H13_R1_IgG

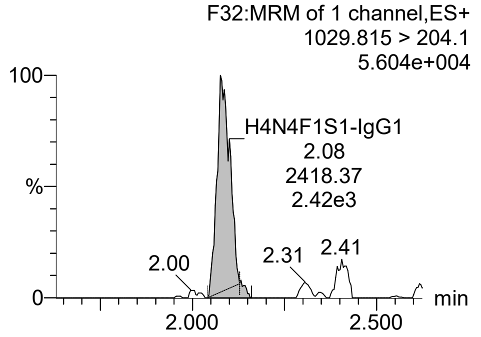

Sample Name: 20211111_A27_R1_IgG

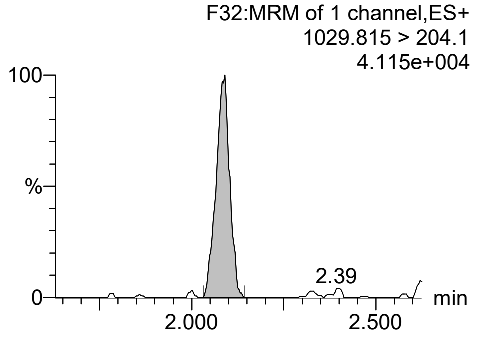

Sample Name: 20211111_A28_R1_IgG

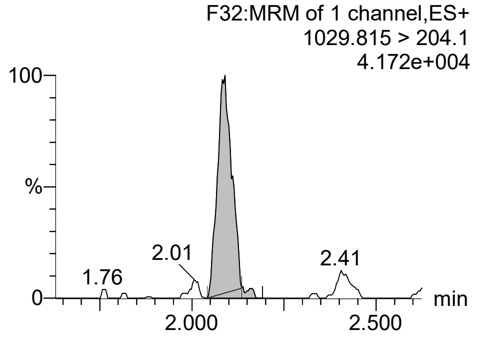

Sample Name: 20211111_L27_R1_IgG

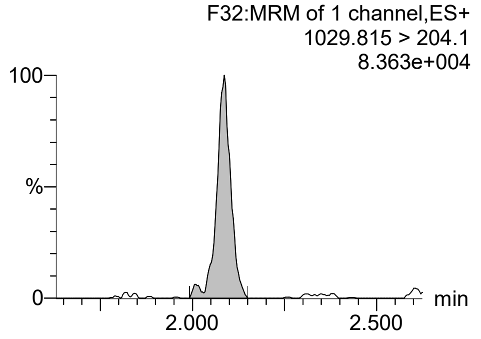

Sample Name: 20211111_L28_R1_IgG

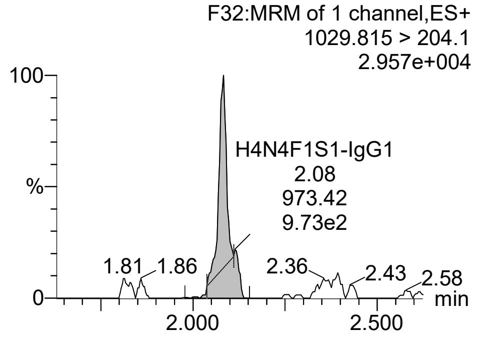

Sample Name: 20211111_H14_R1_IgG

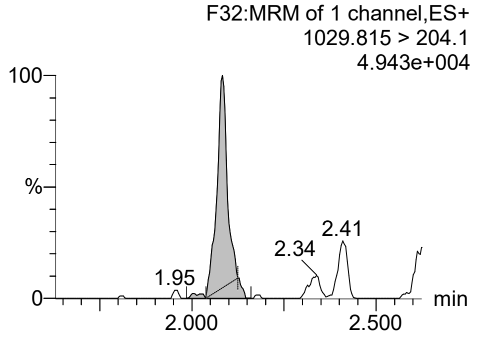

Sample Name: 20211111_A29_R1_IgG

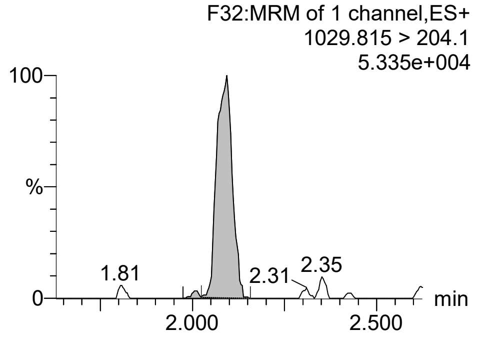

Sample Name: 20211111_A30_R1_IgG

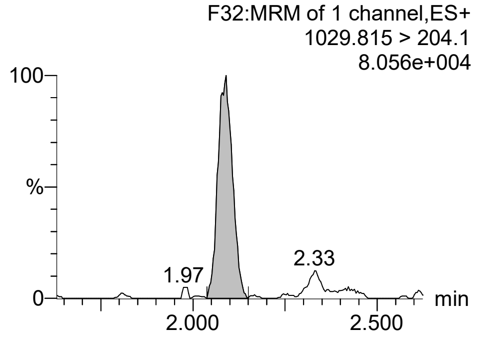

Sample Name: 20211111_QC_R7_IgG

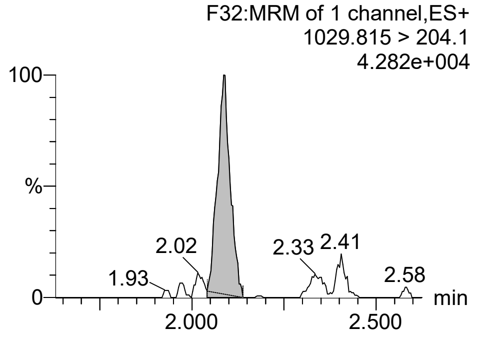

Sample Name: 20211111_L29_R1_IgG

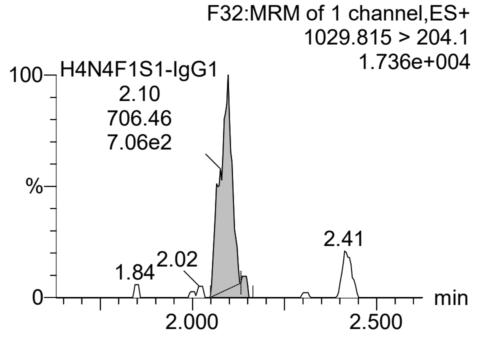

Sample Name: 20211111_L30_R1_IgG

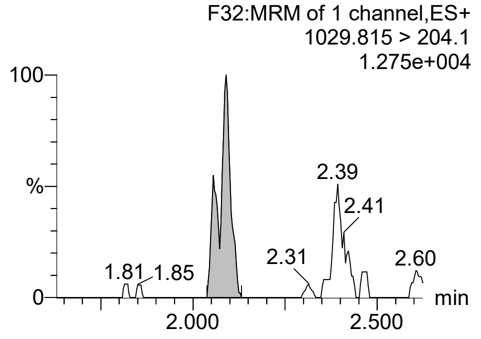

Sample Name: 20211111_H15_R1_IgG

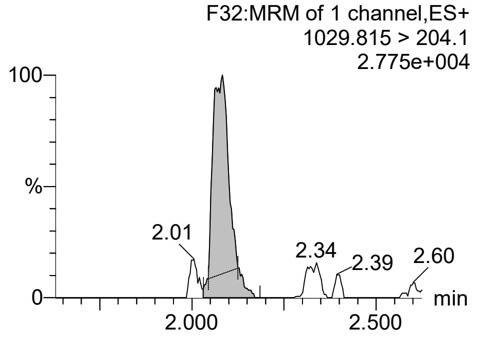

Sample Name: 20211111_A31_R1_IgG

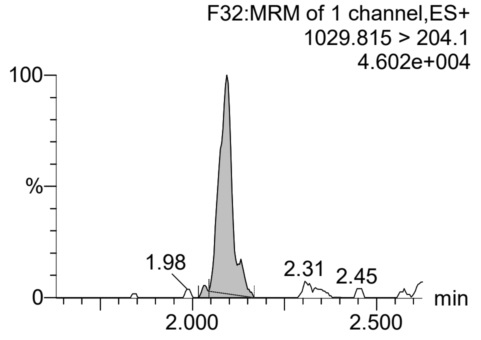

Sample Name: 20211111_A32_R1_IgG

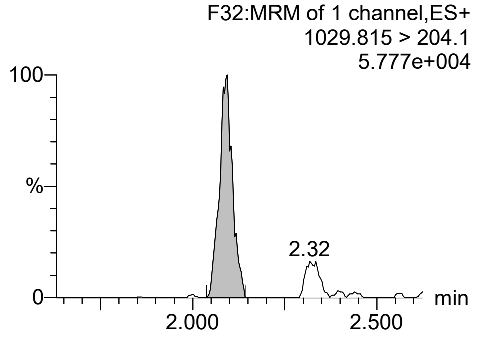

Sample Name: 20211111_L31_R1_IgG

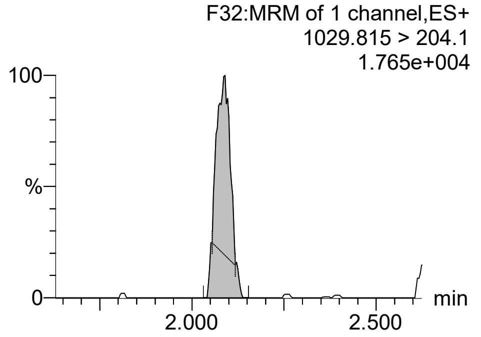

Sample Name: 20211111_L32_R1_IgG

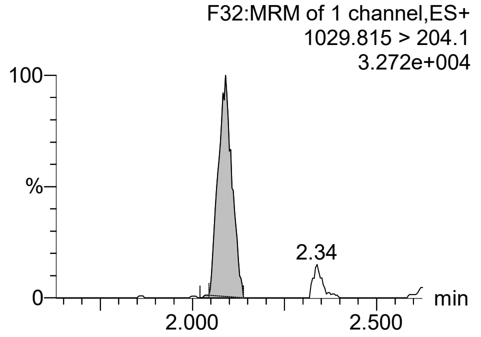

Sample Name: 20211111_H16_R1_IgG

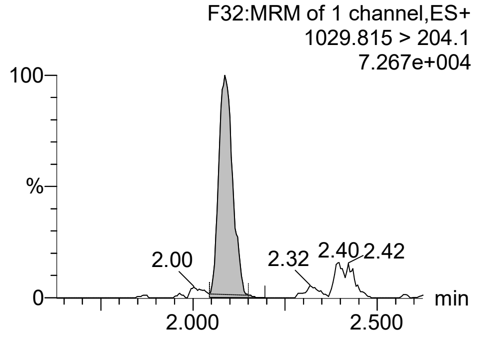

Dataset: C:\MassLynx\10.pro\20211115_clinicalsamples_IgG.qld

Last Altered: Sunday, October 02, 2022 16:02:30 台北標準時間
Printed: Friday, August 01, 2025 16:44:55 台北標準時間

Sample Name: 20211111_A33_R1_IgG
F32:MRM of 1 channel,ES+
1029.815 > 204.1
5.657e+004

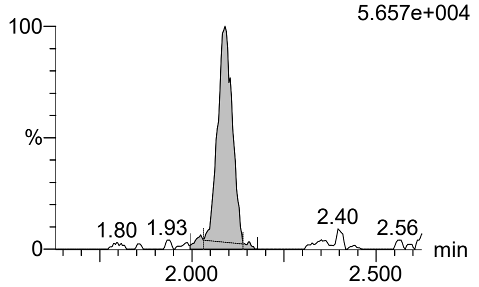

Sample Name: 20211111_A34_R1_IgG
F32:MRM of 1 channel,ES+
1029.815 > 204.1
2.337e+004

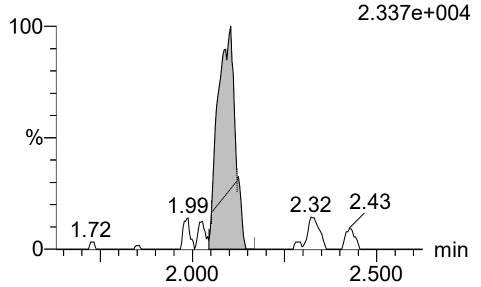

Sample Name: 20211111_H17_R1_IgG
F32:MRM of 1 channel,ES+
1029.815 > 204.1
1.831e+005

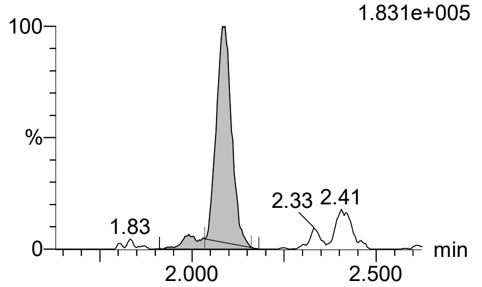

Sample Name: 20211111_A35_R1_IgG
F32:MRM of 1 channel,ES+
1029.815 > 204.1
2.777e+004

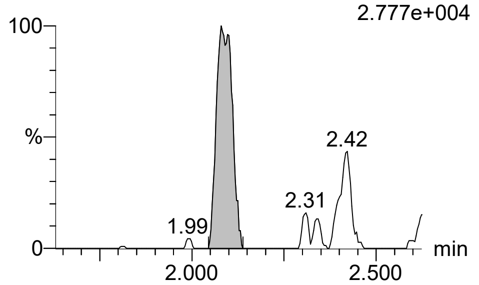

Sample Name: 20211111_QC_R8_IgG
F32:MRM of 1 channel,ES+
1029.815 > 204.1
3.670e+004

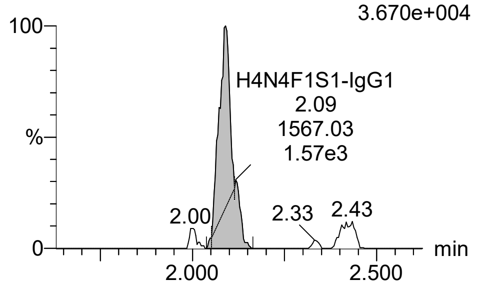

Sample Name: 20211111_A36_R1_IgG
F32:MRM of 1 channel,ES+
1029.815 > 204.1
3.011e+004

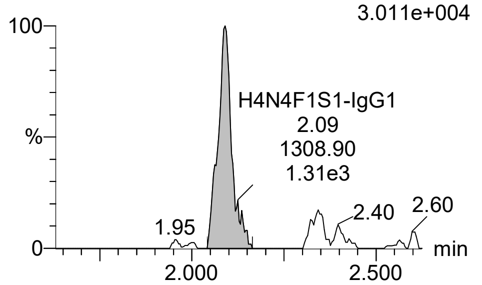

Sample Name: 20211111_H18_R1_IgG
F32:MRM of 1 channel,ES+
1029.815 > 204.1
1.761e+005

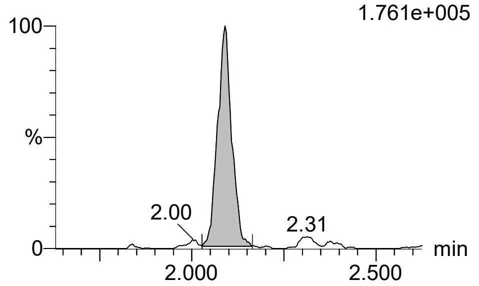

Sample Name: 20211111_A37_R1_IgG
F32:MRM of 1 channel,ES+
1029.815 > 204.1
5.972e+004

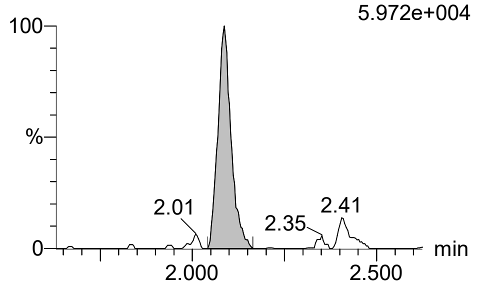

Sample Name: 20211111_A38_R1_IgG
F32:MRM of 1 channel,ES+
1029.815 > 204.1
2.763e+004

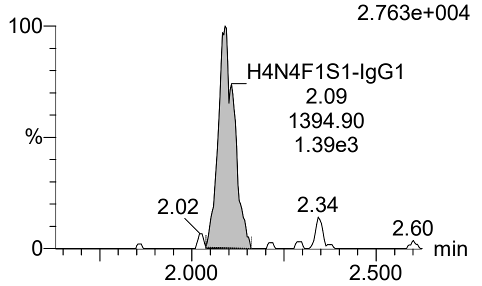

Sample Name: 20211111_H19_R1_IgG
F32:MRM of 1 channel,ES+
1029.815 > 204.1
8.472e+004

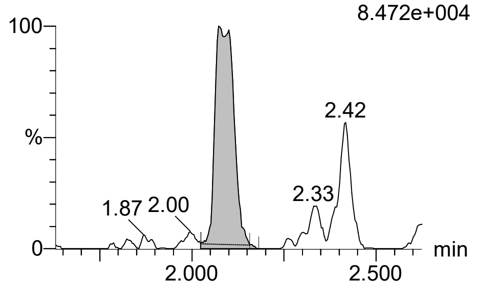

Sample Name: 20211111_A39_R1_IgG
F32:MRM of 1 channel,ES+
1029.815 > 204.1
1.701e+004

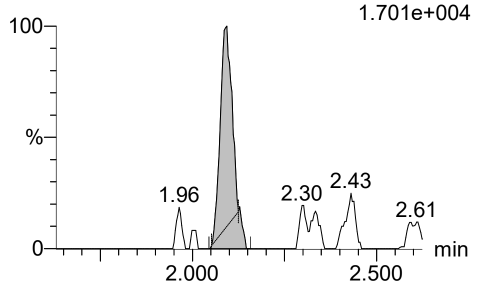

Sample Name: 20211111_A40_R1_IgG
F32:MRM of 1 channel,ES+
1029.815 > 204.1
2.117e+004

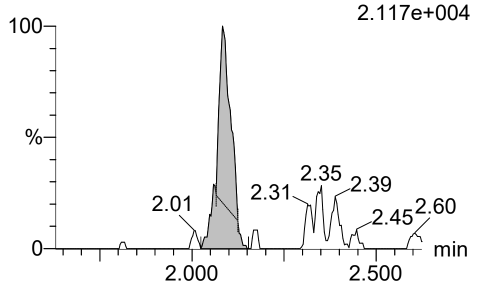

Sample Name: 20211111_H20_R1_IgG
F32:MRM of 1 channel,ES+
1029.815 > 204.1
8.476e+004

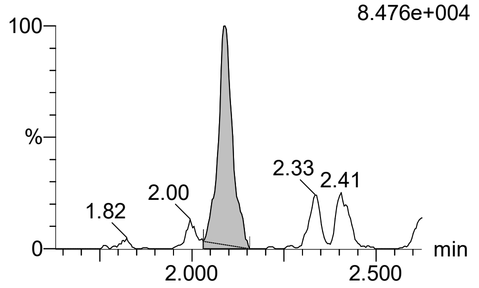

Sample Name: 20211111_A41_R1_IgG
F32:MRM of 1 channel,ES+
1029.815 > 204.1
3.607e+004

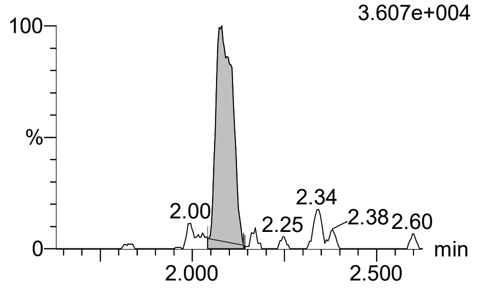

Sample Name: 20211111_A42_R1_IgG
F32:MRM of 1 channel,ES+
1029.815 > 204.1
1.373e+004

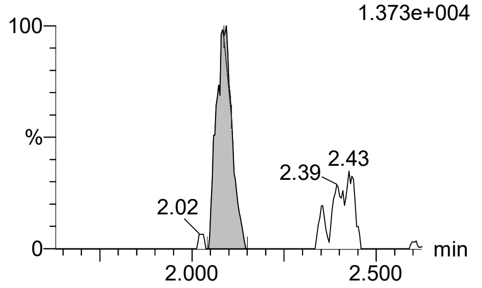

Sample Name: 20211111_A43_R1_IgG

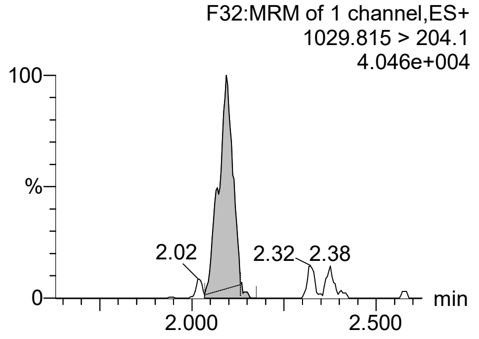

Sample Name: 20211111_A44_R1_IgG

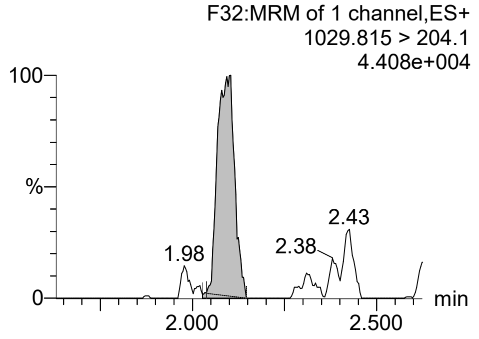

Sample Name: 20211111_QC_R9_IgG

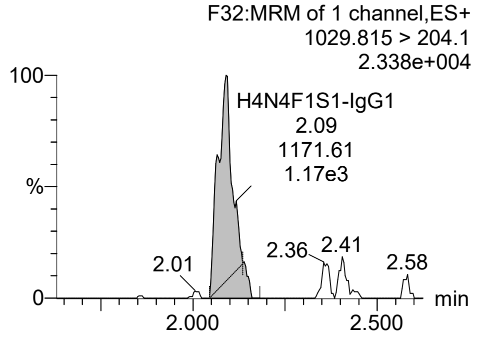

Sample Name: 20211111_A1_R2_IgG

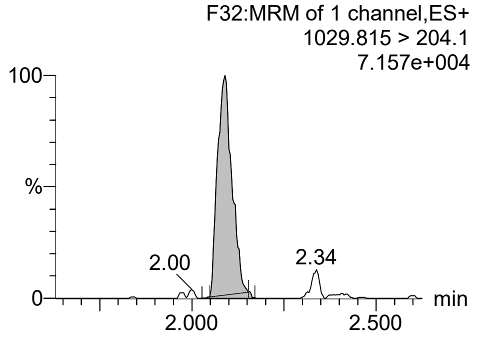

Sample Name: 20211111_A2_R2_IgG

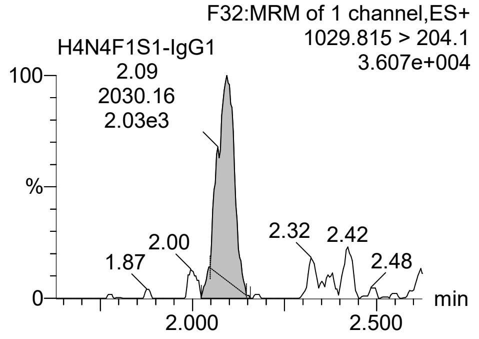

Sample Name: 20211111_L1_R2_IgG

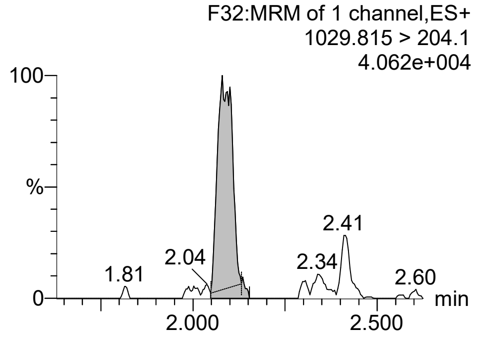

Sample Name: 20211111_L2_R2_IgG

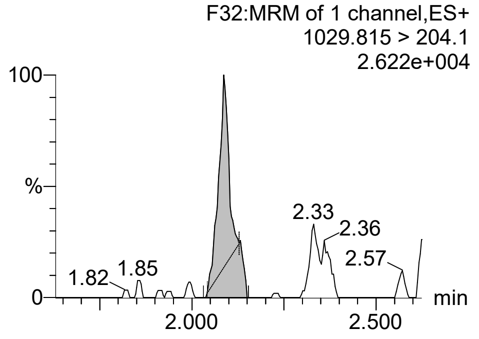

Sample Name: 20211111_H1_R2_IgG

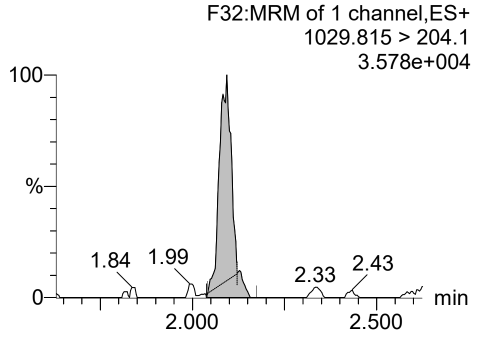

Sample Name: 20211111_A3_R2_IgG

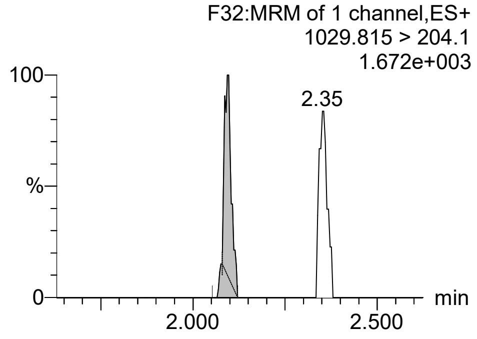

Sample Name: 20211111_A4_R2_IgG

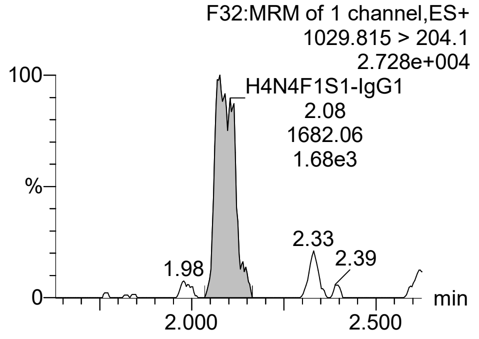

Sample Name: 20211111_L3_R2_IgG

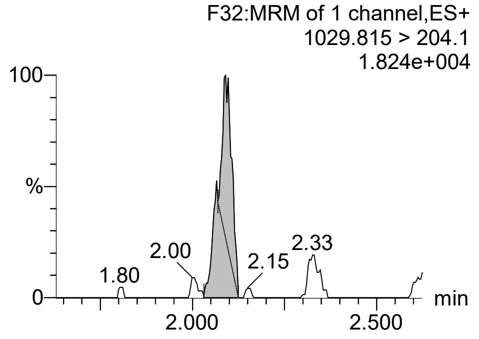

Sample Name: 20211111_L4_R2_IgG

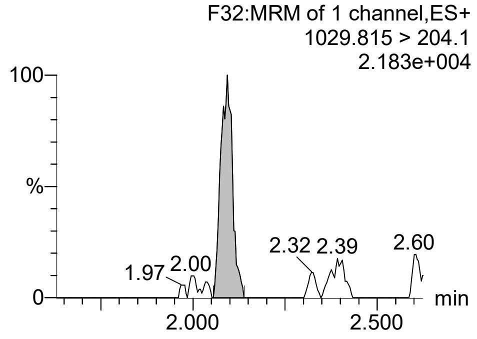

Sample Name: 20211111_H2_R2_IgG

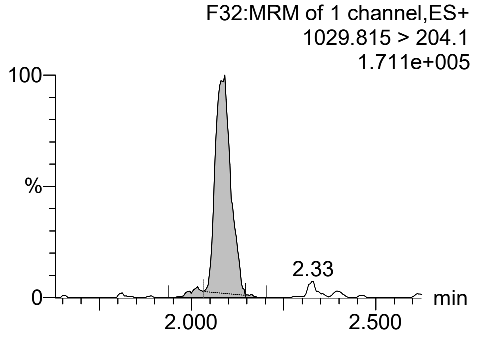

Sample Name: 20211111_A5_R2_IgG

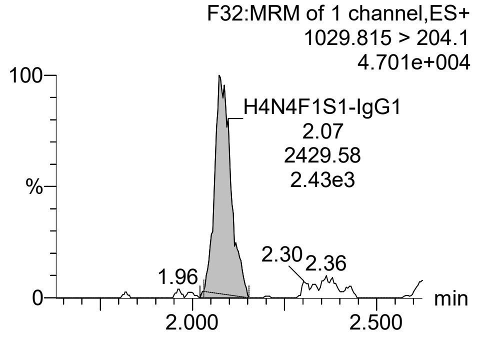

Sample Name: 20211111_A6_R2_IgG

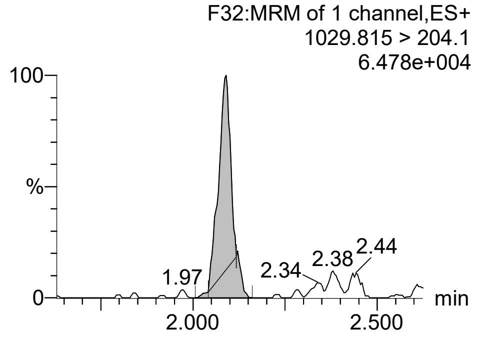

Sample Name: 20211111_QC_R10_IgG
F32:MRM of 1 channel,ES+
1029.815 > 204.1
2.160e+004

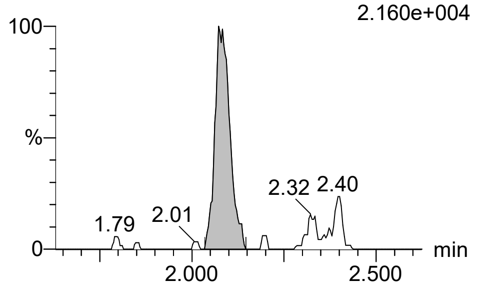

Sample Name: 20211111_L5_R2_IgG
F32:MRM of 1 channel,ES+
1029.815 > 204.1
6.285e+004

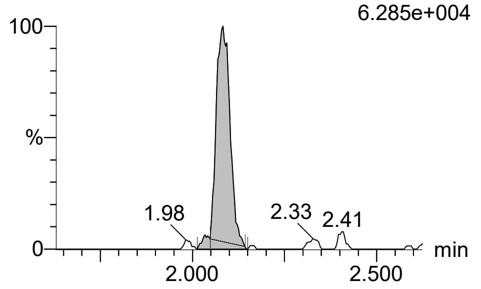

Sample Name: 20211111_L6_R2_IgG
F32:MRM of 1 channel,ES+
1029.815 > 204.1
1.241e+004

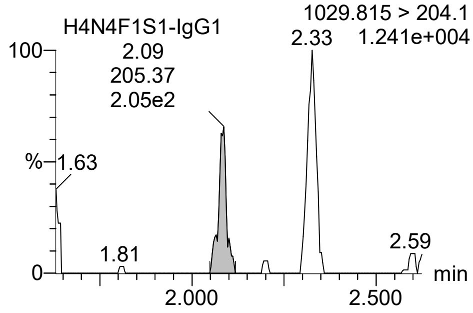

Sample Name: 20211111_H3_R2_IgG
F32:MRM of 1 channel,ES+
1029.815 > 204.1
1.765e+004

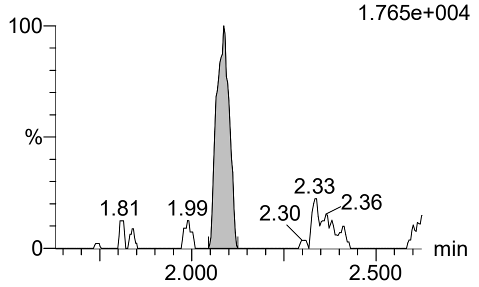

Sample Name: 20211111_A7_R2_IgG
F32:MRM of 1 channel,ES+
1029.815 > 204.1
8.765e+004

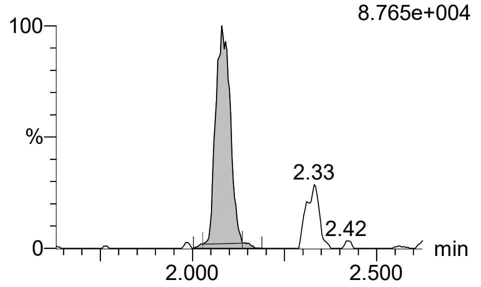

Sample Name: 20211111_A8_R2_IgG
F32:MRM of 1 channel,ES+
1029.815 > 204.1
7.677e+004

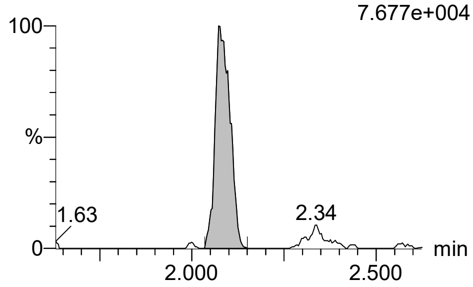

Sample Name: 20211111_L7_R2_IgG
F32:MRM of 1 channel,ES+
1029.815 > 204.1
2.192e+004

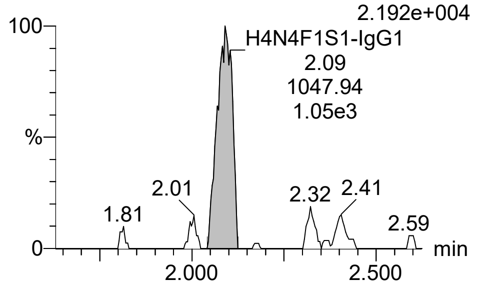

Sample Name: 20211111_L8_R2_IgG
F32:MRM of 1 channel,ES+
1029.815 > 204.1
1.742e+004

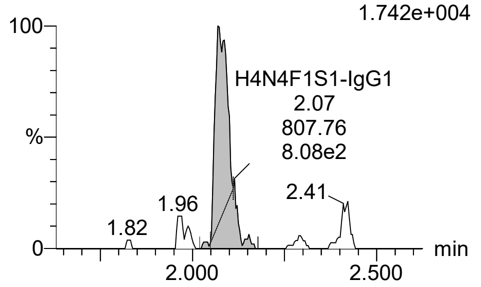

Sample Name: 20211111_H4_R2_IgG
F32:MRM of 1 channel,ES+
1029.815 > 204.1
5.919e+004

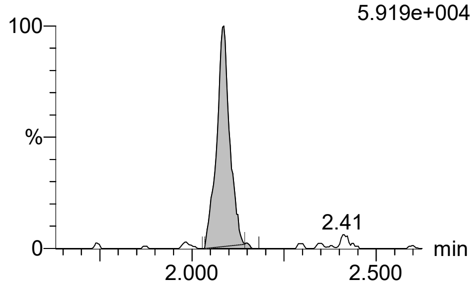

Sample Name: 20211111_A9_R2_IgG
F32:MRM of 1 channel,ES+
1029.815 > 204.1
2.592e+004

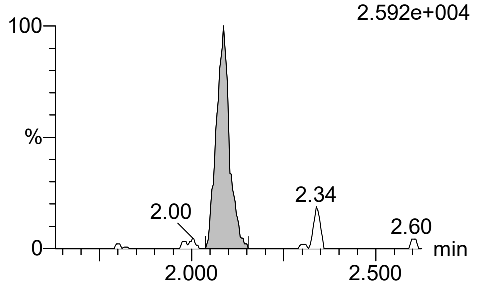

Sample Name: 20211111_A10_R2_IgG
F32:MRM of 1 channel,ES+
1029.815 > 204.1
3.712e+004

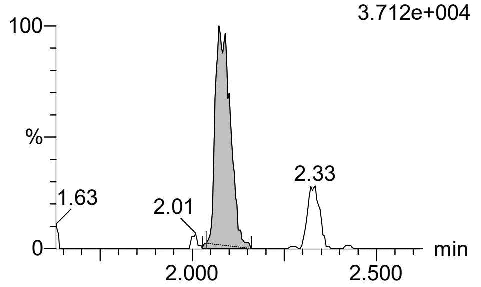

Sample Name: 20211111_L9_R2_IgG
F32:MRM of 1 channel,ES+
1029.815 > 204.1
1.928e+004

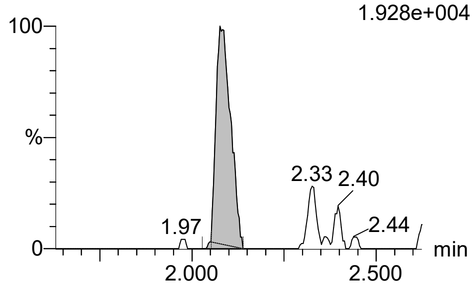

Sample Name: 20211111_L10_R2_IgG
F32:MRM of 1 channel,ES+
1029.815 > 204.1
2.844e+004

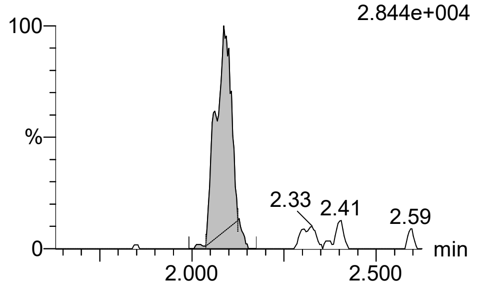

Sample Name: 20211111_QC_R11_IgG
F32:MRM of 1 channel,ES+
1029.815 > 204.1
2.785e+004

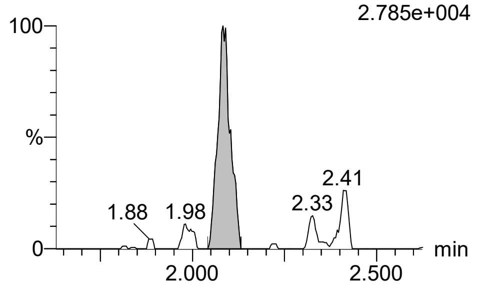

Sample Name: 20211111_H5_R2_IgG
F32:MRM of 1 channel,ES+
1029.815 > 204.1
2.557e+004

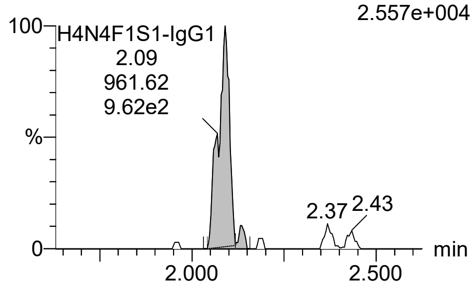

Dataset: C:\MassLynx\10.pro\20211115_clinicalsamples_IgG.qld

Last Altered: Sunday, October 02, 2022 16:02:30 台北標準時間
Printed: Friday, August 01, 2025 16:44:55 台北標準時間

Sample Name: 20211111_A11_R2_IgG

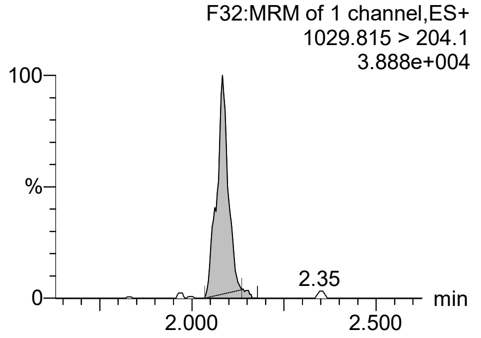

Sample Name: 20211111_A12_R2_IgG

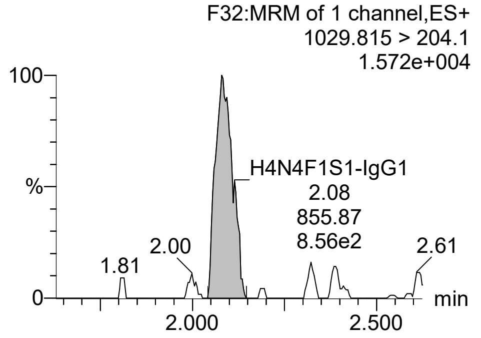

Sample Name: 20211111_L11_R2_IgG

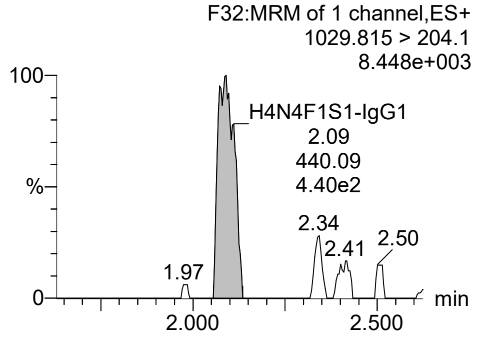

Sample Name: 20211111_L12_R2_IgG

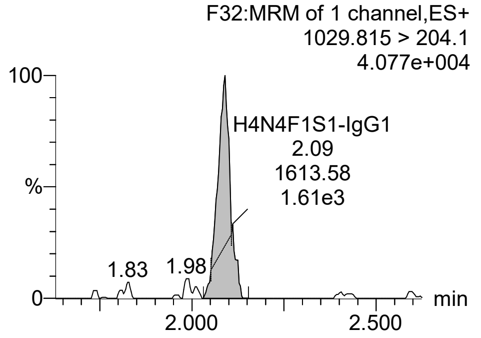

Sample Name: 20211111_H6_R2_IgG

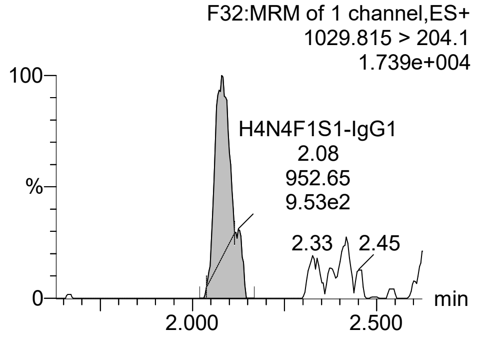

Sample Name: 20211111_A13_R2_IgG

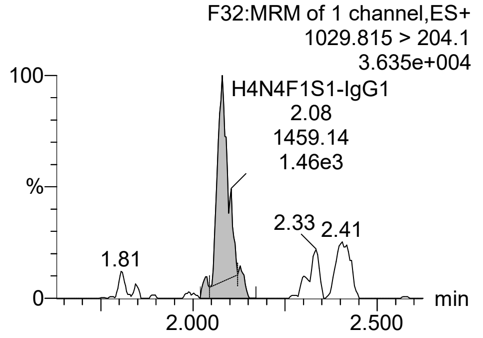

Sample Name: 20211111_A14_R2_IgG

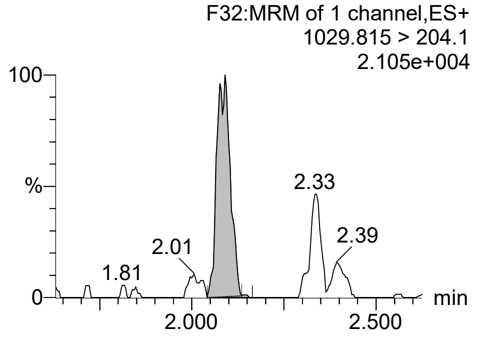

Sample Name: 20211111_L13_R2_IgG

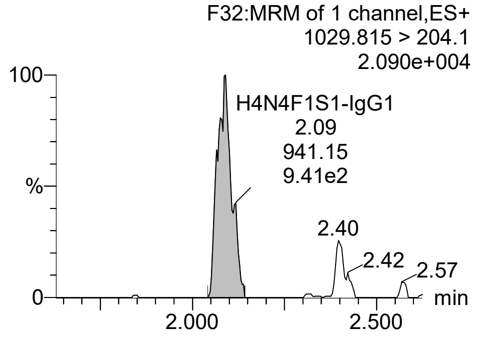

Sample Name: 20211111_L14_R2_IgG

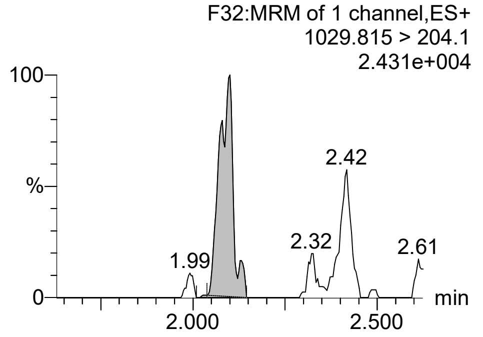

Sample Name: 20211111_H7_R2_IgG

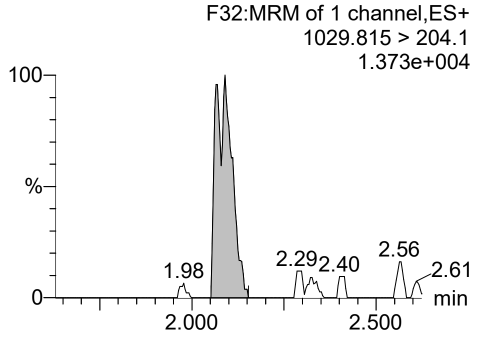

Sample Name: 20211111_A15_R2_IgG

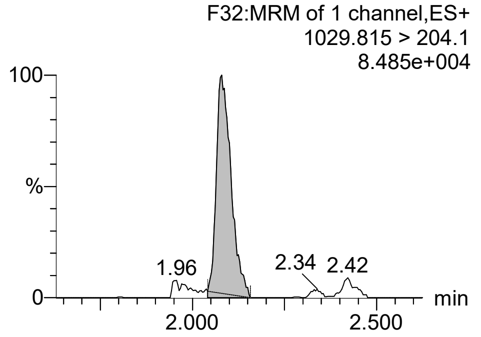

Sample Name: 20211111_QC_R12_IgG

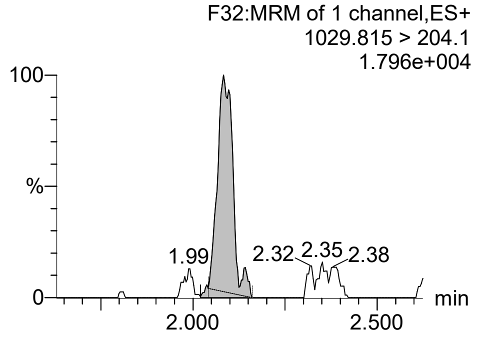

Sample Name: 20211111_A16_R2_IgG

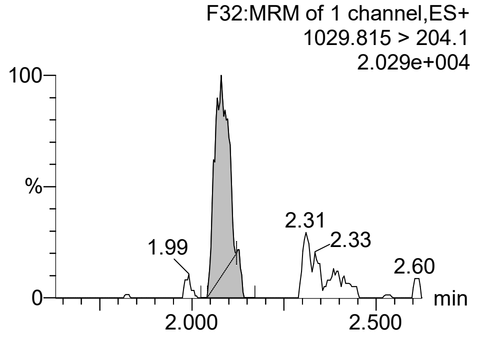

Sample Name: 20211111_L15_R2_IgG

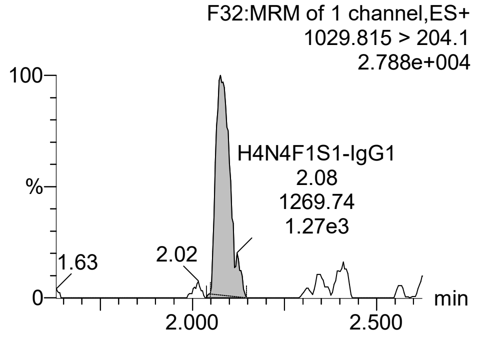

Sample Name: 20211111_L16_R2_IgG

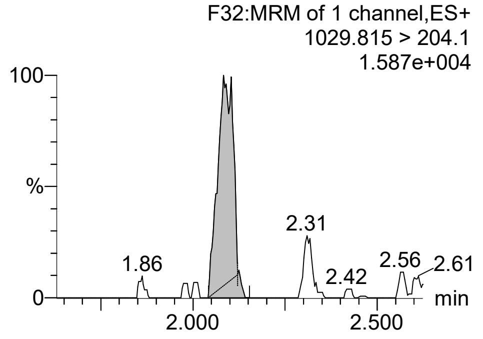

Dataset: C:\MassLynx\10.pro\20211115_clinicalsamples_IgG.qld

Last Altered: Sunday, October 02, 2022 16:02:30 台北標準時間
Printed: Friday, August 01, 2025 16:44:55 台北標準時間

Sample Name: 20211111_H8_R2_IgG

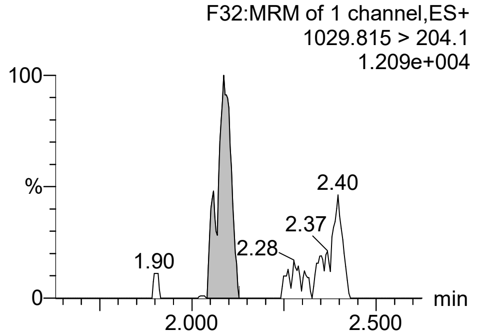

Sample Name: 20211111_A17_R2_IgG

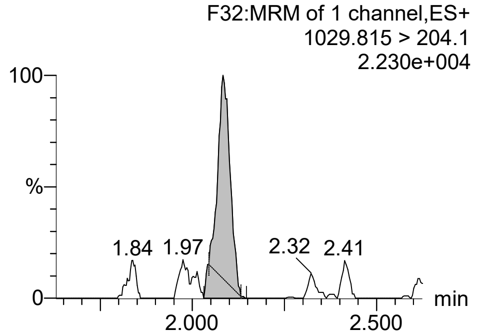

Sample Name: 20211111_A18_R2_IgG

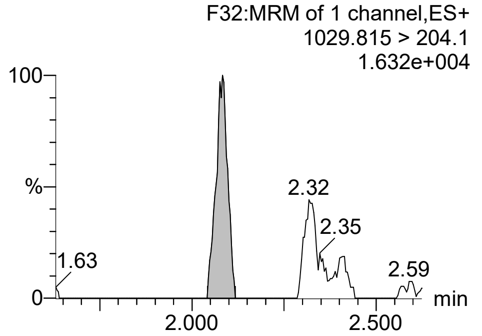

Sample Name: 20211111_L17_R2_IgG

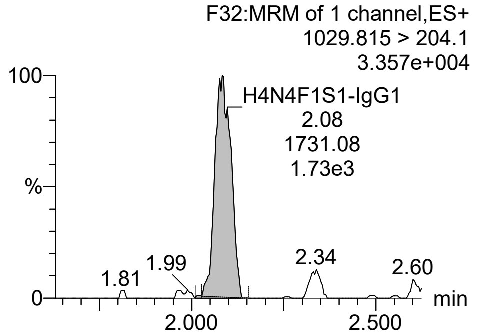

Sample Name: 20211111_L18_R2_IgG

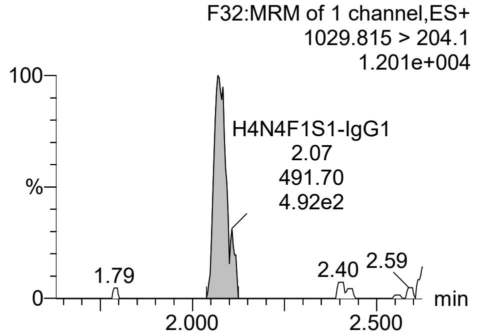

Sample Name: 20211111_H9_R2_IgG

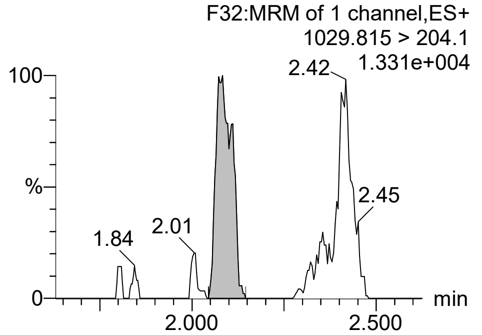

Sample Name: 20211111_A19_R2_IgG

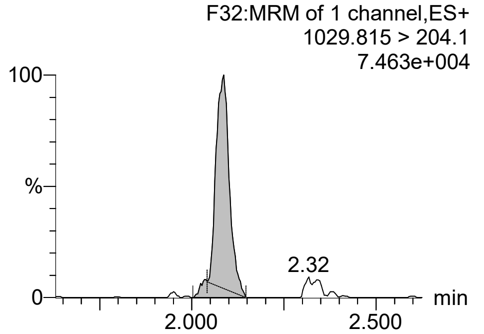

Sample Name: 20211111_A20_R2_IgG

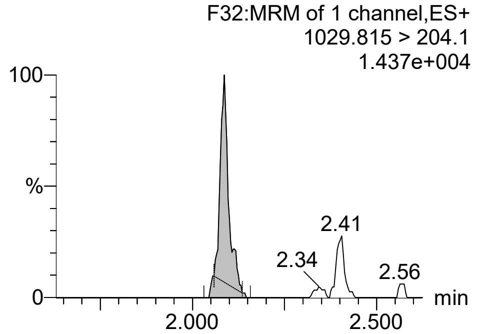

Sample Name: 20211111_L19_R2_IgG

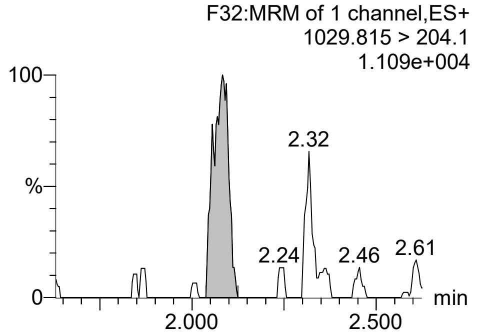

Sample Name: 20211111_QC_R13_IgG

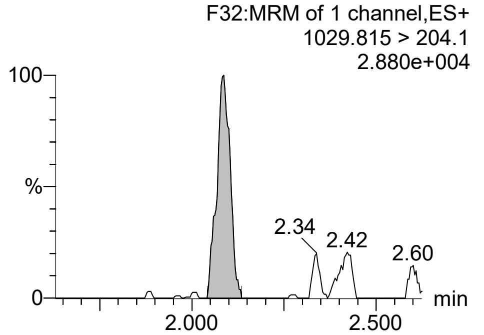

Sample Name: 20211111_L20_R2_IgG

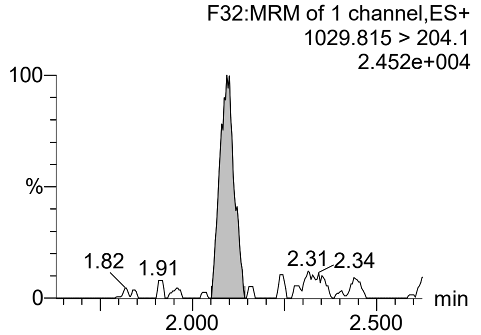

Sample Name: 20211111_H10_R2_IgG

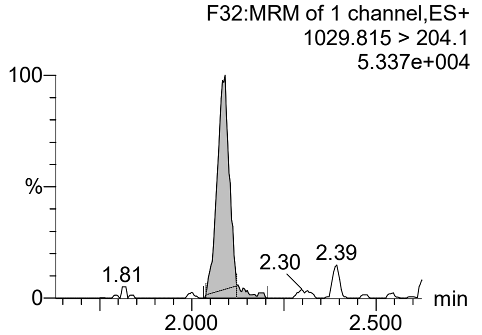

Sample Name: 20211111_A21_R2_IgG

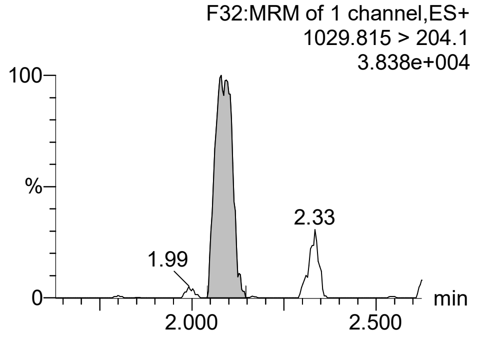

Sample Name: 20211111_A22_R2_IgG

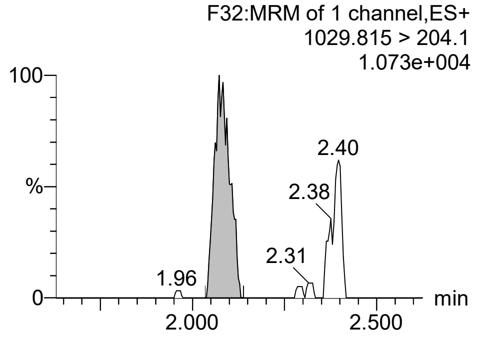

Sample Name: 20211111_L21_R2_IgG

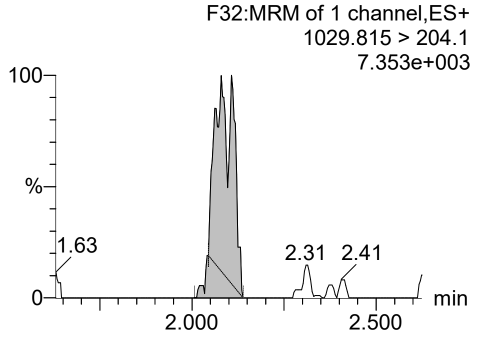

Sample Name: 20211111_L22_R2_IgG
F32:MRM of 1 channel,ES+
1029.815 > 204.1
3.192e+004

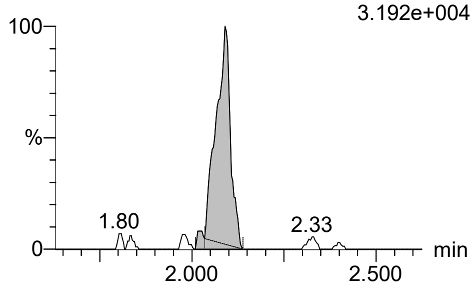

Sample Name: 20211111_H11_R2_IgG
F32:MRM of 1 channel,ES+
1029.815 > 204.1
3.095e+004

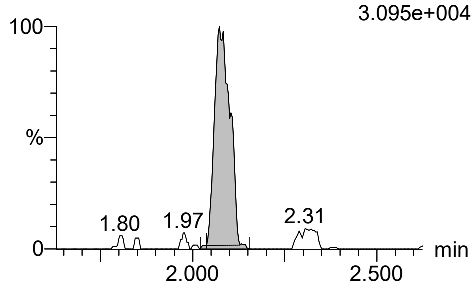

Sample Name: 20211111_A23_R2_IgG
F32:MRM of 1 channel,ES+
1029.815 > 204.1
2.849e+004

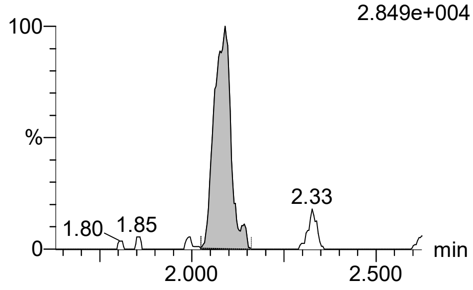

Sample Name: 20211111_A24_R2_IgG
F32:MRM of 1 channel,ES+
1029.815 > 204.1
1.471e+004

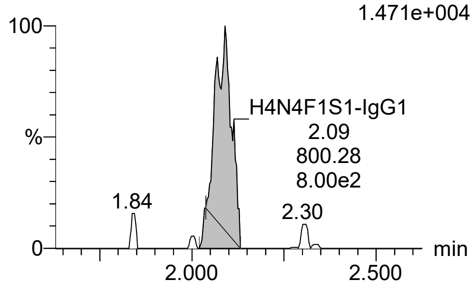

Sample Name: 20211111_L23_R2_IgG
F32:MRM of 1 channel,ES+
1029.815 > 204.1
3.459e+004

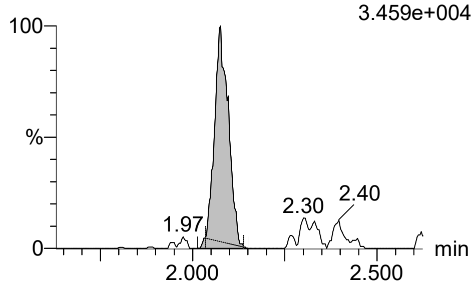

Sample Name: 20211111_L24_R2_IgG
F32:MRM of 1 channel,ES+
1029.815 > 204.1
1.808e+004

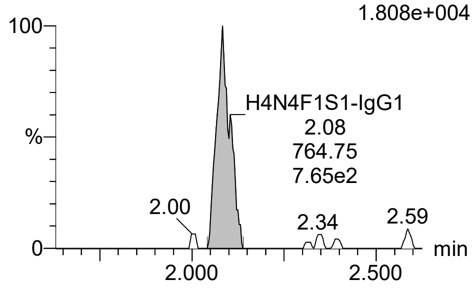

Sample Name: 20211111_H12_R2_IgG
F32:MRM of 1 channel,ES+
1029.815 > 204.1
3.427e+004

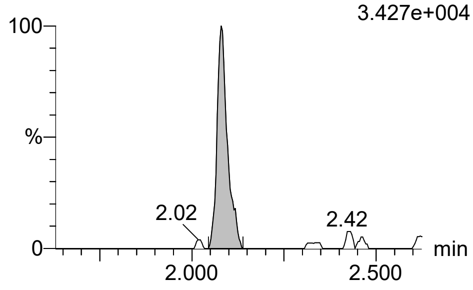

Sample Name: 20211111_QC_R14_IgG
F32:MRM of 1 channel,ES+
1029.815 > 204.1
2.742e+004

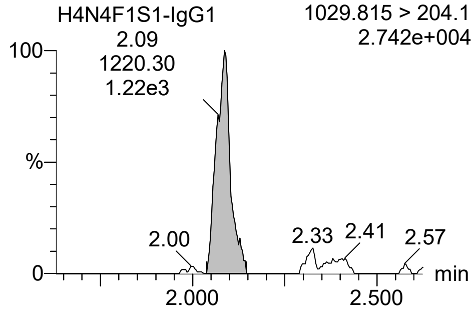

Sample Name: 20211111_A25_R2_IgG
F32:MRM of 1 channel,ES+
1029.815 > 204.1
6.015e+004

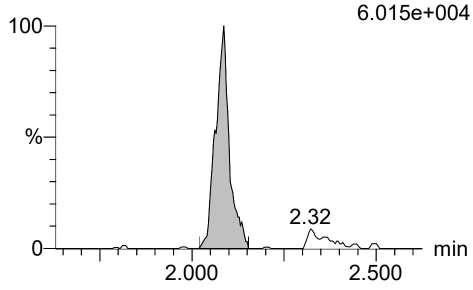

Sample Name: 20211111_A26_R2_IgG
F32:MRM of 1 channel,ES+
1029.815 > 204.1
1.125e+004

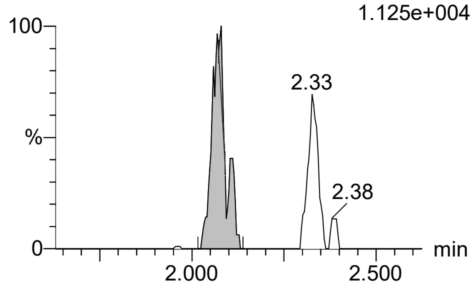

Sample Name: 20211111_L25_R2_IgG
F32:MRM of 1 channel,ES+
1029.815 > 204.1
1.462e+004

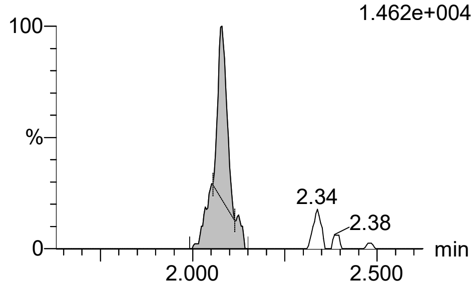

Sample Name: 20211111_L26_R2_IgG
F32:MRM of 1 channel,ES+
1029.815 > 204.1
9.688e+003

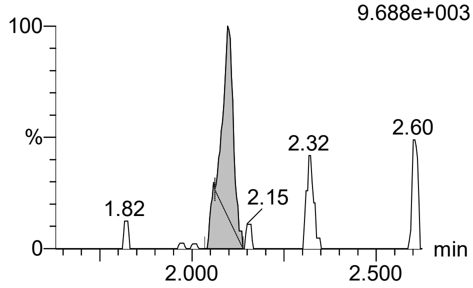

Sample Name: 20211111_H13_R2_IgG
F32:MRM of 1 channel,ES+
1029.815 > 204.1
3.106e+004

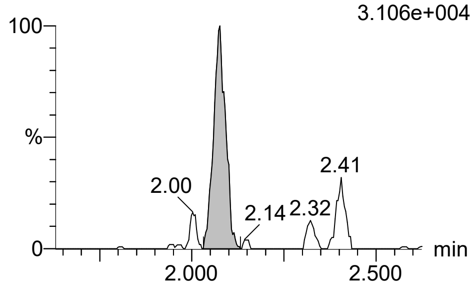

Sample Name: 20211111_A27_R2_IgG
F32:MRM of 1 channel,ES+
1029.815 > 204.1
2.455e+004

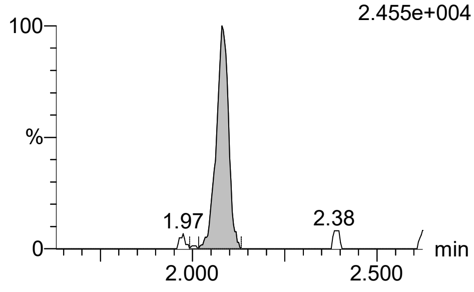

Sample Name: 20211111_A28_R2_IgG
F32:MRM of 1 channel,ES+
1029.815 > 204.1
2.643e+004

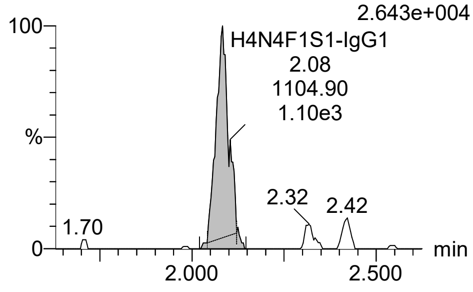

Sample Name: 20211111_L27_R2_IgG

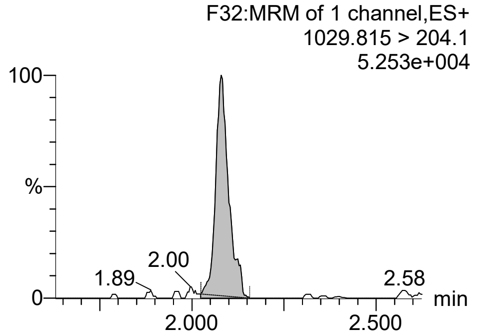

Sample Name: 20211111_L28_R2_IgG

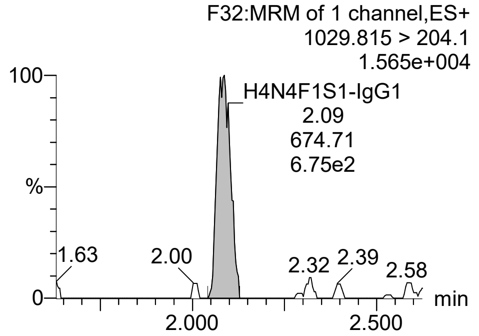

Sample Name: 20211111_H14_R2_IgG

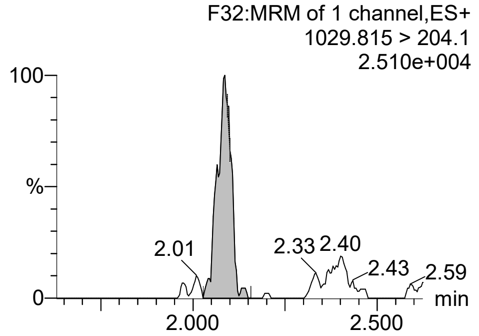

Sample Name: 20211111_A29_R2_IgG

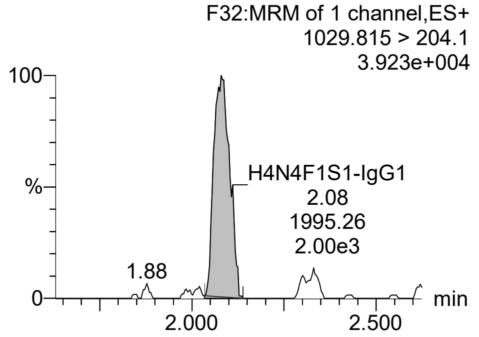

Sample Name: 20211111_A30_R2_IgG

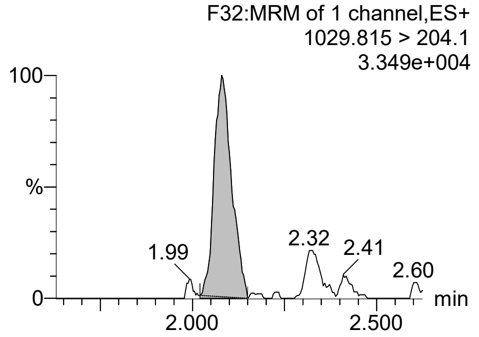

Sample Name: 20211111_QC_R15_IgG

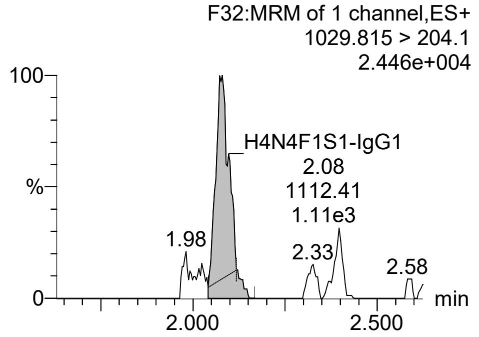

Sample Name: 20211111_L29_R2_IgG

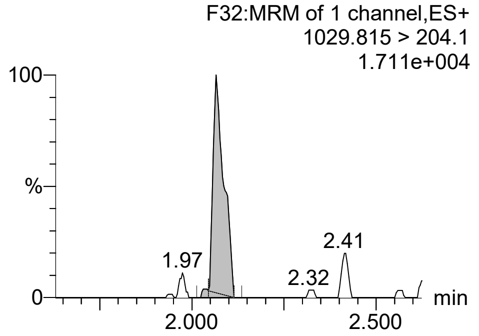

Sample Name: 20211111_L30_R2_IgG

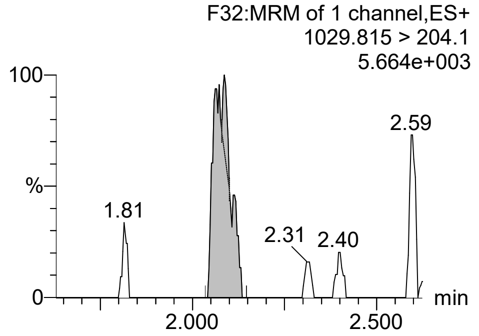

Sample Name: 20211111_H15_R2_IgG

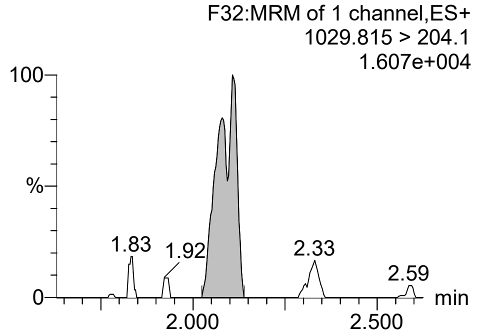

Sample Name: 20211111_A31_R2_IgG

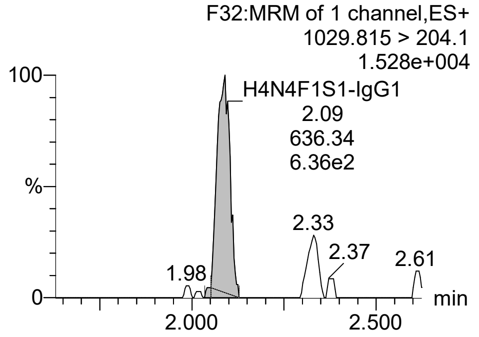

Sample Name: 20211111_A32_R2_IgG

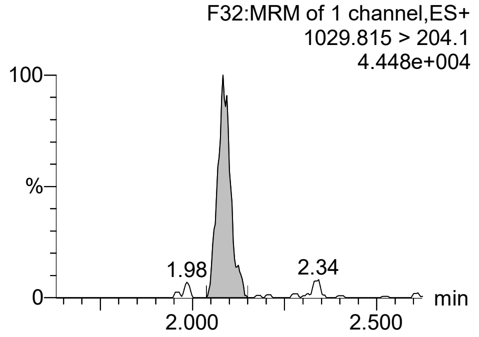

Sample Name: 20211111_L31_R2_IgG

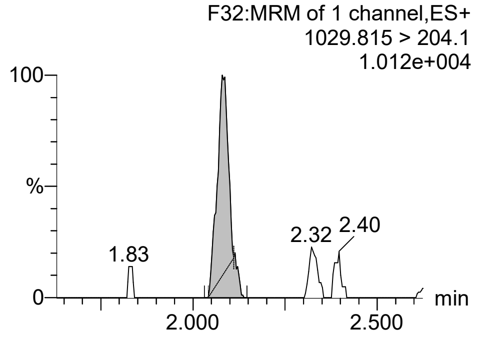

Sample Name: 20211111_L32_R2_IgG

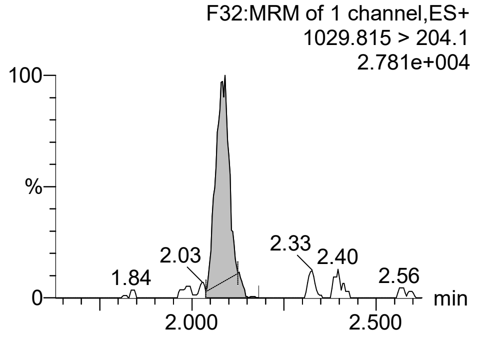

Sample Name: 20211111_H16_R2_IgG

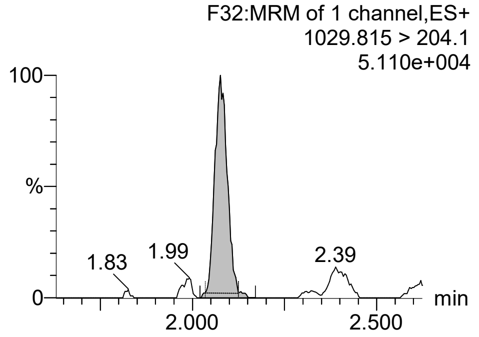

Sample Name: 20211111_A33_R2_IgG

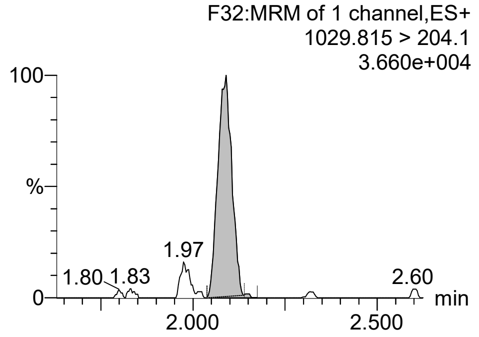

Sample Name: 20211111_A34_R2_IgG
F32:MRM of 1 channel,ES+
1029.815 > 204.1
2.693e+004

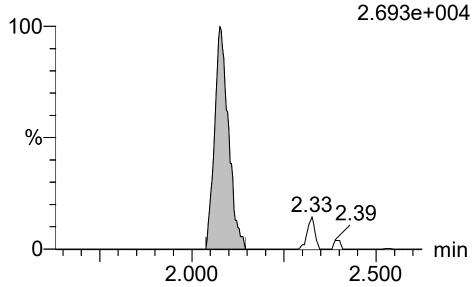

Sample Name: 20211111_H17_R2_IgG
F32:MRM of 1 channel,ES+
1029.815 > 204.1
1.549e+005

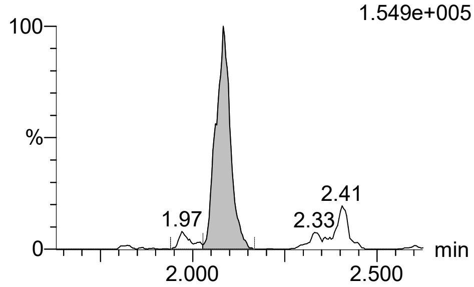

Sample Name: 20211111_A35_R2_IgG
F32:MRM of 1 channel,ES+
1029.815 > 204.1
3.049e+004

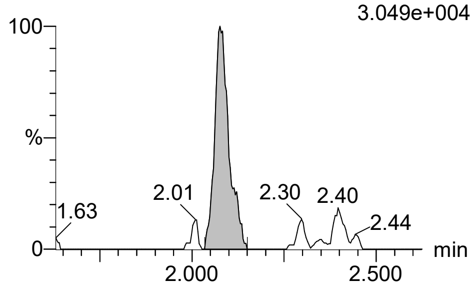

Sample Name: 20211111_QC_R16_IgG
F32:MRM of 1 channel,ES+
1029.815 > 204.1
1.720e+004

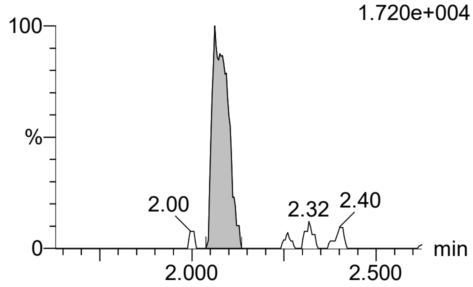

Sample Name: 20211111_A36_R2_IgG
F32:MRM of 1 channel,ES+
1029.815 > 204.1
1.953e+004

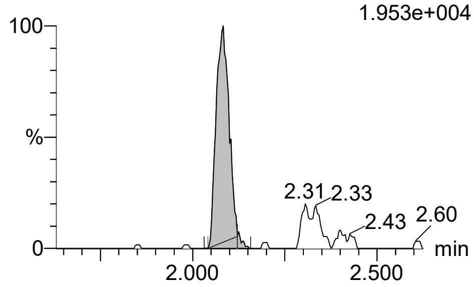

Sample Name: 20211111_H18_R2_IgG
F32:MRM of 1 channel,ES+
1029.815 > 204.1
1.521e+005

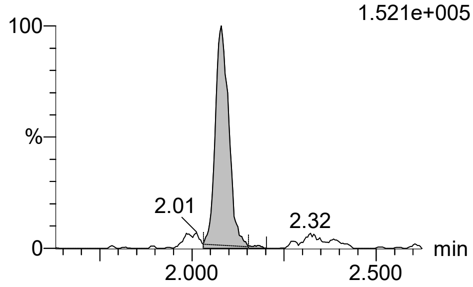

Sample Name: 20211111_A37_R2_IgG
F32:MRM of 1 channel,ES+
1029.815 > 204.1
3.193e+004

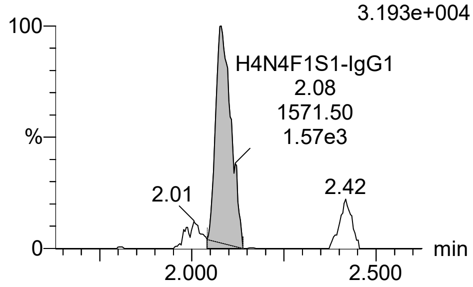

Sample Name: 20211111_A38_R2_IgG
F32:MRM of 1 channel,ES+
1029.815 > 204.1
2.333e+004

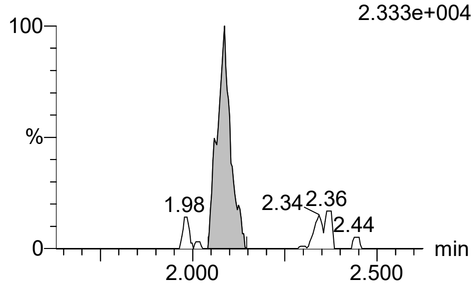

Sample Name: 20211111_H19_R2_IgG
F32:MRM of 1 channel,ES+
1029.815 > 204.1
9.426e+004

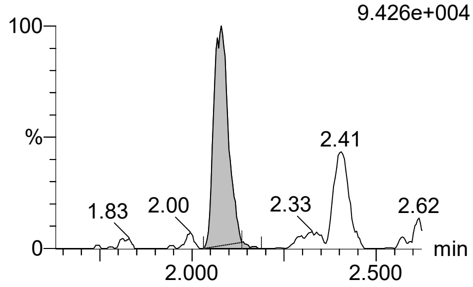

Sample Name: 20211111_A39_R2_IgG
F32:MRM of 1 channel,ES+
1029.815 > 204.1
1.481e+004

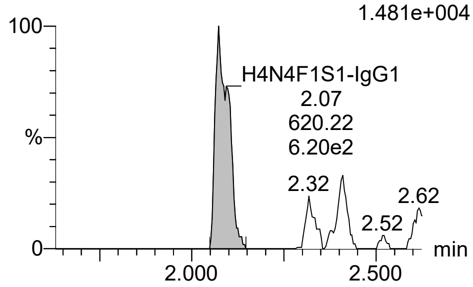

Sample Name: 20211111_A40_R2_IgG
F32:MRM of 1 channel,ES+
1029.815 > 204.1
2.166e+004

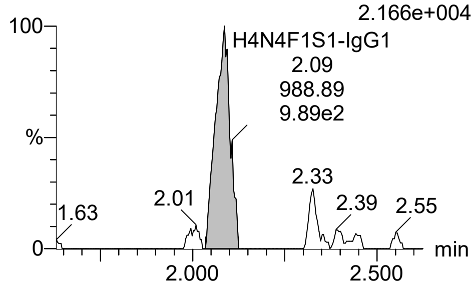

Sample Name: 20211111_H20_R2_IgG
F32:MRM of 1 channel,ES+
1029.815 > 204.1
6.481e+004

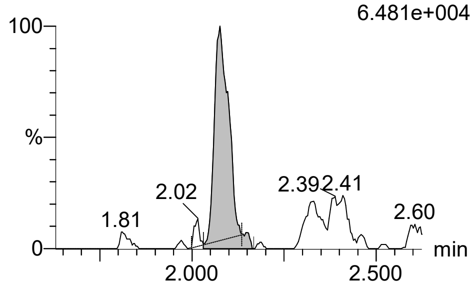

Sample Name: 20211111_A41_R2_IgG
F32:MRM of 1 channel,ES+
1029.815 > 204.1
3.144e+004

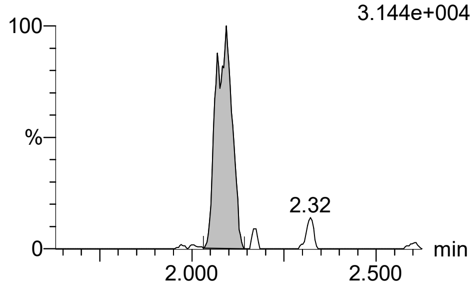

Sample Name: 20211111_A42_R2_IgG
F32:MRM of 1 channel,ES+
1029.815 > 204.1
1.187e+004

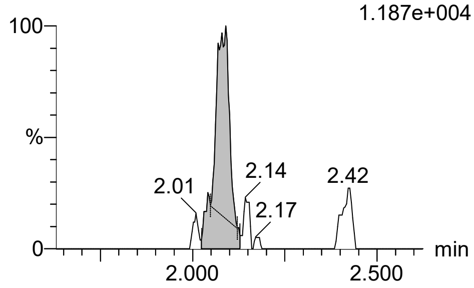

Sample Name: 20211111_A43_R2_IgG
F32:MRM of 1 channel,ES+
1029.815 > 204.1
3.644e+004

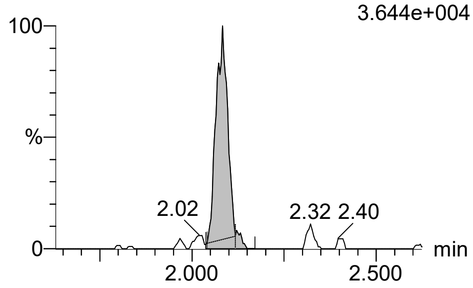

Dataset: C:\MassLynx\10.pro\20211115_clinicalsamples_IgG.qld

Last Altered: Sunday, October 02, 2022 16:02:30 台北標準時間
Printed: Friday, August 01, 2025 16:44:55 台北標準時間

Sample Name: 20211111_A44_R2_IgG

F32:MRM of 1 channel,ES+
1029.815 > 204.1
2.652e+004

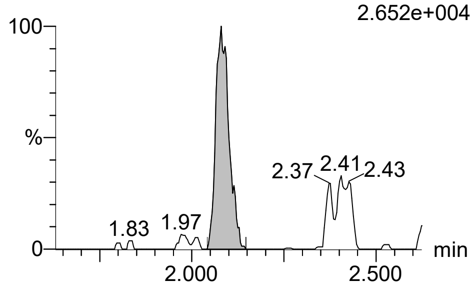

Sample Name: 20211111_QC_R17_IgG

F32:MRM of 1 channel,ES+
1029.815 > 204.1
2.314e+004

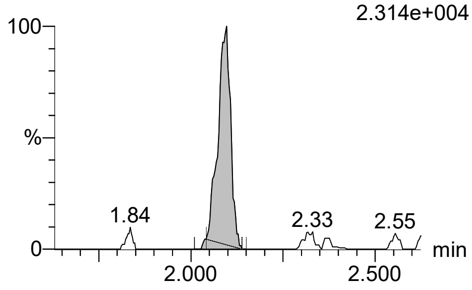

Dataset: C:\MassLynx\10.pro\20211115_clinicalsamples_IgG.qld

Last Altered: Sunday, October 02, 2022 16:02:30 台北標準時間
Printed: Friday, August 01, 2025 16:44:55 台北標準時間

	#	Name	RT	Area	IS Area	Response	Prima...
1	1	20211111_QC_R1_IgG	2.08	2754.4	64968.0	0.042	MM
2	2	20211111_A1_R1_IgG	2.09	5117.7	59059.2	0.087	MM
3	3	20211111_A2_R1_IgG	2.09	2890.5	66621.2	0.043	MM
4	4	20211111_L1_R1_IgG	2.10	3896.9	64201.0	0.061	MM
5	5	20211111_L2_R1_IgG	2.08	1269.0	46950.2	0.027	bb
6	6	20211111_H1_R1_IgG	2.08	1336.7	38648.2	0.035	bb
7	7	20211111_A3_R1_IgG	2.10	6.5	2603.7	0.002	bb
8	8	20211111_A4_R1_IgG	2.09	2857.5	69960.4	0.041	MM
9	9	20211111_L3_R1_IgG	2.08	881.6	38864.7	0.023	MM
10	10	20211111_L4_R1_IgG	2.09	1363.5	43380.5	0.031	MM
11	11	20211111_H2_R1_IgG	2.09	13190.9	71349.2	0.185	MM
12	12	20211111_A5_R1_IgG	2.09	3207.7	65569.7	0.049	MM
13	13	20211111_A6_R1_IgG	2.09	4356.2	53120.7	0.082	bb
14	14	20211111_QC_R2_IgG	2.09	3605.7	81034.1	0.044	MM
15	15	20211111_L5_R1_IgG	2.09	4697.8	50994.6	0.092	MM
16	16	20211111_L6_R1_IgG	2.09	241.2	58058.0	0.004	MM
17	17	20211111_H3_R1_IgG	2.10	721.4	64028.6	0.011	MM
18	18	20211111_A7_R1_IgG	2.09	6491.3	82996.1	0.078	bb
19	19	20211111_A8_R1_IgG	2.09	5047.4	77731.7	0.065	bb
20	20	20211111_L7_R1_IgG	2.08	977.8	38827.3	0.025	bb
21	21	20211111_L8_R1_IgG	2.08	1115.3	44220.1	0.025	MM
22	22	20211111_H4_R1_IgG	2.09	2635.8	65826.3	0.040	MM
23	23	20211111_A9_R1_IgG	2.07	1678.6	60771.1	0.028	MM
24	24	20211111_A10_R1_IgG	2.09	1904.6	48578.7	0.039	bb
25	25	20211111_L9_R1_IgG	2.08	1256.4	50830.7	0.025	bb
26	26	20211111_L10_R1_IgG	2.09	2395.7	38034.2	0.063	MM
27	27	20211111_QC_R3_IgG	2.09	2726.5	67025.5	0.041	MM
28	28	20211111_H5_R1_IgG	2.10	963.2	44544.9	0.022	MM
29	29	20211111_A11_R1_IgG	2.09	2190.7	66408.5	0.033	bb
30	30	20211111_A12_R1_IgG	2.08	1074.8	46624.5	0.023	MM
31	31	20211111_L11_R1_IgG	2.09	818.3	33425.1	0.024	MM
32	32	20211111_L12_R1_IgG	2.09	1097.6	41178.0	0.027	MM
33	33	20211111_H6_R1_IgG	2.09	1697.0	58593.4	0.029	MM
34	34	20211111_A13_R1_IgG	2.09	1734.9	54133.0	0.032	bb
35	35	20211111_A14_R1_IgG	2.10	1649.8	56467.3	0.029	MM
36	36	20211111_L13_R1_IgG	2.09	1055.6	36024.4	0.029	MM
37	37	20211111_L14_R1_IgG	2.09	1573.2	37181.6	0.042	MM
38	38	20211111_H7_R1_IgG	2.08	796.3	42850.6	0.019	bb
39	39	20211111_A15_R1_IgG	2.09	5881.9	62015.1	0.095	MM
40	40	20211111_QC_R4_IgG	2.09	1982.9	58572.5	0.034	MM
41	41	20211111_A16_R1_IgG	2.09	2310.5	78283.8	0.030	MM
42	42	20211111_L15_R1_IgG	2.08	1487.7	44158.2	0.034	MM
43	43	20211111_L16_R1_IgG	2.09	1166.1	55150.8	0.021	MM
44	44	20211111_H8_R1_IgG	2.08	979.0	42965.2	0.023	MM
45	45	20211111_A17_R1_IgG	2.09	1141.7	39717.7	0.029	bb
46	46	20211111_A18_R1_IgG	2.09	995.1	44675.4	0.022	bb
47	47	20211111_L17_R1_IgG	2.08	1575.8	37854.1	0.042	MM
48	48	20211111_L18_R1_IgG	2.08	909.0	50716.9	0.018	bb
49	49	20211111_H9_R1_IgG	2.09	1125.7	36276.0	0.031	MM
50	50	20211111_A19_R1_IgG	2.08	3968.0	42862.5	0.093	MM
51	51	20211111_A20_R1_IgG	2.08	281.2	36542.7	0.008	MM
52	52	20211111_L19_R1_IgG	2.08	899.8	53892.8	0.017	bb
53	53	20211111_QC_R5_IgG	2.09	2182.6	56513.2	0.039	bb
54	54	20211111_L20_R1_IgG	2.07	1911.4	56499.9	0.034	bb
55	55	20211111_H10_R1_IgG	2.09	5222.7	68120.5	0.077	bb
56	56	20211111_A21_R1_IgG	2.07	4128.8	63699.8	0.065	MM

Dataset: C:\MassLynx\10.pro\20211115_clinicalsamples_IgG.qld

Last Altered: Sunday, October 02, 2022 16:02:30 台北標準時間
Printed: Friday, August 01, 2025 16:44:55 台北標準時間

	#	Name	RT	Area	IS Area	Response	Prima...
57	57	20211111_A22_R1_IgG	2.08	1202.1	52222.1	0.023	MM
58	58	20211111_L21_R1_IgG	2.08	1021.5	54372.5	0.019	MM
59	59	20211111_L22_R1_IgG	2.09	2757.8	41353.1	0.067	bb
60	60	20211111_H11_R1_IgG	2.09	3529.2	65579.1	0.054	bb
61	61	20211111_A23_R1_IgG	2.09	3466.2	64131.1	0.054	MM
62	62	20211111_A24_R1_IgG	2.07	957.1	38724.8	0.025	MM
63	63	20211111_L23_R1_IgG	2.08	2391.3	64245.2	0.037	MM
64	64	20211111_L24_R1_IgG	2.08	907.2	45298.0	0.020	MM
65	65	20211111_H12_R1_IgG	2.08	1513.7	44679.3	0.034	bb
66	66	20211111_QC_R6_IgG	2.09	2481.3	48165.0	0.052	MM
67	67	20211111_A25_R1_IgG	2.08	3765.8	57169.9	0.066	MM
68	68	20211111_A26_R1_IgG	2.08	653.5	34254.0	0.019	bb
69	69	20211111_L25_R1_IgG	2.09	1630.5	67491.5	0.024	bb
70	70	20211111_L26_R1_IgG	2.09	815.1	55934.1	0.015	bb
71	71	20211111_H13_R1_IgG	2.08	2418.4	48767.3	0.050	MM
72	72	20211111_A27_R1_IgG	2.09	1751.2	57269.7	0.031	bb
73	73	20211111_A28_R1_IgG	2.09	1975.5	49231.1	0.040	MM
74	74	20211111_L27_R1_IgG	2.09	3692.7	60014.5	0.062	bb
75	75	20211111_L28_R1_IgG	2.08	973.4	35062.6	0.028	MM
76	76	20211111_H14_R1_IgG	2.08	1830.8	62371.9	0.029	MM
77	77	20211111_A29_R1_IgG	2.09	2669.7	43858.2	0.061	MM
78	78	20211111_A30_R1_IgG	2.09	3709.5	62500.4	0.059	bb
79	79	20211111_QC_R7_IgG	2.09	1758.2	43946.9	0.040	MM
80	80	20211111_L29_R1_IgG	2.10	706.5	38728.9	0.018	MM
81	81	20211111_L30_R1_IgG	2.09	468.8	42613.2	0.011	bb
82	82	20211111_H15_R1_IgG	2.08	1518.0	39874.6	0.038	MM
83	83	20211111_A31_R1_IgG	2.09	1992.5	53414.3	0.037	MM
84	84	20211111_A32_R1_IgG	2.09	2373.0	41844.6	0.057	bb
85	85	20211111_L31_R1_IgG	2.09	851.4	40553.7	0.021	MM
86	86	20211111_L32_R1_IgG	2.09	1430.5	49618.7	0.029	MM
87	87	20211111_H16_R1_IgG	2.09	3229.9	60170.9	0.054	MM
88	88	20211111_A33_R1_IgG	2.09	2853.7	48588.2	0.059	MM
89	89	20211111_A34_R1_IgG	2.10	1273.8	43288.0	0.029	MM
90	90	20211111_H17_R1_IgG	2.08	9842.0	47104.5	0.209	MM
91	91	20211111_A35_R1_IgG	2.08	1400.5	41025.9	0.034	bb
92	92	20211111_QC_R8_IgG	2.09	1567.0	40546.3	0.039	MM
93	93	20211111_A36_R1_IgG	2.09	1308.9	44504.4	0.029	bb
94	94	20211111_H18_R1_IgG	2.09	7709.8	34162.2	0.226	bb
95	95	20211111_A37_R1_IgG	2.09	2483.5	39760.0	0.062	bb
96	96	20211111_A38_R1_IgG	2.09	1394.9	43982.6	0.032	MM
97	97	20211111_H19_R1_IgG	2.07	5151.9	45314.8	0.114	MM
98	98	20211111_A39_R1_IgG	2.09	705.6	39388.0	0.018	MM
99	99	20211111_A40_R1_IgG	2.08	921.8	38726.4	0.024	MM
100	1...	20211111_H20_R1_IgG	2.09	3932.1	37814.4	0.104	MM
101	1...	20211111_A41_R1_IgG	2.08	1936.8	48930.3	0.040	MM
102	1...	20211111_A42_R1_IgG	2.09	680.8	40406.8	0.017	MM
103	1...	20211111_A43_R1_IgG	2.09	1880.0	44421.5	0.042	MM
104	1...	20211111_A44_R1_IgG	2.10	2423.5	53128.2	0.046	MM
105	1...	20211111_QC_R9_IgG	2.09	1171.6	37049.0	0.032	MM
106	1...	20211111_A1_R2_IgG	2.09	3343.3	46499.4	0.072	MM
107	1...	20211111_A2_R2_IgG	2.09	2030.2	57260.7	0.035	MM
108	1...	20211111_L1_R2_IgG	2.08	1996.3	48062.9	0.042	MM
109	1...	20211111_L2_R2_IgG	2.09	1092.3	38745.2	0.028	MM
110	1...	20211111_H1_R2_IgG	2.09	1471.3	44273.5	0.033	MM
111	1...	20211111_A3_R2_IgG	2.09	39.9	4755.5	0.008	MM
112	1...	20211111_A4_R2_IgG	2.08	1682.1	50546.7	0.033	bb

Dataset: C:\MassLynx\10.pro\20211115_clinicalsamples_IgG.qld

Last Altered: Sunday, October 02, 2022 16:02:30 台北標準時間
Printed: Friday, August 01, 2025 16:44:55 台北標準時間

	# Name	RT	Area	IS Area	Response	Prima...
113	1... 20211111_L3_R2_IgG	2.09	781.8	34825.8	0.022	MM
114	1... 20211111_L4_R2_IgG	2.09	809.2	39107.7	0.021	bb
115	1... 20211111_H2_R2_IgG	2.09	8928.7	49750.0	0.179	MM
116	1... 20211111_A5_R2_IgG	2.07	2429.6	52536.2	0.046	MM
117	1... 20211111_A6_R2_IgG	2.09	2828.8	42050.0	0.067	MM
118	1... 20211111_QC_R10_IgG	2.07	1024.0	35350.8	0.029	bb
119	1... 20211111_L5_R2_IgG	2.08	2866.5	38039.3	0.075	MM
120	1... 20211111_L6_R2_IgG	2.09	205.4	44907.4	0.005	bb
121	1... 20211111_H3_R2_IgG	2.09	712.0	47729.1	0.015	bb
122	1... 20211111_A7_R2_IgG	2.08	4166.1	57347.1	0.073	MM
123	1... 20211111_A8_R2_IgG	2.07	3619.6	52397.8	0.069	bb
124	1... 20211111_L7_R2_IgG	2.09	1047.9	36318.4	0.029	bb
125	1... 20211111_L8_R2_IgG	2.07	807.8	38131.9	0.021	MM
126	1... 20211111_H4_R2_IgG	2.09	2484.8	46849.6	0.053	MM
127	1... 20211111_A9_R2_IgG	2.09	1058.4	47759.6	0.022	bb
128	1... 20211111_A10_R2_IgG	2.07	1781.7	41366.2	0.043	MM
129	1... 20211111_L9_R2_IgG	2.08	901.4	43052.1	0.021	MM
130	1... 20211111_L10_R2_IgG	2.09	1505.2	31487.4	0.048	MM
131	1... 20211111_QC_R11_IgG	2.08	1111.7	37804.6	0.029	bb
132	1... 20211111_H5_R2_IgG	2.09	961.6	40380.0	0.024	MM
133	1... 20211111_A11_R2_IgG	2.08	1470.8	49533.3	0.030	MM
134	1... 20211111_A12_R2_IgG	2.08	855.9	40226.3	0.021	bb
135	1... 20211111_L11_R2_IgG	2.09	440.1	28884.0	0.015	bb
136	1... 20211111_L12_R2_IgG	2.09	1613.6	34351.3	0.047	MM
137	1... 20211111_H6_R2_IgG	2.08	952.6	40466.6	0.024	MM
138	1... 20211111_A13_R2_IgG	2.08	1459.1	42306.7	0.034	MM
139	1... 20211111_A14_R2_IgG	2.09	905.7	44904.0	0.020	MM
140	1... 20211111_L13_R2_IgG	2.09	941.2	31913.0	0.029	bb
141	1... 20211111_L14_R2_IgG	2.10	1074.9	31527.4	0.034	MM
142	1... 20211111_H7_R2_IgG	2.09	712.7	40838.2	0.017	bb
143	1... 20211111_A15_R2_IgG	2.08	3985.0	49389.7	0.081	MM
144	1... 20211111_QC_R12_IgG	2.08	902.0	35168.7	0.026	MM
145	1... 20211111_A16_R2_IgG	2.08	1012.1	47440.5	0.021	MM
146	1... 20211111_L15_R2_IgG	2.08	1269.7	33145.9	0.038	MM
147	1... 20211111_L16_R2_IgG	2.08	802.9	37596.6	0.021	MM
148	1... 20211111_H8_R2_IgG	2.09	532.9	43933.3	0.012	bb
149	1... 20211111_A17_R2_IgG	2.08	969.9	43605.5	0.022	MM
150	1... 20211111_A18_R2_IgG	2.08	604.2	37581.8	0.016	bb
151	1... 20211111_L17_R2_IgG	2.08	1731.1	33942.5	0.051	MM
152	1... 20211111_L18_R2_IgG	2.07	491.7	37831.5	0.013	bb
153	1... 20211111_H9_R2_IgG	2.08	688.8	30898.4	0.022	bb
154	1... 20211111_A19_R2_IgG	2.09	3388.6	39352.1	0.086	MM
155	1... 20211111_A20_R2_IgG	2.09	422.4	34661.2	0.012	MM
156	1... 20211111_L19_R2_IgG	2.08	545.0	39918.3	0.014	bb
157	1... 20211111_QC_R13_IgG	2.09	1213.7	36239.1	0.033	bb
158	1... 20211111_L20_R2_IgG	2.09	1115.4	32897.7	0.034	bb
159	1... 20211111_H10_R2_IgG	2.09	2181.2	31394.5	0.069	MM
160	1... 20211111_A21_R2_IgG	2.08	2152.5	34863.7	0.062	bb
161	1... 20211111_A22_R2_IgG	2.07	514.0	30342.1	0.017	MM
162	1... 20211111_L21_R2_IgG	2.11	465.5	33706.2	0.014	MM
163	1... 20211111_L22_R2_IgG	2.09	1544.9	31298.6	0.049	MM
164	1... 20211111_H11_R2_IgG	2.07	1538.9	38904.1	0.040	MM
165	1... 20211111_A23_R2_IgG	2.09	1516.3	32382.3	0.047	MM
166	1... 20211111_A24_R2_IgG	2.09	800.3	30261.9	0.026	MM
167	1... 20211111_L23_R2_IgG	2.08	1540.7	43037.5	0.036	MM
168	1... 20211111_L24_R2_IgG	2.08	764.8	37023.3	0.021	bb

Dataset: C:\MassLynx\10.pro\20211115_clinicalsamples_IgG.qld

Last Altered: Sunday, October 02, 2022 16:02:30 台北標準時間
Printed: Friday, August 01, 2025 16:44:55 台北標準時間

	# Name	RT	Area	IS Area	Response	Prima...
169	1... 20211111_H12_R2_IgG	2.08	1122.7	39478.2	0.028	bb
170	1... 20211111_QC_R14_IgG	2.09	1220.3	34463.2	0.035	bb
171	1... 20211111_A25_R2_IgG	2.09	2603.2	39945.3	0.065	bb
172	1... 20211111_A26_R2_IgG	2.08	493.0	29663.2	0.017	MM
173	1... 20211111_L25_R2_IgG	2.08	645.4	38589.3	0.017	MM
174	1... 20211111_L26_R2_IgG	2.10	398.3	38405.2	0.010	MM
175	1... 20211111_H13_R2_IgG	2.08	1175.1	37771.2	0.031	bb
176	1... 20211111_A27_R2_IgG	2.08	945.7	42634.1	0.022	MM
177	1... 20211111_A28_R2_IgG	2.08	1104.9	33231.1	0.033	MM
178	1... 20211111_L27_R2_IgG	2.08	2196.9	41761.1	0.053	MM
179	1... 20211111_L28_R2_IgG	2.09	674.7	26968.6	0.025	bb
180	1... 20211111_H14_R2_IgG	2.09	1105.0	49250.2	0.022	MM
181	1... 20211111_A29_R2_IgG	2.08	1995.3	37601.7	0.053	MM
182	1... 20211111_A30_R2_IgG	2.08	1861.5	40366.8	0.046	MM
183	1... 20211111_QC_R15_IgG	2.08	1112.4	35156.1	0.032	MM
184	1... 20211111_L29_R2_IgG	2.07	596.8	33813.1	0.018	MM
185	1... 20211111_L30_R2_IgG	2.09	308.4	33488.6	0.009	MM
186	1... 20211111_H15_R2_IgG	2.11	944.8	38501.0	0.025	bb
187	1... 20211111_A31_R2_IgG	2.09	636.3	37525.5	0.017	MM
188	1... 20211111_A32_R2_IgG	2.08	1800.9	35770.8	0.050	bb
189	1... 20211111_L31_R2_IgG	2.08	394.9	33282.4	0.012	MM
190	1... 20211111_L32_R2_IgG	2.09	1272.1	42049.6	0.030	MM
191	1... 20211111_H16_R2_IgG	2.08	2053.1	45947.6	0.045	MM
192	1... 20211111_A33_R2_IgG	2.09	1636.1	40202.3	0.041	MM
193	1... 20211111_A34_R2_IgG	2.08	1174.2	36747.0	0.032	bb
194	1... 20211111_H17_R2_IgG	2.08	7078.3	40920.9	0.173	MM
195	1... 20211111_A35_R2_IgG	2.08	1385.4	33689.2	0.041	bb
196	1... 20211111_QC_R16_IgG	2.06	854.4	32783.6	0.026	bb
197	1... 20211111_A36_R2_IgG	2.08	821.8	36822.4	0.022	MM
198	1... 20211111_H18_R2_IgG	2.08	6656.1	31769.3	0.210	MM
199	1... 20211111_A37_R2_IgG	2.08	1571.5	37581.8	0.042	MM
200	2... 20211111_A38_R2_IgG	2.09	983.7	38390.4	0.026	bb
201	2... 20211111_H19_R2_IgG	2.08	4486.2	41217.0	0.109	MM
202	2... 20211111_A39_R2_IgG	2.07	620.2	37392.7	0.017	bb
203	2... 20211111_A40_R2_IgG	2.09	988.9	38295.1	0.026	bb
204	2... 20211111_H20_R2_IgG	2.08	3117.1	35941.8	0.087	MM
205	2... 20211111_A41_R2_IgG	2.09	1603.3	45304.9	0.035	bb
206	2... 20211111_A42_R2_IgG	2.09	565.4	37803.8	0.015	MM
207	2... 20211111_A43_R2_IgG	2.08	1427.8	38161.8	0.037	MM
208	2... 20211111_A44_R2_IgG	2.08	1111.4	37819.8	0.029	bb
209	2... 20211111_QC_R17_IgG	2.10	956.9	31731.8	0.030	MM

Dataset: C:\MassLynx\10.pro\20211115_clinicalsamples_IgG.qld

Last Altered: Sunday, October 02, 2022 16:02:30 台北標準時間
Printed: Friday, August 01, 2025 16:45:25 台北標準時間

Method: C:\MassLynx\10_Pro_IgA.PRO\MethDB\20210926_IgG_DMRM_IgM_IS.mdb 16 Feb 2022 11:58:21
Calibration: 18 Nov 2021 13:56:22

Sample Name: 20211111_QC_R1_IgG

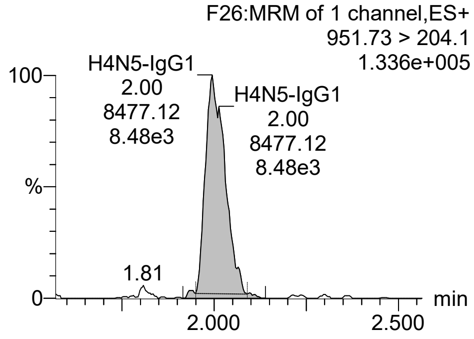

Sample Name: 20211111_A1_R1_IgG

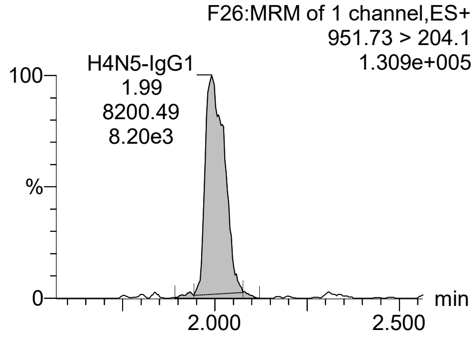

Sample Name: 20211111_A2_R1_IgG

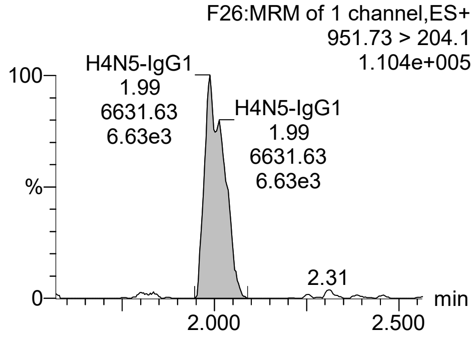

Sample Name: 20211111_L1_R1_IgG

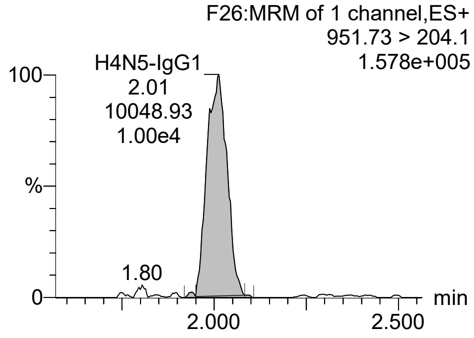

Sample Name: 20211111_L2_R1_IgG

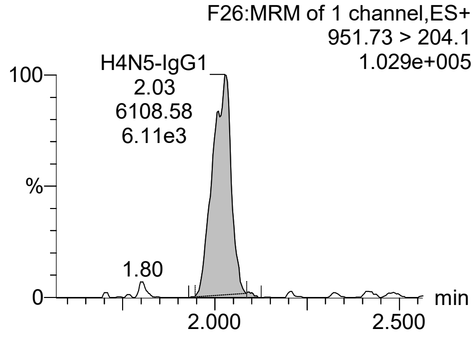

Sample Name: 20211111_H1_R1_IgG

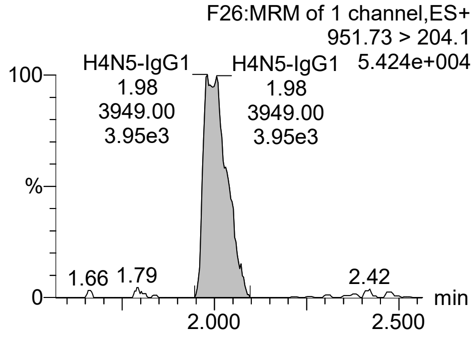

Sample Name: 20211111_A3_R1_IgG

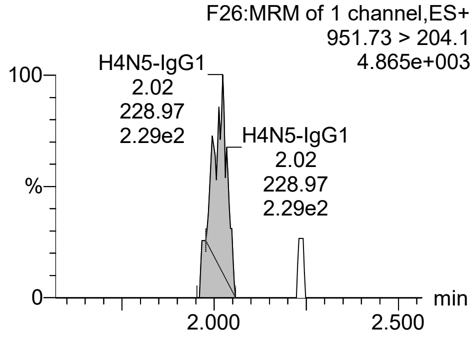

Sample Name: 20211111_A4_R1_IgG

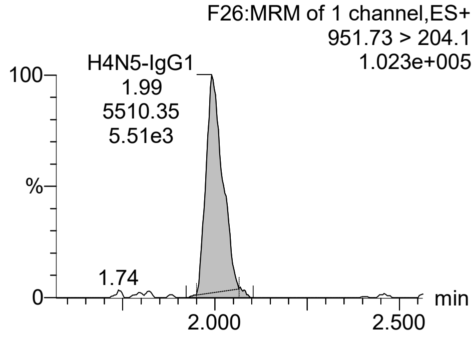

Sample Name: 20211111_L3_R1_IgG

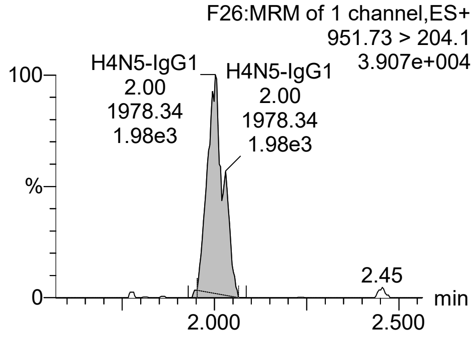

Sample Name: 20211111_L4_R1_IgG

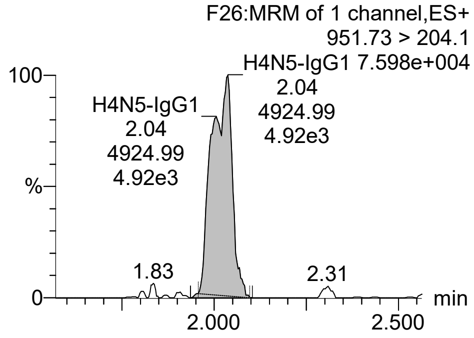

Sample Name: 20211111_H2_R1_IgG

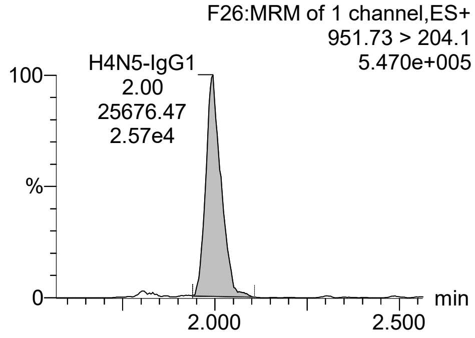

Sample Name: 20211111_A5_R1_IgG

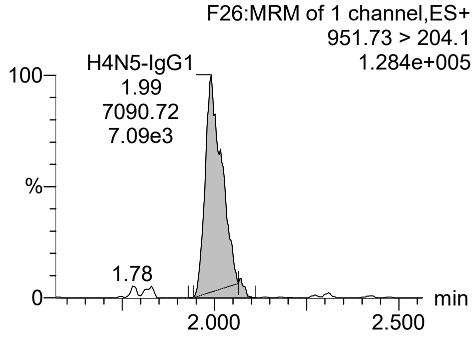

Dataset: C:\MassLynx\10.pro\20211115_clinicalsamples_IgG.qld

Last Altered: Sunday, October 02, 2022 16:02:30 台北標準時間
Printed: Friday, August 01, 2025 16:45:25 台北標準時間

Sample Name: 20211111_A6_R1_IgG

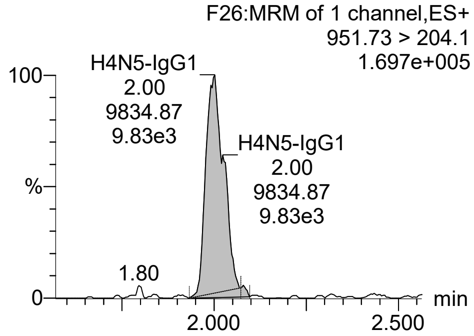

Sample Name: 20211111_QC_R2_IgG

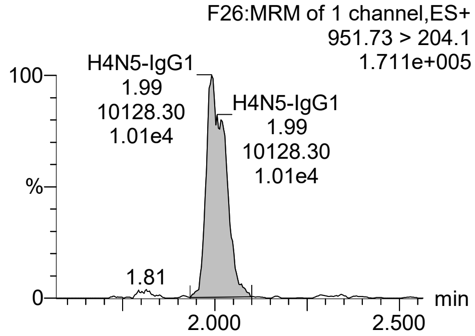

Sample Name: 20211111_L5_R1_IgG

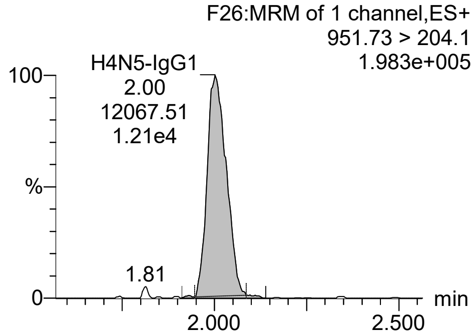

Sample Name: 20211111_L6_R1_IgG

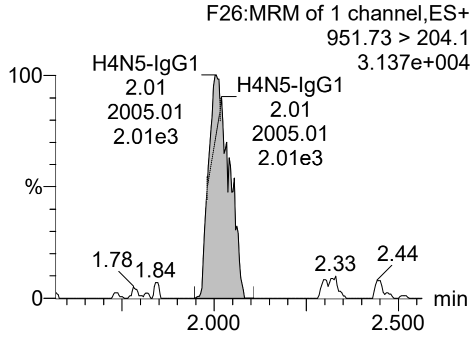

Sample Name: 20211111_H3_R1_IgG

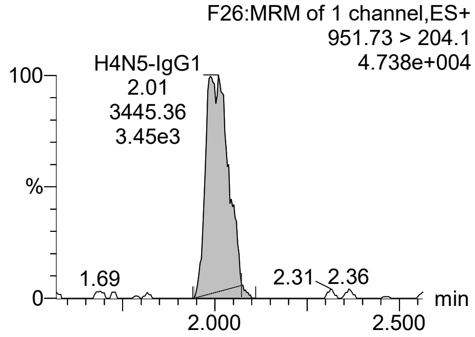

Sample Name: 20211111_A7_R1_IgG

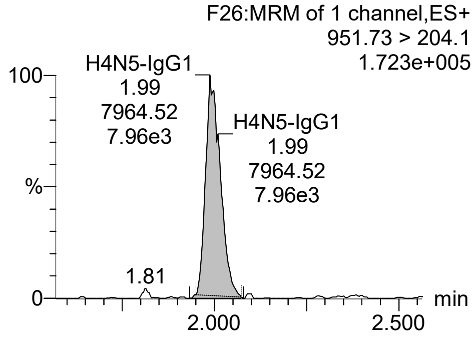

Sample Name: 20211111_A8_R1_IgG

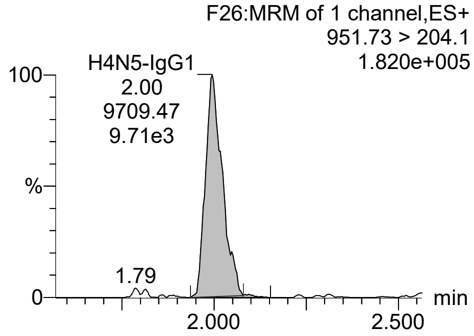

Sample Name: 20211111_L7_R1_IgG

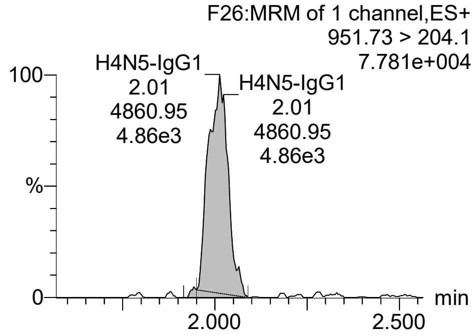

Sample Name: 20211111_L8_R1_IgG

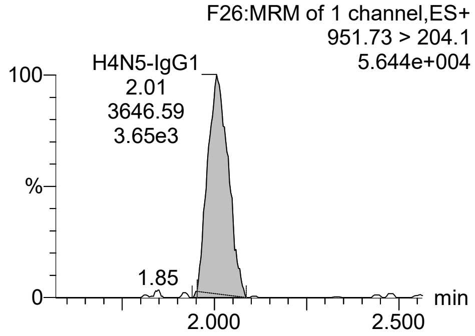

Sample Name: 20211111_H4_R1_IgG

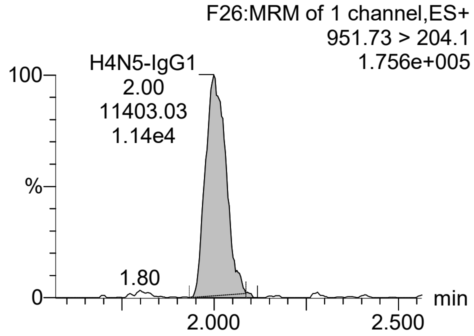

Sample Name: 20211111_A9_R1_IgG

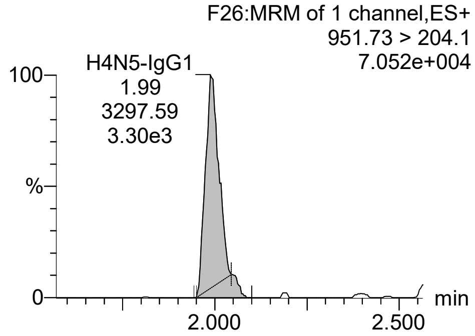

Sample Name: 20211111_A10_R1_IgG

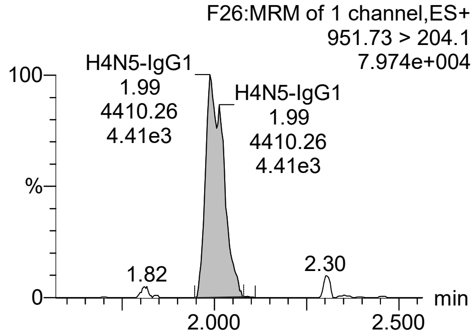

Sample Name: 20211111_L9_R1_IgG

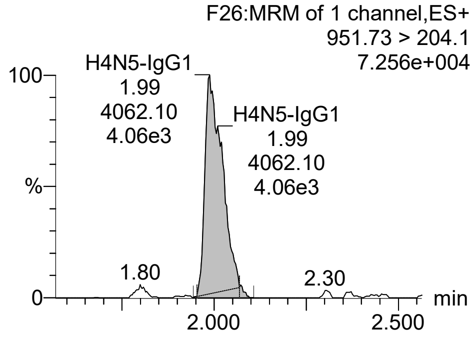

Sample Name: 20211111_L10_R1_IgG

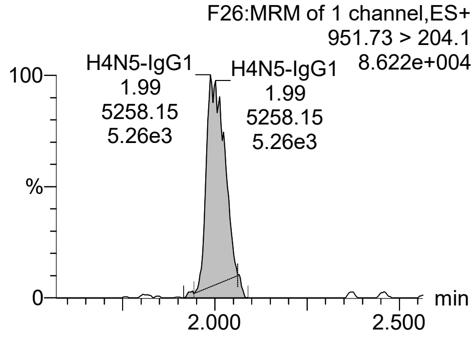

Sample Name: 20211111_QC_R3_IgG

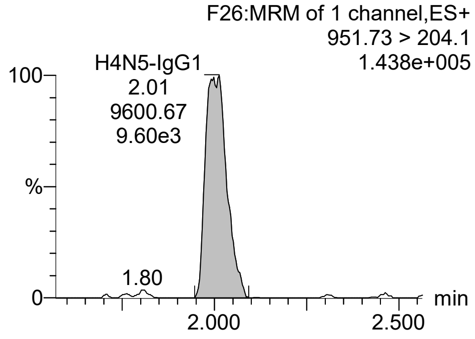

Dataset: C:\MassLynx\10.pro\20211115_clinicalsamples_IgG.qld

Last Altered: Sunday, October 02, 2022 16:02:30 台北標準時間
Printed: Friday, August 01, 2025 16:45:25 台北標準時間

Sample Name: 20211111_H5_R1_IgG
F26:MRM of 1 channel,ES+
951.73 > 204.1
7.367e+004

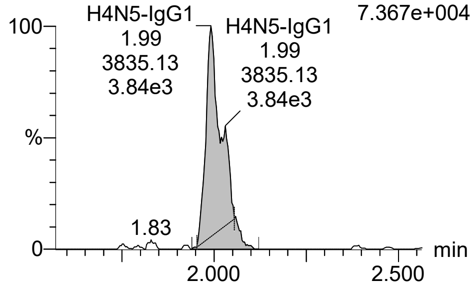

Sample Name: 20211111_A11_R1_IgG
F26:MRM of 1 channel,ES+
951.73 > 204.1
6.199e+004

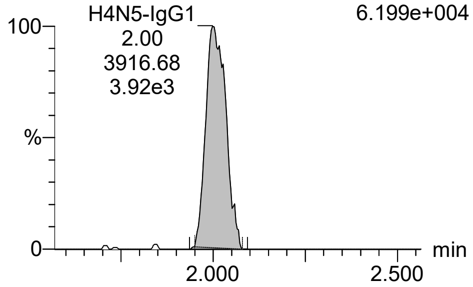

Sample Name: 20211111_A12_R1_IgG
F26:MRM of 1 channel,ES+
951.73 > 204.1
7.913e+004

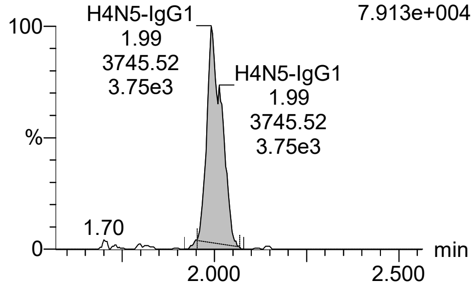

Sample Name: 20211111_L11_R1_IgG
F26:MRM of 1 channel,ES+
951.73 > 204.1
5.554e+004

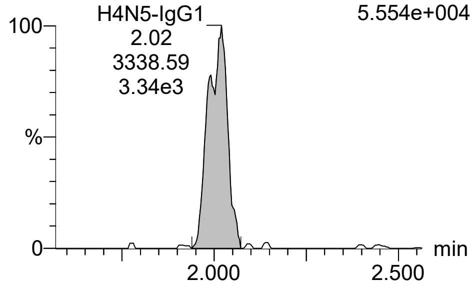

Sample Name: 20211111_L12_R1_IgG
F26:MRM of 1 channel,ES+
951.73 > 204.1
7.444e+004

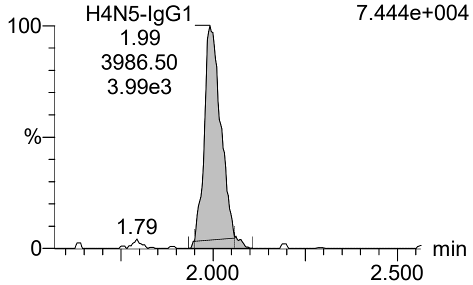

Sample Name: 20211111_H6_R1_IgG
F26:MRM of 1 channel,ES+
951.73 > 204.1
1.581e+005

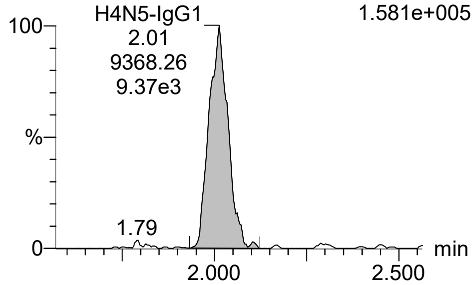

Sample Name: 20211111_A13_R1_IgG
F26:MRM of 1 channel,ES+
951.73 > 204.1
1.430e+005

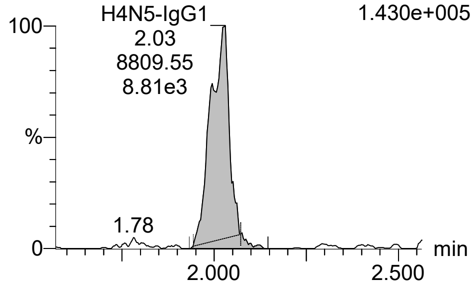

Sample Name: 20211111_A14_R1_IgG
F26:MRM of 1 channel,ES+
951.73 > 204.1
8.500e+004

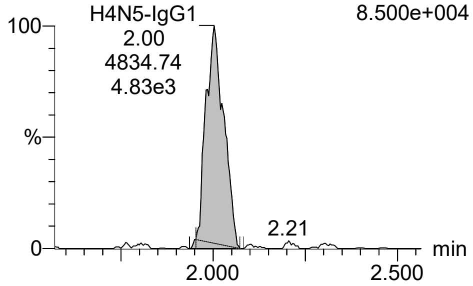

Sample Name: 20211111_L13_R1_IgG
F26:MRM of 1 channel,ES+
951.73 > 204.1
7.109e+004

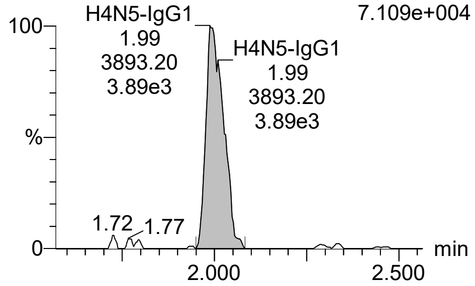

Sample Name: 20211111_L14_R1_IgG
F26:MRM of 1 channel,ES+
951.73 > 204.1
1.123e+005

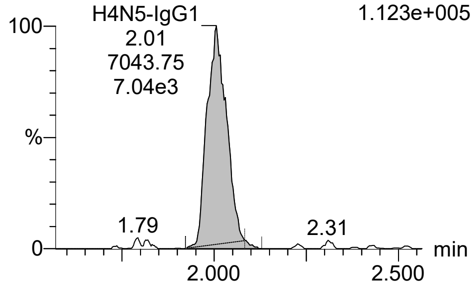

Sample Name: 20211111_H7_R1_IgG
F26:MRM of 1 channel,ES+
951.73 > 204.1
6.374e+004

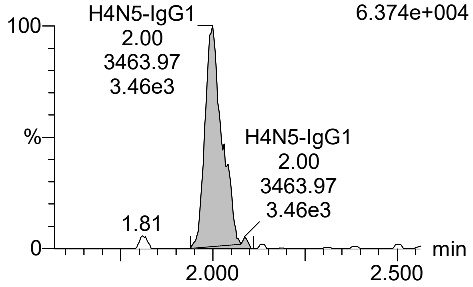

Sample Name: 20211111_A15_R1_IgG
F26:MRM of 1 channel,ES+
951.73 > 204.1
3.117e+005

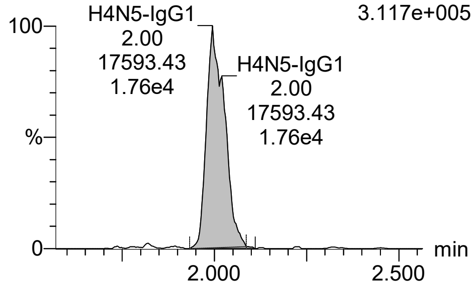

Sample Name: 20211111_QC_R4_IgG
F26:MRM of 1 channel,ES+
951.73 > 204.1
1.429e+005

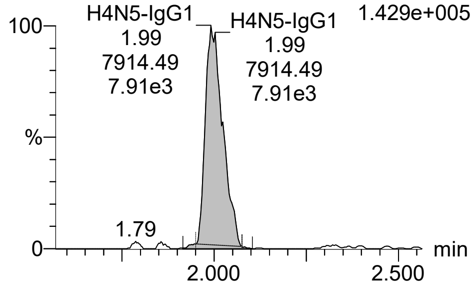

Sample Name: 20211111_A16_R1_IgG
F26:MRM of 1 channel,ES+
951.73 > 204.1
1.100e+005

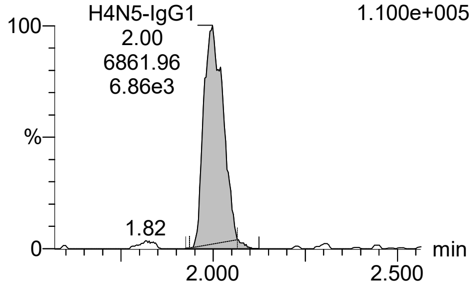

Sample Name: 20211111_L15_R1_IgG
F26:MRM of 1 channel,ES+
951.73 > 204.1
7.507e+004

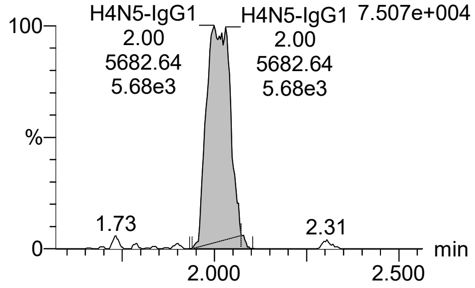

Dataset: C:\MassLynx\10.pro\20211115_clinicalsamples_IgG.qld

Last Altered: Sunday, October 02, 2022 16:02:30 台北標準時間
Printed: Friday, August 01, 2025 16:45:25 台北標準時間

Sample Name: 20211111_L16_R1_IgG

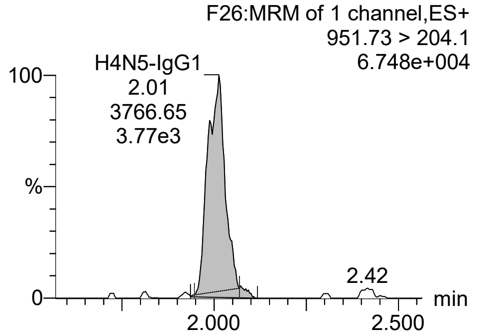

Sample Name: 20211111_H8_R1_IgG

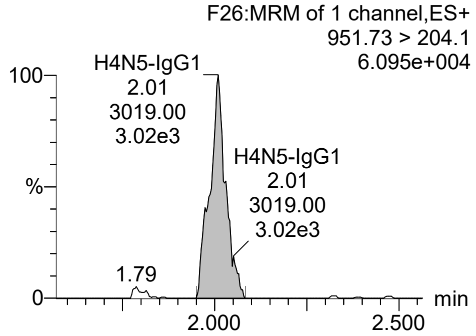

Sample Name: 20211111_A17_R1_IgG

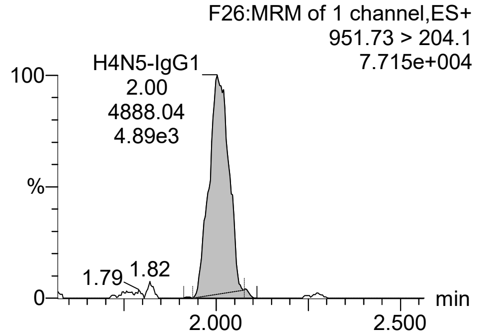

Sample Name: 20211111_A18_R1_IgG

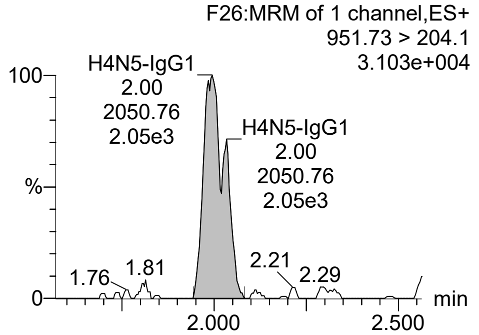

Sample Name: 20211111_L17_R1_IgG

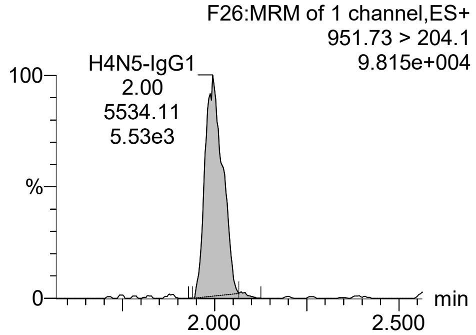

Sample Name: 20211111_L18_R1_IgG

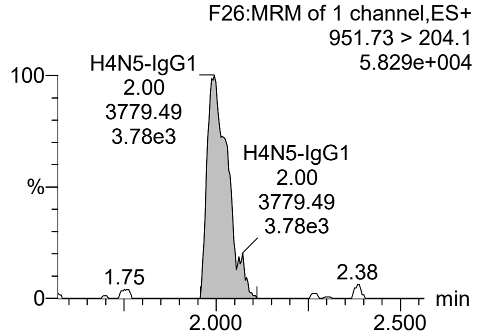

Sample Name: 20211111_H9_R1_IgG

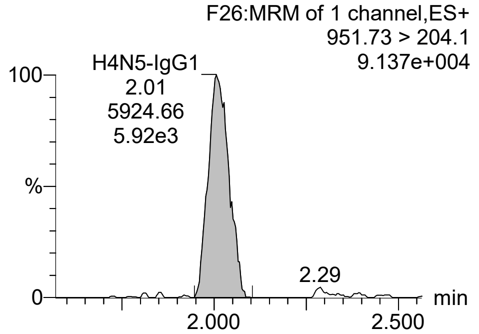

Sample Name: 20211111_A19_R1_IgG

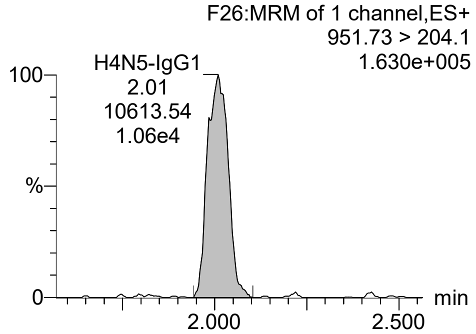

Sample Name: 20211111_A20_R1_IgG

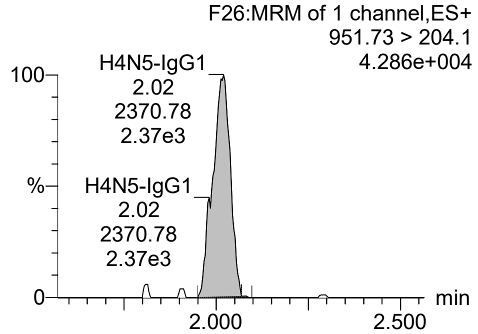

Sample Name: 20211111_L19_R1_IgG

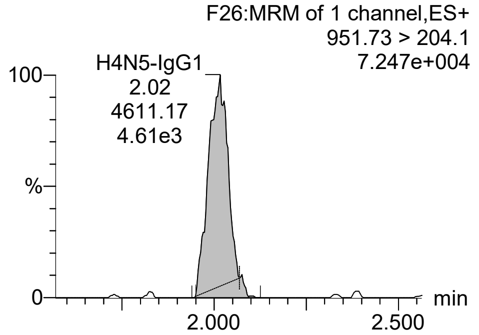

Sample Name: 20211111_QC_R5_IgG

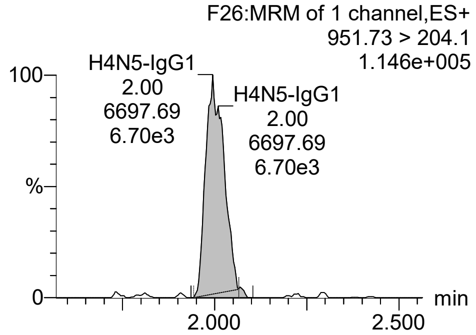

Sample Name: 20211111_L20_R1_IgG

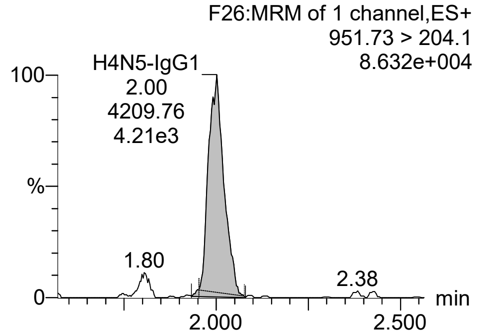

Sample Name: 20211111_H10_R1_IgG

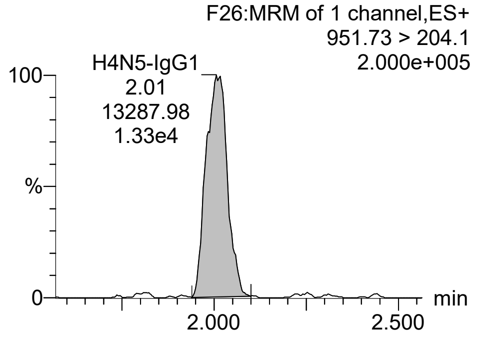

Sample Name: 20211111_A21_R1_IgG

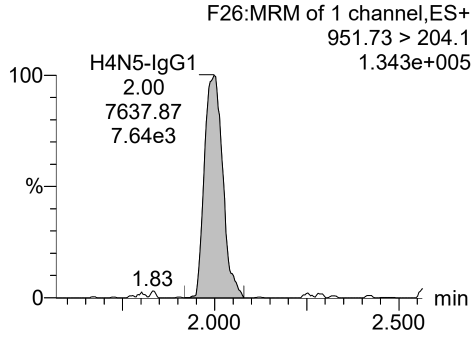

Sample Name: 20211111_A22_R1_IgG

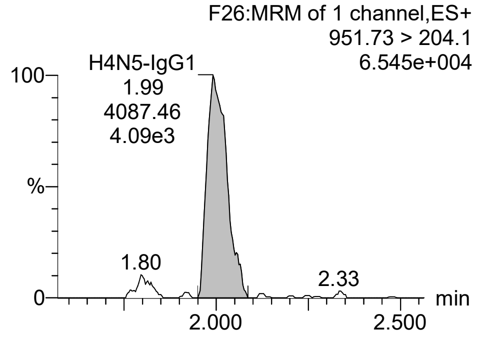

Sample Name: 20211111_L21_R1_IgG

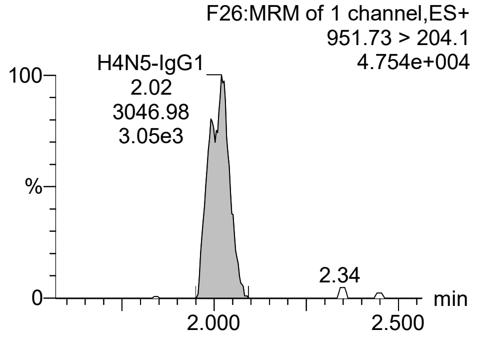

Sample Name: 20211111_L22_R1_IgG

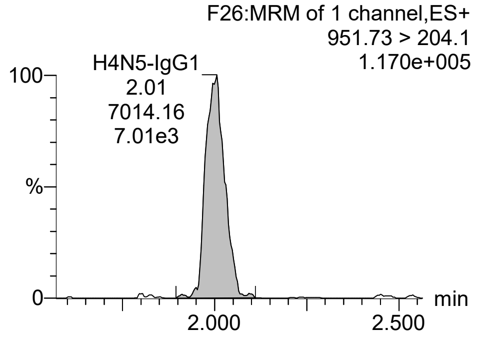

Sample Name: 20211111_H11_R1_IgG

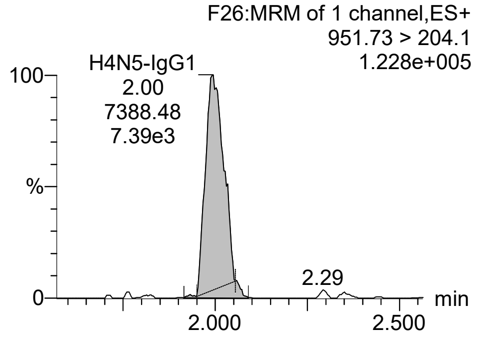

Sample Name: 20211111_A23_R1_IgG

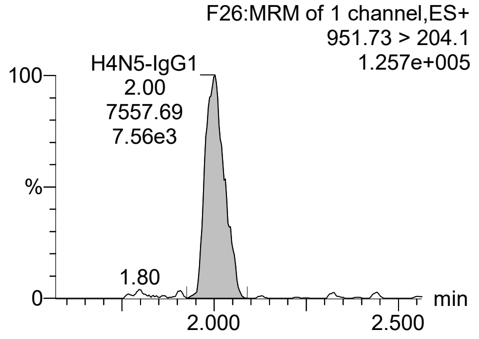

Sample Name: 20211111_A24_R1_IgG

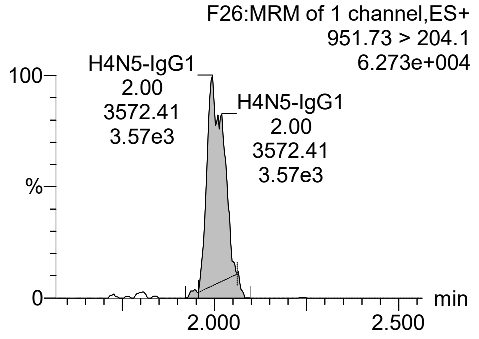

Sample Name: 20211111_L23_R1_IgG

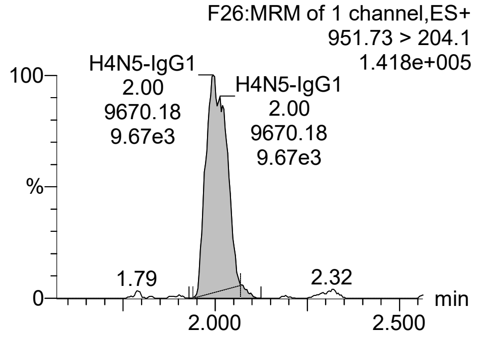

Sample Name: 20211111_L24_R1_IgG

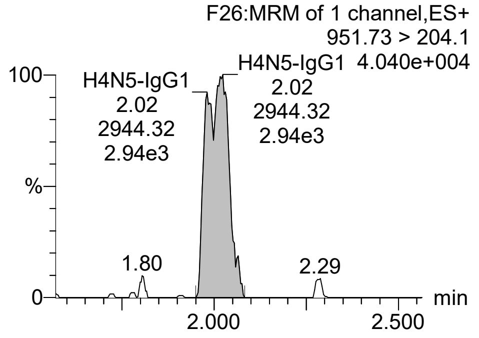

Sample Name: 20211111_H12_R1_IgG

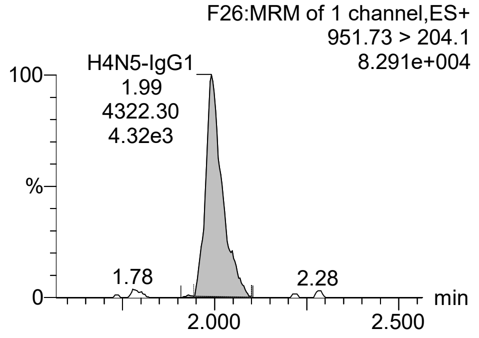

Sample Name: 20211111_QC_R6_IgG

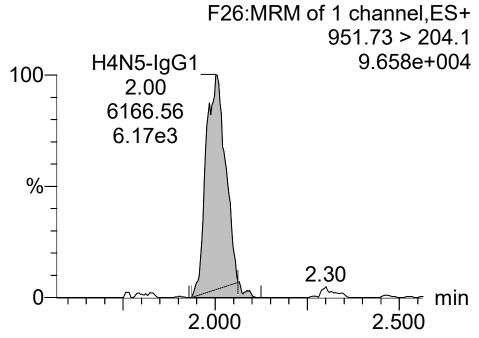

Sample Name: 20211111_A25_R1_IgG

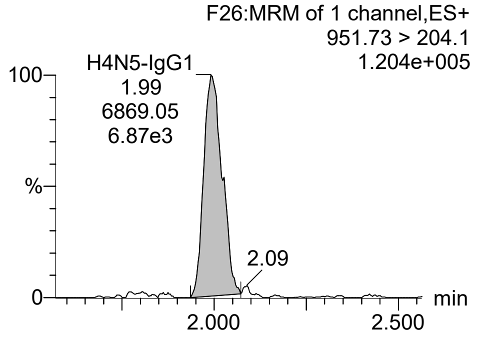

Sample Name: 20211111_A26_R1_IgG

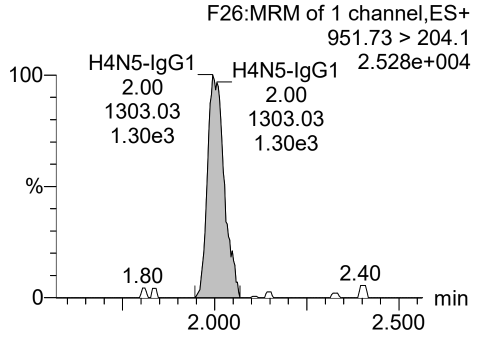

Sample Name: 20211111_L25_R1_IgG

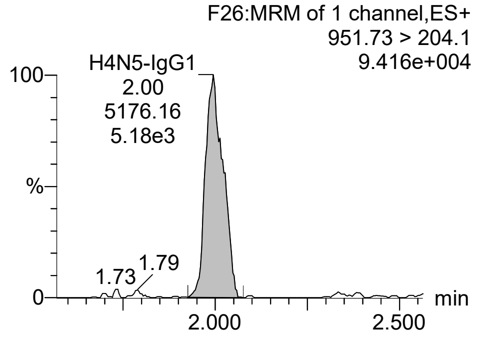

Sample Name: 20211111_L26_R1_IgG

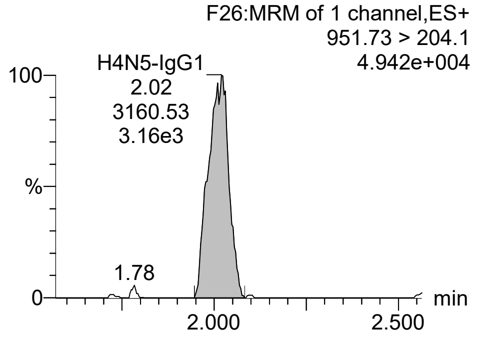

Sample Name: 20211111_H13_R1_IgG

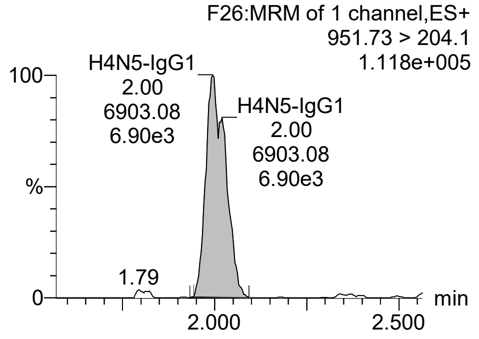

Sample Name: 20211111_A27_R1_IgG

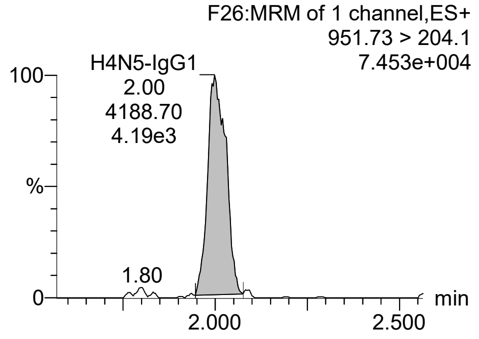

Dataset: C:\MassLynx\10.pro\20211115_clinicalsamples_IgG.qld

Last Altered: Sunday, October 02, 2022 16:02:30 台北標準時間
Printed: Friday, August 01, 2025 16:45:25 台北標準時間

Sample Name: 20211111_A28_R1_IgG

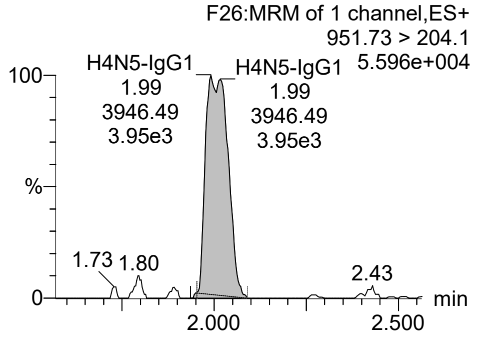

Sample Name: 20211111_L27_R1_IgG

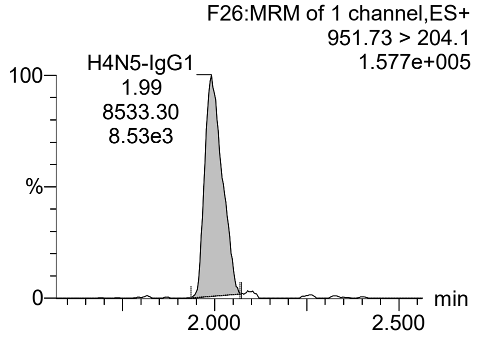

Sample Name: 20211111_L28_R1_IgG

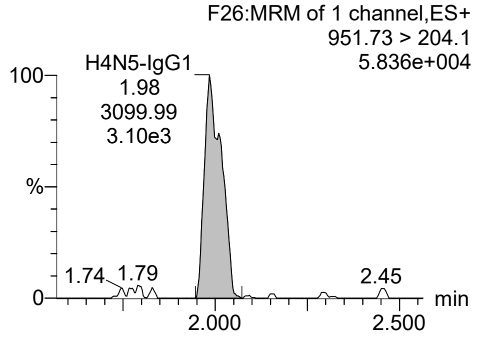

Sample Name: 20211111_H14_R1_IgG

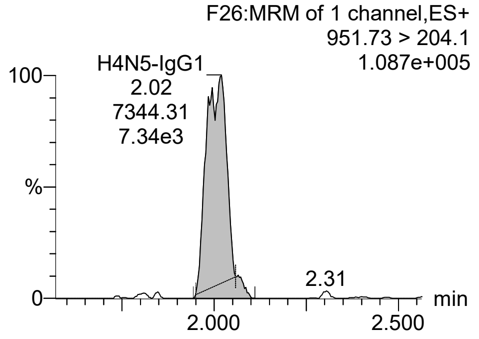

Sample Name: 20211111_A29_R1_IgG

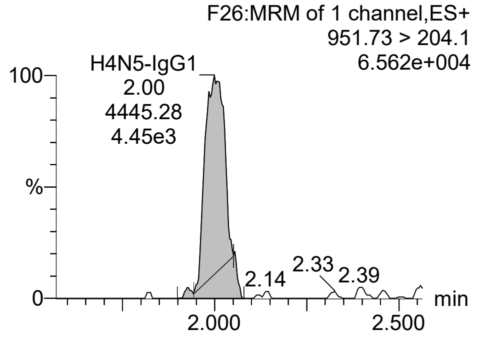

Sample Name: 20211111_A30_R1_IgG

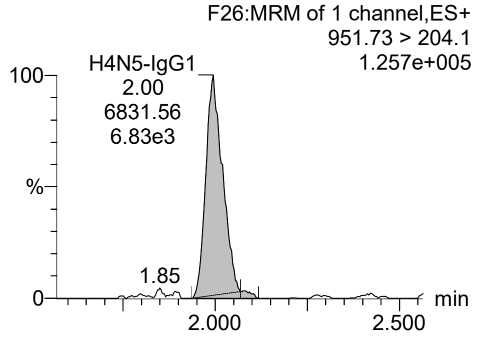

Sample Name: 20211111_QC_R7_IgG

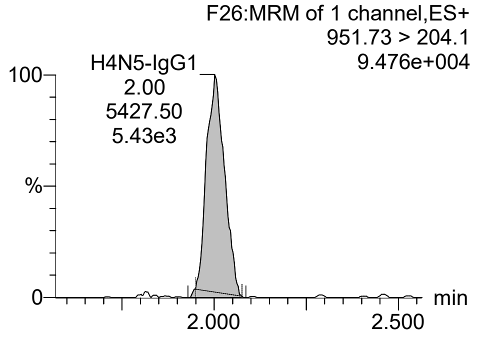

Sample Name: 20211111_L29_R1_IgG

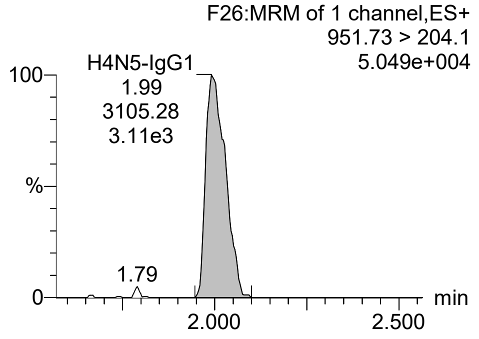

Sample Name: 20211111_L30_R1_IgG

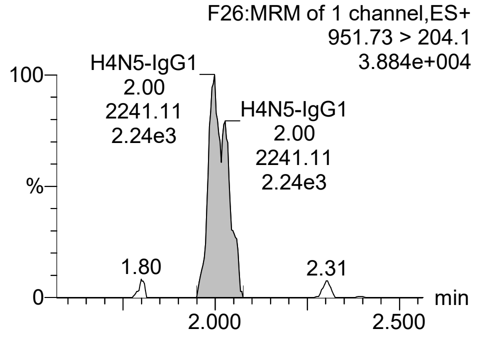

Sample Name: 20211111_H15_R1_IgG

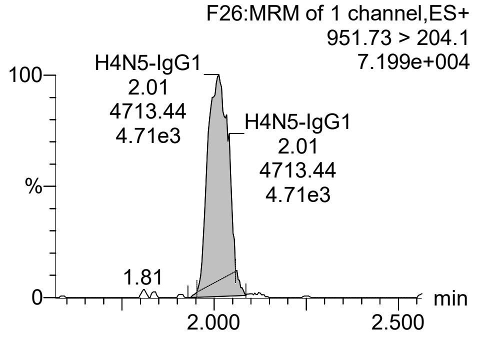

Sample Name: 20211111_A31_R1_IgG

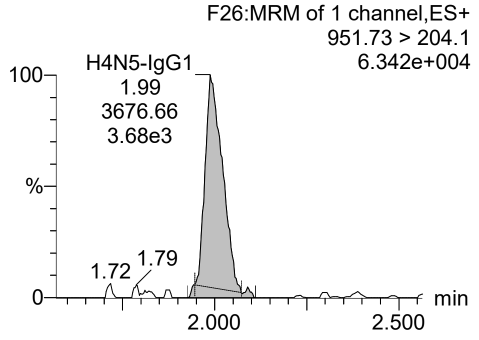

Sample Name: 20211111_A32_R1_IgG

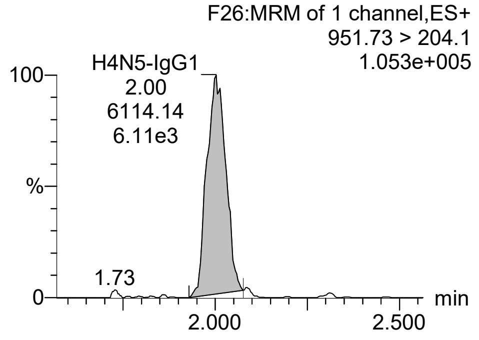

Sample Name: 20211111_L31_R1_IgG

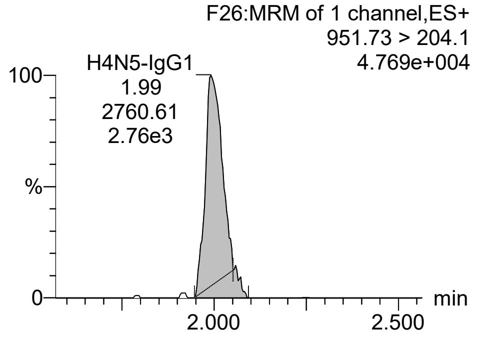

Sample Name: 20211111_L32_R1_IgG

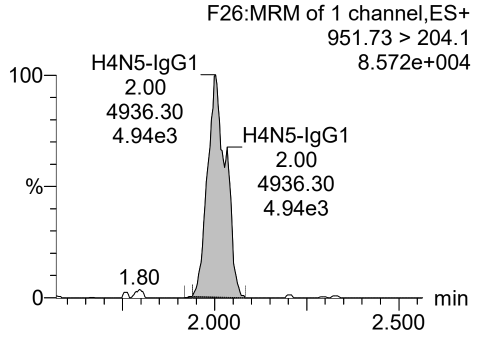

Sample Name: 20211111_H16_R1_IgG

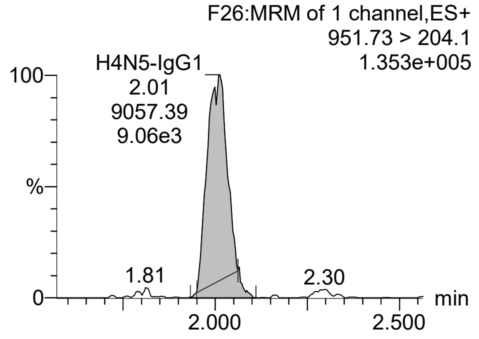

Sample Name: 20211111_A33_R1_IgG

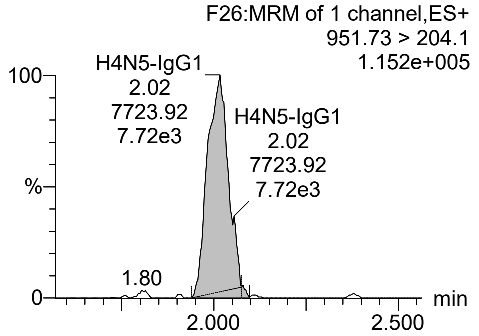

Sample Name: 20211111_A34_R1_IgG

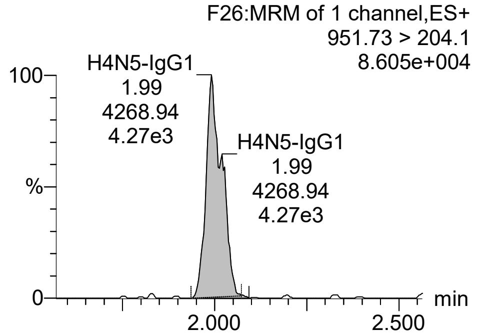

Sample Name: 20211111_H17_R1_IgG

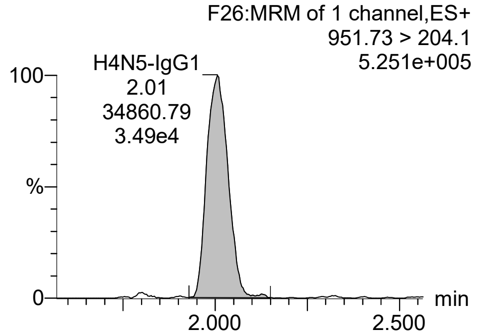

Sample Name: 20211111_A35_R1_IgG

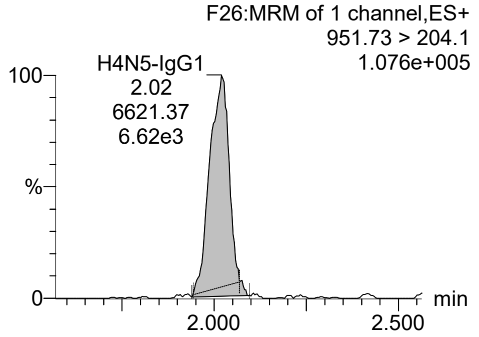

Sample Name: 20211111_QC_R8_IgG

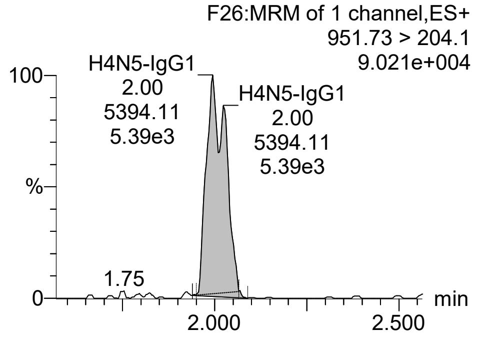

Sample Name: 20211111_A36_R1_IgG

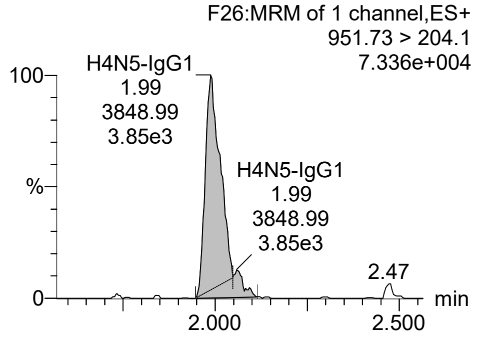

Sample Name: 20211111_H18_R1_IgG

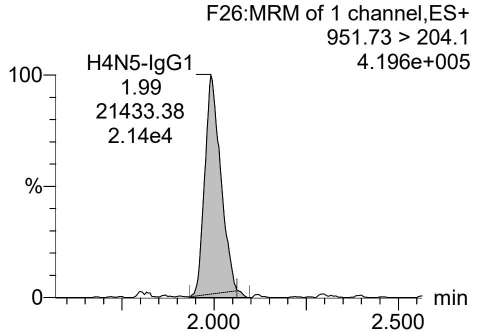

Sample Name: 20211111_A37_R1_IgG

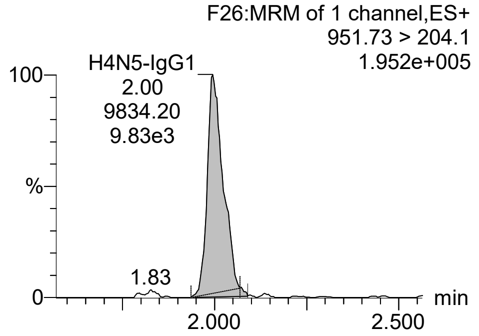

Sample Name: 20211111_A38_R1_IgG

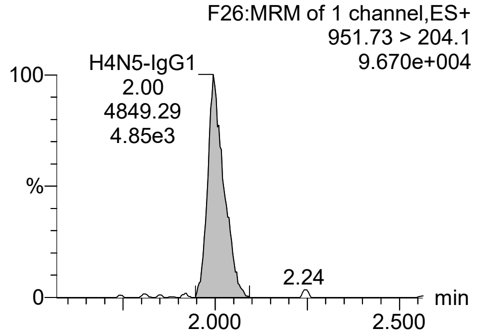

Sample Name: 20211111_H19_R1_IgG

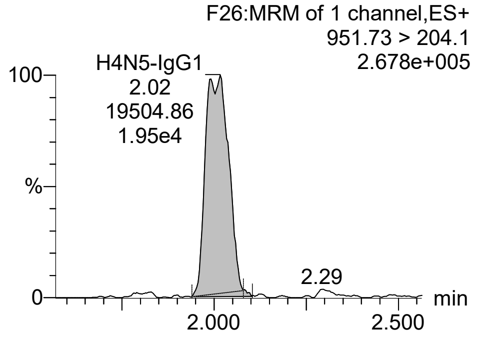

Sample Name: 20211111_A39_R1_IgG

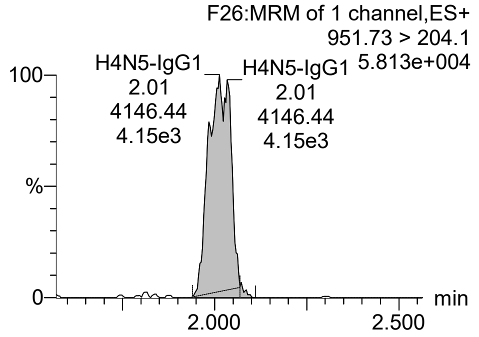

Sample Name: 20211111_A40_R1_IgG

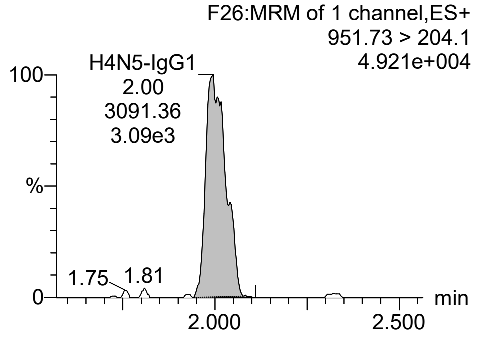

Sample Name: 20211111_H20_R1_IgG

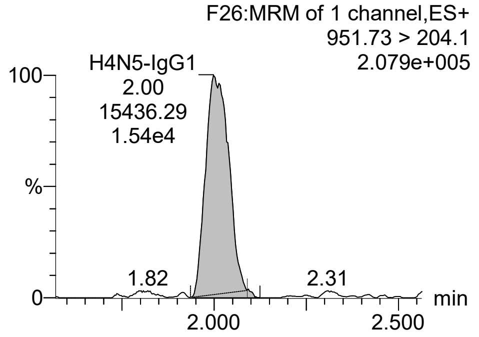

Sample Name: 20211111_A41_R1_IgG

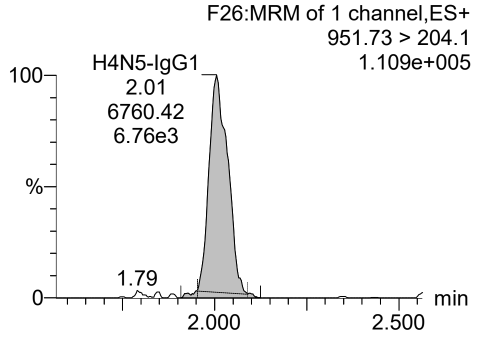

Sample Name: 20211111_A42_R1_IgG

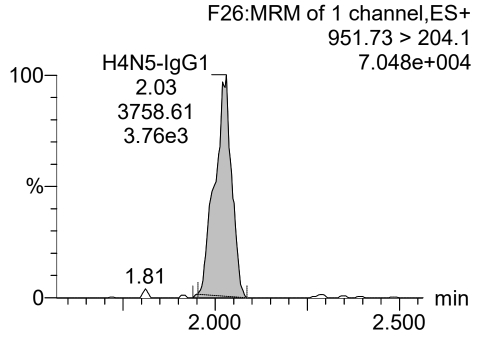

Dataset: C:\MassLynx\10.pro\20211115_clinicalsamples_IgG.qld

Last Altered: Sunday, October 02, 2022 16:02:30 台北標準時間
Printed: Friday, August 01, 2025 16:45:25 台北標準時間

Sample Name: 20211111_A43_R1_IgG

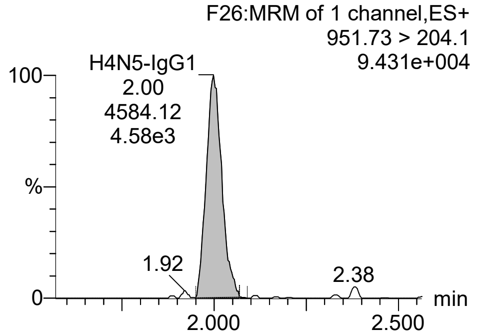

Sample Name: 20211111_A44_R1_IgG

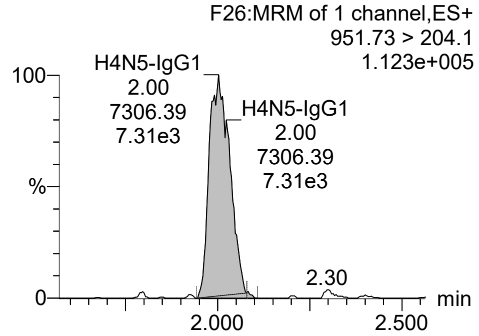

Sample Name: 20211111_QC_R9_IgG

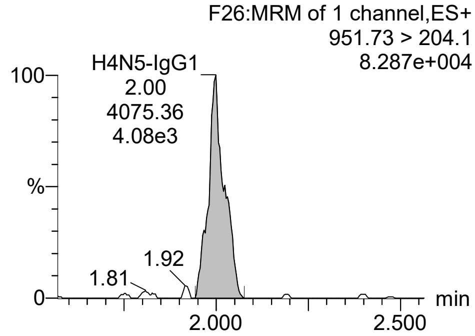

Sample Name: 20211111_A1_R2_IgG

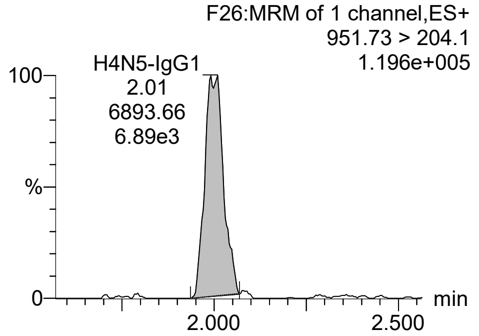

Sample Name: 20211111_A2_R2_IgG

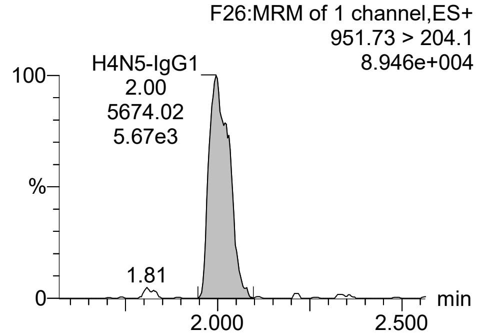

Sample Name: 20211111_L1_R2_IgG

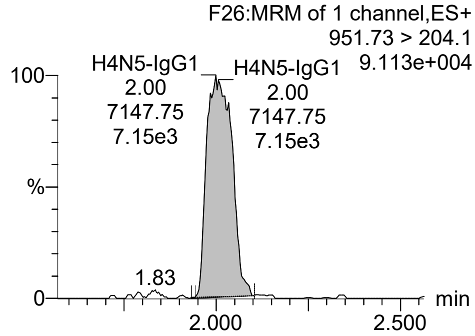

Sample Name: 20211111_L2_R2_IgG

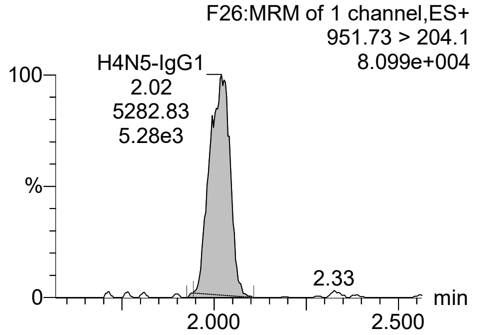

Sample Name: 20211111_H1_R2_IgG

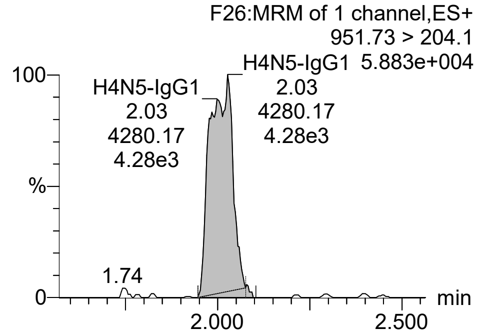

Sample Name: 20211111_A3_R2_IgG

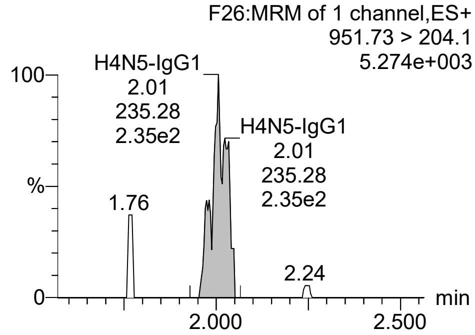

Sample Name: 20211111_A4_R2_IgG

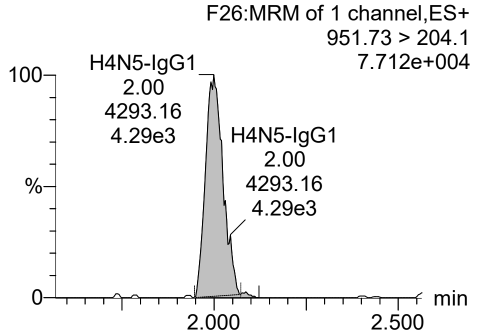

Sample Name: 20211111_L3_R2_IgG

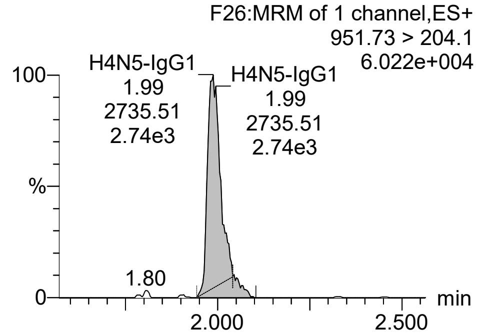

Sample Name: 20211111_L4_R2_IgG

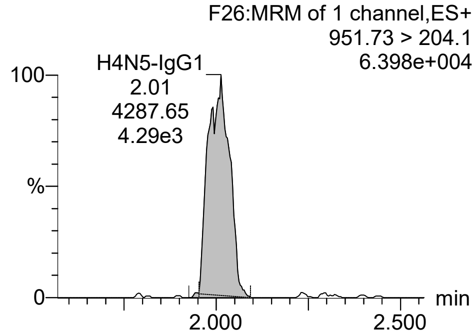

Sample Name: 20211111_H2_R2_IgG

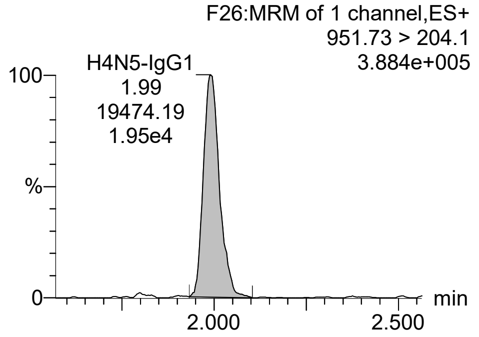

Sample Name: 20211111_A5_R2_IgG

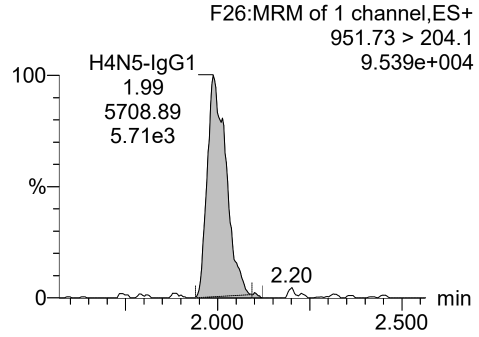

Sample Name: 20211111_A6_R2_IgG

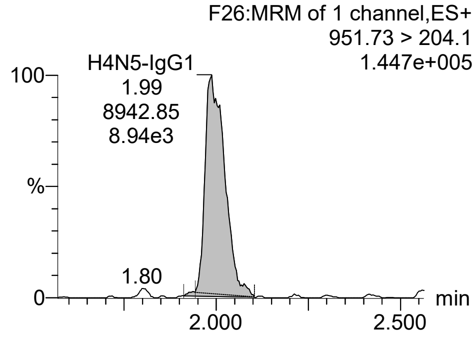

Sample Name: 20211111_QC_R10_IgG

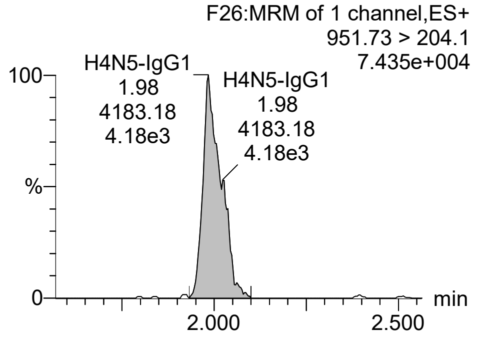

Sample Name: 20211111_L5_R2_IgG

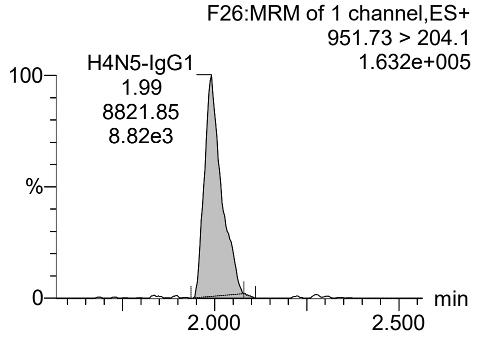

Sample Name: 20211111_L6_R2_IgG

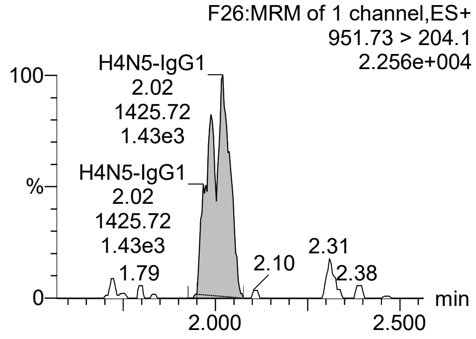

Sample Name: 20211111_H3_R2_IgG

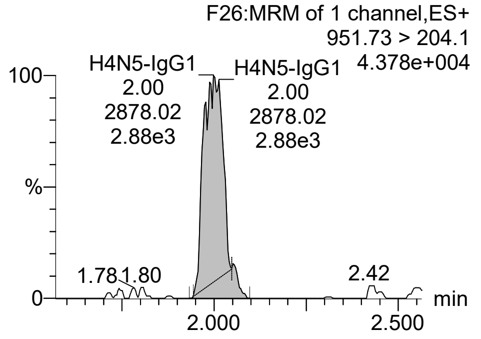

Sample Name: 20211111_A7_R2_IgG

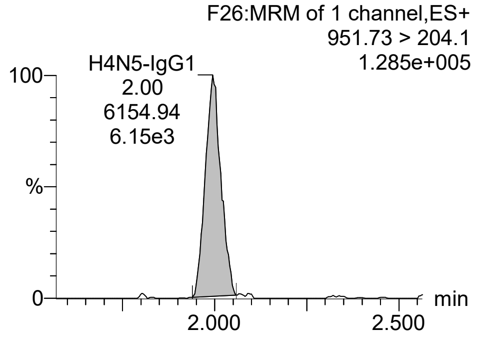

Sample Name: 20211111_A8_R2_IgG

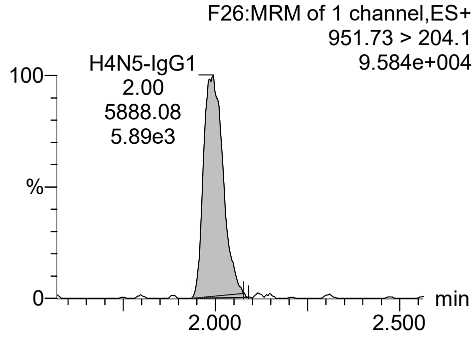

Sample Name: 20211111_L7_R2_IgG

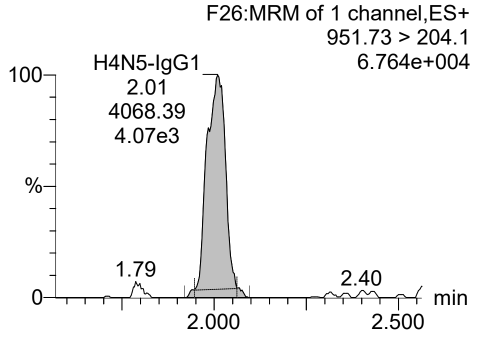

Sample Name: 20211111_L8_R2_IgG

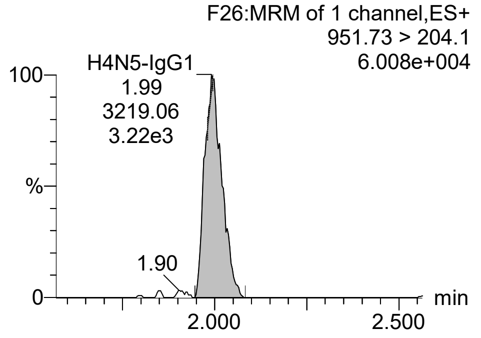

Sample Name: 20211111_H4_R2_IgG

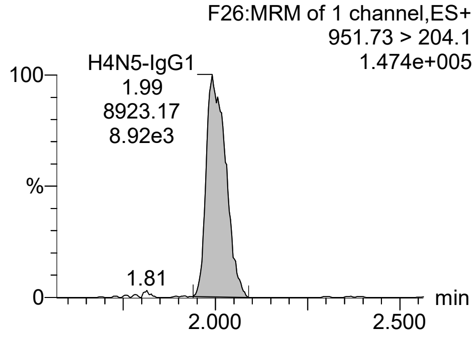

Sample Name: 20211111_A9_R2_IgG

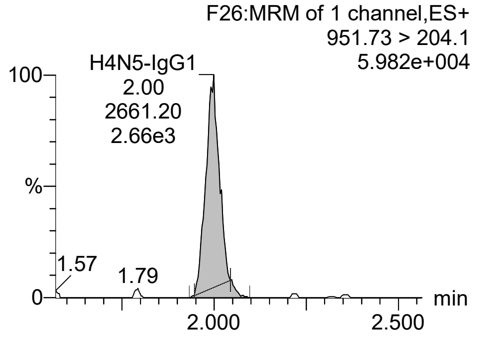

Sample Name: 20211111_A10_R2_IgG

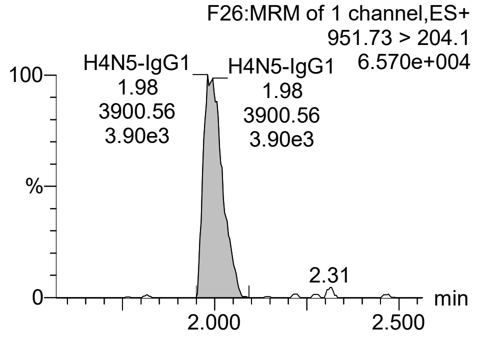

Sample Name: 20211111_L9_R2_IgG

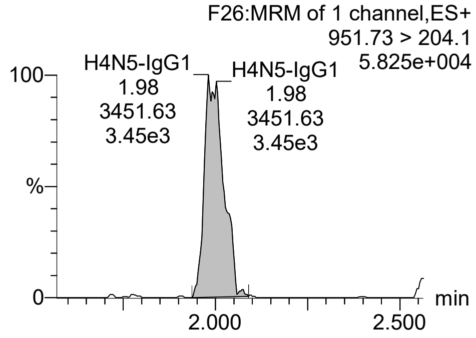

Sample Name: 20211111_L10_R2_IgG

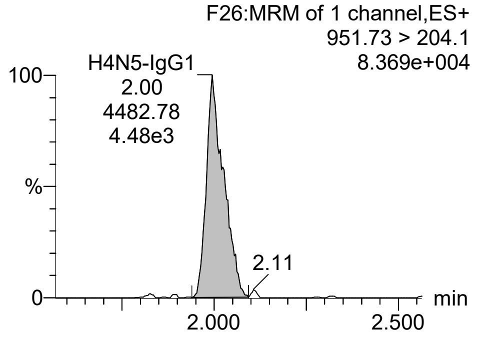

Sample Name: 20211111_QC_R11_IgG

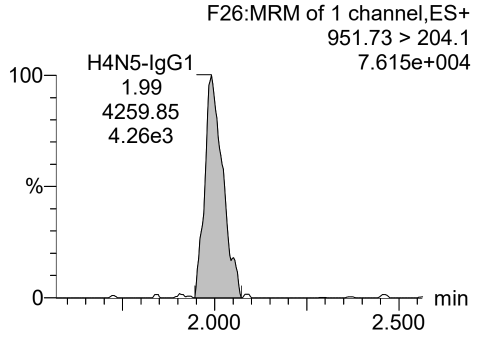

Sample Name: 20211111_H5_R2_IgG

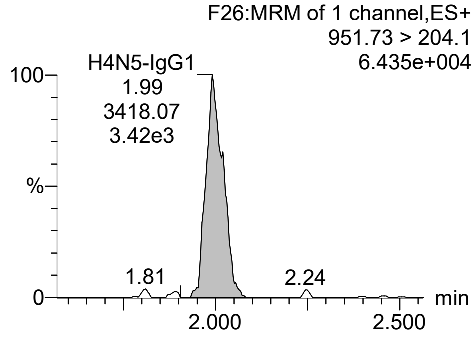

Sample Name: 20211111_A11_R2_IgG

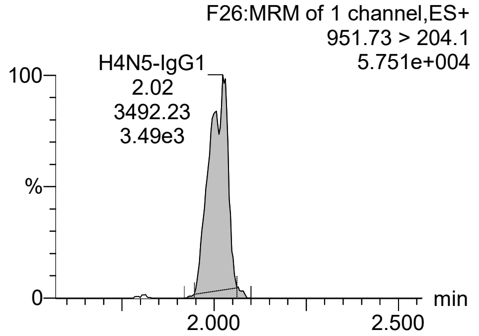

Sample Name: 20211111_A12_R2_IgG

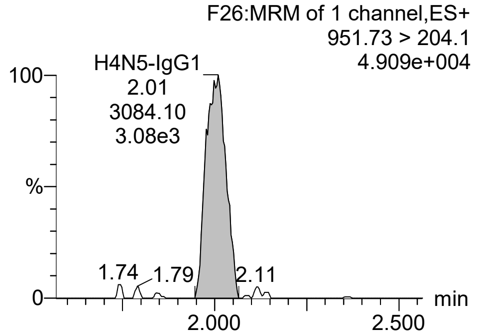

Sample Name: 20211111_L11_R2_IgG

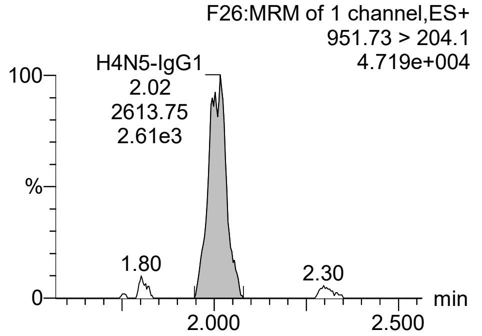

Sample Name: 20211111_L12_R2_IgG

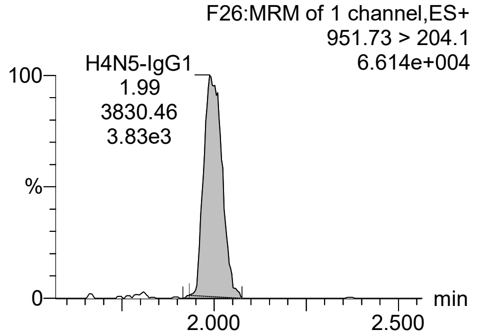

Sample Name: 20211111_H6_R2_IgG

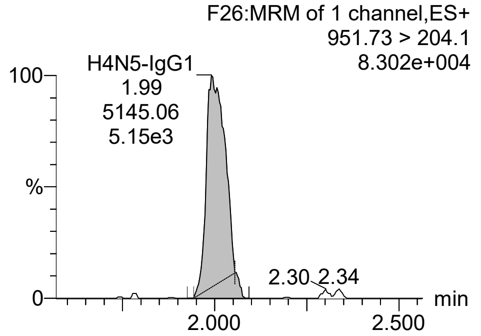

Sample Name: 20211111_A13_R2_IgG

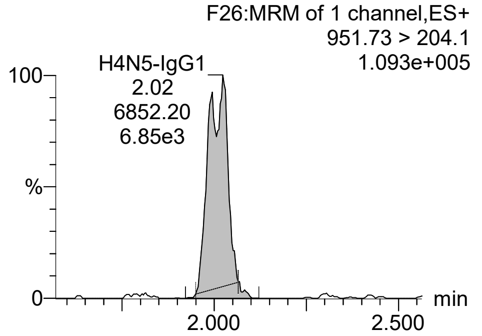

Sample Name: 20211111_A14_R2_IgG

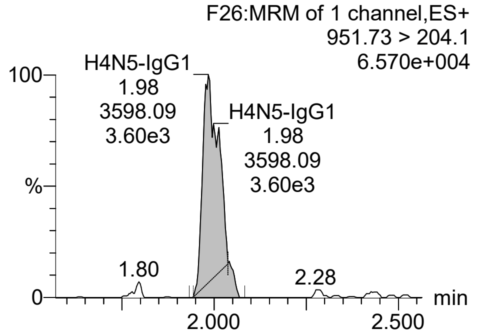

Sample Name: 20211111_L13_R2_IgG

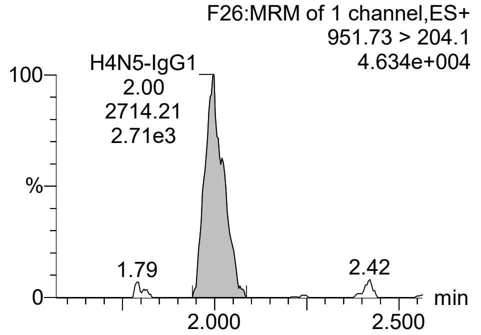

Sample Name: 20211111_L14_R2_IgG

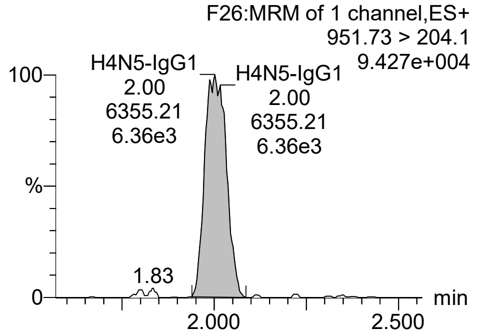

Sample Name: 20211111_H7_R2_IgG

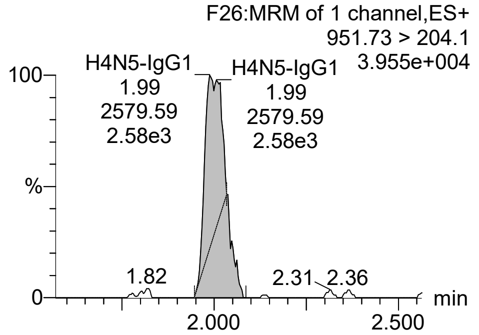

Sample Name: 20211111_A15_R2_IgG

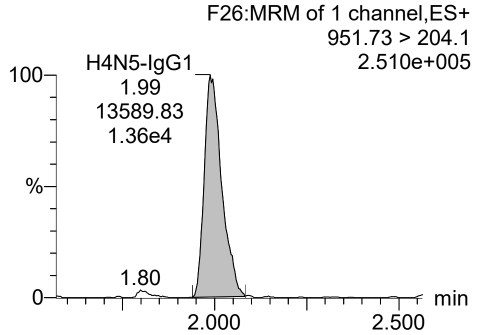

Sample Name: 20211111_QC_R12_IgG

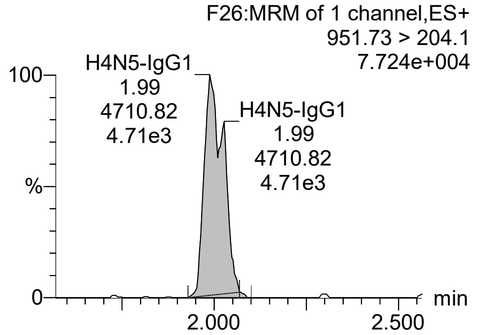

Sample Name: 20211111_A16_R2_IgG

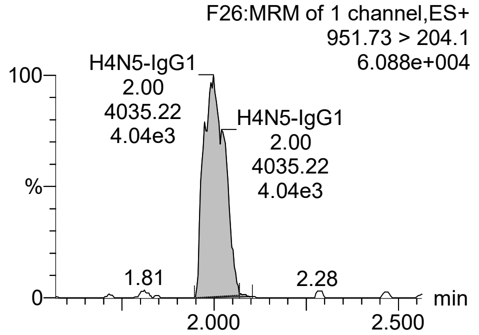

Sample Name: 20211111_L15_R2_IgG

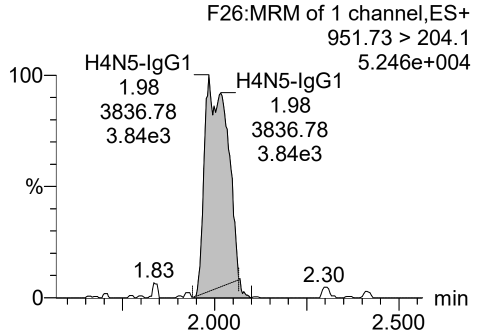

Sample Name: 20211111_L16_R2_IgG

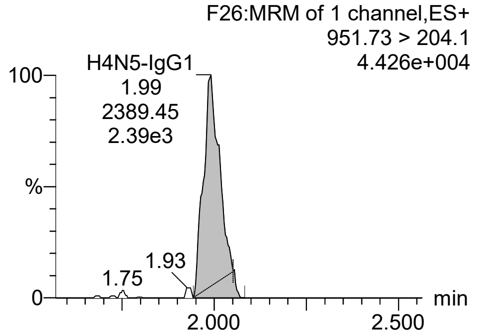

Sample Name: 20211111_H8_R2_IgG

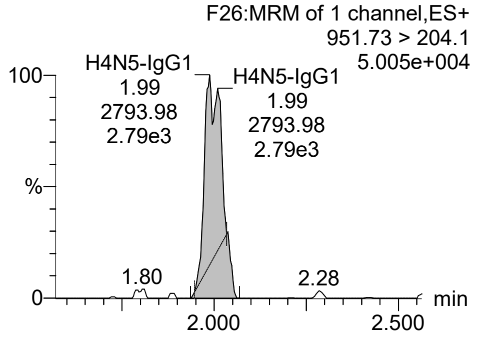

Sample Name: 20211111_A17_R2_IgG

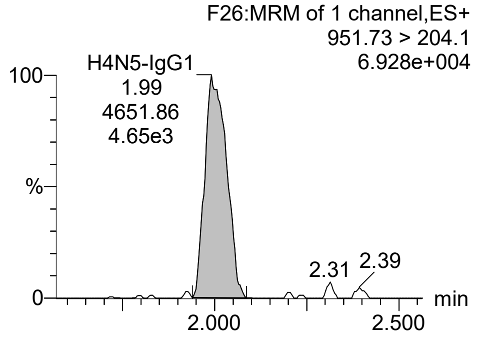

Sample Name: 20211111_A18_R2_IgG

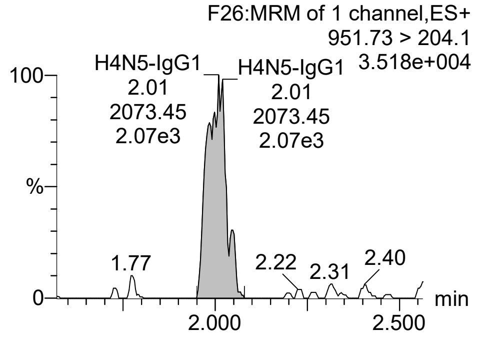

Sample Name: 20211111_L17_R2_IgG

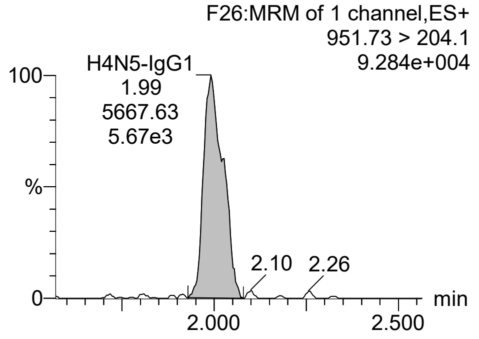

Sample Name: 20211111_L18_R2_IgG

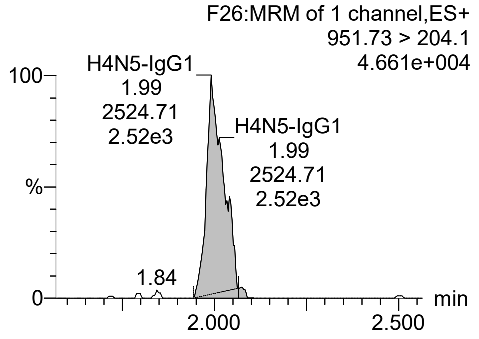

Sample Name: 20211111_H9_R2_IgG

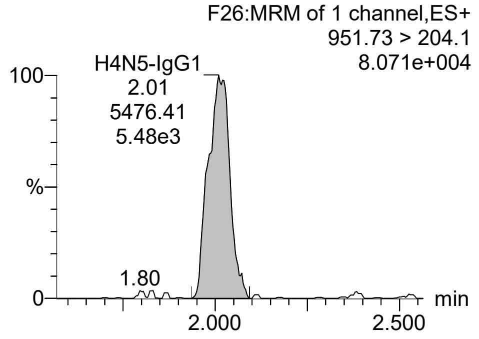

Sample Name: 20211111_A19_R2_IgG

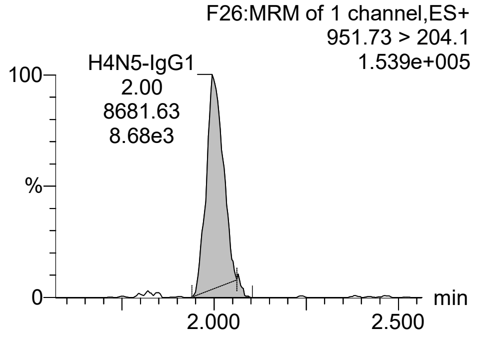

Sample Name: 20211111_A20_R2_IgG

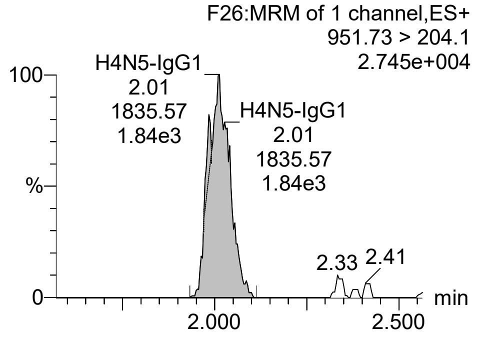

Sample Name: 20211111_L19_R2_IgG

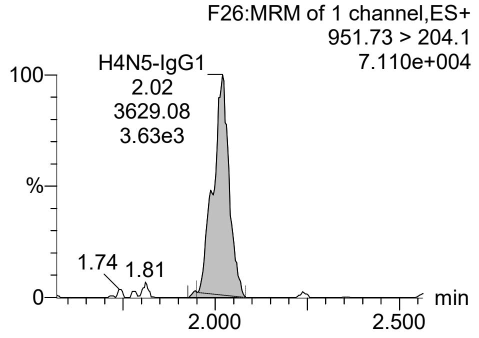

Sample Name: 20211111_QC_R13_IgG

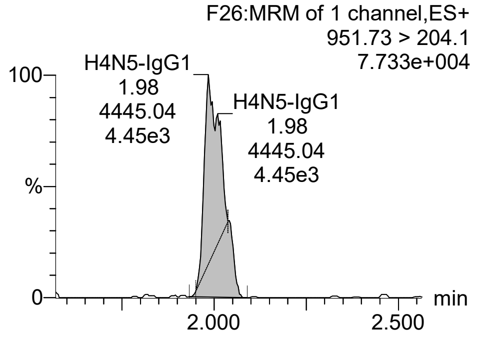

Sample Name: 20211111_L20_R2_IgG

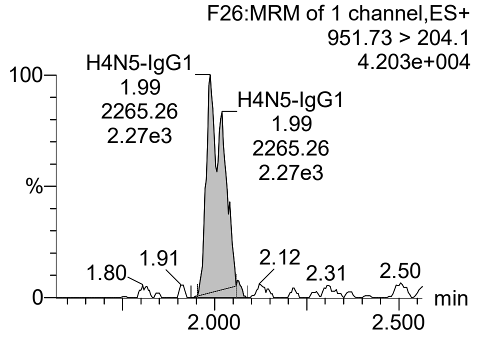

Sample Name: 20211111_H10_R2_IgG

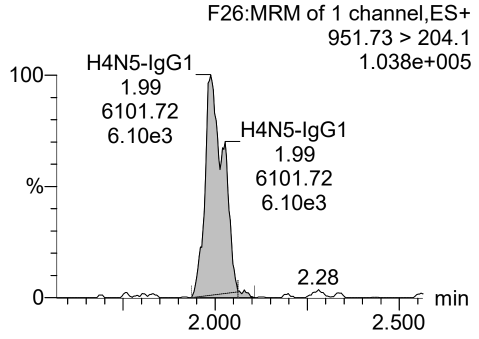

Sample Name: 20211111_A21_R2_IgG

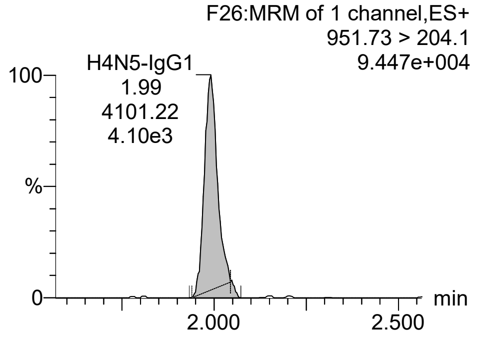

Sample Name: 20211111_A22_R2_IgG

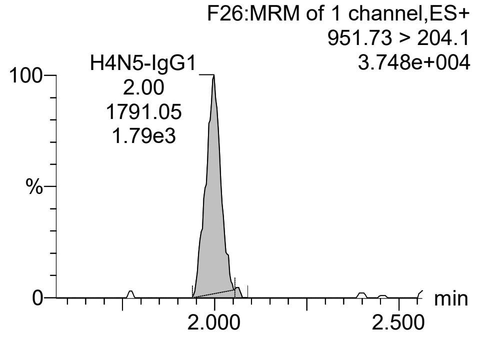

Sample Name: 20211111_L21_R2_IgG

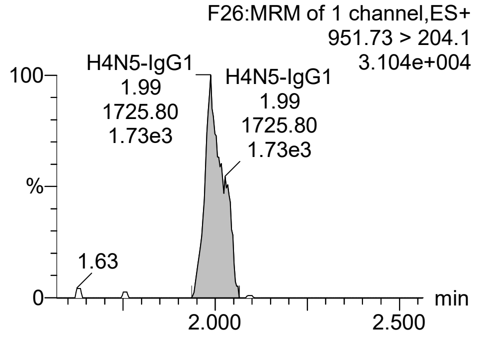

Sample Name: 20211111_L22_R2_IgG

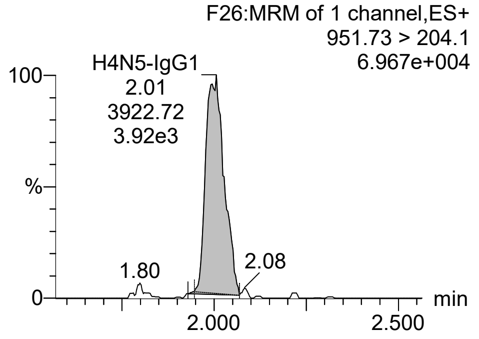

Sample Name: 20211111_H11_R2_IgG

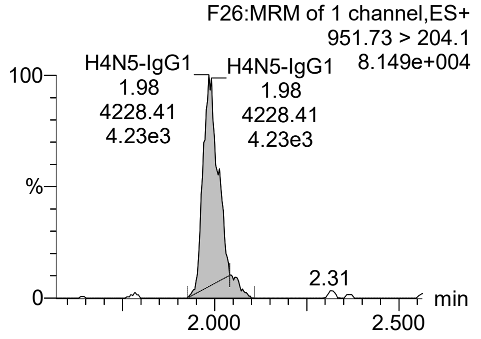

Sample Name: 20211111_A23_R2_IgG

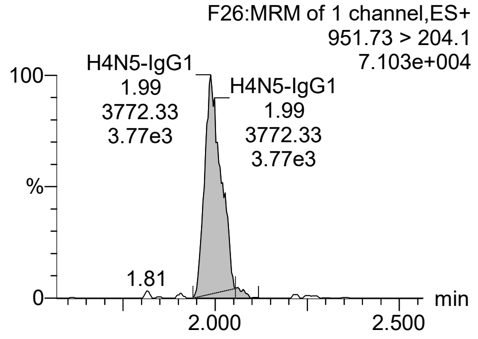

Sample Name: 20211111_A24_R2_IgG

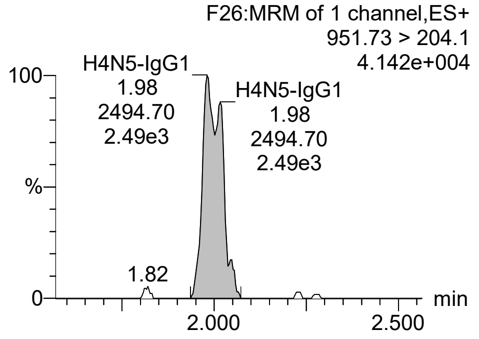

Sample Name: 20211111_L23_R2_IgG

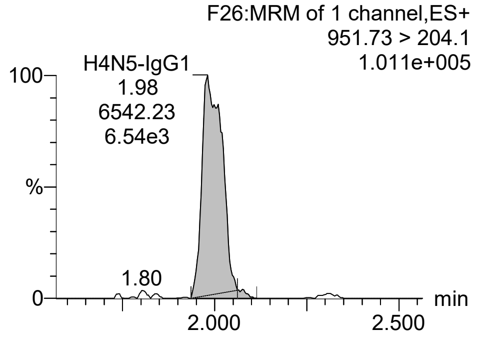

Sample Name: 20211111_L24_R2_IgG

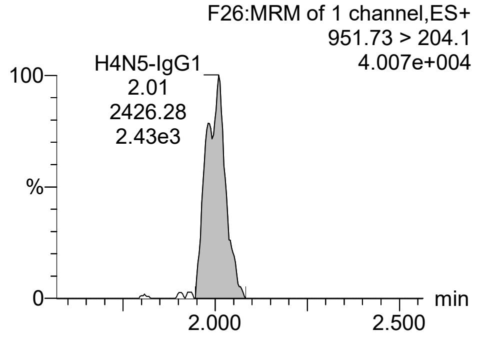

Sample Name: 20211111_H12_R2_IgG

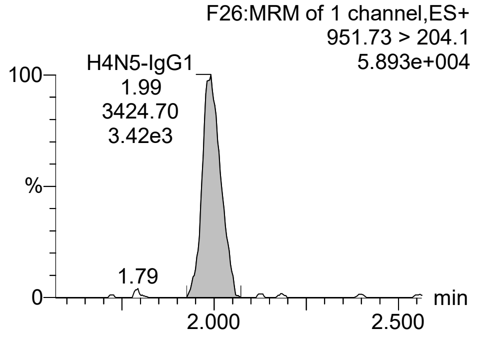

Sample Name: 20211111_QC_R14_IgG

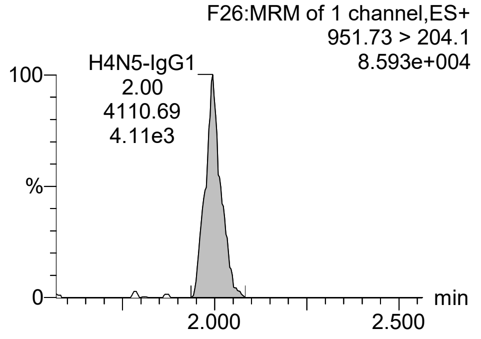

Sample Name: 20211111_A25_R2_IgG

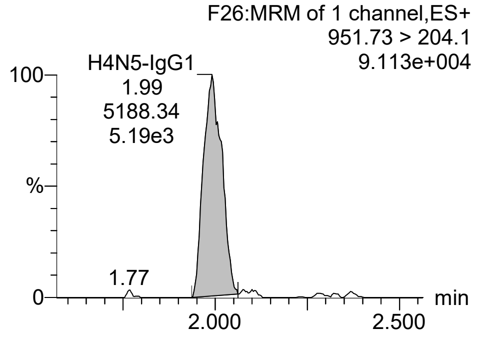

Sample Name: 20211111_A26_R2_IgG

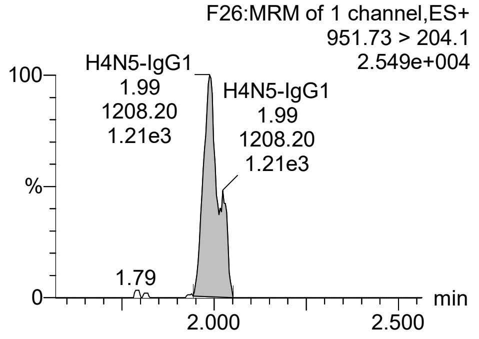

Sample Name: 20211111_L25_R2_IgG

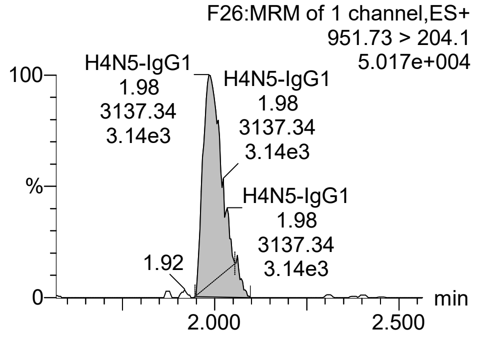

Sample Name: 20211111_L26_R2_IgG

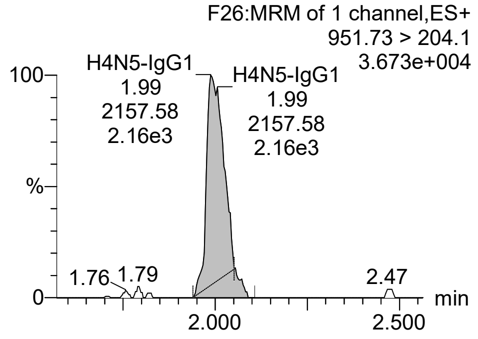

Sample Name: 20211111_H13_R2_IgG

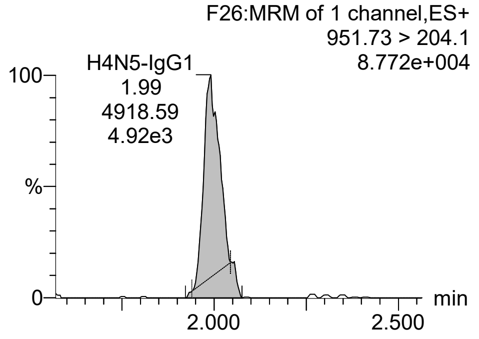

Sample Name: 20211111_A27_R2_IgG

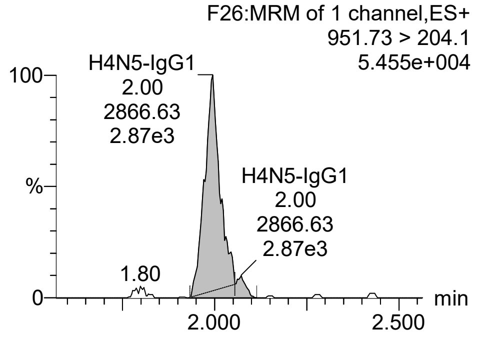

Sample Name: 20211111_A28_R2_IgG

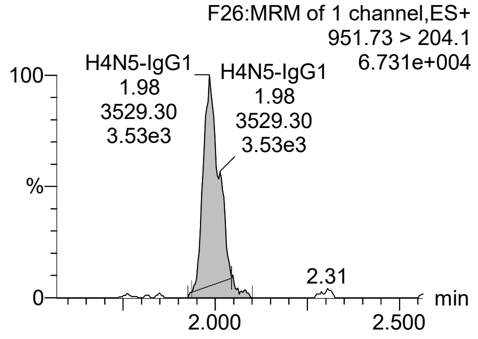

Sample Name: 20211111_L27_R2_IgG
F26:MRM of 1 channel,ES+
951.73 > 204.1

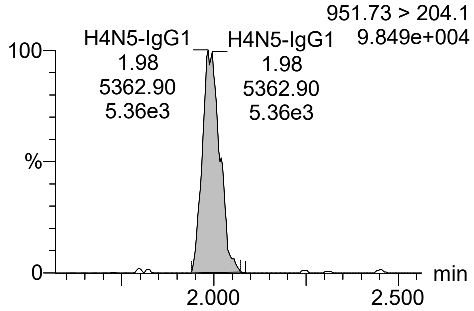

Sample Name: 20211111_L28_R2_IgG
F26:MRM of 1 channel,ES+
951.73 > 204.1

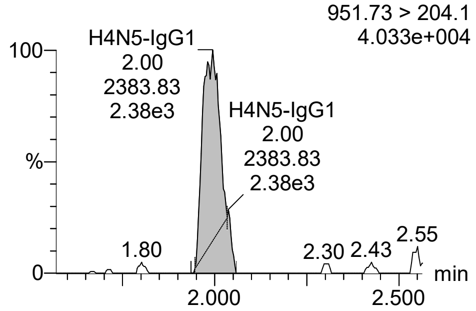

Sample Name: 20211111_H14_R2_IgG
F26:MRM of 1 channel,ES+
951.73 > 204.1

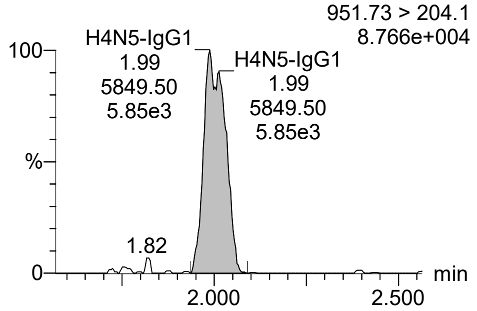

Sample Name: 20211111_A29_R2_IgG
F26:MRM of 1 channel,ES+
951.73 > 204.1

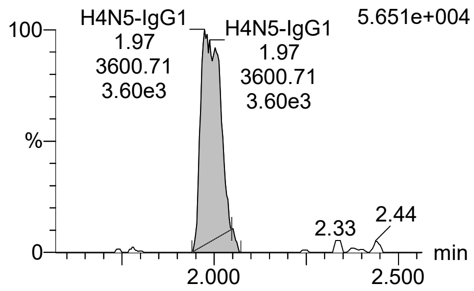

Sample Name: 20211111_A30_R2_IgG
F26:MRM of 1 channel,ES+
951.73 > 204.1

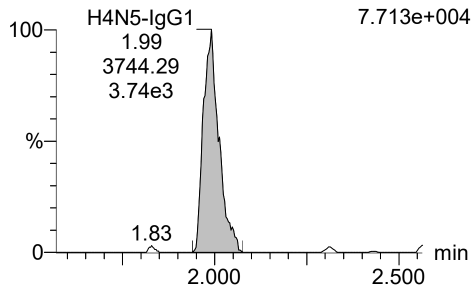

Sample Name: 20211111_QC_R15_IgG
F26:MRM of 1 channel,ES+
951.73 > 204.1

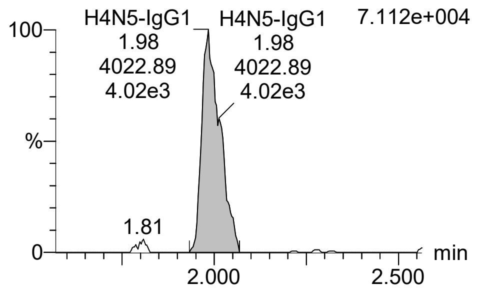

Sample Name: 20211111_L29_R2_IgG
F26:MRM of 1 channel,ES+
951.73 > 204.1

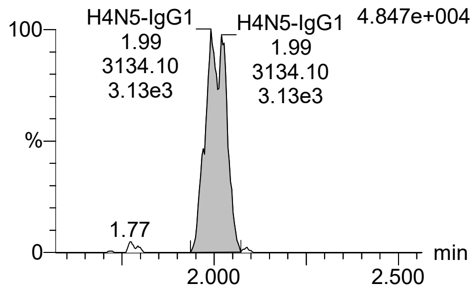

Sample Name: 20211111_L30_R2_IgG
F26:MRM of 1 channel,ES+
951.73 > 204.1

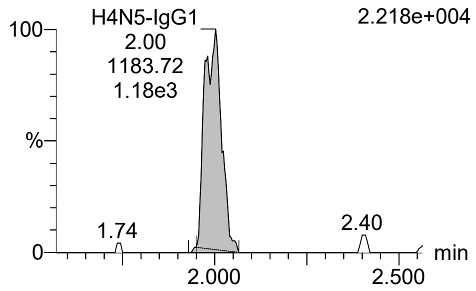

Sample Name: 20211111_H15_R2_IgG
F26:MRM of 1 channel,ES+
951.73 > 204.1

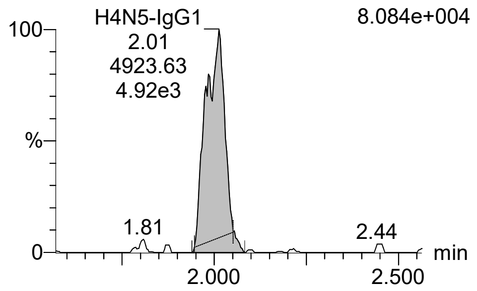

Sample Name: 20211111_A31_R2_IgG
F26:MRM of 1 channel,ES+
951.73 > 204.1

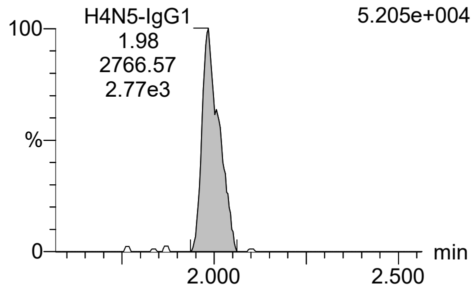

Sample Name: 20211111_A32_R2_IgG
F26:MRM of 1 channel,ES+
951.73 > 204.1

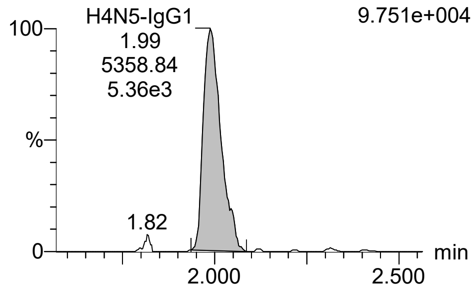

Sample Name: 20211111_L31_R2_IgG
F26:MRM of 1 channel,ES+
951.73 > 204.1

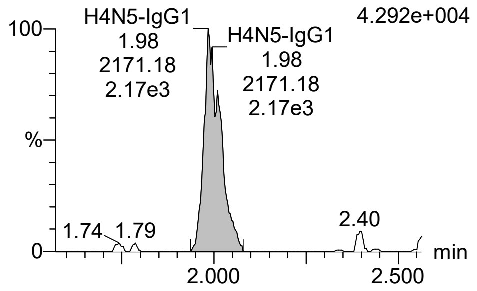

Sample Name: 20211111_L32_R2_IgG
F26:MRM of 1 channel,ES+
951.73 > 204.1

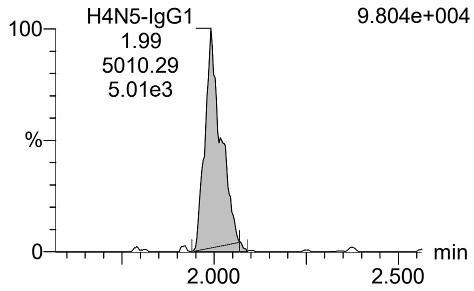

Sample Name: 20211111_H16_R2_IgG
F26:MRM of 1 channel,ES+
951.73 > 204.1

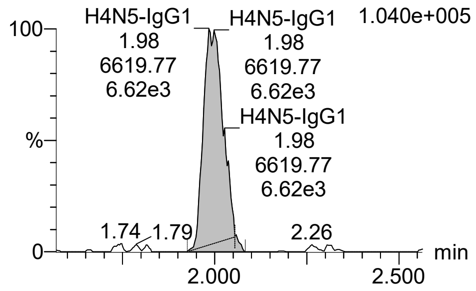

Sample Name: 20211111_A33_R2_IgG
F26:MRM of 1 channel,ES+
951.73 > 204.1

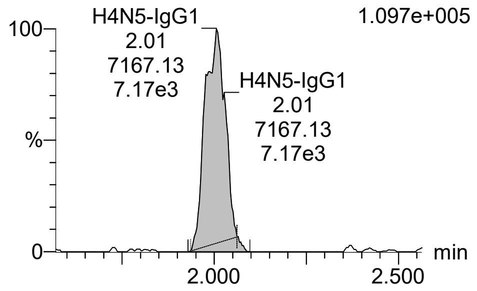

Dataset: C:\MassLynx\10.pro\20211115_clinicalsamples_IgG.qld

Last Altered: Sunday, October 02, 2022 16:02:30 台北標準時間
Printed: Friday, August 01, 2025 16:45:25 台北標準時間

Sample Name: 20211111_A34_R2_IgG

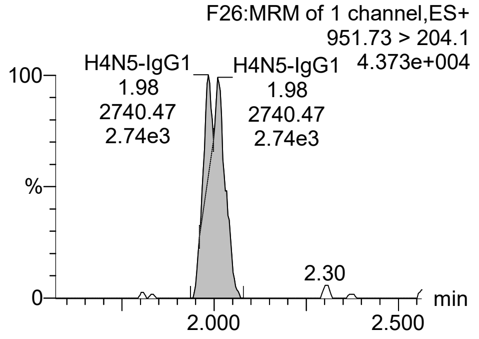

Sample Name: 20211111_H17_R2_IgG

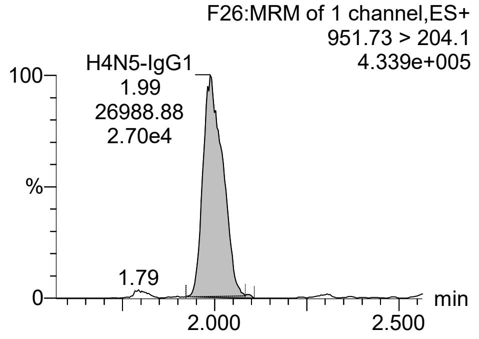

Sample Name: 20211111_A35_R2_IgG

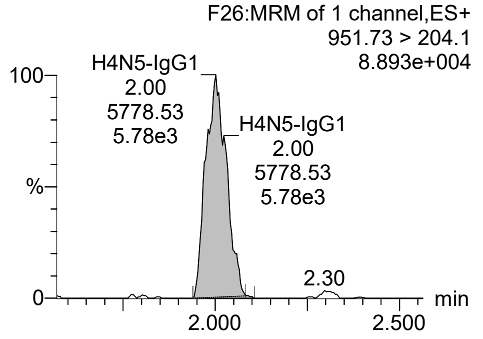

Sample Name: 20211111_QC_R16_IgG

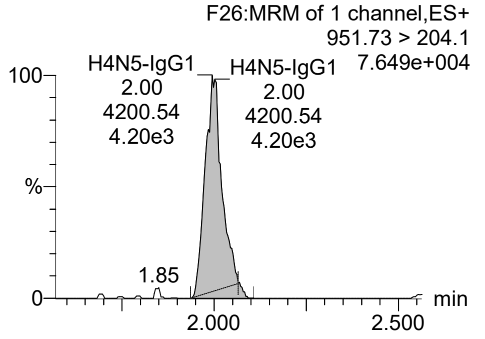

Sample Name: 20211111_A36_R2_IgG

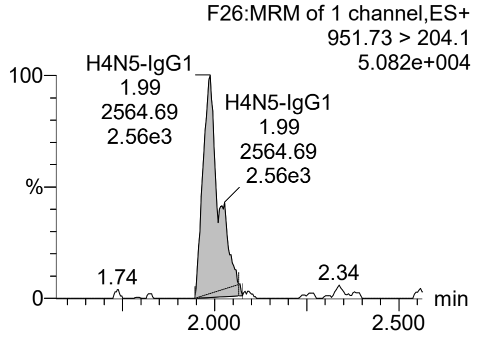

Sample Name: 20211111_H18_R2_IgG

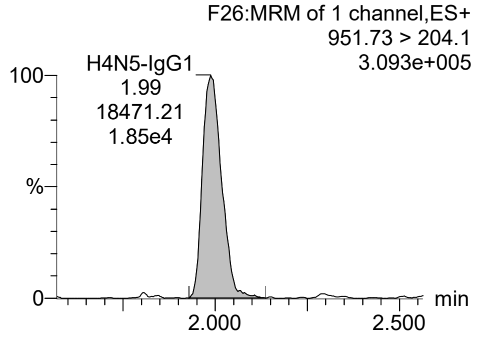

Sample Name: 20211111_A37_R2_IgG

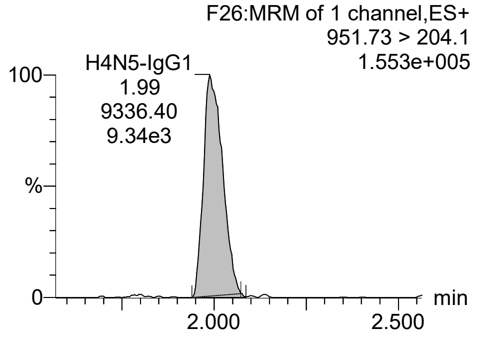

Sample Name: 20211111_A38_R2_IgG

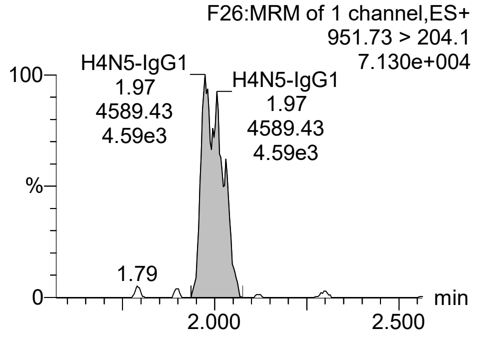

Sample Name: 20211111_H19_R2_IgG

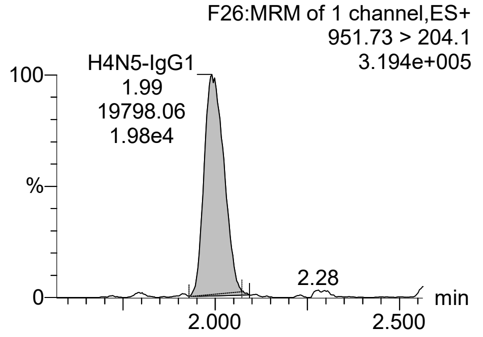

Sample Name: 20211111_A39_R2_IgG

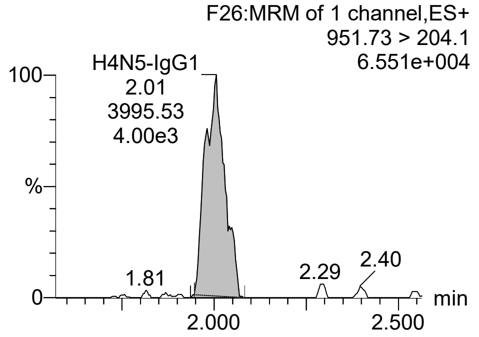

Sample Name: 20211111_A40_R2_IgG

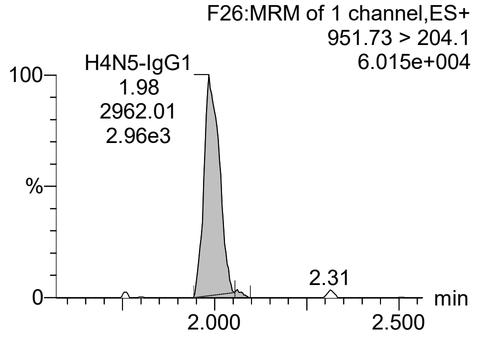

Sample Name: 20211111_H20_R2_IgG

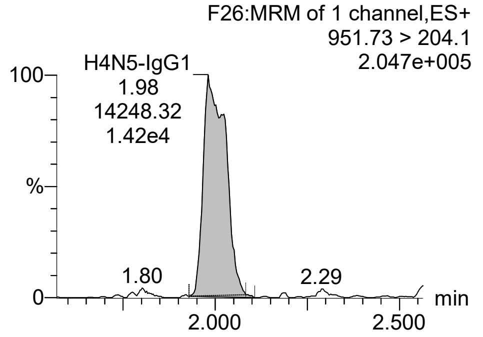

Sample Name: 20211111_A41_R2_IgG

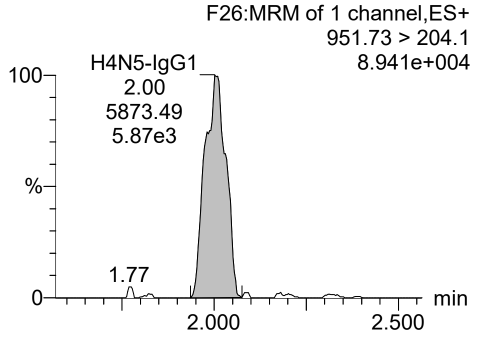

Sample Name: 20211111_A42_R2_IgG

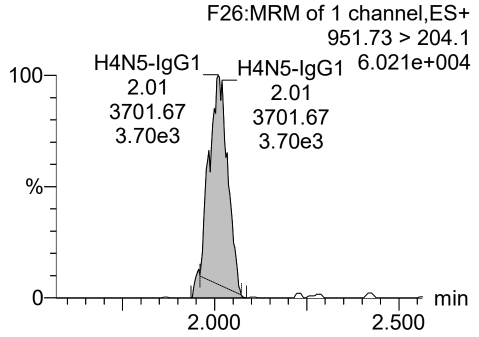

Sample Name: 20211111_A43_R2_IgG

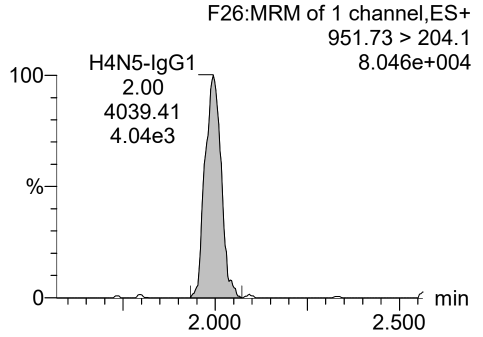

Dataset: C:\MassLynx\10.pro\20211115_clinicalsamples_IgG.qld

Last Altered: Sunday, October 02, 2022 16:02:30 台北標準時間
Printed: Friday, August 01, 2025 16:45:25 台北標準時間

Sample Name: 20211111_A44_R2_IgG

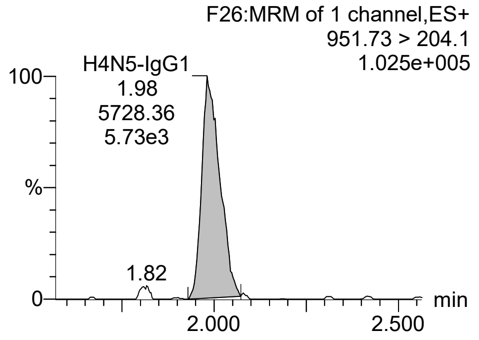

Sample Name: 20211111_QC_R17_IgG

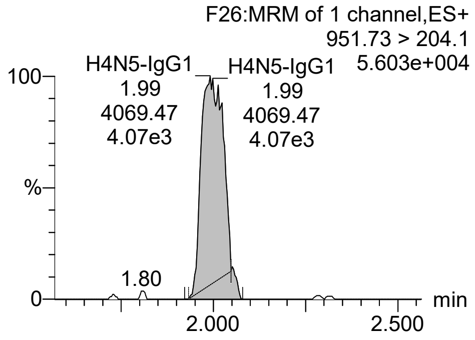

Dataset: C:\MassLynx\10.pro\20211115_clinicalsamples_IgG.qld

Last Altered: Sunday, October 02, 2022 16:02:30 台北標準時間
Printed: Friday, August 01, 2025 16:45:25 台北標準時間

	#	Name	RT	Area	IS Area	Response	Prima...
1	1	20211111_QC_R1_IgG	2.00	8477.1	64968.0	0.130	MM
2	2	20211111_A1_R1_IgG	1.99	8200.5	59059.2	0.139	MM
3	3	20211111_A2_R1_IgG	1.99	6631.6	66621.2	0.100	bb
4	4	20211111_L1_R1_IgG	2.01	10048.9	64201.0	0.157	MM
5	5	20211111_L2_R1_IgG	2.03	6108.6	46950.2	0.130	MM
6	6	20211111_H1_R1_IgG	1.98	3949.0	38648.2	0.102	bb
7	7	20211111_A3_R1_IgG	2.02	229.0	2603.7	0.088	MM
8	8	20211111_A4_R1_IgG	1.99	5510.3	69960.4	0.079	MM
9	9	20211111_L3_R1_IgG	2.00	1978.3	38864.7	0.051	MM
10	10	20211111_L4_R1_IgG	2.04	4925.0	43380.5	0.114	MM
11	11	20211111_H2_R1_IgG	2.00	25676.5	71349.2	0.360	MM
12	12	20211111_A5_R1_IgG	1.99	7090.7	65569.7	0.108	MM
13	13	20211111_A6_R1_IgG	2.00	9834.9	53120.7	0.185	MM
14	14	20211111_QC_R2_IgG	1.99	10128.3	81034.1	0.125	bb
15	15	20211111_L5_R1_IgG	2.00	12067.5	50994.6	0.237	MM
16	16	20211111_L6_R1_IgG	2.01	2005.0	58058.0	0.035	MM
17	17	20211111_H3_R1_IgG	2.01	3445.4	64028.6	0.054	MM
18	18	20211111_A7_R1_IgG	1.99	7964.5	82996.1	0.096	MM
19	19	20211111_A8_R1_IgG	2.00	9709.5	77731.7	0.125	MM
20	20	20211111_L7_R1_IgG	2.01	4860.9	38827.3	0.125	MM
21	21	20211111_L8_R1_IgG	2.01	3646.6	44220.1	0.082	MM
22	22	20211111_H4_R1_IgG	2.00	11403.0	65826.3	0.173	MM
23	23	20211111_A9_R1_IgG	1.99	3297.6	60771.1	0.054	MM
24	24	20211111_A10_R1_IgG	1.99	4410.3	48578.7	0.091	MM
25	25	20211111_L9_R1_IgG	1.99	4062.1	50830.7	0.080	MM
26	26	20211111_L10_R1_IgG	1.99	5258.1	38034.2	0.138	MM
27	27	20211111_QC_R3_IgG	2.01	9600.7	67025.5	0.143	bb
28	28	20211111_H5_R1_IgG	1.99	3835.1	44544.9	0.086	MM
29	29	20211111_A11_R1_IgG	2.00	3916.7	66408.5	0.059	MM
30	30	20211111_A12_R1_IgG	1.99	3745.5	46624.5	0.080	MM
31	31	20211111_L11_R1_IgG	2.02	3338.6	33425.1	0.100	bb
32	32	20211111_L12_R1_IgG	1.99	3986.5	41178.0	0.097	MM
33	33	20211111_H6_R1_IgG	2.01	9368.3	58593.4	0.160	bb
34	34	20211111_A13_R1_IgG	2.03	8809.6	54133.0	0.163	MM
35	35	20211111_A14_R1_IgG	2.00	4834.7	56467.3	0.086	MM
36	36	20211111_L13_R1_IgG	1.99	3893.2	36024.4	0.108	bb
37	37	20211111_L14_R1_IgG	2.01	7043.8	37181.6	0.189	MM
38	38	20211111_H7_R1_IgG	2.00	3464.0	42850.6	0.081	MM
39	39	20211111_A15_R1_IgG	2.00	17593.4	62015.1	0.284	MM
40	40	20211111_QC_R4_IgG	1.99	7914.5	58572.5	0.135	MM
41	41	20211111_A16_R1_IgG	2.00	6862.0	78283.8	0.088	MM
42	42	20211111_L15_R1_IgG	2.00	5682.6	44158.2	0.129	MM
43	43	20211111_L16_R1_IgG	2.01	3766.6	55150.8	0.068	MM
44	44	20211111_H8_R1_IgG	2.01	3019.0	42965.2	0.070	bb
45	45	20211111_A17_R1_IgG	2.00	4888.0	39717.7	0.123	MM
46	46	20211111_A18_R1_IgG	2.00	2050.8	44675.4	0.046	bb
47	47	20211111_L17_R1_IgG	2.00	5534.1	37854.1	0.146	MM
48	48	20211111_L18_R1_IgG	2.00	3779.5	50716.9	0.075	bb
49	49	20211111_H9_R1_IgG	2.01	5924.7	36276.0	0.163	bb
50	50	20211111_A19_R1_IgG	2.01	10613.5	42862.5	0.248	bb
51	51	20211111_A20_R1_IgG	2.02	2370.8	36542.7	0.065	MM
52	52	20211111_L19_R1_IgG	2.02	4611.2	53892.8	0.086	MM
53	53	20211111_QC_R5_IgG	2.00	6697.7	56513.2	0.119	MM
54	54	20211111_L20_R1_IgG	2.00	4209.8	56499.9	0.075	MM
55	55	20211111_H10_R1_IgG	2.01	13288.0	68120.5	0.195	bb
56	56	20211111_A21_R1_IgG	2.00	7637.9	63699.8	0.120	bb

Dataset: C:\MassLynx\10.pro\20211115_clinicalsamples_IgG.qld

Last Altered: Sunday, October 02, 2022 16:02:30 台北標準時間
Printed: Friday, August 01, 2025 16:45:25 台北標準時間

	# Name	RT	Area	IS Area	Response	Prima...
57	57 20211111_A22_R1_IgG	1.99	4087.5	52222.1	0.078	bb
58	58 20211111_L21_R1_IgG	2.02	3047.0	54372.5	0.056	bb
59	59 20211111_L22_R1_IgG	2.01	7014.2	41353.1	0.170	bb
60	60 20211111_H11_R1_IgG	2.00	7388.5	65579.1	0.113	MM
61	61 20211111_A23_R1_IgG	2.00	7557.7	64131.1	0.118	bb
62	62 20211111_A24_R1_IgG	2.00	3572.4	38724.8	0.092	MM
63	63 20211111_L23_R1_IgG	2.00	9670.2	64245.2	0.151	MM
64	64 20211111_L24_R1_IgG	2.02	2944.3	45298.0	0.065	bb
65	65 20211111_H12_R1_IgG	1.99	4322.3	44679.3	0.097	MM
66	66 20211111_QC_R6_IgG	2.00	6166.6	48165.0	0.128	MM
67	67 20211111_A25_R1_IgG	1.99	6869.0	57169.9	0.120	bb
68	68 20211111_A26_R1_IgG	2.00	1303.0	34254.0	0.038	bb
69	69 20211111_L25_R1_IgG	2.00	5176.2	67491.5	0.077	bb
70	70 20211111_L26_R1_IgG	2.02	3160.5	55934.1	0.057	bb
71	71 20211111_H13_R1_IgG	2.00	6903.1	48767.3	0.142	MM
72	72 20211111_A27_R1_IgG	2.00	4188.7	57269.7	0.073	bb
73	73 20211111_A28_R1_IgG	1.99	3946.5	49231.1	0.080	MM
74	74 20211111_L27_R1_IgG	1.99	8533.3	60014.5	0.142	MM
75	75 20211111_L28_R1_IgG	1.98	3100.0	35062.6	0.088	bb
76	76 20211111_H14_R1_IgG	2.02	7344.3	62371.9	0.118	MM
77	77 20211111_A29_R1_IgG	2.00	4445.3	43858.2	0.101	MM
78	78 20211111_A30_R1_IgG	2.00	6831.6	62500.4	0.109	MM
79	79 20211111_QC_R7_IgG	2.00	5427.5	43946.9	0.124	MM
80	80 20211111_L29_R1_IgG	1.99	3105.3	38728.9	0.080	bb
81	81 20211111_L30_R1_IgG	2.00	2241.1	42613.2	0.053	bb
82	82 20211111_H15_R1_IgG	2.01	4713.4	39874.6	0.118	MM
83	83 20211111_A31_R1_IgG	1.99	3676.7	53414.3	0.069	MM
84	84 20211111_A32_R1_IgG	2.00	6114.1	41844.6	0.146	bb
85	85 20211111_L31_R1_IgG	1.99	2760.6	40553.7	0.068	MM
86	86 20211111_L32_R1_IgG	2.00	4936.3	49618.7	0.099	MM
87	87 20211111_H16_R1_IgG	2.01	9057.4	60170.9	0.151	MM
88	88 20211111_A33_R1_IgG	2.02	7723.9	48588.2	0.159	MM
89	89 20211111_A34_R1_IgG	1.99	4268.9	43288.0	0.099	MM
90	90 20211111_H17_R1_IgG	2.01	34860.8	47104.5	0.740	bb
91	91 20211111_A35_R1_IgG	2.02	6621.4	41025.9	0.161	MM
92	92 20211111_QC_R8_IgG	2.00	5394.1	40546.3	0.133	MM
93	93 20211111_A36_R1_IgG	1.99	3849.0	44504.4	0.086	MM
94	94 20211111_H18_R1_IgG	1.99	21433.4	34162.2	0.627	MM
95	95 20211111_A37_R1_IgG	2.00	9834.2	39760.0	0.247	MM
96	96 20211111_A38_R1_IgG	2.00	4849.3	43982.6	0.110	bb
97	97 20211111_H19_R1_IgG	2.02	19504.9	45314.8	0.430	MM
98	98 20211111_A39_R1_IgG	2.01	4146.4	39388.0	0.105	MM
99	99 20211111_A40_R1_IgG	2.00	3091.4	38726.4	0.080	MM
100	1... 20211111_H20_R1_IgG	2.00	15436.3	37814.4	0.408	MM
101	1... 20211111_A41_R1_IgG	2.01	6760.4	48930.3	0.138	MM
102	1... 20211111_A42_R1_IgG	2.03	3758.6	40406.8	0.093	MM
103	1... 20211111_A43_R1_IgG	2.00	4584.1	44421.5	0.103	MM
104	1... 20211111_A44_R1_IgG	2.00	7306.4	53128.2	0.138	MM
105	1... 20211111_QC_R9_IgG	2.00	4075.4	37049.0	0.110	bb
106	1... 20211111_A1_R2_IgG	2.01	6893.7	46499.4	0.148	MM
107	1... 20211111_A2_R2_IgG	2.00	5674.0	57260.7	0.099	bb
108	1... 20211111_L1_R2_IgG	2.00	7147.8	48062.9	0.149	MM
109	1... 20211111_L2_R2_IgG	2.02	5282.8	38745.2	0.136	MM
110	1... 20211111_H1_R2_IgG	2.03	4280.2	44273.5	0.097	MM
111	1... 20211111_A3_R2_IgG	2.01	235.3	4755.5	0.049	MM
112	1... 20211111_A4_R2_IgG	2.00	4293.2	50546.7	0.085	MM

Dataset: C:\MassLynx\10.pro\20211115_clinicalsamples_IgG.qld

Last Altered: Sunday, October 02, 2022 16:02:30 台北標準時間
Printed: Friday, August 01, 2025 16:45:25 台北標準時間

	# Name	RT	Area	IS Area	Response	Prima...
113	1... 20211111_L3_R2_IgG	1.99	2735.5	34825.8	0.079	MM
114	1... 20211111_L4_R2_IgG	2.01	4287.7	39107.7	0.110	MM
115	1... 20211111_H2_R2_IgG	1.99	19474.2	49750.0	0.391	bb
116	1... 20211111_A5_R2_IgG	1.99	5708.9	52536.2	0.109	MM
117	1... 20211111_A6_R2_IgG	1.99	8942.9	42050.0	0.213	MM
118	1... 20211111_QC_R10_IgG	1.98	4183.2	35350.8	0.118	bb
119	1... 20211111_L5_R2_IgG	1.99	8821.8	38039.3	0.232	MM
120	1... 20211111_L6_R2_IgG	2.02	1425.7	44907.4	0.032	MM
121	1... 20211111_H3_R2_IgG	2.00	2878.0	47729.1	0.060	MM
122	1... 20211111_A7_R2_IgG	2.00	6154.9	57347.1	0.107	bb
123	1... 20211111_A8_R2_IgG	2.00	5888.1	52397.8	0.112	MM
124	1... 20211111_L7_R2_IgG	2.01	4068.4	36318.4	0.112	MM
125	1... 20211111_L8_R2_IgG	1.99	3219.1	38131.9	0.084	MM
126	1... 20211111_H4_R2_IgG	1.99	8923.2	46849.6	0.190	bb
127	1... 20211111_A9_R2_IgG	2.00	2661.2	47759.6	0.056	MM
128	1... 20211111_A10_R2_IgG	1.98	3900.6	41366.2	0.094	bb
129	1... 20211111_L9_R2_IgG	1.98	3451.6	43052.1	0.080	bb
130	1... 20211111_L10_R2_IgG	2.00	4482.8	31487.4	0.142	bb
131	1... 20211111_QC_R11_IgG	1.99	4259.8	37804.6	0.113	bb
132	1... 20211111_H5_R2_IgG	1.99	3418.1	40380.0	0.085	bb
133	1... 20211111_A11_R2_IgG	2.02	3492.2	49533.3	0.071	MM
134	1... 20211111_A12_R2_IgG	2.01	3084.1	40226.3	0.077	bb
135	1... 20211111_L11_R2_IgG	2.02	2613.8	28884.0	0.090	bb
136	1... 20211111_L12_R2_IgG	1.99	3830.5	34351.3	0.112	MM
137	1... 20211111_H6_R2_IgG	1.99	5145.1	40466.6	0.127	MM
138	1... 20211111_A13_R2_IgG	2.02	6852.2	42306.7	0.162	MM
139	1... 20211111_A14_R2_IgG	1.98	3598.1	44904.0	0.080	MM
140	1... 20211111_L13_R2_IgG	2.00	2714.2	31913.0	0.085	bb
141	1... 20211111_L14_R2_IgG	2.00	6355.2	31527.4	0.202	bb
142	1... 20211111_H7_R2_IgG	1.99	2579.6	40838.2	0.063	MM
143	1... 20211111_A15_R2_IgG	1.99	13589.8	49389.7	0.275	bb
144	1... 20211111_QC_R12_IgG	1.99	4710.8	35168.7	0.134	MM
145	1... 20211111_A16_R2_IgG	2.00	4035.2	47440.5	0.085	MM
146	1... 20211111_L15_R2_IgG	1.98	3836.8	33145.9	0.116	MM
147	1... 20211111_L16_R2_IgG	1.99	2389.5	37596.6	0.064	MM
148	1... 20211111_H8_R2_IgG	1.99	2794.0	43933.3	0.064	MM
149	1... 20211111_A17_R2_IgG	1.99	4651.9	43605.5	0.107	bb
150	1... 20211111_A18_R2_IgG	2.01	2073.5	37581.8	0.055	bb
151	1... 20211111_L17_R2_IgG	1.99	5667.6	33942.5	0.167	bb
152	1... 20211111_L18_R2_IgG	1.99	2524.7	37831.5	0.067	MM
153	1... 20211111_H9_R2_IgG	2.01	5476.4	30898.4	0.177	bb
154	1... 20211111_A19_R2_IgG	2.00	8681.6	39352.1	0.221	MM
155	1... 20211111_A20_R2_IgG	2.01	1835.6	34661.2	0.053	MM
156	1... 20211111_L19_R2_IgG	2.02	3629.1	39918.3	0.091	MM
157	1... 20211111_QC_R13_IgG	1.98	4445.0	36239.1	0.123	MM
158	1... 20211111_L20_R2_IgG	1.99	2265.3	32897.7	0.069	MM
159	1... 20211111_H10_R2_IgG	1.99	6101.7	31394.5	0.194	MM
160	1... 20211111_A21_R2_IgG	1.99	4101.2	34863.7	0.118	MM
161	1... 20211111_A22_R2_IgG	2.00	1791.0	30342.1	0.059	MM
162	1... 20211111_L21_R2_IgG	1.99	1725.8	33706.2	0.051	bb
163	1... 20211111_L22_R2_IgG	2.01	3922.7	31298.6	0.125	MM
164	1... 20211111_H11_R2_IgG	1.98	4228.4	38904.1	0.109	MM
165	1... 20211111_A23_R2_IgG	1.99	3772.3	32382.3	0.116	MM
166	1... 20211111_A24_R2_IgG	1.98	2494.7	30261.9	0.082	bb
167	1... 20211111_L23_R2_IgG	1.98	6542.2	43037.5	0.152	MM
168	1... 20211111_L24_R2_IgG	2.01	2426.3	37023.3	0.066	bb

Dataset: C:\MassLynx\10.pro\20211115_clinicalsamples_IgG.qld

Last Altered: Sunday, October 02, 2022 16:02:30 台北標準時間
Printed: Friday, August 01, 2025 16:45:25 台北標準時間

	# Name	RT	Area	IS Area	Response	Prima...
169	1... 20211111_H12_R2_IgG	1.99	3424.7	39478.2	0.087	bb
170	1... 20211111_QC_R14_IgG	2.00	4110.7	34463.2	0.119	bb
171	1... 20211111_A25_R2_IgG	1.99	5188.3	39945.3	0.130	bb
172	1... 20211111_A26_R2_IgG	1.99	1208.2	29663.2	0.041	bb
173	1... 20211111_L25_R2_IgG	1.98	3137.3	38589.3	0.081	MM
174	1... 20211111_L26_R2_IgG	1.99	2157.6	38405.2	0.056	MM
175	1... 20211111_H13_R2_IgG	1.99	4918.6	37771.2	0.130	MM
176	1... 20211111_A27_R2_IgG	2.00	2866.6	42634.1	0.067	MM
177	1... 20211111_A28_R2_IgG	1.98	3529.3	33231.1	0.106	MM
178	1... 20211111_L27_R2_IgG	1.98	5362.9	41761.1	0.128	MM
179	1... 20211111_L28_R2_IgG	2.00	2383.8	26968.6	0.088	MM
180	1... 20211111_H14_R2_IgG	1.99	5849.5	49250.2	0.119	bb
181	1... 20211111_A29_R2_IgG	1.97	3600.7	37601.7	0.096	MM
182	1... 20211111_A30_R2_IgG	1.99	3744.3	40366.8	0.093	bb
183	1... 20211111_QC_R15_IgG	1.98	4022.9	35156.1	0.114	bb
184	1... 20211111_L29_R2_IgG	1.99	3134.1	33813.1	0.093	bb
185	1... 20211111_L30_R2_IgG	2.00	1183.7	33488.6	0.035	MM
186	1... 20211111_H15_R2_IgG	2.01	4923.6	38501.0	0.128	MM
187	1... 20211111_A31_R2_IgG	1.98	2766.6	37525.5	0.074	bb
188	1... 20211111_A32_R2_IgG	1.99	5358.8	35770.8	0.150	bb
189	1... 20211111_L31_R2_IgG	1.98	2171.2	33282.4	0.065	bb
190	1... 20211111_L32_R2_IgG	1.99	5010.3	42049.6	0.119	MM
191	1... 20211111_H16_R2_IgG	1.98	6619.8	45947.6	0.144	MM
192	1... 20211111_A33_R2_IgG	2.01	7167.1	40202.3	0.178	MM
193	1... 20211111_A34_R2_IgG	1.98	2740.5	36747.0	0.075	MM
194	1... 20211111_H17_R2_IgG	1.99	26988.9	40920.9	0.660	MM
195	1... 20211111_A35_R2_IgG	2.00	5778.5	33689.2	0.172	MM
196	1... 20211111_QC_R16_IgG	2.00	4200.5	32783.6	0.128	MM
197	1... 20211111_A36_R2_IgG	1.99	2564.7	36822.4	0.070	MM
198	1... 20211111_H18_R2_IgG	1.99	18471.2	31769.3	0.581	bb
199	1... 20211111_A37_R2_IgG	1.99	9336.4	37581.8	0.248	MM
200	2... 20211111_A38_R2_IgG	1.97	4589.4	38390.4	0.120	bb
201	2... 20211111_H19_R2_IgG	1.99	19798.1	41217.0	0.480	MM
202	2... 20211111_A39_R2_IgG	2.01	3995.5	37392.7	0.107	MM
203	2... 20211111_A40_R2_IgG	1.98	2962.0	38295.1	0.077	MM
204	2... 20211111_H20_R2_IgG	1.98	14248.3	35941.8	0.396	MM
205	2... 20211111_A41_R2_IgG	2.00	5873.5	45304.9	0.130	bb
206	2... 20211111_A42_R2_IgG	2.01	3701.7	37803.8	0.098	MM
207	2... 20211111_A43_R2_IgG	2.00	4039.4	38161.8	0.106	bb
208	2... 20211111_A44_R2_IgG	1.98	5728.4	37819.8	0.151	bb
209	2... 20211111_QC_R17_IgG	1.99	4069.5	31731.8	0.128	MM

Dataset: C:\MassLynx\10.pro\20211115_clinicalsamples_IgG.qld

Last Altered: Sunday, October 02, 2022 16:02:30 台北標準時間
Printed: Friday, August 01, 2025 16:45:54 台北標準時間

Method: C:\MassLynx\10_Pro_IgA.PRO\MethDB\20210926_IgG_DMRM_IgM_IS.mdb 16 Feb 2022 11:58:21
Calibration: 18 Nov 2021 13:56:22

Sample Name: 20211111_QC_R1_IgG

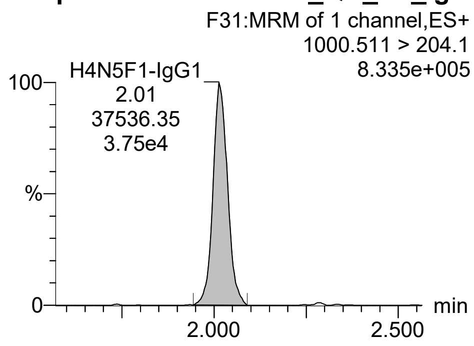

Sample Name: 20211111_A1_R1_IgG

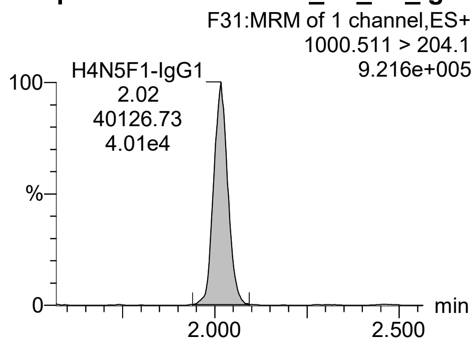

Sample Name: 20211111_A2_R1_IgG

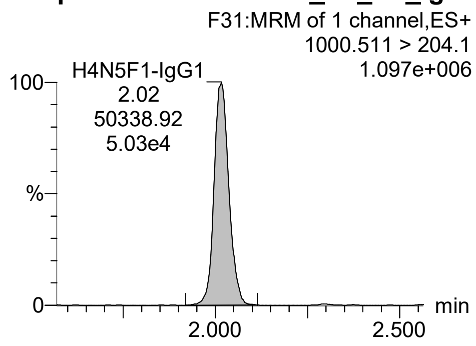

Sample Name: 20211111_L1_R1_IgG

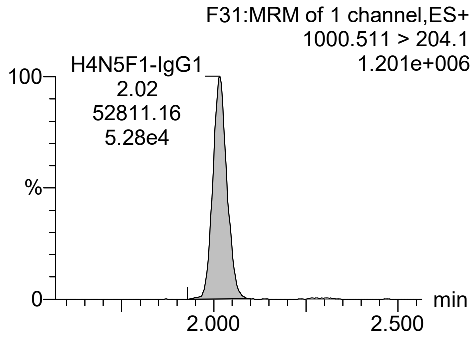

Sample Name: 20211111_L2_R1_IgG

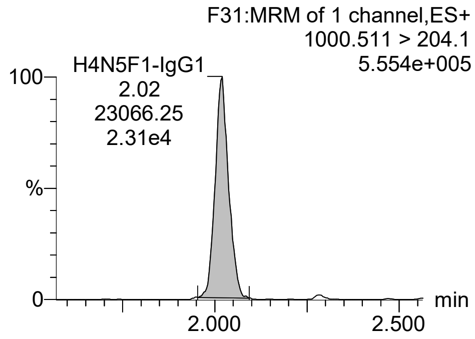

Sample Name: 20211111_H1_R1_IgG

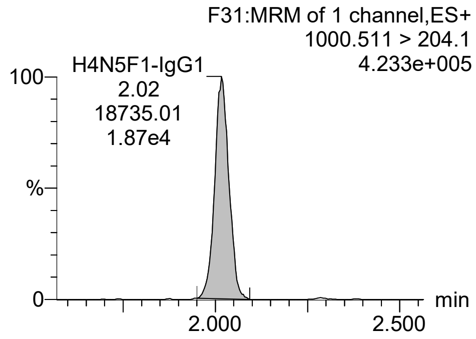

Sample Name: 20211111_A3_R1_IgG

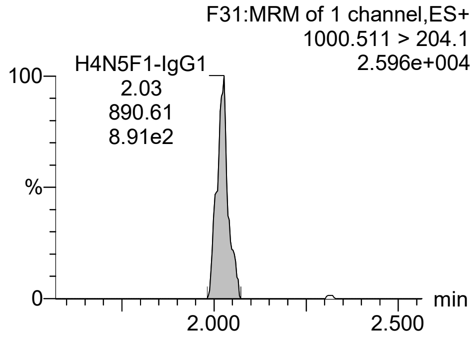

Sample Name: 20211111_A4_R1_IgG

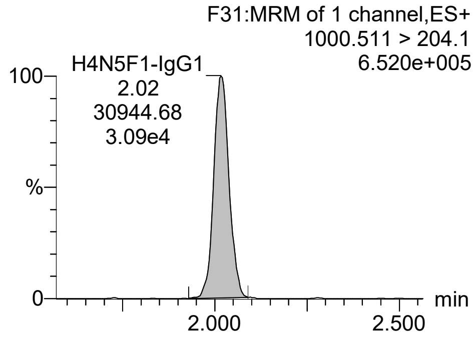

Sample Name: 20211111_L3_R1_IgG

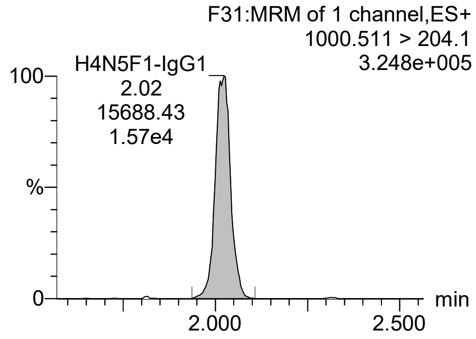

Sample Name: 20211111_L4_R1_IgG

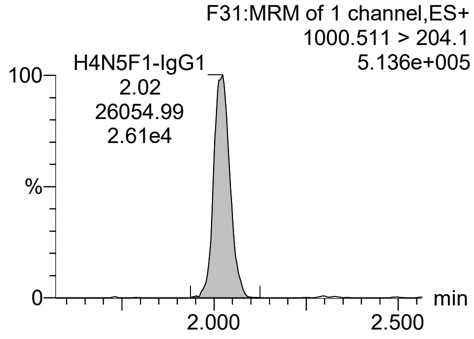

Sample Name: 20211111_H2_R1_IgG

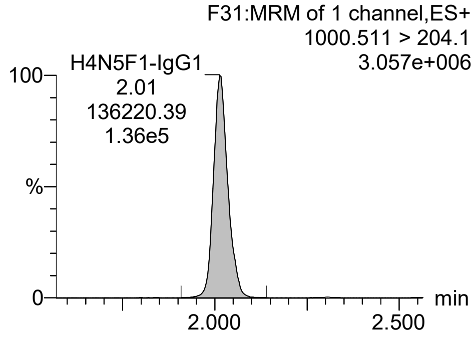

Sample Name: 20211111_A5_R1_IgG

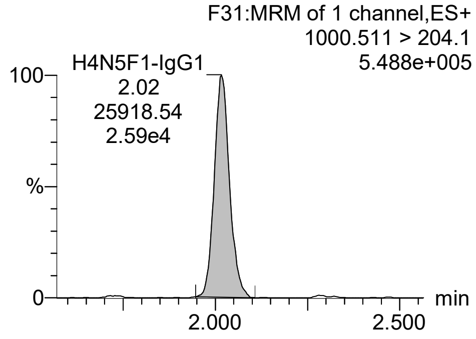

Dataset: C:\MassLynx\10.pro\20211115_clinicalsamples_IgG.qld

Last Altered: Sunday, October 02, 2022 16:02:30 台北標準時間
Printed: Friday, August 01, 2025 16:45:54 台北標準時間

Sample Name: 20211111_A6_R1_IgG

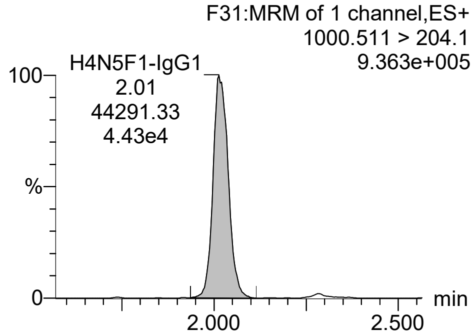

Sample Name: 20211111_QC_R2_IgG

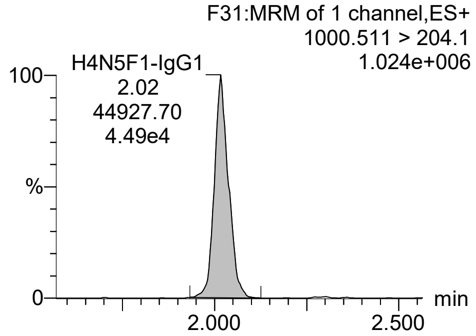

Sample Name: 20211111_L5_R1_IgG

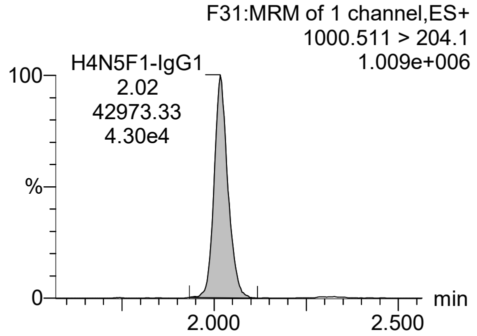

Sample Name: 20211111_L6_R1_IgG

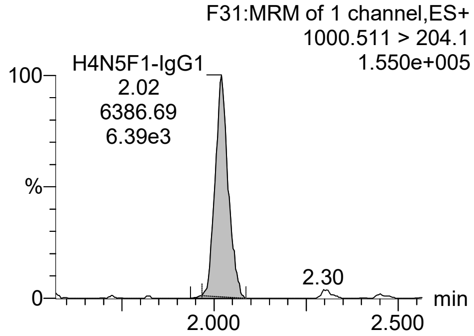

Sample Name: 20211111_H3_R1_IgG

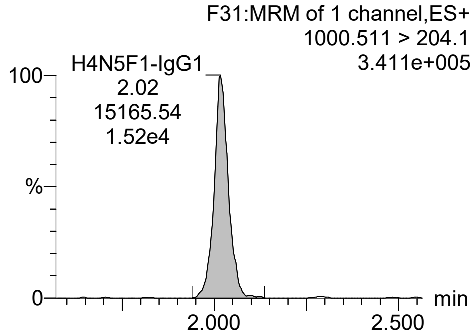

Sample Name: 20211111_A7_R1_IgG

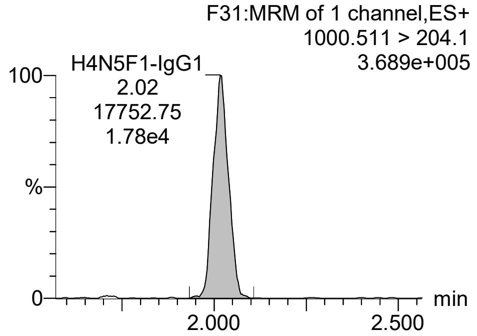

Sample Name: 20211111_A8_R1_IgG

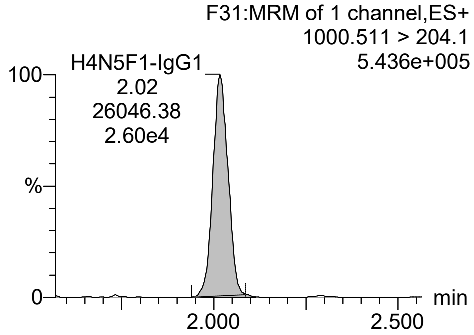

Sample Name: 20211111_L7_R1_IgG

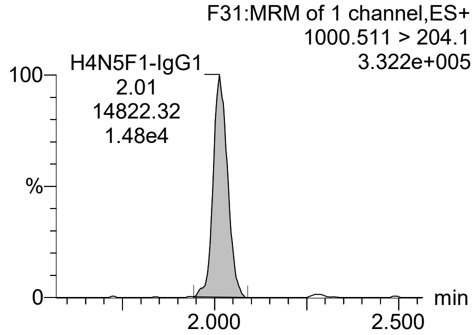

Sample Name: 20211111_L8_R1_IgG

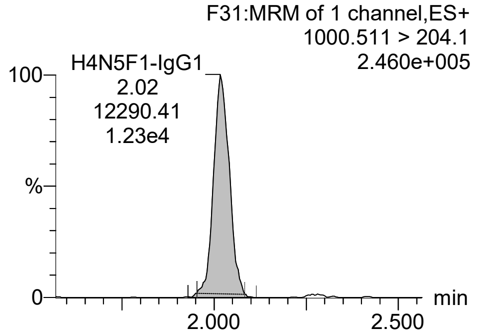

Sample Name: 20211111_H4_R1_IgG

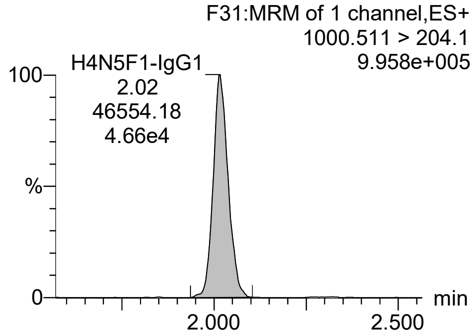

Sample Name: 20211111_A9_R1_IgG

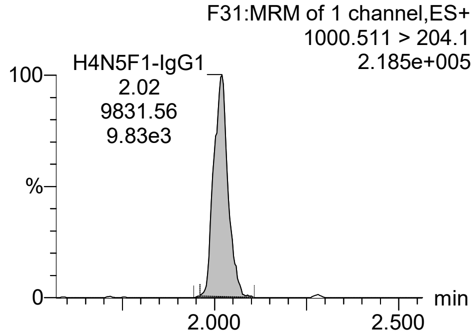

Sample Name: 20211111_A10_R1_IgG

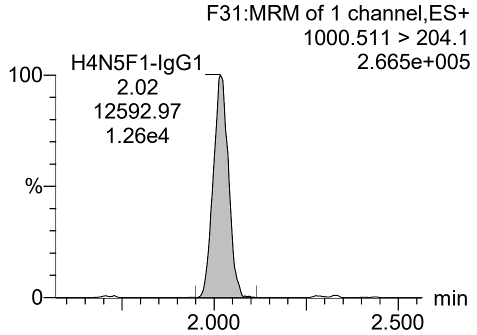

Sample Name: 20211111_L9_R1_IgG

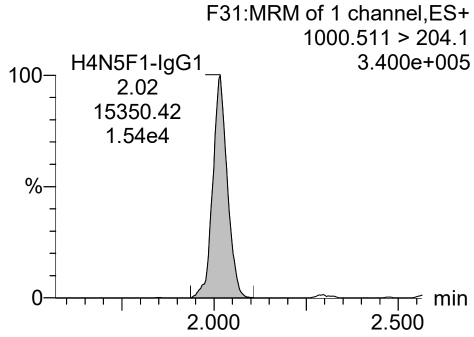

Sample Name: 20211111_L10_R1_IgG

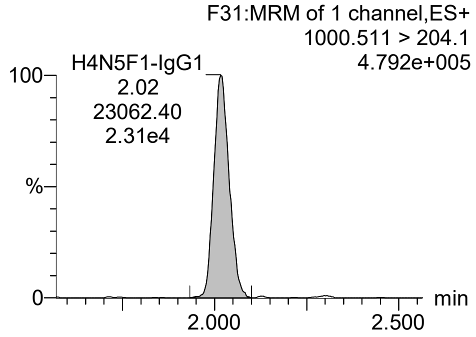

Sample Name: 20211111_QC_R3_IgG

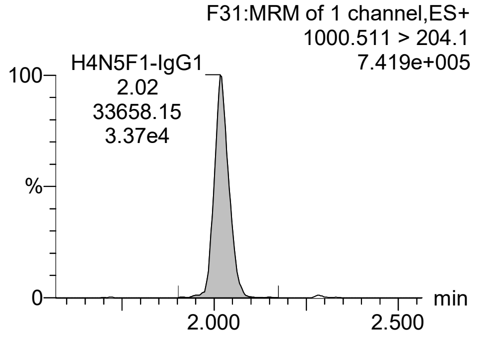

Dataset: C:\MassLynx\10.pro\20211115_clinicalsamples_IgG.qld

Last Altered: Sunday, October 02, 2022 16:02:30 台北標準時間
Printed: Friday, August 01, 2025 16:45:54 台北標準時間

Sample Name: 20211111_H5_R1_IgG

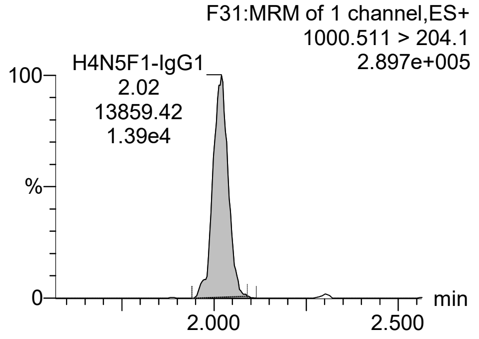

Sample Name: 20211111_A11_R1_IgG

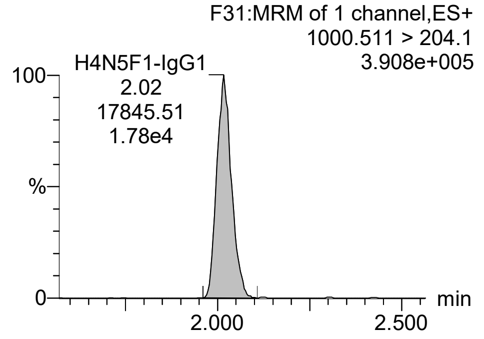

Sample Name: 20211111_A12_R1_IgG

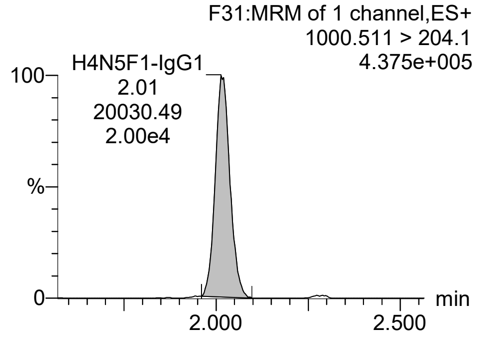

Sample Name: 20211111_L11_R1_IgG

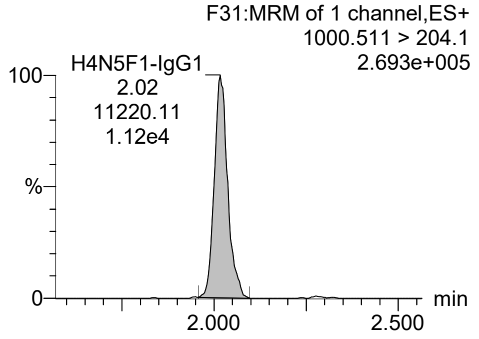

Sample Name: 20211111_L12_R1_IgG

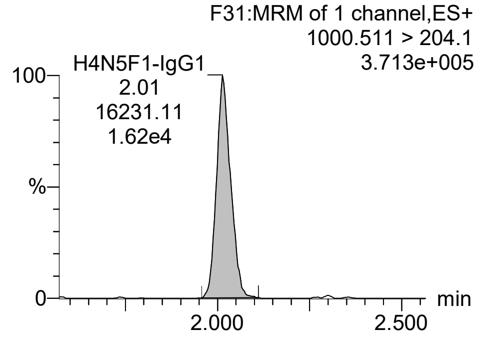

Sample Name: 20211111_H6_R1_IgG

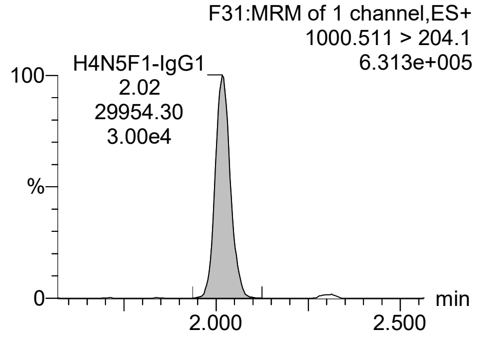

Sample Name: 20211111_A13_R1_IgG

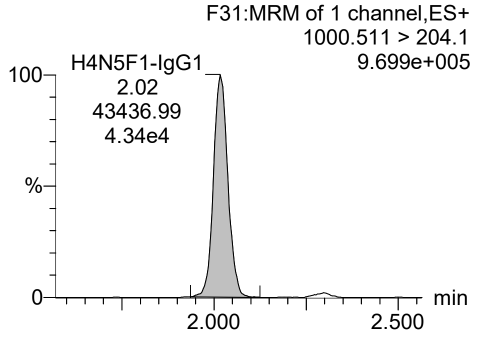

Sample Name: 20211111_A14_R1_IgG

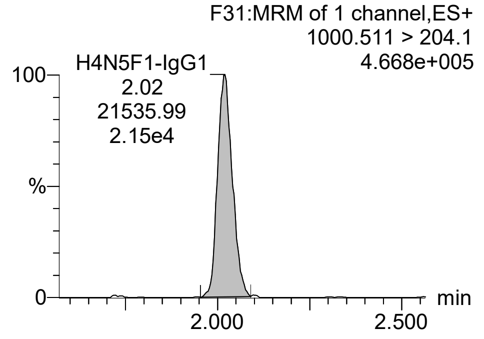

Sample Name: 20211111_L13_R1_IgG

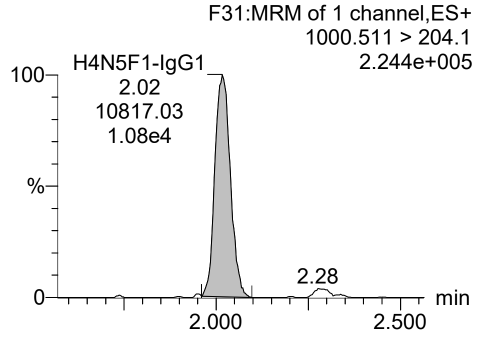

Sample Name: 20211111_L14_R1_IgG

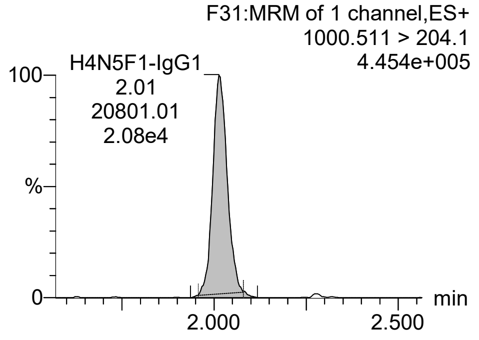

Sample Name: 20211111_H7_R1_IgG

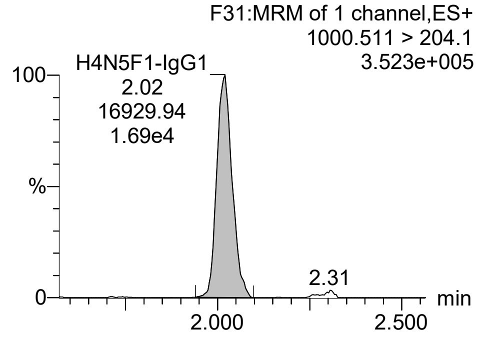

Sample Name: 20211111_A15_R1_IgG

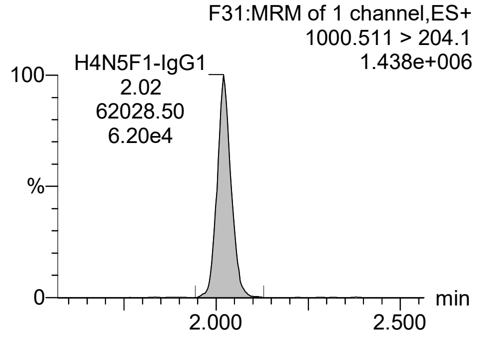

Sample Name: 20211111_QC_R4_IgG

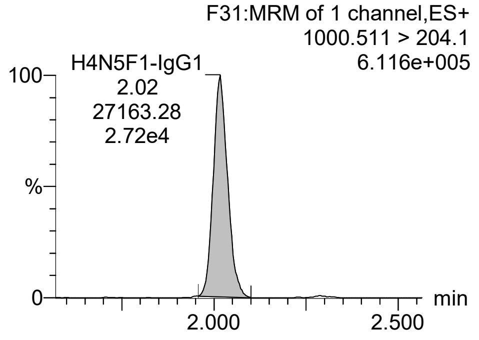

Sample Name: 20211111_A16_R1_IgG

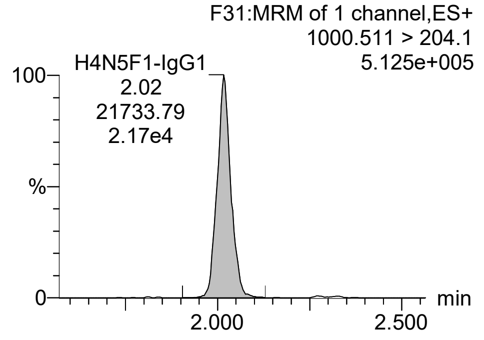

Sample Name: 20211111_L15_R1_IgG

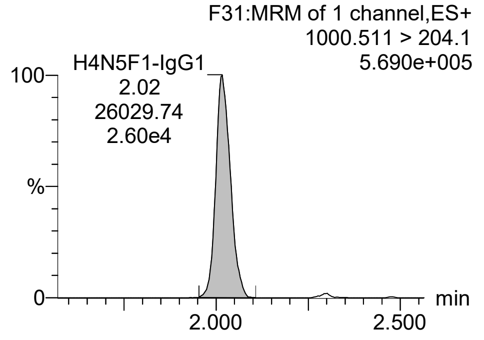

Sample Name: 20211111_L16_R1_IgG
F31:MRM of 1 channel,ES+
1000.511 > 204.1
3.111e+005

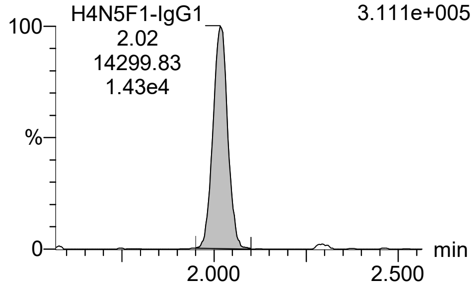

Sample Name: 20211111_H8_R1_IgG
F31:MRM of 1 channel,ES+
1000.511 > 204.1
2.078e+005

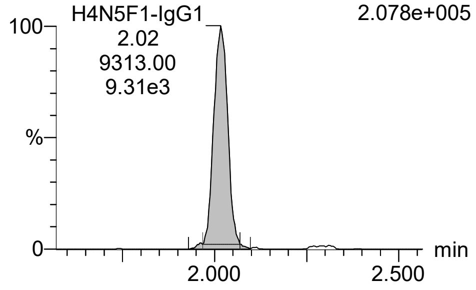

Sample Name: 20211111_A17_R1_IgG
F31:MRM of 1 channel,ES+
1000.511 > 204.1
4.494e+005

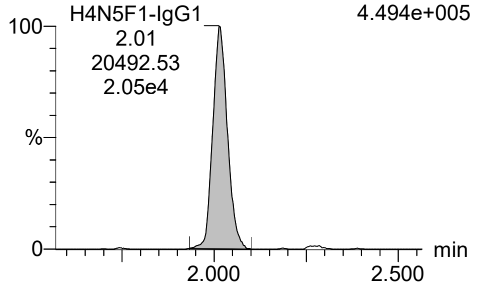

Sample Name: 20211111_A18_R1_IgG
F31:MRM of 1 channel,ES+
1000.511 > 204.1
1.740e+005

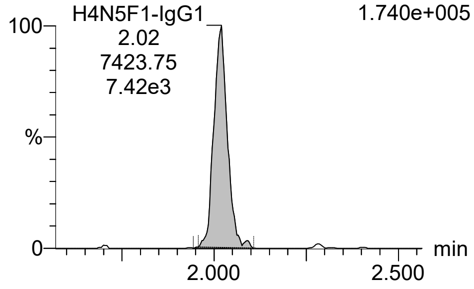

Sample Name: 20211111_L17_R1_IgG
F31:MRM of 1 channel,ES+
1000.511 > 204.1
4.197e+005

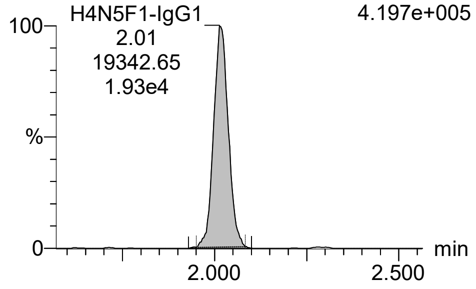

Sample Name: 20211111_L18_R1_IgG
F31:MRM of 1 channel,ES+
1000.511 > 204.1
3.860e+005

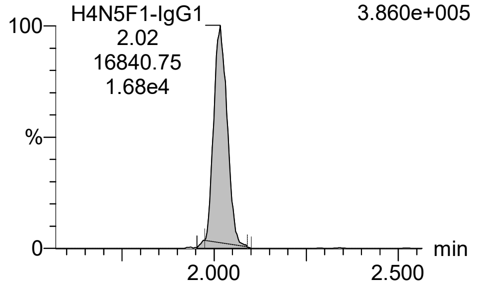

Sample Name: 20211111_H9_R1_IgG
F31:MRM of 1 channel,ES+
1000.511 > 204.1
5.507e+005

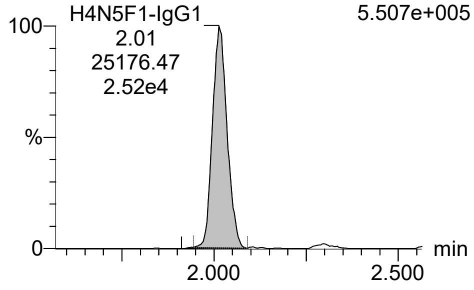

Sample Name: 20211111_A19_R1_IgG
F31:MRM of 1 channel,ES+
1000.511 > 204.1
1.128e+006

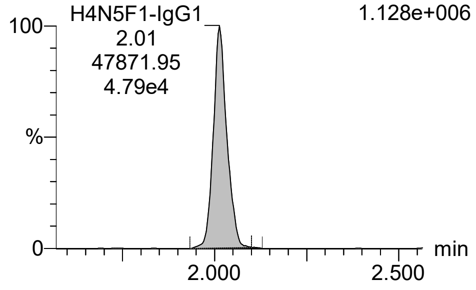

Sample Name: 20211111_A20_R1_IgG
F31:MRM of 1 channel,ES+
1000.511 > 204.1
1.767e+005

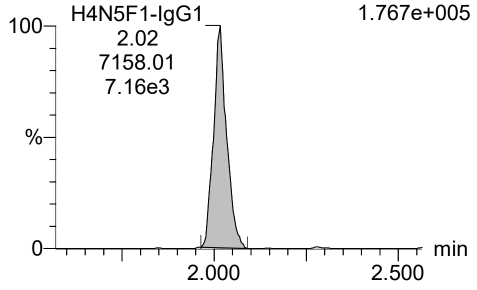

Sample Name: 20211111_L19_R1_IgG
F31:MRM of 1 channel,ES+
1000.511 > 204.1
4.112e+005

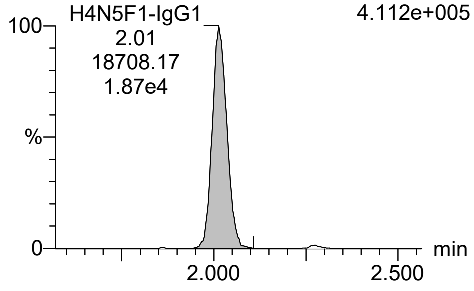

Sample Name: 20211111_QC_R5_IgG
F31:MRM of 1 channel,ES+
1000.511 > 204.1
6.583e+005

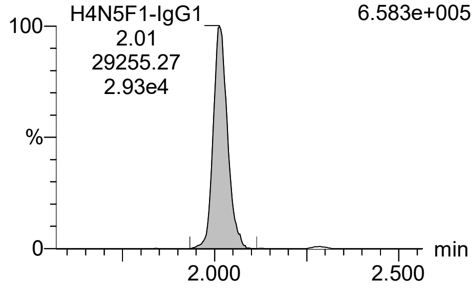

Sample Name: 20211111_L20_R1_IgG
F31:MRM of 1 channel,ES+
1000.511 > 204.1
6.229e+005

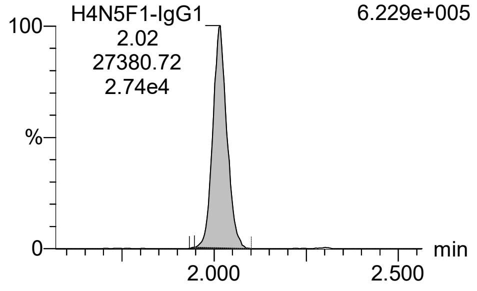

Sample Name: 20211111_H10_R1_IgG
F31:MRM of 1 channel,ES+
1000.511 > 204.1
1.554e+006

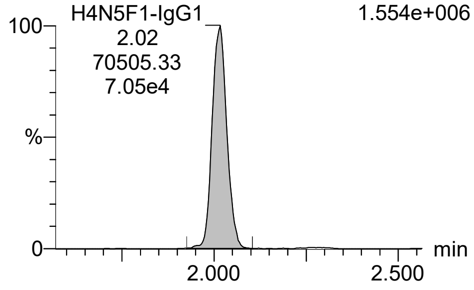

Sample Name: 20211111_A21_R1_IgG
F31:MRM of 1 channel,ES+
1000.511 > 204.1
5.892e+005

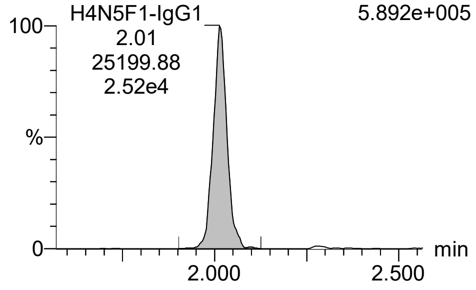

Sample Name: 20211111_A22_R1_IgG
F31:MRM of 1 channel,ES+
1000.511 > 204.1
3.099e+005

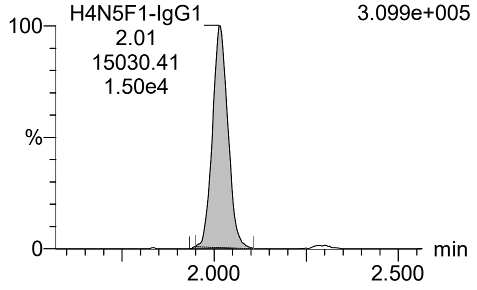

Sample Name: 20211111_L21_R1_IgG

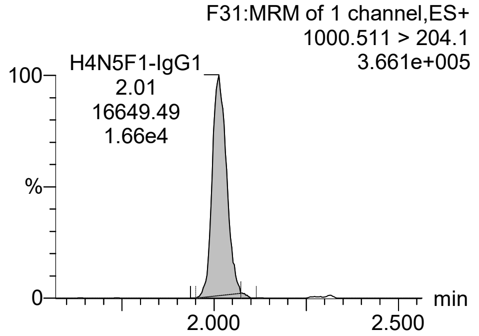

Sample Name: 20211111_L22_R1_IgG

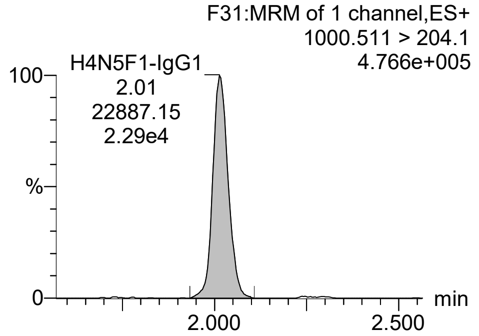

Sample Name: 20211111_H11_R1_IgG

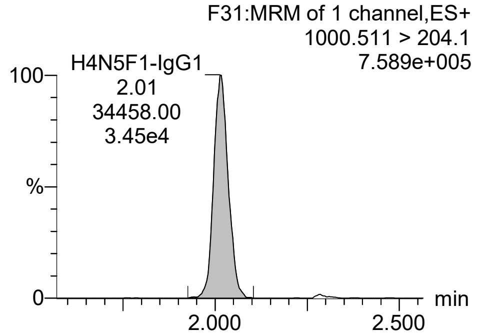

Sample Name: 20211111_A23_R1_IgG

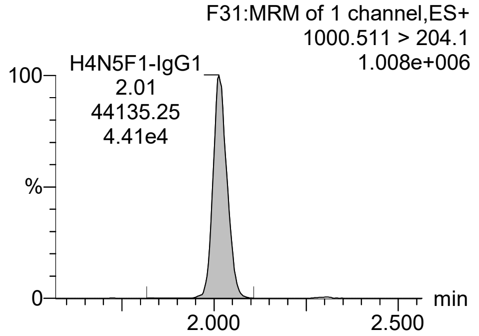

Sample Name: 20211111_A24_R1_IgG

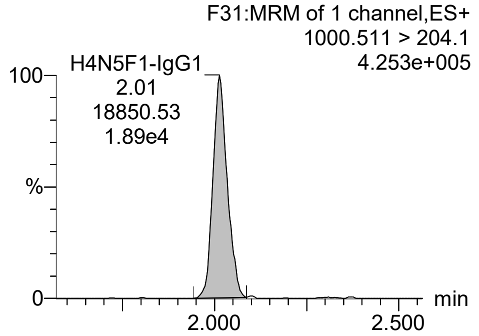

Sample Name: 20211111_L23_R1_IgG

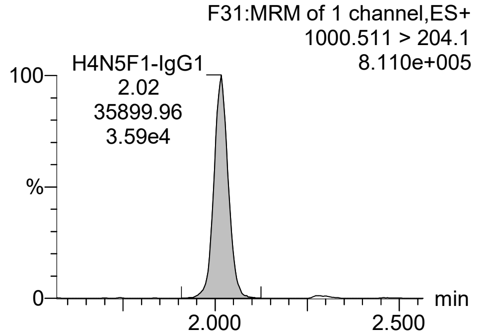

Sample Name: 20211111_L24_R1_IgG

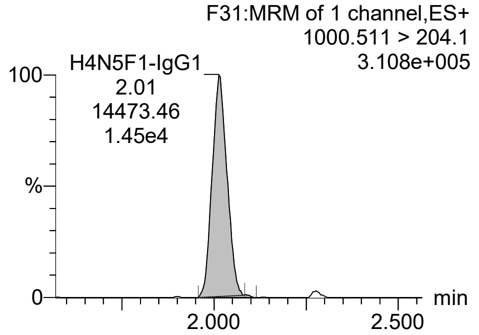

Sample Name: 20211111_H12_R1_IgG

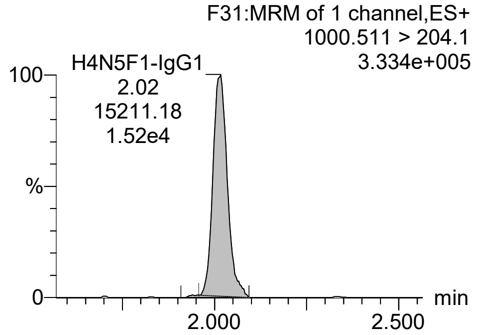

Sample Name: 20211111_QC_R6_IgG

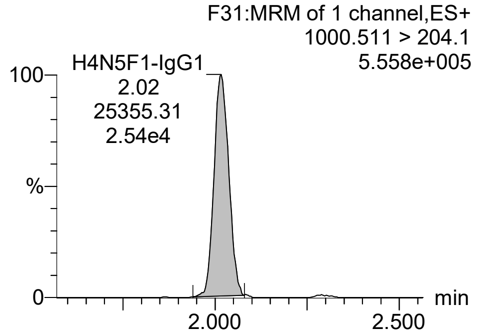

Sample Name: 20211111_A25_R1_IgG

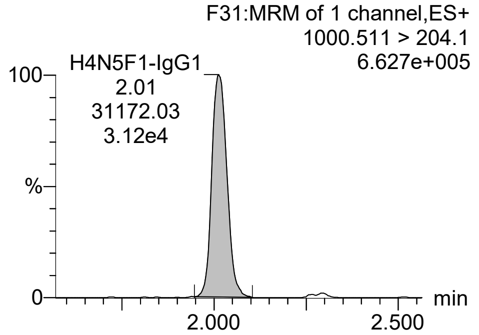

Sample Name: 20211111_A26_R1_IgG

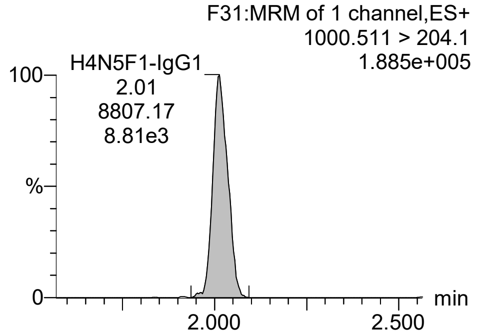

Sample Name: 20211111_L25_R1_IgG

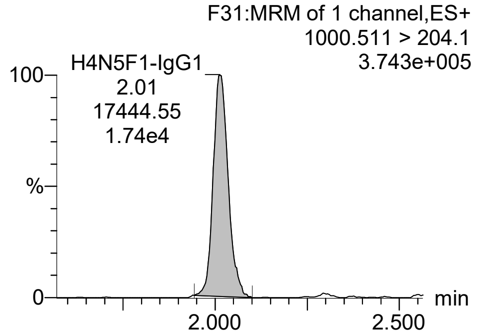

Sample Name: 20211111_L26_R1_IgG

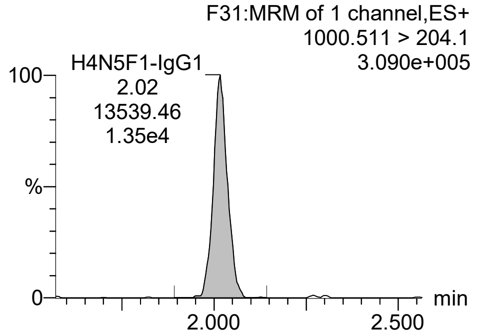

Sample Name: 20211111_H13_R1_IgG

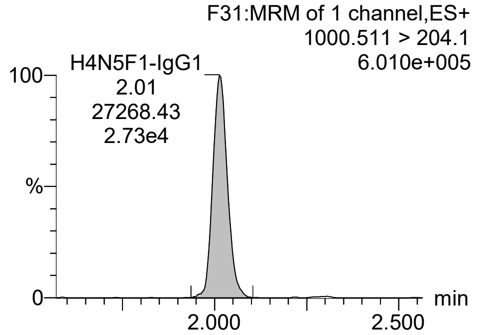

Sample Name: 20211111_A27_R1_IgG

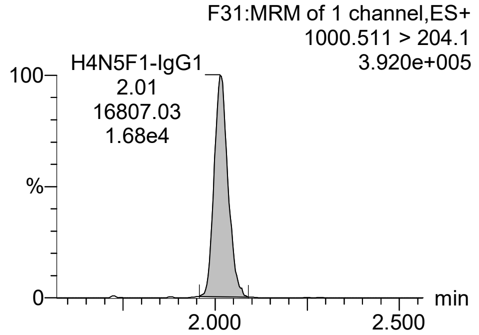

Dataset: C:\MassLynx\10.pro\20211115_clinicalsamples_IgG.qld

Last Altered: Sunday, October 02, 2022 16:02:30 台北標準時間
Printed: Friday, August 01, 2025 16:45:54 台北標準時間

Sample Name: 20211111_A28_R1_IgG

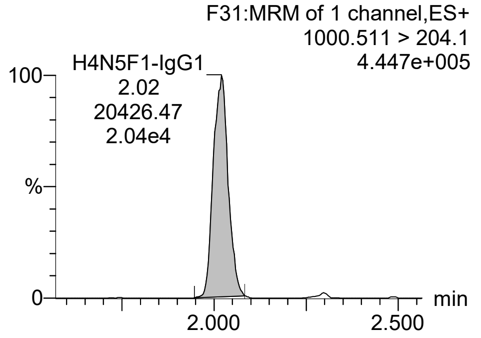

Sample Name: 20211111_L27_R1_IgG

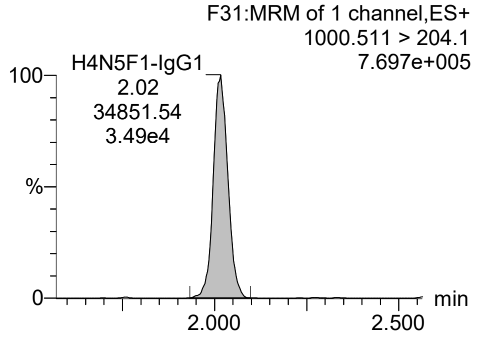

Sample Name: 20211111_L28_R1_IgG

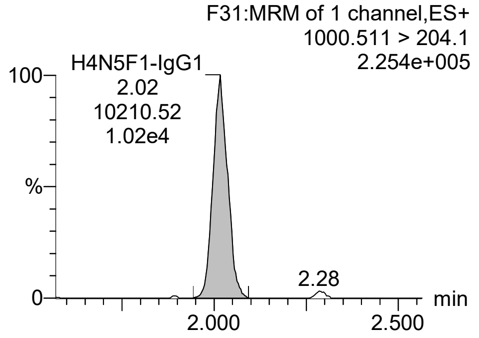

Sample Name: 20211111_H14_R1_IgG

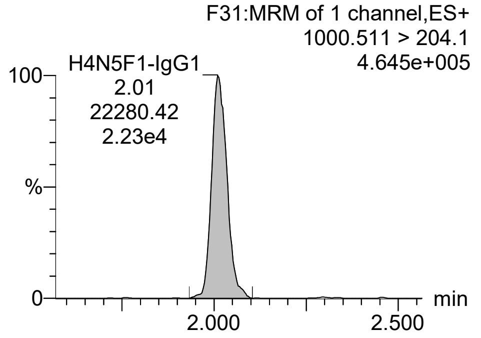

Sample Name: 20211111_A29_R1_IgG

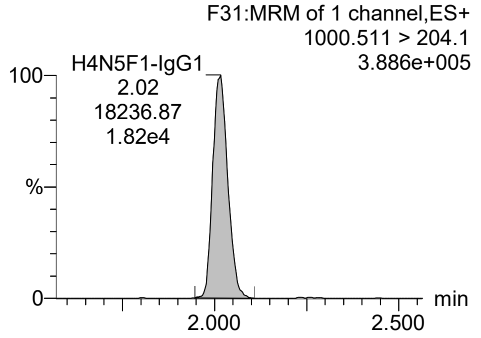

Sample Name: 20211111_A30_R1_IgG

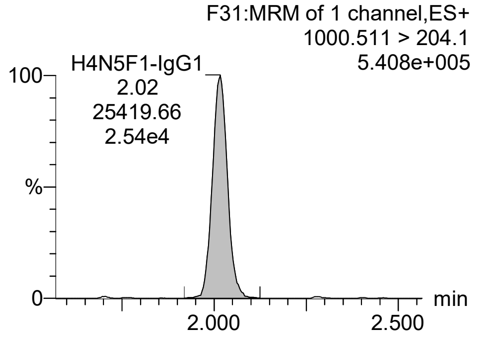

Sample Name: 20211111_QC_R7_IgG

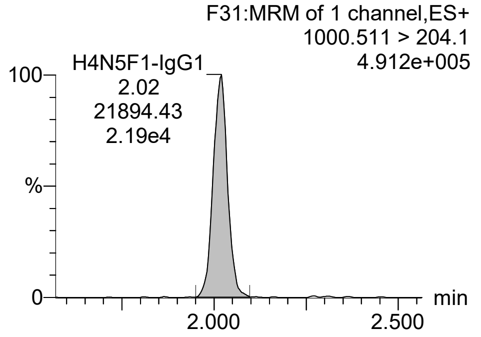

Sample Name: 20211111_L29_R1_IgG

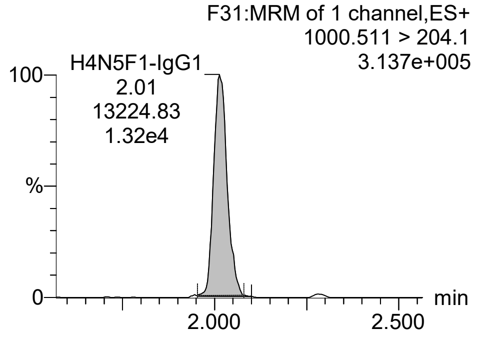

Sample Name: 20211111_L30_R1_IgG

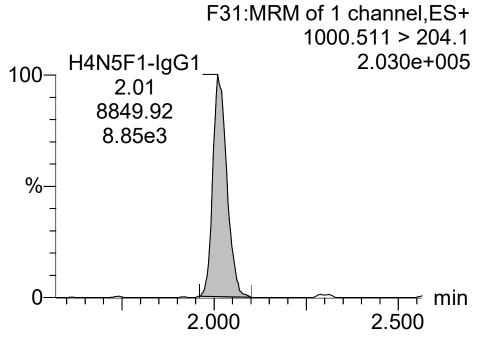

Sample Name: 20211111_H15_R1_IgG

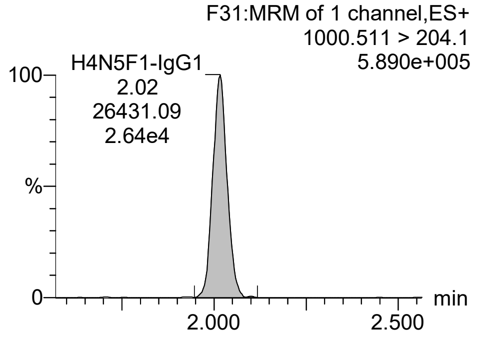

Sample Name: 20211111_A31_R1_IgG

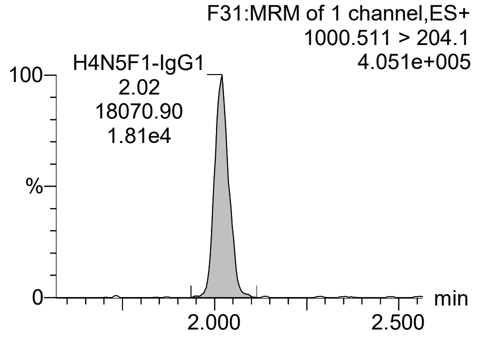

Sample Name: 20211111_A32_R1_IgG

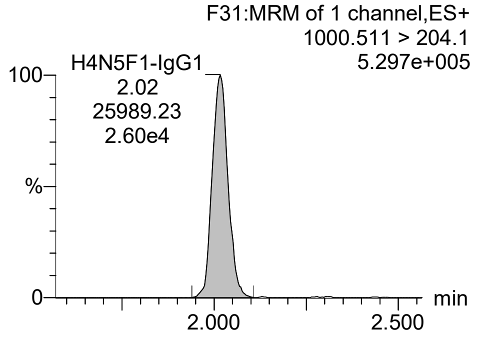

Sample Name: 20211111_L31_R1_IgG

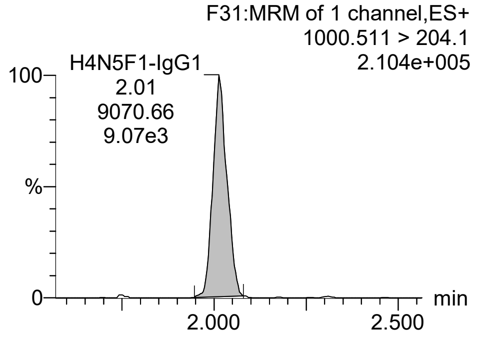

Sample Name: 20211111_L32_R1_IgG

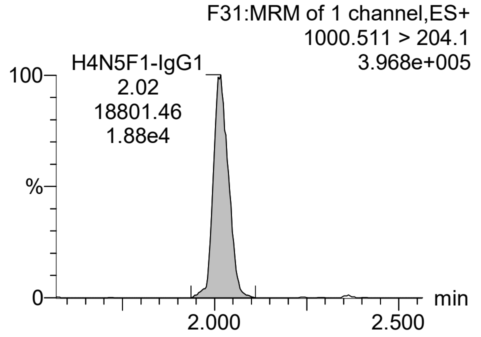

Sample Name: 20211111_H16_R1_IgG

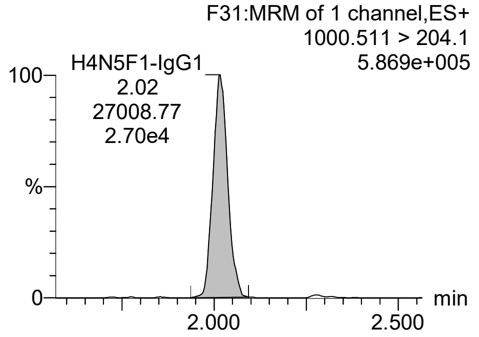

Sample Name: 20211111_A33_R1_IgG

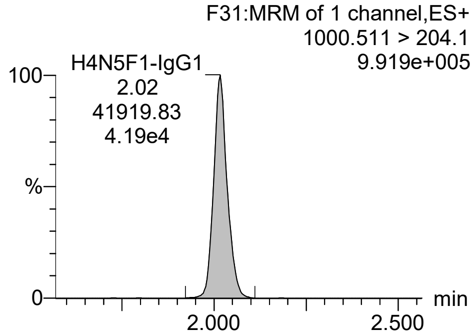

Sample Name: 20211111_A34_R1_IgG

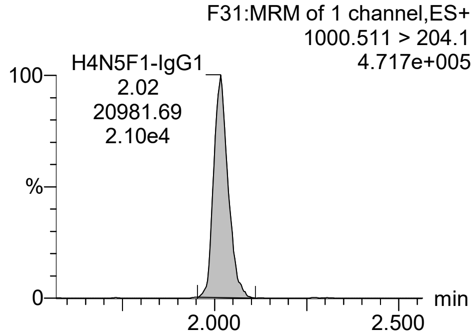

Sample Name: 20211111_H17_R1_IgG

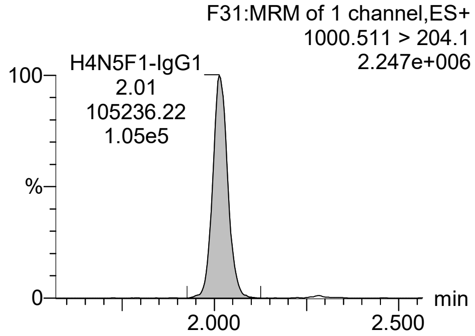

Sample Name: 20211111_A35_R1_IgG

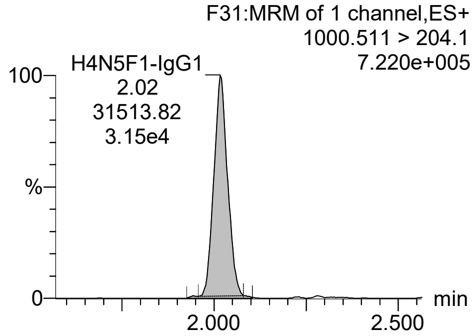

Sample Name: 20211111_QC_R8_IgG

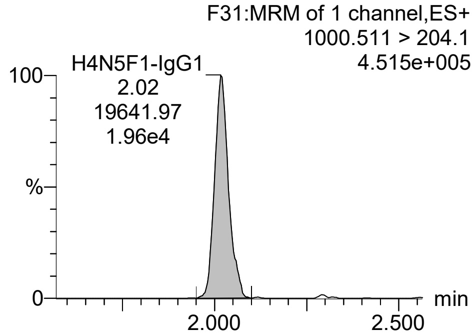

Sample Name: 20211111_A36_R1_IgG

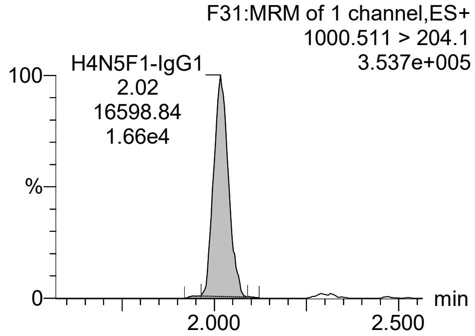

Sample Name: 20211111_H18_R1_IgG

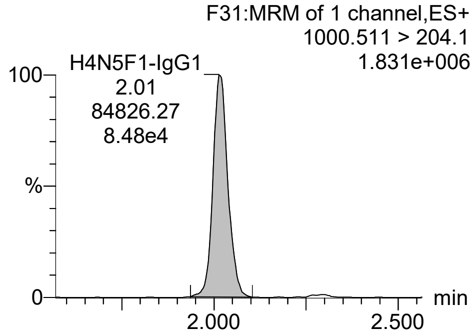

Sample Name: 20211111_A37_R1_IgG

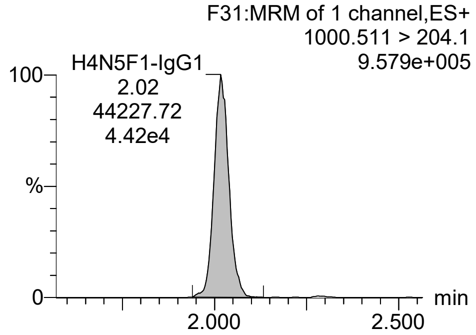

Sample Name: 20211111_A38_R1_IgG

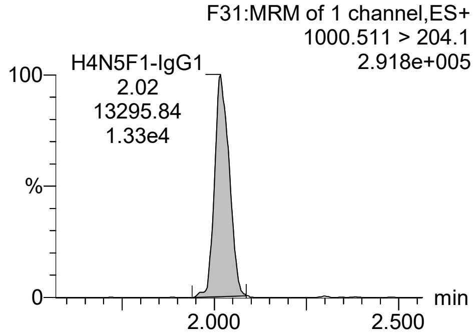

Sample Name: 20211111_H19_R1_IgG

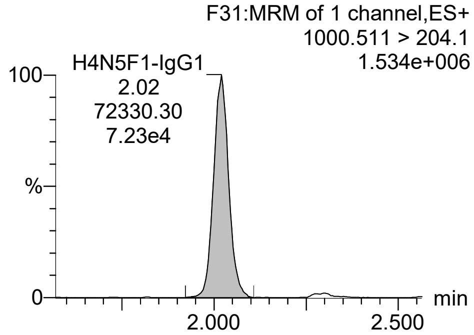

Sample Name: 20211111_A39_R1_IgG

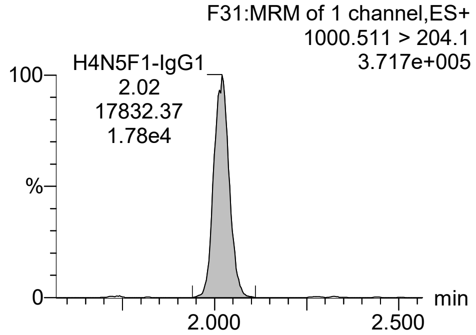

Sample Name: 20211111_A40_R1_IgG

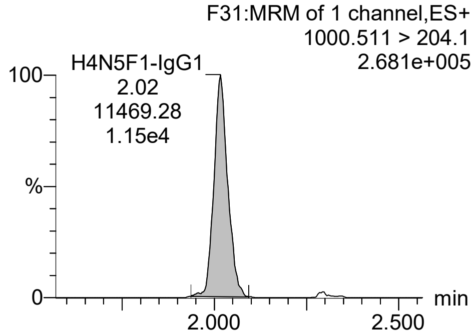

Sample Name: 20211111_H20_R1_IgG

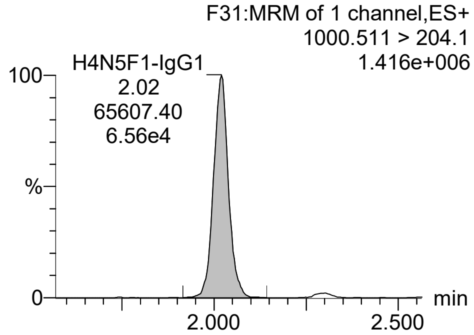

Sample Name: 20211111_A41_R1_IgG

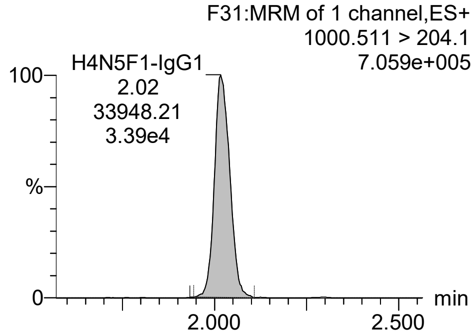

Sample Name: 20211111_A42_R1_IgG

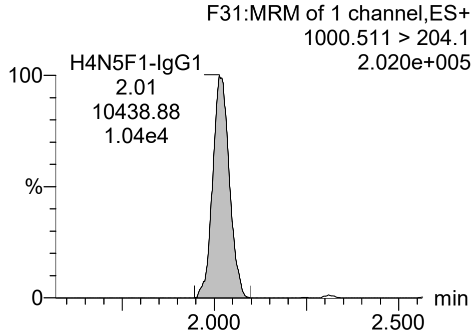

Dataset: C:\MassLynx\10.pro\20211115_clinicalsamples_IgG.qld

Last Altered: Sunday, October 02, 2022 16:02:30 台北標準時間
Printed: Friday, August 01, 2025 16:45:54 台北標準時間

Sample Name: 20211111_A43_R1_IgG

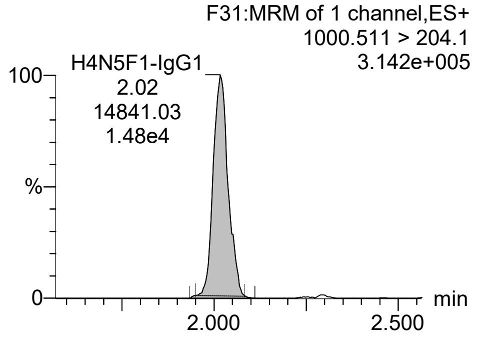

Sample Name: 20211111_A44_R1_IgG

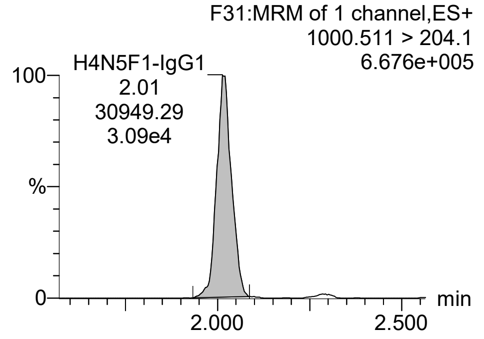

Sample Name: 20211111_QC_R9_IgG

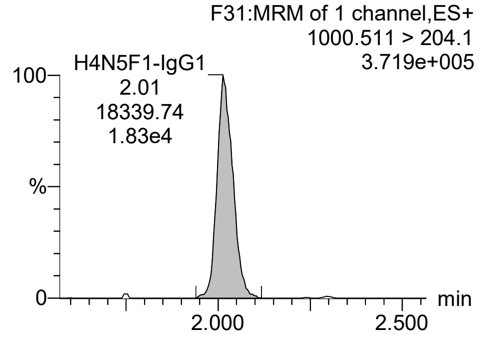

Sample Name: 20211111_A1_R2_IgG

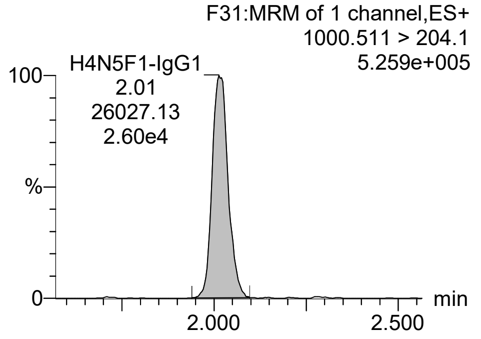

Sample Name: 20211111_A2_R2_IgG

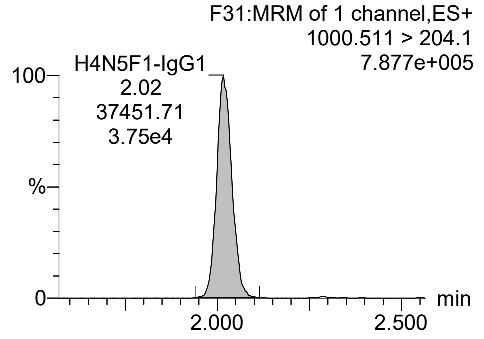

Sample Name: 20211111_L1_R2_IgG

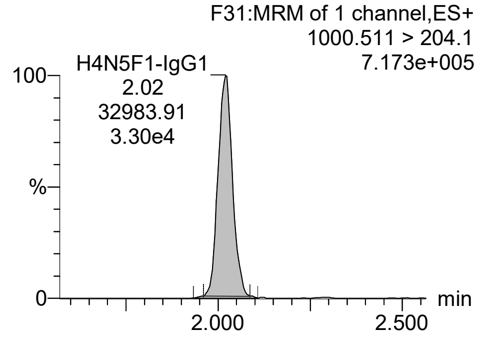

Sample Name: 20211111_L2_R2_IgG

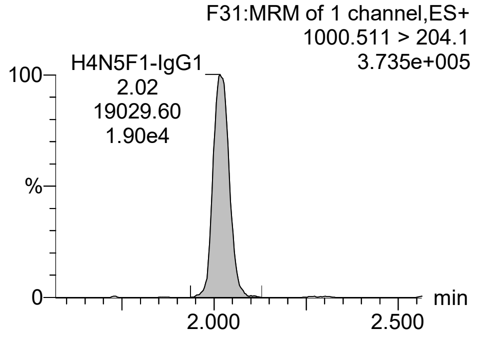

Sample Name: 20211111_H1_R2_IgG

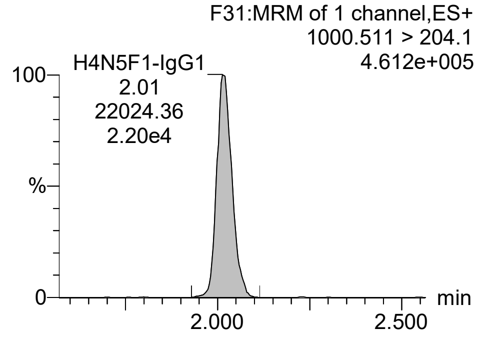

Sample Name: 20211111_A3_R2_IgG

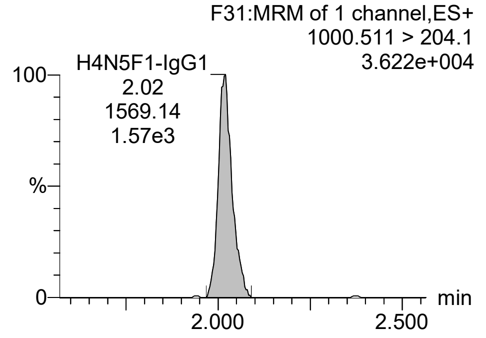

Sample Name: 20211111_A4_R2_IgG

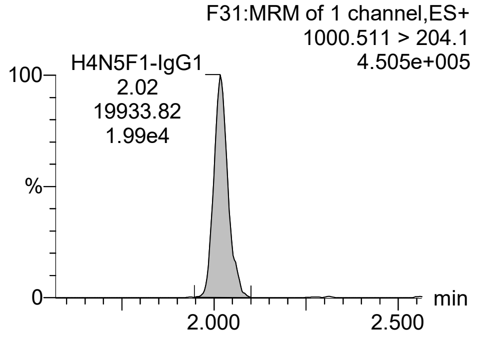

Sample Name: 20211111_L3_R2_IgG

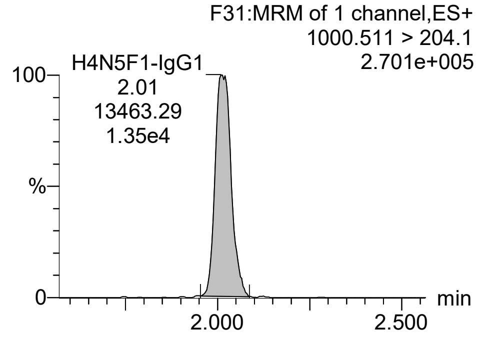

Sample Name: 20211111_L4_R2_IgG

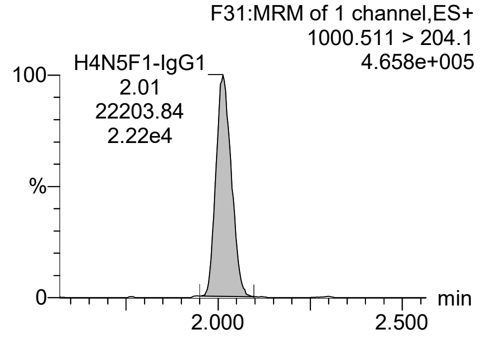

Sample Name: 20211111_H2_R2_IgG

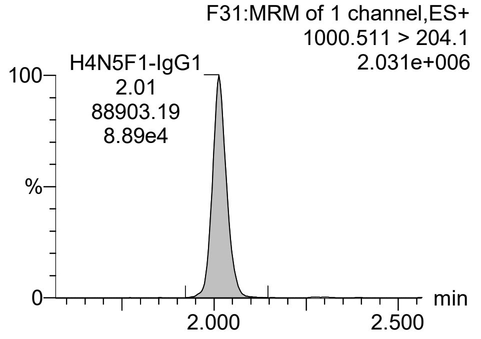

Sample Name: 20211111_A5_R2_IgG

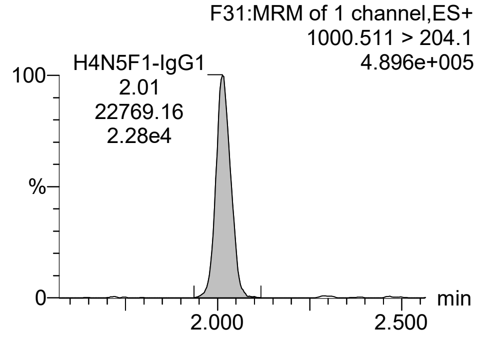

Sample Name: 20211111_A6_R2_IgG

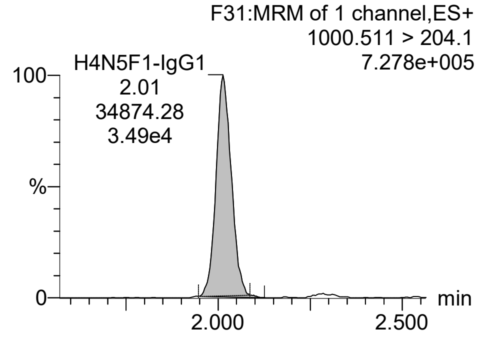

Sample Name: 20211111_QC_R10_IgG

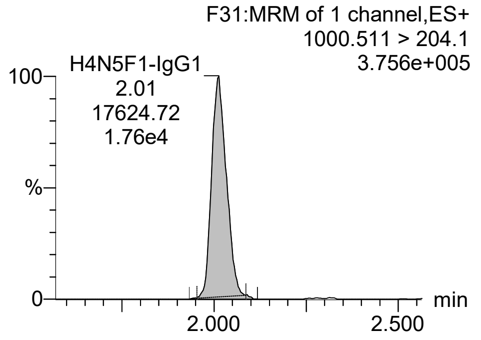

Sample Name: 20211111_L5_R2_IgG

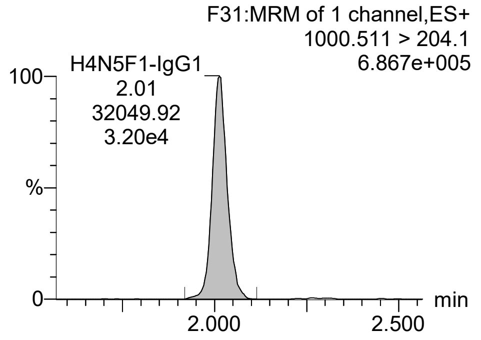

Sample Name: 20211111_L6_R2_IgG

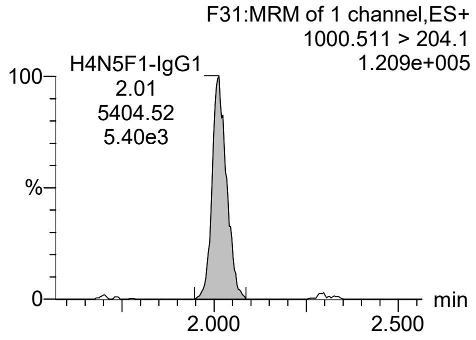

Sample Name: 20211111_H3_R2_IgG

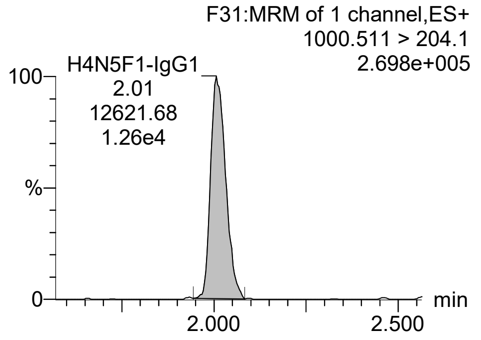

Sample Name: 20211111_A7_R2_IgG

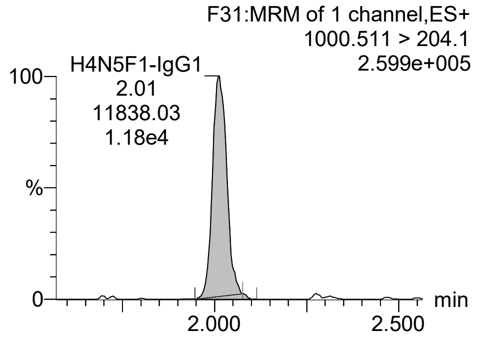

Sample Name: 20211111_A8_R2_IgG

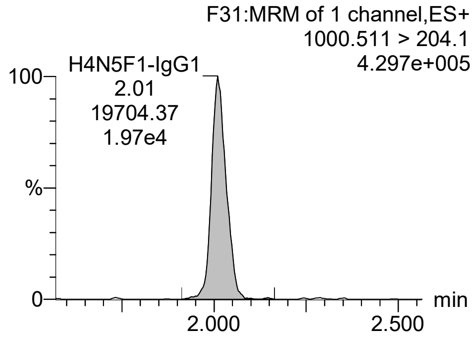

Sample Name: 20211111_L7_R2_IgG

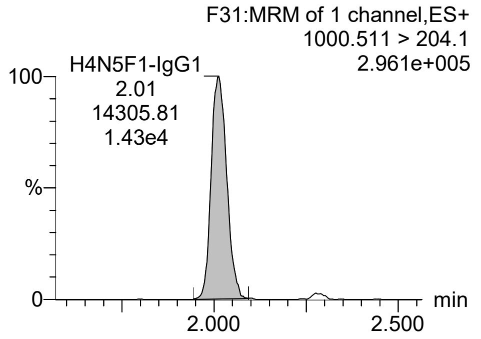

Sample Name: 20211111_L8_R2_IgG

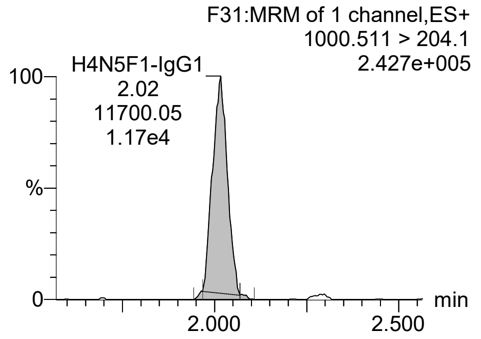

Sample Name: 20211111_H4_R2_IgG

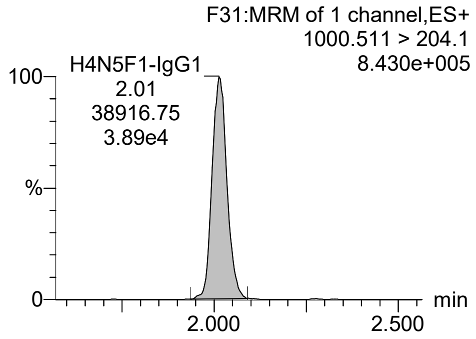

Sample Name: 20211111_A9_R2_IgG

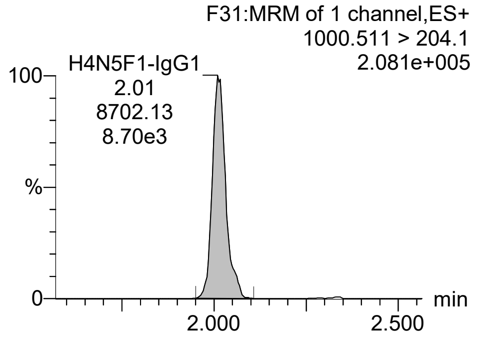

Sample Name: 20211111_A10_R2_IgG

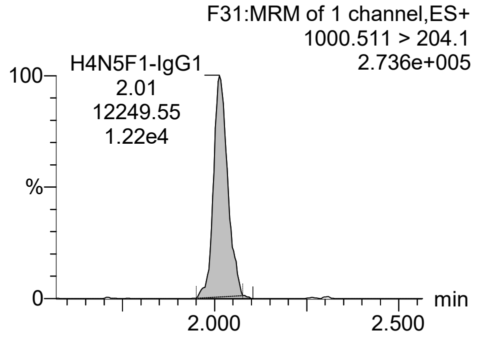

Sample Name: 20211111_L9_R2_IgG

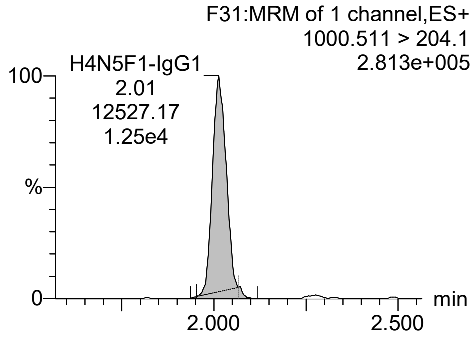

Sample Name: 20211111_L10_R2_IgG

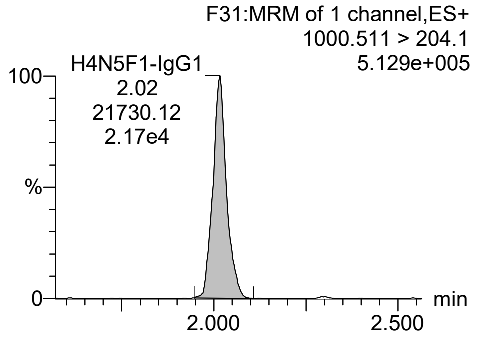

Sample Name: 20211111_QC_R11_IgG

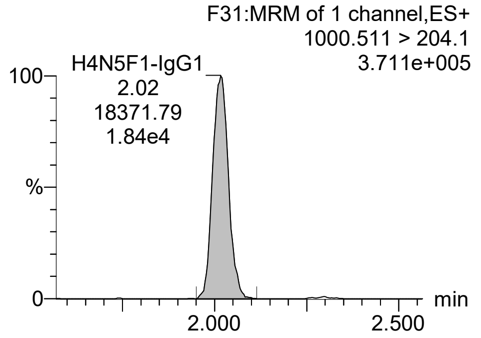

Sample Name: 20211111_H5_R2_IgG

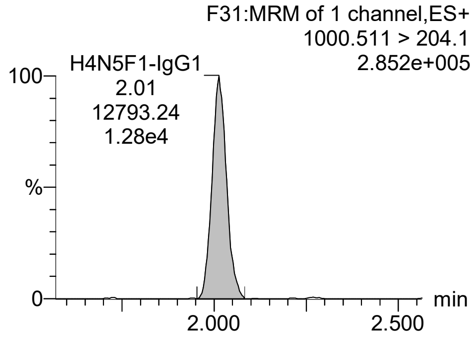

Dataset: C:\MassLynx\10.pro\20211115_clinicalsamples_IgG.qld

Last Altered: Sunday, October 02, 2022 16:02:30 台北標準時間
Printed: Friday, August 01, 2025 16:45:54 台北標準時間

Sample Name: 20211111_A11_R2_IgG

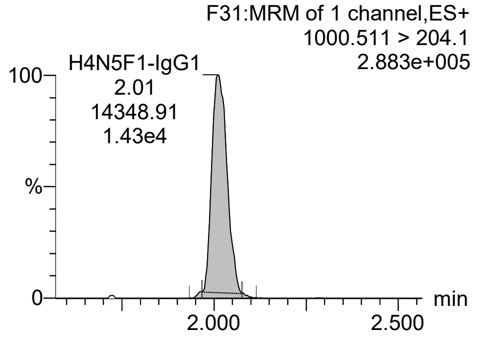

Sample Name: 20211111_A12_R2_IgG

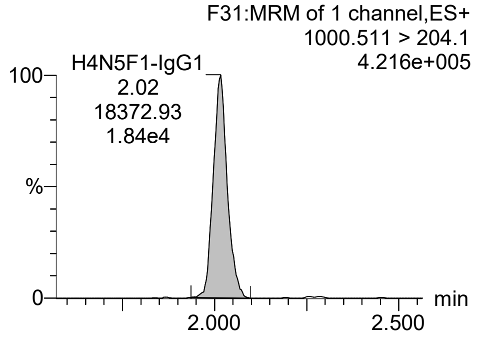

Sample Name: 20211111_L11_R2_IgG

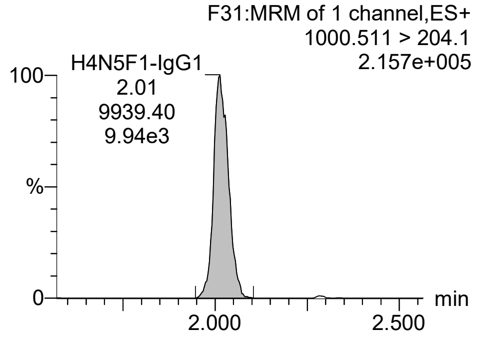

Sample Name: 20211111_L12_R2_IgG

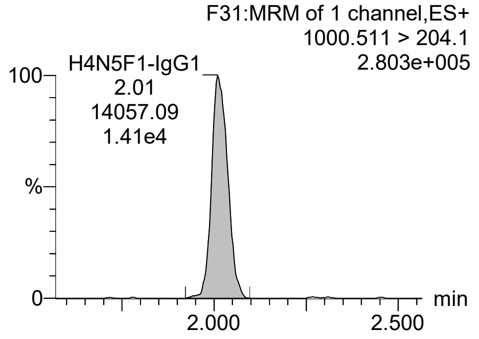

Sample Name: 20211111_H6_R2_IgG

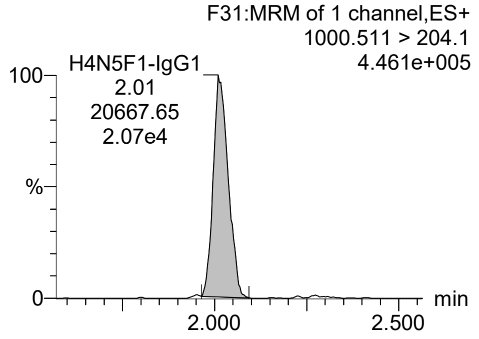

Sample Name: 20211111_A13_R2_IgG

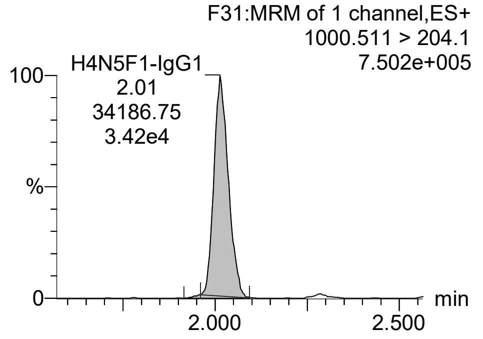

Sample Name: 20211111_A14_R2_IgG

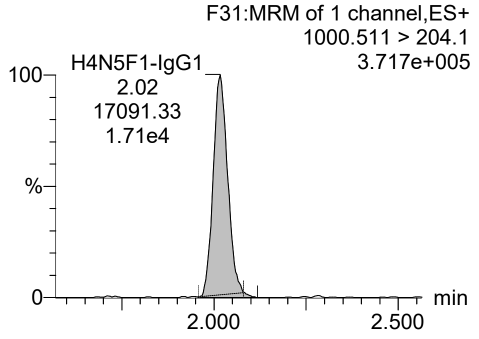

Sample Name: 20211111_L13_R2_IgG

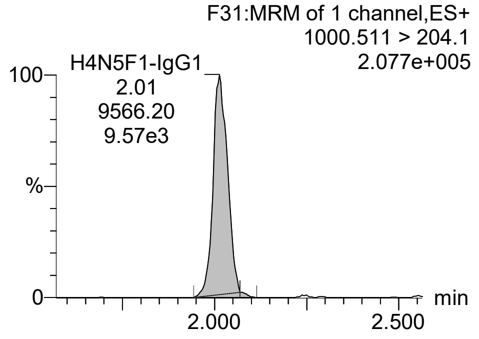

Sample Name: 20211111_L14_R2_IgG

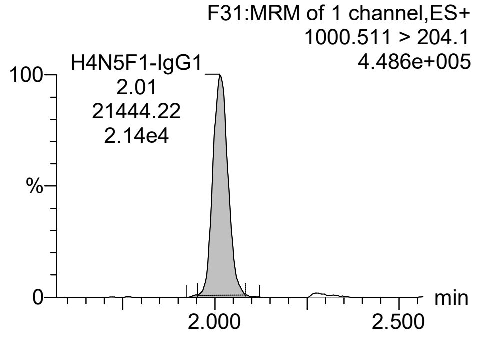

Sample Name: 20211111_H7_R2_IgG

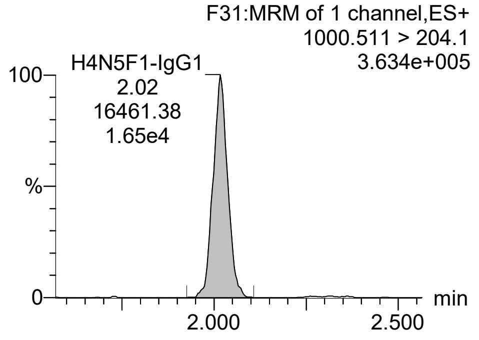

Sample Name: 20211111_A15_R2_IgG

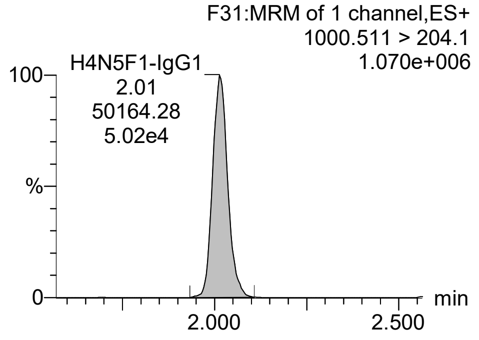

Sample Name: 20211111_QC_R12_IgG

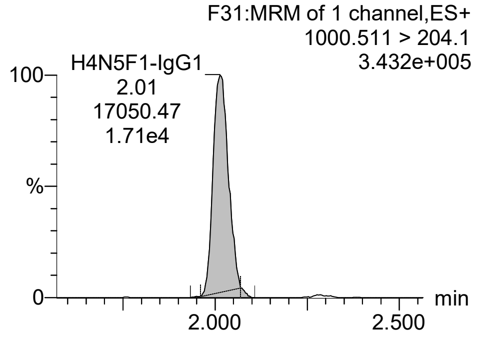

Sample Name: 20211111_A16_R2_IgG

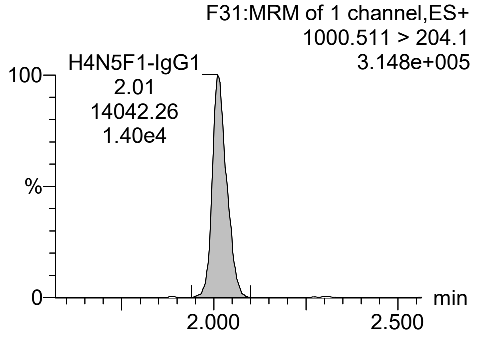

Sample Name: 20211111_L15_R2_IgG

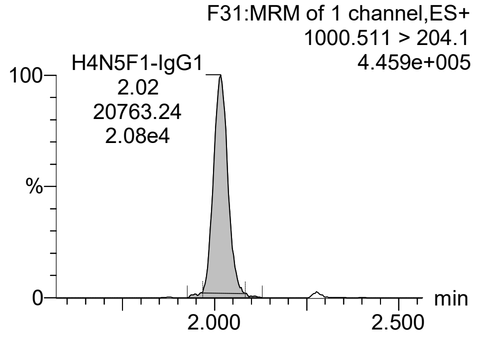

Sample Name: 20211111_L16_R2_IgG

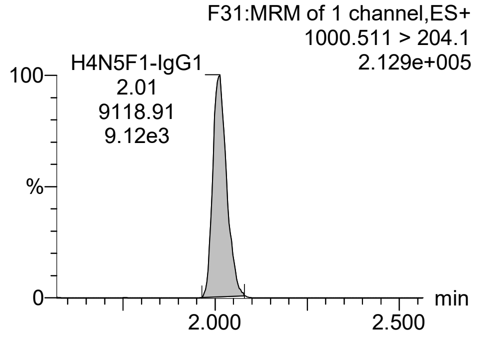

Sample Name: 20211111_H8_R2_IgG

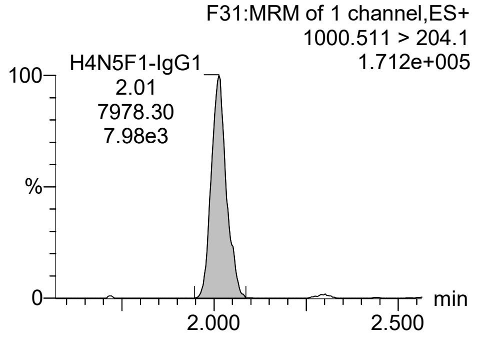

Sample Name: 20211111_A17_R2_IgG

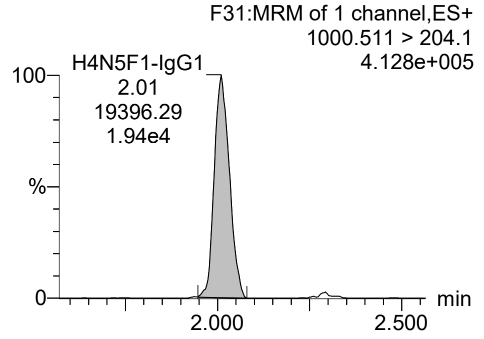

Sample Name: 20211111_A18_R2_IgG

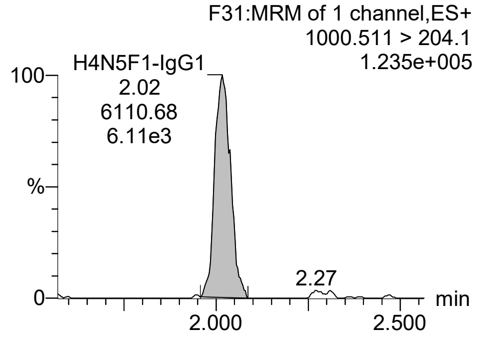

Sample Name: 20211111_L17_R2_IgG

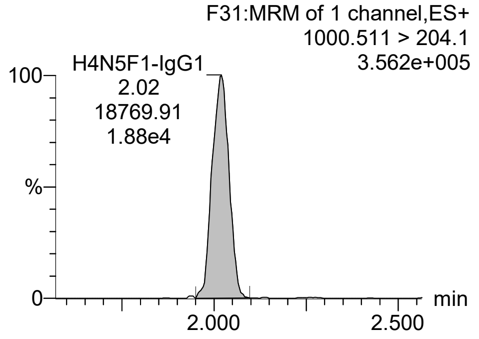

Sample Name: 20211111_L18_R2_IgG

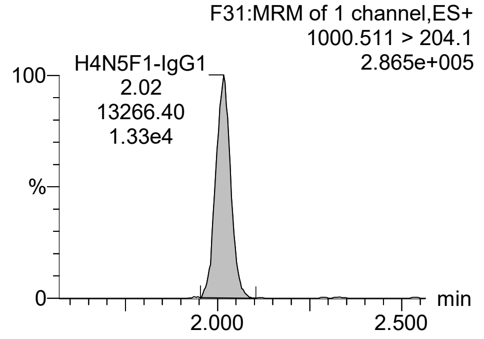

Sample Name: 20211111_H9_R2_IgG

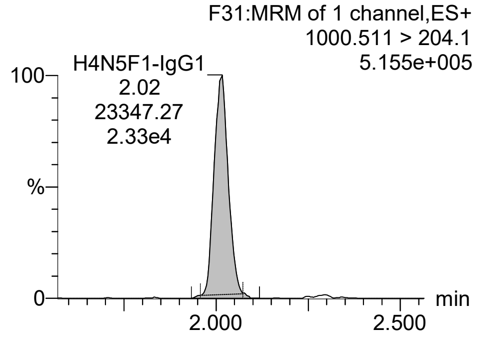

Sample Name: 20211111_A19_R2_IgG

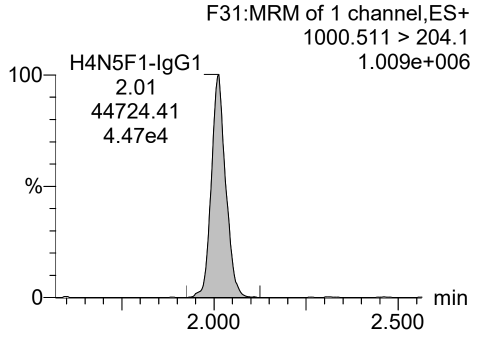

Sample Name: 20211111_A20_R2_IgG

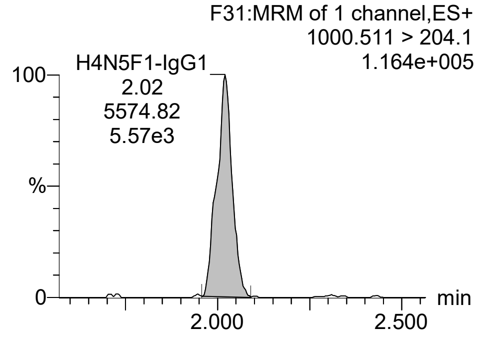

Sample Name: 20211111_L19_R2_IgG

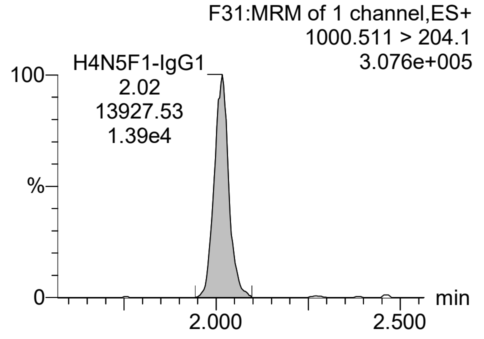

Sample Name: 20211111_QC_R13_IgG

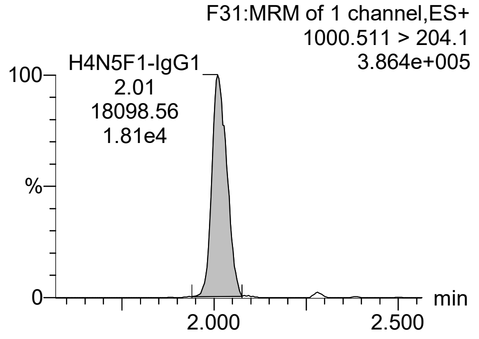

Sample Name: 20211111_L20_R2_IgG

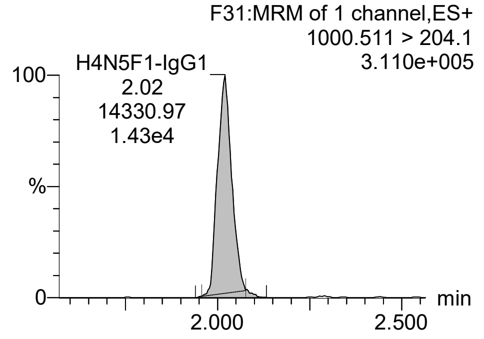

Sample Name: 20211111_H10_R2_IgG

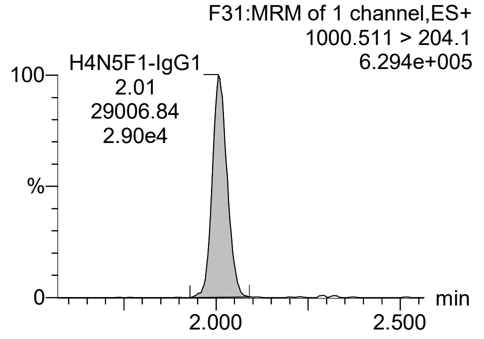

Sample Name: 20211111_A21_R2_IgG

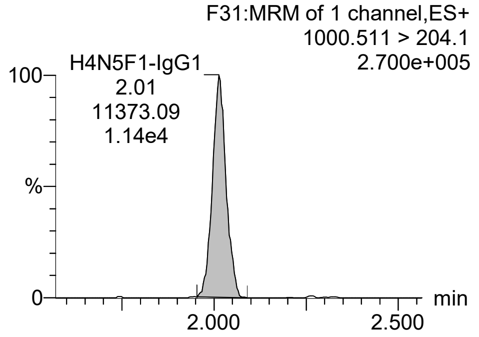

Sample Name: 20211111_A22_R2_IgG

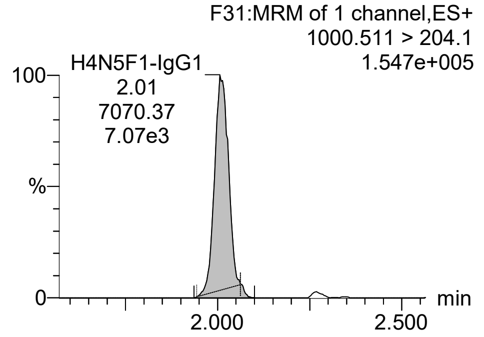

Sample Name: 20211111_L21_R2_IgG

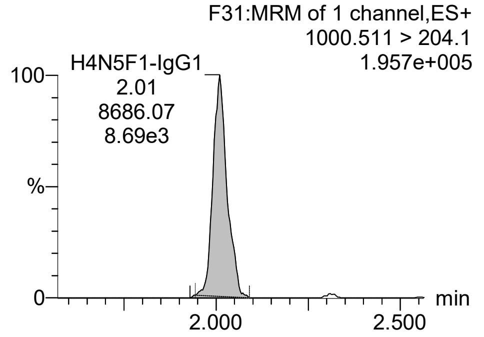

Sample Name: 20211111_L22_R2_IgG

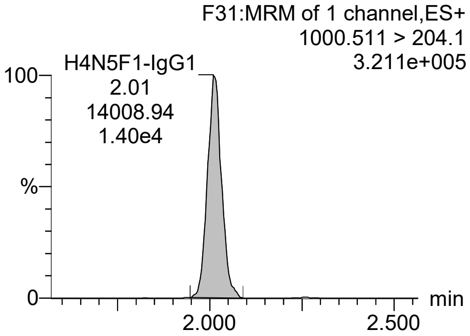

Sample Name: 20211111_H11_R2_IgG

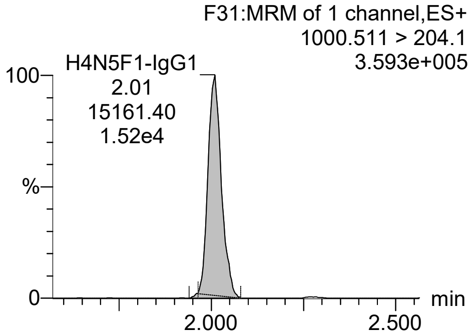

Sample Name: 20211111_A23_R2_IgG

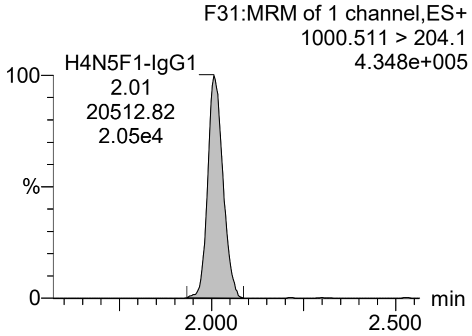

Sample Name: 20211111_A24_R2_IgG

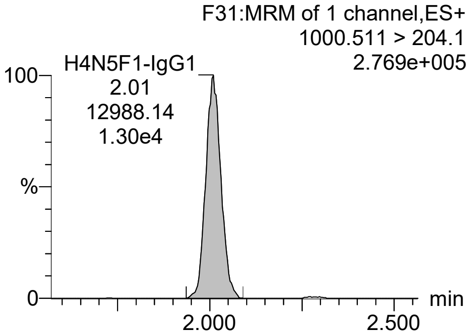

Sample Name: 20211111_L23_R2_IgG

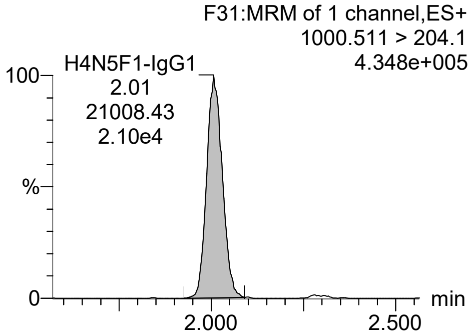

Sample Name: 20211111_L24_R2_IgG

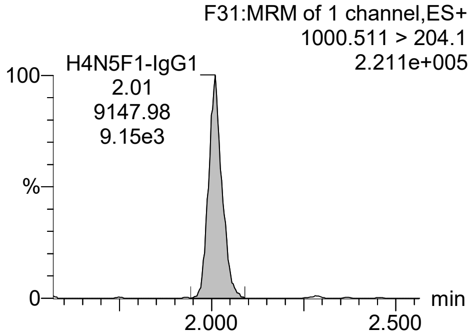

Sample Name: 20211111_H12_R2_IgG

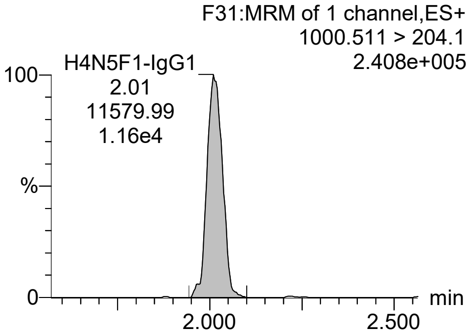

Sample Name: 20211111_QC_R14_IgG

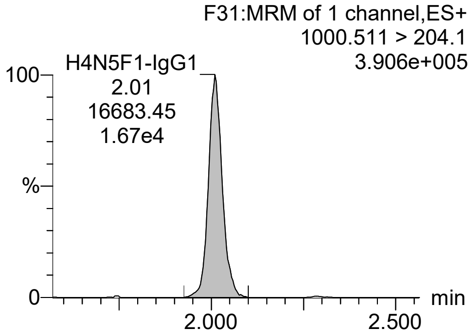

Sample Name: 20211111_A25_R2_IgG

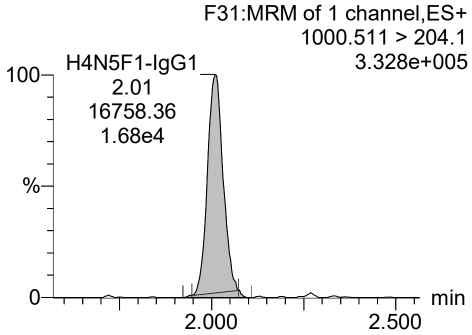

Sample Name: 20211111_A26_R2_IgG

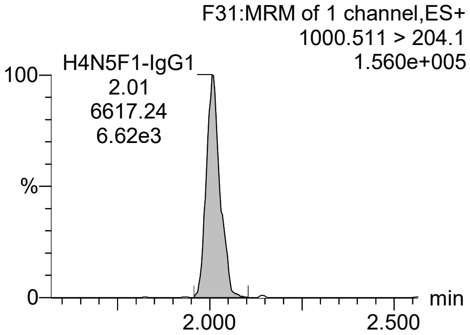

Sample Name: 20211111_L25_R2_IgG

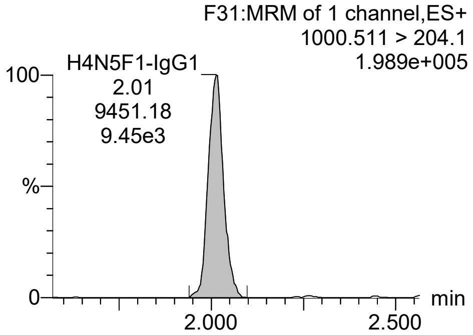

Sample Name: 20211111_L26_R2_IgG

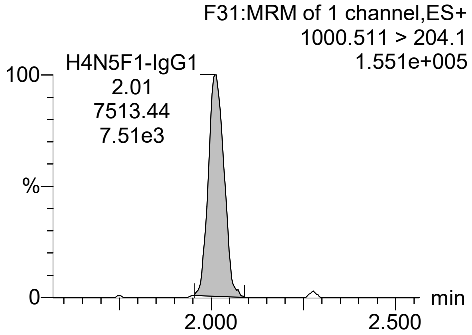

Sample Name: 20211111_H13_R2_IgG

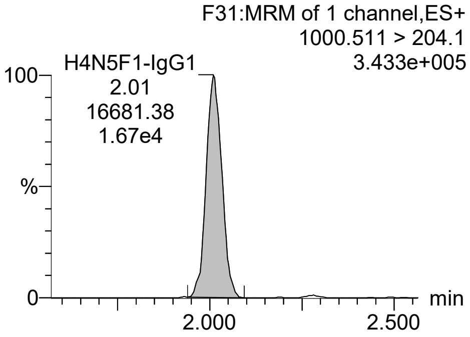

Sample Name: 20211111_A27_R2_IgG

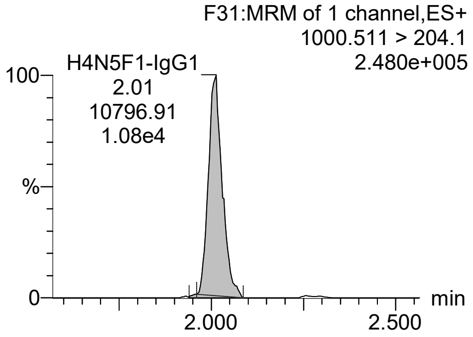

Sample Name: 20211111_A28_R2_IgG

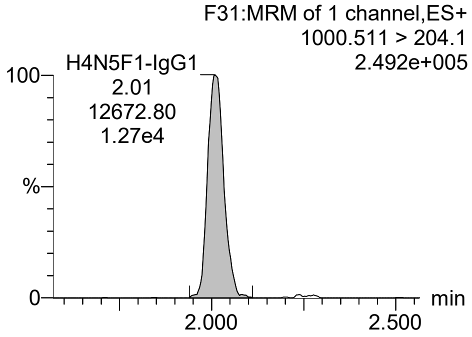

Sample Name: 20211111_L27_R2_IgG

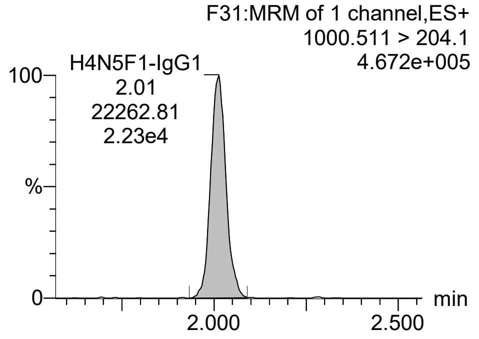

Sample Name: 20211111_L28_R2_IgG

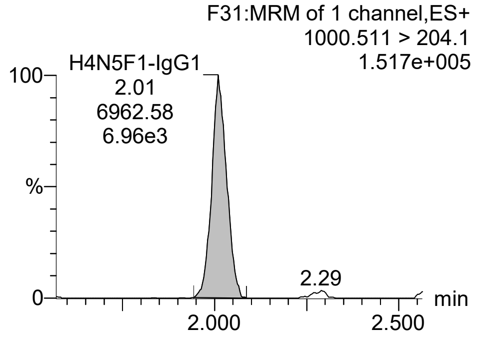

Sample Name: 20211111_H14_R2_IgG

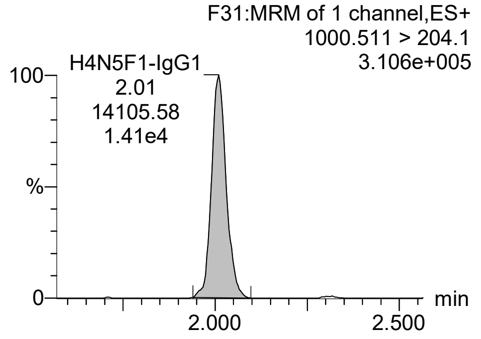

Sample Name: 20211111_A29_R2_IgG

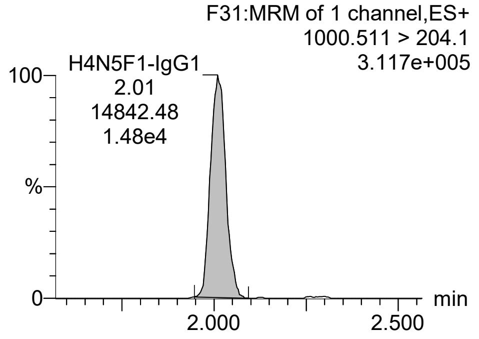

Sample Name: 20211111_A30_R2_IgG

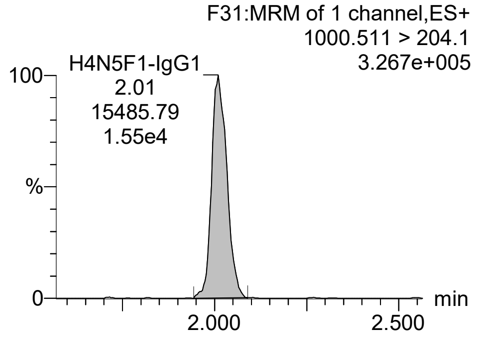

Sample Name: 20211111_QC_R15_IgG

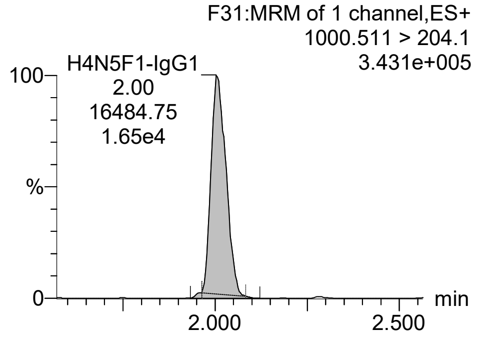

Sample Name: 20211111_L29_R2_IgG

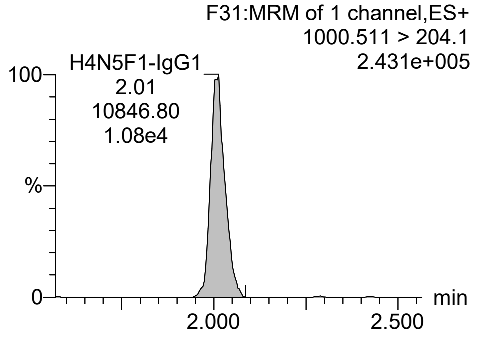

Sample Name: 20211111_L30_R2_IgG

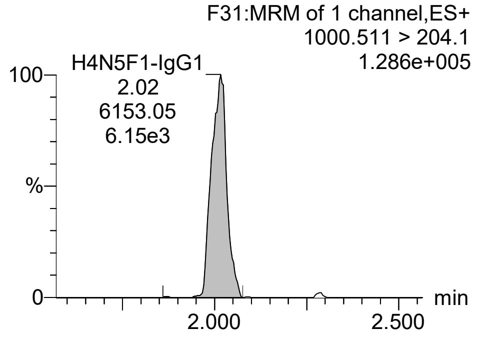

Sample Name: 20211111_H15_R2_IgG

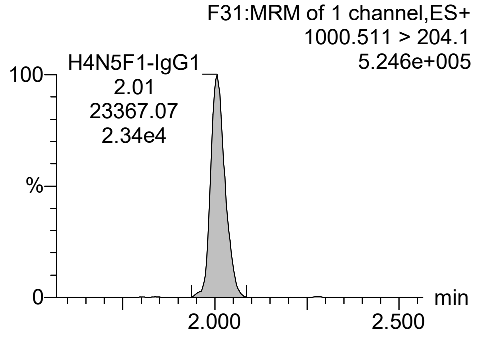

Sample Name: 20211111_A31_R2_IgG

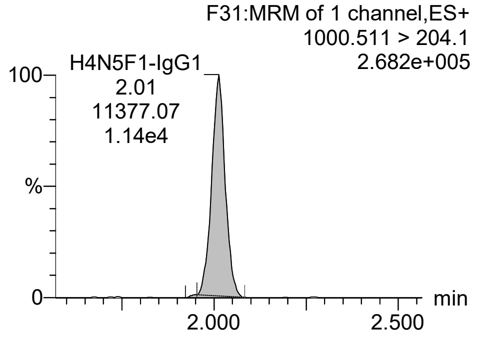

Sample Name: 20211111_A32_R2_IgG

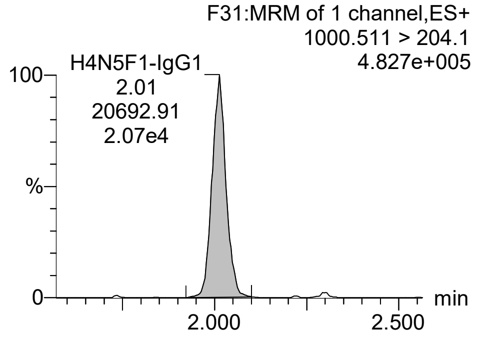

Sample Name: 20211111_L31_R2_IgG

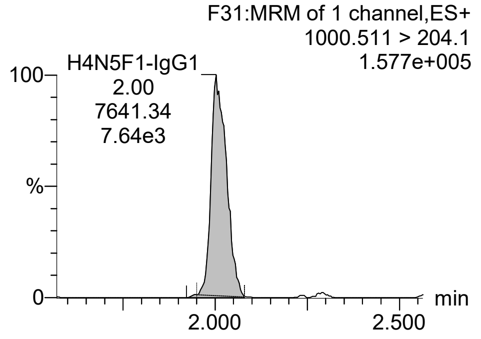

Sample Name: 20211111_L32_R2_IgG

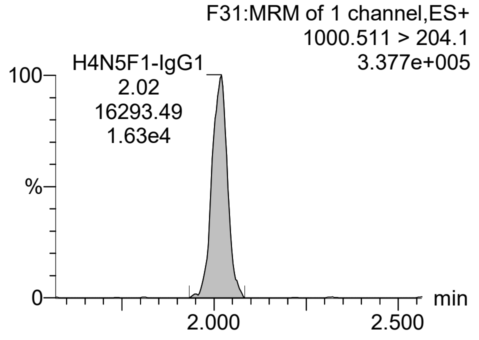

Sample Name: 20211111_H16_R2_IgG

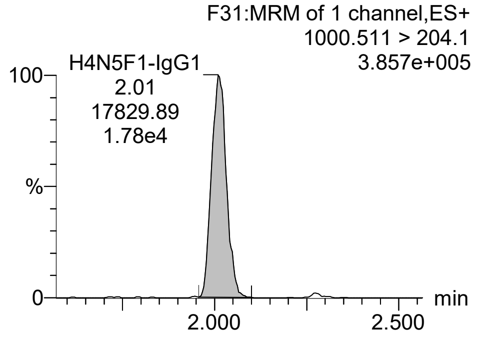

Sample Name: 20211111_A33_R2_IgG

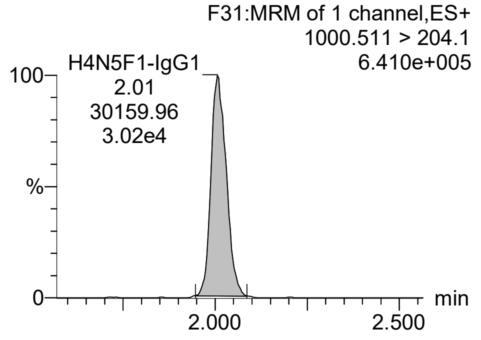

Sample Name: 20211111_A34_R2_IgG

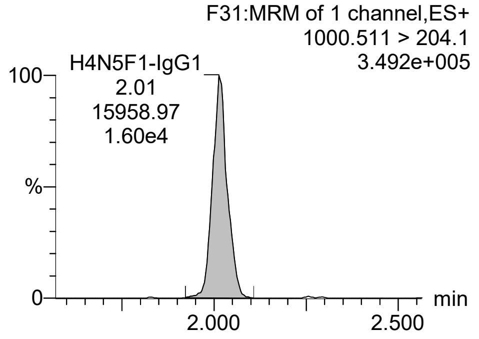

Sample Name: 20211111_H17_R2_IgG

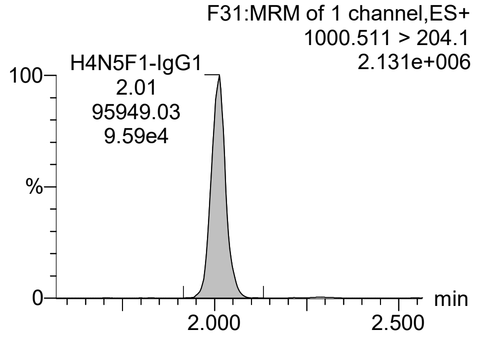

Sample Name: 20211111_A35_R2_IgG

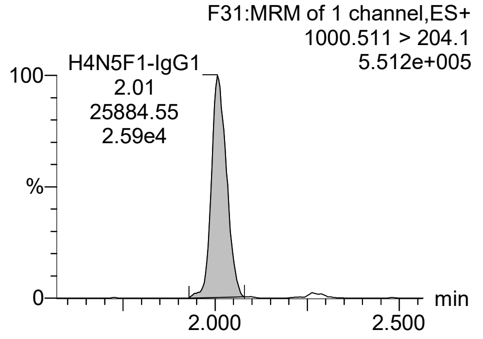

Sample Name: 20211111_QC_R16_IgG

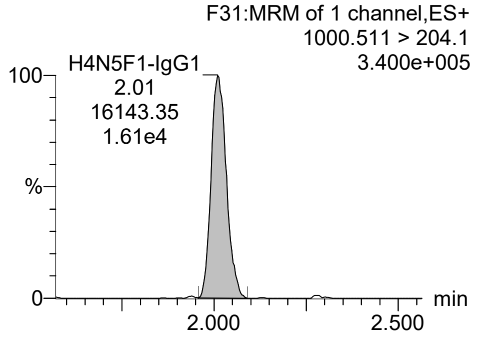

Sample Name: 20211111_A36_R2_IgG

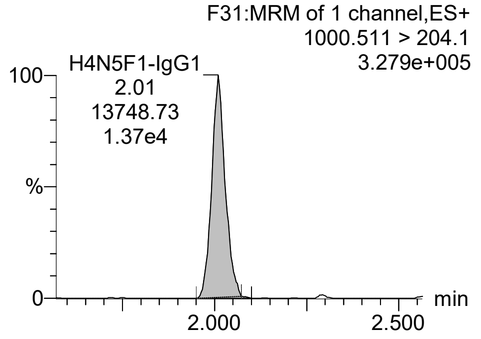

Sample Name: 20211111_H18_R2_IgG

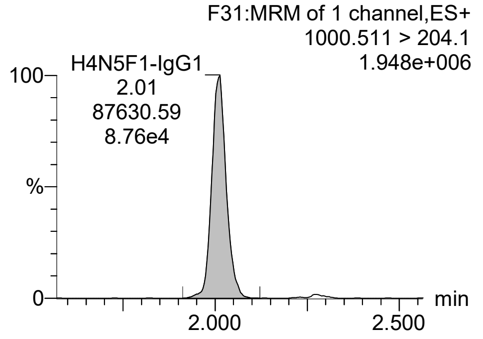

Sample Name: 20211111_A37_R2_IgG

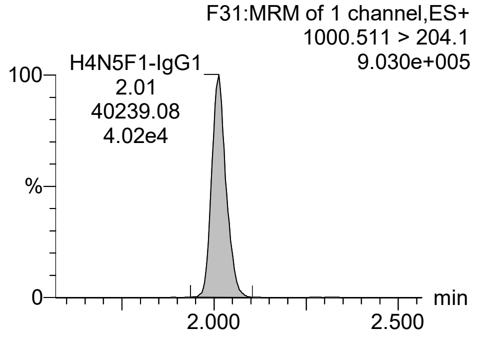

Sample Name: 20211111_A38_R2_IgG

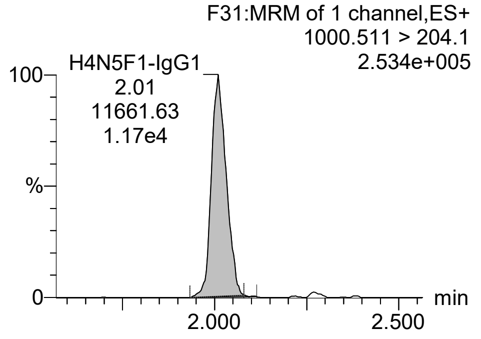

Sample Name: 20211111_H19_R2_IgG

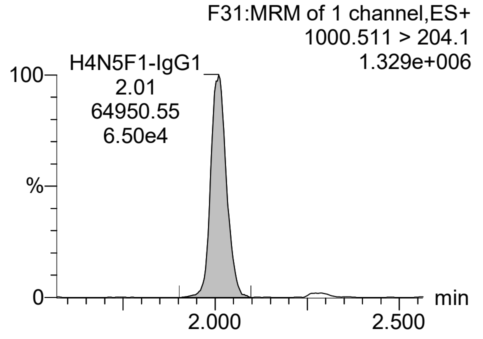

Sample Name: 20211111_A39_R2_IgG

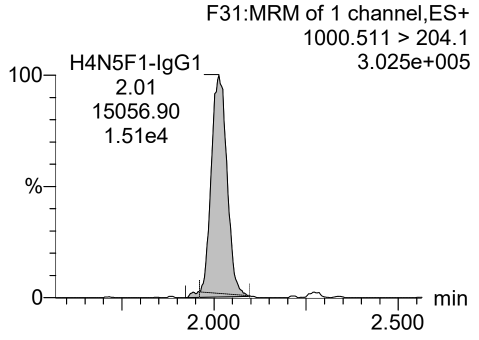

Sample Name: 20211111_A40_R2_IgG

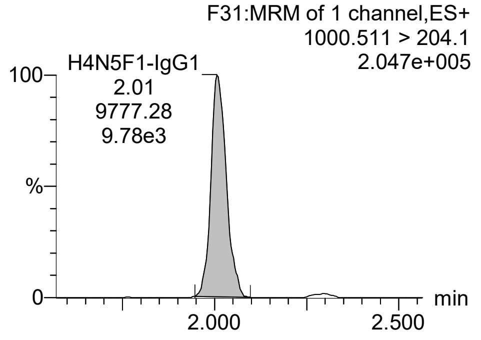

Sample Name: 20211111_H20_R2_IgG

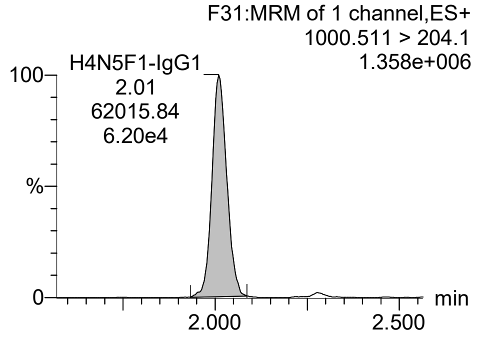

Sample Name: 20211111_A41_R2_IgG

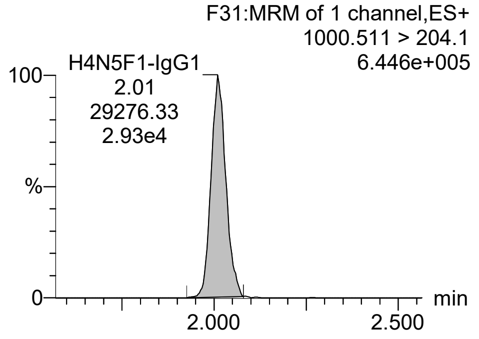

Sample Name: 20211111_A42_R2_IgG

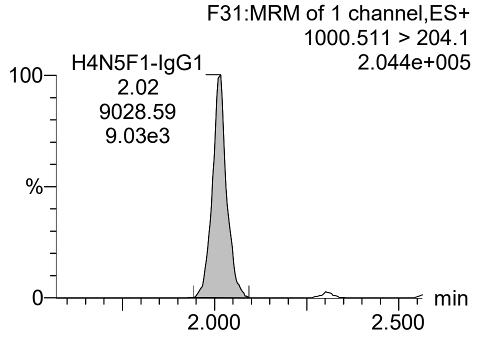

Sample Name: 20211111_A43_R2_IgG

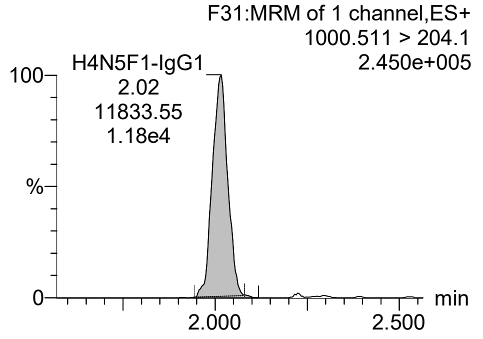

Sample Name: 20211111_A44_R2_IgG

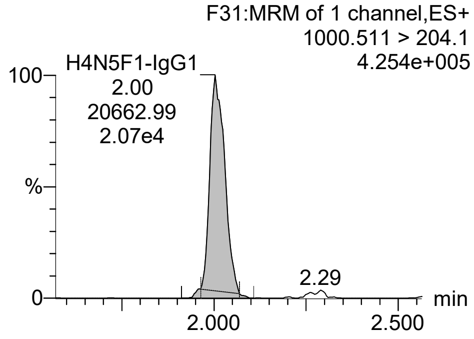

Sample Name: 20211111_QC_R17_IgG

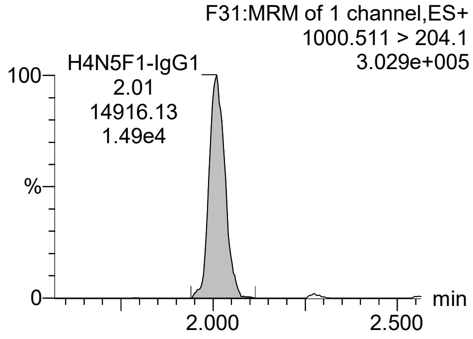

Dataset: C:\MassLynx\10.pro\20211115_clinicalsamples_IgG.qld

Last Altered: Sunday, October 02, 2022 16:02:30 台北標準時間
Printed: Friday, August 01, 2025 16:45:54 台北標準時間

	#	Name	RT	Area	IS Area	Response	Prima...
1	1	20211111_QC_R1_IgG	2.01	37536.3	64968.0	0.578	bb
2	2	20211111_A1_R1_IgG	2.02	40126.7	59059.2	0.679	bb
3	3	20211111_A2_R1_IgG	2.02	50338.9	66621.2	0.756	bb
4	4	20211111_L1_R1_IgG	2.02	52811.2	64201.0	0.823	bb
5	5	20211111_L2_R1_IgG	2.02	23066.3	46950.2	0.491	bb
6	6	20211111_H1_R1_IgG	2.02	18735.0	38648.2	0.485	bb
7	7	20211111_A3_R1_IgG	2.03	890.6	2603.7	0.342	bb
8	8	20211111_A4_R1_IgG	2.02	30944.7	69960.4	0.442	bb
9	9	20211111_L3_R1_IgG	2.02	15688.4	38864.7	0.404	bb
10	10	20211111_L4_R1_IgG	2.02	26055.0	43380.5	0.601	bb
11	11	20211111_H2_R1_IgG	2.01	136220.4	71349.2	1.909	bb
12	12	20211111_A5_R1_IgG	2.02	25918.5	65569.7	0.395	bb
13	13	20211111_A6_R1_IgG	2.01	44291.3	53120.7	0.834	bb
14	14	20211111_QC_R2_IgG	2.02	44927.7	81034.1	0.554	bb
15	15	20211111_L5_R1_IgG	2.02	42973.3	50994.6	0.843	bb
16	16	20211111_L6_R1_IgG	2.02	6386.7	58058.0	0.110	MM
17	17	20211111_H3_R1_IgG	2.02	15165.5	64028.6	0.237	bb
18	18	20211111_A7_R1_IgG	2.02	17752.8	82996.1	0.214	bb
19	19	20211111_A8_R1_IgG	2.02	26046.4	77731.7	0.335	MM
20	20	20211111_L7_R1_IgG	2.01	14822.3	38827.3	0.382	bb
21	21	20211111_L8_R1_IgG	2.02	12290.4	44220.1	0.278	MM
22	22	20211111_H4_R1_IgG	2.02	46554.2	65826.3	0.707	bb
23	23	20211111_A9_R1_IgG	2.02	9831.6	60771.1	0.162	MM
24	24	20211111_A10_R1_IgG	2.02	12593.0	48578.7	0.259	bb
25	25	20211111_L9_R1_IgG	2.02	15350.4	50830.7	0.302	bb
26	26	20211111_L10_R1_IgG	2.02	23062.4	38034.2	0.606	bb
27	27	20211111_QC_R3_IgG	2.02	33658.2	67025.5	0.502	bb
28	28	20211111_H5_R1_IgG	2.02	13859.4	44544.9	0.311	MM
29	29	20211111_A11_R1_IgG	2.02	17845.5	66408.5	0.269	bb
30	30	20211111_A12_R1_IgG	2.01	20030.5	46624.5	0.430	bb
31	31	20211111_L11_R1_IgG	2.02	11220.1	33425.1	0.336	bb
32	32	20211111_L12_R1_IgG	2.01	16231.1	41178.0	0.394	bb
33	33	20211111_H6_R1_IgG	2.02	29954.3	58593.4	0.511	bb
34	34	20211111_A13_R1_IgG	2.02	43437.0	54133.0	0.802	bb
35	35	20211111_A14_R1_IgG	2.02	21536.0	56467.3	0.381	bb
36	36	20211111_L13_R1_IgG	2.02	10817.0	36024.4	0.300	bb
37	37	20211111_L14_R1_IgG	2.01	20801.0	37181.6	0.559	MM
38	38	20211111_H7_R1_IgG	2.02	16929.9	42850.6	0.395	bb
39	39	20211111_A15_R1_IgG	2.02	62028.5	62015.1	1.000	bb
40	40	20211111_QC_R4_IgG	2.02	27163.3	58572.5	0.464	bb
41	41	20211111_A16_R1_IgG	2.02	21733.8	78283.8	0.278	bb
42	42	20211111_L15_R1_IgG	2.02	26029.7	44158.2	0.589	bb
43	43	20211111_L16_R1_IgG	2.02	14299.8	55150.8	0.259	bb
44	44	20211111_H8_R1_IgG	2.02	9313.0	42965.2	0.217	MM
45	45	20211111_A17_R1_IgG	2.01	20492.5	39717.7	0.516	bb
46	46	20211111_A18_R1_IgG	2.02	7423.7	44675.4	0.166	MM
47	47	20211111_L17_R1_IgG	2.01	19342.7	37854.1	0.511	MM
48	48	20211111_L18_R1_IgG	2.02	16840.7	50716.9	0.332	MM
49	49	20211111_H9_R1_IgG	2.01	25176.5	36276.0	0.694	MM
50	50	20211111_A19_R1_IgG	2.01	47871.9	42862.5	1.117	MM
51	51	20211111_A20_R1_IgG	2.02	7158.0	36542.7	0.196	bb
52	52	20211111_L19_R1_IgG	2.01	18708.2	53892.8	0.347	bb
53	53	20211111_QC_R5_IgG	2.01	29255.3	56513.2	0.518	bb
54	54	20211111_L20_R1_IgG	2.02	27380.7	56499.9	0.485	MM
55	55	20211111_H10_R1_IgG	2.02	70505.3	68120.5	1.035	bb
56	56	20211111_A21_R1_IgG	2.01	25199.9	63699.8	0.396	bb

Dataset: C:\MassLynx\10.pro\20211115_clinicalsamples_IgG.qld

Last Altered: Sunday, October 02, 2022 16:02:30 台北標準時間
Printed: Friday, August 01, 2025 16:45:54 台北標準時間

	#	Name	RT	Area	IS Area	Response	Prima...
57	57	20211111_A22_R1_IgG	2.01	15030.4	52222.1	0.288	MM
58	58	20211111_L21_R1_IgG	2.01	16649.5	54372.5	0.306	MM
59	59	20211111_L22_R1_IgG	2.01	22887.1	41353.1	0.553	bb
60	60	20211111_H11_R1_IgG	2.01	34458.0	65579.1	0.525	bb
61	61	20211111_A23_R1_IgG	2.01	44135.2	64131.1	0.688	bb
62	62	20211111_A24_R1_IgG	2.01	18850.5	38724.8	0.487	bb
63	63	20211111_L23_R1_IgG	2.02	35900.0	64245.2	0.559	bb
64	64	20211111_L24_R1_IgG	2.01	14473.5	45298.0	0.320	MM
65	65	20211111_H12_R1_IgG	2.02	15211.2	44679.3	0.340	MM
66	66	20211111_QC_R6_IgG	2.02	25355.3	48165.0	0.526	bb
67	67	20211111_A25_R1_IgG	2.01	31172.0	57169.9	0.545	bb
68	68	20211111_A26_R1_IgG	2.01	8807.2	34254.0	0.257	bb
69	69	20211111_L25_R1_IgG	2.01	17444.6	67491.5	0.258	bb
70	70	20211111_L26_R1_IgG	2.02	13539.5	55934.1	0.242	bb
71	71	20211111_H13_R1_IgG	2.01	27268.4	48767.3	0.559	bb
72	72	20211111_A27_R1_IgG	2.01	16807.0	57269.7	0.293	bb
73	73	20211111_A28_R1_IgG	2.02	20426.5	49231.1	0.415	bb
74	74	20211111_L27_R1_IgG	2.02	34851.5	60014.5	0.581	bb
75	75	20211111_L28_R1_IgG	2.02	10210.5	35062.6	0.291	bb
76	76	20211111_H14_R1_IgG	2.01	22280.4	62371.9	0.357	bb
77	77	20211111_A29_R1_IgG	2.02	18236.9	43858.2	0.416	bb
78	78	20211111_A30_R1_IgG	2.02	25419.7	62500.4	0.407	bb
79	79	20211111_QC_R7_IgG	2.02	21894.4	43946.9	0.498	bb
80	80	20211111_L29_R1_IgG	2.01	13224.8	38728.9	0.341	MM
81	81	20211111_L30_R1_IgG	2.01	8849.9	42613.2	0.208	bb
82	82	20211111_H15_R1_IgG	2.02	26431.1	39874.6	0.663	bb
83	83	20211111_A31_R1_IgG	2.02	18070.9	53414.3	0.338	bb
84	84	20211111_A32_R1_IgG	2.02	25989.2	41844.6	0.621	bb
85	85	20211111_L31_R1_IgG	2.01	9070.7	40553.7	0.224	bb
86	86	20211111_L32_R1_IgG	2.02	18801.5	49618.7	0.379	bb
87	87	20211111_H16_R1_IgG	2.02	27008.8	60170.9	0.449	bb
88	88	20211111_A33_R1_IgG	2.02	41919.8	48588.2	0.863	bb
89	89	20211111_A34_R1_IgG	2.02	20981.7	43288.0	0.485	bb
90	90	20211111_H17_R1_IgG	2.01	105236.2	47104.5	2.234	bb
91	91	20211111_A35_R1_IgG	2.02	31513.8	41025.9	0.768	MM
92	92	20211111_QC_R8_IgG	2.02	19642.0	40546.3	0.484	bb
93	93	20211111_A36_R1_IgG	2.02	16598.8	44504.4	0.373	MM
94	94	20211111_H18_R1_IgG	2.01	84826.3	34162.2	2.483	bb
95	95	20211111_A37_R1_IgG	2.02	44227.7	39760.0	1.112	bb
96	96	20211111_A38_R1_IgG	2.02	13295.8	43982.6	0.302	bb
97	97	20211111_H19_R1_IgG	2.02	72330.3	45314.8	1.596	bb
98	98	20211111_A39_R1_IgG	2.02	17832.4	39388.0	0.453	bb
99	99	20211111_A40_R1_IgG	2.02	11469.3	38726.4	0.296	bb
100	1...	20211111_H20_R1_IgG	2.02	65607.4	37814.4	1.735	bb
101	1...	20211111_A41_R1_IgG	2.02	33948.2	48930.3	0.694	MM
102	1...	20211111_A42_R1_IgG	2.01	10438.9	40406.8	0.258	bb
103	1...	20211111_A43_R1_IgG	2.02	14841.0	44421.5	0.334	MM
104	1...	20211111_A44_R1_IgG	2.01	30949.3	53128.2	0.583	bb
105	1...	20211111_QC_R9_IgG	2.01	18339.7	37049.0	0.495	bb
106	1...	20211111_A1_R2_IgG	2.01	26027.1	46499.4	0.560	bb
107	1...	20211111_A2_R2_IgG	2.02	37451.7	57260.7	0.654	bb
108	1...	20211111_L1_R2_IgG	2.02	32983.9	48062.9	0.686	MM
109	1...	20211111_L2_R2_IgG	2.02	19029.6	38745.2	0.491	bb
110	1...	20211111_H1_R2_IgG	2.01	22024.4	44273.5	0.497	bb
111	1...	20211111_A3_R2_IgG	2.02	1569.1	4755.5	0.330	bb
112	1...	20211111_A4_R2_IgG	2.02	19933.8	50546.7	0.394	bb

Dataset: C:\MassLynx\10.pro\20211115_clinicalsamples_IgG.qld

Last Altered: Sunday, October 02, 2022 16:02:30 台北標準時間
Printed: Friday, August 01, 2025 16:45:54 台北標準時間

	# Name	RT	Area	IS Area	Response	Prima...
113	1... 20211111_L3_R2_IgG	2.01	13463.3	34825.8	0.387	bb
114	1... 20211111_L4_R2_IgG	2.01	22203.8	39107.7	0.568	bb
115	1... 20211111_H2_R2_IgG	2.01	88903.2	49750.0	1.787	bb
116	1... 20211111_A5_R2_IgG	2.01	22769.2	52536.2	0.433	bb
117	1... 20211111_A6_R2_IgG	2.01	34874.3	42050.0	0.829	MM
118	1... 20211111_QC_R10_IgG	2.01	17624.7	35350.8	0.499	MM
119	1... 20211111_L5_R2_IgG	2.01	32049.9	38039.3	0.843	bb
120	1... 20211111_L6_R2_IgG	2.01	5404.5	44907.4	0.120	bb
121	1... 20211111_H3_R2_IgG	2.01	12621.7	47729.1	0.264	bb
122	1... 20211111_A7_R2_IgG	2.01	11838.0	57347.1	0.206	MM
123	1... 20211111_A8_R2_IgG	2.01	19704.4	52397.8	0.376	bb
124	1... 20211111_L7_R2_IgG	2.01	14305.8	36318.4	0.394	bb
125	1... 20211111_L8_R2_IgG	2.02	11700.0	38131.9	0.307	MM
126	1... 20211111_H4_R2_IgG	2.01	38916.7	46849.6	0.831	bb
127	1... 20211111_A9_R2_IgG	2.01	8702.1	47759.6	0.182	bb
128	1... 20211111_A10_R2_IgG	2.01	12249.5	41366.2	0.296	MM
129	1... 20211111_L9_R2_IgG	2.01	12527.2	43052.1	0.291	MM
130	1... 20211111_L10_R2_IgG	2.02	21730.1	31487.4	0.690	bb
131	1... 20211111_QC_R11_IgG	2.02	18371.8	37804.6	0.486	bb
132	1... 20211111_H5_R2_IgG	2.01	12793.2	40380.0	0.317	bb
133	1... 20211111_A11_R2_IgG	2.01	14348.9	49533.3	0.290	MM
134	1... 20211111_A12_R2_IgG	2.02	18372.9	40226.3	0.457	bb
135	1... 20211111_L11_R2_IgG	2.01	9939.4	28884.0	0.344	bb
136	1... 20211111_L12_R2_IgG	2.01	14057.1	34351.3	0.409	bb
137	1... 20211111_H6_R2_IgG	2.01	20667.6	40466.6	0.511	bb
138	1... 20211111_A13_R2_IgG	2.01	34186.7	42306.7	0.808	MM
139	1... 20211111_A14_R2_IgG	2.02	17091.3	44904.0	0.381	MM
140	1... 20211111_L13_R2_IgG	2.01	9566.2	31913.0	0.300	MM
141	1... 20211111_L14_R2_IgG	2.01	21444.2	31527.4	0.680	MM
142	1... 20211111_H7_R2_IgG	2.02	16461.4	40838.2	0.403	bb
143	1... 20211111_A15_R2_IgG	2.01	50164.3	49389.7	1.016	bb
144	1... 20211111_QC_R12_IgG	2.01	17050.5	35168.7	0.485	MM
145	1... 20211111_A16_R2_IgG	2.01	14042.3	47440.5	0.296	bb
146	1... 20211111_L15_R2_IgG	2.02	20763.2	33145.9	0.626	MM
147	1... 20211111_L16_R2_IgG	2.01	9118.9	37596.6	0.243	bb
148	1... 20211111_H8_R2_IgG	2.01	7978.3	43933.3	0.182	bb
149	1... 20211111_A17_R2_IgG	2.01	19396.3	43605.5	0.445	bb
150	1... 20211111_A18_R2_IgG	2.02	6110.7	37581.8	0.163	bb
151	1... 20211111_L17_R2_IgG	2.02	18769.9	33942.5	0.553	bb
152	1... 20211111_L18_R2_IgG	2.02	13266.4	37831.5	0.351	bb
153	1... 20211111_H9_R2_IgG	2.02	23347.3	30898.4	0.756	MM
154	1... 20211111_A19_R2_IgG	2.01	44724.4	39352.1	1.137	bb
155	1... 20211111_A20_R2_IgG	2.02	5574.8	34661.2	0.161	bb
156	1... 20211111_L19_R2_IgG	2.02	13927.5	39918.3	0.349	bb
157	1... 20211111_QC_R13_IgG	2.01	18098.6	36239.1	0.499	bb
158	1... 20211111_L20_R2_IgG	2.02	14331.0	32897.7	0.436	MM
159	1... 20211111_H10_R2_IgG	2.01	29006.8	31394.5	0.924	bb
160	1... 20211111_A21_R2_IgG	2.01	11373.1	34863.7	0.326	bb
161	1... 20211111_A22_R2_IgG	2.01	7070.4	30342.1	0.233	MM
162	1... 20211111_L21_R2_IgG	2.01	8686.1	33706.2	0.258	MM
163	1... 20211111_L22_R2_IgG	2.01	14008.9	31298.6	0.448	bb
164	1... 20211111_H11_R2_IgG	2.01	15161.4	38904.1	0.390	MM
165	1... 20211111_A23_R2_IgG	2.01	20512.8	32382.3	0.633	bb
166	1... 20211111_A24_R2_IgG	2.01	12988.1	30261.9	0.429	bb
167	1... 20211111_L23_R2_IgG	2.01	21008.4	43037.5	0.488	bb
168	1... 20211111_L24_R2_IgG	2.01	9148.0	37023.3	0.247	bb

Dataset: C:\MassLynx\10.pro\20211115_clinicalsamples_IgG.qld

Last Altered: Sunday, October 02, 2022 16:02:30 台北標準時間
Printed: Friday, August 01, 2025 16:45:54 台北標準時間

	# Name	RT	Area	IS Area	Response	Prima...
169	1... 20211111_H12_R2_IgG	2.01	11580.0	39478.2	0.293	bb
170	1... 20211111_QC_R14_IgG	2.01	16683.5	34463.2	0.484	bb
171	1... 20211111_A25_R2_IgG	2.01	16758.4	39945.3	0.420	MM
172	1... 20211111_A26_R2_IgG	2.01	6617.2	29663.2	0.223	bb
173	1... 20211111_L25_R2_IgG	2.01	9451.2	38589.3	0.245	bb
174	1... 20211111_L26_R2_IgG	2.01	7513.4	38405.2	0.196	bb
175	1... 20211111_H13_R2_IgG	2.01	16681.4	37771.2	0.442	bb
176	1... 20211111_A27_R2_IgG	2.01	10796.9	42634.1	0.253	MM
177	1... 20211111_A28_R2_IgG	2.01	12672.8	33231.1	0.381	bb
178	1... 20211111_L27_R2_IgG	2.01	22262.8	41761.1	0.533	bb
179	1... 20211111_L28_R2_IgG	2.01	6962.6	26968.6	0.258	bb
180	1... 20211111_H14_R2_IgG	2.01	14105.6	49250.2	0.286	bb
181	1... 20211111_A29_R2_IgG	2.01	14842.5	37601.7	0.395	bb
182	1... 20211111_A30_R2_IgG	2.01	15485.8	40366.8	0.384	bb
183	1... 20211111_QC_R15_IgG	2.00	16484.7	35156.1	0.469	MM
184	1... 20211111_L29_R2_IgG	2.01	10846.8	33813.1	0.321	bb
185	1... 20211111_L30_R2_IgG	2.02	6153.1	33488.6	0.184	bb
186	1... 20211111_H15_R2_IgG	2.01	23367.1	38501.0	0.607	bb
187	1... 20211111_A31_R2_IgG	2.01	11377.1	37525.5	0.303	MM
188	1... 20211111_A32_R2_IgG	2.01	20692.9	35770.8	0.578	bb
189	1... 20211111_L31_R2_IgG	2.00	7641.3	33282.4	0.230	MM
190	1... 20211111_L32_R2_IgG	2.02	16293.5	42049.6	0.387	bb
191	1... 20211111_H16_R2_IgG	2.01	17829.9	45947.6	0.388	bb
192	1... 20211111_A33_R2_IgG	2.01	30160.0	40202.3	0.750	bb
193	1... 20211111_A34_R2_IgG	2.01	15959.0	36747.0	0.434	bb
194	1... 20211111_H17_R2_IgG	2.01	95949.0	40920.9	2.345	bb
195	1... 20211111_A35_R2_IgG	2.01	25884.6	33689.2	0.768	bb
196	1... 20211111_QC_R16_IgG	2.01	16143.4	32783.6	0.492	bb
197	1... 20211111_A36_R2_IgG	2.01	13748.7	36822.4	0.373	MM
198	1... 20211111_H18_R2_IgG	2.01	87630.6	31769.3	2.758	bb
199	1... 20211111_A37_R2_IgG	2.01	40239.1	37581.8	1.071	bb
200	2... 20211111_A38_R2_IgG	2.01	11661.6	38390.4	0.304	MM
201	2... 20211111_H19_R2_IgG	2.01	64950.5	41217.0	1.576	bb
202	2... 20211111_A39_R2_IgG	2.01	15056.9	37392.7	0.403	MM
203	2... 20211111_A40_R2_IgG	2.01	9777.3	38295.1	0.255	bb
204	2... 20211111_H20_R2_IgG	2.01	62015.8	35941.8	1.725	bb
205	2... 20211111_A41_R2_IgG	2.01	29276.3	45304.9	0.646	bb
206	2... 20211111_A42_R2_IgG	2.02	9028.6	37803.8	0.239	bb
207	2... 20211111_A43_R2_IgG	2.02	11833.6	38161.8	0.310	MM
208	2... 20211111_A44_R2_IgG	2.00	20663.0	37819.8	0.546	MM
209	2... 20211111_QC_R17_IgG	2.01	14916.1	31731.8	0.470	bb

Dataset: C:\MassLynx\10.pro\20211115_clinicalsamples_IgG.qld

Last Altered: Sunday, October 02, 2022 16:02:30 台北標準時間
Printed: Friday, August 01, 2025 16:46:23 台北標準時間

Method: C:\MassLynx\10_Pro_IgA.PRO\MethDB\20210926_IgG_DMRM_IgM_IS.mdb 16 Feb 2022 11:58:21
Calibration: 18 Nov 2021 13:56:22

Sample Name: 20211111_QC_R1_IgG

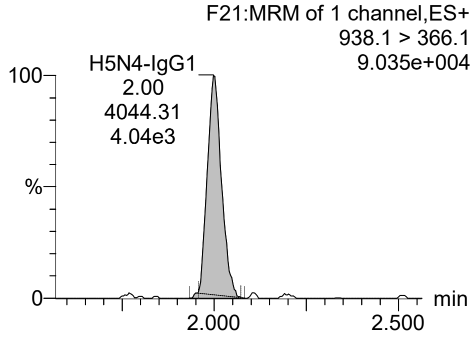

Sample Name: 20211111_A1_R1_IgG

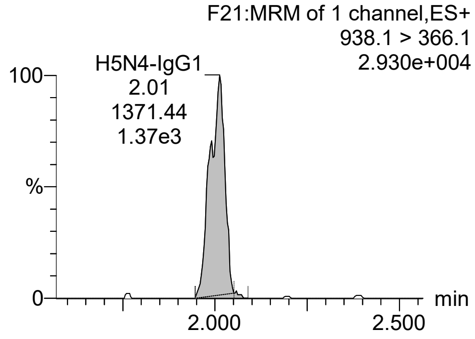

Sample Name: 20211111_A2_R1_IgG

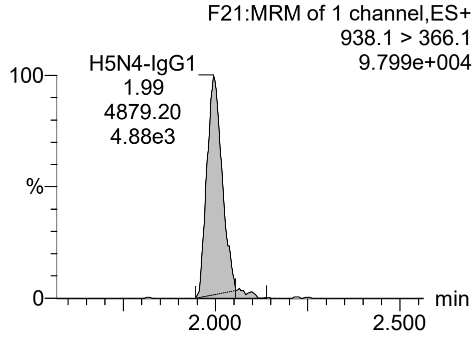

Sample Name: 20211111_L1_R1_IgG

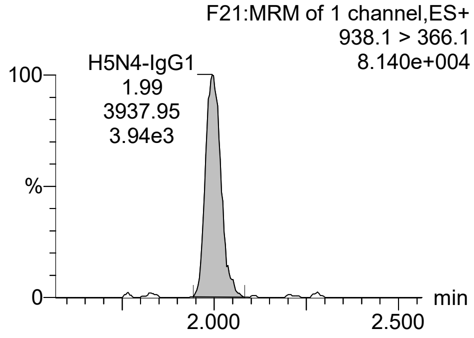

Sample Name: 20211111_L2_R1_IgG

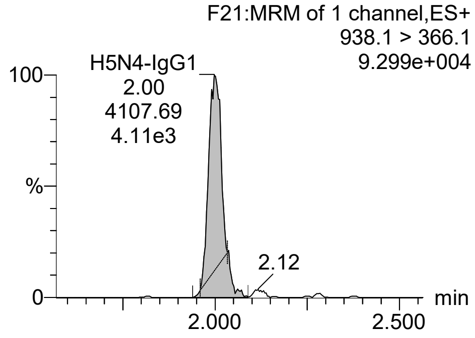

Sample Name: 20211111_H1_R1_IgG

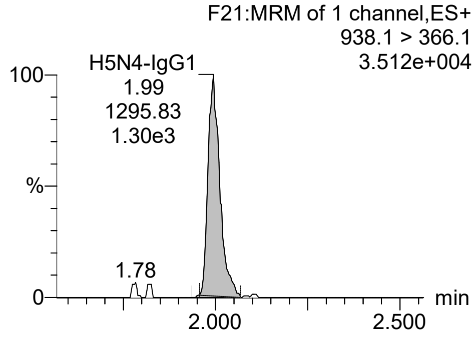

Sample Name: 20211111_A3_R1_IgG

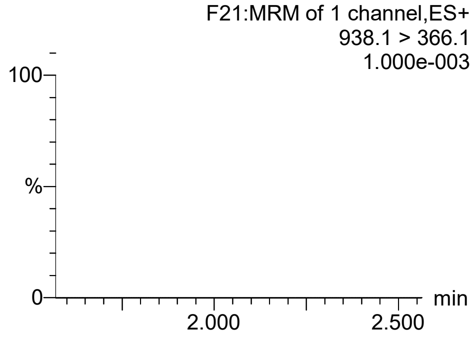

Sample Name: 20211111_A4_R1_IgG

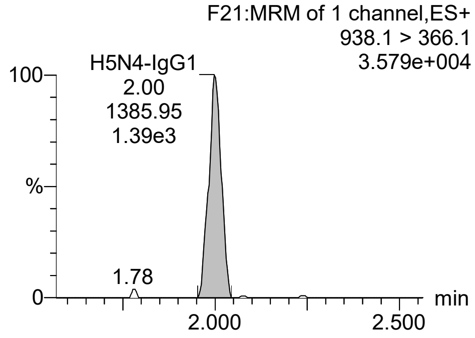

Sample Name: 20211111_L3_R1_IgG

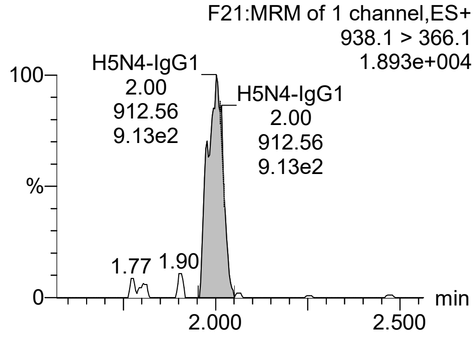

Sample Name: 20211111_L4_R1_IgG

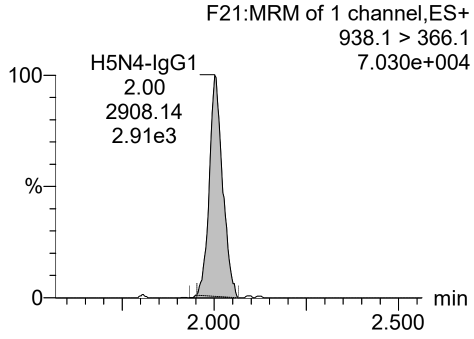

Sample Name: 20211111_H2_R1_IgG

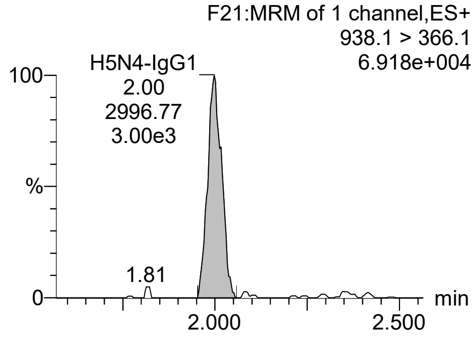

Sample Name: 20211111_A5_R1_IgG

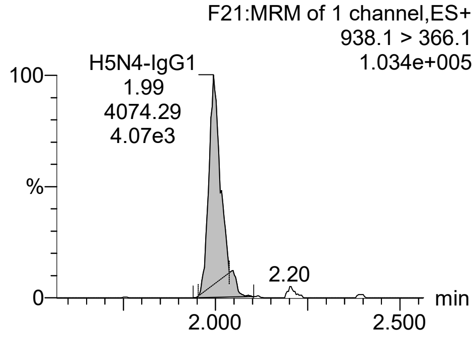

Sample Name: 20211111_A6_R1_IgG

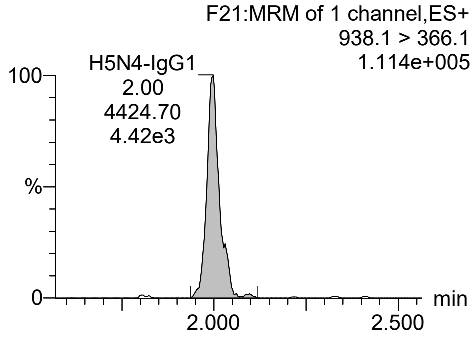

Sample Name: 20211111_QC_R2_IgG

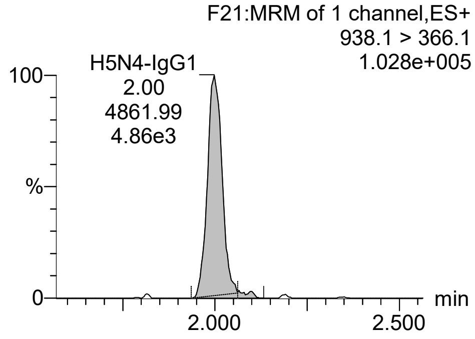

Sample Name: 20211111_L5_R1_IgG

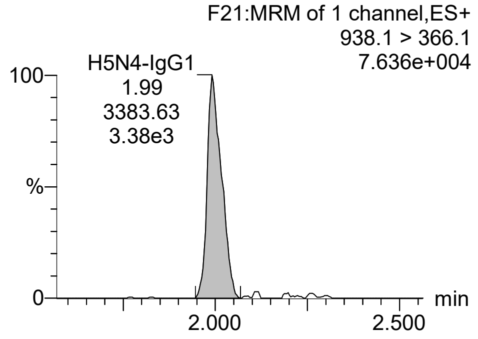

Sample Name: 20211111_L6_R1_IgG

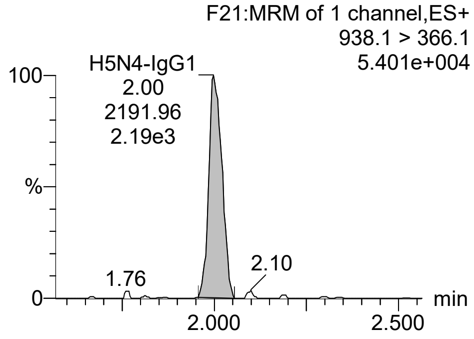

Sample Name: 20211111_H3_R1_IgG

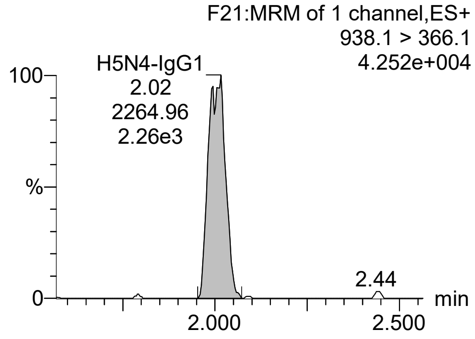

Sample Name: 20211111_A7_R1_IgG

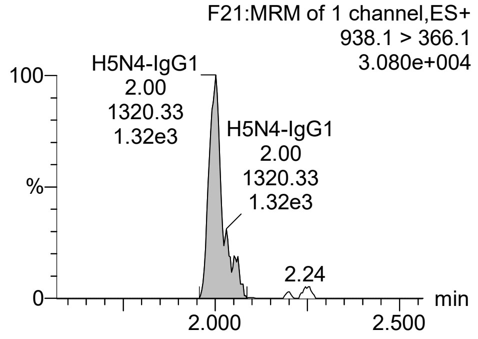

Sample Name: 20211111_A8_R1_IgG

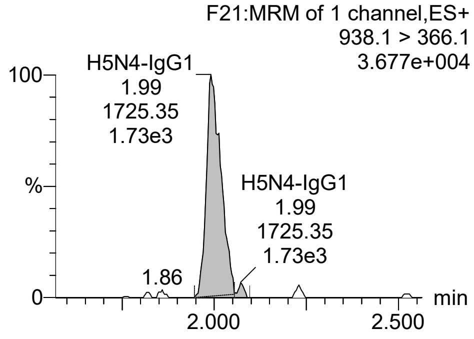

Sample Name: 20211111_L7_R1_IgG

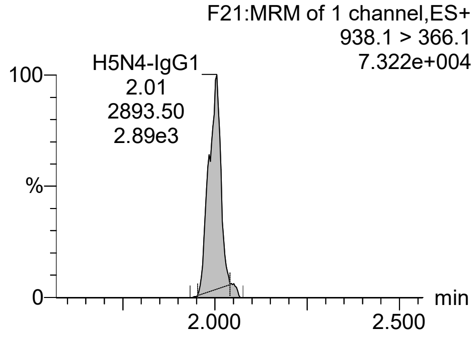

Sample Name: 20211111_L8_R1_IgG

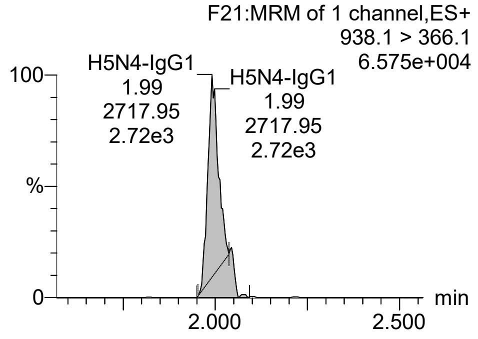

Sample Name: 20211111_H4_R1_IgG

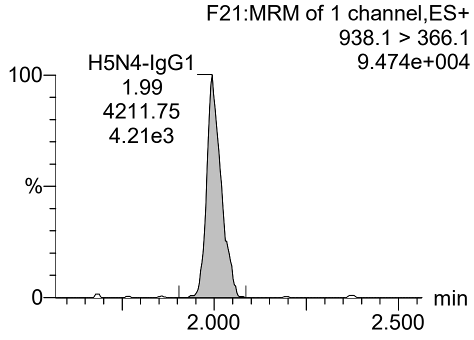

Sample Name: 20211111_A9_R1_IgG

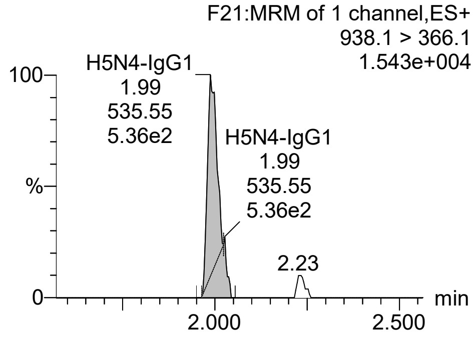

Sample Name: 20211111_A10_R1_IgG

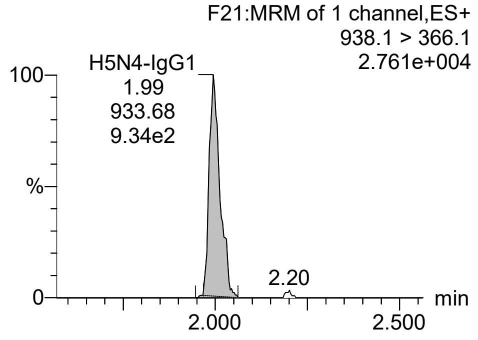

Sample Name: 20211111_L9_R1_IgG

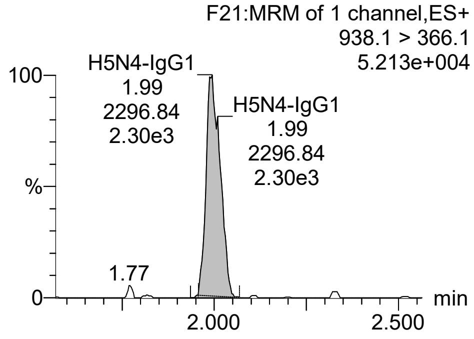

Sample Name: 20211111_L10_R1_IgG

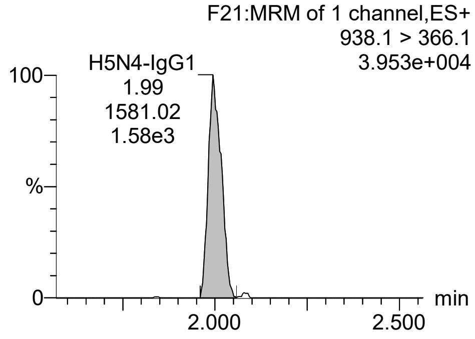

Sample Name: 20211111_QC_R3_IgG

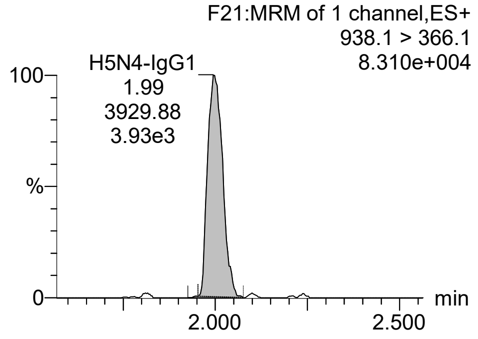

Dataset: C:\MassLynx\10.pro\20211115_clinicalsamples_IgG.qld

Last Altered: Sunday, October 02, 2022 16:02:30 台北標準時間
Printed: Friday, August 01, 2025 16:46:23 台北標準時間

Sample Name: 20211111_H5_R1_IgG

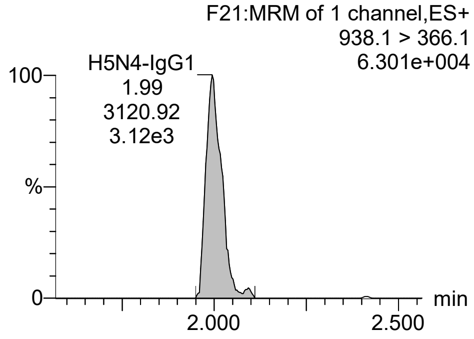

Sample Name: 20211111_A11_R1_IgG

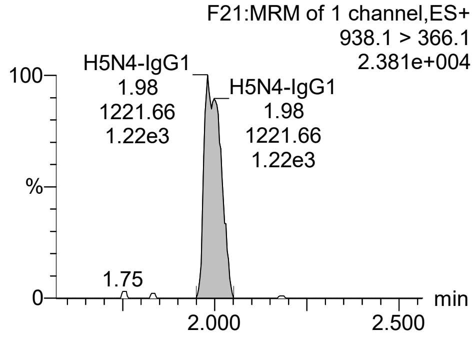

Sample Name: 20211111_A12_R1_IgG

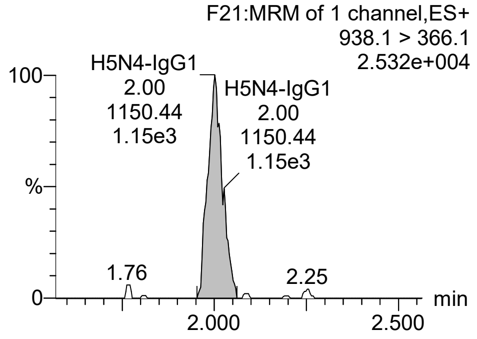

Sample Name: 20211111_L11_R1_IgG

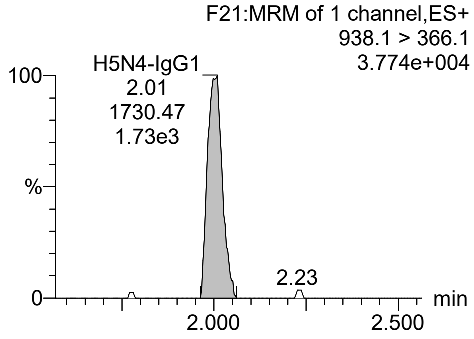

Sample Name: 20211111_L12_R1_IgG

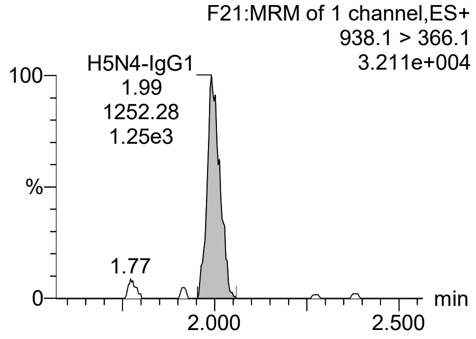

Sample Name: 20211111_H6_R1_IgG

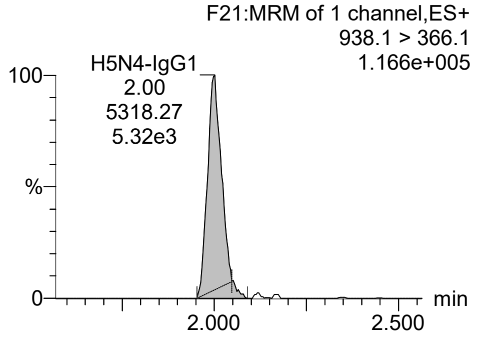

Sample Name: 20211111_A13_R1_IgG

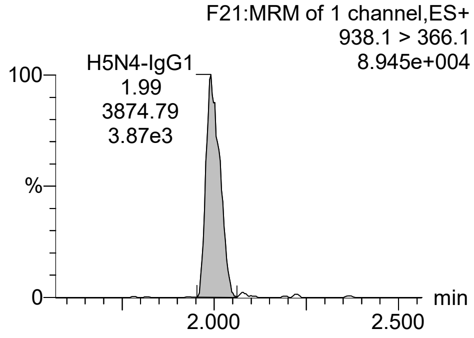

Sample Name: 20211111_A14_R1_IgG

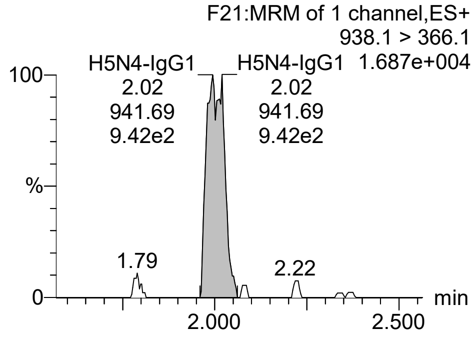

Sample Name: 20211111_L13_R1_IgG

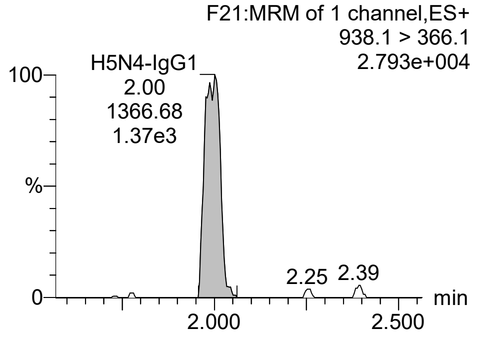

Sample Name: 20211111_L14_R1_IgG

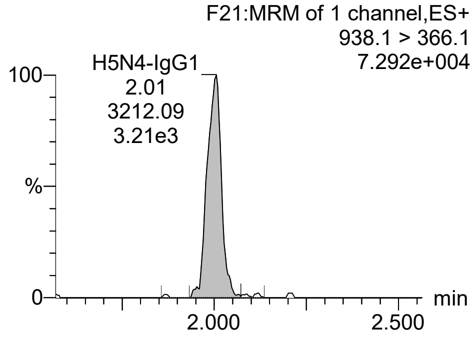

Sample Name: 20211111_H7_R1_IgG

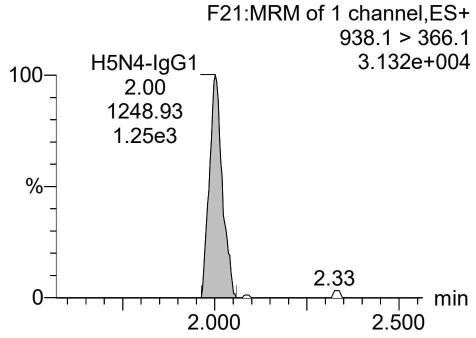

Sample Name: 20211111_A15_R1_IgG

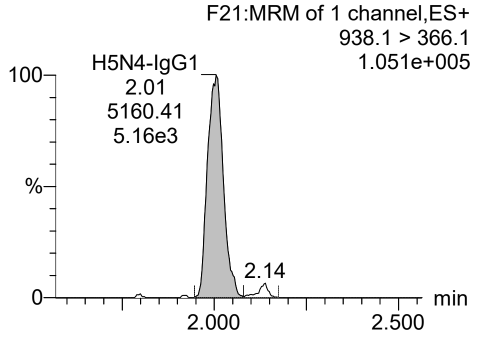

Sample Name: 20211111_QC_R4_IgG

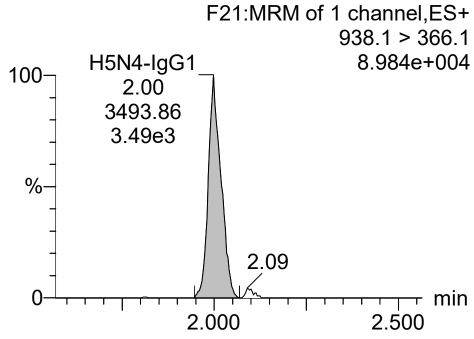

Sample Name: 20211111_A16_R1_IgG

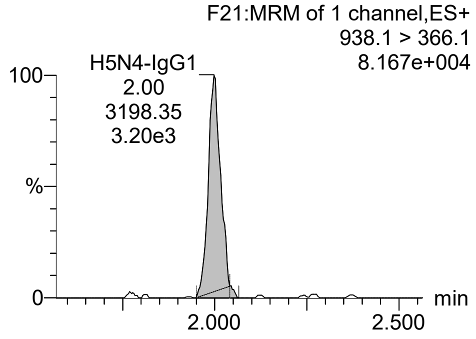

Sample Name: 20211111_L15_R1_IgG

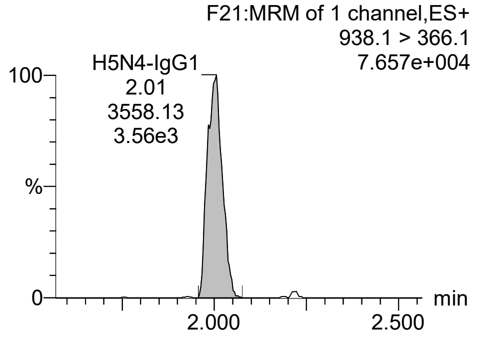

Sample Name: 20211111_L16_R1_IgG

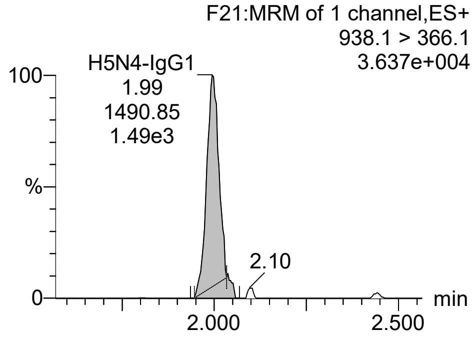

Sample Name: 20211111_H8_R1_IgG

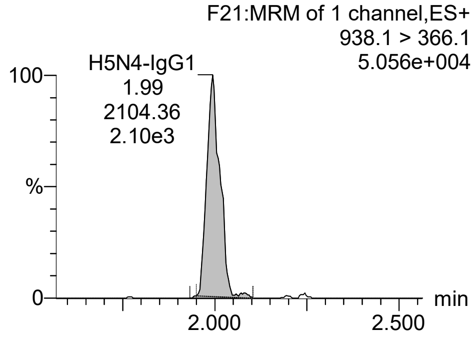

Sample Name: 20211111_A17_R1_IgG

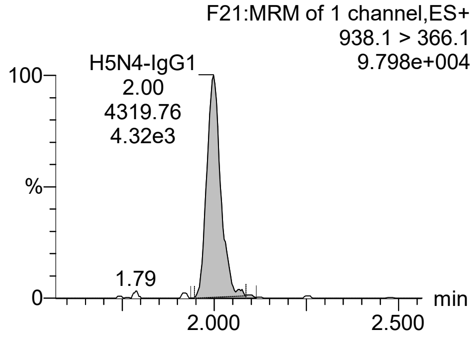

Sample Name: 20211111_A18_R1_IgG

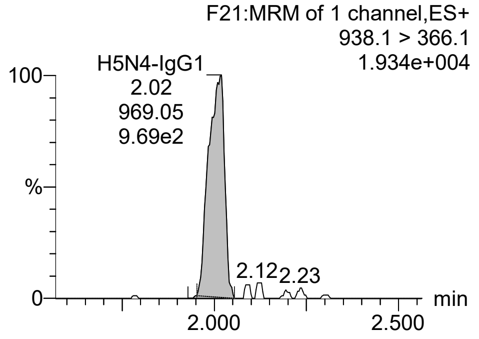

Sample Name: 20211111_L17_R1_IgG

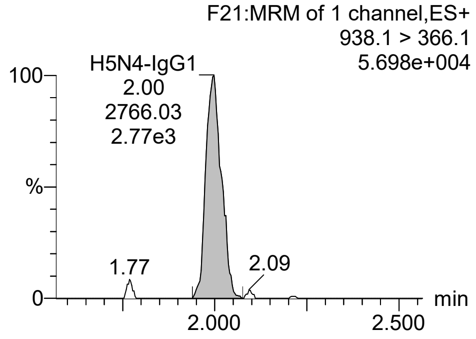

Sample Name: 20211111_L18_R1_IgG

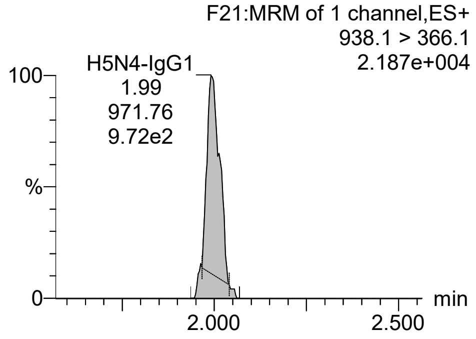

Sample Name: 20211111_H9_R1_IgG

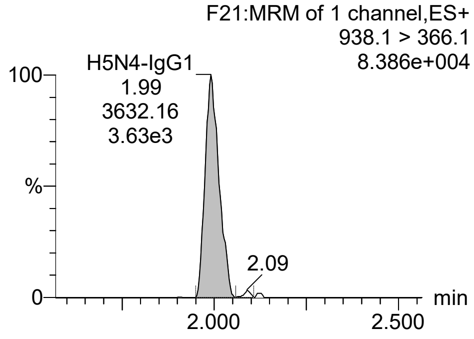

Sample Name: 20211111_A19_R1_IgG

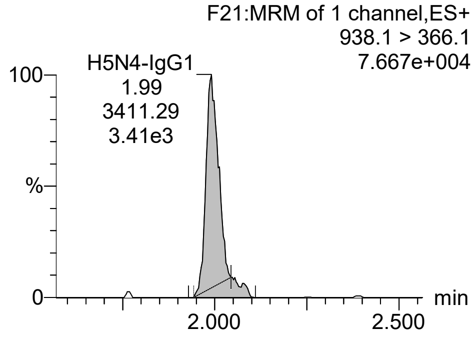

Sample Name: 20211111_A20_R1_IgG

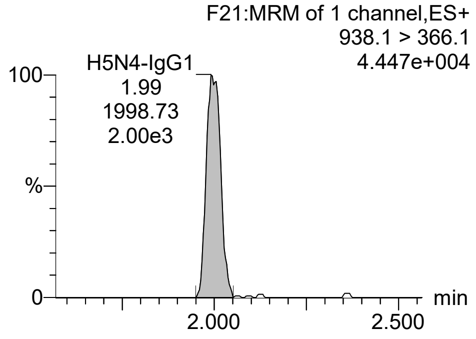

Sample Name: 20211111_L19_R1_IgG

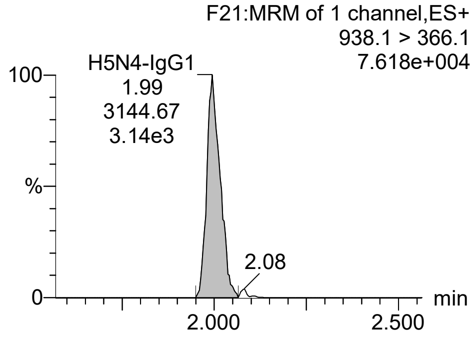

Sample Name: 20211111_QC_R5_IgG

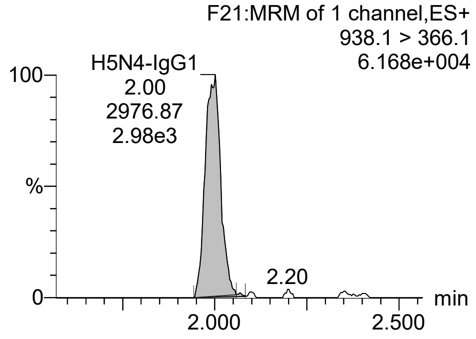

Sample Name: 20211111_L20_R1_IgG

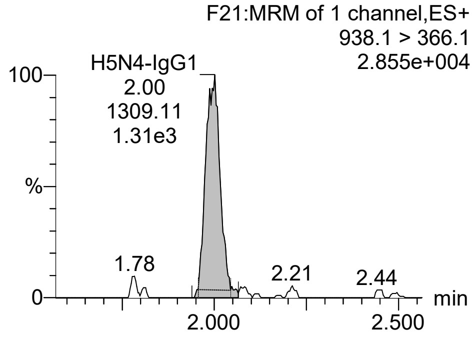

Sample Name: 20211111_H10_R1_IgG

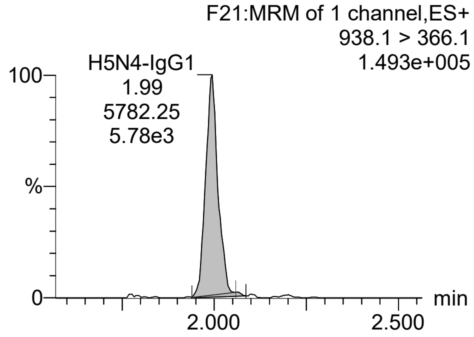

Sample Name: 20211111_A21_R1_IgG

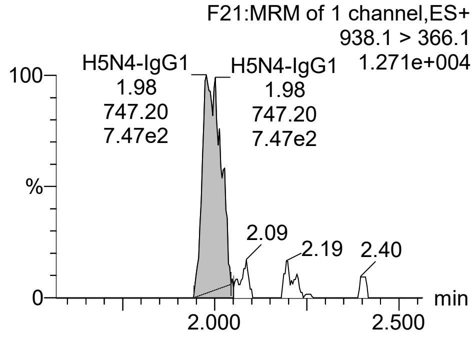

Sample Name: 20211111_A22_R1_IgG

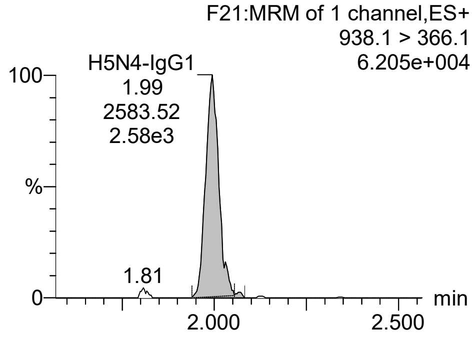

Sample Name: 20211111_L21_R1_IgG

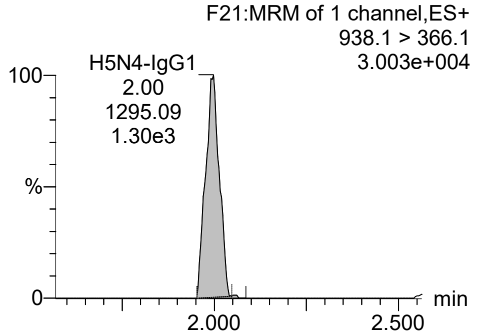

Sample Name: 20211111_L22_R1_IgG

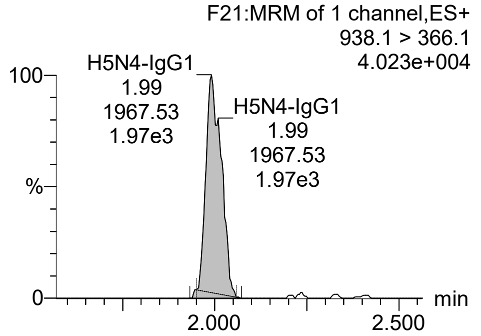

Sample Name: 20211111_H11_R1_IgG

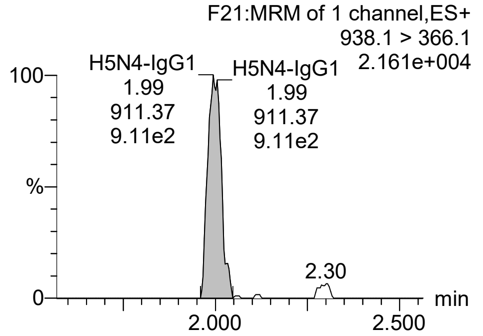

Sample Name: 20211111_A23_R1_IgG

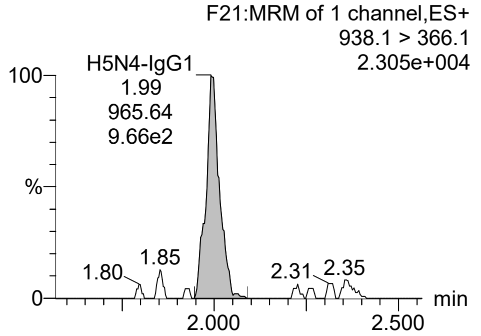

Sample Name: 20211111_A24_R1_IgG

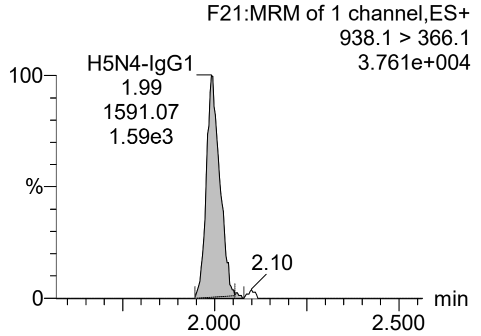

Sample Name: 20211111_L23_R1_IgG

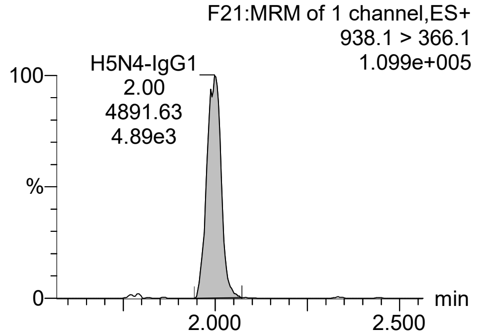

Sample Name: 20211111_L24_R1_IgG

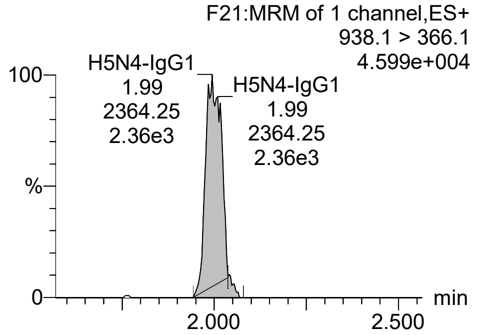

Sample Name: 20211111_H12_R1_IgG

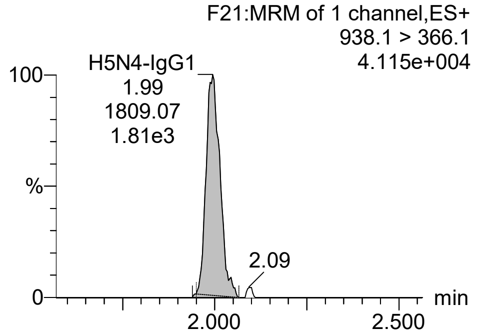

Sample Name: 20211111_QC_R6_IgG

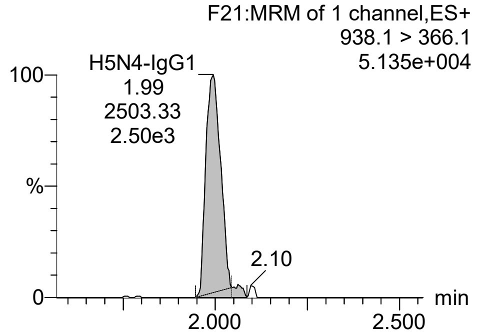

Sample Name: 20211111_A25_R1_IgG

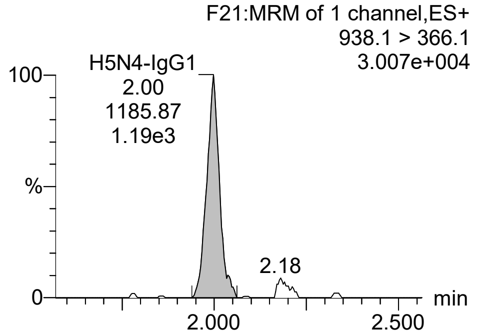

Sample Name: 20211111_A26_R1_IgG

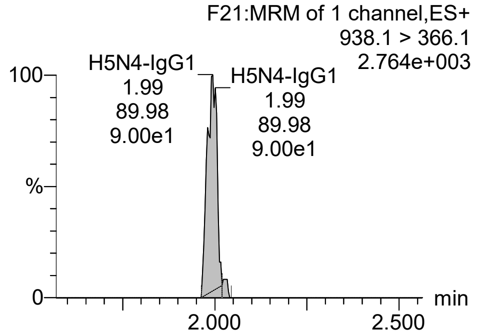

Sample Name: 20211111_L25_R1_IgG

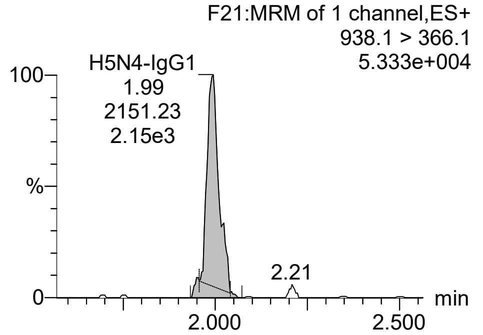

Sample Name: 20211111_L26_R1_IgG

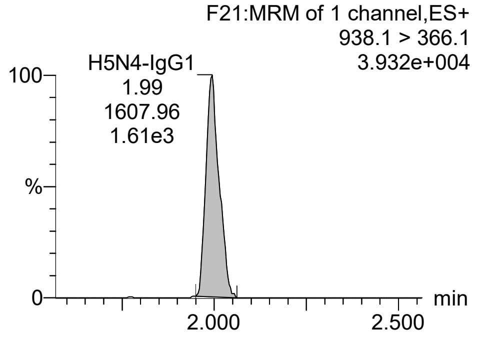

Sample Name: 20211111_H13_R1_IgG

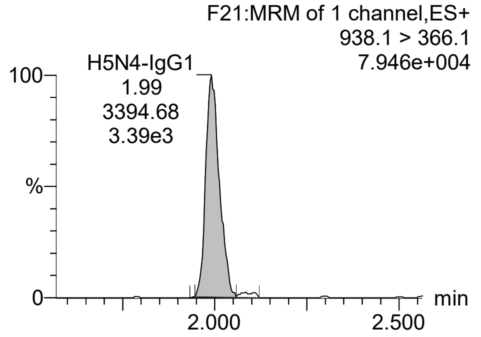

Sample Name: 20211111_A27_R1_IgG

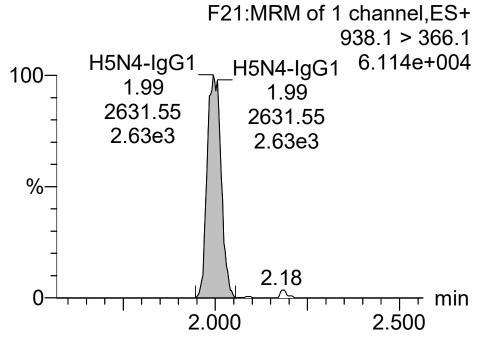

Dataset: C:\MassLynx\10.pro\20211115_clinicalsamples_IgG.qld

Last Altered: Sunday, October 02, 2022 16:02:30 台北標準時間
Printed: Friday, August 01, 2025 16:46:23 台北標準時間

Sample Name: 20211111_A28_R1_IgG

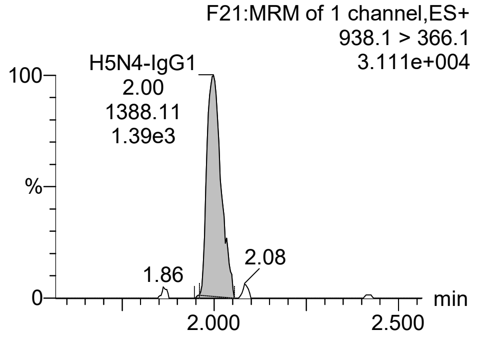

Sample Name: 20211111_L27_R1_IgG

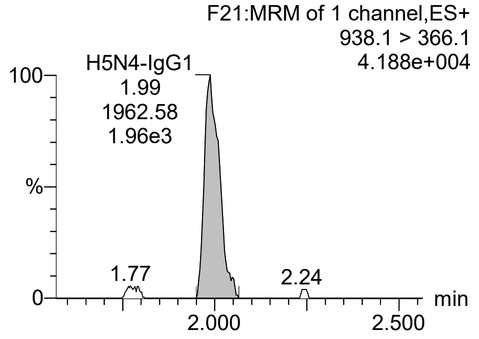

Sample Name: 20211111_L28_R1_IgG

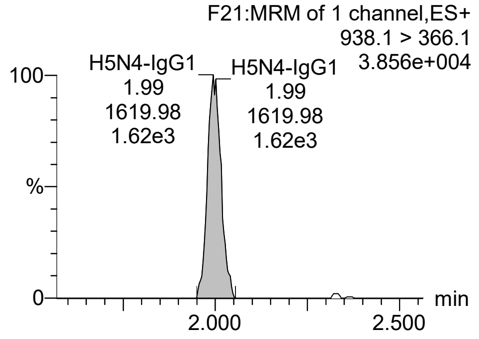

Sample Name: 20211111_H14_R1_IgG

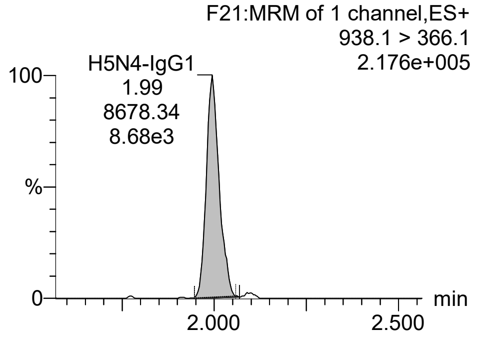

Sample Name: 20211111_A29_R1_IgG

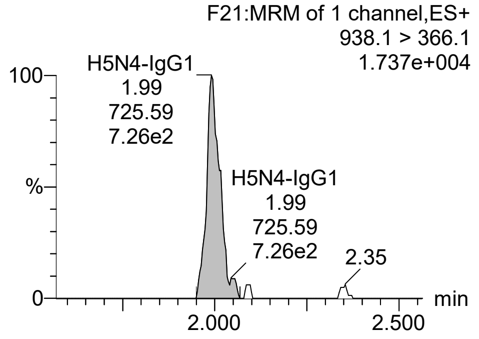

Sample Name: 20211111_A30_R1_IgG

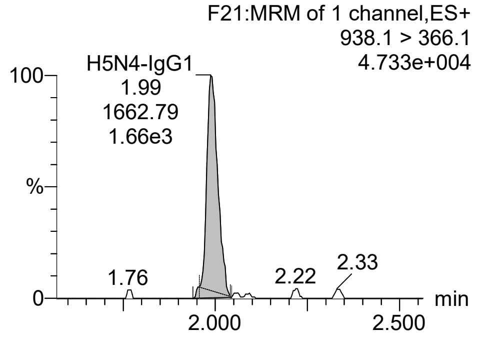

Sample Name: 20211111_QC_R7_IgG

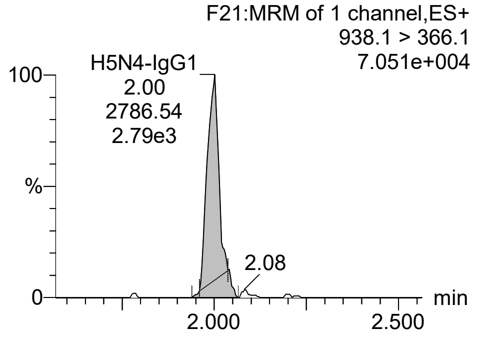

Sample Name: 20211111_L29_R1_IgG

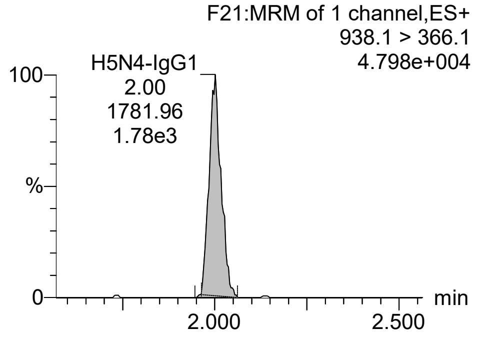

Sample Name: 20211111_L30_R1_IgG

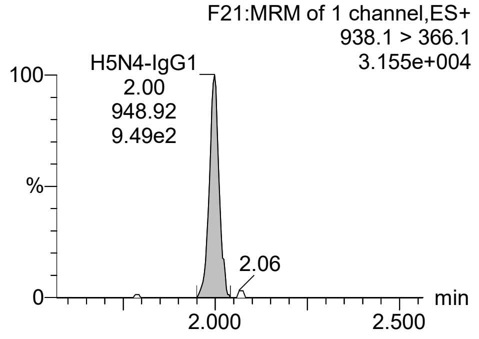

Sample Name: 20211111_H15_R1_IgG

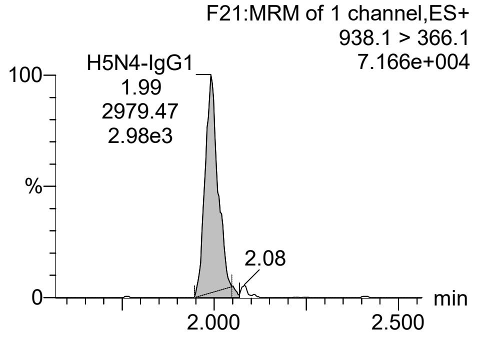

Sample Name: 20211111_A31_R1_IgG

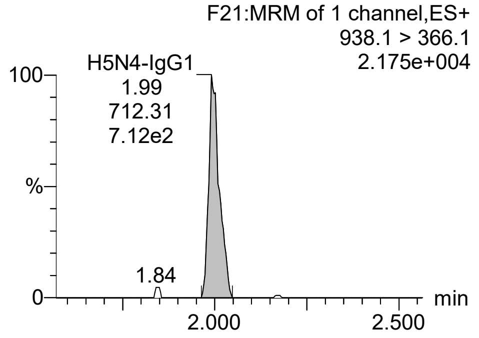

Sample Name: 20211111_A32_R1_IgG

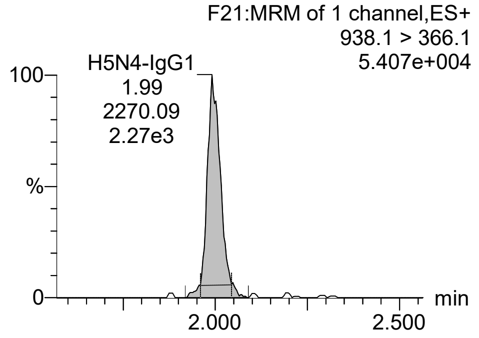

Sample Name: 20211111_L31_R1_IgG

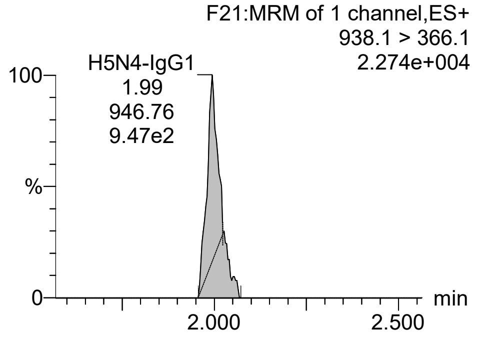

Sample Name: 20211111_L32_R1_IgG

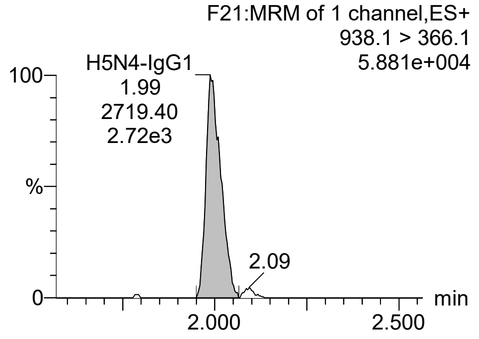

Sample Name: 20211111_H16_R1_IgG

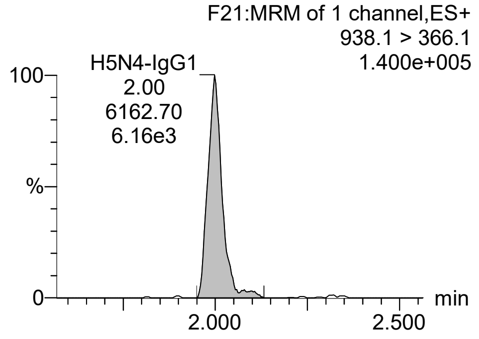

Dataset: C:\MassLynx\10.pro\20211115_clinicalsamples_IgG.qld

Last Altered: Sunday, October 02, 2022 16:02:30 台北標準時間
Printed: Friday, August 01, 2025 16:46:23 台北標準時間

Sample Name: 20211111_A33_R1_IgG

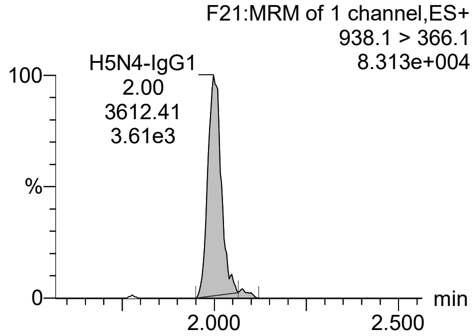

Sample Name: 20211111_A34_R1_IgG

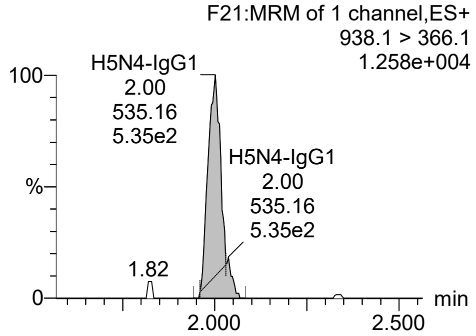

Sample Name: 20211111_H17_R1_IgG

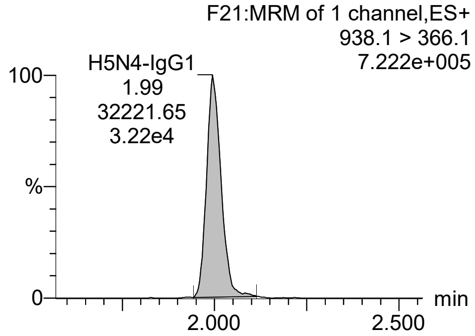

Sample Name: 20211111_A35_R1_IgG

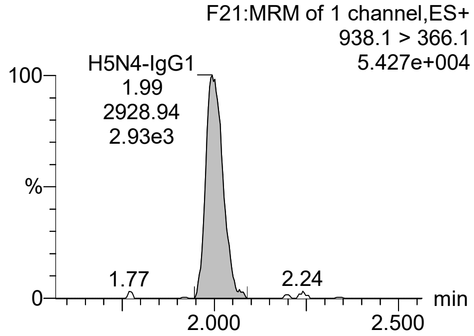

Sample Name: 20211111_QC_R8_IgG

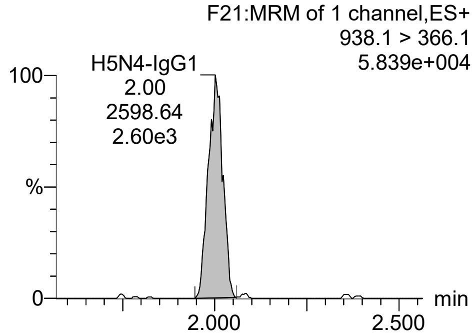

Sample Name: 20211111_A36_R1_IgG

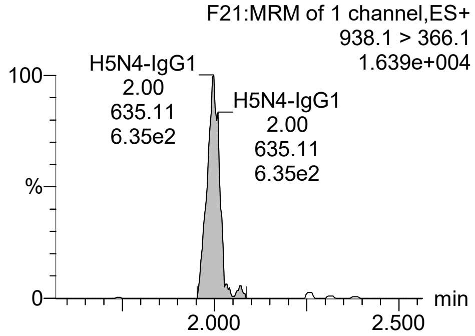

Sample Name: 20211111_H18_R1_IgG

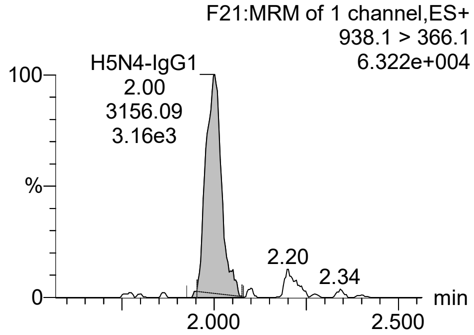

Sample Name: 20211111_A37_R1_IgG

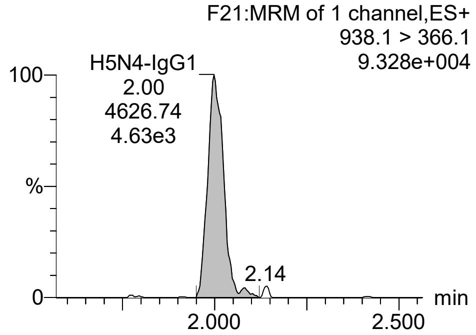

Sample Name: 20211111_A38_R1_IgG

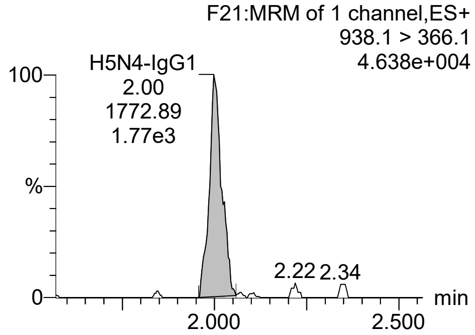

Sample Name: 20211111_H19_R1_IgG

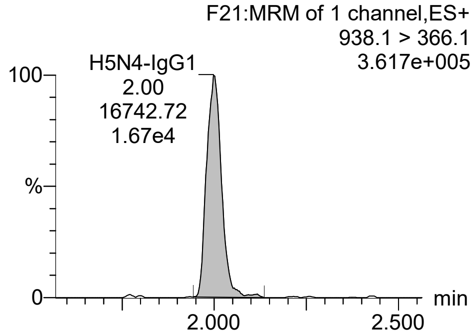

Sample Name: 20211111_A39_R1_IgG

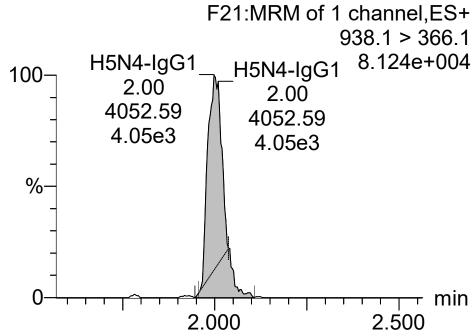

Sample Name: 20211111_A40_R1_IgG

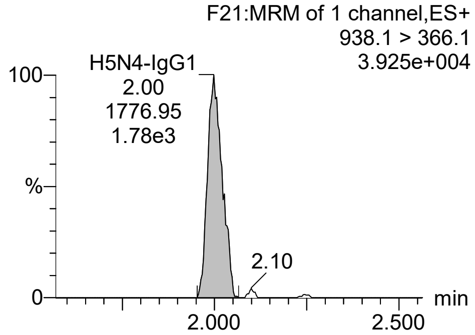

Sample Name: 20211111_H20_R1_IgG

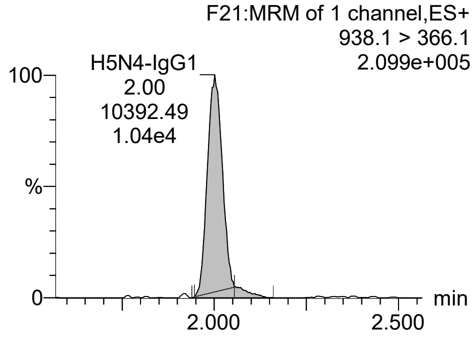

Sample Name: 20211111_A41_R1_IgG

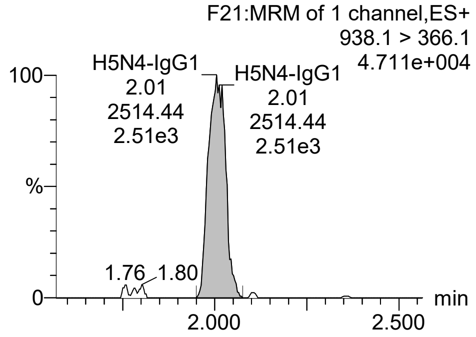

Sample Name: 20211111_A42_R1_IgG

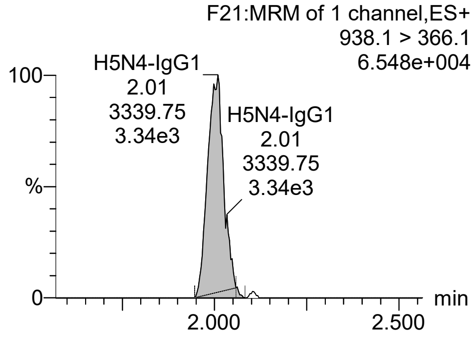

Sample Name: 20211111_A43_R1_IgG

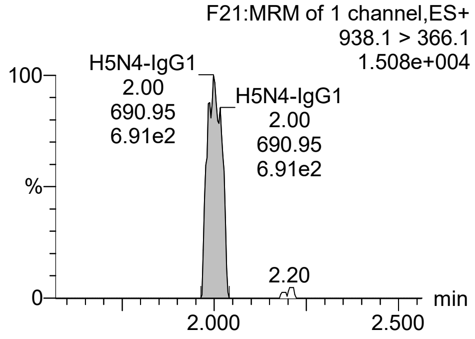

Sample Name: 20211111_A44_R1_IgG

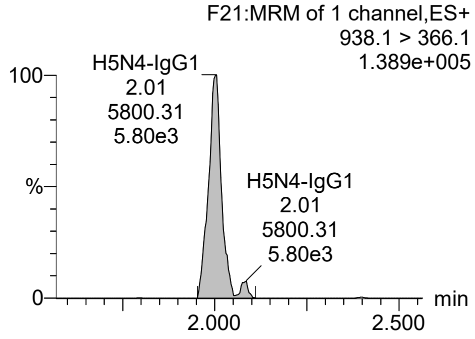

Sample Name: 20211111_QC_R9_IgG

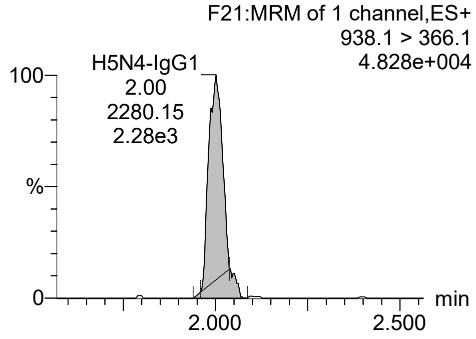

Sample Name: 20211111_A1_R2_IgG

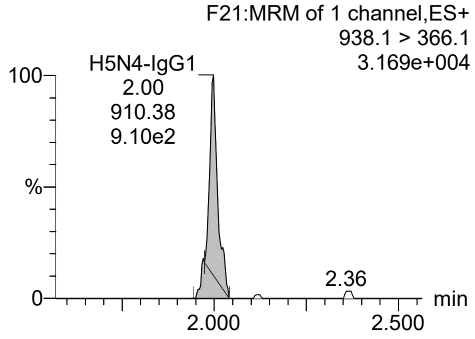

Sample Name: 20211111_A2_R2_IgG

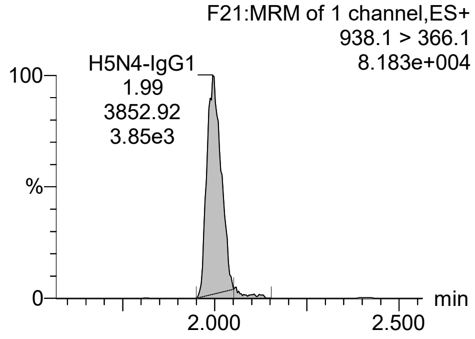

Sample Name: 20211111_L1_R2_IgG

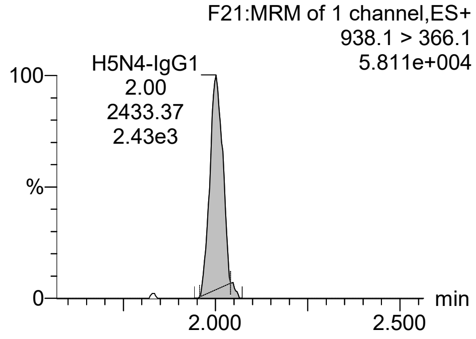

Sample Name: 20211111_L2_R2_IgG

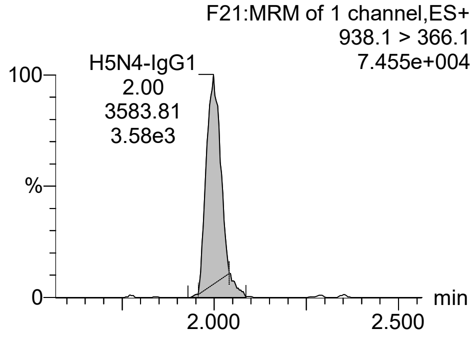

Sample Name: 20211111_H1_R2_IgG

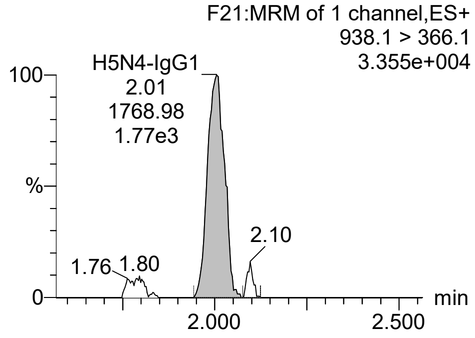

Sample Name: 20211111_A3_R2_IgG

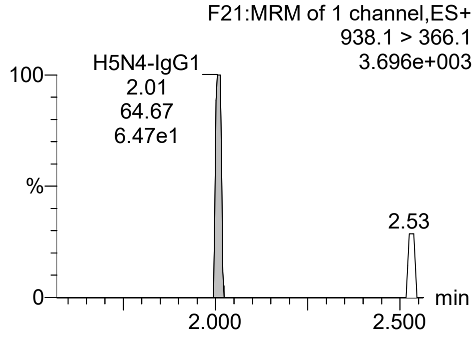

Sample Name: 20211111_A4_R2_IgG

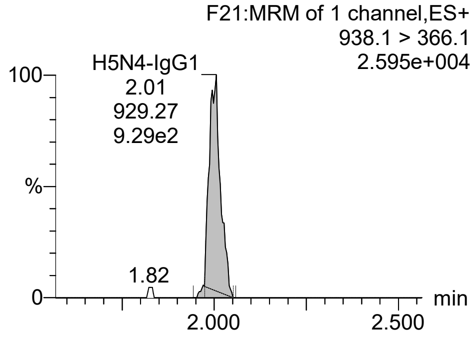

Sample Name: 20211111_L3_R2_IgG

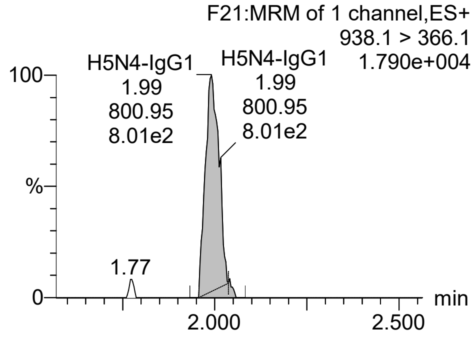

Sample Name: 20211111_L4_R2_IgG

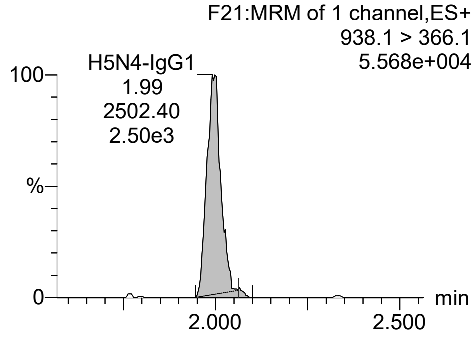

Sample Name: 20211111_H2_R2_IgG

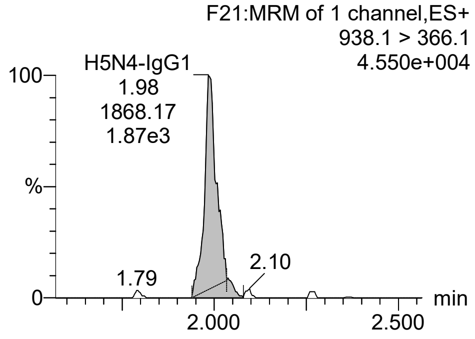

Sample Name: 20211111_A5_R2_IgG

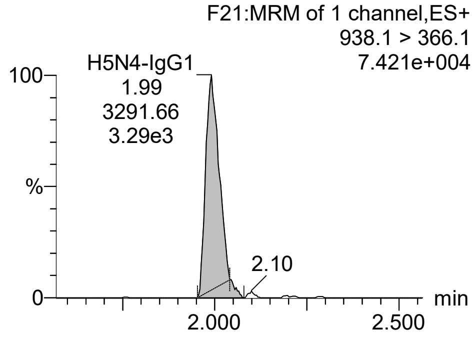

Sample Name: 20211111_A6_R2_IgG

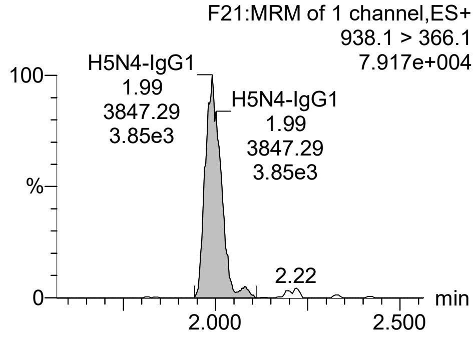

Sample Name: 20211111_QC_R10_IgG

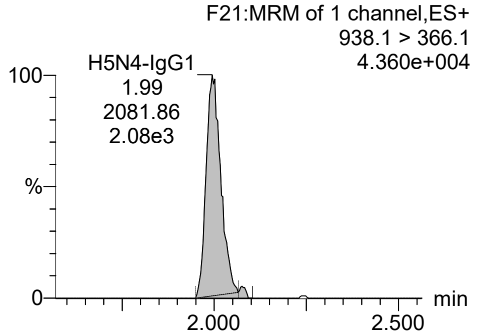

Sample Name: 20211111_L5_R2_IgG

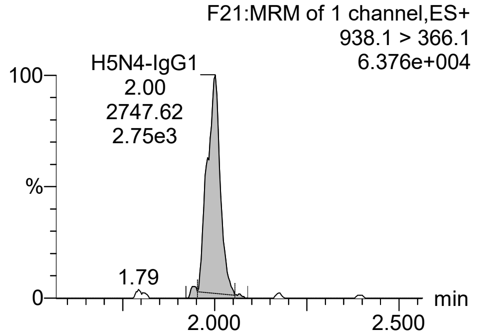

Sample Name: 20211111_L6_R2_IgG

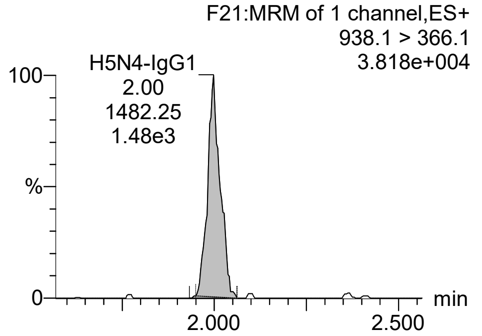

Sample Name: 20211111_H3_R2_IgG

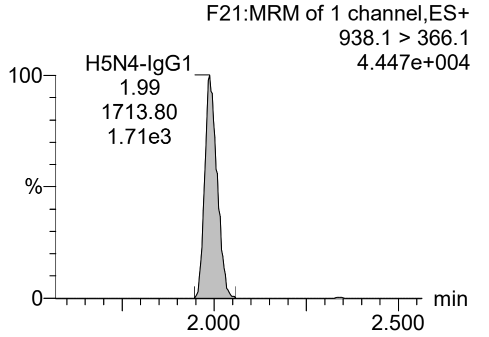

Sample Name: 20211111_A7_R2_IgG

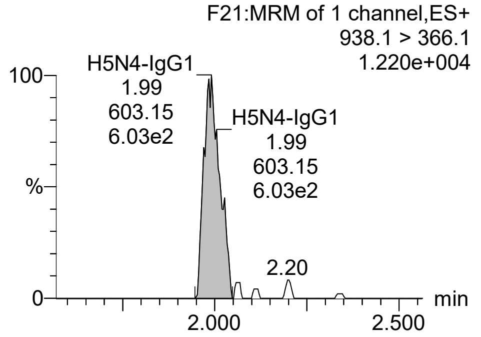

Sample Name: 20211111_A8_R2_IgG

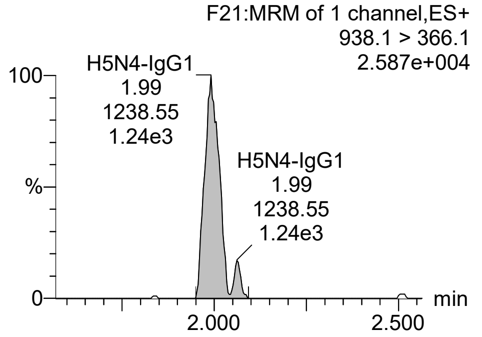

Sample Name: 20211111_L7_R2_IgG

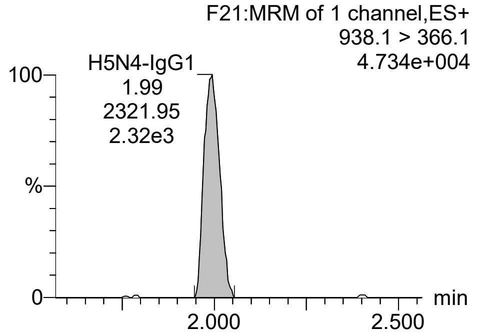

Sample Name: 20211111_L8_R2_IgG

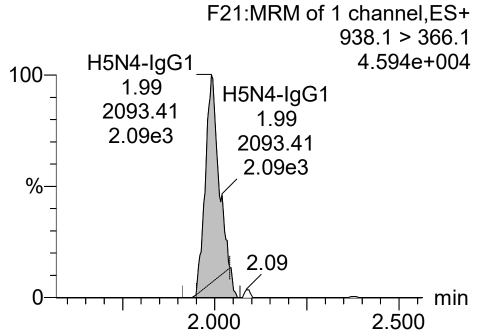

Sample Name: 20211111_H4_R2_IgG

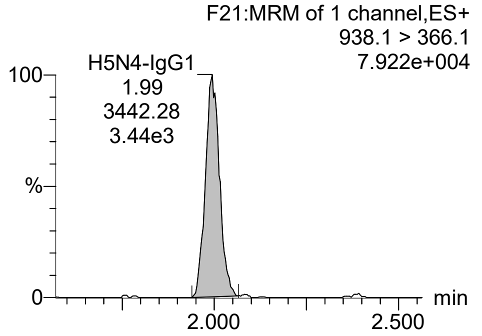

Sample Name: 20211111_A9_R2_IgG

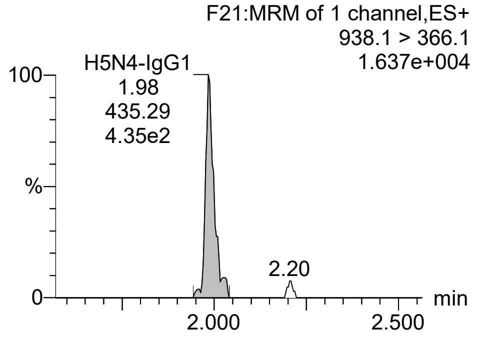

Sample Name: 20211111_A10_R2_IgG

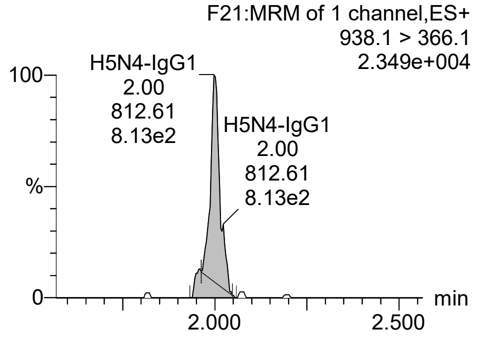

Sample Name: 20211111_L9_R2_IgG

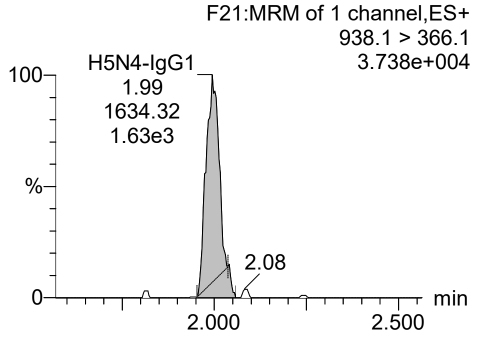

Sample Name: 20211111_L10_R2_IgG

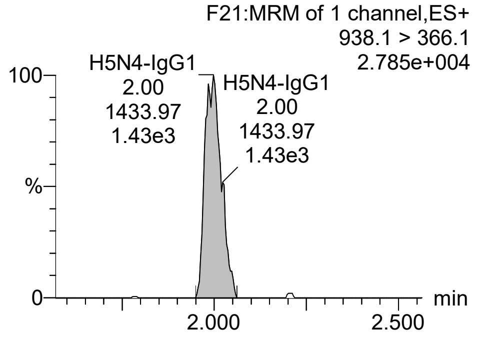

Sample Name: 20211111_QC_R11_IgG

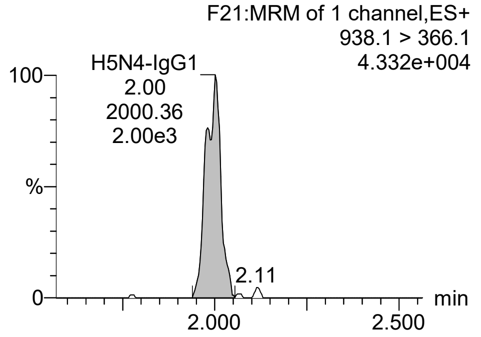

Sample Name: 20211111_H5_R2_IgG

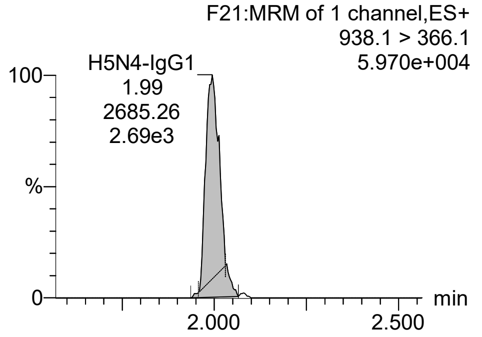

Dataset: C:\MassLynx\10.pro\20211115_clinicalsamples_IgG.qld

Last Altered: Sunday, October 02, 2022 16:02:30 台北標準時間
Printed: Friday, August 01, 2025 16:46:23 台北標準時間

Sample Name: 20211111_A11_R2_IgG

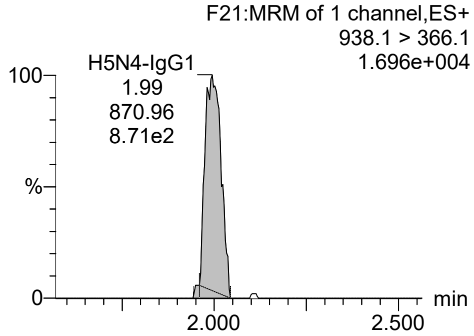

Sample Name: 20211111_A12_R2_IgG

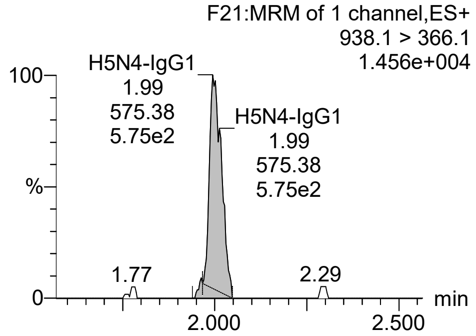

Sample Name: 20211111_L11_R2_IgG

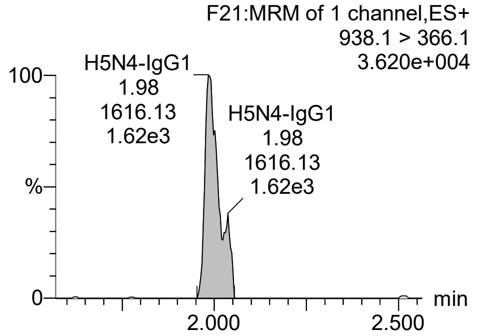

Sample Name: 20211111_L12_R2_IgG

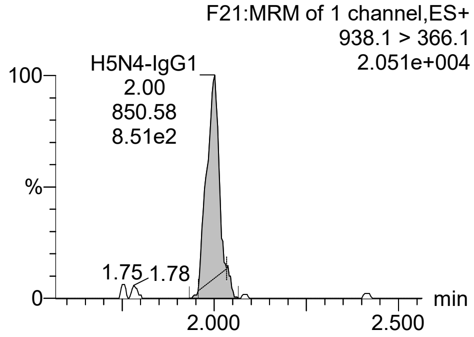

Sample Name: 20211111_H6_R2_IgG

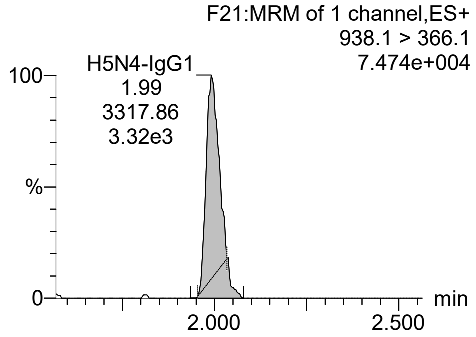

Sample Name: 20211111_A13_R2_IgG

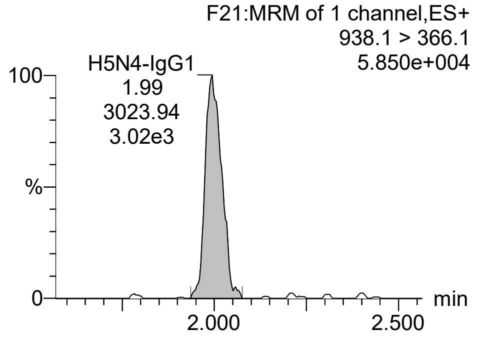

Sample Name: 20211111_A14_R2_IgG

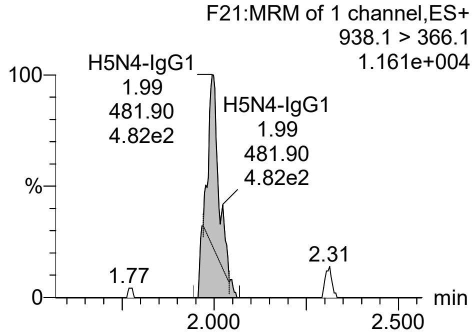

Sample Name: 20211111_L13_R2_IgG

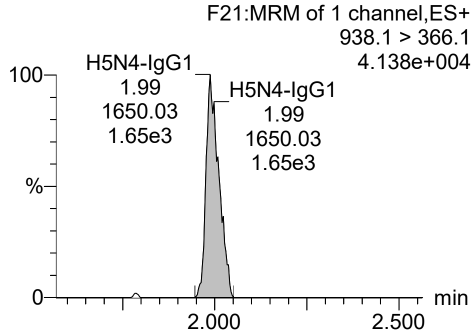

Sample Name: 20211111_L14_R2_IgG

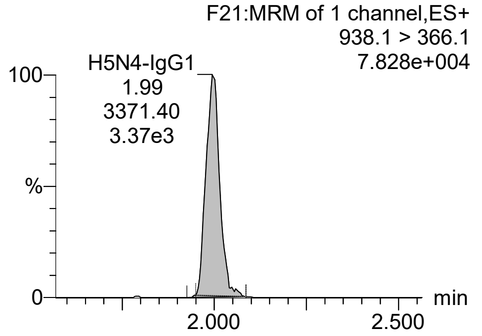

Sample Name: 20211111_H7_R2_IgG

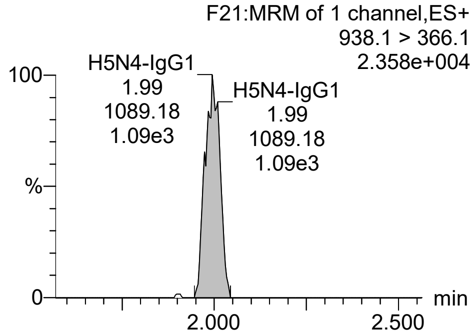

Sample Name: 20211111_A15_R2_IgG

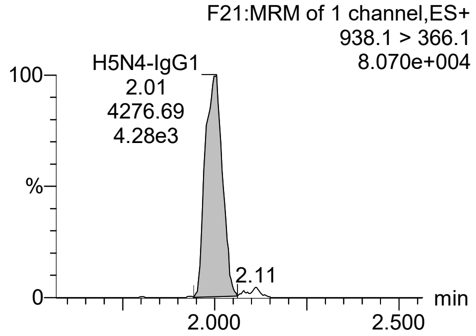

Sample Name: 20211111_QC_R12_IgG

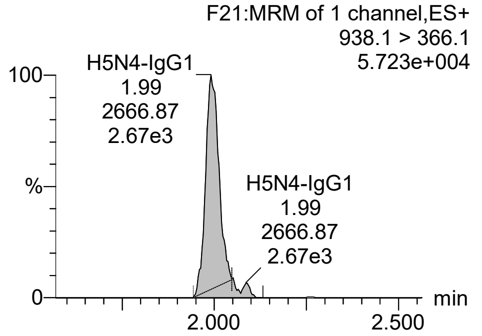

Sample Name: 20211111_A16_R2_IgG

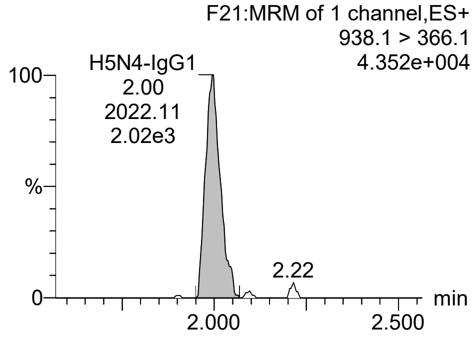

Sample Name: 20211111_L15_R2_IgG

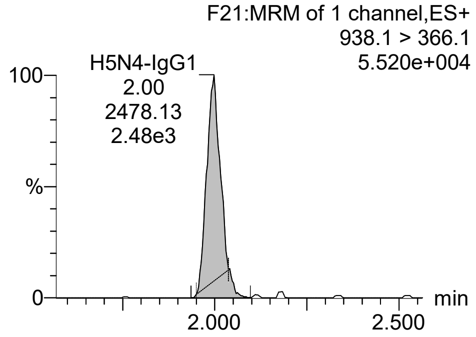

Sample Name: 20211111_L16_R2_IgG

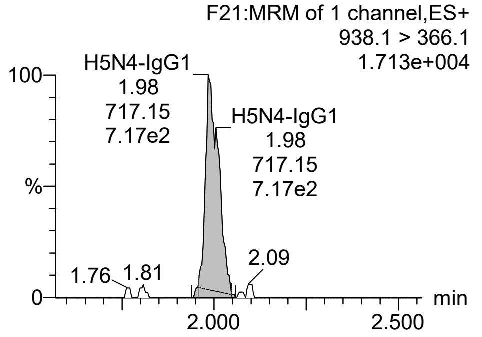

Dataset: C:\MassLynx\10.pro\20211115_clinicalsamples_IgG.qld

Last Altered: Sunday, October 02, 2022 16:02:30 台北標準時間
Printed: Friday, August 01, 2025 16:46:23 台北標準時間

Sample Name: 20211111_H8_R2_IgG

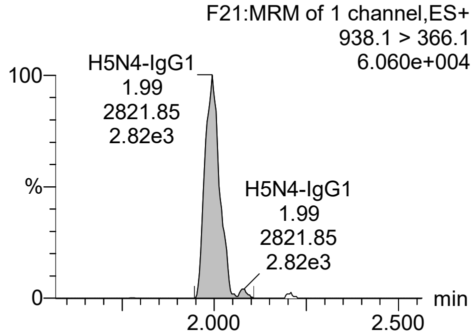

Sample Name: 20211111_A17_R2_IgG

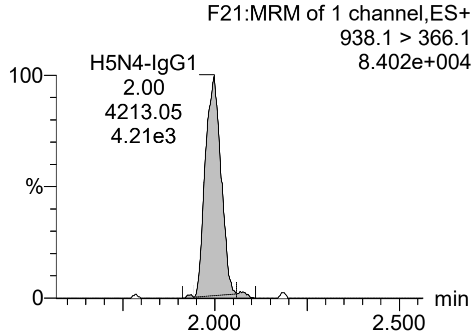

Sample Name: 20211111_A18_R2_IgG

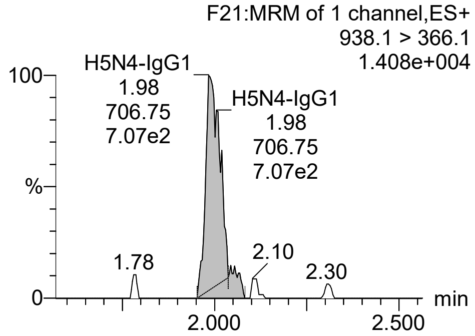

Sample Name: 20211111_L17_R2_IgG

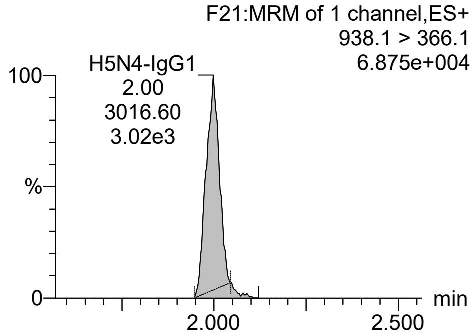

Sample Name: 20211111_L18_R2_IgG

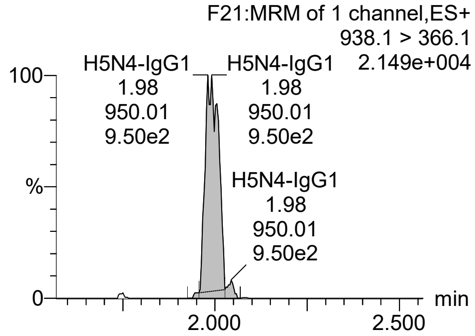

Sample Name: 20211111_H9_R2_IgG

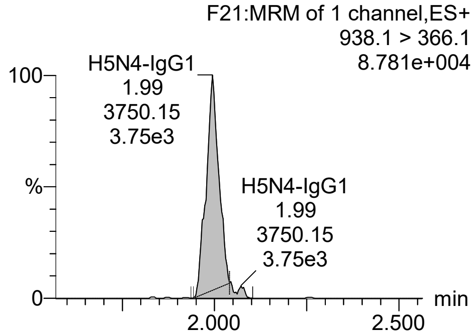

Sample Name: 20211111_A19_R2_IgG

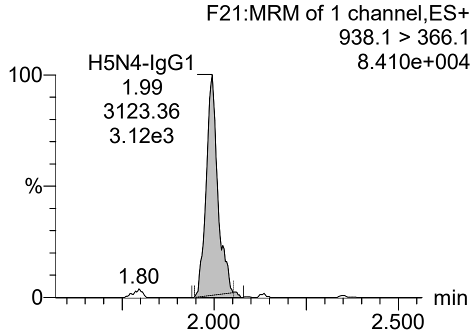

Sample Name: 20211111_A20_R2_IgG

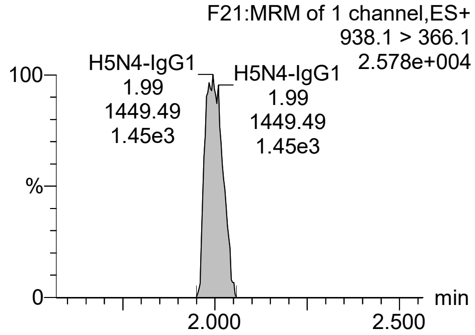

Sample Name: 20211111_L19_R2_IgG

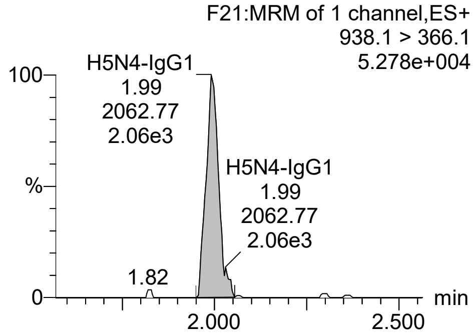

Sample Name: 20211111_QC_R13_IgG

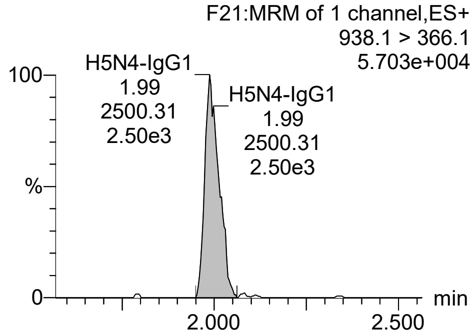

Sample Name: 20211111_L20_R2_IgG

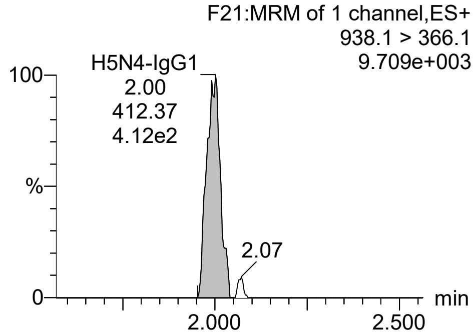

Sample Name: 20211111_H10_R2_IgG

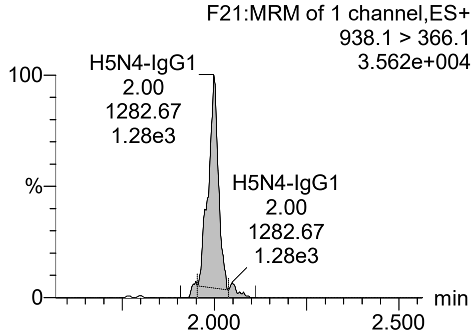

Sample Name: 20211111_A21_R2_IgG

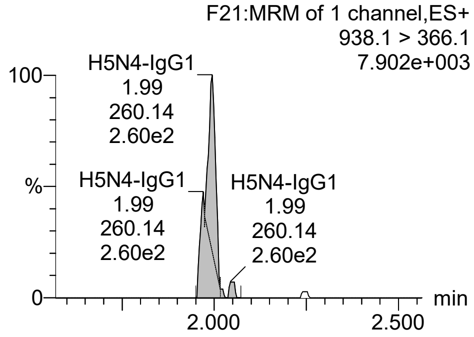

Sample Name: 20211111_A22_R2_IgG

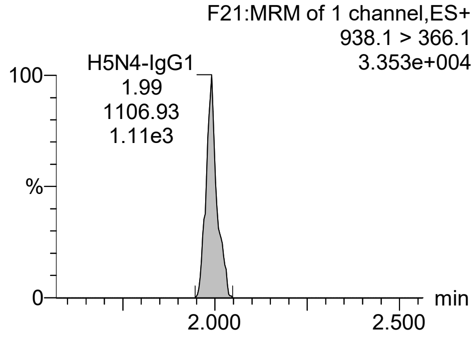

Sample Name: 20211111_L21_R2_IgG

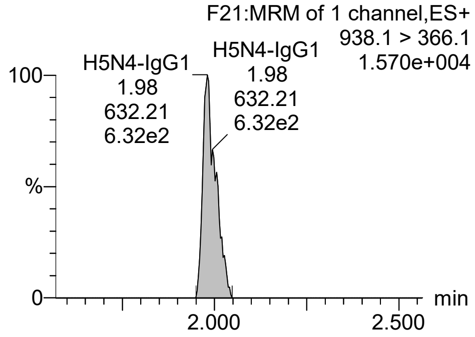

Sample Name: 20211111_L22_R2_IgG

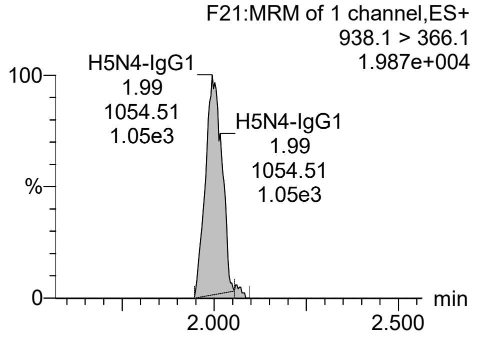

Sample Name: 20211111_H11_R2_IgG

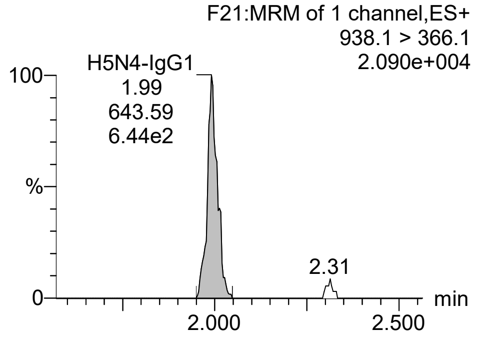

Sample Name: 20211111_A23_R2_IgG

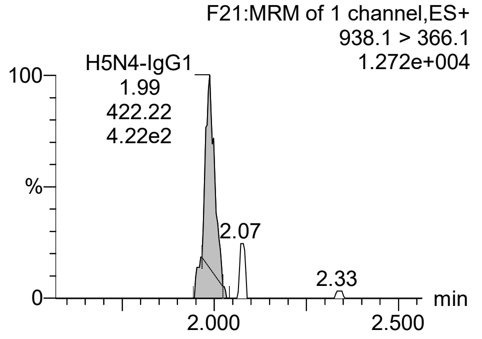

Sample Name: 20211111_A24_R2_IgG

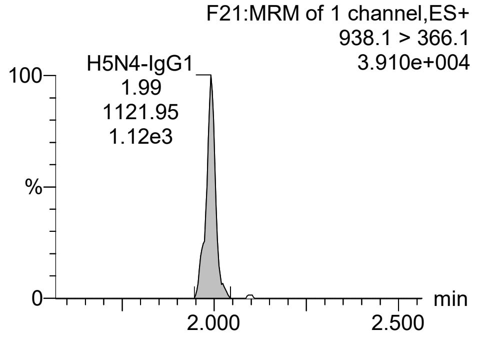

Sample Name: 20211111_L23_R2_IgG

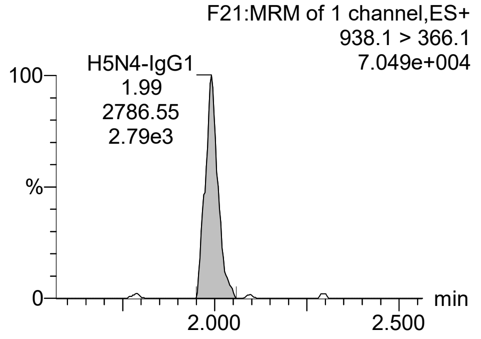

Sample Name: 20211111_L24_R2_IgG

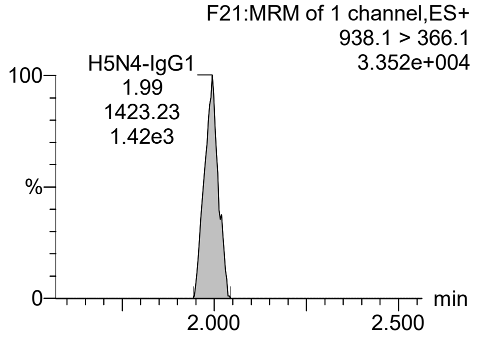

Sample Name: 20211111_H12_R2_IgG

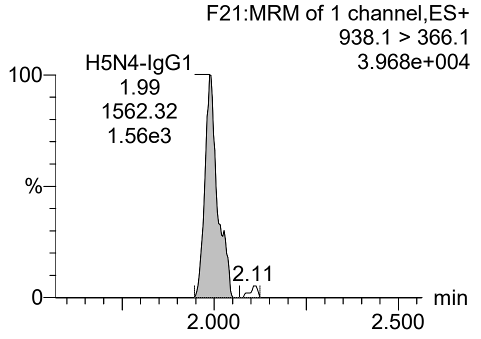

Sample Name: 20211111_QC_R14_IgG

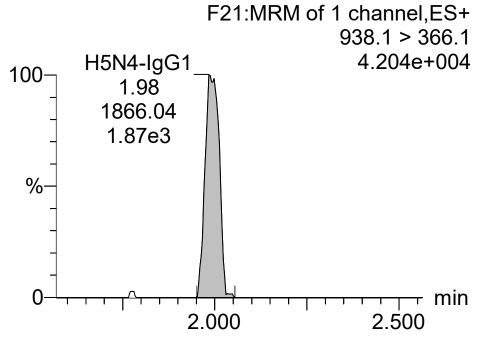

Sample Name: 20211111_A25_R2_IgG

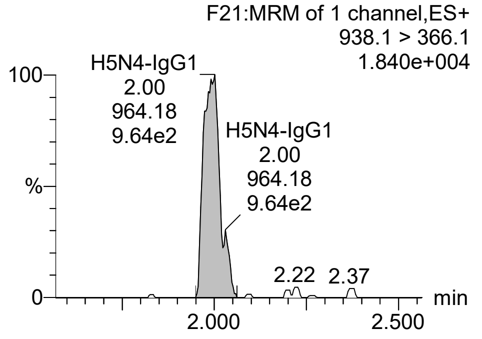

Sample Name: 20211111_A26_R2_IgG

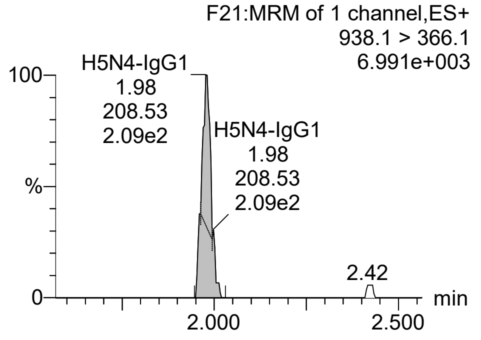

Sample Name: 20211111_L25_R2_IgG

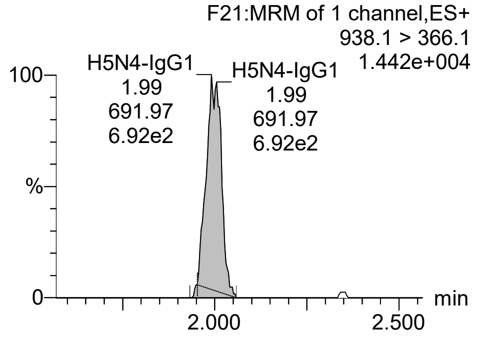

Sample Name: 20211111_L26_R2_IgG

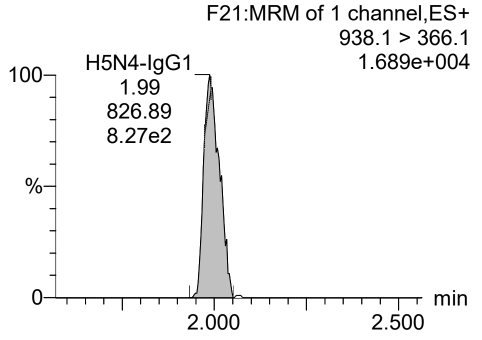

Sample Name: 20211111_H13_R2_IgG

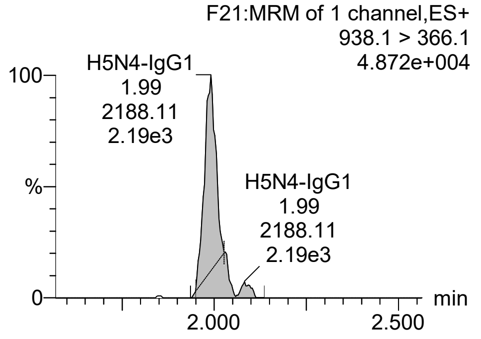

Sample Name: 20211111_A27_R2_IgG

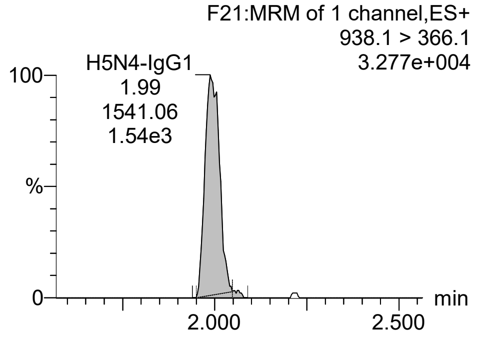

Sample Name: 20211111_A28_R2_IgG

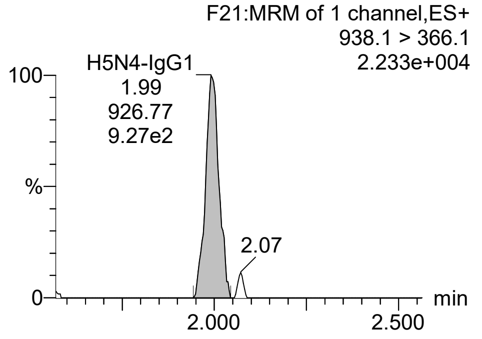

Sample Name: 20211111_L27_R2_IgG

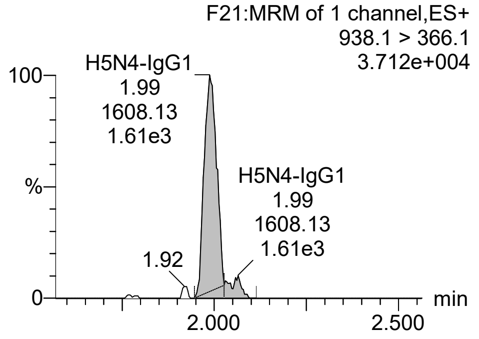

Sample Name: 20211111_L28_R2_IgG

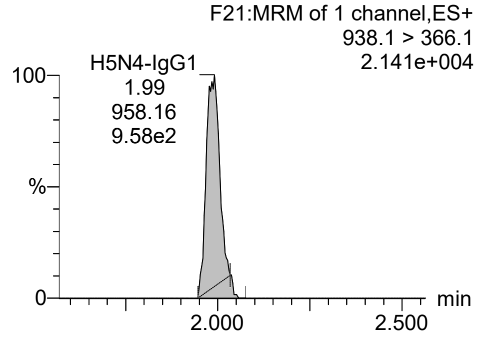

Sample Name: 20211111_H14_R2_IgG

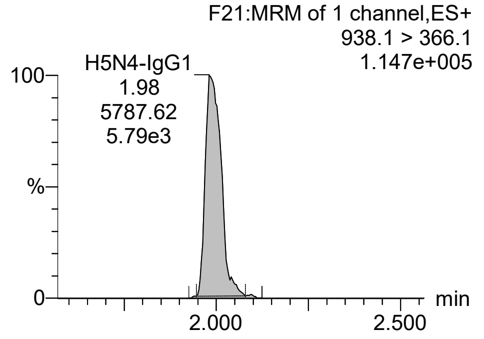

Sample Name: 20211111_A29_R2_IgG

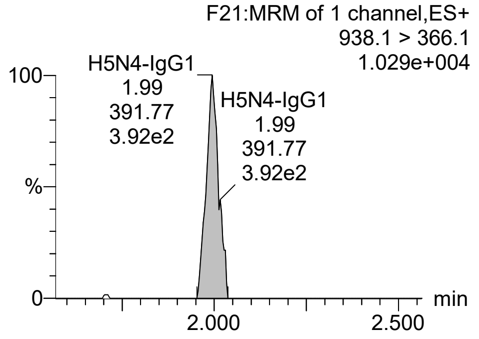

Sample Name: 20211111_A30_R2_IgG

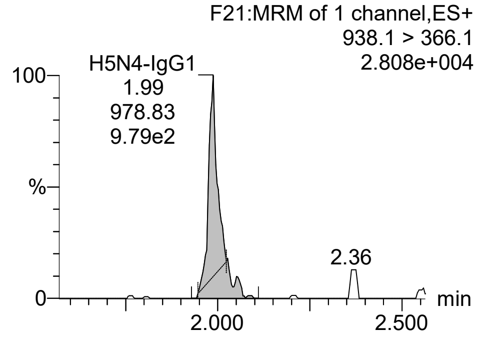

Sample Name: 20211111_QC_R15_IgG

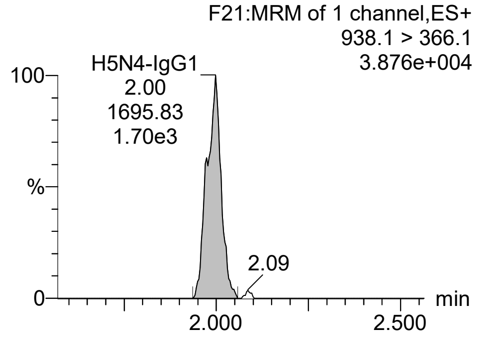

Sample Name: 20211111_L29_R2_IgG

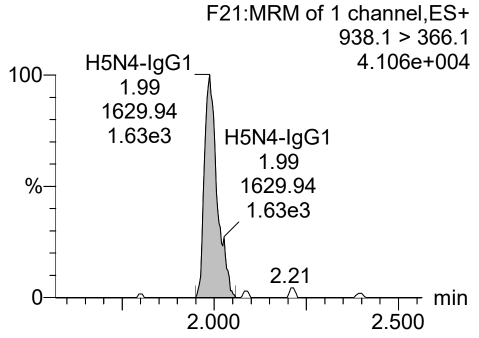

Sample Name: 20211111_L30_R2_IgG

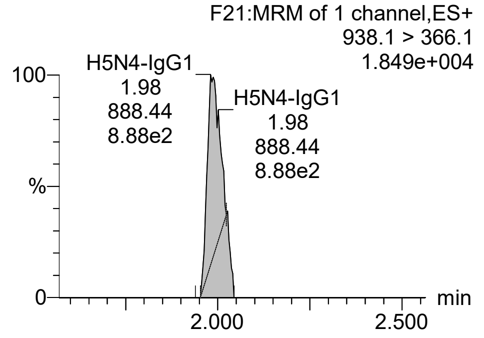

Sample Name: 20211111_H15_R2_IgG

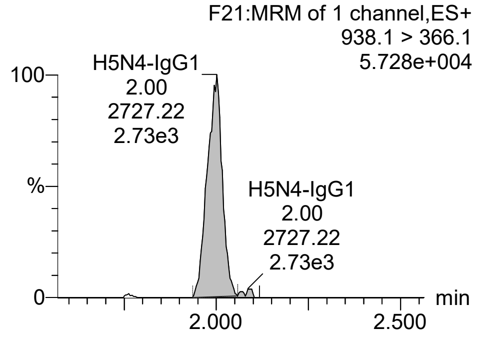

Sample Name: 20211111_A31_R2_IgG

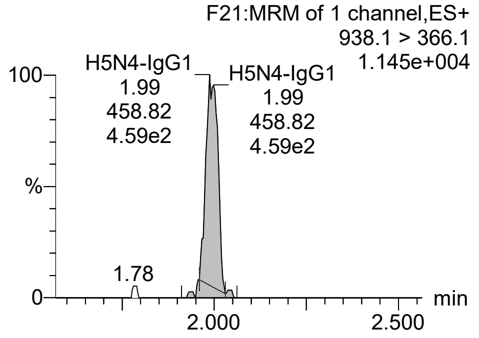

Sample Name: 20211111_A32_R2_IgG

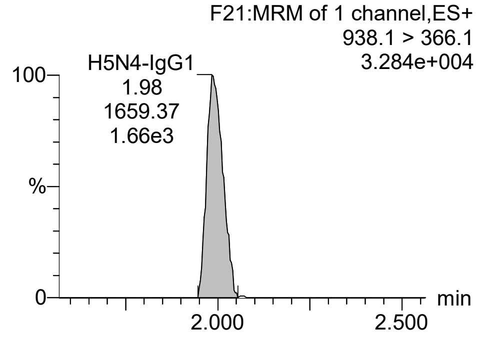

Sample Name: 20211111_L31_R2_IgG

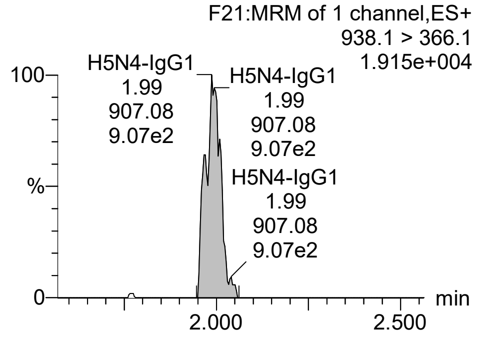

Sample Name: 20211111_L32_R2_IgG

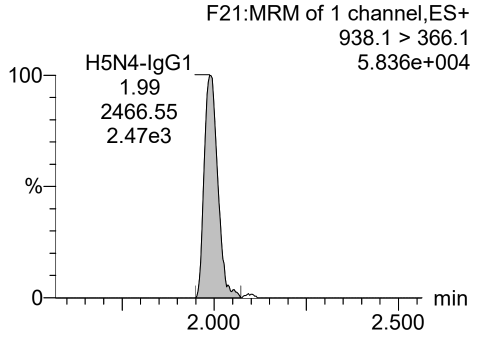

Sample Name: 20211111_H16_R2_IgG

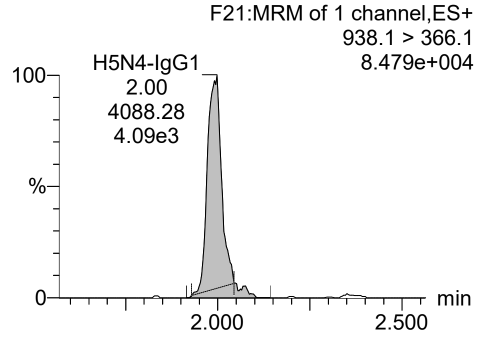

Sample Name: 20211111_A33_R2_IgG

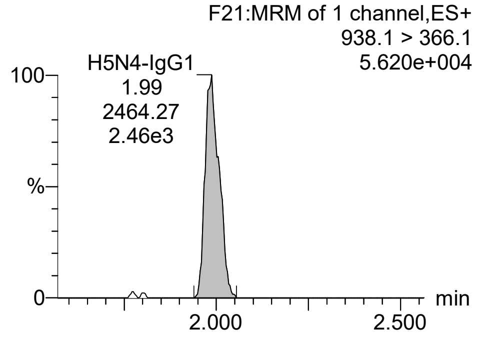

Dataset: C:\MassLynx\10.pro\20211115_clinicalsamples_IgG.qld

Last Altered: Sunday, October 02, 2022 16:02:30 台北標準時間
Printed: Friday, August 01, 2025 16:46:23 台北標準時間

Sample Name: 20211111_A34_R2_IgG

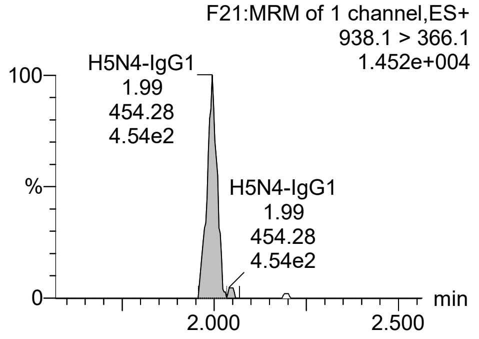

Sample Name: 20211111_H17_R2_IgG

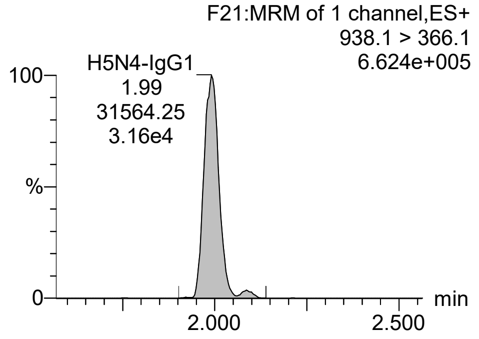

Sample Name: 20211111_A35_R2_IgG

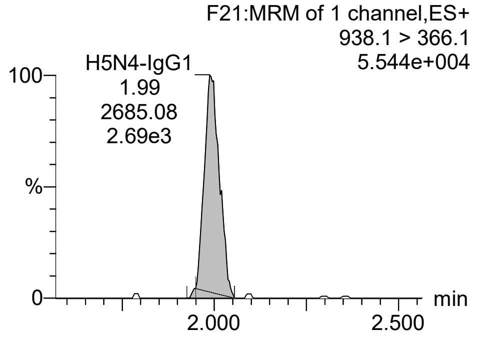

Sample Name: 20211111_QC_R16_IgG

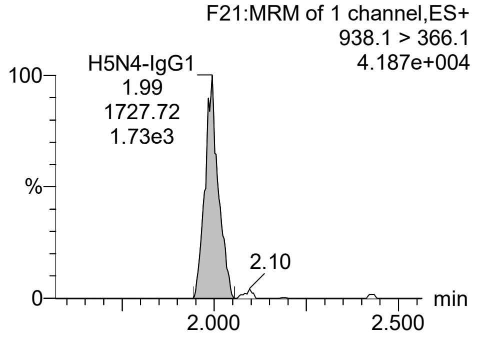

Sample Name: 20211111_A36_R2_IgG

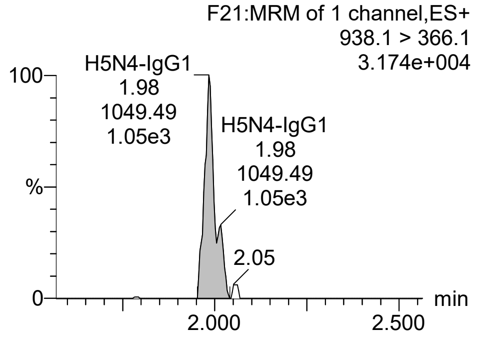

Sample Name: 20211111_H18_R2_IgG

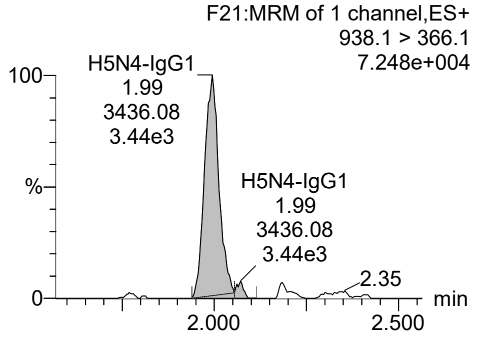

Sample Name: 20211111_A37_R2_IgG

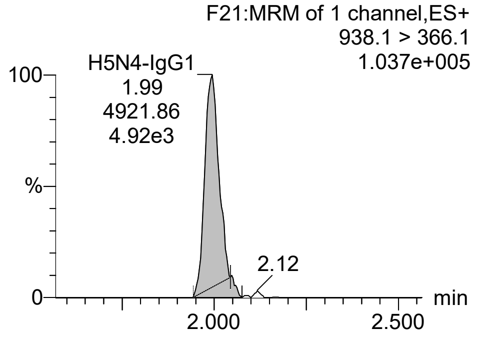

Sample Name: 20211111_A38_R2_IgG

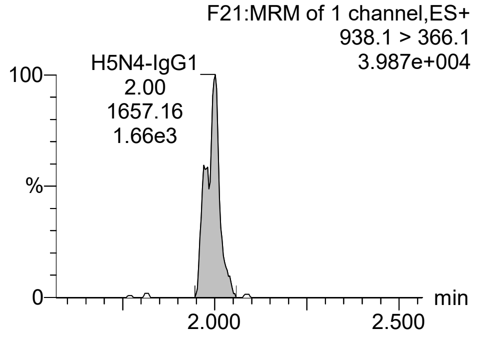

Sample Name: 20211111_H19_R2_IgG

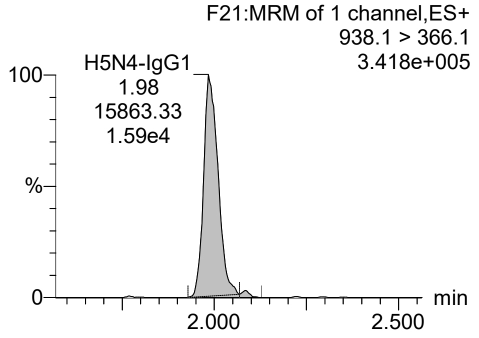

Sample Name: 20211111_A39_R2_IgG

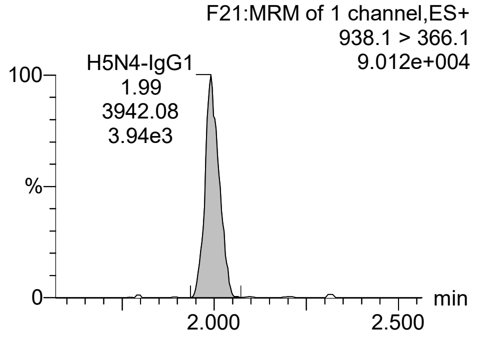

Sample Name: 20211111_A40_R2_IgG

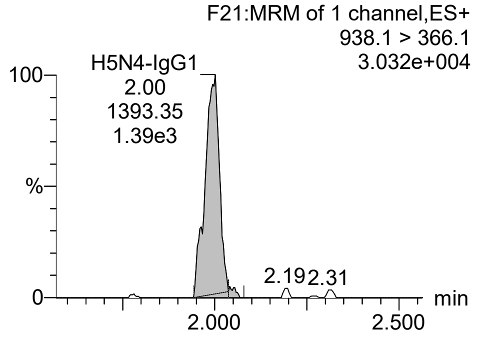

Sample Name: 20211111_H20_R2_IgG

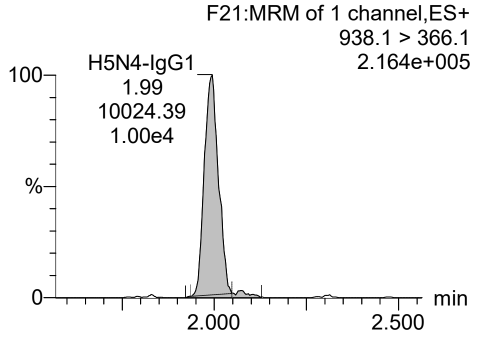

Sample Name: 20211111_A41_R2_IgG

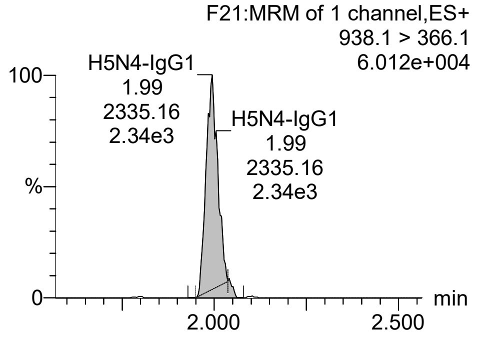

Sample Name: 20211111_A42_R2_IgG

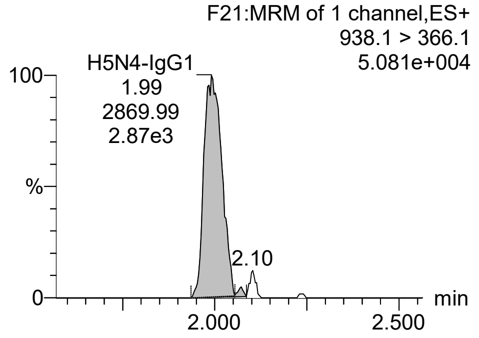

Sample Name: 20211111_A43_R2_IgG

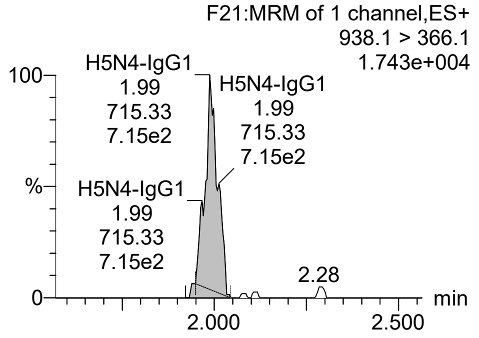

Sample Name: 20211111_A44_R2_IgG

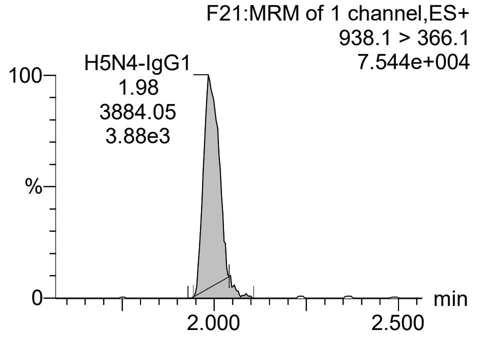

Sample Name: 20211111_QC_R17_IgG

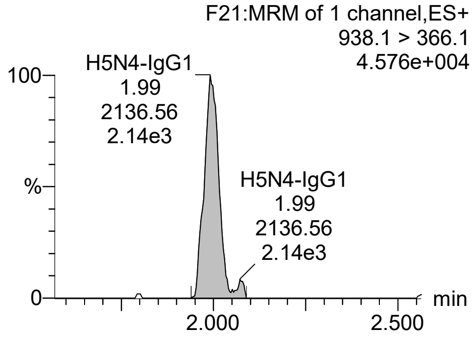

	# Name	RT	Area	IS Area	Response	Prima...
1	1 20211111_QC_R1_IgG	2.00	4044.3	64968.0	0.062	MM
2	2 20211111_A1_R1_IgG	2.01	1371.4	59059.2	0.023	MM
3	3 20211111_A2_R1_IgG	1.99	4879.2	66621.2	0.073	MM
4	4 20211111_L1_R1_IgG	1.99	3937.9	64201.0	0.061	bb
5	5 20211111_L2_R1_IgG	2.00	4107.7	46950.2	0.087	MM
6	6 20211111_H1_R1_IgG	1.99	1295.8	38648.2	0.034	MM
7	7 20211111_A3_R1_IgG			2603.7		
8	8 20211111_A4_R1_IgG	2.00	1385.9	69960.4	0.020	bb
9	9 20211111_L3_R1_IgG	2.00	912.6	38864.7	0.023	MM
10	10 20211111_L4_R1_IgG	2.00	2908.1	43380.5	0.067	MM
11	11 20211111_H2_R1_IgG	2.00	2996.8	71349.2	0.042	bb
12	12 20211111_A5_R1_IgG	1.99	4074.3	65569.7	0.062	MM
13	13 20211111_A6_R1_IgG	2.00	4424.7	53120.7	0.083	bb
14	14 20211111_QC_R2_IgG	2.00	4862.0	81034.1	0.060	MM
15	15 20211111_L5_R1_IgG	1.99	3383.6	50994.6	0.066	bb
16	16 20211111_L6_R1_IgG	2.00	2192.0	58058.0	0.038	bb
17	17 20211111_H3_R1_IgG	2.02	2265.0	64028.6	0.035	bb
18	18 20211111_A7_R1_IgG	2.00	1320.3	82996.1	0.016	bb
19	19 20211111_A8_R1_IgG	1.99	1725.4	77731.7	0.022	MM
20	20 20211111_L7_R1_IgG	2.01	2893.5	38827.3	0.075	MM
21	21 20211111_L8_R1_IgG	1.99	2717.9	44220.1	0.061	MM
22	22 20211111_H4_R1_IgG	1.99	4211.7	65826.3	0.064	bb
23	23 20211111_A9_R1_IgG	1.99	535.5	60771.1	0.009	MM
24	24 20211111_A10_R1_IgG	1.99	933.7	48578.7	0.019	MM
25	25 20211111_L9_R1_IgG	1.99	2296.8	50830.7	0.045	MM
26	26 20211111_L10_R1_IgG	1.99	1581.0	38034.2	0.042	bb
27	27 20211111_QC_R3_IgG	1.99	3929.9	67025.5	0.059	MM
28	28 20211111_H5_R1_IgG	1.99	3120.9	44544.9	0.070	bb
29	29 20211111_A11_R1_IgG	1.98	1221.7	66408.5	0.018	bb
30	30 20211111_A12_R1_IgG	2.00	1150.4	46624.5	0.025	bb
31	31 20211111_L11_R1_IgG	2.01	1730.5	33425.1	0.052	bb
32	32 20211111_L12_R1_IgG	1.99	1252.3	41178.0	0.030	bb
33	33 20211111_H6_R1_IgG	2.00	5318.3	58593.4	0.091	MM
34	34 20211111_A13_R1_IgG	1.99	3874.8	54133.0	0.072	bb
35	35 20211111_A14_R1_IgG	2.02	941.7	56467.3	0.017	bb
36	36 20211111_L13_R1_IgG	2.00	1366.7	36024.4	0.038	bb
37	37 20211111_L14_R1_IgG	2.01	3212.1	37181.6	0.086	MM
38	38 20211111_H7_R1_IgG	2.00	1248.9	42850.6	0.029	bb
39	39 20211111_A15_R1_IgG	2.01	5160.4	62015.1	0.083	MM
40	40 20211111_QC_R4_IgG	2.00	3493.9	58572.5	0.060	bb
41	41 20211111_A16_R1_IgG	2.00	3198.4	78283.8	0.041	MM
42	42 20211111_L15_R1_IgG	2.01	3558.1	44158.2	0.081	bb
43	43 20211111_L16_R1_IgG	1.99	1490.8	55150.8	0.027	MM
44	44 20211111_H8_R1_IgG	1.99	2104.4	42965.2	0.049	MM
45	45 20211111_A17_R1_IgG	2.00	4319.8	39717.7	0.109	MM
46	46 20211111_A18_R1_IgG	2.02	969.0	44675.4	0.022	MM
47	47 20211111_L17_R1_IgG	2.00	2766.0	37854.1	0.073	bb
48	48 20211111_L18_R1_IgG	1.99	971.8	50716.9	0.019	MM
49	49 20211111_H9_R1_IgG	1.99	3632.2	36276.0	0.100	MM
50	50 20211111_A19_R1_IgG	1.99	3411.3	42862.5	0.080	MM
51	51 20211111_A20_R1_IgG	1.99	1998.7	36542.7	0.055	bb
52	52 20211111_L19_R1_IgG	1.99	3144.7	53892.8	0.058	bb
53	53 20211111_QC_R5_IgG	2.00	2976.9	56513.2	0.053	MM
54	54 20211111_L20_R1_IgG	2.00	1309.1	56499.9	0.023	MM
55	55 20211111_H10_R1_IgG	1.99	5782.3	68120.5	0.085	MM
56	56 20211111_A21_R1_IgG	1.98	747.2	63699.8	0.012	MM

Dataset: C:\MassLynx\10.pro\20211115_clinicalsamples_IgG.qld

Last Altered: Sunday, October 02, 2022 16:02:30 台北標準時間
Printed: Friday, August 01, 2025 16:46:23 台北標準時間

	# Name	RT	Area	IS Area	Response	Prima...
57	57 20211111_A22_R1_IgG	1.99	2583.5	52222.1	0.049	MM
58	58 20211111_L21_R1_IgG	2.00	1295.1	54372.5	0.024	MM
59	59 20211111_L22_R1_IgG	1.99	1967.5	41353.1	0.048	MM
60	60 20211111_H11_R1_IgG	1.99	911.4	65579.1	0.014	bb
61	61 20211111_A23_R1_IgG	1.99	965.6	64131.1	0.015	bb
62	62 20211111_A24_R1_IgG	1.99	1591.1	38724.8	0.041	MM
63	63 20211111_L23_R1_IgG	2.00	4891.6	64245.2	0.076	bb
64	64 20211111_L24_R1_IgG	1.99	2364.2	45298.0	0.052	MM
65	65 20211111_H12_R1_IgG	1.99	1809.1	44679.3	0.040	MM
66	66 20211111_QC_R6_IgG	1.99	2503.3	48165.0	0.052	MM
67	67 20211111_A25_R1_IgG	2.00	1185.9	57169.9	0.021	bb
68	68 20211111_A26_R1_IgG	1.99	90.0	34254.0	0.003	MM
69	69 20211111_L25_R1_IgG	1.99	2151.2	67491.5	0.032	MM
70	70 20211111_L26_R1_IgG	1.99	1608.0	55934.1	0.029	bb
71	71 20211111_H13_R1_IgG	1.99	3394.7	48767.3	0.070	MM
72	72 20211111_A27_R1_IgG	1.99	2631.6	57269.7	0.046	bb
73	73 20211111_A28_R1_IgG	2.00	1388.1	49231.1	0.028	MM
74	74 20211111_L27_R1_IgG	1.99	1962.6	60014.5	0.033	bb
75	75 20211111_L28_R1_IgG	1.99	1620.0	35062.6	0.046	bb
76	76 20211111_H14_R1_IgG	1.99	8678.3	62371.9	0.139	MM
77	77 20211111_A29_R1_IgG	1.99	725.6	43858.2	0.017	bb
78	78 20211111_A30_R1_IgG	1.99	1662.8	62500.4	0.027	MM
79	79 20211111_QC_R7_IgG	2.00	2786.5	43946.9	0.063	MM
80	80 20211111_L29_R1_IgG	2.00	1782.0	38728.9	0.046	MM
81	81 20211111_L30_R1_IgG	2.00	948.9	42613.2	0.022	bb
82	82 20211111_H15_R1_IgG	1.99	2979.5	39874.6	0.075	MM
83	83 20211111_A31_R1_IgG	1.99	712.3	53414.3	0.013	bb
84	84 20211111_A32_R1_IgG	1.99	2270.1	41844.6	0.054	MM
85	85 20211111_L31_R1_IgG	1.99	946.8	40553.7	0.023	MM
86	86 20211111_L32_R1_IgG	1.99	2719.4	49618.7	0.055	bb
87	87 20211111_H16_R1_IgG	2.00	6162.7	60170.9	0.102	bb
88	88 20211111_A33_R1_IgG	2.00	3612.4	48588.2	0.074	MM
89	89 20211111_A34_R1_IgG	2.00	535.2	43288.0	0.012	MM
90	90 20211111_H17_R1_IgG	1.99	32221.6	47104.5	0.684	bb
91	91 20211111_A35_R1_IgG	1.99	2928.9	41025.9	0.071	bb
92	92 20211111_QC_R8_IgG	2.00	2598.6	40546.3	0.064	bb
93	93 20211111_A36_R1_IgG	2.00	635.1	44504.4	0.014	bb
94	94 20211111_H18_R1_IgG	2.00	3156.1	34162.2	0.092	MM
95	95 20211111_A37_R1_IgG	2.00	4626.7	39760.0	0.116	bb
96	96 20211111_A38_R1_IgG	2.00	1772.9	43982.6	0.040	bb
97	97 20211111_H19_R1_IgG	2.00	16742.7	45314.8	0.369	bb
98	98 20211111_A39_R1_IgG	2.00	4052.6	39388.0	0.103	MM
99	99 20211111_A40_R1_IgG	2.00	1776.9	38726.4	0.046	bb
100	1... 20211111_H20_R1_IgG	2.00	10392.5	37814.4	0.275	MM
101	1... 20211111_A41_R1_IgG	2.01	2514.4	48930.3	0.051	bb
102	1... 20211111_A42_R1_IgG	2.01	3339.8	40406.8	0.083	MM
103	1... 20211111_A43_R1_IgG	2.00	691.0	44421.5	0.016	bb
104	1... 20211111_A44_R1_IgG	2.01	5800.3	53128.2	0.109	bb
105	1... 20211111_QC_R9_IgG	2.00	2280.1	37049.0	0.062	MM
106	1... 20211111_A1_R2_IgG	2.00	910.4	46499.4	0.020	MM
107	1... 20211111_A2_R2_IgG	1.99	3852.9	57260.7	0.067	MM
108	1... 20211111_L1_R2_IgG	2.00	2433.4	48062.9	0.051	MM
109	1... 20211111_L2_R2_IgG	2.00	3583.8	38745.2	0.092	MM
110	1... 20211111_H1_R2_IgG	2.01	1769.0	44273.5	0.040	MM
111	1... 20211111_A3_R2_IgG	2.01	64.7	4755.5	0.014	bb
112	1... 20211111_A4_R2_IgG	2.01	929.3	50546.7	0.018	MM

Dataset: C:\MassLynx\10.pro\20211115_clinicalsamples_IgG.qld

Last Altered: Sunday, October 02, 2022 16:02:30 台北標準時間
Printed: Friday, August 01, 2025 16:46:23 台北標準時間

	#	Name	RT	Area	IS Area	Response	Prima...
113	1...	20211111_L3_R2_IgG	1.99	801.0	34825.8	0.023	MM
114	1...	20211111_L4_R2_IgG	1.99	2502.4	39107.7	0.064	MM
115	1...	20211111_H2_R2_IgG	1.98	1868.2	49750.0	0.038	MM
116	1...	20211111_A5_R2_IgG	1.99	3291.7	52536.2	0.063	MM
117	1...	20211111_A6_R2_IgG	1.99	3847.3	42050.0	0.091	bb
118	1...	20211111_QC_R10_IgG	1.99	2081.9	35350.8	0.059	MM
119	1...	20211111_L5_R2_IgG	2.00	2747.6	38039.3	0.072	MM
120	1...	20211111_L6_R2_IgG	2.00	1482.3	44907.4	0.033	MM
121	1...	20211111_H3_R2_IgG	1.99	1713.8	47729.1	0.036	bb
122	1...	20211111_A7_R2_IgG	1.99	603.1	57347.1	0.011	bb
123	1...	20211111_A8_R2_IgG	1.99	1238.6	52397.8	0.024	bb
124	1...	20211111_L7_R2_IgG	1.99	2321.9	36318.4	0.064	bb
125	1...	20211111_L8_R2_IgG	1.99	2093.4	38131.9	0.055	MM
126	1...	20211111_H4_R2_IgG	1.99	3442.3	46849.6	0.073	bb
127	1...	20211111_A9_R2_IgG	1.98	435.3	47759.6	0.009	bb
128	1...	20211111_A10_R2_IgG	2.00	812.6	41366.2	0.020	MM
129	1...	20211111_L9_R2_IgG	1.99	1634.3	43052.1	0.038	MM
130	1...	20211111_L10_R2_IgG	2.00	1434.0	31487.4	0.046	bb
131	1...	20211111_QC_R11_IgG	2.00	2000.4	37804.6	0.053	bb
132	1...	20211111_H5_R2_IgG	1.99	2685.3	40380.0	0.066	MM
133	1...	20211111_A11_R2_IgG	1.99	871.0	49533.3	0.018	MM
134	1...	20211111_A12_R2_IgG	1.99	575.4	40226.3	0.014	MM
135	1...	20211111_L11_R2_IgG	1.98	1616.1	28884.0	0.056	bb
136	1...	20211111_L12_R2_IgG	2.00	850.6	34351.3	0.025	MM
137	1...	20211111_H6_R2_IgG	1.99	3317.9	40466.6	0.082	MM
138	1...	20211111_A13_R2_IgG	1.99	3023.9	42306.7	0.071	bb
139	1...	20211111_A14_R2_IgG	1.99	481.9	44904.0	0.011	MM
140	1...	20211111_L13_R2_IgG	1.99	1650.0	31913.0	0.052	bb
141	1...	20211111_L14_R2_IgG	1.99	3371.4	31527.4	0.107	MM
142	1...	20211111_H7_R2_IgG	1.99	1089.2	40838.2	0.027	bb
143	1...	20211111_A15_R2_IgG	2.01	4276.7	49389.7	0.087	bb
144	1...	20211111_QC_R12_IgG	1.99	2666.9	35168.7	0.076	MM
145	1...	20211111_A16_R2_IgG	2.00	2022.1	47440.5	0.043	bb
146	1...	20211111_L15_R2_IgG	2.00	2478.1	33145.9	0.075	MM
147	1...	20211111_L16_R2_IgG	1.98	717.2	37596.6	0.019	MM
148	1...	20211111_H8_R2_IgG	1.99	2821.8	43933.3	0.064	bb
149	1...	20211111_A17_R2_IgG	2.00	4213.1	43605.5	0.097	MM
150	1...	20211111_A18_R2_IgG	1.98	706.7	37581.8	0.019	MM
151	1...	20211111_L17_R2_IgG	2.00	3016.6	33942.5	0.089	MM
152	1...	20211111_L18_R2_IgG	1.98	950.0	37831.5	0.025	MM
153	1...	20211111_H9_R2_IgG	1.99	3750.2	30898.4	0.121	MM
154	1...	20211111_A19_R2_IgG	1.99	3123.4	39352.1	0.079	MM
155	1...	20211111_A20_R2_IgG	1.99	1449.5	34661.2	0.042	bb
156	1...	20211111_L19_R2_IgG	1.99	2062.8	39918.3	0.052	bb
157	1...	20211111_QC_R13_IgG	1.99	2500.3	36239.1	0.069	bb
158	1...	20211111_L20_R2_IgG	2.00	412.4	32897.7	0.013	bb
159	1...	20211111_H10_R2_IgG	2.00	1282.7	31394.5	0.041	MM
160	1...	20211111_A21_R2_IgG	1.99	260.1	34863.7	0.007	MM
161	1...	20211111_A22_R2_IgG	1.99	1106.9	30342.1	0.036	bb
162	1...	20211111_L21_R2_IgG	1.98	632.2	33706.2	0.019	bb
163	1...	20211111_L22_R2_IgG	1.99	1054.5	31298.6	0.034	MM
164	1...	20211111_H11_R2_IgG	1.99	643.6	38904.1	0.017	bb
165	1...	20211111_A23_R2_IgG	1.99	422.2	32382.3	0.013	MM
166	1...	20211111_A24_R2_IgG	1.99	1122.0	30261.9	0.037	bb
167	1...	20211111_L23_R2_IgG	1.99	2786.5	43037.5	0.065	bb
168	1...	20211111_L24_R2_IgG	1.99	1423.2	37023.3	0.038	bb

Dataset: C:\MassLynx\10.pro\20211115_clinicalsamples_IgG.qld

Last Altered: Sunday, October 02, 2022 16:02:30 台北標準時間
Printed: Friday, August 01, 2025 16:46:23 台北標準時間

	# Name	RT	Area	IS Area	Response	Prima...
169	1... 20211111_H12_R2_IgG	1.99	1562.3	39478.2	0.040	MM
170	1... 20211111_QC_R14_IgG	1.98	1866.0	34463.2	0.054	bb
171	1... 20211111_A25_R2_IgG	2.00	964.2	39945.3	0.024	bb
172	1... 20211111_A26_R2_IgG	1.98	208.5	29663.2	0.007	MM
173	1... 20211111_L25_R2_IgG	1.99	692.0	38589.3	0.018	MM
174	1... 20211111_L26_R2_IgG	1.99	826.9	38405.2	0.022	MM
175	1... 20211111_H13_R2_IgG	1.99	2188.1	37771.2	0.058	MM
176	1... 20211111_A27_R2_IgG	1.99	1541.1	42634.1	0.036	MM
177	1... 20211111_A28_R2_IgG	1.99	926.8	33231.1	0.028	bb
178	1... 20211111_L27_R2_IgG	1.99	1608.1	41761.1	0.039	MM
179	1... 20211111_L28_R2_IgG	1.99	958.2	26968.6	0.036	MM
180	1... 20211111_H14_R2_IgG	1.98	5787.6	49250.2	0.118	MM
181	1... 20211111_A29_R2_IgG	1.99	391.8	37601.7	0.010	bb
182	1... 20211111_A30_R2_IgG	1.99	978.8	40366.8	0.024	MM
183	1... 20211111_QC_R15_IgG	2.00	1695.8	35156.1	0.048	bb
184	1... 20211111_L29_R2_IgG	1.99	1629.9	33813.1	0.048	bb
185	1... 20211111_L30_R2_IgG	1.98	888.4	33488.6	0.027	MM
186	1... 20211111_H15_R2_IgG	2.00	2727.2	38501.0	0.071	MM
187	1... 20211111_A31_R2_IgG	1.99	458.8	37525.5	0.012	MM
188	1... 20211111_A32_R2_IgG	1.98	1659.4	35770.8	0.046	bb
189	1... 20211111_L31_R2_IgG	1.99	907.1	33282.4	0.027	MM
190	1... 20211111_L32_R2_IgG	1.99	2466.5	42049.6	0.059	bb
191	1... 20211111_H16_R2_IgG	2.00	4088.3	45947.6	0.089	MM
192	1... 20211111_A33_R2_IgG	1.99	2464.3	40202.3	0.061	bb
193	1... 20211111_A34_R2_IgG	1.99	454.3	36747.0	0.012	MM
194	1... 20211111_H17_R2_IgG	1.99	31564.3	40920.9	0.771	bb
195	1... 20211111_A35_R2_IgG	1.99	2685.1	33689.2	0.080	MM
196	1... 20211111_QC_R16_IgG	1.99	1727.7	32783.6	0.053	bb
197	1... 20211111_A36_R2_IgG	1.98	1049.5	36822.4	0.029	bb
198	1... 20211111_H18_R2_IgG	1.99	3436.1	31769.3	0.108	MM
199	1... 20211111_A37_R2_IgG	1.99	4921.9	37581.8	0.131	MM
200	2... 20211111_A38_R2_IgG	2.00	1657.2	38390.4	0.043	bb
201	2... 20211111_H19_R2_IgG	1.98	15863.3	41217.0	0.385	MM
202	2... 20211111_A39_R2_IgG	1.99	3942.1	37392.7	0.105	bb
203	2... 20211111_A40_R2_IgG	2.00	1393.4	38295.1	0.036	MM
204	2... 20211111_H20_R2_IgG	1.99	10024.4	35941.8	0.279	MM
205	2... 20211111_A41_R2_IgG	1.99	2335.2	45304.9	0.052	MM
206	2... 20211111_A42_R2_IgG	1.99	2870.0	37803.8	0.076	MM
207	2... 20211111_A43_R2_IgG	1.99	715.3	38161.8	0.019	MM
208	2... 20211111_A44_R2_IgG	1.98	3884.1	37819.8	0.103	MM
209	2... 20211111_QC_R17_IgG	1.99	2136.6	31731.8	0.067	bb

Dataset: C:\MassLynx\10.pro\20211115_clinicalsamples_IgG.qld

Last Altered: Sunday, October 02, 2022 16:02:30 台北標準時間
Printed: Friday, August 01, 2025 16:46:52 台北標準時間

Method: C:\MassLynx\10_Pro_IgA.PRO\MethDB\20210926_IgG_DMRM_IgM_IS.mdb 16 Feb 2022 11:58:21
Calibration: 18 Nov 2021 13:56:22

Sample Name: 20211111_QC_R1_IgG

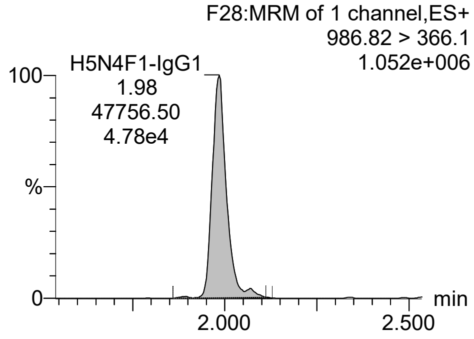

Sample Name: 20211111_A1_R1_IgG

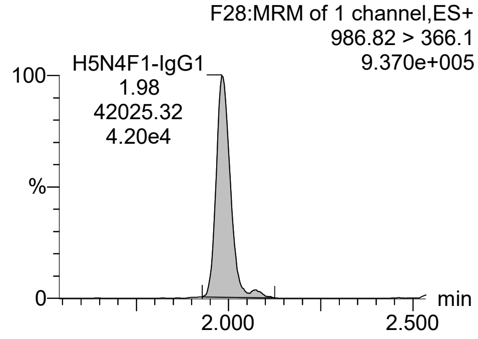

Sample Name: 20211111_A2_R1_IgG

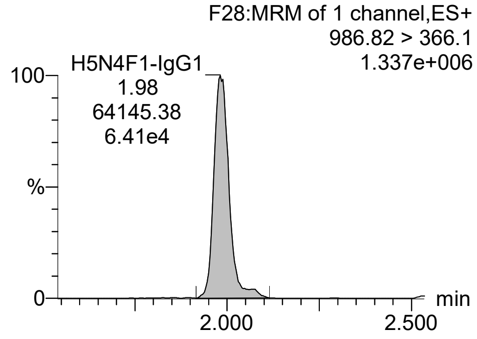

Sample Name: 20211111_L1_R1_IgG

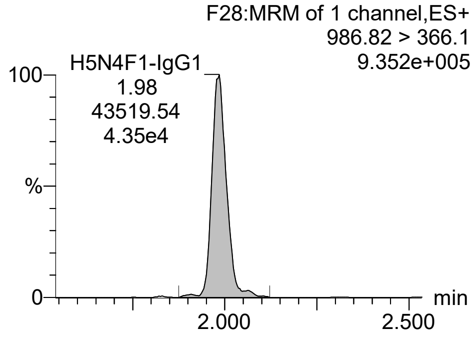

Sample Name: 20211111_L2_R1_IgG

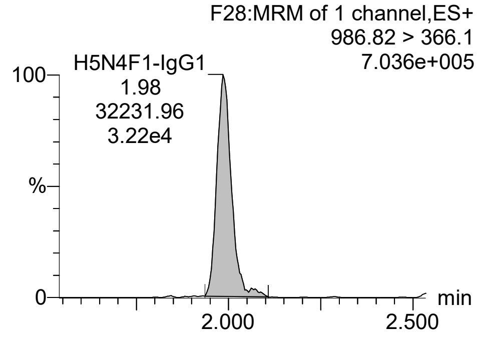

Sample Name: 20211111_H1_R1_IgG

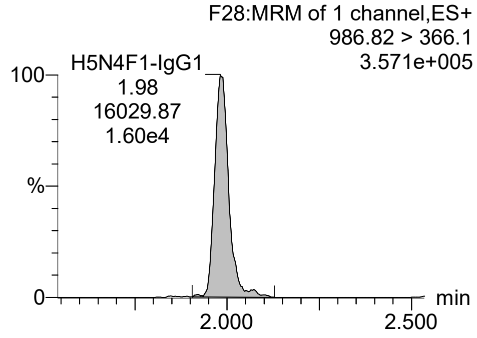

Sample Name: 20211111_A3_R1_IgG

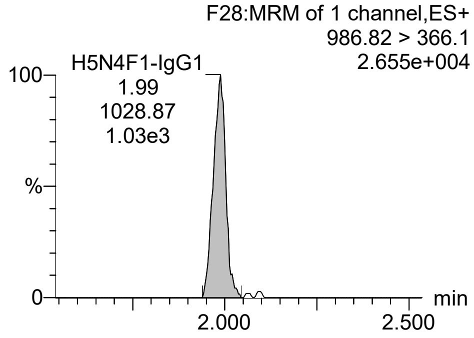

Sample Name: 20211111_A4_R1_IgG

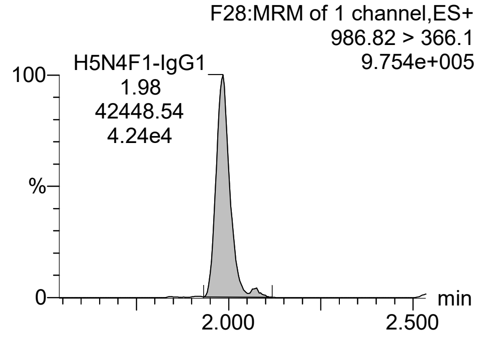

Sample Name: 20211111_L3_R1_IgG

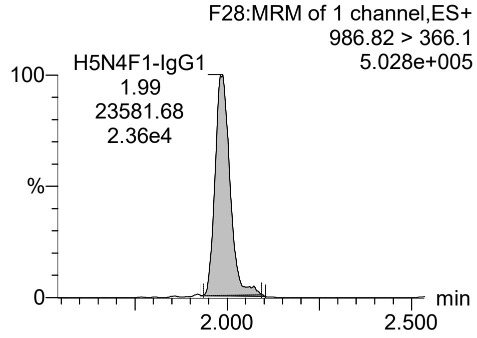

Sample Name: 20211111_L4_R1_IgG

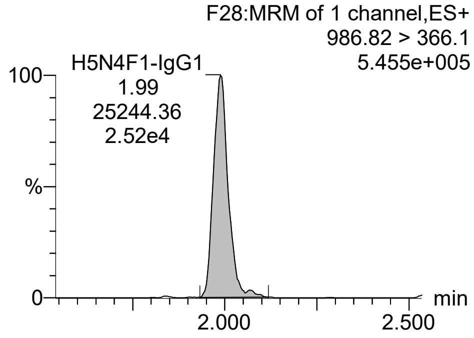

Sample Name: 20211111_H2_R1_IgG

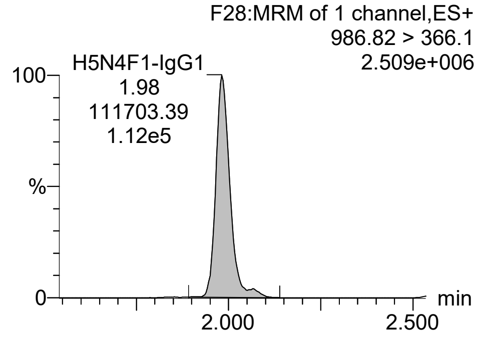

Sample Name: 20211111_A5_R1_IgG

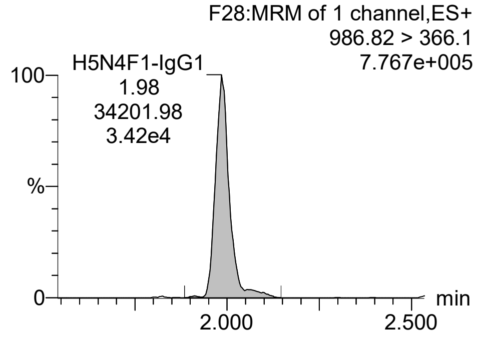

Dataset: C:\MassLynx\10.pro\20211115_clinicalsamples_IgG.qld

Last Altered: Sunday, October 02, 2022 16:02:30 台北標準時間
Printed: Friday, August 01, 2025 16:46:52 台北標準時間

Sample Name: 20211111_A6_R1_IgG

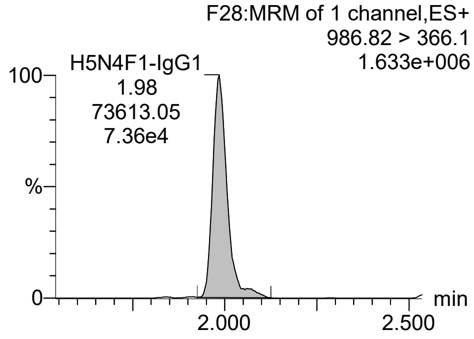

Sample Name: 20211111_QC_R2_IgG

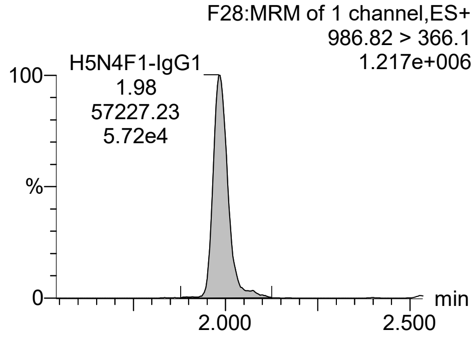

Sample Name: 20211111_L5_R1_IgG

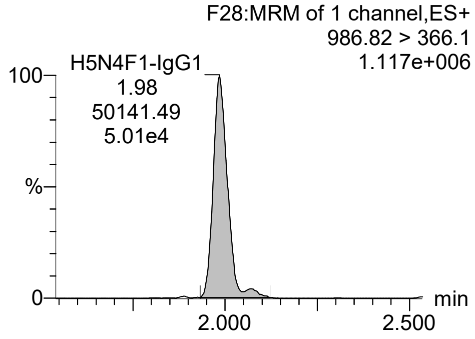

Sample Name: 20211111_L6_R1_IgG

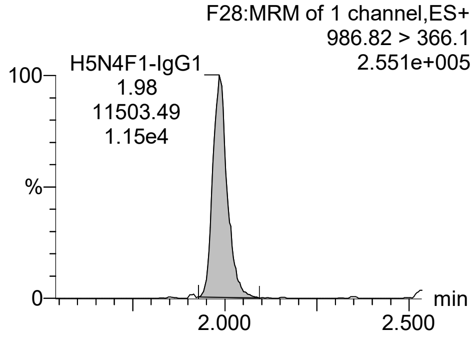

Sample Name: 20211111_H3_R1_IgG

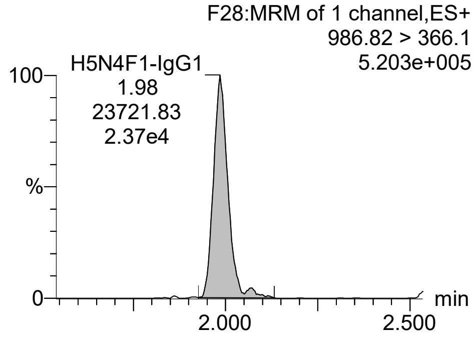

Sample Name: 20211111_A7_R1_IgG

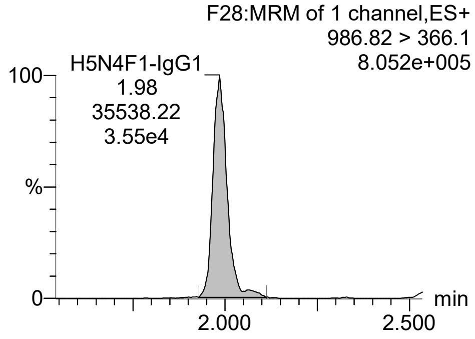

Sample Name: 20211111_A8_R1_IgG

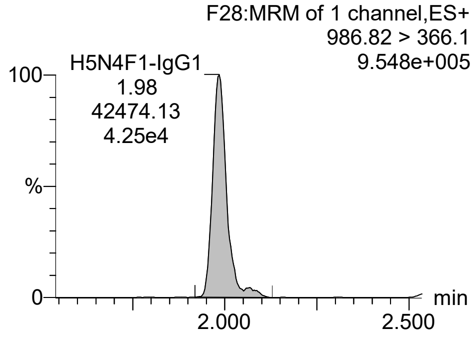

Sample Name: 20211111_L7_R1_IgG

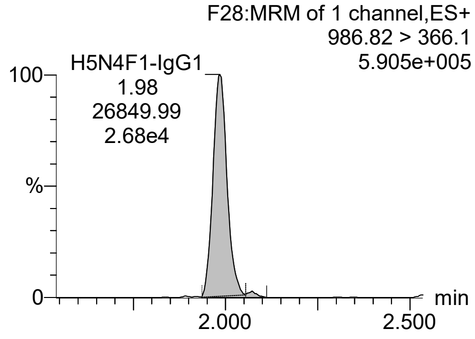

Sample Name: 20211111_L8_R1_IgG

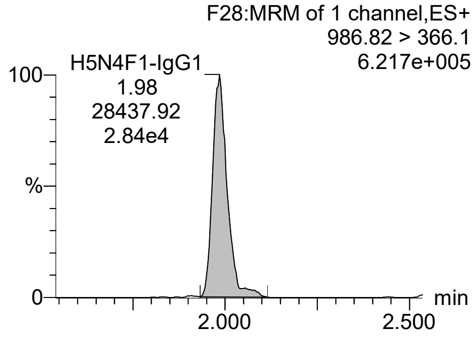

Sample Name: 20211111_H4_R1_IgG

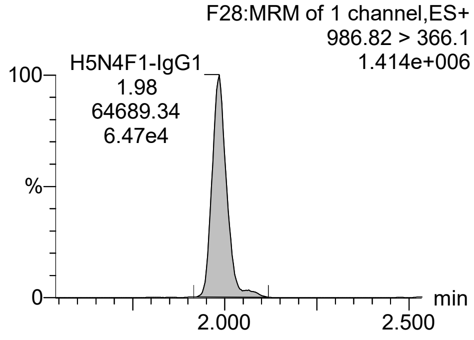

Sample Name: 20211111_A9_R1_IgG

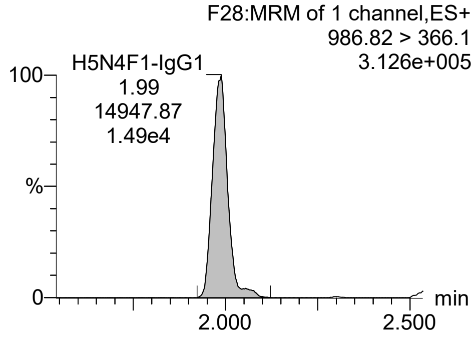

Sample Name: 20211111_A10_R1_IgG

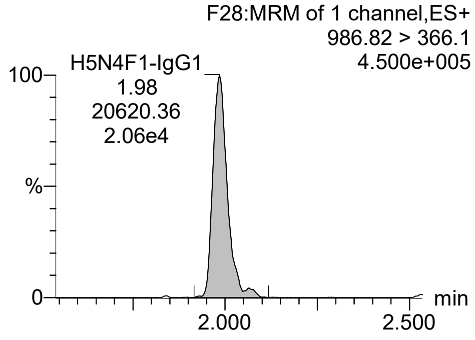

Sample Name: 20211111_L9_R1_IgG

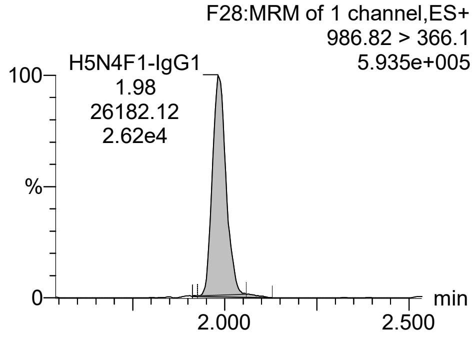

Sample Name: 20211111_L10_R1_IgG

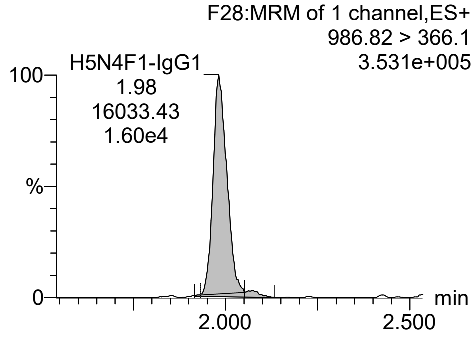

Sample Name: 20211111_QC_R3_IgG

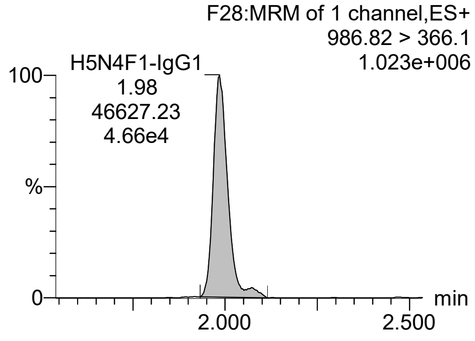

Dataset: C:\MassLynx\10.pro\20211115_clinicalsamples_IgG.qld

Last Altered: Sunday, October 02, 2022 16:02:30 台北標準時間
Printed: Friday, August 01, 2025 16:46:52 台北標準時間

Sample Name: 20211111_H5_R1_IgG

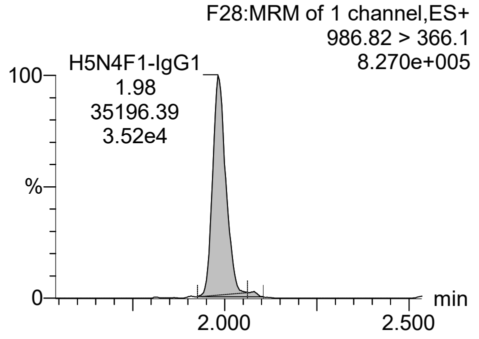

Sample Name: 20211111_A11_R1_IgG

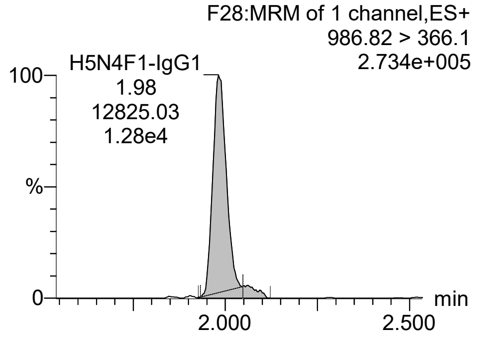

Sample Name: 20211111_A12_R1_IgG

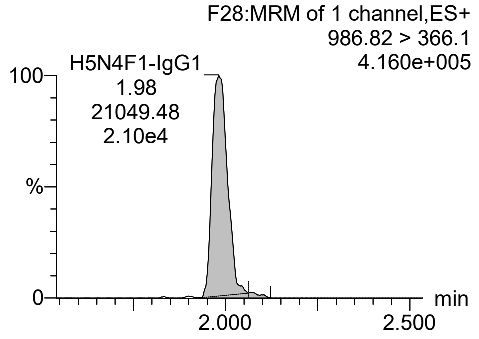

Sample Name: 20211111_L11_R1_IgG

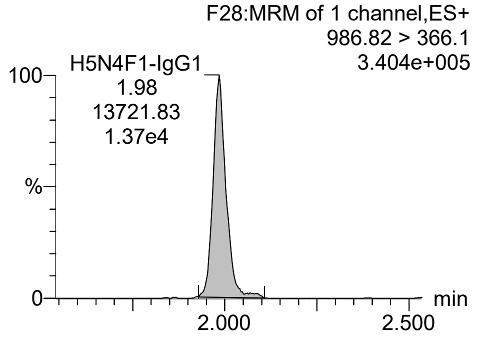

Sample Name: 20211111_L12_R1_IgG

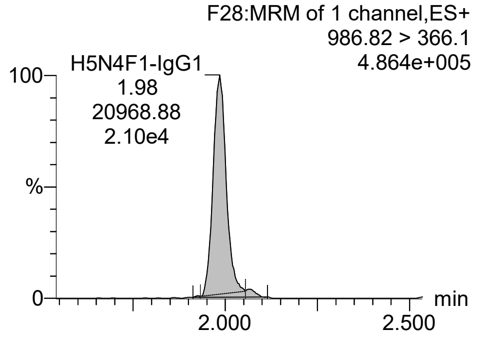

Sample Name: 20211111_H6_R1_IgG

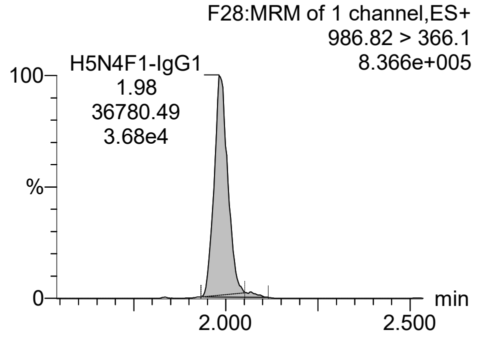

Sample Name: 20211111_A13_R1_IgG

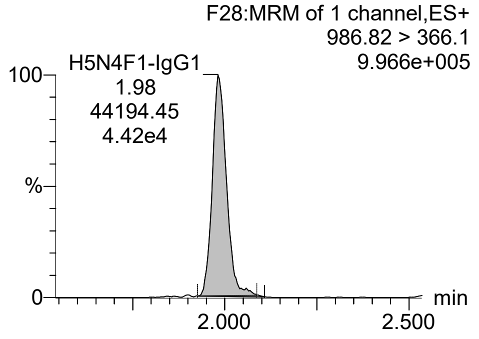

Sample Name: 20211111_A14_R1_IgG

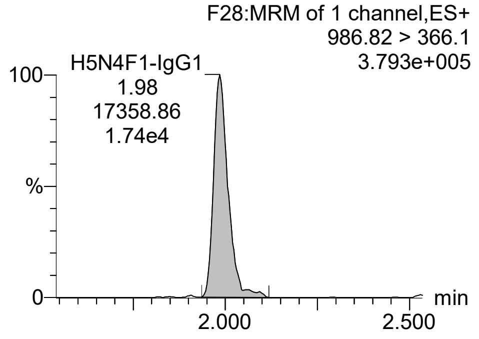

Sample Name: 20211111_L13_R1_IgG

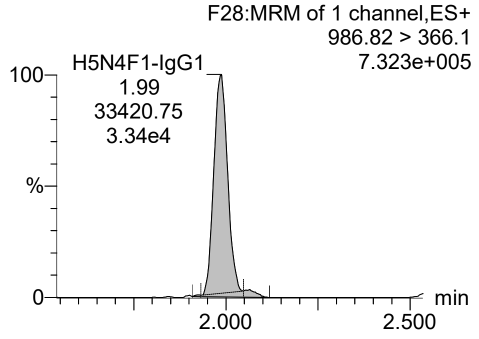

Sample Name: 20211111_L14_R1_IgG

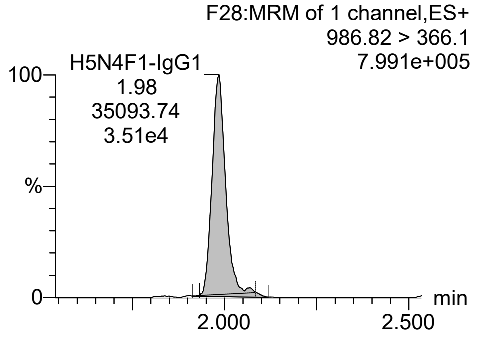

Sample Name: 20211111_H7_R1_IgG

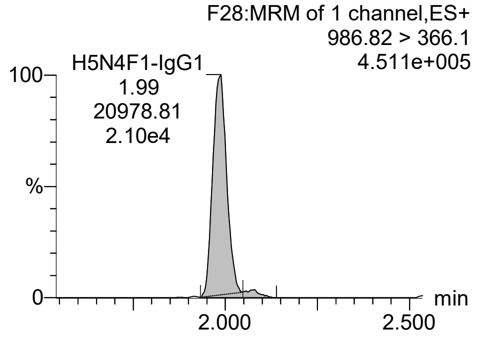

Sample Name: 20211111_A15_R1_IgG

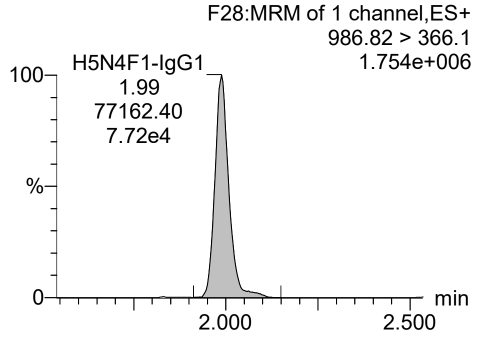

Sample Name: 20211111_QC_R4_IgG

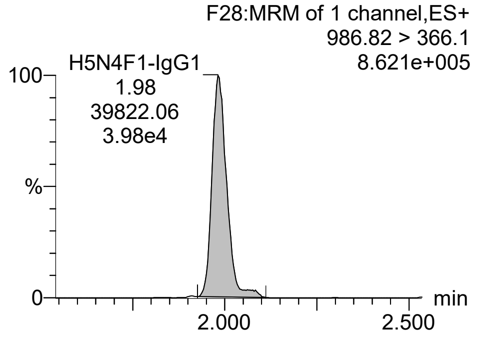

Sample Name: 20211111_A16_R1_IgG

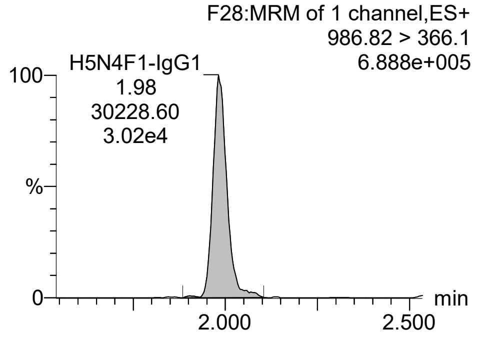

Sample Name: 20211111_L15_R1_IgG

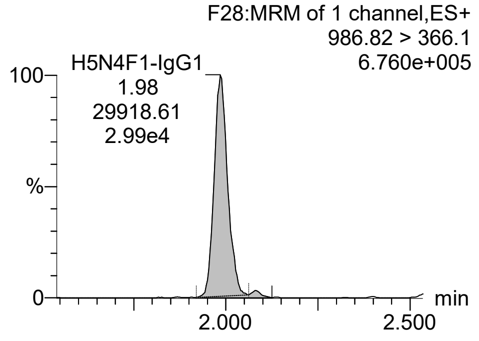

Sample Name: 20211111_L16_R1_IgG

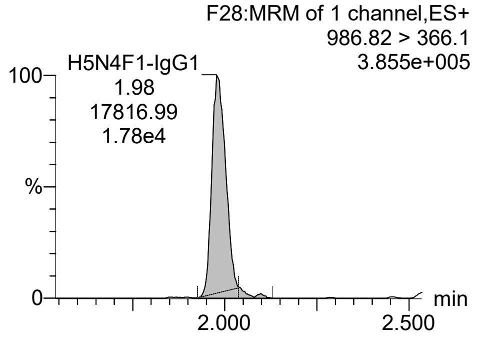

Sample Name: 20211111_H8_R1_IgG

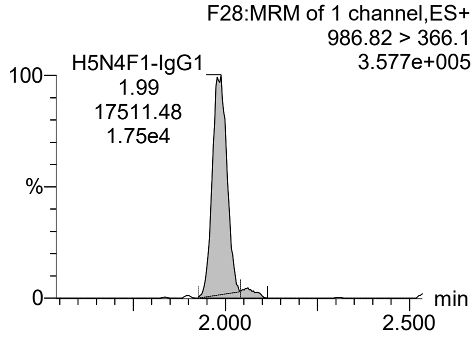

Sample Name: 20211111_A17_R1_IgG

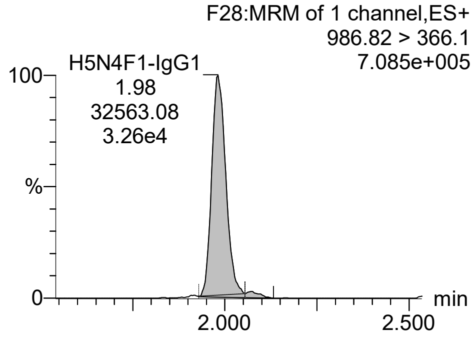

Sample Name: 20211111_A18_R1_IgG

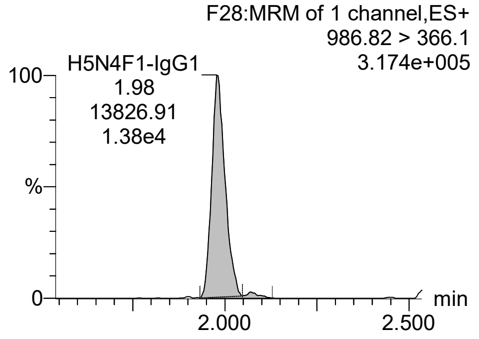

Sample Name: 20211111_L17_R1_IgG

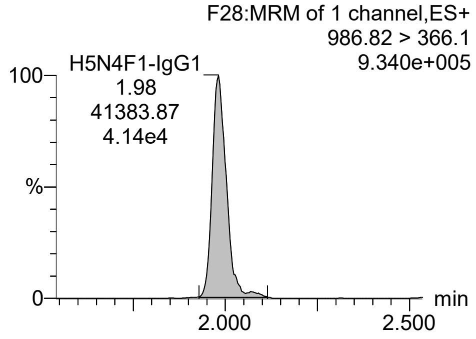

Sample Name: 20211111_L18_R1_IgG

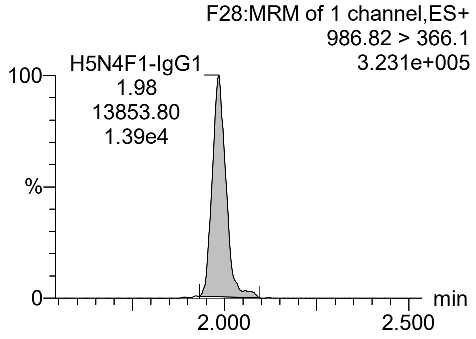

Sample Name: 20211111_H9_R1_IgG

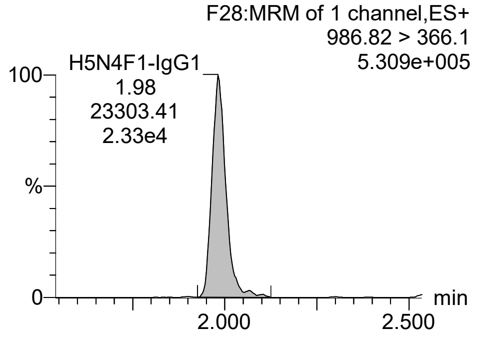

Sample Name: 20211111_A19_R1_IgG

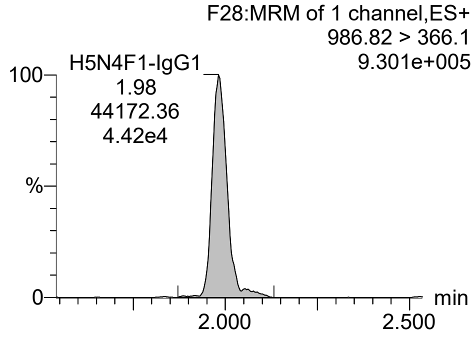

Sample Name: 20211111_A20_R1_IgG

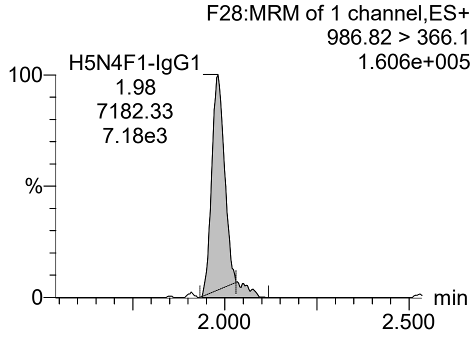

Sample Name: 20211111_L19_R1_IgG

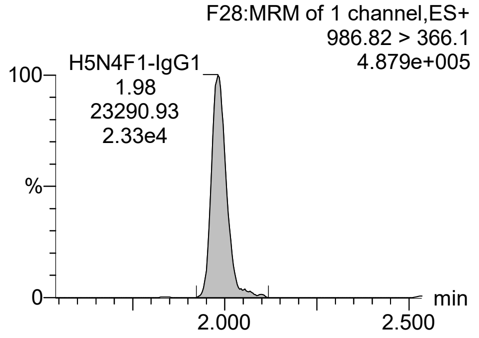

Sample Name: 20211111_QC_R5_IgG

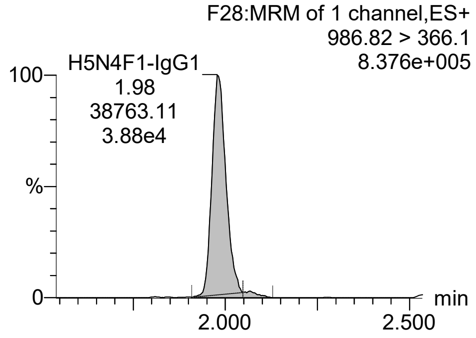

Sample Name: 20211111_L20_R1_IgG

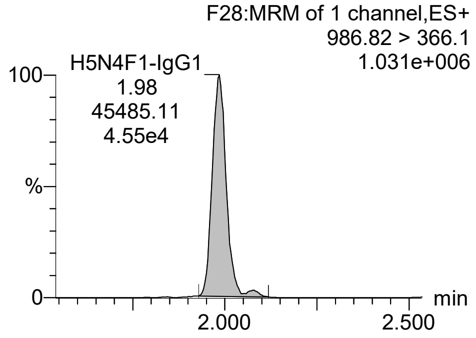

Sample Name: 20211111_H10_R1_IgG

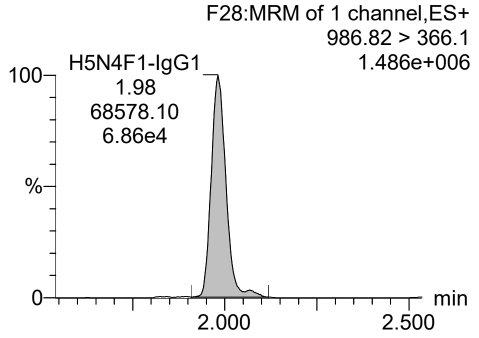

Sample Name: 20211111_A21_R1_IgG

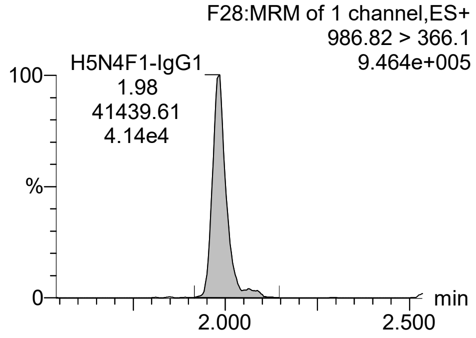

Sample Name: 20211111_A22_R1_IgG

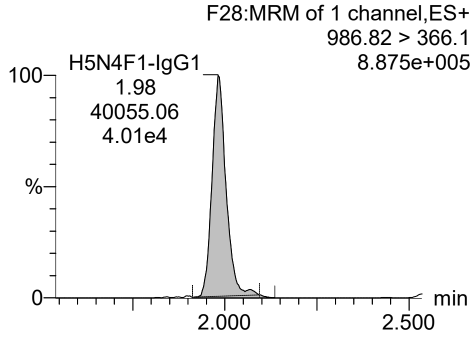

Sample Name: 20211111_L21_R1_IgG
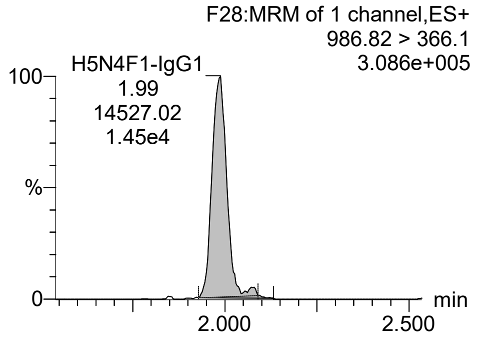

Sample Name: 20211111_L22_R1_IgG
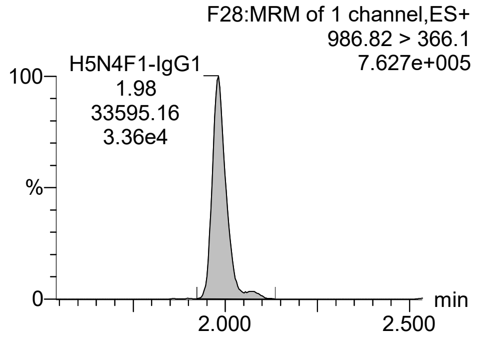

Sample Name: 20211111_H11_R1_IgG
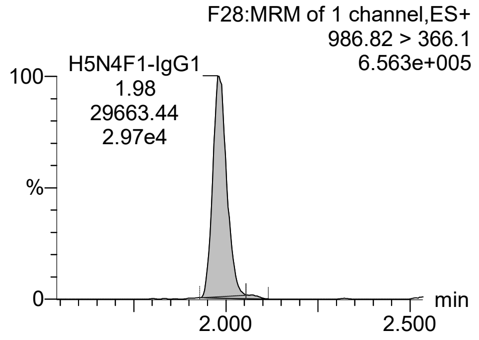

Sample Name: 20211111_A23_R1_IgG
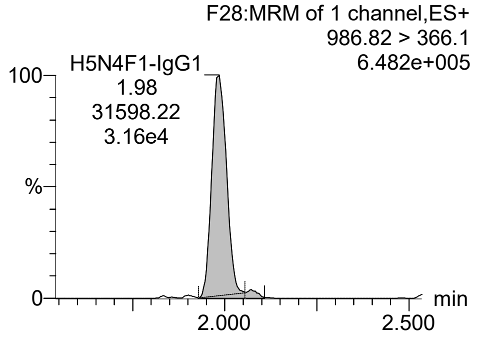

Sample Name: 20211111_A24_R1_IgG
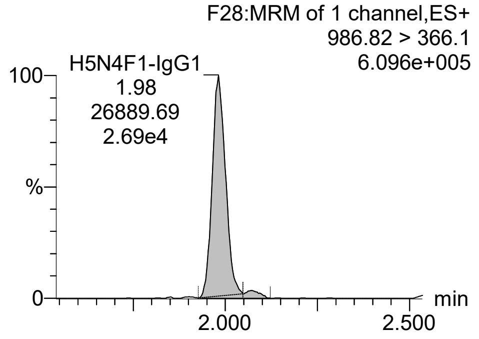

Sample Name: 20211111_L23_R1_IgG
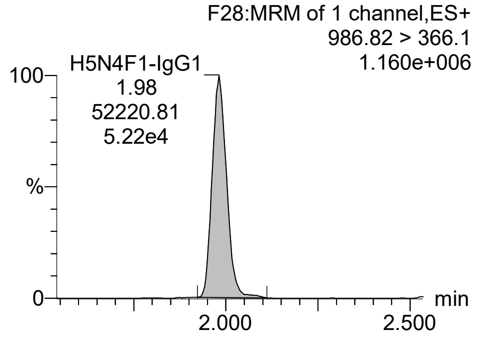

Sample Name: 20211111_L24_R1_IgG
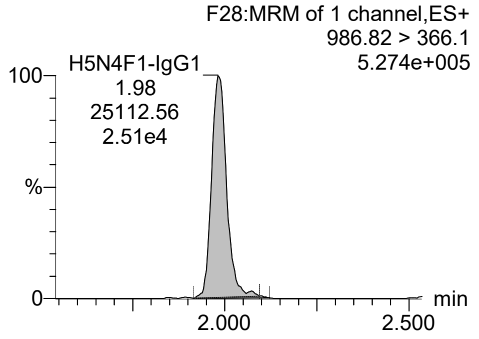

Sample Name: 20211111_H12_R1_IgG
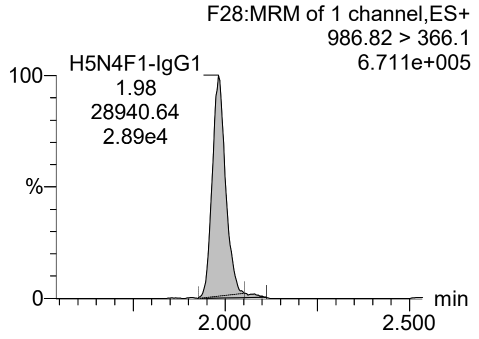

Sample Name: 20211111_QC_R6_IgG
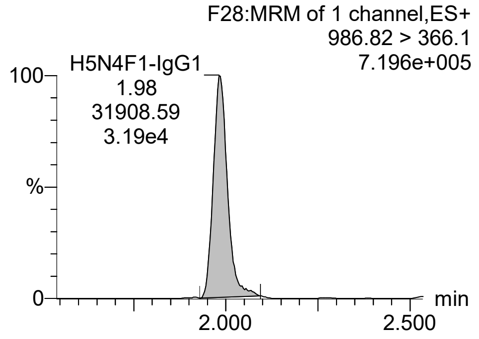

Sample Name: 20211111_A25_R1_IgG
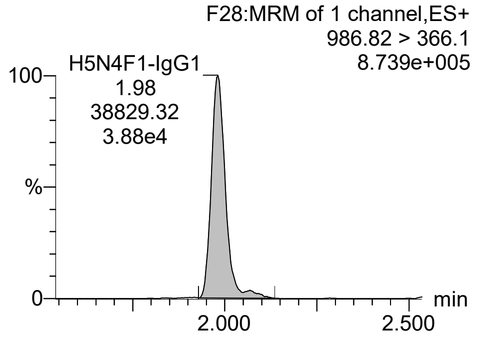

Sample Name: 20211111_A26_R1_IgG
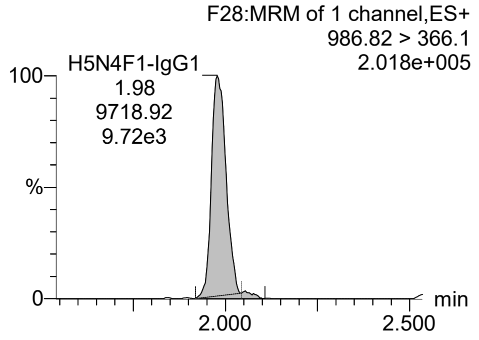

Sample Name: 20211111_L25_R1_IgG
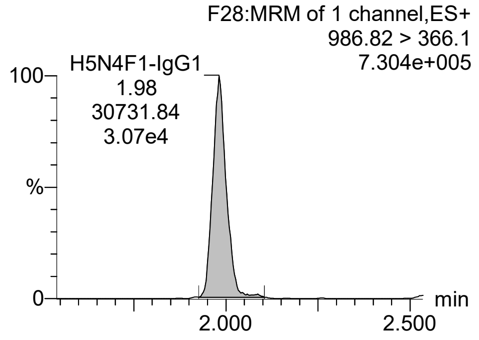

Sample Name: 20211111_L26_R1_IgG
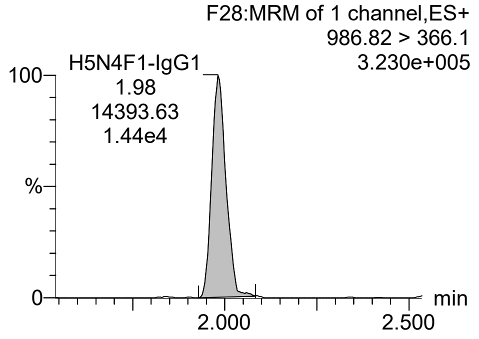

Sample Name: 20211111_H13_R1_IgG
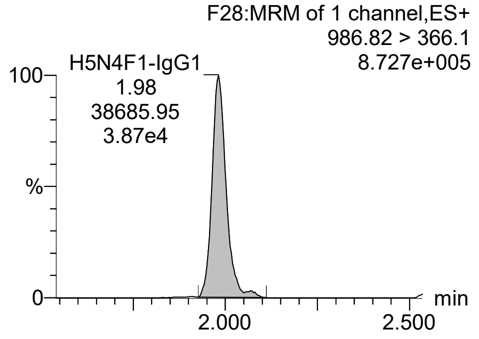

Sample Name: 20211111_A27_R1_IgG
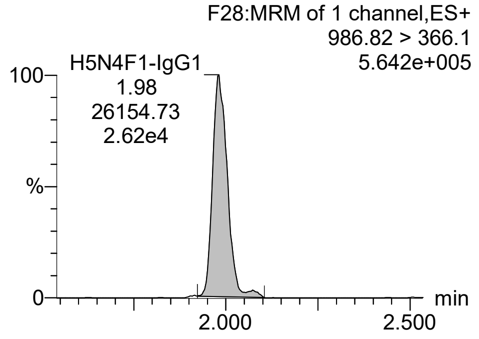

Sample Name: 20211111_A28_R1_IgG

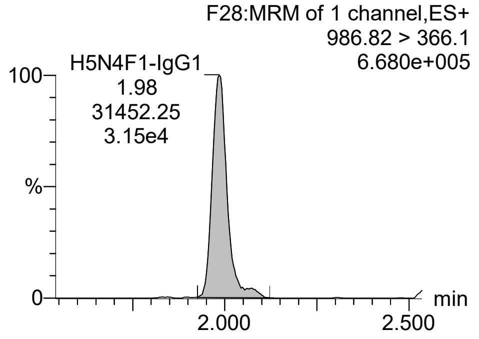

Sample Name: 20211111_L27_R1_IgG

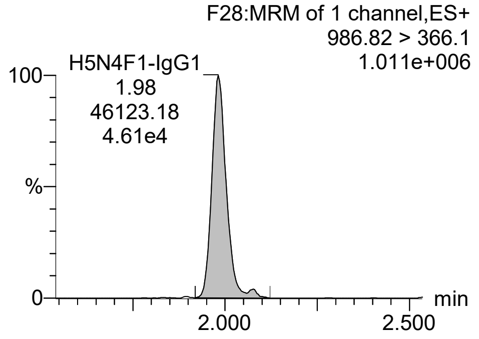

Sample Name: 20211111_L28_R1_IgG

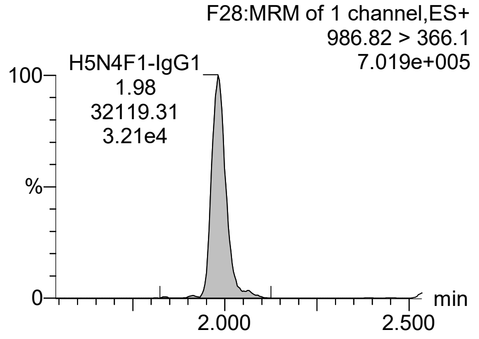

Sample Name: 20211111_H14_R1_IgG

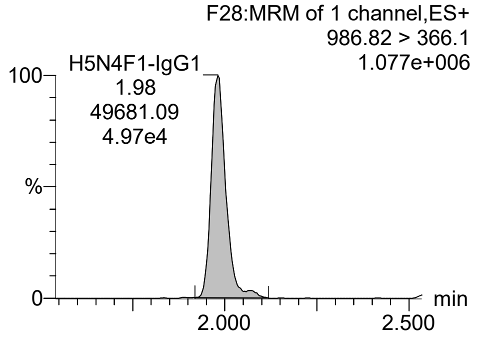

Sample Name: 20211111_A29_R1_IgG

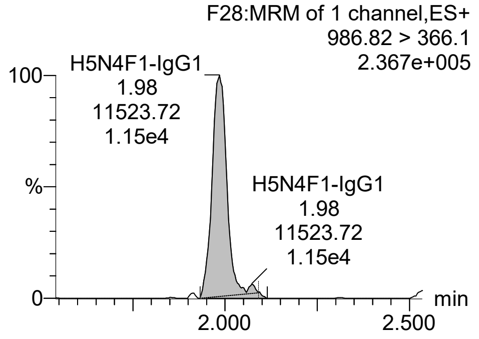

Sample Name: 20211111_A30_R1_IgG

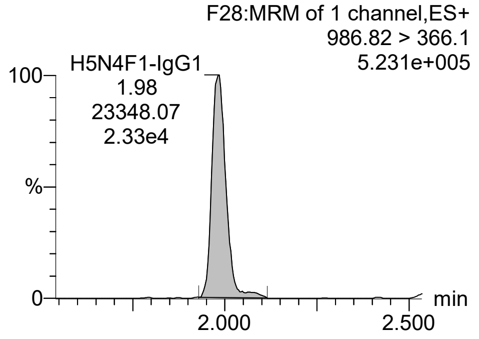

Sample Name: 20211111_QC_R7_IgG

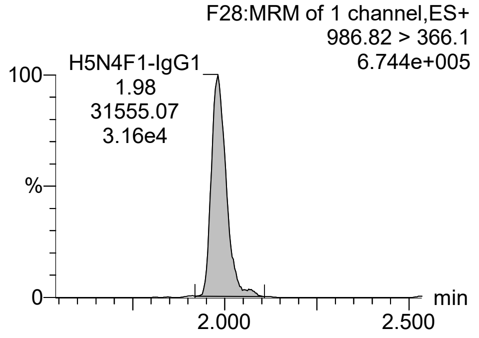

Sample Name: 20211111_L29_R1_IgG

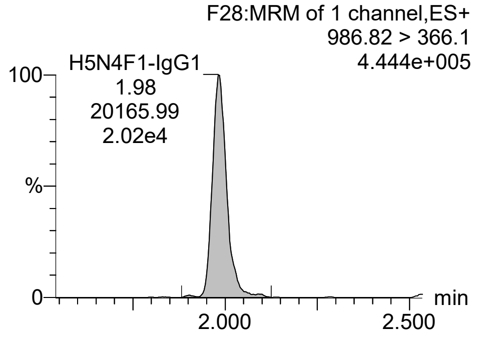

Sample Name: 20211111_L30_R1_IgG

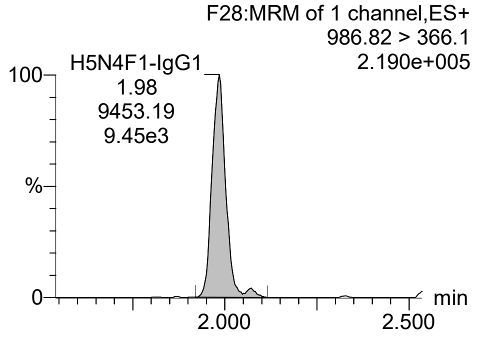

Sample Name: 20211111_H15_R1_IgG

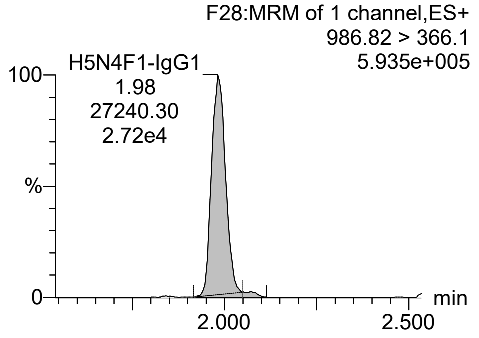

Sample Name: 20211111_A31_R1_IgG

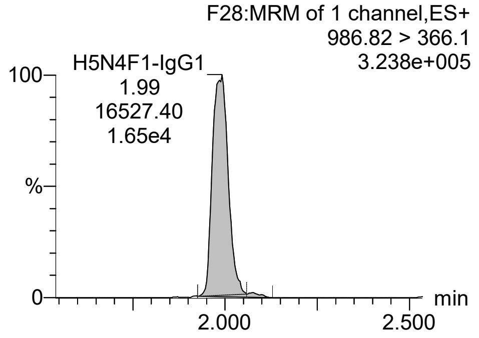

Sample Name: 20211111_A32_R1_IgG

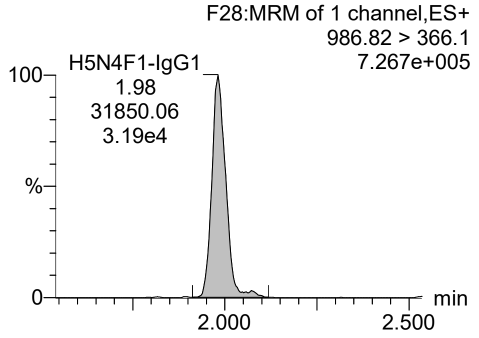

Sample Name: 20211111_L31_R1_IgG

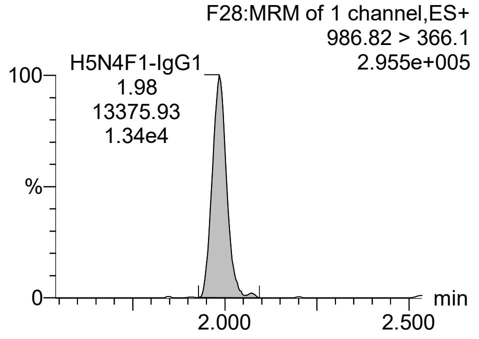

Sample Name: 20211111_L32_R1_IgG

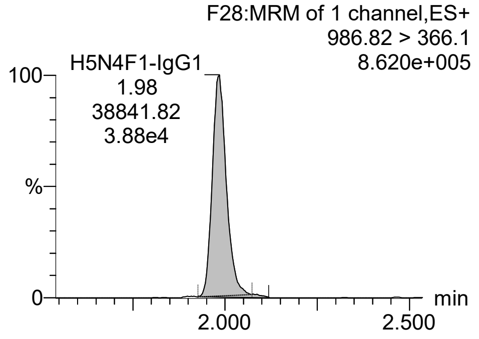

Sample Name: 20211111_H16_R1_IgG

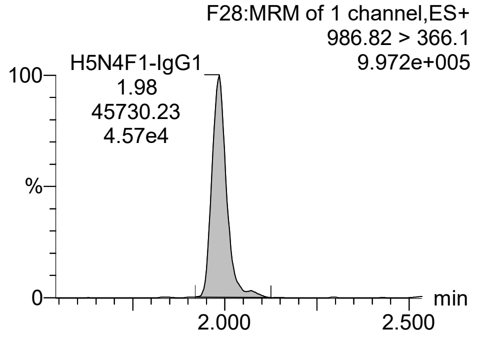

Sample Name: 20211111_A33_R1_IgG

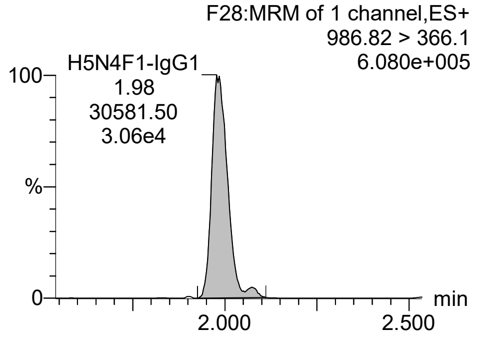

Sample Name: 20211111_A34_R1_IgG

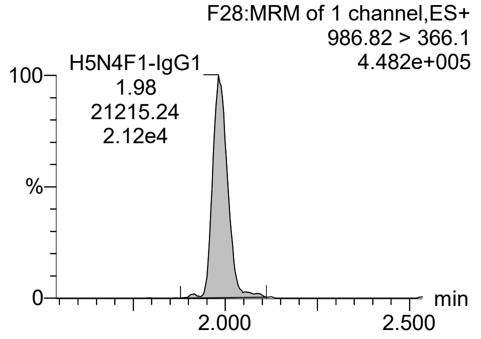

Sample Name: 20211111_H17_R1_IgG

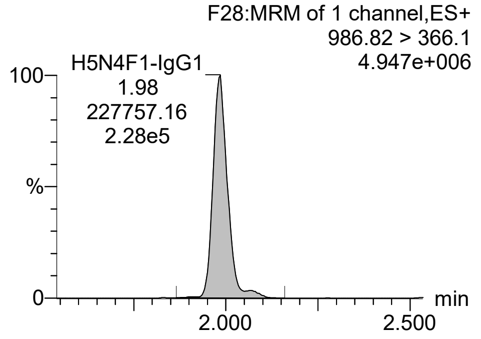

Sample Name: 20211111_A35_R1_IgG

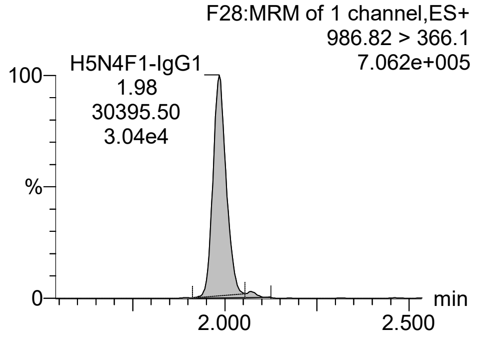

Sample Name: 20211111_QC_R8_IgG

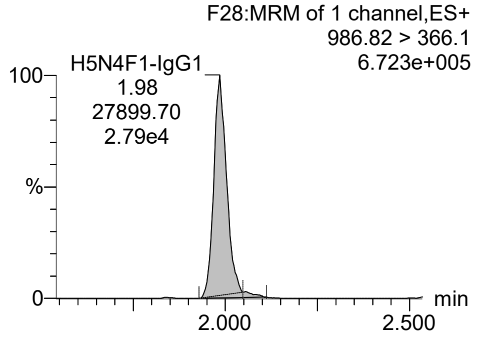

Sample Name: 20211111_A36_R1_IgG

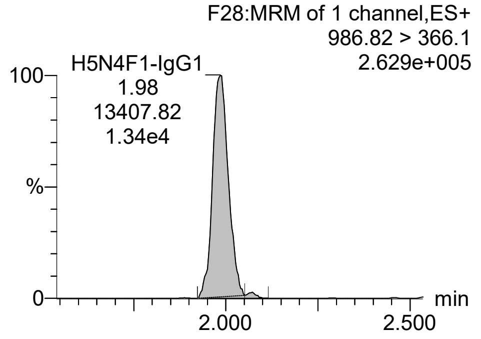

Sample Name: 20211111_H18_R1_IgG

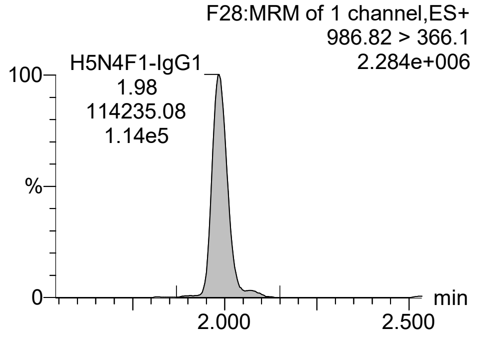

Sample Name: 20211111_A37_R1_IgG

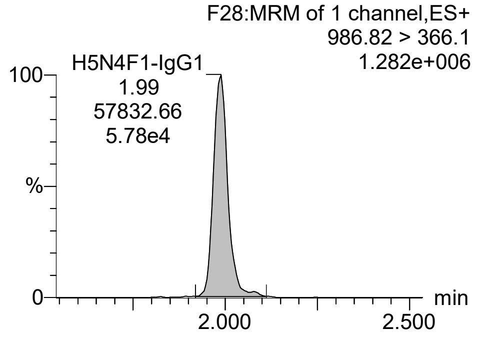

Sample Name: 20211111_A38_R1_IgG

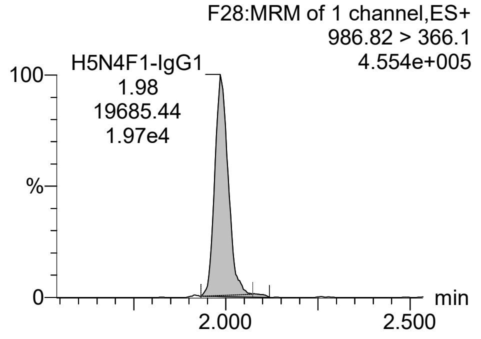

Sample Name: 20211111_H19_R1_IgG

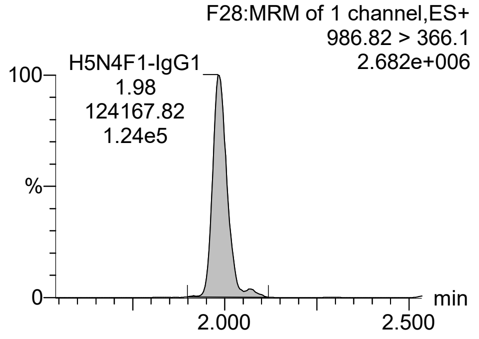

Sample Name: 20211111_A39_R1_IgG

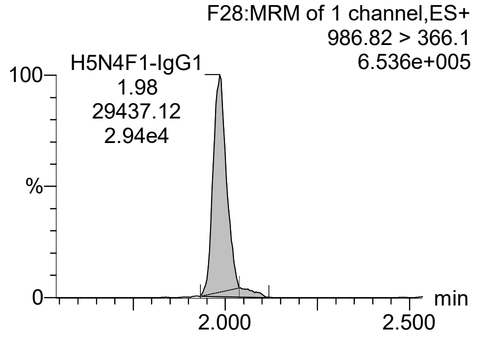

Sample Name: 20211111_A40_R1_IgG

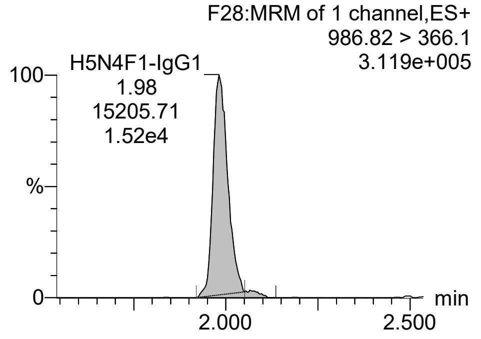

Sample Name: 20211111_H20_R1_IgG

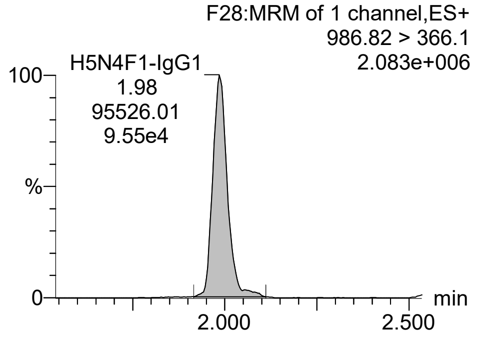

Sample Name: 20211111_A41_R1_IgG

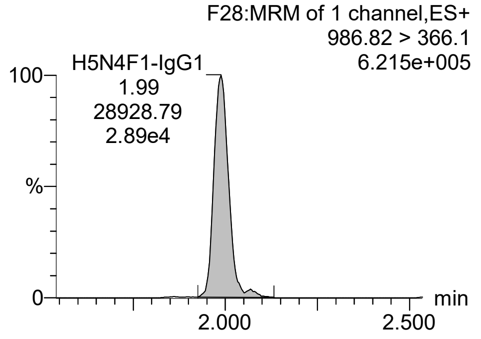

Sample Name: 20211111_A42_R1_IgG

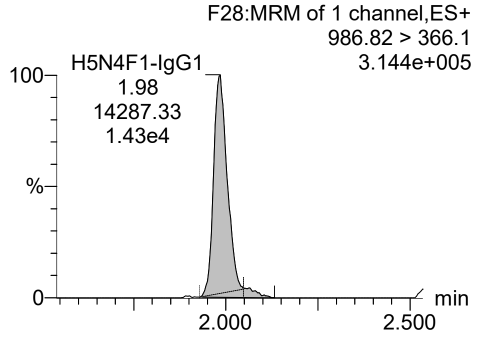

Dataset: C:\MassLynx\10.pro\20211115_clinicalsamples_IgG.qld

Last Altered: Sunday, October 02, 2022 16:02:30 台北標準時間
Printed: Friday, August 01, 2025 16:46:52 台北標準時間

Sample Name: 20211111_A43_R1_IgG

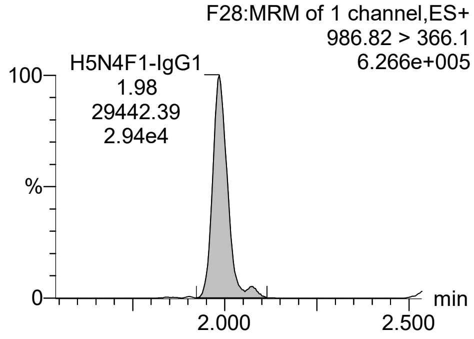

Sample Name: 20211111_A44_R1_IgG

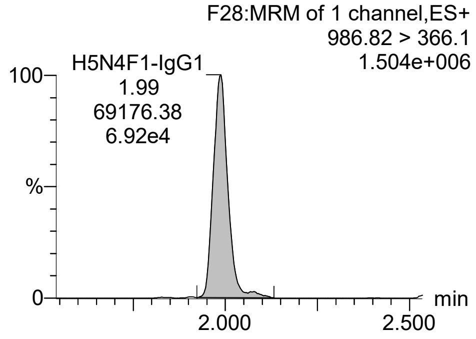

Sample Name: 20211111_QC_R9_IgG

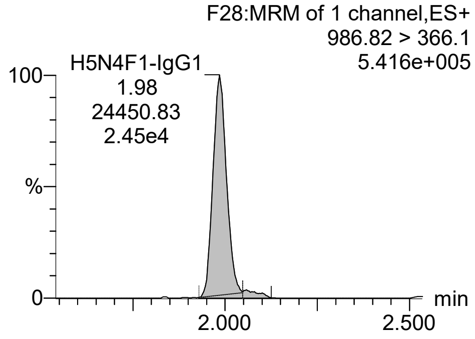

Sample Name: 20211111_A1_R2_IgG

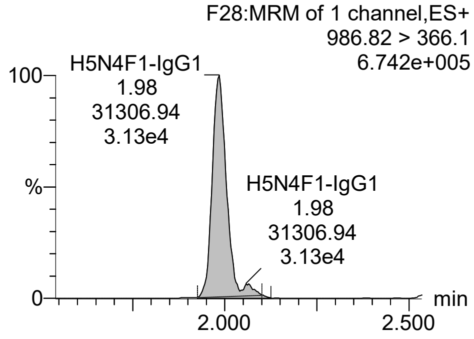

Sample Name: 20211111_A2_R2_IgG

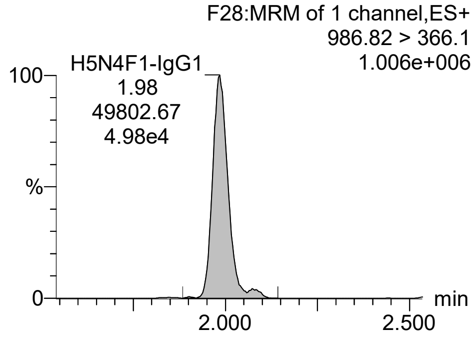

Sample Name: 20211111_L1_R2_IgG

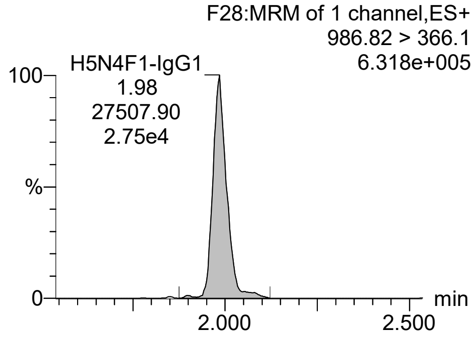

Sample Name: 20211111_L2_R2_IgG

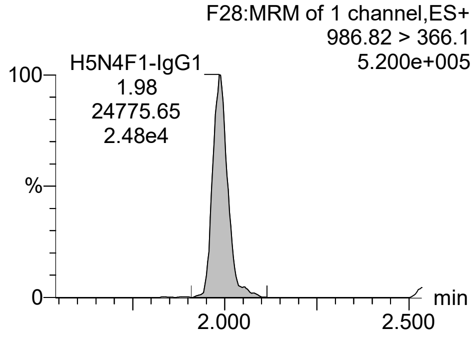

Sample Name: 20211111_H1_R2_IgG

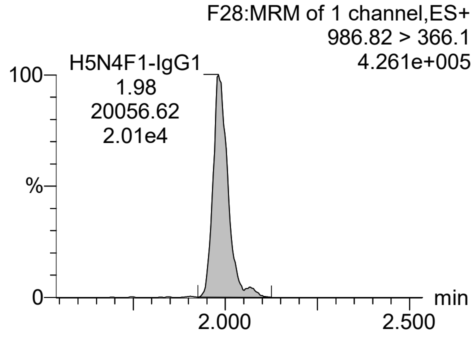

Sample Name: 20211111_A3_R2_IgG

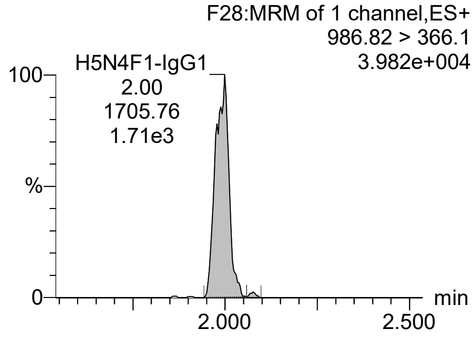

Sample Name: 20211111_A4_R2_IgG

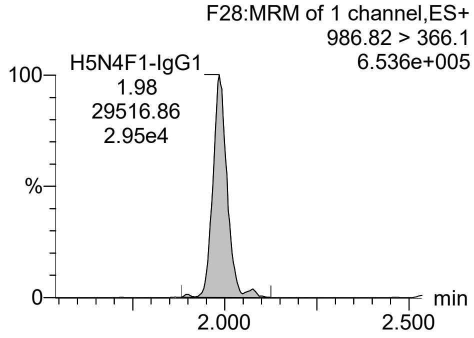

Sample Name: 20211111_L3_R2_IgG

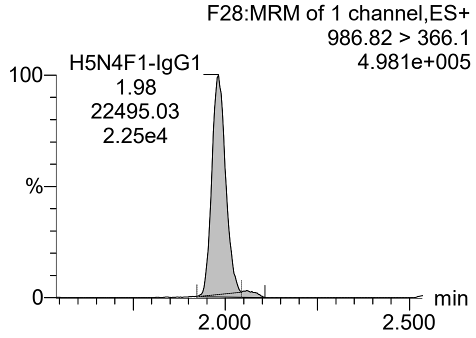

Sample Name: 20211111_L4_R2_IgG

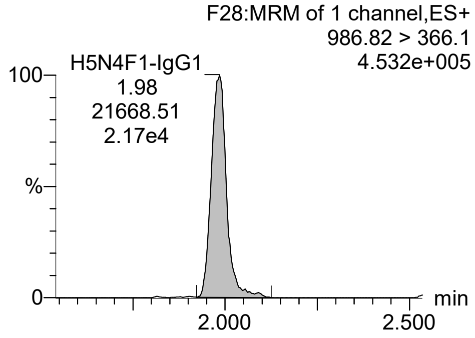

Sample Name: 20211111_H2_R2_IgG

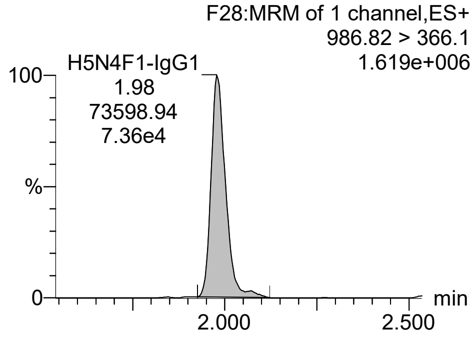

Sample Name: 20211111_A5_R2_IgG

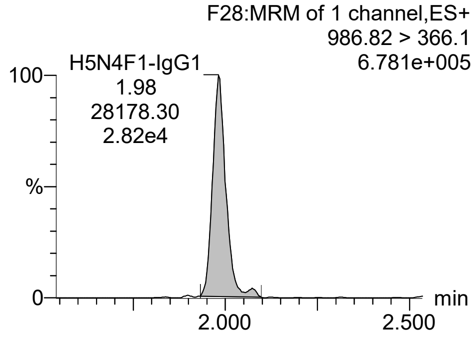

Sample Name: 20211111_A6_R2_IgG

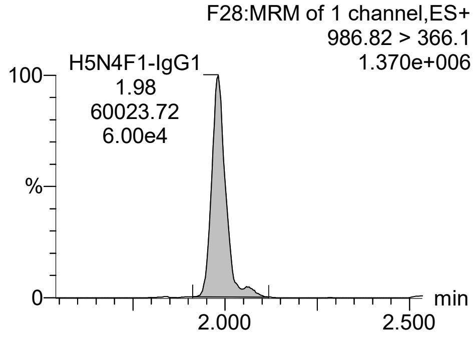

Dataset: C:\MassLynx\10.pro\20211115_clinicalsamples_IgG.qld

Last Altered: Sunday, October 02, 2022 16:02:30 台北標準時間
Printed: Friday, August 01, 2025 16:46:52 台北標準時間

Sample Name: 20211111_QC_R10_IgG

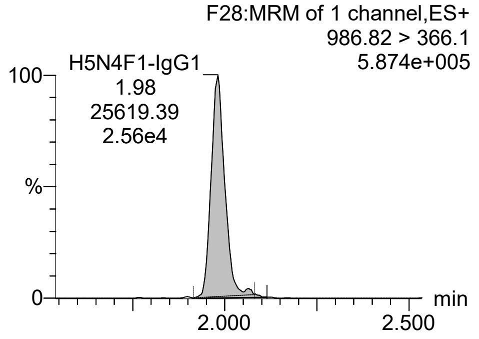

Sample Name: 20211111_L5_R2_IgG

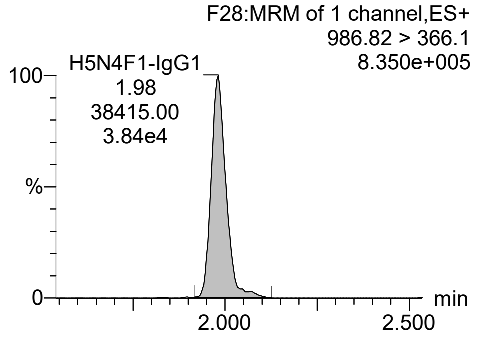

Sample Name: 20211111_L6_R2_IgG

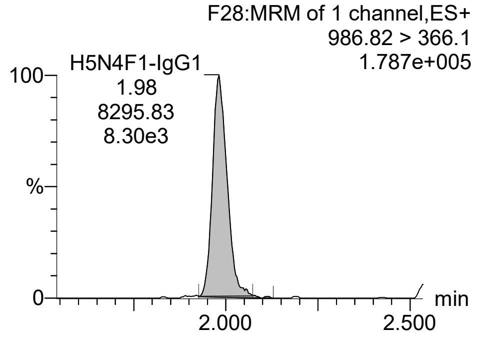

Sample Name: 20211111_H3_R2_IgG

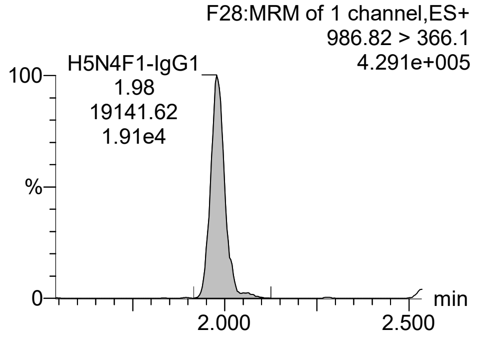

Sample Name: 20211111_A7_R2_IgG

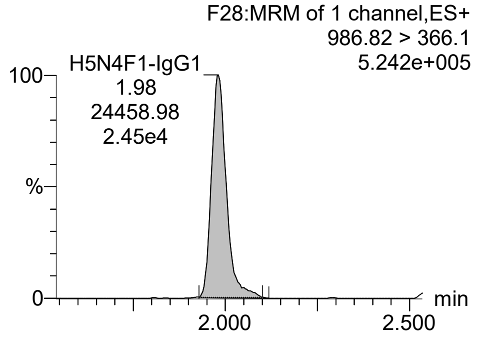

Sample Name: 20211111_A8_R2_IgG

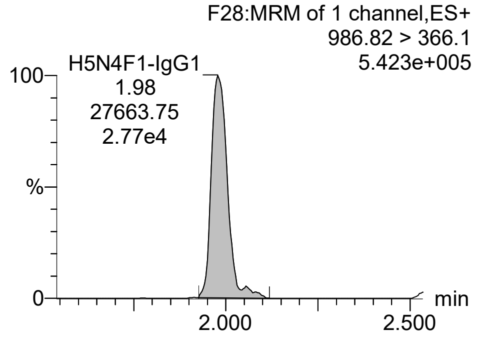

Sample Name: 20211111_L7_R2_IgG

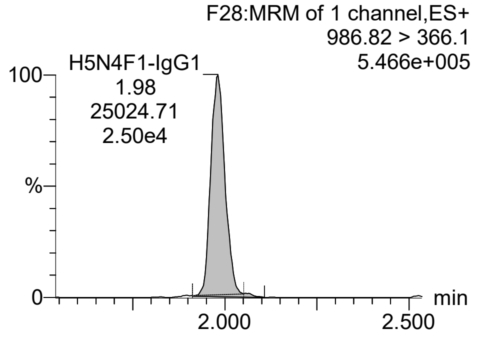

Sample Name: 20211111_L8_R2_IgG

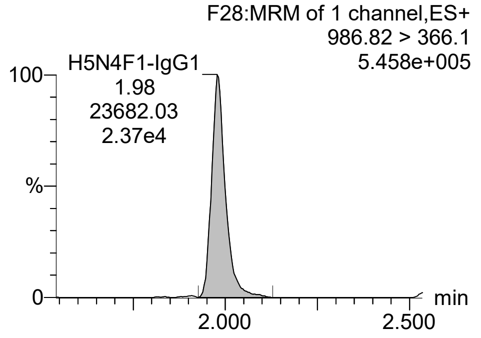

Sample Name: 20211111_H4_R2_IgG

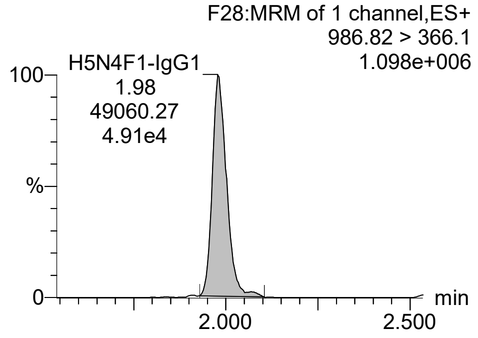

Sample Name: 20211111_A9_R2_IgG

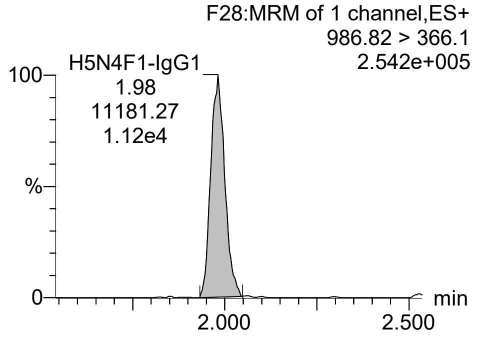

Sample Name: 20211111_A10_R2_IgG

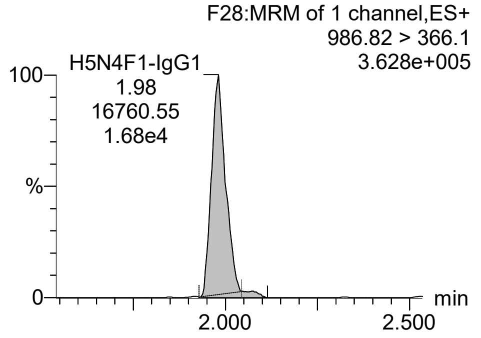

Sample Name: 20211111_L9_R2_IgG

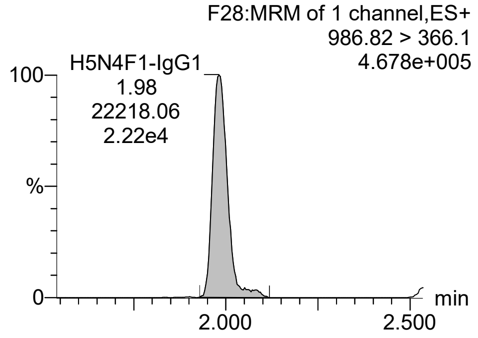

Sample Name: 20211111_L10_R2_IgG

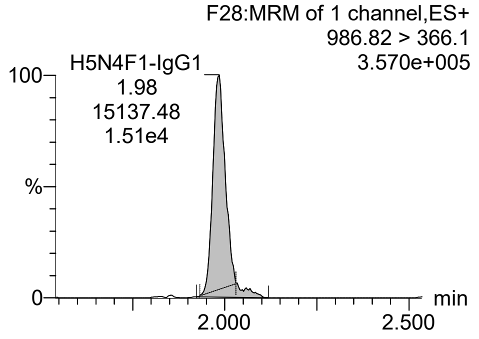

Sample Name: 20211111_QC_R11_IgG

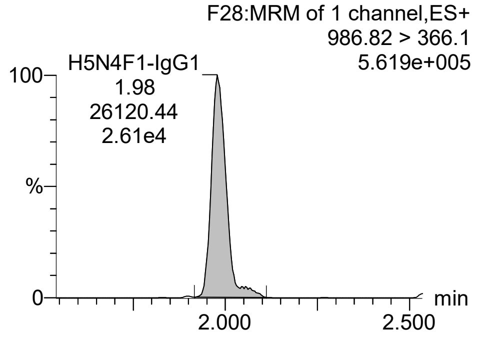

Sample Name: 20211111_H5_R2_IgG

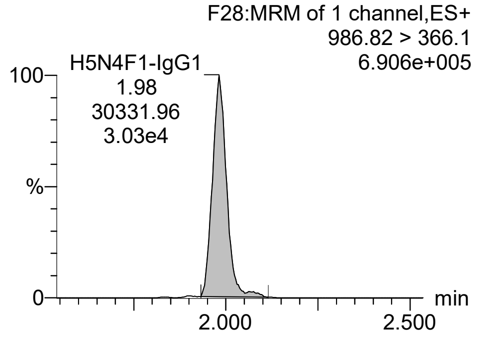

Dataset: C:\MassLynx\10.pro\20211115_clinicalsamples_IgG.qld

Last Altered: Sunday, October 02, 2022 16:02:30 台北標準時間
Printed: Friday, August 01, 2025 16:46:52 台北標準時間

Sample Name: 20211111_A11_R2_IgG
F28:MRM of 1 channel,ES+
986.82 > 366.1
2.037e+005

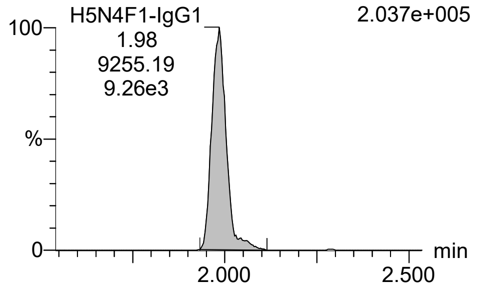

Sample Name: 20211111_A12_R2_IgG
F28:MRM of 1 channel,ES+
986.82 > 366.1
3.730e+005

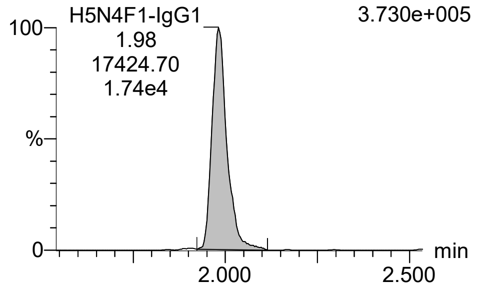

Sample Name: 20211111_L11_R2_IgG
F28:MRM of 1 channel,ES+
986.82 > 366.1
2.508e+005

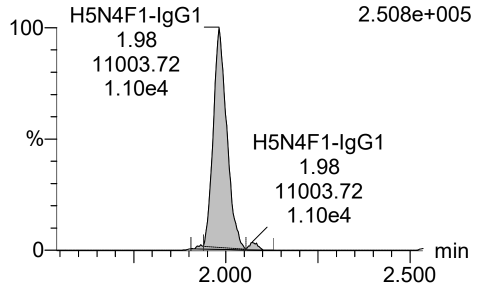

Sample Name: 20211111_L12_R2_IgG
F28:MRM of 1 channel,ES+
986.82 > 366.1
3.902e+005

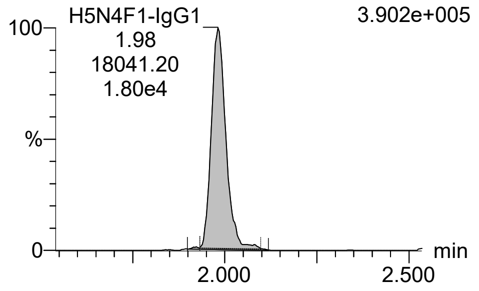

Sample Name: 20211111_H6_R2_IgG
F28:MRM of 1 channel,ES+
986.82 > 366.1
5.109e+005

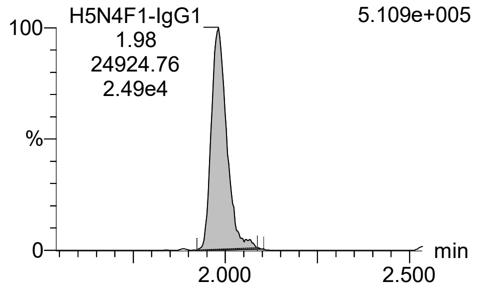

Sample Name: 20211111_A13_R2_IgG
F28:MRM of 1 channel,ES+
986.82 > 366.1
6.145e+005

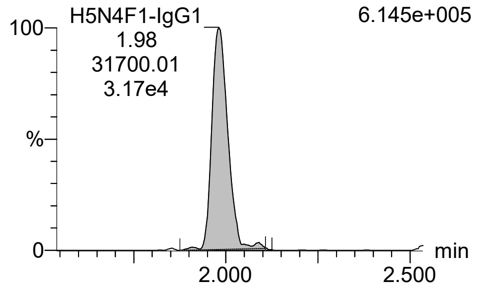

Sample Name: 20211111_A14_R2_IgG
F28:MRM of 1 channel,ES+
986.82 > 366.1
3.014e+005

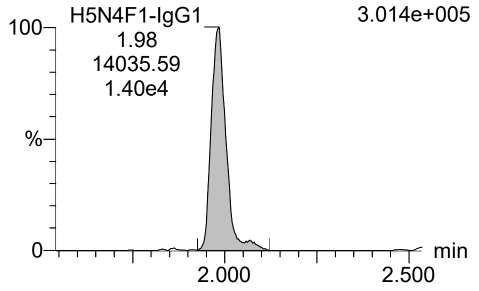

Sample Name: 20211111_L13_R2_IgG
F28:MRM of 1 channel,ES+
986.82 > 366.1
6.290e+005

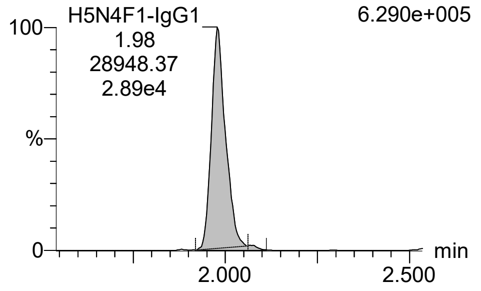

Sample Name: 20211111_L14_R2_IgG
F28:MRM of 1 channel,ES+
986.82 > 366.1
6.713e+005

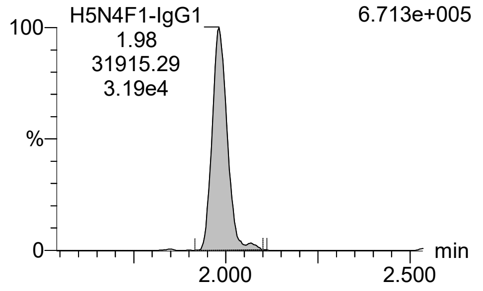

Sample Name: 20211111_H7_R2_IgG
F28:MRM of 1 channel,ES+
986.82 > 366.1
3.964e+005

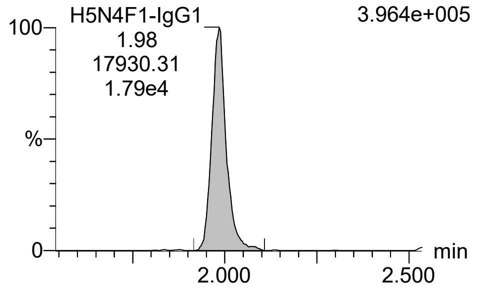

Sample Name: 20211111_A15_R2_IgG
F28:MRM of 1 channel,ES+
986.82 > 366.1
1.402e+006

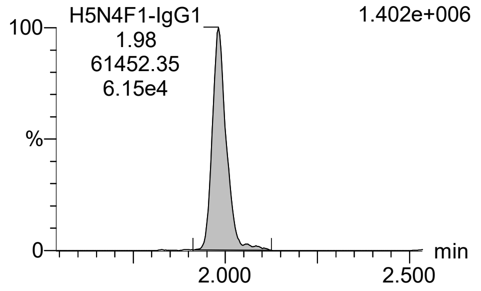

Sample Name: 20211111_QC_R12_IgG
F28:MRM of 1 channel,ES+
986.82 > 366.1
4.795e+005

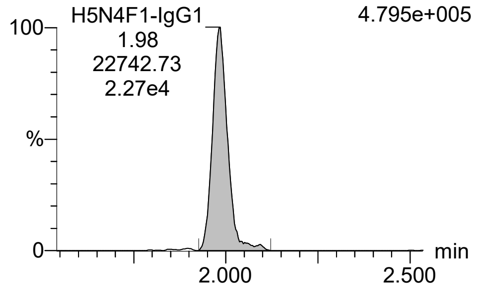

Sample Name: 20211111_A16_R2_IgG
F28:MRM of 1 channel,ES+
986.82 > 366.1
3.258e+005

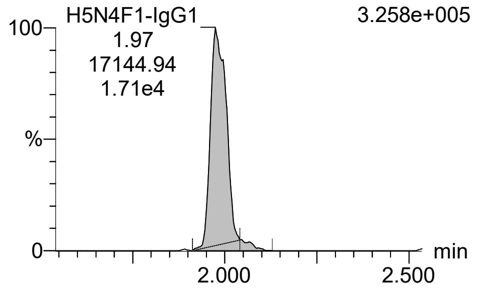

Sample Name: 20211111_L15_R2_IgG
F28:MRM of 1 channel,ES+
986.82 > 366.1
4.775e+005

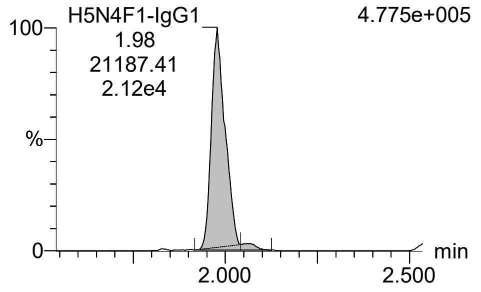

Sample Name: 20211111_L16_R2_IgG
F28:MRM of 1 channel,ES+
986.82 > 366.1
2.395e+005

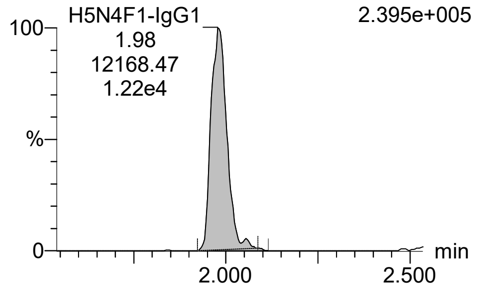

Dataset: C:\MassLynx\10.pro\20211115_clinicalsamples_IgG.qld

Last Altered: Sunday, October 02, 2022 16:02:30 台北標準時間
Printed: Friday, August 01, 2025 16:46:52 台北標準時間

Sample Name: 20211111_H8_R2_IgG

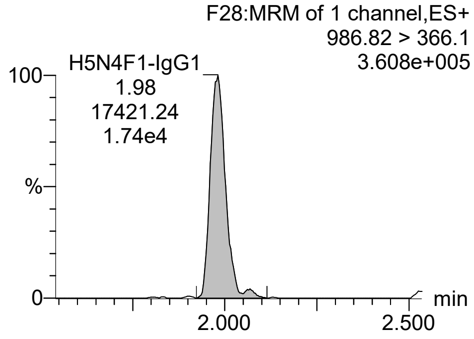

Sample Name: 20211111_A17_R2_IgG

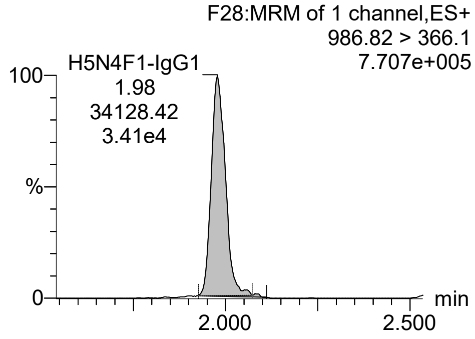

Sample Name: 20211111_A18_R2_IgG

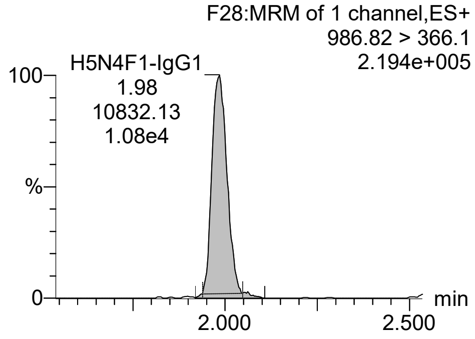

Sample Name: 20211111_L17_R2_IgG

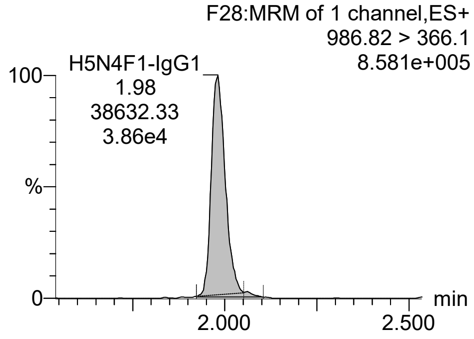

Sample Name: 20211111_L18_R2_IgG

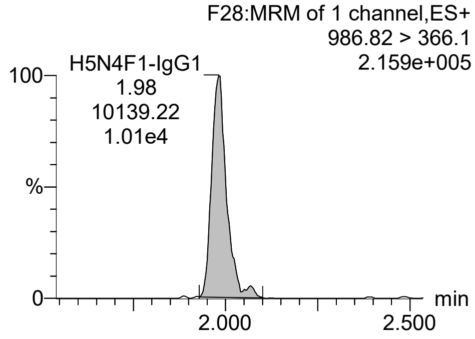

Sample Name: 20211111_H9_R2_IgG

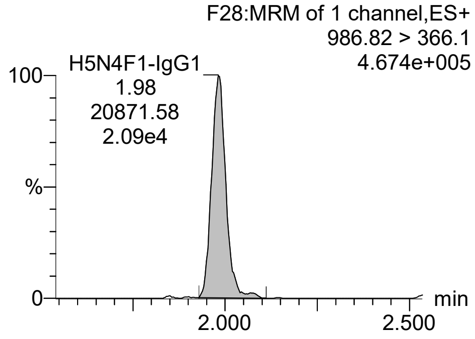

Sample Name: 20211111_A19_R2_IgG

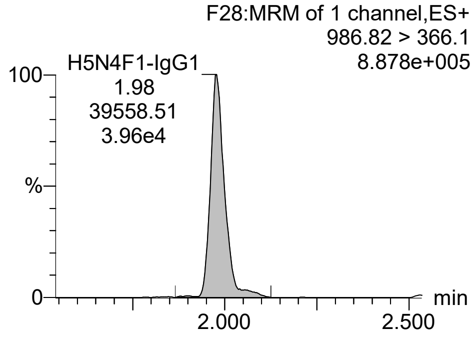

Sample Name: 20211111_A20_R2_IgG

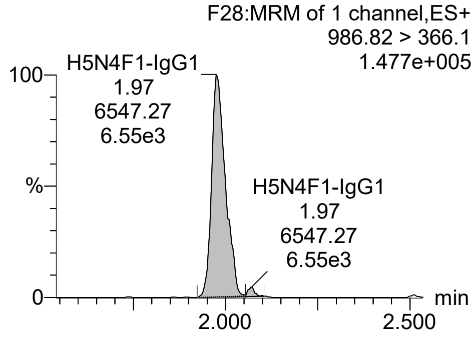

Sample Name: 20211111_L19_R2_IgG

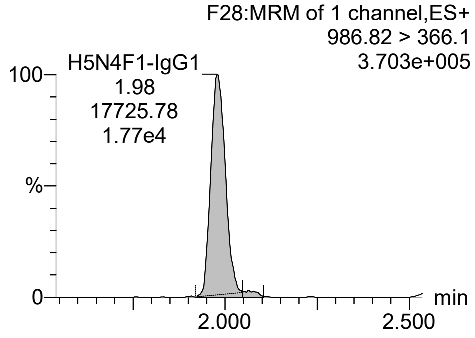

Sample Name: 20211111_QC_R13_IgG

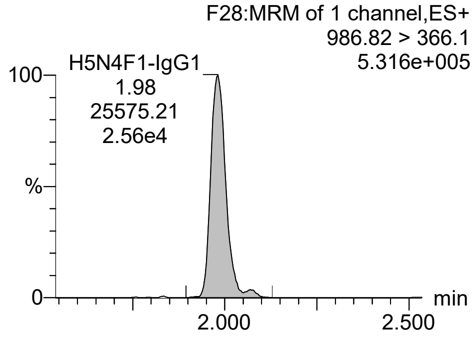

Sample Name: 20211111_L20_R2_IgG

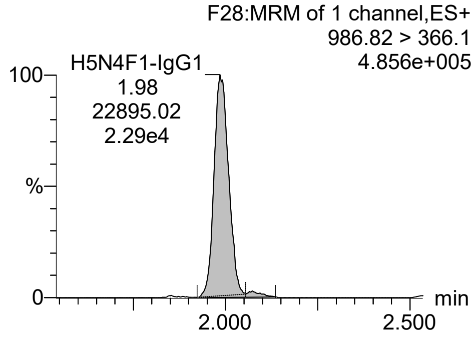

Sample Name: 20211111_H10_R2_IgG

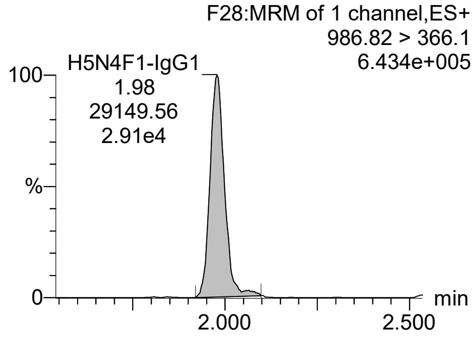

Sample Name: 20211111_A21_R2_IgG

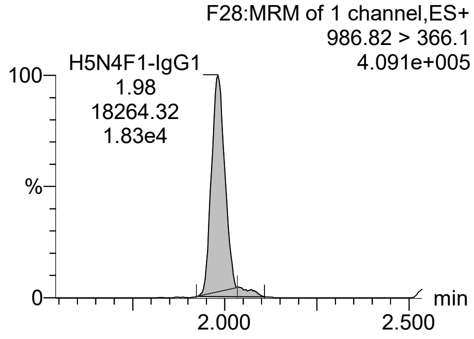

Sample Name: 20211111_A22_R2_IgG

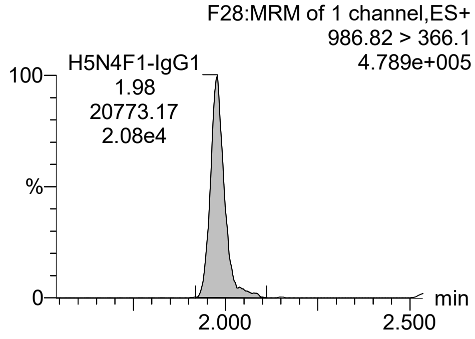

Sample Name: 20211111_L21_R2_IgG

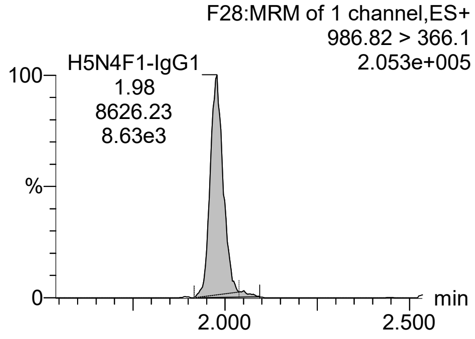

Dataset: C:\MassLynx\10.pro\20211115_clinicalsamples_IgG.qld

Last Altered: Sunday, October 02, 2022 16:02:30 台北標準時間
Printed: Friday, August 01, 2025 16:46:52 台北標準時間

Sample Name: 20211111_L22_R2_IgG
F28:MRM of 1 channel,ES+
986.82 > 366.1

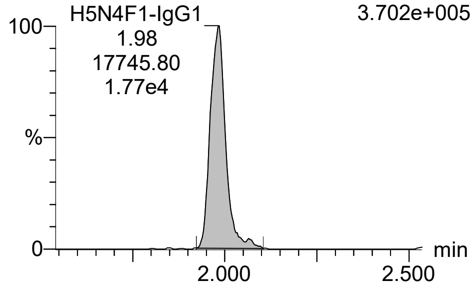

Sample Name: 20211111_H11_R2_IgG
F28:MRM of 1 channel,ES+
986.82 > 366.1

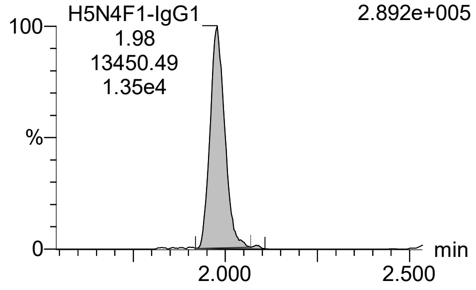

Sample Name: 20211111_A23_R2_IgG
F28:MRM of 1 channel,ES+
986.82 > 366.1

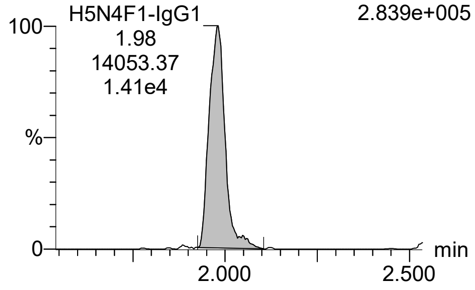

Sample Name: 20211111_A24_R2_IgG
F28:MRM of 1 channel,ES+
986.82 > 366.1

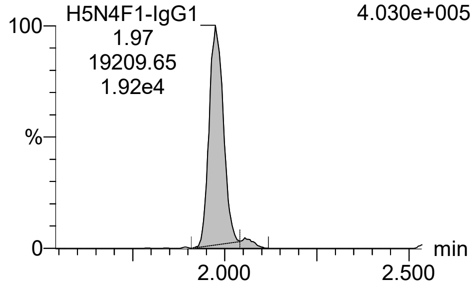

Sample Name: 20211111_L23_R2_IgG
F28:MRM of 1 channel,ES+
986.82 > 366.1

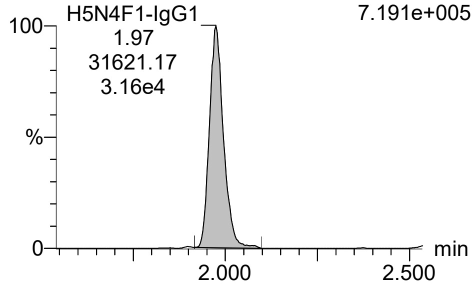

Sample Name: 20211111_L24_R2_IgG
F28:MRM of 1 channel,ES+
986.82 > 366.1

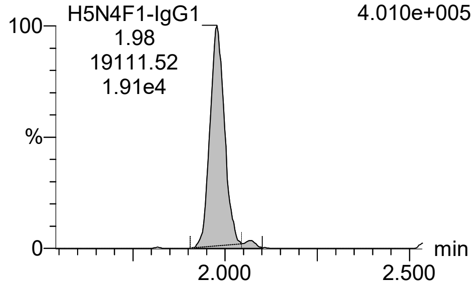

Sample Name: 20211111_H12_R2_IgG
F28:MRM of 1 channel,ES+
986.82 > 366.1

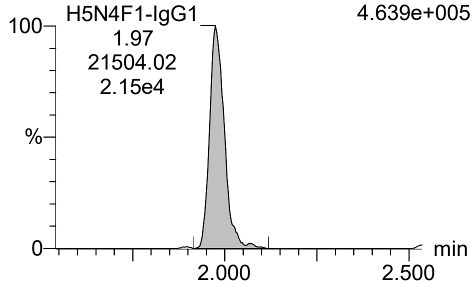

Sample Name: 20211111_QC_R14_IgG
F28:MRM of 1 channel,ES+
986.82 > 366.1

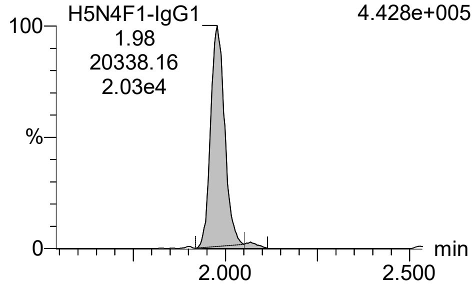

Sample Name: 20211111_A25_R2_IgG
F28:MRM of 1 channel,ES+
986.82 > 366.1

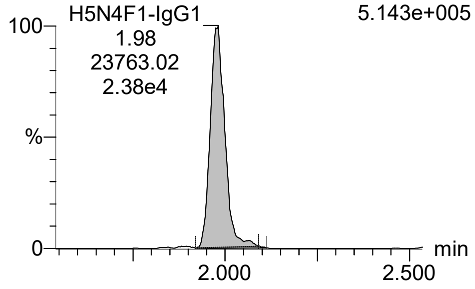

Sample Name: 20211111_A26_R2_IgG
F28:MRM of 1 channel,ES+
986.82 > 366.1

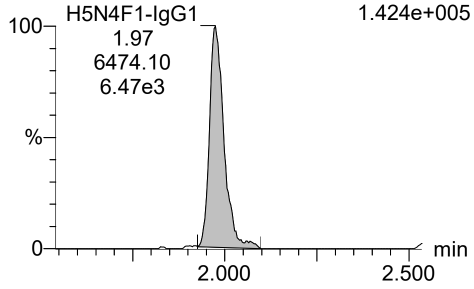

Sample Name: 20211111_L25_R2_IgG
F28:MRM of 1 channel,ES+
986.82 > 366.1

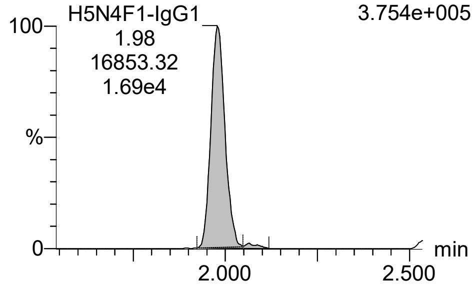

Sample Name: 20211111_L26_R2_IgG
F28:MRM of 1 channel,ES+
986.82 > 366.1

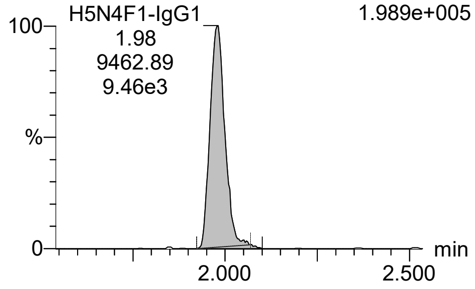

Sample Name: 20211111_H13_R2_IgG
F28:MRM of 1 channel,ES+
986.82 > 366.1

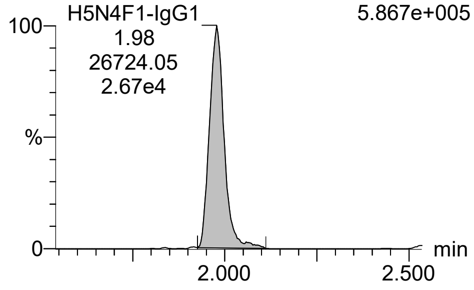

Sample Name: 20211111_A27_R2_IgG
F28:MRM of 1 channel,ES+
986.82 > 366.1

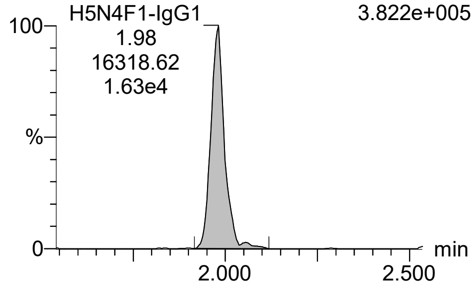

Sample Name: 20211111_A28_R2_IgG
F28:MRM of 1 channel,ES+
986.82 > 366.1

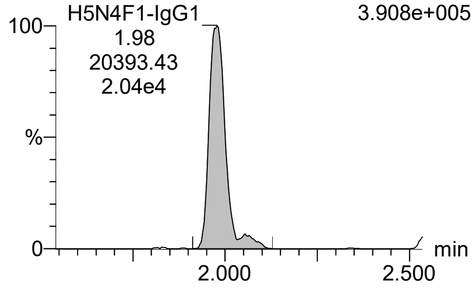

Dataset: C:\MassLynx\10.pro\20211115_clinicalsamples_IgG.qld

Last Altered: Sunday, October 02, 2022 16:02:30 台北標準時間
Printed: Friday, August 01, 2025 16:46:52 台北標準時間

Sample Name: 20211111_L27_R2_IgG

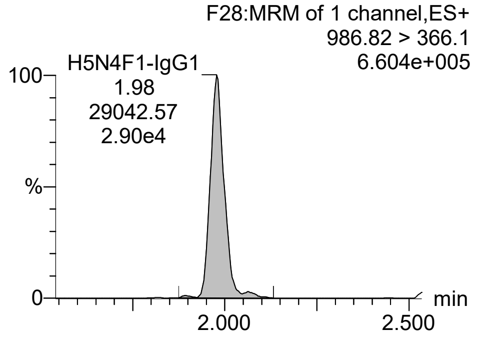

Sample Name: 20211111_L28_R2_IgG

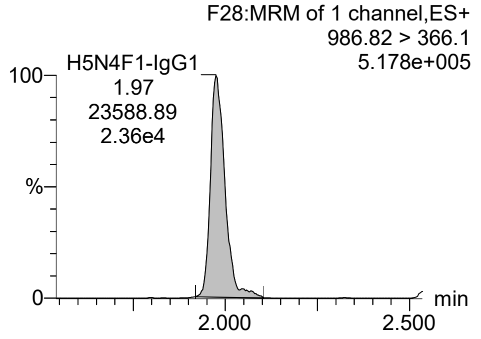

Sample Name: 20211111_H14_R2_IgG

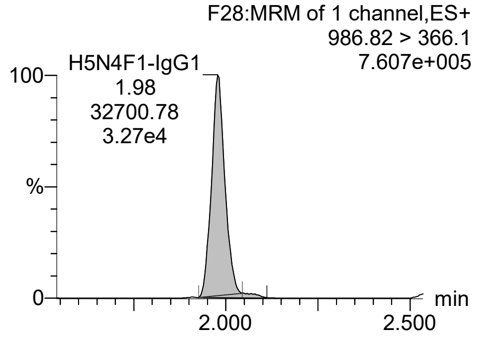

Sample Name: 20211111_A29_R2_IgG

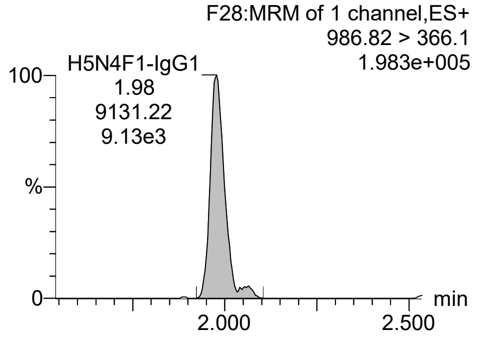

Sample Name: 20211111_A30_R2_IgG

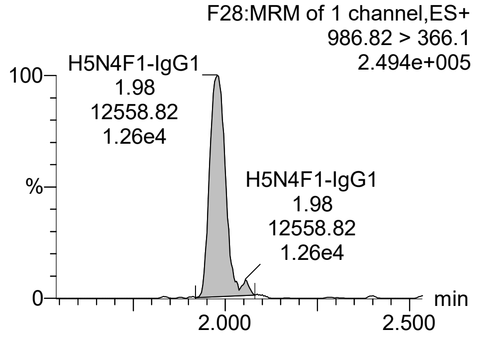

Sample Name: 20211111_QC_R15_IgG

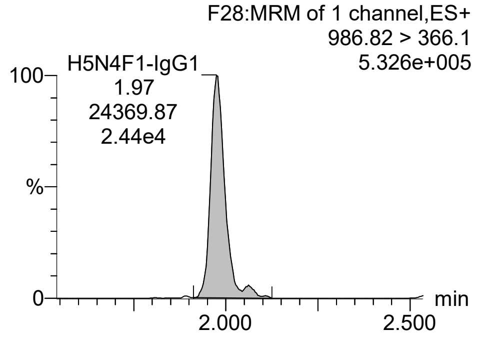

Sample Name: 20211111_L29_R2_IgG

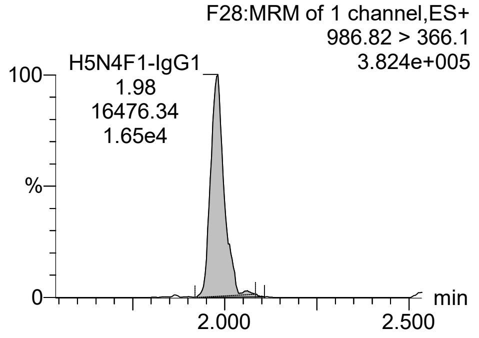

Sample Name: 20211111_L30_R2_IgG

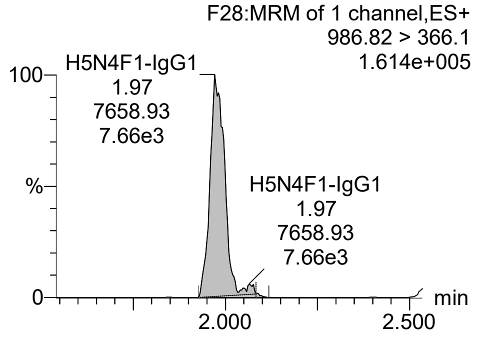

Sample Name: 20211111_H15_R2_IgG

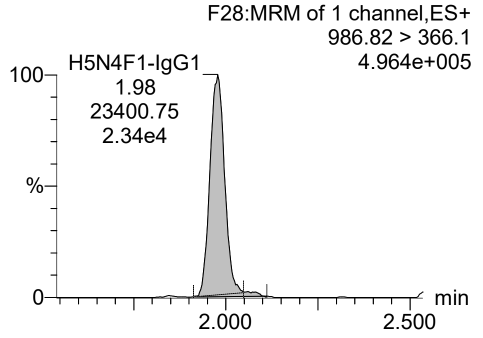

Sample Name: 20211111_A31_R2_IgG

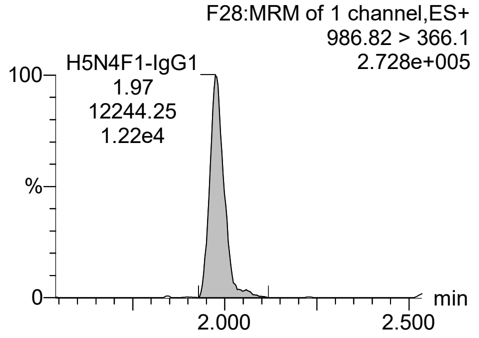

Sample Name: 20211111_A32_R2_IgG

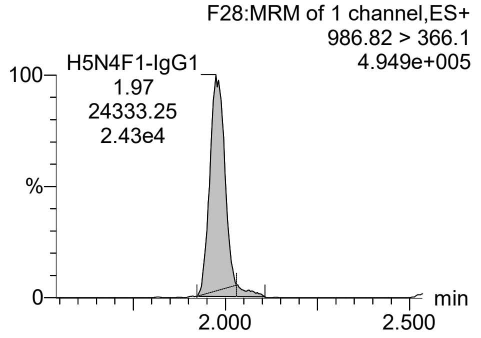

Sample Name: 20211111_L31_R2_IgG

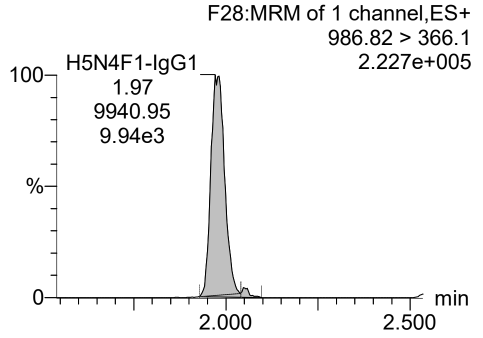

Sample Name: 20211111_L32_R2_IgG

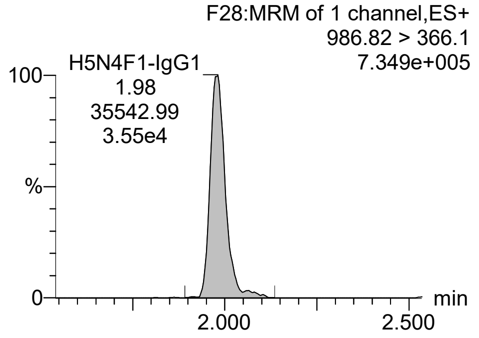

Sample Name: 20211111_H16_R2_IgG

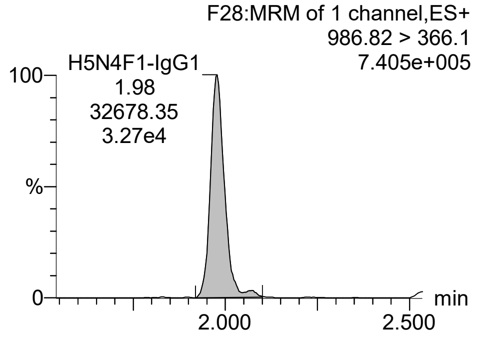

Sample Name: 20211111_A33_R2_IgG

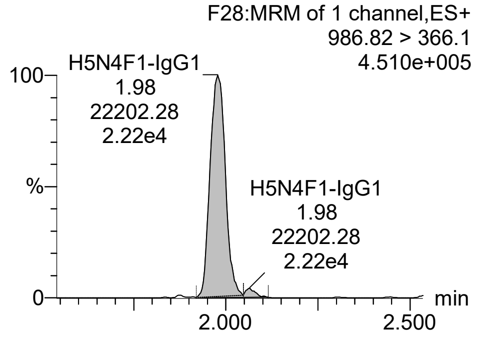

Dataset: C:\MassLynx\10.pro\20211115_clinicalsamples_IgG.qld

Last Altered: Sunday, October 02, 2022 16:02:30 台北標準時間
Printed: Friday, August 01, 2025 16:46:52 台北標準時間

Sample Name: 20211111_A34_R2_IgG

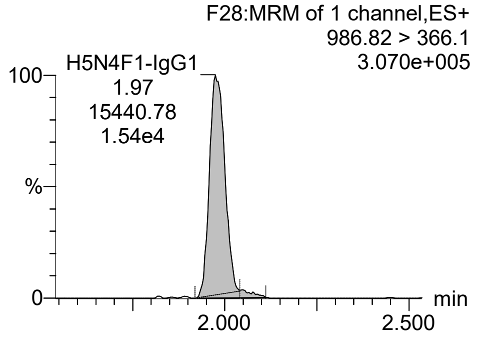

Sample Name: 20211111_H17_R2_IgG

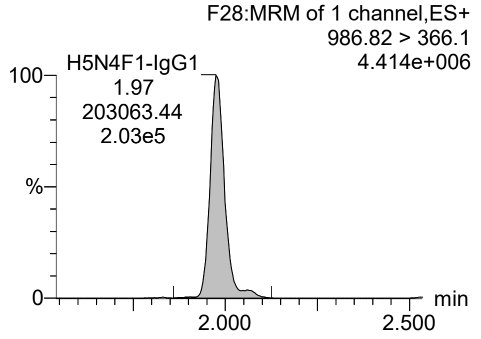

Sample Name: 20211111_A35_R2_IgG

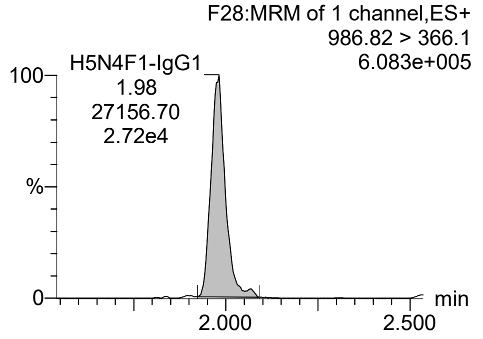

Sample Name: 20211111_QC_R16_IgG

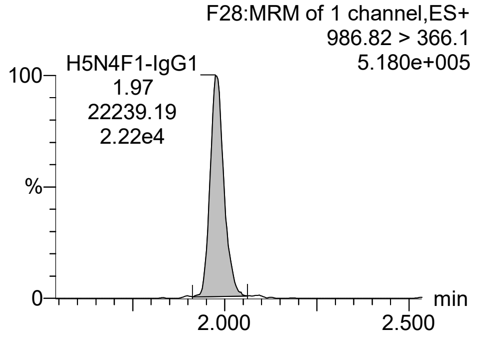

Sample Name: 20211111_A36_R2_IgG

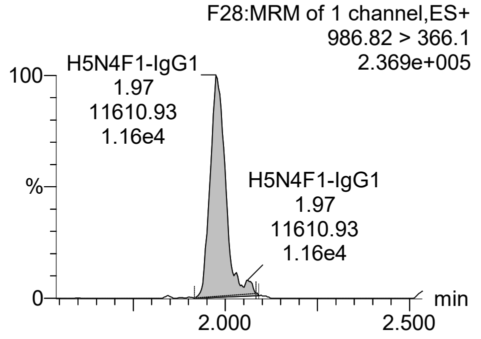

Sample Name: 20211111_H18_R2_IgG

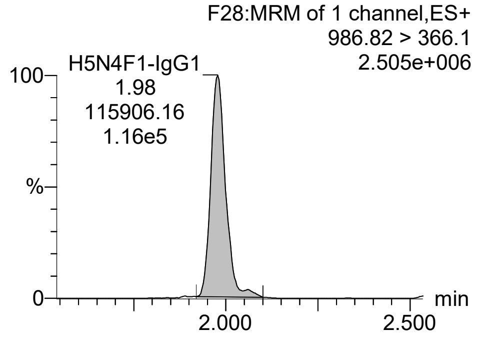

Sample Name: 20211111_A37_R2_IgG

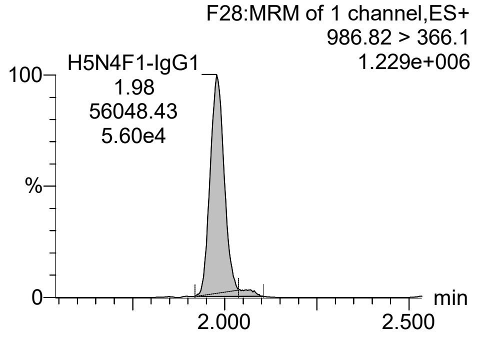

Sample Name: 20211111_A38_R2_IgG

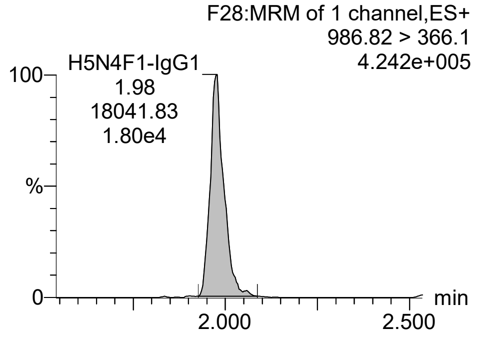

Sample Name: 20211111_H19_R2_IgG

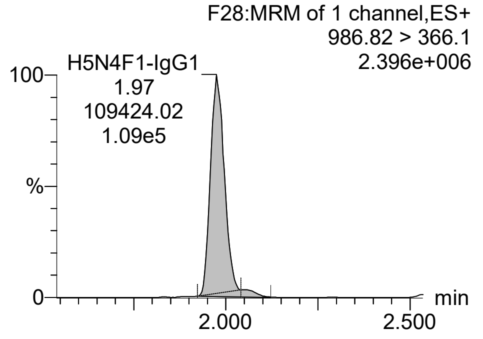

Sample Name: 20211111_A39_R2_IgG

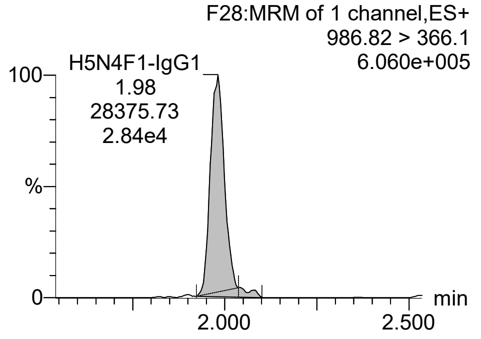

Sample Name: 20211111_A40_R2_IgG

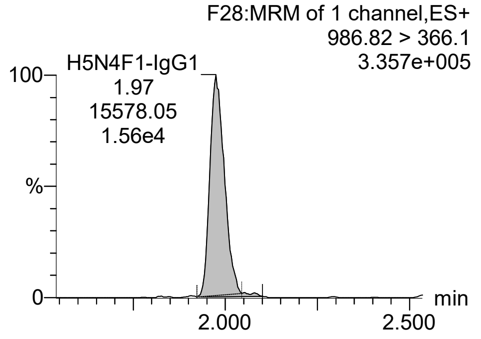

Sample Name: 20211111_H20_R2_IgG

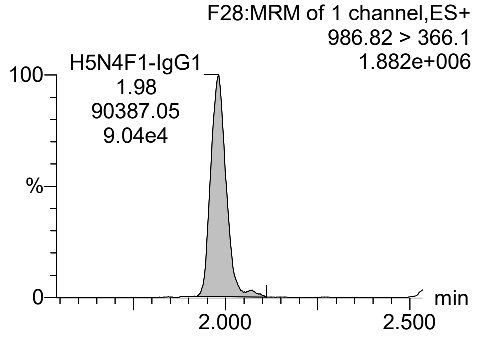

Sample Name: 20211111_A41_R2_IgG

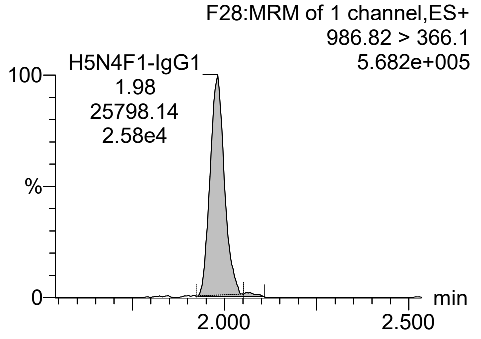

Sample Name: 20211111_A42_R2_IgG

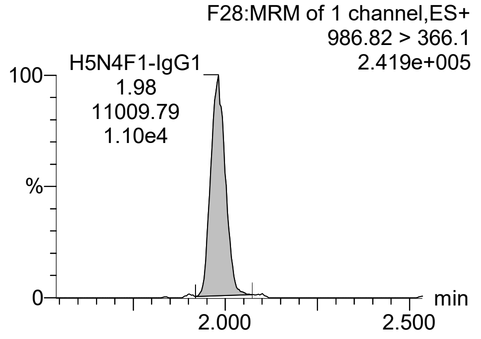

Sample Name: 20211111_A43_R2_IgG

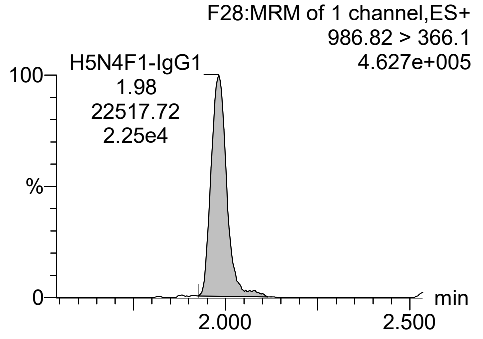

Dataset: C:\MassLynx\10.pro\20211115_clinicalsamples_IgG.qld

Last Altered: Sunday, October 02, 2022 16:02:30 台北標準時間
Printed: Friday, August 01, 2025 16:46:52 台北標準時間

Sample Name: 20211111_A44_R2_IgG

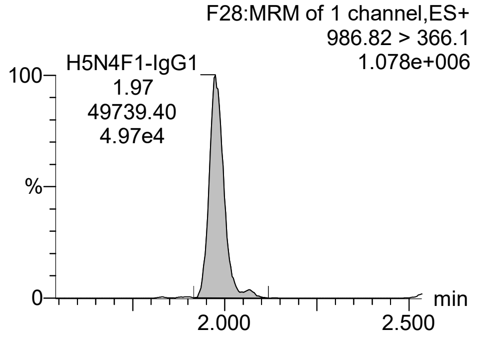

Sample Name: 20211111_QC_R17_IgG

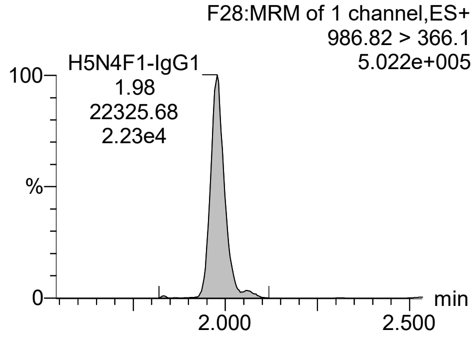

Dataset: C:\MassLynx\10.pro\20211115_clinicalsamples_IgG.qld

Last Altered: Sunday, October 02, 2022 16:02:30 台北標準時間
Printed: Friday, August 01, 2025 16:46:52 台北標準時間

	#	Name	RT	Area	IS Area	Response	Prima...
1	1	20211111_QC_R1_IgG	1.98	47756.5	64968.0	0.735	MM
2	2	20211111_A1_R1_IgG	1.98	42025.3	59059.2	0.712	bb
3	3	20211111_A2_R1_IgG	1.98	64145.4	66621.2	0.963	bb
4	4	20211111_L1_R1_IgG	1.98	43519.5	64201.0	0.678	bb
5	5	20211111_L2_R1_IgG	1.98	32232.0	46950.2	0.687	bb
6	6	20211111_H1_R1_IgG	1.98	16029.9	38648.2	0.415	bb
7	7	20211111_A3_R1_IgG	1.99	1028.9	2603.7	0.395	bb
8	8	20211111_A4_R1_IgG	1.98	42448.5	69960.4	0.607	bb
9	9	20211111_L3_R1_IgG	1.99	23581.7	38864.7	0.607	MM
10	10	20211111_L4_R1_IgG	1.99	25244.4	43380.5	0.582	bb
11	11	20211111_H2_R1_IgG	1.98	111703.4	71349.2	1.566	bb
12	12	20211111_A5_R1_IgG	1.98	34202.0	65569.7	0.522	bb
13	13	20211111_A6_R1_IgG	1.98	73613.1	53120.7	1.386	bb
14	14	20211111_QC_R2_IgG	1.98	57227.2	81034.1	0.706	bb
15	15	20211111_L5_R1_IgG	1.98	50141.5	50994.6	0.983	bb
16	16	20211111_L6_R1_IgG	1.98	11503.5	58058.0	0.198	bb
17	17	20211111_H3_R1_IgG	1.98	23721.8	64028.6	0.370	bb
18	18	20211111_A7_R1_IgG	1.98	35538.2	82996.1	0.428	bb
19	19	20211111_A8_R1_IgG	1.98	42474.1	77731.7	0.546	bb
20	20	20211111_L7_R1_IgG	1.98	26850.0	38827.3	0.692	MM
21	21	20211111_L8_R1_IgG	1.98	28437.9	44220.1	0.643	bb
22	22	20211111_H4_R1_IgG	1.98	64689.3	65826.3	0.983	bb
23	23	20211111_A9_R1_IgG	1.99	14947.9	60771.1	0.246	bb
24	24	20211111_A10_R1_IgG	1.98	20620.4	48578.7	0.424	bb
25	25	20211111_L9_R1_IgG	1.98	26182.1	50830.7	0.515	MM
26	26	20211111_L10_R1_IgG	1.98	16033.4	38034.2	0.422	MM
27	27	20211111_QC_R3_IgG	1.98	46627.2	67025.5	0.696	bb
28	28	20211111_H5_R1_IgG	1.98	35196.4	44544.9	0.790	MM
29	29	20211111_A11_R1_IgG	1.98	12825.0	66408.5	0.193	MM
30	30	20211111_A12_R1_IgG	1.98	21049.5	46624.5	0.451	MM
31	31	20211111_L11_R1_IgG	1.98	13721.8	33425.1	0.411	bb
32	32	20211111_L12_R1_IgG	1.98	20968.9	41178.0	0.509	MM
33	33	20211111_H6_R1_IgG	1.98	36780.5	58593.4	0.628	MM
34	34	20211111_A13_R1_IgG	1.98	44194.4	54133.0	0.816	MM
35	35	20211111_A14_R1_IgG	1.98	17358.9	56467.3	0.307	bb
36	36	20211111_L13_R1_IgG	1.99	33420.8	36024.4	0.928	MM
37	37	20211111_L14_R1_IgG	1.98	35093.7	37181.6	0.944	MM
38	38	20211111_H7_R1_IgG	1.99	20978.8	42850.6	0.490	MM
39	39	20211111_A15_R1_IgG	1.99	77162.4	62015.1	1.244	bb
40	40	20211111_QC_R4_IgG	1.98	39822.1	58572.5	0.680	bb
41	41	20211111_A16_R1_IgG	1.98	30228.6	78283.8	0.386	bb
42	42	20211111_L15_R1_IgG	1.98	29918.6	44158.2	0.678	MM
43	43	20211111_L16_R1_IgG	1.98	17817.0	55150.8	0.323	MM
44	44	20211111_H8_R1_IgG	1.99	17511.5	42965.2	0.408	MM
45	45	20211111_A17_R1_IgG	1.98	32563.1	39717.7	0.820	MM
46	46	20211111_A18_R1_IgG	1.98	13826.9	44675.4	0.309	MM
47	47	20211111_L17_R1_IgG	1.98	41383.9	37854.1	1.093	bb
48	48	20211111_L18_R1_IgG	1.98	13853.8	50716.9	0.273	bb
49	49	20211111_H9_R1_IgG	1.98	23303.4	36276.0	0.642	bb
50	50	20211111_A19_R1_IgG	1.98	44172.4	42862.5	1.031	bb
51	51	20211111_A20_R1_IgG	1.98	7182.3	36542.7	0.197	MM
52	52	20211111_L19_R1_IgG	1.98	23290.9	53892.8	0.432	bb
53	53	20211111_QC_R5_IgG	1.98	38763.1	56513.2	0.686	MM
54	54	20211111_L20_R1_IgG	1.98	45485.1	56499.9	0.805	bb
55	55	20211111_H10_R1_IgG	1.98	68578.1	68120.5	1.007	bb
56	56	20211111_A21_R1_IgG	1.98	41439.6	63699.8	0.651	bb

Dataset: C:\MassLynx\10.pro\20211115_clinicalsamples_IgG.qld

Last Altered: Sunday, October 02, 2022 16:02:30 台北標準時間
Printed: Friday, August 01, 2025 16:46:52 台北標準時間

	# Name	RT	Area	IS Area	Response	Prima...
57	57 20211111_A22_R1_IgG	1.98	40055.1	52222.1	0.767	MM
58	58 20211111_L21_R1_IgG	1.99	14527.0	54372.5	0.267	MM
59	59 20211111_L22_R1_IgG	1.98	33595.2	41353.1	0.812	bb
60	60 20211111_H11_R1_IgG	1.98	29663.4	65579.1	0.452	MM
61	61 20211111_A23_R1_IgG	1.98	31598.2	64131.1	0.493	MM
62	62 20211111_A24_R1_IgG	1.98	26889.7	38724.8	0.694	MM
63	63 20211111_L23_R1_IgG	1.98	52220.8	64245.2	0.813	bb
64	64 20211111_L24_R1_IgG	1.98	25112.6	45298.0	0.554	MM
65	65 20211111_H12_R1_IgG	1.98	28940.6	44679.3	0.648	MM
66	66 20211111_QC_R6_IgG	1.98	31908.6	48165.0	0.662	bb
67	67 20211111_A25_R1_IgG	1.98	38829.3	57169.9	0.679	bb
68	68 20211111_A26_R1_IgG	1.98	9718.9	34254.0	0.284	MM
69	69 20211111_L25_R1_IgG	1.98	30731.8	67491.5	0.455	bb
70	70 20211111_L26_R1_IgG	1.98	14393.6	55934.1	0.257	bb
71	71 20211111_H13_R1_IgG	1.98	38686.0	48767.3	0.793	bb
72	72 20211111_A27_R1_IgG	1.98	26154.7	57269.7	0.457	bb
73	73 20211111_A28_R1_IgG	1.98	31452.3	49231.1	0.639	bb
74	74 20211111_L27_R1_IgG	1.98	46123.2	60014.5	0.769	bb
75	75 20211111_L28_R1_IgG	1.98	32119.3	35062.6	0.916	bb
76	76 20211111_H14_R1_IgG	1.98	49681.1	62371.9	0.797	bb
77	77 20211111_A29_R1_IgG	1.98	11523.7	43858.2	0.263	MM
78	78 20211111_A30_R1_IgG	1.98	23348.1	62500.4	0.374	bb
79	79 20211111_QC_R7_IgG	1.98	31555.1	43946.9	0.718	bb
80	80 20211111_L29_R1_IgG	1.98	20166.0	38728.9	0.521	bb
81	81 20211111_L30_R1_IgG	1.98	9453.2	42613.2	0.222	bb
82	82 20211111_H15_R1_IgG	1.98	27240.3	39874.6	0.683	MM
83	83 20211111_A31_R1_IgG	1.99	16527.4	53414.3	0.309	MM
84	84 20211111_A32_R1_IgG	1.98	31850.1	41844.6	0.761	bb
85	85 20211111_L31_R1_IgG	1.98	13375.9	40553.7	0.330	bb
86	86 20211111_L32_R1_IgG	1.98	38841.8	49618.7	0.783	MM
87	87 20211111_H16_R1_IgG	1.98	45730.2	60170.9	0.760	bb
88	88 20211111_A33_R1_IgG	1.98	30581.5	48588.2	0.629	bb
89	89 20211111_A34_R1_IgG	1.98	21215.2	43288.0	0.490	bb
90	90 20211111_H17_R1_IgG	1.98	227757.2	47104.5	4.835	bb
91	91 20211111_A35_R1_IgG	1.98	30395.5	41025.9	0.741	MM
92	92 20211111_QC_R8_IgG	1.98	27899.7	40546.3	0.688	MM
93	93 20211111_A36_R1_IgG	1.98	13407.8	44504.4	0.301	MM
94	94 20211111_H18_R1_IgG	1.98	114235.1	34162.2	3.344	bb
95	95 20211111_A37_R1_IgG	1.99	57832.7	39760.0	1.455	bb
96	96 20211111_A38_R1_IgG	1.98	19685.4	43982.6	0.448	MM
97	97 20211111_H19_R1_IgG	1.98	124167.8	45314.8	2.740	bb
98	98 20211111_A39_R1_IgG	1.98	29437.1	39388.0	0.747	MM
99	99 20211111_A40_R1_IgG	1.98	15205.7	38726.4	0.393	MM
100	1... 20211111_H20_R1_IgG	1.98	95526.0	37814.4	2.526	bb
101	1... 20211111_A41_R1_IgG	1.99	28928.8	48930.3	0.591	bb
102	1... 20211111_A42_R1_IgG	1.98	14287.3	40406.8	0.354	MM
103	1... 20211111_A43_R1_IgG	1.98	29442.4	44421.5	0.663	bb
104	1... 20211111_A44_R1_IgG	1.99	69176.4	53128.2	1.302	bb
105	1... 20211111_QC_R9_IgG	1.98	24450.8	37049.0	0.660	MM
106	1... 20211111_A1_R2_IgG	1.98	31306.9	46499.4	0.673	MM
107	1... 20211111_A2_R2_IgG	1.98	49802.7	57260.7	0.870	bb
108	1... 20211111_L1_R2_IgG	1.98	27507.9	48062.9	0.572	bb
109	1... 20211111_L2_R2_IgG	1.98	24775.6	38745.2	0.639	bb
110	1... 20211111_H1_R2_IgG	1.98	20056.6	44273.5	0.453	bb
111	1... 20211111_A3_R2_IgG	2.00	1705.8	4755.5	0.359	MM
112	1... 20211111_A4_R2_IgG	1.98	29516.9	50546.7	0.584	bb

Dataset: C:\MassLynx\10.pro\20211115_clinicalsamples_IgG.qld

Last Altered: Sunday, October 02, 2022 16:02:30 台北標準時間
Printed: Friday, August 01, 2025 16:46:52 台北標準時間

	#	Name	RT	Area	IS Area	Response	Prima...
113	1...	20211111_L3_R2_IgG	1.98	22495.0	34825.8	0.646	MM
114	1...	20211111_L4_R2_IgG	1.98	21668.5	39107.7	0.554	bb
115	1...	20211111_H2_R2_IgG	1.98	73598.9	49750.0	1.479	bb
116	1...	20211111_A5_R2_IgG	1.98	28178.3	52536.2	0.536	bb
117	1...	20211111_A6_R2_IgG	1.98	60023.7	42050.0	1.427	bb
118	1...	20211111_QC_R10_IgG	1.98	25619.4	35350.8	0.725	MM
119	1...	20211111_L5_R2_IgG	1.98	38415.0	38039.3	1.010	bb
120	1...	20211111_L6_R2_IgG	1.98	8295.8	44907.4	0.185	MM
121	1...	20211111_H3_R2_IgG	1.98	19141.6	47729.1	0.401	bb
122	1...	20211111_A7_R2_IgG	1.98	24459.0	57347.1	0.427	MM
123	1...	20211111_A8_R2_IgG	1.98	27663.7	52397.8	0.528	bb
124	1...	20211111_L7_R2_IgG	1.98	25024.7	36318.4	0.689	MM
125	1...	20211111_L8_R2_IgG	1.98	23682.0	38131.9	0.621	bb
126	1...	20211111_H4_R2_IgG	1.98	49060.3	46849.6	1.047	bb
127	1...	20211111_A9_R2_IgG	1.98	11181.3	47759.6	0.234	bb
128	1...	20211111_A10_R2_IgG	1.98	16760.5	41366.2	0.405	MM
129	1...	20211111_L9_R2_IgG	1.98	22218.1	43052.1	0.516	bb
130	1...	20211111_L10_R2_IgG	1.98	15137.5	31487.4	0.481	MM
131	1...	20211111_QC_R11_IgG	1.98	26120.4	37804.6	0.691	bb
132	1...	20211111_H5_R2_IgG	1.98	30332.0	40380.0	0.751	bb
133	1...	20211111_A11_R2_IgG	1.98	9255.2	49533.3	0.187	bb
134	1...	20211111_A12_R2_IgG	1.98	17424.7	40226.3	0.433	bb
135	1...	20211111_L11_R2_IgG	1.98	11003.7	28884.0	0.381	MM
136	1...	20211111_L12_R2_IgG	1.98	18041.2	34351.3	0.525	MM
137	1...	20211111_H6_R2_IgG	1.98	24924.8	40466.6	0.616	MM
138	1...	20211111_A13_R2_IgG	1.98	31700.0	42306.7	0.749	MM
139	1...	20211111_A14_R2_IgG	1.98	14035.6	44904.0	0.313	bb
140	1...	20211111_L13_R2_IgG	1.98	28948.4	31913.0	0.907	MM
141	1...	20211111_L14_R2_IgG	1.98	31915.3	31527.4	1.012	MM
142	1...	20211111_H7_R2_IgG	1.98	17930.3	40838.2	0.439	bb
143	1...	20211111_A15_R2_IgG	1.98	61452.4	49389.7	1.244	bb
144	1...	20211111_QC_R12_IgG	1.98	22742.7	35168.7	0.647	bb
145	1...	20211111_A16_R2_IgG	1.97	17144.9	47440.5	0.361	MM
146	1...	20211111_L15_R2_IgG	1.98	21187.4	33145.9	0.639	MM
147	1...	20211111_L16_R2_IgG	1.98	12168.5	37596.6	0.324	MM
148	1...	20211111_H8_R2_IgG	1.98	17421.2	43933.3	0.397	bb
149	1...	20211111_A17_R2_IgG	1.98	34128.4	43605.5	0.783	MM
150	1...	20211111_A18_R2_IgG	1.98	10832.1	37581.8	0.288	MM
151	1...	20211111_L17_R2_IgG	1.98	38632.3	33942.5	1.138	MM
152	1...	20211111_L18_R2_IgG	1.98	10139.2	37831.5	0.268	bb
153	1...	20211111_H9_R2_IgG	1.98	20871.6	30898.4	0.675	bb
154	1...	20211111_A19_R2_IgG	1.98	39558.5	39352.1	1.005	bb
155	1...	20211111_A20_R2_IgG	1.97	6547.3	34661.2	0.189	MM
156	1...	20211111_L19_R2_IgG	1.98	17725.8	39918.3	0.444	MM
157	1...	20211111_QC_R13_IgG	1.98	25575.2	36239.1	0.706	bb
158	1...	20211111_L20_R2_IgG	1.98	22895.0	32897.7	0.696	MM
159	1...	20211111_H10_R2_IgG	1.98	29149.6	31394.5	0.928	bb
160	1...	20211111_A21_R2_IgG	1.98	18264.3	34863.7	0.524	MM
161	1...	20211111_A22_R2_IgG	1.98	20773.2	30342.1	0.685	bb
162	1...	20211111_L21_R2_IgG	1.98	8626.2	33706.2	0.256	MM
163	1...	20211111_L22_R2_IgG	1.98	17745.8	31298.6	0.567	bb
164	1...	20211111_H11_R2_IgG	1.98	13450.5	38904.1	0.346	MM
165	1...	20211111_A23_R2_IgG	1.98	14053.4	32382.3	0.434	bb
166	1...	20211111_A24_R2_IgG	1.97	19209.7	30261.9	0.635	MM
167	1...	20211111_L23_R2_IgG	1.97	31621.2	43037.5	0.735	bb
168	1...	20211111_L24_R2_IgG	1.98	19111.5	37023.3	0.516	MM

Dataset: C:\MassLynx\10.pro\20211115_clinicalsamples_IgG.qld

Last Altered: Sunday, October 02, 2022 16:02:30 台北標準時間
Printed: Friday, August 01, 2025 16:46:52 台北標準時間

	#	Name	RT	Area	IS Area	Response	Prima...
169	1...	20211111_H12_R2_IgG	1.97	21504.0	39478.2	0.545	bb
170	1...	20211111_QC_R14_IgG	1.98	20338.2	34463.2	0.590	MM
171	1...	20211111_A25_R2_IgG	1.98	23763.0	39945.3	0.595	MM
172	1...	20211111_A26_R2_IgG	1.97	6474.1	29663.2	0.218	bb
173	1...	20211111_L25_R2_IgG	1.98	16853.3	38589.3	0.437	MM
174	1...	20211111_L26_R2_IgG	1.98	9462.9	38405.2	0.246	MM
175	1...	20211111_H13_R2_IgG	1.98	26724.0	37771.2	0.708	bb
176	1...	20211111_A27_R2_IgG	1.98	16318.6	42634.1	0.383	bb
177	1...	20211111_A28_R2_IgG	1.98	20393.4	33231.1	0.614	bb
178	1...	20211111_L27_R2_IgG	1.98	29042.6	41761.1	0.695	bb
179	1...	20211111_L28_R2_IgG	1.97	23588.9	26968.6	0.875	bb
180	1...	20211111_H14_R2_IgG	1.98	32700.8	49250.2	0.664	MM
181	1...	20211111_A29_R2_IgG	1.98	9131.2	37601.7	0.243	bb
182	1...	20211111_A30_R2_IgG	1.98	12558.8	40366.8	0.311	bb
183	1...	20211111_QC_R15_IgG	1.97	24369.9	35156.1	0.693	bb
184	1...	20211111_L29_R2_IgG	1.98	16476.3	33813.1	0.487	MM
185	1...	20211111_L30_R2_IgG	1.97	7658.9	33488.6	0.229	MM
186	1...	20211111_H15_R2_IgG	1.98	23400.8	38501.0	0.608	MM
187	1...	20211111_A31_R2_IgG	1.97	12244.3	37525.5	0.326	bb
188	1...	20211111_A32_R2_IgG	1.97	24333.2	35770.8	0.680	MM
189	1...	20211111_L31_R2_IgG	1.97	9941.0	33282.4	0.299	MM
190	1...	20211111_L32_R2_IgG	1.98	35543.0	42049.6	0.845	bb
191	1...	20211111_H16_R2_IgG	1.98	32678.3	45947.6	0.711	bb
192	1...	20211111_A33_R2_IgG	1.98	22202.3	40202.3	0.552	MM
193	1...	20211111_A34_R2_IgG	1.97	15440.8	36747.0	0.420	MM
194	1...	20211111_H17_R2_IgG	1.97	203063.4	40920.9	4.962	bb
195	1...	20211111_A35_R2_IgG	1.98	27156.7	33689.2	0.806	bb
196	1...	20211111_QC_R16_IgG	1.97	22239.2	32783.6	0.678	bb
197	1...	20211111_A36_R2_IgG	1.97	11610.9	36822.4	0.315	MM
198	1...	20211111_H18_R2_IgG	1.98	115906.2	31769.3	3.648	bb
199	1...	20211111_A37_R2_IgG	1.98	56048.4	37581.8	1.491	MM
200	2...	20211111_A38_R2_IgG	1.98	18041.8	38390.4	0.470	bb
201	2...	20211111_H19_R2_IgG	1.97	109424.0	41217.0	2.655	MM
202	2...	20211111_A39_R2_IgG	1.98	28375.7	37392.7	0.759	MM
203	2...	20211111_A40_R2_IgG	1.97	15578.0	38295.1	0.407	MM
204	2...	20211111_H20_R2_IgG	1.98	90387.1	35941.8	2.515	bb
205	2...	20211111_A41_R2_IgG	1.98	25798.1	45304.9	0.569	MM
206	2...	20211111_A42_R2_IgG	1.98	11009.8	37803.8	0.291	bb
207	2...	20211111_A43_R2_IgG	1.98	22517.7	38161.8	0.590	bb
208	2...	20211111_A44_R2_IgG	1.97	49739.4	37819.8	1.315	bb
209	2...	20211111_QC_R17_IgG	1.98	22325.7	31731.8	0.704	bb

Dataset: C:\MassLynx\10.pro\20211115_clinicalsamples_IgG.qld

Last Altered: Sunday, October 02, 2022 16:02:30 台北標準時間
Printed: Friday, August 01, 2025 16:47:29 台北標準時間

Method: C:\MassLynx\10_Pro_IgA.PRO\MethDB\20210926_IgG_DMRM_IgM_IS.mdb 16 Feb 2022 11:58:21
Calibration: 18 Nov 2021 13:56:22

Sample Name: 20211111_QC_R1_IgG
F36:MRM of 1 channel,ES+
1083.83 > 366.1
3.555e+005

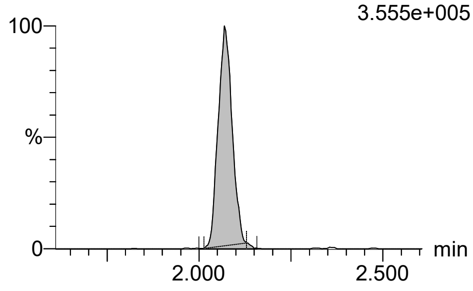

Sample Name: 20211111_A1_R1_IgG
F36:MRM of 1 channel,ES+
1083.83 > 366.1
3.840e+005

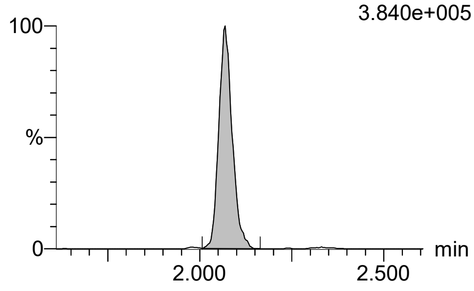

Sample Name: 20211111_A2_R1_IgG
F36:MRM of 1 channel,ES+
1083.83 > 366.1
5.078e+005

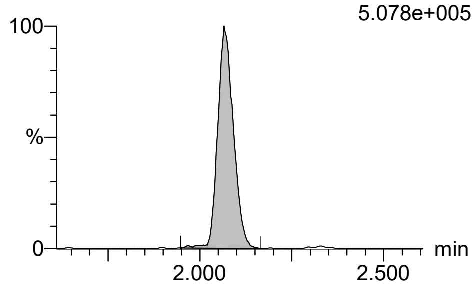

Sample Name: 20211111_L1_R1_IgG
F36:MRM of 1 channel,ES+
1083.83 > 366.1
2.806e+005

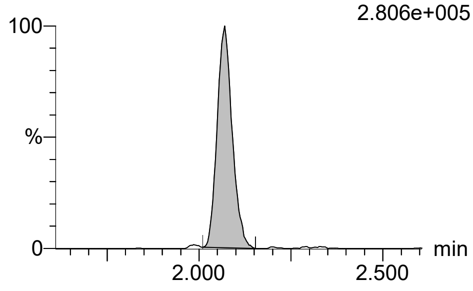

Sample Name: 20211111_L2_R1_IgG
F36:MRM of 1 channel,ES+
1083.83 > 366.1
2.118e+005

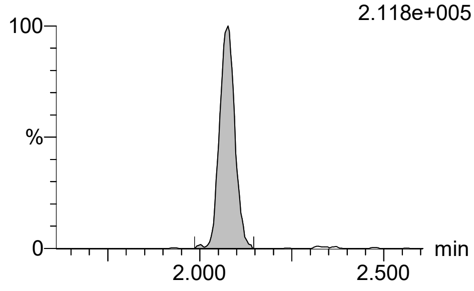

Sample Name: 20211111_H1_R1_IgG
F36:MRM of 1 channel,ES+
1083.83 > 366.1
9.429e+004

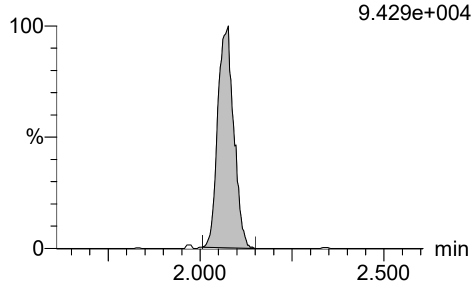

Sample Name: 20211111_A3_R1_IgG
F36:MRM of 1 channel,ES+
1083.83 > 366.1
8.585e+003

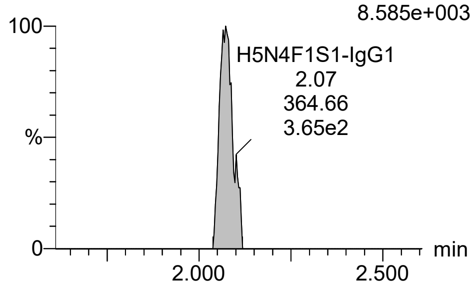

Sample Name: 20211111_A4_R1_IgG
F36:MRM of 1 channel,ES+
1083.83 > 366.1
3.361e+005

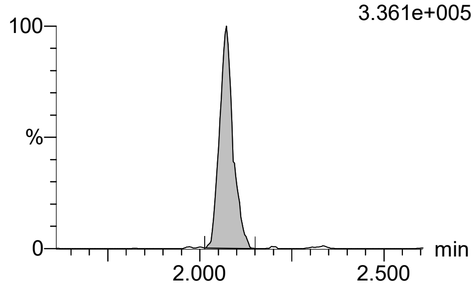

Sample Name: 20211111_L3_R1_IgG
F36:MRM of 1 channel,ES+
1083.83 > 366.1
1.396e+005

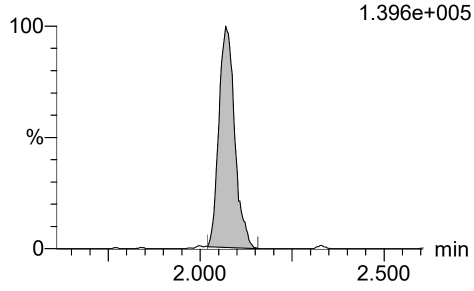

Sample Name: 20211111_L4_R1_IgG
F36:MRM of 1 channel,ES+
1083.83 > 366.1
1.607e+005

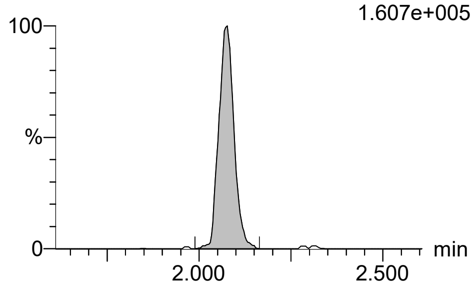

Sample Name: 20211111_H2_R1_IgG
F36:MRM of 1 channel,ES+
1083.83 > 366.1
8.033e+005

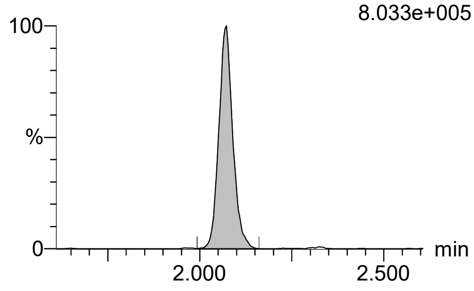

Sample Name: 20211111_A5_R1_IgG
F36:MRM of 1 channel,ES+
1083.83 > 366.1
2.492e+005

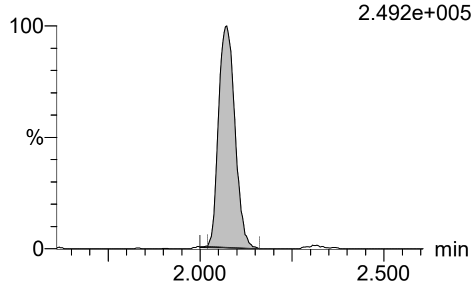

Dataset: C:\MassLynx\10.pro\20211115_clinicalsamples_IgG.qld

Last Altered: Sunday, October 02, 2022 16:02:30 台北標準時間
Printed: Friday, August 01, 2025 16:47:29 台北標準時間

Sample Name: 20211111_A6_R1_IgG

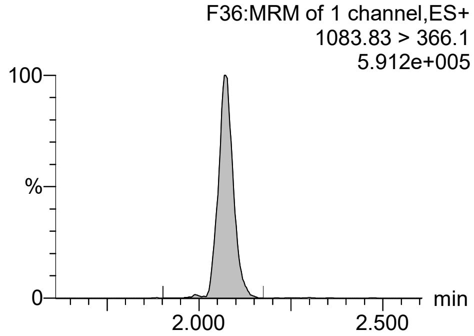

Sample Name: 20211111_QC_R2_IgG

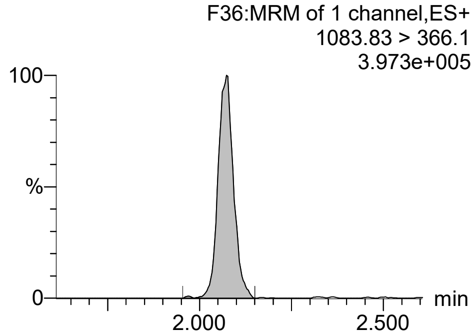

Sample Name: 20211111_L5_R1_IgG

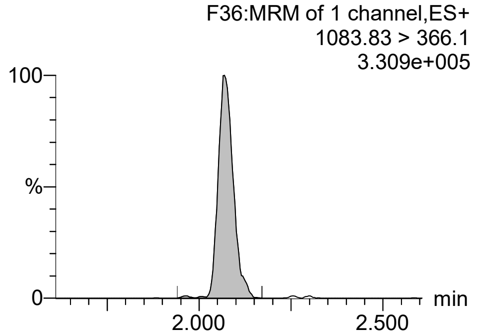

Sample Name: 20211111_L6_R1_IgG

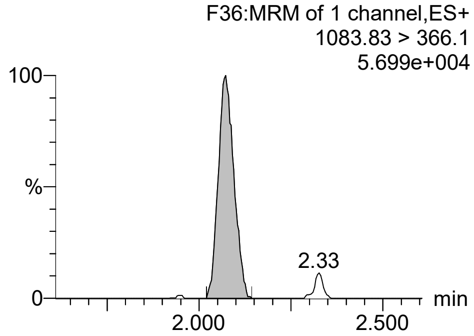

Sample Name: 20211111_H3_R1_IgG

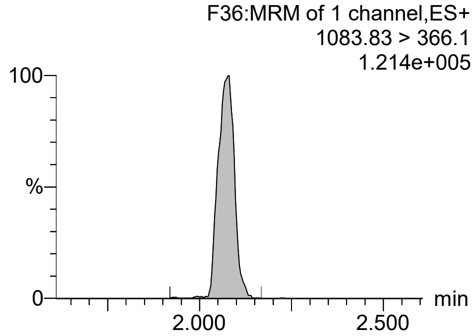

Sample Name: 20211111_A7_R1_IgG

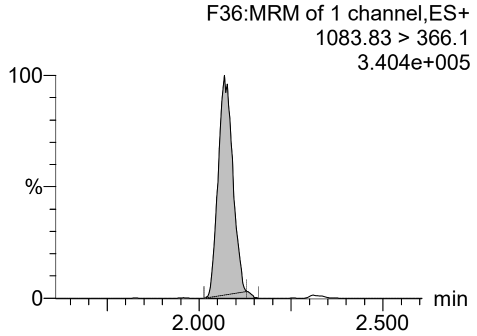

Sample Name: 20211111_A8_R1_IgG

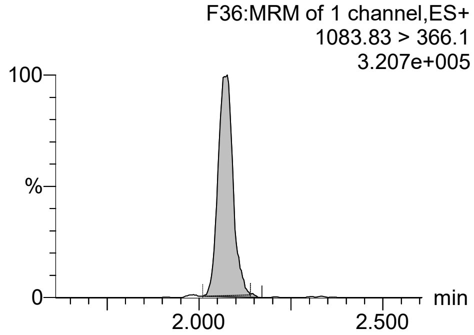

Sample Name: 20211111_L7_R1_IgG

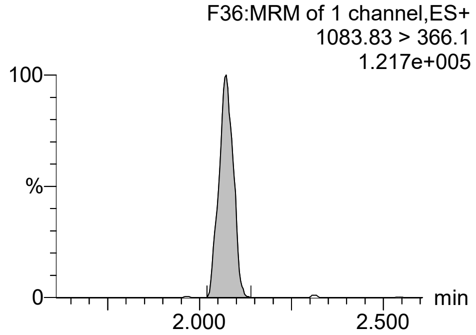

Sample Name: 20211111_L8_R1_IgG

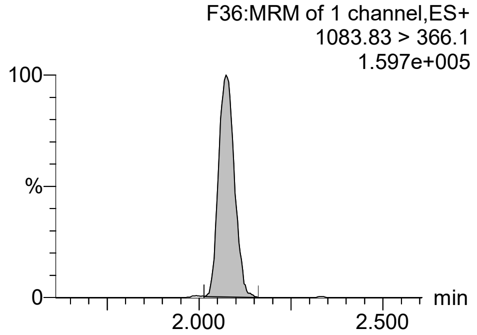

Sample Name: 20211111_H4_R1_IgG

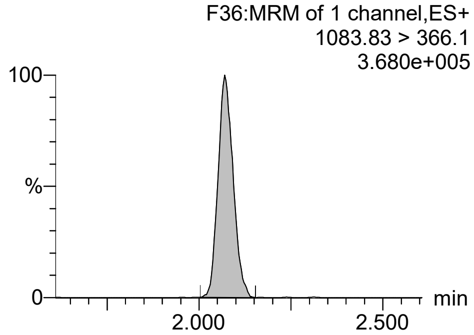

Sample Name: 20211111_A9_R1_IgG

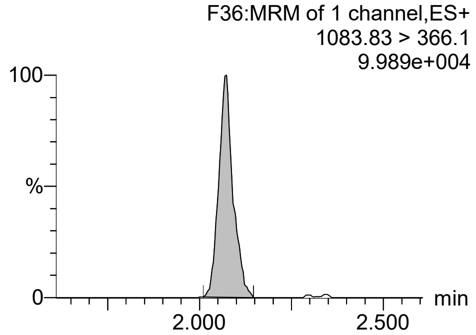

Sample Name: 20211111_A10_R1_IgG

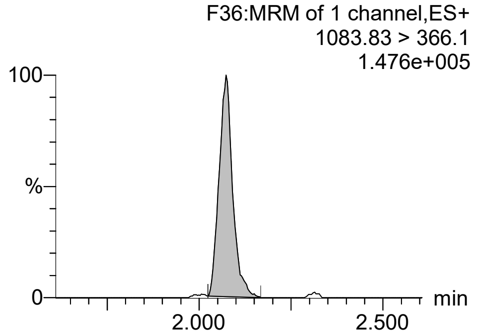

Sample Name: 20211111_L9_R1_IgG

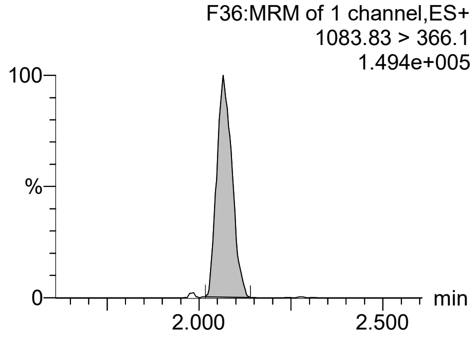

Sample Name: 20211111_L10_R1_IgG

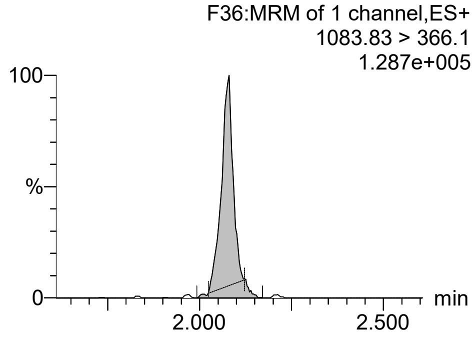

Sample Name: 20211111_QC_R3_IgG

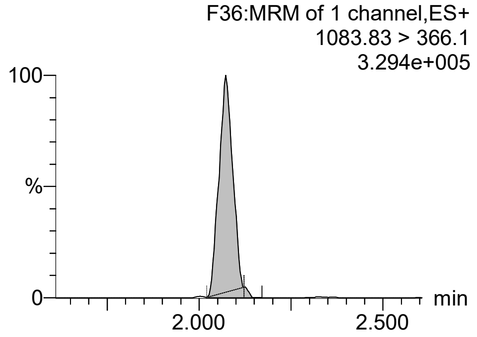

Sample Name: 20211111_H5_R1_IgG
F36:MRM of 1 channel,ES+
1083.83 > 366.1
2.721e+005

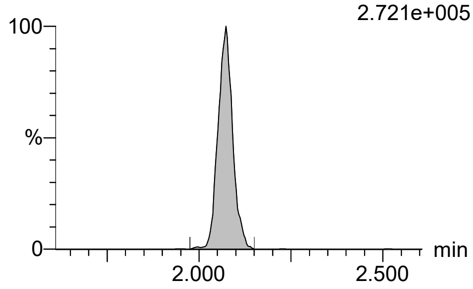

Sample Name: 20211111_A11_R1_IgG
F36:MRM of 1 channel,ES+
1083.83 > 366.1
9.886e+004

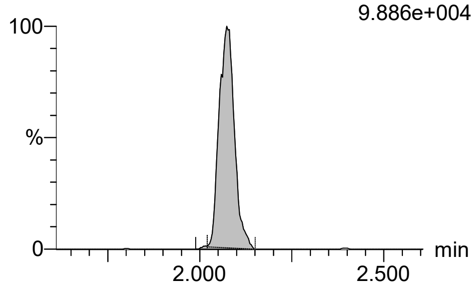

Sample Name: 20211111_A12_R1_IgG
F36:MRM of 1 channel,ES+
1083.83 > 366.1
1.162e+005

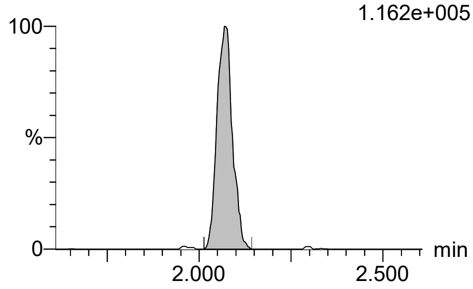

Sample Name: 20211111_L11_R1_IgG
F36:MRM of 1 channel,ES+
1083.83 > 366.1
8.242e+004

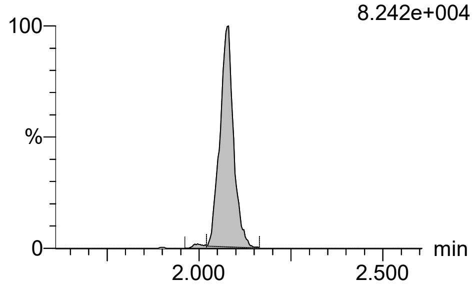

Sample Name: 20211111_L12_R1_IgG
F36:MRM of 1 channel,ES+
1083.83 > 366.1
1.207e+005

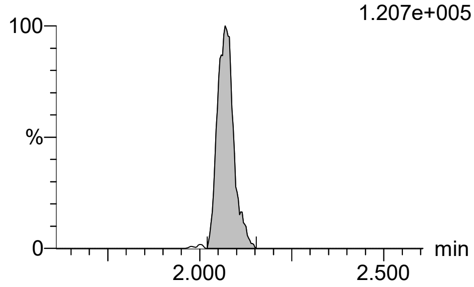

Sample Name: 20211111_H6_R1_IgG
F36:MRM of 1 channel,ES+
1083.83 > 366.1
2.322e+005

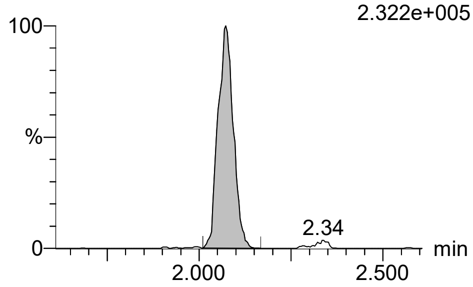

Sample Name: 20211111_A13_R1_IgG
F36:MRM of 1 channel,ES+
1083.83 > 366.1
2.336e+005

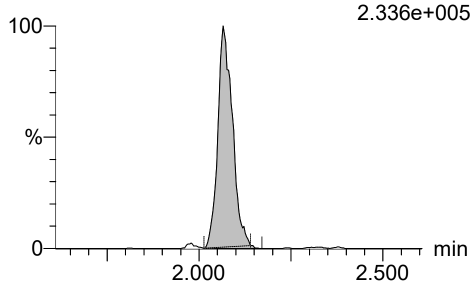

Sample Name: 20211111_A14_R1_IgG
F36:MRM of 1 channel,ES+
1083.83 > 366.1
1.258e+005

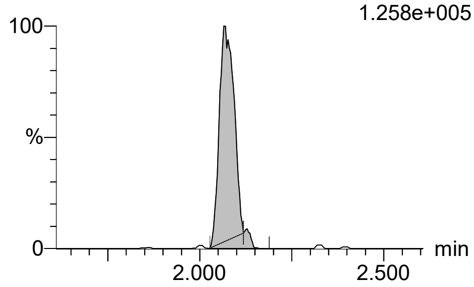

Sample Name: 20211111_L13_R1_IgG
F36:MRM of 1 channel,ES+
1083.83 > 366.1
1.944e+005

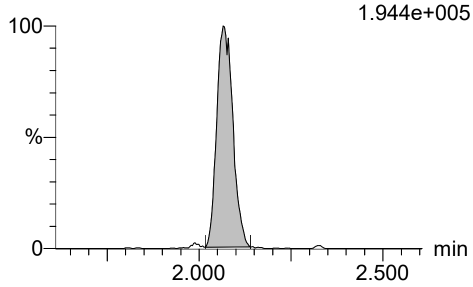

Sample Name: 20211111_L14_R1_IgG
F36:MRM of 1 channel,ES+
1083.83 > 366.1
2.035e+005

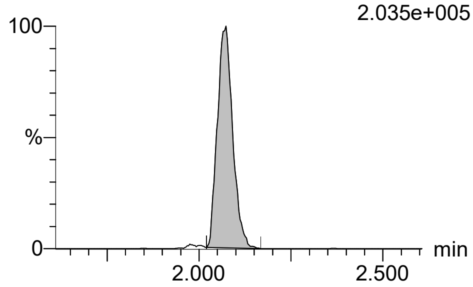

Sample Name: 20211111_H7_R1_IgG
F36:MRM of 1 channel,ES+
1083.83 > 366.1
1.095e+005

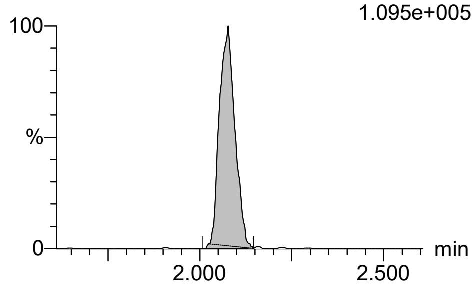

Sample Name: 20211111_A15_R1_IgG
F36:MRM of 1 channel,ES+
1083.83 > 366.1
4.222e+005

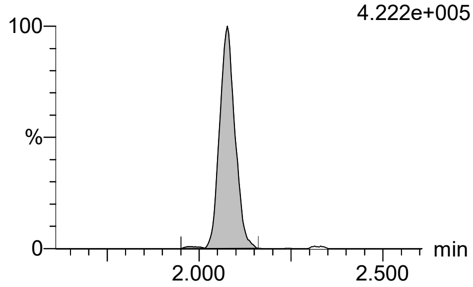

Sample Name: 20211111_QC_R4_IgG
F36:MRM of 1 channel,ES+
1083.83 > 366.1
2.354e+005

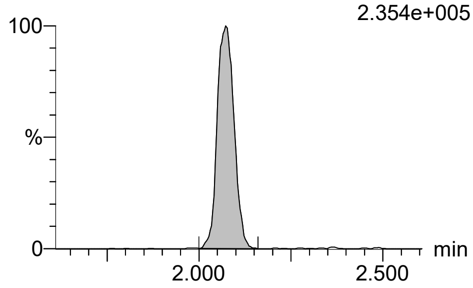

Sample Name: 20211111_A16_R1_IgG
F36:MRM of 1 channel,ES+
1083.83 > 366.1
1.911e+005

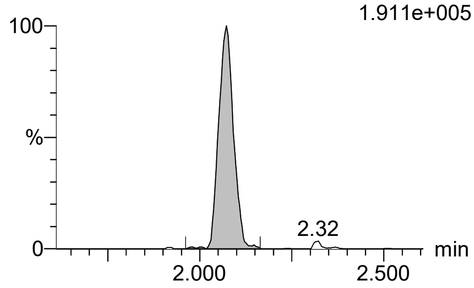

Sample Name: 20211111_L15_R1_IgG
F36:MRM of 1 channel,ES+
1083.83 > 366.1
1.522e+005

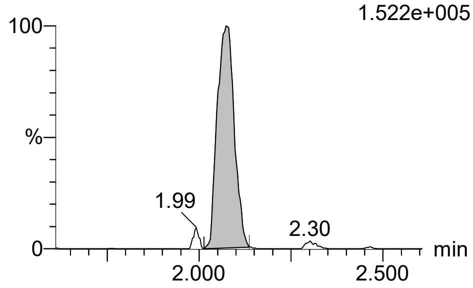

Sample Name: 20211111_L16_R1_IgG
F36:MRM of 1 channel,ES+
1083.83 > 366.1
1.247e+005

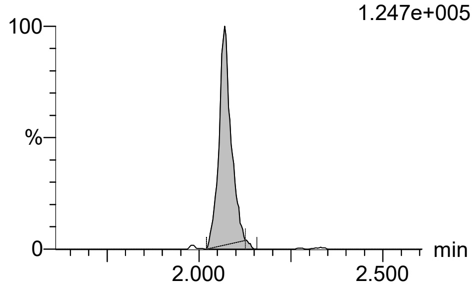

Sample Name: 20211111_H8_R1_IgG
F36:MRM of 1 channel,ES+
1083.83 > 366.1
1.239e+005

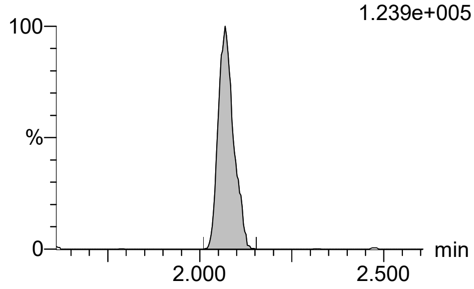

Sample Name: 20211111_A17_R1_IgG
F36:MRM of 1 channel,ES+
1083.83 > 366.1
2.774e+005

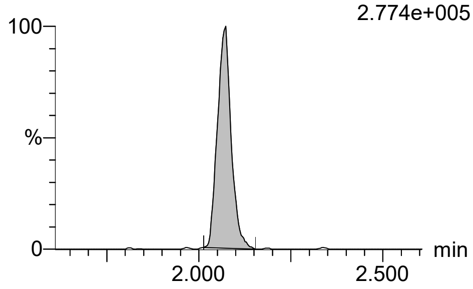

Sample Name: 20211111_A18_R1_IgG
F36:MRM of 1 channel,ES+
1083.83 > 366.1
9.011e+004

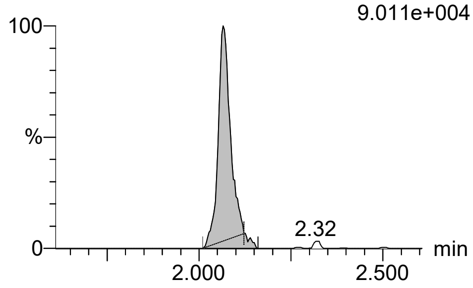

Sample Name: 20211111_L17_R1_IgG
F36:MRM of 1 channel,ES+
1083.83 > 366.1
2.175e+005

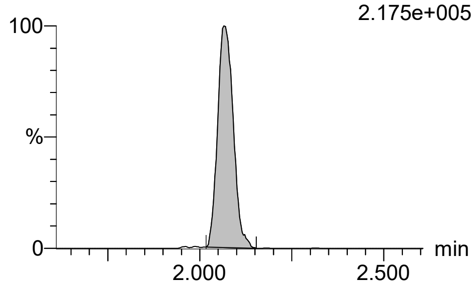

Sample Name: 20211111_L18_R1_IgG
F36:MRM of 1 channel,ES+
1083.83 > 366.1
1.018e+005

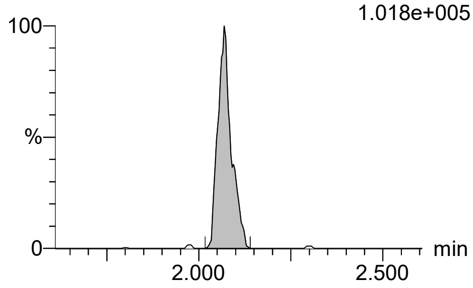

Sample Name: 20211111_H9_R1_IgG
F36:MRM of 1 channel,ES+
1083.83 > 366.1
1.321e+005

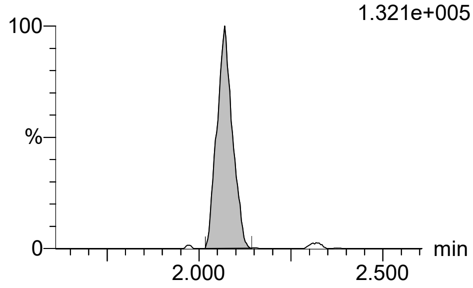

Sample Name: 20211111_A19_R1_IgG
F36:MRM of 1 channel,ES+
1083.83 > 366.1
2.395e+005

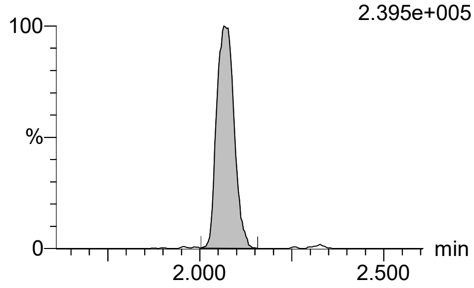

Sample Name: 20211111_A20_R1_IgG
F36:MRM of 1 channel,ES+
1083.83 > 366.1
3.950e+004

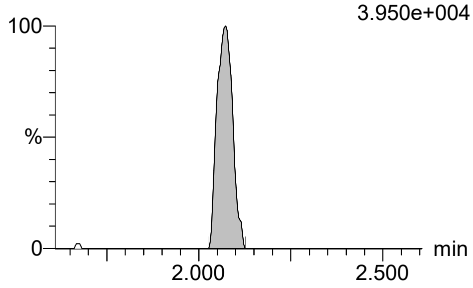

Sample Name: 20211111_L19_R1_IgG
F36:MRM of 1 channel,ES+
1083.83 > 366.1
1.470e+005

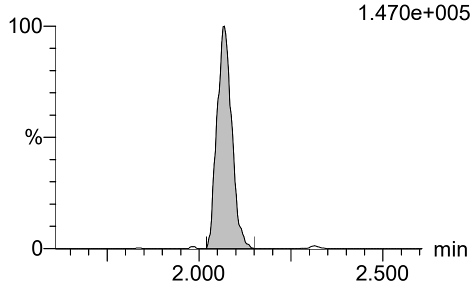

Sample Name: 20211111_QC_R5_IgG
F36:MRM of 1 channel,ES+
1083.83 > 366.1
2.269e+005

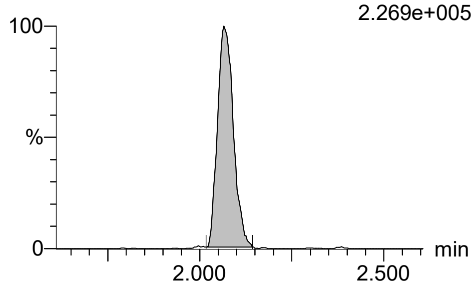

Sample Name: 20211111_L20_R1_IgG
F36:MRM of 1 channel,ES+
1083.83 > 366.1
2.886e+005

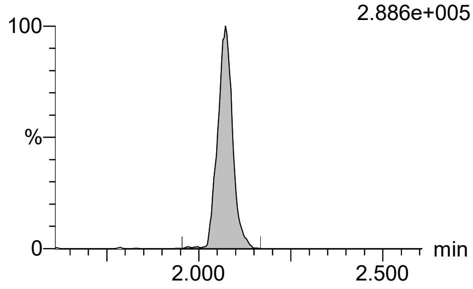

Sample Name: 20211111_H10_R1_IgG
F36:MRM of 1 channel,ES+
1083.83 > 366.1
4.939e+005

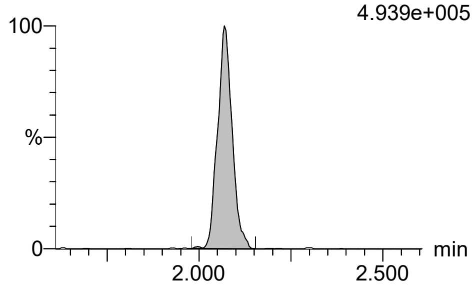

Sample Name: 20211111_A21_R1_IgG
F36:MRM of 1 channel,ES+
1083.83 > 366.1
2.974e+005

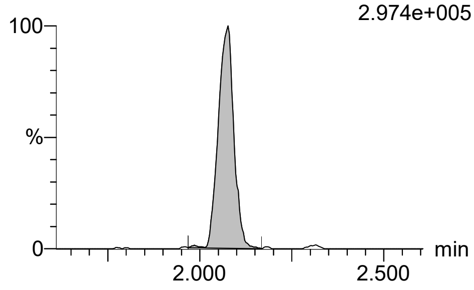

Sample Name: 20211111_A22_R1_IgG
F36:MRM of 1 channel,ES+
1083.83 > 366.1
2.508e+005

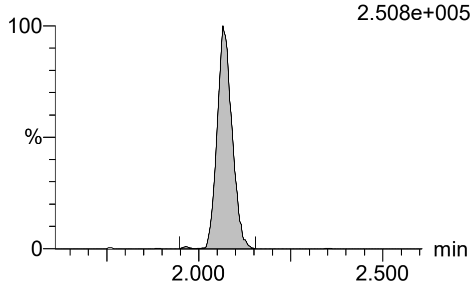

Dataset: C:\MassLynx\10.pro\20211115_clinicalsamples_IgG.qld

Last Altered: Sunday, October 02, 2022 16:02:30 台北標準時間
Printed: Friday, August 01, 2025 16:47:29 台北標準時間

Sample Name: 20211111_L21_R1_IgG
F36:MRM of 1 channel,ES+
1083.83 > 366.1
9.307e+004

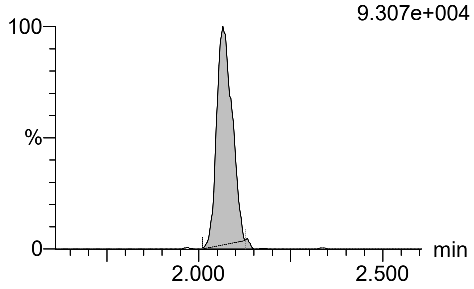

Sample Name: 20211111_L22_R1_IgG
F36:MRM of 1 channel,ES+
1083.83 > 366.1
1.990e+005

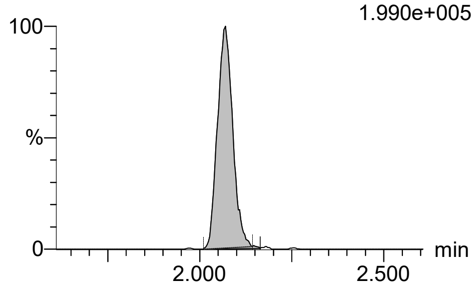

Sample Name: 20211111_H11_R1_IgG
F36:MRM of 1 channel,ES+
1083.83 > 366.1
1.571e+005

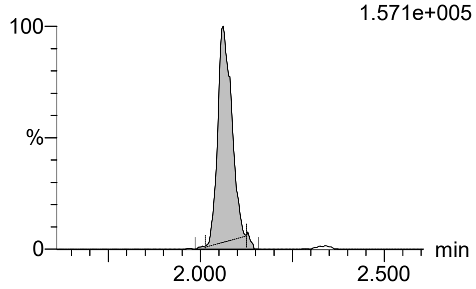

Sample Name: 20211111_A23_R1_IgG
F36:MRM of 1 channel,ES+
1083.83 > 366.1
1.985e+005

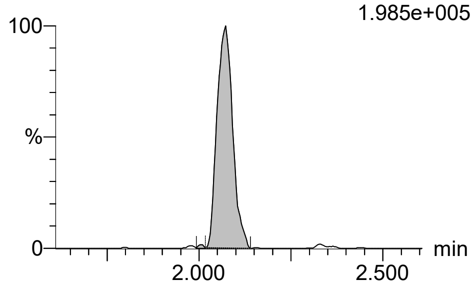

Sample Name: 20211111_A24_R1_IgG
F36:MRM of 1 channel,ES+
1083.83 > 366.1
1.826e+005

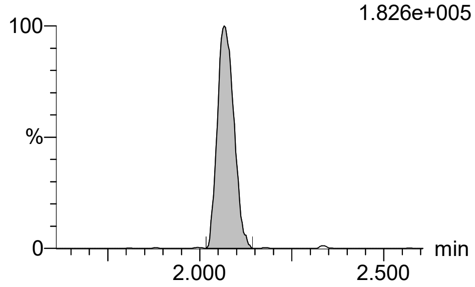

Sample Name: 20211111_L23_R1_IgG
F36:MRM of 1 channel,ES+
1083.83 > 366.1
2.272e+005

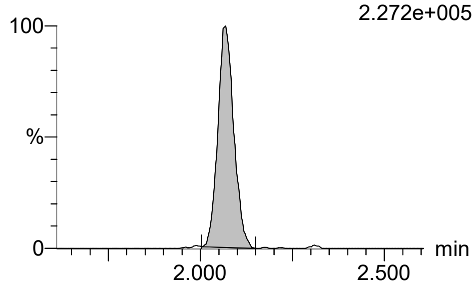

Sample Name: 20211111_L24_R1_IgG
F36:MRM of 1 channel,ES+
1083.83 > 366.1
1.547e+005

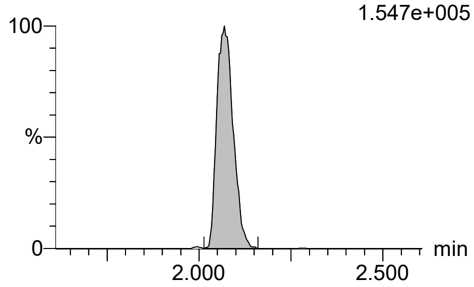

Sample Name: 20211111_H12_R1_IgG
F36:MRM of 1 channel,ES+
1083.83 > 366.1
1.525e+005

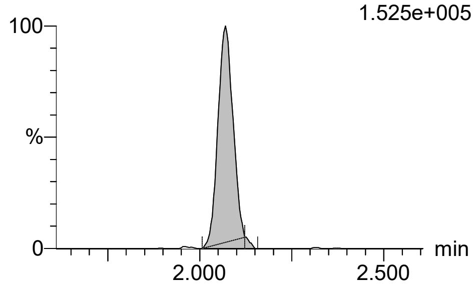

Sample Name: 20211111_QC_R6_IgG
F36:MRM of 1 channel,ES+
1083.83 > 366.1
2.124e+005

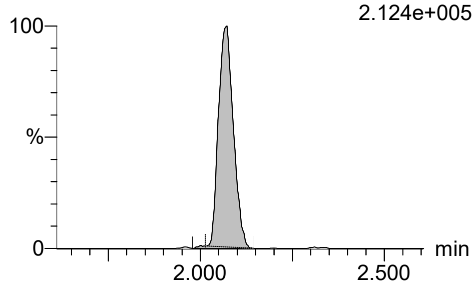

Sample Name: 20211111_A25_R1_IgG
F36:MRM of 1 channel,ES+
1083.83 > 366.1
3.583e+005

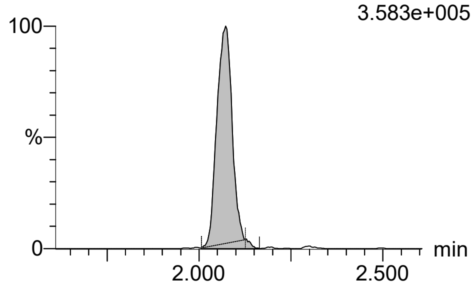

Sample Name: 20211111_A26_R1_IgG
F36:MRM of 1 channel,ES+
1083.83 > 366.1
5.601e+004

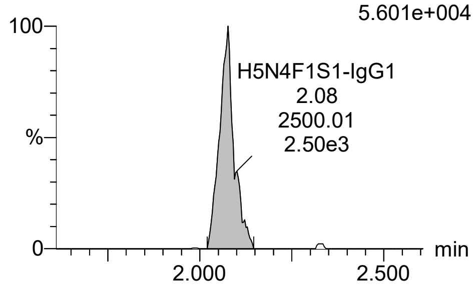

Sample Name: 20211111_L25_R1_IgG
F36:MRM of 1 channel,ES+
1083.83 > 366.1
1.794e+005

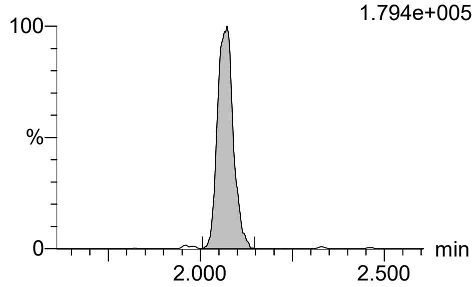

Sample Name: 20211111_L26_R1_IgG
F36:MRM of 1 channel,ES+
1083.83 > 366.1
6.462e+004

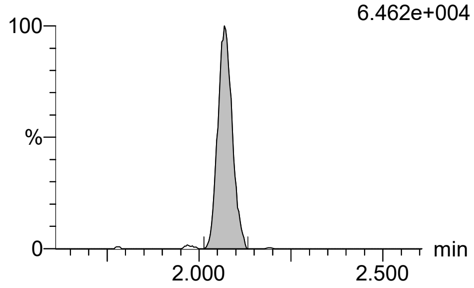

Sample Name: 20211111_H13_R1_IgG
F36:MRM of 1 channel,ES+
1083.83 > 366.1
2.626e+005

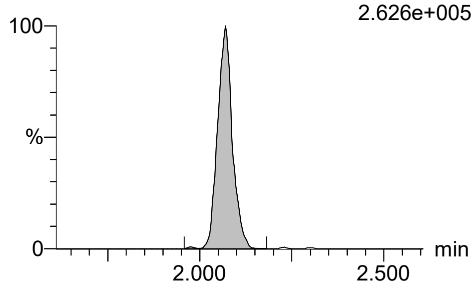

Sample Name: 20211111_A27_R1_IgG
F36:MRM of 1 channel,ES+
1083.83 > 366.1
2.356e+005

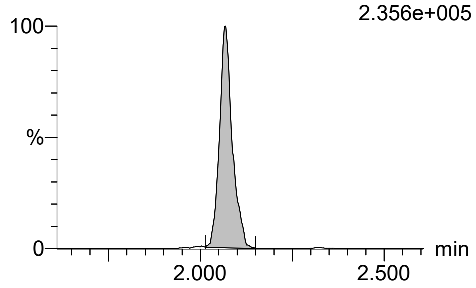

Sample Name: 20211111_A28_R1_IgG
F36:MRM of 1 channel,ES+
1083.83 > 366.1
2.601e+005

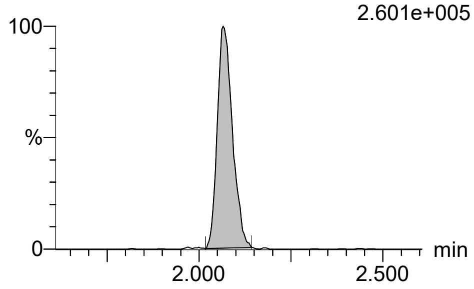

Sample Name: 20211111_L27_R1_IgG
F36:MRM of 1 channel,ES+
1083.83 > 366.1
3.436e+005

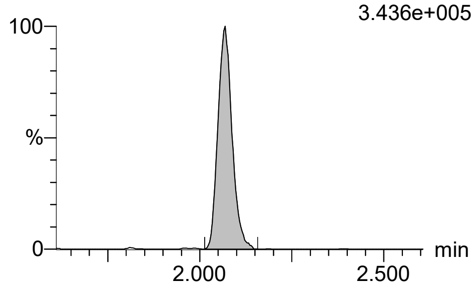

Sample Name: 20211111_L28_R1_IgG
F36:MRM of 1 channel,ES+
1083.83 > 366.1
1.773e+005

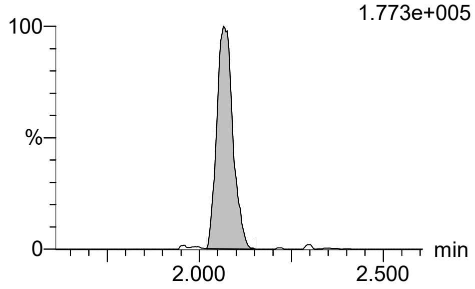

Sample Name: 20211111_H14_R1_IgG
F36:MRM of 1 channel,ES+
1083.83 > 366.1
2.831e+005

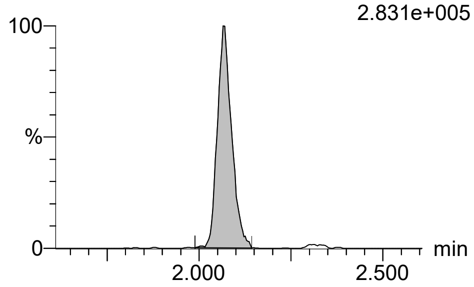

Sample Name: 20211111_A29_R1_IgG
F36:MRM of 1 channel,ES+
1083.83 > 366.1
1.010e+005

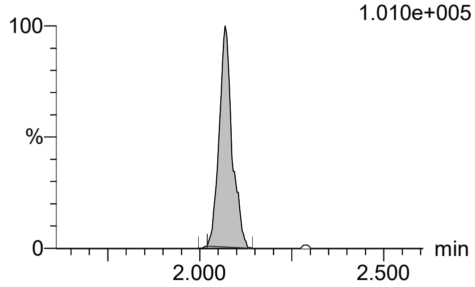

Sample Name: 20211111_A30_R1_IgG
F36:MRM of 1 channel,ES+
1083.83 > 366.1
1.360e+005

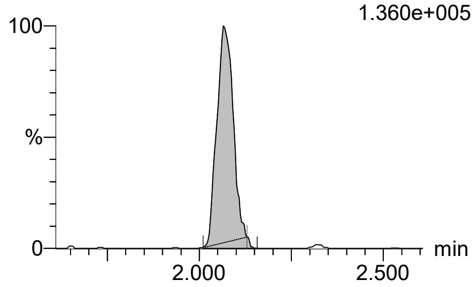

Sample Name: 20211111_QC_R7_IgG
F36:MRM of 1 channel,ES+
1083.83 > 366.1
2.205e+005

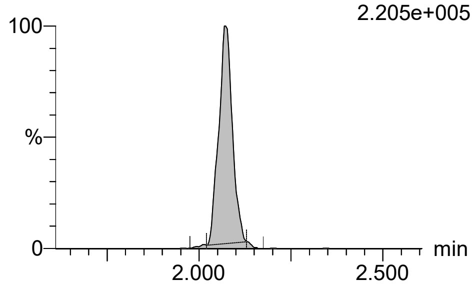

Sample Name: 20211111_L29_R1_IgG
F36:MRM of 1 channel,ES+
1083.83 > 366.1
1.134e+005

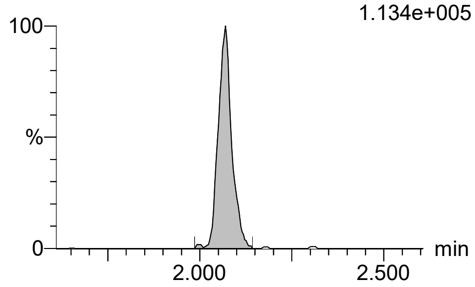

Sample Name: 20211111_L30_R1_IgG
F36:MRM of 1 channel,ES+
1083.83 > 366.1
6.519e+004

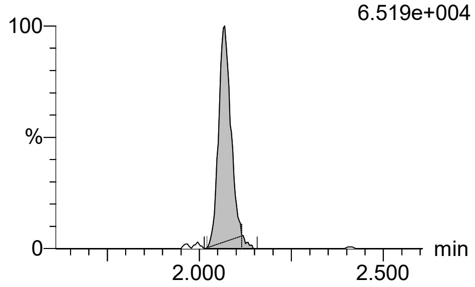

Sample Name: 20211111_H15_R1_IgG
F36:MRM of 1 channel,ES+
1083.83 > 366.1
1.806e+005

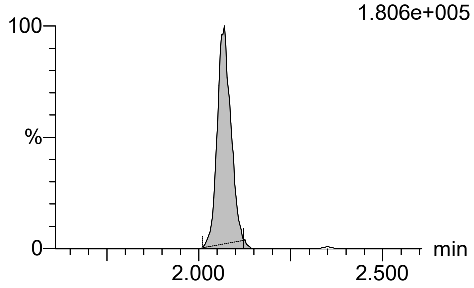

Sample Name: 20211111_A31_R1_IgG
F36:MRM of 1 channel,ES+
1083.83 > 366.1
1.009e+005

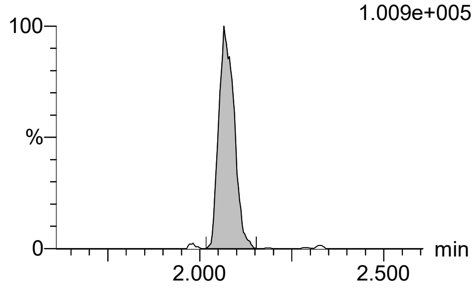

Sample Name: 20211111_A32_R1_IgG
F36:MRM of 1 channel,ES+
1083.83 > 366.1
1.738e+005

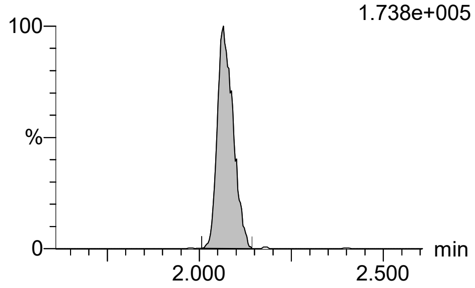

Sample Name: 20211111_L31_R1_IgG
F36:MRM of 1 channel,ES+
1083.83 > 366.1
7.861e+004

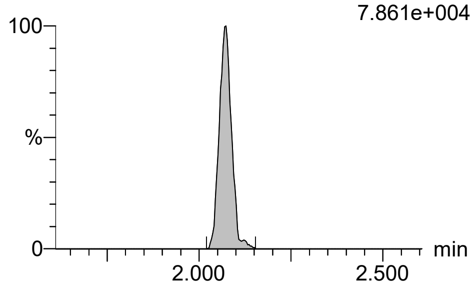

Sample Name: 20211111_L32_R1_IgG
F36:MRM of 1 channel,ES+
1083.83 > 366.1
2.373e+005

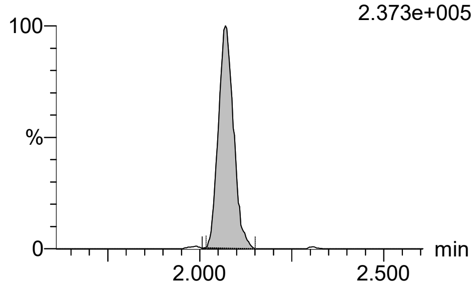

Sample Name: 20211111_H16_R1_IgG
F36:MRM of 1 channel,ES+
1083.83 > 366.1
2.856e+005

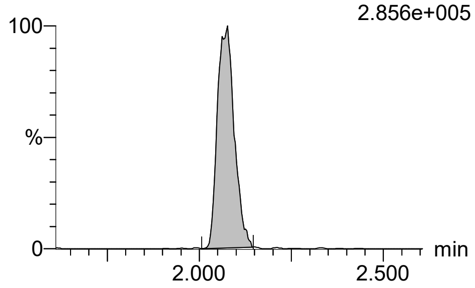

Dataset: C:\MassLynx\10.pro\20211115_clinicalsamples_IgG.qld

Last Altered: Sunday, October 02, 2022 16:02:30 台北標準時間
Printed: Friday, August 01, 2025 16:47:29 台北標準時間

Sample Name: 20211111_A33_R1_IgG
F36:MRM of 1 channel,ES+
1083.83 > 366.1
2.022e+005

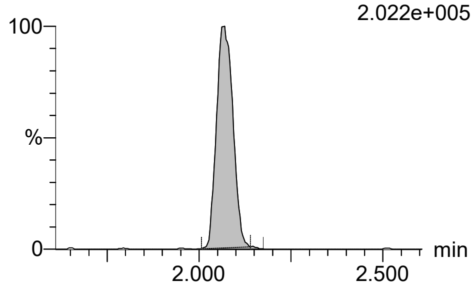

Sample Name: 20211111_A34_R1_IgG
F36:MRM of 1 channel,ES+
1083.83 > 366.1
1.297e+005

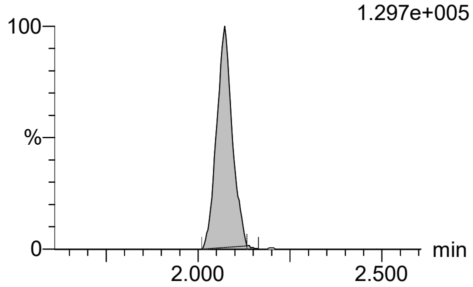

Sample Name: 20211111_H17_R1_IgG
F36:MRM of 1 channel,ES+
1083.83 > 366.1
1.521e+006

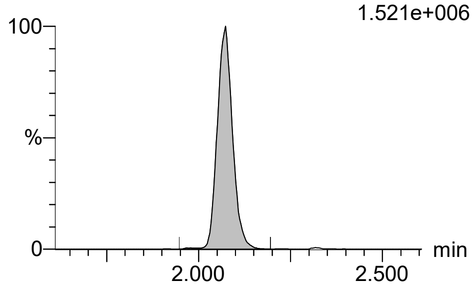

Sample Name: 20211111_A35_R1_IgG
F36:MRM of 1 channel,ES+
1083.83 > 366.1
1.436e+005

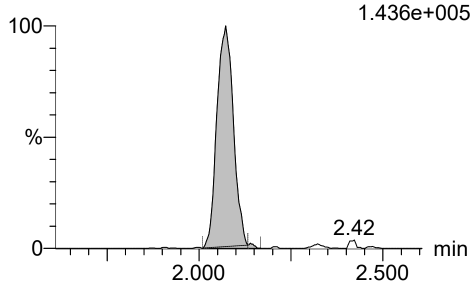

Sample Name: 20211111_QC_R8_IgG
F36:MRM of 1 channel,ES+
1083.83 > 366.1
1.575e+005

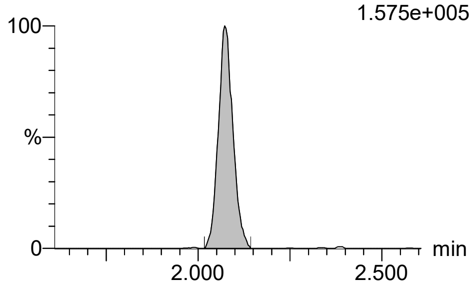

Sample Name: 20211111_A36_R1_IgG
F36:MRM of 1 channel,ES+
1083.83 > 366.1
1.064e+005

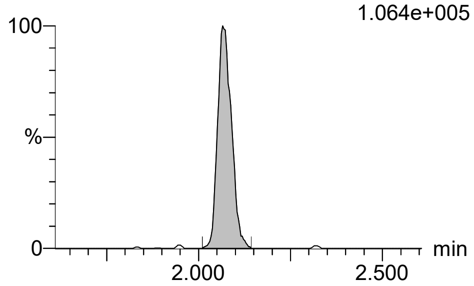

Sample Name: 20211111_H18_R1_IgG
F36:MRM of 1 channel,ES+
1083.83 > 366.1
7.294e+005

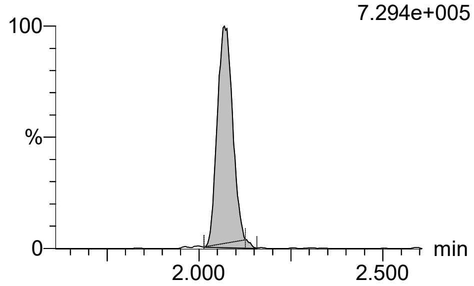

Sample Name: 20211111_A37_R1_IgG
F36:MRM of 1 channel,ES+
1083.83 > 366.1
3.061e+005

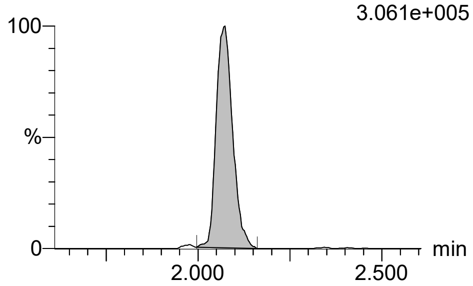

Sample Name: 20211111_A38_R1_IgG
F36:MRM of 1 channel,ES+
1083.83 > 366.1
1.231e+005

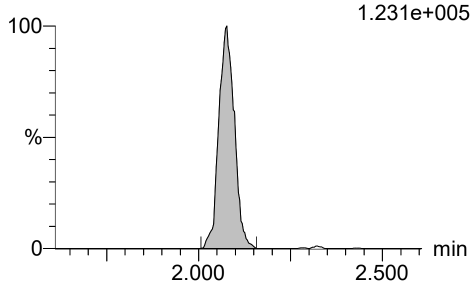

Sample Name: 20211111_H19_R1_IgG
F36:MRM of 1 channel,ES+
1083.83 > 366.1
7.868e+005

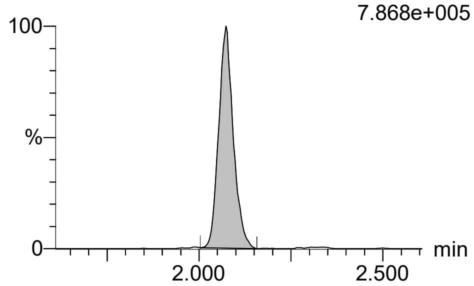

Sample Name: 20211111_A39_R1_IgG
F36:MRM of 1 channel,ES+
1083.83 > 366.1
1.783e+005

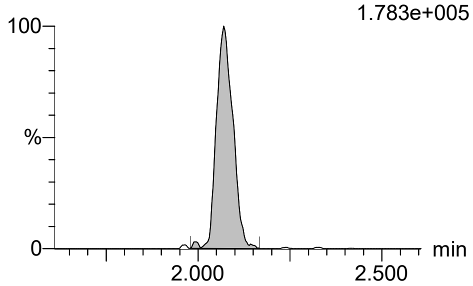

Sample Name: 20211111_A40_R1_IgG
F36:MRM of 1 channel,ES+
1083.83 > 366.1
9.948e+004

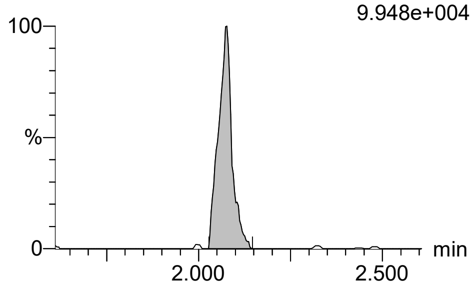

Sample Name: 20211111_H20_R1_IgG
F36:MRM of 1 channel,ES+
1083.83 > 366.1
5.043e+005

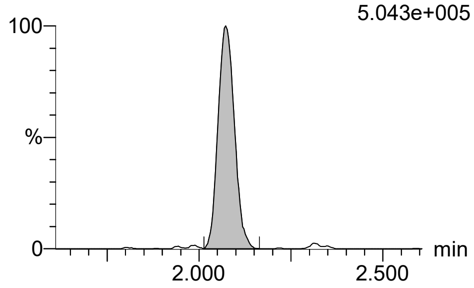

Sample Name: 20211111_A41_R1_IgG
F36:MRM of 1 channel,ES+
1083.83 > 366.1
1.954e+005

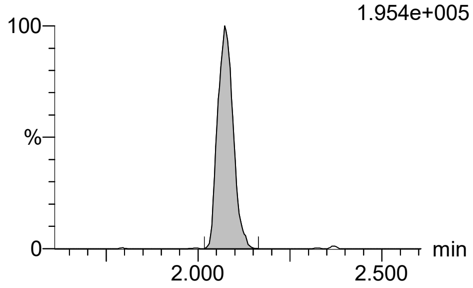

Sample Name: 20211111_A42_R1_IgG
F36:MRM of 1 channel,ES+
1083.83 > 366.1
6.515e+004

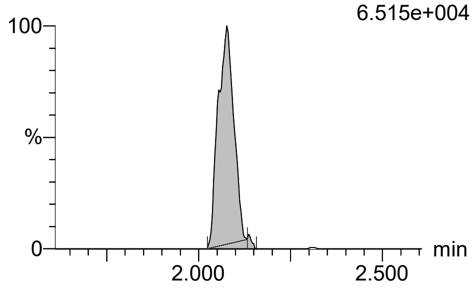

Sample Name: 20211111_A43_R1_IgG
F36:MRM of 1 channel,ES+
1083.83 > 366.1
2.274e+005

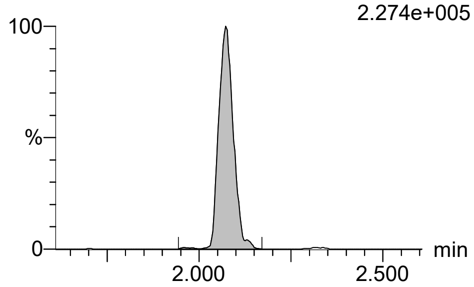

Sample Name: 20211111_A44_R1_IgG
F36:MRM of 1 channel,ES+
1083.83 > 366.1
4.947e+005

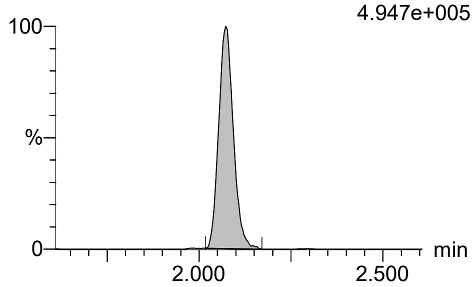

Sample Name: 20211111_QC_R9_IgG
F36:MRM of 1 channel,ES+
1083.83 > 366.1
1.723e+005

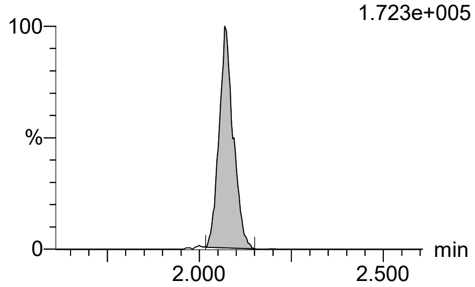

Sample Name: 20211111_A1_R2_IgG
F36:MRM of 1 channel,ES+
1083.83 > 366.1
2.481e+005

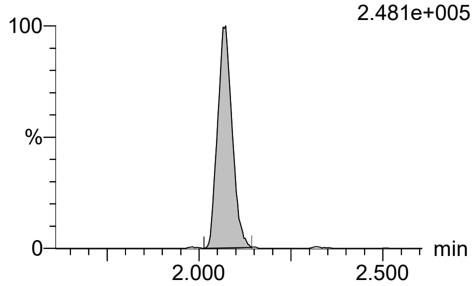

Sample Name: 20211111_A2_R2_IgG
F36:MRM of 1 channel,ES+
1083.83 > 366.1
3.937e+005

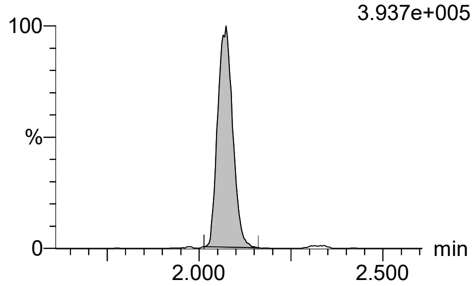

Sample Name: 20211111_L1_R2_IgG
F36:MRM of 1 channel,ES+
1083.83 > 366.1
1.793e+005

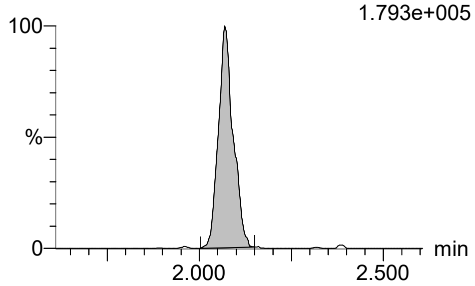

Sample Name: 20211111_L2_R2_IgG
F36:MRM of 1 channel,ES+
1083.83 > 366.1
1.494e+005

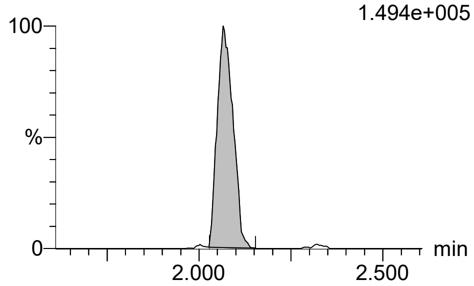

Sample Name: 20211111_H1_R2_IgG
F36:MRM of 1 channel,ES+
1083.83 > 366.1
1.024e+005

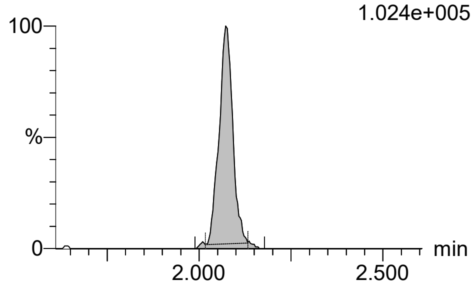

Sample Name: 20211111_A3_R2_IgG
F36:MRM of 1 channel,ES+
1083.83 > 366.1
1.375e+004

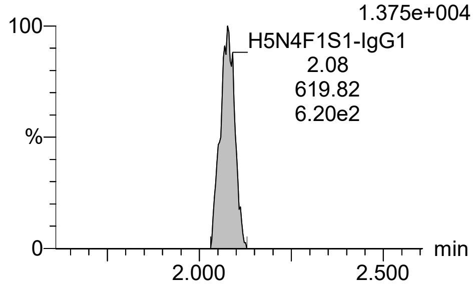

Sample Name: 20211111_A4_R2_IgG
F36:MRM of 1 channel,ES+
1083.83 > 366.1
1.998e+005

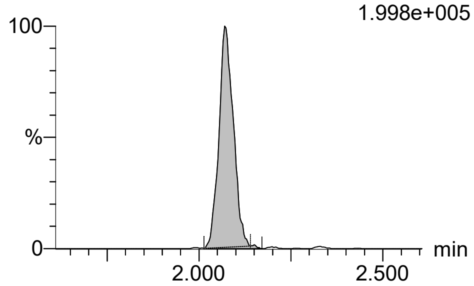

Sample Name: 20211111_L3_R2_IgG
F36:MRM of 1 channel,ES+
1083.83 > 366.1
1.312e+005

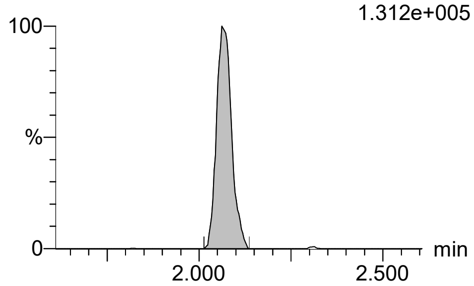

Sample Name: 20211111_L4_R2_IgG
F36:MRM of 1 channel,ES+
1083.83 > 366.1
1.504e+005

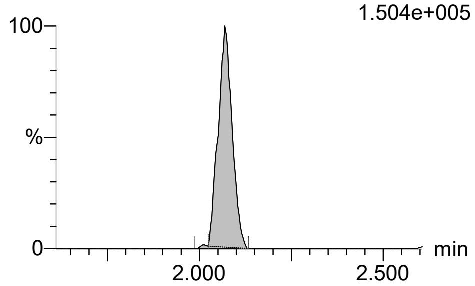

Sample Name: 20211111_H2_R2_IgG
F36:MRM of 1 channel,ES+
1083.83 > 366.1
4.000e+005

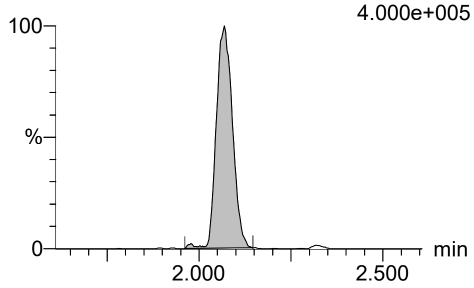

Sample Name: 20211111_A5_R2_IgG
F36:MRM of 1 channel,ES+
1083.83 > 366.1
2.289e+005

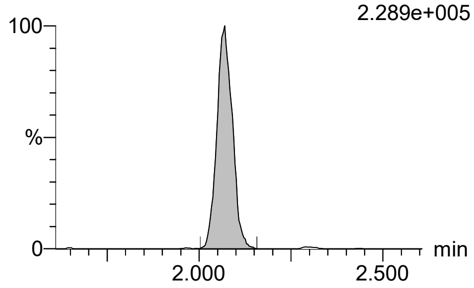

Sample Name: 20211111_A6_R2_IgG
F36:MRM of 1 channel,ES+
1083.83 > 366.1
5.132e+005

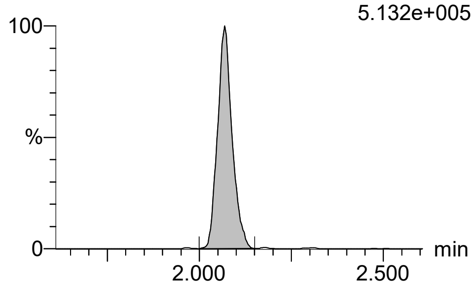

Sample Name: 20211111_QC_R10_IgG
F36:MRM of 1 channel,ES+
1083.83 > 366.1
1.357e+005

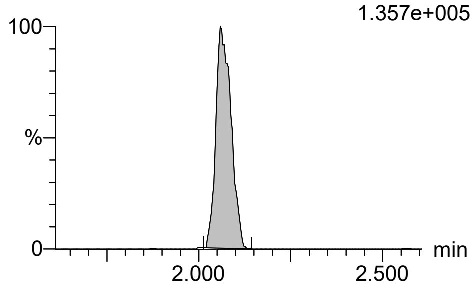

Sample Name: 20211111_L5_R2_IgG
F36:MRM of 1 channel,ES+
1083.83 > 366.1
2.658e+005

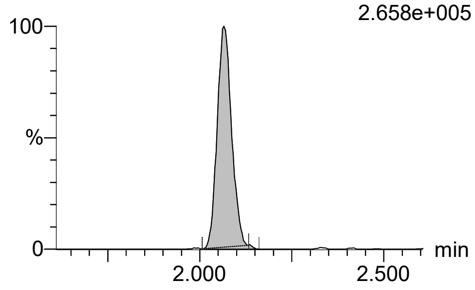

Sample Name: 20211111_L6_R2_IgG
F36:MRM of 1 channel,ES+
1083.83 > 366.1
4.141e+004

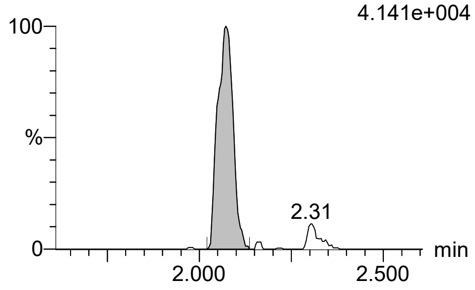

Sample Name: 20211111_H3_R2_IgG
F36:MRM of 1 channel,ES+
1083.83 > 366.1
9.893e+004

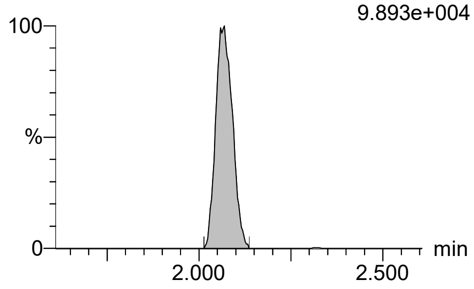

Sample Name: 20211111_A7_R2_IgG
F36:MRM of 1 channel,ES+
1083.83 > 366.1
2.071e+005

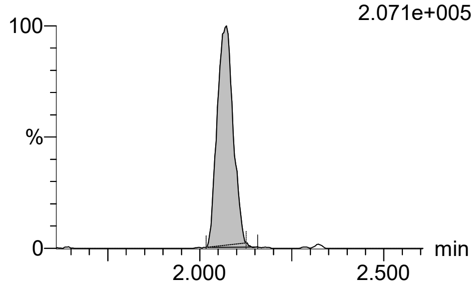

Sample Name: 20211111_A8_R2_IgG
F36:MRM of 1 channel,ES+
1083.83 > 366.1
2.549e+005

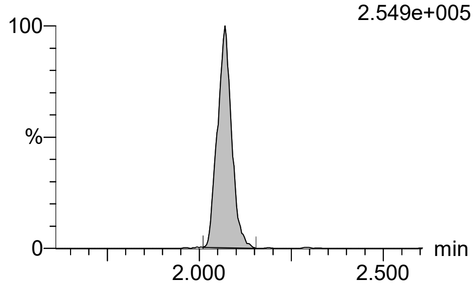

Sample Name: 20211111_L7_R2_IgG
F36:MRM of 1 channel,ES+
1083.83 > 366.1
1.111e+005

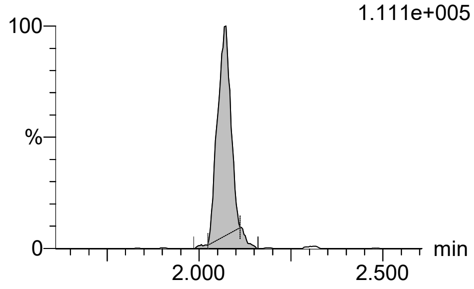

Sample Name: 20211111_L8_R2_IgG
F36:MRM of 1 channel,ES+
1083.83 > 366.1
1.278e+005

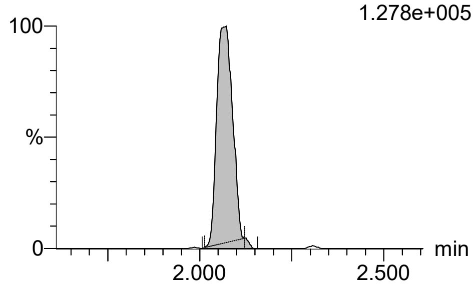

Sample Name: 20211111_H4_R2_IgG
F36:MRM of 1 channel,ES+
1083.83 > 366.1
2.391e+005

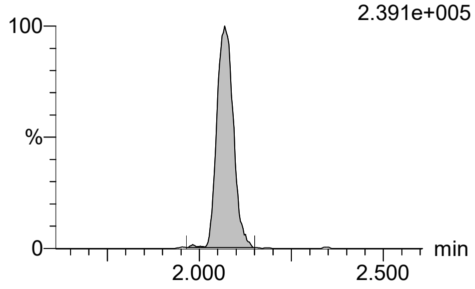

Sample Name: 20211111_A9_R2_IgG
F36:MRM of 1 channel,ES+
1083.83 > 366.1
7.504e+004

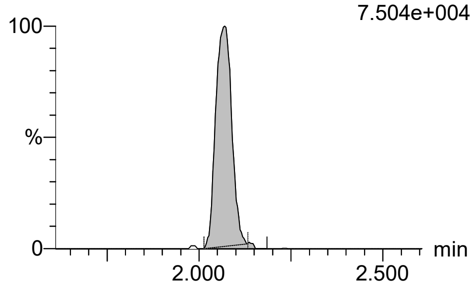

Sample Name: 20211111_A10_R2_IgG
F36:MRM of 1 channel,ES+
1083.83 > 366.1
1.231e+005

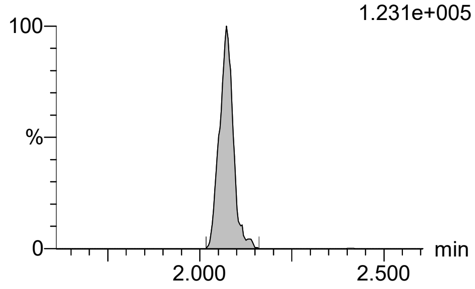

Sample Name: 20211111_L9_R2_IgG
F36:MRM of 1 channel,ES+
1083.83 > 366.1
1.248e+005

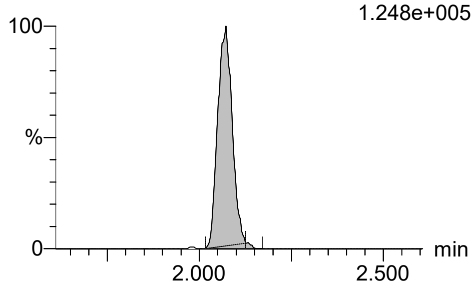

Sample Name: 20211111_L10_R2_IgG
F36:MRM of 1 channel,ES+
1083.83 > 366.1
1.011e+005

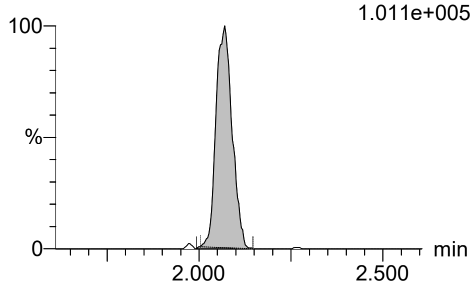

Sample Name: 20211111_QC_R11_IgG
F36:MRM of 1 channel,ES+
1083.83 > 366.1
1.572e+005

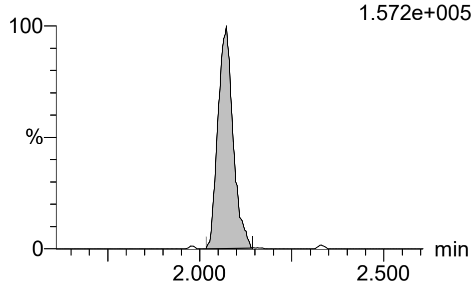

Sample Name: 20211111_H5_R2_IgG
F36:MRM of 1 channel,ES+
1083.83 > 366.1
2.462e+005

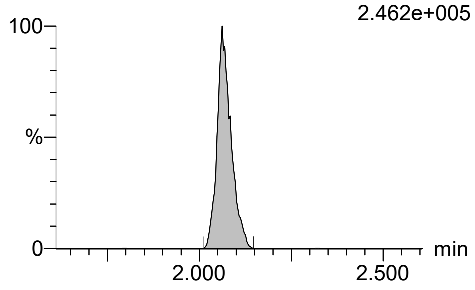

Sample Name: 20211111_A11_R2_IgG
F36:MRM of 1 channel,ES+
1083.83 > 366.1
8.603e+004

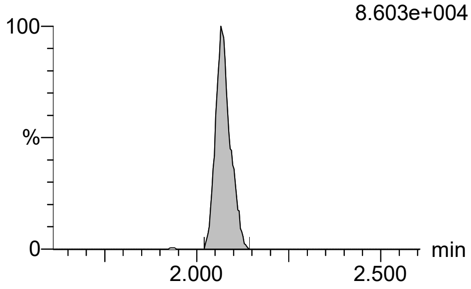

Sample Name: 20211111_A12_R2_IgG
F36:MRM of 1 channel,ES+
1083.83 > 366.1
1.269e+005

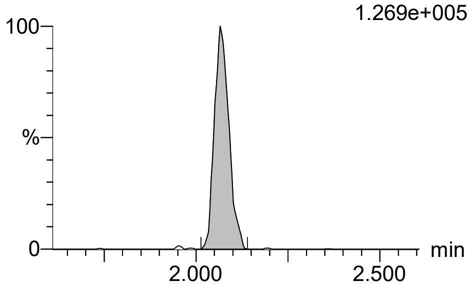

Sample Name: 20211111_L11_R2_IgG
F36:MRM of 1 channel,ES+
1083.83 > 366.1
6.419e+004

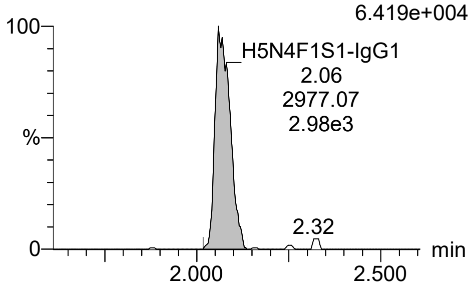

Sample Name: 20211111_L12_R2_IgG
F36:MRM of 1 channel,ES+
1083.83 > 366.1
1.124e+005

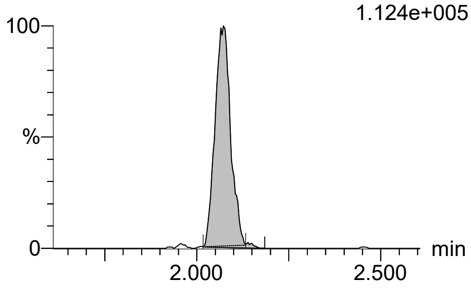

Sample Name: 20211111_H6_R2_IgG
F36:MRM of 1 channel,ES+
1083.83 > 366.1
1.264e+005

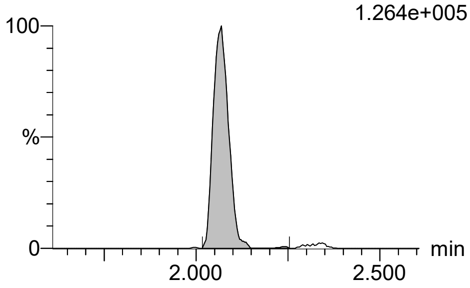

Sample Name: 20211111_A13_R2_IgG
F36:MRM of 1 channel,ES+
1083.83 > 366.1
2.151e+005

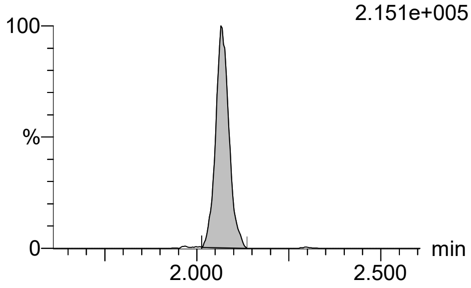

Sample Name: 20211111_A14_R2_IgG
F36:MRM of 1 channel,ES+
1083.83 > 366.1
1.209e+005

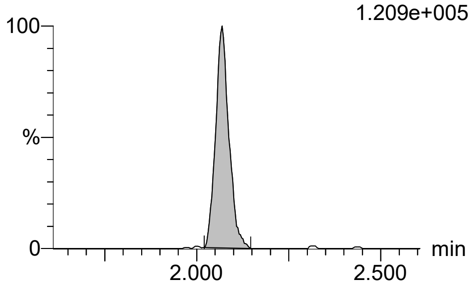

Sample Name: 20211111_L13_R2_IgG
F36:MRM of 1 channel,ES+
1083.83 > 366.1
1.811e+005

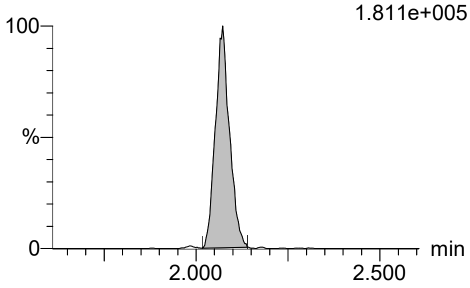

Sample Name: 20211111_L14_R2_IgG
F36:MRM of 1 channel,ES+
1083.83 > 366.1
1.621e+005

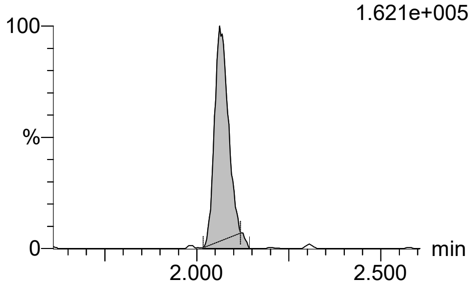

Sample Name: 20211111_H7_R2_IgG
F36:MRM of 1 channel,ES+
1083.83 > 366.1
1.114e+005

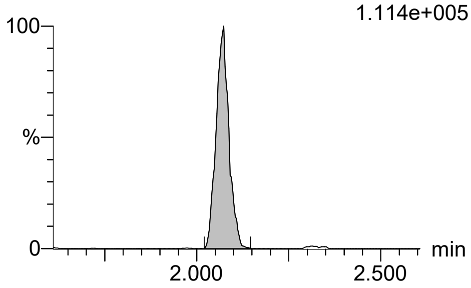

Sample Name: 20211111_A15_R2_IgG
F36:MRM of 1 channel,ES+
1083.83 > 366.1
3.312e+005

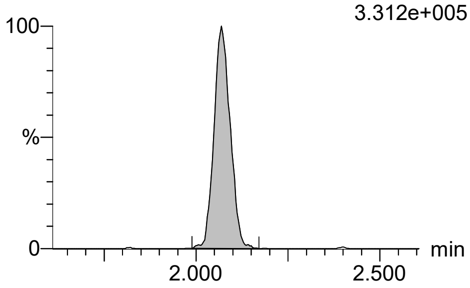

Sample Name: 20211111_QC_R12_IgG
F36:MRM of 1 channel,ES+
1083.83 > 366.1
1.567e+005

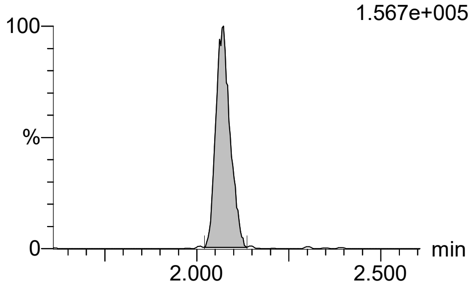

Sample Name: 20211111_A16_R2_IgG
F36:MRM of 1 channel,ES+
1083.83 > 366.1
1.175e+005

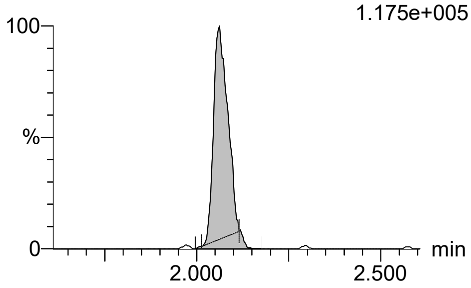

Sample Name: 20211111_L15_R2_IgG
F36:MRM of 1 channel,ES+
1083.83 > 366.1
1.502e+005

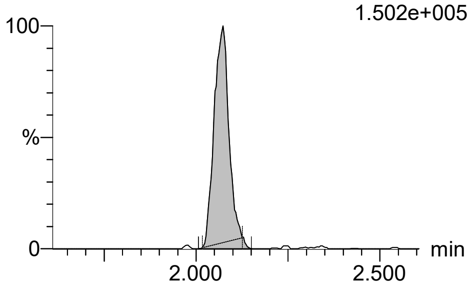

Sample Name: 20211111_L16_R2_IgG
F36:MRM of 1 channel,ES+
1083.83 > 366.1
7.428e+004

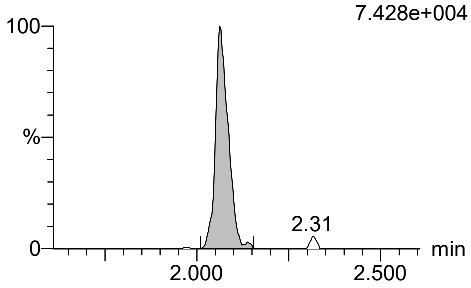

Dataset: C:\MassLynx\10.pro\20211115_clinicalsamples_IgG.qld

Last Altered: Sunday, October 02, 2022 16:02:30 台北標準時間
Printed: Friday, August 01, 2025 16:47:29 台北標準時間

Sample Name: 20211111_H8_R2_IgG
F36:MRM of 1 channel,ES+
1083.83 > 366.1
1.063e+005

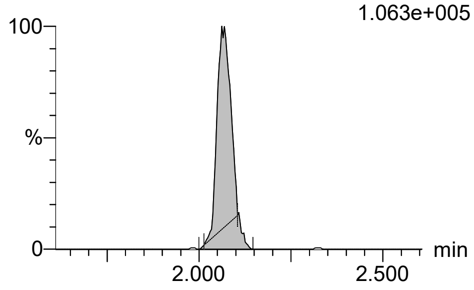

Sample Name: 20211111_A17_R2_IgG
F36:MRM of 1 channel,ES+
1083.83 > 366.1
2.243e+005

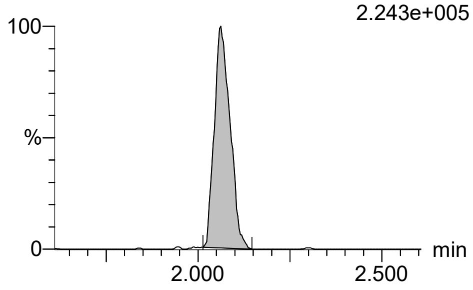

Sample Name: 20211111_A18_R2_IgG
F36:MRM of 1 channel,ES+
1083.83 > 366.1
6.534e+004

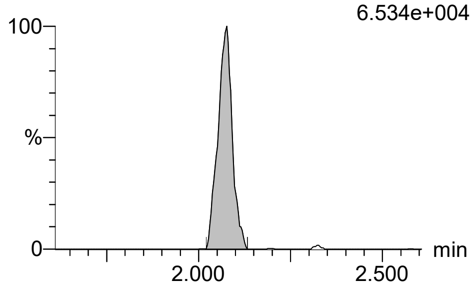

Sample Name: 20211111_L17_R2_IgG
F36:MRM of 1 channel,ES+
1083.83 > 366.1
2.309e+005

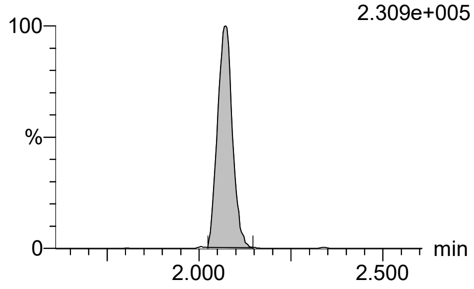

Sample Name: 20211111_L18_R2_IgG
F36:MRM of 1 channel,ES+
1083.83 > 366.1
6.739e+004

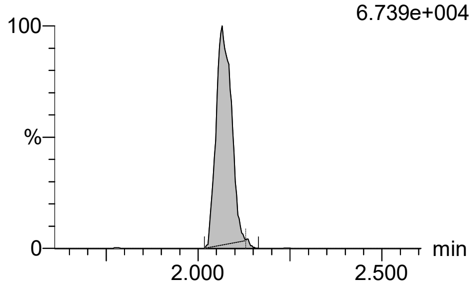

Sample Name: 20211111_H9_R2_IgG
F36:MRM of 1 channel,ES+
1083.83 > 366.1
1.064e+005

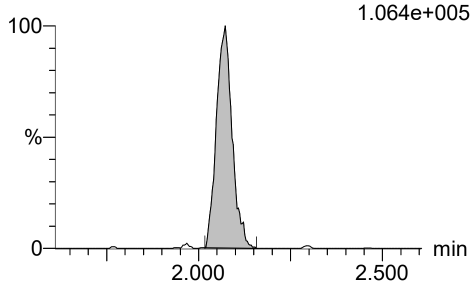

Sample Name: 20211111_A19_R2_IgG
F36:MRM of 1 channel,ES+
1083.83 > 366.1
2.883e+005

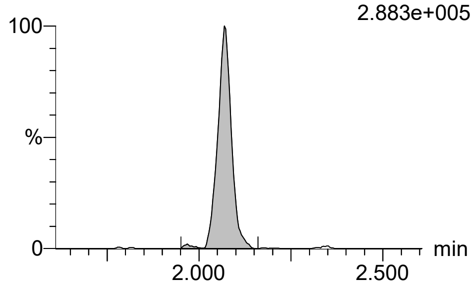

Sample Name: 20211111_A20_R2_IgG
F36:MRM of 1 channel,ES+
1083.83 > 366.1
3.659e+004

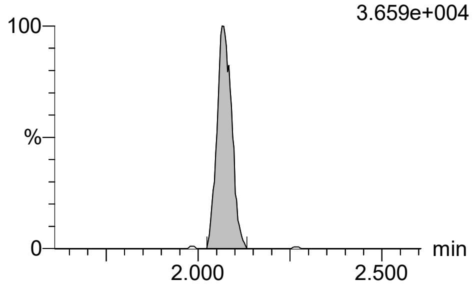

Sample Name: 20211111_L19_R2_IgG
F36:MRM of 1 channel,ES+
1083.83 > 366.1
1.148e+005

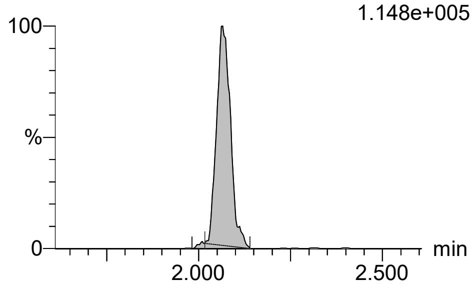

Sample Name: 20211111_QC_R13_IgG
F36:MRM of 1 channel,ES+
1083.83 > 366.1
1.617e+005

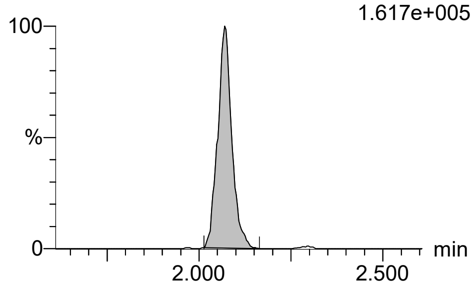

Sample Name: 20211111_L20_R2_IgG
F36:MRM of 1 channel,ES+
1083.83 > 366.1
1.275e+005

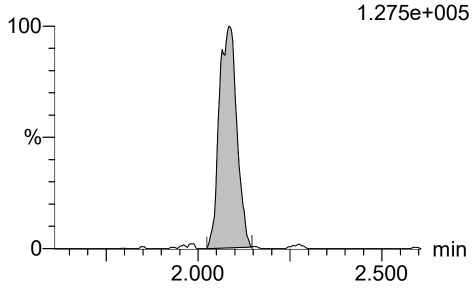

Sample Name: 20211111_H10_R2_IgG
F36:MRM of 1 channel,ES+
1083.83 > 366.1
1.765e+005

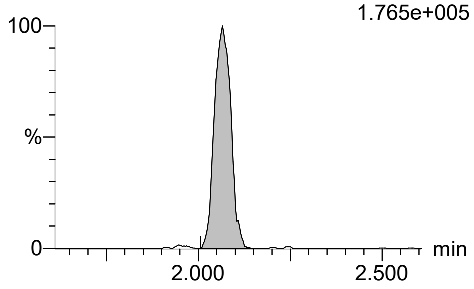

Sample Name: 20211111_A21_R2_IgG
F36:MRM of 1 channel,ES+
1083.83 > 366.1
2.044e+005

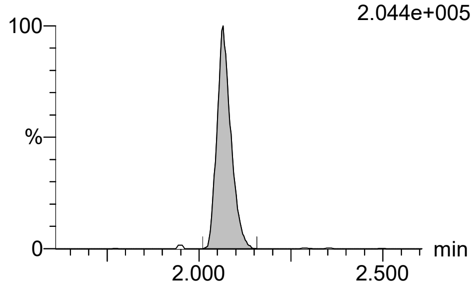

Sample Name: 20211111_A22_R2_IgG
F36:MRM of 1 channel,ES+
1083.83 > 366.1
1.506e+005

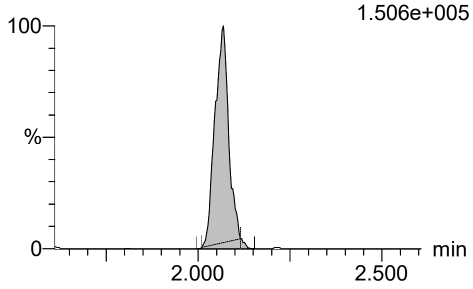

Sample Name: 20211111_L21_R2_IgG
F36:MRM of 1 channel,ES+
1083.83 > 366.1
6.577e+004

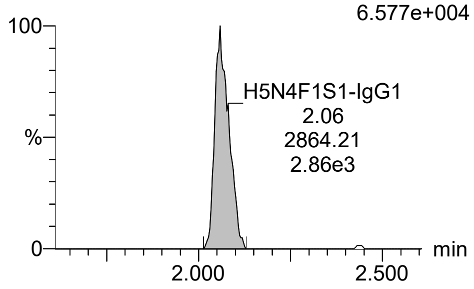

Dataset: C:\MassLynx\10.pro\20211115_clinicalsamples_IgG.qld

Last Altered: Sunday, October 02, 2022 16:02:30 台北標準時間
Printed: Friday, August 01, 2025 16:47:29 台北標準時間

Sample Name: 20211111_L22_R2_IgG
F36:MRM of 1 channel,ES+
1083.83 > 366.1
1.253e+005

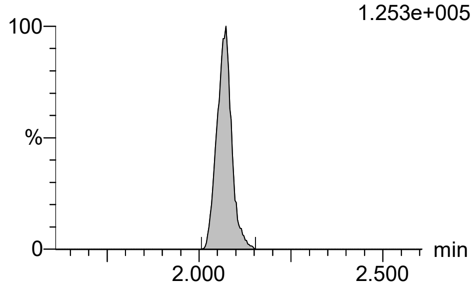

Sample Name: 20211111_H11_R2_IgG
F36:MRM of 1 channel,ES+
1083.83 > 366.1
1.010e+005

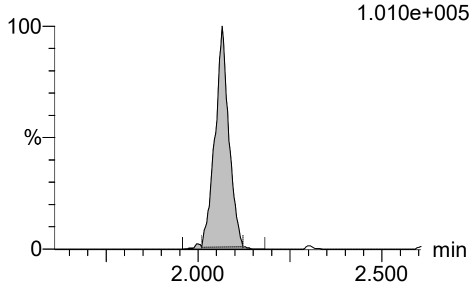

Sample Name: 20211111_A23_R2_IgG
F36:MRM of 1 channel,ES+
1083.83 > 366.1
9.186e+004

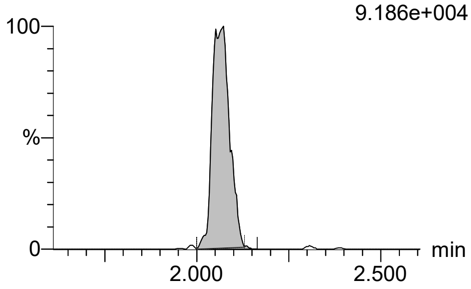

Sample Name: 20211111_A24_R2_IgG
F36:MRM of 1 channel,ES+
1083.83 > 366.1
1.385e+005

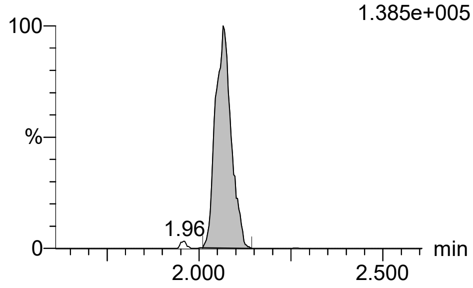

Sample Name: 20211111_L23_R2_IgG
F36:MRM of 1 channel,ES+
1083.83 > 366.1
1.142e+005

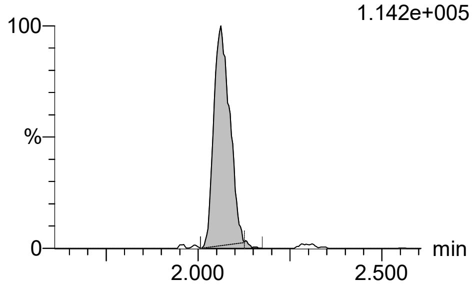

Sample Name: 20211111_L24_R2_IgG
F36:MRM of 1 channel,ES+
1083.83 > 366.1
1.101e+005

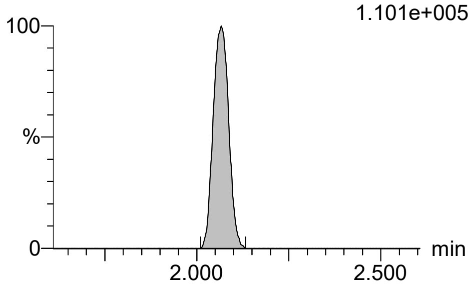

Sample Name: 20211111_H12_R2_IgG
F36:MRM of 1 channel,ES+
1083.83 > 366.1
1.151e+005

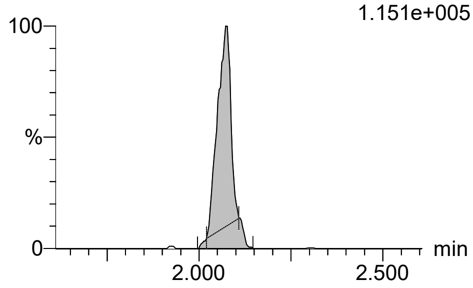

Sample Name: 20211111_QC_R14_IgG
F36:MRM of 1 channel,ES+
1083.83 > 366.1
1.169e+005

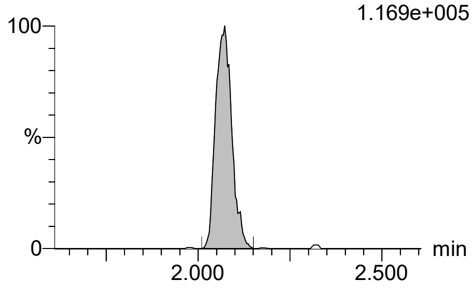

Sample Name: 20211111_A25_R2_IgG
F36:MRM of 1 channel,ES+
1083.83 > 366.1
1.824e+005

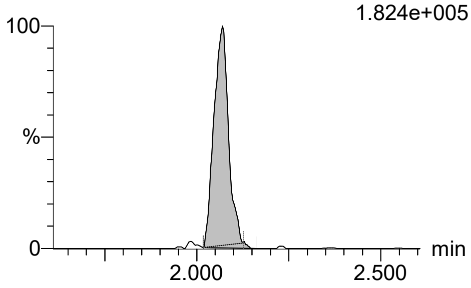

Sample Name: 20211111_A26_R2_IgG
F36:MRM of 1 channel,ES+
1083.83 > 366.1
4.320e+004

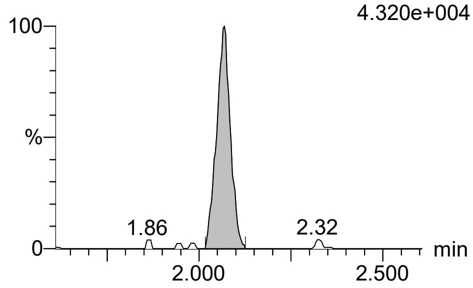

Sample Name: 20211111_L25_R2_IgG
F36:MRM of 1 channel,ES+
1083.83 > 366.1
8.771e+004

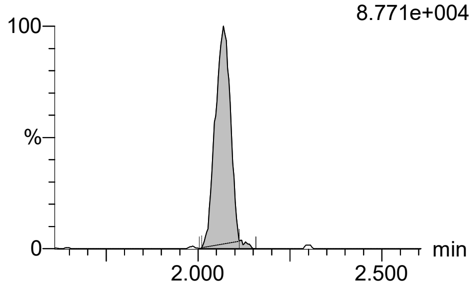

Sample Name: 20211111_L26_R2_IgG
F36:MRM of 1 channel,ES+
1083.83 > 366.1
5.662e+004

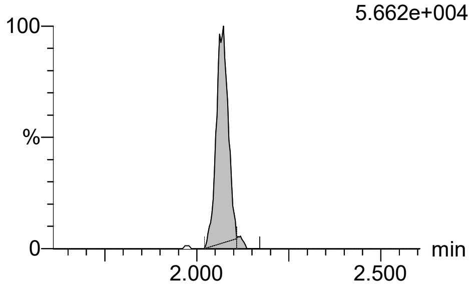

Sample Name: 20211111_H13_R2_IgG
F36:MRM of 1 channel,ES+
1083.83 > 366.1
1.568e+005

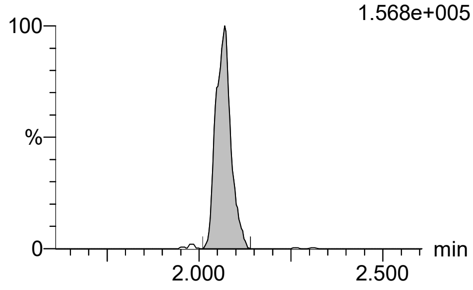

Sample Name: 20211111_A27_R2_IgG
F36:MRM of 1 channel,ES+
1083.83 > 366.1
9.066e+004

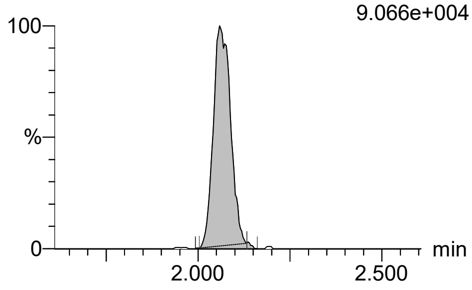

Sample Name: 20211111_A28_R2_IgG
F36:MRM of 1 channel,ES+
1083.83 > 366.1
1.576e+005

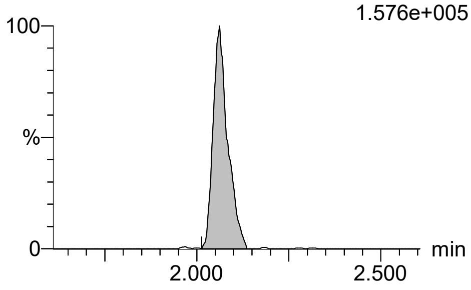

Sample Name: 20211111_L27_R2_IgG
F36:MRM of 1 channel,ES+
1083.83 > 366.1
1.984e+005

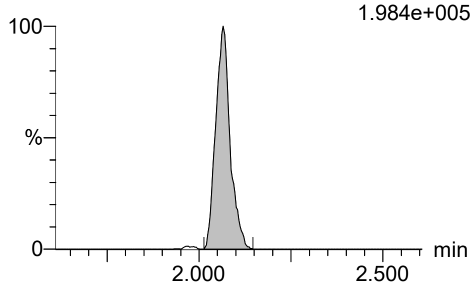

Sample Name: 20211111_L28_R2_IgG
F36:MRM of 1 channel,ES+
1083.83 > 366.1
1.406e+005

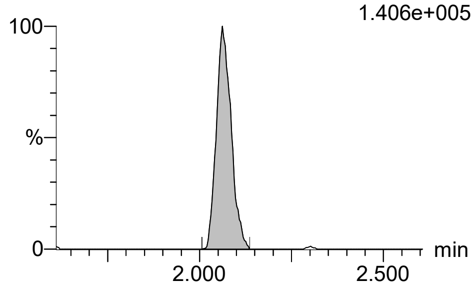

Sample Name: 20211111_H14_R2_IgG
F36:MRM of 1 channel,ES+
1083.83 > 366.1
1.380e+005

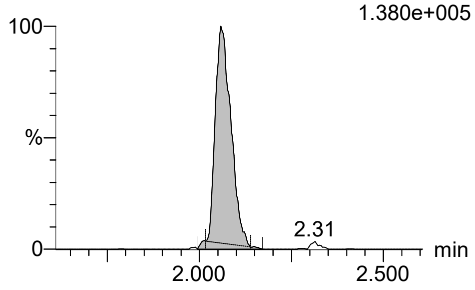

Sample Name: 20211111_A29_R2_IgG
F36:MRM of 1 channel,ES+
1083.83 > 366.1
5.225e+004

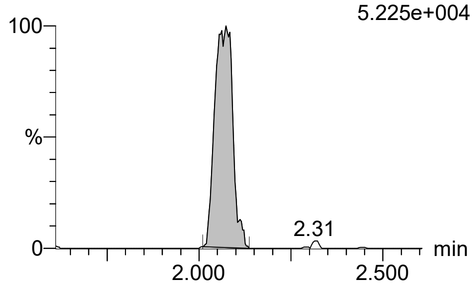

Sample Name: 20211111_A30_R2_IgG
F36:MRM of 1 channel,ES+
1083.83 > 366.1
1.155e+005

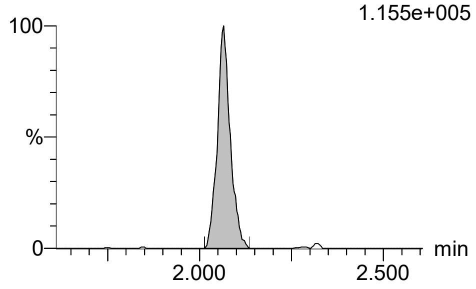

Sample Name: 20211111_QC_R15_IgG
F36:MRM of 1 channel,ES+
1083.83 > 366.1
1.528e+005

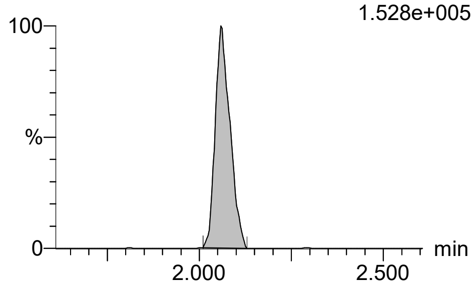

Sample Name: 20211111_L29_R2_IgG
F36:MRM of 1 channel,ES+
1083.83 > 366.1
9.017e+004

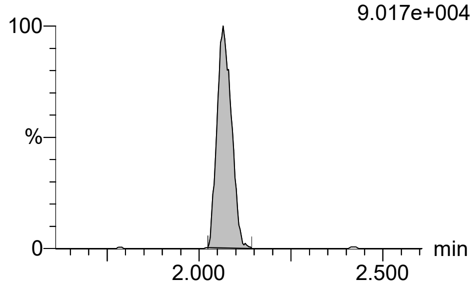

Sample Name: 20211111_L30_R2_IgG
F36:MRM of 1 channel,ES+
1083.83 > 366.1
4.931e+004

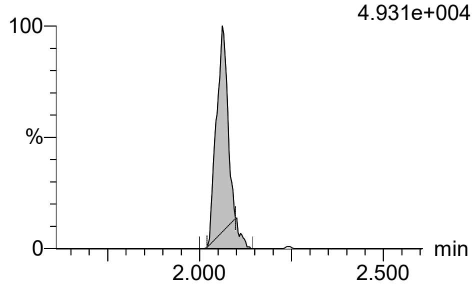

Sample Name: 20211111_H15_R2_IgG
F36:MRM of 1 channel,ES+
1083.83 > 366.1
1.282e+005

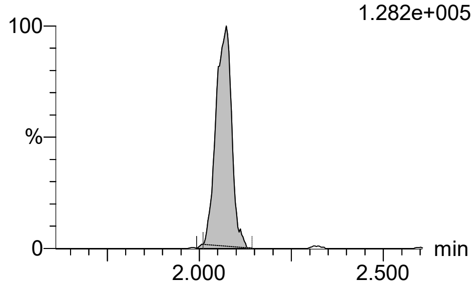

Sample Name: 20211111_A31_R2_IgG
F36:MRM of 1 channel,ES+
1083.83 > 366.1
7.029e+004

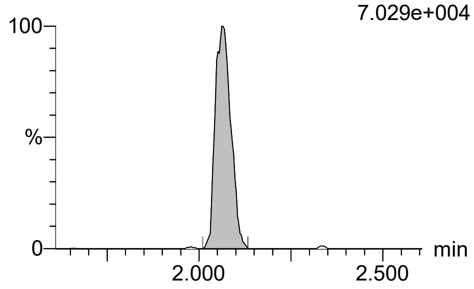

Sample Name: 20211111_A32_R2_IgG
F36:MRM of 1 channel,ES+
1083.83 > 366.1
1.902e+005

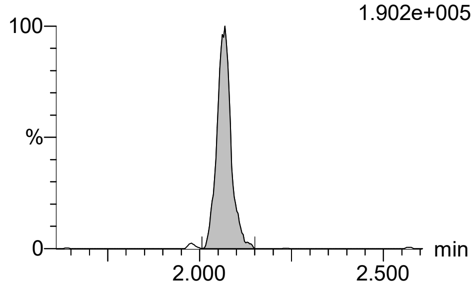

Sample Name: 20211111_L31_R2_IgG
F36:MRM of 1 channel,ES+
1083.83 > 366.1
7.077e+004

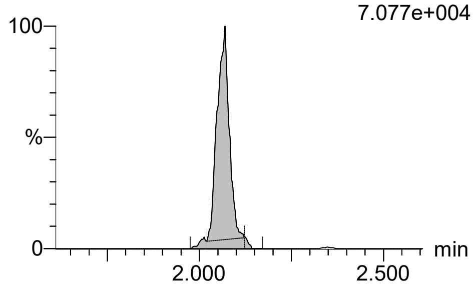

Sample Name: 20211111_L32_R2_IgG
F36:MRM of 1 channel,ES+
1083.83 > 366.1
1.792e+005

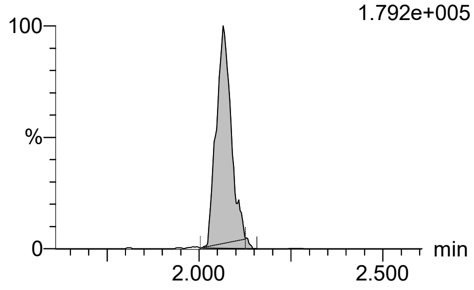

Sample Name: 20211111_H16_R2_IgG
F36:MRM of 1 channel,ES+
1083.83 > 366.1
2.042e+005

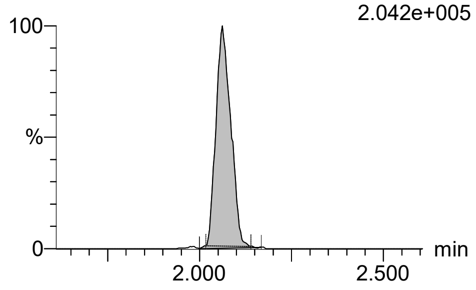

Sample Name: 20211111_A33_R2_IgG
F36:MRM of 1 channel,ES+
1083.83 > 366.1
1.840e+005

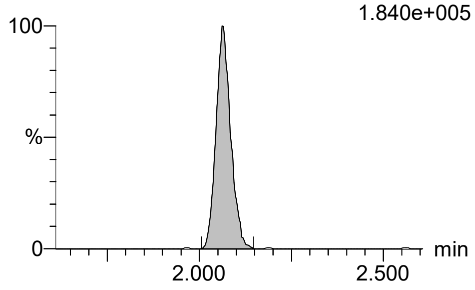

Sample Name: 20211111_A34_R2_IgG

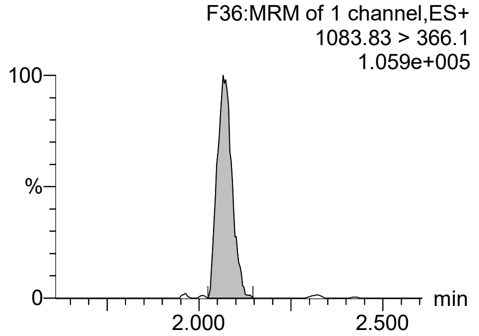

Sample Name: 20211111_H17_R2_IgG

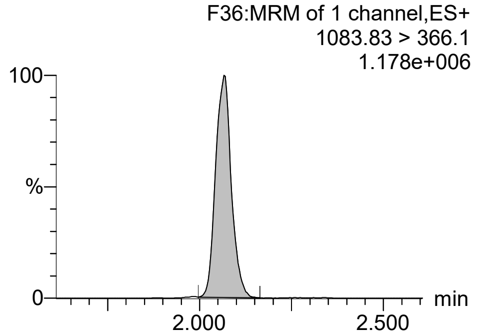

Sample Name: 20211111_A35_R2_IgG

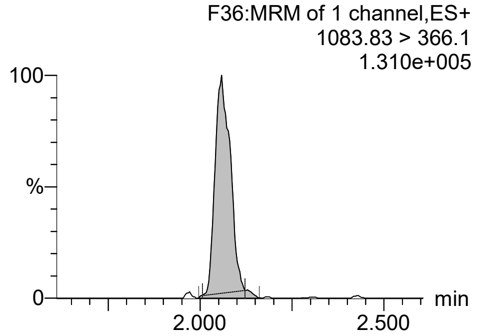

Sample Name: 20211111_QC_R16_IgG

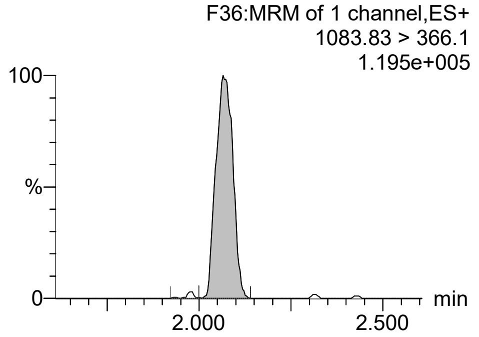

Sample Name: 20211111_A36_R2_IgG

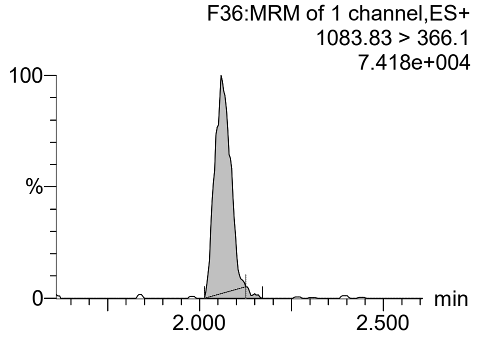

Sample Name: 20211111_H18_R2_IgG

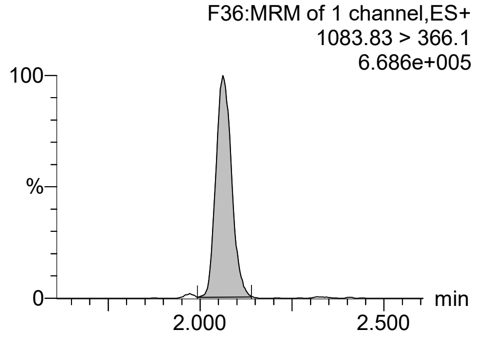

Sample Name: 20211111_A37_R2_IgG

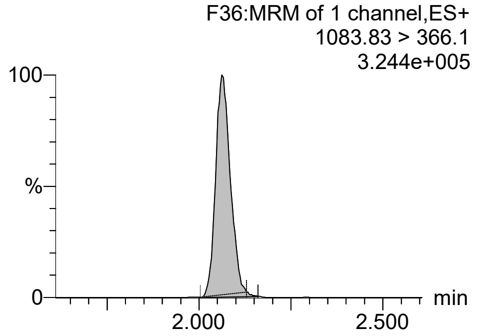

Sample Name: 20211111_A38_R2_IgG

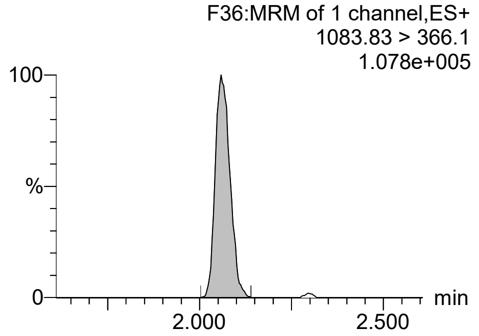

Sample Name: 20211111_H19_R2_IgG

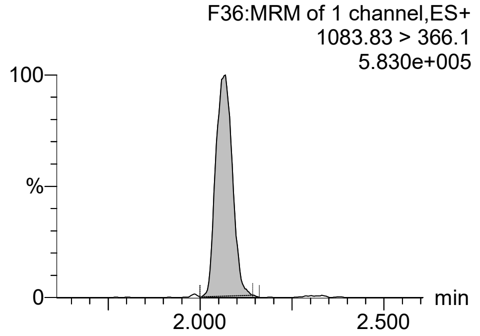

Sample Name: 20211111_A39_R2_IgG

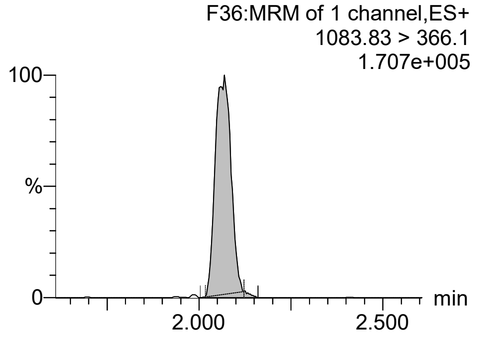

Sample Name: 20211111_A40_R2_IgG

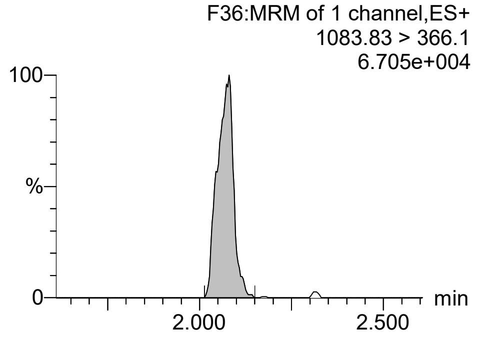

Sample Name: 20211111_H20_R2_IgG

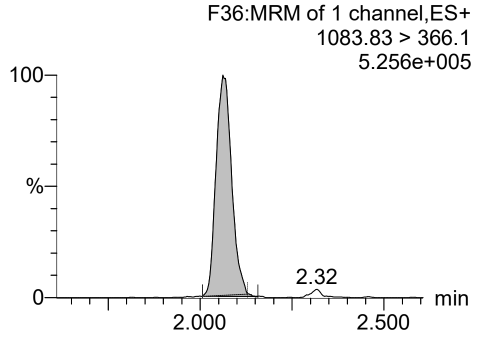

Sample Name: 20211111_A41_R2_IgG

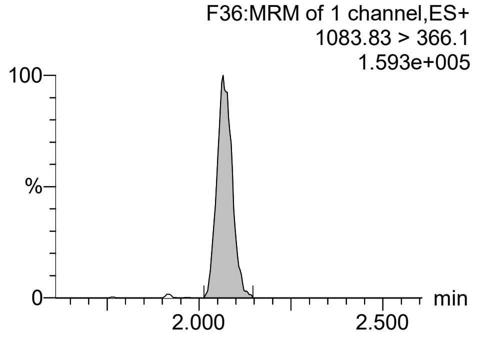

Sample Name: 20211111_A42_R2_IgG

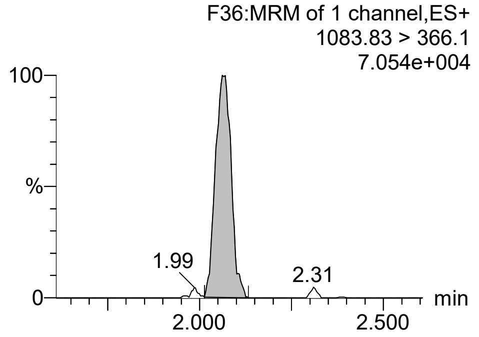

Sample Name: 20211111_A43_R2_IgG

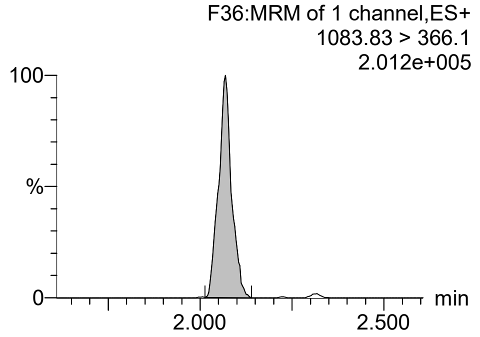

Dataset: C:\MassLynx\10.pro\20211115_clinicalsamples_IgG.qld

Last Altered: Sunday, October 02, 2022 16:02:30 台北標準時間
Printed: Friday, August 01, 2025 16:47:29 台北標準時間

Sample Name: 20211111_A44_R2_IgG

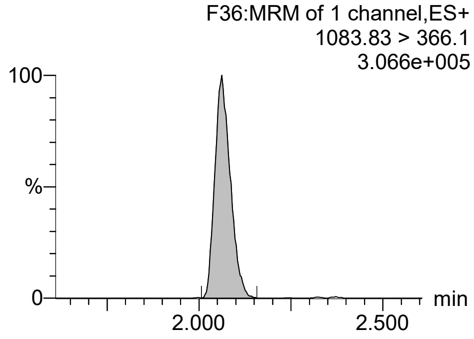

Sample Name: 20211111_QC_R17_IgG

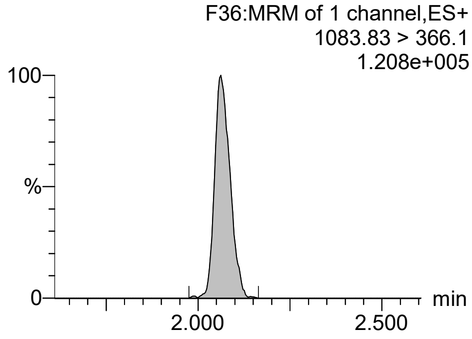

Dataset: C:\MassLynx\10.pro\20211115_clinicalsamples_IgG.qld

Last Altered: Sunday, October 02, 2022 16:02:30 台北標準時間
Printed: Friday, August 01, 2025 16:47:29 台北標準時間

	#	Name	RT	Area	IS Area	Response	Prima...
1	1	20211111_QC_R1_IgG	2.07	15794.2	64968.0	0.243	MM
2	2	20211111_A1_R1_IgG	2.07	15818.1	59059.2	0.268	bb
3	3	20211111_A2_R1_IgG	2.07	24598.5	66621.2	0.369	bb
4	4	20211111_L1_R1_IgG	2.07	13045.3	64201.0	0.203	bb
5	5	20211111_L2_R1_IgG	2.08	10080.9	46950.2	0.215	bb
6	6	20211111_H1_R1_IgG	2.08	4652.2	38648.2	0.120	bb
7	7	20211111_A3_R1_IgG	2.07	364.7	2603.7	0.140	bb
8	8	20211111_A4_R1_IgG	2.07	13957.0	69960.4	0.199	bb
9	9	20211111_L3_R1_IgG	2.07	6801.5	38864.7	0.175	bb
10	10	20211111_L4_R1_IgG	2.08	7571.9	43380.5	0.175	bb
11	11	20211111_H2_R1_IgG	2.07	34421.7	71349.2	0.482	bb
12	12	20211111_A5_R1_IgG	2.07	12681.2	65569.7	0.193	MM
13	13	20211111_A6_R1_IgG	2.07	27483.4	53120.7	0.517	bb
14	14	20211111_QC_R2_IgG	2.07	19027.3	81034.1	0.235	bb
15	15	20211111_L5_R1_IgG	2.07	15580.2	50994.6	0.306	bb
16	16	20211111_L6_R1_IgG	2.07	2776.3	58058.0	0.048	bb
17	17	20211111_H3_R1_IgG	2.08	6198.7	64028.6	0.097	bb
18	18	20211111_A7_R1_IgG	2.07	15868.2	82996.1	0.191	MM
19	19	20211111_A8_R1_IgG	2.08	15009.6	77731.7	0.193	MM
20	20	20211111_L7_R1_IgG	2.07	5434.8	38827.3	0.140	bb
21	21	20211111_L8_R1_IgG	2.07	7854.4	44220.1	0.178	bb
22	22	20211111_H4_R1_IgG	2.07	17193.6	65826.3	0.261	bb
23	23	20211111_A9_R1_IgG	2.07	4380.2	60771.1	0.072	bb
24	24	20211111_A10_R1_IgG	2.07	6160.7	48578.7	0.127	bb
25	25	20211111_L9_R1_IgG	2.07	7031.0	50830.7	0.138	bb
26	26	20211111_L10_R1_IgG	2.08	5147.4	38034.2	0.135	MM
27	27	20211111_QC_R3_IgG	2.07	14547.0	67025.5	0.217	MM
28	28	20211111_H5_R1_IgG	2.07	12240.1	44544.9	0.275	bb
29	29	20211111_A11_R1_IgG	2.07	4736.6	66408.5	0.071	MM
30	30	20211111_A12_R1_IgG	2.07	5566.2	46624.5	0.119	bb
31	31	20211111_L11_R1_IgG	2.08	3559.7	33425.1	0.106	MM
32	32	20211111_L12_R1_IgG	2.07	6139.2	41178.0	0.149	bb
33	33	20211111_H6_R1_IgG	2.07	11017.8	58593.4	0.188	bb
34	34	20211111_A13_R1_IgG	2.07	10741.2	54133.0	0.198	MM
35	35	20211111_A14_R1_IgG	2.07	6158.5	56467.3	0.109	MM
36	36	20211111_L13_R1_IgG	2.07	9765.8	36024.4	0.271	bb
37	37	20211111_L14_R1_IgG	2.07	9608.1	37181.6	0.258	bb
38	38	20211111_H7_R1_IgG	2.08	5299.8	42850.6	0.124	MM
39	39	20211111_A15_R1_IgG	2.08	20242.0	62015.1	0.326	bb
40	40	20211111_QC_R4_IgG	2.07	12343.4	58572.5	0.211	bb
41	41	20211111_A16_R1_IgG	2.07	8769.3	78283.8	0.112	bb
42	42	20211111_L15_R1_IgG	2.07	8037.5	44158.2	0.182	bb
43	43	20211111_L16_R1_IgG	2.07	4855.9	55150.8	0.088	MM
44	44	20211111_H8_R1_IgG	2.07	5889.9	42965.2	0.137	bb
45	45	20211111_A17_R1_IgG	2.07	11671.7	39717.7	0.294	bb
46	46	20211111_A18_R1_IgG	2.07	3771.7	44675.4	0.084	MM
47	47	20211111_L17_R1_IgG	2.07	10517.3	37854.1	0.278	bb
48	48	20211111_L18_R1_IgG	2.07	4178.4	50716.9	0.082	bb
49	49	20211111_H9_R1_IgG	2.07	6294.6	36276.0	0.174	bb
50	50	20211111_A19_R1_IgG	2.07	13070.1	42862.5	0.305	bb
51	51	20211111_A20_R1_IgG	2.07	1942.8	36542.7	0.053	bb
52	52	20211111_L19_R1_IgG	2.07	6739.7	53892.8	0.125	bb
53	53	20211111_QC_R5_IgG	2.07	11056.9	56513.2	0.196	bb
54	54	20211111_L20_R1_IgG	2.07	13288.0	56499.9	0.235	bb
55	55	20211111_H10_R1_IgG	2.07	22239.4	68120.5	0.326	bb
56	56	20211111_A21_R1_IgG	2.08	14322.2	63699.8	0.225	bb

Dataset: C:\MassLynx\10.pro\20211115_clinicalsamples_IgG.qld

Last Altered: Sunday, October 02, 2022 16:02:30 台北標準時間
Printed: Friday, August 01, 2025 16:47:29 台北標準時間

	# Name	RT	Area	IS Area	Response	Prima...
57	57 20211111_A22_R1_IgG	2.07	11526.1	52222.1	0.221	bb
58	58 20211111_L21_R1_IgG	2.07	4656.2	54372.5	0.086	MM
59	59 20211111_L22_R1_IgG	2.07	9418.1	41353.1	0.228	MM
60	60 20211111_H11_R1_IgG	2.06	7342.0	65579.1	0.112	MM
61	61 20211111_A23_R1_IgG	2.07	9731.0	64131.1	0.152	MM
62	62 20211111_A24_R1_IgG	2.07	9424.0	38724.8	0.243	bb
63	63 20211111_L23_R1_IgG	2.07	11187.1	64245.2	0.174	bb
64	64 20211111_L24_R1_IgG	2.07	7895.4	45298.0	0.174	bb
65	65 20211111_H12_R1_IgG	2.07	7411.1	44679.3	0.166	MM
66	66 20211111_QC_R6_IgG	2.07	9942.1	48165.0	0.206	MM
67	67 20211111_A25_R1_IgG	2.07	17577.5	57169.9	0.307	MM
68	68 20211111_A26_R1_IgG	2.08	2500.0	34254.0	0.073	bb
69	69 20211111_L25_R1_IgG	2.07	8808.4	67491.5	0.131	bb
70	70 20211111_L26_R1_IgG	2.07	2897.1	55934.1	0.052	bb
71	71 20211111_H13_R1_IgG	2.07	11961.3	48767.3	0.245	bb
72	72 20211111_A27_R1_IgG	2.07	9272.8	57269.7	0.162	bb
73	73 20211111_A28_R1_IgG	2.07	12131.4	49231.1	0.246	bb
74	74 20211111_L27_R1_IgG	2.07	14970.2	60014.5	0.249	bb
75	75 20211111_L28_R1_IgG	2.07	8849.2	35062.6	0.252	bb
76	76 20211111_H14_R1_IgG	2.07	12485.4	62371.9	0.200	bb
77	77 20211111_A29_R1_IgG	2.07	4043.0	43858.2	0.092	MM
78	78 20211111_A30_R1_IgG	2.07	7150.0	62500.4	0.114	MM
79	79 20211111_QC_R7_IgG	2.07	9824.2	43946.9	0.224	MM
80	80 20211111_L29_R1_IgG	2.07	4719.0	38728.9	0.122	bb
81	81 20211111_L30_R1_IgG	2.07	2585.0	42613.2	0.061	MM
82	82 20211111_H15_R1_IgG	2.07	7795.8	39874.6	0.196	MM
83	83 20211111_A31_R1_IgG	2.07	4822.7	53414.3	0.090	bb
84	84 20211111_A32_R1_IgG	2.07	8518.8	41844.6	0.204	bb
85	85 20211111_L31_R1_IgG	2.07	2975.5	40553.7	0.073	bb
86	86 20211111_L32_R1_IgG	2.07	11138.1	49618.7	0.224	MM
87	87 20211111_H16_R1_IgG	2.08	14898.1	60170.9	0.248	bb
88	88 20211111_A33_R1_IgG	2.07	10488.1	48588.2	0.216	MM
89	89 20211111_A34_R1_IgG	2.07	6350.3	43288.0	0.147	MM
90	90 20211111_H17_R1_IgG	2.07	71613.5	47104.5	1.520	bb
91	91 20211111_A35_R1_IgG	2.07	7485.0	41025.9	0.182	MM
92	92 20211111_QC_R8_IgG	2.07	7107.5	40546.3	0.175	bb
93	93 20211111_A36_R1_IgG	2.07	4583.7	44504.4	0.103	bb
94	94 20211111_H18_R1_IgG	2.07	35738.4	34162.2	1.046	MM
95	95 20211111_A37_R1_IgG	2.07	15597.4	39760.0	0.392	bb
96	96 20211111_A38_R1_IgG	2.08	5959.1	43982.6	0.135	bb
97	97 20211111_H19_R1_IgG	2.07	35365.3	45314.8	0.780	bb
98	98 20211111_A39_R1_IgG	2.07	9164.2	39388.0	0.233	bb
99	99 20211111_A40_R1_IgG	2.08	4166.3	38726.4	0.108	bb
100	1... 20211111_H20_R1_IgG	2.07	25668.5	37814.4	0.679	bb
101	1... 20211111_A41_R1_IgG	2.07	9396.0	48930.3	0.192	bb
102	1... 20211111_A42_R1_IgG	2.08	3310.2	40406.8	0.082	MM
103	1... 20211111_A43_R1_IgG	2.07	10328.3	44421.5	0.233	bb
104	1... 20211111_A44_R1_IgG	2.07	22369.4	53128.2	0.421	bb
105	1... 20211111_QC_R9_IgG	2.07	7440.9	37049.0	0.201	bb
106	1... 20211111_A1_R2_IgG	2.07	11162.7	46499.4	0.240	bb
107	1... 20211111_A2_R2_IgG	2.07	18155.3	57260.7	0.317	bb
108	1... 20211111_L1_R2_IgG	2.07	8172.4	48062.9	0.170	bb
109	1... 20211111_L2_R2_IgG	2.07	7262.8	38745.2	0.187	bb
110	1... 20211111_H1_R2_IgG	2.07	4560.3	44273.5	0.103	MM
111	1... 20211111_A3_R2_IgG	2.08	619.8	4755.5	0.130	bb
112	1... 20211111_A4_R2_IgG	2.07	9054.7	50546.7	0.179	MM

Dataset: C:\MassLynx\10.pro\20211115_clinicalsamples_IgG.qld

Last Altered: Sunday, October 02, 2022 16:02:30 台北標準時間
Printed: Friday, August 01, 2025 16:47:29 台北標準時間

	#	Name	RT	Area	IS Area	Response	Prima...
113	1...	20211111_L3_R2_IgG	2.06	6158.4	34825.8	0.177	bb
114	1...	20211111_L4_R2_IgG	2.07	6552.8	39107.7	0.168	MM
115	1...	20211111_H2_R2_IgG	2.07	19984.0	49750.0	0.402	bb
116	1...	20211111_A5_R2_IgG	2.07	10968.6	52536.2	0.209	bb
117	1...	20211111_A6_R2_IgG	2.07	22921.6	42050.0	0.545	bb
118	1...	20211111_QC_R10_IgG	2.06	6391.5	35350.8	0.181	bb
119	1...	20211111_L5_R2_IgG	2.07	12037.2	38039.3	0.316	MM
120	1...	20211111_L6_R2_IgG	2.07	1990.4	44907.4	0.044	bb
121	1...	20211111_H3_R2_IgG	2.07	5180.0	47729.1	0.109	bb
122	1...	20211111_A7_R2_IgG	2.07	10268.2	57347.1	0.179	MM
123	1...	20211111_A8_R2_IgG	2.07	11050.2	52397.8	0.211	bb
124	1...	20211111_L7_R2_IgG	2.07	5028.9	36318.4	0.138	MM
125	1...	20211111_L8_R2_IgG	2.07	6526.4	38131.9	0.171	MM
126	1...	20211111_H4_R2_IgG	2.07	12167.4	46849.6	0.260	bb
127	1...	20211111_A9_R2_IgG	2.07	3840.4	47759.6	0.080	MM
128	1...	20211111_A10_R2_IgG	2.07	5211.4	41366.2	0.126	bb
129	1...	20211111_L9_R2_IgG	2.07	5731.0	43052.1	0.133	MM
130	1...	20211111_L10_R2_IgG	2.07	5100.7	31487.4	0.162	MM
131	1...	20211111_QC_R11_IgG	2.07	7308.6	37804.6	0.193	bb
132	1...	20211111_H5_R2_IgG	2.06	10452.6	40380.0	0.259	bb
133	1...	20211111_A11_R2_IgG	2.07	3872.0	49533.3	0.078	bb
134	1...	20211111_A12_R2_IgG	2.07	5707.0	40226.3	0.142	bb
135	1...	20211111_L11_R2_IgG	2.06	2977.1	28884.0	0.103	bb
136	1...	20211111_L12_R2_IgG	2.07	5512.8	34351.3	0.160	MM
137	1...	20211111_H6_R2_IgG	2.07	6079.5	40466.6	0.150	bb
138	1...	20211111_A13_R2_IgG	2.07	8870.7	42306.7	0.210	bb
139	1...	20211111_A14_R2_IgG	2.07	4947.3	44904.0	0.110	bb
140	1...	20211111_L13_R2_IgG	2.07	7903.7	31913.0	0.248	bb
141	1...	20211111_L14_R2_IgG	2.06	7386.6	31527.4	0.234	MM
142	1...	20211111_H7_R2_IgG	2.07	4379.1	40838.2	0.107	bb
143	1...	20211111_A15_R2_IgG	2.07	16652.6	49389.7	0.337	bb
144	1...	20211111_QC_R12_IgG	2.07	6890.6	35168.7	0.196	bb
145	1...	20211111_A16_R2_IgG	2.06	5589.7	47440.5	0.118	MM
146	1...	20211111_L15_R2_IgG	2.07	7090.5	33145.9	0.214	MM
147	1...	20211111_L16_R2_IgG	2.06	2949.9	37596.6	0.078	bb
148	1...	20211111_H8_R2_IgG	2.06	5043.6	43933.3	0.115	MM
149	1...	20211111_A17_R2_IgG	2.06	10951.4	43605.5	0.251	bb
150	1...	20211111_A18_R2_IgG	2.08	2880.3	37581.8	0.077	bb
151	1...	20211111_L17_R2_IgG	2.07	10573.9	33942.5	0.312	bb
152	1...	20211111_L18_R2_IgG	2.07	3321.9	37831.5	0.088	MM
153	1...	20211111_H9_R2_IgG	2.07	5158.0	30898.4	0.167	bb
154	1...	20211111_A19_R2_IgG	2.07	12287.2	39352.1	0.312	bb
155	1...	20211111_A20_R2_IgG	2.07	1672.3	34661.2	0.048	bb
156	1...	20211111_L19_R2_IgG	2.07	5107.0	39918.3	0.128	MM
157	1...	20211111_QC_R13_IgG	2.07	7003.5	36239.1	0.193	bb
158	1...	20211111_L20_R2_IgG	2.08	6977.6	32897.7	0.212	bb
159	1...	20211111_H10_R2_IgG	2.07	9066.8	31394.5	0.289	bb
160	1...	20211111_A21_R2_IgG	2.07	8781.7	34863.7	0.252	bb
161	1...	20211111_A22_R2_IgG	2.07	6656.3	30342.1	0.219	MM
162	1...	20211111_L21_R2_IgG	2.06	2864.2	33706.2	0.085	bb
163	1...	20211111_L22_R2_IgG	2.07	5621.8	31298.6	0.180	bb
164	1...	20211111_H11_R2_IgG	2.07	4440.8	38904.1	0.114	MM
165	1...	20211111_A23_R2_IgG	2.07	5102.6	32382.3	0.158	MM
166	1...	20211111_A24_R2_IgG	2.07	6773.9	30261.9	0.224	bb
167	1...	20211111_L23_R2_IgG	2.06	5855.0	43037.5	0.136	MM
168	1...	20211111_L24_R2_IgG	2.07	5300.8	37023.3	0.143	bb

Dataset: C:\MassLynx\10.pro\20211115_clinicalsamples_IgG.qld

Last Altered: Sunday, October 02, 2022 16:02:30 台北標準時間
Printed: Friday, August 01, 2025 16:47:29 台北標準時間

	#	Name	RT	Area	IS Area	Response	Prima...
169	1...	20211111_H12_R2_IgG	2.07	5448.6	39478.2	0.138	MM
170	1...	20211111_QC_R14_IgG	2.07	5879.4	34463.2	0.171	bb
171	1...	20211111_A25_R2_IgG	2.07	8329.4	39945.3	0.209	MM
172	1...	20211111_A26_R2_IgG	2.07	1842.6	29663.2	0.062	bb
173	1...	20211111_L25_R2_IgG	2.07	4253.5	38589.3	0.110	MM
174	1...	20211111_L26_R2_IgG	2.07	2203.8	38405.2	0.057	MM
175	1...	20211111_H13_R2_IgG	2.07	7321.0	37771.2	0.194	bb
176	1...	20211111_A27_R2_IgG	2.06	4988.8	42634.1	0.117	MM
177	1...	20211111_A28_R2_IgG	2.06	7094.0	33231.1	0.213	bb
178	1...	20211111_L27_R2_IgG	2.07	8839.2	41761.1	0.212	bb
179	1...	20211111_L28_R2_IgG	2.06	6431.2	26968.6	0.238	bb
180	1...	20211111_H14_R2_IgG	2.06	7015.3	49250.2	0.142	MM
181	1...	20211111_A29_R2_IgG	2.07	2929.7	37601.7	0.078	bb
182	1...	20211111_A30_R2_IgG	2.07	4586.0	40366.8	0.114	bb
183	1...	20211111_QC_R15_IgG	2.06	6987.8	35156.1	0.199	bb
184	1...	20211111_L29_R2_IgG	2.07	4018.9	33813.1	0.119	bb
185	1...	20211111_L30_R2_IgG	2.06	1942.6	33488.6	0.058	MM
186	1...	20211111_H15_R2_IgG	2.07	6339.8	38501.0	0.165	MM
187	1...	20211111_A31_R2_IgG	2.06	3446.2	37525.5	0.092	bb
188	1...	20211111_A32_R2_IgG	2.07	8242.8	35770.8	0.230	bb
189	1...	20211111_L31_R2_IgG	2.07	2903.9	33282.4	0.087	MM
190	1...	20211111_L32_R2_IgG	2.07	8500.8	42049.6	0.202	MM
191	1...	20211111_H16_R2_IgG	2.06	9450.3	45947.6	0.206	MM
192	1...	20211111_A33_R2_IgG	2.06	8355.5	40202.3	0.208	bb
193	1...	20211111_A34_R2_IgG	2.07	4892.7	36747.0	0.133	bb
194	1...	20211111_H17_R2_IgG	2.07	57137.8	40920.9	1.396	bb
195	1...	20211111_A35_R2_IgG	2.06	6520.6	33689.2	0.194	MM
196	1...	20211111_QC_R16_IgG	2.07	6353.2	32783.6	0.194	MM
197	1...	20211111_A36_R2_IgG	2.06	3839.5	36822.4	0.104	MM
198	1...	20211111_H18_R2_IgG	2.06	32571.2	31769.3	1.025	bb
199	1...	20211111_A37_R2_IgG	2.06	14942.6	37581.8	0.398	MM
200	2...	20211111_A38_R2_IgG	2.06	4942.2	38390.4	0.129	bb
201	2...	20211111_H19_R2_IgG	2.07	30634.6	41217.0	0.743	MM
202	2...	20211111_A39_R2_IgG	2.07	8594.4	37392.7	0.230	MM
203	2...	20211111_A40_R2_IgG	2.08	3345.6	38295.1	0.087	bb
204	2...	20211111_H20_R2_IgG	2.06	25568.4	35941.8	0.711	MM
205	2...	20211111_A41_R2_IgG	2.07	7577.5	45304.9	0.167	bb
206	2...	20211111_A42_R2_IgG	2.06	3464.0	37803.8	0.092	bb
207	2...	20211111_A43_R2_IgG	2.07	8078.2	38161.8	0.212	bb
208	2...	20211111_A44_R2_IgG	2.06	14721.5	37819.8	0.389	bb
209	2...	20211111_QC_R17_IgG	2.06	5824.9	31731.8	0.184	bb

Dataset: C:\MassLynx\10.pro\20211115_clinicalsamples_IgG.qld

Last Altered: Sunday, October 02, 2022 16:02:30 台北標準時間
Printed: Friday, August 01, 2025 16:48:00 台北標準時間

Method: C:\MassLynx\10_Pro_IgA.PRO\MethDB\20210926_IgG_DMRM_IgM_IS.mdb 16 Feb 2022 11:58:21
Calibration: 18 Nov 2021 13:56:22

Sample Name: 20211111_QC_R1_IgG

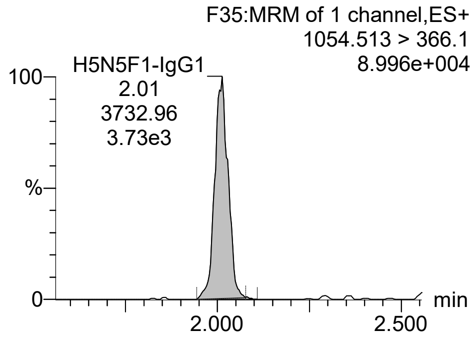

Sample Name: 20211111_A1_R1_IgG

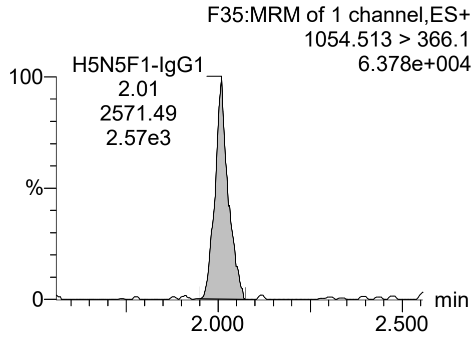

Sample Name: 20211111_A2_R1_IgG

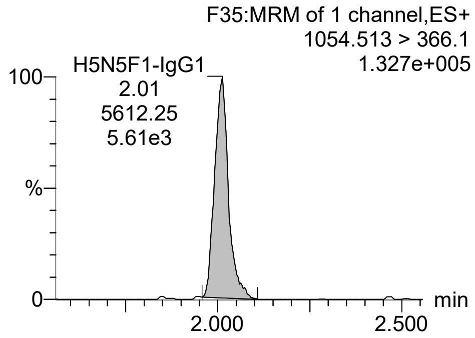

Sample Name: 20211111_L1_R1_IgG

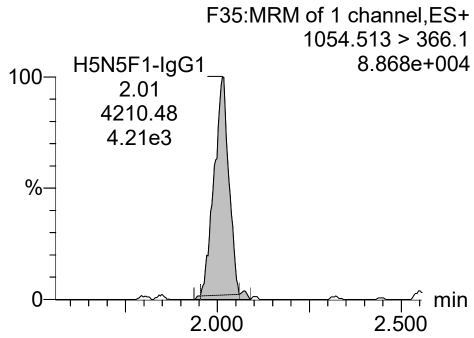

Sample Name: 20211111_L2_R1_IgG

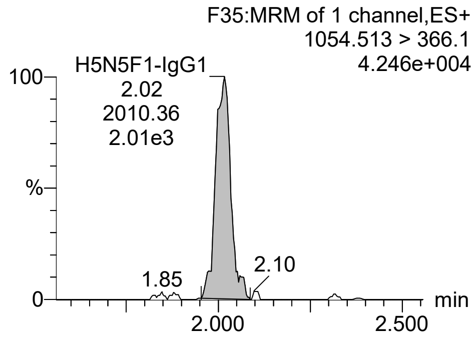

Sample Name: 20211111_H1_R1_IgG

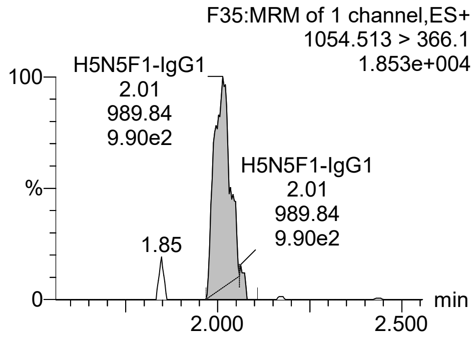

Sample Name: 20211111_A3_R1_IgG

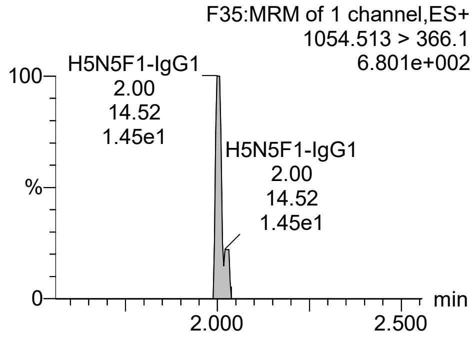

Sample Name: 20211111_A4_R1_IgG

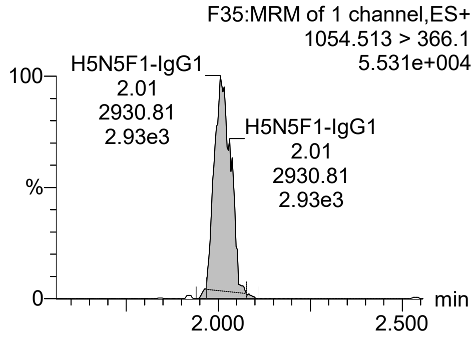

Sample Name: 20211111_L3_R1_IgG

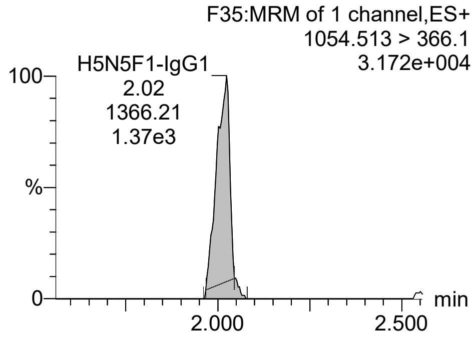

Sample Name: 20211111_L4_R1_IgG

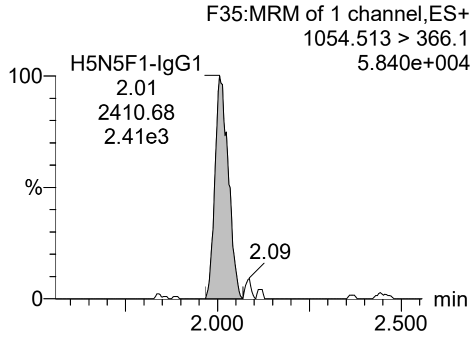

Sample Name: 20211111_H2_R1_IgG

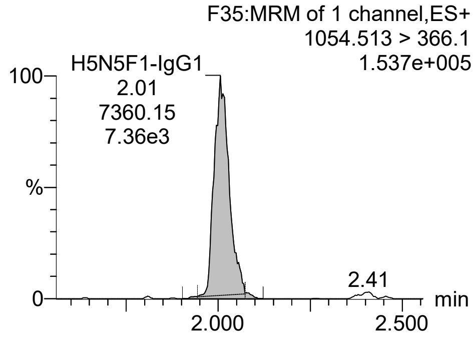

Sample Name: 20211111_A5_R1_IgG

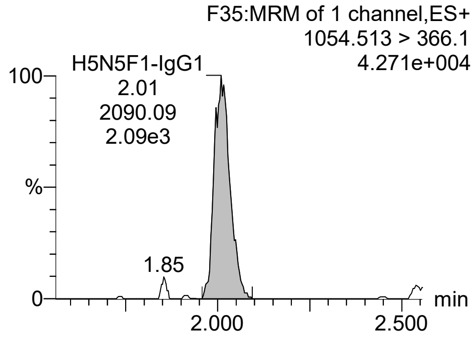

Dataset: C:\MassLynx\10.pro\20211115_clinicalsamples_IgG.qld

Last Altered: Sunday, October 02, 2022 16:02:30 台北標準時間
Printed: Friday, August 01, 2025 16:48:00 台北標準時間

Sample Name: 20211111_A6_R1_IgG

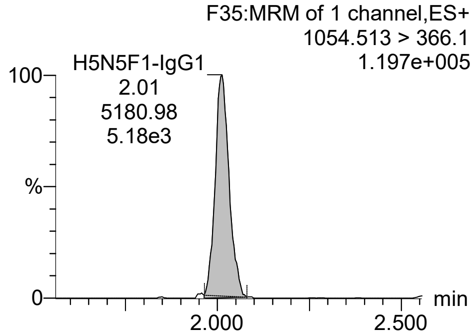

Sample Name: 20211111_QC_R2_IgG

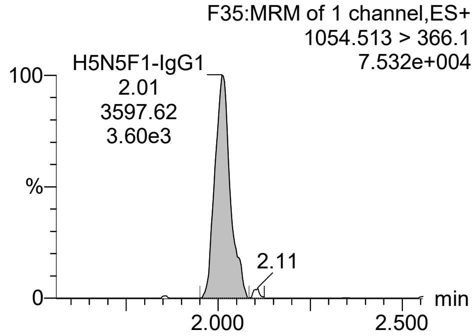

Sample Name: 20211111_L5_R1_IgG

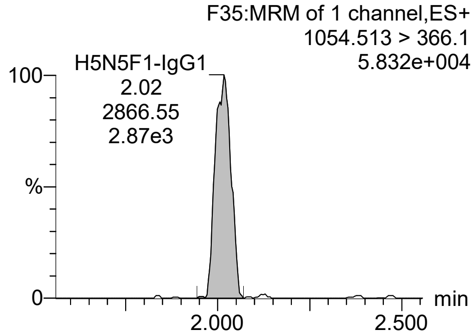

Sample Name: 20211111_L6_R1_IgG

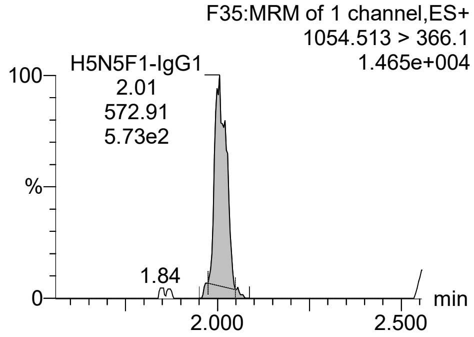

Sample Name: 20211111_H3_R1_IgG

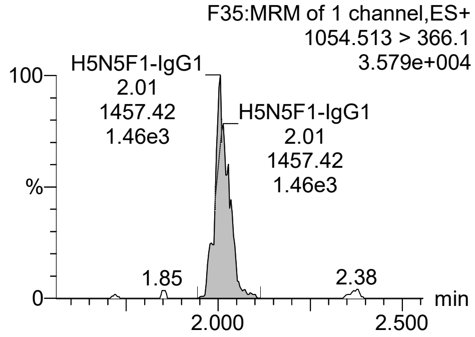

Sample Name: 20211111_A7_R1_IgG

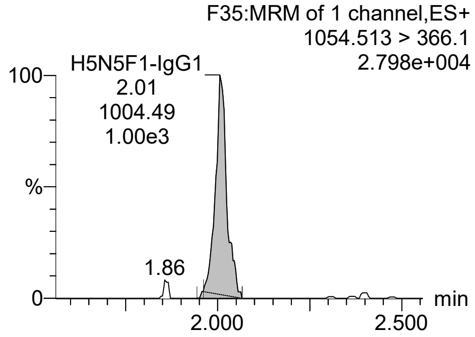

Sample Name: 20211111_A8_R1_IgG

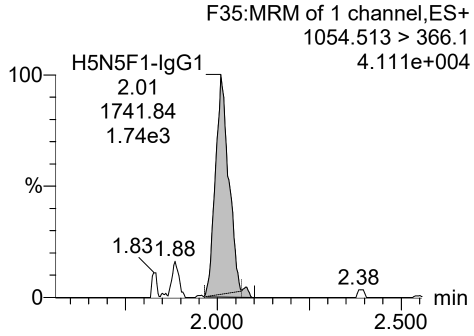

Sample Name: 20211111_L7_R1_IgG

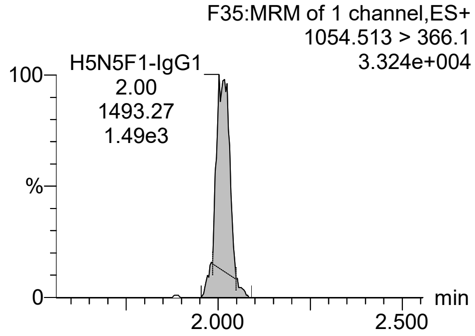

Sample Name: 20211111_L8_R1_IgG

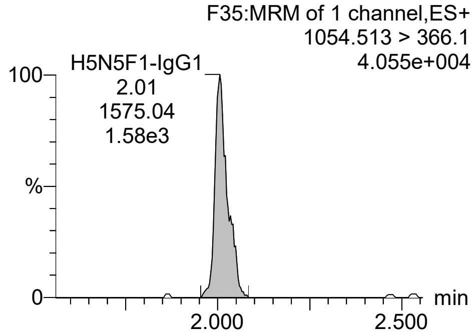

Sample Name: 20211111_H4_R1_IgG

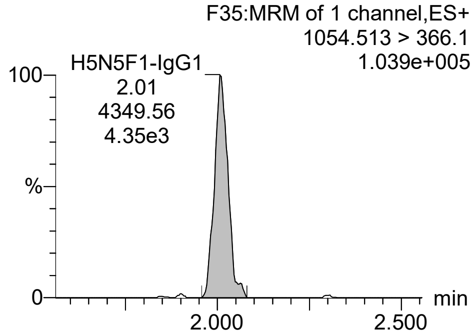

Sample Name: 20211111_A9_R1_IgG

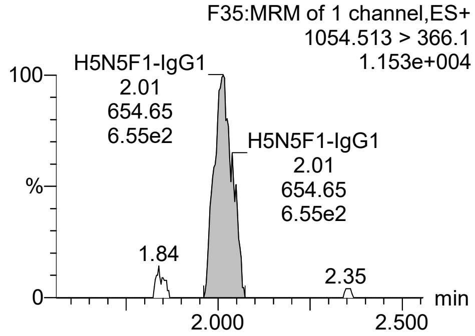

Sample Name: 20211111_A10_R1_IgG

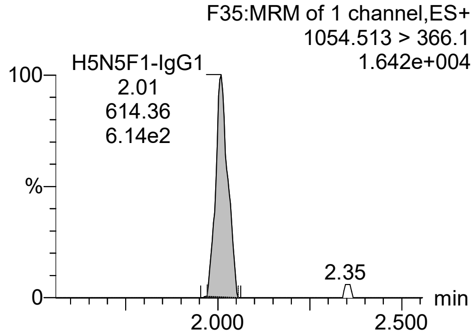

Sample Name: 20211111_L9_R1_IgG

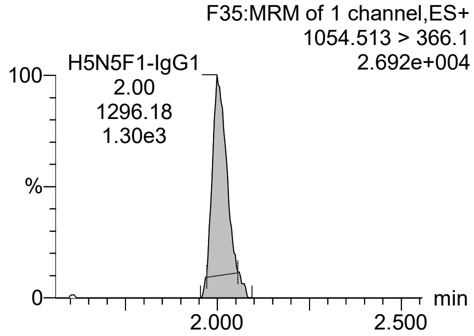

Sample Name: 20211111_L10_R1_IgG

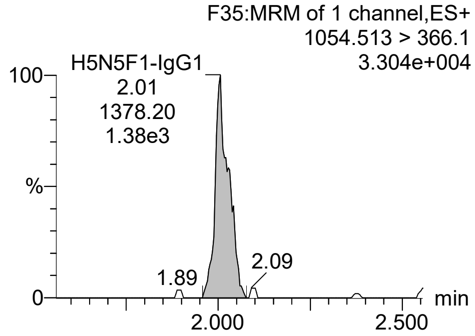

Sample Name: 20211111_QC_R3_IgG

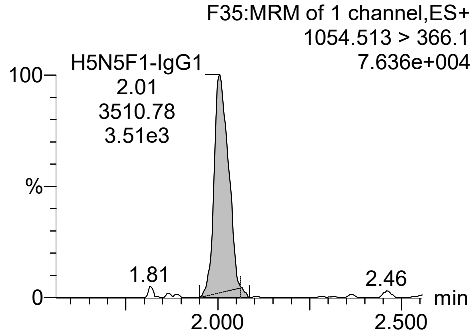

Dataset: C:\MassLynx\10.pro\20211115_clinicalsamples_IgG.qld

Last Altered: Sunday, October 02, 2022 16:02:30 台北標準時間
Printed: Friday, August 01, 2025 16:48:00 台北標準時間

Sample Name: 20211111_H5_R1_IgG

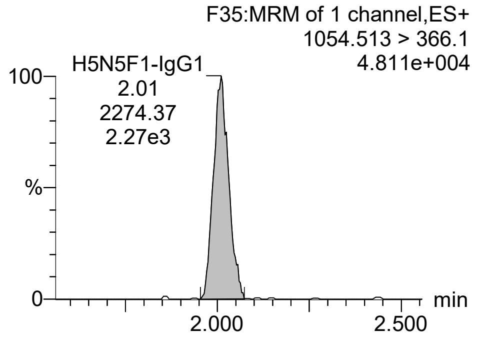

Sample Name: 20211111_A11_R1_IgG

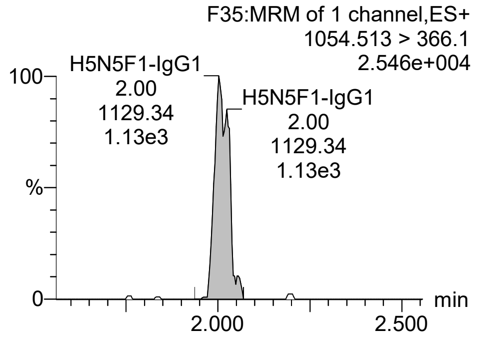

Sample Name: 20211111_A12_R1_IgG

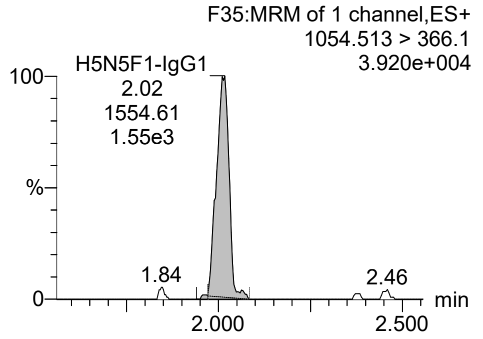

Sample Name: 20211111_L11_R1_IgG

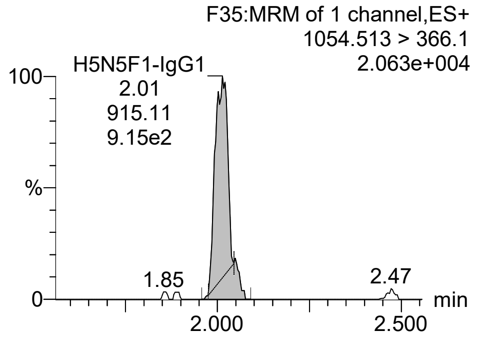

Sample Name: 20211111_L12_R1_IgG

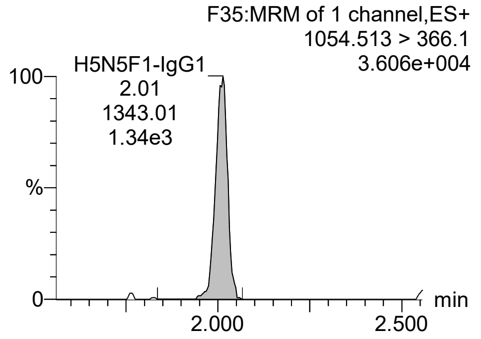

Sample Name: 20211111_H6_R1_IgG

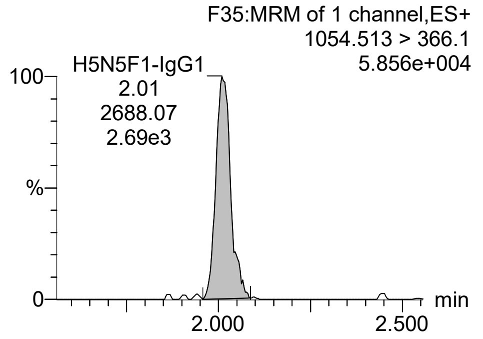

Sample Name: 20211111_A13_R1_IgG

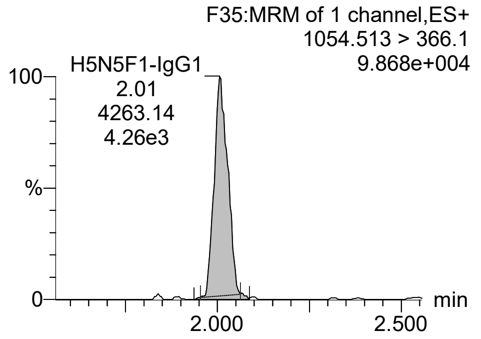

Sample Name: 20211111_A14_R1_IgG

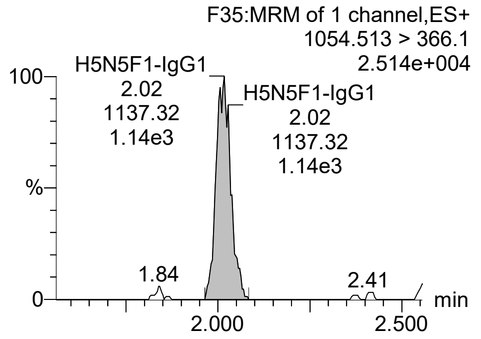

Sample Name: 20211111_L13_R1_IgG

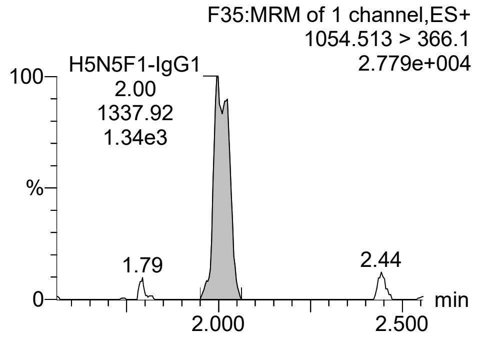

Sample Name: 20211111_L14_R1_IgG

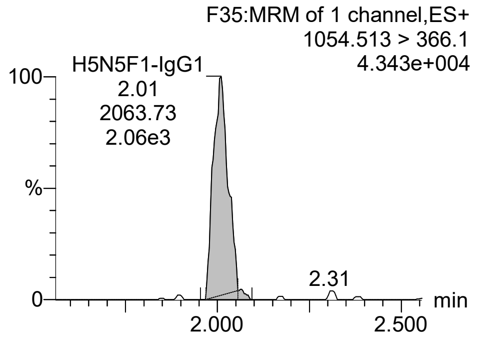

Sample Name: 20211111_H7_R1_IgG

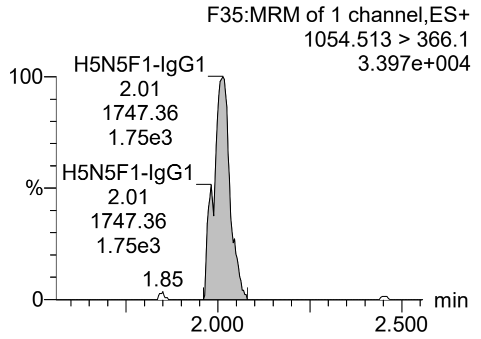

Sample Name: 20211111_A15_R1_IgG

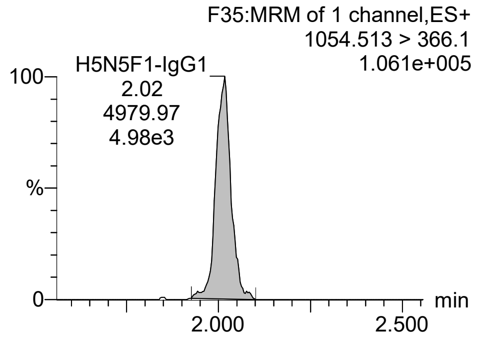

Sample Name: 20211111_QC_R4_IgG

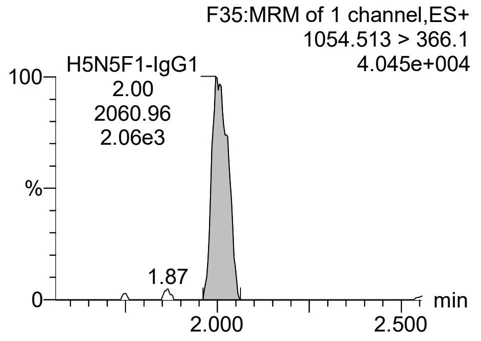

Sample Name: 20211111_A16_R1_IgG

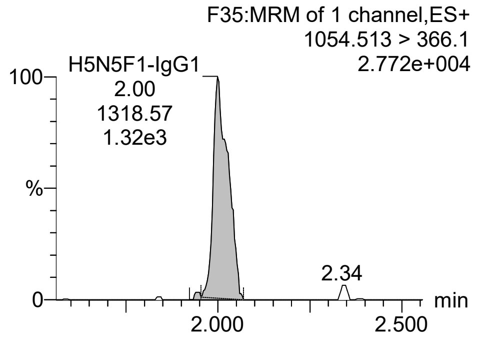

Sample Name: 20211111_L15_R1_IgG

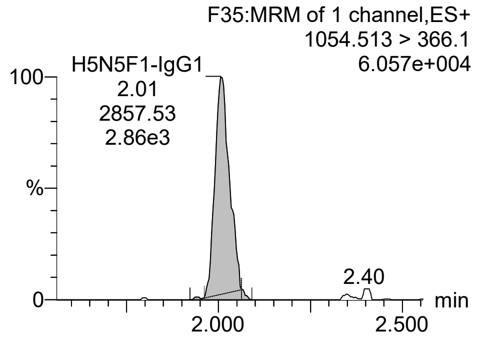

Sample Name: 20211111_L16_R1_IgG

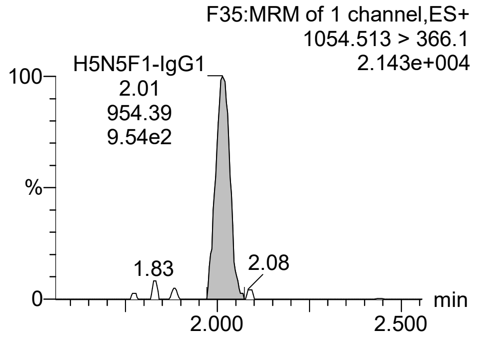

Sample Name: 20211111_H8_R1_IgG

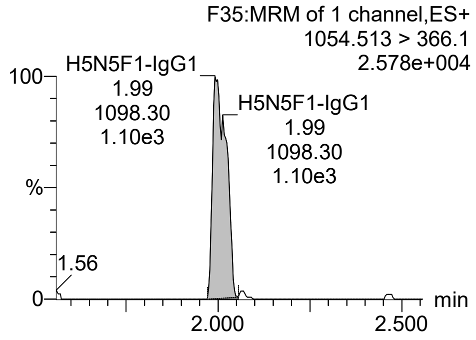

Sample Name: 20211111_A17_R1_IgG

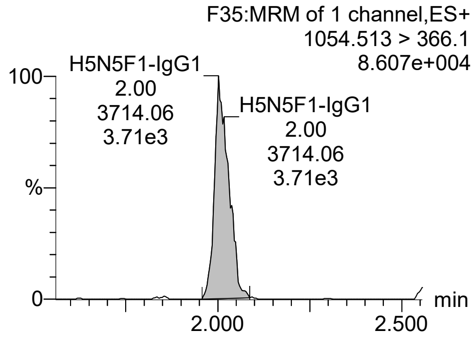

Sample Name: 20211111_A18_R1_IgG

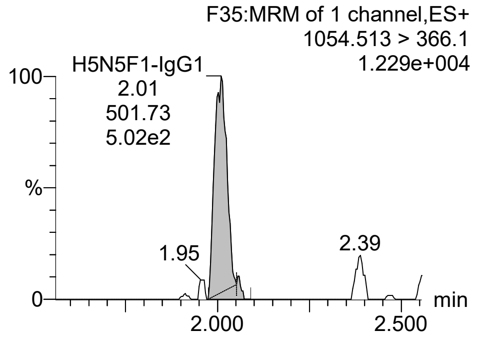

Sample Name: 20211111_L17_R1_IgG

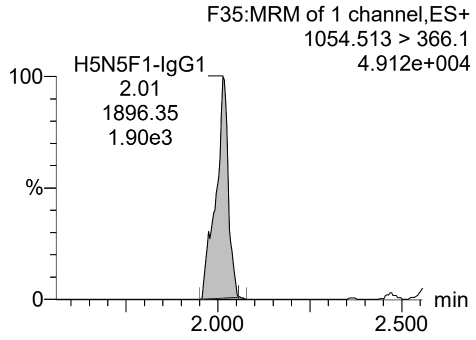

Sample Name: 20211111_L18_R1_IgG

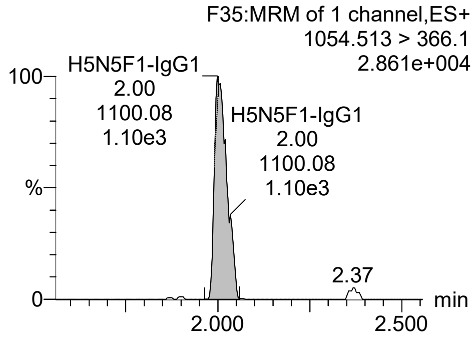

Sample Name: 20211111_H9_R1_IgG

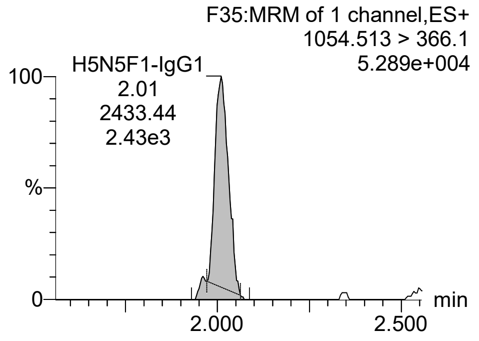

Sample Name: 20211111_A19_R1_IgG

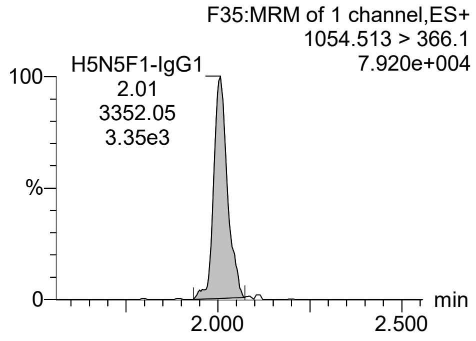

Sample Name: 20211111_A20_R1_IgG

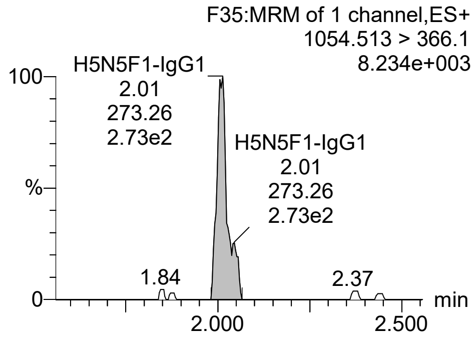

Sample Name: 20211111_L19_R1_IgG

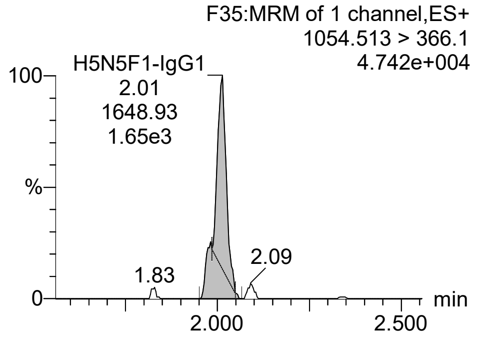

Sample Name: 20211111_QC_R5_IgG

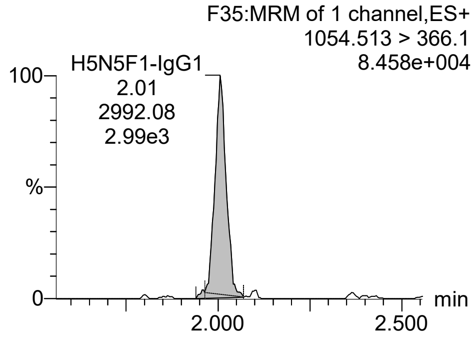

Sample Name: 20211111_L20_R1_IgG

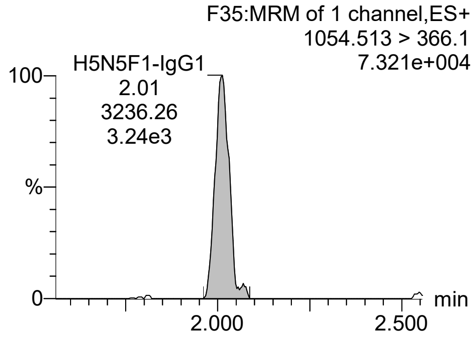

Sample Name: 20211111_H10_R1_IgG

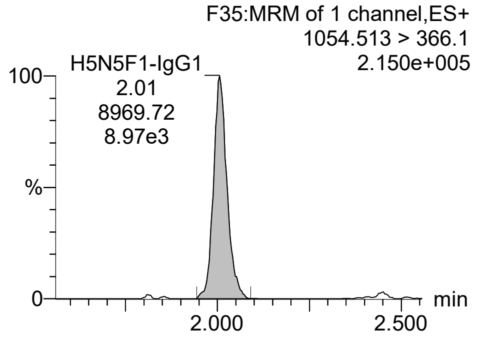

Sample Name: 20211111_A21_R1_IgG

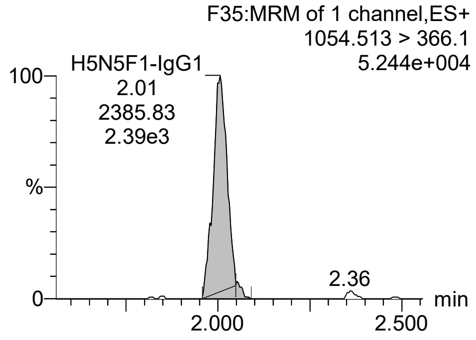

Sample Name: 20211111_A22_R1_IgG

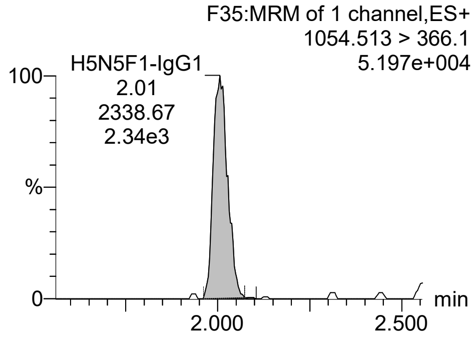

Sample Name: 20211111_L21_R1_IgG

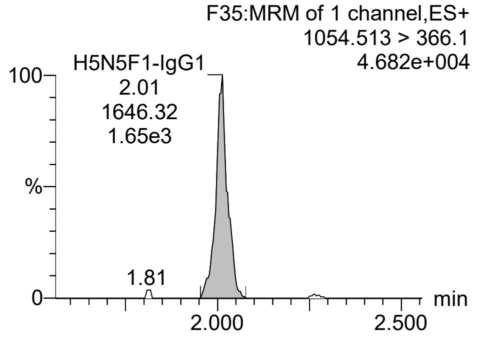

Sample Name: 20211111_L22_R1_IgG

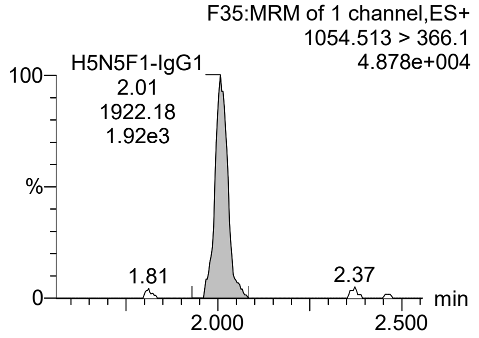

Sample Name: 20211111_H11_R1_IgG

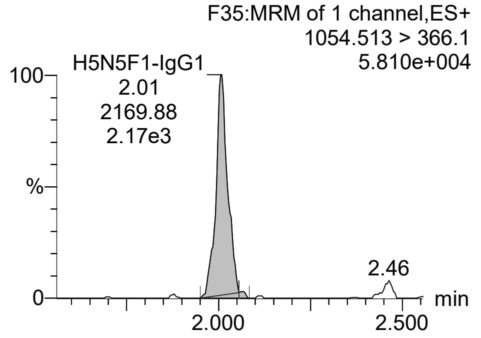

Sample Name: 20211111_A23_R1_IgG

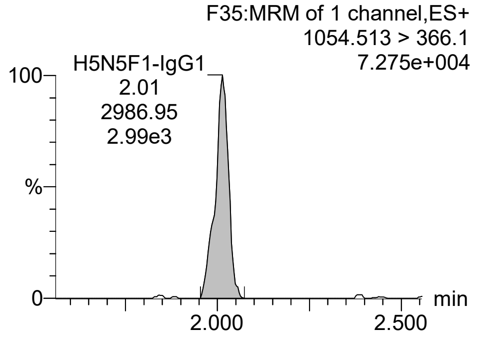

Sample Name: 20211111_A24_R1_IgG

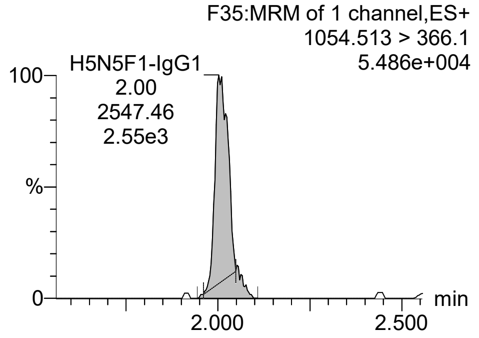

Sample Name: 20211111_L23_R1_IgG

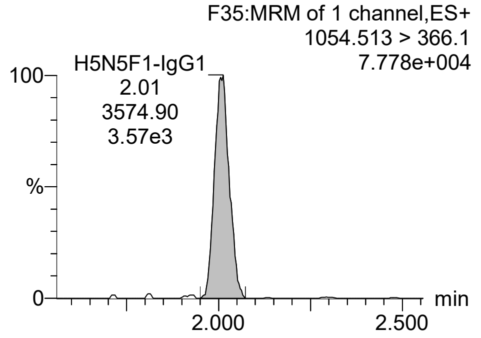

Sample Name: 20211111_L24_R1_IgG

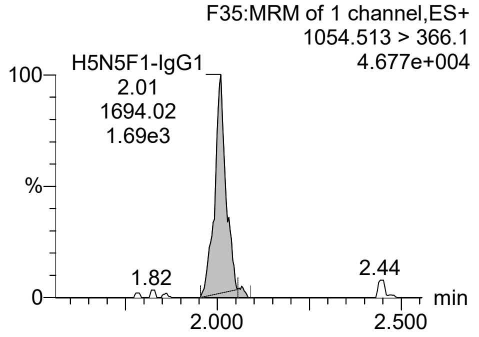

Sample Name: 20211111_H12_R1_IgG

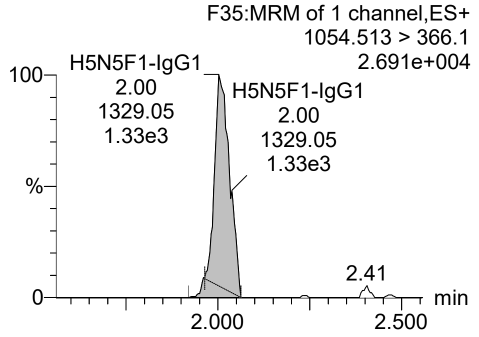

Sample Name: 20211111_QC_R6_IgG

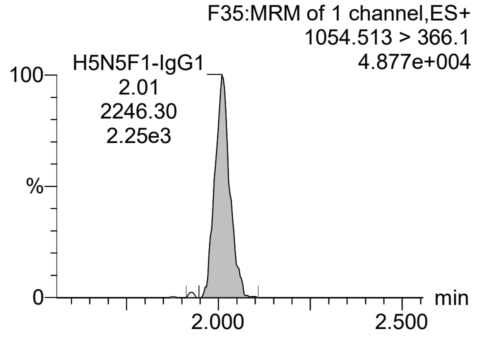

Sample Name: 20211111_A25_R1_IgG

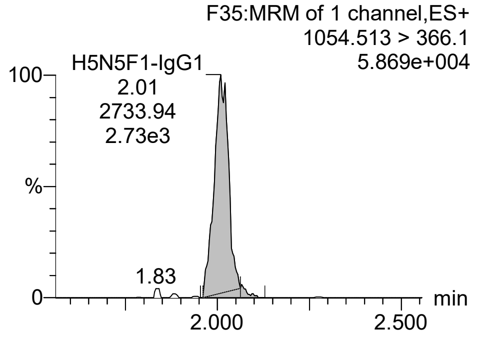

Sample Name: 20211111_A26_R1_IgG

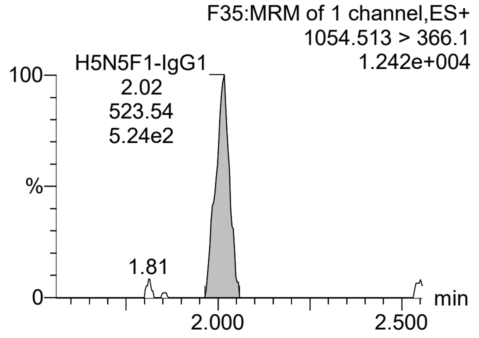

Sample Name: 20211111_L25_R1_IgG

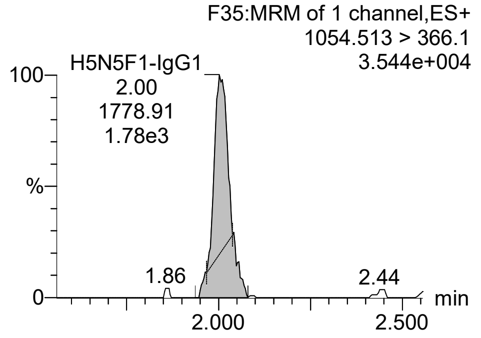

Sample Name: 20211111_L26_R1_IgG

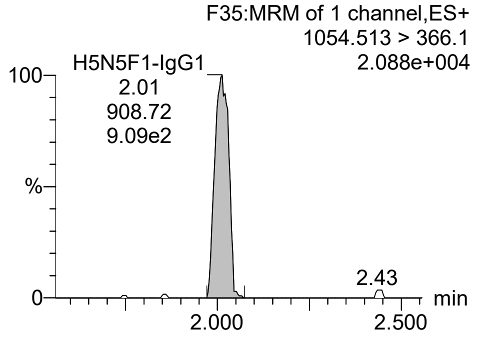

Sample Name: 20211111_H13_R1_IgG

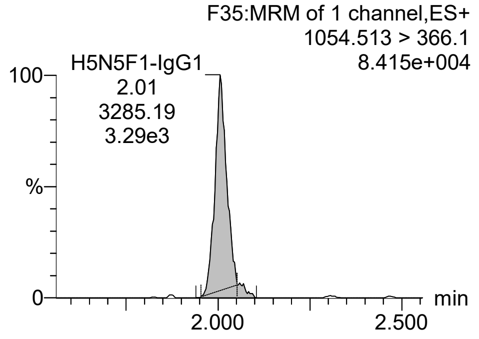

Sample Name: 20211111_A27_R1_IgG

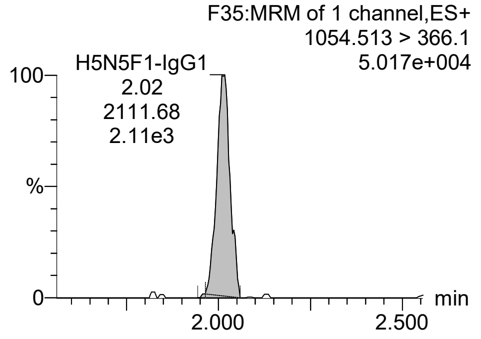

Dataset: C:\MassLynx\10.pro\20211115_clinicalsamples_IgG.qld

Last Altered: Sunday, October 02, 2022 16:02:30 台北標準時間
Printed: Friday, August 01, 2025 16:48:00 台北標準時間

Sample Name: 20211111_A28_R1_IgG
F35:MRM of 1 channel,ES+
1054.513 > 366.1
5.749e+004

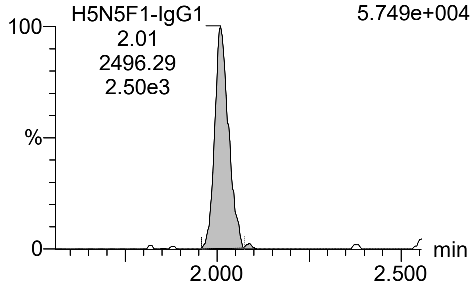

Sample Name: 20211111_L27_R1_IgG
F35:MRM of 1 channel,ES+
1054.513 > 366.1
7.286e+004

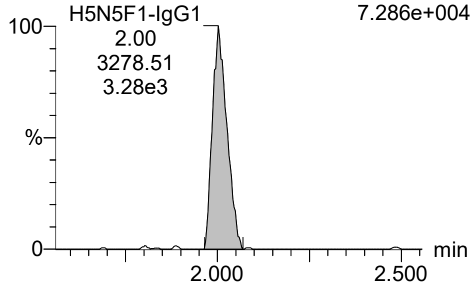

Sample Name: 20211111_L28_R1_IgG
F35:MRM of 1 channel,ES+
1054.513 > 366.1
2.226e+004

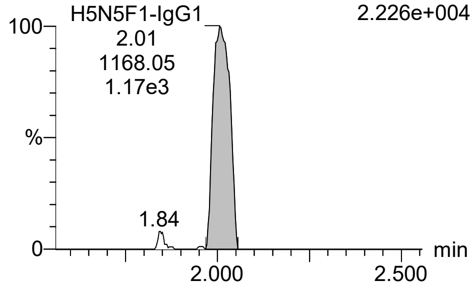

Sample Name: 20211111_H14_R1_IgG
F35:MRM of 1 channel,ES+
1054.513 > 366.1
8.746e+004

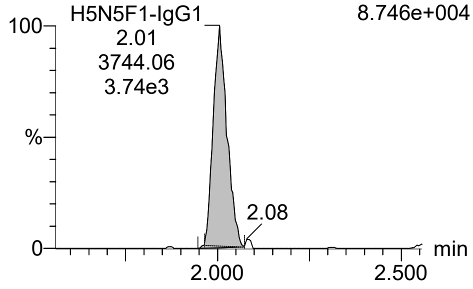

Sample Name: 20211111_A29_R1_IgG
F35:MRM of 1 channel,ES+
1054.513 > 366.1
1.554e+004

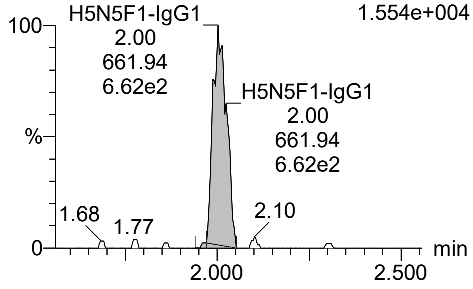

Sample Name: 20211111_A30_R1_IgG
F35:MRM of 1 channel,ES+
1054.513 > 366.1
2.680e+004

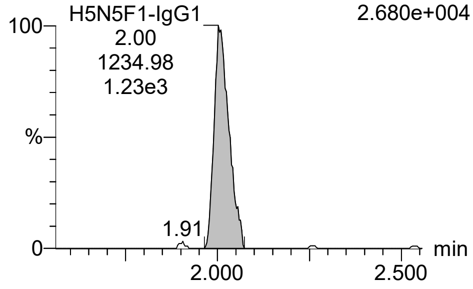

Sample Name: 20211111_QC_R7_IgG
F35:MRM of 1 channel,ES+
1054.513 > 366.1
5.916e+004

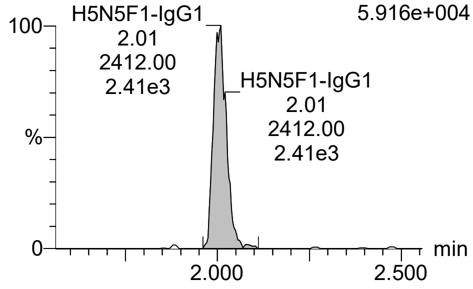

Sample Name: 20211111_L29_R1_IgG
F35:MRM of 1 channel,ES+
1054.513 > 366.1
2.272e+004

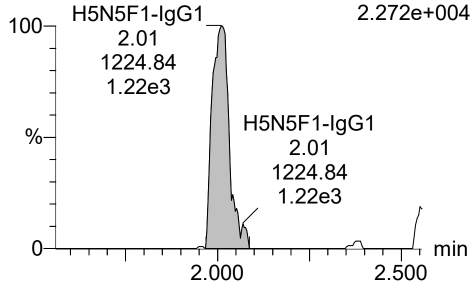

Sample Name: 20211111_L30_R1_IgG
F35:MRM of 1 channel,ES+
1054.513 > 366.1
1.768e+004

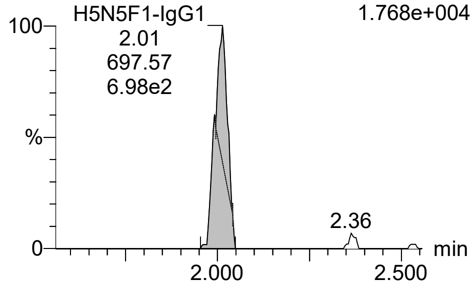

Sample Name: 20211111_H15_R1_IgG
F35:MRM of 1 channel,ES+
1054.513 > 366.1
5.638e+004

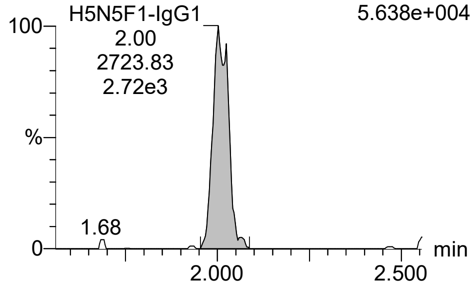

Sample Name: 20211111_A31_R1_IgG
F35:MRM of 1 channel,ES+
1054.513 > 366.1
1.621e+004

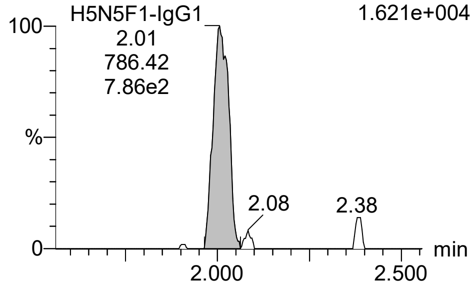

Sample Name: 20211111_A32_R1_IgG
F35:MRM of 1 channel,ES+
1054.513 > 366.1
4.407e+004

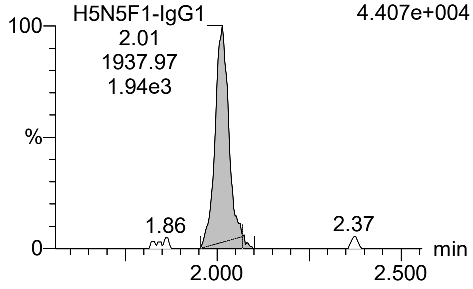

Sample Name: 20211111_L31_R1_IgG
F35:MRM of 1 channel,ES+
1054.513 > 366.1
1.480e+004

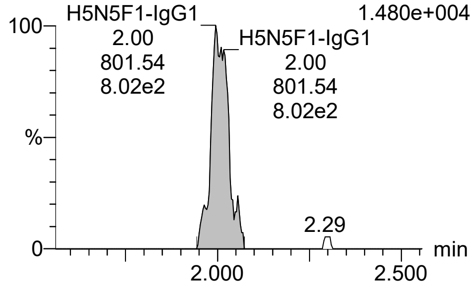

Sample Name: 20211111_L32_R1_IgG
F35:MRM of 1 channel,ES+
1054.513 > 366.1
4.569e+004

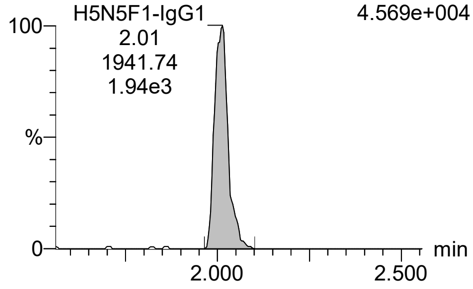

Sample Name: 20211111_H16_R1_IgG
F35:MRM of 1 channel,ES+
1054.513 > 366.1
5.825e+004

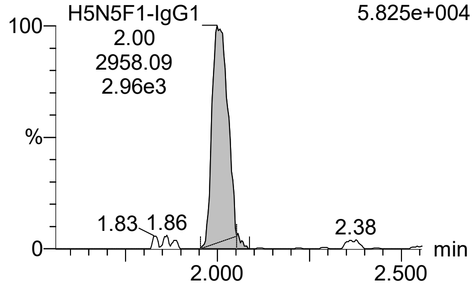

Dataset: C:\MassLynx\10.pro\20211115_clinicalsamples_IgG.qld

Last Altered: Sunday, October 02, 2022 16:02:30 台北標準時間
Printed: Friday, August 01, 2025 16:48:00 台北標準時間

Sample Name: 20211111_A33_R1_IgG

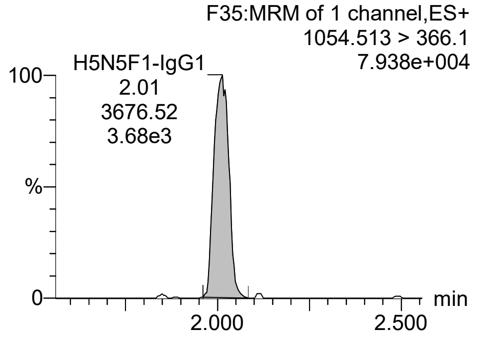

Sample Name: 20211111_A34_R1_IgG

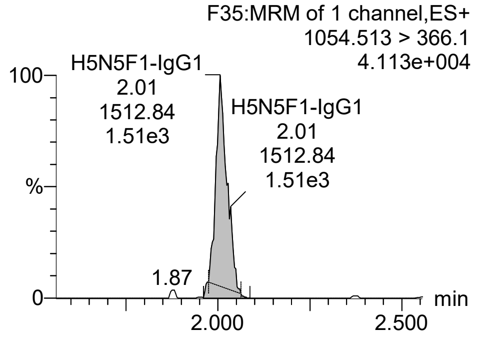

Sample Name: 20211111_H17_R1_IgG

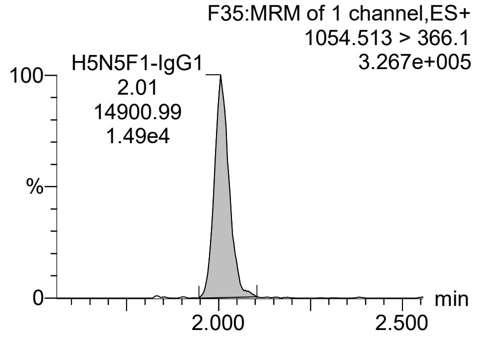

Sample Name: 20211111_A35_R1_IgG

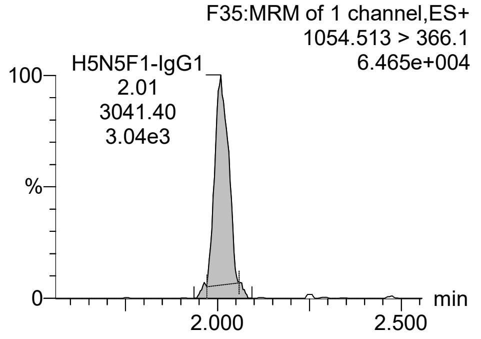

Sample Name: 20211111_QC_R8_IgG

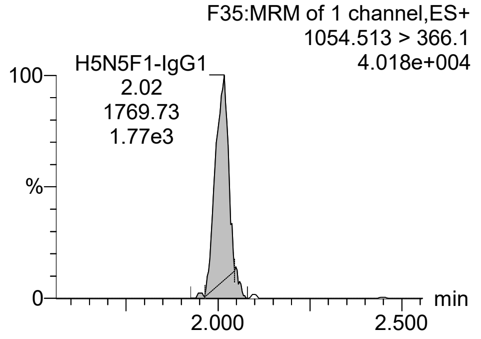

Sample Name: 20211111_A36_R1_IgG

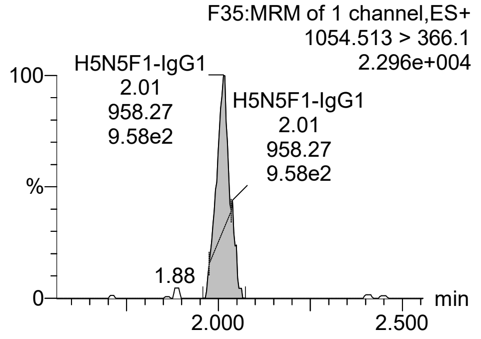

Sample Name: 20211111_H18_R1_IgG

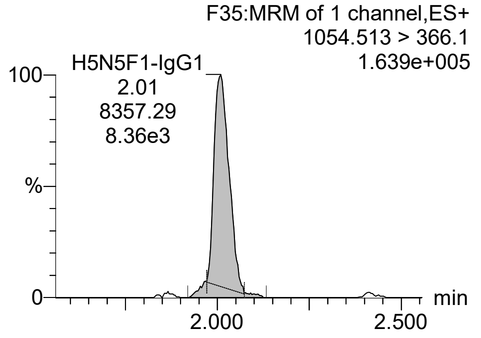

Sample Name: 20211111_A37_R1_IgG

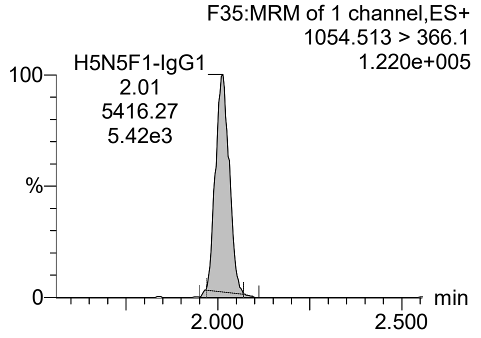

Sample Name: 20211111_A38_R1_IgG

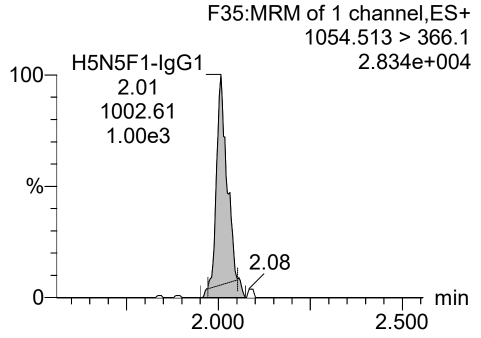

Sample Name: 20211111_H19_R1_IgG

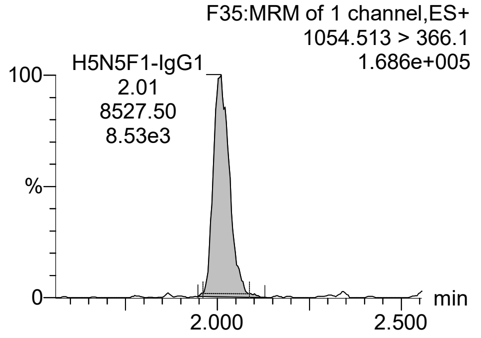

Sample Name: 20211111_A39_R1_IgG

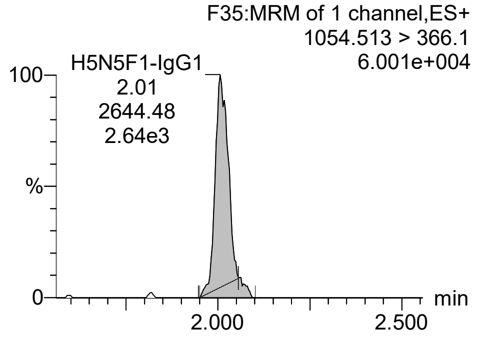

Sample Name: 20211111_A40_R1_IgG

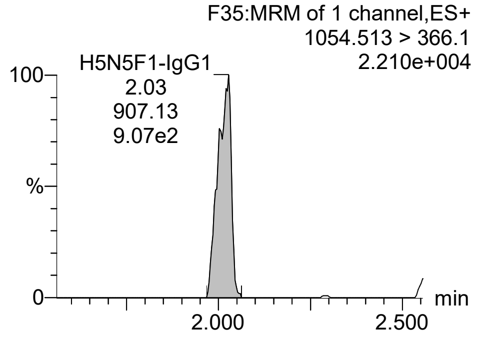

Sample Name: 20211111_H20_R1_IgG

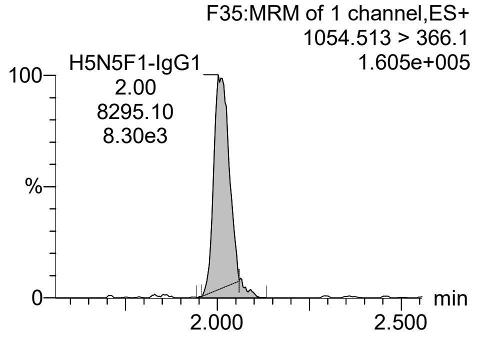

Sample Name: 20211111_A41_R1_IgG

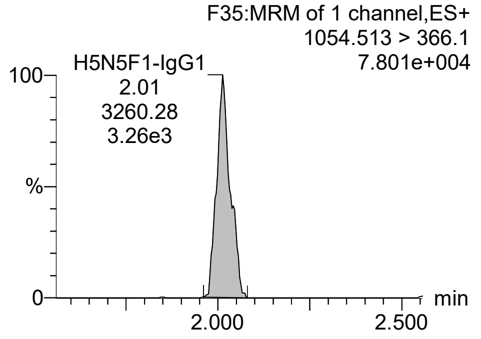

Sample Name: 20211111_A42_R1_IgG

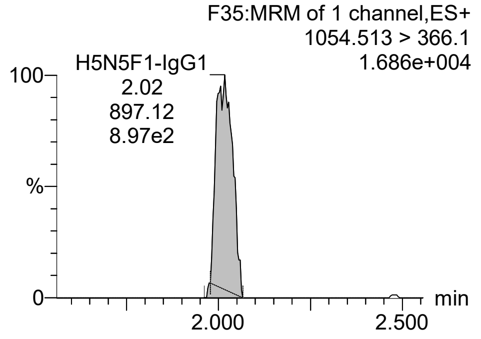

Dataset: C:\MassLynx\10.pro\20211115_clinicalsamples_IgG.qld

Last Altered: Sunday, October 02, 2022 16:02:30 台北標準時間
Printed: Friday, August 01, 2025 16:48:00 台北標準時間

Sample Name: 20211111_A43_R1_IgG

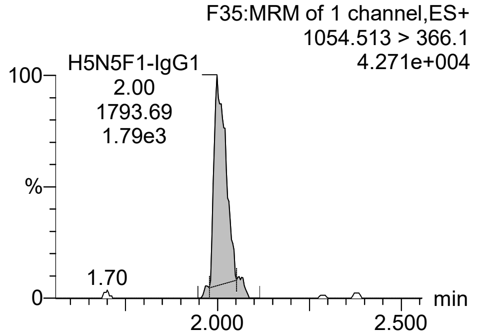

Sample Name: 20211111_A44_R1_IgG

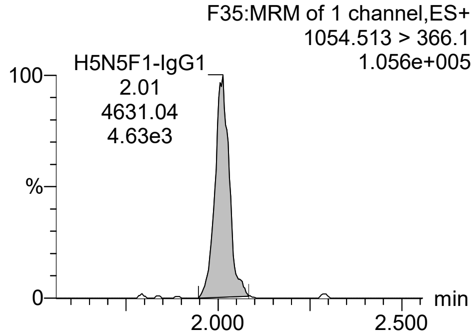

Sample Name: 20211111_QC_R9_IgG

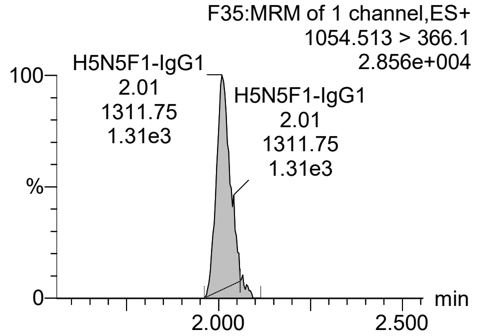

Sample Name: 20211111_A1_R2_IgG

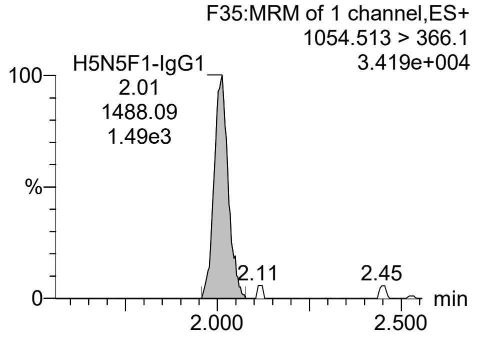

Sample Name: 20211111_A2_R2_IgG

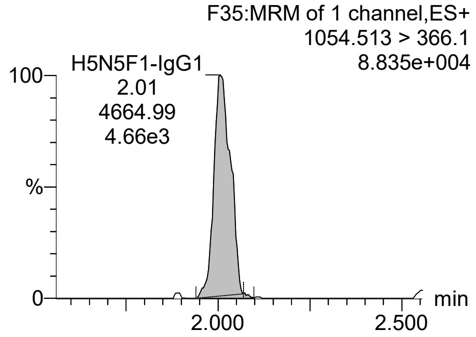

Sample Name: 20211111_L1_R2_IgG

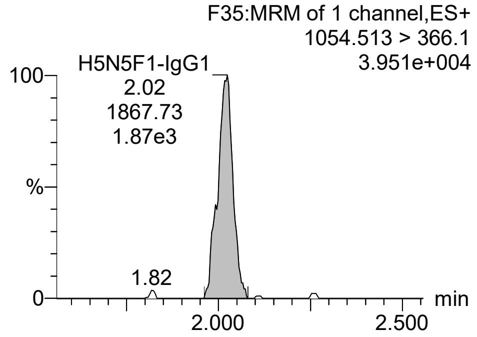

Sample Name: 20211111_L2_R2_IgG

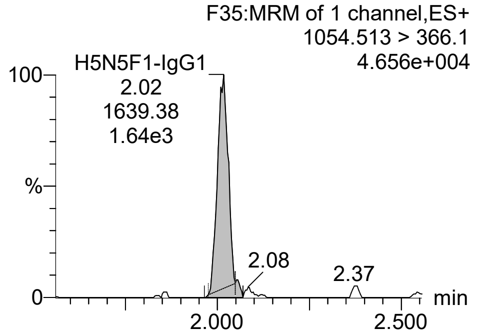

Sample Name: 20211111_H1_R2_IgG

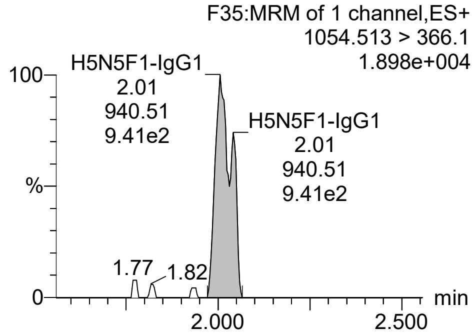

Sample Name: 20211111_A3_R2_IgG

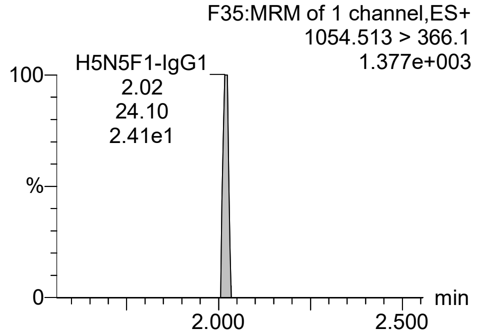

Sample Name: 20211111_A4_R2_IgG

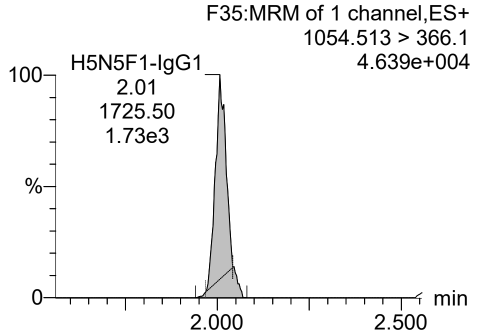

Sample Name: 20211111_L3_R2_IgG

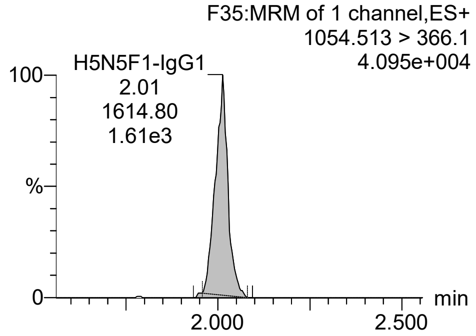

Sample Name: 20211111_L4_R2_IgG

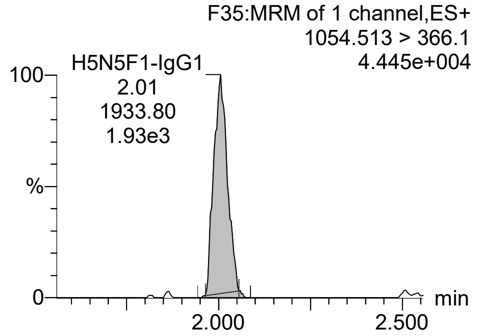

Sample Name: 20211111_H2_R2_IgG

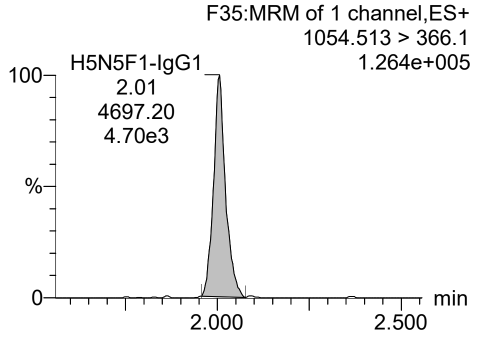

Sample Name: 20211111_A5_R2_IgG

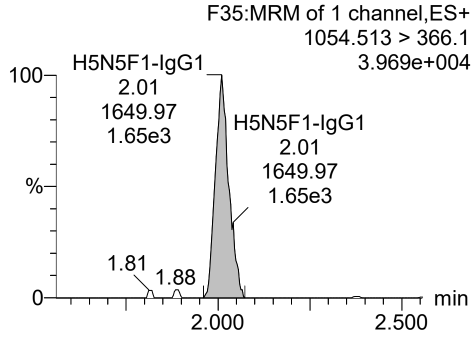

Sample Name: 20211111_A6_R2_IgG

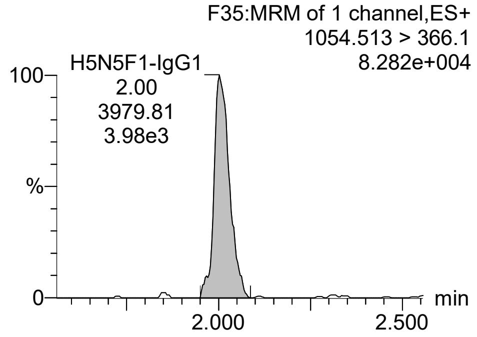

Dataset: C:\MassLynx\10.pro\20211115_clinicalsamples_IgG.qld

Last Altered: Sunday, October 02, 2022 16:02:30 台北標準時間
Printed: Friday, August 01, 2025 16:48:00 台北標準時間

Sample Name: 20211111_QC_R10_IgG
F35:MRM of 1 channel,ES+
1054.513 > 366.1

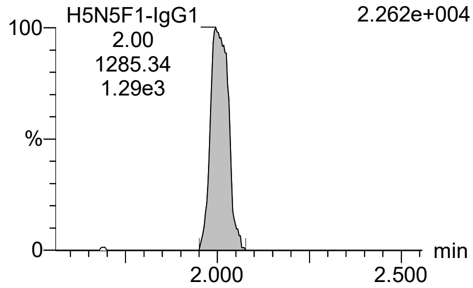

Sample Name: 20211111_L5_R2_IgG
F35:MRM of 1 channel,ES+
1054.513 > 366.1

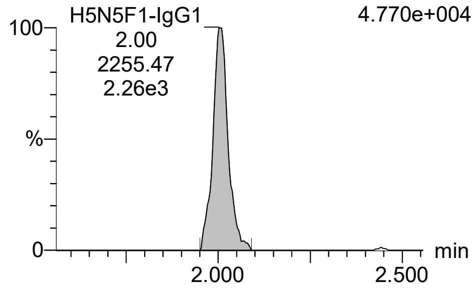

Sample Name: 20211111_L6_R2_IgG
F35:MRM of 1 channel,ES+
1054.513 > 366.1

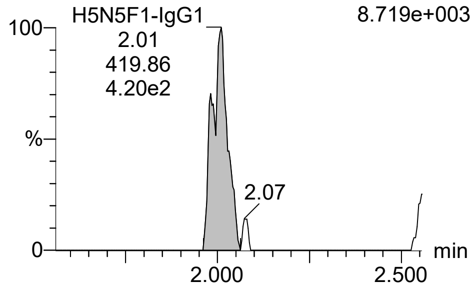

Sample Name: 20211111_H3_R2_IgG
F35:MRM of 1 channel,ES+
1054.513 > 366.1

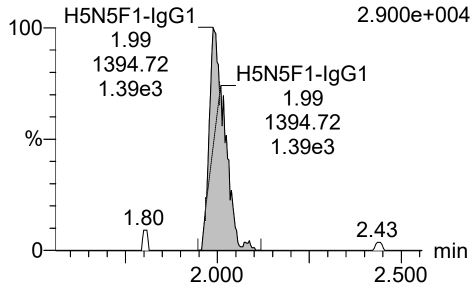

Sample Name: 20211111_A7_R2_IgG
F35:MRM of 1 channel,ES+
1054.513 > 366.1

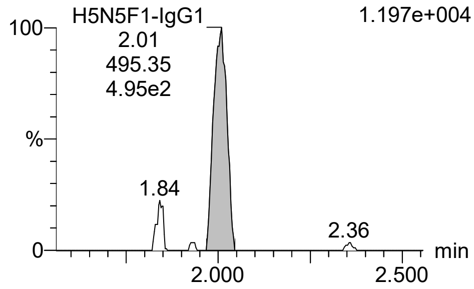

Sample Name: 20211111_A8_R2_IgG
F35:MRM of 1 channel,ES+
1054.513 > 366.1

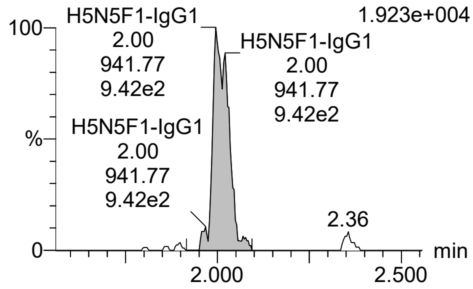

Sample Name: 20211111_L7_R2_IgG
F35:MRM of 1 channel,ES+
1054.513 > 366.1

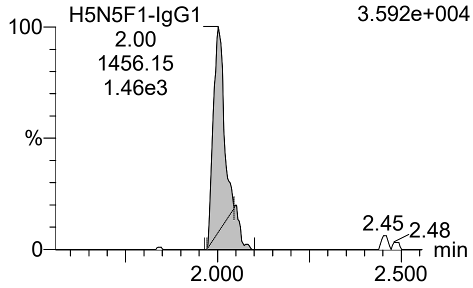

Sample Name: 20211111_L8_R2_IgG
F35:MRM of 1 channel,ES+
1054.513 > 366.1

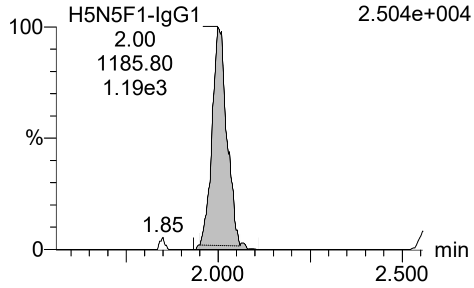

Sample Name: 20211111_H4_R2_IgG
F35:MRM of 1 channel,ES+
1054.513 > 366.1

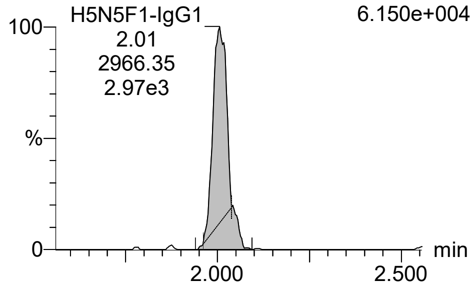

Sample Name: 20211111_A9_R2_IgG
F35:MRM of 1 channel,ES+
1054.513 > 366.1

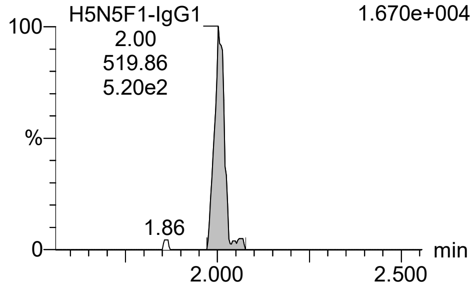

Sample Name: 20211111_A10_R2_IgG
F35:MRM of 1 channel,ES+
1054.513 > 366.1

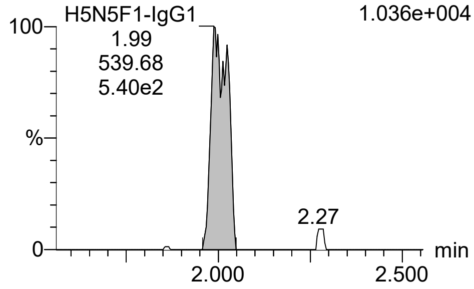

Sample Name: 20211111_L9_R2_IgG
F35:MRM of 1 channel,ES+
1054.513 > 366.1

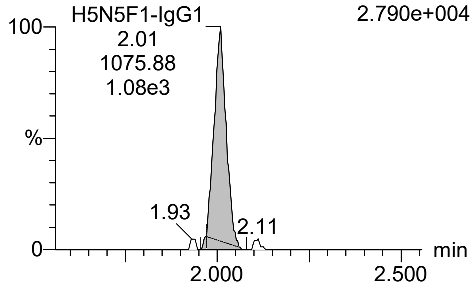

Sample Name: 20211111_L10_R2_IgG
F35:MRM of 1 channel,ES+
1054.513 > 366.1

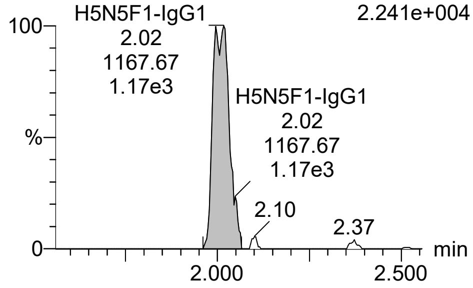

Sample Name: 20211111_QC_R11_IgG
F35:MRM of 1 channel,ES+
1054.513 > 366.1

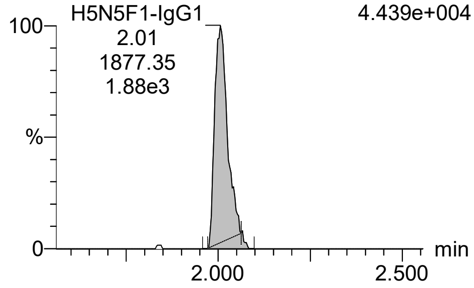

Sample Name: 20211111_H5_R2_IgG
F35:MRM of 1 channel,ES+
1054.513 > 366.1

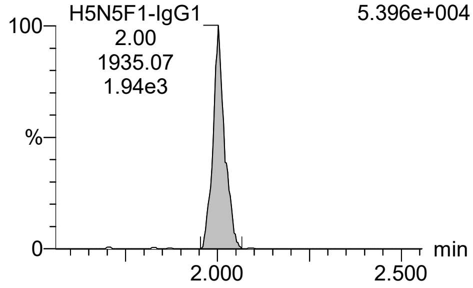

Sample Name: 20211111_A11_R2_IgG

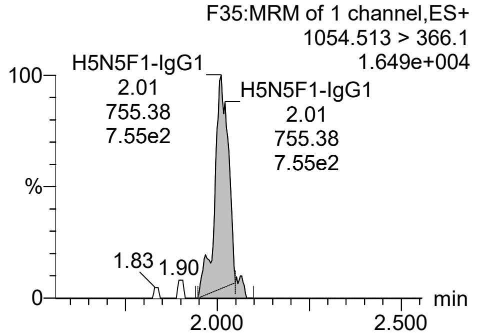

Sample Name: 20211111_A12_R2_IgG

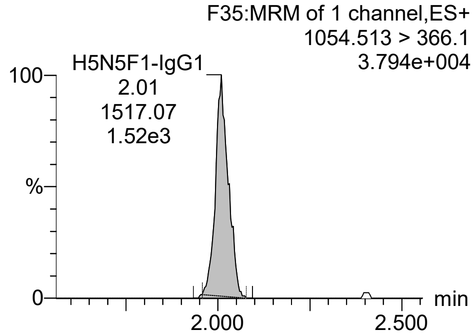

Sample Name: 20211111_L11_R2_IgG

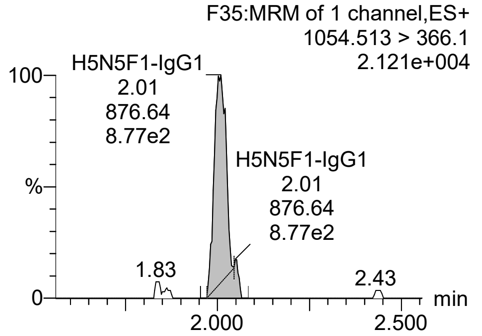

Sample Name: 20211111_L12_R2_IgG

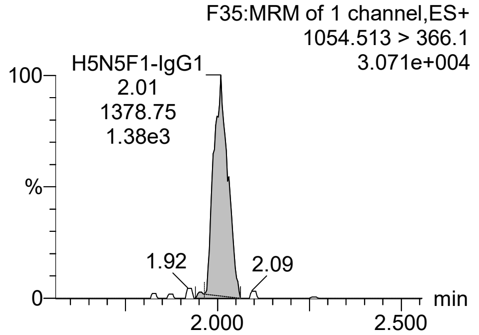

Sample Name: 20211111_H6_R2_IgG

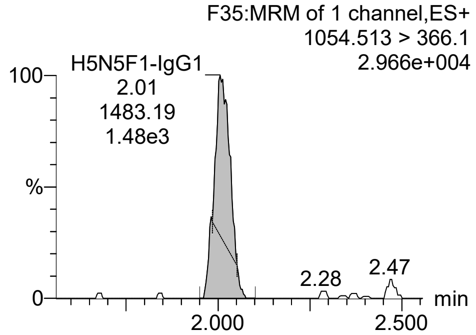

Sample Name: 20211111_A13_R2_IgG

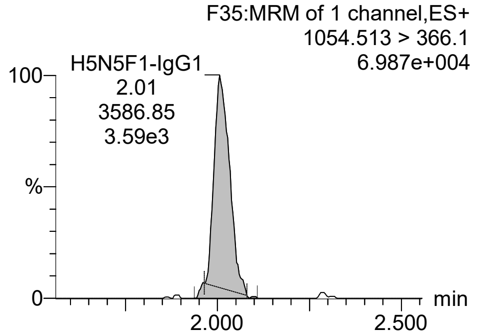

Sample Name: 20211111_A14_R2_IgG

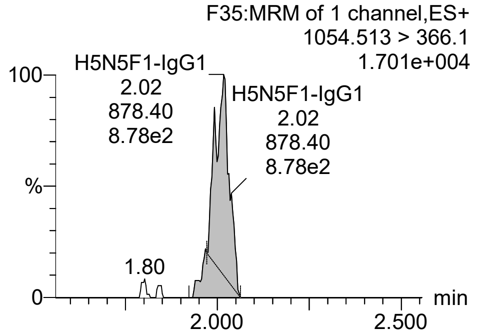

Sample Name: 20211111_L13_R2_IgG

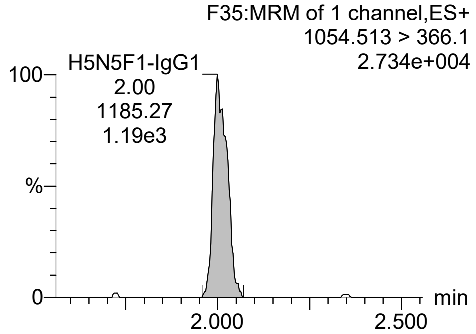

Sample Name: 20211111_L14_R2_IgG

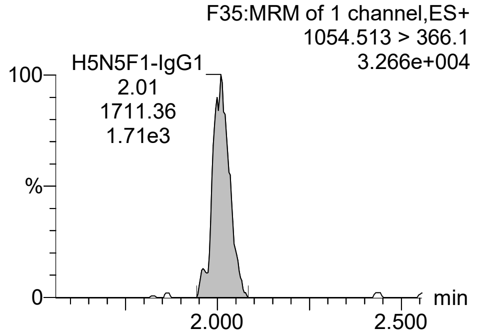

Sample Name: 20211111_H7_R2_IgG

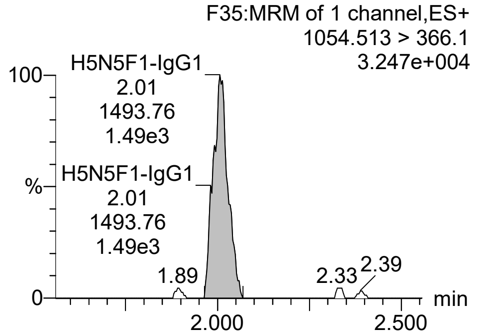

Sample Name: 20211111_A15_R2_IgG

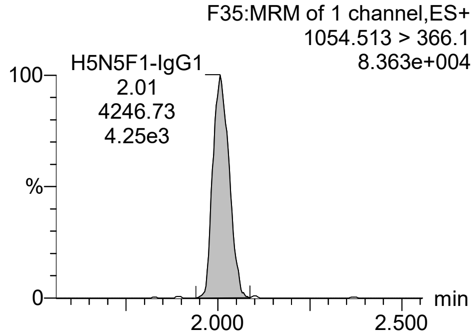

Sample Name: 20211111_QC_R12_IgG

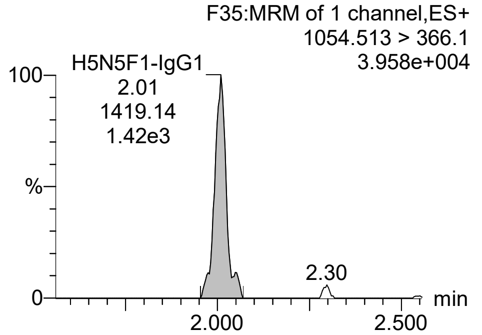

Sample Name: 20211111_A16_R2_IgG

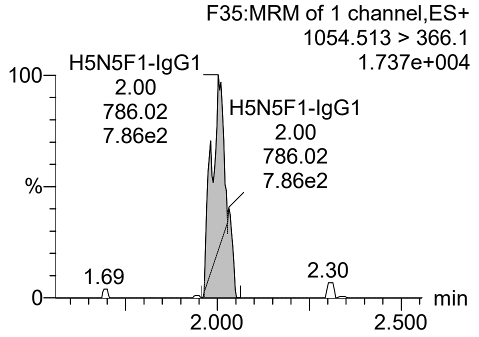

Sample Name: 20211111_L15_R2_IgG

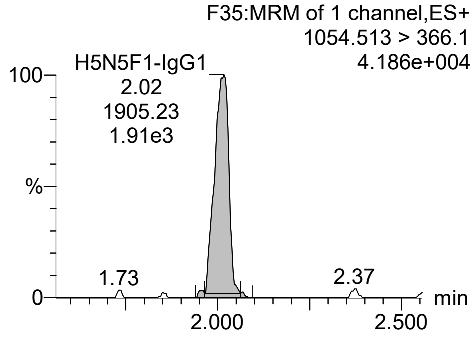

Sample Name: 20211111_L16_R2_IgG

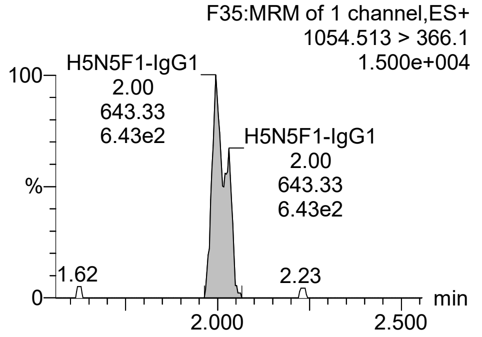

Sample Name: 20211111_H8_R2_IgG

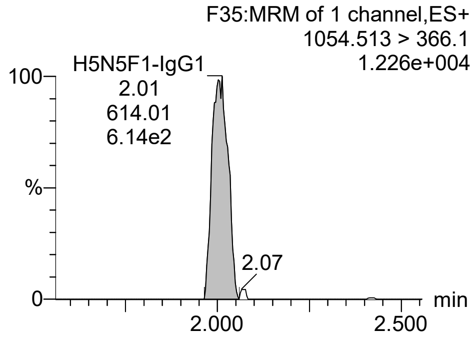

Sample Name: 20211111_A17_R2_IgG

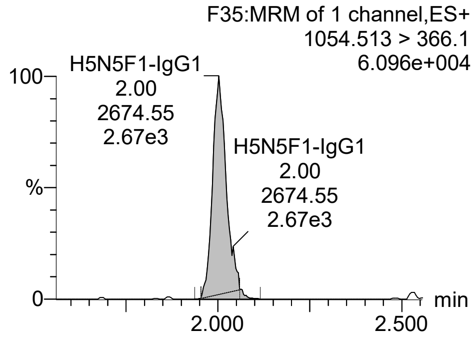

Sample Name: 20211111_A18_R2_IgG

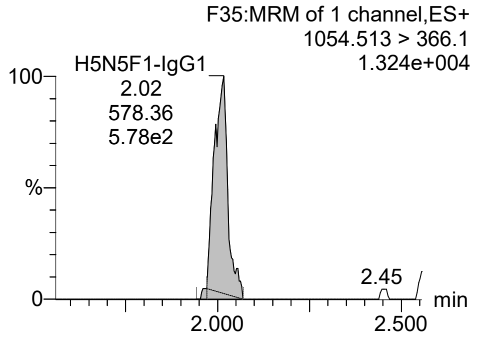

Sample Name: 20211111_L17_R2_IgG

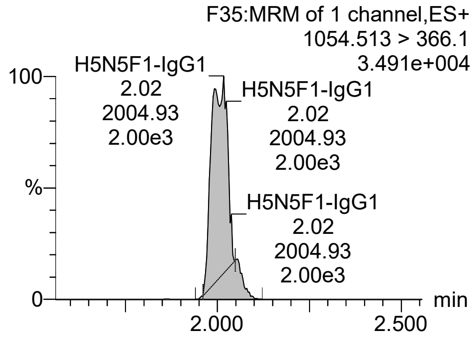

Sample Name: 20211111_L18_R2_IgG

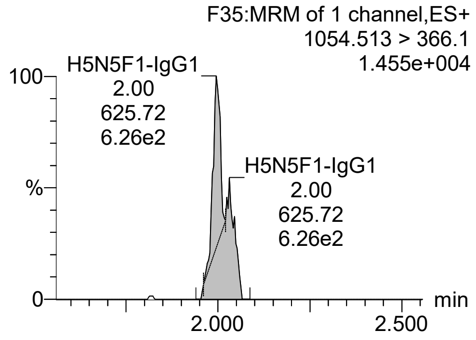

Sample Name: 20211111_H9_R2_IgG

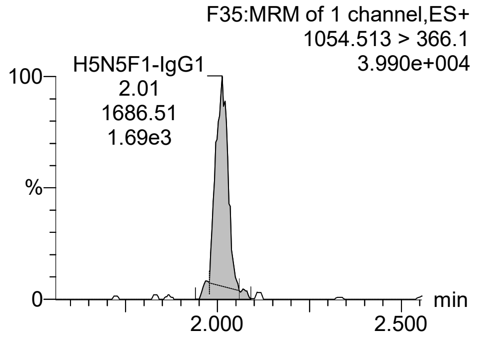

Sample Name: 20211111_A19_R2_IgG

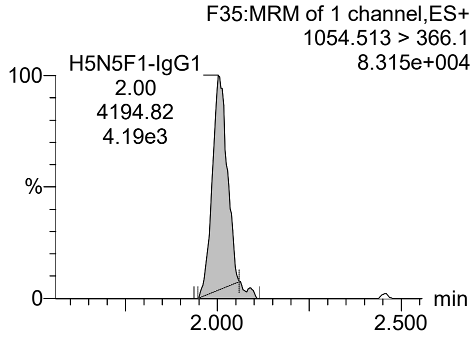

Sample Name: 20211111_A20_R2_IgG

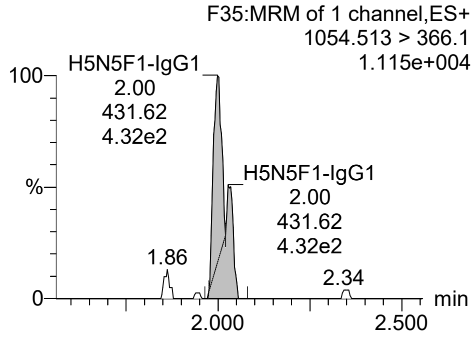

Sample Name: 20211111_L19_R2_IgG

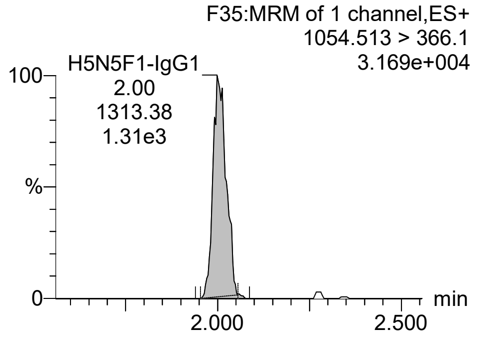

Sample Name: 20211111_QC_R13_IgG

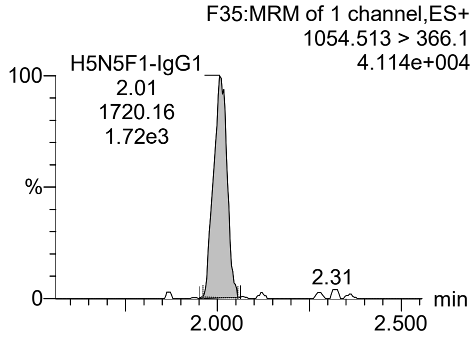

Sample Name: 20211111_L20_R2_IgG

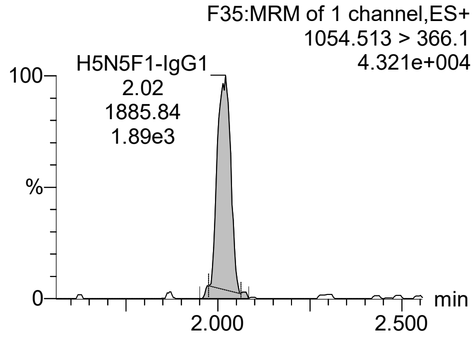

Sample Name: 20211111_H10_R2_IgG

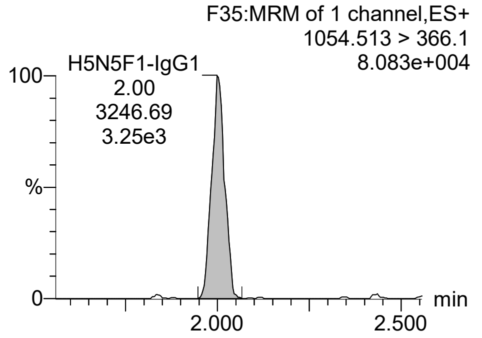

Sample Name: 20211111_A21_R2_IgG

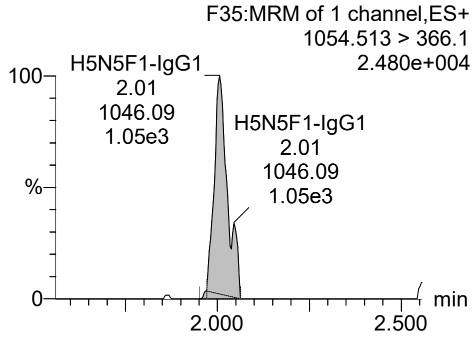

Sample Name: 20211111_A22_R2_IgG

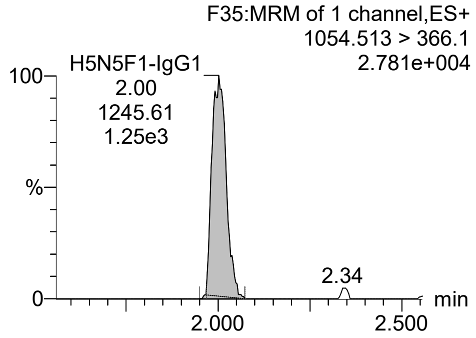

Sample Name: 20211111_L21_R2_IgG

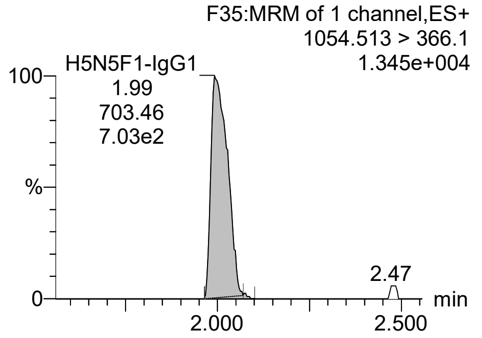

Sample Name: 20211111_L22_R2_IgG

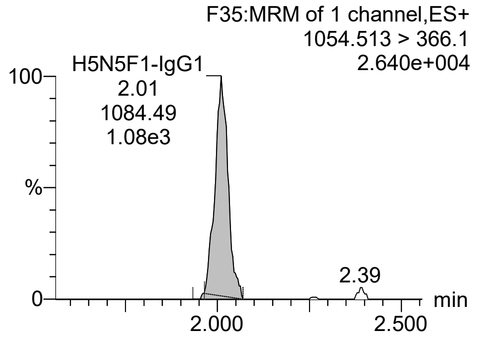

Sample Name: 20211111_H11_R2_IgG

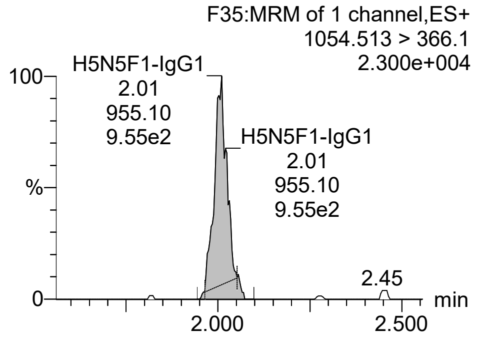

Sample Name: 20211111_A23_R2_IgG

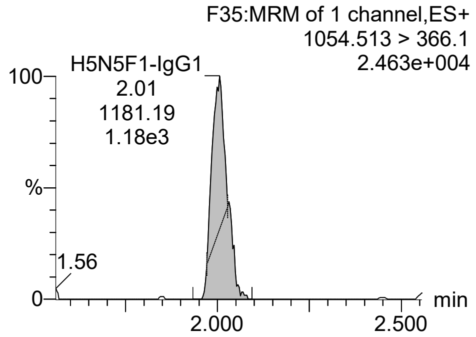

Sample Name: 20211111_A24_R2_IgG

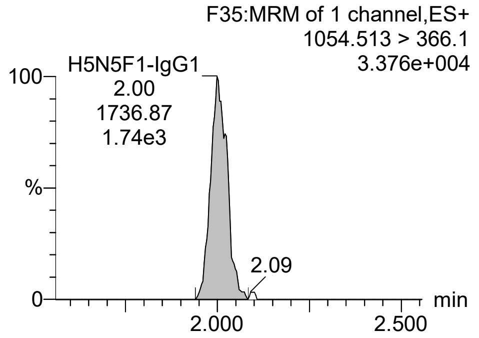

Sample Name: 20211111_L23_R2_IgG

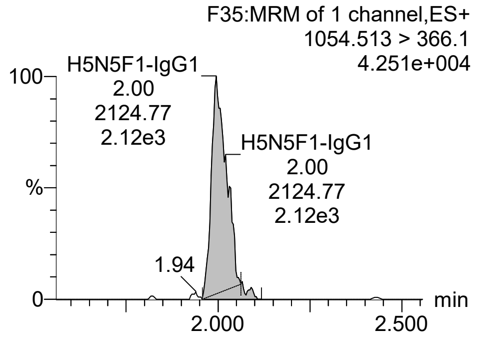

Sample Name: 20211111_L24_R2_IgG

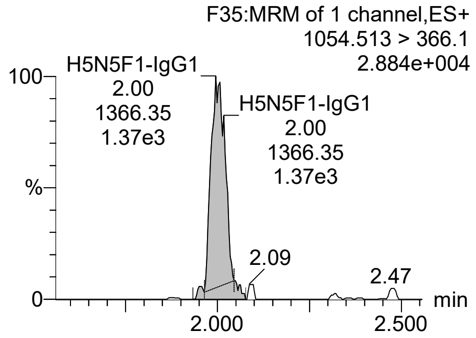

Sample Name: 20211111_H12_R2_IgG

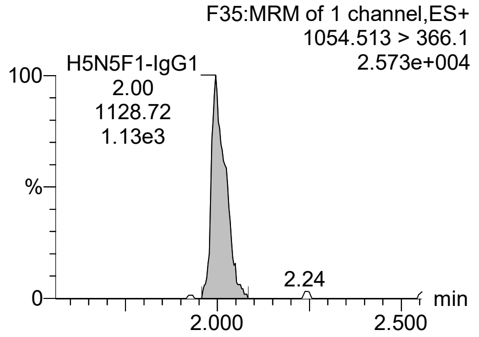

Sample Name: 20211111_QC_R14_IgG

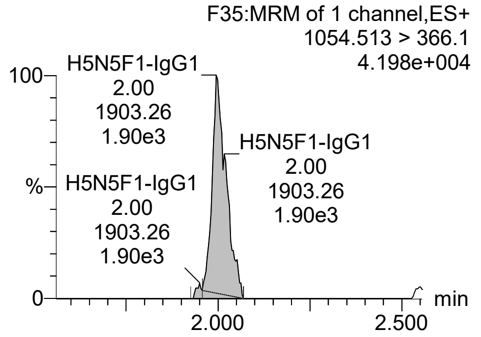

Sample Name: 20211111_A25_R2_IgG

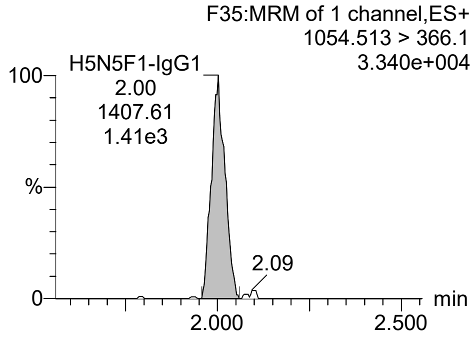

Sample Name: 20211111_A26_R2_IgG

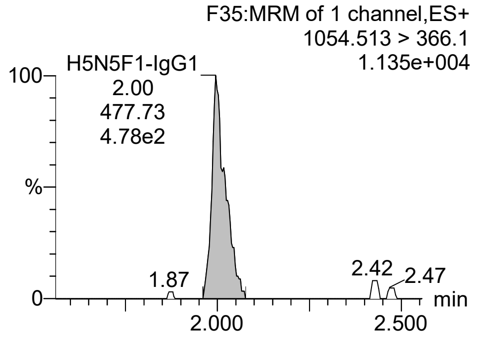

Sample Name: 20211111_L25_R2_IgG

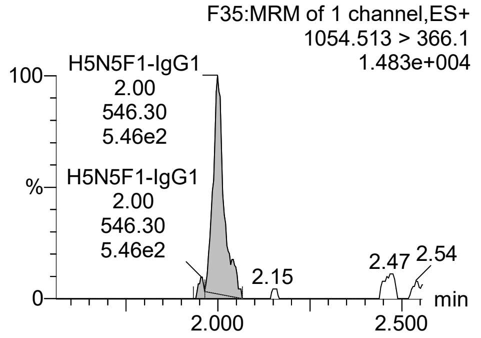

Sample Name: 20211111_L26_R2_IgG

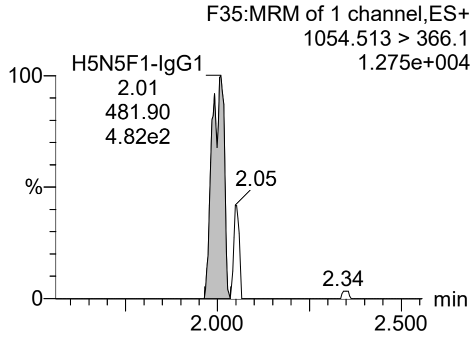

Sample Name: 20211111_H13_R2_IgG

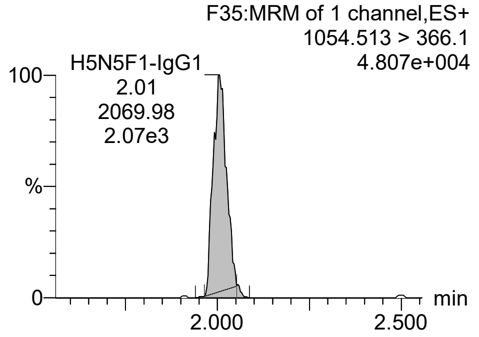

Sample Name: 20211111_A27_R2_IgG

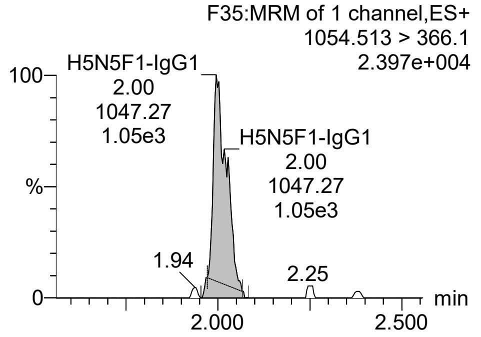

Sample Name: 20211111_A28_R2_IgG

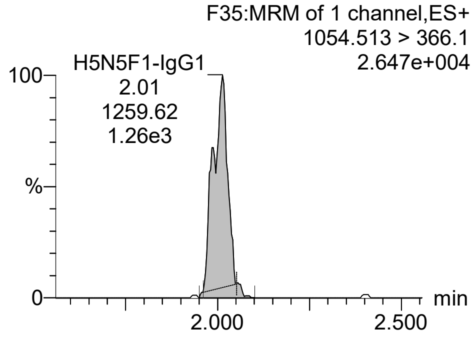

Dataset: C:\MassLynx\10.pro\20211115_clinicalsamples_IgG.qld

Page 203 of 2644.

Last Altered: Sunday, October 02, 2022 16:02:30 台北標準時間
Printed: Friday, August 01, 2025 16:48:00 台北標準時間

Sample Name: 20211111_L27_R2_IgG

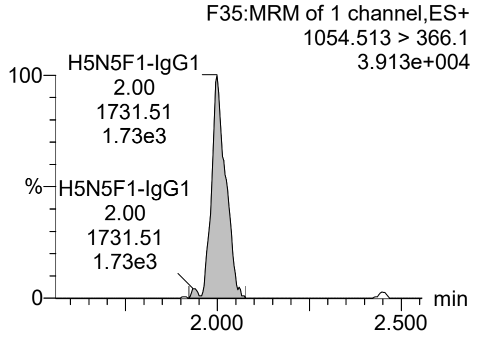

Sample Name: 20211111_L28_R2_IgG

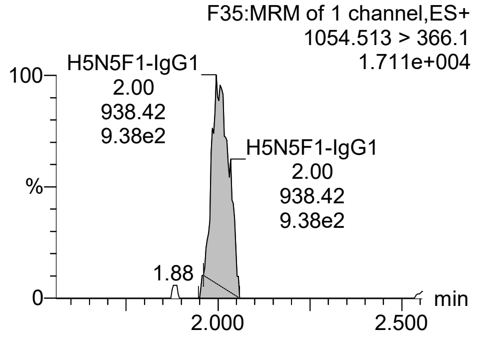

Sample Name: 20211111_H14_R2_IgG

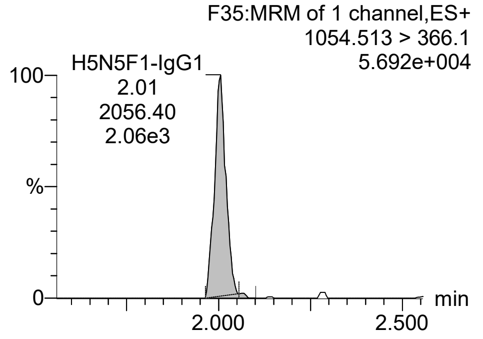

Sample Name: 20211111_A29_R2_IgG

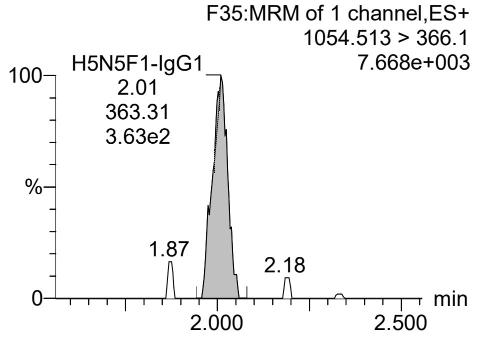

Sample Name: 20211111_A30_R2_IgG

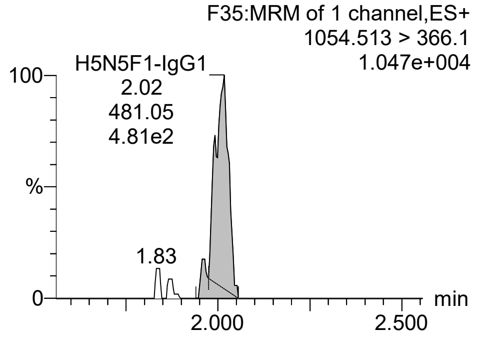

Sample Name: 20211111_QC_R15_IgG

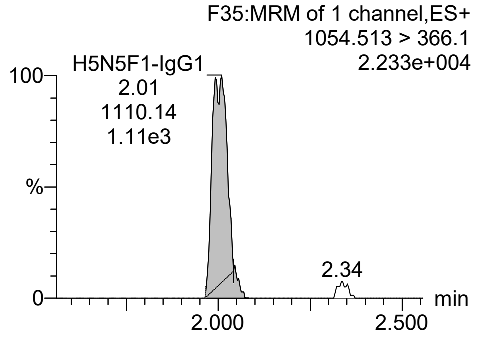

Sample Name: 20211111_L29_R2_IgG

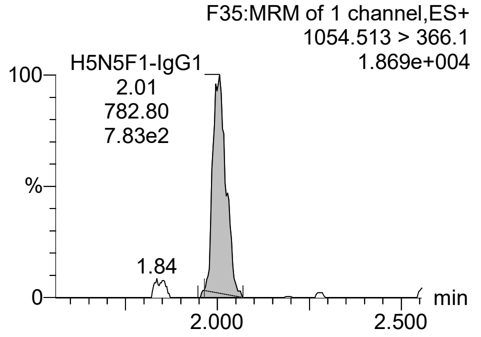

Sample Name: 20211111_L30_R2_IgG

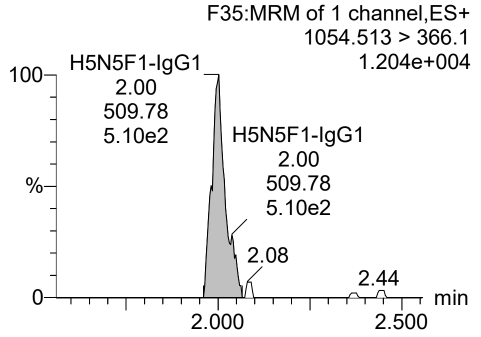

Sample Name: 20211111_H15_R2_IgG

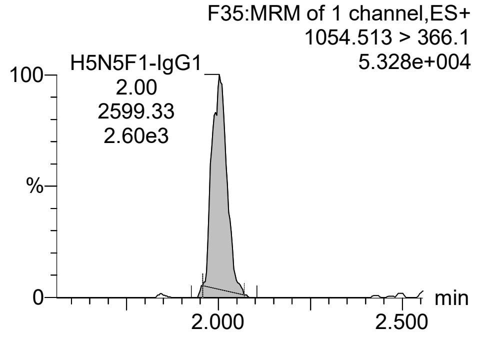

Sample Name: 20211111_A31_R2_IgG

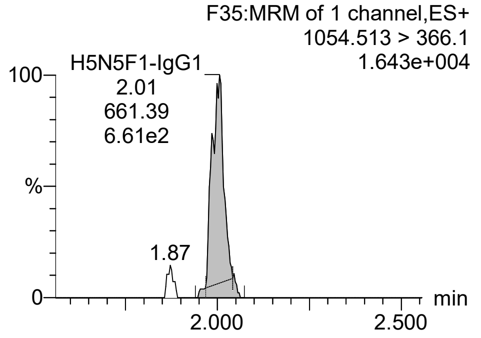

Sample Name: 20211111_A32_R2_IgG

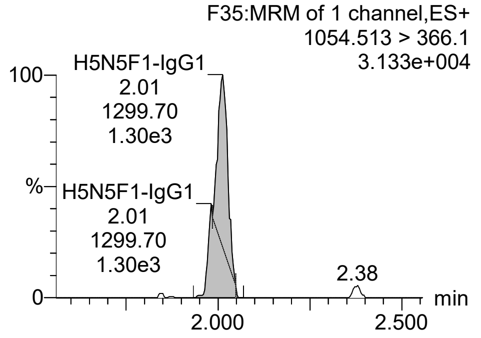

Sample Name: 20211111_L31_R2_IgG

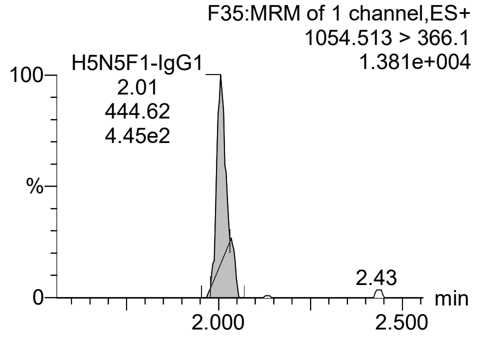

Sample Name: 20211111_L32_R2_IgG

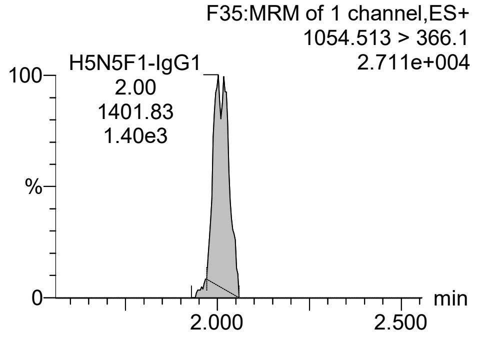

Sample Name: 20211111_H16_R2_IgG

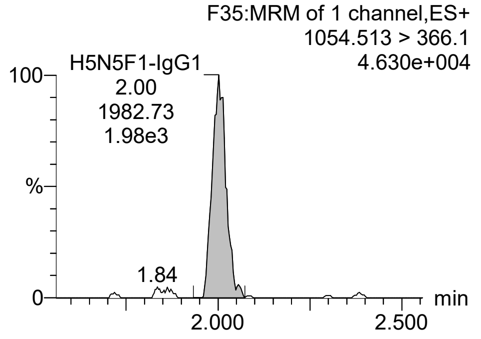

Sample Name: 20211111_A33_R2_IgG

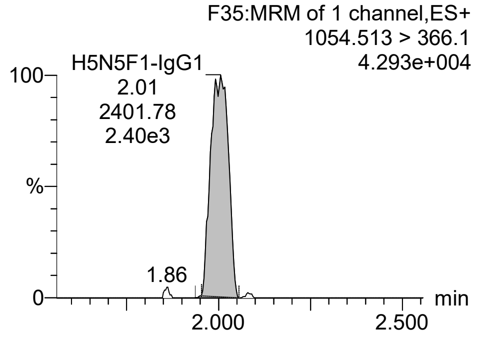

Dataset: C:\MassLynx\10.pro\20211115_clinicalsamples_IgG.qld

Last Altered: Sunday, October 02, 2022 16:02:30 台北標準時間
Printed: Friday, August 01, 2025 16:48:00 台北標準時間

Sample Name: 20211111_A34_R2_IgG

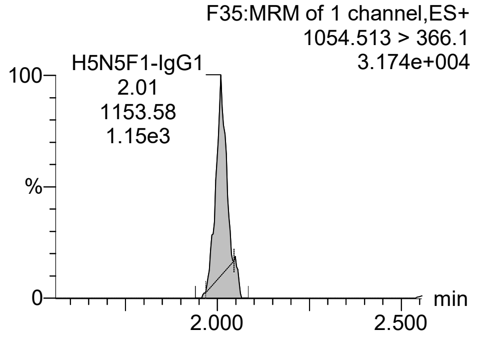

Sample Name: 20211111_H17_R2_IgG

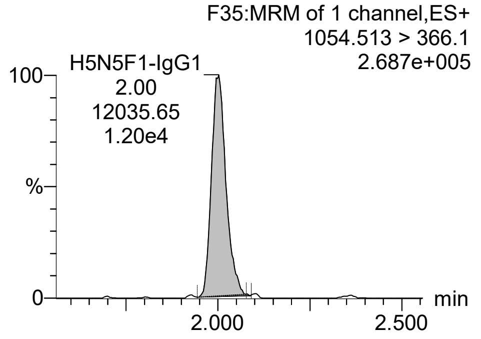

Sample Name: 20211111_A35_R2_IgG

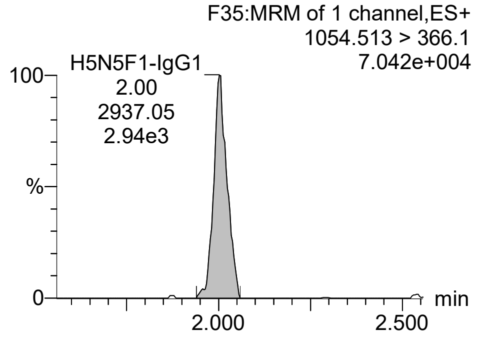

Sample Name: 20211111_QC_R16_IgG

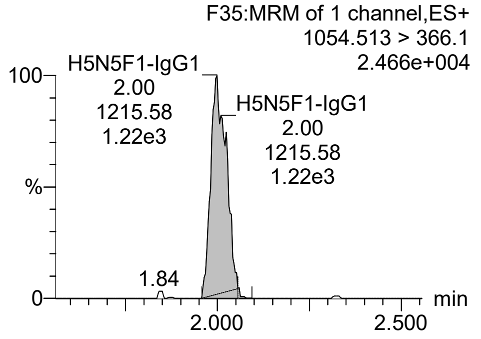

Sample Name: 20211111_A36_R2_IgG

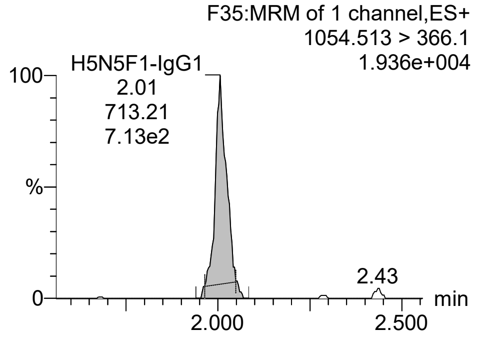

Sample Name: 20211111_H18_R2_IgG

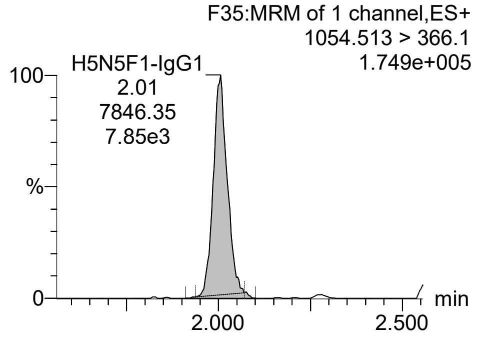

Sample Name: 20211111_A37_R2_IgG

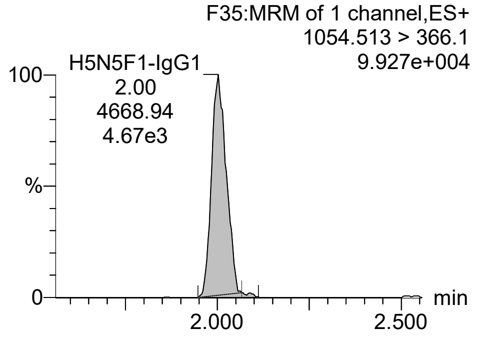

Sample Name: 20211111_A38_R2_IgG

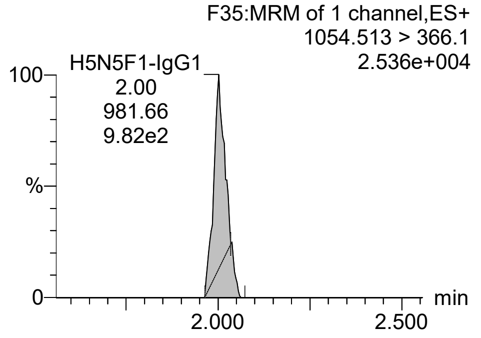

Sample Name: 20211111_H19_R2_IgG

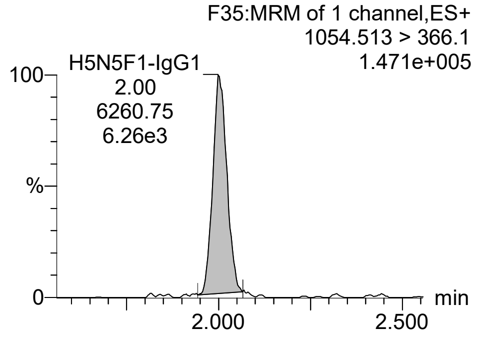

Sample Name: 20211111_A39_R2_IgG

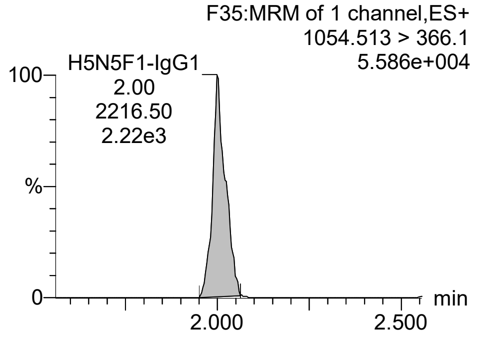

Sample Name: 20211111_A40_R2_IgG

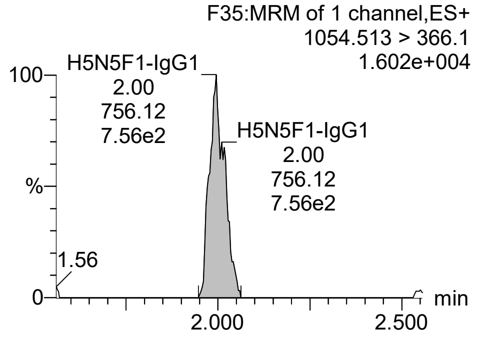

Sample Name: 20211111_H20_R2_IgG

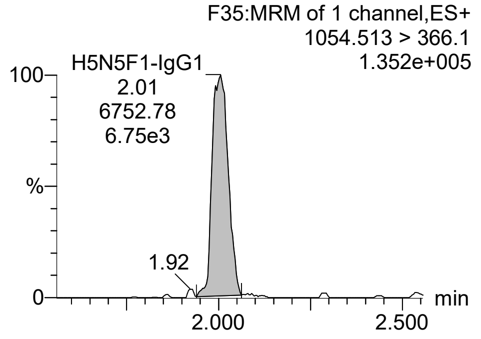

Sample Name: 20211111_A41_R2_IgG

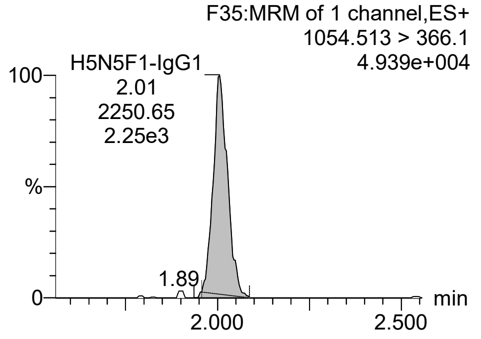

Sample Name: 20211111_A42_R2_IgG

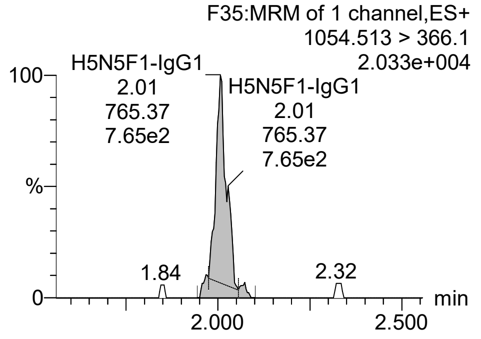

Sample Name: 20211111_A43_R2_IgG

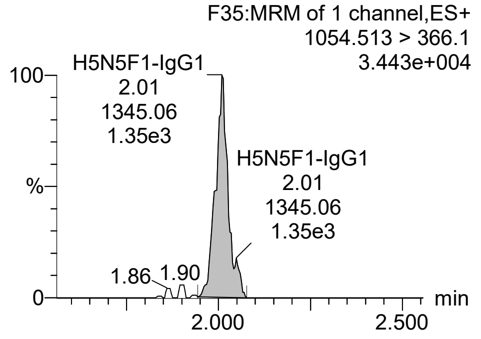

Dataset: C:\MassLynx\10.pro\20211115_clinicalsamples_IgG.qld

Last Altered: Sunday, October 02, 2022 16:02:30 台北標準時間
Printed: Friday, August 01, 2025 16:48:00 台北標準時間

Sample Name: 20211111_A44_R2_IgG

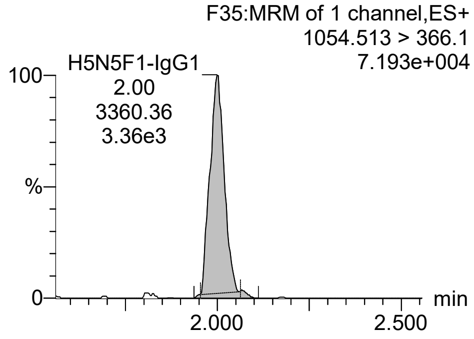

Sample Name: 20211111_QC_R17_IgG

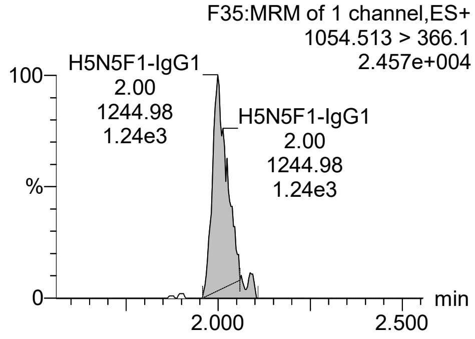

Dataset: C:\MassLynx\10.pro\20211115_clinicalsamples_IgG.qld

Last Altered: Sunday, October 02, 2022 16:02:30 台北標準時間
Printed: Friday, August 01, 2025 16:48:00 台北標準時間

	#	Name	RT	Area	IS Area	Response	Prima...
1	1	20211111_QC_R1_IgG	2.01	3733.0	64968.0	0.057	MM
2	2	20211111_A1_R1_IgG	2.01	2571.5	59059.2	0.044	bb
3	3	20211111_A2_R1_IgG	2.01	5612.3	66621.2	0.084	bb
4	4	20211111_L1_R1_IgG	2.01	4210.5	64201.0	0.066	MM
5	5	20211111_L2_R1_IgG	2.02	2010.4	46950.2	0.043	bb
6	6	20211111_H1_R1_IgG	2.01	989.8	38648.2	0.026	MM
7	7	20211111_A3_R1_IgG	2.00	14.5	2603.7	0.006	bb
8	8	20211111_A4_R1_IgG	2.01	2930.8	69960.4	0.042	MM
9	9	20211111_L3_R1_IgG	2.02	1366.2	38864.7	0.035	MM
10	10	20211111_L4_R1_IgG	2.01	2410.7	43380.5	0.056	bb
11	11	20211111_H2_R1_IgG	2.01	7360.1	71349.2	0.103	MM
12	12	20211111_A5_R1_IgG	2.01	2090.1	65569.7	0.032	bb
13	13	20211111_A6_R1_IgG	2.01	5181.0	53120.7	0.098	MM
14	14	20211111_QC_R2_IgG	2.01	3597.6	81034.1	0.044	MM
15	15	20211111_L5_R1_IgG	2.02	2866.6	50994.6	0.056	bb
16	16	20211111_L6_R1_IgG	2.01	572.9	58058.0	0.010	MM
17	17	20211111_H3_R1_IgG	2.01	1457.4	64028.6	0.023	MM
18	18	20211111_A7_R1_IgG	2.01	1004.5	82996.1	0.012	MM
19	19	20211111_A8_R1_IgG	2.01	1741.8	77731.7	0.022	MM
20	20	20211111_L7_R1_IgG	2.00	1493.3	38827.3	0.038	MM
21	21	20211111_L8_R1_IgG	2.01	1575.0	44220.1	0.036	bb
22	22	20211111_H4_R1_IgG	2.01	4349.6	65826.3	0.066	bb
23	23	20211111_A9_R1_IgG	2.01	654.6	60771.1	0.011	bb
24	24	20211111_A10_R1_IgG	2.01	614.4	48578.7	0.013	MM
25	25	20211111_L9_R1_IgG	2.00	1296.2	50830.7	0.025	MM
26	26	20211111_L10_R1_IgG	2.01	1378.2	38034.2	0.036	bb
27	27	20211111_QC_R3_IgG	2.01	3510.8	67025.5	0.052	MM
28	28	20211111_H5_R1_IgG	2.01	2274.4	44544.9	0.051	bb
29	29	20211111_A11_R1_IgG	2.00	1129.3	66408.5	0.017	bb
30	30	20211111_A12_R1_IgG	2.02	1554.6	46624.5	0.033	MM
31	31	20211111_L11_R1_IgG	2.01	915.1	33425.1	0.027	MM
32	32	20211111_L12_R1_IgG	2.01	1343.0	41178.0	0.033	bb
33	33	20211111_H6_R1_IgG	2.01	2688.1	58593.4	0.046	bb
34	34	20211111_A13_R1_IgG	2.01	4263.1	54133.0	0.079	MM
35	35	20211111_A14_R1_IgG	2.02	1137.3	56467.3	0.020	bb
36	36	20211111_L13_R1_IgG	2.00	1337.9	36024.4	0.037	bb
37	37	20211111_L14_R1_IgG	2.01	2063.7	37181.6	0.056	MM
38	38	20211111_H7_R1_IgG	2.01	1747.4	42850.6	0.041	bb
39	39	20211111_A15_R1_IgG	2.02	4980.0	62015.1	0.080	bb
40	40	20211111_QC_R4_IgG	2.00	2061.0	58572.5	0.035	bb
41	41	20211111_A16_R1_IgG	2.00	1318.6	78283.8	0.017	MM
42	42	20211111_L15_R1_IgG	2.01	2857.5	44158.2	0.065	MM
43	43	20211111_L16_R1_IgG	2.01	954.4	55150.8	0.017	bb
44	44	20211111_H8_R1_IgG	1.99	1098.3	42965.2	0.026	MM
45	45	20211111_A17_R1_IgG	2.00	3714.1	39717.7	0.094	bb
46	46	20211111_A18_R1_IgG	2.01	501.7	44675.4	0.011	MM
47	47	20211111_L17_R1_IgG	2.01	1896.4	37854.1	0.050	MM
48	48	20211111_L18_R1_IgG	2.00	1100.1	50716.9	0.022	MM
49	49	20211111_H9_R1_IgG	2.01	2433.4	36276.0	0.067	MM
50	50	20211111_A19_R1_IgG	2.01	3352.0	42862.5	0.078	bb
51	51	20211111_A20_R1_IgG	2.01	273.3	36542.7	0.007	bb
52	52	20211111_L19_R1_IgG	2.01	1648.9	53892.8	0.031	MM
53	53	20211111_QC_R5_IgG	2.01	2992.1	56513.2	0.053	MM
54	54	20211111_L20_R1_IgG	2.01	3236.3	56499.9	0.057	bb
55	55	20211111_H10_R1_IgG	2.01	8969.7	68120.5	0.132	bb
56	56	20211111_A21_R1_IgG	2.01	2385.8	63699.8	0.037	MM

Dataset: C:\MassLynx\10.pro\20211115_clinicalsamples_IgG.qld

Last Altered: Sunday, October 02, 2022 16:02:30 台北標準時間
Printed: Friday, August 01, 2025 16:48:00 台北標準時間

	# Name	RT	Area	IS Area	Response	Prima...
57	57 20211111_A22_R1_IgG	2.01	2338.7	52222.1	0.045	MM
58	58 20211111_L21_R1_IgG	2.01	1646.3	54372.5	0.030	bb
59	59 20211111_L22_R1_IgG	2.01	1922.2	41353.1	0.046	bb
60	60 20211111_H11_R1_IgG	2.01	2169.9	65579.1	0.033	MM
61	61 20211111_A23_R1_IgG	2.01	2987.0	64131.1	0.047	bb
62	62 20211111_A24_R1_IgG	2.00	2547.5	38724.8	0.066	MM
63	63 20211111_L23_R1_IgG	2.01	3574.9	64245.2	0.056	bb
64	64 20211111_L24_R1_IgG	2.01	1694.0	45298.0	0.037	MM
65	65 20211111_H12_R1_IgG	2.00	1329.0	44679.3	0.030	MM
66	66 20211111_QC_R6_IgG	2.01	2246.3	48165.0	0.047	MM
67	67 20211111_A25_R1_IgG	2.01	2733.9	57169.9	0.048	MM
68	68 20211111_A26_R1_IgG	2.02	523.5	34254.0	0.015	bb
69	69 20211111_L25_R1_IgG	2.00	1778.9	67491.5	0.026	MM
70	70 20211111_L26_R1_IgG	2.01	908.7	55934.1	0.016	bb
71	71 20211111_H13_R1_IgG	2.01	3285.2	48767.3	0.067	MM
72	72 20211111_A27_R1_IgG	2.02	2111.7	57269.7	0.037	MM
73	73 20211111_A28_R1_IgG	2.01	2496.3	49231.1	0.051	MM
74	74 20211111_L27_R1_IgG	2.00	3278.5	60014.5	0.055	bb
75	75 20211111_L28_R1_IgG	2.01	1168.0	35062.6	0.033	bb
76	76 20211111_H14_R1_IgG	2.01	3744.1	62371.9	0.060	MM
77	77 20211111_A29_R1_IgG	2.00	661.9	43858.2	0.015	MM
78	78 20211111_A30_R1_IgG	2.00	1235.0	62500.4	0.020	bb
79	79 20211111_QC_R7_IgG	2.01	2412.0	43946.9	0.055	bb
80	80 20211111_L29_R1_IgG	2.01	1224.8	38728.9	0.032	bb
81	81 20211111_L30_R1_IgG	2.01	697.6	42613.2	0.016	MM
82	82 20211111_H15_R1_IgG	2.00	2723.8	39874.6	0.068	bb
83	83 20211111_A31_R1_IgG	2.01	786.4	53414.3	0.015	bb
84	84 20211111_A32_R1_IgG	2.01	1938.0	41844.6	0.046	MM
85	85 20211111_L31_R1_IgG	2.00	801.5	40553.7	0.020	bb
86	86 20211111_L32_R1_IgG	2.01	1941.7	49618.7	0.039	bb
87	87 20211111_H16_R1_IgG	2.00	2958.1	60170.9	0.049	MM
88	88 20211111_A33_R1_IgG	2.01	3676.5	48588.2	0.076	bb
89	89 20211111_A34_R1_IgG	2.01	1512.8	43288.0	0.035	MM
90	90 20211111_H17_R1_IgG	2.01	14901.0	47104.5	0.316	bb
91	91 20211111_A35_R1_IgG	2.01	3041.4	41025.9	0.074	MM
92	92 20211111_QC_R8_IgG	2.02	1769.7	40546.3	0.044	MM
93	93 20211111_A36_R1_IgG	2.01	958.3	44504.4	0.022	MM
94	94 20211111_H18_R1_IgG	2.01	8357.3	34162.2	0.245	MM
95	95 20211111_A37_R1_IgG	2.01	5416.3	39760.0	0.136	MM
96	96 20211111_A38_R1_IgG	2.01	1002.6	43982.6	0.023	MM
97	97 20211111_H19_R1_IgG	2.01	8527.5	45314.8	0.188	MM
98	98 20211111_A39_R1_IgG	2.01	2644.5	39388.0	0.067	MM
99	99 20211111_A40_R1_IgG	2.03	907.1	38726.4	0.023	bb
100	1... 20211111_H20_R1_IgG	2.00	8295.1	37814.4	0.219	MM
101	1... 20211111_A41_R1_IgG	2.01	3260.3	48930.3	0.067	bb
102	1... 20211111_A42_R1_IgG	2.02	897.1	40406.8	0.022	MM
103	1... 20211111_A43_R1_IgG	2.00	1793.7	44421.5	0.040	MM
104	1... 20211111_A44_R1_IgG	2.01	4631.0	53128.2	0.087	bb
105	1... 20211111_QC_R9_IgG	2.01	1311.7	37049.0	0.035	MM
106	1... 20211111_A1_R2_IgG	2.01	1488.1	46499.4	0.032	bb
107	1... 20211111_A2_R2_IgG	2.01	4665.0	57260.7	0.081	MM
108	1... 20211111_L1_R2_IgG	2.02	1867.7	48062.9	0.039	bb
109	1... 20211111_L2_R2_IgG	2.02	1639.4	38745.2	0.042	MM
110	1... 20211111_H1_R2_IgG	2.01	940.5	44273.5	0.021	bb
111	1... 20211111_A3_R2_IgG	2.02	24.1	4755.5	0.005	bb
112	1... 20211111_A4_R2_IgG	2.01	1725.5	50546.7	0.034	MM

Dataset: C:\MassLynx\10.pro\20211115_clinicalsamples_IgG.qld

Last Altered: Sunday, October 02, 2022 16:02:30 台北標準時間
Printed: Friday, August 01, 2025 16:48:00 台北標準時間

	# Name	RT	Area	IS Area	Response	Prima...
113	1... 20211111_L3_R2_IgG	2.01	1614.8	34825.8	0.046	MM
114	1... 20211111_L4_R2_IgG	2.01	1933.8	39107.7	0.049	MM
115	1... 20211111_H2_R2_IgG	2.01	4697.2	49750.0	0.094	bb
116	1... 20211111_A5_R2_IgG	2.01	1650.0	52536.2	0.031	bb
117	1... 20211111_A6_R2_IgG	2.00	3979.8	42050.0	0.095	bb
118	1... 20211111_QC_R10_IgG	2.00	1285.3	35350.8	0.036	bb
119	1... 20211111_L5_R2_IgG	2.00	2255.5	38039.3	0.059	bb
120	1... 20211111_L6_R2_IgG	2.01	419.9	44907.4	0.009	bb
121	1... 20211111_H3_R2_IgG	1.99	1394.7	47729.1	0.029	MM
122	1... 20211111_A7_R2_IgG	2.01	495.3	57347.1	0.009	bb
123	1... 20211111_A8_R2_IgG	2.00	941.8	52397.8	0.018	bb
124	1... 20211111_L7_R2_IgG	2.00	1456.1	36318.4	0.040	MM
125	1... 20211111_L8_R2_IgG	2.00	1185.8	38131.9	0.031	MM
126	1... 20211111_H4_R2_IgG	2.01	2966.3	46849.6	0.063	MM
127	1... 20211111_A9_R2_IgG	2.00	519.9	47759.6	0.011	bb
128	1... 20211111_A10_R2_IgG	1.99	539.7	41366.2	0.013	bb
129	1... 20211111_L9_R2_IgG	2.01	1075.9	43052.1	0.025	MM
130	1... 20211111_L10_R2_IgG	2.02	1167.7	31487.4	0.037	bb
131	1... 20211111_QC_R11_IgG	2.01	1877.4	37804.6	0.050	MM
132	1... 20211111_H5_R2_IgG	2.00	1935.1	40380.0	0.048	bb
133	1... 20211111_A11_R2_IgG	2.01	755.4	49533.3	0.015	MM
134	1... 20211111_A12_R2_IgG	2.01	1517.1	40226.3	0.038	MM
135	1... 20211111_L11_R2_IgG	2.01	876.6	28884.0	0.030	MM
136	1... 20211111_L12_R2_IgG	2.01	1378.7	34351.3	0.040	MM
137	1... 20211111_H6_R2_IgG	2.01	1483.2	40466.6	0.037	MM
138	1... 20211111_A13_R2_IgG	2.01	3586.9	42306.7	0.085	MM
139	1... 20211111_A14_R2_IgG	2.02	878.4	44904.0	0.020	MM
140	1... 20211111_L13_R2_IgG	2.00	1185.3	31913.0	0.037	bb
141	1... 20211111_L14_R2_IgG	2.01	1711.4	31527.4	0.054	bb
142	1... 20211111_H7_R2_IgG	2.01	1493.8	40838.2	0.037	bb
143	1... 20211111_A15_R2_IgG	2.01	4246.7	49389.7	0.086	bb
144	1... 20211111_QC_R12_IgG	2.01	1419.1	35168.7	0.040	bb
145	1... 20211111_A16_R2_IgG	2.00	786.0	47440.5	0.017	MM
146	1... 20211111_L15_R2_IgG	2.02	1905.2	33145.9	0.057	MM
147	1... 20211111_L16_R2_IgG	2.00	643.3	37596.6	0.017	bb
148	1... 20211111_H8_R2_IgG	2.01	614.0	43933.3	0.014	bb
149	1... 20211111_A17_R2_IgG	2.00	2674.6	43605.5	0.061	MM
150	1... 20211111_A18_R2_IgG	2.02	578.4	37581.8	0.015	MM
151	1... 20211111_L17_R2_IgG	2.02	2004.9	33942.5	0.059	MM
152	1... 20211111_L18_R2_IgG	2.00	625.7	37831.5	0.017	MM
153	1... 20211111_H9_R2_IgG	2.01	1686.5	30898.4	0.055	MM
154	1... 20211111_A19_R2_IgG	2.00	4194.8	39352.1	0.107	MM
155	1... 20211111_A20_R2_IgG	2.00	431.6	34661.2	0.012	MM
156	1... 20211111_L19_R2_IgG	2.00	1313.4	39918.3	0.033	MM
157	1... 20211111_QC_R13_IgG	2.01	1720.2	36239.1	0.047	MM
158	1... 20211111_L20_R2_IgG	2.02	1885.8	32897.7	0.057	MM
159	1... 20211111_H10_R2_IgG	2.00	3246.7	31394.5	0.103	bb
160	1... 20211111_A21_R2_IgG	2.01	1046.1	34863.7	0.030	MM
161	1... 20211111_A22_R2_IgG	2.00	1245.6	30342.1	0.041	MM
162	1... 20211111_L21_R2_IgG	1.99	703.5	33706.2	0.021	MM
163	1... 20211111_L22_R2_IgG	2.01	1084.5	31298.6	0.035	MM
164	1... 20211111_H11_R2_IgG	2.01	955.1	38904.1	0.025	MM
165	1... 20211111_A23_R2_IgG	2.01	1181.2	32382.3	0.036	MM
166	1... 20211111_A24_R2_IgG	2.00	1736.9	30261.9	0.057	bb
167	1... 20211111_L23_R2_IgG	2.00	2124.8	43037.5	0.049	MM
168	1... 20211111_L24_R2_IgG	2.00	1366.3	37023.3	0.037	MM

Dataset: C:\MassLynx\10.pro\20211115_clinicalsamples_IgG.qld

Last Altered: Sunday, October 02, 2022 16:02:30 台北標準時間
Printed: Friday, August 01, 2025 16:48:00 台北標準時間

	# Name	RT	Area	IS Area	Response	Prima...
169	1... 20211111_H12_R2_IgG	2.00	1128.7	39478.2	0.029	bb
170	1... 20211111_QC_R14_IgG	2.00	1903.3	34463.2	0.055	MM
171	1... 20211111_A25_R2_IgG	2.00	1407.6	39945.3	0.035	bb
172	1... 20211111_A26_R2_IgG	2.00	477.7	29663.2	0.016	bb
173	1... 20211111_L25_R2_IgG	2.00	546.3	38589.3	0.014	MM
174	1... 20211111_L26_R2_IgG	2.01	481.9	38405.2	0.013	bb
175	1... 20211111_H13_R2_IgG	2.01	2070.0	37771.2	0.055	MM
176	1... 20211111_A27_R2_IgG	2.00	1047.3	42634.1	0.025	MM
177	1... 20211111_A28_R2_IgG	2.01	1259.6	33231.1	0.038	MM
178	1... 20211111_L27_R2_IgG	2.00	1731.5	41761.1	0.041	bb
179	1... 20211111_L28_R2_IgG	2.00	938.4	26968.6	0.035	MM
180	1... 20211111_H14_R2_IgG	2.01	2056.4	49250.2	0.042	MM
181	1... 20211111_A29_R2_IgG	2.01	363.3	37601.7	0.010	MM
182	1... 20211111_A30_R2_IgG	2.02	481.1	40366.8	0.012	MM
183	1... 20211111_QC_R15_IgG	2.01	1110.1	35156.1	0.032	MM
184	1... 20211111_L29_R2_IgG	2.01	782.8	33813.1	0.023	MM
185	1... 20211111_L30_R2_IgG	2.00	509.8	33488.6	0.015	bb
186	1... 20211111_H15_R2_IgG	2.00	2599.3	38501.0	0.068	MM
187	1... 20211111_A31_R2_IgG	2.01	661.4	37525.5	0.018	MM
188	1... 20211111_A32_R2_IgG	2.01	1299.7	35770.8	0.036	MM
189	1... 20211111_L31_R2_IgG	2.01	444.6	33282.4	0.013	MM
190	1... 20211111_L32_R2_IgG	2.00	1401.8	42049.6	0.033	MM
191	1... 20211111_H16_R2_IgG	2.00	1982.7	45947.6	0.043	bb
192	1... 20211111_A33_R2_IgG	2.01	2401.8	40202.3	0.060	MM
193	1... 20211111_A34_R2_IgG	2.01	1153.6	36747.0	0.031	MM
194	1... 20211111_H17_R2_IgG	2.00	12035.6	40920.9	0.294	MM
195	1... 20211111_A35_R2_IgG	2.00	2937.1	33689.2	0.087	bb
196	1... 20211111_QC_R16_IgG	2.00	1215.6	32783.6	0.037	MM
197	1... 20211111_A36_R2_IgG	2.01	713.2	36822.4	0.019	MM
198	1... 20211111_H18_R2_IgG	2.01	7846.3	31769.3	0.247	MM
199	1... 20211111_A37_R2_IgG	2.00	4668.9	37581.8	0.124	MM
200	2... 20211111_A38_R2_IgG	2.00	981.7	38390.4	0.026	MM
201	2... 20211111_H19_R2_IgG	2.00	6260.8	41217.0	0.152	bb
202	2... 20211111_A39_R2_IgG	2.00	2216.5	37392.7	0.059	bb
203	2... 20211111_A40_R2_IgG	2.00	756.1	38295.1	0.020	bb
204	2... 20211111_H20_R2_IgG	2.01	6752.8	35941.8	0.188	bb
205	2... 20211111_A41_R2_IgG	2.01	2250.6	45304.9	0.050	MM
206	2... 20211111_A42_R2_IgG	2.01	765.4	37803.8	0.020	MM
207	2... 20211111_A43_R2_IgG	2.01	1345.1	38161.8	0.035	bb
208	2... 20211111_A44_R2_IgG	2.00	3360.4	37819.8	0.089	MM
209	2... 20211111_QC_R17_IgG	2.00	1245.0	31731.8	0.039	MM

Dataset: C:\MassLynx\10.pro\20211115_clinicalsamples_IgG.qld

Last Altered: Sunday, October 02, 2022 16:02:30 台北標準時間
Printed: Friday, August 01, 2025 16:48:37 台北標準時間

Method: C:\MassLynx\10_Pro_IgA.PRO\MethDB\20210926_IgG_DMRM_IgM_IS.mdb 16 Feb 2022 11:58:21
Calibration: 18 Nov 2021 13:56:22

Sample Name: 20211111_QC_R1_IgG

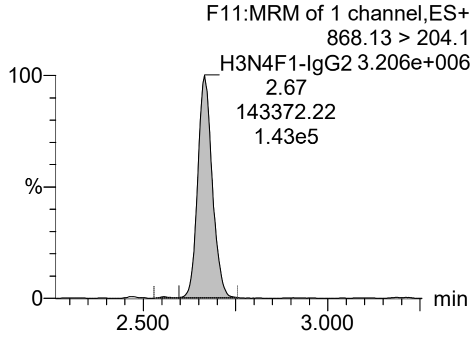

Sample Name: 20211111_A1_R1_IgG

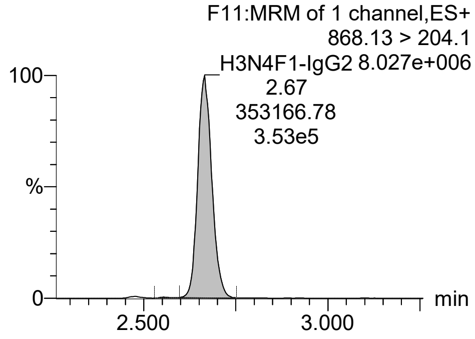

Sample Name: 20211111_A2_R1_IgG

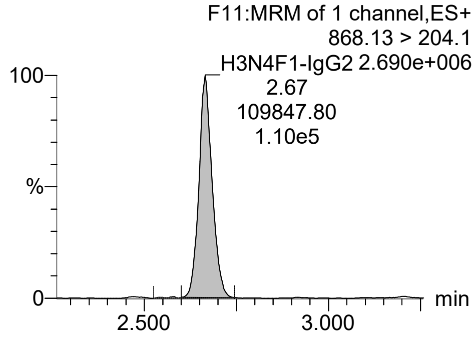

Sample Name: 20211111_L1_R1_IgG

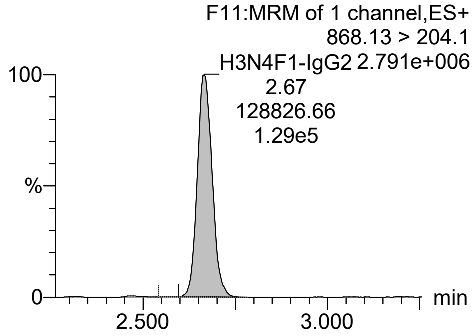

Sample Name: 20211111_L2_R1_IgG

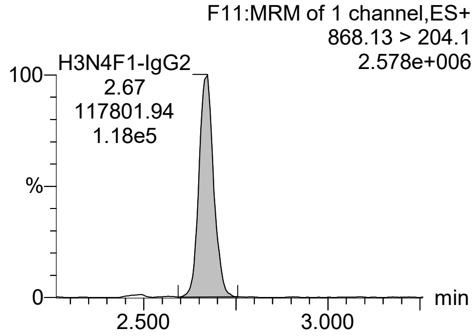

Sample Name: 20211111_H1_R1_IgG

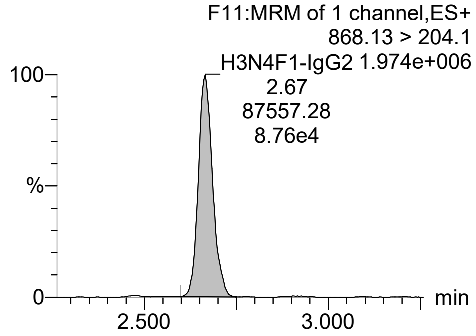

Sample Name: 20211111_A3_R1_IgG

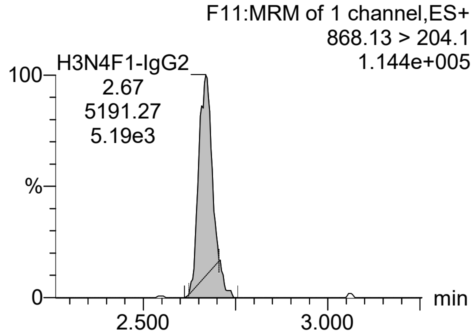

Sample Name: 20211111_A4_R1_IgG

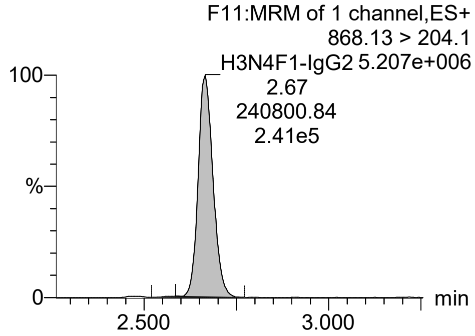

Sample Name: 20211111_L3_R1_IgG

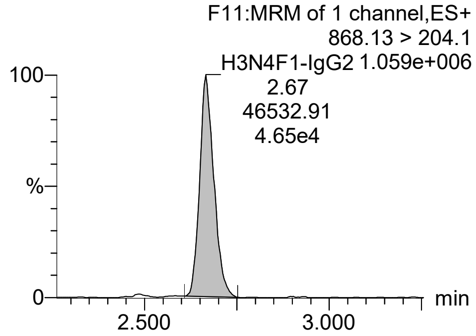

Sample Name: 20211111_L4_R1_IgG

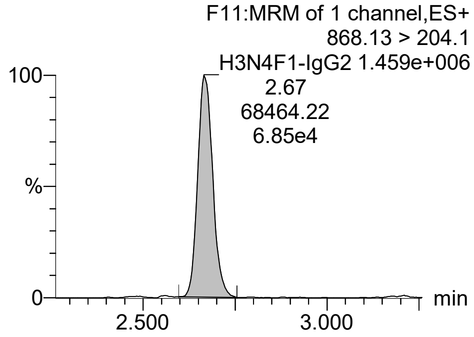

Sample Name: 20211111_H2_R1_IgG

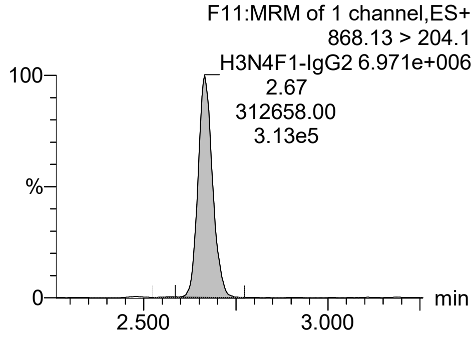

Sample Name: 20211111_A5_R1_IgG

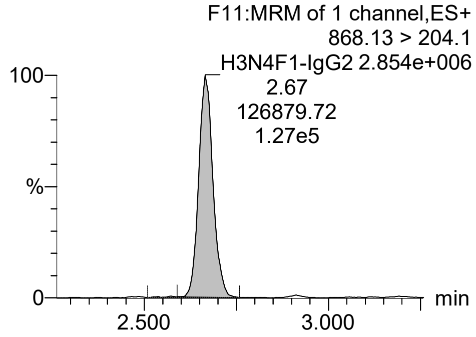

Dataset: C:\MassLynx\10.pro\20211115_clinicalsamples_IgG.qld

Last Altered: Sunday, October 02, 2022 16:02:30 台北標準時間
Printed: Friday, August 01, 2025 16:48:37 台北標準時間

Sample Name: 20211111_A6_R1_IgG

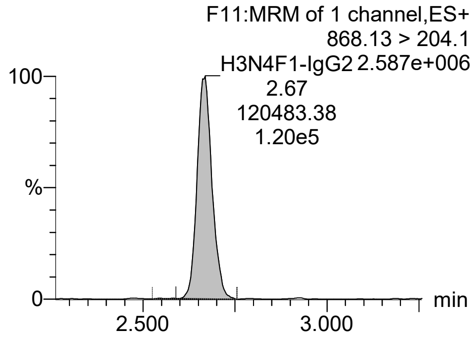

Sample Name: 20211111_QC_R2_IgG

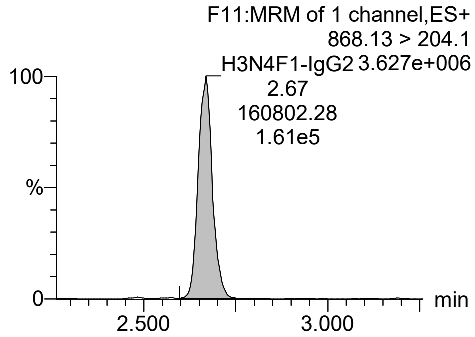

Sample Name: 20211111_L5_R1_IgG

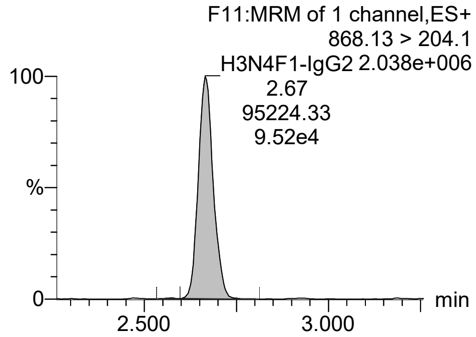

Sample Name: 20211111_L6_R1_IgG

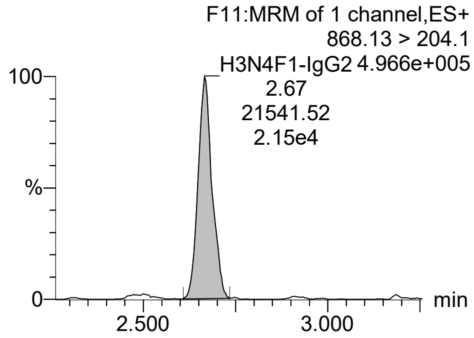

Sample Name: 20211111_H3_R1_IgG

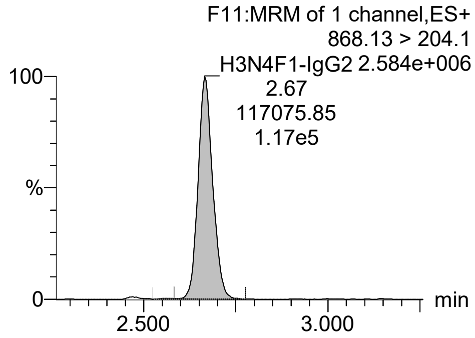

Sample Name: 20211111_A7_R1_IgG

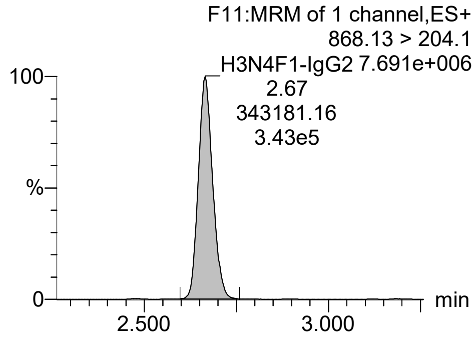

Sample Name: 20211111_A8_R1_IgG

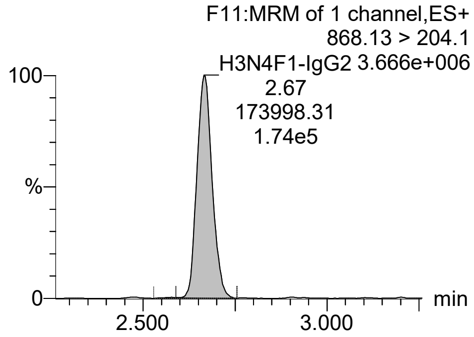

Sample Name: 20211111_L7_R1_IgG

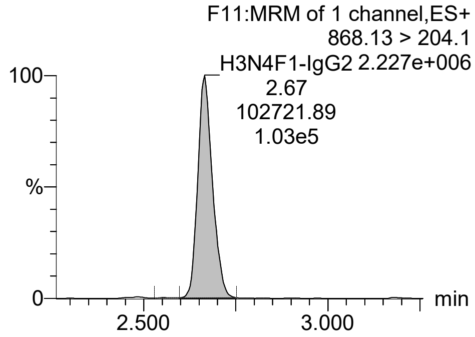

Sample Name: 20211111_L8_R1_IgG

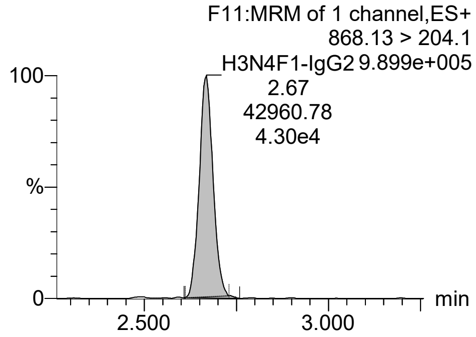

Sample Name: 20211111_H4_R1_IgG

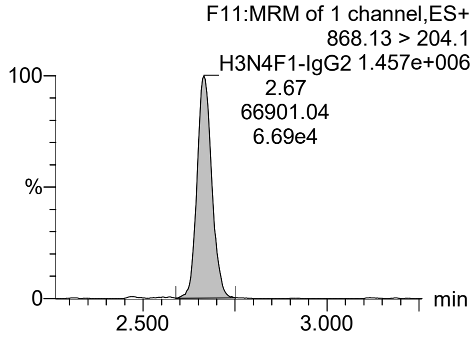

Sample Name: 20211111_A9_R1_IgG

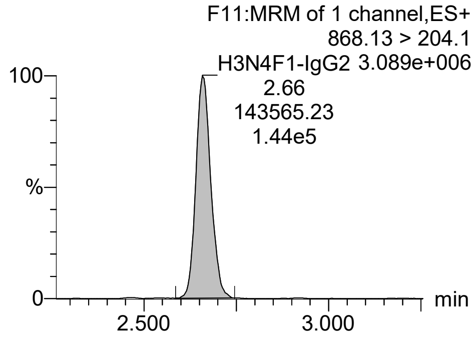

Sample Name: 20211111_A10_R1_IgG

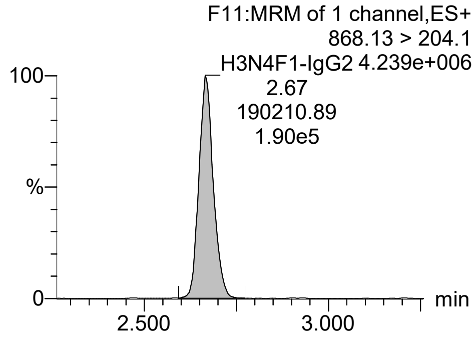

Sample Name: 20211111_L9_R1_IgG

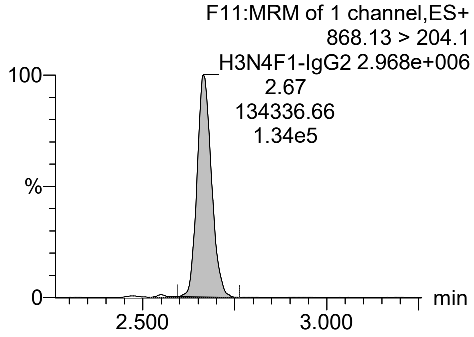

Sample Name: 20211111_L10_R1_IgG

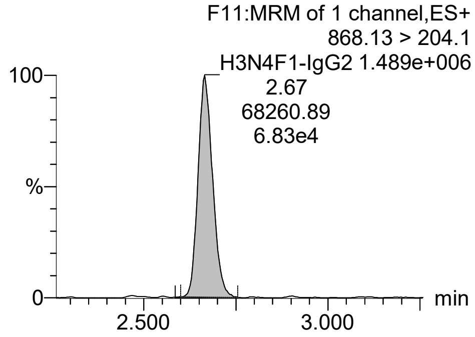

Sample Name: 20211111_QC_R3_IgG

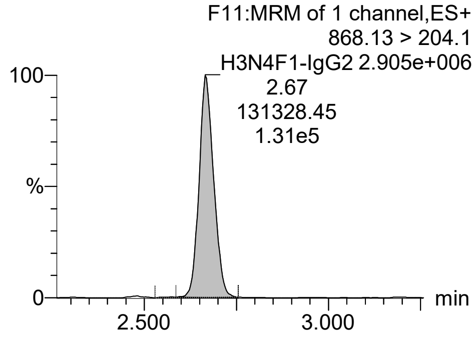

Sample Name: 20211111_H5_R1_IgG

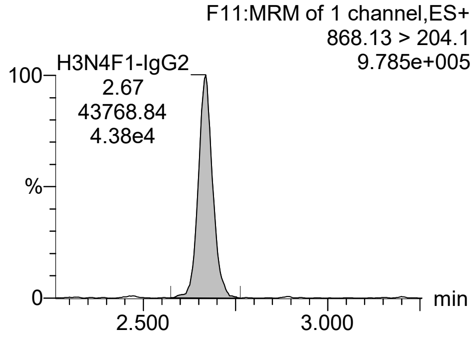

Sample Name: 20211111_A11_R1_IgG

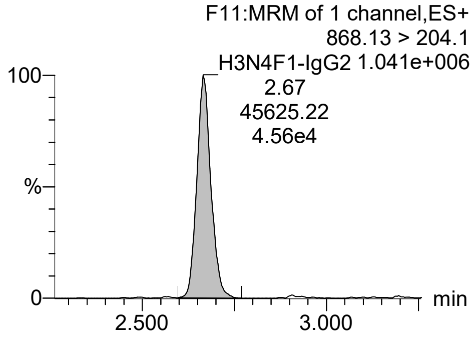

Sample Name: 20211111_A12_R1_IgG

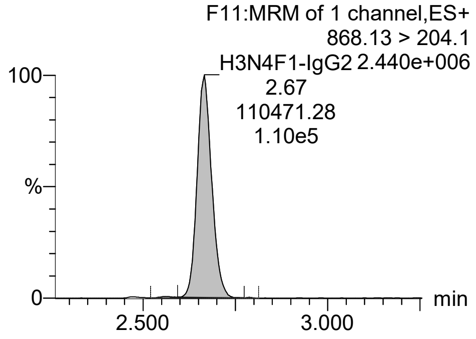

Sample Name: 20211111_L11_R1_IgG

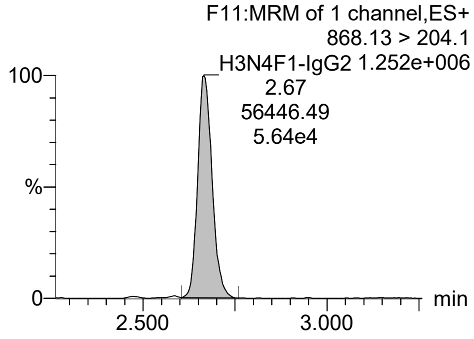

Sample Name: 20211111_L12_R1_IgG

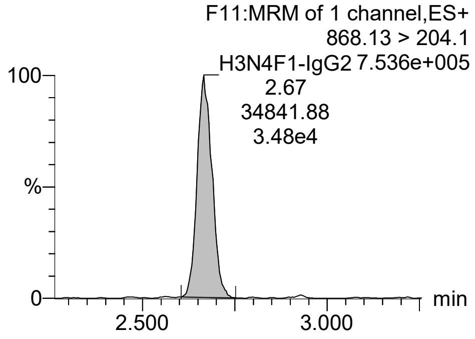

Sample Name: 20211111_H6_R1_IgG

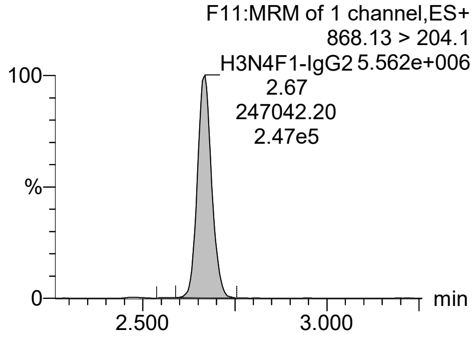

Sample Name: 20211111_A13_R1_IgG

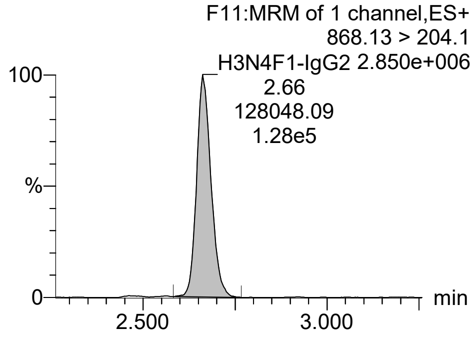

Sample Name: 20211111_A14_R1_IgG

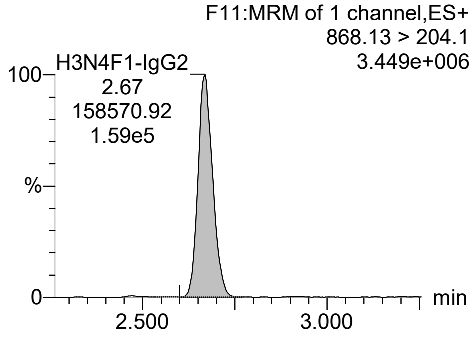

Sample Name: 20211111_L13_R1_IgG

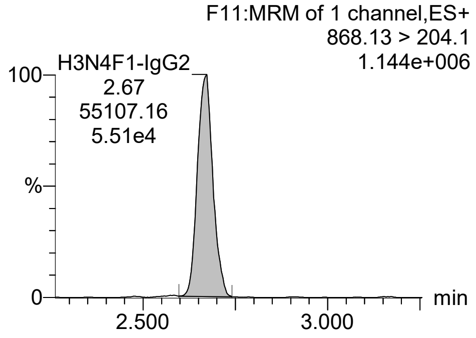

Sample Name: 20211111_L14_R1_IgG

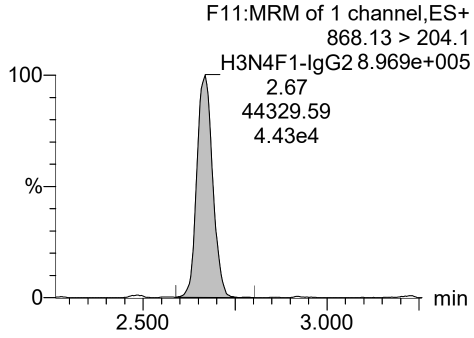

Sample Name: 20211111_H7_R1_IgG

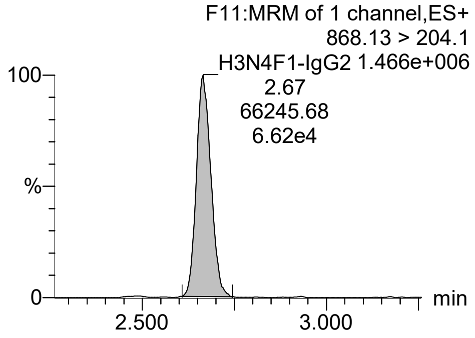

Sample Name: 20211111_A15_R1_IgG

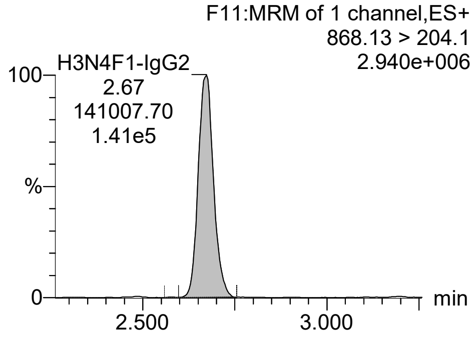

Sample Name: 20211111_QC_R4_IgG

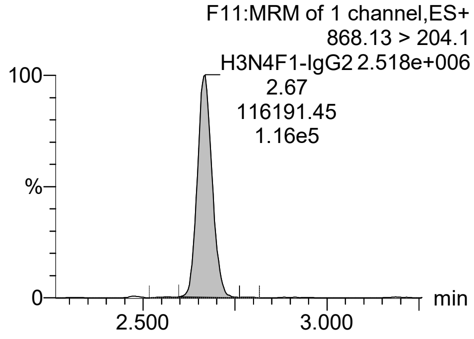

Sample Name: 20211111_A16_R1_IgG

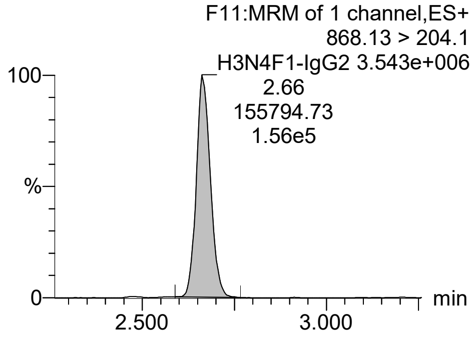

Sample Name: 20211111_L15_R1_IgG

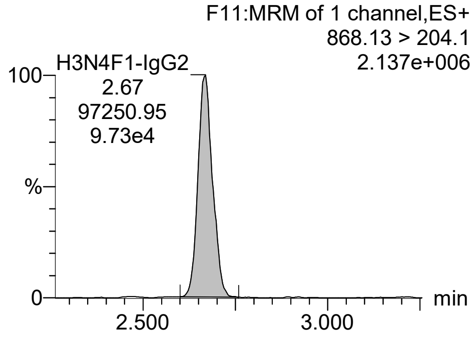

Sample Name: 20211111_L16_R1_IgG

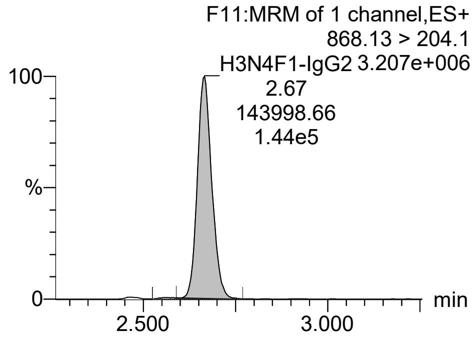

Sample Name: 20211111_H8_R1_IgG

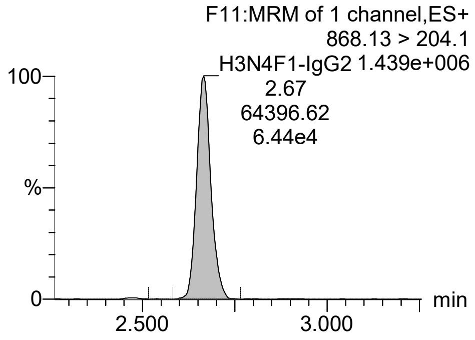

Sample Name: 20211111_A17_R1_IgG

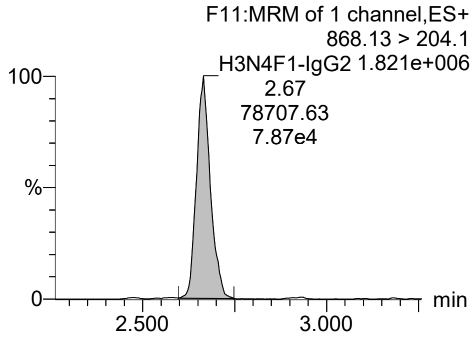

Sample Name: 20211111_A18_R1_IgG

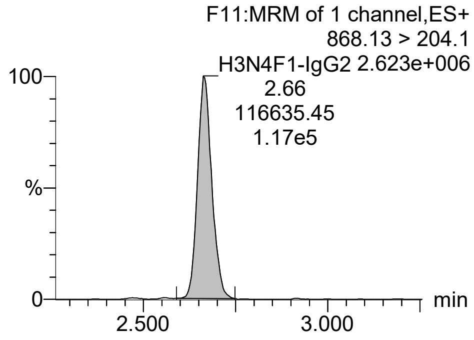

Sample Name: 20211111_L17_R1_IgG

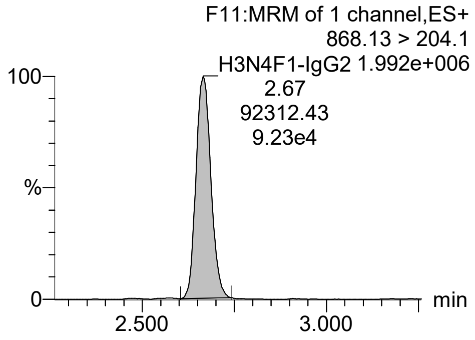

Sample Name: 20211111_L18_R1_IgG

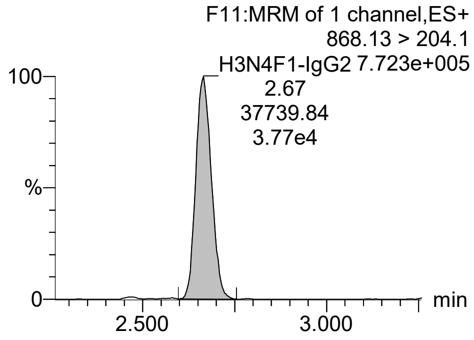

Sample Name: 20211111_H9_R1_IgG

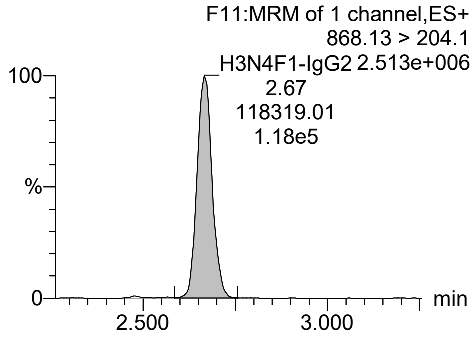

Sample Name: 20211111_A19_R1_IgG

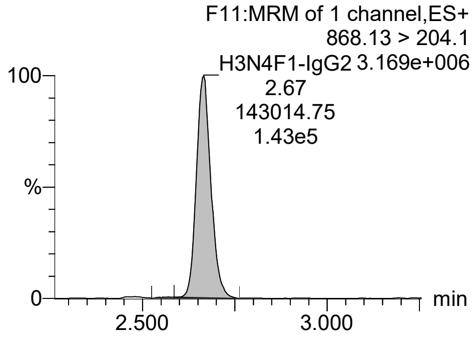

Sample Name: 20211111_A20_R1_IgG

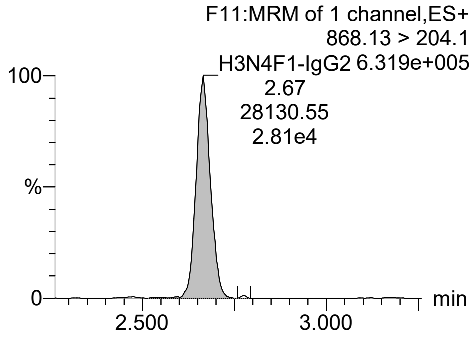

Sample Name: 20211111_L19_R1_IgG

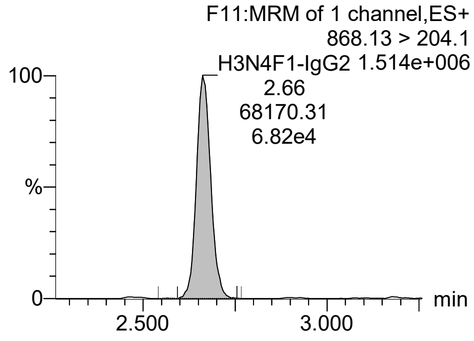

Sample Name: 20211111_QC_R5_IgG

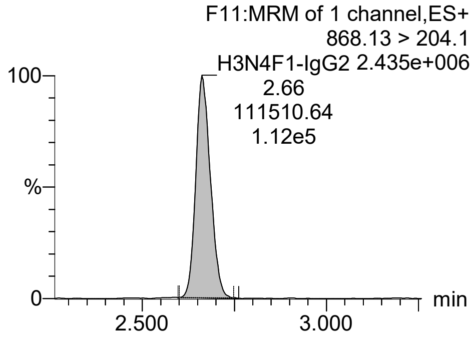

Sample Name: 20211111_L20_R1_IgG

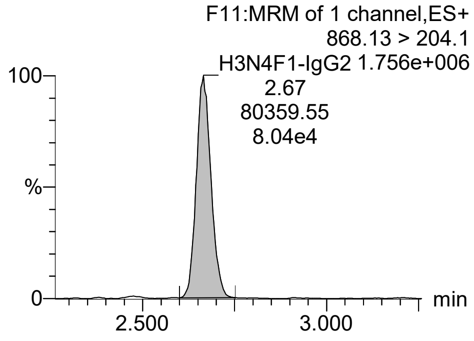

Sample Name: 20211111_H10_R1_IgG

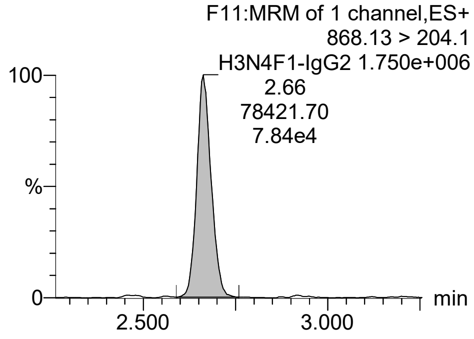

Sample Name: 20211111_A21_R1_IgG

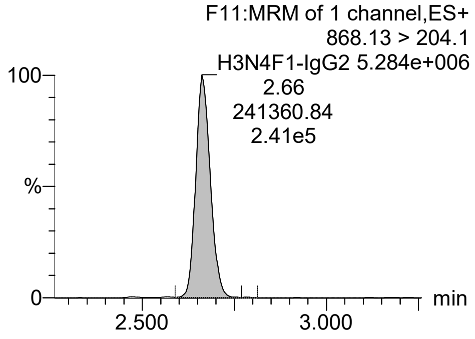

Sample Name: 20211111_A22_R1_IgG

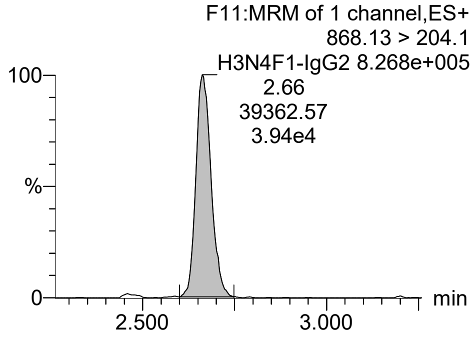

Dataset: C:\MassLynx\10.pro\20211115_clinicalsamples_IgG.qld

Last Altered: Sunday, October 02, 2022 16:02:30 台北標準時間
Printed: Friday, August 01, 2025 16:48:37 台北標準時間

Sample Name: 20211111_L21_R1_IgG

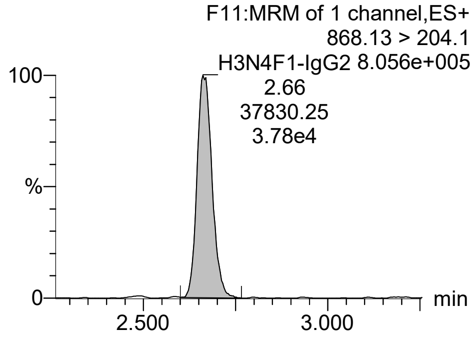

Sample Name: 20211111_L22_R1_IgG

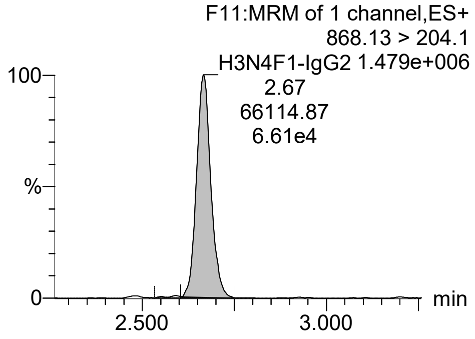

Sample Name: 20211111_H11_R1_IgG

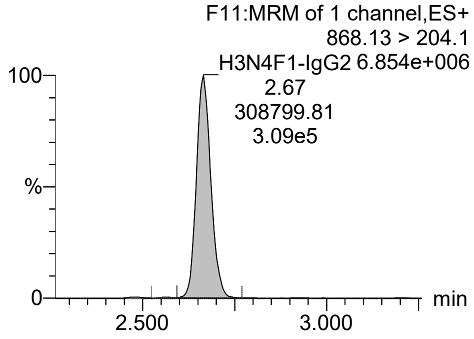

Sample Name: 20211111_A23_R1_IgG

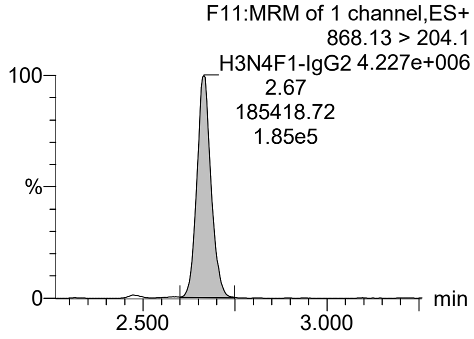

Sample Name: 20211111_A24_R1_IgG

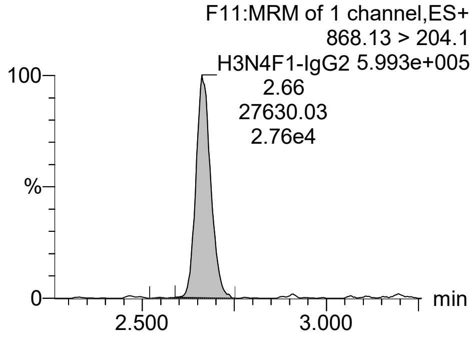

Sample Name: 20211111_L23_R1_IgG

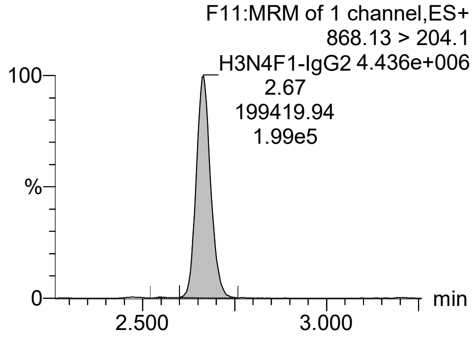

Sample Name: 20211111_L24_R1_IgG

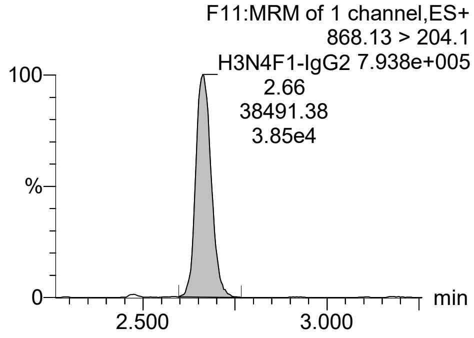

Sample Name: 20211111_H12_R1_IgG

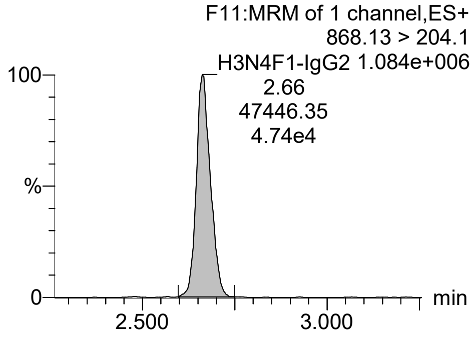

Sample Name: 20211111_QC_R6_IgG

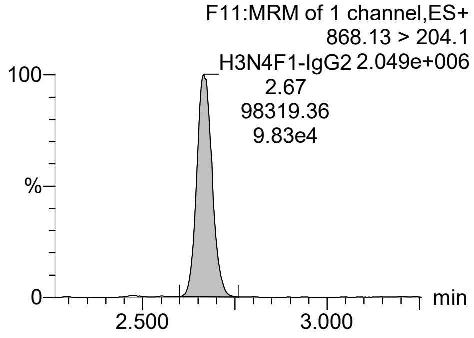

Sample Name: 20211111_A25_R1_IgG

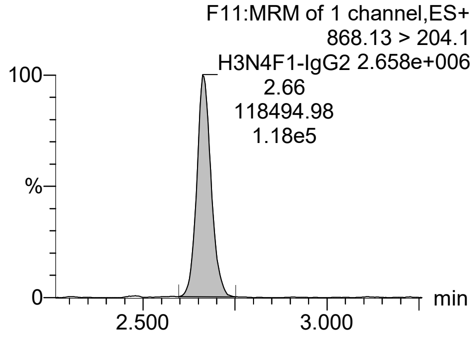

Sample Name: 20211111_A26_R1_IgG

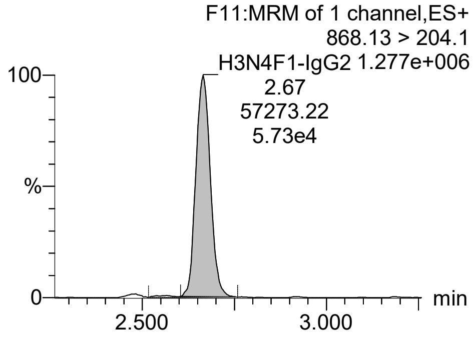

Sample Name: 20211111_L25_R1_IgG

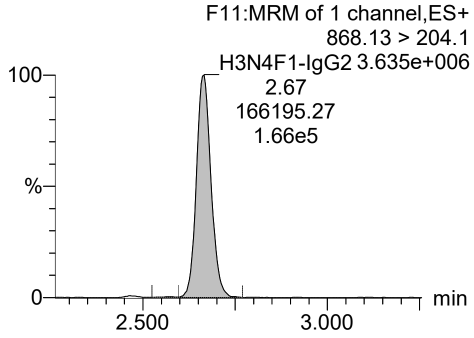

Sample Name: 20211111_L26_R1_IgG

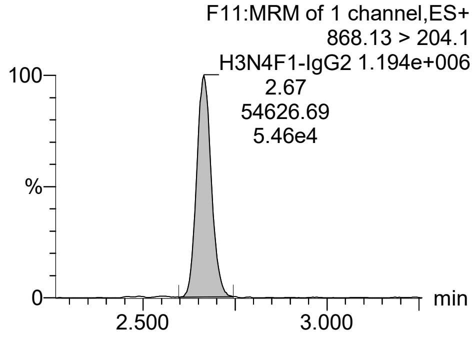

Sample Name: 20211111_H13_R1_IgG

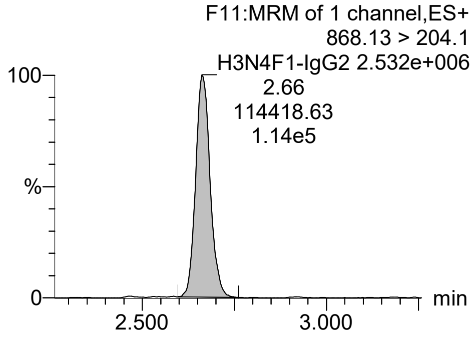

Sample Name: 20211111_A27_R1_IgG

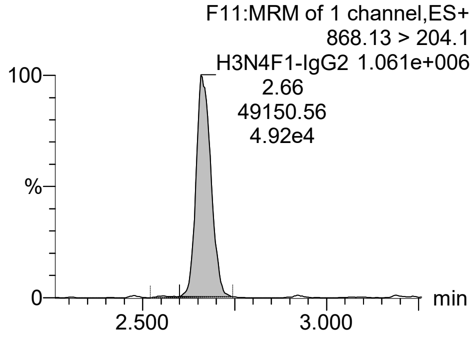

Dataset: C:\MassLynx\10.pro\20211115_clinicalsamples_IgG.qld

Last Altered: Sunday, October 02, 2022 16:02:30 台北標準時間
Printed: Friday, August 01, 2025 16:48:37 台北標準時間

Sample Name: 20211111_A28_R1_IgG

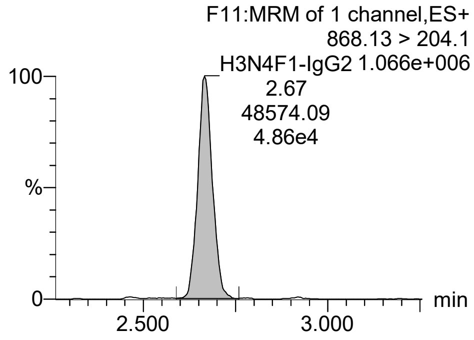

Sample Name: 20211111_L27_R1_IgG

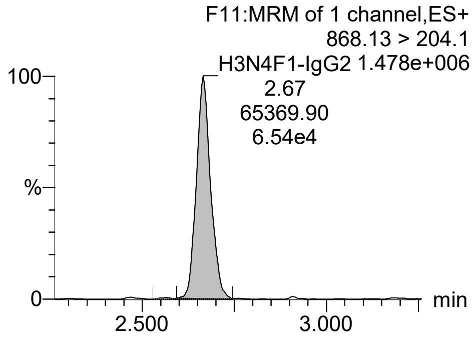

Sample Name: 20211111_L28_R1_IgG

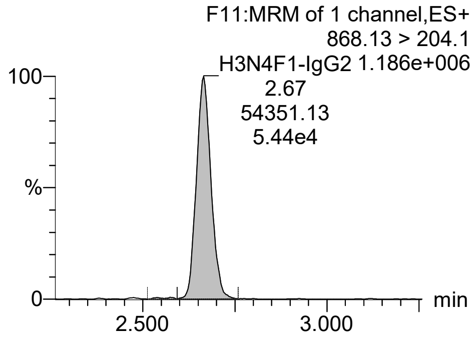

Sample Name: 20211111_H14_R1_IgG

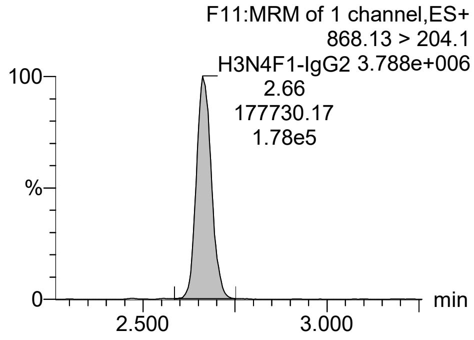

Sample Name: 20211111_A29_R1_IgG

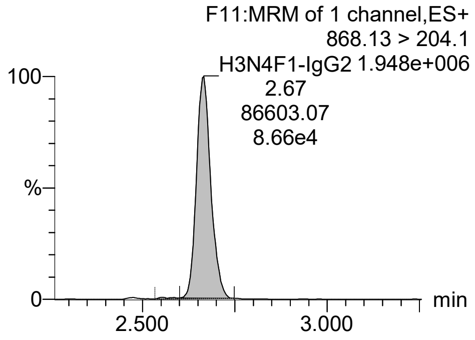

Sample Name: 20211111_A30_R1_IgG

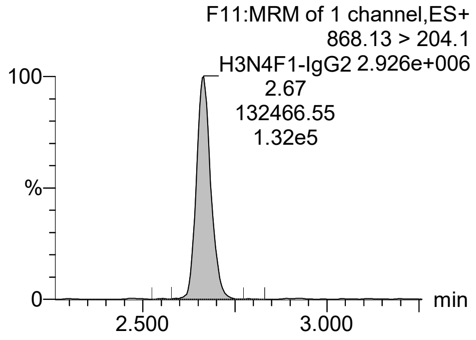

Sample Name: 20211111_QC_R7_IgG

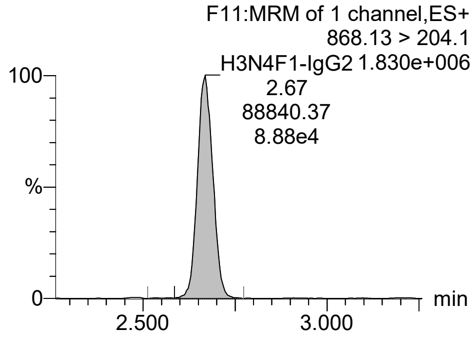

Sample Name: 20211111_L29_R1_IgG

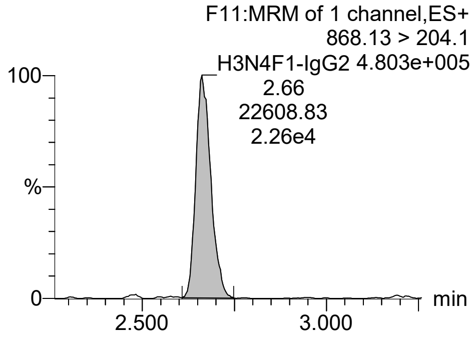

Sample Name: 20211111_L30_R1_IgG

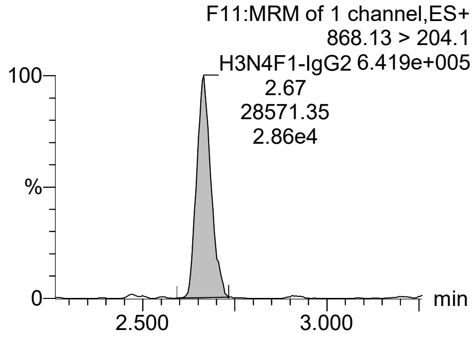

Sample Name: 20211111_H15_R1_IgG

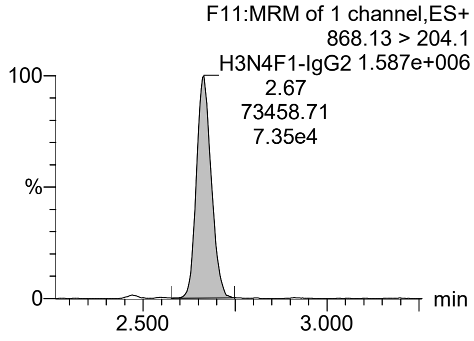

Sample Name: 20211111_A31_R1_IgG

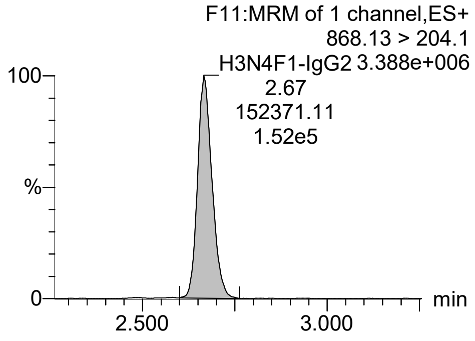

Sample Name: 20211111_A32_R1_IgG

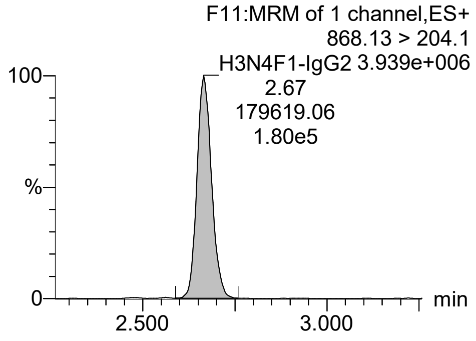

Sample Name: 20211111_L31_R1_IgG

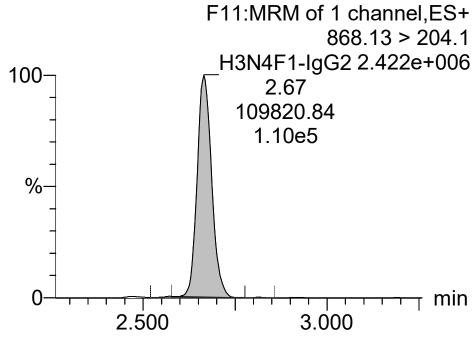

Sample Name: 20211111_L32_R1_IgG

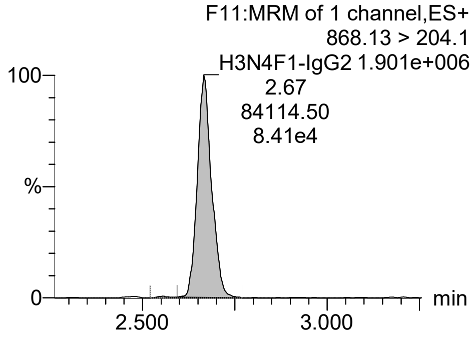

Sample Name: 20211111_H16_R1_IgG

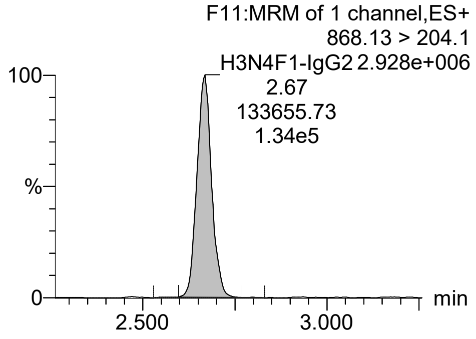

Sample Name: 20211111_A33_R1_IgG

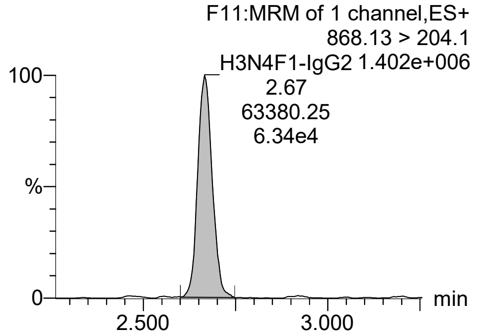

Sample Name: 20211111_A34_R1_IgG

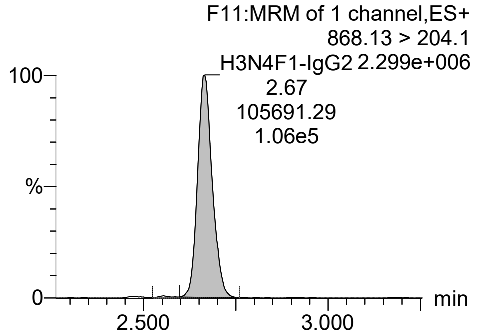

Sample Name: 20211111_H17_R1_IgG

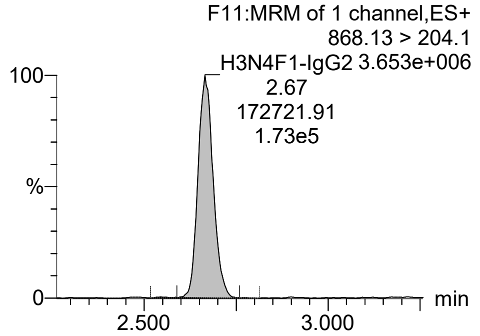

Sample Name: 20211111_A35_R1_IgG

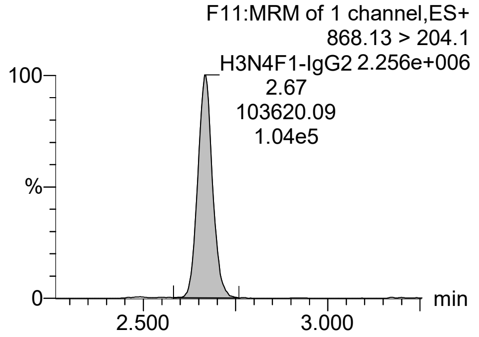

Sample Name: 20211111_QC_R8_IgG

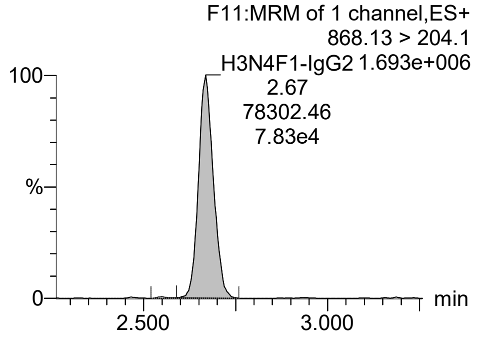

Sample Name: 20211111_A36_R1_IgG

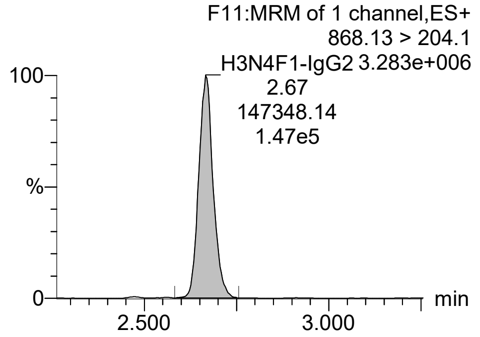

Sample Name: 20211111_H18_R1_IgG

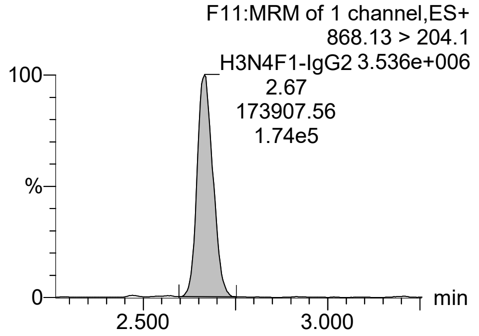

Sample Name: 20211111_A37_R1_IgG

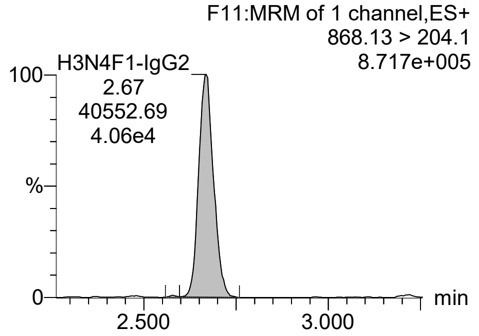

Sample Name: 20211111_A38_R1_IgG

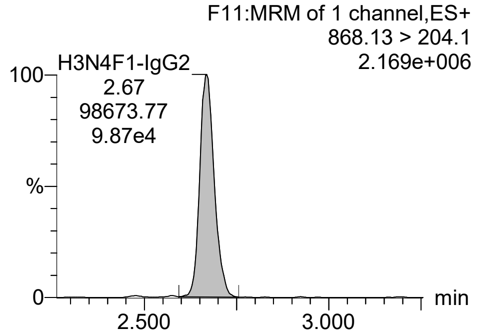

Sample Name: 20211111_H19_R1_IgG

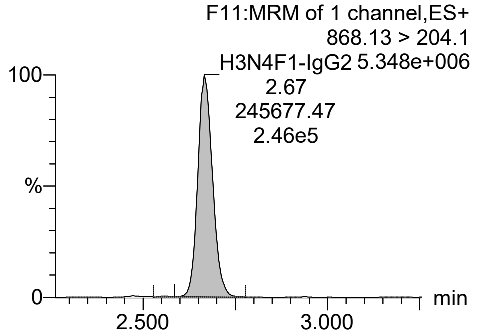

Sample Name: 20211111_A39_R1_IgG

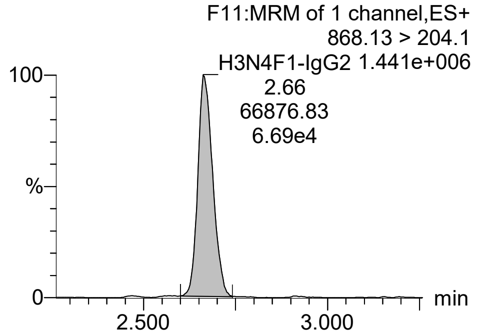

Sample Name: 20211111_A40_R1_IgG

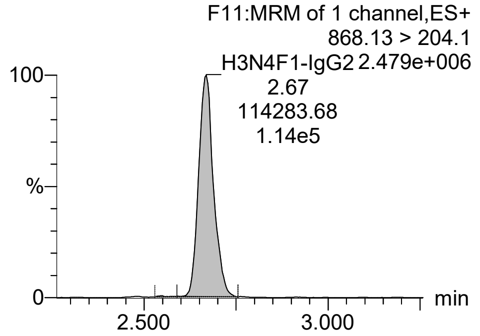

Sample Name: 20211111_H20_R1_IgG

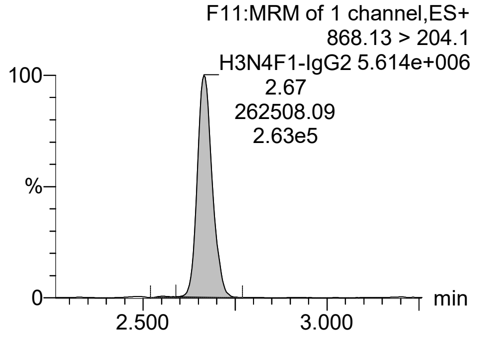

Sample Name: 20211111_A41_R1_IgG

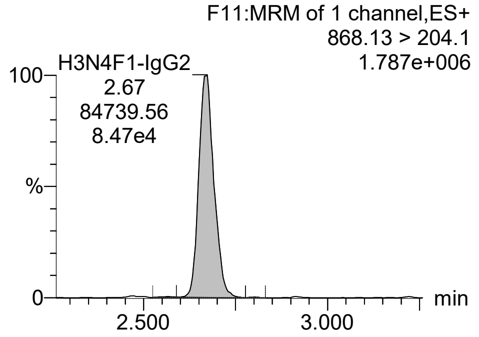

Sample Name: 20211111_A42_R1_IgG

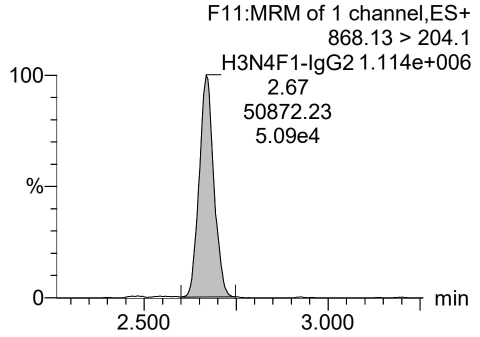

Dataset: C:\MassLynx\10.pro\20211115_clinicalsamples_IgG.qld

Last Altered: Sunday, October 02, 2022 16:02:30 台北標準時間
Printed: Friday, August 01, 2025 16:48:37 台北標準時間

Sample Name: 20211111_A43_R1_IgG

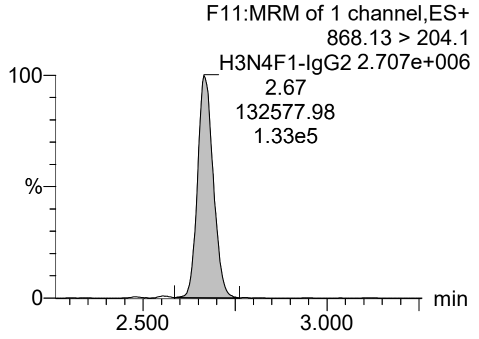

Sample Name: 20211111_A44_R1_IgG

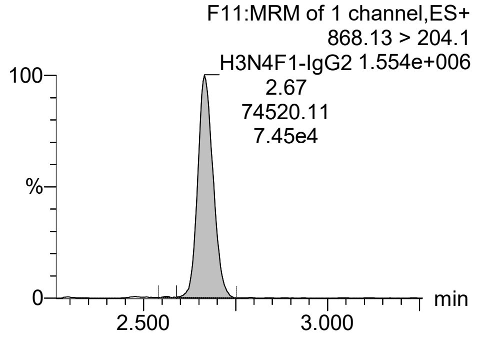

Sample Name: 20211111_QC_R9_IgG

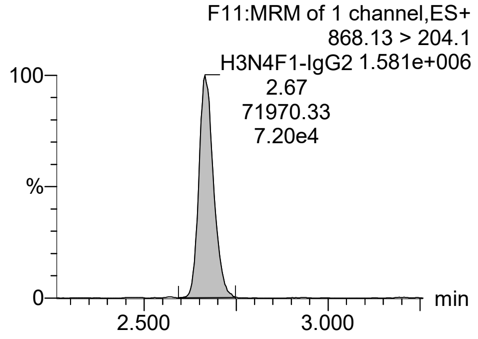

Sample Name: 20211111_A1_R2_IgG

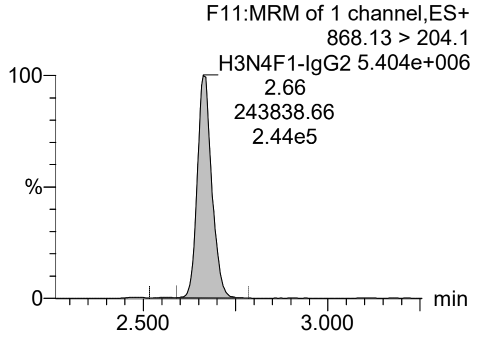

Sample Name: 20211111_A2_R2_IgG

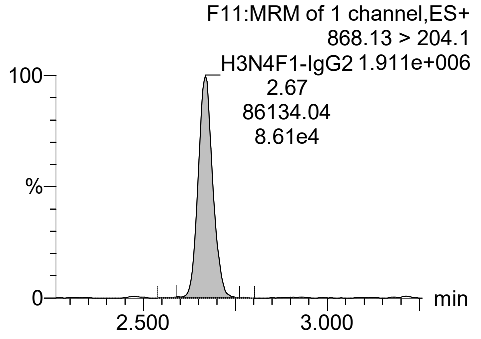

Sample Name: 20211111_L1_R2_IgG

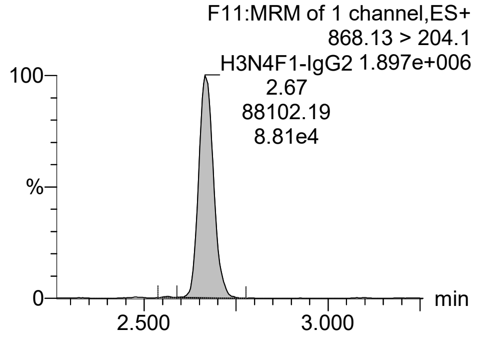

Sample Name: 20211111_L2_R2_IgG

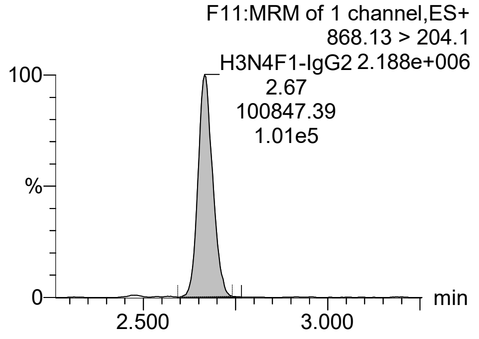

Sample Name: 20211111_H1_R2_IgG

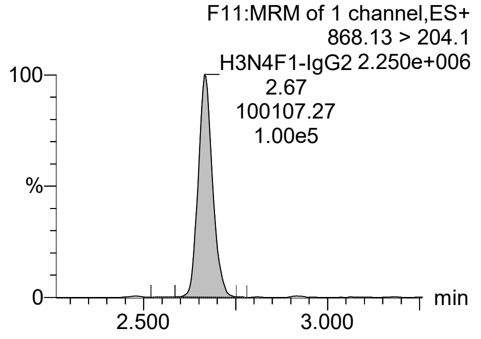

Sample Name: 20211111_A3_R2_IgG

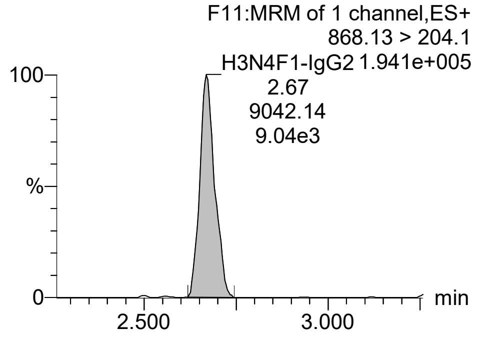

Sample Name: 20211111_A4_R2_IgG

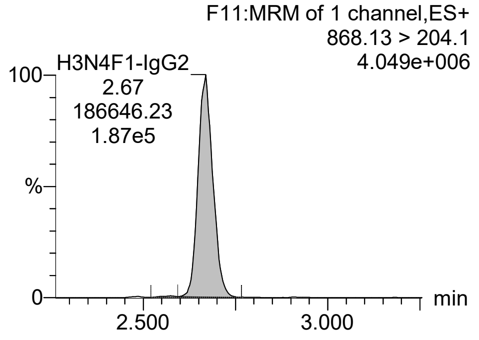

Sample Name: 20211111_L3_R2_IgG

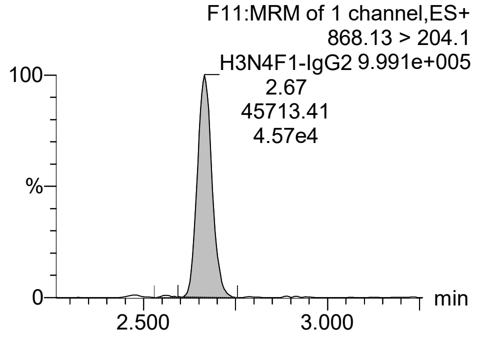

Sample Name: 20211111_L4_R2_IgG

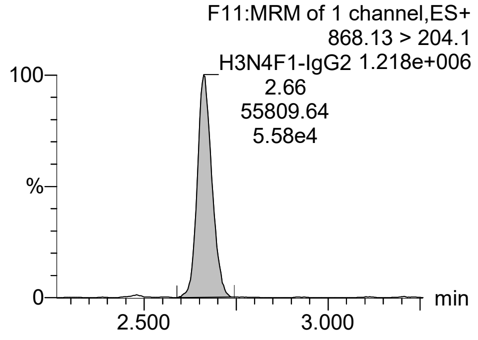

Sample Name: 20211111_H2_R2_IgG

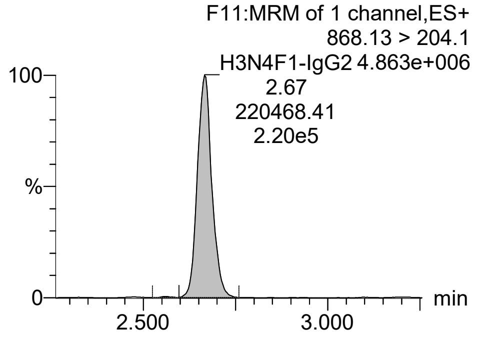

Sample Name: 20211111_A5_R2_IgG

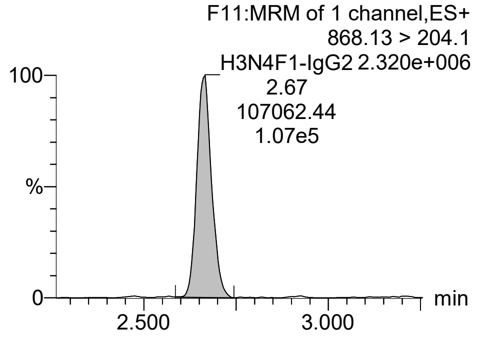

Sample Name: 20211111_A6_R2_IgG

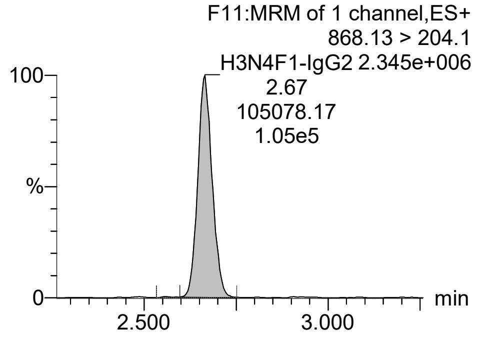

Sample Name: 20211111_QC_R10_IgG

F11:MRM of 1 channel,ES+
868.13 > 204.1
H3N4F1-IgG2 1.646e+006

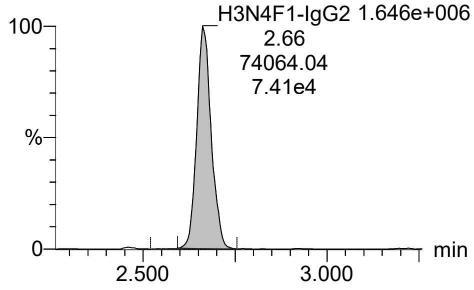

Sample Name: 20211111_L5_R2_IgG

F11:MRM of 1 channel,ES+
868.13 > 204.1
H3N4F1-IgG2 1.797e+006

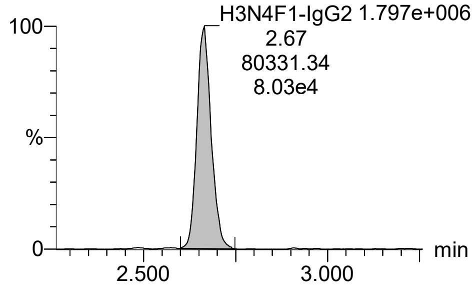

Sample Name: 20211111_L6_R2_IgG

F11:MRM of 1 channel,ES+
868.13 > 204.1
H3N4F1-IgG2 4.018e+005

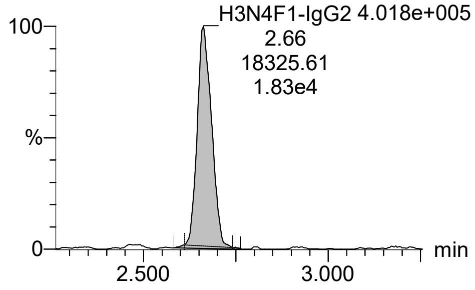

Sample Name: 20211111_H3_R2_IgG

F11:MRM of 1 channel,ES+
868.13 > 204.1
H3N4F1-IgG2 2.086e+006

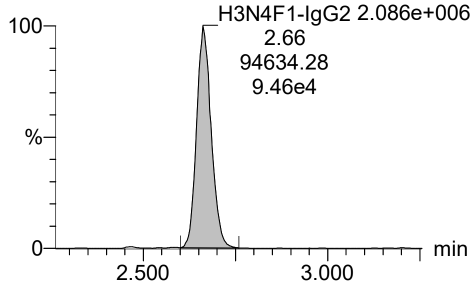

Sample Name: 20211111_A7_R2_IgG

F11:MRM of 1 channel,ES+
868.13 > 204.1
H3N4F1-IgG2 5.088e+006

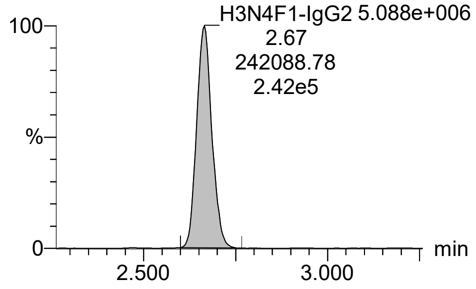

Sample Name: 20211111_A8_R2_IgG

F11:MRM of 1 channel,ES+
868.13 > 204.1
H3N4F1-IgG2 2.871e+006

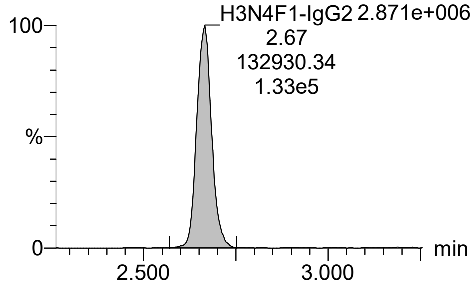

Sample Name: 20211111_L7_R2_IgG

F11:MRM of 1 channel,ES+
868.13 > 204.1
H3N4F1-IgG2 2.008e+006

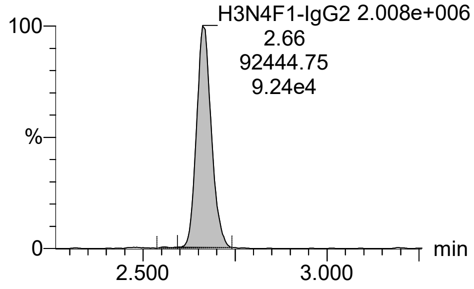

Sample Name: 20211111_L8_R2_IgG

F11:MRM of 1 channel,ES+
868.13 > 204.1
H3N4F1-IgG2 8.219e+005

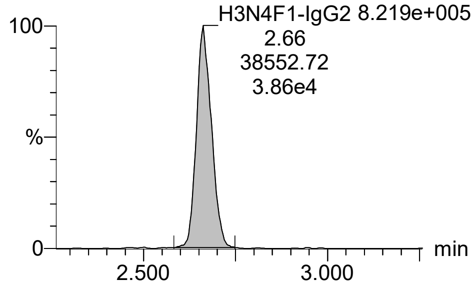

Sample Name: 20211111_H4_R2_IgG

F11:MRM of 1 channel,ES+
868.13 > 204.1
H3N4F1-IgG2 1.146e+006

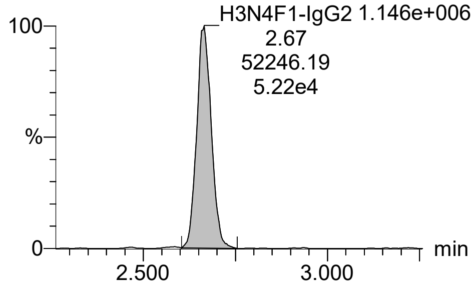

Sample Name: 20211111_A9_R2_IgG

F11:MRM of 1 channel,ES+
868.13 > 204.1
H3N4F1-IgG2 2.371e+006

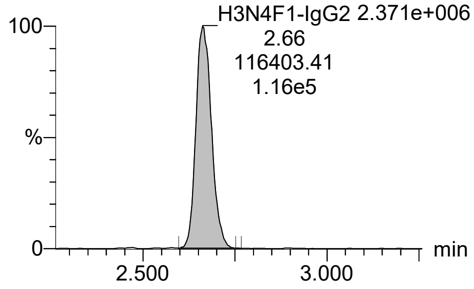

Sample Name: 20211111_A10_R2_IgG

F11:MRM of 1 channel,ES+
868.13 > 204.1
H3N4F1-IgG2 3.694e+006

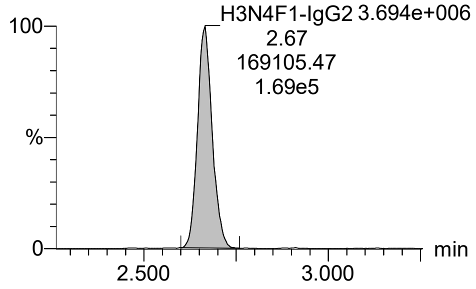

Sample Name: 20211111_L9_R2_IgG

F11:MRM of 1 channel,ES+
868.13 > 204.1
H3N4F1-IgG2 2.400e+006

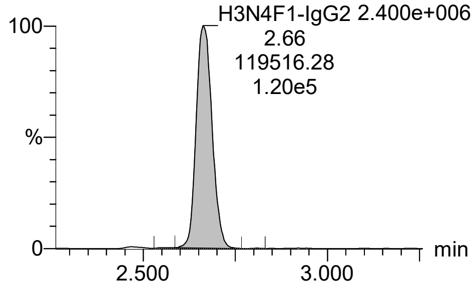

Sample Name: 20211111_L10_R2_IgG

F11:MRM of 1 channel,ES+
868.13 > 204.1
H3N4F1-IgG2 1.351e+006

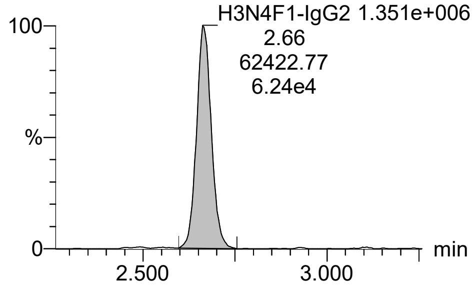

Sample Name: 20211111_QC_R11_IgG

F11:MRM of 1 channel,ES+
868.13 > 204.1
H3N4F1-IgG2 1.573e+006

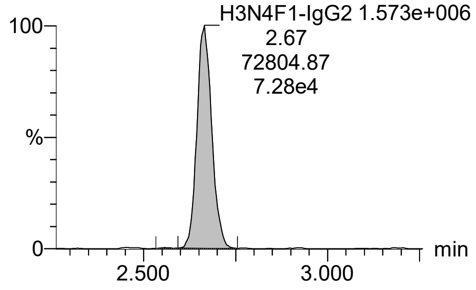

Sample Name: 20211111_H5_R2_IgG

F11:MRM of 1 channel,ES+
868.13 > 204.1
H3N4F1-IgG2 8.347e+005

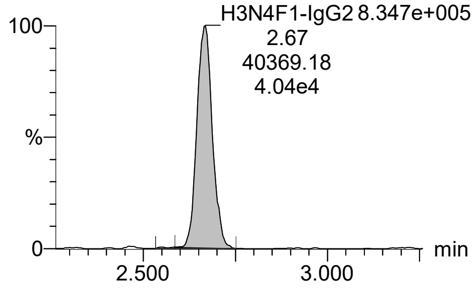

Sample Name: 20211111_A11_R2_IgG

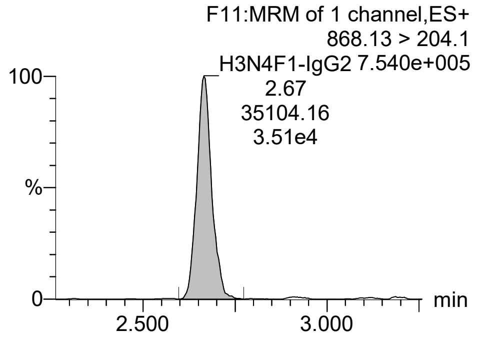

Sample Name: 20211111_A12_R2_IgG

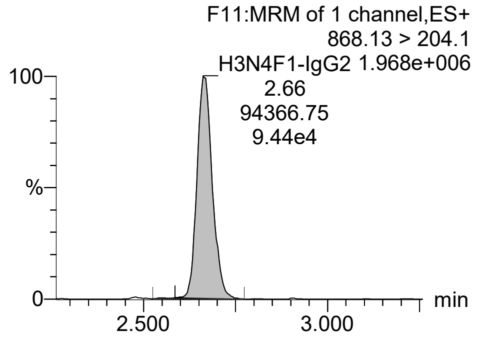

Sample Name: 20211111_L11_R2_IgG

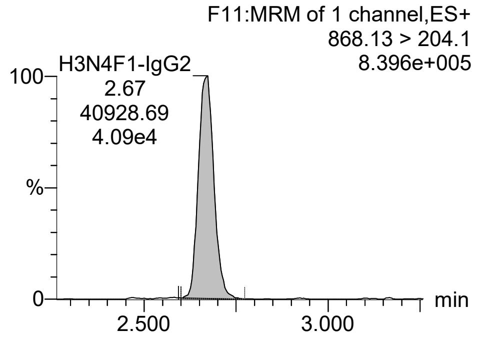

Sample Name: 20211111_L12_R2_IgG

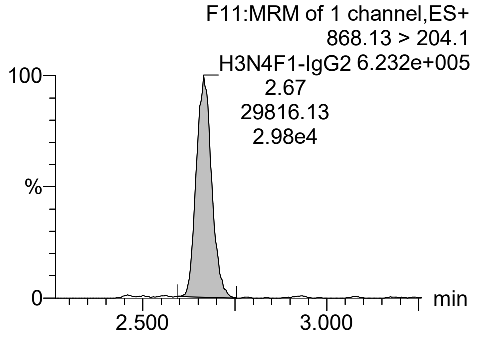

Sample Name: 20211111_H6_R2_IgG

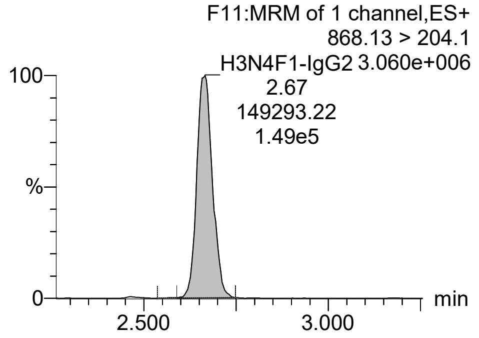

Sample Name: 20211111_A13_R2_IgG

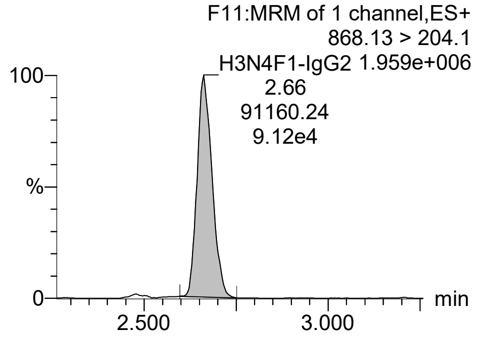

Sample Name: 20211111_A14_R2_IgG

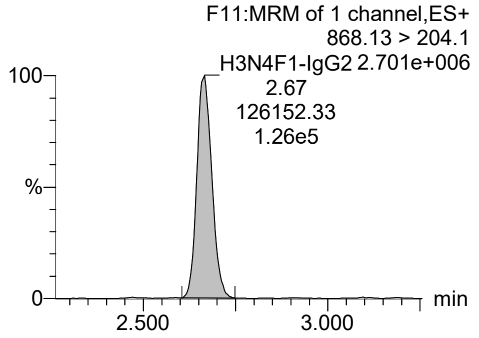

Sample Name: 20211111_L13_R2_IgG

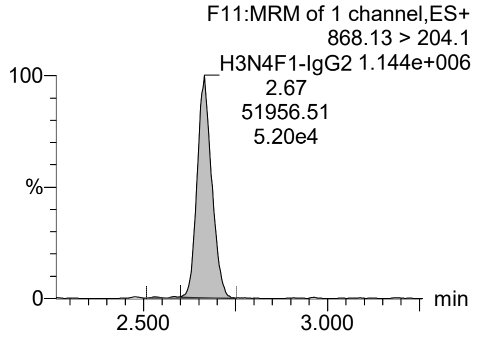

Sample Name: 20211111_L14_R2_IgG

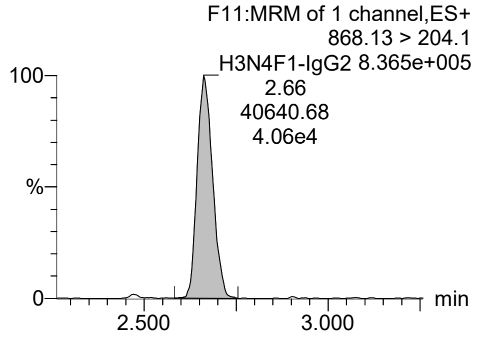

Sample Name: 20211111_H7_R2_IgG

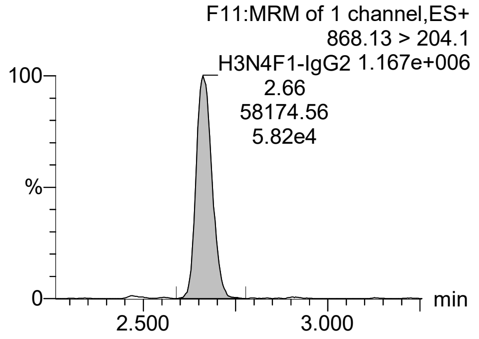

Sample Name: 20211111_A15_R2_IgG

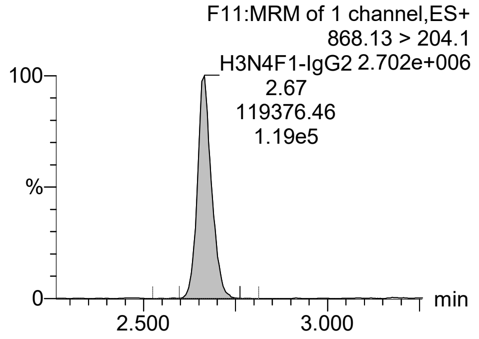

Sample Name: 20211111_QC_R12_IgG

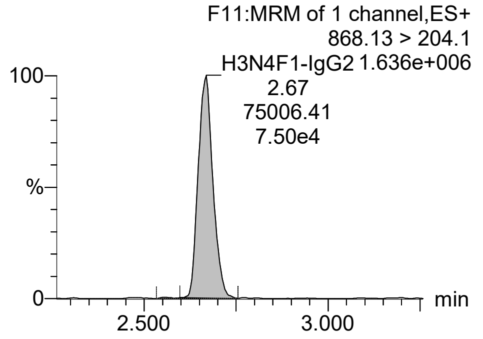

Sample Name: 20211111_A16_R2_IgG

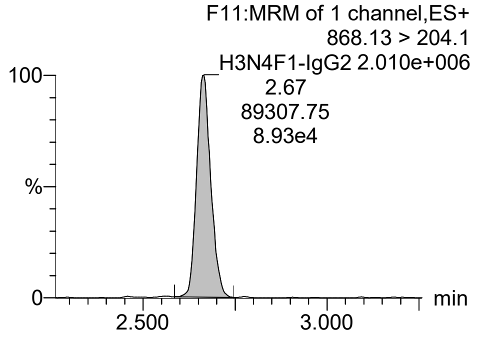

Sample Name: 20211111_L15_R2_IgG

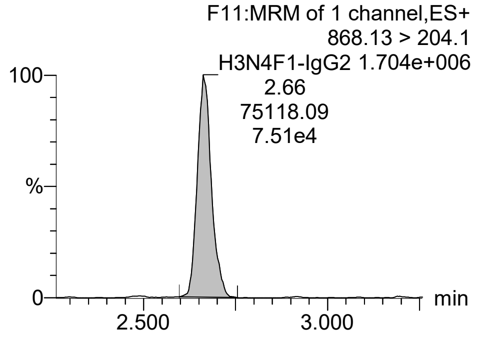

Sample Name: 20211111_L16_R2_IgG

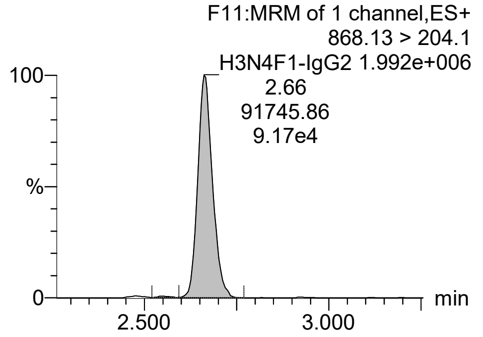

Dataset: C:\MassLynx\10.pro\20211115_clinicalsamples_IgG.qld

Last Altered: Sunday, October 02, 2022 16:02:30 台北標準時間
Printed: Friday, August 01, 2025 16:48:37 台北標準時間

Sample Name: 20211111_H8_R2_IgG

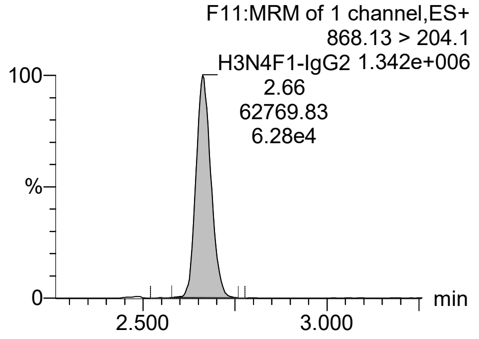

Sample Name: 20211111_A17_R2_IgG

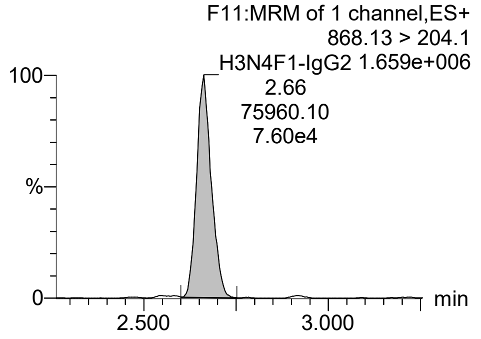

Sample Name: 20211111_A18_R2_IgG

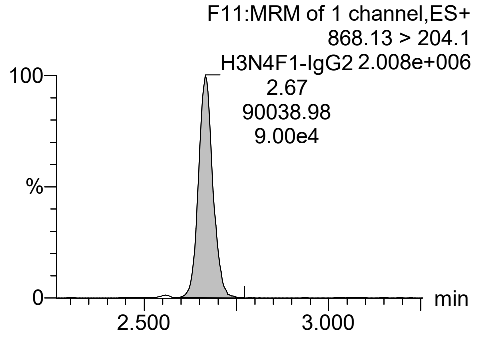

Sample Name: 20211111_L17_R2_IgG

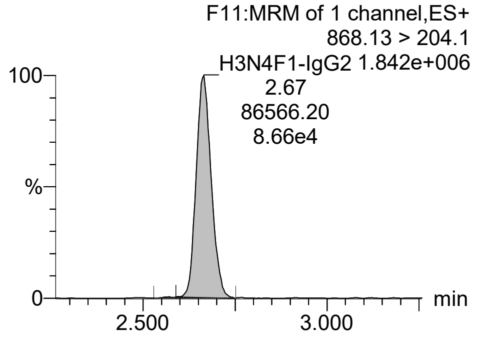

Sample Name: 20211111_L18_R2_IgG

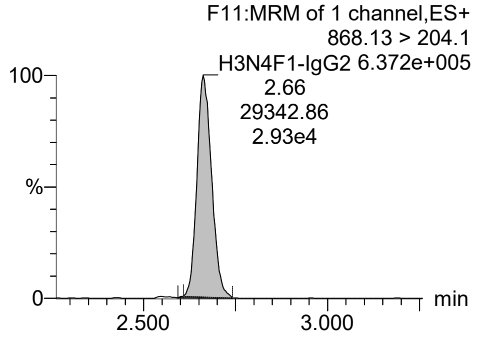

Sample Name: 20211111_H9_R2_IgG

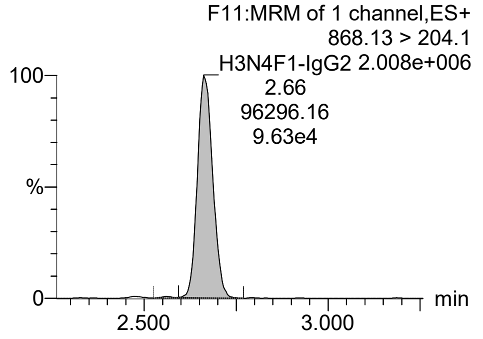

Sample Name: 20211111_A19_R2_IgG

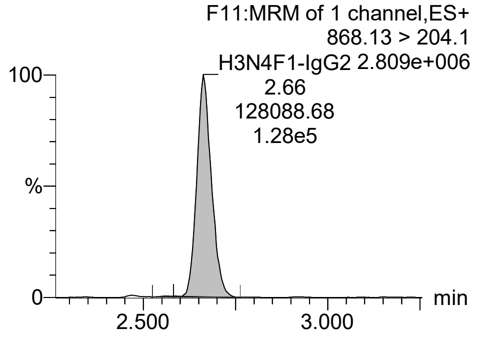

Sample Name: 20211111_A20_R2_IgG

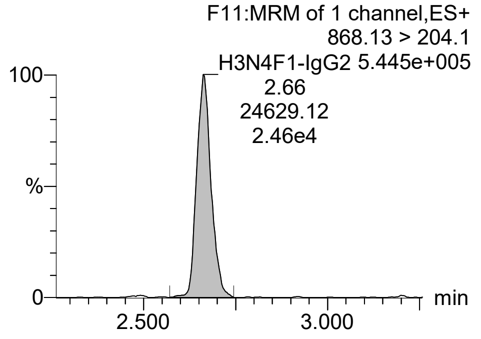

Sample Name: 20211111_L19_R2_IgG

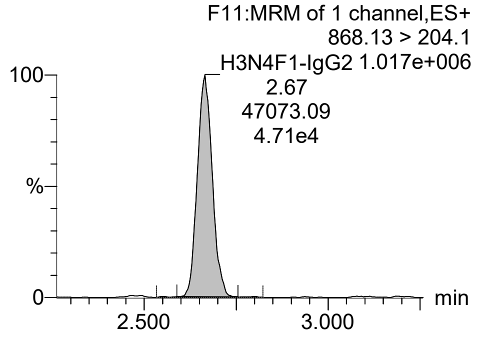

Sample Name: 20211111_QC_R13_IgG

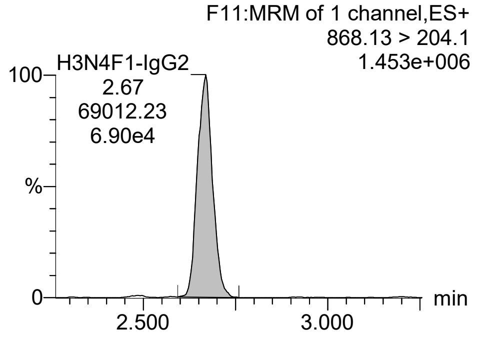

Sample Name: 20211111_L20_R2_IgG

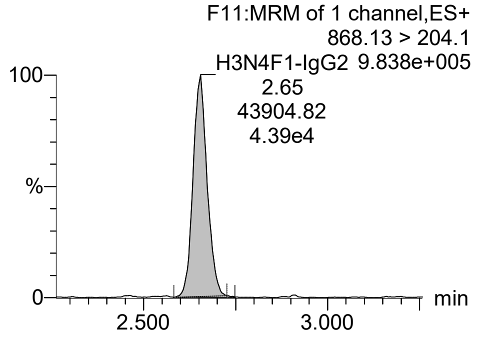

Sample Name: 20211111_H10_R2_IgG

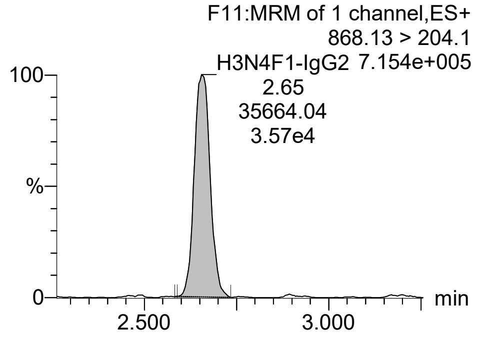

Sample Name: 20211111_A21_R2_IgG

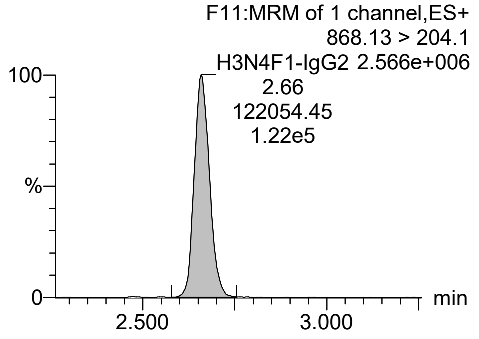

Sample Name: 20211111_A22_R2_IgG

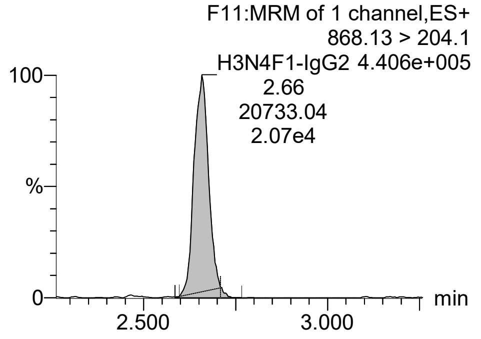

Sample Name: 20211111_L21_R2_IgG

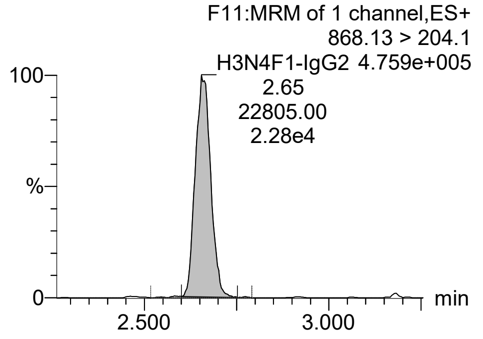

Dataset: C:\MassLynx\10.pro\20211115_clinicalsamples_IgG.qld

Last Altered: Sunday, October 02, 2022 16:02:30 台北標準時間
Printed: Friday, August 01, 2025 16:48:37 台北標準時間

Sample Name: 20211111_L22_R2_IgG

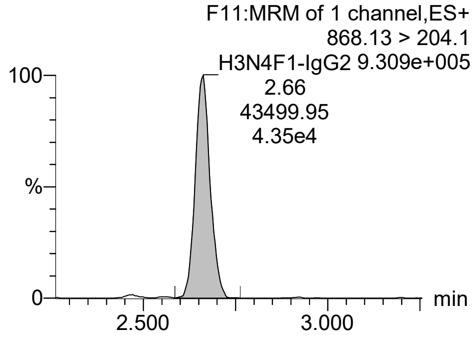

Sample Name: 20211111_H11_R2_IgG

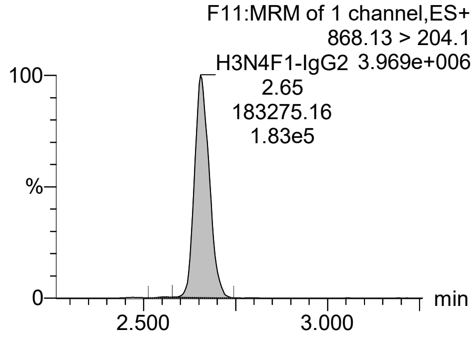

Sample Name: 20211111_A23_R2_IgG

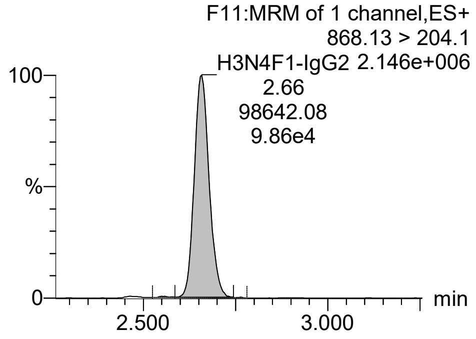

Sample Name: 20211111_A24_R2_IgG

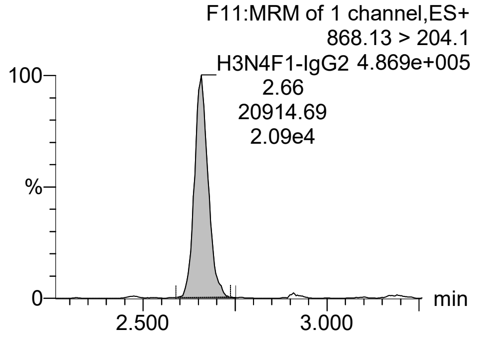

Sample Name: 20211111_L23_R2_IgG

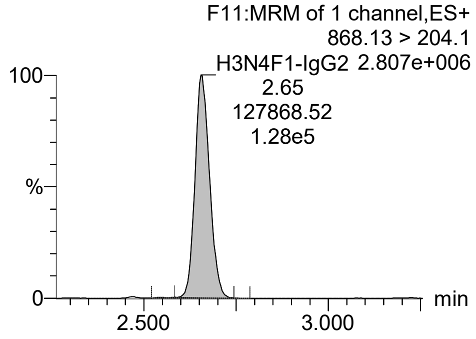

Sample Name: 20211111_L24_R2_IgG

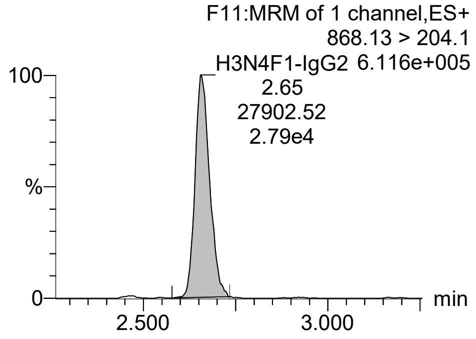

Sample Name: 20211111_H12_R2_IgG

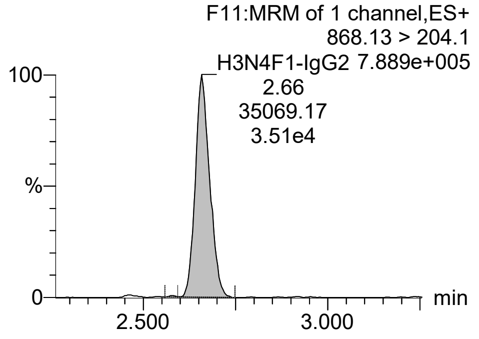

Sample Name: 20211111_QC_R14_IgG

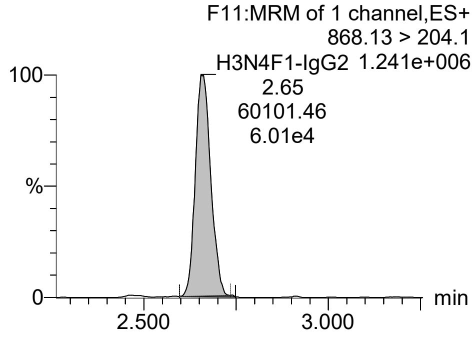

Sample Name: 20211111_A25_R2_IgG

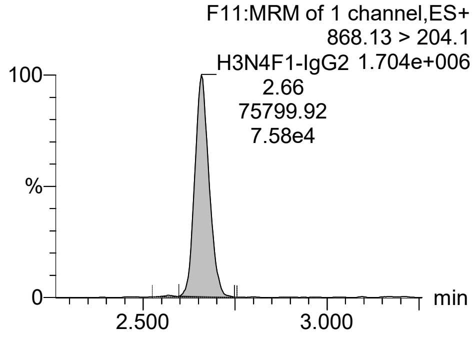

Sample Name: 20211111_A26_R2_IgG

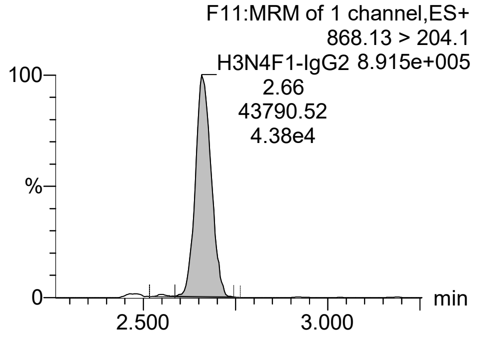

Sample Name: 20211111_L25_R2_IgG

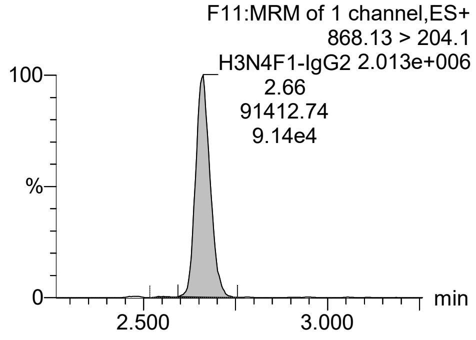

Sample Name: 20211111_L26_R2_IgG

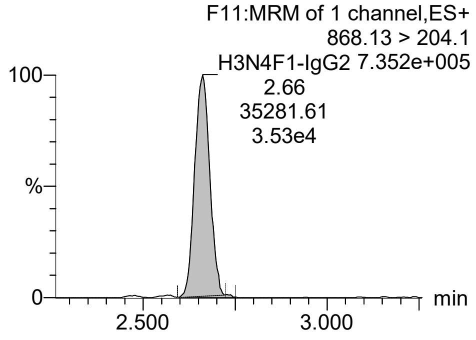

Sample Name: 20211111_H13_R2_IgG

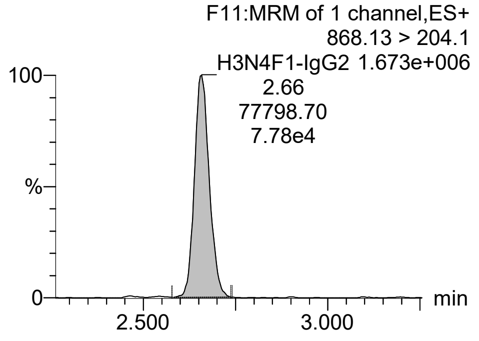

Sample Name: 20211111_A27_R2_IgG

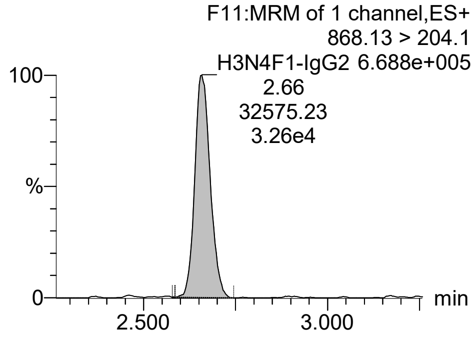

Sample Name: 20211111_A28_R2_IgG

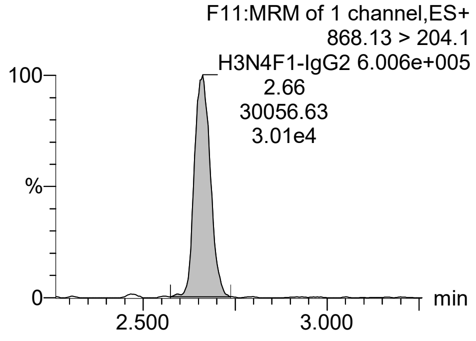

Dataset: C:\MassLynx\10.pro\20211115_clinicalsamples_IgG.qld

Last Altered: Sunday, October 02, 2022 16:02:30 台北標準時間
Printed: Friday, August 01, 2025 16:48:37 台北標準時間

Sample Name: 20211111_L27_R2_IgG

F11:MRM of 1 channel,ES+
868.13 > 204.1
H3N4F1-IgG2 1.041e+006
2.66
46120.96
4.61e4

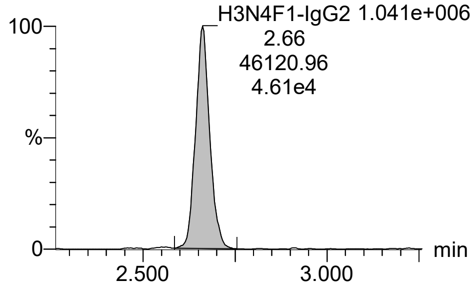

Sample Name: 20211111_L28_R2_IgG

F11:MRM of 1 channel,ES+
868.13 > 204.1
H3N4F1-IgG2 9.320e+005
2.67
42791.06
4.28e4

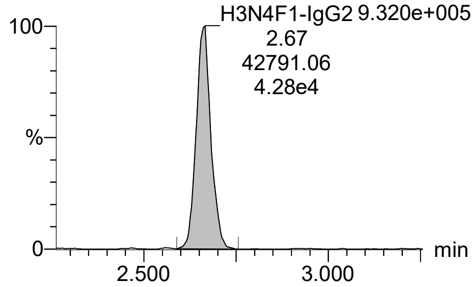

Sample Name: 20211111_H14_R2_IgG

F11:MRM of 1 channel,ES+
868.13 > 204.1
H3N4F1-IgG2 2.483e+006
2.66
116651.52
1.17e5

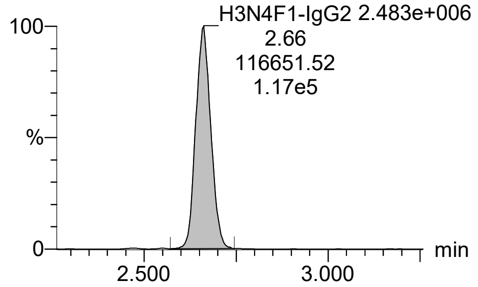

Sample Name: 20211111_A29_R2_IgG

F11:MRM of 1 channel,ES+
868.13 > 204.1
H3N4F1-IgG2 1.436e+006
2.65
70501.89
7.05e4

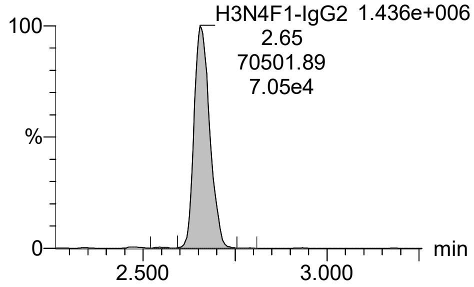

Sample Name: 20211111_A30_R2_IgG

F11:MRM of 1 channel,ES+
868.13 > 204.1
H3N4F1-IgG2 1.592e+006
2.66
79439.46
7.94e4

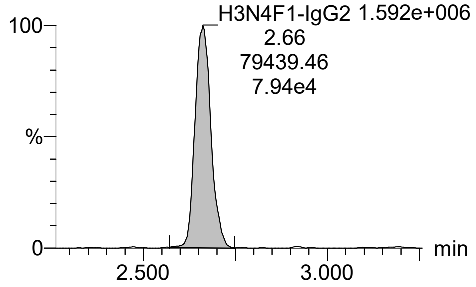

Sample Name: 20211111_QC_R15_IgG

F11:MRM of 1 channel,ES+
868.13 > 204.1
H3N4F1-IgG2 1.498e+006
2.66
67129.66
6.71e4

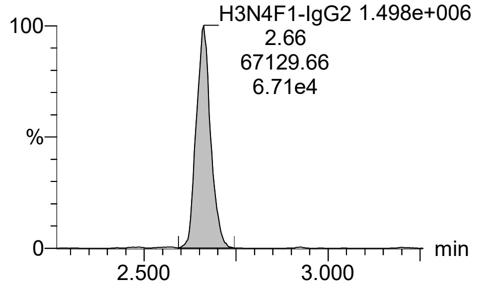

Sample Name: 20211111_L29_R2_IgG

F11:MRM of 1 channel,ES+
868.13 > 204.1
H3N4F1-IgG2 4.091e+005
2.67
18902.93
1.89e4

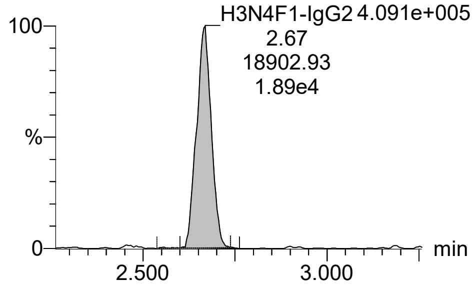

Sample Name: 20211111_L30_R2_IgG

F11:MRM of 1 channel,ES+
868.13 > 204.1
H3N4F1-IgG2 5.288e+005
2.66
23176.60
2.32e4

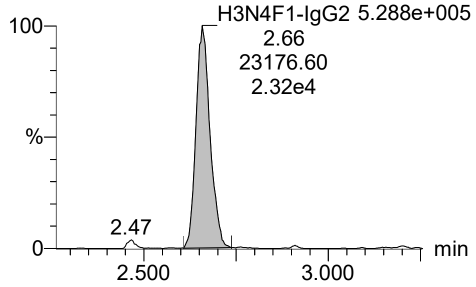

Sample Name: 20211111_H15_R2_IgG

F11:MRM of 1 channel,ES+
868.13 > 204.1
H3N4F1-IgG2 1.491e+006
2.65
66859.55
6.69e4

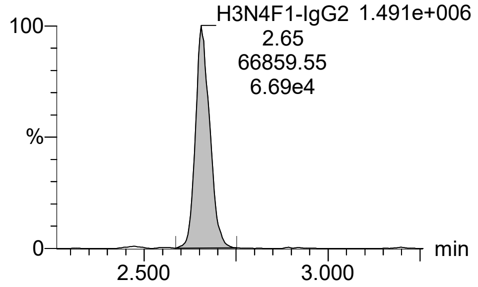

Sample Name: 20211111_A31_R2_IgG

F11:MRM of 1 channel,ES+
868.13 > 204.1
H3N4F1-IgG2 2.454e+006
2.66
105115.01
1.05e5

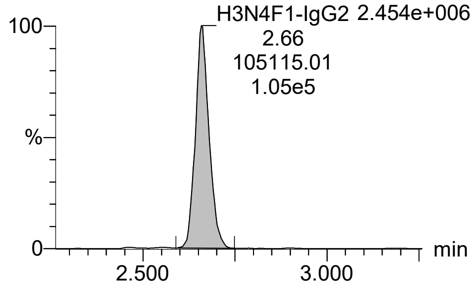

Sample Name: 20211111_A32_R2_IgG

F11:MRM of 1 channel,ES+
868.13 > 204.1
H3N4F1-IgG2 3.040e+006
2.66
149505.67
1.50e5

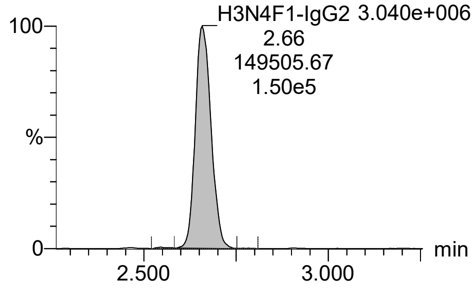

Sample Name: 20211111_L31_R2_IgG

F11:MRM of 1 channel,ES+
868.13 > 204.1
H3N4F1-IgG2 1.731e+006
2.66
81860.96
8.19e4

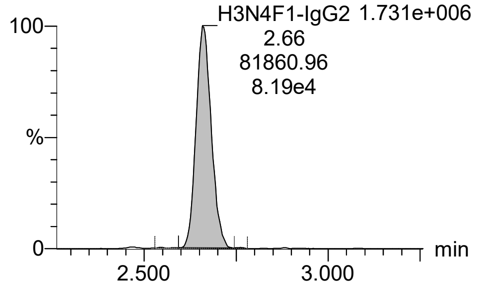

Sample Name: 20211111_L32_R2_IgG

F11:MRM of 1 channel,ES+
868.13 > 204.1
H3N4F1-IgG2 1.702e+006
2.66
78390.73
7.84e4

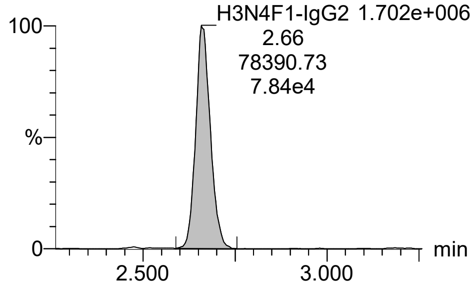

Sample Name: 20211111_H16_R2_IgG

F11:MRM of 1 channel,ES+
868.13 > 204.1
H3N4F1-IgG2 1.947e+006
2.66
89357.48
8.94e4

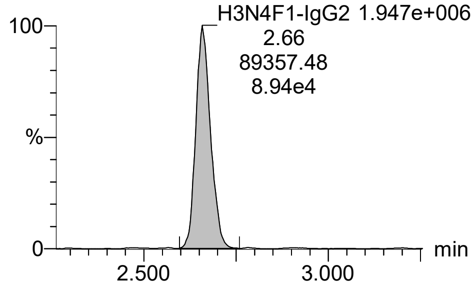

Sample Name: 20211111_A33_R2_IgG

F11:MRM of 1 channel,ES+
868.13 > 204.1
H3N4F1-IgG2 1.086e+006
2.66
52085.04
5.21e4

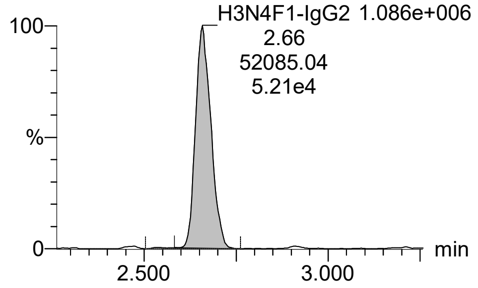

Dataset: C:\MassLynx\10.pro\20211115_clinicalsamples_IgG.qld

Last Altered: Sunday, October 02, 2022 16:02:30 台北標準時間
Printed: Friday, August 01, 2025 16:48:37 台北標準時間

Sample Name: 20211111_A34_R2_IgG

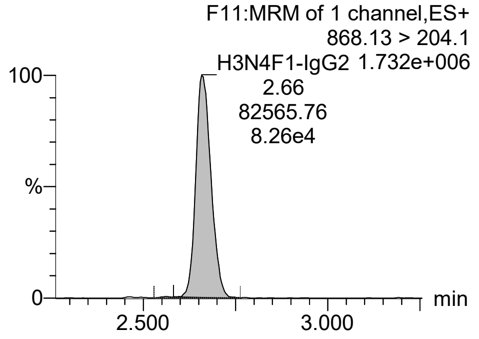

Sample Name: 20211111_H17_R2_IgG

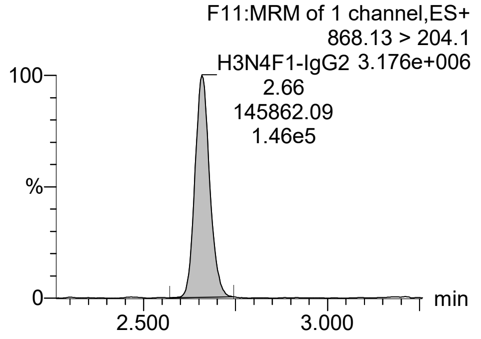

Sample Name: 20211111_A35_R2_IgG

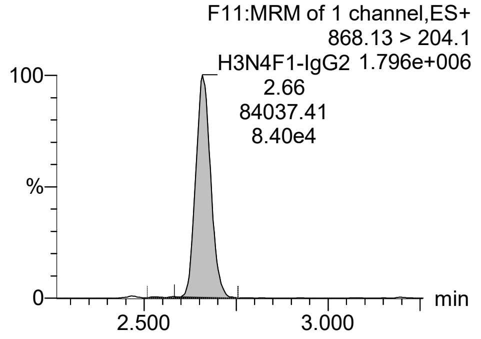

Sample Name: 20211111_QC_R16_IgG

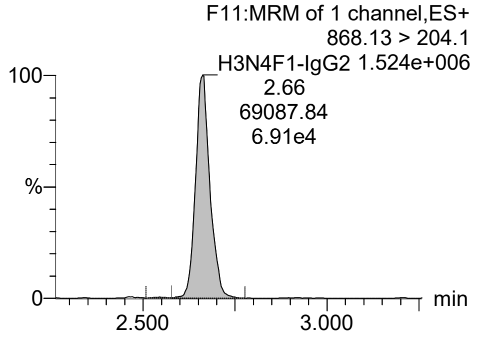

Sample Name: 20211111_A36_R2_IgG

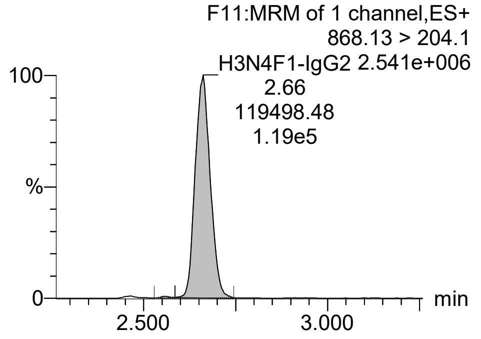

Sample Name: 20211111_H18_R2_IgG

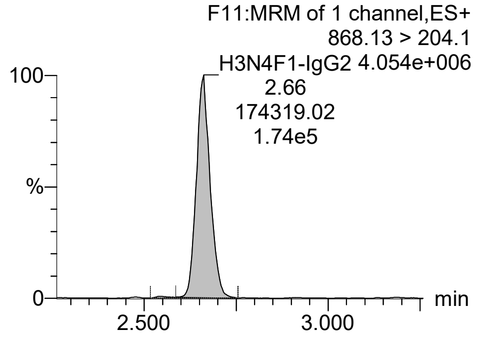

Sample Name: 20211111_A37_R2_IgG

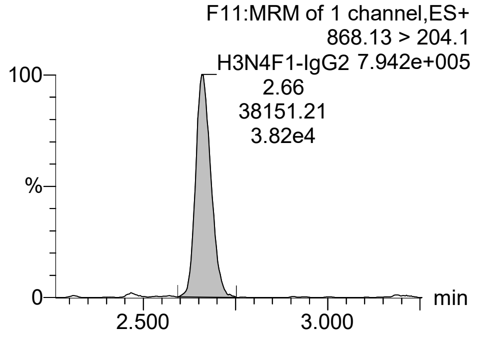

Sample Name: 20211111_A38_R2_IgG

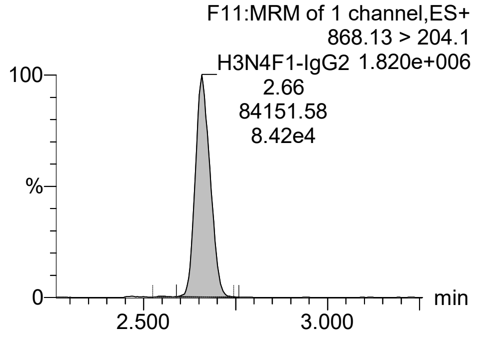

Sample Name: 20211111_H19_R2_IgG

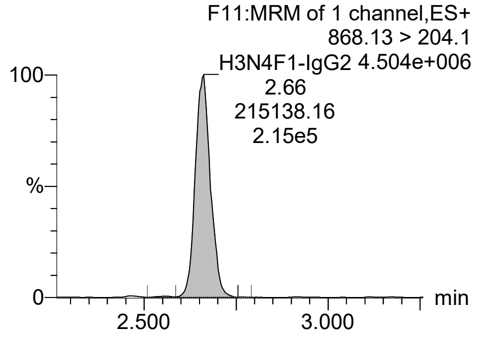

Sample Name: 20211111_A39_R2_IgG

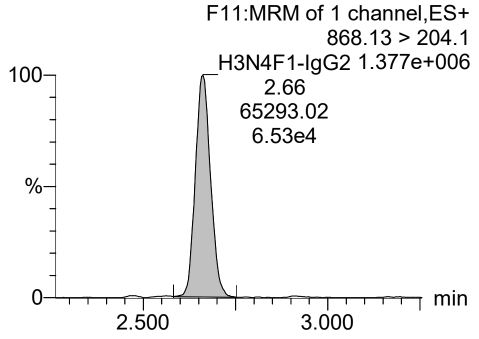

Sample Name: 20211111_A40_R2_IgG

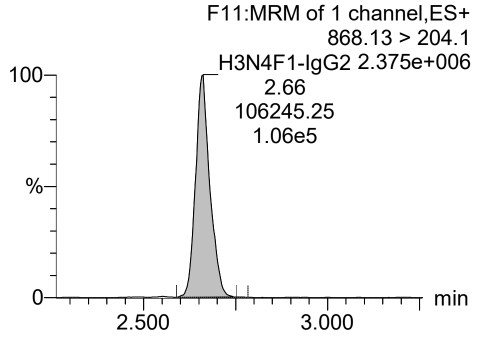

Sample Name: 20211111_H20_R2_IgG

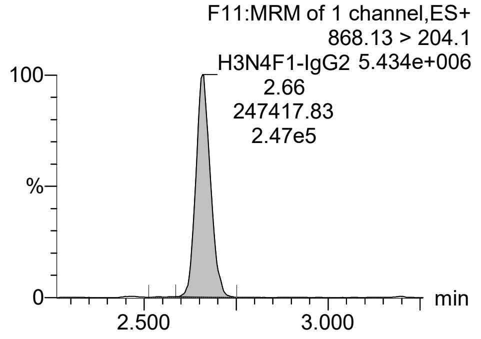

Sample Name: 20211111_A41_R2_IgG

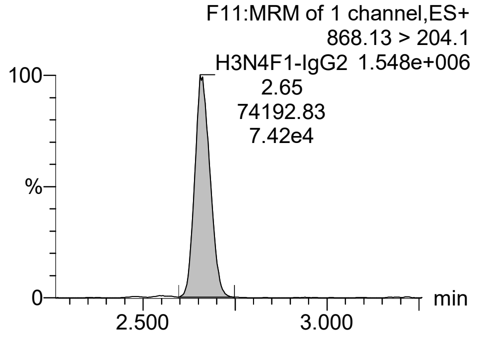

Sample Name: 20211111_A42_R2_IgG

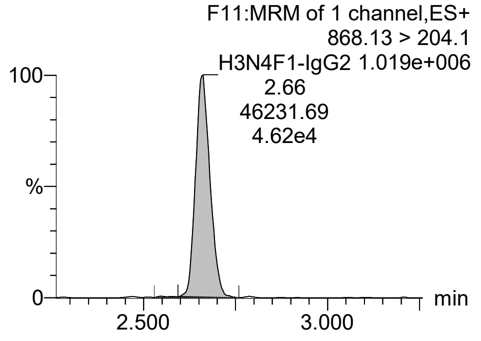

Sample Name: 20211111_A43_R2_IgG

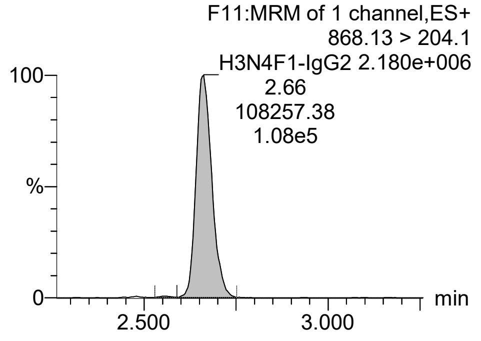

Dataset: C:\MassLynx\10.pro\20211115_clinicalsamples_IgG.qld

Last Altered: Sunday, October 02, 2022 16:02:30 台北標準時間
Printed: Friday, August 01, 2025 16:48:37 台北標準時間

Sample Name: 20211111_A44_R2_IgG

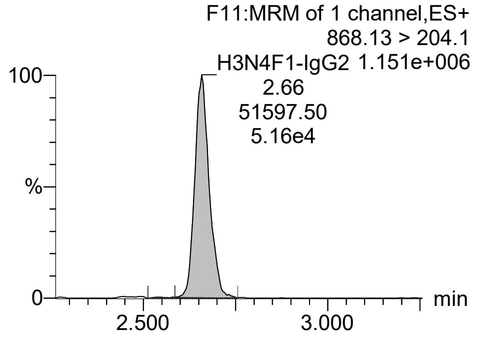

Sample Name: 20211111_QC_R17_IgG

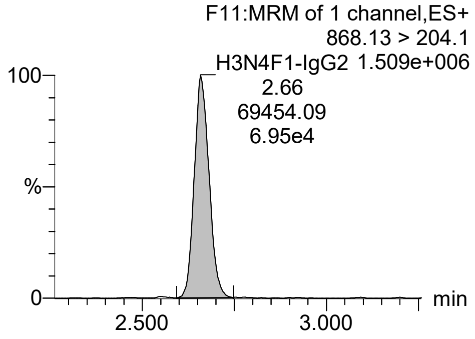

Dataset: C:\MassLynx\10.pro\20211115_clinicalsamples_IgG.qld

Last Altered: Sunday, October 02, 2022 16:02:30 台北標準時間
Printed: Friday, August 01, 2025 16:48:37 台北標準時間

	#	Name	RT	Area	IS Area	Response	Prima...
1	1	20211111_QC_R1_IgG	2.67	143372.2	64968.0	2.207	MM
2	2	20211111_A1_R1_IgG	2.67	353166.8	59059.2	5.980	MM
3	3	20211111_A2_R1_IgG	2.67	109847.8	66621.2	1.649	MM
4	4	20211111_L1_R1_IgG	2.67	128826.7	64201.0	2.007	MM
5	5	20211111_L2_R1_IgG	2.67	117801.9	46950.2	2.509	bb
6	6	20211111_H1_R1_IgG	2.67	87557.3	38648.2	2.265	bb
7	7	20211111_A3_R1_IgG	2.67	5191.3	2603.7	1.994	MM
8	8	20211111_A4_R1_IgG	2.67	240800.8	69960.4	3.442	MM
9	9	20211111_L3_R1_IgG	2.67	46532.9	38864.7	1.197	bb
10	10	20211111_L4_R1_IgG	2.67	68464.2	43380.5	1.578	bb
11	11	20211111_H2_R1_IgG	2.67	312658.0	71349.2	4.382	MM
12	12	20211111_A5_R1_IgG	2.67	126879.7	65569.7	1.935	MM
13	13	20211111_A6_R1_IgG	2.67	120483.4	53120.7	2.268	MM
14	14	20211111_QC_R2_IgG	2.67	160802.3	81034.1	1.984	bb
15	15	20211111_L5_R1_IgG	2.67	95224.3	50994.6	1.867	MM
16	16	20211111_L6_R1_IgG	2.67	21541.5	58058.0	0.371	bb
17	17	20211111_H3_R1_IgG	2.67	117075.9	64028.6	1.828	MM
18	18	20211111_A7_R1_IgG	2.67	343181.2	82996.1	4.135	bb
19	19	20211111_A8_R1_IgG	2.67	173998.3	77731.7	2.238	MM
20	20	20211111_L7_R1_IgG	2.67	102721.9	38827.3	2.646	MM
21	21	20211111_L8_R1_IgG	2.67	42960.8	44220.1	0.972	MM
22	22	20211111_H4_R1_IgG	2.67	66901.0	65826.3	1.016	bb
23	23	20211111_A9_R1_IgG	2.66	143565.2	60771.1	2.362	bb
24	24	20211111_A10_R1_IgG	2.67	190210.9	48578.7	3.916	bb
25	25	20211111_L9_R1_IgG	2.67	134336.7	50830.7	2.643	MM
26	26	20211111_L10_R1_IgG	2.67	68260.9	38034.2	1.795	MM
27	27	20211111_QC_R3_IgG	2.67	131328.5	67025.5	1.959	MM
28	28	20211111_H5_R1_IgG	2.67	43768.8	44544.9	0.983	bb
29	29	20211111_A11_R1_IgG	2.67	45625.2	66408.5	0.687	bb
30	30	20211111_A12_R1_IgG	2.67	110471.3	46624.5	2.369	MM
31	31	20211111_L11_R1_IgG	2.67	56446.5	33425.1	1.689	bb
32	32	20211111_L12_R1_IgG	2.67	34841.9	41178.0	0.846	bb
33	33	20211111_H6_R1_IgG	2.67	247042.2	58593.4	4.216	MM
34	34	20211111_A13_R1_IgG	2.66	128048.1	54133.0	2.365	bb
35	35	20211111_A14_R1_IgG	2.67	158570.9	56467.3	2.808	MM
36	36	20211111_L13_R1_IgG	2.67	55107.2	36024.4	1.530	bb
37	37	20211111_L14_R1_IgG	2.67	44329.6	37181.6	1.192	bb
38	38	20211111_H7_R1_IgG	2.67	66245.7	42850.6	1.546	bb
39	39	20211111_A15_R1_IgG	2.67	141007.7	62015.1	2.274	MM
40	40	20211111_QC_R4_IgG	2.67	116191.5	58572.5	1.984	MM
41	41	20211111_A16_R1_IgG	2.66	155794.7	78283.8	1.990	bb
42	42	20211111_L15_R1_IgG	2.67	97251.0	44158.2	2.202	bb
43	43	20211111_L16_R1_IgG	2.67	143998.7	55150.8	2.611	MM
44	44	20211111_H8_R1_IgG	2.67	64396.6	42965.2	1.499	MM
45	45	20211111_A17_R1_IgG	2.67	78707.6	39717.7	1.982	bb
46	46	20211111_A18_R1_IgG	2.66	116635.5	44675.4	2.611	bb
47	47	20211111_L17_R1_IgG	2.67	92312.4	37854.1	2.439	bb
48	48	20211111_L18_R1_IgG	2.67	37739.8	50716.9	0.744	bb
49	49	20211111_H9_R1_IgG	2.67	118319.0	36276.0	3.262	bb
50	50	20211111_A19_R1_IgG	2.67	143014.8	42862.5	3.337	MM
51	51	20211111_A20_R1_IgG	2.67	28130.6	36542.7	0.770	MM
52	52	20211111_L19_R1_IgG	2.66	68170.3	53892.8	1.265	MM
53	53	20211111_QC_R5_IgG	2.66	111510.6	56513.2	1.973	MM
54	54	20211111_L20_R1_IgG	2.67	80359.5	56499.9	1.422	bb
55	55	20211111_H10_R1_IgG	2.66	78421.7	68120.5	1.151	bb
56	56	20211111_A21_R1_IgG	2.66	241360.8	63699.8	3.789	MM

Dataset: C:\MassLynx\10.pro\20211115_clinicalsamples_IgG.qld

Last Altered: Sunday, October 02, 2022 16:02:30 台北標準時間
Printed: Friday, August 01, 2025 16:48:37 台北標準時間

	# Name	RT	Area	IS Area	Response	Prima...
57	57 20211111_A22_R1_IgG	2.66	39362.6	52222.1	0.754	bb
58	58 20211111_L21_R1_IgG	2.66	37830.2	54372.5	0.696	bb
59	59 20211111_L22_R1_IgG	2.67	66114.9	41353.1	1.599	MM
60	60 20211111_H11_R1_IgG	2.67	308799.8	65579.1	4.709	MM
61	61 20211111_A23_R1_IgG	2.67	185418.7	64131.1	2.891	bb
62	62 20211111_A24_R1_IgG	2.66	27630.0	38724.8	0.713	MM
63	63 20211111_L23_R1_IgG	2.67	199419.9	64245.2	3.104	MM
64	64 20211111_L24_R1_IgG	2.66	38491.4	45298.0	0.850	bb
65	65 20211111_H12_R1_IgG	2.66	47446.3	44679.3	1.062	bb
66	66 20211111_QC_R6_IgG	2.67	98319.4	48165.0	2.041	bb
67	67 20211111_A25_R1_IgG	2.66	118495.0	57169.9	2.073	bb
68	68 20211111_A26_R1_IgG	2.67	57273.2	34254.0	1.672	MM
69	69 20211111_L25_R1_IgG	2.67	166195.3	67491.5	2.462	MM
70	70 20211111_L26_R1_IgG	2.67	54626.7	55934.1	0.977	bb
71	71 20211111_H13_R1_IgG	2.66	114418.6	48767.3	2.346	bb
72	72 20211111_A27_R1_IgG	2.66	49150.6	57269.7	0.858	MM
73	73 20211111_A28_R1_IgG	2.67	48574.1	49231.1	0.987	bb
74	74 20211111_L27_R1_IgG	2.67	65369.9	60014.5	1.089	MM
75	75 20211111_L28_R1_IgG	2.67	54351.1	35062.6	1.550	MM
76	76 20211111_H14_R1_IgG	2.66	177730.2	62371.9	2.850	bb
77	77 20211111_A29_R1_IgG	2.67	86603.1	43858.2	1.975	MM
78	78 20211111_A30_R1_IgG	2.67	132466.5	62500.4	2.119	MM
79	79 20211111_QC_R7_IgG	2.67	88840.4	43946.9	2.022	MM
80	80 20211111_L29_R1_IgG	2.66	22608.8	38728.9	0.584	bb
81	81 20211111_L30_R1_IgG	2.67	28571.3	42613.2	0.670	bb
82	82 20211111_H15_R1_IgG	2.67	73458.7	39874.6	1.842	bb
83	83 20211111_A31_R1_IgG	2.67	152371.1	53414.3	2.853	bb
84	84 20211111_A32_R1_IgG	2.67	179619.1	41844.6	4.293	bb
85	85 20211111_L31_R1_IgG	2.67	109820.8	40553.7	2.708	MM
86	86 20211111_L32_R1_IgG	2.67	84114.5	49618.7	1.695	MM
87	87 20211111_H16_R1_IgG	2.67	133655.7	60170.9	2.221	MM
88	88 20211111_A33_R1_IgG	2.67	63380.3	48588.2	1.304	bb
89	89 20211111_A34_R1_IgG	2.67	105691.3	43288.0	2.442	MM
90	90 20211111_H17_R1_IgG	2.67	172721.9	47104.5	3.667	MM
91	91 20211111_A35_R1_IgG	2.67	103620.1	41025.9	2.526	bb
92	92 20211111_QC_R8_IgG	2.67	78302.5	40546.3	1.931	MM
93	93 20211111_A36_R1_IgG	2.67	147348.1	44504.4	3.311	bb
94	94 20211111_H18_R1_IgG	2.67	173907.6	34162.2	5.091	bb
95	95 20211111_A37_R1_IgG	2.67	40552.7	39760.0	1.020	MM
96	96 20211111_A38_R1_IgG	2.67	98673.8	43982.6	2.243	bb
97	97 20211111_H19_R1_IgG	2.67	245677.5	45314.8	5.422	MM
98	98 20211111_A39_R1_IgG	2.66	66876.8	39388.0	1.698	bb
99	99 20211111_A40_R1_IgG	2.67	114283.7	38726.4	2.951	MM
100	1... 20211111_H20_R1_IgG	2.67	262508.1	37814.4	6.942	MM
101	1... 20211111_A41_R1_IgG	2.67	84739.6	48930.3	1.732	MM
102	1... 20211111_A42_R1_IgG	2.67	50872.2	40406.8	1.259	bb
103	1... 20211111_A43_R1_IgG	2.67	132578.0	44421.5	2.985	bb
104	1... 20211111_A44_R1_IgG	2.67	74520.1	53128.2	1.403	MM
105	1... 20211111_QC_R9_IgG	2.67	71970.3	37049.0	1.943	bb
106	1... 20211111_A1_R2_IgG	2.66	243838.7	46499.4	5.244	MM
107	1... 20211111_A2_R2_IgG	2.67	86134.0	57260.7	1.504	MM
108	1... 20211111_L1_R2_IgG	2.67	88102.2	48062.9	1.833	MM
109	1... 20211111_L2_R2_IgG	2.67	100847.4	38745.2	2.603	MM
110	1... 20211111_H1_R2_IgG	2.67	100107.3	44273.5	2.261	MM
111	1... 20211111_A3_R2_IgG	2.67	9042.1	4755.5	1.901	bb
112	1... 20211111_A4_R2_IgG	2.67	186646.2	50546.7	3.693	MM

Dataset: C:\MassLynx\10.pro\20211115_clinicalsamples_IgG.qld

Last Altered: Sunday, October 02, 2022 16:02:30 台北標準時間
Printed: Friday, August 01, 2025 16:48:37 台北標準時間

	# Name	RT	Area	IS Area	Response	Prima...
113	1... 20211111_L3_R2_IgG	2.67	45713.4	34825.8	1.313	MM
114	1... 20211111_L4_R2_IgG	2.66	55809.6	39107.7	1.427	bb
115	1... 20211111_H2_R2_IgG	2.67	220468.4	49750.0	4.432	MM
116	1... 20211111_A5_R2_IgG	2.67	107062.4	52536.2	2.038	bb
117	1... 20211111_A6_R2_IgG	2.67	105078.2	42050.0	2.499	MM
118	1... 20211111_QC_R10_IgG	2.66	74064.0	35350.8	2.095	MM
119	1... 20211111_L5_R2_IgG	2.67	80331.3	38039.3	2.112	bb
120	1... 20211111_L6_R2_IgG	2.66	18325.6	44907.4	0.408	MM
121	1... 20211111_H3_R2_IgG	2.66	94634.3	47729.1	1.983	bb
122	1... 20211111_A7_R2_IgG	2.67	242088.8	57347.1	4.221	bb
123	1... 20211111_A8_R2_IgG	2.67	132930.3	52397.8	2.537	bb
124	1... 20211111_L7_R2_IgG	2.66	92444.8	36318.4	2.545	MM
125	1... 20211111_L8_R2_IgG	2.66	38552.7	38131.9	1.011	bb
126	1... 20211111_H4_R2_IgG	2.67	52246.2	46849.6	1.115	bb
127	1... 20211111_A9_R2_IgG	2.66	116403.4	47759.6	2.437	MM
128	1... 20211111_A10_R2_IgG	2.67	169105.5	41366.2	4.088	bb
129	1... 20211111_L9_R2_IgG	2.66	119516.3	43052.1	2.776	MM
130	1... 20211111_L10_R2_IgG	2.66	62422.8	31487.4	1.982	bb
131	1... 20211111_QC_R11_IgG	2.67	72804.9	37804.6	1.926	MM
132	1... 20211111_H5_R2_IgG	2.67	40369.2	40380.0	1.000	MM
133	1... 20211111_A11_R2_IgG	2.67	35104.2	49533.3	0.709	bb
134	1... 20211111_A12_R2_IgG	2.66	94366.8	40226.3	2.346	MM
135	1... 20211111_L11_R2_IgG	2.67	40928.7	28884.0	1.417	MM
136	1... 20211111_L12_R2_IgG	2.67	29816.1	34351.3	0.868	bb
137	1... 20211111_H6_R2_IgG	2.67	149293.2	40466.6	3.689	MM
138	1... 20211111_A13_R2_IgG	2.66	91160.2	42306.7	2.155	bb
139	1... 20211111_A14_R2_IgG	2.67	126152.3	44904.0	2.809	bb
140	1... 20211111_L13_R2_IgG	2.67	51956.5	31913.0	1.628	MM
141	1... 20211111_L14_R2_IgG	2.66	40640.7	31527.4	1.289	bb
142	1... 20211111_H7_R2_IgG	2.66	58174.6	40838.2	1.425	bb
143	1... 20211111_A15_R2_IgG	2.67	119376.5	49389.7	2.417	MM
144	1... 20211111_QC_R12_IgG	2.67	75006.4	35168.7	2.133	MM
145	1... 20211111_A16_R2_IgG	2.67	89307.8	47440.5	1.883	bb
146	1... 20211111_L15_R2_IgG	2.66	75118.1	33145.9	2.266	bb
147	1... 20211111_L16_R2_IgG	2.66	91745.9	37596.6	2.440	MM
148	1... 20211111_H8_R2_IgG	2.66	62769.8	43933.3	1.429	MM
149	1... 20211111_A17_R2_IgG	2.66	75960.1	43605.5	1.742	bb
150	1... 20211111_A18_R2_IgG	2.67	90039.0	37581.8	2.396	bb
151	1... 20211111_L17_R2_IgG	2.67	86566.2	33942.5	2.550	MM
152	1... 20211111_L18_R2_IgG	2.66	29342.9	37831.5	0.776	MM
153	1... 20211111_H9_R2_IgG	2.66	96296.2	30898.4	3.117	MM
154	1... 20211111_A19_R2_IgG	2.66	128088.7	39352.1	3.255	MM
155	1... 20211111_A20_R2_IgG	2.66	24629.1	34661.2	0.711	bb
156	1... 20211111_L19_R2_IgG	2.67	47073.1	39918.3	1.179	MM
157	1... 20211111_QC_R13_IgG	2.67	69012.2	36239.1	1.904	bb
158	1... 20211111_L20_R2_IgG	2.65	43904.8	32897.7	1.335	MM
159	1... 20211111_H10_R2_IgG	2.65	35664.0	31394.5	1.136	MM
160	1... 20211111_A21_R2_IgG	2.66	122054.4	34863.7	3.501	bb
161	1... 20211111_A22_R2_IgG	2.66	20733.0	30342.1	0.683	MM
162	1... 20211111_L21_R2_IgG	2.65	22805.0	33706.2	0.677	MM
163	1... 20211111_L22_R2_IgG	2.66	43500.0	31298.6	1.390	bb
164	1... 20211111_H11_R2_IgG	2.65	183275.2	38904.1	4.711	MM
165	1... 20211111_A23_R2_IgG	2.66	98642.1	32382.3	3.046	MM
166	1... 20211111_A24_R2_IgG	2.66	20914.7	30261.9	0.691	MM
167	1... 20211111_L23_R2_IgG	2.65	127868.5	43037.5	2.971	MM
168	1... 20211111_L24_R2_IgG	2.65	27902.5	37023.3	0.754	bb

Dataset: C:\MassLynx\10.pro\20211115_clinicalsamples_IgG.qld

Last Altered: Sunday, October 02, 2022 16:02:30 台北標準時間
Printed: Friday, August 01, 2025 16:48:37 台北標準時間

	#	Name	RT	Area	IS Area	Response	Prima...
169	1...	20211111_H12_R2_IgG	2.66	35069.2	39478.2	0.888	MM
170	1...	20211111_QC_R14_IgG	2.65	60101.5	34463.2	1.744	MM
171	1...	20211111_A25_R2_IgG	2.66	75799.9	39945.3	1.898	MM
172	1...	20211111_A26_R2_IgG	2.66	43790.5	29663.2	1.476	MM
173	1...	20211111_L25_R2_IgG	2.66	91412.7	38589.3	2.369	MM
174	1...	20211111_L26_R2_IgG	2.66	35281.6	38405.2	0.919	MM
175	1...	20211111_H13_R2_IgG	2.66	77798.7	37771.2	2.060	MM
176	1...	20211111_A27_R2_IgG	2.66	32575.2	42634.1	0.764	MM
177	1...	20211111_A28_R2_IgG	2.66	30056.6	33231.1	0.904	bb
178	1...	20211111_L27_R2_IgG	2.66	46121.0	41761.1	1.104	bb
179	1...	20211111_L28_R2_IgG	2.67	42791.1	26968.6	1.587	bb
180	1...	20211111_H14_R2_IgG	2.66	116651.5	49250.2	2.369	bb
181	1...	20211111_A29_R2_IgG	2.65	70501.9	37601.7	1.875	MM
182	1...	20211111_A30_R2_IgG	2.66	79439.5	40366.8	1.968	bb
183	1...	20211111_QC_R15_IgG	2.66	67129.7	35156.1	1.909	bb
184	1...	20211111_L29_R2_IgG	2.67	18902.9	33813.1	0.559	MM
185	1...	20211111_L30_R2_IgG	2.66	23176.6	33488.6	0.692	bb
186	1...	20211111_H15_R2_IgG	2.65	66859.5	38501.0	1.737	bb
187	1...	20211111_A31_R2_IgG	2.66	105115.0	37525.5	2.801	bb
188	1...	20211111_A32_R2_IgG	2.66	149505.7	35770.8	4.180	MM
189	1...	20211111_L31_R2_IgG	2.66	81861.0	33282.4	2.460	MM
190	1...	20211111_L32_R2_IgG	2.66	78390.7	42049.6	1.864	bb
191	1...	20211111_H16_R2_IgG	2.66	89357.5	45947.6	1.945	bb
192	1...	20211111_A33_R2_IgG	2.66	52085.0	40202.3	1.296	MM
193	1...	20211111_A34_R2_IgG	2.66	82565.8	36747.0	2.247	MM
194	1...	20211111_H17_R2_IgG	2.66	145862.1	40920.9	3.564	bb
195	1...	20211111_A35_R2_IgG	2.66	84037.4	33689.2	2.494	MM
196	1...	20211111_QC_R16_IgG	2.66	69087.8	32783.6	2.107	MM
197	1...	20211111_A36_R2_IgG	2.66	119498.5	36822.4	3.245	MM
198	1...	20211111_H18_R2_IgG	2.66	174319.0	31769.3	5.487	MM
199	1...	20211111_A37_R2_IgG	2.66	38151.2	37581.8	1.015	bb
200	2...	20211111_A38_R2_IgG	2.66	84151.6	38390.4	2.192	MM
201	2...	20211111_H19_R2_IgG	2.66	215138.2	41217.0	5.220	MM
202	2...	20211111_A39_R2_IgG	2.66	65293.0	37392.7	1.746	bb
203	2...	20211111_A40_R2_IgG	2.66	106245.3	38295.1	2.774	MM
204	2...	20211111_H20_R2_IgG	2.66	247417.8	35941.8	6.884	MM
205	2...	20211111_A41_R2_IgG	2.65	74192.8	45304.9	1.638	bb
206	2...	20211111_A42_R2_IgG	2.66	46231.7	37803.8	1.223	MM
207	2...	20211111_A43_R2_IgG	2.66	108257.4	38161.8	2.837	MM
208	2...	20211111_A44_R2_IgG	2.66	51597.5	37819.8	1.364	MM
209	2...	20211111_QC_R17_IgG	2.66	69454.1	31731.8	2.189	bb

Dataset: C:\MassLynx\10.pro\20211115_clinicalsamples_IgG.qld

Last Altered: Sunday, October 02, 2022 16:02:30 台北標準時間
Printed: Friday, August 01, 2025 16:49:10 台北標準時間

Method: C:\MassLynx\10_Pro_IgA.PRO\MethDB\20210926_IgG_DMRM_IgM_IS.mdb 16 Feb 2022 11:58:21
Calibration: 18 Nov 2021 13:56:22

Sample Name: 20211111_QC_R1_IgG

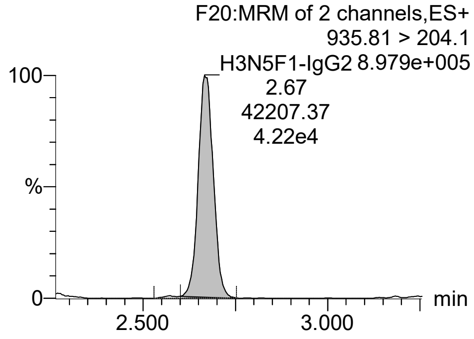

Sample Name: 20211111_QC_R1_IgG

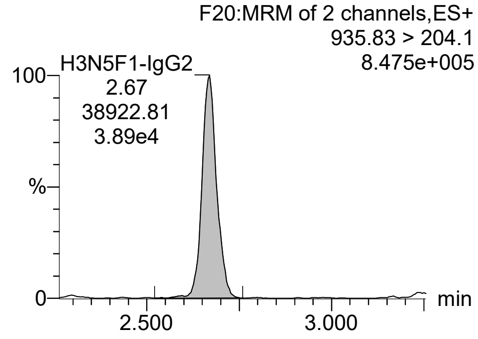

Sample Name: 20211111_A1_R1_IgG

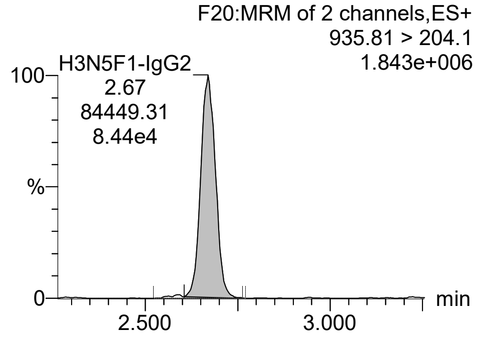

Sample Name: 20211111_A1_R1_IgG

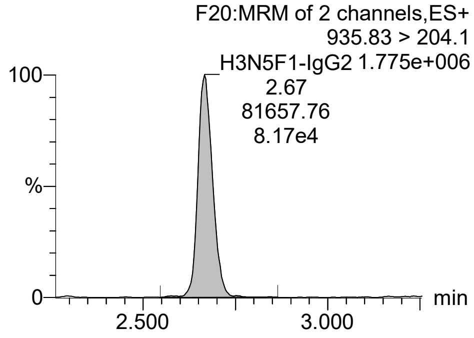

Sample Name: 20211111_A2_R1_IgG

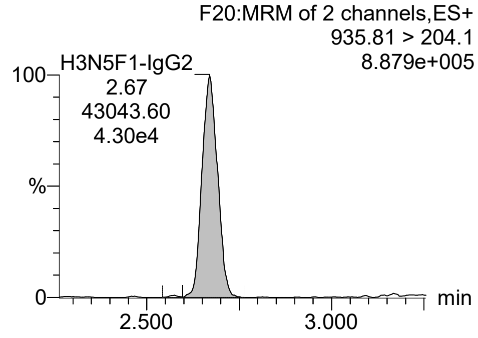

Sample Name: 20211111_A2_R1_IgG

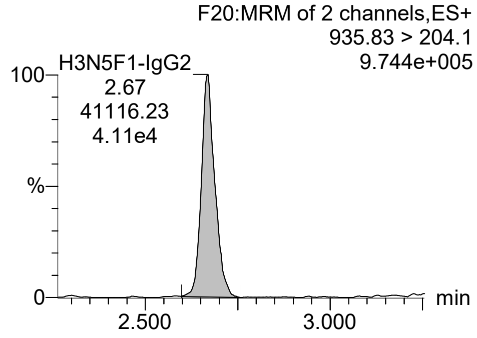

Sample Name: 20211111_L1_R1_IgG

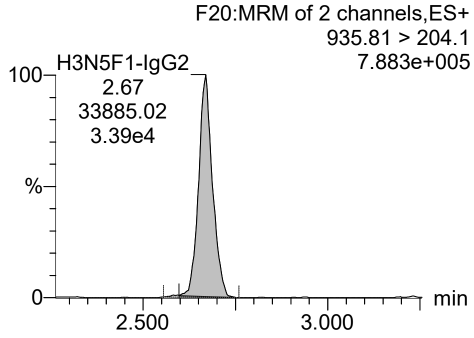

Sample Name: 20211111_L1_R1_IgG

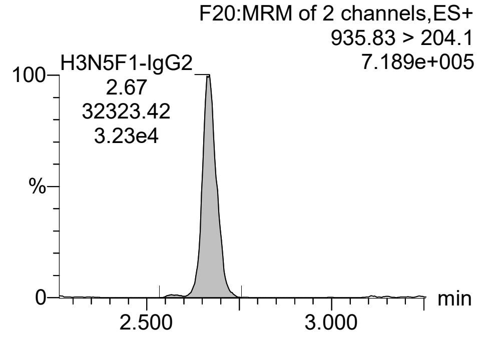

Sample Name: 20211111_L2_R1_IgG

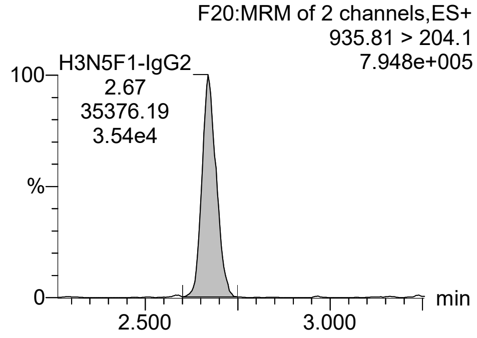

Sample Name: 20211111_L2_R1_IgG

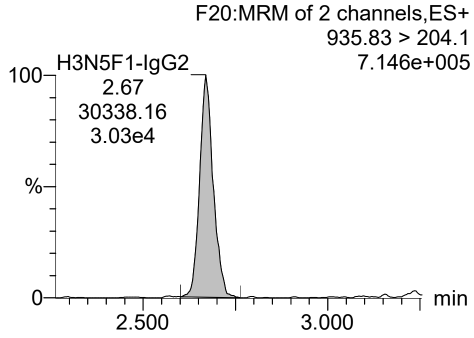

Sample Name: 20211111_H1_R1_IgG

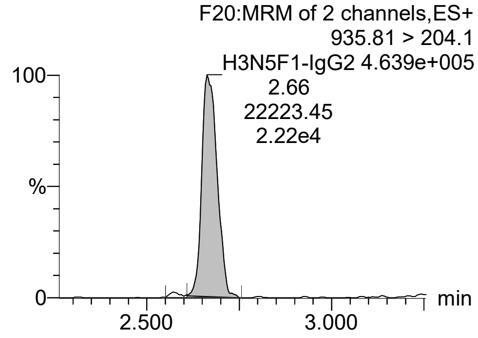

Sample Name: 20211111_H1_R1_IgG

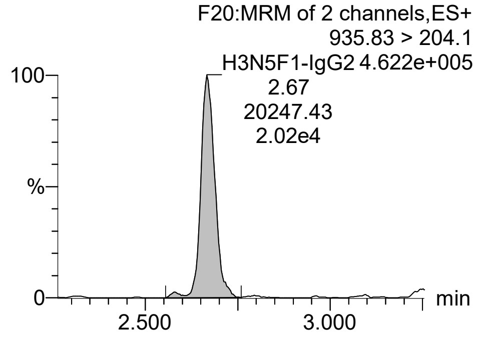

Dataset: C:\MassLynx\10.pro\20211115_clinicalsamples_IgG.qld

Last Altered: Sunday, October 02, 2022 16:02:30 台北標準時間
Printed: Friday, August 01, 2025 16:49:10 台北標準時間

Sample Name: 20211111_A3_R1_IgG
F20:MRM of 2 channels,ES+
935.81 > 204.1

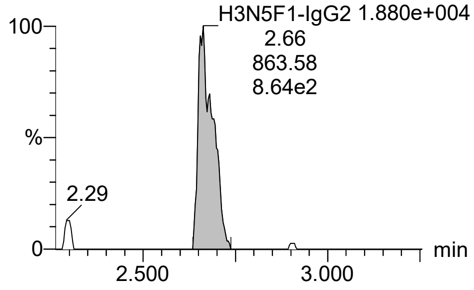

Sample Name: 20211111_A3_R1_IgG
F20:MRM of 2 channels,ES+
935.83 > 204.1

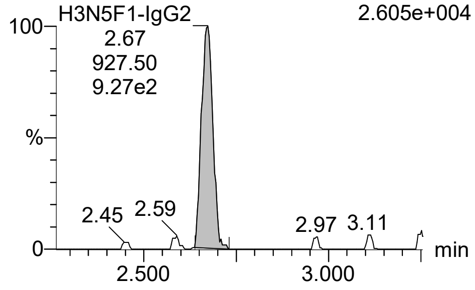

Sample Name: 20211111_A4_R1_IgG
F20:MRM of 2 channels,ES+
935.81 > 204.1

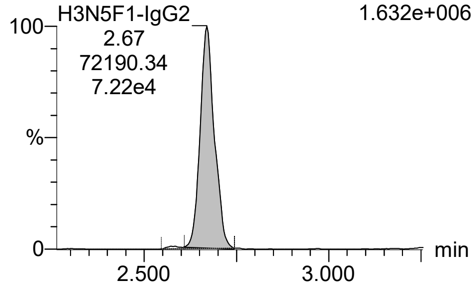

Sample Name: 20211111_A4_R1_IgG
F20:MRM of 2 channels,ES+
935.83 > 204.1

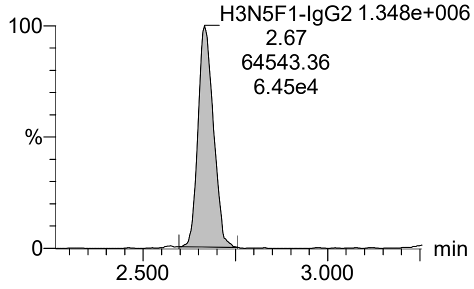

Sample Name: 20211111_L3_R1_IgG
F20:MRM of 2 channels,ES+
935.81 > 204.1

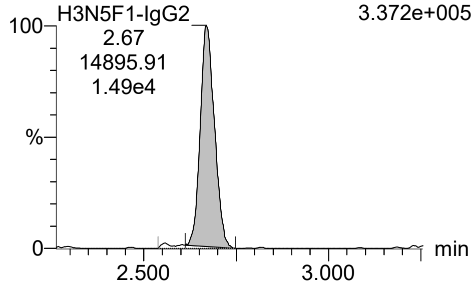

Sample Name: 20211111_L3_R1_IgG
F20:MRM of 2 channels,ES+
935.83 > 204.1

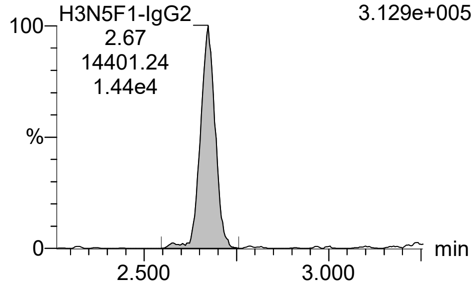

Sample Name: 20211111_L4_R1_IgG
F20:MRM of 2 channels,ES+
935.81 > 204.1

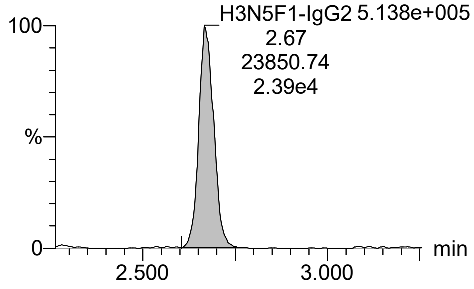

Sample Name: 20211111_L4_R1_IgG
F20:MRM of 2 channels,ES+
935.83 > 204.1

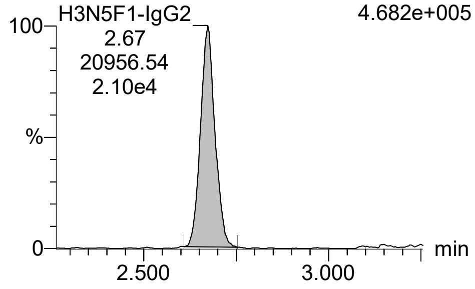

Sample Name: 20211111_H2_R1_IgG
F20:MRM of 2 channels,ES+
935.81 > 204.1

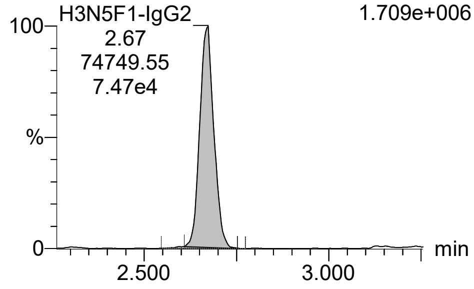

Sample Name: 20211111_H2_R1_IgG
F20:MRM of 2 channels,ES+
935.83 > 204.1

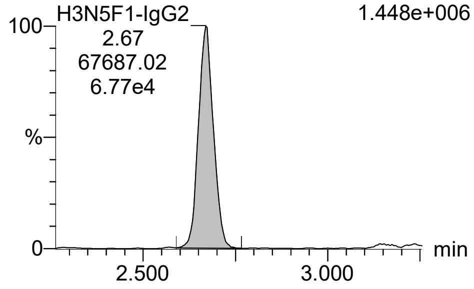

Sample Name: 20211111_A5_R1_IgG
F20:MRM of 2 channels,ES+
935.81 > 204.1

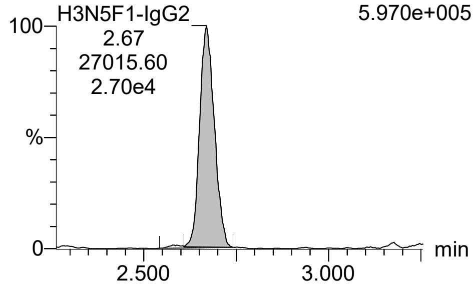

Sample Name: 20211111_A5_R1_IgG
F20:MRM of 2 channels,ES+
935.83 > 204.1

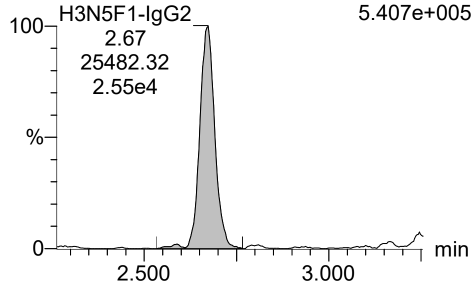

Sample Name: 20211111_A6_R1_IgG
F20:MRM of 2 channels,ES+
935.81 > 204.1

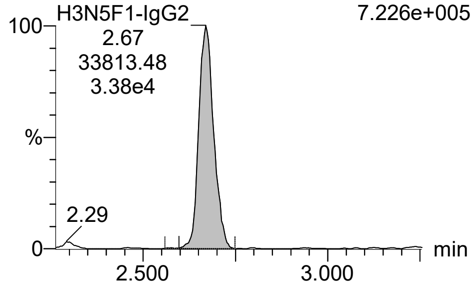

Sample Name: 20211111_A6_R1_IgG
F20:MRM of 2 channels,ES+
935.83 > 204.1

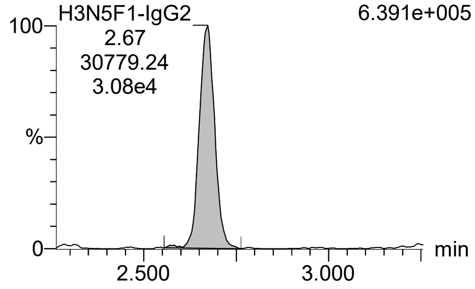

Sample Name: 20211111_QC_R2_IgG
F20:MRM of 2 channels,ES+
935.81 > 204.1

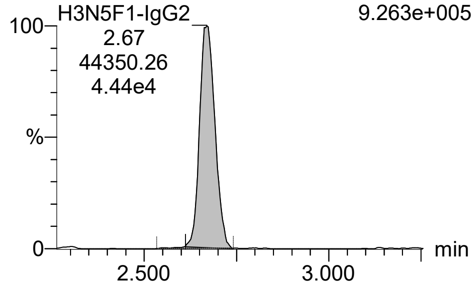

Dataset: C:\MassLynx\10.pro\20211115_clinicalsamples_IgG.qld

Last Altered: Sunday, October 02, 2022 16:02:30 台北標準時間
Printed: Friday, August 01, 2025 16:49:10 台北標準時間

Sample Name: 20211111_QC_R2_IgG

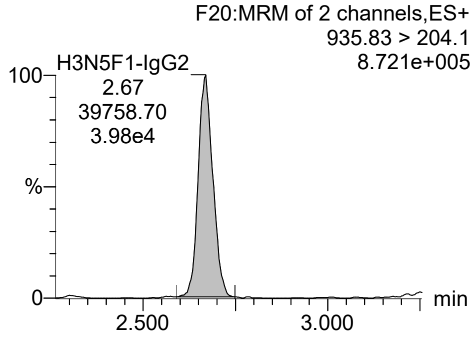

Sample Name: 20211111_L5_R1_IgG

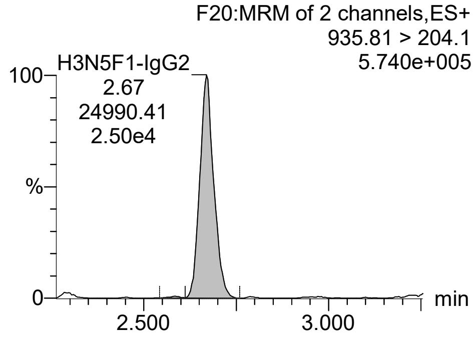

Sample Name: 20211111_L5_R1_IgG

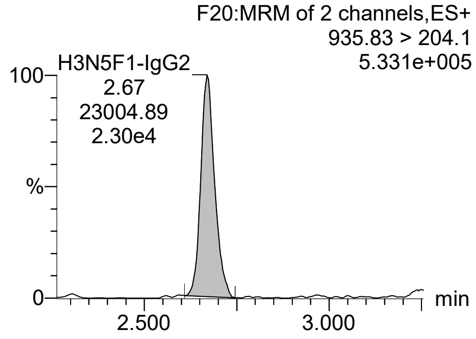

Sample Name: 20211111_L6_R1_IgG

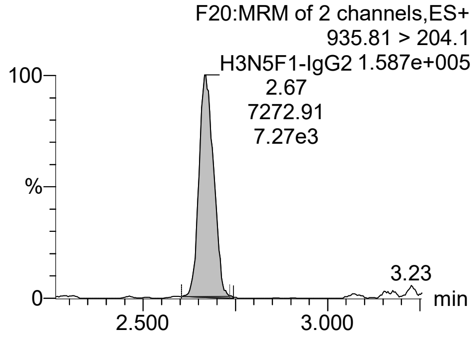

Sample Name: 20211111_L6_R1_IgG

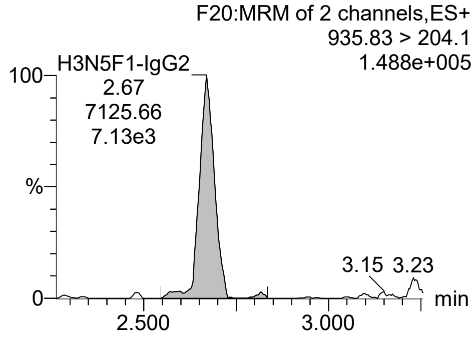

Sample Name: 20211111_H3_R1_IgG

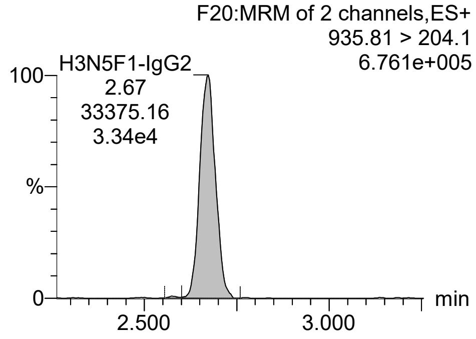

Sample Name: 20211111_H3_R1_IgG

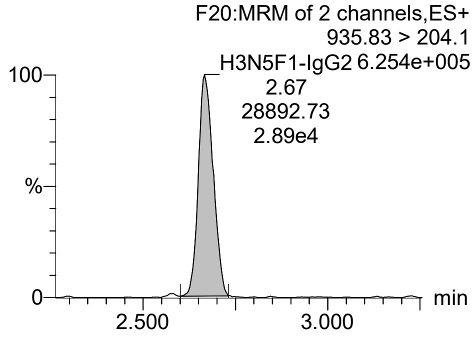

Sample Name: 20211111_A7_R1_IgG

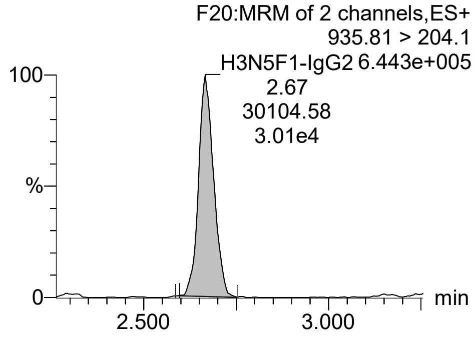

Sample Name: 20211111_A7_R1_IgG

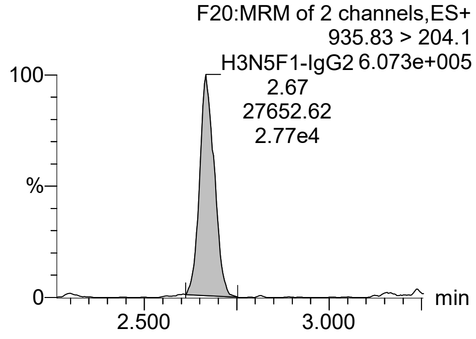

Sample Name: 20211111_A8_R1_IgG

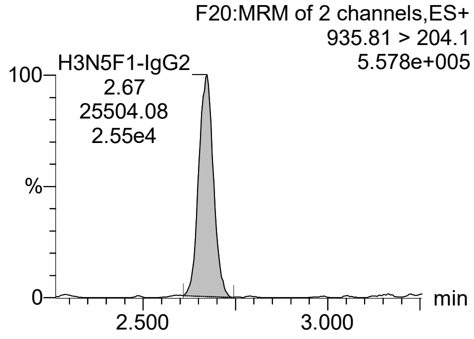

Sample Name: 20211111_A8_R1_IgG

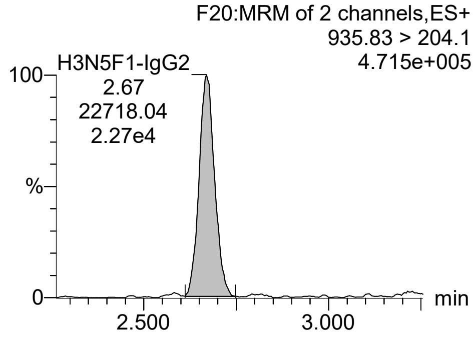

Sample Name: 20211111_L7_R1_IgG

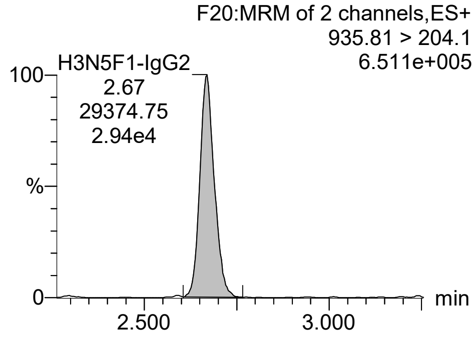

Sample Name: 20211111_L7_R1_IgG

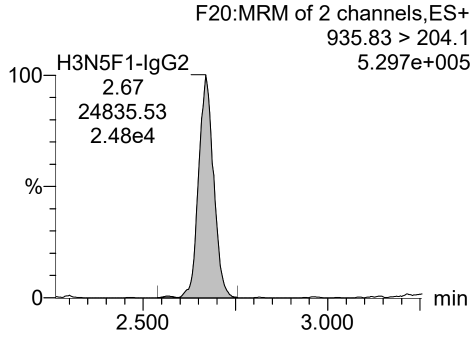

Sample Name: 20211111_L8_R1_IgG

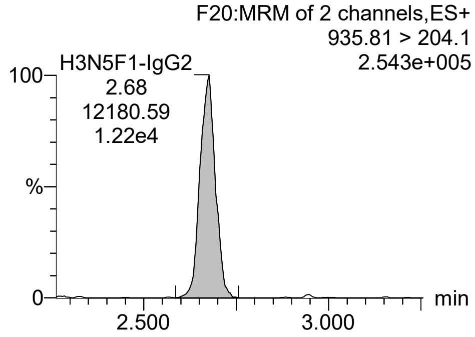

Sample Name: 20211111_L8_R1_IgG

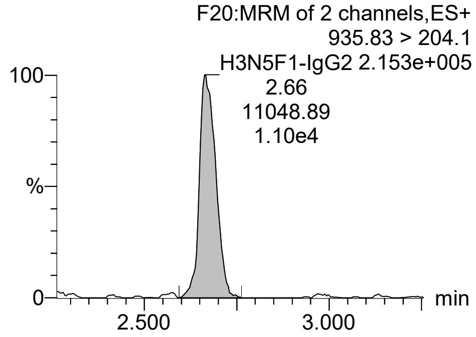

Sample Name: 20211111_H4_R1_IgG

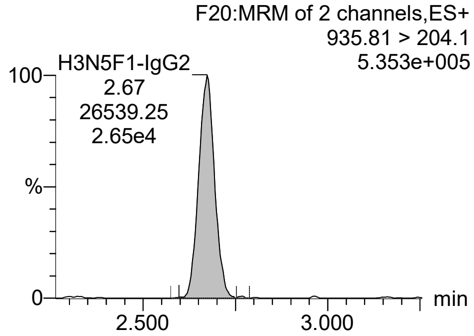

Sample Name: 20211111_H4_R1_IgG

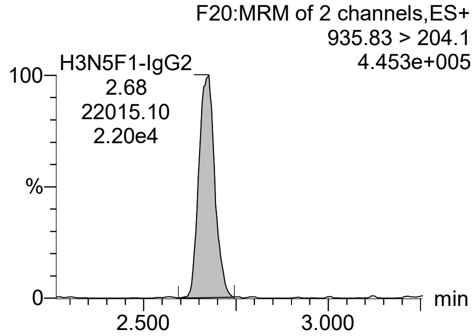

Sample Name: 20211111_A9_R1_IgG

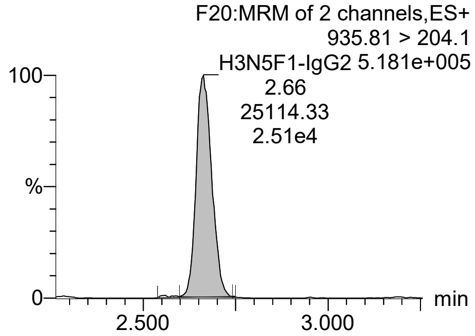

Sample Name: 20211111_A9_R1_IgG

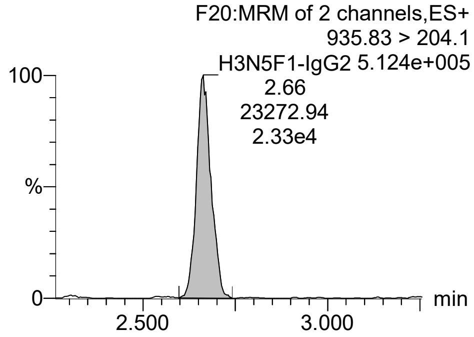

Sample Name: 20211111_A10_R1_IgG

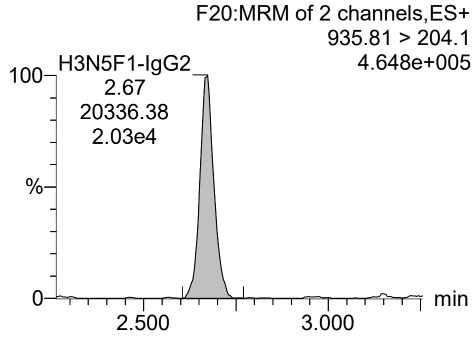

Sample Name: 20211111_A10_R1_IgG

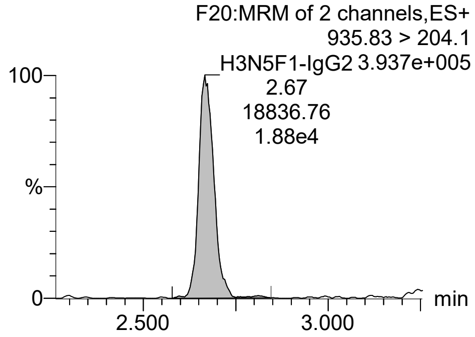

Sample Name: 20211111_L9_R1_IgG

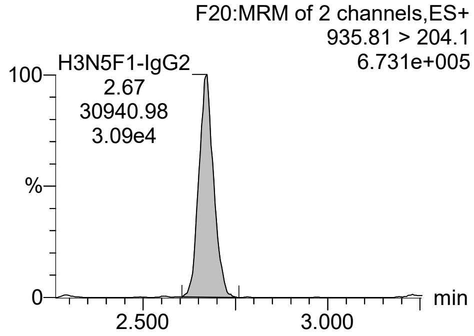

Sample Name: 20211111_L9_R1_IgG

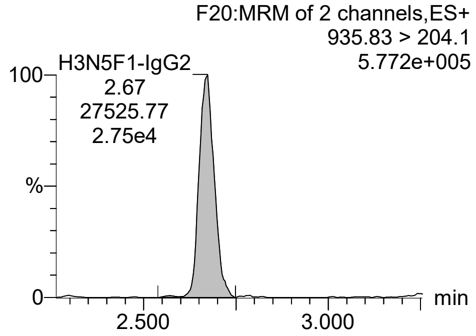

Sample Name: 20211111_L10_R1_IgG

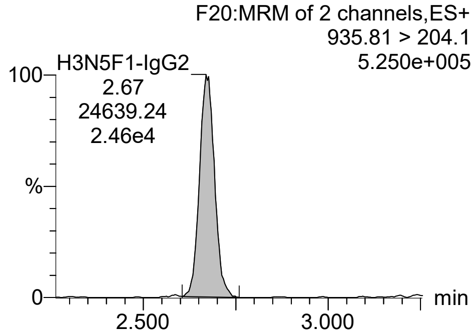

Sample Name: 20211111_L10_R1_IgG

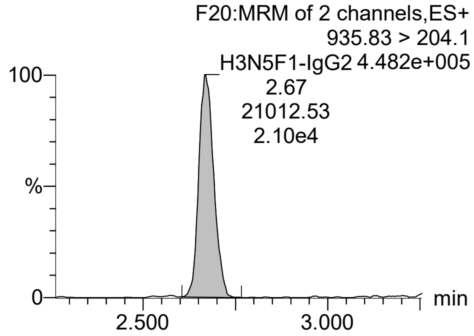

Sample Name: 20211111_QC_R3_IgG

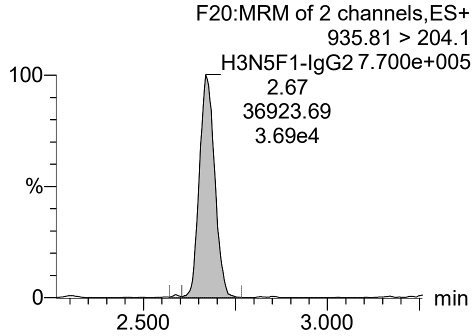

Sample Name: 20211111_QC_R3_IgG

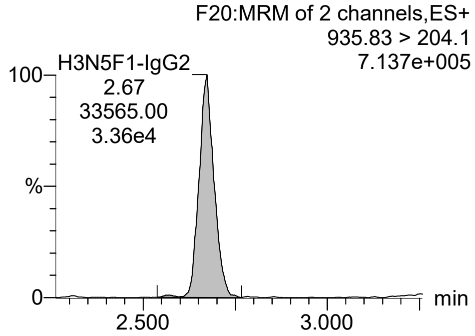

Sample Name: 20211111_H5_R1_IgG

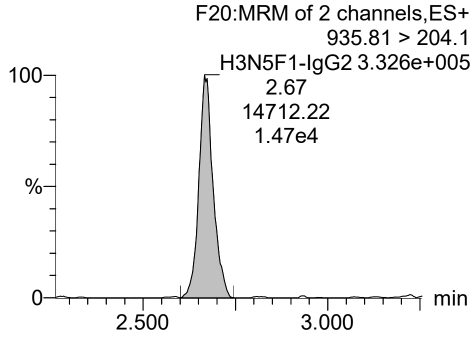

Sample Name: 20211111_H5_R1_IgG

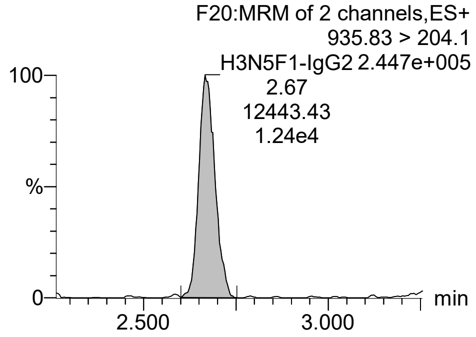

Sample Name: 20211111_A11_R1_IgG

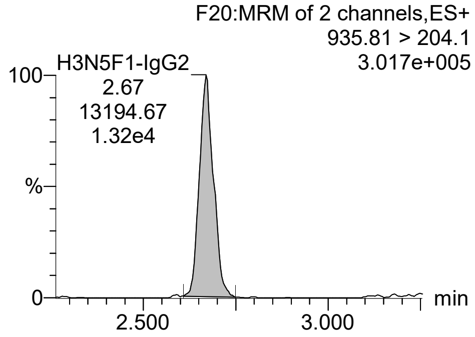

Dataset: C:\MassLynx\10.pro\20211115_clinicalsamples_IgG.qld

Last Altered: Sunday, October 02, 2022 16:02:30 台北標準時間
Printed: Friday, August 01, 2025 16:49:10 台北標準時間

Sample Name: 20211111_A11_R1_IgG

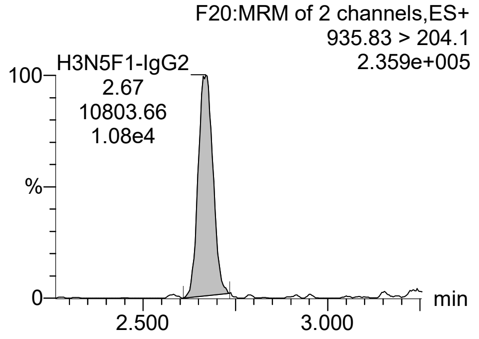

Sample Name: 20211111_A12_R1_IgG

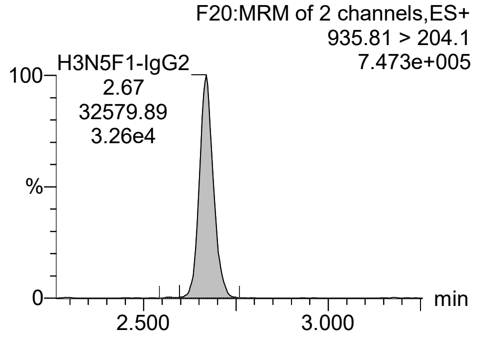

Sample Name: 20211111_A12_R1_IgG

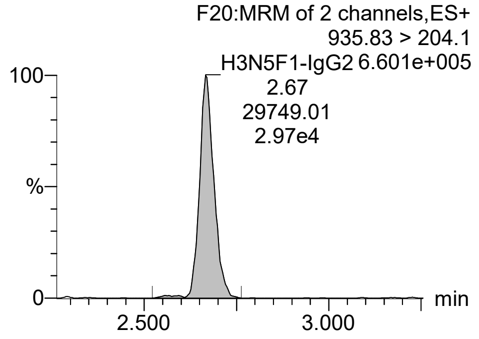

Sample Name: 20211111_L11_R1_IgG

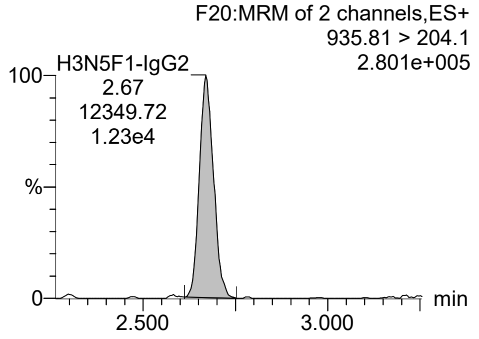

Sample Name: 20211111_L11_R1_IgG

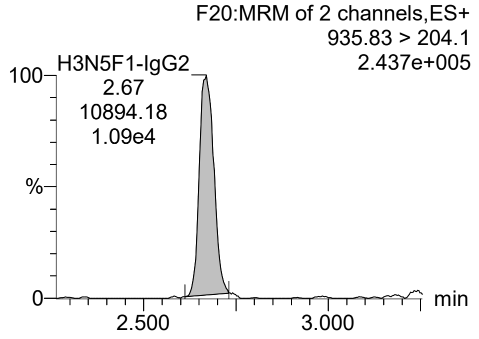

Sample Name: 20211111_L12_R1_IgG

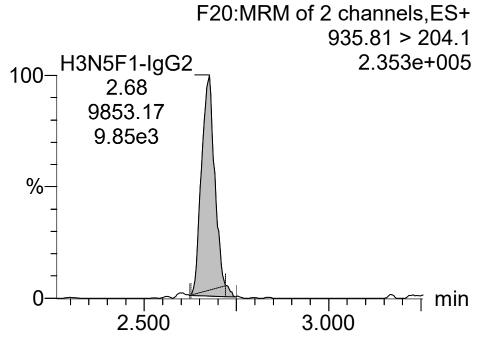

Sample Name: 20211111_L12_R1_IgG

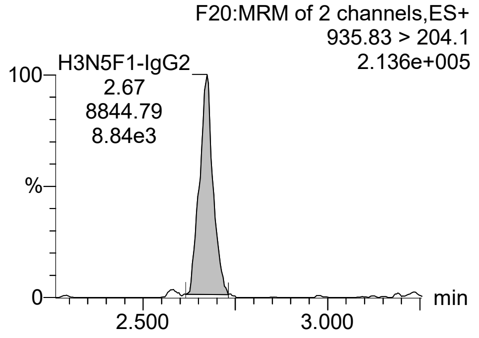

Sample Name: 20211111_H6_R1_IgG

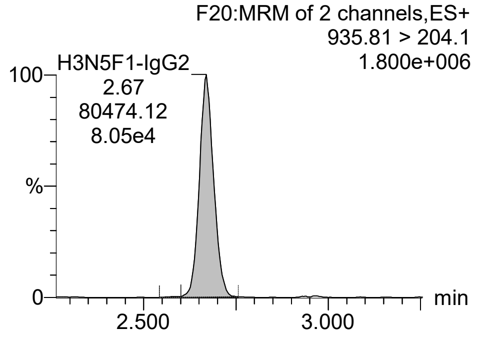

Sample Name: 20211111_H6_R1_IgG

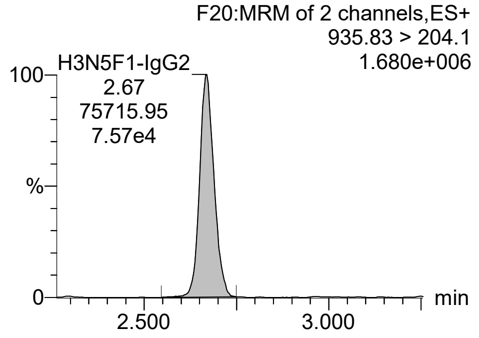

Sample Name: 20211111_A13_R1_IgG

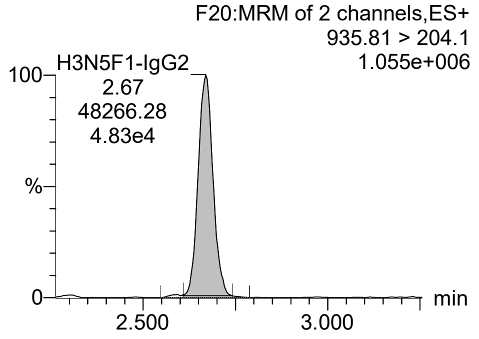

Sample Name: 20211111_A13_R1_IgG

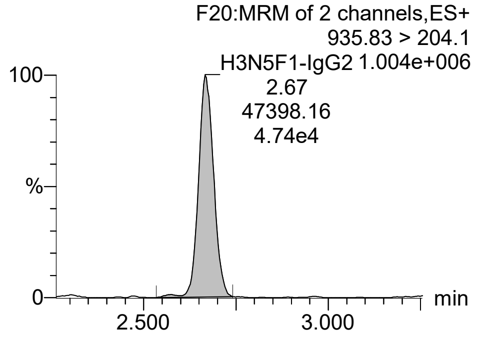

Sample Name: 20211111_A14_R1_IgG

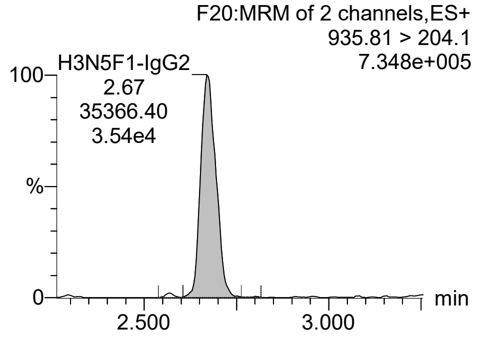

Sample Name: 20211111_A14_R1_IgG

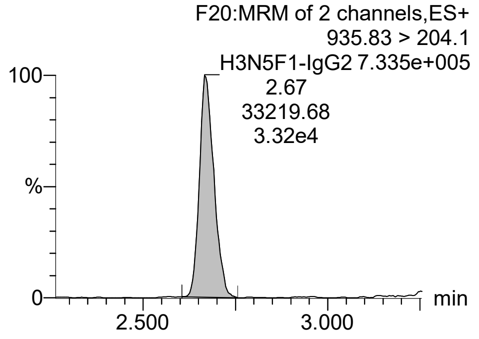

Sample Name: 20211111_L13_R1_IgG

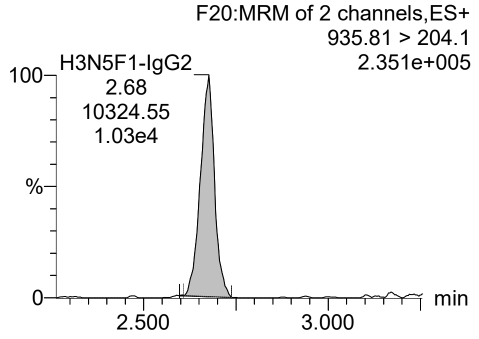

Sample Name: 20211111_L13_R1_IgG

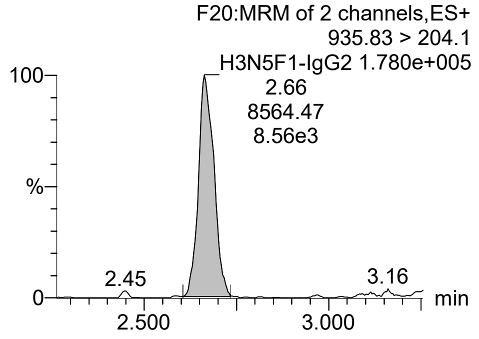

Dataset: C:\MassLynx\10.pro\20211115_clinicalsamples_IgG.qld

Last Altered: Sunday, October 02, 2022 16:02:30 台北標準時間
Printed: Friday, August 01, 2025 16:49:10 台北標準時間

Sample Name: 20211111_L14_R1_IgG

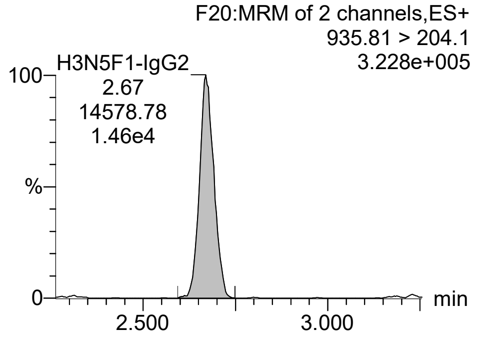

Sample Name: 20211111_L14_R1_IgG

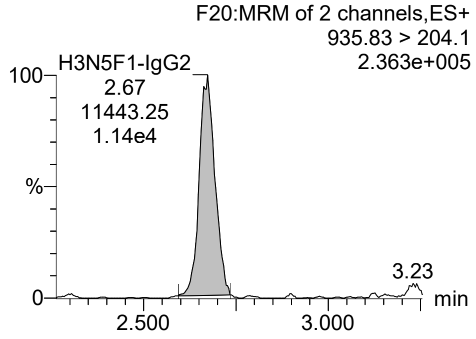

Sample Name: 20211111_H7_R1_IgG

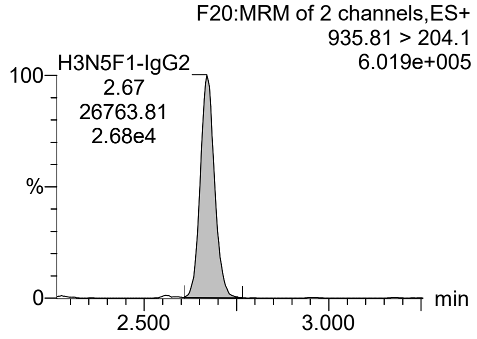

Sample Name: 20211111_H7_R1_IgG

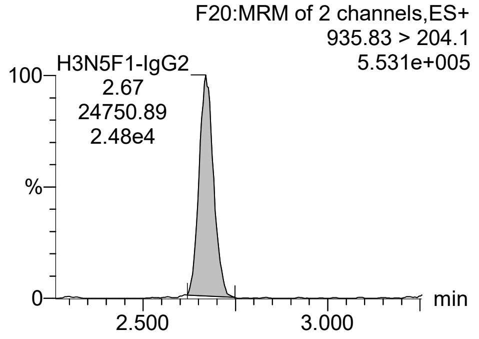

Sample Name: 20211111_A15_R1_IgG

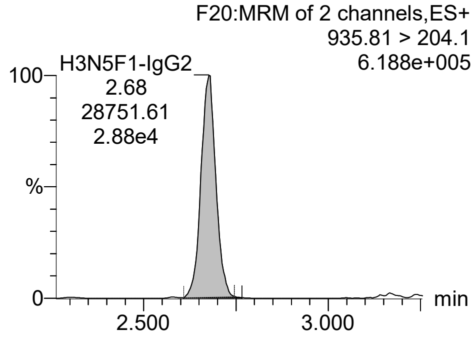

Sample Name: 20211111_A15_R1_IgG

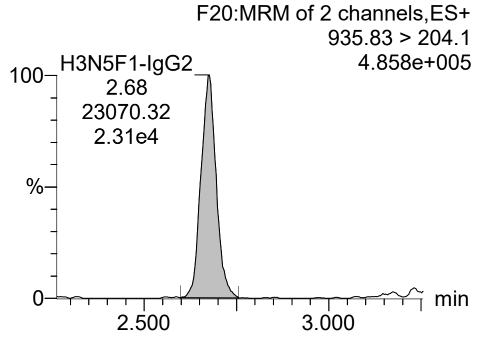

Sample Name: 20211111_QC_R4_IgG

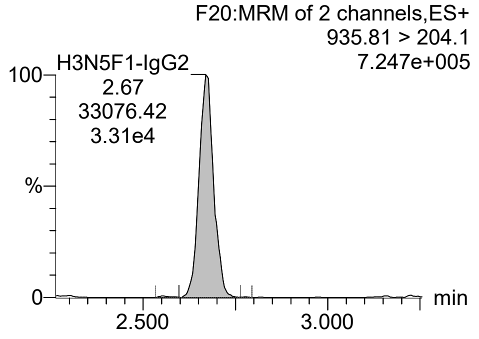

Sample Name: 20211111_QC_R4_IgG

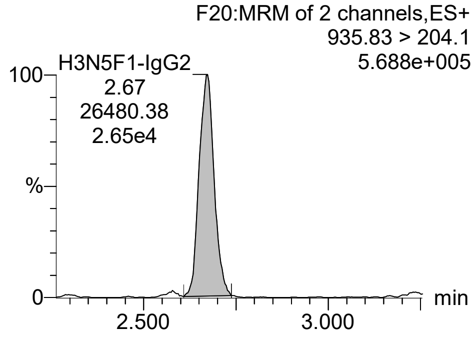

Sample Name: 20211111_A16_R1_IgG

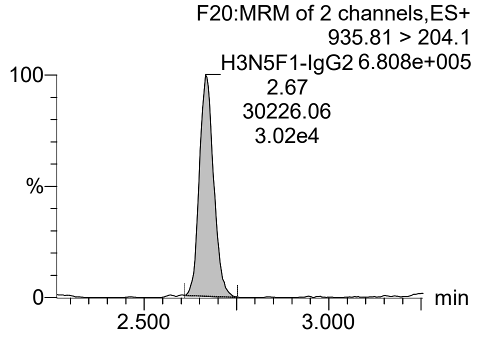

Sample Name: 20211111_A16_R1_IgG

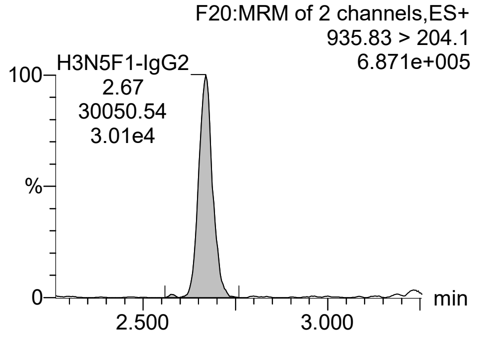

Sample Name: 20211111_L15_R1_IgG

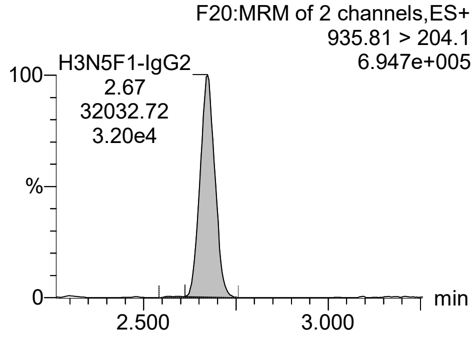

Sample Name: 20211111_L15_R1_IgG

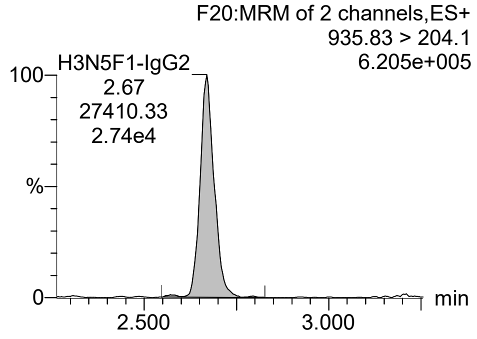

Sample Name: 20211111_L16_R1_IgG

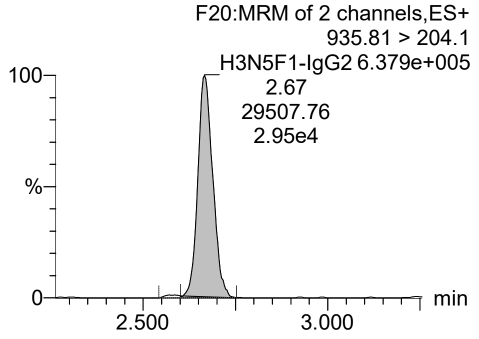

Sample Name: 20211111_L16_R1_IgG

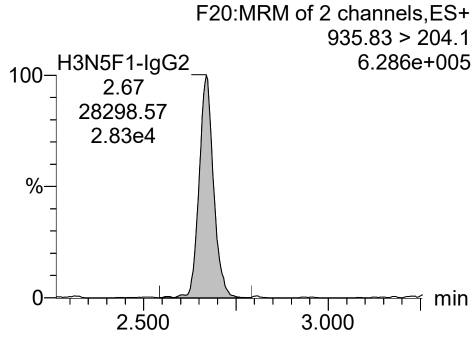

Sample Name: 20211111_H8_R1_IgG

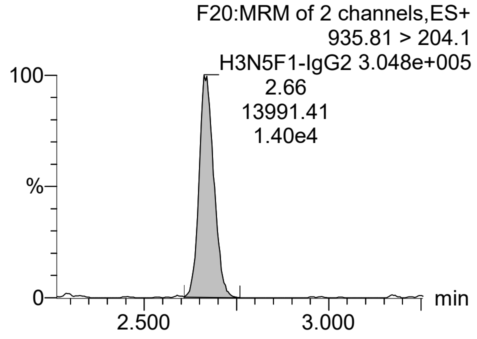

Dataset: C:\MassLynx\10.pro\20211115_clinicalsamples_IgG.qld

Last Altered: Sunday, October 02, 2022 16:02:30 台北標準時間
Printed: Friday, August 01, 2025 16:49:10 台北標準時間

Sample Name: 20211111_H8_R1_IgG

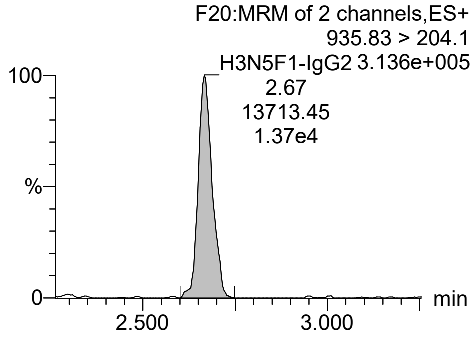

Sample Name: 20211111_A17_R1_IgG

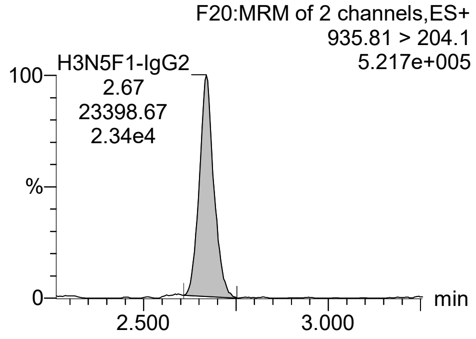

Sample Name: 20211111_A17_R1_IgG

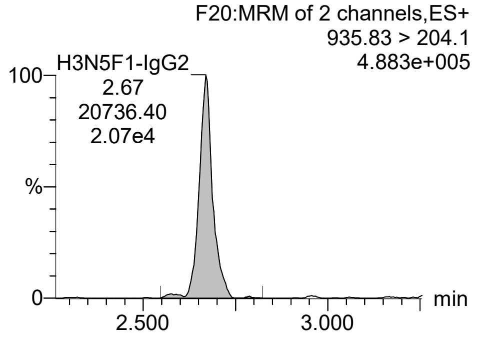

Sample Name: 20211111_A18_R1_IgG

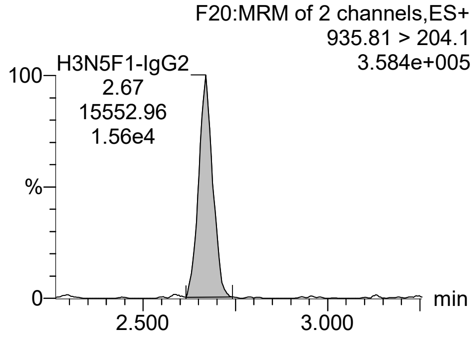

Sample Name: 20211111_A18_R1_IgG

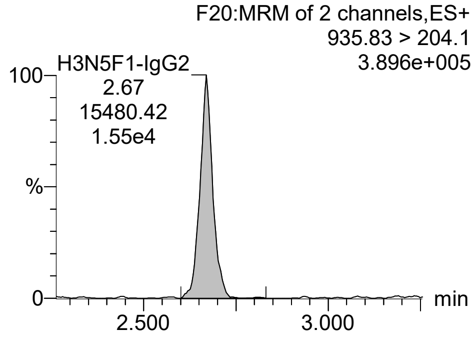

Sample Name: 20211111_L17_R1_IgG

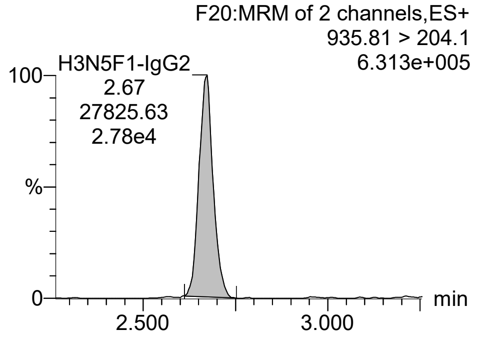

Sample Name: 20211111_L17_R1_IgG

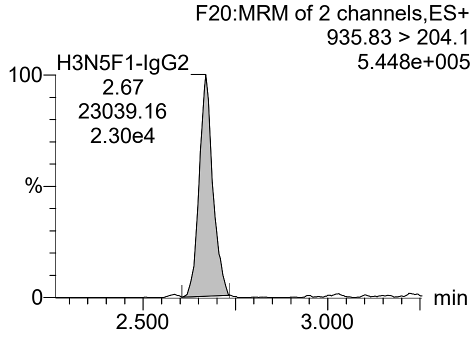

Sample Name: 20211111_L18_R1_IgG

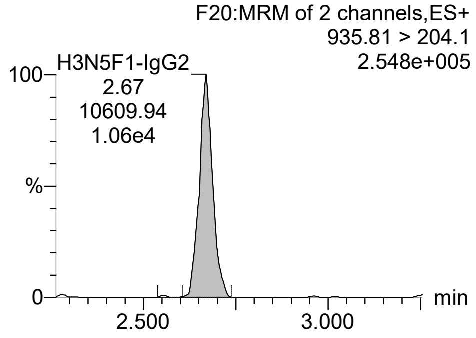

Sample Name: 20211111_L18_R1_IgG

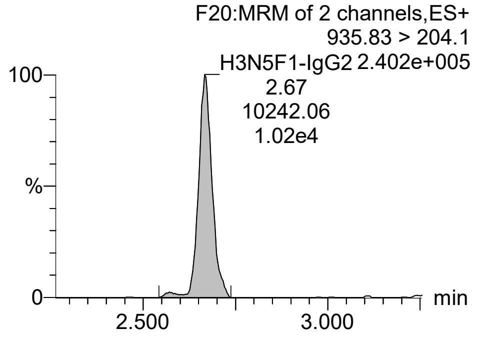

Sample Name: 20211111_H9_R1_IgG

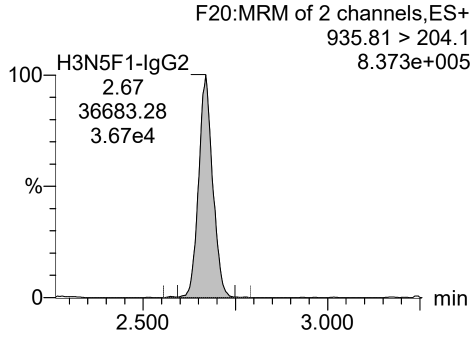

Sample Name: 20211111_H9_R1_IgG

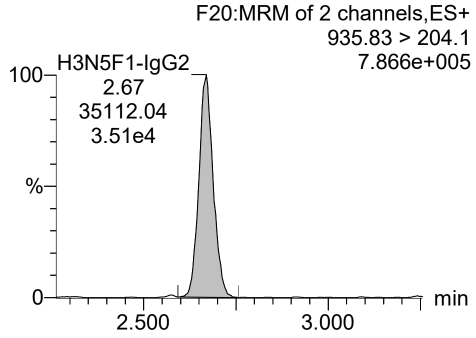

Sample Name: 20211111_A19_R1_IgG

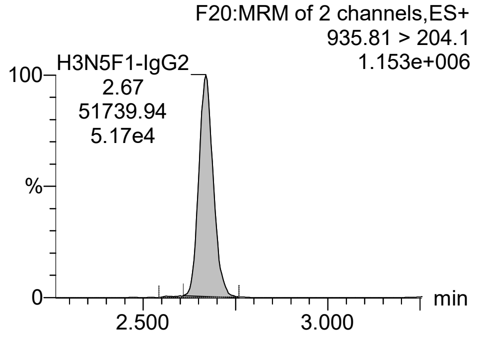

Sample Name: 20211111_A19_R1_IgG

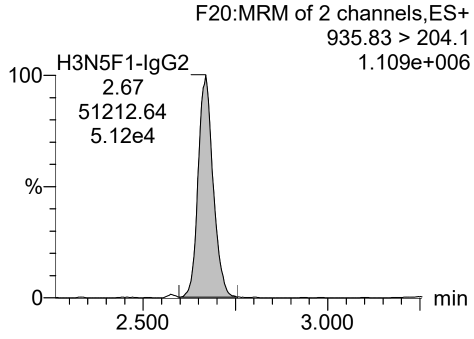

Sample Name: 20211111_A20_R1_IgG

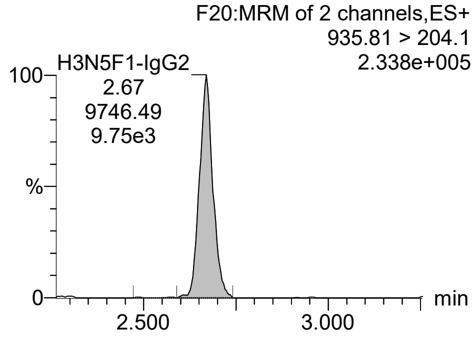

Sample Name: 20211111_A20_R1_IgG

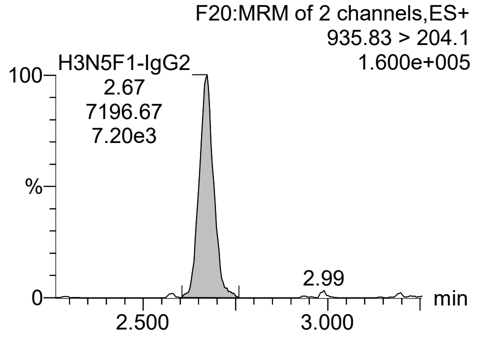

Sample Name: 20211111_L19_R1_IgG
F20:MRM of 2 channels,ES+
935.81 > 204.1

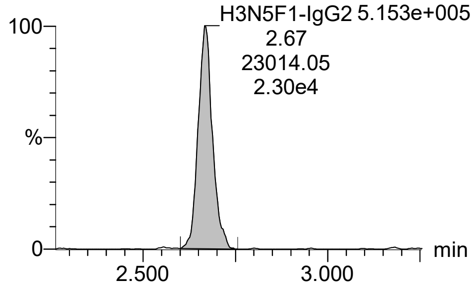

Sample Name: 20211111_L19_R1_IgG
F20:MRM of 2 channels,ES+
935.83 > 204.1

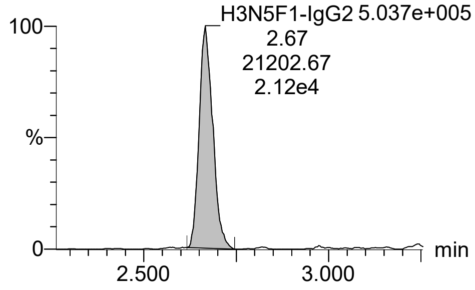

Sample Name: 20211111_QC_R5_IgG
F20:MRM of 2 channels,ES+
935.81 > 204.1

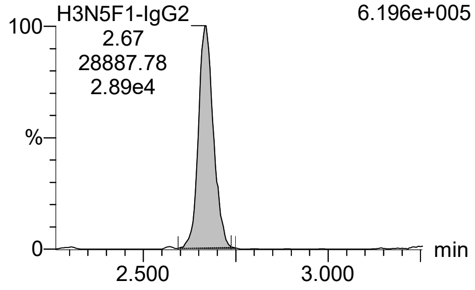

Sample Name: 20211111_QC_R5_IgG
F20:MRM of 2 channels,ES+
935.83 > 204.1

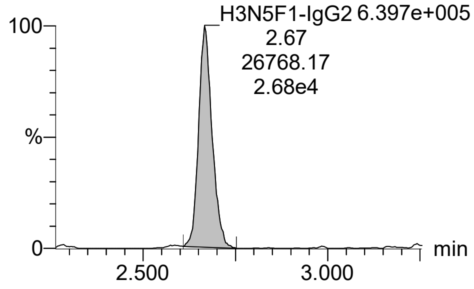

Sample Name: 20211111_L20_R1_IgG
F20:MRM of 2 channels,ES+
935.81 > 204.1

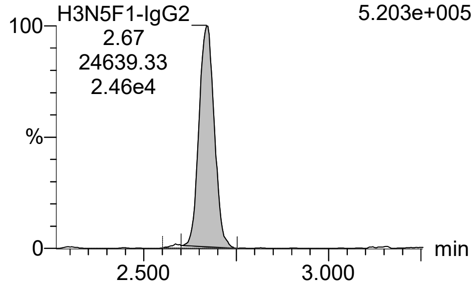

Sample Name: 20211111_L20_R1_IgG
F20:MRM of 2 channels,ES+
935.83 > 204.1

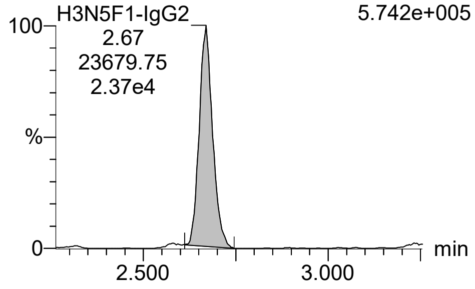

Sample Name: 20211111_H10_R1_IgG
F20:MRM of 2 channels,ES+
935.81 > 204.1

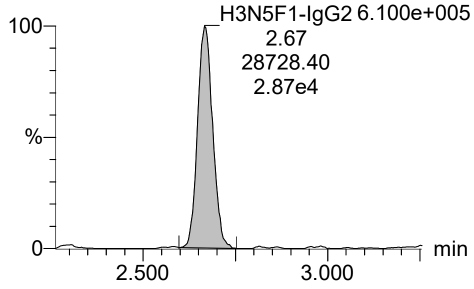

Sample Name: 20211111_H10_R1_IgG
F20:MRM of 2 channels,ES+
935.83 > 204.1

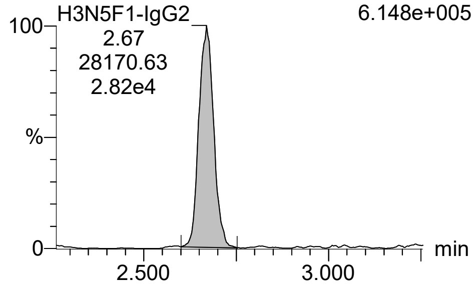

Sample Name: 20211111_A21_R1_IgG
F20:MRM of 2 channels,ES+
935.81 > 204.1

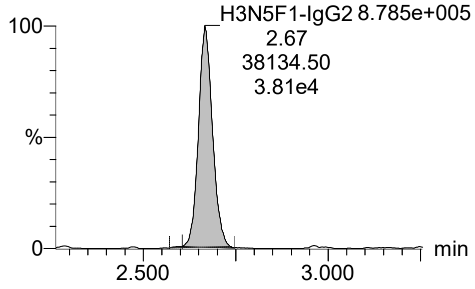

Sample Name: 20211111_A21_R1_IgG
F20:MRM of 2 channels,ES+
935.83 > 204.1

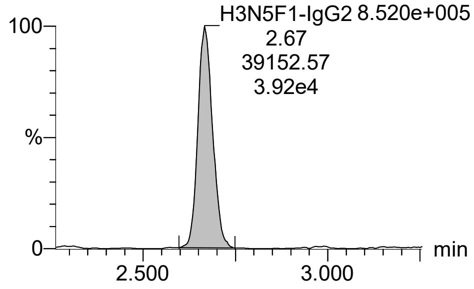

Sample Name: 20211111_A22_R1_IgG
F20:MRM of 2 channels,ES+
935.81 > 204.1

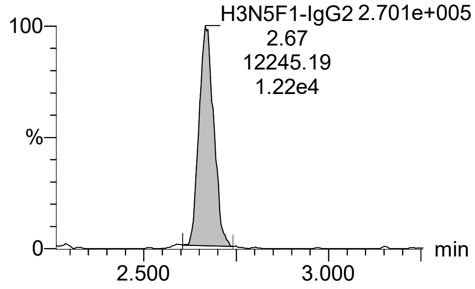

Sample Name: 20211111_A22_R1_IgG
F20:MRM of 2 channels,ES+
935.83 > 204.1

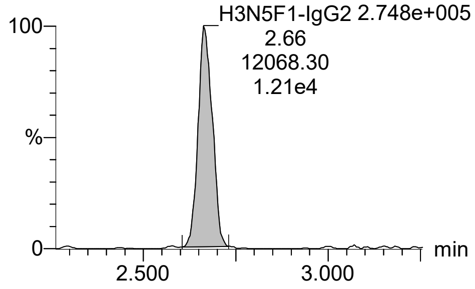

Sample Name: 20211111_L21_R1_IgG
F20:MRM of 2 channels,ES+
935.81 > 204.1

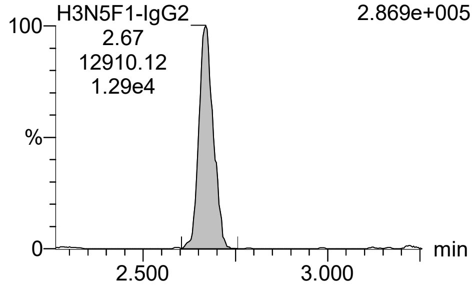

Sample Name: 20211111_L21_R1_IgG
F20:MRM of 2 channels,ES+
935.83 > 204.1

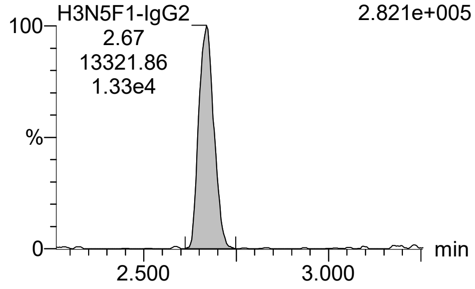

Sample Name: 20211111_L22_R1_IgG
F20:MRM of 2 channels,ES+
935.81 > 204.1

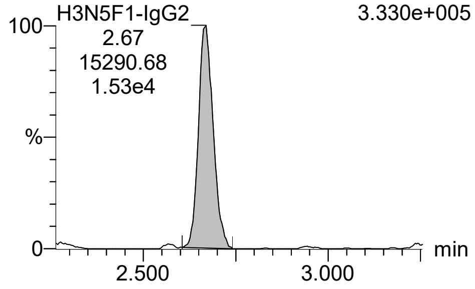

Dataset: C:\MassLynx\10.pro\20211115_clinicalsamples_IgG.qld

Last Altered: Sunday, October 02, 2022 16:02:30 台北標準時間
Printed: Friday, August 01, 2025 16:49:10 台北標準時間

Sample Name: 20211111_L22_R1_IgG

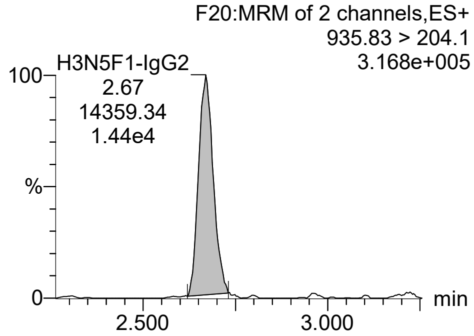

Sample Name: 20211111_H11_R1_IgG

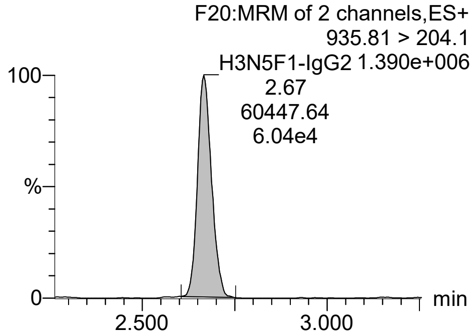

Sample Name: 20211111_H11_R1_IgG

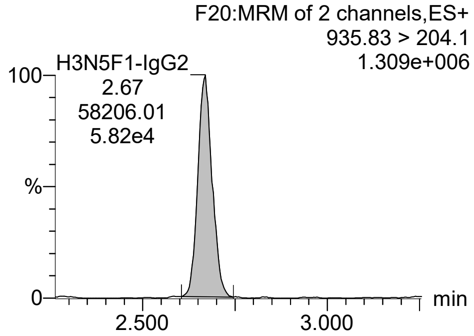

Sample Name: 20211111_A23_R1_IgG

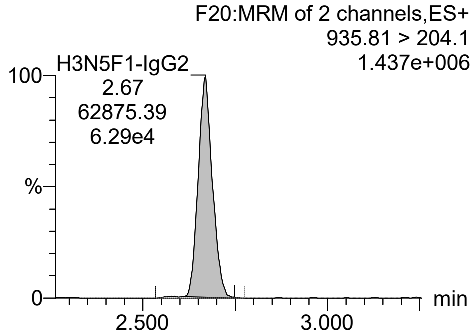

Sample Name: 20211111_A23_R1_IgG

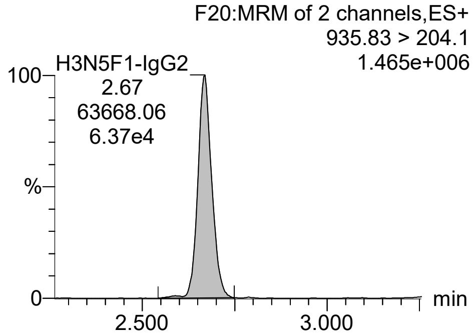

Sample Name: 20211111_A24_R1_IgG

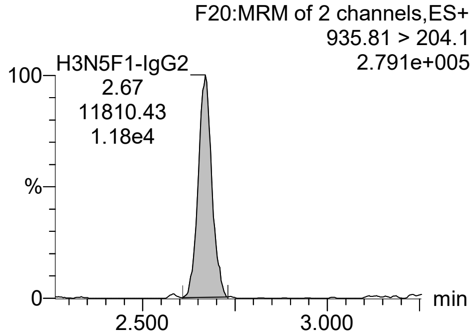

Sample Name: 20211111_A24_R1_IgG

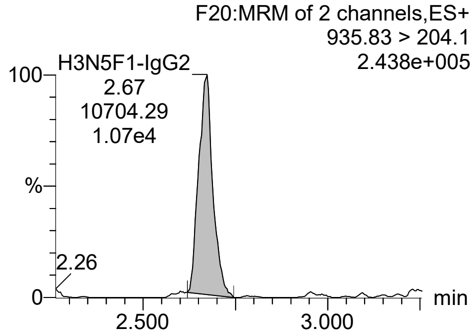

Sample Name: 20211111_L23_R1_IgG

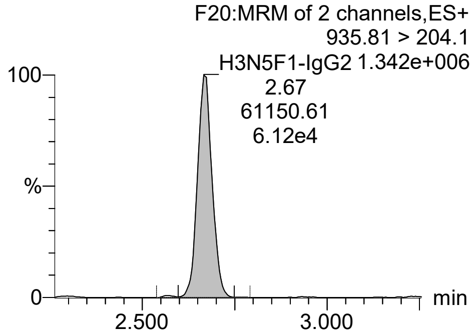

Sample Name: 20211111_L23_R1_IgG

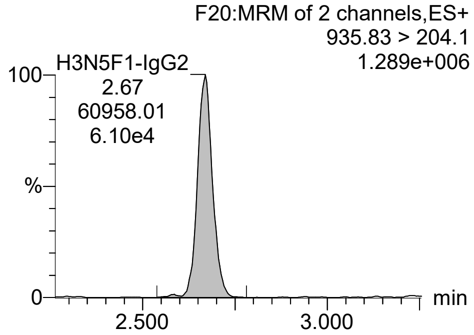

Sample Name: 20211111_L24_R1_IgG

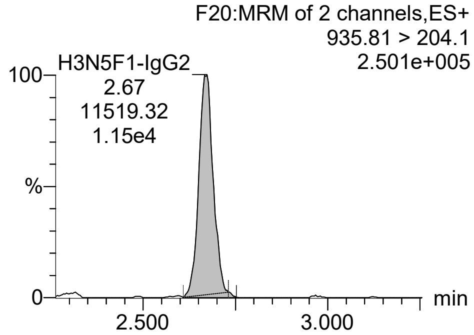

Sample Name: 20211111_L24_R1_IgG

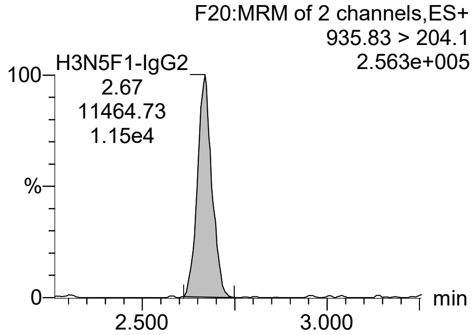

Sample Name: 20211111_H12_R1_IgG

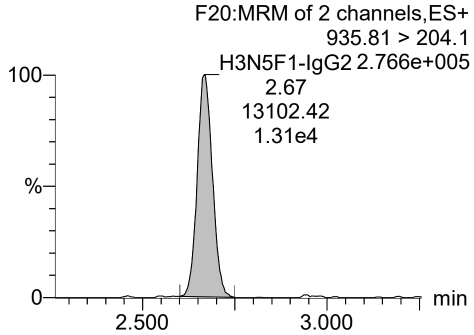

Sample Name: 20211111_H12_R1_IgG

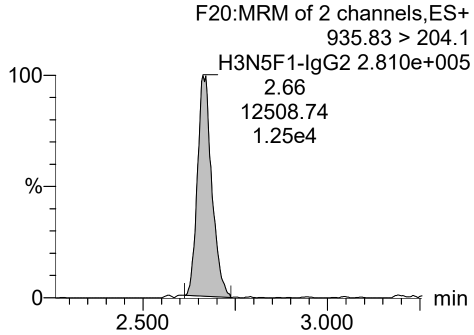

Sample Name: 20211111_QC_R6_IgG

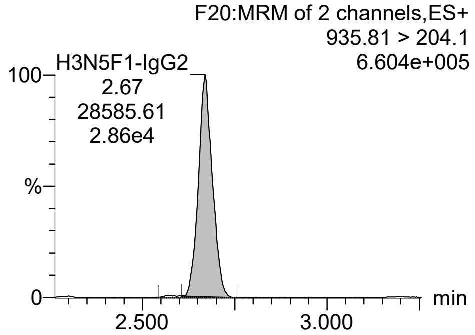

Sample Name: 20211111_QC_R6_IgG

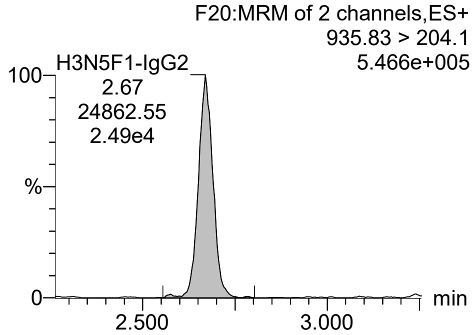

Dataset: C:\MassLynx\10.pro\20211115_clinicalsamples_IgG.qld

Last Altered: Sunday, October 02, 2022 16:02:30 台北標準時間
Printed: Friday, August 01, 2025 16:49:10 台北標準時間

Sample Name: 20211111_A25_R1_IgG

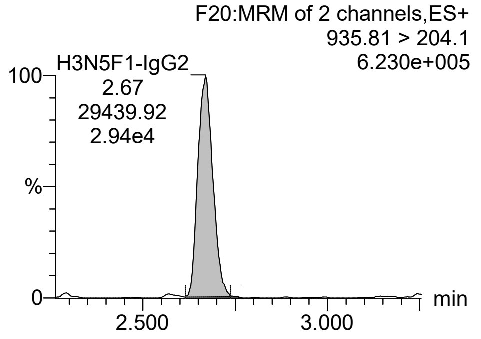

Sample Name: 20211111_A25_R1_IgG

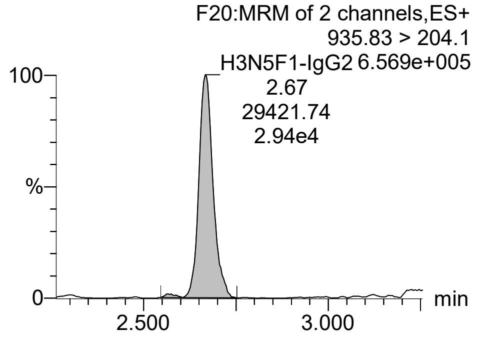

Sample Name: 20211111_A26_R1_IgG

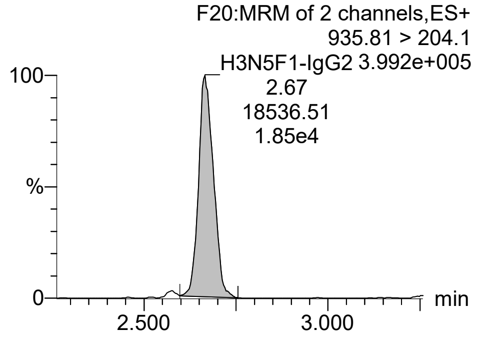

Sample Name: 20211111_A26_R1_IgG

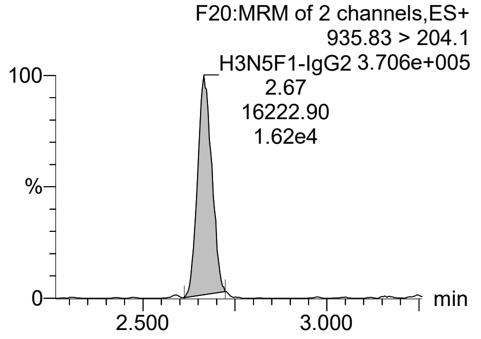

Sample Name: 20211111_L25_R1_IgG

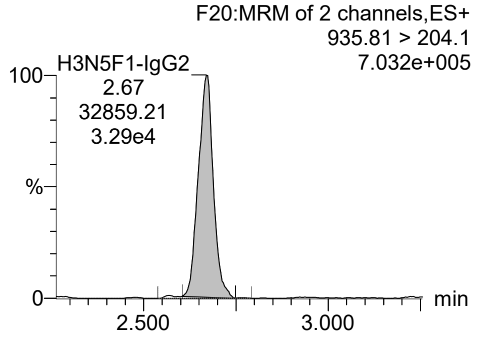

Sample Name: 20211111_L25_R1_IgG

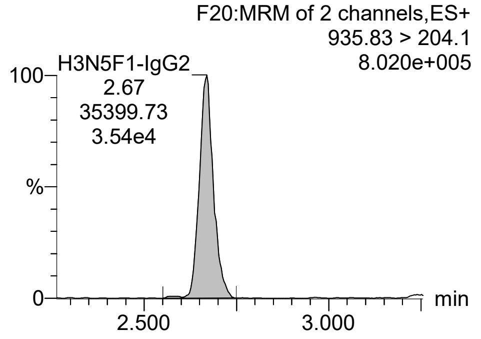

Sample Name: 20211111_L26_R1_IgG

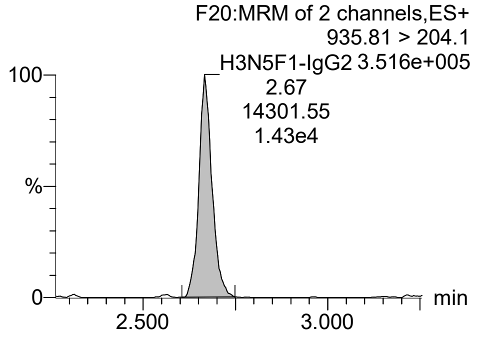

Sample Name: 20211111_L26_R1_IgG

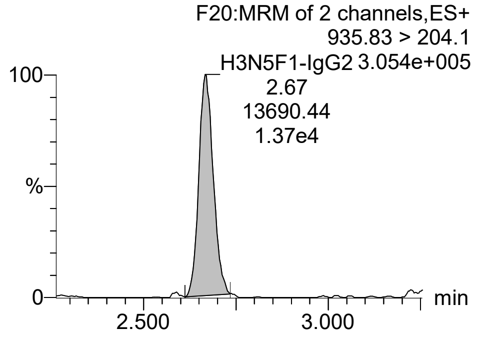

Sample Name: 20211111_H13_R1_IgG

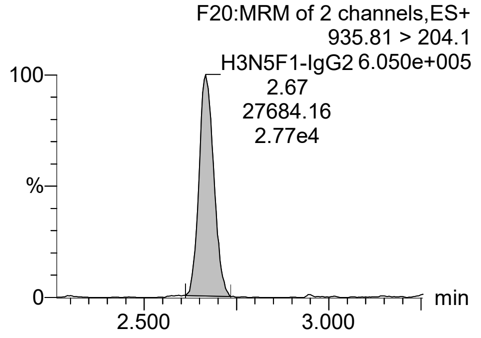

Sample Name: 20211111_H13_R1_IgG

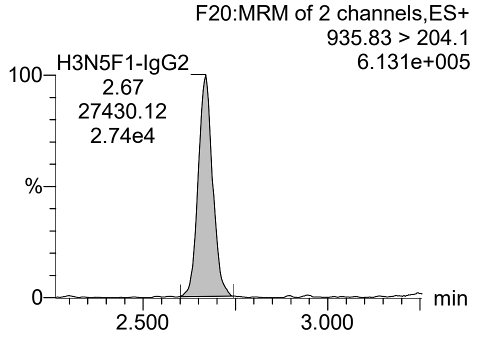

Sample Name: 20211111_A27_R1_IgG

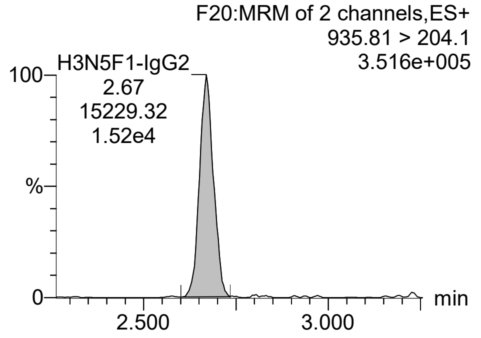

Sample Name: 20211111_A27_R1_IgG

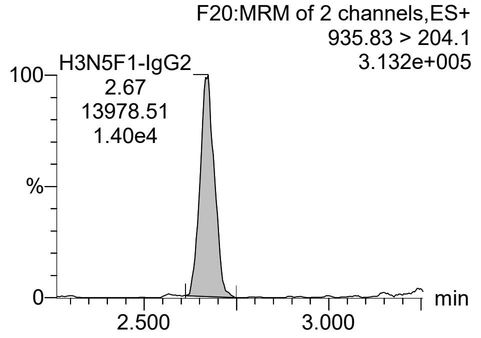

Sample Name: 20211111_A28_R1_IgG

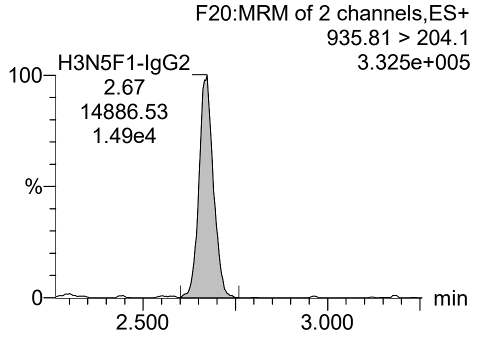

Sample Name: 20211111_A28_R1_IgG

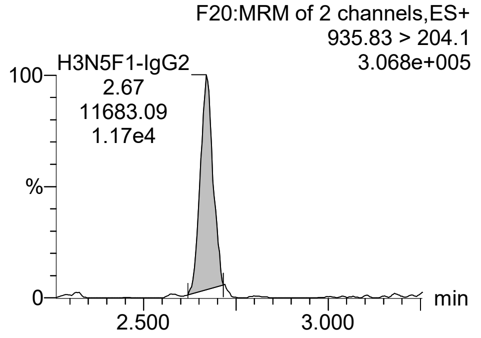

Sample Name: 20211111_L27_R1_IgG

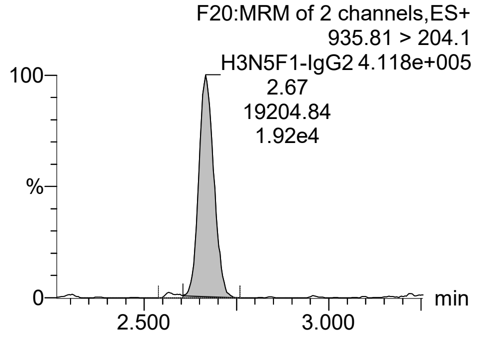

Dataset: C:\MassLynx\10.pro\20211115_clinicalsamples_IgG.qld

Last Altered: Sunday, October 02, 2022 16:02:30 台北標準時間
Printed: Friday, August 01, 2025 16:49:10 台北標準時間

Sample Name: 20211111_L27_R1_IgG

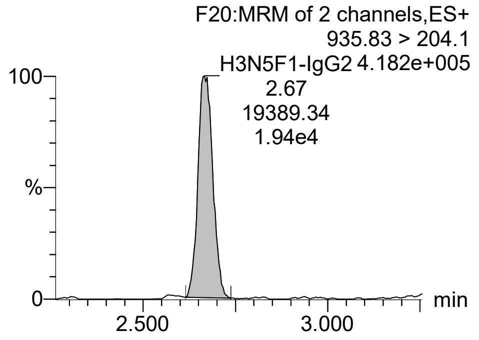

Sample Name: 20211111_L28_R1_IgG

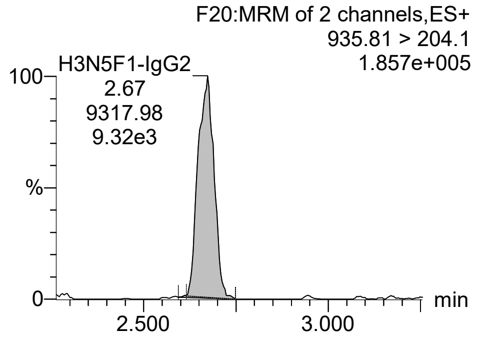

Sample Name: 20211111_L28_R1_IgG

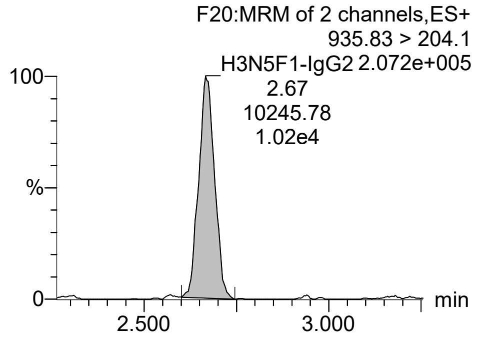

Sample Name: 20211111_H14_R1_IgG

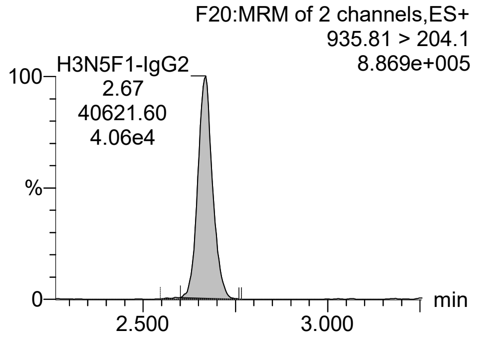

Sample Name: 20211111_H14_R1_IgG

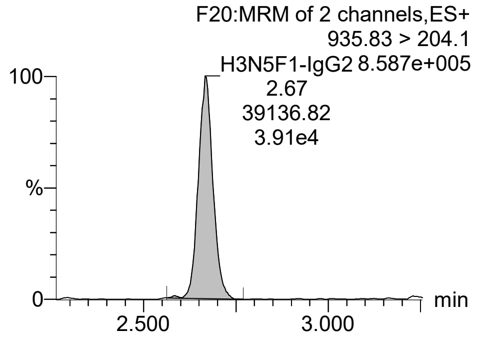

Sample Name: 20211111_A29_R1_IgG

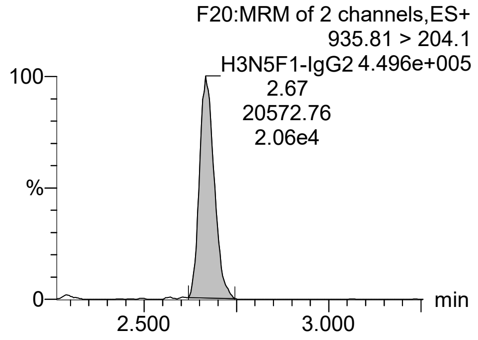

Sample Name: 20211111_A29_R1_IgG

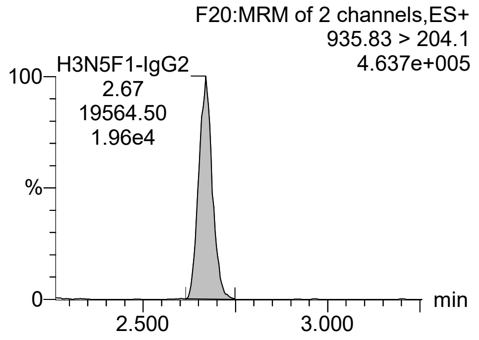

Sample Name: 20211111_A30_R1_IgG

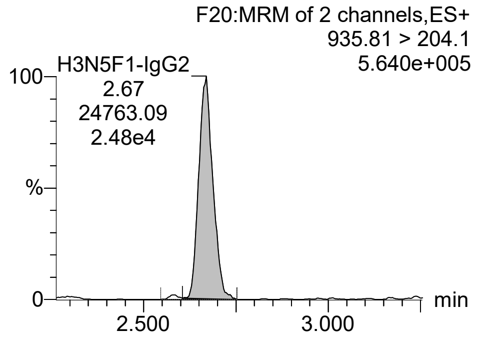

Sample Name: 20211111_A30_R1_IgG

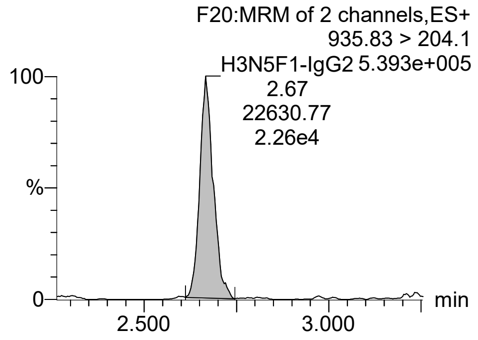

Sample Name: 20211111_QC_R7_IgG

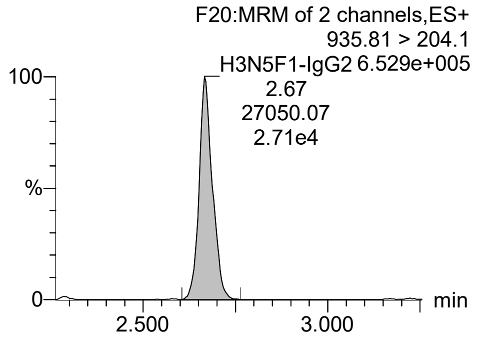

Sample Name: 20211111_QC_R7_IgG

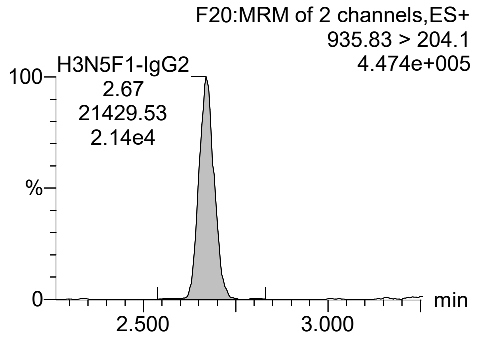

Sample Name: 20211111_L29_R1_IgG

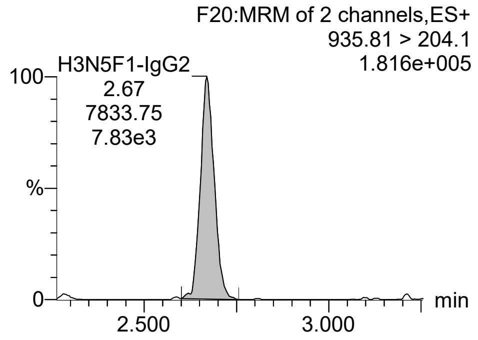

Sample Name: 20211111_L29_R1_IgG

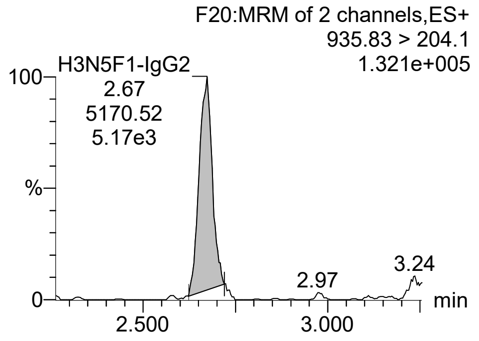

Sample Name: 20211111_L30_R1_IgG

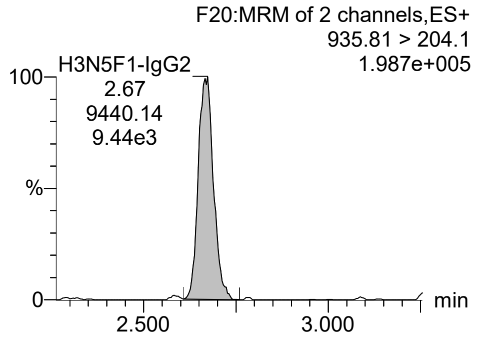

Sample Name: 20211111_L30_R1_IgG

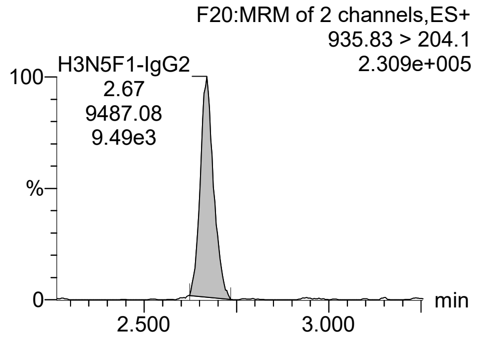

Dataset: C:\MassLynx\10.pro\20211115_clinicalsamples_IgG.qld

Last Altered: Sunday, October 02, 2022 16:02:30 台北標準時間
Printed: Friday, August 01, 2025 16:49:10 台北標準時間

Sample Name: 20211111_H15_R1_IgG

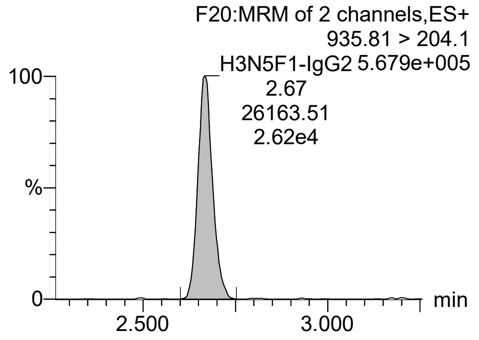

Sample Name: 20211111_H15_R1_IgG

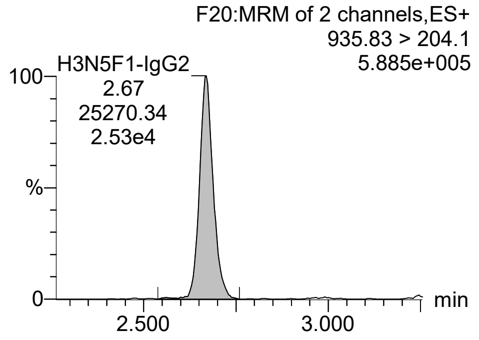

Sample Name: 20211111_A31_R1_IgG

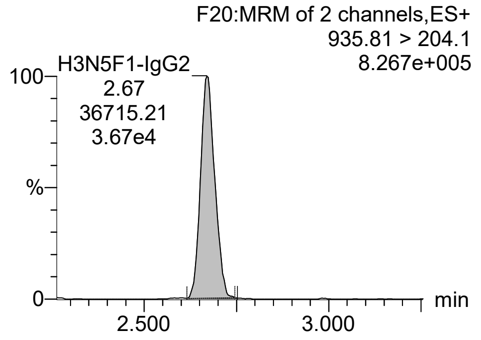

Sample Name: 20211111_A31_R1_IgG

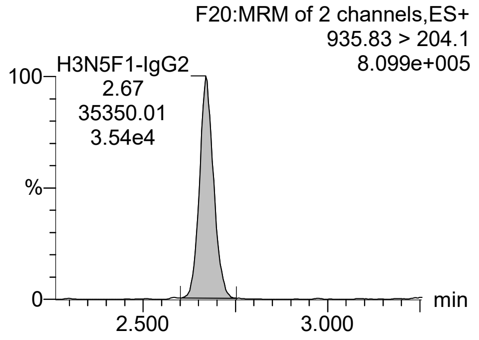

Sample Name: 20211111_A32_R1_IgG

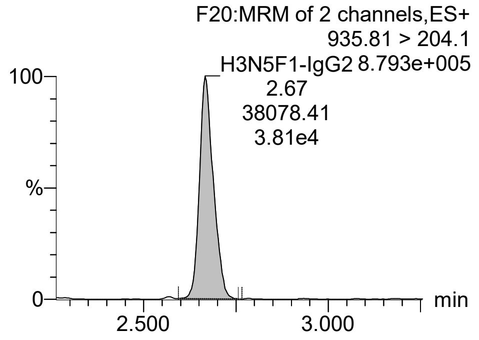

Sample Name: 20211111_A32_R1_IgG

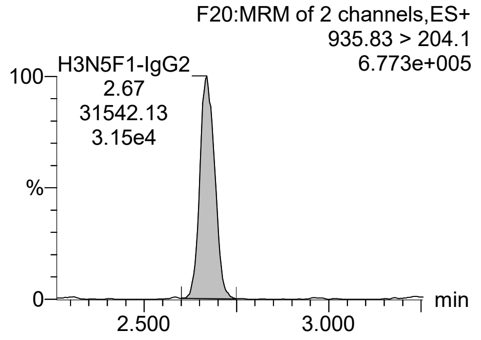

Sample Name: 20211111_L31_R1_IgG

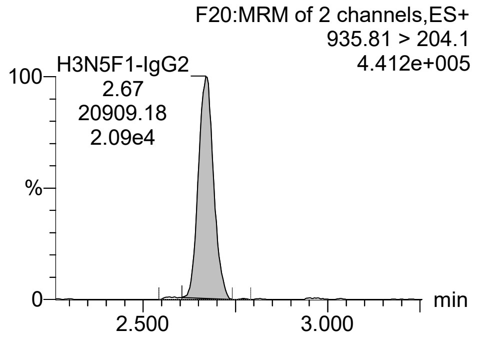

Sample Name: 20211111_L31_R1_IgG

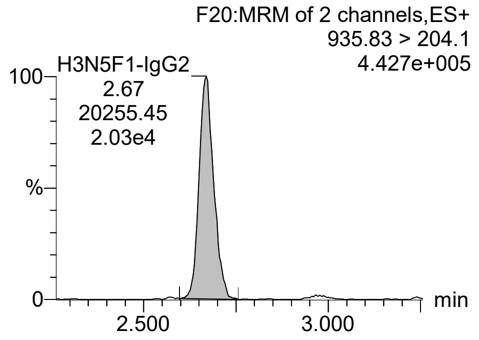

Sample Name: 20211111_L32_R1_IgG

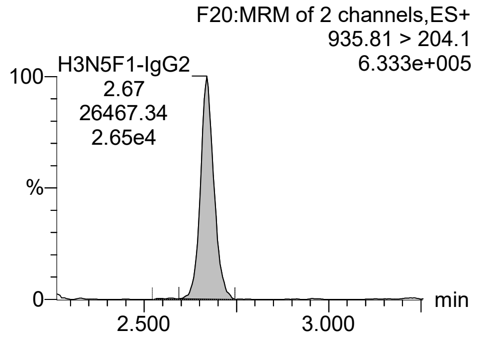

Sample Name: 20211111_L32_R1_IgG

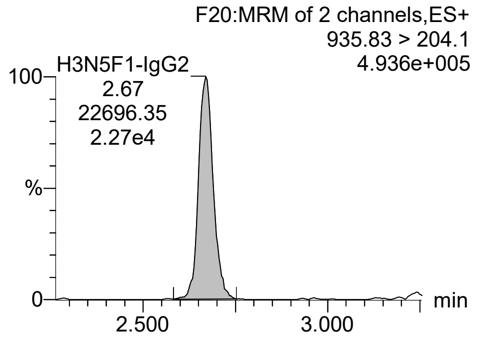

Sample Name: 20211111_H16_R1_IgG

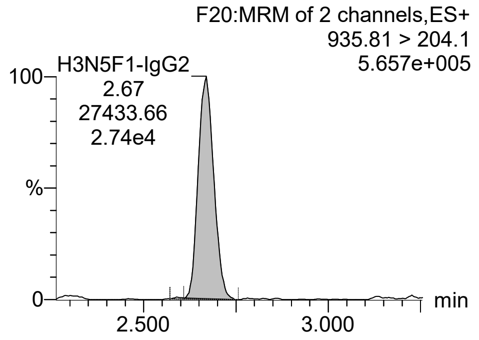

Sample Name: 20211111_H16_R1_IgG

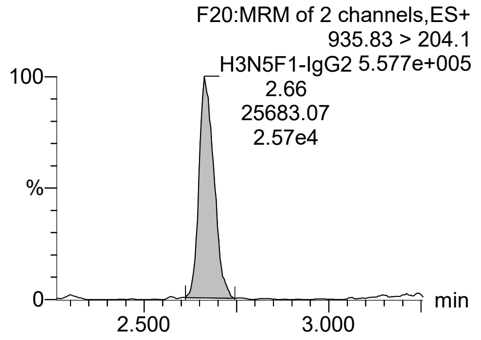

Sample Name: 20211111_A33_R1_IgG

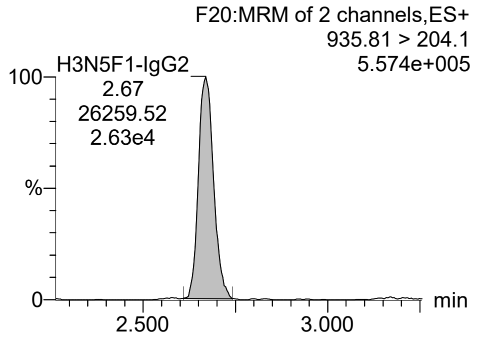

Sample Name: 20211111_A33_R1_IgG

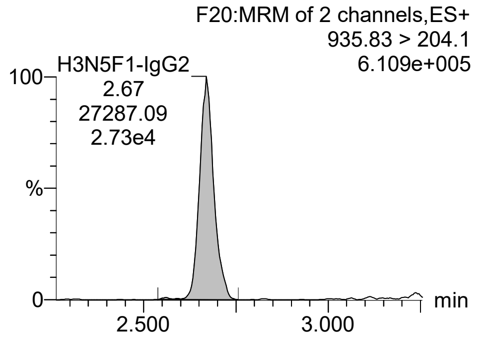

Sample Name: 20211111_A34_R1_IgG

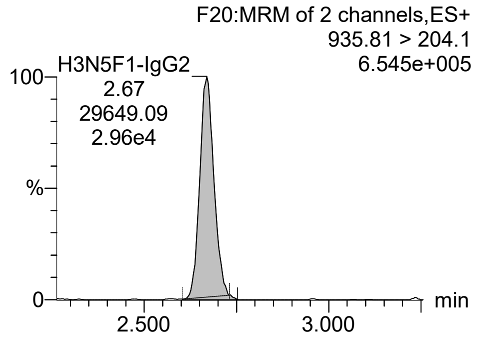

Dataset: C:\MassLynx\10.pro\20211115_clinicalsamples_IgG.qld

Last Altered: Sunday, October 02, 2022 16:02:30 台北標準時間
Printed: Friday, August 01, 2025 16:49:10 台北標準時間

Sample Name: 20211111_A34_R1_IgG

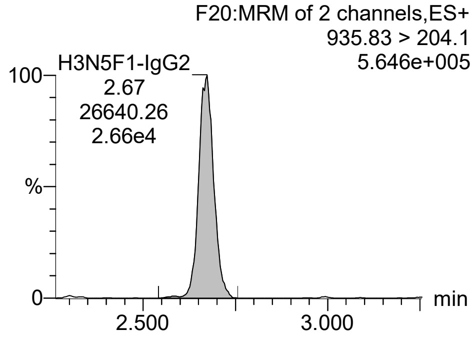

Sample Name: 20211111_H17_R1_IgG

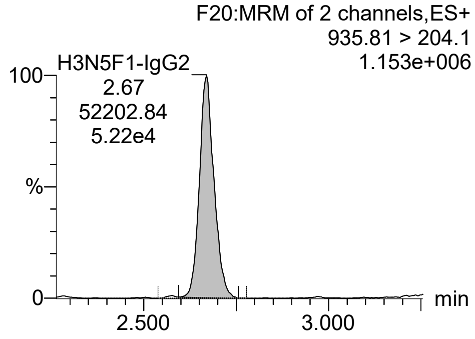

Sample Name: 20211111_H17_R1_IgG

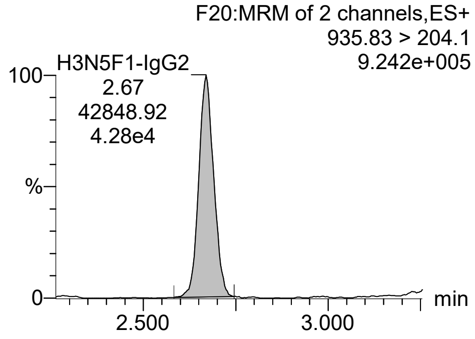

Sample Name: 20211111_A35_R1_IgG

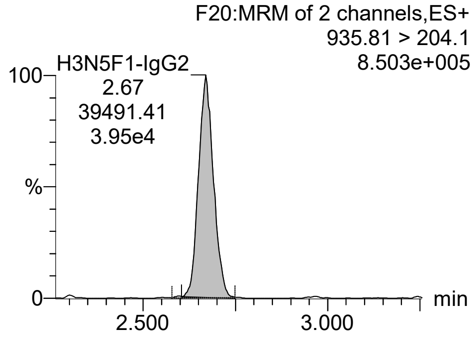

Sample Name: 20211111_A35_R1_IgG

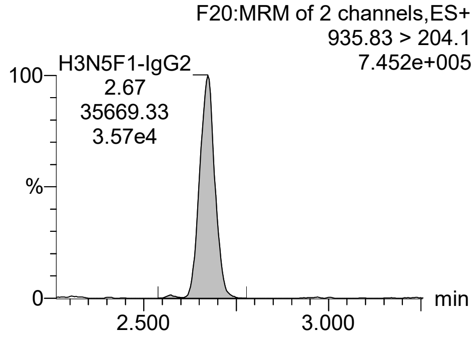

Sample Name: 20211111_QC_R8_IgG

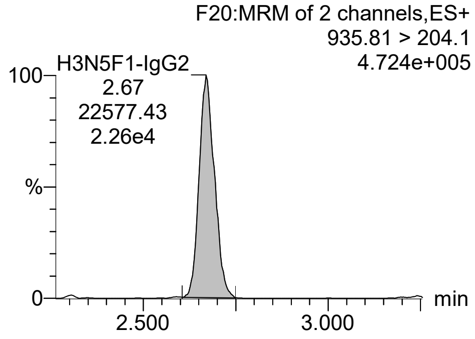

Sample Name: 20211111_QC_R8_IgG

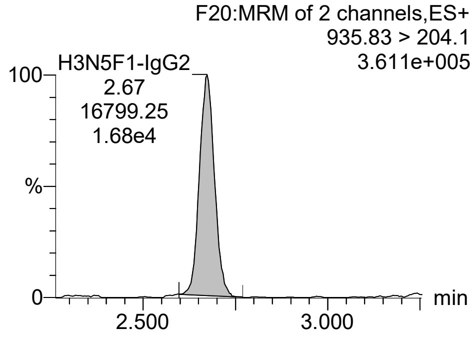

Sample Name: 20211111_A36_R1_IgG

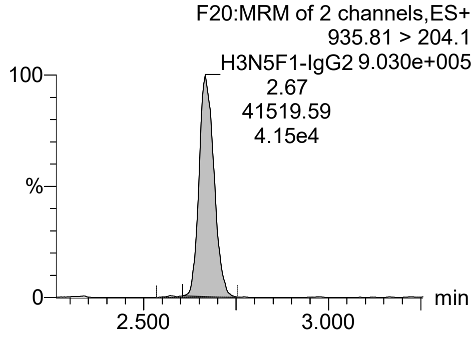

Sample Name: 20211111_A36_R1_IgG

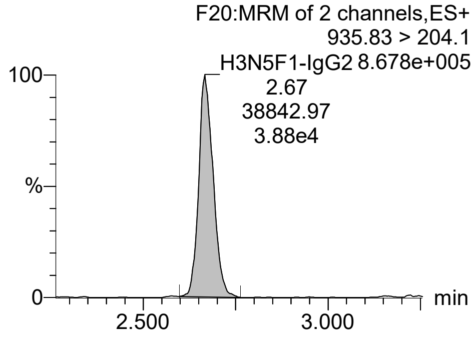

Sample Name: 20211111_H18_R1_IgG

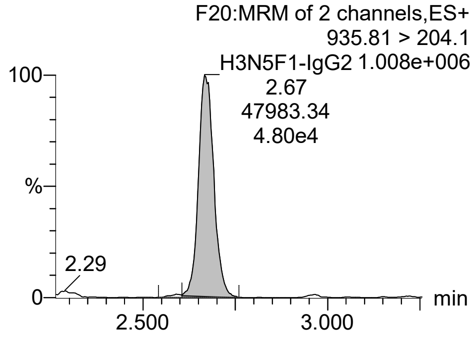

Sample Name: 20211111_H18_R1_IgG

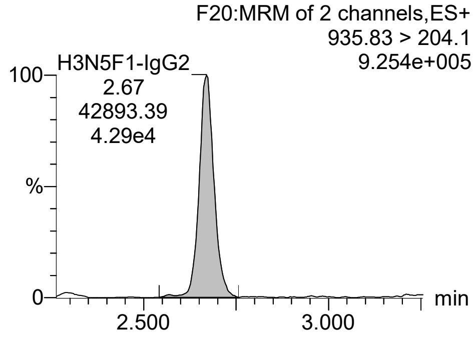

Sample Name: 20211111_A37_R1_IgG

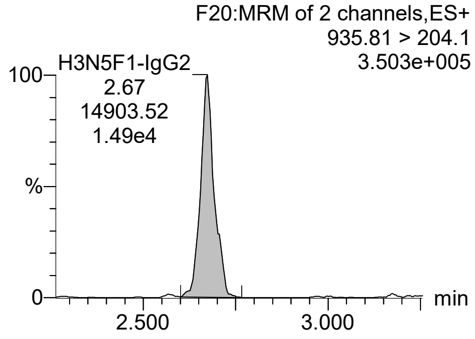

Sample Name: 20211111_A37_R1_IgG

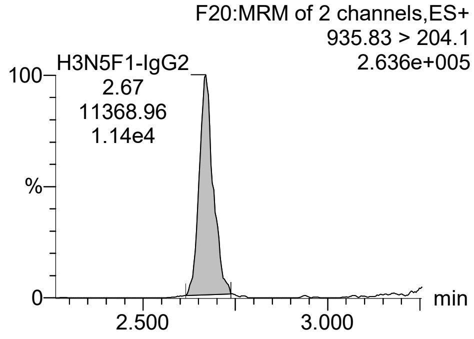

Sample Name: 20211111_A38_R1_IgG

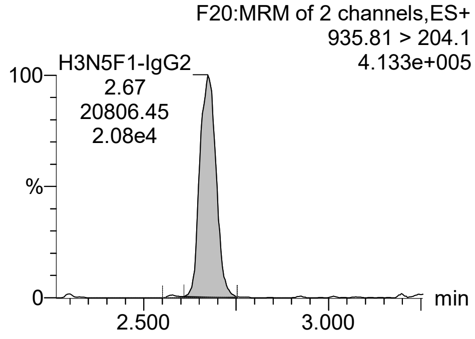

Sample Name: 20211111_A38_R1_IgG

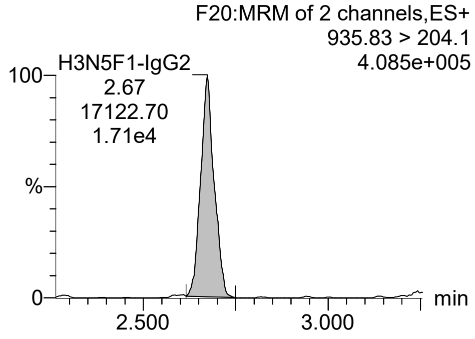

Dataset: C:\MassLynx\10.pro\20211115_clinicalsamples_IgG.qld

Last Altered: Sunday, October 02, 2022 16:02:30 台北標準時間
Printed: Friday, August 01, 2025 16:49:10 台北標準時間

Sample Name: 20211111_H19_R1_IgG

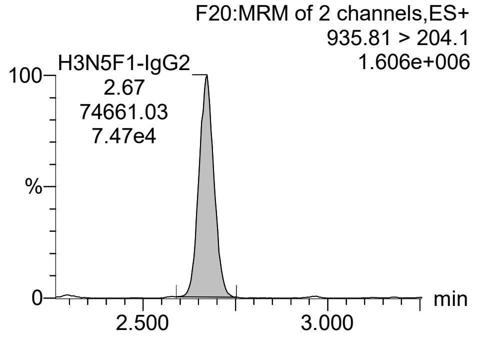

Sample Name: 20211111_H19_R1_IgG

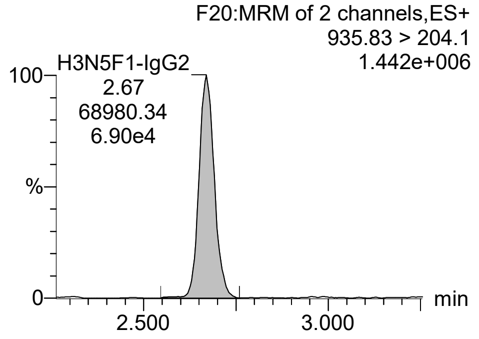

Sample Name: 20211111_A39_R1_IgG

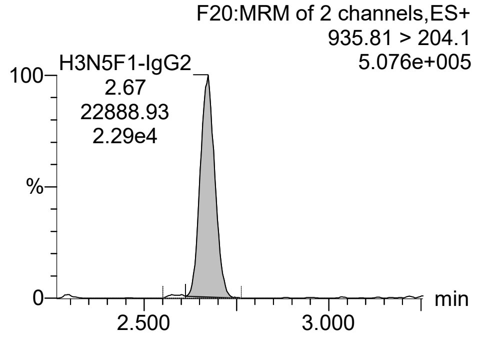

Sample Name: 20211111_A39_R1_IgG

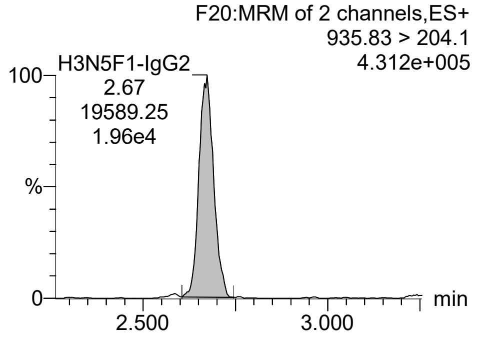

Sample Name: 20211111_A40_R1_IgG

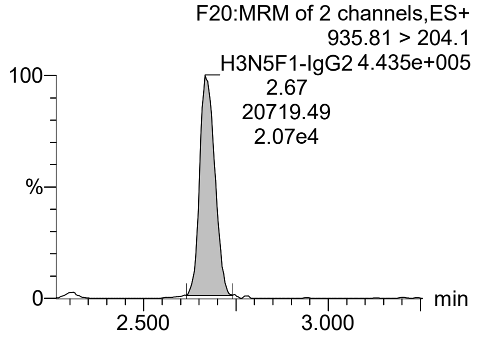

Sample Name: 20211111_A40_R1_IgG

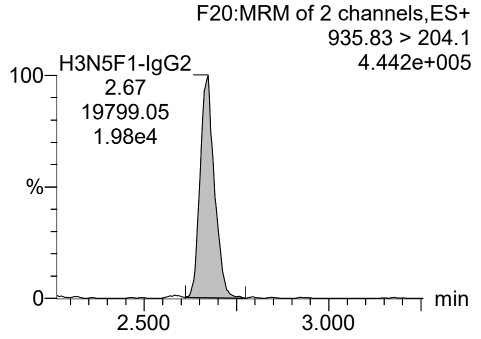

Sample Name: 20211111_H20_R1_IgG

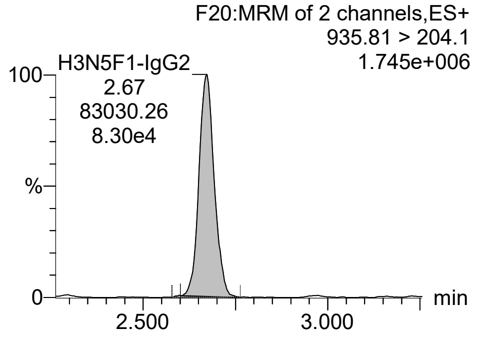

Sample Name: 20211111_H20_R1_IgG

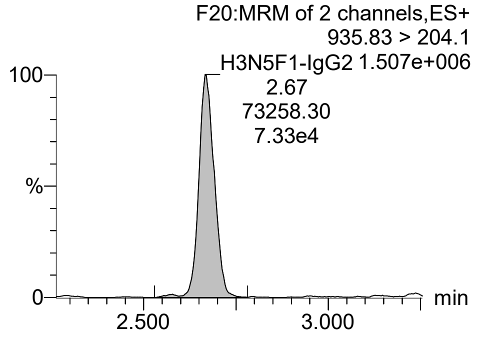

Sample Name: 20211111_A41_R1_IgG

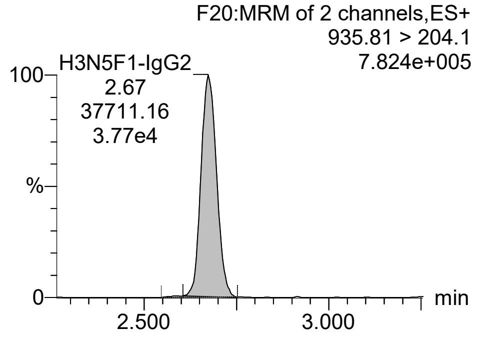

Sample Name: 20211111_A41_R1_IgG

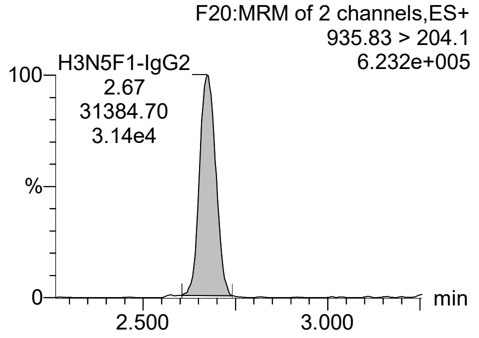

Sample Name: 20211111_A42_R1_IgG

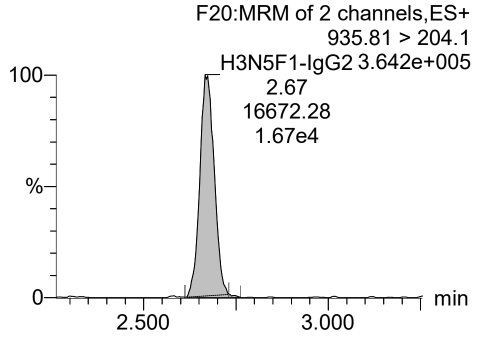

Sample Name: 20211111_A42_R1_IgG

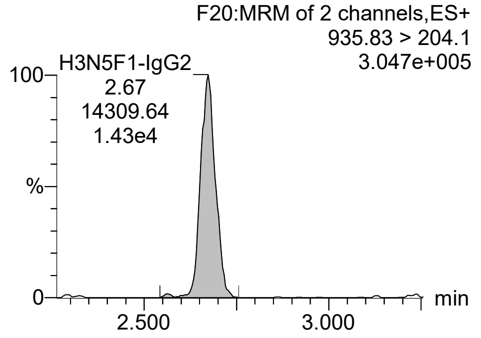

Sample Name: 20211111_A43_R1_IgG

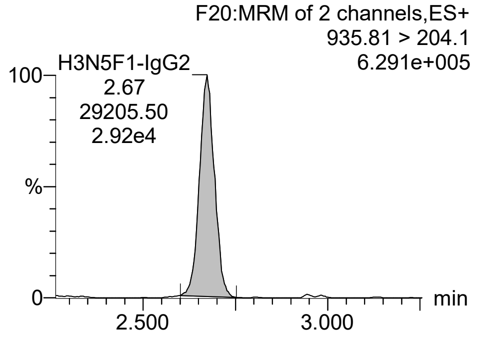

Sample Name: 20211111_A43_R1_IgG

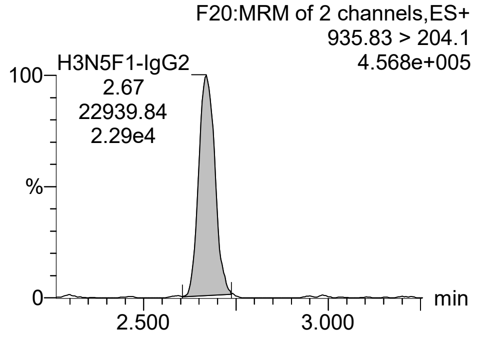

Sample Name: 20211111_A44_R1_IgG

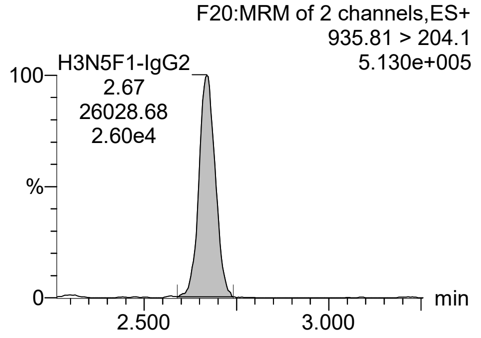

Sample Name: 20211111_A44_R1_IgG

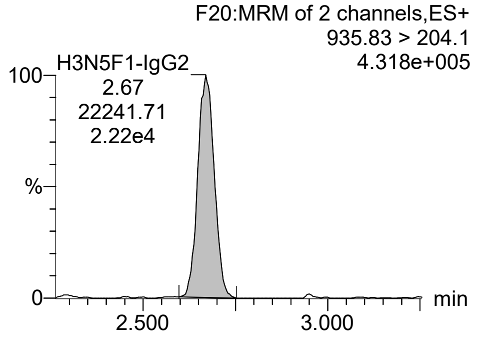

Sample Name: 20211111_QC_R9_IgG

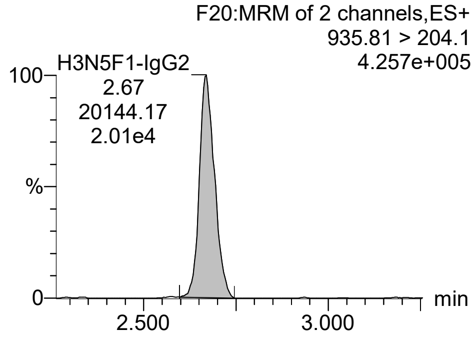

Sample Name: 20211111_QC_R9_IgG

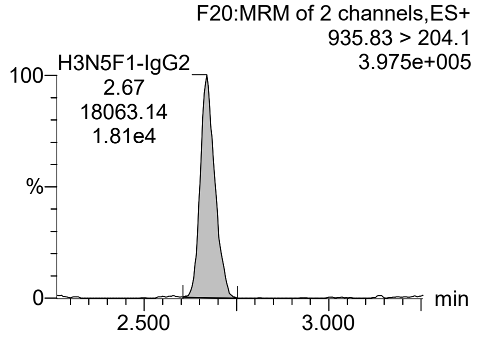

Sample Name: 20211111_A1_R2_IgG

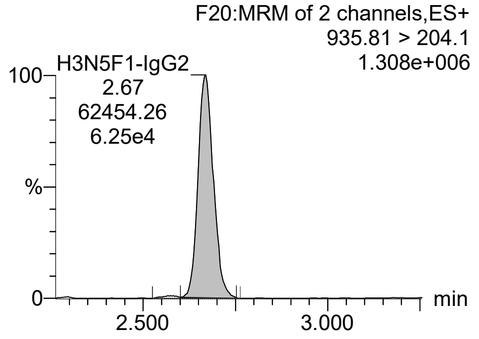

Sample Name: 20211111_A1_R2_IgG

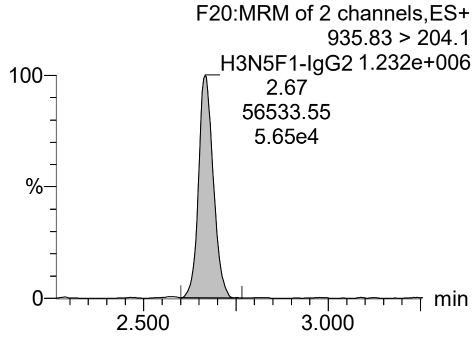

Sample Name: 20211111_A2_R2_IgG

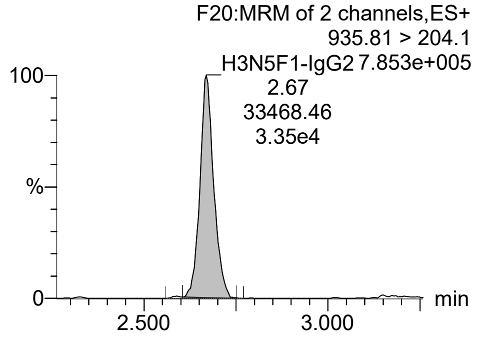

Sample Name: 20211111_A2_R2_IgG

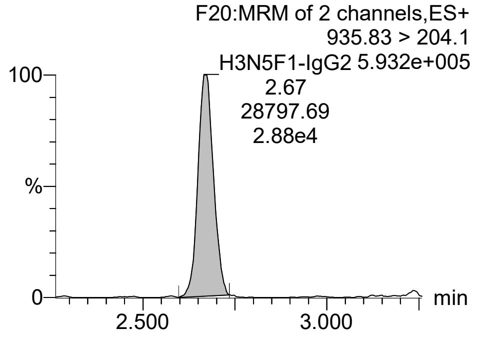

Sample Name: 20211111_L1_R2_IgG

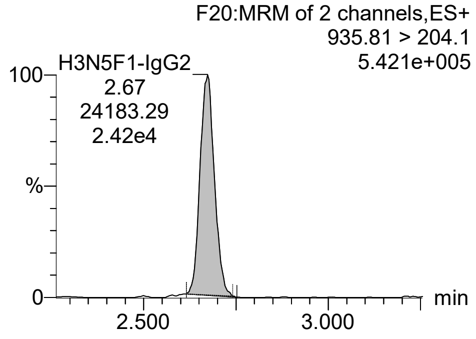

Sample Name: 20211111_L1_R2_IgG

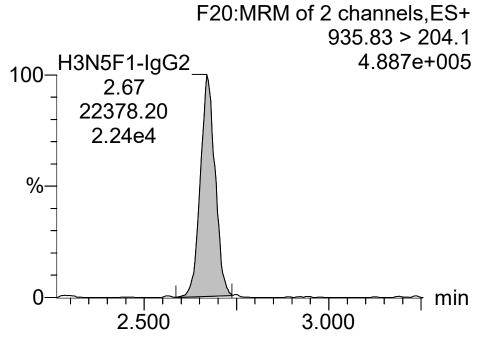

Sample Name: 20211111_L2_R2_IgG

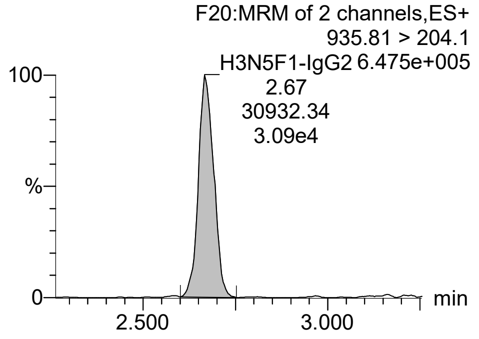

Sample Name: 20211111_L2_R2_IgG

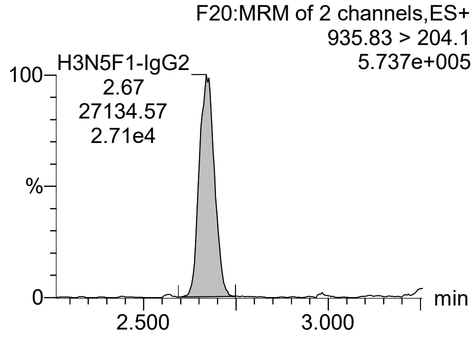

Sample Name: 20211111_H1_R2_IgG

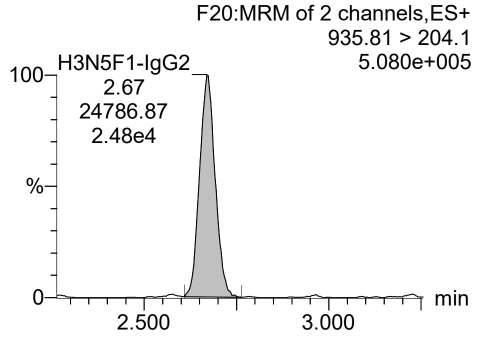

Sample Name: 20211111_H1_R2_IgG

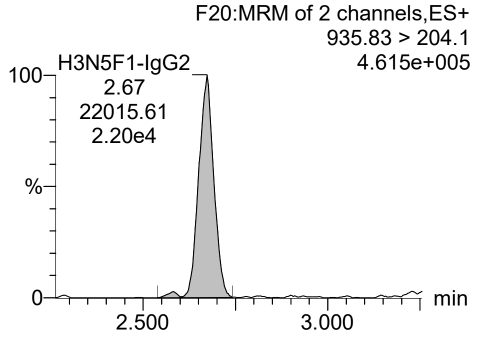

Sample Name: 20211111_A3_R2_IgG

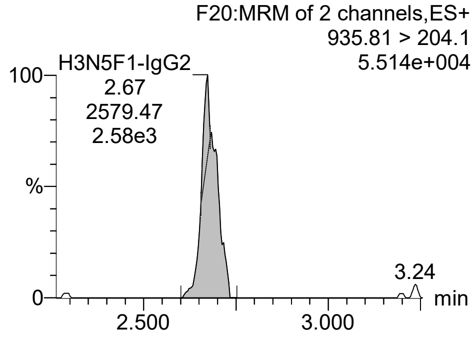

Sample Name: 20211111_A3_R2_IgG

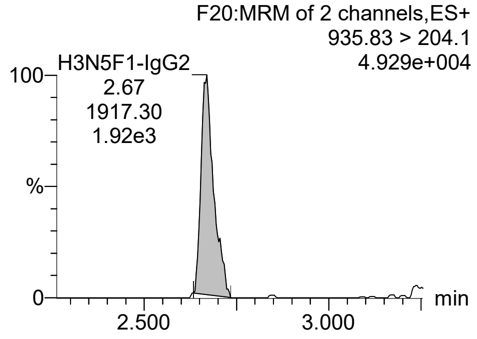

Dataset: C:\MassLynx\10.pro\20211115_clinicalsamples_IgG.qld

Last Altered: Sunday, October 02, 2022 16:02:30 台北標準時間
Printed: Friday, August 01, 2025 16:49:10 台北標準時間

Sample Name: 20211111_A4_R2_IgG

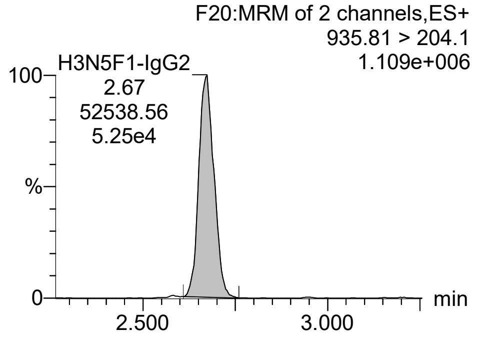

Sample Name: 20211111_A4_R2_IgG

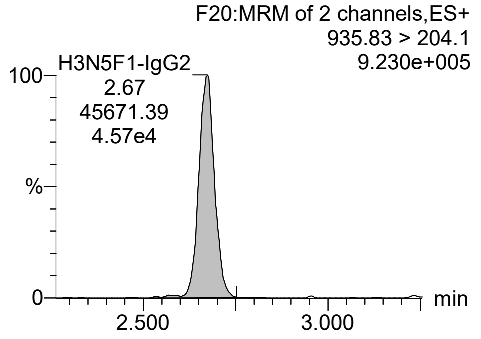

Sample Name: 20211111_L3_R2_IgG

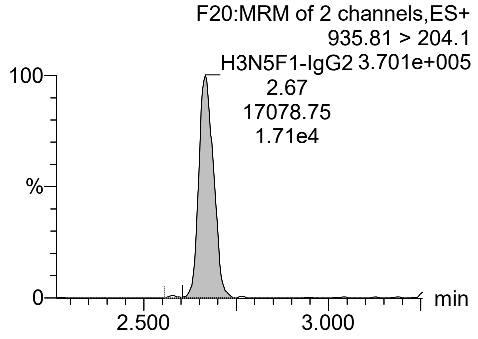

Sample Name: 20211111_L3_R2_IgG

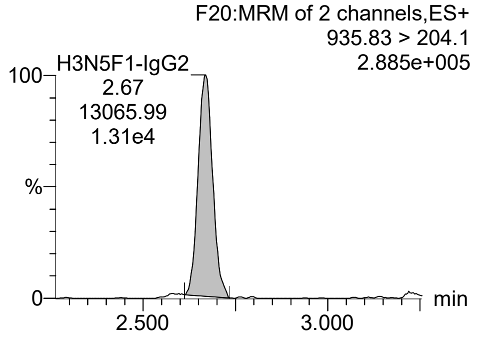

Sample Name: 20211111_L4_R2_IgG

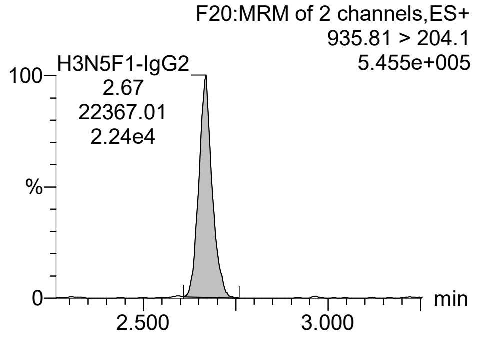

Sample Name: 20211111_L4_R2_IgG

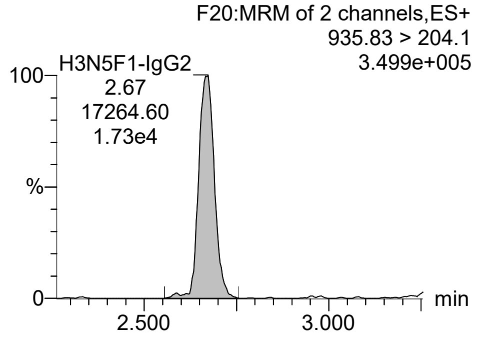

Sample Name: 20211111_H2_R2_IgG

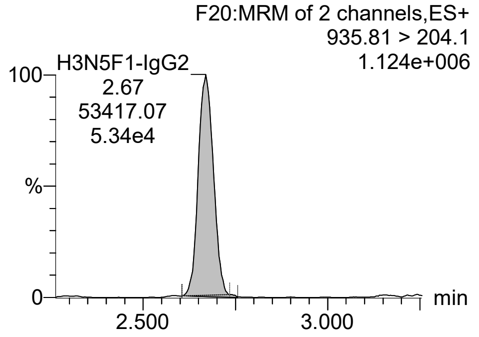

Sample Name: 20211111_H2_R2_IgG

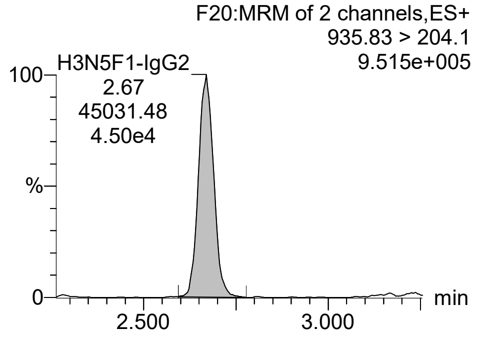

Sample Name: 20211111_A5_R2_IgG

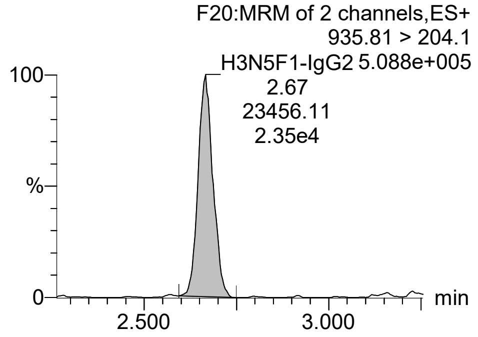

Sample Name: 20211111_A5_R2_IgG

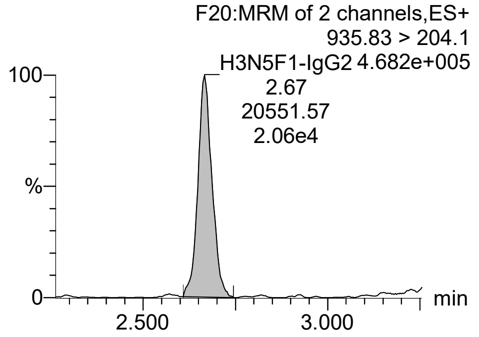

Sample Name: 20211111_A6_R2_IgG

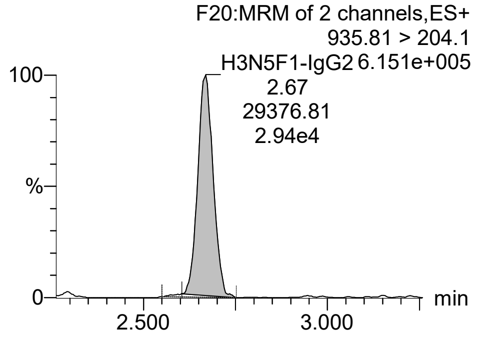

Sample Name: 20211111_A6_R2_IgG

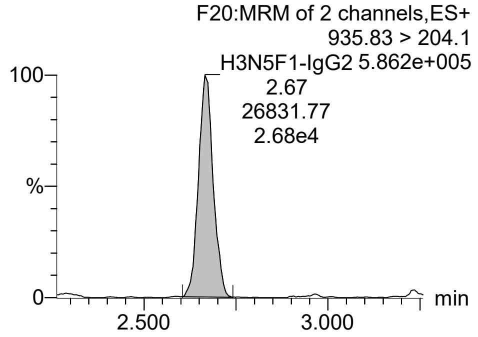

Sample Name: 20211111_QC_R10_IgG

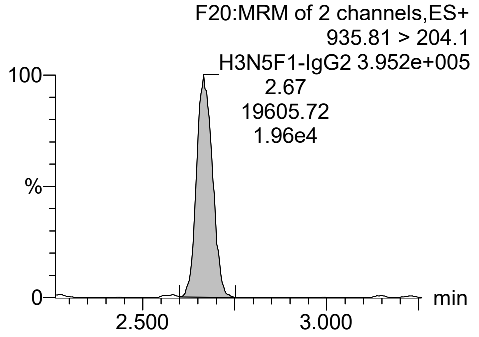

Sample Name: 20211111_QC_R10_IgG

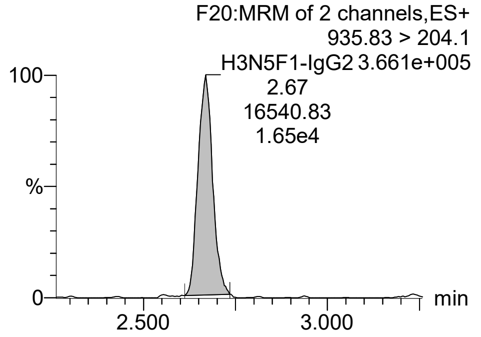

Sample Name: 20211111_L5_R2_IgG

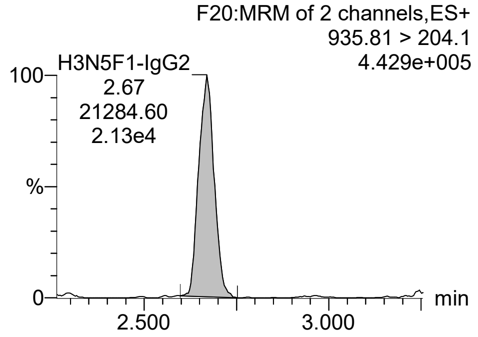

Dataset: C:\MassLynx\10.pro\20211115_clinicalsamples_IgG.qld

Last Altered: Sunday, October 02, 2022 16:02:30 台北標準時間
Printed: Friday, August 01, 2025 16:49:10 台北標準時間

Sample Name: 20211111_L5_R2_IgG

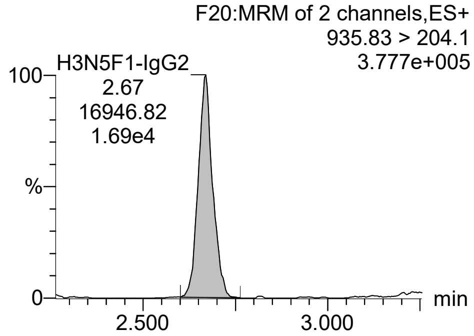

Sample Name: 20211111_L6_R2_IgG

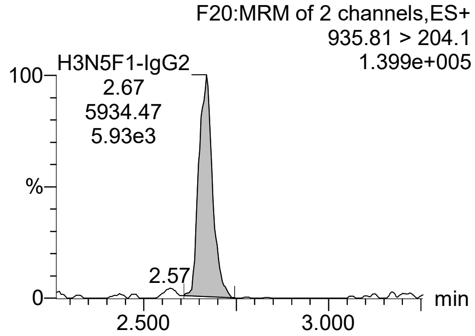

Sample Name: 20211111_L6_R2_IgG

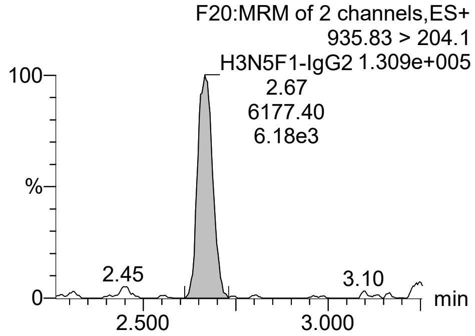

Sample Name: 20211111_H3_R2_IgG

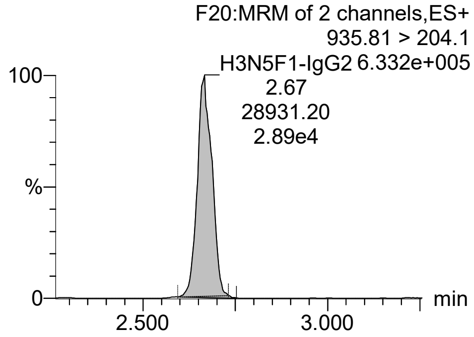

Sample Name: 20211111_H3_R2_IgG

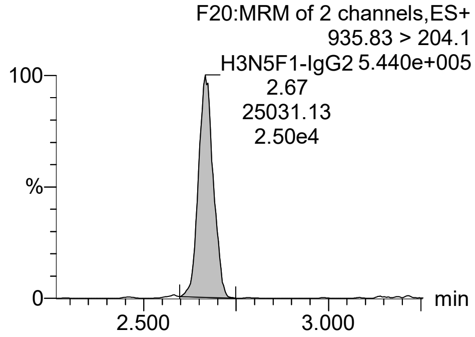

Sample Name: 20211111_A7_R2_IgG

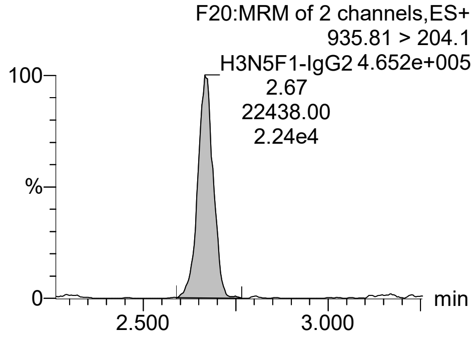

Sample Name: 20211111_A7_R2_IgG

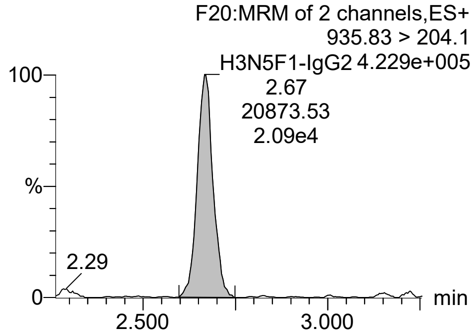

Sample Name: 20211111_A8_R2_IgG

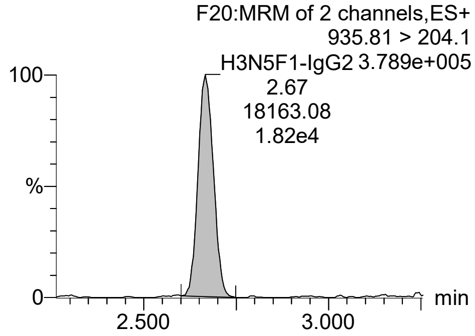

Sample Name: 20211111_A8_R2_IgG

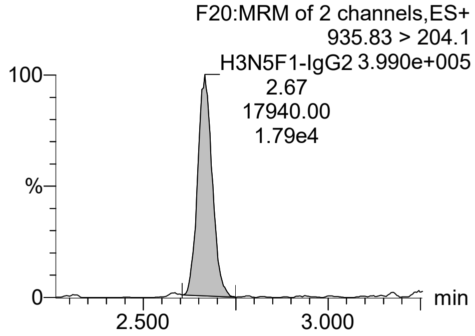

Sample Name: 20211111_L7_R2_IgG

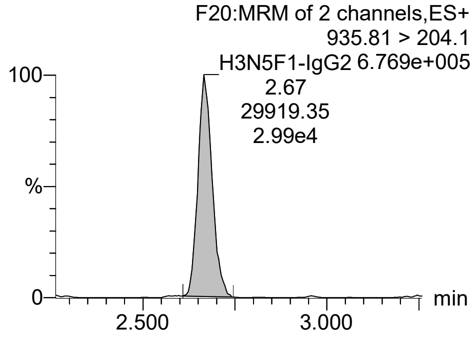

Sample Name: 20211111_L7_R2_IgG

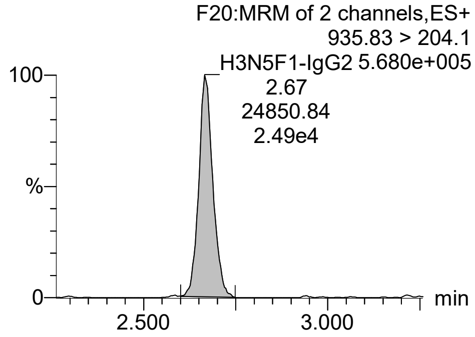

Sample Name: 20211111_L8_R2_IgG

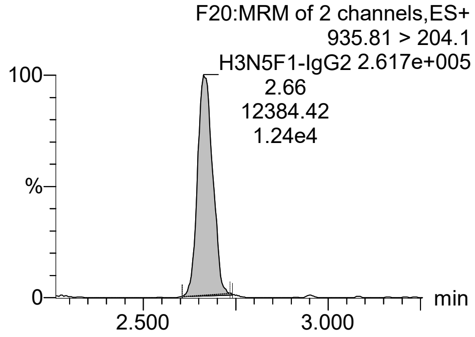

Sample Name: 20211111_L8_R2_IgG

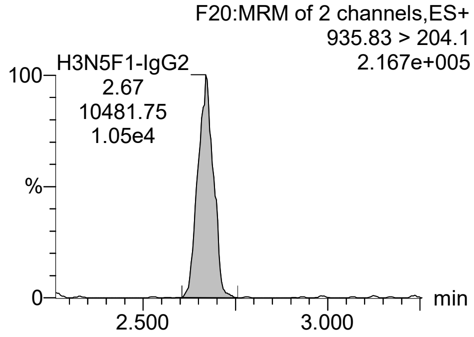

Sample Name: 20211111_H4_R2_IgG

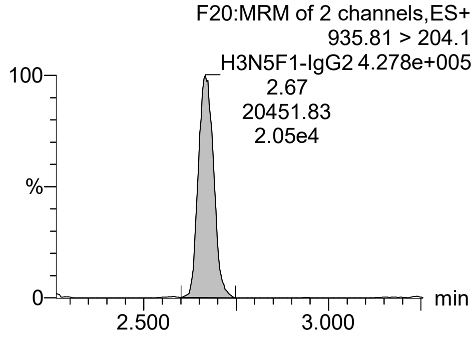

Sample Name: 20211111_H4_R2_IgG

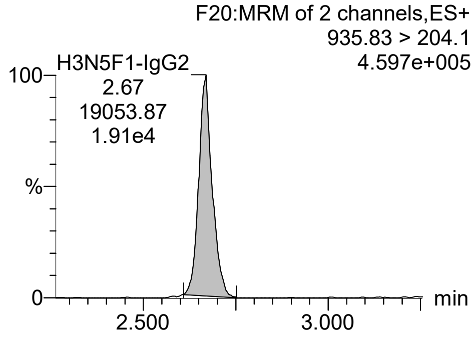

Dataset: C:\MassLynx\10.pro\20211115_clinicalsamples_IgG.qld

Last Altered: Sunday, October 02, 2022 16:02:30 台北標準時間
Printed: Friday, August 01, 2025 16:49:10 台北標準時間

Sample Name: 20211111_A9_R2_IgG

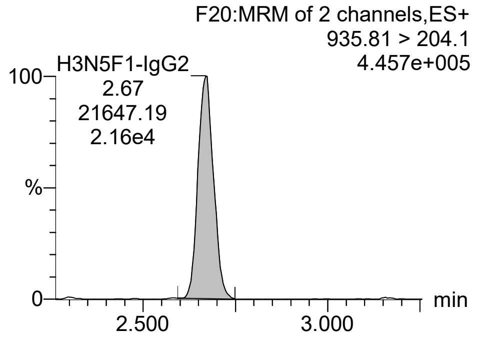

Sample Name: 20211111_A9_R2_IgG

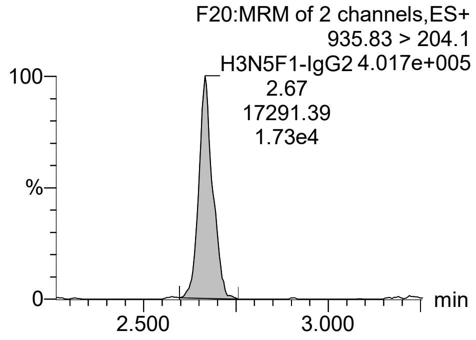

Sample Name: 20211111_A10_R2_IgG

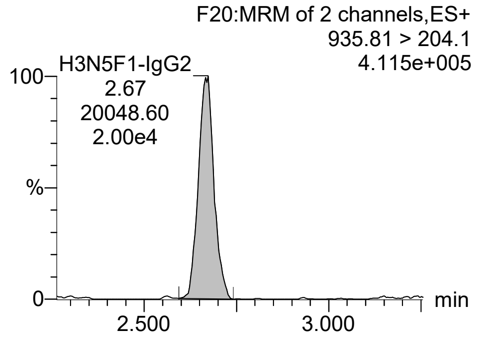

Sample Name: 20211111_A10_R2_IgG

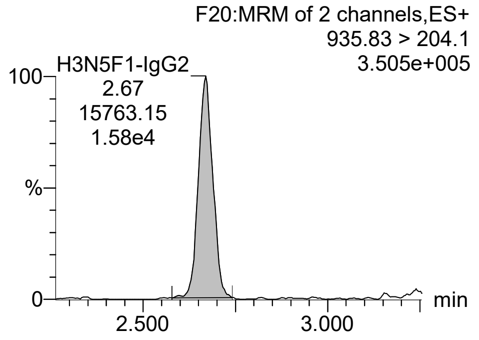

Sample Name: 20211111_L9_R2_IgG

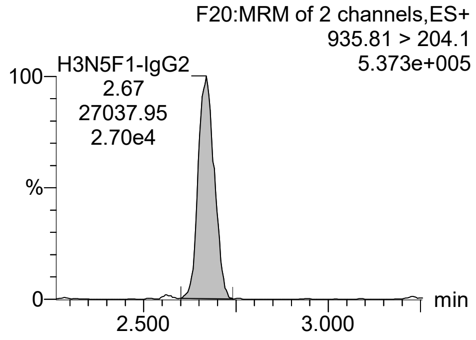

Sample Name: 20211111_L9_R2_IgG

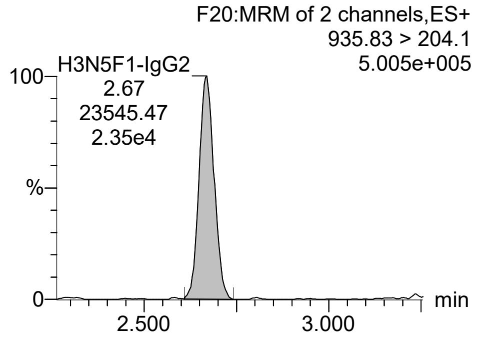

Sample Name: 20211111_L10_R2_IgG

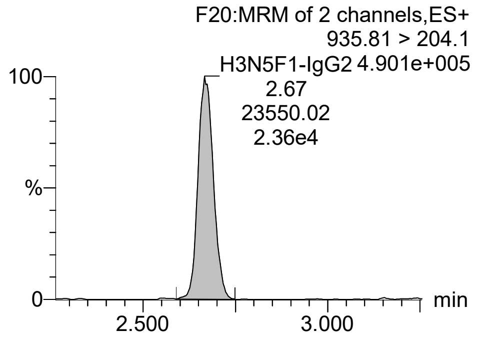

Sample Name: 20211111_L10_R2_IgG

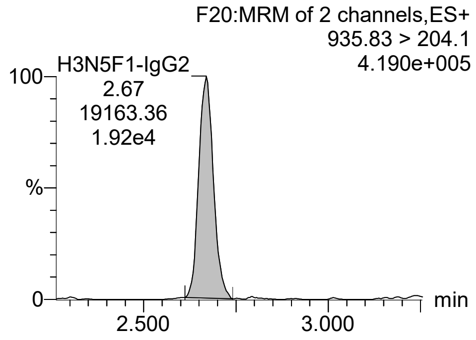

Sample Name: 20211111_QC_R11_IgG

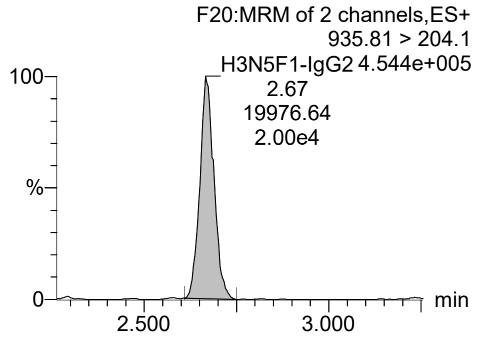

Sample Name: 20211111_QC_R11_IgG

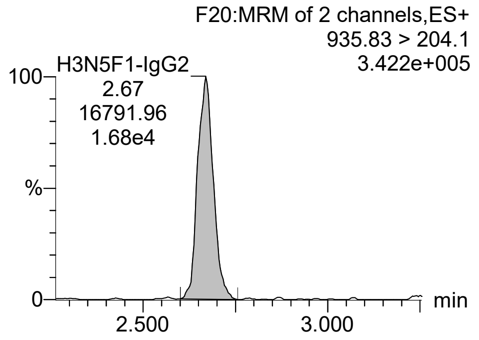

Sample Name: 20211111_H5_R2_IgG

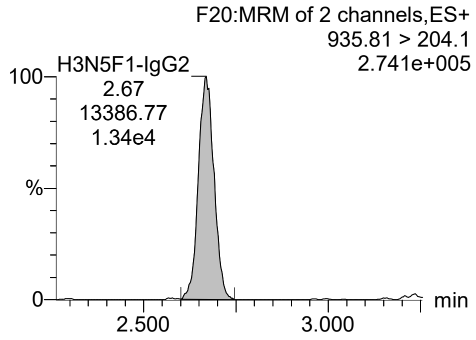

Sample Name: 20211111_H5_R2_IgG

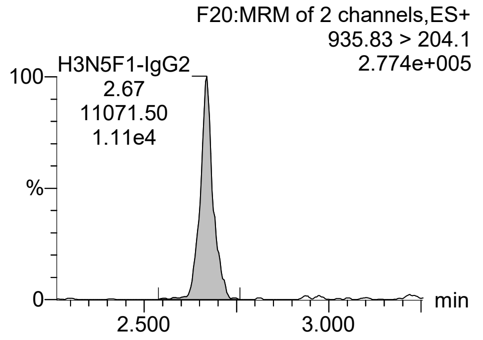

Sample Name: 20211111_A11_R2_IgG

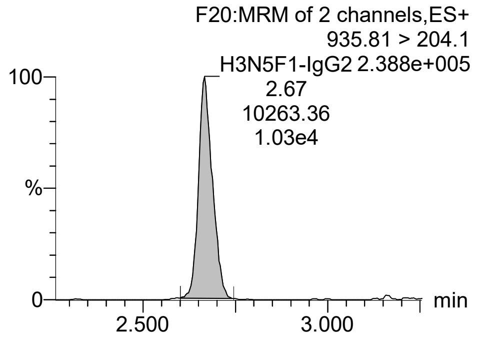

Sample Name: 20211111_A11_R2_IgG

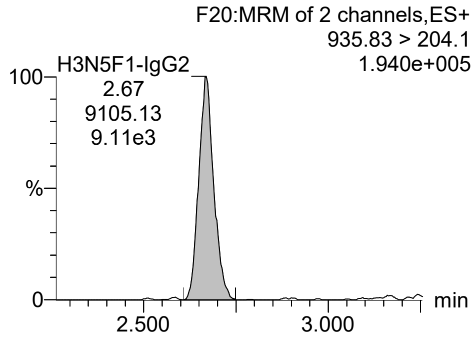

Sample Name: 20211111_A12_R2_IgG

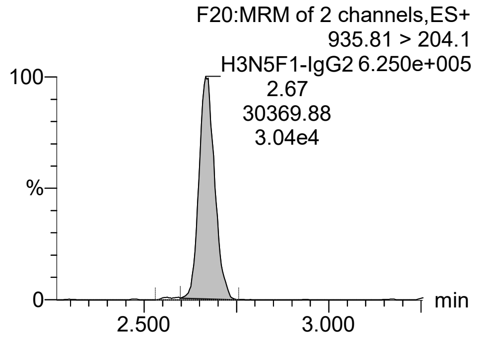

Dataset: C:\MassLynx\10.pro\20211115_clinicalsamples_IgG.qld

Last Altered: Sunday, October 02, 2022 16:02:30 台北標準時間
Printed: Friday, August 01, 2025 16:49:10 台北標準時間

Sample Name: 20211111_A12_R2_IgG

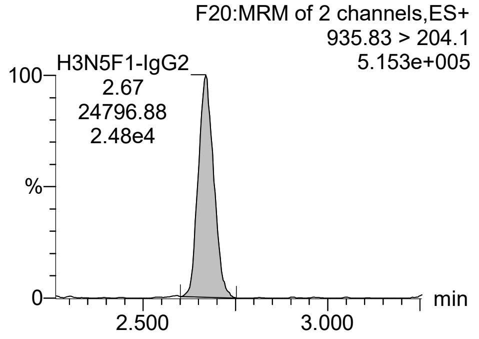

Sample Name: 20211111_L11_R2_IgG

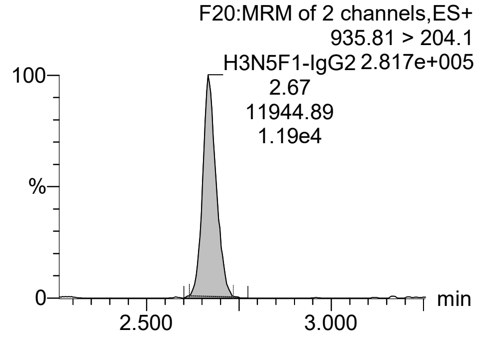

Sample Name: 20211111_L11_R2_IgG

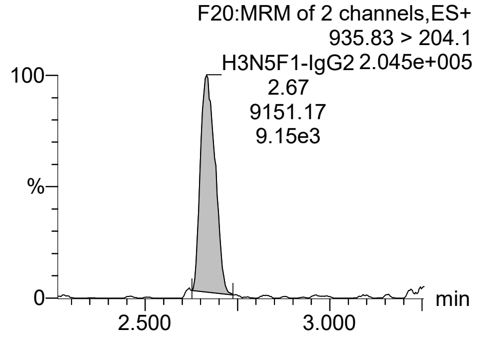

Sample Name: 20211111_L12_R2_IgG

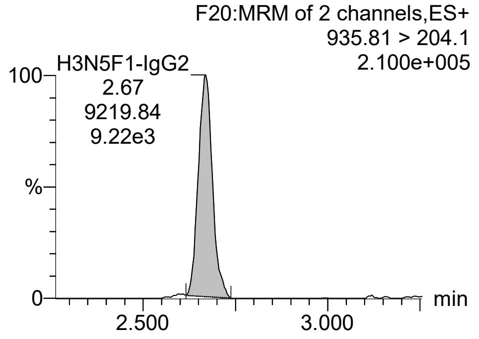

Sample Name: 20211111_L12_R2_IgG

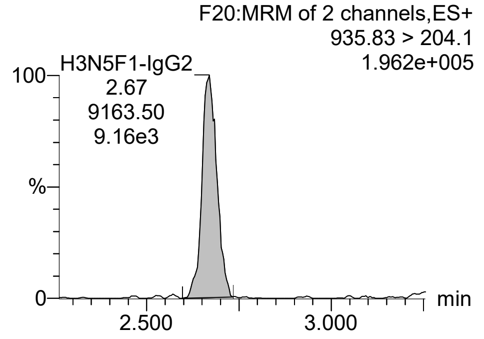

Sample Name: 20211111_H6_R2_IgG

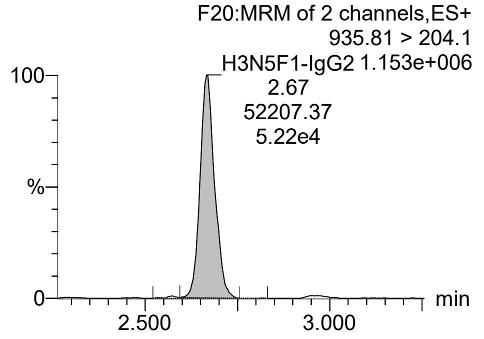

Sample Name: 20211111_H6_R2_IgG

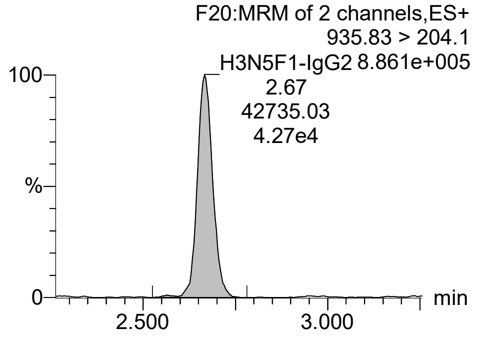

Sample Name: 20211111_A13_R2_IgG

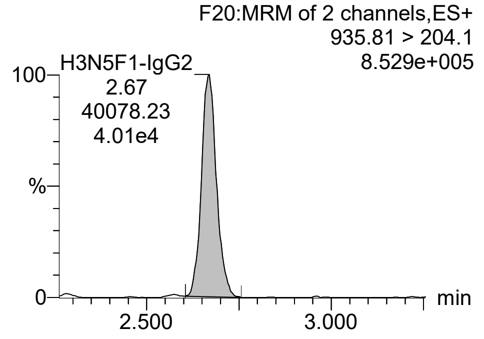

Sample Name: 20211111_A13_R2_IgG

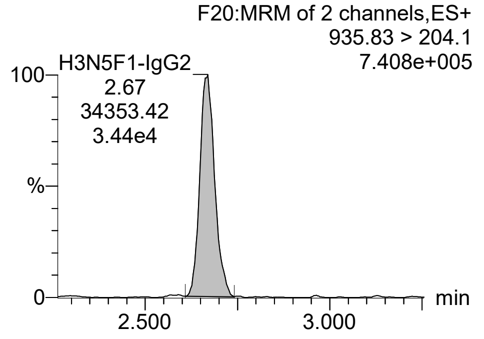

Sample Name: 20211111_A14_R2_IgG

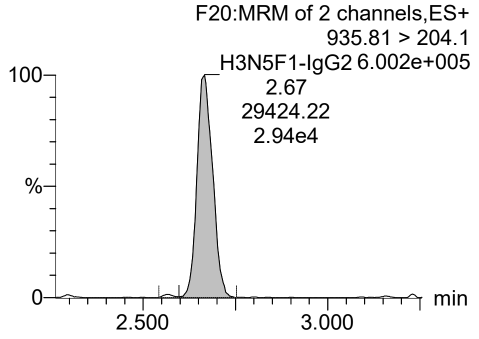

Sample Name: 20211111_A14_R2_IgG

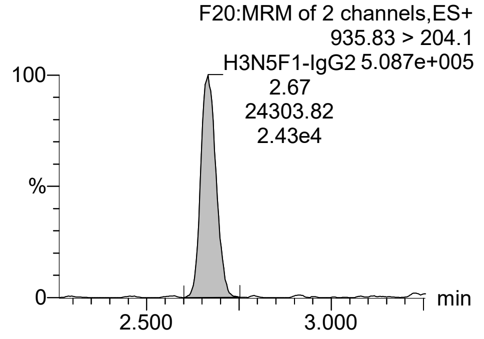

Sample Name: 20211111_L13_R2_IgG

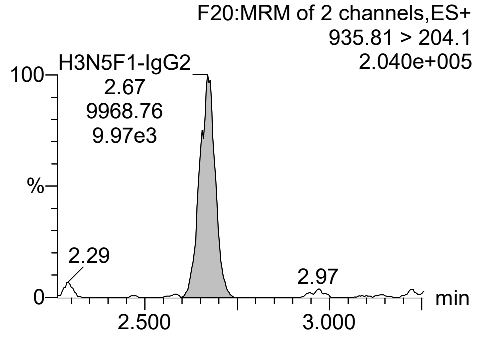

Sample Name: 20211111_L13_R2_IgG

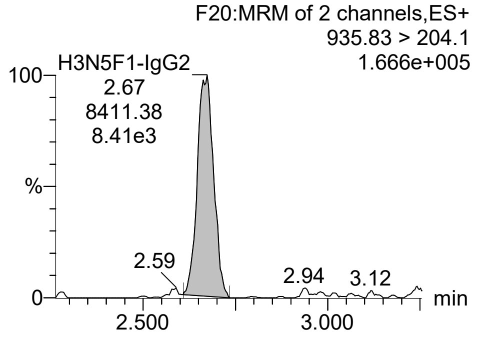

Sample Name: 20211111_L14_R2_IgG

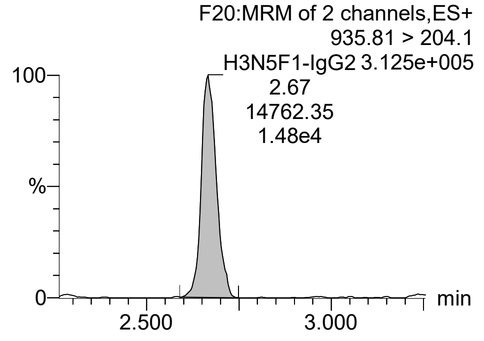

Sample Name: 20211111_L14_R2_IgG

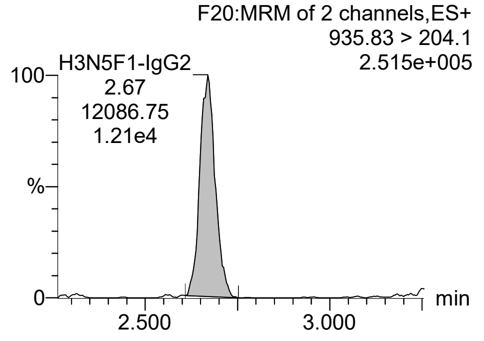

Sample Name: 20211111_H7_R2_IgG

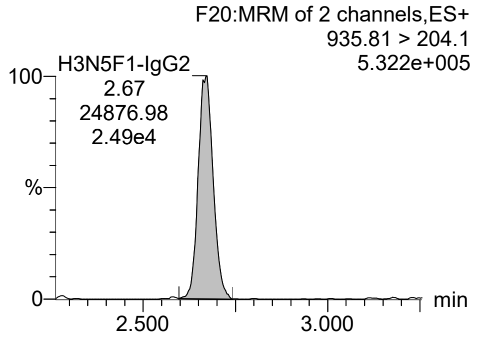

Sample Name: 20211111_H7_R2_IgG

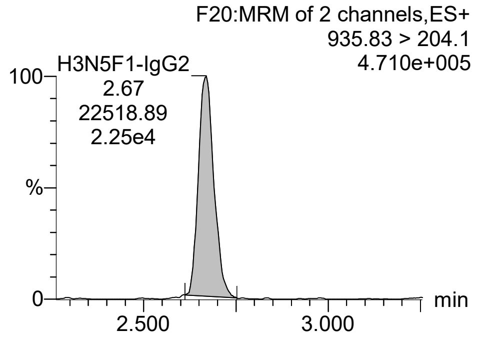

Sample Name: 20211111_A15_R2_IgG

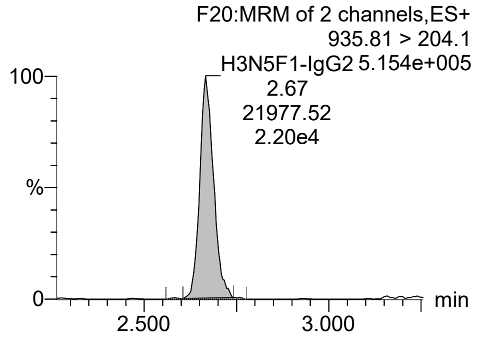

Sample Name: 20211111_A15_R2_IgG

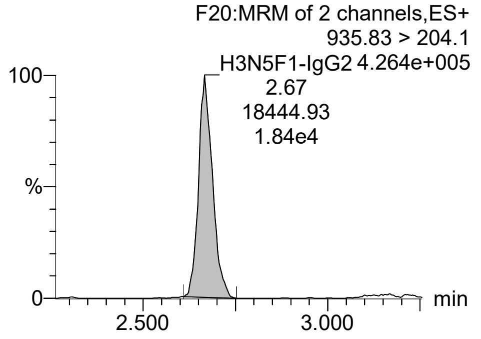

Sample Name: 20211111_QC_R12_IgG

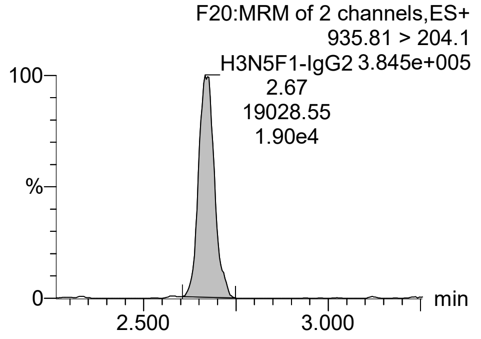

Sample Name: 20211111_QC_R12_IgG

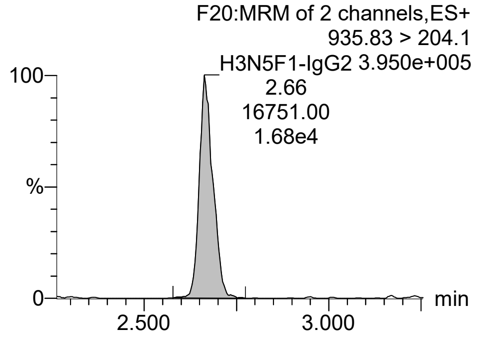

Sample Name: 20211111_A16_R2_IgG

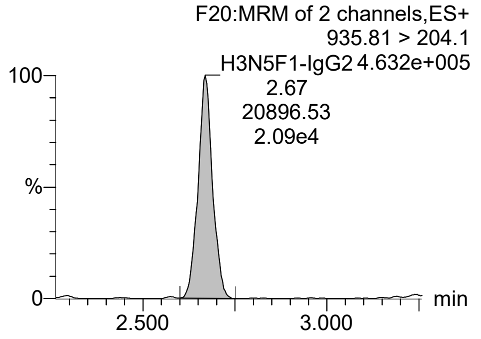

Sample Name: 20211111_A16_R2_IgG

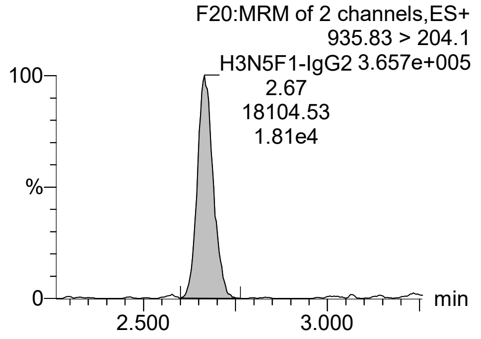

Sample Name: 20211111_L15_R2_IgG

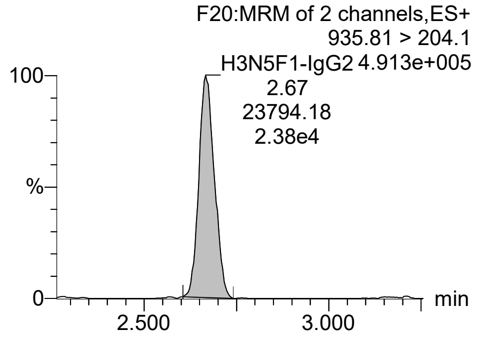

Sample Name: 20211111_L15_R2_IgG

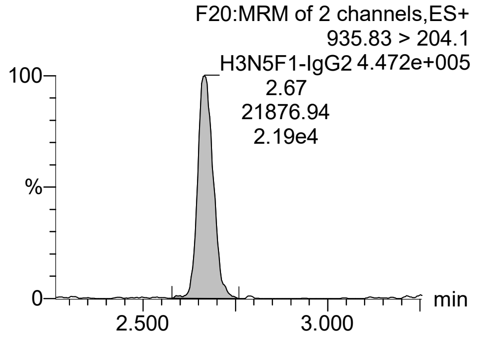

Sample Name: 20211111_L16_R2_IgG

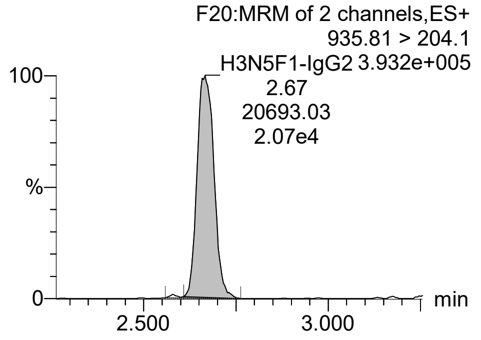

Sample Name: 20211111_L16_R2_IgG

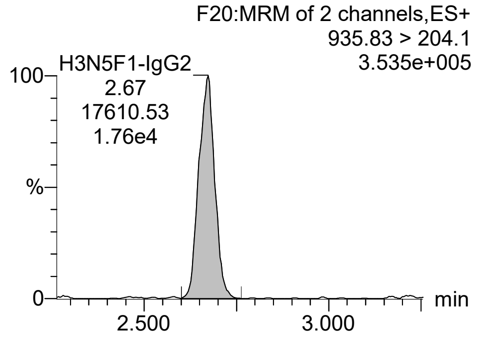

Sample Name: 20211111_H8_R2_IgG

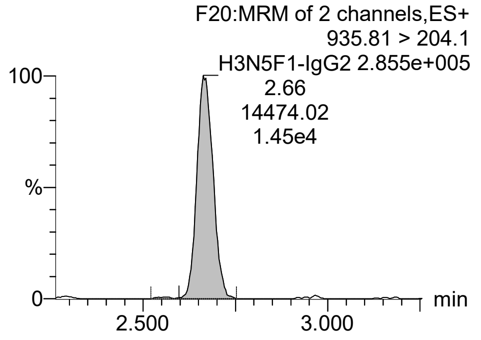

Sample Name: 20211111_H8_R2_IgG

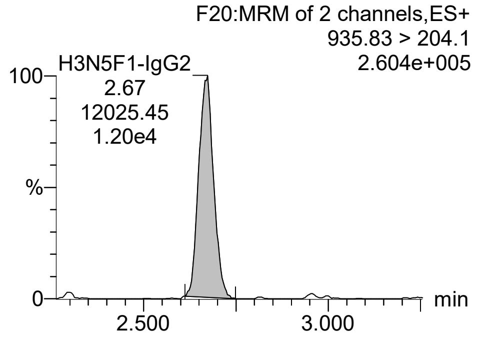

Sample Name: 20211111_A17_R2_IgG

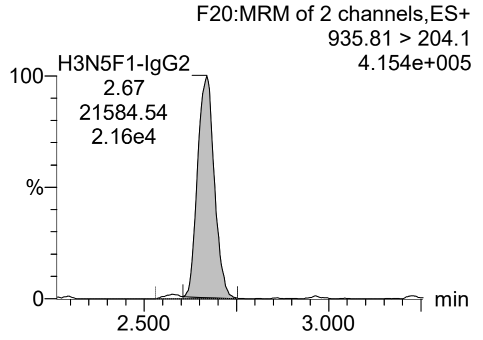

Dataset: C:\MassLynx\10.pro\20211115_clinicalsamples_IgG.qld

Last Altered: Sunday, October 02, 2022 16:02:30 台北標準時間
Printed: Friday, August 01, 2025 16:49:10 台北標準時間

Sample Name: 20211111_A17_R2_IgG

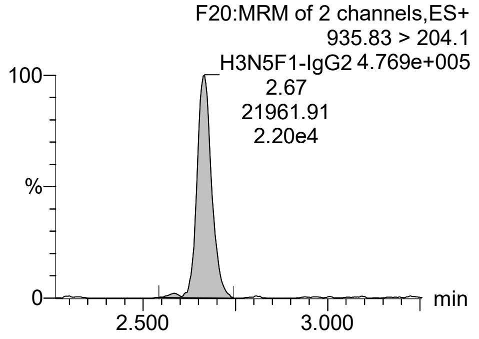

Sample Name: 20211111_A18_R2_IgG

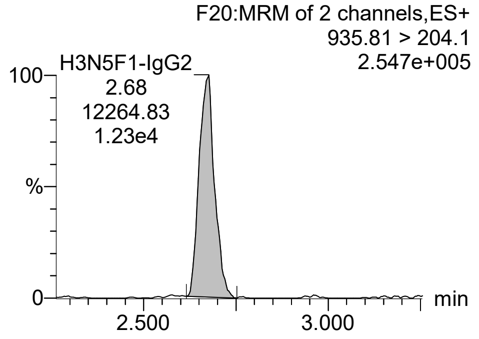

Sample Name: 20211111_A18_R2_IgG

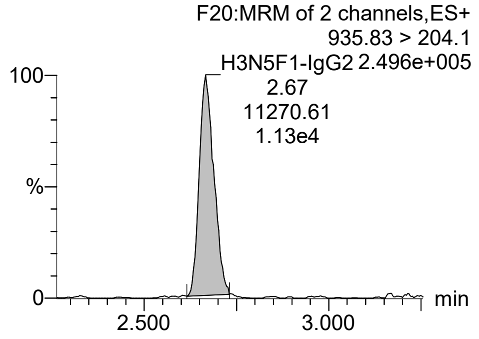

Sample Name: 20211111_L17_R2_IgG

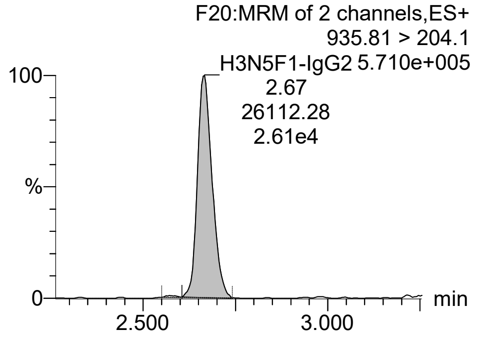

Sample Name: 20211111_L17_R2_IgG

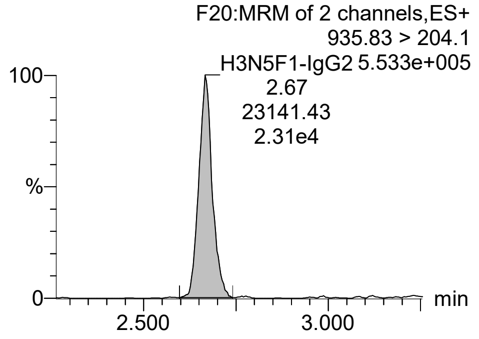

Sample Name: 20211111_L18_R2_IgG

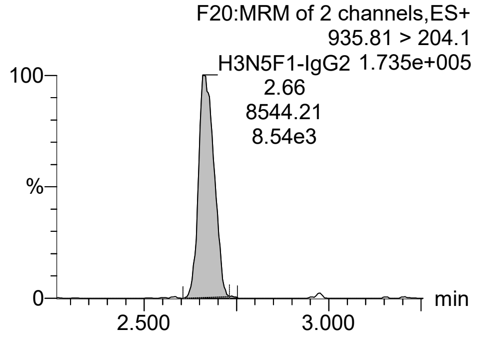

Sample Name: 20211111_L18_R2_IgG

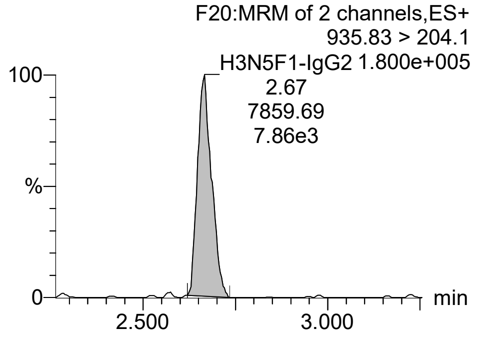

Sample Name: 20211111_H9_R2_IgG

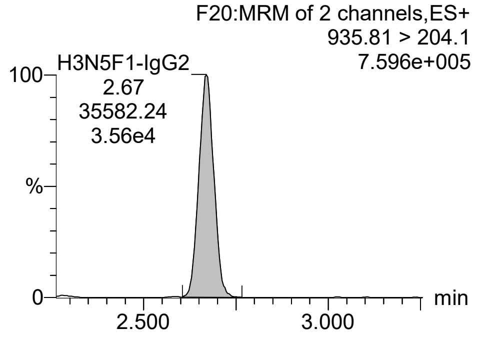

Sample Name: 20211111_H9_R2_IgG

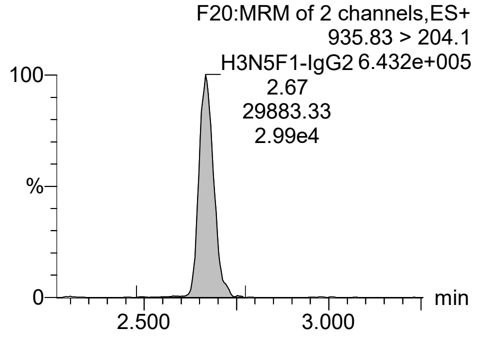

Sample Name: 20211111_A19_R2_IgG

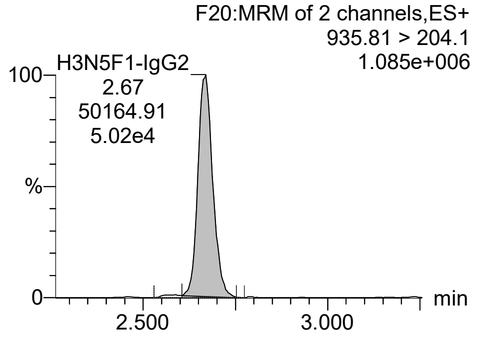

Sample Name: 20211111_A19_R2_IgG

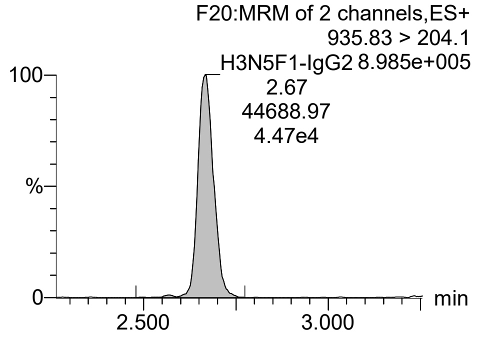

Sample Name: 20211111_A20_R2_IgG

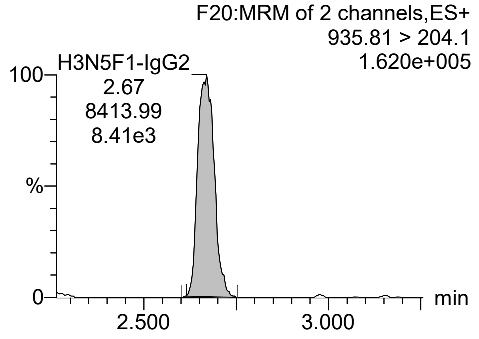

Sample Name: 20211111_A20_R2_IgG

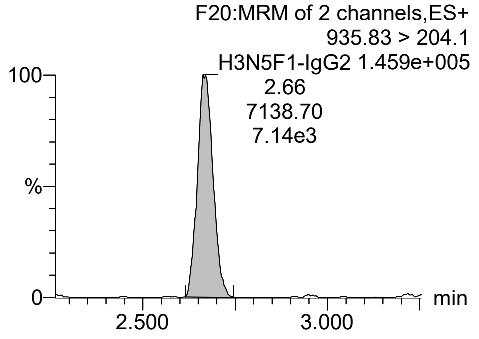

Sample Name: 20211111_L19_R2_IgG

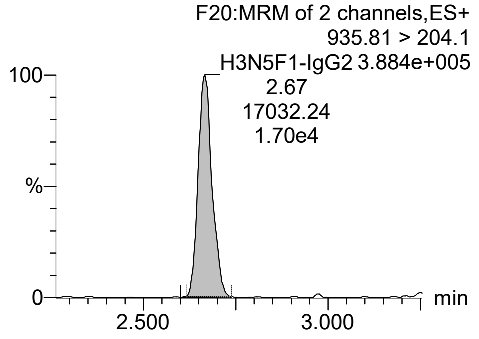

Sample Name: 20211111_L19_R2_IgG

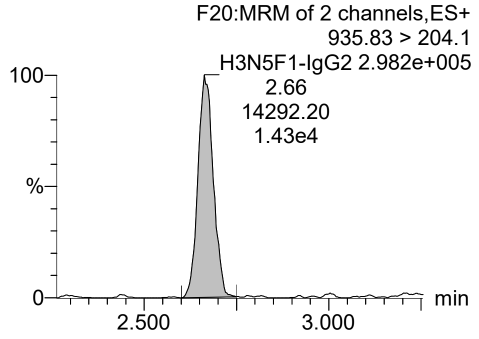

Sample Name: 20211111_QC_R13_IgG

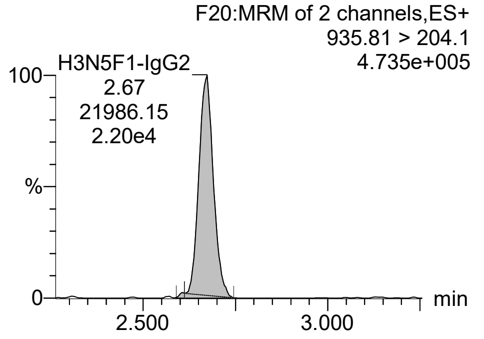

Sample Name: 20211111_QC_R13_IgG

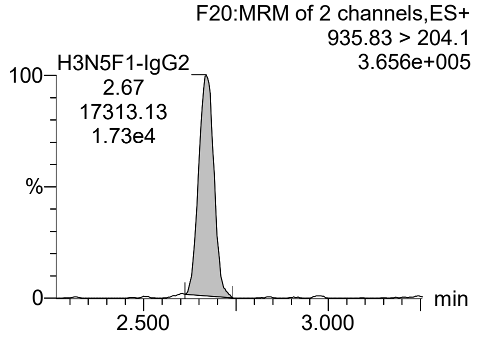

Sample Name: 20211111_L20_R2_IgG

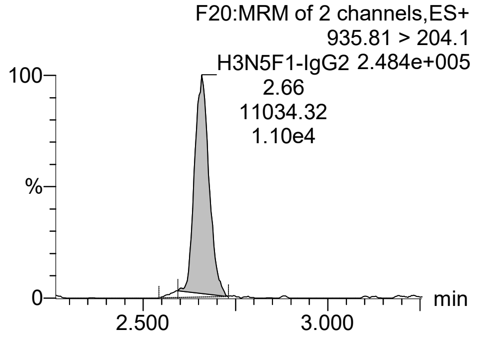

Sample Name: 20211111_L20_R2_IgG

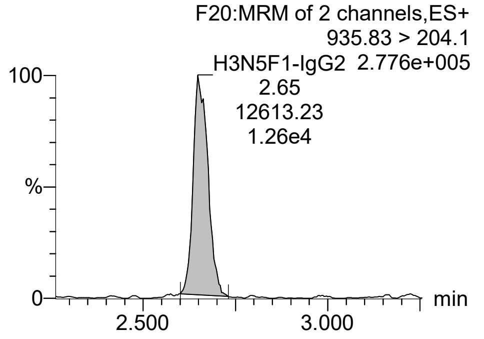

Sample Name: 20211111_H10_R2_IgG

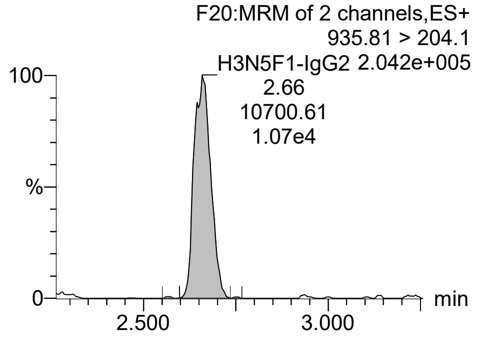

Sample Name: 20211111_H10_R2_IgG

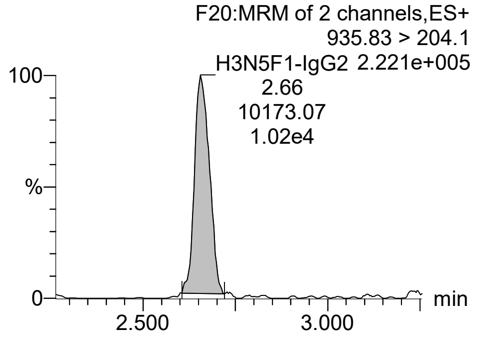

Sample Name: 20211111_A21_R2_IgG

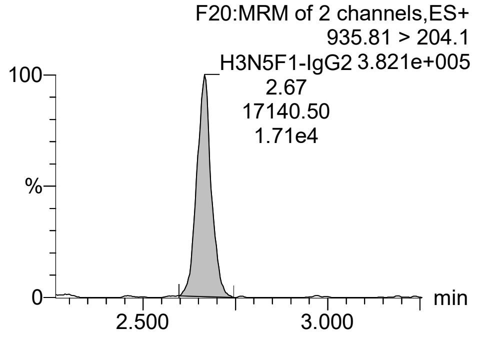

Sample Name: 20211111_A21_R2_IgG

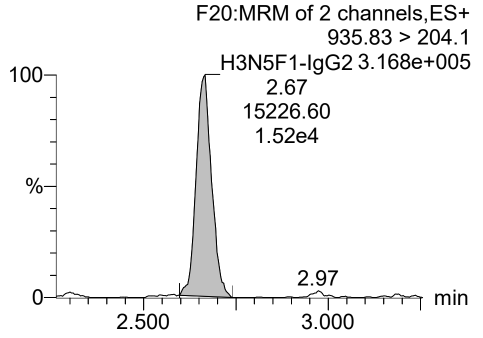

Sample Name: 20211111_A22_R2_IgG

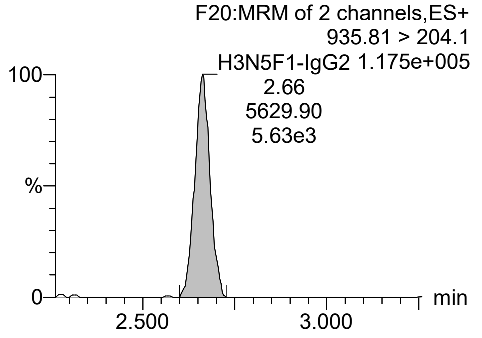

Sample Name: 20211111_A22_R2_IgG

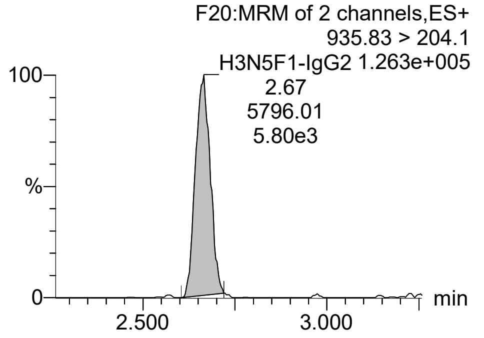

Sample Name: 20211111_L21_R2_IgG

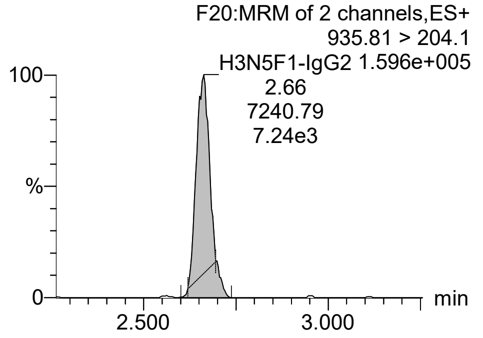

Sample Name: 20211111_L21_R2_IgG

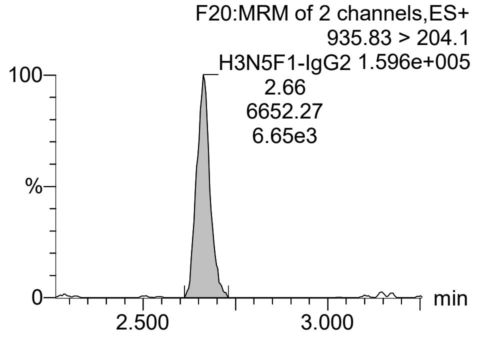

Sample Name: 20211111_L22_R2_IgG

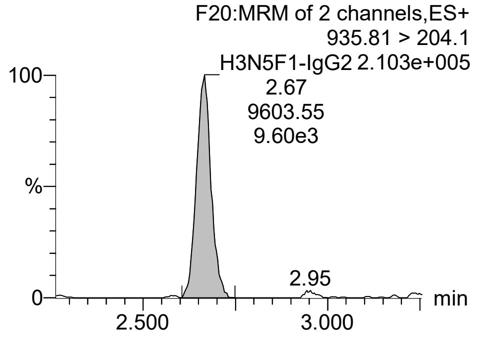

Sample Name: 20211111_L22_R2_IgG

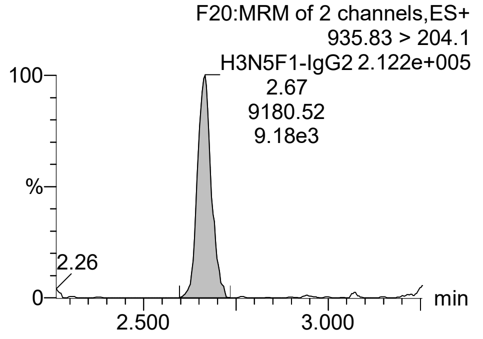

Sample Name: 20211111_H11_R2_IgG

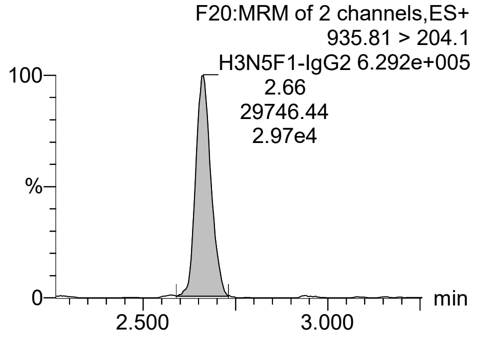

Dataset: C:\MassLynx\10.pro\20211115_clinicalsamples_IgG.qld

Last Altered: Sunday, October 02, 2022 16:02:30 台北標準時間
Printed: Friday, August 01, 2025 16:49:10 台北標準時間

Sample Name: 20211111_H11_R2_IgG

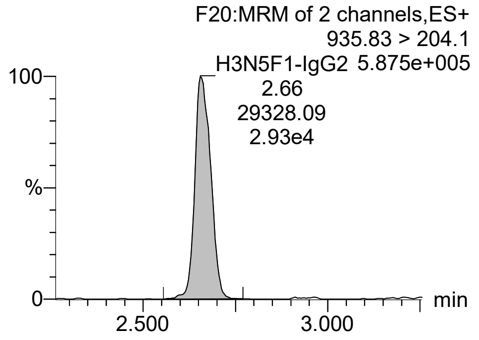

Sample Name: 20211111_A23_R2_IgG

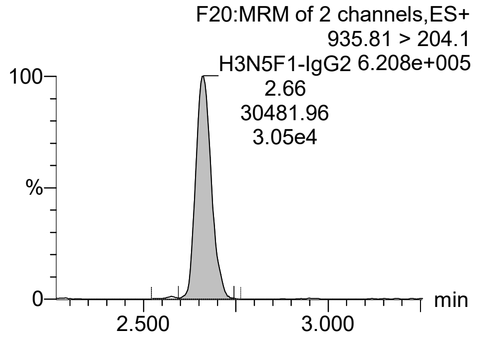

Sample Name: 20211111_A23_R2_IgG

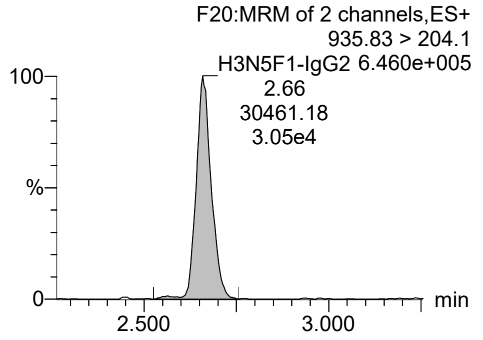

Sample Name: 20211111_A24_R2_IgG

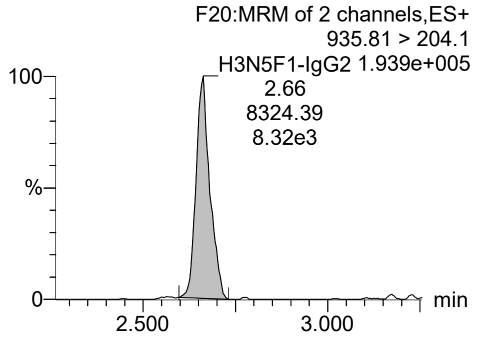

Sample Name: 20211111_A24_R2_IgG

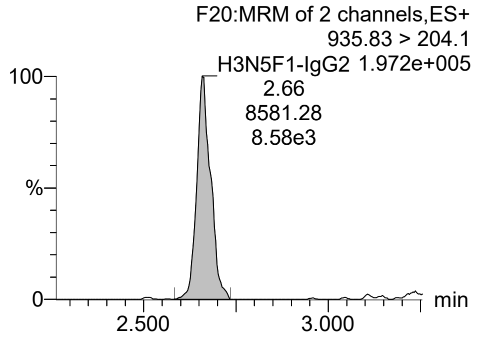

Sample Name: 20211111_L23_R2_IgG

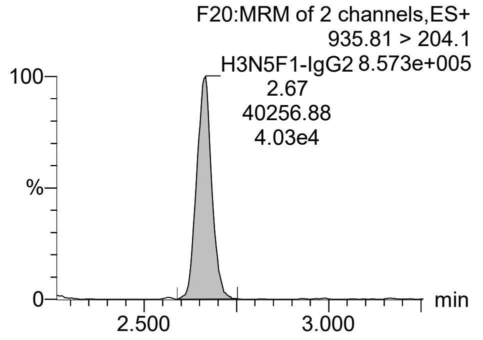

Sample Name: 20211111_L23_R2_IgG

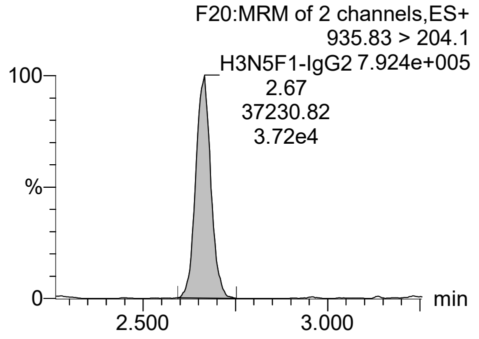

Sample Name: 20211111_L24_R2_IgG

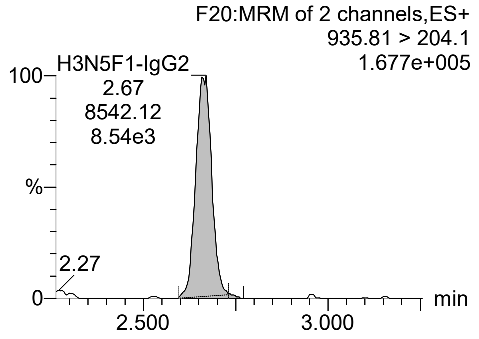

Sample Name: 20211111_L24_R2_IgG

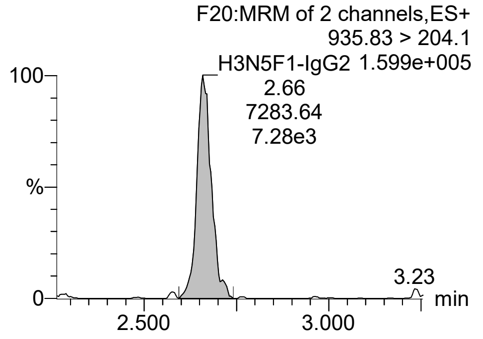

Sample Name: 20211111_H12_R2_IgG

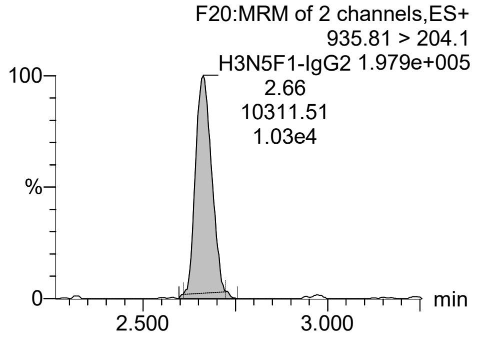

Sample Name: 20211111_H12_R2_IgG

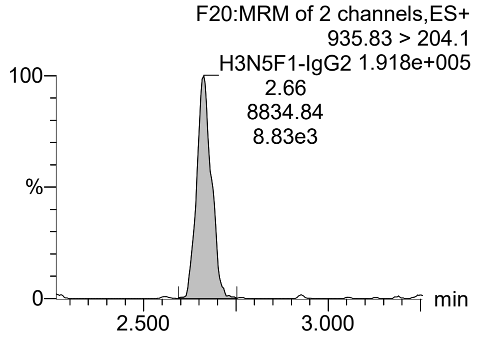

Sample Name: 20211111_QC_R14_IgG

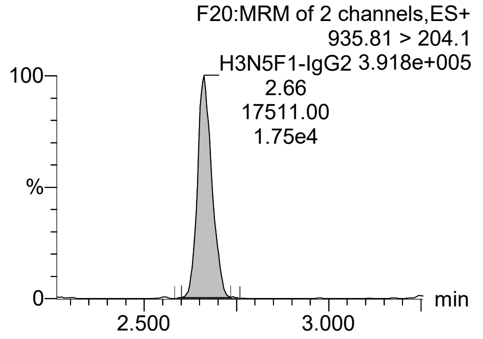

Sample Name: 20211111_QC_R14_IgG

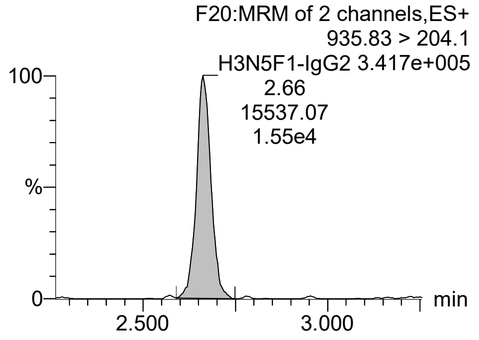

Sample Name: 20211111_A25_R2_IgG

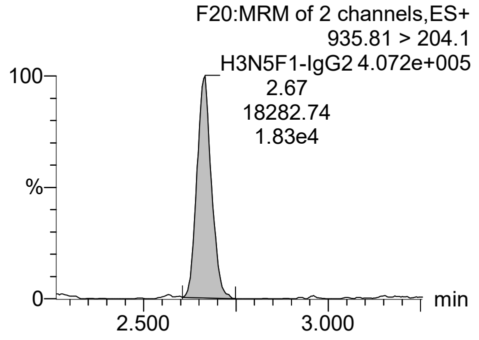

Sample Name: 20211111_A25_R2_IgG

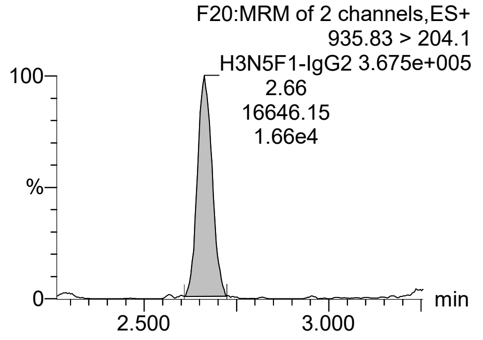

Dataset: C:\MassLynx\10.pro\20211115_clinicalsamples_IgG.qld

Last Altered: Sunday, October 02, 2022 16:02:30 台北標準時間
Printed: Friday, August 01, 2025 16:49:10 台北標準時間

Sample Name: 20211111_A26_R2_IgG

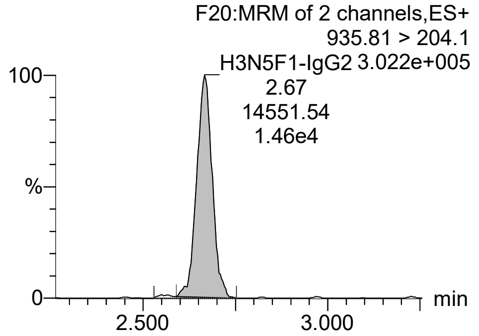

Sample Name: 20211111_A26_R2_IgG

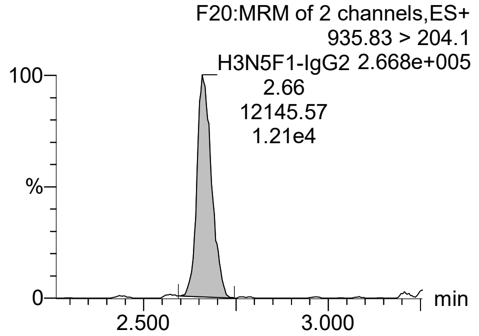

Sample Name: 20211111_L25_R2_IgG

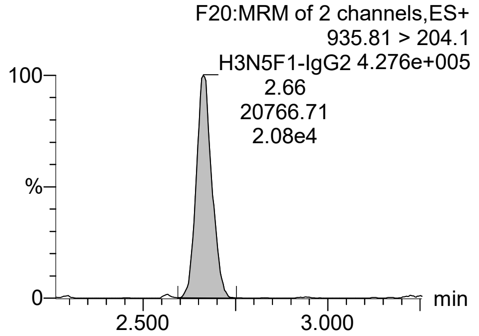

Sample Name: 20211111_L25_R2_IgG

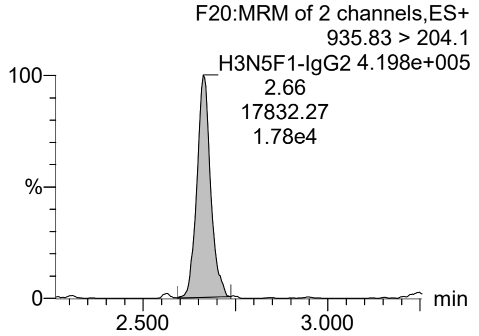

Sample Name: 20211111_L26_R2_IgG

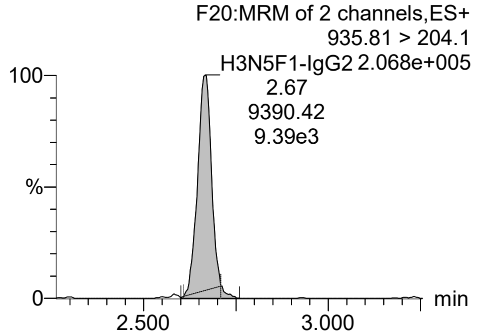

Sample Name: 20211111_L26_R2_IgG

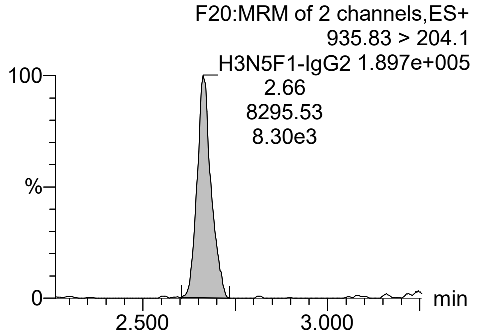

Sample Name: 20211111_H13_R2_IgG

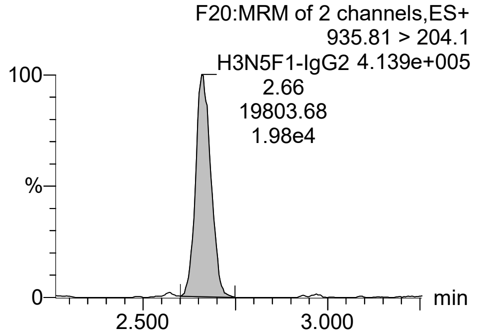

Sample Name: 20211111_H13_R2_IgG

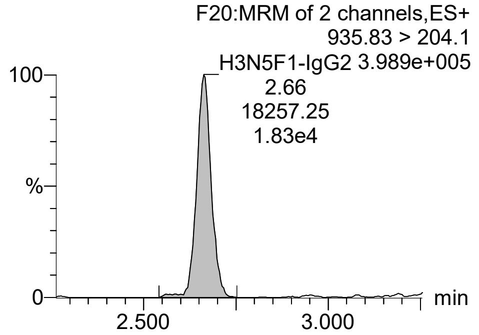

Sample Name: 20211111_A27_R2_IgG

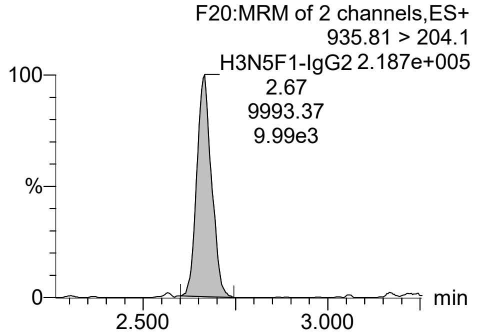

Sample Name: 20211111_A27_R2_IgG

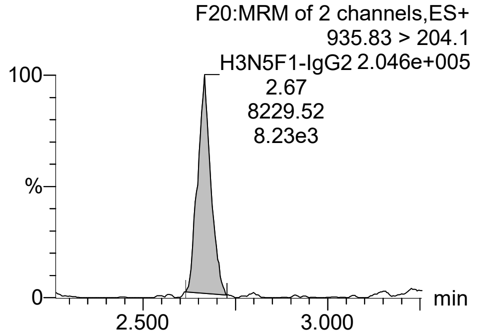

Sample Name: 20211111_A28_R2_IgG

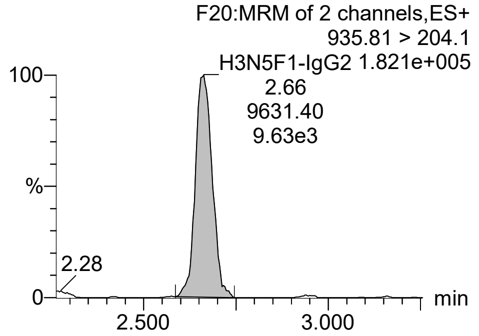

Sample Name: 20211111_A28_R2_IgG

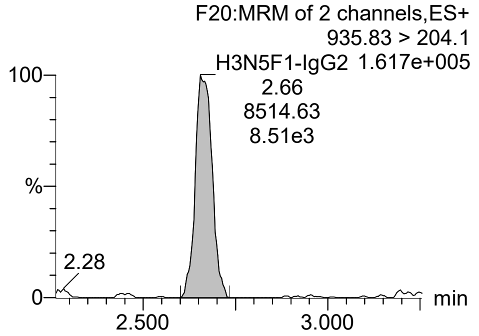

Sample Name: 20211111_L27_R2_IgG

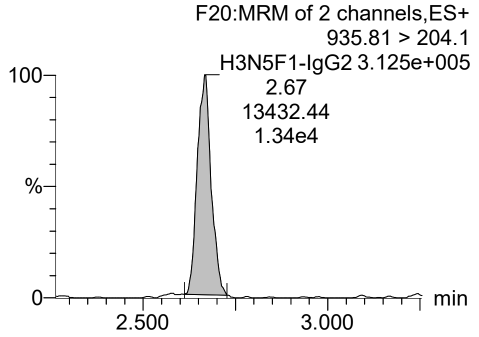

Sample Name: 20211111_L27_R2_IgG

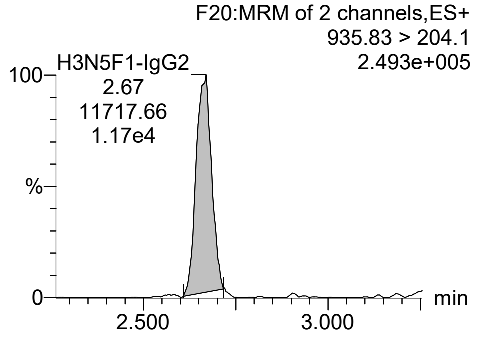

Sample Name: 20211111_L28_R2_IgG

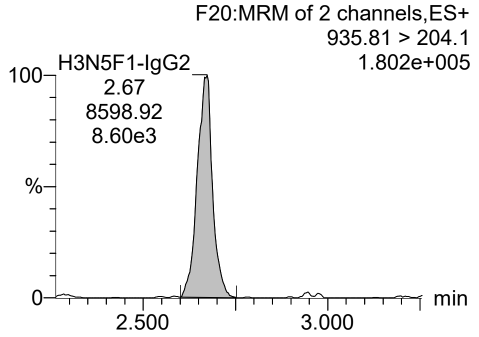

Sample Name: 20211111_L28_R2_IgG

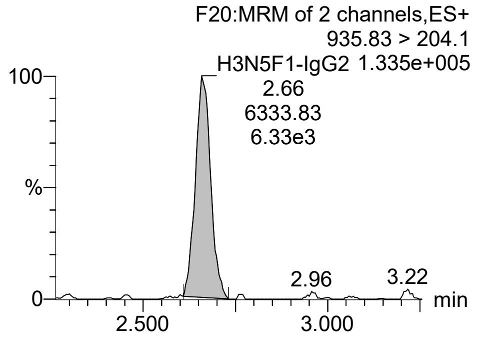

Sample Name: 20211111_H14_R2_IgG

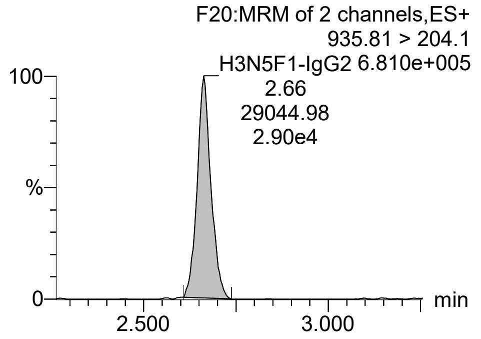

Sample Name: 20211111_H14_R2_IgG

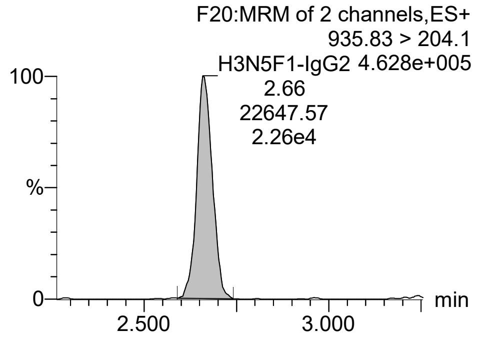

Sample Name: 20211111_A29_R2_IgG

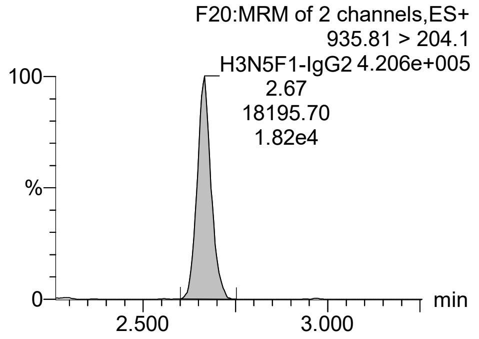

Sample Name: 20211111_A29_R2_IgG

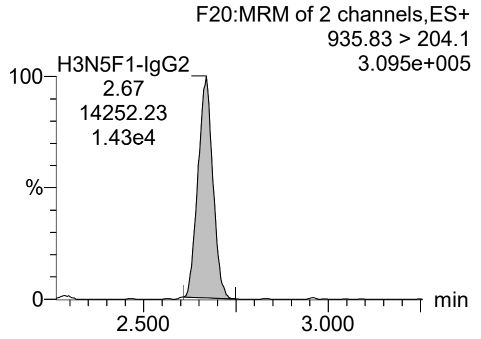

Sample Name: 20211111_A30_R2_IgG

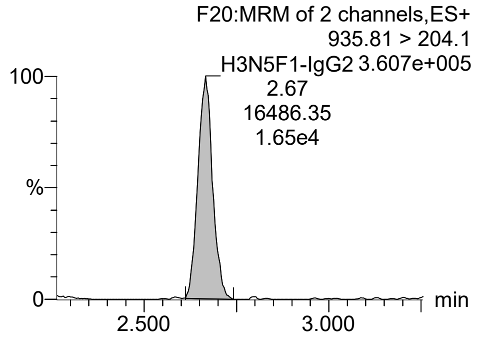

Sample Name: 20211111_A30_R2_IgG

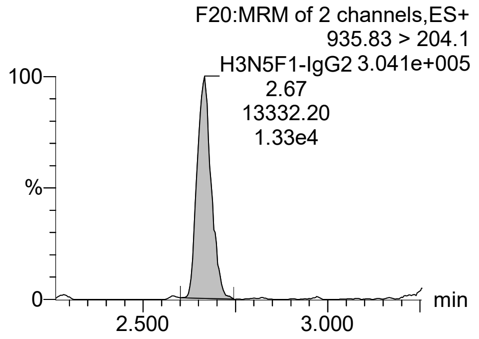

Sample Name: 20211111_QC_R15_IgG

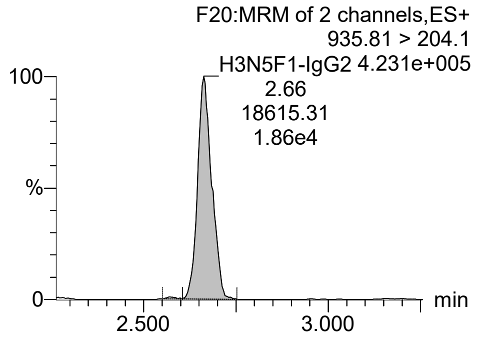

Sample Name: 20211111_QC_R15_IgG

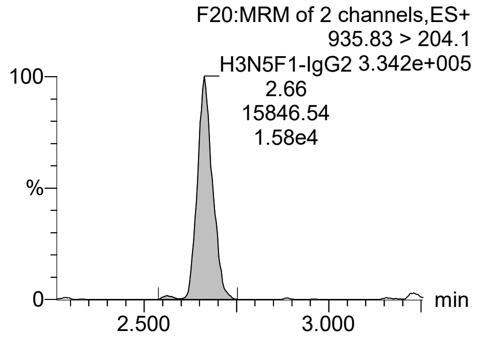

Sample Name: 20211111_L29_R2_IgG

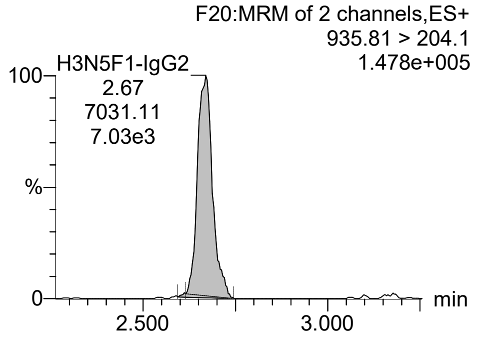

Sample Name: 20211111_L29_R2_IgG

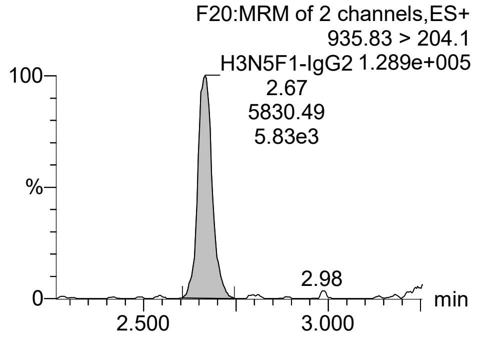

Sample Name: 20211111_L30_R2_IgG

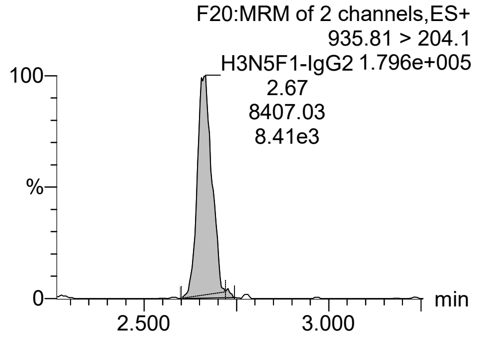

Sample Name: 20211111_L30_R2_IgG

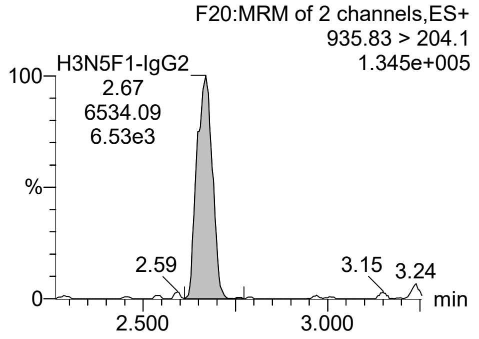

Sample Name: 20211111_H15_R2_IgG

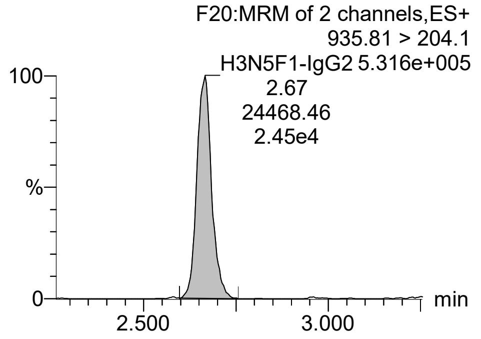

Sample Name: 20211111_H15_R2_IgG

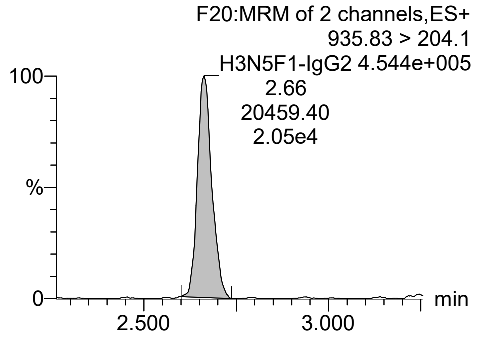

Sample Name: 20211111_A31_R2_IgG

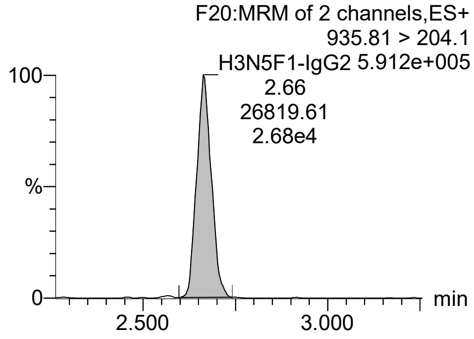

Sample Name: 20211111_A31_R2_IgG

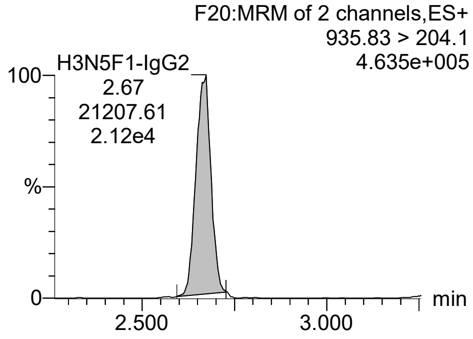

Sample Name: 20211111_A32_R2_IgG

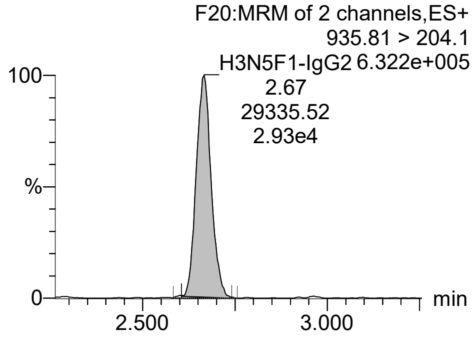

Sample Name: 20211111_A32_R2_IgG

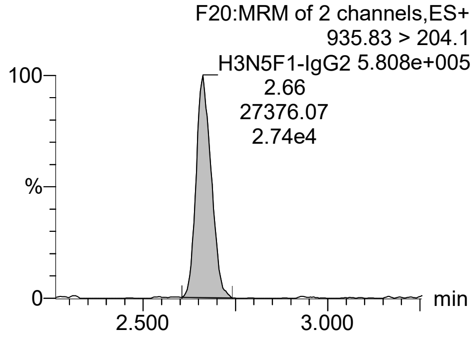

Sample Name: 20211111_L31_R2_IgG

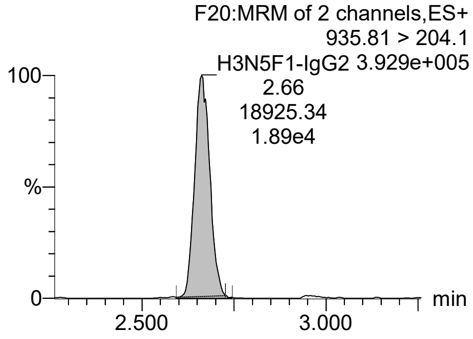

Sample Name: 20211111_L31_R2_IgG

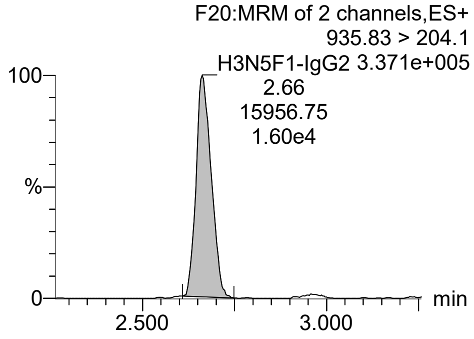

Sample Name: 20211111_L32_R2_IgG

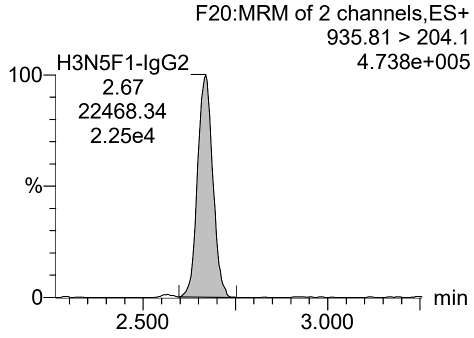

Sample Name: 20211111_L32_R2_IgG

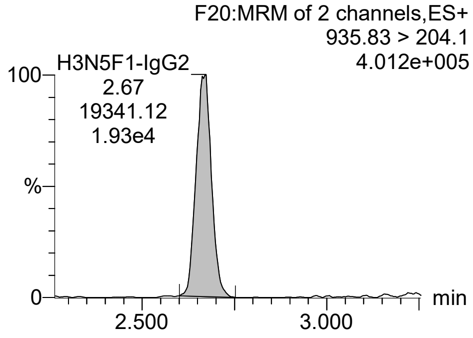

Sample Name: 20211111_H16_R2_IgG

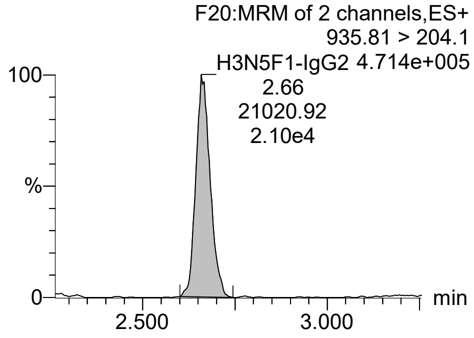

Sample Name: 20211111_H16_R2_IgG

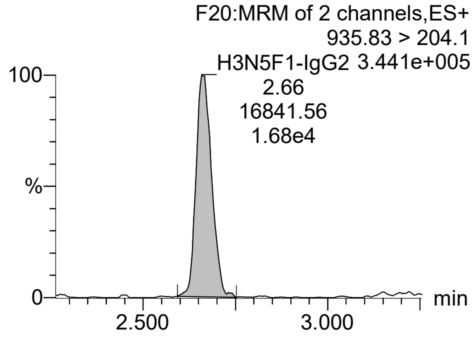

Sample Name: 20211111_A33_R2_IgG

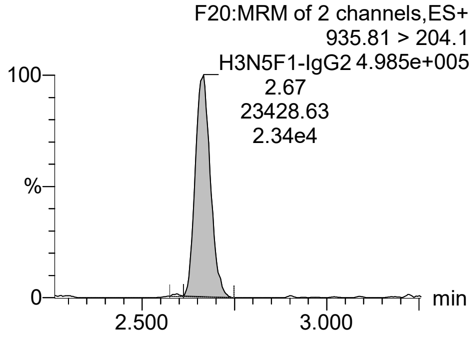

Sample Name: 20211111_A33_R2_IgG

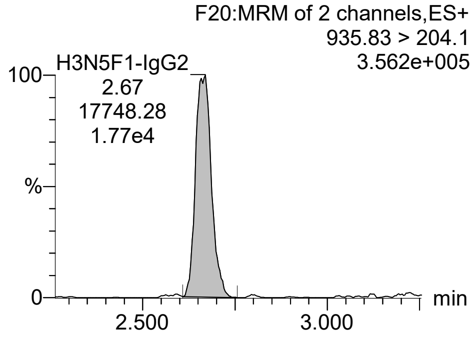

Sample Name: 20211111_A34_R2_IgG

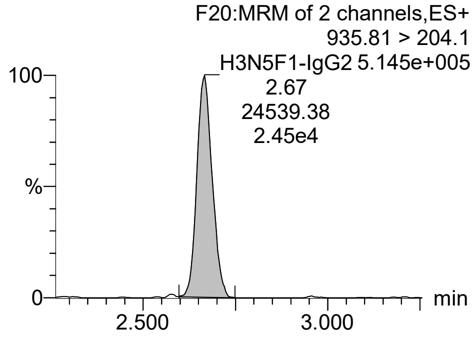

Sample Name: 20211111_A34_R2_IgG

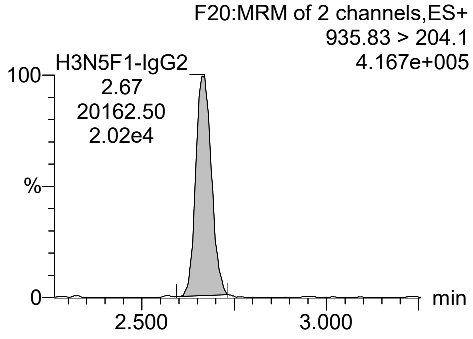

Sample Name: 20211111_H17_R2_IgG

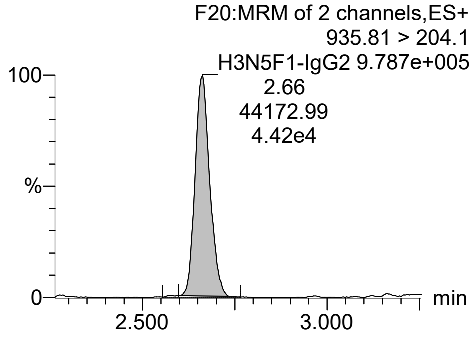

Sample Name: 20211111_H17_R2_IgG

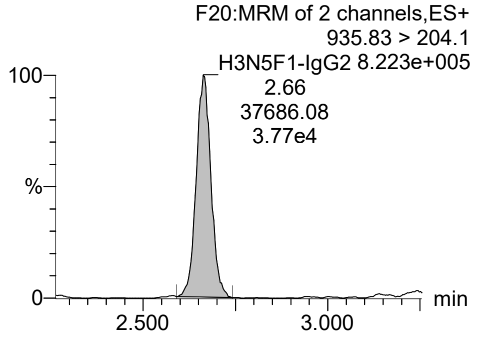

Sample Name: 20211111_A35_R2_IgG

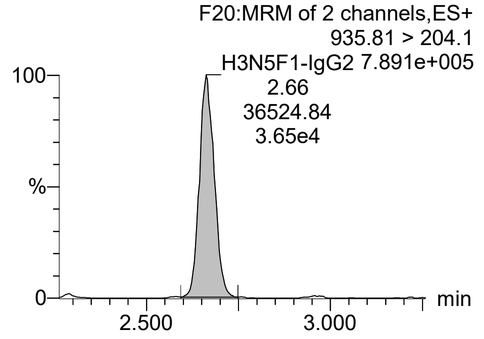

Sample Name: 20211111_A35_R2_IgG

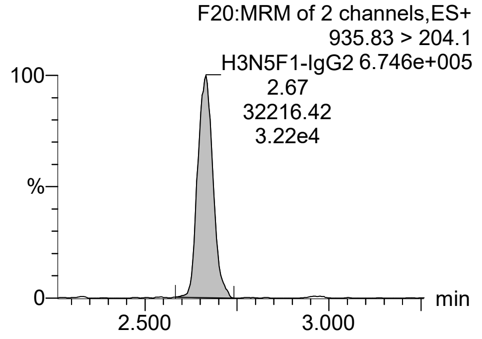

Sample Name: 20211111_QC_R16_IgG

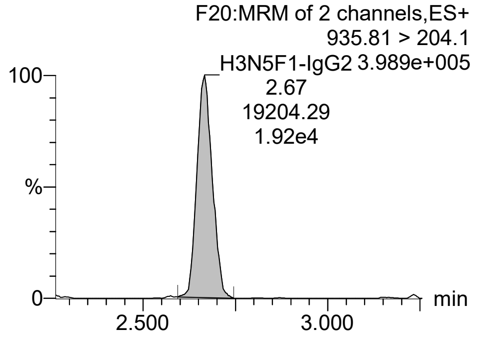

Sample Name: 20211111_QC_R16_IgG

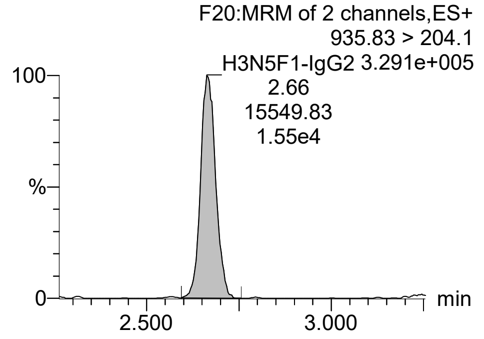

Sample Name: 20211111_A36_R2_IgG

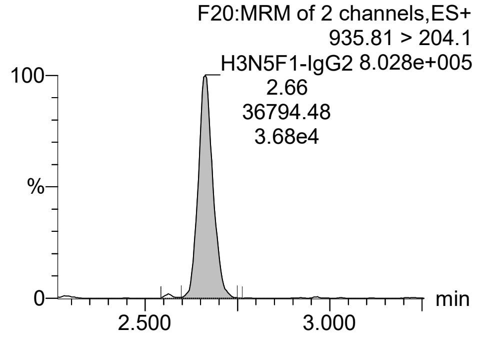

Sample Name: 20211111_A36_R2_IgG

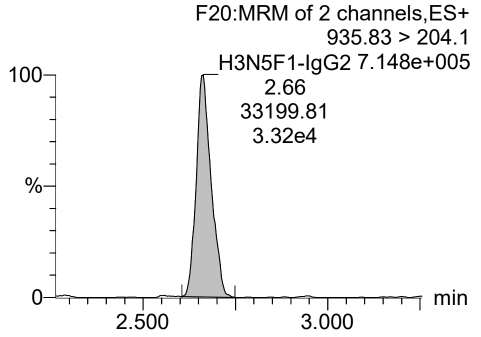

Sample Name: 20211111_H18_R2_IgG

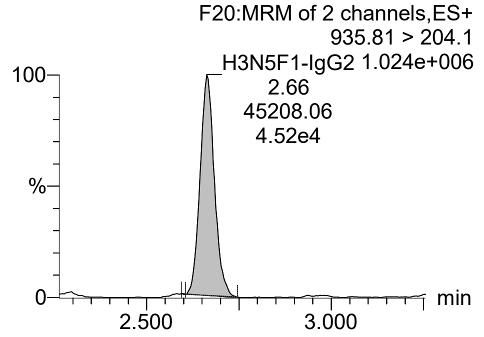

Sample Name: 20211111_H18_R2_IgG

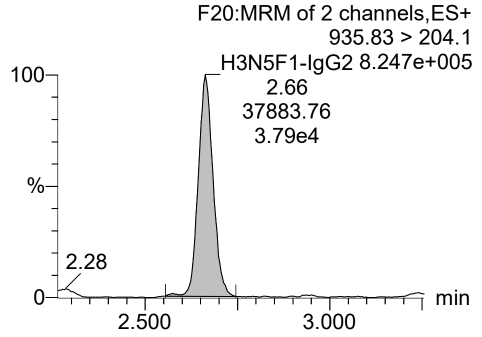

Sample Name: 20211111_A37_R2_IgG

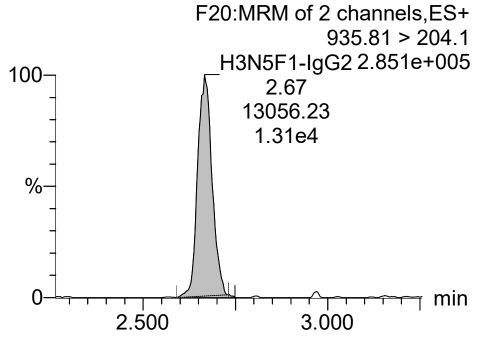

Sample Name: 20211111_A37_R2_IgG

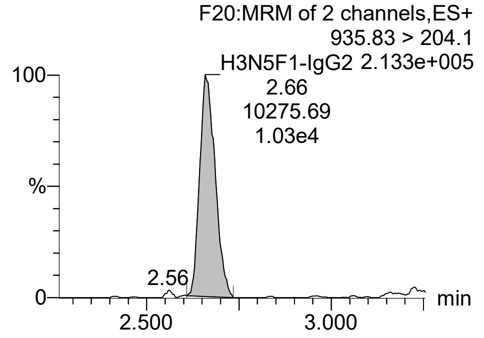

Sample Name: 20211111_A38_R2_IgG

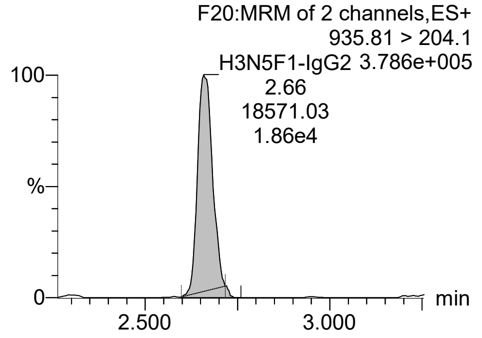

Sample Name: 20211111_A38_R2_IgG

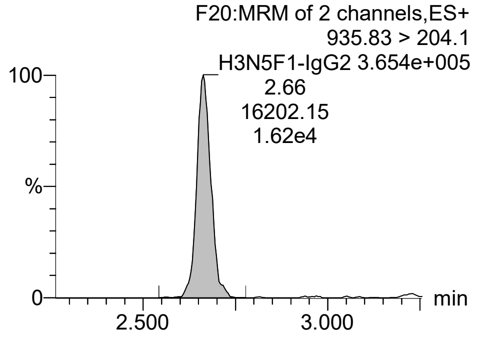

Sample Name: 20211111_H19_R2_IgG

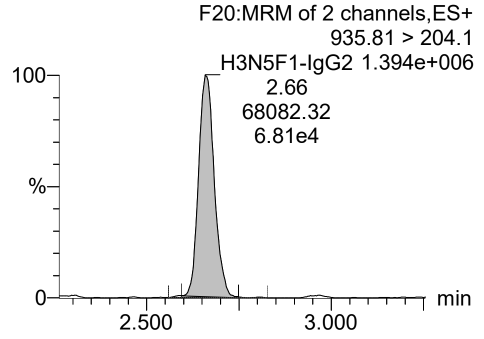

Sample Name: 20211111_H19_R2_IgG

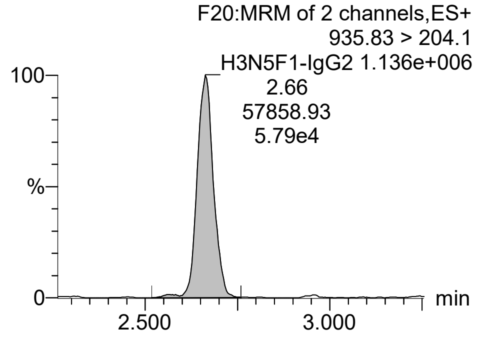

Sample Name: 20211111_A39_R2_IgG

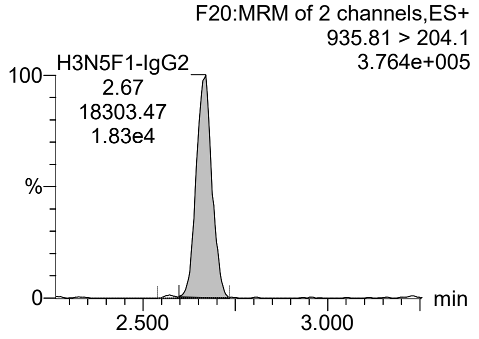

Sample Name: 20211111_A39_R2_IgG

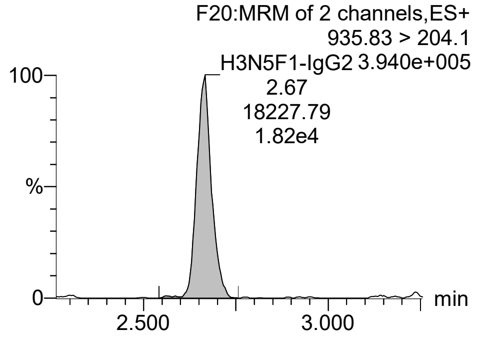

Sample Name: 20211111_A40_R2_IgG

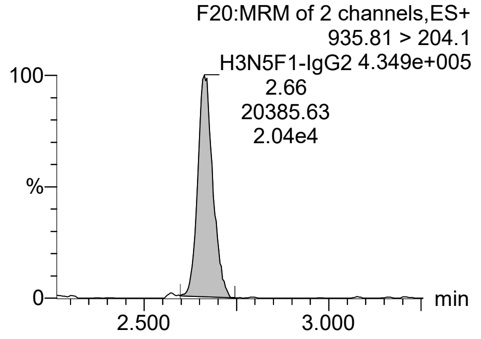

Sample Name: 20211111_A40_R2_IgG

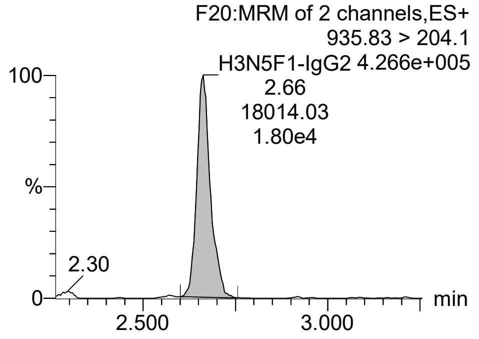

Sample Name: 20211111_H20_R2_IgG

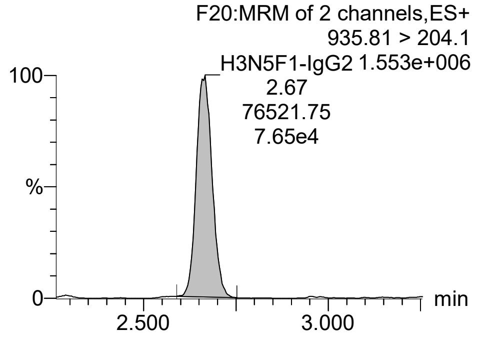

Sample Name: 20211111_H20_R2_IgG

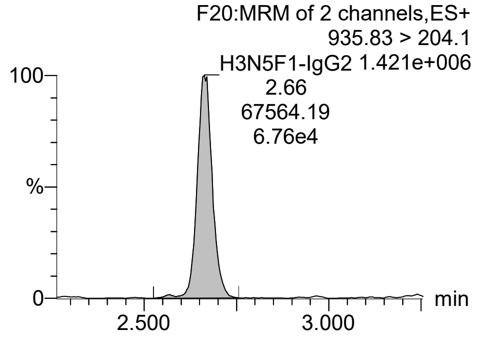

Sample Name: 20211111_A41_R2_IgG

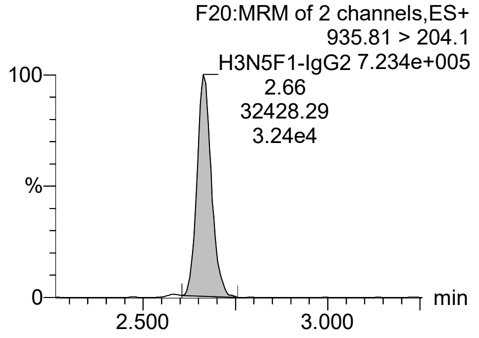

Sample Name: 20211111_A41_R2_IgG

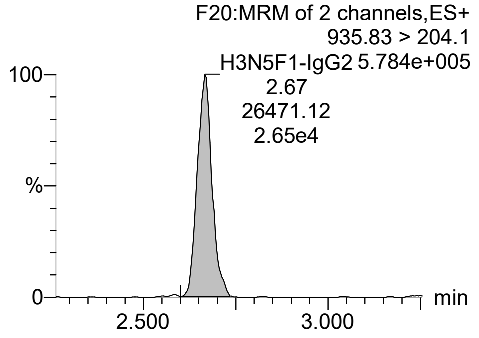

Sample Name: 20211111_A42_R2_IgG

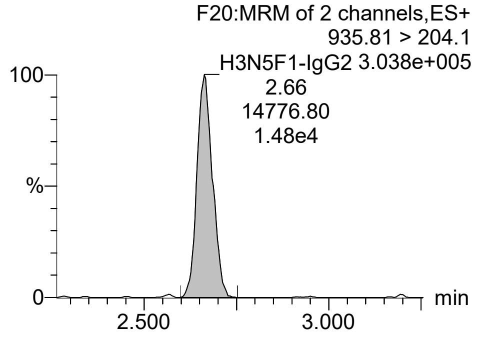

Sample Name: 20211111_A42_R2_IgG

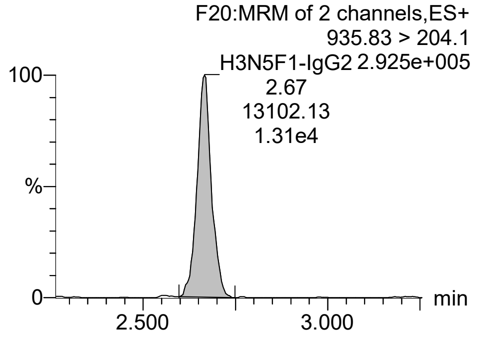

Sample Name: 20211111_A43_R2_IgG

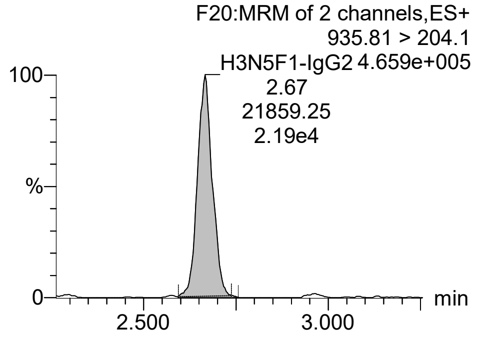

Sample Name: 20211111_A43_R2_IgG

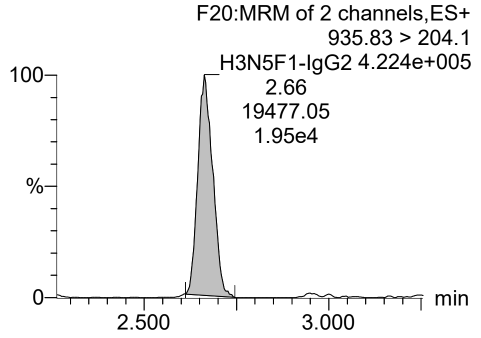

Sample Name: 20211111_A44_R2_IgG

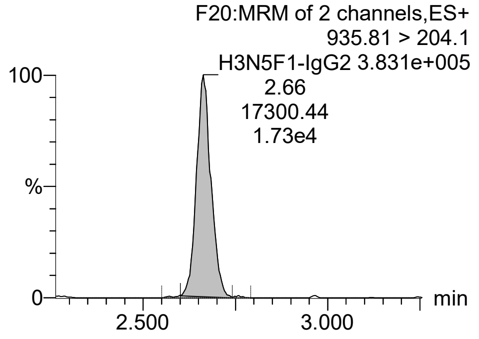

Sample Name: 20211111_A44_R2_IgG

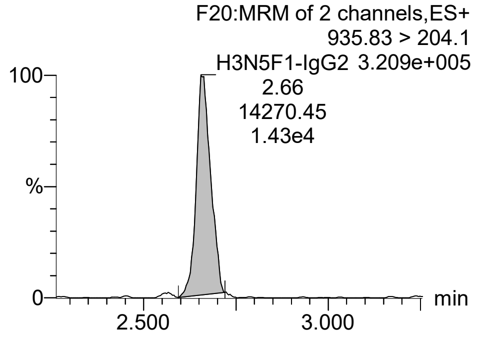

Sample Name: 20211111_QC_R17_IgG

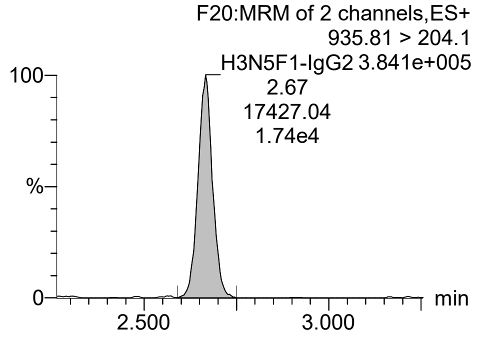

Dataset: C:\MassLynx\10.pro\20211115_clinicalsamples_IgG.qld

Last Altered: Sunday, October 02, 2022 16:02:30 台北標準時間
Printed: Friday, August 01, 2025 16:49:10 台北標準時間

Sample Name: 20211111_QC_R17_IgG

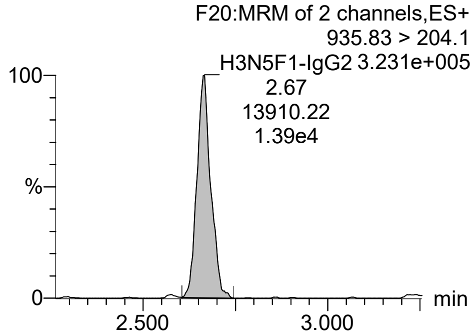

Dataset: C:\MassLynx\10.pro\20211115_clinicalsamples_IgG.qld

Last Altered: Sunday, October 02, 2022 16:02:30 台北標準時間
Printed: Friday, August 01, 2025 16:49:10 台北標準時間

	#	Name	RT	Area	IS Area	Response	Prima...
1	1	20211111_QC_R1_IgG	2.67	42207.4	64968.0	0.650	MM
2	2	20211111_A1_R1_IgG	2.67	84449.3	59059.2	1.430	MM
3	3	20211111_A2_R1_IgG	2.67	43043.6	66621.2	0.646	MM
4	4	20211111_L1_R1_IgG	2.67	33885.0	64201.0	0.528	MM
5	5	20211111_L2_R1_IgG	2.67	35376.2	46950.2	0.753	bb
6	6	20211111_H1_R1_IgG	2.66	22223.5	38648.2	0.575	MM
7	7	20211111_A3_R1_IgG	2.66	863.6	2603.7	0.332	bb
8	8	20211111_A4_R1_IgG	2.67	72190.3	69960.4	1.032	MM
9	9	20211111_L3_R1_IgG	2.67	14895.9	38864.7	0.383	MM
10	10	20211111_L4_R1_IgG	2.67	23850.7	43380.5	0.550	bb
11	11	20211111_H2_R1_IgG	2.67	74749.6	71349.2	1.048	MM
12	12	20211111_A5_R1_IgG	2.67	27015.6	65569.7	0.412	MM
13	13	20211111_A6_R1_IgG	2.67	33813.5	53120.7	0.637	MM
14	14	20211111_QC_R2_IgG	2.67	44350.3	81034.1	0.547	MM
15	15	20211111_L5_R1_IgG	2.67	24990.4	50994.6	0.490	MM
16	16	20211111_L6_R1_IgG	2.67	7272.9	58058.0	0.125	MM
17	17	20211111_H3_R1_IgG	2.67	33375.2	64028.6	0.521	MM
18	18	20211111_A7_R1_IgG	2.67	30104.6	82996.1	0.363	MM
19	19	20211111_A8_R1_IgG	2.67	25504.1	77731.7	0.328	MM
20	20	20211111_L7_R1_IgG	2.67	29374.8	38827.3	0.757	bb
21	21	20211111_L8_R1_IgG	2.68	12180.6	44220.1	0.275	bb
22	22	20211111_H4_R1_IgG	2.67	26539.3	65826.3	0.403	MM
23	23	20211111_A9_R1_IgG	2.66	25114.3	60771.1	0.413	MM
24	24	20211111_A10_R1_IgG	2.67	20336.4	48578.7	0.419	bb
25	25	20211111_L9_R1_IgG	2.67	30941.0	50830.7	0.609	bb
26	26	20211111_L10_R1_IgG	2.67	24639.2	38034.2	0.648	bb
27	27	20211111_QC_R3_IgG	2.67	36923.7	67025.5	0.551	MM
28	28	20211111_H5_R1_IgG	2.67	14712.2	44544.9	0.330	bb
29	29	20211111_A11_R1_IgG	2.67	13194.7	66408.5	0.199	bb
30	30	20211111_A12_R1_IgG	2.67	32579.9	46624.5	0.699	MM
31	31	20211111_L11_R1_IgG	2.67	12349.7	33425.1	0.369	bb
32	32	20211111_L12_R1_IgG	2.68	9853.2	41178.0	0.239	MM
33	33	20211111_H6_R1_IgG	2.67	80474.1	58593.4	1.373	MM
34	34	20211111_A13_R1_IgG	2.67	48266.3	54133.0	0.892	MM
35	35	20211111_A14_R1_IgG	2.67	35366.4	56467.3	0.626	MM
36	36	20211111_L13_R1_IgG	2.68	10324.5	36024.4	0.287	MM
37	37	20211111_L14_R1_IgG	2.67	14578.8	37181.6	0.392	bb
38	38	20211111_H7_R1_IgG	2.67	26763.8	42850.6	0.625	bb
39	39	20211111_A15_R1_IgG	2.68	28751.6	62015.1	0.464	MM
40	40	20211111_QC_R4_IgG	2.67	33076.4	58572.5	0.565	MM
41	41	20211111_A16_R1_IgG	2.67	30226.1	78283.8	0.386	MM
42	42	20211111_L15_R1_IgG	2.67	32032.7	44158.2	0.725	MM
43	43	20211111_L16_R1_IgG	2.67	29507.8	55150.8	0.535	MM
44	44	20211111_H8_R1_IgG	2.66	13991.4	42965.2	0.326	bb
45	45	20211111_A17_R1_IgG	2.67	23398.7	39717.7	0.589	bb
46	46	20211111_A18_R1_IgG	2.67	15553.0	44675.4	0.348	bb
47	47	20211111_L17_R1_IgG	2.67	27825.6	37854.1	0.735	bb
48	48	20211111_L18_R1_IgG	2.67	10609.9	50716.9	0.209	MM
49	49	20211111_H9_R1_IgG	2.67	36683.3	36276.0	1.011	MM
50	50	20211111_A19_R1_IgG	2.67	51739.9	42862.5	1.207	MM
51	51	20211111_A20_R1_IgG	2.67	9746.5	36542.7	0.267	MM
52	52	20211111_L19_R1_IgG	2.67	23014.1	53892.8	0.427	bb
53	53	20211111_QC_R5_IgG	2.67	28887.8	56513.2	0.511	MM
54	54	20211111_L20_R1_IgG	2.67	24639.3	56499.9	0.436	MM
55	55	20211111_H10_R1_IgG	2.67	28728.4	68120.5	0.422	bb
56	56	20211111_A21_R1_IgG	2.67	38134.5	63699.8	0.599	MM

Dataset: C:\MassLynx\10.pro\20211115_clinicalsamples_IgG.qld

Last Altered: Sunday, October 02, 2022 16:02:30 台北標準時間
Printed: Friday, August 01, 2025 16:49:10 台北標準時間

	# Name	RT	Area	IS Area	Response	Prima...
57	57 20211111_A22_R1_IgG	2.67	12245.2	52222.1	0.234	bb
58	58 20211111_L21_R1_IgG	2.67	12910.1	54372.5	0.237	bb
59	59 20211111_L22_R1_IgG	2.67	15290.7	41353.1	0.370	bb
60	60 20211111_H11_R1_IgG	2.67	60447.6	65579.1	0.922	bb
61	61 20211111_A23_R1_IgG	2.67	62875.4	64131.1	0.980	MM
62	62 20211111_A24_R1_IgG	2.67	11810.4	38724.8	0.305	bb
63	63 20211111_L23_R1_IgG	2.67	61150.6	64245.2	0.952	MM
64	64 20211111_L24_R1_IgG	2.67	11519.3	45298.0	0.254	MM
65	65 20211111_H12_R1_IgG	2.67	13102.4	44679.3	0.293	bb
66	66 20211111_QC_R6_IgG	2.67	28585.6	48165.0	0.593	MM
67	67 20211111_A25_R1_IgG	2.67	29439.9	57169.9	0.515	MM
68	68 20211111_A26_R1_IgG	2.67	18536.5	34254.0	0.541	bb
69	69 20211111_L25_R1_IgG	2.67	32859.2	67491.5	0.487	MM
70	70 20211111_L26_R1_IgG	2.67	14301.6	55934.1	0.256	bb
71	71 20211111_H13_R1_IgG	2.67	27684.2	48767.3	0.568	bb
72	72 20211111_A27_R1_IgG	2.67	15229.3	57269.7	0.266	bb
73	73 20211111_A28_R1_IgG	2.67	14886.5	49231.1	0.302	bb
74	74 20211111_L27_R1_IgG	2.67	19204.8	60014.5	0.320	MM
75	75 20211111_L28_R1_IgG	2.67	9318.0	35062.6	0.266	MM
76	76 20211111_H14_R1_IgG	2.67	40621.6	62371.9	0.651	MM
77	77 20211111_A29_R1_IgG	2.67	20572.8	43858.2	0.469	bb
78	78 20211111_A30_R1_IgG	2.67	24763.1	62500.4	0.396	MM
79	79 20211111_QC_R7_IgG	2.67	27050.1	43946.9	0.616	bb
80	80 20211111_L29_R1_IgG	2.67	7833.8	38728.9	0.202	bb
81	81 20211111_L30_R1_IgG	2.67	9440.1	42613.2	0.222	bb
82	82 20211111_H15_R1_IgG	2.67	26163.5	39874.6	0.656	bb
83	83 20211111_A31_R1_IgG	2.67	36715.2	53414.3	0.687	MM
84	84 20211111_A32_R1_IgG	2.67	38078.4	41844.6	0.910	MM
85	85 20211111_L31_R1_IgG	2.67	20909.2	40553.7	0.516	MM
86	86 20211111_L32_R1_IgG	2.67	26467.3	49618.7	0.533	MM
87	87 20211111_H16_R1_IgG	2.67	27433.7	60170.9	0.456	MM
88	88 20211111_A33_R1_IgG	2.67	26259.5	48588.2	0.540	bb
89	89 20211111_A34_R1_IgG	2.67	29649.1	43288.0	0.685	MM
90	90 20211111_H17_R1_IgG	2.67	52202.8	47104.5	1.108	MM
91	91 20211111_A35_R1_IgG	2.67	39491.4	41025.9	0.963	MM
92	92 20211111_QC_R8_IgG	2.67	22577.4	40546.3	0.557	bb
93	93 20211111_A36_R1_IgG	2.67	41519.6	44504.4	0.933	MM
94	94 20211111_H18_R1_IgG	2.67	47983.3	34162.2	1.405	MM
95	95 20211111_A37_R1_IgG	2.67	14903.5	39760.0	0.375	bb
96	96 20211111_A38_R1_IgG	2.67	20806.4	43982.6	0.473	MM
97	97 20211111_H19_R1_IgG	2.67	74661.0	45314.8	1.648	bb
98	98 20211111_A39_R1_IgG	2.67	22888.9	39388.0	0.581	MM
99	99 20211111_A40_R1_IgG	2.67	20719.5	38726.4	0.535	bb
100	1... 20211111_H20_R1_IgG	2.67	83030.3	37814.4	2.196	MM
101	1... 20211111_A41_R1_IgG	2.67	37711.2	48930.3	0.771	MM
102	1... 20211111_A42_R1_IgG	2.67	16672.3	40406.8	0.413	MM
103	1... 20211111_A43_R1_IgG	2.67	29205.5	44421.5	0.657	bb
104	1... 20211111_A44_R1_IgG	2.67	26028.7	53128.2	0.490	bb
105	1... 20211111_QC_R9_IgG	2.67	20144.2	37049.0	0.544	bb
106	1... 20211111_A1_R2_IgG	2.67	62454.3	46499.4	1.343	MM
107	1... 20211111_A2_R2_IgG	2.67	33468.5	57260.7	0.584	MM
108	1... 20211111_L1_R2_IgG	2.67	24183.3	48062.9	0.503	MM
109	1... 20211111_L2_R2_IgG	2.67	30932.3	38745.2	0.798	bb
110	1... 20211111_H1_R2_IgG	2.67	24786.9	44273.5	0.560	bb
111	1... 20211111_A3_R2_IgG	2.67	2579.5	4755.5	0.542	MM
112	1... 20211111_A4_R2_IgG	2.67	52538.6	50546.7	1.039	bb

Dataset: C:\MassLynx\10.pro\20211115_clinicalsamples_IgG.qld

Last Altered: Sunday, October 02, 2022 16:02:30 台北標準時間
Printed: Friday, August 01, 2025 16:49:10 台北標準時間

	# Name	RT	Area	IS Area	Response	Prima...
113	1... 20211111_L3_R2_IgG	2.67	17078.8	34825.8	0.490	MM
114	1... 20211111_L4_R2_IgG	2.67	22367.0	39107.7	0.572	bb
115	1... 20211111_H2_R2_IgG	2.67	53417.1	49750.0	1.074	MM
116	1... 20211111_A5_R2_IgG	2.67	23456.1	52536.2	0.446	bb
117	1... 20211111_A6_R2_IgG	2.67	29376.8	42050.0	0.699	MM
118	1... 20211111_QC_R10_IgG	2.67	19605.7	35350.8	0.555	bb
119	1... 20211111_L5_R2_IgG	2.67	21284.6	38039.3	0.560	bb
120	1... 20211111_L6_R2_IgG	2.67	5934.5	44907.4	0.132	bb
121	1... 20211111_H3_R2_IgG	2.67	28931.2	47729.1	0.606	MM
122	1... 20211111_A7_R2_IgG	2.67	22438.0	57347.1	0.391	bb
123	1... 20211111_A8_R2_IgG	2.67	18163.1	52397.8	0.347	bb
124	1... 20211111_L7_R2_IgG	2.67	29919.4	36318.4	0.824	bb
125	1... 20211111_L8_R2_IgG	2.66	12384.4	38131.9	0.325	MM
126	1... 20211111_H4_R2_IgG	2.67	20451.8	46849.6	0.437	bb
127	1... 20211111_A9_R2_IgG	2.67	21647.2	47759.6	0.453	bb
128	1... 20211111_A10_R2_IgG	2.67	20048.6	41366.2	0.485	bb
129	1... 20211111_L9_R2_IgG	2.67	27037.9	43052.1	0.628	bb
130	1... 20211111_L10_R2_IgG	2.67	23550.0	31487.4	0.748	bb
131	1... 20211111_QC_R11_IgG	2.67	19976.6	37804.6	0.528	bb
132	1... 20211111_H5_R2_IgG	2.67	13386.8	40380.0	0.332	bb
133	1... 20211111_A11_R2_IgG	2.67	10263.4	49533.3	0.207	bb
134	1... 20211111_A12_R2_IgG	2.67	30369.9	40226.3	0.755	MM
135	1... 20211111_L11_R2_IgG	2.67	11944.9	28884.0	0.414	MM
136	1... 20211111_L12_R2_IgG	2.67	9219.8	34351.3	0.268	MM
137	1... 20211111_H6_R2_IgG	2.67	52207.4	40466.6	1.290	MM
138	1... 20211111_A13_R2_IgG	2.67	40078.2	42306.7	0.947	bb
139	1... 20211111_A14_R2_IgG	2.67	29424.2	44904.0	0.655	MM
140	1... 20211111_L13_R2_IgG	2.67	9968.8	31913.0	0.312	bb
141	1... 20211111_L14_R2_IgG	2.67	14762.4	31527.4	0.468	bb
142	1... 20211111_H7_R2_IgG	2.67	24877.0	40838.2	0.609	bb
143	1... 20211111_A15_R2_IgG	2.67	21977.5	49389.7	0.445	MM
144	1... 20211111_QC_R12_IgG	2.67	19028.6	35168.7	0.541	bb
145	1... 20211111_A16_R2_IgG	2.67	20896.5	47440.5	0.440	bb
146	1... 20211111_L15_R2_IgG	2.67	23794.2	33145.9	0.718	bb
147	1... 20211111_L16_R2_IgG	2.67	20693.0	37596.6	0.550	MM
148	1... 20211111_H8_R2_IgG	2.66	14474.0	43933.3	0.329	MM
149	1... 20211111_A17_R2_IgG	2.67	21584.5	43605.5	0.495	MM
150	1... 20211111_A18_R2_IgG	2.68	12264.8	37581.8	0.326	bb
151	1... 20211111_L17_R2_IgG	2.67	26112.3	33942.5	0.769	MM
152	1... 20211111_L18_R2_IgG	2.66	8544.2	37831.5	0.226	MM
153	1... 20211111_H9_R2_IgG	2.67	35582.2	30898.4	1.152	bb
154	1... 20211111_A19_R2_IgG	2.67	50164.9	39352.1	1.275	MM
155	1... 20211111_A20_R2_IgG	2.67	8414.0	34661.2	0.243	MM
156	1... 20211111_L19_R2_IgG	2.67	17032.2	39918.3	0.427	MM
157	1... 20211111_QC_R13_IgG	2.67	21986.1	36239.1	0.607	MM
158	1... 20211111_L20_R2_IgG	2.66	11034.3	32897.7	0.335	MM
159	1... 20211111_H10_R2_IgG	2.66	10700.6	31394.5	0.341	MM
160	1... 20211111_A21_R2_IgG	2.67	17140.5	34863.7	0.492	bb
161	1... 20211111_A22_R2_IgG	2.66	5629.9	30342.1	0.186	bb
162	1... 20211111_L21_R2_IgG	2.66	7240.8	33706.2	0.215	MM
163	1... 20211111_L22_R2_IgG	2.67	9603.5	31298.6	0.307	bb
164	1... 20211111_H11_R2_IgG	2.66	29746.4	38904.1	0.765	bb
165	1... 20211111_A23_R2_IgG	2.66	30482.0	32382.3	0.941	MM
166	1... 20211111_A24_R2_IgG	2.66	8324.4	30261.9	0.275	bb
167	1... 20211111_L23_R2_IgG	2.67	40256.9	43037.5	0.935	bb
168	1... 20211111_L24_R2_IgG	2.67	8542.1	37023.3	0.231	MM

Dataset: C:\MassLynx\10.pro\20211115_clinicalsamples_IgG.qld

Last Altered: Sunday, October 02, 2022 16:02:30 台北標準時間
Printed: Friday, August 01, 2025 16:49:10 台北標準時間

	#	Name	RT	Area	IS Area	Response	Prima...
169	1...	20211111_H12_R2_IgG	2.66	10311.5	39478.2	0.261	MM
170	1...	20211111_QC_R14_IgG	2.66	17511.0	34463.2	0.508	MM
171	1...	20211111_A25_R2_IgG	2.67	18282.7	39945.3	0.458	bb
172	1...	20211111_A26_R2_IgG	2.67	14551.5	29663.2	0.491	MM
173	1...	20211111_L25_R2_IgG	2.66	20766.7	38589.3	0.538	bb
174	1...	20211111_L26_R2_IgG	2.67	9390.4	38405.2	0.245	MM
175	1...	20211111_H13_R2_IgG	2.66	19803.7	37771.2	0.524	bb
176	1...	20211111_A27_R2_IgG	2.67	9993.4	42634.1	0.234	bb
177	1...	20211111_A28_R2_IgG	2.66	9631.4	33231.1	0.290	bb
178	1...	20211111_L27_R2_IgG	2.67	13432.4	41761.1	0.322	bb
179	1...	20211111_L28_R2_IgG	2.67	8598.9	26968.6	0.319	bb
180	1...	20211111_H14_R2_IgG	2.66	29045.0	49250.2	0.590	bb
181	1...	20211111_A29_R2_IgG	2.67	18195.7	37601.7	0.484	bb
182	1...	20211111_A30_R2_IgG	2.67	16486.3	40366.8	0.408	bb
183	1...	20211111_QC_R15_IgG	2.66	18615.3	35156.1	0.530	MM
184	1...	20211111_L29_R2_IgG	2.67	7031.1	33813.1	0.208	MM
185	1...	20211111_L30_R2_IgG	2.67	8407.0	33488.6	0.251	MM
186	1...	20211111_H15_R2_IgG	2.67	24468.5	38501.0	0.636	bb
187	1...	20211111_A31_R2_IgG	2.66	26819.6	37525.5	0.715	bb
188	1...	20211111_A32_R2_IgG	2.67	29335.5	35770.8	0.820	MM
189	1...	20211111_L31_R2_IgG	2.66	18925.3	33282.4	0.569	MM
190	1...	20211111_L32_R2_IgG	2.67	22468.3	42049.6	0.534	bb
191	1...	20211111_H16_R2_IgG	2.66	21020.9	45947.6	0.457	bb
192	1...	20211111_A33_R2_IgG	2.67	23428.6	40202.3	0.583	MM
193	1...	20211111_A34_R2_IgG	2.67	24539.4	36747.0	0.668	bb
194	1...	20211111_H17_R2_IgG	2.66	44173.0	40920.9	1.079	MM
195	1...	20211111_A35_R2_IgG	2.66	36524.8	33689.2	1.084	bb
196	1...	20211111_QC_R16_IgG	2.67	19204.3	32783.6	0.586	bb
197	1...	20211111_A36_R2_IgG	2.66	36794.5	36822.4	0.999	MM
198	1...	20211111_H18_R2_IgG	2.66	45208.1	31769.3	1.423	MM
199	1...	20211111_A37_R2_IgG	2.67	13056.2	37581.8	0.347	MM
200	2...	20211111_A38_R2_IgG	2.66	18571.0	38390.4	0.484	MM
201	2...	20211111_H19_R2_IgG	2.66	68082.3	41217.0	1.652	MM
202	2...	20211111_A39_R2_IgG	2.67	18303.5	37392.7	0.489	MM
203	2...	20211111_A40_R2_IgG	2.66	20385.6	38295.1	0.532	bb
204	2...	20211111_H20_R2_IgG	2.67	76521.8	35941.8	2.129	bb
205	2...	20211111_A41_R2_IgG	2.66	32428.3	45304.9	0.716	bb
206	2...	20211111_A42_R2_IgG	2.66	14776.8	37803.8	0.391	bb
207	2...	20211111_A43_R2_IgG	2.67	21859.2	38161.8	0.573	MM
208	2...	20211111_A44_R2_IgG	2.66	17300.4	37819.8	0.457	MM
209	2...	20211111_QC_R17_IgG	2.67	17427.0	31731.8	0.549	bb

Dataset: C:\MassLynx\10.pro\20211115_clinicalsamples_IgG.qld

Last Altered: Sunday, October 02, 2022 16:02:30 台北標準時間
Printed: Friday, August 01, 2025 16:49:44 台北標準時間

Method: C:\MassLynx\10_Pro_IgA.PRO\MethDB\20210926_IgG_DMRM_IgM_IS.mdb 16 Feb 2022 11:58:21
Calibration: 18 Nov 2021 13:56:22

Sample Name: 20211111_QC_R1_IgG

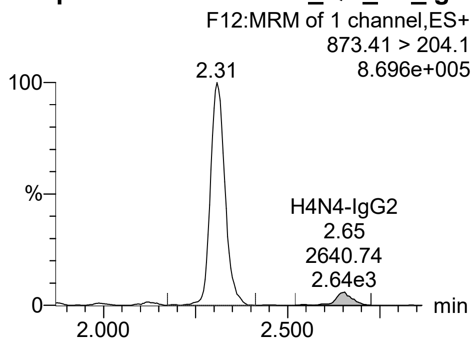

Sample Name: 20211111_A1_R1_IgG

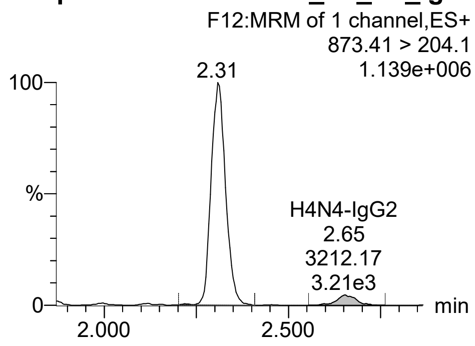

Sample Name: 20211111_A2_R1_IgG

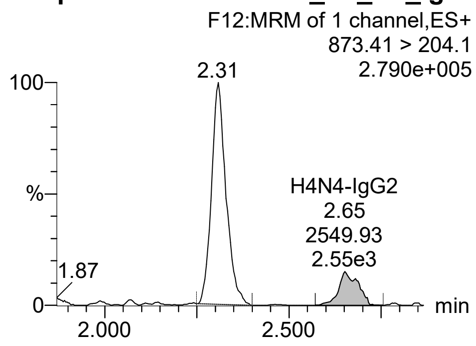

Sample Name: 20211111_L1_R1_IgG

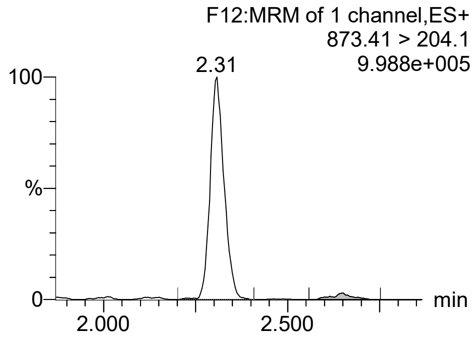

Sample Name: 20211111_L2_R1_IgG

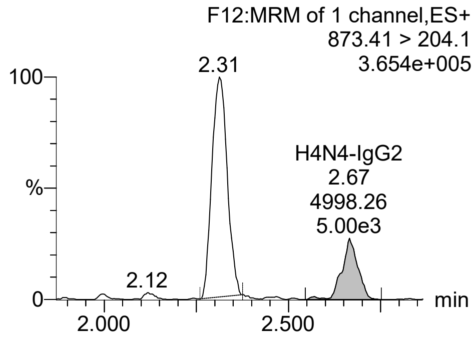

Sample Name: 20211111_H1_R1_IgG

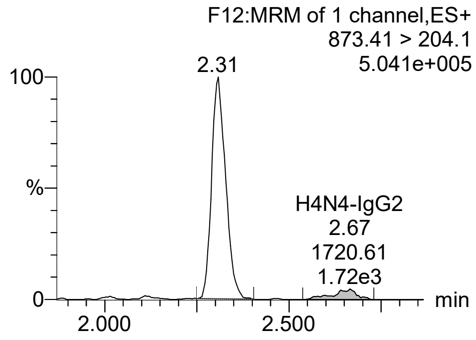

Sample Name: 20211111_A3_R1_IgG

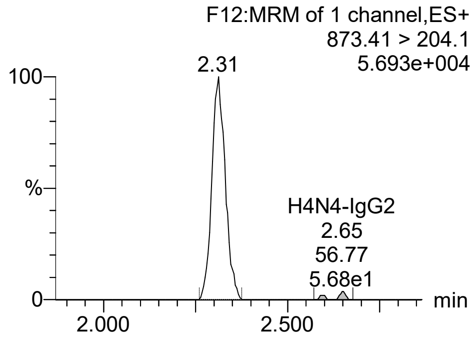

Sample Name: 20211111_A4_R1_IgG

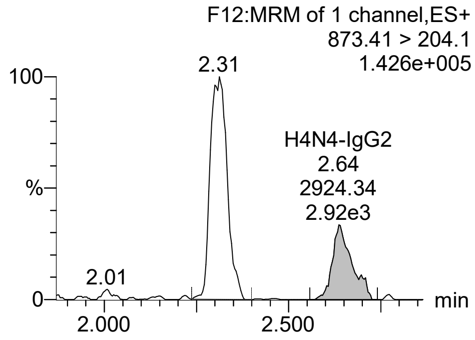

Sample Name: 20211111_L3_R1_IgG

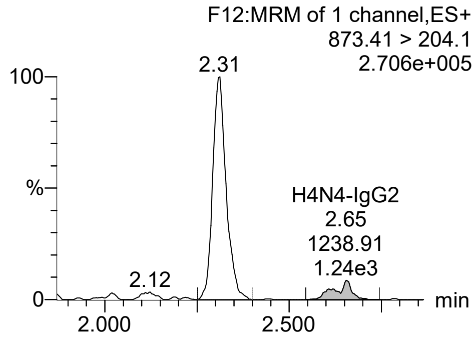

Sample Name: 20211111_L4_R1_IgG

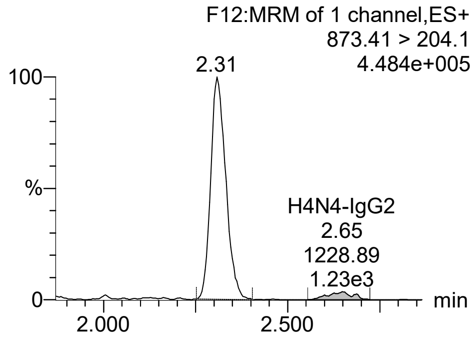

Sample Name: 20211111_H2_R1_IgG

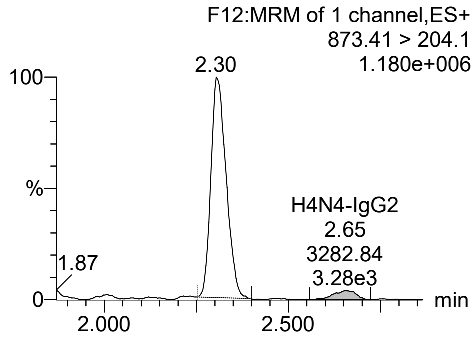

Sample Name: 20211111_A5_R1_IgG

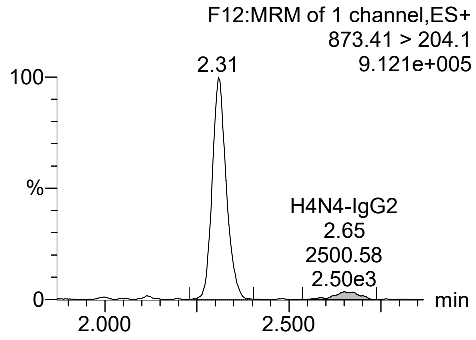

Dataset: C:\MassLynx\10.pro\20211115_clinicalsamples_IgG.qld

Last Altered: Sunday, October 02, 2022 16:02:30 台北標準時間
Printed: Friday, August 01, 2025 16:49:44 台北標準時間

Sample Name: 20211111_A6_R1_IgG

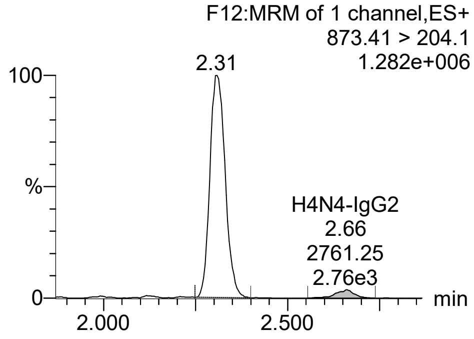

Sample Name: 20211111_QC_R2_IgG

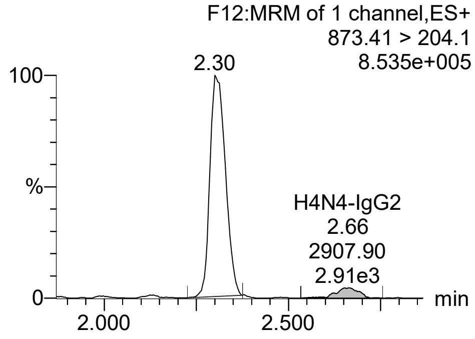

Sample Name: 20211111_L5_R1_IgG

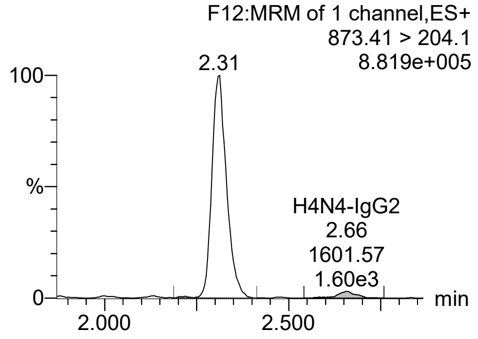

Sample Name: 20211111_L6_R1_IgG

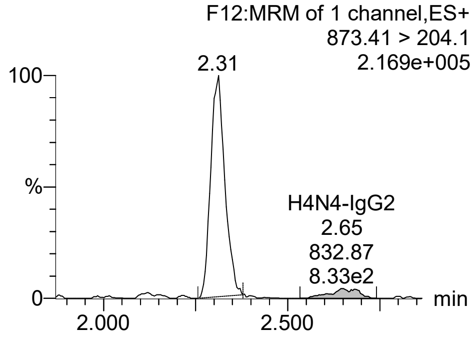

Sample Name: 20211111_H3_R1_IgG

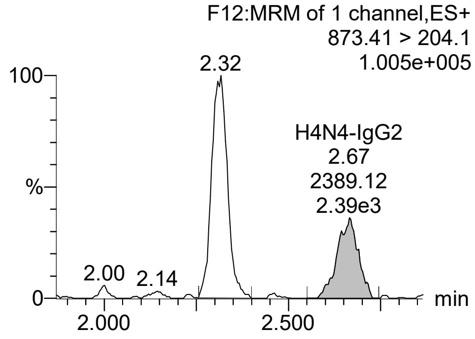

Sample Name: 20211111_A7_R1_IgG

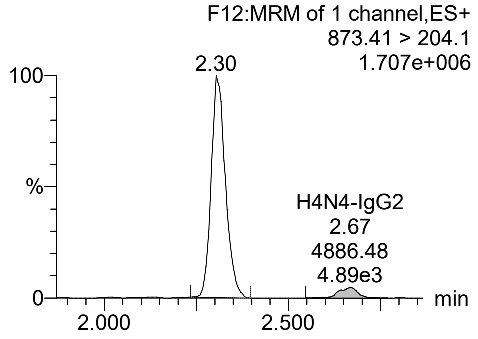

Sample Name: 20211111_A8_R1_IgG

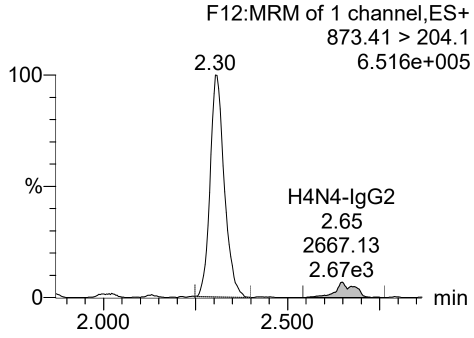

Sample Name: 20211111_L7_R1_IgG

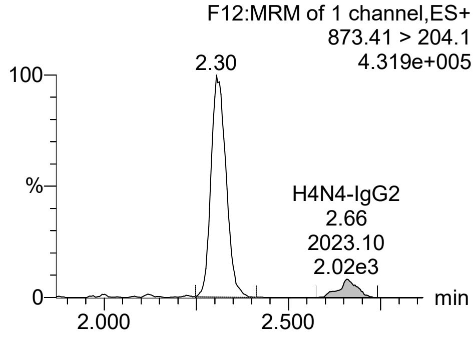

Sample Name: 20211111_L8_R1_IgG

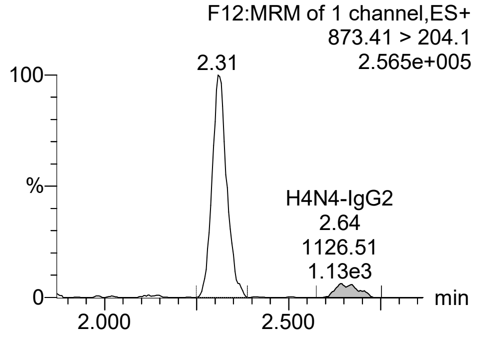

Sample Name: 20211111_H4_R1_IgG

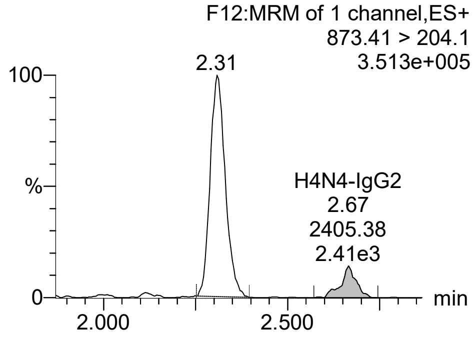

Sample Name: 20211111_A9_R1_IgG

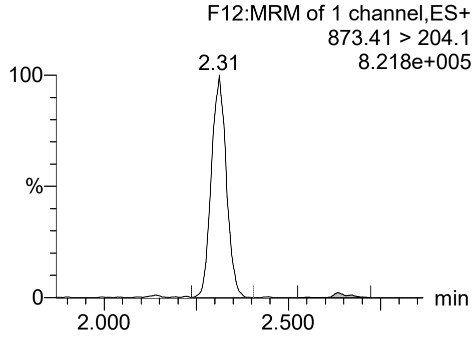

Sample Name: 20211111_A10_R1_IgG

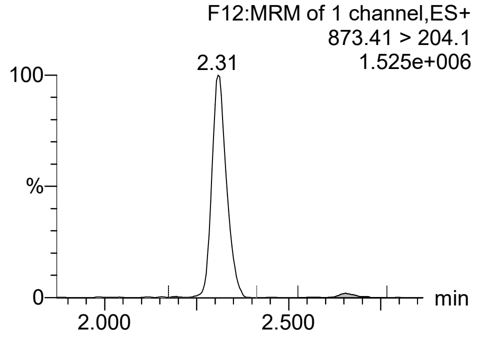

Sample Name: 20211111_L9_R1_IgG

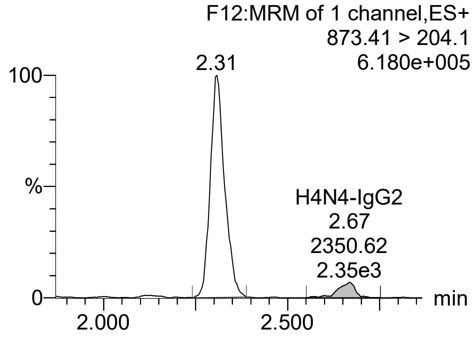

Sample Name: 20211111_L10_R1_IgG

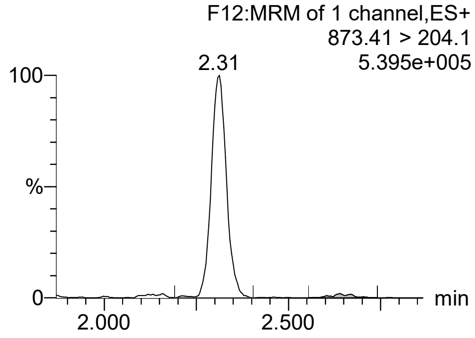

Sample Name: 20211111_QC_R3_IgG

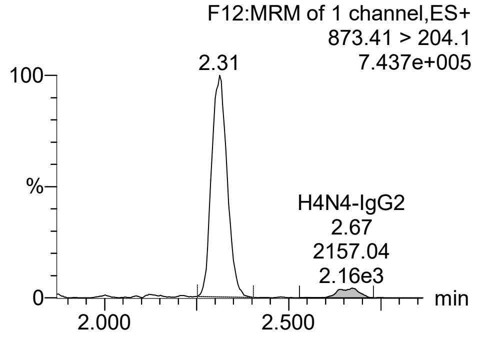

Dataset: C:\MassLynx\10.pro\20211115_clinicalsamples_IgG.qld

Last Altered: Sunday, October 02, 2022 16:02:30 台北標準時間
Printed: Friday, August 01, 2025 16:49:44 台北標準時間

Sample Name: 20211111_H5_R1_IgG
F12:MRM of 1 channel,ES+
873.41 > 204.1
5.881e+004

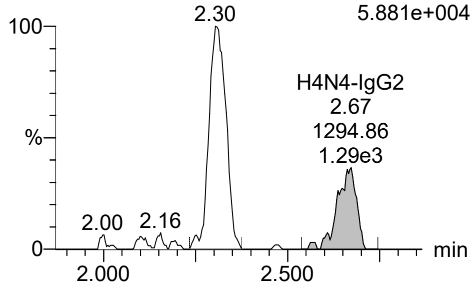

Sample Name: 20211111_A11_R1_IgG
F12:MRM of 1 channel,ES+
873.41 > 204.1
2.425e+005

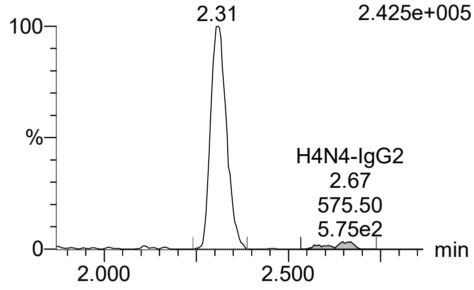

Sample Name: 20211111_A12_R1_IgG
F12:MRM of 1 channel,ES+
873.41 > 204.1
3.505e+005

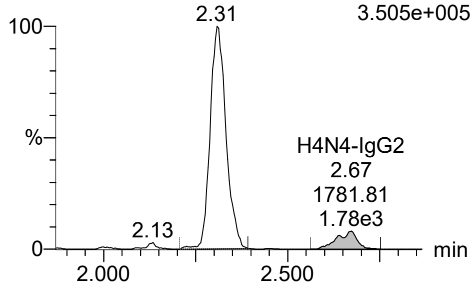

Sample Name: 20211111_L11_R1_IgG
F12:MRM of 1 channel,ES+
873.41 > 204.1
3.564e+005

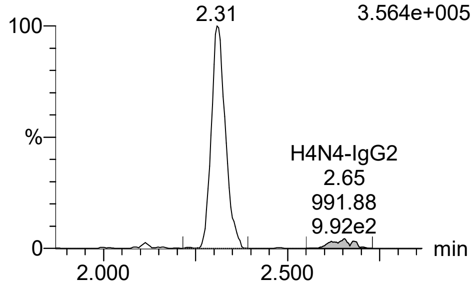

Sample Name: 20211111_L12_R1_IgG
F12:MRM of 1 channel,ES+
873.41 > 204.1
1.473e+005

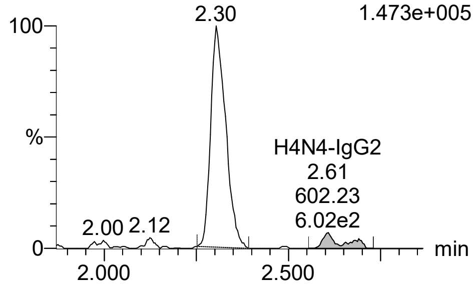

Sample Name: 20211111_H6_R1_IgG
F12:MRM of 1 channel,ES+
873.41 > 204.1
9.235e+005

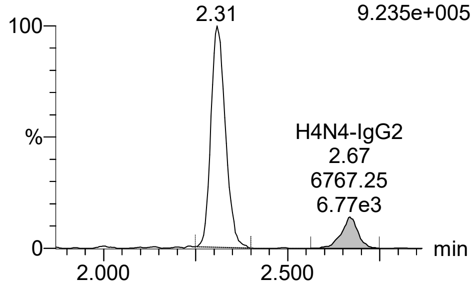

Sample Name: 20211111_A13_R1_IgG
F12:MRM of 1 channel,ES+
873.41 > 204.1
9.224e+005

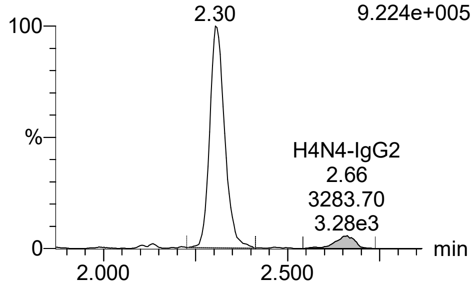

Sample Name: 20211111_A14_R1_IgG
F12:MRM of 1 channel,ES+
873.41 > 204.1
8.818e+005

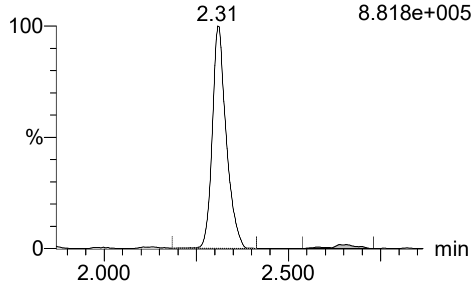

Sample Name: 20211111_L13_R1_IgG
F12:MRM of 1 channel,ES+
873.41 > 204.1
2.063e+005

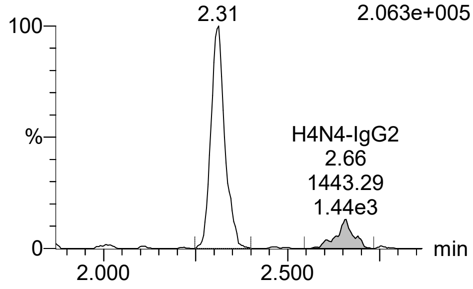

Sample Name: 20211111_L14_R1_IgG
F12:MRM of 1 channel,ES+
873.41 > 204.1
2.691e+005

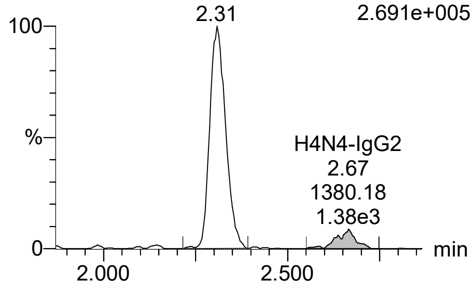

Sample Name: 20211111_H7_R1_IgG
F12:MRM of 1 channel,ES+
873.41 > 204.1
7.048e+005

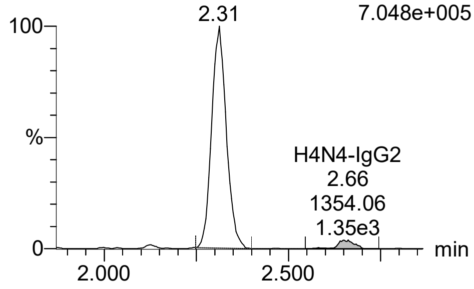

Sample Name: 20211111_A15_R1_IgG
F12:MRM of 1 channel,ES+
873.41 > 204.1
2.379e+005

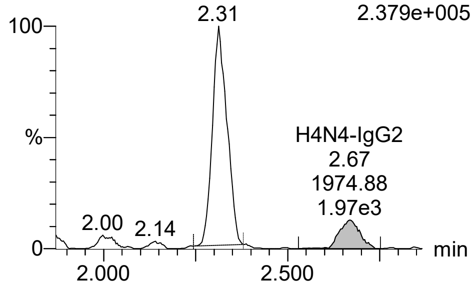

Sample Name: 20211111_QC_R4_IgG
F12:MRM of 1 channel,ES+
873.41 > 204.1
6.558e+005

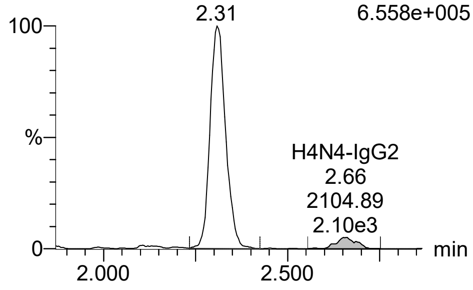

Sample Name: 20211111_A16_R1_IgG
F12:MRM of 1 channel,ES+
873.41 > 204.1
9.579e+005

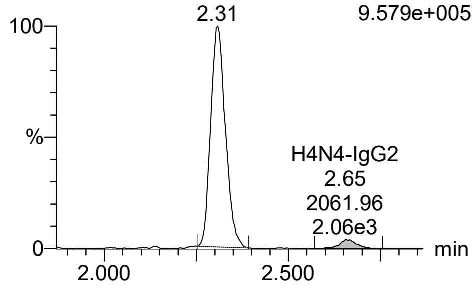

Sample Name: 20211111_L15_R1_IgG
F12:MRM of 1 channel,ES+
873.41 > 204.1
7.034e+005

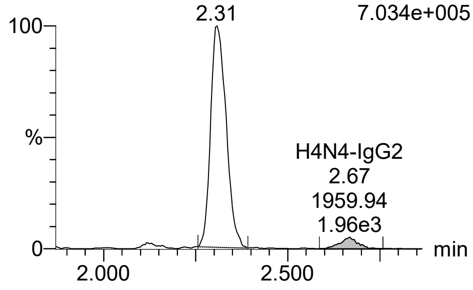

Sample Name: 20211111_L16_R1_IgG

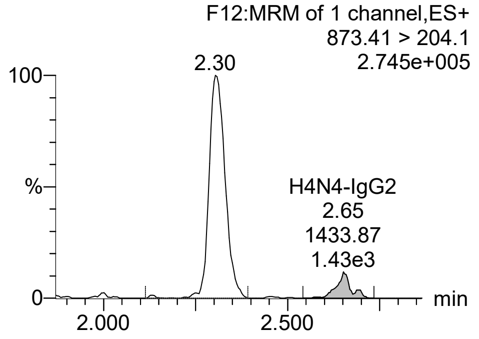

Sample Name: 20211111_H8_R1_IgG

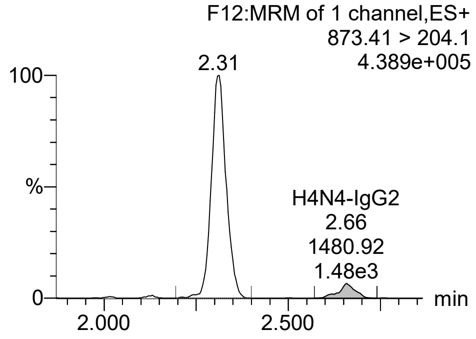

Sample Name: 20211111_A17_R1_IgG

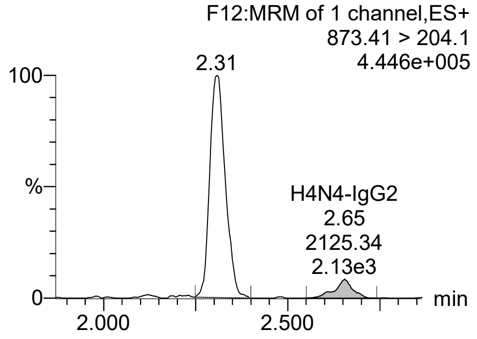

Sample Name: 20211111_A18_R1_IgG

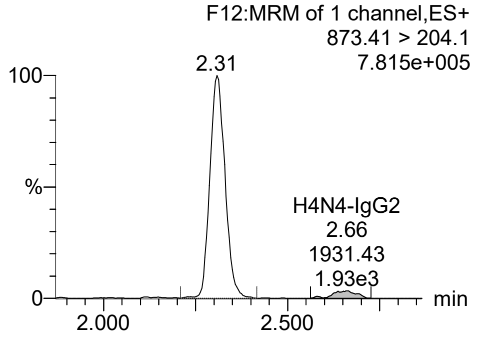

Sample Name: 20211111_L17_R1_IgG

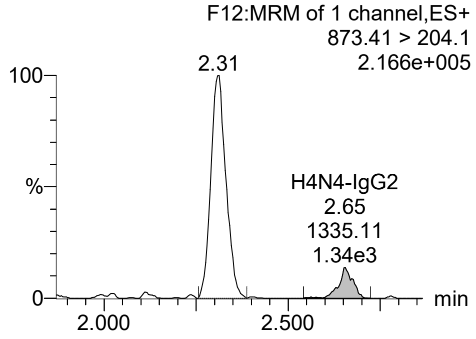

Sample Name: 20211111_L18_R1_IgG

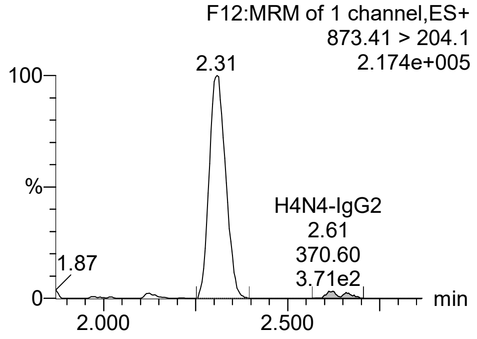

Sample Name: 20211111_H9_R1_IgG

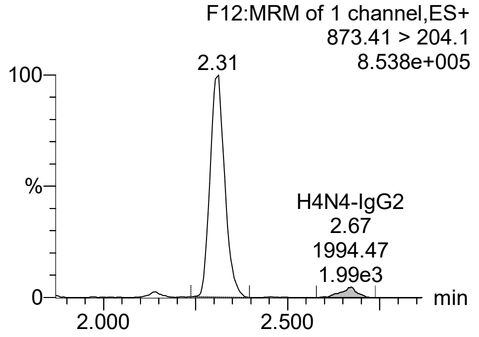

Sample Name: 20211111_A19_R1_IgG

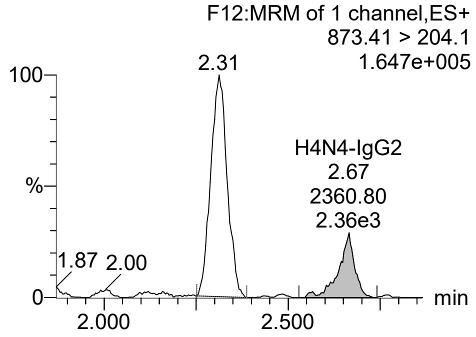

Sample Name: 20211111_A20_R1_IgG

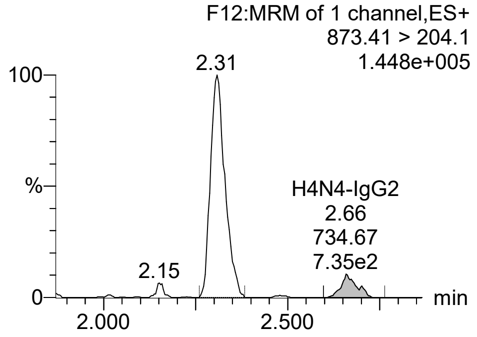

Sample Name: 20211111_L19_R1_IgG

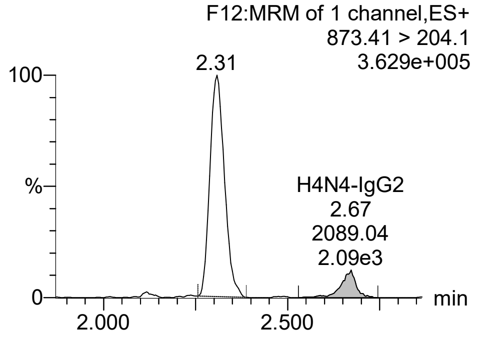

Sample Name: 20211111_QC_R5_IgG

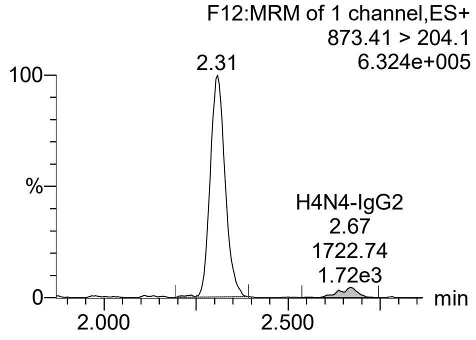

Sample Name: 20211111_L20_R1_IgG

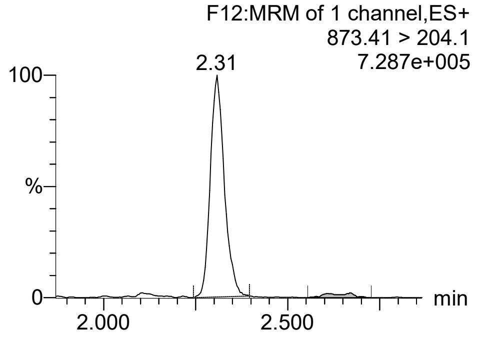

Sample Name: 20211111_H10_R1_IgG

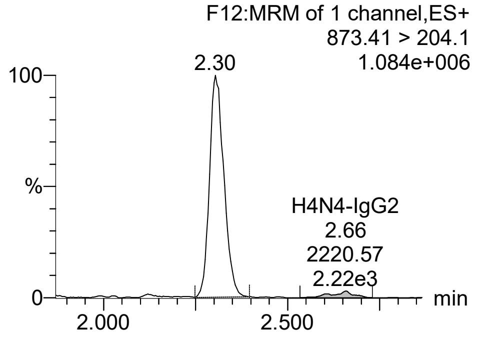

Sample Name: 20211111_A21_R1_IgG

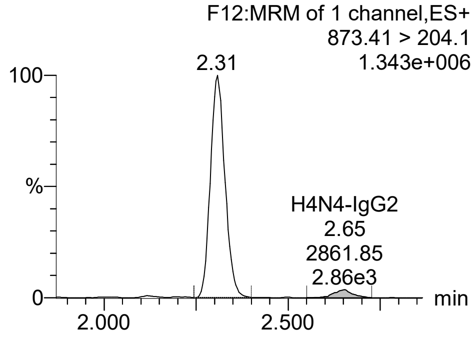

Sample Name: 20211111_A22_R1_IgG

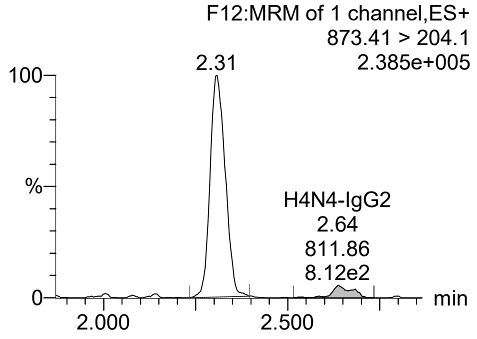

Sample Name: 20211111_L21_R1_IgG

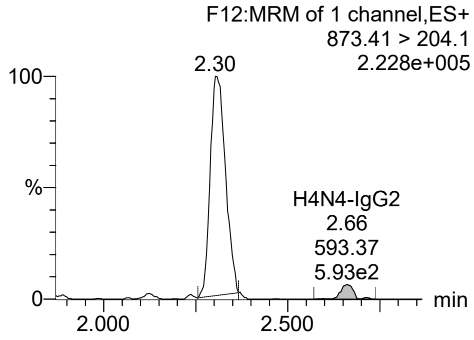

Sample Name: 20211111_L22_R1_IgG

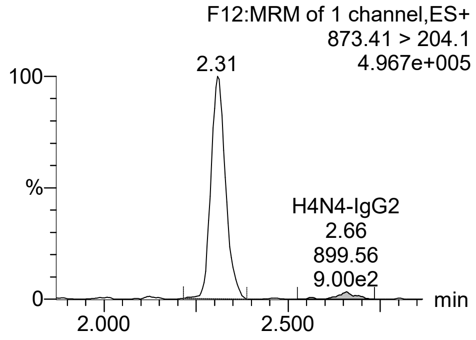

Sample Name: 20211111_H11_R1_IgG

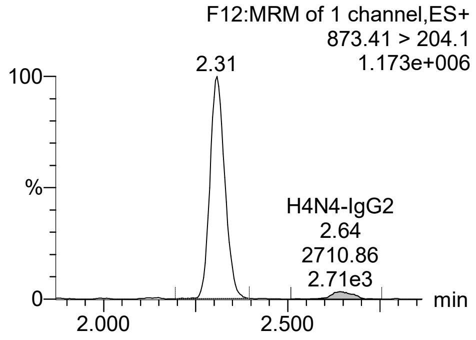

Sample Name: 20211111_A23_R1_IgG

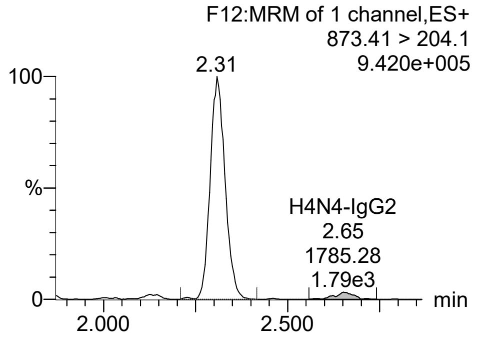

Sample Name: 20211111_A24_R1_IgG

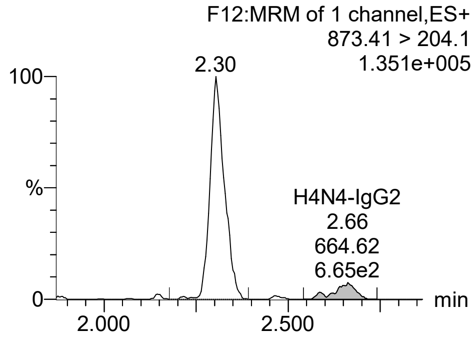

Sample Name: 20211111_L23_R1_IgG

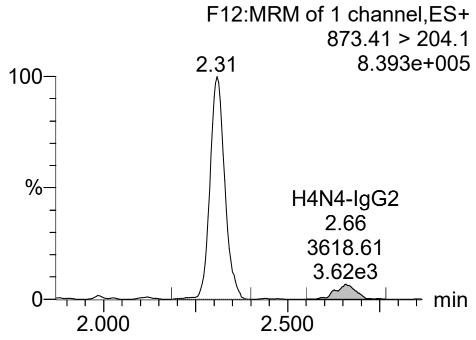

Sample Name: 20211111_L24_R1_IgG

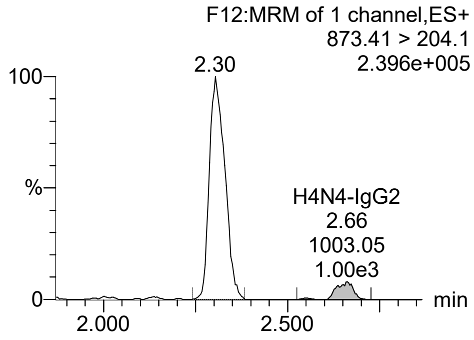

Sample Name: 20211111_H12_R1_IgG

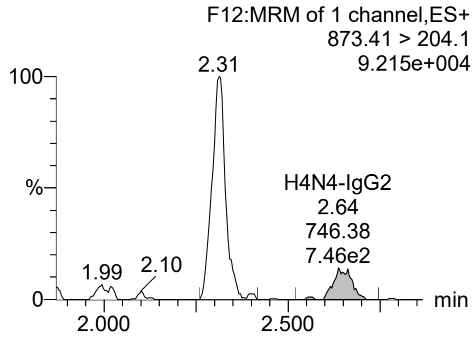

Sample Name: 20211111_QC_R6_IgG

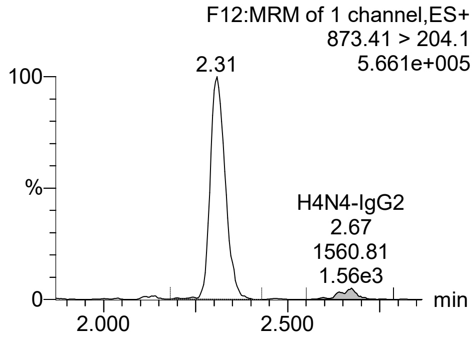

Sample Name: 20211111_A25_R1_IgG

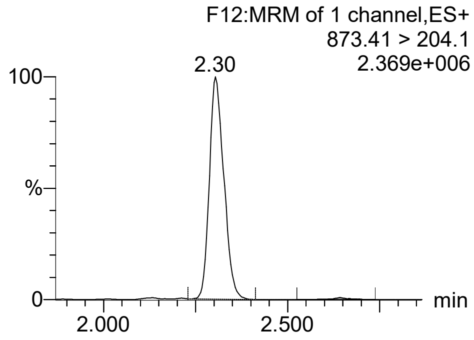

Sample Name: 20211111_A26_R1_IgG

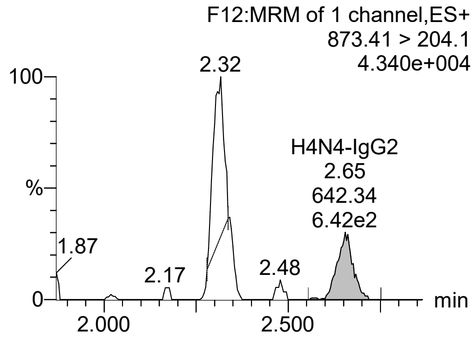

Sample Name: 20211111_L25_R1_IgG

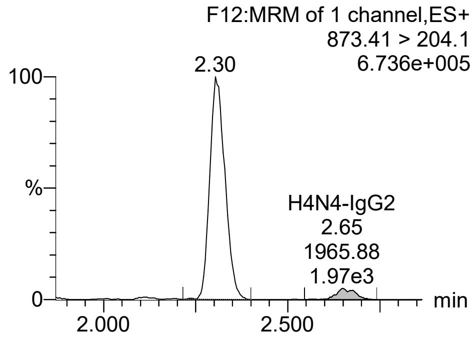

Sample Name: 20211111_L26_R1_IgG

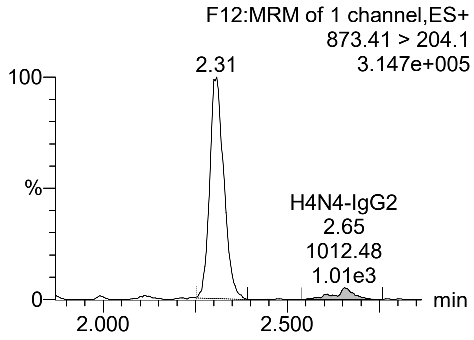

Sample Name: 20211111_H13_R1_IgG

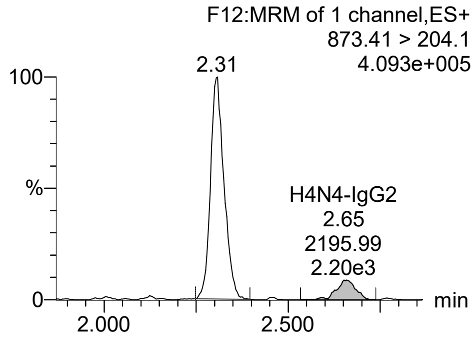

Sample Name: 20211111_A27_R1_IgG

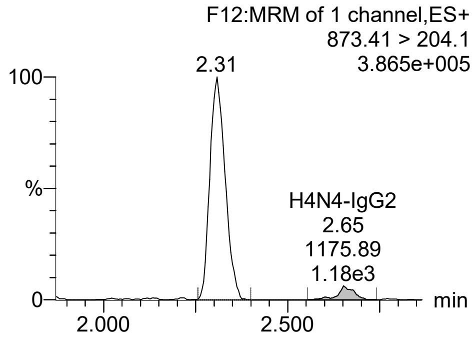

Sample Name: 20211111_A28_R1_IgG
F12:MRM of 1 channel,ES+
873.41 > 204.1
4.807e+005

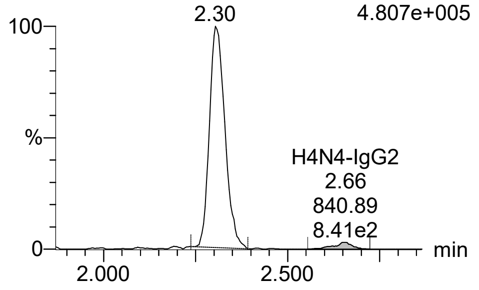

Sample Name: 20211111_L27_R1_IgG
F12:MRM of 1 channel,ES+
873.41 > 204.1
3.032e+005

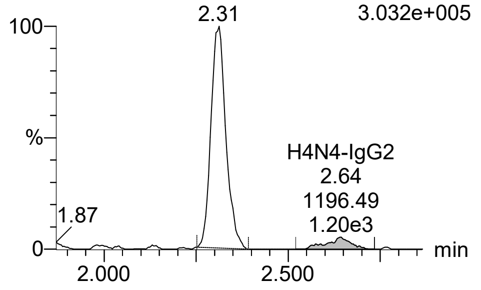

Sample Name: 20211111_L28_R1_IgG
F12:MRM of 1 channel,ES+
873.41 > 204.1
2.123e+005

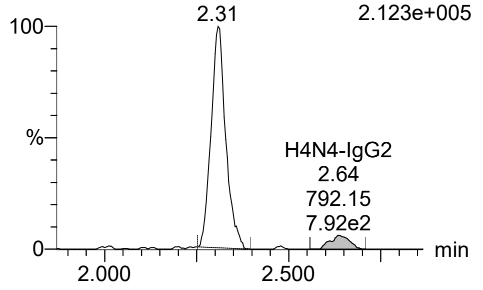

Sample Name: 20211111_H14_R1_IgG
F12:MRM of 1 channel,ES+
873.41 > 204.1
2.169e+005

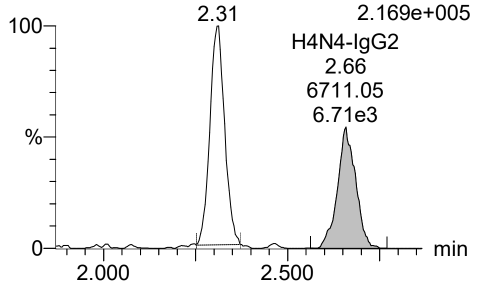

Sample Name: 20211111_A29_R1_IgG
F12:MRM of 1 channel,ES+
873.41 > 204.1
1.056e+006

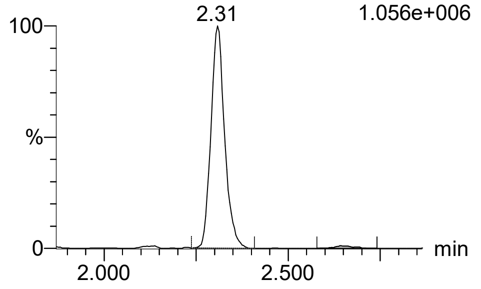

Sample Name: 20211111_A30_R1_IgG
F12:MRM of 1 channel,ES+
873.41 > 204.1
1.424e+006

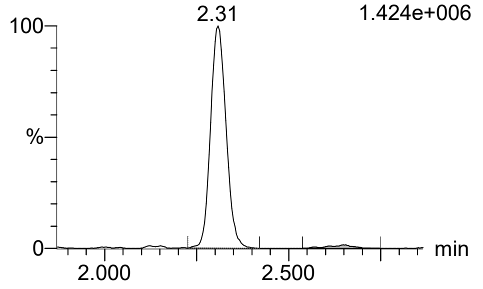

Sample Name: 20211111_QC_R7_IgG
F12:MRM of 1 channel,ES+
873.41 > 204.1
5.170e+005

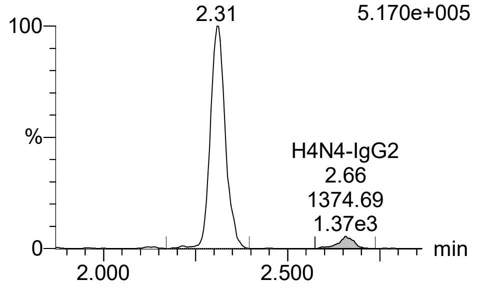

Sample Name: 20211111_L29_R1_IgG
F12:MRM of 1 channel,ES+
873.41 > 204.1
2.100e+005

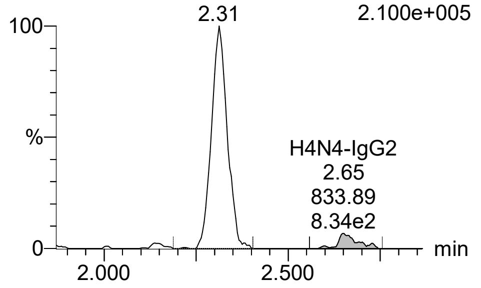

Sample Name: 20211111_L30_R1_IgG
F12:MRM of 1 channel,ES+
873.41 > 204.1
2.308e+005

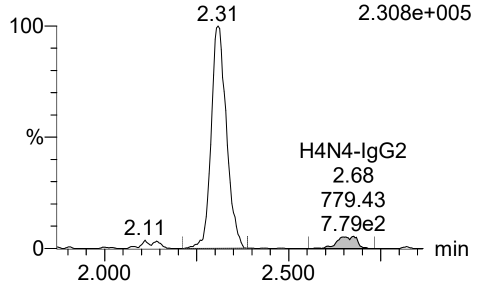

Sample Name: 20211111_H15_R1_IgG
F12:MRM of 1 channel,ES+
873.41 > 204.1
3.923e+004

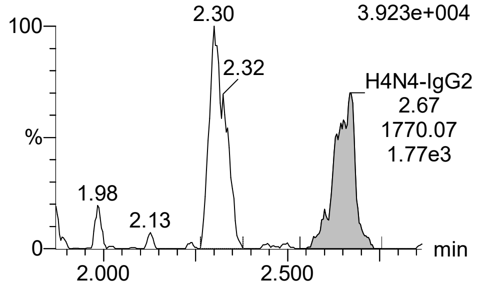

Sample Name: 20211111_A31_R1_IgG
F12:MRM of 1 channel,ES+
873.41 > 204.1
4.656e+005

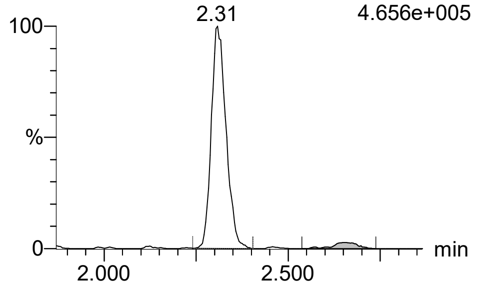

Sample Name: 20211111_A32_R1_IgG
F12:MRM of 1 channel,ES+
873.41 > 204.1
1.670e+006

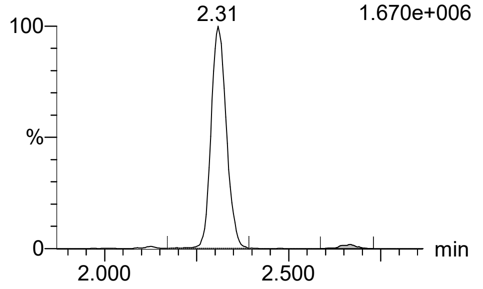

Sample Name: 20211111_L31_R1_IgG
F12:MRM of 1 channel,ES+
873.41 > 204.1
1.773e+005

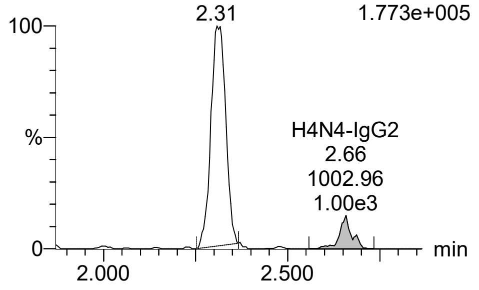

Sample Name: 20211111_L32_R1_IgG
F12:MRM of 1 channel,ES+
873.41 > 204.1
1.926e+005

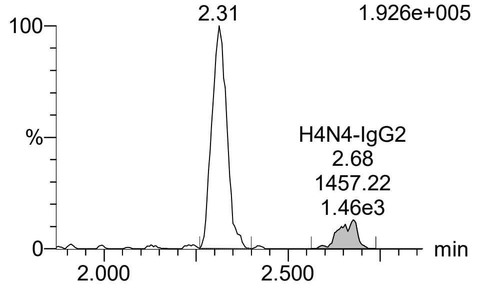

Sample Name: 20211111_H16_R1_IgG
F12:MRM of 1 channel,ES+
873.41 > 204.1
1.401e+006

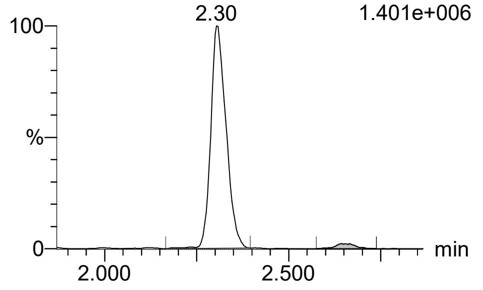

Sample Name: 20211111_A33_R1_IgG

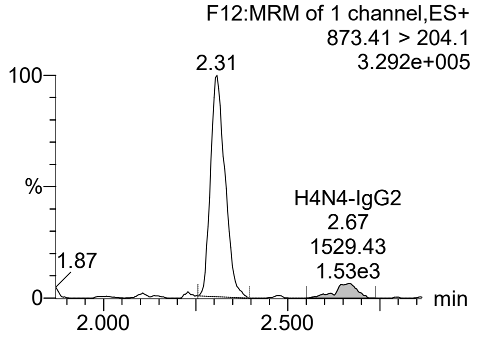

Sample Name: 20211111_A34_R1_IgG

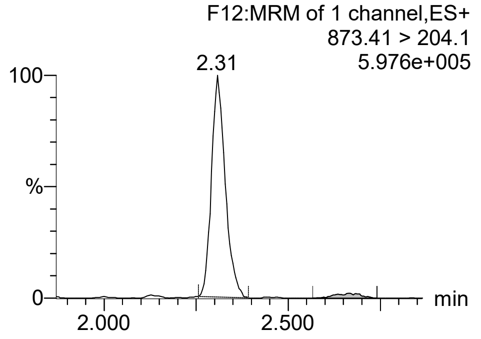

Sample Name: 20211111_H17_R1_IgG

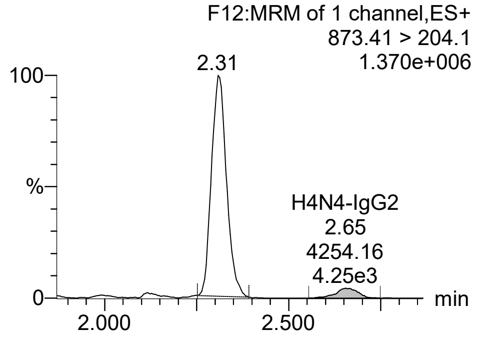

Sample Name: 20211111_A35_R1_IgG

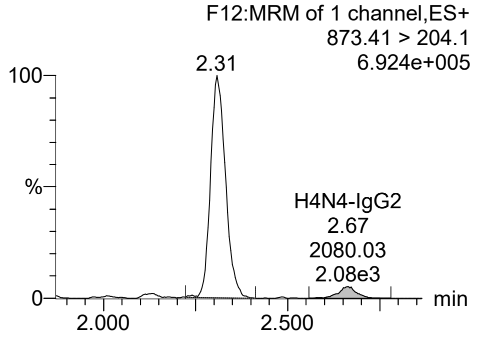

Sample Name: 20211111_QC_R8_IgG

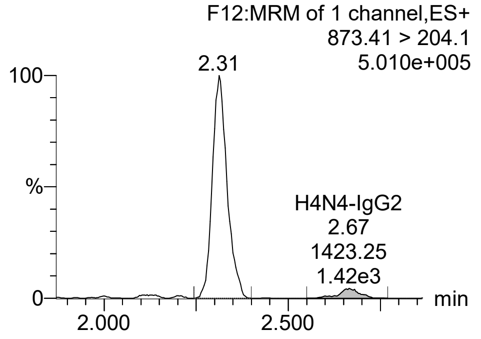

Sample Name: 20211111_A36_R1_IgG

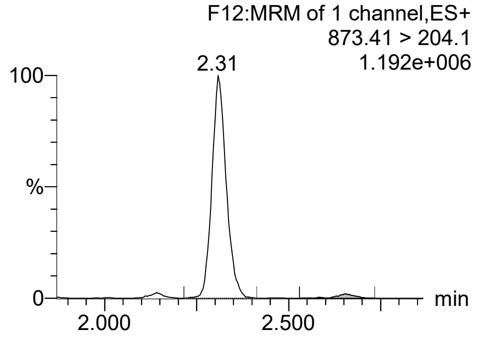

Sample Name: 20211111_H18_R1_IgG

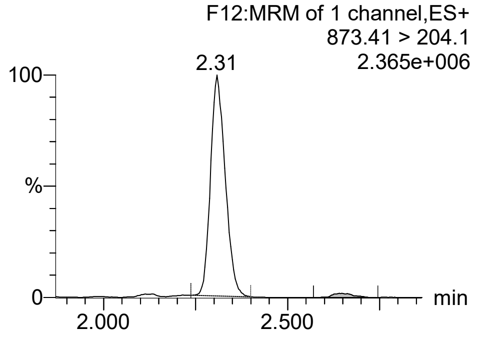

Sample Name: 20211111_A37_R1_IgG

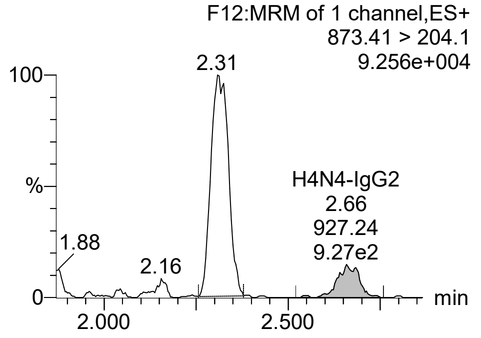

Sample Name: 20211111_A38_R1_IgG

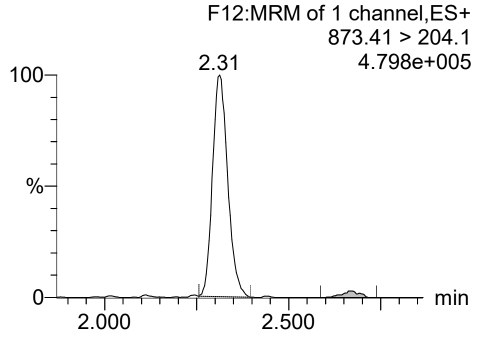

Sample Name: 20211111_H19_R1_IgG

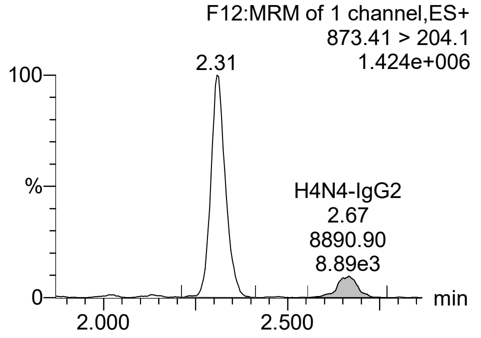

Sample Name: 20211111_A39_R1_IgG

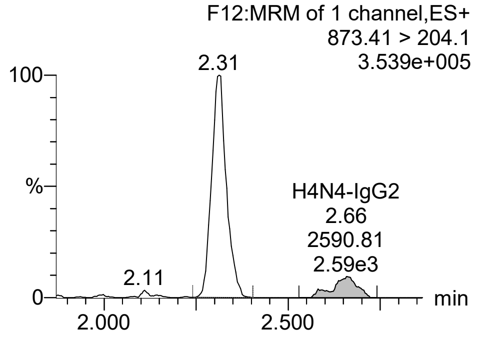

Sample Name: 20211111_A40_R1_IgG

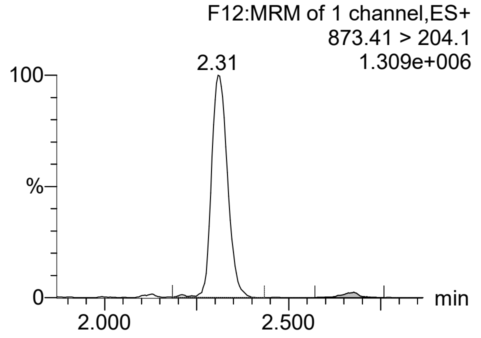

Sample Name: 20211111_H20_R1_IgG

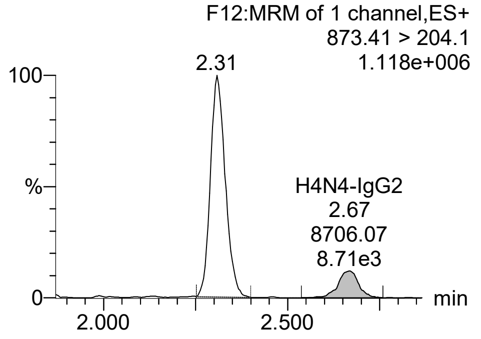

Sample Name: 20211111_A41_R1_IgG

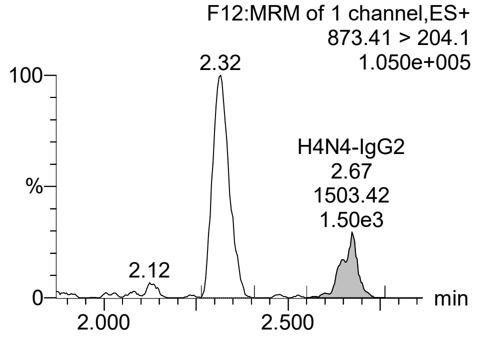

Sample Name: 20211111_A42_R1_IgG

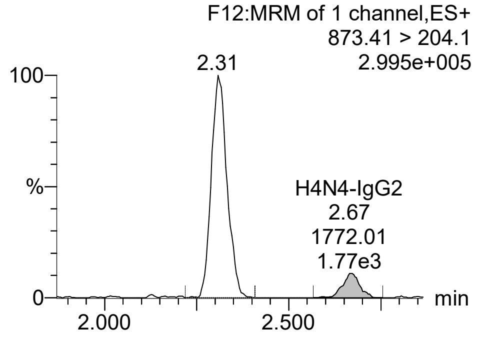

Dataset: C:\MassLynx\10.pro\20211115_clinicalsamples_IgG.qld

Last Altered: Sunday, October 02, 2022 16:02:30 台北標準時間
Printed: Friday, August 01, 2025 16:49:44 台北標準時間

Sample Name: 20211111_A43_R1_IgG

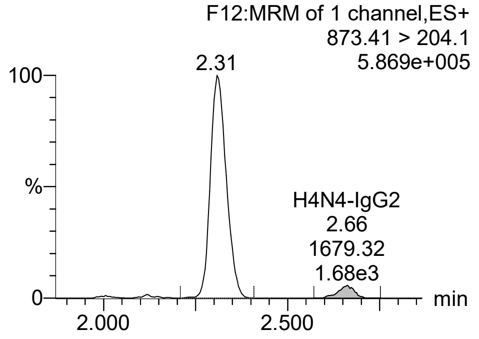

Sample Name: 20211111_A44_R1_IgG

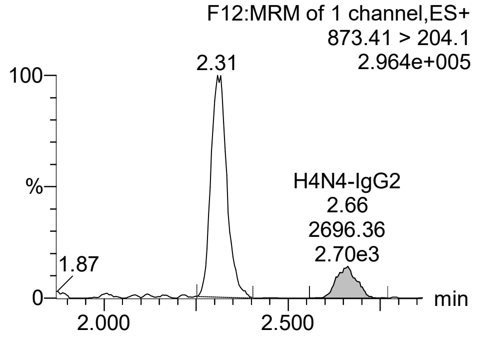

Sample Name: 20211111_QC_R9_IgG

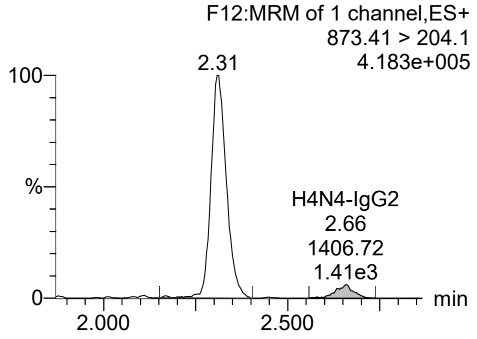

Sample Name: 20211111_A1_R2_IgG

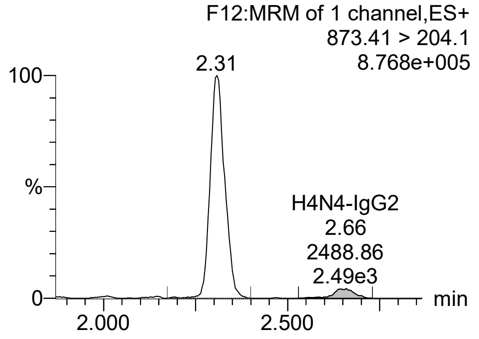

Sample Name: 20211111_A2_R2_IgG

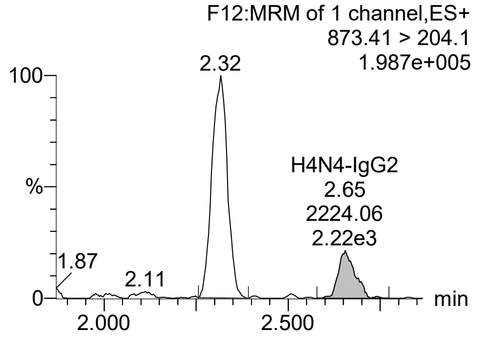

Sample Name: 20211111_L1_R2_IgG

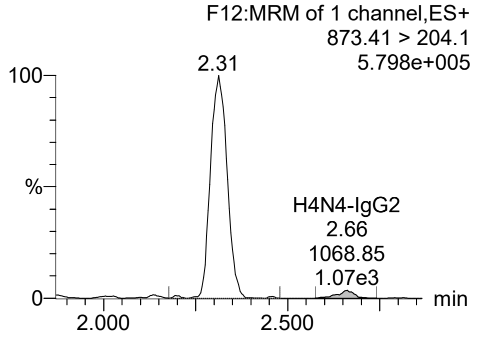

Sample Name: 20211111_L2_R2_IgG

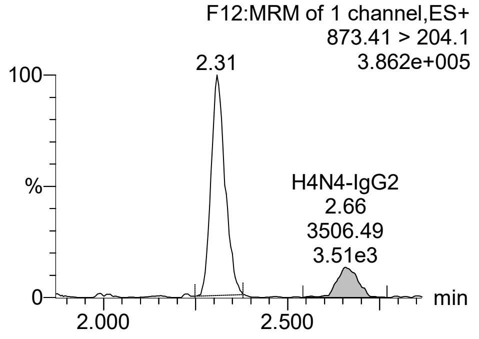

Sample Name: 20211111_H1_R2_IgG

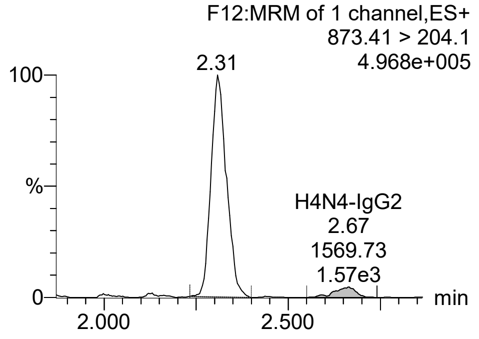

Sample Name: 20211111_A3_R2_IgG

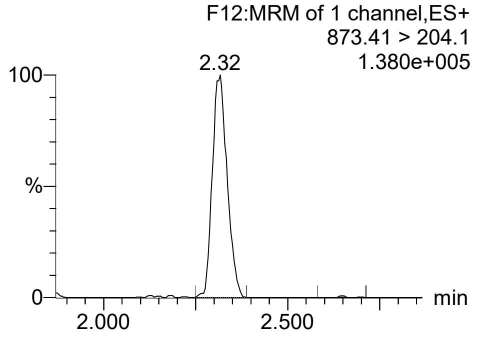

Sample Name: 20211111_A4_R2_IgG

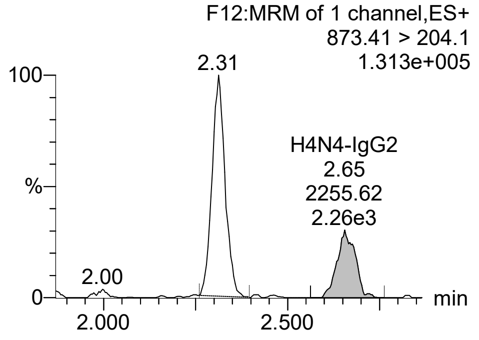

Sample Name: 20211111_L3_R2_IgG

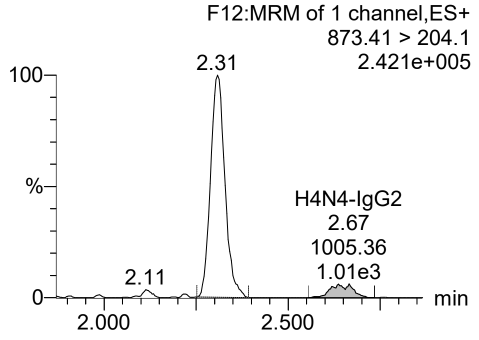

Sample Name: 20211111_L4_R2_IgG

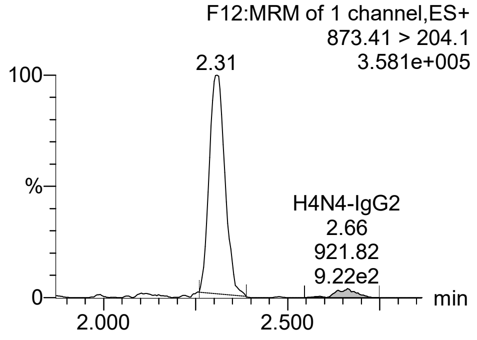

Sample Name: 20211111_H2_R2_IgG

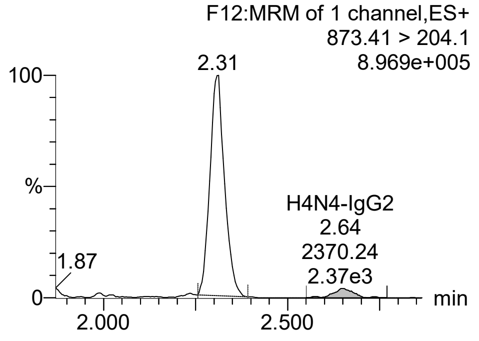

Sample Name: 20211111_A5_R2_IgG

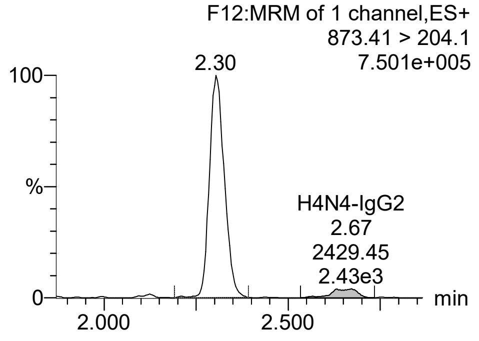

Sample Name: 20211111_A6_R2_IgG

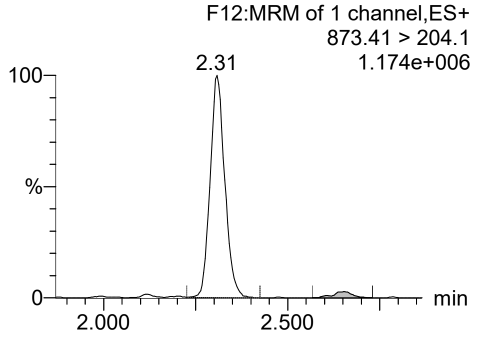

Dataset: C:\MassLynx\10.pro\20211115_clinicalsamples_IgG.qld

Last Altered: Sunday, October 02, 2022 16:02:30 台北標準時間
Printed: Friday, August 01, 2025 16:49:44 台北標準時間

Sample Name: 20211111_QC_R10_IgG

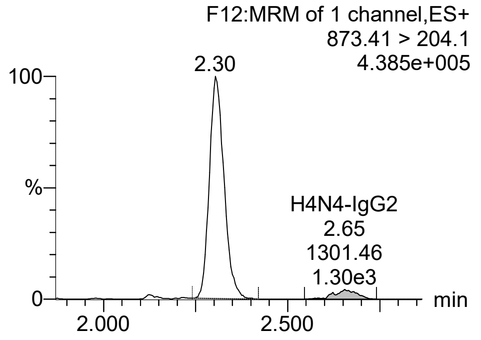

Sample Name: 20211111_L5_R2_IgG

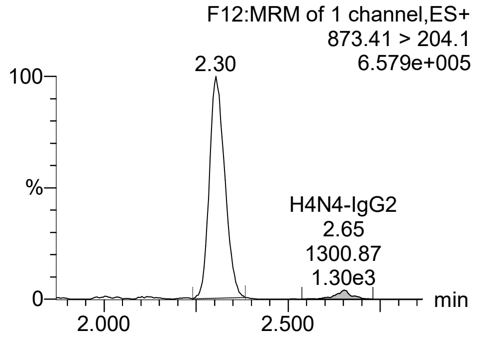

Sample Name: 20211111_L6_R2_IgG

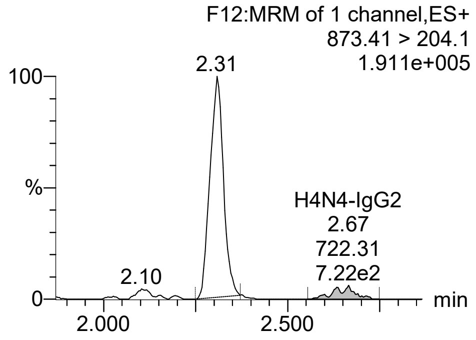

Sample Name: 20211111_H3_R2_IgG

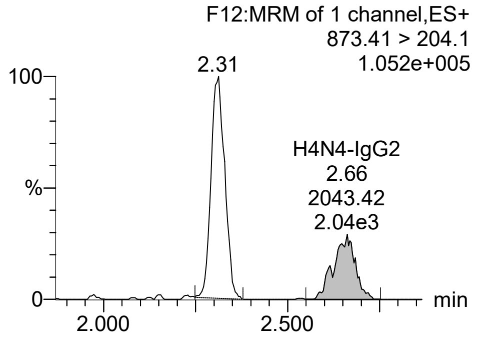

Sample Name: 20211111_A7_R2_IgG

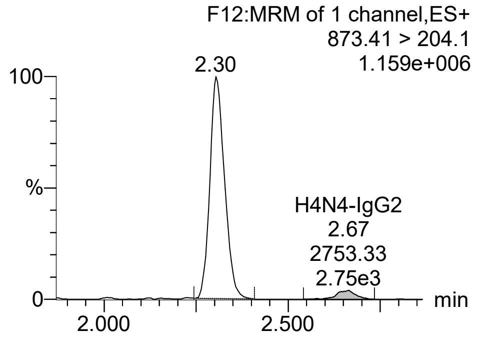

Sample Name: 20211111_A8_R2_IgG

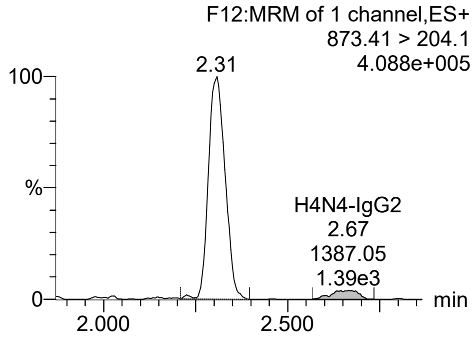

Sample Name: 20211111_L7_R2_IgG

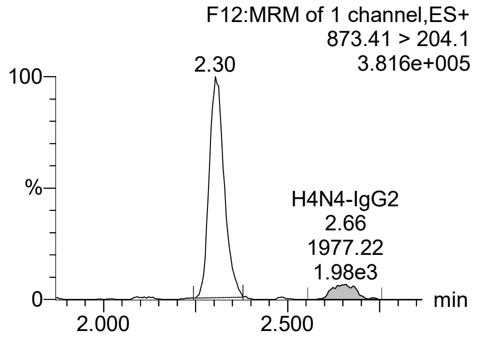

Sample Name: 20211111_L8_R2_IgG

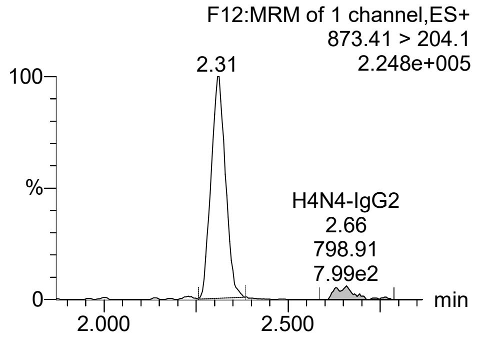

Sample Name: 20211111_H4_R2_IgG

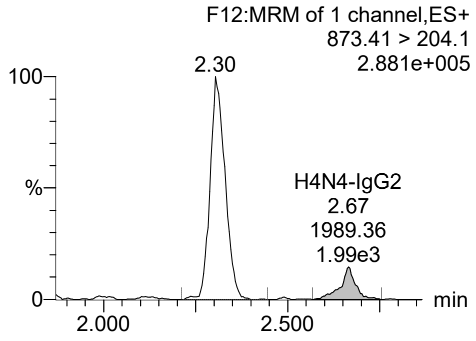

Sample Name: 20211111_A9_R2_IgG

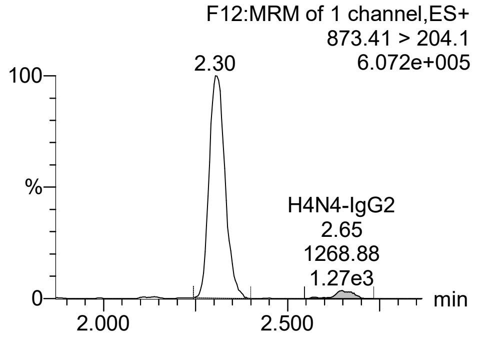

Sample Name: 20211111_A10_R2_IgG

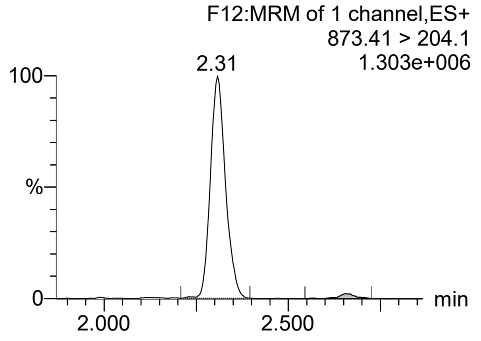

Sample Name: 20211111_L9_R2_IgG

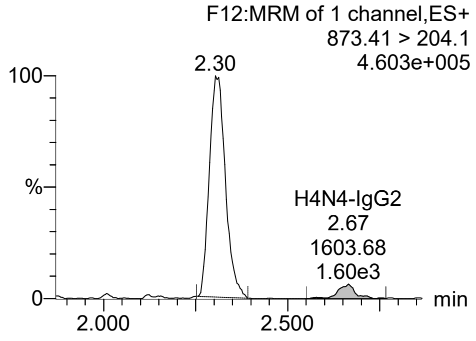

Sample Name: 20211111_L10_R2_IgG

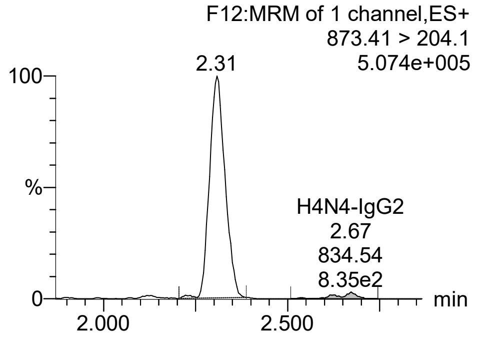

Sample Name: 20211111_QC_R11_IgG

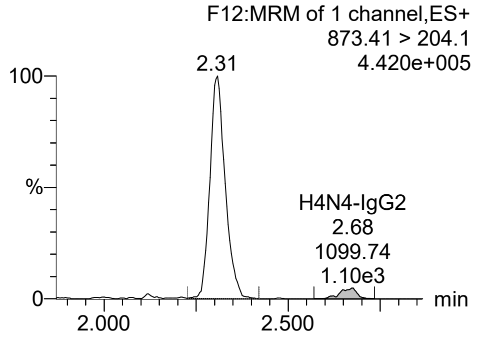

Sample Name: 20211111_H5_R2_IgG

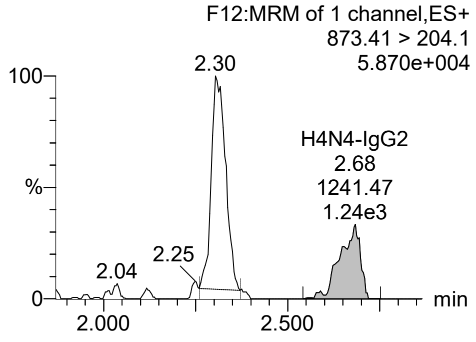

Dataset: C:\MassLynx\10.pro\20211115_clinicalsamples_IgG.qld

Last Altered: Sunday, October 02, 2022 16:02:30 台北標準時間
Printed: Friday, August 01, 2025 16:49:44 台北標準時間

Sample Name: 20211111_A11_R2_IgG
F12:MRM of 1 channel,ES+
873.41 > 204.1
2.025e+005

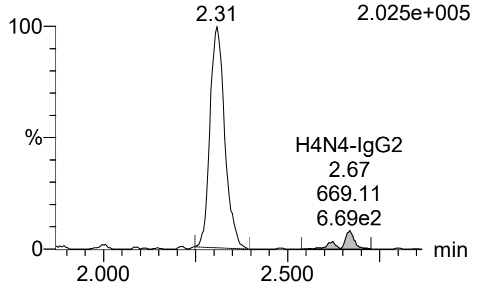

Sample Name: 20211111_A12_R2_IgG
F12:MRM of 1 channel,ES+
873.41 > 204.1
2.928e+005

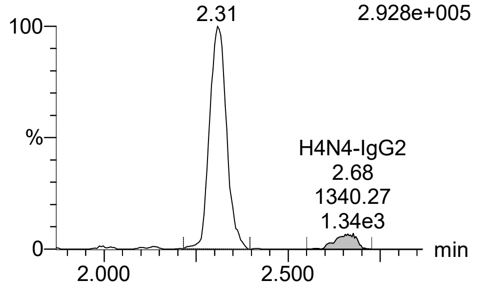

Sample Name: 20211111_L11_R2_IgG
F12:MRM of 1 channel,ES+
873.41 > 204.1
2.327e+005

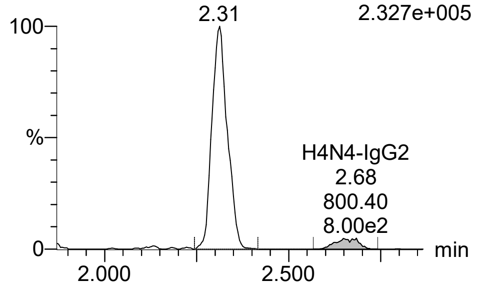

Sample Name: 20211111_L12_R2_IgG
F12:MRM of 1 channel,ES+
873.41 > 204.1
1.328e+005

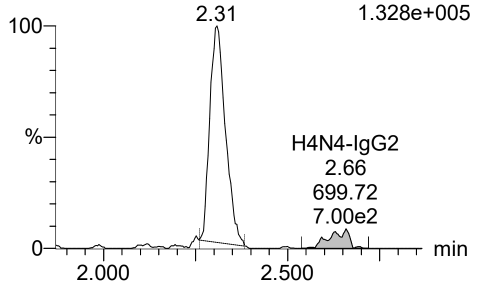

Sample Name: 20211111_H6_R2_IgG
F12:MRM of 1 channel,ES+
873.41 > 204.1
6.057e+005

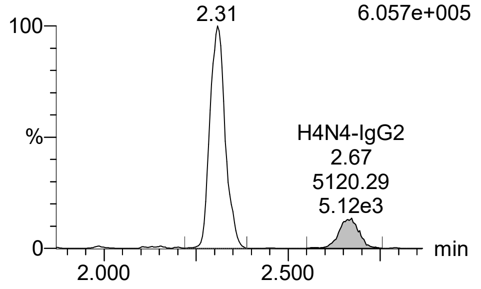

Sample Name: 20211111_A13_R2_IgG
F12:MRM of 1 channel,ES+
873.41 > 204.1
6.679e+005

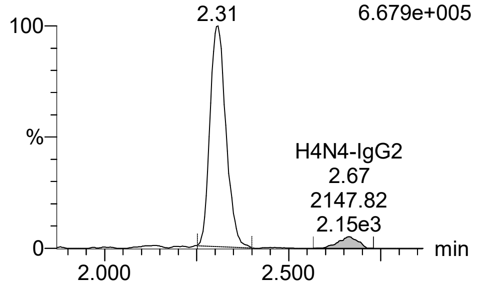

Sample Name: 20211111_A14_R2_IgG
F12:MRM of 1 channel,ES+
873.41 > 204.1
5.824e+005

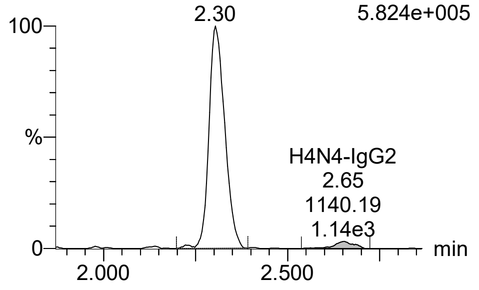

Sample Name: 20211111_L13_R2_IgG
F12:MRM of 1 channel,ES+
873.41 > 204.1
1.400e+005

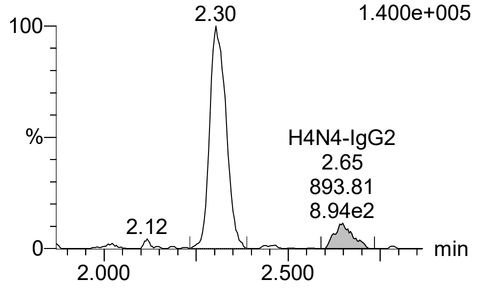

Sample Name: 20211111_L14_R2_IgG
F12:MRM of 1 channel,ES+
873.41 > 204.1
2.917e+005

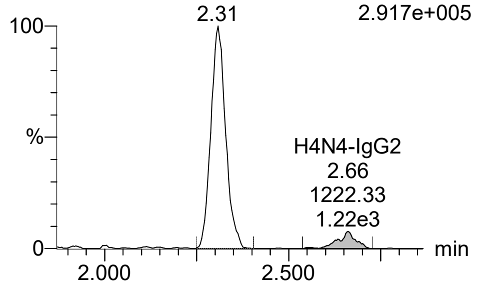

Sample Name: 20211111_H7_R2_IgG
F12:MRM of 1 channel,ES+
873.41 > 204.1
5.989e+005

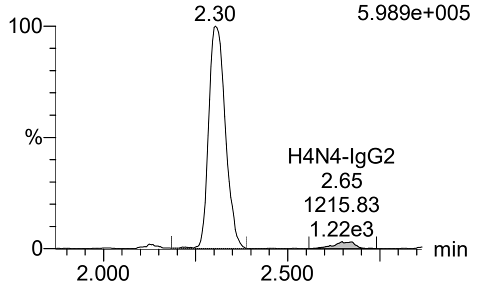

Sample Name: 20211111_A15_R2_IgG
F12:MRM of 1 channel,ES+
873.41 > 204.1
1.934e+005

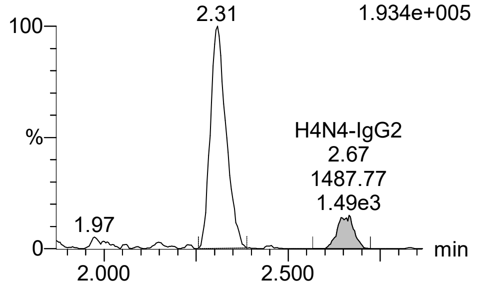

Sample Name: 20211111_QC_R12_IgG
F12:MRM of 1 channel,ES+
873.41 > 204.1
4.965e+005

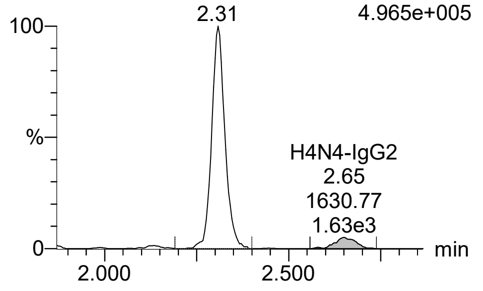

Sample Name: 20211111_A16_R2_IgG
F12:MRM of 1 channel,ES+
873.41 > 204.1
5.331e+005

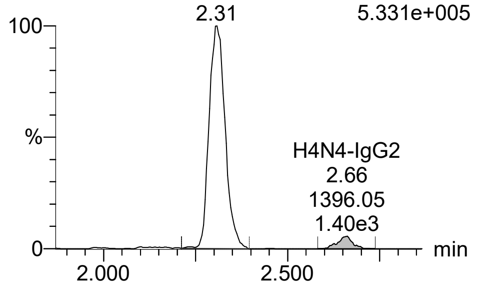

Sample Name: 20211111_L15_R2_IgG
F12:MRM of 1 channel,ES+
873.41 > 204.1
6.288e+005

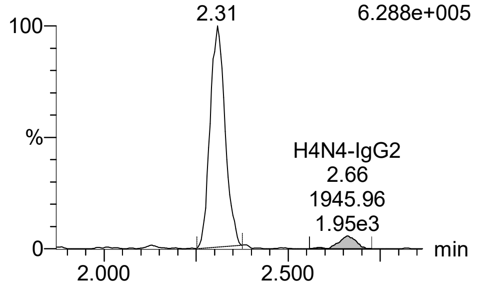

Sample Name: 20211111_L16_R2_IgG
F12:MRM of 1 channel,ES+
873.41 > 204.1
1.996e+005

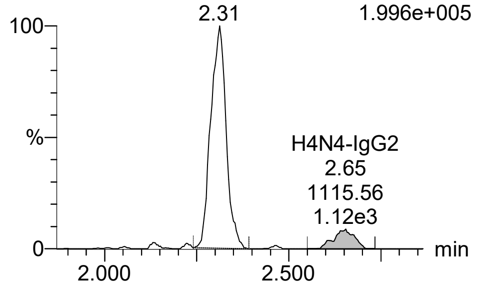

Sample Name: 20211111_H8_R2_IgG

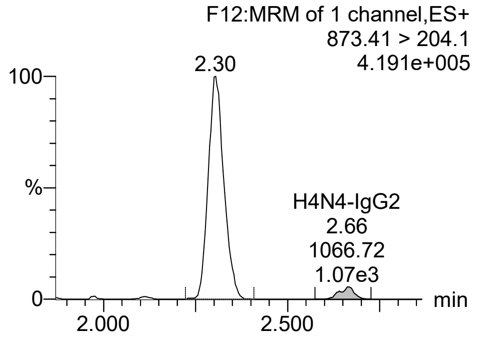

Sample Name: 20211111_A17_R2_IgG

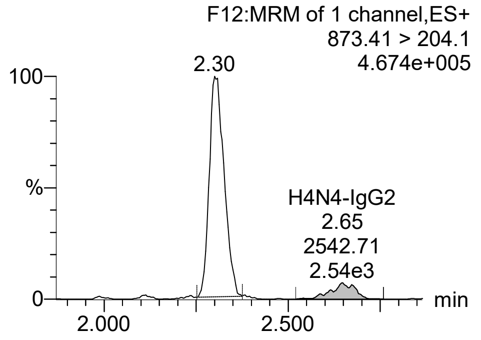

Sample Name: 20211111_A18_R2_IgG

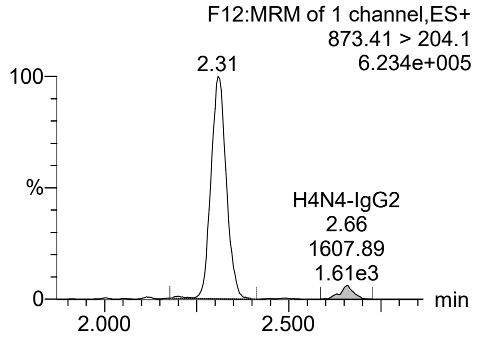

Sample Name: 20211111_L17_R2_IgG

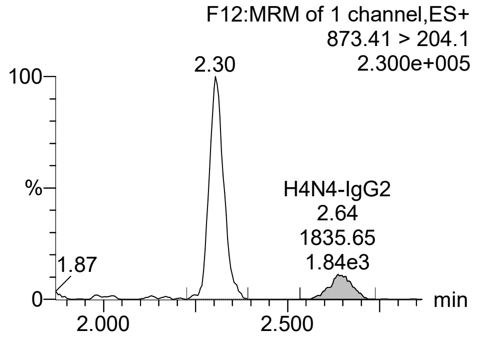

Sample Name: 20211111_L18_R2_IgG

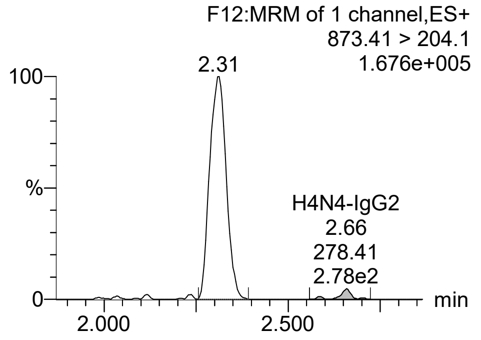

Sample Name: 20211111_H9_R2_IgG

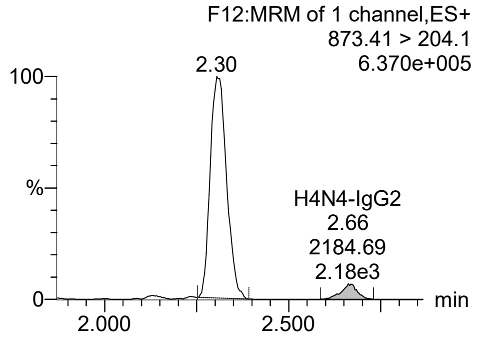

Sample Name: 20211111_A19_R2_IgG

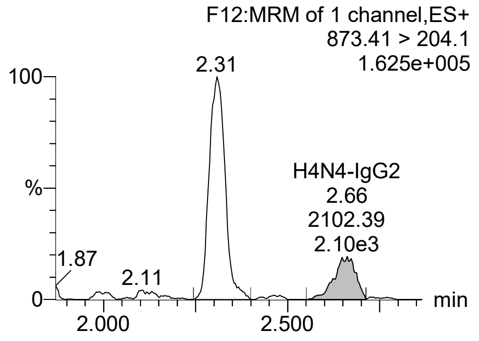

Sample Name: 20211111_A20_R2_IgG

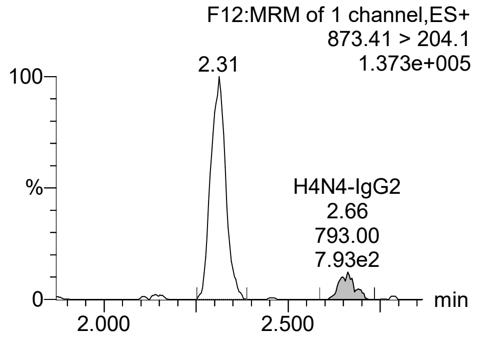

Sample Name: 20211111_L19_R2_IgG

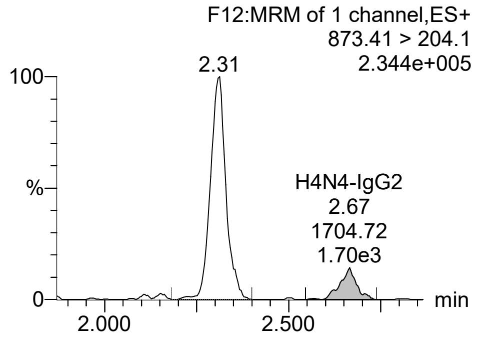

Sample Name: 20211111_QC_R13_IgG

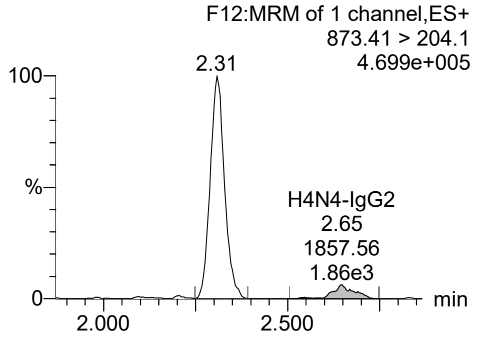

Sample Name: 20211111_L20_R2_IgG

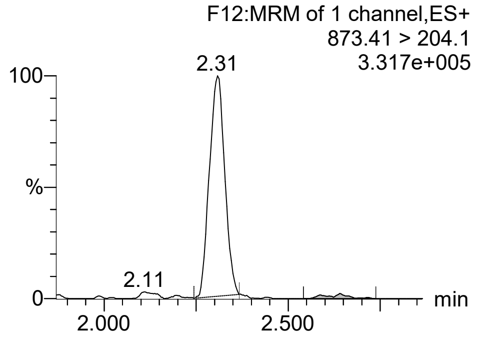

Sample Name: 20211111_H10_R2_IgG

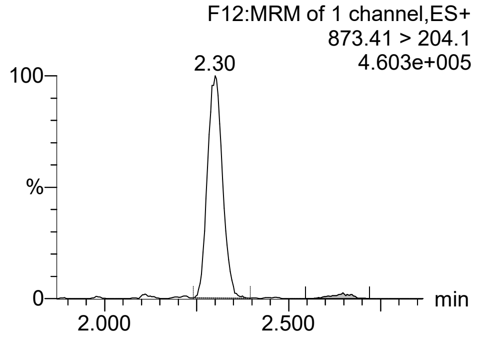

Sample Name: 20211111_A21_R2_IgG

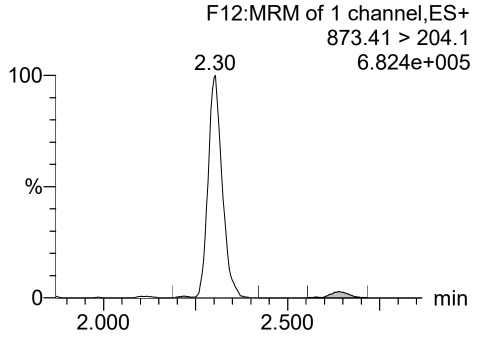

Sample Name: 20211111_A22_R2_IgG

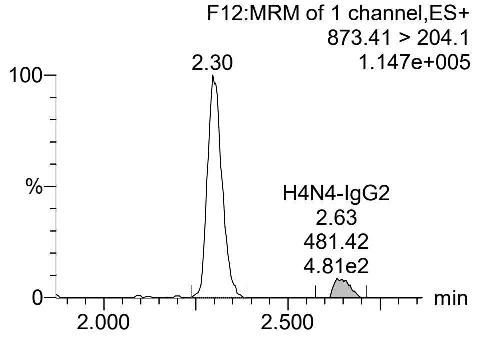

Sample Name: 20211111_L21_R2_IgG

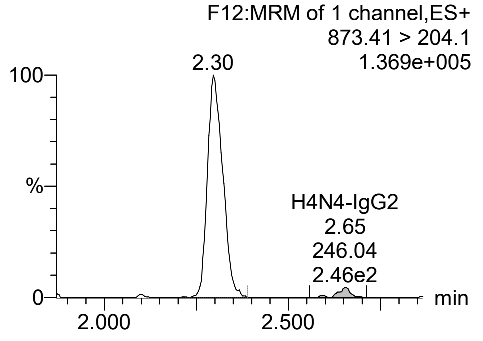

Dataset: C:\MassLynx\10.pro\20211115_clinicalsamples_IgG.qld

Last Altered: Sunday, October 02, 2022 16:02:30 台北標準時間
Printed: Friday, August 01, 2025 16:49:44 台北標準時間

Sample Name: 20211111_L22_R2_IgG

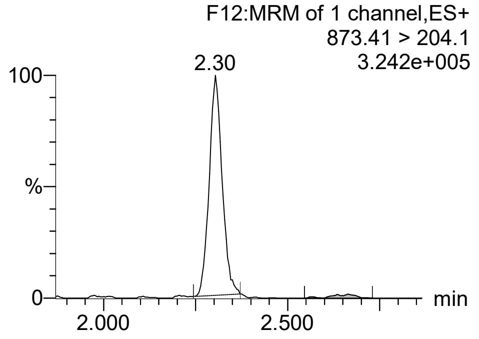

Sample Name: 20211111_H11_R2_IgG

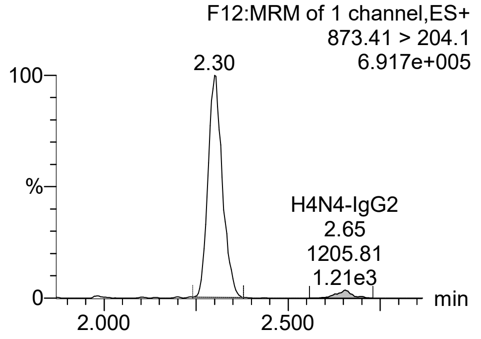

Sample Name: 20211111_A23_R2_IgG

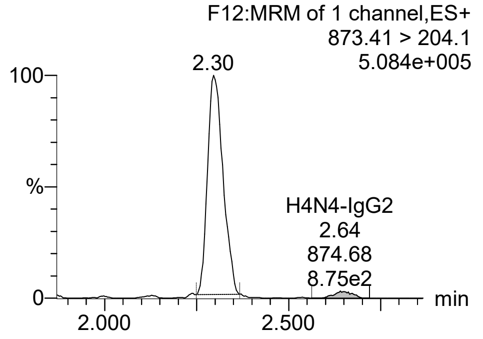

Sample Name: 20211111_A24_R2_IgG

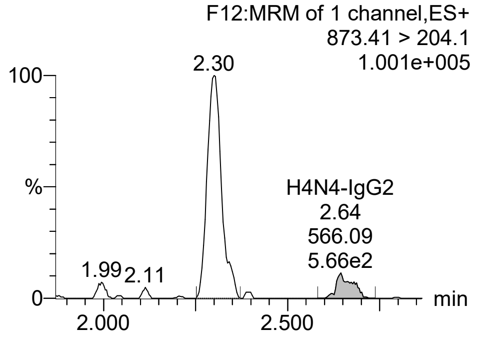

Sample Name: 20211111_L23_R2_IgG

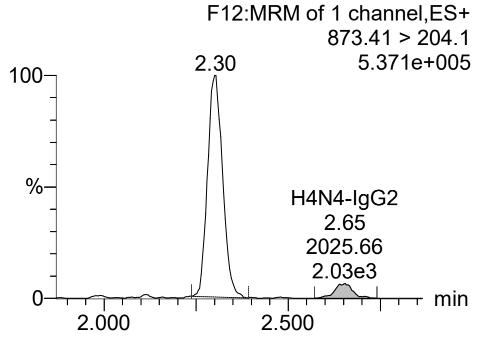

Sample Name: 20211111_L24_R2_IgG

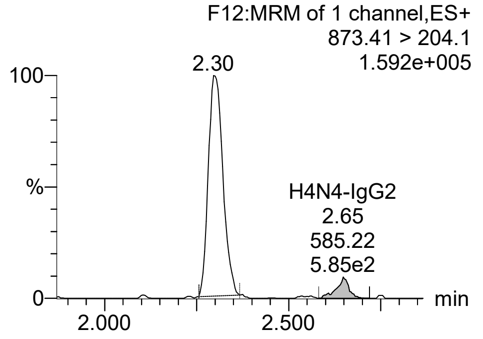

Sample Name: 20211111_H12_R2_IgG

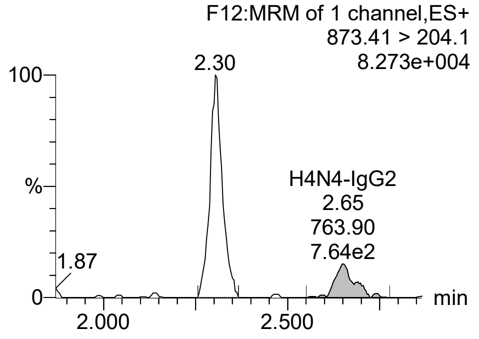

Sample Name: 20211111_QC_R14_IgG

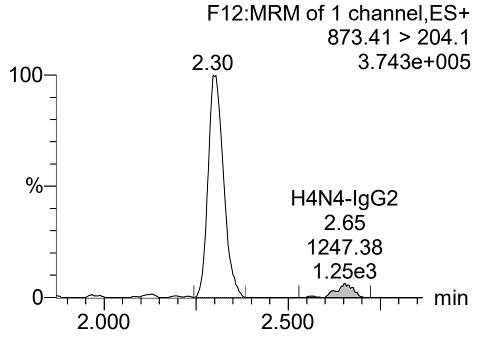

Sample Name: 20211111_A25_R2_IgG

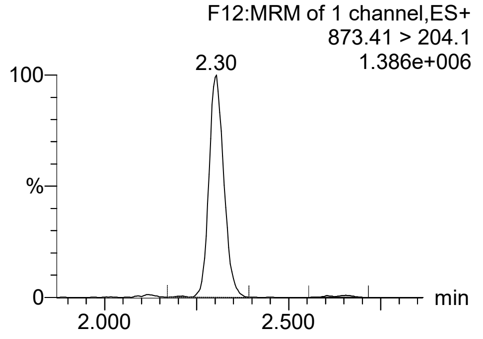

Sample Name: 20211111_A26_R2_IgG

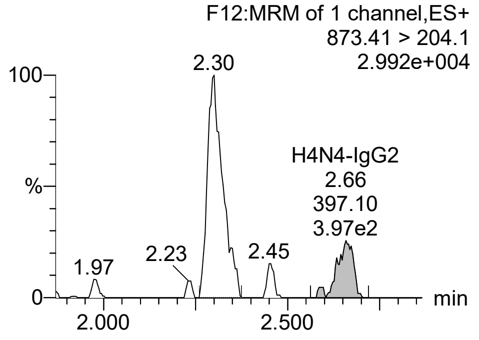

Sample Name: 20211111_L25_R2_IgG

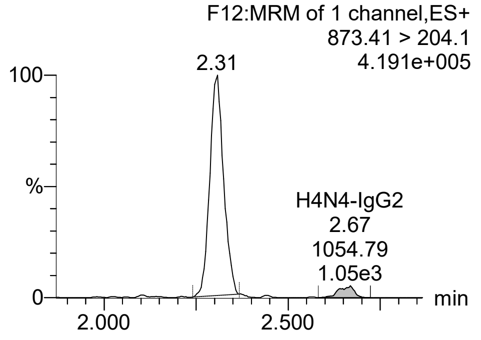

Sample Name: 20211111_L26_R2_IgG

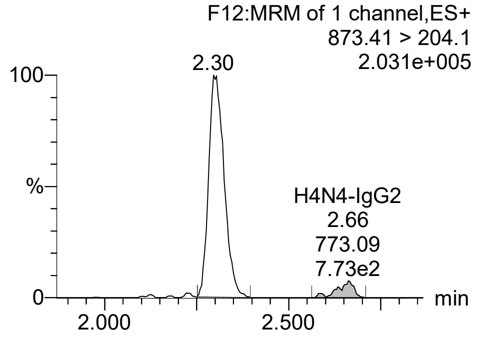

Sample Name: 20211111_H13_R2_IgG

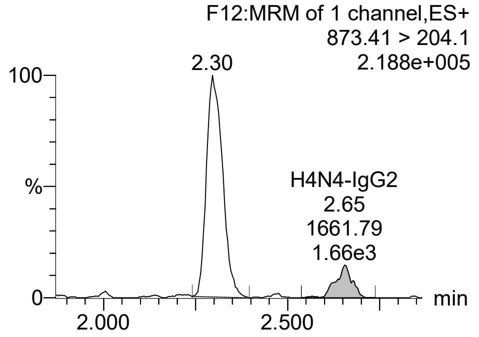

Sample Name: 20211111_A27_R2_IgG

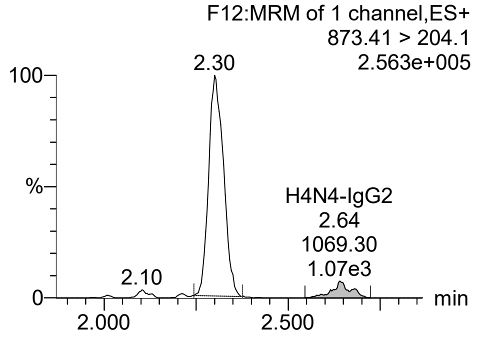

Sample Name: 20211111_A28_R2_IgG

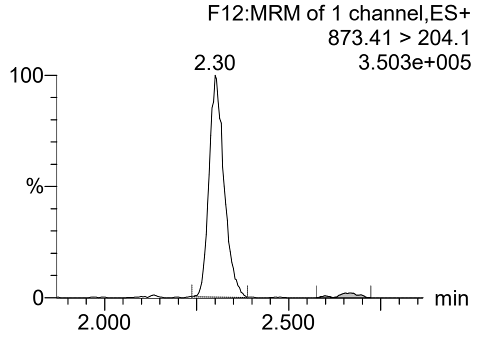

Dataset: C:\MassLynx\10.pro\20211115_clinicalsamples_IgG.qld

Last Altered: Sunday, October 02, 2022 16:02:30 台北標準時間
Printed: Friday, August 01, 2025 16:49:44 台北標準時間

Sample Name: 20211111_L27_R2_IgG

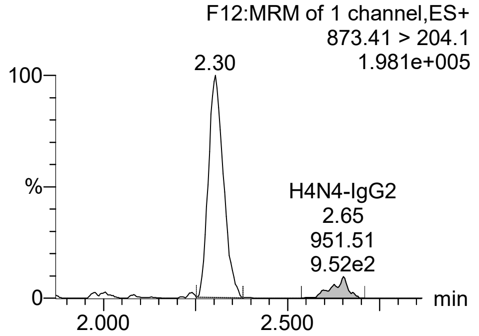

Sample Name: 20211111_L28_R2_IgG

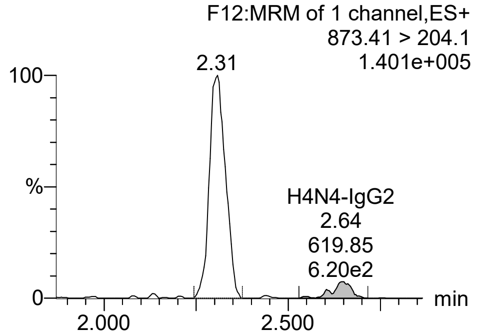

Sample Name: 20211111_H14_R2_IgG

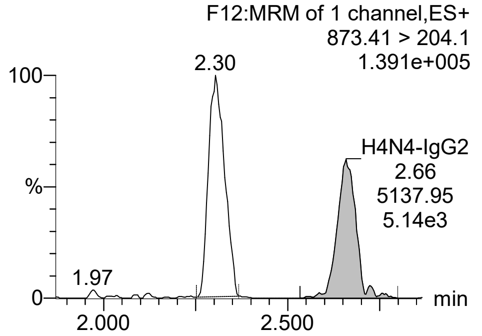

Sample Name: 20211111_A29_R2_IgG

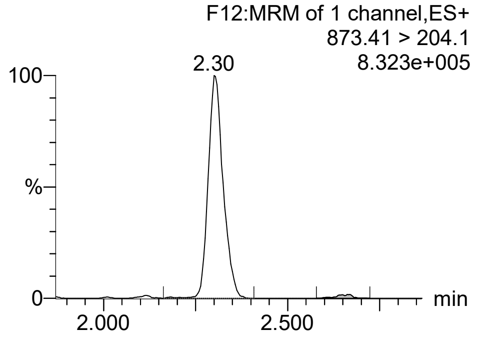

Sample Name: 20211111_A30_R2_IgG

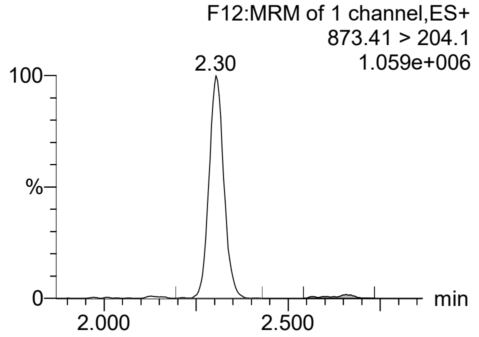

Sample Name: 20211111_QC_R15_IgG

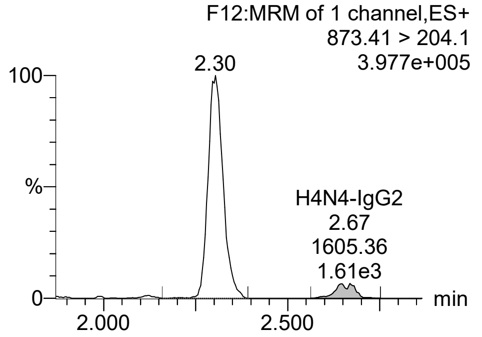

Sample Name: 20211111_L29_R2_IgG

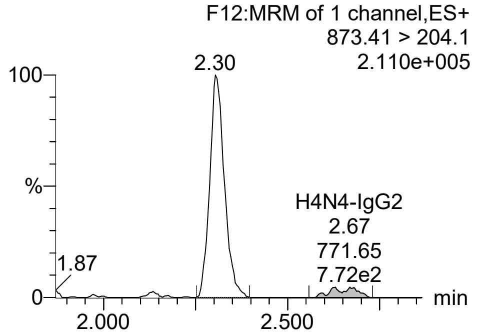

Sample Name: 20211111_L30_R2_IgG

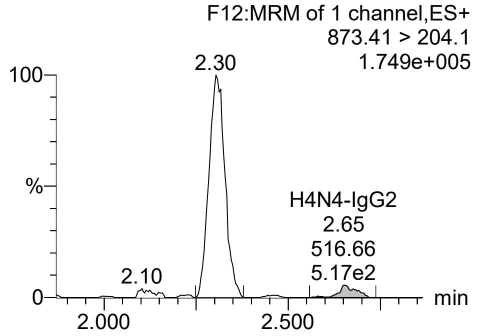

Sample Name: 20211111_H15_R2_IgG

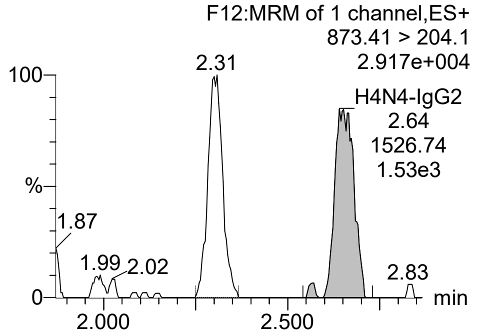

Sample Name: 20211111_A31_R2_IgG

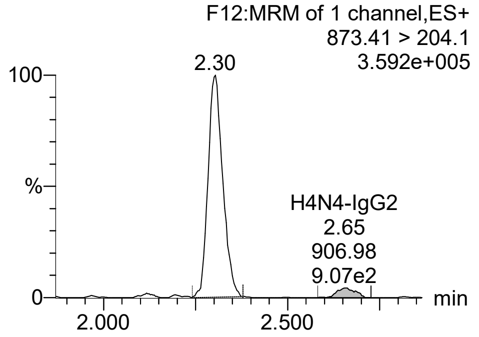

Sample Name: 20211111_A32_R2_IgG

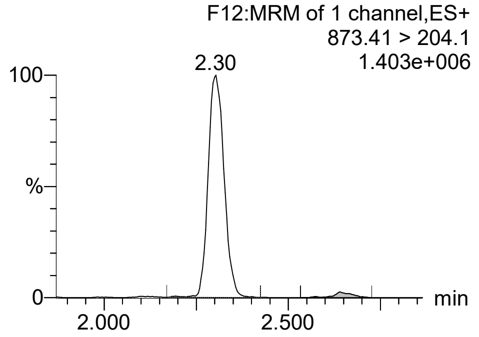

Sample Name: 20211111_L31_R2_IgG

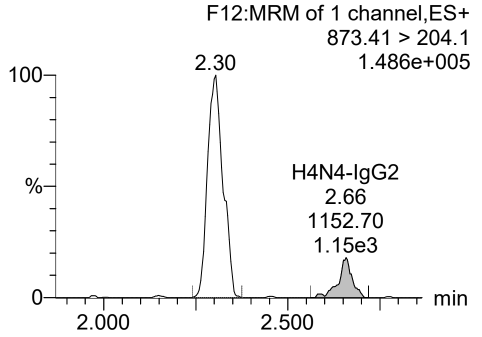

Sample Name: 20211111_L32_R2_IgG

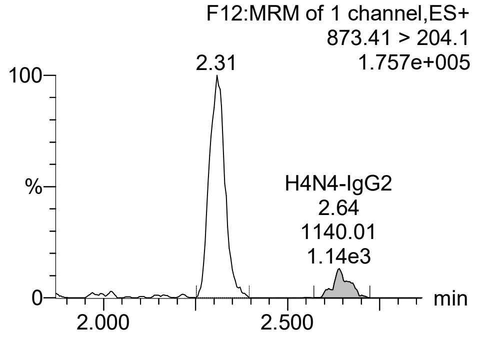

Sample Name: 20211111_H16_R2_IgG

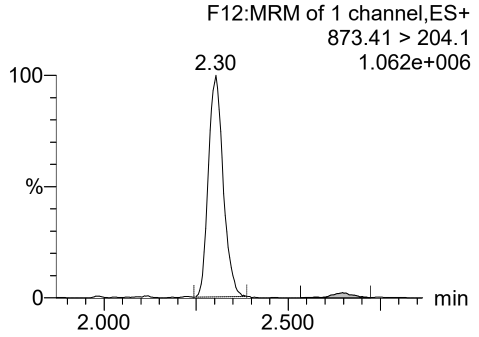

Sample Name: 20211111_A33_R2_IgG

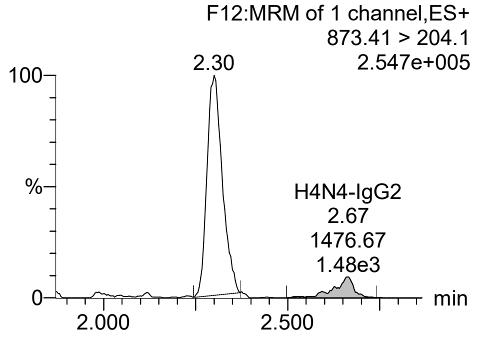

Dataset: C:\MassLynx\10.pro\20211115_clinicalsamples_IgG.qld

Last Altered: Sunday, October 02, 2022 16:02:30 台北標準時間
Printed: Friday, August 01, 2025 16:49:44 台北標準時間

Sample Name: 20211111_A34_R2_IgG

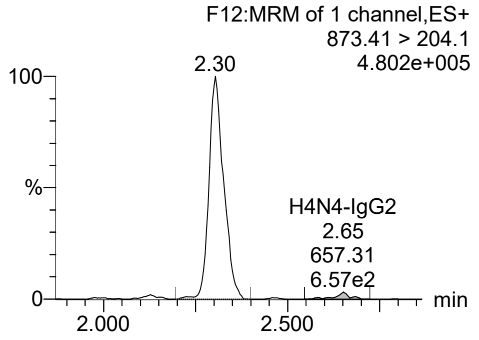

Sample Name: 20211111_H17_R2_IgG

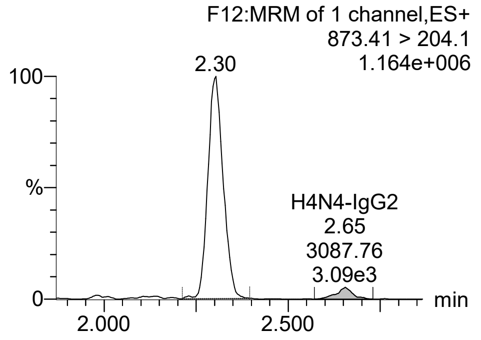

Sample Name: 20211111_A35_R2_IgG

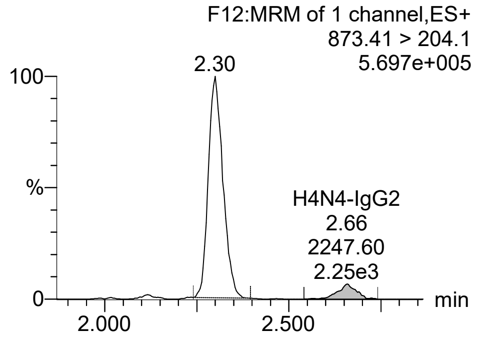

Sample Name: 20211111_QC_R16_IgG

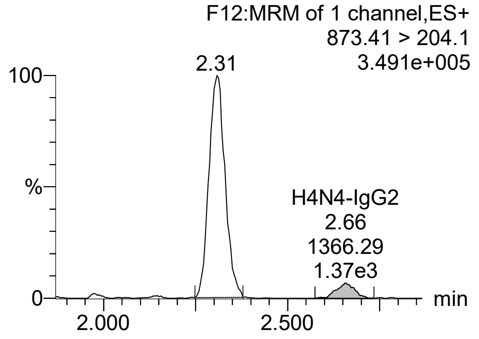

Sample Name: 20211111_A36_R2_IgG

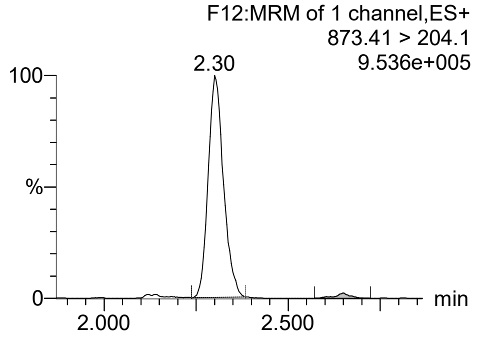

Sample Name: 20211111_H18_R2_IgG

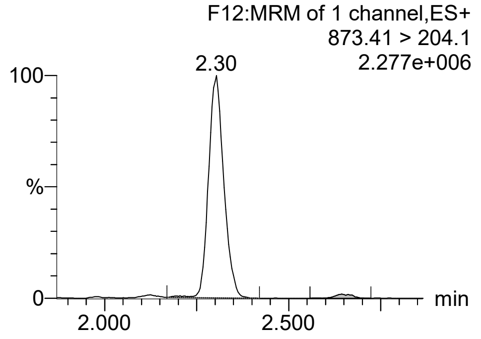

Sample Name: 20211111_A37_R2_IgG

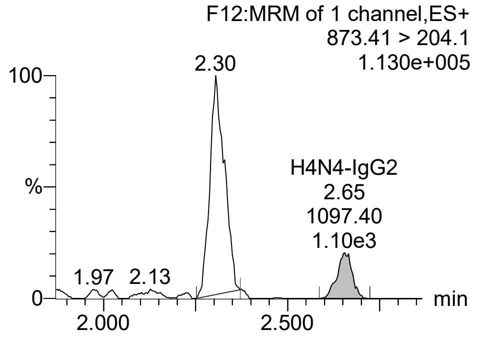

Sample Name: 20211111_A38_R2_IgG

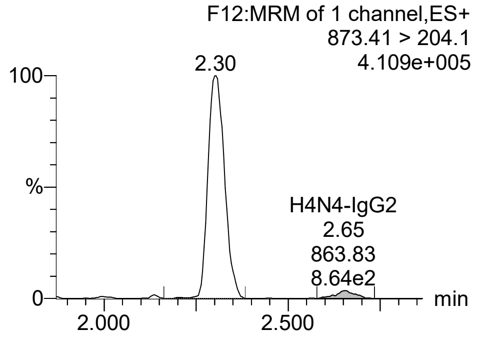

Sample Name: 20211111_H19_R2_IgG

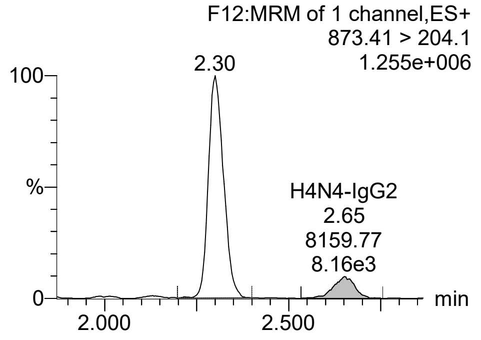

Sample Name: 20211111_A39_R2_IgG

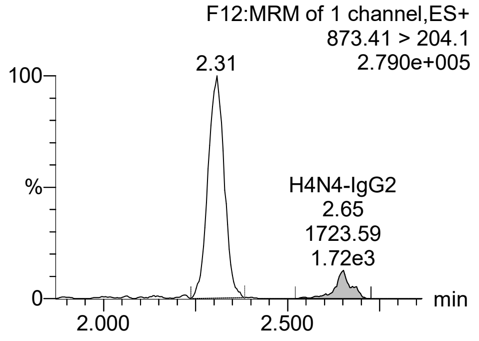

Sample Name: 20211111_A40_R2_IgG

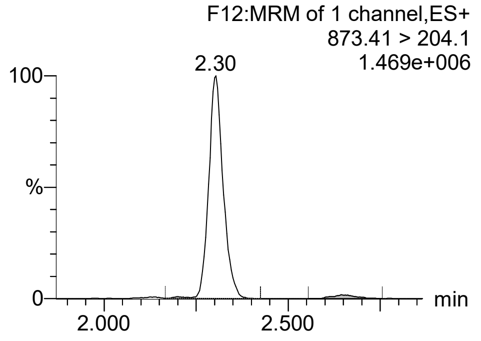

Sample Name: 20211111_H20_R2_IgG

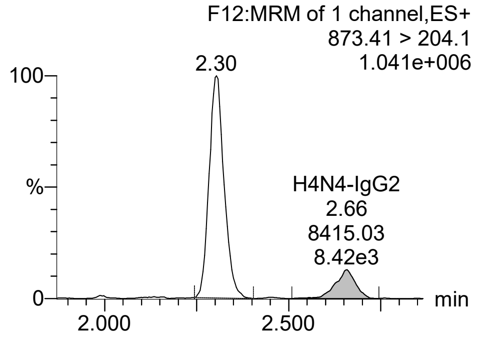

Sample Name: 20211111_A41_R2_IgG

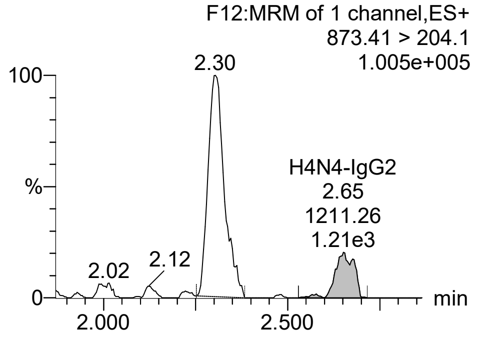

Sample Name: 20211111_A42_R2_IgG

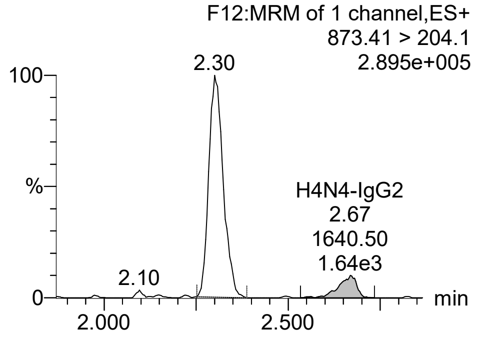

Sample Name: 20211111_A43_R2_IgG

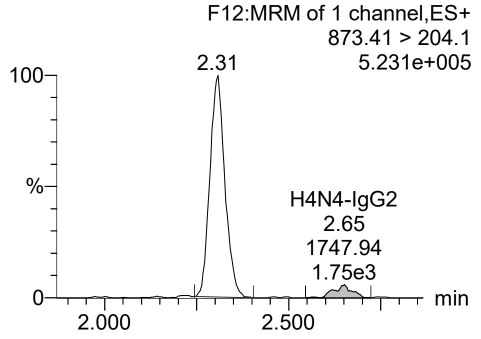

Dataset: C:\MassLynx\10.pro\20211115_clinicalsamples_IgG.qld

Last Altered: Sunday, October 02, 2022 16:02:30 台北標準時間
Printed: Friday, August 01, 2025 16:49:44 台北標準時間

Sample Name: 20211111_A44_R2_IgG

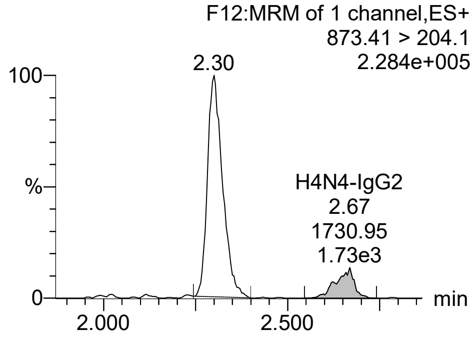

Sample Name: 20211111_QC_R17_IgG

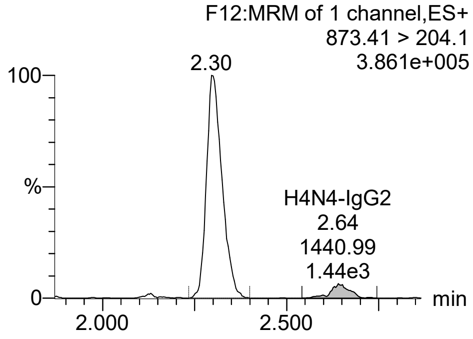

Dataset: C:\MassLynx\10.pro\20211115_clinicalsamples_IgG.qld

Last Altered: Sunday, October 02, 2022 16:02:30 台北標準時間
Printed: Friday, August 01, 2025 16:49:44 台北標準時間

	#	Name	RT	Area	IS Area	Response	Prima...
1	1	20211111_QC_R1_IgG	2.65	2640.7	64968.0	0.041	MM
2	2	20211111_A1_R1_IgG	2.65	3212.2	59059.2	0.054	MM
3	3	20211111_A2_R1_IgG	2.65	2549.9	66621.2	0.038	MM
4	4	20211111_L1_R1_IgG	2.65	1711.8	64201.0	0.027	MM
5	5	20211111_L2_R1_IgG	2.67	4998.3	46950.2	0.106	MM
6	6	20211111_H1_R1_IgG	2.67	1720.6	38648.2	0.045	MM
7	7	20211111_A3_R1_IgG	2.65	56.8	2603.7	0.022	MM
8	8	20211111_A4_R1_IgG	2.64	2924.3	69960.4	0.042	MM
9	9	20211111_L3_R1_IgG	2.65	1238.9	38864.7	0.032	MM
10	10	20211111_L4_R1_IgG	2.65	1228.9	43380.5	0.028	MM
11	11	20211111_H2_R1_IgG	2.65	3282.8	71349.2	0.046	MM
12	12	20211111_A5_R1_IgG	2.65	2500.6	65569.7	0.038	MM
13	13	20211111_A6_R1_IgG	2.66	2761.2	53120.7	0.052	MM
14	14	20211111_QC_R2_IgG	2.66	2907.9	81034.1	0.036	MM
15	15	20211111_L5_R1_IgG	2.66	1601.6	50994.6	0.031	MM
16	16	20211111_L6_R1_IgG	2.65	832.9	58058.0	0.014	MM
17	17	20211111_H3_R1_IgG	2.67	2389.1	64028.6	0.037	MM
18	18	20211111_A7_R1_IgG	2.67	4886.5	82996.1	0.059	MM
19	19	20211111_A8_R1_IgG	2.65	2667.1	77731.7	0.034	MM
20	20	20211111_L7_R1_IgG	2.66	2023.1	38827.3	0.052	MM
21	21	20211111_L8_R1_IgG	2.64	1126.5	44220.1	0.025	MM
22	22	20211111_H4_R1_IgG	2.67	2405.4	65826.3	0.037	MM
23	23	20211111_A9_R1_IgG	2.63	816.6	60771.1	0.013	MM
24	24	20211111_A10_R1_IgG	2.65	1644.9	48578.7	0.034	MM
25	25	20211111_L9_R1_IgG	2.67	2350.6	50830.7	0.046	MM
26	26	20211111_L10_R1_IgG	2.64	620.0	38034.2	0.016	MM
27	27	20211111_QC_R3_IgG	2.67	2157.0	67025.5	0.032	MM
28	28	20211111_H5_R1_IgG	2.67	1294.9	44544.9	0.029	MM
29	29	20211111_A11_R1_IgG	2.67	575.5	66408.5	0.009	MM
30	30	20211111_A12_R1_IgG	2.67	1781.8	46624.5	0.038	MM
31	31	20211111_L11_R1_IgG	2.65	991.9	33425.1	0.030	MM
32	32	20211111_L12_R1_IgG	2.61	602.2	41178.0	0.015	MM
33	33	20211111_H6_R1_IgG	2.67	6767.3	58593.4	0.115	MM
34	34	20211111_A13_R1_IgG	2.66	3283.7	54133.0	0.061	MM
35	35	20211111_A14_R1_IgG	2.64	1188.8	56467.3	0.021	MM
36	36	20211111_L13_R1_IgG	2.66	1443.3	36024.4	0.040	MM
37	37	20211111_L14_R1_IgG	2.67	1380.2	37181.6	0.037	MM
38	38	20211111_H7_R1_IgG	2.66	1354.1	42850.6	0.032	MM
39	39	20211111_A15_R1_IgG	2.67	1974.9	62015.1	0.032	MM
40	40	20211111_QC_R4_IgG	2.66	2104.9	58572.5	0.036	MM
41	41	20211111_A16_R1_IgG	2.65	2062.0	78283.8	0.026	MM
42	42	20211111_L15_R1_IgG	2.67	1959.9	44158.2	0.044	MM
43	43	20211111_L16_R1_IgG	2.65	1433.9	55150.8	0.026	MM
44	44	20211111_H8_R1_IgG	2.66	1480.9	42965.2	0.034	MM
45	45	20211111_A17_R1_IgG	2.65	2125.3	39717.7	0.054	MM
46	46	20211111_A18_R1_IgG	2.66	1931.4	44675.4	0.043	MM
47	47	20211111_L17_R1_IgG	2.65	1335.1	37854.1	0.035	MM
48	48	20211111_L18_R1_IgG	2.61	370.6	50716.9	0.007	MM
49	49	20211111_H9_R1_IgG	2.67	1994.5	36276.0	0.055	MM
50	50	20211111_A19_R1_IgG	2.67	2360.8	42862.5	0.055	MM
51	51	20211111_A20_R1_IgG	2.66	734.7	36542.7	0.020	MM
52	52	20211111_L19_R1_IgG	2.67	2089.0	53892.8	0.039	MM
53	53	20211111_QC_R5_IgG	2.67	1722.7	56513.2	0.030	MM
54	54	20211111_L20_R1_IgG	2.67	1206.8	56499.9	0.021	MM
55	55	20211111_H10_R1_IgG	2.66	2220.6	68120.5	0.033	MM
56	56	20211111_A21_R1_IgG	2.65	2861.9	63699.8	0.045	MM

Dataset: C:\MassLynx\10.pro\20211115_clinicalsamples_IgG.qld

Last Altered: Sunday, October 02, 2022 16:02:30 台北標準時間
Printed: Friday, August 01, 2025 16:49:44 台北標準時間

	# Name	RT	Area	IS Area	Response	Prima...
57	57 20211111_A22_R1_IgG	2.64	811.9	52222.1	0.016	MM
58	58 20211111_L21_R1_IgG	2.66	593.4	54372.5	0.011	MM
59	59 20211111_L22_R1_IgG	2.66	899.6	41353.1	0.022	MM
60	60 20211111_H11_R1_IgG	2.64	2710.9	65579.1	0.041	MM
61	61 20211111_A23_R1_IgG	2.65	1785.3	64131.1	0.028	MM
62	62 20211111_A24_R1_IgG	2.66	664.6	38724.8	0.017	MM
63	63 20211111_L23_R1_IgG	2.66	3618.6	64245.2	0.056	MM
64	64 20211111_L24_R1_IgG	2.66	1003.0	45298.0	0.022	MM
65	65 20211111_H12_R1_IgG	2.64	746.4	44679.3	0.017	MM
66	66 20211111_QC_R6_IgG	2.67	1560.8	48165.0	0.032	MM
67	67 20211111_A25_R1_IgG	2.64	1058.9	57169.9	0.019	MM
68	68 20211111_A26_R1_IgG	2.65	642.3	34254.0	0.019	MM
69	69 20211111_L25_R1_IgG	2.65	1965.9	67491.5	0.029	MM
70	70 20211111_L26_R1_IgG	2.65	1012.5	55934.1	0.018	MM
71	71 20211111_H13_R1_IgG	2.65	2196.0	48767.3	0.045	MM
72	72 20211111_A27_R1_IgG	2.65	1175.9	57269.7	0.021	MM
73	73 20211111_A28_R1_IgG	2.66	840.9	49231.1	0.017	MM
74	74 20211111_L27_R1_IgG	2.64	1196.5	60014.5	0.020	MM
75	75 20211111_L28_R1_IgG	2.64	792.1	35062.6	0.023	MM
76	76 20211111_H14_R1_IgG	2.66	6711.1	62371.9	0.108	MM
77	77 20211111_A29_R1_IgG	2.64	721.8	43858.2	0.016	MM
78	78 20211111_A30_R1_IgG	2.65	1683.5	62500.4	0.027	MM
79	79 20211111_QC_R7_IgG	2.66	1374.7	43946.9	0.031	MM
80	80 20211111_L29_R1_IgG	2.65	833.9	38728.9	0.022	MM
81	81 20211111_L30_R1_IgG	2.68	779.4	42613.2	0.018	MM
82	82 20211111_H15_R1_IgG	2.67	1770.1	39874.6	0.044	MM
83	83 20211111_A31_R1_IgG	2.66	1004.5	53414.3	0.019	MM
84	84 20211111_A32_R1_IgG	2.67	1590.8	41844.6	0.038	MM
85	85 20211111_L31_R1_IgG	2.66	1003.0	40553.7	0.025	MM
86	86 20211111_L32_R1_IgG	2.68	1457.2	49618.7	0.029	MM
87	87 20211111_H16_R1_IgG	2.64	2009.5	60170.9	0.033	MM
88	88 20211111_A33_R1_IgG	2.67	1529.4	48588.2	0.031	MM
89	89 20211111_A34_R1_IgG	2.66	1037.6	43288.0	0.024	MM
90	90 20211111_H17_R1_IgG	2.65	4254.2	47104.5	0.090	MM
91	91 20211111_A35_R1_IgG	2.67	2080.0	41025.9	0.051	MM
92	92 20211111_QC_R8_IgG	2.67	1423.3	40546.3	0.035	MM
93	93 20211111_A36_R1_IgG	2.65	1347.7	44504.4	0.030	MM
94	94 20211111_H18_R1_IgG	2.65	2826.0	34162.2	0.083	MM
95	95 20211111_A37_R1_IgG	2.66	927.2	39760.0	0.023	MM
96	96 20211111_A38_R1_IgG	2.67	749.8	43982.6	0.017	MM
97	97 20211111_H19_R1_IgG	2.67	8890.9	45314.8	0.196	MM
98	98 20211111_A39_R1_IgG	2.66	2590.8	39388.0	0.066	MM
99	99 20211111_A40_R1_IgG	2.68	1735.3	38726.4	0.045	MM
100	1... 20211111_H20_R1_IgG	2.67	8706.1	37814.4	0.230	MM
101	1... 20211111_A41_R1_IgG	2.67	1503.4	48930.3	0.031	MM
102	1... 20211111_A42_R1_IgG	2.67	1772.0	40406.8	0.044	MM
103	1... 20211111_A43_R1_IgG	2.66	1679.3	44421.5	0.038	MM
104	1... 20211111_A44_R1_IgG	2.66	2696.4	53128.2	0.051	MM
105	1... 20211111_QC_R9_IgG	2.66	1406.7	37049.0	0.038	MM
106	1... 20211111_A1_R2_IgG	2.66	2488.9	46499.4	0.054	MM
107	1... 20211111_A2_R2_IgG	2.65	2224.1	57260.7	0.039	MM
108	1... 20211111_L1_R2_IgG	2.66	1068.9	48062.9	0.022	MM
109	1... 20211111_L2_R2_IgG	2.66	3506.5	38745.2	0.091	MM
110	1... 20211111_H1_R2_IgG	2.67	1569.7	44273.5	0.035	MM
111	1... 20211111_A3_R2_IgG	2.64	22.1	4755.5	0.005	MM
112	1... 20211111_A4_R2_IgG	2.65	2255.6	50546.7	0.045	MM

Dataset: C:\MassLynx\10.pro\20211115_clinicalsamples_IgG.qld

Last Altered: Sunday, October 02, 2022 16:02:30 台北標準時間
Printed: Friday, August 01, 2025 16:49:44 台北標準時間

	#	Name	RT	Area	IS Area	Response	Prima...
113	1...	20211111_L3_R2_IgG	2.67	1005.4	34825.8	0.029	MM
114	1...	20211111_L4_R2_IgG	2.66	921.8	39107.7	0.024	MM
115	1...	20211111_H2_R2_IgG	2.64	2370.2	49750.0	0.048	MM
116	1...	20211111_A5_R2_IgG	2.67	2429.4	52536.2	0.046	MM
117	1...	20211111_A6_R2_IgG	2.65	1952.2	42050.0	0.046	MM
118	1...	20211111_QC_R10_IgG	2.65	1301.5	35350.8	0.037	MM
119	1...	20211111_L5_R2_IgG	2.65	1300.9	38039.3	0.034	MM
120	1...	20211111_L6_R2_IgG	2.67	722.3	44907.4	0.016	MM
121	1...	20211111_H3_R2_IgG	2.66	2043.4	47729.1	0.043	MM
122	1...	20211111_A7_R2_IgG	2.67	2753.3	57347.1	0.048	MM
123	1...	20211111_A8_R2_IgG	2.67	1387.0	52397.8	0.026	MM
124	1...	20211111_L7_R2_IgG	2.66	1977.2	36318.4	0.054	MM
125	1...	20211111_L8_R2_IgG	2.66	798.9	38131.9	0.021	MM
126	1...	20211111_H4_R2_IgG	2.67	1989.4	46849.6	0.042	MM
127	1...	20211111_A9_R2_IgG	2.65	1268.9	47759.6	0.027	MM
128	1...	20211111_A10_R2_IgG	2.65	1480.7	41366.2	0.036	MM
129	1...	20211111_L9_R2_IgG	2.67	1603.7	43052.1	0.037	MM
130	1...	20211111_L10_R2_IgG	2.67	834.5	31487.4	0.027	MM
131	1...	20211111_QC_R11_IgG	2.68	1099.7	37804.6	0.029	MM
132	1...	20211111_H5_R2_IgG	2.68	1241.5	40380.0	0.031	MM
133	1...	20211111_A11_R2_IgG	2.67	669.1	49533.3	0.014	MM
134	1...	20211111_A12_R2_IgG	2.68	1340.3	40226.3	0.033	MM
135	1...	20211111_L11_R2_IgG	2.68	800.4	28884.0	0.028	MM
136	1...	20211111_L12_R2_IgG	2.66	699.7	34351.3	0.020	MM
137	1...	20211111_H6_R2_IgG	2.67	5120.3	40466.6	0.127	MM
138	1...	20211111_A13_R2_IgG	2.67	2147.8	42306.7	0.051	MM
139	1...	20211111_A14_R2_IgG	2.65	1140.2	44904.0	0.025	MM
140	1...	20211111_L13_R2_IgG	2.65	893.8	31913.0	0.028	MM
141	1...	20211111_L14_R2_IgG	2.66	1222.3	31527.4	0.039	MM
142	1...	20211111_H7_R2_IgG	2.65	1215.8	40838.2	0.030	MM
143	1...	20211111_A15_R2_IgG	2.67	1487.8	49389.7	0.030	MM
144	1...	20211111_QC_R12_IgG	2.65	1630.8	35168.7	0.046	MM
145	1...	20211111_A16_R2_IgG	2.66	1396.1	47440.5	0.029	MM
146	1...	20211111_L15_R2_IgG	2.66	1946.0	33145.9	0.059	MM
147	1...	20211111_L16_R2_IgG	2.65	1115.6	37596.6	0.030	MM
148	1...	20211111_H8_R2_IgG	2.66	1066.7	43933.3	0.024	MM
149	1...	20211111_A17_R2_IgG	2.65	2542.7	43605.5	0.058	MM
150	1...	20211111_A18_R2_IgG	2.66	1607.9	37581.8	0.043	MM
151	1...	20211111_L17_R2_IgG	2.64	1835.6	33942.5	0.054	MM
152	1...	20211111_L18_R2_IgG	2.66	278.4	37831.5	0.007	MM
153	1...	20211111_H9_R2_IgG	2.66	2184.7	30898.4	0.071	MM
154	1...	20211111_A19_R2_IgG	2.66	2102.4	39352.1	0.053	MM
155	1...	20211111_A20_R2_IgG	2.66	793.0	34661.2	0.023	MM
156	1...	20211111_L19_R2_IgG	2.67	1704.7	39918.3	0.043	MM
157	1...	20211111_QC_R13_IgG	2.65	1857.6	36239.1	0.051	MM
158	1...	20211111_L20_R2_IgG	2.64	486.8	32897.7	0.015	MM
159	1...	20211111_H10_R2_IgG	2.65	649.5	31394.5	0.021	MM
160	1...	20211111_A21_R2_IgG	2.64	1087.4	34863.7	0.031	MM
161	1...	20211111_A22_R2_IgG	2.63	481.4	30342.1	0.016	MM
162	1...	20211111_L21_R2_IgG	2.65	246.0	33706.2	0.007	MM
163	1...	20211111_L22_R2_IgG	2.66	407.5	31298.6	0.013	MM
164	1...	20211111_H11_R2_IgG	2.65	1205.8	38904.1	0.031	MM
165	1...	20211111_A23_R2_IgG	2.64	874.7	32382.3	0.027	MM
166	1...	20211111_A24_R2_IgG	2.64	566.1	30261.9	0.019	MM
167	1...	20211111_L23_R2_IgG	2.65	2025.7	43037.5	0.047	MM
168	1...	20211111_L24_R2_IgG	2.65	585.2	37023.3	0.016	MM

Dataset: C:\MassLynx\10.pro\20211115_clinicalsamples_IgG.qld

Last Altered: Sunday, October 02, 2022 16:02:30 台北標準時間
Printed: Friday, August 01, 2025 16:49:44 台北標準時間

	#	Name	RT	Area	IS Area	Response	Prima...
169	1...	20211111_H12_R2_IgG	2.65	763.9	39478.2	0.019	MM
170	1...	20211111_QC_R14_IgG	2.65	1247.4	34463.2	0.036	MM
171	1...	20211111_A25_R2_IgG	2.65	932.2	39945.3	0.023	MM
172	1...	20211111_A26_R2_IgG	2.66	397.1	29663.2	0.013	MM
173	1...	20211111_L25_R2_IgG	2.67	1054.8	38589.3	0.027	MM
174	1...	20211111_L26_R2_IgG	2.66	773.1	38405.2	0.020	MM
175	1...	20211111_H13_R2_IgG	2.65	1661.8	37771.2	0.044	MM
176	1...	20211111_A27_R2_IgG	2.64	1069.3	42634.1	0.025	MM
177	1...	20211111_A28_R2_IgG	2.67	497.7	33231.1	0.015	MM
178	1...	20211111_L27_R2_IgG	2.65	951.5	41761.1	0.023	MM
179	1...	20211111_L28_R2_IgG	2.64	619.9	26968.6	0.023	MM
180	1...	20211111_H14_R2_IgG	2.66	5138.0	49250.2	0.104	MM
181	1...	20211111_A29_R2_IgG	2.66	719.6	37601.7	0.019	MM
182	1...	20211111_A30_R2_IgG	2.66	1271.1	40366.8	0.031	MM
183	1...	20211111_QC_R15_IgG	2.67	1605.4	35156.1	0.046	MM
184	1...	20211111_L29_R2_IgG	2.67	771.6	33813.1	0.023	MM
185	1...	20211111_L30_R2_IgG	2.65	516.7	33488.6	0.015	MM
186	1...	20211111_H15_R2_IgG	2.64	1526.7	38501.0	0.040	MM
187	1...	20211111_A31_R2_IgG	2.65	907.0	37525.5	0.024	MM
188	1...	20211111_A32_R2_IgG	2.64	2000.5	35770.8	0.056	MM
189	1...	20211111_L31_R2_IgG	2.66	1152.7	33282.4	0.035	MM
190	1...	20211111_L32_R2_IgG	2.64	1140.0	42049.6	0.027	MM
191	1...	20211111_H16_R2_IgG	2.65	1569.8	45947.6	0.034	MM
192	1...	20211111_A33_R2_IgG	2.67	1476.7	40202.3	0.037	MM
193	1...	20211111_A34_R2_IgG	2.65	657.3	36747.0	0.018	MM
194	1...	20211111_H17_R2_IgG	2.65	3087.8	40920.9	0.075	MM
195	1...	20211111_A35_R2_IgG	2.66	2247.6	33689.2	0.067	MM
196	1...	20211111_QC_R16_IgG	2.66	1366.3	32783.6	0.042	MM
197	1...	20211111_A36_R2_IgG	2.65	986.9	36822.4	0.027	MM
198	1...	20211111_H18_R2_IgG	2.64	2302.1	31769.3	0.072	MM
199	1...	20211111_A37_R2_IgG	2.65	1097.4	37581.8	0.029	MM
200	2...	20211111_A38_R2_IgG	2.65	863.8	38390.4	0.023	MM
201	2...	20211111_H19_R2_IgG	2.65	8159.8	41217.0	0.198	MM
202	2...	20211111_A39_R2_IgG	2.65	1723.6	37392.7	0.046	MM
203	2...	20211111_A40_R2_IgG	2.64	1768.4	38295.1	0.046	MM
204	2...	20211111_H20_R2_IgG	2.66	8415.0	35941.8	0.234	MM
205	2...	20211111_A41_R2_IgG	2.65	1211.3	45304.9	0.027	MM
206	2...	20211111_A42_R2_IgG	2.67	1640.5	37803.8	0.043	MM
207	2...	20211111_A43_R2_IgG	2.65	1747.9	38161.8	0.046	MM
208	2...	20211111_A44_R2_IgG	2.67	1731.0	37819.8	0.046	MM
209	2...	20211111_QC_R17_IgG	2.64	1441.0	31731.8	0.045	MM

Dataset: C:\MassLynx\10.pro\20211115_clinicalsamples_IgG.qld

Last Altered: Sunday, October 02, 2022 16:02:30 台北標準時間
Printed: Friday, August 01, 2025 16:50:17 台北標準時間

Method: C:\MassLynx\10_Pro_IgA.PRO\MethDB\20210926_IgG_DMRM_IgM_IS.mdb 16 Feb 2022 11:58:21
Calibration: 18 Nov 2021 13:56:22

Sample Name: 20211111_QC_R1_IgG

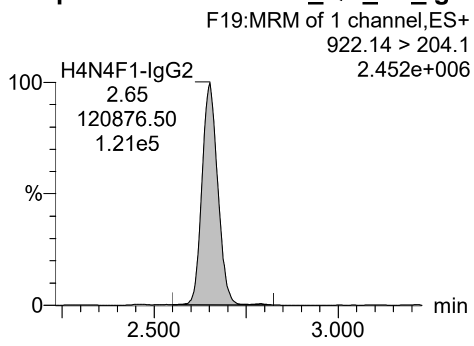

Sample Name: 20211111_A1_R1_IgG

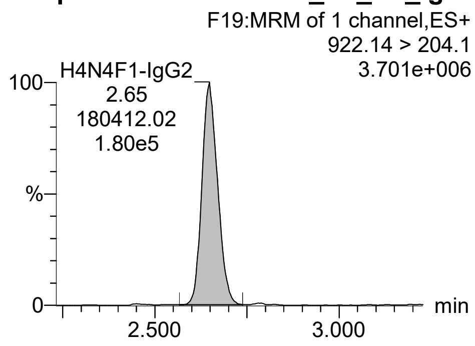

Sample Name: 20211111_A2_R1_IgG

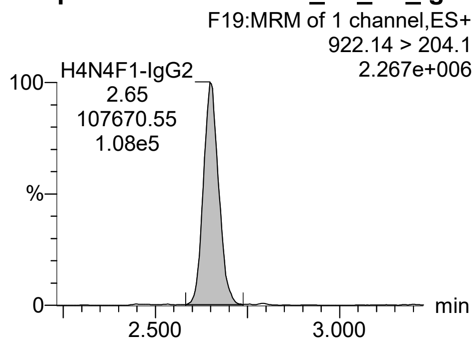

Sample Name: 20211111_L1_R1_IgG

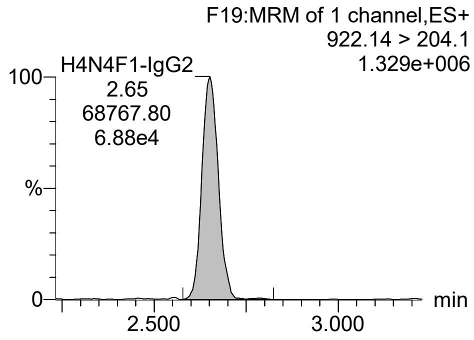

Sample Name: 20211111_L2_R1_IgG

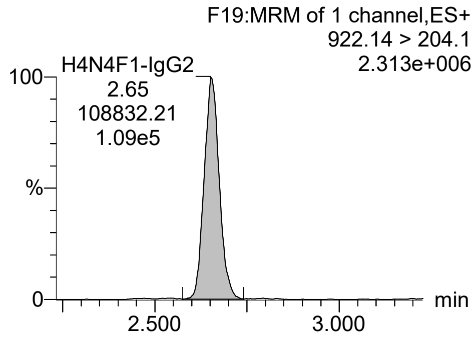

Sample Name: 20211111_H1_R1_IgG

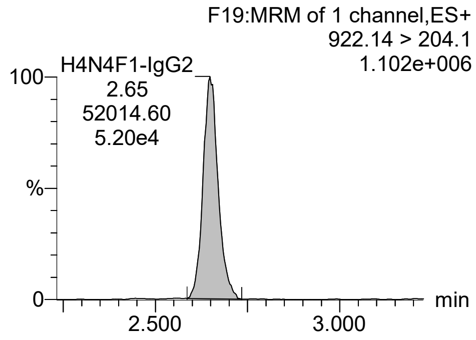

Sample Name: 20211111_A3_R1_IgG

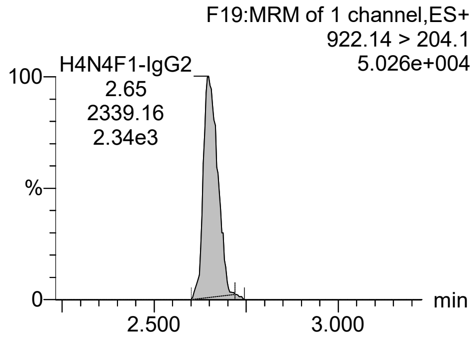

Sample Name: 20211111_A4_R1_IgG

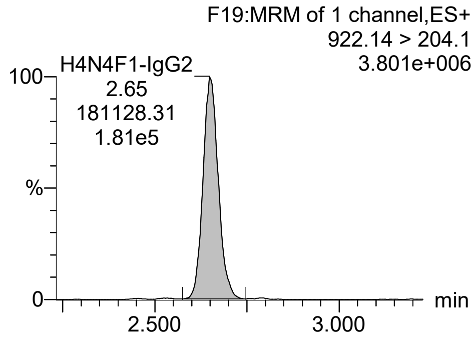

Sample Name: 20211111_L3_R1_IgG

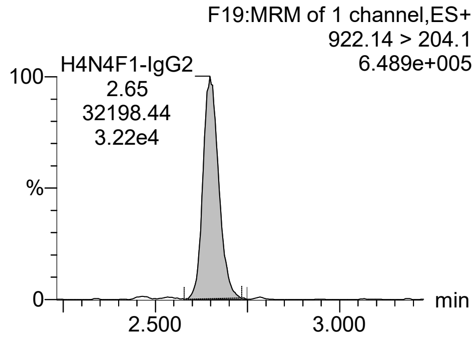

Sample Name: 20211111_L4_R1_IgG

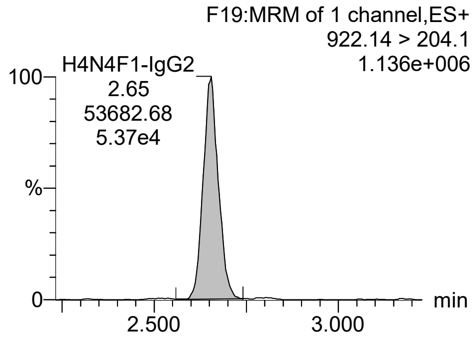

Sample Name: 20211111_H2_R1_IgG

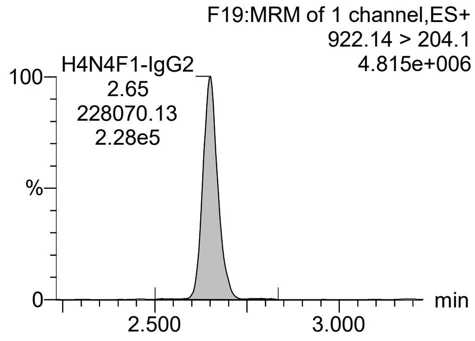

Sample Name: 20211111_A5_R1_IgG

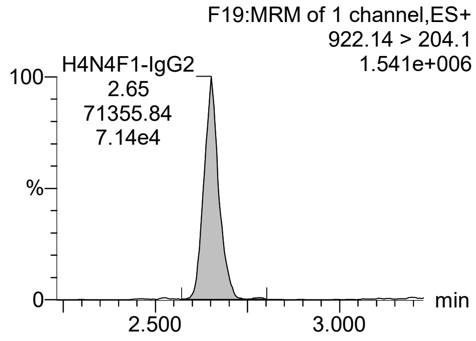

Dataset: C:\MassLynx\10.pro\20211115_clinicalsamples_IgG.qld

Last Altered: Sunday, October 02, 2022 16:02:30 台北標準時間
Printed: Friday, August 01, 2025 16:50:17 台北標準時間

Sample Name: 20211111_A6_R1_IgG

F19:MRM of 1 channel,ES+
922.14 > 204.1
2.887e+006

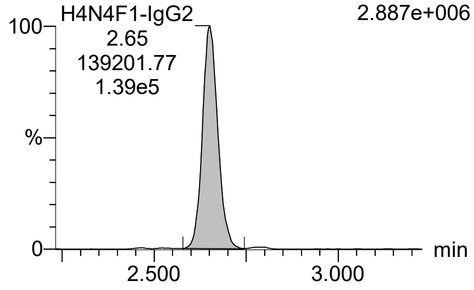

Sample Name: 20211111_QC_R2_IgG

F19:MRM of 1 channel,ES+
922.14 > 204.1
2.534e+006

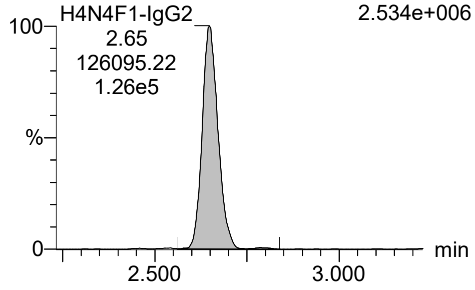

Sample Name: 20211111_L5_R1_IgG

F19:MRM of 1 channel,ES+
922.14 > 204.1
1.553e+006

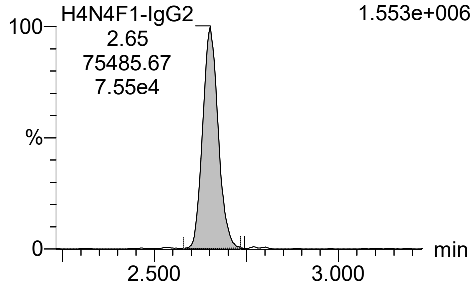

Sample Name: 20211111_L6_R1_IgG

F19:MRM of 1 channel,ES+
922.14 > 204.1
5.256e+005

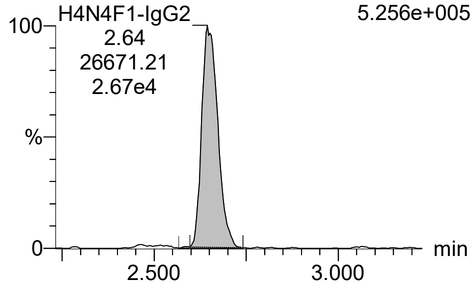

Sample Name: 20211111_H3_R1_IgG

F19:MRM of 1 channel,ES+
922.14 > 204.1
2.123e+006

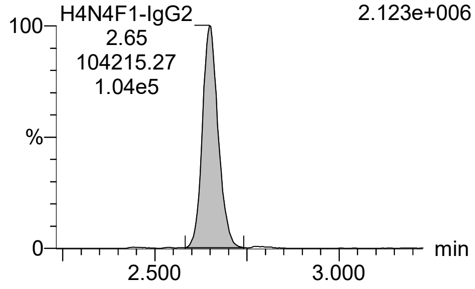

Sample Name: 20211111_A7_R1_IgG

F19:MRM of 1 channel,ES+
922.14 > 204.1
3.949e+006

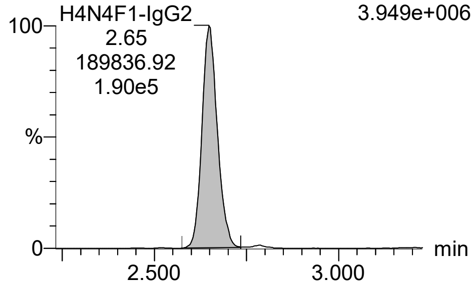

Sample Name: 20211111_A8_R1_IgG

F19:MRM of 1 channel,ES+
922.14 > 204.1
2.095e+006

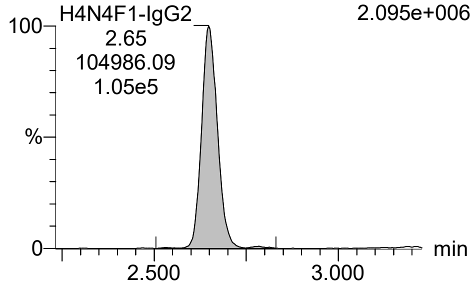

Sample Name: 20211111_L7_R1_IgG

F19:MRM of 1 channel,ES+
922.14 > 204.1
1.419e+006

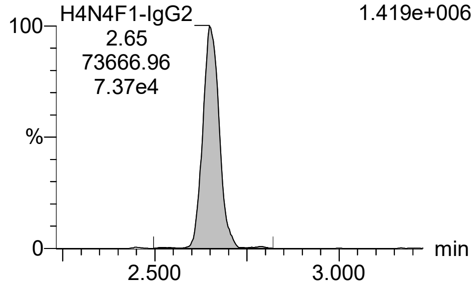

Sample Name: 20211111_L8_R1_IgG

F19:MRM of 1 channel,ES+
922.14 > 204.1
1.123e+006

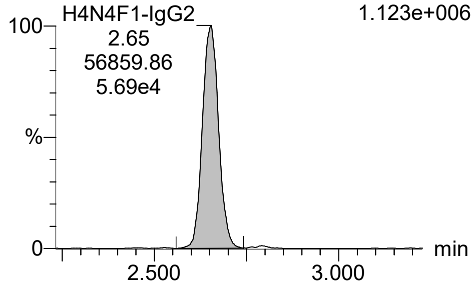

Sample Name: 20211111_H4_R1_IgG

F19:MRM of 1 channel,ES+
922.14 > 204.1
1.232e+006

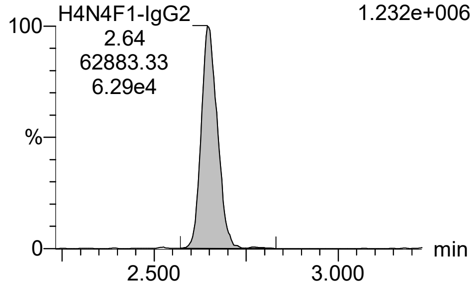

Sample Name: 20211111_A9_R1_IgG

F19:MRM of 1 channel,ES+
922.14 > 204.1
1.404e+006

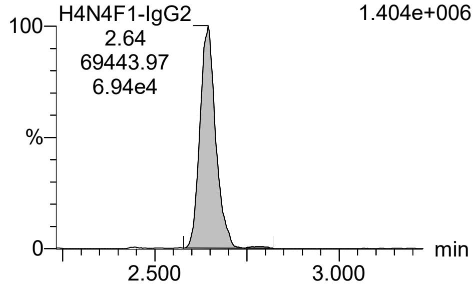

Sample Name: 20211111_A10_R1_IgG

F19:MRM of 1 channel,ES+
922.14 > 204.1
1.576e+006

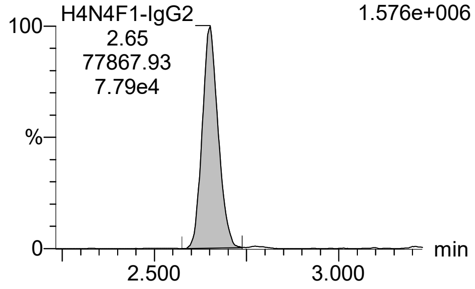

Sample Name: 20211111_L9_R1_IgG

F19:MRM of 1 channel,ES+
922.14 > 204.1
1.892e+006

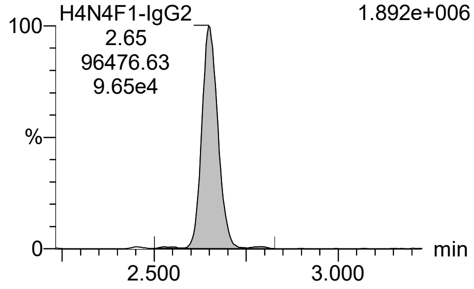

Sample Name: 20211111_L10_R1_IgG

F19:MRM of 1 channel,ES+
922.14 > 204.1
7.469e+005

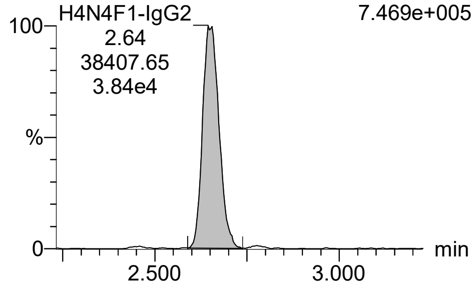

Sample Name: 20211111_QC_R3_IgG

F19:MRM of 1 channel,ES+
922.14 > 204.1
2.176e+006

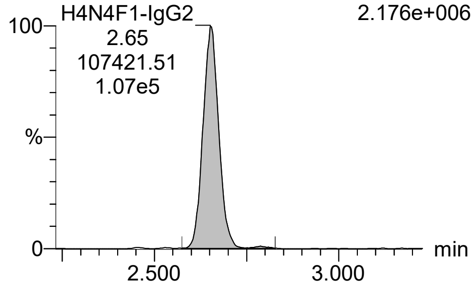

Sample Name: 20211111_H5_R1_IgG

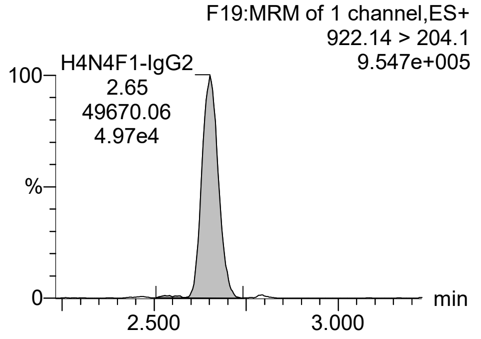

Sample Name: 20211111_A11_R1_IgG

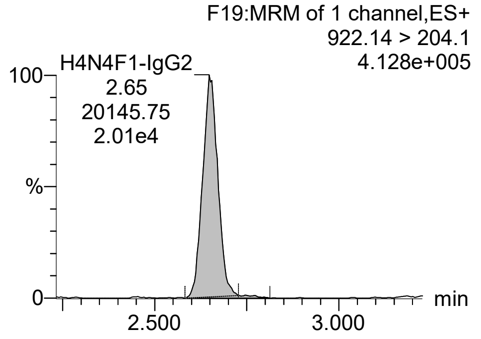

Sample Name: 20211111_A12_R1_IgG

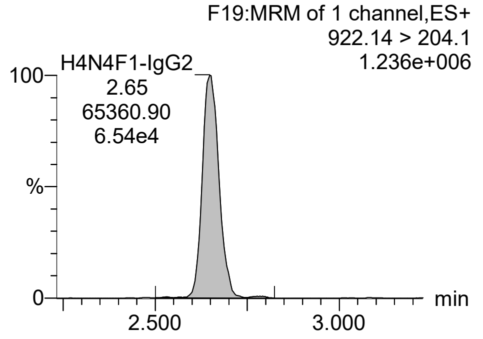

Sample Name: 20211111_L11_R1_IgG

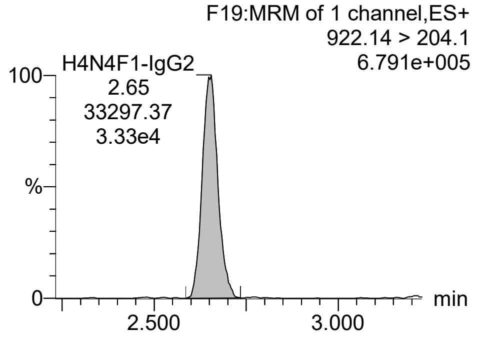

Sample Name: 20211111_L12_R1_IgG

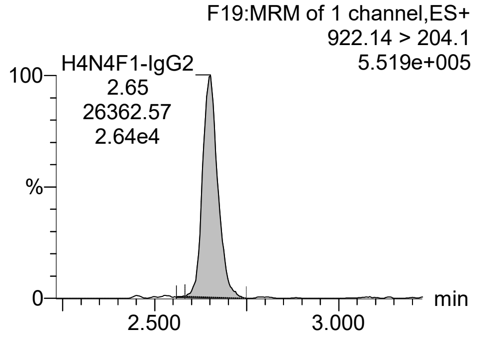

Sample Name: 20211111_H6_R1_IgG

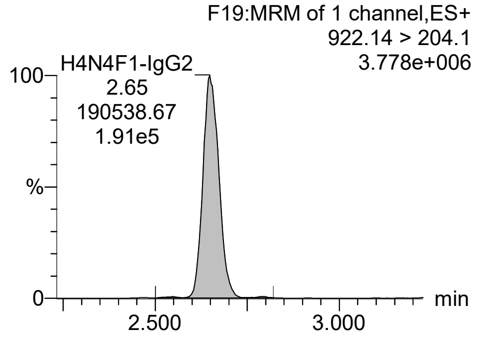

Sample Name: 20211111_A13_R1_IgG

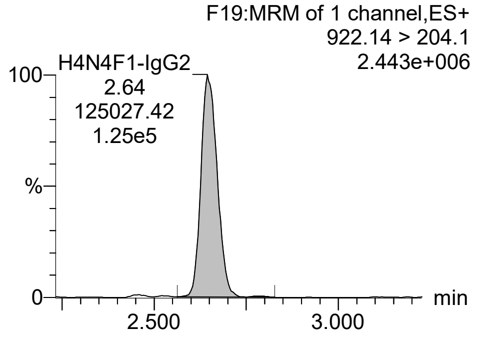

Sample Name: 20211111_A14_R1_IgG

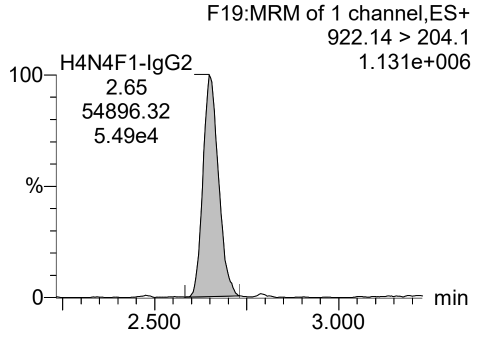

Sample Name: 20211111_L13_R1_IgG

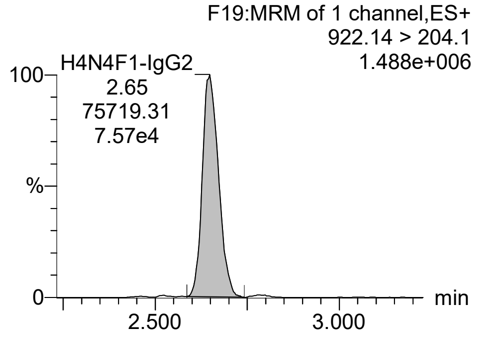

Sample Name: 20211111_L14_R1_IgG

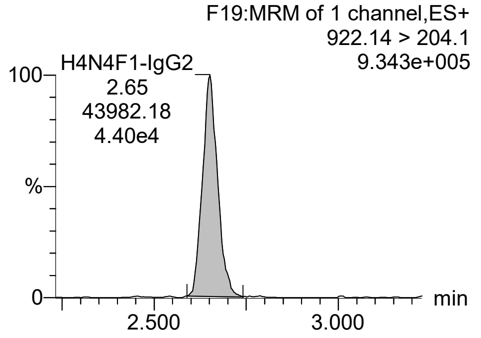

Sample Name: 20211111_H7_R1_IgG

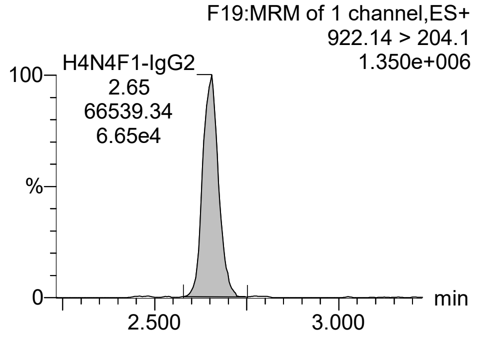

Sample Name: 20211111_A15_R1_IgG

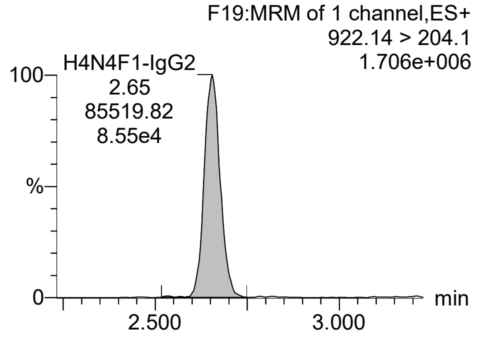

Sample Name: 20211111_QC_R4_IgG

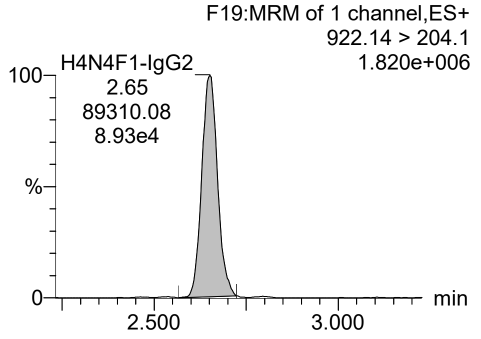

Sample Name: 20211111_A16_R1_IgG

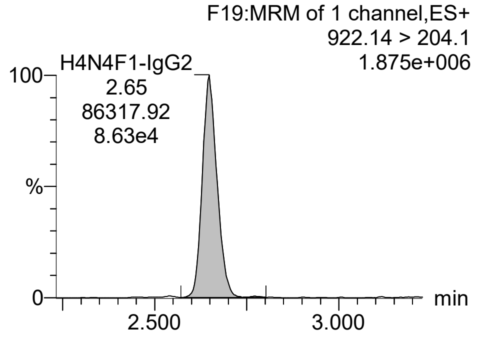

Sample Name: 20211111_L15_R1_IgG

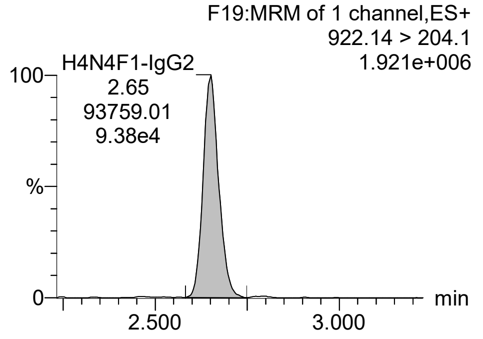

Sample Name: 20211111_L16_R1_IgG

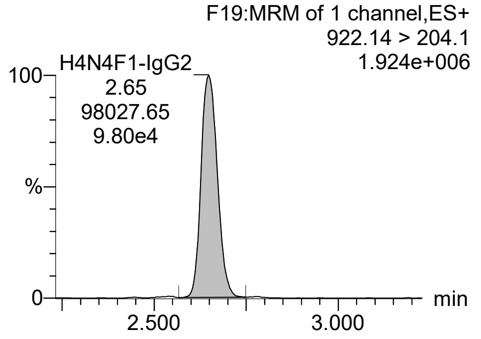

Sample Name: 20211111_H8_R1_IgG

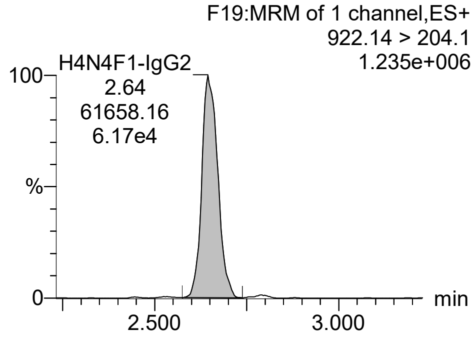

Sample Name: 20211111_A17_R1_IgG

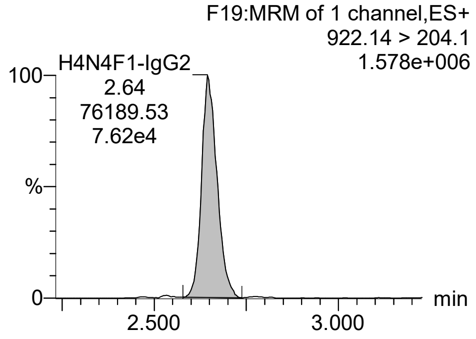

Sample Name: 20211111_A18_R1_IgG

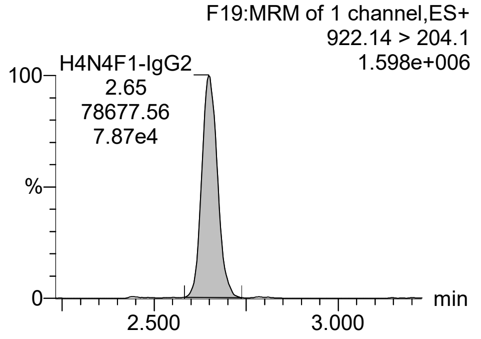

Sample Name: 20211111_L17_R1_IgG

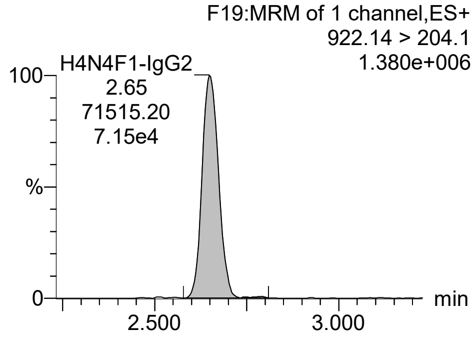

Sample Name: 20211111_L18_R1_IgG

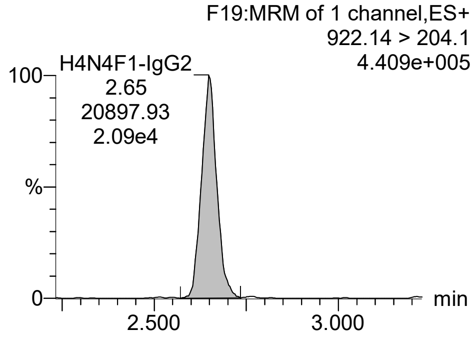

Sample Name: 20211111_H9_R1_IgG

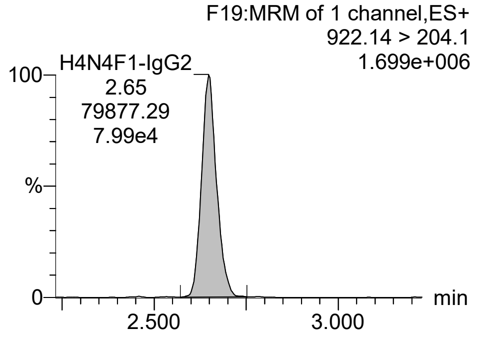

Sample Name: 20211111_A19_R1_IgG

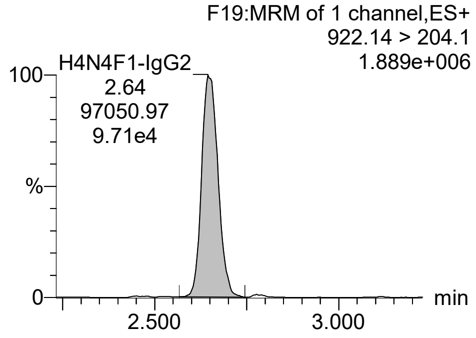

Sample Name: 20211111_A20_R1_IgG

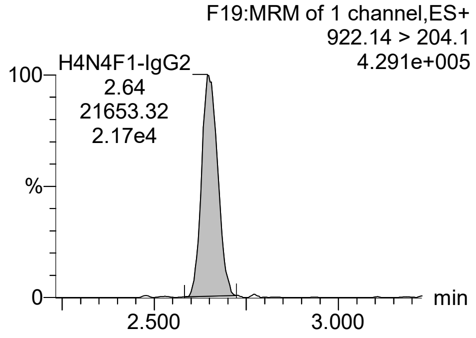

Sample Name: 20211111_L19_R1_IgG

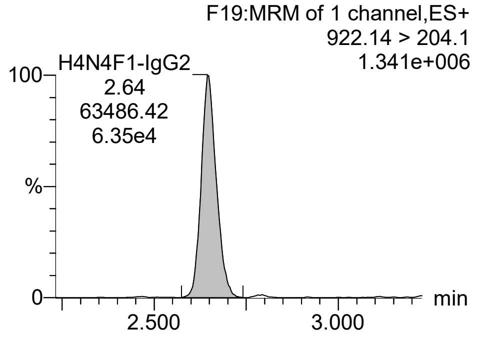

Sample Name: 20211111_QC_R5_IgG

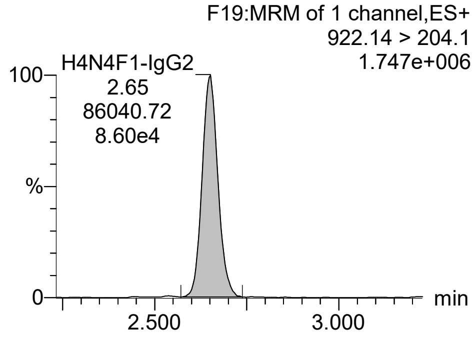

Sample Name: 20211111_L20_R1_IgG

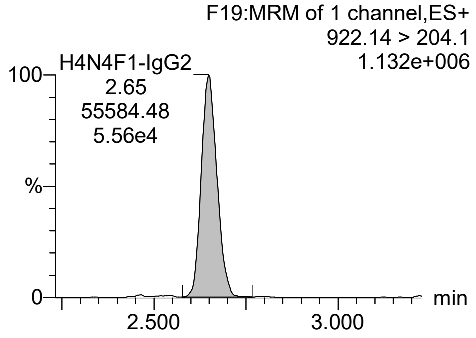

Sample Name: 20211111_H10_R1_IgG

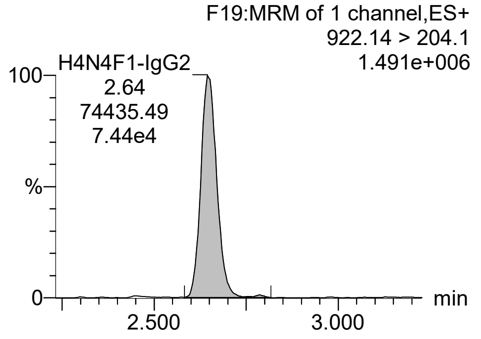

Sample Name: 20211111_A21_R1_IgG

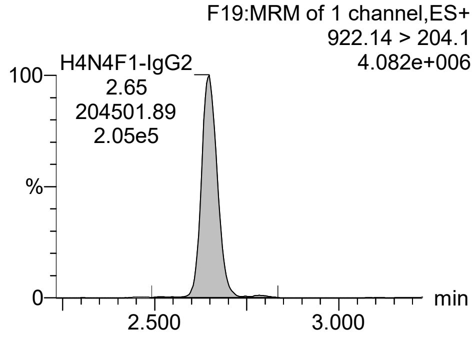

Sample Name: 20211111_A22_R1_IgG

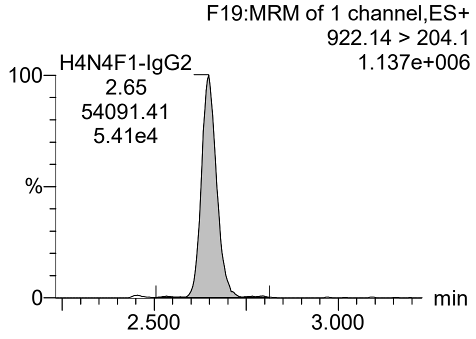

Sample Name: 20211111_L21_R1_IgG

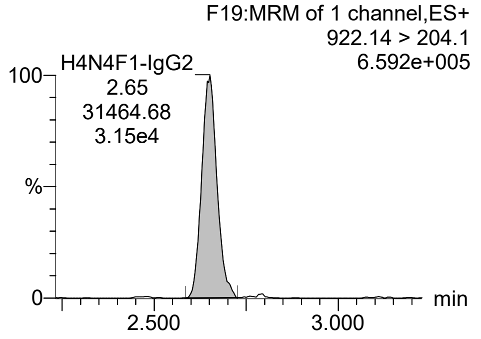

Sample Name: 20211111_L22_R1_IgG

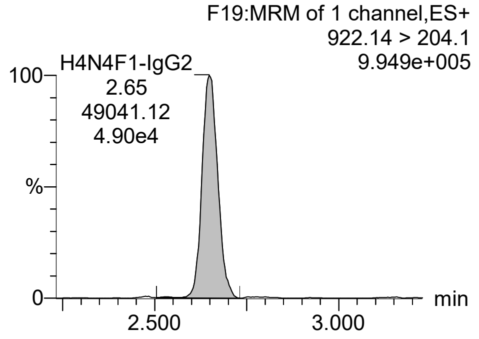

Sample Name: 20211111_H11_R1_IgG

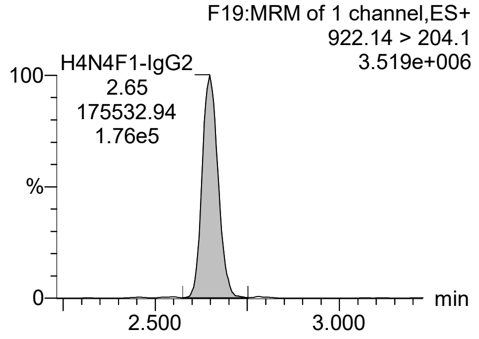

Sample Name: 20211111_A23_R1_IgG

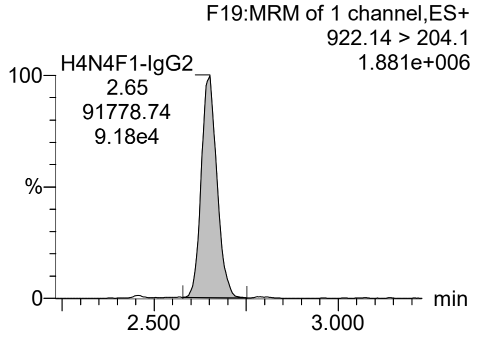

Sample Name: 20211111_A24_R1_IgG

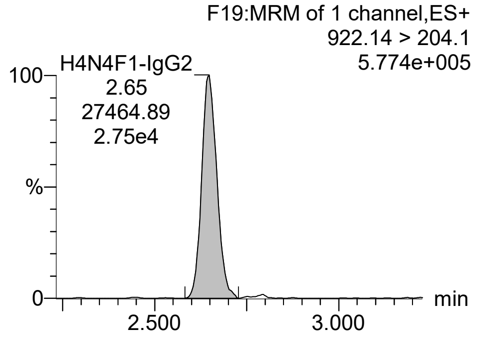

Sample Name: 20211111_L23_R1_IgG

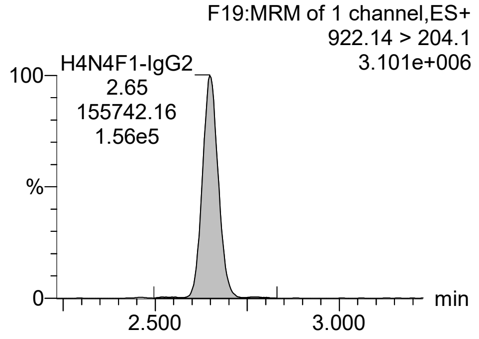

Sample Name: 20211111_L24_R1_IgG

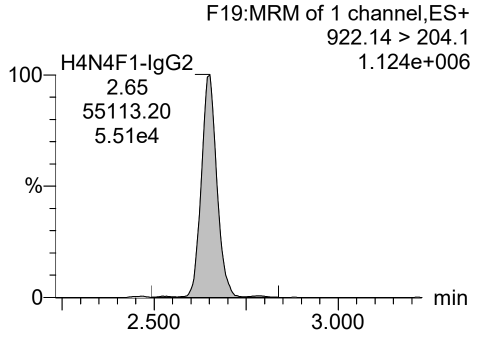

Sample Name: 20211111_H12_R1_IgG

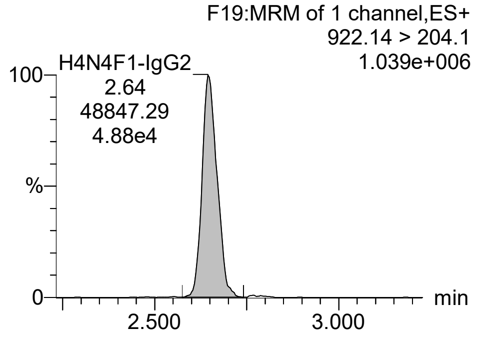

Sample Name: 20211111_QC_R6_IgG

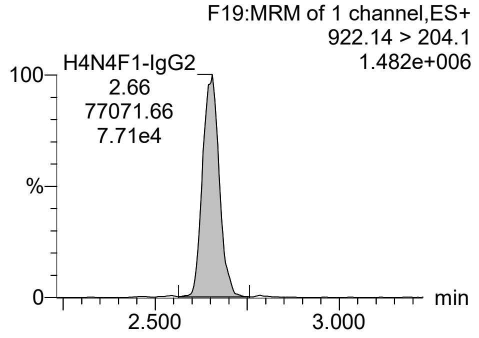

Sample Name: 20211111_A25_R1_IgG

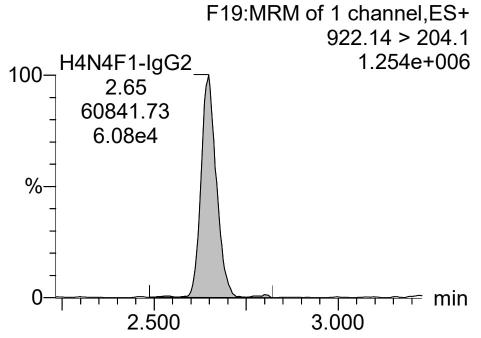

Sample Name: 20211111_A26_R1_IgG

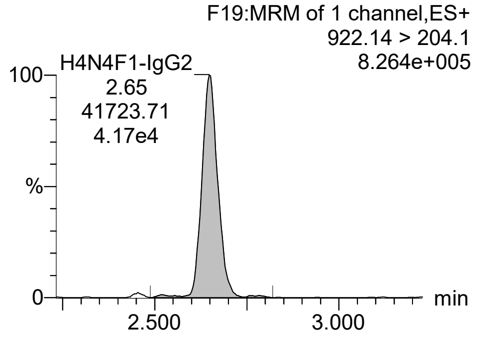

Sample Name: 20211111_L25_R1_IgG

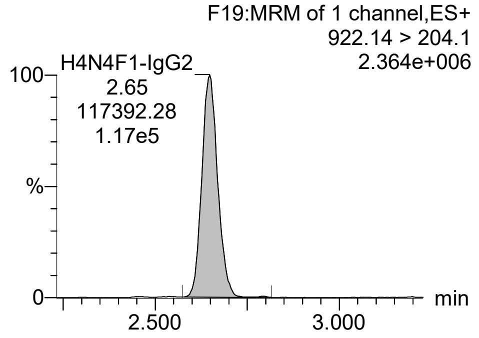

Sample Name: 20211111_L26_R1_IgG

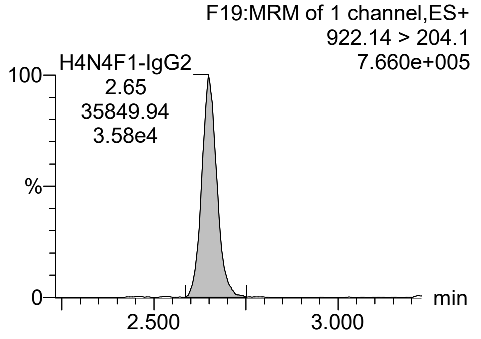

Sample Name: 20211111_H13_R1_IgG

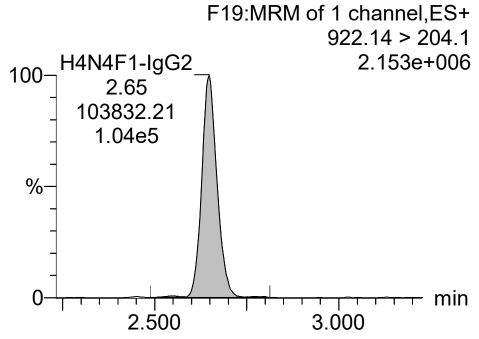

Sample Name: 20211111_A27_R1_IgG

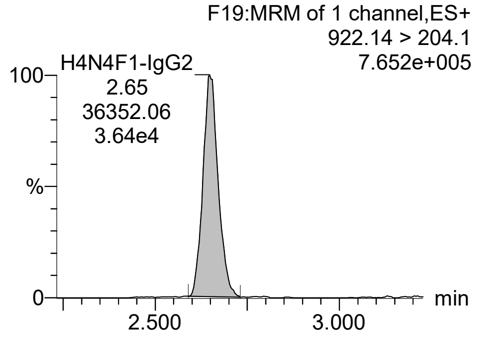

Sample Name: 20211111_A28_R1_IgG

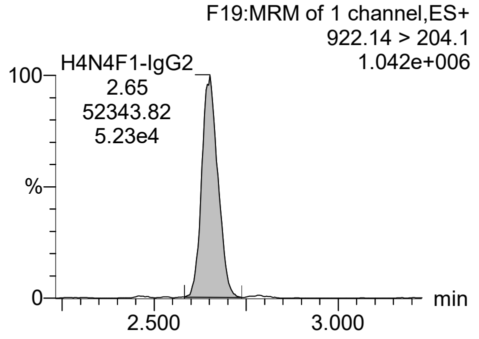

Sample Name: 20211111_L27_R1_IgG

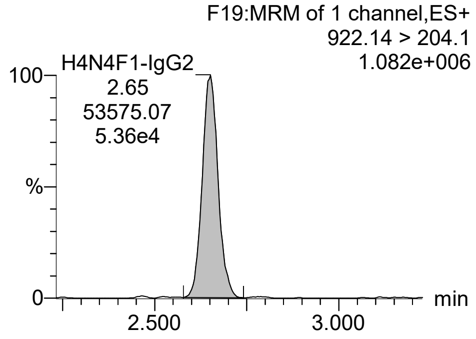

Sample Name: 20211111_L28_R1_IgG

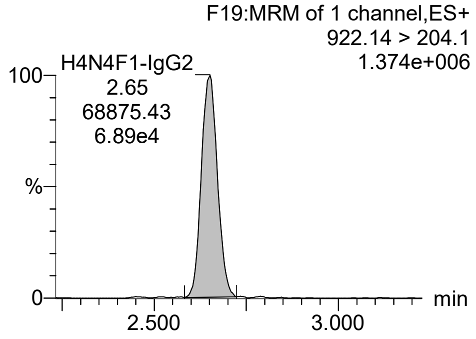

Sample Name: 20211111_H14_R1_IgG

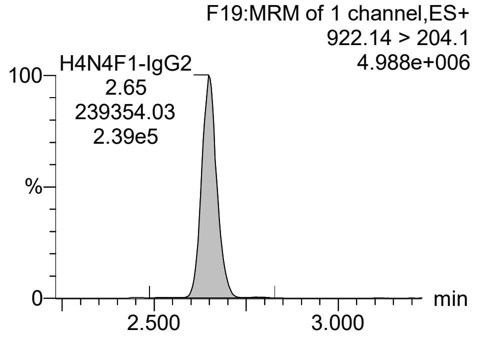

Sample Name: 20211111_A29_R1_IgG

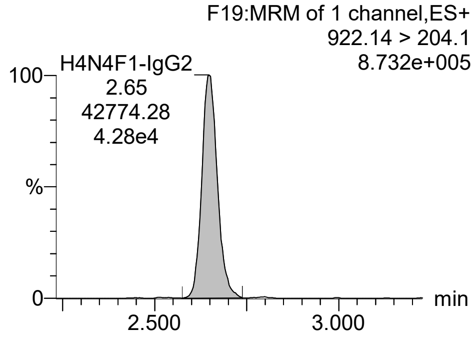

Sample Name: 20211111_A30_R1_IgG

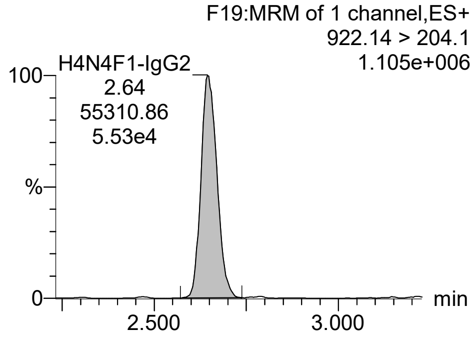

Sample Name: 20211111_QC_R7_IgG

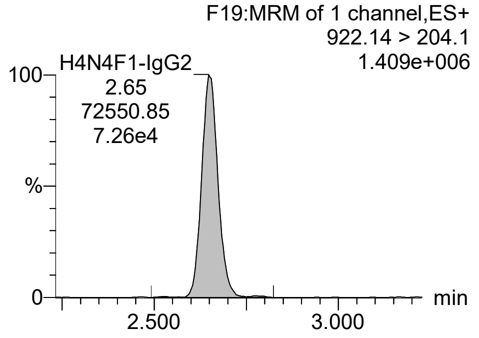

Sample Name: 20211111_L29_R1_IgG

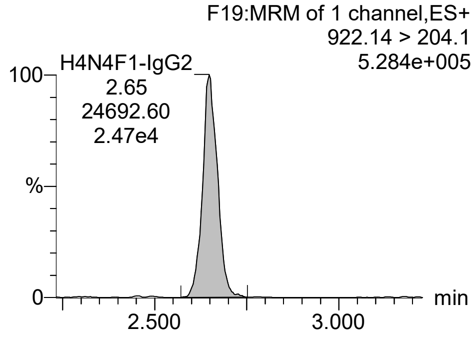

Sample Name: 20211111_L30_R1_IgG

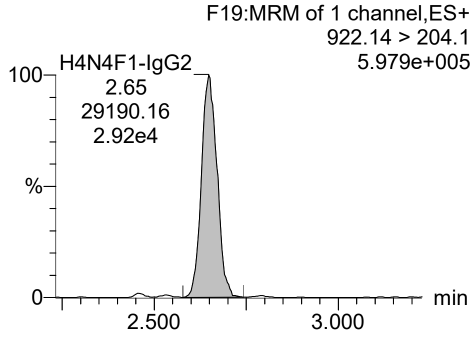

Sample Name: 20211111_H15_R1_IgG

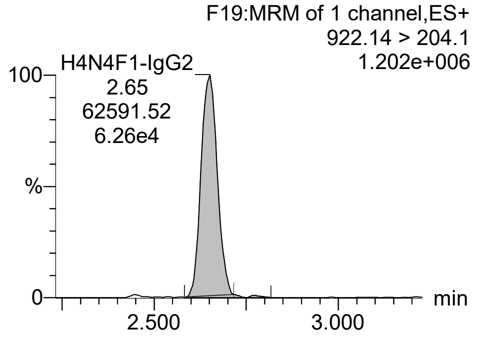

Sample Name: 20211111_A31_R1_IgG

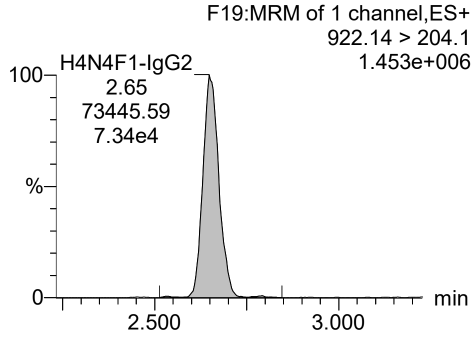

Sample Name: 20211111_A32_R1_IgG

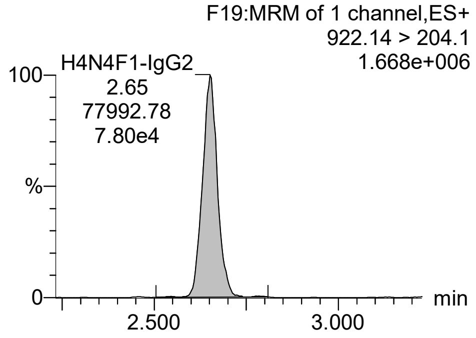

Sample Name: 20211111_L31_R1_IgG

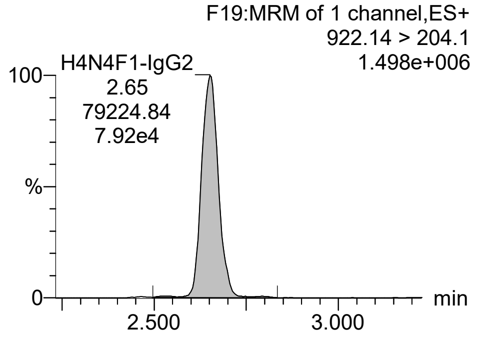

Sample Name: 20211111_L32_R1_IgG

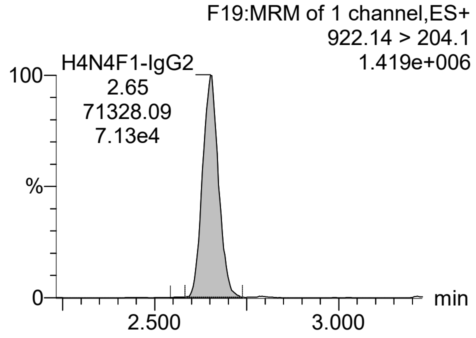

Sample Name: 20211111_H16_R1_IgG

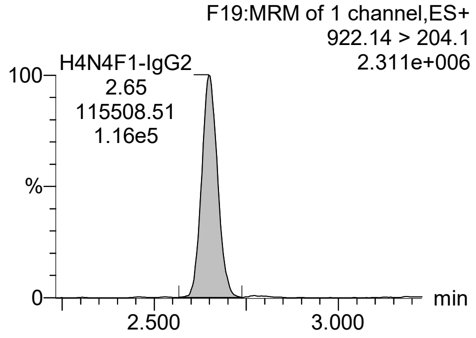

Sample Name: 20211111_A33_R1_IgG

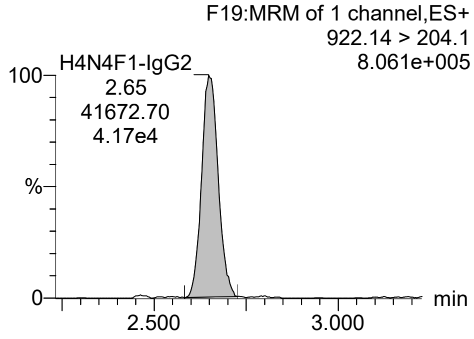

Sample Name: 20211111_A34_R1_IgG

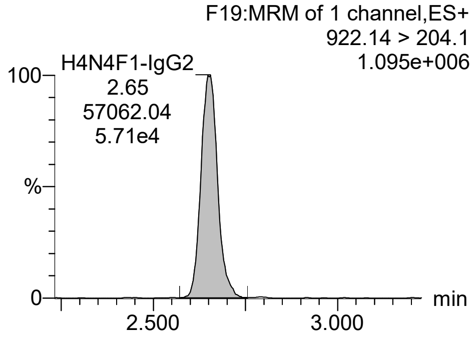

Sample Name: 20211111_H17_R1_IgG

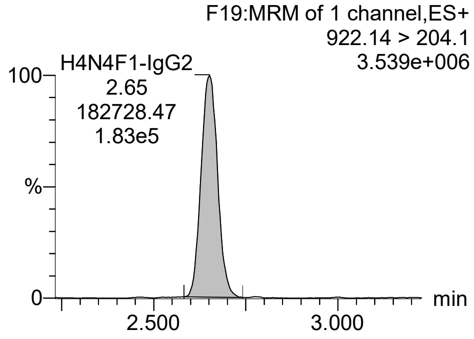

Sample Name: 20211111_A35_R1_IgG

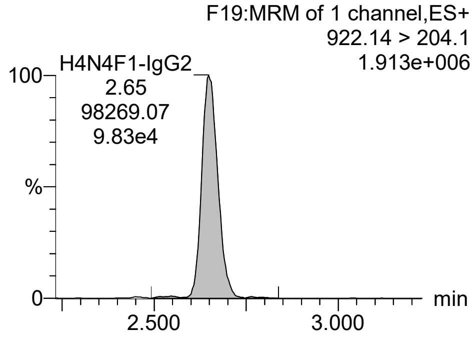

Sample Name: 20211111_QC_R8_IgG

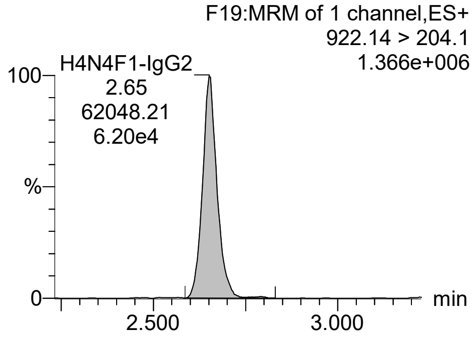

Sample Name: 20211111_A36_R1_IgG

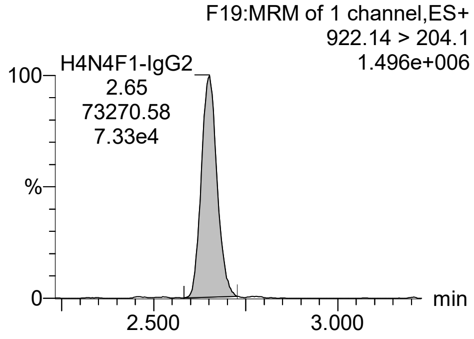

Sample Name: 20211111_H18_R1_IgG

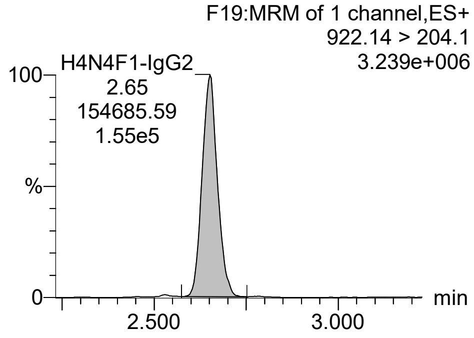

Sample Name: 20211111_A37_R1_IgG

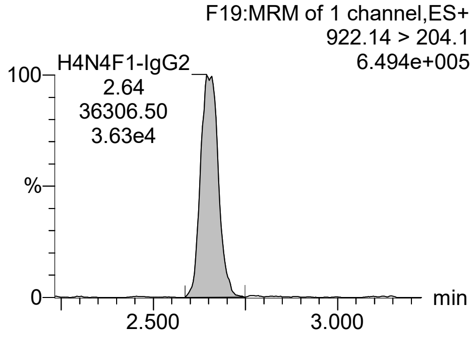

Sample Name: 20211111_A38_R1_IgG

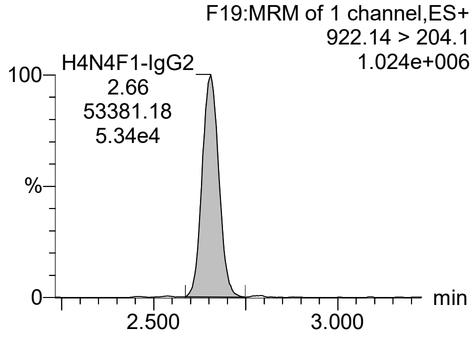

Sample Name: 20211111_H19_R1_IgG

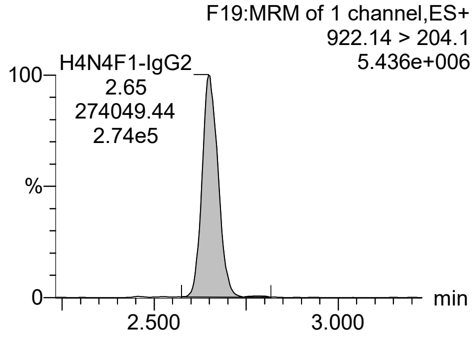

Sample Name: 20211111_A39_R1_IgG

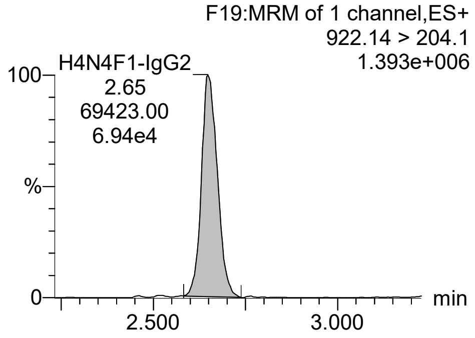

Sample Name: 20211111_A40_R1_IgG

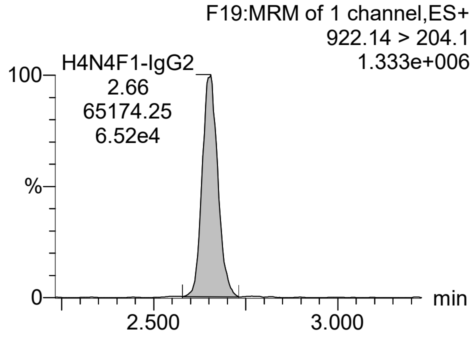

Sample Name: 20211111_H20_R1_IgG

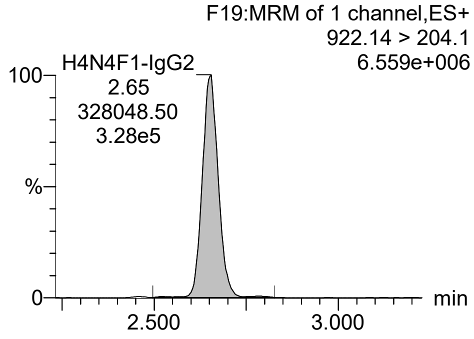

Sample Name: 20211111_A41_R1_IgG

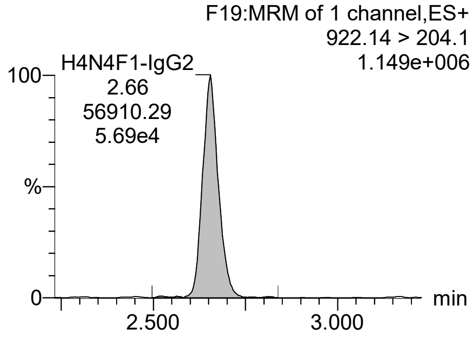

Sample Name: 20211111_A42_R1_IgG

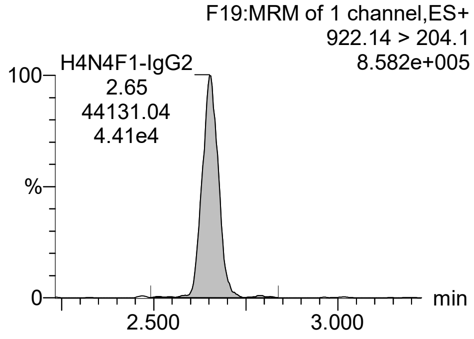

Dataset: C:\MassLynx\10.pro\20211115_clinicalsamples_IgG.qld

Last Altered: Sunday, October 02, 2022 16:02:30 台北標準時間
Printed: Friday, August 01, 2025 16:50:17 台北標準時間

Sample Name: 20211111_A43_R1_IgG

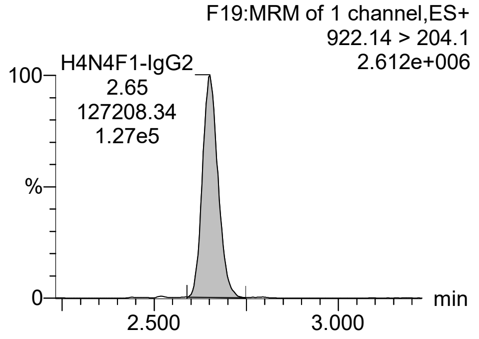

Sample Name: 20211111_A44_R1_IgG

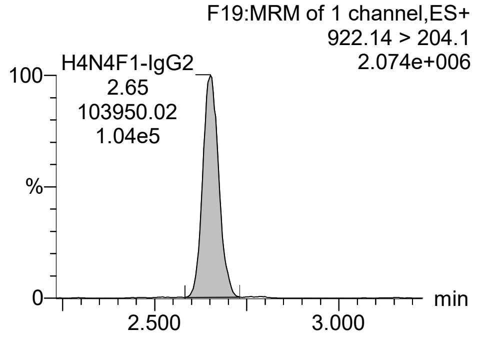

Sample Name: 20211111_QC_R9_IgG

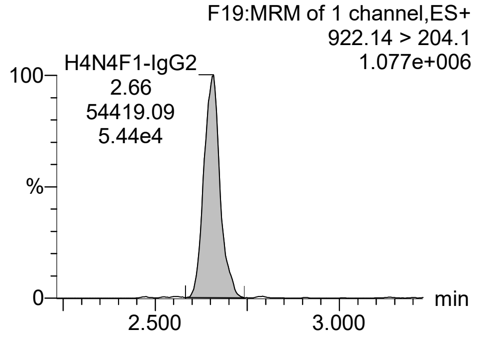

Sample Name: 20211111_A1_R2_IgG

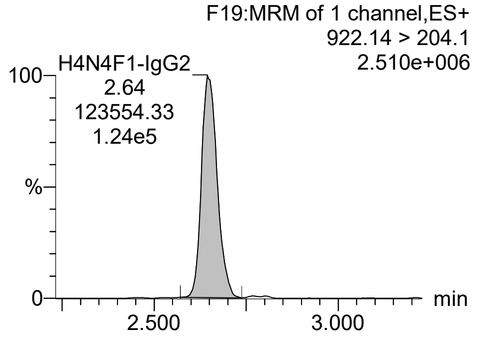

Sample Name: 20211111_A2_R2_IgG

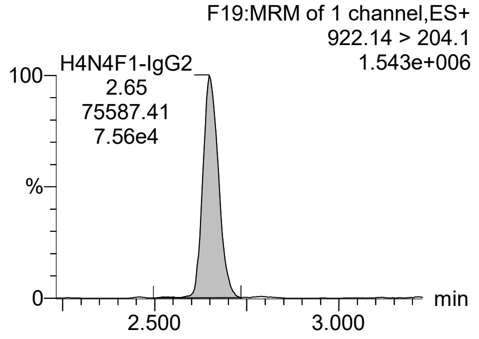

Sample Name: 20211111_L1_R2_IgG

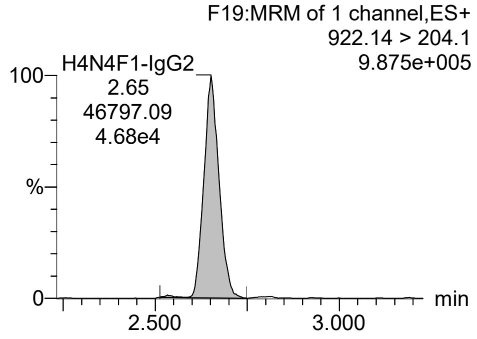

Sample Name: 20211111_L2_R2_IgG

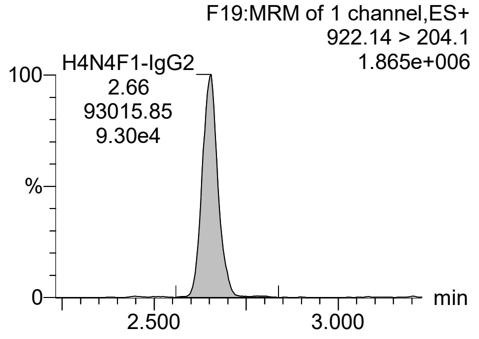

Sample Name: 20211111_H1_R2_IgG

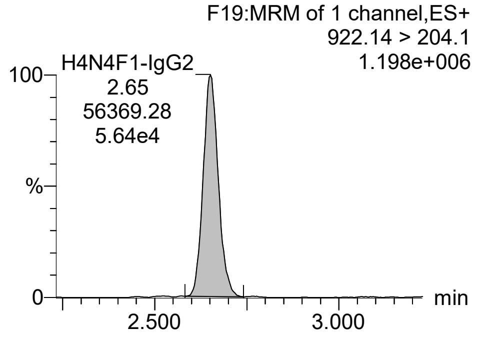

Sample Name: 20211111_A3_R2_IgG

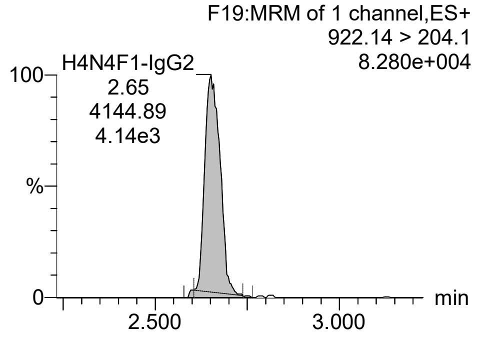

Sample Name: 20211111_A4_R2_IgG

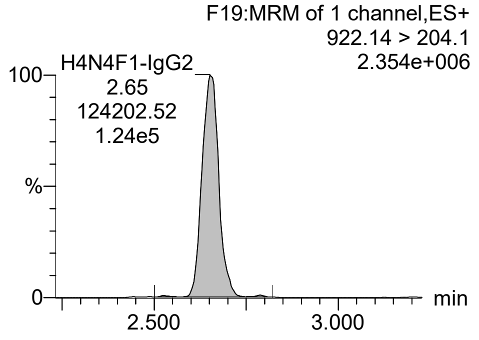

Sample Name: 20211111_L3_R2_IgG

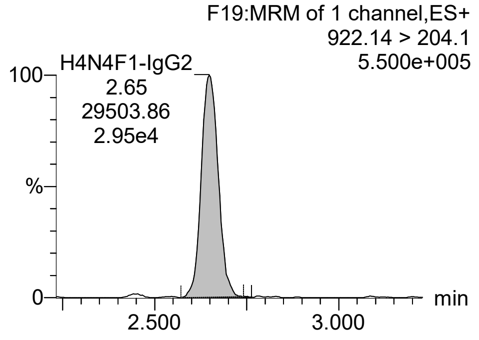

Sample Name: 20211111_L4_R2_IgG

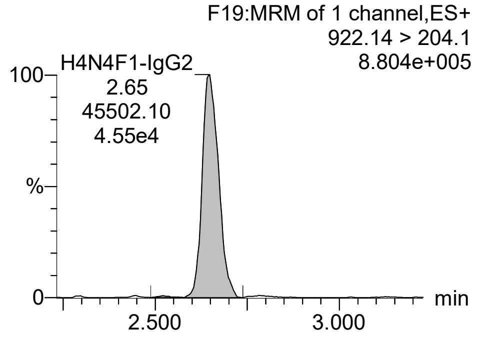

Sample Name: 20211111_H2_R2_IgG

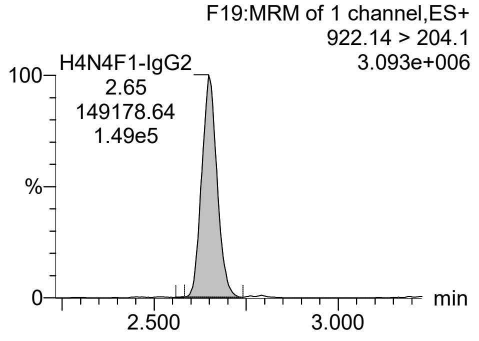

Sample Name: 20211111_A5_R2_IgG

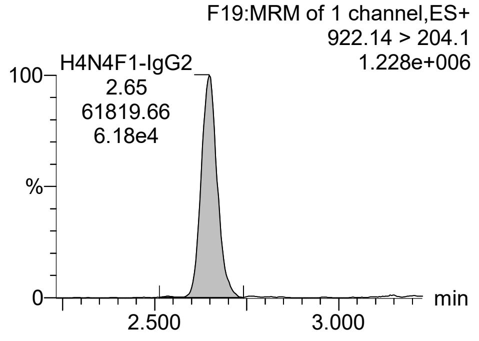

Sample Name: 20211111_A6_R2_IgG

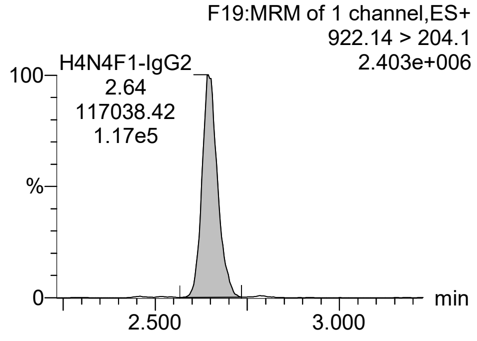

Dataset: C:\MassLynx\10.pro\20211115_clinicalsamples_IgG.qld

Last Altered: Sunday, October 02, 2022 16:02:30 台北標準時間
Printed: Friday, August 01, 2025 16:50:17 台北標準時間

Sample Name: 20211111_QC_R10_IgG
F19:MRM of 1 channel,ES+
922.14 > 204.1
1.154e+006
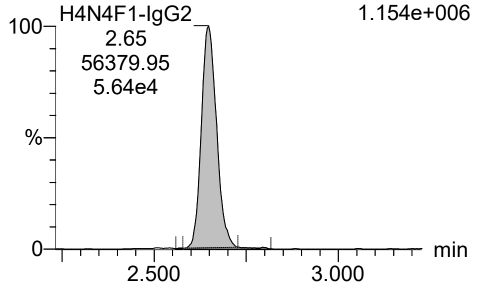

Sample Name: 20211111_L5_R2_IgG
F19:MRM of 1 channel,ES+
922.14 > 204.1
1.095e+006
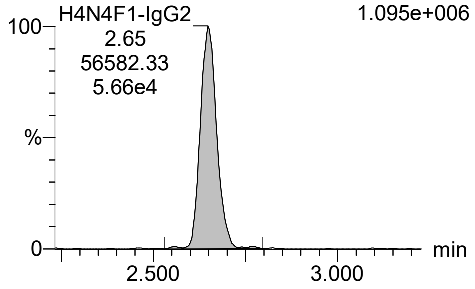

Sample Name: 20211111_L6_R2_IgG
F19:MRM of 1 channel,ES+
922.14 > 204.1
4.077e+005
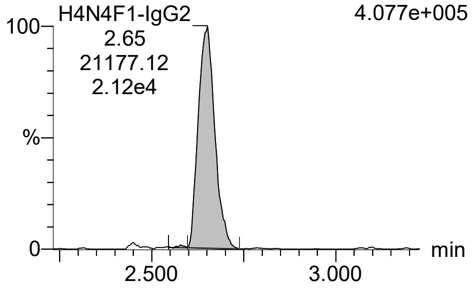

Sample Name: 20211111_H3_R2_IgG
F19:MRM of 1 channel,ES+
922.14 > 204.1
1.756e+006
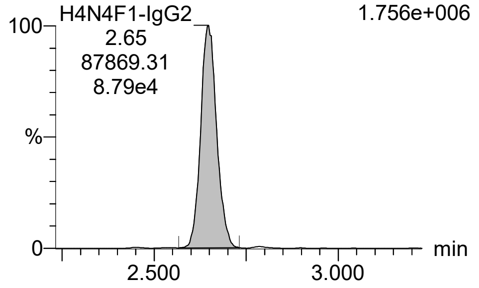

Sample Name: 20211111_A7_R2_IgG
F19:MRM of 1 channel,ES+
922.14 > 204.1
2.801e+006
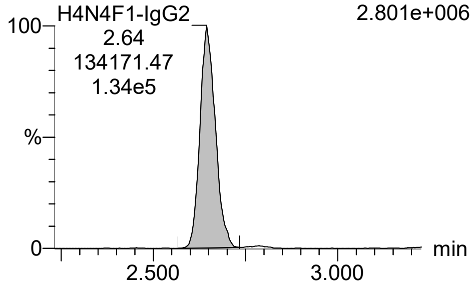

Sample Name: 20211111_A8_R2_IgG
F19:MRM of 1 channel,ES+
922.14 > 204.1
1.618e+006
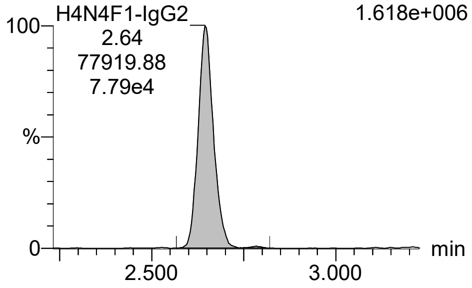

Sample Name: 20211111_L7_R2_IgG
F19:MRM of 1 channel,ES+
922.14 > 204.1
1.259e+006
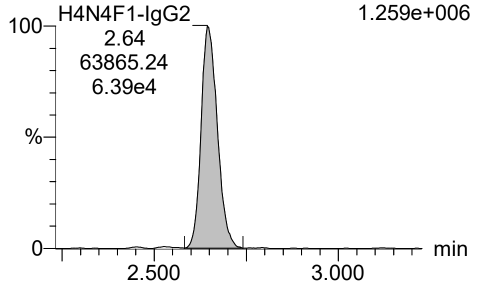

Sample Name: 20211111_L8_R2_IgG
F19:MRM of 1 channel,ES+
922.14 > 204.1
1.119e+006
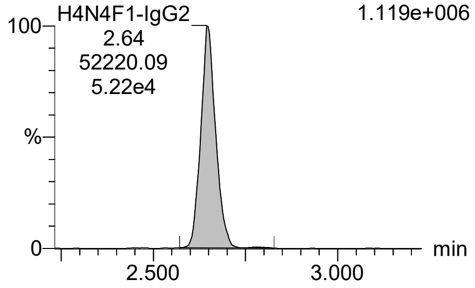

Sample Name: 20211111_H4_R2_IgG
F19:MRM of 1 channel,ES+
922.14 > 204.1
9.430e+005
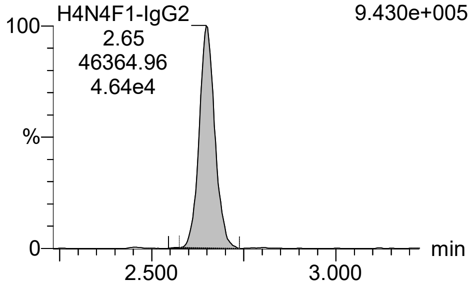

Sample Name: 20211111_A9_R2_IgG
F19:MRM of 1 channel,ES+
922.14 > 204.1
1.131e+006
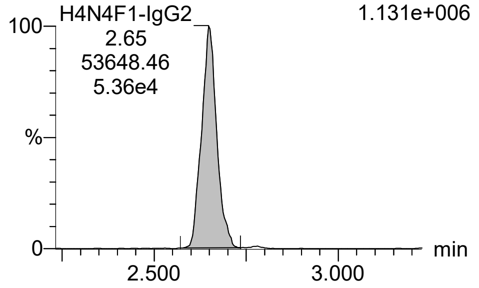

Sample Name: 20211111_A10_R2_IgG
F19:MRM of 1 channel,ES+
922.14 > 204.1
1.380e+006
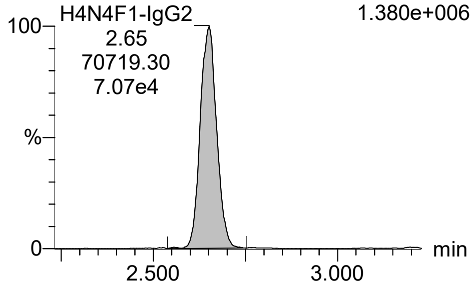

Sample Name: 20211111_L9_R2_IgG
F19:MRM of 1 channel,ES+
922.14 > 204.1
1.620e+006
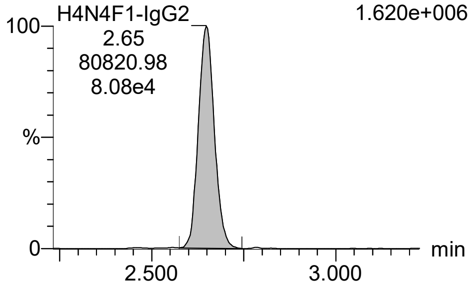

Sample Name: 20211111_L10_R2_IgG
F19:MRM of 1 channel,ES+
922.14 > 204.1
6.202e+005
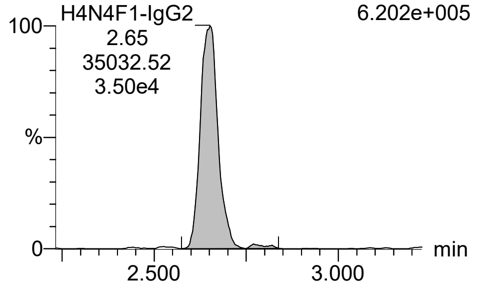

Sample Name: 20211111_QC_R11_IgG
F19:MRM of 1 channel,ES+
922.14 > 204.1
1.052e+006
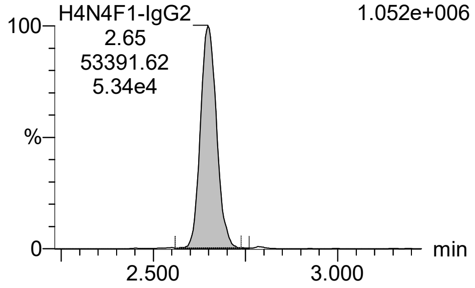

Sample Name: 20211111_H5_R2_IgG
F19:MRM of 1 channel,ES+
922.14 > 204.1
8.937e+005
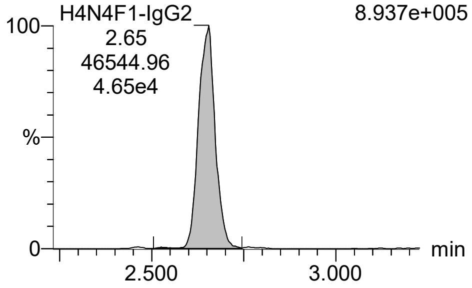

Dataset: C:\MassLynx\10.pro\20211115_clinicalsamples_IgG.qld

Last Altered: Sunday, October 02, 2022 16:02:30 台北標準時間
Printed: Friday, August 01, 2025 16:50:17 台北標準時間

Sample Name: 20211111_A11_R2_IgG

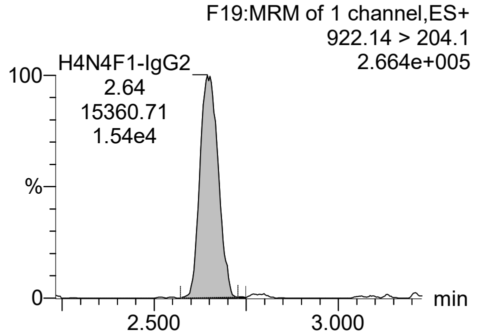

Sample Name: 20211111_A12_R2_IgG

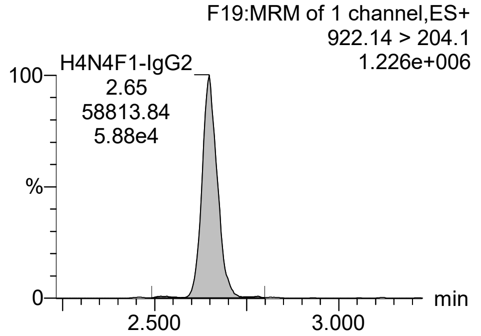

Sample Name: 20211111_L11_R2_IgG

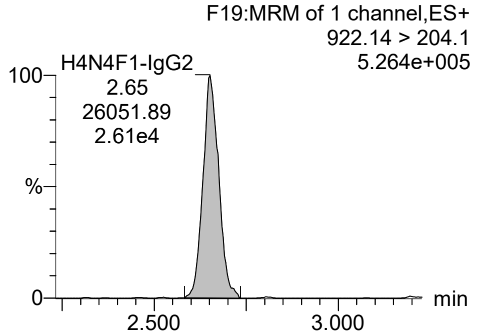

Sample Name: 20211111_L12_R2_IgG

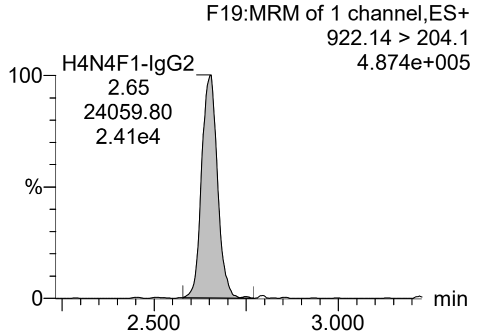

Sample Name: 20211111_H6_R2_IgG

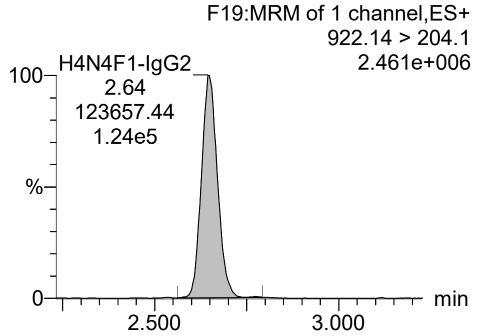

Sample Name: 20211111_A13_R2_IgG

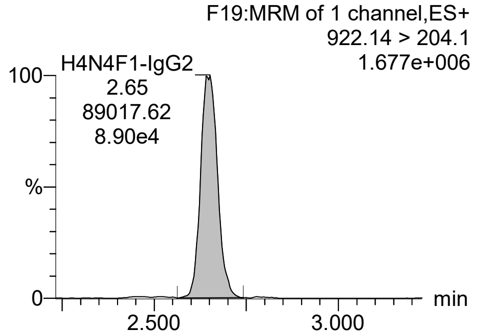

Sample Name: 20211111_A14_R2_IgG

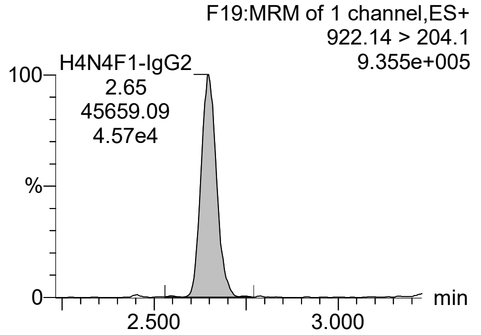

Sample Name: 20211111_L13_R2_IgG

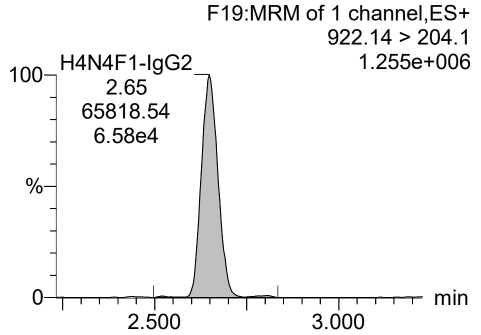

Sample Name: 20211111_L14_R2_IgG

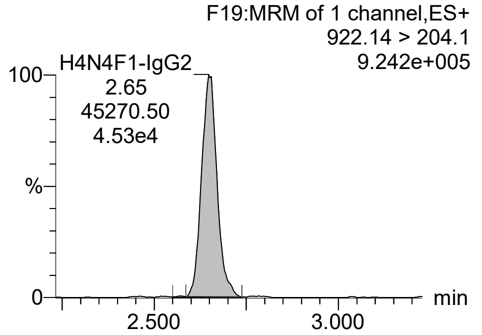

Sample Name: 20211111_H7_R2_IgG

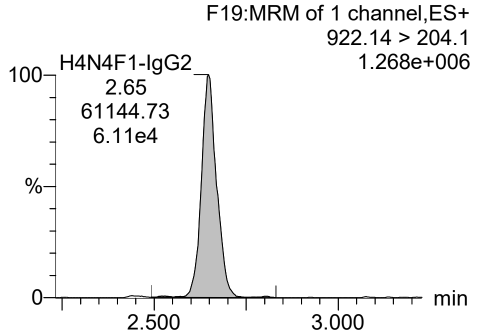

Sample Name: 20211111_A15_R2_IgG

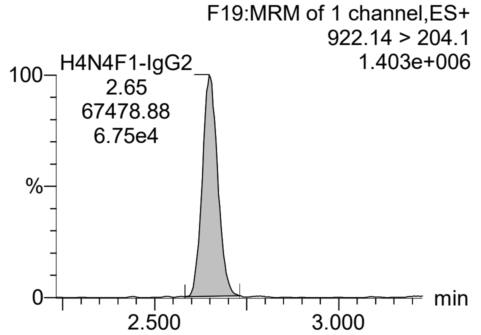

Sample Name: 20211111_QC_R12_IgG

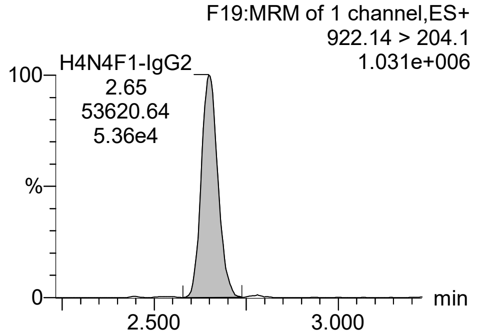

Sample Name: 20211111_A16_R2_IgG

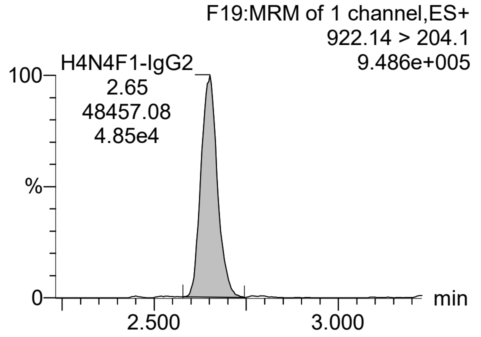

Sample Name: 20211111_L15_R2_IgG

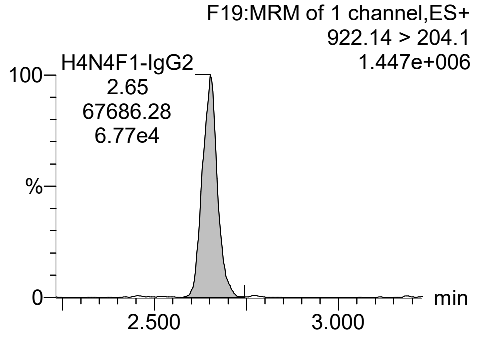

Sample Name: 20211111_L16_R2_IgG

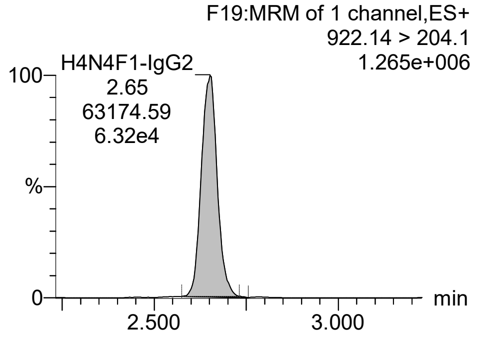

Sample Name: 20211111_H8_R2_IgG

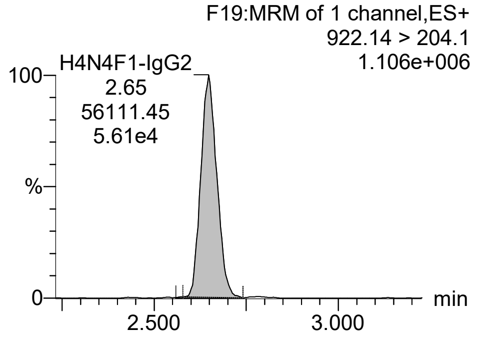

Sample Name: 20211111_A17_R2_IgG

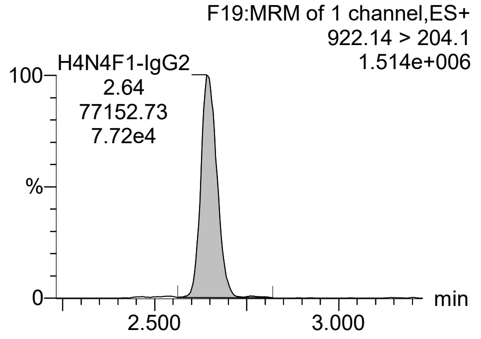

Sample Name: 20211111_A18_R2_IgG

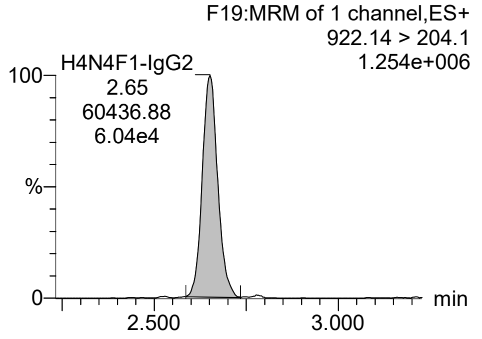

Sample Name: 20211111_L17_R2_IgG

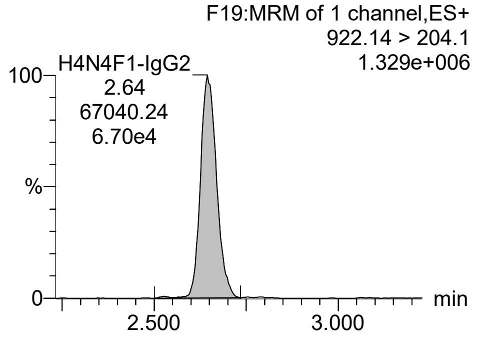

Sample Name: 20211111_L18_R2_IgG

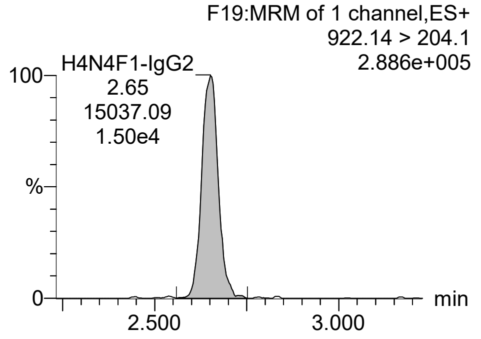

Sample Name: 20211111_H9_R2_IgG

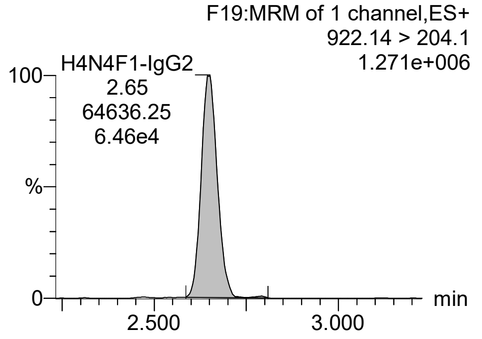

Sample Name: 20211111_A19_R2_IgG

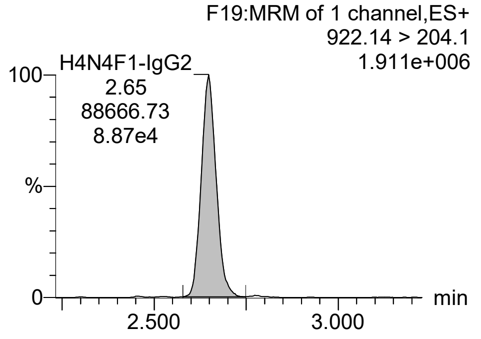

Sample Name: 20211111_A20_R2_IgG

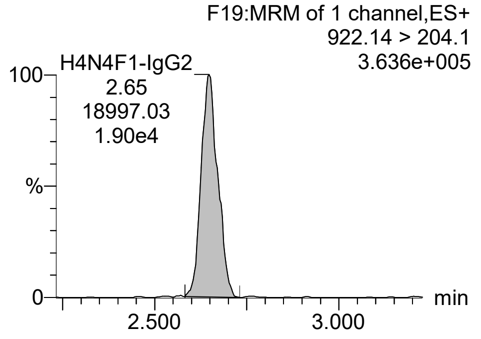

Sample Name: 20211111_L19_R2_IgG

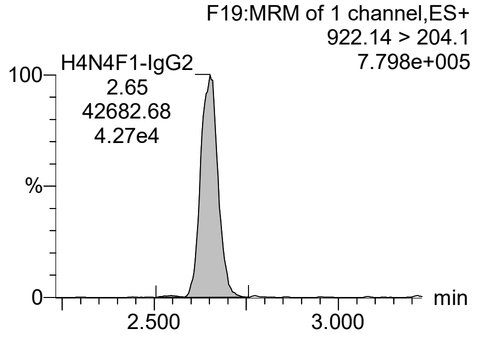

Sample Name: 20211111_QC_R13_IgG

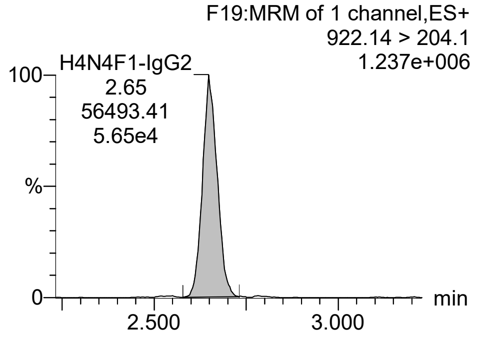

Sample Name: 20211111_L20_R2_IgG

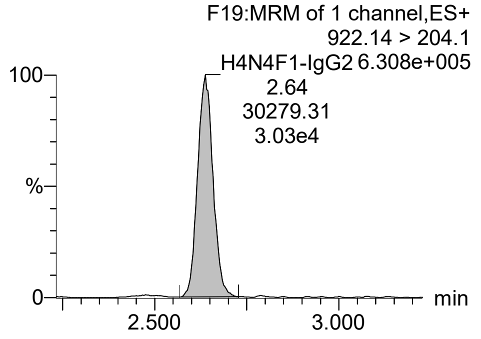

Sample Name: 20211111_H10_R2_IgG

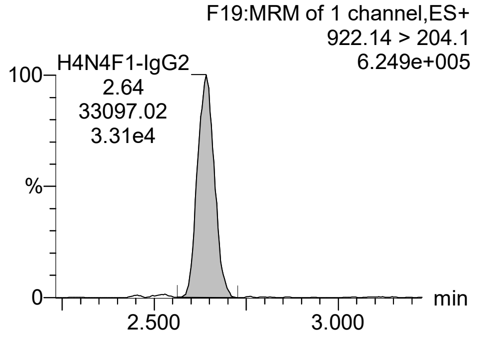

Sample Name: 20211111_A21_R2_IgG

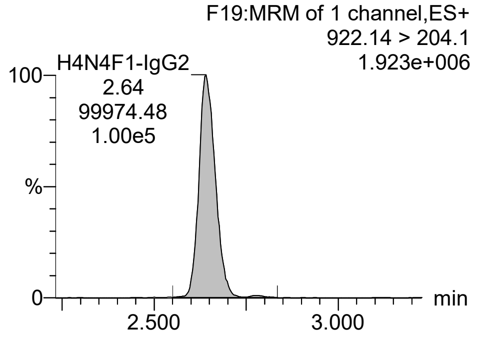

Sample Name: 20211111_A22_R2_IgG

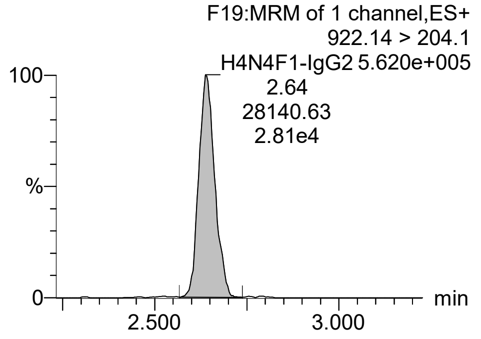

Sample Name: 20211111_L21_R2_IgG

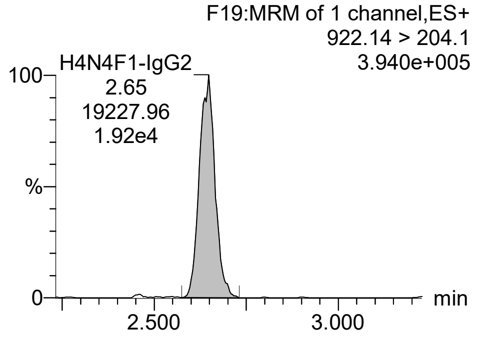

Sample Name: 20211111_L22_R2_IgG

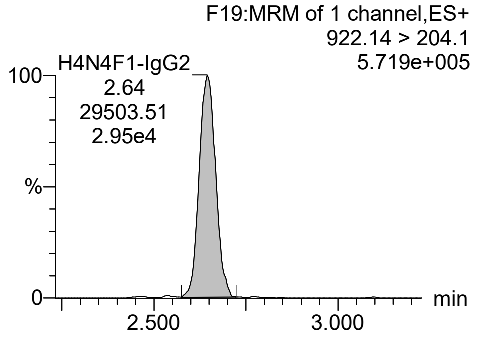

Sample Name: 20211111_H11_R2_IgG

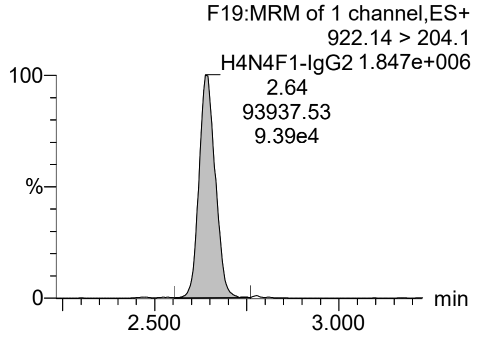

Sample Name: 20211111_A23_R2_IgG

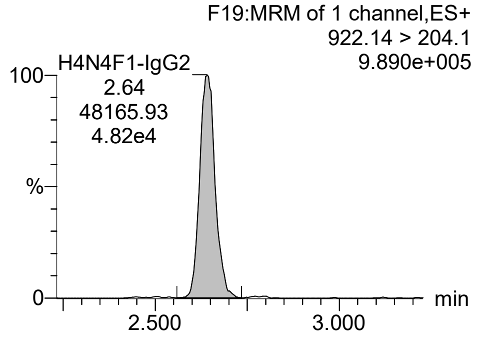

Sample Name: 20211111_A24_R2_IgG

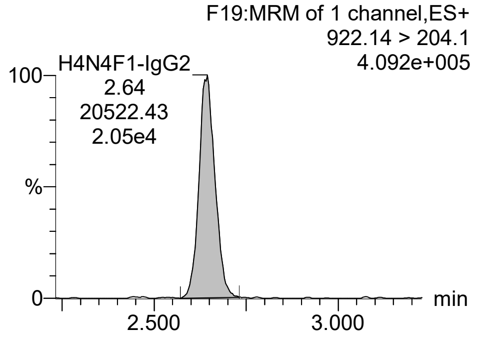

Sample Name: 20211111_L23_R2_IgG

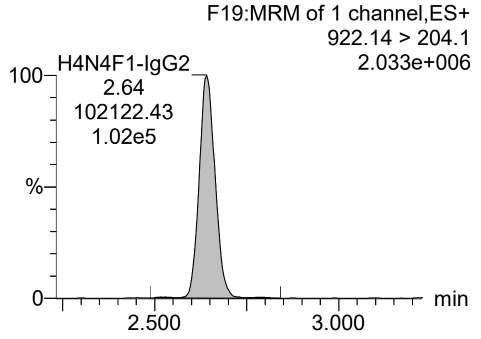

Sample Name: 20211111_L24_R2_IgG

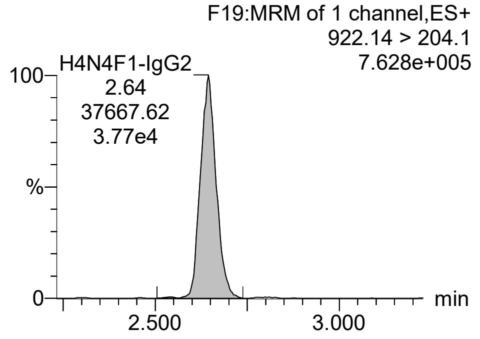

Sample Name: 20211111_H12_R2_IgG

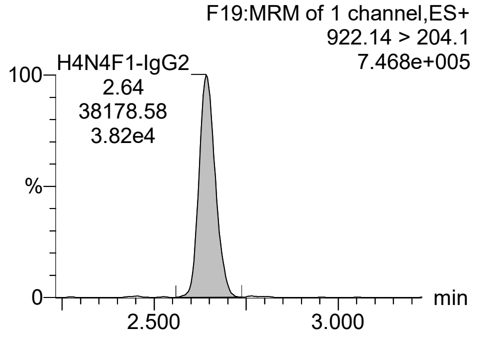

Sample Name: 20211111_QC_R14_IgG

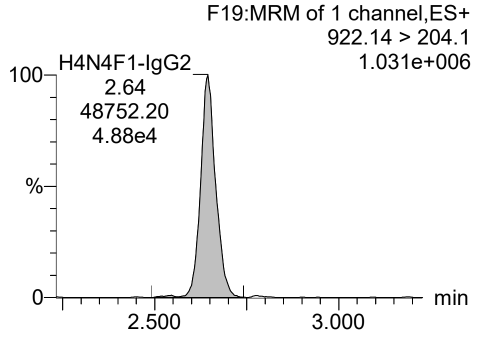

Sample Name: 20211111_A25_R2_IgG

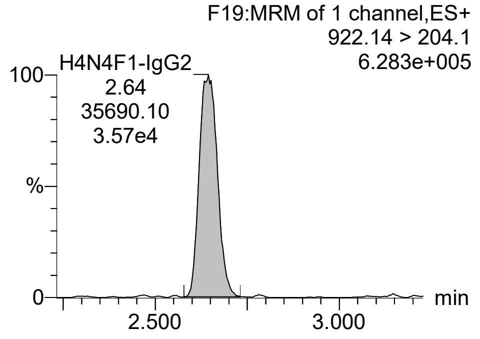

Sample Name: 20211111_A26_R2_IgG

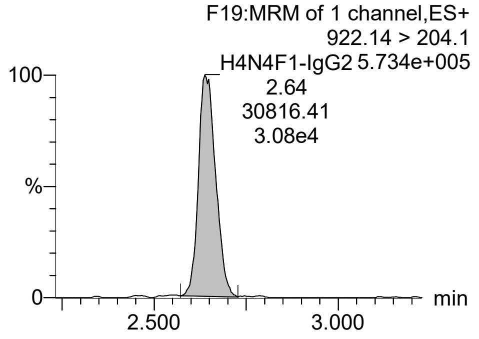

Sample Name: 20211111_L25_R2_IgG

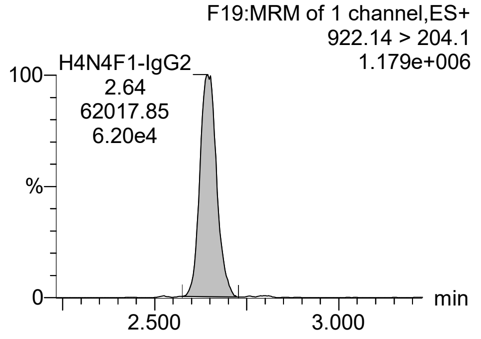

Sample Name: 20211111_L26_R2_IgG

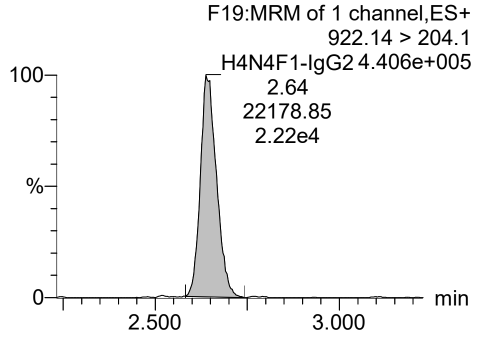

Sample Name: 20211111_H13_R2_IgG

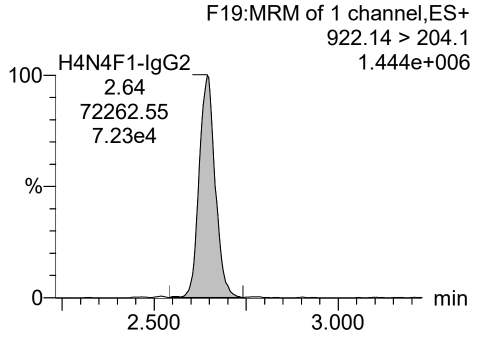

Sample Name: 20211111_A27_R2_IgG

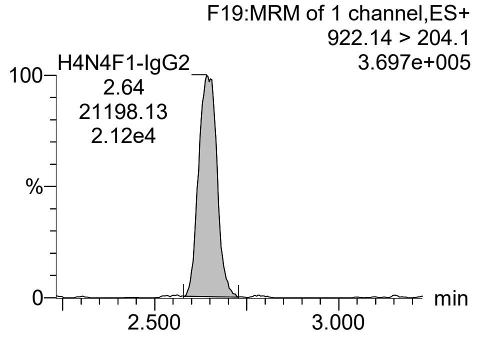

Sample Name: 20211111_A28_R2_IgG

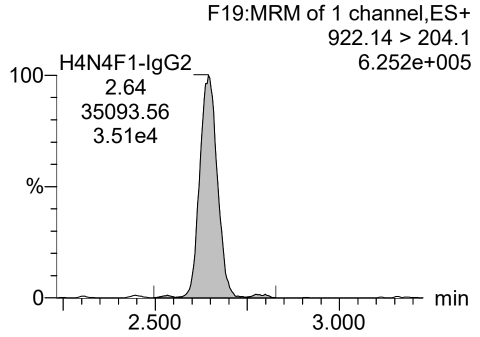

Dataset: C:\MassLynx\10.pro\20211115_clinicalsamples_IgG.qld

Last Altered: Sunday, October 02, 2022 16:02:30 台北標準時間
Printed: Friday, August 01, 2025 16:50:17 台北標準時間

Sample Name: 20211111_L27_R2_IgG

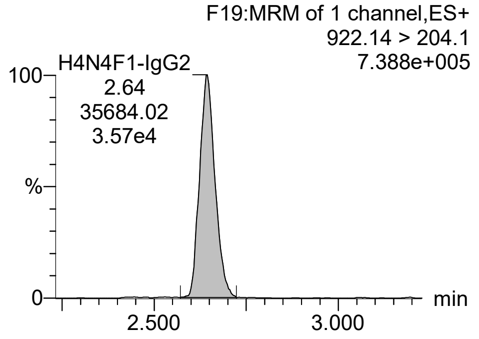

Sample Name: 20211111_L28_R2_IgG

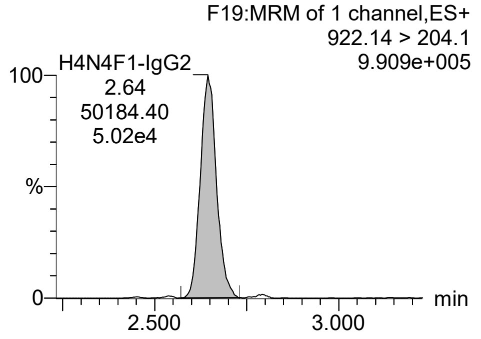

Sample Name: 20211111_H14_R2_IgG

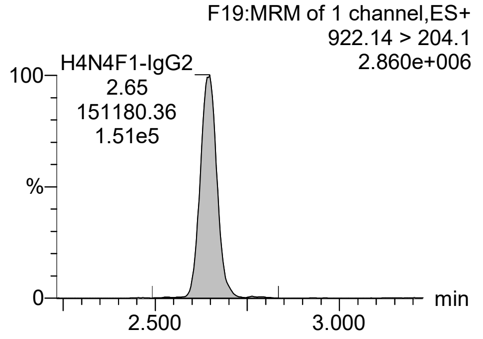

Sample Name: 20211111_A29_R2_IgG

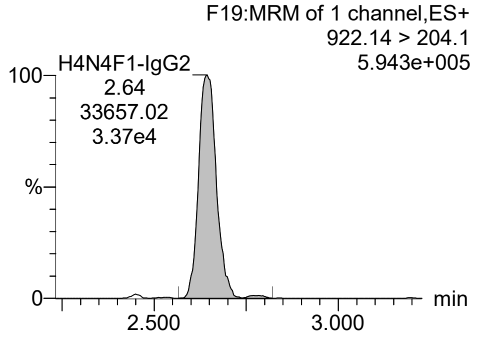

Sample Name: 20211111_A30_R2_IgG

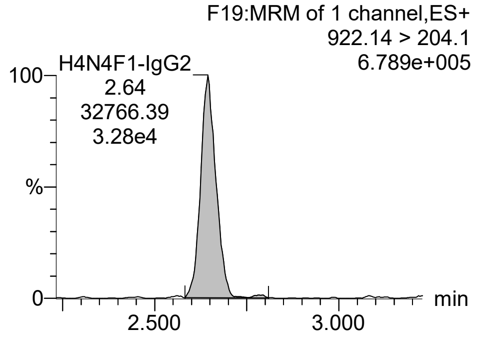

Sample Name: 20211111_QC_R15_IgG

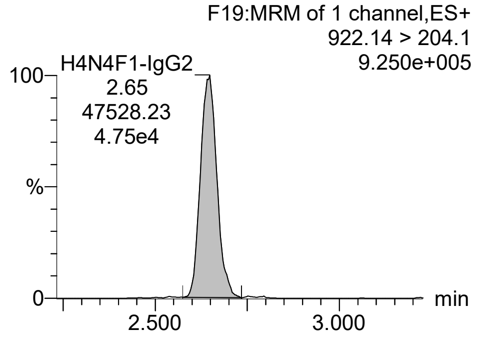

Sample Name: 20211111_L29_R2_IgG

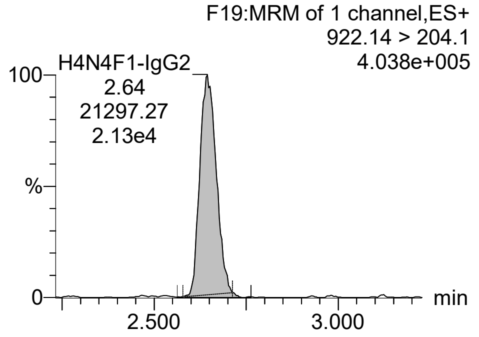

Sample Name: 20211111_L30_R2_IgG

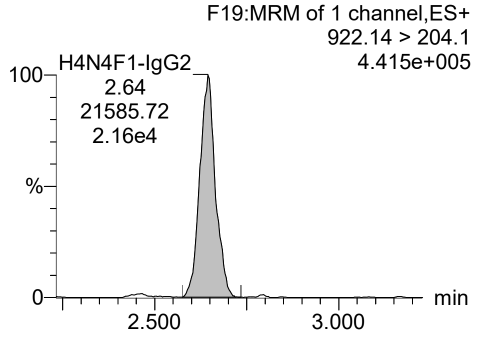

Sample Name: 20211111_H15_R2_IgG

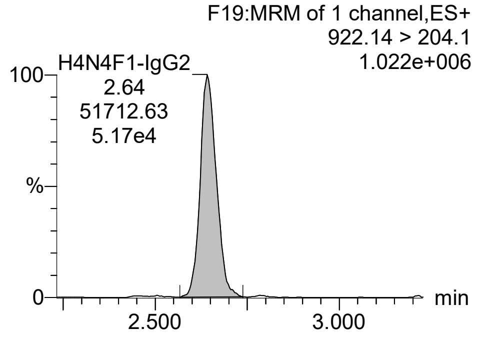

Sample Name: 20211111_A31_R2_IgG

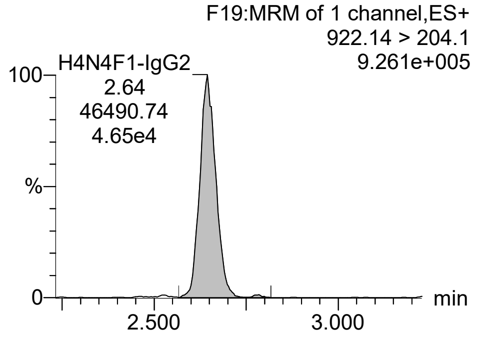

Sample Name: 20211111_A32_R2_IgG

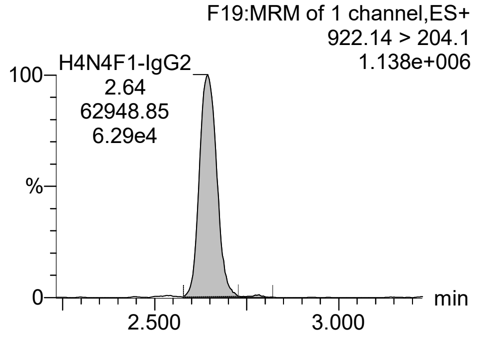

Sample Name: 20211111_L31_R2_IgG

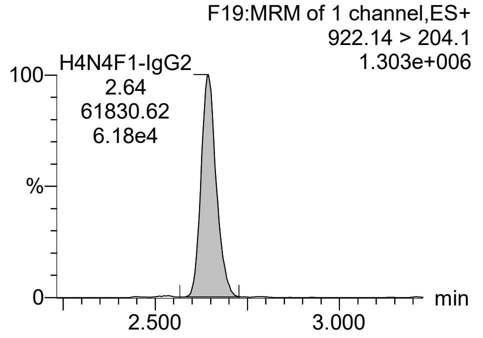

Sample Name: 20211111_L32_R2_IgG

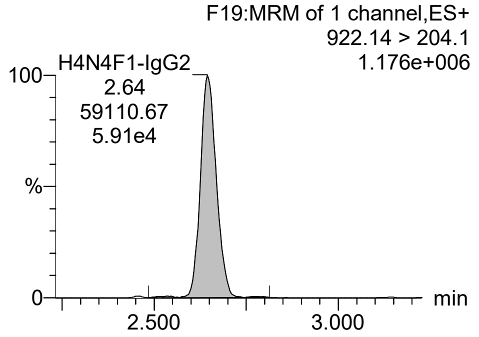

Sample Name: 20211111_H16_R2_IgG

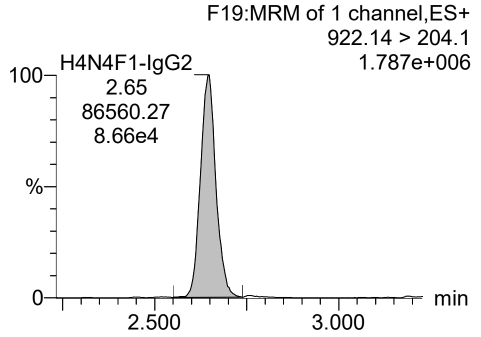

Sample Name: 20211111_A33_R2_IgG

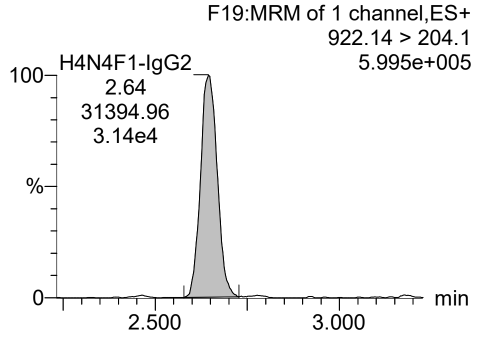

Sample Name: 20211111_A34_R2_IgG

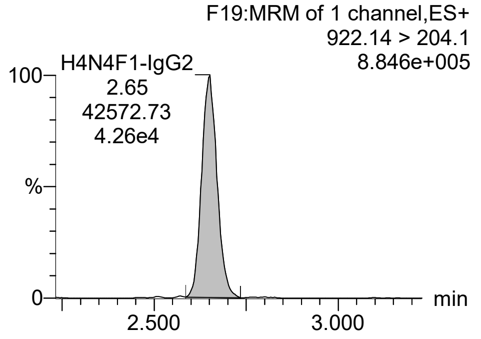

Sample Name: 20211111_H17_R2_IgG

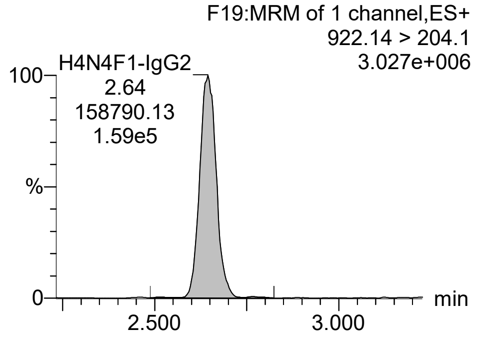

Sample Name: 20211111_A35_R2_IgG

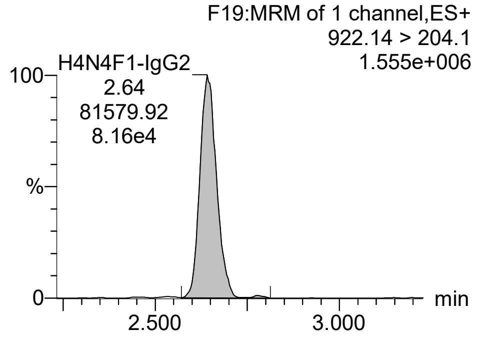

Sample Name: 20211111_QC_R16_IgG

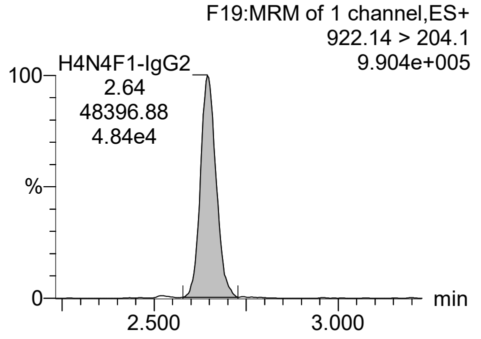

Sample Name: 20211111_A36_R2_IgG

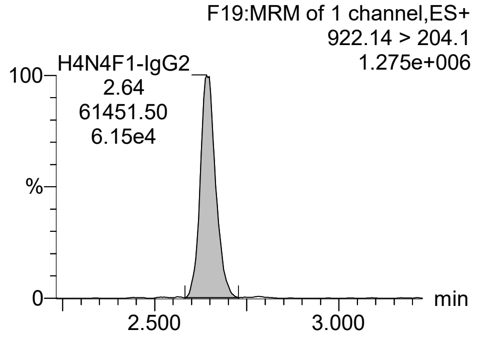

Sample Name: 20211111_H18_R2_IgG

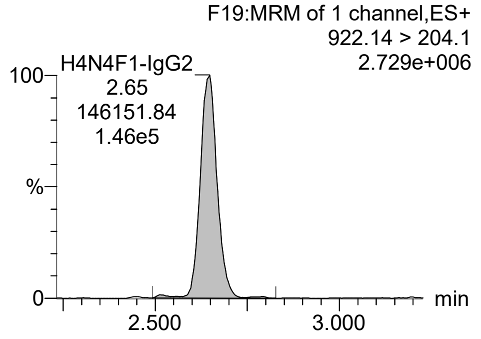

Sample Name: 20211111_A37_R2_IgG

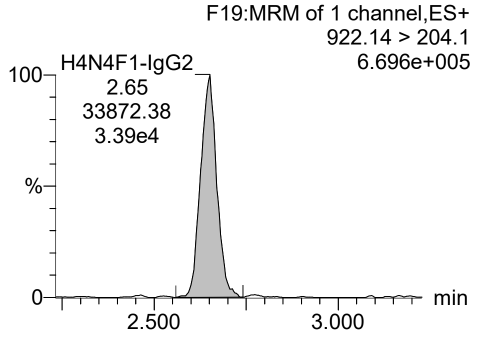

Sample Name: 20211111_A38_R2_IgG

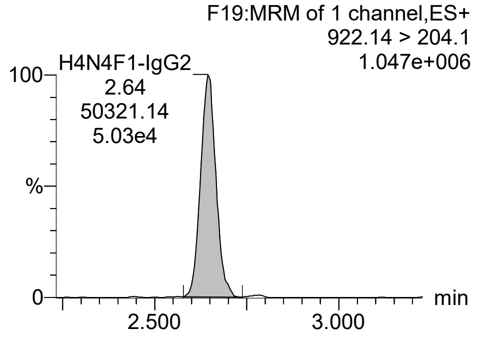

Sample Name: 20211111_H19_R2_IgG

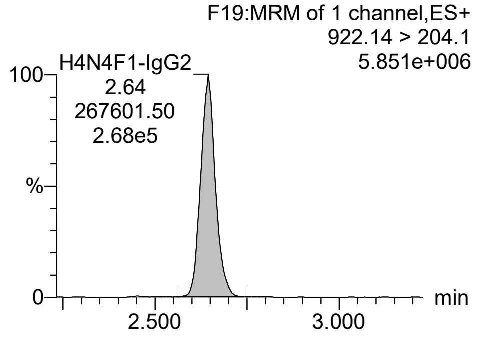

Sample Name: 20211111_A39_R2_IgG

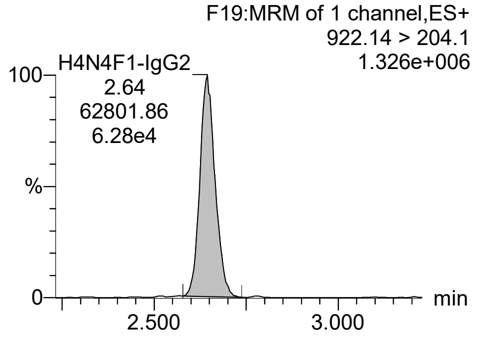

Sample Name: 20211111_A40_R2_IgG

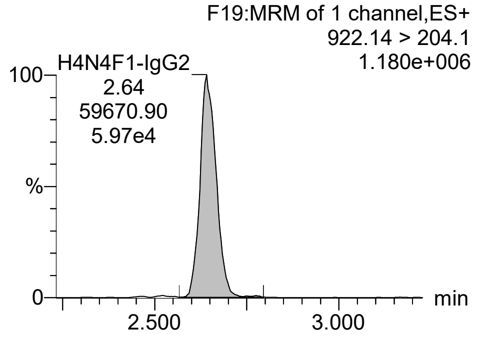

Sample Name: 20211111_H20_R2_IgG

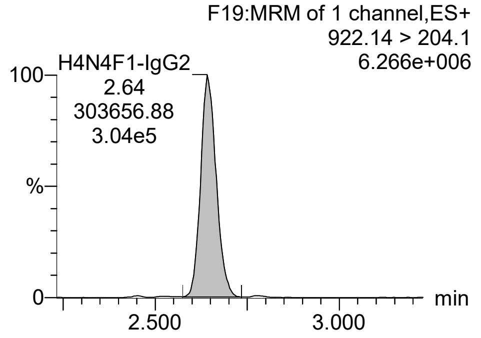

Sample Name: 20211111_A41_R2_IgG

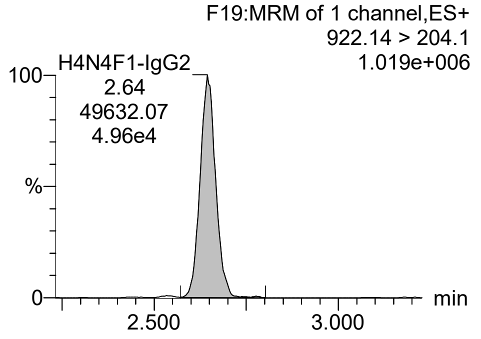

Sample Name: 20211111_A42_R2_IgG

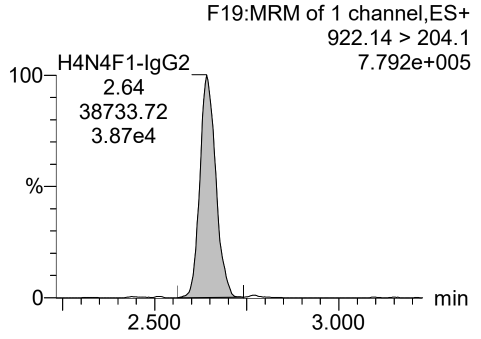

Sample Name: 20211111_A43_R2_IgG

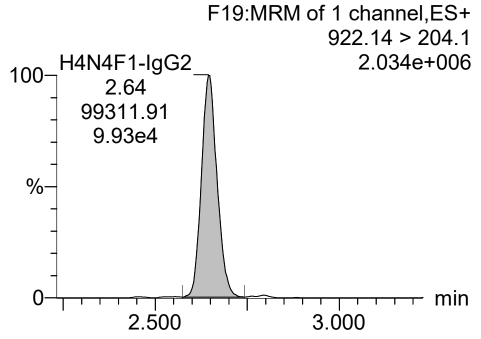

Dataset: C:\MassLynx\10.pro\20211115_clinicalsamples_IgG.qld

Last Altered: Sunday, October 02, 2022 16:02:30 台北標準時間
Printed: Friday, August 01, 2025 16:50:17 台北標準時間

Sample Name: 20211111_A44_R2_IgG

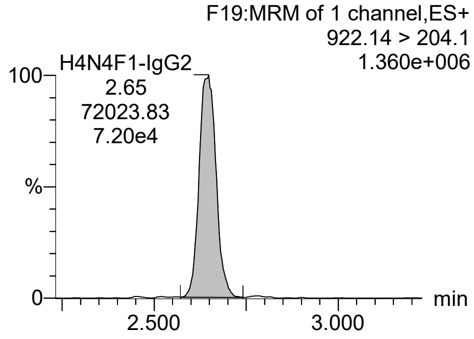

Sample Name: 20211111_QC_R17_IgG

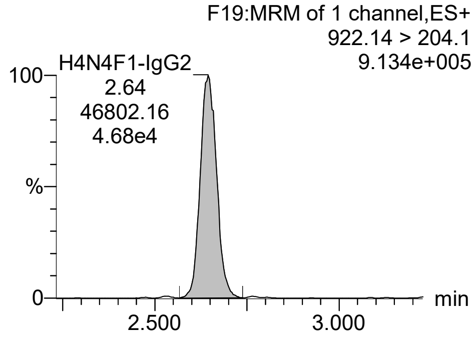

Dataset: C:\MassLynx\10.pro\20211115_clinicalsamples_IgG.qld

Last Altered: Sunday, October 02, 2022 16:02:30 台北標準時間
Printed: Friday, August 01, 2025 16:50:17 台北標準時間

	#	Name	RT	Area	IS Area	Response	Prima...
1	1	20211111_QC_R1_IgG	2.65	120876.5	64968.0	1.861	bb
2	2	20211111_A1_R1_IgG	2.65	180412.0	59059.2	3.055	bb
3	3	20211111_A2_R1_IgG	2.65	107670.5	66621.2	1.616	bb
4	4	20211111_L1_R1_IgG	2.65	68767.8	64201.0	1.071	bb
5	5	20211111_L2_R1_IgG	2.65	108832.2	46950.2	2.318	bb
6	6	20211111_H1_R1_IgG	2.65	52014.6	38648.2	1.346	bb
7	7	20211111_A3_R1_IgG	2.65	2339.2	2603.7	0.898	MM
8	8	20211111_A4_R1_IgG	2.65	181128.3	69960.4	2.589	bb
9	9	20211111_L3_R1_IgG	2.65	32198.4	38864.7	0.828	MM
10	10	20211111_L4_R1_IgG	2.65	53682.7	43380.5	1.237	bb
11	11	20211111_H2_R1_IgG	2.65	228070.1	71349.2	3.197	bb
12	12	20211111_A5_R1_IgG	2.65	71355.8	65569.7	1.088	bb
13	13	20211111_A6_R1_IgG	2.65	139201.8	53120.7	2.620	bb
14	14	20211111_QC_R2_IgG	2.65	126095.2	81034.1	1.556	bb
15	15	20211111_L5_R1_IgG	2.65	75485.7	50994.6	1.480	MM
16	16	20211111_L6_R1_IgG	2.64	26671.2	58058.0	0.459	MM
17	17	20211111_H3_R1_IgG	2.65	104215.3	64028.6	1.628	bb
18	18	20211111_A7_R1_IgG	2.65	189836.9	82996.1	2.287	bb
19	19	20211111_A8_R1_IgG	2.65	104986.1	77731.7	1.351	bb
20	20	20211111_L7_R1_IgG	2.65	73667.0	38827.3	1.897	bb
21	21	20211111_L8_R1_IgG	2.65	56859.9	44220.1	1.286	bb
22	22	20211111_H4_R1_IgG	2.64	62883.3	65826.3	0.955	bb
23	23	20211111_A9_R1_IgG	2.64	69444.0	60771.1	1.143	bb
24	24	20211111_A10_R1_IgG	2.65	77867.9	48578.7	1.603	bb
25	25	20211111_L9_R1_IgG	2.65	96476.6	50830.7	1.898	bb
26	26	20211111_L10_R1_IgG	2.64	38407.6	38034.2	1.010	bb
27	27	20211111_QC_R3_IgG	2.65	107421.5	67025.5	1.603	bb
28	28	20211111_H5_R1_IgG	2.65	49670.1	44544.9	1.115	bb
29	29	20211111_A11_R1_IgG	2.65	20145.7	66408.5	0.303	MM
30	30	20211111_A12_R1_IgG	2.65	65360.9	46624.5	1.402	bb
31	31	20211111_L11_R1_IgG	2.65	33297.4	33425.1	0.996	bb
32	32	20211111_L12_R1_IgG	2.65	26362.6	41178.0	0.640	MM
33	33	20211111_H6_R1_IgG	2.65	190538.7	58593.4	3.252	bb
34	34	20211111_A13_R1_IgG	2.64	125027.4	54133.0	2.310	bb
35	35	20211111_A14_R1_IgG	2.65	54896.3	56467.3	0.972	bb
36	36	20211111_L13_R1_IgG	2.65	75719.3	36024.4	2.102	bb
37	37	20211111_L14_R1_IgG	2.65	43982.2	37181.6	1.183	bb
38	38	20211111_H7_R1_IgG	2.65	66539.3	42850.6	1.553	bb
39	39	20211111_A15_R1_IgG	2.65	85519.8	62015.1	1.379	bb
40	40	20211111_QC_R4_IgG	2.65	89310.1	58572.5	1.525	bb
41	41	20211111_A16_R1_IgG	2.65	86317.9	78283.8	1.103	bb
42	42	20211111_L15_R1_IgG	2.65	93759.0	44158.2	2.123	bb
43	43	20211111_L16_R1_IgG	2.65	98027.6	55150.8	1.777	bb
44	44	20211111_H8_R1_IgG	2.64	61658.2	42965.2	1.435	bb
45	45	20211111_A17_R1_IgG	2.64	76189.5	39717.7	1.918	bb
46	46	20211111_A18_R1_IgG	2.65	78677.6	44675.4	1.761	bb
47	47	20211111_L17_R1_IgG	2.65	71515.2	37854.1	1.889	bb
48	48	20211111_L18_R1_IgG	2.65	20897.9	50716.9	0.412	bb
49	49	20211111_H9_R1_IgG	2.65	79877.3	36276.0	2.202	bb
50	50	20211111_A19_R1_IgG	2.64	97051.0	42862.5	2.264	bb
51	51	20211111_A20_R1_IgG	2.64	21653.3	36542.7	0.593	bb
52	52	20211111_L19_R1_IgG	2.64	63486.4	53892.8	1.178	bb
53	53	20211111_QC_R5_IgG	2.65	86040.7	56513.2	1.522	bb
54	54	20211111_L20_R1_IgG	2.65	55584.5	56499.9	0.984	bb
55	55	20211111_H10_R1_IgG	2.64	74435.5	68120.5	1.093	bb
56	56	20211111_A21_R1_IgG	2.65	204501.9	63699.8	3.210	bb

	# Name	RT	Area	IS Area	Response	Prima...
57	57 20211111_A22_R1_IgG	2.65	54091.4	52222.1	1.036	bb
58	58 20211111_L21_R1_IgG	2.65	31464.7	54372.5	0.579	bb
59	59 20211111_L22_R1_IgG	2.65	49041.1	41353.1	1.186	bb
60	60 20211111_H11_R1_IgG	2.65	175532.9	65579.1	2.677	bb
61	61 20211111_A23_R1_IgG	2.65	91778.7	64131.1	1.431	bb
62	62 20211111_A24_R1_IgG	2.65	27464.9	38724.8	0.709	bb
63	63 20211111_L23_R1_IgG	2.65	155742.2	64245.2	2.424	bb
64	64 20211111_L24_R1_IgG	2.65	55113.2	45298.0	1.217	bb
65	65 20211111_H12_R1_IgG	2.64	48847.3	44679.3	1.093	bb
66	66 20211111_QC_R6_IgG	2.66	77071.7	48165.0	1.600	bb
67	67 20211111_A25_R1_IgG	2.65	60841.7	57169.9	1.064	bb
68	68 20211111_A26_R1_IgG	2.65	41723.7	34254.0	1.218	bb
69	69 20211111_L25_R1_IgG	2.65	117392.3	67491.5	1.739	bb
70	70 20211111_L26_R1_IgG	2.65	35849.9	55934.1	0.641	bb
71	71 20211111_H13_R1_IgG	2.65	103832.2	48767.3	2.129	bb
72	72 20211111_A27_R1_IgG	2.65	36352.1	57269.7	0.635	bb
73	73 20211111_A28_R1_IgG	2.65	52343.8	49231.1	1.063	bb
74	74 20211111_L27_R1_IgG	2.65	53575.1	60014.5	0.893	bb
75	75 20211111_L28_R1_IgG	2.65	68875.4	35062.6	1.964	bb
76	76 20211111_H14_R1_IgG	2.65	239354.0	62371.9	3.838	bb
77	77 20211111_A29_R1_IgG	2.65	42774.3	43858.2	0.975	bb
78	78 20211111_A30_R1_IgG	2.64	55310.9	62500.4	0.885	bb
79	79 20211111_QC_R7_IgG	2.65	72550.9	43946.9	1.651	bb
80	80 20211111_L29_R1_IgG	2.65	24692.6	38728.9	0.638	bb
81	81 20211111_L30_R1_IgG	2.65	29190.2	42613.2	0.685	bb
82	82 20211111_H15_R1_IgG	2.65	62591.5	39874.6	1.570	MM
83	83 20211111_A31_R1_IgG	2.65	73445.6	53414.3	1.375	bb
84	84 20211111_A32_R1_IgG	2.65	77992.8	41844.6	1.864	bb
85	85 20211111_L31_R1_IgG	2.65	79224.8	40553.7	1.954	bb
86	86 20211111_L32_R1_IgG	2.65	71328.1	49618.7	1.438	MM
87	87 20211111_H16_R1_IgG	2.65	115508.5	60170.9	1.920	bb
88	88 20211111_A33_R1_IgG	2.65	41672.7	48588.2	0.858	bb
89	89 20211111_A34_R1_IgG	2.65	57062.0	43288.0	1.318	bb
90	90 20211111_H17_R1_IgG	2.65	182728.5	47104.5	3.879	bb
91	91 20211111_A35_R1_IgG	2.65	98269.1	41025.9	2.395	bb
92	92 20211111_QC_R8_IgG	2.65	62048.2	40546.3	1.530	bb
93	93 20211111_A36_R1_IgG	2.65	73270.6	44504.4	1.646	bb
94	94 20211111_H18_R1_IgG	2.65	154685.6	34162.2	4.528	bb
95	95 20211111_A37_R1_IgG	2.64	36306.5	39760.0	0.913	bb
96	96 20211111_A38_R1_IgG	2.66	53381.2	43982.6	1.214	bb
97	97 20211111_H19_R1_IgG	2.65	274049.4	45314.8	6.048	bb
98	98 20211111_A39_R1_IgG	2.65	69423.0	39388.0	1.763	bb
99	99 20211111_A40_R1_IgG	2.66	65174.2	38726.4	1.683	bb
100	1... 20211111_H20_R1_IgG	2.65	328048.5	37814.4	8.675	bb
101	1... 20211111_A41_R1_IgG	2.66	56910.3	48930.3	1.163	bb
102	1... 20211111_A42_R1_IgG	2.65	44131.0	40406.8	1.092	bb
103	1... 20211111_A43_R1_IgG	2.65	127208.3	44421.5	2.864	bb
104	1... 20211111_A44_R1_IgG	2.65	103950.0	53128.2	1.957	bb
105	1... 20211111_QC_R9_IgG	2.66	54419.1	37049.0	1.469	bb
106	1... 20211111_A1_R2_IgG	2.64	123554.3	46499.4	2.657	bb
107	1... 20211111_A2_R2_IgG	2.65	75587.4	57260.7	1.320	bb
108	1... 20211111_L1_R2_IgG	2.65	46797.1	48062.9	0.974	bb
109	1... 20211111_L2_R2_IgG	2.66	93015.9	38745.2	2.401	bb
110	1... 20211111_H1_R2_IgG	2.65	56369.3	44273.5	1.273	bb
111	1... 20211111_A3_R2_IgG	2.65	4144.9	4755.5	0.872	MM
112	1... 20211111_A4_R2_IgG	2.65	124202.5	50546.7	2.457	bb

Dataset: C:\MassLynx\10.pro\20211115_clinicalsamples_IgG.qld

Last Altered: Sunday, October 02, 2022 16:02:30 台北標準時間
Printed: Friday, August 01, 2025 16:50:17 台北標準時間

	# Name	RT	Area	IS Area	Response	Prima...
113	1... 20211111_L3_R2_IgG	2.65	29503.9	34825.8	0.847	MM
114	1... 20211111_L4_R2_IgG	2.65	45502.1	39107.7	1.164	bb
115	1... 20211111_H2_R2_IgG	2.65	149178.6	49750.0	2.999	MM
116	1... 20211111_A5_R2_IgG	2.65	61819.7	52536.2	1.177	bb
117	1... 20211111_A6_R2_IgG	2.64	117038.4	42050.0	2.783	bb
118	1... 20211111_QC_R10_IgG	2.65	56379.9	35350.8	1.595	MM
119	1... 20211111_L5_R2_IgG	2.65	56582.3	38039.3	1.487	bb
120	1... 20211111_L6_R2_IgG	2.65	21177.1	44907.4	0.472	MM
121	1... 20211111_H3_R2_IgG	2.65	87869.3	47729.1	1.841	bb
122	1... 20211111_A7_R2_IgG	2.64	134171.5	57347.1	2.340	bb
123	1... 20211111_A8_R2_IgG	2.64	77919.9	52397.8	1.487	bb
124	1... 20211111_L7_R2_IgG	2.64	63865.2	36318.4	1.758	bb
125	1... 20211111_L8_R2_IgG	2.64	52220.1	38131.9	1.369	bb
126	1... 20211111_H4_R2_IgG	2.65	46365.0	46849.6	0.990	MM
127	1... 20211111_A9_R2_IgG	2.65	53648.5	47759.6	1.123	bb
128	1... 20211111_A10_R2_IgG	2.65	70719.3	41366.2	1.710	bb
129	1... 20211111_L9_R2_IgG	2.65	80821.0	43052.1	1.877	bb
130	1... 20211111_L10_R2_IgG	2.65	35032.5	31487.4	1.113	bb
131	1... 20211111_QC_R11_IgG	2.65	53391.6	37804.6	1.412	MM
132	1... 20211111_H5_R2_IgG	2.65	46545.0	40380.0	1.153	bb
133	1... 20211111_A11_R2_IgG	2.64	15360.7	49533.3	0.310	MM
134	1... 20211111_A12_R2_IgG	2.65	58813.8	40226.3	1.462	bb
135	1... 20211111_L11_R2_IgG	2.65	26051.9	28884.0	0.902	bb
136	1... 20211111_L12_R2_IgG	2.65	24059.8	34351.3	0.700	bb
137	1... 20211111_H6_R2_IgG	2.64	123657.4	40466.6	3.056	bb
138	1... 20211111_A13_R2_IgG	2.65	89017.6	42306.7	2.104	bb
139	1... 20211111_A14_R2_IgG	2.65	45659.1	44904.0	1.017	bb
140	1... 20211111_L13_R2_IgG	2.65	65818.5	31913.0	2.062	bb
141	1... 20211111_L14_R2_IgG	2.65	45270.5	31527.4	1.436	MM
142	1... 20211111_H7_R2_IgG	2.65	61144.7	40838.2	1.497	bb
143	1... 20211111_A15_R2_IgG	2.65	67478.9	49389.7	1.366	bb
144	1... 20211111_QC_R12_IgG	2.65	53620.6	35168.7	1.525	bb
145	1... 20211111_A16_R2_IgG	2.65	48457.1	47440.5	1.021	bb
146	1... 20211111_L15_R2_IgG	2.65	67686.3	33145.9	2.042	bb
147	1... 20211111_L16_R2_IgG	2.65	63174.6	37596.6	1.680	MM
148	1... 20211111_H8_R2_IgG	2.65	56111.4	43933.3	1.277	MM
149	1... 20211111_A17_R2_IgG	2.64	77152.7	43605.5	1.769	bb
150	1... 20211111_A18_R2_IgG	2.65	60436.9	37581.8	1.608	bb
151	1... 20211111_L17_R2_IgG	2.64	67040.2	33942.5	1.975	bb
152	1... 20211111_L18_R2_IgG	2.65	15037.1	37831.5	0.397	bb
153	1... 20211111_H9_R2_IgG	2.65	64636.3	30898.4	2.092	bb
154	1... 20211111_A19_R2_IgG	2.65	88666.7	39352.1	2.253	bb
155	1... 20211111_A20_R2_IgG	2.65	18997.0	34661.2	0.548	bb
156	1... 20211111_L19_R2_IgG	2.65	42682.7	39918.3	1.069	bb
157	1... 20211111_QC_R13_IgG	2.65	56493.4	36239.1	1.559	bb
158	1... 20211111_L20_R2_IgG	2.64	30279.3	32897.7	0.920	bb
159	1... 20211111_H10_R2_IgG	2.64	33097.0	31394.5	1.054	bb
160	1... 20211111_A21_R2_IgG	2.64	99974.5	34863.7	2.868	bb
161	1... 20211111_A22_R2_IgG	2.64	28140.6	30342.1	0.927	bb
162	1... 20211111_L21_R2_IgG	2.65	19228.0	33706.2	0.570	bb
163	1... 20211111_L22_R2_IgG	2.64	29503.5	31298.6	0.943	bb
164	1... 20211111_H11_R2_IgG	2.64	93937.5	38904.1	2.415	bb
165	1... 20211111_A23_R2_IgG	2.64	48165.9	32382.3	1.487	bb
166	1... 20211111_A24_R2_IgG	2.64	20522.4	30261.9	0.678	bb
167	1... 20211111_L23_R2_IgG	2.64	102122.4	43037.5	2.373	bb
168	1... 20211111_L24_R2_IgG	2.64	37667.6	37023.3	1.017	bb

Dataset: C:\MassLynx\10.pro\20211115_clinicalsamples_IgG.qld

Last Altered: Sunday, October 02, 2022 16:02:30 台北標準時間
Printed: Friday, August 01, 2025 16:50:17 台北標準時間

	# Name	RT	Area	IS Area	Response	Prima...
169	1... 20211111_H12_R2_IgG	2.64	38178.6	39478.2	0.967	bb
170	1... 20211111_QC_R14_IgG	2.64	48752.2	34463.2	1.415	bb
171	1... 20211111_A25_R2_IgG	2.64	35690.1	39945.3	0.893	bb
172	1... 20211111_A26_R2_IgG	2.64	30816.4	29663.2	1.039	bb
173	1... 20211111_L25_R2_IgG	2.64	62017.9	38589.3	1.607	bb
174	1... 20211111_L26_R2_IgG	2.64	22178.9	38405.2	0.577	bb
175	1... 20211111_H13_R2_IgG	2.64	72262.6	37771.2	1.913	bb
176	1... 20211111_A27_R2_IgG	2.64	21198.1	42634.1	0.497	bb
177	1... 20211111_A28_R2_IgG	2.64	35093.6	33231.1	1.056	bb
178	1... 20211111_L27_R2_IgG	2.64	35684.0	41761.1	0.854	bb
179	1... 20211111_L28_R2_IgG	2.64	50184.4	26968.6	1.861	bb
180	1... 20211111_H14_R2_IgG	2.65	151180.4	49250.2	3.070	bb
181	1... 20211111_A29_R2_IgG	2.64	33657.0	37601.7	0.895	bb
182	1... 20211111_A30_R2_IgG	2.64	32766.4	40366.8	0.812	bb
183	1... 20211111_QC_R15_IgG	2.65	47528.2	35156.1	1.352	bb
184	1... 20211111_L29_R2_IgG	2.64	21297.3	33813.1	0.630	MM
185	1... 20211111_L30_R2_IgG	2.64	21585.7	33488.6	0.645	bb
186	1... 20211111_H15_R2_IgG	2.64	51712.6	38501.0	1.343	bb
187	1... 20211111_A31_R2_IgG	2.64	46490.7	37525.5	1.239	bb
188	1... 20211111_A32_R2_IgG	2.64	62948.9	35770.8	1.760	MM
189	1... 20211111_L31_R2_IgG	2.64	61830.6	33282.4	1.858	bb
190	1... 20211111_L32_R2_IgG	2.64	59110.7	42049.6	1.406	bb
191	1... 20211111_H16_R2_IgG	2.65	86560.3	45947.6	1.884	bb
192	1... 20211111_A33_R2_IgG	2.64	31395.0	40202.3	0.781	bb
193	1... 20211111_A34_R2_IgG	2.65	42572.7	36747.0	1.159	bb
194	1... 20211111_H17_R2_IgG	2.64	158790.1	40920.9	3.880	bb
195	1... 20211111_A35_R2_IgG	2.64	81579.9	33689.2	2.422	bb
196	1... 20211111_QC_R16_IgG	2.64	48396.9	32783.6	1.476	bb
197	1... 20211111_A36_R2_IgG	2.64	61451.5	36822.4	1.669	bb
198	1... 20211111_H18_R2_IgG	2.65	146151.8	31769.3	4.600	bb
199	1... 20211111_A37_R2_IgG	2.65	33872.4	37581.8	0.901	bb
200	2... 20211111_A38_R2_IgG	2.64	50321.1	38390.4	1.311	bb
201	2... 20211111_H19_R2_IgG	2.64	267601.5	41217.0	6.492	bb
202	2... 20211111_A39_R2_IgG	2.64	62801.9	37392.7	1.680	bb
203	2... 20211111_A40_R2_IgG	2.64	59670.9	38295.1	1.558	bb
204	2... 20211111_H20_R2_IgG	2.64	303656.9	35941.8	8.449	bb
205	2... 20211111_A41_R2_IgG	2.64	49632.1	45304.9	1.096	bb
206	2... 20211111_A42_R2_IgG	2.64	38733.7	37803.8	1.025	bb
207	2... 20211111_A43_R2_IgG	2.64	99311.9	38161.8	2.602	bb
208	2... 20211111_A44_R2_IgG	2.65	72023.8	37819.8	1.904	bb
209	2... 20211111_QC_R17_IgG	2.64	46802.2	31731.8	1.475	bb

Dataset: C:\MassLynx\10.pro\20211115_clinicalsamples_IgG.qld

Last Altered: Sunday, October 02, 2022 16:02:30 台北標準時間
Printed: Friday, August 01, 2025 16:50:47 台北標準時間

Method: C:\MassLynx\10_Pro_IgA.PRO\MethDB\20210926_IgG_DMRM_IgM_IS.mdb 16 Feb 2022 11:58:21
Calibration: 18 Nov 2021 13:56:22

Sample Name: 20211111_QC_R1_IgG
F30:MRM of 1 channel,ES+
1019.116 > 204.1
1.690e+005

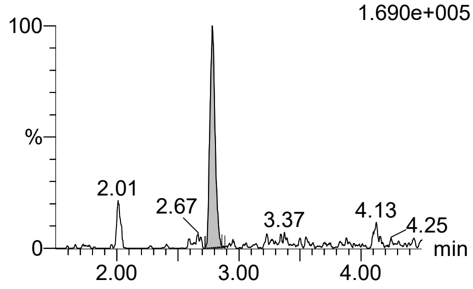

Sample Name: 20211111_A1_R1_IgG
F30:MRM of 1 channel,ES+
1019.116 > 204.1
3.372e+005

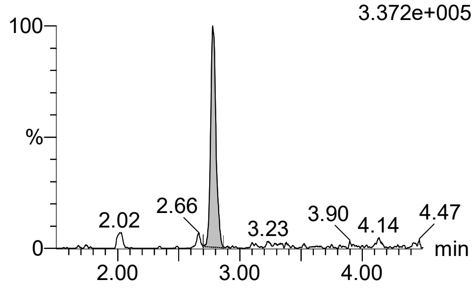

Sample Name: 20211111_A2_R1_IgG
F30:MRM of 1 channel,ES+
1019.116 > 204.1
1.776e+005

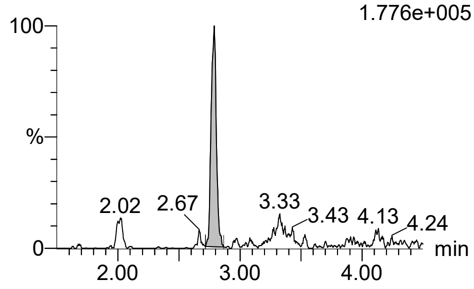

Sample Name: 20211111_L1_R1_IgG
F30:MRM of 1 channel,ES+
1019.116 > 204.1
9.409e+004

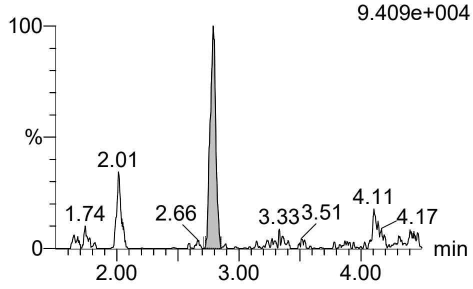

Sample Name: 20211111_L2_R1_IgG
F30:MRM of 1 channel,ES+
1019.116 > 204.1
1.375e+005

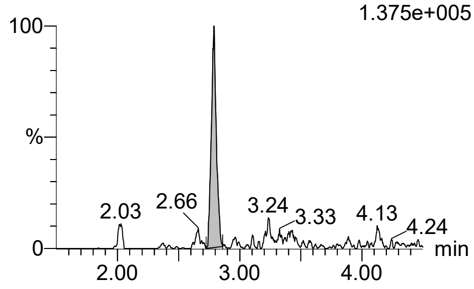

Sample Name: 20211111_H1_R1_IgG
F30:MRM of 1 channel,ES+
1019.116 > 204.1
5.115e+004

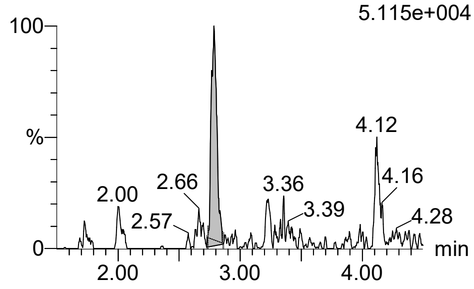

Sample Name: 20211111_A3_R1_IgG
F30:MRM of 1 channel,ES+
1019.116 > 204.1
7.005e+003

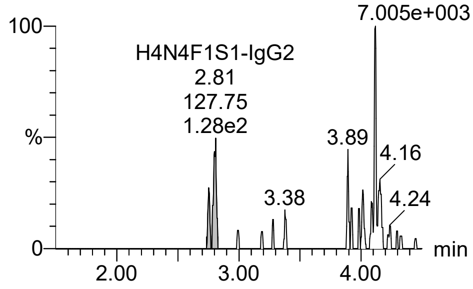

Sample Name: 20211111_A4_R1_IgG
F30:MRM of 1 channel,ES+
1019.116 > 204.1
2.680e+005

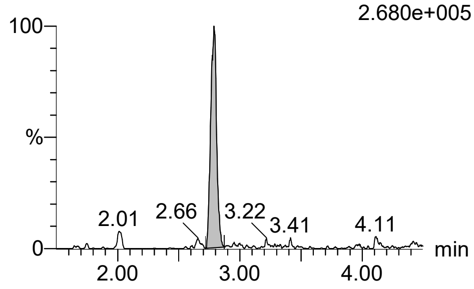

Sample Name: 20211111_L3_R1_IgG
F30:MRM of 1 channel,ES+
1019.116 > 204.1
3.290e+004

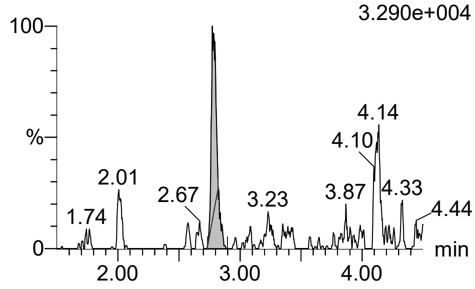

Sample Name: 20211111_L4_R1_IgG
F30:MRM of 1 channel,ES+
1019.116 > 204.1
8.491e+004

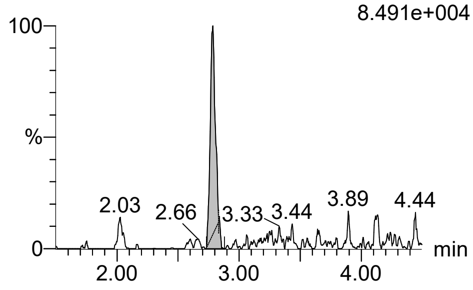

Sample Name: 20211111_H2_R1_IgG
F30:MRM of 1 channel,ES+
1019.116 > 204.1
3.586e+005

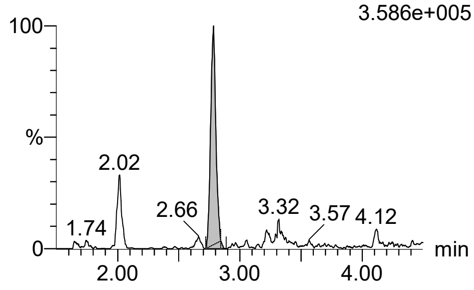

Sample Name: 20211111_A5_R1_IgG
F30:MRM of 1 channel,ES+
1019.116 > 204.1
1.237e+005

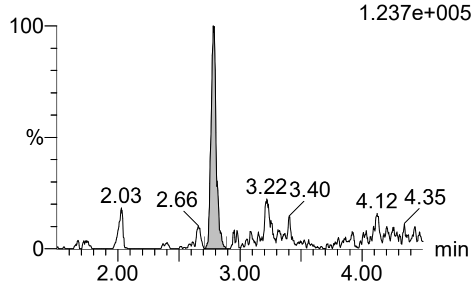

Dataset: C:\MassLynx\10.pro\20211115_clinicalsamples_IgG.qld

Last Altered: Sunday, October 02, 2022 16:02:30 台北標準時間
Printed: Friday, August 01, 2025 16:50:47 台北標準時間

Sample Name: 20211111_A6_R1_IgG

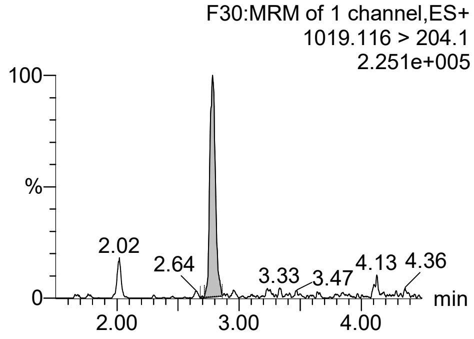

Sample Name: 20211111_QC_R2_IgG

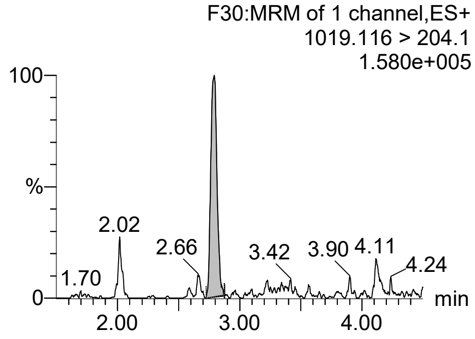

Sample Name: 20211111_L5_R1_IgG

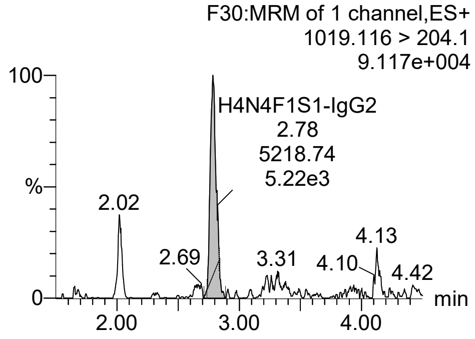

Sample Name: 20211111_L6_R1_IgG

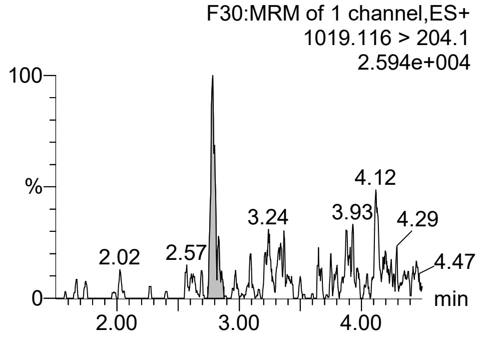

Sample Name: 20211111_H3_R1_IgG

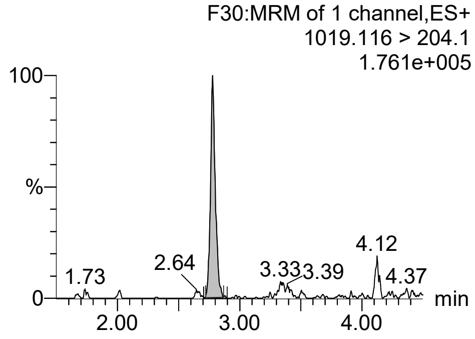

Sample Name: 20211111_A7_R1_IgG

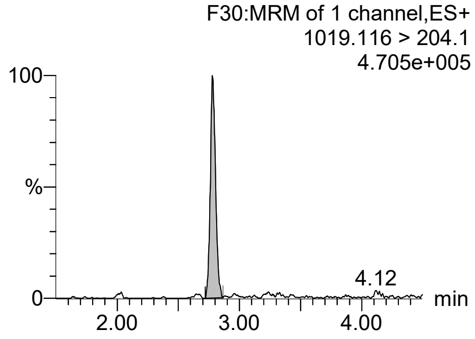

Sample Name: 20211111_A8_R1_IgG

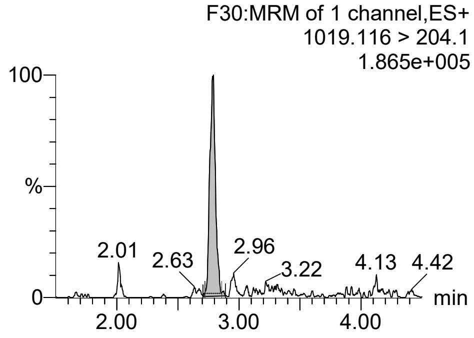

Sample Name: 20211111_L7_R1_IgG

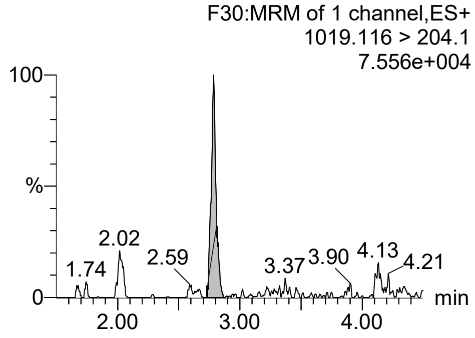

Sample Name: 20211111_L8_R1_IgG

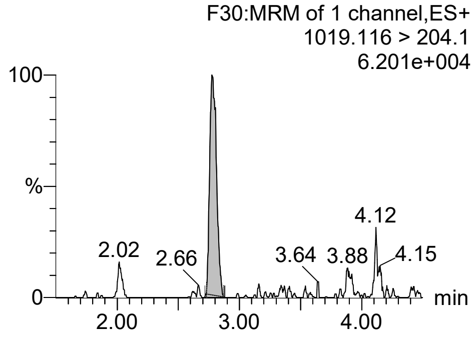

Sample Name: 20211111_H4_R1_IgG

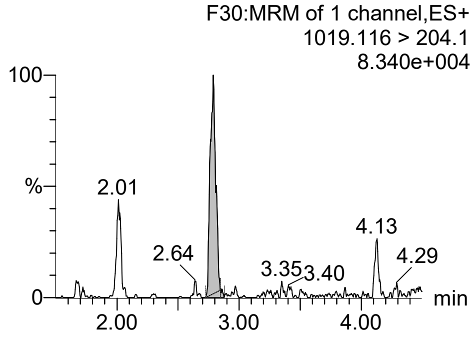

Sample Name: 20211111_A9_R1_IgG

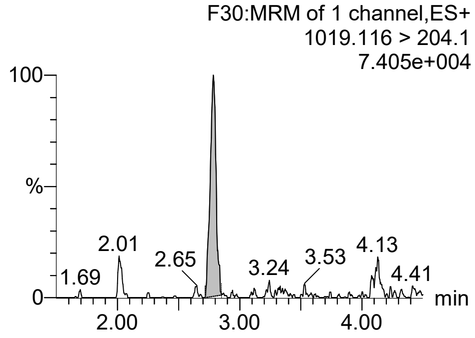

Sample Name: 20211111_A10_R1_IgG

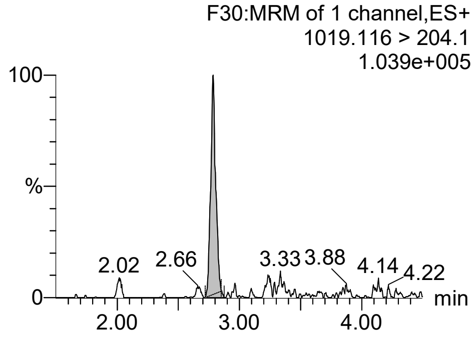

Sample Name: 20211111_L9_R1_IgG

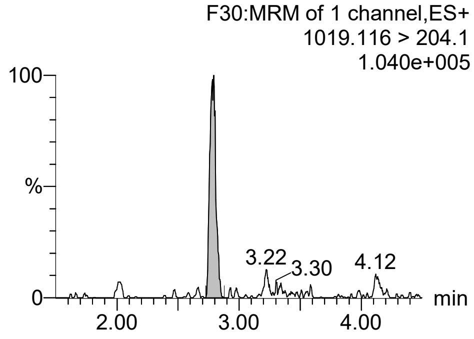

Sample Name: 20211111_L10_R1_IgG

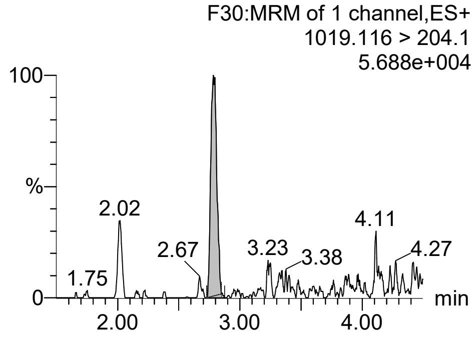

Sample Name: 20211111_QC_R3_IgG

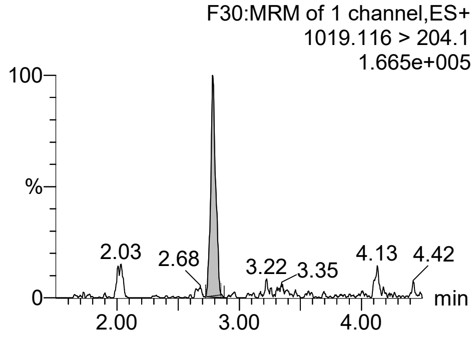

Sample Name: 20211111_H5_R1_IgG
F30:MRM of 1 channel,ES+
1019.116 > 204.1
8.959e+004

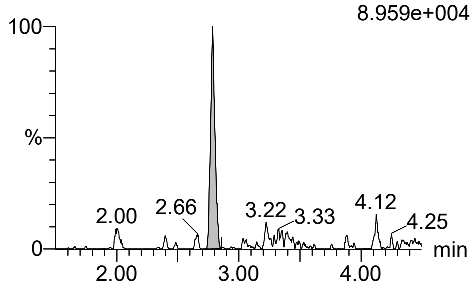

Sample Name: 20211111_A11_R1_IgG
F30:MRM of 1 channel,ES+
1019.116 > 204.1
4.265e+004

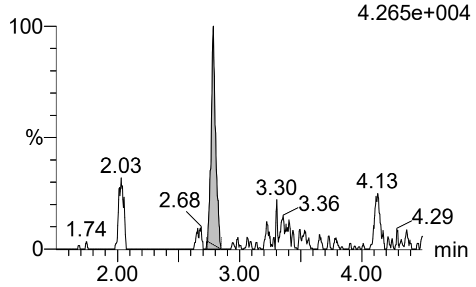

Sample Name: 20211111_A12_R1_IgG
F30:MRM of 1 channel,ES+
1019.116 > 204.1
7.951e+004

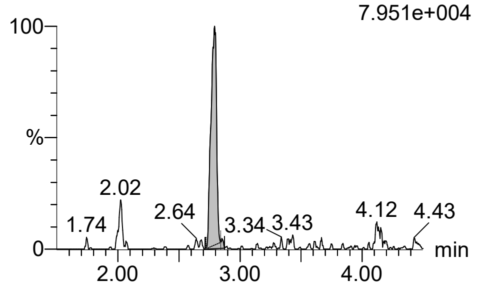

Sample Name: 20211111_L11_R1_IgG
F30:MRM of 1 channel,ES+
1019.116 > 204.1
1.799e+004

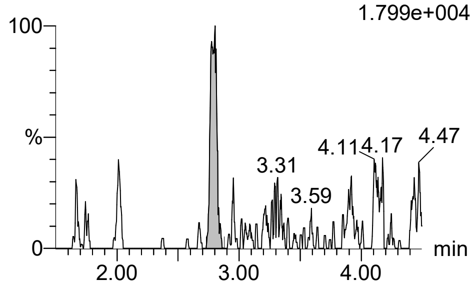

Sample Name: 20211111_L12_R1_IgG
F30:MRM of 1 channel,ES+
1019.116 > 204.1
3.014e+004

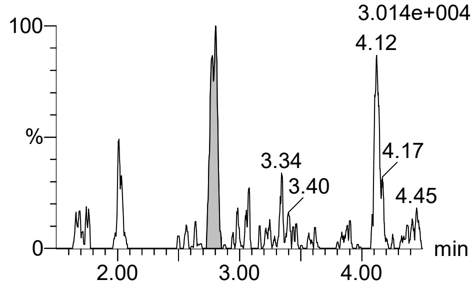

Sample Name: 20211111_H6_R1_IgG
F30:MRM of 1 channel,ES+
1019.116 > 204.1
2.646e+005

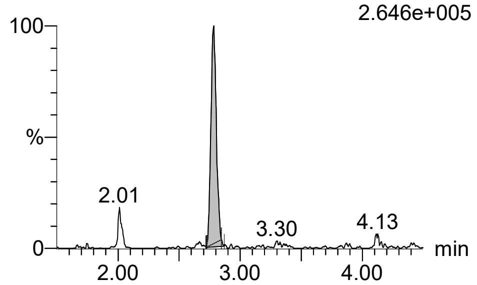

Sample Name: 20211111_A13_R1_IgG
F30:MRM of 1 channel,ES+
1019.116 > 204.1
1.331e+005

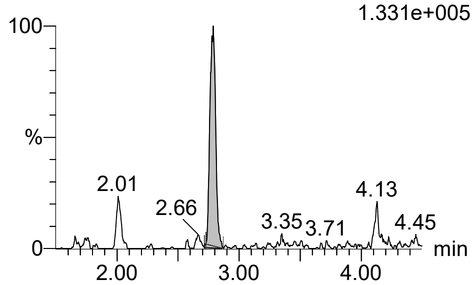

Sample Name: 20211111_A14_R1_IgG
F30:MRM of 1 channel,ES+
1019.116 > 204.1
9.965e+004

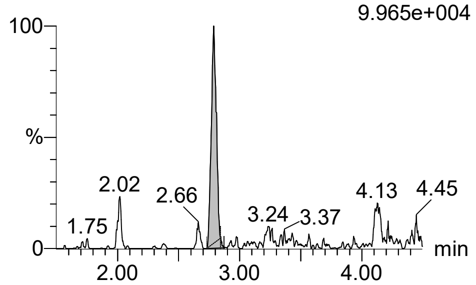

Sample Name: 20211111_L13_R1_IgG
F30:MRM of 1 channel,ES+
1019.116 > 204.1
1.197e+005

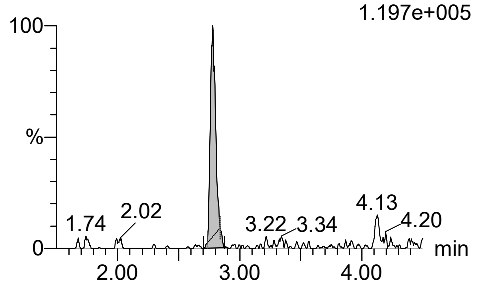

Sample Name: 20211111_L14_R1_IgG
F30:MRM of 1 channel,ES+
1019.116 > 204.1
4.547e+004

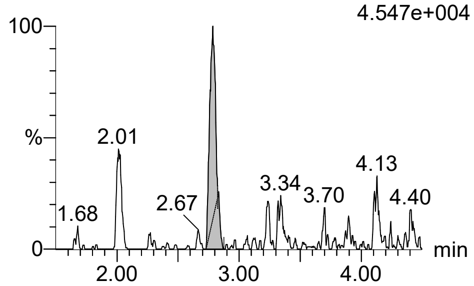

Sample Name: 20211111_H7_R1_IgG
F30:MRM of 1 channel,ES+
1019.116 > 204.1
8.595e+004

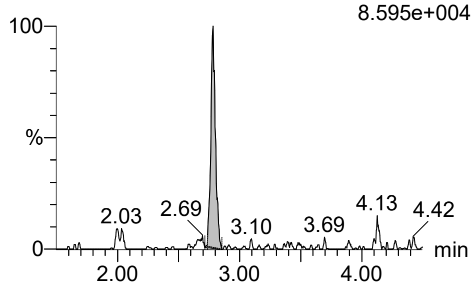

Sample Name: 20211111_A15_R1_IgG
F30:MRM of 1 channel,ES+
1019.116 > 204.1
1.039e+005

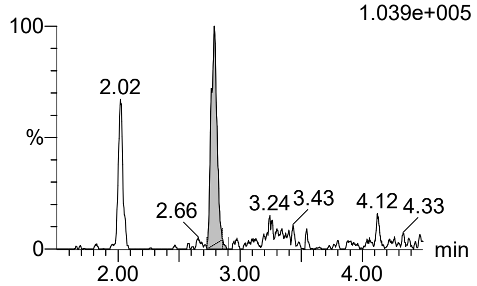

Sample Name: 20211111_QC_R4_IgG
F30:MRM of 1 channel,ES+
1019.116 > 204.1
1.118e+005

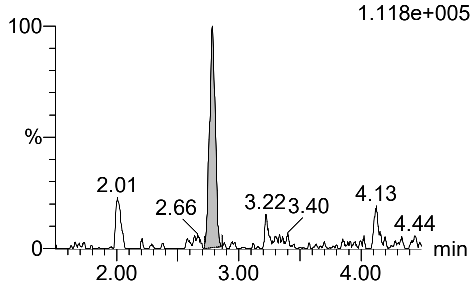

Sample Name: 20211111_A16_R1_IgG
F30:MRM of 1 channel,ES+
1019.116 > 204.1
8.909e+004

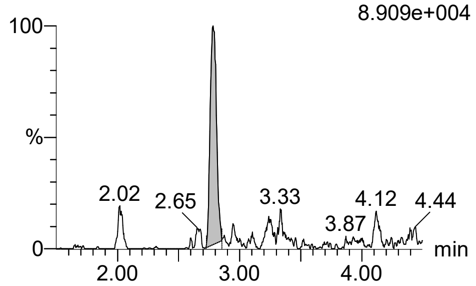

Sample Name: 20211111_L15_R1_IgG
F30:MRM of 1 channel,ES+
1019.116 > 204.1
1.475e+005

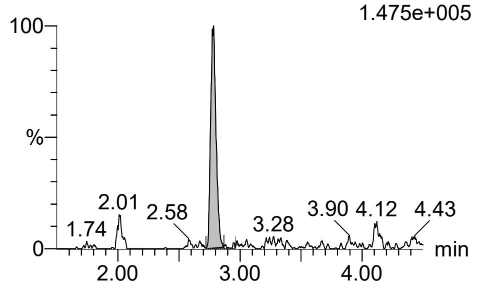

Dataset: C:\MassLynx\10.pro\20211115_clinicalsamples_IgG.qld

Last Altered: Sunday, October 02, 2022 16:02:30 台北標準時間
Printed: Friday, August 01, 2025 16:50:47 台北標準時間

Sample Name: 20211111_L16_R1_IgG
F30:MRM of 1 channel,ES+
1019.116 > 204.1
1.132e+005

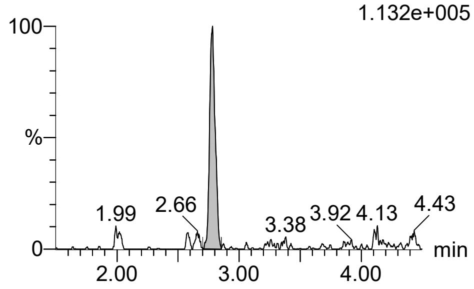

Sample Name: 20211111_H8_R1_IgG
F30:MRM of 1 channel,ES+
1019.116 > 204.1
7.784e+004

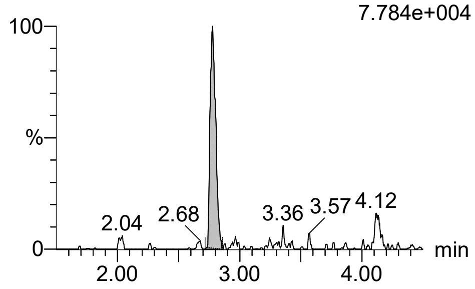

Sample Name: 20211111_A17_R1_IgG
F30:MRM of 1 channel,ES+
1019.116 > 204.1
5.406e+004

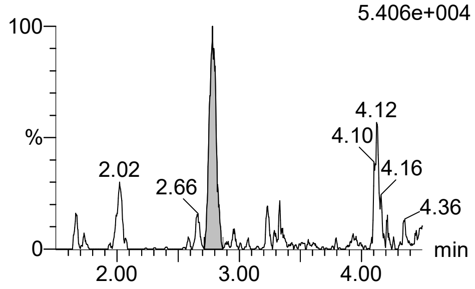

Sample Name: 20211111_A18_R1_IgG
F30:MRM of 1 channel,ES+
1019.116 > 204.1
8.948e+004

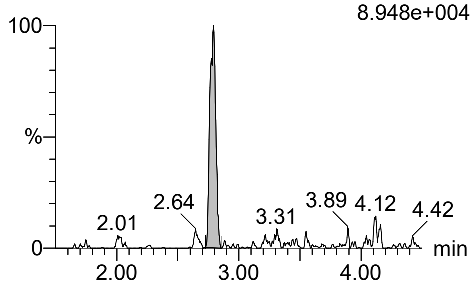

Sample Name: 20211111_L17_R1_IgG
F30:MRM of 1 channel,ES+
1019.116 > 204.1
7.859e+004

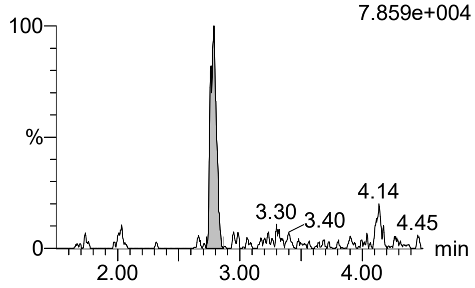

Sample Name: 20211111_L18_R1_IgG
F30:MRM of 1 channel,ES+
1019.116 > 204.1
2.305e+004

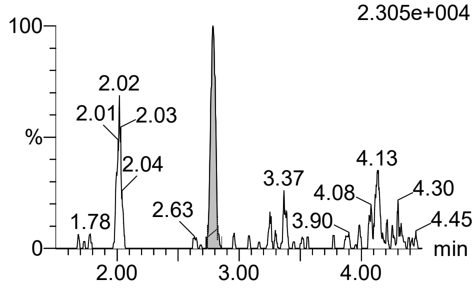

Sample Name: 20211111_H9_R1_IgG
F30:MRM of 1 channel,ES+
1019.116 > 204.1
6.892e+004

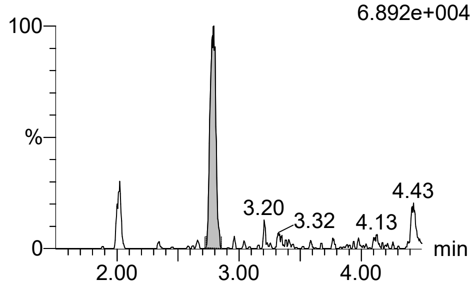

Sample Name: 20211111_A19_R1_IgG
F30:MRM of 1 channel,ES+
1019.116 > 204.1
1.088e+005

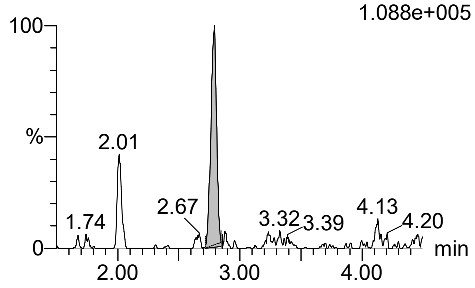

Sample Name: 20211111_A20_R1_IgG
F30:MRM of 1 channel,ES+
1019.116 > 204.1
3.071e+004

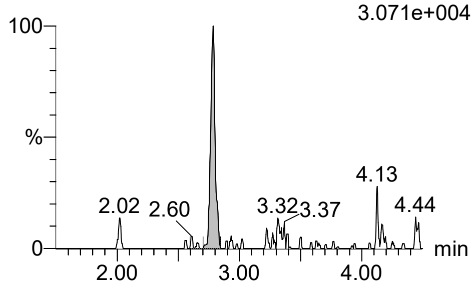

Sample Name: 20211111_L19_R1_IgG
F30:MRM of 1 channel,ES+
1019.116 > 204.1
5.755e+004

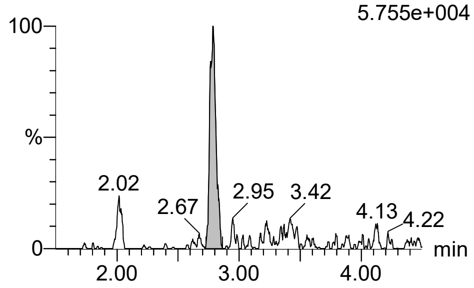

Sample Name: 20211111_QC_R5_IgG
F30:MRM of 1 channel,ES+
1019.116 > 204.1
1.005e+005

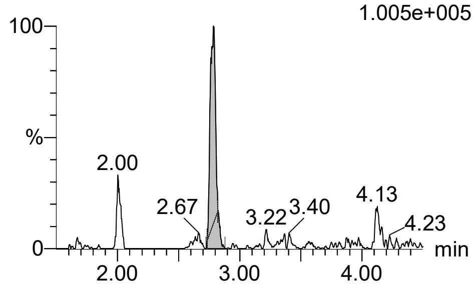

Sample Name: 20211111_L20_R1_IgG
F30:MRM of 1 channel,ES+
1019.116 > 204.1
7.571e+004

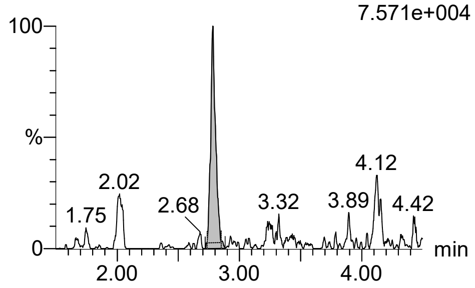

Sample Name: 20211111_H10_R1_IgG
F30:MRM of 1 channel,ES+
1019.116 > 204.1
1.158e+005

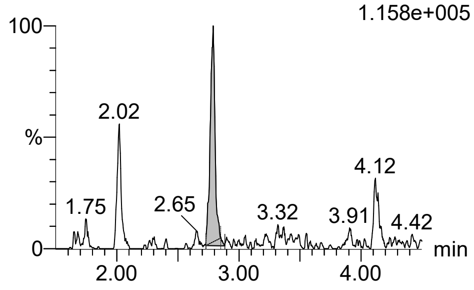

Sample Name: 20211111_A21_R1_IgG
F30:MRM of 1 channel,ES+
1019.116 > 204.1
3.488e+005

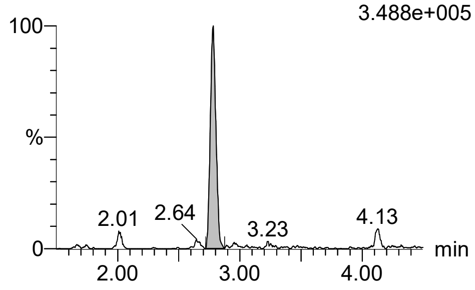

Sample Name: 20211111_A22_R1_IgG
F30:MRM of 1 channel,ES+
1019.116 > 204.1
7.339e+004

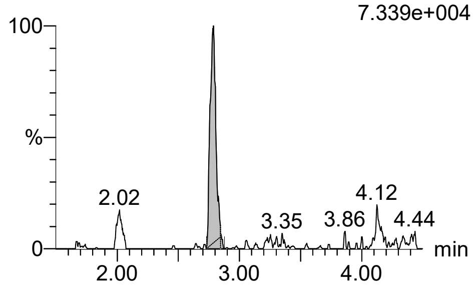

Sample Name: 20211111_L21_R1_IgG
F30:MRM of 1 channel,ES+
1019.116 > 204.1
4.288e+004

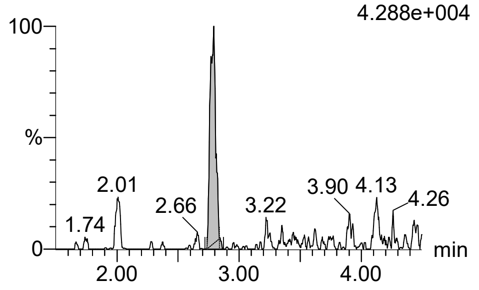

Sample Name: 20211111_L22_R1_IgG
F30:MRM of 1 channel,ES+
1019.116 > 204.1
4.228e+004

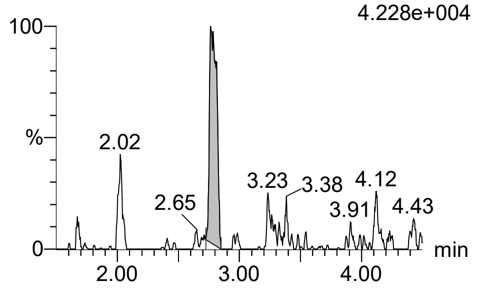

Sample Name: 20211111_H11_R1_IgG
F30:MRM of 1 channel,ES+
1019.116 > 204.1
2.641e+005

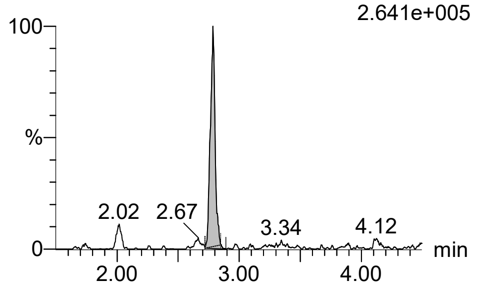

Sample Name: 20211111_A23_R1_IgG
F30:MRM of 1 channel,ES+
1019.116 > 204.1
1.556e+005

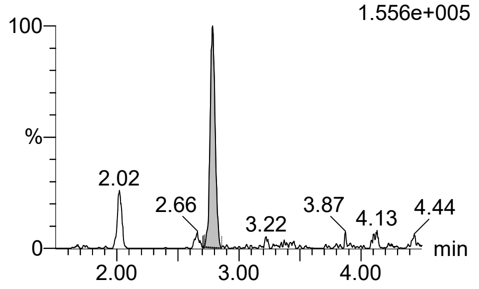

Sample Name: 20211111_A24_R1_IgG
F30:MRM of 1 channel,ES+
1019.116 > 204.1
4.466e+004

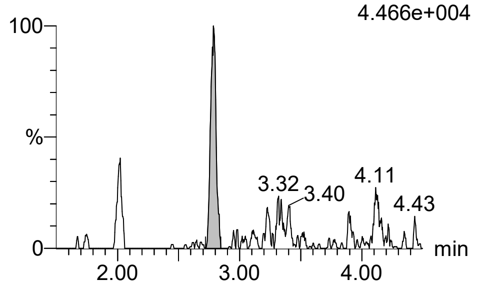

Sample Name: 20211111_L23_R1_IgG
F30:MRM of 1 channel,ES+
1019.116 > 204.1
1.239e+005

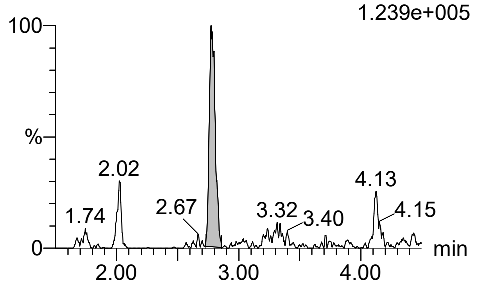

Sample Name: 20211111_L24_R1_IgG
F30:MRM of 1 channel,ES+
1019.116 > 204.1
6.356e+004

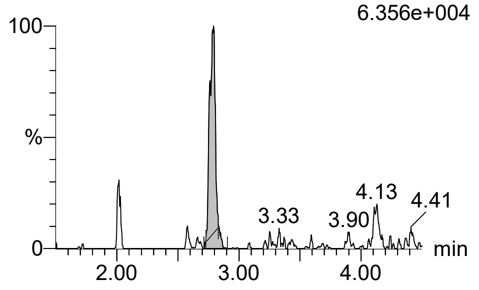

Sample Name: 20211111_H12_R1_IgG
F30:MRM of 1 channel,ES+
1019.116 > 204.1
6.891e+004

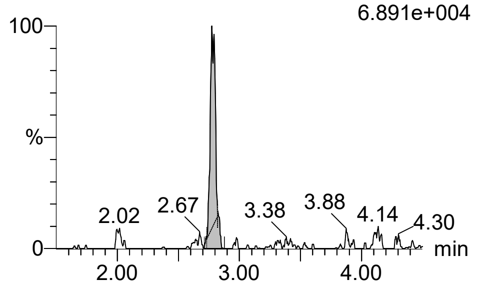

Sample Name: 20211111_QC_R6_IgG
F30:MRM of 1 channel,ES+
1019.116 > 204.1
9.092e+004

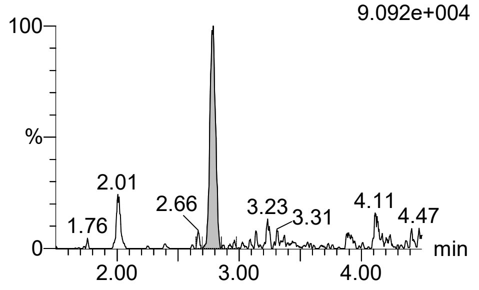

Sample Name: 20211111_A25_R1_IgG
F30:MRM of 1 channel,ES+
1019.116 > 204.1
1.003e+005

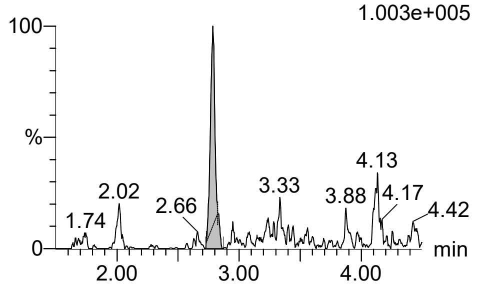

Sample Name: 20211111_A26_R1_IgG
F30:MRM of 1 channel,ES+
1019.116 > 204.1
5.062e+004

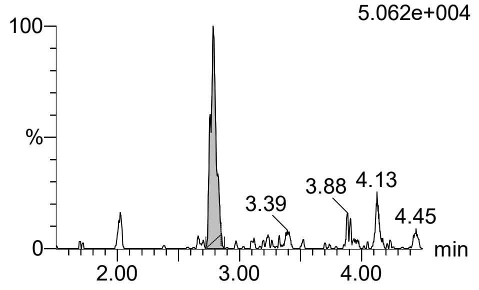

Sample Name: 20211111_L25_R1_IgG
F30:MRM of 1 channel,ES+
1019.116 > 204.1
1.381e+005

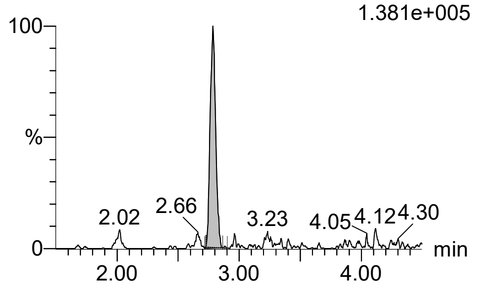

Sample Name: 20211111_L26_R1_IgG
F30:MRM of 1 channel,ES+
1019.116 > 204.1
3.104e+004

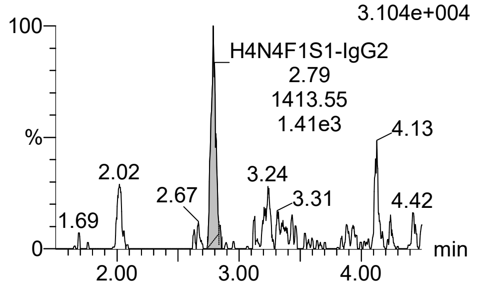

Sample Name: 20211111_H13_R1_IgG
F30:MRM of 1 channel,ES+
1019.116 > 204.1
9.836e+004

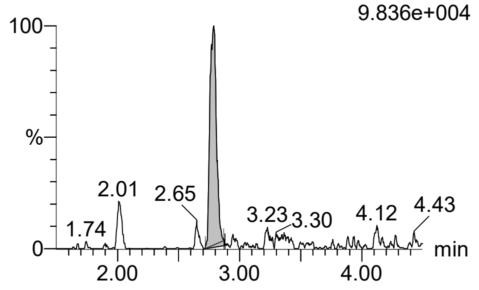

Sample Name: 20211111_A27_R1_IgG
F30:MRM of 1 channel,ES+
1019.116 > 204.1
4.515e+004

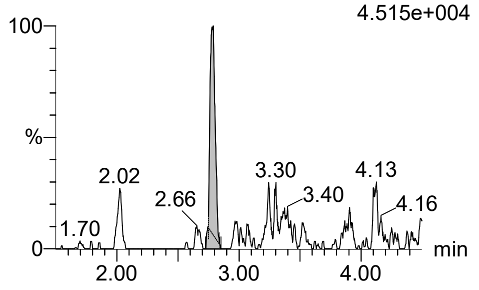

Dataset: C:\MassLynx\10.pro\20211115_clinicalsamples_IgG.qld

Last Altered: Sunday, October 02, 2022 16:02:30 台北標準時間
Printed: Friday, August 01, 2025 16:50:47 台北標準時間

Sample Name: 20211111_A28_R1_IgG

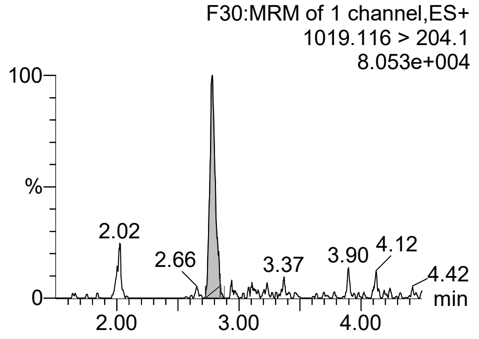

Sample Name: 20211111_L27_R1_IgG

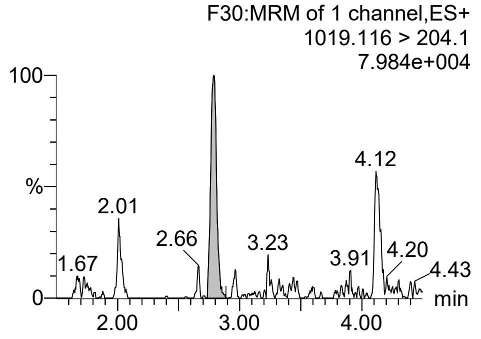

Sample Name: 20211111_L28_R1_IgG

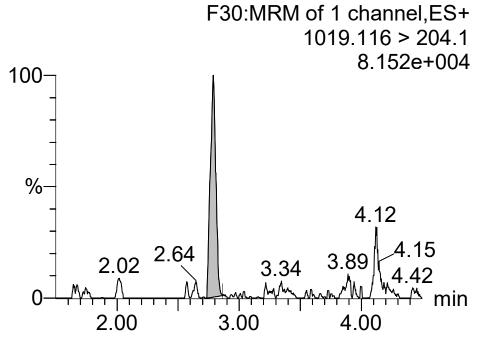

Sample Name: 20211111_H14_R1_IgG

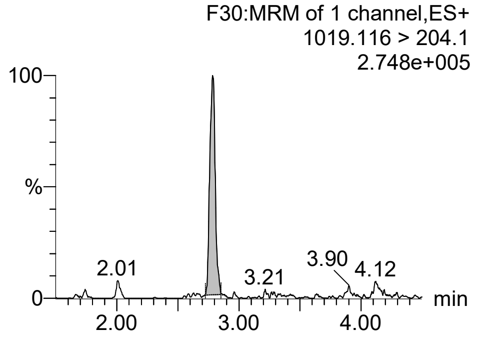

Sample Name: 20211111_A29_R1_IgG

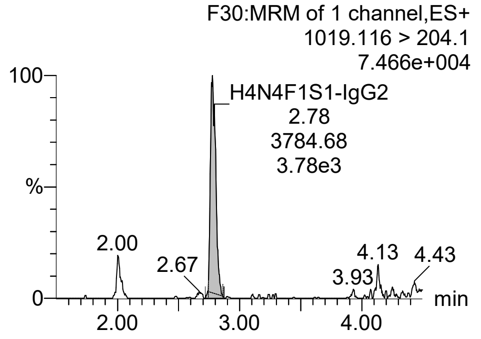

Sample Name: 20211111_A30_R1_IgG

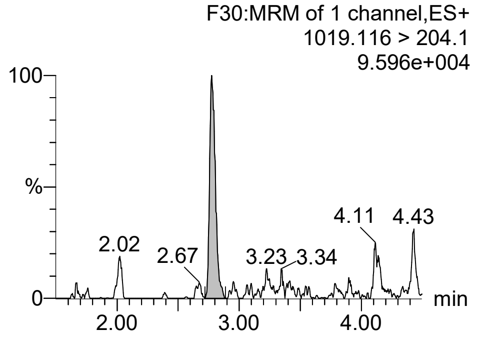

Sample Name: 20211111_QC_R7_IgG

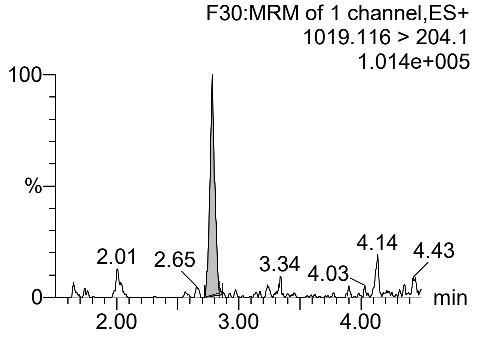

Sample Name: 20211111_L29_R1_IgG

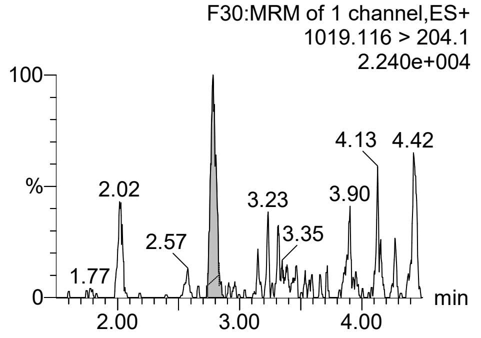

Sample Name: 20211111_L30_R1_IgG

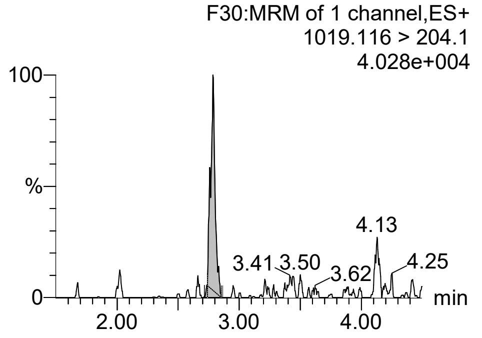

Sample Name: 20211111_H15_R1_IgG

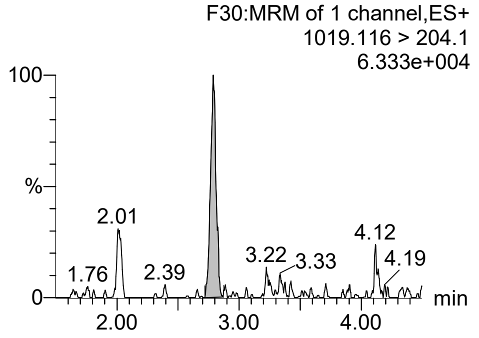

Sample Name: 20211111_A31_R1_IgG

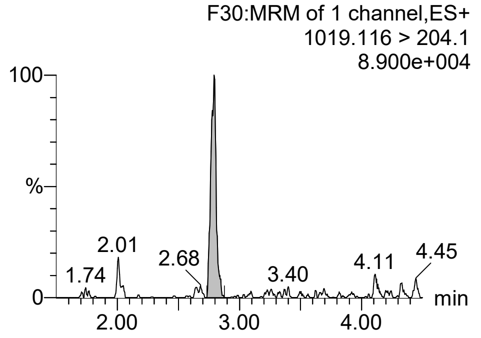

Sample Name: 20211111_A32_R1_IgG

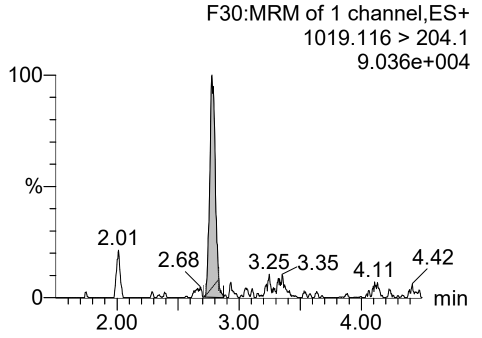

Sample Name: 20211111_L31_R1_IgG

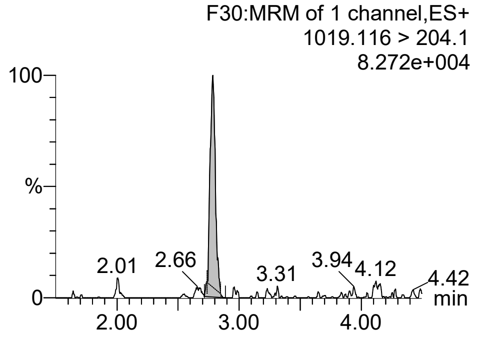

Sample Name: 20211111_L32_R1_IgG

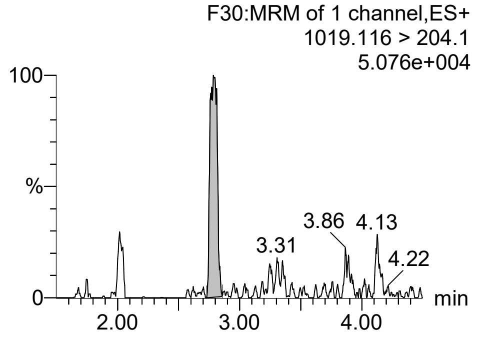

Sample Name: 20211111_H16_R1_IgG

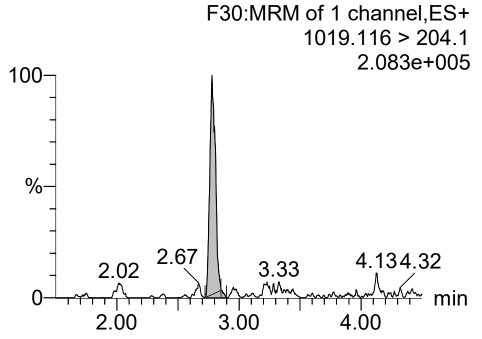

Dataset: C:\MassLynx\10.pro\20211115_clinicalsamples_IgG.qld

Last Altered: Sunday, October 02, 2022 16:02:30 台北標準時間
Printed: Friday, August 01, 2025 16:50:47 台北標準時間

Sample Name: 20211111_A33_R1_IgG
F30:MRM of 1 channel,ES+
1019.116 > 204.1
5.448e+004

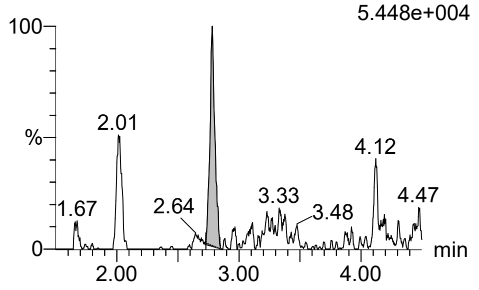

Sample Name: 20211111_A34_R1_IgG
F30:MRM of 1 channel,ES+
1019.116 > 204.1
8.059e+004

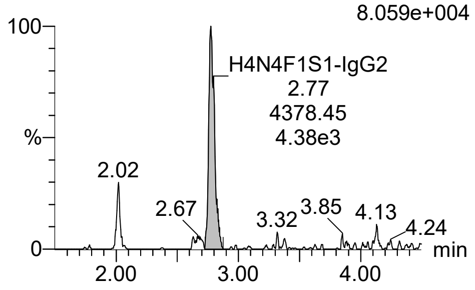

Sample Name: 20211111_H17_R1_IgG
F30:MRM of 1 channel,ES+
1019.116 > 204.1
2.457e+005

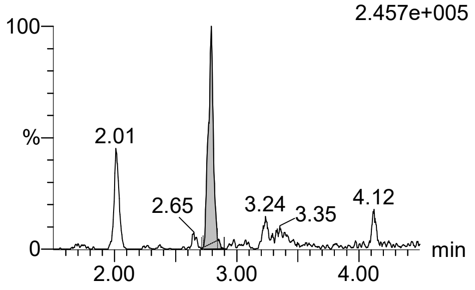

Sample Name: 20211111_A35_R1_IgG
F30:MRM of 1 channel,ES+
1019.116 > 204.1
1.158e+005

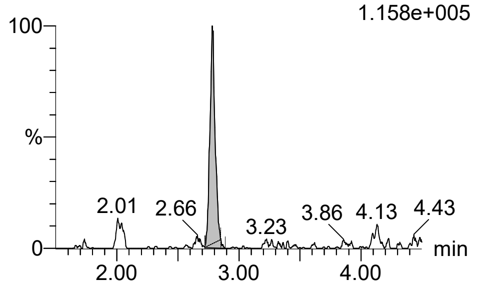

Sample Name: 20211111_QC_R8_IgG
F30:MRM of 1 channel,ES+
1019.116 > 204.1
5.985e+004

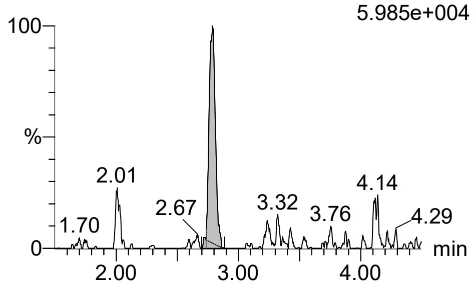

Sample Name: 20211111_A36_R1_IgG
F30:MRM of 1 channel,ES+
1019.116 > 204.1
8.518e+004

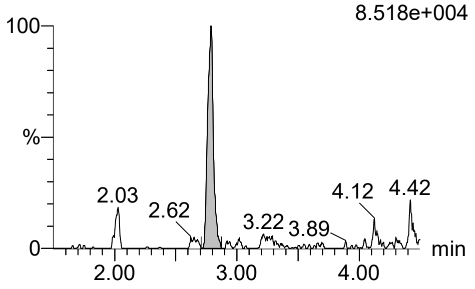

Sample Name: 20211111_H18_R1_IgG
F30:MRM of 1 channel,ES+
1019.116 > 204.1
2.253e+005

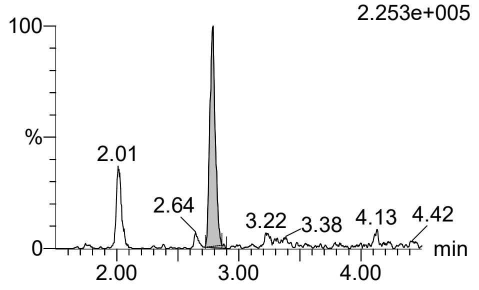

Sample Name: 20211111_A37_R1_IgG
F30:MRM of 1 channel,ES+
1019.116 > 204.1
4.146e+004

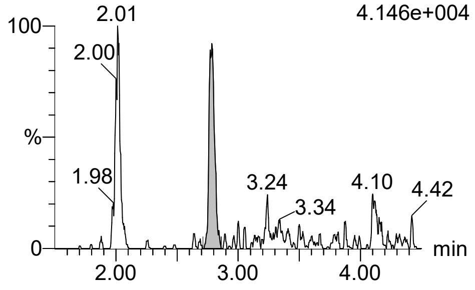

Sample Name: 20211111_A38_R1_IgG
F30:MRM of 1 channel,ES+
1019.116 > 204.1
6.375e+004

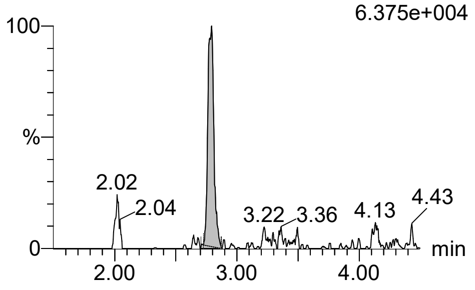

Sample Name: 20211111_H19_R1_IgG
F30:MRM of 1 channel,ES+
1019.116 > 204.1
3.648e+005

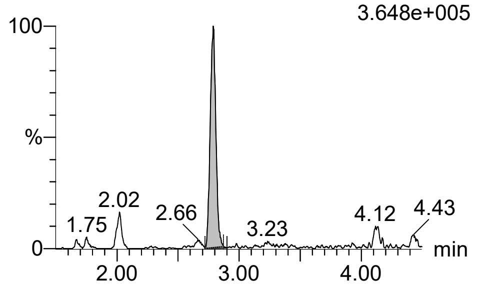

Sample Name: 20211111_A39_R1_IgG
F30:MRM of 1 channel,ES+
1019.116 > 204.1
7.567e+004

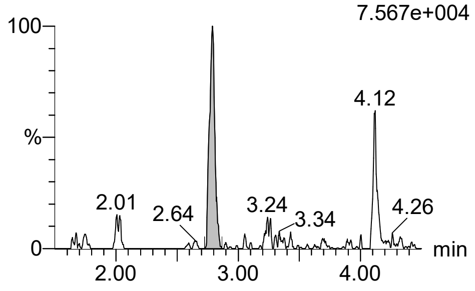

Sample Name: 20211111_A40_R1_IgG
F30:MRM of 1 channel,ES+
1019.116 > 204.1
6.834e+004

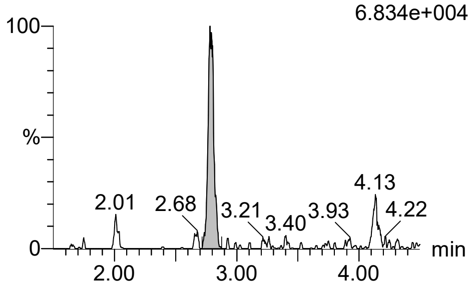

Sample Name: 20211111_H20_R1_IgG
F30:MRM of 1 channel,ES+
1019.116 > 204.1
4.428e+005

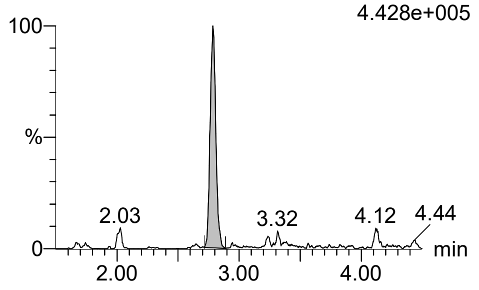

Sample Name: 20211111_A41_R1_IgG
F30:MRM of 1 channel,ES+
1019.116 > 204.1
6.906e+004

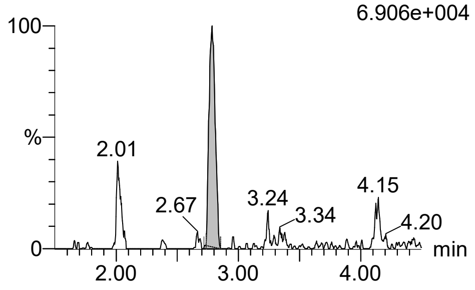

Sample Name: 20211111_A42_R1_IgG
F30:MRM of 1 channel,ES+
1019.116 > 204.1
5.671e+004

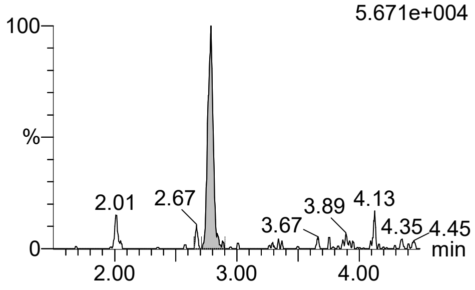

Dataset: C:\MassLynx\10.pro\20211115_clinicalsamples_IgG.qld

Last Altered: Sunday, October 02, 2022 16:02:30 台北標準時間
Printed: Friday, August 01, 2025 16:50:47 台北標準時間

Sample Name: 20211111_A43_R1_IgG
F30:MRM of 1 channel,ES+
1019.116 > 204.1
1.638e+005

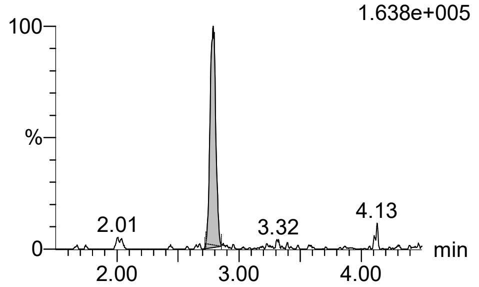

Sample Name: 20211111_A44_R1_IgG
F30:MRM of 1 channel,ES+
1019.116 > 204.1
1.393e+005

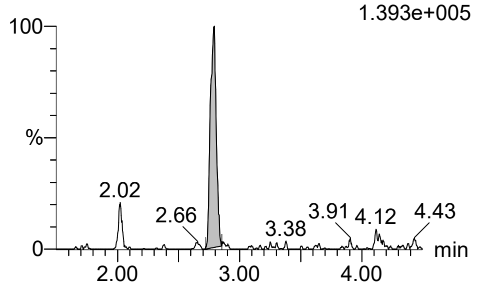

Sample Name: 20211111_QC_R9_IgG
F30:MRM of 1 channel,ES+
1019.116 > 204.1
6.739e+004

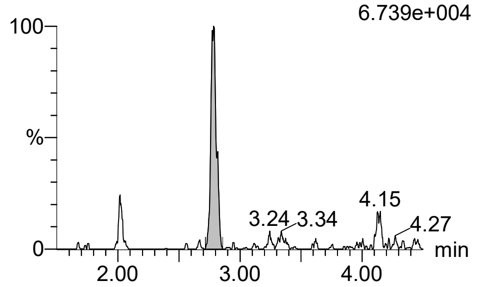

Sample Name: 20211111_A1_R2_IgG
F30:MRM of 1 channel,ES+
1019.116 > 204.1
1.797e+005

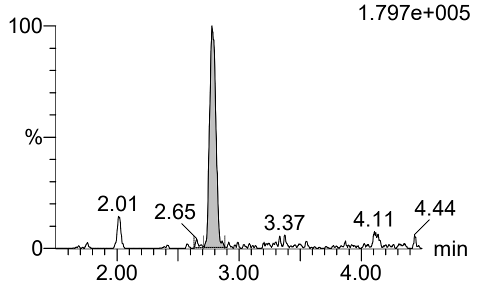

Sample Name: 20211111_A2_R2_IgG
F30:MRM of 1 channel,ES+
1019.116 > 204.1
1.005e+005

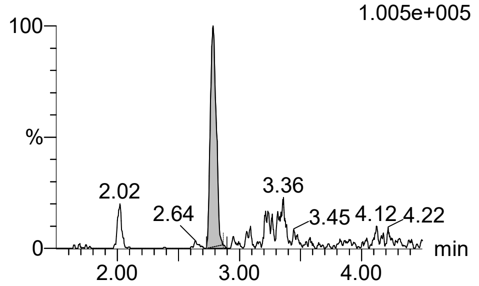

Sample Name: 20211111_L1_R2_IgG
F30:MRM of 1 channel,ES+
1019.116 > 204.1
4.715e+004

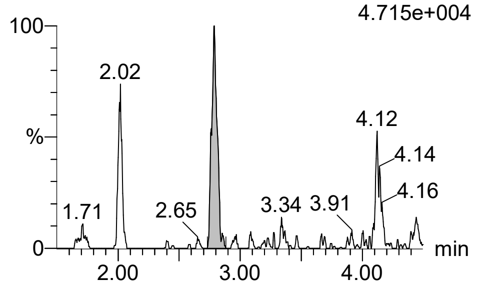

Sample Name: 20211111_L2_R2_IgG
F30:MRM of 1 channel,ES+
1019.116 > 204.1
9.014e+004

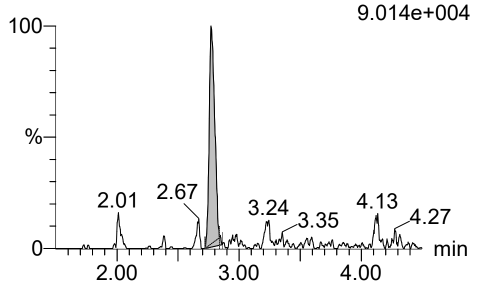

Sample Name: 20211111_H1_R2_IgG
F30:MRM of 1 channel,ES+
1019.116 > 204.1
6.929e+004

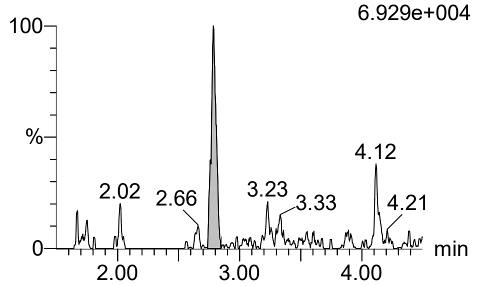

Sample Name: 20211111_A3_R2_IgG
F30:MRM of 1 channel,ES+
1019.116 > 204.1
7.201e+003

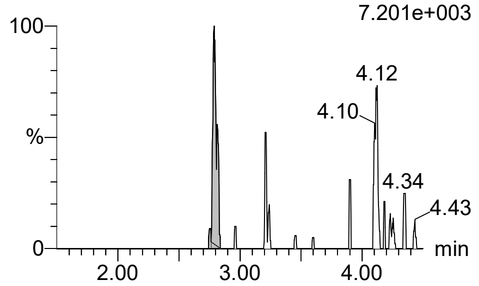

Sample Name: 20211111_A4_R2_IgG
F30:MRM of 1 channel,ES+
1019.116 > 204.1
1.597e+005

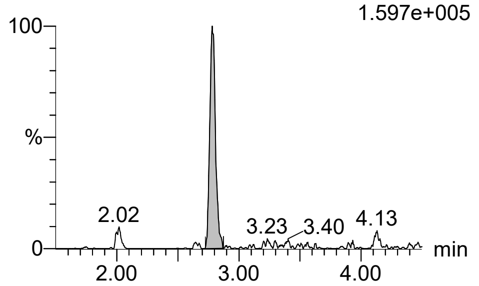

Sample Name: 20211111_L3_R2_IgG
F30:MRM of 1 channel,ES+
1019.116 > 204.1
2.369e+004

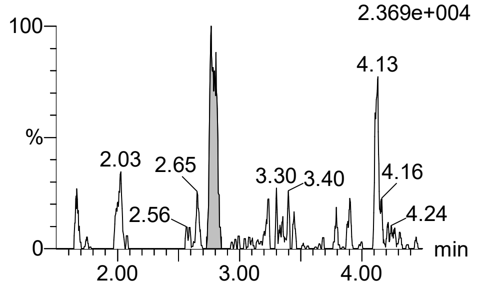

Sample Name: 20211111_L4_R2_IgG
F30:MRM of 1 channel,ES+
1019.116 > 204.1
5.858e+004

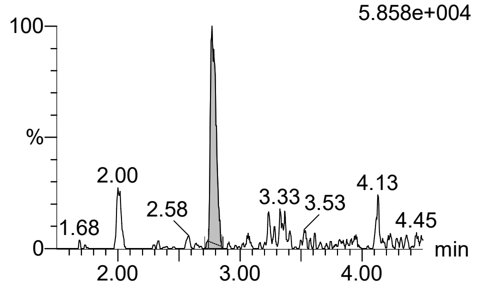

Sample Name: 20211111_H2_R2_IgG
F30:MRM of 1 channel,ES+
1019.116 > 204.1
2.207e+005

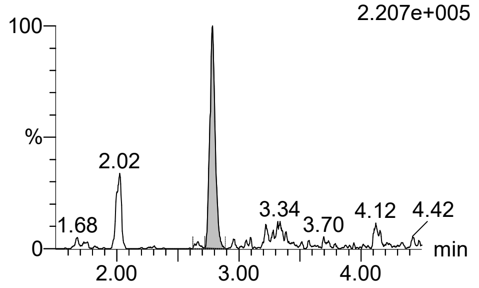

Sample Name: 20211111_A5_R2_IgG
F30:MRM of 1 channel,ES+
1019.116 > 204.1
9.914e+004

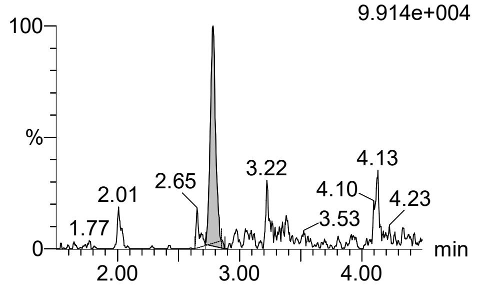

Sample Name: 20211111_A6_R2_IgG
F30:MRM of 1 channel,ES+
1019.116 > 204.1
1.961e+005

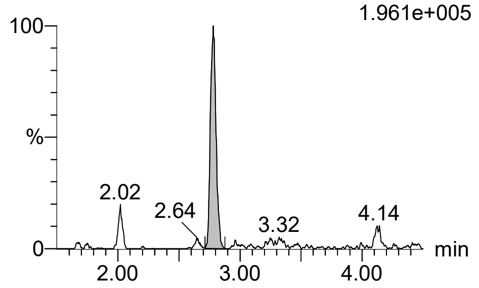

Dataset: C:\MassLynx\10.pro\20211115_clinicalsamples_IgG.qld

Last Altered: Sunday, October 02, 2022 16:02:30 台北標準時間
Printed: Friday, August 01, 2025 16:50:47 台北標準時間

Sample Name: 20211111_QC_R10_IgG
F30:MRM of 1 channel,ES+
1019.116 > 204.1
4.484e+004

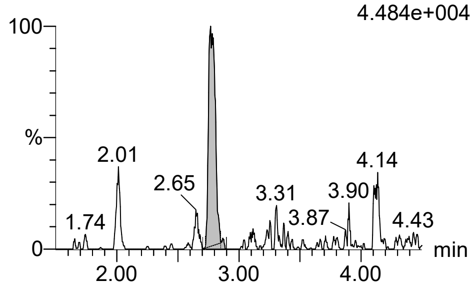

Sample Name: 20211111_L5_R2_IgG
F30:MRM of 1 channel,ES+
1019.116 > 204.1
6.449e+004

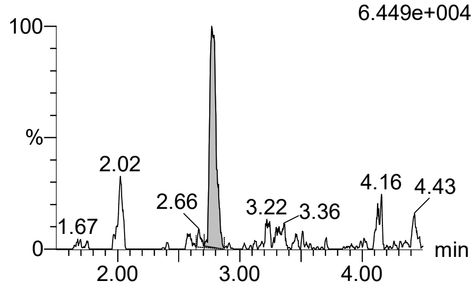

Sample Name: 20211111_L6_R2_IgG
F30:MRM of 1 channel,ES+
1019.116 > 204.1
1.578e+004

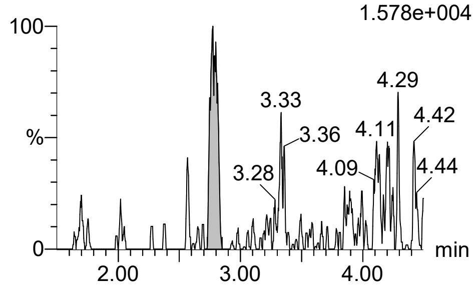

Sample Name: 20211111_H3_R2_IgG
F30:MRM of 1 channel,ES+
1019.116 > 204.1
9.673e+004

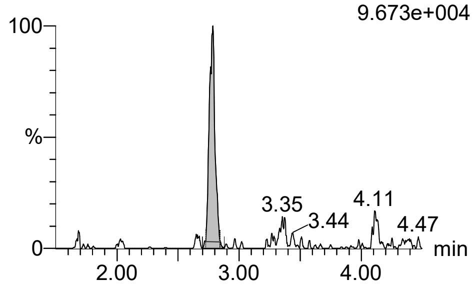

Sample Name: 20211111_A7_R2_IgG
F30:MRM of 1 channel,ES+
1019.116 > 204.1
2.644e+005

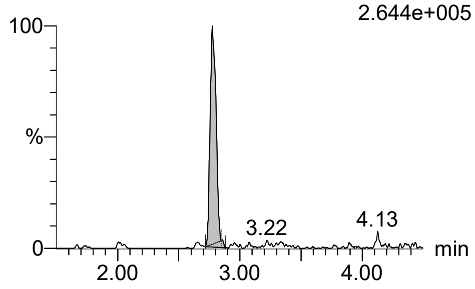

Sample Name: 20211111_A8_R2_IgG
F30:MRM of 1 channel,ES+
1019.116 > 204.1
1.176e+005

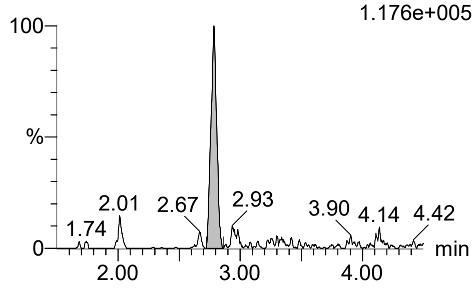

Sample Name: 20211111_L7_R2_IgG
F30:MRM of 1 channel,ES+
1019.116 > 204.1
5.387e+004

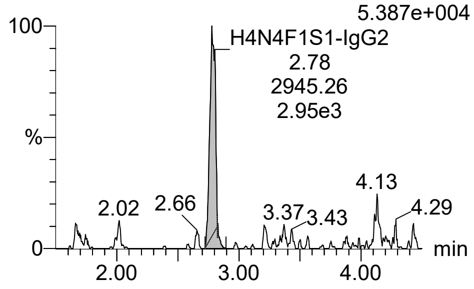

Sample Name: 20211111_L8_R2_IgG
F30:MRM of 1 channel,ES+
1019.116 > 204.1
5.898e+004

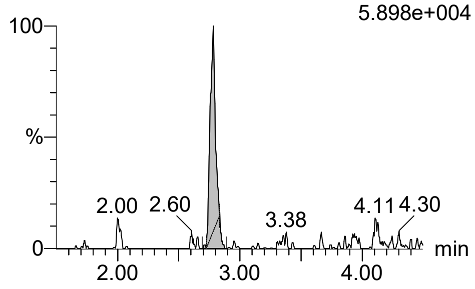

Sample Name: 20211111_H4_R2_IgG
F30:MRM of 1 channel,ES+
1019.116 > 204.1
6.047e+004

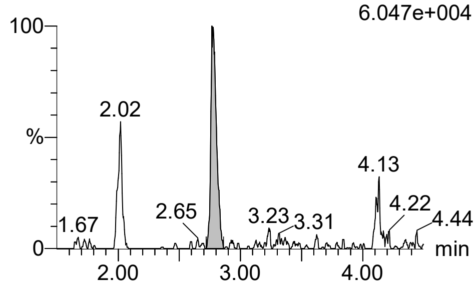

Sample Name: 20211111_A9_R2_IgG
F30:MRM of 1 channel,ES+
1019.116 > 204.1
5.456e+004

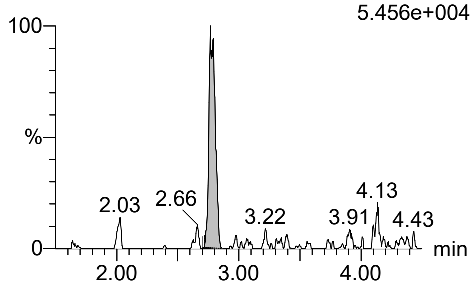

Sample Name: 20211111_A10_R2_IgG
F30:MRM of 1 channel,ES+
1019.116 > 204.1
7.712e+004

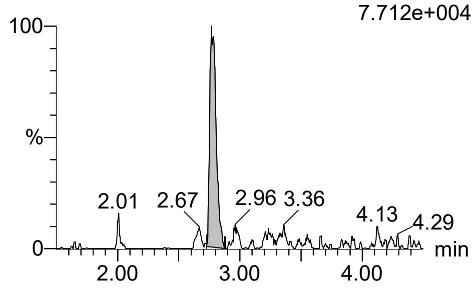

Sample Name: 20211111_L9_R2_IgG
F30:MRM of 1 channel,ES+
1019.116 > 204.1
9.656e+004

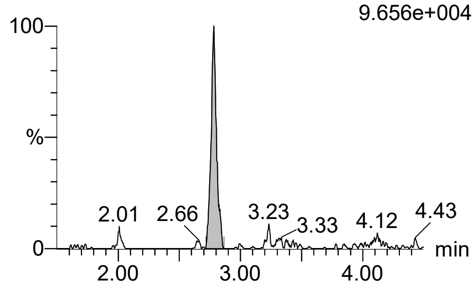

Sample Name: 20211111_L10_R2_IgG
F30:MRM of 1 channel,ES+
1019.116 > 204.1
4.994e+004

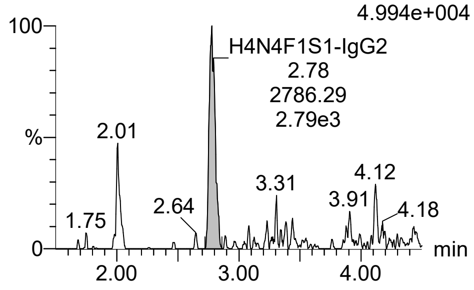

Sample Name: 20211111_QC_R11_IgG
F30:MRM of 1 channel,ES+
1019.116 > 204.1
6.921e+004

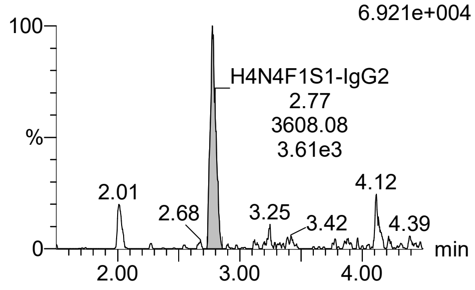

Sample Name: 20211111_H5_R2_IgG
F30:MRM of 1 channel,ES+
1019.116 > 204.1
3.919e+004

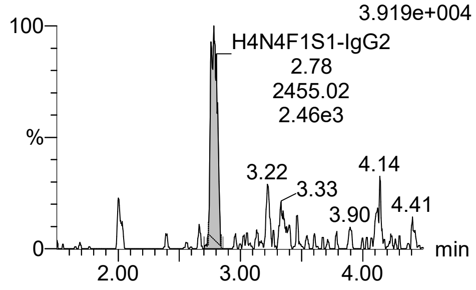

Dataset: C:\MassLynx\10.pro\20211115_clinicalsamples_IgG.qld

Last Altered: Sunday, October 02, 2022 16:02:30 台北標準時間
Printed: Friday, August 01, 2025 16:50:47 台北標準時間

Sample Name: 20211111_A11_R2_IgG
F30:MRM of 1 channel,ES+
1019.116 > 204.1
1.740e+004

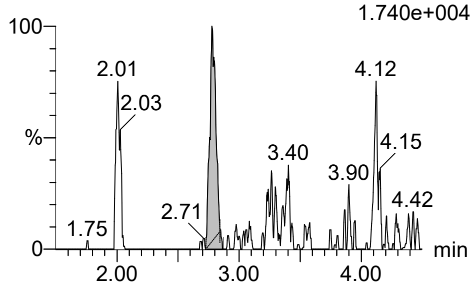

Sample Name: 20211111_A12_R2_IgG
F30:MRM of 1 channel,ES+
1019.116 > 204.1
7.437e+004

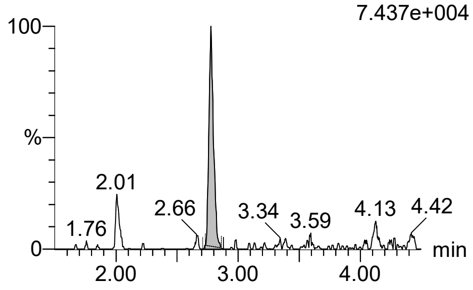

Sample Name: 20211111_L11_R2_IgG
F30:MRM of 1 channel,ES+
1019.116 > 204.1
1.830e+004

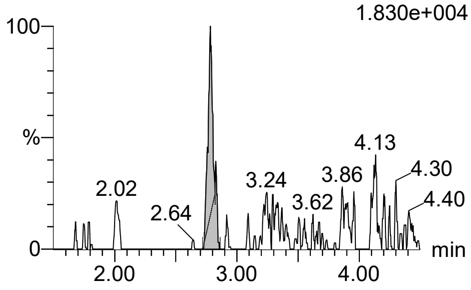

Sample Name: 20211111_L12_R2_IgG
F30:MRM of 1 channel,ES+
1019.116 > 204.1
3.762e+004

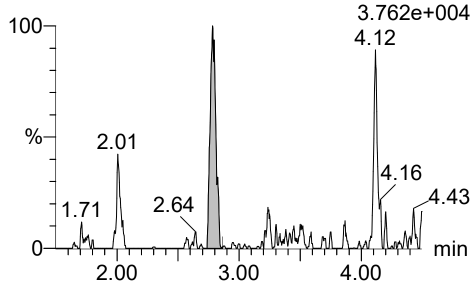

Sample Name: 20211111_H6_R2_IgG
F30:MRM of 1 channel,ES+
1019.116 > 204.1
1.390e+005

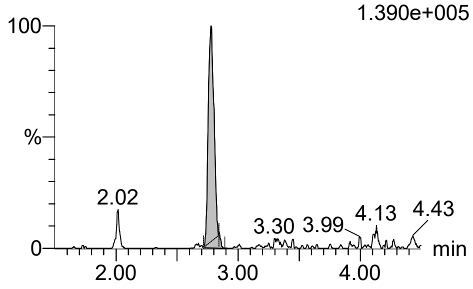

Sample Name: 20211111_A13_R2_IgG
F30:MRM of 1 channel,ES+
1019.116 > 204.1
1.006e+005

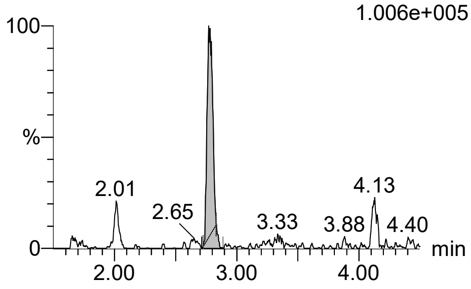

Sample Name: 20211111_A14_R2_IgG
F30:MRM of 1 channel,ES+
1019.116 > 204.1
5.880e+004

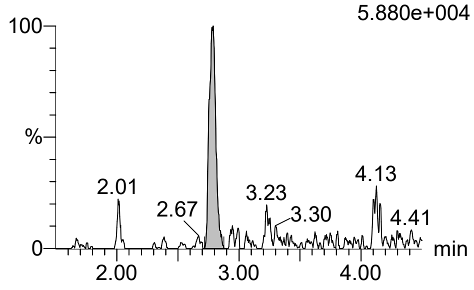

Sample Name: 20211111_L13_R2_IgG
F30:MRM of 1 channel,ES+
1019.116 > 204.1
1.059e+005

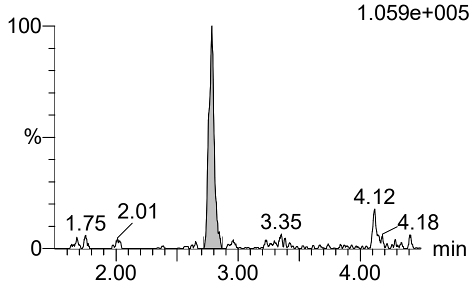

Sample Name: 20211111_L14_R2_IgG
F30:MRM of 1 channel,ES+
1019.116 > 204.1
4.402e+004

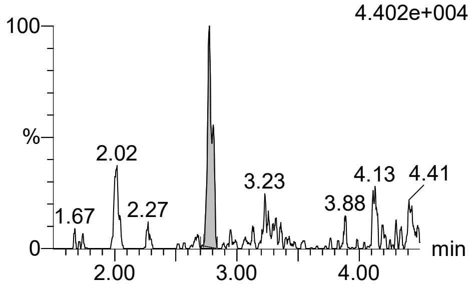

Sample Name: 20211111_H7_R2_IgG
F30:MRM of 1 channel,ES+
1019.116 > 204.1
6.595e+004

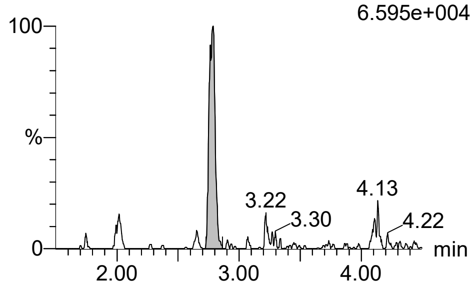

Sample Name: 20211111_A15_R2_IgG
F30:MRM of 1 channel,ES+
1019.116 > 204.1
8.098e+004

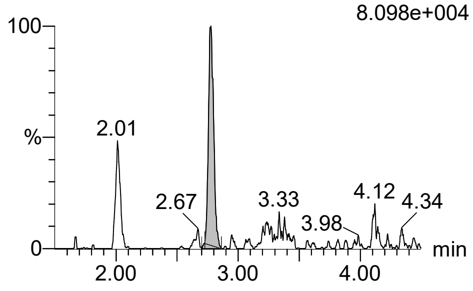

Sample Name: 20211111_QC_R12_IgG
F30:MRM of 1 channel,ES+
1019.116 > 204.1
5.283e+004

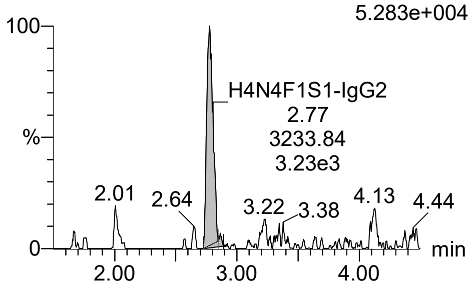

Sample Name: 20211111_A16_R2_IgG
F30:MRM of 1 channel,ES+
1019.116 > 204.1
6.411e+004

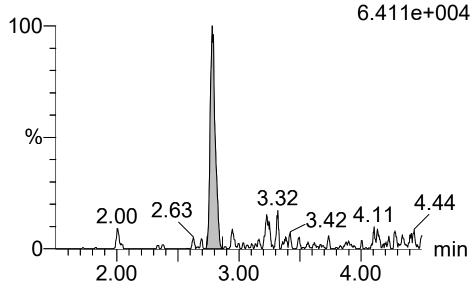

Sample Name: 20211111_L15_R2_IgG
F30:MRM of 1 channel,ES+
1019.116 > 204.1
7.407e+004

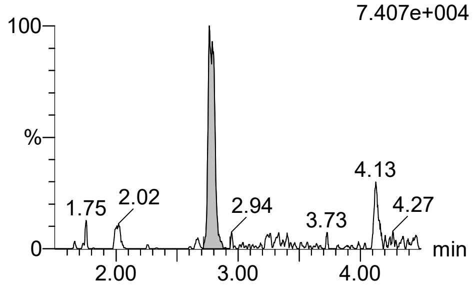

Sample Name: 20211111_L16_R2_IgG
F30:MRM of 1 channel,ES+
1019.116 > 204.1
5.078e+004

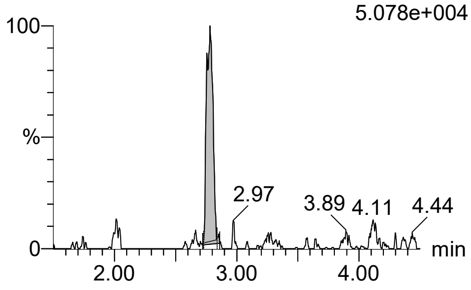

Dataset: C:\MassLynx\10.pro\20211115_clinicalsamples_IgG.qld

Last Altered: Sunday, October 02, 2022 16:02:30 台北標準時間
Printed: Friday, August 01, 2025 16:50:47 台北標準時間

Sample Name: 20211111_H8_R2_IgG
F30:MRM of 1 channel,ES+
1019.116 > 204.1
7.423e+004

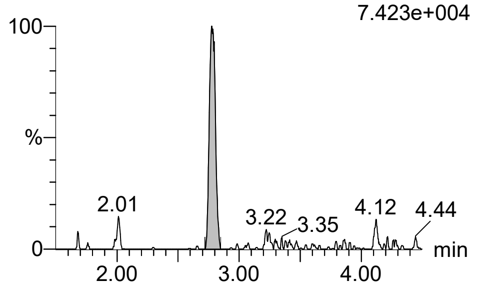

Sample Name: 20211111_A17_R2_IgG
F30:MRM of 1 channel,ES+
1019.116 > 204.1
7.395e+004

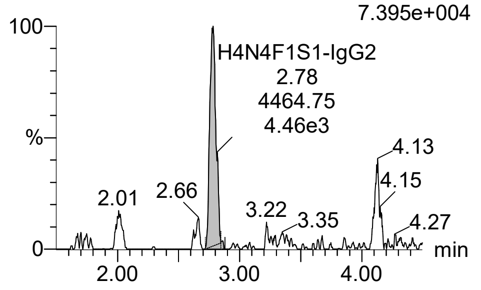

Sample Name: 20211111_A18_R2_IgG
F30:MRM of 1 channel,ES+
1019.116 > 204.1
6.490e+004

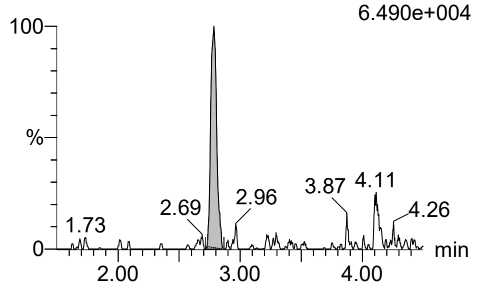

Sample Name: 20211111_L17_R2_IgG
F30:MRM of 1 channel,ES+
1019.116 > 204.1
8.035e+004

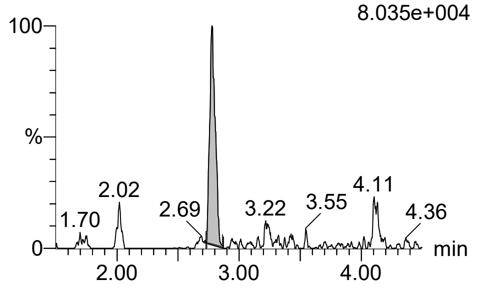

Sample Name: 20211111_L18_R2_IgG
F30:MRM of 1 channel,ES+
1019.116 > 204.1
8.029e+003

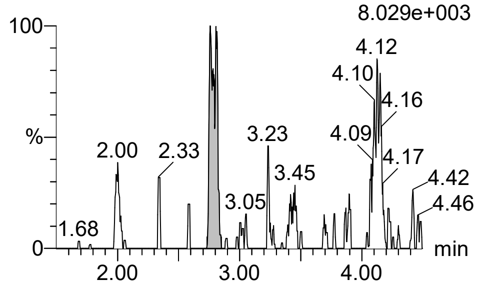

Sample Name: 20211111_H9_R2_IgG
F30:MRM of 1 channel,ES+
1019.116 > 204.1
7.511e+004

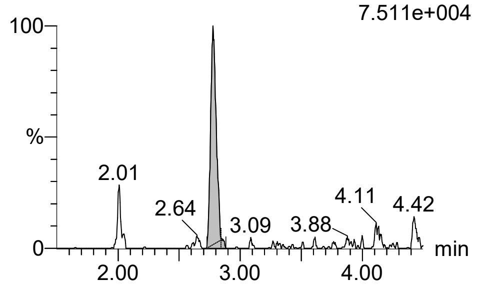

Sample Name: 20211111_A19_R2_IgG
F30:MRM of 1 channel,ES+
1019.116 > 204.1
9.166e+004

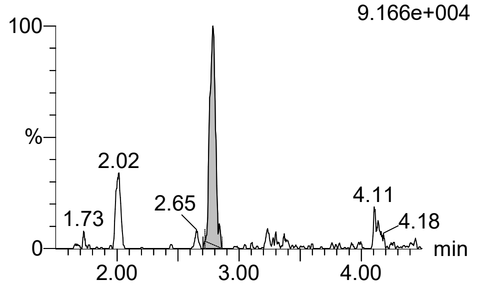

Sample Name: 20211111_A20_R2_IgG
F30:MRM of 1 channel,ES+
1019.116 > 204.1
2.342e+004

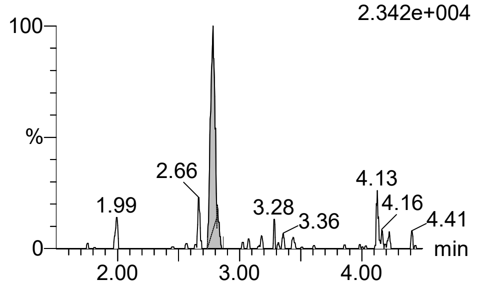

Sample Name: 20211111_L19_R2_IgG
F30:MRM of 1 channel,ES+
1019.116 > 204.1
4.557e+004

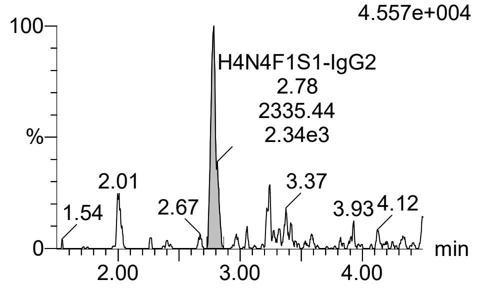

Sample Name: 20211111_QC_R13_IgG
F30:MRM of 1 channel,ES+
1019.116 > 204.1
6.719e+004

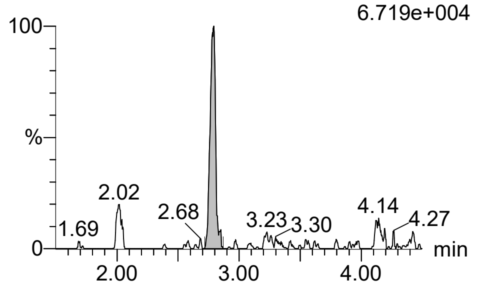

Sample Name: 20211111_L20_R2_IgG
F30:MRM of 1 channel,ES+
1019.116 > 204.1
3.201e+004

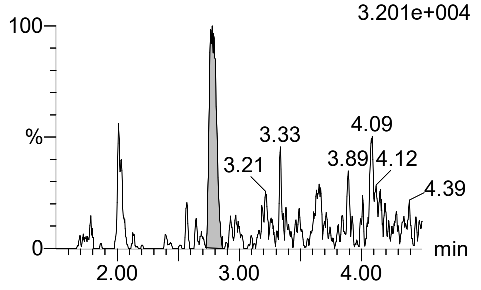

Sample Name: 20211111_H10_R2_IgG
F30:MRM of 1 channel,ES+
1019.116 > 204.1
5.191e+004

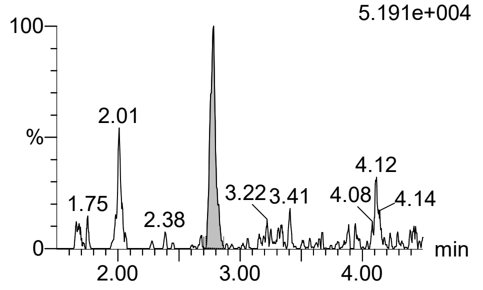

Sample Name: 20211111_A21_R2_IgG
F30:MRM of 1 channel,ES+
1019.116 > 204.1
1.821e+005

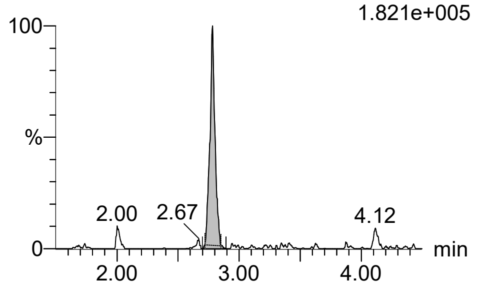

Sample Name: 20211111_A22_R2_IgG
F30:MRM of 1 channel,ES+
1019.116 > 204.1
3.096e+004

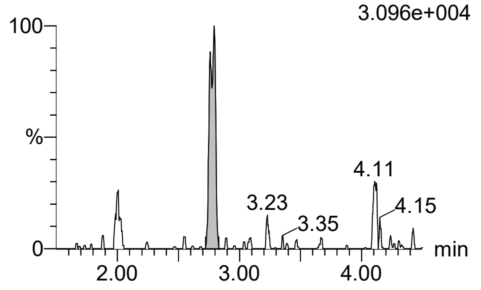

Sample Name: 20211111_L21_R2_IgG
F30:MRM of 1 channel,ES+
1019.116 > 204.1
2.615e+004

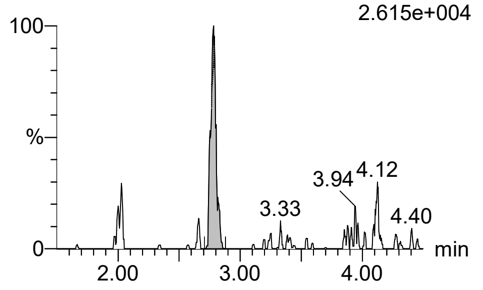

Sample Name: 20211111_L22_R2_IgG
F30:MRM of 1 channel,ES+
1019.116 > 204.1
4.024e+004

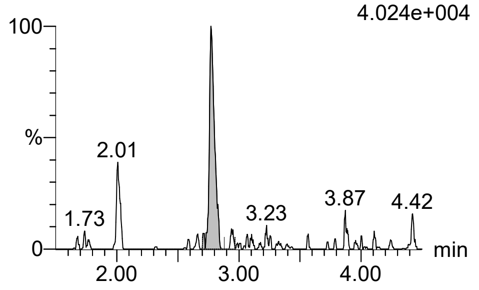

Sample Name: 20211111_H11_R2_IgG
F30:MRM of 1 channel,ES+
1019.116 > 204.1
1.071e+005

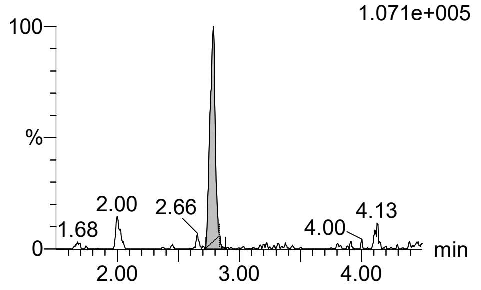

Sample Name: 20211111_A23_R2_IgG
F30:MRM of 1 channel,ES+
1019.116 > 204.1
5.651e+004

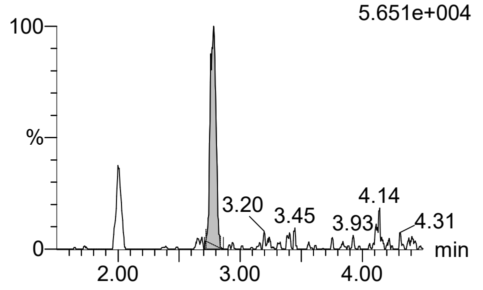

Sample Name: 20211111_A24_R2_IgG
F30:MRM of 1 channel,ES+
1019.116 > 204.1
2.850e+004

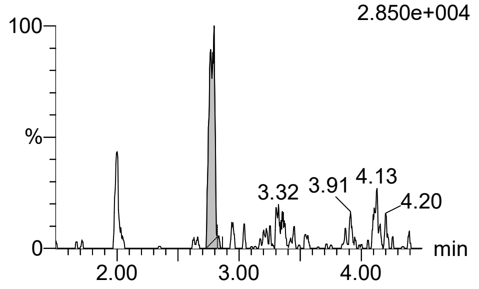

Sample Name: 20211111_L23_R2_IgG
F30:MRM of 1 channel,ES+
1019.116 > 204.1
8.207e+004

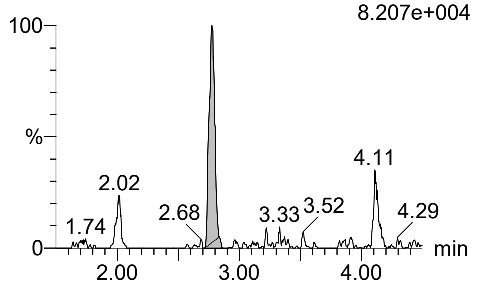

Sample Name: 20211111_L24_R2_IgG
F30:MRM of 1 channel,ES+
1019.116 > 204.1
4.677e+004

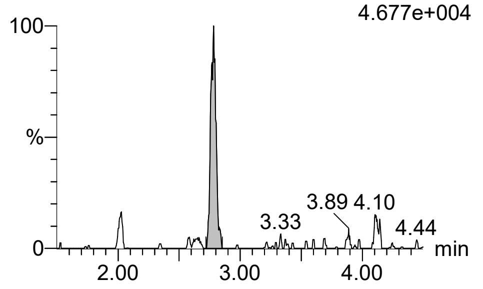

Sample Name: 20211111_H12_R2_IgG
F30:MRM of 1 channel,ES+
1019.116 > 204.1
5.244e+004

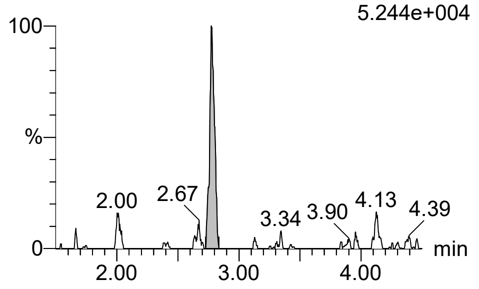

Sample Name: 20211111_QC_R14_IgG
F30:MRM of 1 channel,ES+
1019.116 > 204.1
4.525e+004

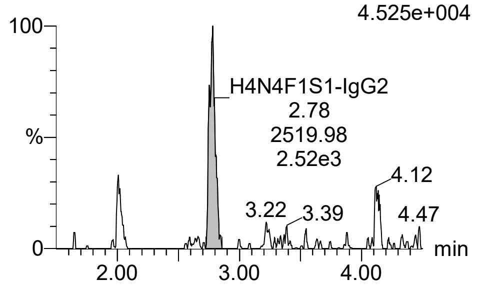

Sample Name: 20211111_A25_R2_IgG
F30:MRM of 1 channel,ES+
1019.116 > 204.1
5.511e+004

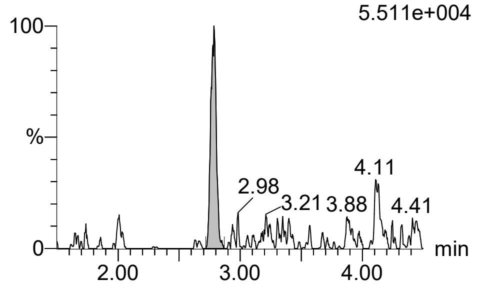

Sample Name: 20211111_A26_R2_IgG
F30:MRM of 1 channel,ES+
1019.116 > 204.1
4.614e+004

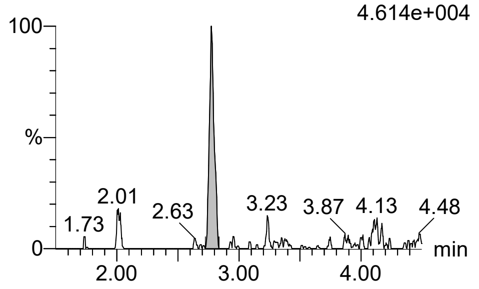

Sample Name: 20211111_L25_R2_IgG
F30:MRM of 1 channel,ES+
1019.116 > 204.1
7.686e+004

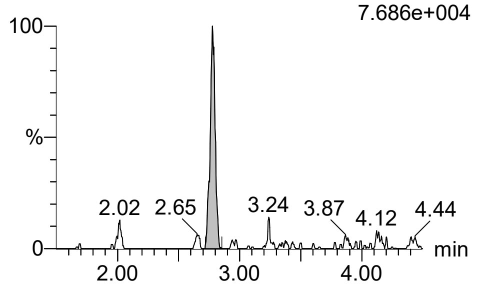

Sample Name: 20211111_L26_R2_IgG
F30:MRM of 1 channel,ES+
1019.116 > 204.1
2.576e+004

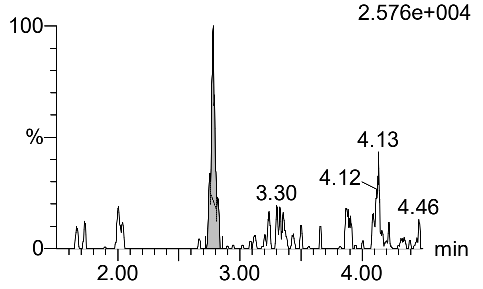

Sample Name: 20211111_H13_R2_IgG
F30:MRM of 1 channel,ES+
1019.116 > 204.1
7.572e+004

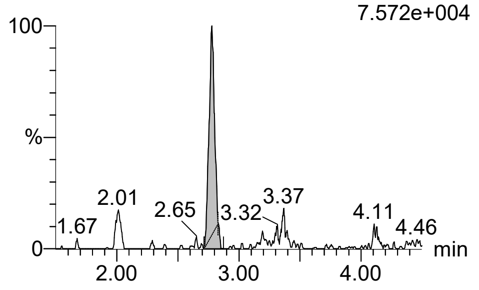

Sample Name: 20211111_A27_R2_IgG
F30:MRM of 1 channel,ES+
1019.116 > 204.1
3.021e+004

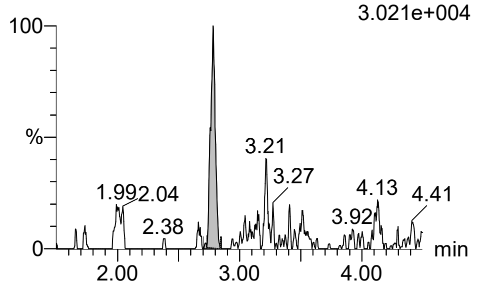

Sample Name: 20211111_A28_R2_IgG
F30:MRM of 1 channel,ES+
1019.116 > 204.1
4.703e+004

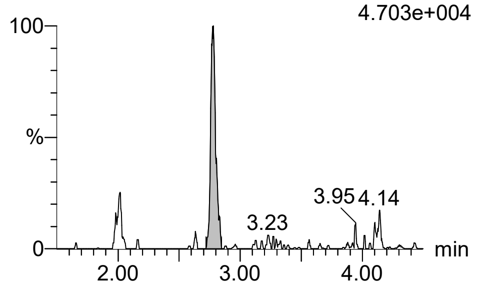

Dataset: C:\MassLynx\10.pro\20211115_clinicalsamples_IgG.qld

Last Altered: Sunday, October 02, 2022 16:02:30 台北標準時間
Printed: Friday, August 01, 2025 16:50:47 台北標準時間

Sample Name: 20211111_L27_R2_IgG

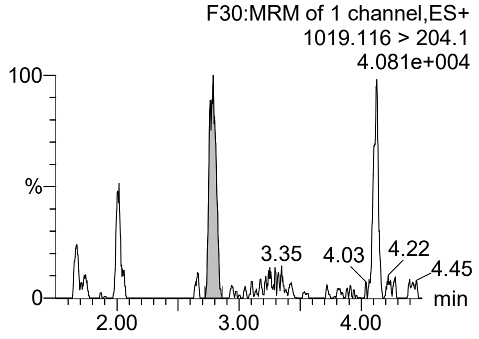

Sample Name: 20211111_L28_R2_IgG

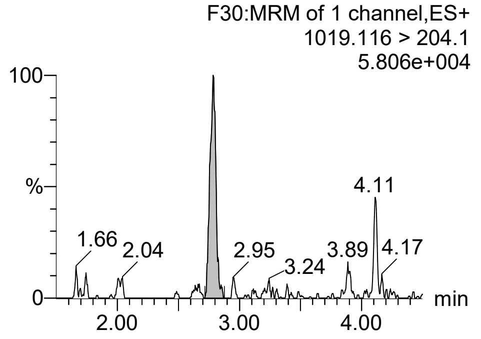

Sample Name: 20211111_H14_R2_IgG

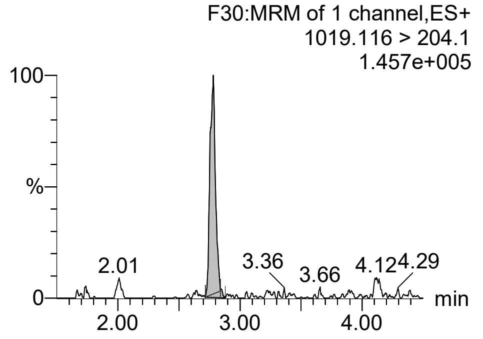

Sample Name: 20211111_A29_R2_IgG

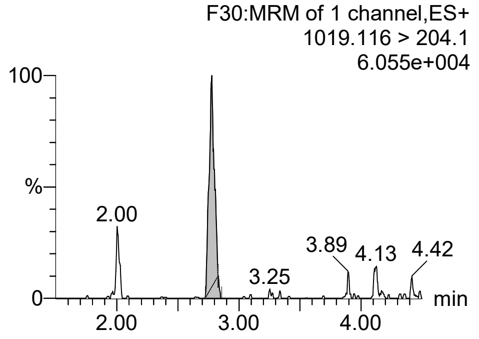

Sample Name: 20211111_A30_R2_IgG

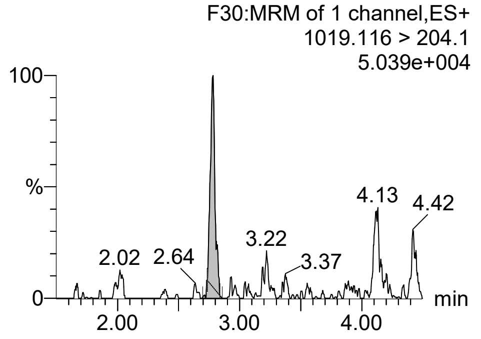

Sample Name: 20211111_QC_R15_IgG

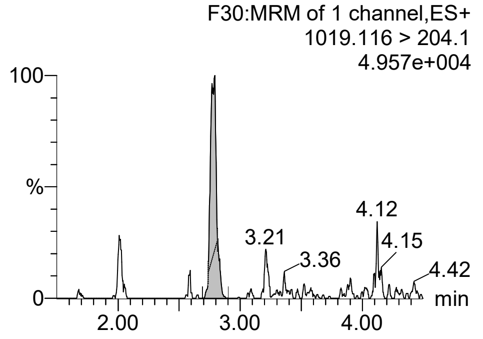

Sample Name: 20211111_L29_R2_IgG

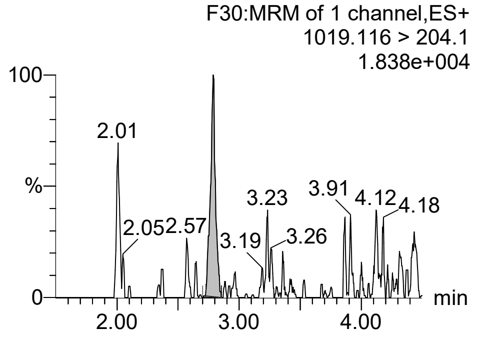

Sample Name: 20211111_L30_R2_IgG

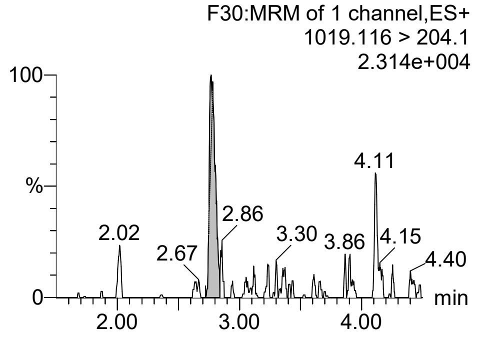

Sample Name: 20211111_H15_R2_IgG

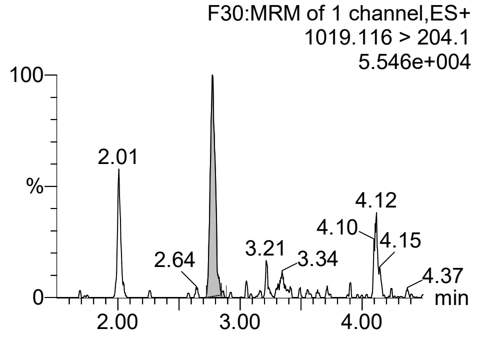

Sample Name: 20211111_A31_R2_IgG

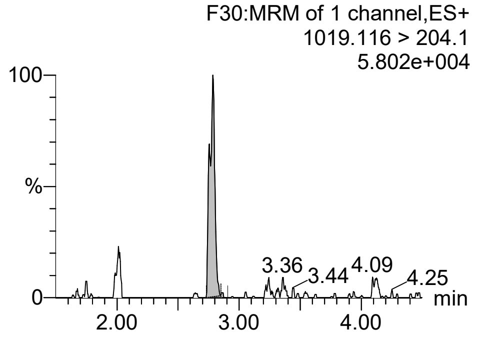

Sample Name: 20211111_A32_R2_IgG

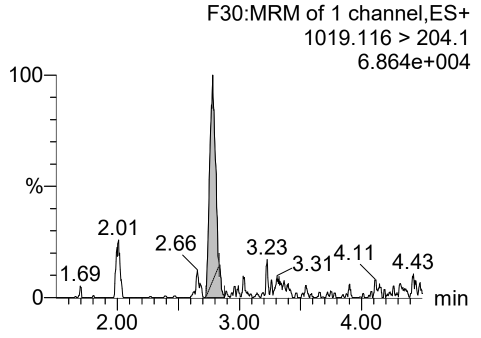

Sample Name: 20211111_L31_R2_IgG

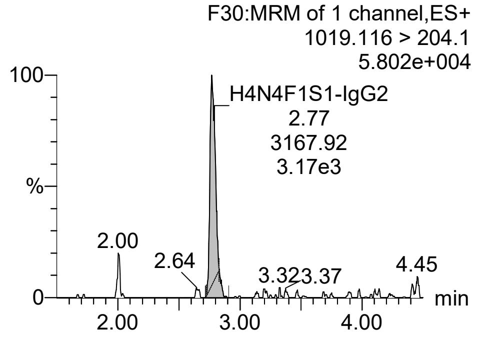

Sample Name: 20211111_L32_R2_IgG

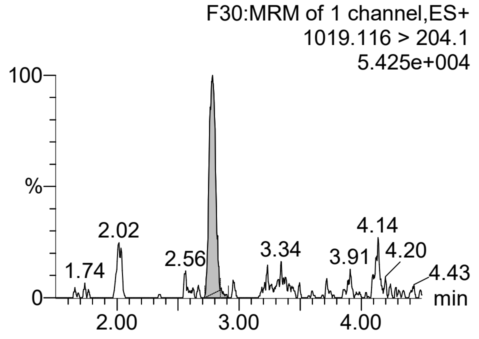

Sample Name: 20211111_H16_R2_IgG

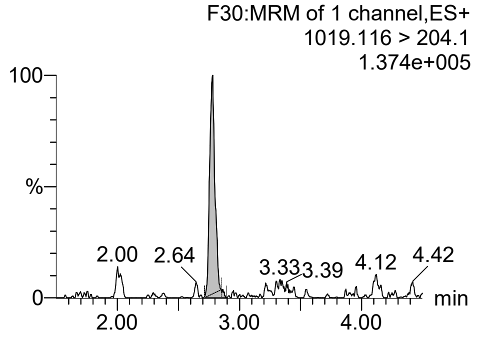

Sample Name: 20211111_A33_R2_IgG

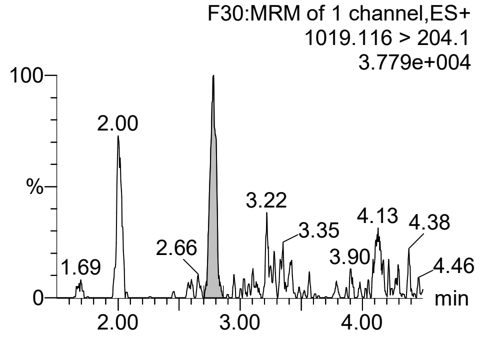

Dataset: C:\MassLynx\10.pro\20211115_clinicalsamples_IgG.qld

Last Altered: Sunday, October 02, 2022 16:02:30 台北標準時間
Printed: Friday, August 01, 2025 16:50:47 台北標準時間

Sample Name: 20211111_A34_R2_IgG

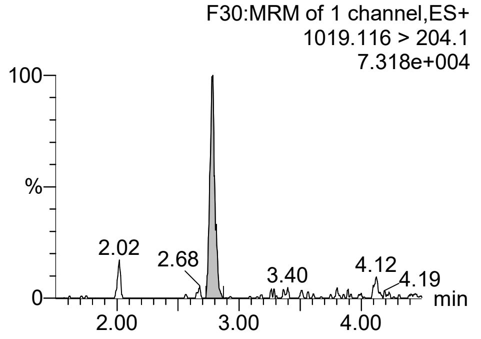

Sample Name: 20211111_H17_R2_IgG

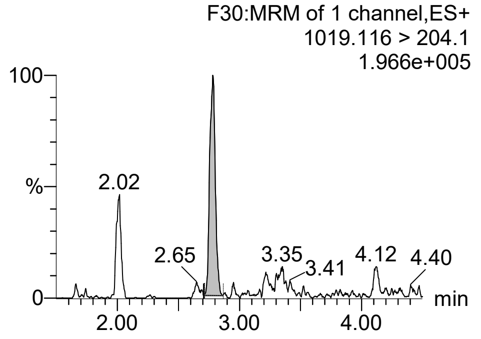

Sample Name: 20211111_A35_R2_IgG

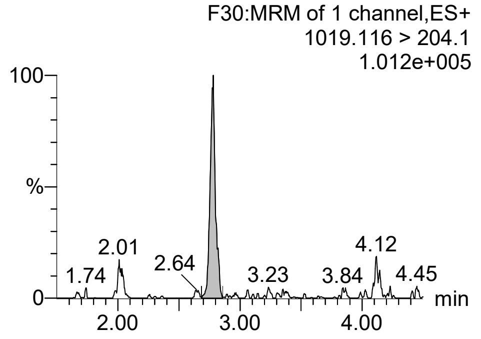

Sample Name: 20211111_QC_R16_IgG

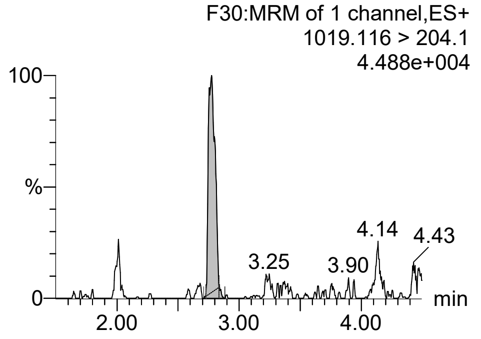

Sample Name: 20211111_A36_R2_IgG

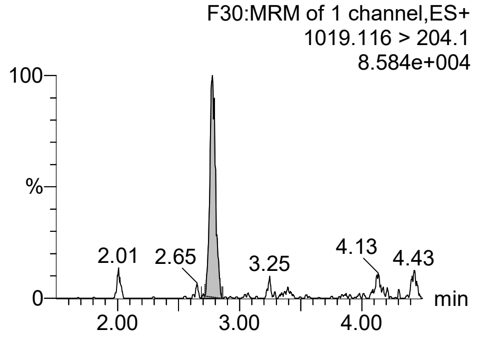

Sample Name: 20211111_H18_R2_IgG

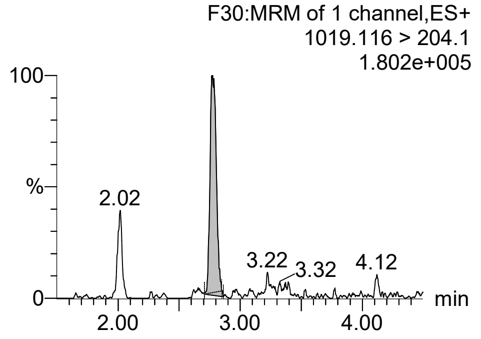

Sample Name: 20211111_A37_R2_IgG

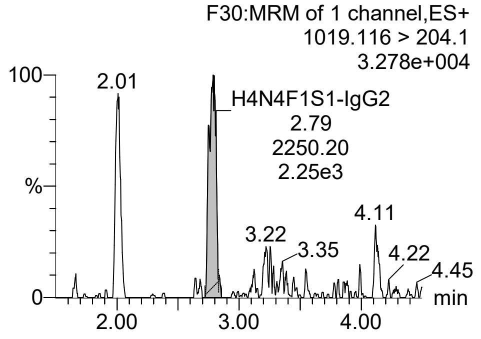

Sample Name: 20211111_A38_R2_IgG

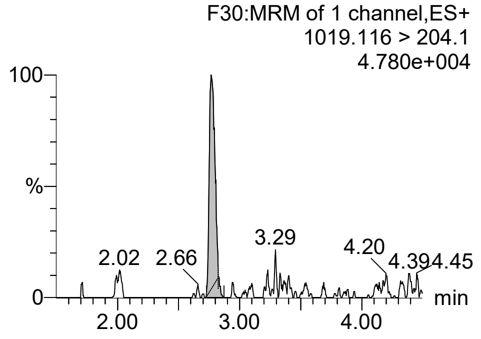

Sample Name: 20211111_H19_R2_IgG

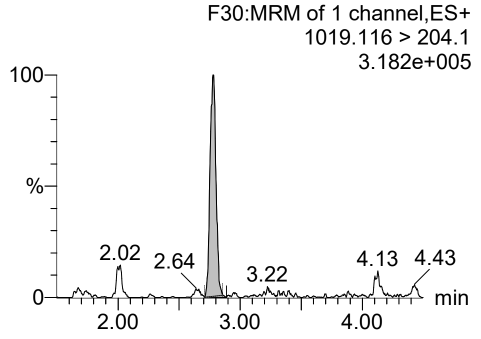

Sample Name: 20211111_A39_R2_IgG

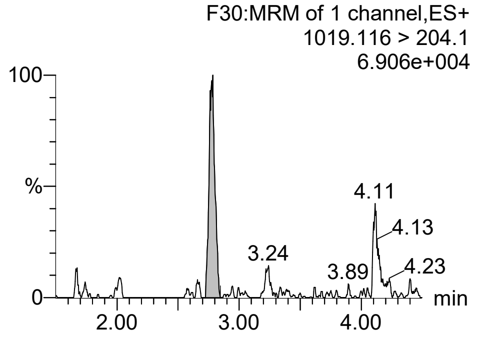

Sample Name: 20211111_A40_R2_IgG

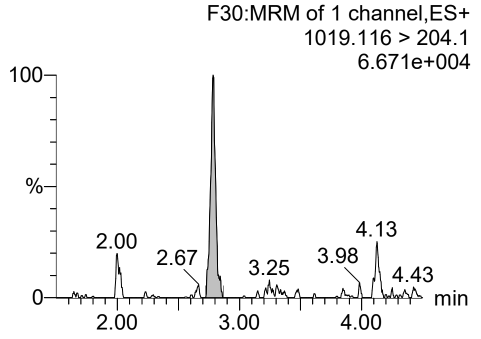

Sample Name: 20211111_H20_R2_IgG

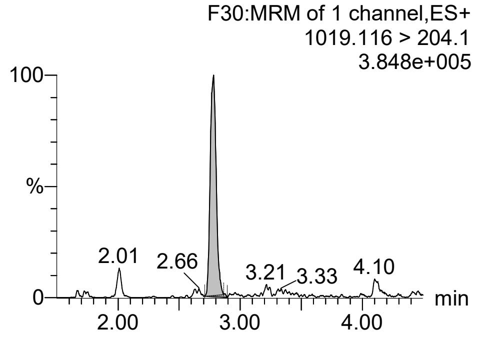

Sample Name: 20211111_A41_R2_IgG

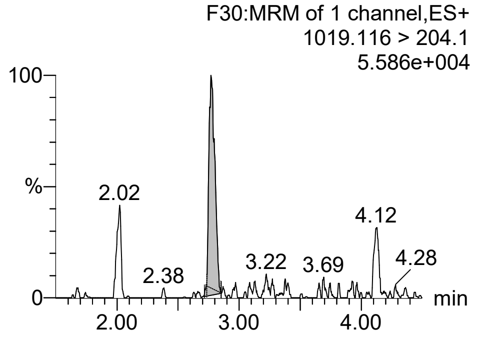

Sample Name: 20211111_A42_R2_IgG

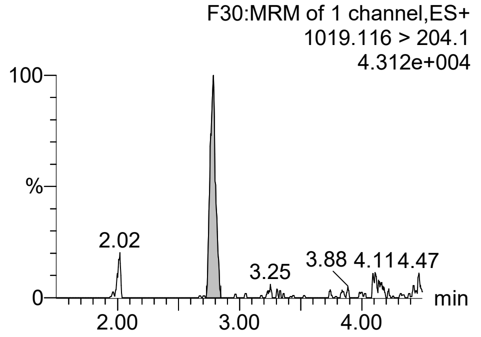

Sample Name: 20211111_A43_R2_IgG

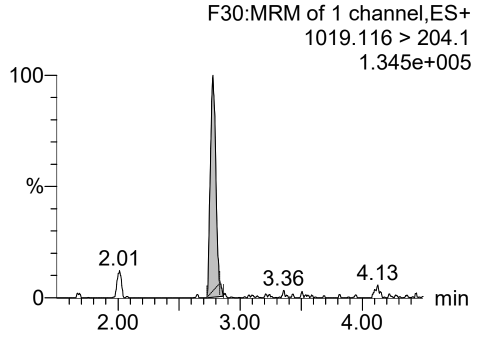

Dataset: C:\MassLynx\10.pro\20211115_clinicalsamples_IgG.qld

Last Altered: Sunday, October 02, 2022 16:02:30 台北標準時間
Printed: Friday, August 01, 2025 16:50:47 台北標準時間

Sample Name: 20211111_A44_R2_IgG

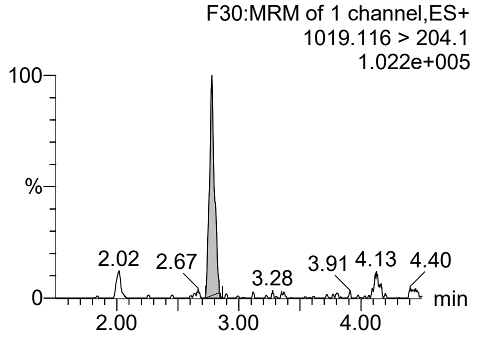

Sample Name: 20211111_QC_R17_IgG

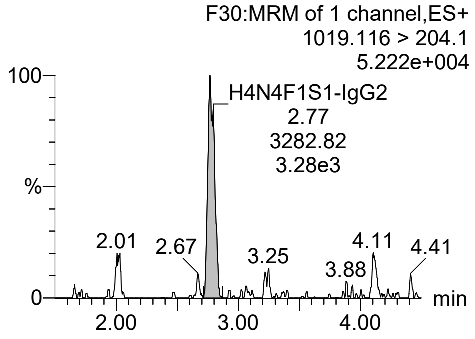

Dataset: C:\MassLynx\10.pro\20211115_clinicalsamples_IgG.qld

Last Altered: Sunday, October 02, 2022 16:02:30 台北標準時間
Printed: Friday, August 01, 2025 16:50:47 台北標準時間

	#	Name	RT	Area	IS Area	Response	Prima...
1	1	20211111_QC_R1_IgG	2.78	8282.9	64968.0	0.127	MM
2	2	20211111_A1_R1_IgG	2.78	16883.3	59059.2	0.286	MM
3	3	20211111_A2_R1_IgG	2.79	8605.3	66621.2	0.129	bb
4	4	20211111_L1_R1_IgG	2.79	4946.7	64201.0	0.077	MM
5	5	20211111_L2_R1_IgG	2.79	6412.6	46950.2	0.137	bb
6	6	20211111_H1_R1_IgG	2.78	2724.7	38648.2	0.071	MM
7	7	20211111_A3_R1_IgG	2.81	127.8	2603.7	0.049	bb
8	8	20211111_A4_R1_IgG	2.79	14785.4	69960.4	0.211	bb
9	9	20211111_L3_R1_IgG	2.77	1730.3	38864.7	0.045	MM
10	10	20211111_L4_R1_IgG	2.78	4364.3	43380.5	0.101	MM
11	11	20211111_H2_R1_IgG	2.78	18025.4	71349.2	0.253	MM
12	12	20211111_A5_R1_IgG	2.78	6036.4	65569.7	0.092	bb
13	13	20211111_A6_R1_IgG	2.78	11879.3	53120.7	0.224	MM
14	14	20211111_QC_R2_IgG	2.79	9247.1	81034.1	0.114	MM
15	15	20211111_L5_R1_IgG	2.78	5218.7	50994.6	0.102	MM
16	16	20211111_L6_R1_IgG	2.78	1192.7	58058.0	0.021	bb
17	17	20211111_H3_R1_IgG	2.78	7973.3	64028.6	0.125	MM
18	18	20211111_A7_R1_IgG	2.78	23137.7	82996.1	0.279	bb
19	19	20211111_A8_R1_IgG	2.79	9895.1	77731.7	0.127	MM
20	20	20211111_L7_R1_IgG	2.78	3426.9	38827.3	0.088	MM
21	21	20211111_L8_R1_IgG	2.77	4035.0	44220.1	0.091	MM
22	22	20211111_H4_R1_IgG	2.79	4465.3	65826.3	0.068	MM
23	23	20211111_A9_R1_IgG	2.78	4080.5	60771.1	0.067	MM
24	24	20211111_A10_R1_IgG	2.78	5016.3	48578.7	0.103	MM
25	25	20211111_L9_R1_IgG	2.79	6059.6	50830.7	0.119	bb
26	26	20211111_L10_R1_IgG	2.78	3282.0	38034.2	0.086	MM
27	27	20211111_QC_R3_IgG	2.78	7854.0	67025.5	0.117	MM
28	28	20211111_H5_R1_IgG	2.78	3672.5	44544.9	0.082	bb
29	29	20211111_A11_R1_IgG	2.78	1810.5	66408.5	0.027	MM
30	30	20211111_A12_R1_IgG	2.79	4312.3	46624.5	0.092	MM
31	31	20211111_L11_R1_IgG	2.80	1078.4	33425.1	0.032	bb
32	32	20211111_L12_R1_IgG	2.80	1970.8	41178.0	0.048	bb
33	33	20211111_H6_R1_IgG	2.78	13691.6	58593.4	0.234	MM
34	34	20211111_A13_R1_IgG	2.79	7252.3	54133.0	0.134	MM
35	35	20211111_A14_R1_IgG	2.79	4841.2	56467.3	0.086	MM
36	36	20211111_L13_R1_IgG	2.78	6293.8	36024.4	0.175	MM
37	37	20211111_L14_R1_IgG	2.78	2771.5	37181.6	0.075	MM
38	38	20211111_H7_R1_IgG	2.78	3709.6	42850.6	0.087	MM
39	39	20211111_A15_R1_IgG	2.79	5876.0	62015.1	0.095	MM
40	40	20211111_QC_R4_IgG	2.78	6043.3	58572.5	0.103	MM
41	41	20211111_A16_R1_IgG	2.78	5242.0	78283.8	0.067	MM
42	42	20211111_L15_R1_IgG	2.78	7449.1	44158.2	0.169	MM
43	43	20211111_L16_R1_IgG	2.78	5957.9	55150.8	0.108	bb
44	44	20211111_H8_R1_IgG	2.78	4074.8	42965.2	0.095	MM
45	45	20211111_A17_R1_IgG	2.78	3453.9	39717.7	0.087	bb
46	46	20211111_A18_R1_IgG	2.79	4971.5	44675.4	0.111	bb
47	47	20211111_L17_R1_IgG	2.79	4478.4	37854.1	0.118	bb
48	48	20211111_L18_R1_IgG	2.78	1093.7	50716.9	0.022	MM
49	49	20211111_H9_R1_IgG	2.79	3791.8	36276.0	0.105	MM
50	50	20211111_A19_R1_IgG	2.79	5806.7	42862.5	0.135	MM
51	51	20211111_A20_R1_IgG	2.78	1306.4	36542.7	0.036	MM
52	52	20211111_L19_R1_IgG	2.78	3354.4	53892.8	0.062	bb
53	53	20211111_QC_R5_IgG	2.78	5531.5	56513.2	0.098	MM
54	54	20211111_L20_R1_IgG	2.78	3583.5	56499.9	0.063	MM
55	55	20211111_H10_R1_IgG	2.79	5268.2	68120.5	0.077	MM
56	56	20211111_A21_R1_IgG	2.78	18357.5	63699.8	0.288	bb

Dataset: C:\MassLynx\10.pro\20211115_clinicalsamples_IgG.qld

Last Altered: Sunday, October 02, 2022 16:02:30 台北標準時間
Printed: Friday, August 01, 2025 16:50:47 台北標準時間

	#	Name	RT	Area	IS Area	Response	Prima...
57	57	20211111_A22_R1_IgG	2.79	4149.2	52222.1	0.079	MM
58	58	20211111_L21_R1_IgG	2.79	2322.4	54372.5	0.043	MM
59	59	20211111_L22_R1_IgG	2.76	2827.8	41353.1	0.068	MM
60	60	20211111_H11_R1_IgG	2.78	11841.2	65579.1	0.181	MM
61	61	20211111_A23_R1_IgG	2.78	7478.2	64131.1	0.117	MM
62	62	20211111_A24_R1_IgG	2.78	2319.7	38724.8	0.060	bb
63	63	20211111_L23_R1_IgG	2.77	6865.3	64245.2	0.107	bb
64	64	20211111_L24_R1_IgG	2.79	3617.6	45298.0	0.080	MM
65	65	20211111_H12_R1_IgG	2.77	3616.5	44679.3	0.081	MM
66	66	20211111_QC_R6_IgG	2.79	4808.7	48165.0	0.100	MM
67	67	20211111_A25_R1_IgG	2.78	4726.5	57169.9	0.083	MM
68	68	20211111_A26_R1_IgG	2.78	2758.9	34254.0	0.081	MM
69	69	20211111_L25_R1_IgG	2.78	7144.4	67491.5	0.106	MM
70	70	20211111_L26_R1_IgG	2.79	1413.6	55934.1	0.025	MM
71	71	20211111_H13_R1_IgG	2.79	6145.8	48767.3	0.126	MM
72	72	20211111_A27_R1_IgG	2.79	2285.7	57269.7	0.040	MM
73	73	20211111_A28_R1_IgG	2.78	4273.0	49231.1	0.087	MM
74	74	20211111_L27_R1_IgG	2.79	4488.2	60014.5	0.075	bb
75	75	20211111_L28_R1_IgG	2.79	4182.8	35062.6	0.119	bb
76	76	20211111_H14_R1_IgG	2.78	13905.1	62371.9	0.223	MM
77	77	20211111_A29_R1_IgG	2.78	3784.7	43858.2	0.086	MM
78	78	20211111_A30_R1_IgG	2.77	5227.4	62500.4	0.084	bb
79	79	20211111_QC_R7_IgG	2.78	4529.2	43946.9	0.103	MM
80	80	20211111_L29_R1_IgG	2.78	1188.5	38728.9	0.031	MM
81	81	20211111_L30_R1_IgG	2.78	1789.6	42613.2	0.042	MM
82	82	20211111_H15_R1_IgG	2.79	3286.8	39874.6	0.082	bb
83	83	20211111_A31_R1_IgG	2.79	4444.7	53414.3	0.083	bb
84	84	20211111_A32_R1_IgG	2.78	4804.2	41844.6	0.115	MM
85	85	20211111_L31_R1_IgG	2.78	4225.5	40553.7	0.104	MM
86	86	20211111_L32_R1_IgG	2.78	3547.8	49618.7	0.072	bb
87	87	20211111_H16_R1_IgG	2.78	11464.1	60170.9	0.191	MM
88	88	20211111_A33_R1_IgG	2.78	2505.6	48588.2	0.052	MM
89	89	20211111_A34_R1_IgG	2.77	4378.5	43288.0	0.101	bb
90	90	20211111_H17_R1_IgG	2.79	11854.3	47104.5	0.252	MM
91	91	20211111_A35_R1_IgG	2.78	5775.7	41025.9	0.141	MM
92	92	20211111_QC_R8_IgG	2.79	3633.6	40546.3	0.090	MM
93	93	20211111_A36_R1_IgG	2.79	4589.5	44504.4	0.103	bb
94	94	20211111_H18_R1_IgG	2.79	11743.3	34162.2	0.344	MM
95	95	20211111_A37_R1_IgG	2.78	2026.2	39760.0	0.051	bb
96	96	20211111_A38_R1_IgG	2.79	3660.8	43982.6	0.083	MM
97	97	20211111_H19_R1_IgG	2.79	19386.7	45314.8	0.428	MM
98	98	20211111_A39_R1_IgG	2.79	3833.3	39388.0	0.097	bb
99	99	20211111_A40_R1_IgG	2.78	3717.3	38726.4	0.096	bb
100	1...	20211111_H20_R1_IgG	2.78	23308.7	37814.4	0.616	bb
101	1...	20211111_A41_R1_IgG	2.78	4085.8	48930.3	0.084	MM
102	1...	20211111_A42_R1_IgG	2.79	2872.3	40406.8	0.071	MM
103	1...	20211111_A43_R1_IgG	2.79	8919.3	44421.5	0.201	MM
104	1...	20211111_A44_R1_IgG	2.79	7665.5	53128.2	0.144	bb
105	1...	20211111_QC_R9_IgG	2.78	3576.2	37049.0	0.097	bb
106	1...	20211111_A1_R2_IgG	2.78	10537.2	46499.4	0.227	MM
107	1...	20211111_A2_R2_IgG	2.78	5677.4	57260.7	0.099	MM
108	1...	20211111_L1_R2_IgG	2.79	2482.1	48062.9	0.052	bb
109	1...	20211111_L2_R2_IgG	2.77	4712.9	38745.2	0.122	MM
110	1...	20211111_H1_R2_IgG	2.78	3228.2	44273.5	0.073	bb
111	1...	20211111_A3_R2_IgG	2.79	286.8	4755.5	0.060	MM
112	1...	20211111_A4_R2_IgG	2.78	8615.5	50546.7	0.170	bb

Dataset: C:\MassLynx\10.pro\20211115_clinicalsamples_IgG.qld

Last Altered: Sunday, October 02, 2022 16:02:30 台北標準時間
Printed: Friday, August 01, 2025 16:50:47 台北標準時間

	# Name	RT	Area	IS Area	Response	Prima...
113	1... 20211111_L3_R2_IgG	2.77	1461.4	34825.8	0.042	bb
114	1... 20211111_L4_R2_IgG	2.77	3129.1	39107.7	0.080	MM
115	1... 20211111_H2_R2_IgG	2.78	11184.4	49750.0	0.225	MM
116	1... 20211111_A5_R2_IgG	2.78	5041.6	52536.2	0.096	MM
117	1... 20211111_A6_R2_IgG	2.78	10316.1	42050.0	0.245	bb
118	1... 20211111_QC_R10_IgG	2.77	2951.5	35350.8	0.083	MM
119	1... 20211111_L5_R2_IgG	2.77	3927.7	38039.3	0.103	MM
120	1... 20211111_L6_R2_IgG	2.77	1079.2	44907.4	0.024	bb
121	1... 20211111_H3_R2_IgG	2.78	5338.8	47729.1	0.112	MM
122	1... 20211111_A7_R2_IgG	2.77	14961.5	57347.1	0.261	MM
123	1... 20211111_A8_R2_IgG	2.78	6400.2	52397.8	0.122	bb
124	1... 20211111_L7_R2_IgG	2.78	2945.3	36318.4	0.081	MM
125	1... 20211111_L8_R2_IgG	2.78	3197.7	38131.9	0.084	MM
126	1... 20211111_H4_R2_IgG	2.77	3227.8	46849.6	0.069	bb
127	1... 20211111_A9_R2_IgG	2.77	3207.5	47759.6	0.067	MM
128	1... 20211111_A10_R2_IgG	2.77	4634.4	41366.2	0.112	MM
129	1... 20211111_L9_R2_IgG	2.78	4944.6	43052.1	0.115	bb
130	1... 20211111_L10_R2_IgG	2.78	2786.3	31487.4	0.088	bb
131	1... 20211111_QC_R11_IgG	2.77	3608.1	37804.6	0.095	bb
132	1... 20211111_H5_R2_IgG	2.78	2455.0	40380.0	0.061	MM
133	1... 20211111_A11_R2_IgG	2.78	1034.5	49533.3	0.021	MM
134	1... 20211111_A12_R2_IgG	2.78	3184.9	40226.3	0.079	MM
135	1... 20211111_L11_R2_IgG	2.78	950.5	28884.0	0.033	MM
136	1... 20211111_L12_R2_IgG	2.78	2027.5	34351.3	0.059	bb
137	1... 20211111_H6_R2_IgG	2.78	7849.6	40466.6	0.194	MM
138	1... 20211111_A13_R2_IgG	2.77	5689.5	42306.7	0.134	MM
139	1... 20211111_A14_R2_IgG	2.79	3699.0	44904.0	0.082	bb
140	1... 20211111_L13_R2_IgG	2.78	5343.7	31913.0	0.167	bb
141	1... 20211111_L14_R2_IgG	2.77	1987.3	31527.4	0.063	MM
142	1... 20211111_H7_R2_IgG	2.79	3756.6	40838.2	0.092	bb
143	1... 20211111_A15_R2_IgG	2.78	4060.1	49389.7	0.082	MM
144	1... 20211111_QC_R12_IgG	2.77	3233.8	35168.7	0.092	MM
145	1... 20211111_A16_R2_IgG	2.78	3062.2	47440.5	0.065	bb
146	1... 20211111_L15_R2_IgG	2.76	4565.3	33145.9	0.138	bb
147	1... 20211111_L16_R2_IgG	2.78	3035.7	37596.6	0.081	MM
148	1... 20211111_H8_R2_IgG	2.78	4291.3	43933.3	0.098	bb
149	1... 20211111_A17_R2_IgG	2.78	4464.7	43605.5	0.102	MM
150	1... 20211111_A18_R2_IgG	2.78	3681.3	37581.8	0.098	MM
151	1... 20211111_L17_R2_IgG	2.78	4132.8	33942.5	0.122	MM
152	1... 20211111_L18_R2_IgG	2.76	533.0	37831.5	0.014	bb
153	1... 20211111_H9_R2_IgG	2.78	4093.8	30898.4	0.132	MM
154	1... 20211111_A19_R2_IgG	2.78	5165.4	39352.1	0.131	MM
155	1... 20211111_A20_R2_IgG	2.78	1069.8	34661.2	0.031	MM
156	1... 20211111_L19_R2_IgG	2.78	2335.4	39918.3	0.059	bb
157	1... 20211111_QC_R13_IgG	2.79	3503.5	36239.1	0.097	bb
158	1... 20211111_L20_R2_IgG	2.78	2185.7	32897.7	0.066	bb
159	1... 20211111_H10_R2_IgG	2.78	2709.9	31394.5	0.086	MM
160	1... 20211111_A21_R2_IgG	2.78	8908.7	34863.7	0.256	MM
161	1... 20211111_A22_R2_IgG	2.79	1676.4	30342.1	0.055	bb
162	1... 20211111_L21_R2_IgG	2.78	1346.5	33706.2	0.040	MM
163	1... 20211111_L22_R2_IgG	2.77	2013.1	31298.6	0.064	MM
164	1... 20211111_H11_R2_IgG	2.79	5508.2	38904.1	0.142	MM
165	1... 20211111_A23_R2_IgG	2.78	3147.8	32382.3	0.097	MM
166	1... 20211111_A24_R2_IgG	2.80	1519.7	30261.9	0.050	MM
167	1... 20211111_L23_R2_IgG	2.77	4536.6	43037.5	0.105	MM
168	1... 20211111_L24_R2_IgG	2.78	2321.3	37023.3	0.063	bb

Dataset: C:\MassLynx\10.pro\20211115_clinicalsamples_IgG.qld

Last Altered: Sunday, October 02, 2022 16:02:30 台北標準時間
Printed: Friday, August 01, 2025 16:50:47 台北標準時間

	#	Name	RT	Area	IS Area	Response	Prima...
169	1...	20211111_H12_R2_IgG	2.77	2409.1	39478.2	0.061	bb
170	1...	20211111_QC_R14_IgG	2.78	2520.0	34463.2	0.073	bb
171	1...	20211111_A25_R2_IgG	2.78	2865.1	39945.3	0.072	bb
172	1...	20211111_A26_R2_IgG	2.77	2063.7	29663.2	0.070	bb
173	1...	20211111_L25_R2_IgG	2.78	3607.0	38589.3	0.093	bb
174	1...	20211111_L26_R2_IgG	2.78	1040.7	38405.2	0.027	MM
175	1...	20211111_H13_R2_IgG	2.78	4158.6	37771.2	0.110	MM
176	1...	20211111_A27_R2_IgG	2.78	1352.9	42634.1	0.032	MM
177	1...	20211111_A28_R2_IgG	2.78	2286.7	33231.1	0.069	bb
178	1...	20211111_L27_R2_IgG	2.79	2607.3	41761.1	0.062	bb
179	1...	20211111_L28_R2_IgG	2.78	3262.6	26968.6	0.121	bb
180	1...	20211111_H14_R2_IgG	2.78	7697.9	49250.2	0.156	MM
181	1...	20211111_A29_R2_IgG	2.78	3179.3	37601.7	0.085	MM
182	1...	20211111_A30_R2_IgG	2.78	2339.6	40366.8	0.058	MM
183	1...	20211111_QC_R15_IgG	2.79	2921.4	35156.1	0.083	MM
184	1...	20211111_L29_R2_IgG	2.79	866.2	33813.1	0.026	MM
185	1...	20211111_L30_R2_IgG	2.77	1293.6	33488.6	0.039	MM
186	1...	20211111_H15_R2_IgG	2.77	2647.1	38501.0	0.069	MM
187	1...	20211111_A31_R2_IgG	2.78	2879.6	37525.5	0.077	MM
188	1...	20211111_A32_R2_IgG	2.78	4052.4	35770.8	0.113	MM
189	1...	20211111_L31_R2_IgG	2.77	3167.9	33282.4	0.095	MM
190	1...	20211111_L32_R2_IgG	2.78	3398.8	42049.6	0.081	MM
191	1...	20211111_H16_R2_IgG	2.78	7119.9	45947.6	0.155	MM
192	1...	20211111_A33_R2_IgG	2.78	2174.6	40202.3	0.054	bb
193	1...	20211111_A34_R2_IgG	2.78	3445.5	36747.0	0.094	bb
194	1...	20211111_H17_R2_IgG	2.78	10243.3	40920.9	0.250	MM
195	1...	20211111_A35_R2_IgG	2.78	5016.5	33689.2	0.149	bb
196	1...	20211111_QC_R16_IgG	2.77	2941.5	32783.6	0.090	MM
197	1...	20211111_A36_R2_IgG	2.78	4699.8	36822.4	0.128	MM
198	1...	20211111_H18_R2_IgG	2.77	10744.3	31769.3	0.338	MM
199	1...	20211111_A37_R2_IgG	2.79	2250.2	37581.8	0.060	MM
200	2...	20211111_A38_R2_IgG	2.77	2708.4	38390.4	0.071	MM
201	2...	20211111_H19_R2_IgG	2.78	17951.0	41217.0	0.436	MM
202	2...	20211111_A39_R2_IgG	2.78	3765.1	37392.7	0.101	bb
203	2...	20211111_A40_R2_IgG	2.78	3173.8	38295.1	0.083	bb
204	2...	20211111_H20_R2_IgG	2.78	21860.7	35941.8	0.608	MM
205	2...	20211111_A41_R2_IgG	2.77	3156.9	45304.9	0.070	MM
206	2...	20211111_A42_R2_IgG	2.78	2240.7	37803.8	0.059	bb
207	2...	20211111_A43_R2_IgG	2.78	7026.1	38161.8	0.184	MM
208	2...	20211111_A44_R2_IgG	2.78	4872.0	37819.8	0.129	MM
209	2...	20211111_QC_R17_IgG	2.77	3282.8	31731.8	0.103	bb

Dataset: C:\MassLynx\10.pro\20211115_clinicalsamples_IgG.qld

Last Altered: Sunday, October 02, 2022 16:02:30 台北標準時間
Printed: Friday, August 01, 2025 16:51:24 台北標準時間

Method: C:\MassLynx\10_Pro_IgA.PRO\MethDB\20210926_IgG_DMRM_IgM_IS.mdb 16 Feb 2022 11:58:21
Calibration: 18 Nov 2021 13:56:22

Sample Name: 20211111_QC_R1_IgG

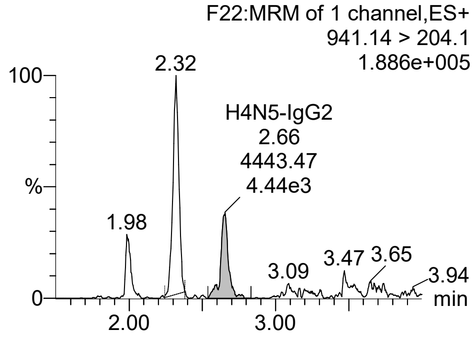

Sample Name: 20211111_A1_R1_IgG

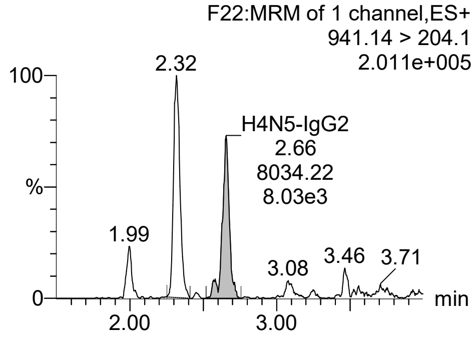

Sample Name: 20211111_A2_R1_IgG

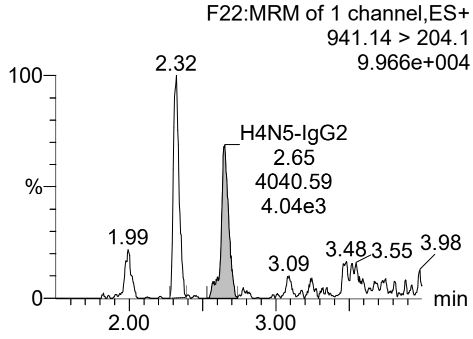

Sample Name: 20211111_L1_R1_IgG

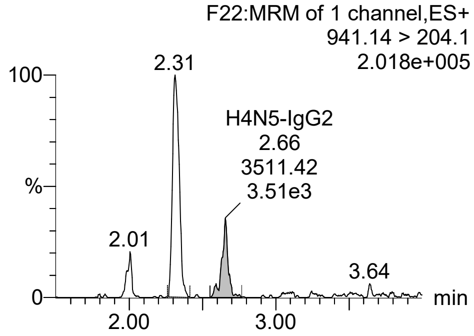

Sample Name: 20211111_L2_R1_IgG

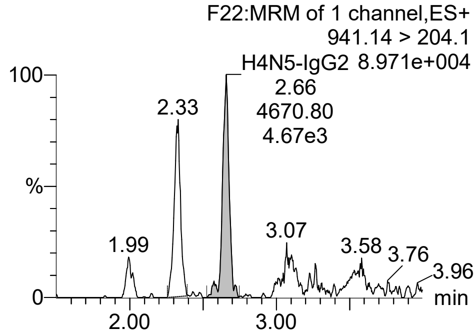

Sample Name: 20211111_H1_R1_IgG

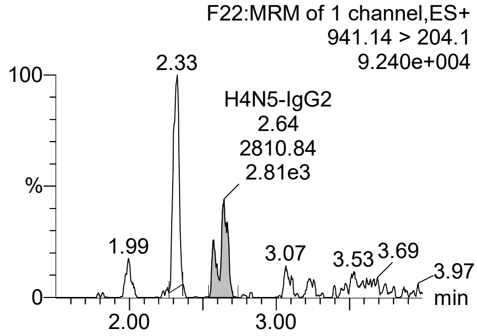

Sample Name: 20211111_A3_R1_IgG

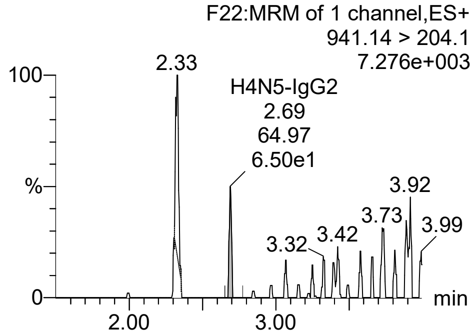

Sample Name: 20211111_A4_R1_IgG

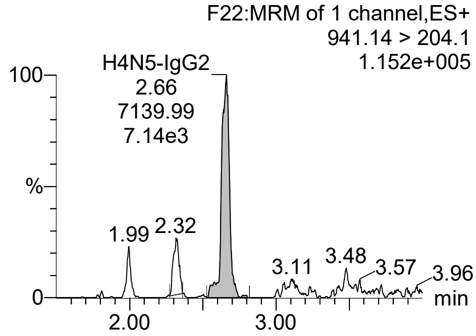

Sample Name: 20211111_L3_R1_IgG

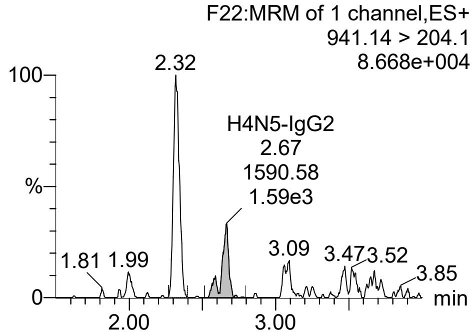

Sample Name: 20211111_L4_R1_IgG

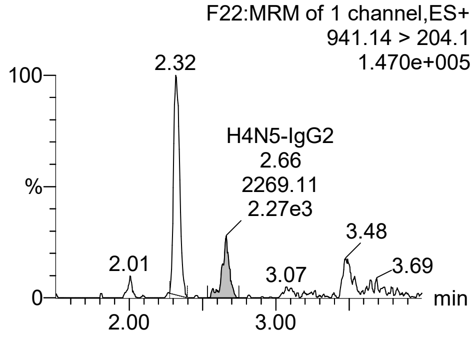

Sample Name: 20211111_H2_R1_IgG

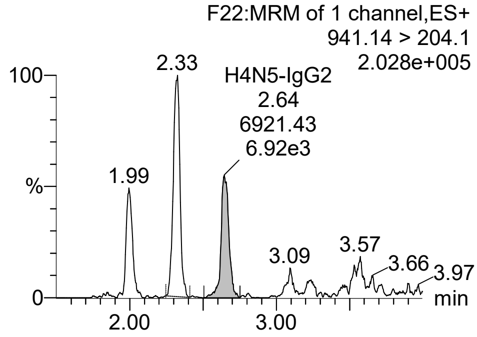

Sample Name: 20211111_A5_R1_IgG

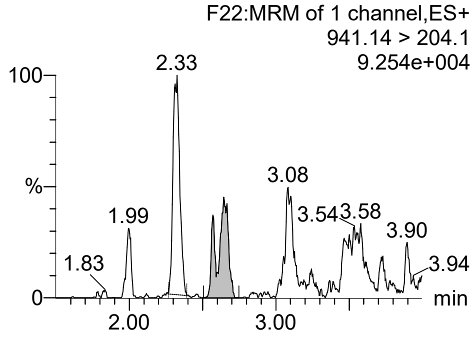

Dataset: C:\MassLynx\10.pro\20211115_clinicalsamples_IgG.qld

Last Altered: Sunday, October 02, 2022 16:02:30 台北標準時間
Printed: Friday, August 01, 2025 16:51:24 台北標準時間

Sample Name: 20211111_A6_R1_IgG
F22:MRM of 1 channel,ES+
941.14 > 204.1

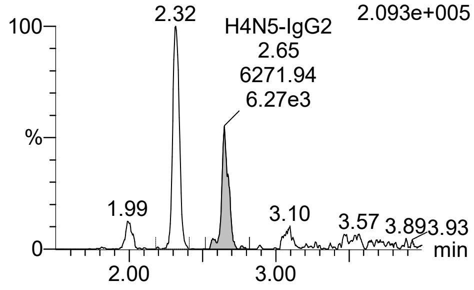

Sample Name: 20211111_QC_R2_IgG
F22:MRM of 1 channel,ES+
941.14 > 204.1

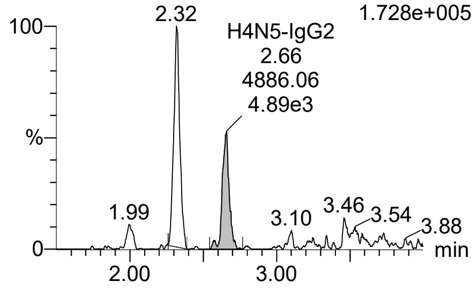

Sample Name: 20211111_L5_R1_IgG
F22:MRM of 1 channel,ES+
941.14 > 204.1

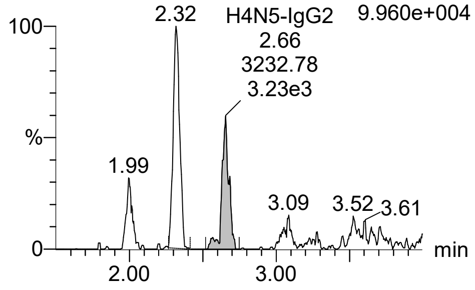

Sample Name: 20211111_L6_R1_IgG
F22:MRM of 1 channel,ES+
941.14 > 204.1

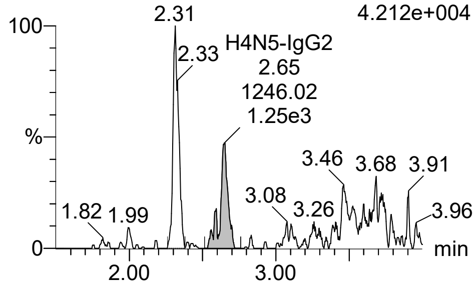

Sample Name: 20211111_H3_R1_IgG
F22:MRM of 1 channel,ES+
941.14 > 204.1

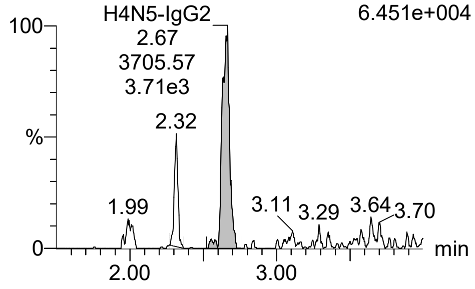

Sample Name: 20211111_A7_R1_IgG
F22:MRM of 1 channel,ES+
941.14 > 204.1

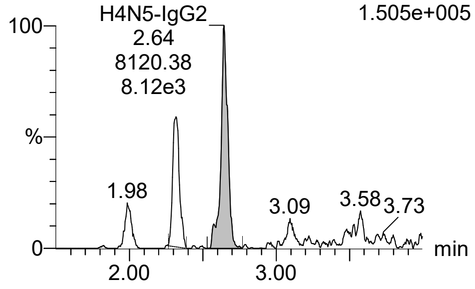

Sample Name: 20211111_A8_R1_IgG
F22:MRM of 1 channel,ES+
941.14 > 204.1

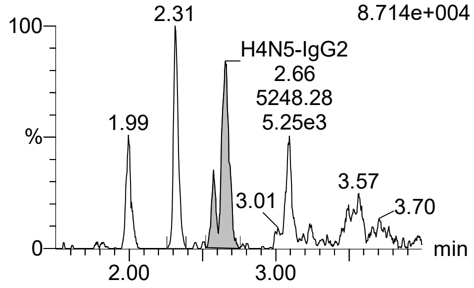

Sample Name: 20211111_L7_R1_IgG
F22:MRM of 1 channel,ES+
941.14 > 204.1

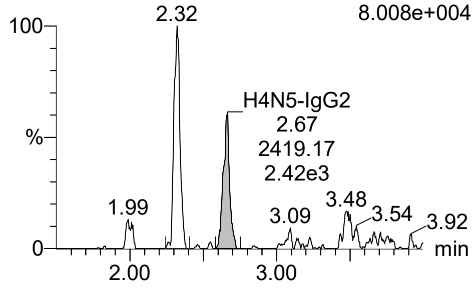

Sample Name: 20211111_L8_R1_IgG
F22:MRM of 1 channel,ES+
941.14 > 204.1

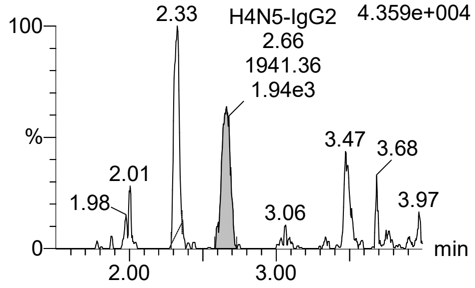

Sample Name: 20211111_H4_R1_IgG
F22:MRM of 1 channel,ES+
941.14 > 204.1

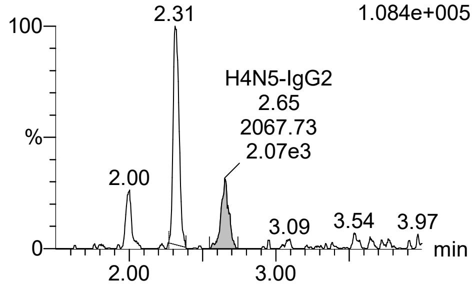

Sample Name: 20211111_A9_R1_IgG
F22:MRM of 1 channel,ES+
941.14 > 204.1

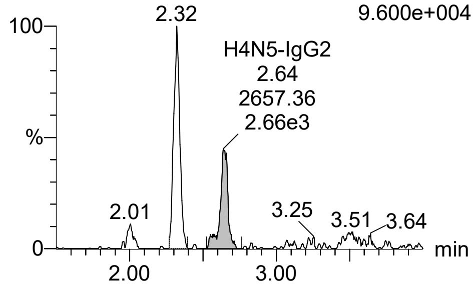

Sample Name: 20211111_A10_R1_IgG
F22:MRM of 1 channel,ES+
941.14 > 204.1

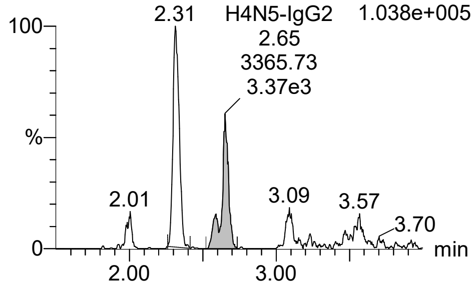

Sample Name: 20211111_L9_R1_IgG
F22:MRM of 1 channel,ES+
941.14 > 204.1

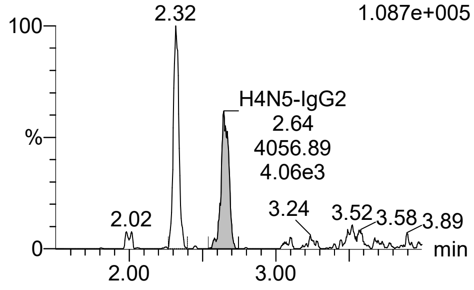

Sample Name: 20211111_L10_R1_IgG
F22:MRM of 1 channel,ES+
941.14 > 204.1

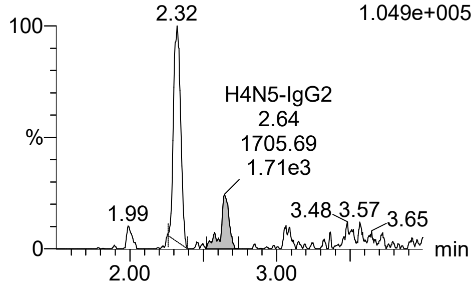

Sample Name: 20211111_QC_R3_IgG
F22:MRM of 1 channel,ES+
941.14 > 204.1

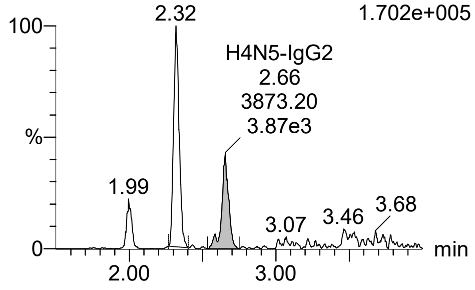

Sample Name: 20211111_H5_R1_IgG

F22:MRM of 1 channel,ES+
941.14 > 204.1
3.543e+004

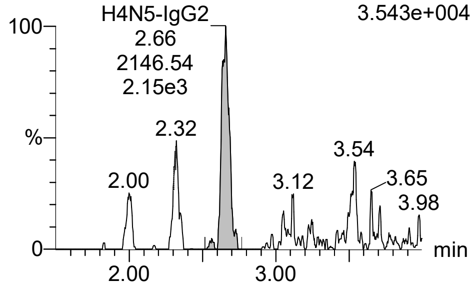

Sample Name: 20211111_A11_R1_IgG

F22:MRM of 1 channel,ES+
941.14 > 204.1
5.241e+004

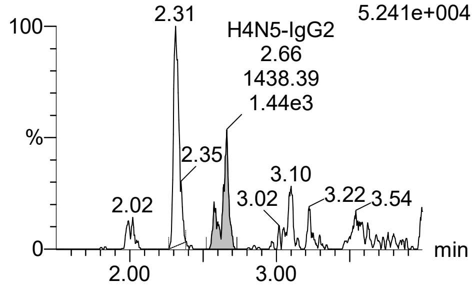

Sample Name: 20211111_A12_R1_IgG

F22:MRM of 1 channel,ES+
941.14 > 204.1
8.773e+004

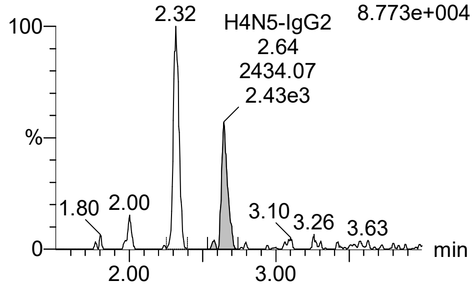

Sample Name: 20211111_L11_R1_IgG

F22:MRM of 1 channel,ES+
941.14 > 204.1
6.671e+004

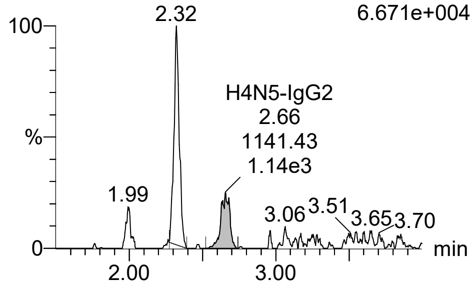

Sample Name: 20211111_L12_R1_IgG

F22:MRM of 1 channel,ES+
941.14 > 204.1
2.507e+004

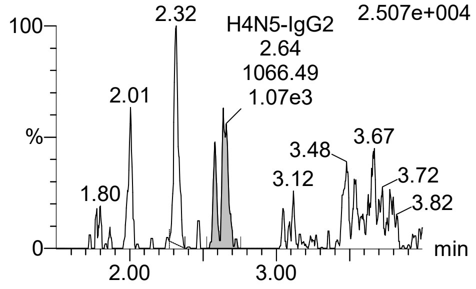

Sample Name: 20211111_H6_R1_IgG

F22:MRM of 1 channel,ES+
941.14 > 204.1
1.629e+005

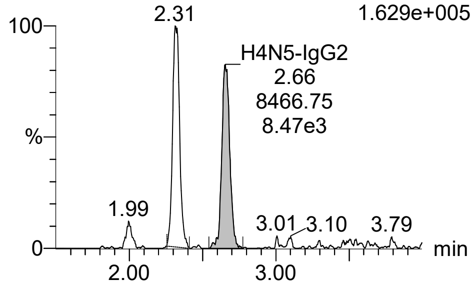

Sample Name: 20211111_A13_R1_IgG

F22:MRM of 1 channel,ES+
941.14 > 204.1
2.306e+005

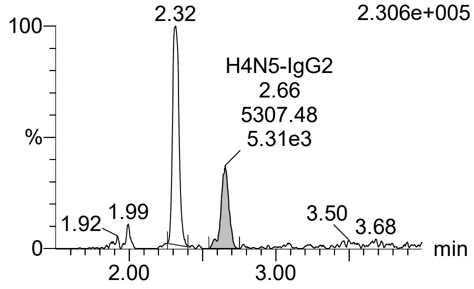

Sample Name: 20211111_A14_R1_IgG

F22:MRM of 1 channel,ES+
941.14 > 204.1
1.468e+005

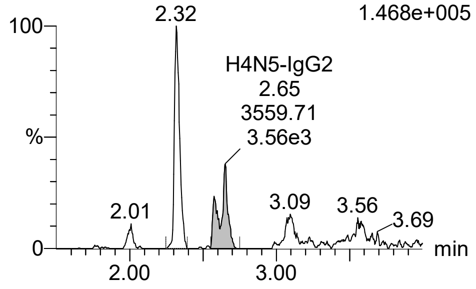

Sample Name: 20211111_L13_R1_IgG

F22:MRM of 1 channel,ES+
941.14 > 204.1
5.735e+004

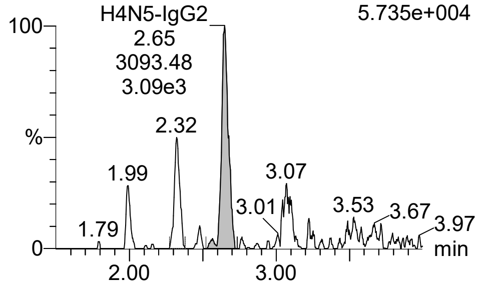

Sample Name: 20211111_L14_R1_IgG

F22:MRM of 1 channel,ES+
941.14 > 204.1
6.610e+004

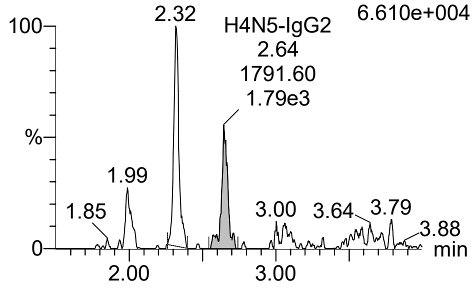

Sample Name: 20211111_H7_R1_IgG

F22:MRM of 1 channel,ES+
941.14 > 204.1
1.854e+005

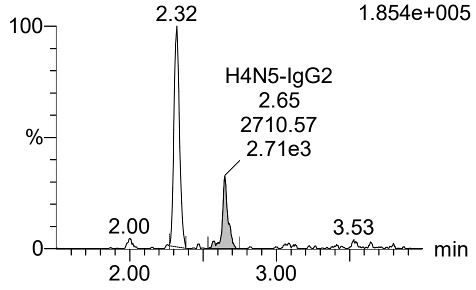

Sample Name: 20211111_A15_R1_IgG

F22:MRM of 1 channel,ES+
941.14 > 204.1
5.015e+004

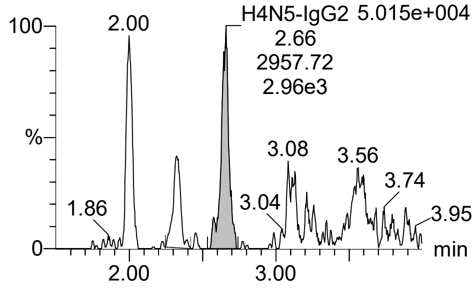

Sample Name: 20211111_QC_R4_IgG

F22:MRM of 1 channel,ES+
941.14 > 204.1
9.982e+004

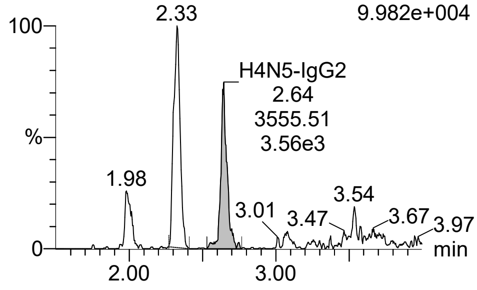

Sample Name: 20211111_A16_R1_IgG

F22:MRM of 1 channel,ES+
941.14 > 204.1
1.156e+005

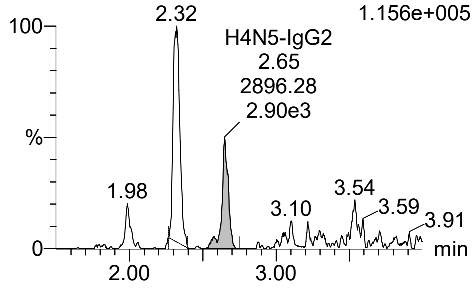

Sample Name: 20211111_L15_R1_IgG

F22:MRM of 1 channel,ES+
941.14 > 204.1
1.755e+005

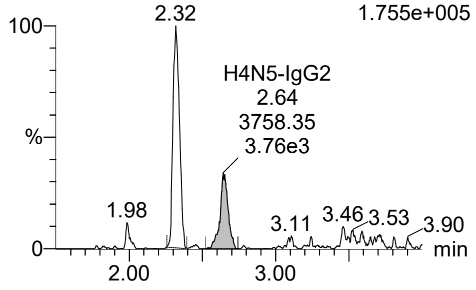

Dataset: C:\MassLynx\10.pro\20211115_clinicalsamples_IgG.qld

Last Altered: Sunday, October 02, 2022 16:02:30 台北標準時間
Printed: Friday, August 01, 2025 16:51:24 台北標準時間

Sample Name: 20211111_L16_R1_IgG

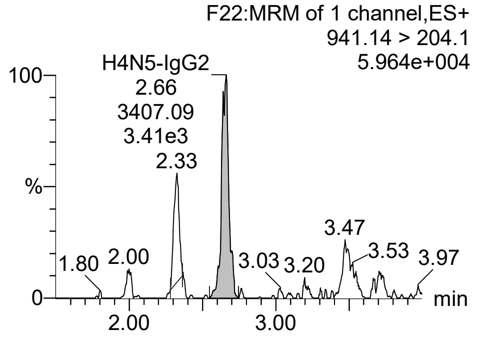

Sample Name: 20211111_H8_R1_IgG

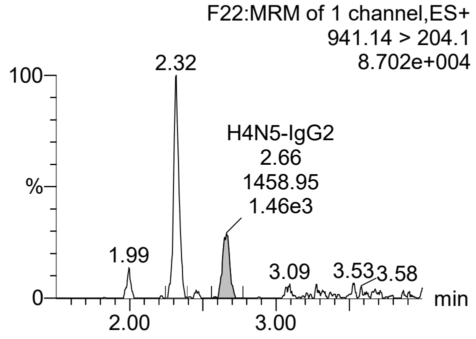

Sample Name: 20211111_A17_R1_IgG

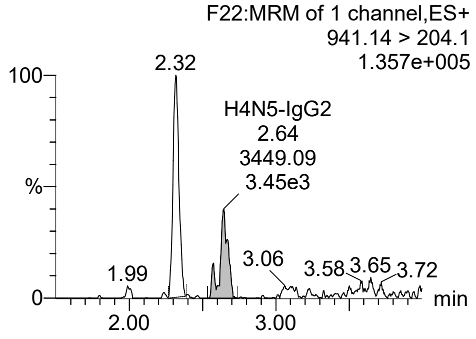

Sample Name: 20211111_A18_R1_IgG

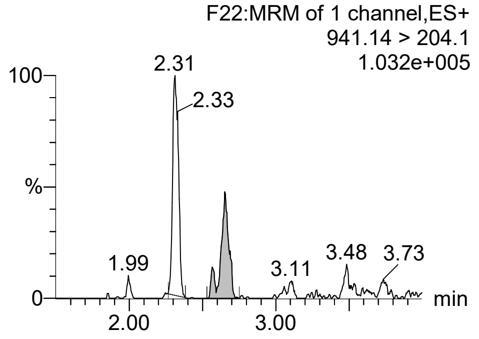

Sample Name: 20211111_L17_R1_IgG

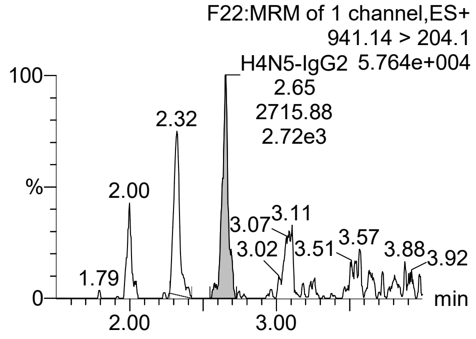

Sample Name: 20211111_L18_R1_IgG

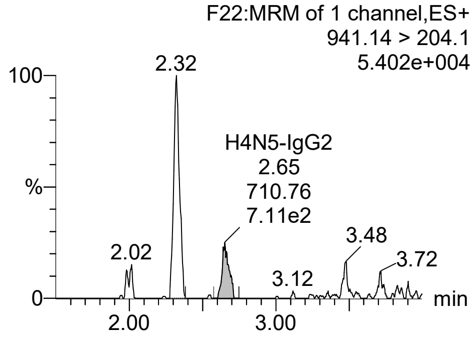

Sample Name: 20211111_H9_R1_IgG

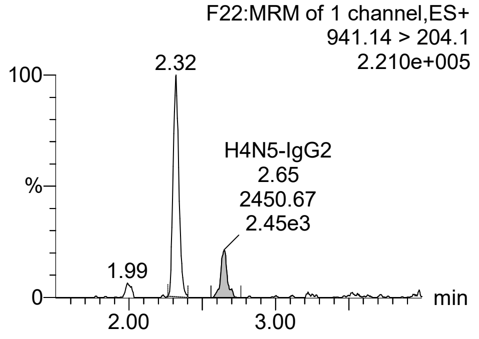

Sample Name: 20211111_A19_R1_IgG

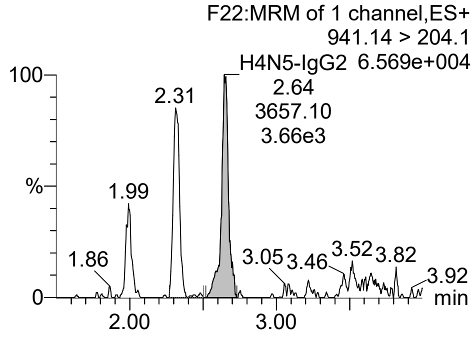

Sample Name: 20211111_A20_R1_IgG

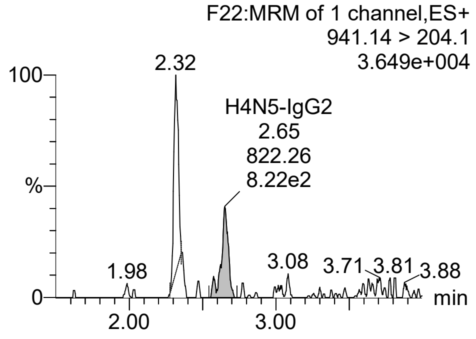

Sample Name: 20211111_L19_R1_IgG

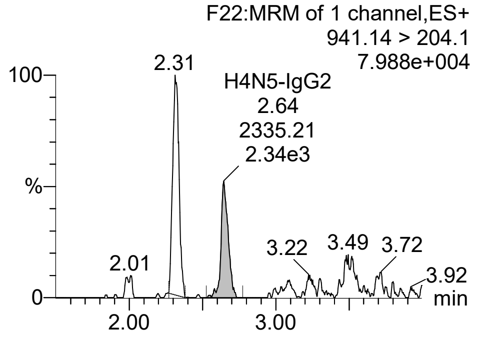

Sample Name: 20211111_QC_R5_IgG

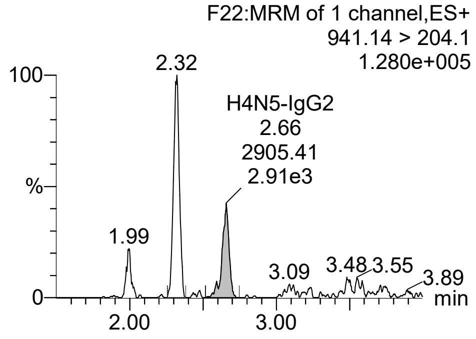

Sample Name: 20211111_L20_R1_IgG

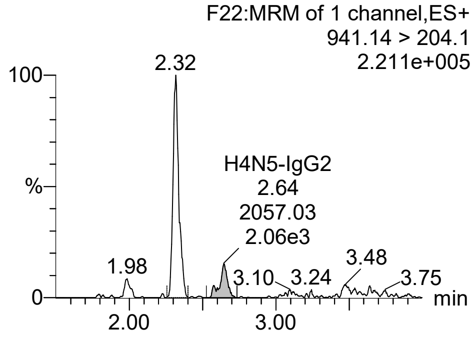

Sample Name: 20211111_H10_R1_IgG

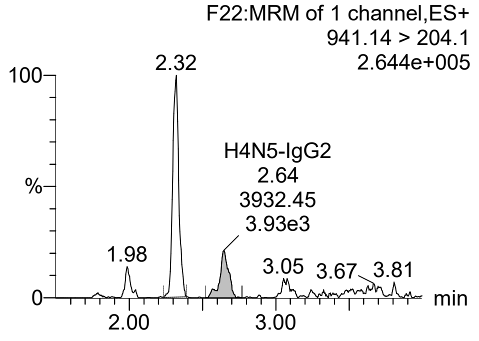

Sample Name: 20211111_A21_R1_IgG

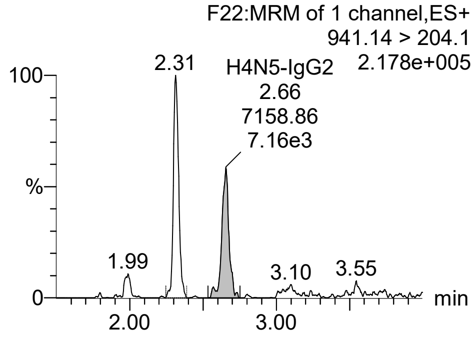

Sample Name: 20211111_A22_R1_IgG

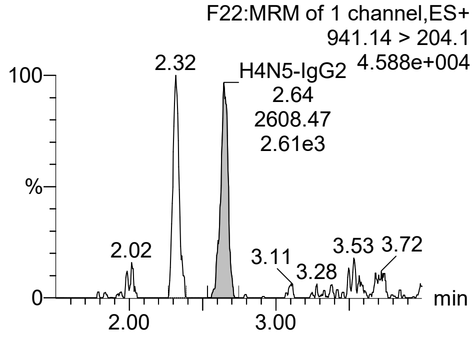

Dataset: C:\MassLynx\10.pro\20211115_clinicalsamples_IgG.qld

Last Altered: Sunday, October 02, 2022 16:02:30 台北標準時間
Printed: Friday, August 01, 2025 16:51:24 台北標準時間

Sample Name: 20211111_L21_R1_IgG

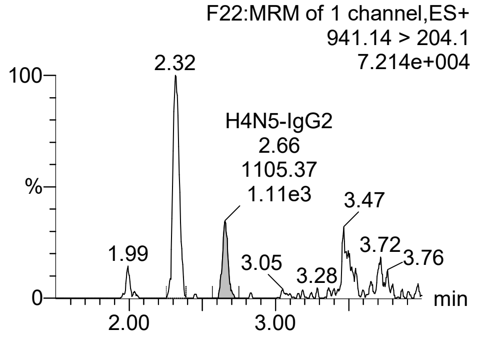

Sample Name: 20211111_L22_R1_IgG

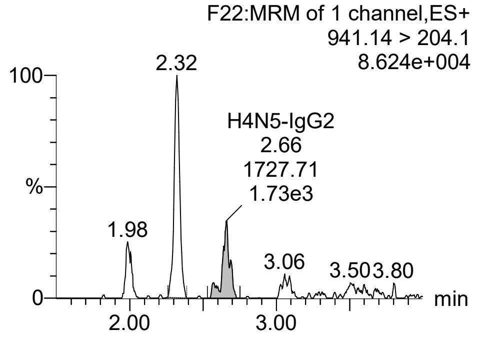

Sample Name: 20211111_H11_R1_IgG

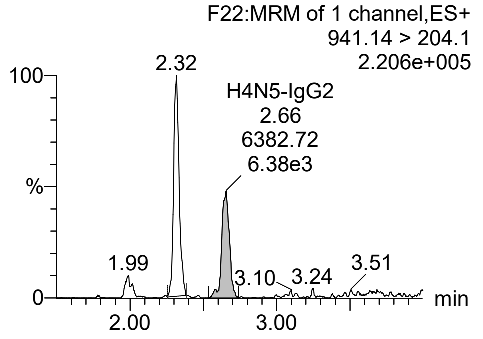

Sample Name: 20211111_A23_R1_IgG

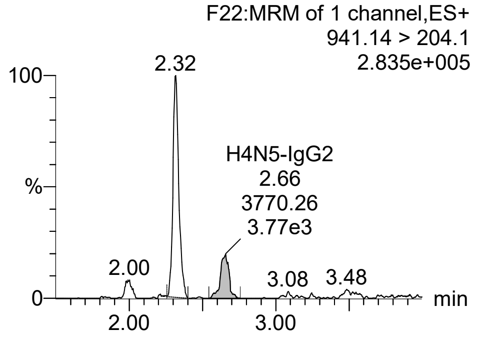

Sample Name: 20211111_A24_R1_IgG

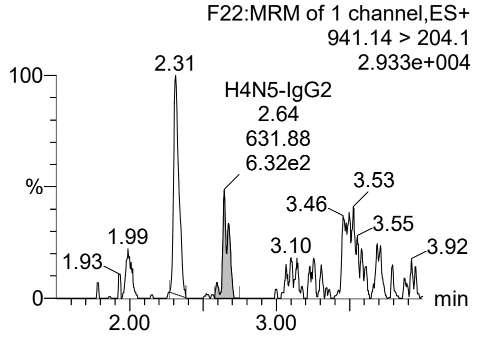

Sample Name: 20211111_L23_R1_IgG

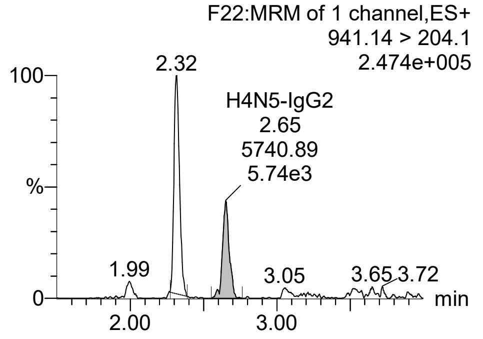

Sample Name: 20211111_L24_R1_IgG

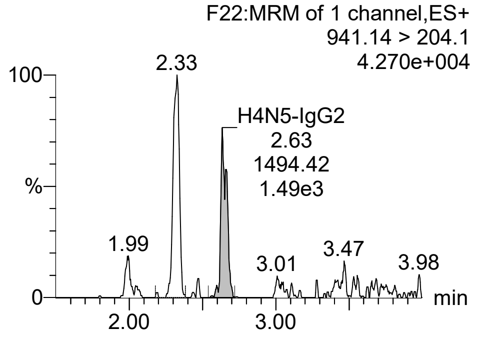

Sample Name: 20211111_H12_R1_IgG

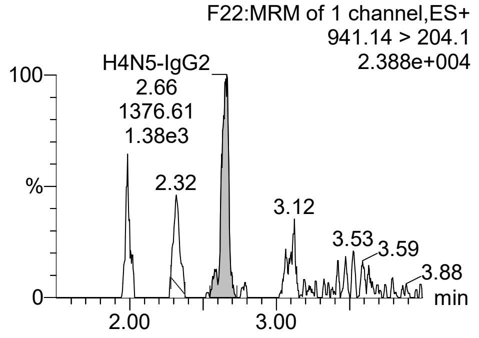

Sample Name: 20211111_QC_R6_IgG

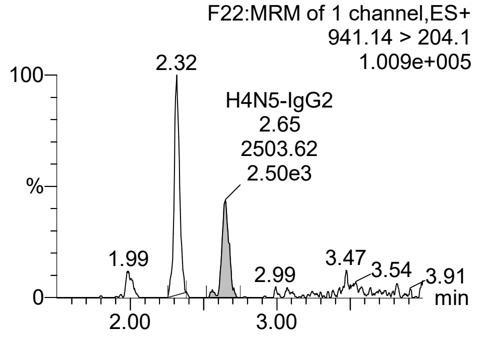

Sample Name: 20211111_A25_R1_IgG

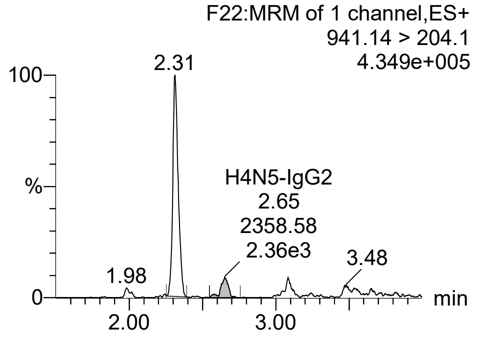

Sample Name: 20211111_A26_R1_IgG

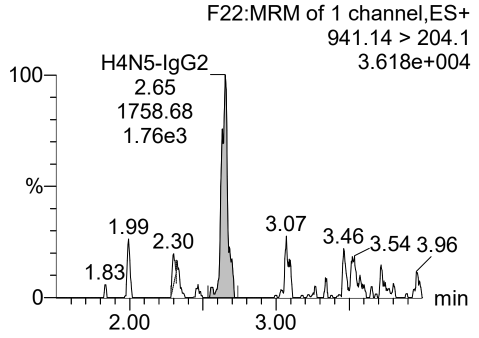

Sample Name: 20211111_L25_R1_IgG

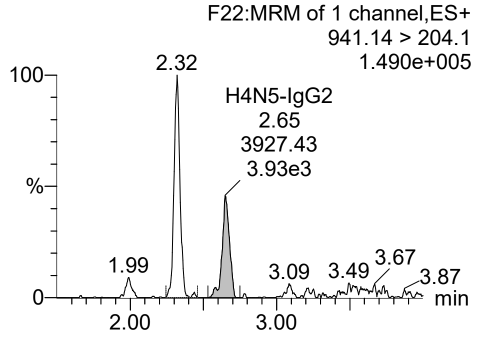

Sample Name: 20211111_L26_R1_IgG

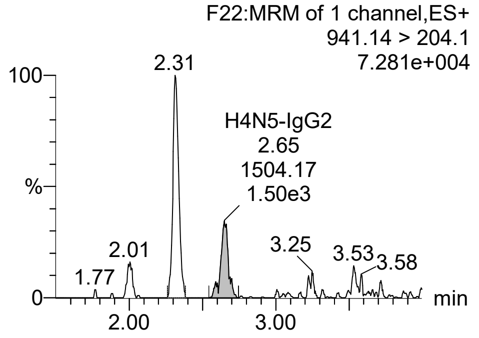

Sample Name: 20211111_H13_R1_IgG

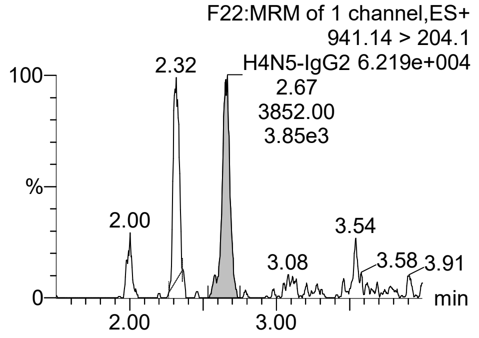

Sample Name: 20211111_A27_R1_IgG

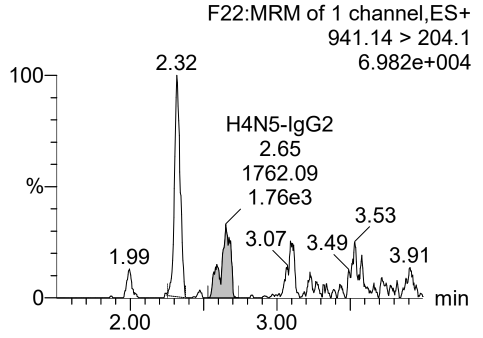

Dataset: C:\MassLynx\10.pro\20211115_clinicalsamples_IgG.qld

Last Altered: Sunday, October 02, 2022 16:02:30 台北標準時間
Printed: Friday, August 01, 2025 16:51:24 台北標準時間

Sample Name: 20211111_A28_R1_IgG

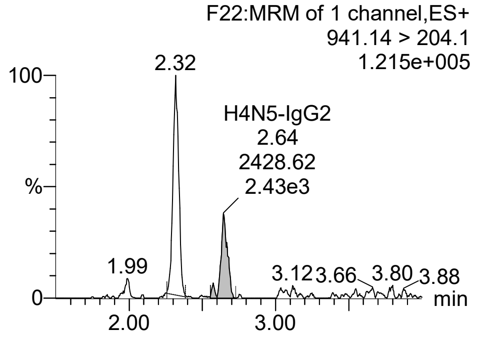

Sample Name: 20211111_L27_R1_IgG

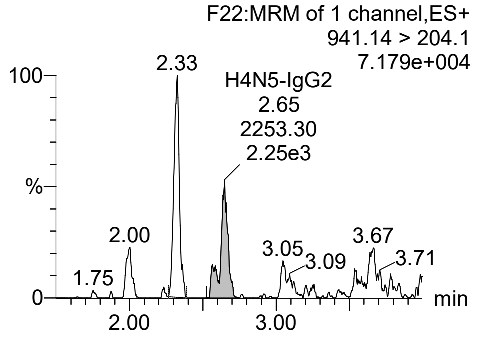

Sample Name: 20211111_L28_R1_IgG

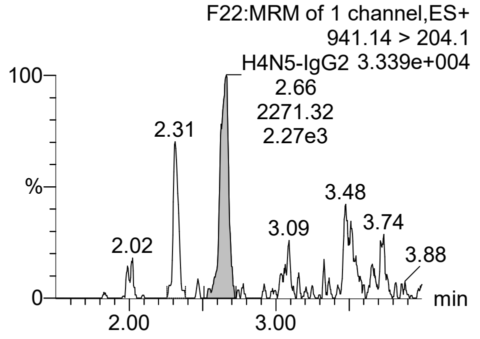

Sample Name: 20211111_H14_R1_IgG

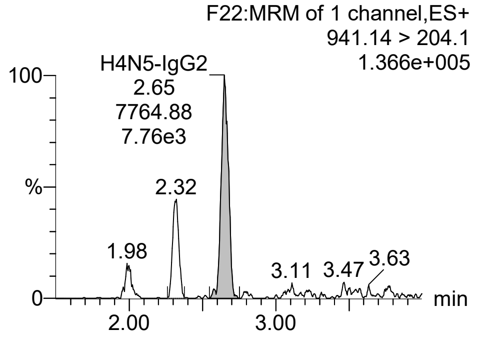

Sample Name: 20211111_A29_R1_IgG

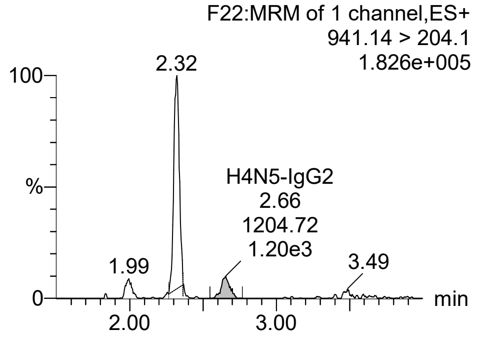

Sample Name: 20211111_A30_R1_IgG

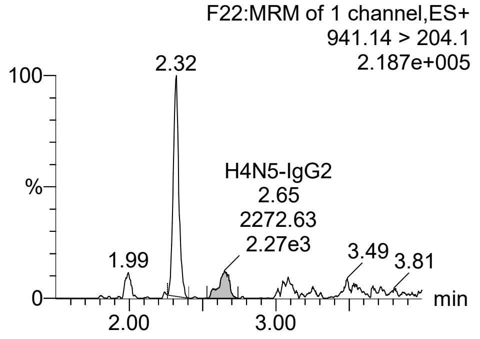

Sample Name: 20211111_QC_R7_IgG

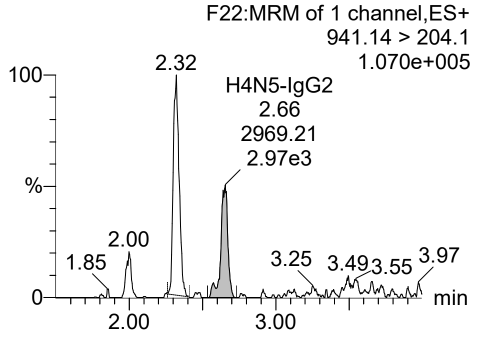

Sample Name: 20211111_L29_R1_IgG

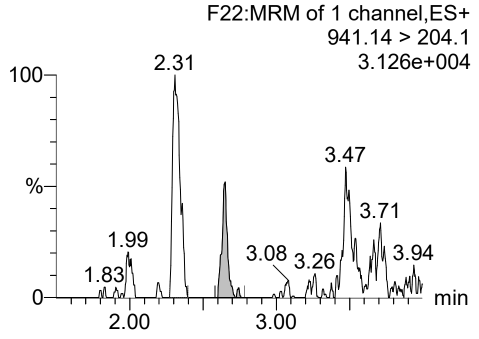

Sample Name: 20211111_L30_R1_IgG

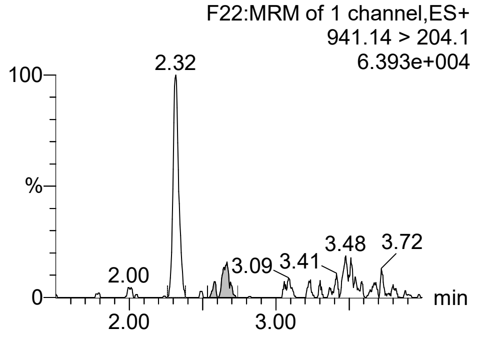

Sample Name: 20211111_H15_R1_IgG

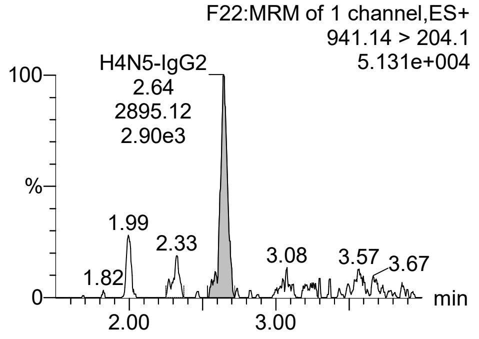

Sample Name: 20211111_A31_R1_IgG

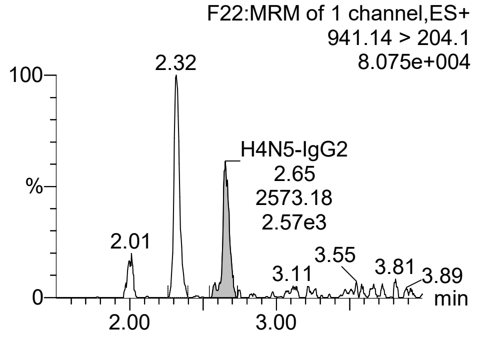

Sample Name: 20211111_A32_R1_IgG

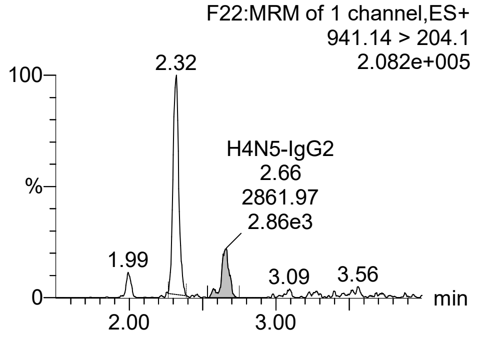

Sample Name: 20211111_L31_R1_IgG

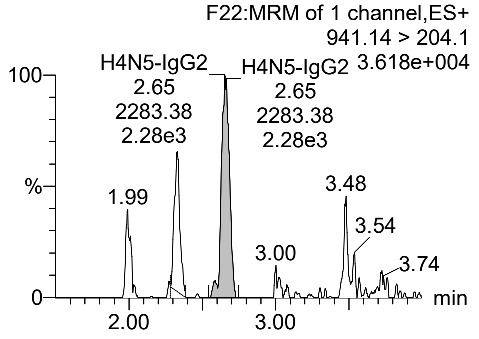

Sample Name: 20211111_L32_R1_IgG

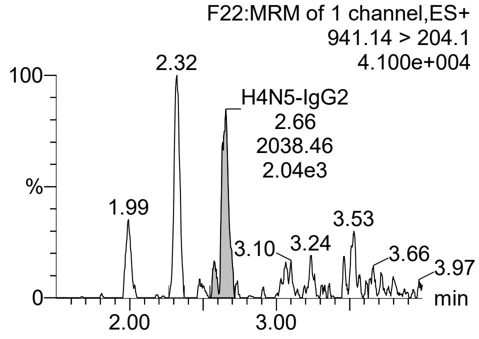

Sample Name: 20211111_H16_R1_IgG

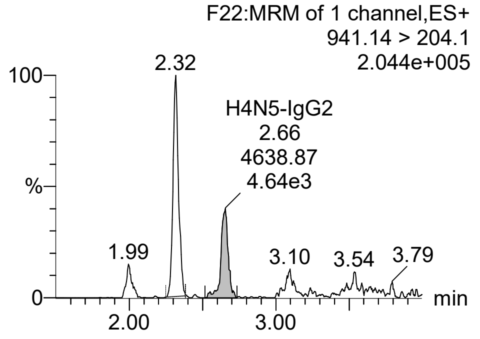

Dataset: C:\MassLynx\10.pro\20211115_clinicalsamples_IgG.qld

Last Altered: Sunday, October 02, 2022 16:02:30 台北標準時間
Printed: Friday, August 01, 2025 16:51:24 台北標準時間

Sample Name: 20211111_A33_R1_IgG

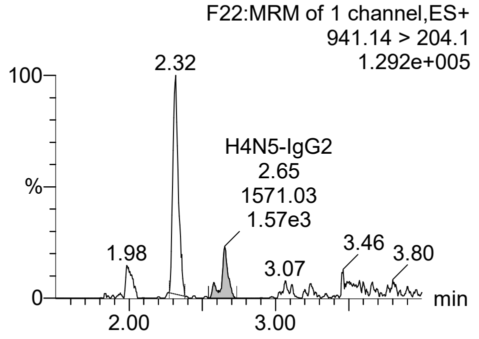

Sample Name: 20211111_A34_R1_IgG

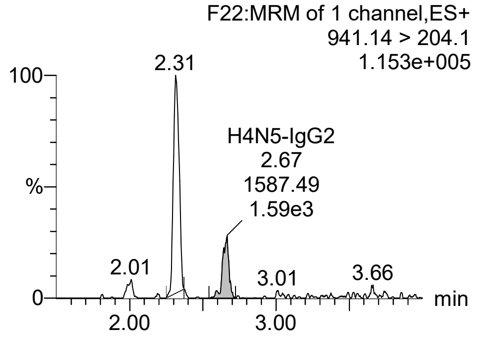

Sample Name: 20211111_H17_R1_IgG

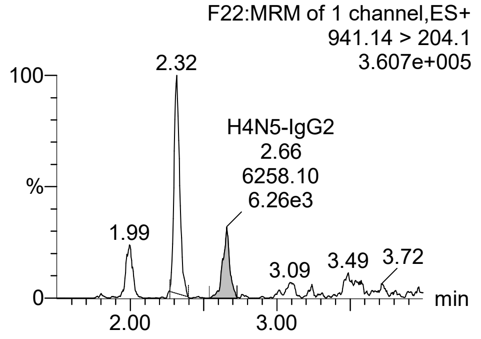

Sample Name: 20211111_A35_R1_IgG

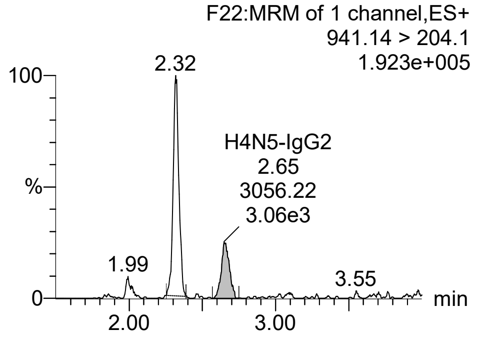

Sample Name: 20211111_QC_R8_IgG

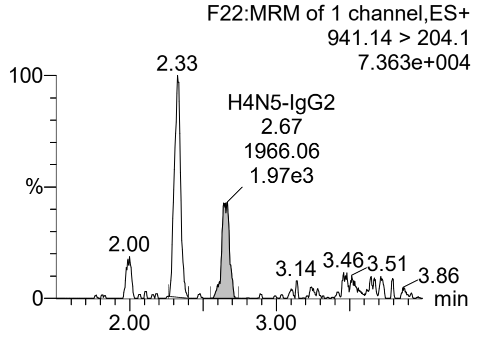

Sample Name: 20211111_A36_R1_IgG

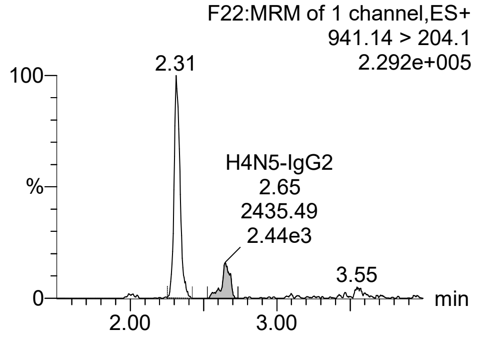

Sample Name: 20211111_H18_R1_IgG

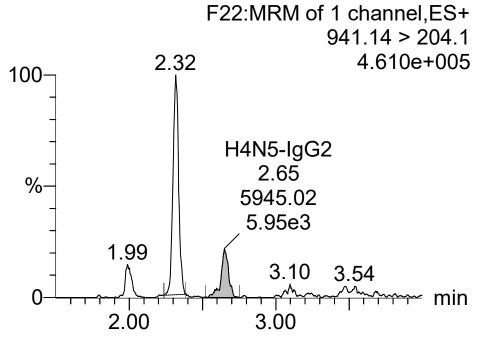

Sample Name: 20211111_A37_R1_IgG

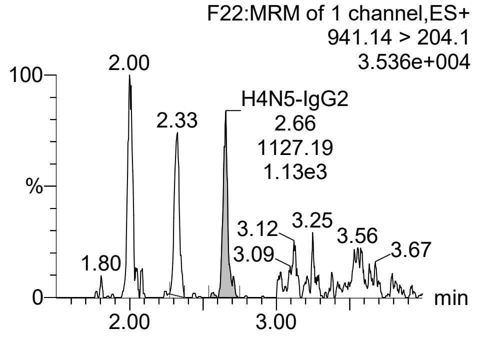

Sample Name: 20211111_A38_R1_IgG

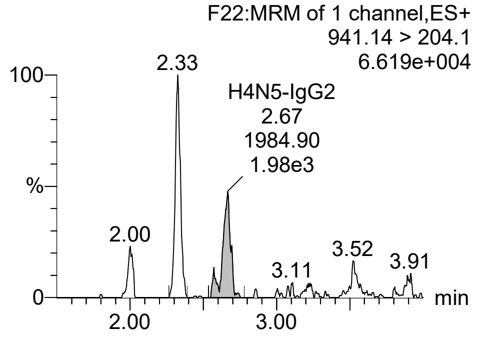

Sample Name: 20211111_H19_R1_IgG

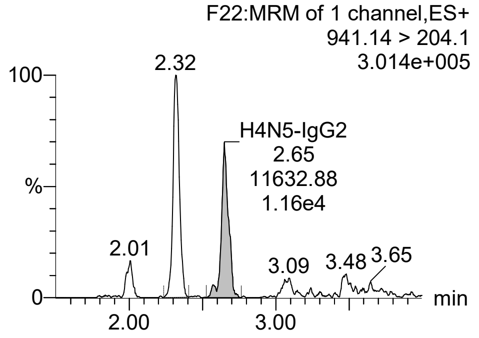

Sample Name: 20211111_A39_R1_IgG

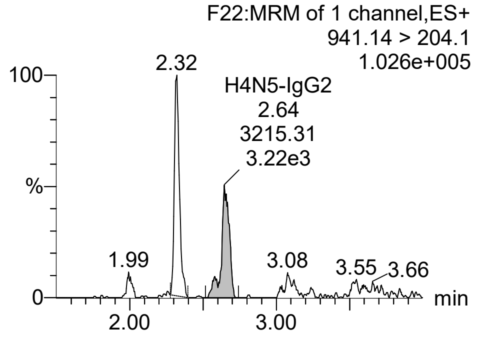

Sample Name: 20211111_A40_R1_IgG

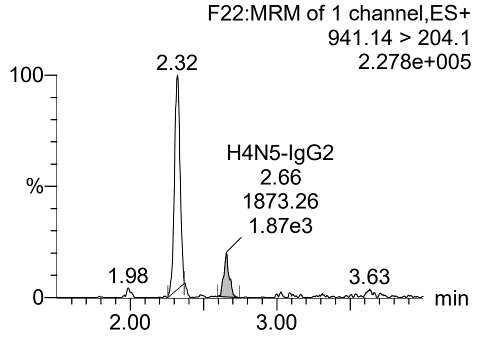

Sample Name: 20211111_H20_R1_IgG

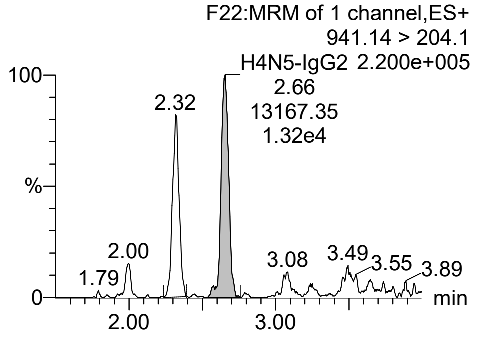

Sample Name: 20211111_A41_R1_IgG

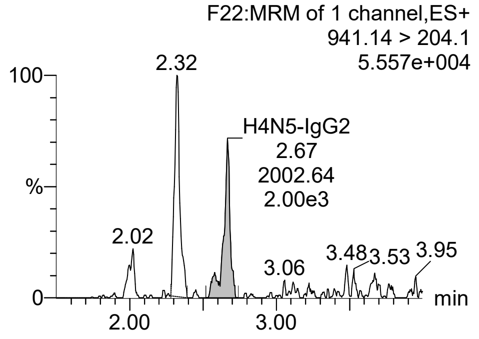

Sample Name: 20211111_A42_R1_IgG

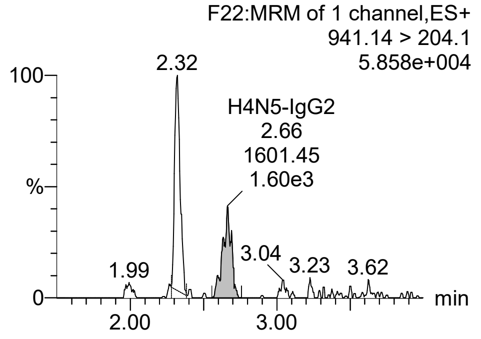

Dataset: C:\MassLynx\10.pro\20211115_clinicalsamples_IgG.qld

Last Altered: Sunday, October 02, 2022 16:02:30 台北標準時間
Printed: Friday, August 01, 2025 16:51:24 台北標準時間

Sample Name: 20211111_A43_R1_IgG

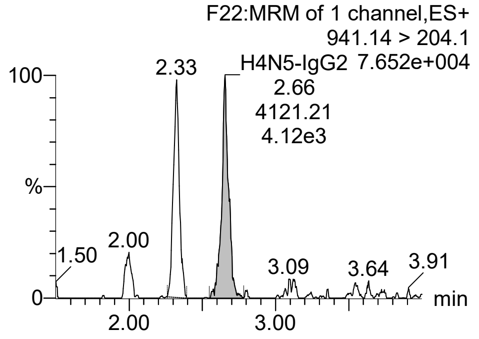

Sample Name: 20211111_A44_R1_IgG

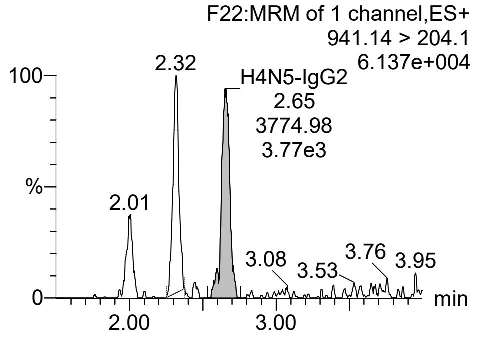

Sample Name: 20211111_QC_R9_IgG

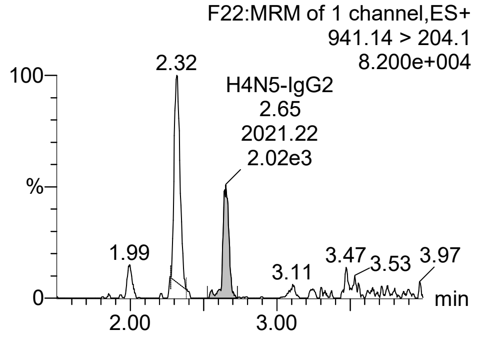

Sample Name: 20211111_A1_R2_IgG

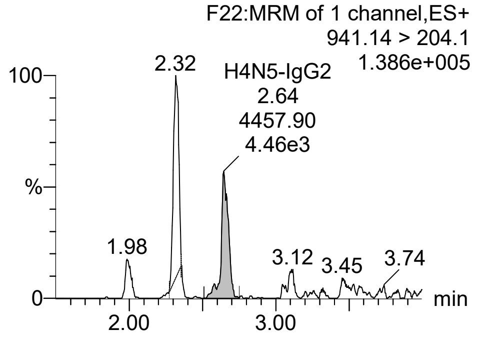

Sample Name: 20211111_A2_R2_IgG

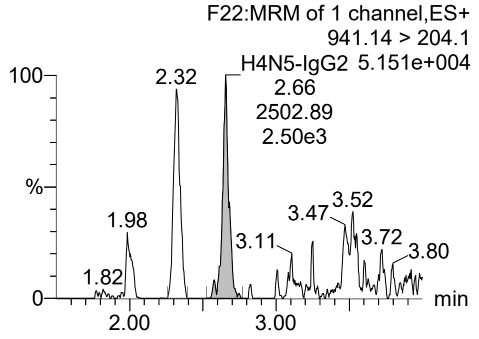

Sample Name: 20211111_L1_R2_IgG

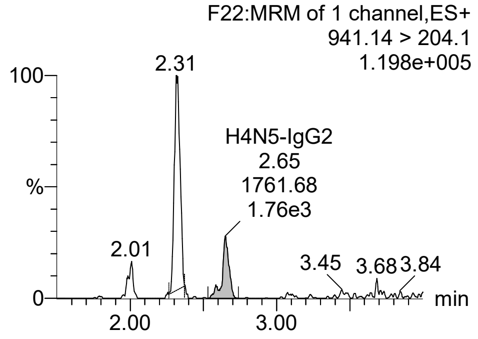

Sample Name: 20211111_L2_R2_IgG

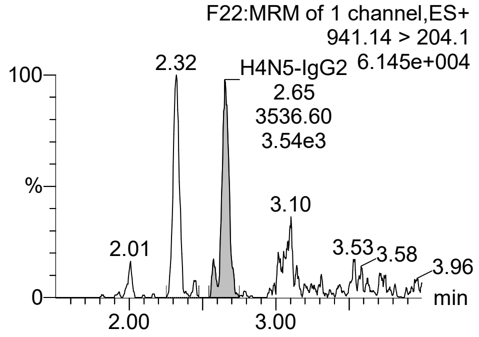

Sample Name: 20211111_H1_R2_IgG

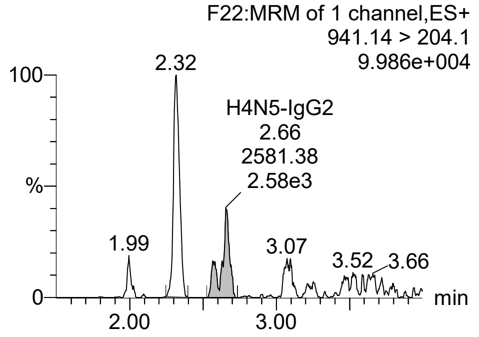

Sample Name: 20211111_A3_R2_IgG

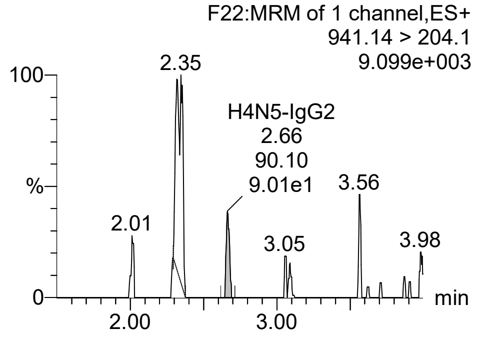

Sample Name: 20211111_A4_R2_IgG

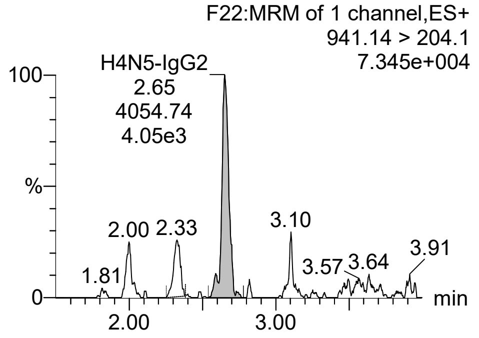

Sample Name: 20211111_L3_R2_IgG

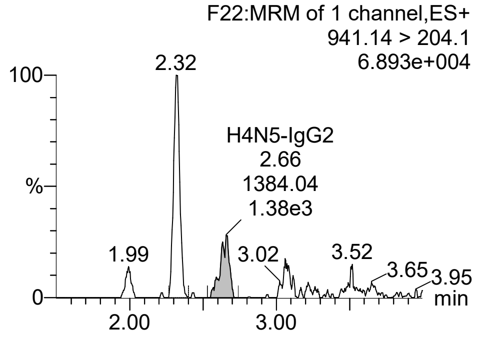

Sample Name: 20211111_L4_R2_IgG

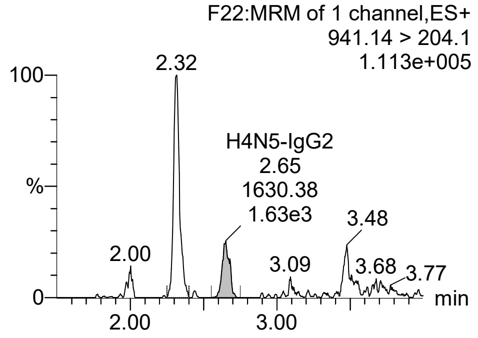

Sample Name: 20211111_H2_R2_IgG

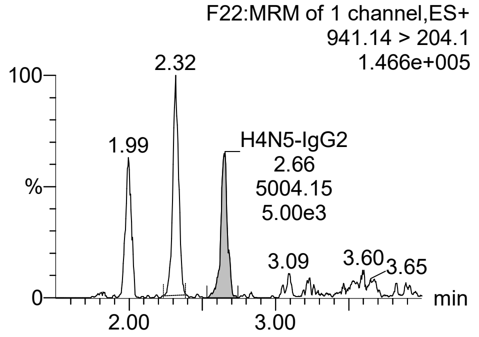

Sample Name: 20211111_A5_R2_IgG

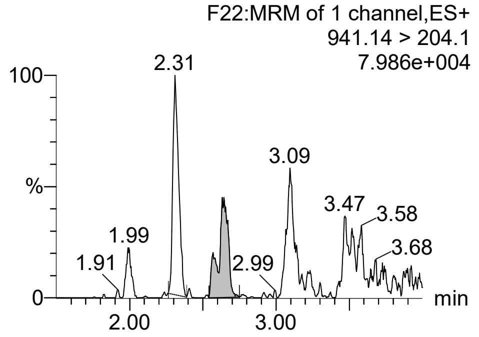

Sample Name: 20211111_A6_R2_IgG

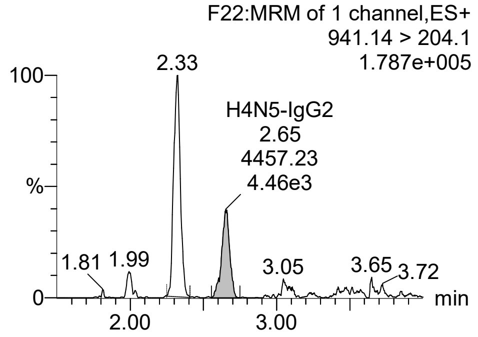

Sample Name: 20211111_QC_R10_IgG
F22:MRM of 1 channel,ES+
941.14 > 204.1
1.114e+005

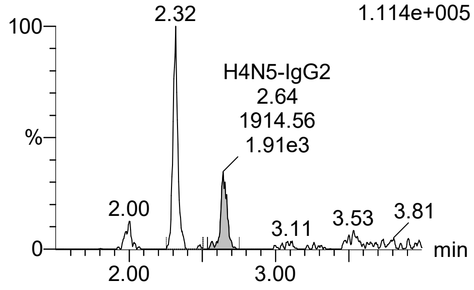

Sample Name: 20211111_L5_R2_IgG
F22:MRM of 1 channel,ES+
941.14 > 204.1
8.965e+004

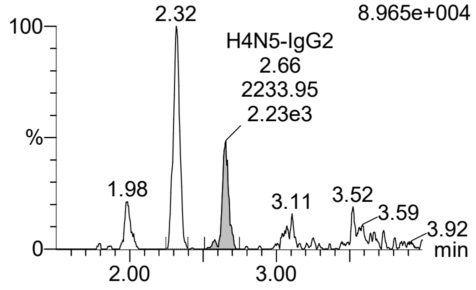

Sample Name: 20211111_L6_R2_IgG
F22:MRM of 1 channel,ES+
941.14 > 204.1
2.481e+004

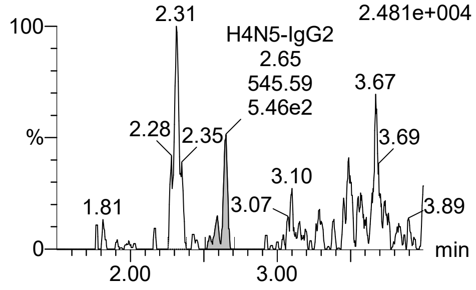

Sample Name: 20211111_H3_R2_IgG
F22:MRM of 1 channel,ES+
941.14 > 204.1
5.452e+004

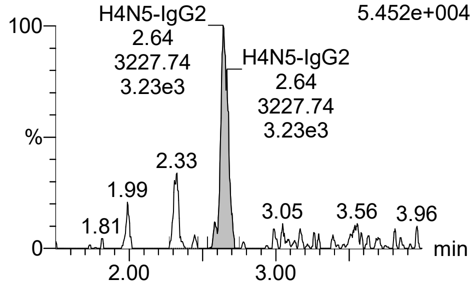

Sample Name: 20211111_A7_R2_IgG
F22:MRM of 1 channel,ES+
941.14 > 204.1
7.622e+004

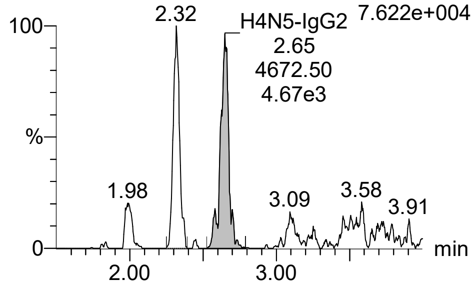

Sample Name: 20211111_A8_R2_IgG
F22:MRM of 1 channel,ES+
941.14 > 204.1
6.001e+004

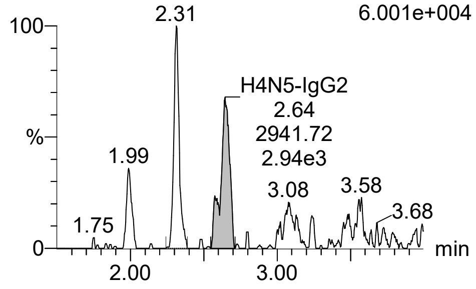

Sample Name: 20211111_L7_R2_IgG
F22:MRM of 1 channel,ES+
941.14 > 204.1
1.088e+005

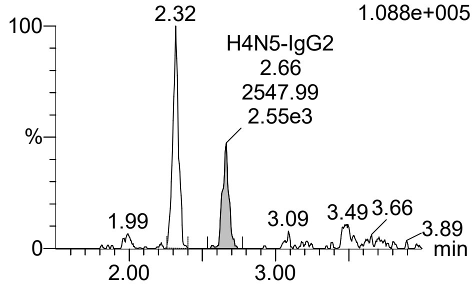

Sample Name: 20211111_L8_R2_IgG
F22:MRM of 1 channel,ES+
941.14 > 204.1
3.405e+004

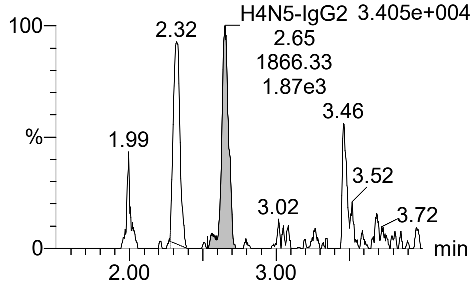

Sample Name: 20211111_H4_R2_IgG
F22:MRM of 1 channel,ES+
941.14 > 204.1
8.064e+004

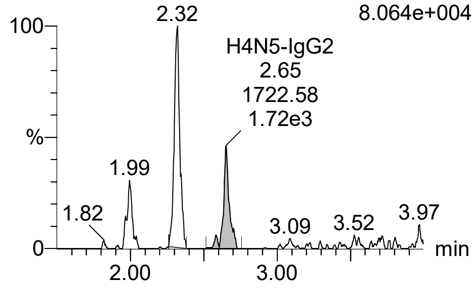

Sample Name: 20211111_A9_R2_IgG
F22:MRM of 1 channel,ES+
941.14 > 204.1
8.629e+004

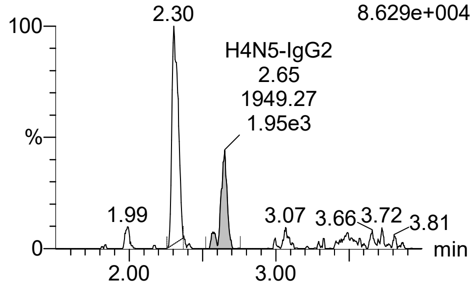

Sample Name: 20211111_A10_R2_IgG
F22:MRM of 1 channel,ES+
941.14 > 204.1
7.180e+004

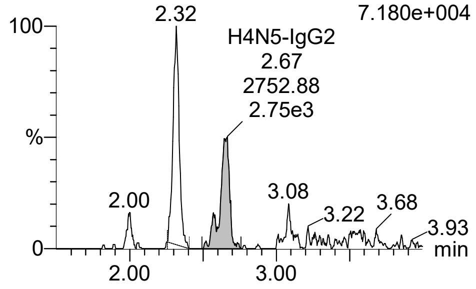

Sample Name: 20211111_L9_R2_IgG
F22:MRM of 1 channel,ES+
941.14 > 204.1
8.675e+004

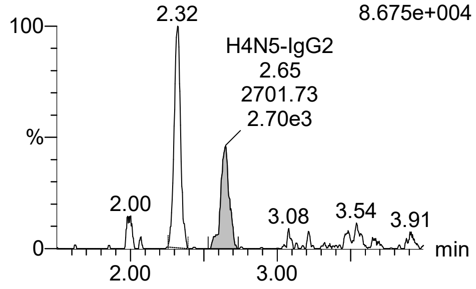

Sample Name: 20211111_L10_R2_IgG
F22:MRM of 1 channel,ES+
941.14 > 204.1
1.412e+005

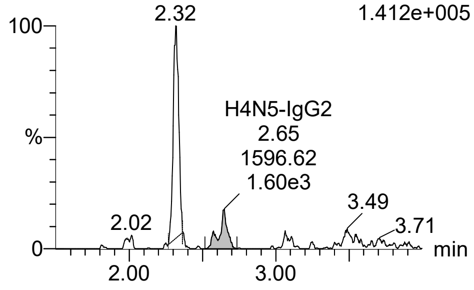

Sample Name: 20211111_QC_R11_IgG
F22:MRM of 1 channel,ES+
941.14 > 204.1
7.068e+004

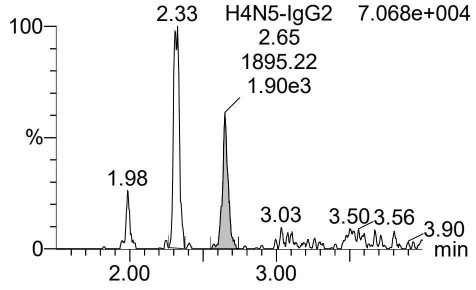

Sample Name: 20211111_H5_R2_IgG
F22:MRM of 1 channel,ES+
941.14 > 204.1
2.150e+004

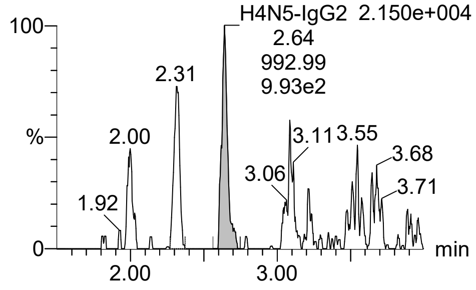

Dataset: C:\MassLynx\10.pro\20211115_clinicalsamples_IgG.qld

Last Altered: Sunday, October 02, 2022 16:02:30 台北標準時間
Printed: Friday, August 01, 2025 16:51:24 台北標準時間

Sample Name: 20211111_A11_R2_IgG

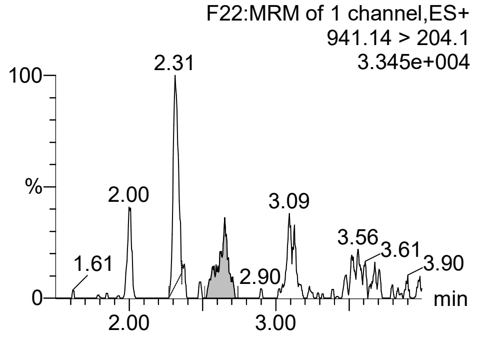

Sample Name: 20211111_A12_R2_IgG

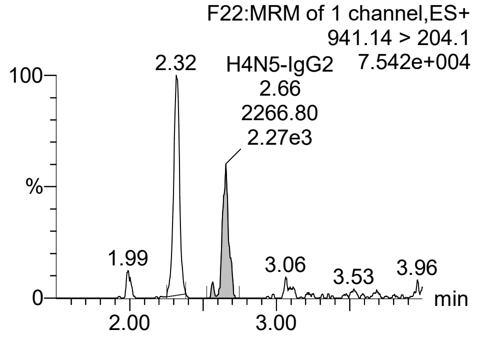

Sample Name: 20211111_L11_R2_IgG

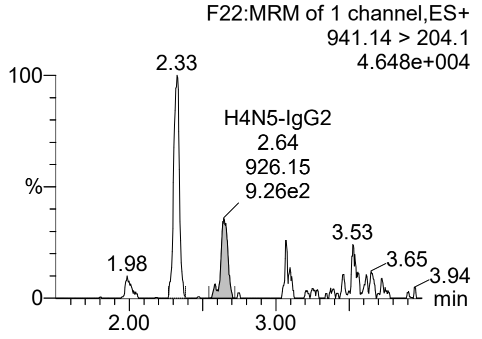

Sample Name: 20211111_L12_R2_IgG

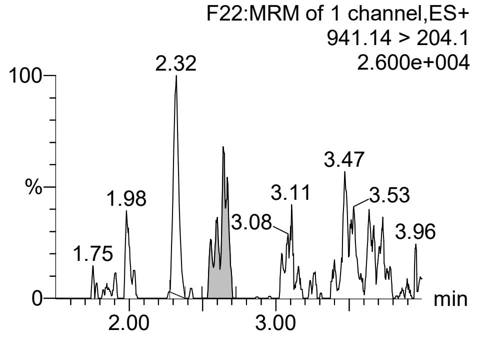

Sample Name: 20211111_H6_R2_IgG

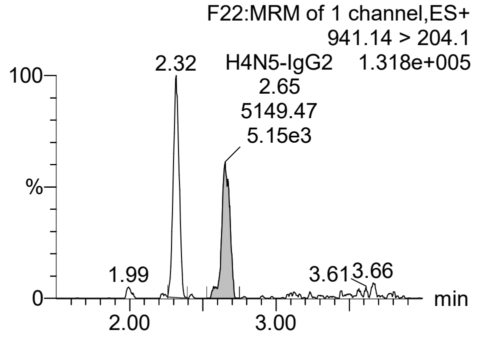

Sample Name: 20211111_A13_R2_IgG

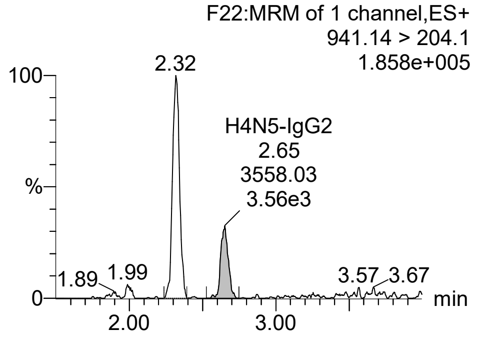

Sample Name: 20211111_A14_R2_IgG

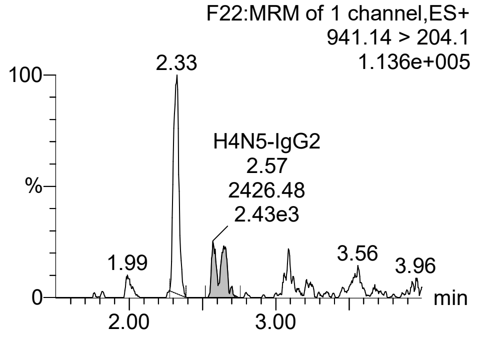

Sample Name: 20211111_L13_R2_IgG

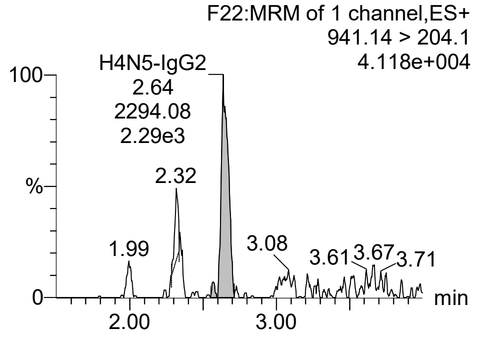

Sample Name: 20211111_L14_R2_IgG

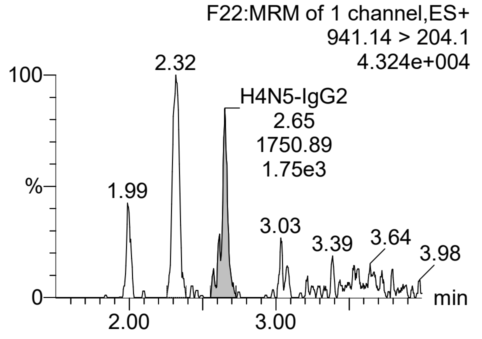

Sample Name: 20211111_H7_R2_IgG

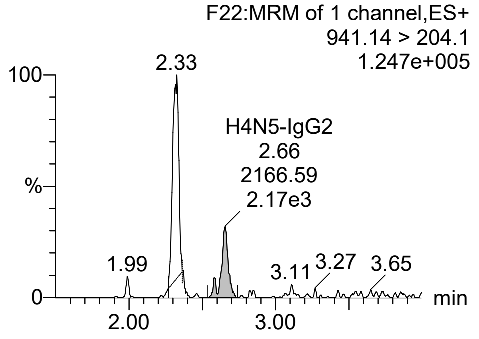

Sample Name: 20211111_A15_R2_IgG

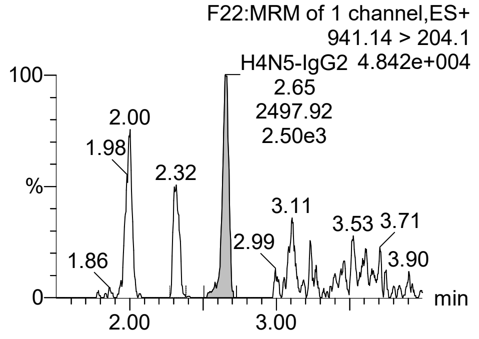

Sample Name: 20211111_QC_R12_IgG

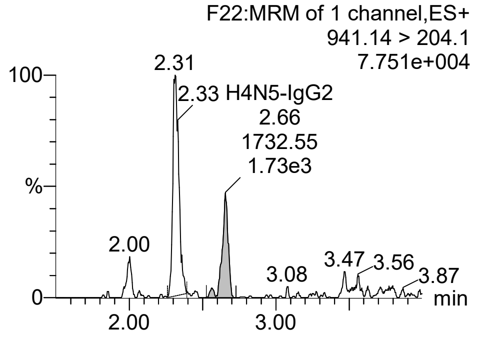

Sample Name: 20211111_A16_R2_IgG

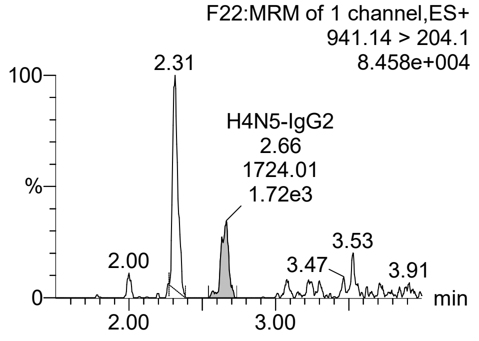

Sample Name: 20211111_L15_R2_IgG

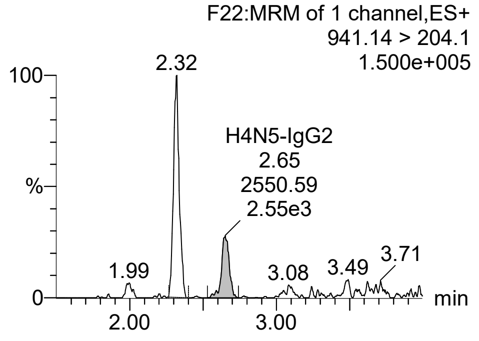

Sample Name: 20211111_L16_R2_IgG

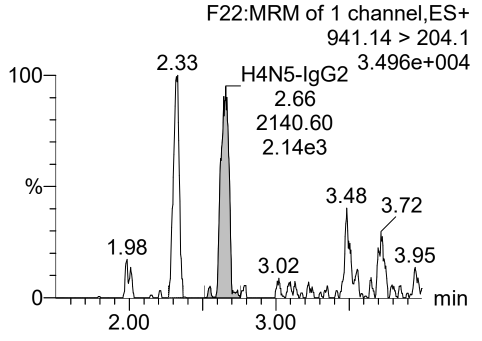

Sample Name: 20211111_H8_R2_IgG

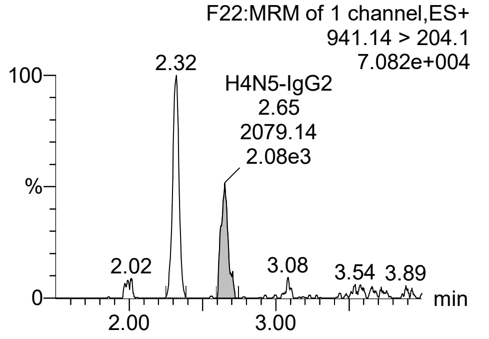

Sample Name: 20211111_A17_R2_IgG

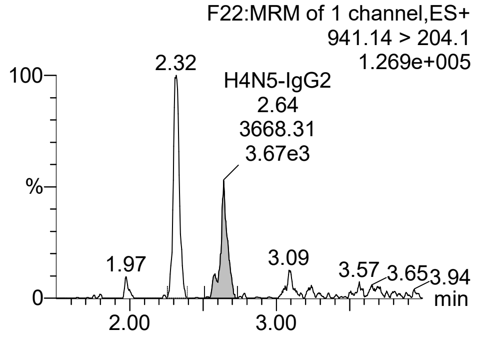

Sample Name: 20211111_A18_R2_IgG

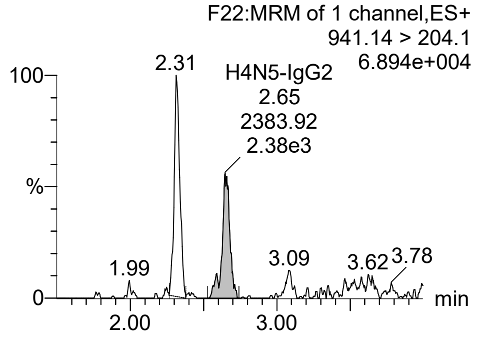

Sample Name: 20211111_L17_R2_IgG

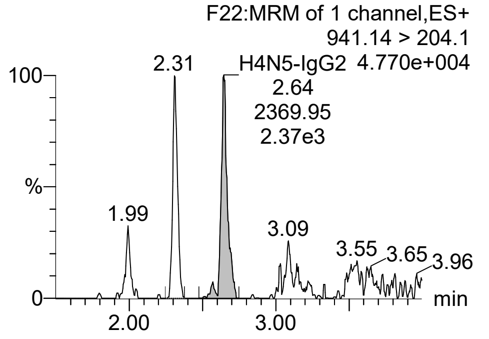

Sample Name: 20211111_L18_R2_IgG

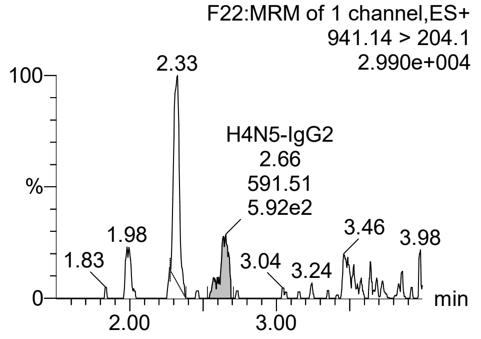

Sample Name: 20211111_H9_R2_IgG

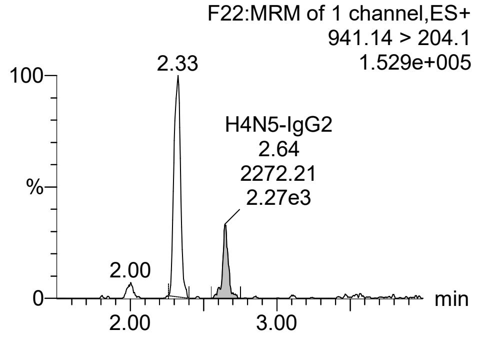

Sample Name: 20211111_A19_R2_IgG

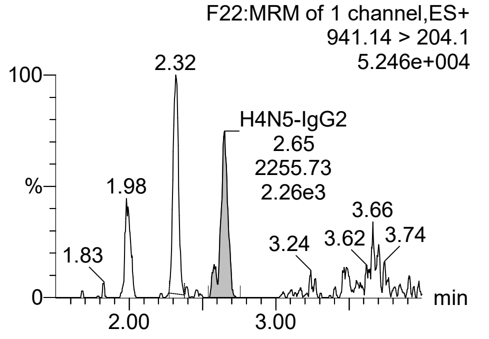

Sample Name: 20211111_A20_R2_IgG

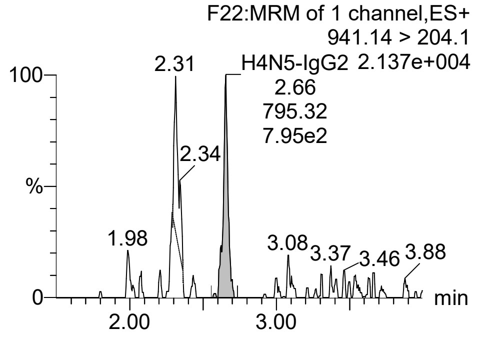

Sample Name: 20211111_L19_R2_IgG

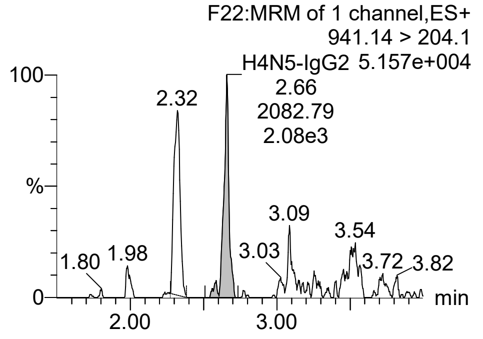

Sample Name: 20211111_QC_R13_IgG

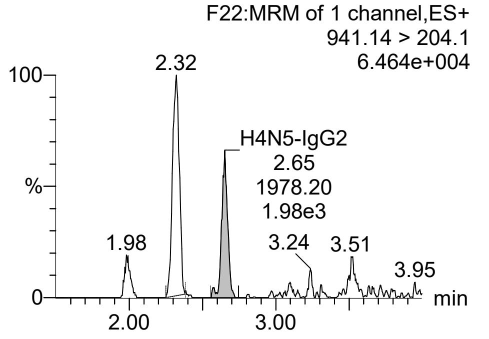

Sample Name: 20211111_L20_R2_IgG

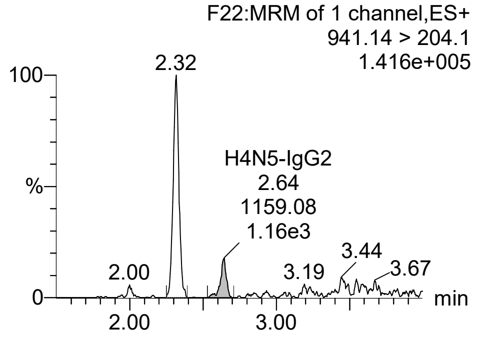

Sample Name: 20211111_H10_R2_IgG

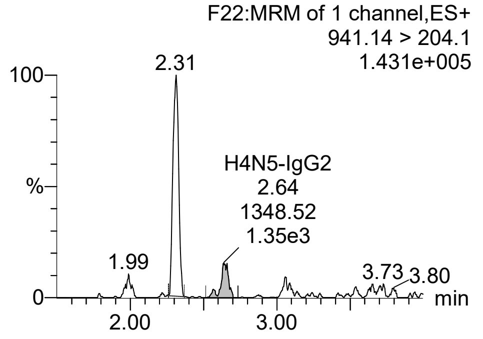

Sample Name: 20211111_A21_R2_IgG

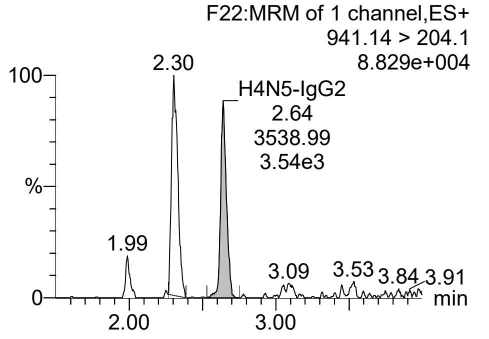

Sample Name: 20211111_A22_R2_IgG

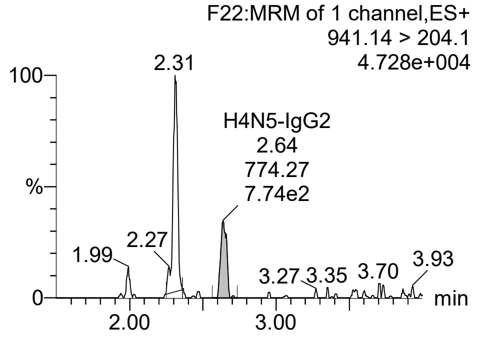

Sample Name: 20211111_L21_R2_IgG

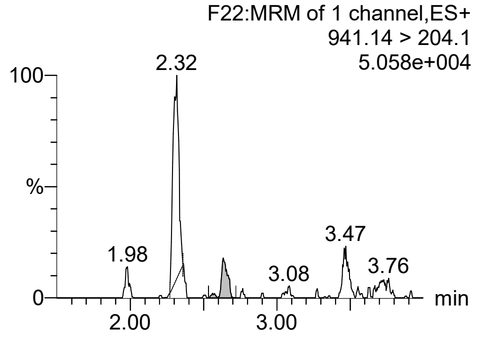

Sample Name: 20211111_L22_R2_IgG

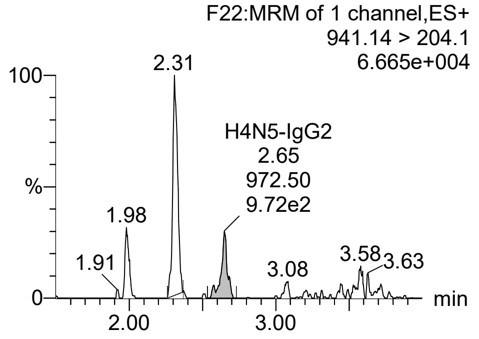

Sample Name: 20211111_H11_R2_IgG

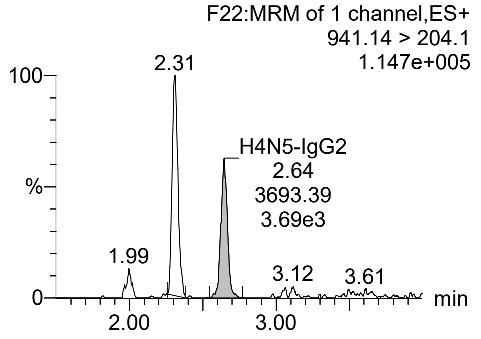

Sample Name: 20211111_A23_R2_IgG

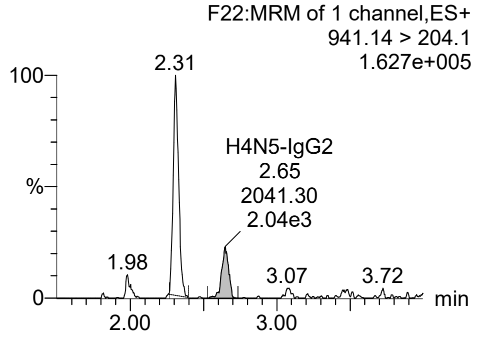

Sample Name: 20211111_A24_R2_IgG

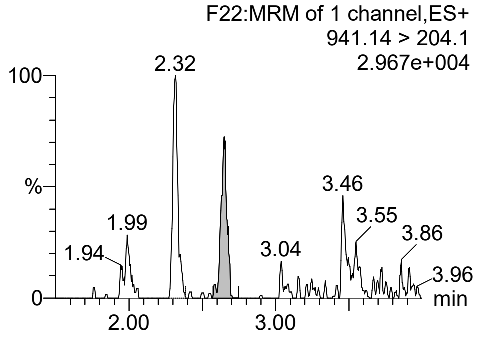

Sample Name: 20211111_L23_R2_IgG

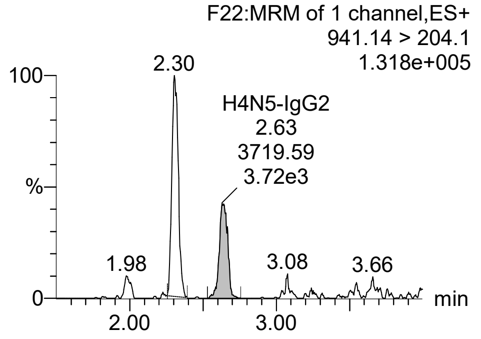

Sample Name: 20211111_L24_R2_IgG

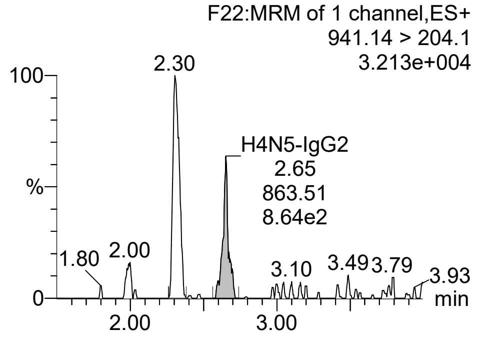

Sample Name: 20211111_H12_R2_IgG

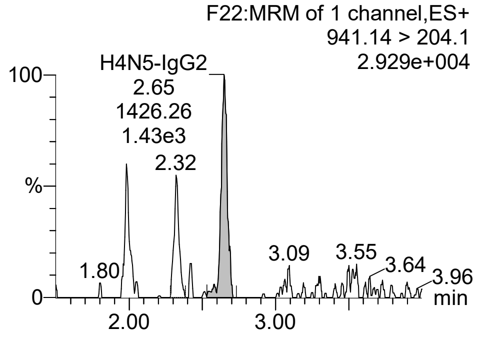

Sample Name: 20211111_QC_R14_IgG

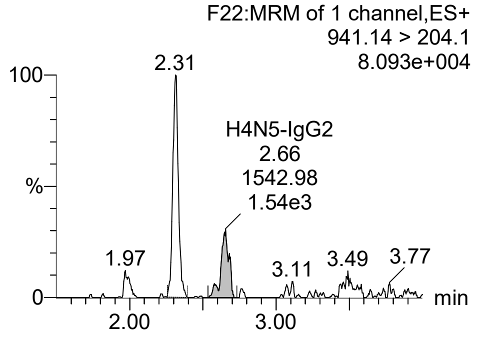

Sample Name: 20211111_A25_R2_IgG

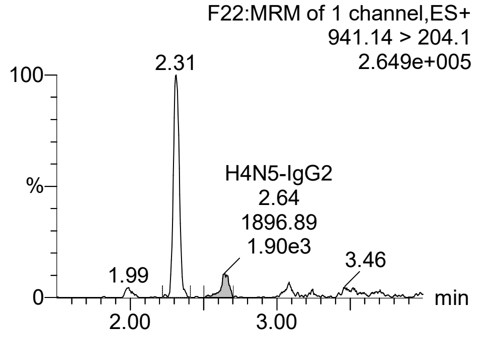

Sample Name: 20211111_A26_R2_IgG

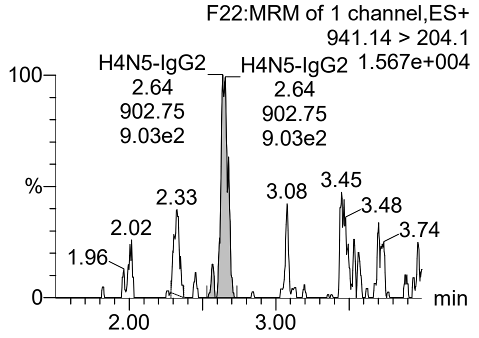

Sample Name: 20211111_L25_R2_IgG

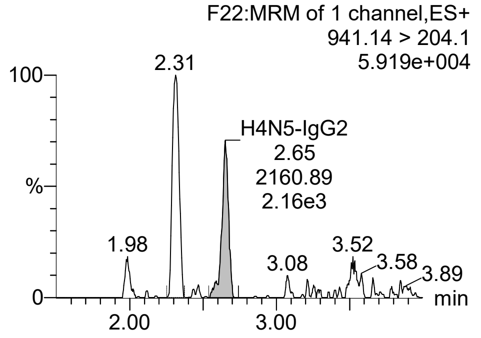

Sample Name: 20211111_L26_R2_IgG

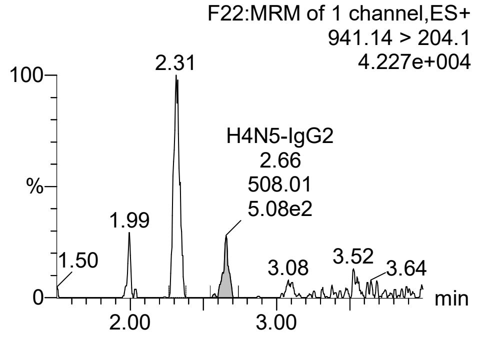

Sample Name: 20211111_H13_R2_IgG

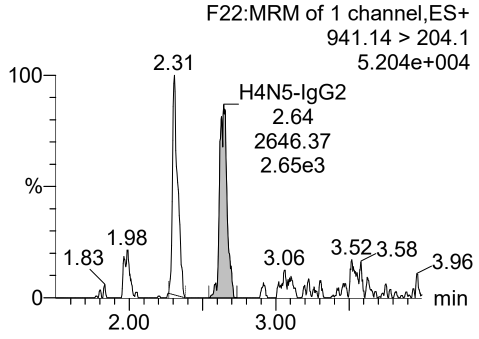

Sample Name: 20211111_A27_R2_IgG

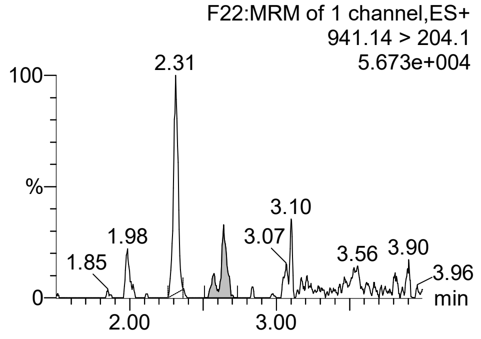

Sample Name: 20211111_A28_R2_IgG

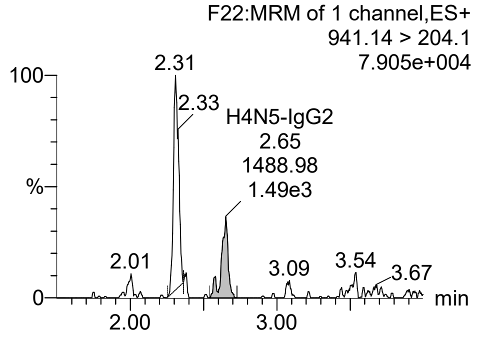

Dataset: C:\MassLynx\10.pro\20211115_clinicalsamples_IgG.qld

Last Altered: Sunday, October 02, 2022 16:02:30 台北標準時間
Printed: Friday, August 01, 2025 16:51:24 台北標準時間

Sample Name: 20211111_L27_R2_IgG

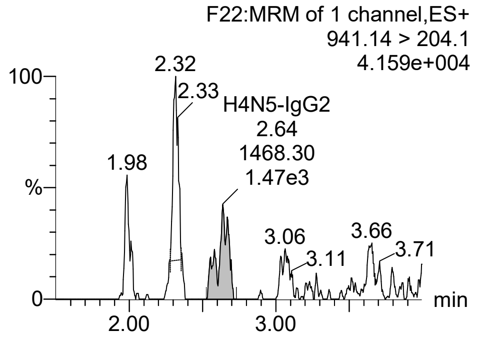

Sample Name: 20211111_L28_R2_IgG

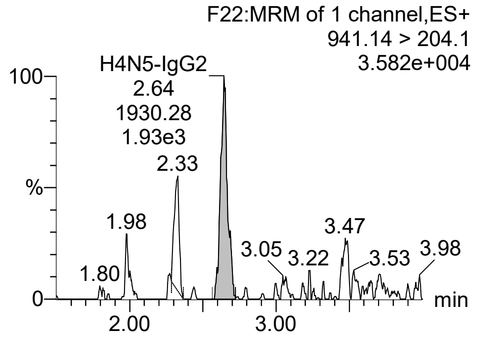

Sample Name: 20211111_H14_R2_IgG

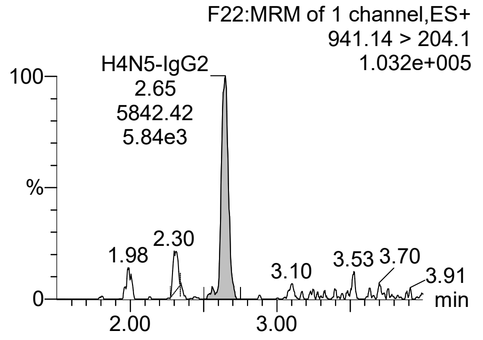

Sample Name: 20211111_A29_R2_IgG

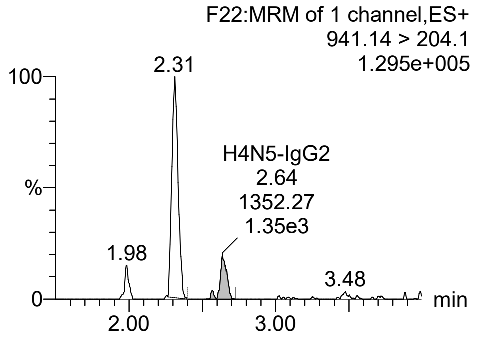

Sample Name: 20211111_A30_R2_IgG

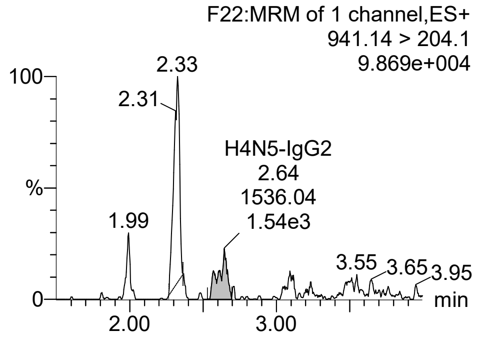

Sample Name: 20211111_QC_R15_IgG

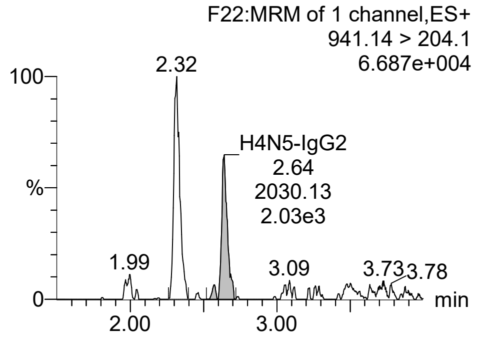

Sample Name: 20211111_L29_R2_IgG

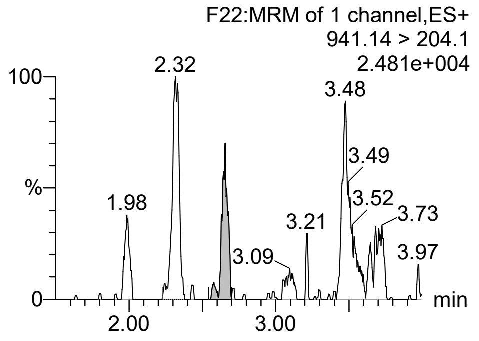

Sample Name: 20211111_L30_R2_IgG

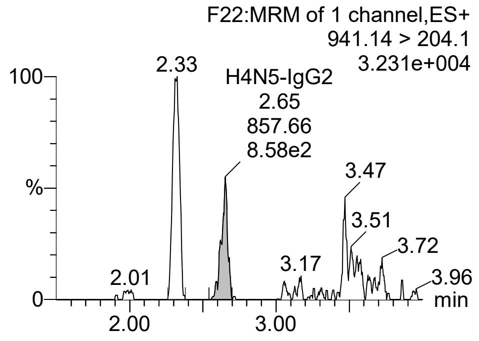

Sample Name: 20211111_H15_R2_IgG

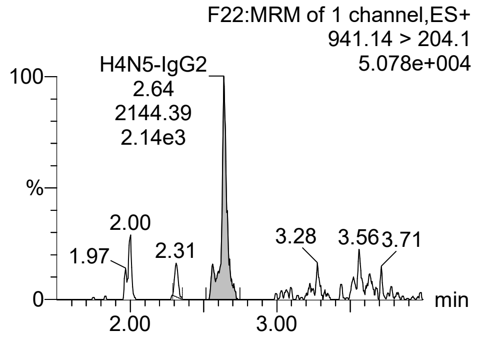

Sample Name: 20211111_A31_R2_IgG

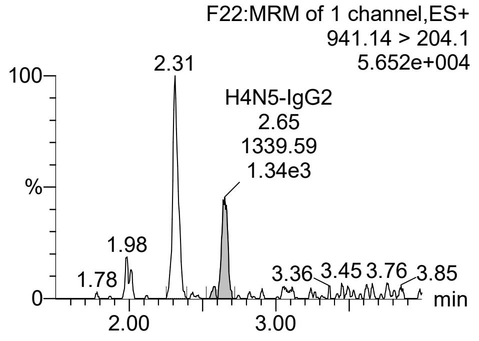

Sample Name: 20211111_A32_R2_IgG

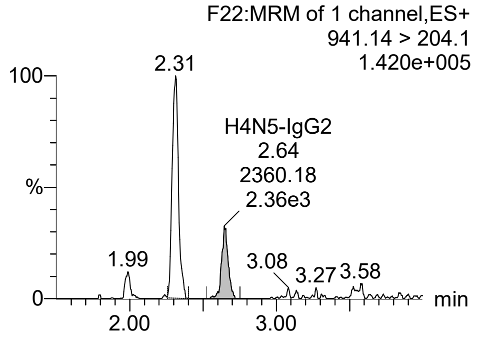

Sample Name: 20211111_L31_R2_IgG

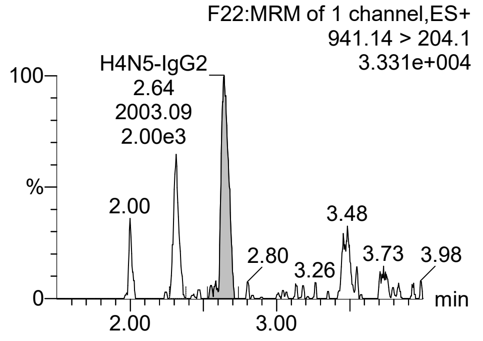

Sample Name: 20211111_L32_R2_IgG

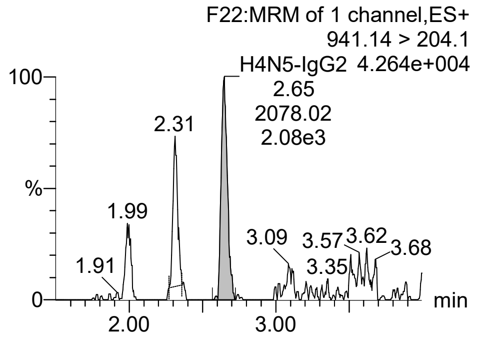

Sample Name: 20211111_H16_R2_IgG

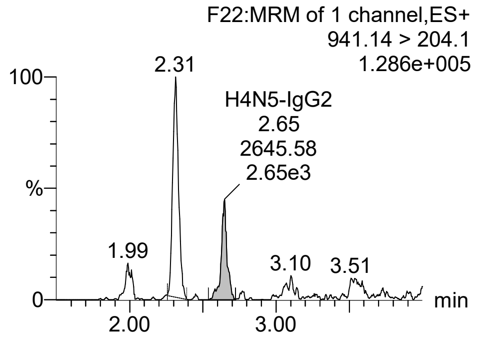

Sample Name: 20211111_A33_R2_IgG

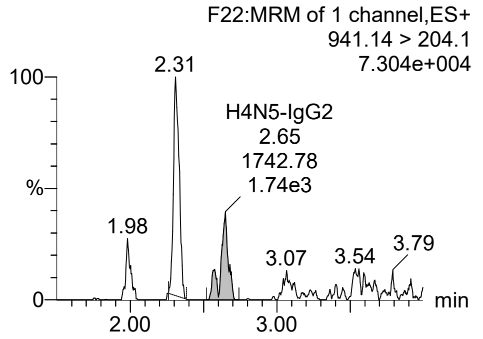

Sample Name: 20211111_A34_R2_IgG

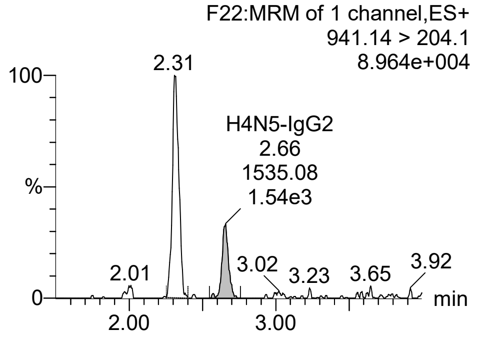

Sample Name: 20211111_H17_R2_IgG

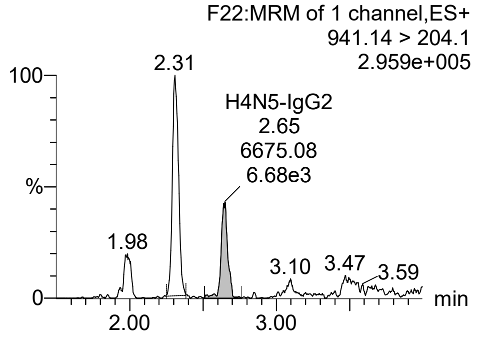

Sample Name: 20211111_A35_R2_IgG

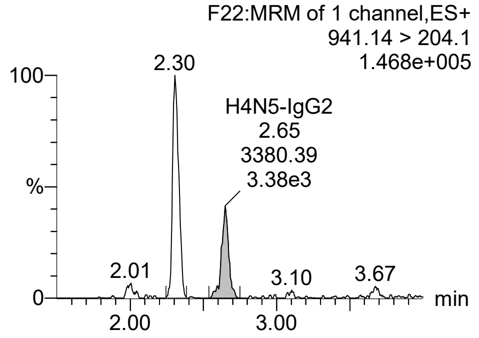

Sample Name: 20211111_QC_R16_IgG

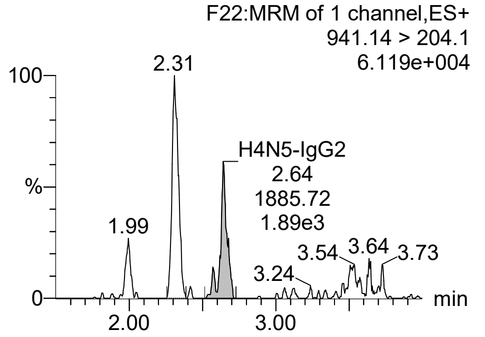

Sample Name: 20211111_A36_R2_IgG

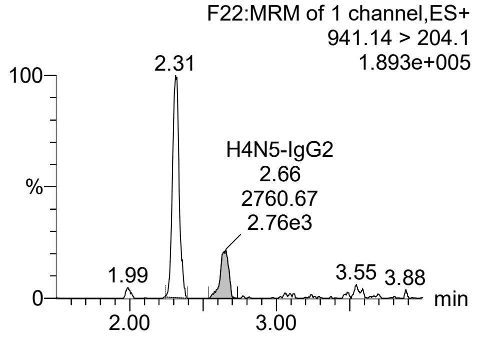

Sample Name: 20211111_H18_R2_IgG

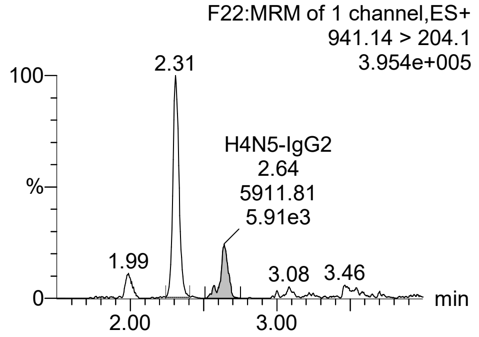

Sample Name: 20211111_A37_R2_IgG

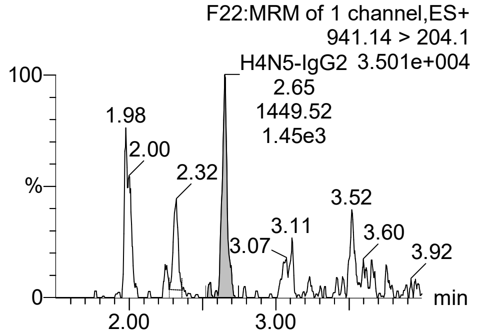

Sample Name: 20211111_A38_R2_IgG

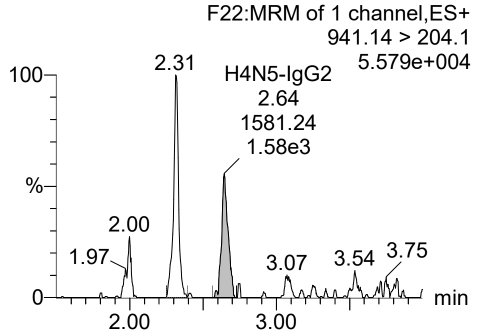

Sample Name: 20211111_H19_R2_IgG

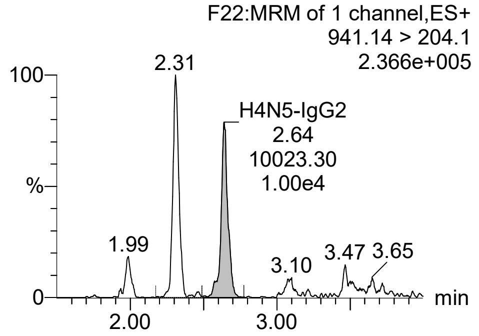

Sample Name: 20211111_A39_R2_IgG

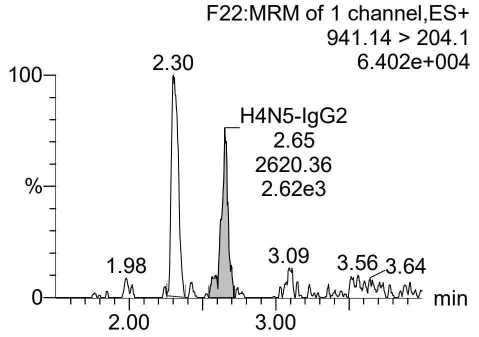

Sample Name: 20211111_A40_R2_IgG

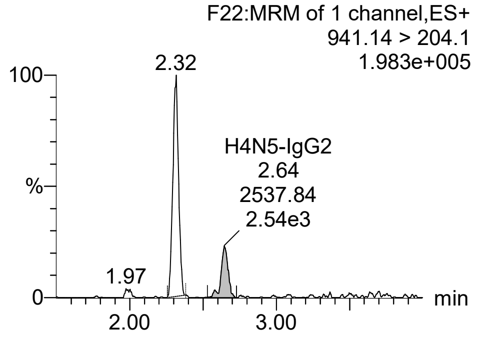

Sample Name: 20211111_H20_R2_IgG

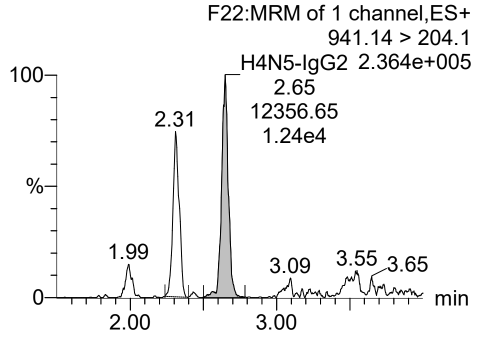

Sample Name: 20211111_A41_R2_IgG

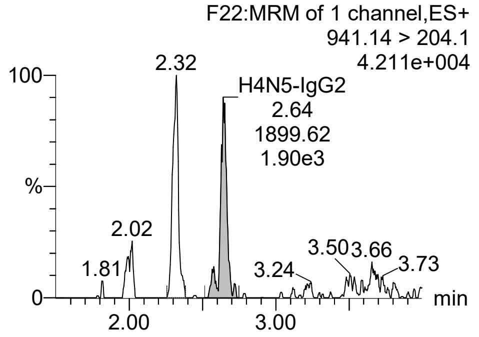

Sample Name: 20211111_A42_R2_IgG

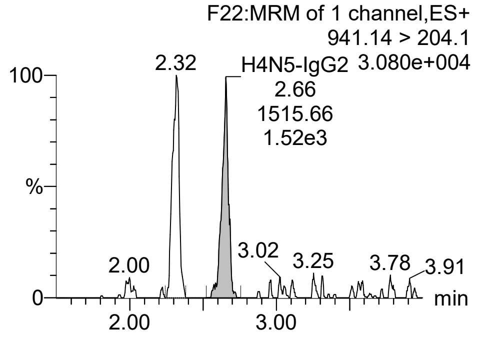

Sample Name: 20211111_A43_R2_IgG

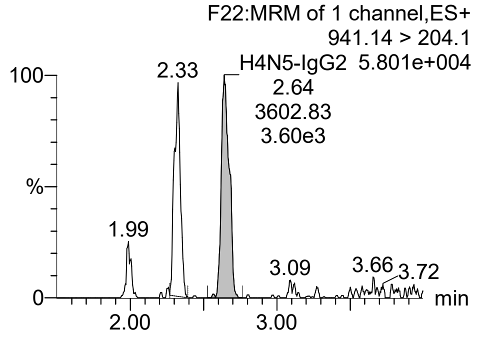

Dataset: C:\MassLynx\10.pro\20211115_clinicalsamples_IgG.qld

Last Altered: Sunday, October 02, 2022 16:02:30 台北標準時間
Printed: Friday, August 01, 2025 16:51:24 台北標準時間

Sample Name: 20211111_A44_R2_IgG

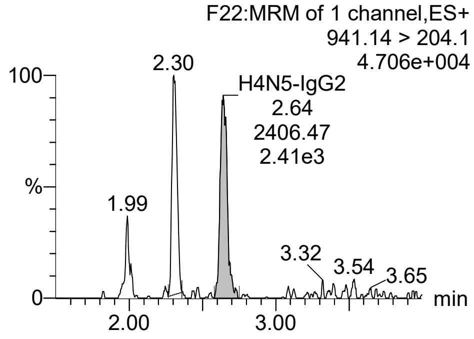

Sample Name: 20211111_QC_R17_IgG

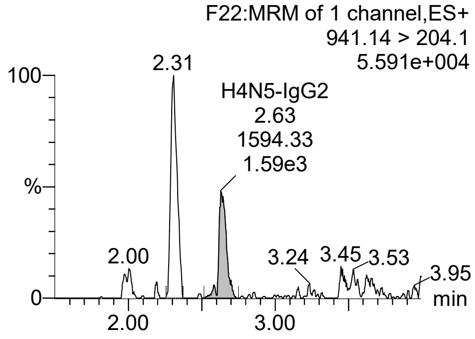

Dataset: C:\MassLynx\10.pro\20211115_clinicalsamples_IgG.qld

Last Altered: Sunday, October 02, 2022 16:02:30 台北標準時間
Printed: Friday, August 01, 2025 16:51:24 台北標準時間

	#	Name	RT	Area	IS Area	Response	Prima...
1	1	20211111_QC_R1_IgG	2.66	4443.5	64968.0	0.068	MM
2	2	20211111_A1_R1_IgG	2.66	8034.2	59059.2	0.136	MM
3	3	20211111_A2_R1_IgG	2.65	4040.6	66621.2	0.061	MM
4	4	20211111_L1_R1_IgG	2.66	3511.4	64201.0	0.055	MM
5	5	20211111_L2_R1_IgG	2.66	4670.8	46950.2	0.099	MM
6	6	20211111_H1_R1_IgG	2.64	2810.8	38648.2	0.073	MM
7	7	20211111_A3_R1_IgG	2.69	65.0	2603.7	0.025	MM
8	8	20211111_A4_R1_IgG	2.66	7140.0	69960.4	0.102	MM
9	9	20211111_L3_R1_IgG	2.67	1590.6	38864.7	0.041	MM
10	10	20211111_L4_R1_IgG	2.66	2269.1	43380.5	0.052	MM
11	11	20211111_H2_R1_IgG	2.64	6921.4	71349.2	0.097	MM
12	12	20211111_A5_R1_IgG	2.64	3628.0	65569.7	0.055	MM
13	13	20211111_A6_R1_IgG	2.65	6271.9	53120.7	0.118	MM
14	14	20211111_QC_R2_IgG	2.66	4886.1	81034.1	0.060	MM
15	15	20211111_L5_R1_IgG	2.66	3232.8	50994.6	0.063	MM
16	16	20211111_L6_R1_IgG	2.65	1246.0	58058.0	0.021	MM
17	17	20211111_H3_R1_IgG	2.67	3705.6	64028.6	0.058	MM
18	18	20211111_A7_R1_IgG	2.64	8120.4	82996.1	0.098	MM
19	19	20211111_A8_R1_IgG	2.66	5248.3	77731.7	0.068	MM
20	20	20211111_L7_R1_IgG	2.67	2419.2	38827.3	0.062	MM
21	21	20211111_L8_R1_IgG	2.66	1941.4	44220.1	0.044	MM
22	22	20211111_H4_R1_IgG	2.65	2067.7	65826.3	0.031	MM
23	23	20211111_A9_R1_IgG	2.64	2657.4	60771.1	0.044	MM
24	24	20211111_A10_R1_IgG	2.65	3365.7	48578.7	0.069	MM
25	25	20211111_L9_R1_IgG	2.64	4056.9	50830.7	0.080	MM
26	26	20211111_L10_R1_IgG	2.64	1705.7	38034.2	0.045	MM
27	27	20211111_QC_R3_IgG	2.66	3873.2	67025.5	0.058	MM
28	28	20211111_H5_R1_IgG	2.66	2146.5	44544.9	0.048	MM
29	29	20211111_A11_R1_IgG	2.66	1438.4	66408.5	0.022	MM
30	30	20211111_A12_R1_IgG	2.64	2434.1	46624.5	0.052	MM
31	31	20211111_L11_R1_IgG	2.66	1141.4	33425.1	0.034	MM
32	32	20211111_L12_R1_IgG	2.64	1066.5	41178.0	0.026	MM
33	33	20211111_H6_R1_IgG	2.66	8466.8	58593.4	0.145	MM
34	34	20211111_A13_R1_IgG	2.66	5307.5	54133.0	0.098	MM
35	35	20211111_A14_R1_IgG	2.65	3559.7	56467.3	0.063	MM
36	36	20211111_L13_R1_IgG	2.65	3093.5	36024.4	0.086	MM
37	37	20211111_L14_R1_IgG	2.64	1791.6	37181.6	0.048	MM
38	38	20211111_H7_R1_IgG	2.65	2710.6	42850.6	0.063	MM
39	39	20211111_A15_R1_IgG	2.66	2957.7	62015.1	0.048	MM
40	40	20211111_QC_R4_IgG	2.64	3555.5	58572.5	0.061	MM
41	41	20211111_A16_R1_IgG	2.65	2896.3	78283.8	0.037	MM
42	42	20211111_L15_R1_IgG	2.64	3758.4	44158.2	0.085	MM
43	43	20211111_L16_R1_IgG	2.66	3407.1	55150.8	0.062	MM
44	44	20211111_H8_R1_IgG	2.66	1458.9	42965.2	0.034	MM
45	45	20211111_A17_R1_IgG	2.64	3449.1	39717.7	0.087	MM
46	46	20211111_A18_R1_IgG	2.65	3125.6	44675.4	0.070	MM
47	47	20211111_L17_R1_IgG	2.65	2715.9	37854.1	0.072	MM
48	48	20211111_L18_R1_IgG	2.65	710.8	50716.9	0.014	MM
49	49	20211111_H9_R1_IgG	2.65	2450.7	36276.0	0.068	MM
50	50	20211111_A19_R1_IgG	2.64	3657.1	42862.5	0.085	MM
51	51	20211111_A20_R1_IgG	2.65	822.3	36542.7	0.023	MM
52	52	20211111_L19_R1_IgG	2.64	2335.2	53892.8	0.043	MM
53	53	20211111_QC_R5_IgG	2.66	2905.4	56513.2	0.051	MM
54	54	20211111_L20_R1_IgG	2.64	2057.0	56499.9	0.036	MM
55	55	20211111_H10_R1_IgG	2.64	3932.4	68120.5	0.058	MM
56	56	20211111_A21_R1_IgG	2.66	7158.9	63699.8	0.112	MM

Dataset: C:\MassLynx\10.pro\20211115_clinicalsamples_IgG.qld

Last Altered: Sunday, October 02, 2022 16:02:30 台北標準時間
Printed: Friday, August 01, 2025 16:51:24 台北標準時間

	# Name	RT	Area	IS Area	Response	Prima...
57	57 20211111_A22_R1_IgG	2.64	2608.5	52222.1	0.050	MM
58	58 20211111_L21_R1_IgG	2.66	1105.4	54372.5	0.020	MM
59	59 20211111_L22_R1_IgG	2.66	1727.7	41353.1	0.042	MM
60	60 20211111_H11_R1_IgG	2.66	6382.7	65579.1	0.097	MM
61	61 20211111_A23_R1_IgG	2.66	3770.3	64131.1	0.059	MM
62	62 20211111_A24_R1_IgG	2.64	631.9	38724.8	0.016	MM
63	63 20211111_L23_R1_IgG	2.65	5740.9	64245.2	0.089	MM
64	64 20211111_L24_R1_IgG	2.63	1494.4	45298.0	0.033	MM
65	65 20211111_H12_R1_IgG	2.66	1376.6	44679.3	0.031	MM
66	66 20211111_QC_R6_IgG	2.65	2503.6	48165.0	0.052	MM
67	67 20211111_A25_R1_IgG	2.65	2358.6	57169.9	0.041	MM
68	68 20211111_A26_R1_IgG	2.65	1758.7	34254.0	0.051	MM
69	69 20211111_L25_R1_IgG	2.65	3927.4	67491.5	0.058	MM
70	70 20211111_L26_R1_IgG	2.65	1504.2	55934.1	0.027	MM
71	71 20211111_H13_R1_IgG	2.67	3852.0	48767.3	0.079	MM
72	72 20211111_A27_R1_IgG	2.65	1762.1	57269.7	0.031	MM
73	73 20211111_A28_R1_IgG	2.64	2428.6	49231.1	0.049	MM
74	74 20211111_L27_R1_IgG	2.65	2253.3	60014.5	0.038	MM
75	75 20211111_L28_R1_IgG	2.66	2271.3	35062.6	0.065	MM
76	76 20211111_H14_R1_IgG	2.65	7764.9	62371.9	0.124	MM
77	77 20211111_A29_R1_IgG	2.66	1204.7	43858.2	0.027	MM
78	78 20211111_A30_R1_IgG	2.65	2272.6	62500.4	0.036	MM
79	79 20211111_QC_R7_IgG	2.66	2969.2	43946.9	0.068	MM
80	80 20211111_L29_R1_IgG	2.65	719.7	38728.9	0.019	MM
81	81 20211111_L30_R1_IgG	2.67	670.4	42613.2	0.016	MM
82	82 20211111_H15_R1_IgG	2.64	2895.1	39874.6	0.073	MM
83	83 20211111_A31_R1_IgG	2.65	2573.2	53414.3	0.048	MM
84	84 20211111_A32_R1_IgG	2.66	2862.0	41844.6	0.068	MM
85	85 20211111_L31_R1_IgG	2.65	2283.4	40553.7	0.056	MM
86	86 20211111_L32_R1_IgG	2.66	2038.5	49618.7	0.041	MM
87	87 20211111_H16_R1_IgG	2.66	4638.9	60170.9	0.077	MM
88	88 20211111_A33_R1_IgG	2.65	1571.0	48588.2	0.032	MM
89	89 20211111_A34_R1_IgG	2.67	1587.5	43288.0	0.037	MM
90	90 20211111_H17_R1_IgG	2.66	6258.1	47104.5	0.133	MM
91	91 20211111_A35_R1_IgG	2.65	3056.2	41025.9	0.074	MM
92	92 20211111_QC_R8_IgG	2.67	1966.1	40546.3	0.048	MM
93	93 20211111_A36_R1_IgG	2.65	2435.5	44504.4	0.055	MM
94	94 20211111_H18_R1_IgG	2.65	5945.0	34162.2	0.174	MM
95	95 20211111_A37_R1_IgG	2.66	1127.2	39760.0	0.028	MM
96	96 20211111_A38_R1_IgG	2.67	1984.9	43982.6	0.045	MM
97	97 20211111_H19_R1_IgG	2.65	11632.9	45314.8	0.257	MM
98	98 20211111_A39_R1_IgG	2.64	3215.3	39388.0	0.082	MM
99	99 20211111_A40_R1_IgG	2.66	1873.3	38726.4	0.048	MM
100	1... 20211111_H20_R1_IgG	2.66	13167.3	37814.4	0.348	MM
101	1... 20211111_A41_R1_IgG	2.67	2002.6	48930.3	0.041	MM
102	1... 20211111_A42_R1_IgG	2.66	1601.5	40406.8	0.040	MM
103	1... 20211111_A43_R1_IgG	2.66	4121.2	44421.5	0.093	MM
104	1... 20211111_A44_R1_IgG	2.65	3775.0	53128.2	0.071	MM
105	1... 20211111_QC_R9_IgG	2.65	2021.2	37049.0	0.055	MM
106	1... 20211111_A1_R2_IgG	2.64	4457.9	46499.4	0.096	MM
107	1... 20211111_A2_R2_IgG	2.66	2502.9	57260.7	0.044	MM
108	1... 20211111_L1_R2_IgG	2.65	1761.7	48062.9	0.037	MM
109	1... 20211111_L2_R2_IgG	2.65	3536.6	38745.2	0.091	MM
110	1... 20211111_H1_R2_IgG	2.66	2581.4	44273.5	0.058	MM
111	1... 20211111_A3_R2_IgG	2.66	90.1	4755.5	0.019	MM
112	1... 20211111_A4_R2_IgG	2.65	4054.7	50546.7	0.080	MM

Dataset: C:\MassLynx\10.pro\20211115_clinicalsamples_IgG.qld

Last Altered: Sunday, October 02, 2022 16:02:30 台北標準時間
Printed: Friday, August 01, 2025 16:51:24 台北標準時間

	# Name	RT	Area	IS Area	Response	Prima...
113	1... 20211111_L3_R2_IgG	2.66	1384.0	34825.8	0.040	MM
114	1... 20211111_L4_R2_IgG	2.65	1630.4	39107.7	0.042	MM
115	1... 20211111_H2_R2_IgG	2.66	5004.2	49750.0	0.101	MM
116	1... 20211111_A5_R2_IgG	2.64	2815.8	52536.2	0.054	MM
117	1... 20211111_A6_R2_IgG	2.65	4457.2	42050.0	0.106	MM
118	1... 20211111_QC_R10_IgG	2.64	1914.6	35350.8	0.054	MM
119	1... 20211111_L5_R2_IgG	2.66	2234.0	38039.3	0.059	MM
120	1... 20211111_L6_R2_IgG	2.65	545.6	44907.4	0.012	MM
121	1... 20211111_H3_R2_IgG	2.64	3227.7	47729.1	0.068	MM
122	1... 20211111_A7_R2_IgG	2.65	4672.5	57347.1	0.081	MM
123	1... 20211111_A8_R2_IgG	2.64	2941.7	52397.8	0.056	MM
124	1... 20211111_L7_R2_IgG	2.66	2548.0	36318.4	0.070	MM
125	1... 20211111_L8_R2_IgG	2.65	1866.3	38131.9	0.049	MM
126	1... 20211111_H4_R2_IgG	2.65	1722.6	46849.6	0.037	MM
127	1... 20211111_A9_R2_IgG	2.65	1949.3	47759.6	0.041	MM
128	1... 20211111_A10_R2_IgG	2.67	2752.9	41366.2	0.067	MM
129	1... 20211111_L9_R2_IgG	2.65	2701.7	43052.1	0.063	MM
130	1... 20211111_L10_R2_IgG	2.65	1596.6	31487.4	0.051	MM
131	1... 20211111_QC_R11_IgG	2.65	1895.2	37804.6	0.050	MM
132	1... 20211111_H5_R2_IgG	2.64	993.0	40380.0	0.025	MM
133	1... 20211111_A11_R2_IgG	2.65	916.1	49533.3	0.018	MM
134	1... 20211111_A12_R2_IgG	2.66	2266.8	40226.3	0.056	MM
135	1... 20211111_L11_R2_IgG	2.64	926.2	28884.0	0.032	MM
136	1... 20211111_L12_R2_IgG	2.64	1256.2	34351.3	0.037	MM
137	1... 20211111_H6_R2_IgG	2.65	5149.5	40466.6	0.127	MM
138	1... 20211111_A13_R2_IgG	2.65	3558.0	42306.7	0.084	MM
139	1... 20211111_A14_R2_IgG	2.57	2426.5	44904.0	0.054	MM
140	1... 20211111_L13_R2_IgG	2.64	2294.1	31913.0	0.072	MM
141	1... 20211111_L14_R2_IgG	2.65	1750.9	31527.4	0.056	MM
142	1... 20211111_H7_R2_IgG	2.66	2166.6	40838.2	0.053	MM
143	1... 20211111_A15_R2_IgG	2.65	2497.9	49389.7	0.051	MM
144	1... 20211111_QC_R12_IgG	2.66	1732.5	35168.7	0.049	MM
145	1... 20211111_A16_R2_IgG	2.66	1724.0	47440.5	0.036	MM
146	1... 20211111_L15_R2_IgG	2.65	2550.6	33145.9	0.077	MM
147	1... 20211111_L16_R2_IgG	2.66	2140.6	37596.6	0.057	MM
148	1... 20211111_H8_R2_IgG	2.65	2079.1	43933.3	0.047	MM
149	1... 20211111_A17_R2_IgG	2.64	3668.3	43605.5	0.084	MM
150	1... 20211111_A18_R2_IgG	2.65	2383.9	37581.8	0.063	MM
151	1... 20211111_L17_R2_IgG	2.64	2369.9	33942.5	0.070	MM
152	1... 20211111_L18_R2_IgG	2.66	591.5	37831.5	0.016	MM
153	1... 20211111_H9_R2_IgG	2.64	2272.2	30898.4	0.074	MM
154	1... 20211111_A19_R2_IgG	2.65	2255.7	39352.1	0.057	MM
155	1... 20211111_A20_R2_IgG	2.66	795.3	34661.2	0.023	MM
156	1... 20211111_L19_R2_IgG	2.66	2082.8	39918.3	0.052	MM
157	1... 20211111_QC_R13_IgG	2.65	1978.2	36239.1	0.055	MM
158	1... 20211111_L20_R2_IgG	2.64	1159.1	32897.7	0.035	MM
159	1... 20211111_H10_R2_IgG	2.64	1348.5	31394.5	0.043	MM
160	1... 20211111_A21_R2_IgG	2.64	3539.0	34863.7	0.102	MM
161	1... 20211111_A22_R2_IgG	2.64	774.3	30342.1	0.026	MM
162	1... 20211111_L21_R2_IgG	2.63	444.5	33706.2	0.013	MM
163	1... 20211111_L22_R2_IgG	2.65	972.5	31298.6	0.031	MM
164	1... 20211111_H11_R2_IgG	2.64	3693.4	38904.1	0.095	MM
165	1... 20211111_A23_R2_IgG	2.65	2041.3	32382.3	0.063	MM
166	1... 20211111_A24_R2_IgG	2.65	1004.3	30261.9	0.033	MM
167	1... 20211111_L23_R2_IgG	2.63	3719.6	43037.5	0.086	MM
168	1... 20211111_L24_R2_IgG	2.65	863.5	37023.3	0.023	MM

Dataset: C:\MassLynx\10.pro\20211115_clinicalsamples_IgG.qld

Last Altered: Sunday, October 02, 2022 16:02:30 台北標準時間
Printed: Friday, August 01, 2025 16:51:24 台北標準時間

	# Name	RT	Area	IS Area	Response	Prima...
169	1... 20211111_H12_R2_IgG	2.65	1426.3	39478.2	0.036	MM
170	1... 20211111_QC_R14_IgG	2.66	1543.0	34463.2	0.045	MM
171	1... 20211111_A25_R2_IgG	2.64	1896.9	39945.3	0.047	MM
172	1... 20211111_A26_R2_IgG	2.64	902.8	29663.2	0.030	MM
173	1... 20211111_L25_R2_IgG	2.65	2160.9	38589.3	0.056	MM
174	1... 20211111_L26_R2_IgG	2.66	508.0	38405.2	0.013	MM
175	1... 20211111_H13_R2_IgG	2.64	2646.4	37771.2	0.070	MM
176	1... 20211111_A27_R2_IgG	2.64	968.5	42634.1	0.023	MM
177	1... 20211111_A28_R2_IgG	2.65	1489.0	33231.1	0.045	MM
178	1... 20211111_L27_R2_IgG	2.64	1468.3	41761.1	0.035	MM
179	1... 20211111_L28_R2_IgG	2.64	1930.3	26968.6	0.072	MM
180	1... 20211111_H14_R2_IgG	2.65	5842.4	49250.2	0.119	MM
181	1... 20211111_A29_R2_IgG	2.64	1352.3	37601.7	0.036	MM
182	1... 20211111_A30_R2_IgG	2.64	1536.0	40366.8	0.038	MM
183	1... 20211111_QC_R15_IgG	2.64	2030.1	35156.1	0.058	MM
184	1... 20211111_L29_R2_IgG	2.66	803.5	33813.1	0.024	MM
185	1... 20211111_L30_R2_IgG	2.65	857.7	33488.6	0.026	MM
186	1... 20211111_H15_R2_IgG	2.64	2144.4	38501.0	0.056	MM
187	1... 20211111_A31_R2_IgG	2.65	1339.6	37525.5	0.036	MM
188	1... 20211111_A32_R2_IgG	2.64	2360.2	35770.8	0.066	MM
189	1... 20211111_L31_R2_IgG	2.64	2003.1	33282.4	0.060	MM
190	1... 20211111_L32_R2_IgG	2.65	2078.0	42049.6	0.049	MM
191	1... 20211111_H16_R2_IgG	2.65	2645.6	45947.6	0.058	MM
192	1... 20211111_A33_R2_IgG	2.65	1742.8	40202.3	0.043	MM
193	1... 20211111_A34_R2_IgG	2.66	1535.1	36747.0	0.042	MM
194	1... 20211111_H17_R2_IgG	2.65	6675.1	40920.9	0.163	MM
195	1... 20211111_A35_R2_IgG	2.65	3380.4	33689.2	0.100	MM
196	1... 20211111_QC_R16_IgG	2.64	1885.7	32783.6	0.058	MM
197	1... 20211111_A36_R2_IgG	2.66	2760.7	36822.4	0.075	MM
198	1... 20211111_H18_R2_IgG	2.64	5911.8	31769.3	0.186	MM
199	1... 20211111_A37_R2_IgG	2.65	1449.5	37581.8	0.039	MM
200	2... 20211111_A38_R2_IgG	2.64	1581.2	38390.4	0.041	MM
201	2... 20211111_H19_R2_IgG	2.64	10023.3	41217.0	0.243	MM
202	2... 20211111_A39_R2_IgG	2.65	2620.4	37392.7	0.070	MM
203	2... 20211111_A40_R2_IgG	2.64	2537.8	38295.1	0.066	MM
204	2... 20211111_H20_R2_IgG	2.65	12356.7	35941.8	0.344	MM
205	2... 20211111_A41_R2_IgG	2.64	1899.6	45304.9	0.042	MM
206	2... 20211111_A42_R2_IgG	2.66	1515.7	37803.8	0.040	MM
207	2... 20211111_A43_R2_IgG	2.64	3602.8	38161.8	0.094	MM
208	2... 20211111_A44_R2_IgG	2.64	2406.5	37819.8	0.064	MM
209	2... 20211111_QC_R17_IgG	2.63	1594.3	31731.8	0.050	MM

Dataset: C:\MassLynx\10.pro\20211115_clinicalsamples_IgG.qld

Last Altered: Sunday, October 02, 2022 16:02:30 台北標準時間
Printed: Friday, August 01, 2025 16:51:59 台北標準時間

Method: C:\MassLynx\10_Pro_IgA.PRO\MethDB\20210926_IgG_DMRM_IgM_IS.mdb 16 Feb 2022 11:58:21
Calibration: 18 Nov 2021 13:56:22

Sample Name: 20211111_QC_R1_IgG

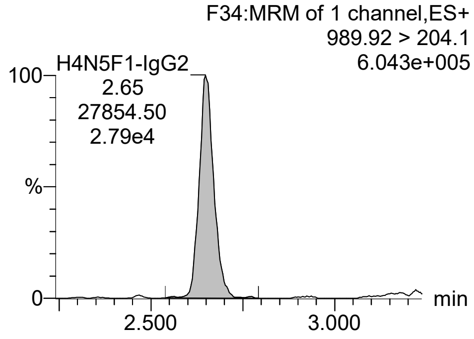

Sample Name: 20211111_A1_R1_IgG

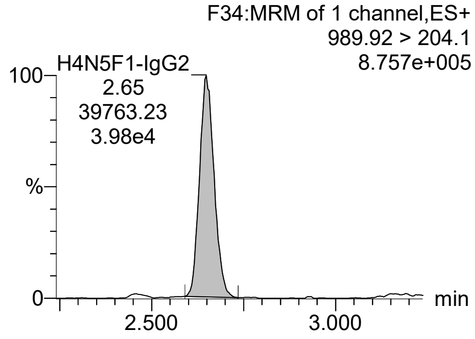

Sample Name: 20211111_A2_R1_IgG

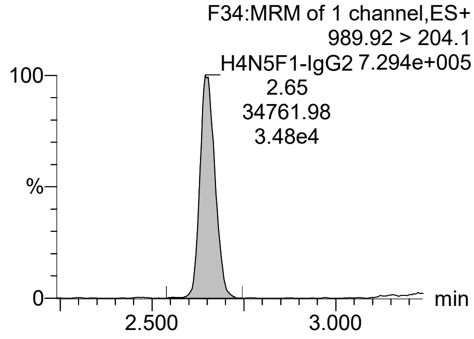

Sample Name: 20211111_L1_R1_IgG

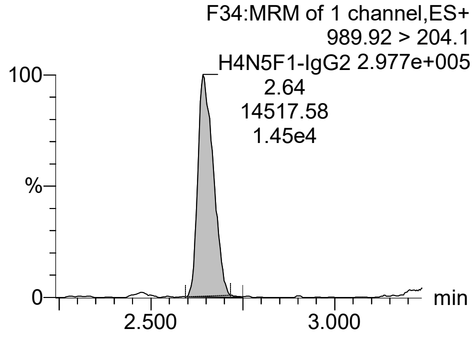

Sample Name: 20211111_L2_R1_IgG

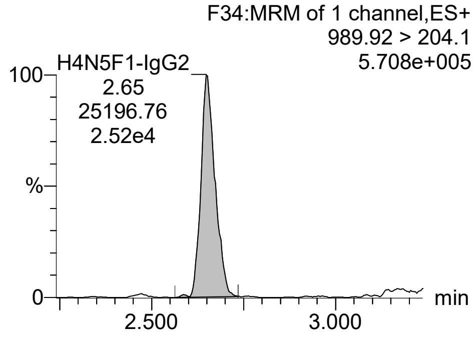

Sample Name: 20211111_H1_R1_IgG

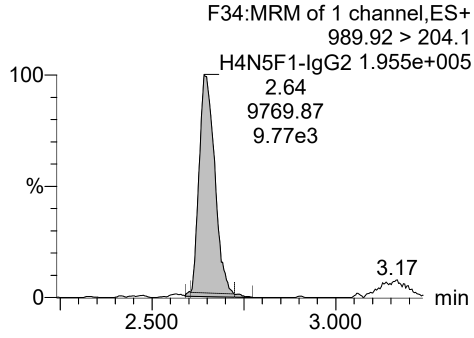

Sample Name: 20211111_A3_R1_IgG

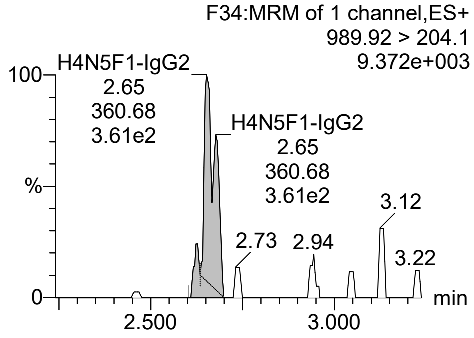

Sample Name: 20211111_A4_R1_IgG

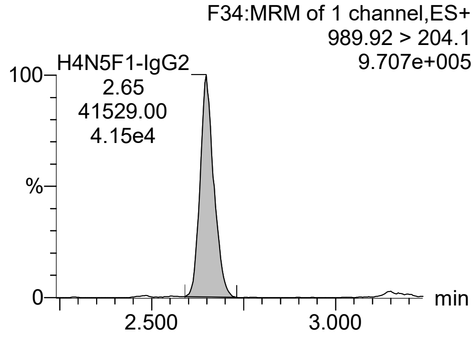

Sample Name: 20211111_L3_R1_IgG

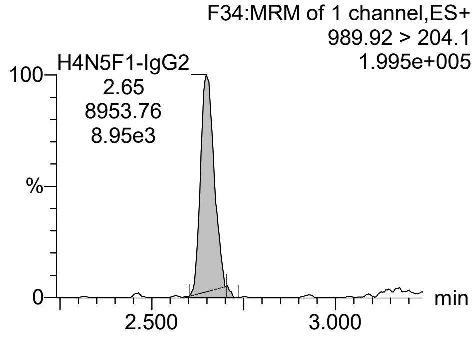

Sample Name: 20211111_L4_R1_IgG

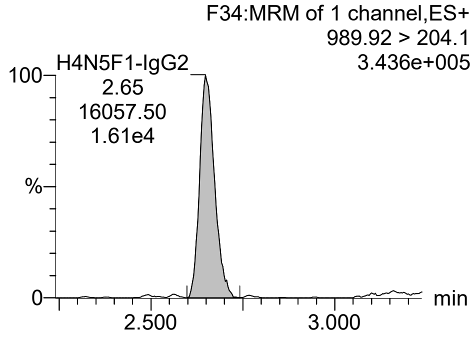

Sample Name: 20211111_H2_R1_IgG

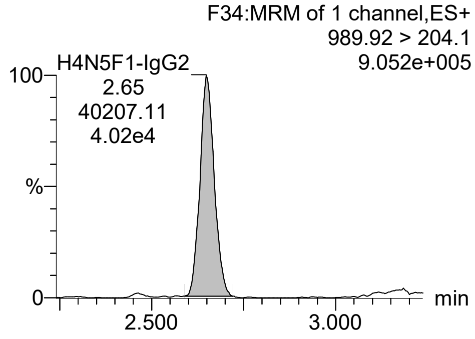

Sample Name: 20211111_A5_R1_IgG

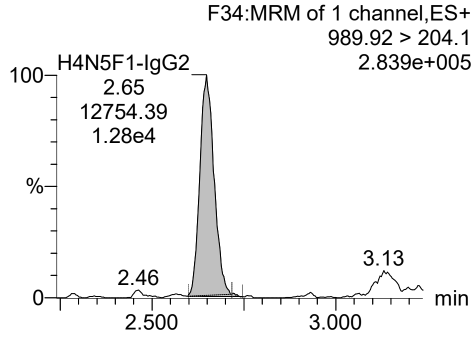

Sample Name: 20211111_A6_R1_IgG

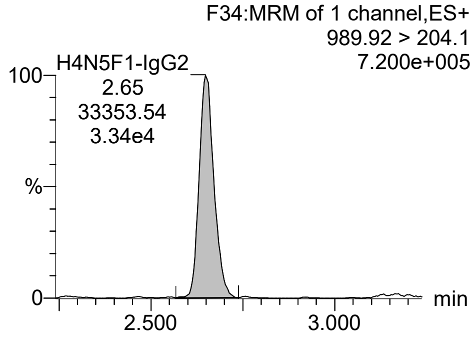

Sample Name: 20211111_QC_R2_IgG

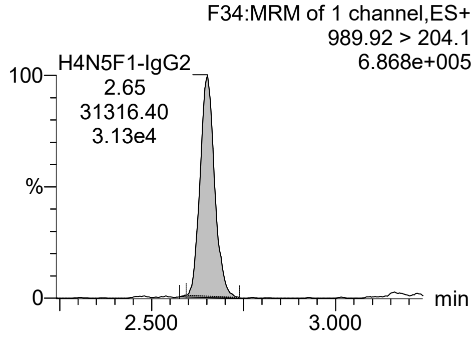

Sample Name: 20211111_L5_R1_IgG

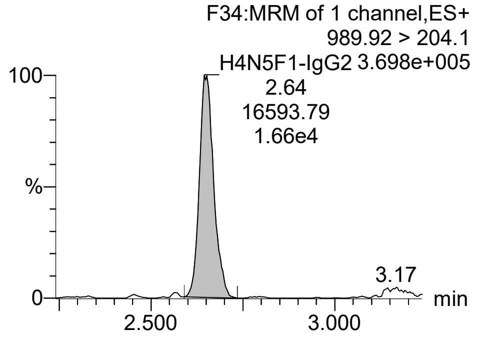

Sample Name: 20211111_L6_R1_IgG

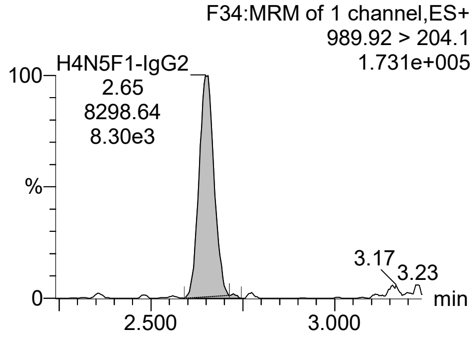

Sample Name: 20211111_H3_R1_IgG

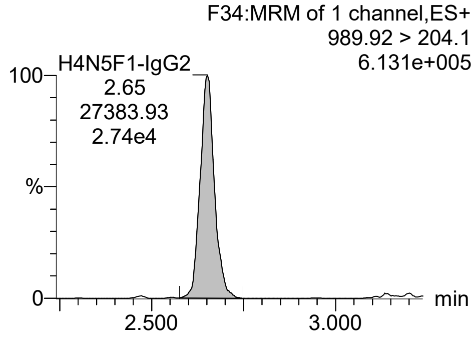

Sample Name: 20211111_A7_R1_IgG

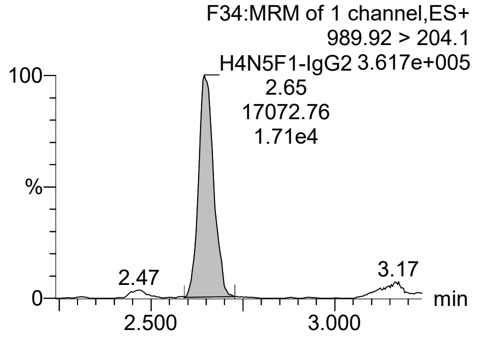

Sample Name: 20211111_A8_R1_IgG

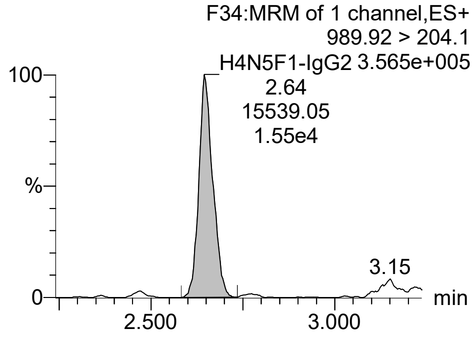

Sample Name: 20211111_L7_R1_IgG

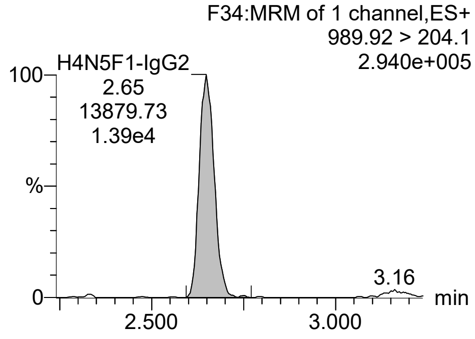

Sample Name: 20211111_L8_R1_IgG

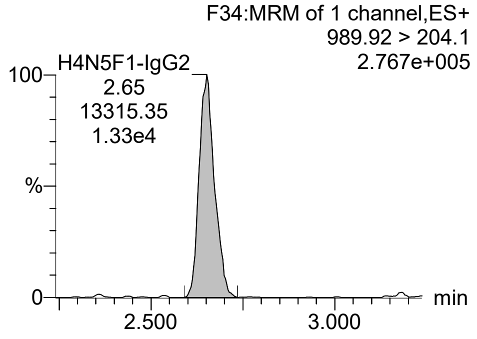

Sample Name: 20211111_H4_R1_IgG

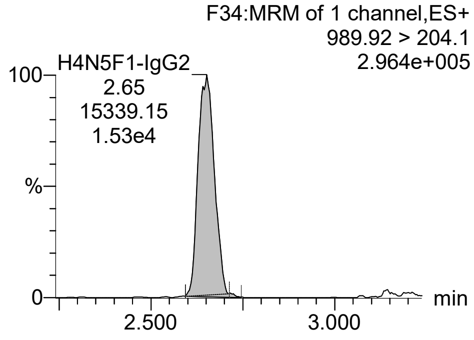

Sample Name: 20211111_A9_R1_IgG

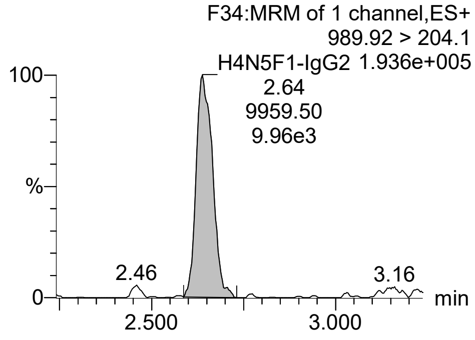

Sample Name: 20211111_A10_R1_IgG

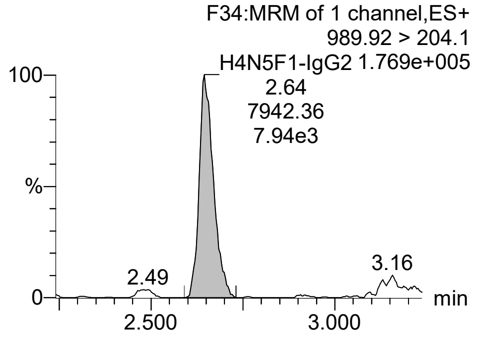

Sample Name: 20211111_L9_R1_IgG

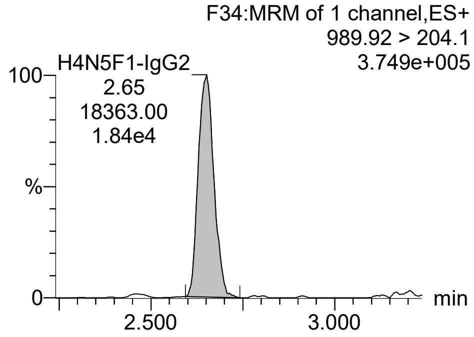

Sample Name: 20211111_L10_R1_IgG

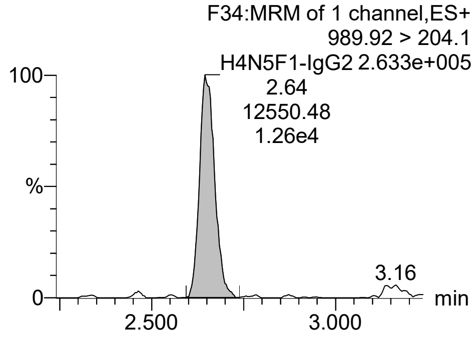

Sample Name: 20211111_QC_R3_IgG

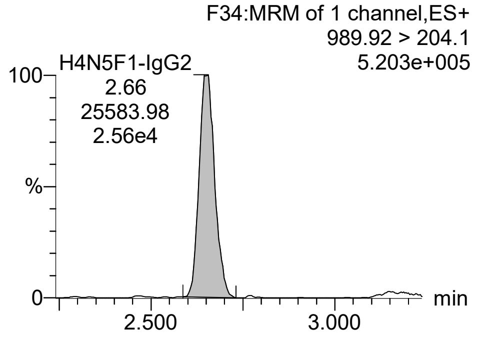

Sample Name: 20211111_H5_R1_IgG

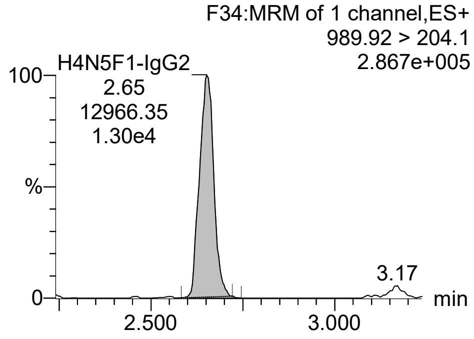

Sample Name: 20211111_A11_R1_IgG

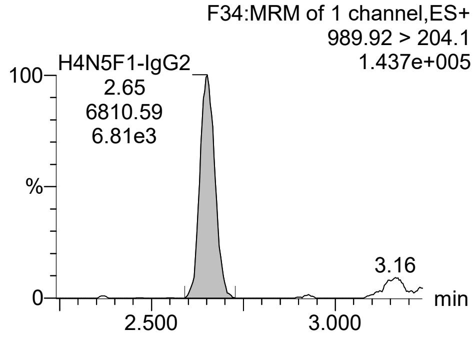

Sample Name: 20211111_A12_R1_IgG

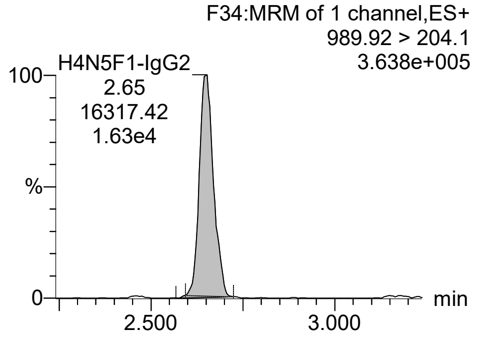

Sample Name: 20211111_L11_R1_IgG

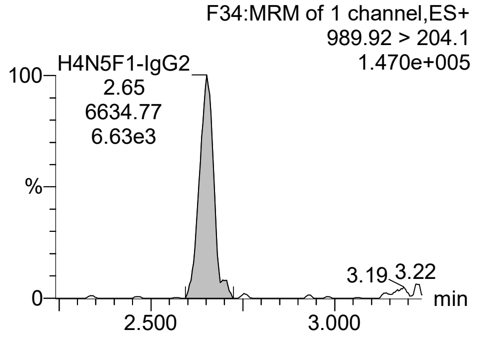

Sample Name: 20211111_L12_R1_IgG

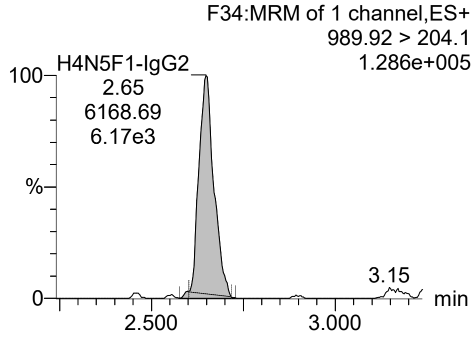

Sample Name: 20211111_H6_R1_IgG

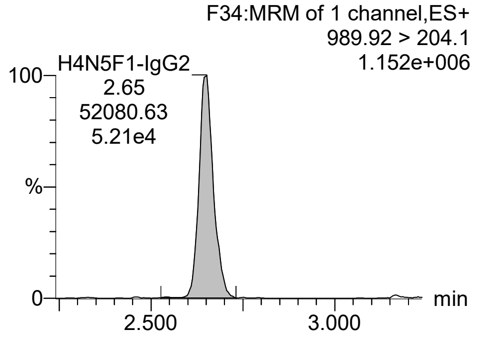

Sample Name: 20211111_A13_R1_IgG

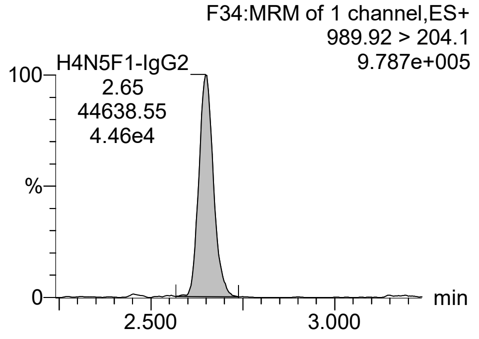

Sample Name: 20211111_A14_R1_IgG

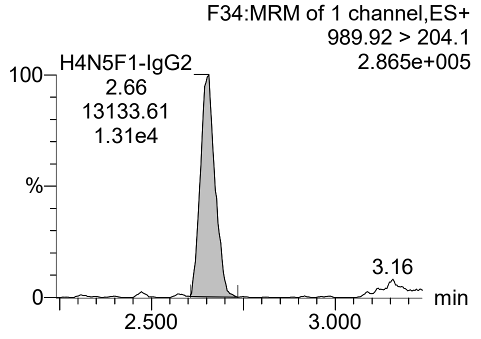

Sample Name: 20211111_L13_R1_IgG

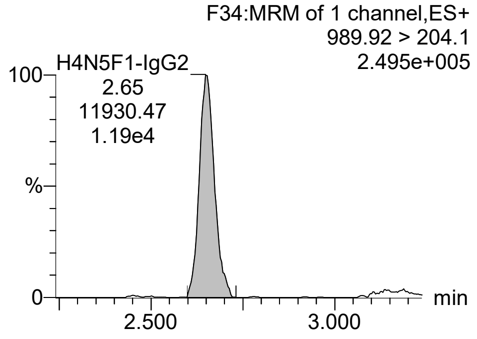

Sample Name: 20211111_L14_R1_IgG

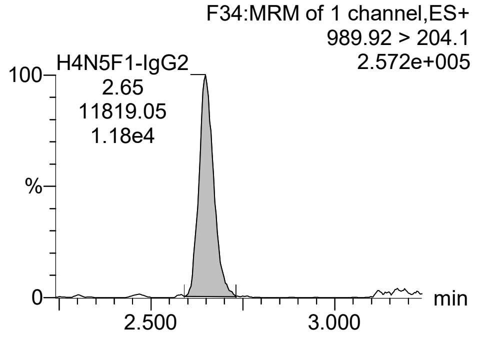

Sample Name: 20211111_H7_R1_IgG

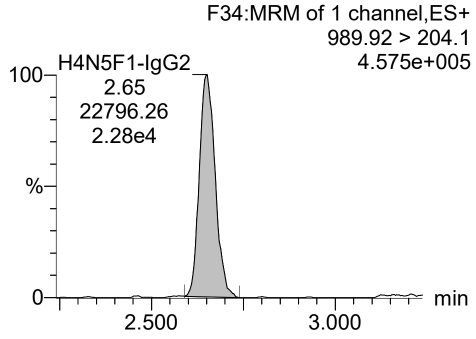

Sample Name: 20211111_A15_R1_IgG

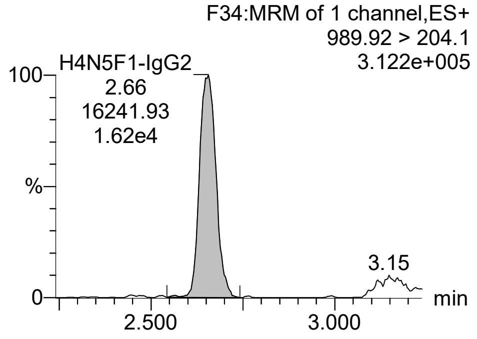

Sample Name: 20211111_QC_R4_IgG

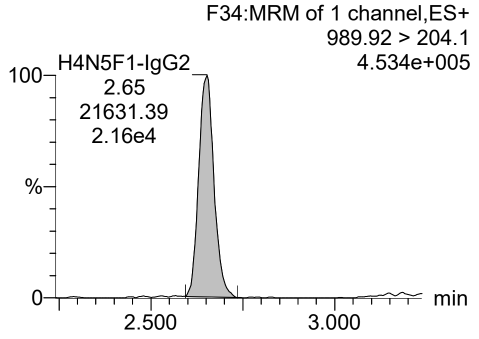

Sample Name: 20211111_A16_R1_IgG

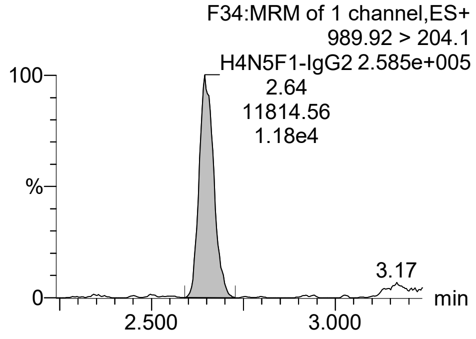

Sample Name: 20211111_L15_R1_IgG

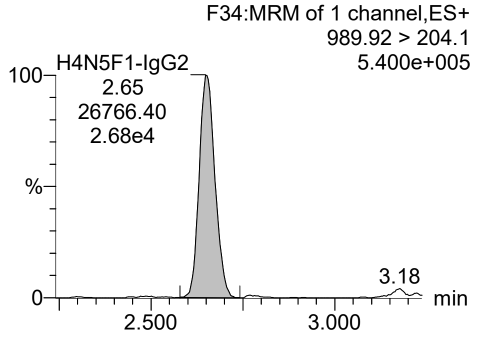

Sample Name: 20211111_L16_R1_IgG

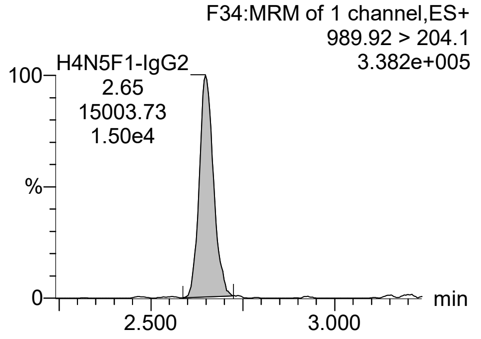

Sample Name: 20211111_H8_R1_IgG

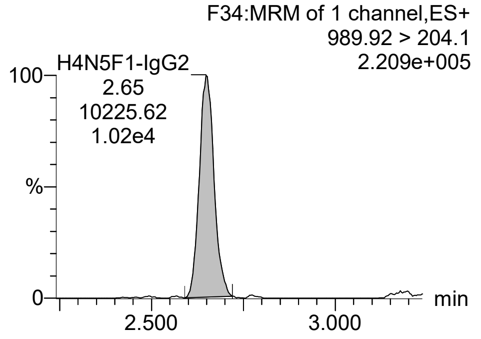

Sample Name: 20211111_A17_R1_IgG

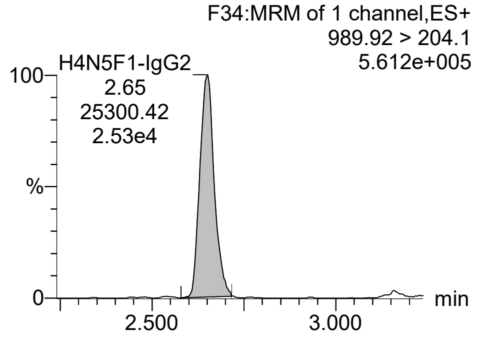

Sample Name: 20211111_A18_R1_IgG

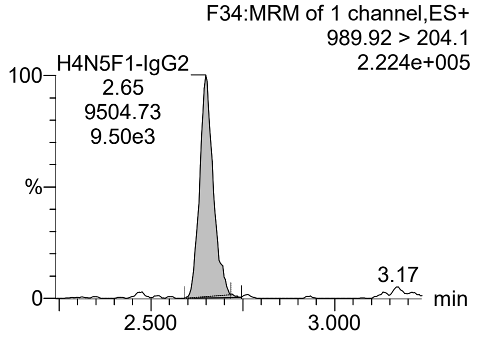

Sample Name: 20211111_L17_R1_IgG

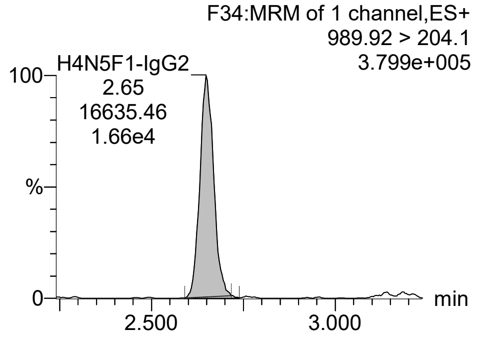

Sample Name: 20211111_L18_R1_IgG

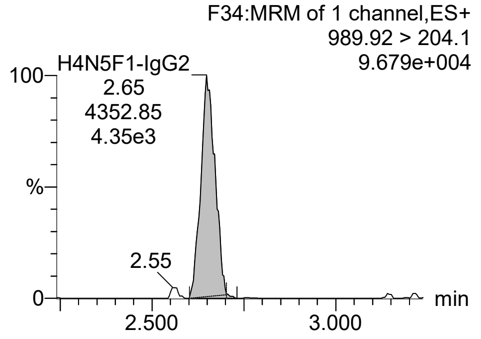

Sample Name: 20211111_H9_R1_IgG

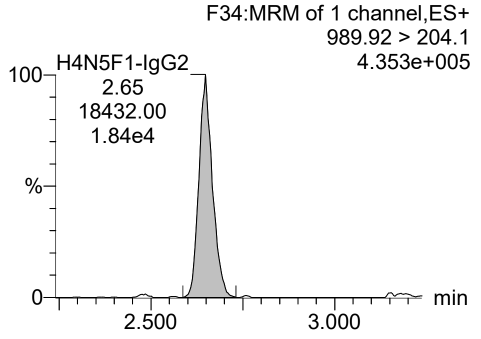

Sample Name: 20211111_A19_R1_IgG

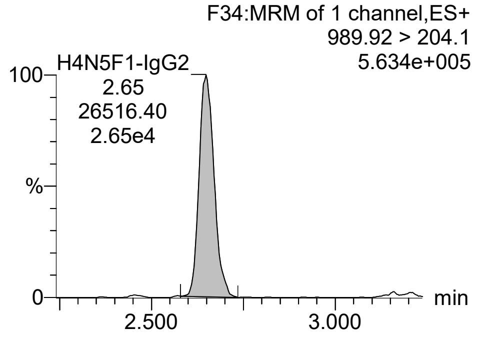

Sample Name: 20211111_A20_R1_IgG

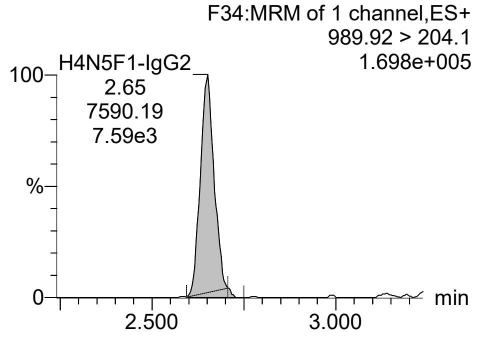

Sample Name: 20211111_L19_R1_IgG

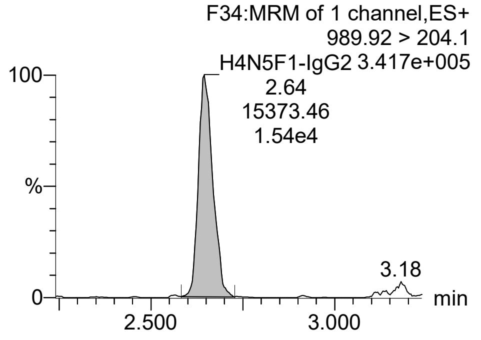

Sample Name: 20211111_QC_R5_IgG

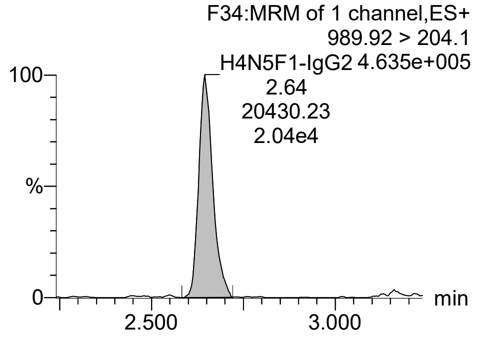

Sample Name: 20211111_L20_R1_IgG

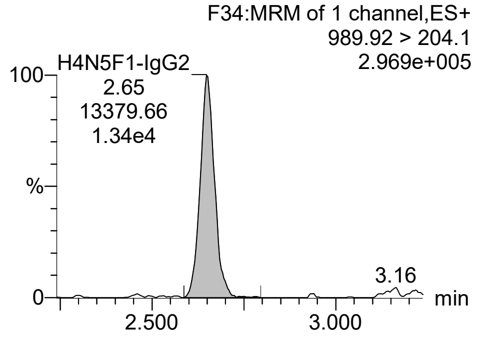

Sample Name: 20211111_H10_R1_IgG

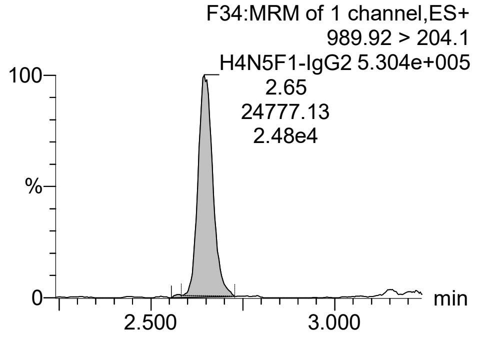

Sample Name: 20211111_A21_R1_IgG

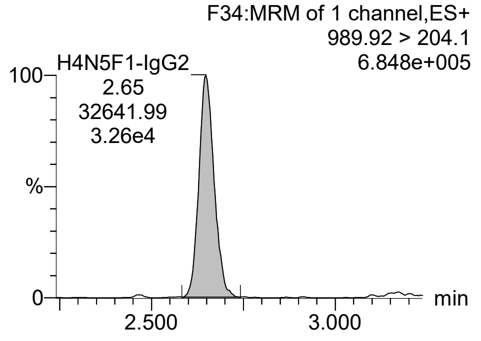

Sample Name: 20211111_A22_R1_IgG

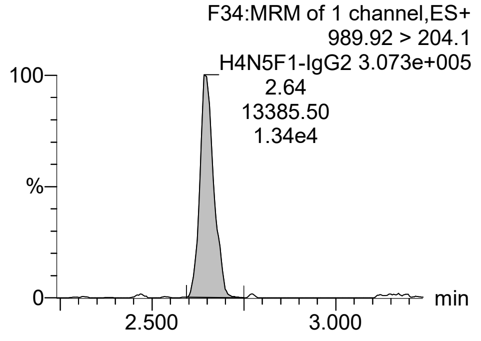

Dataset: C:\MassLynx\10.pro\20211115_clinicalsamples_IgG.qld

Last Altered: Sunday, October 02, 2022 16:02:30 台北標準時間
Printed: Friday, August 01, 2025 16:51:59 台北標準時間

Sample Name: 20211111_L21_R1_IgG

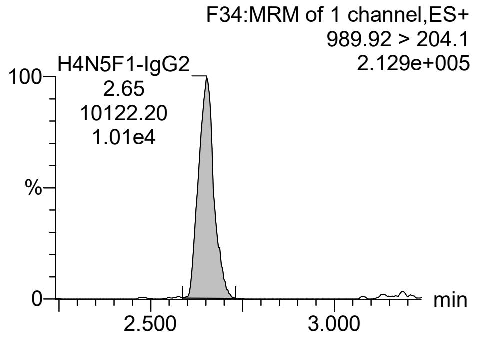

Sample Name: 20211111_L22_R1_IgG

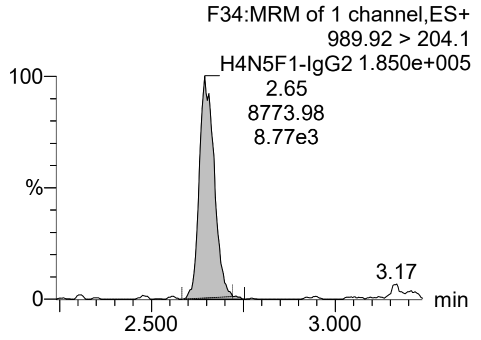

Sample Name: 20211111_H11_R1_IgG

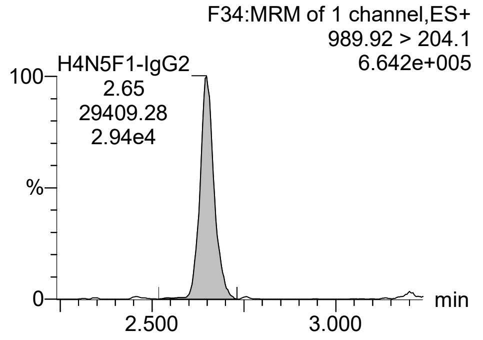

Sample Name: 20211111_A23_R1_IgG

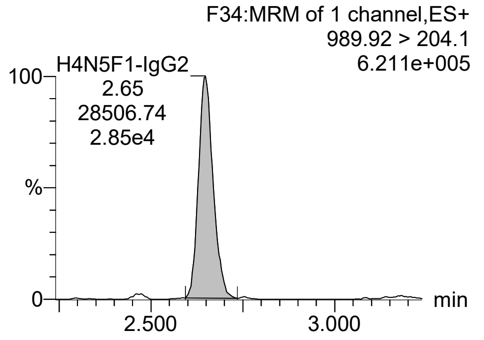

Sample Name: 20211111_A24_R1_IgG

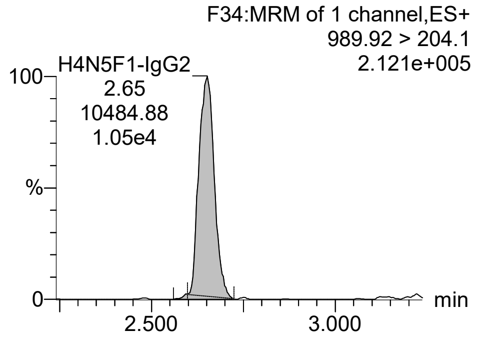

Sample Name: 20211111_L23_R1_IgG

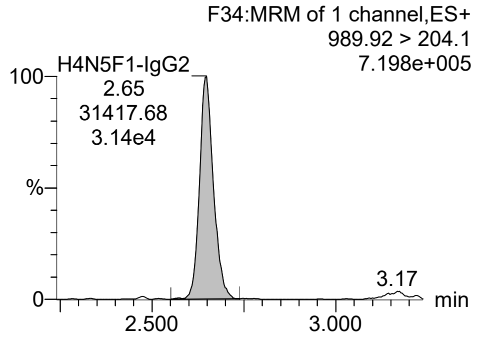

Sample Name: 20211111_L24_R1_IgG

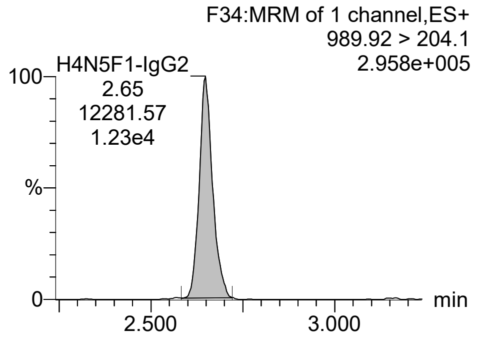

Sample Name: 20211111_H12_R1_IgG

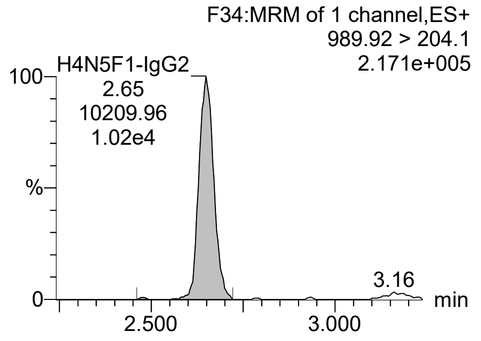

Sample Name: 20211111_QC_R6_IgG

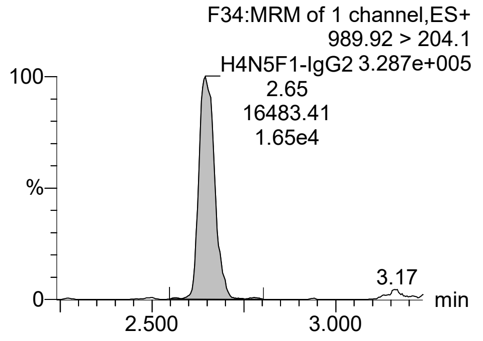

Sample Name: 20211111_A25_R1_IgG

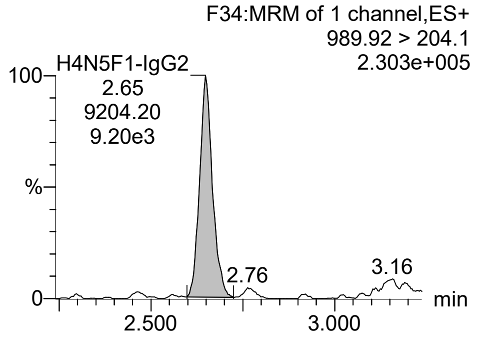

Sample Name: 20211111_A26_R1_IgG

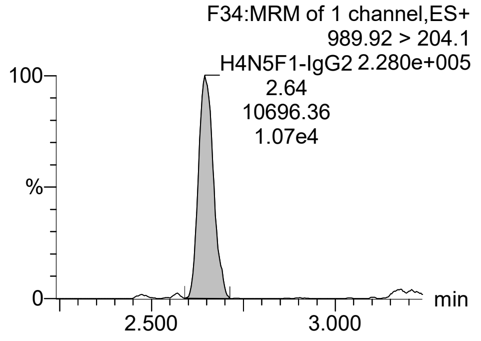

Sample Name: 20211111_L25_R1_IgG

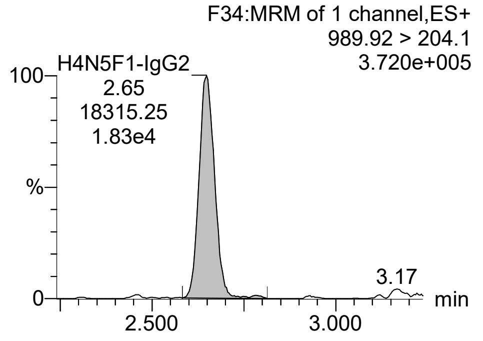

Sample Name: 20211111_L26_R1_IgG

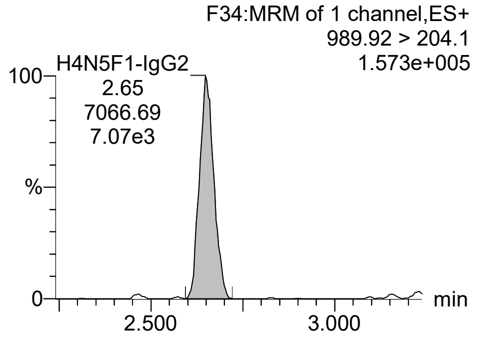

Sample Name: 20211111_H13_R1_IgG

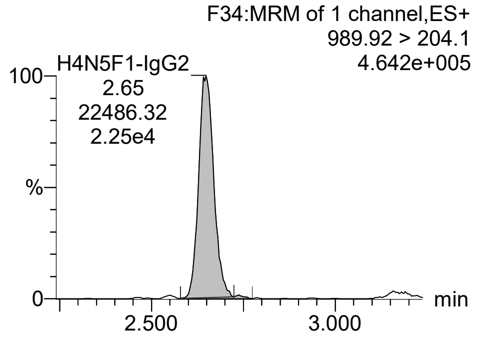

Sample Name: 20211111_A27_R1_IgG

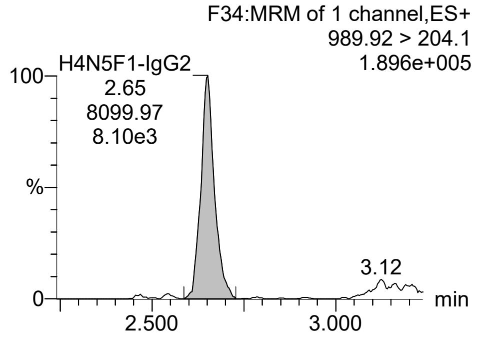

Dataset: C:\MassLynx\10.pro\20211115_clinicalsamples_IgG.qld

Last Altered: Sunday, October 02, 2022 16:02:30 台北標準時間
Printed: Friday, August 01, 2025 16:51:59 台北標準時間

Sample Name: 20211111_A28_R1_IgG
F34:MRM of 1 channel,ES+
989.92 > 204.1

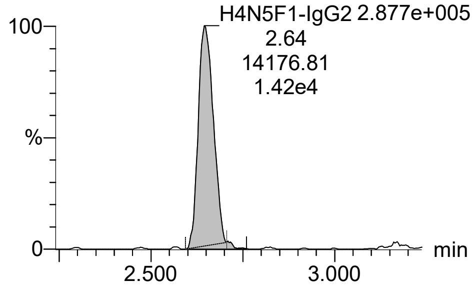

Sample Name: 20211111_L27_R1_IgG
F34:MRM of 1 channel,ES+
989.92 > 204.1

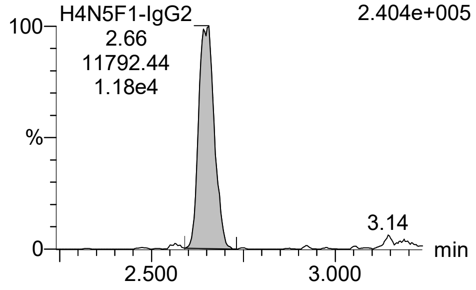

Sample Name: 20211111_L28_R1_IgG
F34:MRM of 1 channel,ES+
989.92 > 204.1

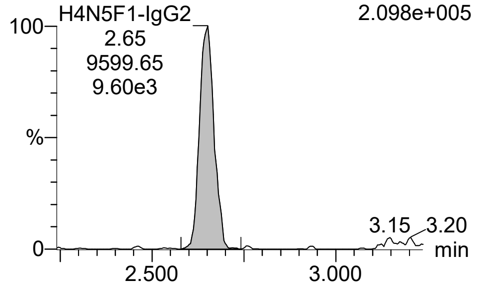

Sample Name: 20211111_H14_R1_IgG
F34:MRM of 1 channel,ES+
989.92 > 204.1

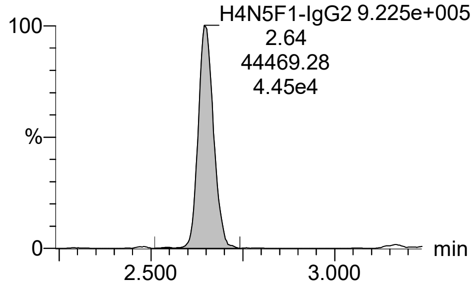

Sample Name: 20211111_A29_R1_IgG
F34:MRM of 1 channel,ES+
989.92 > 204.1

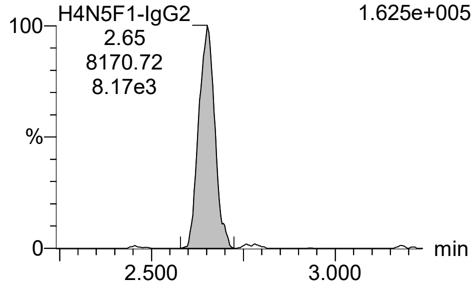

Sample Name: 20211111_A30_R1_IgG
F34:MRM of 1 channel,ES+
989.92 > 204.1

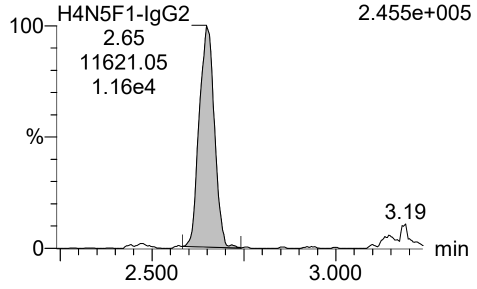

Sample Name: 20211111_QC_R7_IgG
F34:MRM of 1 channel,ES+
989.92 > 204.1

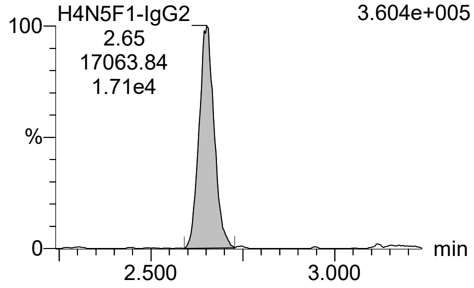

Sample Name: 20211111_L29_R1_IgG
F34:MRM of 1 channel,ES+
989.92 > 204.1

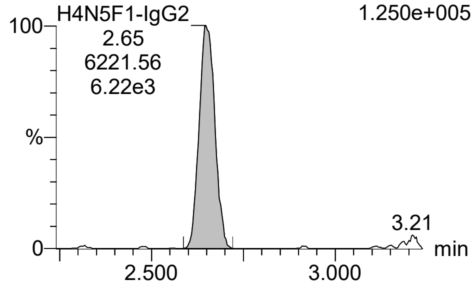

Sample Name: 20211111_L30_R1_IgG
F34:MRM of 1 channel,ES+
989.92 > 204.1

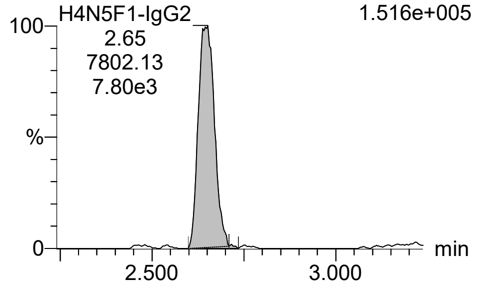

Sample Name: 20211111_H15_R1_IgG
F34:MRM of 1 channel,ES+
989.92 > 204.1

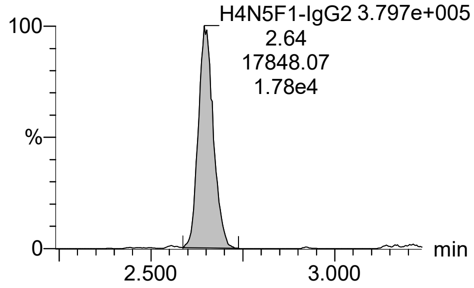

Sample Name: 20211111_A31_R1_IgG
F34:MRM of 1 channel,ES+
989.92 > 204.1

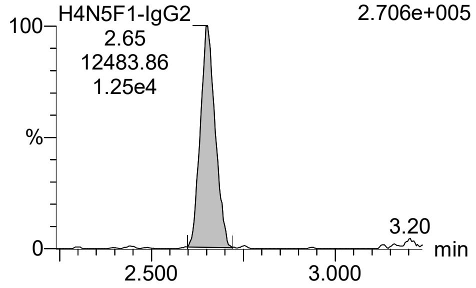

Sample Name: 20211111_A32_R1_IgG
F34:MRM of 1 channel,ES+
989.92 > 204.1

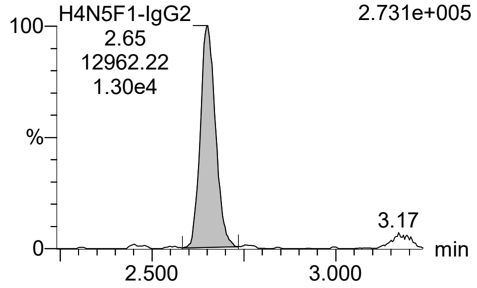

Sample Name: 20211111_L31_R1_IgG
F34:MRM of 1 channel,ES+
989.92 > 204.1

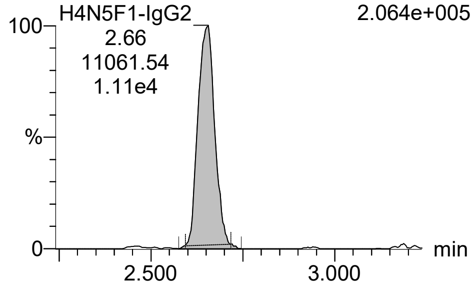

Sample Name: 20211111_L32_R1_IgG
F34:MRM of 1 channel,ES+
989.92 > 204.1

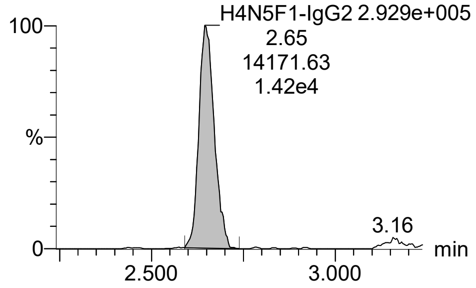

Sample Name: 20211111_H16_R1_IgG
F34:MRM of 1 channel,ES+
989.92 > 204.1

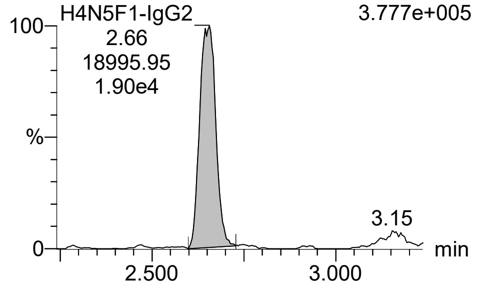

Dataset: C:\MassLynx\10.pro\20211115_clinicalsamples_IgG.qld

Last Altered: Sunday, October 02, 2022 16:02:30 台北標準時間
Printed: Friday, August 01, 2025 16:51:59 台北標準時間

Sample Name: 20211111_A33_R1_IgG

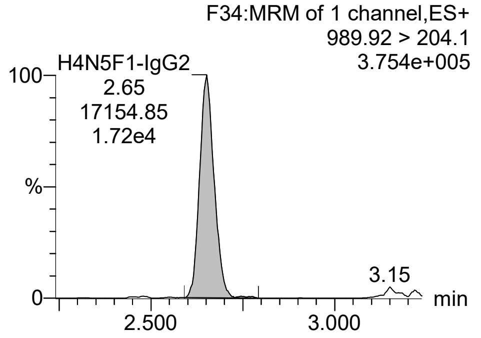

Sample Name: 20211111_A34_R1_IgG

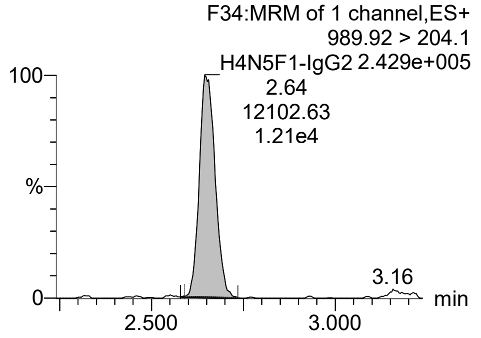

Sample Name: 20211111_H17_R1_IgG

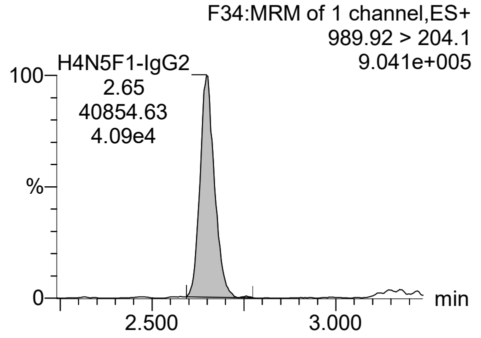

Sample Name: 20211111_A35_R1_IgG

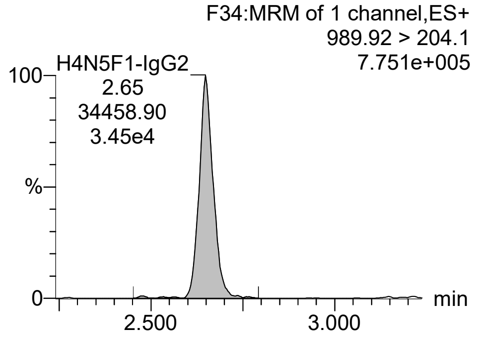

Sample Name: 20211111_QC_R8_IgG

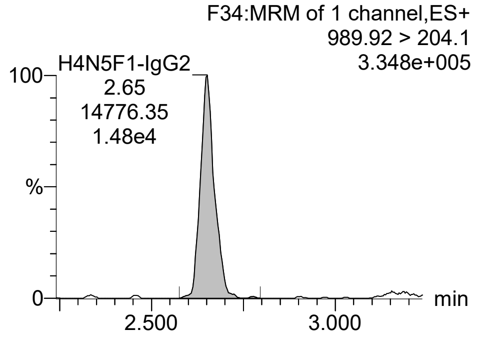

Sample Name: 20211111_A36_R1_IgG

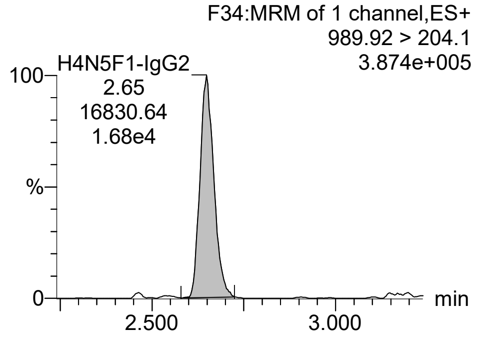

Sample Name: 20211111_H18_R1_IgG

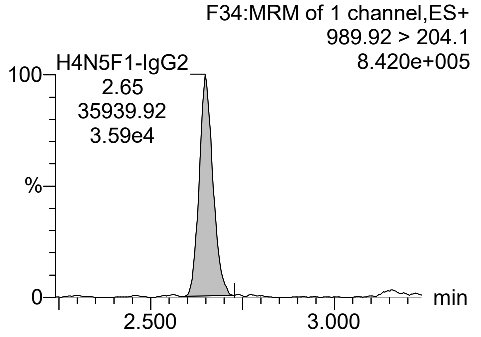

Sample Name: 20211111_A37_R1_IgG

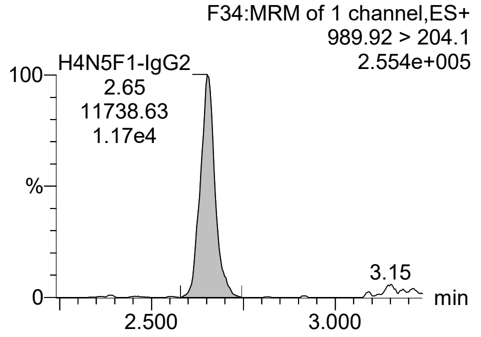

Sample Name: 20211111_A38_R1_IgG

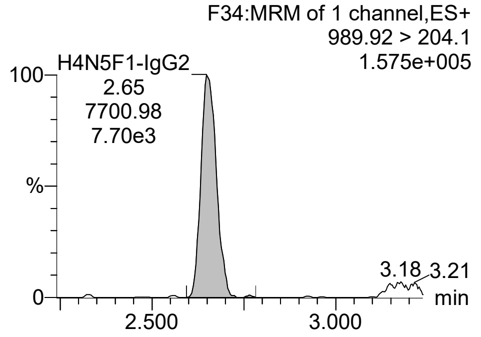

Sample Name: 20211111_H19_R1_IgG

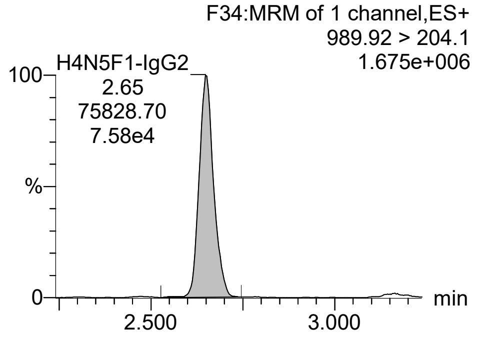

Sample Name: 20211111_A39_R1_IgG

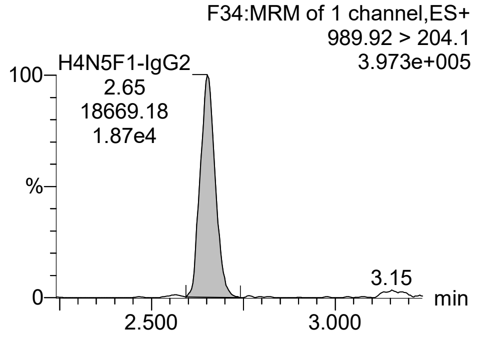

Sample Name: 20211111_A40_R1_IgG

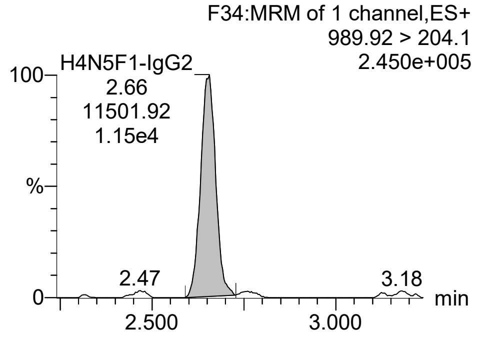

Sample Name: 20211111_H20_R1_IgG

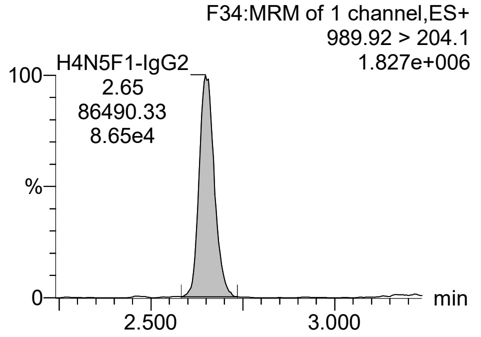

Sample Name: 20211111_A41_R1_IgG

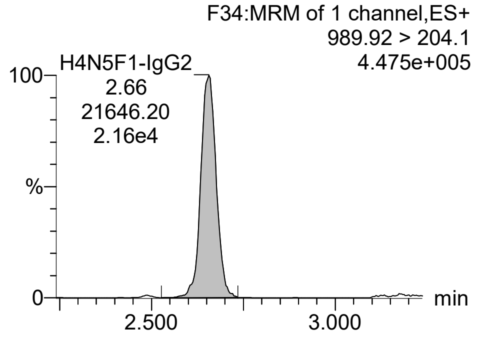

Sample Name: 20211111_A42_R1_IgG

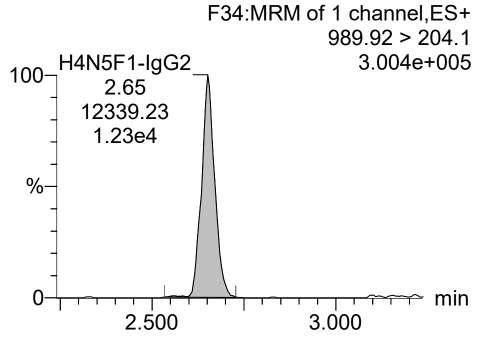

Sample Name: 20211111_A43_R1_IgG

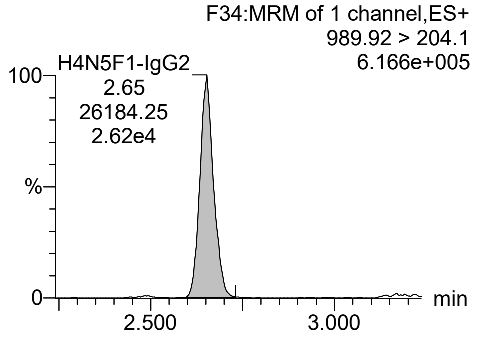

Sample Name: 20211111_A44_R1_IgG

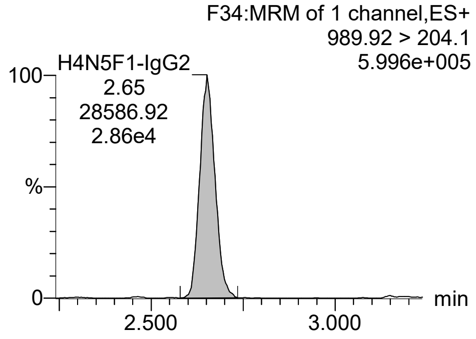

Sample Name: 20211111_QC_R9_IgG

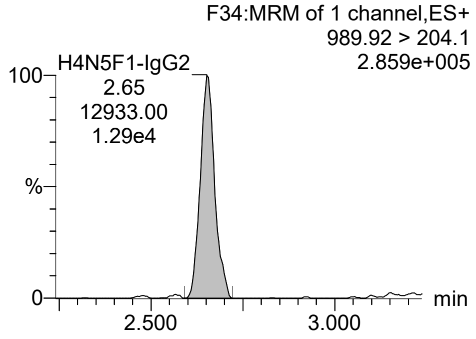

Sample Name: 20211111_A1_R2_IgG

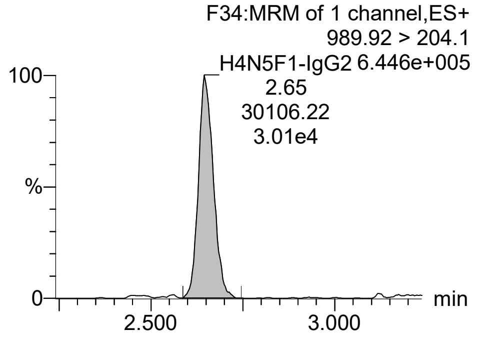

Sample Name: 20211111_A2_R2_IgG

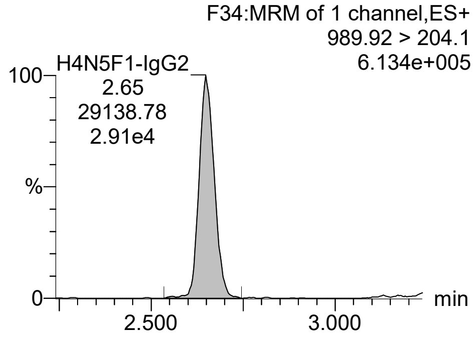

Sample Name: 20211111_L1_R2_IgG

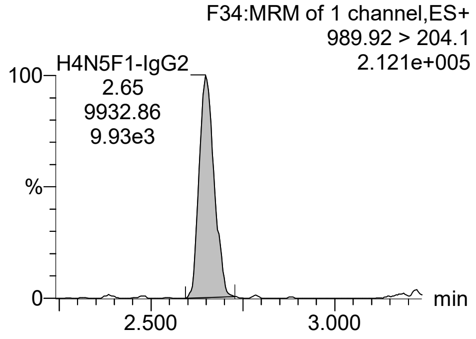

Sample Name: 20211111_L2_R2_IgG

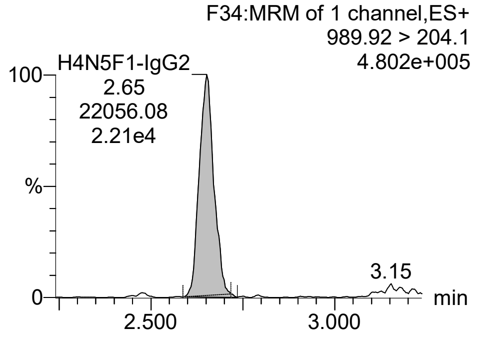

Sample Name: 20211111_H1_R2_IgG

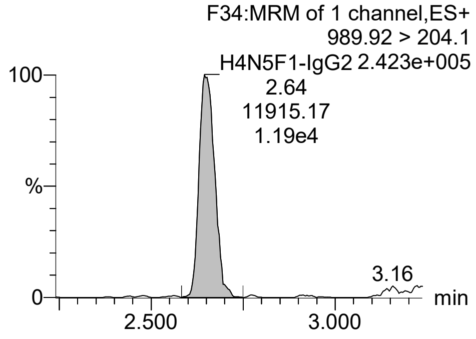

Sample Name: 20211111_A3_R2_IgG

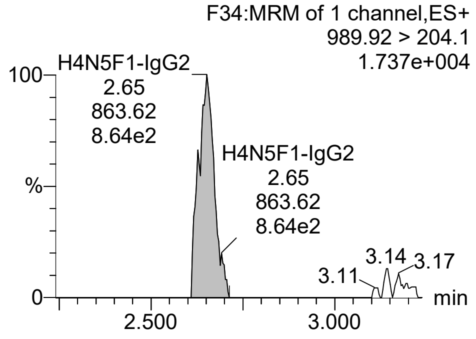

Sample Name: 20211111_A4_R2_IgG

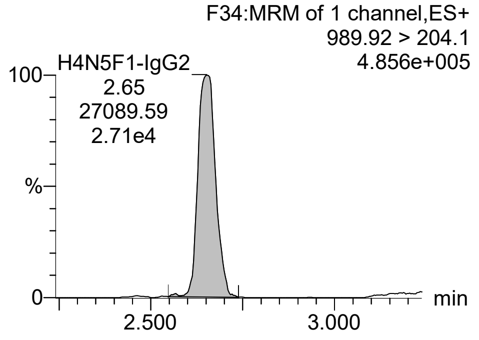

Sample Name: 20211111_L3_R2_IgG

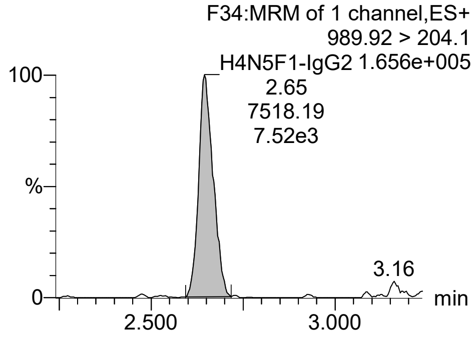

Sample Name: 20211111_L4_R2_IgG

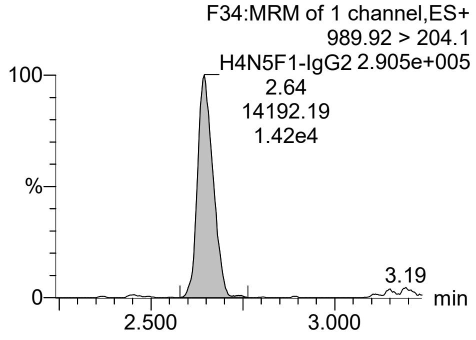

Sample Name: 20211111_H2_R2_IgG

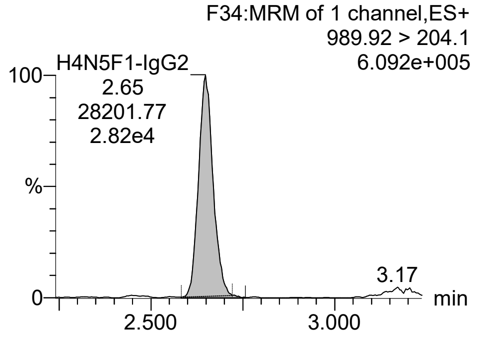

Sample Name: 20211111_A5_R2_IgG

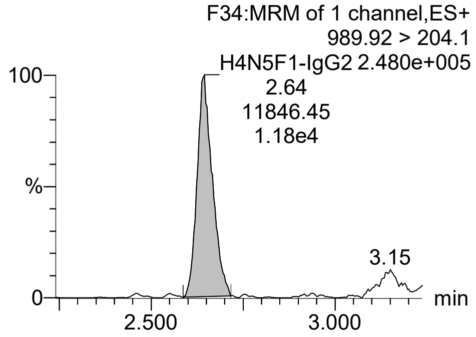

Sample Name: 20211111_A6_R2_IgG

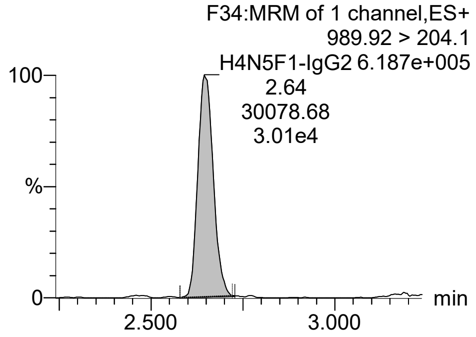

Dataset: C:\MassLynx\10.pro\20211115_clinicalsamples_IgG.qld

Last Altered: Sunday, October 02, 2022 16:02:30 台北標準時間
Printed: Friday, August 01, 2025 16:51:59 台北標準時間

Sample Name: 20211111_QC_R10_IgG

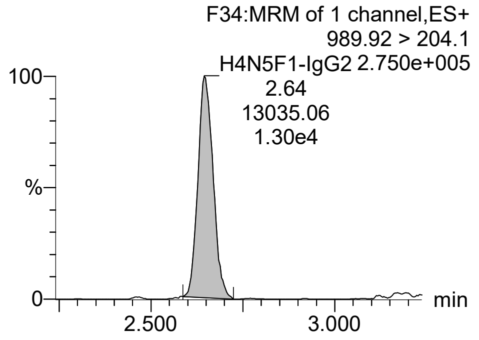

Sample Name: 20211111_L5_R2_IgG

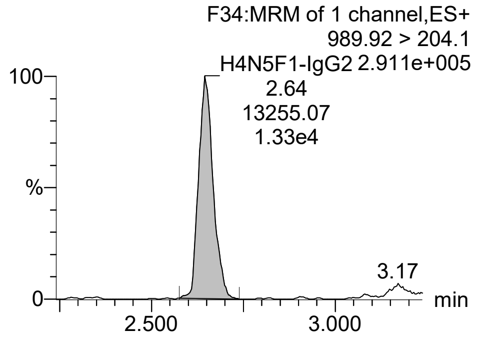

Sample Name: 20211111_L6_R2_IgG

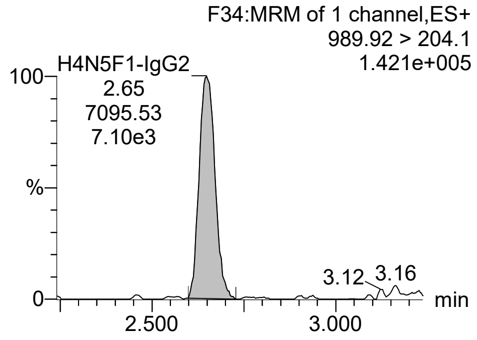

Sample Name: 20211111_H3_R2_IgG

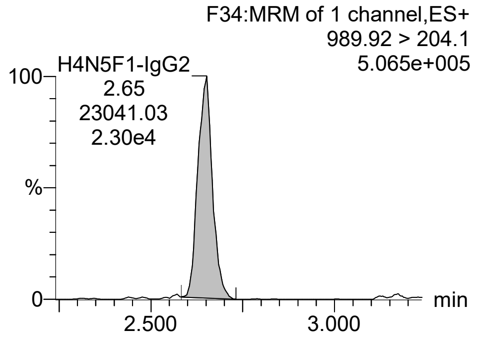

Sample Name: 20211111_A7_R2_IgG

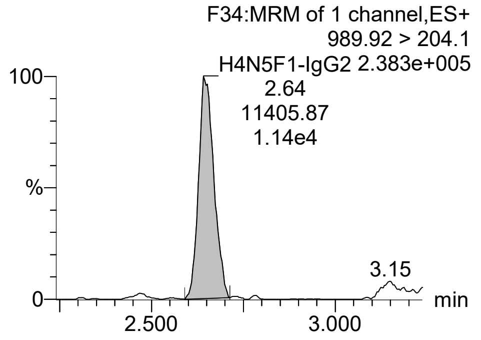

Sample Name: 20211111_A8_R2_IgG

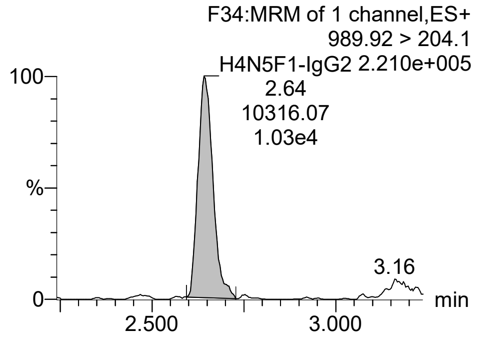

Sample Name: 20211111_L7_R2_IgG

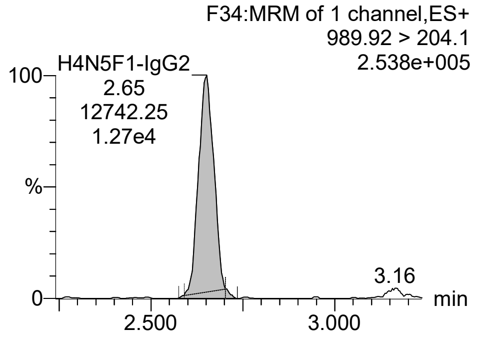

Sample Name: 20211111_L8_R2_IgG

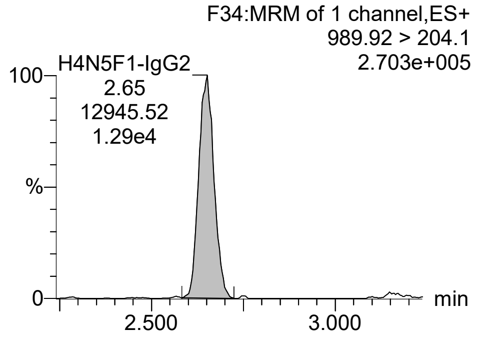

Sample Name: 20211111_H4_R2_IgG

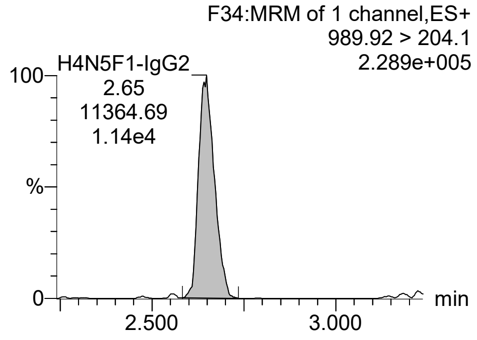

Sample Name: 20211111_A9_R2_IgG

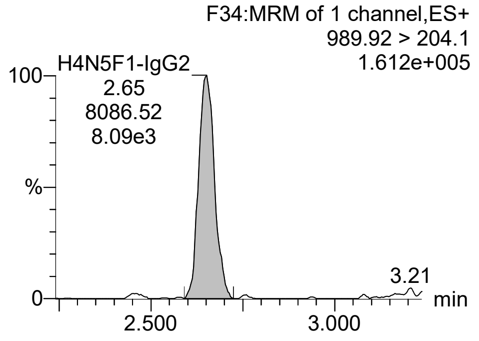

Sample Name: 20211111_A10_R2_IgG

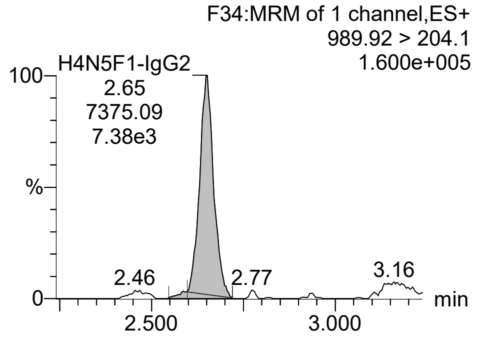

Sample Name: 20211111_L9_R2_IgG

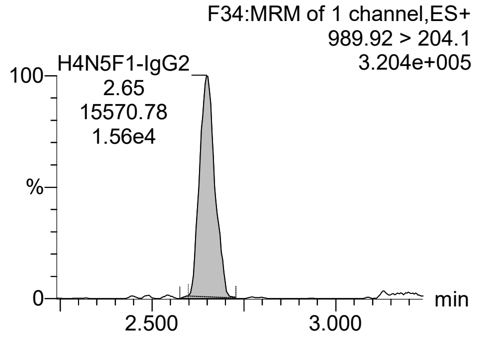

Sample Name: 20211111_L10_R2_IgG

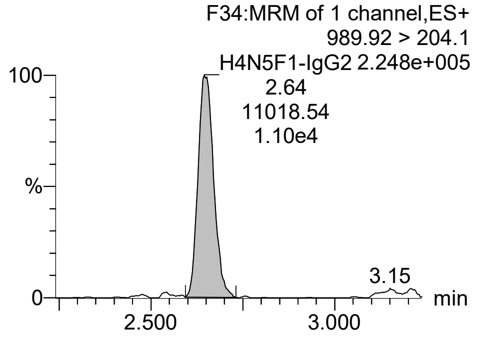

Sample Name: 20211111_QC_R11_IgG

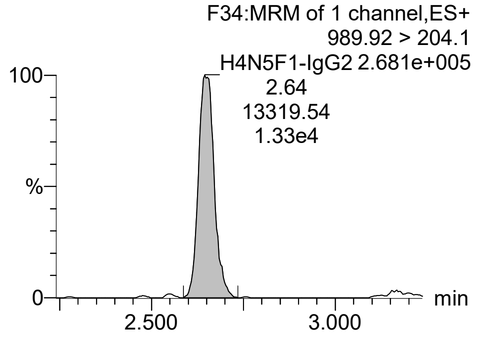

Sample Name: 20211111_H5_R2_IgG

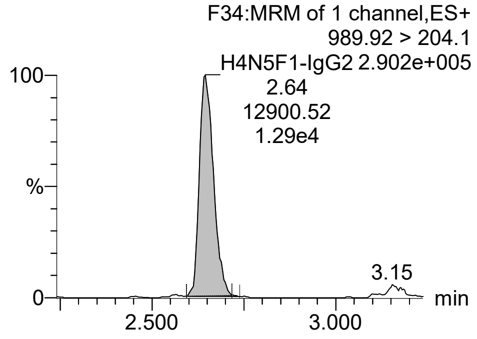

Dataset: C:\MassLynx\10.pro\20211115_clinicalsamples_IgG.qld

Last Altered: Sunday, October 02, 2022 16:02:30 台北標準時間
Printed: Friday, August 01, 2025 16:51:59 台北標準時間

Sample Name: 20211111_A11_R2_IgG

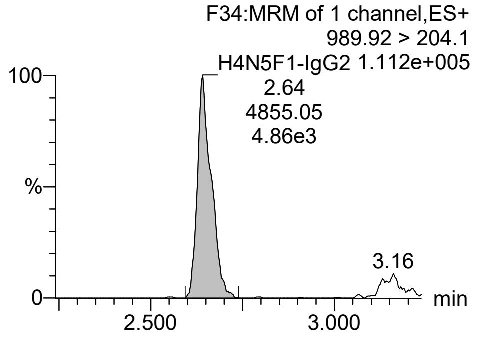

Sample Name: 20211111_A12_R2_IgG

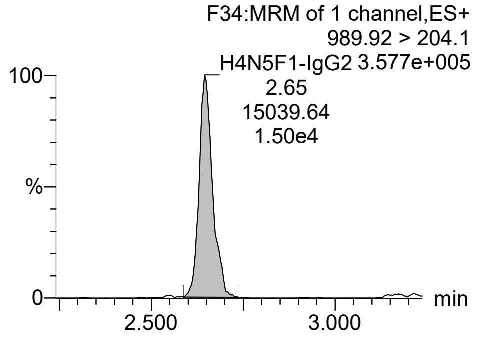

Sample Name: 20211111_L11_R2_IgG

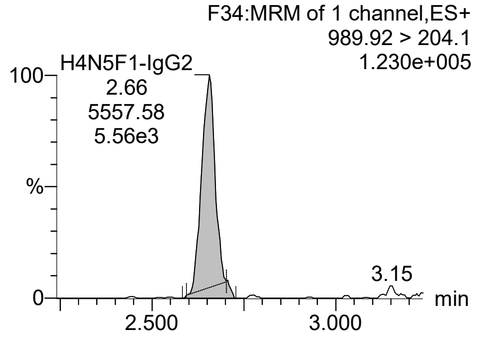

Sample Name: 20211111_L12_R2_IgG

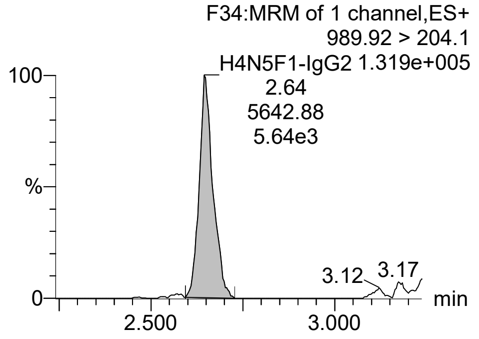

Sample Name: 20211111_H6_R2_IgG

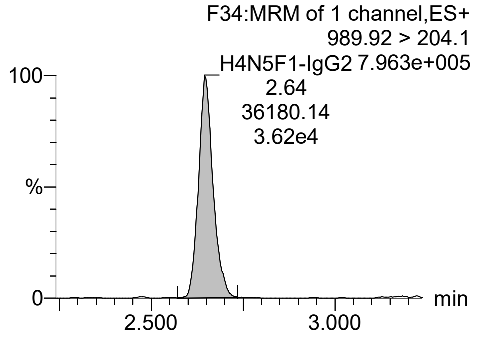

Sample Name: 20211111_A13_R2_IgG

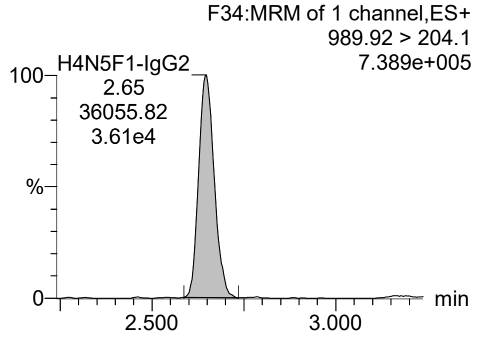

Sample Name: 20211111_A14_R2_IgG

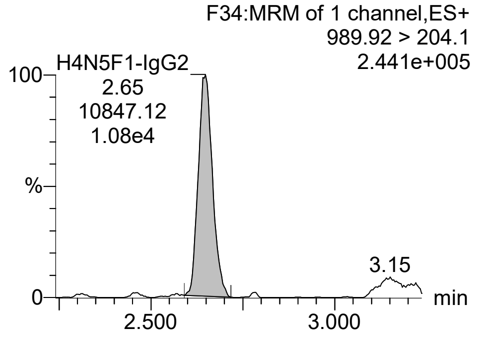

Sample Name: 20211111_L13_R2_IgG

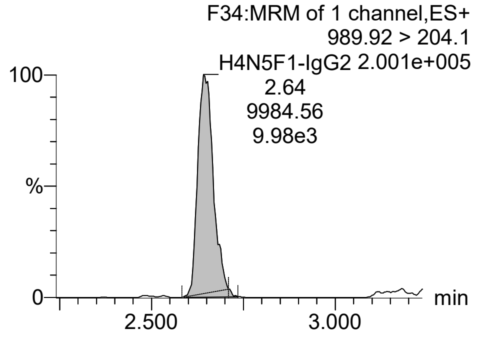

Sample Name: 20211111_L14_R2_IgG

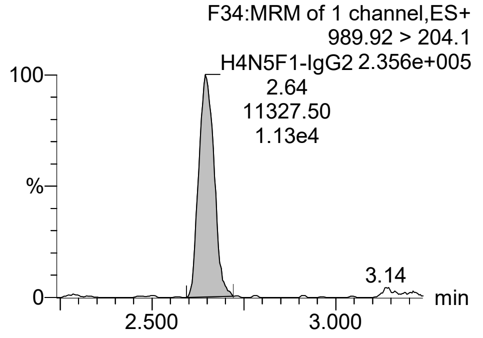

Sample Name: 20211111_H7_R2_IgG

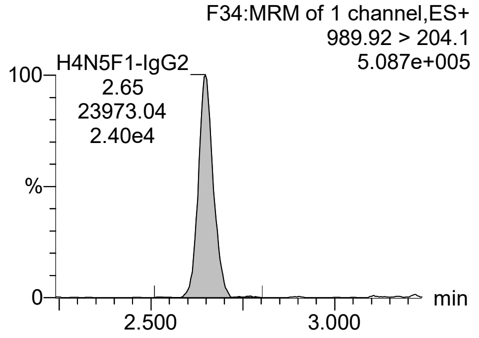

Sample Name: 20211111_A15_R2_IgG

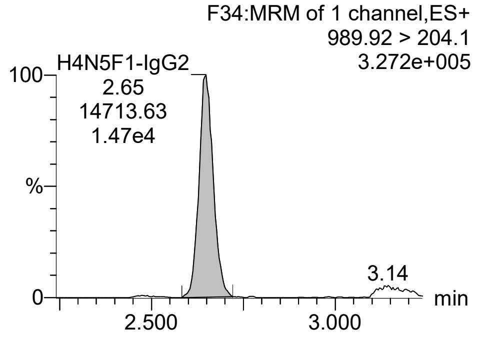

Sample Name: 20211111_QC_R12_IgG

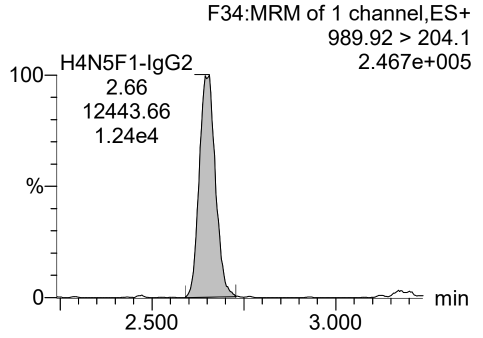

Sample Name: 20211111_A16_R2_IgG

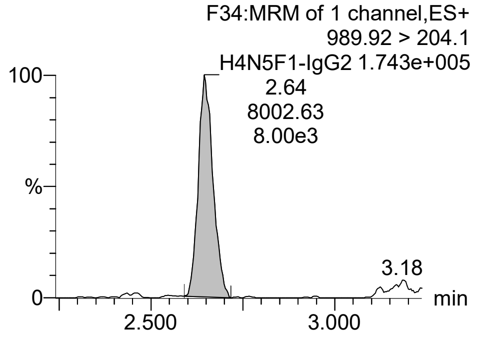

Sample Name: 20211111_L15_R2_IgG

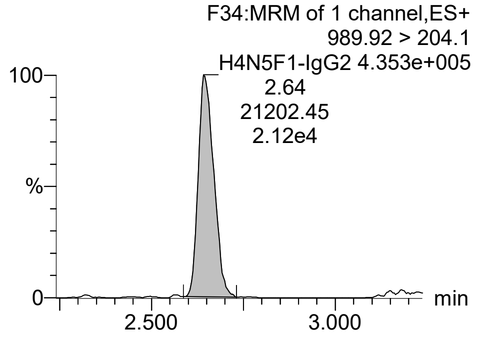

Sample Name: 20211111_L16_R2_IgG

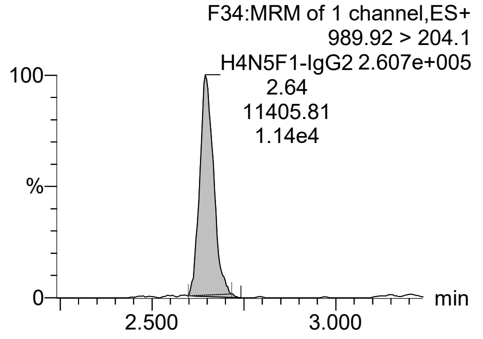

Dataset: C:\MassLynx\10.pro\20211115_clinicalsamples_IgG.qld

Last Altered: Sunday, October 02, 2022 16:02:30 台北標準時間
Printed: Friday, August 01, 2025 16:51:59 台北標準時間

Sample Name: 20211111_H8_R2_IgG

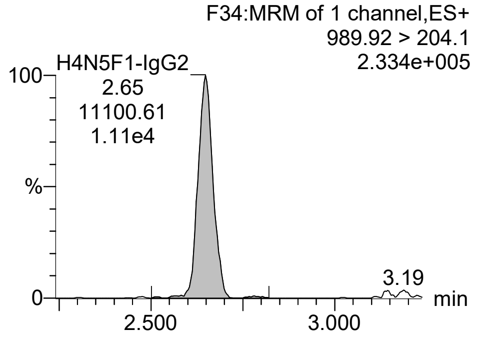

Sample Name: 20211111_A17_R2_IgG

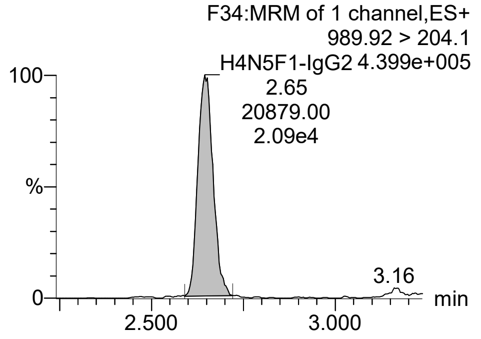

Sample Name: 20211111_A18_R2_IgG

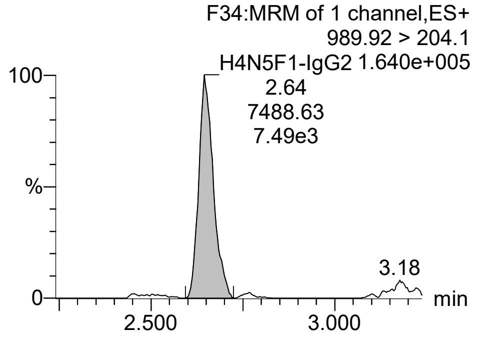

Sample Name: 20211111_L17_R2_IgG

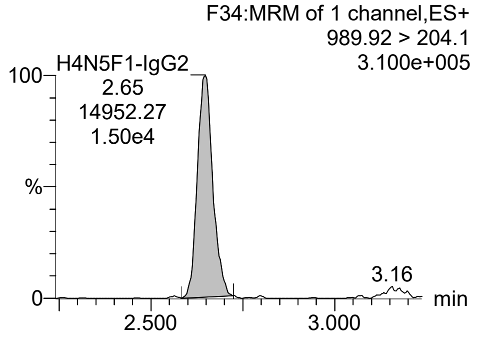

Sample Name: 20211111_L18_R2_IgG

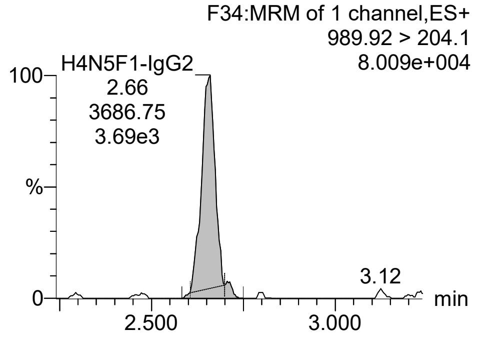

Sample Name: 20211111_H9_R2_IgG

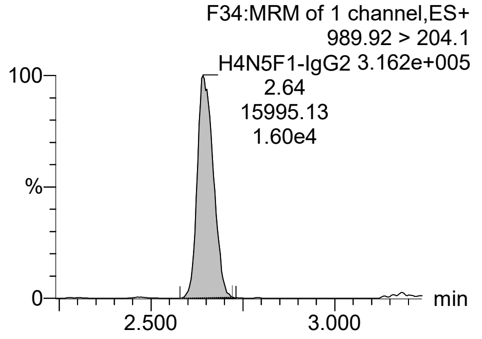

Sample Name: 20211111_A19_R2_IgG

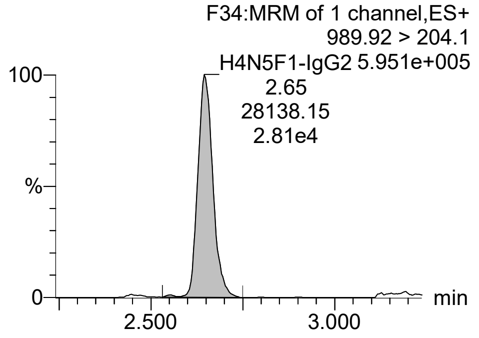

Sample Name: 20211111_A20_R2_IgG

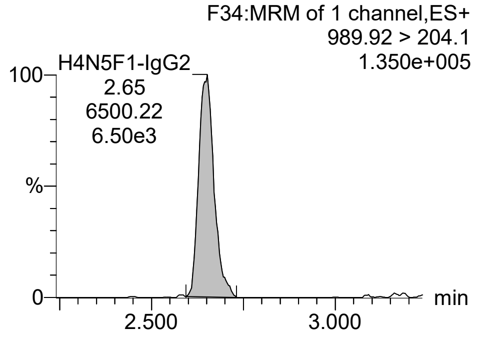

Sample Name: 20211111_L19_R2_IgG

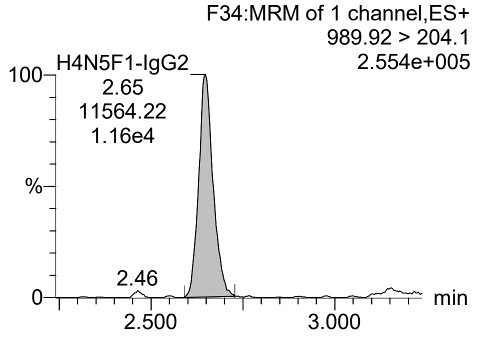

Sample Name: 20211111_QC_R13_IgG

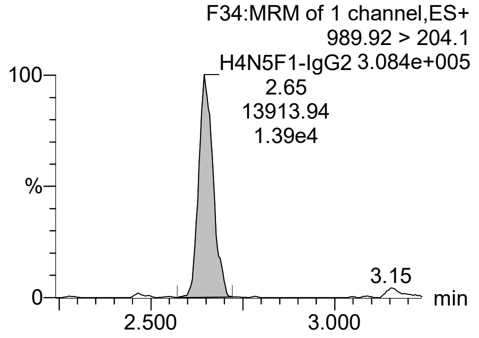

Sample Name: 20211111_L20_R2_IgG

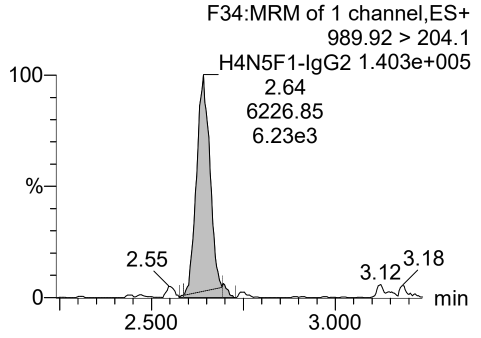

Sample Name: 20211111_H10_R2_IgG

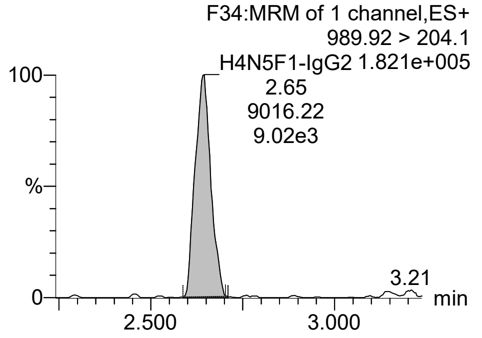

Sample Name: 20211111_A21_R2_IgG

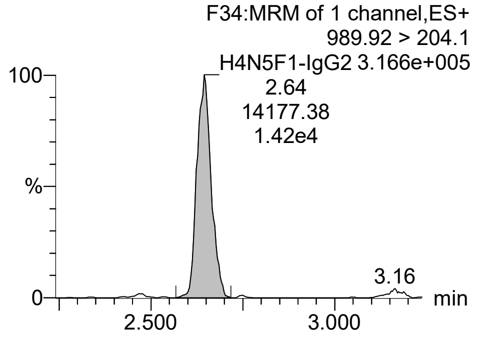

Sample Name: 20211111_A22_R2_IgG

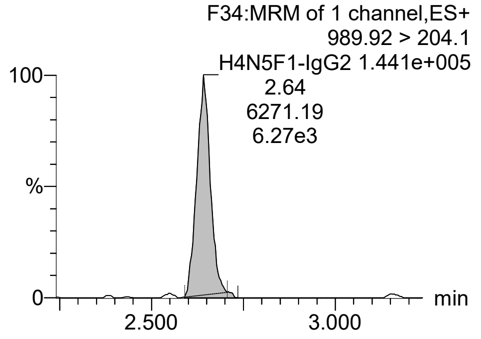

Sample Name: 20211111_L21_R2_IgG

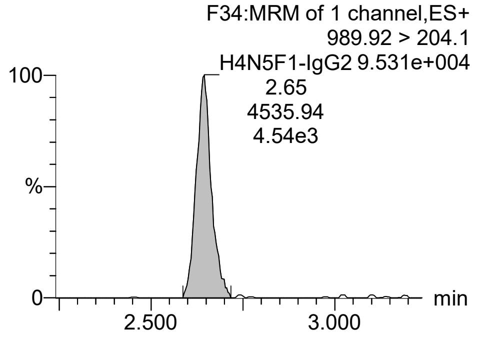

Dataset: C:\MassLynx\10.pro\20211115_clinicalsamples_IgG.qld

Last Altered: Sunday, October 02, 2022 16:02:30 台北標準時間
Printed: Friday, August 01, 2025 16:51:59 台北標準時間

Sample Name: 20211111_L22_R2_IgG

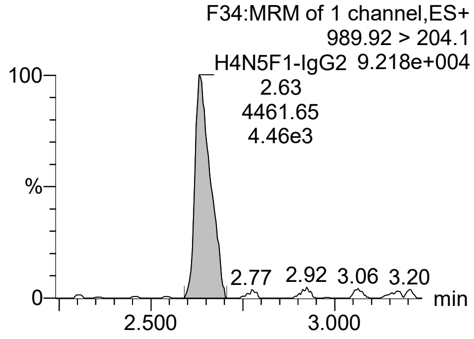

Sample Name: 20211111_H11_R2_IgG

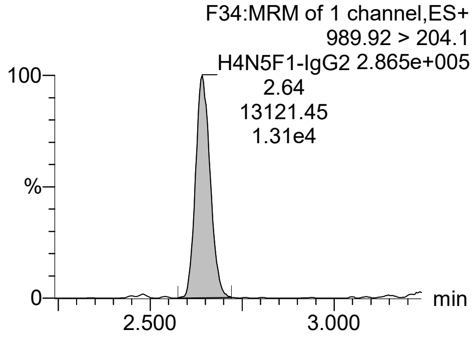

Sample Name: 20211111_A23_R2_IgG

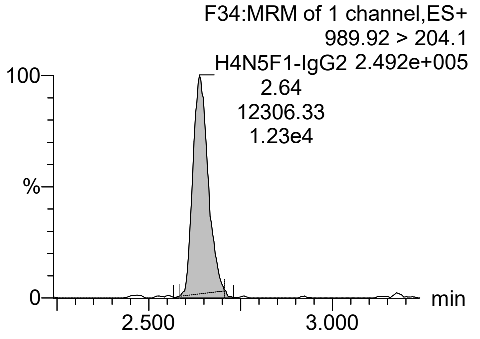

Sample Name: 20211111_A24_R2_IgG

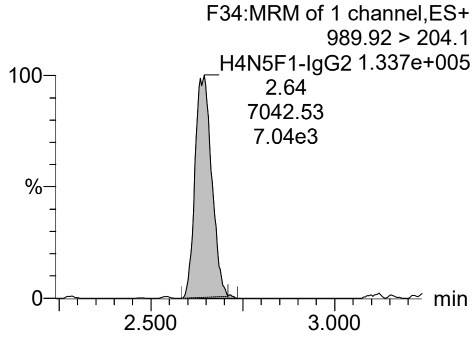

Sample Name: 20211111_L23_R2_IgG

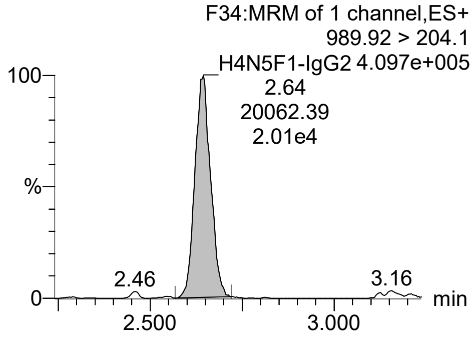

Sample Name: 20211111_L24_R2_IgG

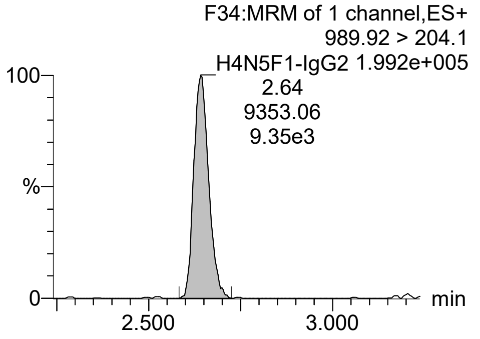

Sample Name: 20211111_H12_R2_IgG

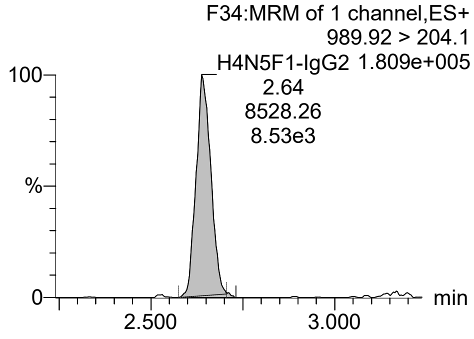

Sample Name: 20211111_QC_R14_IgG

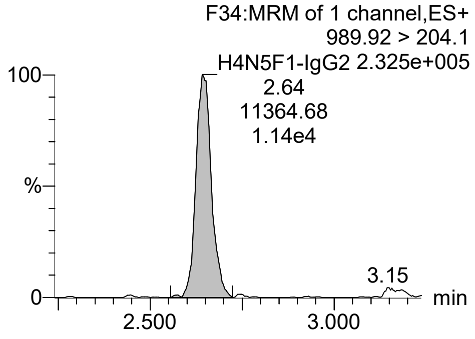

Sample Name: 20211111_A25_R2_IgG

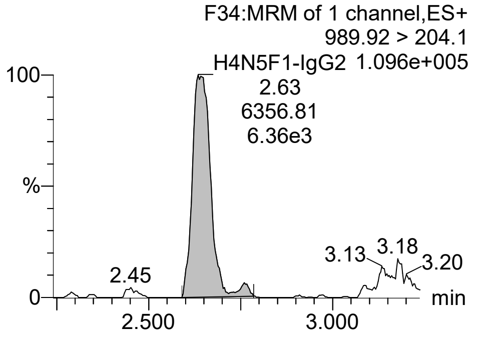

Sample Name: 20211111_A26_R2_IgG

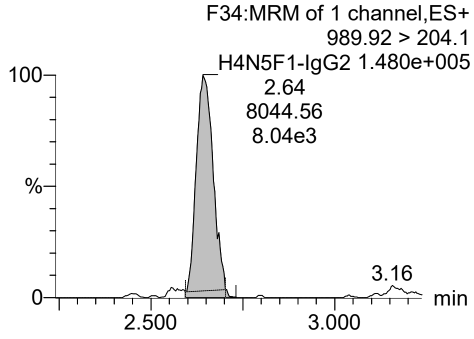

Sample Name: 20211111_L25_R2_IgG

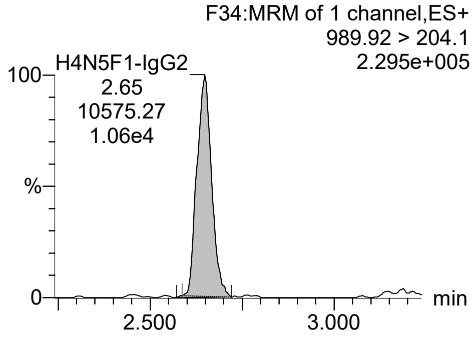

Sample Name: 20211111_L26_R2_IgG

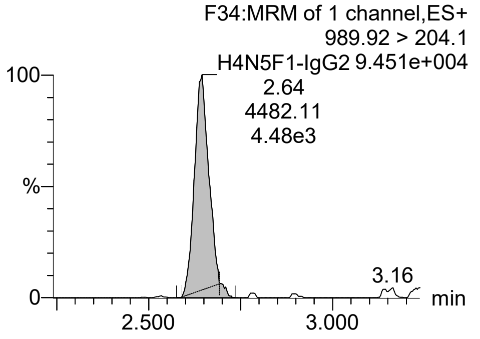

Sample Name: 20211111_H13_R2_IgG

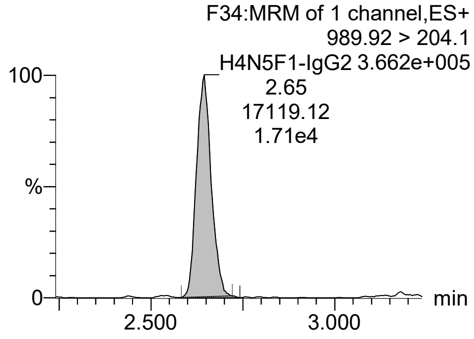

Sample Name: 20211111_A27_R2_IgG

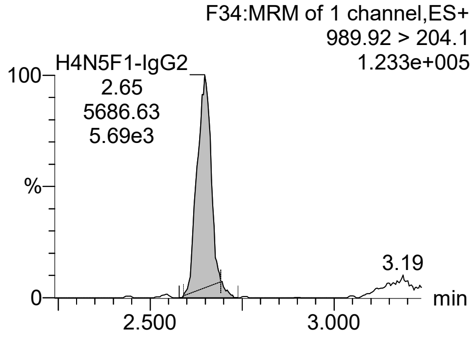

Sample Name: 20211111_A28_R2_IgG

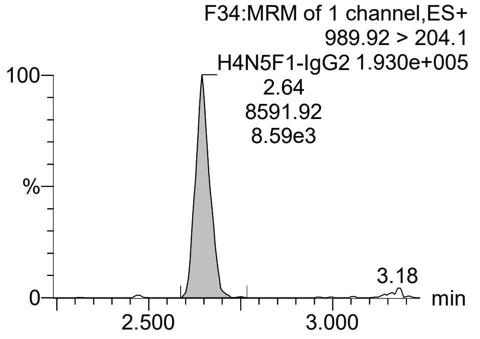

Sample Name: 20211111_L27_R2_IgG

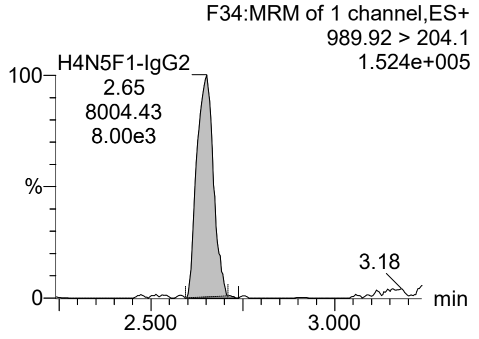

Sample Name: 20211111_L28_R2_IgG

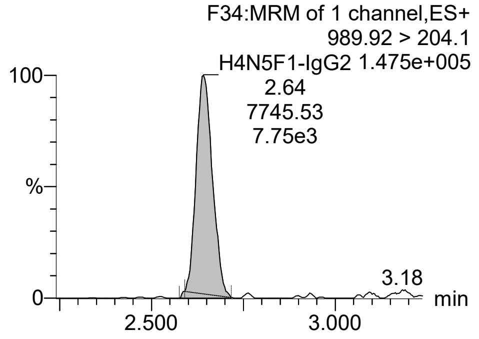

Sample Name: 20211111_H14_R2_IgG

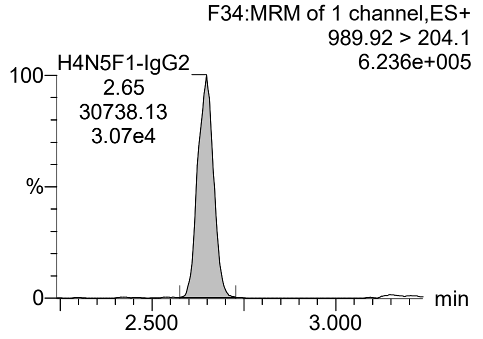

Sample Name: 20211111_A29_R2_IgG

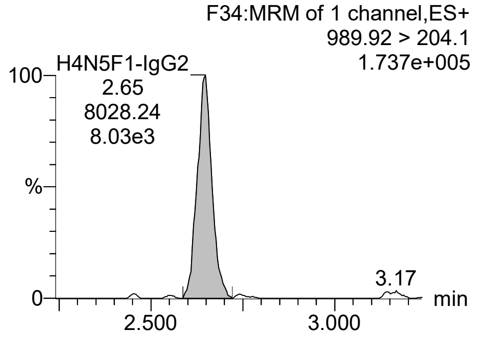

Sample Name: 20211111_A30_R2_IgG

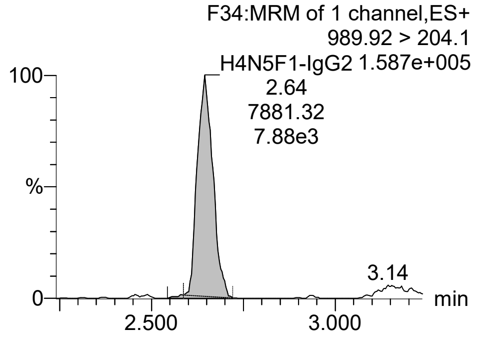

Sample Name: 20211111_QC_R15_IgG

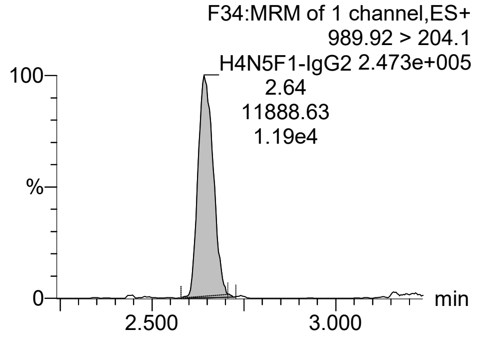

Sample Name: 20211111_L29_R2_IgG

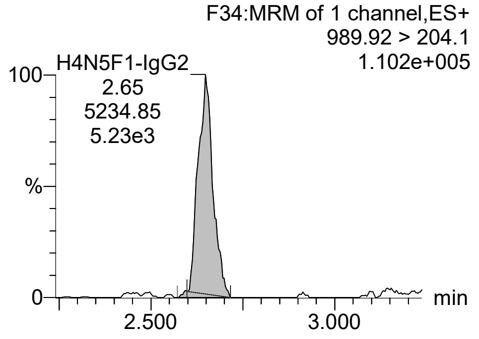

Sample Name: 20211111_L30_R2_IgG

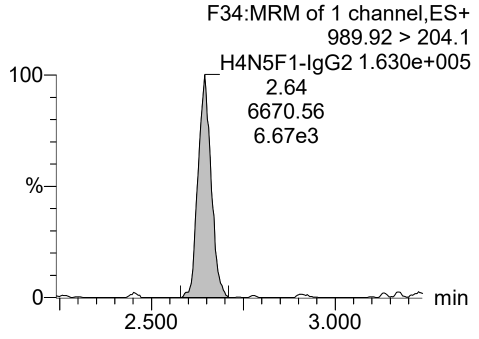

Sample Name: 20211111_H15_R2_IgG

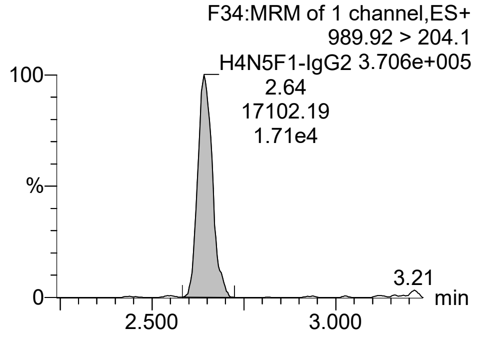

Sample Name: 20211111_A31_R2_IgG

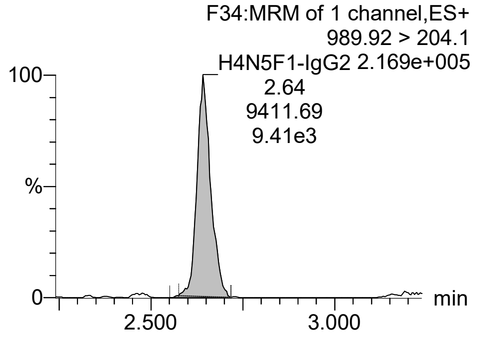

Sample Name: 20211111_A32_R2_IgG

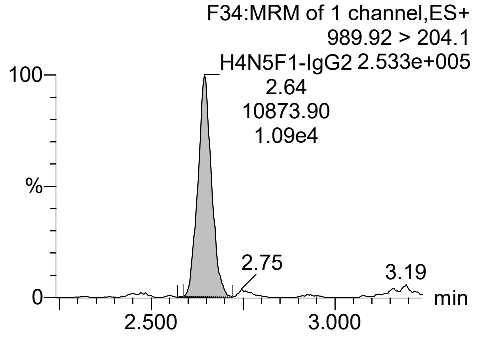

Sample Name: 20211111_L31_R2_IgG

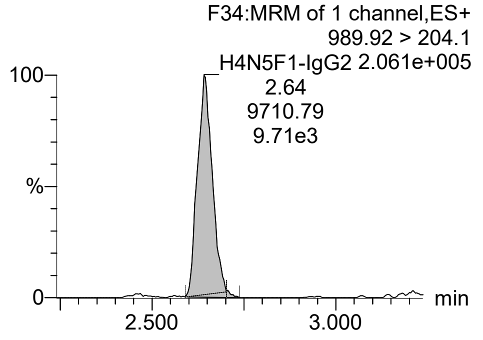

Sample Name: 20211111_L32_R2_IgG

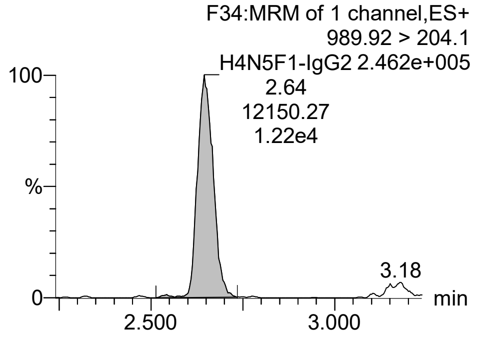

Sample Name: 20211111_H16_R2_IgG

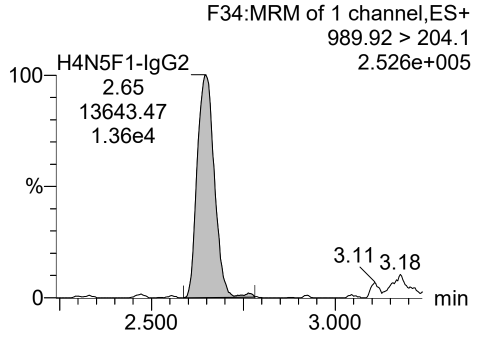

Sample Name: 20211111_A33_R2_IgG

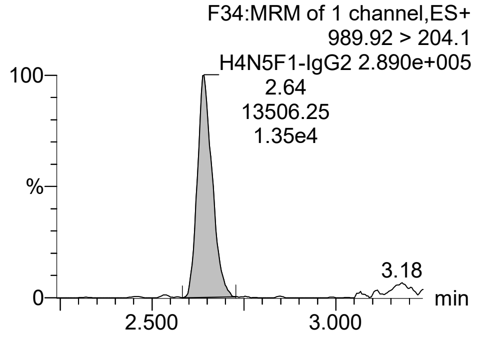

Dataset: C:\MassLynx\10.pro\20211115_clinicalsamples_IgG.qld

Last Altered: Sunday, October 02, 2022 16:02:30 台北標準時間
Printed: Friday, August 01, 2025 16:51:59 台北標準時間

Sample Name: 20211111_A34_R2_IgG

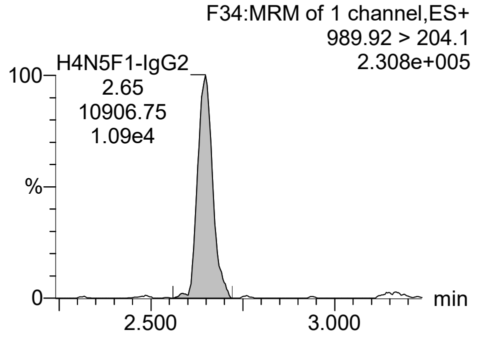

Sample Name: 20211111_H17_R2_IgG

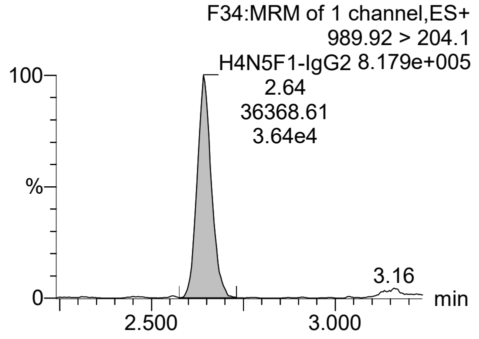

Sample Name: 20211111_A35_R2_IgG

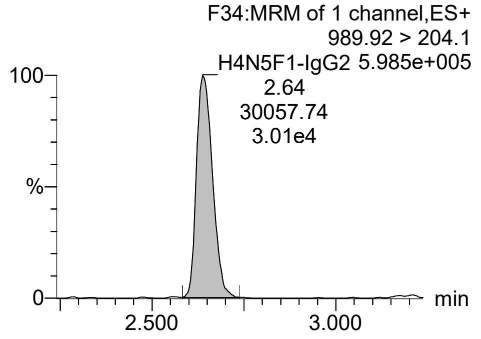

Sample Name: 20211111_QC_R16_IgG

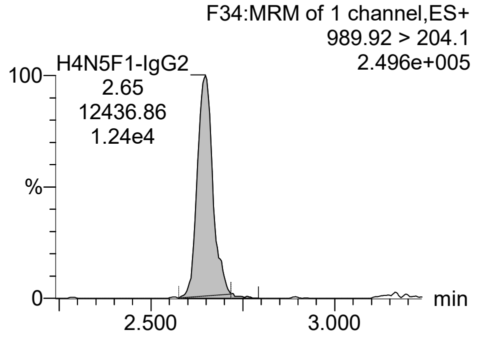

Sample Name: 20211111_A36_R2_IgG

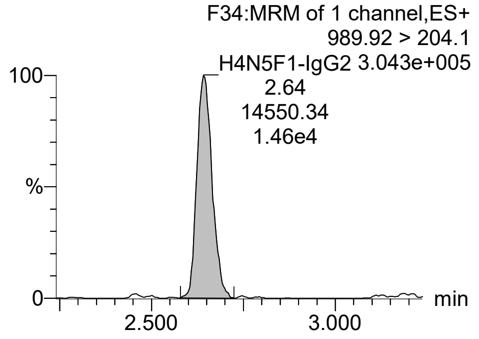

Sample Name: 20211111_H18_R2_IgG

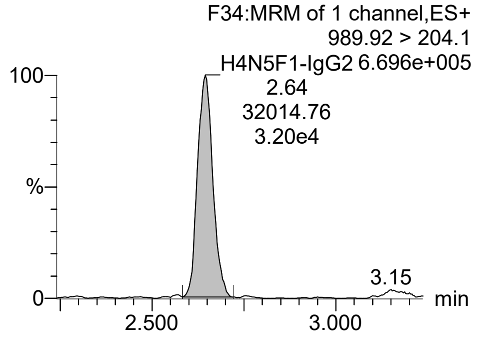

Sample Name: 20211111_A37_R2_IgG

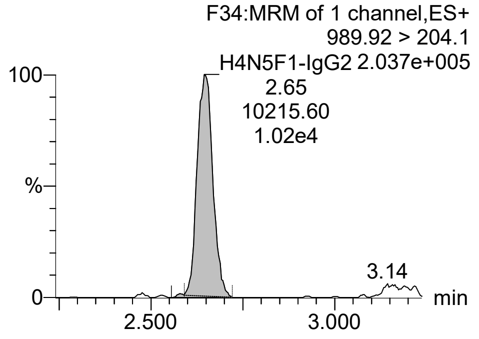

Sample Name: 20211111_A38_R2_IgG

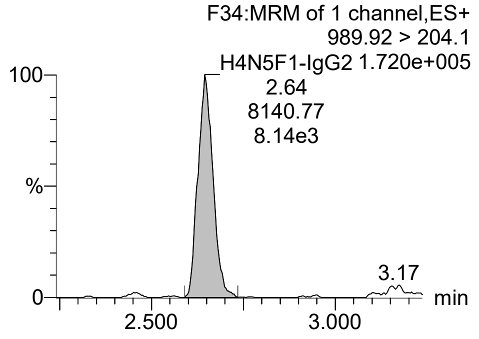

Sample Name: 20211111_H19_R2_IgG

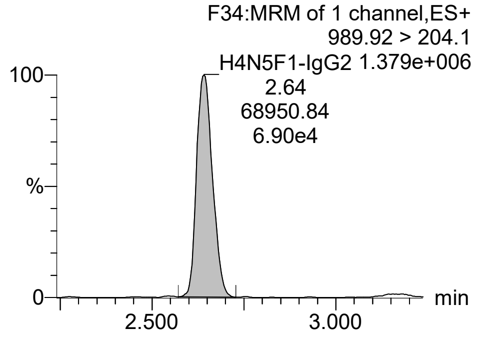

Sample Name: 20211111_A39_R2_IgG

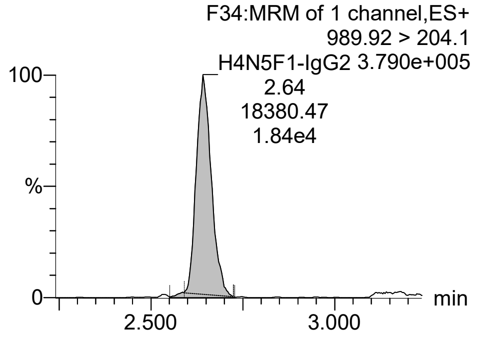

Sample Name: 20211111_A40_R2_IgG

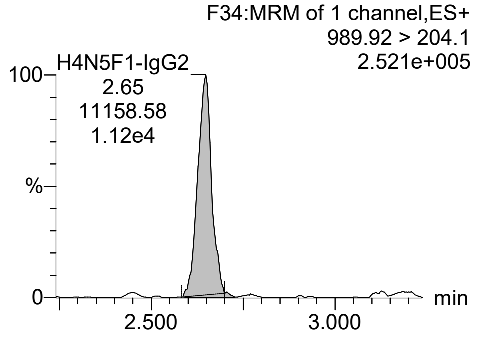

Sample Name: 20211111_H20_R2_IgG

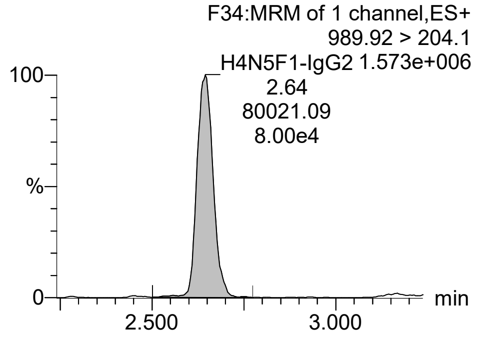

Sample Name: 20211111_A41_R2_IgG

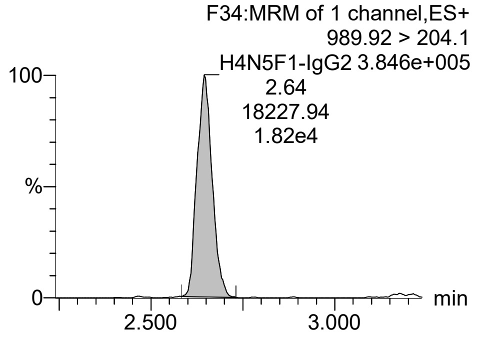

Sample Name: 20211111_A42_R2_IgG

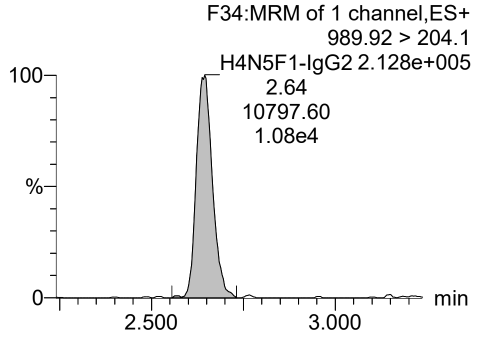

Sample Name: 20211111_A43_R2_IgG

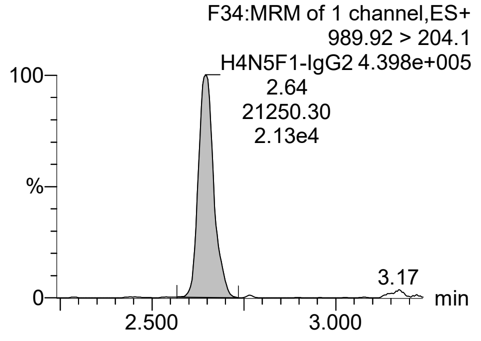

Dataset: C:\MassLynx\10.pro\20211115_clinicalsamples_IgG.qld

Last Altered: Sunday, October 02, 2022 16:02:30 台北標準時間
Printed: Friday, August 01, 2025 16:51:59 台北標準時間

Sample Name: 20211111_A44_R2_IgG

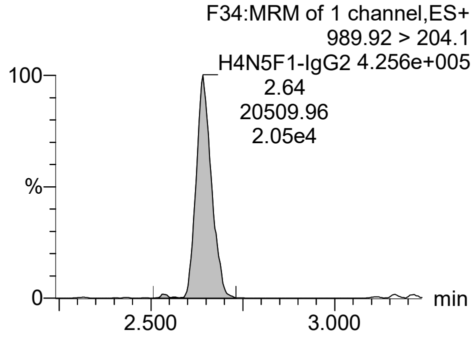

Sample Name: 20211111_QC_R17_IgG

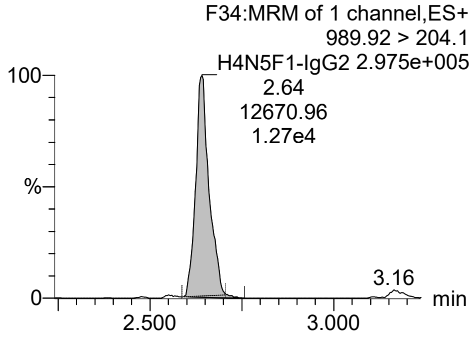

Dataset: C:\MassLynx\10.pro\20211115_clinicalsamples_IgG.qld

Last Altered: Sunday, October 02, 2022 16:02:30 台北標準時間
Printed: Friday, August 01, 2025 16:51:59 台北標準時間

	#	Name	RT	Area	IS Area	Response	Prima...
1	1	20211111_QC_R1_IgG	2.65	27854.5	64968.0	0.429	bb
2	2	20211111_A1_R1_IgG	2.65	39763.2	59059.2	0.673	bb
3	3	20211111_A2_R1_IgG	2.65	34762.0	66621.2	0.522	bb
4	4	20211111_L1_R1_IgG	2.64	14517.6	64201.0	0.226	MM
5	5	20211111_L2_R1_IgG	2.65	25196.8	46950.2	0.537	bb
6	6	20211111_H1_R1_IgG	2.64	9769.9	38648.2	0.253	MM
7	7	20211111_A3_R1_IgG	2.65	360.7	2603.7	0.139	MM
8	8	20211111_A4_R1_IgG	2.65	41529.0	69960.4	0.594	bb
9	9	20211111_L3_R1_IgG	2.65	8953.8	38864.7	0.230	MM
10	10	20211111_L4_R1_IgG	2.65	16057.5	43380.5	0.370	bb
11	11	20211111_H2_R1_IgG	2.65	40207.1	71349.2	0.564	bb
12	12	20211111_A5_R1_IgG	2.65	12754.4	65569.7	0.195	MM
13	13	20211111_A6_R1_IgG	2.65	33353.5	53120.7	0.628	bb
14	14	20211111_QC_R2_IgG	2.65	31316.4	81034.1	0.386	MM
15	15	20211111_L5_R1_IgG	2.64	16593.8	50994.6	0.325	bb
16	16	20211111_L6_R1_IgG	2.65	8298.6	58058.0	0.143	MM
17	17	20211111_H3_R1_IgG	2.65	27383.9	64028.6	0.428	bb
18	18	20211111_A7_R1_IgG	2.65	17072.8	82996.1	0.206	bb
19	19	20211111_A8_R1_IgG	2.64	15539.0	77731.7	0.200	bb
20	20	20211111_L7_R1_IgG	2.65	13879.7	38827.3	0.357	bb
21	21	20211111_L8_R1_IgG	2.65	13315.4	44220.1	0.301	bb
22	22	20211111_H4_R1_IgG	2.65	15339.1	65826.3	0.233	MM
23	23	20211111_A9_R1_IgG	2.64	9959.5	60771.1	0.164	bb
24	24	20211111_A10_R1_IgG	2.64	7942.4	48578.7	0.163	bb
25	25	20211111_L9_R1_IgG	2.65	18363.0	50830.7	0.361	bb
26	26	20211111_L10_R1_IgG	2.64	12550.5	38034.2	0.330	bb
27	27	20211111_QC_R3_IgG	2.66	25584.0	67025.5	0.382	bb
28	28	20211111_H5_R1_IgG	2.65	12966.3	44544.9	0.291	MM
29	29	20211111_A11_R1_IgG	2.65	6810.6	66408.5	0.103	bb
30	30	20211111_A12_R1_IgG	2.65	16317.4	46624.5	0.350	MM
31	31	20211111_L11_R1_IgG	2.65	6634.8	33425.1	0.198	bb
32	32	20211111_L12_R1_IgG	2.65	6168.7	41178.0	0.150	MM
33	33	20211111_H6_R1_IgG	2.65	52080.6	58593.4	0.889	bb
34	34	20211111_A13_R1_IgG	2.65	44638.6	54133.0	0.825	bb
35	35	20211111_A14_R1_IgG	2.66	13133.6	56467.3	0.233	bb
36	36	20211111_L13_R1_IgG	2.65	11930.5	36024.4	0.331	bb
37	37	20211111_L14_R1_IgG	2.65	11819.1	37181.6	0.318	bb
38	38	20211111_H7_R1_IgG	2.65	22796.3	42850.6	0.532	bb
39	39	20211111_A15_R1_IgG	2.66	16241.9	62015.1	0.262	bb
40	40	20211111_QC_R4_IgG	2.65	21631.4	58572.5	0.369	bb
41	41	20211111_A16_R1_IgG	2.64	11814.6	78283.8	0.151	bb
42	42	20211111_L15_R1_IgG	2.65	26766.4	44158.2	0.606	bb
43	43	20211111_L16_R1_IgG	2.65	15003.7	55150.8	0.272	bb
44	44	20211111_H8_R1_IgG	2.65	10225.6	42965.2	0.238	bb
45	45	20211111_A17_R1_IgG	2.65	25300.4	39717.7	0.637	bb
46	46	20211111_A18_R1_IgG	2.65	9504.7	44675.4	0.213	MM
47	47	20211111_L17_R1_IgG	2.65	16635.5	37854.1	0.439	MM
48	48	20211111_L18_R1_IgG	2.65	4352.8	50716.9	0.086	MM
49	49	20211111_H9_R1_IgG	2.65	18432.0	36276.0	0.508	bb
50	50	20211111_A19_R1_IgG	2.65	26516.4	42862.5	0.619	bb
51	51	20211111_A20_R1_IgG	2.65	7590.2	36542.7	0.208	MM
52	52	20211111_L19_R1_IgG	2.64	15373.5	53892.8	0.285	bb
53	53	20211111_QC_R5_IgG	2.64	20430.2	56513.2	0.362	bb
54	54	20211111_L20_R1_IgG	2.65	13379.7	56499.9	0.237	bb
55	55	20211111_H10_R1_IgG	2.65	24777.1	68120.5	0.364	MM
56	56	20211111_A21_R1_IgG	2.65	32642.0	63699.8	0.512	bb

Dataset: C:\MassLynx\10.pro\20211115_clinicalsamples_IgG.qld

Last Altered: Sunday, October 02, 2022 16:02:30 台北標準時間
Printed: Friday, August 01, 2025 16:51:59 台北標準時間

	# Name	RT	Area	IS Area	Response	Prima...
57	57 20211111_A22_R1_IgG	2.64	13385.5	52222.1	0.256	bb
58	58 20211111_L21_R1_IgG	2.65	10122.2	54372.5	0.186	bb
59	59 20211111_L22_R1_IgG	2.65	8774.0	41353.1	0.212	MM
60	60 20211111_H11_R1_IgG	2.65	29409.3	65579.1	0.448	bb
61	61 20211111_A23_R1_IgG	2.65	28506.7	64131.1	0.445	bb
62	62 20211111_A24_R1_IgG	2.65	10484.9	38724.8	0.271	MM
63	63 20211111_L23_R1_IgG	2.65	31417.7	64245.2	0.489	bb
64	64 20211111_L24_R1_IgG	2.65	12281.6	45298.0	0.271	bb
65	65 20211111_H12_R1_IgG	2.65	10210.0	44679.3	0.229	bb
66	66 20211111_QC_R6_IgG	2.65	16483.4	48165.0	0.342	bb
67	67 20211111_A25_R1_IgG	2.65	9204.2	57169.9	0.161	bb
68	68 20211111_A26_R1_IgG	2.64	10696.4	34254.0	0.312	bb
69	69 20211111_L25_R1_IgG	2.65	18315.3	67491.5	0.271	bb
70	70 20211111_L26_R1_IgG	2.65	7066.7	55934.1	0.126	bb
71	71 20211111_H13_R1_IgG	2.65	22486.3	48767.3	0.461	MM
72	72 20211111_A27_R1_IgG	2.65	8100.0	57269.7	0.141	bb
73	73 20211111_A28_R1_IgG	2.64	14176.8	49231.1	0.288	MM
74	74 20211111_L27_R1_IgG	2.66	11792.4	60014.5	0.196	bb
75	75 20211111_L28_R1_IgG	2.65	9599.6	35062.6	0.274	bb
76	76 20211111_H14_R1_IgG	2.64	44469.3	62371.9	0.713	bb
77	77 20211111_A29_R1_IgG	2.65	8170.7	43858.2	0.186	bb
78	78 20211111_A30_R1_IgG	2.65	11621.1	62500.4	0.186	bb
79	79 20211111_QC_R7_IgG	2.65	17063.8	43946.9	0.388	bb
80	80 20211111_L29_R1_IgG	2.65	6221.6	38728.9	0.161	bb
81	81 20211111_L30_R1_IgG	2.65	7802.1	42613.2	0.183	MM
82	82 20211111_H15_R1_IgG	2.64	17848.1	39874.6	0.448	bb
83	83 20211111_A31_R1_IgG	2.65	12483.9	53414.3	0.234	bb
84	84 20211111_A32_R1_IgG	2.65	12962.2	41844.6	0.310	bb
85	85 20211111_L31_R1_IgG	2.66	11061.5	40553.7	0.273	MM
86	86 20211111_L32_R1_IgG	2.65	14171.6	49618.7	0.286	bb
87	87 20211111_H16_R1_IgG	2.66	18996.0	60170.9	0.316	bb
88	88 20211111_A33_R1_IgG	2.65	17154.9	48588.2	0.353	bb
89	89 20211111_A34_R1_IgG	2.64	12102.6	43288.0	0.280	MM
90	90 20211111_H17_R1_IgG	2.65	40854.6	47104.5	0.867	bb
91	91 20211111_A35_R1_IgG	2.65	34458.9	41025.9	0.840	bb
92	92 20211111_QC_R8_IgG	2.65	14776.3	40546.3	0.364	bb
93	93 20211111_A36_R1_IgG	2.65	16830.6	44504.4	0.378	bb
94	94 20211111_H18_R1_IgG	2.65	35939.9	34162.2	1.052	bb
95	95 20211111_A37_R1_IgG	2.65	11738.6	39760.0	0.295	bb
96	96 20211111_A38_R1_IgG	2.65	7701.0	43982.6	0.175	bb
97	97 20211111_H19_R1_IgG	2.65	75828.7	45314.8	1.673	bb
98	98 20211111_A39_R1_IgG	2.65	18669.2	39388.0	0.474	bb
99	99 20211111_A40_R1_IgG	2.66	11501.9	38726.4	0.297	bb
100	1... 20211111_H20_R1_IgG	2.65	86490.3	37814.4	2.287	bb
101	1... 20211111_A41_R1_IgG	2.66	21646.2	48930.3	0.442	bb
102	1... 20211111_A42_R1_IgG	2.65	12339.2	40406.8	0.305	bb
103	1... 20211111_A43_R1_IgG	2.65	26184.3	44421.5	0.589	bb
104	1... 20211111_A44_R1_IgG	2.65	28586.9	53128.2	0.538	bb
105	1... 20211111_QC_R9_IgG	2.65	12933.0	37049.0	0.349	bb
106	1... 20211111_A1_R2_IgG	2.65	30106.2	46499.4	0.647	bb
107	1... 20211111_A2_R2_IgG	2.65	29138.8	57260.7	0.509	bb
108	1... 20211111_L1_R2_IgG	2.65	9932.9	48062.9	0.207	bb
109	1... 20211111_L2_R2_IgG	2.65	22056.1	38745.2	0.569	MM
110	1... 20211111_H1_R2_IgG	2.64	11915.2	44273.5	0.269	bb
111	1... 20211111_A3_R2_IgG	2.65	863.6	4755.5	0.182	bb
112	1... 20211111_A4_R2_IgG	2.65	27089.6	50546.7	0.536	bb

Dataset: C:\MassLynx\10.pro\20211115_clinicalsamples_IgG.qld

Last Altered: Sunday, October 02, 2022 16:02:30 台北標準時間
Printed: Friday, August 01, 2025 16:51:59 台北標準時間

	#	Name	RT	Area	IS Area	Response	Prima...
113	1...	20211111_L3_R2_IgG	2.65	7518.2	34825.8	0.216	bb
114	1...	20211111_L4_R2_IgG	2.64	14192.2	39107.7	0.363	bb
115	1...	20211111_H2_R2_IgG	2.65	28201.8	49750.0	0.567	MM
116	1...	20211111_A5_R2_IgG	2.64	11846.5	52536.2	0.225	bb
117	1...	20211111_A6_R2_IgG	2.64	30078.7	42050.0	0.715	MM
118	1...	20211111_QC_R10_IgG	2.64	13035.1	35350.8	0.369	bb
119	1...	20211111_L5_R2_IgG	2.64	13255.1	38039.3	0.348	bb
120	1...	20211111_L6_R2_IgG	2.65	7095.5	44907.4	0.158	bb
121	1...	20211111_H3_R2_IgG	2.65	23041.0	47729.1	0.483	bb
122	1...	20211111_A7_R2_IgG	2.64	11405.9	57347.1	0.199	bb
123	1...	20211111_A8_R2_IgG	2.64	10316.1	52397.8	0.197	bb
124	1...	20211111_L7_R2_IgG	2.65	12742.2	36318.4	0.351	MM
125	1...	20211111_L8_R2_IgG	2.65	12945.5	38131.9	0.339	bb
126	1...	20211111_H4_R2_IgG	2.65	11364.7	46849.6	0.243	bb
127	1...	20211111_A9_R2_IgG	2.65	8086.5	47759.6	0.169	bb
128	1...	20211111_A10_R2_IgG	2.65	7375.1	41366.2	0.178	MM
129	1...	20211111_L9_R2_IgG	2.65	15570.8	43052.1	0.362	MM
130	1...	20211111_L10_R2_IgG	2.64	11018.5	31487.4	0.350	bb
131	1...	20211111_QC_R11_IgG	2.64	13319.5	37804.6	0.352	bb
132	1...	20211111_H5_R2_IgG	2.64	12900.5	40380.0	0.319	MM
133	1...	20211111_A11_R2_IgG	2.64	4855.1	49533.3	0.098	bb
134	1...	20211111_A12_R2_IgG	2.65	15039.6	40226.3	0.374	bb
135	1...	20211111_L11_R2_IgG	2.66	5557.6	28884.0	0.192	MM
136	1...	20211111_L12_R2_IgG	2.64	5642.9	34351.3	0.164	bb
137	1...	20211111_H6_R2_IgG	2.64	36180.1	40466.6	0.894	bb
138	1...	20211111_A13_R2_IgG	2.65	36055.8	42306.7	0.852	bb
139	1...	20211111_A14_R2_IgG	2.65	10847.1	44904.0	0.242	bb
140	1...	20211111_L13_R2_IgG	2.64	9984.6	31913.0	0.313	MM
141	1...	20211111_L14_R2_IgG	2.64	11327.5	31527.4	0.359	bb
142	1...	20211111_H7_R2_IgG	2.65	23973.0	40838.2	0.587	bb
143	1...	20211111_A15_R2_IgG	2.65	14713.6	49389.7	0.298	bb
144	1...	20211111_QC_R12_IgG	2.66	12443.7	35168.7	0.354	bb
145	1...	20211111_A16_R2_IgG	2.64	8002.6	47440.5	0.169	bb
146	1...	20211111_L15_R2_IgG	2.64	21202.4	33145.9	0.640	bb
147	1...	20211111_L16_R2_IgG	2.64	11405.8	37596.6	0.303	MM
148	1...	20211111_H8_R2_IgG	2.65	11100.6	43933.3	0.253	bb
149	1...	20211111_A17_R2_IgG	2.65	20879.0	43605.5	0.479	bb
150	1...	20211111_A18_R2_IgG	2.64	7488.6	37581.8	0.199	bb
151	1...	20211111_L17_R2_IgG	2.65	14952.3	33942.5	0.441	bb
152	1...	20211111_L18_R2_IgG	2.66	3686.7	37831.5	0.097	MM
153	1...	20211111_H9_R2_IgG	2.64	15995.1	30898.4	0.518	MM
154	1...	20211111_A19_R2_IgG	2.65	28138.1	39352.1	0.715	bb
155	1...	20211111_A20_R2_IgG	2.65	6500.2	34661.2	0.188	bb
156	1...	20211111_L19_R2_IgG	2.65	11564.2	39918.3	0.290	bb
157	1...	20211111_QC_R13_IgG	2.65	13913.9	36239.1	0.384	bb
158	1...	20211111_L20_R2_IgG	2.64	6226.9	32897.7	0.189	MM
159	1...	20211111_H10_R2_IgG	2.65	9016.2	31394.5	0.287	MM
160	1...	20211111_A21_R2_IgG	2.64	14177.4	34863.7	0.407	bb
161	1...	20211111_A22_R2_IgG	2.64	6271.2	30342.1	0.207	MM
162	1...	20211111_L21_R2_IgG	2.65	4535.9	33706.2	0.135	bb
163	1...	20211111_L22_R2_IgG	2.63	4461.7	31298.6	0.143	bb
164	1...	20211111_H11_R2_IgG	2.64	13121.4	38904.1	0.337	bb
165	1...	20211111_A23_R2_IgG	2.64	12306.3	32382.3	0.380	MM
166	1...	20211111_A24_R2_IgG	2.64	7042.5	30261.9	0.233	MM
167	1...	20211111_L23_R2_IgG	2.64	20062.4	43037.5	0.466	bb
168	1...	20211111_L24_R2_IgG	2.64	9353.1	37023.3	0.253	bb

Dataset: C:\MassLynx\10.pro\20211115_clinicalsamples_IgG.qld

Last Altered: Sunday, October 02, 2022 16:02:30 台北標準時間
Printed: Friday, August 01, 2025 16:51:59 台北標準時間

	# Name	RT	Area	IS Area	Response	Prima...
169	1... 20211111_H12_R2_IgG	2.64	8528.3	39478.2	0.216	MM
170	1... 20211111_QC_R14_IgG	2.64	11364.7	34463.2	0.330	bb
171	1... 20211111_A25_R2_IgG	2.63	6356.8	39945.3	0.159	bb
172	1... 20211111_A26_R2_IgG	2.64	8044.6	29663.2	0.271	MM
173	1... 20211111_L25_R2_IgG	2.65	10575.3	38589.3	0.274	MM
174	1... 20211111_L26_R2_IgG	2.64	4482.1	38405.2	0.117	MM
175	1... 20211111_H13_R2_IgG	2.65	17119.1	37771.2	0.453	MM
176	1... 20211111_A27_R2_IgG	2.65	5686.6	42634.1	0.133	MM
177	1... 20211111_A28_R2_IgG	2.64	8591.9	33231.1	0.259	bb
178	1... 20211111_L27_R2_IgG	2.65	8004.4	41761.1	0.192	MM
179	1... 20211111_L28_R2_IgG	2.64	7745.5	26968.6	0.287	MM
180	1... 20211111_H14_R2_IgG	2.65	30738.1	49250.2	0.624	bb
181	1... 20211111_A29_R2_IgG	2.65	8028.2	37601.7	0.214	bb
182	1... 20211111_A30_R2_IgG	2.64	7881.3	40366.8	0.195	MM
183	1... 20211111_QC_R15_IgG	2.64	11888.6	35156.1	0.338	MM
184	1... 20211111_L29_R2_IgG	2.65	5234.9	33813.1	0.155	MM
185	1... 20211111_L30_R2_IgG	2.64	6670.6	33488.6	0.199	bb
186	1... 20211111_H15_R2_IgG	2.64	17102.2	38501.0	0.444	bb
187	1... 20211111_A31_R2_IgG	2.64	9411.7	37525.5	0.251	MM
188	1... 20211111_A32_R2_IgG	2.64	10873.9	35770.8	0.304	MM
189	1... 20211111_L31_R2_IgG	2.64	9710.8	33282.4	0.292	MM
190	1... 20211111_L32_R2_IgG	2.64	12150.3	42049.6	0.289	bb
191	1... 20211111_H16_R2_IgG	2.65	13643.5	45947.6	0.297	bb
192	1... 20211111_A33_R2_IgG	2.64	13506.3	40202.3	0.336	bb
193	1... 20211111_A34_R2_IgG	2.65	10906.7	36747.0	0.297	bb
194	1... 20211111_H17_R2_IgG	2.64	36368.6	40920.9	0.889	bb
195	1... 20211111_A35_R2_IgG	2.64	30057.7	33689.2	0.892	bb
196	1... 20211111_QC_R16_IgG	2.65	12436.9	32783.6	0.379	MM
197	1... 20211111_A36_R2_IgG	2.64	14550.3	36822.4	0.395	bb
198	1... 20211111_H18_R2_IgG	2.64	32014.8	31769.3	1.008	bb
199	1... 20211111_A37_R2_IgG	2.65	10215.6	37581.8	0.272	MM
200	2... 20211111_A38_R2_IgG	2.64	8140.8	38390.4	0.212	bb
201	2... 20211111_H19_R2_IgG	2.64	68950.8	41217.0	1.673	bb
202	2... 20211111_A39_R2_IgG	2.64	18380.5	37392.7	0.492	MM
203	2... 20211111_A40_R2_IgG	2.65	11158.6	38295.1	0.291	MM
204	2... 20211111_H20_R2_IgG	2.64	80021.1	35941.8	2.226	bb
205	2... 20211111_A41_R2_IgG	2.64	18227.9	45304.9	0.402	bb
206	2... 20211111_A42_R2_IgG	2.64	10797.6	37803.8	0.286	bb
207	2... 20211111_A43_R2_IgG	2.64	21250.3	38161.8	0.557	bb
208	2... 20211111_A44_R2_IgG	2.64	20510.0	37819.8	0.542	bb
209	2... 20211111_QC_R17_IgG	2.64	12671.0	31731.8	0.399	MM

Dataset: C:\MassLynx\10.pro\20211115_clinicalsamples_IgG.qld

Last Altered: Sunday, October 02, 2022 16:02:30 台北標準時間
Printed: Friday, August 01, 2025 16:52:28 台北標準時間

Method: C:\MassLynx\10_Pro_IgA.PRO\MethDB\20210926_IgG_DMRM_IgM_IS.mdb 16 Feb 2022 11:58:21
Calibration: 18 Nov 2021 13:56:22

Sample Name: 20211111_QC_R1_IgG

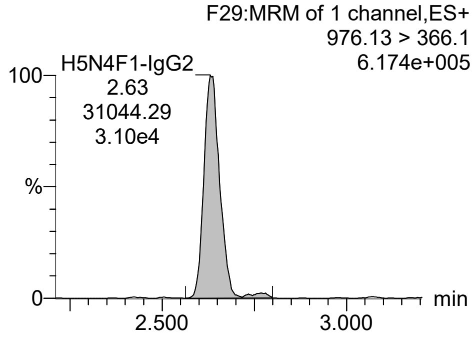

Sample Name: 20211111_A1_R1_IgG

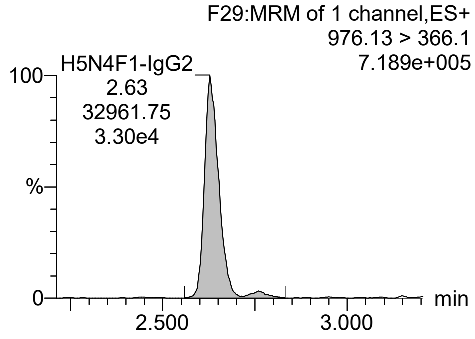

Sample Name: 20211111_A2_R1_IgG

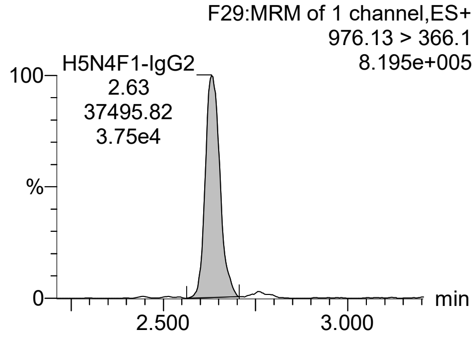

Sample Name: 20211111_L1_R1_IgG

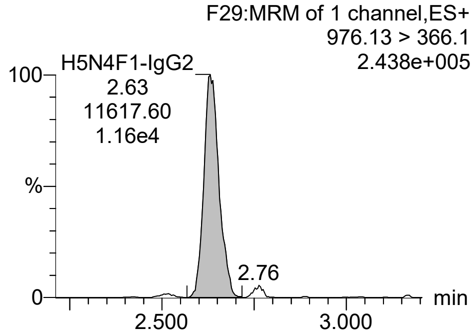

Sample Name: 20211111_L2_R1_IgG

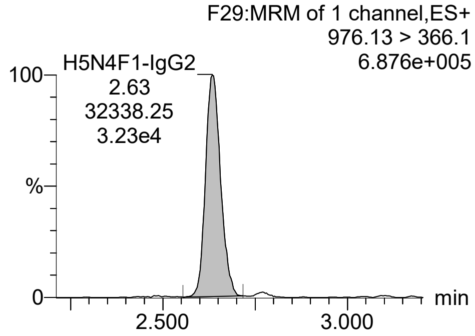

Sample Name: 20211111_H1_R1_IgG

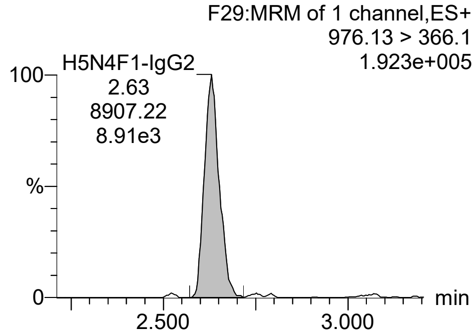

Sample Name: 20211111_A3_R1_IgG

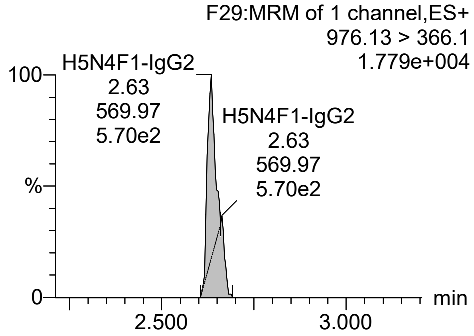

Sample Name: 20211111_A4_R1_IgG

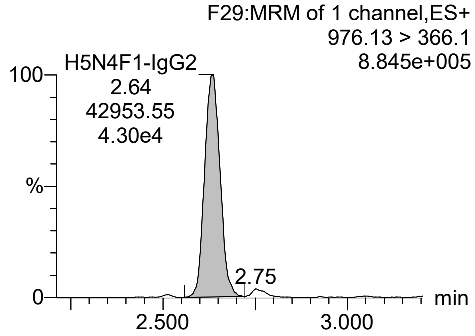

Sample Name: 20211111_L3_R1_IgG

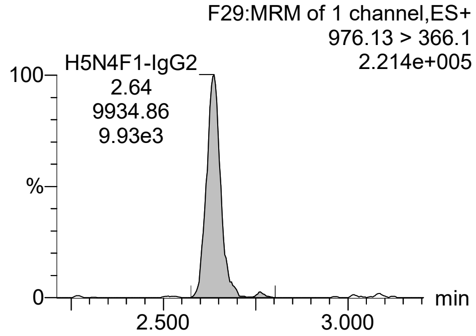

Sample Name: 20211111_L4_R1_IgG

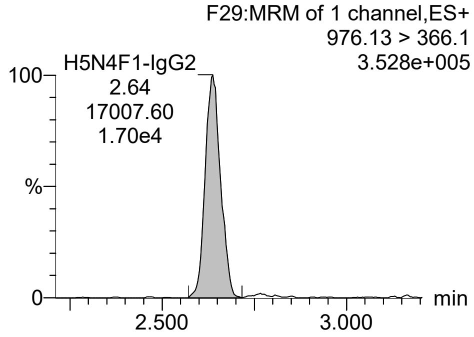

Sample Name: 20211111_H2_R1_IgG

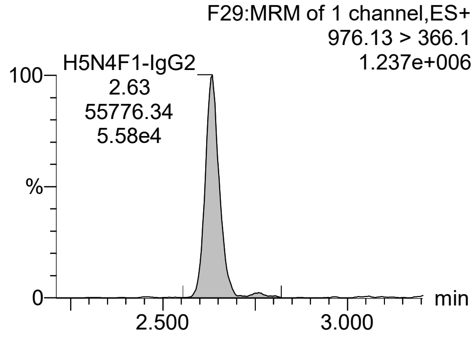

Sample Name: 20211111_A5_R1_IgG

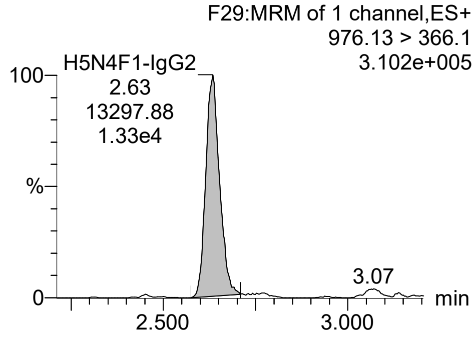

Dataset: C:\MassLynx\10.pro\20211115_clinicalsamples_IgG.qld

Last Altered: Sunday, October 02, 2022 16:02:30 台北標準時間
Printed: Friday, August 01, 2025 16:52:28 台北標準時間

Sample Name: 20211111_A6_R1_IgG

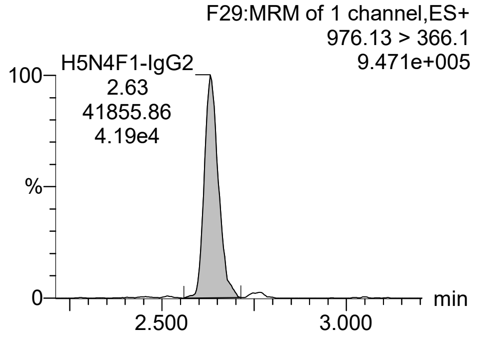

Sample Name: 20211111_QC_R2_IgG

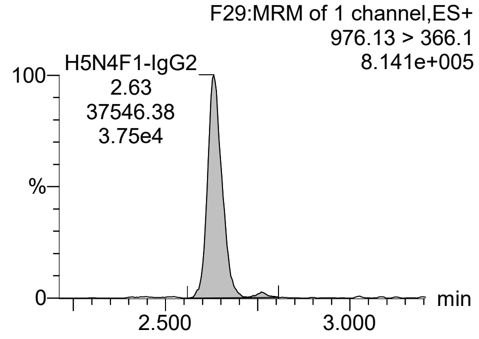

Sample Name: 20211111_L5_R1_IgG

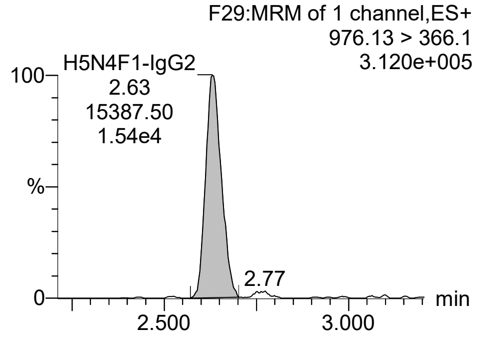

Sample Name: 20211111_L6_R1_IgG

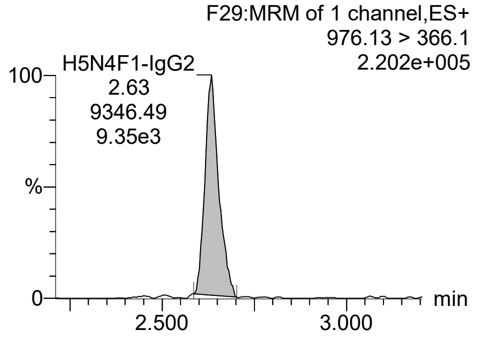

Sample Name: 20211111_H3_R1_IgG

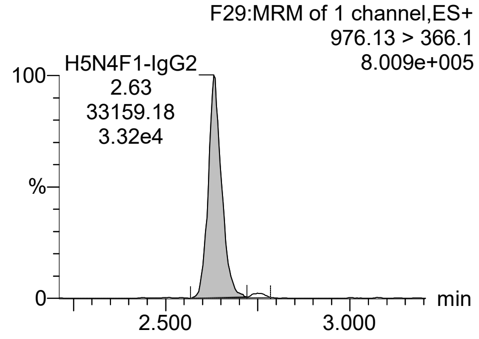

Sample Name: 20211111_A7_R1_IgG

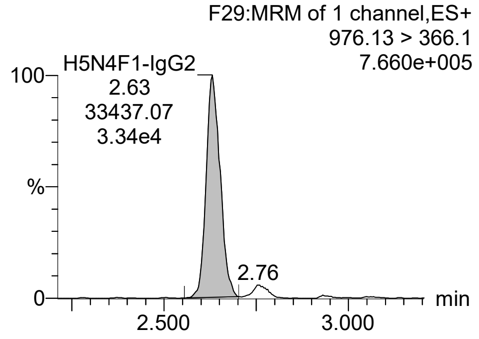

Sample Name: 20211111_A8_R1_IgG

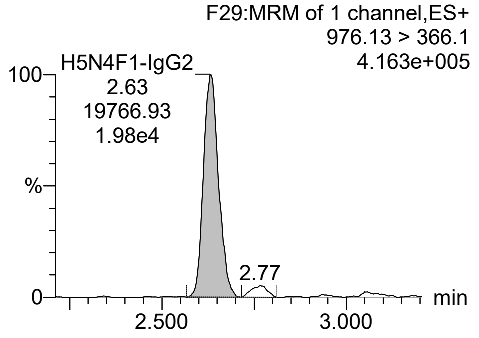

Sample Name: 20211111_L7_R1_IgG

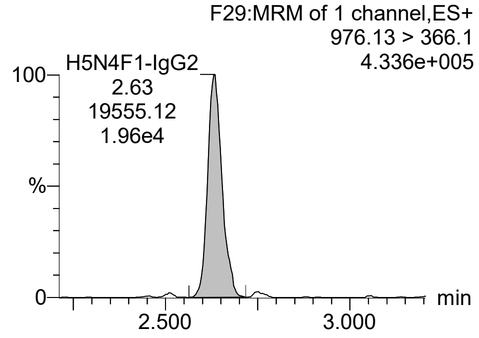

Sample Name: 20211111_L8_R1_IgG

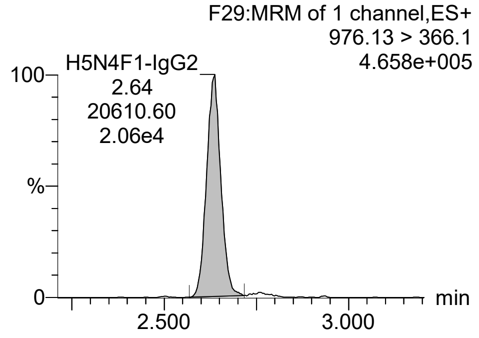

Sample Name: 20211111_H4_R1_IgG

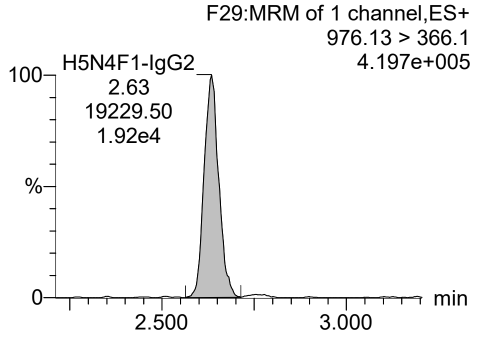

Sample Name: 20211111_A9_R1_IgG

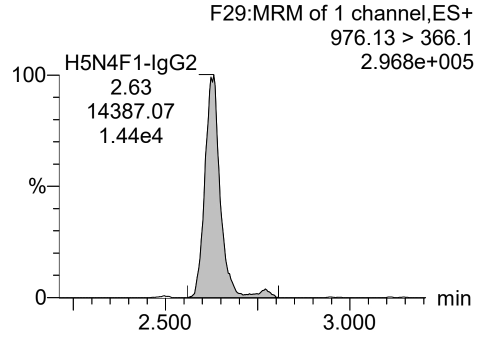

Sample Name: 20211111_A10_R1_IgG

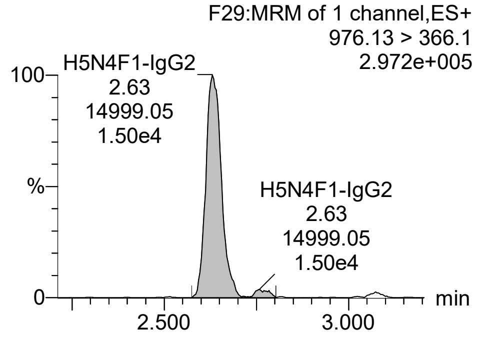

Sample Name: 20211111_L9_R1_IgG

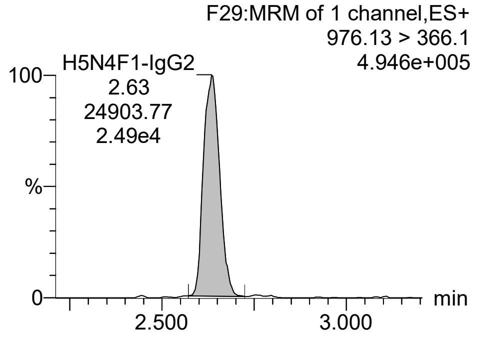

Sample Name: 20211111_L10_R1_IgG

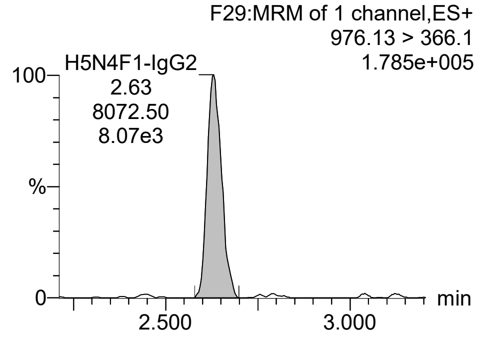

Sample Name: 20211111_QC_R3_IgG

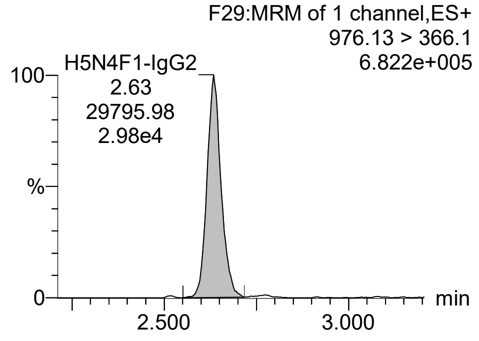

Sample Name: 20211111_H5_R1_IgG

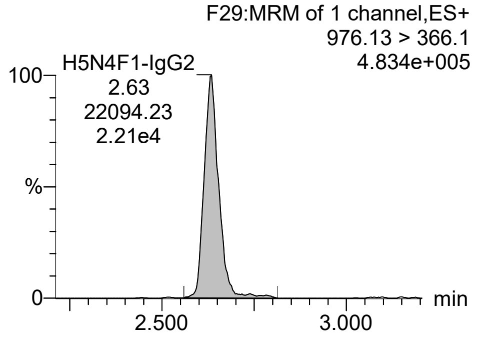

Sample Name: 20211111_A11_R1_IgG

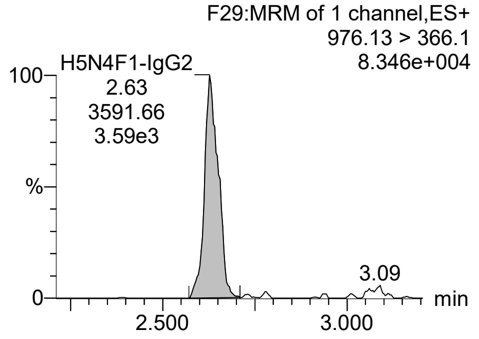

Sample Name: 20211111_A12_R1_IgG

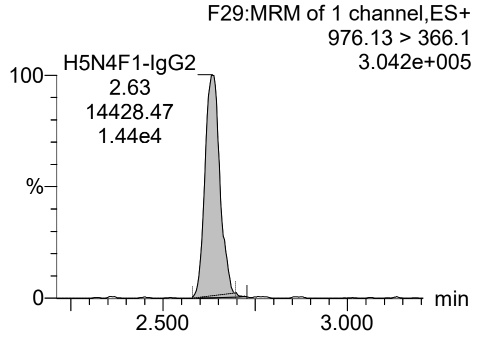

Sample Name: 20211111_L11_R1_IgG

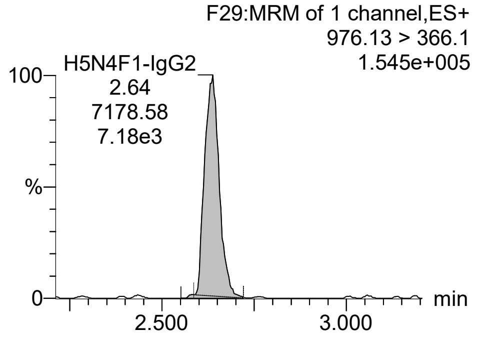

Sample Name: 20211111_L12_R1_IgG

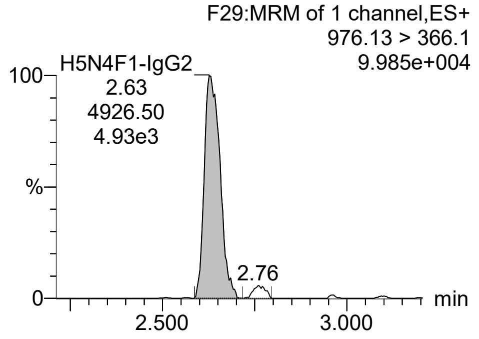

Sample Name: 20211111_H6_R1_IgG

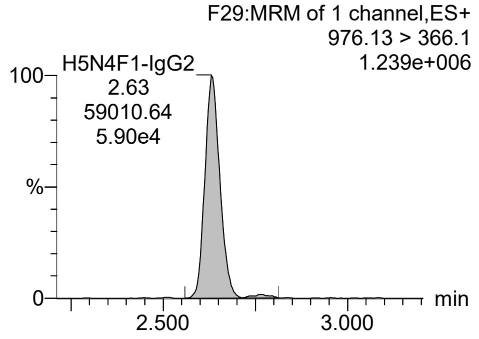

Sample Name: 20211111_A13_R1_IgG

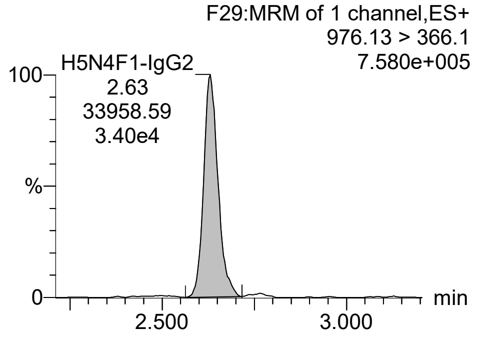

Sample Name: 20211111_A14_R1_IgG

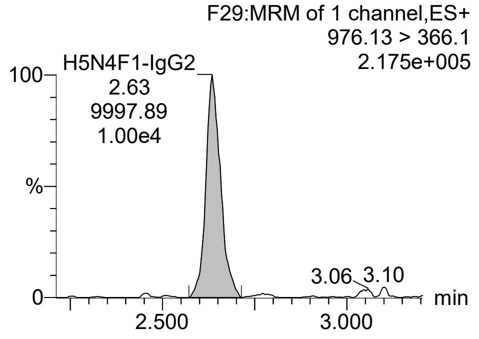

Sample Name: 20211111_L13_R1_IgG

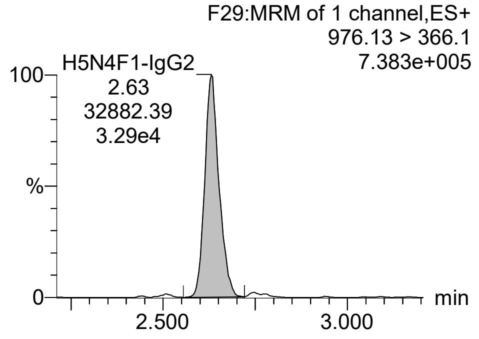

Sample Name: 20211111_L14_R1_IgG

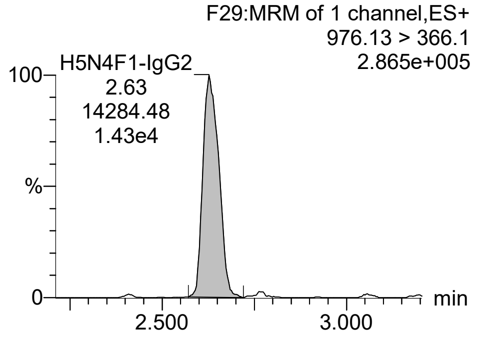

Sample Name: 20211111_H7_R1_IgG

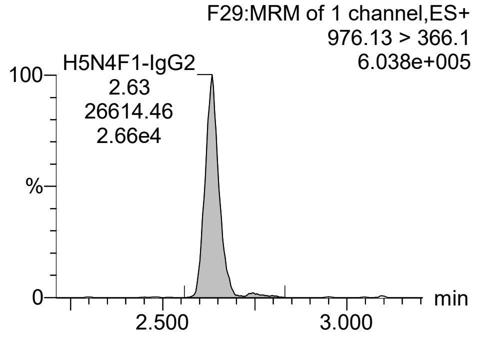

Sample Name: 20211111_A15_R1_IgG

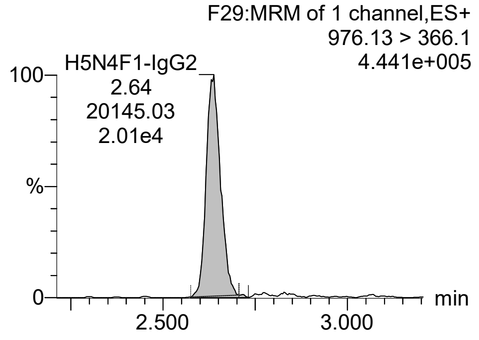

Sample Name: 20211111_QC_R4_IgG

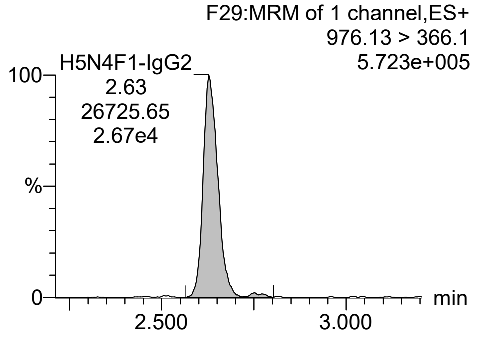

Sample Name: 20211111_A16_R1_IgG

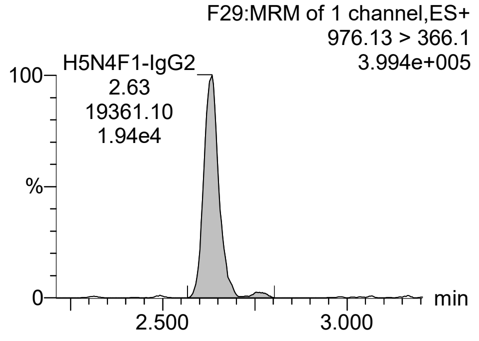

Sample Name: 20211111_L15_R1_IgG

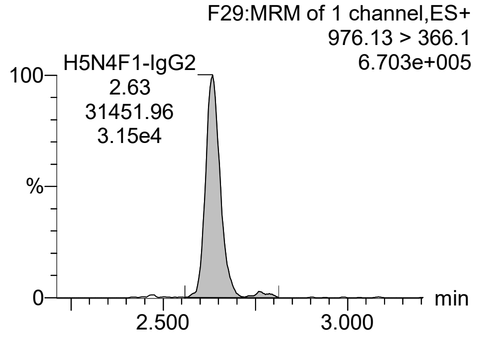

Dataset: C:\MassLynx\10.pro\20211115_clinicalsamples_IgG.qld

Last Altered: Sunday, October 02, 2022 16:02:30 台北標準時間
Printed: Friday, August 01, 2025 16:52:28 台北標準時間

Sample Name: 20211111_L16_R1_IgG

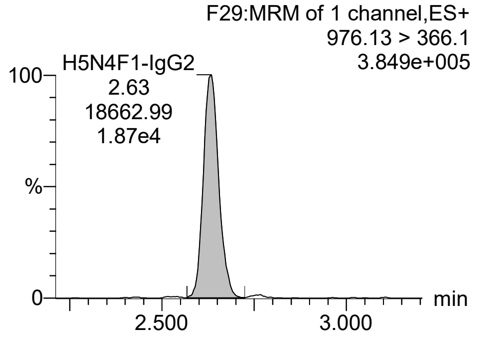

Sample Name: 20211111_H8_R1_IgG

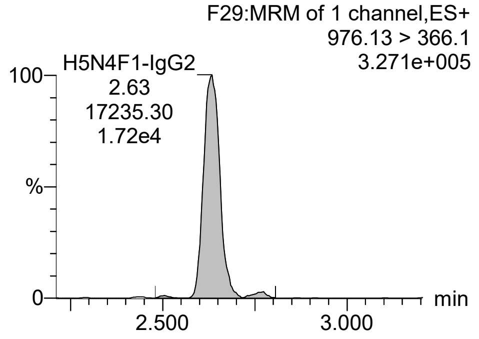

Sample Name: 20211111_A17_R1_IgG

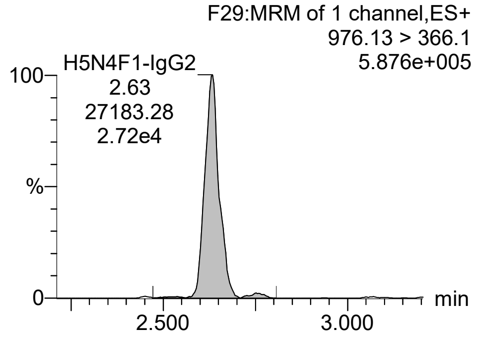

Sample Name: 20211111_A18_R1_IgG

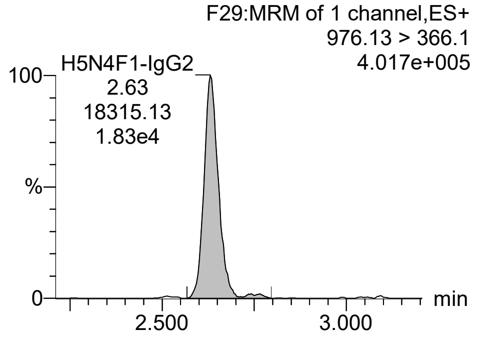

Sample Name: 20211111_L17_R1_IgG

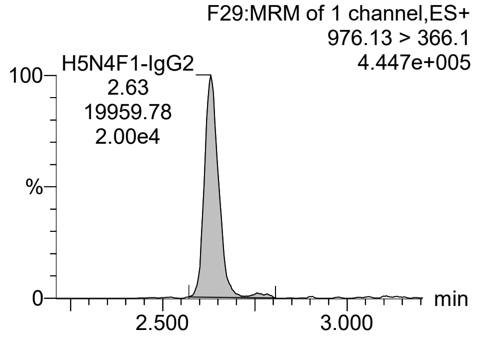

Sample Name: 20211111_L18_R1_IgG

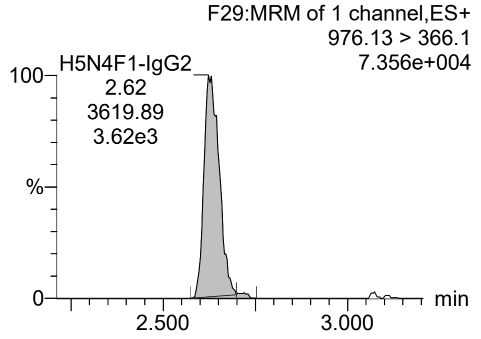

Sample Name: 20211111_H9_R1_IgG

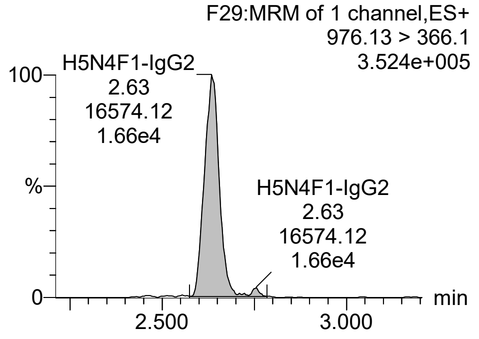

Sample Name: 20211111_A19_R1_IgG

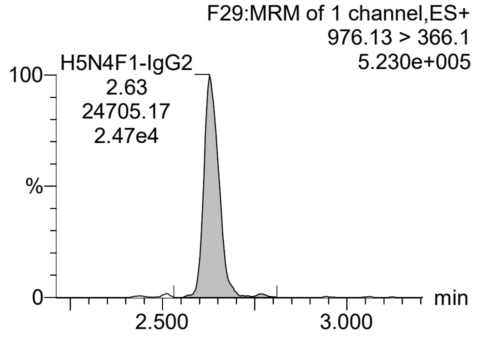

Sample Name: 20211111_A20_R1_IgG

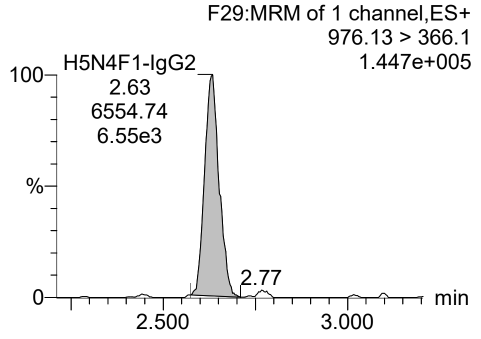

Sample Name: 20211111_L19_R1_IgG

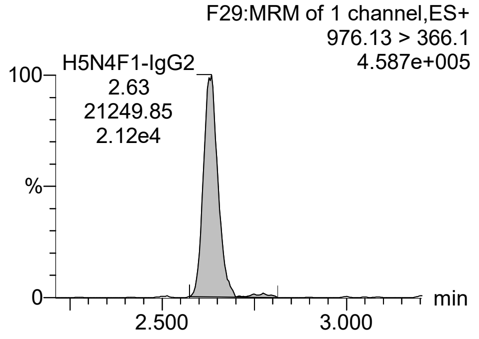

Sample Name: 20211111_QC_R5_IgG

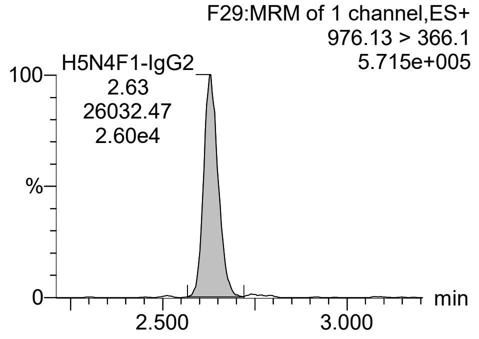

Sample Name: 20211111_L20_R1_IgG

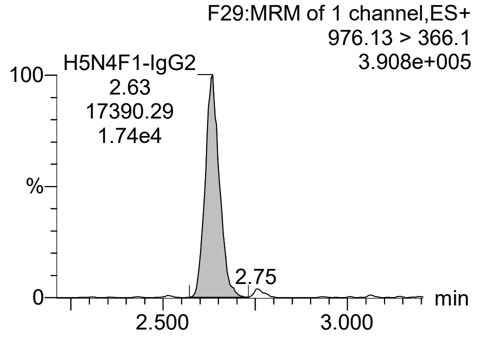

Sample Name: 20211111_H10_R1_IgG

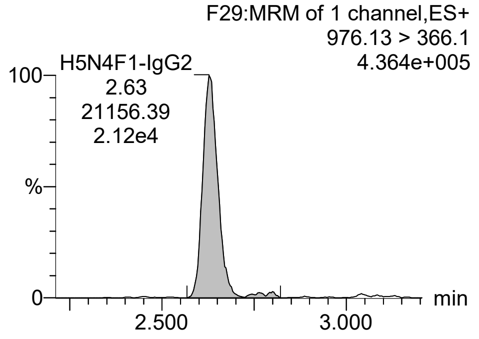

Sample Name: 20211111_A21_R1_IgG

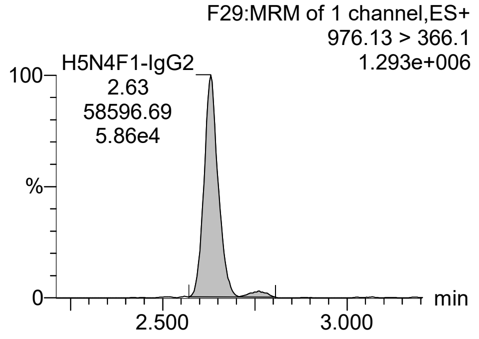

Sample Name: 20211111_A22_R1_IgG

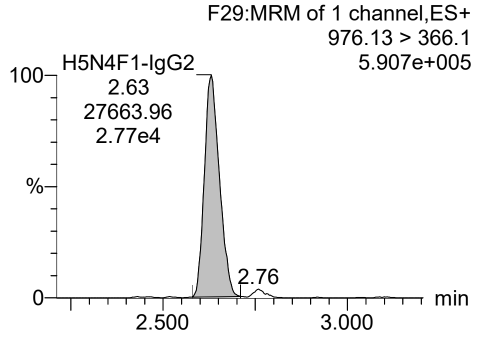

Dataset: C:\MassLynx\10.pro\20211115_clinicalsamples_IgG.qld

Last Altered: Sunday, October 02, 2022 16:02:30 台北標準時間
Printed: Friday, August 01, 2025 16:52:28 台北標準時間

Sample Name: 20211111_L21_R1_IgG
F29:MRM of 1 channel,ES+
976.13 > 366.1
2.077e+005

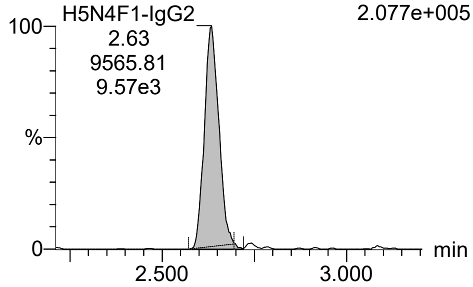

Sample Name: 20211111_L22_R1_IgG
F29:MRM of 1 channel,ES+
976.13 > 366.1
2.262e+005

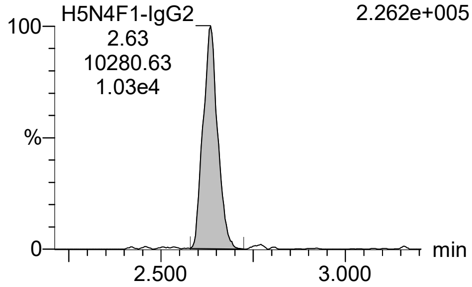

Sample Name: 20211111_H11_R1_IgG
F29:MRM of 1 channel,ES+
976.13 > 366.1
7.500e+005

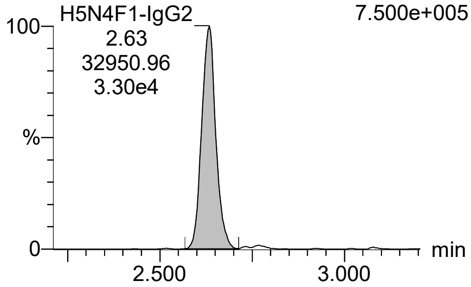

Sample Name: 20211111_A23_R1_IgG
F29:MRM of 1 channel,ES+
976.13 > 366.1
4.164e+005

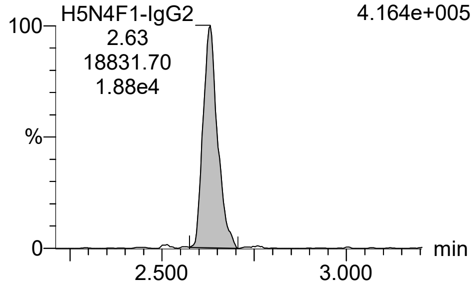

Sample Name: 20211111_A24_R1_IgG
F29:MRM of 1 channel,ES+
976.13 > 366.1
2.427e+005

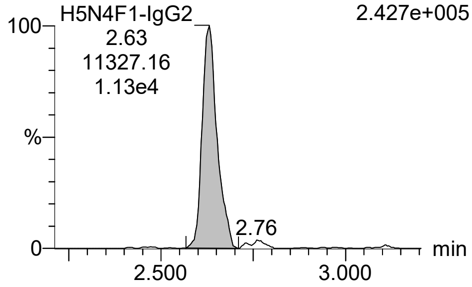

Sample Name: 20211111_L23_R1_IgG
F29:MRM of 1 channel,ES+
976.13 > 366.1
9.772e+005

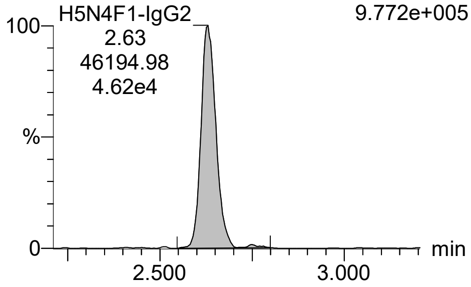

Sample Name: 20211111_L24_R1_IgG
F29:MRM of 1 channel,ES+
976.13 > 366.1
5.002e+005

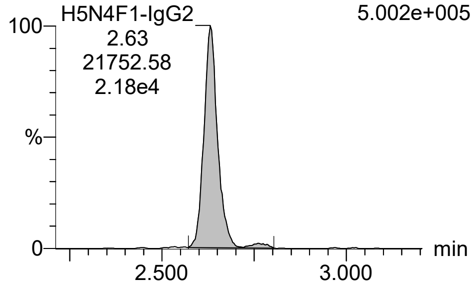

Sample Name: 20211111_H12_R1_IgG
F29:MRM of 1 channel,ES+
976.13 > 366.1
3.588e+005

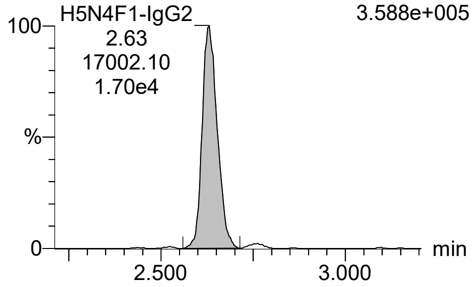

Sample Name: 20211111_QC_R6_IgG
F29:MRM of 1 channel,ES+
976.13 > 366.1
4.823e+005

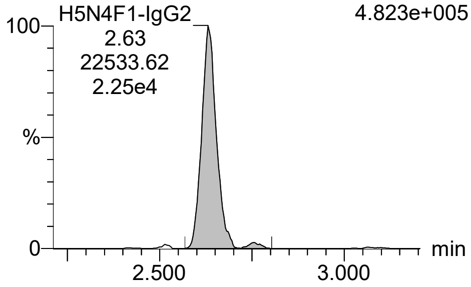

Sample Name: 20211111_A25_R1_IgG
F29:MRM of 1 channel,ES+
976.13 > 366.1
3.392e+005

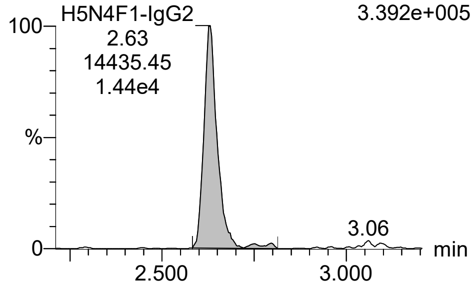

Sample Name: 20211111_A26_R1_IgG
F29:MRM of 1 channel,ES+
976.13 > 366.1
2.072e+005

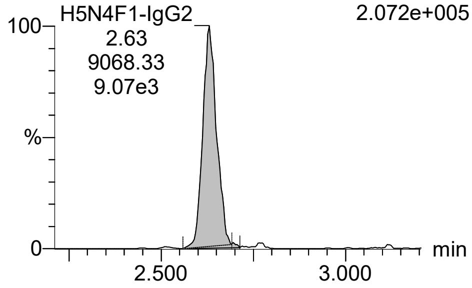

Sample Name: 20211111_L25_R1_IgG
F29:MRM of 1 channel,ES+
976.13 > 366.1
6.569e+005

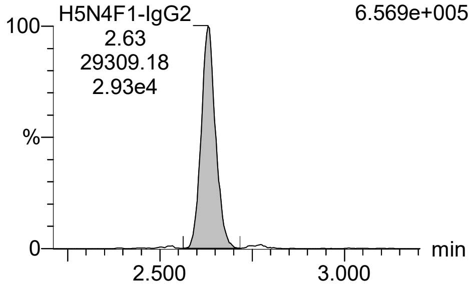

Sample Name: 20211111_L26_R1_IgG
F29:MRM of 1 channel,ES+
976.13 > 366.1
1.656e+005

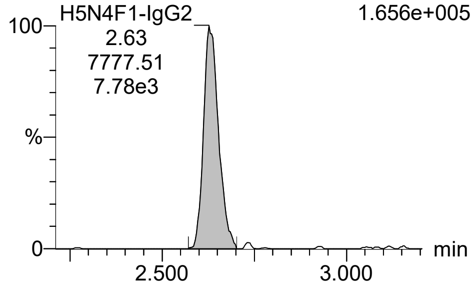

Sample Name: 20211111_H13_R1_IgG
F29:MRM of 1 channel,ES+
976.13 > 366.1
7.100e+005

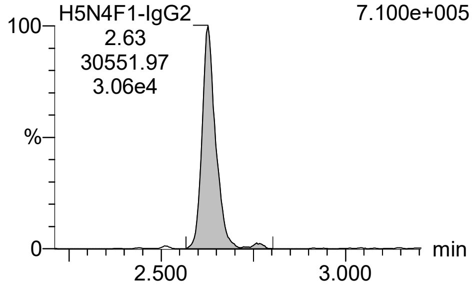

Sample Name: 20211111_A27_R1_IgG
F29:MRM of 1 channel,ES+
976.13 > 366.1
1.855e+005

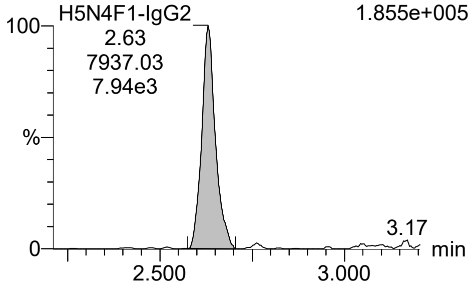

Dataset: C:\MassLynx\10.pro\20211115_clinicalsamples_IgG.qld

Last Altered: Sunday, October 02, 2022 16:02:30 台北標準時間
Printed: Friday, August 01, 2025 16:52:28 台北標準時間

Sample Name: 20211111_A28_R1_IgG

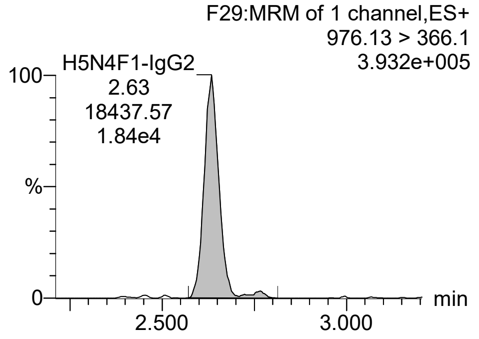

Sample Name: 20211111_L27_R1_IgG

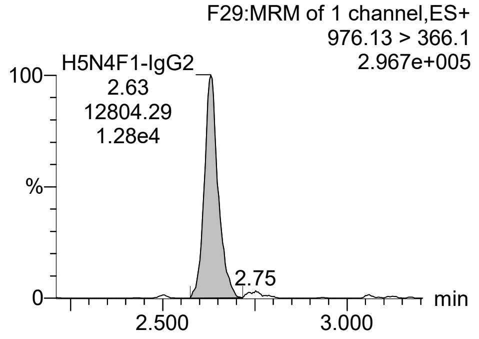

Sample Name: 20211111_L28_R1_IgG

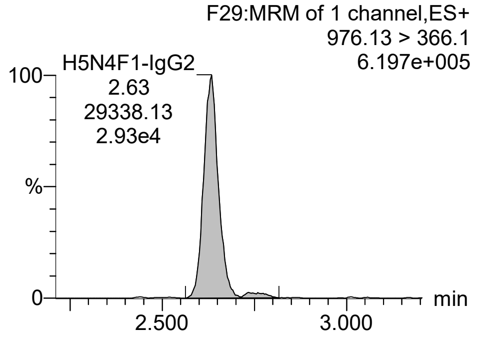

Sample Name: 20211111_H14_R1_IgG

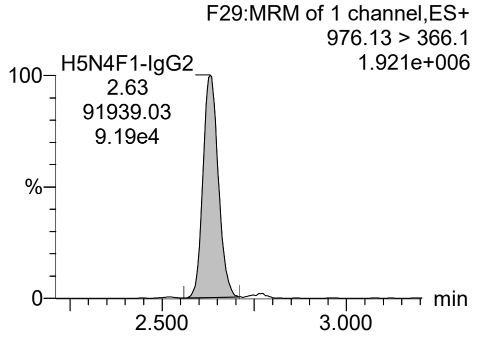

Sample Name: 20211111_A29_R1_IgG

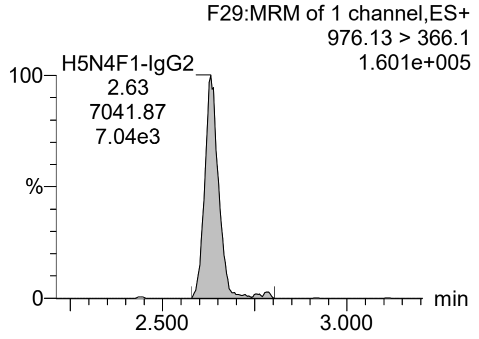

Sample Name: 20211111_A30_R1_IgG

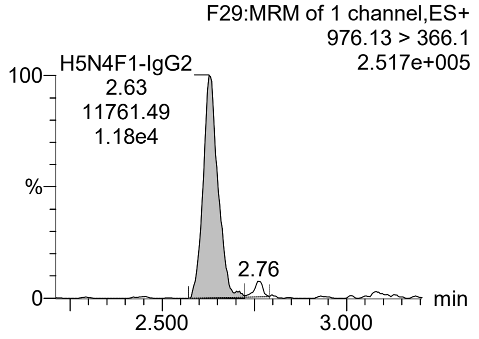

Sample Name: 20211111_QC_R7_IgG

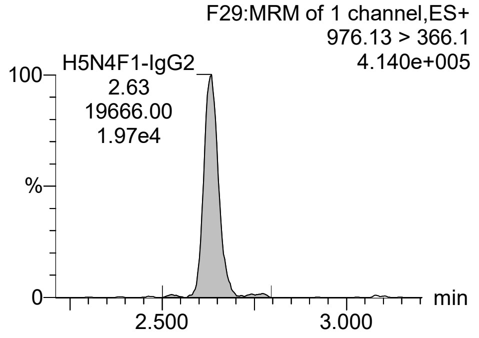

Sample Name: 20211111_L29_R1_IgG

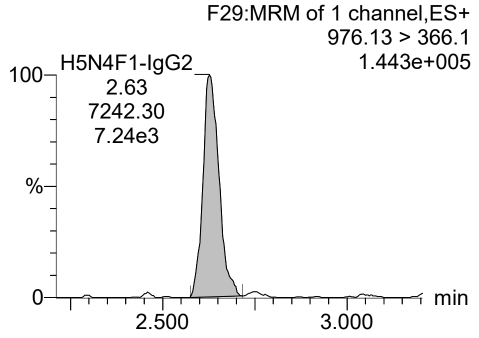

Sample Name: 20211111_L30_R1_IgG

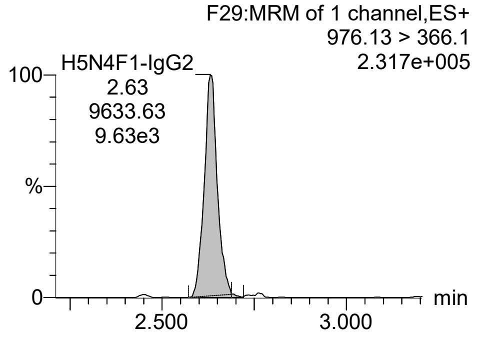

Sample Name: 20211111_H15_R1_IgG

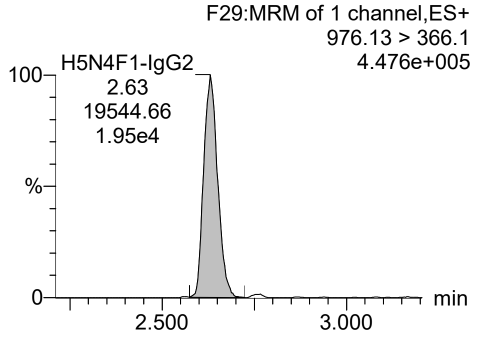

Sample Name: 20211111_A31_R1_IgG

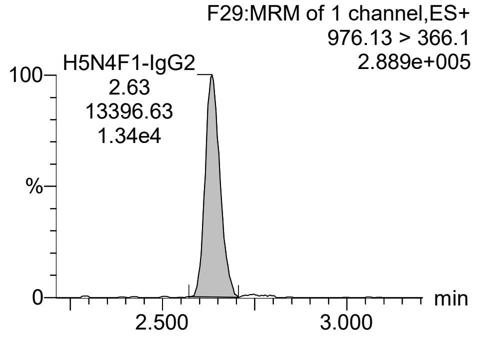

Sample Name: 20211111_A32_R1_IgG

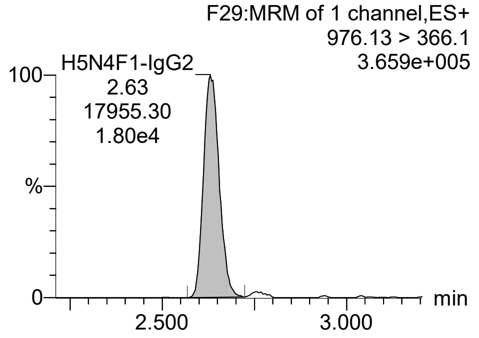

Sample Name: 20211111_L31_R1_IgG

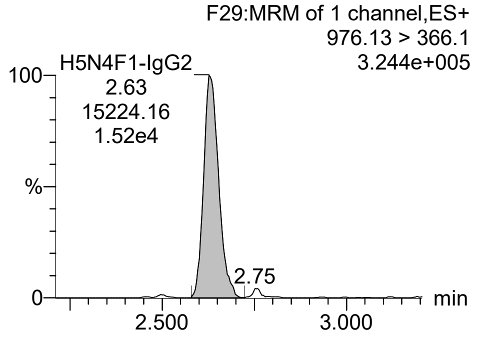

Sample Name: 20211111_L32_R1_IgG

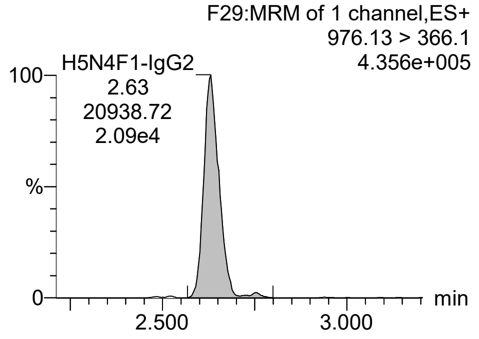

Sample Name: 20211111_H16_R1_IgG

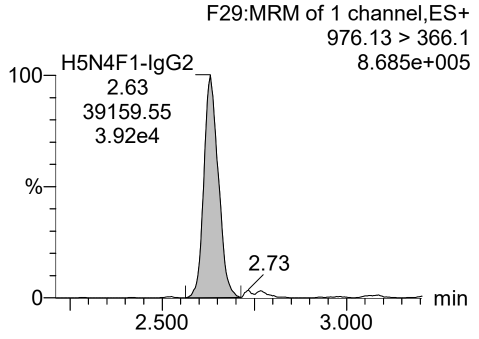

Dataset: C:\MassLynx\10.pro\20211115_clinicalsamples_IgG.qld

Last Altered: Sunday, October 02, 2022 16:02:30 台北標準時間
Printed: Friday, August 01, 2025 16:52:28 台北標準時間

Sample Name: 20211111_A33_R1_IgG

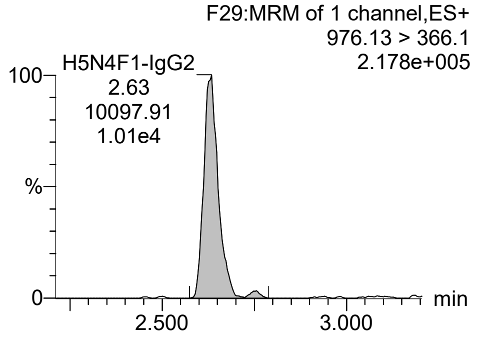

Sample Name: 20211111_A34_R1_IgG

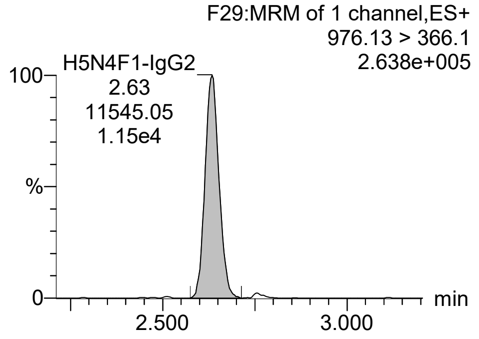

Sample Name: 20211111_H17_R1_IgG

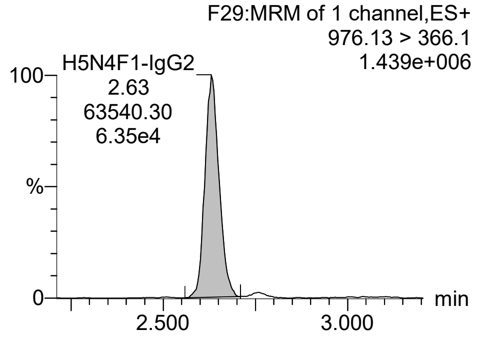

Sample Name: 20211111_A35_R1_IgG

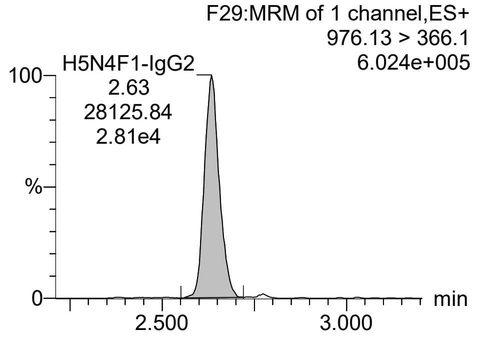

Sample Name: 20211111_QC_R8_IgG

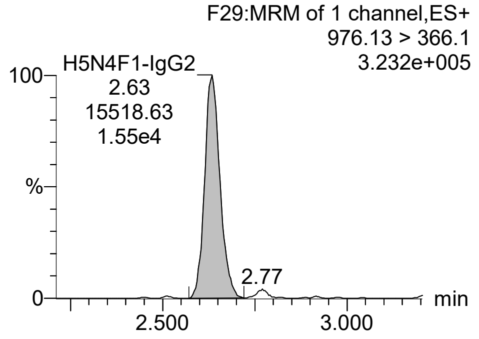

Sample Name: 20211111_A36_R1_IgG

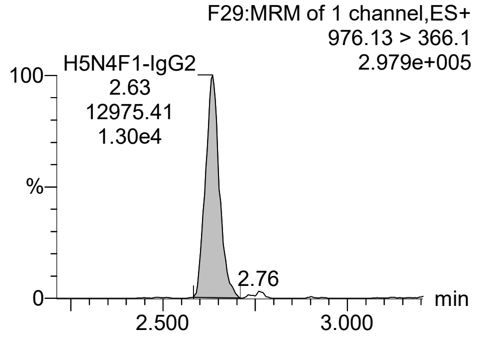

Sample Name: 20211111_H18_R1_IgG

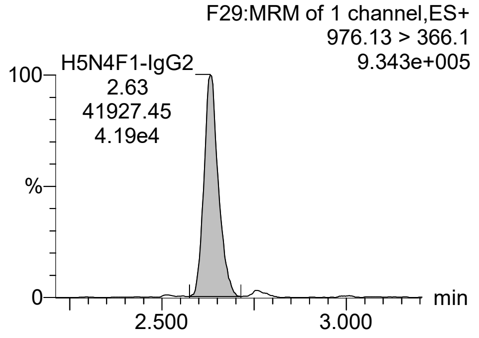

Sample Name: 20211111_A37_R1_IgG

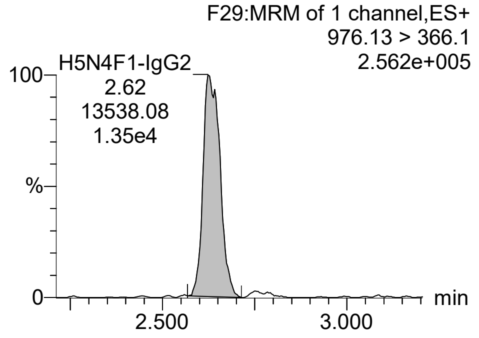

Sample Name: 20211111_A38_R1_IgG

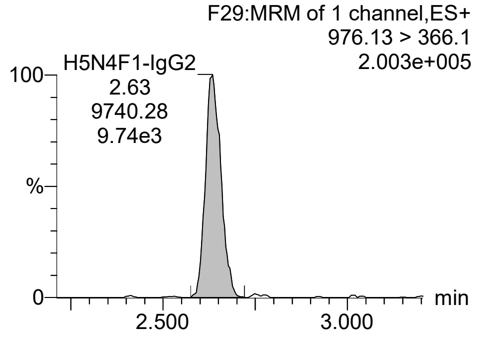

Sample Name: 20211111_H19_R1_IgG

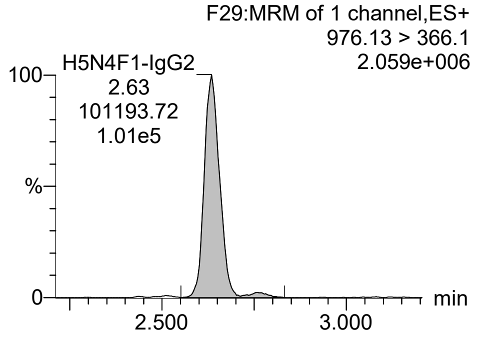

Sample Name: 20211111_A39_R1_IgG

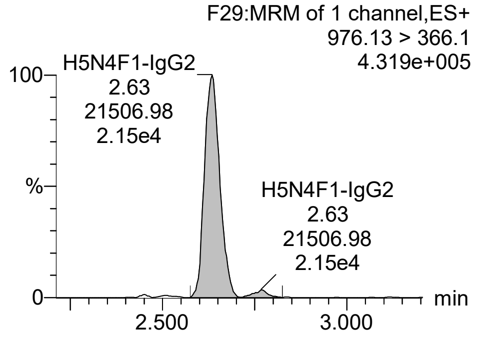

Sample Name: 20211111_A40_R1_IgG

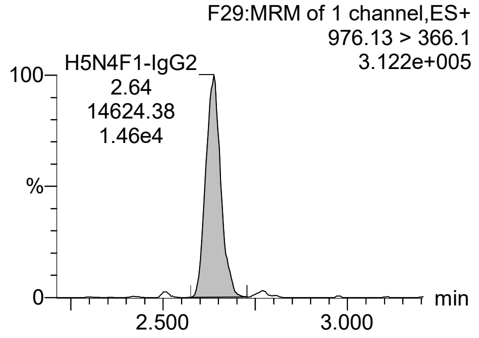

Sample Name: 20211111_H20_R1_IgG

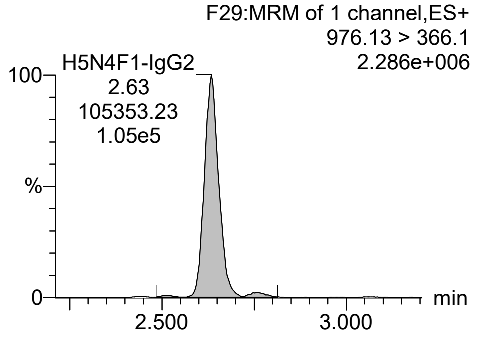

Sample Name: 20211111_A41_R1_IgG

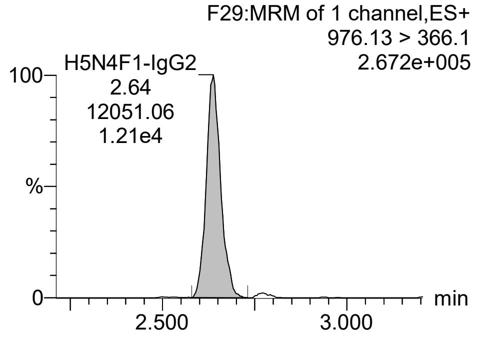

Sample Name: 20211111_A42_R1_IgG

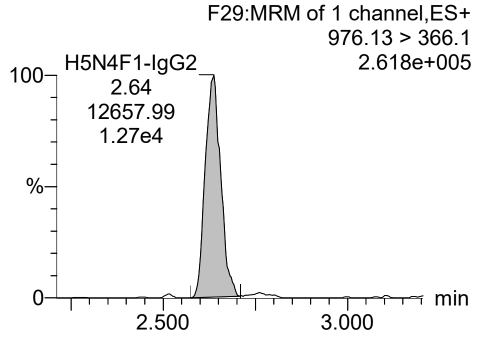

Sample Name: 20211111_A43_R1_IgG

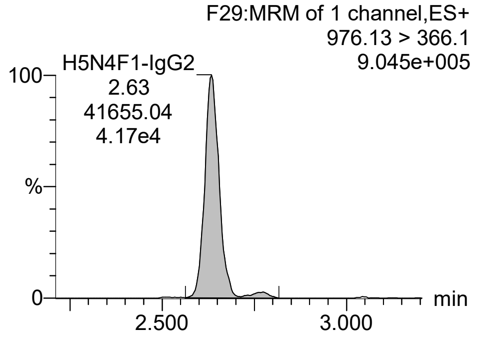

Sample Name: 20211111_A44_R1_IgG

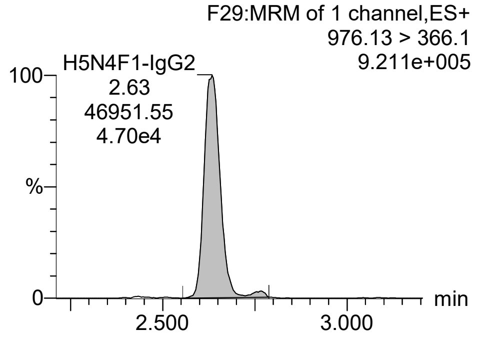

Sample Name: 20211111_QC_R9_IgG

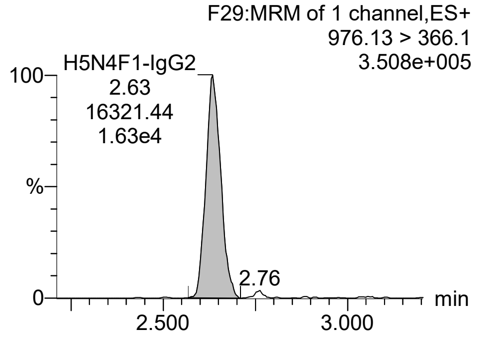

Sample Name: 20211111_A1_R2_IgG

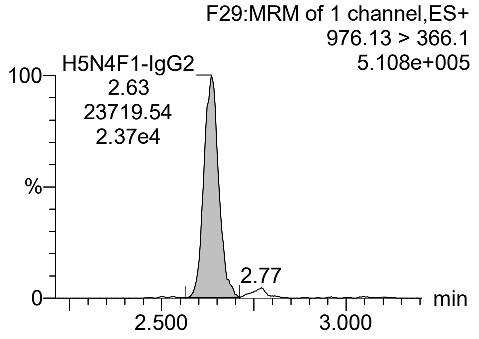

Sample Name: 20211111_A2_R2_IgG

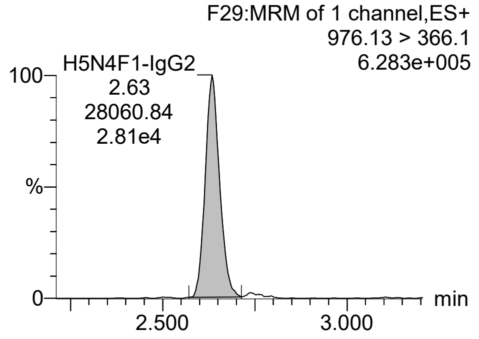

Sample Name: 20211111_L1_R2_IgG

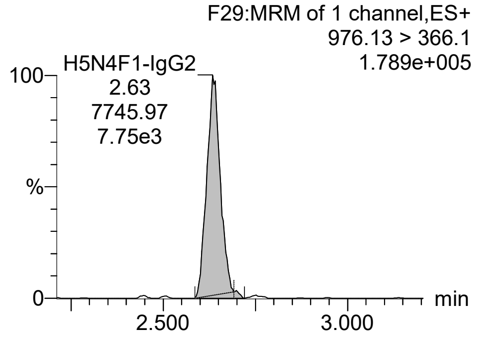

Sample Name: 20211111_L2_R2_IgG

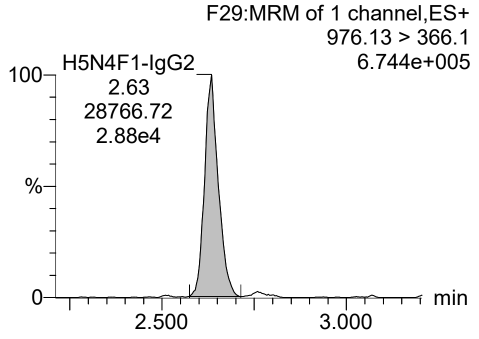

Sample Name: 20211111_H1_R2_IgG

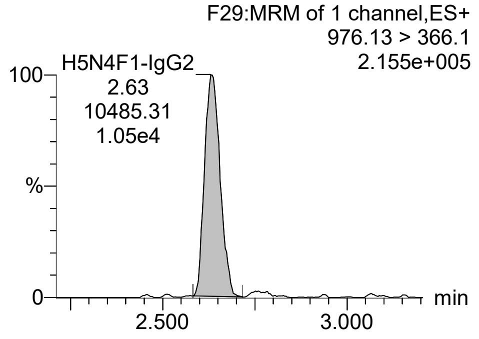

Sample Name: 20211111_A3_R2_IgG

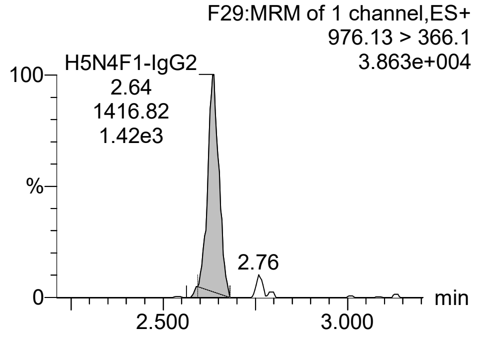

Sample Name: 20211111_A4_R2_IgG

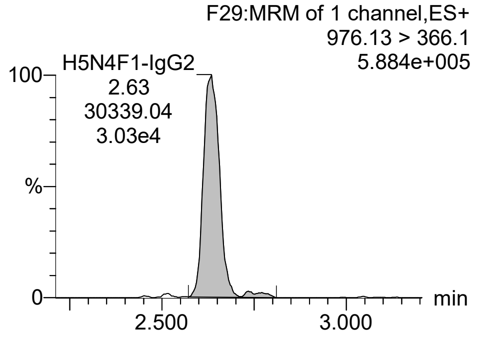

Sample Name: 20211111_L3_R2_IgG

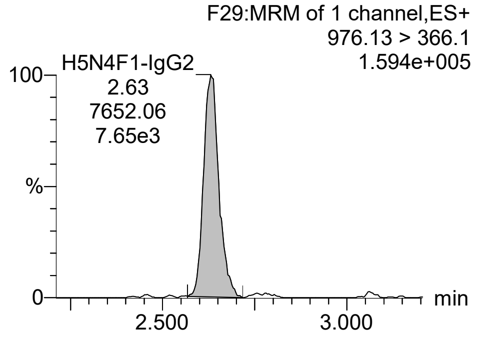

Sample Name: 20211111_L4_R2_IgG

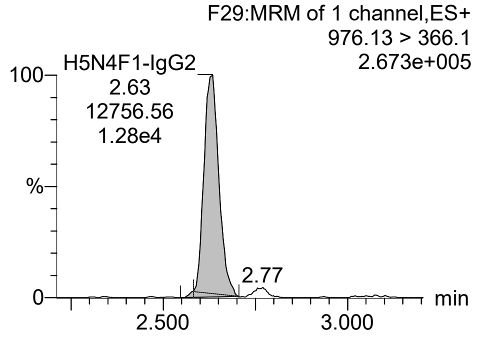

Sample Name: 20211111_H2_R2_IgG

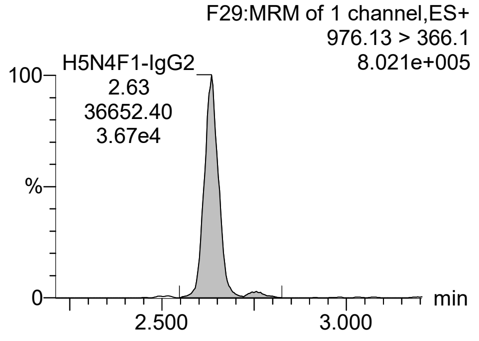

Sample Name: 20211111_A5_R2_IgG

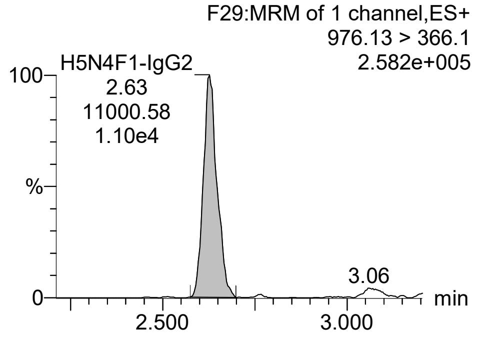

Sample Name: 20211111_A6_R2_IgG

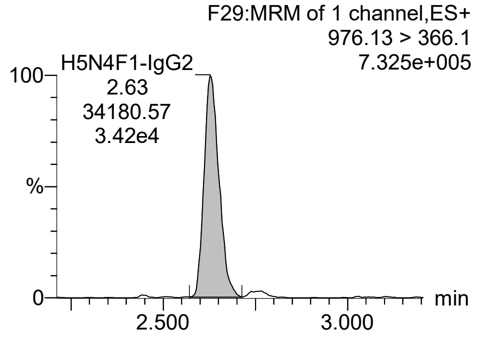

Sample Name: 20211111_QC_R10_IgG

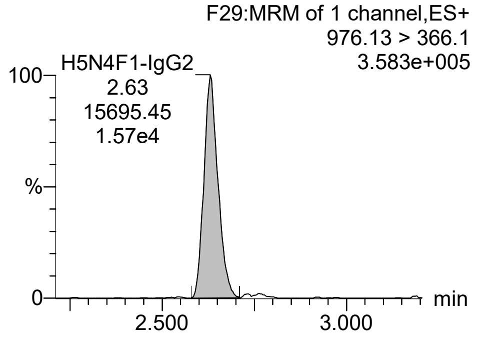

Sample Name: 20211111_L5_R2_IgG

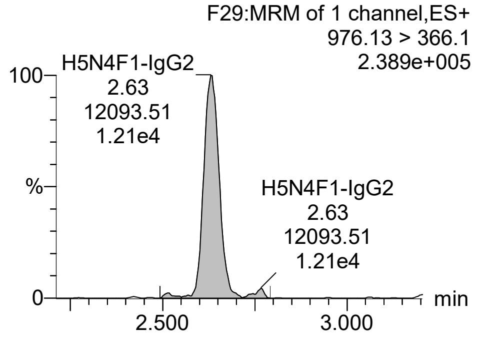

Sample Name: 20211111_L6_R2_IgG

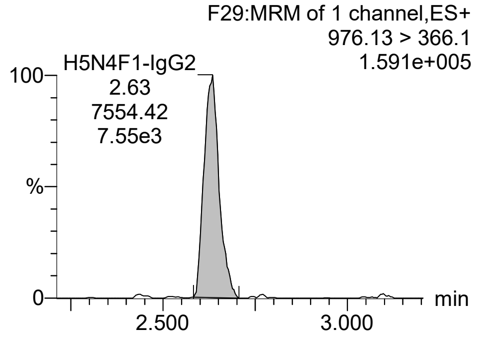

Sample Name: 20211111_H3_R2_IgG

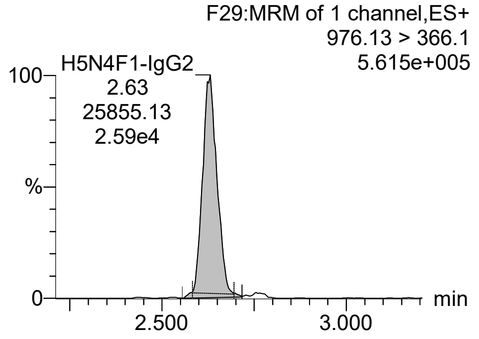

Sample Name: 20211111_A7_R2_IgG

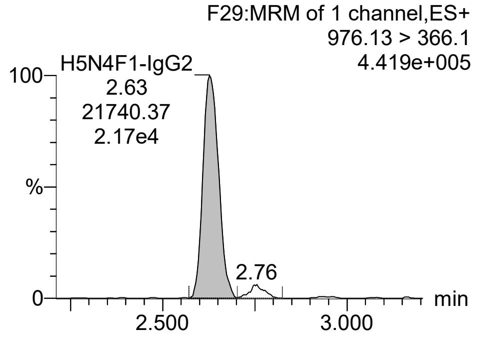

Sample Name: 20211111_A8_R2_IgG

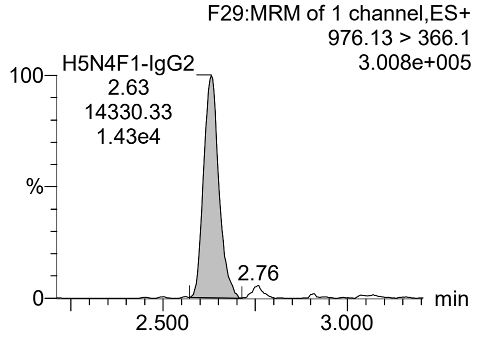

Sample Name: 20211111_L7_R2_IgG

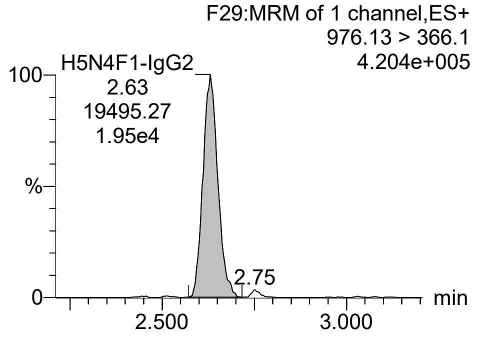

Sample Name: 20211111_L8_R2_IgG

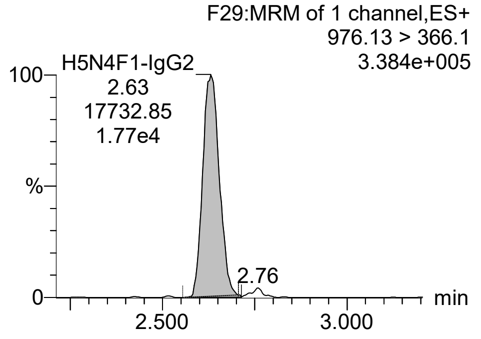

Sample Name: 20211111_H4_R2_IgG

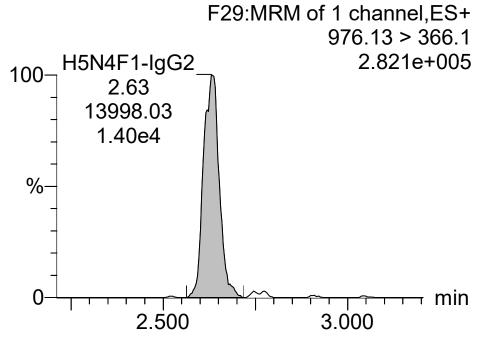

Sample Name: 20211111_A9_R2_IgG

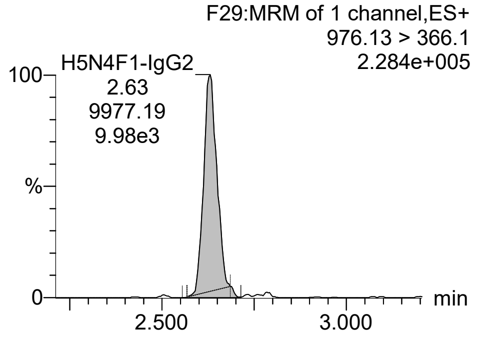

Sample Name: 20211111_A10_R2_IgG

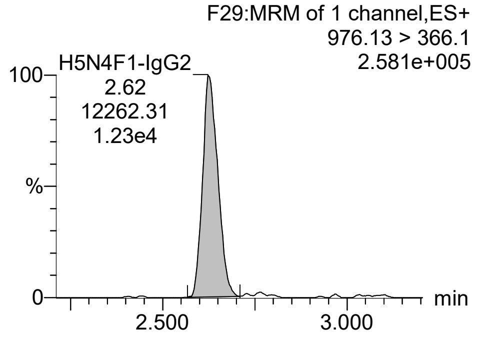

Sample Name: 20211111_L9_R2_IgG

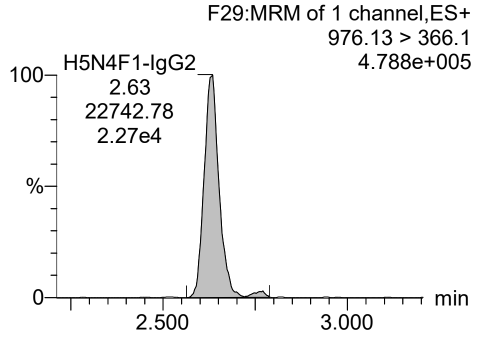

Sample Name: 20211111_L10_R2_IgG

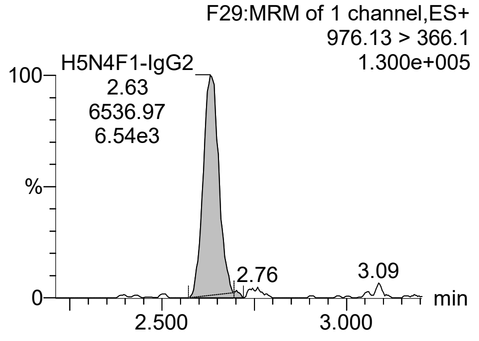

Sample Name: 20211111_QC_R11_IgG

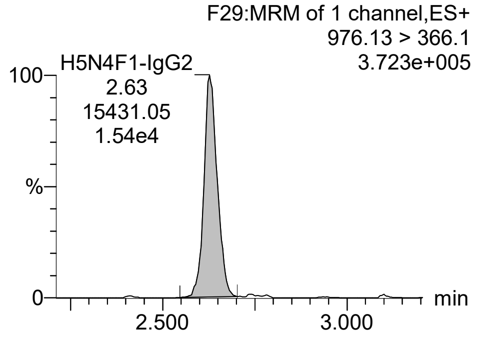

Sample Name: 20211111_H5_R2_IgG

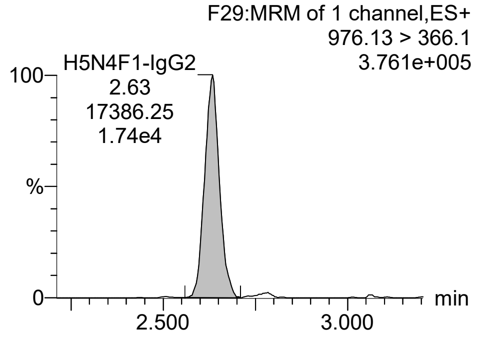

Dataset: C:\MassLynx\10.pro\20211115_clinicalsamples_IgG.qld

Last Altered: Sunday, October 02, 2022 16:02:30 台北標準時間
Printed: Friday, August 01, 2025 16:52:28 台北標準時間

Sample Name: 20211111_A11_R2_IgG

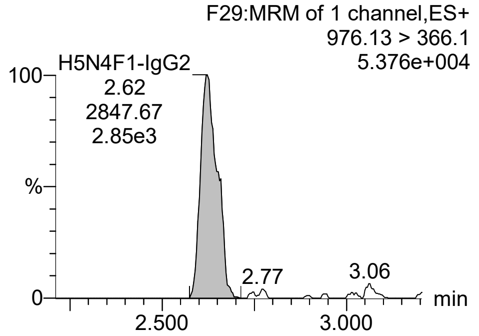

Sample Name: 20211111_A12_R2_IgG

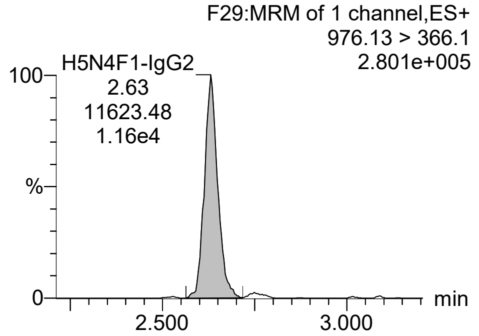

Sample Name: 20211111_L11_R2_IgG

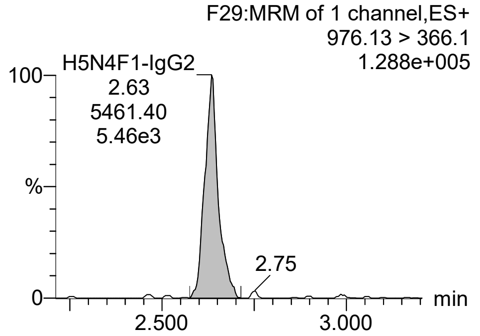

Sample Name: 20211111_L12_R2_IgG

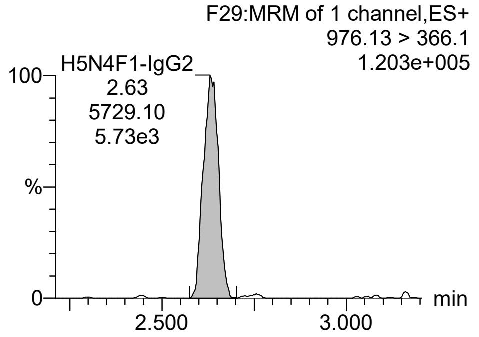

Sample Name: 20211111_H6_R2_IgG

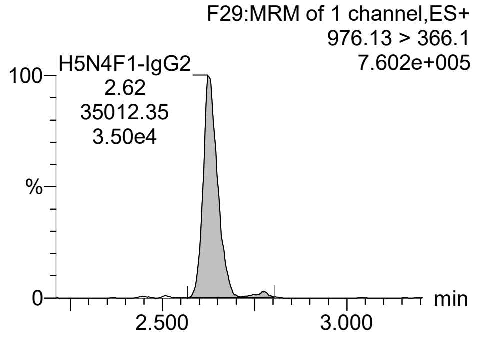

Sample Name: 20211111_A13_R2_IgG

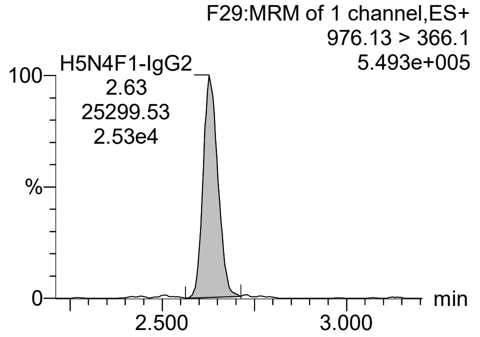

Sample Name: 20211111_A14_R2_IgG

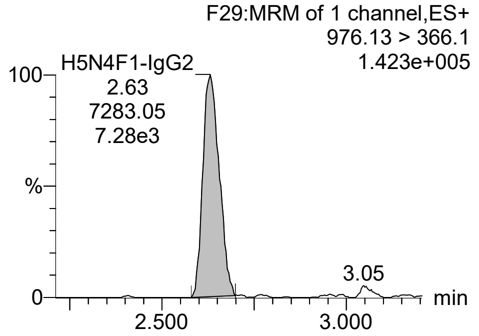

Sample Name: 20211111_L13_R2_IgG

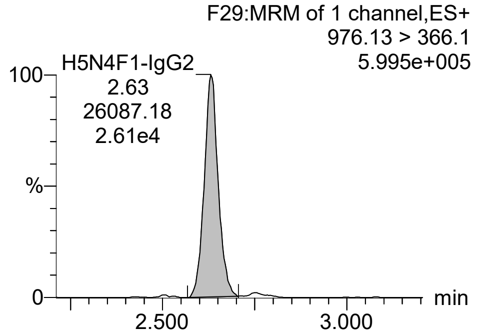

Sample Name: 20211111_L14_R2_IgG

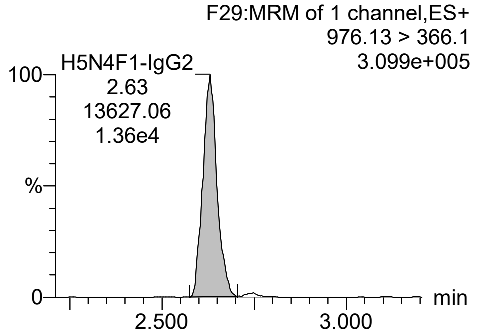

Sample Name: 20211111_H7_R2_IgG

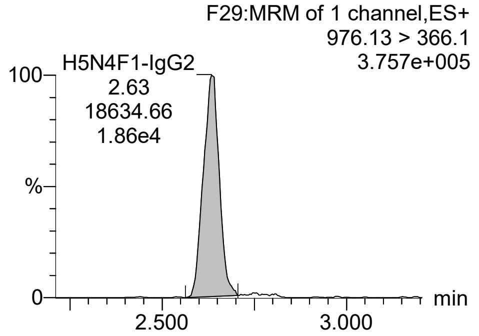

Sample Name: 20211111_A15_R2_IgG

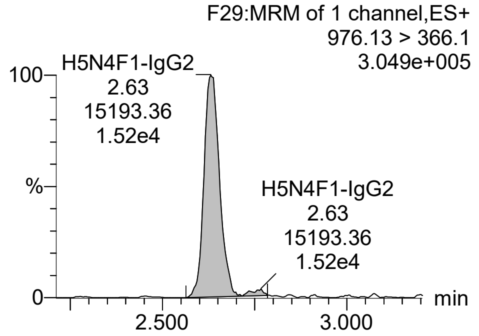

Sample Name: 20211111_QC_R12_IgG

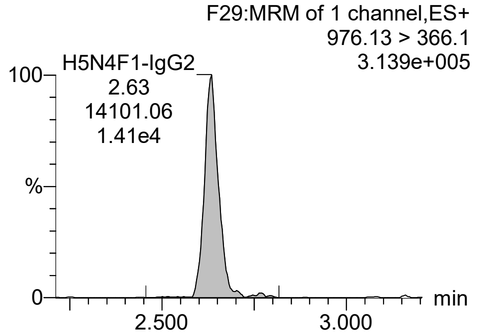

Sample Name: 20211111_A16_R2_IgG

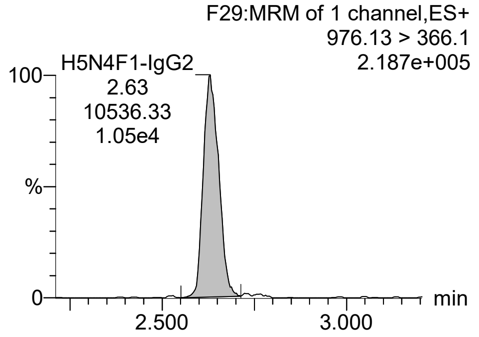

Sample Name: 20211111_L15_R2_IgG

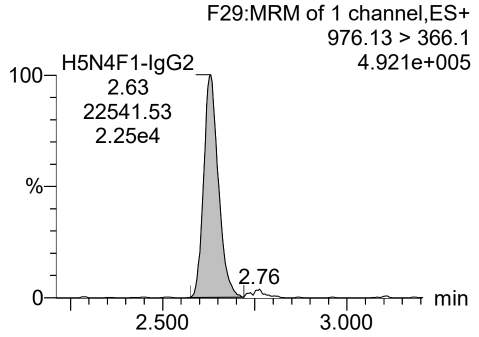

Sample Name: 20211111_L16_R2_IgG

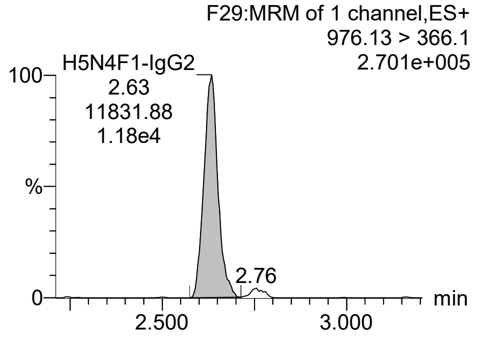

Dataset: C:\MassLynx\10.pro\20211115_clinicalsamples_IgG.qld

Last Altered: Sunday, October 02, 2022 16:02:30 台北標準時間
Printed: Friday, August 01, 2025 16:52:28 台北標準時間

Sample Name: 20211111_H8_R2_IgG

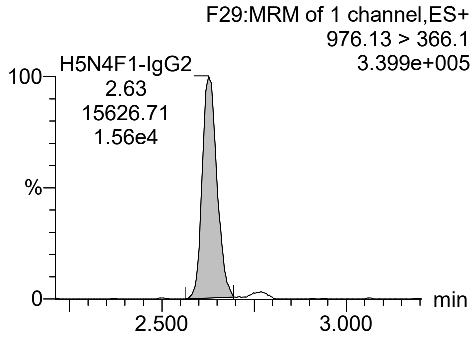

Sample Name: 20211111_A17_R2_IgG

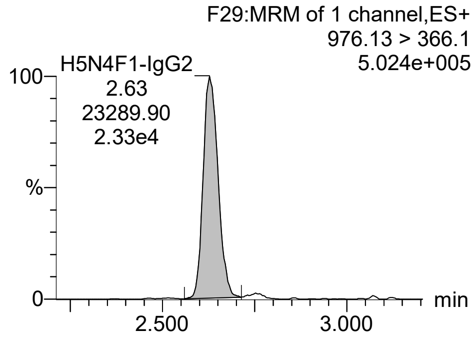

Sample Name: 20211111_A18_R2_IgG

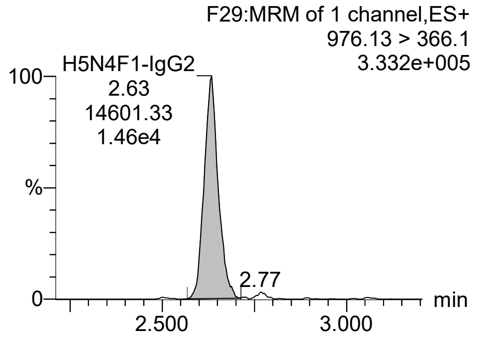

Sample Name: 20211111_L17_R2_IgG

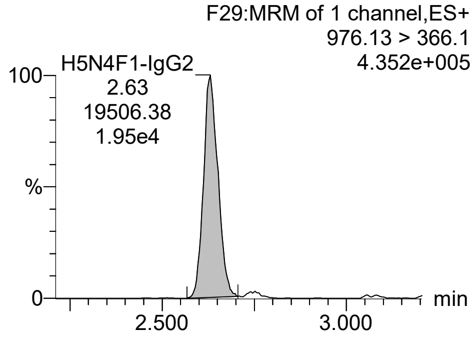

Sample Name: 20211111_L18_R2_IgG

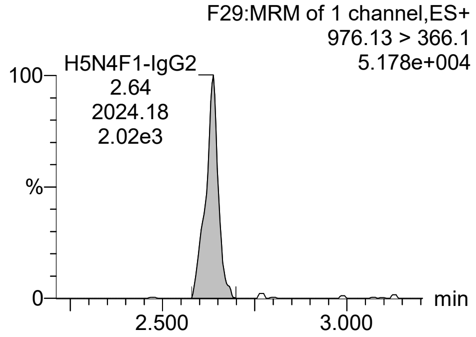

Sample Name: 20211111_H9_R2_IgG

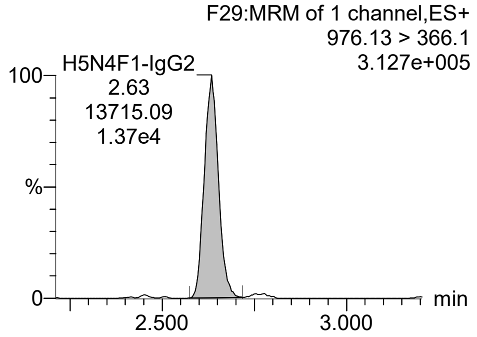

Sample Name: 20211111_A19_R2_IgG

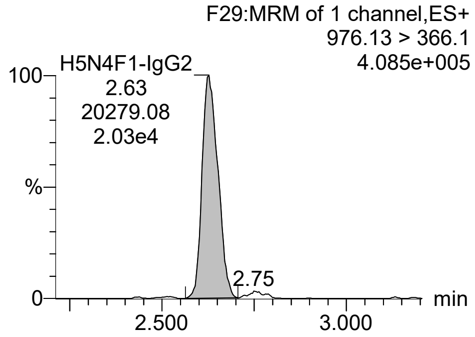

Sample Name: 20211111_A20_R2_IgG

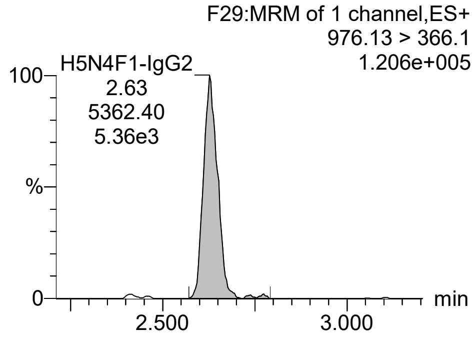

Sample Name: 20211111_L19_R2_IgG

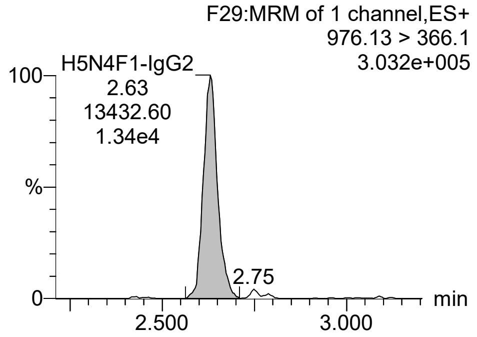

Sample Name: 20211111_QC_R13_IgG

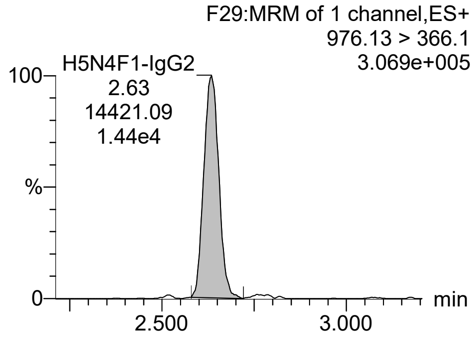

Sample Name: 20211111_L20_R2_IgG

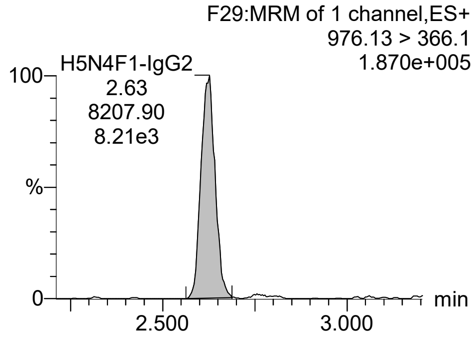

Sample Name: 20211111_H10_R2_IgG

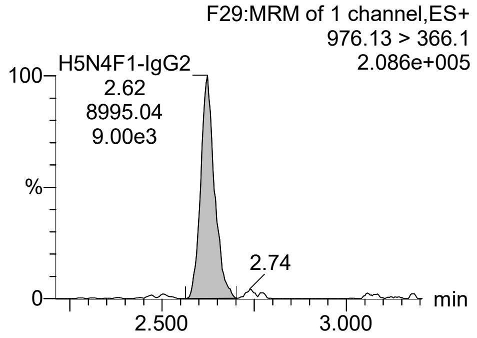

Sample Name: 20211111_A21_R2_IgG

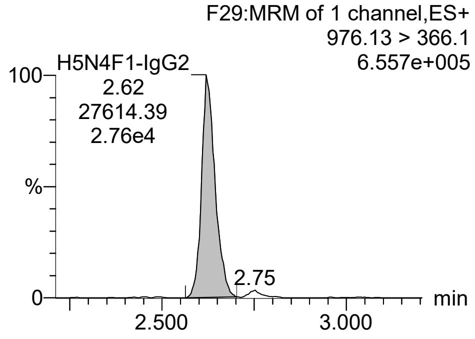

Sample Name: 20211111_A22_R2_IgG

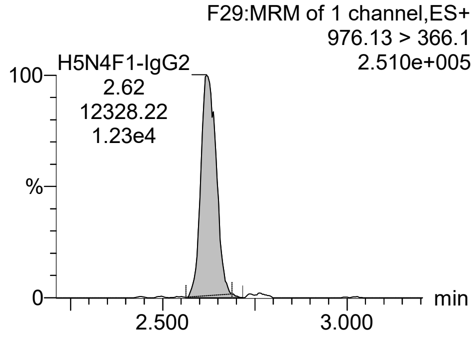

Sample Name: 20211111_L21_R2_IgG

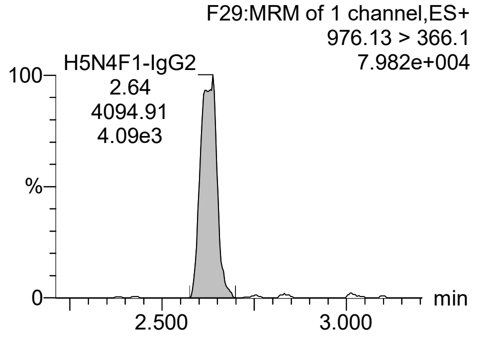

Dataset: C:\MassLynx\10.pro\20211115_clinicalsamples_IgG.qld

Last Altered: Sunday, October 02, 2022 16:02:30 台北標準時間
Printed: Friday, August 01, 2025 16:52:28 台北標準時間

Sample Name: 20211111_L22_R2_IgG

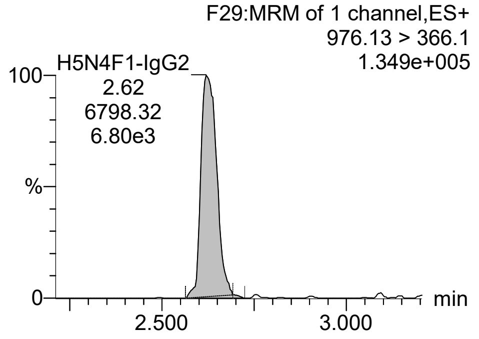

Sample Name: 20211111_H11_R2_IgG

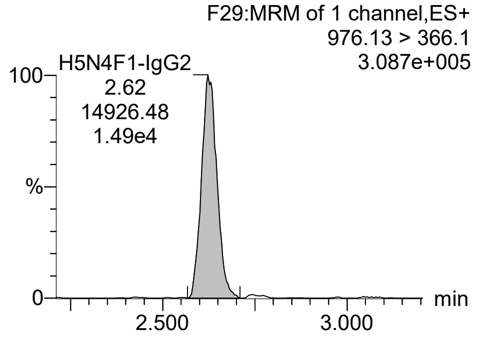

Sample Name: 20211111_A23_R2_IgG

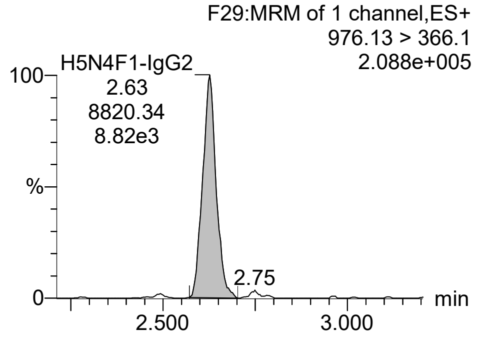

Sample Name: 20211111_A24_R2_IgG

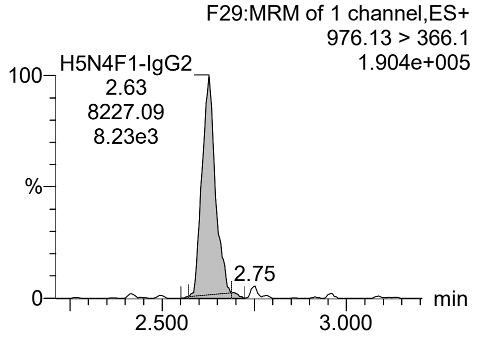

Sample Name: 20211111_L23_R2_IgG

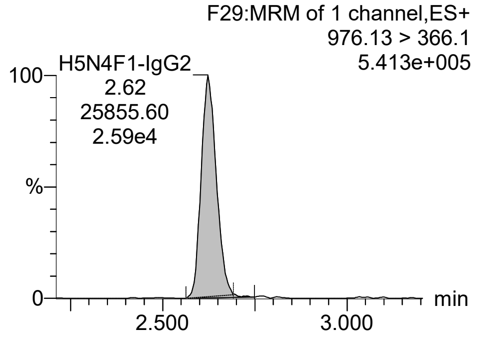

Sample Name: 20211111_L24_R2_IgG

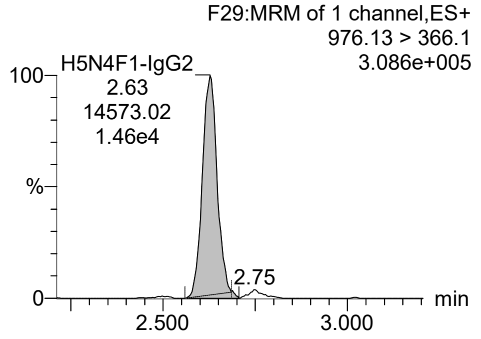

Sample Name: 20211111_H12_R2_IgG

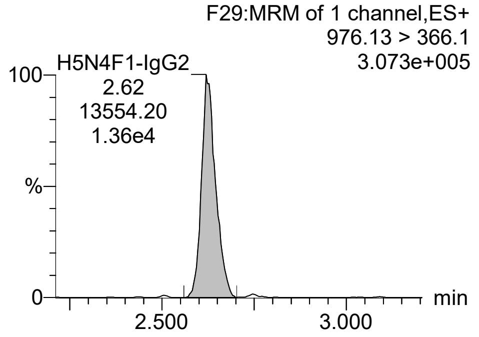

Sample Name: 20211111_QC_R14_IgG

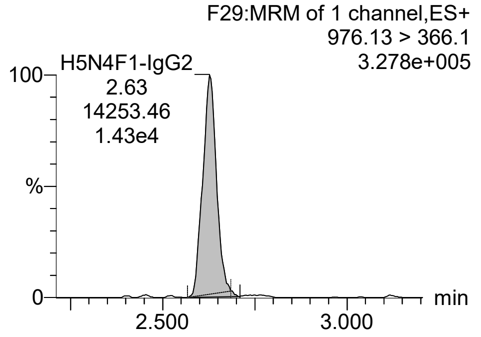

Sample Name: 20211111_A25_R2_IgG

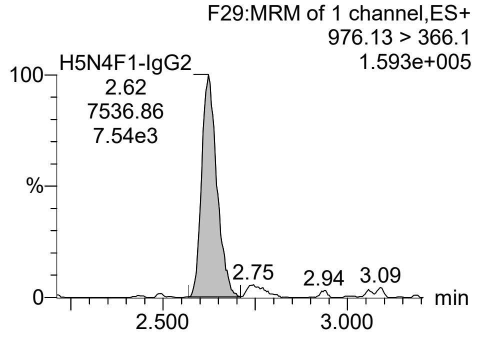

Sample Name: 20211111_A26_R2_IgG

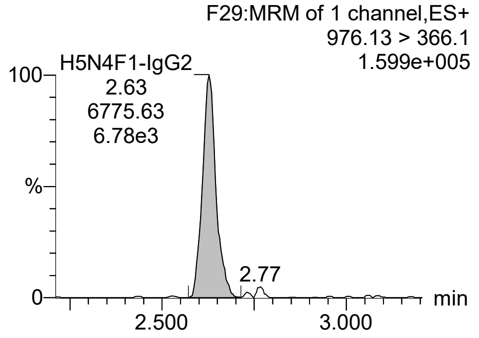

Sample Name: 20211111_L25_R2_IgG

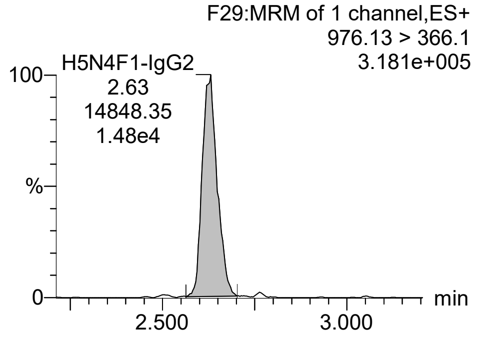

Sample Name: 20211111_L26_R2_IgG

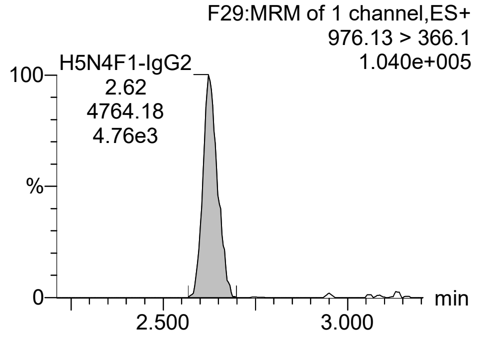

Sample Name: 20211111_H13_R2_IgG

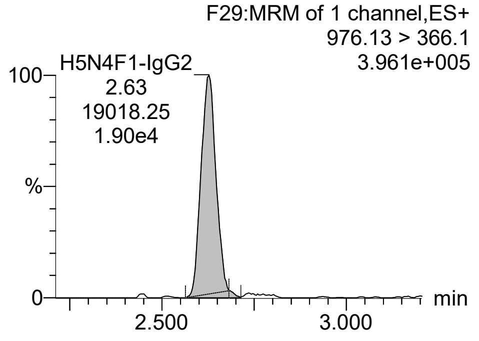

Sample Name: 20211111_A27_R2_IgG

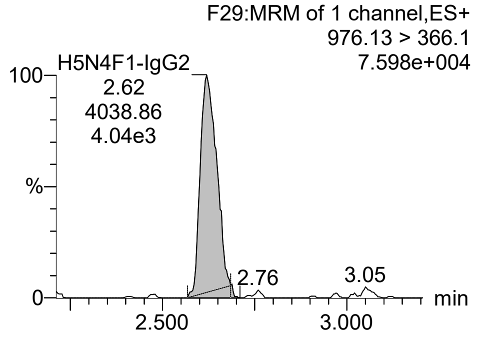

Sample Name: 20211111_A28_R2_IgG

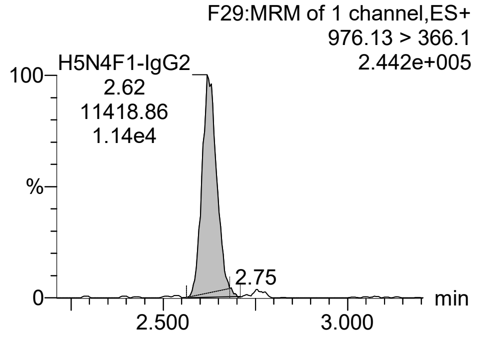

Dataset: C:\MassLynx\10.pro\20211115_clinicalsamples_IgG.qld

Last Altered: Sunday, October 02, 2022 16:02:30 台北標準時間
Printed: Friday, August 01, 2025 16:52:28 台北標準時間

Sample Name: 20211111_L27_R2_IgG

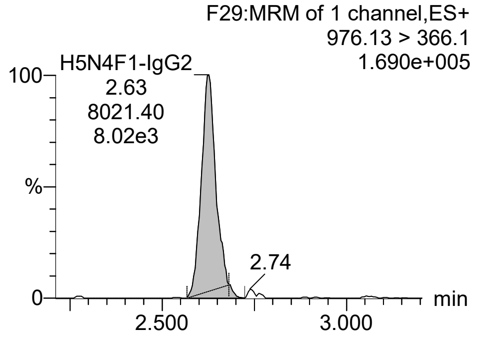

Sample Name: 20211111_L28_R2_IgG

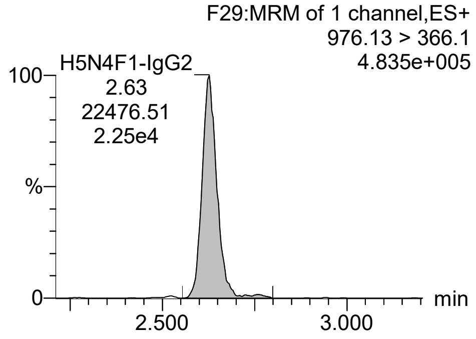

Sample Name: 20211111_H14_R2_IgG

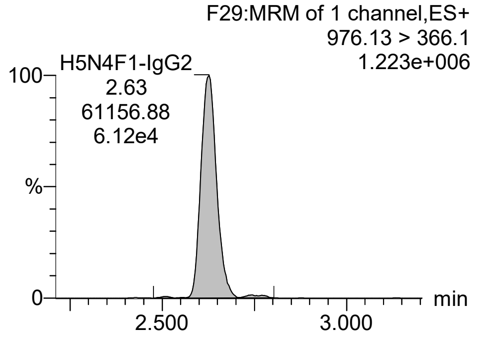

Sample Name: 20211111_A29_R2_IgG

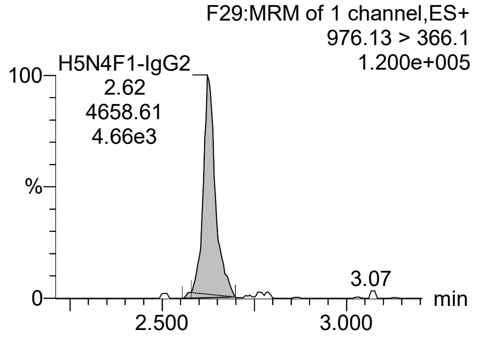

Sample Name: 20211111_A30_R2_IgG

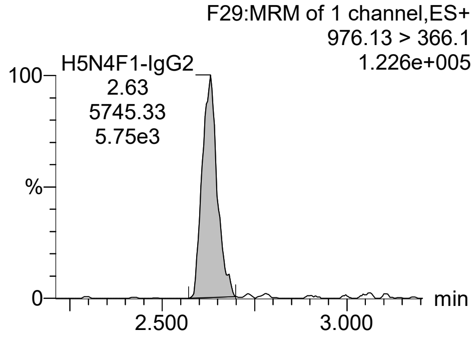

Sample Name: 20211111_QC_R15_IgG

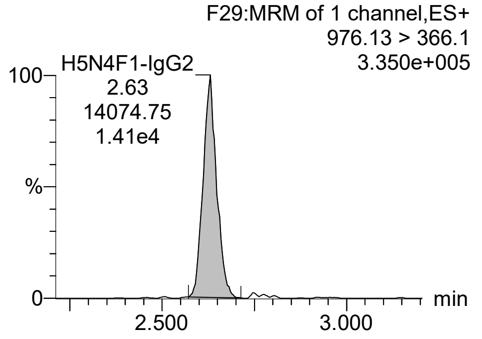

Sample Name: 20211111_L29_R2_IgG

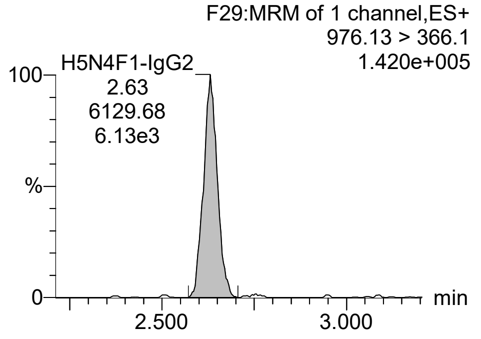

Sample Name: 20211111_L30_R2_IgG

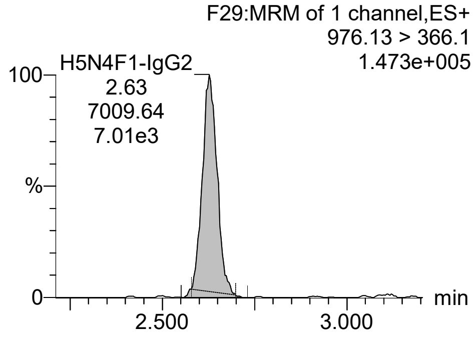

Sample Name: 20211111_H15_R2_IgG

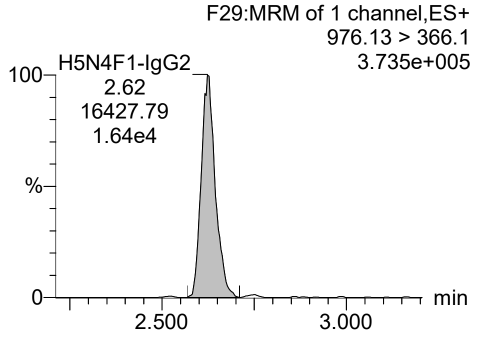

Sample Name: 20211111_A31_R2_IgG

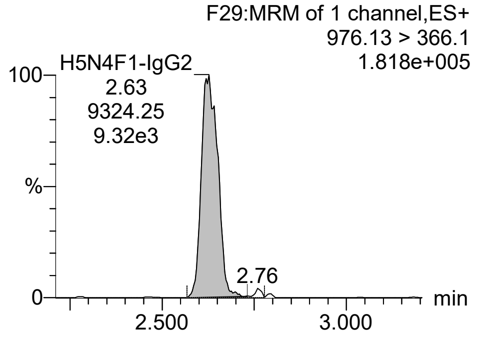

Sample Name: 20211111_A32_R2_IgG

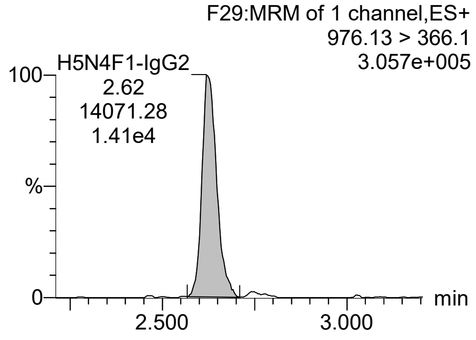

Sample Name: 20211111_L31_R2_IgG

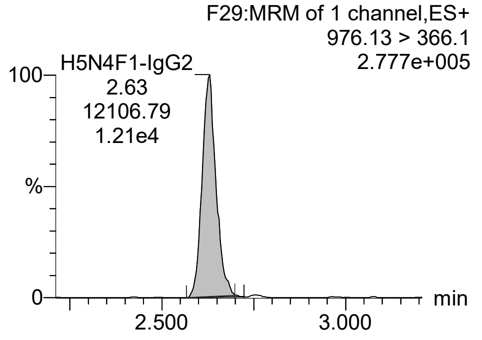

Sample Name: 20211111_L32_R2_IgG

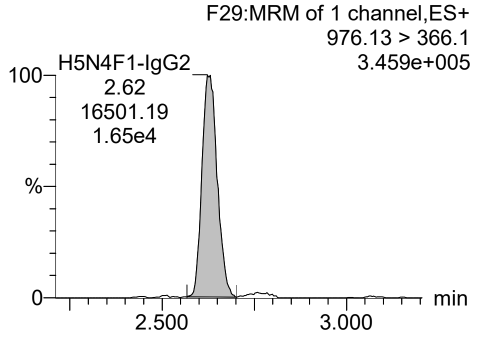

Sample Name: 20211111_H16_R2_IgG

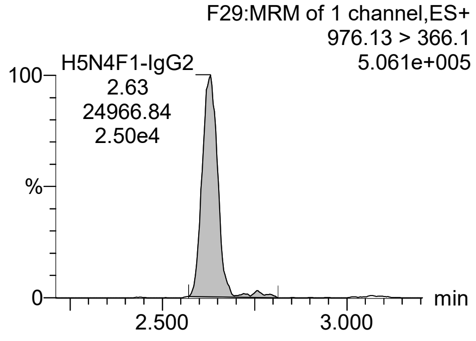

Sample Name: 20211111_A33_R2_IgG

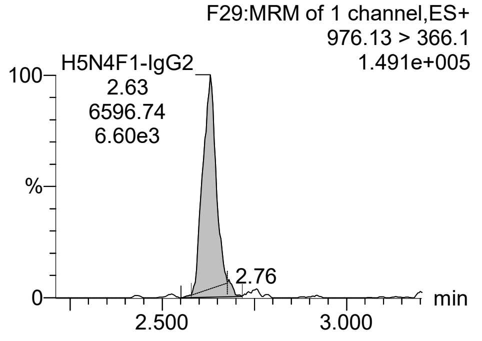

Dataset: C:\MassLynx\10.pro\20211115_clinicalsamples_IgG.qld

Last Altered: Sunday, October 02, 2022 16:02:30 台北標準時間
Printed: Friday, August 01, 2025 16:52:28 台北標準時間

Sample Name: 20211111_A34_R2_IgG

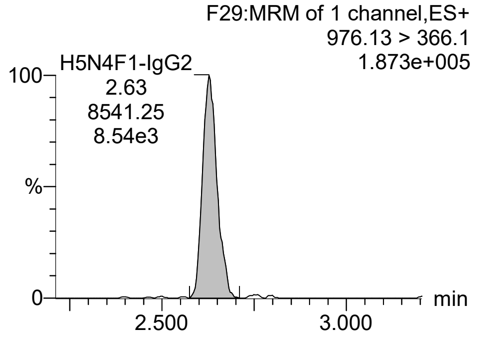

Sample Name: 20211111_H17_R2_IgG

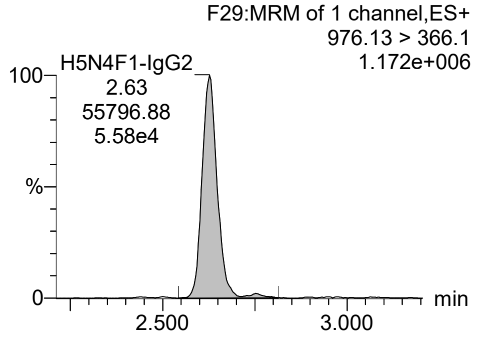

Sample Name: 20211111_A35_R2_IgG

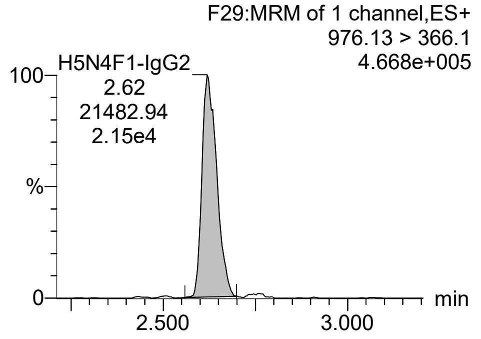

Sample Name: 20211111_QC_R16_IgG

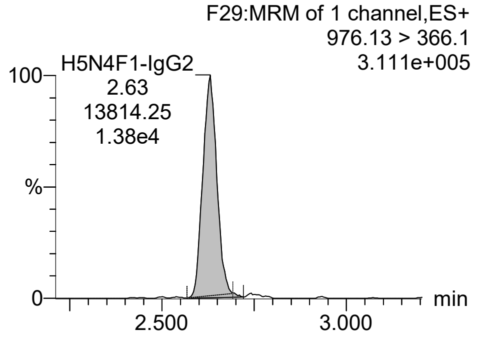

Sample Name: 20211111_A36_R2_IgG

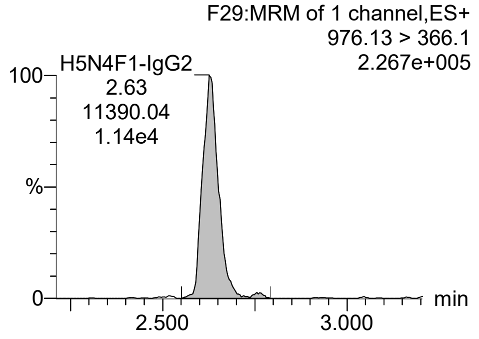

Sample Name: 20211111_H18_R2_IgG

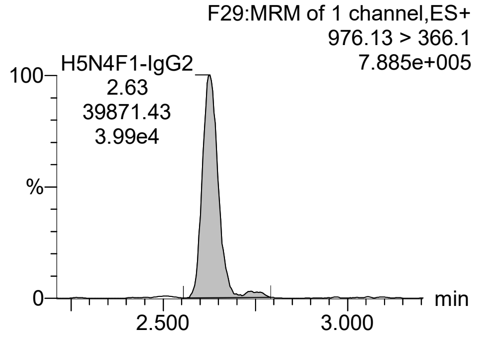

Sample Name: 20211111_A37_R2_IgG

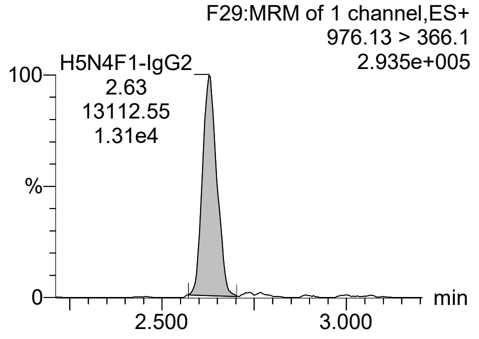

Sample Name: 20211111_A38_R2_IgG

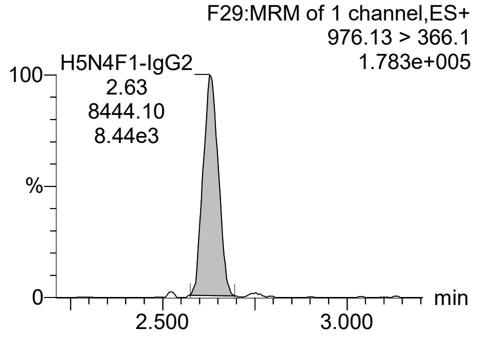

Sample Name: 20211111_H19_R2_IgG

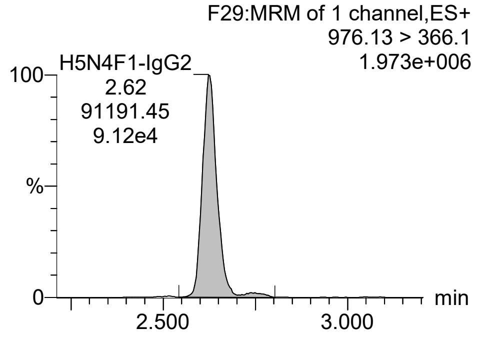

Sample Name: 20211111_A39_R2_IgG

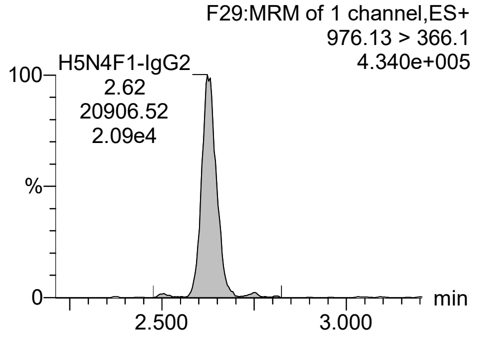

Sample Name: 20211111_A40_R2_IgG

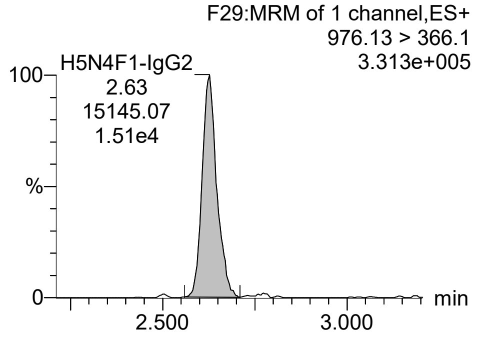

Sample Name: 20211111_H20_R2_IgG

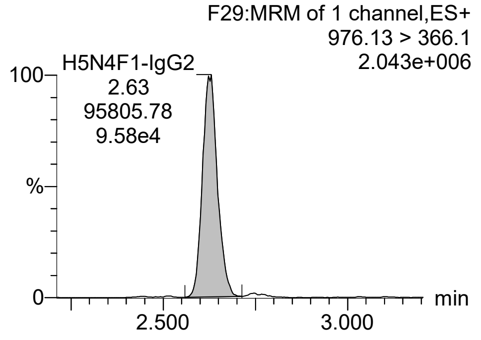

Sample Name: 20211111_A41_R2_IgG

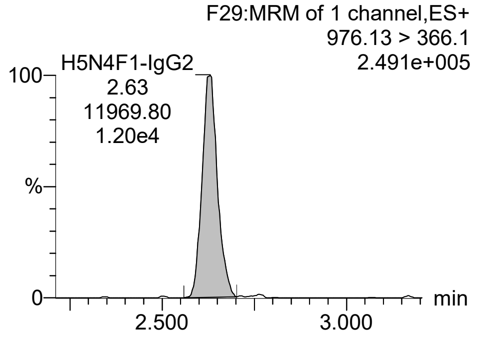

Sample Name: 20211111_A42_R2_IgG

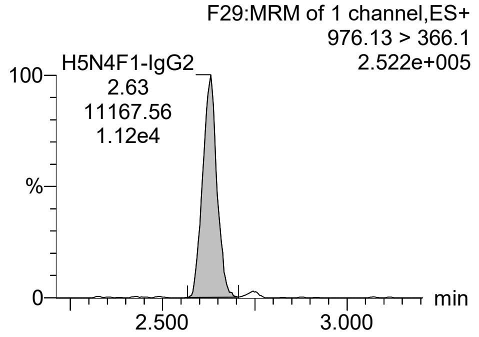

Sample Name: 20211111_A43_R2_IgG

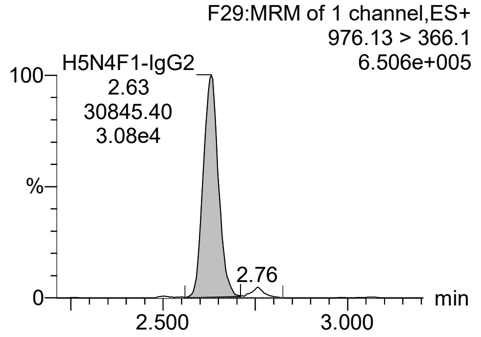

Sample Name: 20211111_A44_R2_IgG

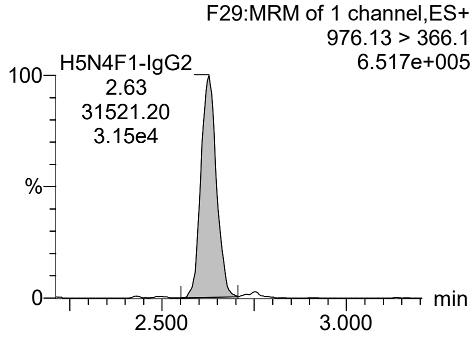

Sample Name: 20211111_QC_R17_IgG

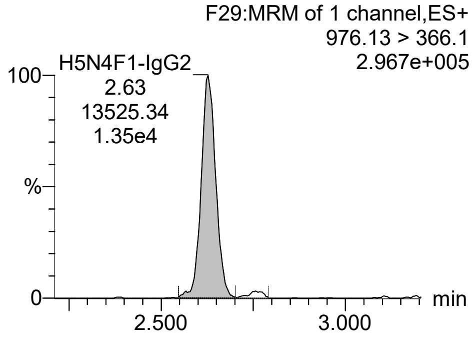

Dataset: C:\MassLynx\10.pro\20211115_clinicalsamples_IgG.qld

Last Altered: Sunday, October 02, 2022 16:02:30 台北標準時間
Printed: Friday, August 01, 2025 16:52:28 台北標準時間

	#	Name	RT	Area	IS Area	Response	Prima...
1	1	20211111_QC_R1_IgG	2.63	31044.3	64968.0	0.478	bb
2	2	20211111_A1_R1_IgG	2.63	32961.8	59059.2	0.558	bb
3	3	20211111_A2_R1_IgG	2.63	37495.8	66621.2	0.563	bb
4	4	20211111_L1_R1_IgG	2.63	11617.6	64201.0	0.181	bb
5	5	20211111_L2_R1_IgG	2.63	32338.3	46950.2	0.689	bb
6	6	20211111_H1_R1_IgG	2.63	8907.2	38648.2	0.230	bb
7	7	20211111_A3_R1_IgG	2.63	570.0	2603.7	0.219	MM
8	8	20211111_A4_R1_IgG	2.64	42953.6	69960.4	0.614	bb
9	9	20211111_L3_R1_IgG	2.64	9934.9	38864.7	0.256	bb
10	10	20211111_L4_R1_IgG	2.64	17007.6	43380.5	0.392	bb
11	11	20211111_H2_R1_IgG	2.63	55776.3	71349.2	0.782	bb
12	12	20211111_A5_R1_IgG	2.63	13297.9	65569.7	0.203	bb
13	13	20211111_A6_R1_IgG	2.63	41855.9	53120.7	0.788	bb
14	14	20211111_QC_R2_IgG	2.63	37546.4	81034.1	0.463	bb
15	15	20211111_L5_R1_IgG	2.63	15387.5	50994.6	0.302	bb
16	16	20211111_L6_R1_IgG	2.63	9346.5	58058.0	0.161	bb
17	17	20211111_H3_R1_IgG	2.63	33159.2	64028.6	0.518	MM
18	18	20211111_A7_R1_IgG	2.63	33437.1	82996.1	0.403	bb
19	19	20211111_A8_R1_IgG	2.63	19766.9	77731.7	0.254	MM
20	20	20211111_L7_R1_IgG	2.63	19555.1	38827.3	0.504	bb
21	21	20211111_L8_R1_IgG	2.64	20610.6	44220.1	0.466	bb
22	22	20211111_H4_R1_IgG	2.63	19229.5	65826.3	0.292	bb
23	23	20211111_A9_R1_IgG	2.63	14387.1	60771.1	0.237	bb
24	24	20211111_A10_R1_IgG	2.63	14999.0	48578.7	0.309	bb
25	25	20211111_L9_R1_IgG	2.63	24903.8	50830.7	0.490	bb
26	26	20211111_L10_R1_IgG	2.63	8072.5	38034.2	0.212	bb
27	27	20211111_QC_R3_IgG	2.63	29796.0	67025.5	0.445	bb
28	28	20211111_H5_R1_IgG	2.63	22094.2	44544.9	0.496	bb
29	29	20211111_A11_R1_IgG	2.63	3591.7	66408.5	0.054	bb
30	30	20211111_A12_R1_IgG	2.63	14428.5	46624.5	0.309	MM
31	31	20211111_L11_R1_IgG	2.64	7178.6	33425.1	0.215	MM
32	32	20211111_L12_R1_IgG	2.63	4926.5	41178.0	0.120	MM
33	33	20211111_H6_R1_IgG	2.63	59010.6	58593.4	1.007	bb
34	34	20211111_A13_R1_IgG	2.63	33958.6	54133.0	0.627	bb
35	35	20211111_A14_R1_IgG	2.63	9997.9	56467.3	0.177	bb
36	36	20211111_L13_R1_IgG	2.63	32882.4	36024.4	0.913	bb
37	37	20211111_L14_R1_IgG	2.63	14284.5	37181.6	0.384	bb
38	38	20211111_H7_R1_IgG	2.63	26614.5	42850.6	0.621	bb
39	39	20211111_A15_R1_IgG	2.64	20145.0	62015.1	0.325	MM
40	40	20211111_QC_R4_IgG	2.63	26725.6	58572.5	0.456	bb
41	41	20211111_A16_R1_IgG	2.63	19361.1	78283.8	0.247	bb
42	42	20211111_L15_R1_IgG	2.63	31452.0	44158.2	0.712	bb
43	43	20211111_L16_R1_IgG	2.63	18663.0	55150.8	0.338	bb
44	44	20211111_H8_R1_IgG	2.63	17235.3	42965.2	0.401	bb
45	45	20211111_A17_R1_IgG	2.63	27183.3	39717.7	0.684	bb
46	46	20211111_A18_R1_IgG	2.63	18315.1	44675.4	0.410	bb
47	47	20211111_L17_R1_IgG	2.63	19959.8	37854.1	0.527	bb
48	48	20211111_L18_R1_IgG	2.62	3619.9	50716.9	0.071	MM
49	49	20211111_H9_R1_IgG	2.63	16574.1	36276.0	0.457	bb
50	50	20211111_A19_R1_IgG	2.63	24705.2	42862.5	0.576	bb
51	51	20211111_A20_R1_IgG	2.63	6554.7	36542.7	0.179	bb
52	52	20211111_L19_R1_IgG	2.63	21249.8	53892.8	0.394	bb
53	53	20211111_QC_R5_IgG	2.63	26032.5	56513.2	0.461	bb
54	54	20211111_L20_R1_IgG	2.63	17390.3	56499.9	0.308	bb
55	55	20211111_H10_R1_IgG	2.63	21156.4	68120.5	0.311	bb
56	56	20211111_A21_R1_IgG	2.63	58596.7	63699.8	0.920	bb

Dataset: C:\MassLynx\10.pro\20211115_clinicalsamples_IgG.qld

Last Altered: Sunday, October 02, 2022 16:02:30 台北標準時間
Printed: Friday, August 01, 2025 16:52:28 台北標準時間

	# Name	RT	Area	IS Area	Response	Prima...
57	57 20211111_A22_R1_IgG	2.63	27664.0	52222.1	0.530	bb
58	58 20211111_L21_R1_IgG	2.63	9565.8	54372.5	0.176	MM
59	59 20211111_L22_R1_IgG	2.63	10280.6	41353.1	0.249	bb
60	60 20211111_H11_R1_IgG	2.63	32951.0	65579.1	0.502	bb
61	61 20211111_A23_R1_IgG	2.63	18831.7	64131.1	0.294	bb
62	62 20211111_A24_R1_IgG	2.63	11327.2	38724.8	0.293	bb
63	63 20211111_L23_R1_IgG	2.63	46195.0	64245.2	0.719	bb
64	64 20211111_L24_R1_IgG	2.63	21752.6	45298.0	0.480	bb
65	65 20211111_H12_R1_IgG	2.63	17002.1	44679.3	0.381	bb
66	66 20211111_QC_R6_IgG	2.63	22533.6	48165.0	0.468	bb
67	67 20211111_A25_R1_IgG	2.63	14435.4	57169.9	0.253	bb
68	68 20211111_A26_R1_IgG	2.63	9068.3	34254.0	0.265	MM
69	69 20211111_L25_R1_IgG	2.63	29309.2	67491.5	0.434	bb
70	70 20211111_L26_R1_IgG	2.63	7777.5	55934.1	0.139	bb
71	71 20211111_H13_R1_IgG	2.63	30552.0	48767.3	0.626	bb
72	72 20211111_A27_R1_IgG	2.63	7937.0	57269.7	0.139	bb
73	73 20211111_A28_R1_IgG	2.63	18437.6	49231.1	0.375	bb
74	74 20211111_L27_R1_IgG	2.63	12804.3	60014.5	0.213	bb
75	75 20211111_L28_R1_IgG	2.63	29338.1	35062.6	0.837	bb
76	76 20211111_H14_R1_IgG	2.63	91939.0	62371.9	1.474	bb
77	77 20211111_A29_R1_IgG	2.63	7041.9	43858.2	0.161	bb
78	78 20211111_A30_R1_IgG	2.63	11761.5	62500.4	0.188	MM
79	79 20211111_QC_R7_IgG	2.63	19666.0	43946.9	0.447	bb
80	80 20211111_L29_R1_IgG	2.63	7242.3	38728.9	0.187	bb
81	81 20211111_L30_R1_IgG	2.63	9633.6	42613.2	0.226	MM
82	82 20211111_H15_R1_IgG	2.63	19544.7	39874.6	0.490	bb
83	83 20211111_A31_R1_IgG	2.63	13396.6	53414.3	0.251	bb
84	84 20211111_A32_R1_IgG	2.63	17955.3	41844.6	0.429	bb
85	85 20211111_L31_R1_IgG	2.63	15224.2	40553.7	0.375	bb
86	86 20211111_L32_R1_IgG	2.63	20938.7	49618.7	0.422	bb
87	87 20211111_H16_R1_IgG	2.63	39159.6	60170.9	0.651	bb
88	88 20211111_A33_R1_IgG	2.63	10097.9	48588.2	0.208	bb
89	89 20211111_A34_R1_IgG	2.63	11545.0	43288.0	0.267	bb
90	90 20211111_H17_R1_IgG	2.63	63540.3	47104.5	1.349	bb
91	91 20211111_A35_R1_IgG	2.63	28125.8	41025.9	0.686	bb
92	92 20211111_QC_R8_IgG	2.63	15518.6	40546.3	0.383	bb
93	93 20211111_A36_R1_IgG	2.63	12975.4	44504.4	0.292	bb
94	94 20211111_H18_R1_IgG	2.63	41927.4	34162.2	1.227	bb
95	95 20211111_A37_R1_IgG	2.62	13538.1	39760.0	0.340	bb
96	96 20211111_A38_R1_IgG	2.63	9740.3	43982.6	0.221	bb
97	97 20211111_H19_R1_IgG	2.63	101193.7	45314.8	2.233	bb
98	98 20211111_A39_R1_IgG	2.63	21507.0	39388.0	0.546	bb
99	99 20211111_A40_R1_IgG	2.64	14624.4	38726.4	0.378	bb
100	1... 20211111_H20_R1_IgG	2.63	105353.2	37814.4	2.786	bb
101	1... 20211111_A41_R1_IgG	2.64	12051.1	48930.3	0.246	bb
102	1... 20211111_A42_R1_IgG	2.64	12658.0	40406.8	0.313	bb
103	1... 20211111_A43_R1_IgG	2.63	41655.0	44421.5	0.938	bb
104	1... 20211111_A44_R1_IgG	2.63	46951.6	53128.2	0.884	bb
105	1... 20211111_QC_R9_IgG	2.63	16321.4	37049.0	0.441	bb
106	1... 20211111_A1_R2_IgG	2.63	23719.5	46499.4	0.510	bb
107	1... 20211111_A2_R2_IgG	2.63	28060.8	57260.7	0.490	bb
108	1... 20211111_L1_R2_IgG	2.63	7746.0	48062.9	0.161	MM
109	1... 20211111_L2_R2_IgG	2.63	28766.7	38745.2	0.742	bb
110	1... 20211111_H1_R2_IgG	2.63	10485.3	44273.5	0.237	bb
111	1... 20211111_A3_R2_IgG	2.64	1416.8	4755.5	0.298	MM
112	1... 20211111_A4_R2_IgG	2.63	30339.0	50546.7	0.600	bb

Dataset: C:\MassLynx\10.pro\20211115_clinicalsamples_IgG.qld

Last Altered: Sunday, October 02, 2022 16:02:30 台北標準時間
Printed: Friday, August 01, 2025 16:52:28 台北標準時間

	# Name	RT	Area	IS Area	Response	Prima...
113	1... 20211111_L3_R2_IgG	2.63	7652.1	34825.8	0.220	bb
114	1... 20211111_L4_R2_IgG	2.63	12756.6	39107.7	0.326	MM
115	1... 20211111_H2_R2_IgG	2.63	36652.4	49750.0	0.737	bb
116	1... 20211111_A5_R2_IgG	2.63	11000.6	52536.2	0.209	bb
117	1... 20211111_A6_R2_IgG	2.63	34180.6	42050.0	0.813	bb
118	1... 20211111_QC_R10_IgG	2.63	15695.5	35350.8	0.444	bb
119	1... 20211111_L5_R2_IgG	2.63	12093.5	38039.3	0.318	bb
120	1... 20211111_L6_R2_IgG	2.63	7554.4	44907.4	0.168	bb
121	1... 20211111_H3_R2_IgG	2.63	25855.1	47729.1	0.542	MM
122	1... 20211111_A7_R2_IgG	2.63	21740.4	57347.1	0.379	MM
123	1... 20211111_A8_R2_IgG	2.63	14330.3	52397.8	0.273	bb
124	1... 20211111_L7_R2_IgG	2.63	19495.3	36318.4	0.537	bb
125	1... 20211111_L8_R2_IgG	2.63	17732.9	38131.9	0.465	MM
126	1... 20211111_H4_R2_IgG	2.63	13998.0	46849.6	0.299	bb
127	1... 20211111_A9_R2_IgG	2.63	9977.2	47759.6	0.209	MM
128	1... 20211111_A10_R2_IgG	2.62	12262.3	41366.2	0.296	bb
129	1... 20211111_L9_R2_IgG	2.63	22742.8	43052.1	0.528	bb
130	1... 20211111_L10_R2_IgG	2.63	6537.0	31487.4	0.208	MM
131	1... 20211111_QC_R11_IgG	2.63	15431.1	37804.6	0.408	bb
132	1... 20211111_H5_R2_IgG	2.63	17386.2	40380.0	0.431	bb
133	1... 20211111_A11_R2_IgG	2.62	2847.7	49533.3	0.057	bb
134	1... 20211111_A12_R2_IgG	2.63	11623.5	40226.3	0.289	bb
135	1... 20211111_L11_R2_IgG	2.63	5461.4	28884.0	0.189	bb
136	1... 20211111_L12_R2_IgG	2.63	5729.1	34351.3	0.167	bb
137	1... 20211111_H6_R2_IgG	2.62	35012.3	40466.6	0.865	bb
138	1... 20211111_A13_R2_IgG	2.63	25299.5	42306.7	0.598	bb
139	1... 20211111_A14_R2_IgG	2.63	7283.1	44904.0	0.162	bb
140	1... 20211111_L13_R2_IgG	2.63	26087.2	31913.0	0.817	bb
141	1... 20211111_L14_R2_IgG	2.63	13627.1	31527.4	0.432	bb
142	1... 20211111_H7_R2_IgG	2.63	18634.7	40838.2	0.456	bb
143	1... 20211111_A15_R2_IgG	2.63	15193.4	49389.7	0.308	bb
144	1... 20211111_QC_R12_IgG	2.63	14101.1	35168.7	0.401	bb
145	1... 20211111_A16_R2_IgG	2.63	10536.3	47440.5	0.222	bb
146	1... 20211111_L15_R2_IgG	2.63	22541.5	33145.9	0.680	bb
147	1... 20211111_L16_R2_IgG	2.63	11831.9	37596.6	0.315	bb
148	1... 20211111_H8_R2_IgG	2.63	15626.7	43933.3	0.356	bb
149	1... 20211111_A17_R2_IgG	2.63	23289.9	43605.5	0.534	bb
150	1... 20211111_A18_R2_IgG	2.63	14601.3	37581.8	0.389	bb
151	1... 20211111_L17_R2_IgG	2.63	19506.4	33942.5	0.575	bb
152	1... 20211111_L18_R2_IgG	2.64	2024.2	37831.5	0.054	bb
153	1... 20211111_H9_R2_IgG	2.63	13715.1	30898.4	0.444	bb
154	1... 20211111_A19_R2_IgG	2.63	20279.1	39352.1	0.515	bb
155	1... 20211111_A20_R2_IgG	2.63	5362.4	34661.2	0.155	bb
156	1... 20211111_L19_R2_IgG	2.63	13432.6	39918.3	0.337	bb
157	1... 20211111_QC_R13_IgG	2.63	14421.1	36239.1	0.398	bb
158	1... 20211111_L20_R2_IgG	2.63	8207.9	32897.7	0.249	bb
159	1... 20211111_H10_R2_IgG	2.62	8995.0	31394.5	0.287	bb
160	1... 20211111_A21_R2_IgG	2.62	27614.4	34863.7	0.792	bb
161	1... 20211111_A22_R2_IgG	2.62	12328.2	30342.1	0.406	MM
162	1... 20211111_L21_R2_IgG	2.64	4094.9	33706.2	0.121	bb
163	1... 20211111_L22_R2_IgG	2.62	6798.3	31298.6	0.217	MM
164	1... 20211111_H11_R2_IgG	2.62	14926.5	38904.1	0.384	bb
165	1... 20211111_A23_R2_IgG	2.63	8820.3	32382.3	0.272	bb
166	1... 20211111_A24_R2_IgG	2.63	8227.1	30261.9	0.272	MM
167	1... 20211111_L23_R2_IgG	2.62	25855.6	43037.5	0.601	MM
168	1... 20211111_L24_R2_IgG	2.63	14573.0	37023.3	0.394	MM

Dataset: C:\MassLynx\10.pro\20211115_clinicalsamples_IgG.qld

Last Altered: Sunday, October 02, 2022 16:02:30 台北標準時間
Printed: Friday, August 01, 2025 16:52:28 台北標準時間

	#	Name	RT	Area	IS Area	Response	Prima...
169	1...	20211111_H12_R2_IgG	2.62	13554.2	39478.2	0.343	bb
170	1...	20211111_QC_R14_IgG	2.63	14253.5	34463.2	0.414	MM
171	1...	20211111_A25_R2_IgG	2.62	7536.9	39945.3	0.189	bb
172	1...	20211111_A26_R2_IgG	2.63	6775.6	29663.2	0.228	bb
173	1...	20211111_L25_R2_IgG	2.63	14848.3	38589.3	0.385	bb
174	1...	20211111_L26_R2_IgG	2.62	4764.2	38405.2	0.124	bb
175	1...	20211111_H13_R2_IgG	2.63	19018.3	37771.2	0.504	MM
176	1...	20211111_A27_R2_IgG	2.62	4038.9	42634.1	0.095	MM
177	1...	20211111_A28_R2_IgG	2.62	11418.9	33231.1	0.344	MM
178	1...	20211111_L27_R2_IgG	2.63	8021.4	41761.1	0.192	MM
179	1...	20211111_L28_R2_IgG	2.63	22476.5	26968.6	0.833	bb
180	1...	20211111_H14_R2_IgG	2.63	61156.9	49250.2	1.242	bb
181	1...	20211111_A29_R2_IgG	2.62	4658.6	37601.7	0.124	MM
182	1...	20211111_A30_R2_IgG	2.63	5745.3	40366.8	0.142	bb
183	1...	20211111_QC_R15_IgG	2.63	14074.7	35156.1	0.400	bb
184	1...	20211111_L29_R2_IgG	2.63	6129.7	33813.1	0.181	bb
185	1...	20211111_L30_R2_IgG	2.63	7009.6	33488.6	0.209	MM
186	1...	20211111_H15_R2_IgG	2.62	16427.8	38501.0	0.427	bb
187	1...	20211111_A31_R2_IgG	2.63	9324.2	37525.5	0.248	MM
188	1...	20211111_A32_R2_IgG	2.62	14071.3	35770.8	0.393	bb
189	1...	20211111_L31_R2_IgG	2.63	12106.8	33282.4	0.364	MM
190	1...	20211111_L32_R2_IgG	2.62	16501.2	42049.6	0.392	bb
191	1...	20211111_H16_R2_IgG	2.63	24966.8	45947.6	0.543	bb
192	1...	20211111_A33_R2_IgG	2.63	6596.7	40202.3	0.164	MM
193	1...	20211111_A34_R2_IgG	2.63	8541.3	36747.0	0.232	bb
194	1...	20211111_H17_R2_IgG	2.63	55796.9	40920.9	1.364	bb
195	1...	20211111_A35_R2_IgG	2.62	21482.9	33689.2	0.638	bb
196	1...	20211111_QC_R16_IgG	2.63	13814.2	32783.6	0.421	MM
197	1...	20211111_A36_R2_IgG	2.63	11390.0	36822.4	0.309	bb
198	1...	20211111_H18_R2_IgG	2.63	39871.4	31769.3	1.255	bb
199	1...	20211111_A37_R2_IgG	2.63	13112.6	37581.8	0.349	bb
200	2...	20211111_A38_R2_IgG	2.63	8444.1	38390.4	0.220	bb
201	2...	20211111_H19_R2_IgG	2.62	91191.4	41217.0	2.212	bb
202	2...	20211111_A39_R2_IgG	2.62	20906.5	37392.7	0.559	bb
203	2...	20211111_A40_R2_IgG	2.63	15145.1	38295.1	0.395	bb
204	2...	20211111_H20_R2_IgG	2.63	95805.8	35941.8	2.666	bb
205	2...	20211111_A41_R2_IgG	2.63	11969.8	45304.9	0.264	bb
206	2...	20211111_A42_R2_IgG	2.63	11167.6	37803.8	0.295	bb
207	2...	20211111_A43_R2_IgG	2.63	30845.4	38161.8	0.808	MM
208	2...	20211111_A44_R2_IgG	2.63	31521.2	37819.8	0.833	bb
209	2...	20211111_QC_R17_IgG	2.63	13525.3	31731.8	0.426	MM

Dataset: C:\MassLynx\10.pro\20211115_clinicalsamples_IgG.qld

Last Altered: Sunday, October 02, 2022 16:02:30 台北標準時間
Printed: Friday, August 01, 2025 16:53:01 台北標準時間

Method: C:\MassLynx\10_Pro_IgA.PRO\MethDB\20210926_IgG_DMRM_IgM_IS.mdb 16 Feb 2022 11:58:21
Calibration: 18 Nov 2021 13:56:22

Sample Name: 20211111_QC_R1_IgG

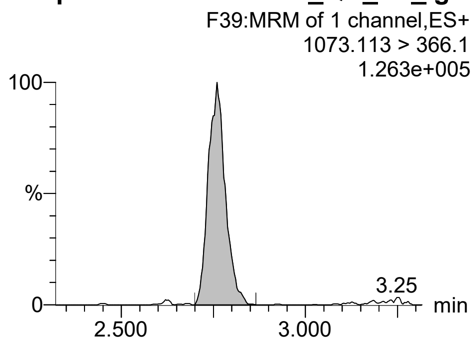

Sample Name: 20211111_A1_R1_IgG

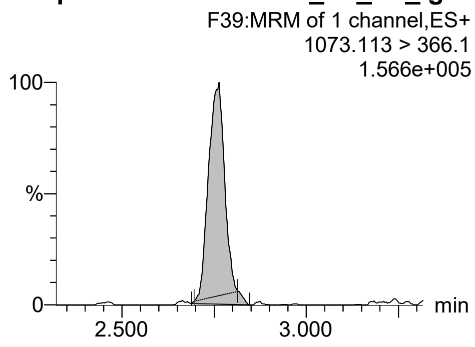

Sample Name: 20211111_A2_R1_IgG

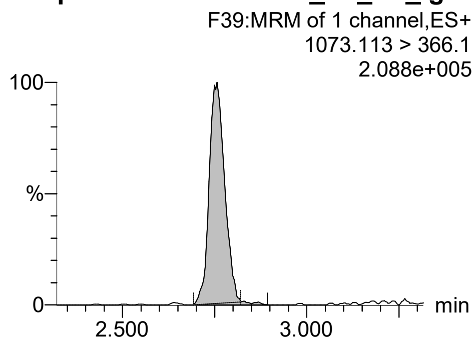

Sample Name: 20211111_L1_R1_IgG

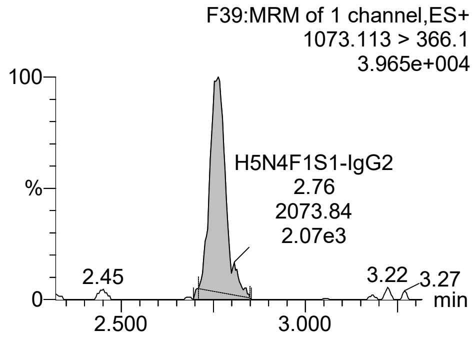

Sample Name: 20211111_L2_R1_IgG

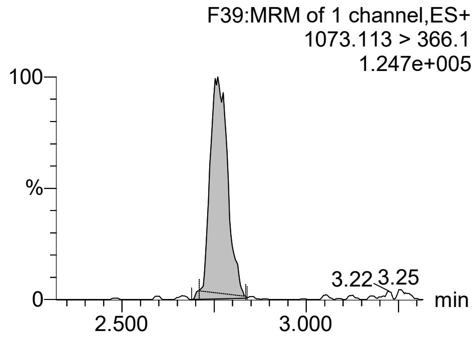

Sample Name: 20211111_H1_R1_IgG

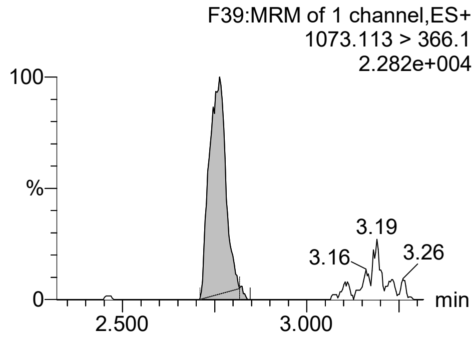

Sample Name: 20211111_A3_R1_IgG

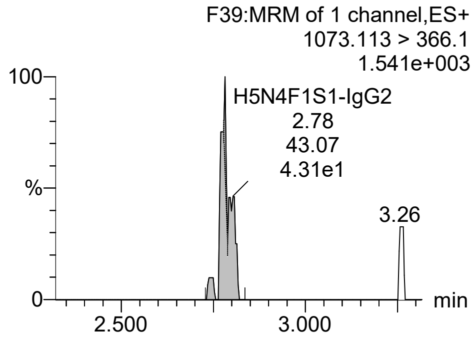

Sample Name: 20211111_A4_R1_IgG

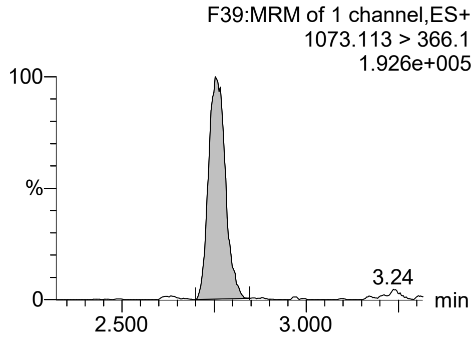

Sample Name: 20211111_L3_R1_IgG

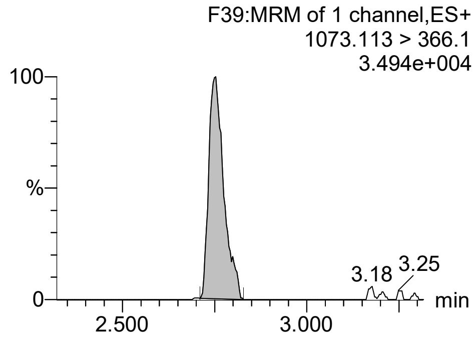

Sample Name: 20211111_L4_R1_IgG

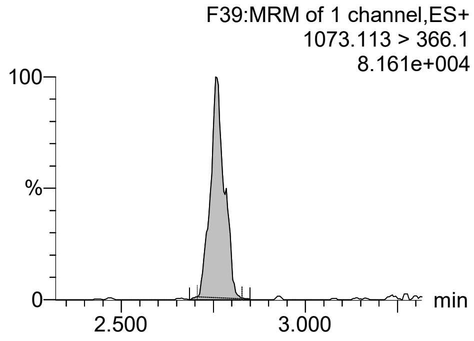

Sample Name: 20211111_H2_R1_IgG

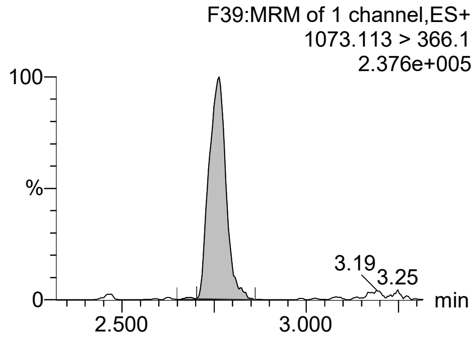

Sample Name: 20211111_A5_R1_IgG

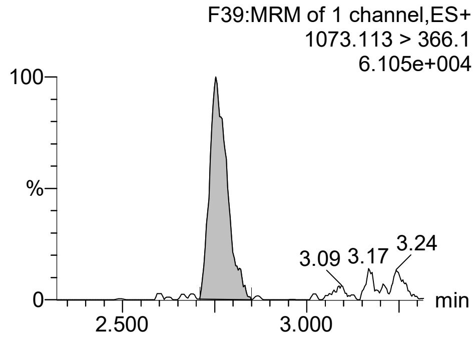

Sample Name: 20211111_A6_R1_IgG

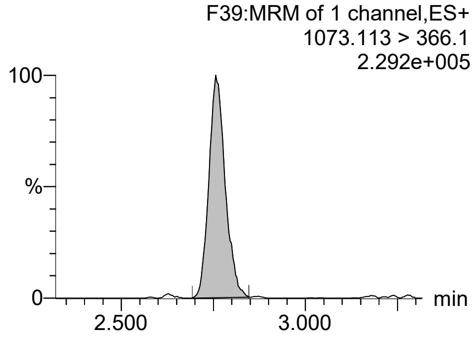

Sample Name: 20211111_QC_R2_IgG

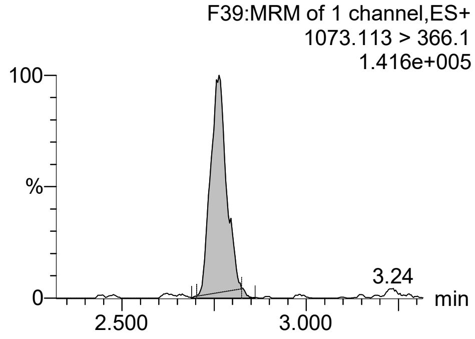

Sample Name: 20211111_L5_R1_IgG

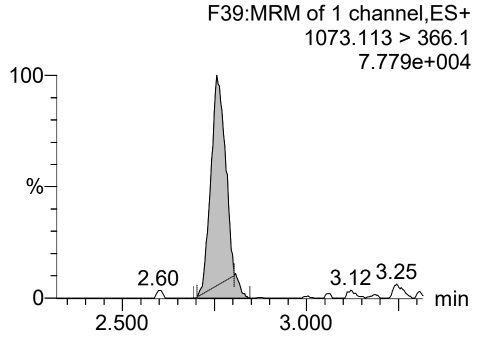

Sample Name: 20211111_L6_R1_IgG

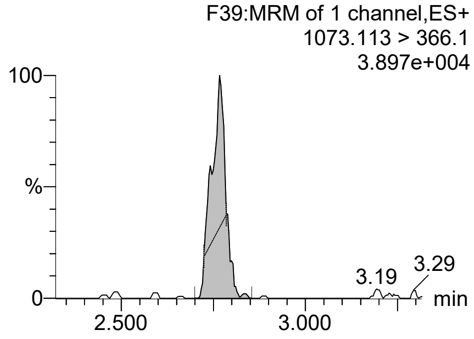

Sample Name: 20211111_H3_R1_IgG

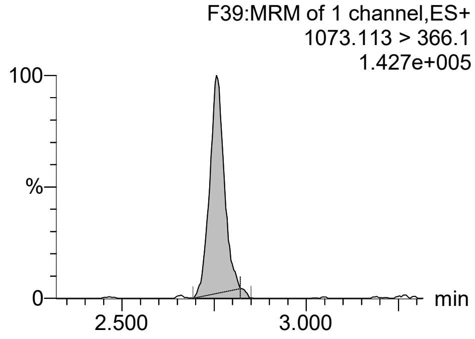

Sample Name: 20211111_A7_R1_IgG

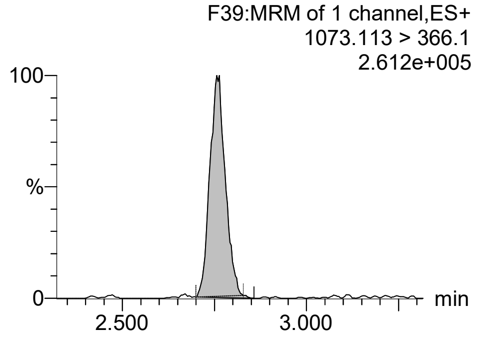

Sample Name: 20211111_A8_R1_IgG

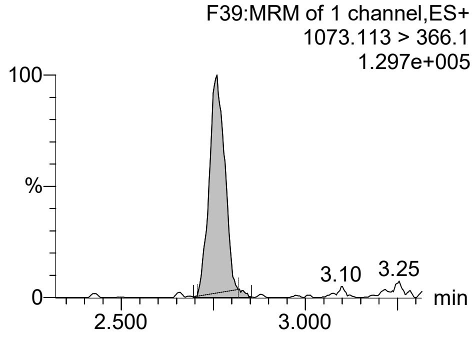

Sample Name: 20211111_L7_R1_IgG

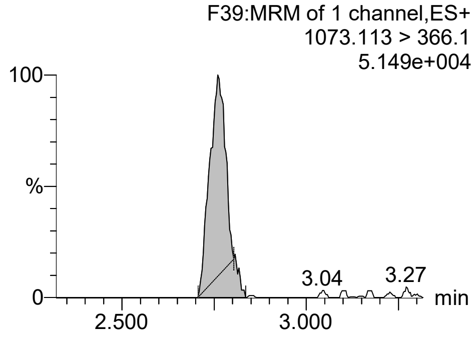

Sample Name: 20211111_L8_R1_IgG

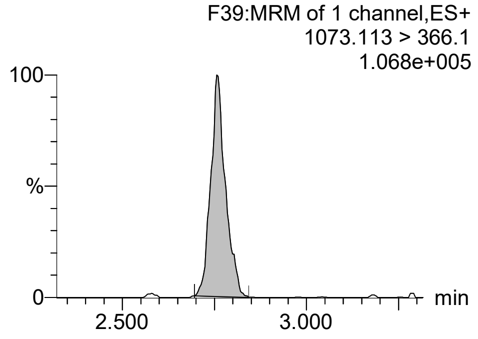

Sample Name: 20211111_H4_R1_IgG

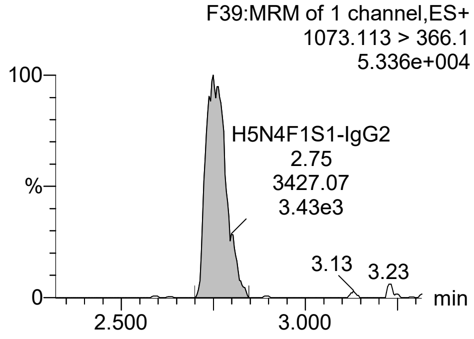

Sample Name: 20211111_A9_R1_IgG

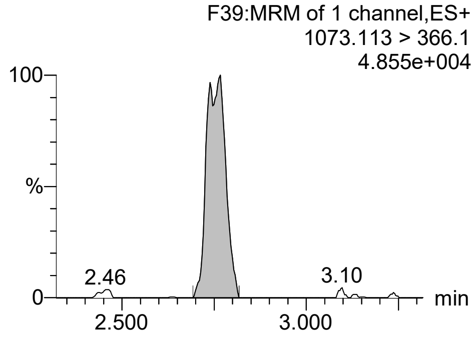

Sample Name: 20211111_A10_R1_IgG

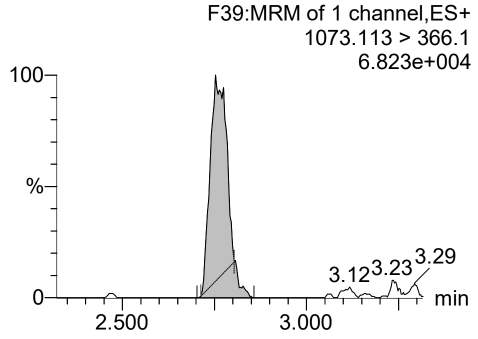

Sample Name: 20211111_L9_R1_IgG

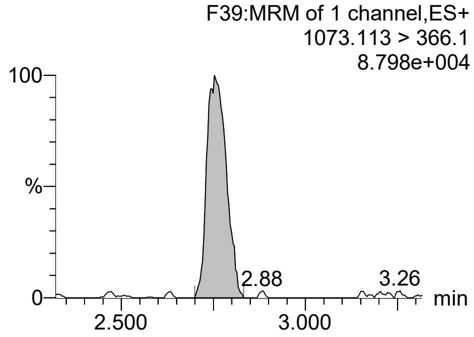

Sample Name: 20211111_L10_R1_IgG

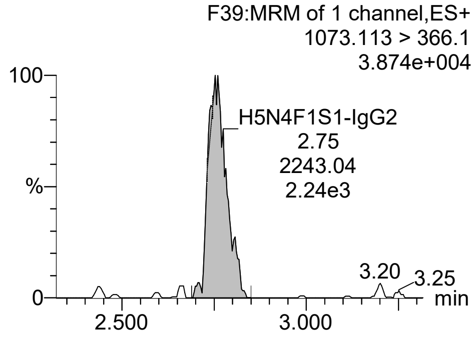

Sample Name: 20211111_QC_R3_IgG

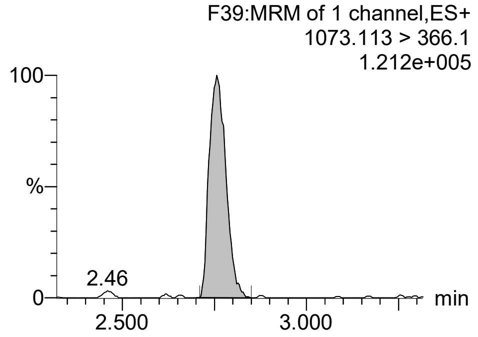

Dataset: C:\MassLynx\10.pro\20211115_clinicalsamples_IgG.qld

Last Altered: Sunday, October 02, 2022 16:02:30 台北標準時間
Printed: Friday, August 01, 2025 16:53:01 台北標準時間

Sample Name: 20211111_H5_R1_IgG
F39:MRM of 1 channel,ES+
1073.113 > 366.1
8.615e+004

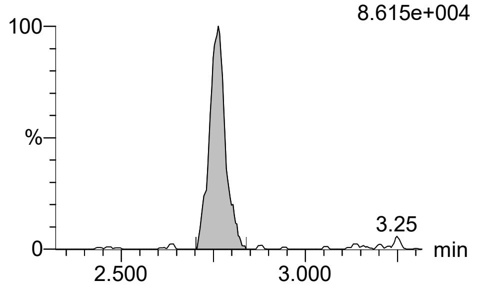

Sample Name: 20211111_A11_R1_IgG
F39:MRM of 1 channel,ES+
1073.113 > 366.1
2.088e+004

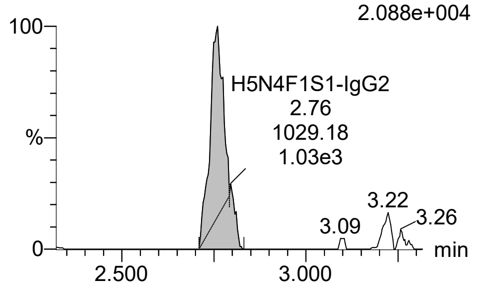

Sample Name: 20211111_A12_R1_IgG
F39:MRM of 1 channel,ES+
1073.113 > 366.1
6.128e+004

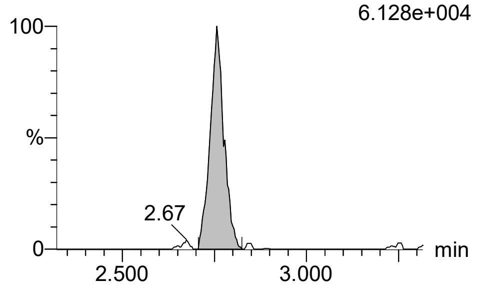

Sample Name: 20211111_L11_R1_IgG
F39:MRM of 1 channel,ES+
1073.113 > 366.1
1.567e+004

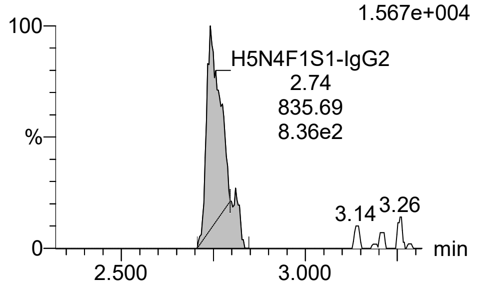

Sample Name: 20211111_L12_R1_IgG
F39:MRM of 1 channel,ES+
1073.113 > 366.1
2.371e+004

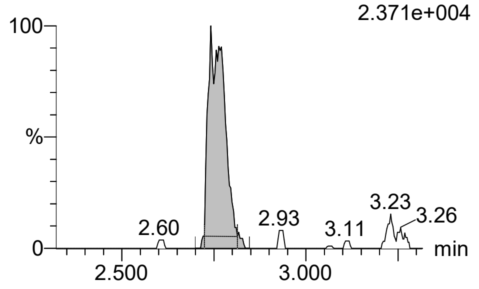

Sample Name: 20211111_H6_R1_IgG
F39:MRM of 1 channel,ES+
1073.113 > 366.1
1.984e+005

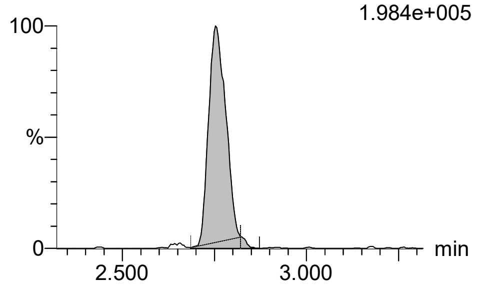

Sample Name: 20211111_A13_R1_IgG
F39:MRM of 1 channel,ES+
1073.113 > 366.1
9.907e+004

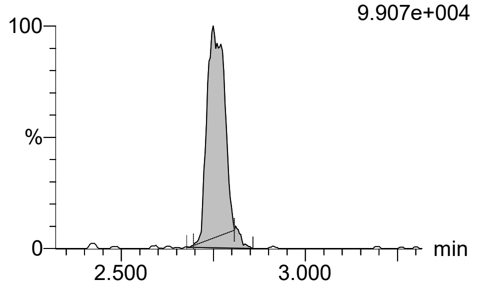

Sample Name: 20211111_A14_R1_IgG
F39:MRM of 1 channel,ES+
1073.113 > 366.1
4.569e+004

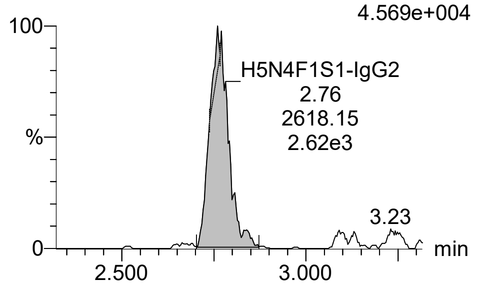

Sample Name: 20211111_L13_R1_IgG
F39:MRM of 1 channel,ES+
1073.113 > 366.1
1.373e+005

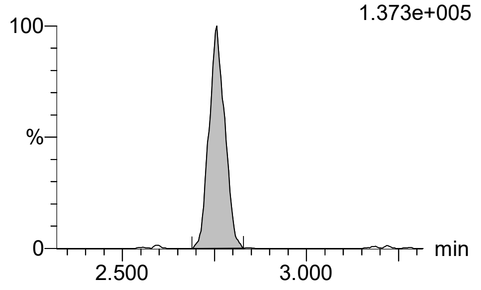

Sample Name: 20211111_L14_R1_IgG
F39:MRM of 1 channel,ES+
1073.113 > 366.1
5.393e+004

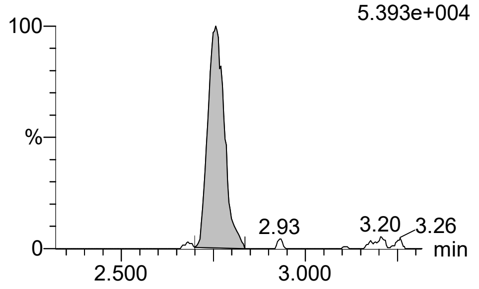

Sample Name: 20211111_H7_R1_IgG
F39:MRM of 1 channel,ES+
1073.113 > 366.1
8.743e+004

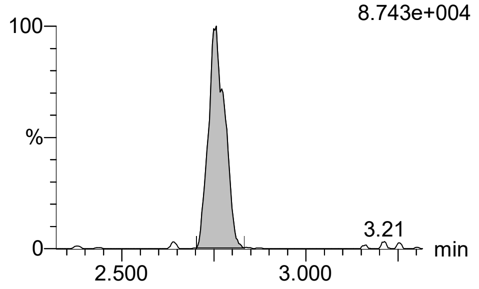

Sample Name: 20211111_A15_R1_IgG
F39:MRM of 1 channel,ES+
1073.113 > 366.1
9.761e+004

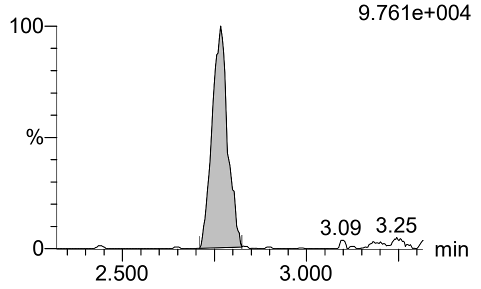

Sample Name: 20211111_QC_R4_IgG
F39:MRM of 1 channel,ES+
1073.113 > 366.1
9.879e+004

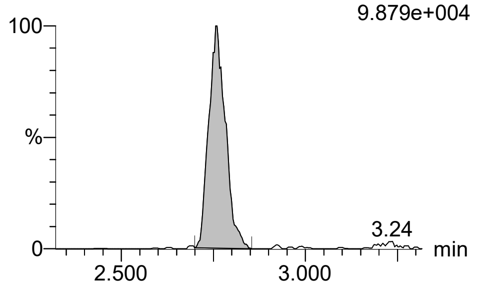

Sample Name: 20211111_A16_R1_IgG
F39:MRM of 1 channel,ES+
1073.113 > 366.1
5.581e+004

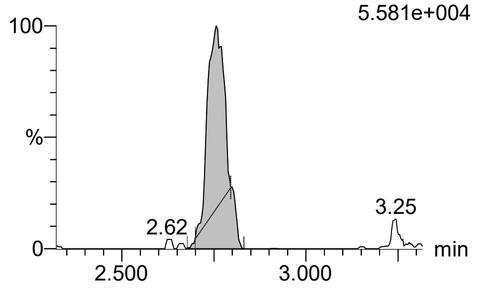

Sample Name: 20211111_L15_R1_IgG
F39:MRM of 1 channel,ES+
1073.113 > 366.1
1.166e+005

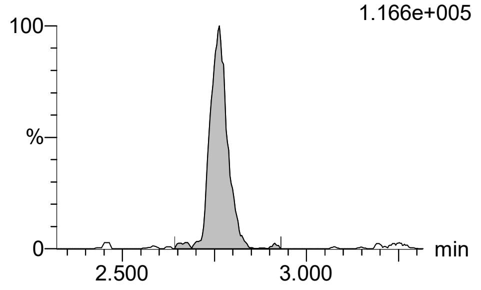

Sample Name: 20211111_L16_R1_IgG
F39:MRM of 1 channel,ES+
1073.113 > 366.1
4.998e+004

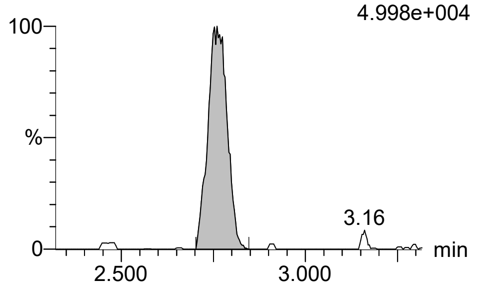

Sample Name: 20211111_H8_R1_IgG
F39:MRM of 1 channel,ES+
1073.113 > 366.1
8.222e+004

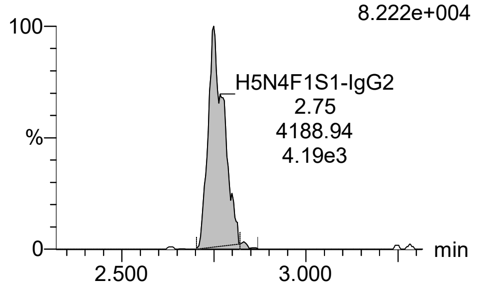

Sample Name: 20211111_A17_R1_IgG
F39:MRM of 1 channel,ES+
1073.113 > 366.1
1.003e+005

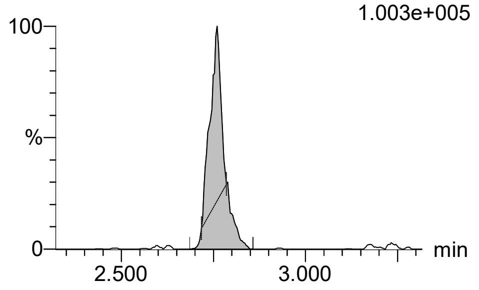

Sample Name: 20211111_A18_R1_IgG
F39:MRM of 1 channel,ES+
1073.113 > 366.1
7.417e+004

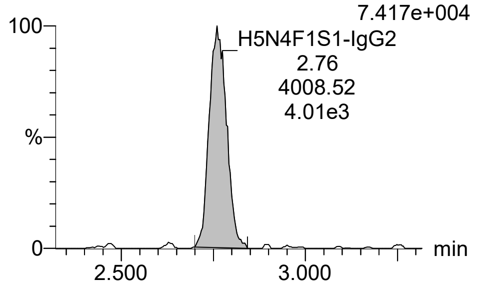

Sample Name: 20211111_L17_R1_IgG
F39:MRM of 1 channel,ES+
1073.113 > 366.1
8.351e+004

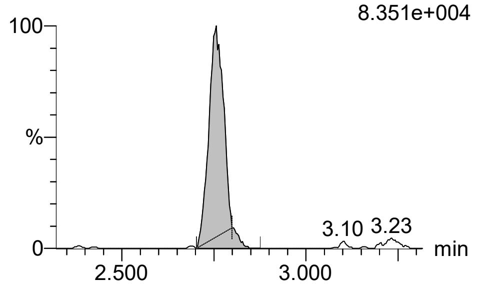

Sample Name: 20211111_L18_R1_IgG
F39:MRM of 1 channel,ES+
1073.113 > 366.1
1.098e+004

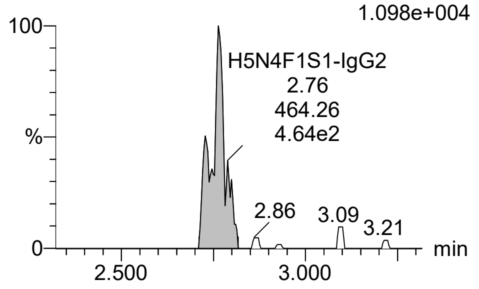

Sample Name: 20211111_H9_R1_IgG
F39:MRM of 1 channel,ES+
1073.113 > 366.1
5.596e+004

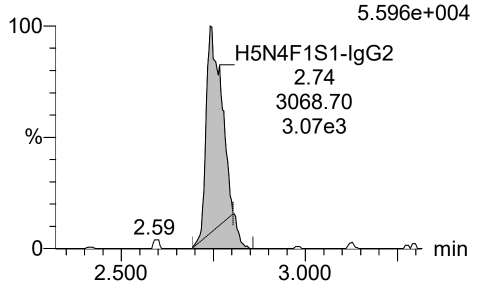

Sample Name: 20211111_A19_R1_IgG
F39:MRM of 1 channel,ES+
1073.113 > 366.1
8.420e+004

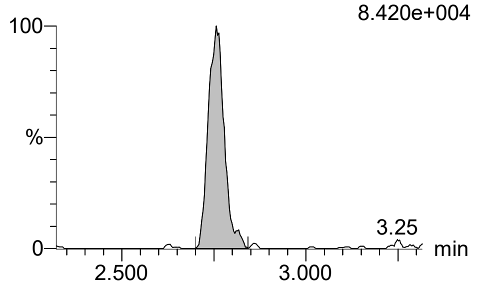

Sample Name: 20211111_A20_R1_IgG
F39:MRM of 1 channel,ES+
1073.113 > 366.1
2.716e+004

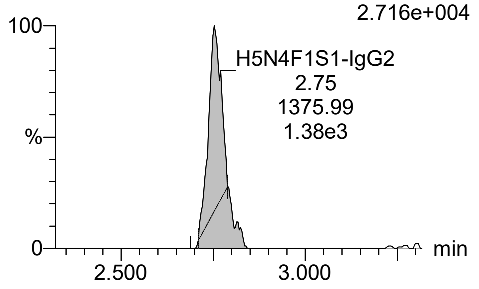

Sample Name: 20211111_L19_R1_IgG
F39:MRM of 1 channel,ES+
1073.113 > 366.1
6.794e+004

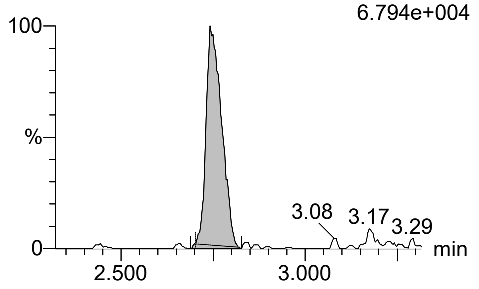

Sample Name: 20211111_QC_R5_IgG
F39:MRM of 1 channel,ES+
1073.113 > 366.1
7.469e+004

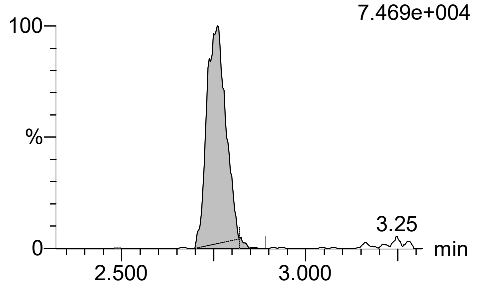

Sample Name: 20211111_L20_R1_IgG
F39:MRM of 1 channel,ES+
1073.113 > 366.1
6.178e+004

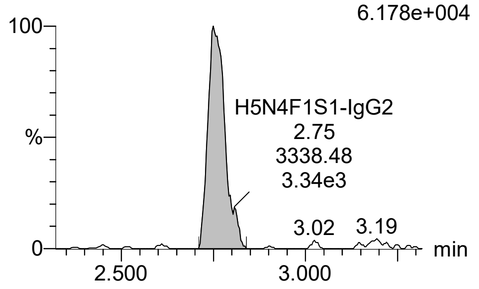

Sample Name: 20211111_H10_R1_IgG
F39:MRM of 1 channel,ES+
1073.113 > 366.1
8.120e+004

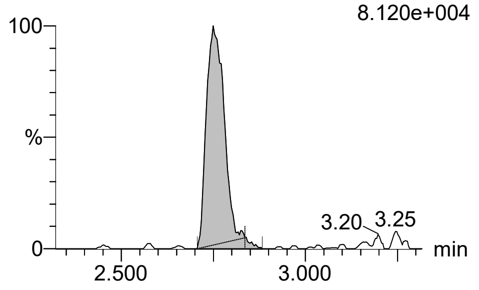

Sample Name: 20211111_A21_R1_IgG
F39:MRM of 1 channel,ES+
1073.113 > 366.1
2.469e+005

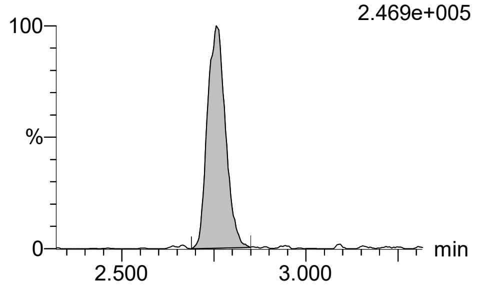

Sample Name: 20211111_A22_R1_IgG
F39:MRM of 1 channel,ES+
1073.113 > 366.1
1.328e+005

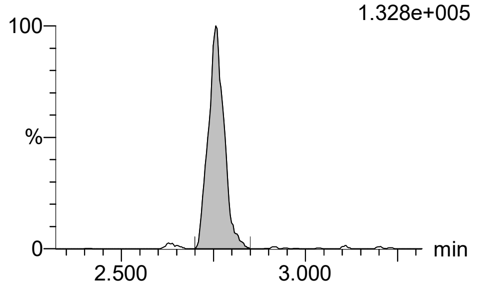

Sample Name: 20211111_L21_R1_IgG

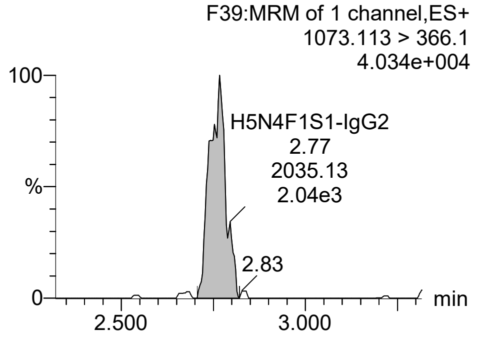

Sample Name: 20211111_L22_R1_IgG

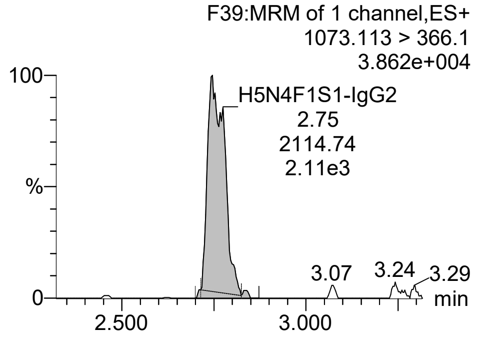

Sample Name: 20211111_H11_R1_IgG

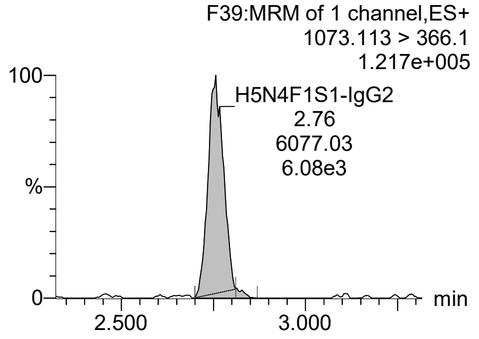

Sample Name: 20211111_A23_R1_IgG

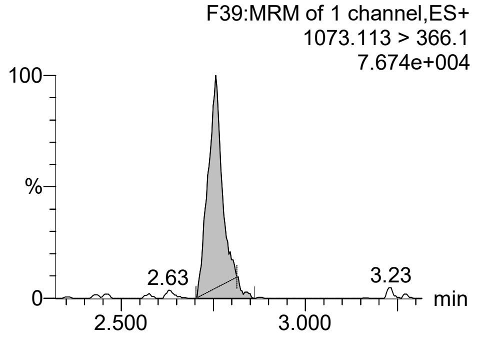

Sample Name: 20211111_A24_R1_IgG

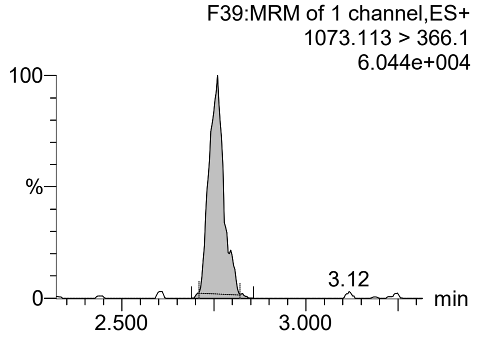

Sample Name: 20211111_L23_R1_IgG

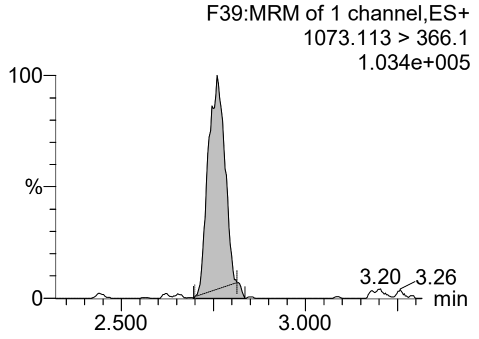

Sample Name: 20211111_L24_R1_IgG

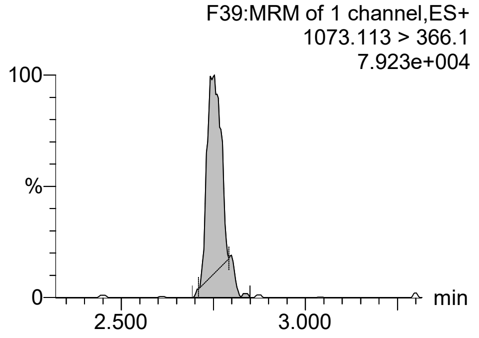

Sample Name: 20211111_H12_R1_IgG

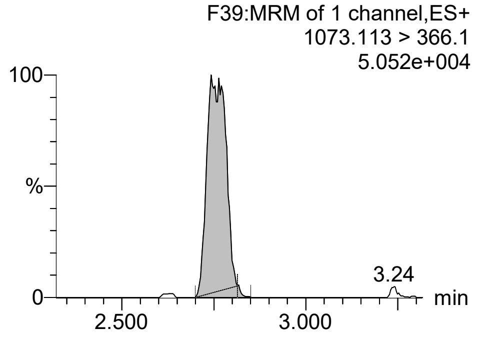

Sample Name: 20211111_QC_R6_IgG

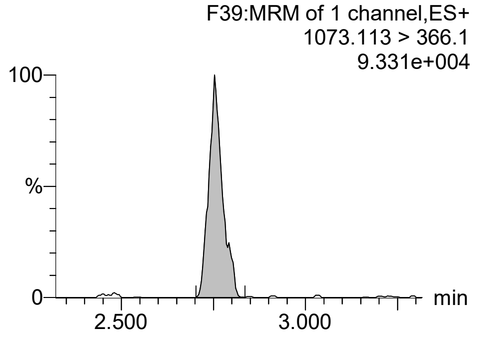

Sample Name: 20211111_A25_R1_IgG

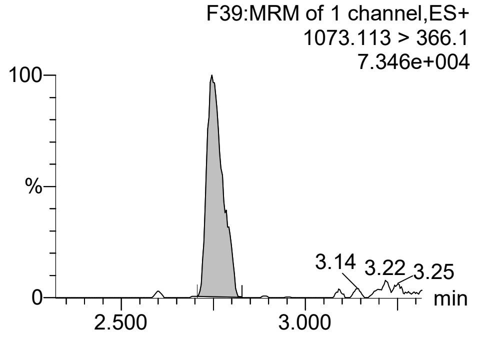

Sample Name: 20211111_A26_R1_IgG

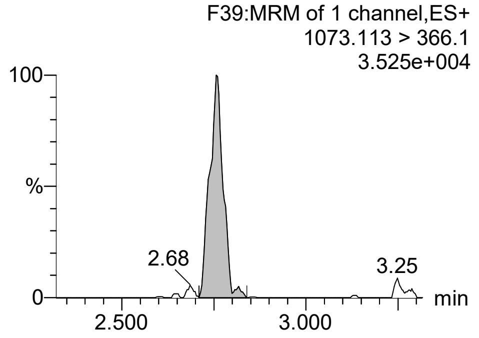

Sample Name: 20211111_L25_R1_IgG

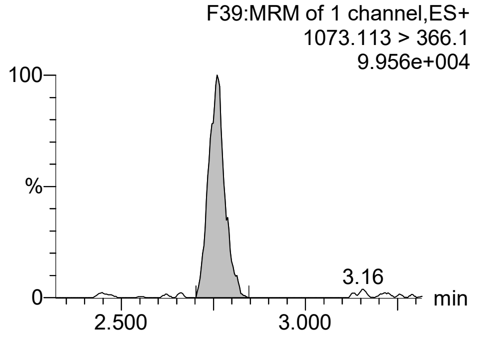

Sample Name: 20211111_L26_R1_IgG

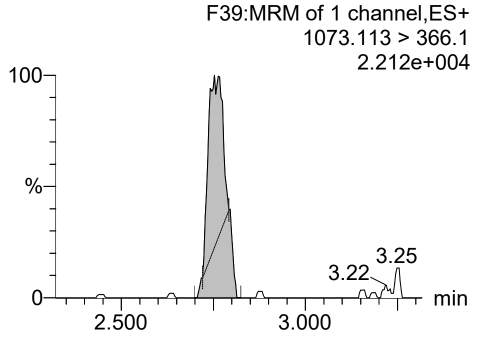

Sample Name: 20211111_H13_R1_IgG

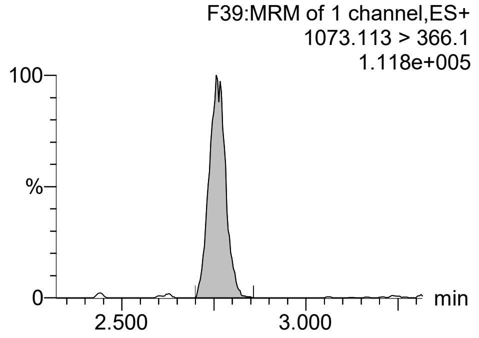

Sample Name: 20211111_A27_R1_IgG

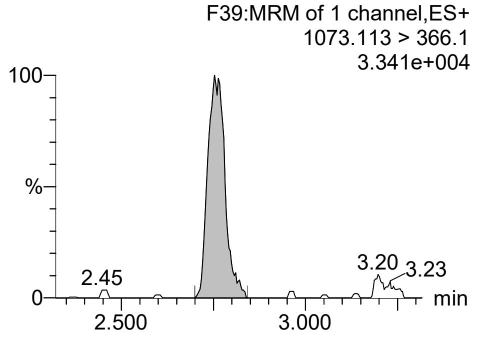

Sample Name: 20211111_A28_R1_IgG

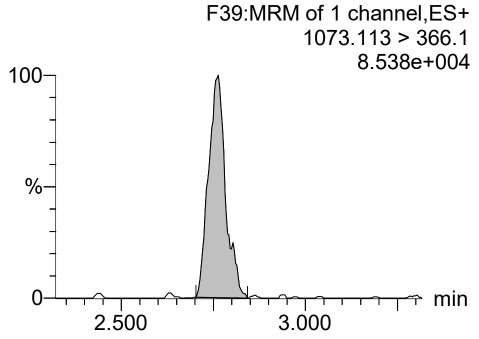

Sample Name: 20211111_L27_R1_IgG

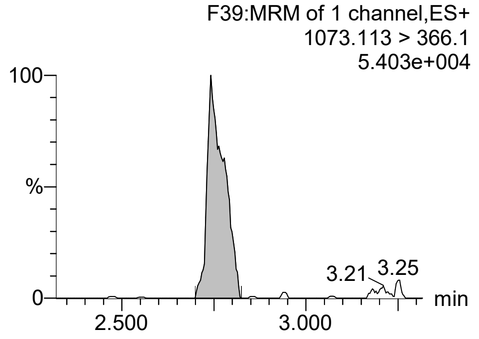

Sample Name: 20211111_L28_R1_IgG

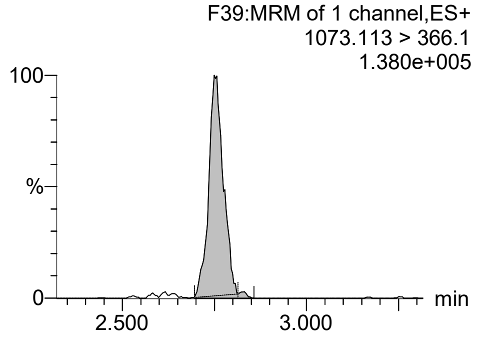

Sample Name: 20211111_H14_R1_IgG

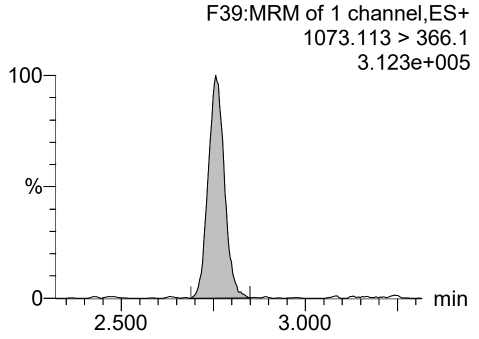

Sample Name: 20211111_A29_R1_IgG

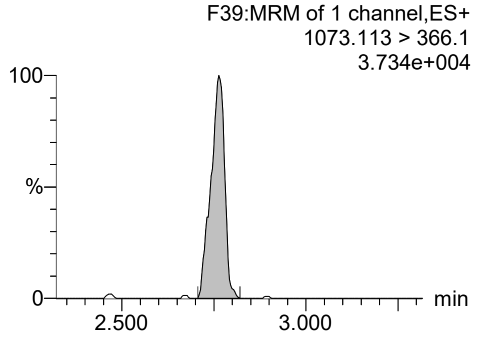

Sample Name: 20211111_A30_R1_IgG

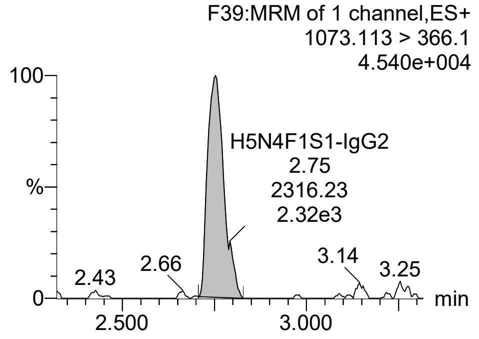

Sample Name: 20211111_QC_R7_IgG

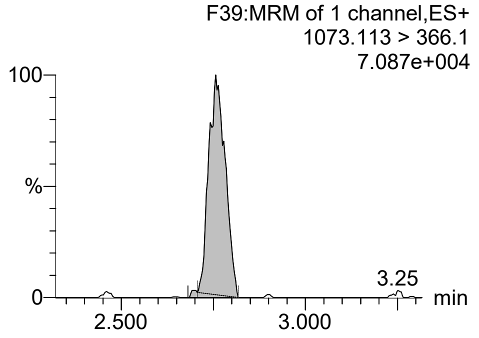

Sample Name: 20211111_L29_R1_IgG

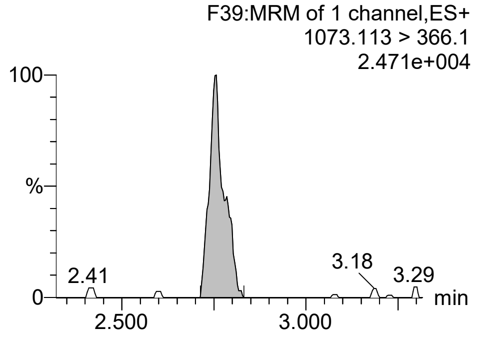

Sample Name: 20211111_L30_R1_IgG

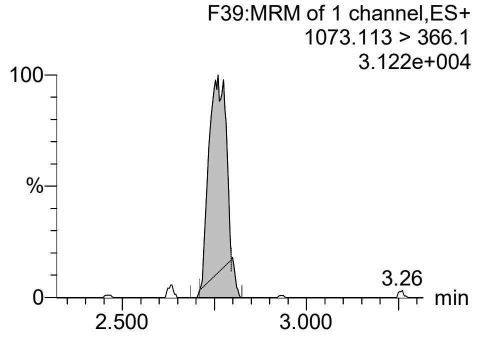

Sample Name: 20211111_H15_R1_IgG

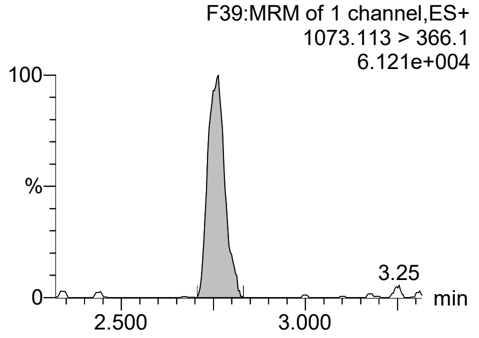

Sample Name: 20211111_A31_R1_IgG

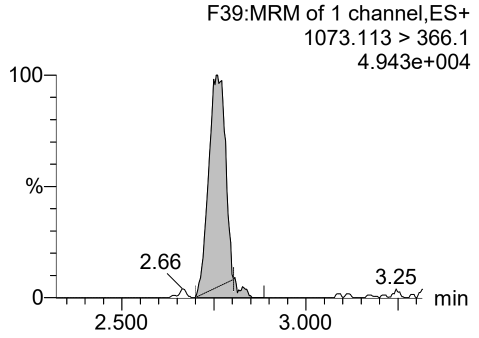

Sample Name: 20211111_A32_R1_IgG

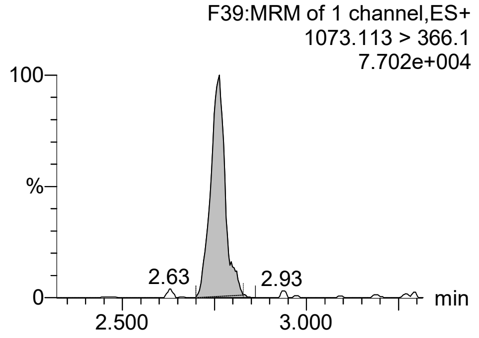

Sample Name: 20211111_L31_R1_IgG

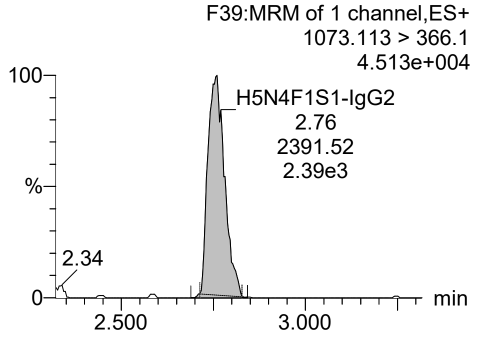

Sample Name: 20211111_L32_R1_IgG

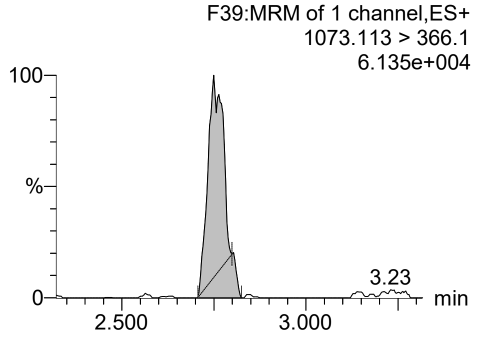

Sample Name: 20211111_H16_R1_IgG

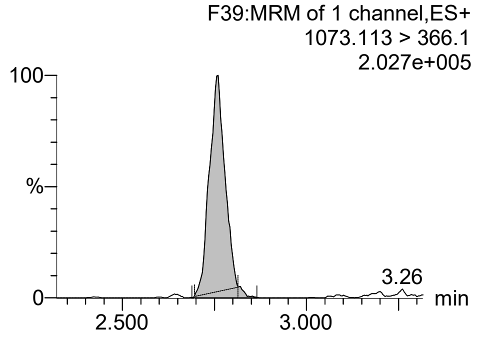

Sample Name: 20211111_A33_R1_IgG
F39:MRM of 1 channel,ES+
1073.113 > 366.1
4.665e+004

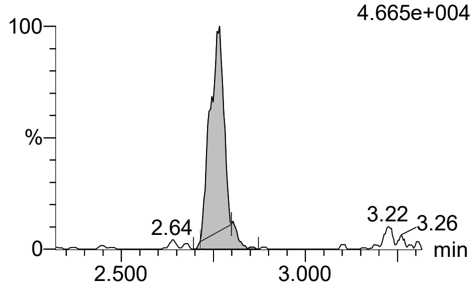

Sample Name: 20211111_A34_R1_IgG
F39:MRM of 1 channel,ES+
1073.113 > 366.1
5.264e+004

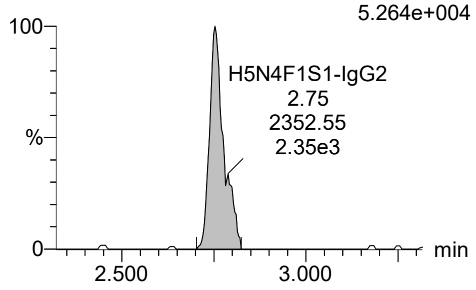

Sample Name: 20211111_H17_R1_IgG
F39:MRM of 1 channel,ES+
1073.113 > 366.1
2.432e+005

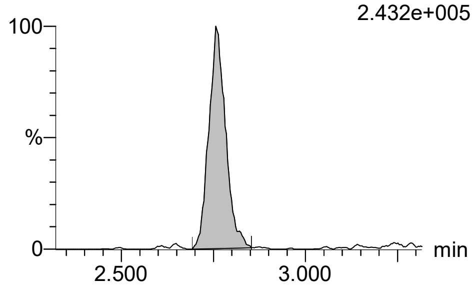

Sample Name: 20211111_A35_R1_IgG
F39:MRM of 1 channel,ES+
1073.113 > 366.1
8.717e+004

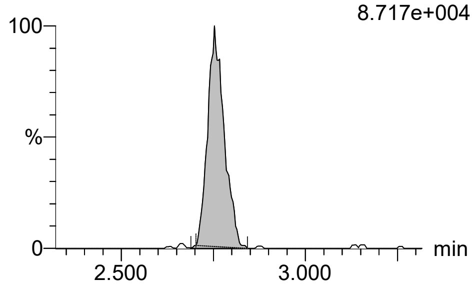

Sample Name: 20211111_QC_R8_IgG
F39:MRM of 1 channel,ES+
1073.113 > 366.1
5.577e+004

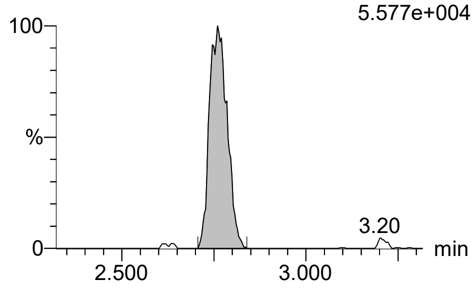

Sample Name: 20211111_A36_R1_IgG
F39:MRM of 1 channel,ES+
1073.113 > 366.1
6.160e+004

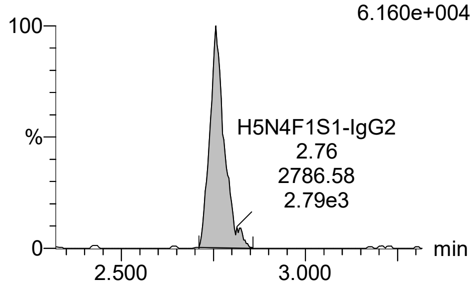

Sample Name: 20211111_H18_R1_IgG
F39:MRM of 1 channel,ES+
1073.113 > 366.1
1.624e+005

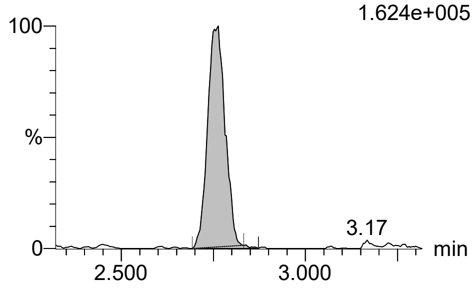

Sample Name: 20211111_A37_R1_IgG
F39:MRM of 1 channel,ES+
1073.113 > 366.1
6.765e+004

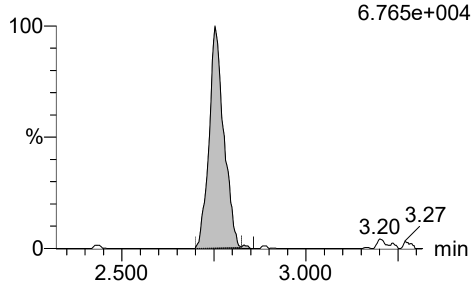

Sample Name: 20211111_A38_R1_IgG
F39:MRM of 1 channel,ES+
1073.113 > 366.1
3.210e+004

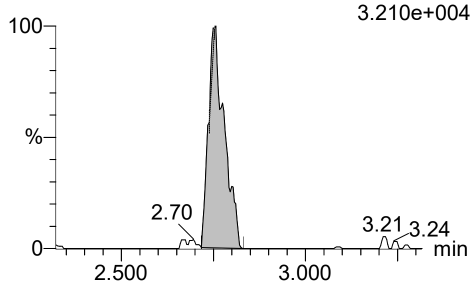

Sample Name: 20211111_H19_R1_IgG
F39:MRM of 1 channel,ES+
1073.113 > 366.1
3.988e+005

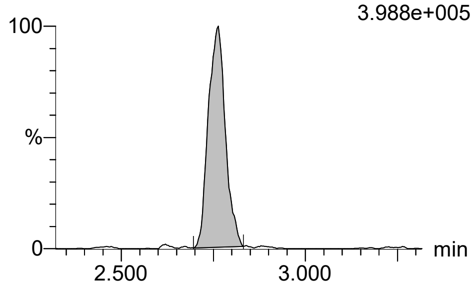

Sample Name: 20211111_A39_R1_IgG
F39:MRM of 1 channel,ES+
1073.113 > 366.1
6.810e+004

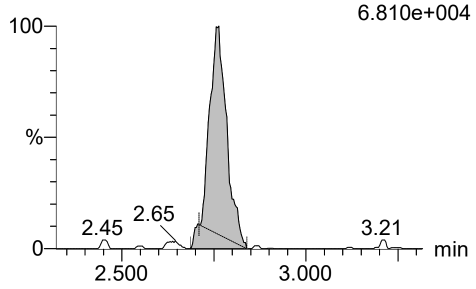

Sample Name: 20211111_A40_R1_IgG
F39:MRM of 1 channel,ES+
1073.113 > 366.1
4.777e+004

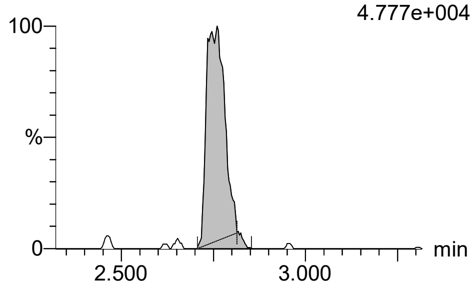

Sample Name: 20211111_H20_R1_IgG
F39:MRM of 1 channel,ES+
1073.113 > 366.1
3.859e+005

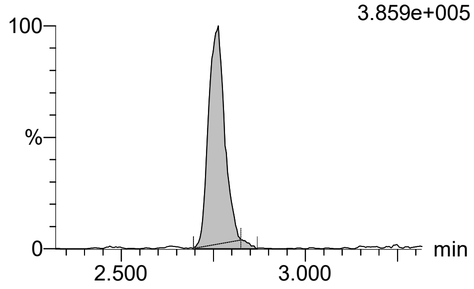

Sample Name: 20211111_A41_R1_IgG
F39:MRM of 1 channel,ES+
1073.113 > 366.1
5.816e+004

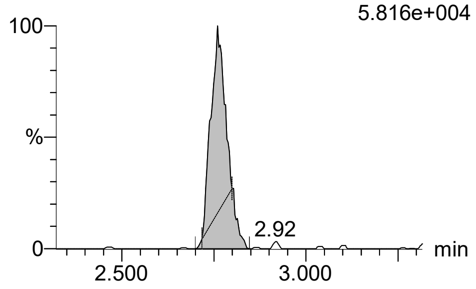

Sample Name: 20211111_A42_R1_IgG
F39:MRM of 1 channel,ES+
1073.113 > 366.1
6.990e+004

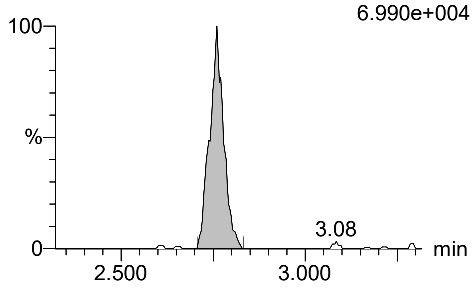

Sample Name: 20211111_A43_R1_IgG
F39:MRM of 1 channel,ES+
1073.113 > 366.1
1.910e+005

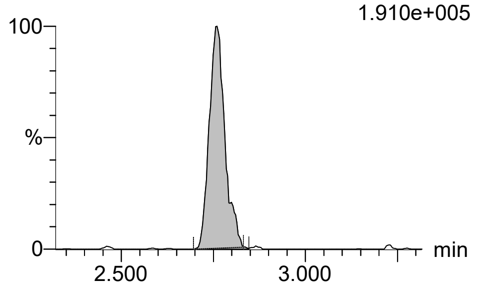

Sample Name: 20211111_A44_R1_IgG
F39:MRM of 1 channel,ES+
1073.113 > 366.1
1.872e+005

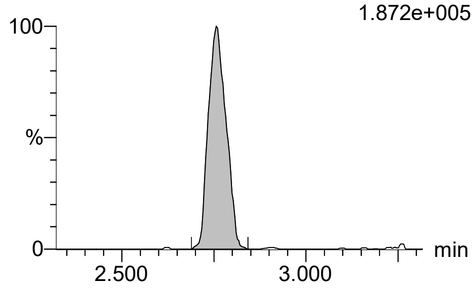

Sample Name: 20211111_QC_R9_IgG
F39:MRM of 1 channel,ES+
1073.113 > 366.1
7.163e+004

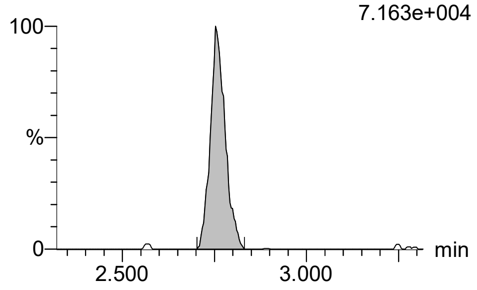

Sample Name: 20211111_A1_R2_IgG
F39:MRM of 1 channel,ES+
1073.113 > 366.1
1.124e+005

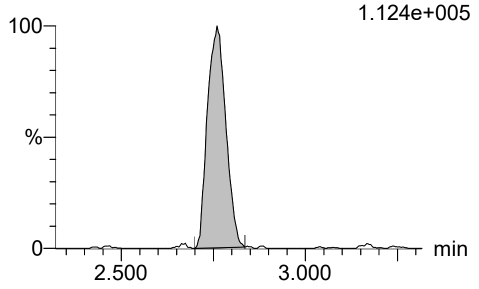

Sample Name: 20211111_A2_R2_IgG
F39:MRM of 1 channel,ES+
1073.113 > 366.1
1.762e+005

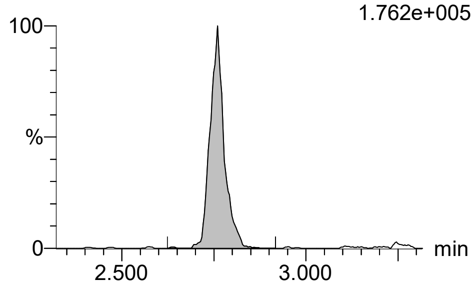

Sample Name: 20211111_L1_R2_IgG
F39:MRM of 1 channel,ES+
1073.113 > 366.1
3.355e+004

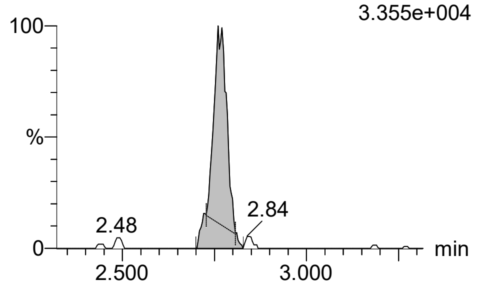

Sample Name: 20211111_L2_R2_IgG
F39:MRM of 1 channel,ES+
1073.113 > 366.1
1.091e+005

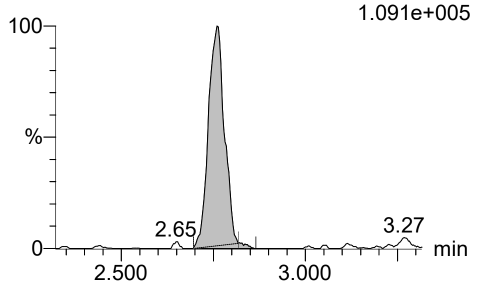

Sample Name: 20211111_H1_R2_IgG
F39:MRM of 1 channel,ES+
1073.113 > 366.1
3.449e+004

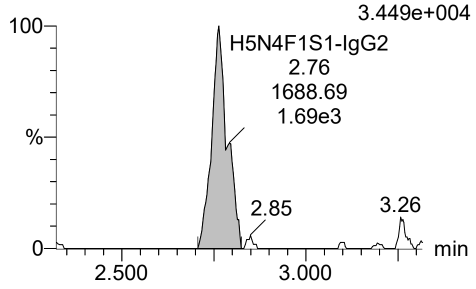

Sample Name: 20211111_A3_R2_IgG
F39:MRM of 1 channel,ES+
1073.113 > 366.1
5.253e+003

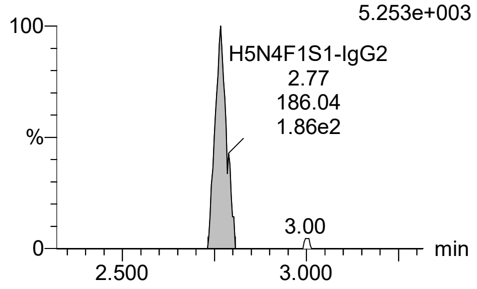

Sample Name: 20211111_A4_R2_IgG
F39:MRM of 1 channel,ES+
1073.113 > 366.1
1.340e+005

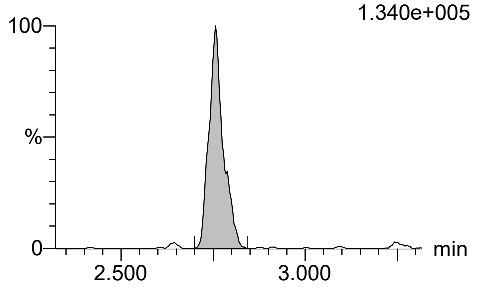

Sample Name: 20211111_L3_R2_IgG
F39:MRM of 1 channel,ES+
1073.113 > 366.1
2.422e+004

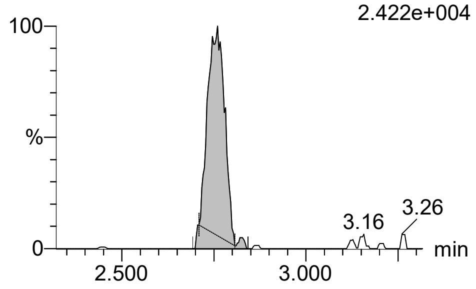

Sample Name: 20211111_L4_R2_IgG
F39:MRM of 1 channel,ES+
1073.113 > 366.1
4.093e+004

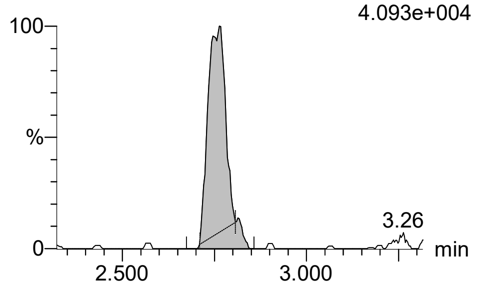

Sample Name: 20211111_H2_R2_IgG
F39:MRM of 1 channel,ES+
1073.113 > 366.1
1.454e+005

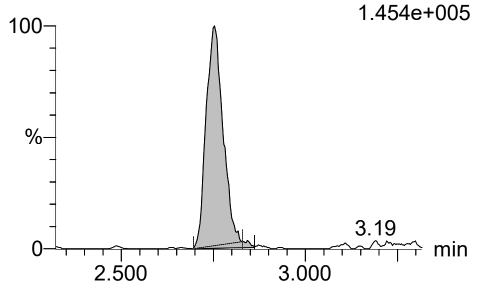

Sample Name: 20211111_A5_R2_IgG
F39:MRM of 1 channel,ES+
1073.113 > 366.1
4.839e+004

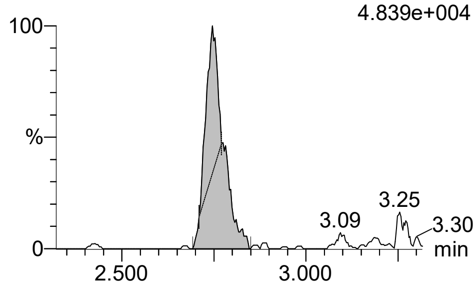

Sample Name: 20211111_A6_R2_IgG
F39:MRM of 1 channel,ES+
1073.113 > 366.1
1.482e+005

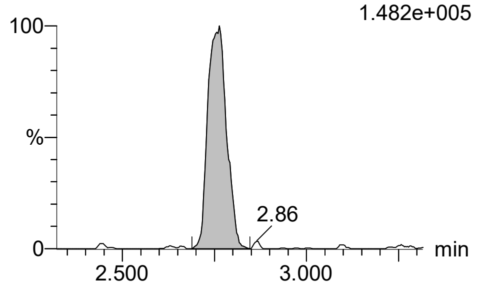

Dataset: C:\MassLynx\10.pro\20211115_clinicalsamples_IgG.qld

Last Altered: Sunday, October 02, 2022 16:02:30 台北標準時間
Printed: Friday, August 01, 2025 16:53:01 台北標準時間

Sample Name: 20211111_QC_R10_IgG
F39:MRM of 1 channel,ES+
1073.113 > 366.1
5.616e+004

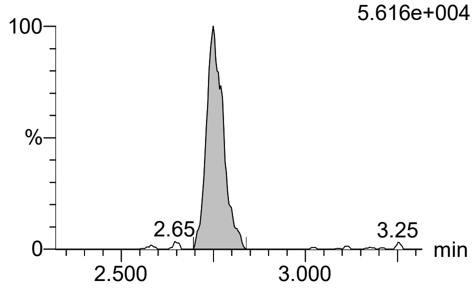

Sample Name: 20211111_L5_R2_IgG
F39:MRM of 1 channel,ES+
1073.113 > 366.1
5.325e+004

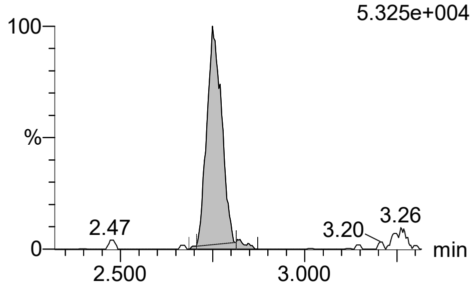

Sample Name: 20211111_L6_R2_IgG
F39:MRM of 1 channel,ES+
1073.113 > 366.1
2.575e+004

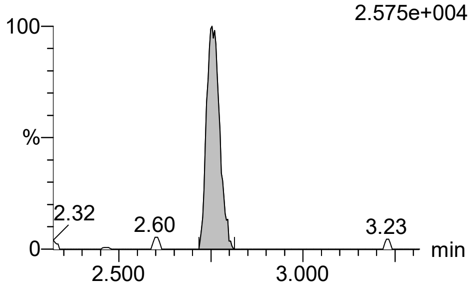

Sample Name: 20211111_H3_R2_IgG
F39:MRM of 1 channel,ES+
1073.113 > 366.1
9.447e+004

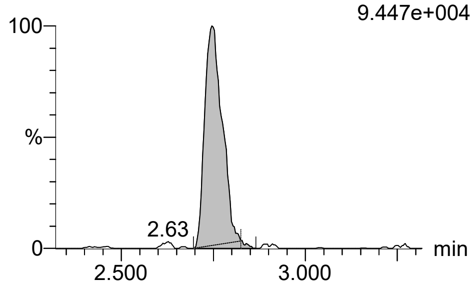

Sample Name: 20211111_A7_R2_IgG
F39:MRM of 1 channel,ES+
1073.113 > 366.1
1.588e+005

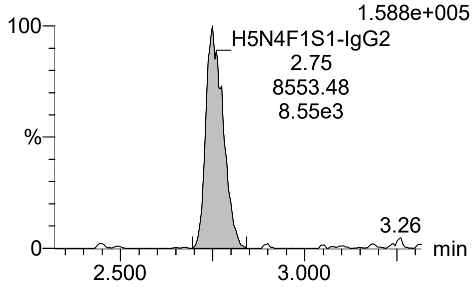

Sample Name: 20211111_A8_R2_IgG
F39:MRM of 1 channel,ES+
1073.113 > 366.1
1.012e+005

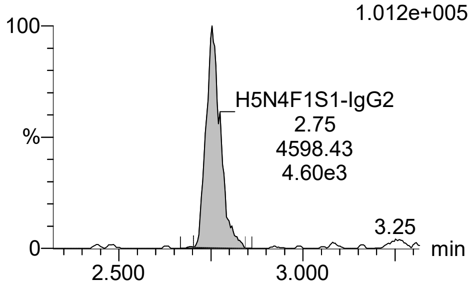

Sample Name: 20211111_L7_R2_IgG
F39:MRM of 1 channel,ES+
1073.113 > 366.1
6.380e+004

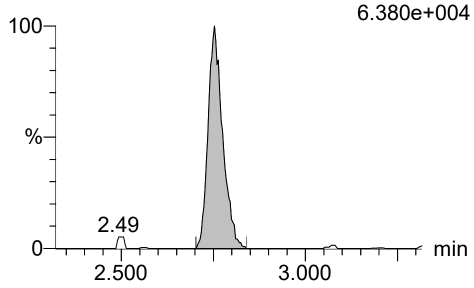

Sample Name: 20211111_L8_R2_IgG
F39:MRM of 1 channel,ES+
1073.113 > 366.1
8.769e+004

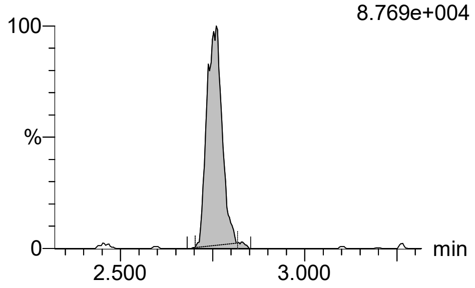

Sample Name: 20211111_H4_R2_IgG
F39:MRM of 1 channel,ES+
1073.113 > 366.1
4.751e+004

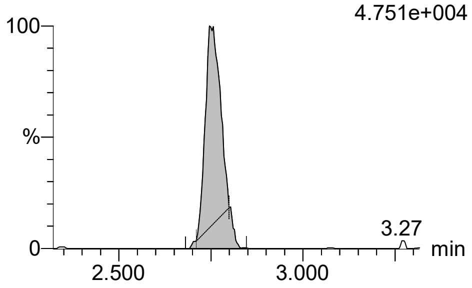

Sample Name: 20211111_A9_R2_IgG
F39:MRM of 1 channel,ES+
1073.113 > 366.1
4.080e+004

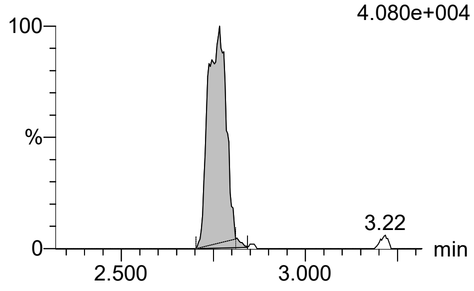

Sample Name: 20211111_A10_R2_IgG
F39:MRM of 1 channel,ES+
1073.113 > 366.1
6.265e+004

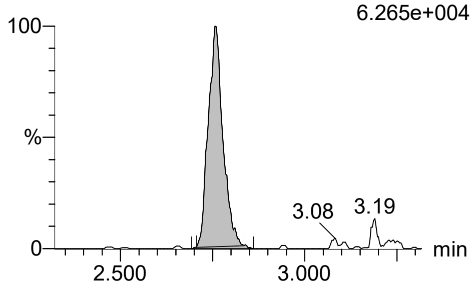

Sample Name: 20211111_L9_R2_IgG
F39:MRM of 1 channel,ES+
1073.113 > 366.1
7.535e+004

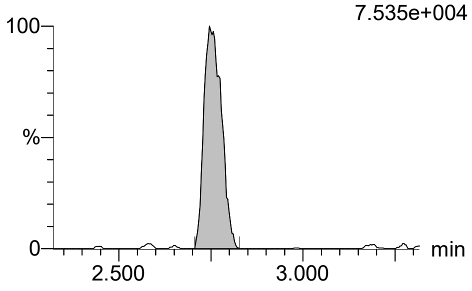

Sample Name: 20211111_L10_R2_IgG
F39:MRM of 1 channel,ES+
1073.113 > 366.1
4.801e+004

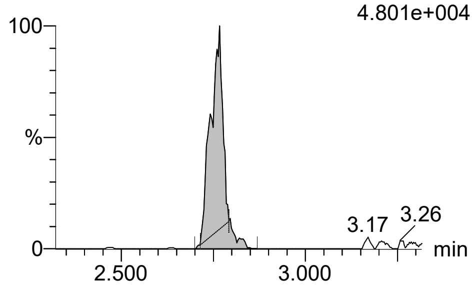

Sample Name: 20211111_QC_R11_IgG
F39:MRM of 1 channel,ES+
1073.113 > 366.1
6.217e+004

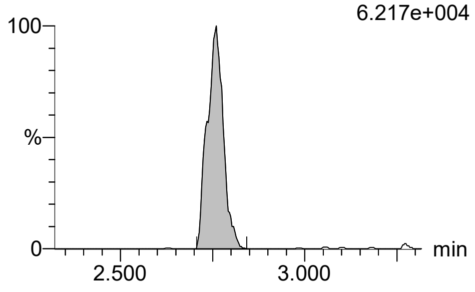

Sample Name: 20211111_H5_R2_IgG
F39:MRM of 1 channel,ES+
1073.113 > 366.1
7.676e+004

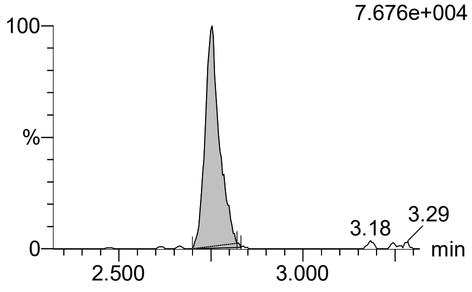

Dataset: C:\MassLynx\10.pro\20211115_clinicalsamples_IgG.qld

Last Altered: Sunday, October 02, 2022 16:02:30 台北標準時間
Printed: Friday, August 01, 2025 16:53:01 台北標準時間

Sample Name: 20211111_A11_R2_IgG
F39:MRM of 1 channel,ES+
1073.113 > 366.1
1.002e+004

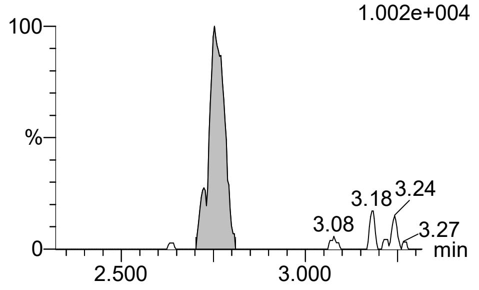

Sample Name: 20211111_A12_R2_IgG
F39:MRM of 1 channel,ES+
1073.113 > 366.1
3.086e+004

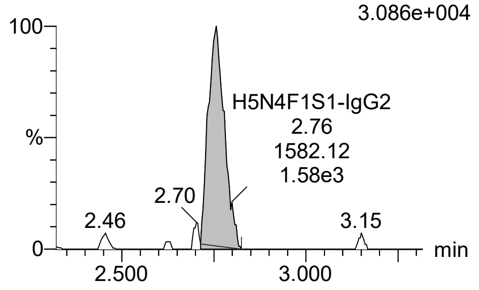

Sample Name: 20211111_L11_R2_IgG
F39:MRM of 1 channel,ES+
1073.113 > 366.1
2.145e+004

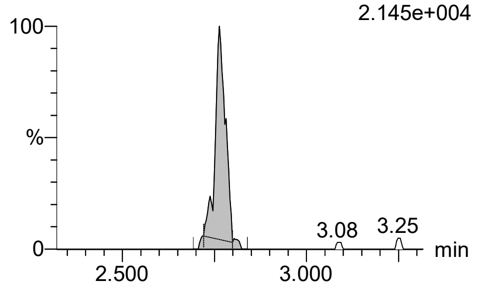

Sample Name: 20211111_L12_R2_IgG
F39:MRM of 1 channel,ES+
1073.113 > 366.1
2.331e+004

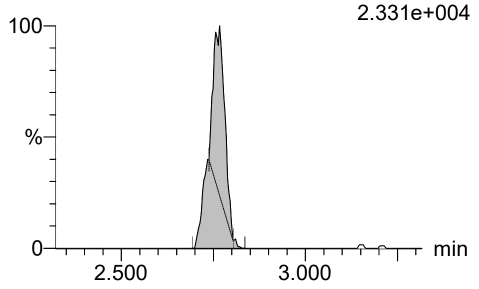

Sample Name: 20211111_H6_R2_IgG
F39:MRM of 1 channel,ES+
1073.113 > 366.1
1.453e+005

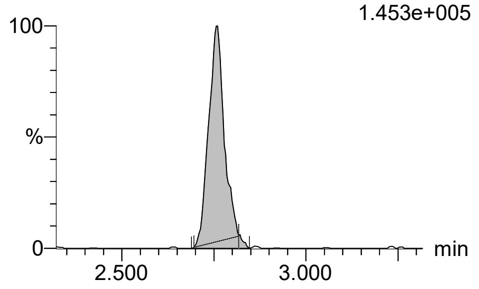

Sample Name: 20211111_A13_R2_IgG
F39:MRM of 1 channel,ES+
1073.113 > 366.1
9.400e+004

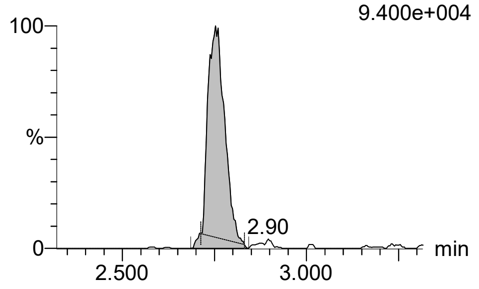

Sample Name: 20211111_A14_R2_IgG
F39:MRM of 1 channel,ES+
1073.113 > 366.1
3.183e+004

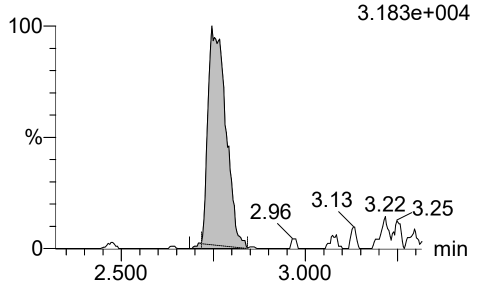

Sample Name: 20211111_L13_R2_IgG
F39:MRM of 1 channel,ES+
1073.113 > 366.1
1.166e+005

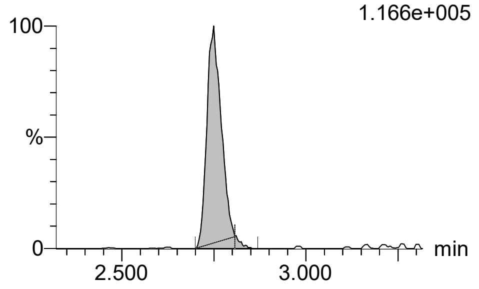

Sample Name: 20211111_L14_R2_IgG
F39:MRM of 1 channel,ES+
1073.113 > 366.1
4.428e+004

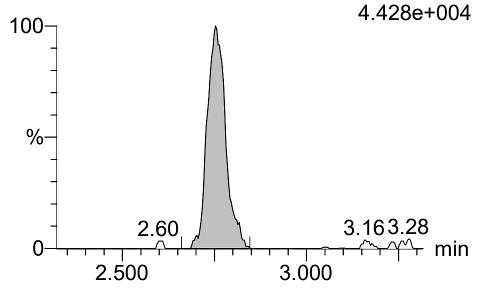

Sample Name: 20211111_H7_R2_IgG
F39:MRM of 1 channel,ES+
1073.113 > 366.1
7.456e+004

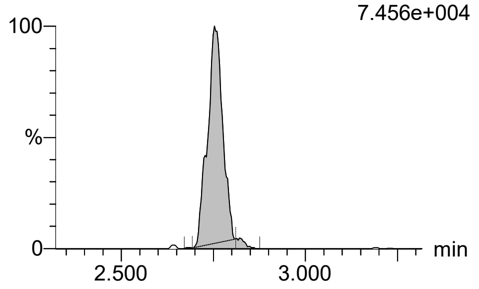

Sample Name: 20211111_A15_R2_IgG
F39:MRM of 1 channel,ES+
1073.113 > 366.1
5.605e+004

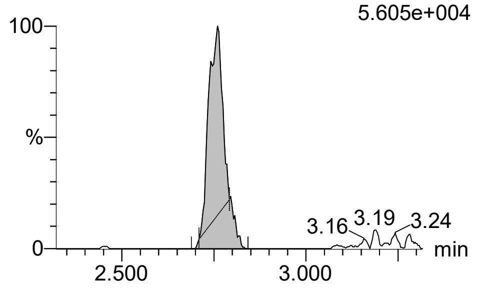

Sample Name: 20211111_QC_R12_IgG
F39:MRM of 1 channel,ES+
1073.113 > 366.1
6.213e+004

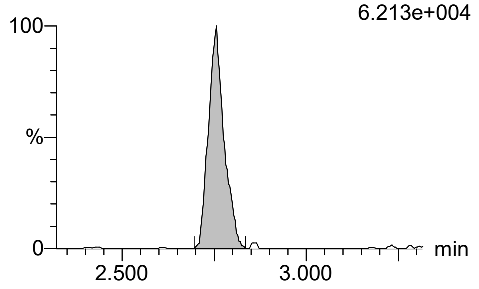

Sample Name: 20211111_A16_R2_IgG
F39:MRM of 1 channel,ES+
1073.113 > 366.1
4.774e+004

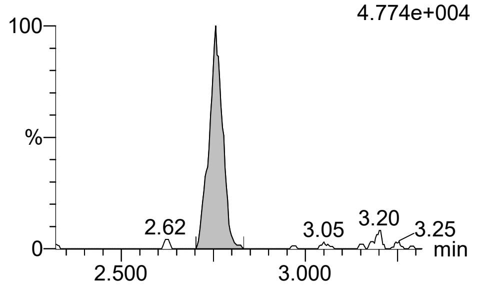

Sample Name: 20211111_L15_R2_IgG
F39:MRM of 1 channel,ES+
1073.113 > 366.1
1.055e+005

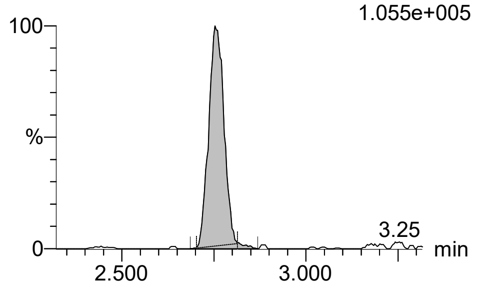

Sample Name: 20211111_L16_R2_IgG
F39:MRM of 1 channel,ES+
1073.113 > 366.1
3.618e+004

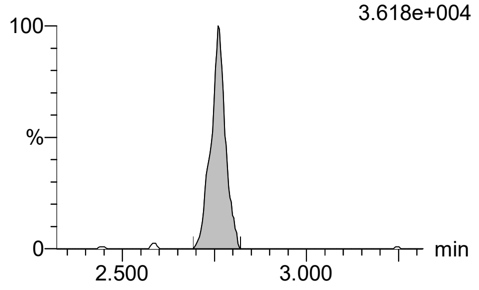

Dataset: C:\MassLynx\10.pro\20211115_clinicalsamples_IgG.qld

Last Altered: Sunday, October 02, 2022 16:02:30 台北標準時間
Printed: Friday, August 01, 2025 16:53:01 台北標準時間

Sample Name: 20211111_H8_R2_IgG
F39:MRM of 1 channel,ES+
1073.113 > 366.1
8.024e+004

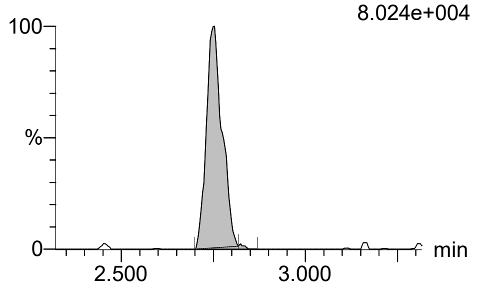

Sample Name: 20211111_A17_R2_IgG
F39:MRM of 1 channel,ES+
1073.113 > 366.1
8.845e+004

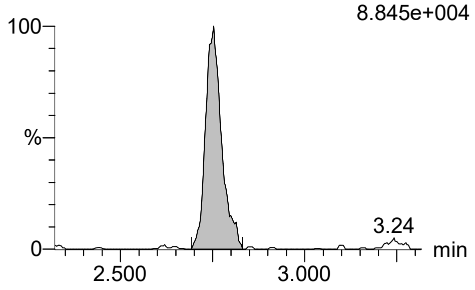

Sample Name: 20211111_A18_R2_IgG
F39:MRM of 1 channel,ES+
1073.113 > 366.1
4.993e+004

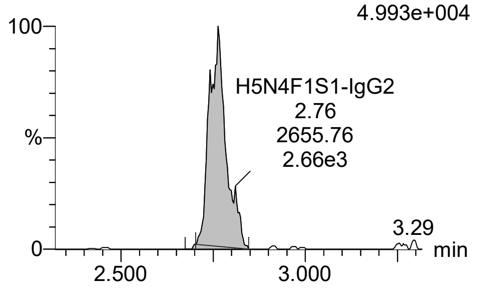

Sample Name: 20211111_L17_R2_IgG
F39:MRM of 1 channel,ES+
1073.113 > 366.1
8.981e+004

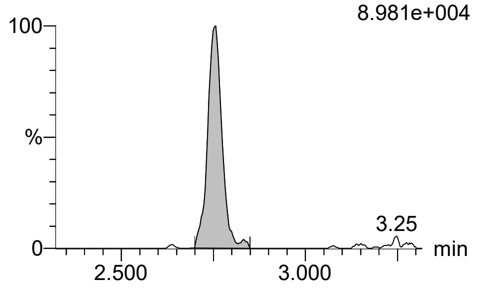

Sample Name: 20211111_L18_R2_IgG
F39:MRM of 1 channel,ES+
1073.113 > 366.1
9.316e+003

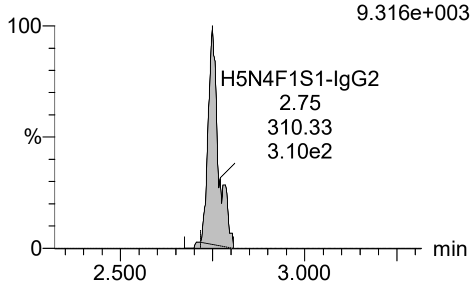

Sample Name: 20211111_H9_R2_IgG
F39:MRM of 1 channel,ES+
1073.113 > 366.1
4.426e+004

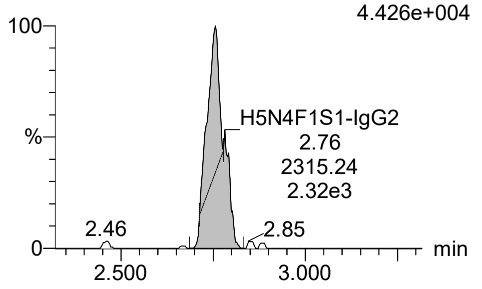

Sample Name: 20211111_A19_R2_IgG
F39:MRM of 1 channel,ES+
1073.113 > 366.1
4.708e+004

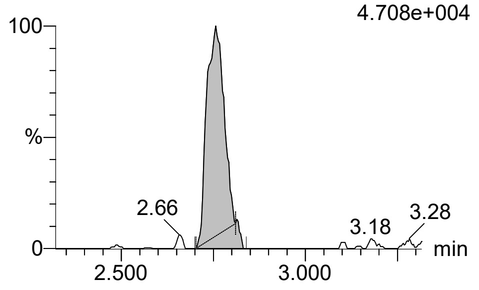

Sample Name: 20211111_A20_R2_IgG
F39:MRM of 1 channel,ES+
1073.113 > 366.1
3.241e+004

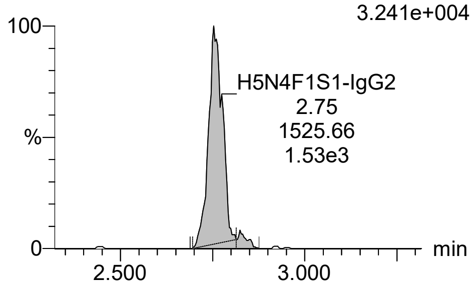

Sample Name: 20211111_L19_R2_IgG
F39:MRM of 1 channel,ES+
1073.113 > 366.1
3.453e+004

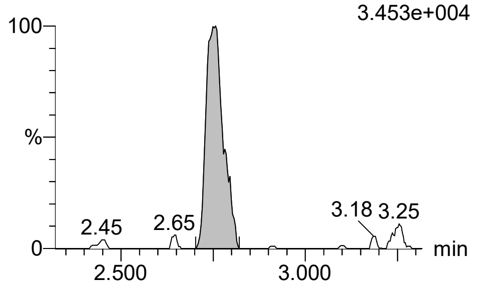

Sample Name: 20211111_QC_R13_IgG
F39:MRM of 1 channel,ES+
1073.113 > 366.1
5.829e+004

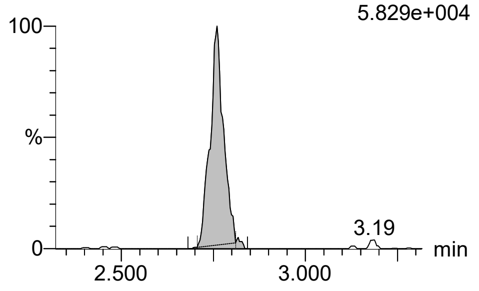

Sample Name: 20211111_L20_R2_IgG
F39:MRM of 1 channel,ES+
1073.113 > 366.1
4.825e+004

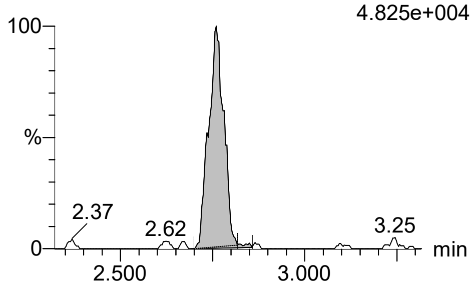

Sample Name: 20211111_H10_R2_IgG
F39:MRM of 1 channel,ES+
1073.113 > 366.1
3.641e+004

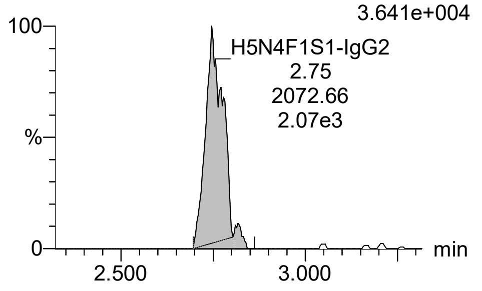

Sample Name: 20211111_A21_R2_IgG
F39:MRM of 1 channel,ES+
1073.113 > 366.1
1.283e+005

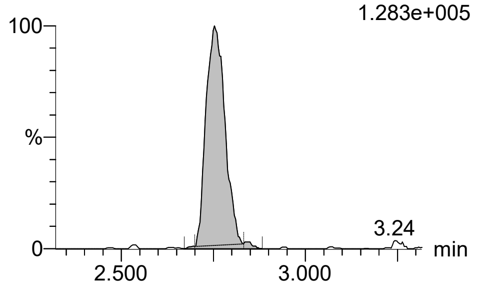

Sample Name: 20211111_A22_R2_IgG
F39:MRM of 1 channel,ES+
1073.113 > 366.1
5.405e+004

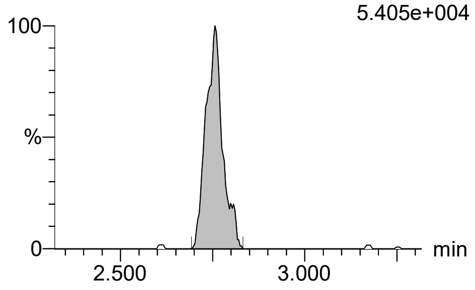

Sample Name: 20211111_L21_R2_IgG
F39:MRM of 1 channel,ES+
1073.113 > 366.1
1.984e+004

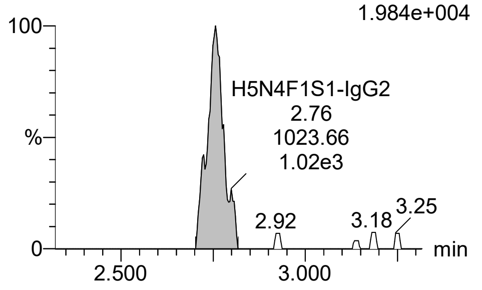

Dataset: C:\MassLynx\10.pro\20211115_clinicalsamples_IgG.qld

Last Altered: Sunday, October 02, 2022 16:02:30 台北標準時間
Printed: Friday, August 01, 2025 16:53:01 台北標準時間

Sample Name: 20211111_L22_R2_IgG
F39:MRM of 1 channel,ES+
1073.113 > 366.1
2.688e+004

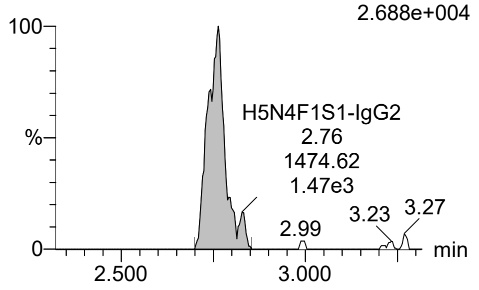

Sample Name: 20211111_H11_R2_IgG
F39:MRM of 1 channel,ES+
1073.113 > 366.1
6.012e+004

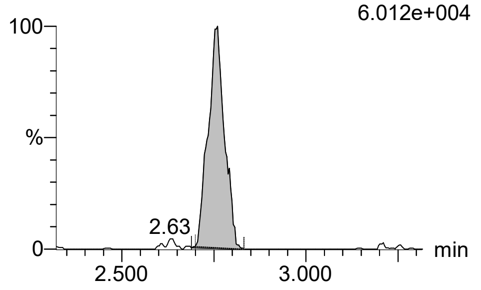

Sample Name: 20211111_A23_R2_IgG
F39:MRM of 1 channel,ES+
1073.113 > 366.1
2.855e+004

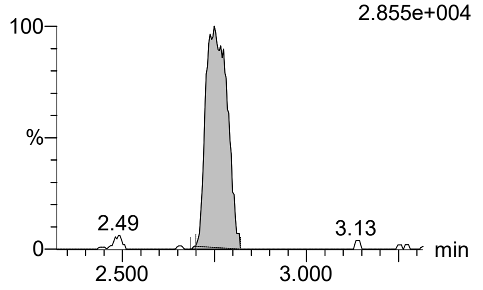

Sample Name: 20211111_A24_R2_IgG
F39:MRM of 1 channel,ES+
1073.113 > 366.1
3.634e+004

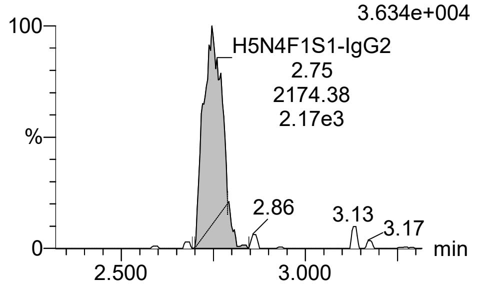

Sample Name: 20211111_L23_R2_IgG
F39:MRM of 1 channel,ES+
1073.113 > 366.1
6.073e+004

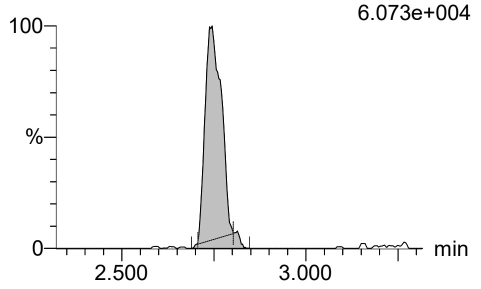

Sample Name: 20211111_L24_R2_IgG
F39:MRM of 1 channel,ES+
1073.113 > 366.1
6.087e+004

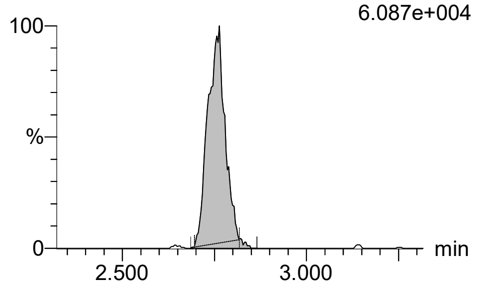

Sample Name: 20211111_H12_R2_IgG
F39:MRM of 1 channel,ES+
1073.113 > 366.1
4.590e+004

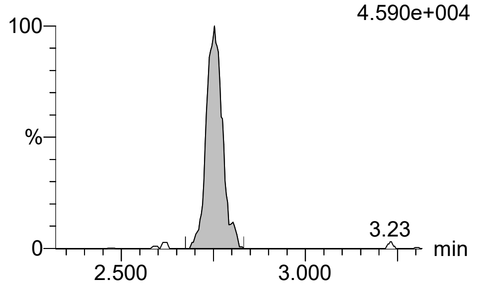

Sample Name: 20211111_QC_R14_IgG
F39:MRM of 1 channel,ES+
1073.113 > 366.1
4.061e+004

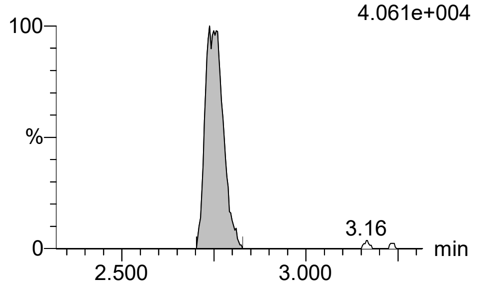

Sample Name: 20211111_A25_R2_IgG
F39:MRM of 1 channel,ES+
1073.113 > 366.1
4.580e+004

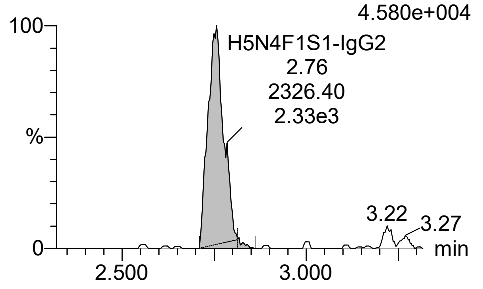

Sample Name: 20211111_A26_R2_IgG
F39:MRM of 1 channel,ES+
1073.113 > 366.1
2.466e+004

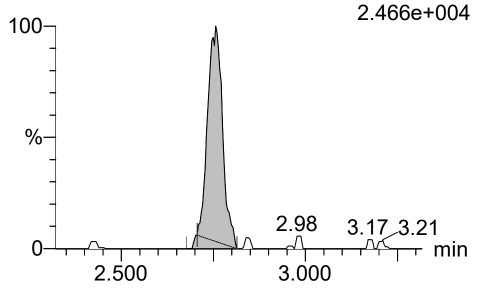

Sample Name: 20211111_L25_R2_IgG
F39:MRM of 1 channel,ES+
1073.113 > 366.1
6.519e+004

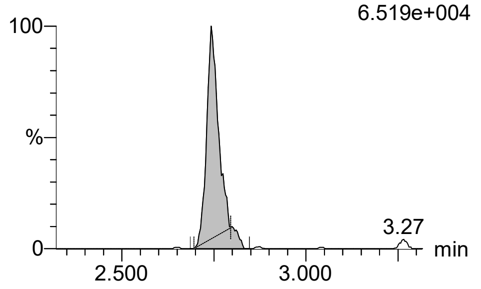

Sample Name: 20211111_L26_R2_IgG
F39:MRM of 1 channel,ES+
1073.113 > 366.1
1.056e+004

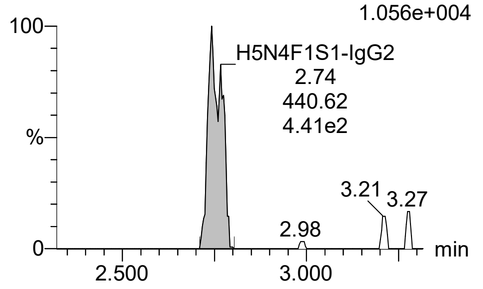

Sample Name: 20211111_H13_R2_IgG
F39:MRM of 1 channel,ES+
1073.113 > 366.1
6.794e+004

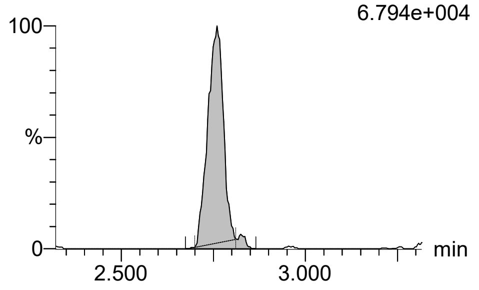

Sample Name: 20211111_A27_R2_IgG
F39:MRM of 1 channel,ES+
1073.113 > 366.1
9.919e+003

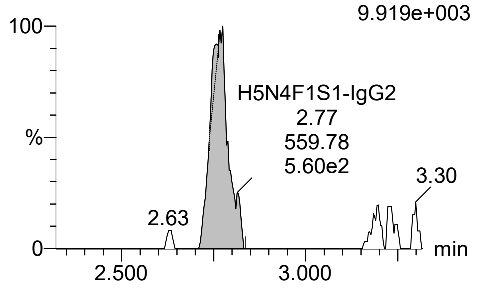

Sample Name: 20211111_A28_R2_IgG
F39:MRM of 1 channel,ES+
1073.113 > 366.1
4.979e+004

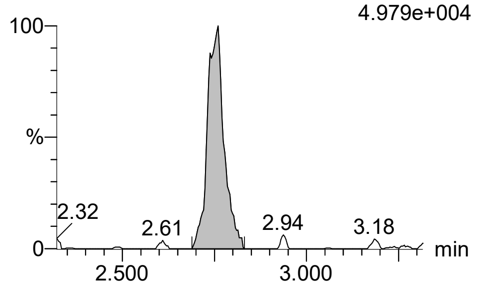

Sample Name: 20211111_L27_R2_IgG

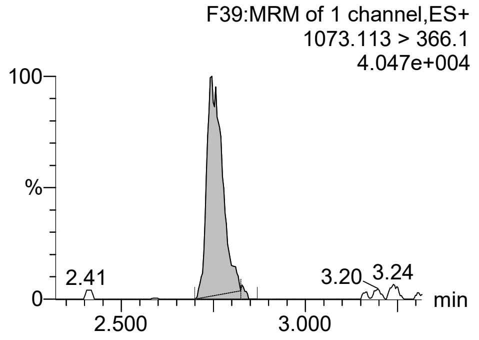

Sample Name: 20211111_L28_R2_IgG

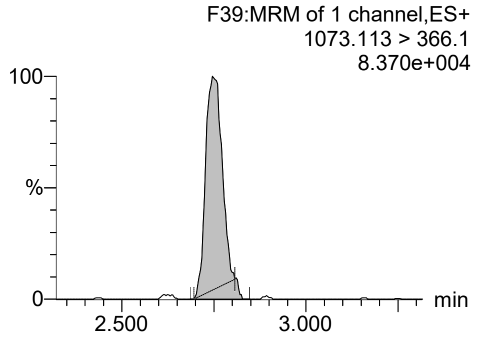

Sample Name: 20211111_H14_R2_IgG

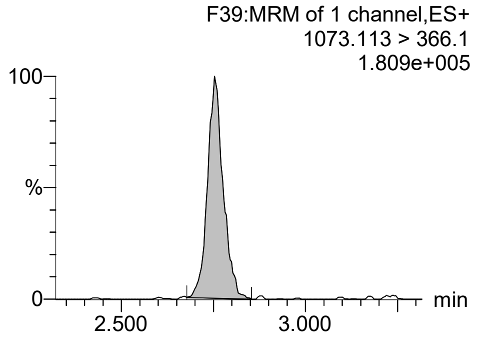

Sample Name: 20211111_A29_R2_IgG

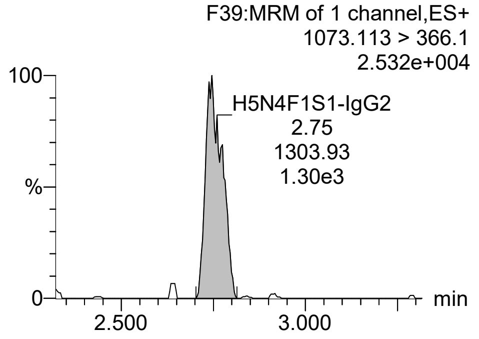

Sample Name: 20211111_A30_R2_IgG

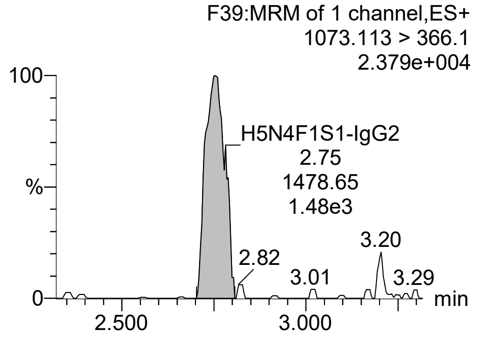

Sample Name: 20211111_QC_R15_IgG

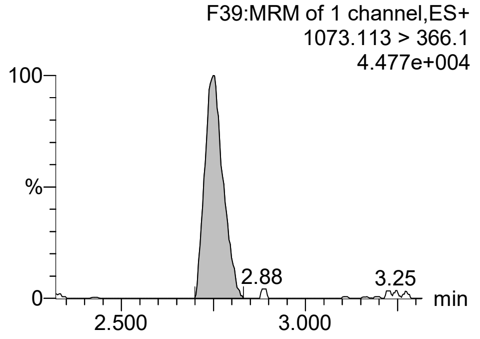

Sample Name: 20211111_L29_R2_IgG

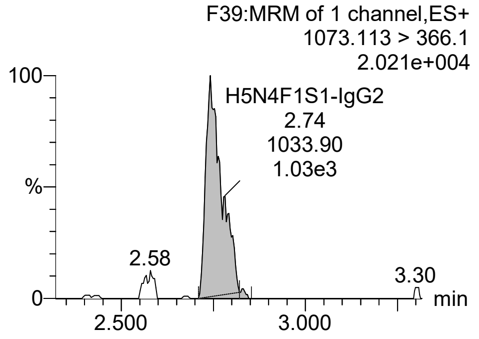

Sample Name: 20211111_L30_R2_IgG

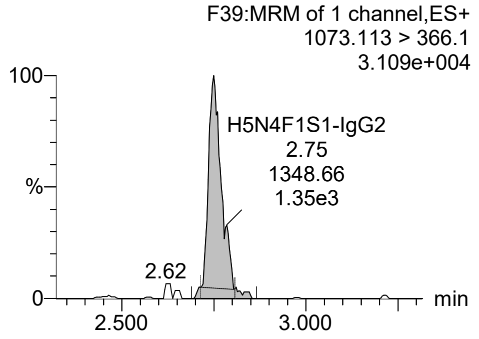

Sample Name: 20211111_H15_R2_IgG

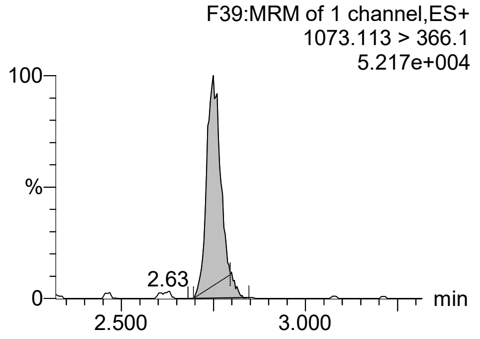

Sample Name: 20211111_A31_R2_IgG

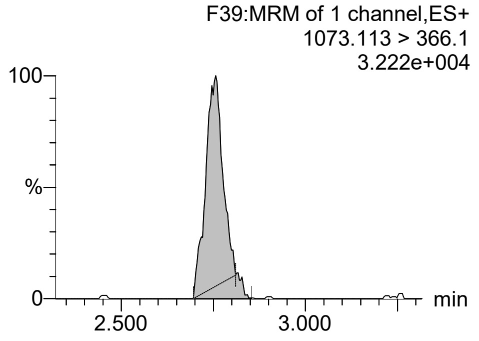

Sample Name: 20211111_A32_R2_IgG

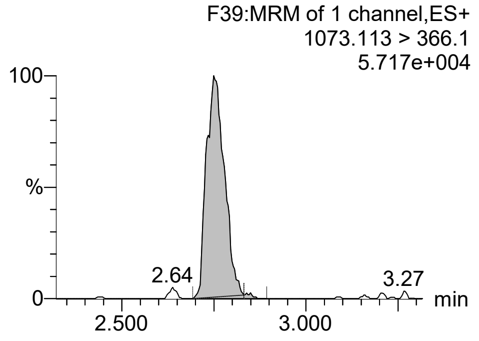

Sample Name: 20211111_L31_R2_IgG

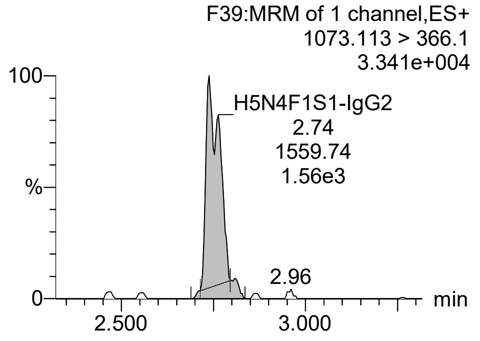

Sample Name: 20211111_L32_R2_IgG

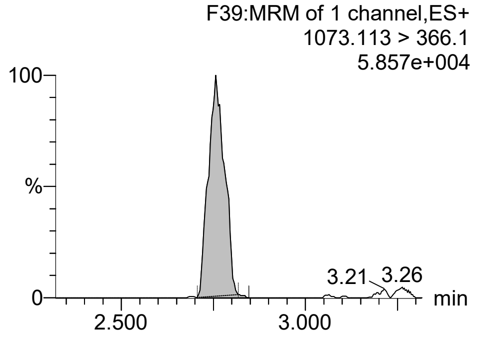

Sample Name: 20211111_H16_R2_IgG

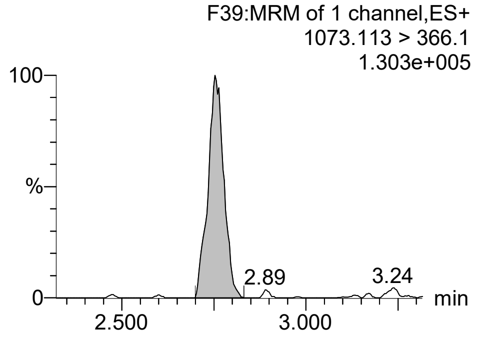

Sample Name: 20211111_A33_R2_IgG

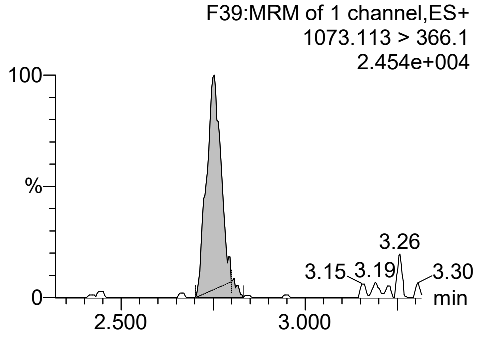

Sample Name: 20211111_A34_R2_IgG
F39:MRM of 1 channel,ES+
1073.113 > 366.1
3.676e+004

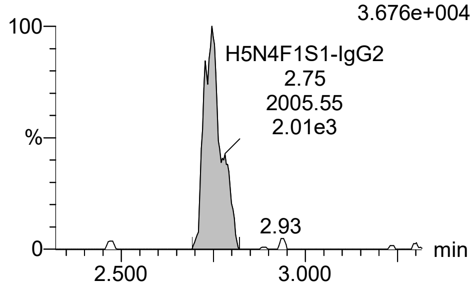

Sample Name: 20211111_H17_R2_IgG
F39:MRM of 1 channel,ES+
1073.113 > 366.1
2.090e+005

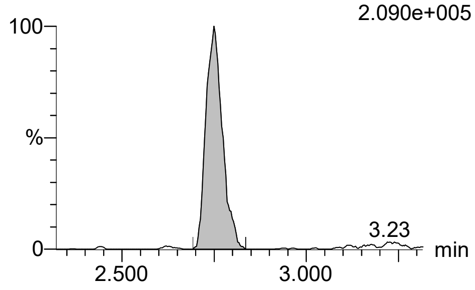

Sample Name: 20211111_A35_R2_IgG
F39:MRM of 1 channel,ES+
1073.113 > 366.1
8.629e+004

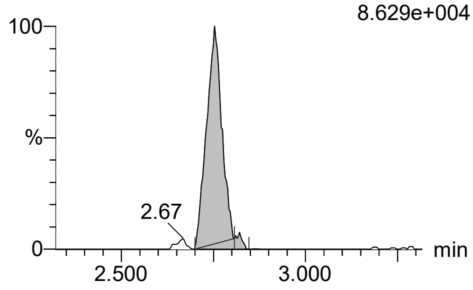

Sample Name: 20211111_QC_R16_IgG
F39:MRM of 1 channel,ES+
1073.113 > 366.1
6.504e+004

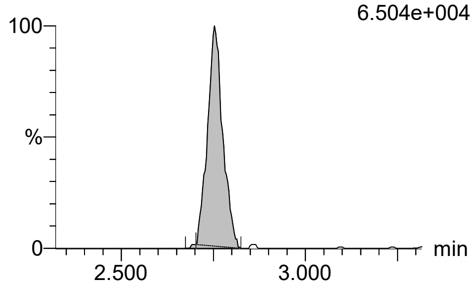

Sample Name: 20211111_A36_R2_IgG
F39:MRM of 1 channel,ES+
1073.113 > 366.1
4.052e+004

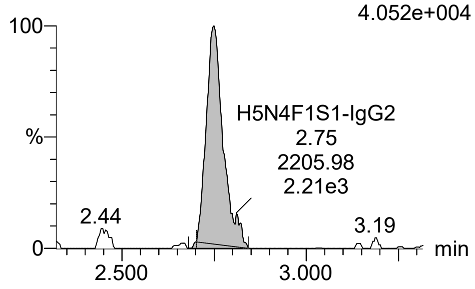

Sample Name: 20211111_H18_R2_IgG
F39:MRM of 1 channel,ES+
1073.113 > 366.1
1.557e+005

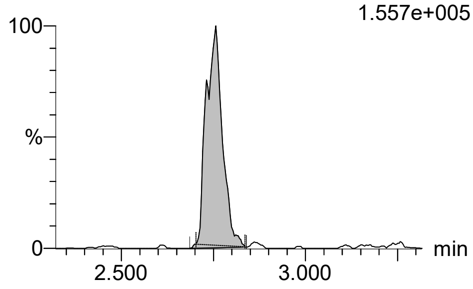

Sample Name: 20211111_A37_R2_IgG
F39:MRM of 1 channel,ES+
1073.113 > 366.1
4.534e+004

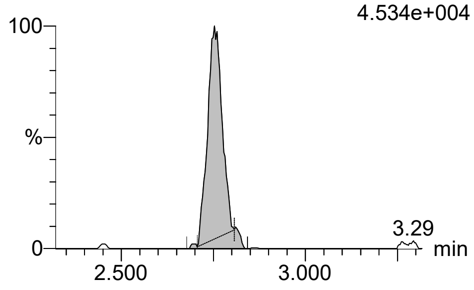

Sample Name: 20211111_A38_R2_IgG
F39:MRM of 1 channel,ES+
1073.113 > 366.1
3.439e+004

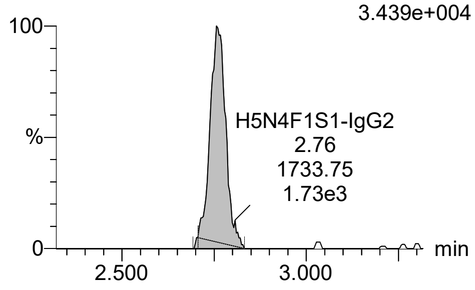

Sample Name: 20211111_H19_R2_IgG
F39:MRM of 1 channel,ES+
1073.113 > 366.1
3.525e+005

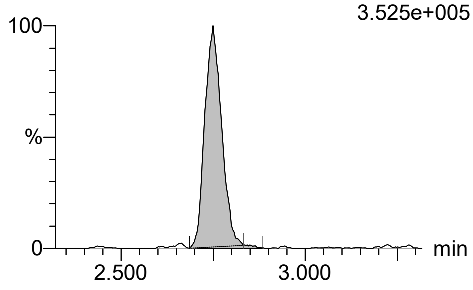

Sample Name: 20211111_A39_R2_IgG
F39:MRM of 1 channel,ES+
1073.113 > 366.1
5.898e+004

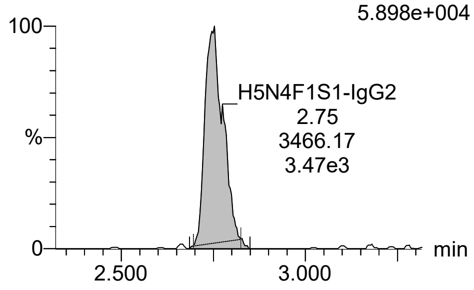

Sample Name: 20211111_A40_R2_IgG
F39:MRM of 1 channel,ES+
1073.113 > 366.1
5.517e+004

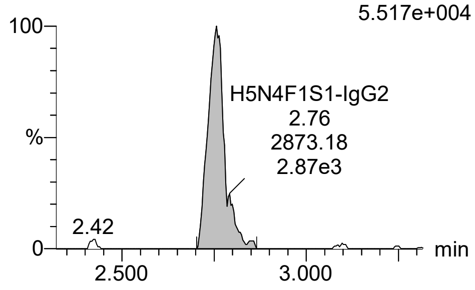

Sample Name: 20211111_H20_R2_IgG
F39:MRM of 1 channel,ES+
1073.113 > 366.1
3.462e+005

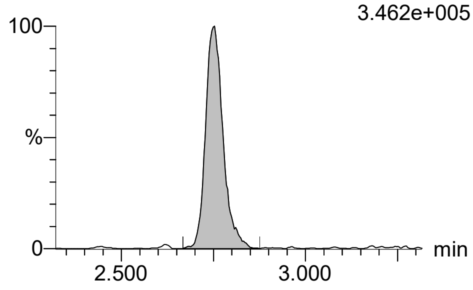

Sample Name: 20211111_A41_R2_IgG
F39:MRM of 1 channel,ES+
1073.113 > 366.1
4.529e+004

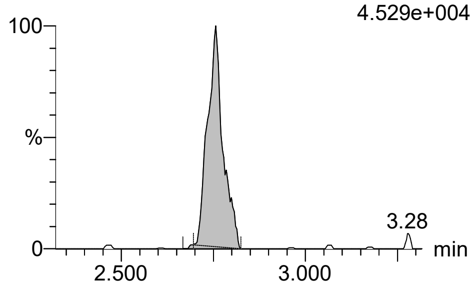

Sample Name: 20211111_A42_R2_IgG
F39:MRM of 1 channel,ES+
1073.113 > 366.1
5.285e+004

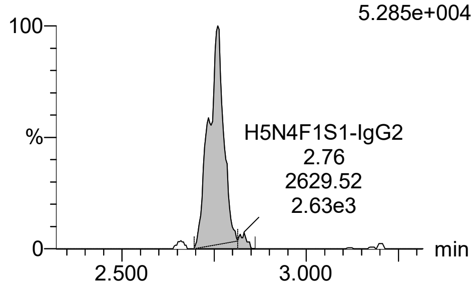

Sample Name: 20211111_A43_R2_IgG
F39:MRM of 1 channel,ES+
1073.113 > 366.1
1.315e+005

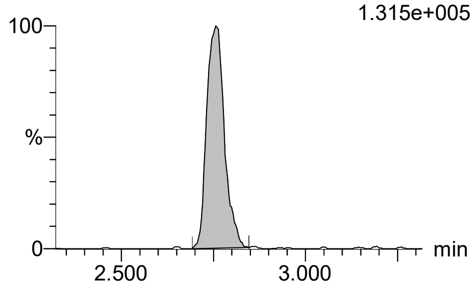

Dataset: C:\MassLynx\10.pro\20211115_clinicalsamples_IgG.qld

Last Altered: Sunday, October 02, 2022 16:02:30 台北標準時間
Printed: Friday, August 01, 2025 16:53:01 台北標準時間

Sample Name: 20211111_A44_R2_IgG

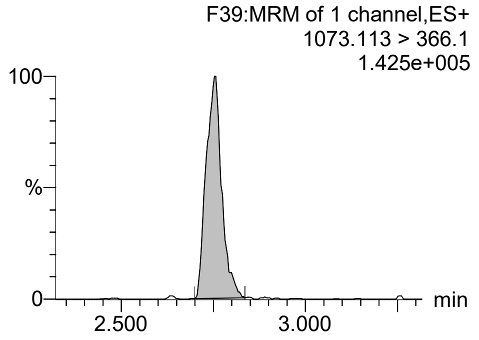

Sample Name: 20211111_QC_R17_IgG

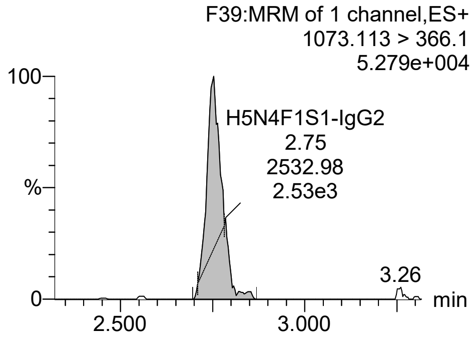

Dataset: C:\MassLynx\10.pro\20211115_clinicalsamples_IgG.qld

Last Altered: Sunday, October 02, 2022 16:02:30 台北標準時間
Printed: Friday, August 01, 2025 16:53:01 台北標準時間

	#	Name	RT	Area	IS Area	Response	Prima...
1	1	20211111_QC_R1_IgG	2.76	6730.5	64968.0	0.104	bb
2	2	20211111_A1_R1_IgG	2.76	8264.6	59059.2	0.140	MM
3	3	20211111_A2_R1_IgG	2.76	10360.8	66621.2	0.156	MM
4	4	20211111_L1_R1_IgG	2.76	2073.8	64201.0	0.032	MM
5	5	20211111_L2_R1_IgG	2.76	7002.2	46950.2	0.149	MM
6	6	20211111_H1_R1_IgG	2.76	1228.8	38648.2	0.032	MM
7	7	20211111_A3_R1_IgG	2.78	43.1	2603.7	0.017	MM
8	8	20211111_A4_R1_IgG	2.75	10136.2	69960.4	0.145	bb
9	9	20211111_L3_R1_IgG	2.75	1639.7	38864.7	0.042	bb
10	10	20211111_L4_R1_IgG	2.76	3631.2	43380.5	0.084	MM
11	11	20211111_H2_R1_IgG	2.76	12750.7	71349.2	0.179	MM
12	12	20211111_A5_R1_IgG	2.75	3333.8	65569.7	0.051	bb
13	13	20211111_A6_R1_IgG	2.76	11680.8	53120.7	0.220	bb
14	14	20211111_QC_R2_IgG	2.76	7368.4	81034.1	0.091	MM
15	15	20211111_L5_R1_IgG	2.76	3848.4	50994.6	0.075	MM
16	16	20211111_L6_R1_IgG	2.77	1746.7	58058.0	0.030	MM
17	17	20211111_H3_R1_IgG	2.76	6835.0	64028.6	0.107	MM
18	18	20211111_A7_R1_IgG	2.76	12785.3	82996.1	0.154	MM
19	19	20211111_A8_R1_IgG	2.76	6515.6	77731.7	0.084	MM
20	20	20211111_L7_R1_IgG	2.76	2852.4	38827.3	0.073	MM
21	21	20211111_L8_R1_IgG	2.76	4909.4	44220.1	0.111	bb
22	22	20211111_H4_R1_IgG	2.75	3427.1	65826.3	0.052	bb
23	23	20211111_A9_R1_IgG	2.77	2935.7	60771.1	0.048	bb
24	24	20211111_A10_R1_IgG	2.75	3806.3	48578.7	0.078	MM
25	25	20211111_L9_R1_IgG	2.75	5375.8	50830.7	0.106	bb
26	26	20211111_L10_R1_IgG	2.75	2243.0	38034.2	0.059	MM
27	27	20211111_QC_R3_IgG	2.76	6361.7	67025.5	0.095	bb
28	28	20211111_H5_R1_IgG	2.76	4314.6	44544.9	0.097	bb
29	29	20211111_A11_R1_IgG	2.76	1029.2	66408.5	0.015	MM
30	30	20211111_A12_R1_IgG	2.76	2638.7	46624.5	0.057	bb
31	31	20211111_L11_R1_IgG	2.74	835.7	33425.1	0.025	MM
32	32	20211111_L12_R1_IgG	2.74	1242.7	41178.0	0.030	MM
33	33	20211111_H6_R1_IgG	2.75	11223.9	58593.4	0.192	MM
34	34	20211111_A13_R1_IgG	2.75	5816.7	54133.0	0.107	MM
35	35	20211111_A14_R1_IgG	2.76	2618.2	56467.3	0.046	MM
36	36	20211111_L13_R1_IgG	2.76	6767.7	36024.4	0.188	bb
37	37	20211111_L14_R1_IgG	2.76	2809.4	37181.6	0.076	bb
38	38	20211111_H7_R1_IgG	2.76	4514.0	42850.6	0.105	bb
39	39	20211111_A15_R1_IgG	2.77	4722.9	62015.1	0.076	bb
40	40	20211111_QC_R4_IgG	2.76	5194.6	58572.5	0.089	bb
41	41	20211111_A16_R1_IgG	2.76	3402.9	78283.8	0.043	MM
42	42	20211111_L15_R1_IgG	2.76	6417.6	44158.2	0.145	bb
43	43	20211111_L16_R1_IgG	2.76	3019.7	55150.8	0.055	bb
44	44	20211111_H8_R1_IgG	2.75	4188.9	42965.2	0.097	MM
45	45	20211111_A17_R1_IgG	2.76	4586.3	39717.7	0.115	MM
46	46	20211111_A18_R1_IgG	2.76	4008.5	44675.4	0.090	bb
47	47	20211111_L17_R1_IgG	2.76	4138.9	37854.1	0.109	MM
48	48	20211111_L18_R1_IgG	2.76	464.3	50716.9	0.009	bb
49	49	20211111_H9_R1_IgG	2.74	3068.7	36276.0	0.085	MM
50	50	20211111_A19_R1_IgG	2.76	4211.9	42862.5	0.098	bb
51	51	20211111_A20_R1_IgG	2.75	1376.0	36542.7	0.038	MM
52	52	20211111_L19_R1_IgG	2.74	3351.1	53892.8	0.062	MM
53	53	20211111_QC_R5_IgG	2.76	4622.8	56513.2	0.082	MM
54	54	20211111_L20_R1_IgG	2.75	3338.5	56499.9	0.059	bb
55	55	20211111_H10_R1_IgG	2.75	4754.1	68120.5	0.070	MM
56	56	20211111_A21_R1_IgG	2.76	13950.1	63699.8	0.219	bb

Dataset: C:\MassLynx\10.pro\20211115_clinicalsamples_IgG.qld

Last Altered: Sunday, October 02, 2022 16:02:30 台北標準時間
Printed: Friday, August 01, 2025 16:53:01 台北標準時間

	# Name	RT	Area	IS Area	Response	Prima...
57	57 20211111_A22_R1_IgG	2.76	6561.4	52222.1	0.126	bb
58	58 20211111_L21_R1_IgG	2.77	2035.1	54372.5	0.037	bb
59	59 20211111_L22_R1_IgG	2.75	2114.7	41353.1	0.051	MM
60	60 20211111_H11_R1_IgG	2.76	6077.0	65579.1	0.093	MM
61	61 20211111_A23_R1_IgG	2.76	3661.7	64131.1	0.057	MM
62	62 20211111_A24_R1_IgG	2.76	2834.2	38724.8	0.073	MM
63	63 20211111_L23_R1_IgG	2.76	5709.0	64245.2	0.089	MM
64	64 20211111_L24_R1_IgG	2.75	4116.6	45298.0	0.091	MM
65	65 20211111_H12_R1_IgG	2.74	3117.4	44679.3	0.070	MM
66	66 20211111_QC_R6_IgG	2.75	3948.8	48165.0	0.082	bb
67	67 20211111_A25_R1_IgG	2.75	3651.7	57169.9	0.064	bb
68	68 20211111_A26_R1_IgG	2.76	1502.5	34254.0	0.044	bb
69	69 20211111_L25_R1_IgG	2.76	5011.0	67491.5	0.074	bb
70	70 20211111_L26_R1_IgG	2.75	1250.8	55934.1	0.022	MM
71	71 20211111_H13_R1_IgG	2.76	5778.3	48767.3	0.118	bb
72	72 20211111_A27_R1_IgG	2.75	1808.1	57269.7	0.032	bb
73	73 20211111_A28_R1_IgG	2.76	4578.3	49231.1	0.093	bb
74	74 20211111_L27_R1_IgG	2.74	2843.5	60014.5	0.047	bb
75	75 20211111_L28_R1_IgG	2.75	6511.5	35062.6	0.186	MM
76	76 20211111_H14_R1_IgG	2.76	15831.1	62371.9	0.254	bb
77	77 20211111_A29_R1_IgG	2.76	1614.7	43858.2	0.037	bb
78	78 20211111_A30_R1_IgG	2.75	2316.2	62500.4	0.037	bb
79	79 20211111_QC_R7_IgG	2.76	3774.9	43946.9	0.086	MM
80	80 20211111_L29_R1_IgG	2.76	1144.4	38728.9	0.030	bb
81	81 20211111_L30_R1_IgG	2.76	1794.2	42613.2	0.042	MM
82	82 20211111_H15_R1_IgG	2.76	3259.5	39874.6	0.082	bb
83	83 20211111_A31_R1_IgG	2.76	2765.6	53414.3	0.052	MM
84	84 20211111_A32_R1_IgG	2.76	3528.9	41844.6	0.084	MM
85	85 20211111_L31_R1_IgG	2.76	2391.5	40553.7	0.059	MM
86	86 20211111_L32_R1_IgG	2.75	3311.1	49618.7	0.067	MM
87	87 20211111_H16_R1_IgG	2.76	10368.8	60170.9	0.172	MM
88	88 20211111_A33_R1_IgG	2.77	2226.9	48588.2	0.046	MM
89	89 20211111_A34_R1_IgG	2.75	2352.5	43288.0	0.054	bb
90	90 20211111_H17_R1_IgG	2.76	12764.3	47104.5	0.271	bb
91	91 20211111_A35_R1_IgG	2.75	4519.5	41025.9	0.110	MM
92	92 20211111_QC_R8_IgG	2.76	3107.0	40546.3	0.077	bb
93	93 20211111_A36_R1_IgG	2.76	2786.6	44504.4	0.063	bb
94	94 20211111_H18_R1_IgG	2.76	9045.9	34162.2	0.265	MM
95	95 20211111_A37_R1_IgG	2.75	3132.8	39760.0	0.079	MM
96	96 20211111_A38_R1_IgG	2.76	1599.8	43982.6	0.036	MM
97	97 20211111_H19_R1_IgG	2.76	21133.4	45314.8	0.466	bb
98	98 20211111_A39_R1_IgG	2.76	3922.2	39388.0	0.100	MM
99	99 20211111_A40_R1_IgG	2.76	2908.6	38726.4	0.075	MM
100	1... 20211111_H20_R1_IgG	2.76	20387.0	37814.4	0.539	MM
101	1... 20211111_A41_R1_IgG	2.76	2995.0	48930.3	0.061	MM
102	1... 20211111_A42_R1_IgG	2.76	3029.9	40406.8	0.075	bb
103	1... 20211111_A43_R1_IgG	2.76	9470.6	44421.5	0.213	MM
104	1... 20211111_A44_R1_IgG	2.76	10463.5	53128.2	0.197	bb
105	1... 20211111_QC_R9_IgG	2.75	3301.8	37049.0	0.089	bb
106	1... 20211111_A1_R2_IgG	2.76	6455.8	46499.4	0.139	bb
107	1... 20211111_A2_R2_IgG	2.76	7708.8	57260.7	0.135	bb
108	1... 20211111_L1_R2_IgG	2.76	1562.7	48062.9	0.033	MM
109	1... 20211111_L2_R2_IgG	2.76	5609.8	38745.2	0.145	MM
110	1... 20211111_H1_R2_IgG	2.76	1688.7	44273.5	0.038	bb
111	1... 20211111_A3_R2_IgG	2.77	186.0	4755.5	0.039	bb
112	1... 20211111_A4_R2_IgG	2.76	5964.3	50546.7	0.118	bb

Dataset: C:\MassLynx\10.pro\20211115_clinicalsamples_IgG.qld

Last Altered: Sunday, October 02, 2022 16:02:30 台北標準時間
Printed: Friday, August 01, 2025 16:53:01 台北標準時間

	# Name	RT	Area	IS Area	Response	Prima...
113	1... 20211111_L3_R2_IgG	2.76	1385.1	34825.8	0.040	MM
114	1... 20211111_L4_R2_IgG	2.76	2477.3	39107.7	0.063	MM
115	1... 20211111_H2_R2_IgG	2.75	7911.2	49750.0	0.159	MM
116	1... 20211111_A5_R2_IgG	2.75	2732.3	52536.2	0.052	MM
117	1... 20211111_A6_R2_IgG	2.76	8693.8	42050.0	0.207	bb
118	1... 20211111_QC_R10_IgG	2.75	2956.0	35350.8	0.084	bb
119	1... 20211111_L5_R2_IgG	2.75	2590.7	38039.3	0.068	MM
120	1... 20211111_L6_R2_IgG	2.75	1065.3	44907.4	0.024	bb
121	1... 20211111_H3_R2_IgG	2.75	5380.9	47729.1	0.113	MM
122	1... 20211111_A7_R2_IgG	2.75	8553.5	57347.1	0.149	bb
123	1... 20211111_A8_R2_IgG	2.75	4598.4	52397.8	0.088	MM
124	1... 20211111_L7_R2_IgG	2.75	2906.8	36318.4	0.080	bb
125	1... 20211111_L8_R2_IgG	2.76	4324.8	38131.9	0.113	MM
126	1... 20211111_H4_R2_IgG	2.75	2566.5	46849.6	0.055	MM
127	1... 20211111_A9_R2_IgG	2.77	2358.5	47759.6	0.049	MM
128	1... 20211111_A10_R2_IgG	2.76	2932.3	41366.2	0.071	MM
129	1... 20211111_L9_R2_IgG	2.75	4189.1	43052.1	0.097	bb
130	1... 20211111_L10_R2_IgG	2.77	2034.8	31487.4	0.065	MM
131	1... 20211111_QC_R11_IgG	2.76	3122.8	37804.6	0.083	bb
132	1... 20211111_H5_R2_IgG	2.75	3486.3	40380.0	0.086	MM
133	1... 20211111_A11_R2_IgG	2.75	494.4	49533.3	0.010	bb
134	1... 20211111_A12_R2_IgG	2.76	1582.1	40226.3	0.039	MM
135	1... 20211111_L11_R2_IgG	2.76	760.5	28884.0	0.026	MM
136	1... 20211111_L12_R2_IgG	2.77	1143.4	34351.3	0.033	MM
137	1... 20211111_H6_R2_IgG	2.76	7475.9	40466.6	0.185	MM
138	1... 20211111_A13_R2_IgG	2.75	5162.4	42306.7	0.122	MM
139	1... 20211111_A14_R2_IgG	2.75	1797.4	44904.0	0.040	MM
140	1... 20211111_L13_R2_IgG	2.75	5499.7	31913.0	0.172	MM
141	1... 20211111_L14_R2_IgG	2.75	2554.5	31527.4	0.081	bb
142	1... 20211111_H7_R2_IgG	2.75	3768.2	40838.2	0.092	MM
143	1... 20211111_A15_R2_IgG	2.76	2866.6	49389.7	0.058	MM
144	1... 20211111_QC_R12_IgG	2.76	3019.9	35168.7	0.086	bb
145	1... 20211111_A16_R2_IgG	2.76	2003.0	47440.5	0.042	bb
146	1... 20211111_L15_R2_IgG	2.75	5189.9	33145.9	0.157	MM
147	1... 20211111_L16_R2_IgG	2.76	1631.1	37596.6	0.043	bb
148	1... 20211111_H8_R2_IgG	2.75	4026.5	43933.3	0.092	MM
149	1... 20211111_A17_R2_IgG	2.75	4440.6	43605.5	0.102	bb
150	1... 20211111_A18_R2_IgG	2.76	2655.8	37581.8	0.071	MM
151	1... 20211111_L17_R2_IgG	2.76	4048.4	33942.5	0.119	bb
152	1... 20211111_L18_R2_IgG	2.75	310.3	37831.5	0.008	MM
153	1... 20211111_H9_R2_IgG	2.76	2315.2	30898.4	0.075	MM
154	1... 20211111_A19_R2_IgG	2.76	2776.6	39352.1	0.071	MM
155	1... 20211111_A20_R2_IgG	2.75	1525.7	34661.2	0.044	MM
156	1... 20211111_L19_R2_IgG	2.76	1890.1	39918.3	0.047	bb
157	1... 20211111_QC_R13_IgG	2.76	2551.6	36239.1	0.070	MM
158	1... 20211111_L20_R2_IgG	2.76	2328.6	32897.7	0.071	MM
159	1... 20211111_H10_R2_IgG	2.75	2072.7	31394.5	0.066	MM
160	1... 20211111_A21_R2_IgG	2.75	7882.3	34863.7	0.226	MM
161	1... 20211111_A22_R2_IgG	2.76	2832.2	30342.1	0.093	bb
162	1... 20211111_L21_R2_IgG	2.76	1023.7	33706.2	0.030	bb
163	1... 20211111_L22_R2_IgG	2.76	1474.6	31298.6	0.047	bb
164	1... 20211111_H11_R2_IgG	2.76	3080.8	38904.1	0.079	MM
165	1... 20211111_A23_R2_IgG	2.75	1946.4	32382.3	0.060	MM
166	1... 20211111_A24_R2_IgG	2.75	2174.4	30261.9	0.072	MM
167	1... 20211111_L23_R2_IgG	2.75	3314.8	43037.5	0.077	MM
168	1... 20211111_L24_R2_IgG	2.76	3378.8	37023.3	0.091	MM

Dataset: C:\MassLynx\10.pro\20211115_clinicalsamples_IgG.qld

Last Altered: Sunday, October 02, 2022 16:02:30 台北標準時間
Printed: Friday, August 01, 2025 16:53:01 台北標準時間

	#	Name	RT	Area	IS Area	Response	Prima...
169	1...	20211111_H12_R2_IgG	2.75	2324.4	39478.2	0.059	bb
170	1...	20211111_QC_R14_IgG	2.74	2325.7	34463.2	0.067	bb
171	1...	20211111_A25_R2_IgG	2.76	2326.4	39945.3	0.058	MM
172	1...	20211111_A26_R2_IgG	2.76	1198.7	29663.2	0.040	MM
173	1...	20211111_L25_R2_IgG	2.74	2703.9	38589.3	0.070	MM
174	1...	20211111_L26_R2_IgG	2.74	440.6	38405.2	0.011	bb
175	1...	20211111_H13_R2_IgG	2.76	3518.5	37771.2	0.093	MM
176	1...	20211111_A27_R2_IgG	2.77	559.8	42634.1	0.013	MM
177	1...	20211111_A28_R2_IgG	2.76	2591.7	33231.1	0.078	bb
178	1...	20211111_L27_R2_IgG	2.75	2090.8	41761.1	0.050	MM
179	1...	20211111_L28_R2_IgG	2.75	4647.2	26968.6	0.172	MM
180	1...	20211111_H14_R2_IgG	2.75	8809.5	49250.2	0.179	bb
181	1...	20211111_A29_R2_IgG	2.75	1303.9	37601.7	0.035	bb
182	1...	20211111_A30_R2_IgG	2.75	1478.7	40366.8	0.037	bb
183	1...	20211111_QC_R15_IgG	2.75	2587.9	35156.1	0.074	bb
184	1...	20211111_L29_R2_IgG	2.74	1033.9	33813.1	0.031	MM
185	1...	20211111_L30_R2_IgG	2.75	1348.7	33488.6	0.040	MM
186	1...	20211111_H15_R2_IgG	2.75	2389.8	38501.0	0.062	MM
187	1...	20211111_A31_R2_IgG	2.76	1912.5	37525.5	0.051	MM
188	1...	20211111_A32_R2_IgG	2.75	3316.4	35770.8	0.093	MM
189	1...	20211111_L31_R2_IgG	2.74	1559.7	33282.4	0.047	MM
190	1...	20211111_L32_R2_IgG	2.76	3007.5	42049.6	0.072	MM
191	1...	20211111_H16_R2_IgG	2.75	6413.1	45947.6	0.140	bb
192	1...	20211111_A33_R2_IgG	2.75	1185.1	40202.3	0.029	MM
193	1...	20211111_A34_R2_IgG	2.75	2005.5	36747.0	0.055	bb
194	1...	20211111_H17_R2_IgG	2.75	10775.6	40920.9	0.263	bb
195	1...	20211111_A35_R2_IgG	2.75	4345.2	33689.2	0.129	MM
196	1...	20211111_QC_R16_IgG	2.75	3154.5	32783.6	0.096	MM
197	1...	20211111_A36_R2_IgG	2.75	2206.0	36822.4	0.060	MM
198	1...	20211111_H18_R2_IgG	2.76	8004.8	31769.3	0.252	MM
199	1...	20211111_A37_R2_IgG	2.75	2273.0	37581.8	0.060	MM
200	2...	20211111_A38_R2_IgG	2.76	1733.8	38390.4	0.045	MM
201	2...	20211111_H19_R2_IgG	2.75	18926.4	41217.0	0.459	MM
202	2...	20211111_A39_R2_IgG	2.75	3466.2	37392.7	0.093	MM
203	2...	20211111_A40_R2_IgG	2.76	2873.2	38295.1	0.075	bb
204	2...	20211111_H20_R2_IgG	2.75	18988.7	35941.8	0.528	bb
205	2...	20211111_A41_R2_IgG	2.76	2222.2	45304.9	0.049	MM
206	2...	20211111_A42_R2_IgG	2.76	2629.5	37803.8	0.070	MM
207	2...	20211111_A43_R2_IgG	2.76	7287.7	38161.8	0.191	bb
208	2...	20211111_A44_R2_IgG	2.75	6923.9	37819.8	0.183	bb
209	2...	20211111_QC_R17_IgG	2.75	2533.0	31731.8	0.080	MM

Dataset: C:\MassLynx\10.pro\20211115_clinicalsamples_IgG.qld

Last Altered: Sunday, October 02, 2022 16:02:30 台北標準時間
Printed: Friday, August 01, 2025 16:53:36 台北標準時間

Method: C:\MassLynx\10_Pro_IgA.PRO\MethDB\20210926_IgG_DMRM_IgM_IS.mdb 16 Feb 2022 11:58:21
Calibration: 18 Nov 2021 13:56:22

Sample Name: 20211111_QC_R1_IgG

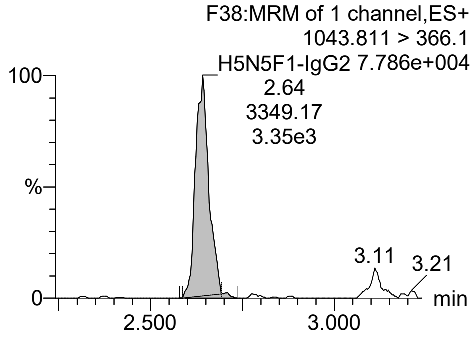

Sample Name: 20211111_A1_R1_IgG

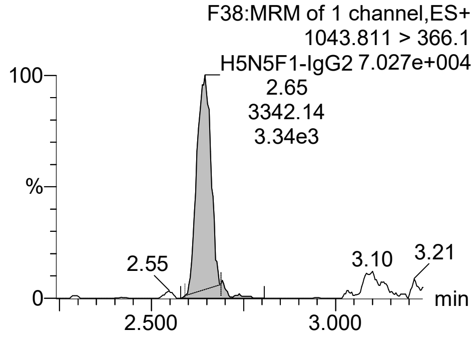

Sample Name: 20211111_A2_R1_IgG

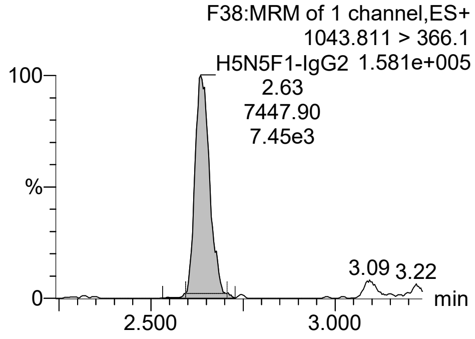

Sample Name: 20211111_L1_R1_IgG

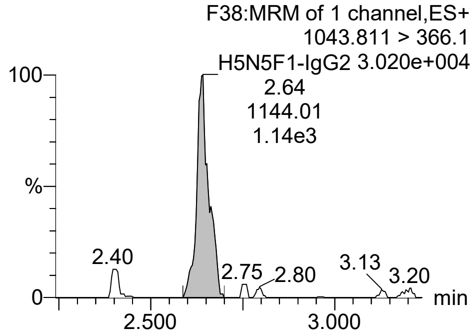

Sample Name: 20211111_L2_R1_IgG

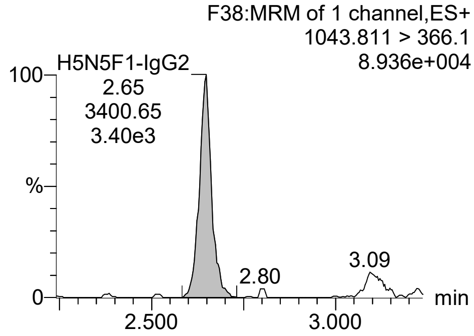

Sample Name: 20211111_H1_R1_IgG

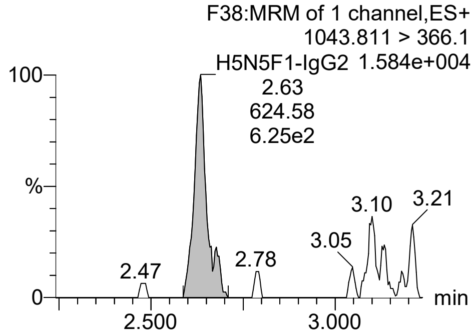

Sample Name: 20211111_A3_R1_IgG

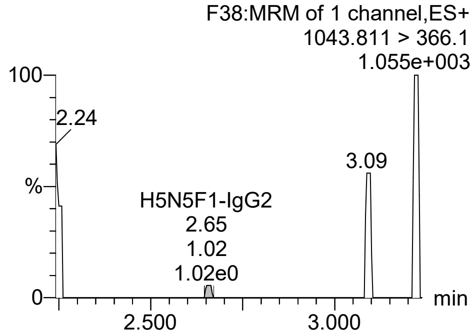

Sample Name: 20211111_A4_R1_IgG

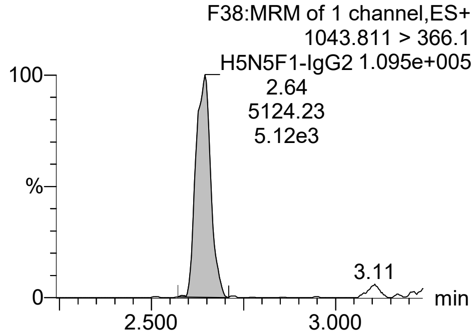

Sample Name: 20211111_L3_R1_IgG

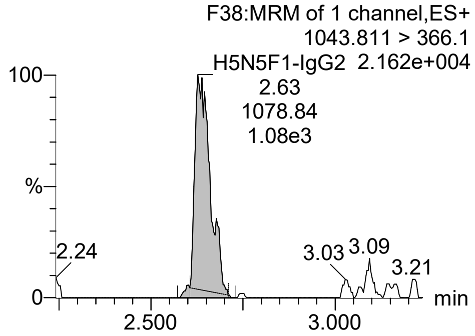

Sample Name: 20211111_L4_R1_IgG

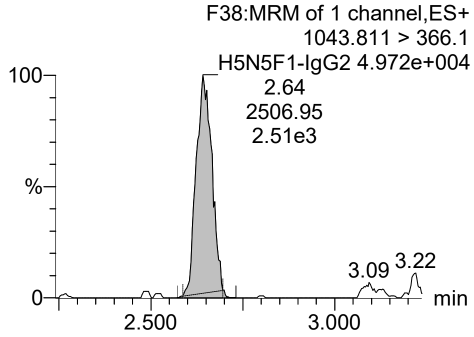

Sample Name: 20211111_H2_R1_IgG

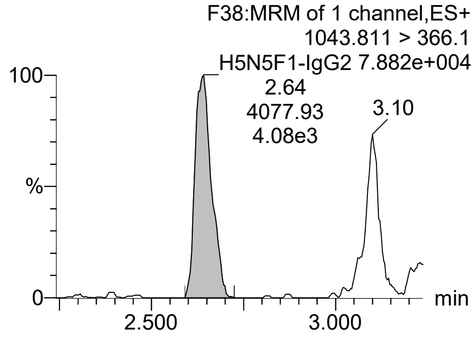

Sample Name: 20211111_A5_R1_IgG

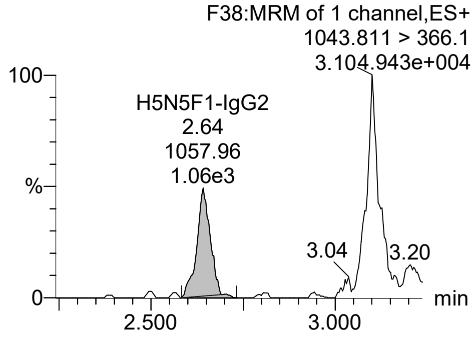

Sample Name: 20211111_A6_R1_IgG

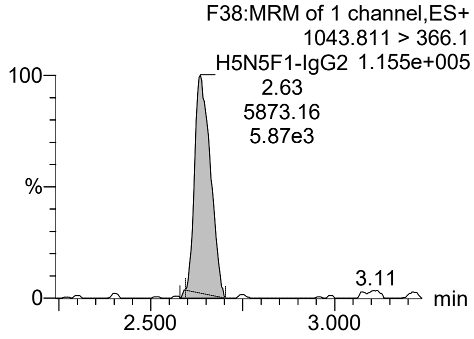

Sample Name: 20211111_QC_R2_IgG

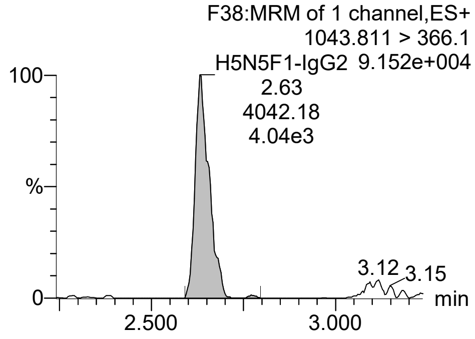

Sample Name: 20211111_L5_R1_IgG

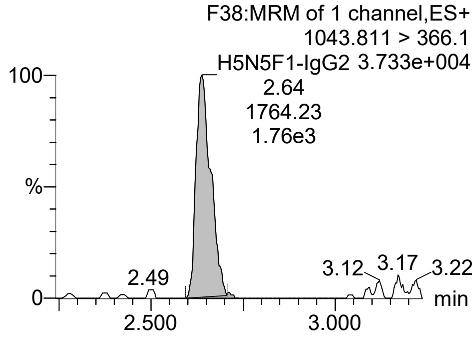

Sample Name: 20211111_L6_R1_IgG

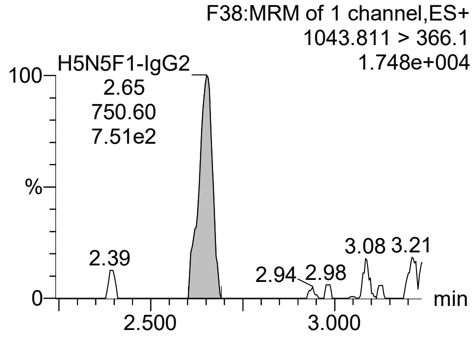

Sample Name: 20211111_H3_R1_IgG

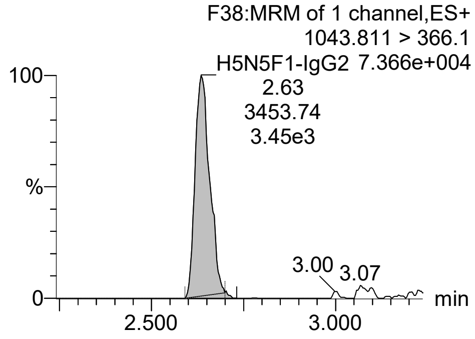

Sample Name: 20211111_A7_R1_IgG

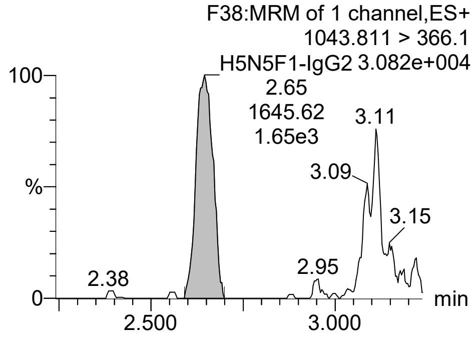

Sample Name: 20211111_A8_R1_IgG

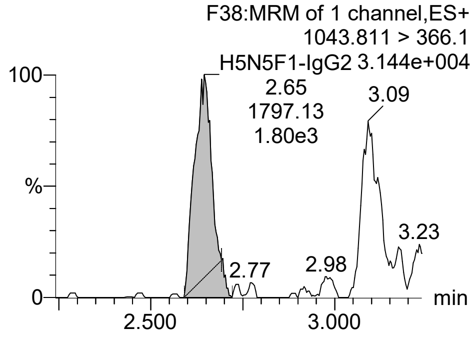

Sample Name: 20211111_L7_R1_IgG

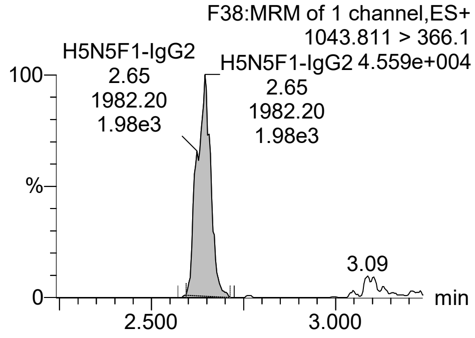

Sample Name: 20211111_L8_R1_IgG

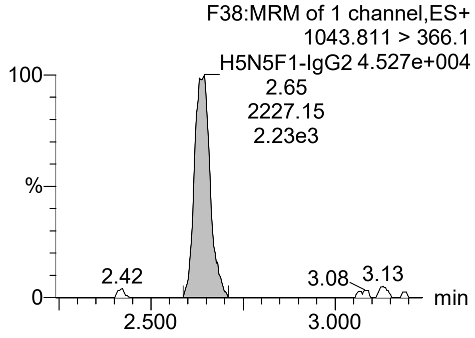

Sample Name: 20211111_H4_R1_IgG

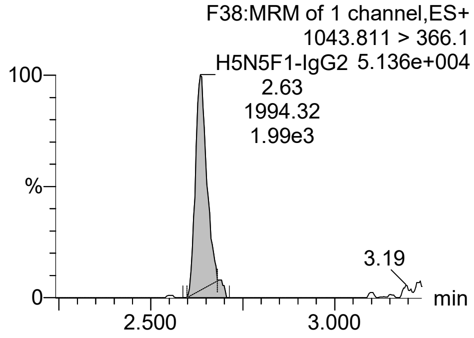

Sample Name: 20211111_A9_R1_IgG

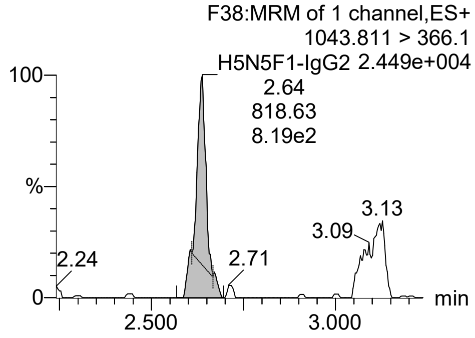

Sample Name: 20211111_A10_R1_IgG

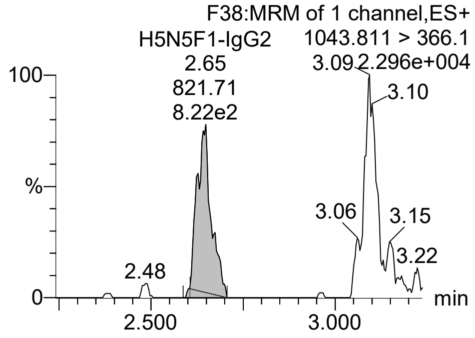

Sample Name: 20211111_L9_R1_IgG

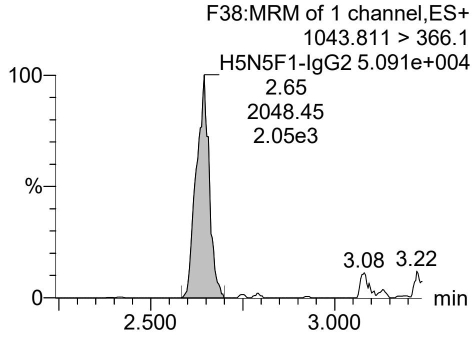

Sample Name: 20211111_L10_R1_IgG

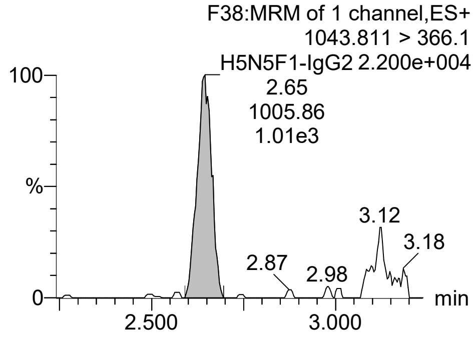

Sample Name: 20211111_QC_R3_IgG

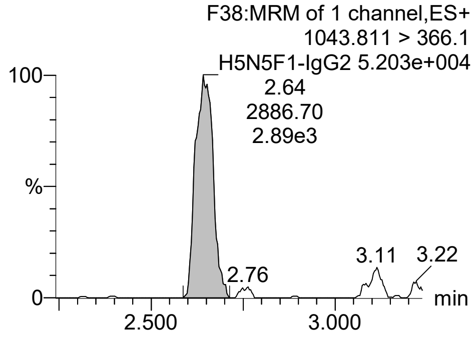

Sample Name: 20211111_H5_R1_IgG

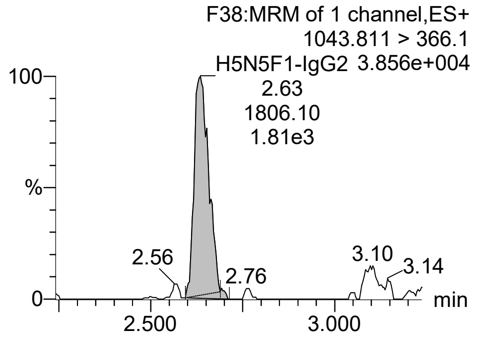

Sample Name: 20211111_A11_R1_IgG

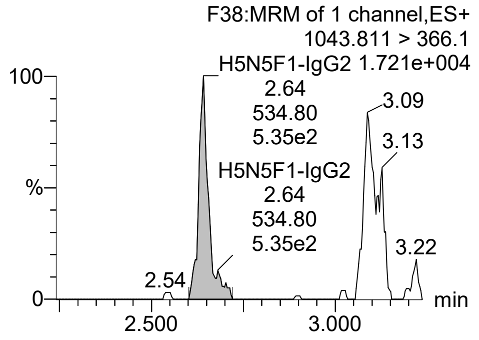

Sample Name: 20211111_A12_R1_IgG

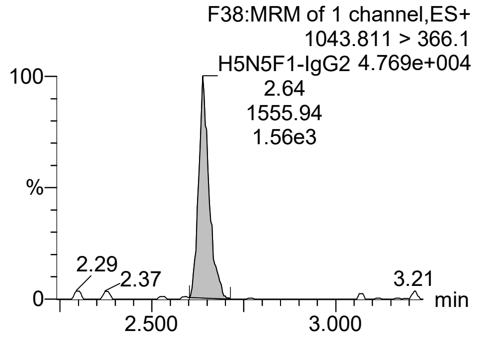

Sample Name: 20211111_L11_R1_IgG

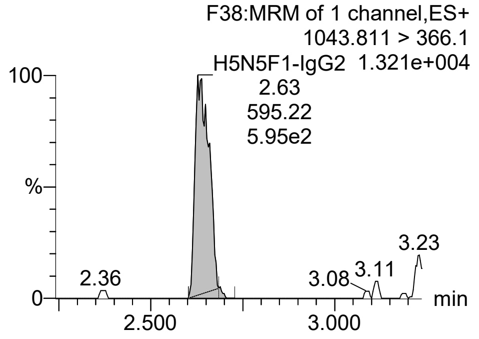

Sample Name: 20211111_L12_R1_IgG

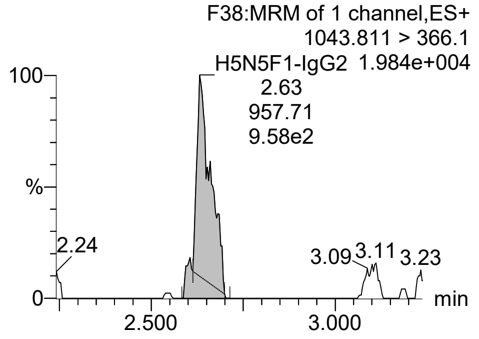

Sample Name: 20211111_H6_R1_IgG

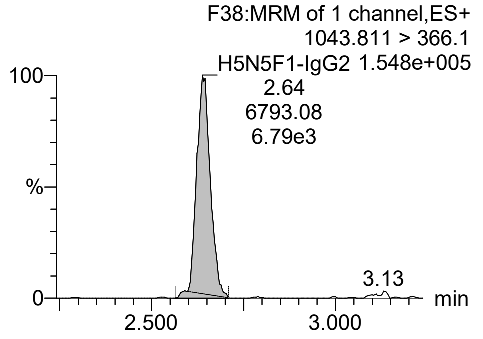

Sample Name: 20211111_A13_R1_IgG

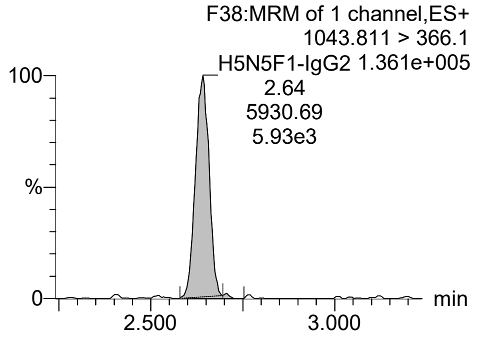

Sample Name: 20211111_A14_R1_IgG

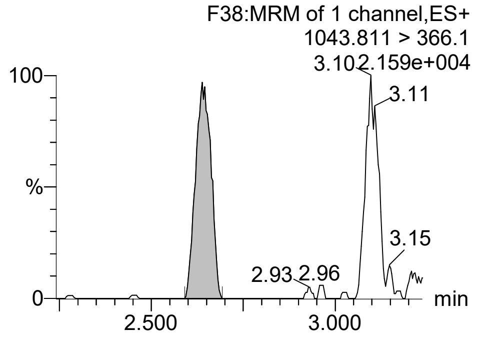

Sample Name: 20211111_L13_R1_IgG

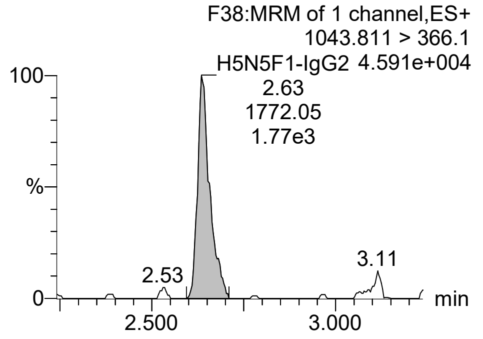

Sample Name: 20211111_L14_R1_IgG

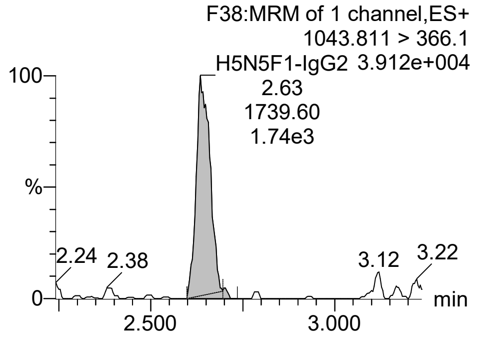

Sample Name: 20211111_H7_R1_IgG

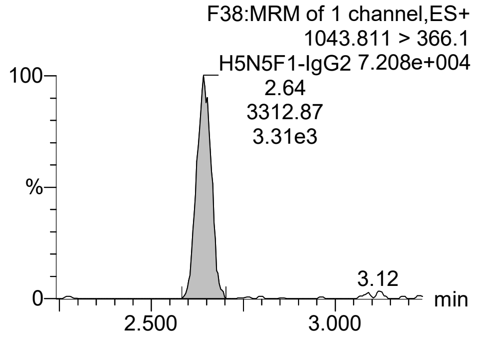

Sample Name: 20211111_A15_R1_IgG

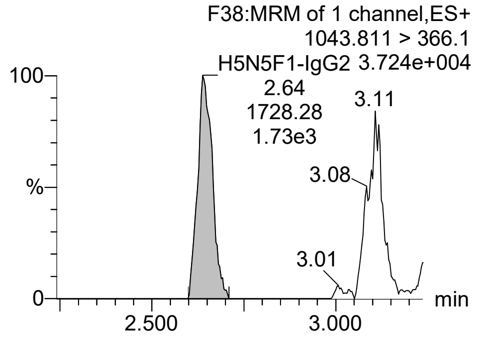

Sample Name: 20211111_QC_R4_IgG

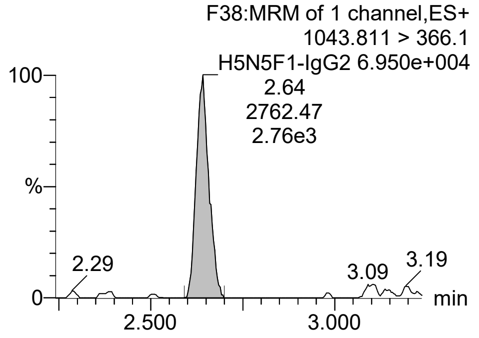

Sample Name: 20211111_A16_R1_IgG

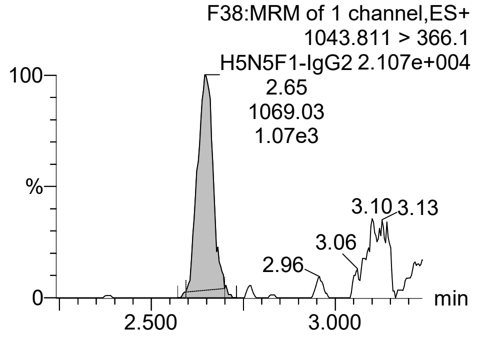

Sample Name: 20211111_L15_R1_IgG

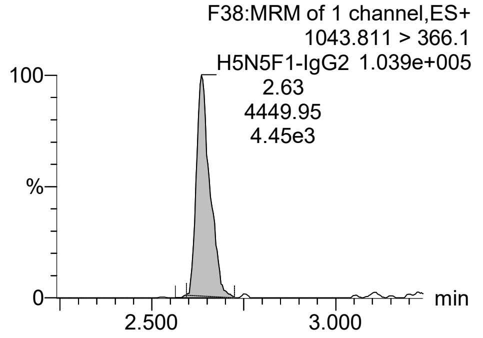

Dataset: C:\MassLynx\10.pro\20211115_clinicalsamples_IgG.qld

Last Altered: Sunday, October 02, 2022 16:02:30 台北標準時間
Printed: Friday, August 01, 2025 16:53:36 台北標準時間

Sample Name: 20211111_L16_R1_IgG

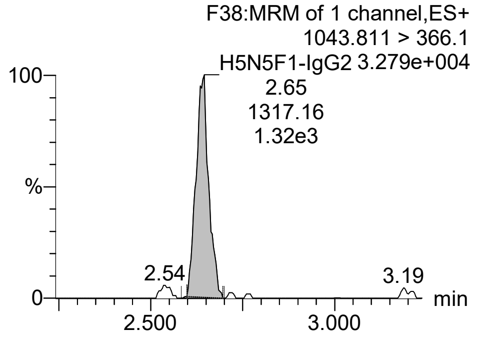

Sample Name: 20211111_H8_R1_IgG

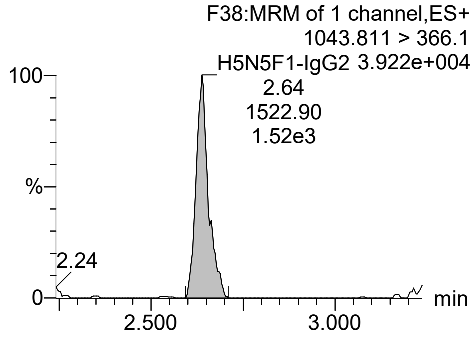

Sample Name: 20211111_A17_R1_IgG

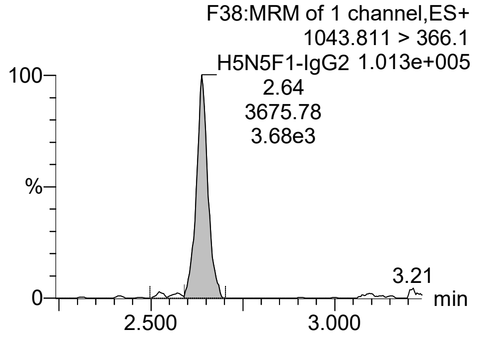

Sample Name: 20211111_A18_R1_IgG

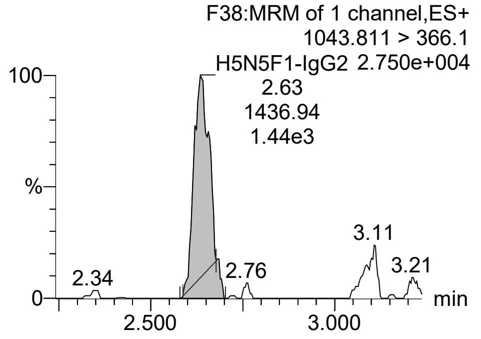

Sample Name: 20211111_L17_R1_IgG

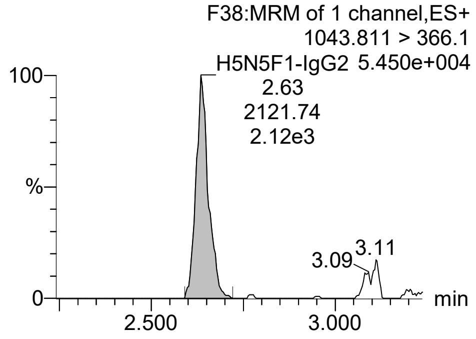

Sample Name: 20211111_L18_R1_IgG

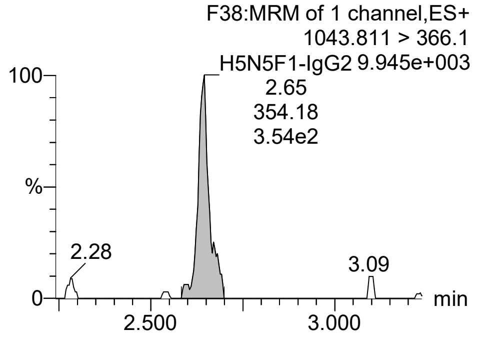

Sample Name: 20211111_H9_R1_IgG

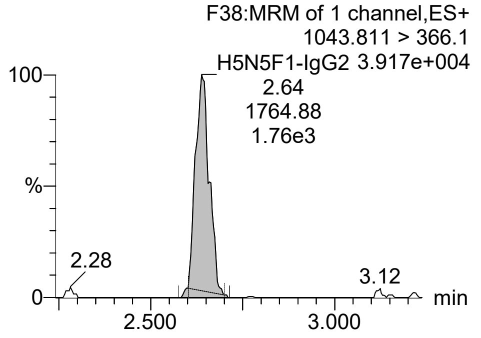

Sample Name: 20211111_A19_R1_IgG

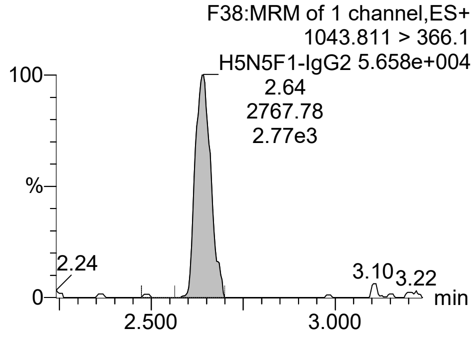

Sample Name: 20211111_A20_R1_IgG

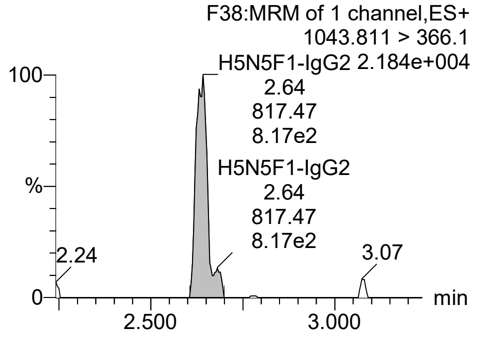

Sample Name: 20211111_L19_R1_IgG

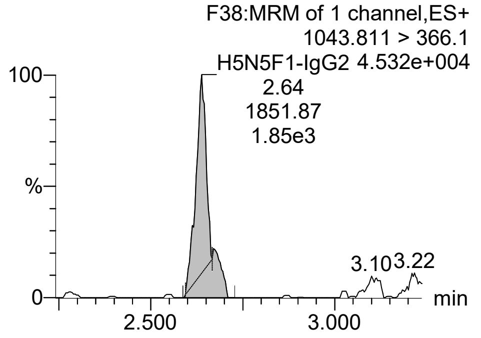

Sample Name: 20211111_QC_R5_IgG

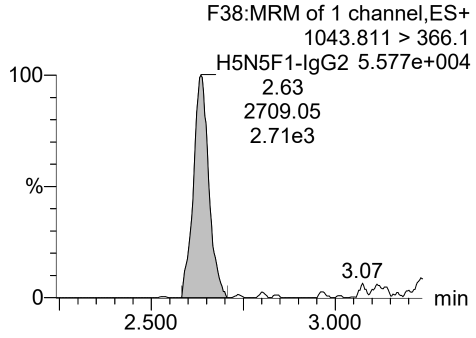

Sample Name: 20211111_L20_R1_IgG

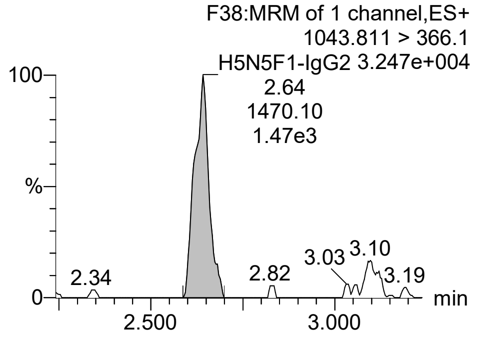

Sample Name: 20211111_H10_R1_IgG

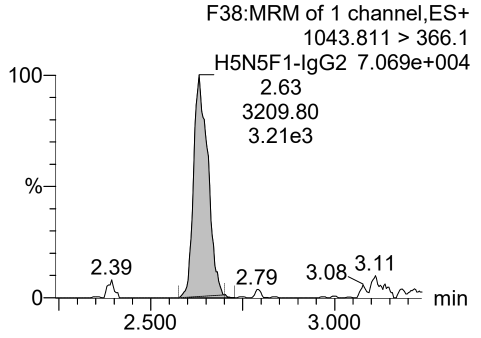

Sample Name: 20211111_A21_R1_IgG

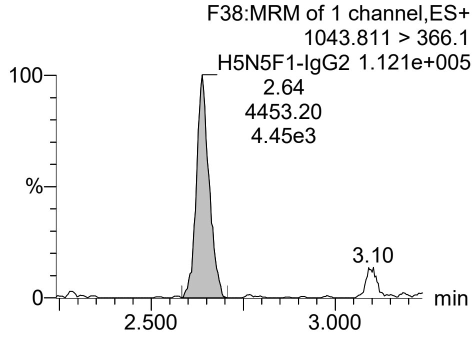

Sample Name: 20211111_A22_R1_IgG

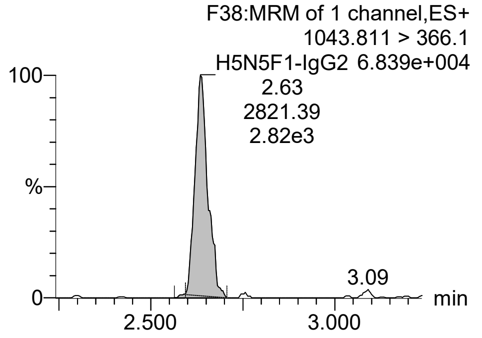

Sample Name: 20211111_L21_R1_IgG
F38:MRM of 1 channel,ES+
1043.811 > 366.1

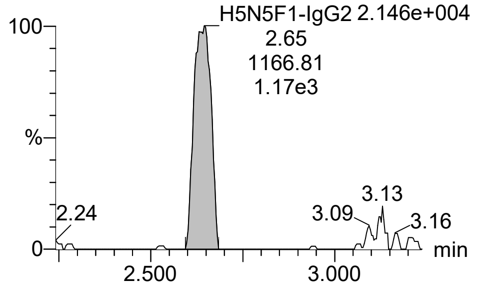

Sample Name: 20211111_L22_R1_IgG
F38:MRM of 1 channel,ES+
1043.811 > 366.1

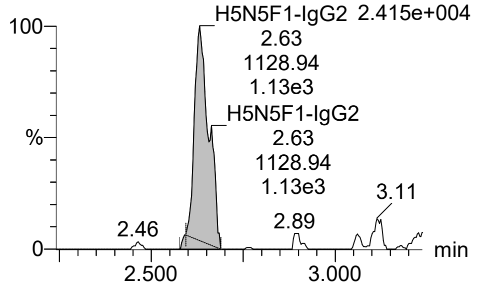

Sample Name: 20211111_H11_R1_IgG
F38:MRM of 1 channel,ES+
1043.811 > 366.1

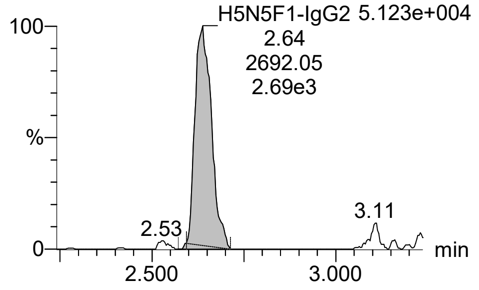

Sample Name: 20211111_A23_R1_IgG
F38:MRM of 1 channel,ES+
1043.811 > 366.1

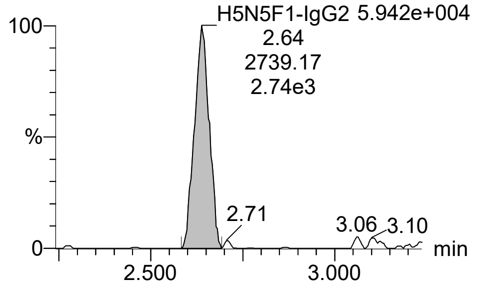

Sample Name: 20211111_A24_R1_IgG
F38:MRM of 1 channel,ES+
1043.811 > 366.1

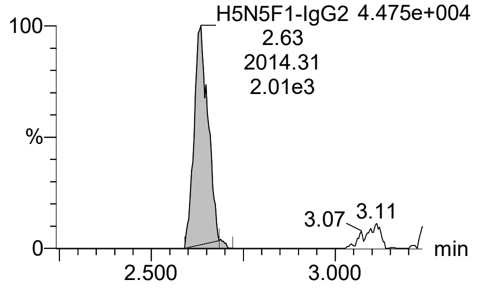

Sample Name: 20211111_L23_R1_IgG
F38:MRM of 1 channel,ES+
1043.811 > 366.1

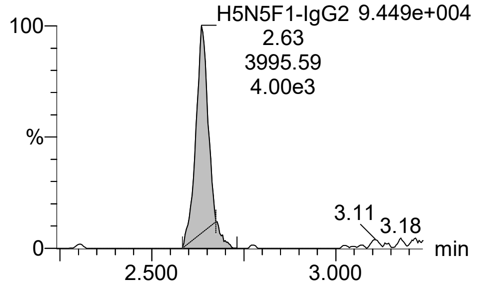

Sample Name: 20211111_L24_R1_IgG
F38:MRM of 1 channel,ES+
1043.811 > 366.1

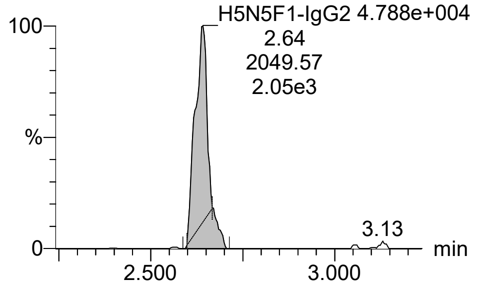

Sample Name: 20211111_H12_R1_IgG
F38:MRM of 1 channel,ES+
1043.811 > 366.1

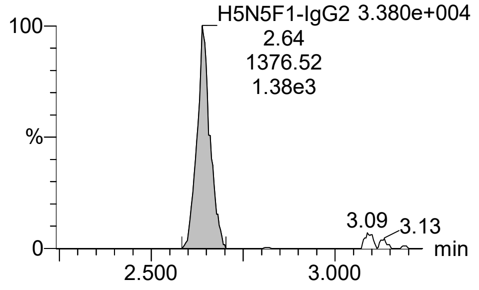

Sample Name: 20211111_QC_R6_IgG
F38:MRM of 1 channel,ES+
1043.811 > 366.1

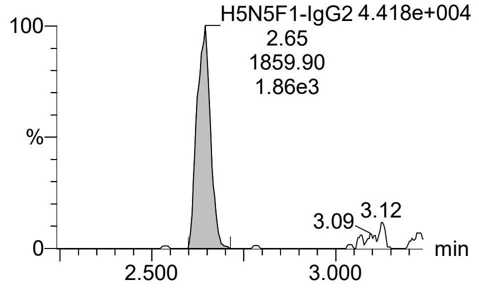

Sample Name: 20211111_A25_R1_IgG
F38:MRM of 1 channel,ES+
1043.811 > 366.1

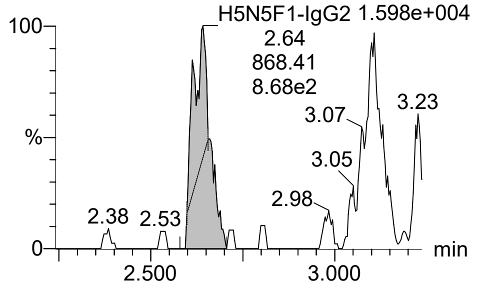

Sample Name: 20211111_A26_R1_IgG
F38:MRM of 1 channel,ES+
1043.811 > 366.1

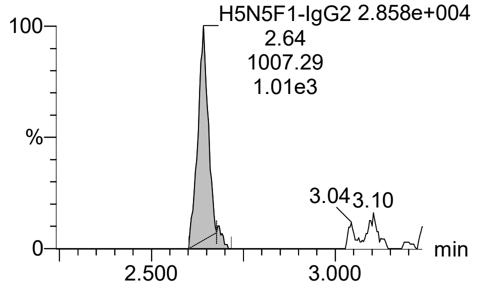

Sample Name: 20211111_L25_R1_IgG
F38:MRM of 1 channel,ES+
1043.811 > 366.1

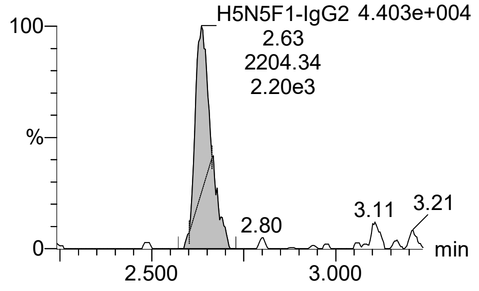

Sample Name: 20211111_L26_R1_IgG
F38:MRM of 1 channel,ES+
1043.811 > 366.1

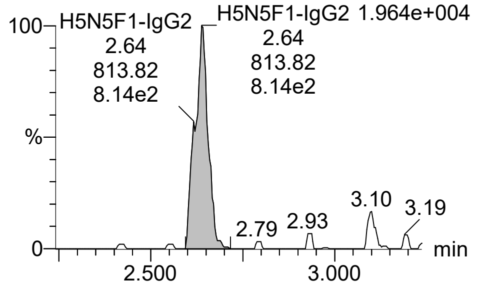

Sample Name: 20211111_H13_R1_IgG
F38:MRM of 1 channel,ES+
1043.811 > 366.1

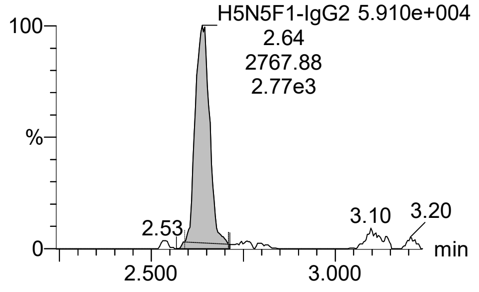

Sample Name: 20211111_A27_R1_IgG
F38:MRM of 1 channel,ES+
1043.811 > 366.1

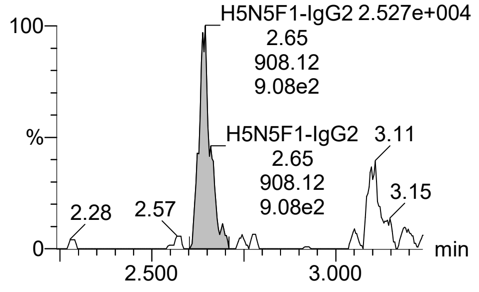

Dataset: C:\MassLynx\10.pro\20211115_clinicalsamples_IgG.qld

Last Altered: Sunday, October 02, 2022 16:02:30 台北標準時間
Printed: Friday, August 01, 2025 16:53:36 台北標準時間

Sample Name: 20211111_A28_R1_IgG

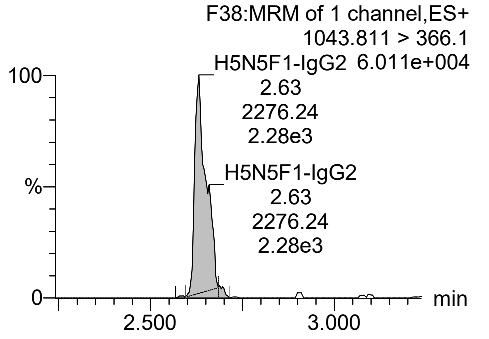

Sample Name: 20211111_L27_R1_IgG

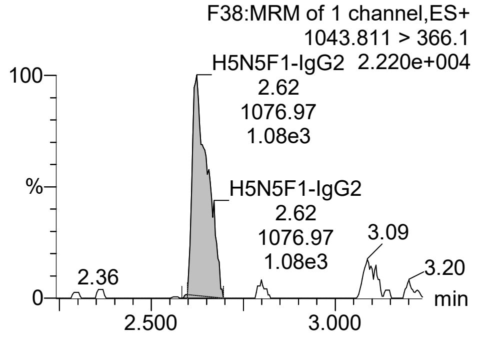

Sample Name: 20211111_L28_R1_IgG

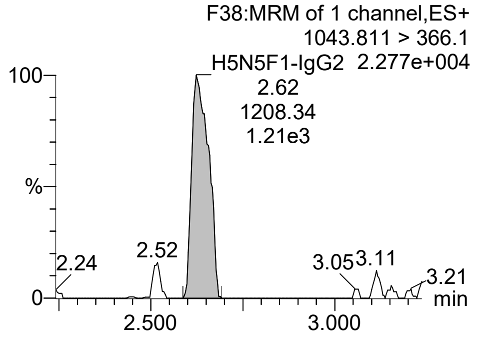

Sample Name: 20211111_H14_R1_IgG

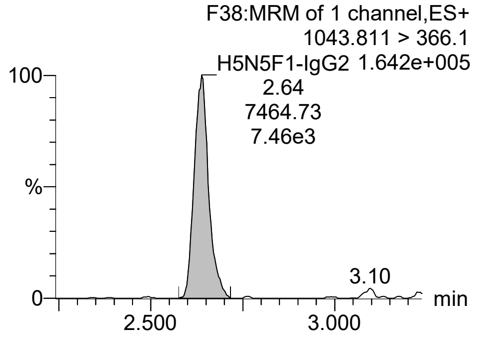

Sample Name: 20211111_A29_R1_IgG

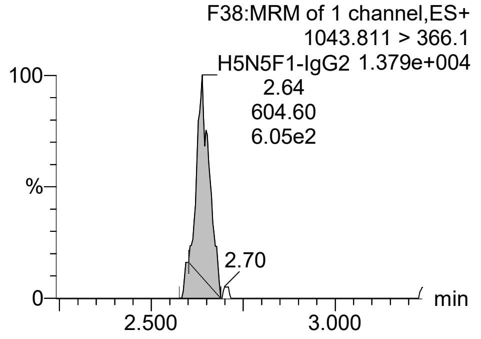

Sample Name: 20211111_A30_R1_IgG

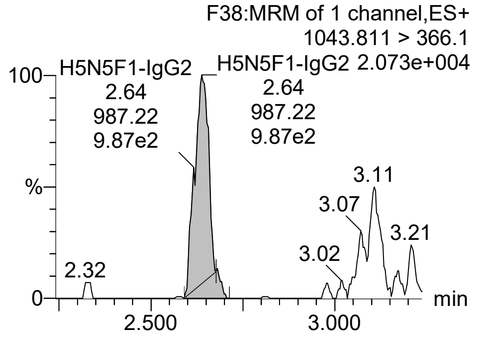

Sample Name: 20211111_QC_R7_IgG

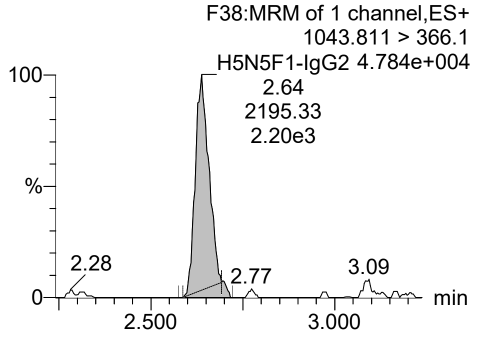

Sample Name: 20211111_L29_R1_IgG

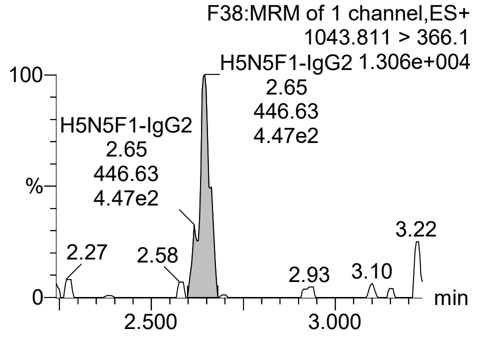

Sample Name: 20211111_L30_R1_IgG

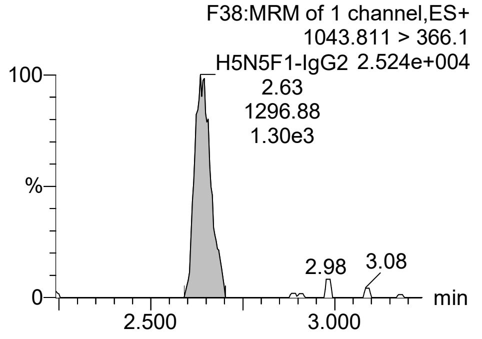

Sample Name: 20211111_H15_R1_IgG

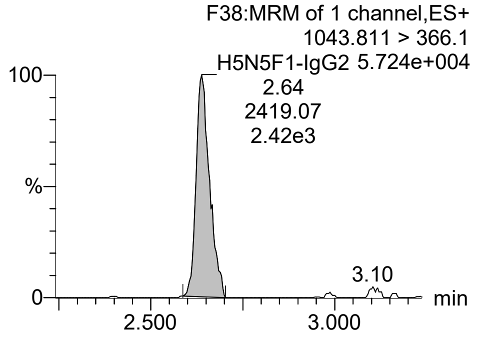

Sample Name: 20211111_A31_R1_IgG

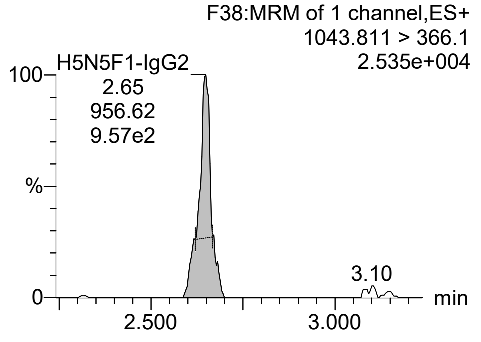

Sample Name: 20211111_A32_R1_IgG

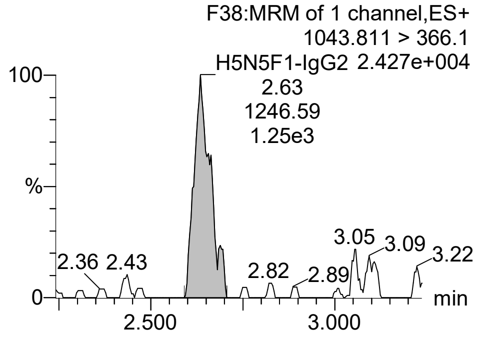

Sample Name: 20211111_L31_R1_IgG

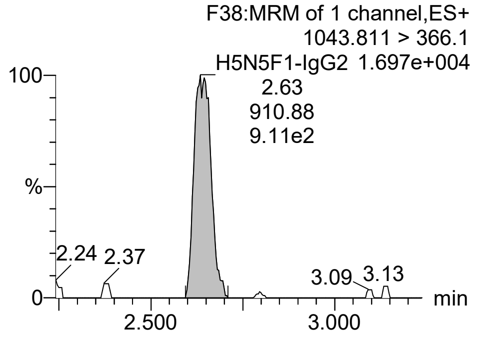

Sample Name: 20211111_L32_R1_IgG

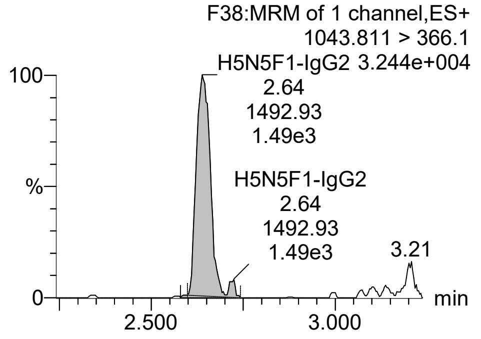

Sample Name: 20211111_H16_R1_IgG

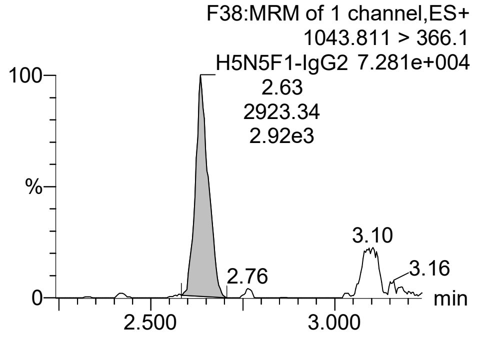

Dataset: C:\MassLynx\10.pro\20211115_clinicalsamples_IgG.qld

Last Altered: Sunday, October 02, 2022 16:02:30 台北標準時間
Printed: Friday, August 01, 2025 16:53:36 台北標準時間

Sample Name: 20211111_A33_R1_IgG
F38:MRM of 1 channel,ES+
1043.811 > 366.1

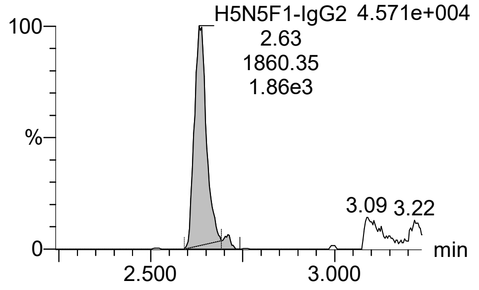

Sample Name: 20211111_A34_R1_IgG
F38:MRM of 1 channel,ES+
1043.811 > 366.1

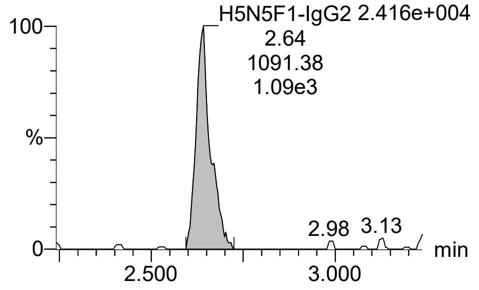

Sample Name: 20211111_H17_R1_IgG
F38:MRM of 1 channel,ES+
1043.811 > 366.1

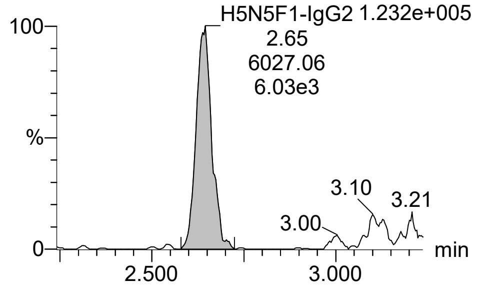

Sample Name: 20211111_A35_R1_IgG
F38:MRM of 1 channel,ES+
1043.811 > 366.1

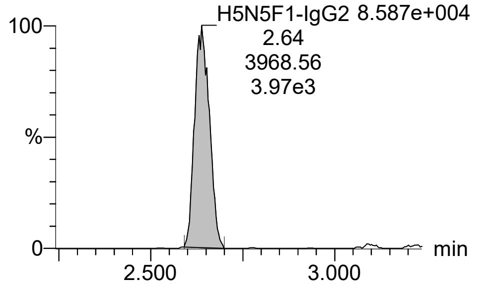

Sample Name: 20211111_QC_R8_IgG
F38:MRM of 1 channel,ES+
1043.811 > 366.1

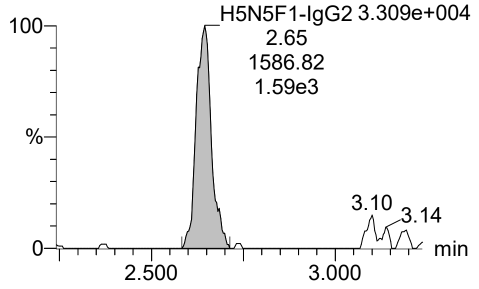

Sample Name: 20211111_A36_R1_IgG
F38:MRM of 1 channel,ES+
1043.811 > 366.1

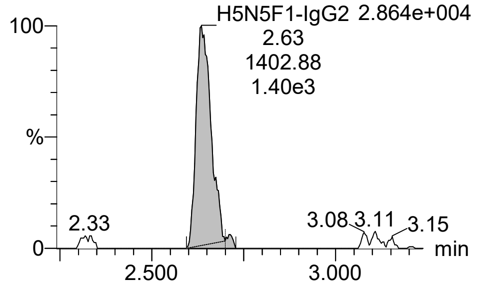

Sample Name: 20211111_H18_R1_IgG
F38:MRM of 1 channel,ES+
1043.811 > 366.1

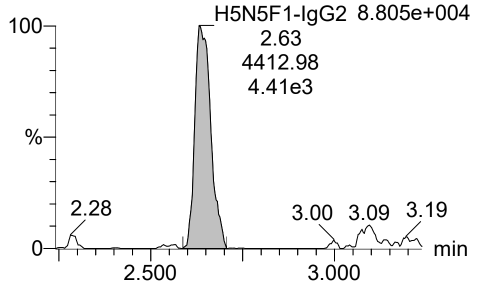

Sample Name: 20211111_A37_R1_IgG
F38:MRM of 1 channel,ES+
1043.811 > 366.1

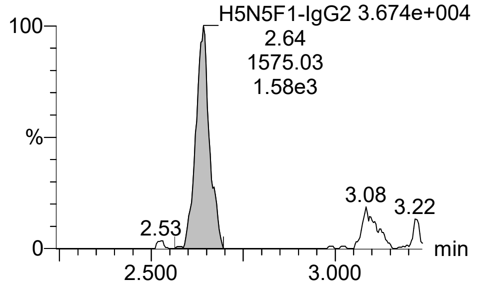

Sample Name: 20211111_A38_R1_IgG
F38:MRM of 1 channel,ES+
1043.811 > 366.1

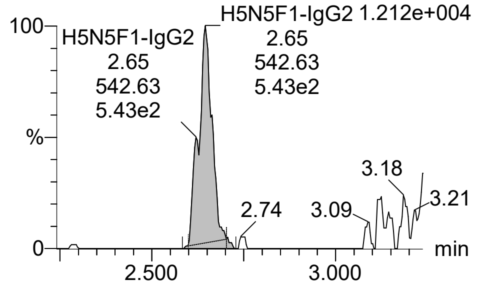

Sample Name: 20211111_H19_R1_IgG
F38:MRM of 1 channel,ES+
1043.811 > 366.1

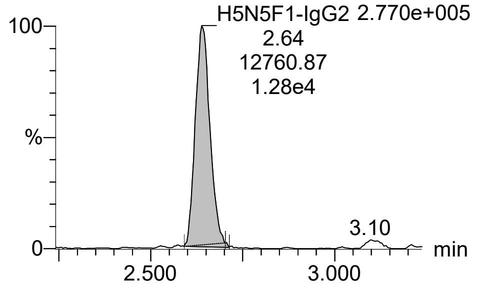

Sample Name: 20211111_A39_R1_IgG
F38:MRM of 1 channel,ES+
1043.811 > 366.1

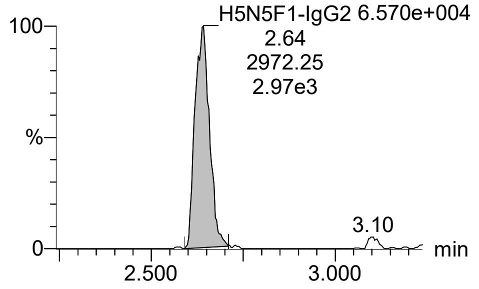

Sample Name: 20211111_A40_R1_IgG
F38:MRM of 1 channel,ES+
1043.811 > 366.1

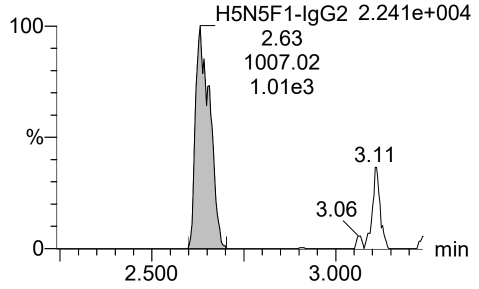

Sample Name: 20211111_H20_R1_IgG
F38:MRM of 1 channel,ES+
1043.811 > 366.1

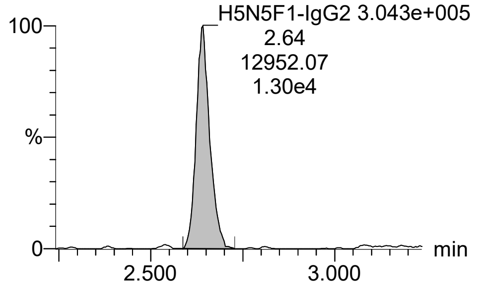

Sample Name: 20211111_A41_R1_IgG
F38:MRM of 1 channel,ES+
1043.811 > 366.1

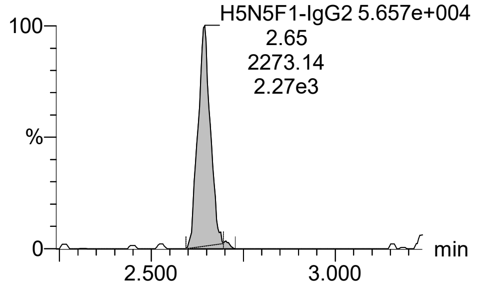

Sample Name: 20211111_A42_R1_IgG
F38:MRM of 1 channel,ES+
1043.811 > 366.1

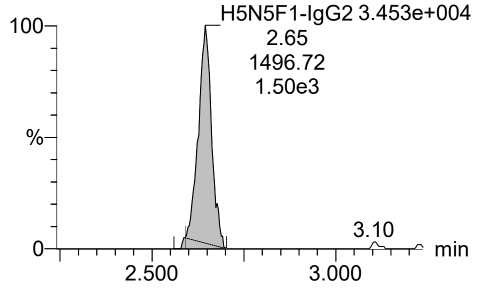

Sample Name: 20211111_A43_R1_IgG

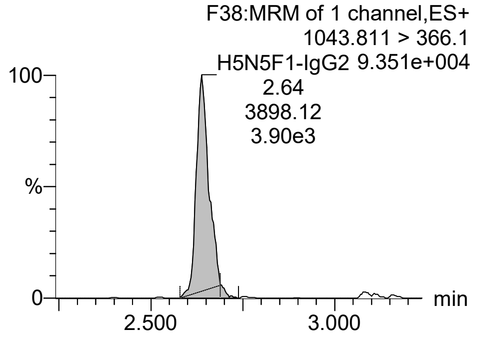

Sample Name: 20211111_A44_R1_IgG

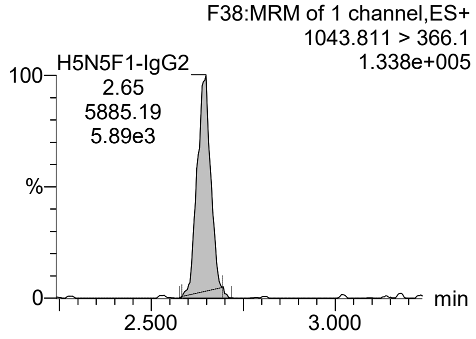

Sample Name: 20211111_QC_R9_IgG

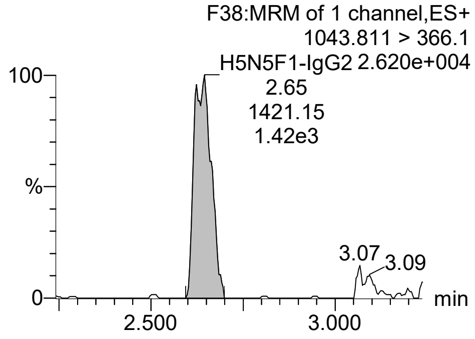

Sample Name: 20211111_A1_R2_IgG

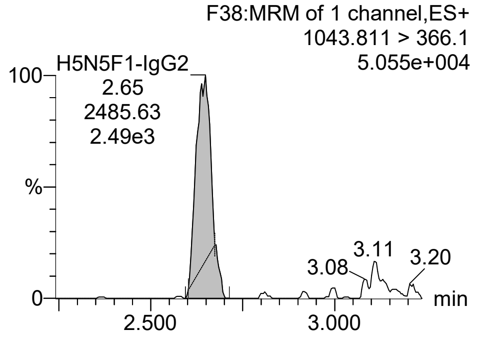

Sample Name: 20211111_A2_R2_IgG

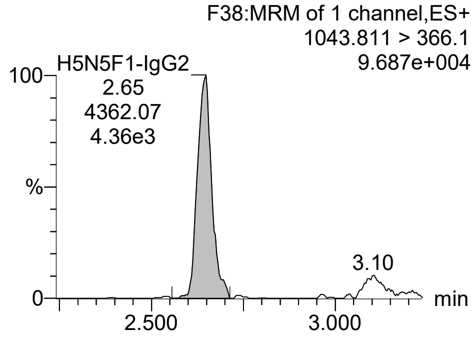

Sample Name: 20211111_L1_R2_IgG

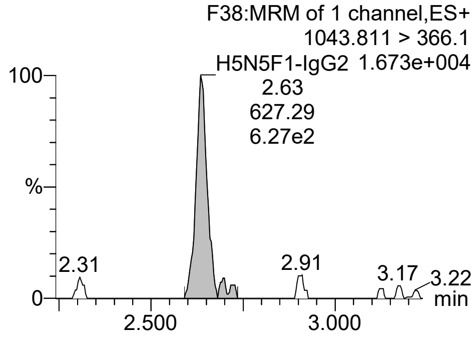

Sample Name: 20211111_L2_R2_IgG

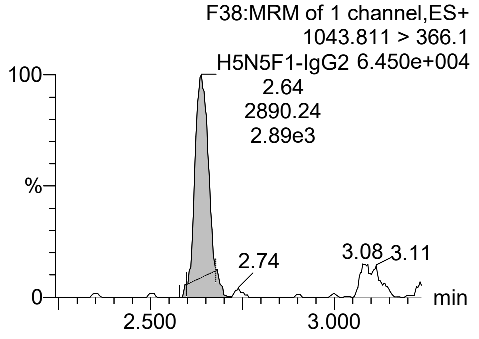

Sample Name: 20211111_H1_R2_IgG

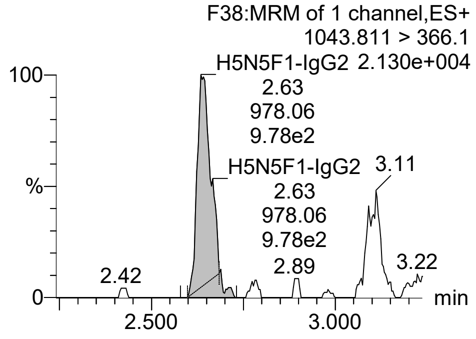

Sample Name: 20211111_A3_R2_IgG

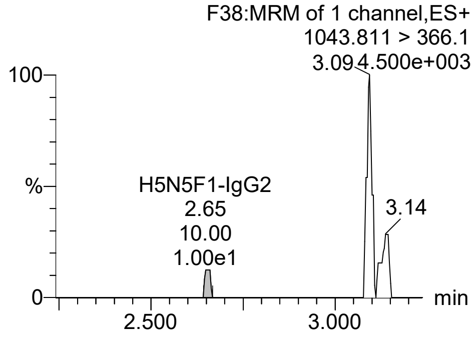

Sample Name: 20211111_A4_R2_IgG

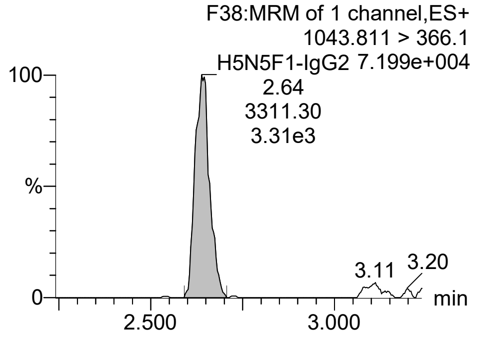

Sample Name: 20211111_L3_R2_IgG

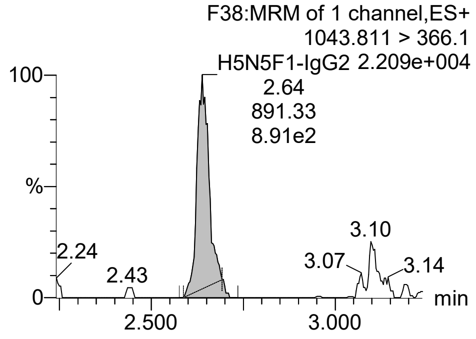

Sample Name: 20211111_L4_R2_IgG

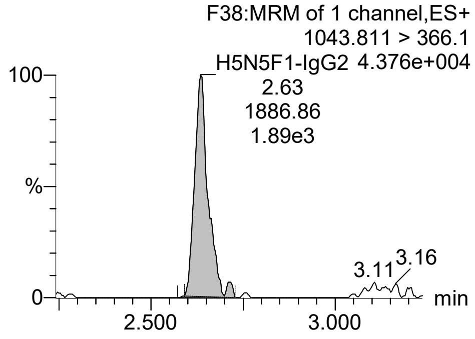

Sample Name: 20211111_H2_R2_IgG

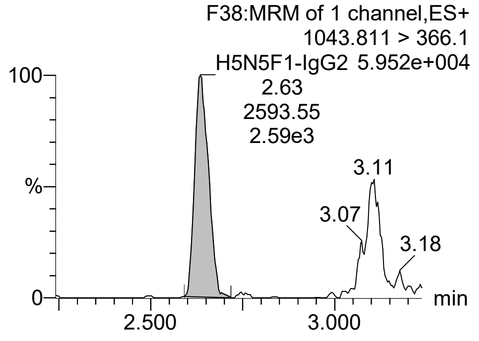

Sample Name: 20211111_A5_R2_IgG

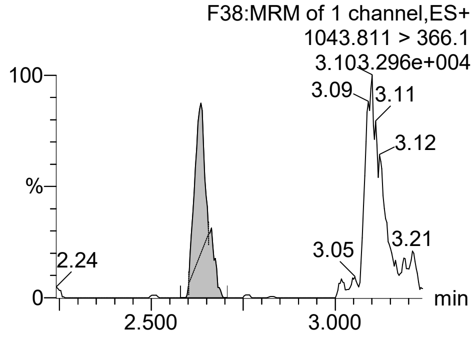

Sample Name: 20211111_A6_R2_IgG

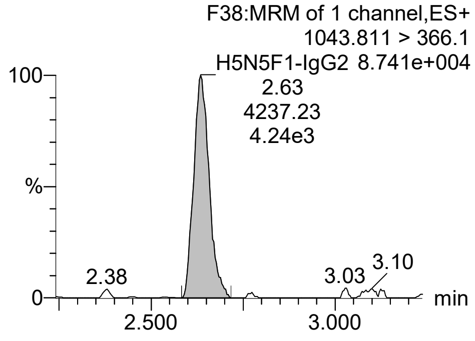

Dataset: C:\MassLynx\10.pro\20211115_clinicalsamples_IgG.qld

Last Altered: Sunday, October 02, 2022 16:02:30 台北標準時間
Printed: Friday, August 01, 2025 16:53:36 台北標準時間

Sample Name: 20211111_QC_R10_IgG

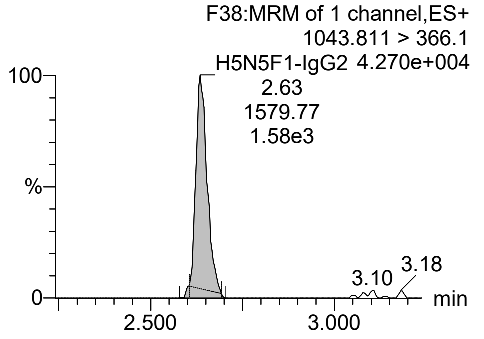

Sample Name: 20211111_L5_R2_IgG

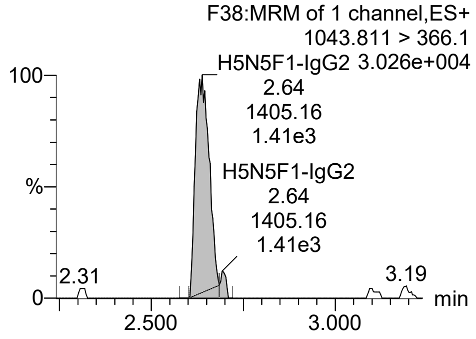

Sample Name: 20211111_L6_R2_IgG

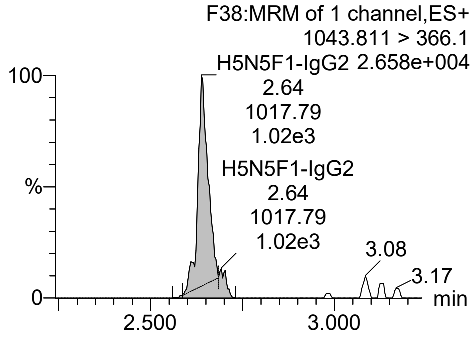

Sample Name: 20211111_H3_R2_IgG

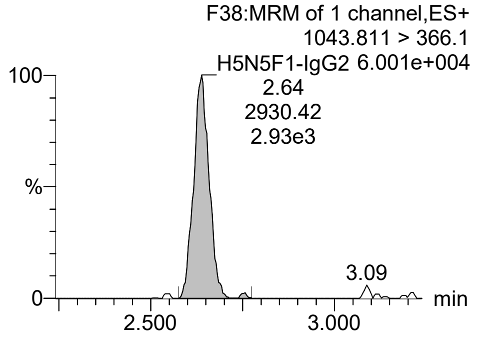

Sample Name: 20211111_A7_R2_IgG

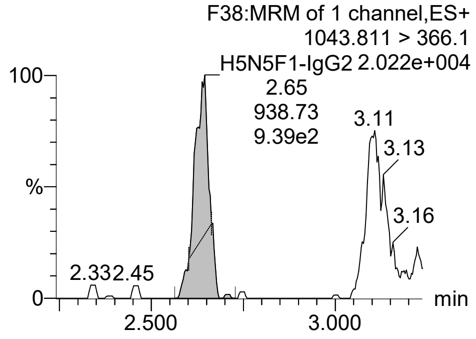

Sample Name: 20211111_A8_R2_IgG

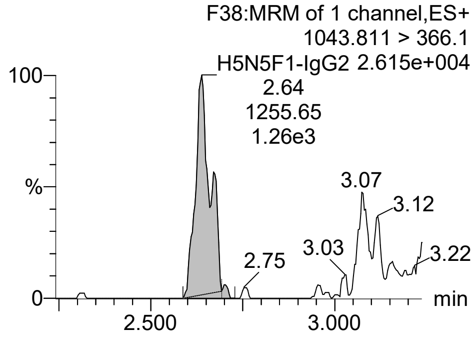

Sample Name: 20211111_L7_R2_IgG

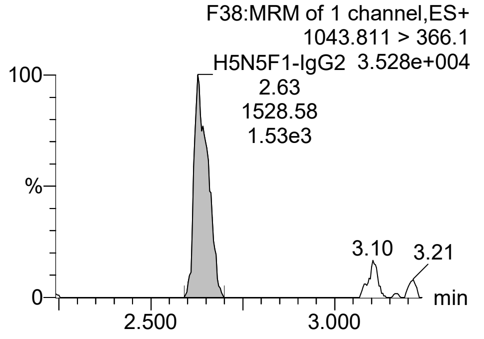

Sample Name: 20211111_L8_R2_IgG

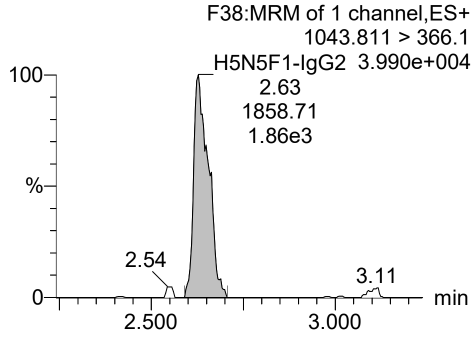

Sample Name: 20211111_H4_R2_IgG

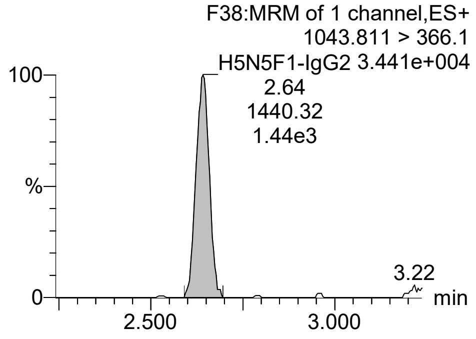

Sample Name: 20211111_A9_R2_IgG

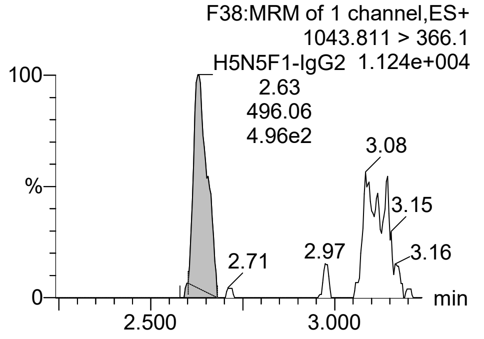

Sample Name: 20211111_A10_R2_IgG

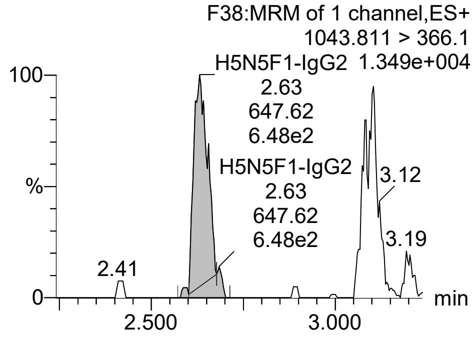

Sample Name: 20211111_L9_R2_IgG

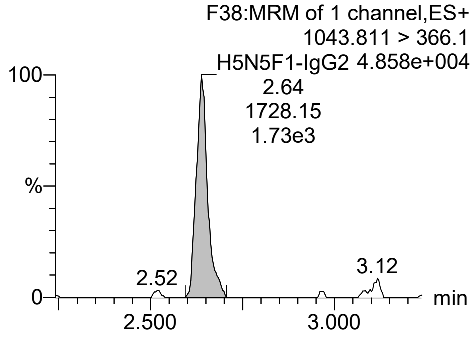

Sample Name: 20211111_L10_R2_IgG

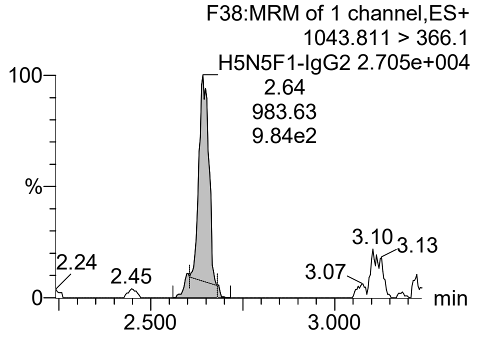

Sample Name: 20211111_QC_R11_IgG

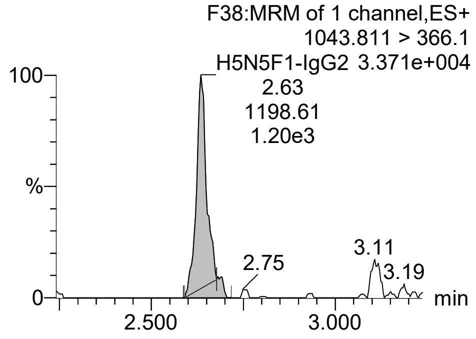

Sample Name: 20211111_H5_R2_IgG

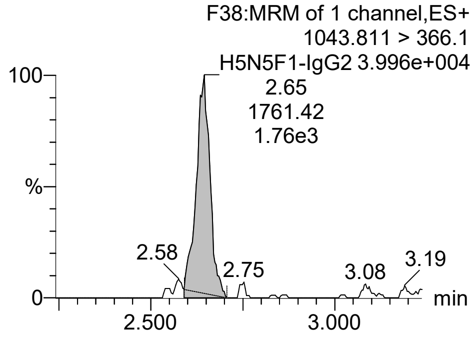

Dataset: C:\MassLynx\10.pro\20211115_clinicalsamples_IgG.qld

Last Altered: Sunday, October 02, 2022 16:02:30 台北標準時間
Printed: Friday, August 01, 2025 16:53:36 台北標準時間

Sample Name: 20211111_A11_R2_IgG

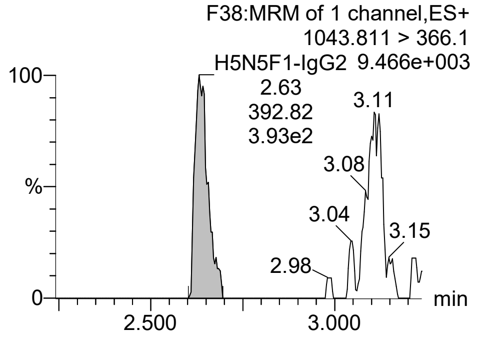

Sample Name: 20211111_A12_R2_IgG

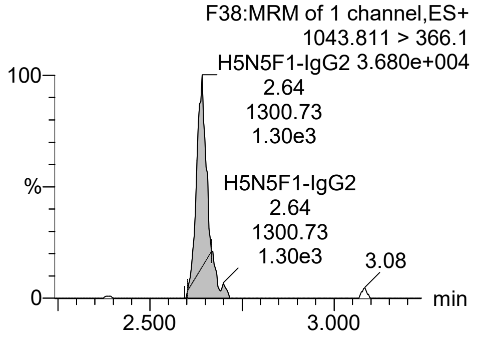

Sample Name: 20211111_L11_R2_IgG

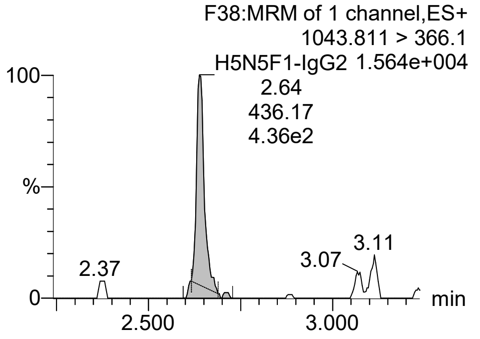

Sample Name: 20211111_L12_R2_IgG

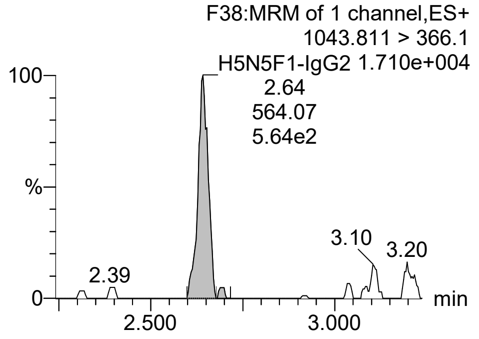

Sample Name: 20211111_H6_R2_IgG

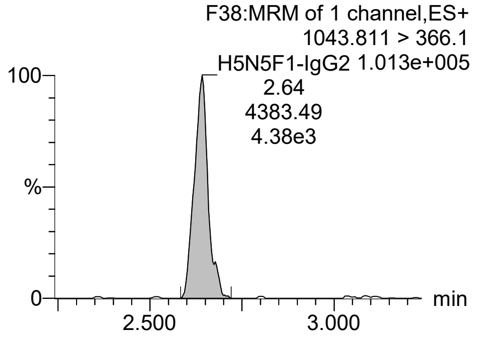

Sample Name: 20211111_A13_R2_IgG

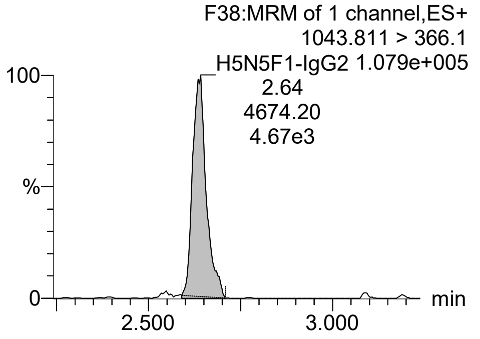

Sample Name: 20211111_A14_R2_IgG

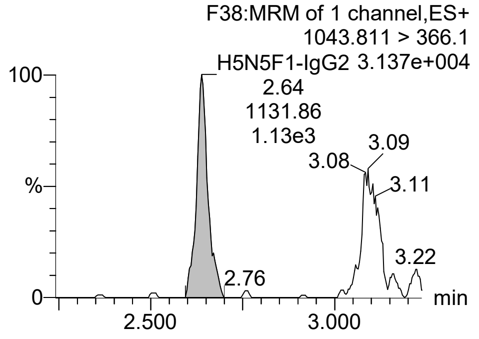

Sample Name: 20211111_L13_R2_IgG

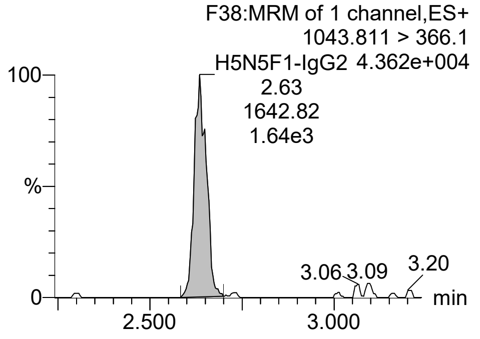

Sample Name: 20211111_L14_R2_IgG

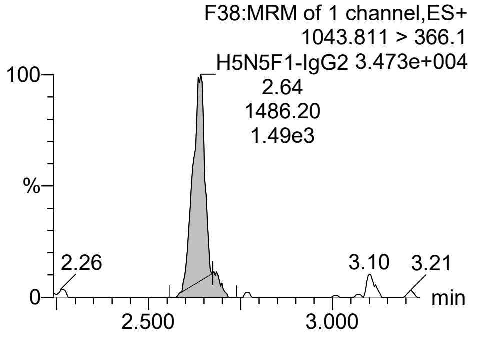

Sample Name: 20211111_H7_R2_IgG

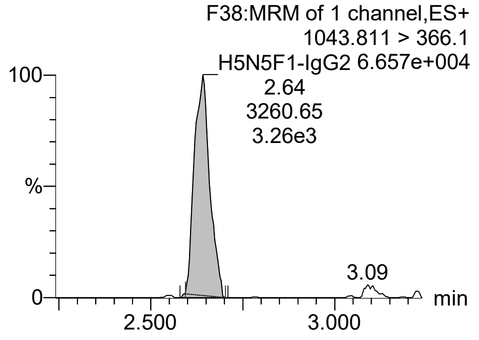

Sample Name: 20211111_A15_R2_IgG

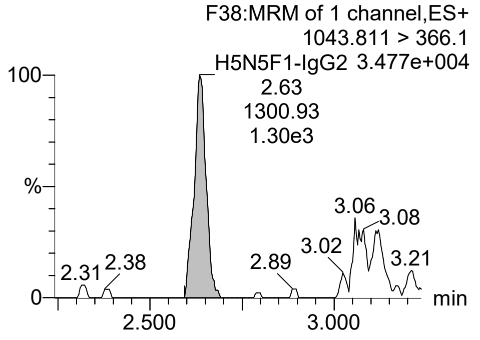

Sample Name: 20211111_QC_R12_IgG

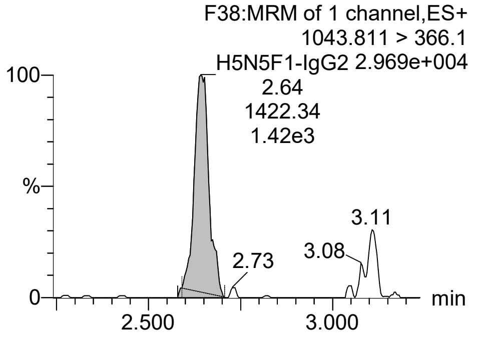

Sample Name: 20211111_A16_R2_IgG

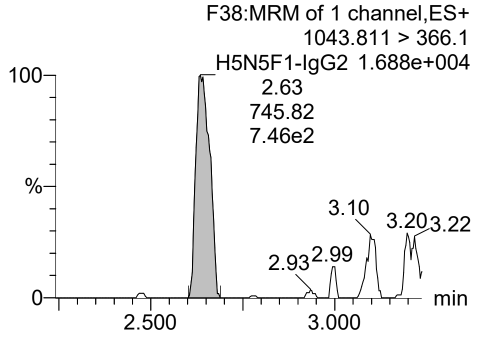

Sample Name: 20211111_L15_R2_IgG

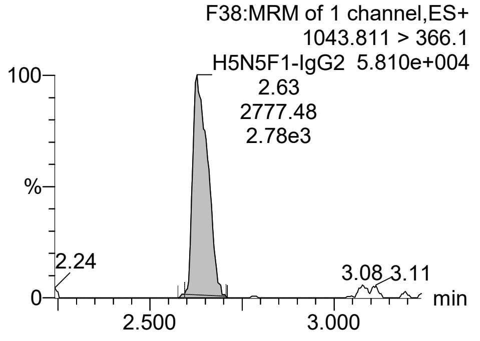

Sample Name: 20211111_L16_R2_IgG

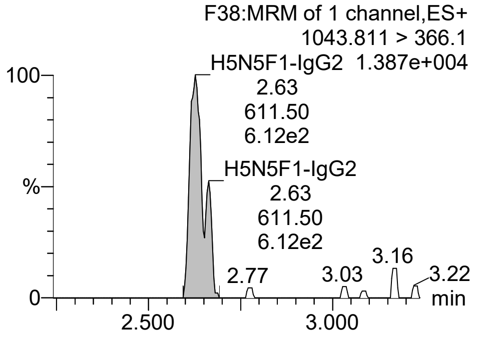

Dataset: C:\MassLynx\10.pro\20211115_clinicalsamples_IgG.qld

Last Altered: Sunday, October 02, 2022 16:02:30 台北標準時間
Printed: Friday, August 01, 2025 16:53:36 台北標準時間

Sample Name: 20211111_H8_R2_IgG

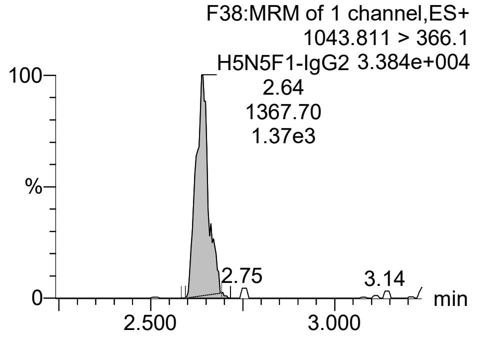

Sample Name: 20211111_A17_R2_IgG

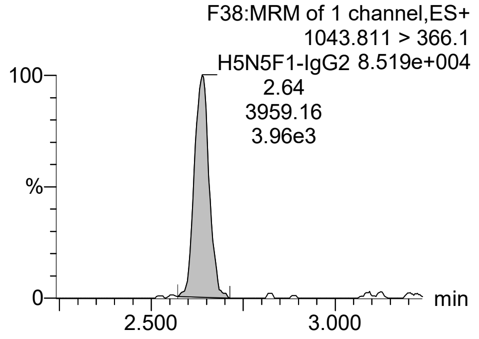

Sample Name: 20211111_A18_R2_IgG

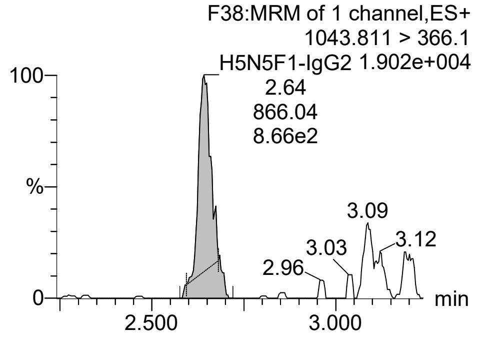

Sample Name: 20211111_L17_R2_IgG

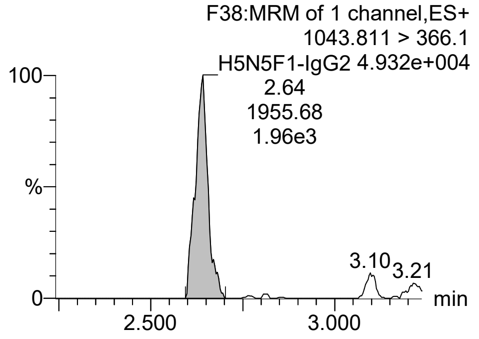

Sample Name: 20211111_L18_R2_IgG

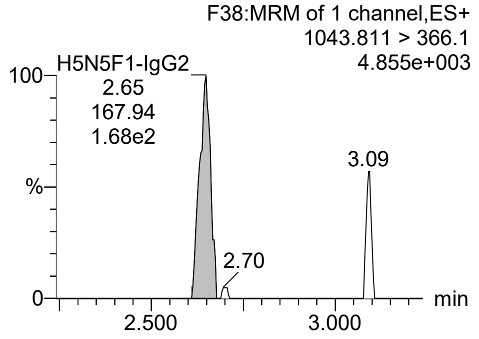

Sample Name: 20211111_H9_R2_IgG

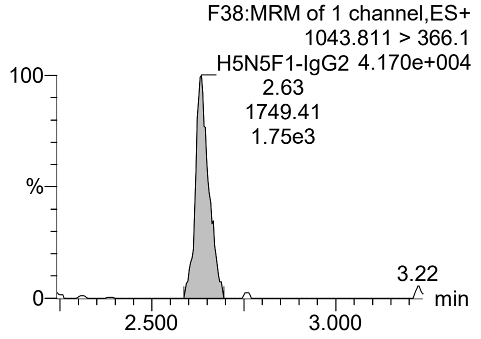

Sample Name: 20211111_A19_R2_IgG

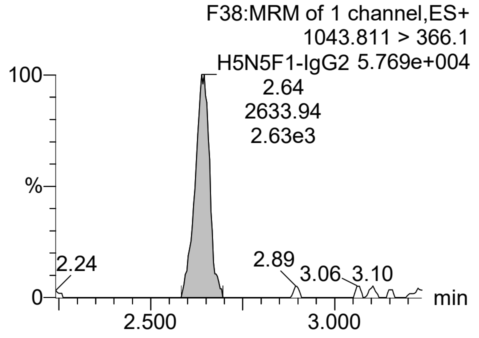

Sample Name: 20211111_A20_R2_IgG

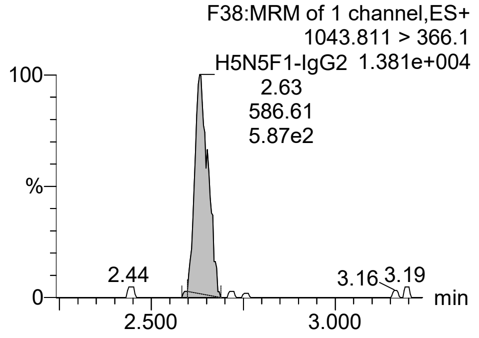

Sample Name: 20211111_L19_R2_IgG

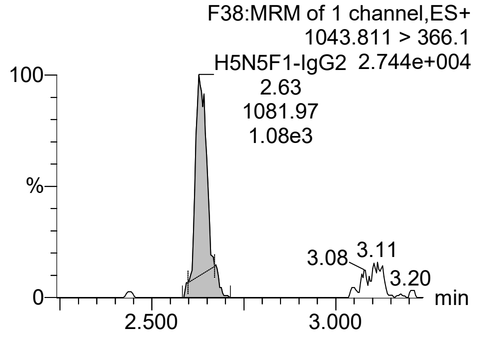

Sample Name: 20211111_QC_R13_IgG

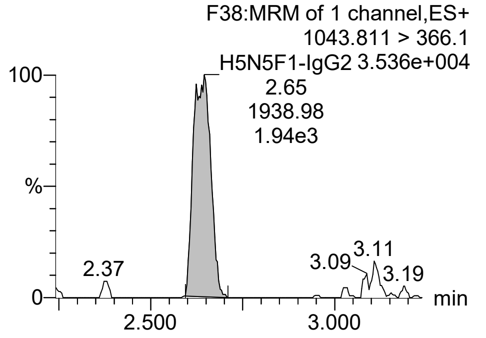

Sample Name: 20211111_L20_R2_IgG

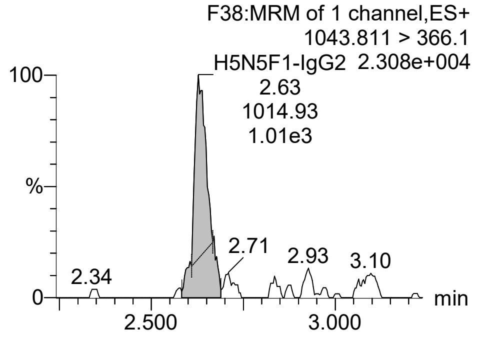

Sample Name: 20211111_H10_R2_IgG

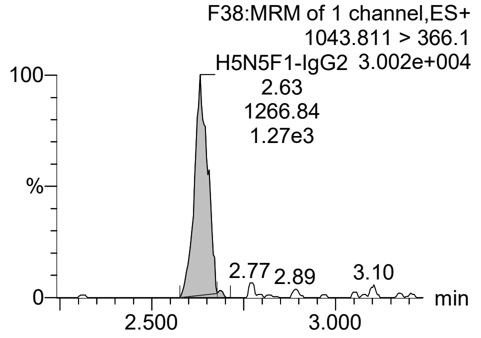

Sample Name: 20211111_A21_R2_IgG

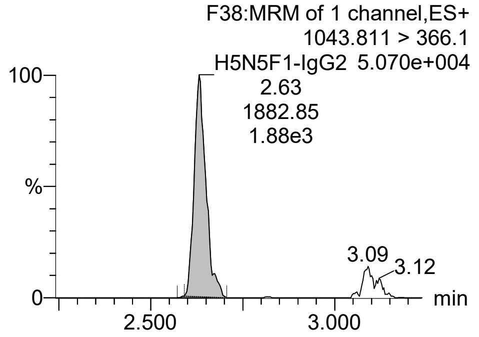

Sample Name: 20211111_A22_R2_IgG

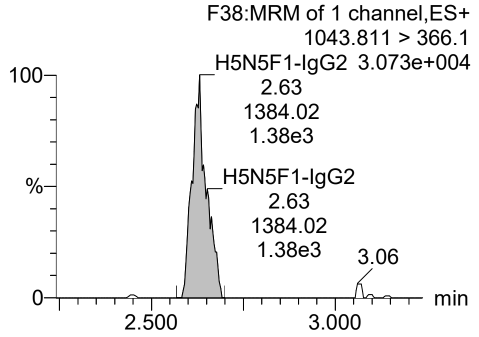

Sample Name: 20211111_L21_R2_IgG

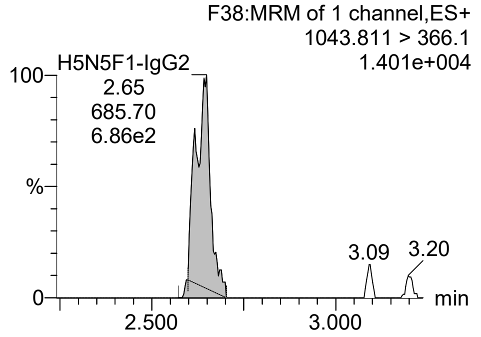

Dataset: C:\MassLynx\10.pro\20211115_clinicalsamples_IgG.qld

Last Altered: Sunday, October 02, 2022 16:02:30 台北標準時間
Printed: Friday, August 01, 2025 16:53:36 台北標準時間

Sample Name: 20211111_L22_R2_IgG

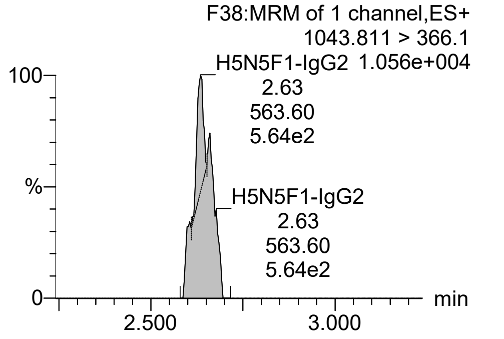

Sample Name: 20211111_H11_R2_IgG

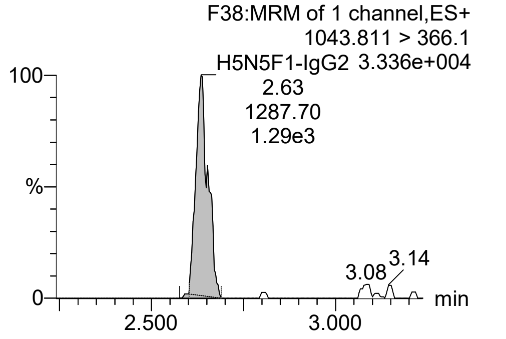

Sample Name: 20211111_A23_R2_IgG

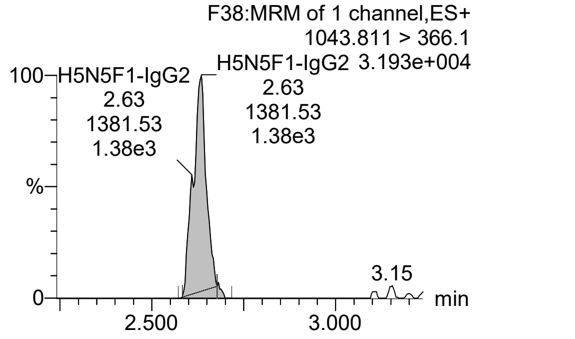

Sample Name: 20211111_A24_R2_IgG

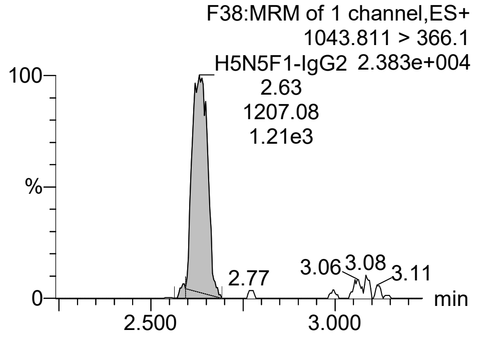

Sample Name: 20211111_L23_R2_IgG

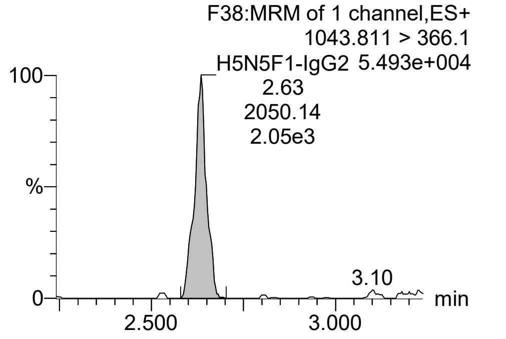

Sample Name: 20211111_L24_R2_IgG

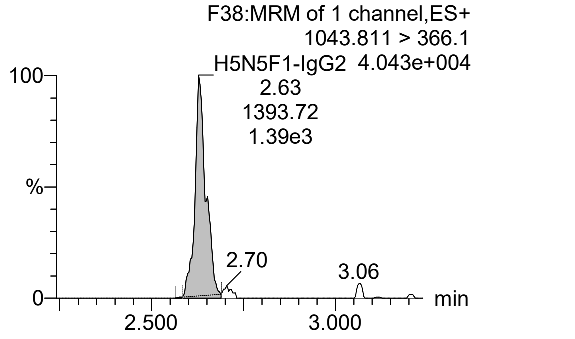

Sample Name: 20211111_H12_R2_IgG

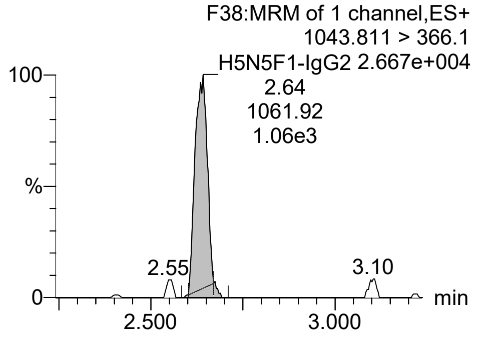

Sample Name: 20211111_QC_R14_IgG

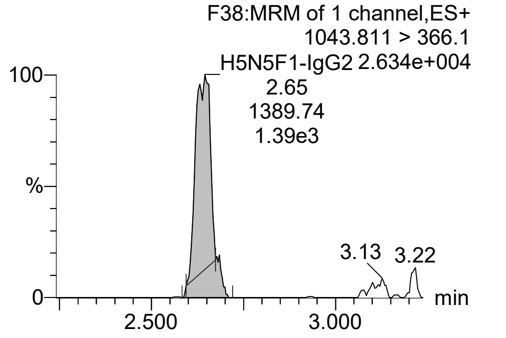

Sample Name: 20211111_A25_R2_IgG

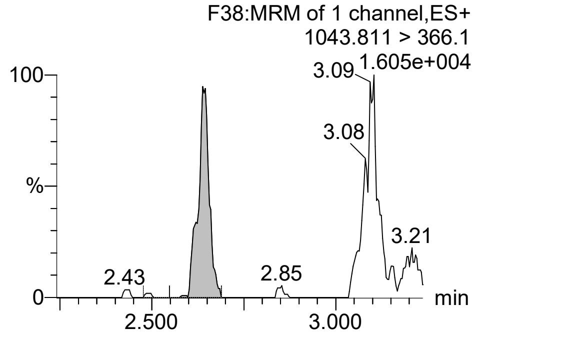

Sample Name: 20211111_A26_R2_IgG

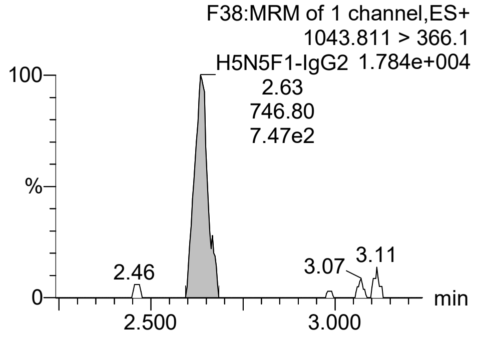

Sample Name: 20211111_L25_R2_IgG

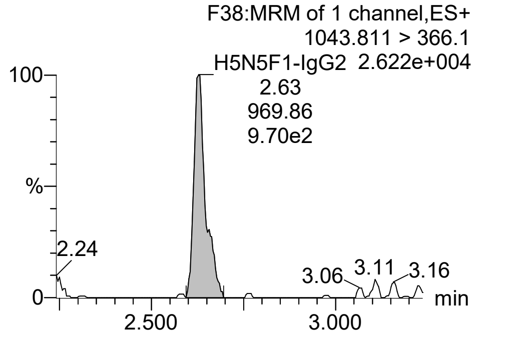

Sample Name: 20211111_L26_R2_IgG

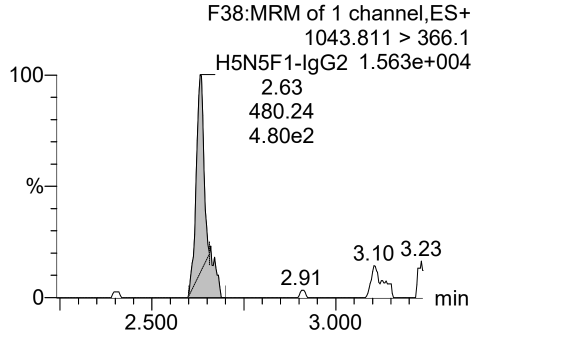

Sample Name: 20211111_H13_R2_IgG

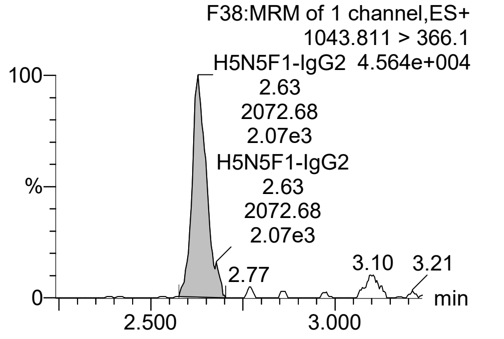

Sample Name: 20211111_A27_R2_IgG

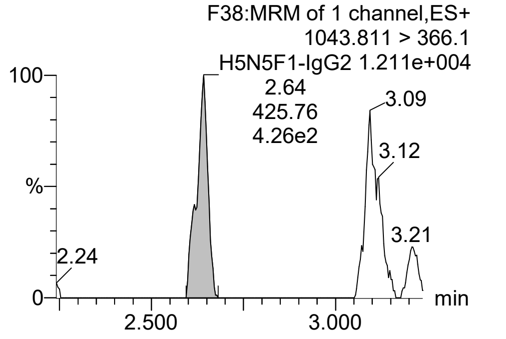

Sample Name: 20211111_A28_R2_IgG

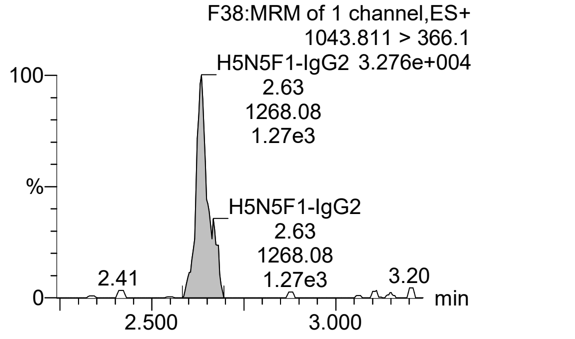

Sample Name: 20211111_L27_R2_IgG

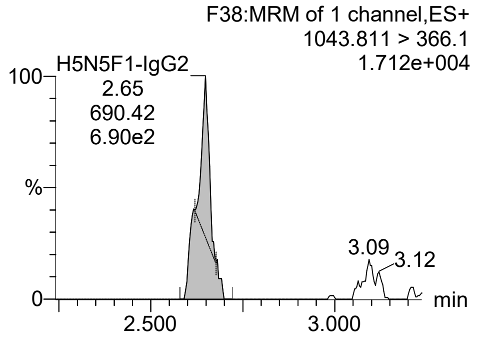

Sample Name: 20211111_L28_R2_IgG

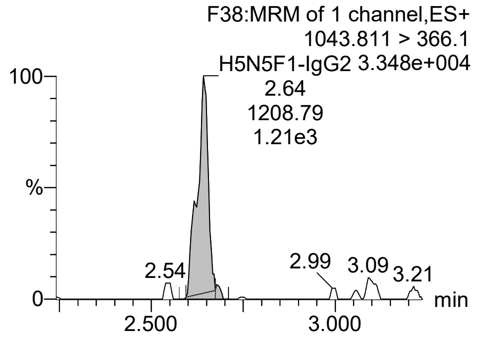

Sample Name: 20211111_H14_R2_IgG

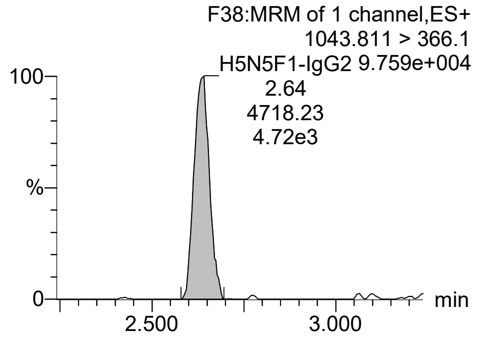

Sample Name: 20211111_A29_R2_IgG

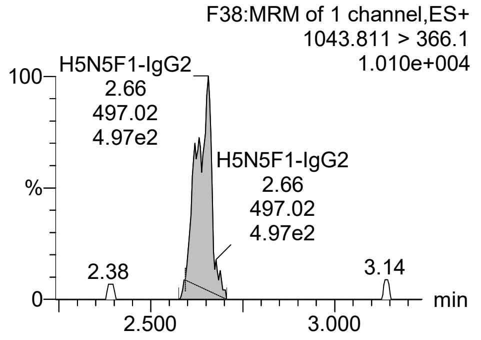

Sample Name: 20211111_A30_R2_IgG

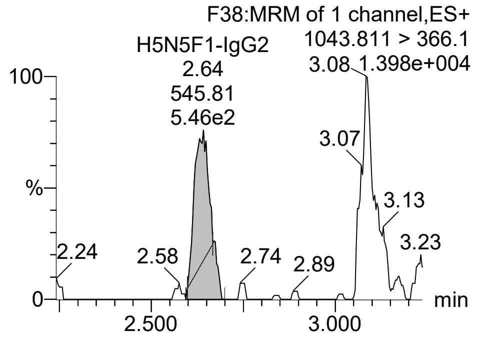

Sample Name: 20211111_QC_R15_IgG

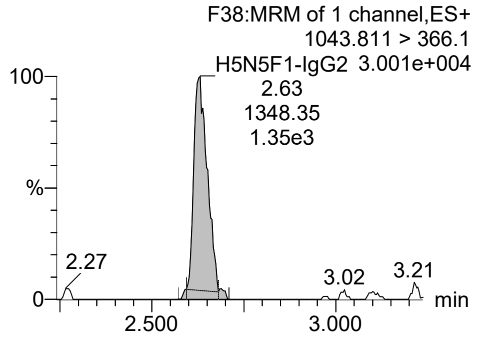

Sample Name: 20211111_L29_R2_IgG

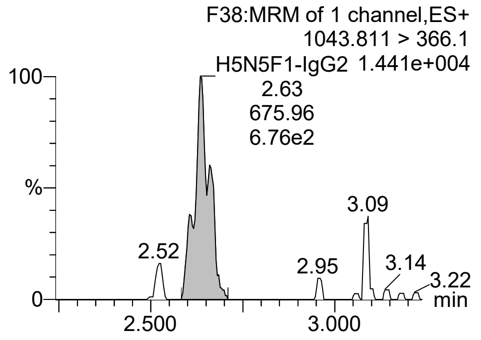

Sample Name: 20211111_L30_R2_IgG

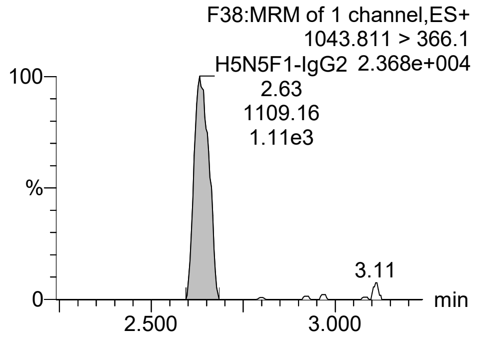

Sample Name: 20211111_H15_R2_IgG

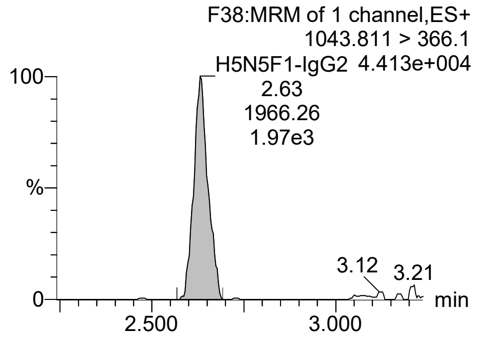

Sample Name: 20211111_A31_R2_IgG

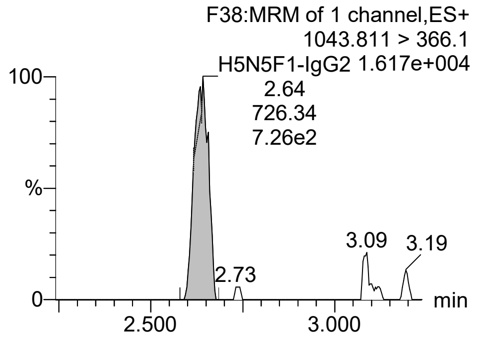

Sample Name: 20211111_A32_R2_IgG

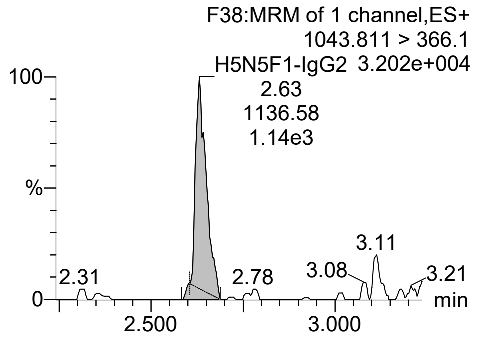

Sample Name: 20211111_L31_R2_IgG

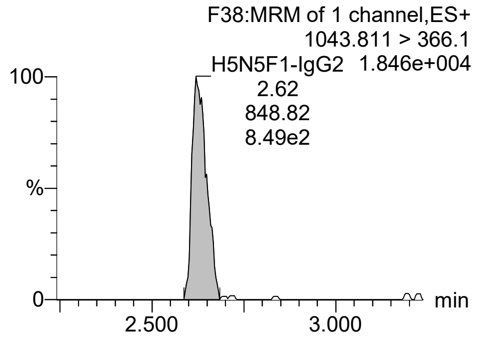

Sample Name: 20211111_L32_R2_IgG

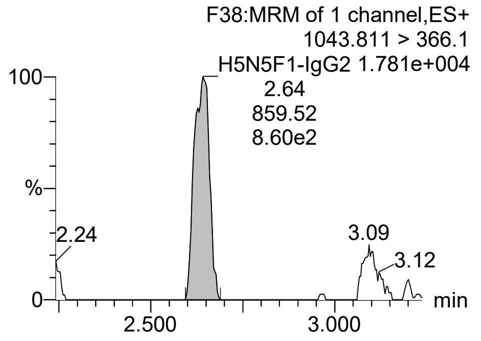

Sample Name: 20211111_H16_R2_IgG

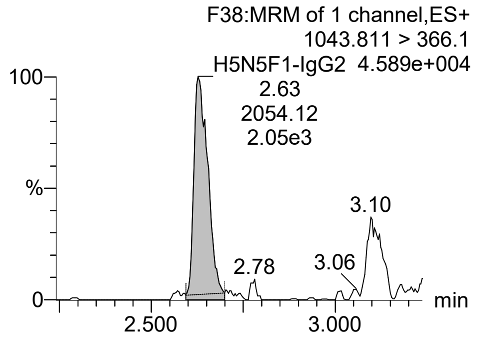

Sample Name: 20211111_A33_R2_IgG

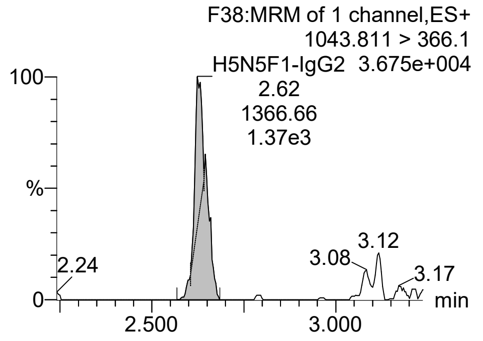

Dataset: C:\MassLynx\10.pro\20211115_clinicalsamples_IgG.qld

Last Altered: Sunday, October 02, 2022 16:02:30 台北標準時間
Printed: Friday, August 01, 2025 16:53:36 台北標準時間

Sample Name: 20211111_A34_R2_IgG
F38:MRM of 1 channel,ES+
1043.811 > 366.1

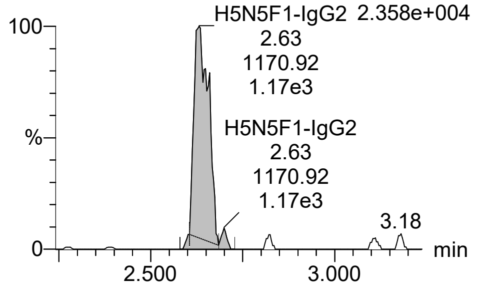

Sample Name: 20211111_H17_R2_IgG
F38:MRM of 1 channel,ES+
1043.811 > 366.1

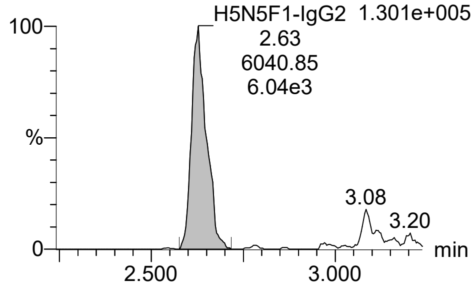

Sample Name: 20211111_A35_R2_IgG
F38:MRM of 1 channel,ES+
1043.811 > 366.1

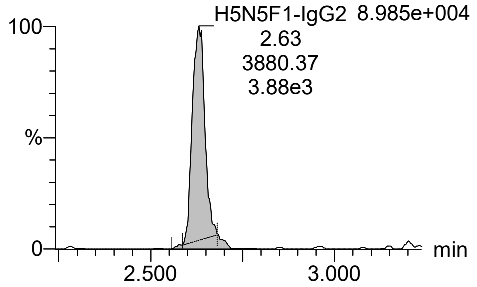

Sample Name: 20211111_QC_R16_IgG
F38:MRM of 1 channel,ES+
1043.811 > 366.1

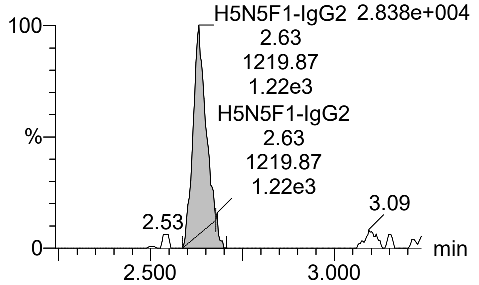

Sample Name: 20211111_A36_R2_IgG
F38:MRM of 1 channel,ES+
1043.811 > 366.1

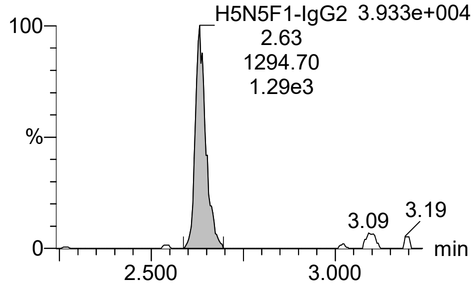

Sample Name: 20211111_H18_R2_IgG
F38:MRM of 1 channel,ES+
1043.811 > 366.1

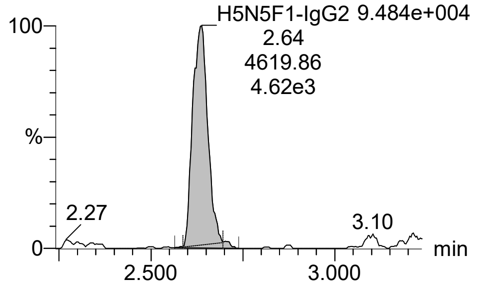

Sample Name: 20211111_A37_R2_IgG
F38:MRM of 1 channel,ES+
1043.811 > 366.1

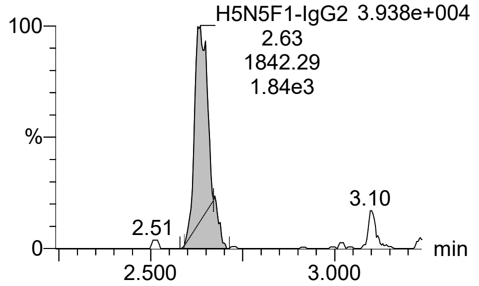

Sample Name: 20211111_A38_R2_IgG
F38:MRM of 1 channel,ES+
1043.811 > 366.1

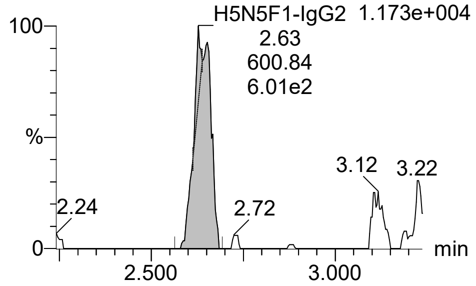

Sample Name: 20211111_H19_R2_IgG
F38:MRM of 1 channel,ES+
1043.811 > 366.1

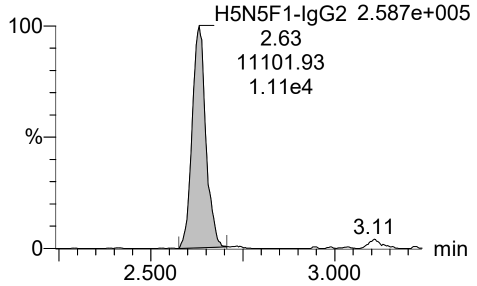

Sample Name: 20211111_A39_R2_IgG
F38:MRM of 1 channel,ES+
1043.811 > 366.1

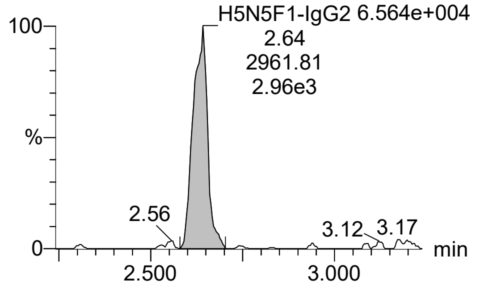

Sample Name: 20211111_A40_R2_IgG
F38:MRM of 1 channel,ES+
1043.811 > 366.1

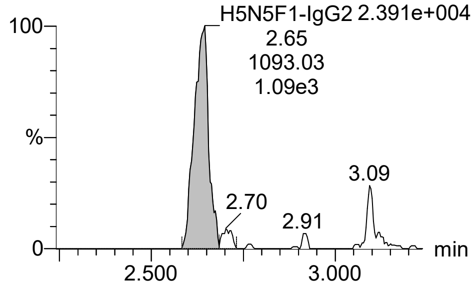

Sample Name: 20211111_H20_R2_IgG
F38:MRM of 1 channel,ES+
1043.811 > 366.1

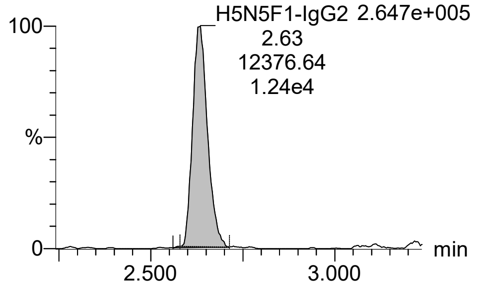

Sample Name: 20211111_A41_R2_IgG
F38:MRM of 1 channel,ES+
1043.811 > 366.1

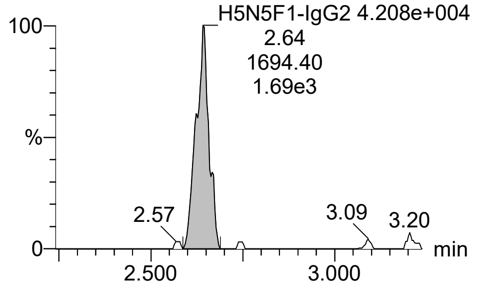

Sample Name: 20211111_A42_R2_IgG
F38:MRM of 1 channel,ES+
1043.811 > 366.1

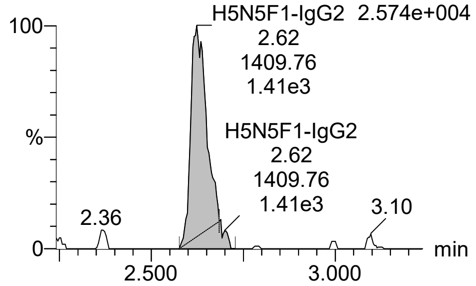

Sample Name: 20211111_A43_R2_IgG
F38:MRM of 1 channel,ES+
1043.811 > 366.1

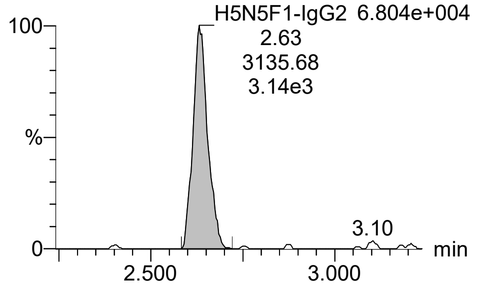

Dataset: C:\MassLynx\10.pro\20211115_clinicalsamples_IgG.qld

Last Altered: Sunday, October 02, 2022 16:02:30 台北標準時間
Printed: Friday, August 01, 2025 16:53:36 台北標準時間

Sample Name: 20211111_A44_R2_IgG

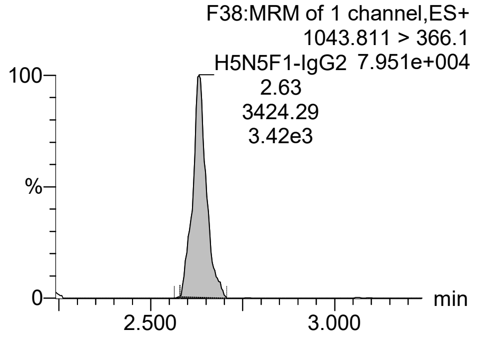

Sample Name: 20211111_QC_R17_IgG

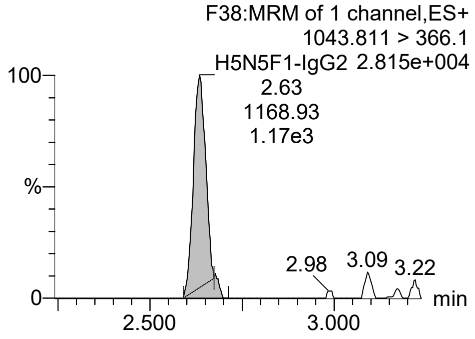

Dataset: C:\MassLynx\10.pro\20211115_clinicalsamples_IgG.qld

Last Altered: Sunday, October 02, 2022 16:02:30 台北標準時間
Printed: Friday, August 01, 2025 16:53:36 台北標準時間

	#	Name	RT	Area	IS Area	Response	Prima...
1	1	20211111_QC_R1_IgG	2.64	3349.2	64968.0	0.052	MM
2	2	20211111_A1_R1_IgG	2.65	3342.1	59059.2	0.057	MM
3	3	20211111_A2_R1_IgG	2.63	7447.9	66621.2	0.112	MM
4	4	20211111_L1_R1_IgG	2.64	1144.0	64201.0	0.018	bb
5	5	20211111_L2_R1_IgG	2.65	3400.6	46950.2	0.072	bb
6	6	20211111_H1_R1_IgG	2.63	624.6	38648.2	0.016	bb
7	7	20211111_A3_R1_IgG	2.65	1.0	2603.7	0.000	bb
8	8	20211111_A4_R1_IgG	2.64	5124.2	69960.4	0.073	bb
9	9	20211111_L3_R1_IgG	2.63	1078.8	38864.7	0.028	MM
10	10	20211111_L4_R1_IgG	2.64	2507.0	43380.5	0.058	MM
11	11	20211111_H2_R1_IgG	2.64	4077.9	71349.2	0.057	bb
12	12	20211111_A5_R1_IgG	2.64	1058.0	65569.7	0.016	MM
13	13	20211111_A6_R1_IgG	2.63	5873.2	53120.7	0.111	MM
14	14	20211111_QC_R2_IgG	2.63	4042.2	81034.1	0.050	bb
15	15	20211111_L5_R1_IgG	2.64	1764.2	50994.6	0.035	MM
16	16	20211111_L6_R1_IgG	2.65	750.6	58058.0	0.013	bb
17	17	20211111_H3_R1_IgG	2.63	3453.7	64028.6	0.054	MM
18	18	20211111_A7_R1_IgG	2.65	1645.6	82996.1	0.020	bb
19	19	20211111_A8_R1_IgG	2.65	1797.1	77731.7	0.023	MM
20	20	20211111_L7_R1_IgG	2.65	1982.2	38827.3	0.051	MM
21	21	20211111_L8_R1_IgG	2.65	2227.2	44220.1	0.050	bb
22	22	20211111_H4_R1_IgG	2.63	1994.3	65826.3	0.030	MM
23	23	20211111_A9_R1_IgG	2.64	818.6	60771.1	0.013	MM
24	24	20211111_A10_R1_IgG	2.65	821.7	48578.7	0.017	MM
25	25	20211111_L9_R1_IgG	2.65	2048.5	50830.7	0.040	bb
26	26	20211111_L10_R1_IgG	2.65	1005.9	38034.2	0.026	bb
27	27	20211111_QC_R3_IgG	2.64	2886.7	67025.5	0.043	bb
28	28	20211111_H5_R1_IgG	2.63	1806.1	44544.9	0.041	MM
29	29	20211111_A11_R1_IgG	2.64	534.8	66408.5	0.008	bb
30	30	20211111_A12_R1_IgG	2.64	1555.9	46624.5	0.033	bb
31	31	20211111_L11_R1_IgG	2.63	595.2	33425.1	0.018	MM
32	32	20211111_L12_R1_IgG	2.63	957.7	41178.0	0.023	MM
33	33	20211111_H6_R1_IgG	2.64	6793.1	58593.4	0.116	MM
34	34	20211111_A13_R1_IgG	2.64	5930.7	54133.0	0.110	MM
35	35	20211111_A14_R1_IgG	2.64	1020.6	56467.3	0.018	bb
36	36	20211111_L13_R1_IgG	2.63	1772.0	36024.4	0.049	bb
37	37	20211111_L14_R1_IgG	2.63	1739.6	37181.6	0.047	MM
38	38	20211111_H7_R1_IgG	2.64	3312.9	42850.6	0.077	bb
39	39	20211111_A15_R1_IgG	2.64	1728.3	62015.1	0.028	bb
40	40	20211111_QC_R4_IgG	2.64	2762.5	58572.5	0.047	bb
41	41	20211111_A16_R1_IgG	2.65	1069.0	78283.8	0.014	MM
42	42	20211111_L15_R1_IgG	2.63	4449.9	44158.2	0.101	MM
43	43	20211111_L16_R1_IgG	2.65	1317.2	55150.8	0.024	MM
44	44	20211111_H8_R1_IgG	2.64	1522.9	42965.2	0.035	bb
45	45	20211111_A17_R1_IgG	2.64	3675.8	39717.7	0.093	MM
46	46	20211111_A18_R1_IgG	2.63	1436.9	44675.4	0.032	MM
47	47	20211111_L17_R1_IgG	2.63	2121.7	37854.1	0.056	bb
48	48	20211111_L18_R1_IgG	2.65	354.2	50716.9	0.007	bb
49	49	20211111_H9_R1_IgG	2.64	1764.9	36276.0	0.049	MM
50	50	20211111_A19_R1_IgG	2.64	2767.8	42862.5	0.065	MM
51	51	20211111_A20_R1_IgG	2.64	817.5	36542.7	0.022	bb
52	52	20211111_L19_R1_IgG	2.64	1851.9	53892.8	0.034	MM
53	53	20211111_QC_R5_IgG	2.63	2709.1	56513.2	0.048	bb
54	54	20211111_L20_R1_IgG	2.64	1470.1	56499.9	0.026	bb
55	55	20211111_H10_R1_IgG	2.63	3209.8	68120.5	0.047	MM
56	56	20211111_A21_R1_IgG	2.64	4453.2	63699.8	0.070	bb

Dataset: C:\MassLynx\10.pro\20211115_clinicalsamples_IgG.qld

Last Altered: Sunday, October 02, 2022 16:02:30 台北標準時間
Printed: Friday, August 01, 2025 16:53:36 台北標準時間

	#	Name	RT	Area	IS Area	Response	Prima...
57	57	20211111_A22_R1_IgG	2.63	2821.4	52222.1	0.054	MM
58	58	20211111_L21_R1_IgG	2.65	1166.8	54372.5	0.021	bb
59	59	20211111_L22_R1_IgG	2.63	1128.9	41353.1	0.027	MM
60	60	20211111_H11_R1_IgG	2.64	2692.0	65579.1	0.041	MM
61	61	20211111_A23_R1_IgG	2.64	2739.2	64131.1	0.043	bb
62	62	20211111_A24_R1_IgG	2.63	2014.3	38724.8	0.052	MM
63	63	20211111_L23_R1_IgG	2.63	3995.6	64245.2	0.062	MM
64	64	20211111_L24_R1_IgG	2.64	2049.6	45298.0	0.045	MM
65	65	20211111_H12_R1_IgG	2.64	1376.5	44679.3	0.031	bb
66	66	20211111_QC_R6_IgG	2.65	1859.9	48165.0	0.039	bb
67	67	20211111_A25_R1_IgG	2.64	868.4	57169.9	0.015	MM
68	68	20211111_A26_R1_IgG	2.64	1007.3	34254.0	0.029	MM
69	69	20211111_L25_R1_IgG	2.63	2204.3	67491.5	0.033	MM
70	70	20211111_L26_R1_IgG	2.64	813.8	55934.1	0.015	bb
71	71	20211111_H13_R1_IgG	2.64	2767.9	48767.3	0.057	MM
72	72	20211111_A27_R1_IgG	2.65	908.1	57269.7	0.016	bb
73	73	20211111_A28_R1_IgG	2.63	2276.2	49231.1	0.046	MM
74	74	20211111_L27_R1_IgG	2.62	1077.0	60014.5	0.018	MM
75	75	20211111_L28_R1_IgG	2.62	1208.3	35062.6	0.034	bb
76	76	20211111_H14_R1_IgG	2.64	7464.7	62371.9	0.120	bb
77	77	20211111_A29_R1_IgG	2.64	604.6	43858.2	0.014	MM
78	78	20211111_A30_R1_IgG	2.64	987.2	62500.4	0.016	MM
79	79	20211111_QC_R7_IgG	2.64	2195.3	43946.9	0.050	MM
80	80	20211111_L29_R1_IgG	2.65	446.6	38728.9	0.012	bb
81	81	20211111_L30_R1_IgG	2.63	1296.9	42613.2	0.030	bb
82	82	20211111_H15_R1_IgG	2.64	2419.1	39874.6	0.061	bb
83	83	20211111_A31_R1_IgG	2.65	956.6	53414.3	0.018	MM
84	84	20211111_A32_R1_IgG	2.63	1246.6	41844.6	0.030	bb
85	85	20211111_L31_R1_IgG	2.63	910.9	40553.7	0.022	bb
86	86	20211111_L32_R1_IgG	2.64	1492.9	49618.7	0.030	MM
87	87	20211111_H16_R1_IgG	2.63	2923.3	60170.9	0.049	bb
88	88	20211111_A33_R1_IgG	2.63	1860.3	48588.2	0.038	MM
89	89	20211111_A34_R1_IgG	2.64	1091.4	43288.0	0.025	bb
90	90	20211111_H17_R1_IgG	2.65	6027.1	47104.5	0.128	bb
91	91	20211111_A35_R1_IgG	2.64	3968.6	41025.9	0.097	bb
92	92	20211111_QC_R8_IgG	2.65	1586.8	40546.3	0.039	bb
93	93	20211111_A36_R1_IgG	2.63	1402.9	44504.4	0.032	MM
94	94	20211111_H18_R1_IgG	2.63	4413.0	34162.2	0.129	bb
95	95	20211111_A37_R1_IgG	2.64	1575.0	39760.0	0.040	bb
96	96	20211111_A38_R1_IgG	2.65	542.6	43982.6	0.012	MM
97	97	20211111_H19_R1_IgG	2.64	12760.9	45314.8	0.282	MM
98	98	20211111_A39_R1_IgG	2.64	2972.2	39388.0	0.075	bb
99	99	20211111_A40_R1_IgG	2.63	1007.0	38726.4	0.026	bb
100	1...	20211111_H20_R1_IgG	2.64	12952.1	37814.4	0.343	bb
101	1...	20211111_A41_R1_IgG	2.65	2273.1	48930.3	0.046	MM
102	1...	20211111_A42_R1_IgG	2.65	1496.7	40406.8	0.037	MM
103	1...	20211111_A43_R1_IgG	2.64	3898.1	44421.5	0.088	MM
104	1...	20211111_A44_R1_IgG	2.65	5885.2	53128.2	0.111	MM
105	1...	20211111_QC_R9_IgG	2.65	1421.1	37049.0	0.038	bb
106	1...	20211111_A1_R2_IgG	2.65	2485.6	46499.4	0.053	MM
107	1...	20211111_A2_R2_IgG	2.65	4362.1	57260.7	0.076	bb
108	1...	20211111_L1_R2_IgG	2.63	627.3	48062.9	0.013	bb
109	1...	20211111_L2_R2_IgG	2.64	2890.2	38745.2	0.075	MM
110	1...	20211111_H1_R2_IgG	2.63	978.1	44273.5	0.022	MM
111	1...	20211111_A3_R2_IgG	2.65	10.0	4755.5	0.002	bb
112	1...	20211111_A4_R2_IgG	2.64	3311.3	50546.7	0.066	bb

Dataset: C:\MassLynx\10.pro\20211115_clinicalsamples_IgG.qld

Last Altered: Sunday, October 02, 2022 16:02:30 台北標準時間
Printed: Friday, August 01, 2025 16:53:36 台北標準時間

	# Name	RT	Area	IS Area	Response	Prima...
113	1... 20211111_L3_R2_IgG	2.64	891.3	34825.8	0.026	MM
114	1... 20211111_L4_R2_IgG	2.63	1886.9	39107.7	0.048	MM
115	1... 20211111_H2_R2_IgG	2.63	2593.5	49750.0	0.052	bb
116	1... 20211111_A5_R2_IgG	2.63	1173.3	52536.2	0.022	MM
117	1... 20211111_A6_R2_IgG	2.63	4237.2	42050.0	0.101	bb
118	1... 20211111_QC_R10_IgG	2.63	1579.8	35350.8	0.045	MM
119	1... 20211111_L5_R2_IgG	2.64	1405.2	38039.3	0.037	MM
120	1... 20211111_L6_R2_IgG	2.64	1017.8	44907.4	0.023	MM
121	1... 20211111_H3_R2_IgG	2.64	2930.4	47729.1	0.061	bb
122	1... 20211111_A7_R2_IgG	2.65	938.7	57347.1	0.016	MM
123	1... 20211111_A8_R2_IgG	2.64	1255.6	52397.8	0.024	MM
124	1... 20211111_L7_R2_IgG	2.63	1528.6	36318.4	0.042	bb
125	1... 20211111_L8_R2_IgG	2.63	1858.7	38131.9	0.049	bb
126	1... 20211111_H4_R2_IgG	2.64	1440.3	46849.6	0.031	bb
127	1... 20211111_A9_R2_IgG	2.63	496.1	47759.6	0.010	MM
128	1... 20211111_A10_R2_IgG	2.63	647.6	41366.2	0.016	MM
129	1... 20211111_L9_R2_IgG	2.64	1728.2	43052.1	0.040	bb
130	1... 20211111_L10_R2_IgG	2.64	983.6	31487.4	0.031	MM
131	1... 20211111_QC_R11_IgG	2.63	1198.6	37804.6	0.032	MM
132	1... 20211111_H5_R2_IgG	2.65	1761.4	40380.0	0.044	MM
133	1... 20211111_A11_R2_IgG	2.63	392.8	49533.3	0.008	bb
134	1... 20211111_A12_R2_IgG	2.64	1300.7	40226.3	0.032	MM
135	1... 20211111_L11_R2_IgG	2.64	436.2	28884.0	0.015	MM
136	1... 20211111_L12_R2_IgG	2.64	564.1	34351.3	0.016	MM
137	1... 20211111_H6_R2_IgG	2.64	4383.5	40466.6	0.108	bb
138	1... 20211111_A13_R2_IgG	2.64	4674.2	42306.7	0.110	MM
139	1... 20211111_A14_R2_IgG	2.64	1131.9	44904.0	0.025	bb
140	1... 20211111_L13_R2_IgG	2.63	1642.8	31913.0	0.051	bb
141	1... 20211111_L14_R2_IgG	2.64	1486.2	31527.4	0.047	MM
142	1... 20211111_H7_R2_IgG	2.64	3260.6	40838.2	0.080	MM
143	1... 20211111_A15_R2_IgG	2.63	1300.9	49389.7	0.026	bb
144	1... 20211111_QC_R12_IgG	2.64	1422.3	35168.7	0.040	MM
145	1... 20211111_A16_R2_IgG	2.63	745.8	47440.5	0.016	bb
146	1... 20211111_L15_R2_IgG	2.63	2777.5	33145.9	0.084	MM
147	1... 20211111_L16_R2_IgG	2.63	611.5	37596.6	0.016	bb
148	1... 20211111_H8_R2_IgG	2.64	1367.7	43933.3	0.031	MM
149	1... 20211111_A17_R2_IgG	2.64	3959.2	43605.5	0.091	bb
150	1... 20211111_A18_R2_IgG	2.64	866.0	37581.8	0.023	MM
151	1... 20211111_L17_R2_IgG	2.64	1955.7	33942.5	0.058	bb
152	1... 20211111_L18_R2_IgG	2.65	167.9	37831.5	0.004	bb
153	1... 20211111_H9_R2_IgG	2.63	1749.4	30898.4	0.057	bb
154	1... 20211111_A19_R2_IgG	2.64	2633.9	39352.1	0.067	bb
155	1... 20211111_A20_R2_IgG	2.63	586.6	34661.2	0.017	MM
156	1... 20211111_L19_R2_IgG	2.63	1082.0	39918.3	0.027	MM
157	1... 20211111_QC_R13_IgG	2.65	1939.0	36239.1	0.054	bb
158	1... 20211111_L20_R2_IgG	2.63	1014.9	32897.7	0.031	MM
159	1... 20211111_H10_R2_IgG	2.63	1266.8	31394.5	0.040	MM
160	1... 20211111_A21_R2_IgG	2.63	1882.9	34863.7	0.054	MM
161	1... 20211111_A22_R2_IgG	2.63	1384.0	30342.1	0.046	MM
162	1... 20211111_L21_R2_IgG	2.65	685.7	33706.2	0.020	MM
163	1... 20211111_L22_R2_IgG	2.63	563.6	31298.6	0.018	MM
164	1... 20211111_H11_R2_IgG	2.63	1287.7	38904.1	0.033	MM
165	1... 20211111_A23_R2_IgG	2.63	1381.5	32382.3	0.043	MM
166	1... 20211111_A24_R2_IgG	2.63	1207.1	30261.9	0.040	MM
167	1... 20211111_L23_R2_IgG	2.63	2050.1	43037.5	0.048	bb
168	1... 20211111_L24_R2_IgG	2.63	1393.7	37023.3	0.038	MM

Dataset: C:\MassLynx\10.pro\20211115_clinicalsamples_IgG.qld

Last Altered: Sunday, October 02, 2022 16:02:30 台北標準時間
Printed: Friday, August 01, 2025 16:53:36 台北標準時間

	# Name	RT	Area	IS Area	Response	Prima...
169	1... 20211111_H12_R2_IgG	2.64	1061.9	39478.2	0.027	MM
170	1... 20211111_QC_R14_IgG	2.65	1389.7	34463.2	0.040	MM
171	1... 20211111_A25_R2_IgG	2.64	535.0	39945.3	0.013	MM
172	1... 20211111_A26_R2_IgG	2.63	746.8	29663.2	0.025	bb
173	1... 20211111_L25_R2_IgG	2.63	969.9	38589.3	0.025	bb
174	1... 20211111_L26_R2_IgG	2.63	480.2	38405.2	0.013	MM
175	1... 20211111_H13_R2_IgG	2.63	2072.7	37771.2	0.055	bb
176	1... 20211111_A27_R2_IgG	2.64	425.8	42634.1	0.010	bb
177	1... 20211111_A28_R2_IgG	2.63	1268.1	33231.1	0.038	bb
178	1... 20211111_L27_R2_IgG	2.65	690.4	41761.1	0.017	MM
179	1... 20211111_L28_R2_IgG	2.64	1208.8	26968.6	0.045	MM
180	1... 20211111_H14_R2_IgG	2.64	4718.2	49250.2	0.096	bb
181	1... 20211111_A29_R2_IgG	2.66	497.0	37601.7	0.013	MM
182	1... 20211111_A30_R2_IgG	2.64	545.8	40366.8	0.014	MM
183	1... 20211111_QC_R15_IgG	2.63	1348.3	35156.1	0.038	MM
184	1... 20211111_L29_R2_IgG	2.63	676.0	33813.1	0.020	bb
185	1... 20211111_L30_R2_IgG	2.63	1109.2	33488.6	0.033	bb
186	1... 20211111_H15_R2_IgG	2.63	1966.3	38501.0	0.051	bb
187	1... 20211111_A31_R2_IgG	2.64	726.3	37525.5	0.019	MM
188	1... 20211111_A32_R2_IgG	2.63	1136.6	35770.8	0.032	MM
189	1... 20211111_L31_R2_IgG	2.62	848.8	33282.4	0.026	bb
190	1... 20211111_L32_R2_IgG	2.64	859.5	42049.6	0.020	bb
191	1... 20211111_H16_R2_IgG	2.63	2054.1	45947.6	0.045	MM
192	1... 20211111_A33_R2_IgG	2.62	1366.7	40202.3	0.034	MM
193	1... 20211111_A34_R2_IgG	2.63	1170.9	36747.0	0.032	MM
194	1... 20211111_H17_R2_IgG	2.63	6040.9	40920.9	0.148	bb
195	1... 20211111_A35_R2_IgG	2.63	3880.4	33689.2	0.115	MM
196	1... 20211111_QC_R16_IgG	2.63	1219.9	32783.6	0.037	MM
197	1... 20211111_A36_R2_IgG	2.63	1294.7	36822.4	0.035	bb
198	1... 20211111_H18_R2_IgG	2.64	4619.9	31769.3	0.145	MM
199	1... 20211111_A37_R2_IgG	2.63	1842.3	37581.8	0.049	MM
200	2... 20211111_A38_R2_IgG	2.63	600.8	38390.4	0.016	MM
201	2... 20211111_H19_R2_IgG	2.63	11101.9	41217.0	0.269	bb
202	2... 20211111_A39_R2_IgG	2.64	2961.8	37392.7	0.079	bb
203	2... 20211111_A40_R2_IgG	2.65	1093.0	38295.1	0.029	MM
204	2... 20211111_H20_R2_IgG	2.63	12376.6	35941.8	0.344	MM
205	2... 20211111_A41_R2_IgG	2.64	1694.4	45304.9	0.037	bb
206	2... 20211111_A42_R2_IgG	2.62	1409.8	37803.8	0.037	MM
207	2... 20211111_A43_R2_IgG	2.63	3135.7	38161.8	0.082	bb
208	2... 20211111_A44_R2_IgG	2.63	3424.3	37819.8	0.091	MM
209	2... 20211111_QC_R17_IgG	2.63	1168.9	31731.8	0.037	MM

Dataset: C:\MassLynx\10.pro\20211115_clinicalsamples_IgG.qld

Last Altered: Sunday, October 02, 2022 16:02:30 台北標準時間
Printed: Friday, August 01, 2025 16:54:07 台北標準時間

Method: C:\MassLynx\10_Pro_IgA.PRO\MethDB\20210926_IgG_DMRM_IgM_IS.mdb 16 Feb 2022 11:58:21
Calibration: 18 Nov 2021 13:56:22

Sample Name: 20211111_QC_R1_IgG

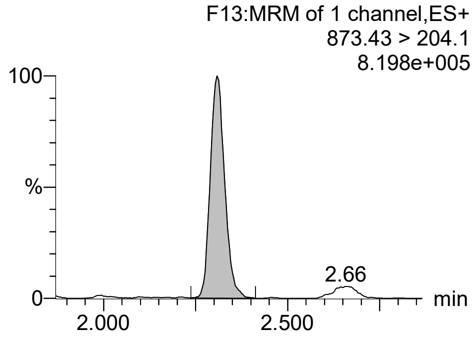

Sample Name: 20211111_A1_R1_IgG

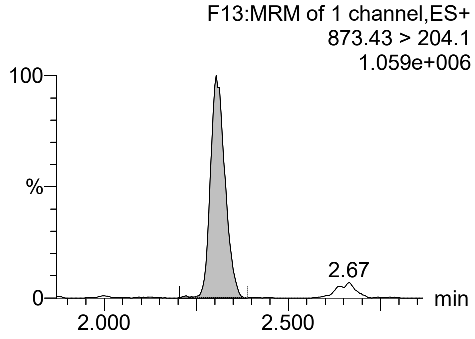

Sample Name: 20211111_A2_R1_IgG

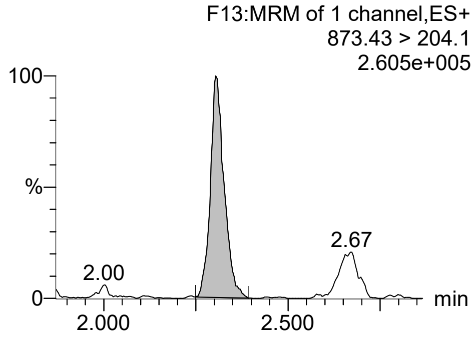

Sample Name: 20211111_L1_R1_IgG

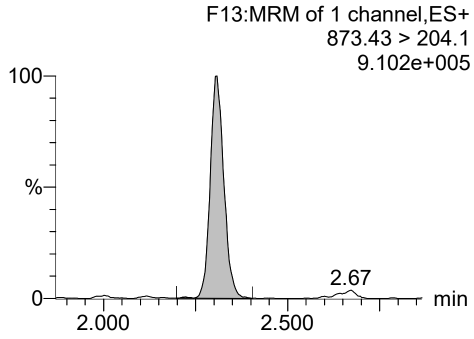

Sample Name: 20211111_L2_R1_IgG

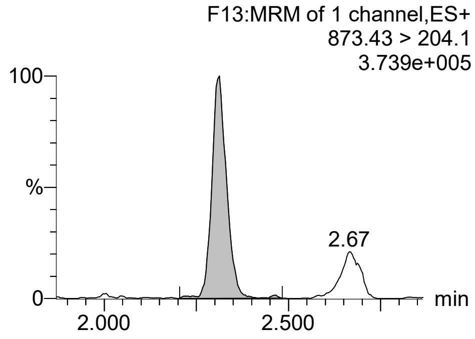

Sample Name: 20211111_H1_R1_IgG

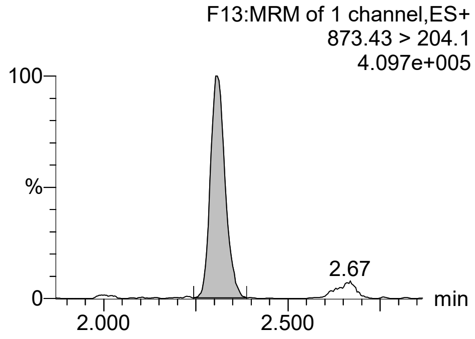

Sample Name: 20211111_A3_R1_IgG

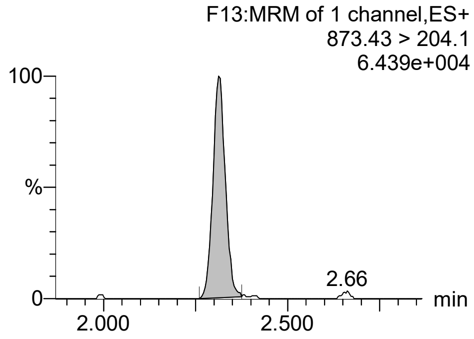

Sample Name: 20211111_A4_R1_IgG

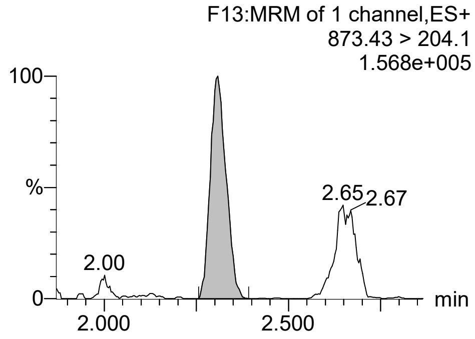

Sample Name: 20211111_L3_R1_IgG

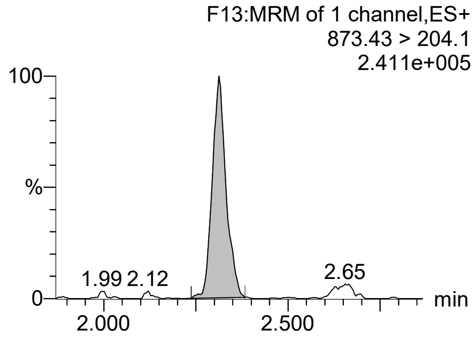

Sample Name: 20211111_L4_R1_IgG

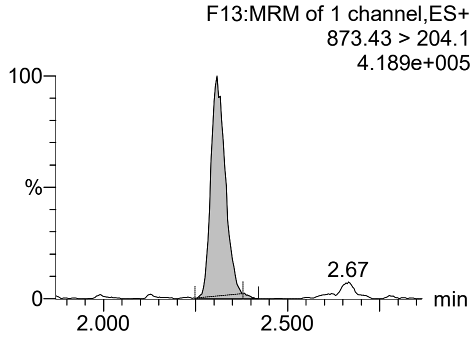

Sample Name: 20211111_H2_R1_IgG

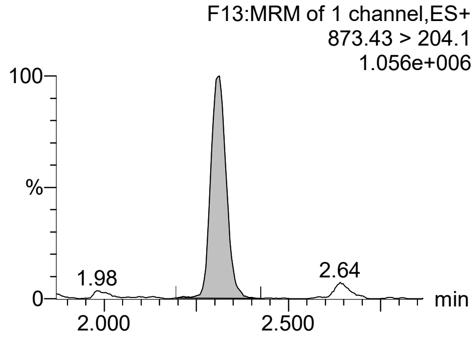

Sample Name: 20211111_A5_R1_IgG

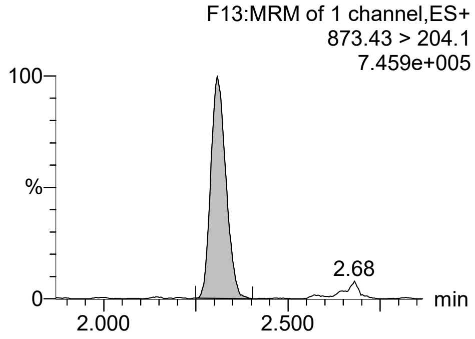

Sample Name: 20211111_A6_R1_IgG
F13:MRM of 1 channel,ES+
873.43 > 204.1
1.020e+006

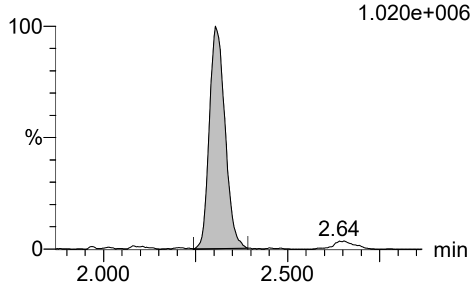

Sample Name: 20211111_QC_R2_IgG
F13:MRM of 1 channel,ES+
873.43 > 204.1
8.573e+005

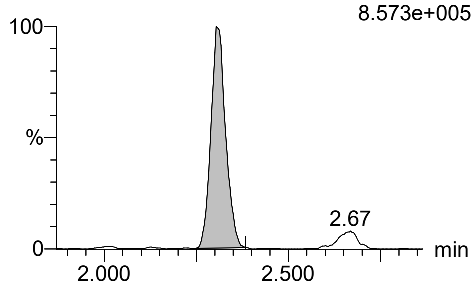

Sample Name: 20211111_L5_R1_IgG
F13:MRM of 1 channel,ES+
873.43 > 204.1
7.944e+005

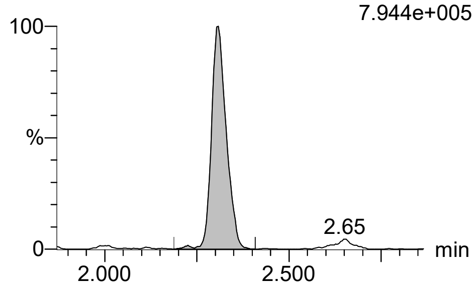

Sample Name: 20211111_L6_R1_IgG
F13:MRM of 1 channel,ES+
873.43 > 204.1
1.728e+005

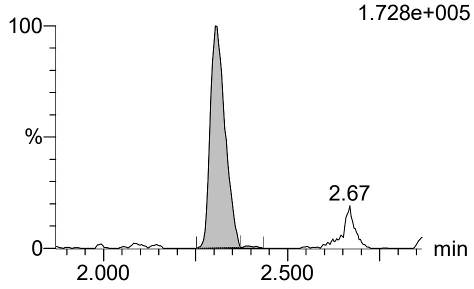

Sample Name: 20211111_H3_R1_IgG
F13:MRM of 1 channel,ES+
873.43 > 204.1
1.240e+005

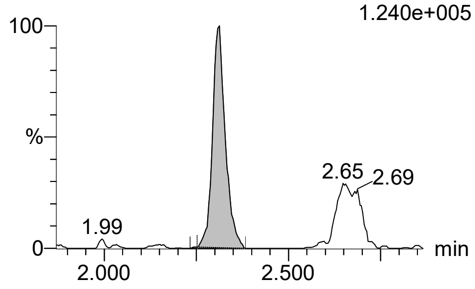

Sample Name: 20211111_A7_R1_IgG
F13:MRM of 1 channel,ES+
873.43 > 204.1
1.408e+006

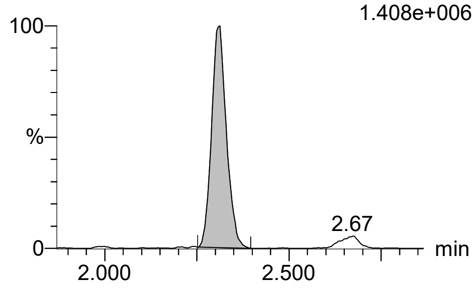

Sample Name: 20211111_A8_R1_IgG
F13:MRM of 1 channel,ES+
873.43 > 204.1
5.291e+005

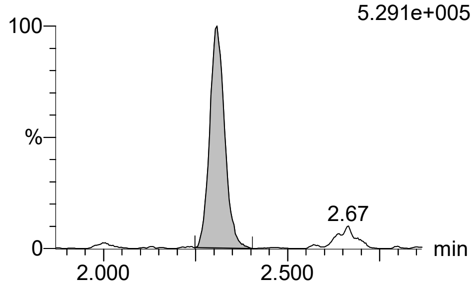

Sample Name: 20211111_L7_R1_IgG
F13:MRM of 1 channel,ES+
873.43 > 204.1
3.600e+005

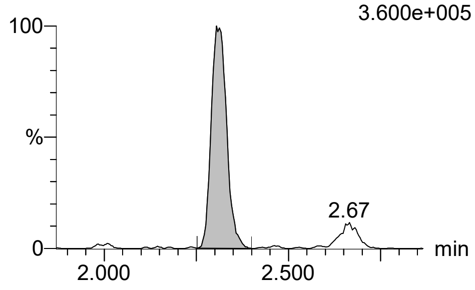

Sample Name: 20211111_L8_R1_IgG
F13:MRM of 1 channel,ES+
873.43 > 204.1
2.579e+005

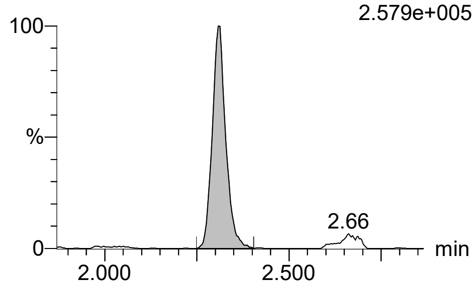

Sample Name: 20211111_H4_R1_IgG
F13:MRM of 1 channel,ES+
873.43 > 204.1
3.000e+005

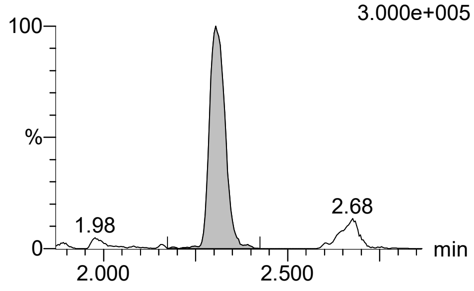

Sample Name: 20211111_A9_R1_IgG
F13:MRM of 1 channel,ES+
873.43 > 204.1
7.893e+005

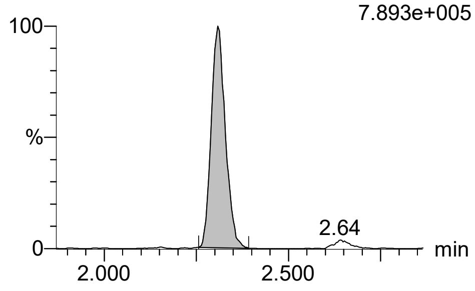

Sample Name: 20211111_A10_R1_IgG
F13:MRM of 1 channel,ES+
873.43 > 204.1
1.157e+006

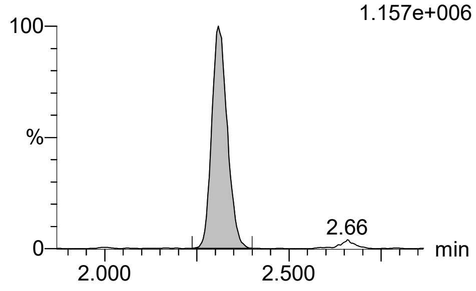

Sample Name: 20211111_L9_R1_IgG
F13:MRM of 1 channel,ES+
873.43 > 204.1
4.874e+005

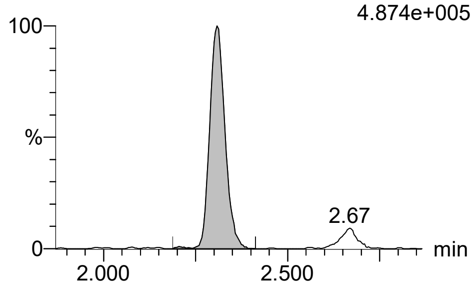

Sample Name: 20211111_L10_R1_IgG
F13:MRM of 1 channel,ES+
873.43 > 204.1
4.775e+005

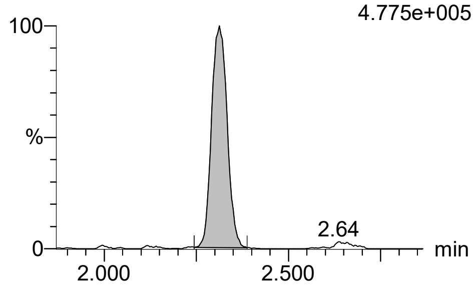

Sample Name: 20211111_QC_R3_IgG
F13:MRM of 1 channel,ES+
873.43 > 204.1
7.226e+005

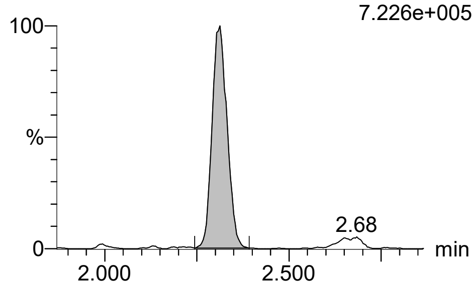

Dataset: C:\MassLynx\10.pro\20211115_clinicalsamples_IgG.qld

Last Altered: Sunday, October 02, 2022 16:02:30 台北標準時間
Printed: Friday, August 01, 2025 16:54:07 台北標準時間

Sample Name: 20211111_H5_R1_IgG
F13:MRM of 1 channel,ES+
873.43 > 204.1
6.679e+004

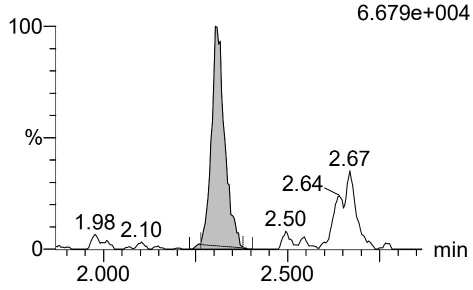

Sample Name: 20211111_A11_R1_IgG
F13:MRM of 1 channel,ES+
873.43 > 204.1
2.361e+005

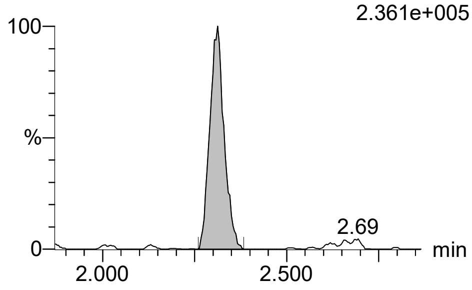

Sample Name: 20211111_A12_R1_IgG
F13:MRM of 1 channel,ES+
873.43 > 204.1
3.392e+005

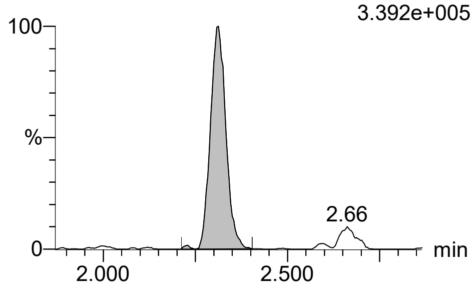

Sample Name: 20211111_L11_R1_IgG
F13:MRM of 1 channel,ES+
873.43 > 204.1
3.059e+005

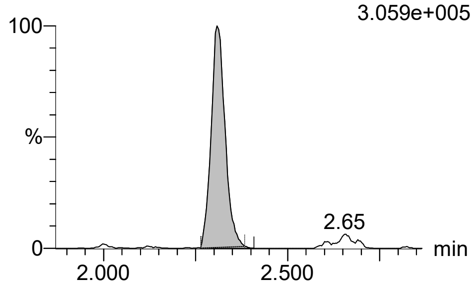

Sample Name: 20211111_L12_R1_IgG
F13:MRM of 1 channel,ES+
873.43 > 204.1
1.073e+005

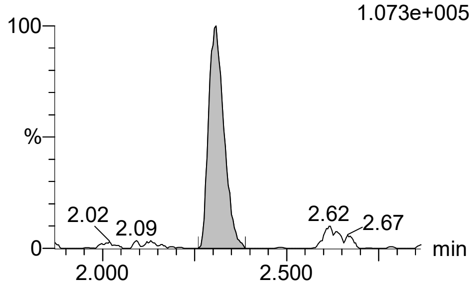

Sample Name: 20211111_H6_R1_IgG
F13:MRM of 1 channel,ES+
873.43 > 204.1
8.117e+005

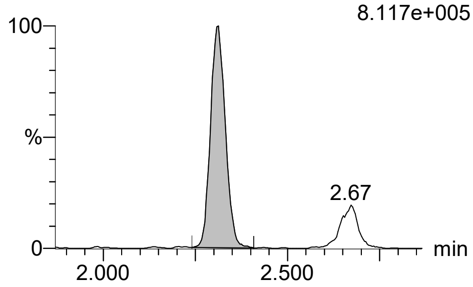

Sample Name: 20211111_A13_R1_IgG
F13:MRM of 1 channel,ES+
873.43 > 204.1
8.589e+005

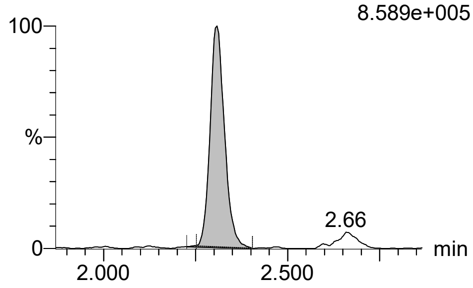

Sample Name: 20211111_A14_R1_IgG
F13:MRM of 1 channel,ES+
873.43 > 204.1
7.229e+005

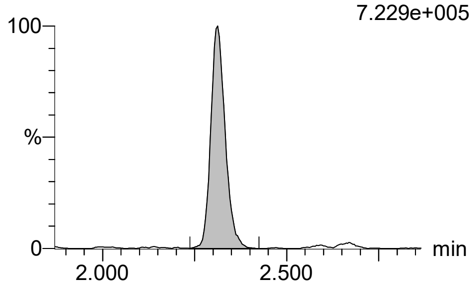

Sample Name: 20211111_L13_R1_IgG
F13:MRM of 1 channel,ES+
873.43 > 204.1
1.707e+005

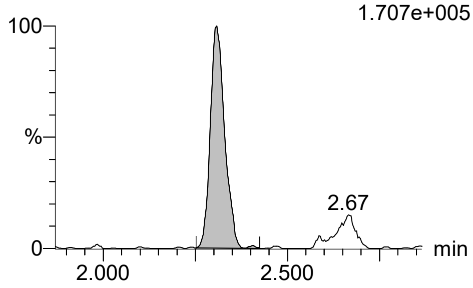

Sample Name: 20211111_L14_R1_IgG
F13:MRM of 1 channel,ES+
873.43 > 204.1
2.112e+005

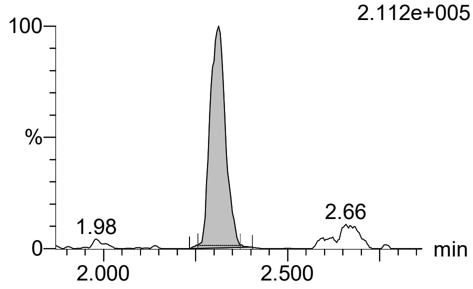

Sample Name: 20211111_H7_R1_IgG
F13:MRM of 1 channel,ES+
873.43 > 204.1
6.074e+005

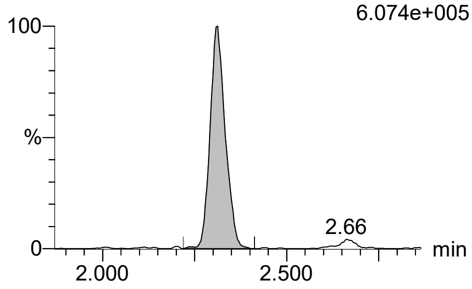

Sample Name: 20211111_A15_R1_IgG
F13:MRM of 1 channel,ES+
873.43 > 204.1
1.794e+005

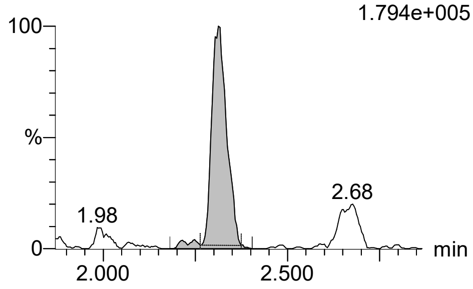

Sample Name: 20211111_QC_R4_IgG
F13:MRM of 1 channel,ES+
873.43 > 204.1
5.611e+005

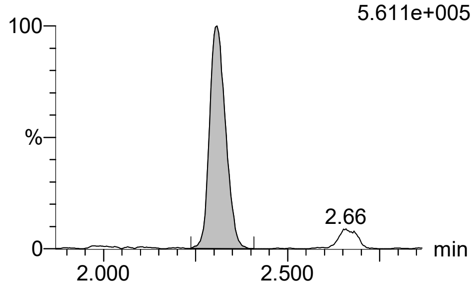

Sample Name: 20211111_A16_R1_IgG
F13:MRM of 1 channel,ES+
873.43 > 204.1
8.759e+005

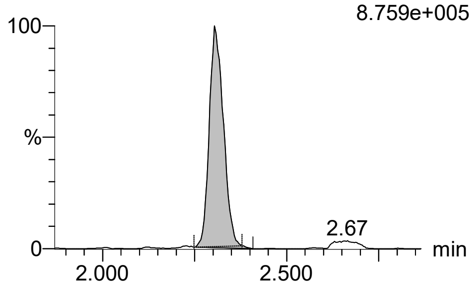

Sample Name: 20211111_L15_R1_IgG
F13:MRM of 1 channel,ES+
873.43 > 204.1
7.141e+005

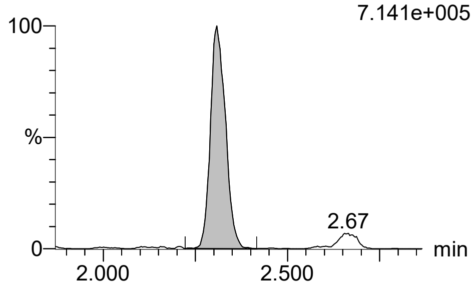

Sample Name: 20211111_L16_R1_IgG
F13:MRM of 1 channel,ES+
873.43 > 204.1
2.281e+005

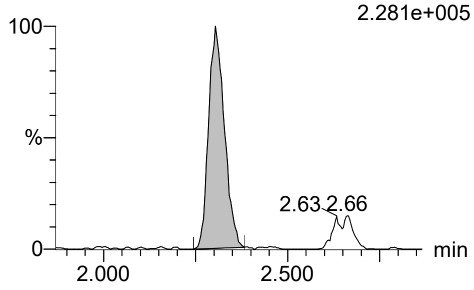

Sample Name: 20211111_H8_R1_IgG
F13:MRM of 1 channel,ES+
873.43 > 204.1
3.155e+005

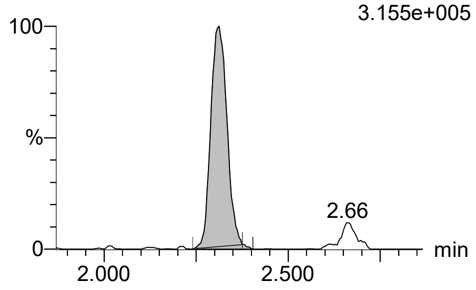

Sample Name: 20211111_A17_R1_IgG
F13:MRM of 1 channel,ES+
873.43 > 204.1
3.950e+005

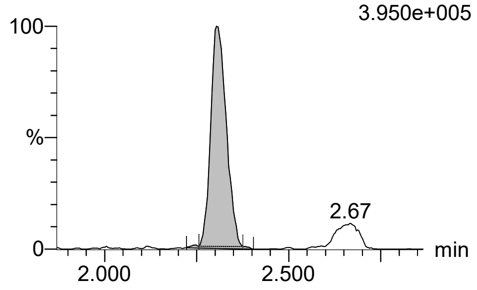

Sample Name: 20211111_A18_R1_IgG
F13:MRM of 1 channel,ES+
873.43 > 204.1
6.819e+005

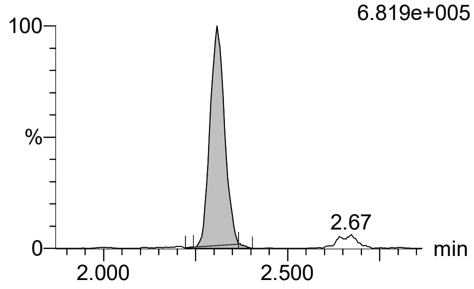

Sample Name: 20211111_L17_R1_IgG
F13:MRM of 1 channel,ES+
873.43 > 204.1
2.079e+005

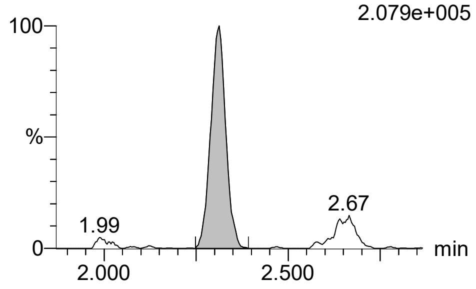

Sample Name: 20211111_L18_R1_IgG
F13:MRM of 1 channel,ES+
873.43 > 204.1
2.020e+005

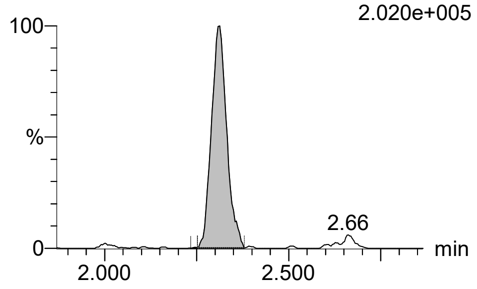

Sample Name: 20211111_H9_R1_IgG
F13:MRM of 1 channel,ES+
873.43 > 204.1
7.272e+005

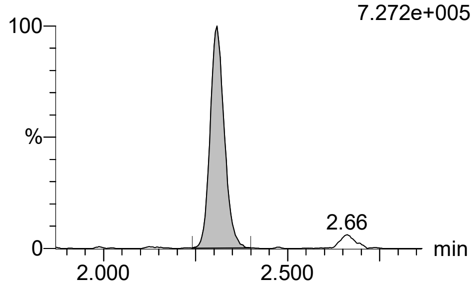

Sample Name: 20211111_A19_R1_IgG
F13:MRM of 1 channel,ES+
873.43 > 204.1
1.611e+005

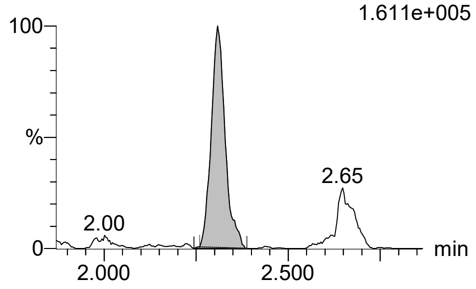

Sample Name: 20211111_A20_R1_IgG
F13:MRM of 1 channel,ES+
873.43 > 204.1
1.475e+005

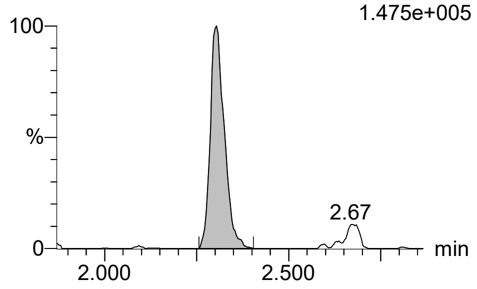

Sample Name: 20211111_L19_R1_IgG
F13:MRM of 1 channel,ES+
873.43 > 204.1
3.221e+005

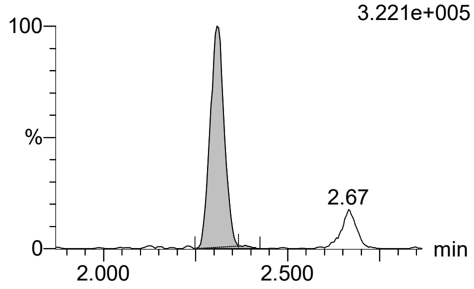

Sample Name: 20211111_QC_R5_IgG
F13:MRM of 1 channel,ES+
873.43 > 204.1
5.535e+005

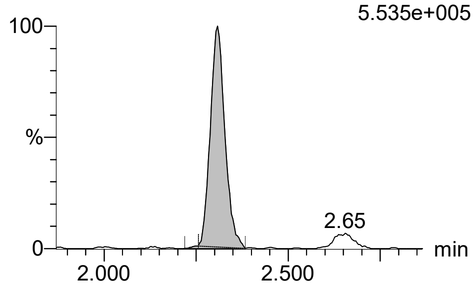

Sample Name: 20211111_L20_R1_IgG
F13:MRM of 1 channel,ES+
873.43 > 204.1
5.387e+005

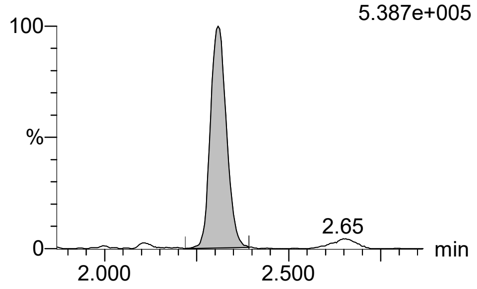

Sample Name: 20211111_H10_R1_IgG
F13:MRM of 1 channel,ES+
873.43 > 204.1
1.092e+006

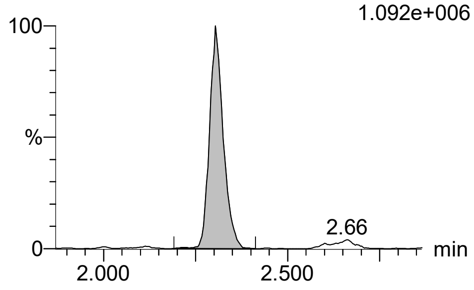

Sample Name: 20211111_A21_R1_IgG
F13:MRM of 1 channel,ES+
873.43 > 204.1
1.312e+006

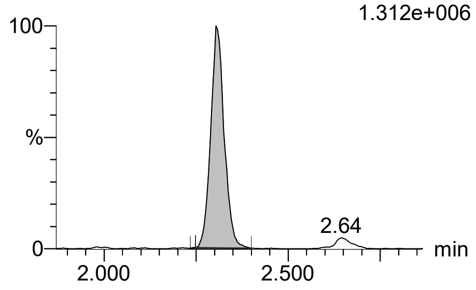

Sample Name: 20211111_A22_R1_IgG
F13:MRM of 1 channel,ES+
873.43 > 204.1
1.896e+005

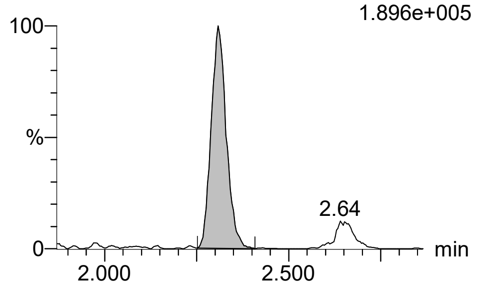

Dataset: C:\MassLynx\10.pro\20211115_clinicalsamples_IgG.qld

Last Altered: Sunday, October 02, 2022 16:02:30 台北標準時間
Printed: Friday, August 01, 2025 16:54:07 台北標準時間

Sample Name: 20211111_L21_R1_IgG
F13:MRM of 1 channel,ES+
873.43 > 204.1
2.275e+005

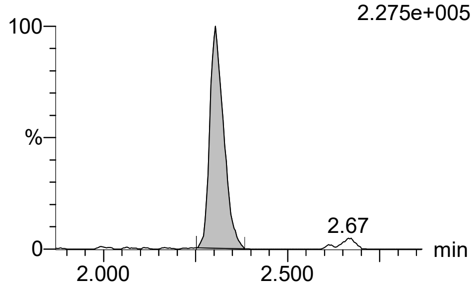

Sample Name: 20211111_L22_R1_IgG
F13:MRM of 1 channel,ES+
873.43 > 204.1
3.786e+005

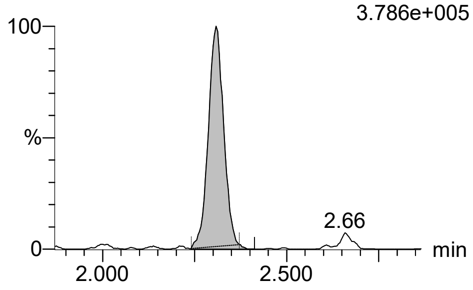

Sample Name: 20211111_H11_R1_IgG
F13:MRM of 1 channel,ES+
873.43 > 204.1
1.038e+006

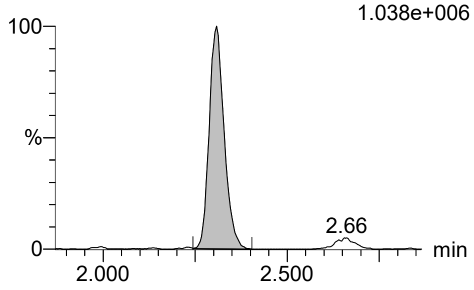

Sample Name: 20211111_A23_R1_IgG
F13:MRM of 1 channel,ES+
873.43 > 204.1
7.761e+005

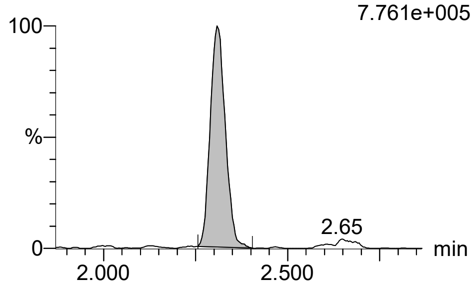

Sample Name: 20211111_A24_R1_IgG
F13:MRM of 1 channel,ES+
873.43 > 204.1
1.189e+005

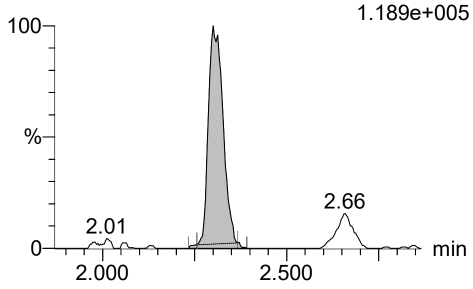

Sample Name: 20211111_L23_R1_IgG
F13:MRM of 1 channel,ES+
873.43 > 204.1
7.700e+005

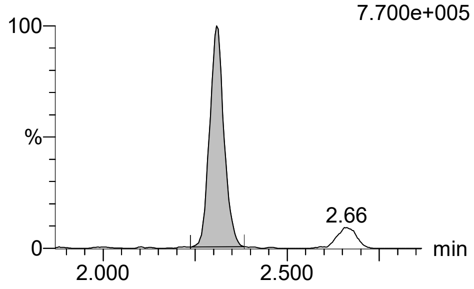

Sample Name: 20211111_L24_R1_IgG
F13:MRM of 1 channel,ES+
873.43 > 204.1
1.865e+005

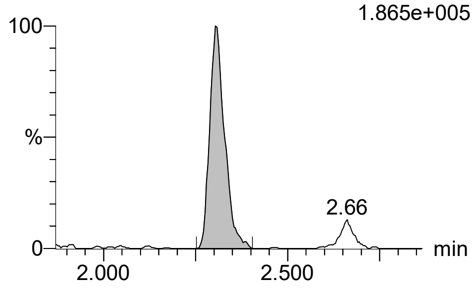

Sample Name: 20211111_H12_R1_IgG
F13:MRM of 1 channel,ES+
873.43 > 204.1
8.524e+004

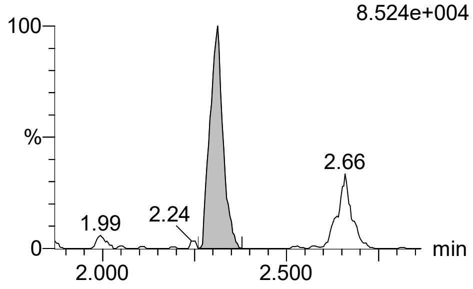

Sample Name: 20211111_QC_R6_IgG
F13:MRM of 1 channel,ES+
873.43 > 204.1
4.876e+005

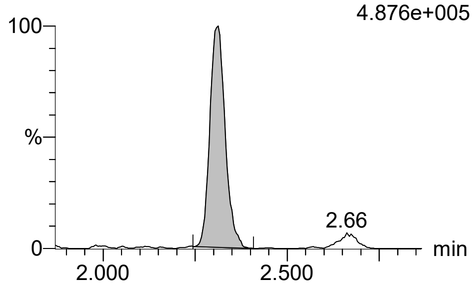

Sample Name: 20211111_A25_R1_IgG
F13:MRM of 1 channel,ES+
873.43 > 204.1
2.184e+006

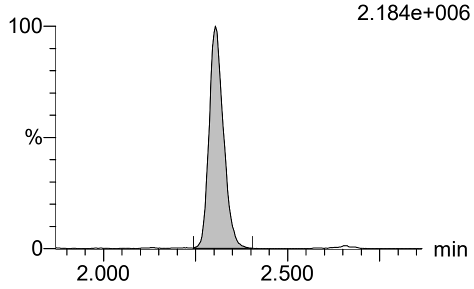

Sample Name: 20211111_A26_R1_IgG
F13:MRM of 1 channel,ES+
873.43 > 204.1
2.898e+004

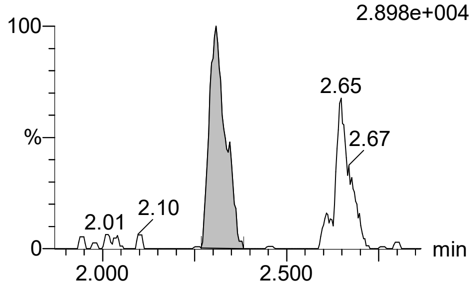

Sample Name: 20211111_L25_R1_IgG
F13:MRM of 1 channel,ES+
873.43 > 204.1
5.086e+005

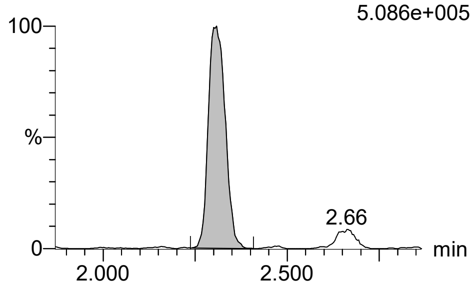

Sample Name: 20211111_L26_R1_IgG
F13:MRM of 1 channel,ES+
873.43 > 204.1
2.757e+005

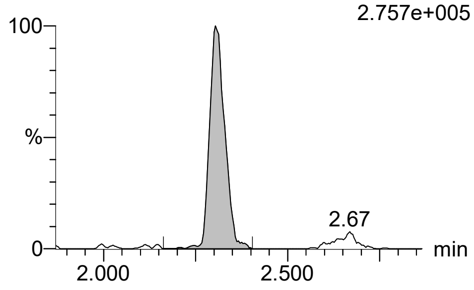

Sample Name: 20211111_H13_R1_IgG
F13:MRM of 1 channel,ES+
873.43 > 204.1
3.124e+005

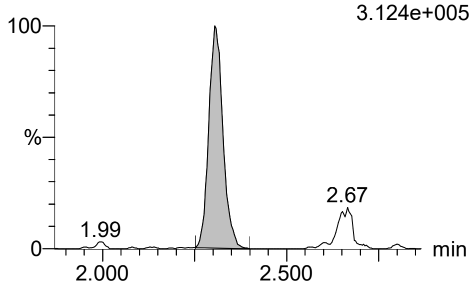

Sample Name: 20211111_A27_R1_IgG
F13:MRM of 1 channel,ES+
873.43 > 204.1
3.308e+005

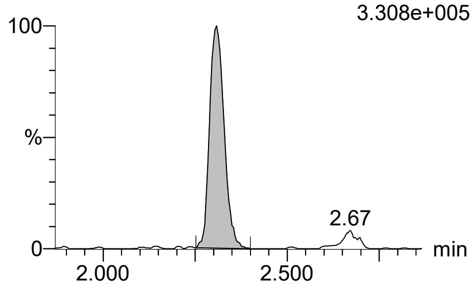

Dataset: C:\MassLynx\10.pro\20211115_clinicalsamples_IgG.qld

Last Altered: Sunday, October 02, 2022 16:02:30 台北標準時間
Printed: Friday, August 01, 2025 16:54:07 台北標準時間

Sample Name: 20211111_A28_R1_IgG
F13:MRM of 1 channel,ES+
873.43 > 204.1
4.686e+005

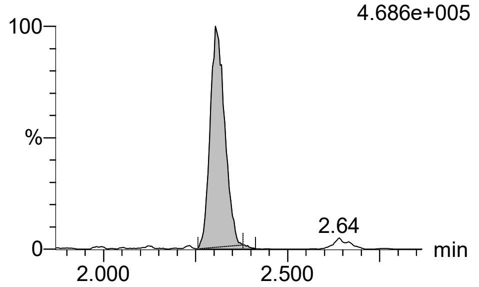

Sample Name: 20211111_L27_R1_IgG
F13:MRM of 1 channel,ES+
873.43 > 204.1
3.018e+005

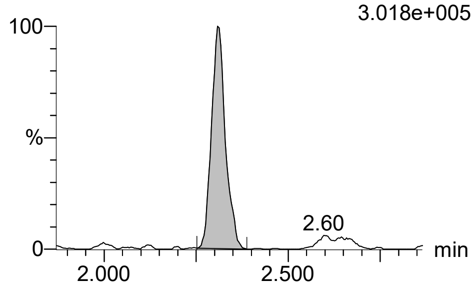

Sample Name: 20211111_L28_R1_IgG
F13:MRM of 1 channel,ES+
873.43 > 204.1
1.648e+005

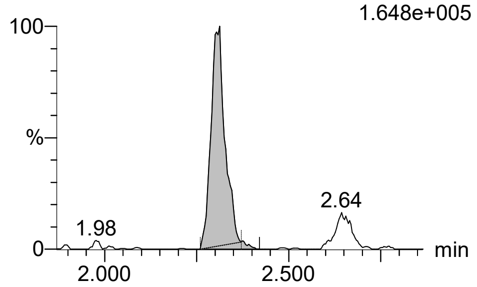

Sample Name: 20211111_H14_R1_IgG
F13:MRM of 1 channel,ES+
873.43 > 204.1
2.206e+005

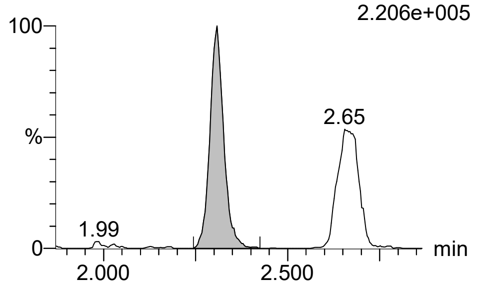

Sample Name: 20211111_A29_R1_IgG
F13:MRM of 1 channel,ES+
873.43 > 204.1
8.572e+005

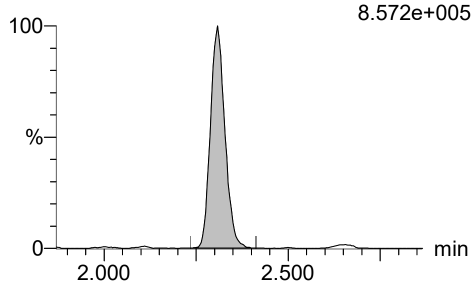

Sample Name: 20211111_A30_R1_IgG
F13:MRM of 1 channel,ES+
873.43 > 204.1
1.263e+006

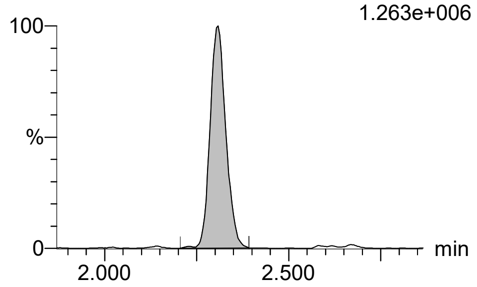

Sample Name: 20211111_QC_R7_IgG
F13:MRM of 1 channel,ES+
873.43 > 204.1
4.555e+005

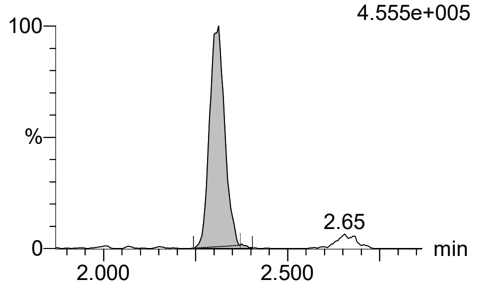

Sample Name: 20211111_L29_R1_IgG
F13:MRM of 1 channel,ES+
873.43 > 204.1
2.220e+005

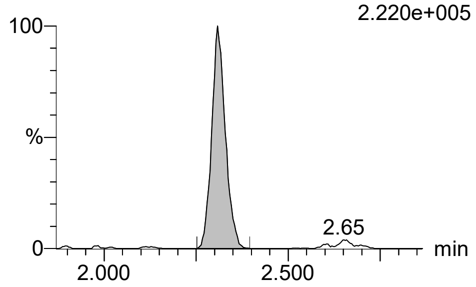

Sample Name: 20211111_L30_R1_IgG
F13:MRM of 1 channel,ES+
873.43 > 204.1
1.703e+005

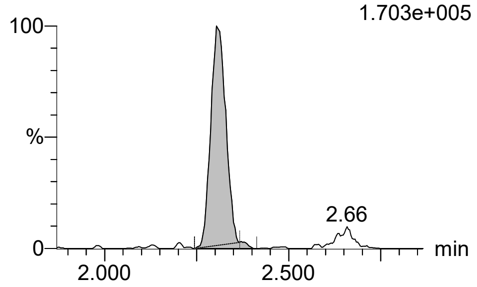

Sample Name: 20211111_H15_R1_IgG
F13:MRM of 1 channel,ES+
873.43 > 204.1
2.566e+004

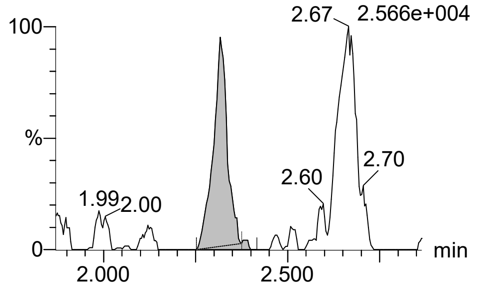

Sample Name: 20211111_A31_R1_IgG
F13:MRM of 1 channel,ES+
873.43 > 204.1
3.926e+005

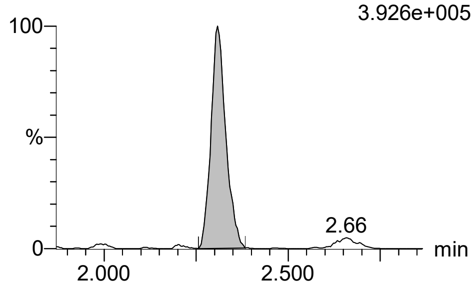

Sample Name: 20211111_A32_R1_IgG
F13:MRM of 1 channel,ES+
873.43 > 204.1
1.348e+006

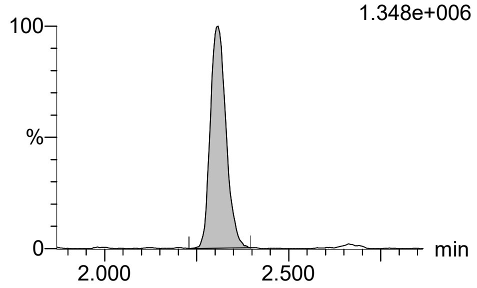

Sample Name: 20211111_L31_R1_IgG
F13:MRM of 1 channel,ES+
873.43 > 204.1
1.890e+005

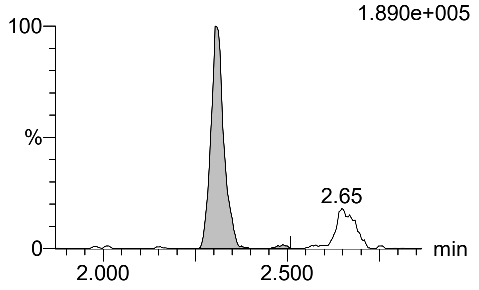

Sample Name: 20211111_L32_R1_IgG
F13:MRM of 1 channel,ES+
873.43 > 204.1
1.619e+005

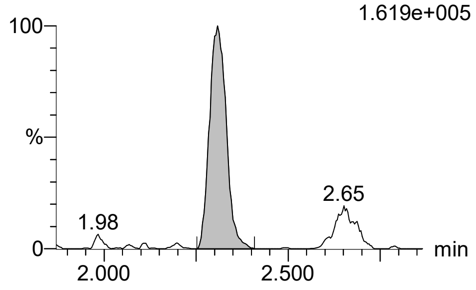

Sample Name: 20211111_H16_R1_IgG
F13:MRM of 1 channel,ES+
873.43 > 204.1
1.200e+006

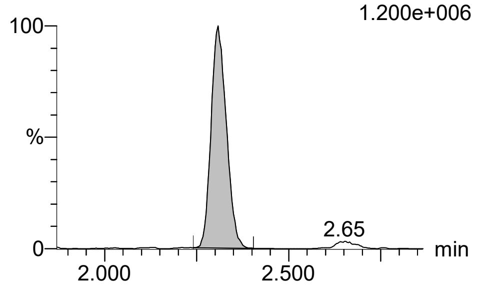

Dataset: C:\MassLynx\10.pro\20211115_clinicalsamples_IgG.qld

Last Altered: Sunday, October 02, 2022 16:02:30 台北標準時間
Printed: Friday, August 01, 2025 16:54:07 台北標準時間

Sample Name: 20211111_A33_R1_IgG
F13:MRM of 1 channel,ES+
873.43 > 204.1
2.899e+005

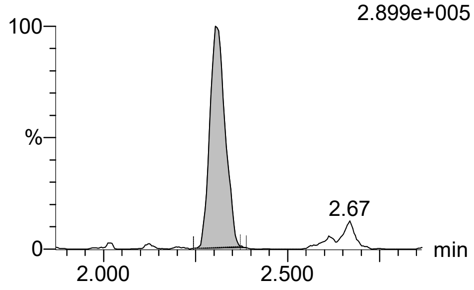

Sample Name: 20211111_A34_R1_IgG
F13:MRM of 1 channel,ES+
873.43 > 204.1
4.601e+005

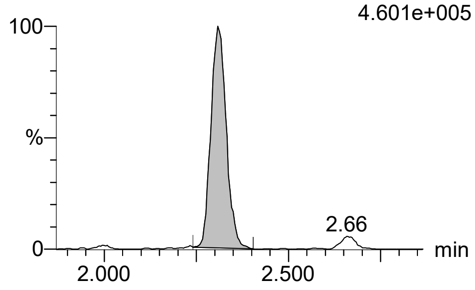

Sample Name: 20211111_H17_R1_IgG
F13:MRM of 1 channel,ES+
873.43 > 204.1
1.126e+006

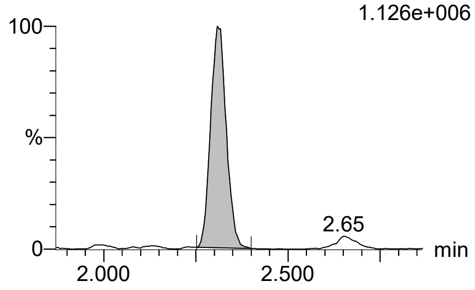

Sample Name: 20211111_A35_R1_IgG
F13:MRM of 1 channel,ES+
873.43 > 204.1
6.317e+005

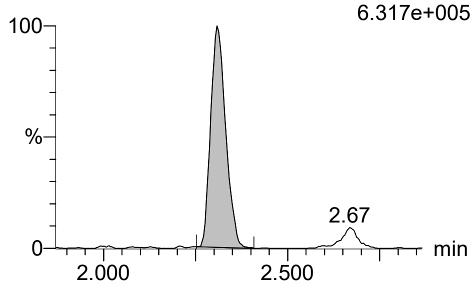

Sample Name: 20211111_QC_R8_IgG
F13:MRM of 1 channel,ES+
873.43 > 204.1
4.056e+005

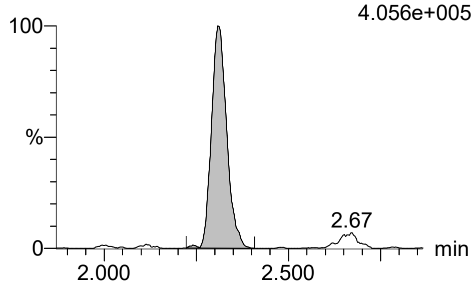

Sample Name: 20211111_A36_R1_IgG
F13:MRM of 1 channel,ES+
873.43 > 204.1
9.410e+005

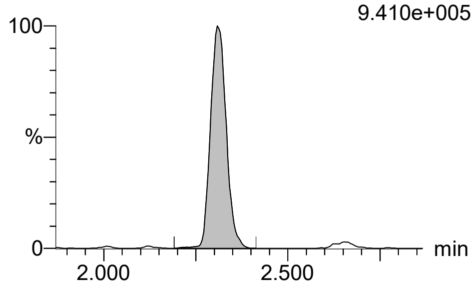

Sample Name: 20211111_H18_R1_IgG
F13:MRM of 1 channel,ES+
873.43 > 204.1
2.151e+006

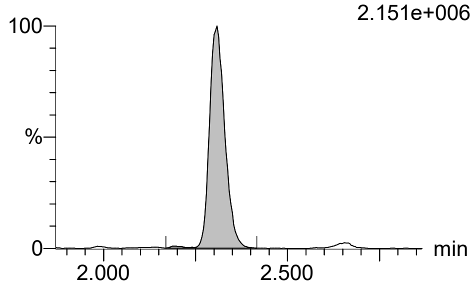

Sample Name: 20211111_A37_R1_IgG
F13:MRM of 1 channel,ES+
873.43 > 204.1
1.059e+005

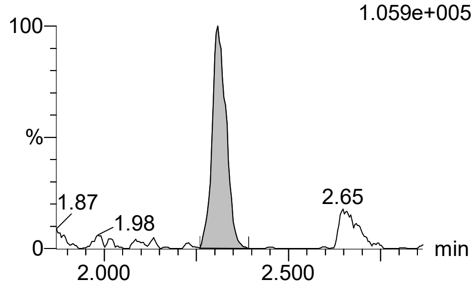

Sample Name: 20211111_A38_R1_IgG
F13:MRM of 1 channel,ES+
873.43 > 204.1
3.916e+005

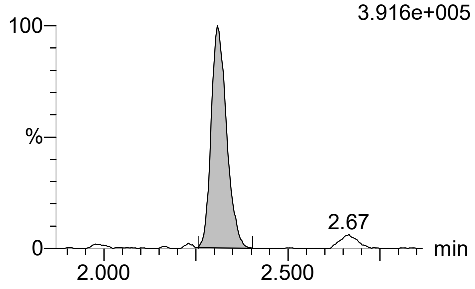

Sample Name: 20211111_H19_R1_IgG
F13:MRM of 1 channel,ES+
873.43 > 204.1
1.076e+006

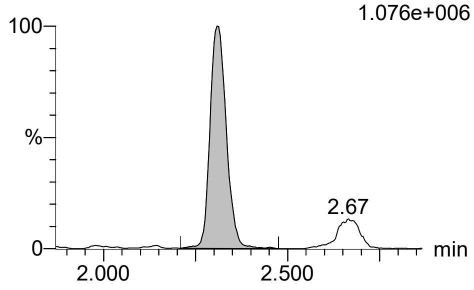

Sample Name: 20211111_A39_R1_IgG
F13:MRM of 1 channel,ES+
873.43 > 204.1
3.290e+005

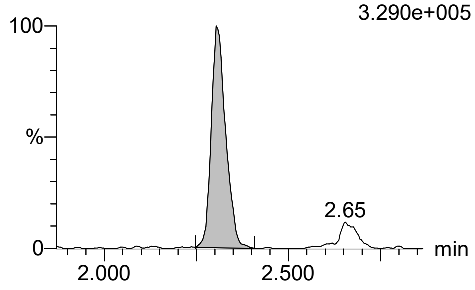

Sample Name: 20211111_A40_R1_IgG
F13:MRM of 1 channel,ES+
873.43 > 204.1
1.288e+006

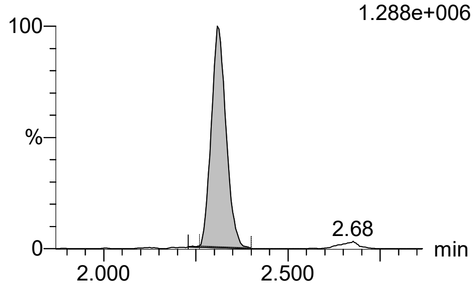

Sample Name: 20211111_H20_R1_IgG
F13:MRM of 1 channel,ES+
873.43 > 204.1
9.854e+005

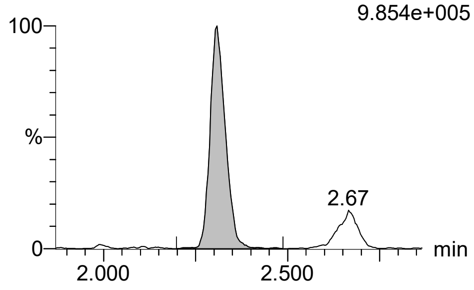

Sample Name: 20211111_A41_R1_IgG
F13:MRM of 1 channel,ES+
873.43 > 204.1
9.431e+004

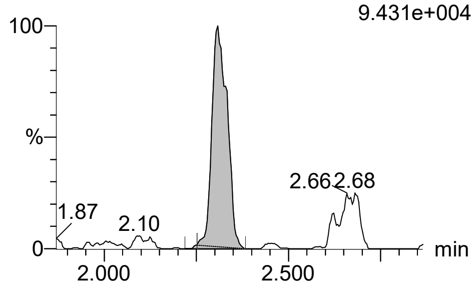

Sample Name: 20211111_A42_R1_IgG
F13:MRM of 1 channel,ES+
873.43 > 204.1
2.798e+005

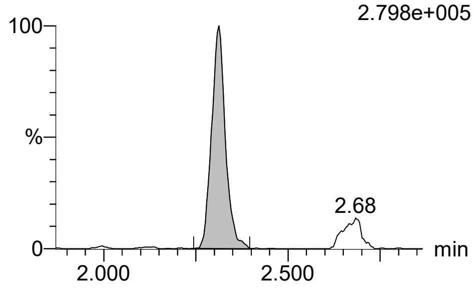

Sample Name: 20211111_A43_R1_IgG
F13:MRM of 1 channel,ES+
873.43 > 204.1
5.771e+005

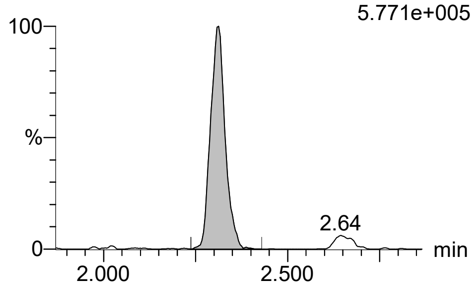

Sample Name: 20211111_A44_R1_IgG
F13:MRM of 1 channel,ES+
873.43 > 204.1
2.454e+005

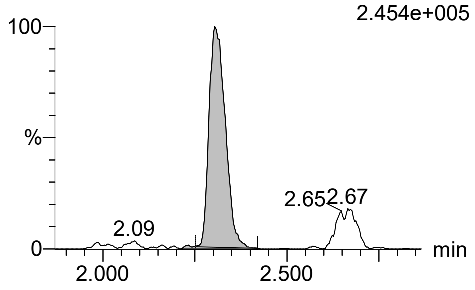

Sample Name: 20211111_QC_R9_IgG
F13:MRM of 1 channel,ES+
873.43 > 204.1
3.524e+005

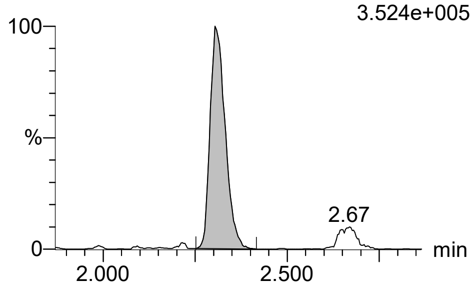

Sample Name: 20211111_A1_R2_IgG
F13:MRM of 1 channel,ES+
873.43 > 204.1
7.175e+005

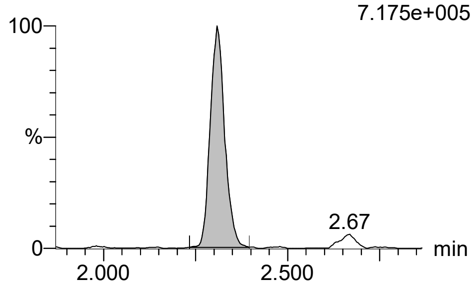

Sample Name: 20211111_A2_R2_IgG
F13:MRM of 1 channel,ES+
873.43 > 204.1
2.118e+005

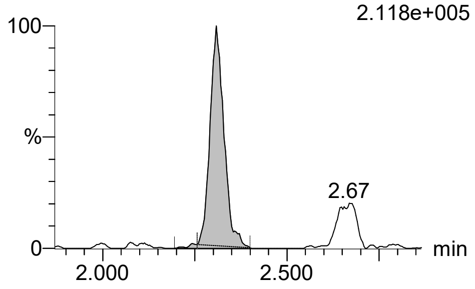

Sample Name: 20211111_L1_R2_IgG
F13:MRM of 1 channel,ES+
873.43 > 204.1
5.436e+005

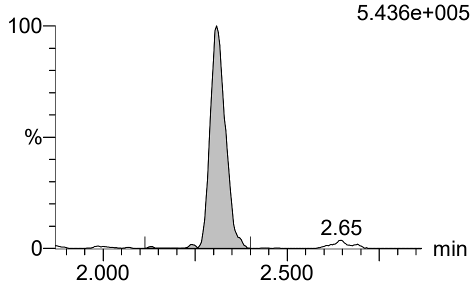

Sample Name: 20211111_L2_R2_IgG
F13:MRM of 1 channel,ES+
873.43 > 204.1
3.242e+005

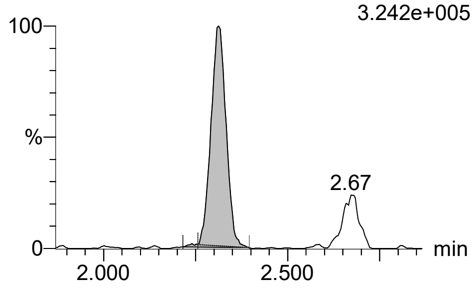

Sample Name: 20211111_H1_R2_IgG
F13:MRM of 1 channel,ES+
873.43 > 204.1
4.284e+005

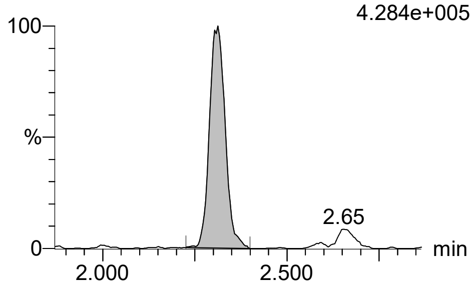

Sample Name: 20211111_A3_R2_IgG
F13:MRM of 1 channel,ES+
873.43 > 204.1
1.021e+005

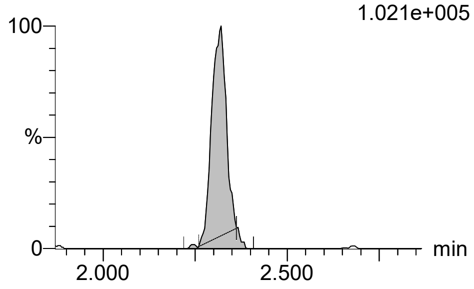

Sample Name: 20211111_A4_R2_IgG
F13:MRM of 1 channel,ES+
873.43 > 204.1
9.265e+004

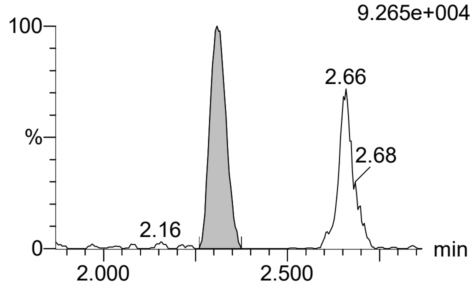

Sample Name: 20211111_L3_R2_IgG
F13:MRM of 1 channel,ES+
873.43 > 204.1
2.086e+005

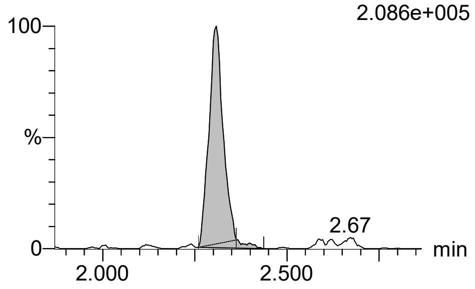

Sample Name: 20211111_L4_R2_IgG
F13:MRM of 1 channel,ES+
873.43 > 204.1
3.461e+005

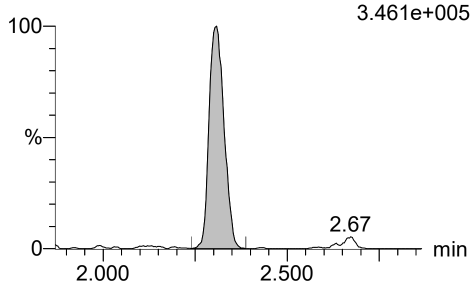

Sample Name: 20211111_H2_R2_IgG
F13:MRM of 1 channel,ES+
873.43 > 204.1
6.733e+005

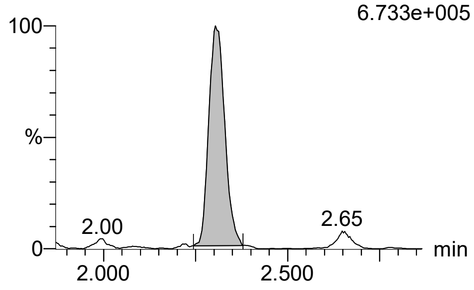

Sample Name: 20211111_A5_R2_IgG
F13:MRM of 1 channel,ES+
873.43 > 204.1
6.769e+005

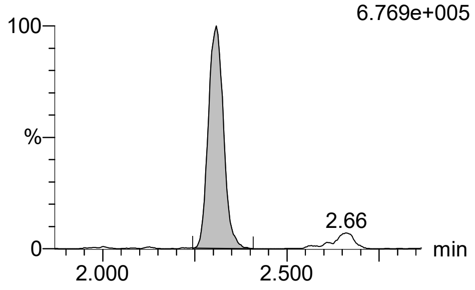

Sample Name: 20211111_A6_R2_IgG
F13:MRM of 1 channel,ES+
873.43 > 204.1
1.043e+006

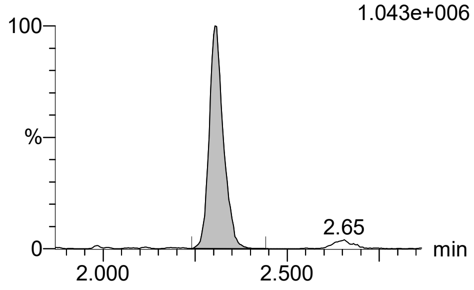

Sample Name: 20211111_QC_R10_IgG
F13:MRM of 1 channel,ES+
873.43 > 204.1
3.404e+005

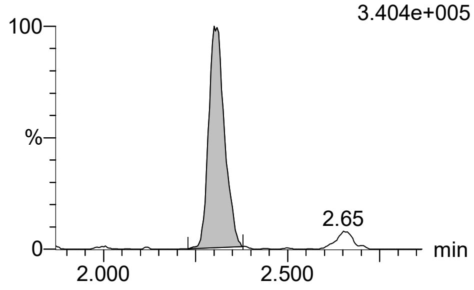

Sample Name: 20211111_L5_R2_IgG
F13:MRM of 1 channel,ES+
873.43 > 204.1
5.486e+005

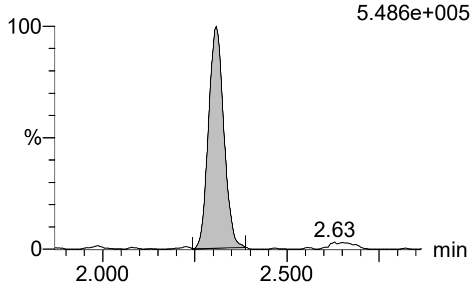

Sample Name: 20211111_L6_R2_IgG
F13:MRM of 1 channel,ES+
873.43 > 204.1
1.446e+005

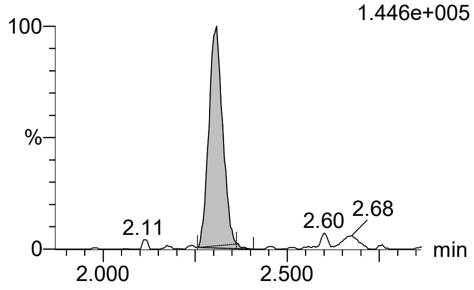

Sample Name: 20211111_H3_R2_IgG
F13:MRM of 1 channel,ES+
873.43 > 204.1
6.258e+004

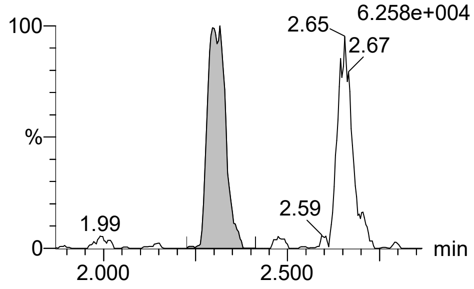

Sample Name: 20211111_A7_R2_IgG
F13:MRM of 1 channel,ES+
873.43 > 204.1
9.159e+005

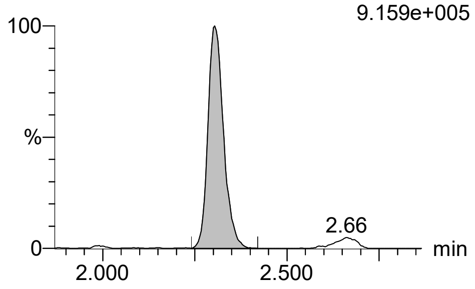

Sample Name: 20211111_A8_R2_IgG
F13:MRM of 1 channel,ES+
873.43 > 204.1
3.805e+005

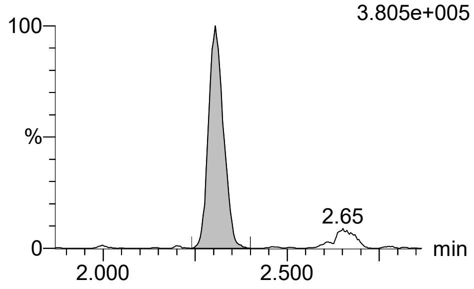

Sample Name: 20211111_L7_R2_IgG
F13:MRM of 1 channel,ES+
873.43 > 204.1
3.685e+005

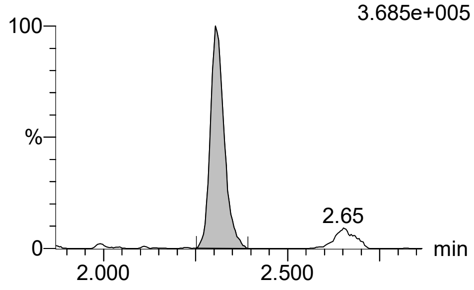

Sample Name: 20211111_L8_R2_IgG
F13:MRM of 1 channel,ES+
873.43 > 204.1
1.940e+005

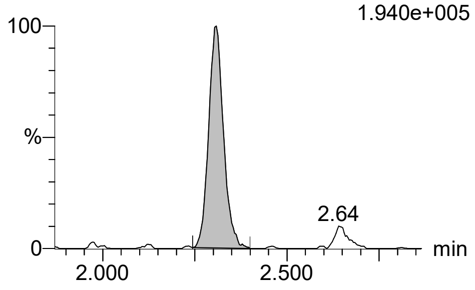

Sample Name: 20211111_H4_R2_IgG
F13:MRM of 1 channel,ES+
873.43 > 204.1
2.152e+005

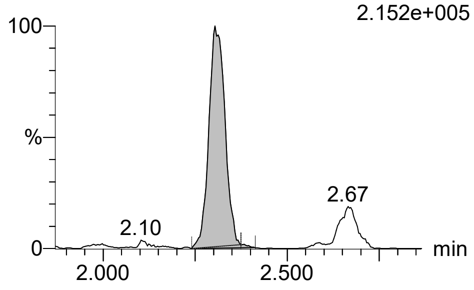

Sample Name: 20211111_A9_R2_IgG
F13:MRM of 1 channel,ES+
873.43 > 204.1
5.299e+005

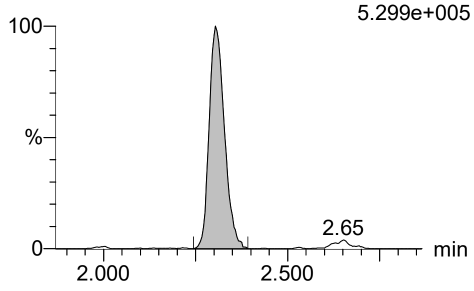

Sample Name: 20211111_A10_R2_IgG
F13:MRM of 1 channel,ES+
873.43 > 204.1
1.046e+006

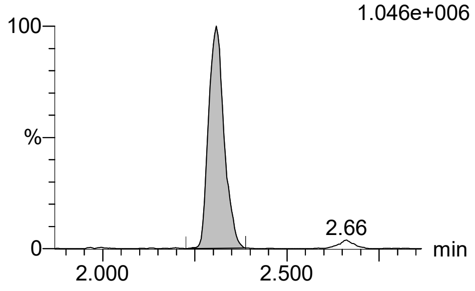

Sample Name: 20211111_L9_R2_IgG
F13:MRM of 1 channel,ES+
873.43 > 204.1
4.719e+005

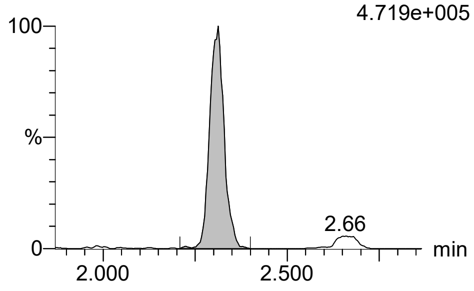

Sample Name: 20211111_L10_R2_IgG
F13:MRM of 1 channel,ES+
873.43 > 204.1
3.830e+005

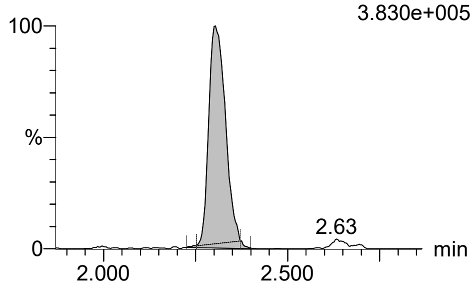

Sample Name: 20211111_QC_R11_IgG
F13:MRM of 1 channel,ES+
873.43 > 204.1
3.666e+005

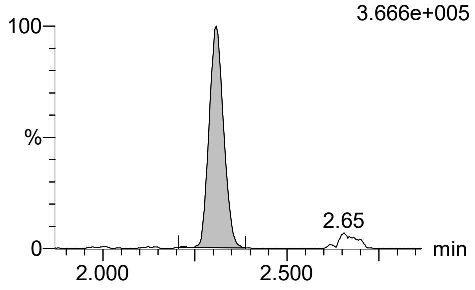

Sample Name: 20211111_H5_R2_IgG
F13:MRM of 1 channel,ES+
873.43 > 204.1
5.763e+004

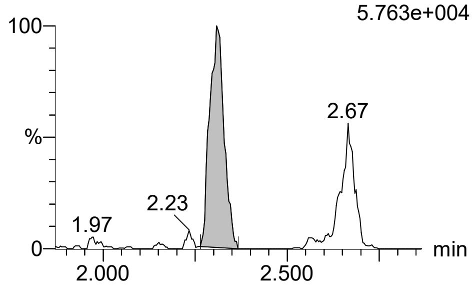

Dataset: C:\MassLynx\10.pro\20211115_clinicalsamples_IgG.qld

Last Altered: Sunday, October 02, 2022 16:02:30 台北標準時間
Printed: Friday, August 01, 2025 16:54:07 台北標準時間

Sample Name: 20211111_A11_R2_IgG
F13:MRM of 1 channel,ES+
873.43 > 204.1
1.598e+005

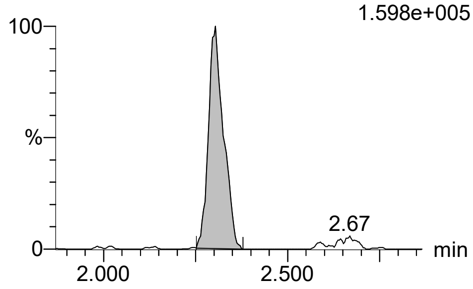

Sample Name: 20211111_A12_R2_IgG
F13:MRM of 1 channel,ES+
873.43 > 204.1
2.600e+005

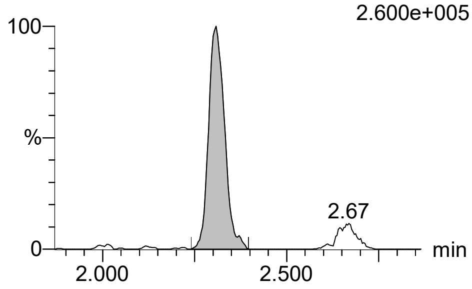

Sample Name: 20211111_L11_R2_IgG
F13:MRM of 1 channel,ES+
873.43 > 204.1
1.923e+005

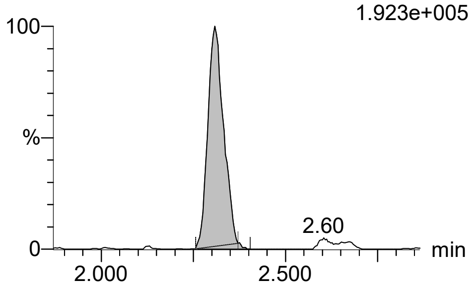

Sample Name: 20211111_L12_R2_IgG
F13:MRM of 1 channel,ES+
873.43 > 204.1
9.064e+004

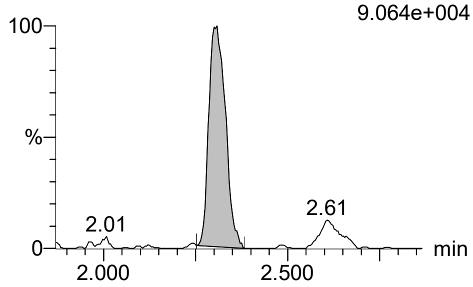

Sample Name: 20211111_H6_R2_IgG
F13:MRM of 1 channel,ES+
873.43 > 204.1
5.237e+005

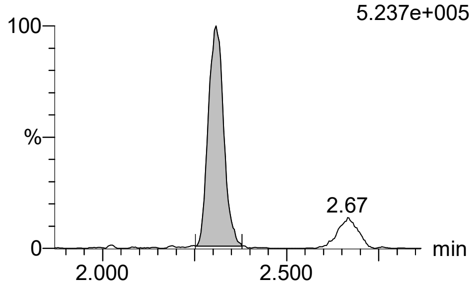

Sample Name: 20211111_A13_R2_IgG
F13:MRM of 1 channel,ES+
873.43 > 204.1
6.287e+005

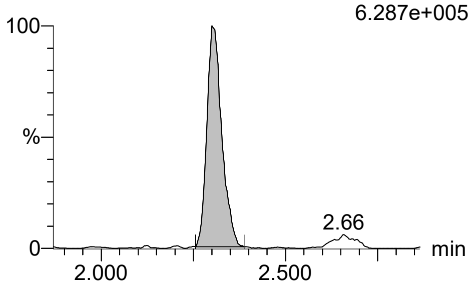

Sample Name: 20211111_A14_R2_IgG
F13:MRM of 1 channel,ES+
873.43 > 204.1
5.830e+005

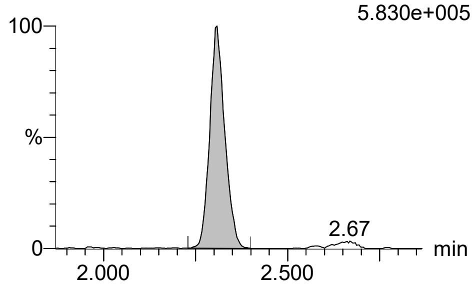

Sample Name: 20211111_L13_R2_IgG
F13:MRM of 1 channel,ES+
873.43 > 204.1
1.010e+005

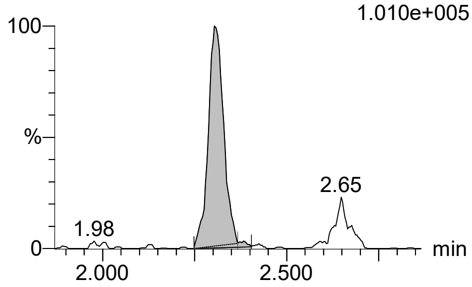

Sample Name: 20211111_L14_R2_IgG
F13:MRM of 1 channel,ES+
873.43 > 204.1
2.169e+005

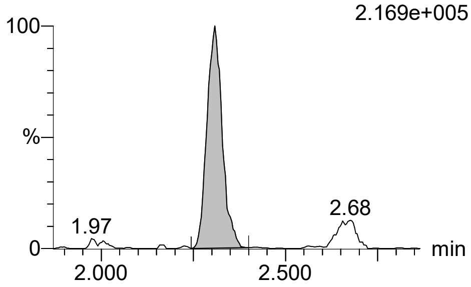

Sample Name: 20211111_H7_R2_IgG
F13:MRM of 1 channel,ES+
873.43 > 204.1
5.043e+005

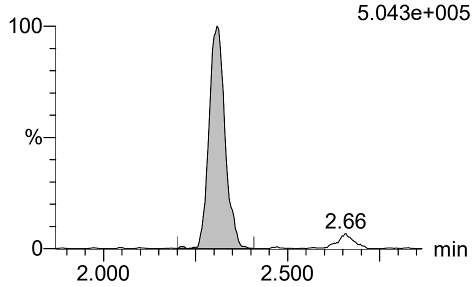

Sample Name: 20211111_A15_R2_IgG
F13:MRM of 1 channel,ES+
873.43 > 204.1
1.427e+005

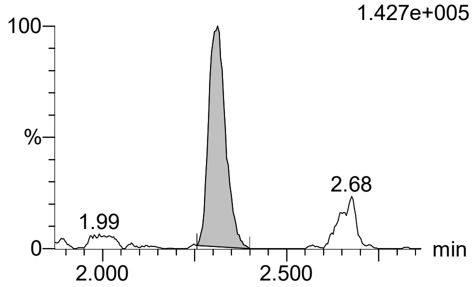

Sample Name: 20211111_QC_R12_IgG
F13:MRM of 1 channel,ES+
873.43 > 204.1
3.620e+005

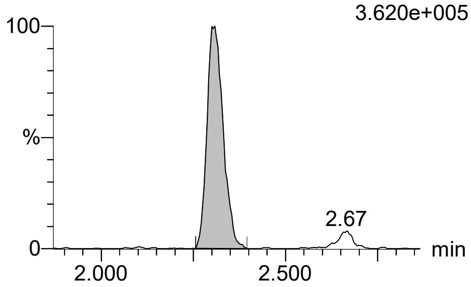

Sample Name: 20211111_A16_R2_IgG
F13:MRM of 1 channel,ES+
873.43 > 204.1
4.958e+005

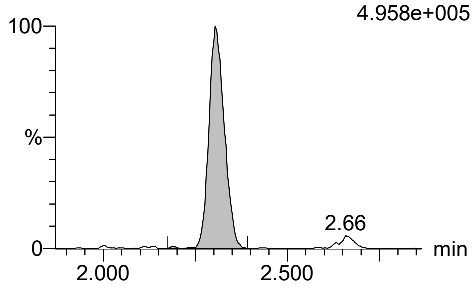

Sample Name: 20211111_L15_R2_IgG
F13:MRM of 1 channel,ES+
873.43 > 204.1
5.421e+005

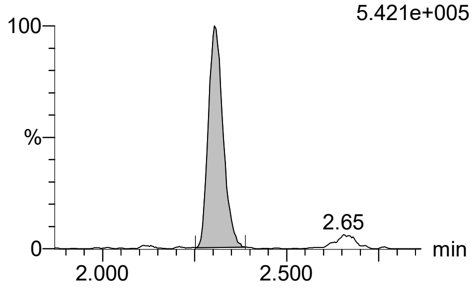

Sample Name: 20211111_L16_R2_IgG
F13:MRM of 1 channel,ES+
873.43 > 204.1
1.793e+005

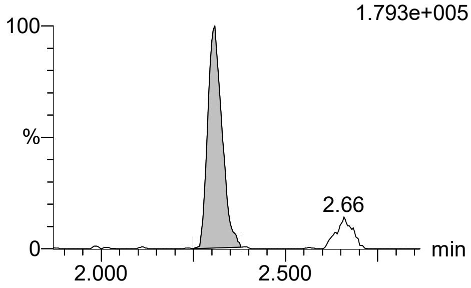

Dataset: C:\MassLynx\10.pro\20211115_clinicalsamples_IgG.qld

Last Altered: Sunday, October 02, 2022 16:02:30 台北標準時間
Printed: Friday, August 01, 2025 16:54:07 台北標準時間

Sample Name: 20211111_H8_R2_IgG
F13:MRM of 1 channel,ES+
873.43 > 204.1
3.462e+005

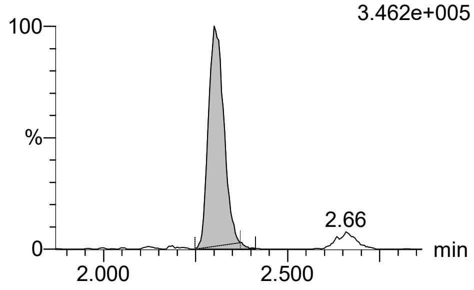

Sample Name: 20211111_A17_R2_IgG
F13:MRM of 1 channel,ES+
873.43 > 204.1
3.848e+005

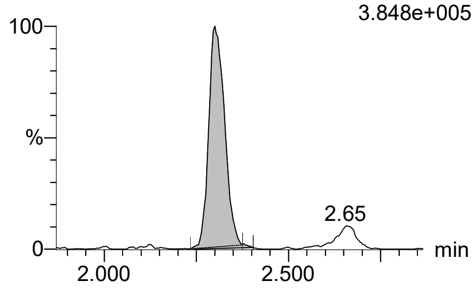

Sample Name: 20211111_A18_R2_IgG
F13:MRM of 1 channel,ES+
873.43 > 204.1
5.695e+005

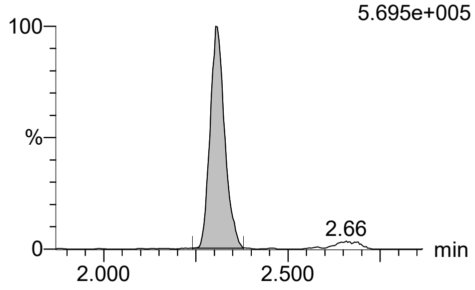

Sample Name: 20211111_L17_R2_IgG
F13:MRM of 1 channel,ES+
873.43 > 204.1
1.513e+005

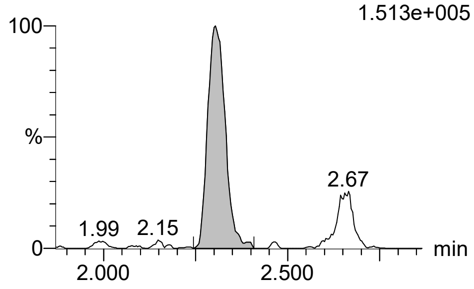

Sample Name: 20211111_L18_R2_IgG
F13:MRM of 1 channel,ES+
873.43 > 204.1
1.656e+005

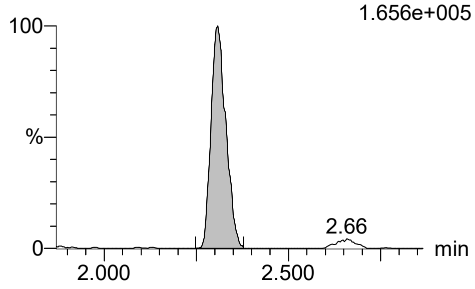

Sample Name: 20211111_H9_R2_IgG
F13:MRM of 1 channel,ES+
873.43 > 204.1
6.148e+005

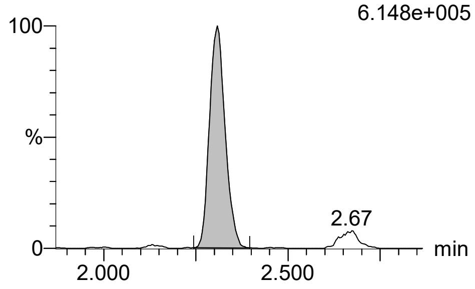

Sample Name: 20211111_A19_R2_IgG
F13:MRM of 1 channel,ES+
873.43 > 204.1
1.229e+005

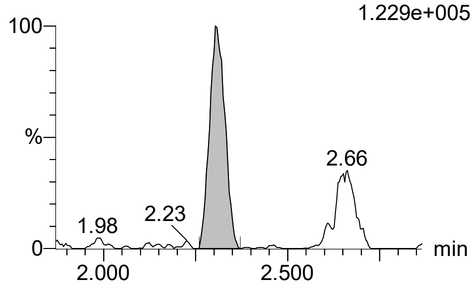

Sample Name: 20211111_A20_R2_IgG
F13:MRM of 1 channel,ES+
873.43 > 204.1
1.188e+005

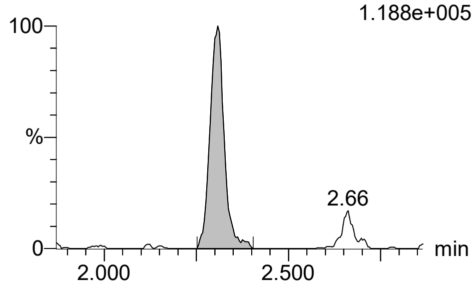

Sample Name: 20211111_L19_R2_IgG
F13:MRM of 1 channel,ES+
873.43 > 204.1
2.041e+005

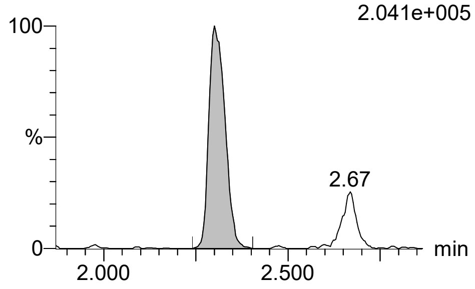

Sample Name: 20211111_QC_R13_IgG
F13:MRM of 1 channel,ES+
873.43 > 204.1
3.736e+005

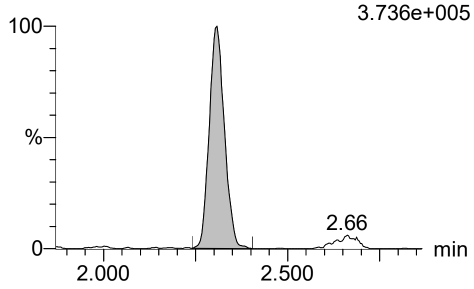

Sample Name: 20211111_L20_R2_IgG
F13:MRM of 1 channel,ES+
873.43 > 204.1
3.784e+005

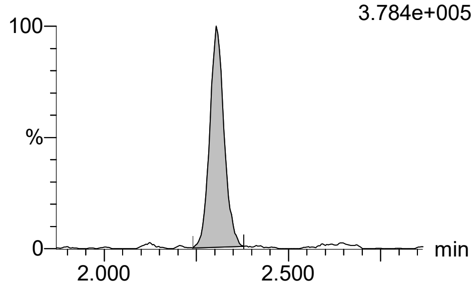

Sample Name: 20211111_H10_R2_IgG
F13:MRM of 1 channel,ES+
873.43 > 204.1
3.752e+005

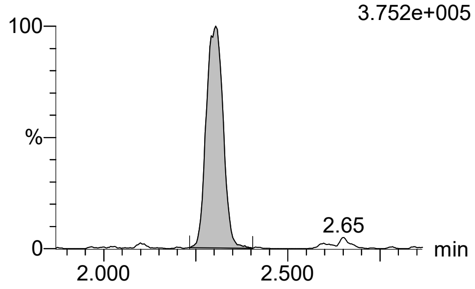

Sample Name: 20211111_A21_R2_IgG
F13:MRM of 1 channel,ES+
873.43 > 204.1
6.783e+005

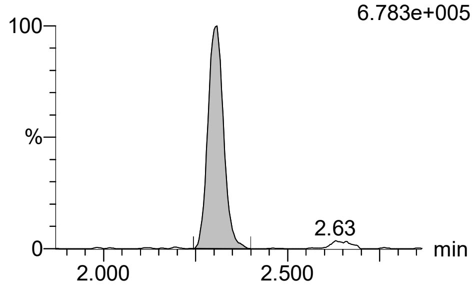

Sample Name: 20211111_A22_R2_IgG
F13:MRM of 1 channel,ES+
873.43 > 204.1
1.351e+005

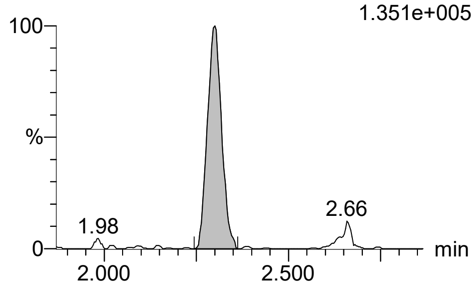

Sample Name: 20211111_L21_R2_IgG
F13:MRM of 1 channel,ES+
873.43 > 204.1
1.701e+005

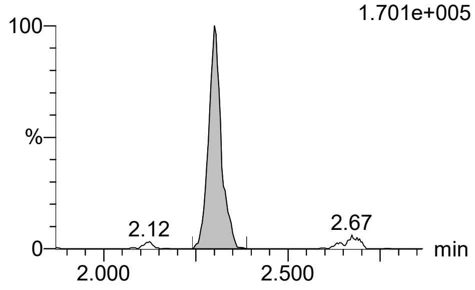

Dataset: C:\MassLynx\10.pro\20211115_clinicalsamples_IgG.qld

Last Altered: Sunday, October 02, 2022 16:02:30 台北標準時間
Printed: Friday, August 01, 2025 16:54:07 台北標準時間

Sample Name: 20211111_L22_R2_IgG
F13:MRM of 1 channel,ES+
873.43 > 204.1
2.873e+005

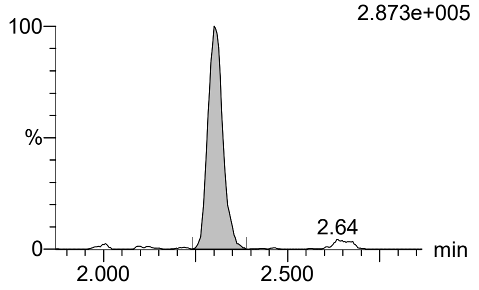

Sample Name: 20211111_H11_R2_IgG
F13:MRM of 1 channel,ES+
873.43 > 204.1
6.824e+005

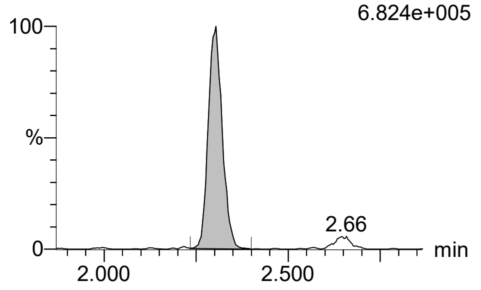

Sample Name: 20211111_A23_R2_IgG
F13:MRM of 1 channel,ES+
873.43 > 204.1
4.475e+005

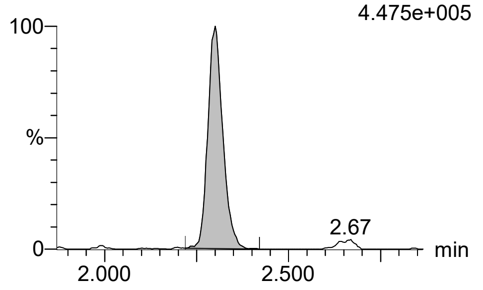

Sample Name: 20211111_A24_R2_IgG
F13:MRM of 1 channel,ES+
873.43 > 204.1
8.048e+004

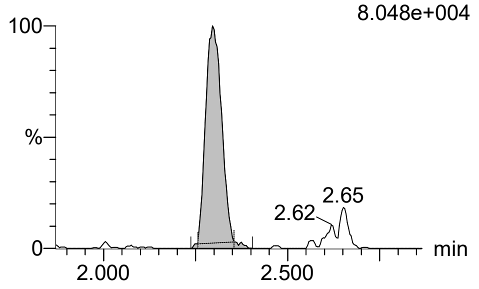

Sample Name: 20211111_L23_R2_IgG
F13:MRM of 1 channel,ES+
873.43 > 204.1
4.922e+005

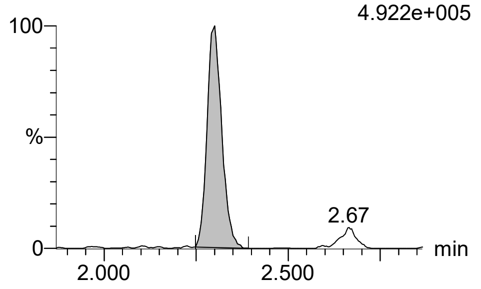

Sample Name: 20211111_L24_R2_IgG
F13:MRM of 1 channel,ES+
873.43 > 204.1
1.693e+005

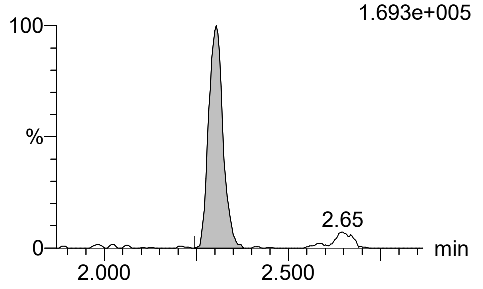

Sample Name: 20211111_H12_R2_IgG
F13:MRM of 1 channel,ES+
873.43 > 204.1
6.044e+004

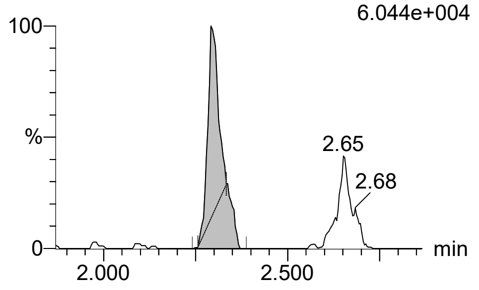

Sample Name: 20211111_QC_R14_IgG
F13:MRM of 1 channel,ES+
873.43 > 204.1
2.940e+005

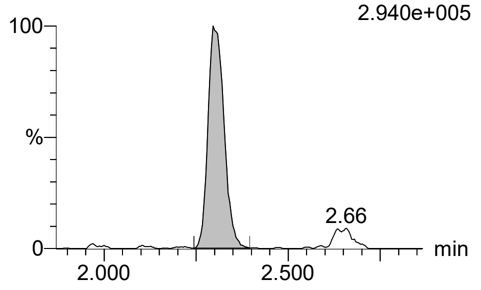

Sample Name: 20211111_A25_R2_IgG
F13:MRM of 1 channel,ES+
873.43 > 204.1
1.156e+006

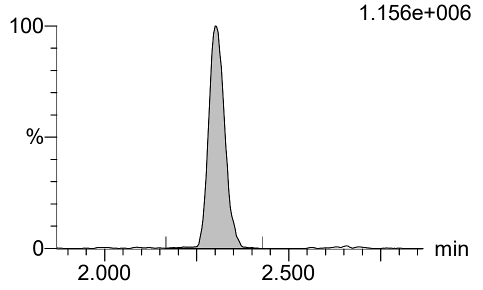

Sample Name: 20211111_A26_R2_IgG
F13:MRM of 1 channel,ES+
873.43 > 204.1
2.619e+004

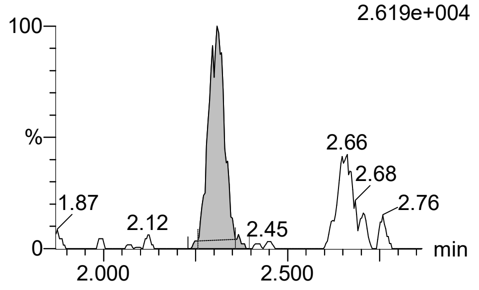

Sample Name: 20211111_L25_R2_IgG
F13:MRM of 1 channel,ES+
873.43 > 204.1
3.946e+005

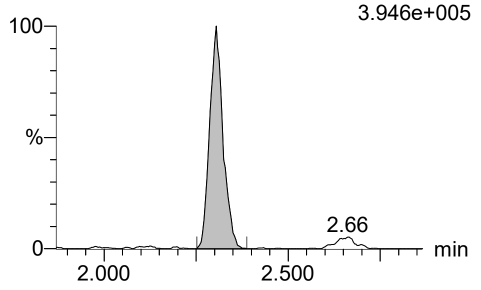

Sample Name: 20211111_L26_R2_IgG
F13:MRM of 1 channel,ES+
873.43 > 204.1
1.881e+005

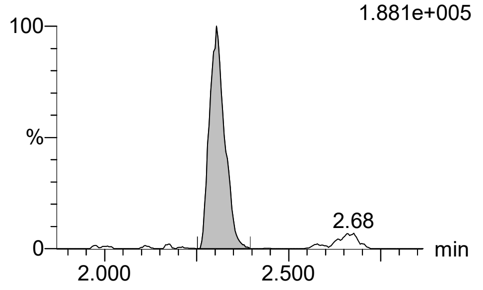

Sample Name: 20211111_H13_R2_IgG
F13:MRM of 1 channel,ES+
873.43 > 204.1
2.018e+005

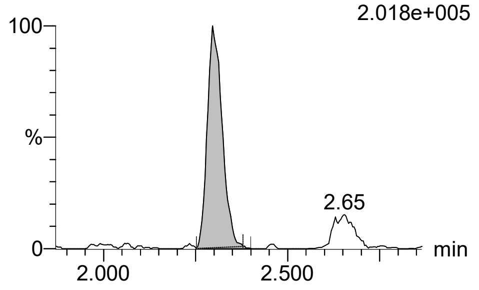

Sample Name: 20211111_A27_R2_IgG
F13:MRM of 1 channel,ES+
873.43 > 204.1
2.736e+005

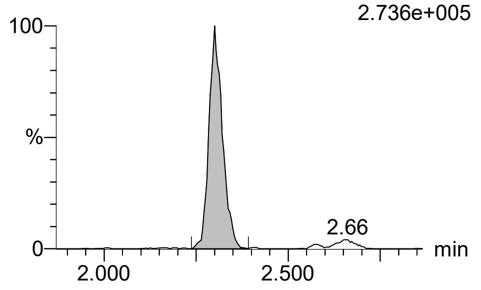

Sample Name: 20211111_A28_R2_IgG
F13:MRM of 1 channel,ES+
873.43 > 204.1
2.543e+005

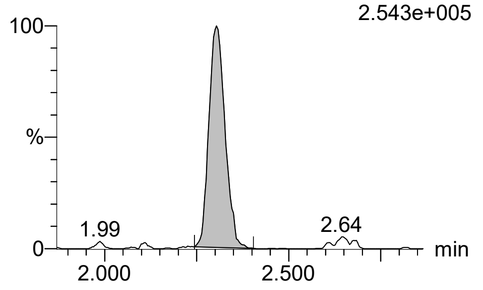

Sample Name: 20211111_L27_R2_IgG
F13:MRM of 1 channel,ES+
873.43 > 204.1
1.373e+005

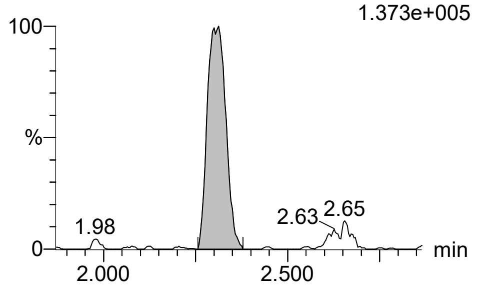

Sample Name: 20211111_L28_R2_IgG
F13:MRM of 1 channel,ES+
873.43 > 204.1
8.429e+004

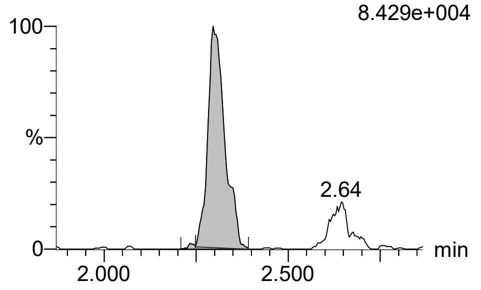

Sample Name: 20211111_H14_R2_IgG
F13:MRM of 1 channel,ES+
873.43 > 204.1
1.415e+005

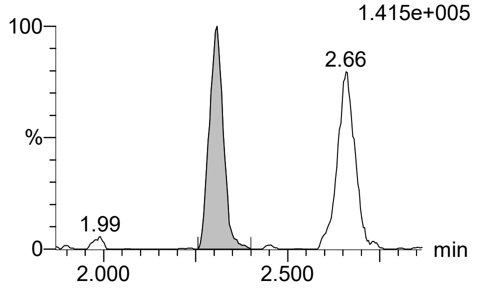

Sample Name: 20211111_A29_R2_IgG
F13:MRM of 1 channel,ES+
873.43 > 204.1
6.415e+005

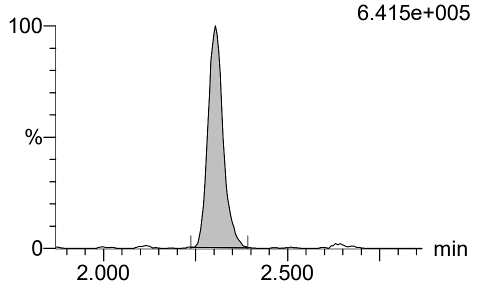

Sample Name: 20211111_A30_R2_IgG
F13:MRM of 1 channel,ES+
873.43 > 204.1
8.695e+005

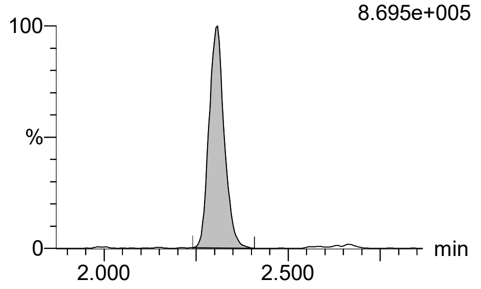

Sample Name: 20211111_QC_R15_IgG
F13:MRM of 1 channel,ES+
873.43 > 204.1
3.101e+005

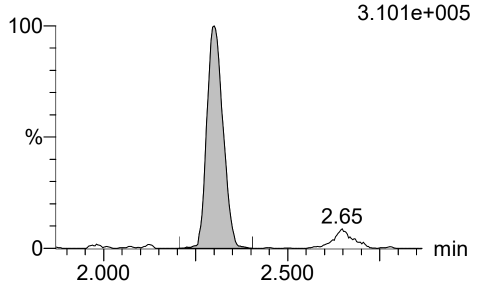

Sample Name: 20211111_L29_R2_IgG
F13:MRM of 1 channel,ES+
873.43 > 204.1
1.801e+005

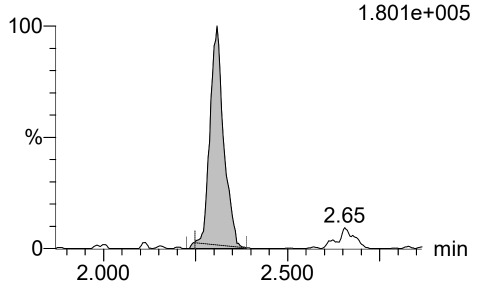

Sample Name: 20211111_L30_R2_IgG
F13:MRM of 1 channel,ES+
873.43 > 204.1
1.399e+005

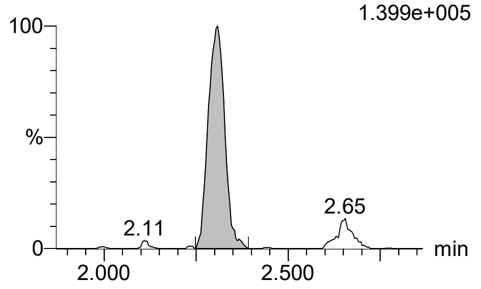

Sample Name: 20211111_H15_R2_IgG
F13:MRM of 1 channel,ES+
873.43 > 204.1
3.065e+004

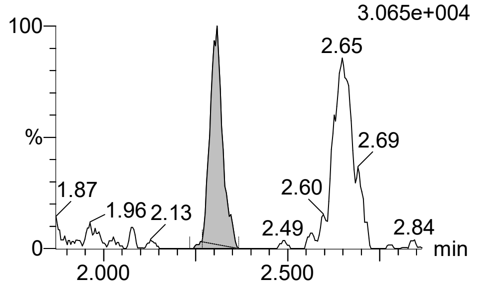

Sample Name: 20211111_A31_R2_IgG
F13:MRM of 1 channel,ES+
873.43 > 204.1
2.470e+005

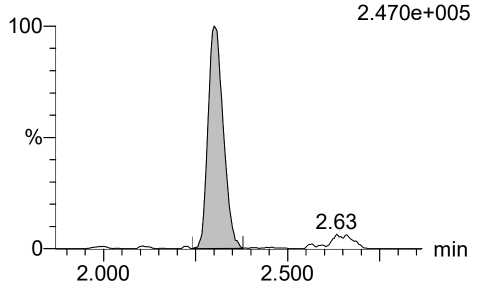

Sample Name: 20211111_A32_R2_IgG
F13:MRM of 1 channel,ES+
873.43 > 204.1
1.322e+006

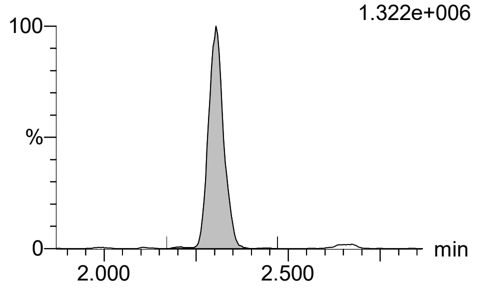

Sample Name: 20211111_L31_R2_IgG
F13:MRM of 1 channel,ES+
873.43 > 204.1
1.533e+005

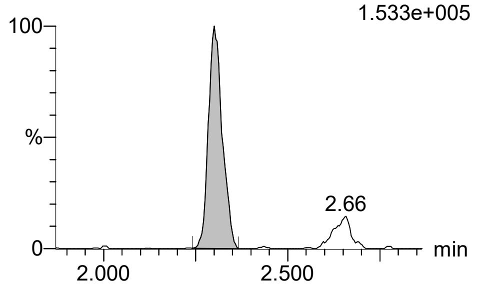

Sample Name: 20211111_L32_R2_IgG
F13:MRM of 1 channel,ES+
873.43 > 204.1
1.452e+005

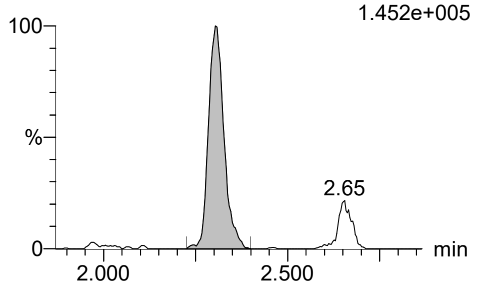

Sample Name: 20211111_H16_R2_IgG
F13:MRM of 1 channel,ES+
873.43 > 204.1
9.171e+005

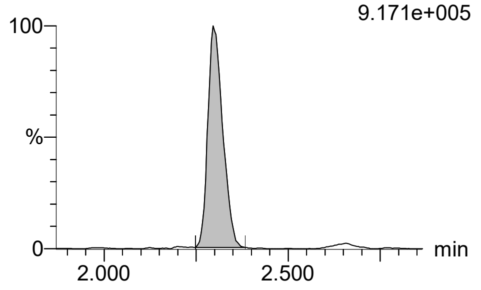

Sample Name: 20211111_A33_R2_IgG
F13:MRM of 1 channel,ES+
873.43 > 204.1
2.508e+005

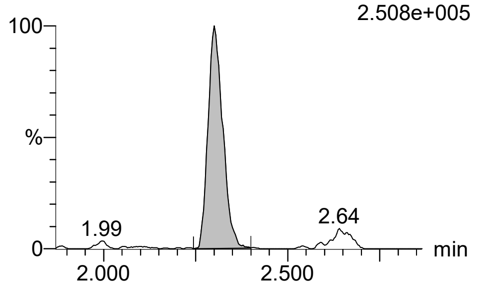

Sample Name: 20211111_A34_R2_IgG
F13:MRM of 1 channel,ES+
873.43 > 204.1
4.135e+005

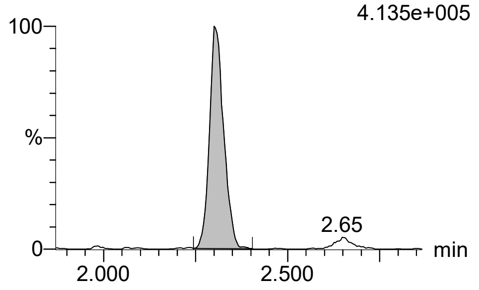

Sample Name: 20211111_H17_R2_IgG
F13:MRM of 1 channel,ES+
873.43 > 204.1
9.942e+005

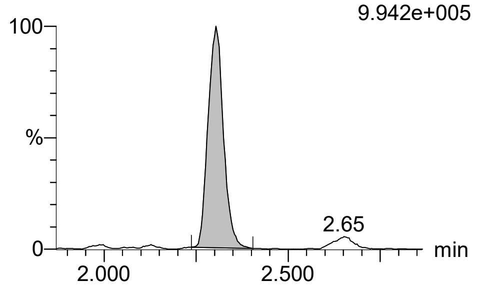

Sample Name: 20211111_A35_R2_IgG
F13:MRM of 1 channel,ES+
873.43 > 204.1
4.421e+005

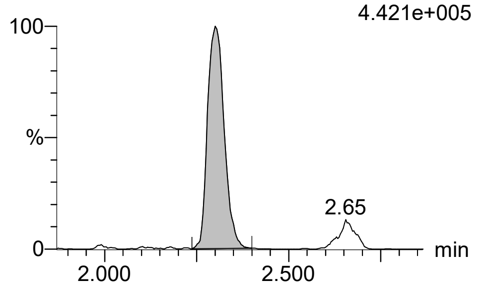

Sample Name: 20211111_QC_R16_IgG
F13:MRM of 1 channel,ES+
873.43 > 204.1
3.146e+005

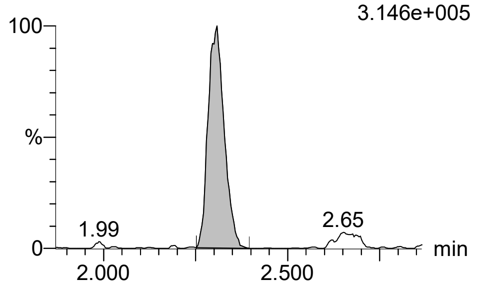

Sample Name: 20211111_A36_R2_IgG
F13:MRM of 1 channel,ES+
873.43 > 204.1
8.069e+005

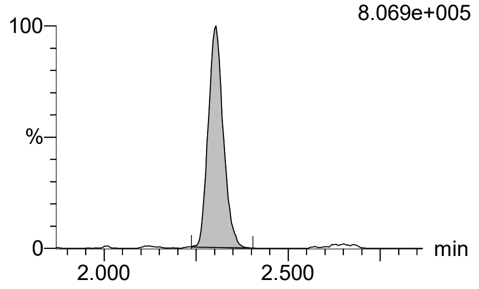

Sample Name: 20211111_H18_R2_IgG
F13:MRM of 1 channel,ES+
873.43 > 204.1
1.851e+006

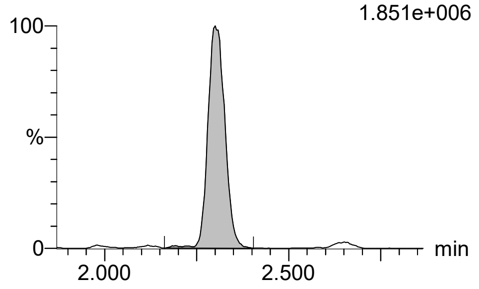

Sample Name: 20211111_A37_R2_IgG
F13:MRM of 1 channel,ES+
873.43 > 204.1
6.587e+004

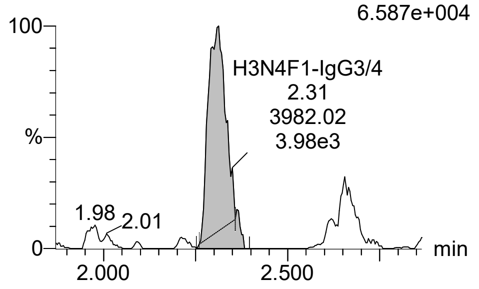

Sample Name: 20211111_A38_R2_IgG
F13:MRM of 1 channel,ES+
873.43 > 204.1
3.936e+005

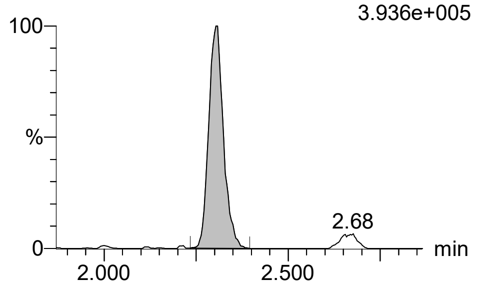

Sample Name: 20211111_H19_R2_IgG
F13:MRM of 1 channel,ES+
873.43 > 204.1
1.155e+006

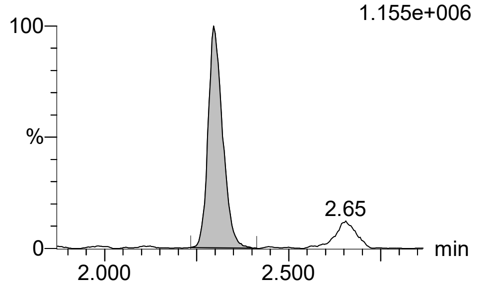

Sample Name: 20211111_A39_R2_IgG
F13:MRM of 1 channel,ES+
873.43 > 204.1
2.806e+005

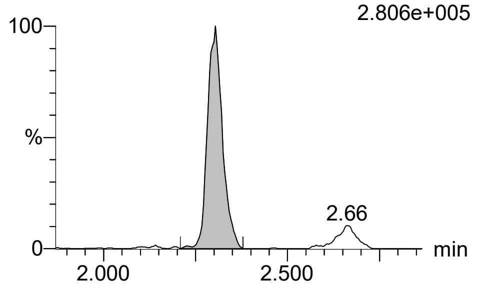

Sample Name: 20211111_A40_R2_IgG
F13:MRM of 1 channel,ES+
873.43 > 204.1
1.196e+006

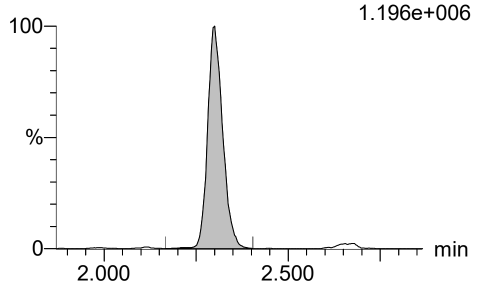

Sample Name: 20211111_H20_R2_IgG
F13:MRM of 1 channel,ES+
873.43 > 204.1
9.492e+005

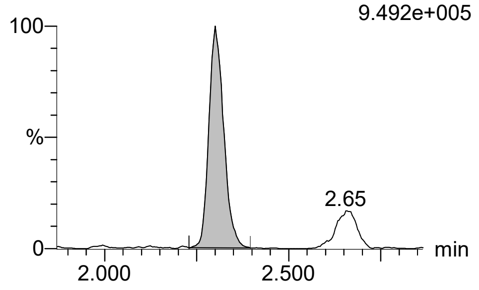

Sample Name: 20211111_A41_R2_IgG
F13:MRM of 1 channel,ES+
873.43 > 204.1
7.129e+004

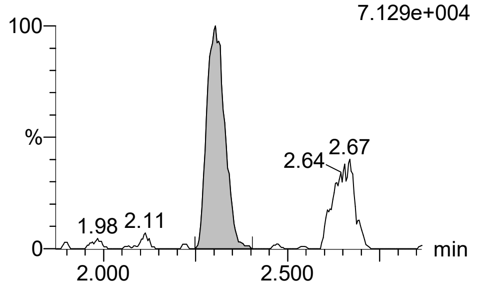

Sample Name: 20211111_A42_R2_IgG
F13:MRM of 1 channel,ES+
873.43 > 204.1
2.196e+005

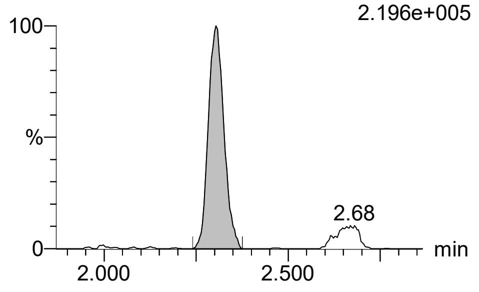

Sample Name: 20211111_A43_R2_IgG
F13:MRM of 1 channel,ES+
873.43 > 204.1
4.233e+005

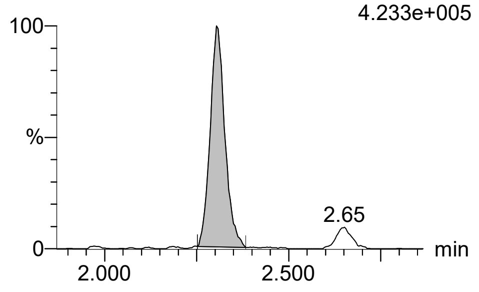

Sample Name: 20211111_A44_R2_IgG

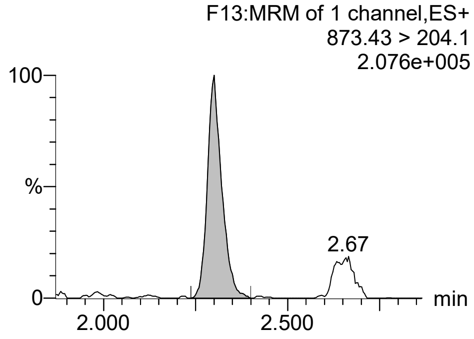

Sample Name: 20211111_QC_R17_IgG

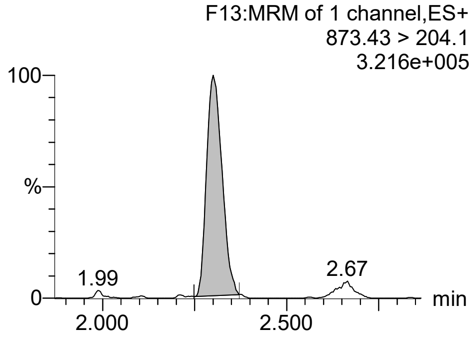

	#	Name	RT	Area	IS Area	Response	Prima...
1	1	20211111_QC_R1_IgG	2.31	36992.9	64968.0	0.569	bb
2	2	20211111_A1_R1_IgG	2.30	48573.7	59059.2	0.822	MM
3	3	20211111_A2_R1_IgG	2.30	11168.0	66621.2	0.168	bb
4	4	20211111_L1_R1_IgG	2.31	38768.8	64201.0	0.604	bb
5	5	20211111_L2_R1_IgG	2.31	17388.4	46950.2	0.370	bb
6	6	20211111_H1_R1_IgG	2.31	18930.4	38648.2	0.490	bb
7	7	20211111_A3_R1_IgG	2.31	2482.7	2603.7	0.954	bb
8	8	20211111_A4_R1_IgG	2.31	7766.9	69960.4	0.111	bb
9	9	20211111_L3_R1_IgG	2.31	10341.2	38864.7	0.266	bb
10	10	20211111_L4_R1_IgG	2.31	19375.2	43380.5	0.447	MM
11	11	20211111_H2_R1_IgG	2.31	51281.0	71349.2	0.719	bb
12	12	20211111_A5_R1_IgG	2.31	35087.0	65569.7	0.535	bb
13	13	20211111_A6_R1_IgG	2.30	49795.0	53120.7	0.937	bb
14	14	20211111_QC_R2_IgG	2.30	39317.9	81034.1	0.485	bb
15	15	20211111_L5_R1_IgG	2.31	36897.3	50994.6	0.724	bb
16	16	20211111_L6_R1_IgG	2.30	8129.0	58058.0	0.140	MM
17	17	20211111_H3_R1_IgG	2.31	4983.8	64028.6	0.078	MM
18	18	20211111_A7_R1_IgG	2.31	64830.4	82996.1	0.781	bb
19	19	20211111_A8_R1_IgG	2.31	24698.0	77731.7	0.318	bb
20	20	20211111_L7_R1_IgG	2.30	17390.8	38827.3	0.448	bb
21	21	20211111_L8_R1_IgG	2.31	10846.5	44220.1	0.245	bb
22	22	20211111_H4_R1_IgG	2.30	14904.2	65826.3	0.226	bb
23	23	20211111_A9_R1_IgG	2.31	34666.8	60771.1	0.570	bb
24	24	20211111_A10_R1_IgG	2.31	56235.6	48578.7	1.158	bb
25	25	20211111_L9_R1_IgG	2.31	23396.5	50830.7	0.460	bb
26	26	20211111_L10_R1_IgG	2.31	23149.7	38034.2	0.609	bb
27	27	20211111_QC_R3_IgG	2.31	33800.7	67025.5	0.504	bb
28	28	20211111_H5_R1_IgG	2.30	2823.9	44544.9	0.063	MM
29	29	20211111_A11_R1_IgG	2.31	10572.4	66408.5	0.159	bb
30	30	20211111_A12_R1_IgG	2.31	16496.8	46624.5	0.354	bb
31	31	20211111_L11_R1_IgG	2.31	13474.1	33425.1	0.403	MM
32	32	20211111_L12_R1_IgG	2.31	5182.8	41178.0	0.126	bb
33	33	20211111_H6_R1_IgG	2.31	37581.1	58593.4	0.641	bb
34	34	20211111_A13_R1_IgG	2.31	38999.7	54133.0	0.720	MM
35	35	20211111_A14_R1_IgG	2.31	32495.5	56467.3	0.575	bb
36	36	20211111_L13_R1_IgG	2.31	7703.5	36024.4	0.214	bb
37	37	20211111_L14_R1_IgG	2.31	10276.4	37181.6	0.276	MM
38	38	20211111_H7_R1_IgG	2.31	28875.9	42850.6	0.674	bb
39	39	20211111_A15_R1_IgG	2.31	8898.5	62015.1	0.143	MM
40	40	20211111_QC_R4_IgG	2.31	28197.9	58572.5	0.481	bb
41	41	20211111_A16_R1_IgG	2.30	39002.1	78283.8	0.498	MM
42	42	20211111_L15_R1_IgG	2.31	33618.4	44158.2	0.761	bb
43	43	20211111_L16_R1_IgG	2.30	11236.1	55150.8	0.204	bb
44	44	20211111_H8_R1_IgG	2.31	16301.1	42965.2	0.379	MM
45	45	20211111_A17_R1_IgG	2.30	19603.1	39717.7	0.494	MM
46	46	20211111_A18_R1_IgG	2.31	31839.0	44675.4	0.713	MM
47	47	20211111_L17_R1_IgG	2.31	9787.4	37854.1	0.259	bb
48	48	20211111_L18_R1_IgG	2.31	9715.1	50716.9	0.192	MM
49	49	20211111_H9_R1_IgG	2.31	32057.2	36276.0	0.884	bb
50	50	20211111_A19_R1_IgG	2.31	7045.7	42862.5	0.164	MM
51	51	20211111_A20_R1_IgG	2.30	6504.7	36542.7	0.178	bb
52	52	20211111_L19_R1_IgG	2.31	14505.0	53892.8	0.269	MM
53	53	20211111_QC_R5_IgG	2.31	25613.2	56513.2	0.453	MM
54	54	20211111_L20_R1_IgG	2.31	26990.5	56499.9	0.478	bb
55	55	20211111_H10_R1_IgG	2.30	46685.6	68120.5	0.685	bb
56	56	20211111_A21_R1_IgG	2.30	55869.8	63699.8	0.877	MM

Dataset: C:\MassLynx\10.pro\20211115_clinicalsamples_IgG.qld

Last Altered: Sunday, October 02, 2022 16:02:30 台北標準時間
Printed: Friday, August 01, 2025 16:54:07 台北標準時間

	# Name	RT	Area	IS Area	Response	Prima...
57	57 20211111_A22_R1_IgG	2.31	8926.0	52222.1	0.171	bb
58	58 20211111_L21_R1_IgG	2.30	9870.7	54372.5	0.182	bb
59	59 20211111_L22_R1_IgG	2.31	18456.6	41353.1	0.446	MM
60	60 20211111_H11_R1_IgG	2.31	48291.2	65579.1	0.736	bb
61	61 20211111_A23_R1_IgG	2.31	36478.7	64131.1	0.569	bb
62	62 20211111_A24_R1_IgG	2.30	5621.6	38724.8	0.145	MM
63	63 20211111_L23_R1_IgG	2.31	34129.8	64245.2	0.531	bb
64	64 20211111_L24_R1_IgG	2.30	8698.7	45298.0	0.192	bb
65	65 20211111_H12_R1_IgG	2.31	3478.9	44679.3	0.078	bb
66	66 20211111_QC_R6_IgG	2.31	23272.9	48165.0	0.483	bb
67	67 20211111_A25_R1_IgG	2.30	97505.2	57169.9	1.706	bb
68	68 20211111_A26_R1_IgG	2.31	1472.4	34254.0	0.043	bb
69	69 20211111_L25_R1_IgG	2.31	27060.5	67491.5	0.401	bb
70	70 20211111_L26_R1_IgG	2.30	13159.1	55934.1	0.235	bb
71	71 20211111_H13_R1_IgG	2.30	13780.3	48767.3	0.283	bb
72	72 20211111_A27_R1_IgG	2.31	15097.3	57269.7	0.264	bb
73	73 20211111_A28_R1_IgG	2.30	21971.4	49231.1	0.446	MM
74	74 20211111_L27_R1_IgG	2.31	13098.6	60014.5	0.218	bb
75	75 20211111_L28_R1_IgG	2.31	7518.7	35062.6	0.214	MM
76	76 20211111_H14_R1_IgG	2.31	9520.3	62371.9	0.153	bb
77	77 20211111_A29_R1_IgG	2.31	38758.4	43858.2	0.884	bb
78	78 20211111_A30_R1_IgG	2.31	62377.6	62500.4	0.998	bb
79	79 20211111_QC_R7_IgG	2.31	21971.7	43946.9	0.500	MM
80	80 20211111_L29_R1_IgG	2.31	9422.6	38728.9	0.243	bb
81	81 20211111_L30_R1_IgG	2.30	8301.2	42613.2	0.195	MM
82	82 20211111_H15_R1_IgG	2.32	1112.9	39874.6	0.028	MM
83	83 20211111_A31_R1_IgG	2.31	18156.8	53414.3	0.340	bb
84	84 20211111_A32_R1_IgG	2.31	66365.9	41844.6	1.586	bb
85	85 20211111_L31_R1_IgG	2.30	7761.2	40553.7	0.191	bb
86	86 20211111_L32_R1_IgG	2.31	8539.1	49618.7	0.172	bb
87	87 20211111_H16_R1_IgG	2.31	57254.2	60170.9	0.952	bb
88	88 20211111_A33_R1_IgG	2.30	13839.0	48588.2	0.285	MM
89	89 20211111_A34_R1_IgG	2.31	21898.6	43288.0	0.506	bb
90	90 20211111_H17_R1_IgG	2.31	54661.6	47104.5	1.160	bb
91	91 20211111_A35_R1_IgG	2.31	29584.0	41025.9	0.721	bb
92	92 20211111_QC_R8_IgG	2.31	19396.7	40546.3	0.478	bb
93	93 20211111_A36_R1_IgG	2.31	46278.7	44504.4	1.040	bb
94	94 20211111_H18_R1_IgG	2.31	103786.4	34162.2	3.038	bb
95	95 20211111_A37_R1_IgG	2.31	4640.1	39760.0	0.117	bb
96	96 20211111_A38_R1_IgG	2.31	18874.1	43982.6	0.429	bb
97	97 20211111_H19_R1_IgG	2.31	55089.6	45314.8	1.216	bb
98	98 20211111_A39_R1_IgG	2.30	14794.7	39388.0	0.376	bb
99	99 20211111_A40_R1_IgG	2.31	59737.4	38726.4	1.543	MM
100	1... 20211111_H20_R1_IgG	2.31	47474.8	37814.4	1.255	bb
101	1... 20211111_A41_R1_IgG	2.31	4564.6	48930.3	0.093	MM
102	1... 20211111_A42_R1_IgG	2.31	12021.8	40406.8	0.298	bb
103	1... 20211111_A43_R1_IgG	2.31	26528.3	44421.5	0.597	bb
104	1... 20211111_A44_R1_IgG	2.30	12754.4	53128.2	0.240	MM
105	1... 20211111_QC_R9_IgG	2.30	16655.4	37049.0	0.450	bb
106	1... 20211111_A1_R2_IgG	2.31	32221.1	46499.4	0.693	bb
107	1... 20211111_A2_R2_IgG	2.31	9479.6	57260.7	0.166	MM
108	1... 20211111_L1_R2_IgG	2.31	26353.7	48062.9	0.548	bb
109	1... 20211111_L2_R2_IgG	2.31	15363.3	38745.2	0.397	MM
110	1... 20211111_H1_R2_IgG	2.31	21130.4	44273.5	0.477	bb
111	1... 20211111_A3_R2_IgG	2.32	5049.2	4755.5	1.062	MM
112	1... 20211111_A4_R2_IgG	2.31	4774.1	50546.7	0.094	bb

Dataset: C:\MassLynx\10.pro\20211115_clinicalsamples_IgG.qld

Last Altered: Sunday, October 02, 2022 16:02:30 台北標準時間
Printed: Friday, August 01, 2025 16:54:07 台北標準時間

	# Name	RT	Area	IS Area	Response	Prima...
113	1... 20211111_L3_R2_IgG	2.31	9629.9	34825.8	0.277	MM
114	1... 20211111_L4_R2_IgG	2.31	16316.5	39107.7	0.417	bb
115	1... 20211111_H2_R2_IgG	2.30	33269.1	49750.0	0.669	bb
116	1... 20211111_A5_R2_IgG	2.31	32091.8	52536.2	0.611	bb
117	1... 20211111_A6_R2_IgG	2.30	46153.9	42050.0	1.098	bb
118	1... 20211111_QC_R10_IgG	2.30	16792.0	35350.8	0.475	bb
119	1... 20211111_L5_R2_IgG	2.31	26134.9	38039.3	0.687	bb
120	1... 20211111_L6_R2_IgG	2.31	6303.7	44907.4	0.140	MM
121	1... 20211111_H3_R2_IgG	2.32	3610.3	47729.1	0.076	bb
122	1... 20211111_A7_R2_IgG	2.30	44653.3	57347.1	0.779	bb
123	1... 20211111_A8_R2_IgG	2.30	17867.6	52397.8	0.341	bb
124	1... 20211111_L7_R2_IgG	2.30	15942.5	36318.4	0.439	bb
125	1... 20211111_L8_R2_IgG	2.31	9000.7	38131.9	0.236	bb
126	1... 20211111_H4_R2_IgG	2.30	10952.7	46849.6	0.234	MM
127	1... 20211111_A9_R2_IgG	2.30	25353.1	47759.6	0.531	bb
128	1... 20211111_A10_R2_IgG	2.31	50586.5	41366.2	1.223	bb
129	1... 20211111_L9_R2_IgG	2.31	21335.1	43052.1	0.496	bb
130	1... 20211111_L10_R2_IgG	2.30	20516.2	31487.4	0.652	MM
131	1... 20211111_QC_R11_IgG	2.31	16594.8	37804.6	0.439	bb
132	1... 20211111_H5_R2_IgG	2.31	2579.1	40380.0	0.064	bb
133	1... 20211111_A11_R2_IgG	2.30	7494.2	49533.3	0.151	bb
134	1... 20211111_A12_R2_IgG	2.31	13031.3	40226.3	0.324	bb
135	1... 20211111_L11_R2_IgG	2.31	9678.2	28884.0	0.335	MM
136	1... 20211111_L12_R2_IgG	2.31	4711.1	34351.3	0.137	bb
137	1... 20211111_H6_R2_IgG	2.31	24921.4	40466.6	0.616	bb
138	1... 20211111_A13_R2_IgG	2.30	29088.5	42306.7	0.688	bb
139	1... 20211111_A14_R2_IgG	2.31	27436.6	44904.0	0.611	bb
140	1... 20211111_L13_R2_IgG	2.30	4968.0	31913.0	0.156	MM
141	1... 20211111_L14_R2_IgG	2.31	10346.1	31527.4	0.328	bb
142	1... 20211111_H7_R2_IgG	2.31	25292.0	40838.2	0.619	bb
143	1... 20211111_A15_R2_IgG	2.31	7518.1	49389.7	0.152	bb
144	1... 20211111_QC_R12_IgG	2.31	18326.4	35168.7	0.521	bb
145	1... 20211111_A16_R2_IgG	2.30	24026.0	47440.5	0.506	bb
146	1... 20211111_L15_R2_IgG	2.30	24835.1	33145.9	0.749	bb
147	1... 20211111_L16_R2_IgG	2.31	7983.0	37596.6	0.212	bb
148	1... 20211111_H8_R2_IgG	2.30	17191.4	43933.3	0.391	MM
149	1... 20211111_A17_R2_IgG	2.30	19420.5	43605.5	0.445	MM
150	1... 20211111_A18_R2_IgG	2.30	25769.8	37581.8	0.686	bb
151	1... 20211111_L17_R2_IgG	2.30	8382.9	33942.5	0.247	bb
152	1... 20211111_L18_R2_IgG	2.31	7701.1	37831.5	0.204	bb
153	1... 20211111_H9_R2_IgG	2.31	30041.6	30898.4	0.972	bb
154	1... 20211111_A19_R2_IgG	2.30	5938.0	39352.1	0.151	bb
155	1... 20211111_A20_R2_IgG	2.31	5284.4	34661.2	0.152	bb
156	1... 20211111_L19_R2_IgG	2.30	10355.5	39918.3	0.259	bb
157	1... 20211111_QC_R13_IgG	2.31	17763.9	36239.1	0.490	bb
158	1... 20211111_L20_R2_IgG	2.30	16809.4	32897.7	0.511	bb
159	1... 20211111_H10_R2_IgG	2.30	19798.1	31394.5	0.631	bb
160	1... 20211111_A21_R2_IgG	2.31	33265.8	34863.7	0.954	bb
161	1... 20211111_A22_R2_IgG	2.30	6024.9	30342.1	0.199	bb
162	1... 20211111_L21_R2_IgG	2.30	6725.3	33706.2	0.200	bb
163	1... 20211111_L22_R2_IgG	2.30	13485.1	31298.6	0.431	bb
164	1... 20211111_H11_R2_IgG	2.30	29824.9	38904.1	0.767	bb
165	1... 20211111_A23_R2_IgG	2.30	20968.8	32382.3	0.648	bb
166	1... 20211111_A24_R2_IgG	2.30	4177.0	30261.9	0.138	MM
167	1... 20211111_L23_R2_IgG	2.30	22612.4	43037.5	0.525	bb
168	1... 20211111_L24_R2_IgG	2.30	7594.1	37023.3	0.205	bb

Dataset: C:\MassLynx\10.pro\20211115_clinicalsamples_IgG.qld

Last Altered: Sunday, October 02, 2022 16:02:30 台北標準時間
Printed: Friday, August 01, 2025 16:54:07 台北標準時間

	#	Name	RT	Area	IS Area	Response	Prima...
169	1...	20211111_H12_R2_IgG	2.29	2700.4	39478.2	0.068	MM
170	1...	20211111_QC_R14_IgG	2.30	14775.7	34463.2	0.429	bb
171	1...	20211111_A25_R2_IgG	2.30	58738.5	39945.3	1.470	bb
172	1...	20211111_A26_R2_IgG	2.31	1356.3	29663.2	0.046	MM
173	1...	20211111_L25_R2_IgG	2.30	16432.9	38589.3	0.426	bb
174	1...	20211111_L26_R2_IgG	2.30	8950.0	38405.2	0.233	bb
175	1...	20211111_H13_R2_IgG	2.30	9474.4	37771.2	0.251	MM
176	1...	20211111_A27_R2_IgG	2.30	11488.4	42634.1	0.269	bb
177	1...	20211111_A28_R2_IgG	2.30	12802.7	33231.1	0.385	bb
178	1...	20211111_L27_R2_IgG	2.31	8014.3	41761.1	0.192	bb
179	1...	20211111_L28_R2_IgG	2.30	4559.5	26968.6	0.169	MM
180	1...	20211111_H14_R2_IgG	2.31	6229.3	49250.2	0.126	bb
181	1...	20211111_A29_R2_IgG	2.30	30299.6	37601.7	0.806	bb
182	1...	20211111_A30_R2_IgG	2.31	41129.8	40366.8	1.019	bb
183	1...	20211111_QC_R15_IgG	2.30	15743.2	35156.1	0.448	bb
184	1...	20211111_L29_R2_IgG	2.31	7906.8	33813.1	0.234	MM
185	1...	20211111_L30_R2_IgG	2.31	7233.7	33488.6	0.216	bb
186	1...	20211111_H15_R2_IgG	2.31	1221.5	38501.0	0.032	MM
187	1...	20211111_A31_R2_IgG	2.30	11808.6	37525.5	0.315	bb
188	1...	20211111_A32_R2_IgG	2.30	63121.3	35770.8	1.765	bb
189	1...	20211111_L31_R2_IgG	2.30	6637.1	33282.4	0.199	bb
190	1...	20211111_L32_R2_IgG	2.30	7034.3	42049.6	0.167	bb
191	1...	20211111_H16_R2_IgG	2.30	42590.8	45947.6	0.927	bb
192	1...	20211111_A33_R2_IgG	2.30	11583.1	40202.3	0.288	bb
193	1...	20211111_A34_R2_IgG	2.30	18384.6	36747.0	0.500	bb
194	1...	20211111_H17_R2_IgG	2.30	47465.1	40920.9	1.160	bb
195	1...	20211111_A35_R2_IgG	2.30	23038.3	33689.2	0.684	bb
196	1...	20211111_QC_R16_IgG	2.31	15869.9	32783.6	0.484	bb
197	1...	20211111_A36_R2_IgG	2.30	37692.2	36822.4	1.024	bb
198	1...	20211111_H18_R2_IgG	2.30	98630.4	31769.3	3.105	bb
199	1...	20211111_A37_R2_IgG	2.31	3982.0	37581.8	0.106	MM
200	2...	20211111_A38_R2_IgG	2.30	18132.9	38390.4	0.472	bb
201	2...	20211111_H19_R2_IgG	2.30	52256.4	41217.0	1.268	bb
202	2...	20211111_A39_R2_IgG	2.30	13149.3	37392.7	0.352	bb
203	2...	20211111_A40_R2_IgG	2.30	56320.5	38295.1	1.471	bb
204	2...	20211111_H20_R2_IgG	2.30	43984.0	35941.8	1.224	bb
205	2...	20211111_A41_R2_IgG	2.30	4042.3	45304.9	0.089	bb
206	2...	20211111_A42_R2_IgG	2.30	10764.3	37803.8	0.285	bb
207	2...	20211111_A43_R2_IgG	2.30	19290.4	38161.8	0.505	bb
208	2...	20211111_A44_R2_IgG	2.30	9344.2	37819.8	0.247	bb
209	2...	20211111_QC_R17_IgG	2.30	15442.1	31731.8	0.487	bb

Dataset: C:\MassLynx\10.pro\20211115_clinicalsamples_IgG.qld

Last Altered: Sunday, October 02, 2022 16:02:30 台北標準時間
Printed: Friday, August 01, 2025 16:54:36 台北標準時間

Method: C:\MassLynx\10_Pro_IgA.PRO\MethDB\20210926_IgG_DMRM_IgM_IS.mdb 16 Feb 2022 11:58:21
Calibration: 18 Nov 2021 13:56:22

Sample Name: 20211111_QC_R1_IgG
F24:MRM of 1 channel,ES+
941.1 > 204.1
2.372e+005

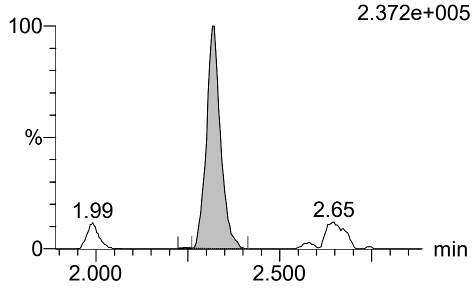

Sample Name: 20211111_A1_R1_IgG
F24:MRM of 1 channel,ES+
941.1 > 204.1
2.454e+005

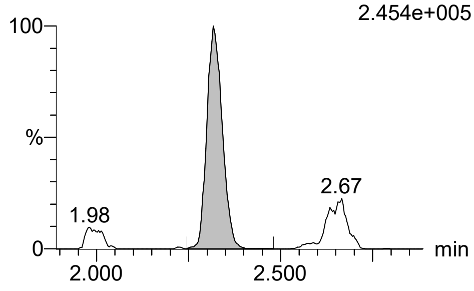

Sample Name: 20211111_A2_R1_IgG
F24:MRM of 1 channel,ES+
941.1 > 204.1
1.143e+005

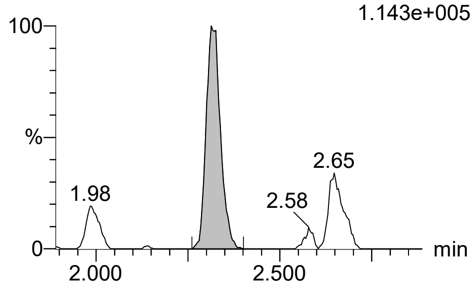

Sample Name: 20211111_L1_R1_IgG
F24:MRM of 1 channel,ES+
941.1 > 204.1
2.612e+005

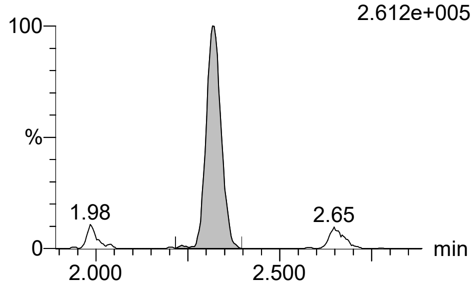

Sample Name: 20211111_L2_R1_IgG
F24:MRM of 1 channel,ES+
941.1 > 204.1
9.098e+004

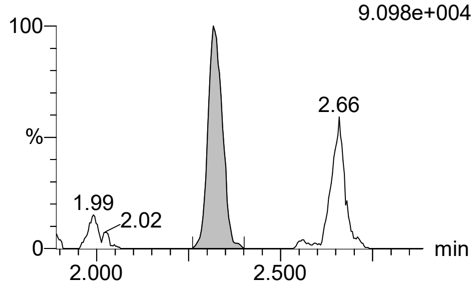

Sample Name: 20211111_H1_R1_IgG
F24:MRM of 1 channel,ES+
941.1 > 204.1
1.149e+005

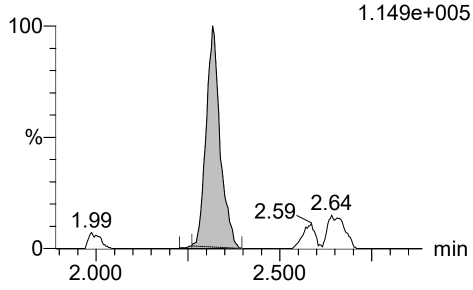

Sample Name: 20211111_A3_R1_IgG
F24:MRM of 1 channel,ES+
941.1 > 204.1
1.243e+004

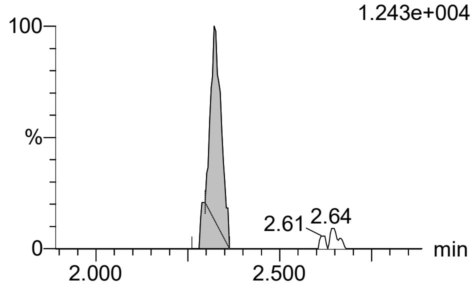

Sample Name: 20211111_A4_R1_IgG
F24:MRM of 1 channel,ES+
941.1 > 204.1
2.657.218e+004

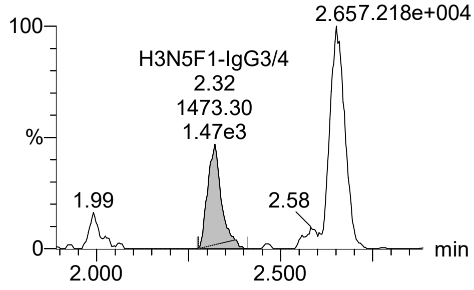

Sample Name: 20211111_L3_R1_IgG
F24:MRM of 1 channel,ES+
941.1 > 204.1
1.265e+005

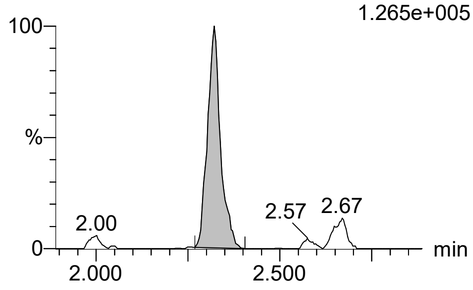

Sample Name: 20211111_L4_R1_IgG
F24:MRM of 1 channel,ES+
941.1 > 204.1
1.887e+005

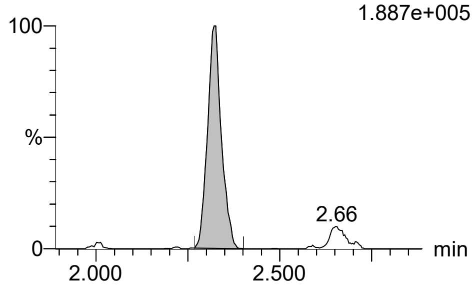

Sample Name: 20211111_H2_R1_IgG
F24:MRM of 1 channel,ES+
941.1 > 204.1
2.925e+005

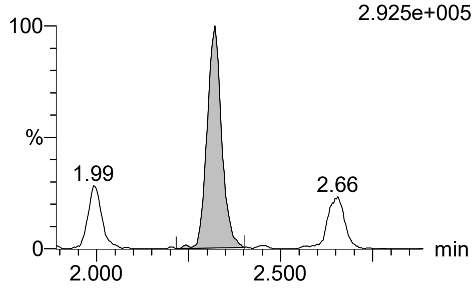

Sample Name: 20211111_A5_R1_IgG
F24:MRM of 1 channel,ES+
941.1 > 204.1
1.077e+005

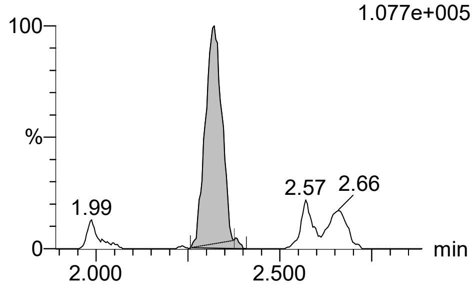

Sample Name: 20211111_A6_R1_IgG

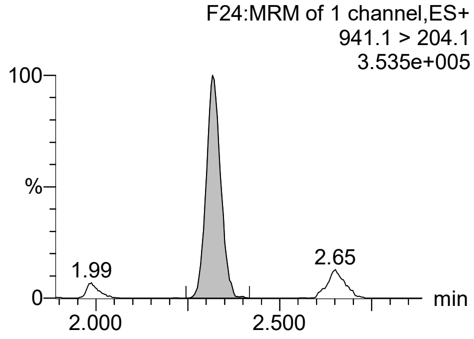

Sample Name: 20211111_QC_R2_IgG

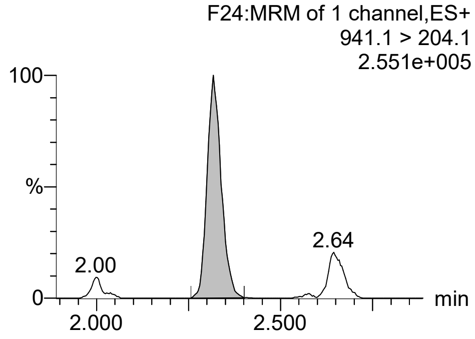

Sample Name: 20211111_L5_R1_IgG

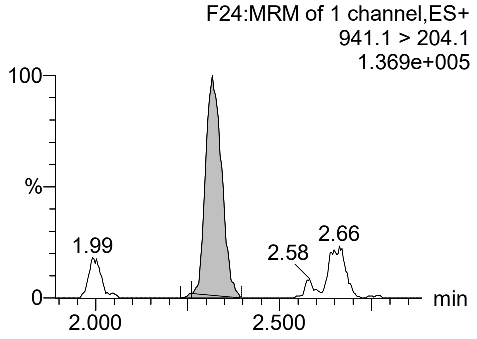

Sample Name: 20211111_L6_R1_IgG

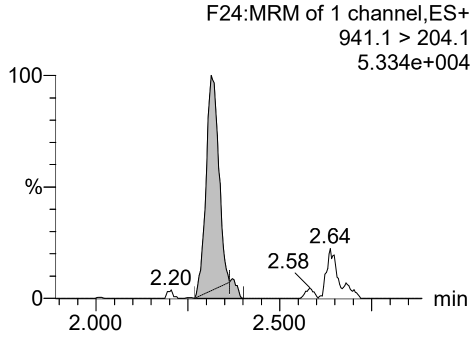

Sample Name: 20211111_H3_R1_IgG

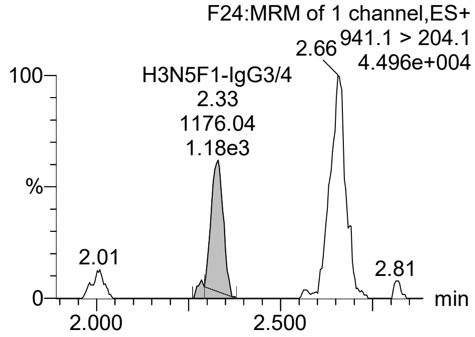

Sample Name: 20211111_A7_R1_IgG

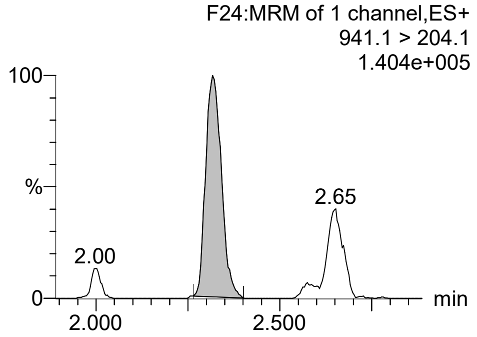

Sample Name: 20211111_A8_R1_IgG

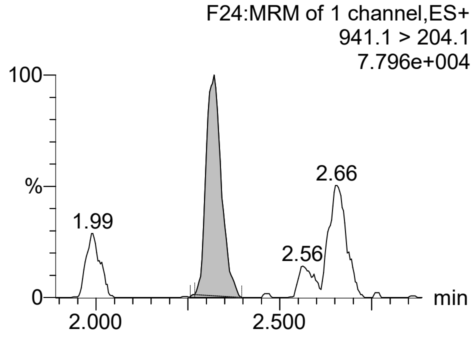

Sample Name: 20211111_L7_R1_IgG

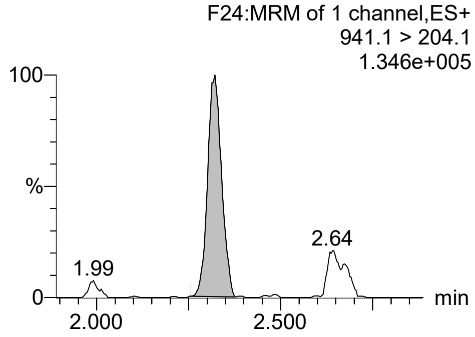

Sample Name: 20211111_L8_R1_IgG

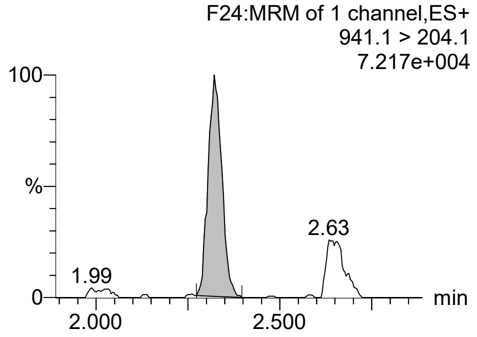

Sample Name: 20211111_H4_R1_IgG

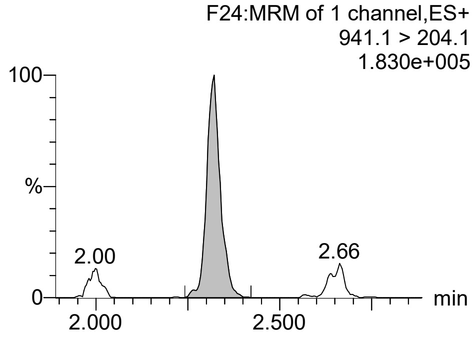

Sample Name: 20211111_A9_R1_IgG

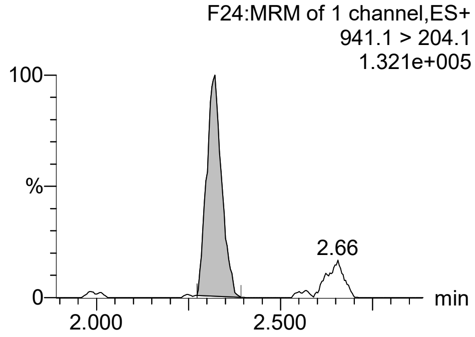

Sample Name: 20211111_A10_R1_IgG

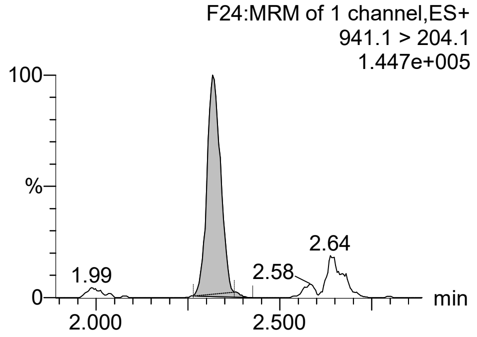

Sample Name: 20211111_L9_R1_IgG

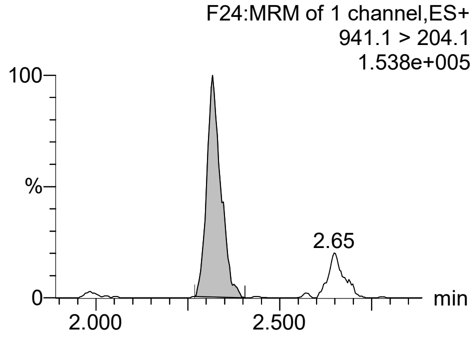

Sample Name: 20211111_L10_R1_IgG

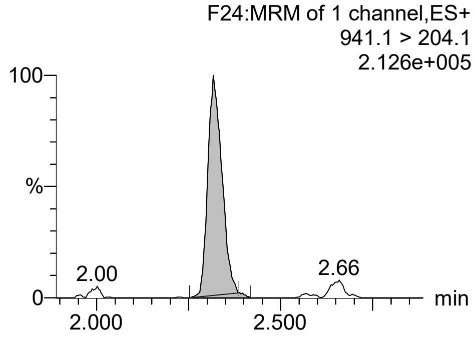

Sample Name: 20211111_QC_R3_IgG

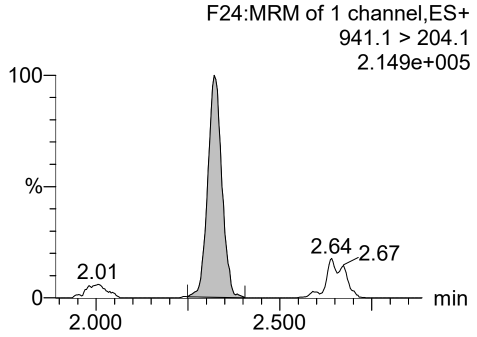

Sample Name: 20211111_H5_R1_IgG

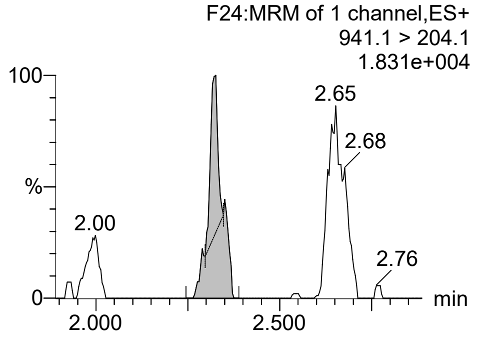

Sample Name: 20211111_A11_R1_IgG

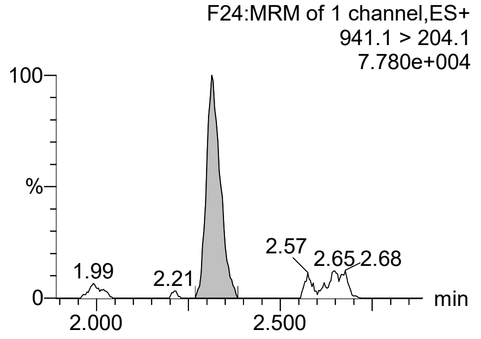

Sample Name: 20211111_A12_R1_IgG

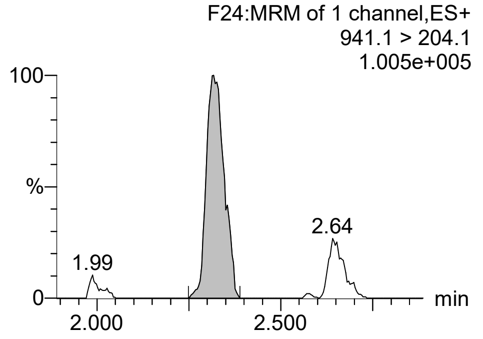

Sample Name: 20211111_L11_R1_IgG

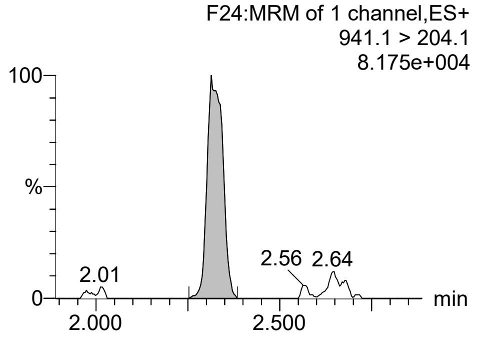

Sample Name: 20211111_L12_R1_IgG

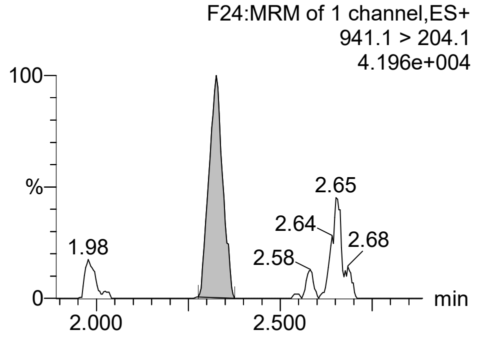

Sample Name: 20211111_H6_R1_IgG

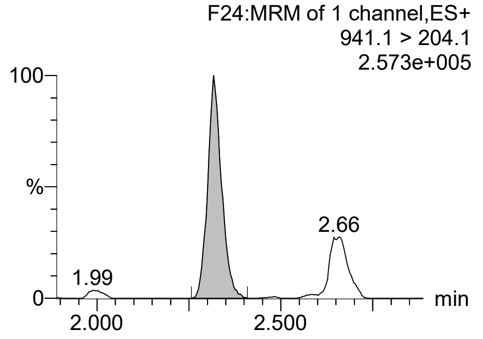

Sample Name: 20211111_A13_R1_IgG

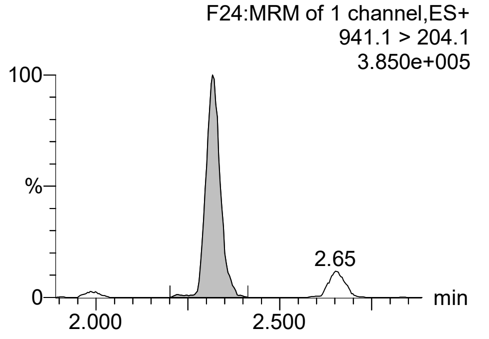

Sample Name: 20211111_A14_R1_IgG

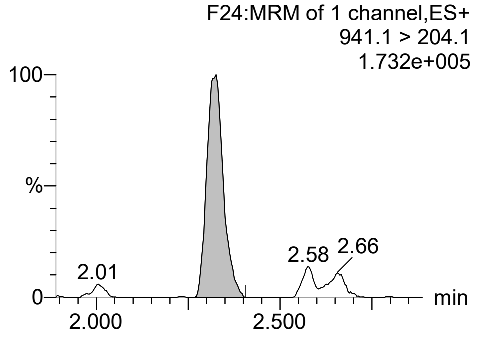

Sample Name: 20211111_L13_R1_IgG

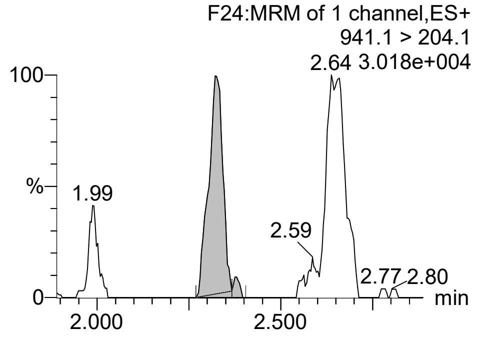

Sample Name: 20211111_L14_R1_IgG

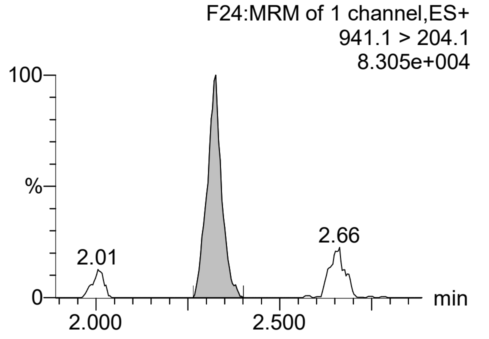

Sample Name: 20211111_H7_R1_IgG

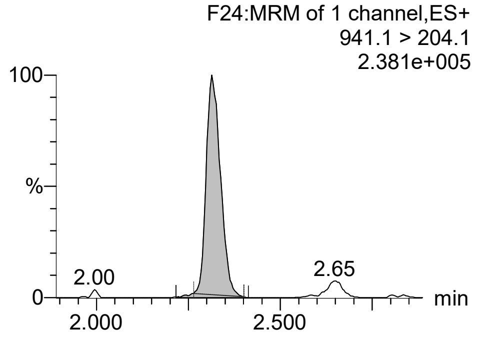

Sample Name: 20211111_A15_R1_IgG

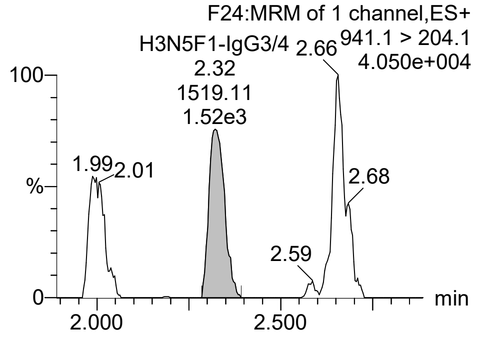

Sample Name: 20211111_QC_R4_IgG

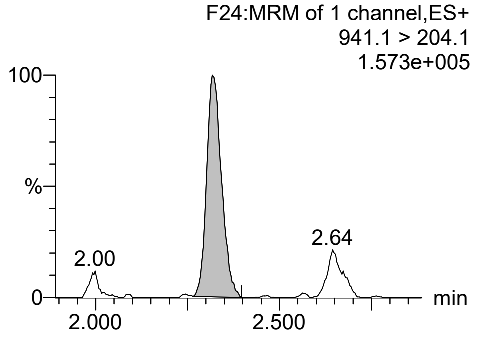

Sample Name: 20211111_A16_R1_IgG

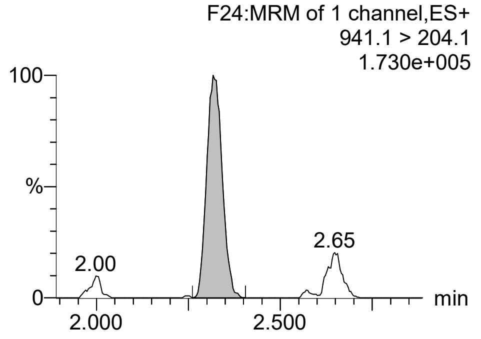

Sample Name: 20211111_L15_R1_IgG

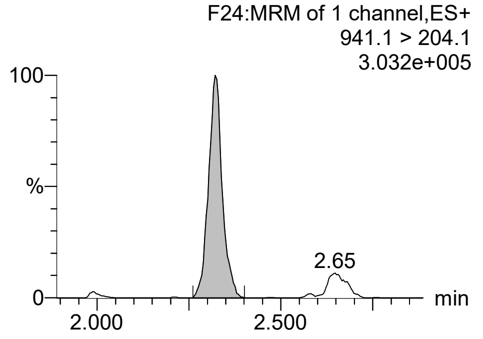

Sample Name: 20211111_L16_R1_IgG
F24:MRM of 1 channel,ES+
941.1 > 204.1
6.652e+004

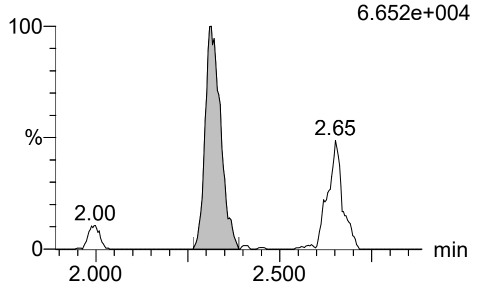

Sample Name: 20211111_H8_R1_IgG
F24:MRM of 1 channel,ES+
941.1 > 204.1
1.142e+005

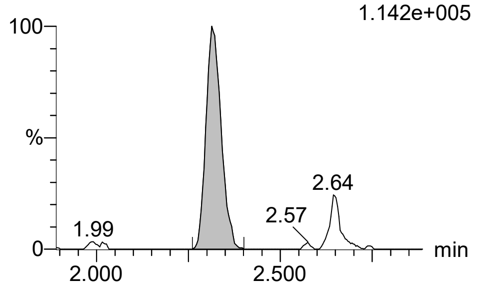

Sample Name: 20211111_A17_R1_IgG
F24:MRM of 1 channel,ES+
941.1 > 204.1
1.706e+005

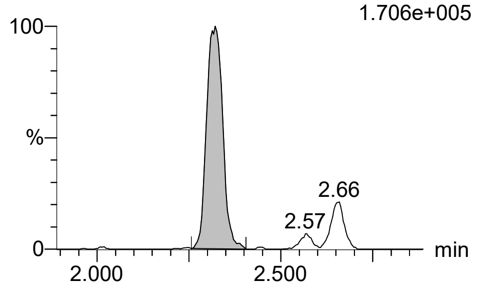

Sample Name: 20211111_A18_R1_IgG
F24:MRM of 1 channel,ES+
941.1 > 204.1
1.074e+005

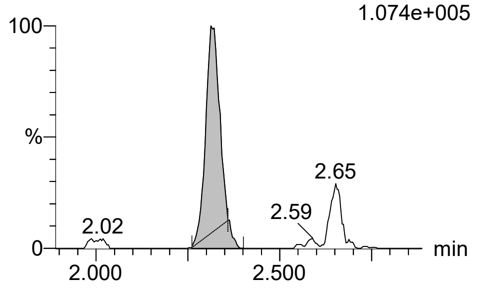

Sample Name: 20211111_L17_R1_IgG
F24:MRM of 1 channel,ES+
941.1 > 204.1
7.194e+004

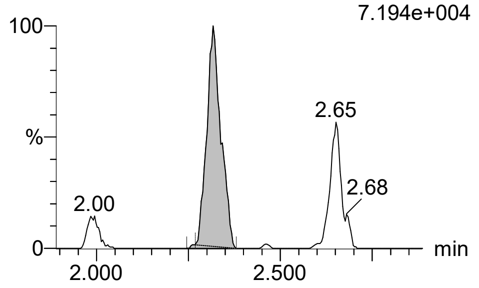

Sample Name: 20211111_L18_R1_IgG
F24:MRM of 1 channel,ES+
941.1 > 204.1
6.998e+004

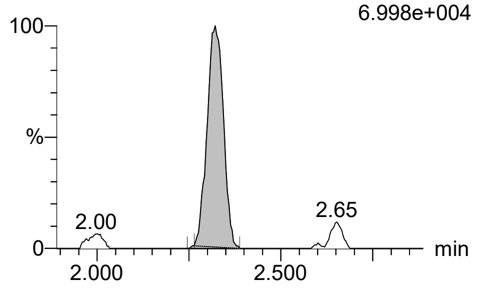

Sample Name: 20211111_H9_R1_IgG
F24:MRM of 1 channel,ES+
941.1 > 204.1
2.710e+005

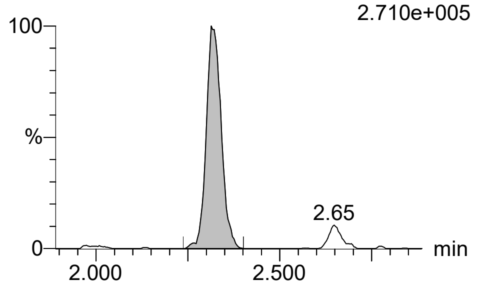

Sample Name: 20211111_A19_R1_IgG
F24:MRM of 1 channel,ES+
941.1 > 204.1
7.517e+004

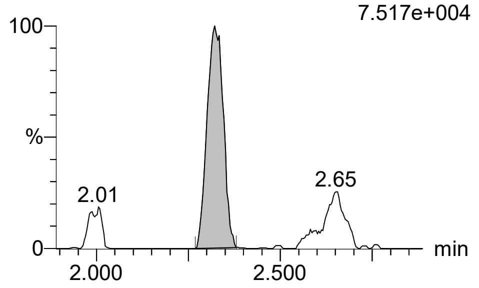

Sample Name: 20211111_A20_R1_IgG
F24:MRM of 1 channel,ES+
941.1 > 204.1
5.961e+004

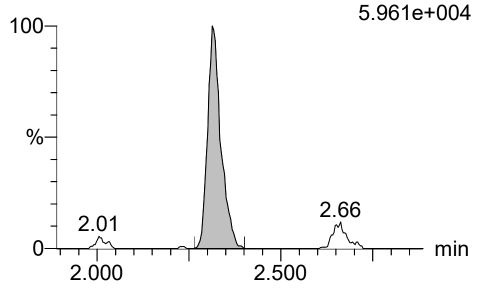

Sample Name: 20211111_L19_R1_IgG
F24:MRM of 1 channel,ES+
941.1 > 204.1
1.016e+005

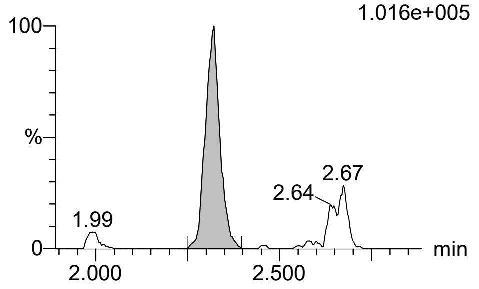

Sample Name: 20211111_QC_R5_IgG
F24:MRM of 1 channel,ES+
941.1 > 204.1
1.590e+005

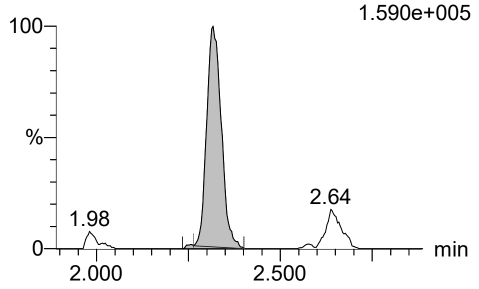

Sample Name: 20211111_L20_R1_IgG
F24:MRM of 1 channel,ES+
941.1 > 204.1
2.967e+005

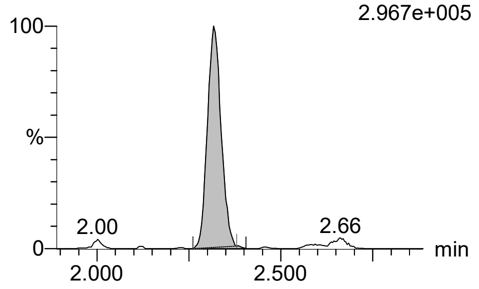

Sample Name: 20211111_H10_R1_IgG
F24:MRM of 1 channel,ES+
941.1 > 204.1
3.304e+005

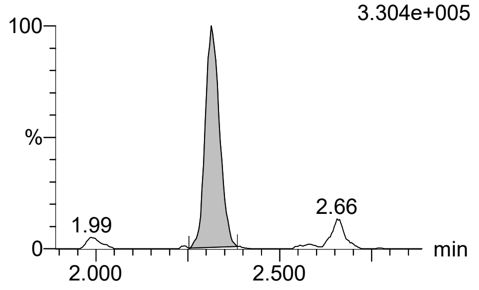

Sample Name: 20211111_A21_R1_IgG
F24:MRM of 1 channel,ES+
941.1 > 204.1
2.586e+005

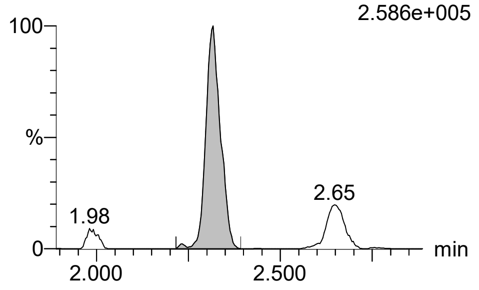

Sample Name: 20211111_A22_R1_IgG
F24:MRM of 1 channel,ES+
941.1 > 204.1
7.454e+004

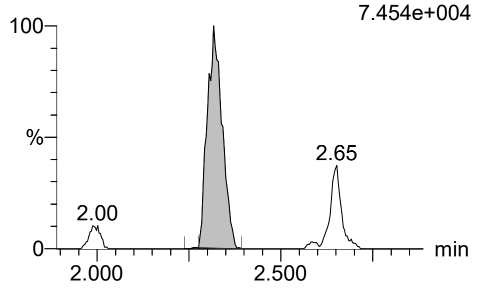

Dataset: C:\MassLynx\10.pro\20211115_clinicalsamples_IgG.qld

Last Altered: Sunday, October 02, 2022 16:02:30 台北標準時間
Printed: Friday, August 01, 2025 16:54:36 台北標準時間

Sample Name: 20211111_L21_R1_IgG
F24:MRM of 1 channel,ES+
941.1 > 204.1
9.849e+004

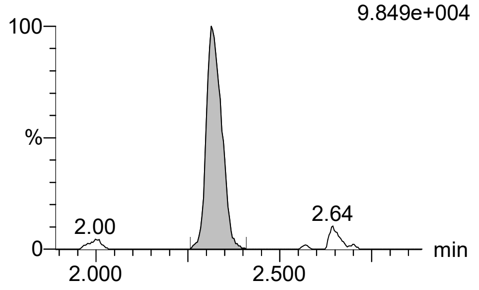

Sample Name: 20211111_L22_R1_IgG
F24:MRM of 1 channel,ES+
941.1 > 204.1
7.247e+004

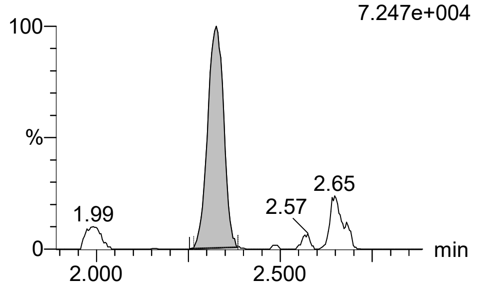

Sample Name: 20211111_H11_R1_IgG
F24:MRM of 1 channel,ES+
941.1 > 204.1
2.089e+005

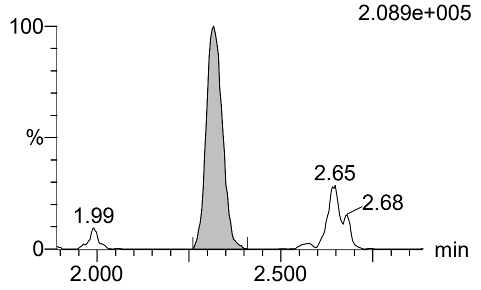

Sample Name: 20211111_A23_R1_IgG
F24:MRM of 1 channel,ES+
941.1 > 204.1
3.218e+005

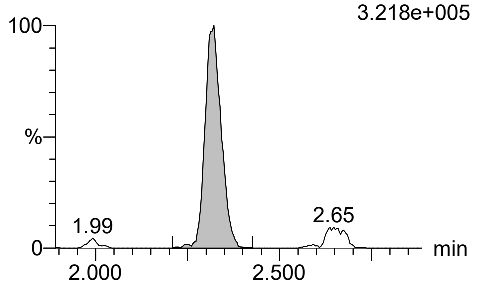

Sample Name: 20211111_A24_R1_IgG
F24:MRM of 1 channel,ES+
941.1 > 204.1
4.584e+004

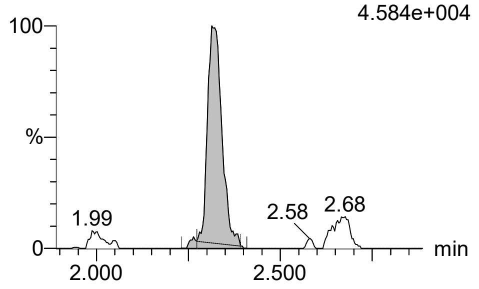

Sample Name: 20211111_L23_R1_IgG
F24:MRM of 1 channel,ES+
941.1 > 204.1
2.898e+005

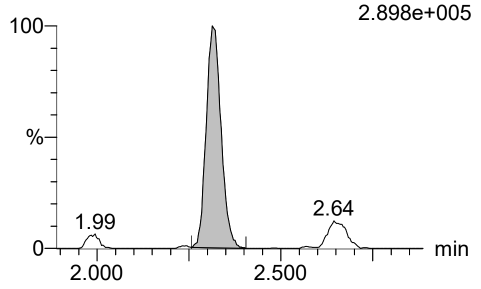

Sample Name: 20211111_L24_R1_IgG
F24:MRM of 1 channel,ES+
941.1 > 204.1
5.272e+004

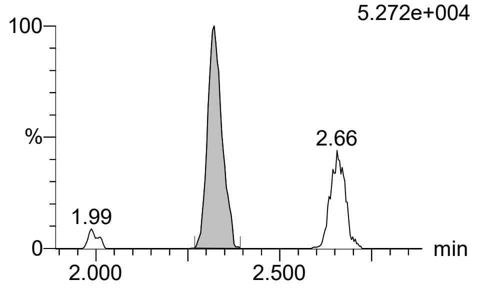

Sample Name: 20211111_H12_R1_IgG
F24:MRM of 1 channel,ES+
941.1 > 204.1
1.479e+004

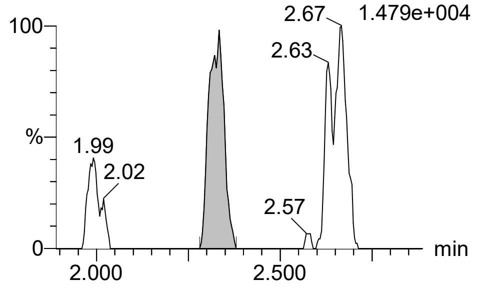

Sample Name: 20211111_QC_R6_IgG
F24:MRM of 1 channel,ES+
941.1 > 204.1
1.438e+005

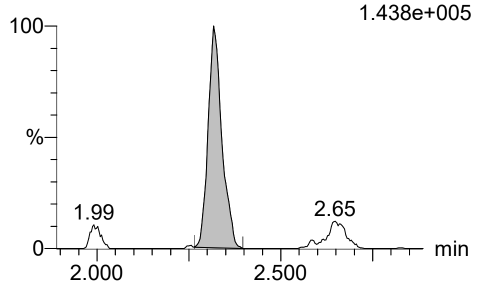

Sample Name: 20211111_A25_R1_IgG
F24:MRM of 1 channel,ES+
941.1 > 204.1
4.809e+005

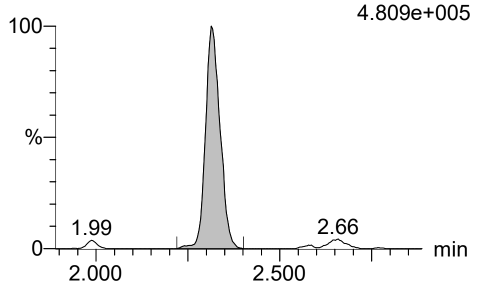

Sample Name: 20211111_A26_R1_IgG
F24:MRM of 1 channel,ES+
941.1 > 204.1
1.559e+004

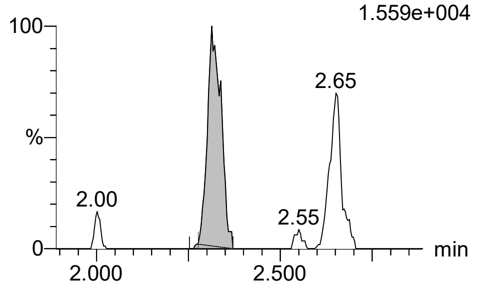

Sample Name: 20211111_L25_R1_IgG
F24:MRM of 1 channel,ES+
941.1 > 204.1
2.006e+005

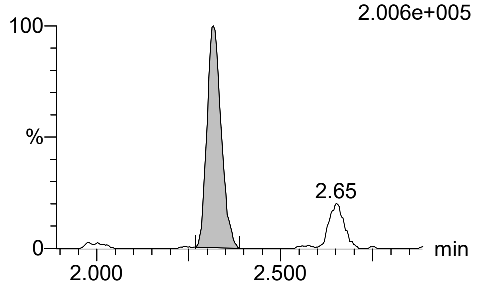

Sample Name: 20211111_L26_R1_IgG
F24:MRM of 1 channel,ES+
941.1 > 204.1
8.789e+004

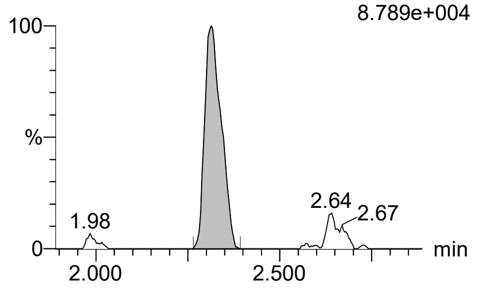

Sample Name: 20211111_H13_R1_IgG
F24:MRM of 1 channel,ES+
941.1 > 204.1
8.674e+004

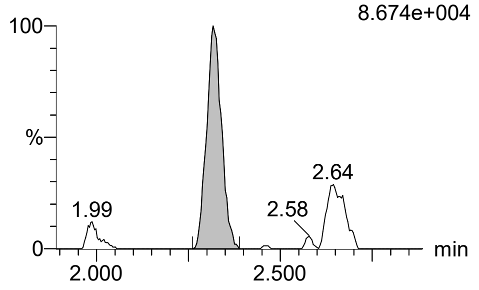

Sample Name: 20211111_A27_R1_IgG
F24:MRM of 1 channel,ES+
941.1 > 204.1
9.141e+004

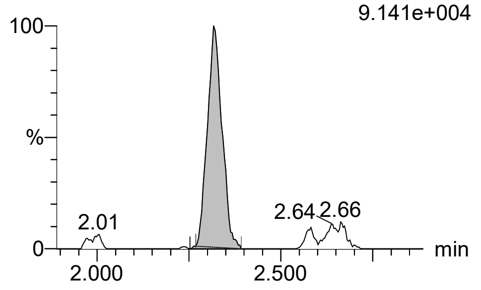

Dataset: C:\MassLynx\10.pro\20211115_clinicalsamples_IgG.qld

Last Altered: Sunday, October 02, 2022 16:02:30 台北標準時間
Printed: Friday, August 01, 2025 16:54:36 台北標準時間

Sample Name: 20211111_A28_R1_IgG
F24:MRM of 1 channel,ES+
941.1 > 204.1
1.555e+005

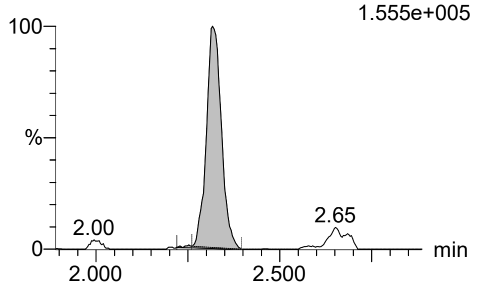

Sample Name: 20211111_L27_R1_IgG
F24:MRM of 1 channel,ES+
941.1 > 204.1
8.754e+004

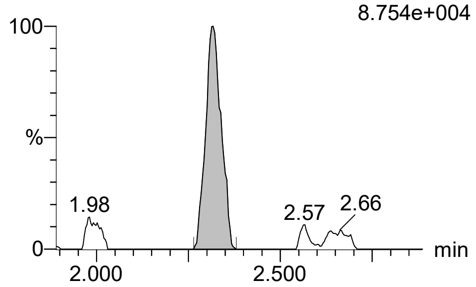

Sample Name: 20211111_L28_R1_IgG
F24:MRM of 1 channel,ES+
941.1 > 204.1
3.672e+004

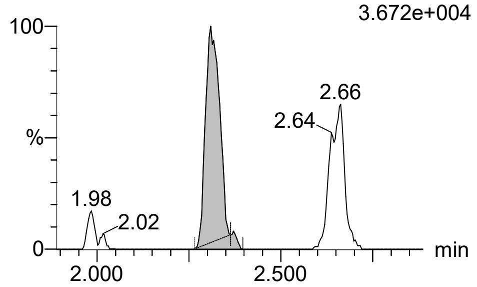

Sample Name: 20211111_H14_R1_IgG
F24:MRM of 1 channel,ES+
941.1 > 204.1
7.069e+004

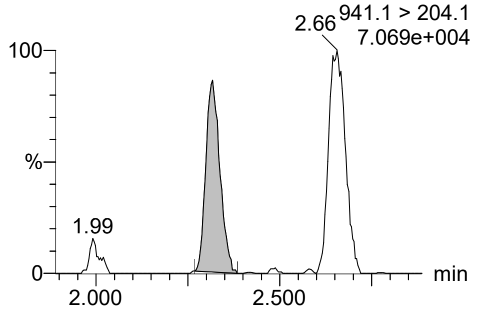

Sample Name: 20211111_A29_R1_IgG
F24:MRM of 1 channel,ES+
941.1 > 204.1
2.269e+005

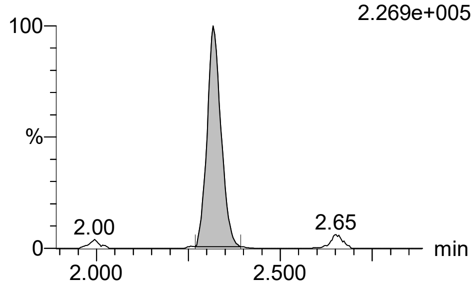

Sample Name: 20211111_A30_R1_IgG
F24:MRM of 1 channel,ES+
941.1 > 204.1
2.617e+005

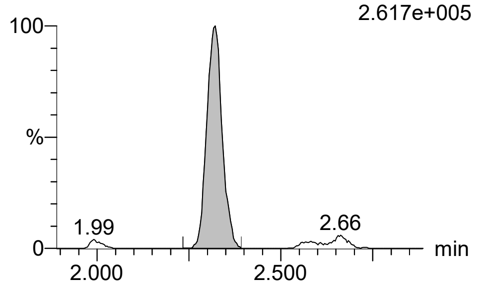

Sample Name: 20211111_QC_R7_IgG
F24:MRM of 1 channel,ES+
941.1 > 204.1
1.031e+005

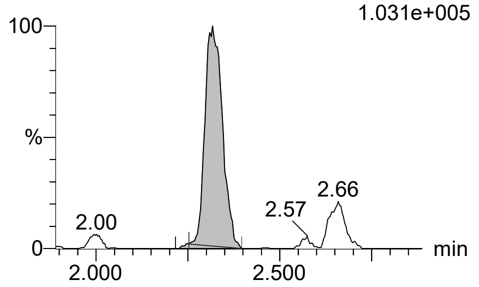

Sample Name: 20211111_L29_R1_IgG
F24:MRM of 1 channel,ES+
941.1 > 204.1
6.024e+004

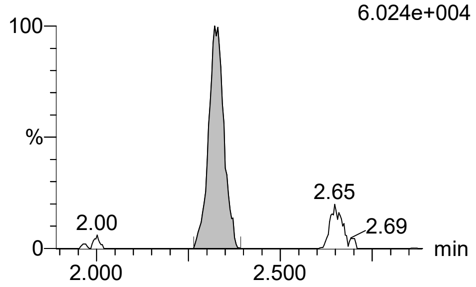

Sample Name: 20211111_L30_R1_IgG
F24:MRM of 1 channel,ES+
941.1 > 204.1
8.908e+004

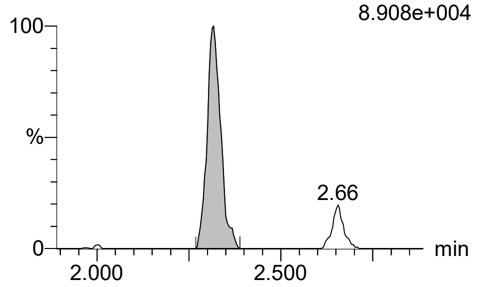

Sample Name: 20211111_H15_R1_IgG
F24:MRM of 1 channel,ES+
941.1 > 204.1
1.760e+004

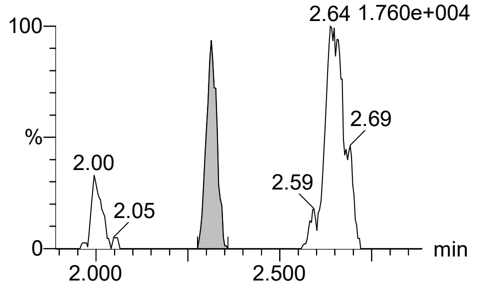

Sample Name: 20211111_A31_R1_IgG
F24:MRM of 1 channel,ES+
941.1 > 204.1
1.058e+005

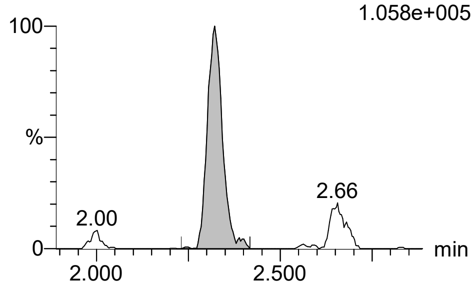

Sample Name: 20211111_A32_R1_IgG
F24:MRM of 1 channel,ES+
941.1 > 204.1
2.647e+005

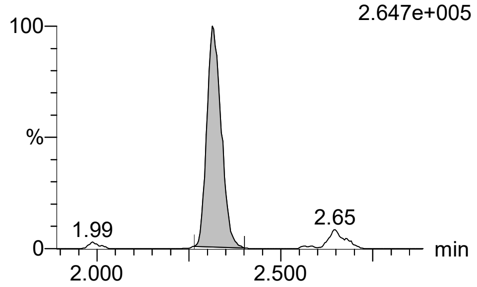

Sample Name: 20211111_L31_R1_IgG
F24:MRM of 1 channel,ES+
941.1 > 204.1
3.177e+004

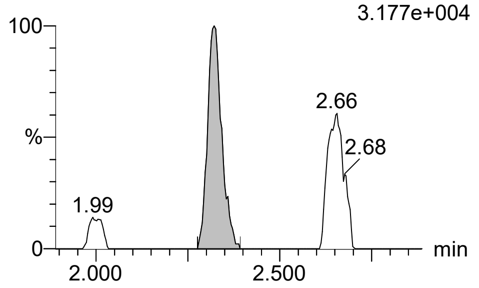

Sample Name: 20211111_L32_R1_IgG
F24:MRM of 1 channel,ES+
941.1 > 204.1
6.381e+004

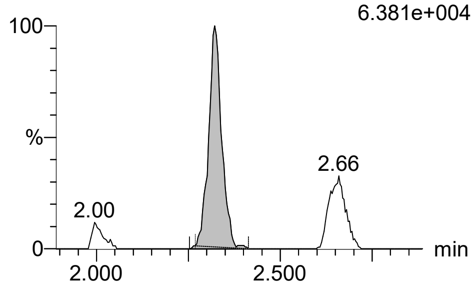

Sample Name: 20211111_H16_R1_IgG
F24:MRM of 1 channel,ES+
941.1 > 204.1
2.494e+005

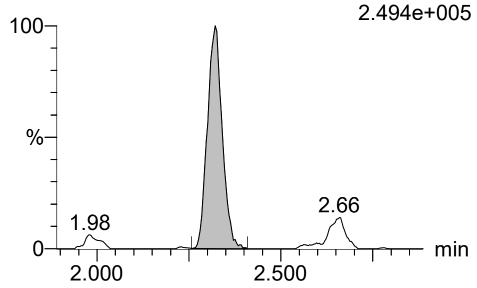

Dataset: C:\MassLynx\10.pro\20211115_clinicalsamples_IgG.qld

Last Altered: Sunday, October 02, 2022 16:02:30 台北標準時間
Printed: Friday, August 01, 2025 16:54:36 台北標準時間

Sample Name: 20211111_A33_R1_IgG
F24:MRM of 1 channel,ES+
941.1 > 204.1
1.207e+005

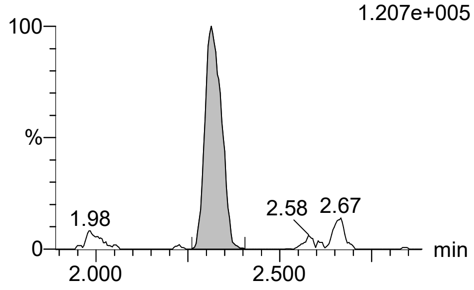

Sample Name: 20211111_A34_R1_IgG
F24:MRM of 1 channel,ES+
941.1 > 204.1
1.872e+005

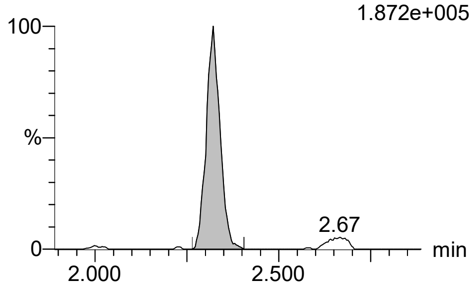

Sample Name: 20211111_H17_R1_IgG
F24:MRM of 1 channel,ES+
941.1 > 204.1
4.787e+005

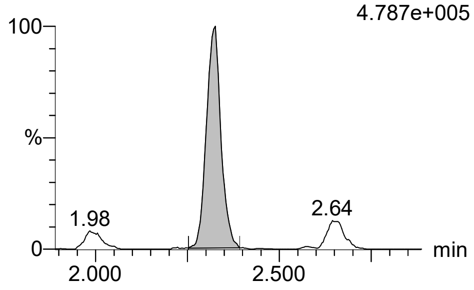

Sample Name: 20211111_A35_R1_IgG
F24:MRM of 1 channel,ES+
941.1 > 204.1
2.534e+005

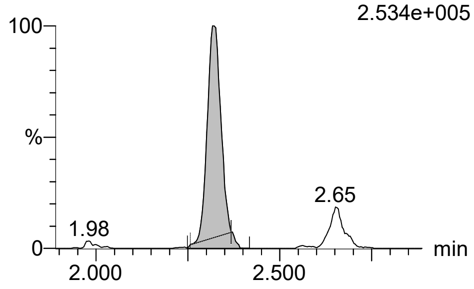

Sample Name: 20211111_QC_R8_IgG
F24:MRM of 1 channel,ES+
941.1 > 204.1
1.122e+005

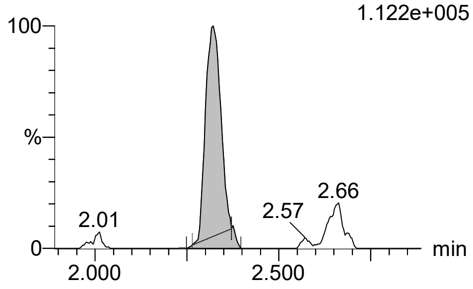

Sample Name: 20211111_A36_R1_IgG
F24:MRM of 1 channel,ES+
941.1 > 204.1
3.064e+005

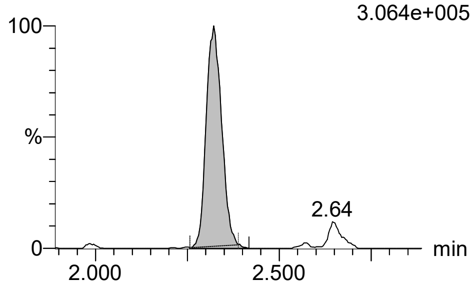

Sample Name: 20211111_H18_R1_IgG
F24:MRM of 1 channel,ES+
941.1 > 204.1
5.084e+005

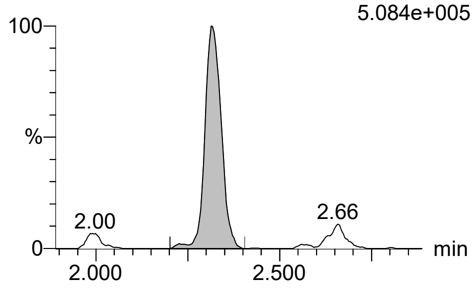

Sample Name: 20211111_A37_R1_IgG
F24:MRM of 1 channel,ES+
941.1 > 204.1
4.140e+004

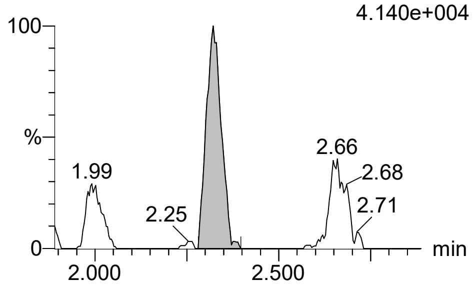

Sample Name: 20211111_A38_R1_IgG
F24:MRM of 1 channel,ES+
941.1 > 204.1
6.825e+004

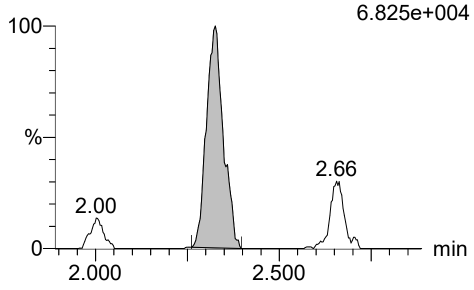

Sample Name: 20211111_H19_R1_IgG
F24:MRM of 1 channel,ES+
941.1 > 204.1
3.782e+005

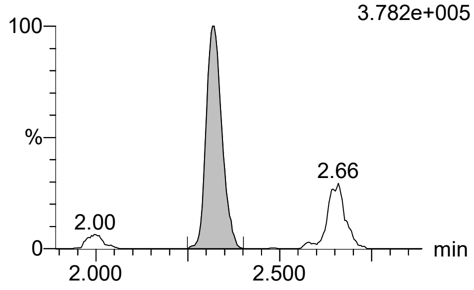

Sample Name: 20211111_A39_R1_IgG
F24:MRM of 1 channel,ES+
941.1 > 204.1
1.250e+005

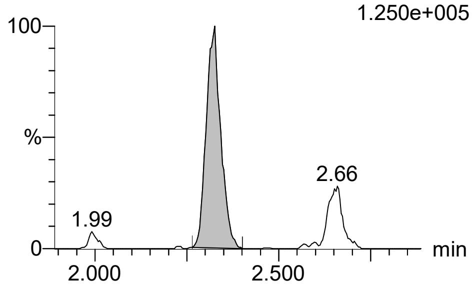

Sample Name: 20211111_A40_R1_IgG
F24:MRM of 1 channel,ES+
941.1 > 204.1
3.134e+005

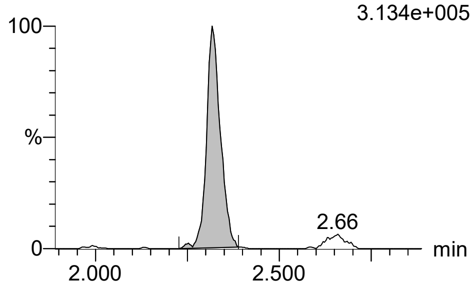

Sample Name: 20211111_H20_R1_IgG
F24:MRM of 1 channel,ES+
941.1 > 204.1
2.521e+005

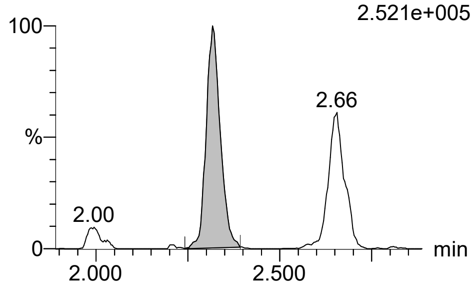

Sample Name: 20211111_A41_R1_IgG
F24:MRM of 1 channel,ES+
941.1 > 204.1
6.894e+004

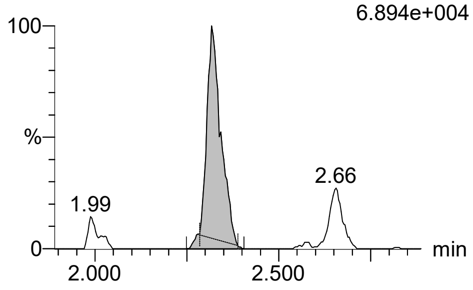

Sample Name: 20211111_A42_R1_IgG
F24:MRM of 1 channel,ES+
941.1 > 204.1
7.858e+004

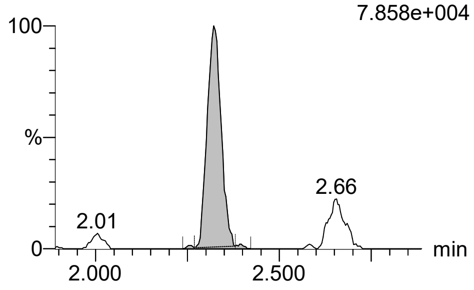

Dataset: C:\MassLynx\10.pro\20211115_clinicalsamples_IgG.qld

Last Altered: Sunday, October 02, 2022 16:02:30 台北標準時間
Printed: Friday, August 01, 2025 16:54:36 台北標準時間

Sample Name: 20211111_A43_R1_IgG
F24:MRM of 1 channel,ES+
941.1 > 204.1
9.384e+004

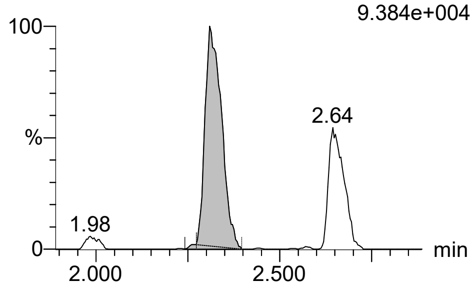

Sample Name: 20211111_A44_R1_IgG
F24:MRM of 1 channel,ES+
941.1 > 204.1
9.071e+004

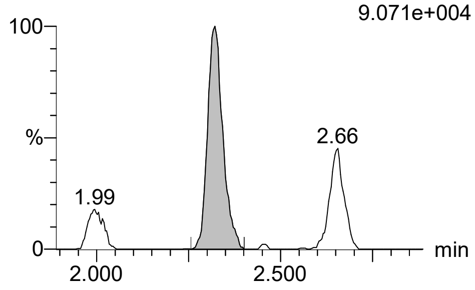

Sample Name: 20211111_QC_R9_IgG
F24:MRM of 1 channel,ES+
941.1 > 204.1
9.653e+004

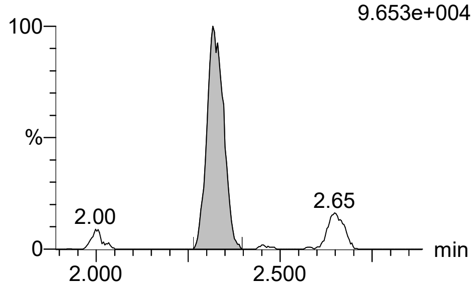

Sample Name: 20211111_A1_R2_IgG
F24:MRM of 1 channel,ES+
941.1 > 204.1
1.970e+005

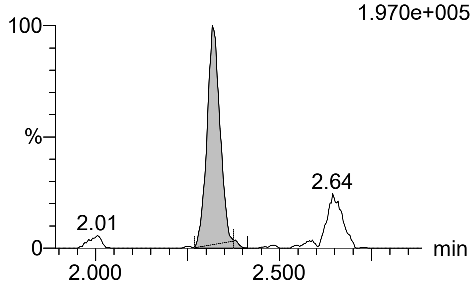

Sample Name: 20211111_A2_R2_IgG
F24:MRM of 1 channel,ES+
941.1 > 204.1
9.288e+004

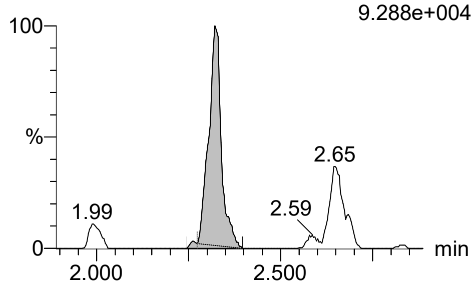

Sample Name: 20211111_L1_R2_IgG
F24:MRM of 1 channel,ES+
941.1 > 204.1
1.729e+005

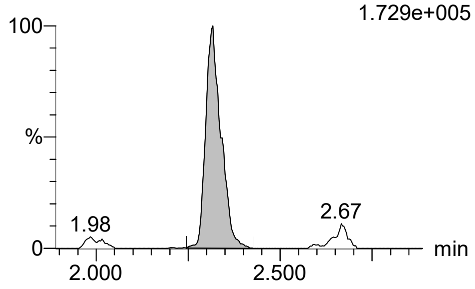

Sample Name: 20211111_L2_R2_IgG
F24:MRM of 1 channel,ES+
941.1 > 204.1
9.744e+004

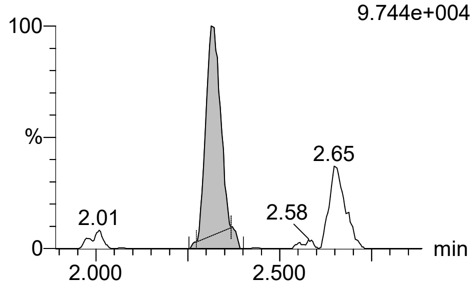

Sample Name: 20211111_H1_R2_IgG
F24:MRM of 1 channel,ES+
941.1 > 204.1
1.394e+005

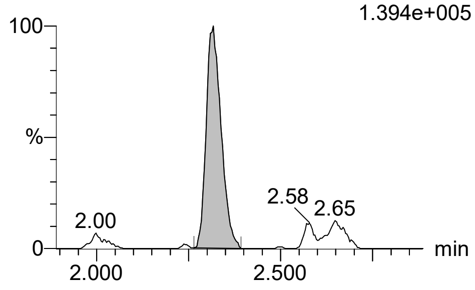

Sample Name: 20211111_A3_R2_IgG
F24:MRM of 1 channel,ES+
941.1 > 204.1
1.867e+004

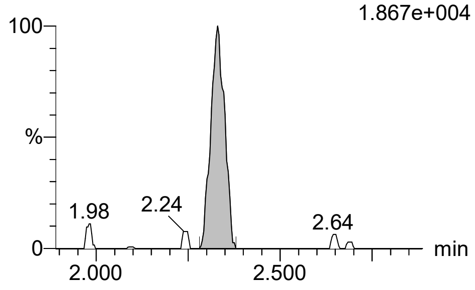

Sample Name: 20211111_A4_R2_IgG
F24:MRM of 1 channel,ES+
941.1 > 204.1
5.570e+004

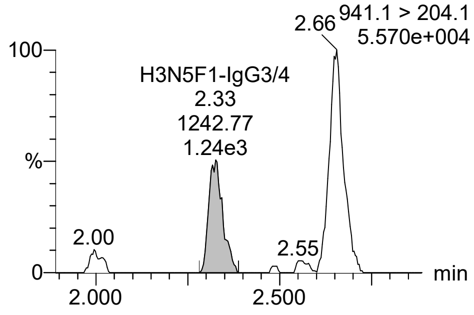

Sample Name: 20211111_L3_R2_IgG
F24:MRM of 1 channel,ES+
941.1 > 204.1
1.068e+005

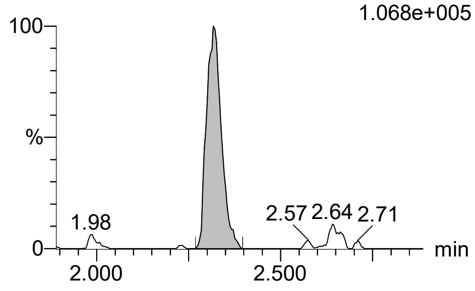

Sample Name: 20211111_L4_R2_IgG
F24:MRM of 1 channel,ES+
941.1 > 204.1
1.529e+005

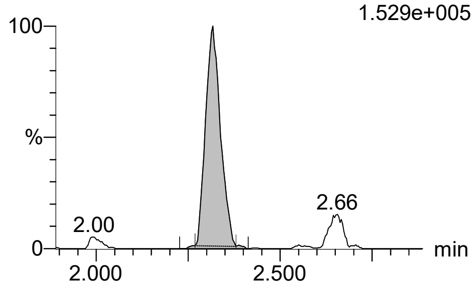

Sample Name: 20211111_H2_R2_IgG
F24:MRM of 1 channel,ES+
941.1 > 204.1
1.789e+005

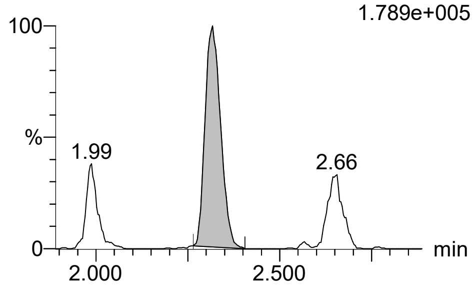

Sample Name: 20211111_A5_R2_IgG
F24:MRM of 1 channel,ES+
941.1 > 204.1
1.088e+005

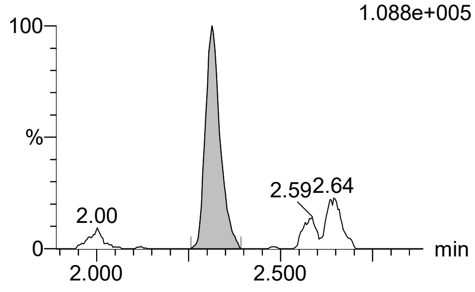

Sample Name: 20211111_A6_R2_IgG
F24:MRM of 1 channel,ES+
941.1 > 204.1
2.732e+005

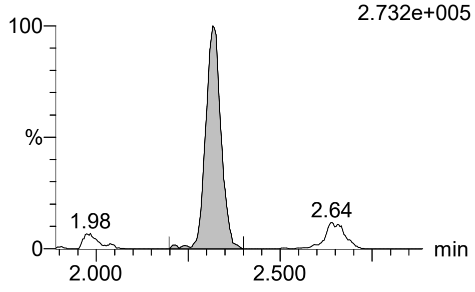

Dataset: C:\MassLynx\10.pro\20211115_clinicalsamples_IgG.qld

Last Altered: Sunday, October 02, 2022 16:02:30 台北標準時間
Printed: Friday, August 01, 2025 16:54:36 台北標準時間

Sample Name: 20211111_QC_R10_IgG
F24:MRM of 1 channel,ES+
941.1 > 204.1
1.077e+005

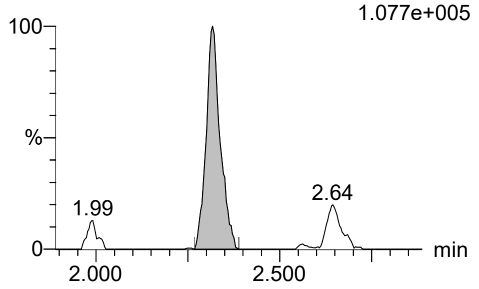

Sample Name: 20211111_L5_R2_IgG
F24:MRM of 1 channel,ES+
941.1 > 204.1
1.234e+005

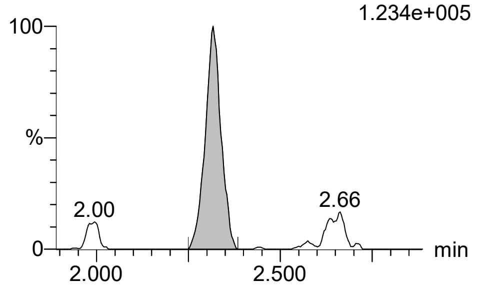

Sample Name: 20211111_L6_R2_IgG
F24:MRM of 1 channel,ES+
941.1 > 204.1
3.560e+004

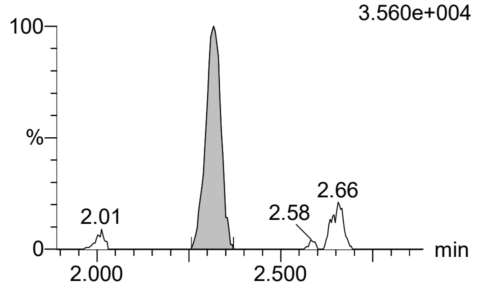

Sample Name: 20211111_H3_R2_IgG
F24:MRM of 1 channel,ES+
941.1 > 204.1
2.654.576e+004

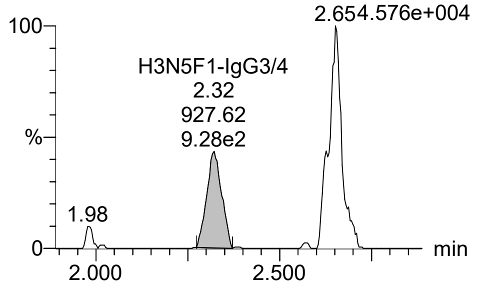

Sample Name: 20211111_A7_R2_IgG
F24:MRM of 1 channel,ES+
941.1 > 204.1
1.075e+005

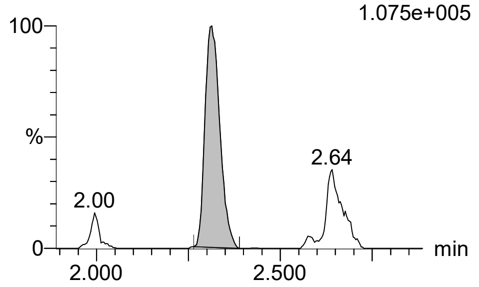

Sample Name: 20211111_A8_R2_IgG
F24:MRM of 1 channel,ES+
941.1 > 204.1
4.993e+004

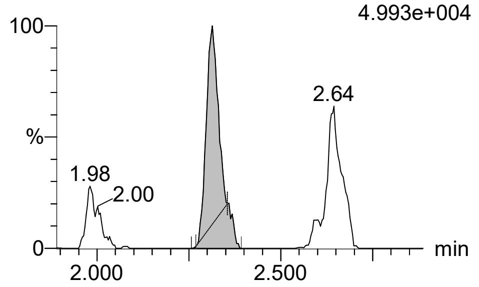

Sample Name: 20211111_L7_R2_IgG
F24:MRM of 1 channel,ES+
941.1 > 204.1
1.334e+005

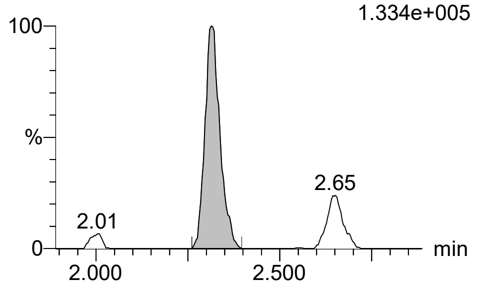

Sample Name: 20211111_L8_R2_IgG
F24:MRM of 1 channel,ES+
941.1 > 204.1
6.308e+004

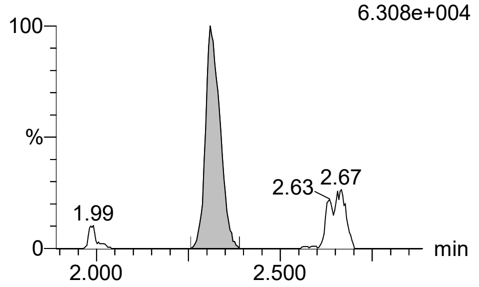

Sample Name: 20211111_H4_R2_IgG
F24:MRM of 1 channel,ES+
941.1 > 204.1
1.073e+005

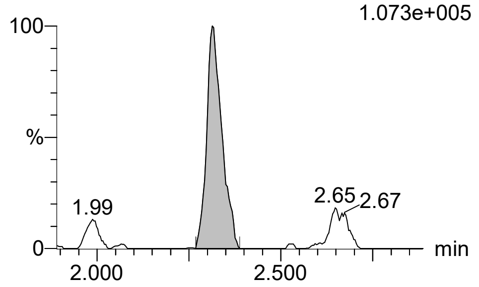

Sample Name: 20211111_A9_R2_IgG
F24:MRM of 1 channel,ES+
941.1 > 204.1
1.055e+005

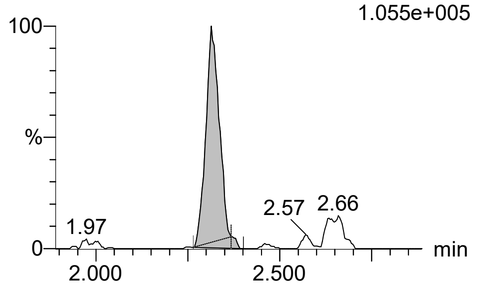

Sample Name: 20211111_A10_R2_IgG
F24:MRM of 1 channel,ES+
941.1 > 204.1
1.306e+005

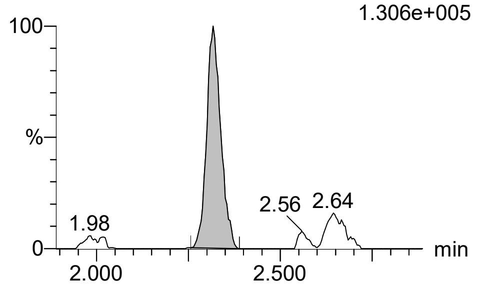

Sample Name: 20211111_L9_R2_IgG
F24:MRM of 1 channel,ES+
941.1 > 204.1
1.016e+005

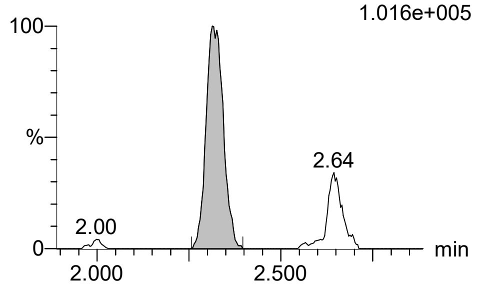

Sample Name: 20211111_L10_R2_IgG
F24:MRM of 1 channel,ES+
941.1 > 204.1
1.595e+005

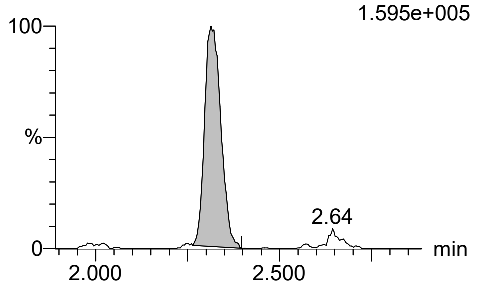

Sample Name: 20211111_QC_R11_IgG
F24:MRM of 1 channel,ES+
941.1 > 204.1
1.112e+005

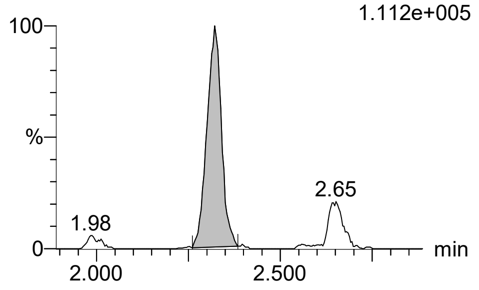

Sample Name: 20211111_H5_R2_IgG
F24:MRM of 1 channel,ES+
941.1 > 204.1
2.65 1.838e+004

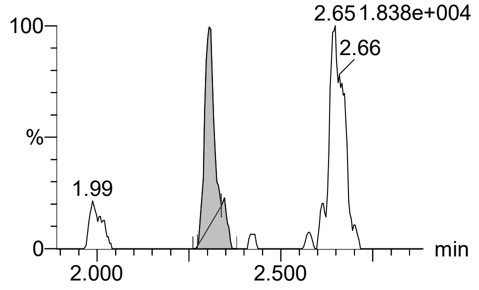

Sample Name: 20211111_A11_R2_IgG
F24:MRM of 1 channel,ES+
941.1 > 204.1
6.606e+004

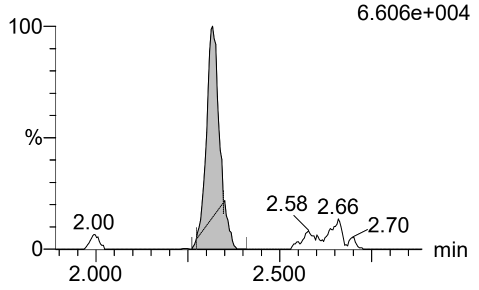

Sample Name: 20211111_A12_R2_IgG
F24:MRM of 1 channel,ES+
941.1 > 204.1
9.018e+004

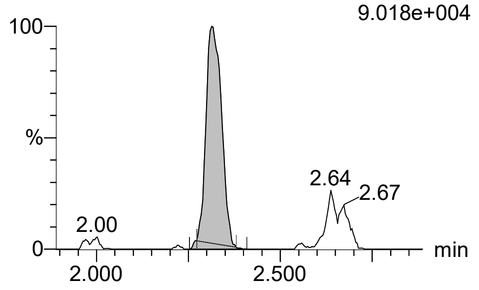

Sample Name: 20211111_L11_R2_IgG
F24:MRM of 1 channel,ES+
941.1 > 204.1
8.160e+004

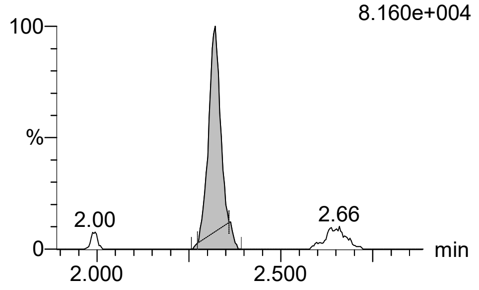

Sample Name: 20211111_L12_R2_IgG
F24:MRM of 1 channel,ES+
941.1 > 204.1
3.429e+004

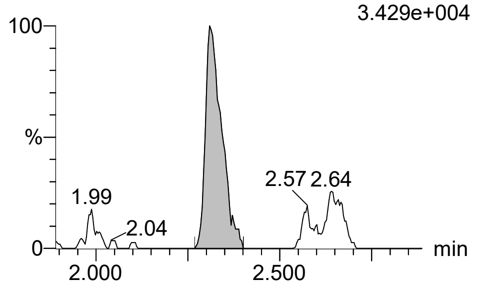

Sample Name: 20211111_H6_R2_IgG
F24:MRM of 1 channel,ES+
941.1 > 204.1
1.382e+005

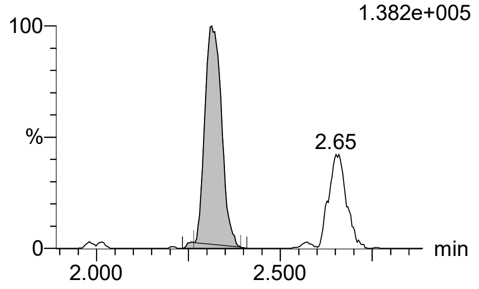

Sample Name: 20211111_A13_R2_IgG
F24:MRM of 1 channel,ES+
941.1 > 204.1
2.721e+005

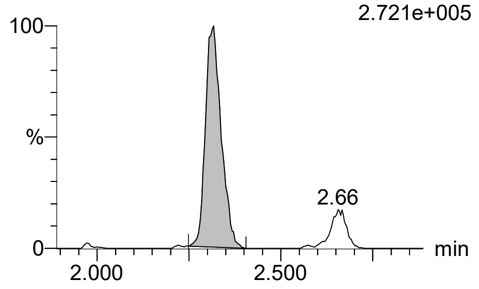

Sample Name: 20211111_A14_R2_IgG
F24:MRM of 1 channel,ES+
941.1 > 204.1
1.347e+005

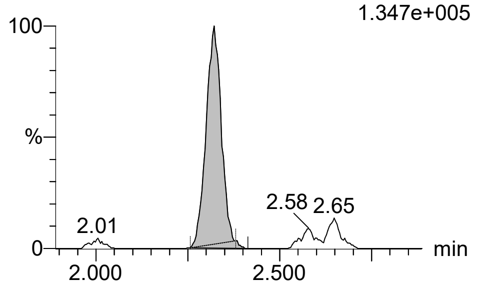

Sample Name: 20211111_L13_R2_IgG
F24:MRM of 1 channel,ES+
941.1 > 204.1
2.196e+004

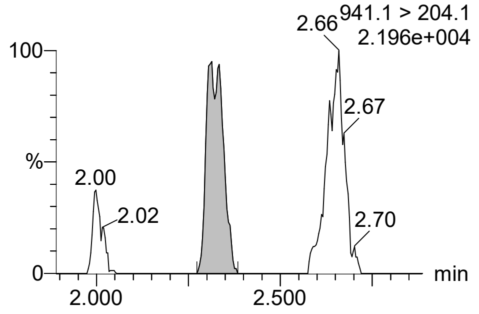

Sample Name: 20211111_L14_R2_IgG
F24:MRM of 1 channel,ES+
941.1 > 204.1
7.854e+004

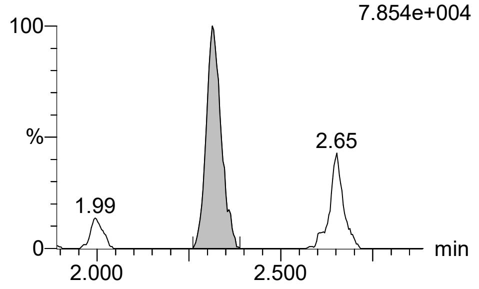

Sample Name: 20211111_H7_R2_IgG
F24:MRM of 1 channel,ES+
941.1 > 204.1
1.662e+005

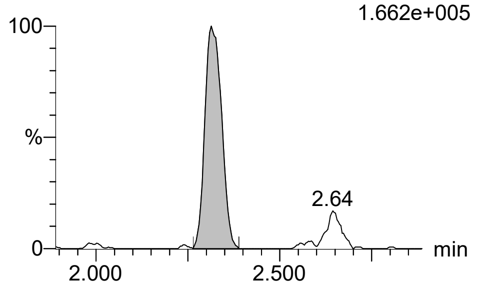

Sample Name: 20211111_A15_R2_IgG
F24:MRM of 1 channel,ES+
941.1 > 204.1
2.805e+004

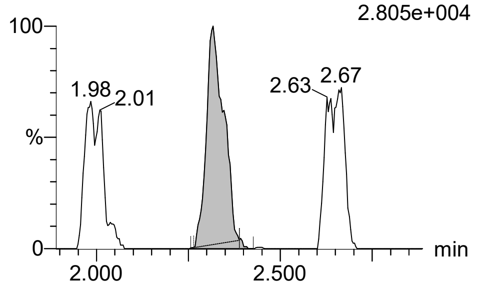

Sample Name: 20211111_QC_R12_IgG
F24:MRM of 1 channel,ES+
941.1 > 204.1
8.811e+004

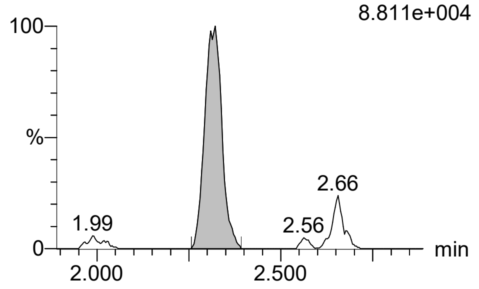

Sample Name: 20211111_A16_R2_IgG
F24:MRM of 1 channel,ES+
941.1 > 204.1
1.030e+005

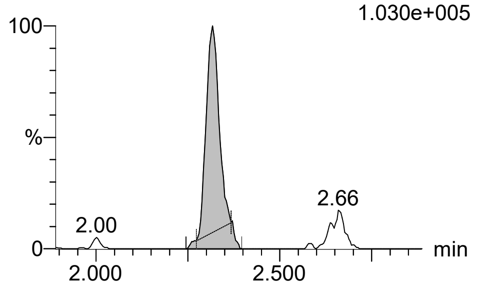

Sample Name: 20211111_L15_R2_IgG
F24:MRM of 1 channel,ES+
941.1 > 204.1
1.924e+005

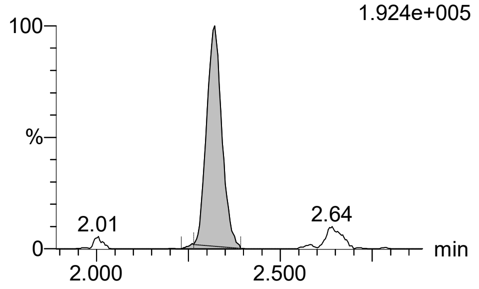

Sample Name: 20211111_L16_R2_IgG
F24:MRM of 1 channel,ES+
941.1 > 204.1
3.316e+004

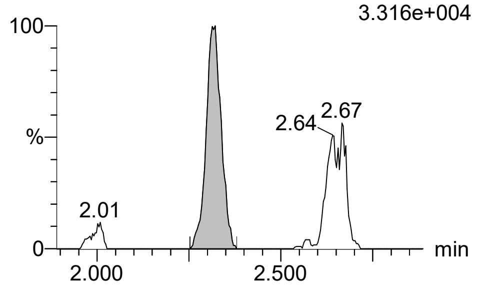

Dataset: C:\MassLynx\10.pro\20211115_clinicalsamples_IgG.qld

Last Altered: Sunday, October 02, 2022 16:02:30 台北標準時間
Printed: Friday, August 01, 2025 16:54:36 台北標準時間

Sample Name: 20211111_H8_R2_IgG
F24:MRM of 1 channel,ES+
941.1 > 204.1
7.980e+004

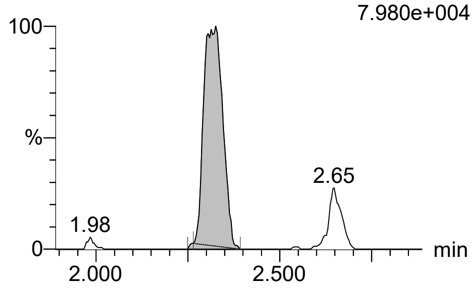

Sample Name: 20211111_A17_R2_IgG
F24:MRM of 1 channel,ES+
941.1 > 204.1
1.675e+005

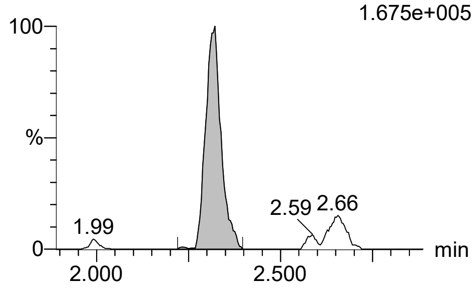

Sample Name: 20211111_A18_R2_IgG
F24:MRM of 1 channel,ES+
941.1 > 204.1
1.094e+005

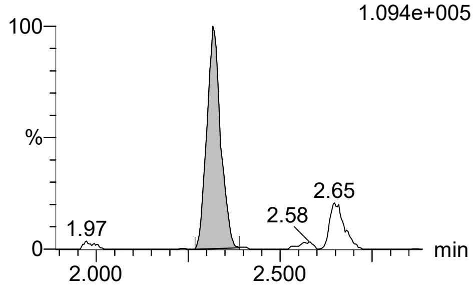

Sample Name: 20211111_L17_R2_IgG
F24:MRM of 1 channel,ES+
941.1 > 204.1
5.544e+004

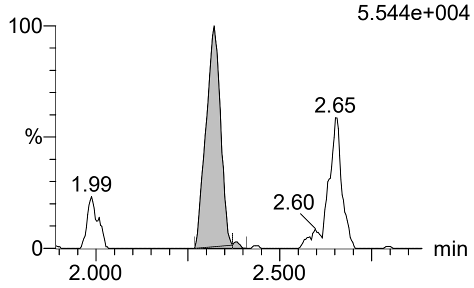

Sample Name: 20211111_L18_R2_IgG
F24:MRM of 1 channel,ES+
941.1 > 204.1
6.002e+004

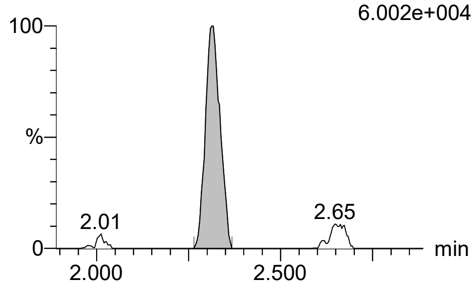

Sample Name: 20211111_H9_R2_IgG
F24:MRM of 1 channel,ES+
941.1 > 204.1
2.375e+005

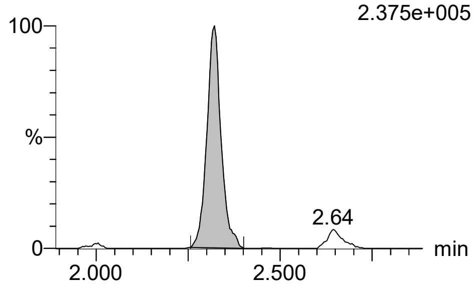

Sample Name: 20211111_A19_R2_IgG
F24:MRM of 1 channel,ES+
941.1 > 204.1
8.499e+004

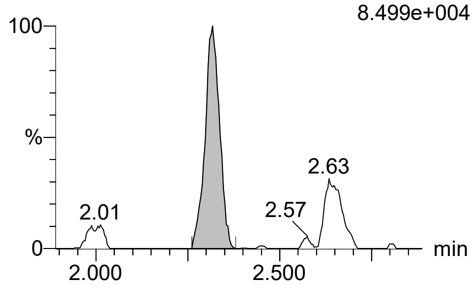

Sample Name: 20211111_A20_R2_IgG
F24:MRM of 1 channel,ES+
941.1 > 204.1
3.387e+004

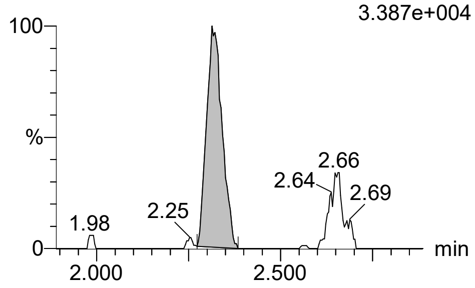

Sample Name: 20211111_L19_R2_IgG
F24:MRM of 1 channel,ES+
941.1 > 204.1
7.625e+004

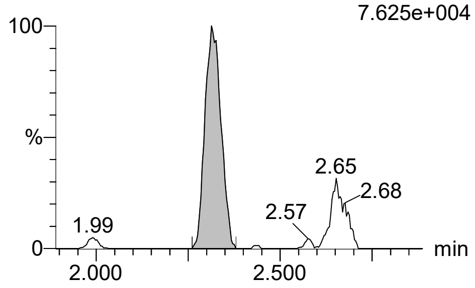

Sample Name: 20211111_QC_R13_IgG
F24:MRM of 1 channel,ES+
941.1 > 204.1
1.026e+005

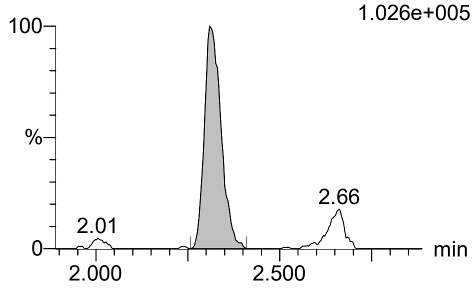

Sample Name: 20211111_L20_R2_IgG
F24:MRM of 1 channel,ES+
941.1 > 204.1
1.186e+005

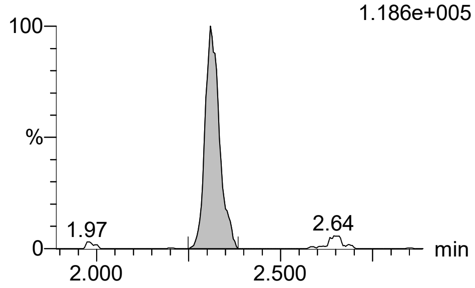

Sample Name: 20211111_H10_R2_IgG
F24:MRM of 1 channel,ES+
941.1 > 204.1
1.231e+005

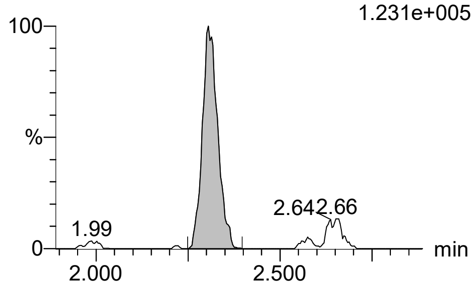

Sample Name: 20211111_A21_R2_IgG
F24:MRM of 1 channel,ES+
941.1 > 204.1
9.952e+004

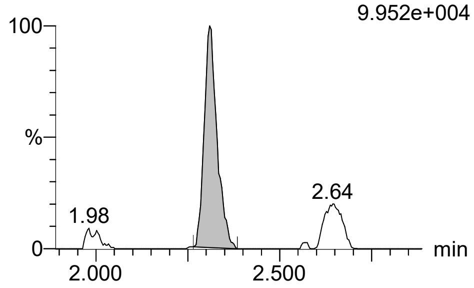

Sample Name: 20211111_A22_R2_IgG
F24:MRM of 1 channel,ES+
941.1 > 204.1
2.180e+004

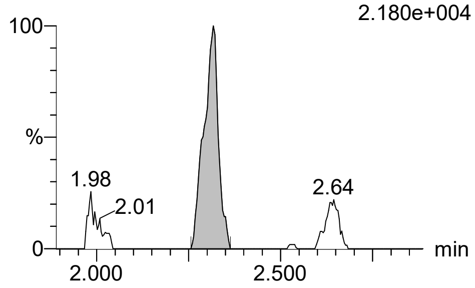

Sample Name: 20211111_L21_R2_IgG
F24:MRM of 1 channel,ES+
941.1 > 204.1
4.656e+004

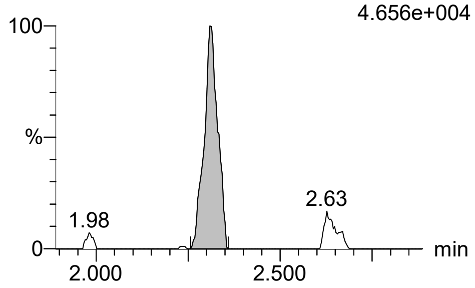

Sample Name: 20211111_L22_R2_IgG
F24:MRM of 1 channel,ES+
941.1 > 204.1
3.925e+004

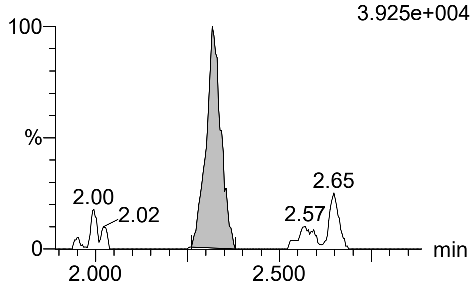

Sample Name: 20211111_H11_R2_IgG
F24:MRM of 1 channel,ES+
941.1 > 204.1
9.608e+004

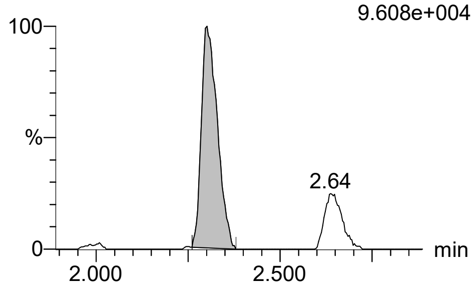

Sample Name: 20211111_A23_R2_IgG
F24:MRM of 1 channel,ES+
941.1 > 204.1
1.275e+005

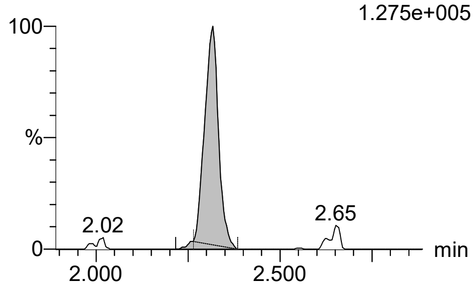

Sample Name: 20211111_A24_R2_IgG
F24:MRM of 1 channel,ES+
941.1 > 204.1
2.296e+004

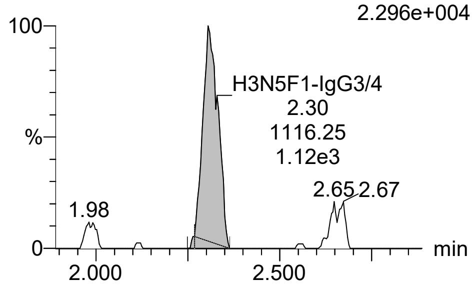

Sample Name: 20211111_L23_R2_IgG
F24:MRM of 1 channel,ES+
941.1 > 204.1
1.505e+005

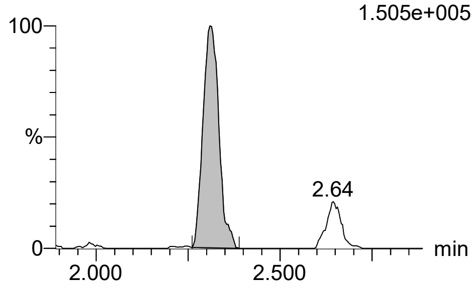

Sample Name: 20211111_L24_R2_IgG
F24:MRM of 1 channel,ES+
941.1 > 204.1
3.688e+004

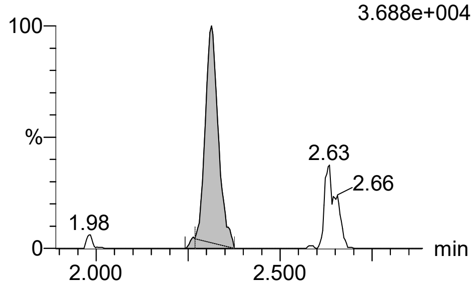

Sample Name: 20211111_H12_R2_IgG
F24:MRM of 1 channel,ES+
941.1 > 204.1
1.782e+004

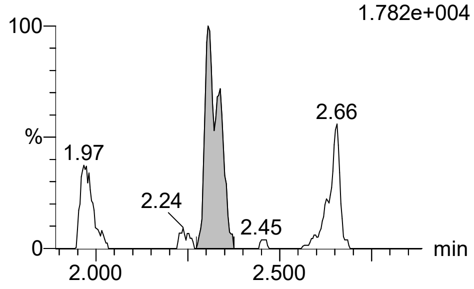

Sample Name: 20211111_QC_R14_IgG
F24:MRM of 1 channel,ES+
941.1 > 204.1
9.031e+004

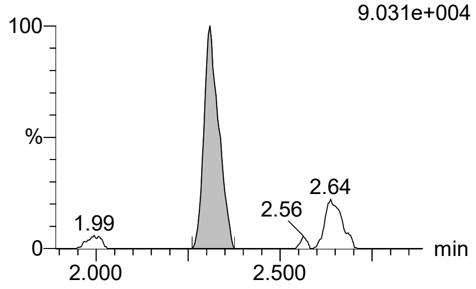

Sample Name: 20211111_A25_R2_IgG
F24:MRM of 1 channel,ES+
941.1 > 204.1
3.013e+005

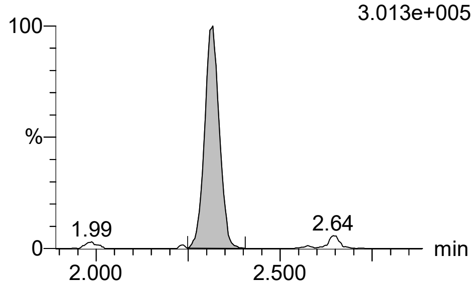

Sample Name: 20211111_A26_R2_IgG
F24:MRM of 1 channel,ES+
941.1 > 204.1
1.096e+004

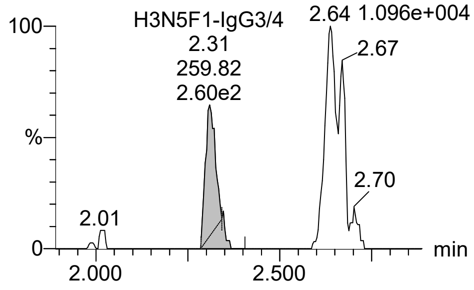

Sample Name: 20211111_L25_R2_IgG
F24:MRM of 1 channel,ES+
941.1 > 204.1
1.033e+005

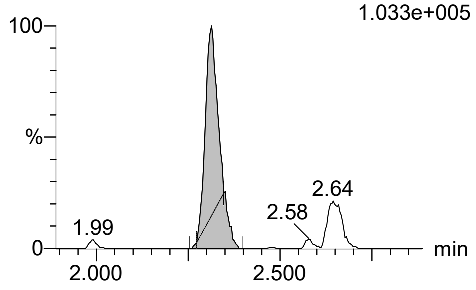

Sample Name: 20211111_L26_R2_IgG
F24:MRM of 1 channel,ES+
941.1 > 204.1
7.288e+004

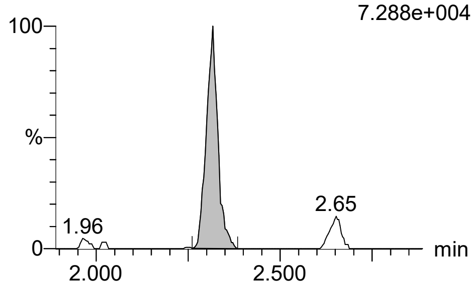

Sample Name: 20211111_H13_R2_IgG
F24:MRM of 1 channel,ES+
941.1 > 204.1
5.573e+004

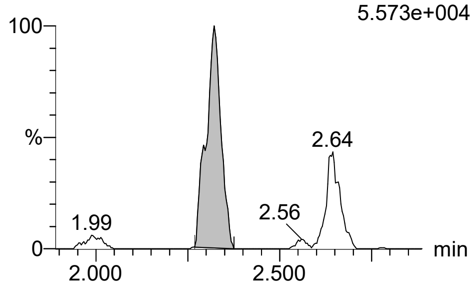

Sample Name: 20211111_A27_R2_IgG
F24:MRM of 1 channel,ES+
941.1 > 204.1
5.790e+004

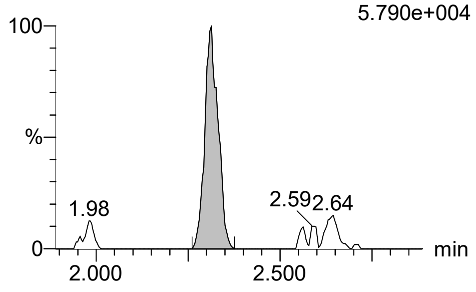

Sample Name: 20211111_A28_R2_IgG
F24:MRM of 1 channel,ES+
941.1 > 204.1
9.782e+004

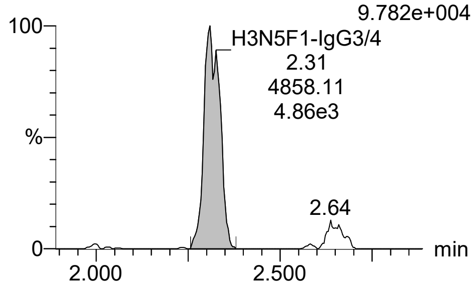

Sample Name: 20211111_L27_R2_IgG
F24:MRM of 1 channel,ES+
941.1 > 204.1
6.368e+004

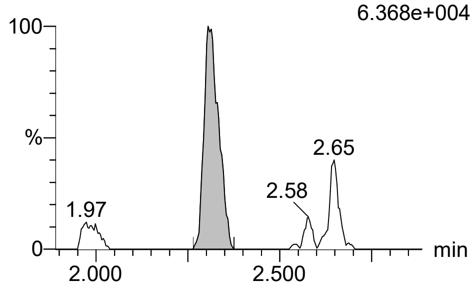

Sample Name: 20211111_L28_R2_IgG
F24:MRM of 1 channel,ES+
941.1 > 204.1
2.087e+004

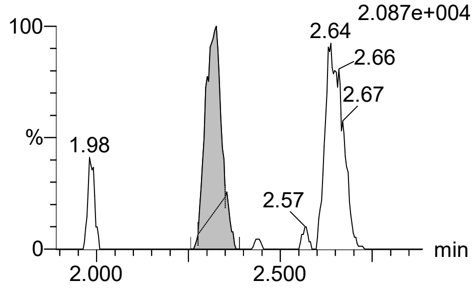

Sample Name: 20211111_H14_R2_IgG
F24:MRM of 1 channel,ES+
941.1 > 204.1
6.225e+004

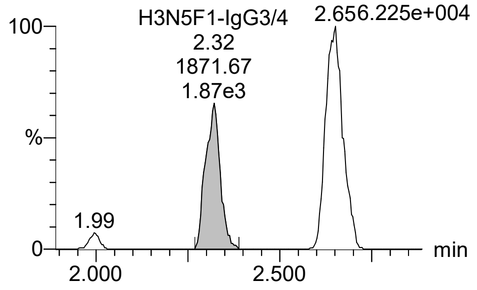

Sample Name: 20211111_A29_R2_IgG
F24:MRM of 1 channel,ES+
941.1 > 204.1
1.752e+005

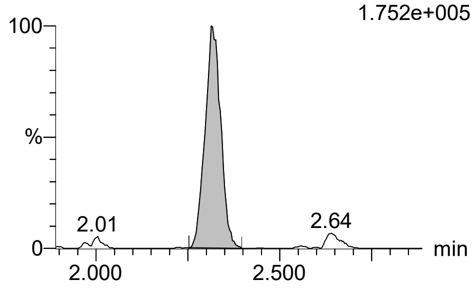

Sample Name: 20211111_A30_R2_IgG
F24:MRM of 1 channel,ES+
941.1 > 204.1
1.604e+005

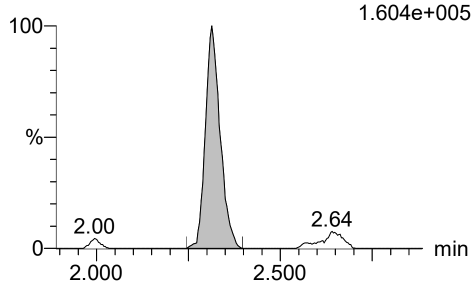

Sample Name: 20211111_QC_R15_IgG
F24:MRM of 1 channel,ES+
941.1 > 204.1
1.066e+005

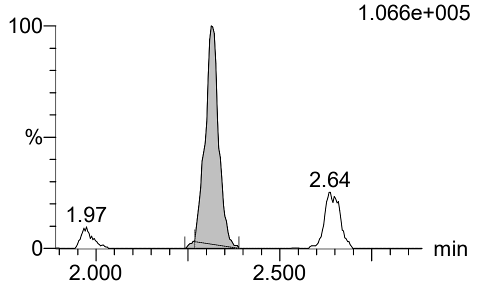

Sample Name: 20211111_L29_R2_IgG
F24:MRM of 1 channel,ES+
941.1 > 204.1
5.394e+004

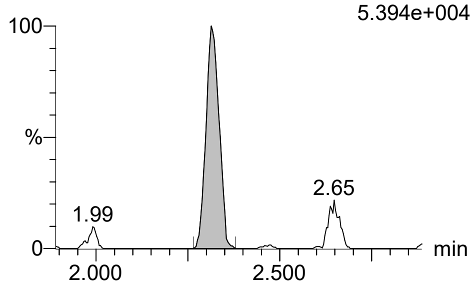

Sample Name: 20211111_L30_R2_IgG
F24:MRM of 1 channel,ES+
941.1 > 204.1
4.990e+004

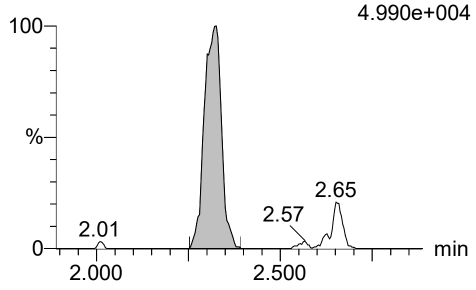

Sample Name: 20211111_H15_R2_IgG
F24:MRM of 1 channel,ES+
941.1 > 204.1
2.407e+004

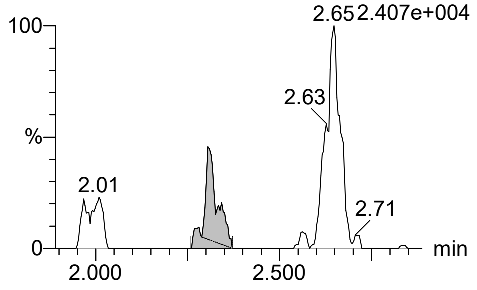

Sample Name: 20211111_A31_R2_IgG
F24:MRM of 1 channel,ES+
941.1 > 204.1
8.823e+004

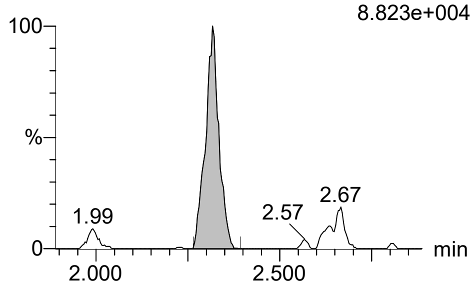

Sample Name: 20211111_A32_R2_IgG
F24:MRM of 1 channel,ES+
941.1 > 204.1
1.890e+005

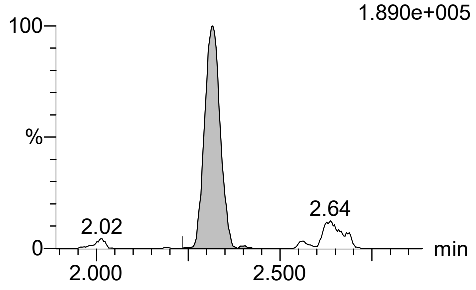

Sample Name: 20211111_L31_R2_IgG
F24:MRM of 1 channel,ES+
941.1 > 204.1
2.493e+004

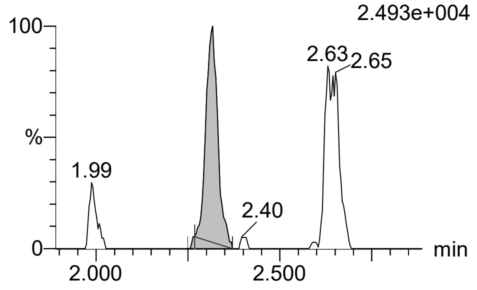

Sample Name: 20211111_L32_R2_IgG
F24:MRM of 1 channel,ES+
941.1 > 204.1
4.717e+004

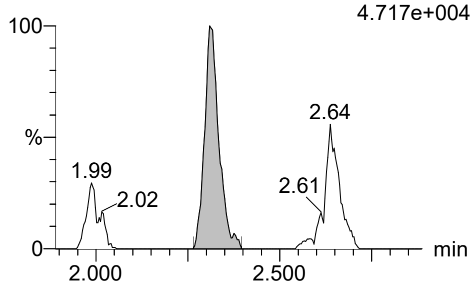

Sample Name: 20211111_H16_R2_IgG
F24:MRM of 1 channel,ES+
941.1 > 204.1
1.873e+005

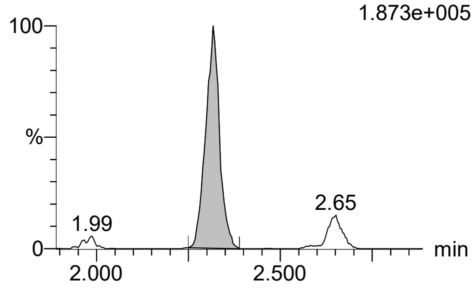

Sample Name: 20211111_A33_R2_IgG
F24:MRM of 1 channel,ES+
941.1 > 204.1
9.702e+004

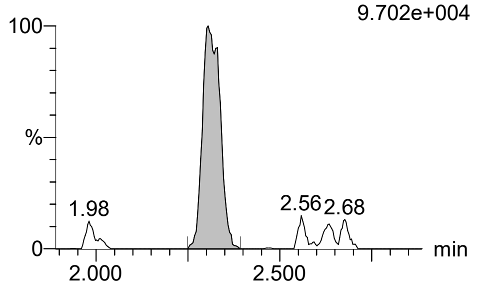

Dataset: C:\MassLynx\10.pro\20211115_clinicalsamples_IgG.qld

Last Altered: Sunday, October 02, 2022 16:02:30 台北標準時間
Printed: Friday, August 01, 2025 16:54:36 台北標準時間

Sample Name: 20211111_A34_R2_IgG
F24:MRM of 1 channel,ES+
941.1 > 204.1
1.279e+005

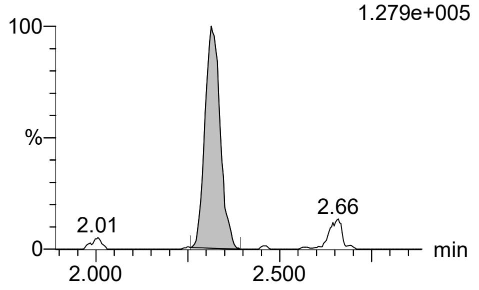

Sample Name: 20211111_H17_R2_IgG
F24:MRM of 1 channel,ES+
941.1 > 204.1
3.678e+005

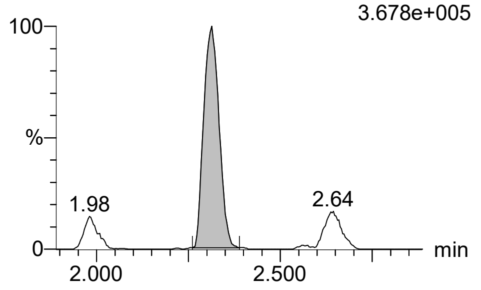

Sample Name: 20211111_A35_R2_IgG
F24:MRM of 1 channel,ES+
941.1 > 204.1
2.144e+005

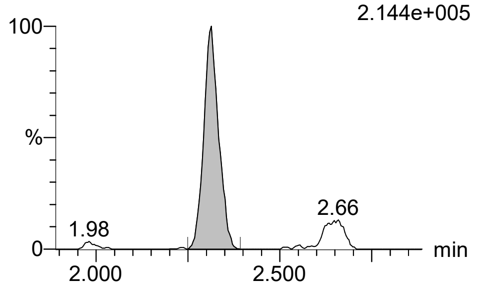

Sample Name: 20211111_QC_R16_IgG
F24:MRM of 1 channel,ES+
941.1 > 204.1
7.075e+004

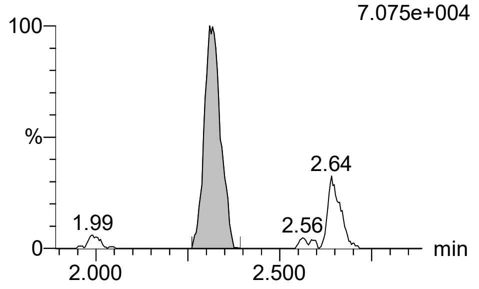

Sample Name: 20211111_A36_R2_IgG
F24:MRM of 1 channel,ES+
941.1 > 204.1
2.731e+005

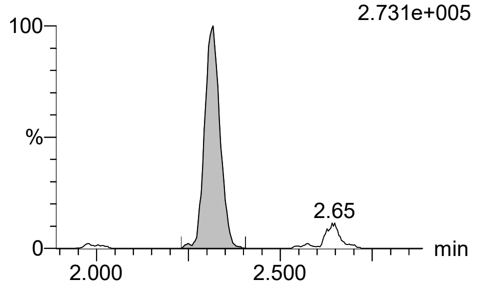

Sample Name: 20211111_H18_R2_IgG
F24:MRM of 1 channel,ES+
941.1 > 204.1
5.136e+005

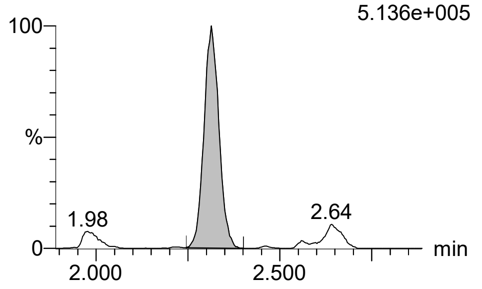

Sample Name: 20211111_A37_R2_IgG
F24:MRM of 1 channel,ES+
941.1 > 204.1
3.821e+004

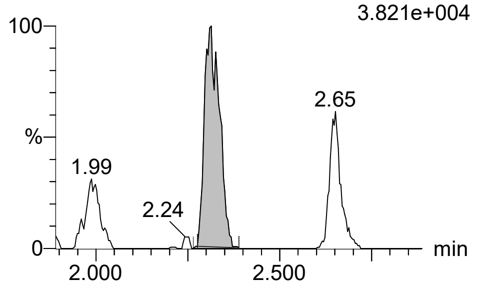

Sample Name: 20211111_A38_R2_IgG
F24:MRM of 1 channel,ES+
941.1 > 204.1
7.520e+004

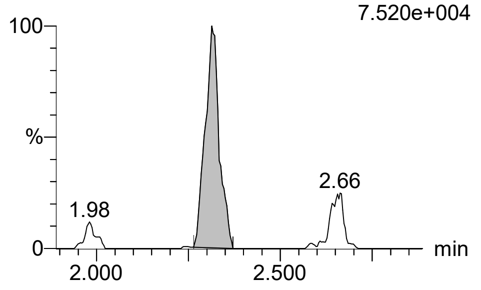

Sample Name: 20211111_H19_R2_IgG
F24:MRM of 1 channel,ES+
941.1 > 204.1
3.539e+005

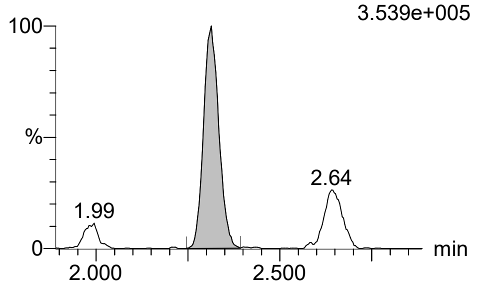

Sample Name: 20211111_A39_R2_IgG
F24:MRM of 1 channel,ES+
941.1 > 204.1
1.258e+005

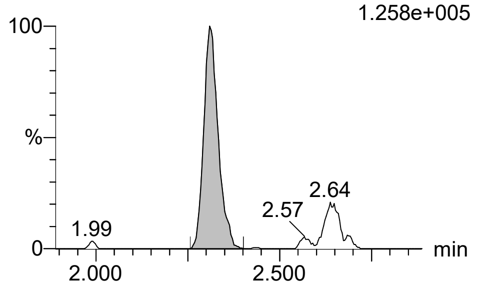

Sample Name: 20211111_A40_R2_IgG
F24:MRM of 1 channel,ES+
941.1 > 204.1
1.986e+005

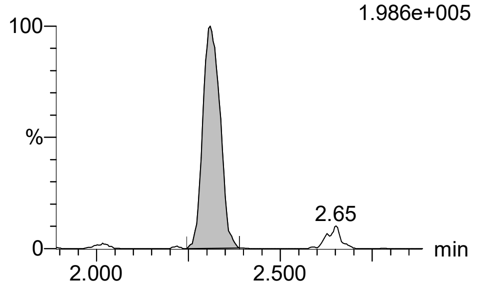

Sample Name: 20211111_H20_R2_IgG
F24:MRM of 1 channel,ES+
941.1 > 204.1
2.192e+005

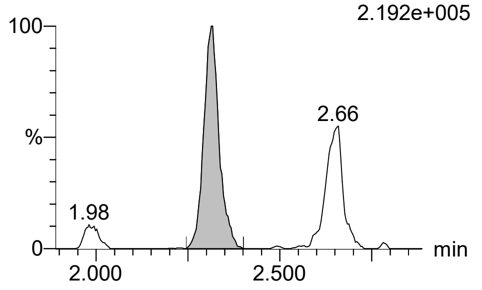

Sample Name: 20211111_A41_R2_IgG
F24:MRM of 1 channel,ES+
941.1 > 204.1
6.078e+004

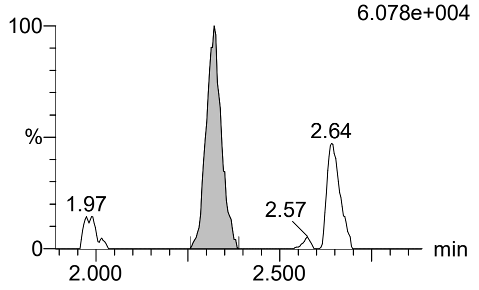

Sample Name: 20211111_A42_R2_IgG
F24:MRM of 1 channel,ES+
941.1 > 204.1
5.400e+004

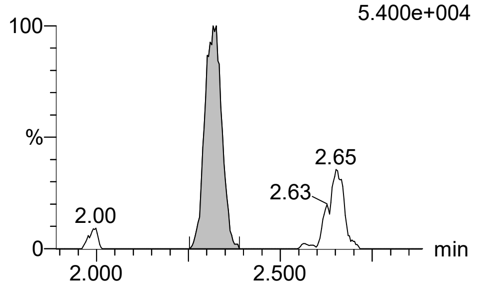

Sample Name: 20211111_A43_R2_IgG
F24:MRM of 1 channel,ES+
941.1 > 204.1
1.060e+005

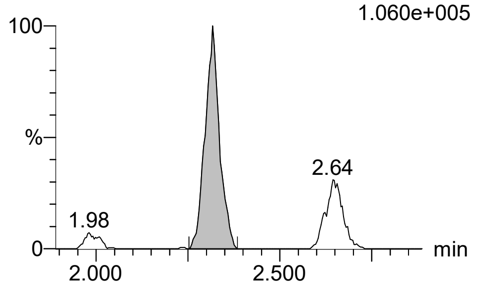

Dataset: C:\MassLynx\10.pro\20211115_clinicalsamples_IgG.qld

Last Altered: Sunday, October 02, 2022 16:02:30 台北標準時間
Printed: Friday, August 01, 2025 16:54:36 台北標準時間

Sample Name: 20211111_A44_R2_IgG

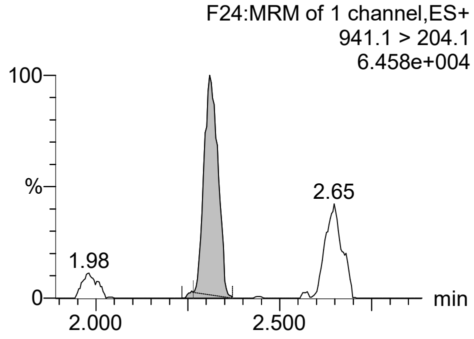

Sample Name: 20211111_QC_R17_IgG

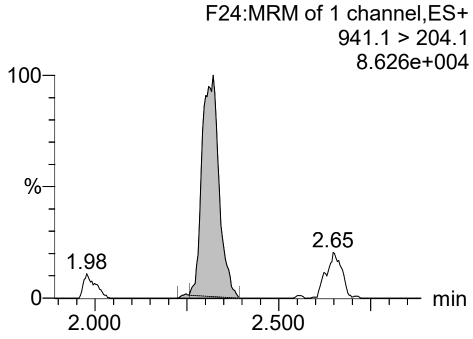

Dataset: C:\MassLynx\10.pro\20211115_clinicalsamples_IgG.qld

Last Altered: Sunday, October 02, 2022 16:02:30 台北標準時間
Printed: Friday, August 01, 2025 16:54:36 台北標準時間

	#	Name	RT	Area	IS Area	Response	Prima...
1	1	20211111_QC_R1_IgG	2.32	10529.4	64968.0	0.162	MM
2	2	20211111_A1_R1_IgG	2.32	11857.0	59059.2	0.201	bb
3	3	20211111_A2_R1_IgG	2.31	5150.1	66621.2	0.077	bb
4	4	20211111_L1_R1_IgG	2.32	12328.9	64201.0	0.192	bb
5	5	20211111_L2_R1_IgG	2.32	4141.1	46950.2	0.088	bb
6	6	20211111_H1_R1_IgG	2.32	5090.1	38648.2	0.132	MM
7	7	20211111_A3_R1_IgG	2.32	479.0	2603.7	0.184	MM
8	8	20211111_A4_R1_IgG	2.32	1473.3	69960.4	0.021	MM
9	9	20211111_L3_R1_IgG	2.32	5133.7	38864.7	0.132	bb
10	10	20211111_L4_R1_IgG	2.32	8188.1	43380.5	0.189	bb
11	11	20211111_H2_R1_IgG	2.32	13274.2	71349.2	0.186	bb
12	12	20211111_A5_R1_IgG	2.32	5743.4	65569.7	0.088	MM
13	13	20211111_A6_R1_IgG	2.32	16021.5	53120.7	0.302	bb
14	14	20211111_QC_R2_IgG	2.32	11270.1	81034.1	0.139	bb
15	15	20211111_L5_R1_IgG	2.32	7043.4	50994.6	0.138	MM
16	16	20211111_L6_R1_IgG	2.31	2327.2	58058.0	0.040	MM
17	17	20211111_H3_R1_IgG	2.33	1176.0	64028.6	0.018	MM
18	18	20211111_A7_R1_IgG	2.32	6897.8	82996.1	0.083	bb
19	19	20211111_A8_R1_IgG	2.32	3808.5	77731.7	0.049	MM
20	20	20211111_L7_R1_IgG	2.32	5867.7	38827.3	0.151	bb
21	21	20211111_L8_R1_IgG	2.32	3075.2	44220.1	0.070	bb
22	22	20211111_H4_R1_IgG	2.32	7838.7	65826.3	0.119	bb
23	23	20211111_A9_R1_IgG	2.32	6146.4	60771.1	0.101	bb
24	24	20211111_A10_R1_IgG	2.32	6313.2	48578.7	0.130	MM
25	25	20211111_L9_R1_IgG	2.32	6753.4	50830.7	0.133	bb
26	26	20211111_L10_R1_IgG	2.32	9648.4	38034.2	0.254	MM
27	27	20211111_QC_R3_IgG	2.32	9477.8	67025.5	0.141	bb
28	28	20211111_H5_R1_IgG	2.33	750.5	44544.9	0.017	MM
29	29	20211111_A11_R1_IgG	2.31	3388.9	66408.5	0.051	bb
30	30	20211111_A12_R1_IgG	2.32	5669.8	46624.5	0.122	bb
31	31	20211111_L11_R1_IgG	2.31	4003.0	33425.1	0.120	bb
32	32	20211111_L12_R1_IgG	2.33	1789.3	41178.0	0.043	bb
33	33	20211111_H6_R1_IgG	2.32	11165.0	58593.4	0.191	bb
34	34	20211111_A13_R1_IgG	2.32	17448.6	54133.0	0.322	bb
35	35	20211111_A14_R1_IgG	2.33	9040.2	56467.3	0.160	bb
36	36	20211111_L13_R1_IgG	2.32	1415.5	36024.4	0.039	MM
37	37	20211111_L14_R1_IgG	2.33	3730.9	37181.6	0.100	bb
38	38	20211111_H7_R1_IgG	2.31	10961.0	42850.6	0.256	MM
39	39	20211111_A15_R1_IgG	2.32	1519.1	62015.1	0.024	bb
40	40	20211111_QC_R4_IgG	2.32	7332.3	58572.5	0.125	bb
41	41	20211111_A16_R1_IgG	2.32	8479.8	78283.8	0.108	bb
42	42	20211111_L15_R1_IgG	2.32	13062.5	44158.2	0.296	bb
43	43	20211111_L16_R1_IgG	2.31	3247.6	55150.8	0.059	bb
44	44	20211111_H8_R1_IgG	2.31	5452.8	42965.2	0.127	bb
45	45	20211111_A17_R1_IgG	2.32	8651.1	39717.7	0.218	bb
46	46	20211111_A18_R1_IgG	2.31	5098.3	44675.4	0.114	MM
47	47	20211111_L17_R1_IgG	2.32	3222.8	37854.1	0.085	MM
48	48	20211111_L18_R1_IgG	2.32	3626.1	50716.9	0.071	MM
49	49	20211111_H9_R1_IgG	2.31	12723.6	36276.0	0.351	bb
50	50	20211111_A19_R1_IgG	2.32	3834.2	42862.5	0.089	bb
51	51	20211111_A20_R1_IgG	2.31	2483.7	36542.7	0.068	bb
52	52	20211111_L19_R1_IgG	2.32	4580.7	53892.8	0.085	bb
53	53	20211111_QC_R5_IgG	2.32	7450.7	56513.2	0.132	MM
54	54	20211111_L20_R1_IgG	2.32	12941.2	56499.9	0.229	MM
55	55	20211111_H10_R1_IgG	2.31	14785.9	68120.5	0.217	bb
56	56	20211111_A21_R1_IgG	2.32	11893.5	63699.8	0.187	bb

Dataset: C:\MassLynx\10.pro\20211115_clinicalsamples_IgG.qld

Last Altered: Sunday, October 02, 2022 16:02:30 台北標準時間
Printed: Friday, August 01, 2025 16:54:36 台北標準時間

	# Name	RT	Area	IS Area	Response	Prima...
57	57 20211111_A22_R1_IgG	2.32	3503.0	52222.1	0.067	MM
58	58 20211111_L21_R1_IgG	2.31	4756.9	54372.5	0.087	bb
59	59 20211111_L22_R1_IgG	2.33	3652.5	41353.1	0.088	MM
60	60 20211111_H11_R1_IgG	2.32	10662.9	65579.1	0.163	bb
61	61 20211111_A23_R1_IgG	2.32	16380.7	64131.1	0.255	bb
62	62 20211111_A24_R1_IgG	2.31	2176.9	38724.8	0.056	MM
63	63 20211111_L23_R1_IgG	2.31	13459.7	64245.2	0.210	bb
64	64 20211111_L24_R1_IgG	2.32	2404.6	45298.0	0.053	bb
65	65 20211111_H12_R1_IgG	2.33	693.9	44679.3	0.016	bb
66	66 20211111_QC_R6_IgG	2.32	6182.2	48165.0	0.128	bb
67	67 20211111_A25_R1_IgG	2.31	22033.5	57169.9	0.385	bb
68	68 20211111_A26_R1_IgG	2.31	674.7	34254.0	0.020	MM
69	69 20211111_L25_R1_IgG	2.32	8880.5	67491.5	0.132	bb
70	70 20211111_L26_R1_IgG	2.31	4593.3	55934.1	0.082	bb
71	71 20211111_H13_R1_IgG	2.32	4055.6	48767.3	0.083	bb
72	72 20211111_A27_R1_IgG	2.32	4236.2	57269.7	0.074	MM
73	73 20211111_A28_R1_IgG	2.32	7393.1	49231.1	0.150	MM
74	74 20211111_L27_R1_IgG	2.32	4294.3	60014.5	0.072	bb
75	75 20211111_L28_R1_IgG	2.31	1723.5	35062.6	0.049	MM
76	76 20211111_H14_R1_IgG	2.32	2696.5	62371.9	0.043	bb
77	77 20211111_A29_R1_IgG	2.32	9794.4	43858.2	0.223	bb
78	78 20211111_A30_R1_IgG	2.32	12672.6	62500.4	0.203	bb
79	79 20211111_QC_R7_IgG	2.32	5765.4	43946.9	0.131	MM
80	80 20211111_L29_R1_IgG	2.32	2893.0	38728.9	0.075	bb
81	81 20211111_L30_R1_IgG	2.32	3805.5	42613.2	0.089	bb
82	82 20211111_H15_R1_IgG	2.31	548.2	39874.6	0.014	bb
83	83 20211111_A31_R1_IgG	2.32	5068.0	53414.3	0.095	bb
84	84 20211111_A32_R1_IgG	2.31	11866.1	41844.6	0.284	bb
85	85 20211111_L31_R1_IgG	2.32	1422.1	40553.7	0.035	bb
86	86 20211111_L32_R1_IgG	2.32	2642.6	49618.7	0.053	MM
87	87 20211111_H16_R1_IgG	2.32	11586.8	60170.9	0.193	bb
88	88 20211111_A33_R1_IgG	2.31	6330.7	48588.2	0.130	bb
89	89 20211111_A34_R1_IgG	2.32	8037.3	43288.0	0.186	bb
90	90 20211111_H17_R1_IgG	2.33	21642.7	47104.5	0.459	bb
91	91 20211111_A35_R1_IgG	2.32	11698.0	41025.9	0.285	MM
92	92 20211111_QC_R8_IgG	2.32	5810.1	40546.3	0.143	MM
93	93 20211111_A36_R1_IgG	2.32	15083.2	44504.4	0.339	MM
94	94 20211111_H18_R1_IgG	2.31	26112.1	34162.2	0.764	bb
95	95 20211111_A37_R1_IgG	2.32	1967.6	39760.0	0.049	bb
96	96 20211111_A38_R1_IgG	2.33	3611.8	43982.6	0.082	bb
97	97 20211111_H19_R1_IgG	2.32	18748.0	45314.8	0.414	bb
98	98 20211111_A39_R1_IgG	2.33	5647.2	39388.0	0.143	bb
99	99 20211111_A40_R1_IgG	2.32	13577.8	38726.4	0.351	bb
100	1... 20211111_H20_R1_IgG	2.32	11099.5	37814.4	0.294	bb
101	1... 20211111_A41_R1_IgG	2.32	3190.7	48930.3	0.065	MM
102	1... 20211111_A42_R1_IgG	2.32	3525.8	40406.8	0.087	MM
103	1... 20211111_A43_R1_IgG	2.31	4947.9	44421.5	0.111	MM
104	1... 20211111_A44_R1_IgG	2.32	4359.5	53128.2	0.082	bb
105	1... 20211111_QC_R9_IgG	2.32	5073.0	37049.0	0.137	bb
106	1... 20211111_A1_R2_IgG	2.32	8267.6	46499.4	0.178	MM
107	1... 20211111_A2_R2_IgG	2.32	3801.8	57260.7	0.066	MM
108	1... 20211111_L1_R2_IgG	2.32	8272.3	48062.9	0.172	bb
109	1... 20211111_L2_R2_IgG	2.31	4893.1	38745.2	0.126	MM
110	1... 20211111_H1_R2_IgG	2.32	6520.8	44273.5	0.147	bb
111	1... 20211111_A3_R2_IgG	2.33	808.1	4755.5	0.170	bb
112	1... 20211111_A4_R2_IgG	2.33	1242.8	50546.7	0.025	bb

Dataset: C:\MassLynx\10.pro\20211115_clinicalsamples_IgG.qld

Last Altered: Sunday, October 02, 2022 16:02:30 台北標準時間
Printed: Friday, August 01, 2025 16:54:36 台北標準時間

	#	Name	RT	Area	IS Area	Response	Prima...
113	1...	20211111_L3_R2_IgG	2.32	5002.6	34825.8	0.144	bb
114	1...	20211111_L4_R2_IgG	2.32	7323.4	39107.7	0.187	MM
115	1...	20211111_H2_R2_IgG	2.32	8689.4	49750.0	0.175	bb
116	1...	20211111_A5_R2_IgG	2.31	5020.0	52536.2	0.096	bb
117	1...	20211111_A6_R2_IgG	2.32	13084.7	42050.0	0.311	bb
118	1...	20211111_QC_R10_IgG	2.32	4739.9	35350.8	0.134	bb
119	1...	20211111_L5_R2_IgG	2.32	6097.4	38039.3	0.160	bb
120	1...	20211111_L6_R2_IgG	2.32	1722.7	44907.4	0.038	bb
121	1...	20211111_H3_R2_IgG	2.32	927.6	47729.1	0.019	bb
122	1...	20211111_A7_R2_IgG	2.31	5024.0	57347.1	0.088	bb
123	1...	20211111_A8_R2_IgG	2.31	2234.7	52397.8	0.043	MM
124	1...	20211111_L7_R2_IgG	2.31	6326.4	36318.4	0.174	bb
125	1...	20211111_L8_R2_IgG	2.31	2871.7	38131.9	0.075	bb
126	1...	20211111_H4_R2_IgG	2.31	5127.6	46849.6	0.109	bb
127	1...	20211111_A9_R2_IgG	2.31	4598.3	47759.6	0.096	MM
128	1...	20211111_A10_R2_IgG	2.32	5761.4	41366.2	0.139	bb
129	1...	20211111_L9_R2_IgG	2.31	5539.8	43052.1	0.129	bb
130	1...	20211111_L10_R2_IgG	2.31	8100.8	31487.4	0.257	bb
131	1...	20211111_QC_R11_IgG	2.32	4996.5	37804.6	0.132	bb
132	1...	20211111_H5_R2_IgG	2.30	657.2	40380.0	0.016	MM
133	1...	20211111_A11_R2_IgG	2.32	2784.5	49533.3	0.056	MM
134	1...	20211111_A12_R2_IgG	2.31	4538.9	40226.3	0.113	MM
135	1...	20211111_L11_R2_IgG	2.32	3340.1	28884.0	0.116	MM
136	1...	20211111_L12_R2_IgG	2.31	1750.0	34351.3	0.051	bb
137	1...	20211111_H6_R2_IgG	2.31	7470.1	40466.6	0.185	MM
138	1...	20211111_A13_R2_IgG	2.32	13408.3	42306.7	0.317	bb
139	1...	20211111_A14_R2_IgG	2.32	6521.5	44904.0	0.145	MM
140	1...	20211111_L13_R2_IgG	2.31	1137.2	31913.0	0.036	bb
141	1...	20211111_L14_R2_IgG	2.31	3702.9	31527.4	0.117	bb
142	1...	20211111_H7_R2_IgG	2.31	8771.8	40838.2	0.215	bb
143	1...	20211111_A15_R2_IgG	2.32	1582.7	49389.7	0.032	MM
144	1...	20211111_QC_R12_IgG	2.32	4762.2	35168.7	0.135	bb
145	1...	20211111_A16_R2_IgG	2.32	4775.1	47440.5	0.101	MM
146	1...	20211111_L15_R2_IgG	2.32	9447.7	33145.9	0.285	MM
147	1...	20211111_L16_R2_IgG	2.32	1572.8	37596.6	0.042	bb
148	1...	20211111_H8_R2_IgG	2.33	4884.6	43933.3	0.111	MM
149	1...	20211111_A17_R2_IgG	2.32	8122.4	43605.5	0.186	bb
150	1...	20211111_A18_R2_IgG	2.32	4700.5	37581.8	0.125	bb
151	1...	20211111_L17_R2_IgG	2.32	2569.7	33942.5	0.076	MM
152	1...	20211111_L18_R2_IgG	2.31	2775.4	37831.5	0.073	bb
153	1...	20211111_H9_R2_IgG	2.32	10548.5	30898.4	0.341	bb
154	1...	20211111_A19_R2_IgG	2.32	3863.1	39352.1	0.098	bb
155	1...	20211111_A20_R2_IgG	2.31	1644.9	34661.2	0.047	bb
156	1...	20211111_L19_R2_IgG	2.31	3846.4	39918.3	0.096	bb
157	1...	20211111_QC_R13_IgG	2.31	5395.1	36239.1	0.149	bb
158	1...	20211111_L20_R2_IgG	2.31	5360.3	32897.7	0.163	bb
159	1...	20211111_H10_R2_IgG	2.31	5924.8	31394.5	0.189	bb
160	1...	20211111_A21_R2_IgG	2.31	3985.8	34863.7	0.114	bb
161	1...	20211111_A22_R2_IgG	2.32	983.0	30342.1	0.032	bb
162	1...	20211111_L21_R2_IgG	2.31	1987.5	33706.2	0.059	bb
163	1...	20211111_L22_R2_IgG	2.32	1807.7	31298.6	0.058	bb
164	1...	20211111_H11_R2_IgG	2.30	4801.3	38904.1	0.123	bb
165	1...	20211111_A23_R2_IgG	2.32	5772.1	32382.3	0.178	MM
166	1...	20211111_A24_R2_IgG	2.30	1116.3	30261.9	0.037	MM
167	1...	20211111_L23_R2_IgG	2.31	7486.9	43037.5	0.174	bb
168	1...	20211111_L24_R2_IgG	2.31	1578.6	37023.3	0.043	MM

Dataset: C:\MassLynx\10.pro\20211115_clinicalsamples_IgG.qld

Last Altered: Sunday, October 02, 2022 16:02:30 台北標準時間
Printed: Friday, August 01, 2025 16:54:36 台北標準時間

	# Name	RT	Area	IS Area	Response	Prima...
169	1... 20211111_H12_R2_IgG	2.31	802.6	39478.2	0.020	bb
170	1... 20211111_QC_R14_IgG	2.31	4190.9	34463.2	0.122	bb
171	1... 20211111_A25_R2_IgG	2.32	13917.5	39945.3	0.348	bb
172	1... 20211111_A26_R2_IgG	2.31	259.8	29663.2	0.009	MM
173	1... 20211111_L25_R2_IgG	2.31	4621.8	38589.3	0.120	MM
174	1... 20211111_L26_R2_IgG	2.32	2609.0	38405.2	0.068	bb
175	1... 20211111_H13_R2_IgG	2.32	2594.1	37771.2	0.069	bb
176	1... 20211111_A27_R2_IgG	2.31	2400.1	42634.1	0.056	bb
177	1... 20211111_A28_R2_IgG	2.31	4858.1	33231.1	0.146	bb
178	1... 20211111_L27_R2_IgG	2.30	2952.5	41761.1	0.071	bb
179	1... 20211111_L28_R2_IgG	2.33	1082.1	26968.6	0.040	MM
180	1... 20211111_H14_R2_IgG	2.32	1871.7	49250.2	0.038	bb
181	1... 20211111_A29_R2_IgG	2.31	8616.9	37601.7	0.229	bb
182	1... 20211111_A30_R2_IgG	2.31	7418.5	40366.8	0.184	bb
183	1... 20211111_QC_R15_IgG	2.31	4661.9	35156.1	0.133	MM
184	1... 20211111_L29_R2_IgG	2.31	2219.1	33813.1	0.066	bb
185	1... 20211111_L30_R2_IgG	2.33	2694.4	33488.6	0.080	bb
186	1... 20211111_H15_R2_IgG	2.31	456.0	38501.0	0.012	MM
187	1... 20211111_A31_R2_IgG	2.32	3689.2	37525.5	0.098	bb
188	1... 20211111_A32_R2_IgG	2.32	9320.0	35770.8	0.261	bb
189	1... 20211111_L31_R2_IgG	2.32	981.2	33282.4	0.029	MM
190	1... 20211111_L32_R2_IgG	2.31	2122.2	42049.6	0.050	bb
191	1... 20211111_H16_R2_IgG	2.32	8108.6	45947.6	0.176	bb
192	1... 20211111_A33_R2_IgG	2.30	5415.9	40202.3	0.135	bb
193	1... 20211111_A34_R2_IgG	2.31	6128.4	36747.0	0.167	bb
194	1... 20211111_H17_R2_IgG	2.31	17663.8	40920.9	0.432	bb
195	1... 20211111_A35_R2_IgG	2.31	9990.4	33689.2	0.297	bb
196	1... 20211111_QC_R16_IgG	2.31	3638.6	32783.6	0.111	bb
197	1... 20211111_A36_R2_IgG	2.32	13232.4	36822.4	0.359	bb
198	1... 20211111_H18_R2_IgG	2.31	24068.1	31769.3	0.758	bb
199	1... 20211111_A37_R2_IgG	2.31	1820.5	37581.8	0.048	MM
200	2... 20211111_A38_R2_IgG	2.31	3305.8	38390.4	0.086	bb
201	2... 20211111_H19_R2_IgG	2.31	17021.3	41217.0	0.413	bb
202	2... 20211111_A39_R2_IgG	2.31	5688.2	37392.7	0.152	bb
203	2... 20211111_A40_R2_IgG	2.31	10818.1	38295.1	0.282	bb
204	2... 20211111_H20_R2_IgG	2.32	10579.5	35941.8	0.294	bb
205	2... 20211111_A41_R2_IgG	2.32	2884.0	45304.9	0.064	bb
206	2... 20211111_A42_R2_IgG	2.33	2920.0	37803.8	0.077	bb
207	2... 20211111_A43_R2_IgG	2.32	4744.6	38161.8	0.124	bb
208	2... 20211111_A44_R2_IgG	2.31	2907.8	37819.8	0.077	MM
209	2... 20211111_QC_R17_IgG	2.32	4670.0	31731.8	0.147	MM

Dataset: C:\MassLynx\10.pro\20211115_clinicalsamples_IgG.qld

Last Altered: Sunday, October 02, 2022 16:02:30 台北標準時間
Printed: Friday, August 01, 2025 16:55:04 台北標準時間

Method: C:\MassLynx\10_Pro_IgA.PRO\MethDB\20210926_IgG_DMRM_IgM_IS.mdb 16 Feb 2022 11:58:21
Calibration: 18 Nov 2021 13:56:22

Sample Name: 20211111_QC_R1_IgG

F18:MRM of 1 channel,ES+
927.44 > 204.1
5.870e+005

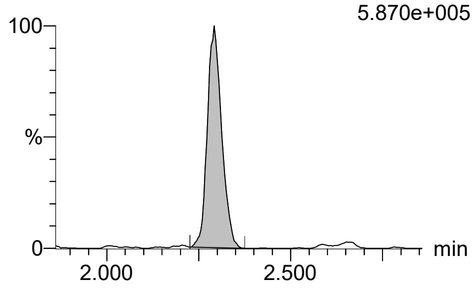

Sample Name: 20211111_A1_R1_IgG

F18:MRM of 1 channel,ES+
927.44 > 204.1
4.946e+005

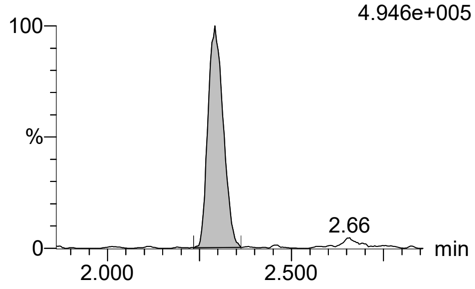

Sample Name: 20211111_A2_R1_IgG

F18:MRM of 1 channel,ES+
927.44 > 204.1
2.157e+005

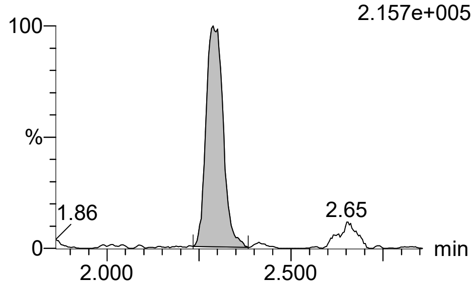

Sample Name: 20211111_L1_R1_IgG

F18:MRM of 1 channel,ES+
927.44 > 204.1
3.852e+005

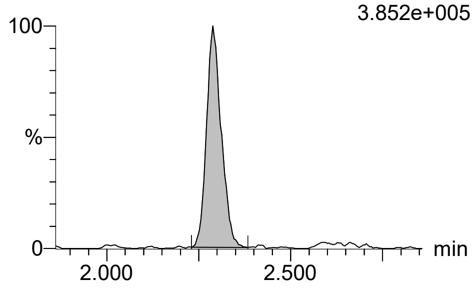

Sample Name: 20211111_L2_R1_IgG

F18:MRM of 1 channel,ES+
927.44 > 204.1
2.985e+005

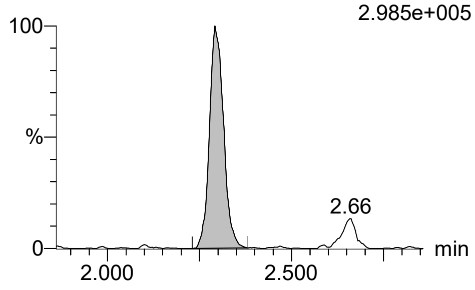

Sample Name: 20211111_H1_R1_IgG

F18:MRM of 1 channel,ES+
927.44 > 204.1
1.562e+005

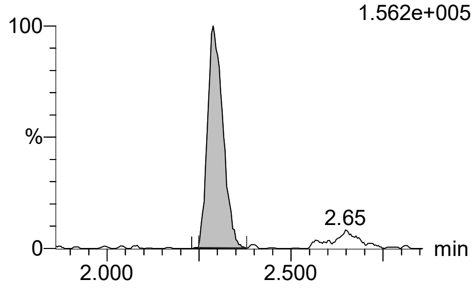

Sample Name: 20211111_A3_R1_IgG

F18:MRM of 1 channel,ES+
927.44 > 204.1
3.071e+004

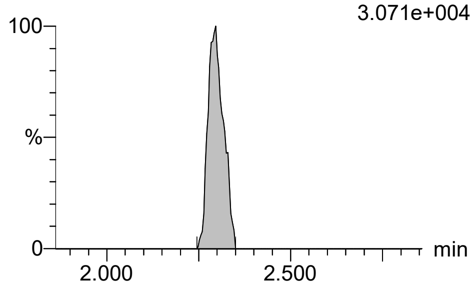

Sample Name: 20211111_A4_R1_IgG

F18:MRM of 1 channel,ES+
927.44 > 204.1
2.262e+005

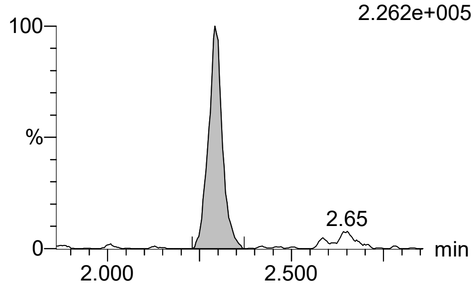

Sample Name: 20211111_L3_R1_IgG

F18:MRM of 1 channel,ES+
927.44 > 204.1
9.574e+004

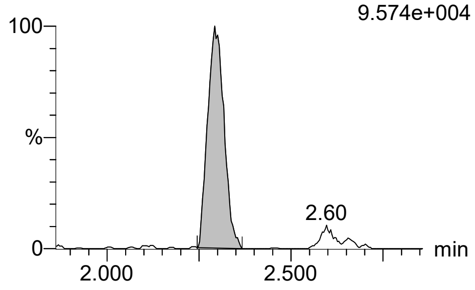

Sample Name: 20211111_L4_R1_IgG

F18:MRM of 1 channel,ES+
927.44 > 204.1
2.776e+005

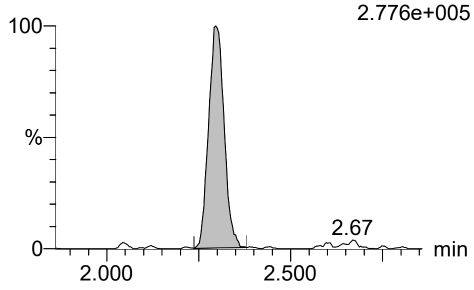

Sample Name: 20211111_H2_R1_IgG

F18:MRM of 1 channel,ES+
927.44 > 204.1
8.190e+005

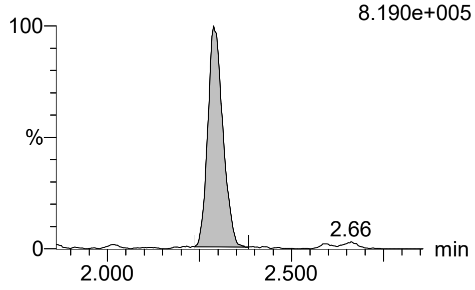

Sample Name: 20211111_A5_R1_IgG

F18:MRM of 1 channel,ES+
927.44 > 204.1
4.816e+005

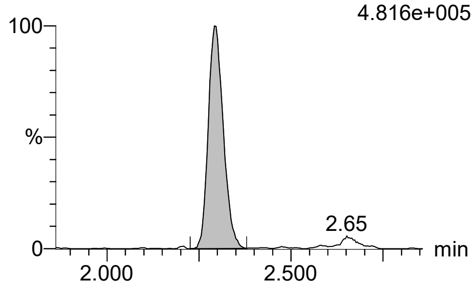

Dataset: C:\MassLynx\10.pro\20211115_clinicalsamples_IgG.qld

Last Altered: Sunday, October 02, 2022 16:02:30 台北標準時間
Printed: Friday, August 01, 2025 16:55:04 台北標準時間

Sample Name: 20211111_A6_R1_IgG

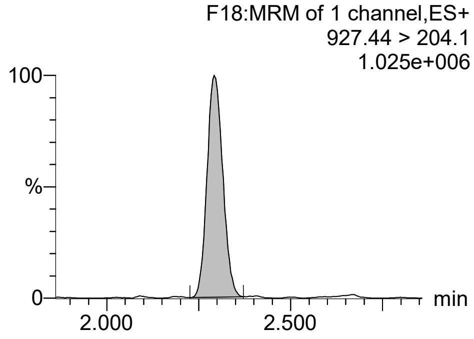

Sample Name: 20211111_QC_R2_IgG

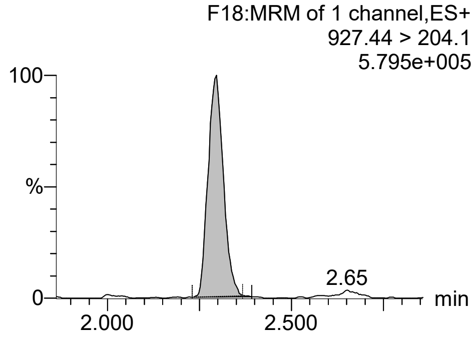

Sample Name: 20211111_L5_R1_IgG

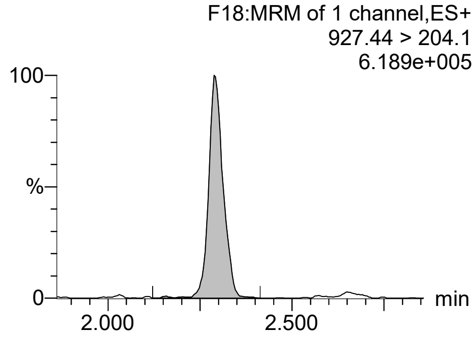

Sample Name: 20211111_L6_R1_IgG

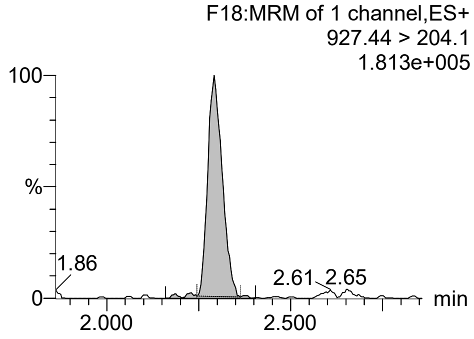

Sample Name: 20211111_H3_R1_IgG

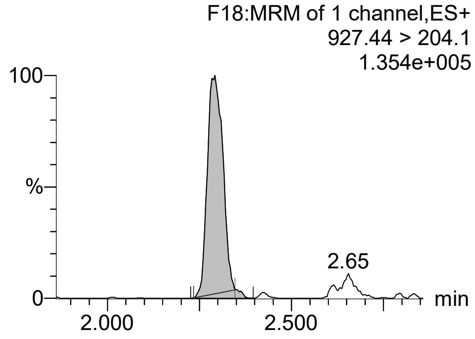

Sample Name: 20211111_A7_R1_IgG

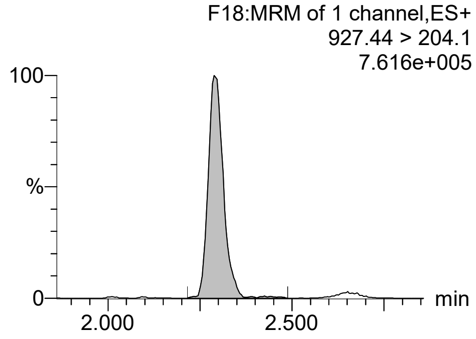

Sample Name: 20211111_A8_R1_IgG

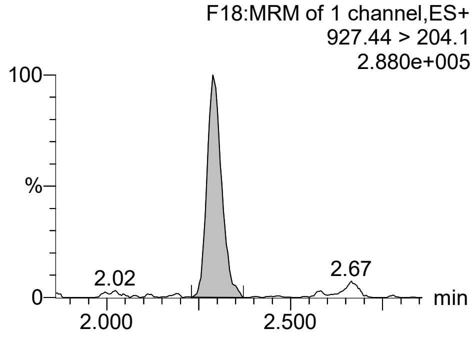

Sample Name: 20211111_L7_R1_IgG

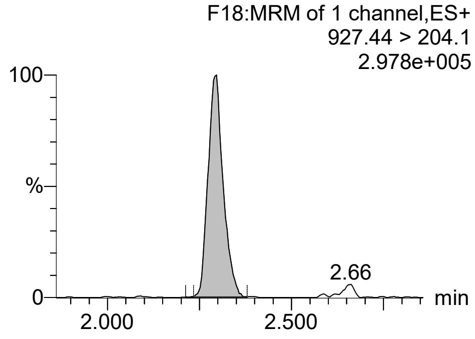

Sample Name: 20211111_L8_R1_IgG

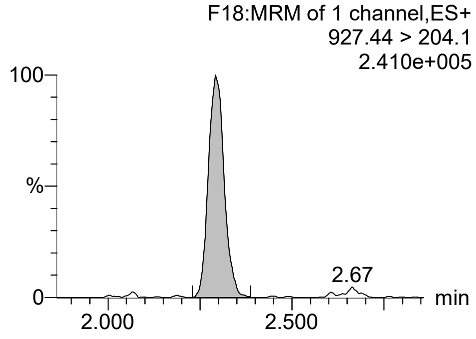

Sample Name: 20211111_H4_R1_IgG

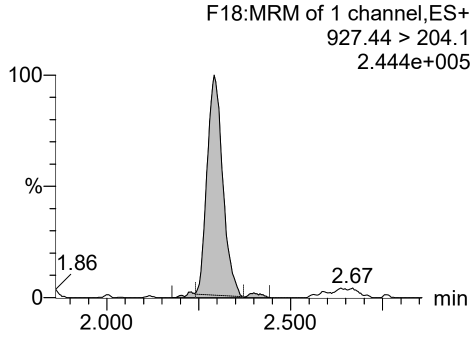

Sample Name: 20211111_A9_R1_IgG

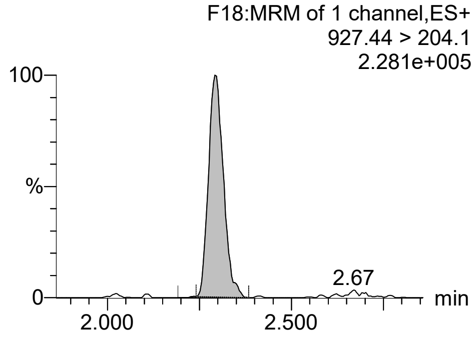

Sample Name: 20211111_A10_R1_IgG

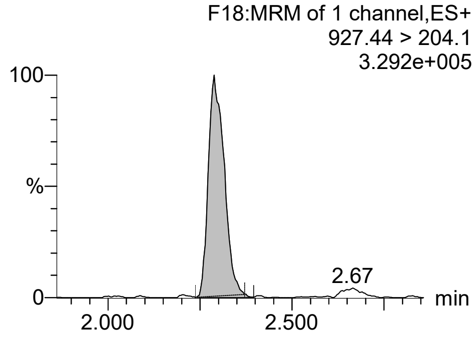

Sample Name: 20211111_L9_R1_IgG

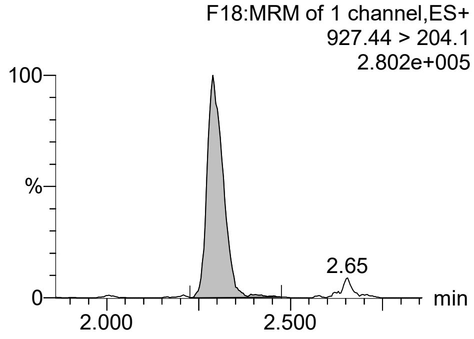

Sample Name: 20211111_L10_R1_IgG

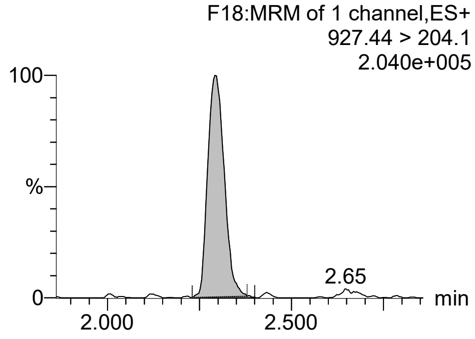

Sample Name: 20211111_QC_R3_IgG

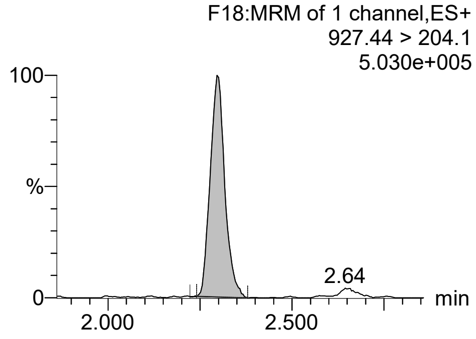

Sample Name: 20211111_H5_R1_IgG
F18:MRM of 1 channel,ES+
927.44 > 204.1
8.102e+004

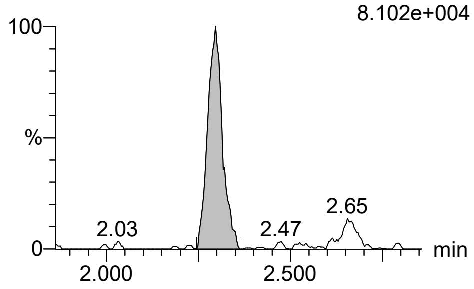

Sample Name: 20211111_A11_R1_IgG
F18:MRM of 1 channel,ES+
927.44 > 204.1
8.485e+004

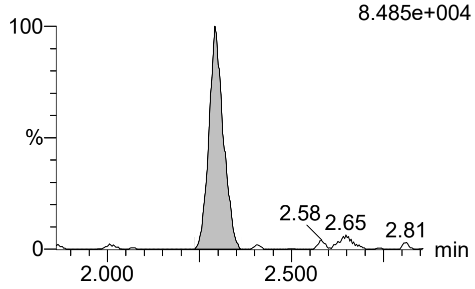

Sample Name: 20211111_A12_R1_IgG
F18:MRM of 1 channel,ES+
927.44 > 204.1
2.088e+005

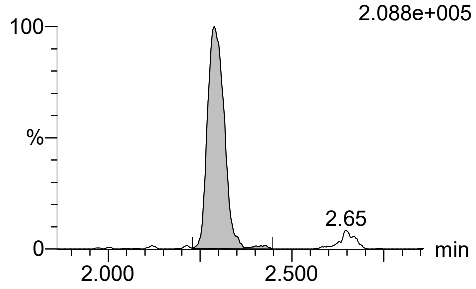

Sample Name: 20211111_L11_R1_IgG
F18:MRM of 1 channel,ES+
927.44 > 204.1
1.890e+005

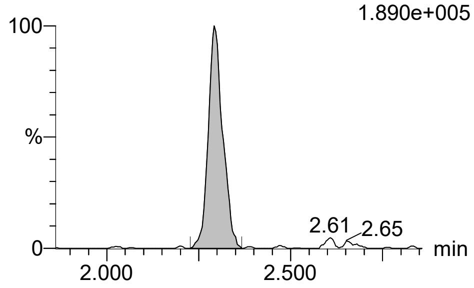

Sample Name: 20211111_L12_R1_IgG
F18:MRM of 1 channel,ES+
927.44 > 204.1
1.224e+005

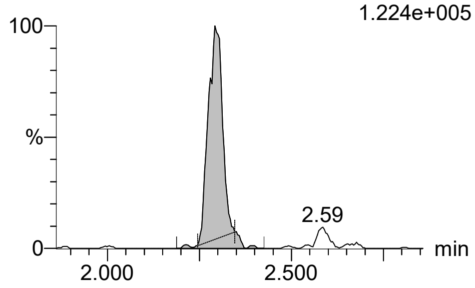

Sample Name: 20211111_H6_R1_IgG
F18:MRM of 1 channel,ES+
927.44 > 204.1
6.441e+005

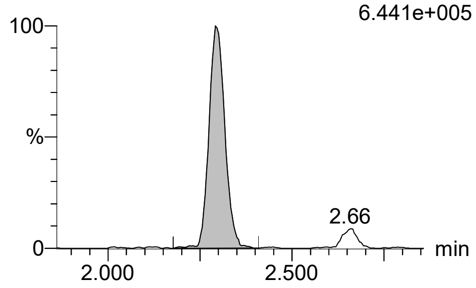

Sample Name: 20211111_A13_R1_IgG
F18:MRM of 1 channel,ES+
927.44 > 204.1
9.796e+005

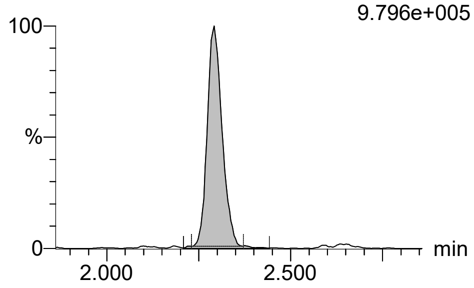

Sample Name: 20211111_A14_R1_IgG
F18:MRM of 1 channel,ES+
927.44 > 204.1
2.610e+005

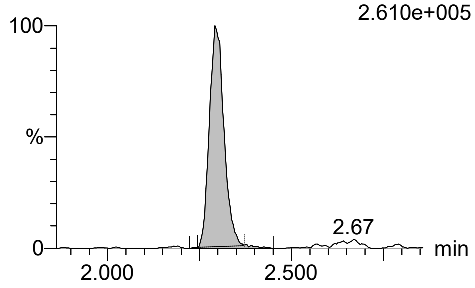

Sample Name: 20211111_L13_R1_IgG
F18:MRM of 1 channel,ES+
927.44 > 204.1
2.294e+005

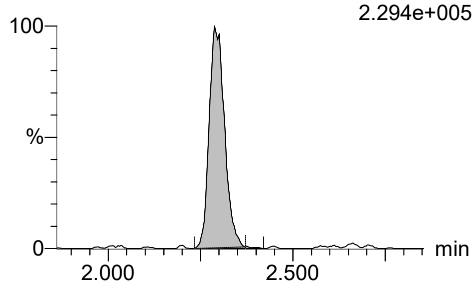

Sample Name: 20211111_L14_R1_IgG
F18:MRM of 1 channel,ES+
927.44 > 204.1
3.084e+005

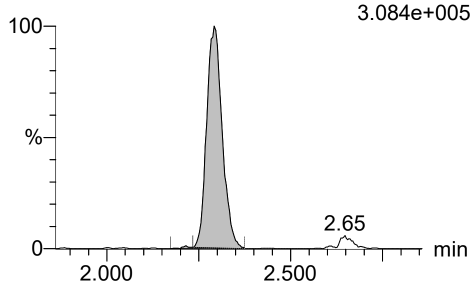

Sample Name: 20211111_H7_R1_IgG
F18:MRM of 1 channel,ES+
927.44 > 204.1
3.517e+005

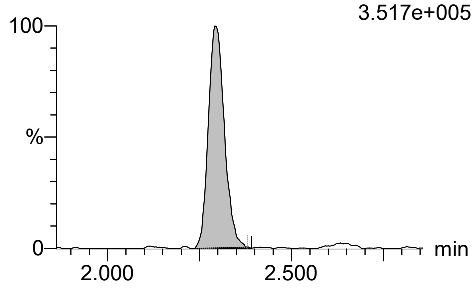

Sample Name: 20211111_A15_R1_IgG
F18:MRM of 1 channel,ES+
927.44 > 204.1
1.389e+005

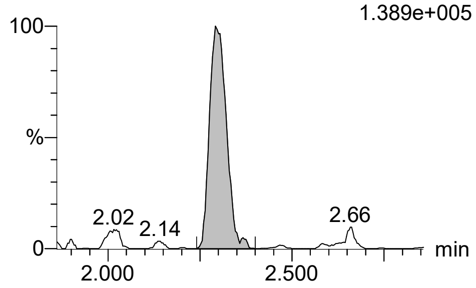

Sample Name: 20211111_QC_R4_IgG
F18:MRM of 1 channel,ES+
927.44 > 204.1
4.560e+005

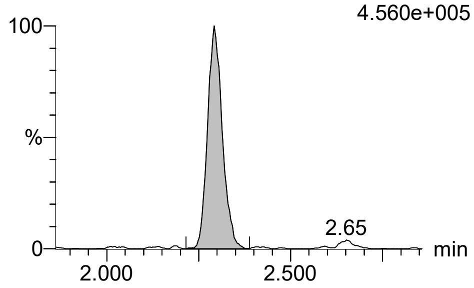

Sample Name: 20211111_A16_R1_IgG
F18:MRM of 1 channel,ES+
927.44 > 204.1
5.790e+005

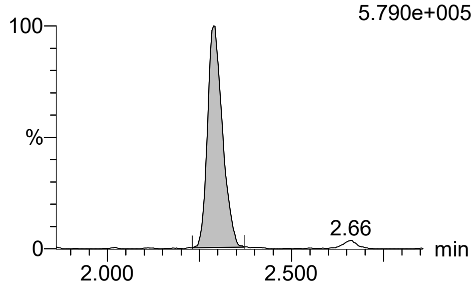

Sample Name: 20211111_L15_R1_IgG
F18:MRM of 1 channel,ES+
927.44 > 204.1
4.534e+005

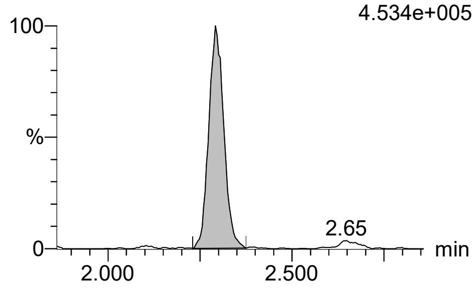

Dataset: C:\MassLynx\10.pro\20211115_clinicalsamples_IgG.qld

Last Altered: Sunday, October 02, 2022 16:02:30 台北標準時間
Printed: Friday, August 01, 2025 16:55:04 台北標準時間

Sample Name: 20211111_L16_R1_IgG

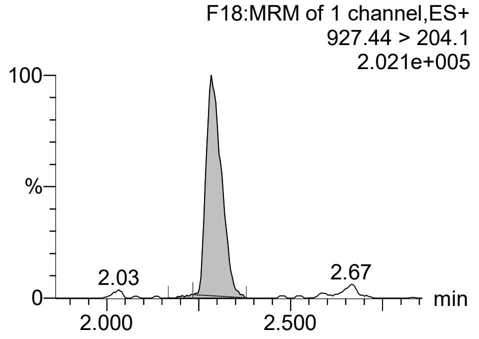

Sample Name: 20211111_H8_R1_IgG

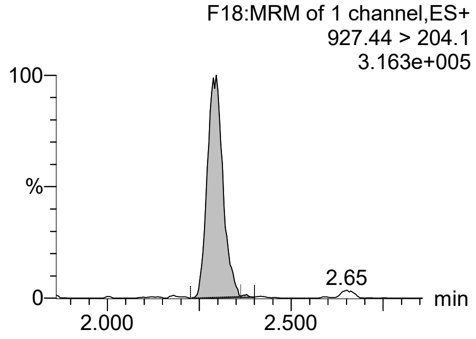

Sample Name: 20211111_A17_R1_IgG

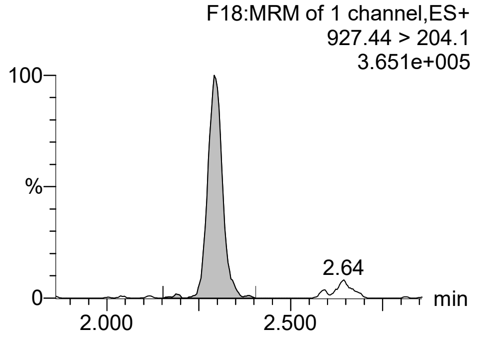

Sample Name: 20211111_A18_R1_IgG

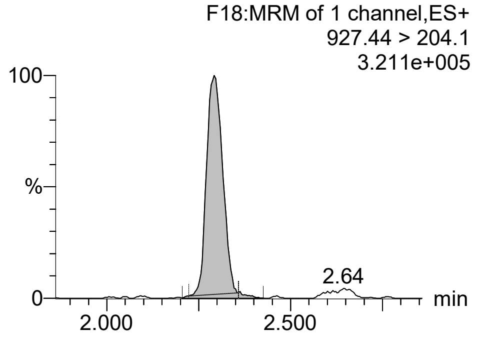

Sample Name: 20211111_L17_R1_IgG

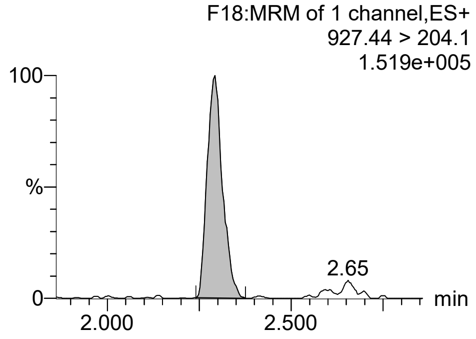

Sample Name: 20211111_L18_R1_IgG

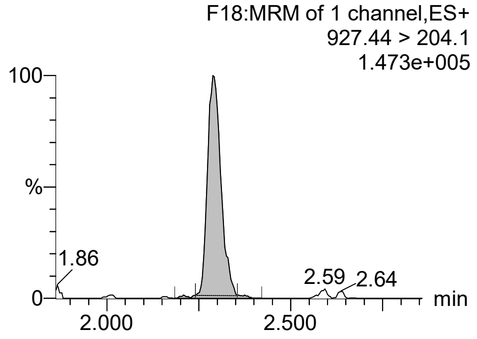

Sample Name: 20211111_H9_R1_IgG

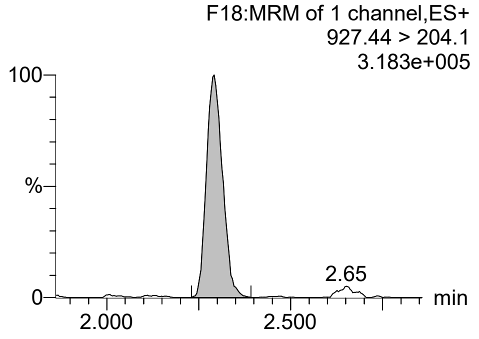

Sample Name: 20211111_A19_R1_IgG

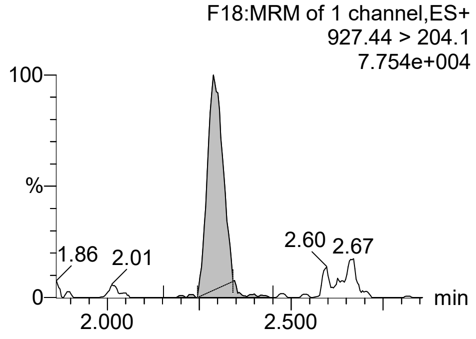

Sample Name: 20211111_A20_R1_IgG

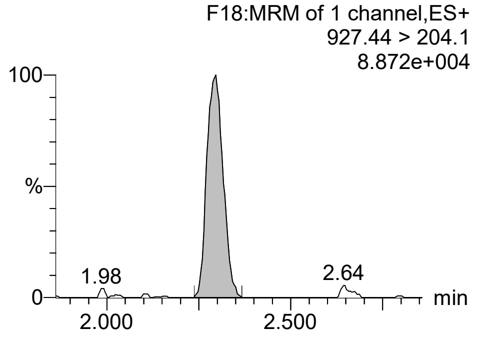

Sample Name: 20211111_L19_R1_IgG

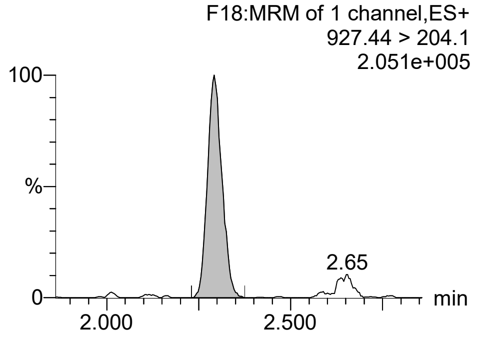

Sample Name: 20211111_QC_R5_IgG

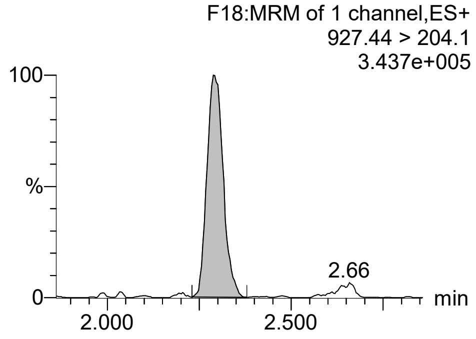

Sample Name: 20211111_L20_R1_IgG

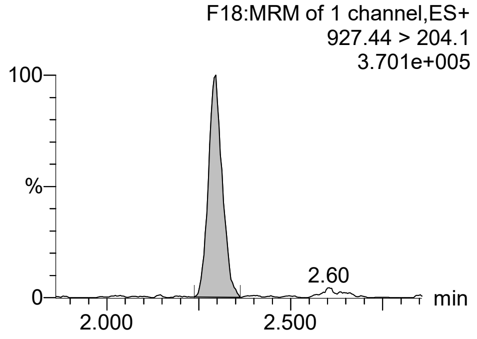

Sample Name: 20211111_H10_R1_IgG

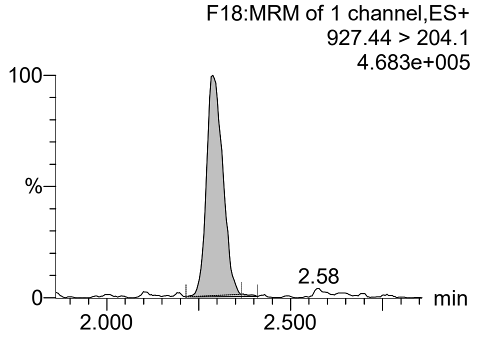

Sample Name: 20211111_A21_R1_IgG

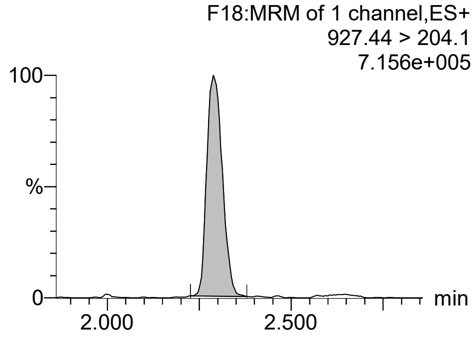

Sample Name: 20211111_A22_R1_IgG

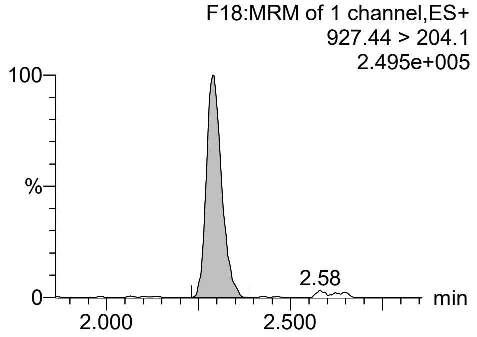

Sample Name: 20211111_L21_R1_IgG
F18:MRM of 1 channel,ES+
927.44 > 204.1
1.713e+005

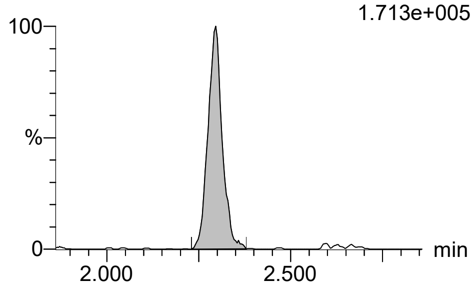

Sample Name: 20211111_L22_R1_IgG
F18:MRM of 1 channel,ES+
927.44 > 204.1
2.879e+005

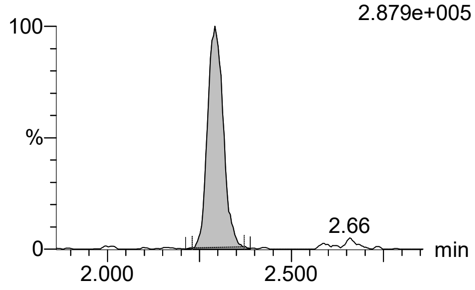

Sample Name: 20211111_H11_R1_IgG
F18:MRM of 1 channel,ES+
927.44 > 204.1
6.936e+005

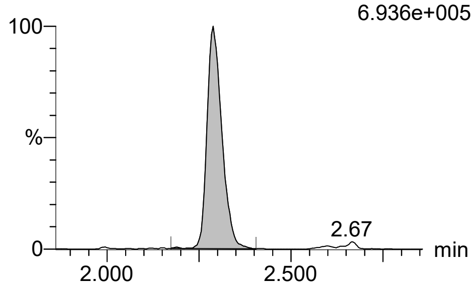

Sample Name: 20211111_A23_R1_IgG
F18:MRM of 1 channel,ES+
927.44 > 204.1
2.700e+005

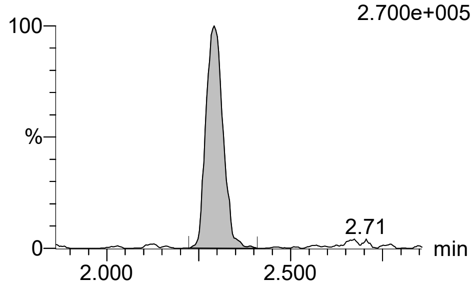

Sample Name: 20211111_A24_R1_IgG
F18:MRM of 1 channel,ES+
927.44 > 204.1
1.052e+005

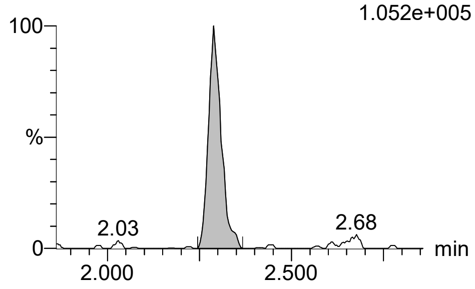

Sample Name: 20211111_L23_R1_IgG
F18:MRM of 1 channel,ES+
927.44 > 204.1
7.536e+005

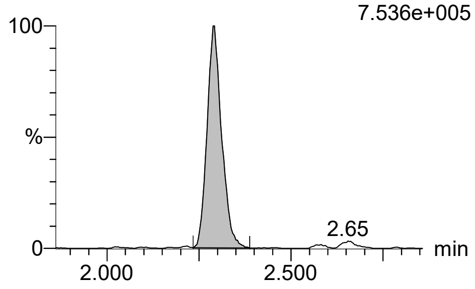

Sample Name: 20211111_L24_R1_IgG
F18:MRM of 1 channel,ES+
927.44 > 204.1
2.056e+005

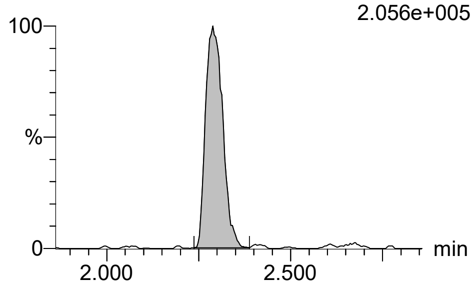

Sample Name: 20211111_H12_R1_IgG
F18:MRM of 1 channel,ES+
927.44 > 204.1
4.908e+004

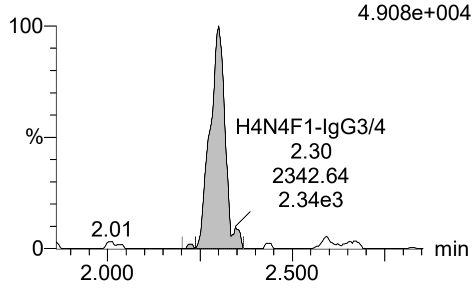

Sample Name: 20211111_QC_R6_IgG
F18:MRM of 1 channel,ES+
927.44 > 204.1
3.581e+005

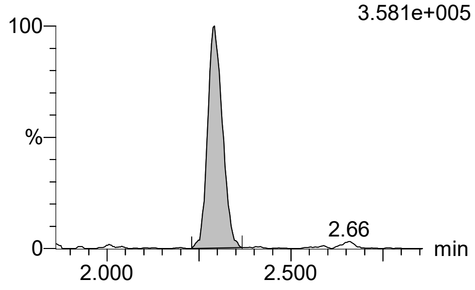

Sample Name: 20211111_A25_R1_IgG
F18:MRM of 1 channel,ES+
927.44 > 204.1
9.756e+005

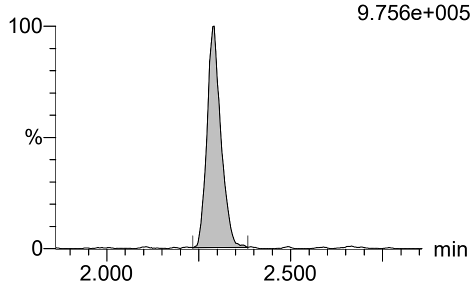

Sample Name: 20211111_A26_R1_IgG
F18:MRM of 1 channel,ES+
927.44 > 204.1
7.356e+004

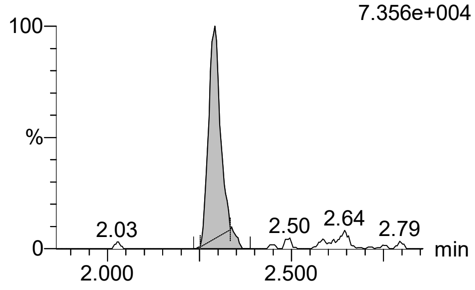

Sample Name: 20211111_L25_R1_IgG
F18:MRM of 1 channel,ES+
927.44 > 204.1
4.247e+005

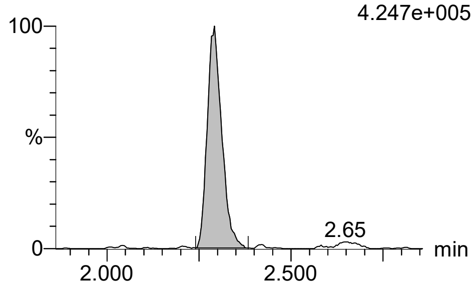

Sample Name: 20211111_L26_R1_IgG
F18:MRM of 1 channel,ES+
927.44 > 204.1
1.705e+005

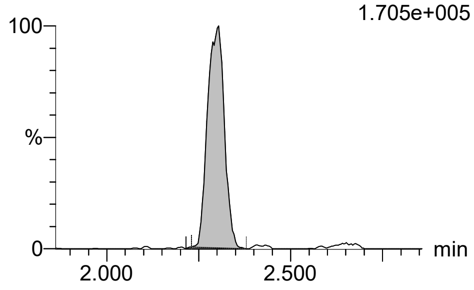

Sample Name: 20211111_H13_R1_IgG
F18:MRM of 1 channel,ES+
927.44 > 204.1
2.557e+005

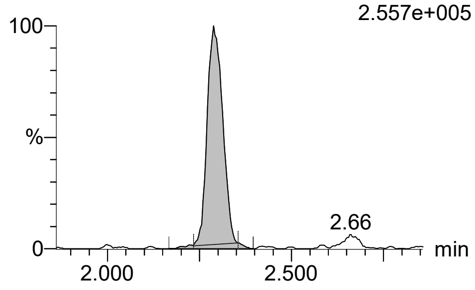

Sample Name: 20211111_A27_R1_IgG
F18:MRM of 1 channel,ES+
927.44 > 204.1
2.605e+005

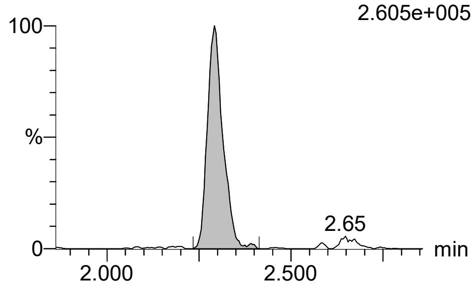

Dataset: C:\MassLynx\10.pro\20211115_clinicalsamples_IgG.qld

Last Altered: Sunday, October 02, 2022 16:02:30 台北標準時間
Printed: Friday, August 01, 2025 16:55:04 台北標準時間

Sample Name: 20211111_A28_R1_IgG

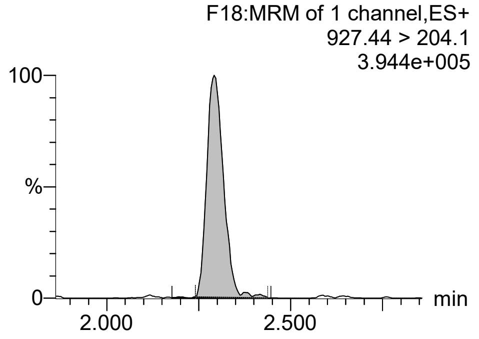

Sample Name: 20211111_L27_R1_IgG

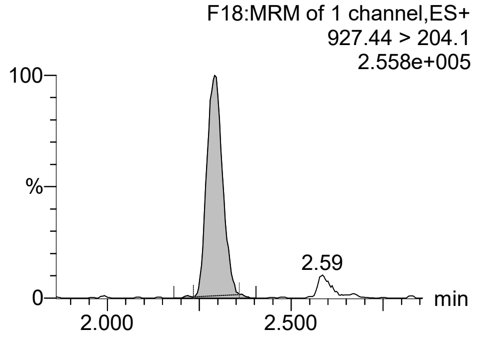

Sample Name: 20211111_L28_R1_IgG

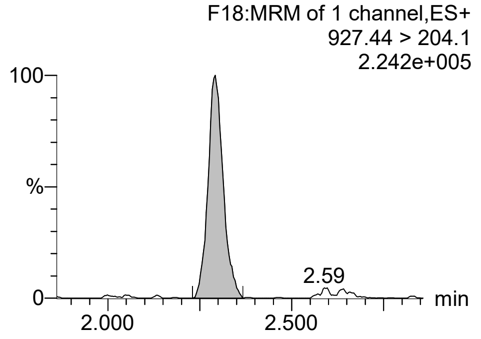

Sample Name: 20211111_H14_R1_IgG

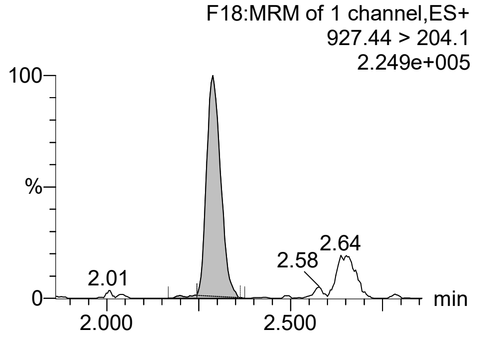

Sample Name: 20211111_A29_R1_IgG

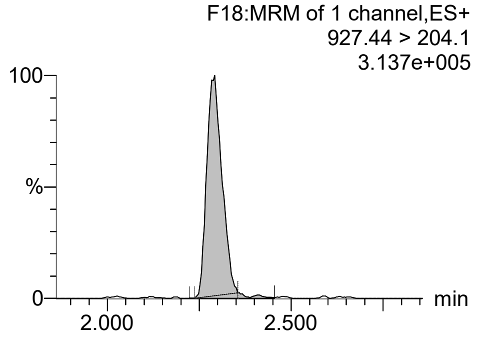

Sample Name: 20211111_A30_R1_IgG

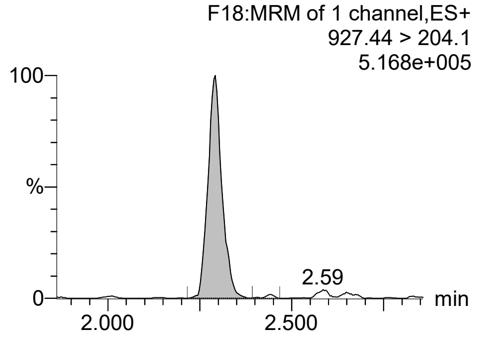

Sample Name: 20211111_QC_R7_IgG

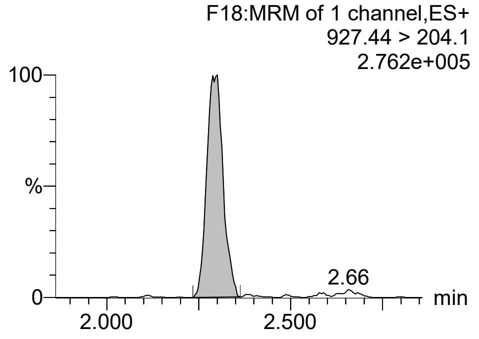

Sample Name: 20211111_L29_R1_IgG

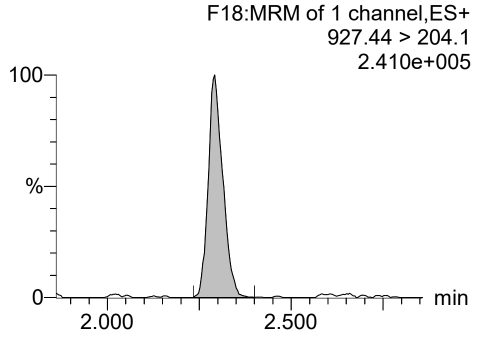

Sample Name: 20211111_L30_R1_IgG

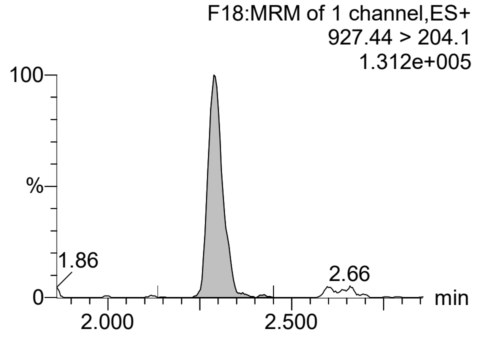

Sample Name: 20211111_H15_R1_IgG

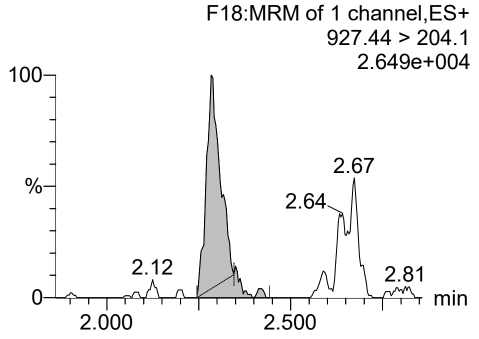

Sample Name: 20211111_A31_R1_IgG

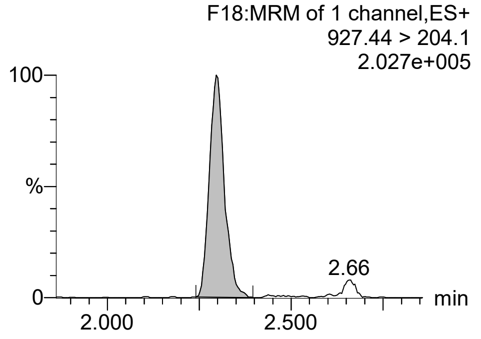

Sample Name: 20211111_A32_R1_IgG

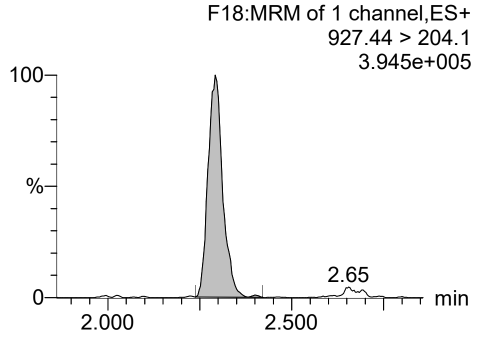

Sample Name: 20211111_L31_R1_IgG

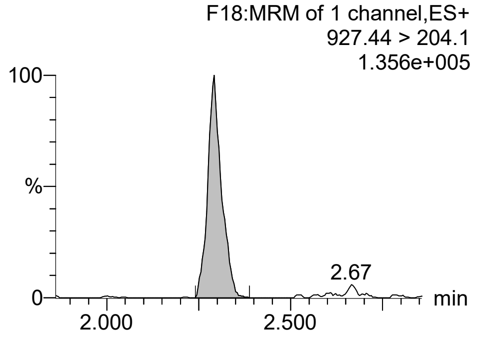

Sample Name: 20211111_L32_R1_IgG

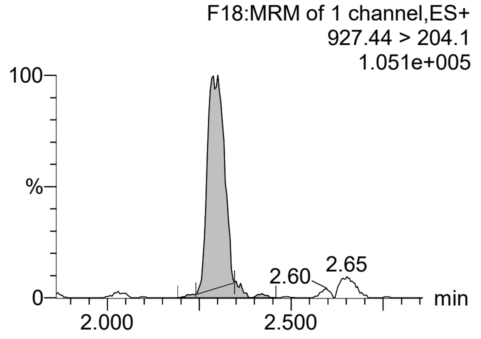

Sample Name: 20211111_H16_R1_IgG

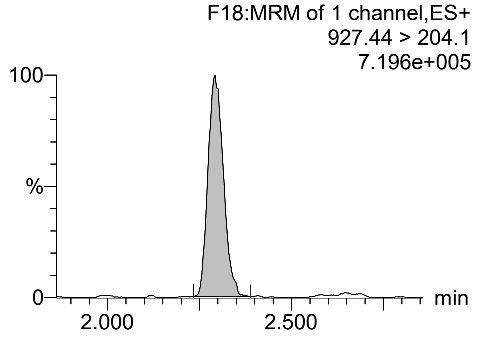

Dataset: C:\MassLynx\10.pro\20211115_clinicalsamples_IgG.qld

Last Altered: Sunday, October 02, 2022 16:02:30 台北標準時間
Printed: Friday, August 01, 2025 16:55:04 台北標準時間

Sample Name: 20211111_A33_R1_IgG
F18:MRM of 1 channel,ES+
927.44 > 204.1
1.641e+005

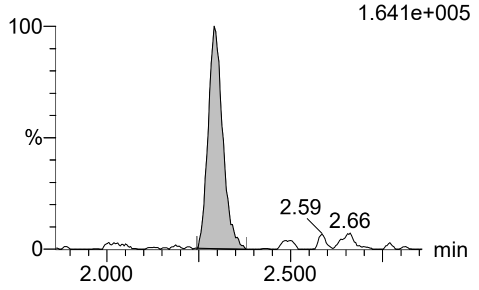

Sample Name: 20211111_A34_R1_IgG
F18:MRM of 1 channel,ES+
927.44 > 204.1
2.424e+005

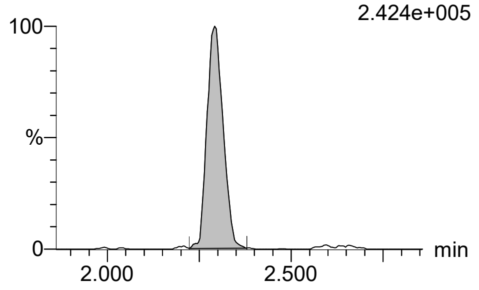

Sample Name: 20211111_H17_R1_IgG
F18:MRM of 1 channel,ES+
927.44 > 204.1
5.829e+005

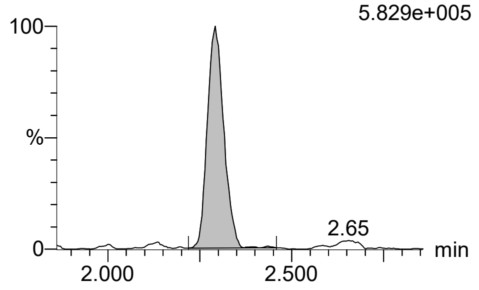

Sample Name: 20211111_A35_R1_IgG
F18:MRM of 1 channel,ES+
927.44 > 204.1
5.723e+005

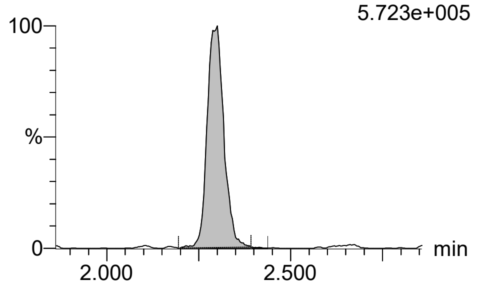

Sample Name: 20211111_QC_R8_IgG
F18:MRM of 1 channel,ES+
927.44 > 204.1
2.710e+005

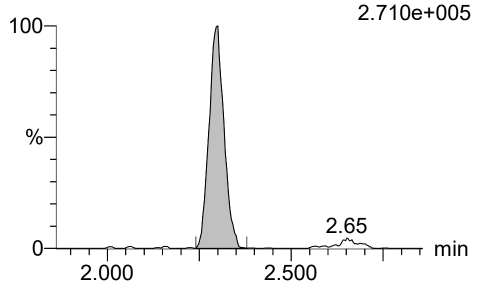

Sample Name: 20211111_A36_R1_IgG
F18:MRM of 1 channel,ES+
927.44 > 204.1
4.501e+005

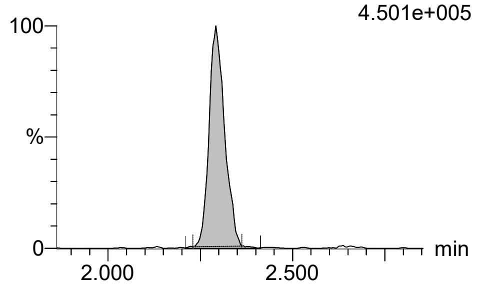

Sample Name: 20211111_H18_R1_IgG
F18:MRM of 1 channel,ES+
927.44 > 204.1
2.214e+006

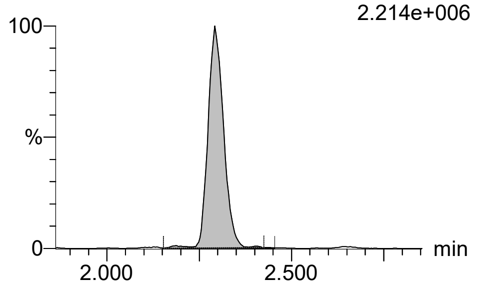

Sample Name: 20211111_A37_R1_IgG
F18:MRM of 1 channel,ES+
927.44 > 204.1
7.308e+004

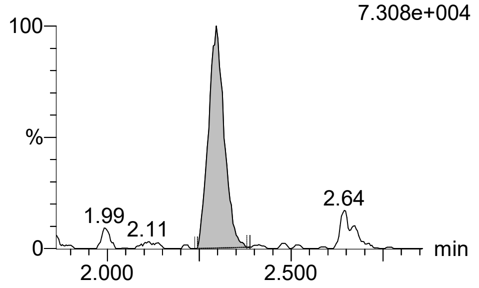

Sample Name: 20211111_A38_R1_IgG
F18:MRM of 1 channel,ES+
927.44 > 204.1
2.863e+005

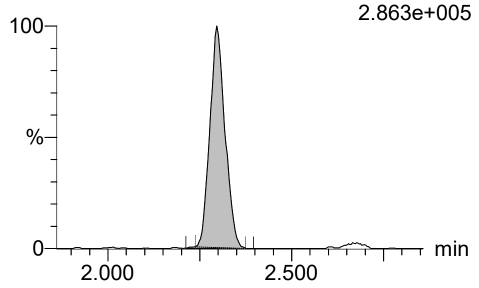

Sample Name: 20211111_H19_R1_IgG
F18:MRM of 1 channel,ES+
927.44 > 204.1
1.457e+006

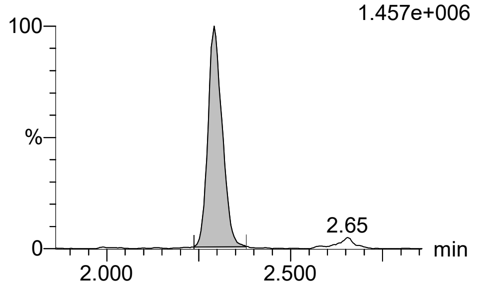

Sample Name: 20211111_A39_R1_IgG
F18:MRM of 1 channel,ES+
927.44 > 204.1
2.622e+005

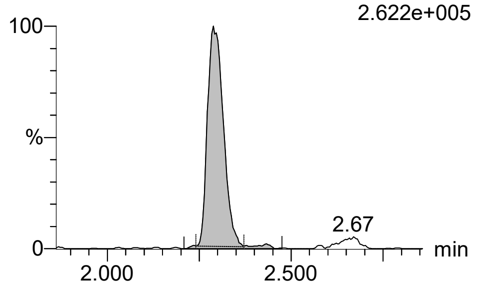

Sample Name: 20211111_A40_R1_IgG
F18:MRM of 1 channel,ES+
927.44 > 204.1
4.388e+005

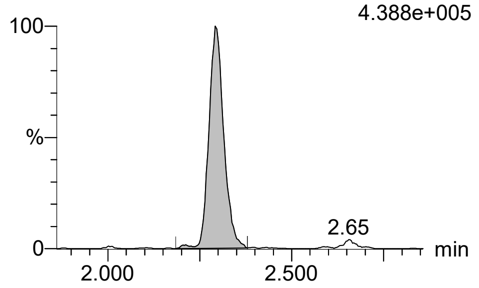

Sample Name: 20211111_H20_R1_IgG
F18:MRM of 1 channel,ES+
927.44 > 204.1
1.316e+006

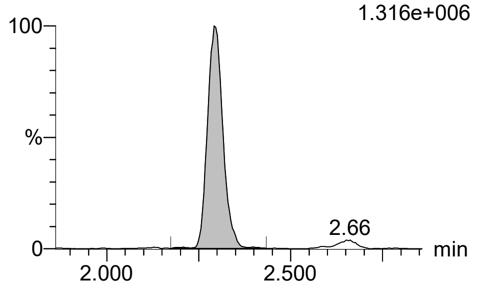

Sample Name: 20211111_A41_R1_IgG
F18:MRM of 1 channel,ES+
927.44 > 204.1
5.153e+004

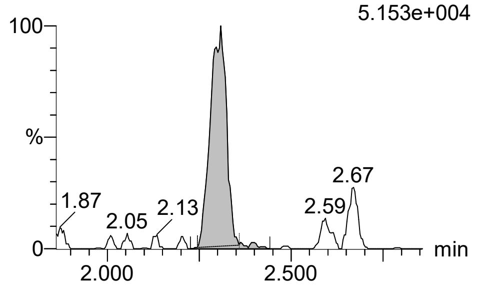

Sample Name: 20211111_A42_R1_IgG
F18:MRM of 1 channel,ES+
927.44 > 204.1
1.823e+005

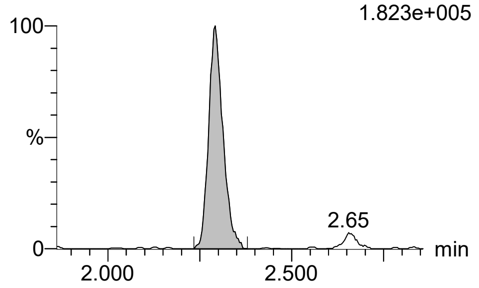

Sample Name: 20211111_A43_R1_IgG
F18:MRM of 1 channel,ES+
927.44 > 204.1
3.510e+005

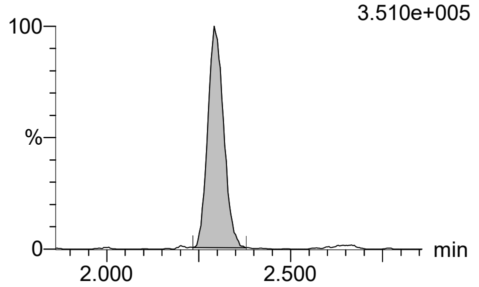

Sample Name: 20211111_A44_R1_IgG
F18:MRM of 1 channel,ES+
927.44 > 204.1
4.253e+005

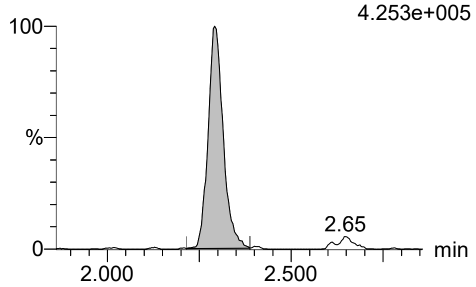

Sample Name: 20211111_QC_R9_IgG
F18:MRM of 1 channel,ES+
927.44 > 204.1
2.530e+005

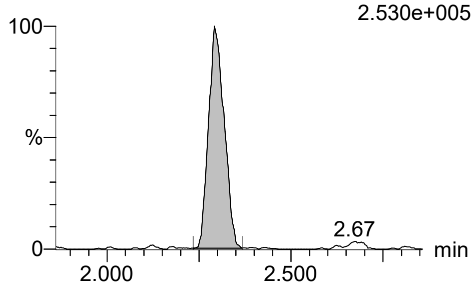

Sample Name: 20211111_A1_R2_IgG
F18:MRM of 1 channel,ES+
927.44 > 204.1
2.789e+005

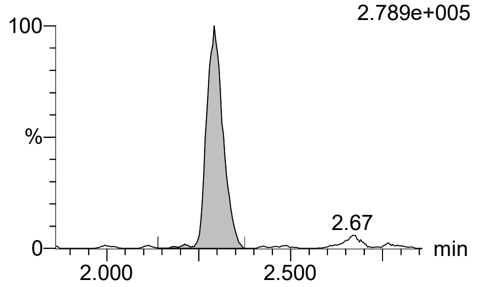

Sample Name: 20211111_A2_R2_IgG
F18:MRM of 1 channel,ES+
927.44 > 204.1
1.711e+005

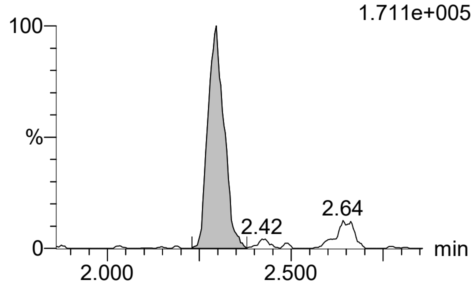

Sample Name: 20211111_L1_R2_IgG
F18:MRM of 1 channel,ES+
927.44 > 204.1
2.116e+005

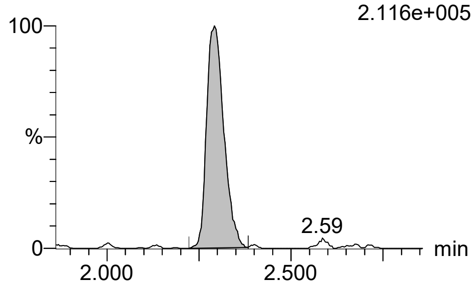

Sample Name: 20211111_L2_R2_IgG
F18:MRM of 1 channel,ES+
927.44 > 204.1
1.624e+005

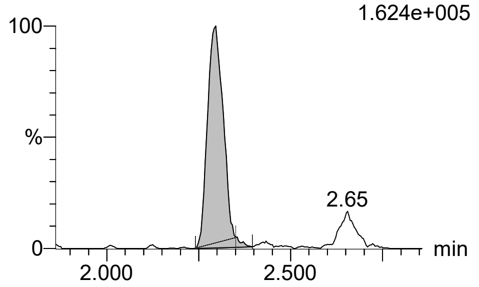

Sample Name: 20211111_H1_R2_IgG
F18:MRM of 1 channel,ES+
927.44 > 204.1
1.834e+005

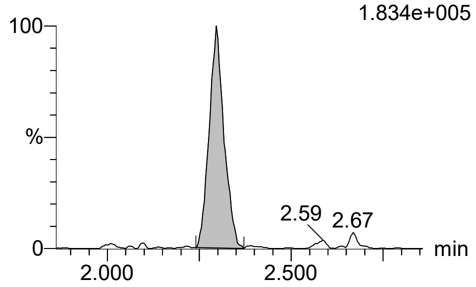

Sample Name: 20211111_A3_R2_IgG
F18:MRM of 1 channel,ES+
927.44 > 204.1
4.015e+004

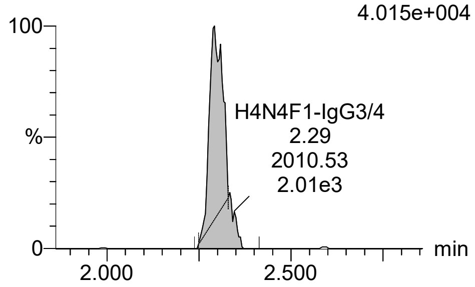

Sample Name: 20211111_A4_R2_IgG
F18:MRM of 1 channel,ES+
927.44 > 204.1
1.360e+005

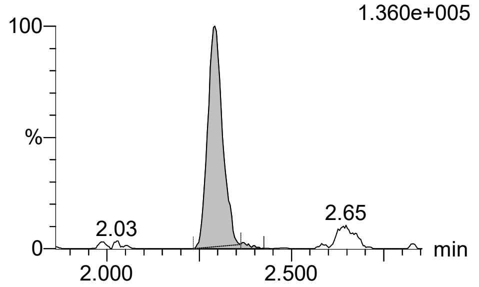

Sample Name: 20211111_L3_R2_IgG
F18:MRM of 1 channel,ES+
927.44 > 204.1
8.664e+004

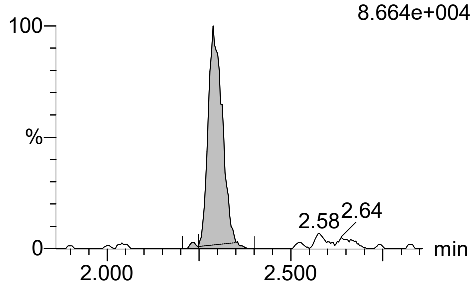

Sample Name: 20211111_L4_R2_IgG
F18:MRM of 1 channel,ES+
927.44 > 204.1
2.566e+005

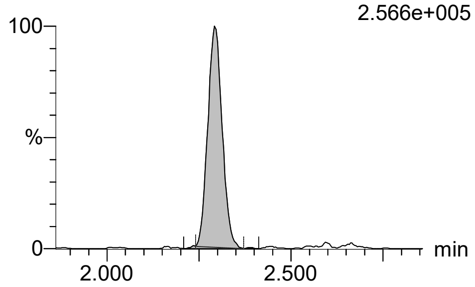

Sample Name: 20211111_H2_R2_IgG
F18:MRM of 1 channel,ES+
927.44 > 204.1
5.110e+005

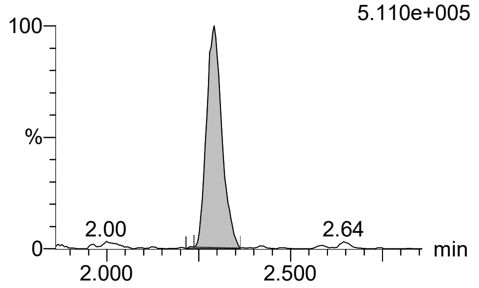

Sample Name: 20211111_A5_R2_IgG
F18:MRM of 1 channel,ES+
927.44 > 204.1
4.191e+005

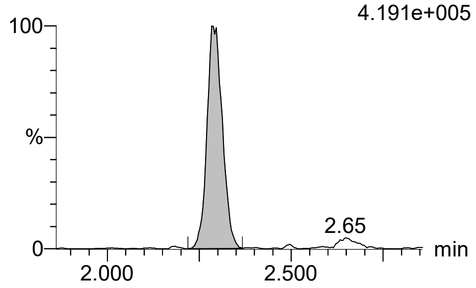

Sample Name: 20211111_A6_R2_IgG
F18:MRM of 1 channel,ES+
927.44 > 204.1
1.050e+006

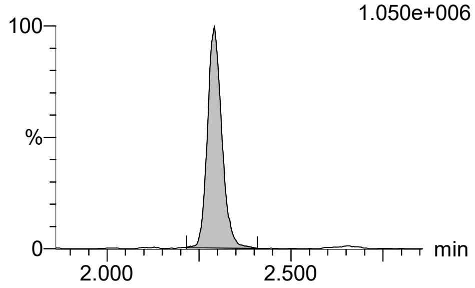

Dataset: C:\MassLynx\10.pro\20211115_clinicalsamples_IgG.qld

Last Altered: Sunday, October 02, 2022 16:02:30 台北標準時間
Printed: Friday, August 01, 2025 16:55:04 台北標準時間

Sample Name: 20211111_QC_R10_IgG

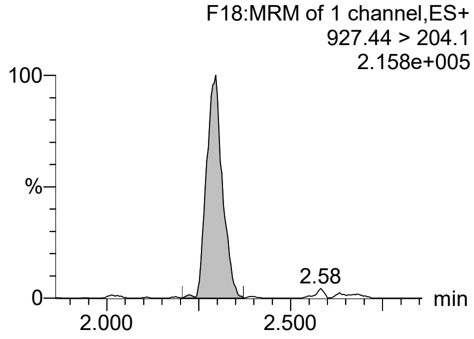

Sample Name: 20211111_L5_R2_IgG

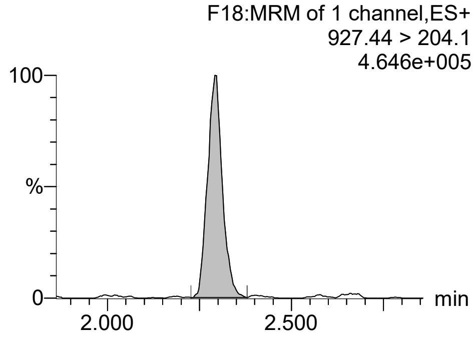

Sample Name: 20211111_L6_R2_IgG

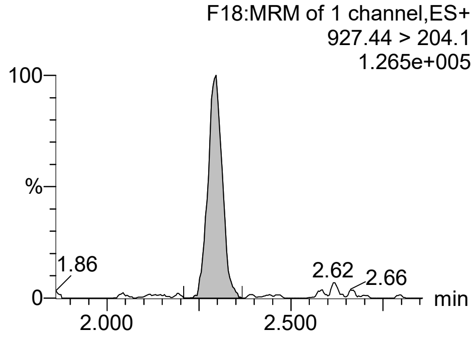

Sample Name: 20211111_H3_R2_IgG

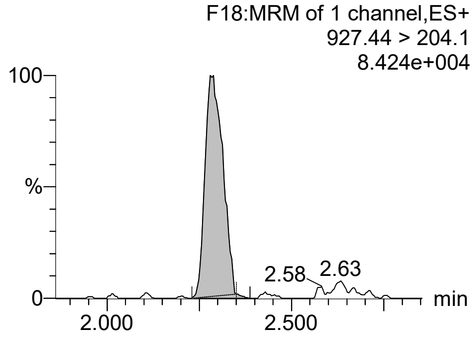

Sample Name: 20211111_A7_R2_IgG

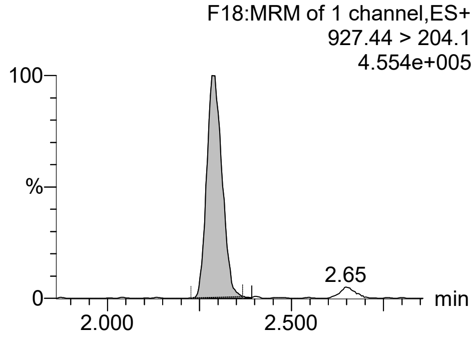

Sample Name: 20211111_A8_R2_IgG

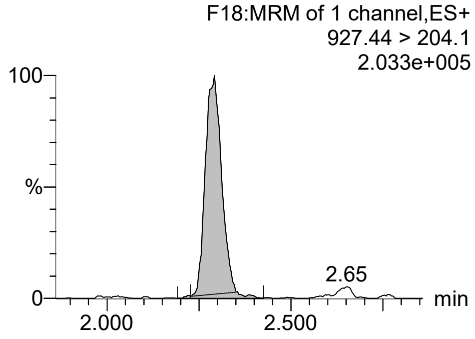

Sample Name: 20211111_L7_R2_IgG

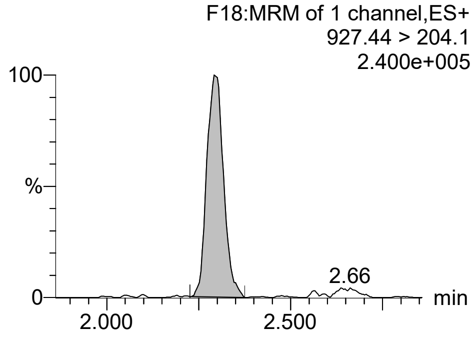

Sample Name: 20211111_L8_R2_IgG

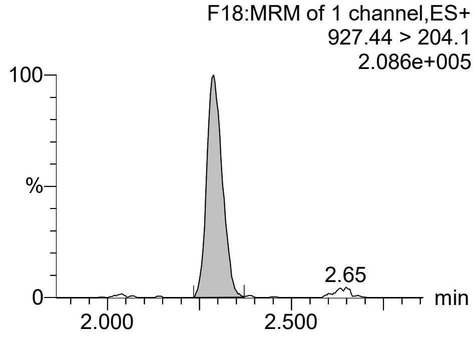

Sample Name: 20211111_H4_R2_IgG

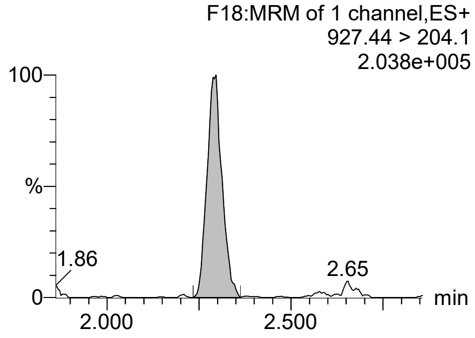

Sample Name: 20211111_A9_R2_IgG

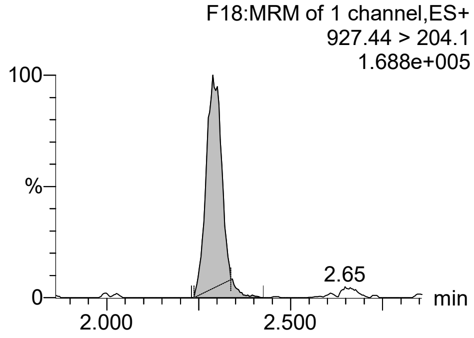

Sample Name: 20211111_A10_R2_IgG

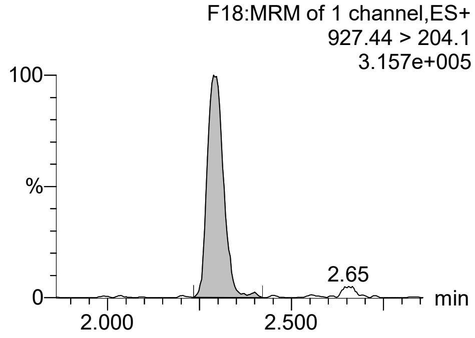

Sample Name: 20211111_L9_R2_IgG

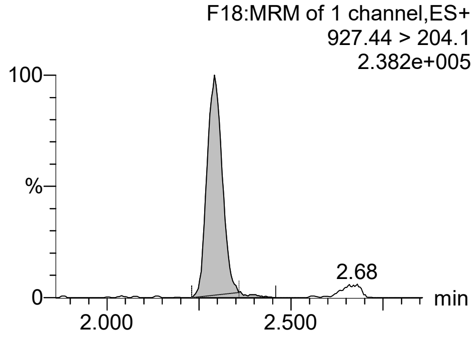

Sample Name: 20211111_L10_R2_IgG

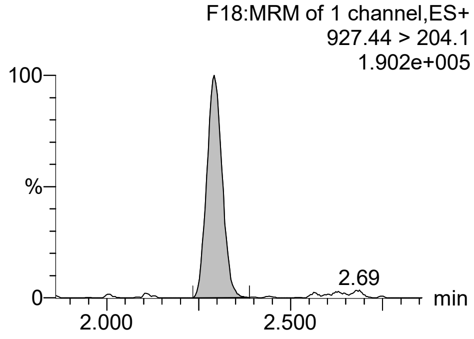

Sample Name: 20211111_QC_R11_IgG

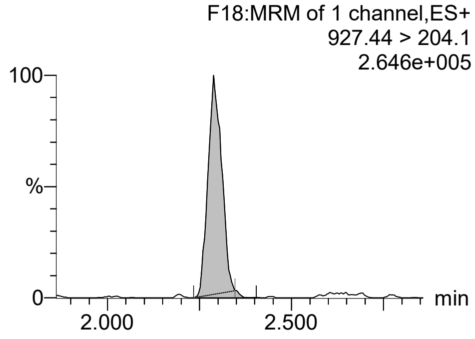

Sample Name: 20211111_H5_R2_IgG

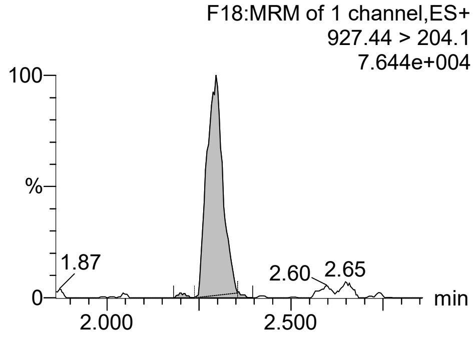

Sample Name: 20211111_A11_R2_IgG
F18:MRM of 1 channel,ES+
927.44 > 204.1
5.073e+004

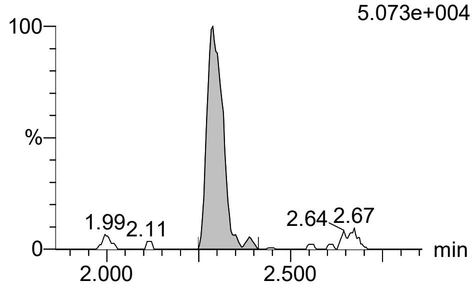

Sample Name: 20211111_A12_R2_IgG
F18:MRM of 1 channel,ES+
927.44 > 204.1
1.925e+005

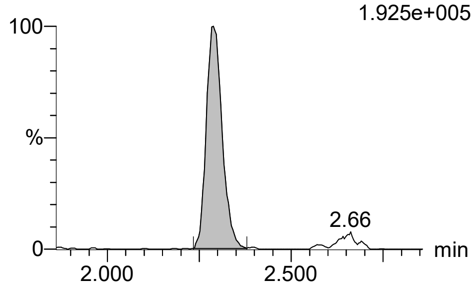

Sample Name: 20211111_L11_R2_IgG
F18:MRM of 1 channel,ES+
927.44 > 204.1
1.560e+005

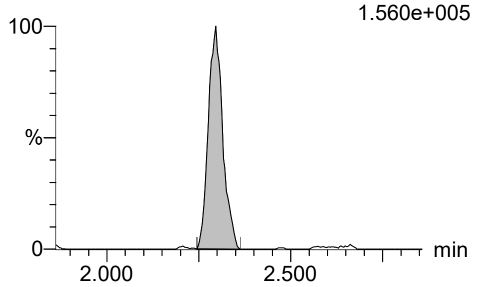

Sample Name: 20211111_L12_R2_IgG
F18:MRM of 1 channel,ES+
927.44 > 204.1
1.069e+005

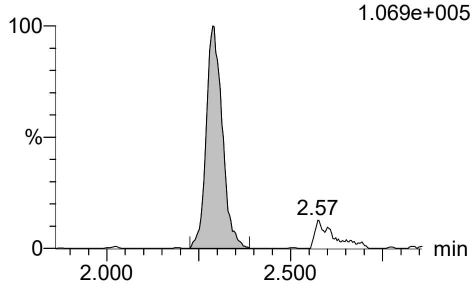

Sample Name: 20211111_H6_R2_IgG
F18:MRM of 1 channel,ES+
927.44 > 204.1
4.205e+005

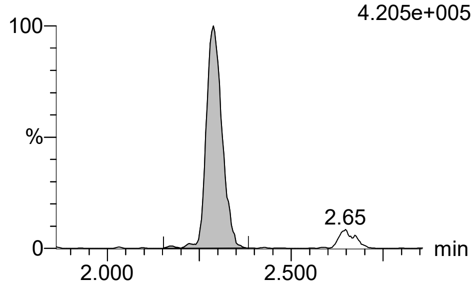

Sample Name: 20211111_A13_R2_IgG
F18:MRM of 1 channel,ES+
927.44 > 204.1
5.993e+005

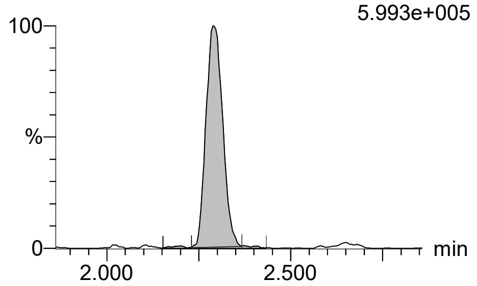

Sample Name: 20211111_A14_R2_IgG
F18:MRM of 1 channel,ES+
927.44 > 204.1
2.023e+005

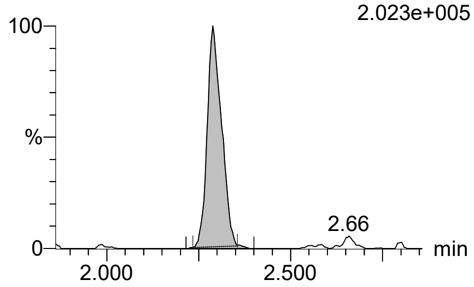

Sample Name: 20211111_L13_R2_IgG
F18:MRM of 1 channel,ES+
927.44 > 204.1
1.865e+005

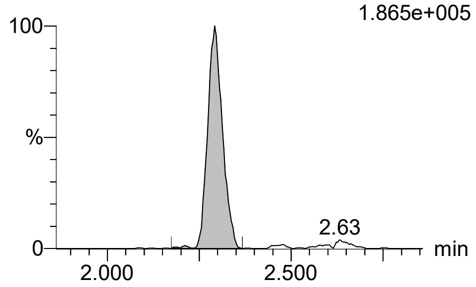

Sample Name: 20211111_L14_R2_IgG
F18:MRM of 1 channel,ES+
927.44 > 204.1
3.048e+005

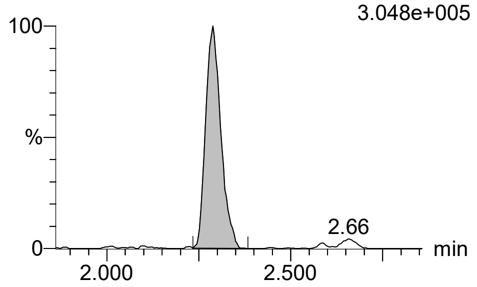

Sample Name: 20211111_H7_R2_IgG
F18:MRM of 1 channel,ES+
927.44 > 204.1
3.603e+005

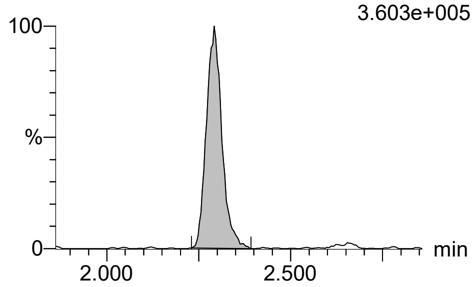

Sample Name: 20211111_A15_R2_IgG
F18:MRM of 1 channel,ES+
927.44 > 204.1
1.435e+005

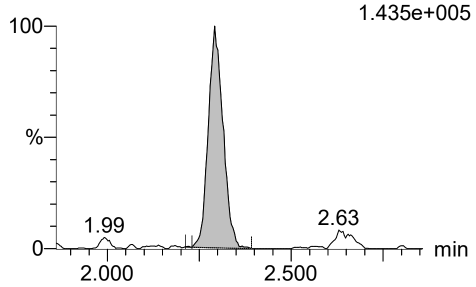

Sample Name: 20211111_QC_R12_IgG
F18:MRM of 1 channel,ES+
927.44 > 204.1
2.541e+005

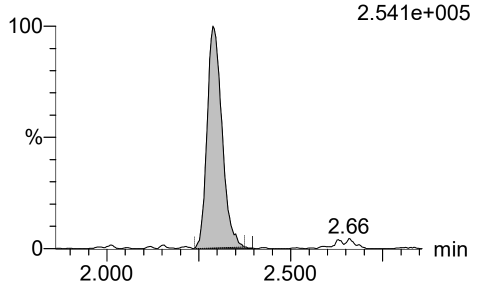

Sample Name: 20211111_A16_R2_IgG
F18:MRM of 1 channel,ES+
927.44 > 204.1
3.147e+005

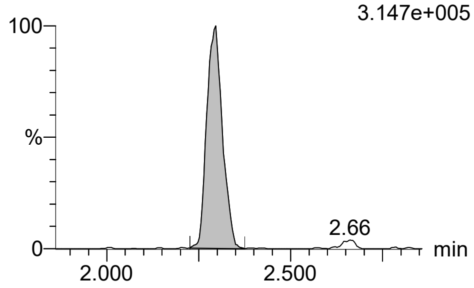

Sample Name: 20211111_L15_R2_IgG
F18:MRM of 1 channel,ES+
927.44 > 204.1
3.071e+005

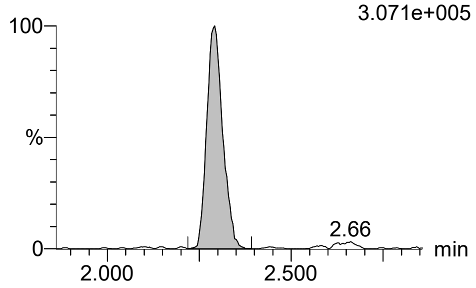

Sample Name: 20211111_L16_R2_IgG
F18:MRM of 1 channel,ES+
927.44 > 204.1
1.072e+005

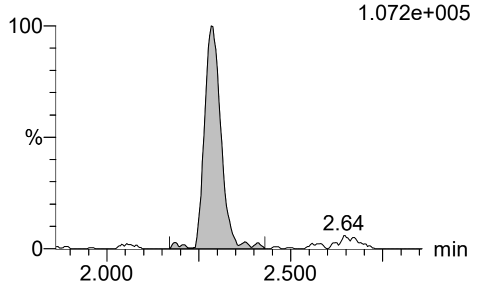

Dataset: C:\MassLynx\10.pro\20211115_clinicalsamples_IgG.qld

Last Altered: Sunday, October 02, 2022 16:02:30 台北標準時間
Printed: Friday, August 01, 2025 16:55:04 台北標準時間

Sample Name: 20211111_H8_R2_IgG
F18:MRM of 1 channel,ES+
927.44 > 204.1
2.567e+005

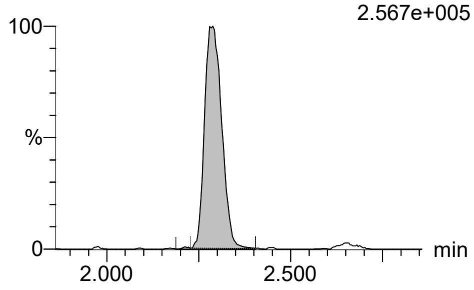

Sample Name: 20211111_A17_R2_IgG
F18:MRM of 1 channel,ES+
927.44 > 204.1
3.171e+005

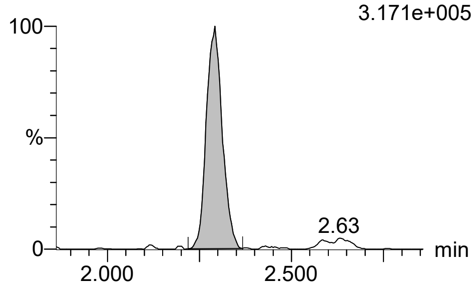

Sample Name: 20211111_A18_R2_IgG
F18:MRM of 1 channel,ES+
927.44 > 204.1
2.559e+005

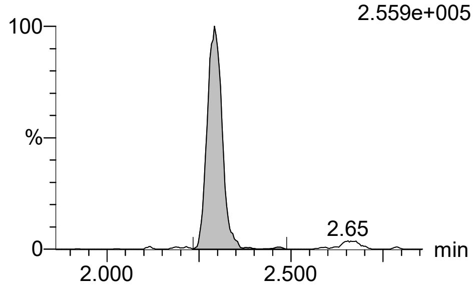

Sample Name: 20211111_L17_R2_IgG
F18:MRM of 1 channel,ES+
927.44 > 204.1
1.389e+005

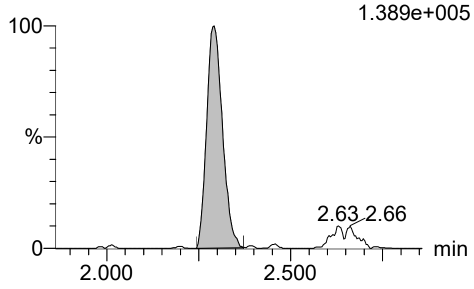

Sample Name: 20211111_L18_R2_IgG
F18:MRM of 1 channel,ES+
927.44 > 204.1
9.054e+004

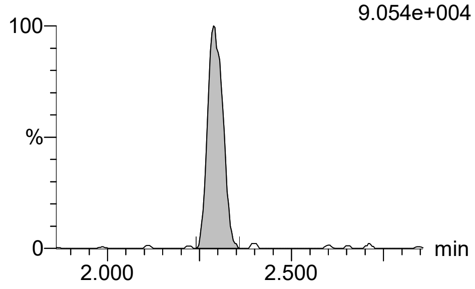

Sample Name: 20211111_H9_R2_IgG
F18:MRM of 1 channel,ES+
927.44 > 204.1
2.687e+005

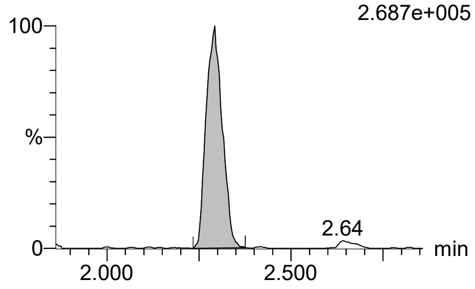

Sample Name: 20211111_A19_R2_IgG
F18:MRM of 1 channel,ES+
927.44 > 204.1
1.142e+005

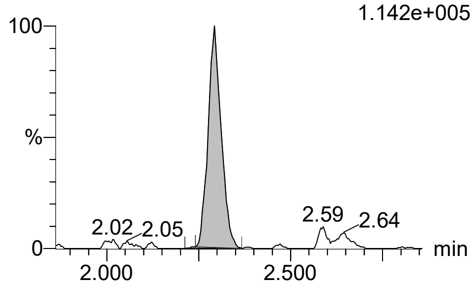

Sample Name: 20211111_A20_R2_IgG
F18:MRM of 1 channel,ES+
927.44 > 204.1
7.023e+004

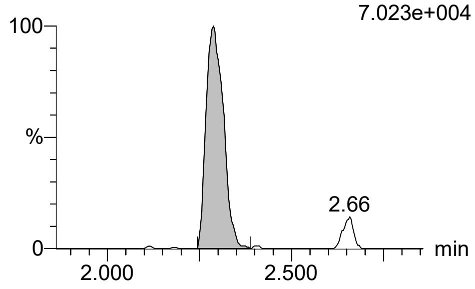

Sample Name: 20211111_L19_R2_IgG
F18:MRM of 1 channel,ES+
927.44 > 204.1
1.575e+005

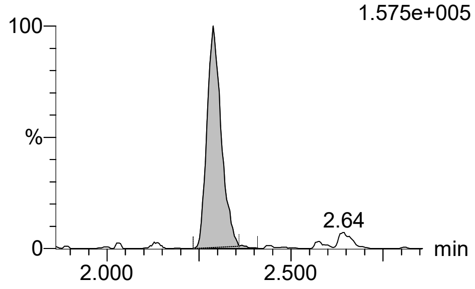

Sample Name: 20211111_QC_R13_IgG
F18:MRM of 1 channel,ES+
927.44 > 204.1
2.195e+005

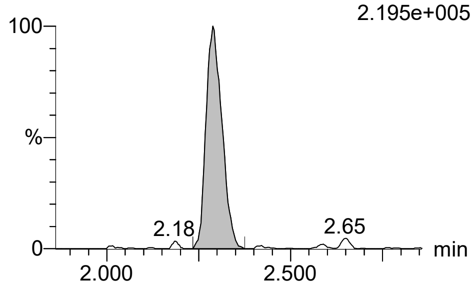

Sample Name: 20211111_L20_R2_IgG
F18:MRM of 1 channel,ES+
927.44 > 204.1
2.039e+005

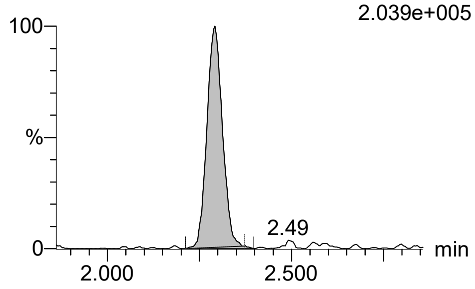

Sample Name: 20211111_H10_R2_IgG
F18:MRM of 1 channel,ES+
927.44 > 204.1
2.299e+005

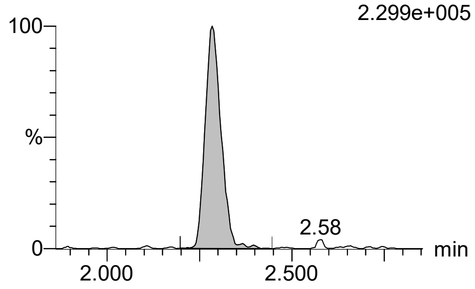

Sample Name: 20211111_A21_R2_IgG
F18:MRM of 1 channel,ES+
927.44 > 204.1
3.685e+005

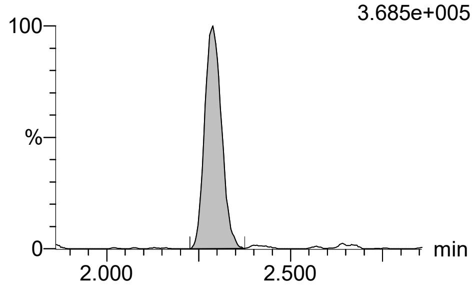

Sample Name: 20211111_A22_R2_IgG
F18:MRM of 1 channel,ES+
927.44 > 204.1
1.567e+005

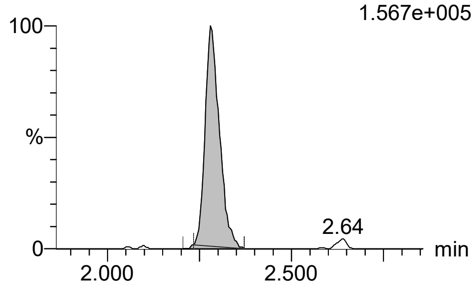

Sample Name: 20211111_L21_R2_IgG
F18:MRM of 1 channel,ES+
927.44 > 204.1
1.030e+005

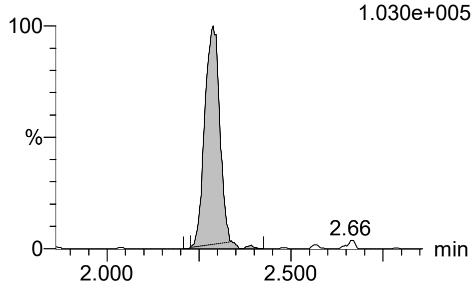

Dataset: C:\MassLynx\10.pro\20211115_clinicalsamples_IgG.qld

Last Altered: Sunday, October 02, 2022 16:02:30 台北標準時間
Printed: Friday, August 01, 2025 16:55:04 台北標準時間

Sample Name: 20211111_L22_R2_IgG
F18:MRM of 1 channel,ES+
927.44 > 204.1
1.655e+005

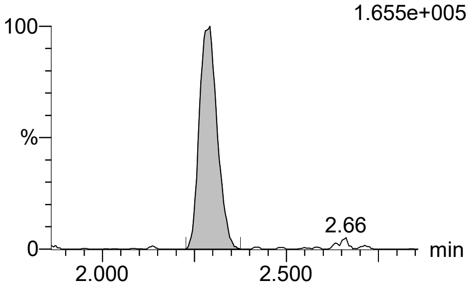

Sample Name: 20211111_H11_R2_IgG
F18:MRM of 1 channel,ES+
927.44 > 204.1
4.262e+005

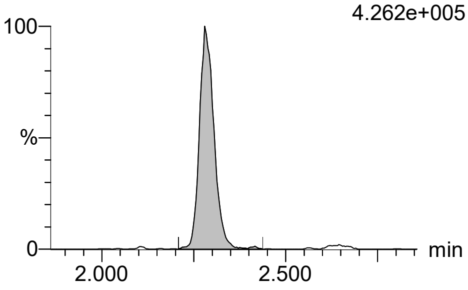

Sample Name: 20211111_A23_R2_IgG
F18:MRM of 1 channel,ES+
927.44 > 204.1
1.817e+005

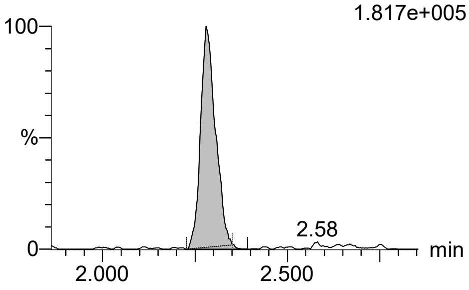

Sample Name: 20211111_A24_R2_IgG
F18:MRM of 1 channel,ES+
927.44 > 204.1
7.143e+004

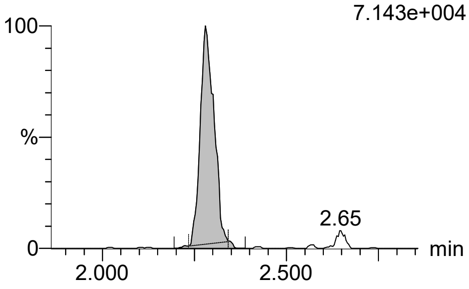

Sample Name: 20211111_L23_R2_IgG
F18:MRM of 1 channel,ES+
927.44 > 204.1
4.434e+005

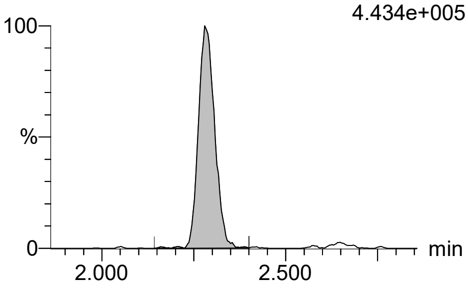

Sample Name: 20211111_L24_R2_IgG
F18:MRM of 1 channel,ES+
927.44 > 204.1
1.790e+005

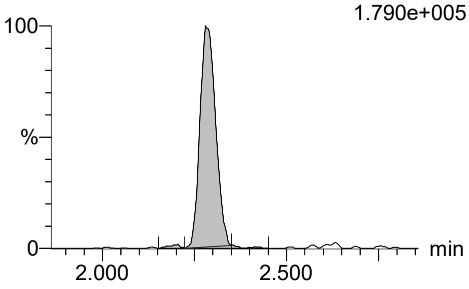

Sample Name: 20211111_H12_R2_IgG
F18:MRM of 1 channel,ES+
927.44 > 204.1
3.802e+004

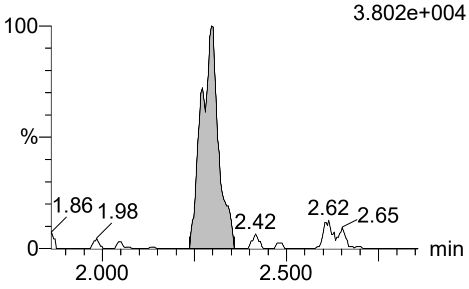

Sample Name: 20211111_QC_R14_IgG
F18:MRM of 1 channel,ES+
927.44 > 204.1
2.157e+005

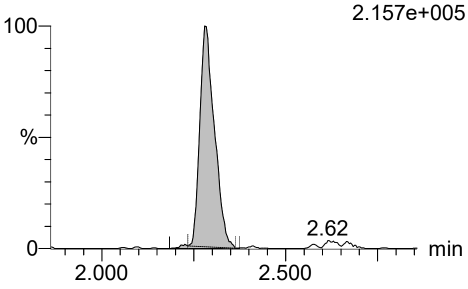

Sample Name: 20211111_A25_R2_IgG
F18:MRM of 1 channel,ES+
927.44 > 204.1
5.584e+005

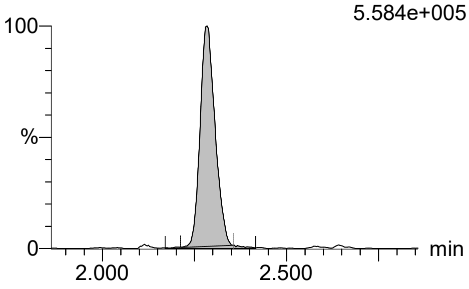

Sample Name: 20211111_A26_R2_IgG
F18:MRM of 1 channel,ES+
927.44 > 204.1
4.537e+004

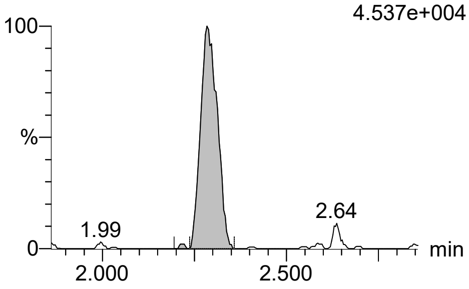

Sample Name: 20211111_L25_R2_IgG
F18:MRM of 1 channel,ES+
927.44 > 204.1
2.229e+005

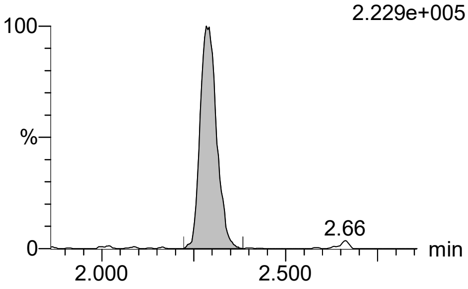

Sample Name: 20211111_L26_R2_IgG
F18:MRM of 1 channel,ES+
927.44 > 204.1
1.294e+005

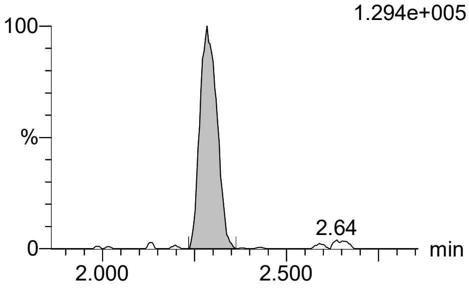

Sample Name: 20211111_H13_R2_IgG
F18:MRM of 1 channel,ES+
927.44 > 204.1
1.743e+005

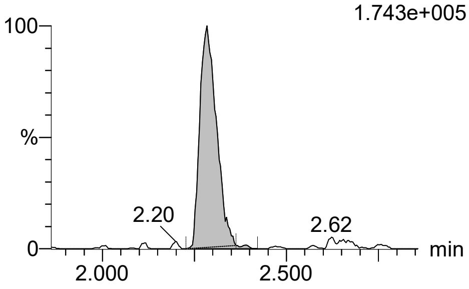

Sample Name: 20211111_A27_R2_IgG
F18:MRM of 1 channel,ES+
927.44 > 204.1
1.673e+005

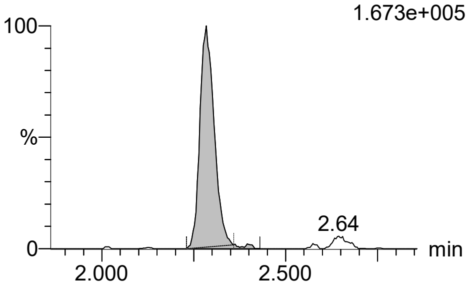

Sample Name: 20211111_A28_R2_IgG
F18:MRM of 1 channel,ES+
927.44 > 204.1
2.615e+005

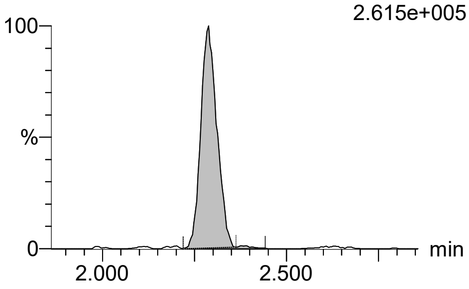

Dataset: C:\MassLynx\10.pro\20211115_clinicalsamples_IgG.qld

Last Altered: Sunday, October 02, 2022 16:02:30 台北標準時間
Printed: Friday, August 01, 2025 16:55:04 台北標準時間

Sample Name: 20211111_L27_R2_IgG

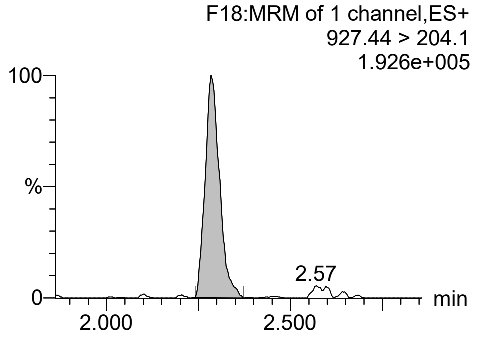

Sample Name: 20211111_L28_R2_IgG

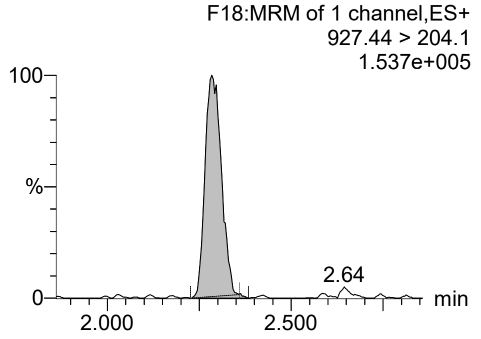

Sample Name: 20211111_H14_R2_IgG

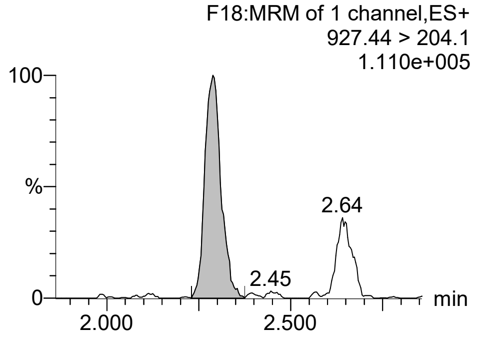

Sample Name: 20211111_A29_R2_IgG

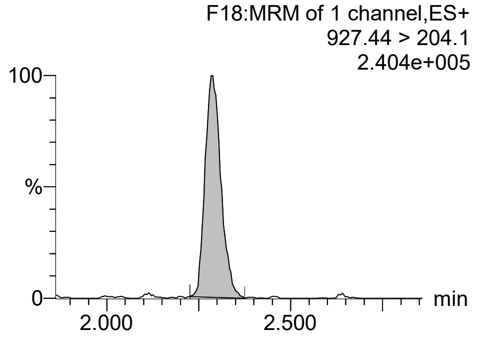

Sample Name: 20211111_A30_R2_IgG

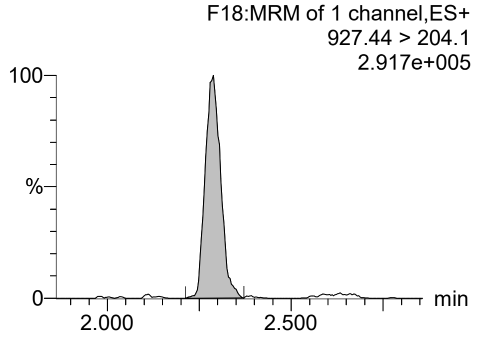

Sample Name: 20211111_QC_R15_IgG

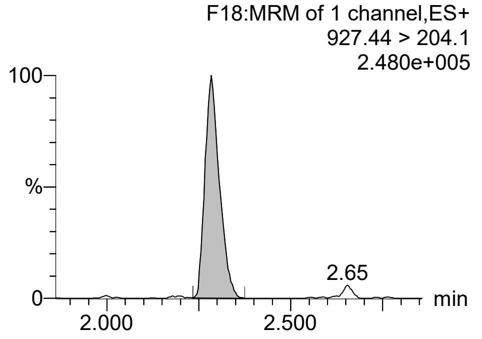

Sample Name: 20211111_L29_R2_IgG

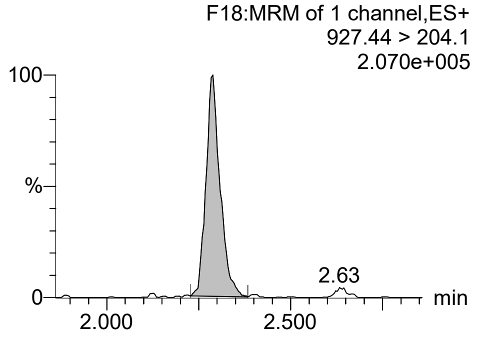

Sample Name: 20211111_L30_R2_IgG

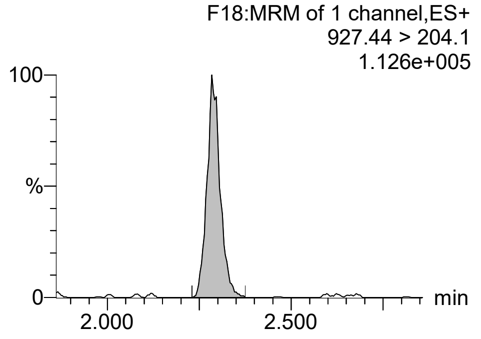

Sample Name: 20211111_H15_R2_IgG

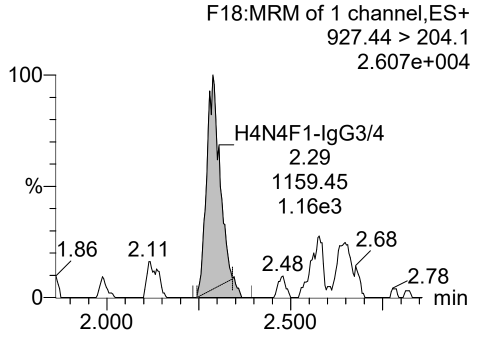

Sample Name: 20211111_A31_R2_IgG

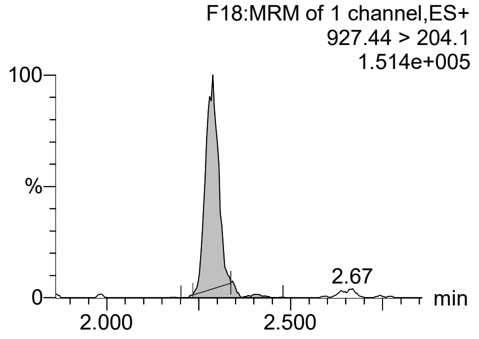

Sample Name: 20211111_A32_R2_IgG

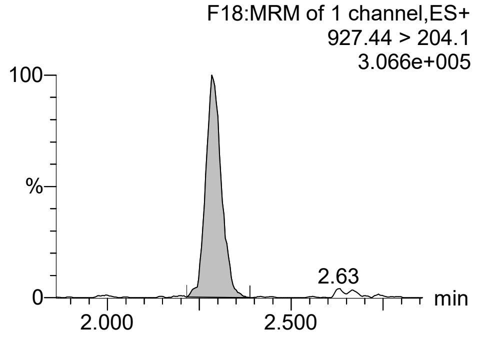

Sample Name: 20211111_L31_R2_IgG

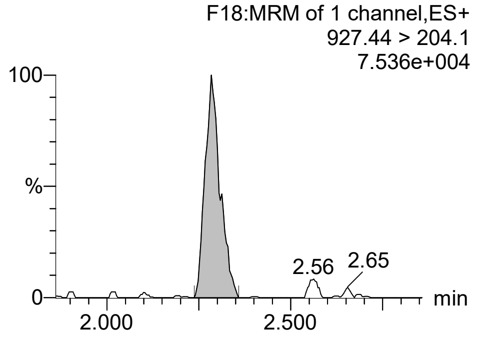

Sample Name: 20211111_L32_R2_IgG

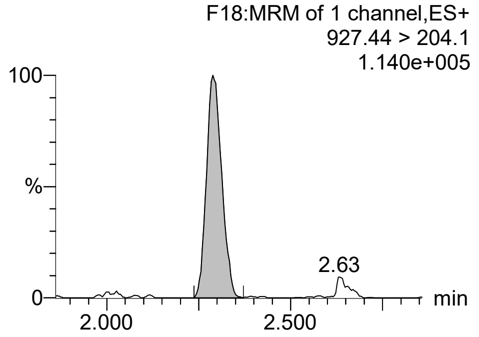

Sample Name: 20211111_H16_R2_IgG

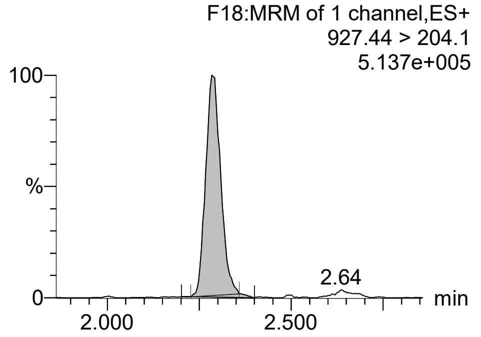

Sample Name: 20211111_A33_R2_IgG

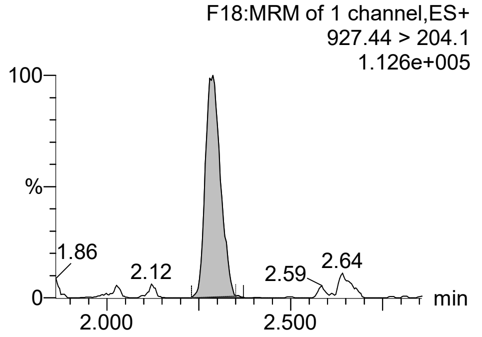

Dataset: C:\MassLynx\10.pro\20211115_clinicalsamples_IgG.qld

Last Altered: Sunday, October 02, 2022 16:02:30 台北標準時間
Printed: Friday, August 01, 2025 16:55:04 台北標準時間

Sample Name: 20211111_A34_R2_IgG
F18:MRM of 1 channel,ES+
927.44 > 204.1
2.077e+005

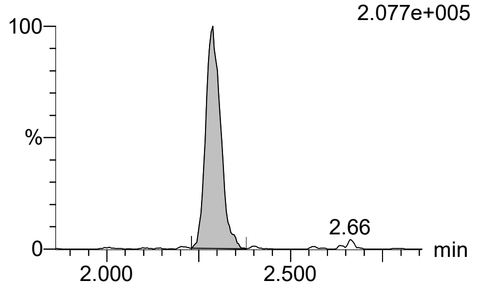

Sample Name: 20211111_H17_R2_IgG
F18:MRM of 1 channel,ES+
927.44 > 204.1
5.245e+005

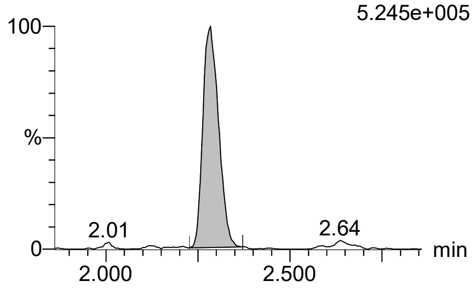

Sample Name: 20211111_A35_R2_IgG
F18:MRM of 1 channel,ES+
927.44 > 204.1
5.587e+005

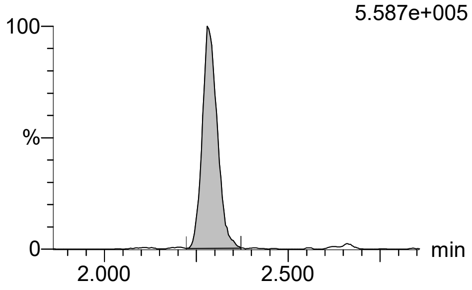

Sample Name: 20211111_QC_R16_IgG
F18:MRM of 1 channel,ES+
927.44 > 204.1
2.375e+005

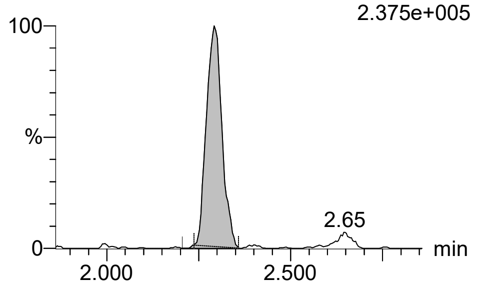

Sample Name: 20211111_A36_R2_IgG
F18:MRM of 1 channel,ES+
927.44 > 204.1
3.866e+005

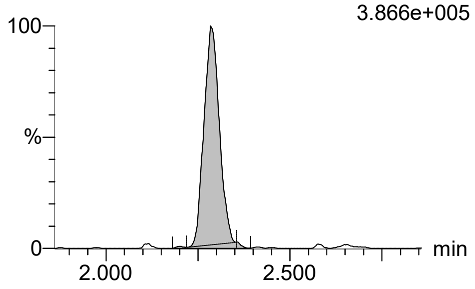

Sample Name: 20211111_H18_R2_IgG
F18:MRM of 1 channel,ES+
927.44 > 204.1
2.041e+006

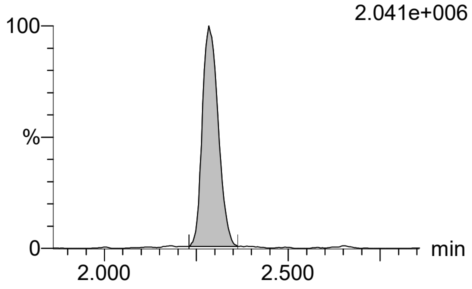

Sample Name: 20211111_A37_R2_IgG
F18:MRM of 1 channel,ES+
927.44 > 204.1
7.902e+004

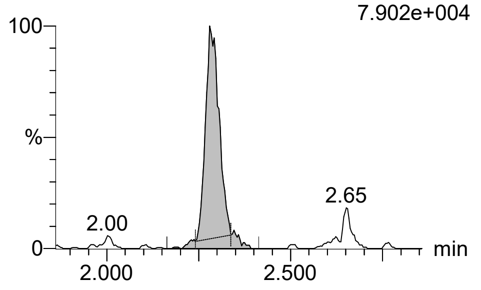

Sample Name: 20211111_A38_R2_IgG
F18:MRM of 1 channel,ES+
927.44 > 204.1
2.207e+005

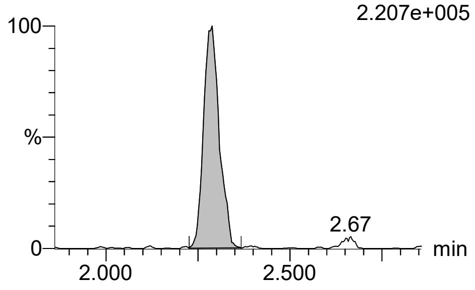

Sample Name: 20211111_H19_R2_IgG
F18:MRM of 1 channel,ES+
927.44 > 204.1
1.191e+006

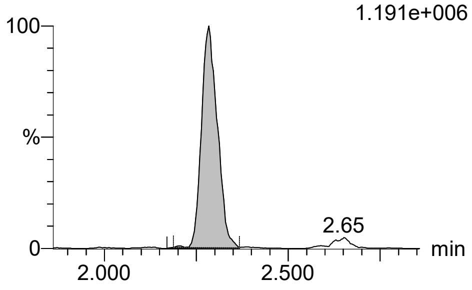

Sample Name: 20211111_A39_R2_IgG
F18:MRM of 1 channel,ES+
927.44 > 204.1
2.441e+005

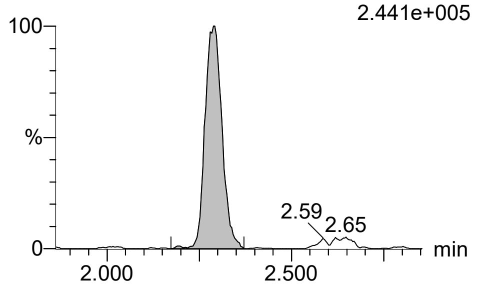

Sample Name: 20211111_A40_R2_IgG
F18:MRM of 1 channel,ES+
927.44 > 204.1
3.615e+005

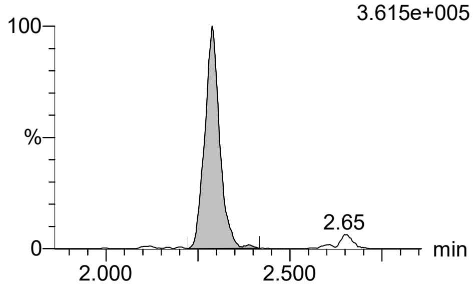

Sample Name: 20211111_H20_R2_IgG
F18:MRM of 1 channel,ES+
927.44 > 204.1
1.188e+006

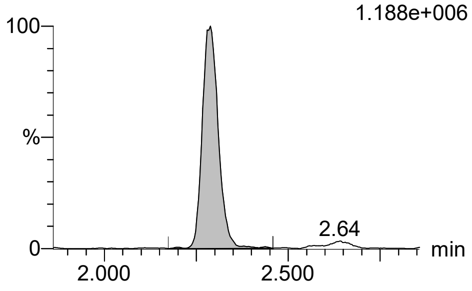

Sample Name: 20211111_A41_R2_IgG
F18:MRM of 1 channel,ES+
927.44 > 204.1
5.095e+004

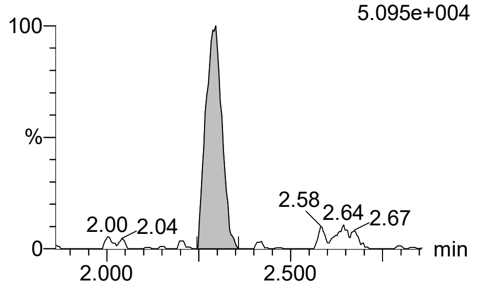

Sample Name: 20211111_A42_R2_IgG
F18:MRM of 1 channel,ES+
927.44 > 204.1
1.217e+005

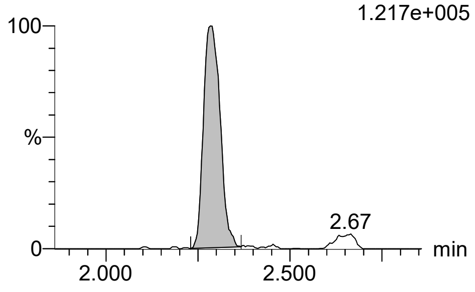

Sample Name: 20211111_A43_R2_IgG
F18:MRM of 1 channel,ES+
927.44 > 204.1
3.131e+005

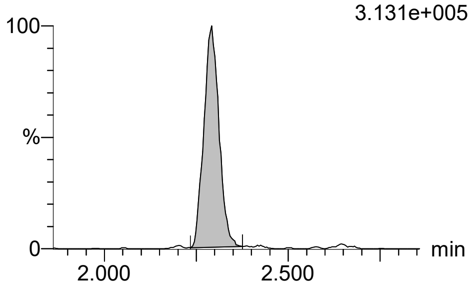

Sample Name: 20211111_A44_R2_IgG

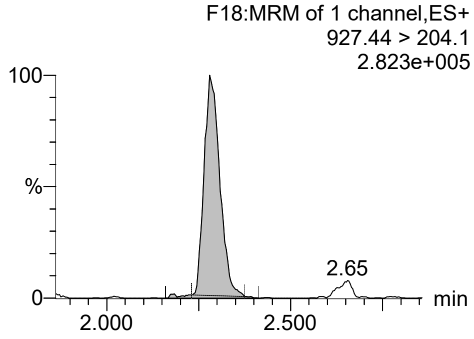

Sample Name: 20211111_QC_R17_IgG

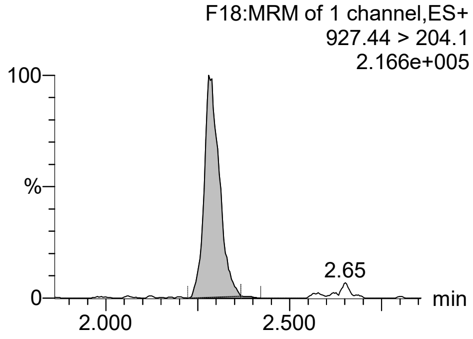

Dataset: C:\MassLynx\10.pro\20211115_clinicalsamples_IgG.qld

Last Altered: Sunday, October 02, 2022 16:02:30 台北標準時間
Printed: Friday, August 01, 2025 16:55:04 台北標準時間

	#	Name	RT	Area	IS Area	Response	Prima...
1	1	20211111_QC_R1_IgG	2.29	26257.3	64968.0	0.404	bb
2	2	20211111_A1_R1_IgG	2.29	23602.2	59059.2	0.400	bb
3	3	20211111_A2_R1_IgG	2.29	11512.3	66621.2	0.173	bb
4	4	20211111_L1_R1_IgG	2.29	17652.3	64201.0	0.275	bb
5	5	20211111_L2_R1_IgG	2.29	13111.2	46950.2	0.279	bb
6	6	20211111_H1_R1_IgG	2.29	7548.2	38648.2	0.195	MM
7	7	20211111_A3_R1_IgG	2.30	1512.5	2603.7	0.581	bb
8	8	20211111_A4_R1_IgG	2.29	9839.6	69960.4	0.141	bb
9	9	20211111_L3_R1_IgG	2.29	4903.3	38864.7	0.126	bb
10	10	20211111_L4_R1_IgG	2.30	13442.8	43380.5	0.310	bb
11	11	20211111_H2_R1_IgG	2.29	38231.1	71349.2	0.536	bb
12	12	20211111_A5_R1_IgG	2.29	22682.0	65569.7	0.346	bb
13	13	20211111_A6_R1_IgG	2.29	50377.5	53120.7	0.948	bb
14	14	20211111_QC_R2_IgG	2.30	27499.6	81034.1	0.339	MM
15	15	20211111_L5_R1_IgG	2.29	27996.2	50994.6	0.549	bb
16	16	20211111_L6_R1_IgG	2.29	8562.6	58058.0	0.147	MM
17	17	20211111_H3_R1_IgG	2.29	6868.4	64028.6	0.107	MM
18	18	20211111_A7_R1_IgG	2.29	36568.7	82996.1	0.441	bb
19	19	20211111_A8_R1_IgG	2.29	13183.4	77731.7	0.170	bb
20	20	20211111_L7_R1_IgG	2.30	14160.7	38827.3	0.365	MM
21	21	20211111_L8_R1_IgG	2.29	11943.0	44220.1	0.270	bb
22	22	20211111_H4_R1_IgG	2.29	12546.8	65826.3	0.191	MM
23	23	20211111_A9_R1_IgG	2.29	10756.0	60771.1	0.177	MM
24	24	20211111_A10_R1_IgG	2.29	16564.7	48578.7	0.341	MM
25	25	20211111_L9_R1_IgG	2.29	14133.1	50830.7	0.278	bb
26	26	20211111_L10_R1_IgG	2.29	10684.2	38034.2	0.281	MM
27	27	20211111_QC_R3_IgG	2.30	22892.1	67025.5	0.342	MM
28	28	20211111_H5_R1_IgG	2.30	3749.1	44544.9	0.084	bb
29	29	20211111_A11_R1_IgG	2.29	3806.4	66408.5	0.057	bb
30	30	20211111_A12_R1_IgG	2.29	11058.4	46624.5	0.237	bb
31	31	20211111_L11_R1_IgG	2.29	8555.5	33425.1	0.256	bb
32	32	20211111_L12_R1_IgG	2.29	5981.2	41178.0	0.145	MM
33	33	20211111_H6_R1_IgG	2.29	32139.8	58593.4	0.549	bb
34	34	20211111_A13_R1_IgG	2.29	46650.1	54133.0	0.862	MM
35	35	20211111_A14_R1_IgG	2.29	12195.1	56467.3	0.216	MM
36	36	20211111_L13_R1_IgG	2.29	11105.8	36024.4	0.308	MM
37	37	20211111_L14_R1_IgG	2.29	15147.6	37181.6	0.407	MM
38	38	20211111_H7_R1_IgG	2.29	17837.2	42850.6	0.416	MM
39	39	20211111_A15_R1_IgG	2.29	7403.6	62015.1	0.119	bb
40	40	20211111_QC_R4_IgG	2.29	21084.2	58572.5	0.360	bb
41	41	20211111_A16_R1_IgG	2.29	26871.1	78283.8	0.343	bb
42	42	20211111_L15_R1_IgG	2.29	21153.7	44158.2	0.479	bb
43	43	20211111_L16_R1_IgG	2.28	10016.5	55150.8	0.182	MM
44	44	20211111_H8_R1_IgG	2.30	15447.8	42965.2	0.360	MM
45	45	20211111_A17_R1_IgG	2.29	17010.0	39717.7	0.428	bb
46	46	20211111_A18_R1_IgG	2.29	16987.6	44675.4	0.380	MM
47	47	20211111_L17_R1_IgG	2.29	7298.4	37854.1	0.193	bb
48	48	20211111_L18_R1_IgG	2.29	6454.3	50716.9	0.127	MM
49	49	20211111_H9_R1_IgG	2.29	16120.7	36276.0	0.444	bb
50	50	20211111_A19_R1_IgG	2.29	4067.0	42862.5	0.095	MM
51	51	20211111_A20_R1_IgG	2.30	4555.3	36542.7	0.125	bb
52	52	20211111_L19_R1_IgG	2.29	9318.0	53892.8	0.173	bb
53	53	20211111_QC_R5_IgG	2.29	17542.6	56513.2	0.310	bb
54	54	20211111_L20_R1_IgG	2.30	16025.1	56499.9	0.284	bb
55	55	20211111_H10_R1_IgG	2.29	24067.9	68120.5	0.353	MM
56	56	20211111_A21_R1_IgG	2.29	34742.4	63699.8	0.545	bb

Dataset: C:\MassLynx\10.pro\20211115_clinicalsamples_IgG.qld

Last Altered: Sunday, October 02, 2022 16:02:30 台北標準時間
Printed: Friday, August 01, 2025 16:55:04 台北標準時間

	# Name	RT	Area	IS Area	Response	Prima...
57	57 20211111_A22_R1_IgG	2.29	11977.1	52222.1	0.229	bb
58	58 20211111_L21_R1_IgG	2.30	7654.3	54372.5	0.141	bb
59	59 20211111_L22_R1_IgG	2.29	14404.9	41353.1	0.348	MM
60	60 20211111_H11_R1_IgG	2.29	32254.6	65579.1	0.492	bb
61	61 20211111_A23_R1_IgG	2.29	14494.3	64131.1	0.226	bb
62	62 20211111_A24_R1_IgG	2.29	4195.3	38724.8	0.108	bb
63	63 20211111_L23_R1_IgG	2.29	34372.2	64245.2	0.535	bb
64	64 20211111_L24_R1_IgG	2.29	11023.8	45298.0	0.243	bb
65	65 20211111_H12_R1_IgG	2.30	2342.6	44679.3	0.052	MM
66	66 20211111_QC_R6_IgG	2.29	16586.1	48165.0	0.344	bb
67	67 20211111_A25_R1_IgG	2.29	42153.3	57169.9	0.737	bb
68	68 20211111_A26_R1_IgG	2.29	2979.8	34254.0	0.087	MM
69	69 20211111_L25_R1_IgG	2.29	19193.2	67491.5	0.284	bb
70	70 20211111_L26_R1_IgG	2.30	9081.2	55934.1	0.162	MM
71	71 20211111_H13_R1_IgG	2.29	12989.2	48767.3	0.266	MM
72	72 20211111_A27_R1_IgG	2.29	12734.2	57269.7	0.222	bb
73	73 20211111_A28_R1_IgG	2.29	20968.6	49231.1	0.426	MM
74	74 20211111_L27_R1_IgG	2.29	12997.5	60014.5	0.217	MM
75	75 20211111_L28_R1_IgG	2.29	10686.3	35062.6	0.305	bb
76	76 20211111_H14_R1_IgG	2.29	10175.7	62371.9	0.163	MM
77	77 20211111_A29_R1_IgG	2.29	15549.1	43858.2	0.355	MM
78	78 20211111_A30_R1_IgG	2.29	22688.2	62500.4	0.363	MM
79	79 20211111_QC_R7_IgG	2.30	14178.3	43946.9	0.323	bb
80	80 20211111_L29_R1_IgG	2.29	11022.3	38728.9	0.285	bb
81	81 20211111_L30_R1_IgG	2.29	6162.6	42613.2	0.145	bb
82	82 20211111_H15_R1_IgG	2.28	1328.8	39874.6	0.033	MM
83	83 20211111_A31_R1_IgG	2.30	9552.0	53414.3	0.179	bb
84	84 20211111_A32_R1_IgG	2.29	18191.9	41844.6	0.435	bb
85	85 20211111_L31_R1_IgG	2.29	5826.6	40553.7	0.144	bb
86	86 20211111_L32_R1_IgG	2.30	6034.8	49618.7	0.122	MM
87	87 20211111_H16_R1_IgG	2.29	34765.2	60170.9	0.578	bb
88	88 20211111_A33_R1_IgG	2.29	7430.2	48588.2	0.153	bb
89	89 20211111_A34_R1_IgG	2.29	12256.4	43288.0	0.283	bb
90	90 20211111_H17_R1_IgG	2.29	30008.9	47104.5	0.637	bb
91	91 20211111_A35_R1_IgG	2.30	30261.6	41025.9	0.738	MM
92	92 20211111_QC_R8_IgG	2.30	12724.7	40546.3	0.314	bb
93	93 20211111_A36_R1_IgG	2.29	22330.3	44504.4	0.502	MM
94	94 20211111_H18_R1_IgG	2.29	109277.6	34162.2	3.199	MM
95	95 20211111_A37_R1_IgG	2.30	3571.0	39760.0	0.090	MM
96	96 20211111_A38_R1_IgG	2.30	13668.3	43982.6	0.311	MM
97	97 20211111_H19_R1_IgG	2.29	64474.0	45314.8	1.423	bb
98	98 20211111_A39_R1_IgG	2.29	13623.5	39388.0	0.346	MM
99	99 20211111_A40_R1_IgG	2.29	20435.8	38726.4	0.528	bb
100	1... 20211111_H20_R1_IgG	2.29	64033.3	37814.4	1.693	bb
101	1... 20211111_A41_R1_IgG	2.31	2698.2	48930.3	0.055	MM
102	1... 20211111_A42_R1_IgG	2.29	8198.8	40406.8	0.203	bb
103	1... 20211111_A43_R1_IgG	2.29	17273.0	44421.5	0.389	bb
104	1... 20211111_A44_R1_IgG	2.29	20040.9	53128.2	0.377	bb
105	1... 20211111_QC_R9_IgG	2.29	12345.0	37049.0	0.333	bb
106	1... 20211111_A1_R2_IgG	2.29	14441.2	46499.4	0.311	bb
107	1... 20211111_A2_R2_IgG	2.30	8685.4	57260.7	0.152	bb
108	1... 20211111_L1_R2_IgG	2.29	11417.3	48062.9	0.238	bb
109	1... 20211111_L2_R2_IgG	2.30	8206.6	38745.2	0.212	MM
110	1... 20211111_H1_R2_IgG	2.30	8458.8	44273.5	0.191	bb
111	1... 20211111_A3_R2_IgG	2.29	2010.5	4755.5	0.423	MM
112	1... 20211111_A4_R2_IgG	2.29	6523.7	50546.7	0.129	MM

Dataset: C:\MassLynx\10.pro\20211115_clinicalsamples_IgG.qld

Last Altered: Sunday, October 02, 2022 16:02:30 台北標準時間
Printed: Friday, August 01, 2025 16:55:04 台北標準時間

	# Name	RT	Area	IS Area	Response	Prima...
113	1... 20211111_L3_R2_IgG	2.29	3952.5	34825.8	0.113	MM
114	1... 20211111_L4_R2_IgG	2.29	11770.3	39107.7	0.301	MM
115	1... 20211111_H2_R2_IgG	2.29	24895.6	49750.0	0.500	MM
116	1... 20211111_A5_R2_IgG	2.28	20206.1	52536.2	0.385	bb
117	1... 20211111_A6_R2_IgG	2.29	46703.7	42050.0	1.111	bb
118	1... 20211111_QC_R10_IgG	2.30	10745.2	35350.8	0.304	bb
119	1... 20211111_L5_R2_IgG	2.29	21373.9	38039.3	0.562	bb
120	1... 20211111_L6_R2_IgG	2.30	5676.8	44907.4	0.126	bb
121	1... 20211111_H3_R2_IgG	2.28	4793.8	47729.1	0.100	MM
122	1... 20211111_A7_R2_IgG	2.28	22140.9	57347.1	0.386	MM
123	1... 20211111_A8_R2_IgG	2.29	10744.9	52397.8	0.205	MM
124	1... 20211111_L7_R2_IgG	2.29	12638.0	36318.4	0.348	bb
125	1... 20211111_L8_R2_IgG	2.29	10199.7	38131.9	0.267	bb
126	1... 20211111_H4_R2_IgG	2.30	9571.0	46849.6	0.204	bb
127	1... 20211111_A9_R2_IgG	2.29	8651.9	47759.6	0.181	MM
128	1... 20211111_A10_R2_IgG	2.29	16224.2	41366.2	0.392	bb
129	1... 20211111_L9_R2_IgG	2.29	11781.8	43052.1	0.274	MM
130	1... 20211111_L10_R2_IgG	2.29	9165.0	31487.4	0.291	bb
131	1... 20211111_QC_R11_IgG	2.29	11717.7	37804.6	0.310	MM
132	1... 20211111_H5_R2_IgG	2.30	3945.0	40380.0	0.098	MM
133	1... 20211111_A11_R2_IgG	2.29	2566.7	49533.3	0.052	bb
134	1... 20211111_A12_R2_IgG	2.29	9557.5	40226.3	0.238	bb
135	1... 20211111_L11_R2_IgG	2.30	7050.5	28884.0	0.244	bb
136	1... 20211111_L12_R2_IgG	2.29	5379.2	34351.3	0.157	bb
137	1... 20211111_H6_R2_IgG	2.29	20812.5	40466.6	0.514	bb
138	1... 20211111_A13_R2_IgG	2.29	32330.7	42306.7	0.764	MM
139	1... 20211111_A14_R2_IgG	2.29	9230.2	44904.0	0.206	MM
140	1... 20211111_L13_R2_IgG	2.29	8697.3	31913.0	0.273	bb
141	1... 20211111_L14_R2_IgG	2.29	14566.0	31527.4	0.462	bb
142	1... 20211111_H7_R2_IgG	2.29	17424.5	40838.2	0.427	bb
143	1... 20211111_A15_R2_IgG	2.29	6603.6	49389.7	0.134	MM
144	1... 20211111_QC_R12_IgG	2.29	11923.8	35168.7	0.339	MM
145	1... 20211111_A16_R2_IgG	2.30	15481.7	47440.5	0.326	bb
146	1... 20211111_L15_R2_IgG	2.29	15402.3	33145.9	0.465	bb
147	1... 20211111_L16_R2_IgG	2.28	5651.5	37596.6	0.150	bb
148	1... 20211111_H8_R2_IgG	2.29	13997.6	43933.3	0.319	MM
149	1... 20211111_A17_R2_IgG	2.29	15781.5	43605.5	0.362	bb
150	1... 20211111_A18_R2_IgG	2.29	12070.5	37581.8	0.321	bb
151	1... 20211111_L17_R2_IgG	2.29	6885.4	33942.5	0.203	bb
152	1... 20211111_L18_R2_IgG	2.29	4385.4	37831.5	0.116	bb
153	1... 20211111_H9_R2_IgG	2.29	13547.3	30898.4	0.438	bb
154	1... 20211111_A19_R2_IgG	2.29	4703.3	39352.1	0.120	MM
155	1... 20211111_A20_R2_IgG	2.29	3827.8	34661.2	0.110	bb
156	1... 20211111_L19_R2_IgG	2.29	7128.7	39918.3	0.179	MM
157	1... 20211111_QC_R13_IgG	2.29	11023.5	36239.1	0.304	bb
158	1... 20211111_L20_R2_IgG	2.29	10044.8	32897.7	0.305	MM
159	1... 20211111_H10_R2_IgG	2.28	11500.6	31394.5	0.366	bb
160	1... 20211111_A21_R2_IgG	2.29	19555.1	34863.7	0.561	bb
161	1... 20211111_A22_R2_IgG	2.28	7132.4	30342.1	0.235	MM
162	1... 20211111_L21_R2_IgG	2.29	4992.9	33706.2	0.148	MM
163	1... 20211111_L22_R2_IgG	2.29	9318.3	31298.6	0.298	bb
164	1... 20211111_H11_R2_IgG	2.28	19459.1	38904.1	0.500	bb
165	1... 20211111_A23_R2_IgG	2.28	8606.9	32382.3	0.266	MM
166	1... 20211111_A24_R2_IgG	2.28	3195.6	30261.9	0.106	MM
167	1... 20211111_L23_R2_IgG	2.28	22734.3	43037.5	0.528	bb
168	1... 20211111_L24_R2_IgG	2.28	9114.4	37023.3	0.246	MM

Dataset: C:\MassLynx\10.pro\20211115_clinicalsamples_IgG.qld

Last Altered: Sunday, October 02, 2022 16:02:30 台北標準時間
Printed: Friday, August 01, 2025 16:55:04 台北標準時間

	#	Name	RT	Area	IS Area	Response	Prima...
169	1...	20211111_H12_R2_IgG	2.30	2055.9	39478.2	0.052	bb
170	1...	20211111_QC_R14_IgG	2.28	10079.4	34463.2	0.292	MM
171	1...	20211111_A25_R2_IgG	2.28	27024.8	39945.3	0.677	MM
172	1...	20211111_A26_R2_IgG	2.28	2408.4	29663.2	0.081	MM
173	1...	20211111_L25_R2_IgG	2.28	11597.8	38589.3	0.301	bb
174	1...	20211111_L26_R2_IgG	2.28	6950.2	38405.2	0.181	bb
175	1...	20211111_H13_R2_IgG	2.28	9254.0	37771.2	0.245	MM
176	1...	20211111_A27_R2_IgG	2.28	7728.9	42634.1	0.181	MM
177	1...	20211111_A28_R2_IgG	2.29	13427.0	33231.1	0.404	MM
178	1...	20211111_L27_R2_IgG	2.28	8743.7	41761.1	0.209	bb
179	1...	20211111_L28_R2_IgG	2.28	8037.2	26968.6	0.298	MM
180	1...	20211111_H14_R2_IgG	2.29	5670.0	49250.2	0.115	bb
181	1...	20211111_A29_R2_IgG	2.28	11959.3	37601.7	0.318	bb
182	1...	20211111_A30_R2_IgG	2.29	14178.2	40366.8	0.351	bb
183	1...	20211111_QC_R15_IgG	2.28	11256.3	35156.1	0.320	bb
184	1...	20211111_L29_R2_IgG	2.29	9084.7	33813.1	0.269	bb
185	1...	20211111_L30_R2_IgG	2.28	4590.6	33488.6	0.137	bb
186	1...	20211111_H15_R2_IgG	2.29	1159.4	38501.0	0.030	MM
187	1...	20211111_A31_R2_IgG	2.29	6694.6	37525.5	0.178	MM
188	1...	20211111_A32_R2_IgG	2.28	15045.8	35770.8	0.421	bb
189	1...	20211111_L31_R2_IgG	2.28	3549.0	33282.4	0.107	bb
190	1...	20211111_L32_R2_IgG	2.29	5473.1	42049.6	0.130	bb
191	1...	20211111_H16_R2_IgG	2.28	24591.6	45947.6	0.535	MM
192	1...	20211111_A33_R2_IgG	2.29	5407.5	40202.3	0.135	MM
193	1...	20211111_A34_R2_IgG	2.29	9721.4	36747.0	0.265	bb
194	1...	20211111_H17_R2_IgG	2.28	25680.6	40920.9	0.628	bb
195	1...	20211111_A35_R2_IgG	2.28	26205.9	33689.2	0.778	bb
196	1...	20211111_QC_R16_IgG	2.29	11754.1	32783.6	0.359	MM
197	1...	20211111_A36_R2_IgG	2.28	19337.5	36822.4	0.525	MM
198	1...	20211111_H18_R2_IgG	2.28	101335.1	31769.3	3.190	bb
199	1...	20211111_A37_R2_IgG	2.28	3791.0	37581.8	0.101	MM
200	2...	20211111_A38_R2_IgG	2.29	10895.6	38390.4	0.284	bb
201	2...	20211111_H19_R2_IgG	2.28	59914.8	41217.0	1.454	MM
202	2...	20211111_A39_R2_IgG	2.29	12961.1	37392.7	0.347	bb
203	2...	20211111_A40_R2_IgG	2.29	16874.7	38295.1	0.441	bb
204	2...	20211111_H20_R2_IgG	2.29	59972.6	35941.8	1.669	bb
205	2...	20211111_A41_R2_IgG	2.30	2577.0	45304.9	0.057	bb
206	2...	20211111_A42_R2_IgG	2.28	6338.6	37803.8	0.168	bb
207	2...	20211111_A43_R2_IgG	2.29	14788.1	38161.8	0.388	bb
208	2...	20211111_A44_R2_IgG	2.28	14450.7	37819.8	0.382	MM
209	2...	20211111_QC_R17_IgG	2.28	10514.8	31731.8	0.331	MM

Dataset: C:\MassLynx\10.pro\20211115_clinicalsamples_IgG.qld

Last Altered: Sunday, October 02, 2022 16:02:30 台北標準時間
Printed: Friday, August 01, 2025 16:55:36 台北標準時間

Method: C:\MassLynx\10_Pro_IgA.PRO\MethDB\20210926_IgG_DMRM_IgM_IS.mdb 16 Feb 2022 11:58:21
Calibration: 18 Nov 2021 13:56:22

Sample Name: 20211111_QC_R1_IgG
F33:MRM of 1 channel,ES+
995.113 > 204.1
1.717e+005

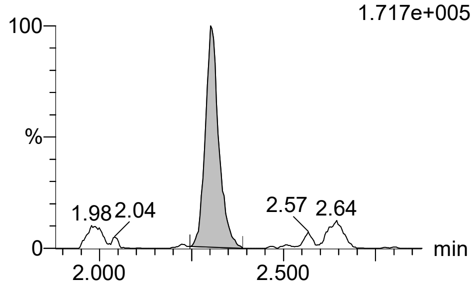

Sample Name: 20211111_A1_R1_IgG
F33:MRM of 1 channel,ES+
995.113 > 204.1
1.436e+005

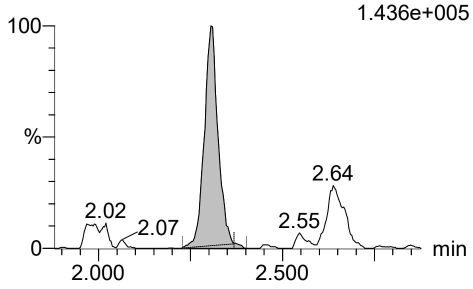

Sample Name: 20211111_A2_R1_IgG
F33:MRM of 1 channel,ES+
995.113 > 204.1
1.220e+005

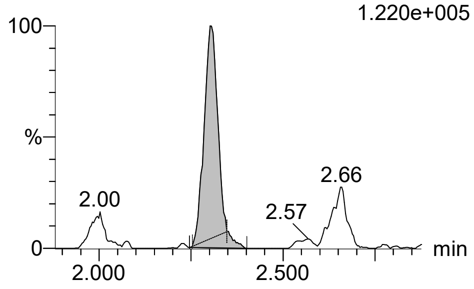

Sample Name: 20211111_L1_R1_IgG
F33:MRM of 1 channel,ES+
995.113 > 204.1
1.092e+005

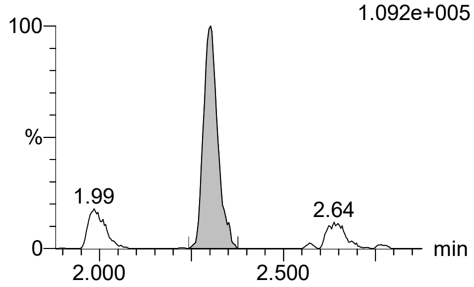

Sample Name: 20211111_L2_R1_IgG
F33:MRM of 1 channel,ES+
995.113 > 204.1
7.385e+004

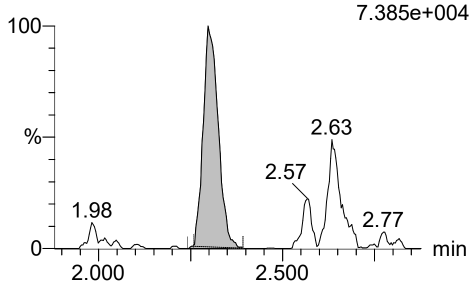

Sample Name: 20211111_H1_R1_IgG
F33:MRM of 1 channel,ES+
995.113 > 204.1
3.656e+004

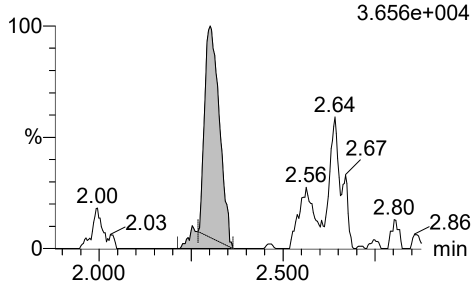

Sample Name: 20211111_A3_R1_IgG
F33:MRM of 1 channel,ES+
995.113 > 204.1
6.937e+003

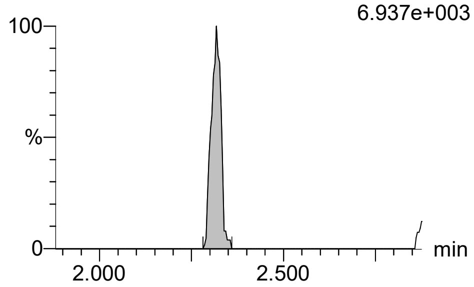

Sample Name: 20211111_A4_R1_IgG
F33:MRM of 1 channel,ES+
995.113 > 204.1
7.855e+004

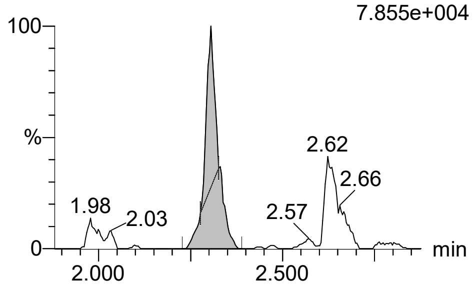

Sample Name: 20211111_L3_R1_IgG
F33:MRM of 1 channel,ES+
995.113 > 204.1
5.130e+004

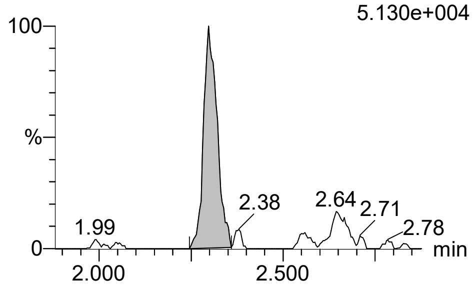

Sample Name: 20211111_L4_R1_IgG
F33:MRM of 1 channel,ES+
995.113 > 204.1
1.061e+005

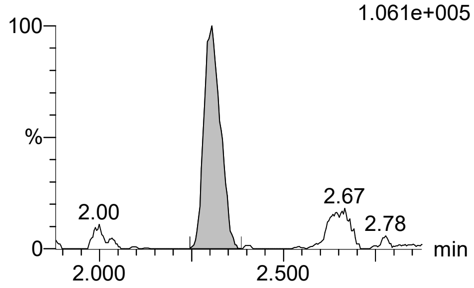

Sample Name: 20211111_H2_R1_IgG
F33:MRM of 1 channel,ES+
995.113 > 204.1
2.042e+005

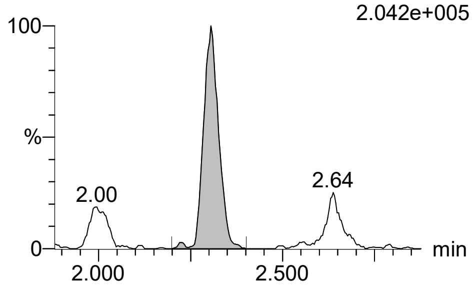

Sample Name: 20211111_A5_R1_IgG
F33:MRM of 1 channel,ES+
995.113 > 204.1
1.078e+005

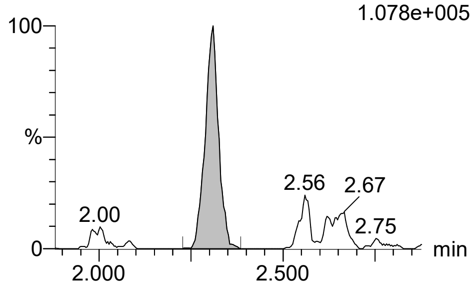

Sample Name: 20211111_A6_R1_IgG
F33:MRM of 1 channel,ES+
995.113 > 204.1
2.683e+005

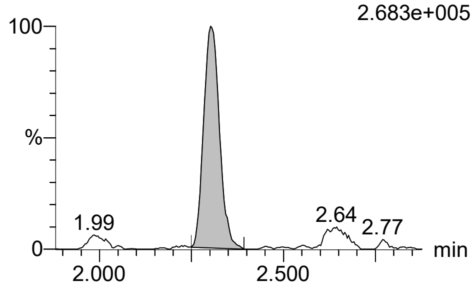

Sample Name: 20211111_QC_R2_IgG
F33:MRM of 1 channel,ES+
995.113 > 204.1
1.439e+005

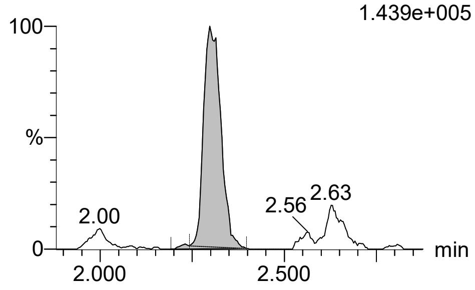

Sample Name: 20211111_L5_R1_IgG
F33:MRM of 1 channel,ES+
995.113 > 204.1
1.133e+005

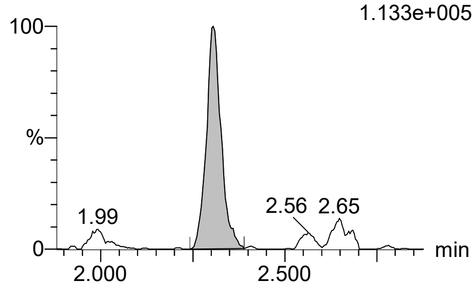

Sample Name: 20211111_L6_R1_IgG
F33:MRM of 1 channel,ES+
995.113 > 204.1
6.198e+004

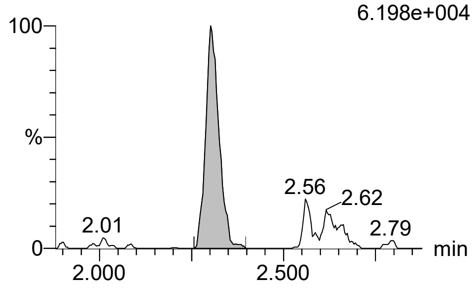

Sample Name: 20211111_H3_R1_IgG
F33:MRM of 1 channel,ES+
995.113 > 204.1
4.142e+004

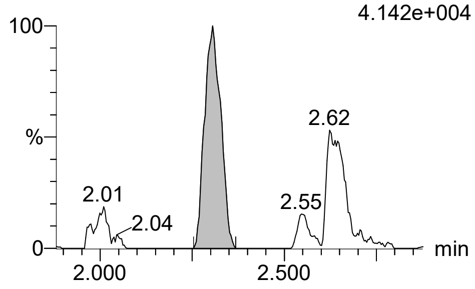

Sample Name: 20211111_A7_R1_IgG
F33:MRM of 1 channel,ES+
995.113 > 204.1
1.103e+005

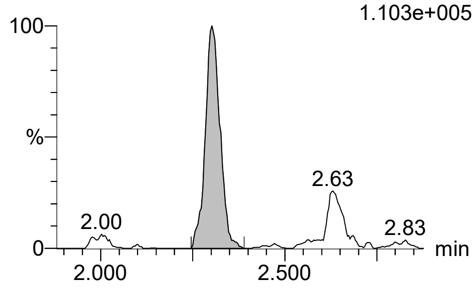

Sample Name: 20211111_A8_R1_IgG
F33:MRM of 1 channel,ES+
995.113 > 204.1
4.408e+004

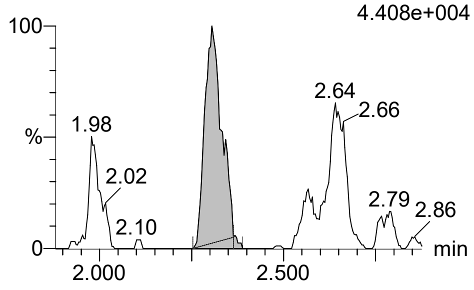

Sample Name: 20211111_L7_R1_IgG
F33:MRM of 1 channel,ES+
995.113 > 204.1
6.875e+004

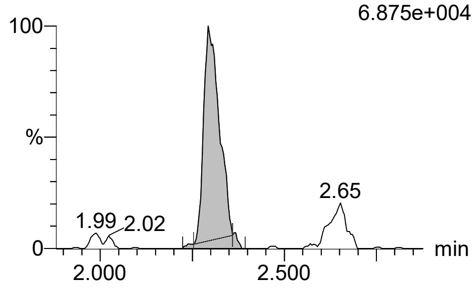

Sample Name: 20211111_L8_R1_IgG
F33:MRM of 1 channel,ES+
995.113 > 204.1
7.157e+004

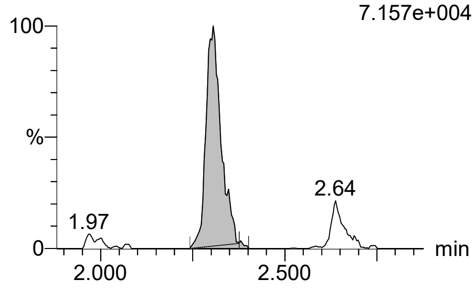

Sample Name: 20211111_H4_R1_IgG
F33:MRM of 1 channel,ES+
995.113 > 204.1
1.041e+005

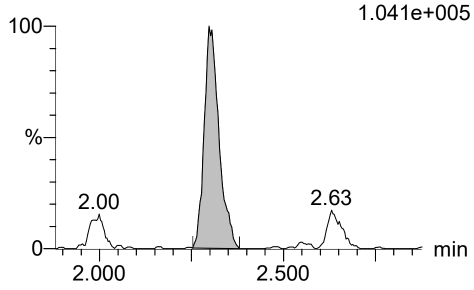

Sample Name: 20211111_A9_R1_IgG
F33:MRM of 1 channel,ES+
995.113 > 204.1
3.902e+004

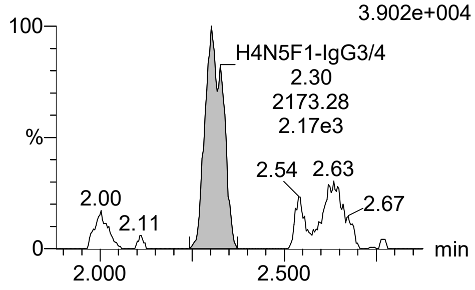

Sample Name: 20211111_A10_R1_IgG
F33:MRM of 1 channel,ES+
995.113 > 204.1
3.867e+004

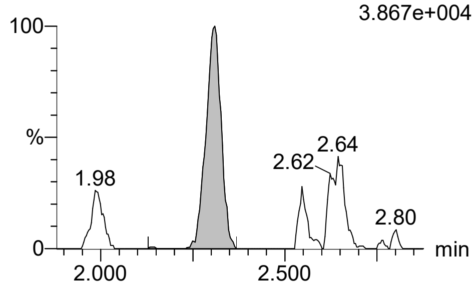

Sample Name: 20211111_L9_R1_IgG
F33:MRM of 1 channel,ES+
995.113 > 204.1
6.124e+004

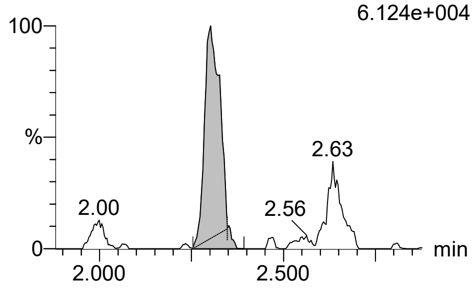

Sample Name: 20211111_L10_R1_IgG
F33:MRM of 1 channel,ES+
995.113 > 204.1
9.884e+004

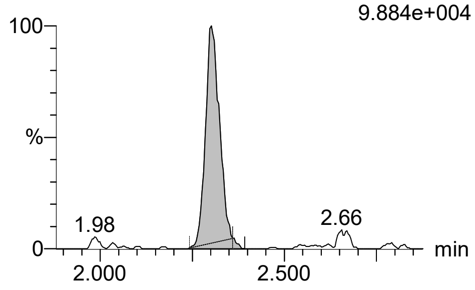

Sample Name: 20211111_QC_R3_IgG
F33:MRM of 1 channel,ES+
995.113 > 204.1
1.600e+005

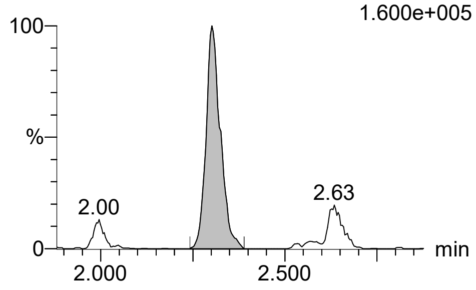

Dataset: C:\MassLynx\10.pro\20211115_clinicalsamples_IgG.qld

Last Altered: Sunday, October 02, 2022 16:02:30 台北標準時間
Printed: Friday, August 01, 2025 16:55:36 台北標準時間

Sample Name: 20211111_H5_R1_IgG

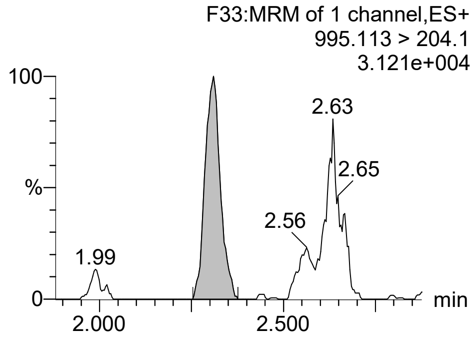

Sample Name: 20211111_A11_R1_IgG

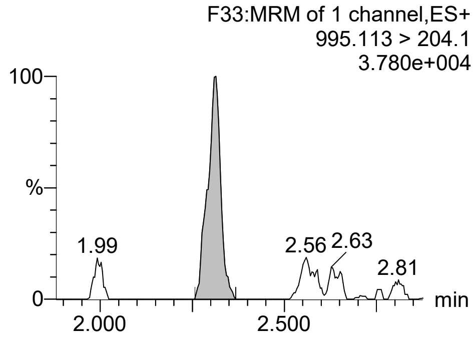

Sample Name: 20211111_A12_R1_IgG

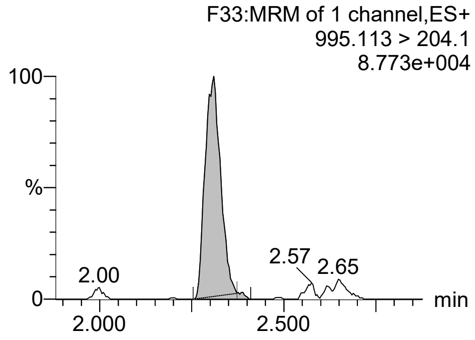

Sample Name: 20211111_L11_R1_IgG

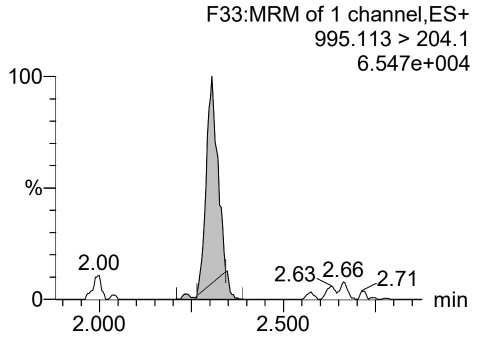

Sample Name: 20211111_L12_R1_IgG

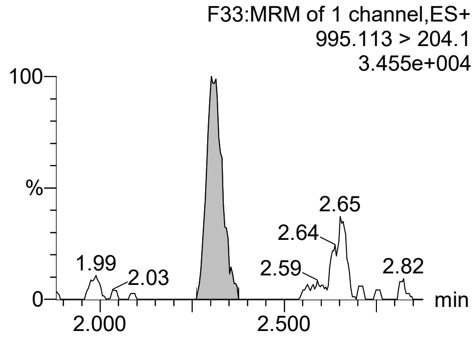

Sample Name: 20211111_H6_R1_IgG

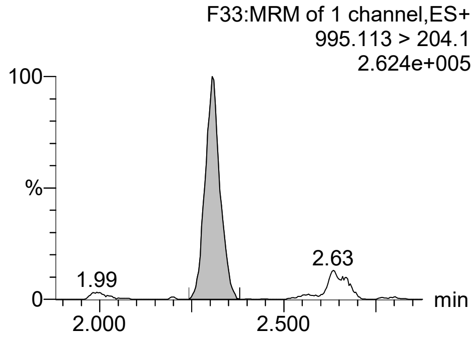

Sample Name: 20211111_A13_R1_IgG

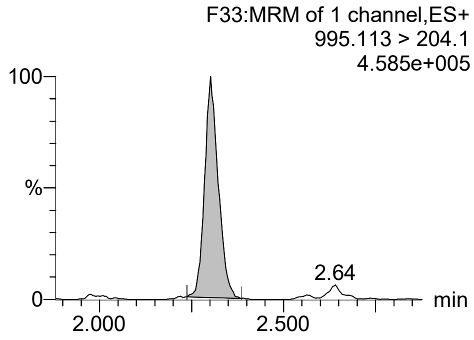

Sample Name: 20211111_A14_R1_IgG

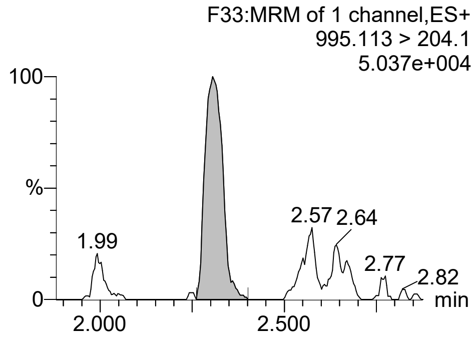

Sample Name: 20211111_L13_R1_IgG

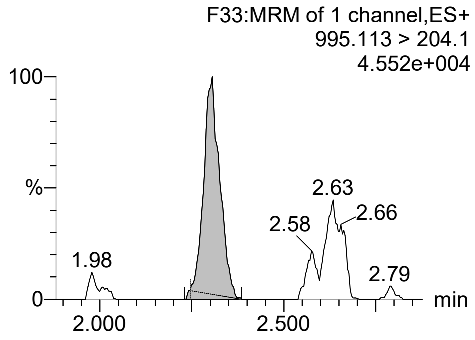

Sample Name: 20211111_L14_R1_IgG

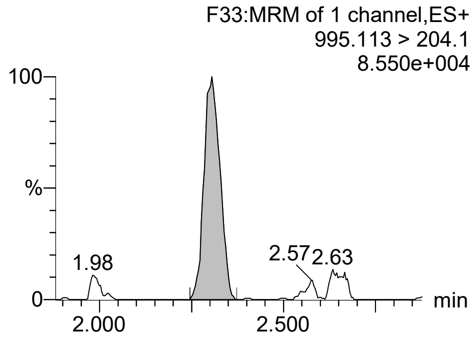

Sample Name: 20211111_H7_R1_IgG

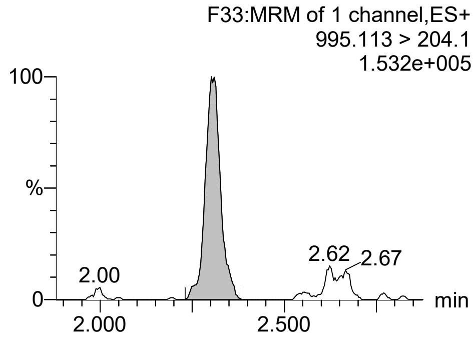

Sample Name: 20211111_A15_R1_IgG

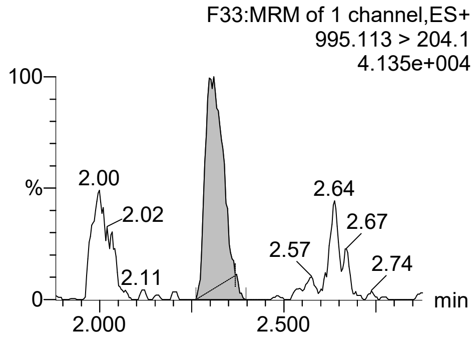

Sample Name: 20211111_QC_R4_IgG

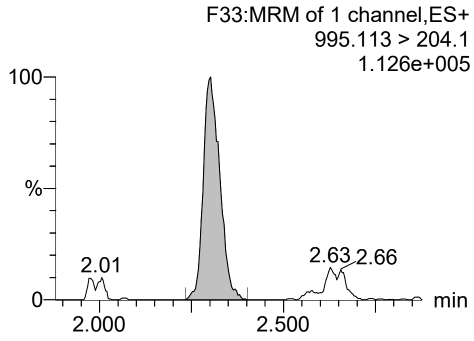

Sample Name: 20211111_A16_R1_IgG

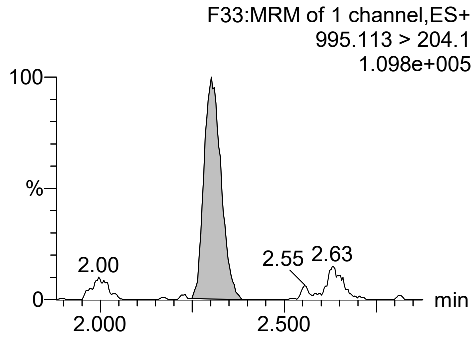

Sample Name: 20211111_L15_R1_IgG

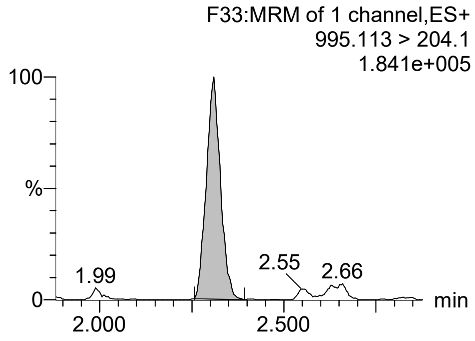

Sample Name: 20211111_L16_R1_IgG

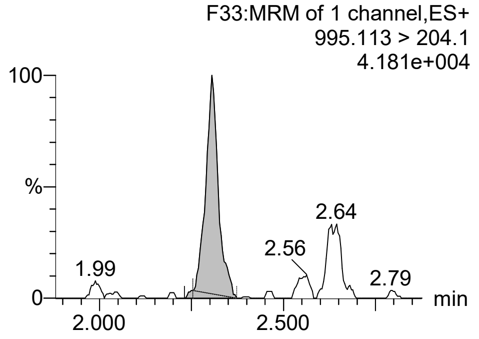

Sample Name: 20211111_H8_R1_IgG

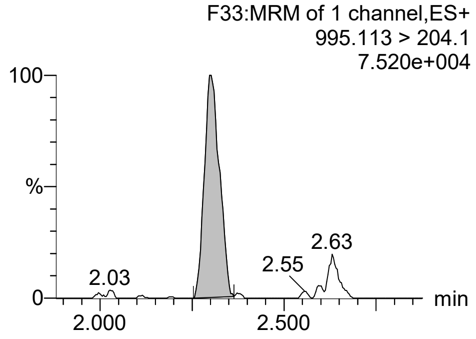

Sample Name: 20211111_A17_R1_IgG

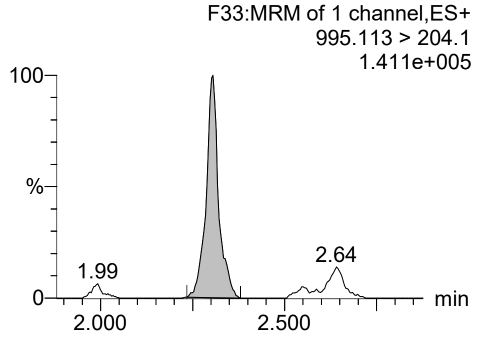

Sample Name: 20211111_A18_R1_IgG

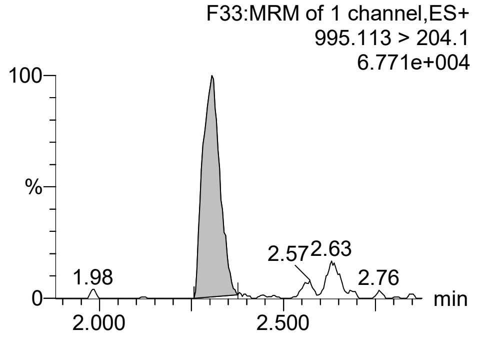

Sample Name: 20211111_L17_R1_IgG

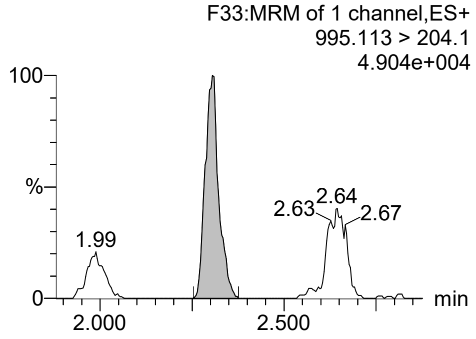

Sample Name: 20211111_L18_R1_IgG

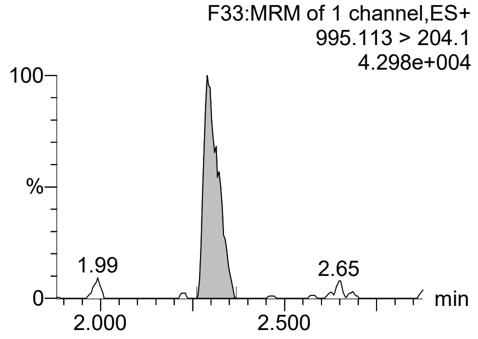

Sample Name: 20211111_H9_R1_IgG

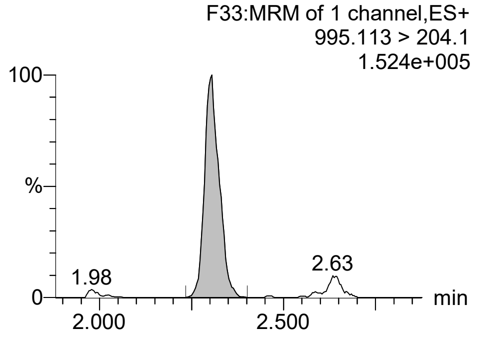

Sample Name: 20211111_A19_R1_IgG

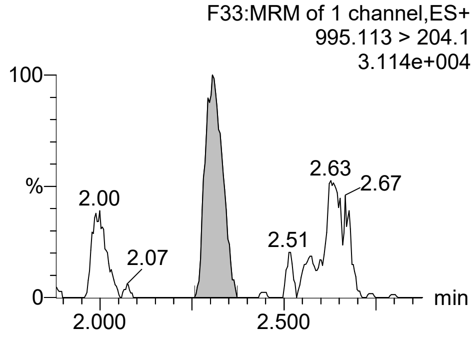

Sample Name: 20211111_A20_R1_IgG

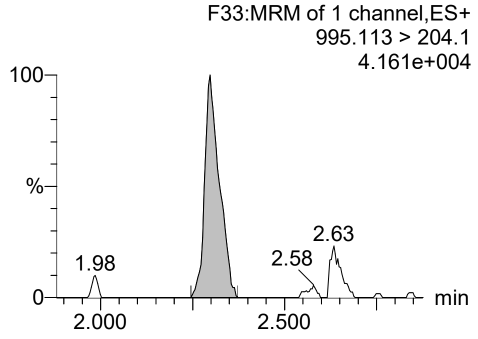

Sample Name: 20211111_L19_R1_IgG

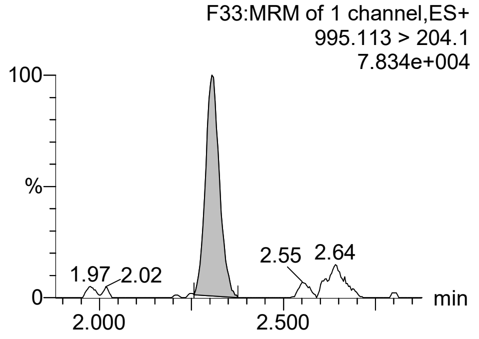

Sample Name: 20211111_QC_R5_IgG

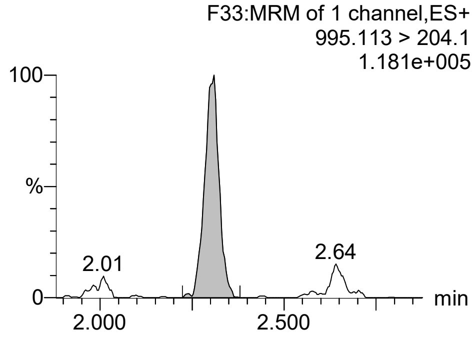

Sample Name: 20211111_L20_R1_IgG

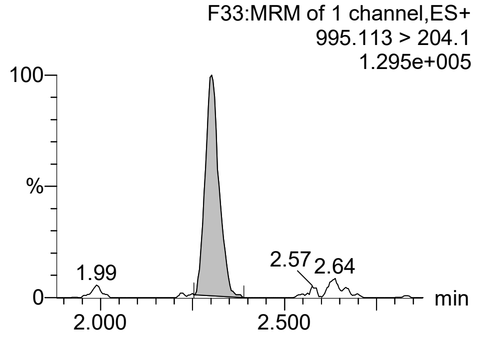

Sample Name: 20211111_H10_R1_IgG

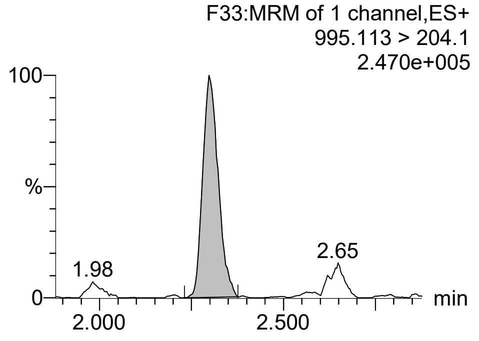

Sample Name: 20211111_A21_R1_IgG

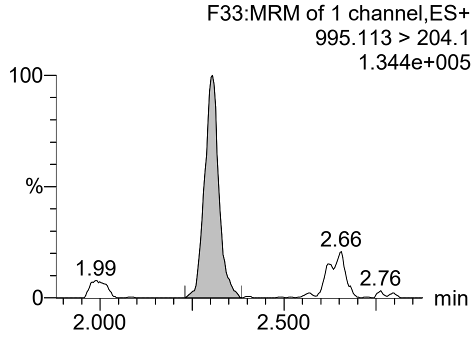

Sample Name: 20211111_A22_R1_IgG

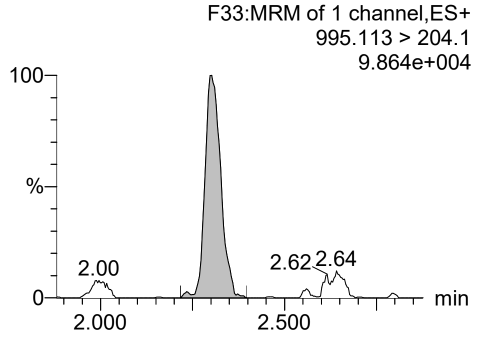

Dataset: C:\MassLynx\10.pro\20211115_clinicalsamples_IgG.qld

Last Altered: Sunday, October 02, 2022 16:02:30 台北標準時間
Printed: Friday, August 01, 2025 16:55:36 台北標準時間

Sample Name: 20211111_L21_R1_IgG
F33:MRM of 1 channel,ES+
995.113 > 204.1
9.241e+004

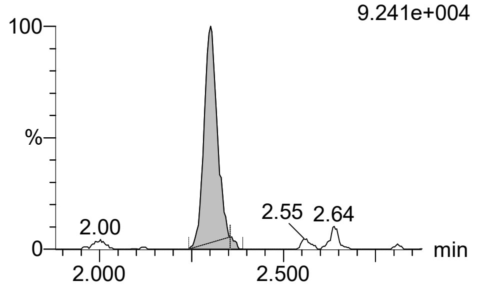

Sample Name: 20211111_L22_R1_IgG
F33:MRM of 1 channel,ES+
995.113 > 204.1
5.989e+004

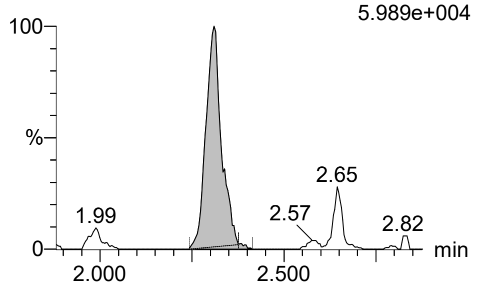

Sample Name: 20211111_H11_R1_IgG
F33:MRM of 1 channel,ES+
995.113 > 204.1
1.578e+005

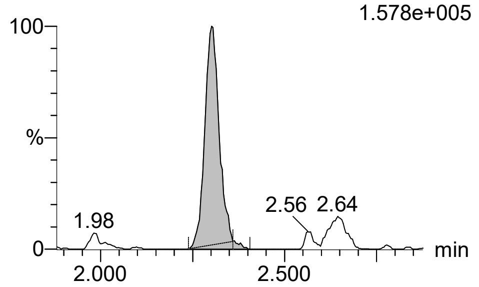

Sample Name: 20211111_A23_R1_IgG
F33:MRM of 1 channel,ES+
995.113 > 204.1
1.498e+005

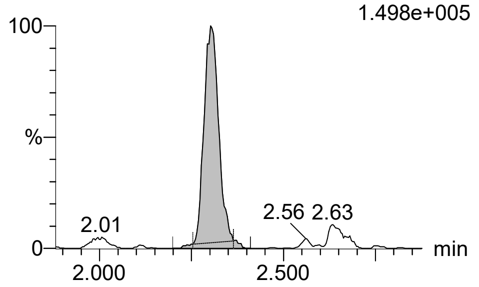

Sample Name: 20211111_A24_R1_IgG
F33:MRM of 1 channel,ES+
995.113 > 204.1
5.089e+004

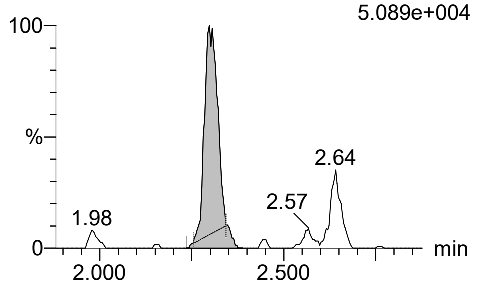

Sample Name: 20211111_L23_R1_IgG
F33:MRM of 1 channel,ES+
995.113 > 204.1
1.439e+005

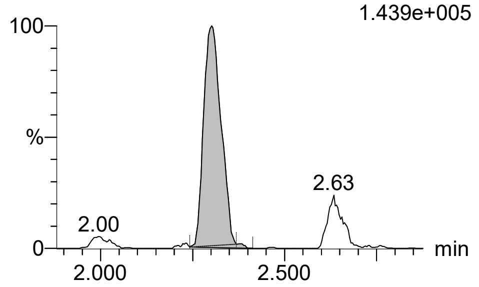

Sample Name: 20211111_L24_R1_IgG
F33:MRM of 1 channel,ES+
995.113 > 204.1
9.243e+004

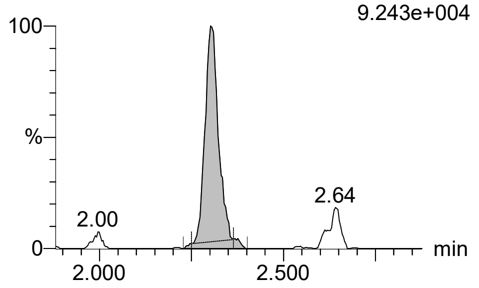

Sample Name: 20211111_H12_R1_IgG
F33:MRM of 1 channel,ES+
995.113 > 204.1
2.089e+004

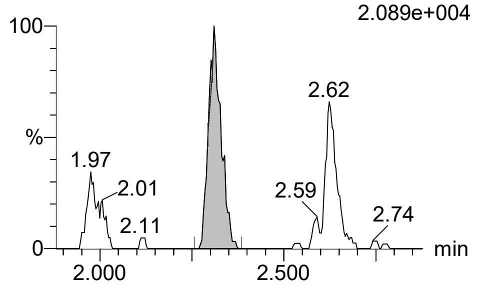

Sample Name: 20211111_QC_R6_IgG
F33:MRM of 1 channel,ES+
995.113 > 204.1
1.168e+005

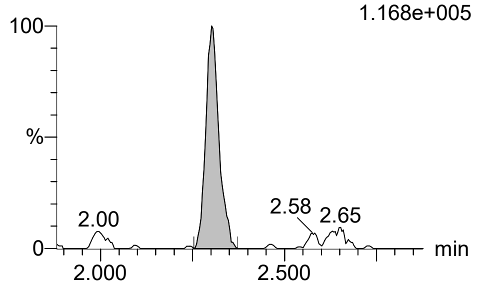

Sample Name: 20211111_A25_R1_IgG
F33:MRM of 1 channel,ES+
995.113 > 204.1
2.326e+005

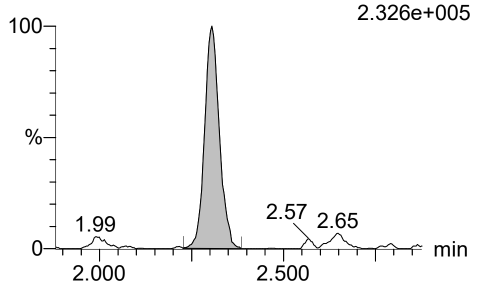

Sample Name: 20211111_A26_R1_IgG
F33:MRM of 1 channel,ES+
995.113 > 204.1
2.405e+004

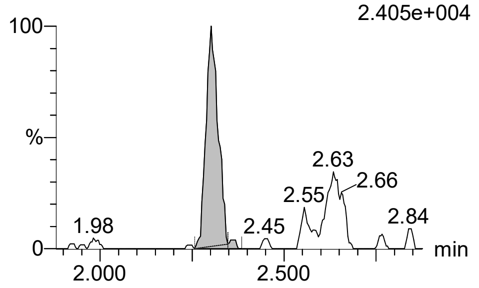

Sample Name: 20211111_L25_R1_IgG
F33:MRM of 1 channel,ES+
995.113 > 204.1
9.982e+004

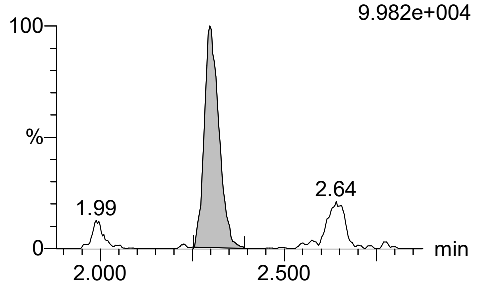

Sample Name: 20211111_L26_R1_IgG
F33:MRM of 1 channel,ES+
995.113 > 204.1
6.708e+004

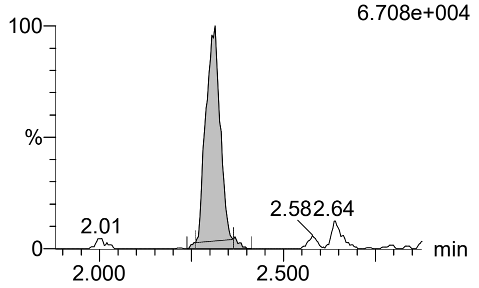

Sample Name: 20211111_H13_R1_IgG
F33:MRM of 1 channel,ES+
995.113 > 204.1
8.056e+004

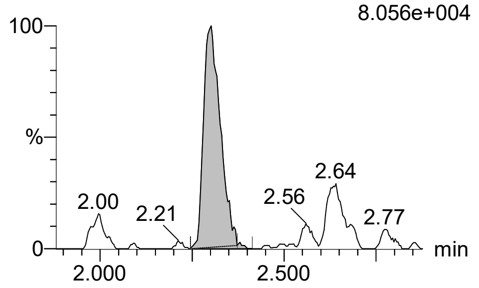

Sample Name: 20211111_A27_R1_IgG
F33:MRM of 1 channel,ES+
995.113 > 204.1
6.654e+004

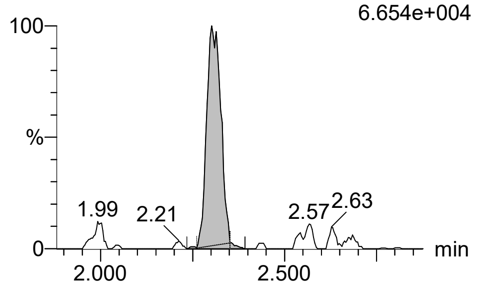

Sample Name: 20211111_A28_R1_IgG
F33:MRM of 1 channel,ES+
995.113 > 204.1
1.566e+005

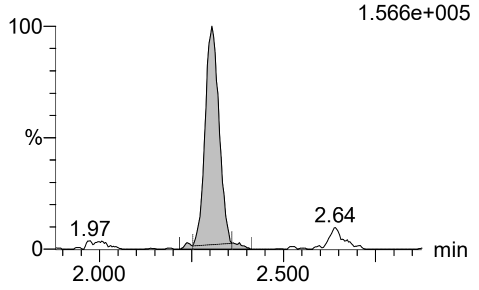

Sample Name: 20211111_L27_R1_IgG
F33:MRM of 1 channel,ES+
995.113 > 204.1
9.587e+004

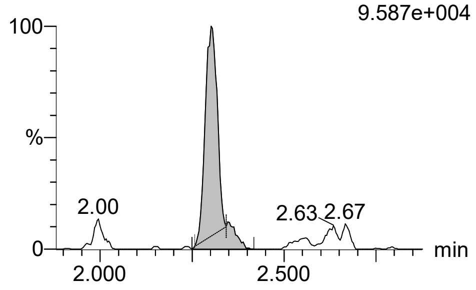

Sample Name: 20211111_L28_R1_IgG
F33:MRM of 1 channel,ES+
995.113 > 204.1
3.925e+004

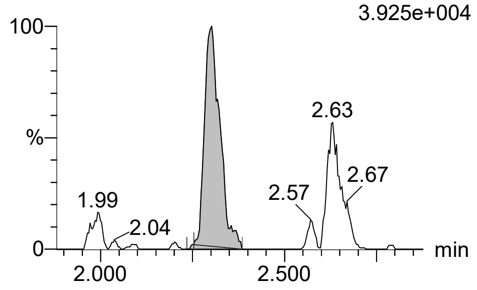

Sample Name: 20211111_H14_R1_IgG
F33:MRM of 1 channel,ES+
995.113 > 204.1
5.485e+004

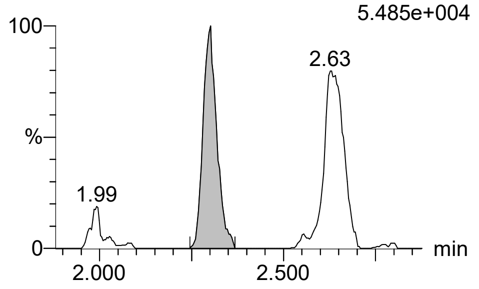

Sample Name: 20211111_A29_R1_IgG
F33:MRM of 1 channel,ES+
995.113 > 204.1
8.623e+004

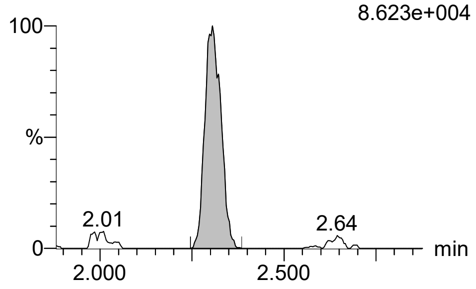

Sample Name: 20211111_A30_R1_IgG
F33:MRM of 1 channel,ES+
995.113 > 204.1
1.453e+005

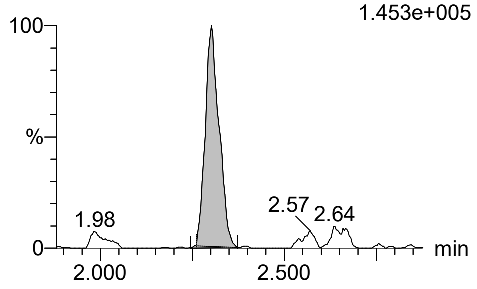

Sample Name: 20211111_QC_R7_IgG
F33:MRM of 1 channel,ES+
995.113 > 204.1
1.026e+005

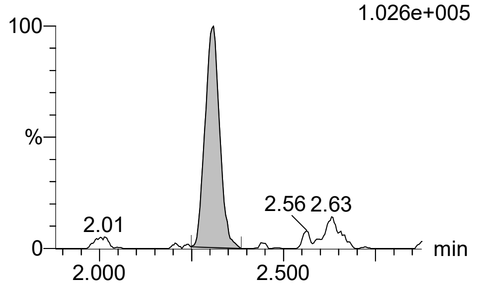

Sample Name: 20211111_L29_R1_IgG
F33:MRM of 1 channel,ES+
995.113 > 204.1
7.519e+004

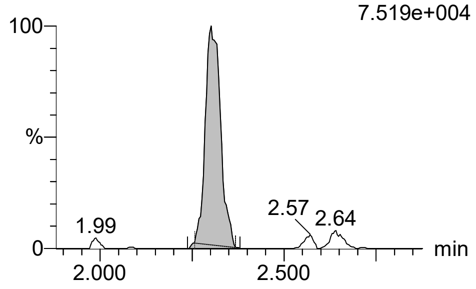

Sample Name: 20211111_L30_R1_IgG
F33:MRM of 1 channel,ES+
995.113 > 204.1
5.097e+004

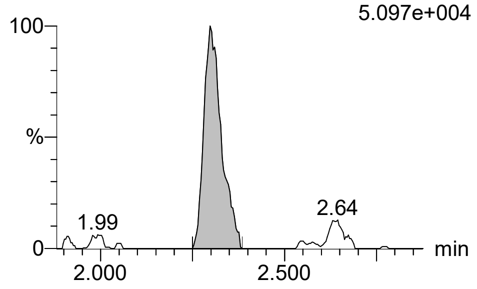

Sample Name: 20211111_H15_R1_IgG
F33:MRM of 1 channel,ES+
995.113 > 204.1
1.480e+004

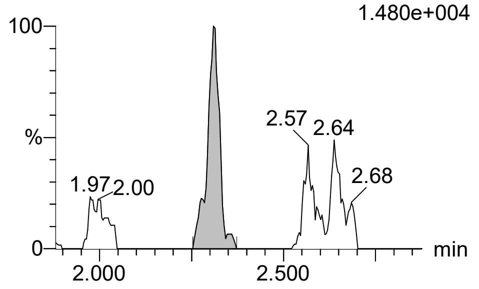

Sample Name: 20211111_A31_R1_IgG
F33:MRM of 1 channel,ES+
995.113 > 204.1
5.331e+004

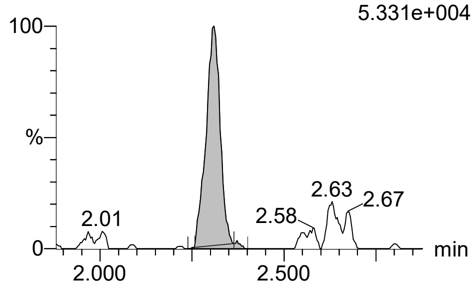

Sample Name: 20211111_A32_R1_IgG
F33:MRM of 1 channel,ES+
995.113 > 204.1
7.394e+004

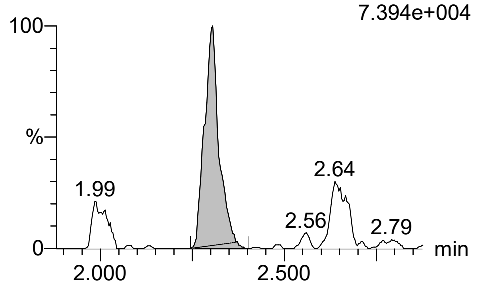

Sample Name: 20211111_L31_R1_IgG
F33:MRM of 1 channel,ES+
995.113 > 204.1
1.804e+004

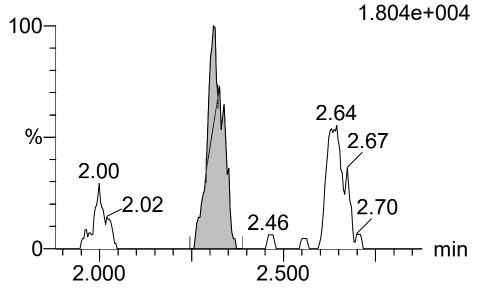

Sample Name: 20211111_L32_R1_IgG
F33:MRM of 1 channel,ES+
995.113 > 204.1
3.362e+004

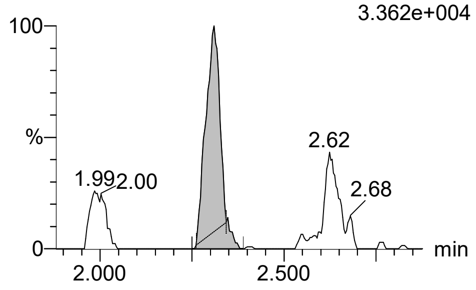

Sample Name: 20211111_H16_R1_IgG
F33:MRM of 1 channel,ES+
995.113 > 204.1
1.853e+005

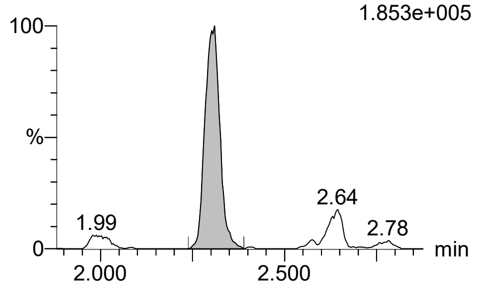

Dataset: C:\MassLynx\10.pro\20211115_clinicalsamples_IgG.qld

Last Altered: Sunday, October 02, 2022 16:02:30 台北標準時間
Printed: Friday, August 01, 2025 16:55:36 台北標準時間

Sample Name: 20211111_A33_R1_IgG
F33:MRM of 1 channel,ES+
995.113 > 204.1
8.158e+004

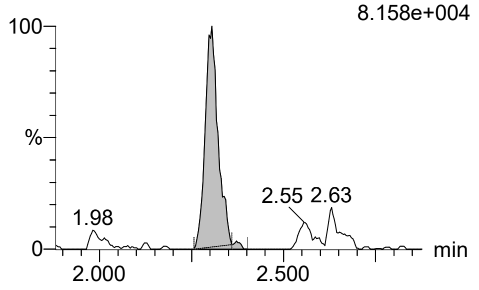

Sample Name: 20211111_A34_R1_IgG
F33:MRM of 1 channel,ES+
995.113 > 204.1
9.860e+004

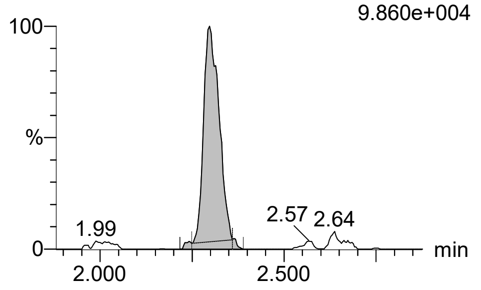

Sample Name: 20211111_H17_R1_IgG
F33:MRM of 1 channel,ES+
995.113 > 204.1
1.901e+005

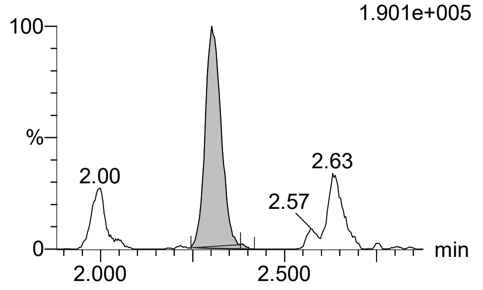

Sample Name: 20211111_A35_R1_IgG
F33:MRM of 1 channel,ES+
995.113 > 204.1
2.683e+005

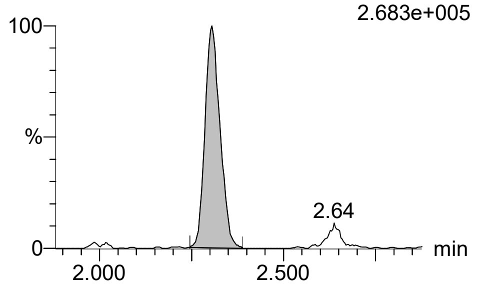

Sample Name: 20211111_QC_R8_IgG
F33:MRM of 1 channel,ES+
995.113 > 204.1
7.207e+004

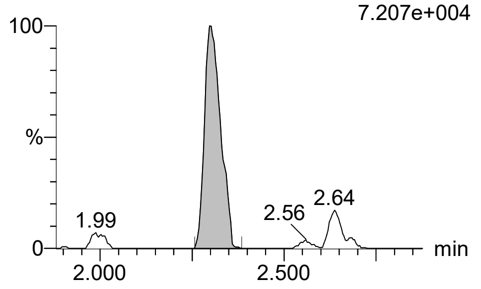

Sample Name: 20211111_A36_R1_IgG
F33:MRM of 1 channel,ES+
995.113 > 204.1
1.740e+005

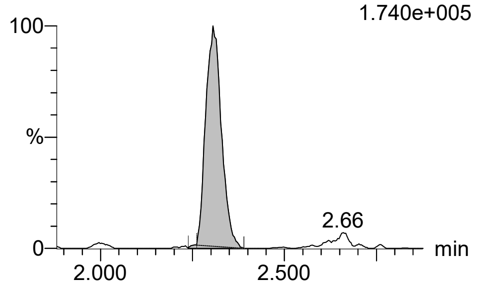

Sample Name: 20211111_H18_R1_IgG
F33:MRM of 1 channel,ES+
995.113 > 204.1
4.272e+005

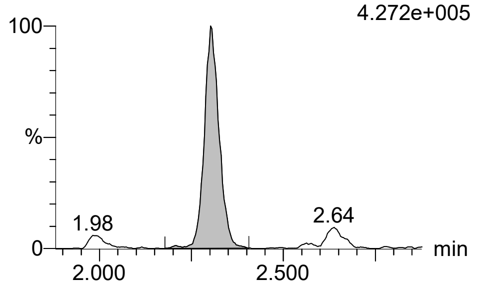

Sample Name: 20211111_A37_R1_IgG
F33:MRM of 1 channel,ES+
995.113 > 204.1
4.019e+004

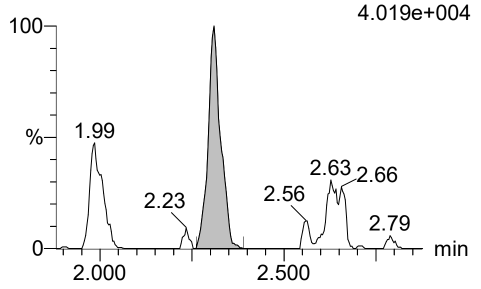

Sample Name: 20211111_A38_R1_IgG
F33:MRM of 1 channel,ES+
995.113 > 204.1
5.971e+004

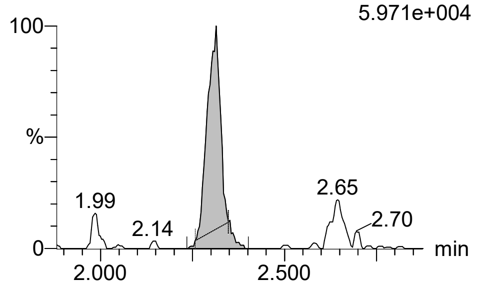

Sample Name: 20211111_H19_R1_IgG
F33:MRM of 1 channel,ES+
995.113 > 204.1
4.701e+005

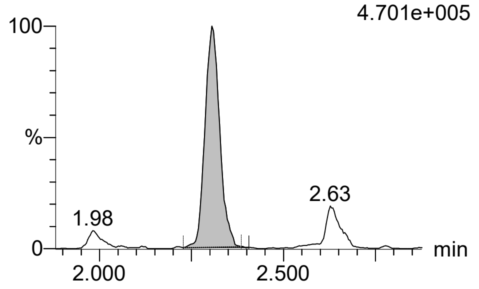

Sample Name: 20211111_A39_R1_IgG
F33:MRM of 1 channel,ES+
995.113 > 204.1
1.015e+005

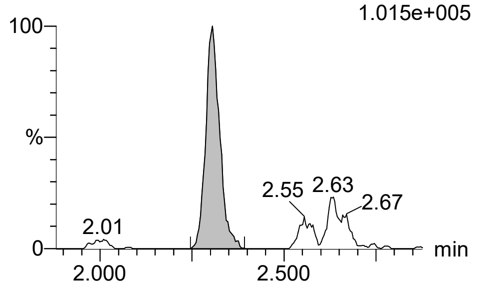

Sample Name: 20211111_A40_R1_IgG
F33:MRM of 1 channel,ES+
995.113 > 204.1
1.151e+005

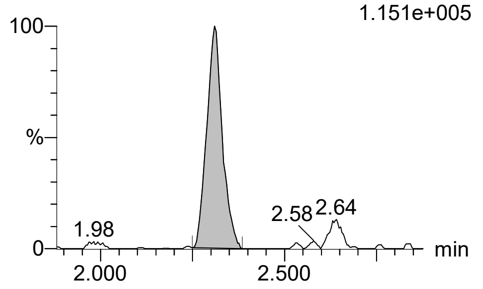

Sample Name: 20211111_H20_R1_IgG
F33:MRM of 1 channel,ES+
995.113 > 204.1
3.920e+005

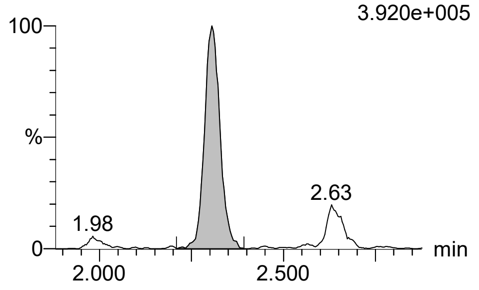

Sample Name: 20211111_A41_R1_IgG
F33:MRM of 1 channel,ES+
995.113 > 204.1
2.749e+004

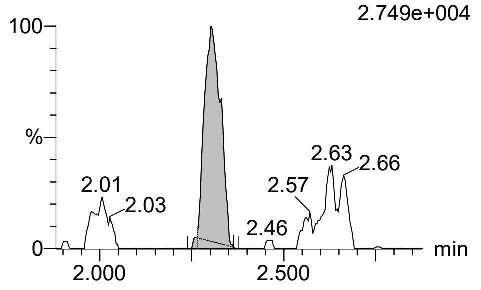

Sample Name: 20211111_A42_R1_IgG
F33:MRM of 1 channel,ES+
995.113 > 204.1
5.378e+004

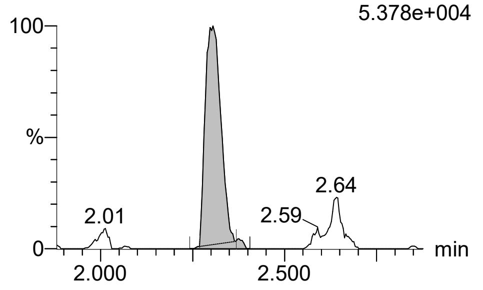

Sample Name: 20211111_A43_R1_IgG

F33:MRM of 1 channel,ES+
995.113 > 204.1
7.359e+004

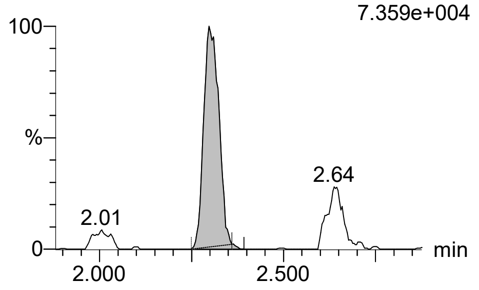

Sample Name: 20211111_A44_R1_IgG

F33:MRM of 1 channel,ES+
995.113 > 204.1
1.533e+005

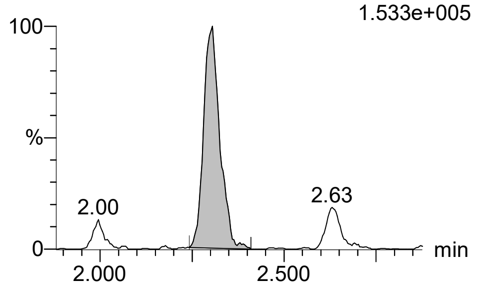

Sample Name: 20211111_QC_R9_IgG

F33:MRM of 1 channel,ES+
995.113 > 204.1
6.780e+004

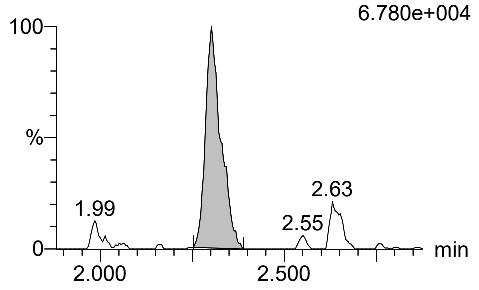

Sample Name: 20211111_A1_R2_IgG

F33:MRM of 1 channel,ES+
995.113 > 204.1
8.832e+004

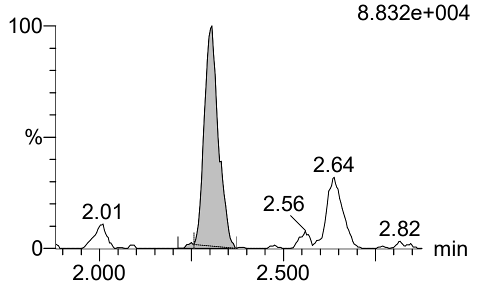

Sample Name: 20211111_A2_R2_IgG

F33:MRM of 1 channel,ES+
995.113 > 204.1
9.117e+004

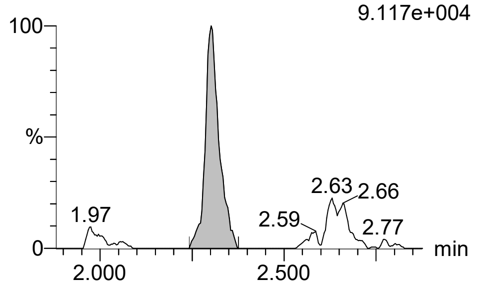

Sample Name: 20211111_L1_R2_IgG

F33:MRM of 1 channel,ES+
995.113 > 204.1
6.557e+004

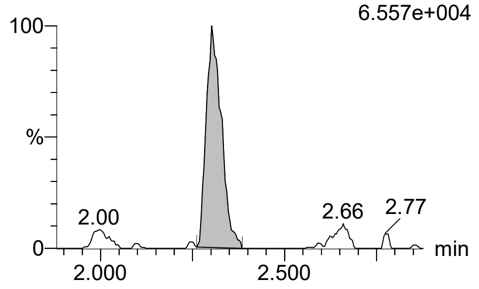

Sample Name: 20211111_L2_R2_IgG

F33:MRM of 1 channel,ES+
995.113 > 204.1
5.129e+004

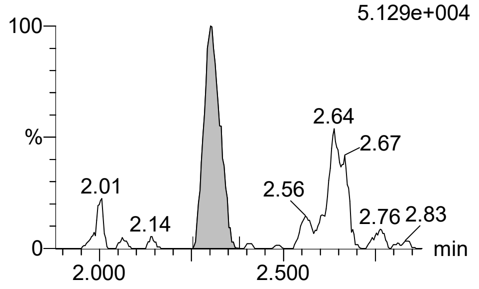

Sample Name: 20211111_H1_R2_IgG

F33:MRM of 1 channel,ES+
995.113 > 204.1
3.461e+004

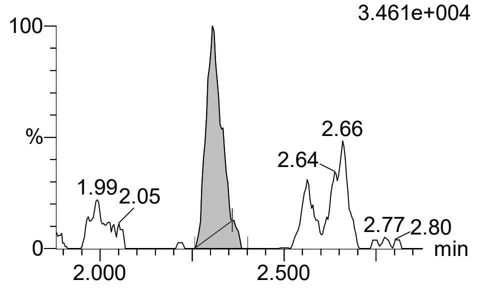

Sample Name: 20211111_A3_R2_IgG

F33:MRM of 1 channel,ES+
995.113 > 204.1
1.068e+004

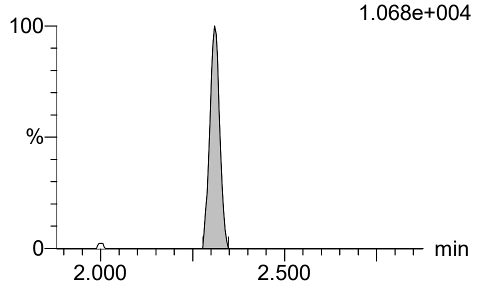

Sample Name: 20211111_A4_R2_IgG

F33:MRM of 1 channel,ES+
995.113 > 204.1
3.879e+004

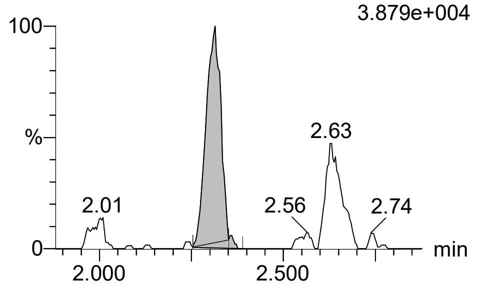

Sample Name: 20211111_L3_R2_IgG

F33:MRM of 1 channel,ES+
995.113 > 204.1
3.436e+004

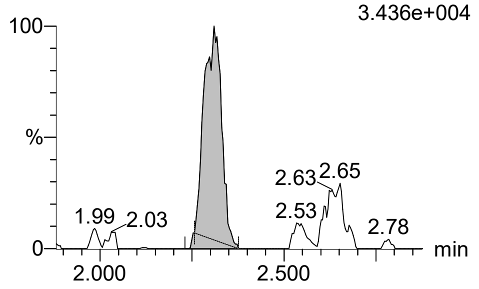

Sample Name: 20211111_L4_R2_IgG

F33:MRM of 1 channel,ES+
995.113 > 204.1
9.097e+004

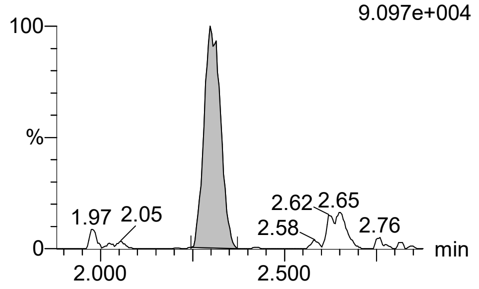

Sample Name: 20211111_H2_R2_IgG

F33:MRM of 1 channel,ES+
995.113 > 204.1
1.351e+005

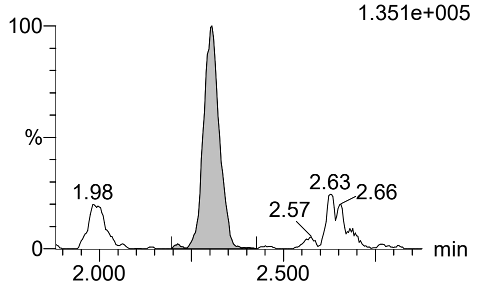

Sample Name: 20211111_A5_R2_IgG

F33:MRM of 1 channel,ES+
995.113 > 204.1
7.974e+004

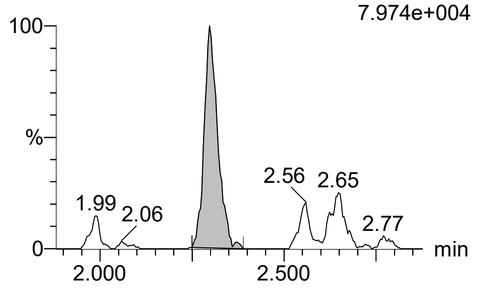

Sample Name: 20211111_A6_R2_IgG

F33:MRM of 1 channel,ES+
995.113 > 204.1
2.496e+005

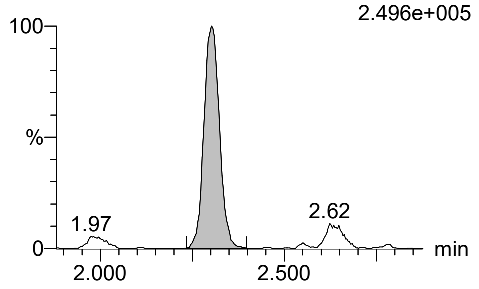

Sample Name: 20211111_QC_R10_IgG

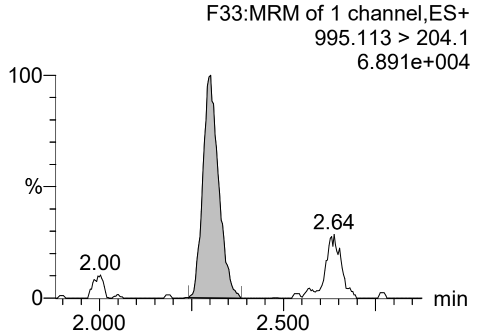

Sample Name: 20211111_L5_R2_IgG

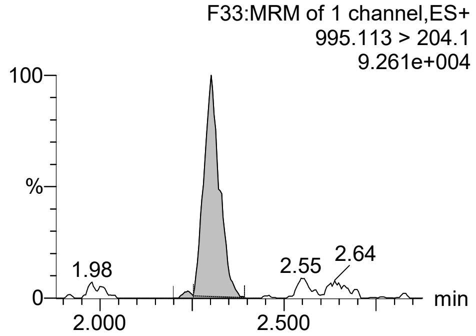

Sample Name: 20211111_L6_R2_IgG

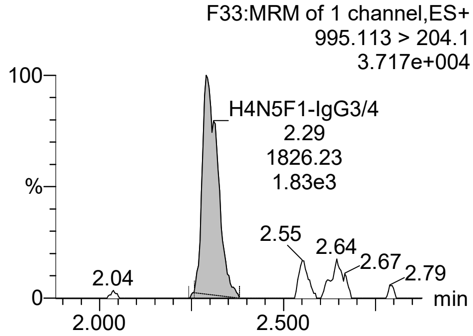

Sample Name: 20211111_H3_R2_IgG

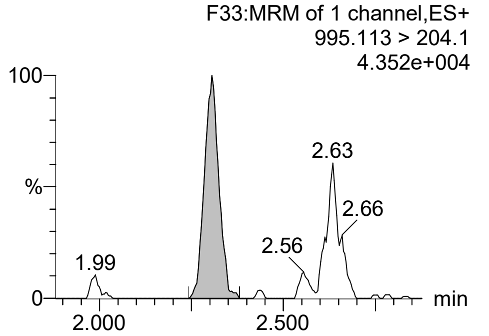

Sample Name: 20211111_A7_R2_IgG

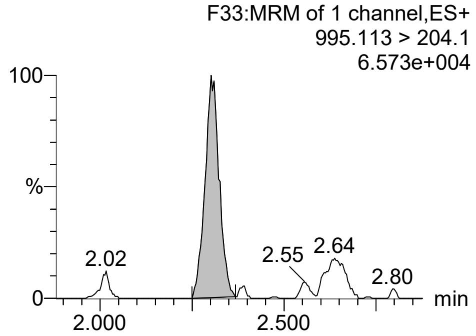

Sample Name: 20211111_A8_R2_IgG

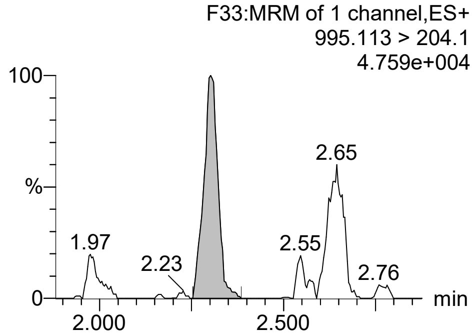

Sample Name: 20211111_L7_R2_IgG

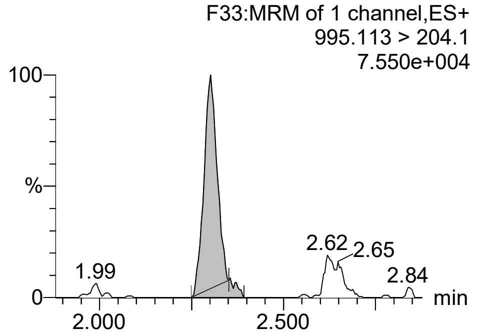

Sample Name: 20211111_L8_R2_IgG

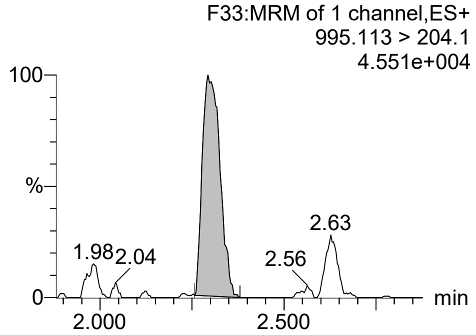

Sample Name: 20211111_H4_R2_IgG

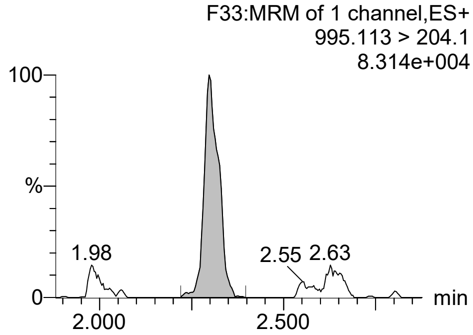

Sample Name: 20211111_A9_R2_IgG

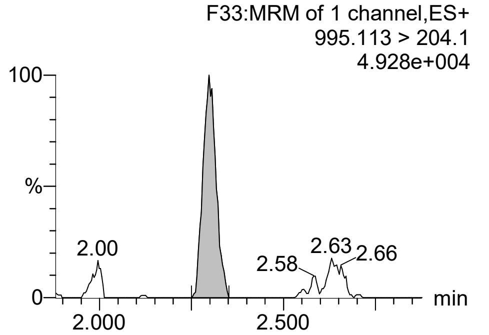

Sample Name: 20211111_A10_R2_IgG

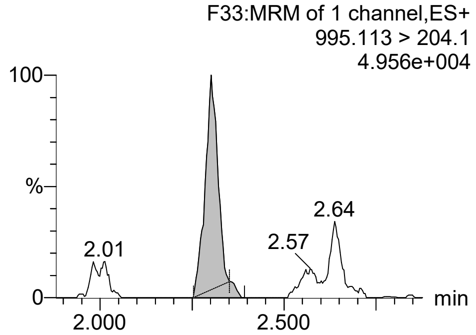

Sample Name: 20211111_L9_R2_IgG

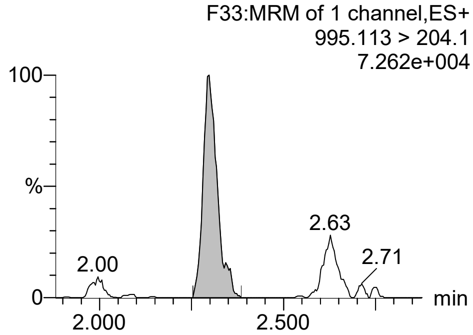

Sample Name: 20211111_L10_R2_IgG

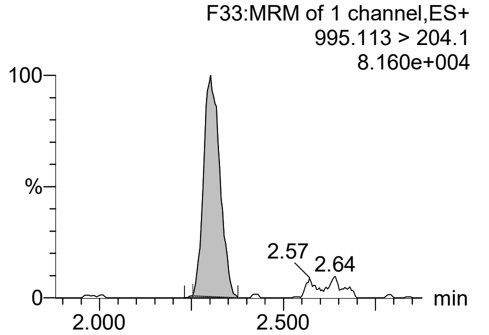

Sample Name: 20211111_QC_R11_IgG

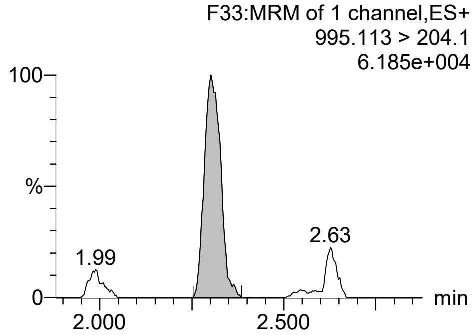

Sample Name: 20211111_H5_R2_IgG

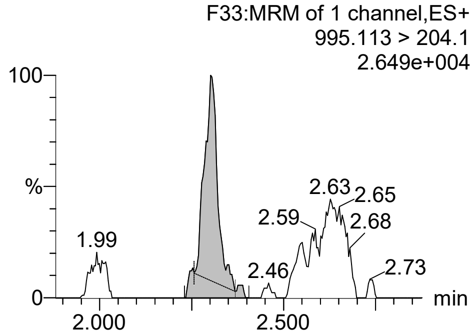

Dataset: C:\MassLynx\10.pro\20211115_clinicalsamples_IgG.qld

Last Altered: Sunday, October 02, 2022 16:02:30 台北標準時間
Printed: Friday, August 01, 2025 16:55:36 台北標準時間

Sample Name: 20211111_A11_R2_IgG
F33:MRM of 1 channel,ES+
995.113 > 204.1
3.769e+004

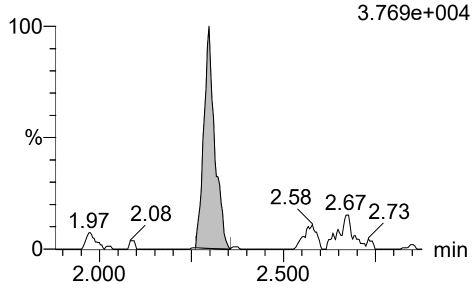

Sample Name: 20211111_A12_R2_IgG
F33:MRM of 1 channel,ES+
995.113 > 204.1
7.777e+004

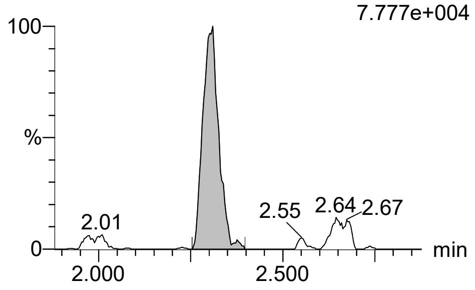

Sample Name: 20211111_L11_R2_IgG
F33:MRM of 1 channel,ES+
995.113 > 204.1
3.867e+004

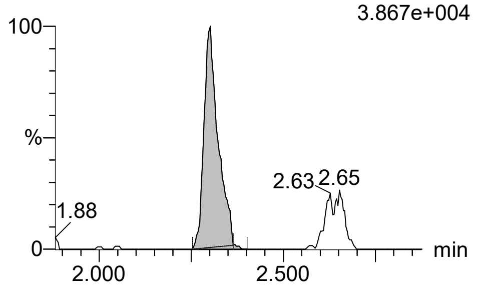

Sample Name: 20211111_L12_R2_IgG
F33:MRM of 1 channel,ES+
995.113 > 204.1
2.447e+004

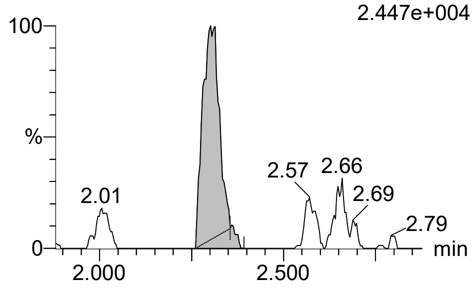

Sample Name: 20211111_H6_R2_IgG
F33:MRM of 1 channel,ES+
995.113 > 204.1
1.504e+005

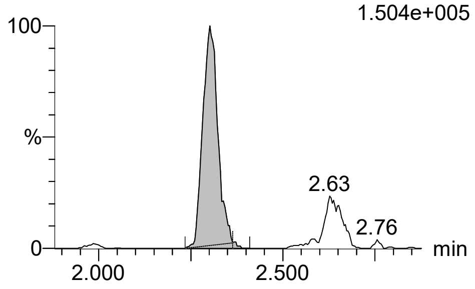

Sample Name: 20211111_A13_R2_IgG
F33:MRM of 1 channel,ES+
995.113 > 204.1
3.457e+005

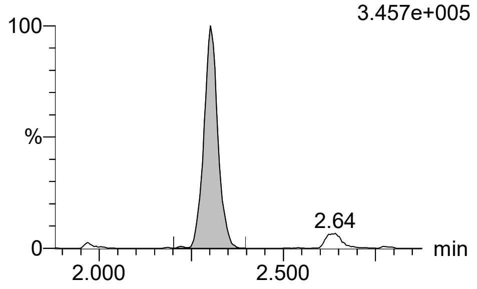

Sample Name: 20211111_A14_R2_IgG
F33:MRM of 1 channel,ES+
995.113 > 204.1
4.904e+004

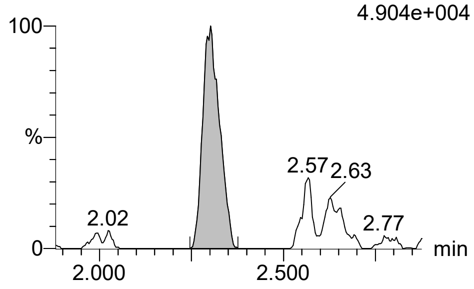

Sample Name: 20211111_L13_R2_IgG
F33:MRM of 1 channel,ES+
995.113 > 204.1
4.273e+004

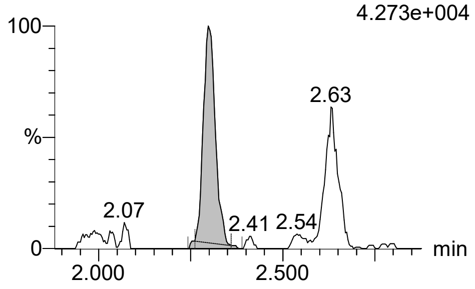

Sample Name: 20211111_L14_R2_IgG
F33:MRM of 1 channel,ES+
995.113 > 204.1
1.148e+005

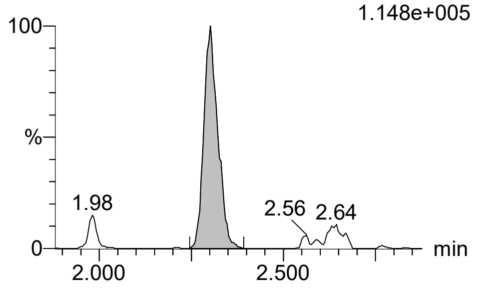

Sample Name: 20211111_H7_R2_IgG
F33:MRM of 1 channel,ES+
995.113 > 204.1
1.346e+005

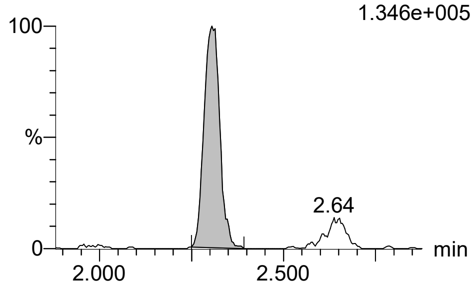

Sample Name: 20211111_A15_R2_IgG
F33:MRM of 1 channel,ES+
995.113 > 204.1
3.311e+004

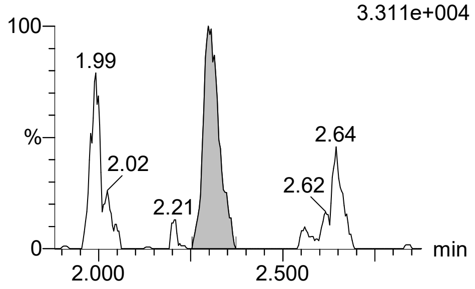

Sample Name: 20211111_QC_R12_IgG
F33:MRM of 1 channel,ES+
995.113 > 204.1
6.504e+004

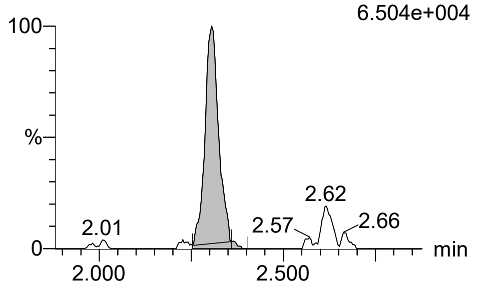

Sample Name: 20211111_A16_R2_IgG
F33:MRM of 1 channel,ES+
995.113 > 204.1
6.275e+004

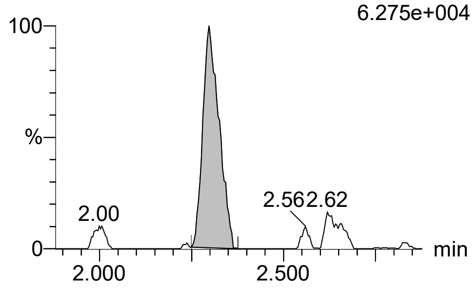

Sample Name: 20211111_L15_R2_IgG
F33:MRM of 1 channel,ES+
995.113 > 204.1
1.238e+005

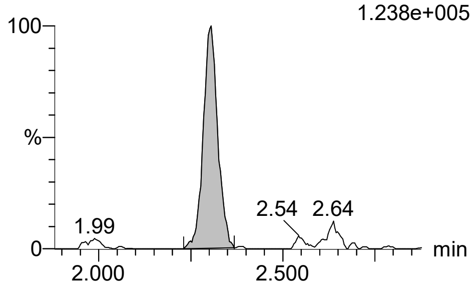

Sample Name: 20211111_L16_R2_IgG
F33:MRM of 1 channel,ES+
995.113 > 204.1
2.146e+004

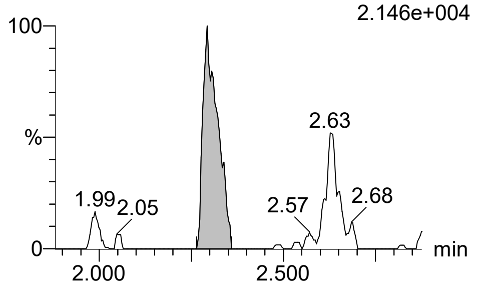

Sample Name: 20211111_H8_R2_IgG
F33:MRM of 1 channel,ES+
995.113 > 204.1
9.141e+004

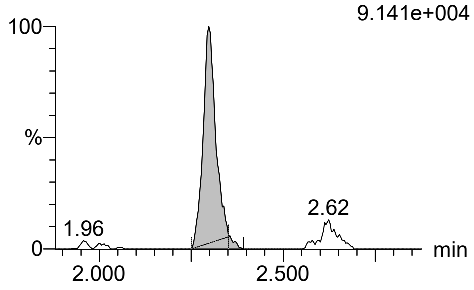

Sample Name: 20211111_A17_R2_IgG
F33:MRM of 1 channel,ES+
995.113 > 204.1
1.253e+005

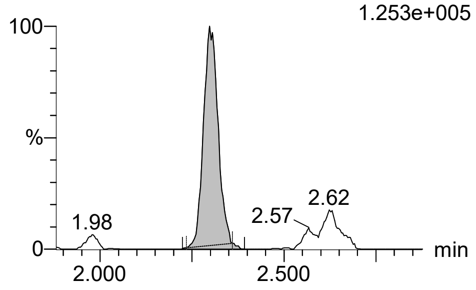

Sample Name: 20211111_A18_R2_IgG
F33:MRM of 1 channel,ES+
995.113 > 204.1
7.624e+004

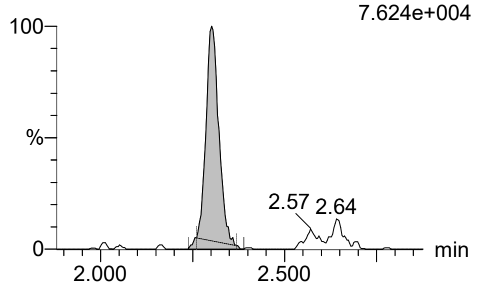

Sample Name: 20211111_L17_R2_IgG
F33:MRM of 1 channel,ES+
995.113 > 204.1
3.673e+004

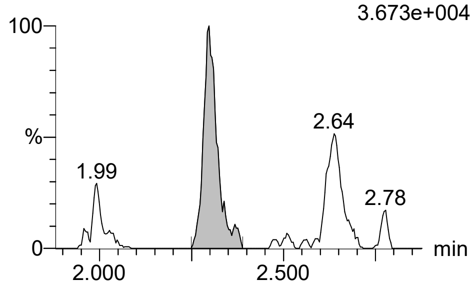

Sample Name: 20211111_L18_R2_IgG
F33:MRM of 1 channel,ES+
995.113 > 204.1
2.675e+004

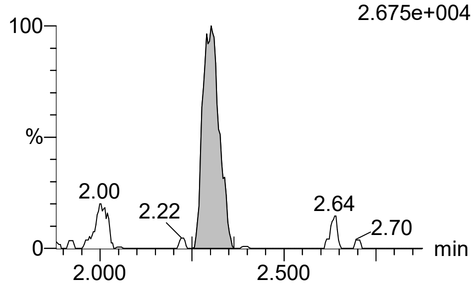

Sample Name: 20211111_H9_R2_IgG
F33:MRM of 1 channel,ES+
995.113 > 204.1
1.225e+005

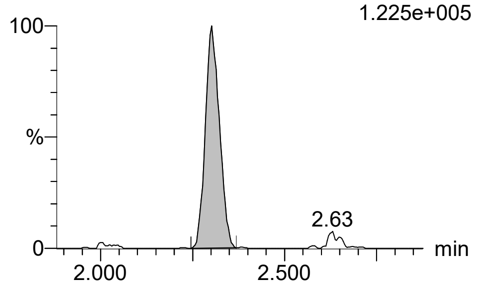

Sample Name: 20211111_A19_R2_IgG
F33:MRM of 1 channel,ES+
995.113 > 204.1
4.079e+004

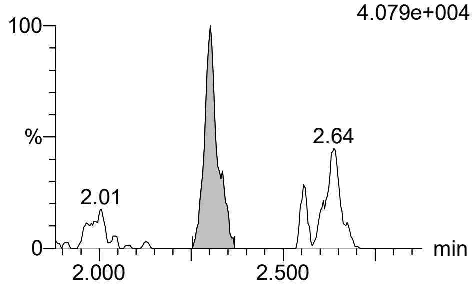

Sample Name: 20211111_A20_R2_IgG
F33:MRM of 1 channel,ES+
995.113 > 204.1
3.258e+004

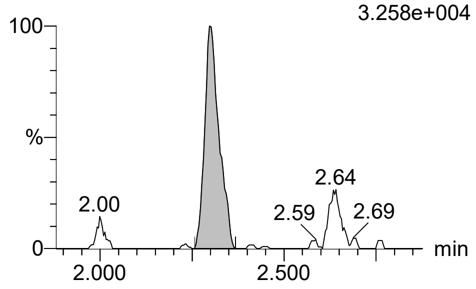

Sample Name: 20211111_L19_R2_IgG
F33:MRM of 1 channel,ES+
995.113 > 204.1
4.828e+004

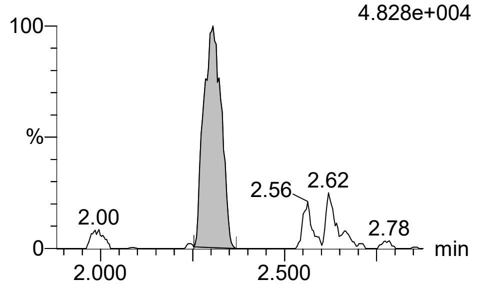

Sample Name: 20211111_QC_R13_IgG
F33:MRM of 1 channel,ES+
995.113 > 204.1
7.669e+004

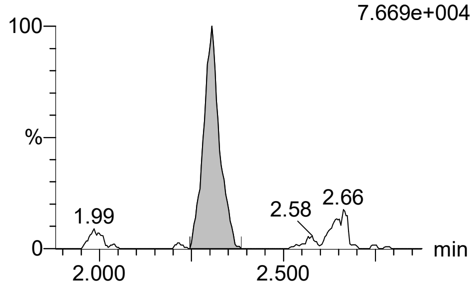

Sample Name: 20211111_L20_R2_IgG
F33:MRM of 1 channel,ES+
995.113 > 204.1
6.280e+004

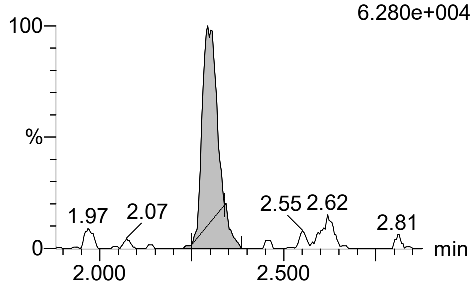

Sample Name: 20211111_H10_R2_IgG
F33:MRM of 1 channel,ES+
995.113 > 204.1
9.937e+004

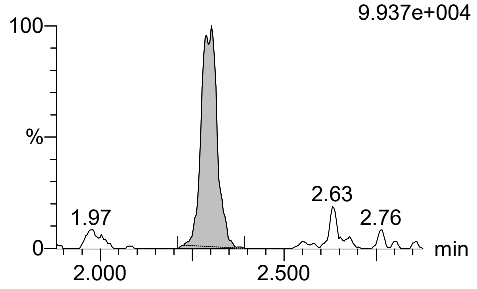

Sample Name: 20211111_A21_R2_IgG
F33:MRM of 1 channel,ES+
995.113 > 204.1
7.290e+004

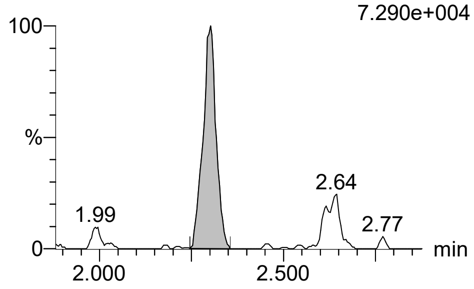

Sample Name: 20211111_A22_R2_IgG
F33:MRM of 1 channel,ES+
995.113 > 204.1
4.106e+004

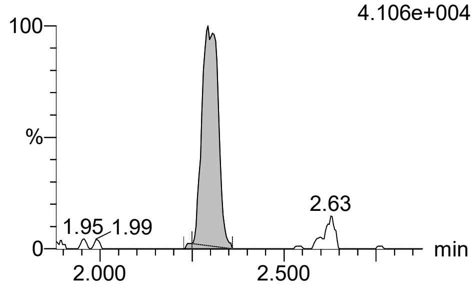

Sample Name: 20211111_L21_R2_IgG
F33:MRM of 1 channel,ES+
995.113 > 204.1
3.596e+004

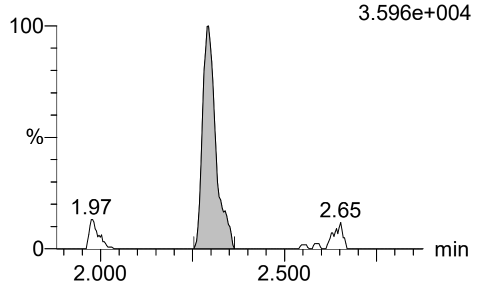

Sample Name: 20211111_L22_R2_IgG
F33:MRM of 1 channel,ES+
995.113 > 204.1
2.811e+004

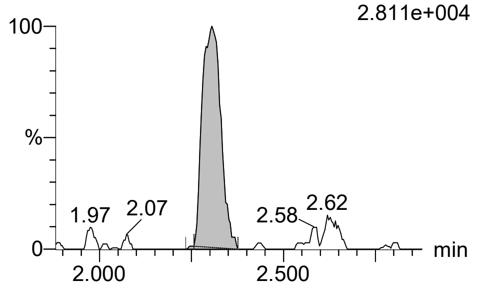

Sample Name: 20211111_H11_R2_IgG
F33:MRM of 1 channel,ES+
995.113 > 204.1
7.289e+004

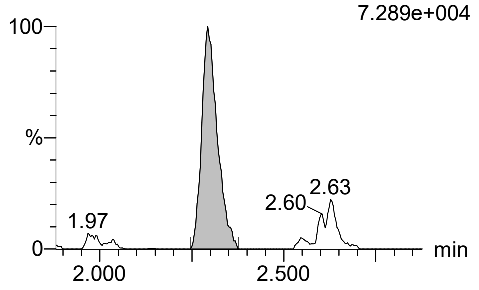

Sample Name: 20211111_A23_R2_IgG
F33:MRM of 1 channel,ES+
995.113 > 204.1
6.517e+004

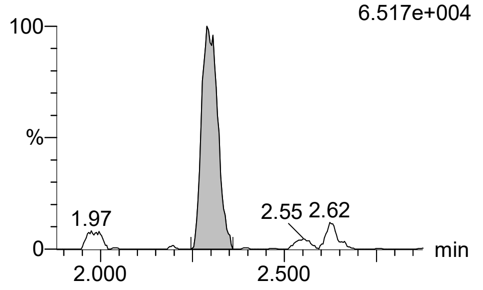

Sample Name: 20211111_A24_R2_IgG
F33:MRM of 1 channel,ES+
995.113 > 204.1
2.464e+004

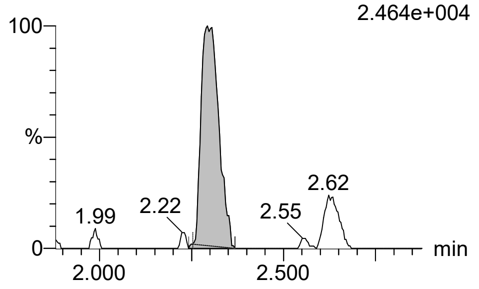

Sample Name: 20211111_L23_R2_IgG
F33:MRM of 1 channel,ES+
995.113 > 204.1
1.183e+005

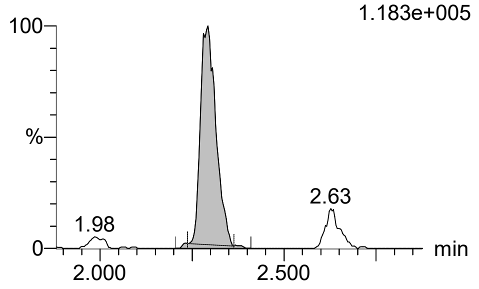

Sample Name: 20211111_L24_R2_IgG
F33:MRM of 1 channel,ES+
995.113 > 204.1
6.024e+004

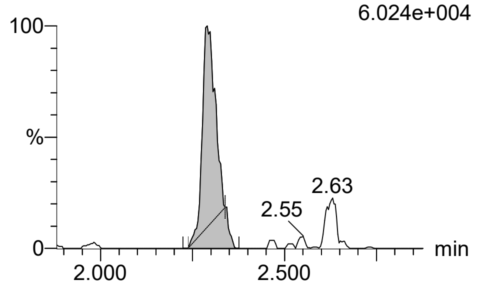

Sample Name: 20211111_H12_R2_IgG
F33:MRM of 1 channel,ES+
995.113 > 204.1
1.345e+004

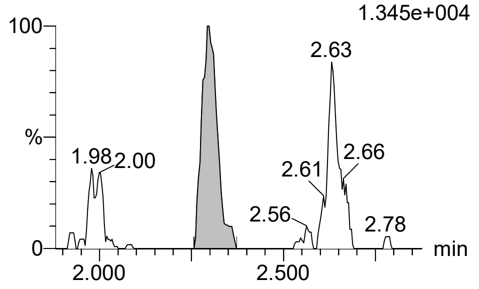

Sample Name: 20211111_QC_R14_IgG
F33:MRM of 1 channel,ES+
995.113 > 204.1
5.462e+004

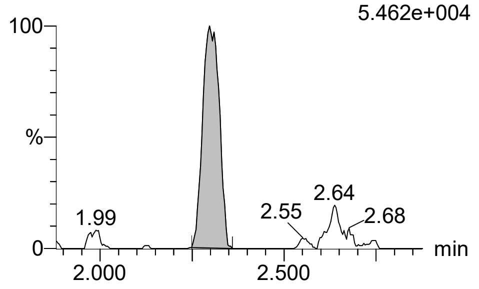

Sample Name: 20211111_A25_R2_IgG
F33:MRM of 1 channel,ES+
995.113 > 204.1
1.361e+005

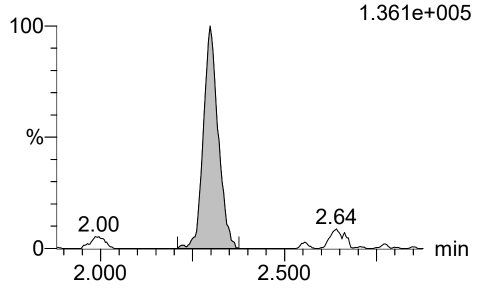

Sample Name: 20211111_A26_R2_IgG
F33:MRM of 1 channel,ES+
995.113 > 204.1
1.680e+004

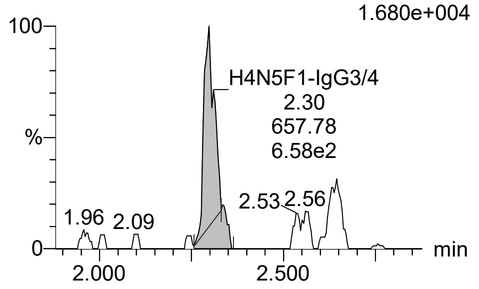

Sample Name: 20211111_L25_R2_IgG
F33:MRM of 1 channel,ES+
995.113 > 204.1
3.431e+004

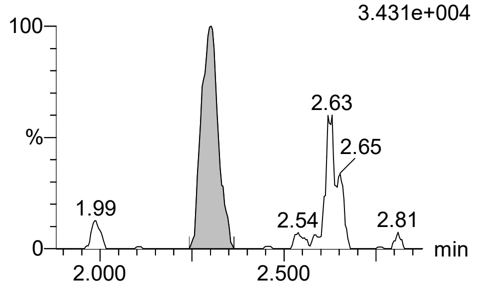

Sample Name: 20211111_L26_R2_IgG
F33:MRM of 1 channel,ES+
995.113 > 204.1
3.816e+004

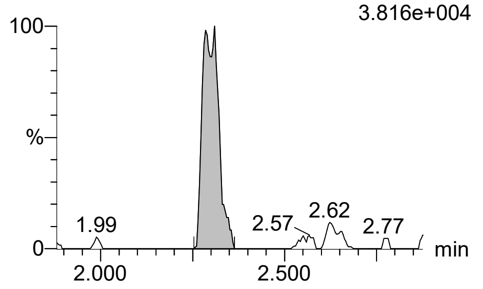

Sample Name: 20211111_H13_R2_IgG
F33:MRM of 1 channel,ES+
995.113 > 204.1
5.112e+004

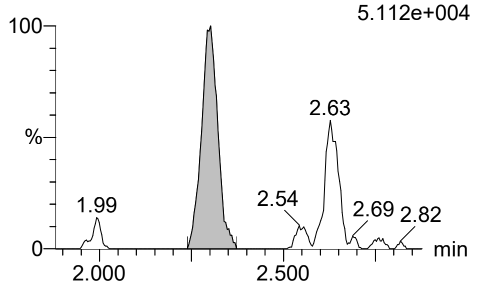

Sample Name: 20211111_A27_R2_IgG
F33:MRM of 1 channel,ES+
995.113 > 204.1
2.999e+004

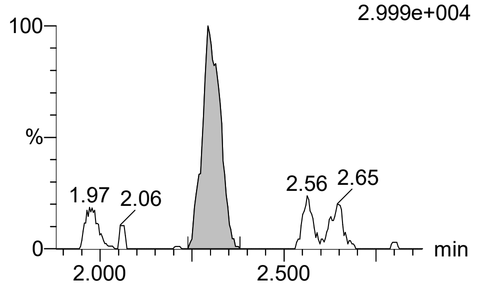

Sample Name: 20211111_A28_R2_IgG
F33:MRM of 1 channel,ES+
995.113 > 204.1
6.945e+004

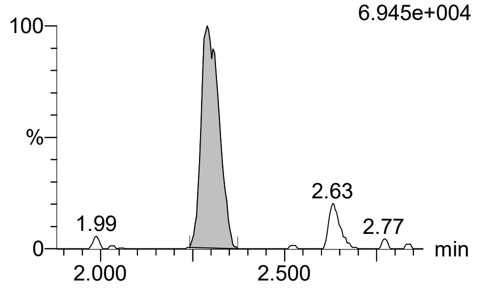

Dataset: C:\MassLynx\10.pro\20211115_clinicalsamples_IgG.qld

Last Altered: Sunday, October 02, 2022 16:02:30 台北標準時間
Printed: Friday, August 01, 2025 16:55:36 台北標準時間

Sample Name: 20211111_L27_R2_IgG

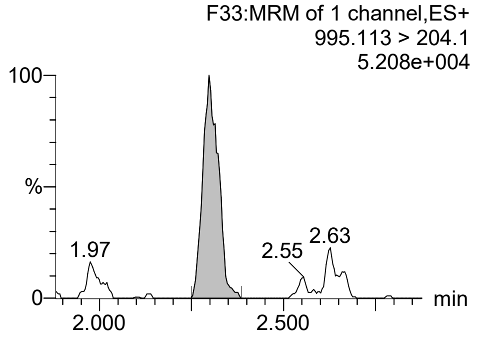

Sample Name: 20211111_L28_R2_IgG

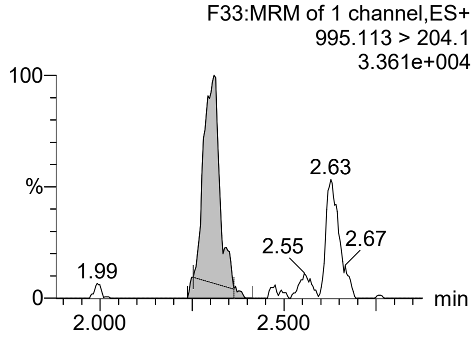

Sample Name: 20211111_H14_R2_IgG

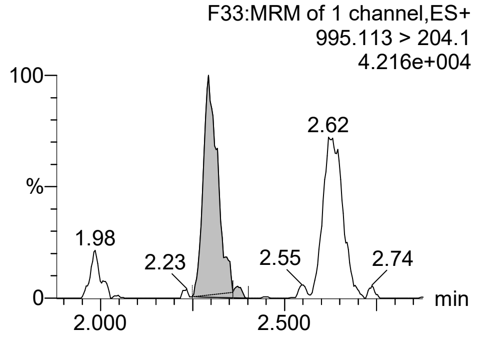

Sample Name: 20211111_A29_R2_IgG

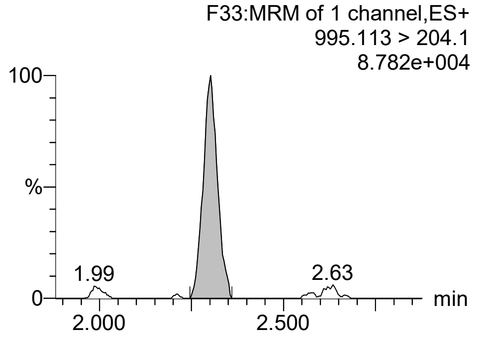

Sample Name: 20211111_A30_R2_IgG

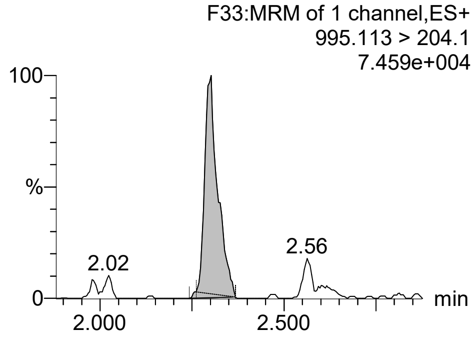

Sample Name: 20211111_QC_R15_IgG

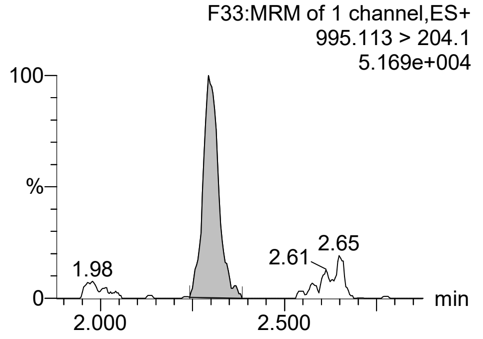

Sample Name: 20211111_L29_R2_IgG

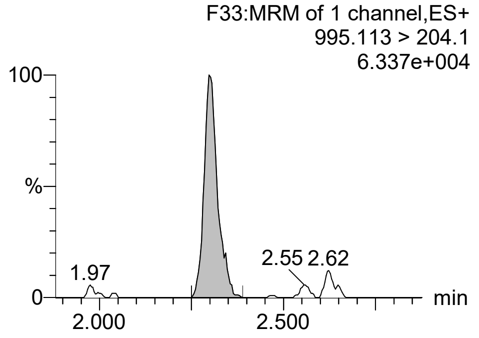

Sample Name: 20211111_L30_R2_IgG

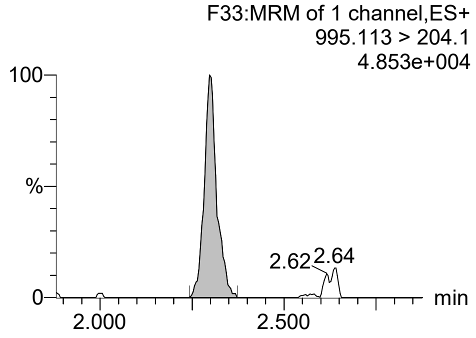

Sample Name: 20211111_H15_R2_IgG

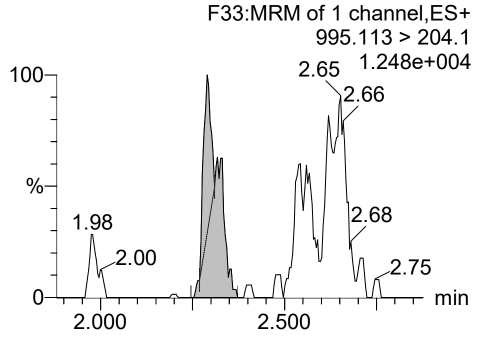

Sample Name: 20211111_A31_R2_IgG

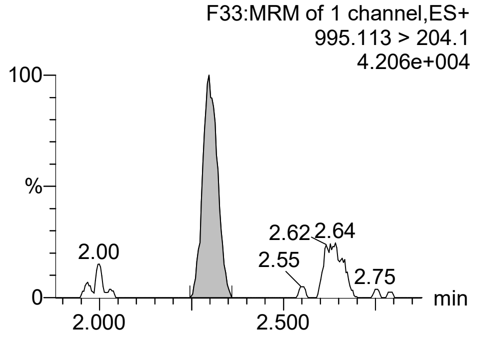

Sample Name: 20211111_A32_R2_IgG

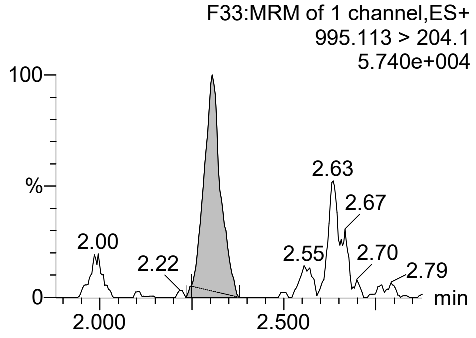

Sample Name: 20211111_L31_R2_IgG

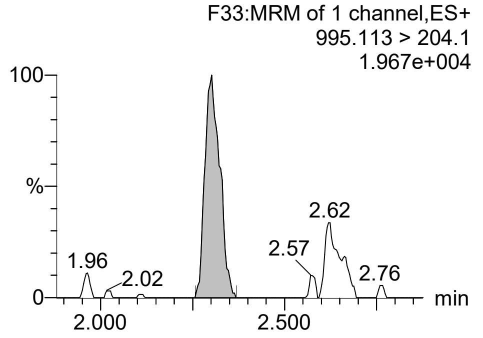

Sample Name: 20211111_L32_R2_IgG

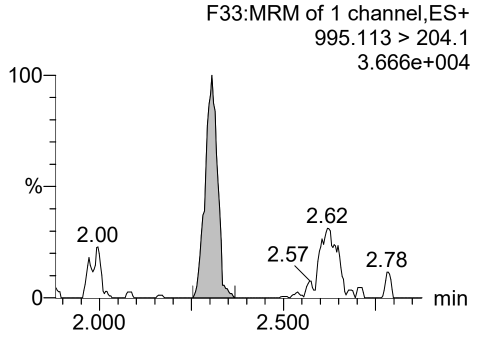

Sample Name: 20211111_H16_R2_IgG

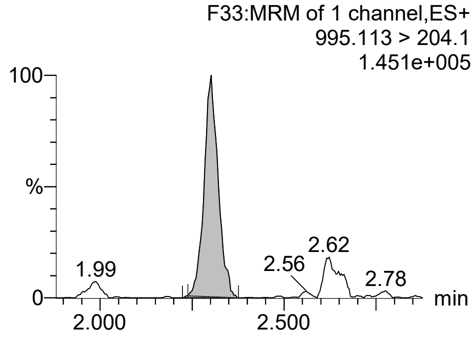

Sample Name: 20211111_A33_R2_IgG

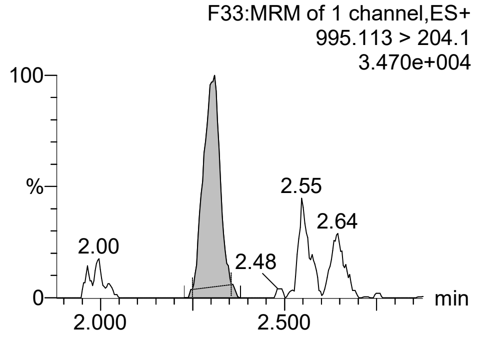

Sample Name: 20211111_A34_R2_IgG
F33:MRM of 1 channel,ES+
995.113 > 204.1
7.264e+004

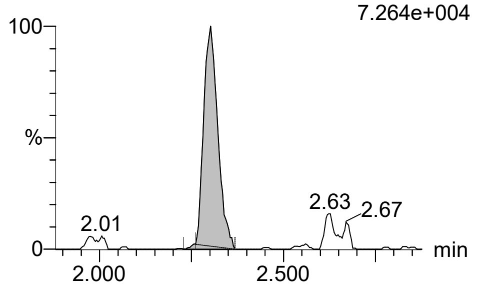

Sample Name: 20211111_H17_R2_IgG
F33:MRM of 1 channel,ES+
995.113 > 204.1
1.738e+005

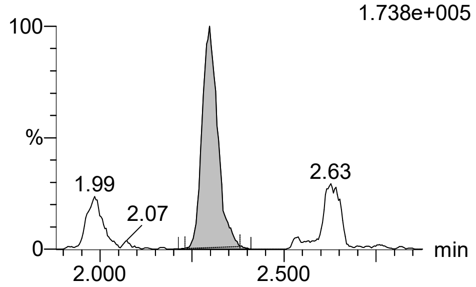

Sample Name: 20211111_A35_R2_IgG
F33:MRM of 1 channel,ES+
995.113 > 204.1
2.238e+005

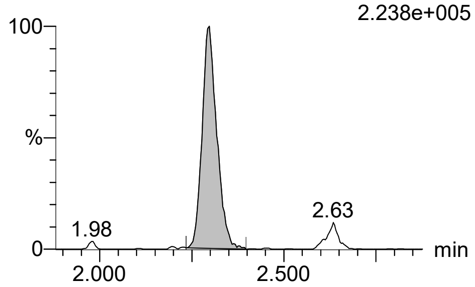

Sample Name: 20211111_QC_R16_IgG
F33:MRM of 1 channel,ES+
995.113 > 204.1
5.692e+004

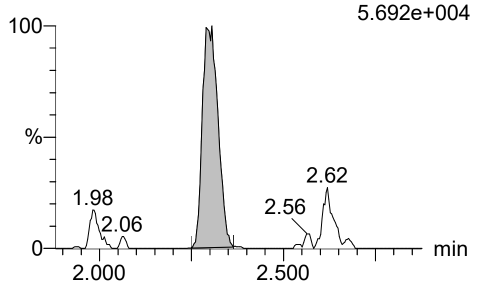

Sample Name: 20211111_A36_R2_IgG
F33:MRM of 1 channel,ES+
995.113 > 204.1
1.670e+005

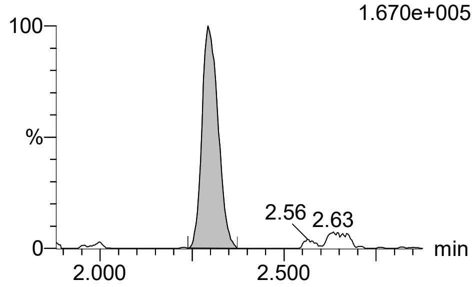

Sample Name: 20211111_H18_R2_IgG
F33:MRM of 1 channel,ES+
995.113 > 204.1
4.433e+005

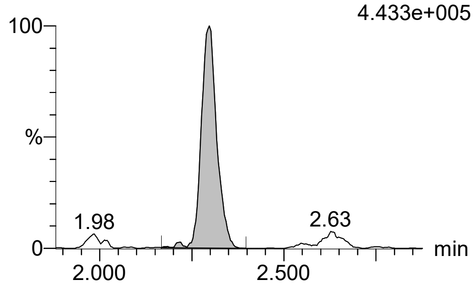

Sample Name: 20211111_A37_R2_IgG
F33:MRM of 1 channel,ES+
995.113 > 204.1
2.300e+004

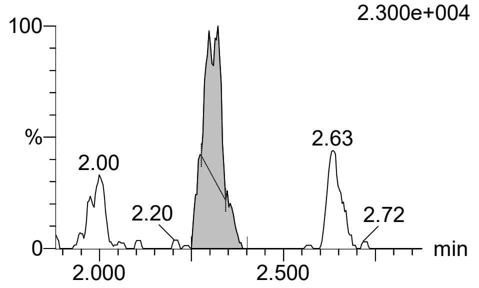

Sample Name: 20211111_A38_R2_IgG
F33:MRM of 1 channel,ES+
995.113 > 204.1
3.656e+004

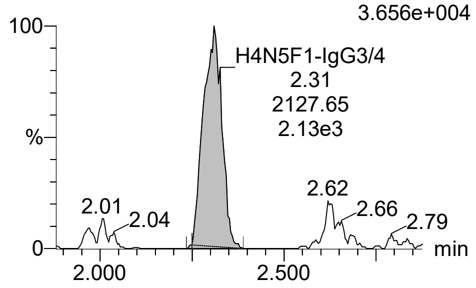

Sample Name: 20211111_H19_R2_IgG
F33:MRM of 1 channel,ES+
995.113 > 204.1
3.516e+005

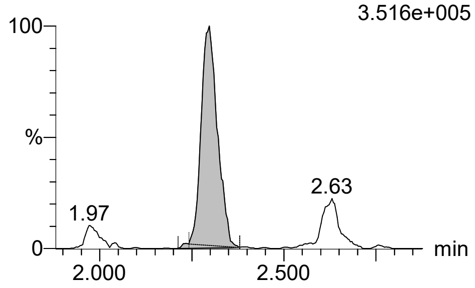

Sample Name: 20211111_A39_R2_IgG
F33:MRM of 1 channel,ES+
995.113 > 204.1
8.804e+004

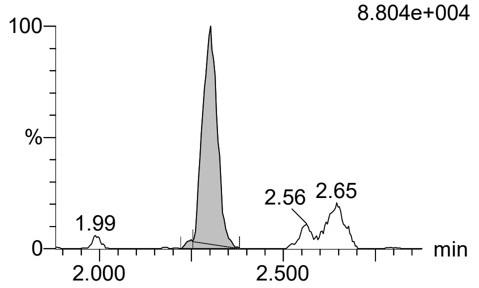

Sample Name: 20211111_A40_R2_IgG
F33:MRM of 1 channel,ES+
995.113 > 204.1
9.631e+004

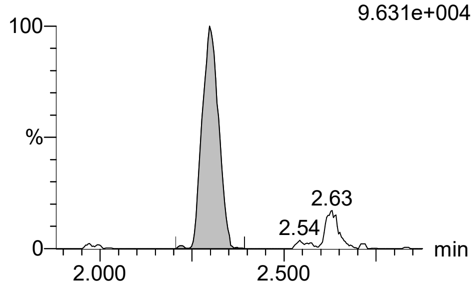

Sample Name: 20211111_H20_R2_IgG
F33:MRM of 1 channel,ES+
995.113 > 204.1
3.531e+005

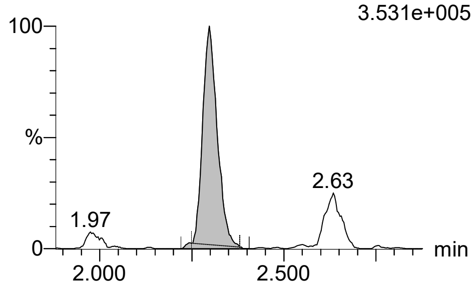

Sample Name: 20211111_A41_R2_IgG
F33:MRM of 1 channel,ES+
995.113 > 204.1
2.354e+004

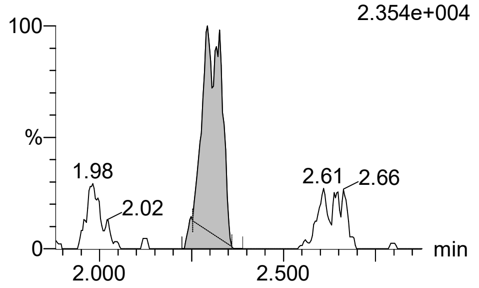

Sample Name: 20211111_A42_R2_IgG
F33:MRM of 1 channel,ES+
995.113 > 204.1
4.598e+004

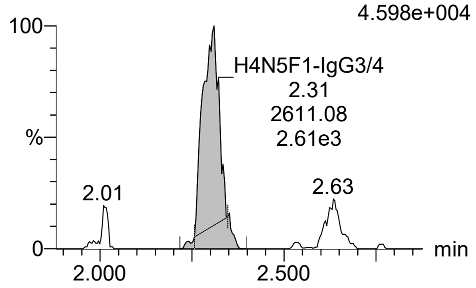

Sample Name: 20211111_A43_R2_IgG
F33:MRM of 1 channel,ES+
995.113 > 204.1
6.393e+004

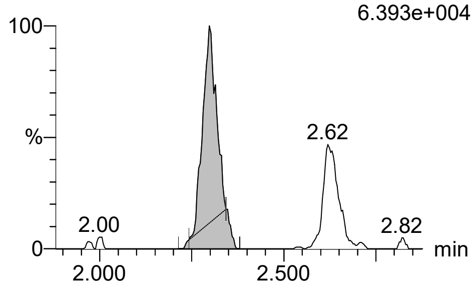

Dataset: C:\MassLynx\10.pro\20211115_clinicalsamples_IgG.qld

Last Altered: Sunday, October 02, 2022 16:02:30 台北標準時間
Printed: Friday, August 01, 2025 16:55:36 台北標準時間

Sample Name: 20211111_A44_R2_IgG

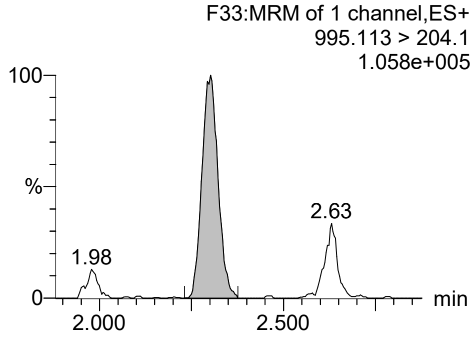

Sample Name: 20211111_QC_R17_IgG

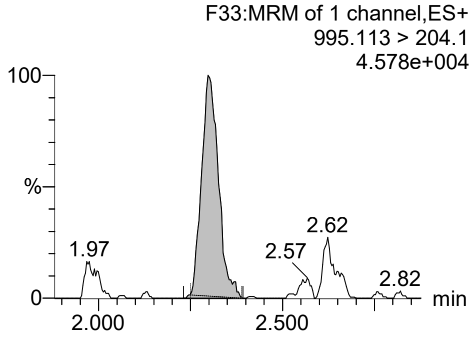

Dataset: C:\MassLynx\10.pro\20211115_clinicalsamples_IgG.qld

Last Altered: Sunday, October 02, 2022 16:02:30 台北標準時間
Printed: Friday, August 01, 2025 16:55:36 台北標準時間

	# Name	RT	Area	IS Area	Response	Prima...
1	1 20211111_QC_R1_IgG	2.30	7226.3	64968.0	0.111	bb
2	2 20211111_A1_R1_IgG	2.31	6335.5	59059.2	0.107	MM
3	3 20211111_A2_R1_IgG	2.30	5524.7	66621.2	0.083	MM
4	4 20211111_L1_R1_IgG	2.30	4909.0	64201.0	0.076	bb
5	5 20211111_L2_R1_IgG	2.30	3521.0	46950.2	0.075	MM
6	6 20211111_H1_R1_IgG	2.30	1848.5	38648.2	0.048	MM
7	7 20211111_A3_R1_IgG	2.32	212.4	2603.7	0.082	bb
8	8 20211111_A4_R1_IgG	2.31	3120.6	69960.4	0.045	MM
9	9 20211111_L3_R1_IgG	2.30	2177.7	38864.7	0.056	bb
10	10 20211111_L4_R1_IgG	2.31	5542.2	43380.5	0.128	bb
11	11 20211111_H2_R1_IgG	2.31	9717.6	71349.2	0.136	bb
12	12 20211111_A5_R1_IgG	2.31	4520.1	65569.7	0.069	bb
13	13 20211111_A6_R1_IgG	2.30	13273.3	53120.7	0.250	bb
14	14 20211111_QC_R2_IgG	2.30	7872.8	81034.1	0.097	MM
15	15 20211111_L5_R1_IgG	2.31	5228.5	50994.6	0.103	bb
16	16 20211111_L6_R1_IgG	2.30	2598.5	58058.0	0.045	bb
17	17 20211111_H3_R1_IgG	2.31	2113.0	64028.6	0.033	bb
18	18 20211111_A7_R1_IgG	2.30	5108.3	82996.1	0.062	bb
19	19 20211111_A8_R1_IgG	2.31	2421.3	77731.7	0.031	MM
20	20 20211111_L7_R1_IgG	2.29	3618.6	38827.3	0.093	MM
21	21 20211111_L8_R1_IgG	2.31	3485.7	44220.1	0.079	MM
22	22 20211111_H4_R1_IgG	2.30	4822.9	65826.3	0.073	bb
23	23 20211111_A9_R1_IgG	2.30	2173.3	60771.1	0.036	bb
24	24 20211111_A10_R1_IgG	2.31	1855.8	48578.7	0.038	bb
25	25 20211111_L9_R1_IgG	2.30	3097.3	50830.7	0.061	MM
26	26 20211111_L10_R1_IgG	2.30	4515.7	38034.2	0.119	MM
27	27 20211111_QC_R3_IgG	2.30	7191.7	67025.5	0.107	bb
28	28 20211111_H5_R1_IgG	2.31	1507.7	44544.9	0.034	bb
29	29 20211111_A11_R1_IgG	2.31	1541.9	66408.5	0.023	bb
30	30 20211111_A12_R1_IgG	2.31	4401.6	46624.5	0.094	MM
31	31 20211111_L11_R1_IgG	2.31	2632.7	33425.1	0.079	MM
32	32 20211111_L12_R1_IgG	2.30	1738.5	41178.0	0.042	bb
33	33 20211111_H6_R1_IgG	2.31	12233.4	58593.4	0.209	bb
34	34 20211111_A13_R1_IgG	2.30	19417.5	54133.0	0.359	bb
35	35 20211111_A14_R1_IgG	2.31	2810.4	56467.3	0.050	bb
36	36 20211111_L13_R1_IgG	2.31	2352.3	36024.4	0.065	MM
37	37 20211111_L14_R1_IgG	2.31	4316.0	37181.6	0.116	bb
38	38 20211111_H7_R1_IgG	2.30	7382.7	42850.6	0.172	bb
39	39 20211111_A15_R1_IgG	2.31	2454.7	62015.1	0.040	MM
40	40 20211111_QC_R4_IgG	2.30	5823.8	58572.5	0.099	bb
41	41 20211111_A16_R1_IgG	2.30	5868.3	78283.8	0.075	bb
42	42 20211111_L15_R1_IgG	2.31	8068.7	44158.2	0.183	bb
43	43 20211111_L16_R1_IgG	2.31	1716.3	55150.8	0.031	MM
44	44 20211111_H8_R1_IgG	2.30	3567.3	42965.2	0.083	bb
45	45 20211111_A17_R1_IgG	2.31	5493.8	39717.7	0.138	bb
46	46 20211111_A18_R1_IgG	2.31	3636.8	44675.4	0.081	bb
47	47 20211111_L17_R1_IgG	2.31	2087.0	37854.1	0.055	bb
48	48 20211111_L18_R1_IgG	2.29	1978.2	50716.9	0.039	bb
49	49 20211111_H9_R1_IgG	2.31	7015.9	36276.0	0.193	bb
50	50 20211111_A19_R1_IgG	2.31	1723.7	42862.5	0.040	bb
51	51 20211111_A20_R1_IgG	2.30	1913.4	36542.7	0.052	bb
52	52 20211111_L19_R1_IgG	2.31	3428.8	53892.8	0.064	bb
53	53 20211111_QC_R5_IgG	2.31	5374.1	56513.2	0.095	bb
54	54 20211111_L20_R1_IgG	2.30	5618.4	56499.9	0.099	bb
55	55 20211111_H10_R1_IgG	2.30	12000.6	68120.5	0.176	bb
56	56 20211111_A21_R1_IgG	2.31	6037.5	63699.8	0.095	bb

Dataset: C:\MassLynx\10.pro\20211115_clinicalsamples_IgG.qld

Last Altered: Sunday, October 02, 2022 16:02:30 台北標準時間
Printed: Friday, August 01, 2025 16:55:36 台北標準時間

	# Name	RT	Area	IS Area	Response	Prima...
57	57 20211111_A22_R1_IgG	2.30	4916.9	52222.1	0.094	bb
58	58 20211111_L21_R1_IgG	2.30	4028.7	54372.5	0.074	MM
59	59 20211111_L22_R1_IgG	2.31	2982.9	41353.1	0.072	MM
60	60 20211111_H11_R1_IgG	2.30	7403.5	65579.1	0.113	MM
61	61 20211111_A23_R1_IgG	2.30	7260.2	64131.1	0.113	MM
62	62 20211111_A24_R1_IgG	2.30	2332.7	38724.8	0.060	MM
63	63 20211111_L23_R1_IgG	2.30	7948.0	64245.2	0.124	MM
64	64 20211111_L24_R1_IgG	2.30	4158.1	45298.0	0.092	MM
65	65 20211111_H12_R1_IgG	2.31	816.4	44679.3	0.018	MM
66	66 20211111_QC_R6_IgG	2.30	4836.6	48165.0	0.100	bb
67	67 20211111_A25_R1_IgG	2.31	10779.4	57169.9	0.189	bb
68	68 20211111_A26_R1_IgG	2.30	942.6	34254.0	0.028	MM
69	69 20211111_L25_R1_IgG	2.30	4583.5	67491.5	0.068	bb
70	70 20211111_L26_R1_IgG	2.31	3168.3	55934.1	0.057	MM
71	71 20211111_H13_R1_IgG	2.30	4335.6	48767.3	0.089	MM
72	72 20211111_A27_R1_IgG	2.30	3040.9	57269.7	0.053	MM
73	73 20211111_A28_R1_IgG	2.31	7244.6	49231.1	0.147	MM
74	74 20211111_L27_R1_IgG	2.30	4248.9	60014.5	0.071	MM
75	75 20211111_L28_R1_IgG	2.30	1940.8	35062.6	0.055	MM
76	76 20211111_H14_R1_IgG	2.30	2362.1	62371.9	0.038	bb
77	77 20211111_A29_R1_IgG	2.31	4394.0	43858.2	0.100	bb
78	78 20211111_A30_R1_IgG	2.30	6295.6	62500.4	0.101	MM
79	79 20211111_QC_R7_IgG	2.31	4682.4	43946.9	0.107	bb
80	80 20211111_L29_R1_IgG	2.30	3677.3	38728.9	0.095	MM
81	81 20211111_L30_R1_IgG	2.30	2821.4	42613.2	0.066	bb
82	82 20211111_H15_R1_IgG	2.31	565.1	39874.6	0.014	bb
83	83 20211111_A31_R1_IgG	2.31	2431.2	53414.3	0.046	MM
84	84 20211111_A32_R1_IgG	2.31	3410.5	41844.6	0.082	MM
85	85 20211111_L31_R1_IgG	2.31	884.8	40553.7	0.022	MM
86	86 20211111_L32_R1_IgG	2.31	1594.8	49618.7	0.032	MM
87	87 20211111_H16_R1_IgG	2.31	8622.4	60170.9	0.143	bb
88	88 20211111_A33_R1_IgG	2.31	3415.5	48588.2	0.070	MM
89	89 20211111_A34_R1_IgG	2.30	5112.3	43288.0	0.118	MM
90	90 20211111_H17_R1_IgG	2.30	9580.9	47104.5	0.203	MM
91	91 20211111_A35_R1_IgG	2.31	12657.0	41025.9	0.309	bb
92	92 20211111_QC_R8_IgG	2.30	3650.0	40546.3	0.090	bb
93	93 20211111_A36_R1_IgG	2.31	8678.9	44504.4	0.195	MM
94	94 20211111_H18_R1_IgG	2.30	19792.9	34162.2	0.579	bb
95	95 20211111_A37_R1_IgG	2.31	1579.7	39760.0	0.040	bb
96	96 20211111_A38_R1_IgG	2.31	2714.2	43982.6	0.062	MM
97	97 20211111_H19_R1_IgG	2.31	21657.8	45314.8	0.478	MM
98	98 20211111_A39_R1_IgG	2.31	4447.0	39388.0	0.113	bb
99	99 20211111_A40_R1_IgG	2.31	5610.3	38726.4	0.145	bb
100	1... 20211111_H20_R1_IgG	2.31	19214.9	37814.4	0.508	bb
101	1... 20211111_A41_R1_IgG	2.30	1447.2	48930.3	0.030	MM
102	1... 20211111_A42_R1_IgG	2.31	2764.7	40406.8	0.068	MM
103	1... 20211111_A43_R1_IgG	2.30	3563.0	44421.5	0.080	MM
104	1... 20211111_A44_R1_IgG	2.31	7784.8	53128.2	0.147	bb
105	1... 20211111_QC_R9_IgG	2.30	3260.2	37049.0	0.088	bb
106	1... 20211111_A1_R2_IgG	2.31	4007.3	46499.4	0.086	MM
107	1... 20211111_A2_R2_IgG	2.30	3989.5	57260.7	0.070	bb
108	1... 20211111_L1_R2_IgG	2.30	3123.3	48062.9	0.065	bb
109	1... 20211111_L2_R2_IgG	2.30	2626.0	38745.2	0.068	bb
110	1... 20211111_H1_R2_IgG	2.31	1754.3	44273.5	0.040	MM
111	1... 20211111_A3_R2_IgG	2.31	332.5	4755.5	0.070	bb
112	1... 20211111_A4_R2_IgG	2.31	1839.3	50546.7	0.036	MM

Dataset: C:\MassLynx\10.pro\20211115_clinicalsamples_IgG.qld

Last Altered: Sunday, October 02, 2022 16:02:30 台北標準時間
Printed: Friday, August 01, 2025 16:55:36 台北標準時間

	#	Name	RT	Area	IS Area	Response	Prima...
113	1...	20211111_L3_R2_IgG	2.31	1969.7	34825.8	0.057	MM
114	1...	20211111_L4_R2_IgG	2.30	4693.0	39107.7	0.120	bb
115	1...	20211111_H2_R2_IgG	2.31	6783.4	49750.0	0.136	bb
116	1...	20211111_A5_R2_IgG	2.30	3359.1	52536.2	0.064	bb
117	1...	20211111_A6_R2_IgG	2.30	12192.6	42050.0	0.290	bb
118	1...	20211111_QC_R10_IgG	2.30	3399.8	35350.8	0.096	bb
119	1...	20211111_L5_R2_IgG	2.30	4651.7	38039.3	0.122	MM
120	1...	20211111_L6_R2_IgG	2.29	1826.2	44907.4	0.041	MM
121	1...	20211111_H3_R2_IgG	2.31	1971.8	47729.1	0.041	bb
122	1...	20211111_A7_R2_IgG	2.30	2913.6	57347.1	0.051	bb
123	1...	20211111_A8_R2_IgG	2.30	2134.2	52397.8	0.041	bb
124	1...	20211111_L7_R2_IgG	2.30	3423.3	36318.4	0.094	MM
125	1...	20211111_L8_R2_IgG	2.29	2505.8	38131.9	0.066	bb
126	1...	20211111_H4_R2_IgG	2.30	3815.3	46849.6	0.081	bb
127	1...	20211111_A9_R2_IgG	2.30	2022.9	47759.6	0.042	bb
128	1...	20211111_A10_R2_IgG	2.30	2116.7	41366.2	0.051	MM
129	1...	20211111_L9_R2_IgG	2.30	3227.3	43052.1	0.075	bb
130	1...	20211111_L10_R2_IgG	2.30	4199.7	31487.4	0.133	MM
131	1...	20211111_QC_R11_IgG	2.30	3177.6	37804.6	0.084	bb
132	1...	20211111_H5_R2_IgG	2.30	1305.3	40380.0	0.032	MM
133	1...	20211111_A11_R2_IgG	2.30	1274.9	49533.3	0.026	bb
134	1...	20211111_A12_R2_IgG	2.31	3872.6	40226.3	0.096	bb
135	1...	20211111_L11_R2_IgG	2.30	1739.5	28884.0	0.060	MM
136	1...	20211111_L12_R2_IgG	2.30	1374.6	34351.3	0.040	MM
137	1...	20211111_H6_R2_IgG	2.30	7259.5	40466.6	0.179	MM
138	1...	20211111_A13_R2_IgG	2.30	15122.6	42306.7	0.357	bb
139	1...	20211111_A14_R2_IgG	2.30	2617.4	44904.0	0.058	bb
140	1...	20211111_L13_R2_IgG	2.30	1679.1	31913.0	0.053	MM
141	1...	20211111_L14_R2_IgG	2.30	5029.1	31527.4	0.160	bb
142	1...	20211111_H7_R2_IgG	2.31	6669.3	40838.2	0.163	bb
143	1...	20211111_A15_R2_IgG	2.30	1674.4	49389.7	0.034	bb
144	1...	20211111_QC_R12_IgG	2.31	2896.1	35168.7	0.082	MM
145	1...	20211111_A16_R2_IgG	2.30	3185.9	47440.5	0.067	bb
146	1...	20211111_L15_R2_IgG	2.31	5618.0	33145.9	0.169	bb
147	1...	20211111_L16_R2_IgG	2.29	992.4	37596.6	0.026	bb
148	1...	20211111_H8_R2_IgG	2.30	3849.5	43933.3	0.088	MM
149	1...	20211111_A17_R2_IgG	2.30	5882.5	43605.5	0.135	MM
150	1...	20211111_A18_R2_IgG	2.30	3253.7	37581.8	0.087	MM
151	1...	20211111_L17_R2_IgG	2.30	1604.8	33942.5	0.047	bb
152	1...	20211111_L18_R2_IgG	2.30	1399.5	37831.5	0.037	bb
153	1...	20211111_H9_R2_IgG	2.30	5401.0	30898.4	0.175	bb
154	1...	20211111_A19_R2_IgG	2.30	1673.6	39352.1	0.043	bb
155	1...	20211111_A20_R2_IgG	2.30	1474.5	34661.2	0.043	bb
156	1...	20211111_L19_R2_IgG	2.31	2666.9	39918.3	0.067	bb
157	1...	20211111_QC_R13_IgG	2.31	3833.6	36239.1	0.106	bb
158	1...	20211111_L20_R2_IgG	2.29	3241.6	32897.7	0.099	MM
159	1...	20211111_H10_R2_IgG	2.30	5070.6	31394.5	0.162	MM
160	1...	20211111_A21_R2_IgG	2.30	2992.0	34863.7	0.086	bb
161	1...	20211111_A22_R2_IgG	2.29	2146.0	30342.1	0.071	MM
162	1...	20211111_L21_R2_IgG	2.29	1507.7	33706.2	0.045	bb
163	1...	20211111_L22_R2_IgG	2.31	1668.2	31298.6	0.053	MM
164	1...	20211111_H11_R2_IgG	2.29	3580.1	38904.1	0.092	bb
165	1...	20211111_A23_R2_IgG	2.29	3312.4	32382.3	0.102	bb
166	1...	20211111_A24_R2_IgG	2.29	1385.1	30261.9	0.046	MM
167	1...	20211111_L23_R2_IgG	2.29	5985.0	43037.5	0.139	MM
168	1...	20211111_L24_R2_IgG	2.29	2861.6	37023.3	0.077	MM

Dataset: C:\MassLynx\10.pro\20211115_clinicalsamples_IgG.qld

Last Altered: Sunday, October 02, 2022 16:02:30 台北標準時間
Printed: Friday, August 01, 2025 16:55:36 台北標準時間

	#	Name	RT	Area	IS Area	Response	Prima...
169	1...	20211111_H12_R2_IgG	2.30	659.1	39478.2	0.017	bb
170	1...	20211111_QC_R14_IgG	2.30	2849.4	34463.2	0.083	bb
171	1...	20211111_A25_R2_IgG	2.30	6316.7	39945.3	0.158	bb
172	1...	20211111_A26_R2_IgG	2.30	657.8	29663.2	0.022	MM
173	1...	20211111_L25_R2_IgG	2.30	1835.0	38589.3	0.048	bb
174	1...	20211111_L26_R2_IgG	2.31	1976.7	38405.2	0.051	bb
175	1...	20211111_H13_R2_IgG	2.30	2634.8	37771.2	0.070	bb
176	1...	20211111_A27_R2_IgG	2.29	1701.6	42634.1	0.040	bb
177	1...	20211111_A28_R2_IgG	2.29	3920.8	33231.1	0.118	bb
178	1...	20211111_L27_R2_IgG	2.30	2536.5	41761.1	0.061	bb
179	1...	20211111_L28_R2_IgG	2.31	1868.0	26968.6	0.069	MM
180	1...	20211111_H14_R2_IgG	2.29	1998.8	49250.2	0.041	MM
181	1...	20211111_A29_R2_IgG	2.30	3944.2	37601.7	0.105	bb
182	1...	20211111_A30_R2_IgG	2.30	3126.9	40366.8	0.077	MM
183	1...	20211111_QC_R15_IgG	2.29	2536.2	35156.1	0.072	bb
184	1...	20211111_L29_R2_IgG	2.30	2698.0	33813.1	0.080	bb
185	1...	20211111_L30_R2_IgG	2.30	1905.9	33488.6	0.057	bb
186	1...	20211111_H15_R2_IgG	2.29	595.2	38501.0	0.015	MM
187	1...	20211111_A31_R2_IgG	2.30	1958.8	37525.5	0.052	bb
188	1...	20211111_A32_R2_IgG	2.31	3043.9	35770.8	0.085	MM
189	1...	20211111_L31_R2_IgG	2.30	927.6	33282.4	0.028	bb
190	1...	20211111_L32_R2_IgG	2.31	1413.3	42049.6	0.034	bb
191	1...	20211111_H16_R2_IgG	2.30	6452.2	45947.6	0.140	MM
192	1...	20211111_A33_R2_IgG	2.31	1882.3	40202.3	0.047	MM
193	1...	20211111_A34_R2_IgG	2.30	3278.1	36747.0	0.089	MM
194	1...	20211111_H17_R2_IgG	2.30	8653.3	40920.9	0.211	MM
195	1...	20211111_A35_R2_IgG	2.30	10295.9	33689.2	0.306	bb
196	1...	20211111_QC_R16_IgG	2.31	2777.5	32783.6	0.085	bb
197	1...	20211111_A36_R2_IgG	2.29	8455.2	36822.4	0.230	bb
198	1...	20211111_H18_R2_IgG	2.30	21575.1	31769.3	0.679	bb
199	1...	20211111_A37_R2_IgG	2.32	1440.5	37581.8	0.038	MM
200	2...	20211111_A38_R2_IgG	2.31	2127.6	38390.4	0.055	MM
201	2...	20211111_H19_R2_IgG	2.30	18287.6	41217.0	0.444	MM
202	2...	20211111_A39_R2_IgG	2.30	4181.0	37392.7	0.112	MM
203	2...	20211111_A40_R2_IgG	2.30	5003.1	38295.1	0.131	bb
204	2...	20211111_H20_R2_IgG	2.30	16544.3	35941.8	0.460	MM
205	2...	20211111_A41_R2_IgG	2.29	1530.8	45304.9	0.034	MM
206	2...	20211111_A42_R2_IgG	2.31	2611.1	37803.8	0.069	MM
207	2...	20211111_A43_R2_IgG	2.30	3238.5	38161.8	0.085	MM
208	2...	20211111_A44_R2_IgG	2.30	5355.3	37819.8	0.142	bb
209	2...	20211111_QC_R17_IgG	2.30	2448.5	31731.8	0.077	MM

Dataset: C:\MassLynx\10.pro\20211115_clinicalsamples_IgG.qld

Last Altered: Sunday, October 02, 2022 16:02:30 台北標準時間
Printed: Friday, August 01, 2025 16:56:05 台北標準時間

Method: C:\MassLynx\10_Pro_IgA.PRO\MethDB\20210926_IgG_DMRM_IgM_IS.mdb 16 Feb 2022 11:58:21
Calibration: 18 Nov 2021 13:56:22

Sample Name: 20211111_QC_R1_IgG

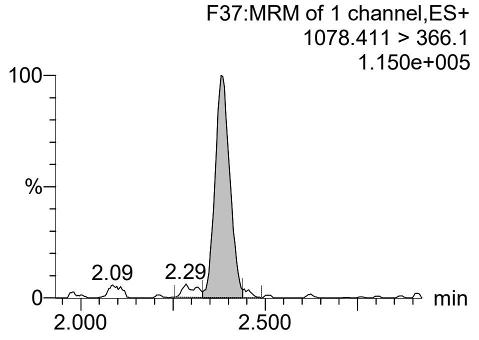

Sample Name: 20211111_A1_R1_IgG

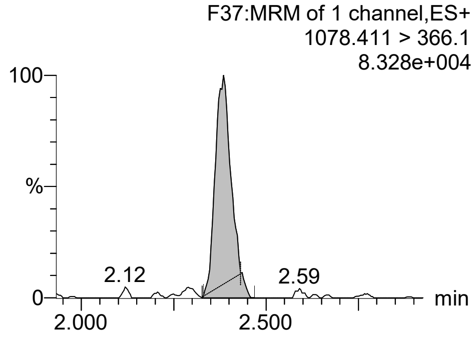

Sample Name: 20211111_A2_R1_IgG

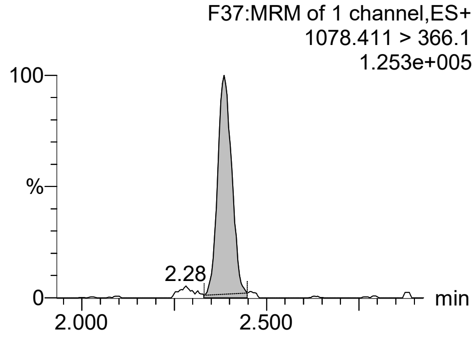

Sample Name: 20211111_L1_R1_IgG

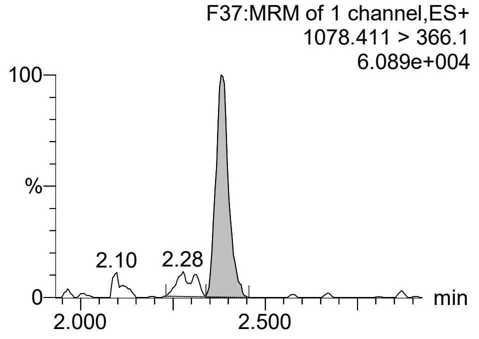

Sample Name: 20211111_L2_R1_IgG

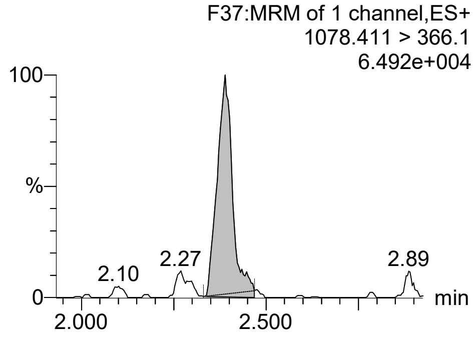

Sample Name: 20211111_H1_R1_IgG

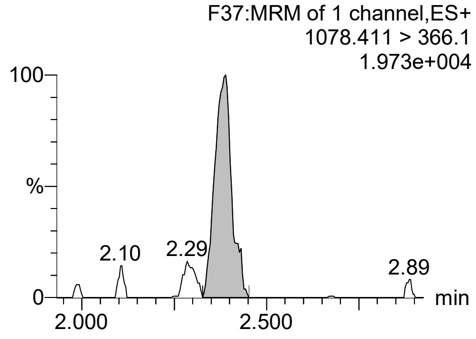

Sample Name: 20211111_A3_R1_IgG

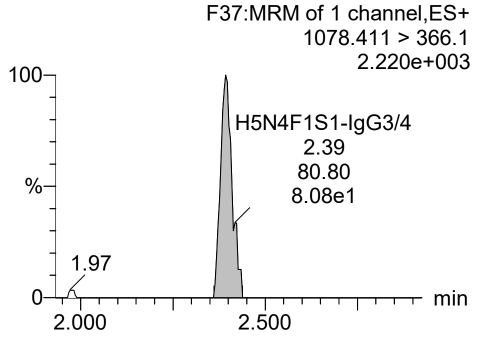

Sample Name: 20211111_A4_R1_IgG

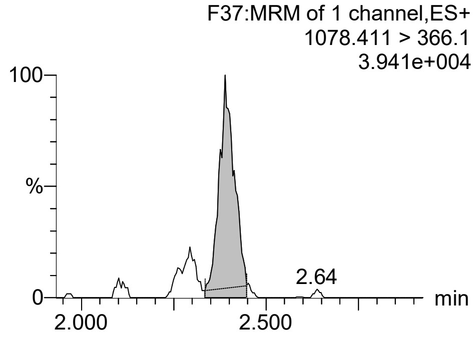

Sample Name: 20211111_L3_R1_IgG

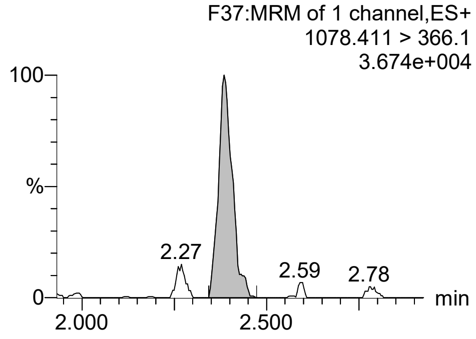

Sample Name: 20211111_L4_R1_IgG

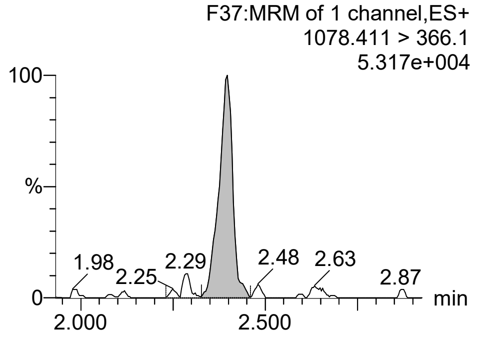

Sample Name: 20211111_H2_R1_IgG

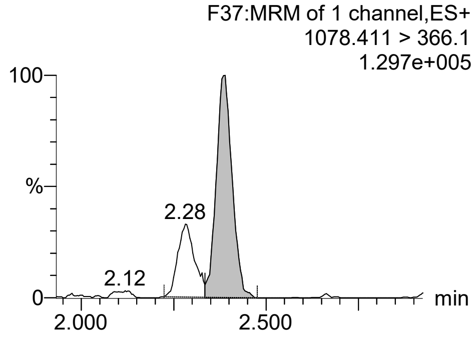

Sample Name: 20211111_A5_R1_IgG

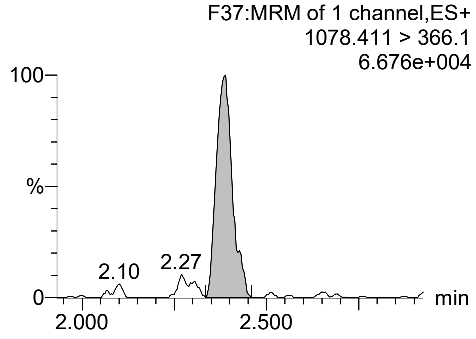

Dataset: C:\MassLynx\10.pro\20211115_clinicalsamples_IgG.qld

Last Altered: Sunday, October 02, 2022 16:02:30 台北標準時間
Printed: Friday, August 01, 2025 16:56:05 台北標準時間

Sample Name: 20211111_A6_R1_IgG

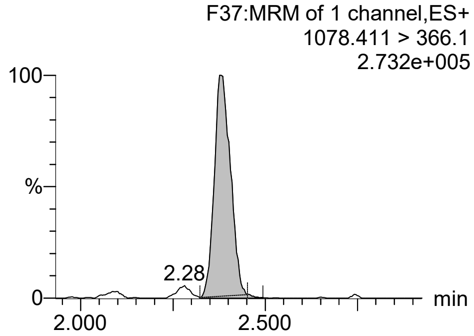

Sample Name: 20211111_QC_R2_IgG

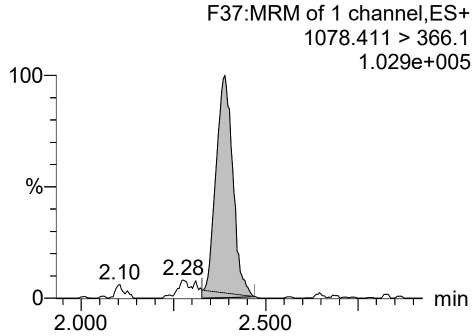

Sample Name: 20211111_L5_R1_IgG

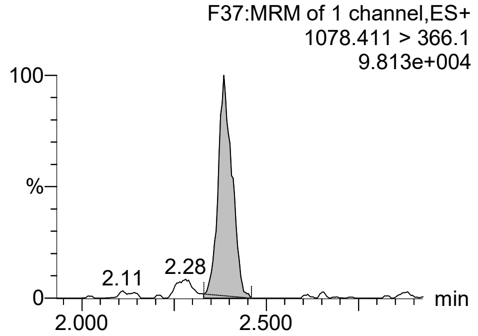

Sample Name: 20211111_L6_R1_IgG

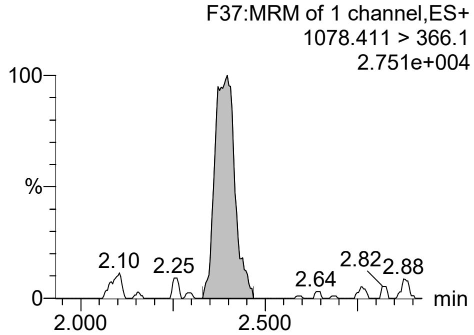

Sample Name: 20211111_H3_R1_IgG

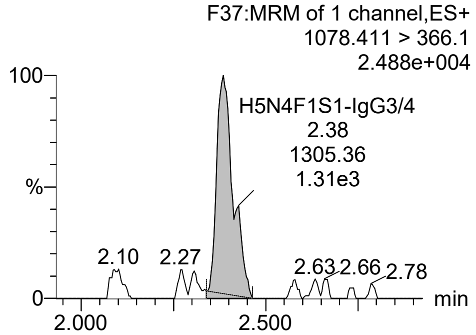

Sample Name: 20211111_A7_R1_IgG

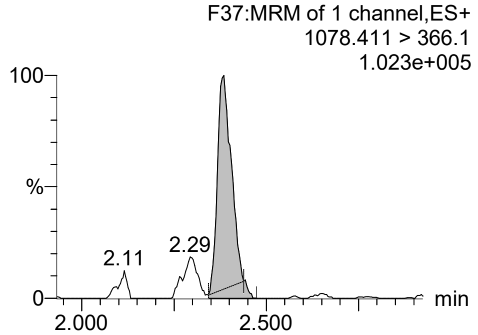

Sample Name: 20211111_A8_R1_IgG

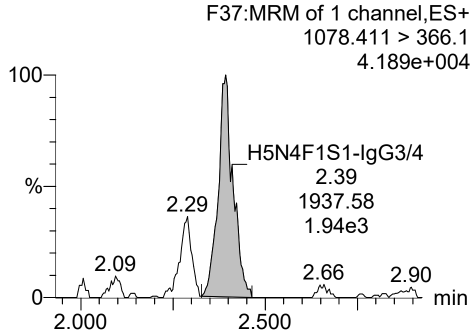

Sample Name: 20211111_L7_R1_IgG

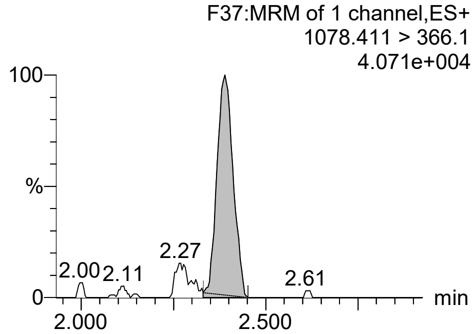

Sample Name: 20211111_L8_R1_IgG

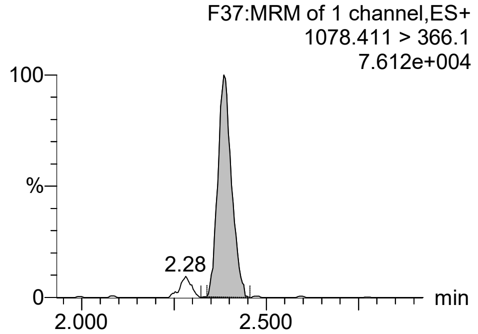

Sample Name: 20211111_H4_R1_IgG

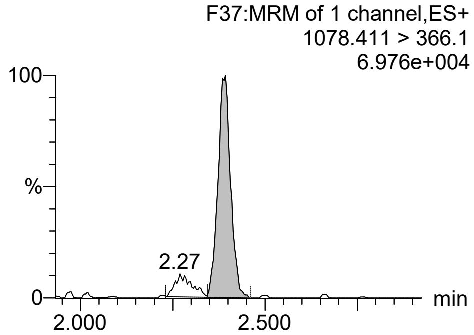

Sample Name: 20211111_A9_R1_IgG

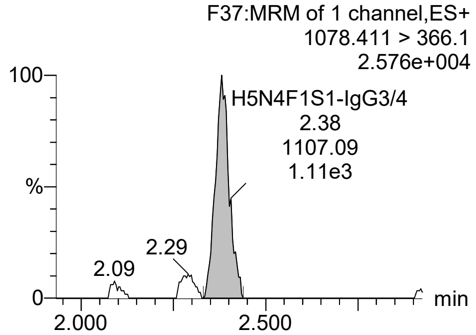

Sample Name: 20211111_A10_R1_IgG

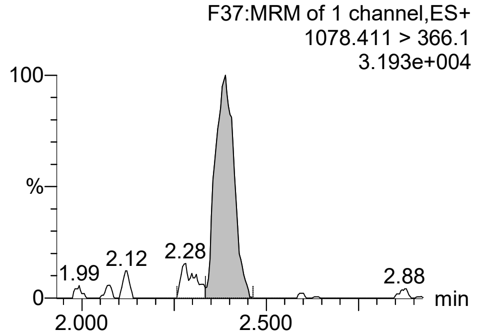

Sample Name: 20211111_L9_R1_IgG

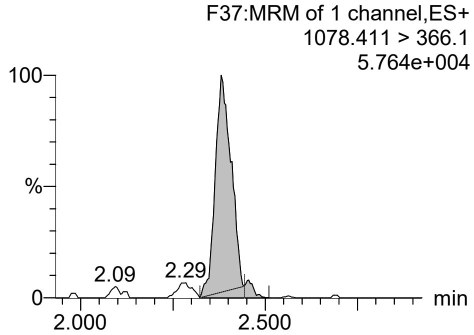

Sample Name: 20211111_L10_R1_IgG

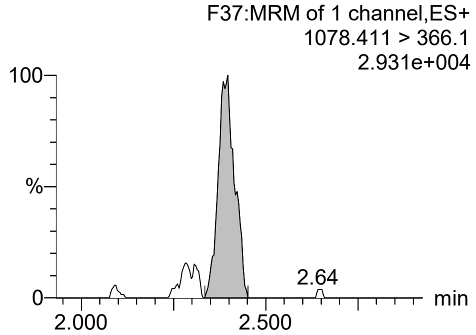

Sample Name: 20211111_QC_R3_IgG

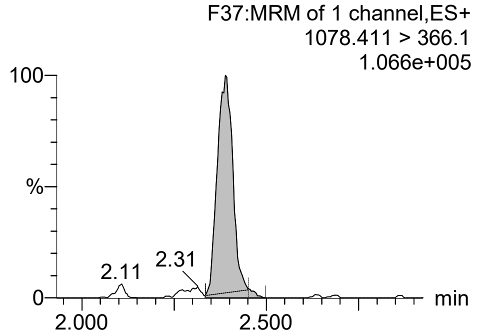

Dataset: C:\MassLynx\10.pro\20211115_clinicalsamples_IgG.qld

Last Altered: Sunday, October 02, 2022 16:02:30 台北標準時間
Printed: Friday, August 01, 2025 16:56:05 台北標準時間

Sample Name: 20211111_H5_R1_IgG
F37:MRM of 1 channel,ES+
1078.411 > 366.1
4.941e+004

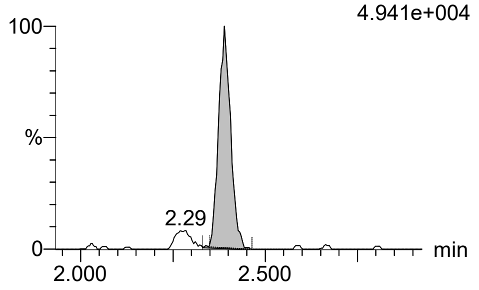

Sample Name: 20211111_A11_R1_IgG
F37:MRM of 1 channel,ES+
1078.411 > 366.1
1.602e+004

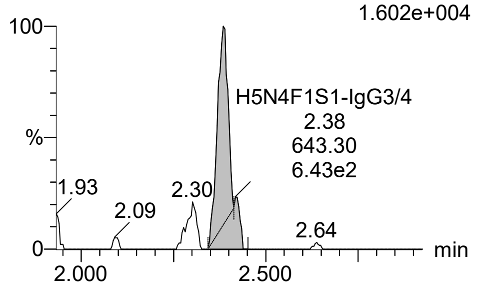

Sample Name: 20211111_A12_R1_IgG
F37:MRM of 1 channel,ES+
1078.411 > 366.1
4.236e+004

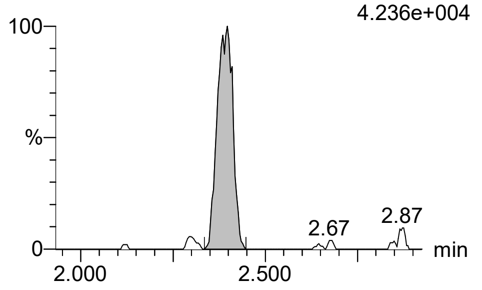

Sample Name: 20211111_L11_R1_IgG
F37:MRM of 1 channel,ES+
1078.411 > 366.1
1.446e+004

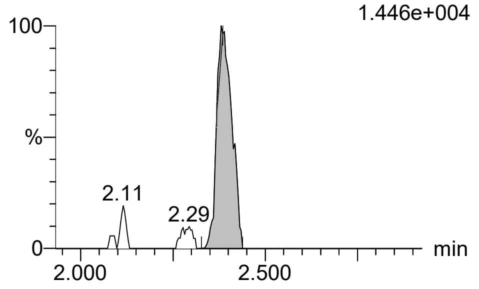

Sample Name: 20211111_L12_R1_IgG
F37:MRM of 1 channel,ES+
1078.411 > 366.1
2.390e+004

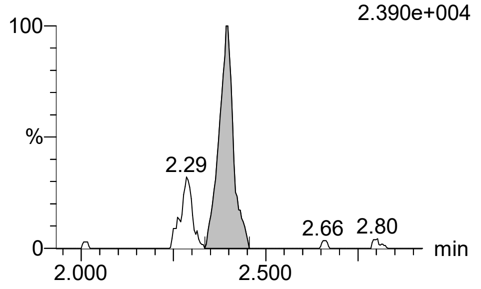

Sample Name: 20211111_H6_R1_IgG
F37:MRM of 1 channel,ES+
1078.411 > 366.1
1.396e+005

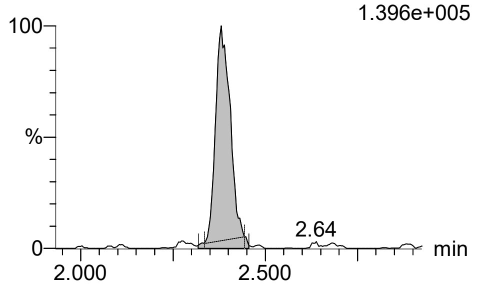

Sample Name: 20211111_A13_R1_IgG
F37:MRM of 1 channel,ES+
1078.411 > 366.1
1.902e+005

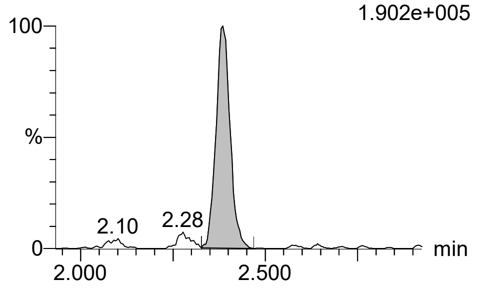

Sample Name: 20211111_A14_R1_IgG
F37:MRM of 1 channel,ES+
1078.411 > 366.1
3.822e+004

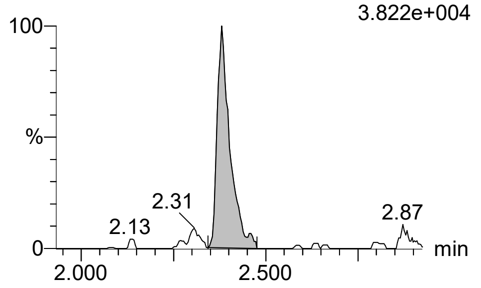

Sample Name: 20211111_L13_R1_IgG
F37:MRM of 1 channel,ES+
1078.411 > 366.1
7.306e+004

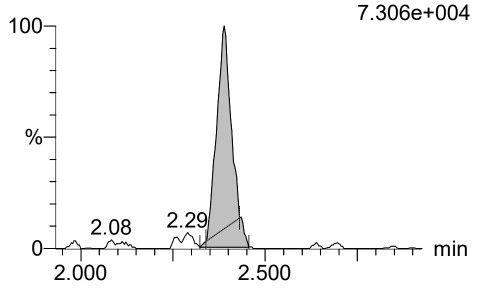

Sample Name: 20211111_L14_R1_IgG
F37:MRM of 1 channel,ES+
1078.411 > 366.1
7.183e+004

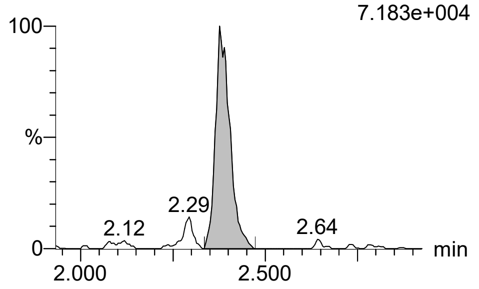

Sample Name: 20211111_H7_R1_IgG
F37:MRM of 1 channel,ES+
1078.411 > 366.1
4.812e+004

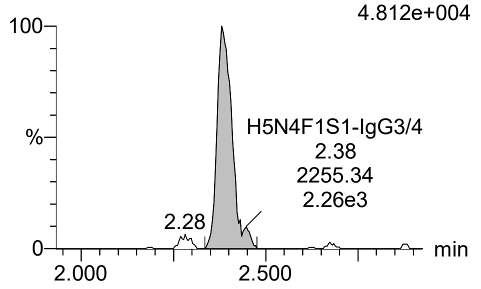

Sample Name: 20211111_A15_R1_IgG
F37:MRM of 1 channel,ES+
1078.411 > 366.1
2.490e+004

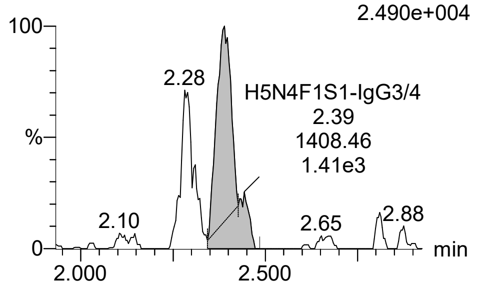

Sample Name: 20211111_QC_R4_IgG
F37:MRM of 1 channel,ES+
1078.411 > 366.1
8.940e+004

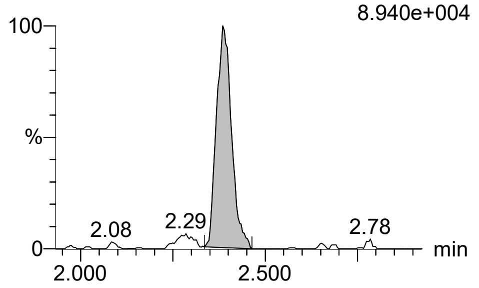

Sample Name: 20211111_A16_R1_IgG
F37:MRM of 1 channel,ES+
1078.411 > 366.1
1.073e+005

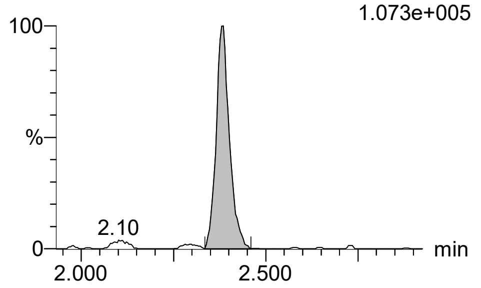

Sample Name: 20211111_L15_R1_IgG
F37:MRM of 1 channel,ES+
1078.411 > 366.1
9.512e+004

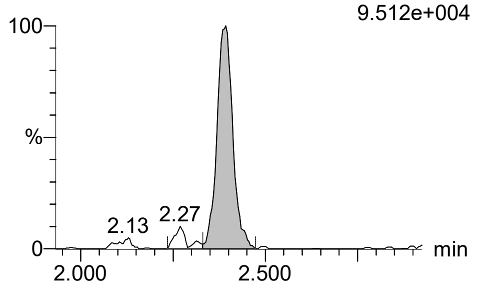

Dataset: C:\MassLynx\10.pro\20211115_clinicalsamples_IgG.qld

Last Altered: Sunday, October 02, 2022 16:02:30 台北標準時間
Printed: Friday, August 01, 2025 16:56:05 台北標準時間

Sample Name: 20211111_L16_R1_IgG
F37:MRM of 1 channel,ES+
1078.411 > 366.1
2.001e+004

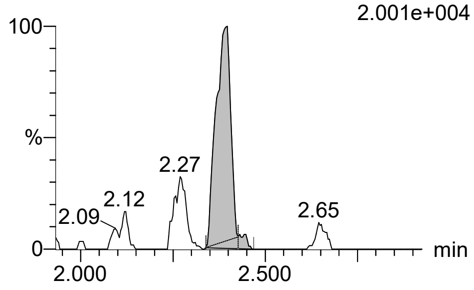

Sample Name: 20211111_H8_R1_IgG
F37:MRM of 1 channel,ES+
1078.411 > 366.1
7.836e+004

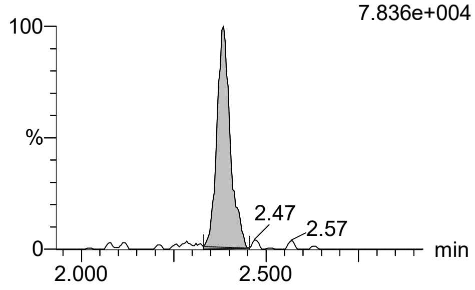

Sample Name: 20211111_A17_R1_IgG
F37:MRM of 1 channel,ES+
1078.411 > 366.1
7.082e+004

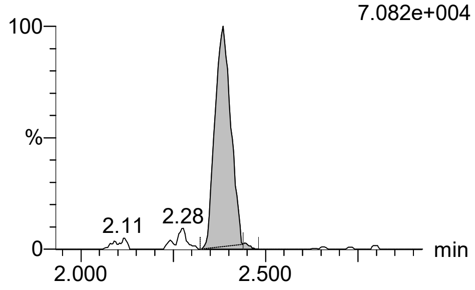

Sample Name: 20211111_A18_R1_IgG
F37:MRM of 1 channel,ES+
1078.411 > 366.1
6.281e+004

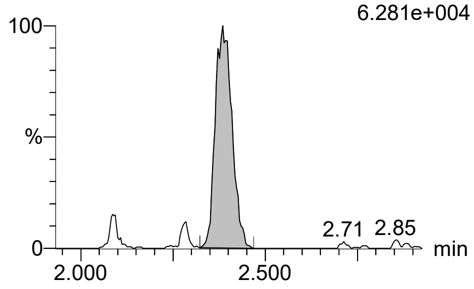

Sample Name: 20211111_L17_R1_IgG
F37:MRM of 1 channel,ES+
1078.411 > 366.1
4.503e+004

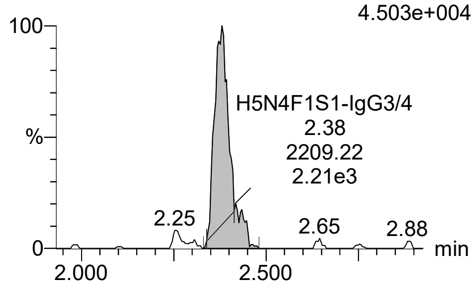

Sample Name: 20211111_L18_R1_IgG
F37:MRM of 1 channel,ES+
1078.411 > 366.1
1.207e+004

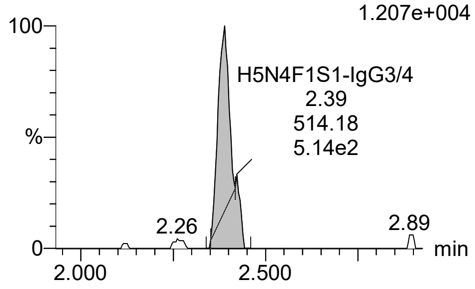

Sample Name: 20211111_H9_R1_IgG
F37:MRM of 1 channel,ES+
1078.411 > 366.1
5.611e+004

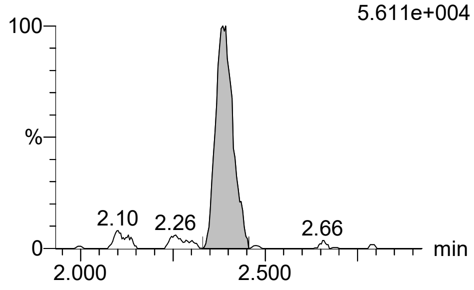

Sample Name: 20211111_A19_R1_IgG
F37:MRM of 1 channel,ES+
1078.411 > 366.1
2.573e+004

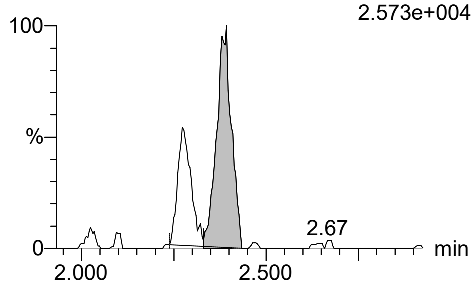

Sample Name: 20211111_A20_R1_IgG
F37:MRM of 1 channel,ES+
1078.411 > 366.1
1.952e+004

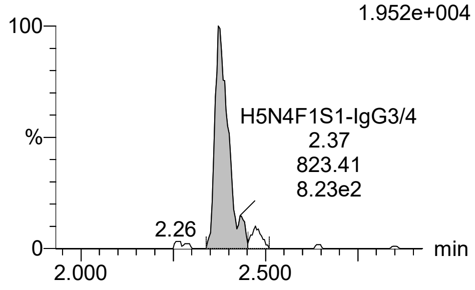

Sample Name: 20211111_L19_R1_IgG
F37:MRM of 1 channel,ES+
1078.411 > 366.1
7.166e+004

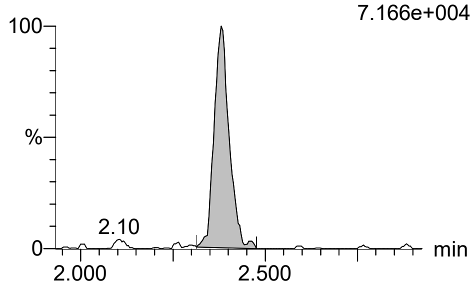

Sample Name: 20211111_QC_R5_IgG
F37:MRM of 1 channel,ES+
1078.411 > 366.1
6.964e+004

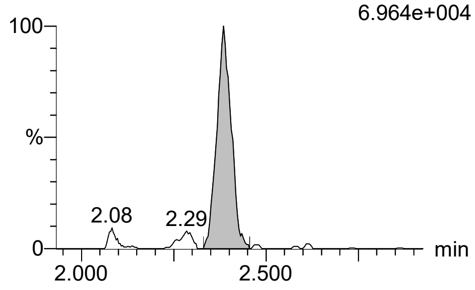

Sample Name: 20211111_L20_R1_IgG
F37:MRM of 1 channel,ES+
1078.411 > 366.1
1.330e+005

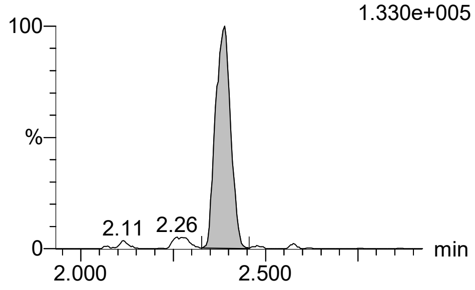

Sample Name: 20211111_H10_R1_IgG
F37:MRM of 1 channel,ES+
1078.411 > 366.1
1.003e+005

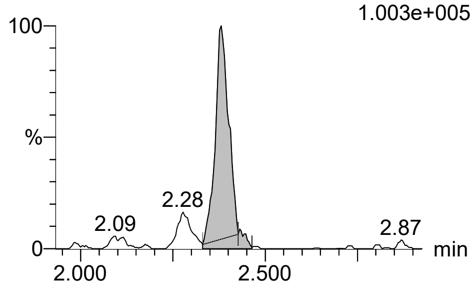

Sample Name: 20211111_A21_R1_IgG
F37:MRM of 1 channel,ES+
1078.411 > 366.1
1.193e+005

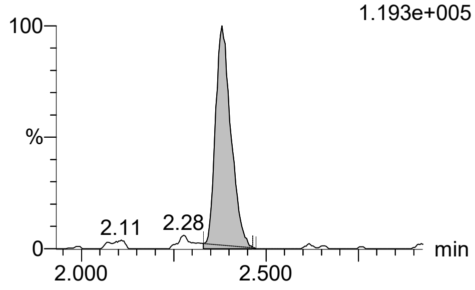

Sample Name: 20211111_A22_R1_IgG
F37:MRM of 1 channel,ES+
1078.411 > 366.1
1.634e+005

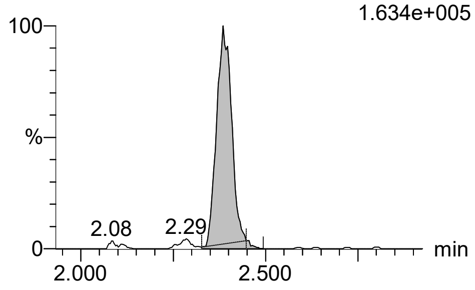

Sample Name: 20211111_L21_R1_IgG

F37:MRM of 1 channel,ES+
1078.411 > 366.1
3.328e+004

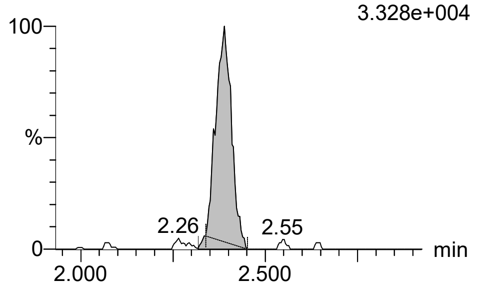

Sample Name: 20211111_L22_R1_IgG

F37:MRM of 1 channel,ES+
1078.411 > 366.1
2.826e+004

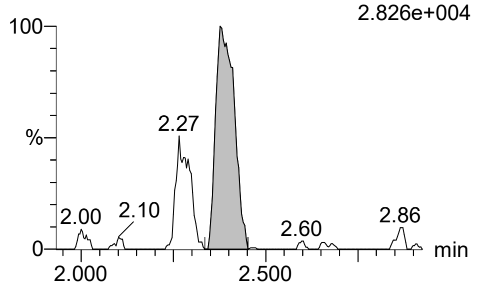

Sample Name: 20211111_H11_R1_IgG

F37:MRM of 1 channel,ES+
1078.411 > 366.1
6.094e+004

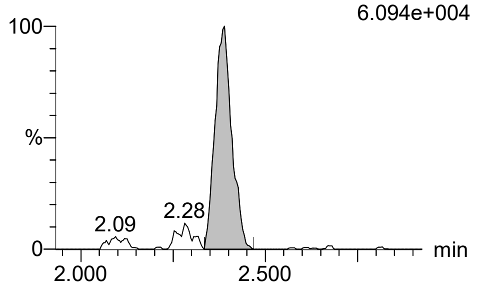

Sample Name: 20211111_A23_R1_IgG

F37:MRM of 1 channel,ES+
1078.411 > 366.1
5.054e+004

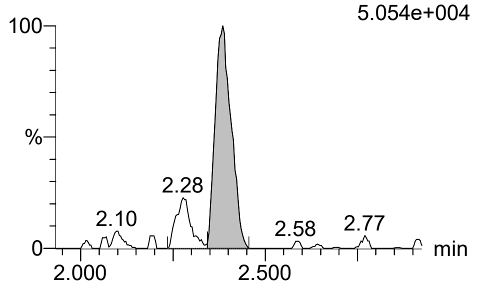

Sample Name: 20211111_A24_R1_IgG

F37:MRM of 1 channel,ES+
1078.411 > 366.1
4.744e+004

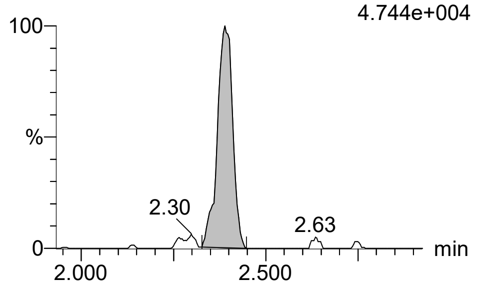

Sample Name: 20211111_L23_R1_IgG

F37:MRM of 1 channel,ES+
1078.411 > 366.1
9.935e+004

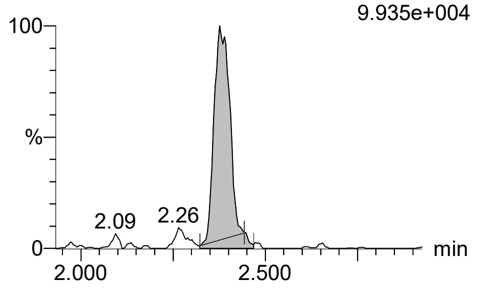

Sample Name: 20211111_L24_R1_IgG

F37:MRM of 1 channel,ES+
1078.411 > 366.1
8.996e+004

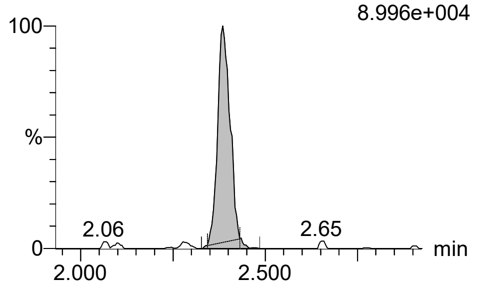

Sample Name: 20211111_H12_R1_IgG

F37:MRM of 1 channel,ES+
1078.411 > 366.1
1.698e+004

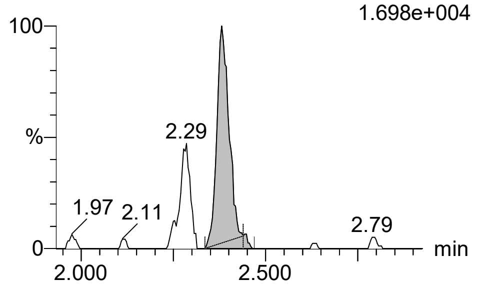

Sample Name: 20211111_QC_R6_IgG

F37:MRM of 1 channel,ES+
1078.411 > 366.1
6.034e+004

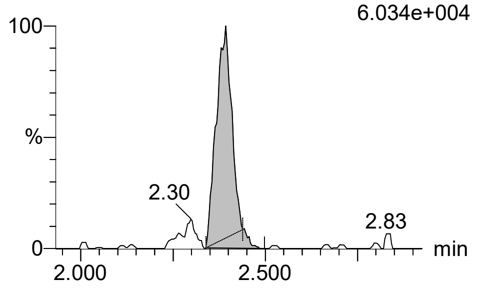

Sample Name: 20211111_A25_R1_IgG

F37:MRM of 1 channel,ES+
1078.411 > 366.1
1.969e+005

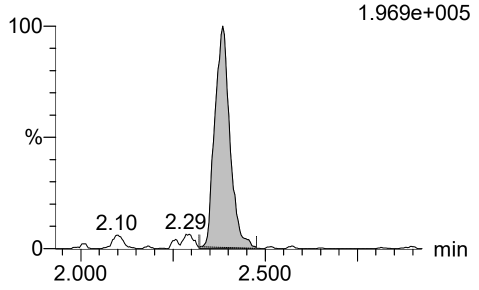

Sample Name: 20211111_A26_R1_IgG

F37:MRM of 1 channel,ES+
1078.411 > 366.1
8.809e+003

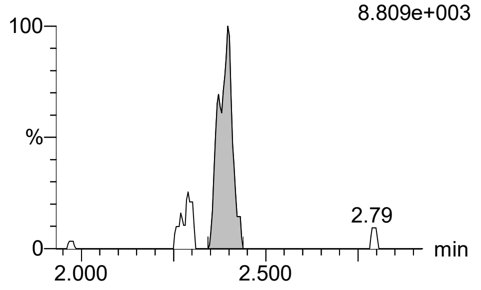

Sample Name: 20211111_L25_R1_IgG

F37:MRM of 1 channel,ES+
1078.411 > 366.1
5.769e+004

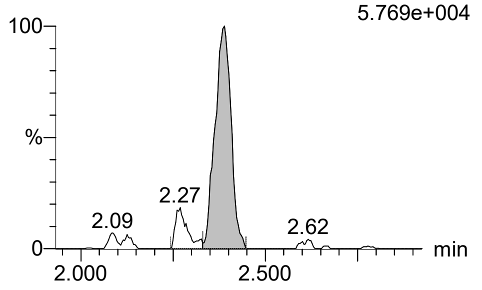

Sample Name: 20211111_L26_R1_IgG

F37:MRM of 1 channel,ES+
1078.411 > 366.1
3.329e+004

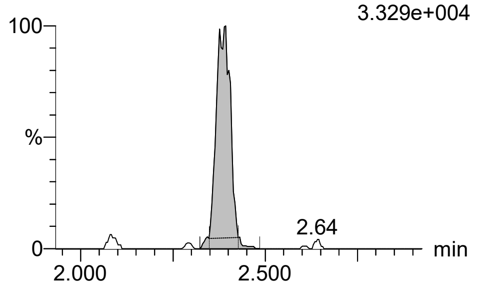

Sample Name: 20211111_H13_R1_IgG

F37:MRM of 1 channel,ES+
1078.411 > 366.1
5.613e+004

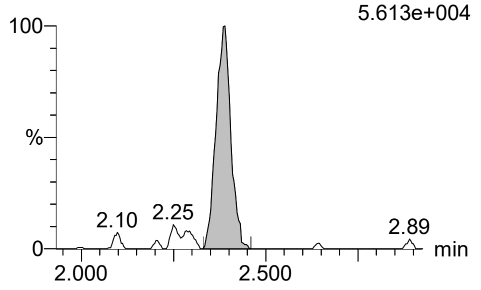

Sample Name: 20211111_A27_R1_IgG

F37:MRM of 1 channel,ES+
1078.411 > 366.1
5.071e+004

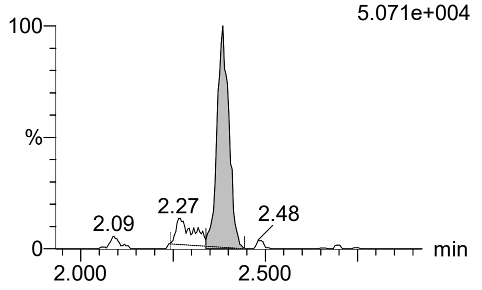

Dataset: C:\MassLynx\10.pro\20211115_clinicalsamples_IgG.qld

Last Altered: Sunday, October 02, 2022 16:02:30 台北標準時間
Printed: Friday, August 01, 2025 16:56:05 台北標準時間

Sample Name: 20211111_A28_R1_IgG
F37:MRM of 1 channel,ES+
1078.411 > 366.1
1.144e+005

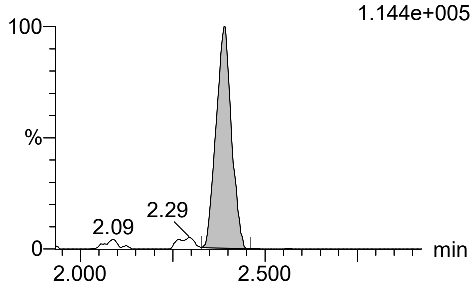

Sample Name: 20211111_L27_R1_IgG
F37:MRM of 1 channel,ES+
1078.411 > 366.1
4.917e+004

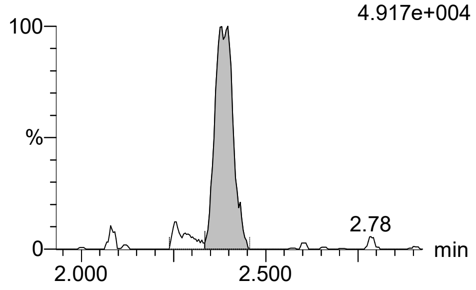

Sample Name: 20211111_L28_R1_IgG
F37:MRM of 1 channel,ES+
1078.411 > 366.1
7.697e+004

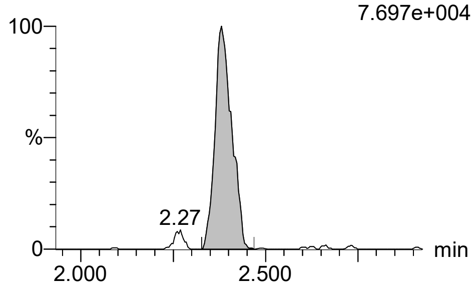

Sample Name: 20211111_H14_R1_IgG
F37:MRM of 1 channel,ES+
1078.411 > 366.1
4.606e+004

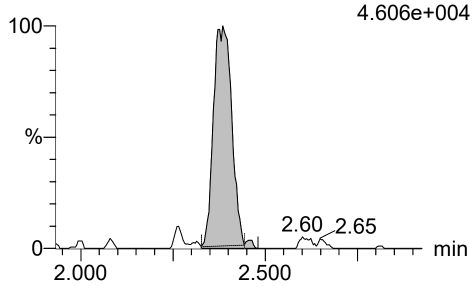

Sample Name: 20211111_A29_R1_IgG
F37:MRM of 1 channel,ES+
1078.411 > 366.1
2.484e+004

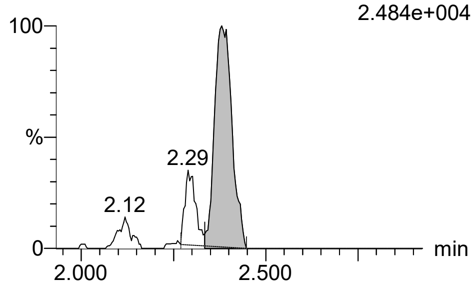

Sample Name: 20211111_A30_R1_IgG
F37:MRM of 1 channel,ES+
1078.411 > 366.1
5.753e+004

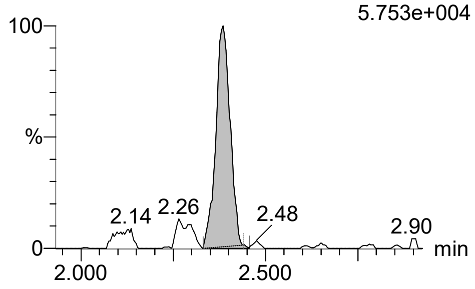

Sample Name: 20211111_QC_R7_IgG
F37:MRM of 1 channel,ES+
1078.411 > 366.1
7.044e+004

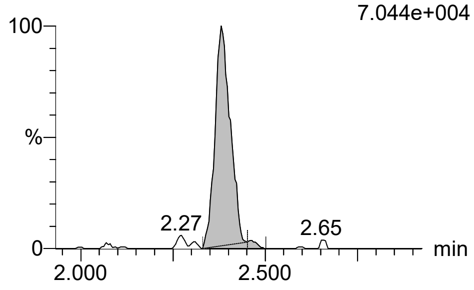

Sample Name: 20211111_L29_R1_IgG
F37:MRM of 1 channel,ES+
1078.411 > 366.1
5.008e+004

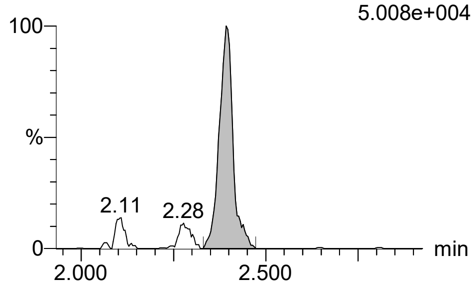

Sample Name: 20211111_L30_R1_IgG
F37:MRM of 1 channel,ES+
1078.411 > 366.1
3.193e+004

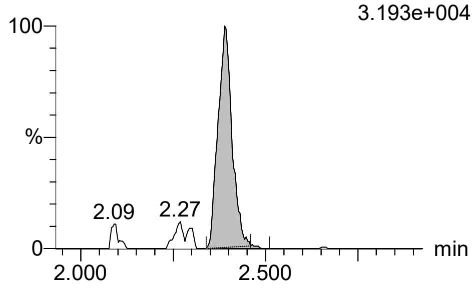

Sample Name: 20211111_H15_R1_IgG
F37:MRM of 1 channel,ES+
1078.411 > 366.1
9.728e+003

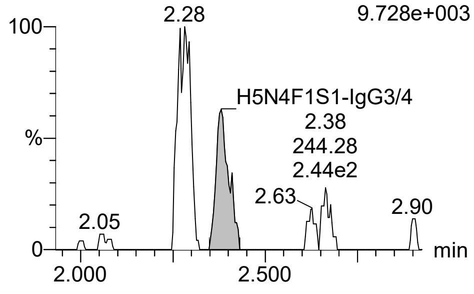

Sample Name: 20211111_A31_R1_IgG
F37:MRM of 1 channel,ES+
1078.411 > 366.1
2.298e+004

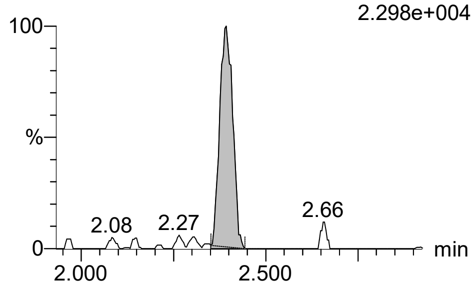

Sample Name: 20211111_A32_R1_IgG
F37:MRM of 1 channel,ES+
1078.411 > 366.1
5.326e+004

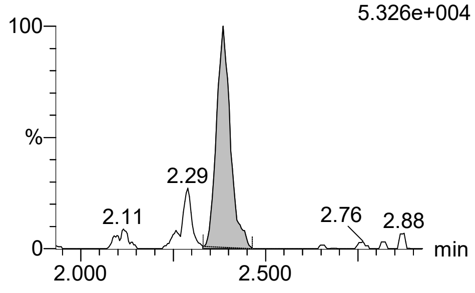

Sample Name: 20211111_L31_R1_IgG
F37:MRM of 1 channel,ES+
1078.411 > 366.1
1.347e+004

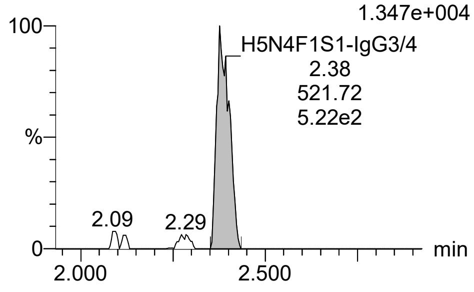

Sample Name: 20211111_L32_R1_IgG
F37:MRM of 1 channel,ES+
1078.411 > 366.1
3.821e+004

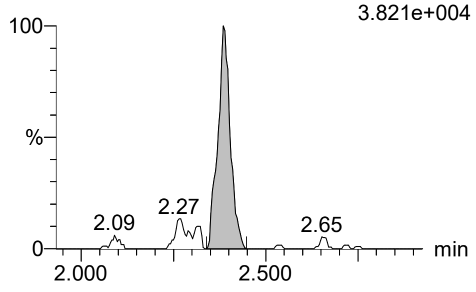

Sample Name: 20211111_H16_R1_IgG
F37:MRM of 1 channel,ES+
1078.411 > 366.1
1.346e+005

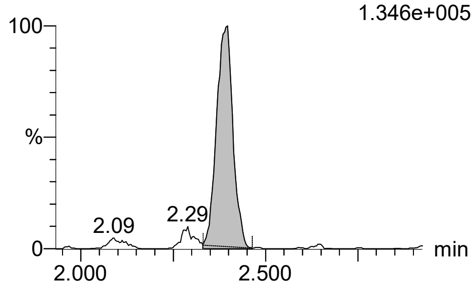

Sample Name: 20211111_A33_R1_IgG
F37:MRM of 1 channel,ES+
1078.411 > 366.1
2.063e+004

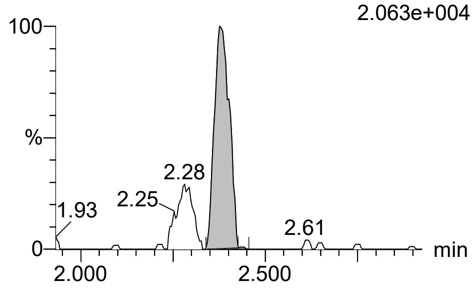

Sample Name: 20211111_A34_R1_IgG
F37:MRM of 1 channel,ES+
1078.411 > 366.1
7.613e+004

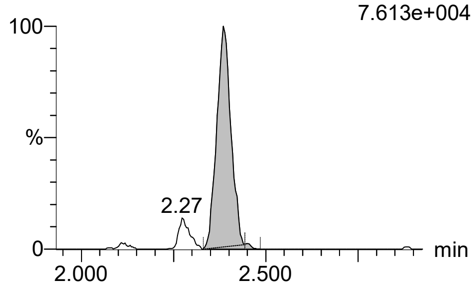

Sample Name: 20211111_H17_R1_IgG
F37:MRM of 1 channel,ES+
1078.411 > 366.1
1.762e+005

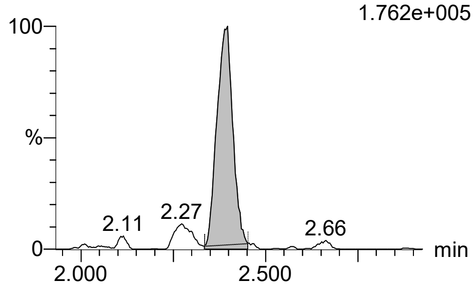

Sample Name: 20211111_A35_R1_IgG
F37:MRM of 1 channel,ES+
1078.411 > 366.1
8.564e+004

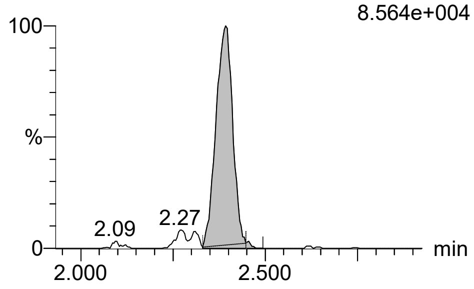

Sample Name: 20211111_QC_R8_IgG
F37:MRM of 1 channel,ES+
1078.411 > 366.1
4.340e+004

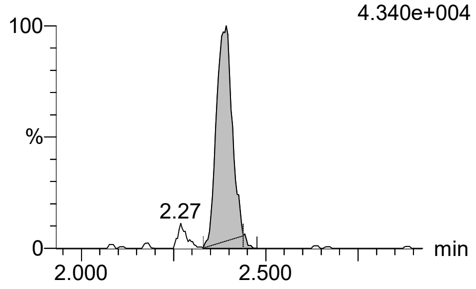

Sample Name: 20211111_A36_R1_IgG
F37:MRM of 1 channel,ES+
1078.411 > 366.1
5.526e+004

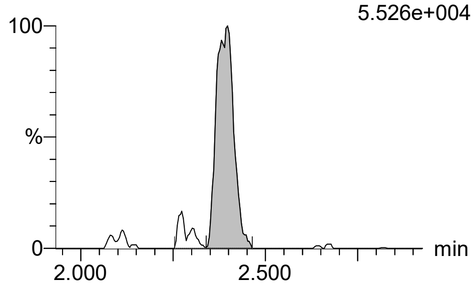

Sample Name: 20211111_H18_R1_IgG
F37:MRM of 1 channel,ES+
1078.411 > 366.1
3.470e+005

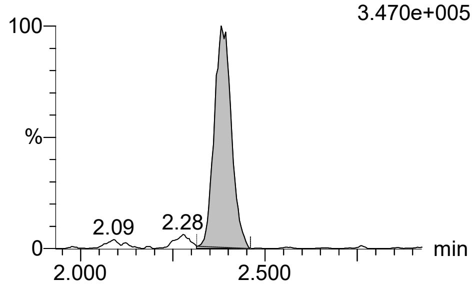

Sample Name: 20211111_A37_R1_IgG
F37:MRM of 1 channel,ES+
1078.411 > 366.1
3.430e+004

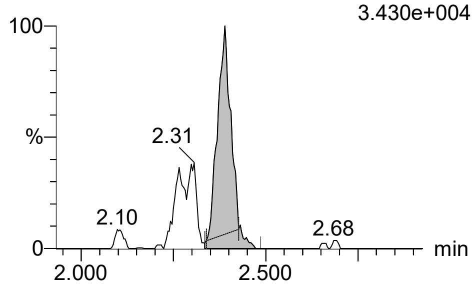

Sample Name: 20211111_A38_R1_IgG
F37:MRM of 1 channel,ES+
1078.411 > 366.1
3.382e+004

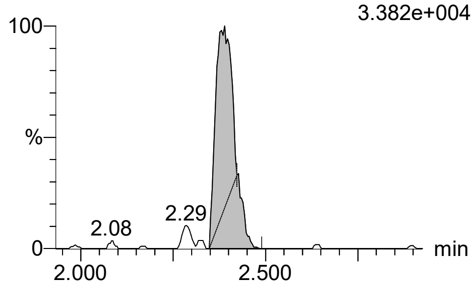

Sample Name: 20211111_H19_R1_IgG
F37:MRM of 1 channel,ES+
1078.411 > 366.1
2.726e+005

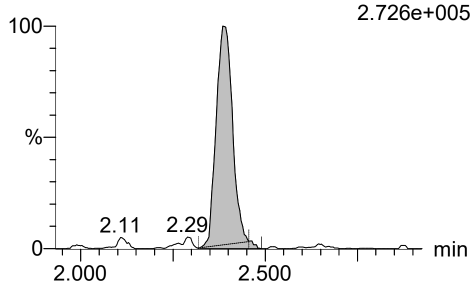

Sample Name: 20211111_A39_R1_IgG
F37:MRM of 1 channel,ES+
1078.411 > 366.1
7.418e+004

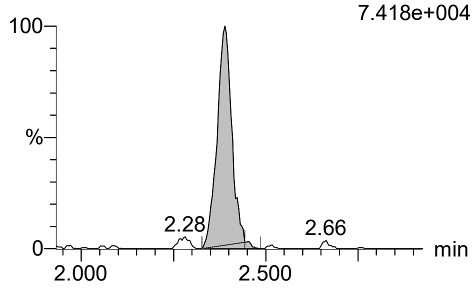

Sample Name: 20211111_A40_R1_IgG
F37:MRM of 1 channel,ES+
1078.411 > 366.1
6.482e+004

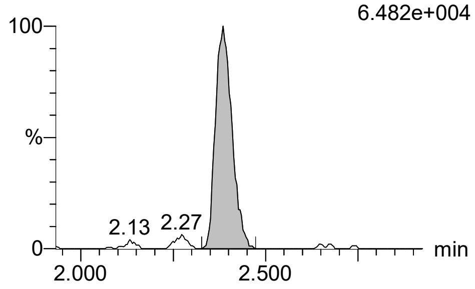

Sample Name: 20211111_H20_R1_IgG
F37:MRM of 1 channel,ES+
1078.411 > 366.1
2.349e+005

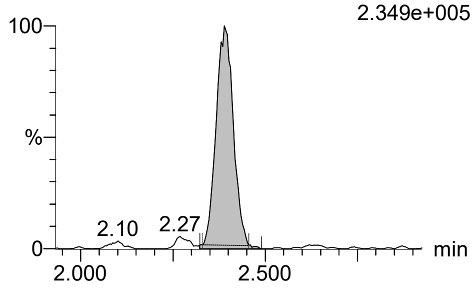

Sample Name: 20211111_A41_R1_IgG
F37:MRM of 1 channel,ES+
1078.411 > 366.1
1.956e+004

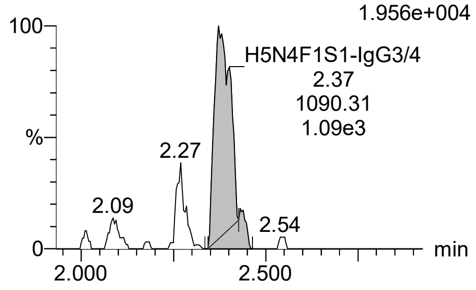

Sample Name: 20211111_A42_R1_IgG
F37:MRM of 1 channel,ES+
1078.411 > 366.1
2.731e+004

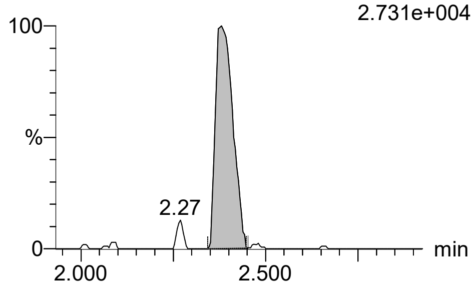

Sample Name: 20211111_A43_R1_IgG
F37:MRM of 1 channel,ES+
1078.411 > 366.1
8.278e+004

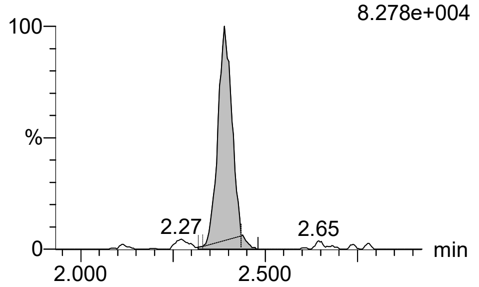

Sample Name: 20211111_A44_R1_IgG
F37:MRM of 1 channel,ES+
1078.411 > 366.1
2.228e+005

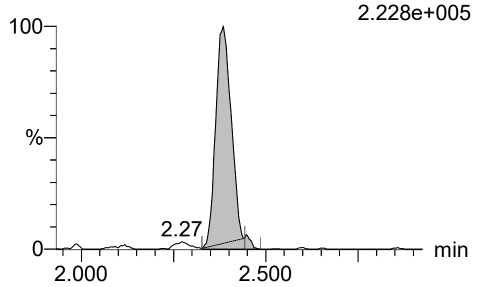

Sample Name: 20211111_QC_R9_IgG
F37:MRM of 1 channel,ES+
1078.411 > 366.1
4.263e+004

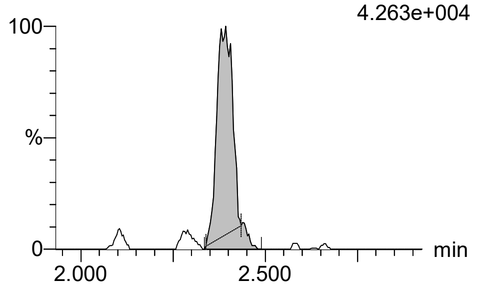

Sample Name: 20211111_A1_R2_IgG
F37:MRM of 1 channel,ES+
1078.411 > 366.1
4.522e+004

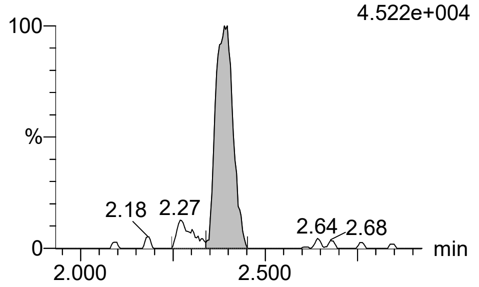

Sample Name: 20211111_A2_R2_IgG
F37:MRM of 1 channel,ES+
1078.411 > 366.1
7.664e+004

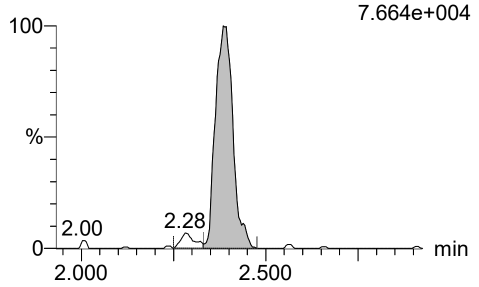

Sample Name: 20211111_L1_R2_IgG
F37:MRM of 1 channel,ES+
1078.411 > 366.1
4.081e+004

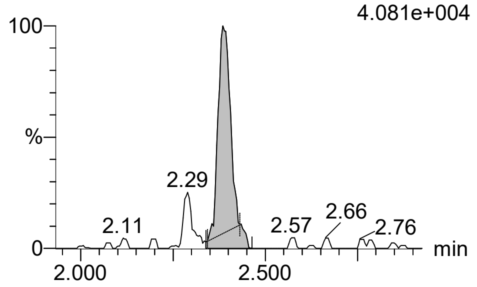

Sample Name: 20211111_L2_R2_IgG
F37:MRM of 1 channel,ES+
1078.411 > 366.1
5.538e+004

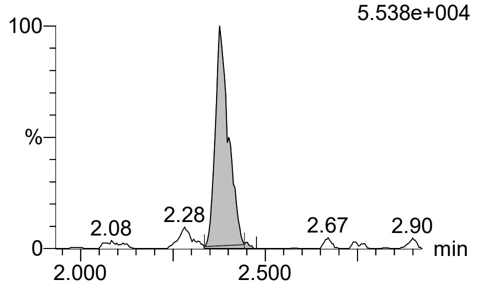

Sample Name: 20211111_H1_R2_IgG
F37:MRM of 1 channel,ES+
1078.411 > 366.1
2.322e+004

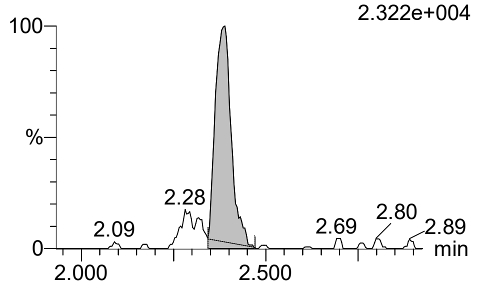

Sample Name: 20211111_A3_R2_IgG
F37:MRM of 1 channel,ES+
1078.411 > 366.1
8.740e+003

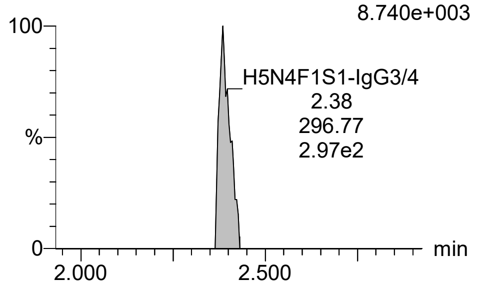

Sample Name: 20211111_A4_R2_IgG
F37:MRM of 1 channel,ES+
1078.411 > 366.1
3.269e+004

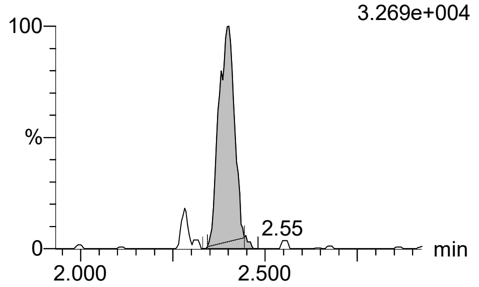

Sample Name: 20211111_L3_R2_IgG
F37:MRM of 1 channel,ES+
1078.411 > 366.1
3.000e+004

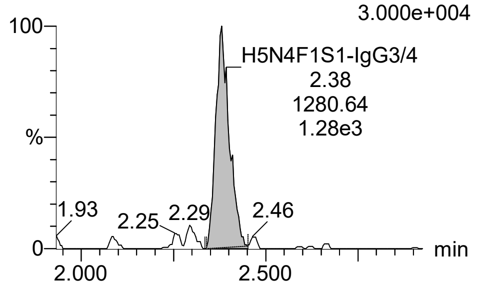

Sample Name: 20211111_L4_R2_IgG
F37:MRM of 1 channel,ES+
1078.411 > 366.1
3.378e+004

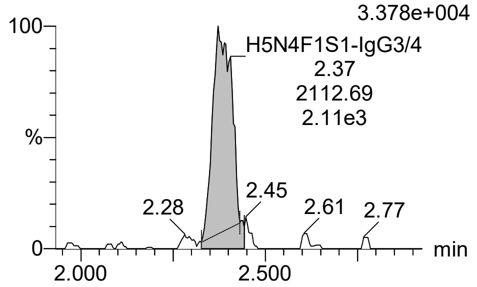

Sample Name: 20211111_H2_R2_IgG
F37:MRM of 1 channel,ES+
1078.411 > 366.1
1.100e+005

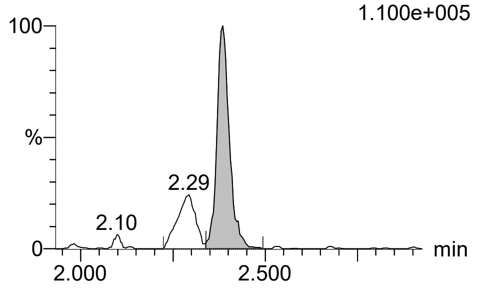

Sample Name: 20211111_A5_R2_IgG
F37:MRM of 1 channel,ES+
1078.411 > 366.1
6.489e+004

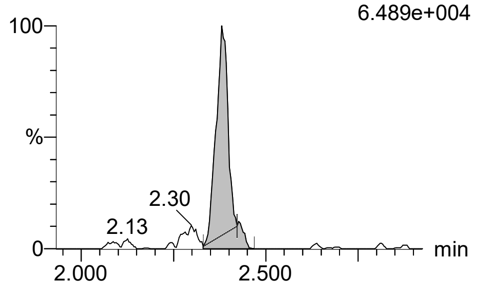

Sample Name: 20211111_A6_R2_IgG
F37:MRM of 1 channel,ES+
1078.411 > 366.1
2.115e+005

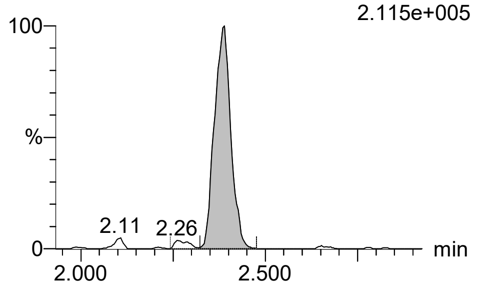

Dataset: C:\MassLynx\10.pro\20211115_clinicalsamples_IgG.qld

Last Altered: Sunday, October 02, 2022 16:02:30 台北標準時間
Printed: Friday, August 01, 2025 16:56:05 台北標準時間

Sample Name: 20211111_QC_R10_IgG
F37:MRM of 1 channel,ES+
1078.411 > 366.1
5.714e+004

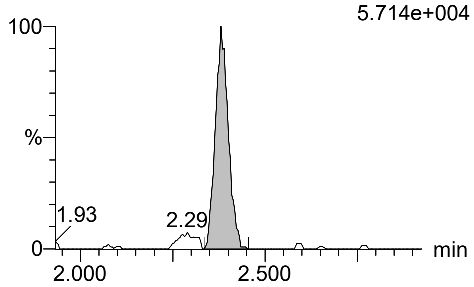

Sample Name: 20211111_L5_R2_IgG
F37:MRM of 1 channel,ES+
1078.411 > 366.1
5.126e+004

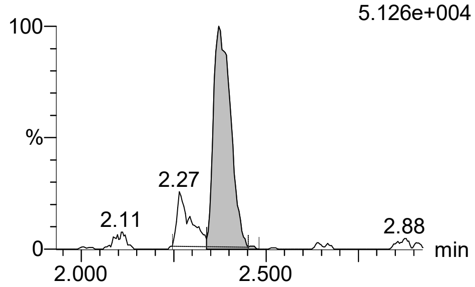

Sample Name: 20211111_L6_R2_IgG
F37:MRM of 1 channel,ES+
1078.411 > 366.1
3.154e+004

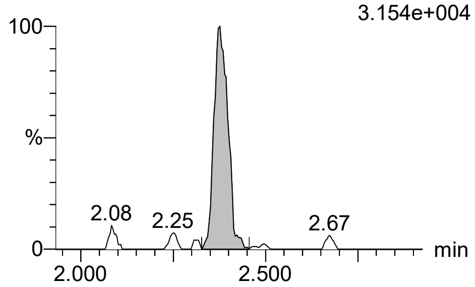

Sample Name: 20211111_H3_R2_IgG
F37:MRM of 1 channel,ES+
1078.411 > 366.1
1.961e+004

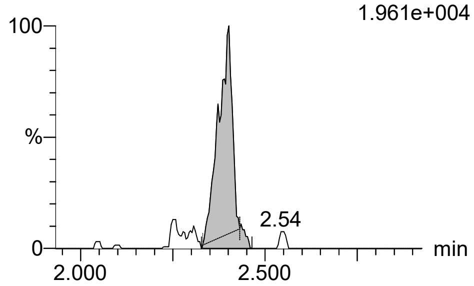

Sample Name: 20211111_A7_R2_IgG
F37:MRM of 1 channel,ES+
1078.411 > 366.1
6.876e+004

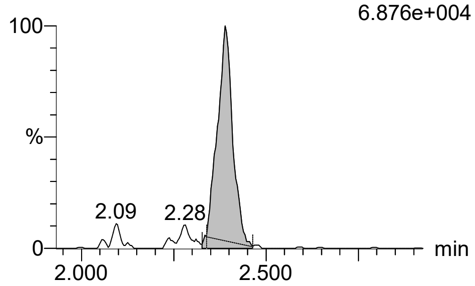

Sample Name: 20211111_A8_R2_IgG
F37:MRM of 1 channel,ES+
1078.411 > 366.1
2.553e+004

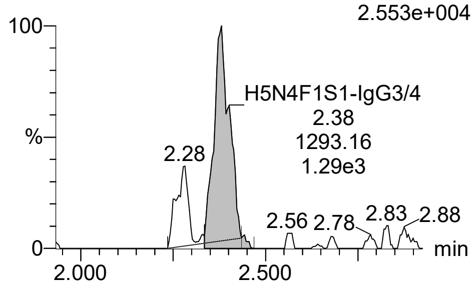

Sample Name: 20211111_L7_R2_IgG
F37:MRM of 1 channel,ES+
1078.411 > 366.1
3.990e+004

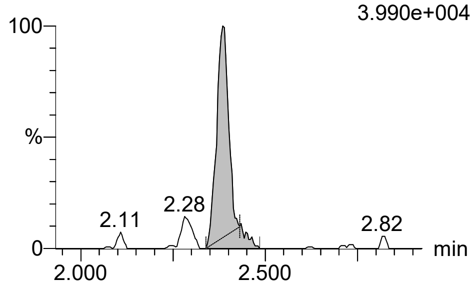

Sample Name: 20211111_L8_R2_IgG
F37:MRM of 1 channel,ES+
1078.411 > 366.1
8.588e+004

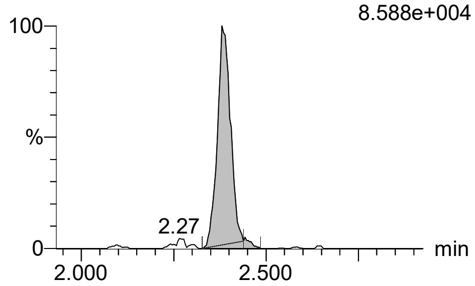

Sample Name: 20211111_H4_R2_IgG
F37:MRM of 1 channel,ES+
1078.411 > 366.1
3.644e+004

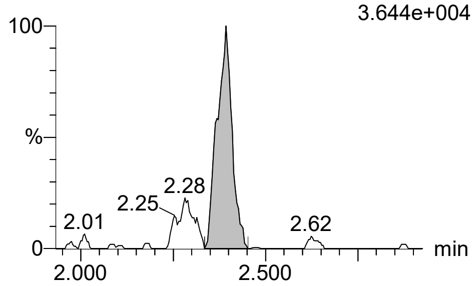

Sample Name: 20211111_A9_R2_IgG
F37:MRM of 1 channel,ES+
1078.411 > 366.1
3.169e+004

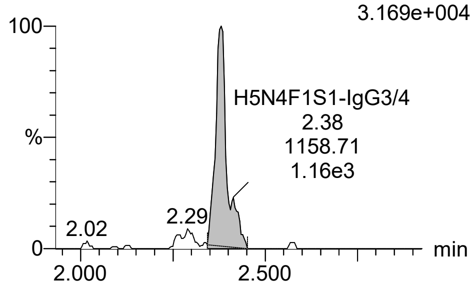

Sample Name: 20211111_A10_R2_IgG
F37:MRM of 1 channel,ES+
1078.411 > 366.1
3.487e+004

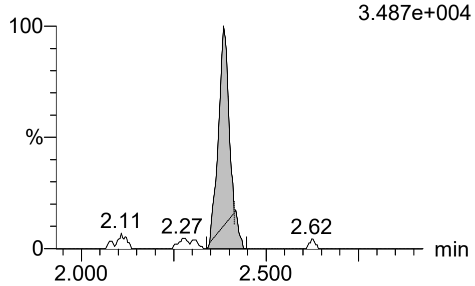

Sample Name: 20211111_L9_R2_IgG
F37:MRM of 1 channel,ES+
1078.411 > 366.1
5.355e+004

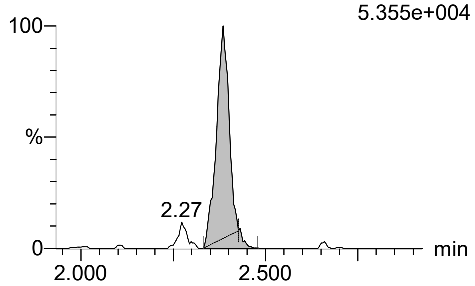

Sample Name: 20211111_L10_R2_IgG
F37:MRM of 1 channel,ES+
1078.411 > 366.1
3.002e+004

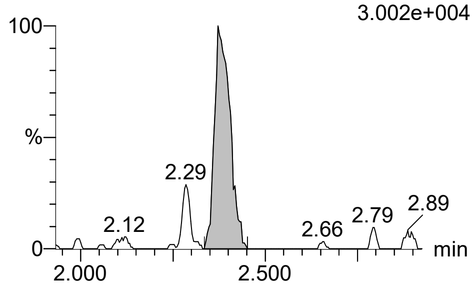

Sample Name: 20211111_QC_R11_IgG
F37:MRM of 1 channel,ES+
1078.411 > 366.1
3.591e+004

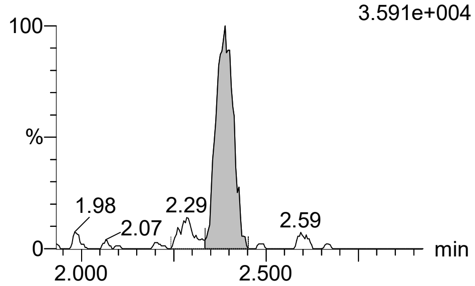

Sample Name: 20211111_H5_R2_IgG
F37:MRM of 1 channel,ES+
1078.411 > 366.1
3.097e+004

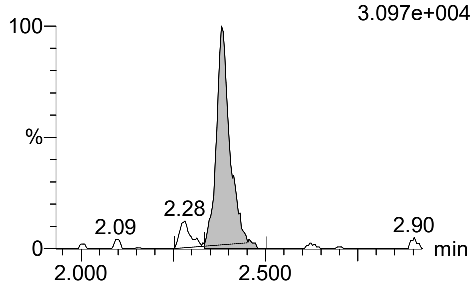

Sample Name: 20211111_A11_R2_IgG
F37:MRM of 1 channel,ES+
1078.411 > 366.1
4.794e+003

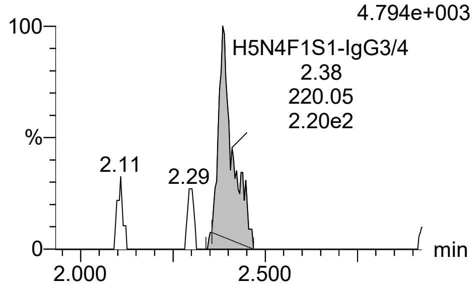

Sample Name: 20211111_A12_R2_IgG
F37:MRM of 1 channel,ES+
1078.411 > 366.1
3.982e+004

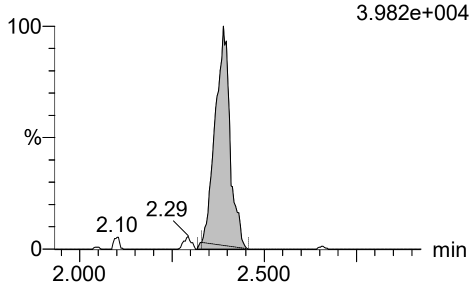

Sample Name: 20211111_L11_R2_IgG
F37:MRM of 1 channel,ES+
1078.411 > 366.1
2.209e+004

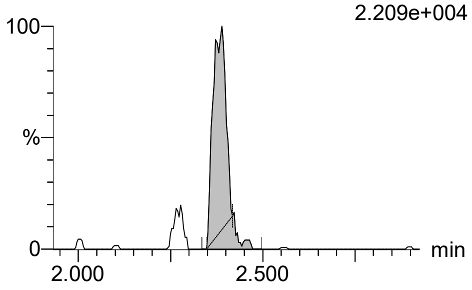

Sample Name: 20211111_L12_R2_IgG
F37:MRM of 1 channel,ES+
1078.411 > 366.1
2.399e+004

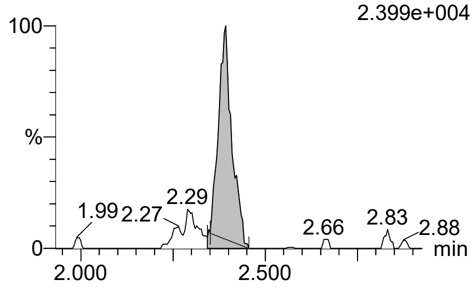

Sample Name: 20211111_H6_R2_IgG
F37:MRM of 1 channel,ES+
1078.411 > 366.1
7.564e+004

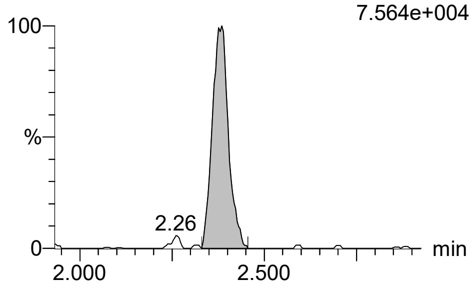

Sample Name: 20211111_A13_R2_IgG
F37:MRM of 1 channel,ES+
1078.411 > 366.1
1.093e+005

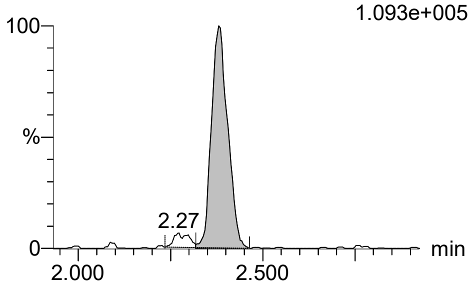

Sample Name: 20211111_A14_R2_IgG
F37:MRM of 1 channel,ES+
1078.411 > 366.1
2.127e+004

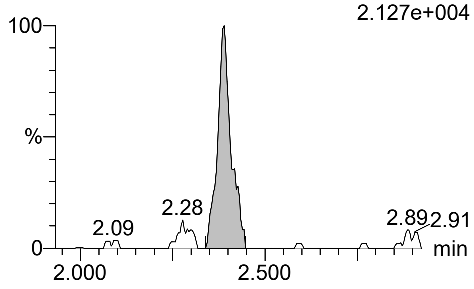

Sample Name: 20211111_L13_R2_IgG
F37:MRM of 1 channel,ES+
1078.411 > 366.1
6.161e+004

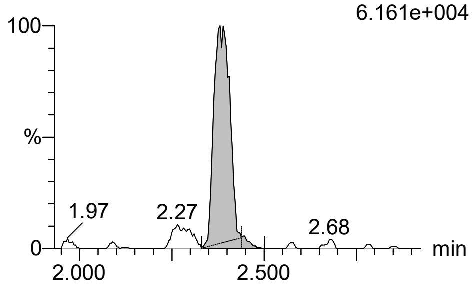

Sample Name: 20211111_L14_R2_IgG
F37:MRM of 1 channel,ES+
1078.411 > 366.1
5.804e+004

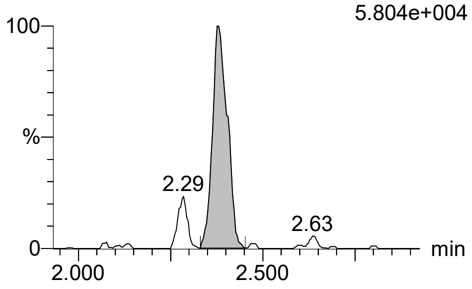

Sample Name: 20211111_H7_R2_IgG
F37:MRM of 1 channel,ES+
1078.411 > 366.1
7.263e+004

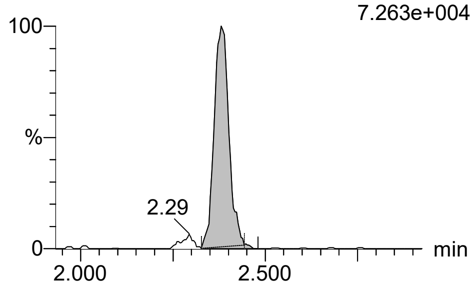

Sample Name: 20211111_A15_R2_IgG
F37:MRM of 1 channel,ES+
1078.411 > 366.1
1.994e+004

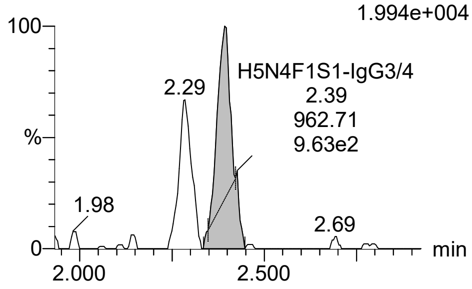

Sample Name: 20211111_QC_R12_IgG
F37:MRM of 1 channel,ES+
1078.411 > 366.1
3.860e+004

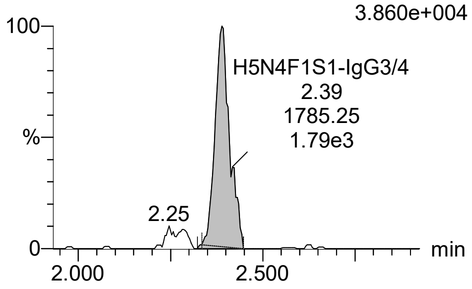

Sample Name: 20211111_A16_R2_IgG
F37:MRM of 1 channel,ES+
1078.411 > 366.1
5.293e+004

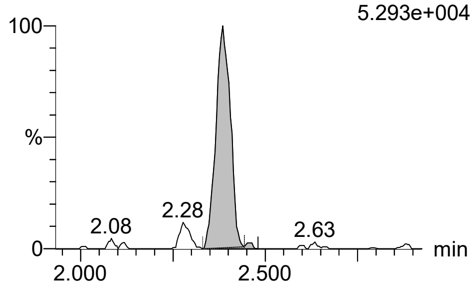

Sample Name: 20211111_L15_R2_IgG
F37:MRM of 1 channel,ES+
1078.411 > 366.1
6.573e+004

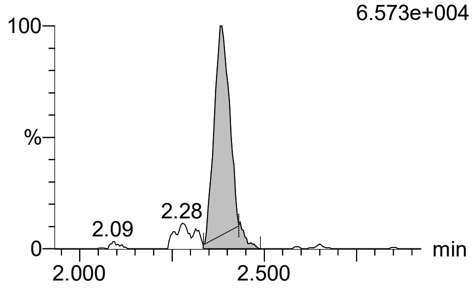

Sample Name: 20211111_L16_R2_IgG
F37:MRM of 1 channel,ES+
1078.411 > 366.1
1.661e+004

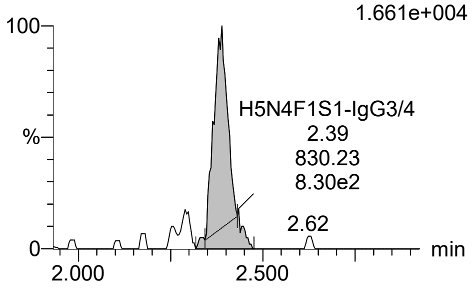

Dataset: C:\MassLynx\10.pro\20211115_clinicalsamples_IgG.qld

Last Altered: Sunday, October 02, 2022 16:02:30 台北標準時間
Printed: Friday, August 01, 2025 16:56:05 台北標準時間

Sample Name: 20211111_H8_R2_IgG
F37:MRM of 1 channel,ES+
1078.411 > 366.1
5.918e+004

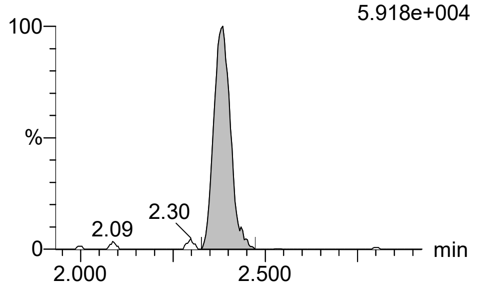

Sample Name: 20211111_A17_R2_IgG
F37:MRM of 1 channel,ES+
1078.411 > 366.1
7.897e+004

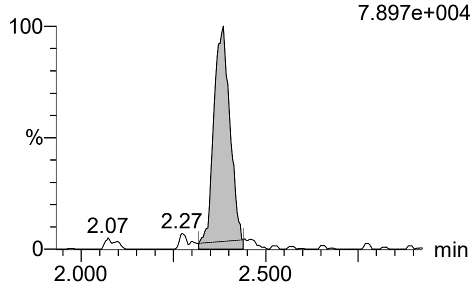

Sample Name: 20211111_A18_R2_IgG
F37:MRM of 1 channel,ES+
1078.411 > 366.1
4.142e+004

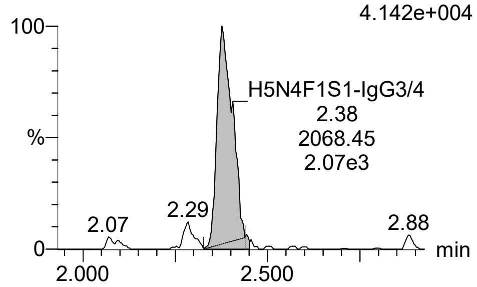

Sample Name: 20211111_L17_R2_IgG
F37:MRM of 1 channel,ES+
1078.411 > 366.1
4.667e+004

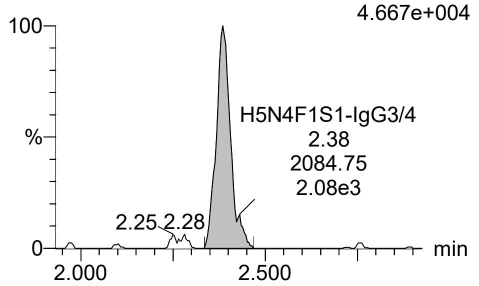

Sample Name: 20211111_L18_R2_IgG
F37:MRM of 1 channel,ES+
1078.411 > 366.1
1.457e+004

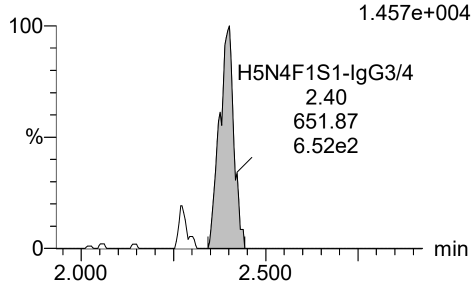

Sample Name: 20211111_H9_R2_IgG
F37:MRM of 1 channel,ES+
1078.411 > 366.1
6.228e+004

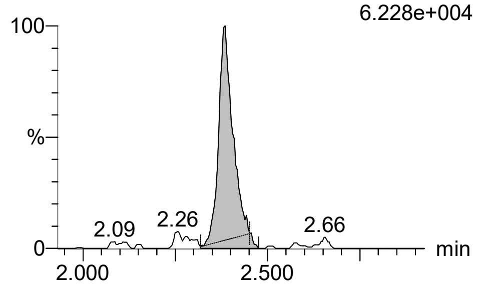

Sample Name: 20211111_A19_R2_IgG
F37:MRM of 1 channel,ES+
1078.411 > 366.1
1.822e+004

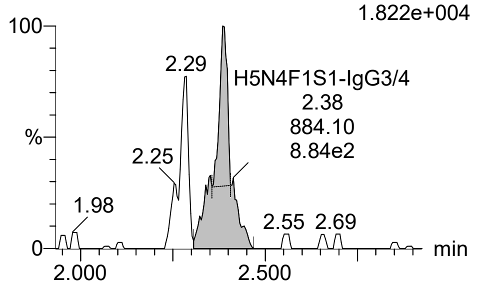

Sample Name: 20211111_A20_R2_IgG
F37:MRM of 1 channel,ES+
1078.411 > 366.1
1.500e+004

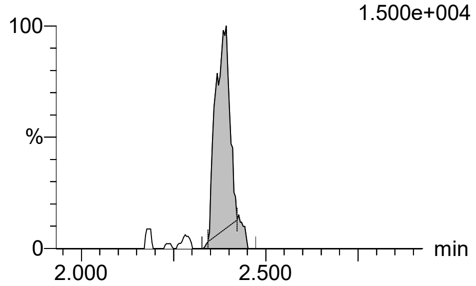

Sample Name: 20211111_L19_R2_IgG
F37:MRM of 1 channel,ES+
1078.411 > 366.1
5.859e+004

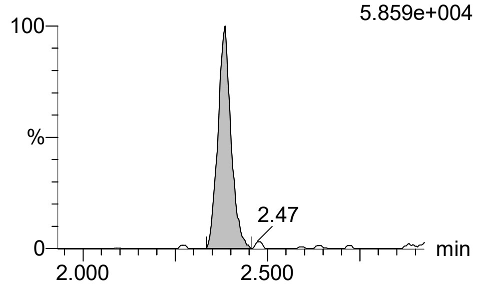

Sample Name: 20211111_QC_R13_IgG
F37:MRM of 1 channel,ES+
1078.411 > 366.1
5.058e+004

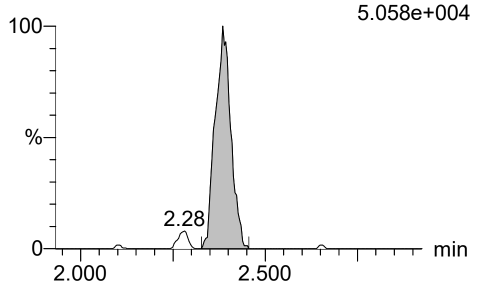

Sample Name: 20211111_L20_R2_IgG
F37:MRM of 1 channel,ES+
1078.411 > 366.1
6.233e+004

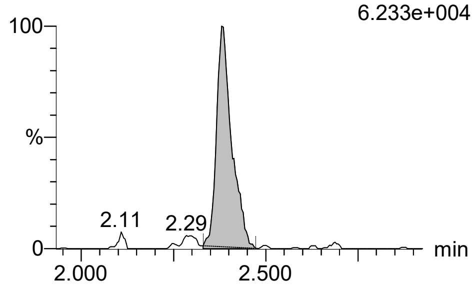

Sample Name: 20211111_H10_R2_IgG
F37:MRM of 1 channel,ES+
1078.411 > 366.1
3.563e+004

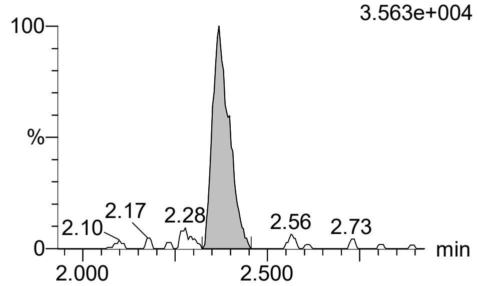

Sample Name: 20211111_A21_R2_IgG
F37:MRM of 1 channel,ES+
1078.411 > 366.1
5.125e+004

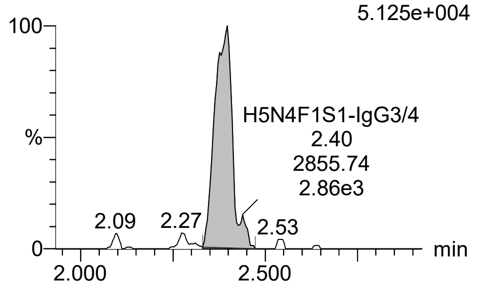

Sample Name: 20211111_A22_R2_IgG
F37:MRM of 1 channel,ES+
1078.411 > 366.1
6.887e+004

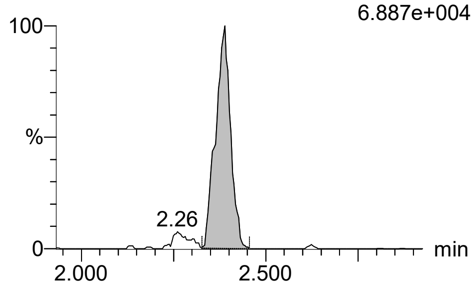

Sample Name: 20211111_L21_R2_IgG
F37:MRM of 1 channel,ES+
1078.411 > 366.1
1.574e+004

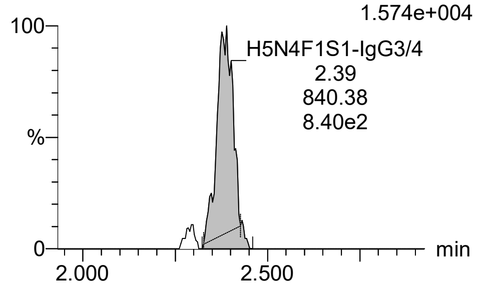

Dataset: C:\MassLynx\10.pro\20211115_clinicalsamples_IgG.qld

Last Altered: Sunday, October 02, 2022 16:02:30 台北標準時間
Printed: Friday, August 01, 2025 16:56:05 台北標準時間

Sample Name: 20211111_L22_R2_IgG

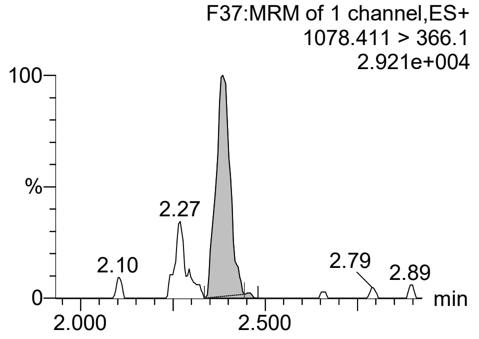

Sample Name: 20211111_H11_R2_IgG

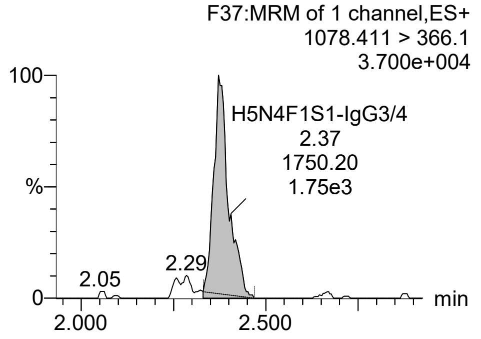

Sample Name: 20211111_A23_R2_IgG

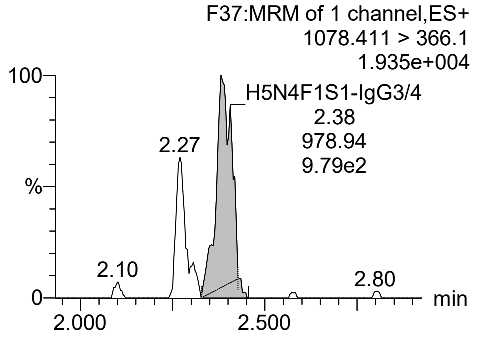

Sample Name: 20211111_A24_R2_IgG

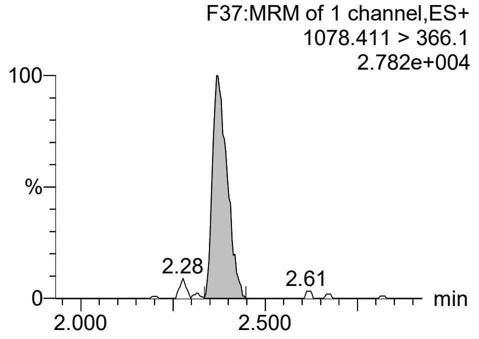

Sample Name: 20211111_L23_R2_IgG

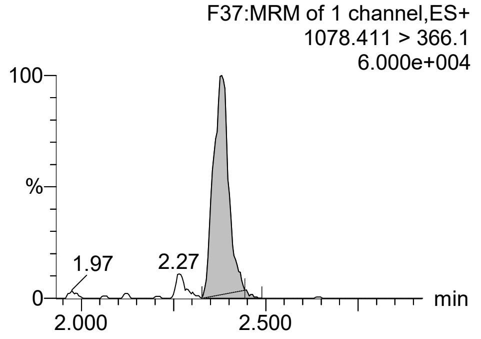

Sample Name: 20211111_L24_R2_IgG

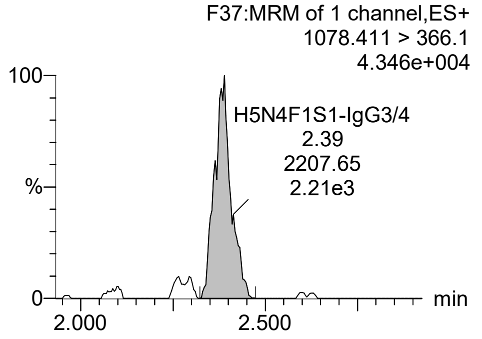

Sample Name: 20211111_H12_R2_IgG

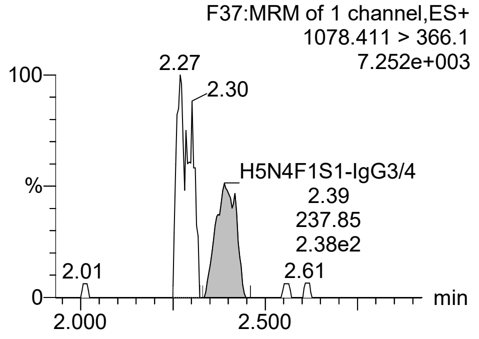

Sample Name: 20211111_QC_R14_IgG

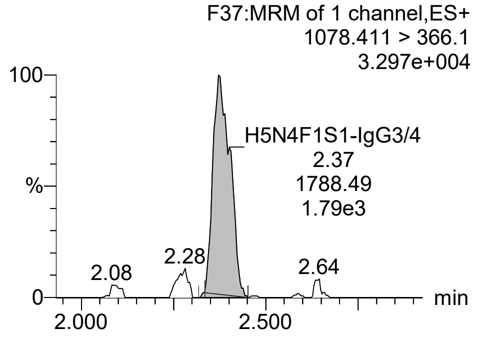

Sample Name: 20211111_A25_R2_IgG

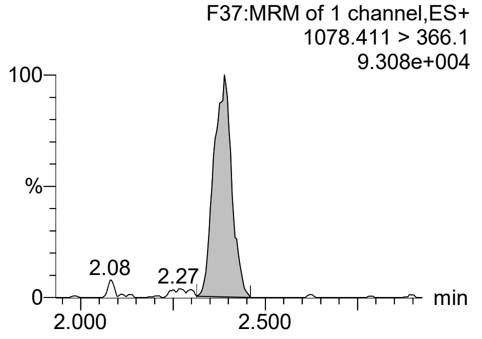

Sample Name: 20211111_A26_R2_IgG

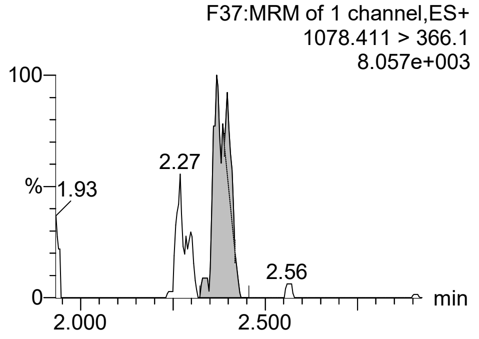

Sample Name: 20211111_L25_R2_IgG

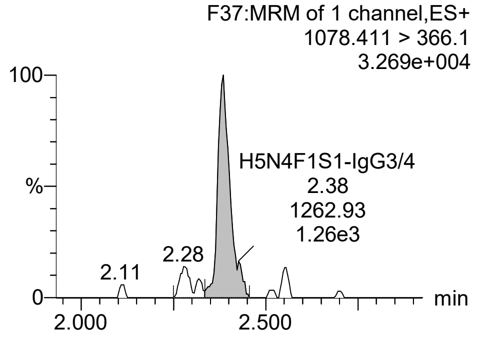

Sample Name: 20211111_L26_R2_IgG

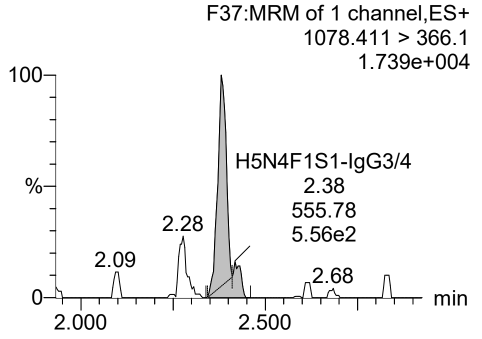

Sample Name: 20211111_H13_R2_IgG

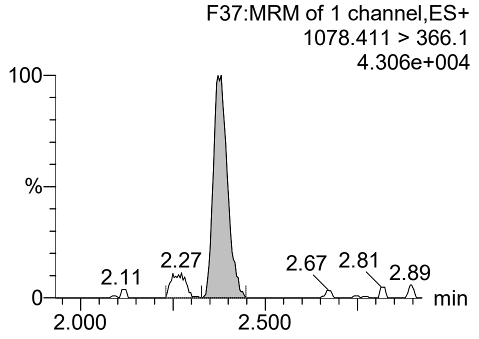

Sample Name: 20211111_A27_R2_IgG

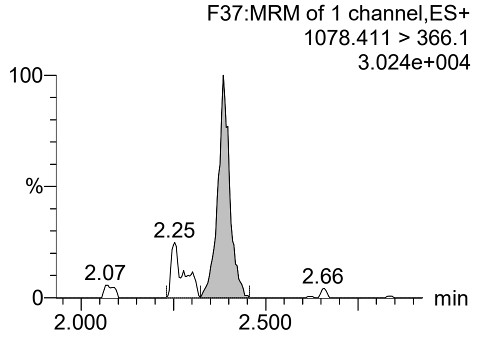

Sample Name: 20211111_A28_R2_IgG

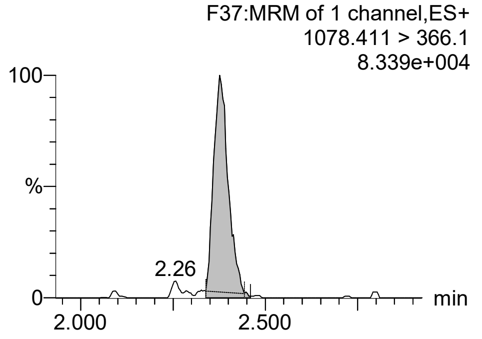

Sample Name: 20211111_L27_R2_IgG
F37:MRM of 1 channel,ES+
1078.411 > 366.1
3.370e+004

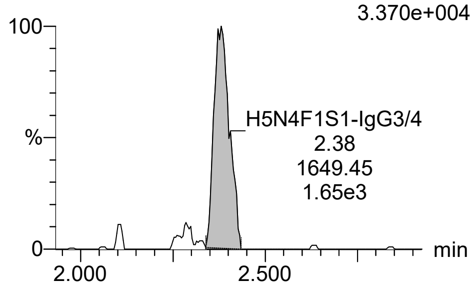

Sample Name: 20211111_L28_R2_IgG
F37:MRM of 1 channel,ES+
1078.411 > 366.1
4.209e+004

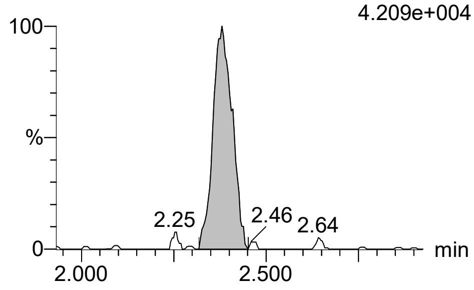

Sample Name: 20211111_H14_R2_IgG
F37:MRM of 1 channel,ES+
1078.411 > 366.1
2.556e+004

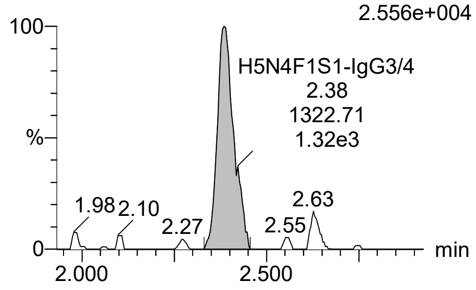

Sample Name: 20211111_A29_R2_IgG
F37:MRM of 1 channel,ES+
1078.411 > 366.1
1.943e+004

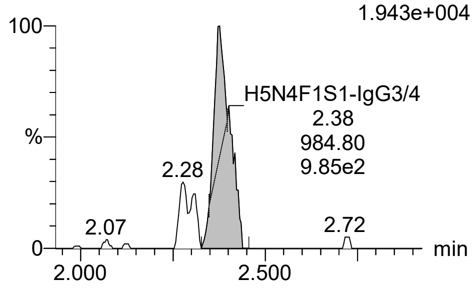

Sample Name: 20211111_A30_R2_IgG
F37:MRM of 1 channel,ES+
1078.411 > 366.1
3.585e+004

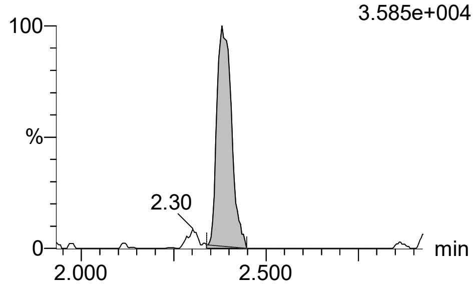

Sample Name: 20211111_QC_R15_IgG
F37:MRM of 1 channel,ES+
1078.411 > 366.1
5.727e+004

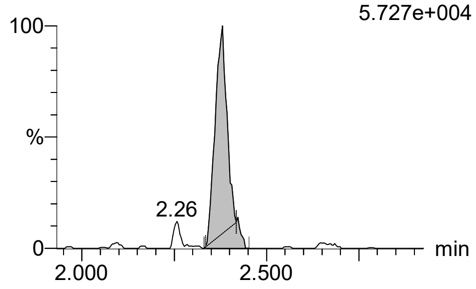

Sample Name: 20211111_L29_R2_IgG
F37:MRM of 1 channel,ES+
1078.411 > 366.1
3.008e+004

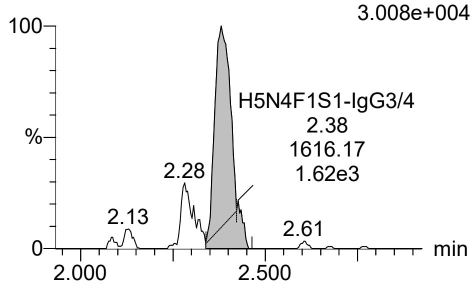

Sample Name: 20211111_L30_R2_IgG
F37:MRM of 1 channel,ES+
1078.411 > 366.1
1.899e+004

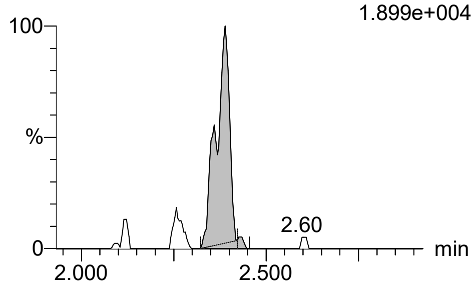

Sample Name: 20211111_H15_R2_IgG
F37:MRM of 1 channel,ES+
1078.411 > 366.1
7.124e+003

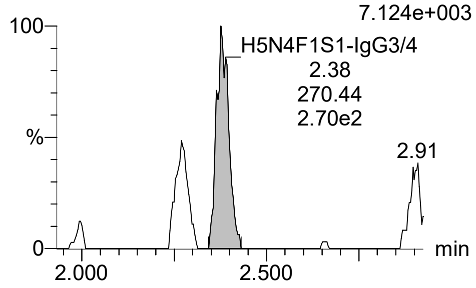

Sample Name: 20211111_A31_R2_IgG
F37:MRM of 1 channel,ES+
1078.411 > 366.1
1.217e+004

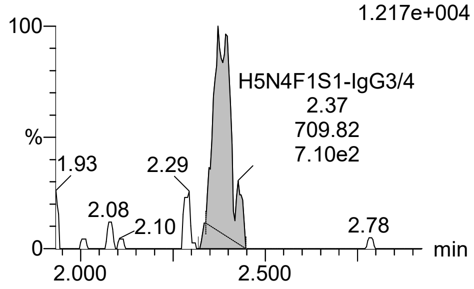

Sample Name: 20211111_A32_R2_IgG
F37:MRM of 1 channel,ES+
1078.411 > 366.1
3.051e+004

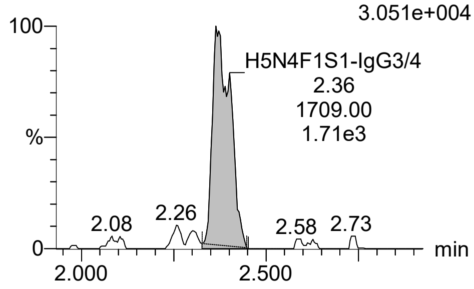

Sample Name: 20211111_L31_R2_IgG
F37:MRM of 1 channel,ES+
1078.411 > 366.1
5.989e+003

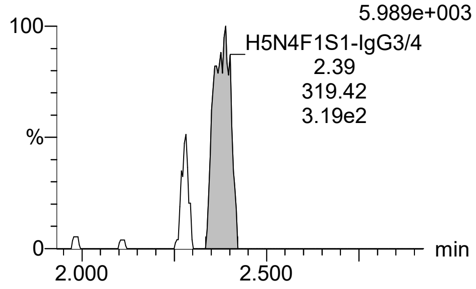

Sample Name: 20211111_L32_R2_IgG
F37:MRM of 1 channel,ES+
1078.411 > 366.1
3.574e+004

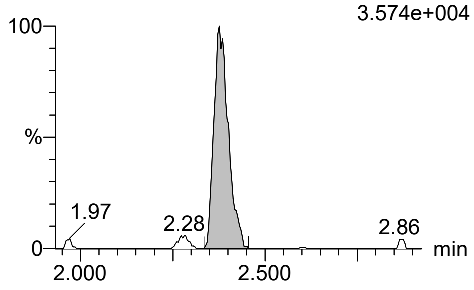

Sample Name: 20211111_H16_R2_IgG
F37:MRM of 1 channel,ES+
1078.411 > 366.1
1.132e+005

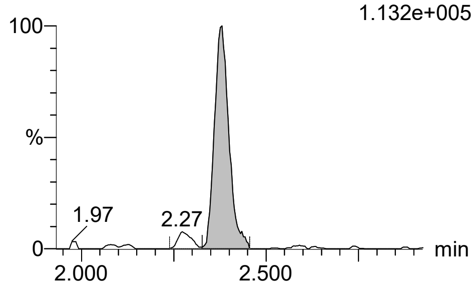

Sample Name: 20211111_A33_R2_IgG
F37:MRM of 1 channel,ES+
1078.411 > 366.1
1.635e+004

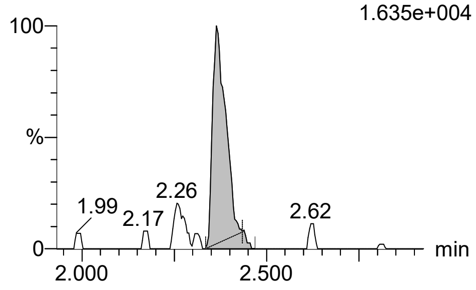

Dataset: C:\MassLynx\10.pro\20211115_clinicalsamples_IgG.qld

Last Altered: Sunday, October 02, 2022 16:02:30 台北標準時間
Printed: Friday, August 01, 2025 16:56:05 台北標準時間

Sample Name: 20211111_A34_R2_IgG
F37:MRM of 1 channel,ES+
1078.411 > 366.1
4.913e+004

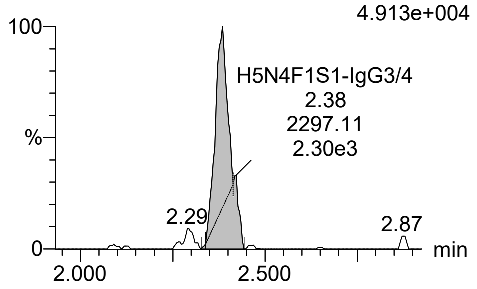

Sample Name: 20211111_H17_R2_IgG
F37:MRM of 1 channel,ES+
1078.411 > 366.1
1.701e+005

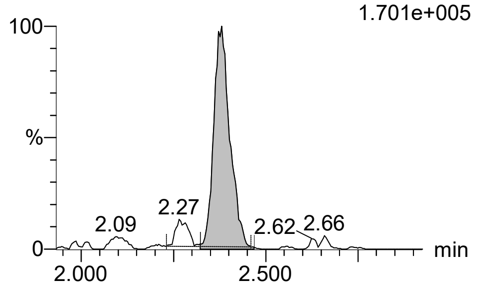

Sample Name: 20211111_A35_R2_IgG
F37:MRM of 1 channel,ES+
1078.411 > 366.1
8.488e+004

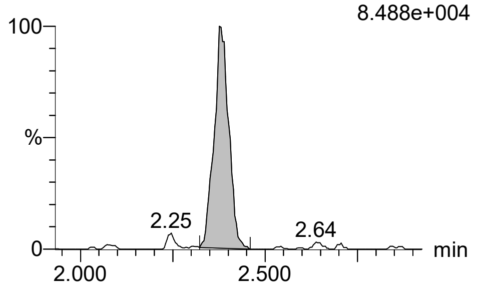

Sample Name: 20211111_QC_R16_IgG
F37:MRM of 1 channel,ES+
1078.411 > 366.1
5.044e+004

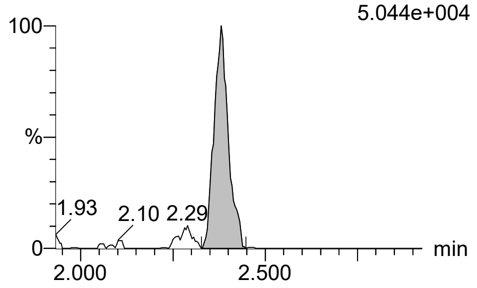

Sample Name: 20211111_A36_R2_IgG
F37:MRM of 1 channel,ES+
1078.411 > 366.1
4.788e+004

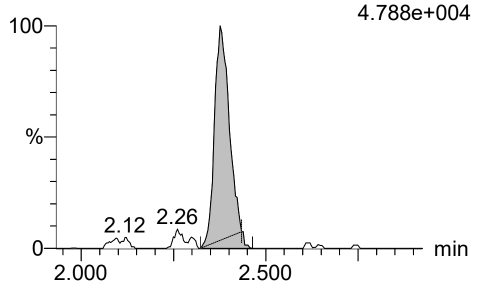

Sample Name: 20211111_H18_R2_IgG
F37:MRM of 1 channel,ES+
1078.411 > 366.1
3.016e+005

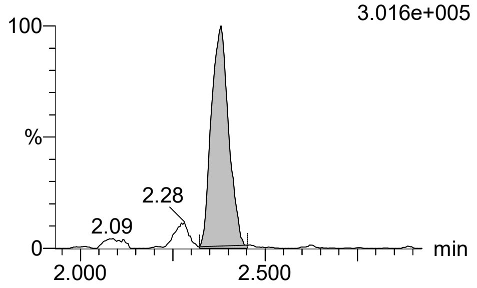

Sample Name: 20211111_A37_R2_IgG
F37:MRM of 1 channel,ES+
1078.411 > 366.1
1.885e+004

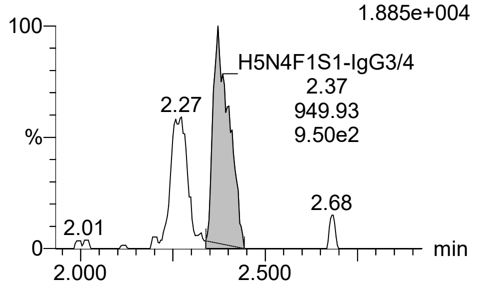

Sample Name: 20211111_A38_R2_IgG
F37:MRM of 1 channel,ES+
1078.411 > 366.1
3.649e+004

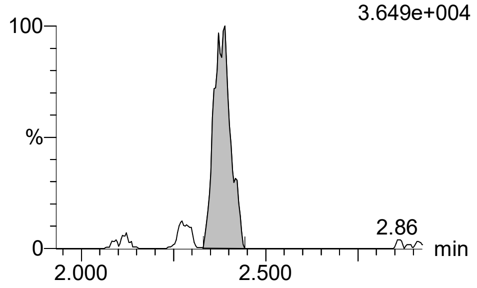

Sample Name: 20211111_H19_R2_IgG
F37:MRM of 1 channel,ES+
1078.411 > 366.1
2.574e+005

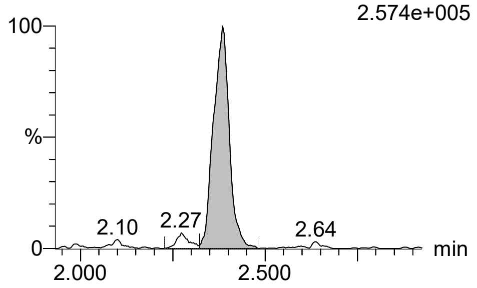

Sample Name: 20211111_A39_R2_IgG
F37:MRM of 1 channel,ES+
1078.411 > 366.1
7.228e+004

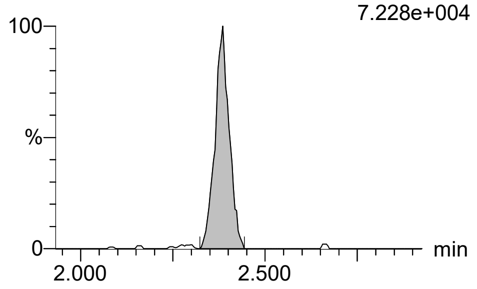

Sample Name: 20211111_A40_R2_IgG
F37:MRM of 1 channel,ES+
1078.411 > 366.1
7.240e+004

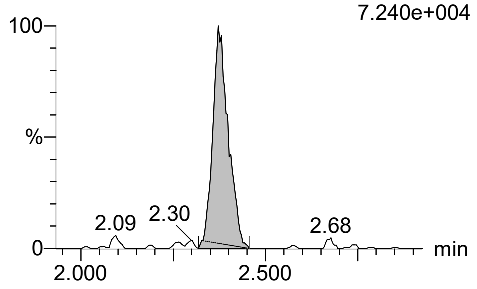

Sample Name: 20211111_H20_R2_IgG
F37:MRM of 1 channel,ES+
1078.411 > 366.1
1.774e+005

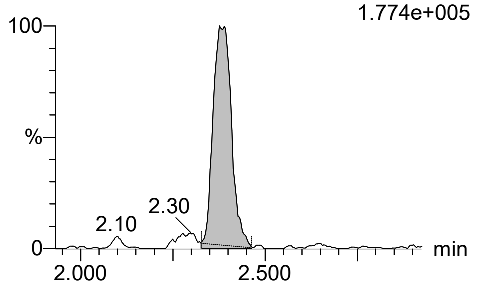

Sample Name: 20211111_A41_R2_IgG
F37:MRM of 1 channel,ES+
1078.411 > 366.1
8.205e+003

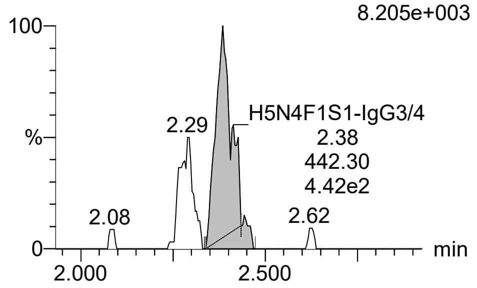

Sample Name: 20211111_A42_R2_IgG
F37:MRM of 1 channel,ES+
1078.411 > 366.1
3.583e+004

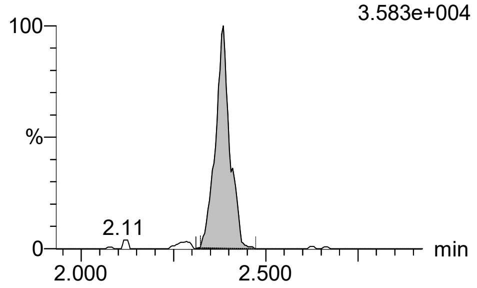

Sample Name: 20211111_A43_R2_IgG
F37:MRM of 1 channel,ES+
1078.411 > 366.1
5.606e+004

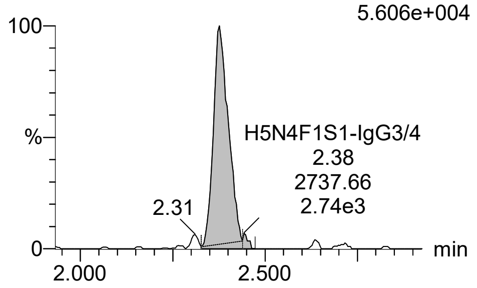

Sample Name: 20211111_A44_R2_IgG

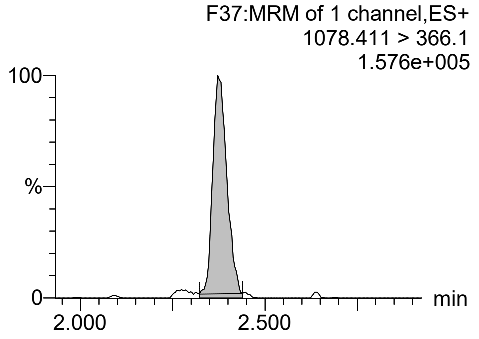

Sample Name: 20211111_QC_R17_IgG

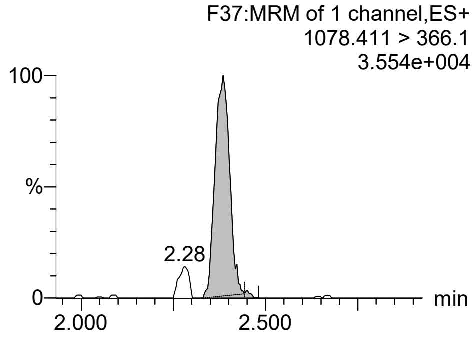

Dataset: C:\MassLynx\10.pro\20211115_clinicalsamples_IgG.qld

Last Altered: Sunday, October 02, 2022 16:02:30 台北標準時間
Printed: Friday, August 01, 2025 16:56:05 台北標準時間

	#	Name	RT	Area	IS Area	Response	Prima...
1	1	20211111_QC_R1_IgG	2.38	5323.2	64968.0	0.082	MM
2	2	20211111_A1_R1_IgG	2.38	4262.1	59059.2	0.072	MM
3	3	20211111_A2_R1_IgG	2.38	5631.6	66621.2	0.085	MM
4	4	20211111_L1_R1_IgG	2.38	2445.8	64201.0	0.038	MM
5	5	20211111_L2_R1_IgG	2.39	3109.7	46950.2	0.066	MM
6	6	20211111_H1_R1_IgG	2.39	1048.8	38648.2	0.027	bb
7	7	20211111_A3_R1_IgG	2.39	80.8	2603.7	0.031	bb
8	8	20211111_A4_R1_IgG	2.39	1898.4	69960.4	0.027	MM
9	9	20211111_L3_R1_IgG	2.38	1594.9	38864.7	0.041	bb
10	10	20211111_L4_R1_IgG	2.40	2290.8	43380.5	0.053	MM
11	11	20211111_H2_R1_IgG	2.39	6459.0	71349.2	0.091	MM
12	12	20211111_A5_R1_IgG	2.39	3359.2	65569.7	0.051	bb
13	13	20211111_A6_R1_IgG	2.38	14126.4	53120.7	0.266	MM
14	14	20211111_QC_R2_IgG	2.39	5493.3	81034.1	0.068	MM
15	15	20211111_L5_R1_IgG	2.38	4302.7	50994.6	0.084	MM
16	16	20211111_L6_R1_IgG	2.40	1734.5	58058.0	0.030	bb
17	17	20211111_H3_R1_IgG	2.38	1305.4	64028.6	0.020	MM
18	18	20211111_A7_R1_IgG	2.38	4791.3	82996.1	0.058	MM
19	19	20211111_A8_R1_IgG	2.39	1937.6	77731.7	0.025	bb
20	20	20211111_L7_R1_IgG	2.39	2077.6	38827.3	0.054	MM
21	21	20211111_L8_R1_IgG	2.38	3281.7	44220.1	0.074	MM
22	22	20211111_H4_R1_IgG	2.39	2765.3	65826.3	0.042	MM
23	23	20211111_A9_R1_IgG	2.38	1107.1	60771.1	0.018	bb
24	24	20211111_A10_R1_IgG	2.39	1873.1	48578.7	0.039	MM
25	25	20211111_L9_R1_IgG	2.38	3073.9	50830.7	0.060	MM
26	26	20211111_L10_R1_IgG	2.40	1514.2	38034.2	0.040	bb
27	27	20211111_QC_R3_IgG	2.39	5375.6	67025.5	0.080	MM
28	28	20211111_H5_R1_IgG	2.39	1848.8	44544.9	0.042	MM
29	29	20211111_A11_R1_IgG	2.38	643.3	66408.5	0.010	MM
30	30	20211111_A12_R1_IgG	2.40	2087.7	46624.5	0.045	bb
31	31	20211111_L11_R1_IgG	2.38	686.3	33425.1	0.021	MM
32	32	20211111_L12_R1_IgG	2.39	1075.5	41178.0	0.026	bb
33	33	20211111_H6_R1_IgG	2.38	6745.1	58593.4	0.115	MM
34	34	20211111_A13_R1_IgG	2.38	8400.3	54133.0	0.155	bb
35	35	20211111_A14_R1_IgG	2.38	1527.5	56467.3	0.027	bb
36	36	20211111_L13_R1_IgG	2.39	3511.3	36024.4	0.097	MM
37	37	20211111_L14_R1_IgG	2.38	3218.1	37181.6	0.087	bb
38	38	20211111_H7_R1_IgG	2.38	2255.3	42850.6	0.053	bb
39	39	20211111_A15_R1_IgG	2.39	1408.5	62015.1	0.023	MM
40	40	20211111_QC_R4_IgG	2.38	4202.5	58572.5	0.072	bb
41	41	20211111_A16_R1_IgG	2.38	4454.3	78283.8	0.057	bb
42	42	20211111_L15_R1_IgG	2.39	4723.6	44158.2	0.107	MM
43	43	20211111_L16_R1_IgG	2.40	965.8	55150.8	0.018	MM
44	44	20211111_H8_R1_IgG	2.38	3272.6	42965.2	0.076	MM
45	45	20211111_A17_R1_IgG	2.38	3587.7	39717.7	0.090	MM
46	46	20211111_A18_R1_IgG	2.38	3283.2	44675.4	0.073	bb
47	47	20211111_L17_R1_IgG	2.38	2209.2	37854.1	0.058	MM
48	48	20211111_L18_R1_IgG	2.39	514.2	50716.9	0.010	MM
49	49	20211111_H9_R1_IgG	2.39	3098.5	36276.0	0.085	bb
50	50	20211111_A19_R1_IgG	2.39	1191.1	42862.5	0.028	MM
51	51	20211111_A20_R1_IgG	2.37	823.4	36542.7	0.023	MM
52	52	20211111_L19_R1_IgG	2.38	3367.7	53892.8	0.062	bb
53	53	20211111_QC_R5_IgG	2.38	3125.4	56513.2	0.055	bb
54	54	20211111_L20_R1_IgG	2.39	6297.2	56499.9	0.111	bb
55	55	20211111_H10_R1_IgG	2.38	4566.3	68120.5	0.067	MM
56	56	20211111_A21_R1_IgG	2.38	5838.8	63699.8	0.092	MM

Dataset: C:\MassLynx\10.pro\20211115_clinicalsamples_IgG.qld

Last Altered: Sunday, October 02, 2022 16:02:30 台北標準時間
Printed: Friday, August 01, 2025 16:56:05 台北標準時間

	# Name	RT	Area	IS Area	Response	Prima...
57	57 20211111_A22_R1_IgG	2.38	7923.7	52222.1	0.152	MM
58	58 20211111_L21_R1_IgG	2.39	1701.0	54372.5	0.031	MM
59	59 20211111_L22_R1_IgG	2.38	1643.4	41353.1	0.040	bb
60	60 20211111_H11_R1_IgG	2.39	3194.0	65579.1	0.049	bb
61	61 20211111_A23_R1_IgG	2.38	2544.4	64131.1	0.040	MM
62	62 20211111_A24_R1_IgG	2.39	2275.3	38724.8	0.059	bb
63	63 20211111_L23_R1_IgG	2.38	5195.5	64245.2	0.081	MM
64	64 20211111_L24_R1_IgG	2.38	3632.8	45298.0	0.080	MM
65	65 20211111_H12_R1_IgG	2.38	685.1	44679.3	0.015	MM
66	66 20211111_QC_R6_IgG	2.39	3090.7	48165.0	0.064	MM
67	67 20211111_A25_R1_IgG	2.38	9350.8	57169.9	0.164	MM
68	68 20211111_A26_R1_IgG	2.40	377.8	34254.0	0.011	bb
69	69 20211111_L25_R1_IgG	2.39	2889.6	67491.5	0.043	MM
70	70 20211111_L26_R1_IgG	2.39	1543.0	55934.1	0.028	MM
71	71 20211111_H13_R1_IgG	2.39	2598.2	48767.3	0.053	bb
72	72 20211111_A27_R1_IgG	2.38	1885.8	57269.7	0.033	MM
73	73 20211111_A28_R1_IgG	2.39	5464.6	49231.1	0.111	bb
74	74 20211111_L27_R1_IgG	2.40	2844.7	60014.5	0.047	MM
75	75 20211111_L28_R1_IgG	2.38	4048.9	35062.6	0.115	bb
76	76 20211111_H14_R1_IgG	2.38	2694.6	62371.9	0.043	MM
77	77 20211111_A29_R1_IgG	2.38	1365.0	43858.2	0.031	MM
78	78 20211111_A30_R1_IgG	2.38	2604.0	62500.4	0.042	MM
79	79 20211111_QC_R7_IgG	2.38	3499.9	43946.9	0.080	MM
80	80 20211111_L29_R1_IgG	2.39	2144.7	38728.9	0.055	bb
81	81 20211111_L30_R1_IgG	2.39	1415.1	42613.2	0.033	MM
82	82 20211111_H15_R1_IgG	2.38	244.3	39874.6	0.006	bb
83	83 20211111_A31_R1_IgG	2.39	941.6	53414.3	0.018	MM
84	84 20211111_A32_R1_IgG	2.38	2328.2	41844.6	0.056	MM
85	85 20211111_L31_R1_IgG	2.38	521.7	40553.7	0.013	bb
86	86 20211111_L32_R1_IgG	2.38	1484.3	49618.7	0.030	bb
87	87 20211111_H16_R1_IgG	2.40	6944.9	60170.9	0.115	MM
88	88 20211111_A33_R1_IgG	2.38	974.6	48588.2	0.020	MM
89	89 20211111_A34_R1_IgG	2.38	3447.2	43288.0	0.080	MM
90	90 20211111_H17_R1_IgG	2.40	8905.1	47104.5	0.189	MM
91	91 20211111_A35_R1_IgG	2.39	4563.7	41025.9	0.111	MM
92	92 20211111_QC_R8_IgG	2.39	2222.8	40546.3	0.055	MM
93	93 20211111_A36_R1_IgG	2.40	3057.1	44504.4	0.069	MM
94	94 20211111_H18_R1_IgG	2.38	18714.8	34162.2	0.548	MM
95	95 20211111_A37_R1_IgG	2.39	1535.3	39760.0	0.039	MM
96	96 20211111_A38_R1_IgG	2.39	2003.2	43982.6	0.046	MM
97	97 20211111_H19_R1_IgG	2.38	14456.6	45314.8	0.319	MM
98	98 20211111_A39_R1_IgG	2.39	3488.1	39388.0	0.089	MM
99	99 20211111_A40_R1_IgG	2.38	3357.2	38726.4	0.087	bb
100	1... 20211111_H20_R1_IgG	2.39	12912.6	37814.4	0.341	MM
101	1... 20211111_A41_R1_IgG	2.37	1090.3	48930.3	0.022	MM
102	1... 20211111_A42_R1_IgG	2.38	1495.5	40406.8	0.037	MM
103	1... 20211111_A43_R1_IgG	2.39	3828.9	44421.5	0.086	MM
104	1... 20211111_A44_R1_IgG	2.38	11362.9	53128.2	0.214	MM
105	1... 20211111_QC_R9_IgG	2.39	2307.2	37049.0	0.062	MM
106	1... 20211111_A1_R2_IgG	2.40	2486.4	46499.4	0.053	MM
107	1... 20211111_A2_R2_IgG	2.38	4202.6	57260.7	0.073	MM
108	1... 20211111_L1_R2_IgG	2.38	1764.4	48062.9	0.037	MM
109	1... 20211111_L2_R2_IgG	2.38	2337.1	38745.2	0.060	MM
110	1... 20211111_H1_R2_IgG	2.39	1186.1	44273.5	0.027	MM
111	1... 20211111_A3_R2_IgG	2.38	296.8	4755.5	0.062	bb
112	1... 20211111_A4_R2_IgG	2.40	1655.6	50546.7	0.033	MM

Dataset: C:\MassLynx\10.pro\20211115_clinicalsamples_IgG.qld

Last Altered: Sunday, October 02, 2022 16:02:30 台北標準時間
Printed: Friday, August 01, 2025 16:56:05 台北標準時間

	# Name	RT	Area	IS Area	Response	Prima...
113	1... 20211111_L3_R2_IgG	2.38	1280.6	34825.8	0.037	MM
114	1... 20211111_L4_R2_IgG	2.37	2112.7	39107.7	0.054	MM
115	1... 20211111_H2_R2_IgG	2.38	4297.3	49750.0	0.086	MM
116	1... 20211111_A5_R2_IgG	2.38	2709.2	52536.2	0.052	MM
117	1... 20211111_A6_R2_IgG	2.39	10810.4	42050.0	0.257	MM
118	1... 20211111_QC_R10_IgG	2.38	2307.3	35350.8	0.065	bb
119	1... 20211111_L5_R2_IgG	2.37	2780.7	38039.3	0.073	MM
120	1... 20211111_L6_R2_IgG	2.38	1381.5	44907.4	0.031	bb
121	1... 20211111_H3_R2_IgG	2.40	929.4	47729.1	0.019	MM
122	1... 20211111_A7_R2_IgG	2.39	3414.4	57347.1	0.060	MM
123	1... 20211111_A8_R2_IgG	2.38	1293.2	52397.8	0.025	MM
124	1... 20211111_L7_R2_IgG	2.38	1690.6	36318.4	0.047	MM
125	1... 20211111_L8_R2_IgG	2.38	3690.3	38131.9	0.097	MM
126	1... 20211111_H4_R2_IgG	2.39	1696.0	46849.6	0.036	bb
127	1... 20211111_A9_R2_IgG	2.38	1158.7	47759.6	0.024	MM
128	1... 20211111_A10_R2_IgG	2.38	1259.6	41366.2	0.030	MM
129	1... 20211111_L9_R2_IgG	2.38	2259.1	43052.1	0.052	MM
130	1... 20211111_L10_R2_IgG	2.37	1455.7	31487.4	0.046	bb
131	1... 20211111_QC_R11_IgG	2.39	1948.6	37804.6	0.052	MM
132	1... 20211111_H5_R2_IgG	2.38	1339.6	40380.0	0.033	MM
133	1... 20211111_A11_R2_IgG	2.38	220.1	49533.3	0.004	MM
134	1... 20211111_A12_R2_IgG	2.39	1890.1	40226.3	0.047	MM
135	1... 20211111_L11_R2_IgG	2.39	1007.4	28884.0	0.035	MM
136	1... 20211111_L12_R2_IgG	2.39	1043.7	34351.3	0.030	MM
137	1... 20211111_H6_R2_IgG	2.38	3826.7	40466.6	0.095	bb
138	1... 20211111_A13_R2_IgG	2.38	5583.7	42306.7	0.132	MM
139	1... 20211111_A14_R2_IgG	2.39	899.9	44904.0	0.020	bb
140	1... 20211111_L13_R2_IgG	2.38	3241.2	31913.0	0.102	MM
141	1... 20211111_L14_R2_IgG	2.38	2698.1	31527.4	0.086	bb
142	1... 20211111_H7_R2_IgG	2.38	3424.8	40838.2	0.084	MM
143	1... 20211111_A15_R2_IgG	2.39	962.7	49389.7	0.019	MM
144	1... 20211111_QC_R12_IgG	2.39	1785.2	35168.7	0.051	MM
145	1... 20211111_A16_R2_IgG	2.38	2430.4	47440.5	0.051	MM
146	1... 20211111_L15_R2_IgG	2.38	3440.0	33145.9	0.104	MM
147	1... 20211111_L16_R2_IgG	2.39	830.2	37596.6	0.022	MM
148	1... 20211111_H8_R2_IgG	2.38	3100.4	43933.3	0.071	bb
149	1... 20211111_A17_R2_IgG	2.38	4099.4	43605.5	0.094	MM
150	1... 20211111_A18_R2_IgG	2.38	2068.5	37581.8	0.055	MM
151	1... 20211111_L17_R2_IgG	2.38	2084.8	33942.5	0.061	bb
152	1... 20211111_L18_R2_IgG	2.40	651.9	37831.5	0.017	bb
153	1... 20211111_H9_R2_IgG	2.38	2972.5	30898.4	0.096	MM
154	1... 20211111_A19_R2_IgG	2.38	884.1	39352.1	0.022	MM
155	1... 20211111_A20_R2_IgG	2.39	746.1	34661.2	0.022	MM
156	1... 20211111_L19_R2_IgG	2.38	2380.9	39918.3	0.060	bb
157	1... 20211111_QC_R13_IgG	2.38	2474.0	36239.1	0.068	bb
158	1... 20211111_L20_R2_IgG	2.38	2999.8	32897.7	0.091	MM
159	1... 20211111_H10_R2_IgG	2.37	1866.6	31394.5	0.059	bb
160	1... 20211111_A21_R2_IgG	2.40	2855.7	34863.7	0.082	MM
161	1... 20211111_A22_R2_IgG	2.39	3219.2	30342.1	0.106	MM
162	1... 20211111_L21_R2_IgG	2.39	840.4	33706.2	0.025	MM
163	1... 20211111_L22_R2_IgG	2.38	1329.3	31298.6	0.042	MM
164	1... 20211111_H11_R2_IgG	2.37	1750.2	38904.1	0.045	MM
165	1... 20211111_A23_R2_IgG	2.38	978.9	32382.3	0.030	MM
166	1... 20211111_A24_R2_IgG	2.37	1282.7	30261.9	0.042	bb
167	1... 20211111_L23_R2_IgG	2.38	2997.7	43037.5	0.070	MM
168	1... 20211111_L24_R2_IgG	2.39	2207.7	37023.3	0.060	bb

Dataset: C:\MassLynx\10.pro\20211115_clinicalsamples_IgG.qld

Last Altered: Sunday, October 02, 2022 16:02:30 台北標準時間
Printed: Friday, August 01, 2025 16:56:05 台北標準時間

	#	Name	RT	Area	IS Area	Response	Prima...
169	1...	20211111_H12_R2_IgG	2.39	237.9	39478.2	0.006	MM
170	1...	20211111_QC_R14_IgG	2.37	1788.5	34463.2	0.052	MM
171	1...	20211111_A25_R2_IgG	2.39	5190.1	39945.3	0.130	bb
172	1...	20211111_A26_R2_IgG	2.37	397.7	29663.2	0.013	MM
173	1...	20211111_L25_R2_IgG	2.38	1262.9	38589.3	0.033	MM
174	1...	20211111_L26_R2_IgG	2.38	555.8	38405.2	0.014	MM
175	1...	20211111_H13_R2_IgG	2.38	1907.2	37771.2	0.050	MM
176	1...	20211111_A27_R2_IgG	2.38	1120.3	42634.1	0.026	MM
177	1...	20211111_A28_R2_IgG	2.38	3893.2	33231.1	0.117	MM
178	1...	20211111_L27_R2_IgG	2.38	1649.4	41761.1	0.039	MM
179	1...	20211111_L28_R2_IgG	2.38	2532.2	26968.6	0.094	bb
180	1...	20211111_H14_R2_IgG	2.38	1322.7	49250.2	0.027	bb
181	1...	20211111_A29_R2_IgG	2.38	984.8	37601.7	0.026	MM
182	1...	20211111_A30_R2_IgG	2.38	1653.4	40366.8	0.041	MM
183	1...	20211111_QC_R15_IgG	2.38	2355.2	35156.1	0.067	MM
184	1...	20211111_L29_R2_IgG	2.38	1616.2	33813.1	0.048	MM
185	1...	20211111_L30_R2_IgG	2.39	827.1	33488.6	0.025	MM
186	1...	20211111_H15_R2_IgG	2.38	270.4	38501.0	0.007	bb
187	1...	20211111_A31_R2_IgG	2.37	709.8	37525.5	0.019	MM
188	1...	20211111_A32_R2_IgG	2.36	1709.0	35770.8	0.048	MM
189	1...	20211111_L31_R2_IgG	2.39	319.4	33282.4	0.010	bb
190	1...	20211111_L32_R2_IgG	2.38	1610.1	42049.6	0.038	bb
191	1...	20211111_H16_R2_IgG	2.38	5124.4	45947.6	0.112	MM
192	1...	20211111_A33_R2_IgG	2.36	729.7	40202.3	0.018	MM
193	1...	20211111_A34_R2_IgG	2.38	2297.1	36747.0	0.063	MM
194	1...	20211111_H17_R2_IgG	2.38	8668.7	40920.9	0.212	MM
195	1...	20211111_A35_R2_IgG	2.38	3866.9	33689.2	0.115	bb
196	1...	20211111_QC_R16_IgG	2.38	2252.7	32783.6	0.069	bb
197	1...	20211111_A36_R2_IgG	2.38	2318.7	36822.4	0.063	MM
198	1...	20211111_H18_R2_IgG	2.38	16409.4	31769.3	0.517	MM
199	1...	20211111_A37_R2_IgG	2.37	949.9	37581.8	0.025	MM
200	2...	20211111_A38_R2_IgG	2.39	1930.9	38390.4	0.050	bb
201	2...	20211111_H19_R2_IgG	2.38	12678.2	41217.0	0.308	MM
202	2...	20211111_A39_R2_IgG	2.38	3194.3	37392.7	0.085	bb
203	2...	20211111_A40_R2_IgG	2.37	3520.2	38295.1	0.092	MM
204	2...	20211111_H20_R2_IgG	2.38	10320.9	35941.8	0.287	MM
205	2...	20211111_A41_R2_IgG	2.38	442.3	45304.9	0.010	MM
206	2...	20211111_A42_R2_IgG	2.38	1603.9	37803.8	0.042	MM
207	2...	20211111_A43_R2_IgG	2.38	2737.7	38161.8	0.072	MM
208	2...	20211111_A44_R2_IgG	2.37	7126.4	37819.8	0.188	MM
209	2...	20211111_QC_R17_IgG	2.38	1663.4	31731.8	0.052	MM

Dataset: C:\MassLynx\10.pro\20211111_clinicalsamples_IgM_IgA.qld

Last Altered: Thursday, March 09, 2023 19:58:25 台北標準時間
Printed: Friday, August 01, 2025 15:00:43 台北標準時間

Method: C:\MassLynx\10_Pro_IgA.PRO\MethDB\20211111_IgM_IgA_DMRM_new.mdb 24 Feb 2022 13:44:14
Calibration: 15 Nov 2021 14:42:11

Sample Name: 20211112_QC_R001_IgM

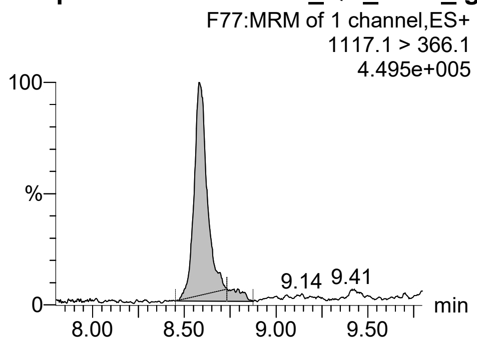

Sample Name: 20211112_QC_R002_IgM

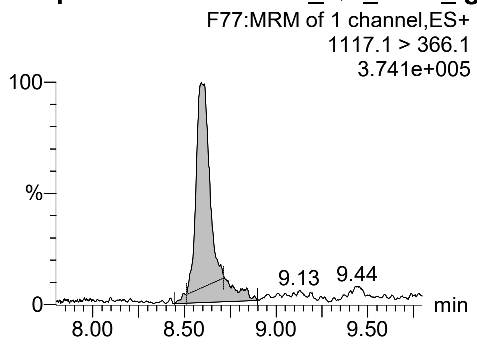

Sample Name: 20211111_A1_R2_IgM

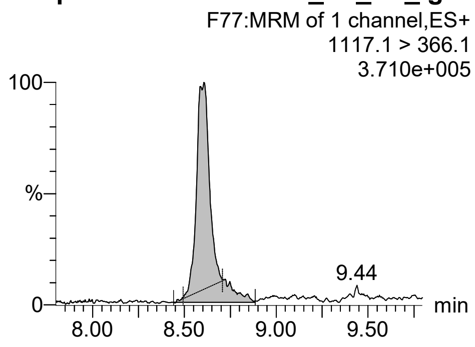

Sample Name: 20211111_A1_R3_IgM

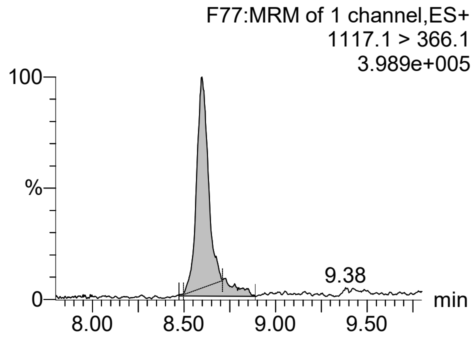

Sample Name: 20211111_A2_R2_IgM

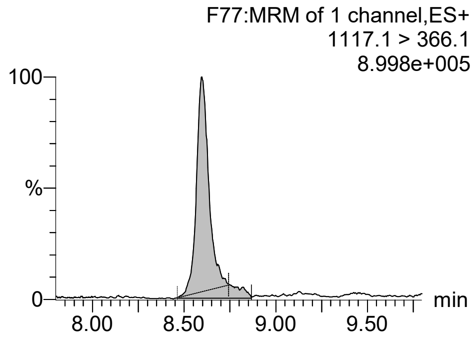

Sample Name: 20211111_A2_R3_IgM

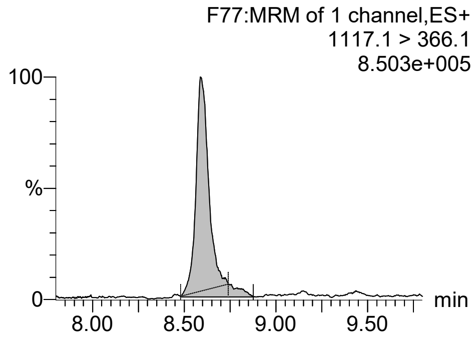

Sample Name: 20211111_L1_R2_IgM

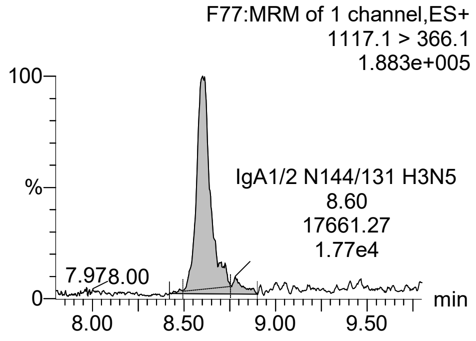

Sample Name: 20211111_L1_R3_IgM

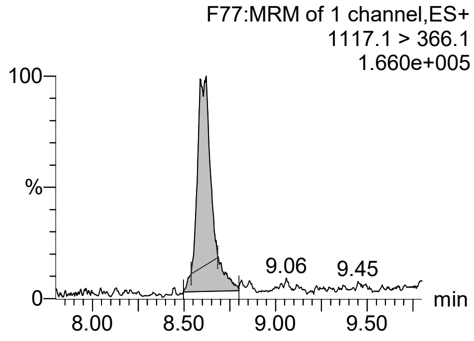

Sample Name: 20211111_L2_R2_IgM

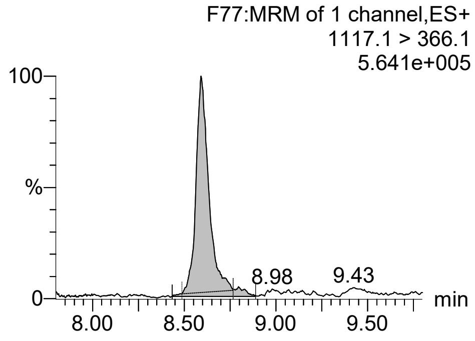

Sample Name: 20211111_H1_R2_IgM

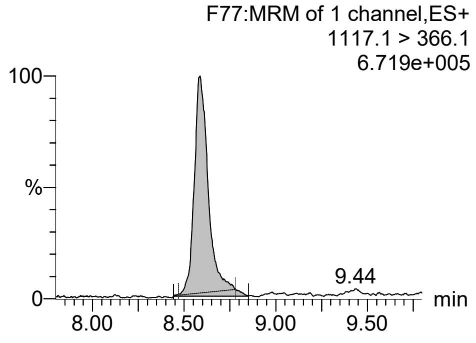

Sample Name: 20211111_A3_R2_IgM

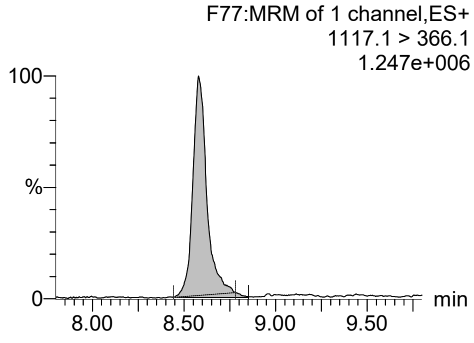

Sample Name: 20211111_A4_R2_IgM

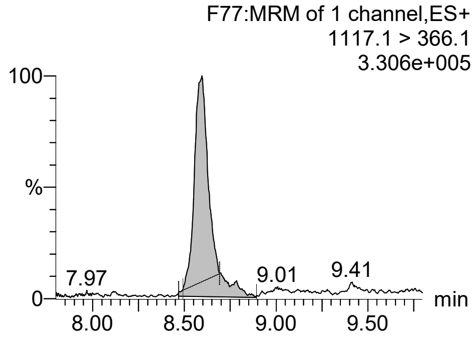

Dataset: C:\MassLynx\10.pro\20211111_clinicalsamples_IgM_IgA.qld

Last Altered: Thursday, March 09, 2023 19:58:25 台北標準時間
Printed: Friday, August 01, 2025 15:00:43 台北標準時間

Sample Name: 20211111_L3_R2_IgM
F77:MRM of 1 channel,ES+
1117.1 > 366.1
2.398e+005

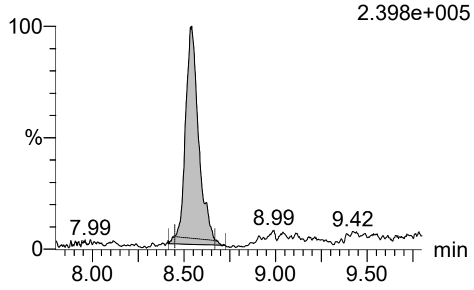

Sample Name: 20211111_L4_R2_IgM
F77:MRM of 1 channel,ES+
1117.1 > 366.1
4.795e+005

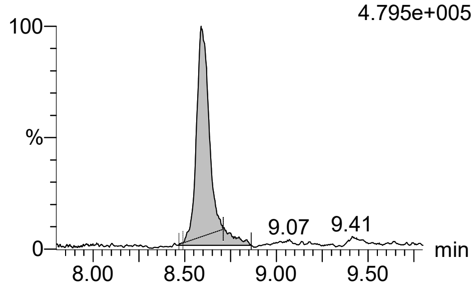

Sample Name: 20211111_H2_R2_IgM
F77:MRM of 1 channel,ES+
1117.1 > 366.1
1.659e+006

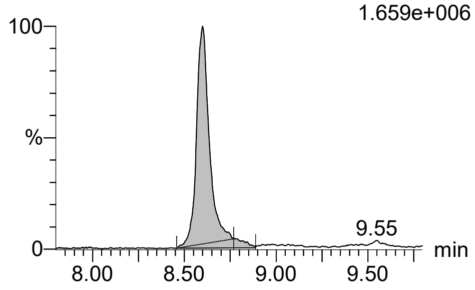

Sample Name: 20211111_A5_R2_IgM
F77:MRM of 1 channel,ES+
1117.1 > 366.1
1.870e+006

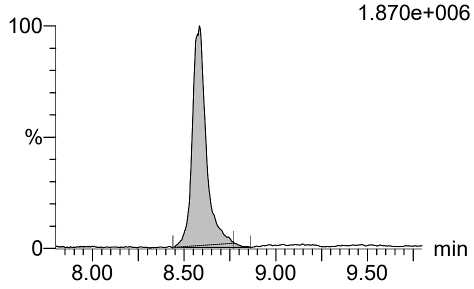

Sample Name: 20211111_A6_R2_IgM
F77:MRM of 1 channel,ES+
1117.1 > 366.1
3.862e+005

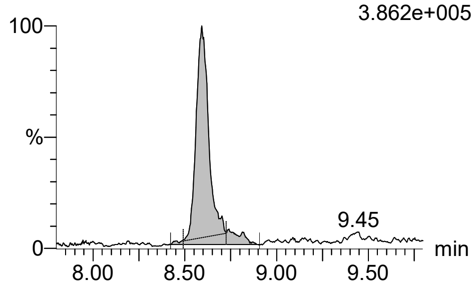

Sample Name: 20211111_QC_R10_IgM
F77:MRM of 1 channel,ES+
1117.1 > 366.1
4.377e+005

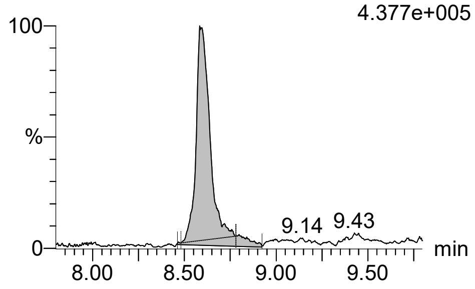

Sample Name: 20211111_L5_R2_IgM
F77:MRM of 1 channel,ES+
1117.1 > 366.1
4.690e+005

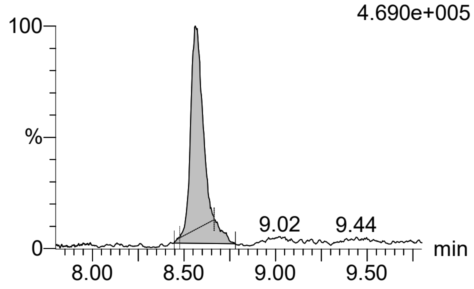

Sample Name: 20211111_L6_R2_IgM
F77:MRM of 1 channel,ES+
1117.1 > 366.1
3.714e+005

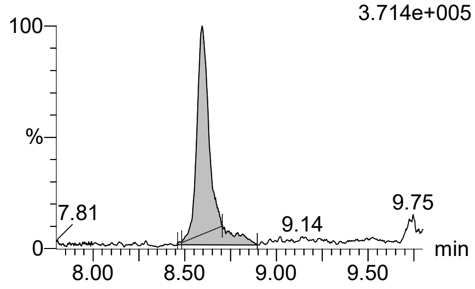

Sample Name: 20211111_H3_R2_IgM
F77:MRM of 1 channel,ES+
1117.1 > 366.1
2.996e+005

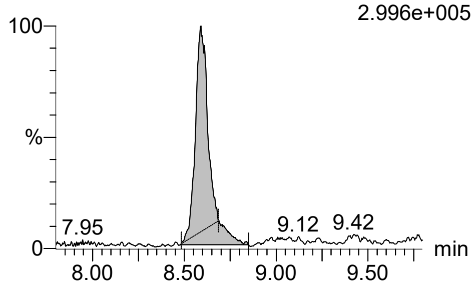

Sample Name: 20211111_A7_R2_IgM
F77:MRM of 1 channel,ES+
1117.1 > 366.1
7.271e+005

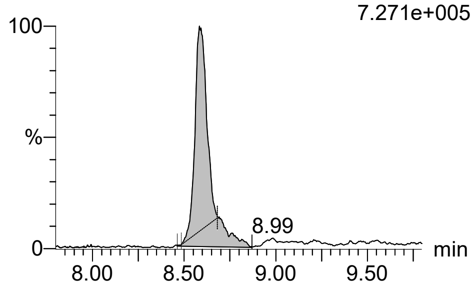

Sample Name: 20211111_A8_R2_IgM
F77:MRM of 1 channel,ES+
1117.1 > 366.1
1.130e+006

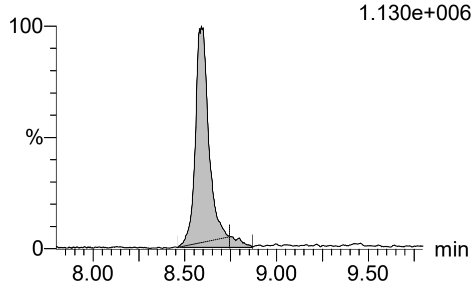

Sample Name: 20211111_L7_R2_IgM
F77:MRM of 1 channel,ES+
1117.1 > 366.1
1.866e+005

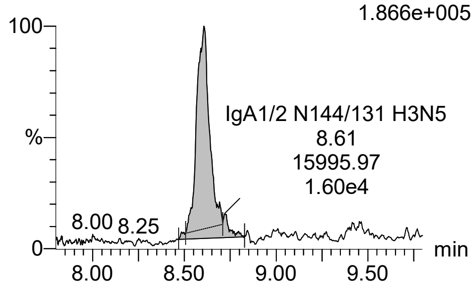

Sample Name: 20211111_L8_R2_IgM
F77:MRM of 1 channel,ES+
1117.1 > 366.1
8.653e+004

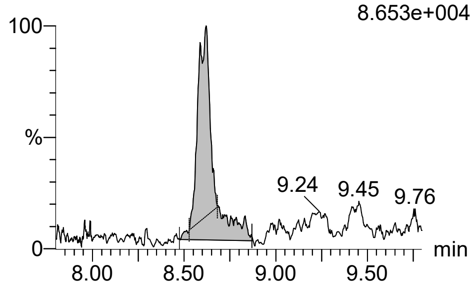

Sample Name: 20211111_H4_R2_IgM
F77:MRM of 1 channel,ES+
1117.1 > 366.1
2.512e+005

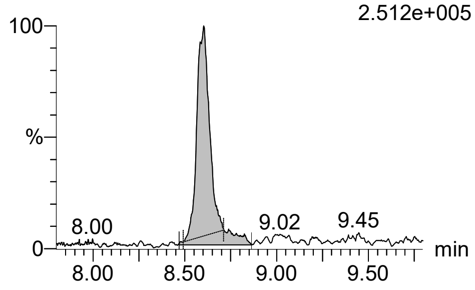

Sample Name: 20211111_A9_R2_IgM
F77:MRM of 1 channel,ES+
1117.1 > 366.1
4.165e+005

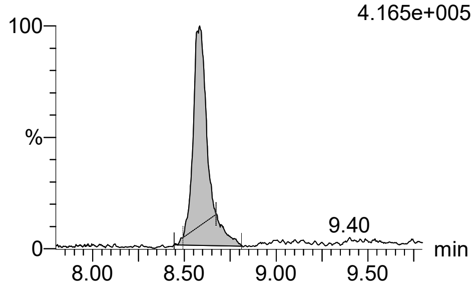

Sample Name: 20211111_A10_R2_IgM

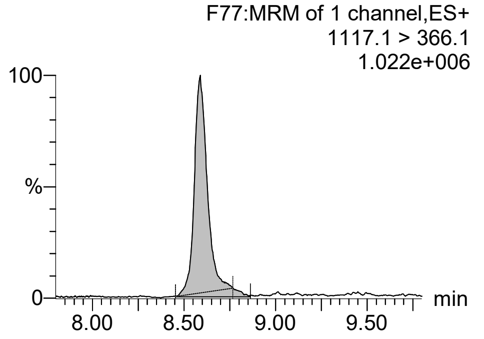

Sample Name: 20211111_L9_R2_IgM

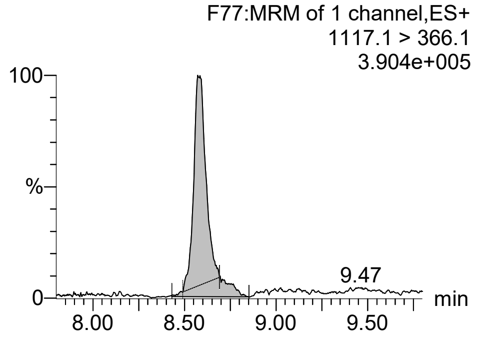

Sample Name: 20211111_L10_R2_IgM

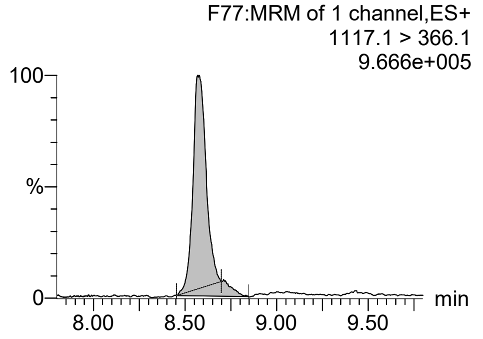

Sample Name: 20211111_QC_R11_IgM

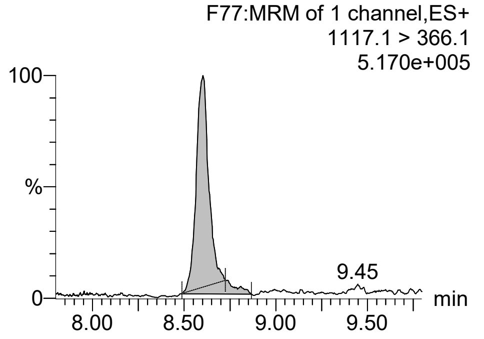

Sample Name: 20211111_H5_R2_IgM

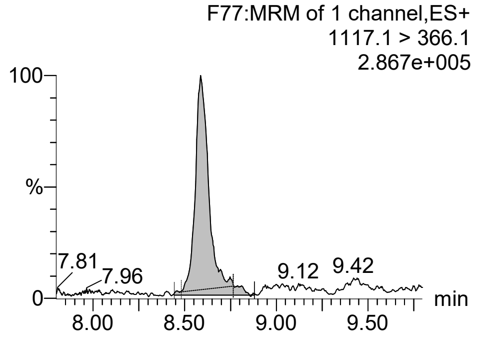

Sample Name: 20211111_A11_R2_IgM

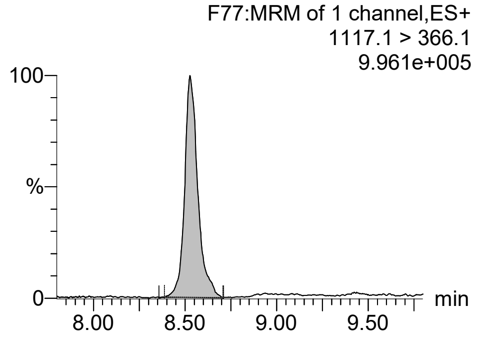

Sample Name: 20211111_A12_R2_IgM

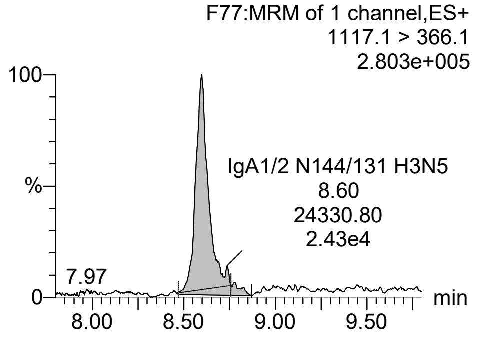

Sample Name: 20211111_L11_R2_IgM

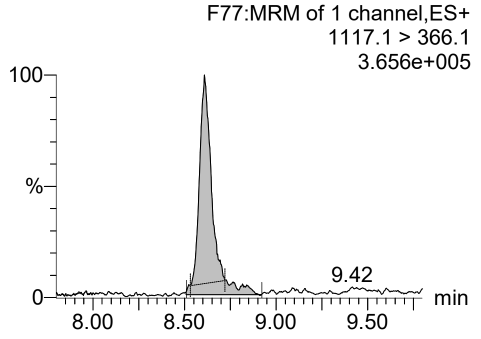

Sample Name: 20211111_L12_R2_IgM

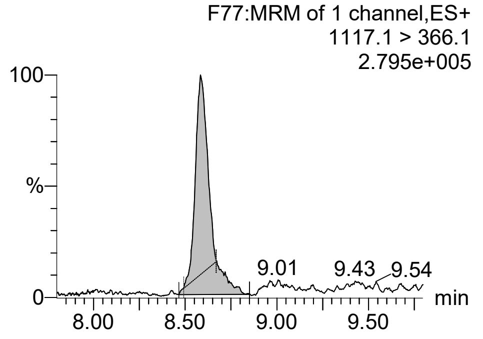

Sample Name: 20211111_H6_R2_IgM

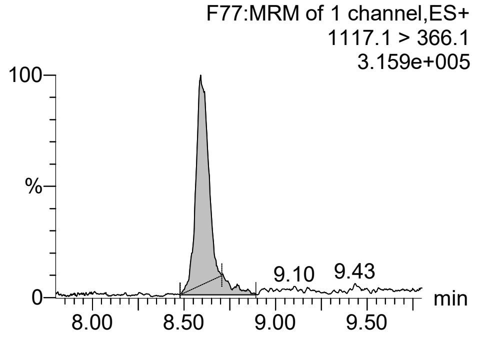

Sample Name: 20211111_A13_R2_IgM

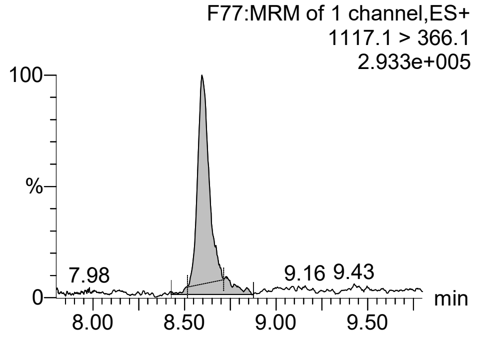

Sample Name: 20211111_A14_R2_IgM

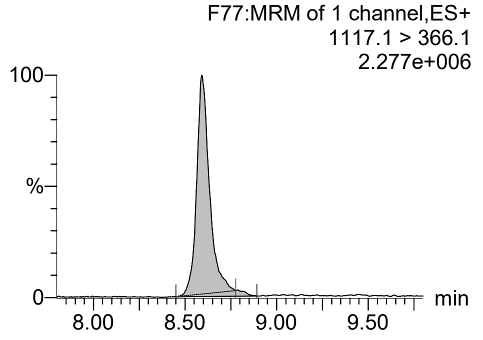

Sample Name: 20211111_L13_R2_IgM

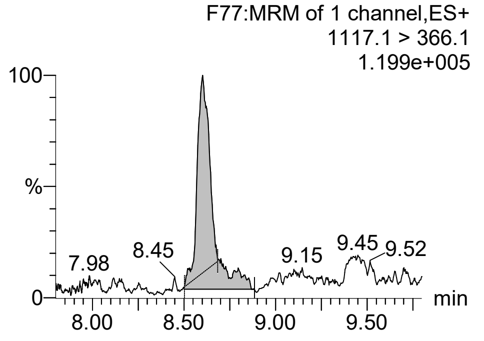

Sample Name: 20211111_L14_R2_IgM

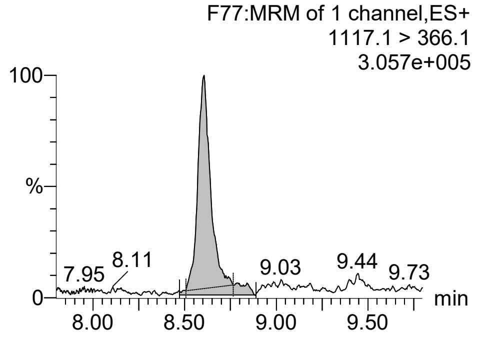

Sample Name: 20211111_H7_R2_IgM

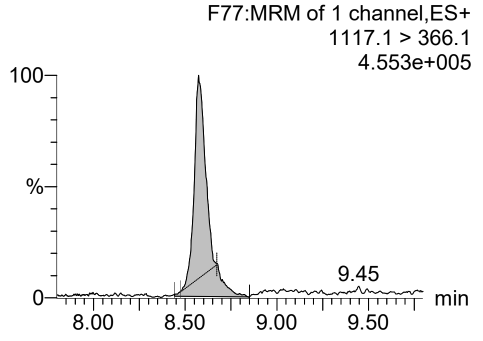

Sample Name: 20211111_A15_R2_IgM
F77:MRM of 1 channel,ES+
1117.1 > 366.1
2.451e+006

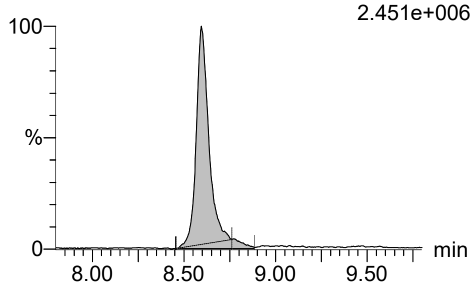

Sample Name: 20211111_QC_R12_IgM
F77:MRM of 1 channel,ES+
1117.1 > 366.1
5.553e+005

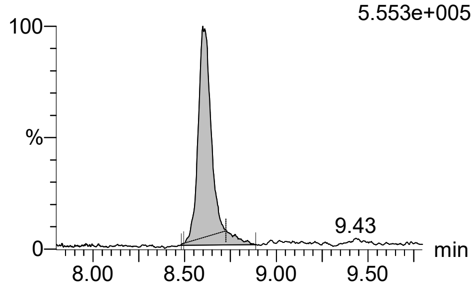

Sample Name: 20211111_A16_R2_IgM
F77:MRM of 1 channel,ES+
1117.1 > 366.1
1.141e+006

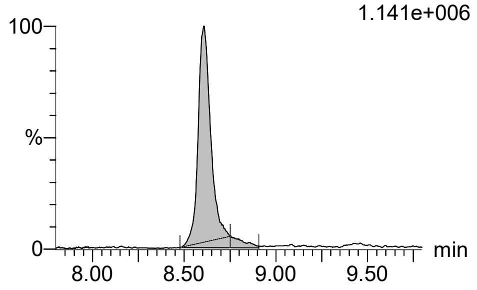

Sample Name: 20211111_L15_R2_IgM
F77:MRM of 1 channel,ES+
1117.1 > 366.1
7.484e+005

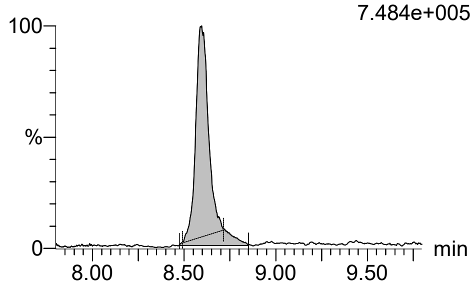

Sample Name: 20211111_L16_R2_IgM
F77:MRM of 1 channel,ES+
1117.1 > 366.1
1.532e+005

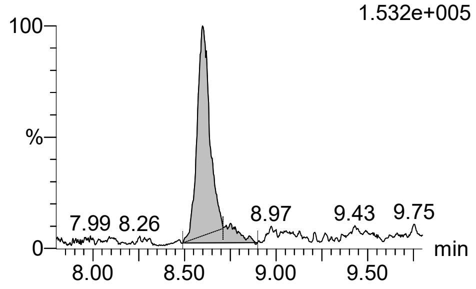

Sample Name: 20211111_H8_R2_IgM
F77:MRM of 1 channel,ES+
1117.1 > 366.1
1.727e+005

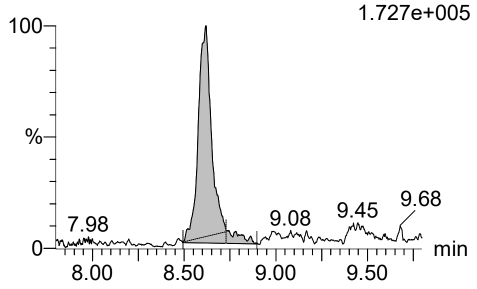

Sample Name: 20211111_A17_R2_IgM
F77:MRM of 1 channel,ES+
1117.1 > 366.1
6.088e+005

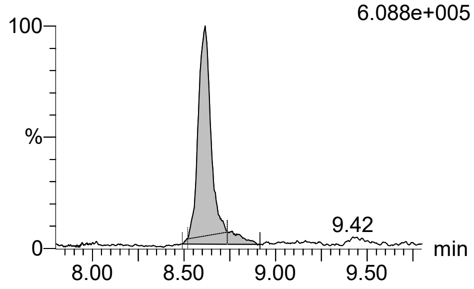

Sample Name: 20211111_A18_R2_IgM
F77:MRM of 1 channel,ES+
1117.1 > 366.1
4.378e+005

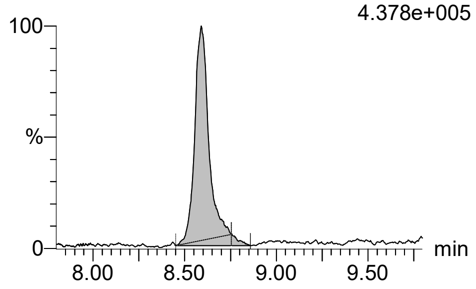

Sample Name: 20211111_L17_R2_IgM
F77:MRM of 1 channel,ES+
1117.1 > 366.1
5.614e+005

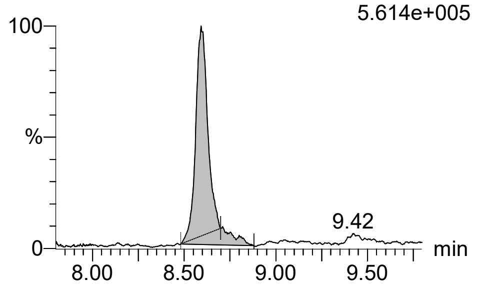

Sample Name: 20211111_L18_R2_IgM
F77:MRM of 1 channel,ES+
1117.1 > 366.1
1.277e+005

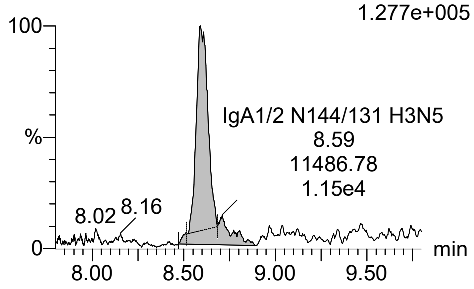

Sample Name: 20211111_H9_R2_IgM
F77:MRM of 1 channel,ES+
1117.1 > 366.1
2.896e+005

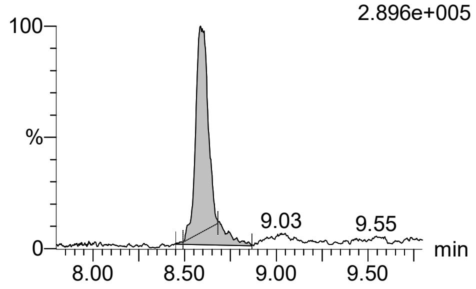

Sample Name: 20211111_A19_R2_IgM
F77:MRM of 1 channel,ES+
1117.1 > 366.1
4.979e+005

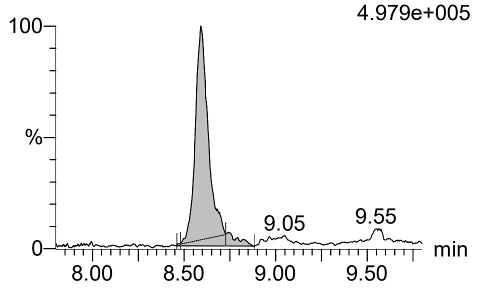

Sample Name: 20211111_A20_R2_IgM
F77:MRM of 1 channel,ES+
1117.1 > 366.1
4.009e+005

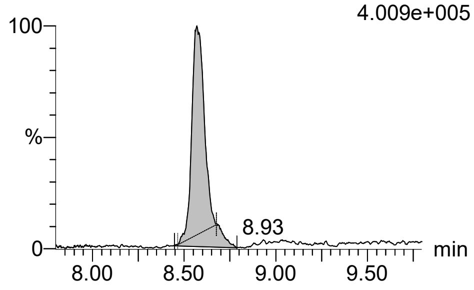

Sample Name: 20211111_L19_R2_IgM
F77:MRM of 1 channel,ES+
1117.1 > 366.1
3.803e+005

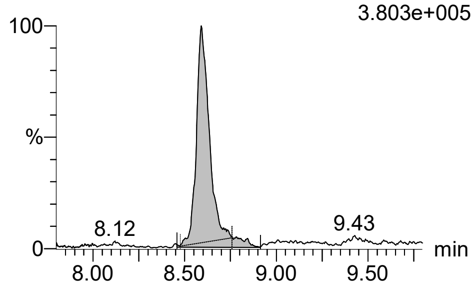

Sample Name: 20211111_QC_R13_IgM
F77:MRM of 1 channel,ES+
1117.1 > 366.1
5.499e+005

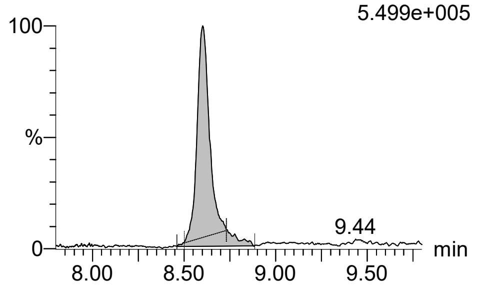

Sample Name: 20211111_L20_R2_IgM

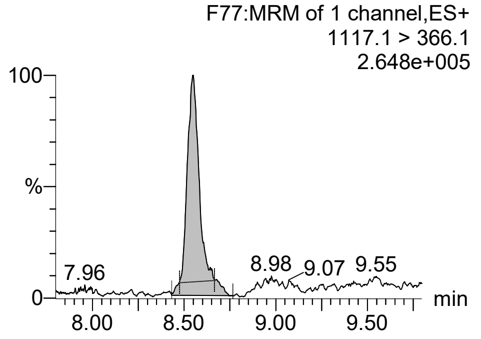

Sample Name: 20211111_H10_R2_IgM

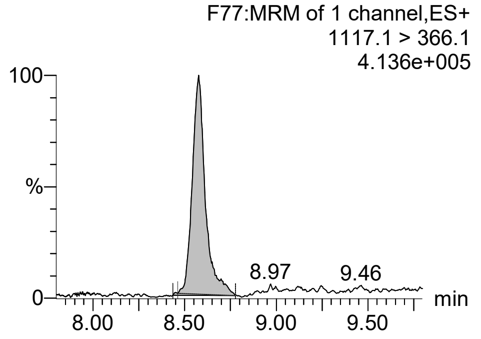

Sample Name: 20211111_A21_R2_IgM

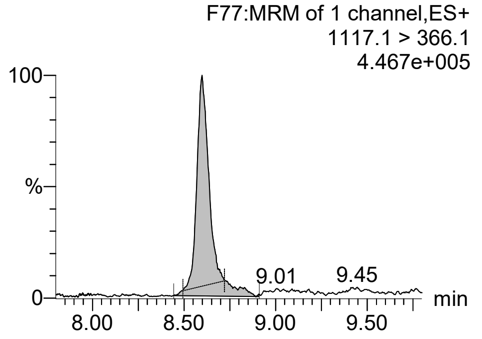

Sample Name: 20211111_A22_R2_IgM

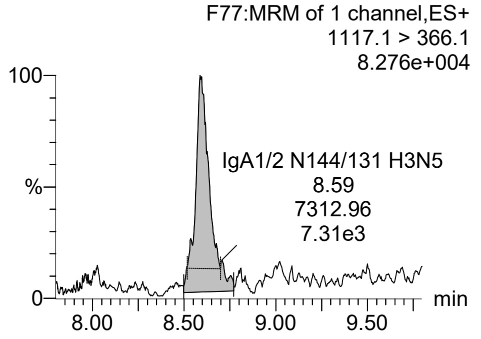

Sample Name: 20211111_L21_R2_IgM

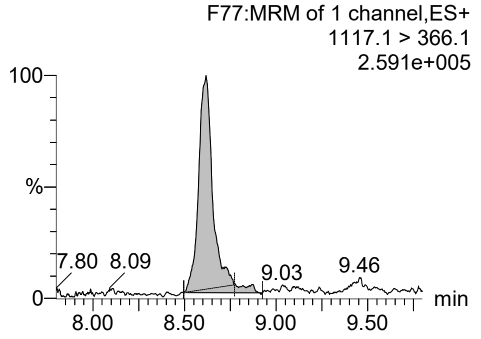

Sample Name: 20211111_L22_R2_IgM

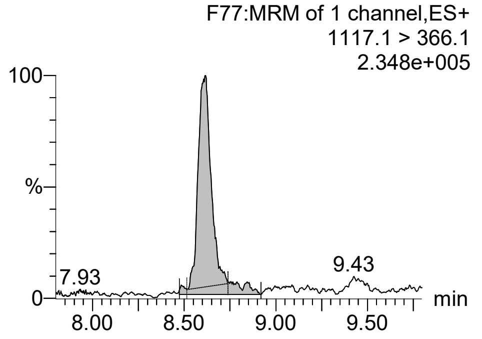

Sample Name: 20211111_H11_R2_IgM

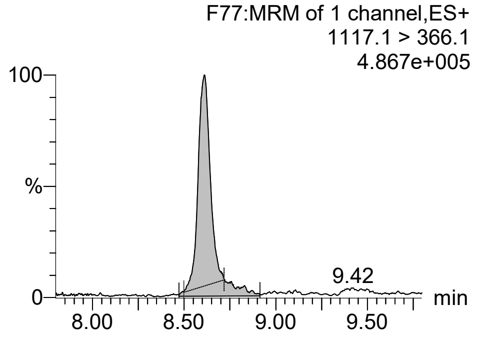

Sample Name: 20211111_A23_R2_IgM

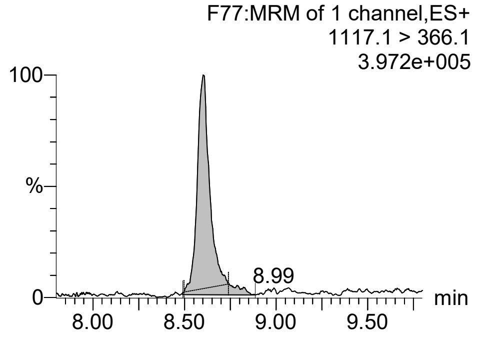

Sample Name: 20211111_A24_R2_IgM

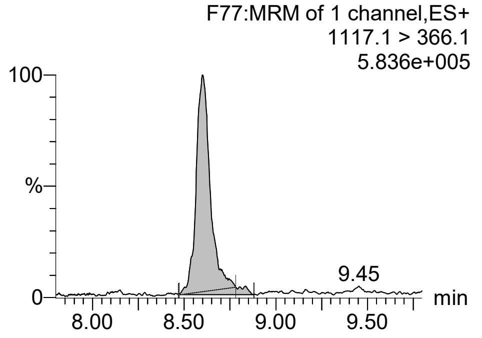

Sample Name: 20211111_L23_R2_IgM

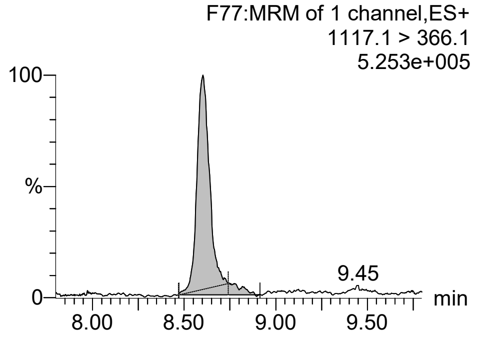

Sample Name: 20211111_L24_R2_IgM

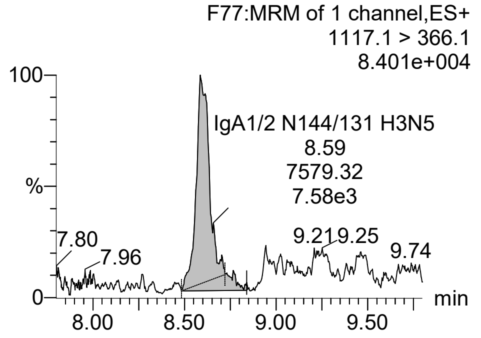

Sample Name: 20211111_H12_R2_IgM

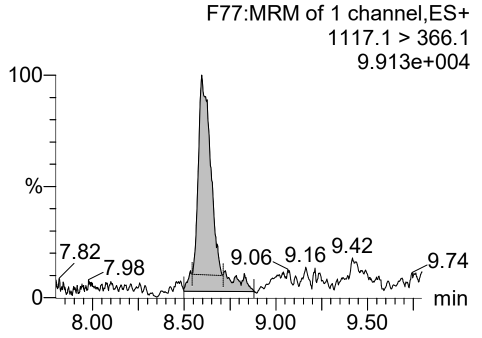

Sample Name: 20211111_QC_R14_IgM

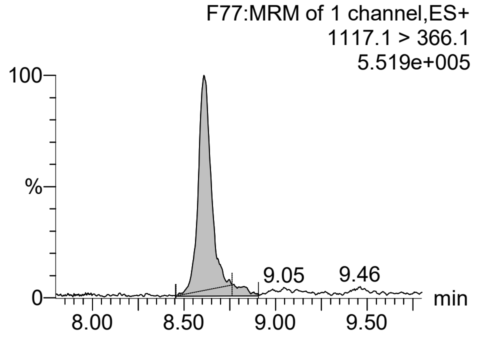

Sample Name: 20211111_A25_R2_IgM

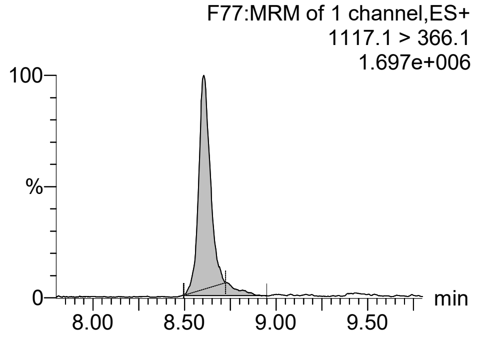

Sample Name: 20211111_A26_R2_IgM

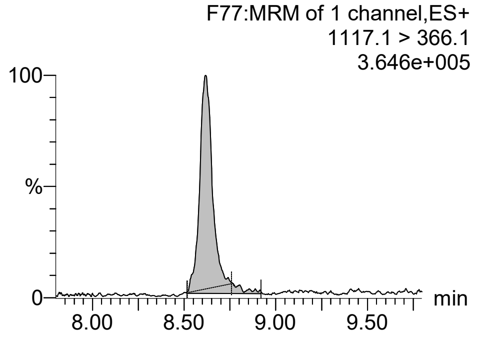

Sample Name: 20211111_L25_R2_IgM
F77:MRM of 1 channel,ES+
1117.1 > 366.1
3.452e+005

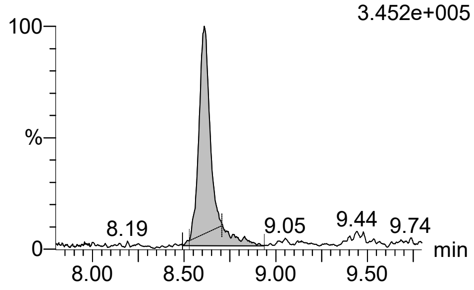

Sample Name: 20211111_L26_R2_IgM
F77:MRM of 1 channel,ES+
1117.1 > 366.1
2.600e+005

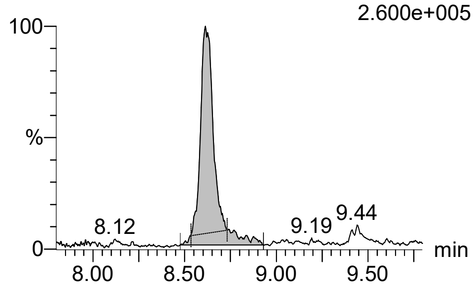

Sample Name: 20211111_H13_R2_IgM
F77:MRM of 1 channel,ES+
1117.1 > 366.1
6.901e+005

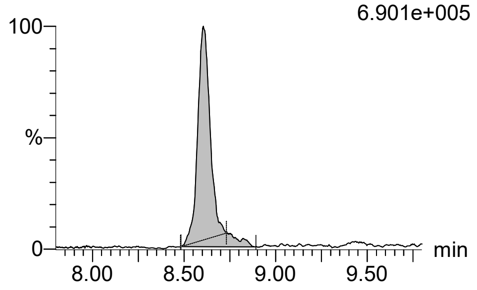

Sample Name: 20211111_A27_R2_IgM
F77:MRM of 1 channel,ES+
1117.1 > 366.1
4.162e+005

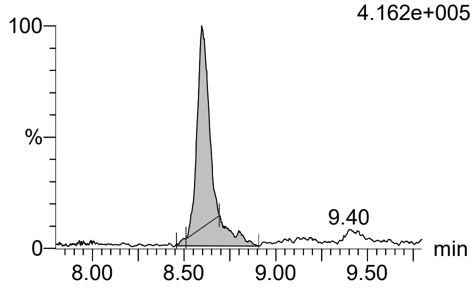

Sample Name: 20211111_A28_R2_IgM
F77:MRM of 1 channel,ES+
1117.1 > 366.1
1.741e+005

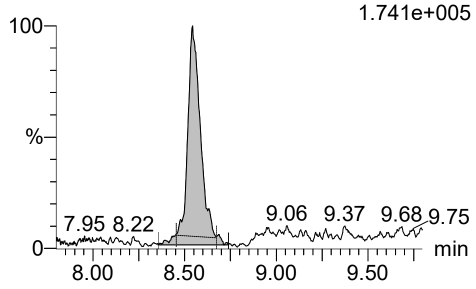

Sample Name: 20211111_L27_R2_IgM
F77:MRM of 1 channel,ES+
1117.1 > 366.1
4.855e+005

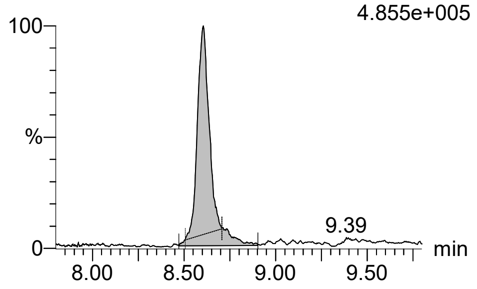

Sample Name: 20211111_L28_R2_IgM
F77:MRM of 1 channel,ES+
1117.1 > 366.1
1.148e+005

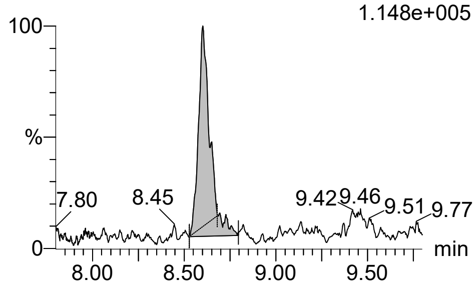

Sample Name: 20211111_H14_R2_IgM
F77:MRM of 1 channel,ES+
1117.1 > 366.1
3.411e+005

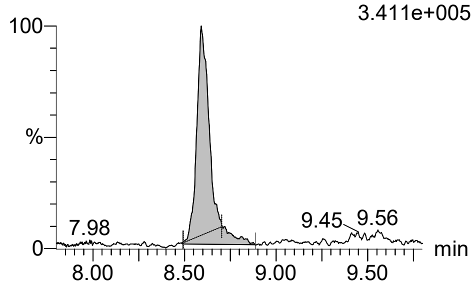

Sample Name: 20211111_A29_R2_IgM
F77:MRM of 1 channel,ES+
1117.1 > 366.1
1.165e+005

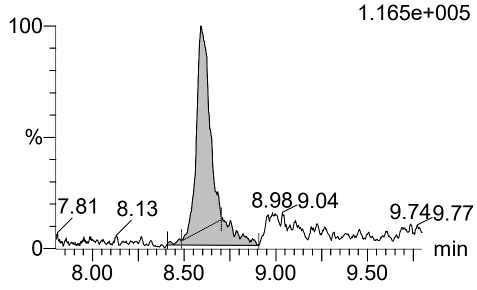

Sample Name: 20211111_A30_R2_IgM
F77:MRM of 1 channel,ES+
1117.1 > 366.1
1.482e+006

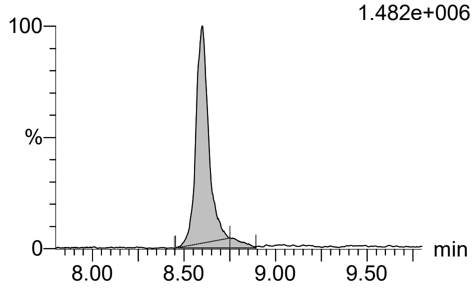

Sample Name: 20211111_QC_R15_IgM
F77:MRM of 1 channel,ES+
1117.1 > 366.1
5.039e+005

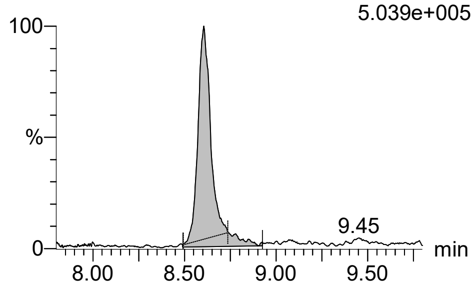

Sample Name: 20211111_L29_R2_IgM
F77:MRM of 1 channel,ES+
1117.1 > 366.1
1.124e+005

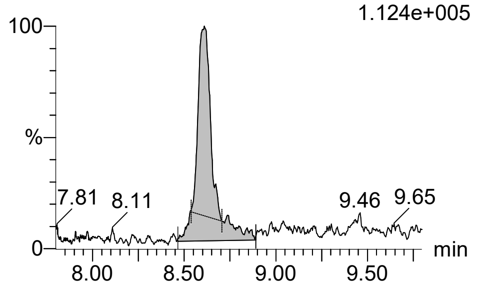

Sample Name: 20211111_L30_R2_IgM
F77:MRM of 1 channel,ES+
1117.1 > 366.1
1.337e+005

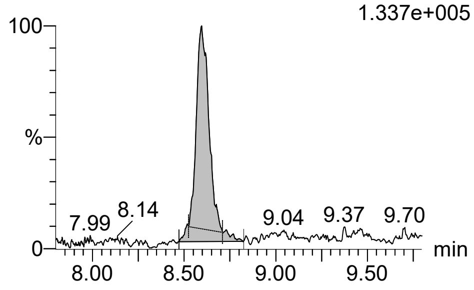

Sample Name: 20211111_H15_R2_IgM
F77:MRM of 1 channel,ES+
1117.1 > 366.1
2.668e+005

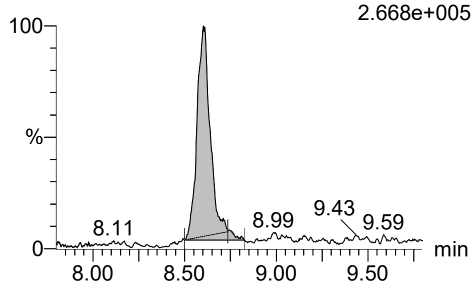

Sample Name: 20211111_A31_R2_IgM
F77:MRM of 1 channel,ES+
1117.1 > 366.1
3.462e+005

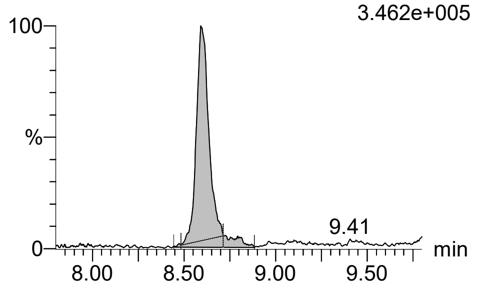

Dataset: C:\MassLynx\10.pro\20211111_clinicalsamples_IgM_IgA.qld

Last Altered: Thursday, March 09, 2023 19:58:25 台北標準時間
Printed: Friday, August 01, 2025 15:00:43 台北標準時間

Sample Name: 20211111_A32_R2_IgM

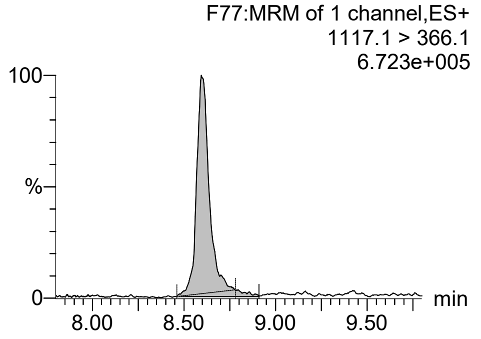

Sample Name: 20211111_L31_R2_IgM

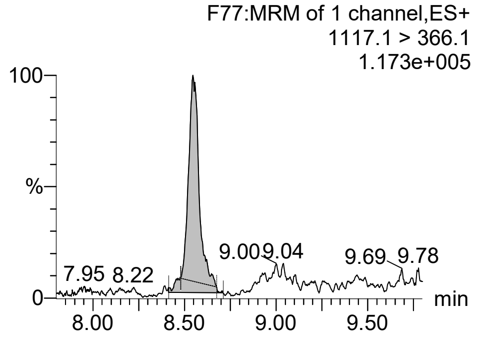

Sample Name: 20211111_L32_R2_IgM

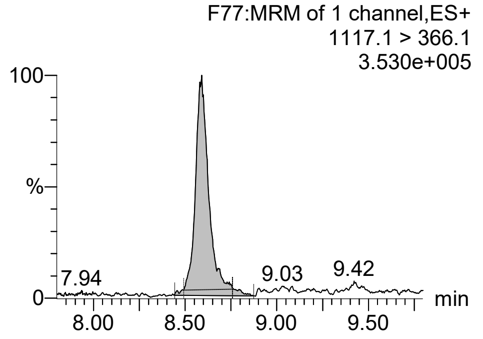

Sample Name: 20211111_H16_R2_IgM

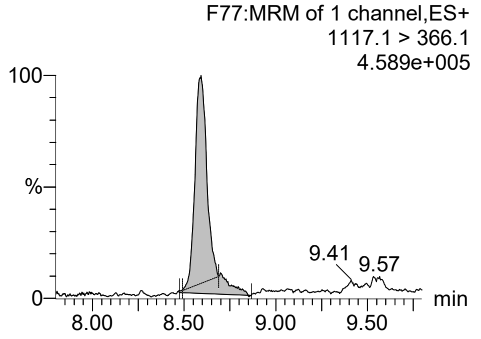

Sample Name: 20211111_A33_R2_IgM

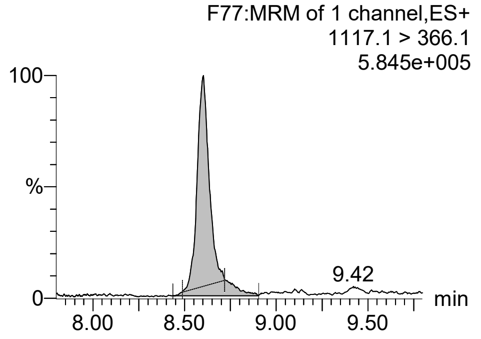

Sample Name: 20211111_A34_R2_IgM

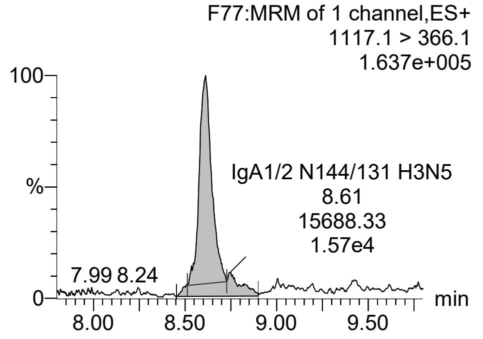

Sample Name: 20211111_H17_R2_IgM

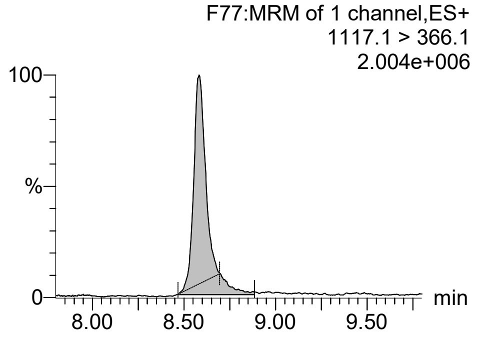

Sample Name: 20211111_A35_R2_IgM

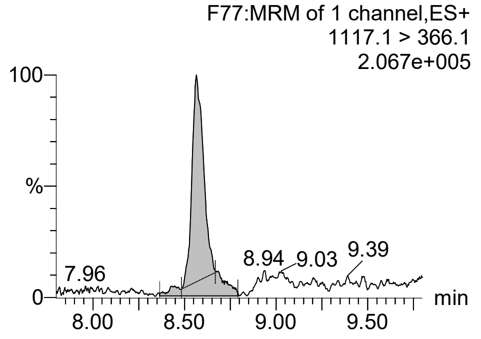

Sample Name: 20211111_QC_R16_IgM

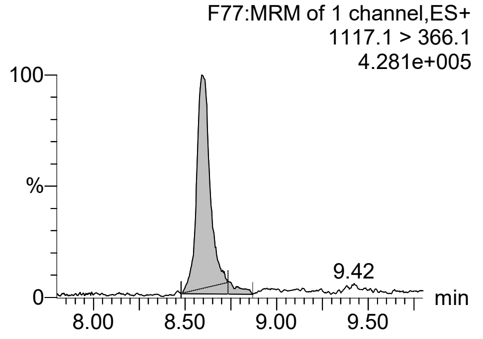

Sample Name: 20211111_A36_R2_IgM

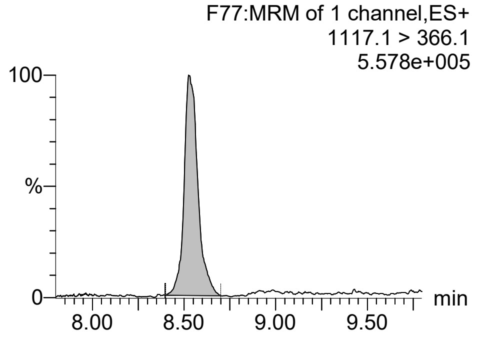

Sample Name: 20211111_H18_R2_IgM

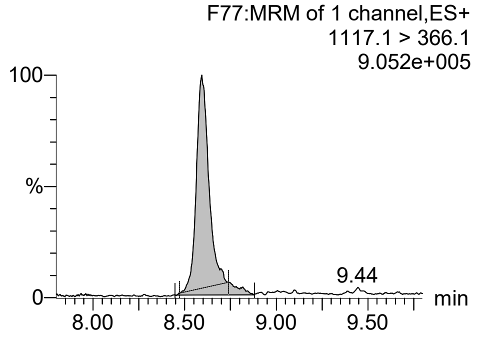

Sample Name: 20211111_A37_R2_IgM

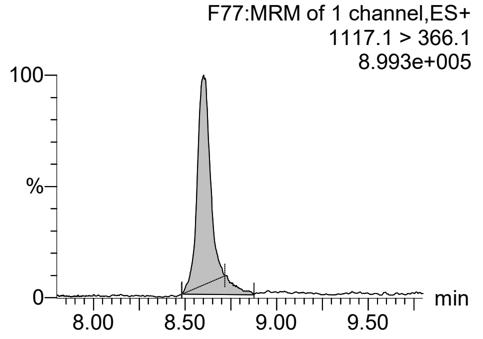

Sample Name: 20211111_A38_R2_IgM

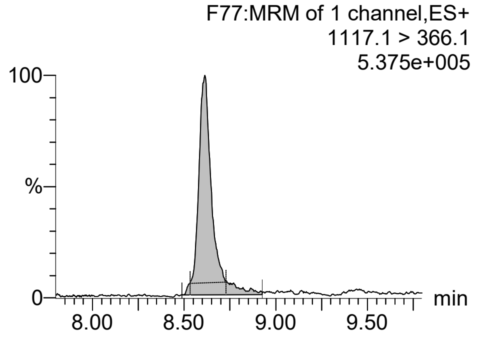

Sample Name: 20211111_H19_R2_IgM

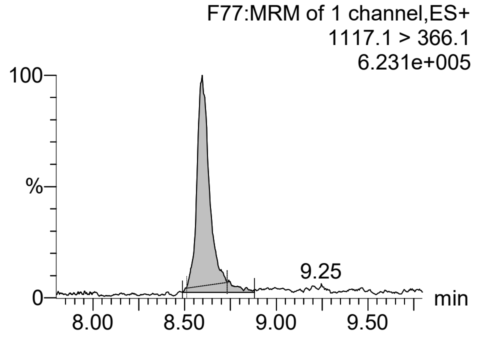

Sample Name: 20211111_A39_R2_IgM

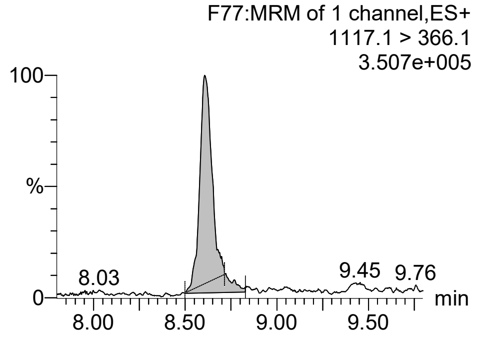

Dataset: C:\MassLynx\10.pro\20211111_clinicalsamples_IgM_IgA.qld

Last Altered: Thursday, March 09, 2023 19:58:25 台北標準時間
Printed: Friday, August 01, 2025 15:00:43 台北標準時間

Sample Name: 20211111_A40_R2_IgM
F77:MRM of 1 channel,ES+
1117.1 > 366.1
2.476e+005

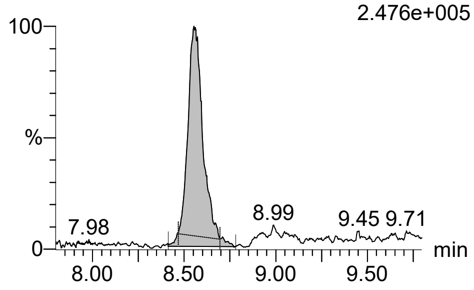

Sample Name: 20211111_H20_R2_IgM
F77:MRM of 1 channel,ES+
1117.1 > 366.1
1.530e+006

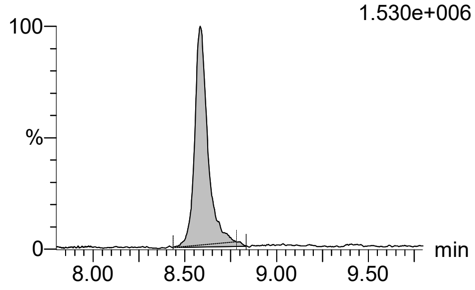

Sample Name: 20211111_A41_R2_IgM
F77:MRM of 1 channel,ES+
1117.1 > 366.1
2.690e+005

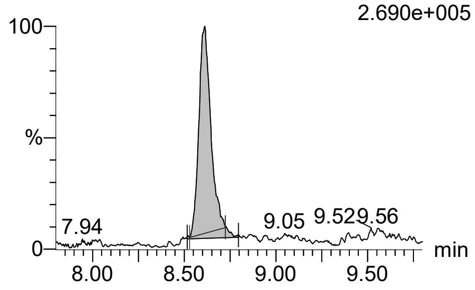

Sample Name: 20211111_A42_R2_IgM
F77:MRM of 1 channel,ES+
1117.1 > 366.1
1.830e+005

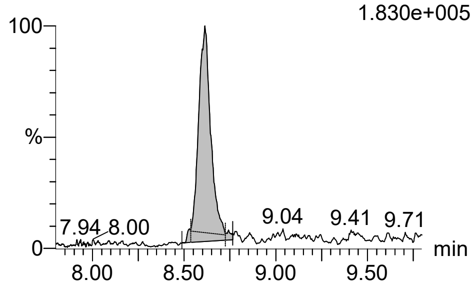

Sample Name: 20211111_A43_R2_IgM
F77:MRM of 1 channel,ES+
1117.1 > 366.1
1.410e+005

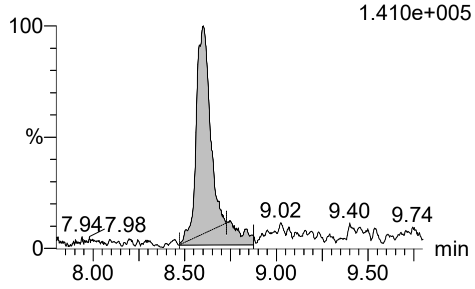

Sample Name: 20211111_A44_R2_IgM
F77:MRM of 1 channel,ES+
1117.1 > 366.1
1.235e+005

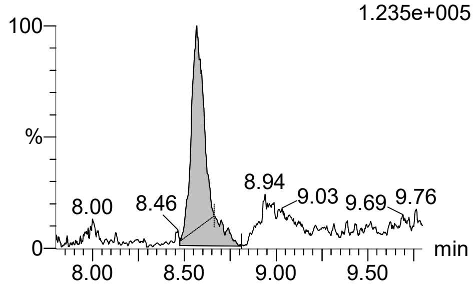

Sample Name: 20211111_QC_R17_IgM
F77:MRM of 1 channel,ES+
1117.1 > 366.1
3.910e+005

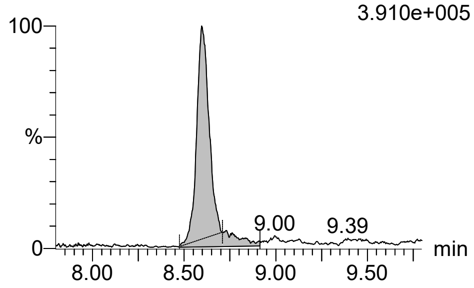

Sample Name: 20211111_L2_R3_IgM
F77:MRM of 1 channel,ES+
1117.1 > 366.1
5.761e+005

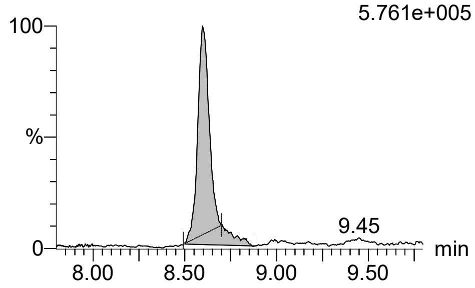

Sample Name: 20211111_H1_R3_IgM
F77:MRM of 1 channel,ES+
1117.1 > 366.1
6.684e+005

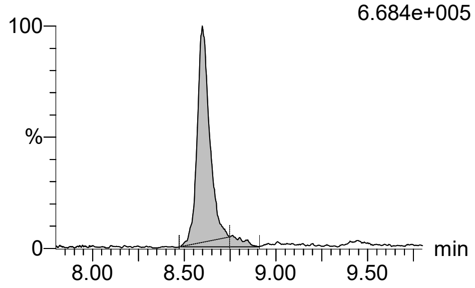

Sample Name: 20211111_A3_R3_IgM
F77:MRM of 1 channel,ES+
1117.1 > 366.1
1.113e+006

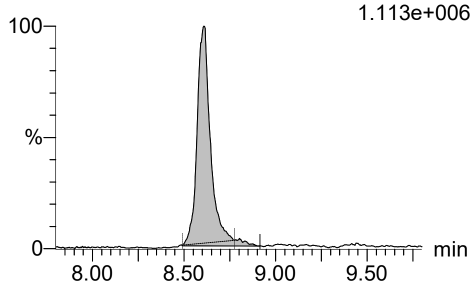

Sample Name: 20211111_A4_R3_IgM
F77:MRM of 1 channel,ES+
1117.1 > 366.1
3.400e+005

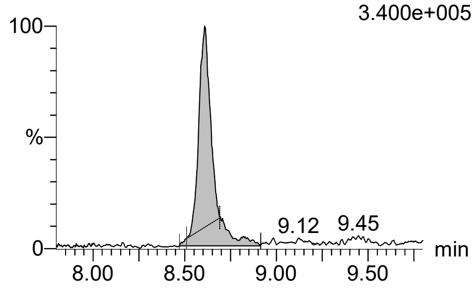

Sample Name: 20211111_L3_R3_IgM
F77:MRM of 1 channel,ES+
1117.1 > 366.1
2.128e+005

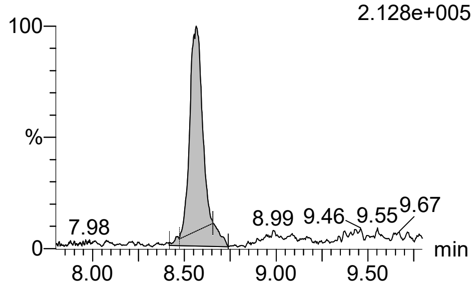

Sample Name: 20211111_L4_R3_IgM
F77:MRM of 1 channel,ES+
1117.1 > 366.1
3.999e+005

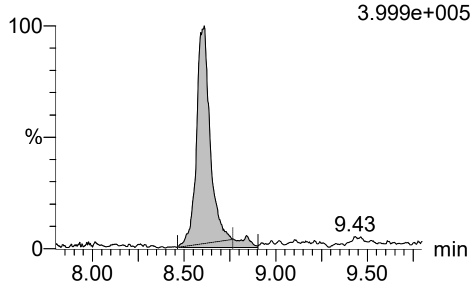

Sample Name: 20211111_H2_R3_IgM
F77:MRM of 1 channel,ES+
1117.1 > 366.1
1.635e+006

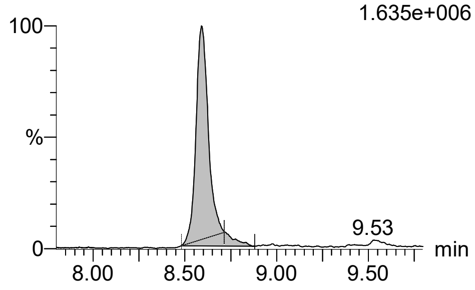

Sample Name: 20211111_A5_R3_IgM
F77:MRM of 1 channel,ES+
1117.1 > 366.1
1.695e+006

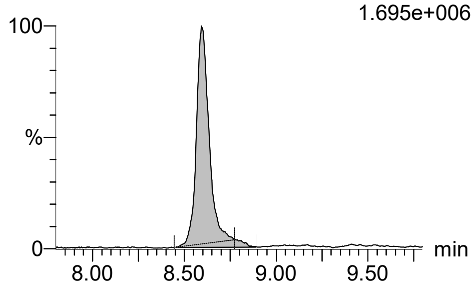

Sample Name: 20211111_A6_R3_IgM
F77:MRM of 1 channel,ES+
1117.1 > 366.1
3.259e+005

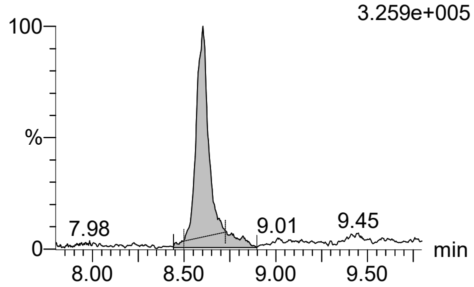

Sample Name: 20211111_QC_R18_IgM
F77:MRM of 1 channel,ES+
1117.1 > 366.1
3.797e+005

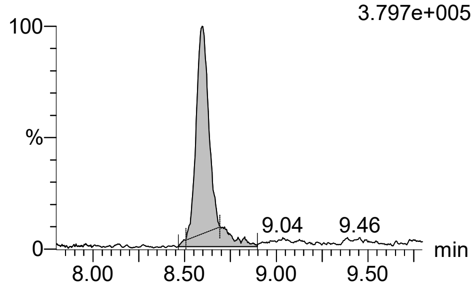

Sample Name: 20211111_L5_R3_IgM
F77:MRM of 1 channel,ES+
1117.1 > 366.1
3.439e+005

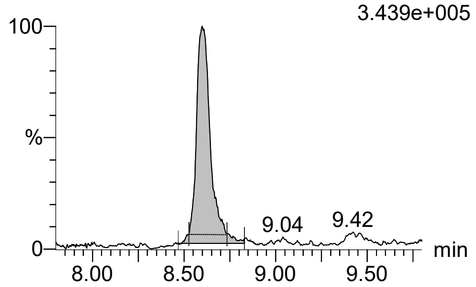

Sample Name: 20211111_L6_R3_IgM
F77:MRM of 1 channel,ES+
1117.1 > 366.1
3.294e+005

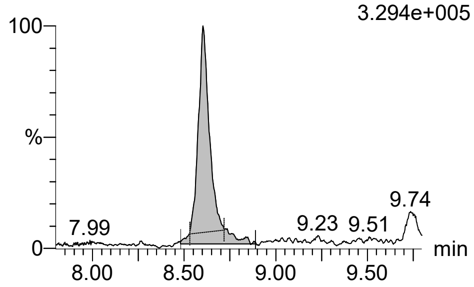

Sample Name: 20211111_H3_R3_IgM
F77:MRM of 1 channel,ES+
1117.1 > 366.1
2.527e+005

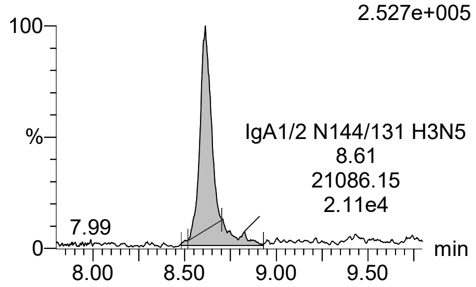

Sample Name: 20211111_A7_R3_IgM
F77:MRM of 1 channel,ES+
1117.1 > 366.1
7.263e+005

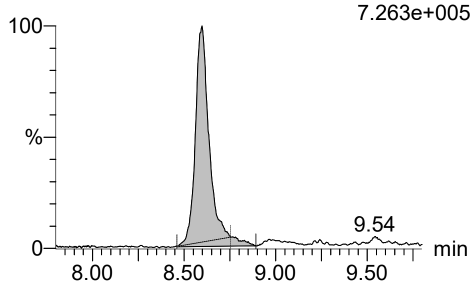

Sample Name: 20211111_A8_R3_IgM
F77:MRM of 1 channel,ES+
1117.1 > 366.1
9.232e+005

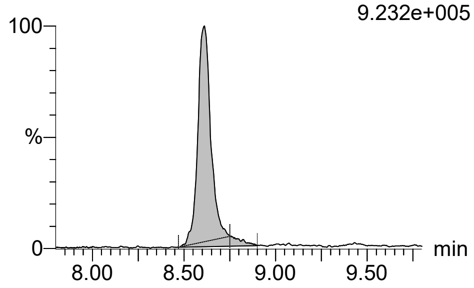

Sample Name: 20211111_L7_R3_IgM
F77:MRM of 1 channel,ES+
1117.1 > 366.1
1.829e+005

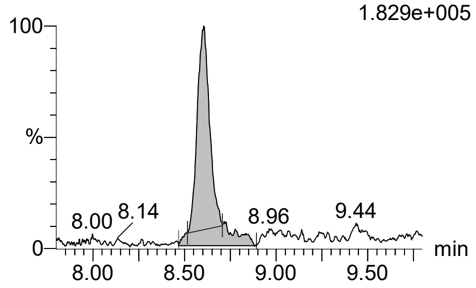

Sample Name: 20211111_L8_R3_IgM
F77:MRM of 1 channel,ES+
1117.1 > 366.1
8.297e+004

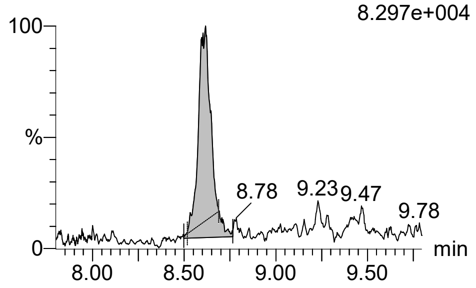

Sample Name: 20211111_H4_R3_IgM
F77:MRM of 1 channel,ES+
1117.1 > 366.1
1.918e+005

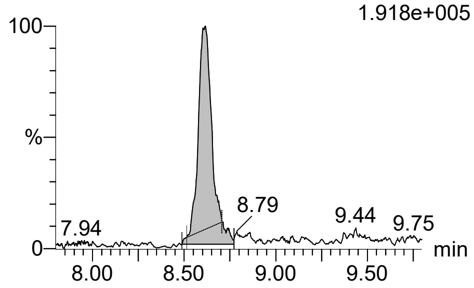

Sample Name: 20211111_A9_R3_IgM
F77:MRM of 1 channel,ES+
1117.1 > 366.1
3.634e+005

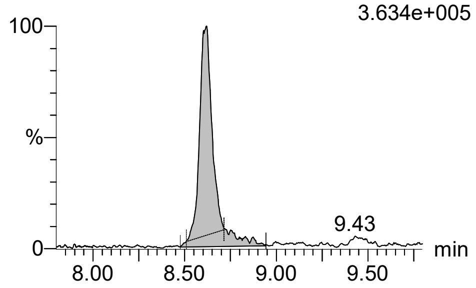

Sample Name: 20211111_A10_R3_IgM
F77:MRM of 1 channel,ES+
1117.1 > 366.1
9.435e+005

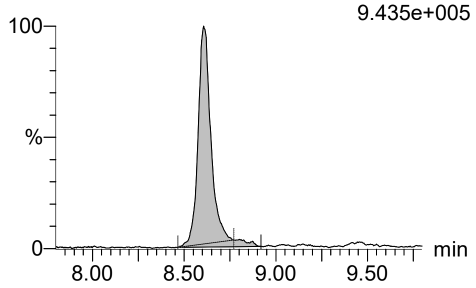

Sample Name: 20211111_L9_R3_IgM
F77:MRM of 1 channel,ES+
1117.1 > 366.1
3.728e+005

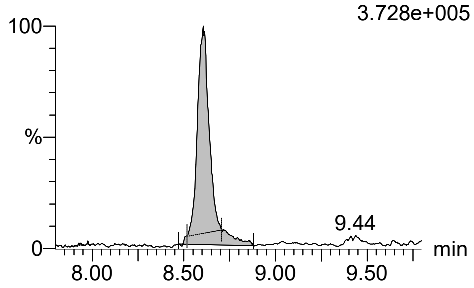

Sample Name: 20211111_L10_R3_IgM
F77:MRM of 1 channel,ES+
1117.1 > 366.1
8.313e+005

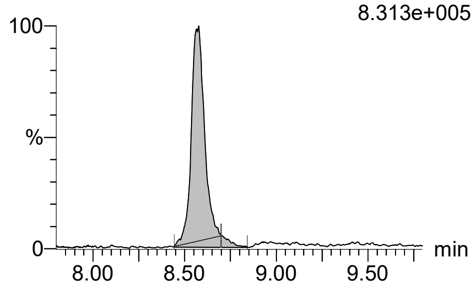

Sample Name: 20211111_QC_R19_IgM
F77:MRM of 1 channel,ES+
1117.1 > 366.1
4.158e+005

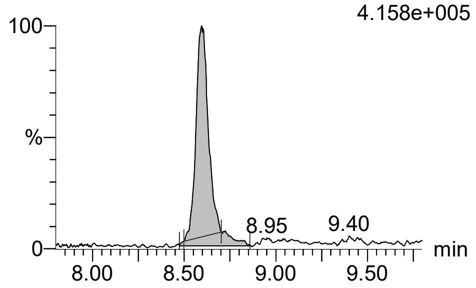

Sample Name: 20211111_H5_R3_IgM
F77:MRM of 1 channel,ES+
1117.1 > 366.1
1.749e+005

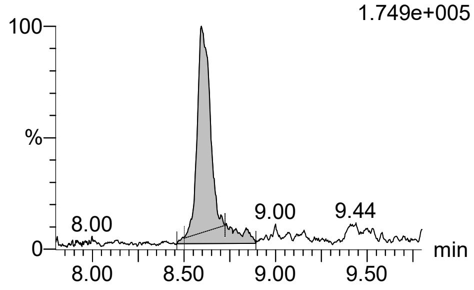

Sample Name: 20211111_A11_R3_IgM
F77:MRM of 1 channel,ES+
1117.1 > 366.1
7.156e+005

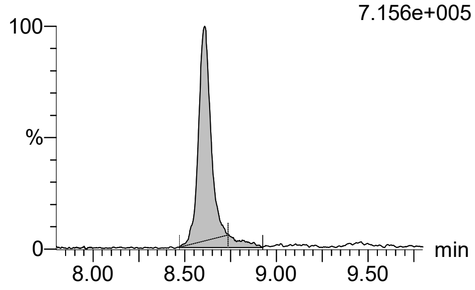

Sample Name: 20211111_A12_R3_IgM
F77:MRM of 1 channel,ES+
1117.1 > 366.1
2.538e+005

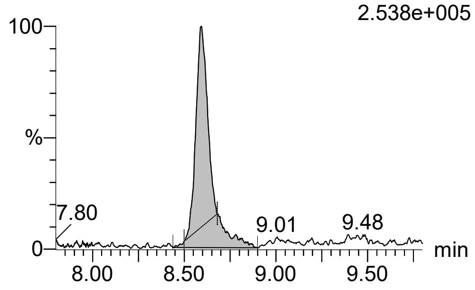

Sample Name: 20211111_L11_R3_IgM
F77:MRM of 1 channel,ES+
1117.1 > 366.1
2.626e+005

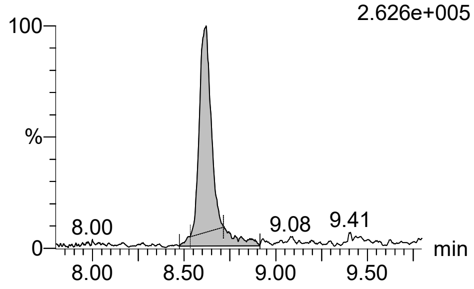

Sample Name: 20211111_L12_R3_IgM
F77:MRM of 1 channel,ES+
1117.1 > 366.1
2.350e+005

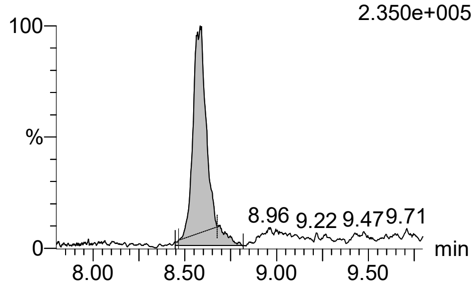

Sample Name: 20211111_H6_R3_IgM
F77:MRM of 1 channel,ES+
1117.1 > 366.1
3.106e+005

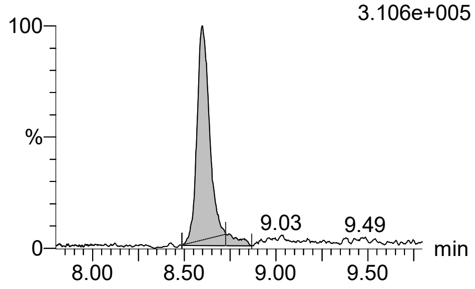

Sample Name: 20211111_A13_R3_IgM
F77:MRM of 1 channel,ES+
1117.1 > 366.1
2.620e+005

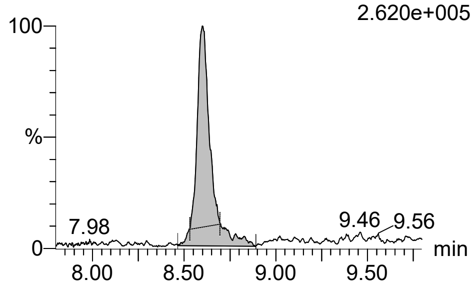

Sample Name: 20211111_A14_R3_IgM
F77:MRM of 1 channel,ES+
1117.1 > 366.1
1.828e+006

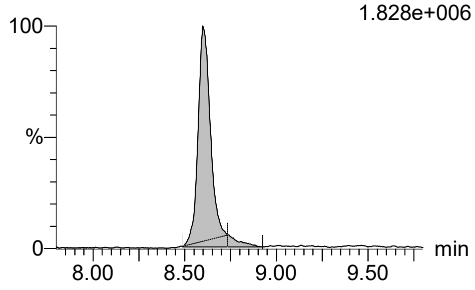

Sample Name: 20211111_L13_R3_IgM
F77:MRM of 1 channel,ES+
1117.1 > 366.1
1.117e+005

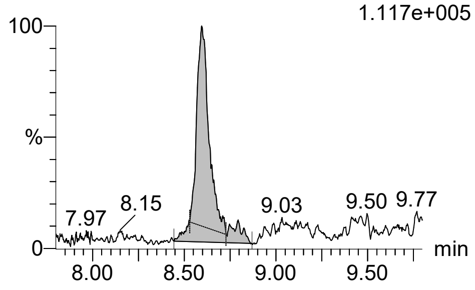

Sample Name: 20211111_L14_R3_IgM
F77:MRM of 1 channel,ES+
1117.1 > 366.1
2.423e+005

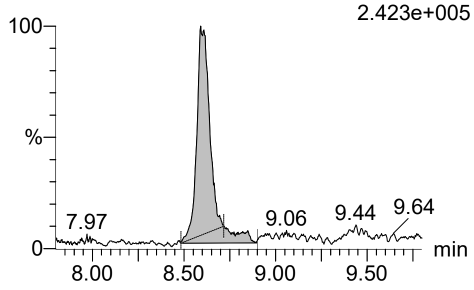

Sample Name: 20211111_H7_R3_IgM
F77:MRM of 1 channel,ES+
1117.1 > 366.1
3.416e+005

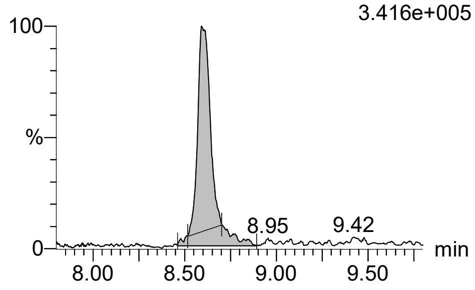

Sample Name: 20211111_A15_R3_IgM
F77:MRM of 1 channel,ES+
1117.1 > 366.1
2.006e+006

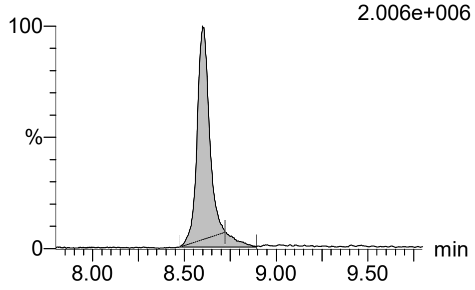

Sample Name: 20211111_QC_R20_IgM
F77:MRM of 1 channel,ES+
1117.1 > 366.1
4.740e+005

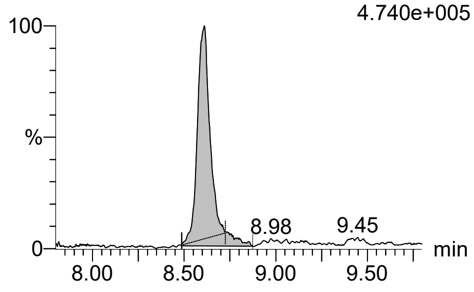

Sample Name: 20211111_A16_R3_IgM
F77:MRM of 1 channel,ES+
1117.1 > 366.1
9.736e+005

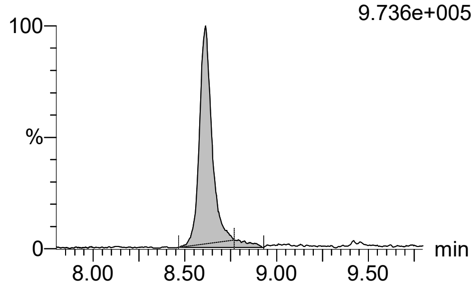

Sample Name: 20211111_L15_R3_IgM
F77:MRM of 1 channel,ES+
1117.1 > 366.1
7.110e+005

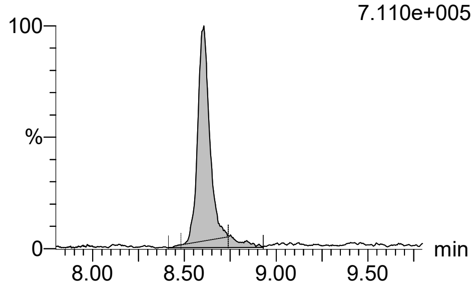

Dataset: C:\MassLynx\10.pro\20211111_clinicalsamples_IgM_IgA.qld

Last Altered: Thursday, March 09, 2023 19:58:25 台北標準時間
Printed: Friday, August 01, 2025 15:00:43 台北標準時間

Sample Name: 20211111_L16_R3_IgM
F77:MRM of 1 channel,ES+
1117.1 > 366.1
1.430e+005

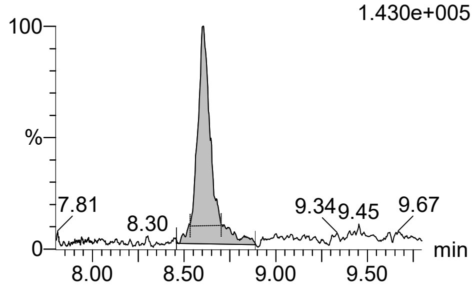

Sample Name: 20211111_H8_R3_IgM
F77:MRM of 1 channel,ES+
1117.1 > 366.1
1.704e+005

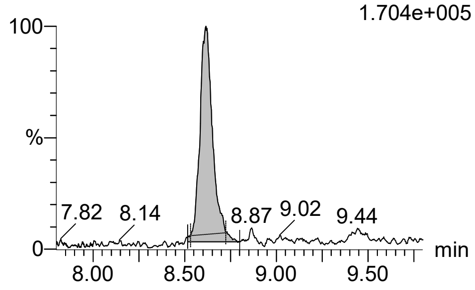

Sample Name: 20211111_A17_R3_IgM
F77:MRM of 1 channel,ES+
1117.1 > 366.1
5.238e+005

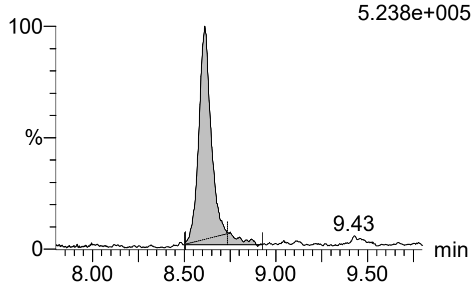

Sample Name: 20211111_A18_R3_IgM
F77:MRM of 1 channel,ES+
1117.1 > 366.1
3.726e+005

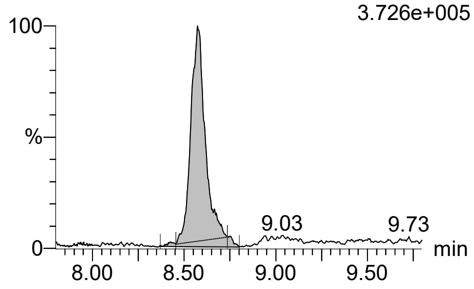

Sample Name: 20211111_L17_R3_IgM
F77:MRM of 1 channel,ES+
1117.1 > 366.1
4.752e+005

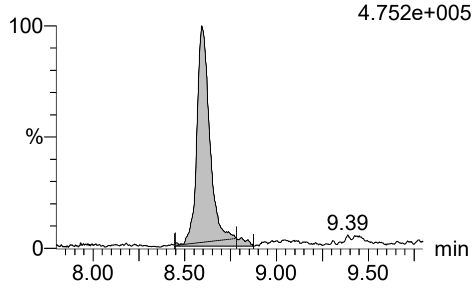

Sample Name: 20211111_L18_R3_IgM
F77:MRM of 1 channel,ES+
1117.1 > 366.1
1.213e+005

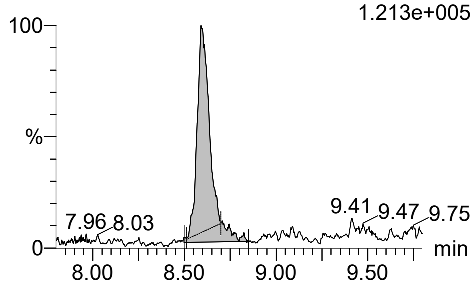

Sample Name: 20211111_H9_R3_IgM
F77:MRM of 1 channel,ES+
1117.1 > 366.1
2.586e+005

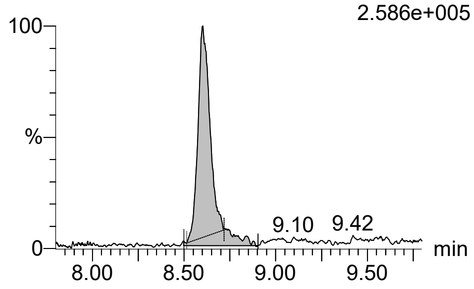

Sample Name: 20211111_A19_R3_IgM
F77:MRM of 1 channel,ES+
1117.1 > 366.1
4.619e+005

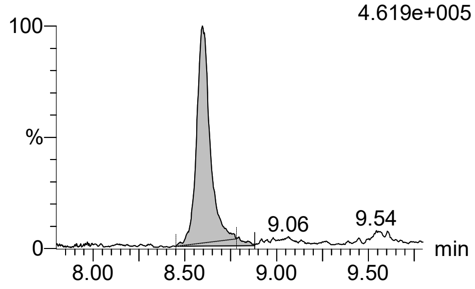

Sample Name: 20211111_A20_R3_IgM
F77:MRM of 1 channel,ES+
1117.1 > 366.1
3.889e+005

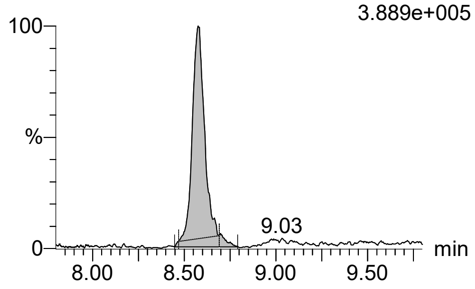

Sample Name: 20211111_L19_R3_IgM
F77:MRM of 1 channel,ES+
1117.1 > 366.1
4.106e+005

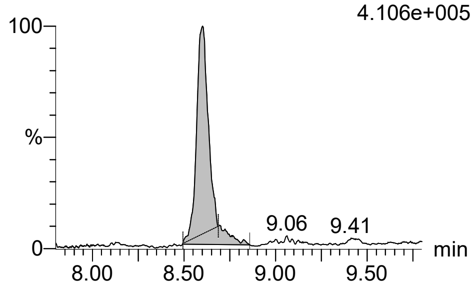

Sample Name: 20211111_QC_R21_IgM
F77:MRM of 1 channel,ES+
1117.1 > 366.1
4.798e+005

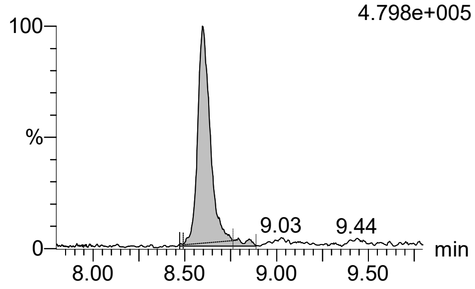

Sample Name: 20211111_L20_R3_IgM
F77:MRM of 1 channel,ES+
1117.1 > 366.1
1.952e+005

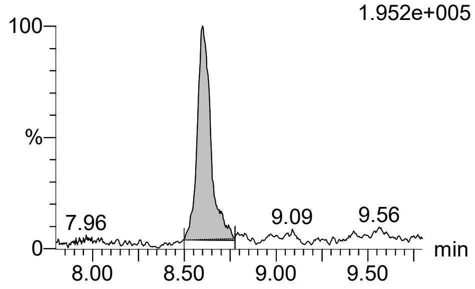

Sample Name: 20211111_H10_R3_IgM
F77:MRM of 1 channel,ES+
1117.1 > 366.1
3.716e+005

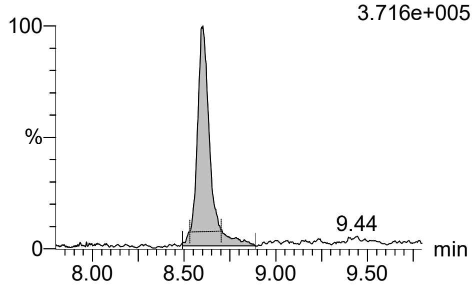

Sample Name: 20211111_A21_R3_IgM
F77:MRM of 1 channel,ES+
1117.1 > 366.1
4.190e+005

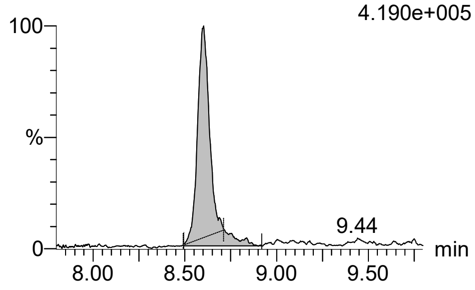

Sample Name: 20211111_A22_R3_IgM
F77:MRM of 1 channel,ES+
1117.1 > 366.1
7.178e+004

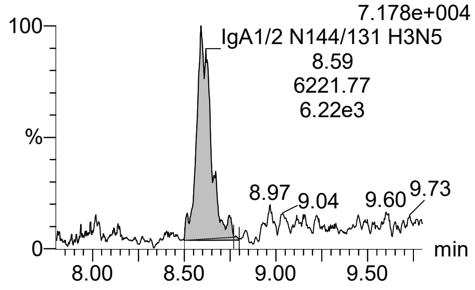

Sample Name: 20211111_L21_R3_IgM
F77:MRM of 1 channel,ES+
1117.1 > 366.1
2.276e+005

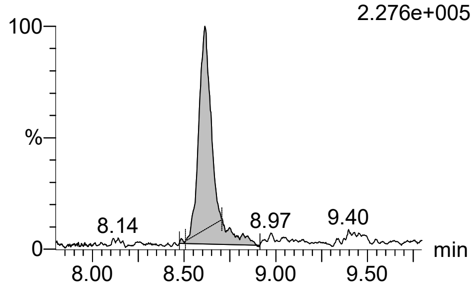

Sample Name: 20211111_L22_R3_IgM
F77:MRM of 1 channel,ES+
1117.1 > 366.1
2.289e+005

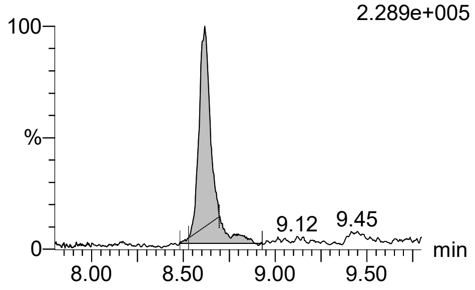

Sample Name: 20211111_H11_R3_IgM
F77:MRM of 1 channel,ES+
1117.1 > 366.1
4.693e+005

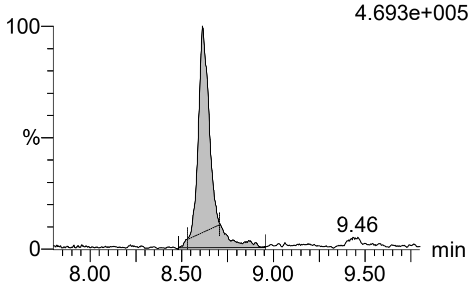

Sample Name: 20211111_A23_R3_IgM
F77:MRM of 1 channel,ES+
1117.1 > 366.1
3.592e+005

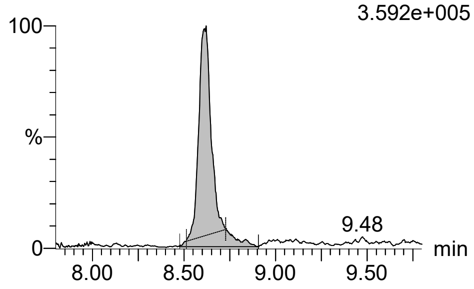

Sample Name: 20211111_A24_R3_IgM
F77:MRM of 1 channel,ES+
1117.1 > 366.1
5.668e+005

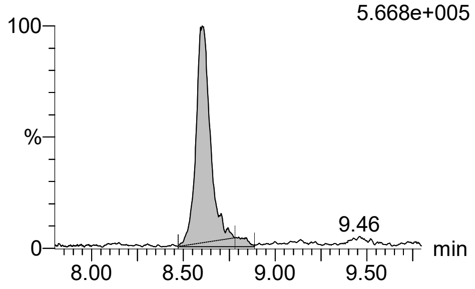

Sample Name: 20211111_L23_R3_IgM
F77:MRM of 1 channel,ES+
1117.1 > 366.1
4.654e+005

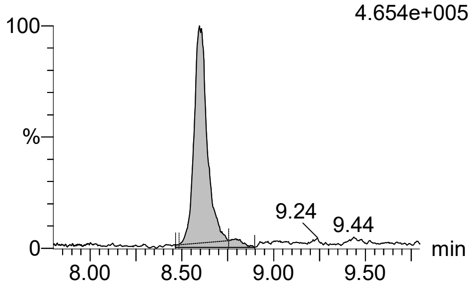

Sample Name: 20211111_L24_R3_IgM
F77:MRM of 1 channel,ES+
1117.1 > 366.1
7.929e+004

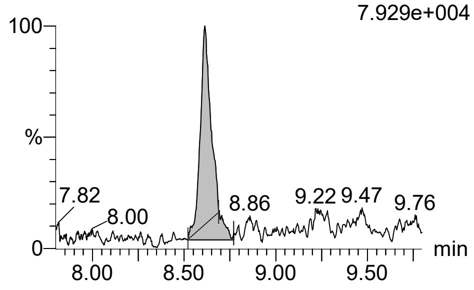

Sample Name: 20211111_H12_R3_IgM
F77:MRM of 1 channel,ES+
1117.1 > 366.1
9.337e+004

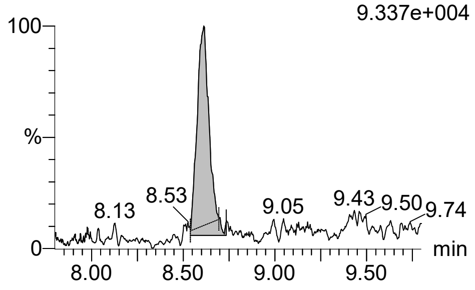

Sample Name: 20211111_QC_R22_IgM
F77:MRM of 1 channel,ES+
1117.1 > 366.1
4.613e+005

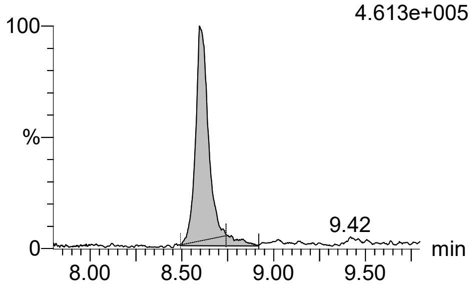

Sample Name: 20211111_A25_R3_IgM
F77:MRM of 1 channel,ES+
1117.1 > 366.1
1.265e+006

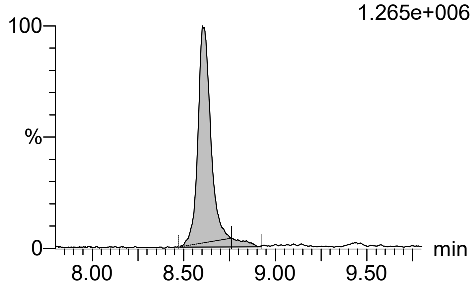

Sample Name: 20211111_A26_R3_IgM
F77:MRM of 1 channel,ES+
1117.1 > 366.1
3.590e+005

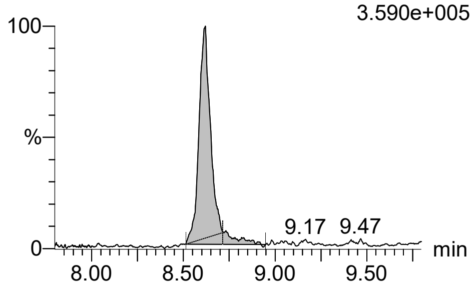

Sample Name: 20211111_L25_R3_IgM
F77:MRM of 1 channel,ES+
1117.1 > 366.1
2.827e+005

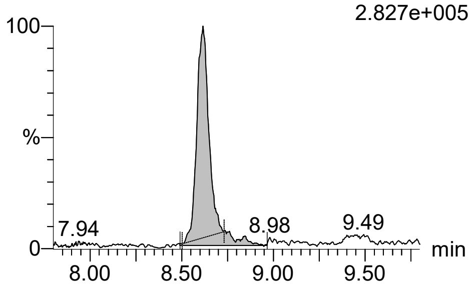

Sample Name: 20211111_L26_R3_IgM
F77:MRM of 1 channel,ES+
1117.1 > 366.1
2.283e+005

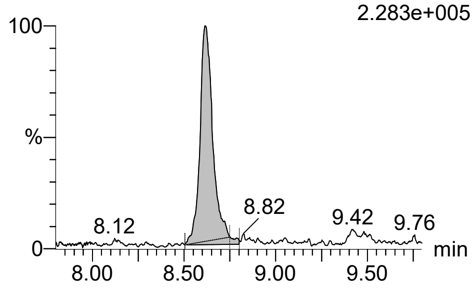

Sample Name: 20211111_H13_R3_IgM
F77:MRM of 1 channel,ES+
1117.1 > 366.1
6.888e+005

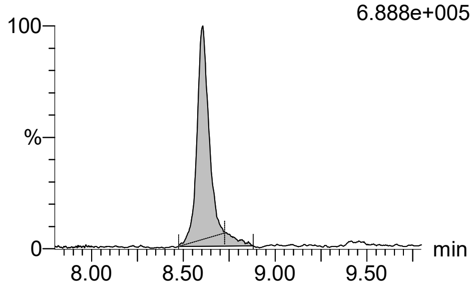

Sample Name: 20211111_A27_R3_IgM
F77:MRM of 1 channel,ES+
1117.1 > 366.1
3.396e+005

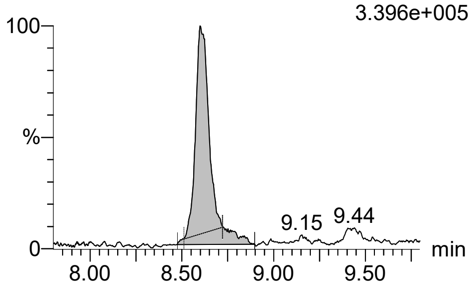

Dataset: C:\MassLynx\10.pro\20211111_clinicalsamples_IgM_IgA.qld

Last Altered: Thursday, March 09, 2023 19:58:25 台北標準時間
Printed: Friday, August 01, 2025 15:00:43 台北標準時間

Sample Name: 20211111_A28_R3_IgM
F77:MRM of 1 channel,ES+
1117.1 > 366.1
1.600e+005

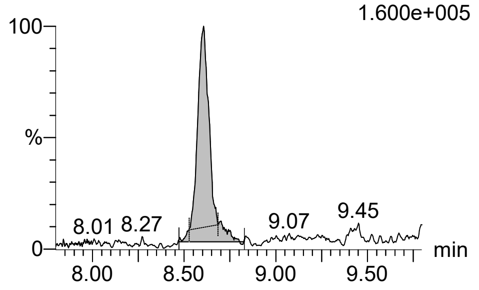

Sample Name: 20211111_L27_R3_IgM
F77:MRM of 1 channel,ES+
1117.1 > 366.1
4.643e+005

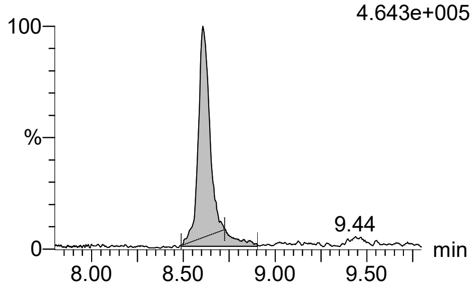

Sample Name: 20211111_L28_R3_IgM
F77:MRM of 1 channel,ES+
1117.1 > 366.1
1.093e+005

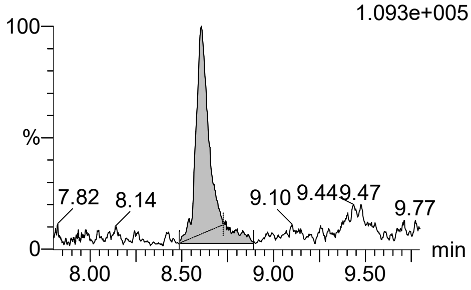

Sample Name: 20211111_H14_R3_IgM
F77:MRM of 1 channel,ES+
1117.1 > 366.1
3.899e+005

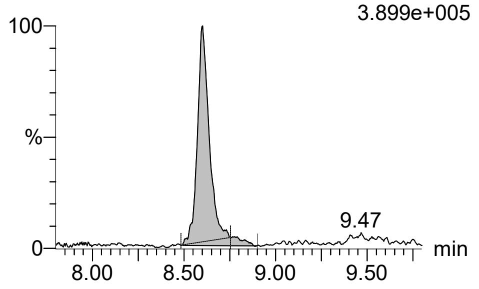

Sample Name: 20211111_A29_R3_IgM
F77:MRM of 1 channel,ES+
1117.1 > 366.1
1.211e+005

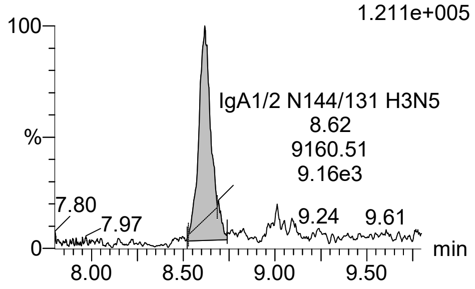

Sample Name: 20211111_A30_R3_IgM
F77:MRM of 1 channel,ES+
1117.1 > 366.1
1.414e+006

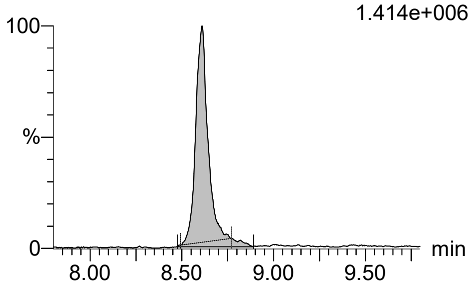

Sample Name: 20211111_QC_R23_IgM
F77:MRM of 1 channel,ES+
1117.1 > 366.1
4.235e+005

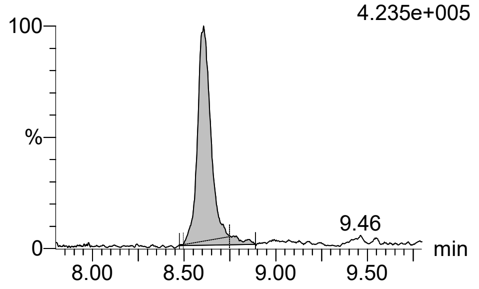

Sample Name: 20211111_L29_R3_IgM
F77:MRM of 1 channel,ES+
1117.1 > 366.1
1.250e+005

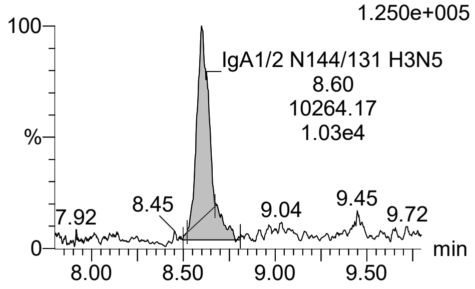

Sample Name: 20211111_L30_R3_IgM
F77:MRM of 1 channel,ES+
1117.1 > 366.1
1.234e+005

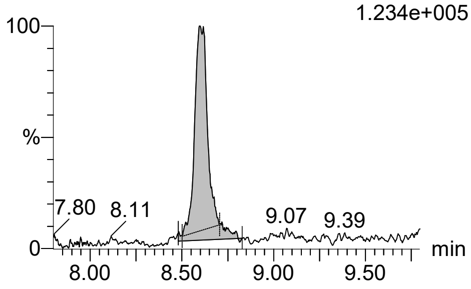

Sample Name: 20211111_H15_R3_IgM
F77:MRM of 1 channel,ES+
1117.1 > 366.1
2.393e+005

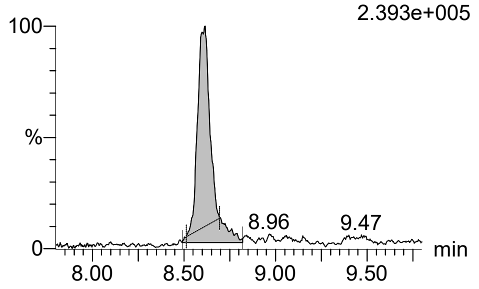

Sample Name: 20211111_A31_R3_IgM
F77:MRM of 1 channel,ES+
1117.1 > 366.1
3.256e+005

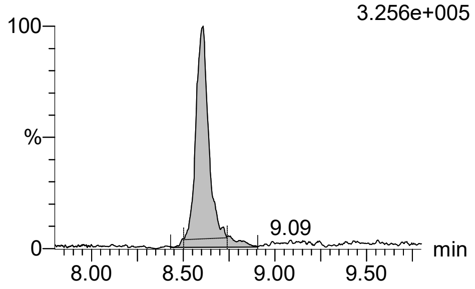

Sample Name: 20211111_A32_R3_IgM
F77:MRM of 1 channel,ES+
1117.1 > 366.1
5.923e+005

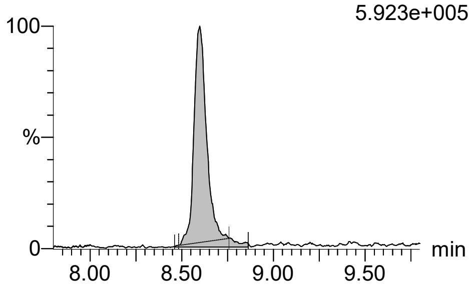

Sample Name: 20211111_L31_R3_IgM
F77:MRM of 1 channel,ES+
1117.1 > 366.1
8.477e+004

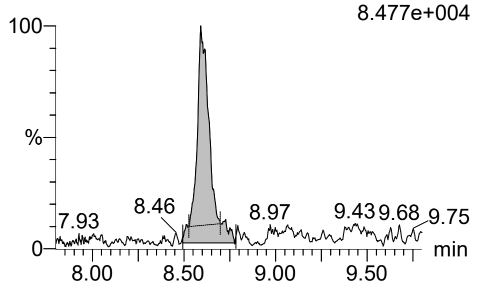

Sample Name: 20211111_L32_R3_IgM
F77:MRM of 1 channel,ES+
1117.1 > 366.1
2.956e+005

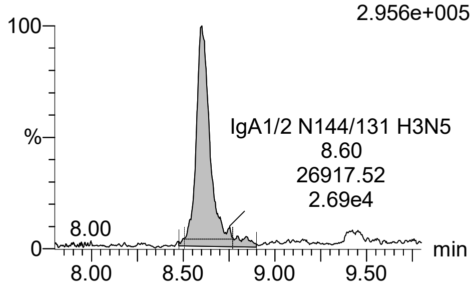

Sample Name: 20211111_H16_R3_IgM
F77:MRM of 1 channel,ES+
1117.1 > 366.1
4.237e+005

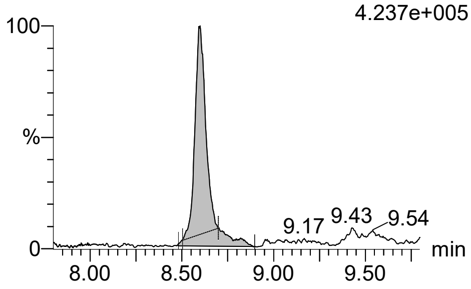

Sample Name: 20211111_A33_R3_IgM

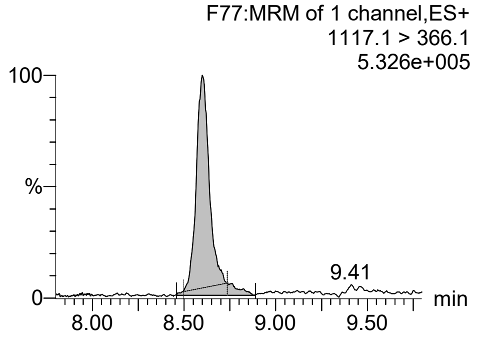

Sample Name: 20211111_A34_R3_IgM

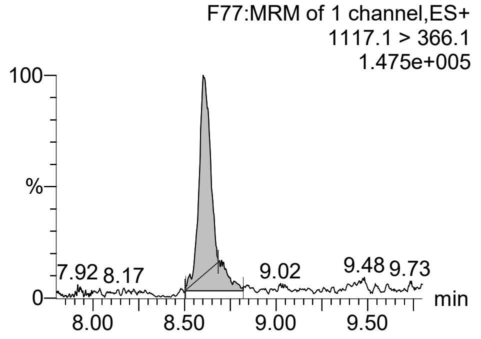

Sample Name: 20211111_H17_R3_IgM

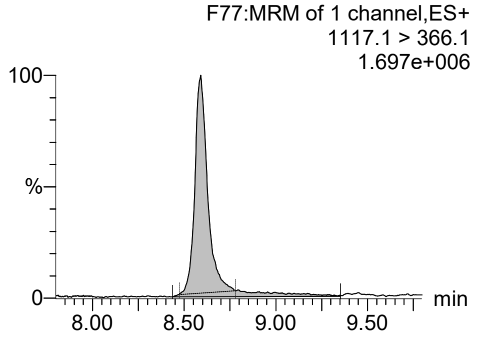

Sample Name: 20211111_A35_R3_IgM

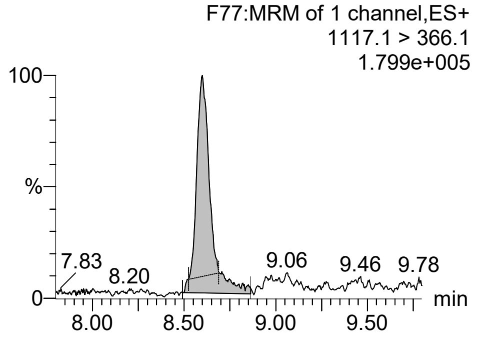

Sample Name: 20211111_QC_R24_IgM

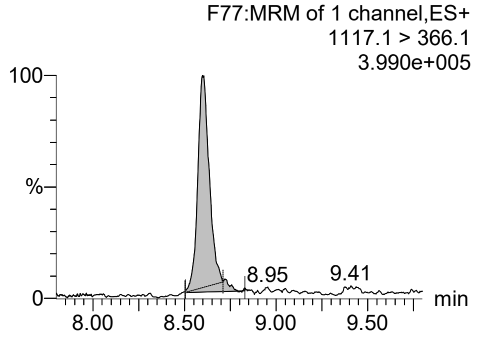

Sample Name: 20211111_A36_R3_IgM

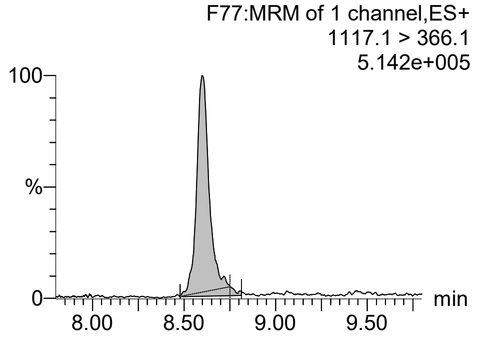

Sample Name: 20211111_H18_R3_IgM

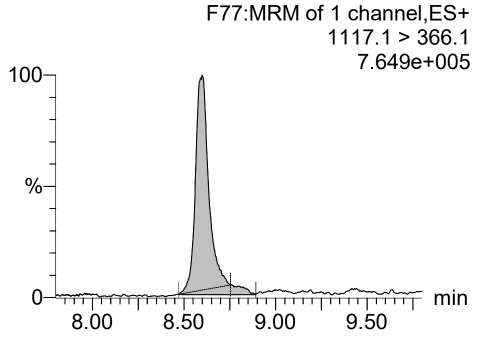

Sample Name: 20211111_A37_R3_IgM

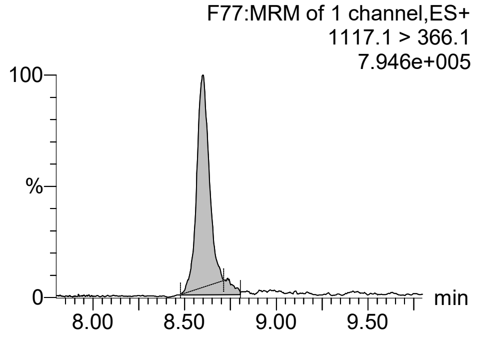

Sample Name: 20211111_A38_R3_IgM

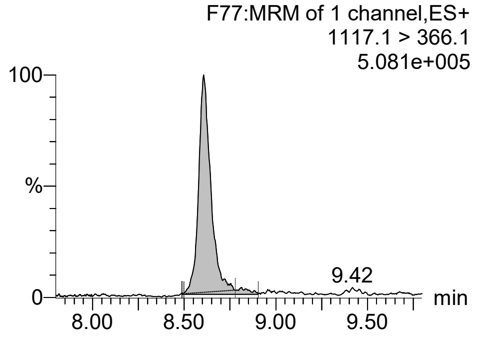

Sample Name: 20211111_H19_R3_IgM

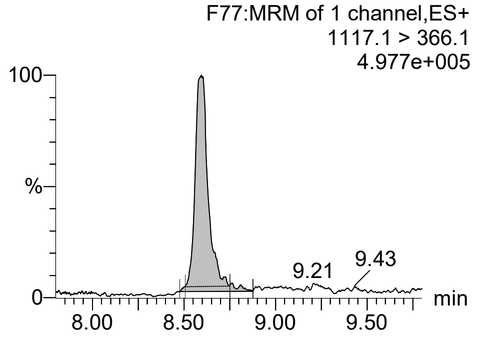

Sample Name: 20211111_A39_R3_IgM

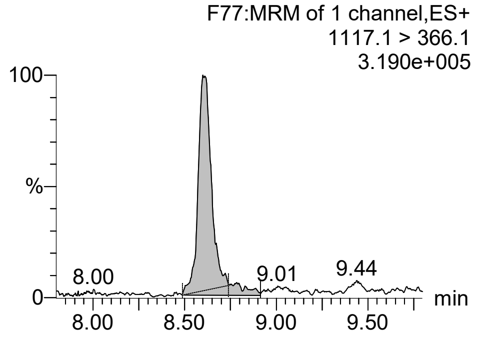

Sample Name: 20211111_A40_R3_IgM

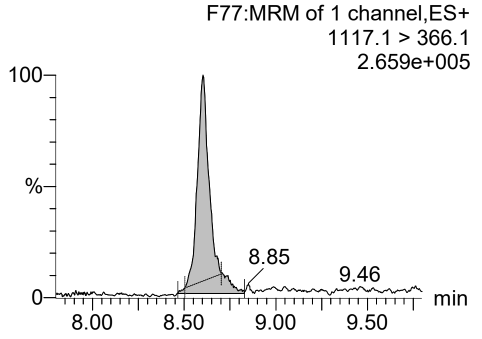

Sample Name: 20211111_H20_R3_IgM

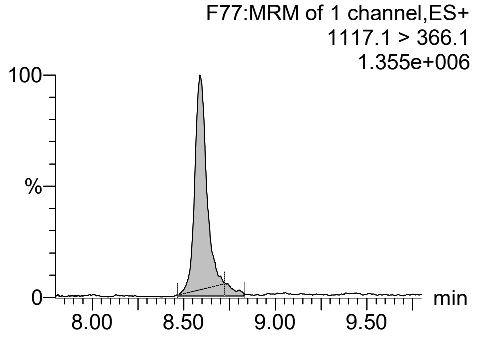

Sample Name: 20211111_A41_R3_IgM

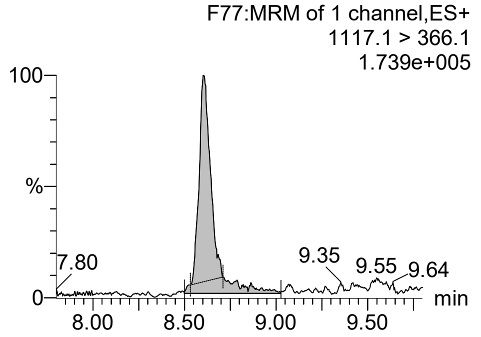

Sample Name: 20211111_A42_R3_IgM

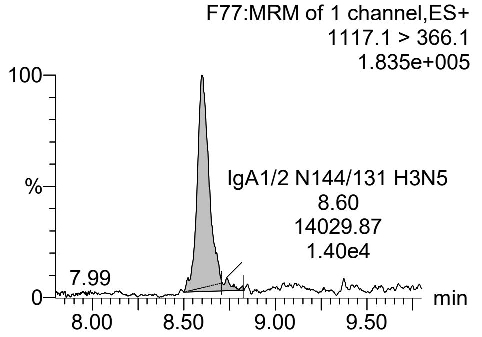

Dataset: C:\MassLynx\10.pro\20211111_clinicalsamples_IgM_IgA.qld

Last Altered: Thursday, March 09, 2023 19:58:25 台北標準時間
Printed: Friday, August 01, 2025 15:00:43 台北標準時間

Sample Name: 20211111_A43_R3_IgM

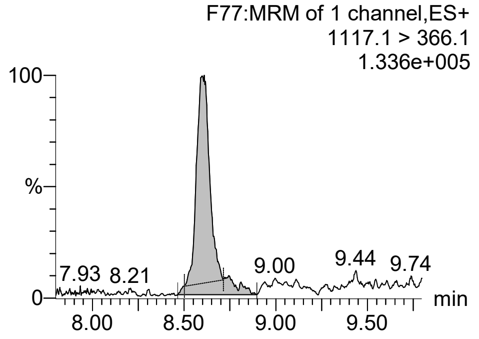

Sample Name: 20211111_A44_R3_IgM

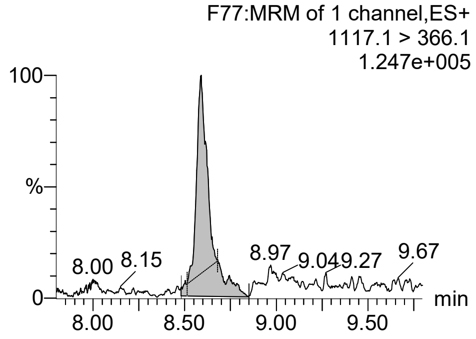

Sample Name: 20211111_QC_R25_IgM

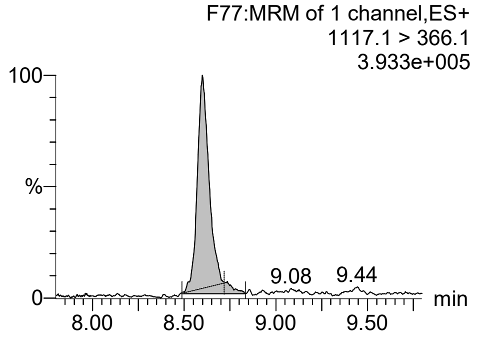

Dataset: C:\MassLynx\10.pro\20211111_clinicalsamples_IgM_IgA.qld

Last Altered: Thursday, March 09, 2023 19:58:25 台北標準時間
Printed: Friday, August 01, 2025 15:00:43 台北標準時間

	# Name	RT	Area	IS Area	Response	Prima...
1	1 20211112_QC_R001_IgM	8.58	40760.6	15421.4	2.643	MM
2	2 20211112_QC_R002_IgM	8.59	38128.3	13188.8	2.891	MM
3	3 20211111_A1_R2_IgM	8.61	35490.2	11871.0	2.990	MM
4	4 20211111_A1_R3_IgM	8.60	33711.1	12030.5	2.802	MM
5	5 20211111_A2_R2_IgM	8.60	79768.6	11446.8	6.969	MM
6	6 20211111_A2_R3_IgM	8.59	75161.2	11098.4	6.772	MM
7	7 20211111_L1_R2_IgM	8.60	17661.3	11779.4	1.499	MM
8	8 20211111_L1_R3_IgM	8.62	14858.8	11176.2	1.330	MM
9	9 20211111_L2_R2_IgM	8.59	48422.5	11375.0	4.257	MM
10	10 20211111_H1_R2_IgM	8.59	61060.7	13595.2	4.491	MM
11	11 20211111_A3_R2_IgM	8.58	112868.8	14112.3	7.998	MM
12	12 20211111_A4_R2_IgM	8.60	32984.4	11977.5	2.754	MM
13	13 20211111_L3_R2_IgM	8.54	19795.3	12242.1	1.617	MM
14	14 20211111_L4_R2_IgM	8.59	41608.7	12797.0	3.251	MM
15	15 20211111_H2_R2_IgM	8.60	144481.9	10006.0	14.439	MM
16	16 20211111_A5_R2_IgM	8.58	167704.3	11434.6	14.666	MM
17	17 20211111_A6_R2_IgM	8.59	34429.4	13193.7	2.610	MM
18	18 20211111_QC_R10_IgM	8.58	41365.2	12566.1	3.292	MM
19	19 20211111_L5_R2_IgM	8.56	39848.7	13616.4	2.927	MM
20	20 20211111_L6_R2_IgM	8.59	31857.7	13698.5	2.326	MM
21	21 20211111_H3_R2_IgM	8.59	26169.0	15793.1	1.657	MM
22	22 20211111_A7_R2_IgM	8.58	66321.1	12936.9	5.127	MM
23	23 20211111_A8_R2_IgM	8.59	102439.2	13108.0	7.815	MM
24	24 20211111_L7_R2_IgM	8.61	15996.0	13278.5	1.205	MM
25	25 20211111_L8_R2_IgM	8.62	8210.9	15233.6	0.539	MM
26	26 20211111_H4_R2_IgM	8.60	22515.0	16521.9	1.363	MM
27	27 20211111_A9_R2_IgM	8.58	38768.7	16451.6	2.357	MM
28	28 20211111_A10_R2_IgM	8.59	88971.1	13036.0	6.825	MM
29	29 20211111_L9_R2_IgM	8.57	35367.6	14554.1	2.430	MM
30	30 20211111_L10_R2_IgM	8.58	88967.1	12250.4	7.262	MM
31	31 20211111_QC_R11_IgM	8.60	44605.9	11459.2	3.893	MM
32	32 20211111_H5_R2_IgM	8.59	25817.1	14358.2	1.798	MM
33	33 20211111_A11_R2_IgM	8.53	82573.7	16184.1	5.102	MM
34	34 20211111_A12_R2_IgM	8.60	24330.8	13465.4	1.807	MM
35	35 20211111_L11_R2_IgM	8.61	30145.7	13277.9	2.270	MM
36	36 20211111_L12_R2_IgM	8.58	25705.7	15958.8	1.611	MM
37	37 20211111_H6_R2_IgM	8.59	28246.7	14003.5	2.017	MM
38	38 20211111_A13_R2_IgM	8.60	24671.8	13656.1	1.807	MM
39	39 20211111_A14_R2_IgM	8.59	192983.1	14071.3	13.715	MM
40	40 20211111_L13_R2_IgM	8.60	10972.5	14785.6	0.742	MM
41	41 20211111_L14_R2_IgM	8.61	27259.3	14772.2	1.845	MM
42	42 20211111_H7_R2_IgM	8.57	38782.6	15164.4	2.557	MM
43	43 20211111_A15_R2_IgM	8.60	210990.8	15721.5	13.421	MM
44	44 20211111_QC_R12_IgM	8.60	48237.7	13711.5	3.518	MM
45	45 20211111_A16_R2_IgM	8.61	99732.3	14275.6	6.986	MM
46	46 20211111_L15_R2_IgM	8.60	66897.1	12935.8	5.171	MM
47	47 20211111_L16_R2_IgM	8.60	13658.5	13342.8	1.024	MM
48	48 20211111_H8_R2_IgM	8.62	15445.5	15835.1	0.975	MM
49	49 20211111_A17_R2_IgM	8.61	53142.4	15786.7	3.366	MM
50	50 20211111_A18_R2_IgM	8.59	41099.4	16986.8	2.419	MM
51	51 20211111_L17_R2_IgM	8.59	48112.1	13342.7	3.606	MM
52	52 20211111_L18_R2_IgM	8.59	11486.8	14692.0	0.782	MM
53	53 20211111_H9_R2_IgM	8.59	25883.9	15639.7	1.655	MM
54	54 20211111_A19_R2_IgM	8.59	44157.8	14198.7	3.110	MM
55	55 20211111_A20_R2_IgM	8.57	35549.9	14162.7	2.510	MM
56	56 20211111_L19_R2_IgM	8.59	33650.5	9293.4	3.621	MM

	# Name	RT	Area	IS Area	Response	Prima...
57	57 20211111_QC_R13_IgM	8.60	48215.7	13903.0	3.468	MM
58	58 20211111_L20_R2_IgM	8.55	21702.9	12781.6	1.698	MM
59	59 20211111_H10_R2_IgM	8.58	33806.8	14796.4	2.285	MM
60	60 20211111_A21_R2_IgM	8.60	38405.9	13669.0	2.810	MM
61	61 20211111_A22_R2_IgM	8.59	7313.0	14098.3	0.519	MM
62	62 20211111_L21_R2_IgM	8.62	23588.1	15678.2	1.505	MM
63	63 20211111_L22_R2_IgM	8.62	21740.9	15724.5	1.383	MM
64	64 20211111_H11_R2_IgM	8.61	43461.0	15890.2	2.735	MM
65	65 20211111_A23_R2_IgM	8.60	33351.4	13423.2	2.485	MM
66	66 20211111_A24_R2_IgM	8.60	54396.7	12438.0	4.373	MM
67	67 20211111_L23_R2_IgM	8.60	45494.4	14075.0	3.232	MM
68	68 20211111_L24_R2_IgM	8.59	7579.3	15972.1	0.475	MM
69	69 20211111_H12_R2_IgM	8.60	8950.3	16270.9	0.550	MM
70	70 20211111_QC_R14_IgM	8.61	50055.9	15806.8	3.167	MM
71	71 20211111_A25_R2_IgM	8.60	138413.9	14552.1	9.512	MM
72	72 20211111_A26_R2_IgM	8.62	30946.8	11492.7	2.693	MM
73	73 20211111_L25_R2_IgM	8.61	27715.0	14403.3	1.924	MM
74	74 20211111_L26_R2_IgM	8.61	23480.8	16533.9	1.420	MM
75	75 20211111_H13_R2_IgM	8.61	59240.9	15520.1	3.817	MM
76	76 20211111_A27_R2_IgM	8.60	38337.0	13337.4	2.874	MM
77	77 20211111_A28_R2_IgM	8.54	14451.7	11895.6	1.215	MM
78	78 20211111_L27_R2_IgM	8.60	40996.7	14125.7	2.902	MM
79	79 20211111_L28_R2_IgM	8.60	8016.7	11807.5	0.679	MM
80	80 20211111_H14_R2_IgM	8.59	28713.9	15035.7	1.910	MM
81	81 20211111_A29_R2_IgM	8.59	11755.3	13554.4	0.867	MM
82	82 20211111_A30_R2_IgM	8.60	130829.6	12984.1	10.076	MM
83	83 20211111_QC_R15_IgM	8.60	44804.3	12299.5	3.643	MM
84	84 20211111_L29_R2_IgM	8.61	10866.0	13590.7	0.800	MM
85	85 20211111_L30_R2_IgM	8.60	11416.5	12511.4	0.912	MM
86	86 20211111_H15_R2_IgM	8.60	22060.5	11885.0	1.856	MM
87	87 20211111_A31_R2_IgM	8.59	30271.1	13422.1	2.255	MM
88	88 20211111_A32_R2_IgM	8.59	56839.8	11461.3	4.959	MM
89	89 20211111_L31_R2_IgM	8.55	8381.0	13753.4	0.609	MM
90	90 20211111_L32_R2_IgM	8.59	30049.2	12949.0	2.321	MM
91	91 20211111_H16_R2_IgM	8.59	38823.3	11465.4	3.386	MM
92	92 20211111_A33_R2_IgM	8.60	50547.3	10551.5	4.791	MM
93	93 20211111_A34_R2_IgM	8.61	15688.3	12409.9	1.264	MM
94	94 20211111_H17_R2_IgM	8.58	172426.8	10160.9	16.970	MM
95	95 20211111_A35_R2_IgM	8.57	18701.9	10460.6	1.788	MM
96	96 20211111_QC_R16_IgM	8.59	38927.8	10433.5	3.731	MM
97	97 20211111_A36_R2_IgM	8.52	47994.0	12504.8	3.838	MM
98	98 20211111_H18_R2_IgM	8.59	78512.0	9140.4	8.590	MM
99	99 20211111_A37_R2_IgM	8.60	82361.1	9796.9	8.407	MM
100	1... 20211111_A38_R2_IgM	8.61	45285.8	11224.3	4.035	MM
101	1... 20211111_H19_R2_IgM	8.60	50937.5	11739.2	4.339	MM
102	1... 20211111_A39_R2_IgM	8.61	29685.6	12718.9	2.334	MM
103	1... 20211111_A40_R2_IgM	8.55	24351.7	10006.1	2.434	MM
104	1... 20211111_H20_R2_IgM	8.58	129659.3	7988.2	16.231	MM
105	1... 20211111_A41_R2_IgM	8.61	20374.6	11513.3	1.770	MM
106	1... 20211111_A42_R2_IgM	8.61	14241.9	10238.5	1.391	MM
107	1... 20211111_A43_R2_IgM	8.60	14611.7	11098.9	1.317	MM
108	1... 20211111_A44_R2_IgM	8.57	11394.2	10969.9	1.039	MM
109	1... 20211111_QC_R17_IgM	8.60	35008.3	9700.8	3.609	MM
110	1... 20211111_L2_R3_IgM	8.60	47360.5	8528.0	5.554	MM
111	1... 20211111_H1_R3_IgM	8.60	57629.1	9438.2	6.106	MM
112	1... 20211111_A3_R3_IgM	8.61	96014.5	11598.6	8.278	MM

Dataset: C:\MassLynx\10.pro\20211111_clinicalsamples_IgM_IgA.qld

Last Altered: Thursday, March 09, 2023 19:58:25 台北標準時間
Printed: Friday, August 01, 2025 15:00:43 台北標準時間

	#	Name	RT	Area	IS Area	Response	Prima...
113	1...	20211111_A4_R3_IgM	8.61	29930.5	11364.1	2.634	MM
114	1...	20211111_L3_R3_IgM	8.56	19055.6	10753.4	1.772	MM
115	1...	20211111_L4_R3_IgM	8.61	37054.8	10291.6	3.601	MM
116	1...	20211111_H2_R3_IgM	8.59	138701.8	9315.8	14.889	MM
117	1...	20211111_A5_R3_IgM	8.59	143888.9	9102.2	15.808	MM
118	1...	20211111_A6_R3_IgM	8.60	28919.8	10356.4	2.792	MM
119	1...	20211111_QC_R18_IgM	8.60	34947.7	8398.3	4.161	MM
120	1...	20211111_L5_R3_IgM	8.60	30002.5	9786.7	3.066	MM
121	1...	20211111_L6_R3_IgM	8.60	26025.8	9911.3	2.626	MM
122	1...	20211111_H3_R3_IgM	8.61	21086.1	12155.5	1.735	MM
123	1...	20211111_A7_R3_IgM	8.60	65547.3	11804.8	5.553	MM
124	1...	20211111_A8_R3_IgM	8.61	81445.8	10510.6	7.749	MM
125	1...	20211111_L7_R3_IgM	8.60	17505.8	12177.9	1.438	MM
126	1...	20211111_L8_R3_IgM	8.62	6569.5	11668.6	0.563	MM
127	1...	20211111_H4_R3_IgM	8.62	16965.9	11192.0	1.516	MM
128	1...	20211111_A9_R3_IgM	8.62	33077.5	14077.4	2.350	MM
129	1...	20211111_A10_R3_IgM	8.61	79933.5	11190.4	7.143	MM
130	1...	20211111_L9_R3_IgM	8.61	30554.3	14649.3	2.086	MM
131	1...	20211111_L10_R3_IgM	8.58	74431.9	9361.3	7.951	MM
132	1...	20211111_QC_R19_IgM	8.60	36054.6	10125.5	3.561	MM
133	1...	20211111_H5_R3_IgM	8.59	16515.2	10010.9	1.650	MM
134	1...	20211111_A11_R3_IgM	8.61	62304.2	12697.2	4.907	MM
135	1...	20211111_A12_R3_IgM	8.59	23537.8	13148.5	1.790	MM
136	1...	20211111_L11_R3_IgM	8.62	22879.0	10786.6	2.121	MM
137	1...	20211111_L12_R3_IgM	8.58	21533.6	11681.8	1.843	MM
138	1...	20211111_H6_R3_IgM	8.60	26483.1	12095.5	2.190	MM
139	1...	20211111_A13_R3_IgM	8.60	23067.3	12908.3	1.787	MM
140	1...	20211111_A14_R3_IgM	8.60	154670.1	11322.6	13.660	MM
141	1...	20211111_L13_R3_IgM	8.60	10133.3	11516.1	0.880	MM
142	1...	20211111_L14_R3_IgM	8.59	21751.2	10790.7	2.016	MM
143	1...	20211111_H7_R3_IgM	8.59	31619.6	11572.1	2.732	MM
144	1...	20211111_A15_R3_IgM	8.60	173569.6	12434.4	13.959	MM
145	1...	20211111_QC_R20_IgM	8.61	40709.4	10701.0	3.804	MM
146	1...	20211111_A16_R3_IgM	8.61	81865.5	11452.1	7.149	MM
147	1...	20211111_L15_R3_IgM	8.61	60050.0	10531.1	5.702	MM
148	1...	20211111_L16_R3_IgM	8.60	12478.8	12049.4	1.036	MM
149	1...	20211111_H8_R3_IgM	8.61	13403.9	13434.9	0.998	MM
150	1...	20211111_A17_R3_IgM	8.61	43343.3	12452.0	3.481	MM
151	1...	20211111_A18_R3_IgM	8.57	36119.3	12506.5	2.888	MM
152	1...	20211111_L17_R3_IgM	8.59	40810.3	9948.9	4.102	MM
153	1...	20211111_L18_R3_IgM	8.59	10020.0	13462.1	0.744	MM
154	1...	20211111_H9_R3_IgM	8.60	22312.3	13178.7	1.693	MM
155	1...	20211111_A19_R3_IgM	8.60	41092.9	11298.6	3.637	MM
156	1...	20211111_A20_R3_IgM	8.58	32227.9	11175.6	2.884	MM
157	1...	20211111_L19_R3_IgM	8.60	33571.1	11558.2	2.905	MM
158	1...	20211111_QC_R21_IgM	8.60	39735.9	9977.0	3.983	MM
159	1...	20211111_L20_R3_IgM	8.60	16139.2	13508.1	1.195	MM
160	1...	20211111_H10_R3_IgM	8.60	29490.4	12374.8	2.383	MM
161	1...	20211111_A21_R3_IgM	8.60	34999.9	12443.6	2.813	MM
162	1...	20211111_A22_R3_IgM	8.59	6221.8	11589.3	0.537	MM
163	1...	20211111_L21_R3_IgM	8.61	19757.8	13107.4	1.507	MM
164	1...	20211111_L22_R3_IgM	8.62	18436.1	12021.5	1.534	MM
165	1...	20211111_H11_R3_IgM	8.61	38083.6	12687.8	3.002	MM
166	1...	20211111_A23_R3_IgM	8.62	32022.2	11611.8	2.758	MM
167	1...	20211111_A24_R3_IgM	8.60	55203.4	10476.8	5.269	MM
168	1...	20211111_L23_R3_IgM	8.60	41478.2	11873.1	3.493	MM

Dataset: C:\MassLynx\10.pro\20211111_clinicalsamples_IgM_IgA.qld

Last Altered: Thursday, March 09, 2023 19:58:25 台北標準時間
Printed: Friday, August 01, 2025 15:00:43 台北標準時間

	# Name	RT	Area	IS Area	Response	Prima...
169	1... 20211111_L24_R3_IgM	8.61	5695.0	13468.0	0.423	MM
170	1... 20211111_H12_R3_IgM	8.61	6787.3	14229.1	0.477	MM
171	1... 20211111_QC_R22_IgM	8.60	39737.0	11264.4	3.528	MM
172	1... 20211111_A25_R3_IgM	8.60	109420.2	11775.7	9.292	MM
173	1... 20211111_A26_R3_IgM	8.62	27833.4	10473.9	2.657	MM
174	1... 20211111_L25_R3_IgM	8.61	23872.3	13256.8	1.801	MM
175	1... 20211111_L26_R3_IgM	8.61	18552.7	13565.0	1.368	MM
176	1... 20211111_H13_R3_IgM	8.61	56458.8	13146.0	4.295	MM
177	1... 20211111_A27_R3_IgM	8.60	31824.8	12025.6	2.646	MM
178	1... 20211111_A28_R3_IgM	8.61	12313.2	11229.1	1.097	MM
179	1... 20211111_L27_R3_IgM	8.61	37872.8	14960.0	2.532	MM
180	1... 20211111_L28_R3_IgM	8.61	9741.6	11359.0	0.858	MM
181	1... 20211111_H14_R3_IgM	8.60	30968.4	12830.8	2.414	MM
182	1... 20211111_A29_R3_IgM	8.62	9160.5	13434.3	0.682	MM
183	1... 20211111_A30_R3_IgM	8.61	117290.4	11272.2	10.405	MM
184	1... 20211111_QC_R23_IgM	8.61	37088.5	11872.2	3.124	MM
185	1... 20211111_L29_R3_IgM	8.60	10264.2	12948.7	0.793	MM
186	1... 20211111_L30_R3_IgM	8.60	10598.1	11026.6	0.961	MM
187	1... 20211111_H15_R3_IgM	8.61	20815.5	10786.3	1.930	MM
188	1... 20211111_A31_R3_IgM	8.61	28928.7	12010.5	2.409	MM
189	1... 20211111_A32_R3_IgM	8.60	50576.3	11107.7	4.553	MM
190	1... 20211111_L31_R3_IgM	8.59	6853.8	11021.8	0.622	MM
191	1... 20211111_L32_R3_IgM	8.60	26917.5	11683.8	2.304	MM
192	1... 20211111_H16_R3_IgM	8.60	33781.6	9831.7	3.436	MM
193	1... 20211111_A33_R3_IgM	8.60	47962.6	11213.1	4.277	MM
194	1... 20211111_A34_R3_IgM	8.60	12076.3	11550.1	1.046	MM
195	1... 20211111_H17_R3_IgM	8.59	155593.3	8874.4	17.533	MM
196	1... 20211111_A35_R3_IgM	8.60	15625.6	9856.3	1.585	MM
197	1... 20211111_QC_R24_IgM	8.60	30046.0	7561.8	3.973	MM
198	1... 20211111_A36_R3_IgM	8.60	42643.7	9562.8	4.459	MM
199	1... 20211111_H18_R3_IgM	8.60	69066.5	8165.3	8.459	MM
200	2... 20211111_A37_R3_IgM	8.60	68802.2	9278.8	7.415	MM
201	2... 20211111_A38_R3_IgM	8.61	40099.6	10920.3	3.672	MM
202	2... 20211111_H19_R3_IgM	8.60	41641.9	9285.3	4.485	MM
203	2... 20211111_A39_R3_IgM	8.60	29311.6	9083.4	3.227	MM
204	2... 20211111_A40_R3_IgM	8.60	21614.5	8382.4	2.579	MM
205	2... 20211111_H20_R3_IgM	8.59	112151.4	7807.9	14.364	MM
206	2... 20211111_A41_R3_IgM	8.61	14816.7	5925.2	2.501	MM
207	2... 20211111_A42_R3_IgM	8.60	14029.9	9352.6	1.500	MM
208	2... 20211111_A43_R3_IgM	8.61	12775.1	10216.3	1.250	MM
209	2... 20211111_A44_R3_IgM	8.59	10474.8	10262.7	1.021	MM
210	2... 20211111_QC_R25_IgM	8.60	30845.5	8575.3	3.597	MM

Dataset: C:\MassLynx\10.pro\20211111_clinicalsamples_IgM_IgA.qld

Last Altered: Thursday, March 09, 2023 19:58:25 台北標準時間
Printed: Friday, August 01, 2025 15:01:57 台北標準時間

Method: C:\MassLynx\10_Pro_IgA.PRO\MethDB\20211111_IgM_IgA_DMRM_new.mdb 24 Feb 2022 13:44:14
Calibration: 15 Nov 2021 14:42:11

Sample Name: 20211112_QC_R001_IgM
F39:MRM of 2 channels,ES+
943.9 > 366.1
1.889e+005

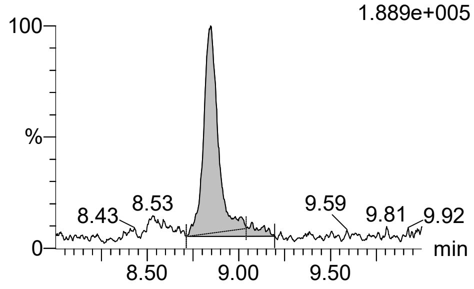

Sample Name: 20211112_QC_R001_IgM
F39:MRM of 2 channels,ES+
1179.6 > 204.1
1.841e+006

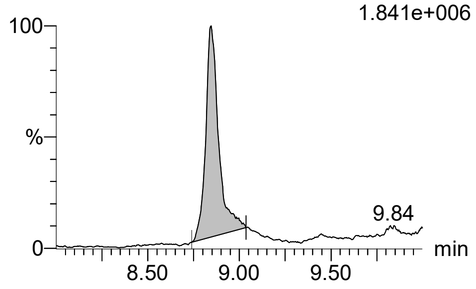

Sample Name: 20211112_QC_R002_IgM
F39:MRM of 2 channels,ES+
943.9 > 366.1
2.177e+005

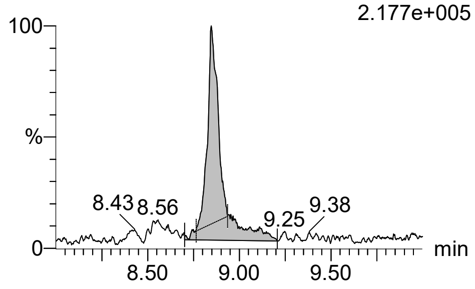

Sample Name: 20211112_QC_R002_IgM
F39:MRM of 2 channels,ES+
1179.6 > 204.1
1.972e+006

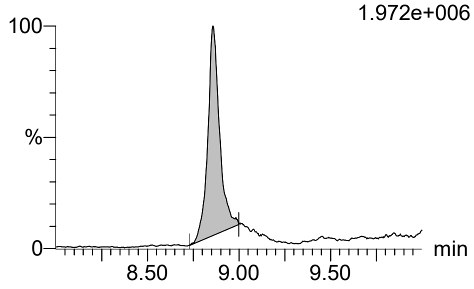

Sample Name: 20211111_A1_R2_IgM
F39:MRM of 2 channels,ES+
943.9 > 366.1
1.598e+005

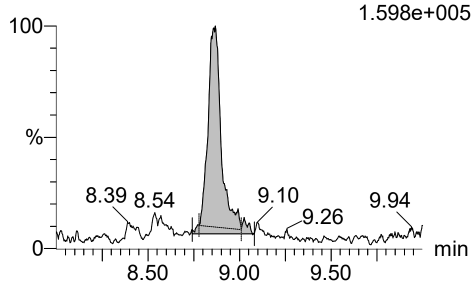

Sample Name: 20211111_A1_R2_IgM
F39:MRM of 2 channels,ES+
1179.6 > 204.1
1.750e+006

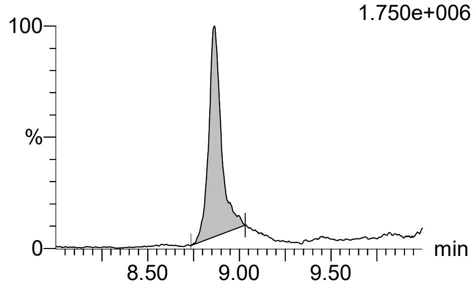

Sample Name: 20211111_A1_R3_IgM
F39:MRM of 2 channels,ES+
943.9 > 366.1
1.616e+005

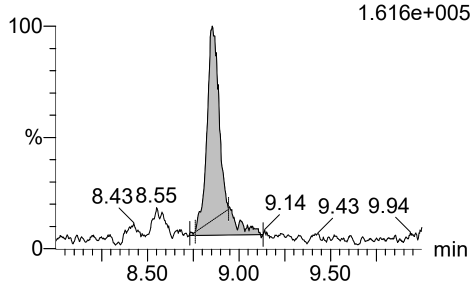

Sample Name: 20211111_A1_R3_IgM
F39:MRM of 2 channels,ES+
1179.6 > 204.1
1.724e+006

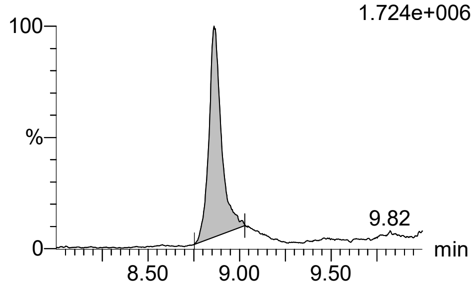

Sample Name: 20211111_A2_R2_IgM
F39:MRM of 2 channels,ES+
943.9 > 366.1
2.012e+005

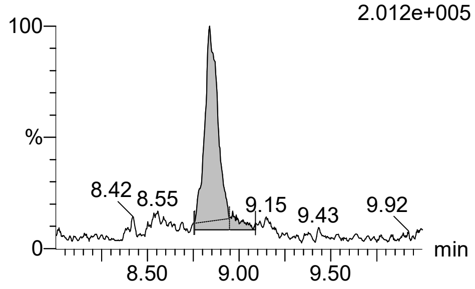

Sample Name: 20211111_A2_R2_IgM
F39:MRM of 2 channels,ES+
1179.6 > 204.1
1.973e+006

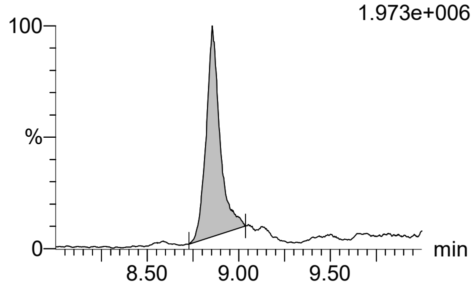

Sample Name: 20211111_A2_R3_IgM
F39:MRM of 2 channels,ES+
943.9 > 366.1
2.200e+005

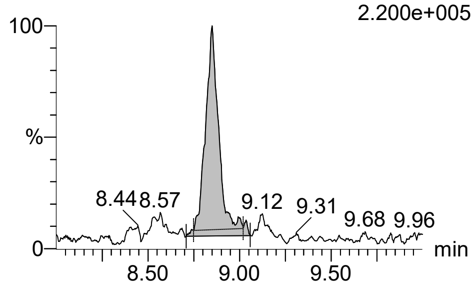

Sample Name: 20211111_A2_R3_IgM
F39:MRM of 2 channels,ES+
1179.6 > 204.1
1.953e+006

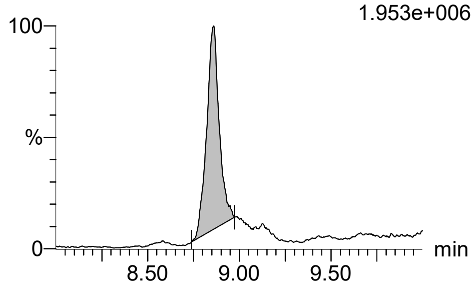

Sample Name: 20211111_L1_R2_IgM
F39:MRM of 2 channels,ES+
943.9 > 366.1
6.883e+004

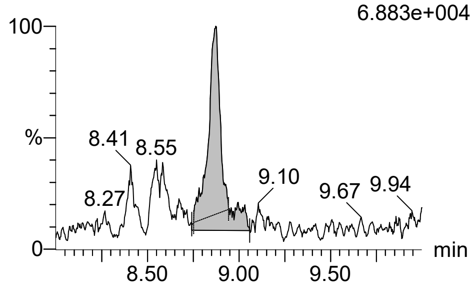

Sample Name: 20211111_L1_R2_IgM
F39:MRM of 2 channels,ES+
1179.6 > 204.1
6.762e+005

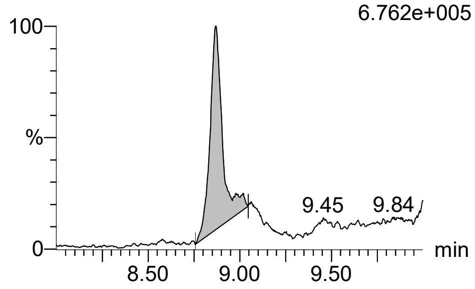

Sample Name: 20211111_L1_R3_IgM
F39:MRM of 2 channels,ES+
943.9 > 366.1
6.819e+004

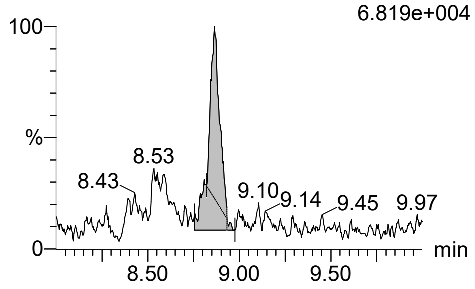

Sample Name: 20211111_L1_R3_IgM
F39:MRM of 2 channels,ES+
1179.6 > 204.1
6.410e+005

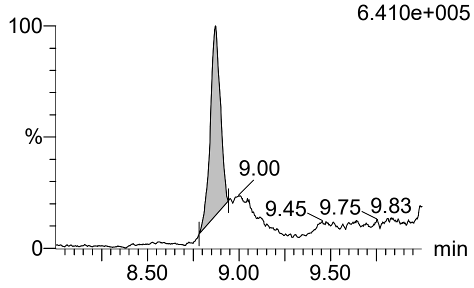

Sample Name: 20211111_L2_R2_IgM
F39:MRM of 2 channels,ES+
943.9 > 366.1
1.708e+005

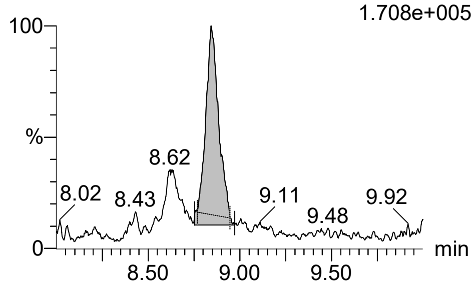

Sample Name: 20211111_L2_R2_IgM
F39:MRM of 2 channels,ES+
1179.6 > 204.1
1.428e+006

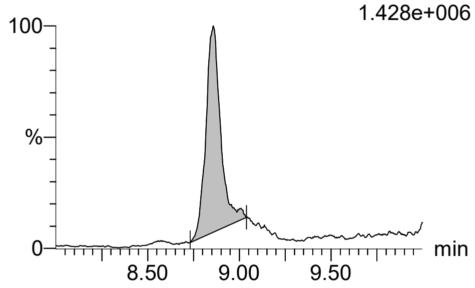

Sample Name: 20211111_H1_R2_IgM
F39:MRM of 2 channels,ES+
943.9 > 366.1
2.571e+005

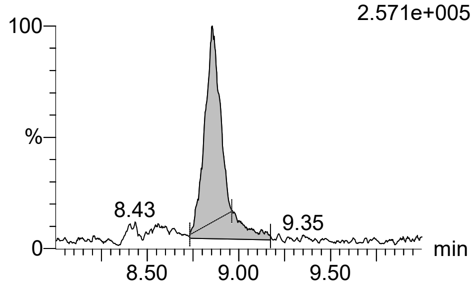

Sample Name: 20211111_H1_R2_IgM
F39:MRM of 2 channels,ES+
1179.6 > 204.1
3.053e+006

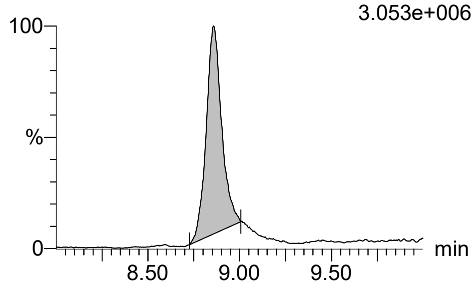

Sample Name: 20211111_A3_R2_IgM
F39:MRM of 2 channels,ES+
943.9 > 366.1
2.228e+005

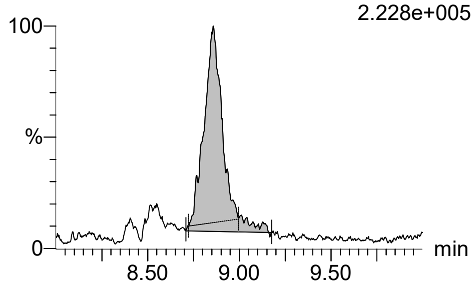

Sample Name: 20211111_A3_R2_IgM
F39:MRM of 2 channels,ES+
1179.6 > 204.1
2.598e+006

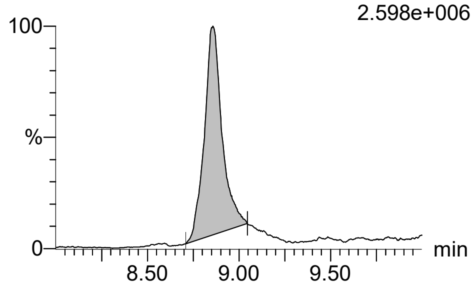

Sample Name: 20211111_A4_R2_IgM
F39:MRM of 2 channels,ES+
943.9 > 366.1
1.160e+005

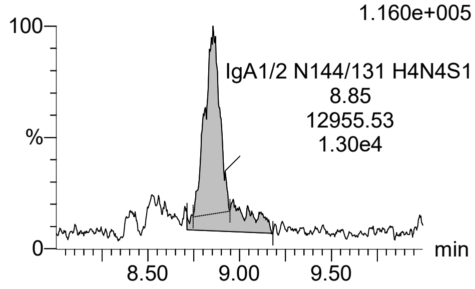

Sample Name: 20211111_A4_R2_IgM
F39:MRM of 2 channels,ES+
1179.6 > 204.1
1.252e+006

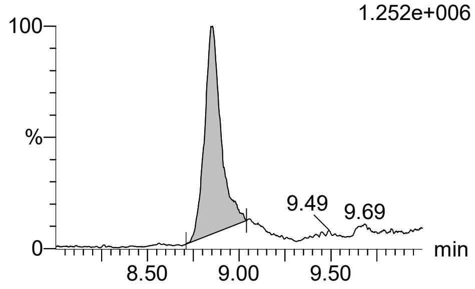

Sample Name: 20211111_L3_R2_IgM
F39:MRM of 2 channels,ES+
943.9 > 366.1
5.395e+004

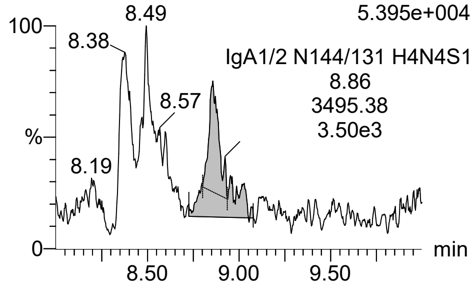

Sample Name: 20211111_L3_R2_IgM
F39:MRM of 2 channels,ES+
1179.6 > 204.1
3.703e+005

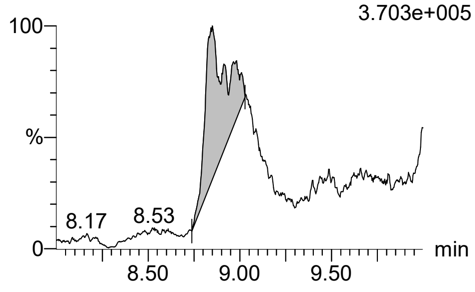

Sample Name: 20211111_L4_R2_IgM
F39:MRM of 2 channels,ES+
943.9 > 366.1
1.088e+005

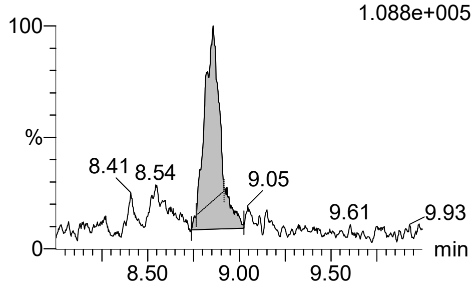

Dataset: C:\MassLynx\10.pro\20211111_clinicalsamples_IgM_IgA.qld

Last Altered: Thursday, March 09, 2023 19:58:25 台北標準時間
Printed: Friday, August 01, 2025 15:01:57 台北標準時間

Sample Name: 20211111_L4_R2_IgM
F39:MRM of 2 channels,ES+
1179.6 > 204.1
1.039e+006

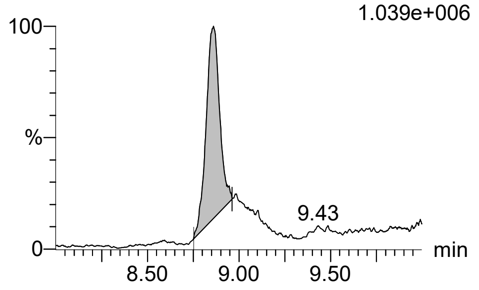

Sample Name: 20211111_H2_R2_IgM
F39:MRM of 2 channels,ES+
943.9 > 366.1
6.675e+005

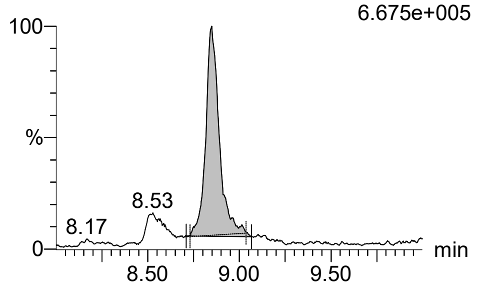

Sample Name: 20211111_H2_R2_IgM
F39:MRM of 2 channels,ES+
1179.6 > 204.1
7.045e+006

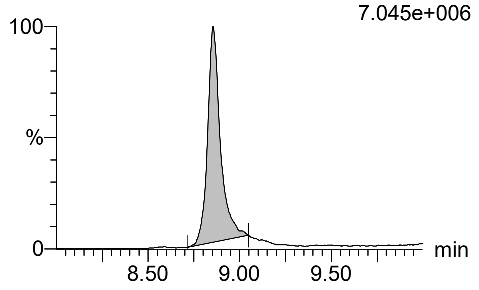

Sample Name: 20211111_A5_R2_IgM
F39:MRM of 2 channels,ES+
943.9 > 366.1
4.607e+005

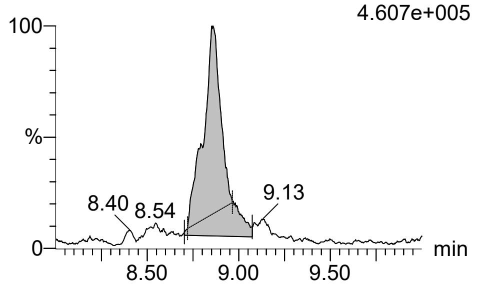

Sample Name: 20211111_A5_R2_IgM
F39:MRM of 2 channels,ES+
1179.6 > 204.1
4.974e+006

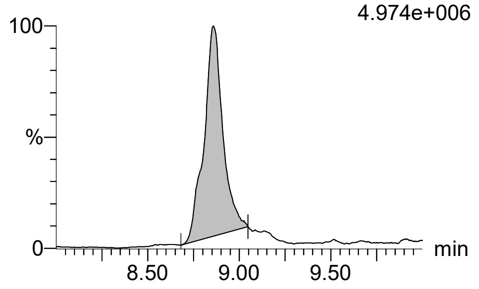

Sample Name: 20211111_A6_R2_IgM
F39:MRM of 2 channels,ES+
943.9 > 366.1
1.719e+005

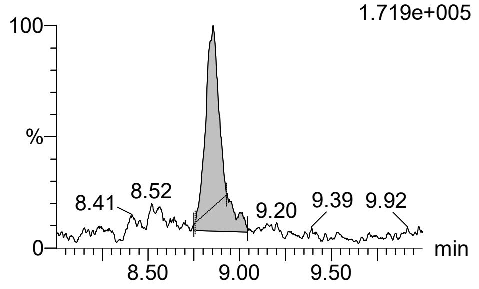

Sample Name: 20211111_A6_R2_IgM
F39:MRM of 2 channels,ES+
1179.6 > 204.1
1.848e+006

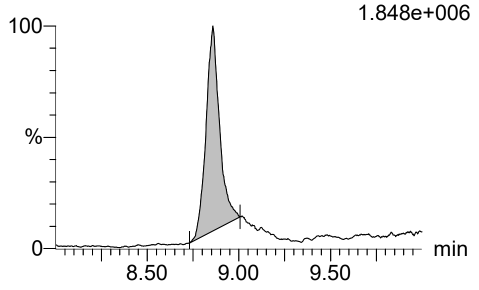

Sample Name: 20211111_QC_R10_IgM
F39:MRM of 2 channels,ES+
943.9 > 366.1
2.069e+005

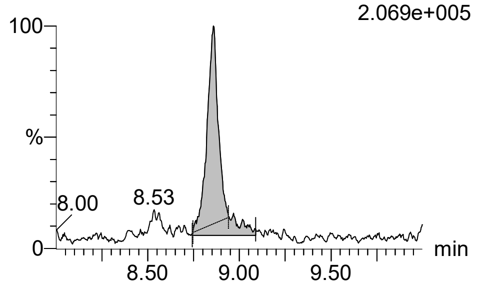

Sample Name: 20211111_QC_R10_IgM
F39:MRM of 2 channels,ES+
1179.6 > 204.1
1.882e+006

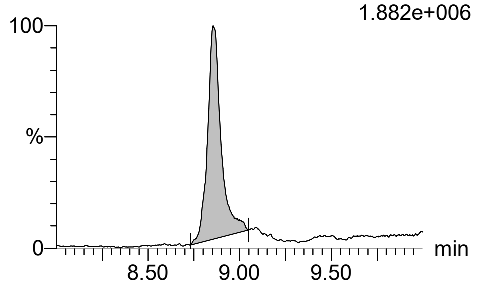

Sample Name: 20211111_L5_R2_IgM
F39:MRM of 2 channels,ES+
943.9 > 366.1
1.233e+005

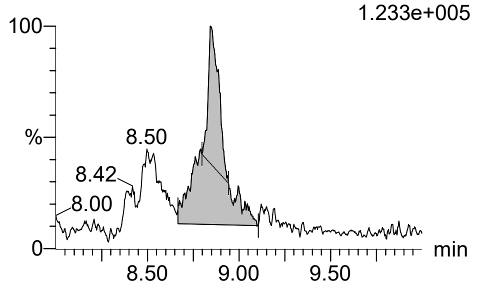

Sample Name: 20211111_L5_R2_IgM
F39:MRM of 2 channels,ES+
1179.6 > 204.1
1.503e+006

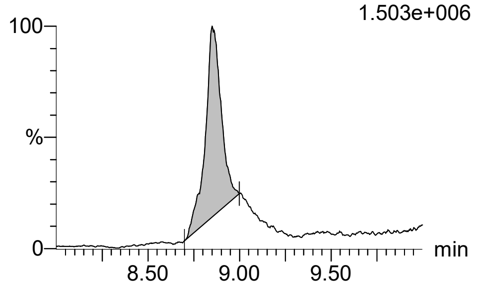

Sample Name: 20211111_L6_R2_IgM
F39:MRM of 2 channels,ES+
943.9 > 366.1
1.557e+005

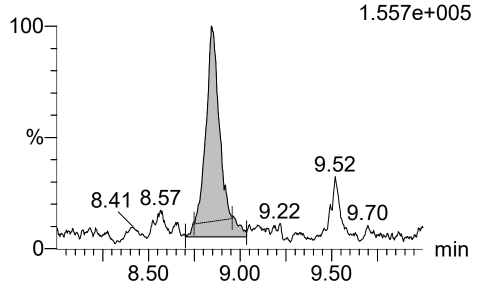

Sample Name: 20211111_L6_R2_IgM
F39:MRM of 2 channels,ES+
1179.6 > 204.1
1.585e+006

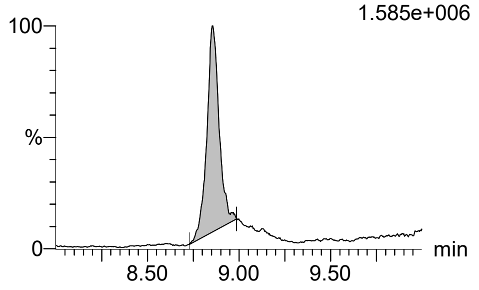

Sample Name: 20211111_H3_R2_IgM
F39:MRM of 2 channels,ES+
943.9 > 366.1
5.204e+004

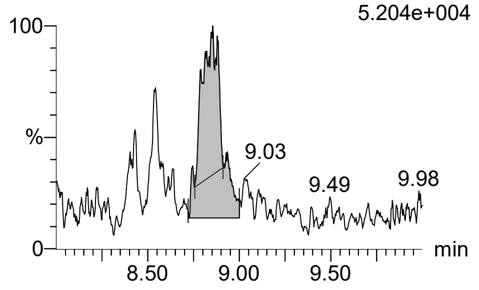

Sample Name: 20211111_H3_R2_IgM
F39:MRM of 2 channels,ES+
1179.6 > 204.1
5.215e+005

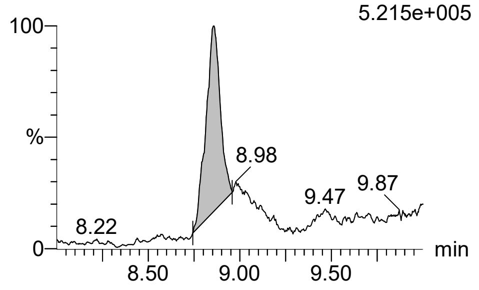

Dataset: C:\MassLynx\10.pro\20211111_clinicalsamples_IgM_IgA.qld

Last Altered: Thursday, March 09, 2023 19:58:25 台北標準時間
Printed: Friday, August 01, 2025 15:01:57 台北標準時間

Sample Name: 20211111_A7_R2_IgM
F39:MRM of 2 channels,ES+
943.9 > 366.1
5.747e+005

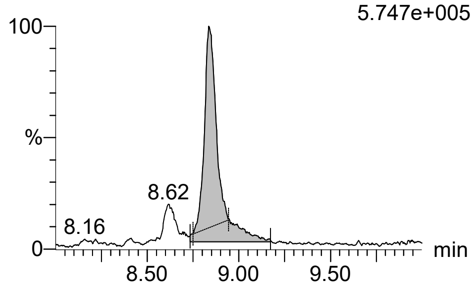

Sample Name: 20211111_A7_R2_IgM
F39:MRM of 2 channels,ES+
1179.6 > 204.1
6.173e+006

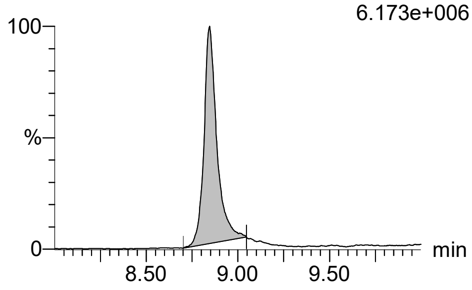

Sample Name: 20211111_A8_R2_IgM
F39:MRM of 2 channels,ES+
943.9 > 366.1
2.695e+005

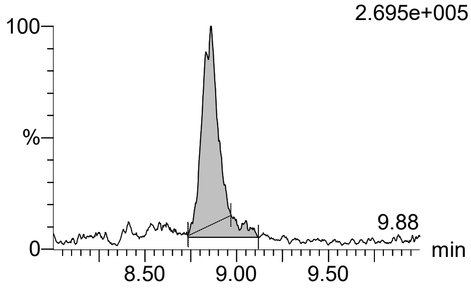

Sample Name: 20211111_A8_R2_IgM
F39:MRM of 2 channels,ES+
1179.6 > 204.1
3.217e+006

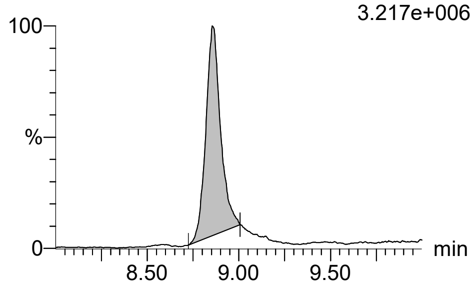

Sample Name: 20211111_L7_R2_IgM
F39:MRM of 2 channels,ES+
943.9 > 366.1
1.115e+005

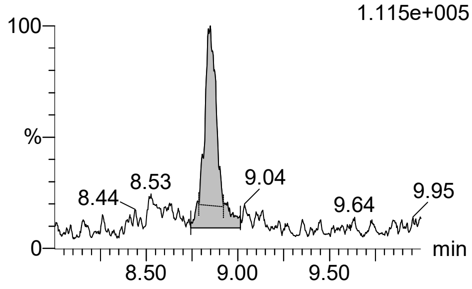

Sample Name: 20211111_L7_R2_IgM
F39:MRM of 2 channels,ES+
1179.6 > 204.1
1.128e+006

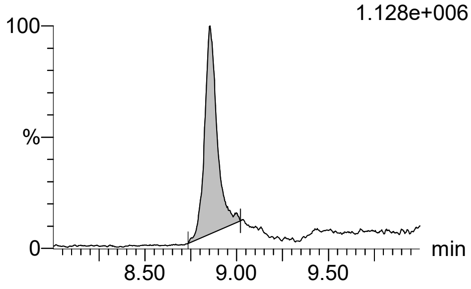

Sample Name: 20211111_L8_R2_IgM
F39:MRM of 2 channels,ES+
943.9 > 366.1
3.799e+004

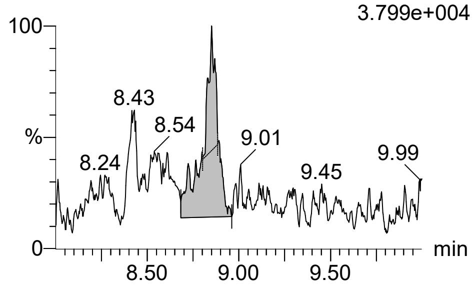

Sample Name: 20211111_L8_R2_IgM
F39:MRM of 2 channels,ES+
1179.6 > 204.1
2.503e+005

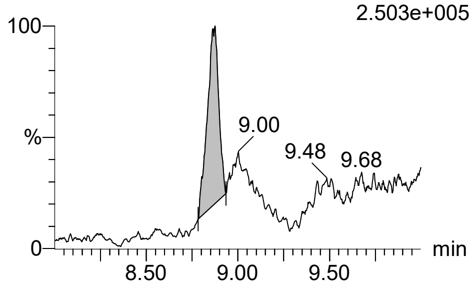

Sample Name: 20211111_H4_R2_IgM
F39:MRM of 2 channels,ES+
943.9 > 366.1
6.250e+004

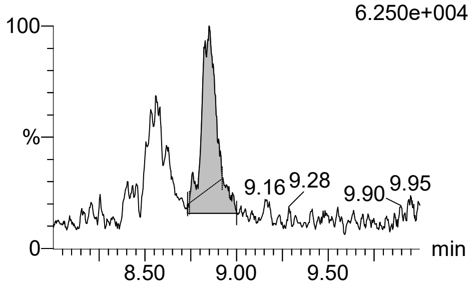

Sample Name: 20211111_H4_R2_IgM
F39:MRM of 2 channels,ES+
1179.6 > 204.1
5.448e+005

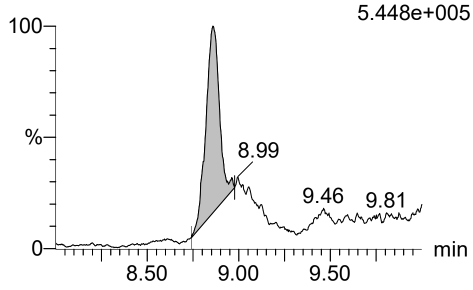

Sample Name: 20211111_A9_R2_IgM
F39:MRM of 2 channels,ES+
943.9 > 366.1
9.467e+004

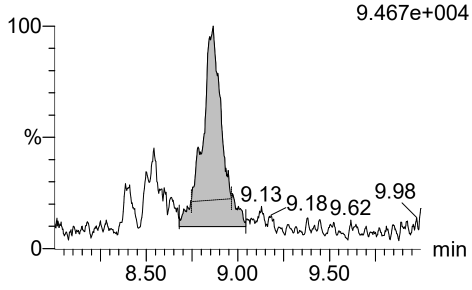

Sample Name: 20211111_A9_R2_IgM
F39:MRM of 2 channels,ES+
1179.6 > 204.1
1.118e+006

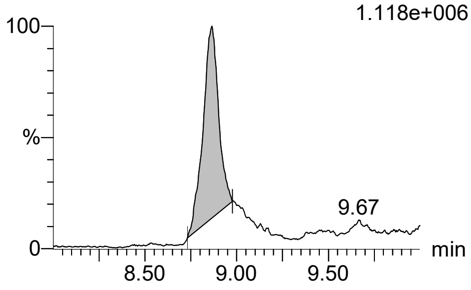

Sample Name: 20211111_A10_R2_IgM
F39:MRM of 2 channels,ES+
943.9 > 366.1
3.359e+005

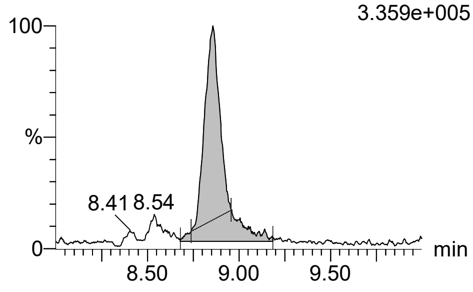

Sample Name: 20211111_A10_R2_IgM
F39:MRM of 2 channels,ES+
1179.6 > 204.1
4.095e+006

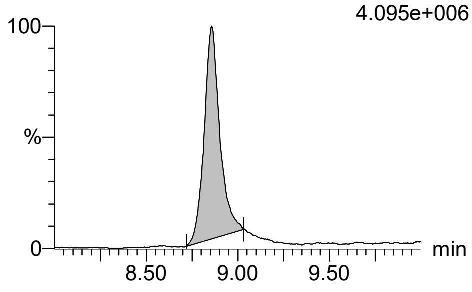

Sample Name: 20211111_L9_R2_IgM
F39:MRM of 2 channels,ES+
943.9 > 366.1
7.878e+004

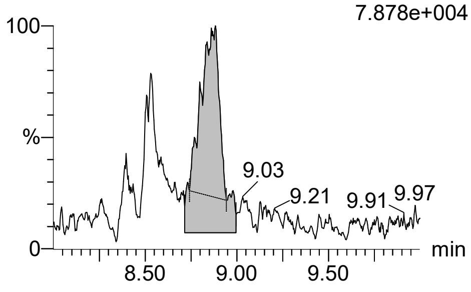

Dataset: C:\MassLynx\10.pro\20211111_clinicalsamples_IgM_IgA.qld

Last Altered: Thursday, March 09, 2023 19:58:25 台北標準時間
Printed: Friday, August 01, 2025 15:01:57 台北標準時間

Sample Name: 20211111_L9_R2_IgM
F39:MRM of 2 channels,ES+
1179.6 > 204.1
1.014e+006

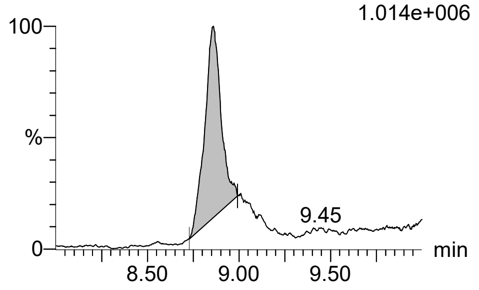

Sample Name: 20211111_L10_R2_IgM
F39:MRM of 2 channels,ES+
943.9 > 366.1
1.421e+005

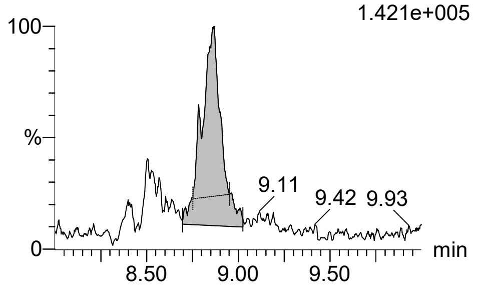

Sample Name: 20211111_L10_R2_IgM
F39:MRM of 2 channels,ES+
1179.6 > 204.1
1.659e+006

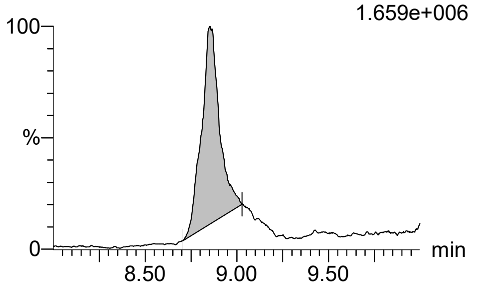

Sample Name: 20211111_QC_R11_IgM
F39:MRM of 2 channels,ES+
943.9 > 366.1
2.023e+005

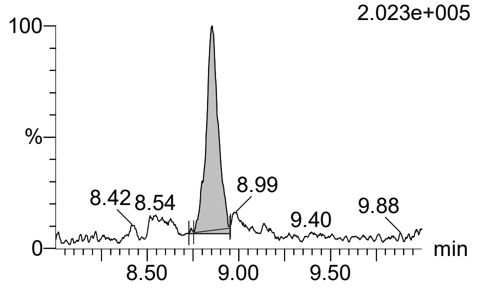

Sample Name: 20211111_QC_R11_IgM
F39:MRM of 2 channels,ES+
1179.6 > 204.1
2.139e+006

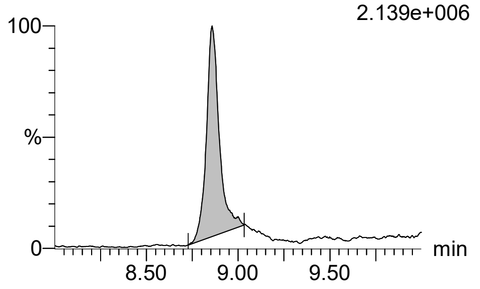

Sample Name: 20211111_H5_R2_IgM
F39:MRM of 2 channels,ES+
943.9 > 366.1
7.227e+004

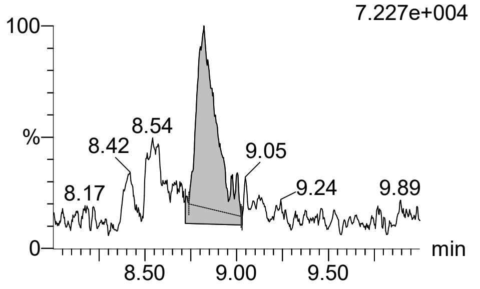

Sample Name: 20211111_H5_R2_IgM
F39:MRM of 2 channels,ES+
1179.6 > 204.1
7.252e+005

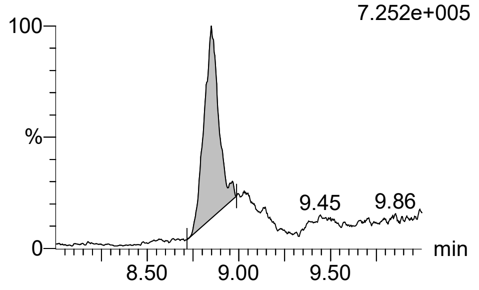

Sample Name: 20211111_A11_R2_IgM
F39:MRM of 2 channels,ES+
943.9 > 366.1
1.037e+005

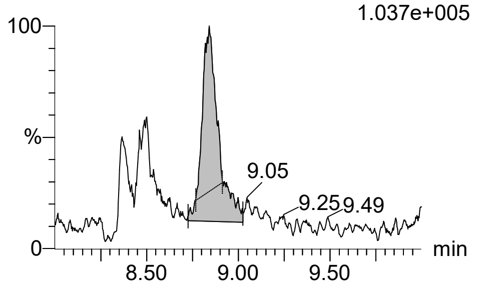

Sample Name: 20211111_A11_R2_IgM
F39:MRM of 2 channels,ES+
1179.6 > 204.1
1.187e+006

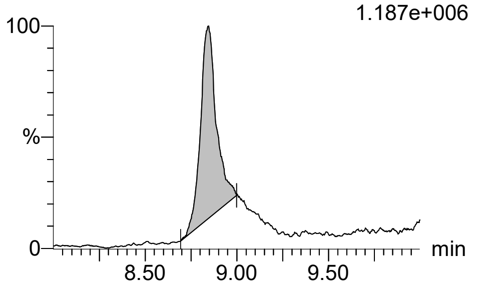

Sample Name: 20211111_A12_R2_IgM
F39:MRM of 2 channels,ES+
943.9 > 366.1
6.720e+004

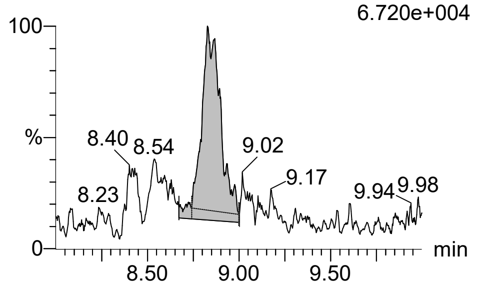

Sample Name: 20211111_A12_R2_IgM
F39:MRM of 2 channels,ES+
1179.6 > 204.1
8.041e+005

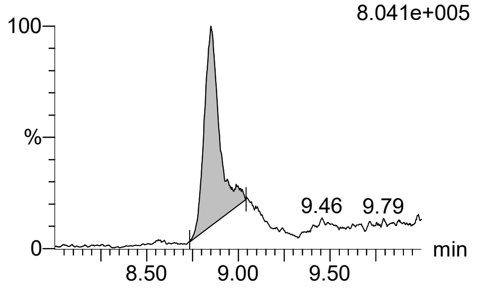

Sample Name: 20211111_L11_R2_IgM
F39:MRM of 2 channels,ES+
943.9 > 366.1
1.707e+005

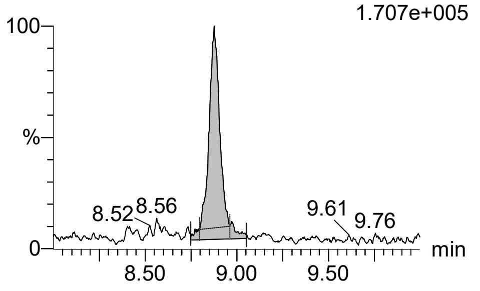

Sample Name: 20211111_L11_R2_IgM
F39:MRM of 2 channels,ES+
1179.6 > 204.1
1.470e+006

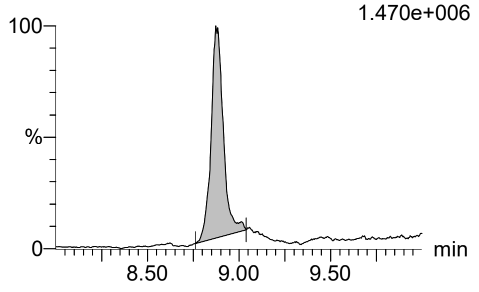

Sample Name: 20211111_L12_R2_IgM
F39:MRM of 2 channels,ES+
943.9 > 366.1
6.650e+004

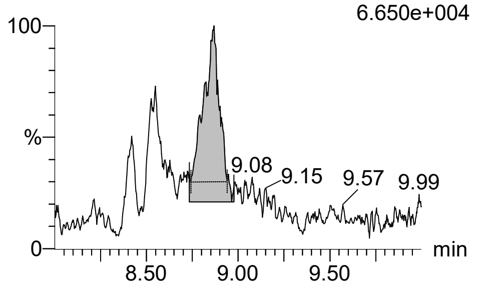

Sample Name: 20211111_L12_R2_IgM
F39:MRM of 2 channels,ES+
1179.6 > 204.1
7.310e+005

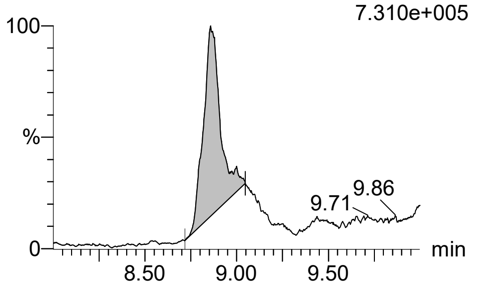

Dataset: C:\MassLynx\10.pro\20211111_clinicalsamples_IgM_IgA.qld

Last Altered: Thursday, March 09, 2023 19:58:25 台北標準時間
Printed: Friday, August 01, 2025 15:01:57 台北標準時間

Sample Name: 20211111_H6_R2_IgM
F39:MRM of 2 channels,ES+
943.9 > 366.1
4.950e+004

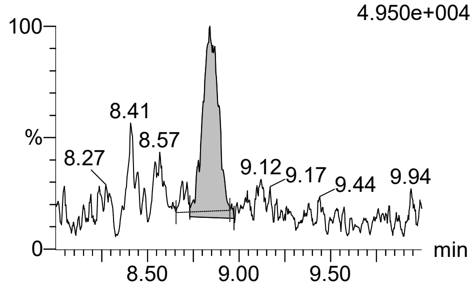

Sample Name: 20211111_H6_R2_IgM
F39:MRM of 2 channels,ES+
1179.6 > 204.1
5.208e+005

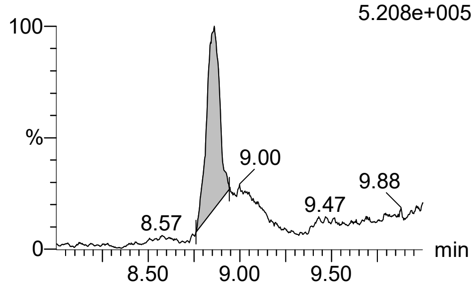

Sample Name: 20211111_A13_R2_IgM
F39:MRM of 2 channels,ES+
943.9 > 366.1
6.977e+004

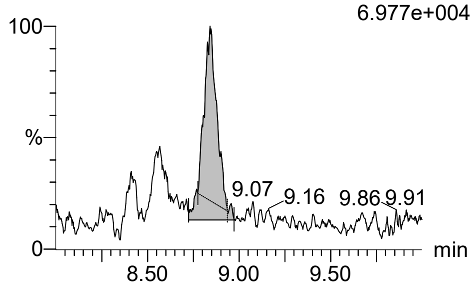

Sample Name: 20211111_A13_R2_IgM
F39:MRM of 2 channels,ES+
1179.6 > 204.1
6.124e+005

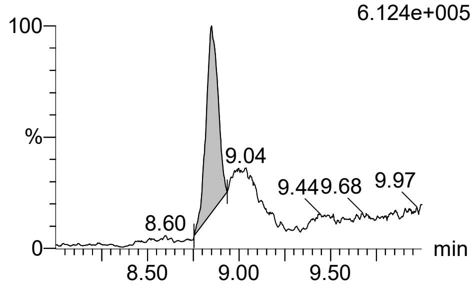

Sample Name: 20211111_A14_R2_IgM
F39:MRM of 2 channels,ES+
943.9 > 366.1
4.586e+005

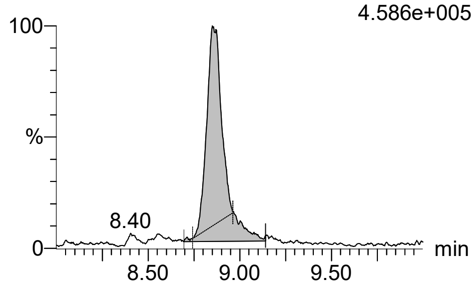

Sample Name: 20211111_A14_R2_IgM
F39:MRM of 2 channels,ES+
1179.6 > 204.1
5.698e+006

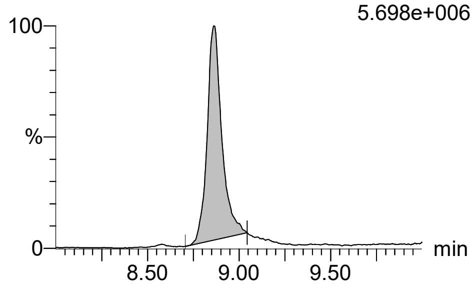

Sample Name: 20211111_L13_R2_IgM
F39:MRM of 2 channels,ES+
943.9 > 366.1
9.248e+004

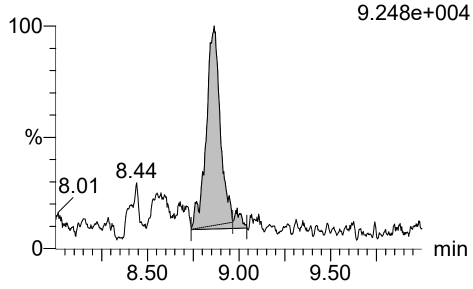

Sample Name: 20211111_L13_R2_IgM
F39:MRM of 2 channels,ES+
1179.6 > 204.1
9.199e+005

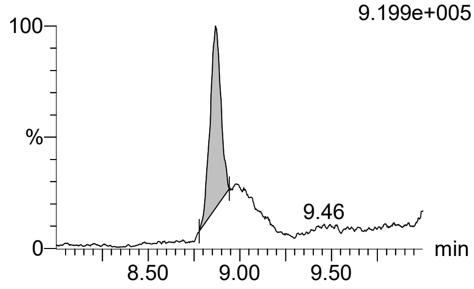

Sample Name: 20211111_L14_R2_IgM
F39:MRM of 2 channels,ES+
943.9 > 366.1
1.201e+005

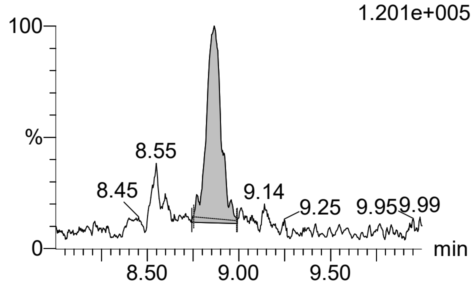

Sample Name: 20211111_L14_R2_IgM
F39:MRM of 2 channels,ES+
1179.6 > 204.1
1.379e+006

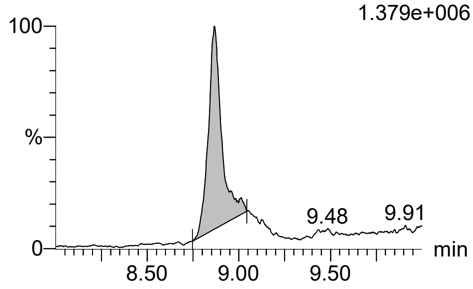

Sample Name: 20211111_H7_R2_IgM
F39:MRM of 2 channels,ES+
943.9 > 366.1
4.965e+004

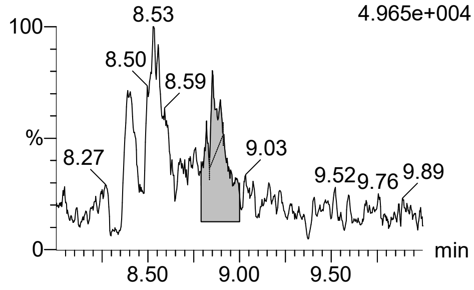

Sample Name: 20211111_H7_R2_IgM
F39:MRM of 2 channels,ES+
1179.6 > 204.1
3.724e+005

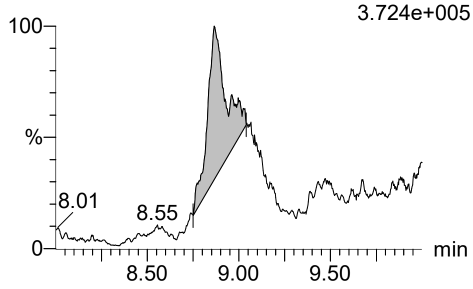

Sample Name: 20211111_A15_R2_IgM
F39:MRM of 2 channels,ES+
943.9 > 366.1
5.314e+005

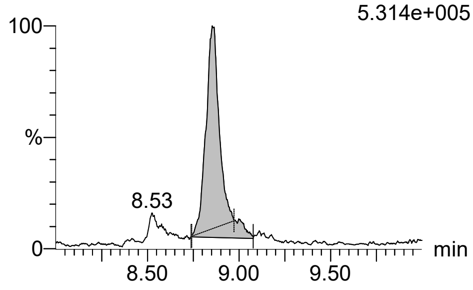

Sample Name: 20211111_A15_R2_IgM
F39:MRM of 2 channels,ES+
1179.6 > 204.1
5.731e+006

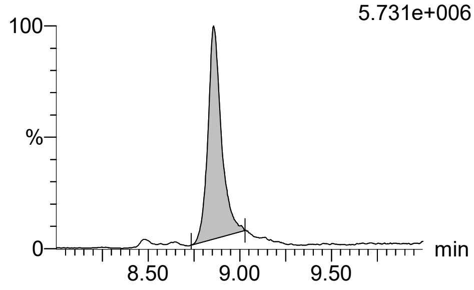

Sample Name: 20211111_QC_R12_IgM
F39:MRM of 2 channels,ES+
943.9 > 366.1
2.179e+005

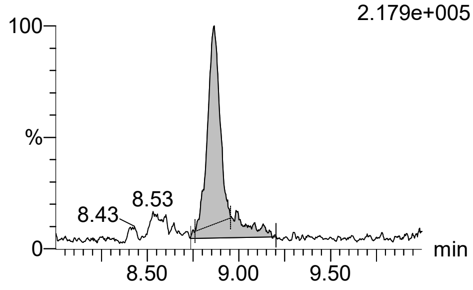

Sample Name: 20211111_QC_R12_IgM
F39:MRM of 2 channels,ES+
1179.6 > 204.1
2.437e+006

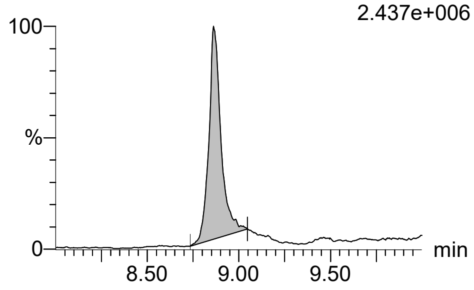

Sample Name: 20211111_A16_R2_IgM
F39:MRM of 2 channels,ES+
943.9 > 366.1
4.791e+005

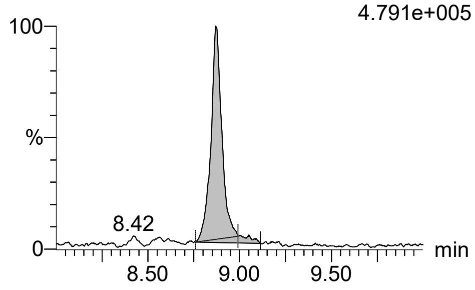

Sample Name: 20211111_A16_R2_IgM
F39:MRM of 2 channels,ES+
1179.6 > 204.1
4.855e+006

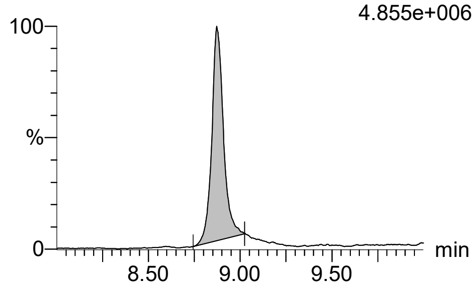

Sample Name: 20211111_L15_R2_IgM
F39:MRM of 2 channels,ES+
943.9 > 366.1
1.052e+005

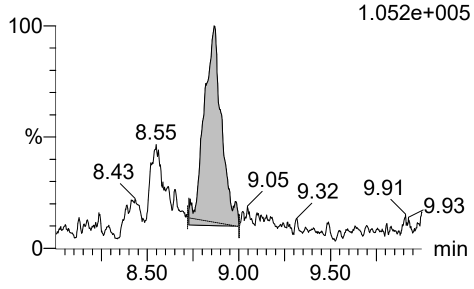

Sample Name: 20211111_L15_R2_IgM
F39:MRM of 2 channels,ES+
1179.6 > 204.1
1.212e+006

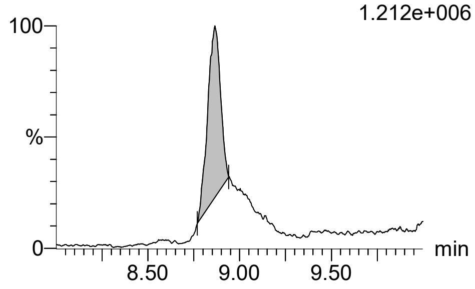

Sample Name: 20211111_L16_R2_IgM
F39:MRM of 2 channels,ES+
943.9 > 366.1
7.030e+004

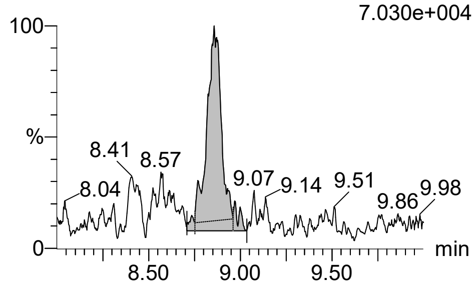

Sample Name: 20211111_L16_R2_IgM
F39:MRM of 2 channels,ES+
1179.6 > 204.1
7.556e+005

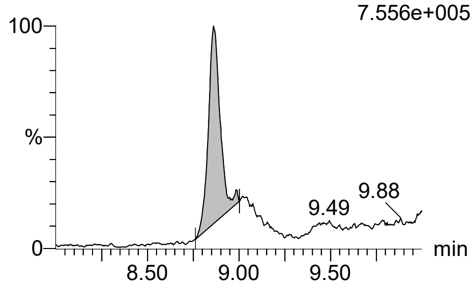

Sample Name: 20211111_H8_R2_IgM
F39:MRM of 2 channels,ES+
943.9 > 366.1
1.337e+005

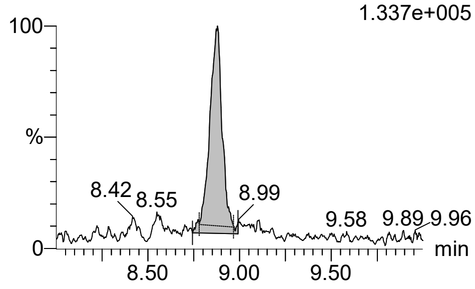

Sample Name: 20211111_H8_R2_IgM
F39:MRM of 2 channels,ES+
1179.6 > 204.1
1.426e+006

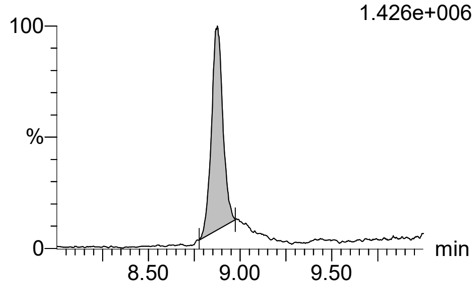

Sample Name: 20211111_A17_R2_IgM
F39:MRM of 2 channels,ES+
943.9 > 366.1
3.112e+005

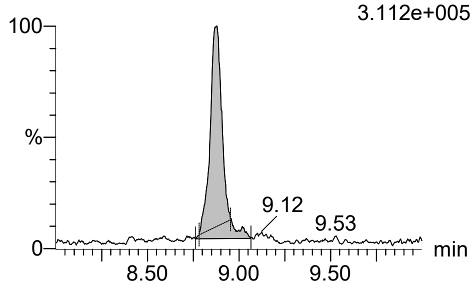

Sample Name: 20211111_A17_R2_IgM
F39:MRM of 2 channels,ES+
1179.6 > 204.1
3.512e+006

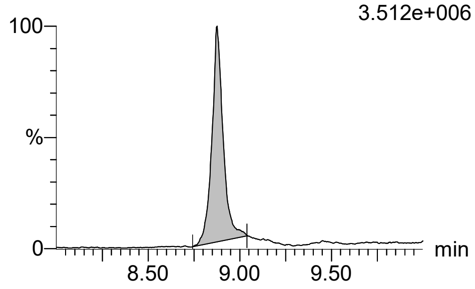

Sample Name: 20211111_A18_R2_IgM
F39:MRM of 2 channels,ES+
943.9 > 366.1
9.586e+004

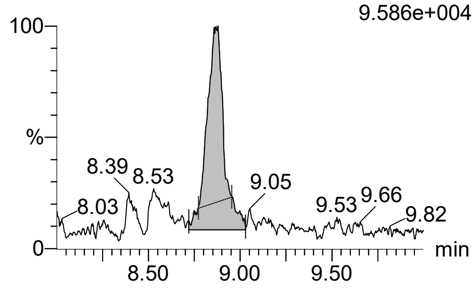

Sample Name: 20211111_A18_R2_IgM
F39:MRM of 2 channels,ES+
1179.6 > 204.1
1.103e+006

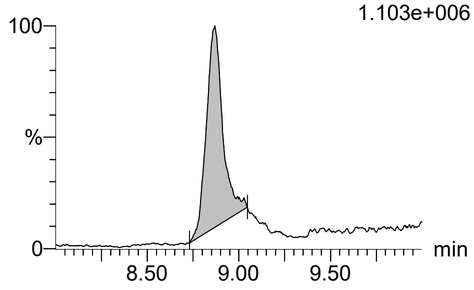

Sample Name: 20211111_L17_R2_IgM
F39:MRM of 2 channels,ES+
943.9 > 366.1
1.646e+005

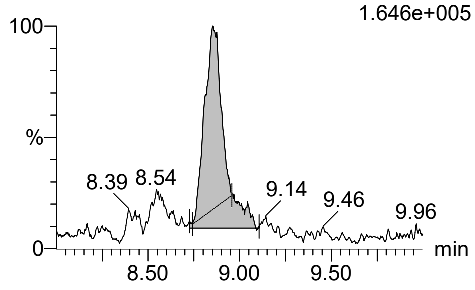

Sample Name: 20211111_L17_R2_IgM
F39:MRM of 2 channels,ES+
1179.6 > 204.1
1.971e+006

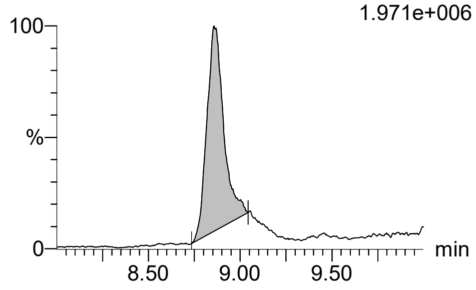

Sample Name: 20211111_L18_R2_IgM
F39:MRM of 2 channels,ES+
943.9 > 366.1
3.481e+004

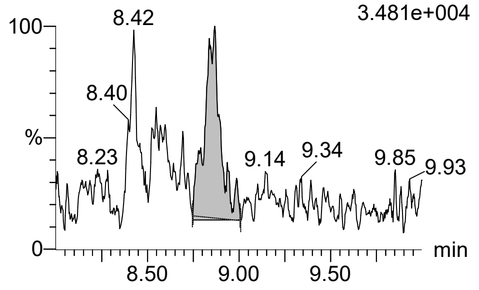

Sample Name: 20211111_L18_R2_IgM
F39:MRM of 2 channels,ES+
1179.6 > 204.1
3.401e+005

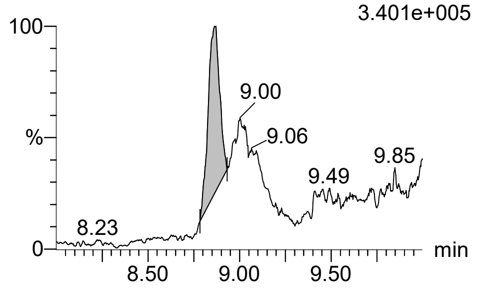

Sample Name: 20211111_H9_R2_IgM
F39:MRM of 2 channels,ES+
943.9 > 366.1
4.412e+004

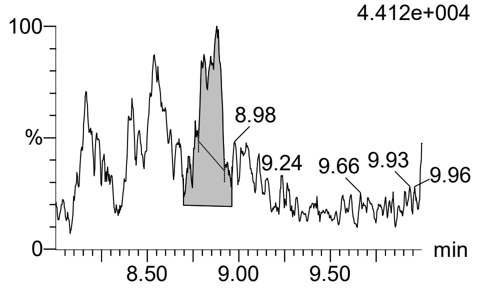

Sample Name: 20211111_H9_R2_IgM
F39:MRM of 2 channels,ES+
1179.6 > 204.1
5.417e+005

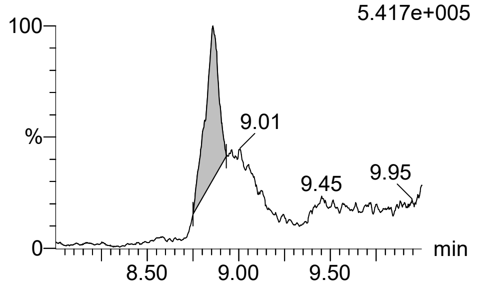

Sample Name: 20211111_A19_R2_IgM
F39:MRM of 2 channels,ES+
943.9 > 366.1
1.259e+005

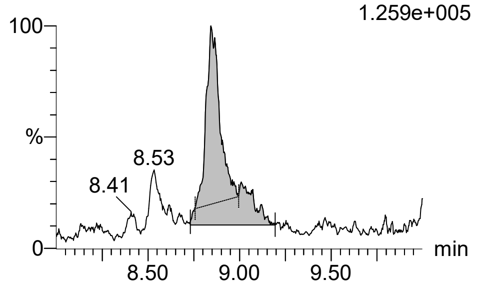

Sample Name: 20211111_A19_R2_IgM
F39:MRM of 2 channels,ES+
1179.6 > 204.1
1.403e+006

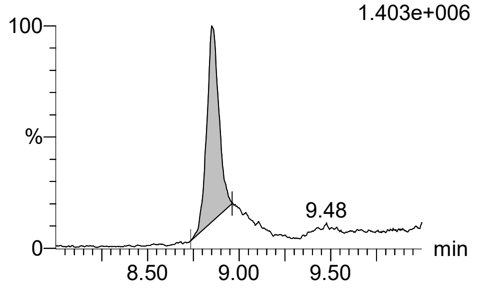

Sample Name: 20211111_A20_R2_IgM
F39:MRM of 2 channels,ES+
943.9 > 366.1
3.672e+004

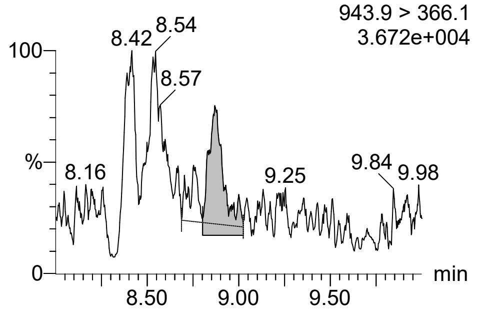

Sample Name: 20211111_A20_R2_IgM
F39:MRM of 2 channels,ES+
1179.6 > 204.1
3.520e+005

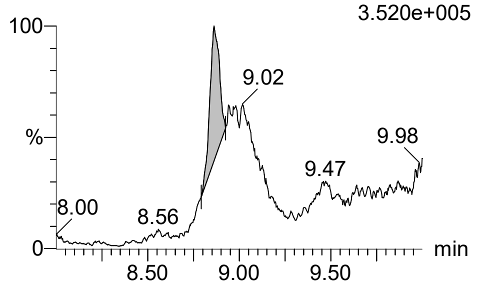

Sample Name: 20211111_L19_R2_IgM
F39:MRM of 2 channels,ES+
943.9 > 366.1
7.738e+004

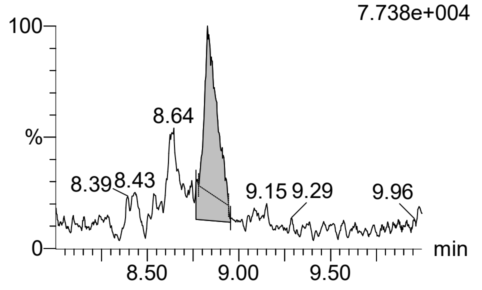

Sample Name: 20211111_L19_R2_IgM
F39:MRM of 2 channels,ES+
1179.6 > 204.1
6.953e+005

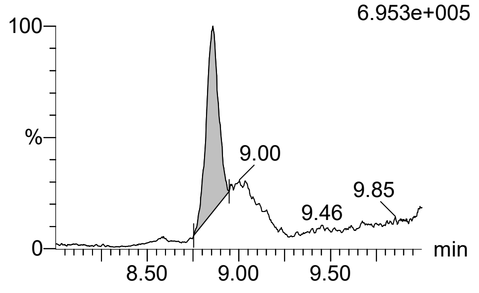

Sample Name: 20211111_QC_R13_IgM
F39:MRM of 2 channels,ES+
943.9 > 366.1
1.972e+005

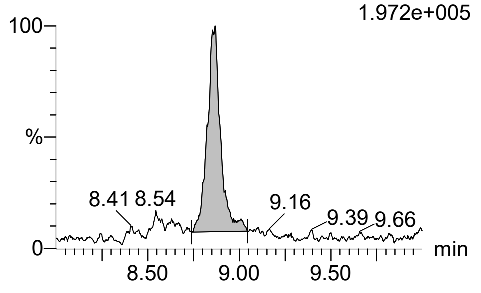

Sample Name: 20211111_QC_R13_IgM
F39:MRM of 2 channels,ES+
1179.6 > 204.1
2.130e+006

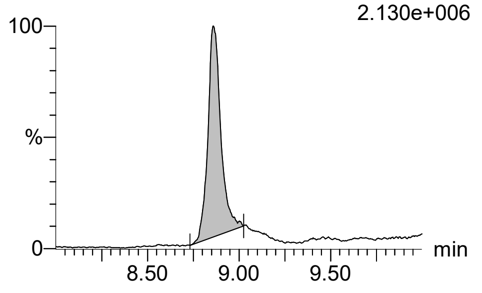

Sample Name: 20211111_L20_R2_IgM
F39:MRM of 2 channels,ES+
943.9 > 366.1
6.195e+004

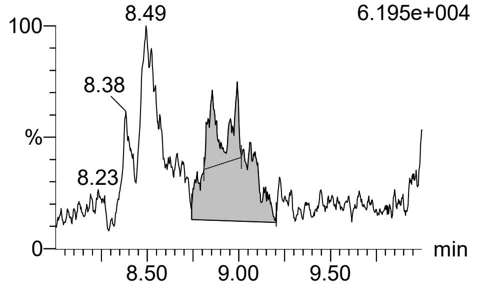

Sample Name: 20211111_L20_R2_IgM
F39:MRM of 2 channels,ES+
1179.6 > 204.1
3.886e+005

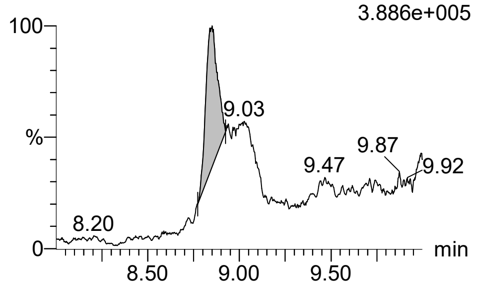

Sample Name: 20211111_H10_R2_IgM
F39:MRM of 2 channels,ES+
943.9 > 366.1
4.173e+004

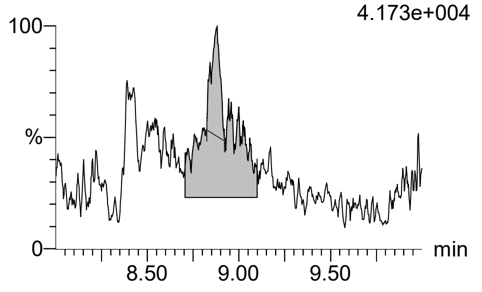

Sample Name: 20211111_H10_R2_IgM
F39:MRM of 2 channels,ES+
1179.6 > 204.1
4.664e+005

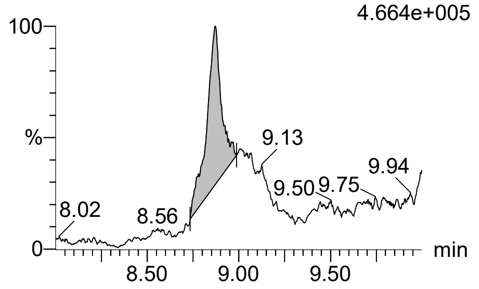

Sample Name: 20211111_A21_R2_IgM
F39:MRM of 2 channels,ES+
943.9 > 366.1
1.508e+005

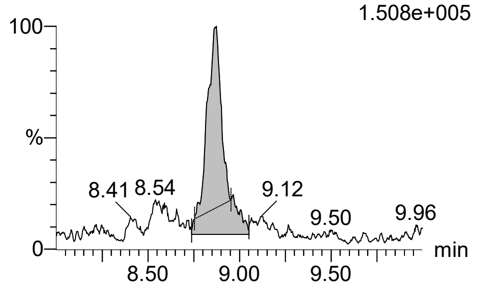

Sample Name: 20211111_A21_R2_IgM
F39:MRM of 2 channels,ES+
1179.6 > 204.1
1.861e+006

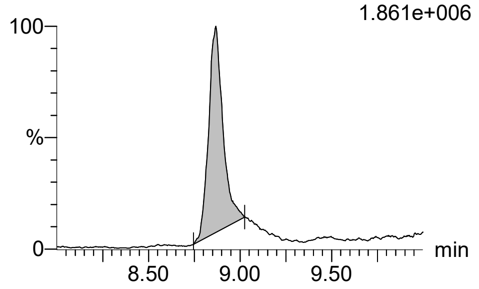

Sample Name: 20211111_A22_R2_IgM
F39:MRM of 2 channels,ES+
943.9 > 366.1
2.776e+004

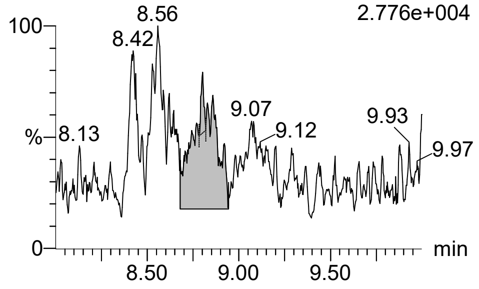

Sample Name: 20211111_A22_R2_IgM
F39:MRM of 2 channels,ES+
1179.6 > 204.1
1.671e+005

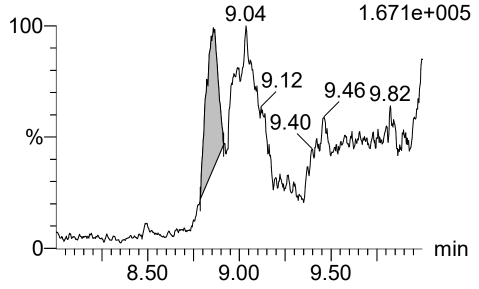

Sample Name: 20211111_L21_R2_IgM
F39:MRM of 2 channels,ES+
943.9 > 366.1
1.171e+005

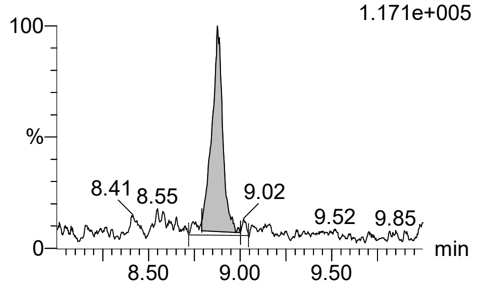

Sample Name: 20211111_L21_R2_IgM
F39:MRM of 2 channels,ES+
1179.6 > 204.1
9.058e+005

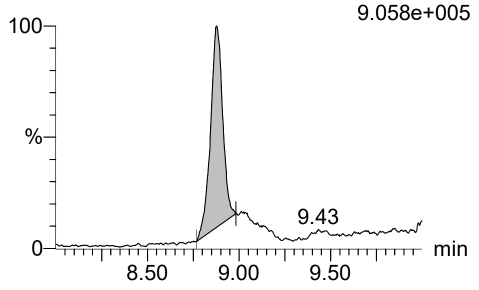

Sample Name: 20211111_L22_R2_IgM
F39:MRM of 2 channels,ES+
943.9 > 366.1
1.893e+005

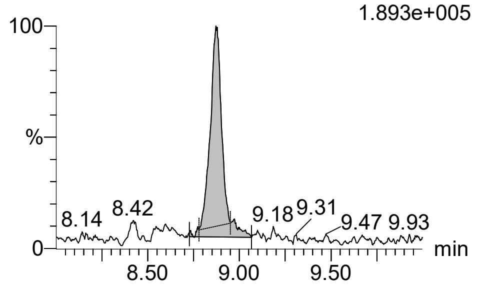

Sample Name: 20211111_L22_R2_IgM
F39:MRM of 2 channels,ES+
1179.6 > 204.1
1.857e+006

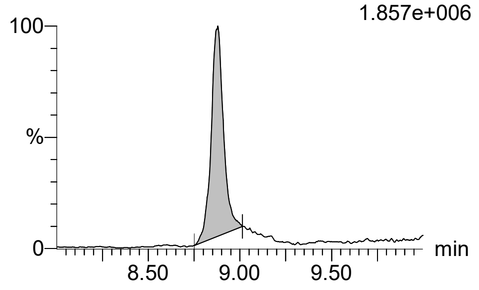

Sample Name: 20211111_H11_R2_IgM
F39:MRM of 2 channels,ES+
943.9 > 366.1
3.321e+005

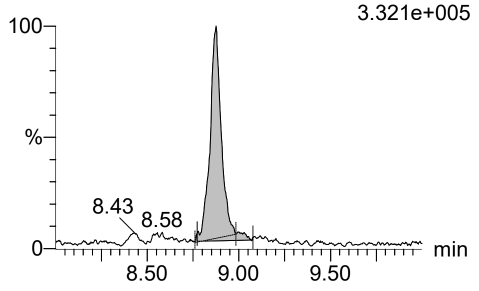

Sample Name: 20211111_H11_R2_IgM
F39:MRM of 2 channels,ES+
1179.6 > 204.1
3.190e+006

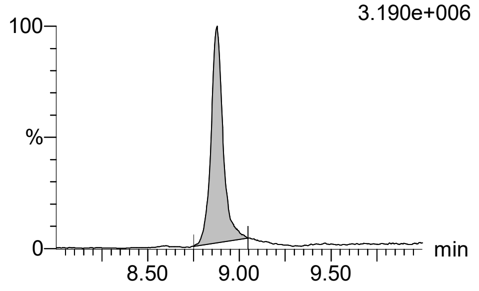

Sample Name: 20211111_A23_R2_IgM
F39:MRM of 2 channels,ES+
943.9 > 366.1
6.122e+004

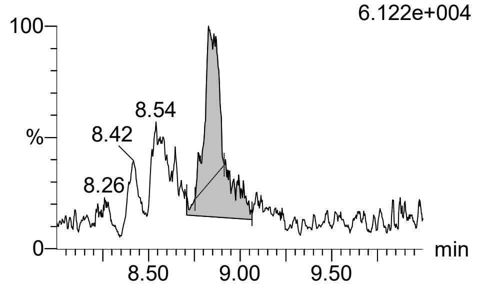

Sample Name: 20211111_A23_R2_IgM
F39:MRM of 2 channels,ES+
1179.6 > 204.1
6.837e+005

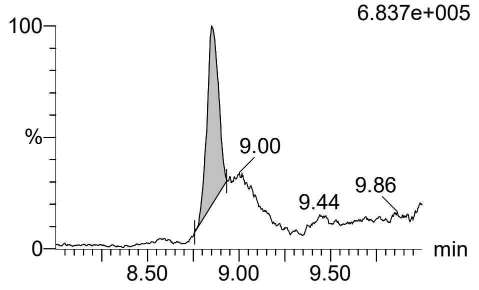

Sample Name: 20211111_A24_R2_IgM
F39:MRM of 2 channels,ES+
943.9 > 366.1
1.376e+005

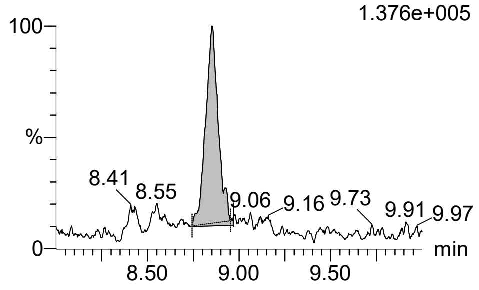

Sample Name: 20211111_A24_R2_IgM
F39:MRM of 2 channels,ES+
1179.6 > 204.1
1.271e+006

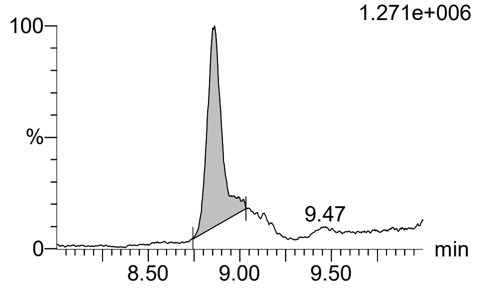

Sample Name: 20211111_L23_R2_IgM
F39:MRM of 2 channels,ES+
943.9 > 366.1
2.327e+005

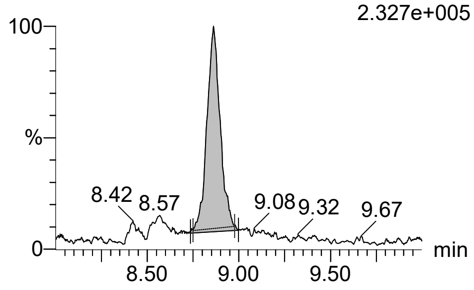

Sample Name: 20211111_L23_R2_IgM
F39:MRM of 2 channels,ES+
1179.6 > 204.1
2.571e+006

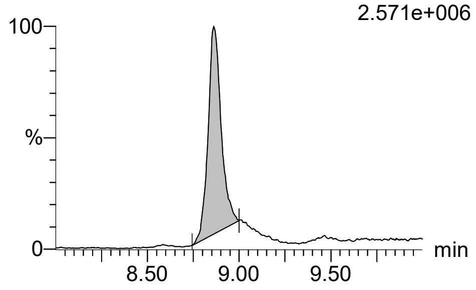

Sample Name: 20211111_L24_R2_IgM
F39:MRM of 2 channels,ES+
943.9 > 366.1
3.625e+004

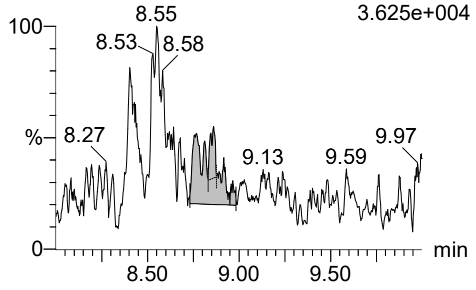

Sample Name: 20211111_L24_R2_IgM
F39:MRM of 2 channels,ES+
1179.6 > 204.1
1.801e+005

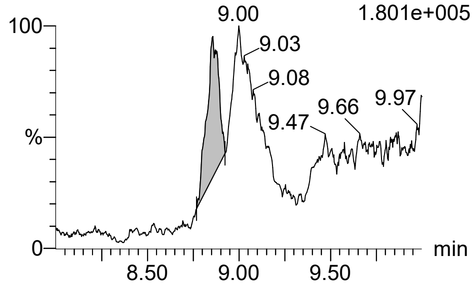

Sample Name: 20211111_H12_R2_IgM
F39:MRM of 2 channels,ES+
943.9 > 366.1
6.164e+004

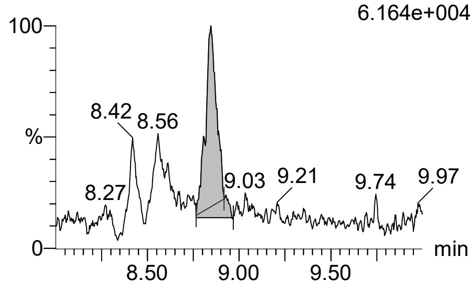

Sample Name: 20211111_H12_R2_IgM
F39:MRM of 2 channels,ES+
1179.6 > 204.1
5.722e+005

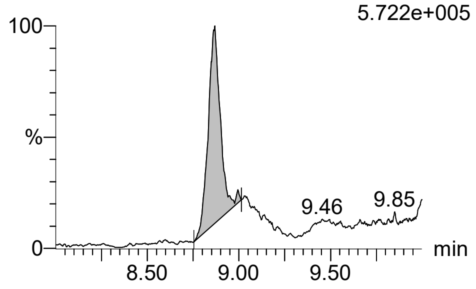

Sample Name: 20211111_QC_R14_IgM
F39:MRM of 2 channels,ES+
943.9 > 366.1
2.201e+005

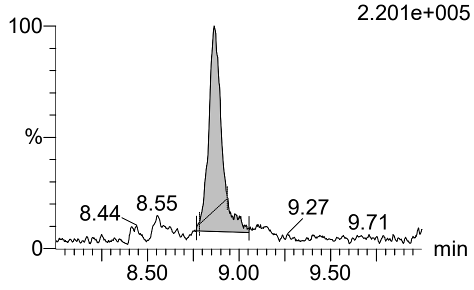

Sample Name: 20211111_QC_R14_IgM
F39:MRM of 2 channels,ES+
1179.6 > 204.1
2.444e+006

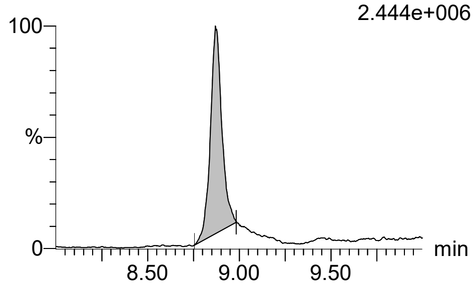

Sample Name: 20211111_A25_R2_IgM
F39:MRM of 2 channels,ES+
943.9 > 366.1
6.287e+005

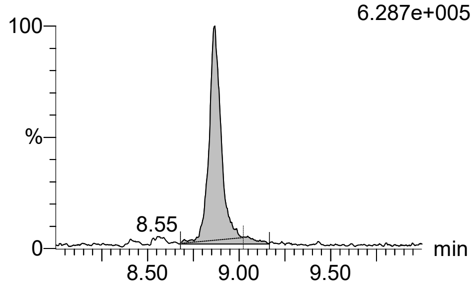

Sample Name: 20211111_A25_R2_IgM
F39:MRM of 2 channels,ES+
1179.6 > 204.1
6.227e+006

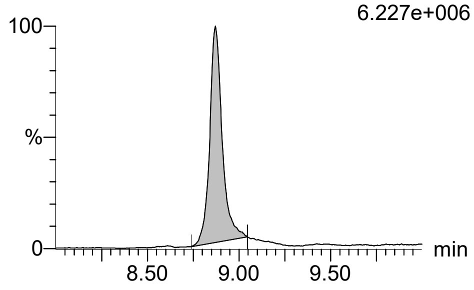

Sample Name: 20211111_A26_R2_IgM
F39:MRM of 2 channels,ES+
943.9 > 366.1
2.602e+005

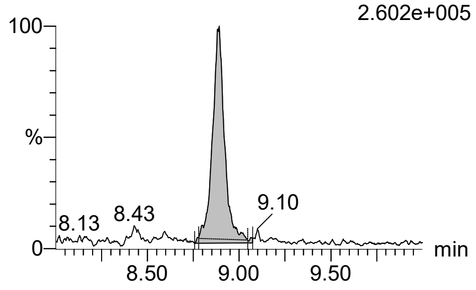

Sample Name: 20211111_A26_R2_IgM
F39:MRM of 2 channels,ES+
1179.6 > 204.1
2.313e+006

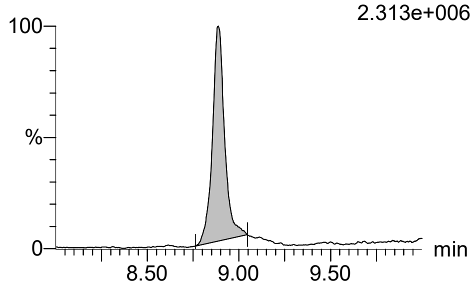

Sample Name: 20211111_L25_R2_IgM
F39:MRM of 2 channels,ES+
943.9 > 366.1
1.948e+005

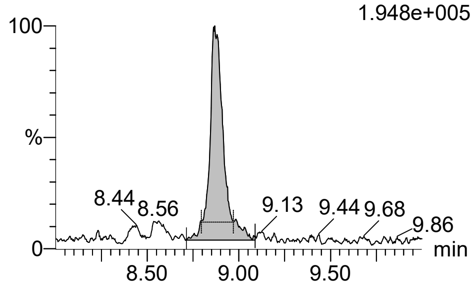

Sample Name: 20211111_L25_R2_IgM
F39:MRM of 2 channels,ES+
1179.6 > 204.1
2.312e+006

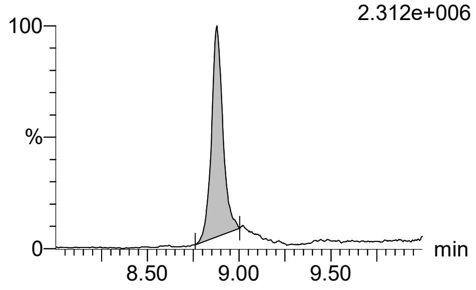

Sample Name: 20211111_L26_R2_IgM
F39:MRM of 2 channels,ES+
943.9 > 366.1
1.808e+005

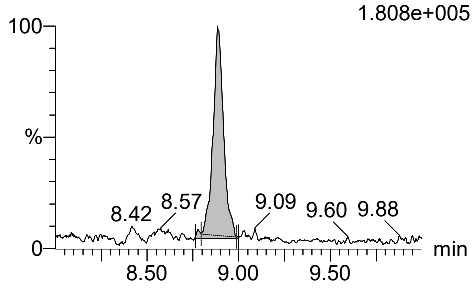

Dataset: C:\MassLynx\10.pro\20211111_clinicalsamples_IgM_IgA.qld

Last Altered: Thursday, March 09, 2023 19:58:25 台北標準時間
Printed: Friday, August 01, 2025 15:01:57 台北標準時間

Sample Name: 20211111_L26_R2_IgM
F39:MRM of 2 channels,ES+
1179.6 > 204.1
1.600e+006

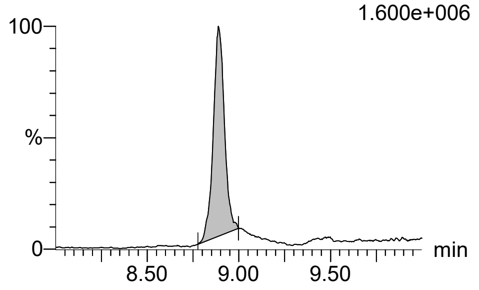

Sample Name: 20211111_H13_R2_IgM
F39:MRM of 2 channels,ES+
943.9 > 366.1
1.868e+005

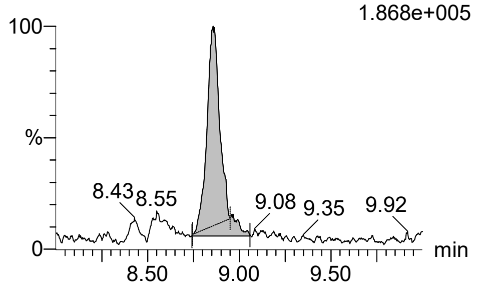

Sample Name: 20211111_H13_R2_IgM
F39:MRM of 2 channels,ES+
1179.6 > 204.1
2.046e+006

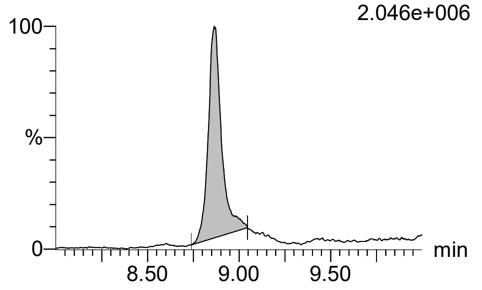

Sample Name: 20211111_A27_R2_IgM
F39:MRM of 2 channels,ES+
943.9 > 366.1
1.917e+005

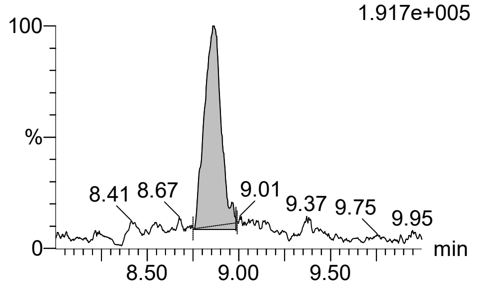

Sample Name: 20211111_A27_R2_IgM
F39:MRM of 2 channels,ES+
1179.6 > 204.1
2.312e+006

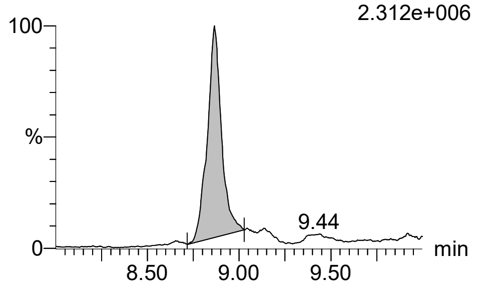

Sample Name: 20211111_A28_R2_IgM
F39:MRM of 2 channels,ES+
943.9 > 366.1
5.011e+004

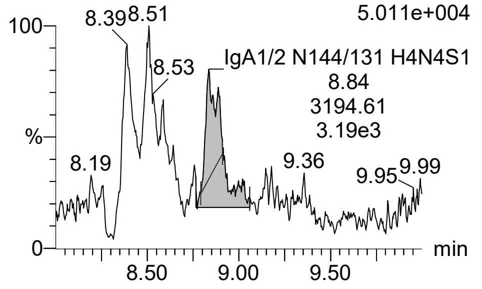

Sample Name: 20211111_A28_R2_IgM
F39:MRM of 2 channels,ES+
1179.6 > 204.1
4.389e+005

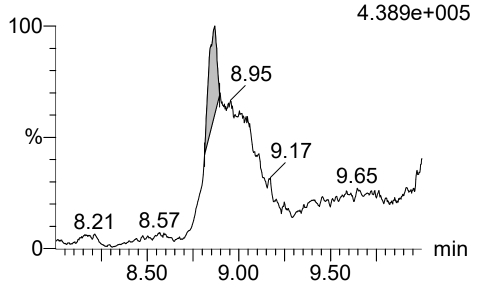

Sample Name: 20211111_L27_R2_IgM
F39:MRM of 2 channels,ES+
943.9 > 366.1
1.815e+005

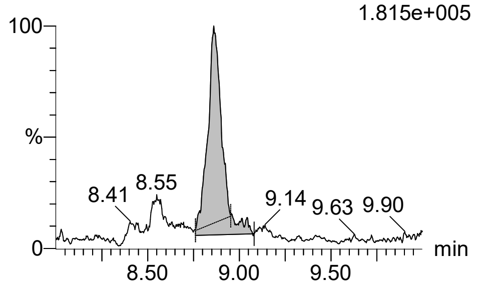

Sample Name: 20211111_L27_R2_IgM
F39:MRM of 2 channels,ES+
1179.6 > 204.1
1.899e+006

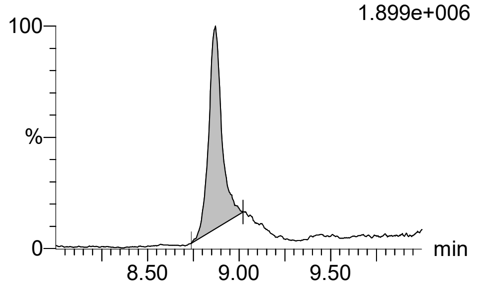

Sample Name: 20211111_L28_R2_IgM
F39:MRM of 2 channels,ES+
943.9 > 366.1
8.335e+004

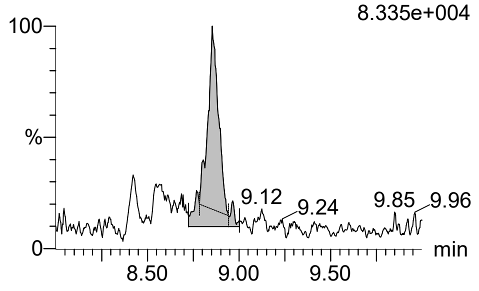

Sample Name: 20211111_L28_R2_IgM
F39:MRM of 2 channels,ES+
1179.6 > 204.1
7.644e+005

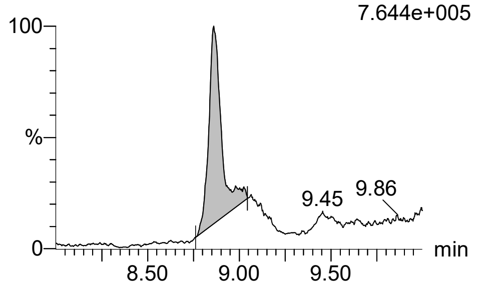

Sample Name: 20211111_H14_R2_IgM
F39:MRM of 2 channels,ES+
943.9 > 366.1
1.723e+005

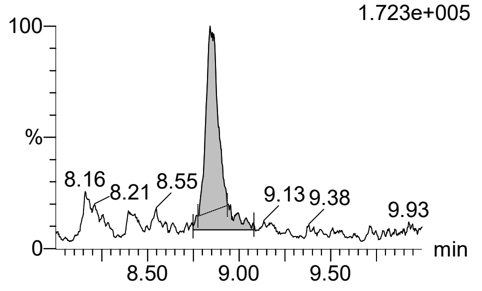

Sample Name: 20211111_H14_R2_IgM
F39:MRM of 2 channels,ES+
1179.6 > 204.1
1.709e+006

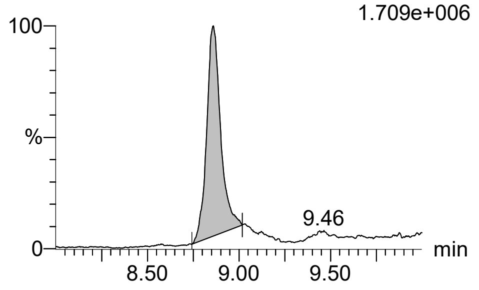

Sample Name: 20211111_A29_R2_IgM
F39:MRM of 2 channels,ES+
943.9 > 366.1
7.501e+004

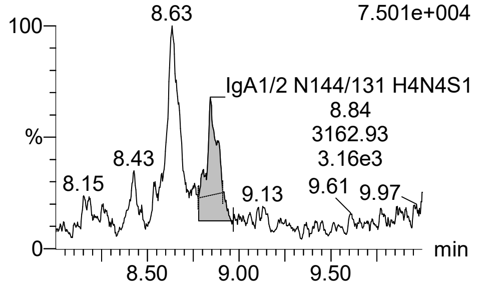

Sample Name: 20211111_A29_R2_IgM
F39:MRM of 2 channels,ES+
1179.6 > 204.1
2.579e+005

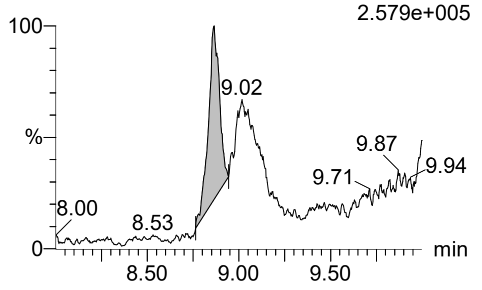

Dataset: C:\MassLynx\10.pro\20211111_clinicalsamples_IgM_IgA.qld

Last Altered: Thursday, March 09, 2023 19:58:25 台北標準時間
Printed: Friday, August 01, 2025 15:01:57 台北標準時間

Sample Name: 20211111_A30_R2_IgM

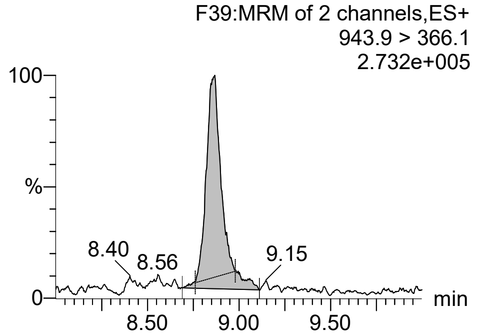

Sample Name: 20211111_A30_R2_IgM

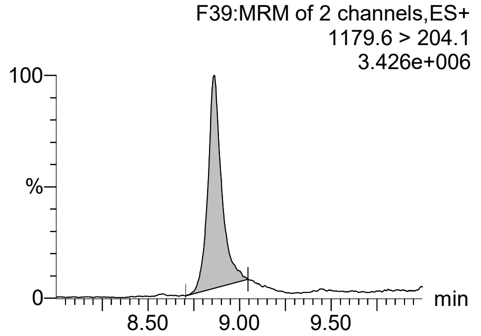

Sample Name: 20211111_QC_R15_IgM

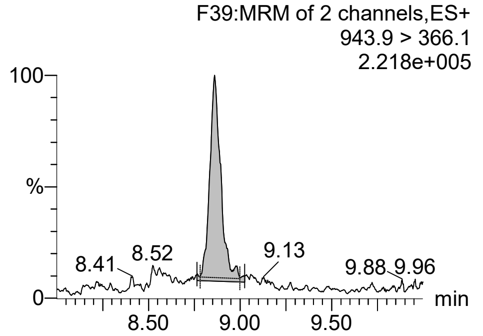

Sample Name: 20211111_QC_R15_IgM

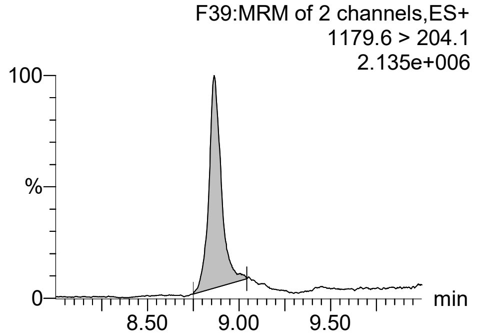

Sample Name: 20211111_L29_R2_IgM

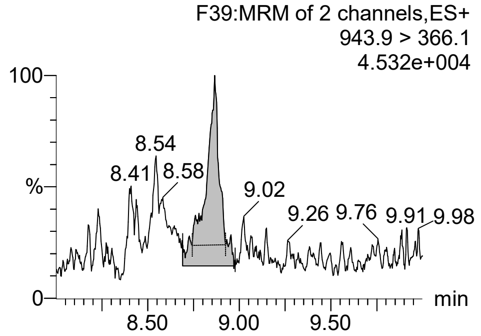

Sample Name: 20211111_L29_R2_IgM

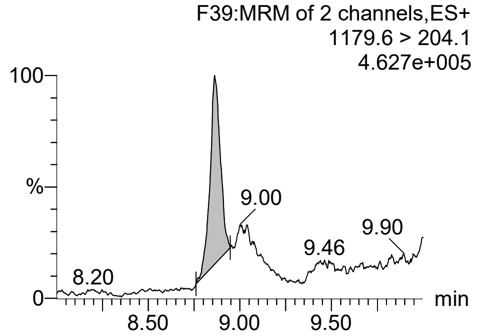

Sample Name: 20211111_L30_R2_IgM

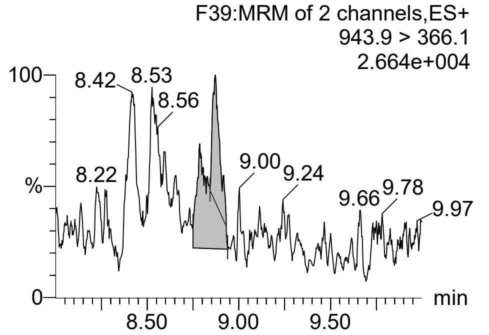

Sample Name: 20211111_L30_R2_IgM

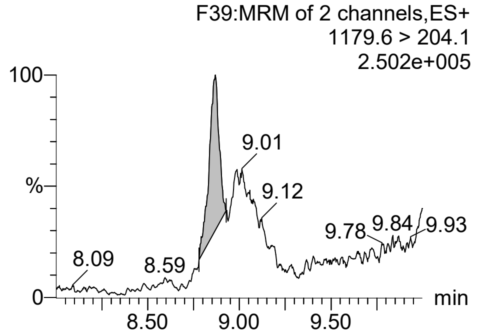

Sample Name: 20211111_H15_R2_IgM

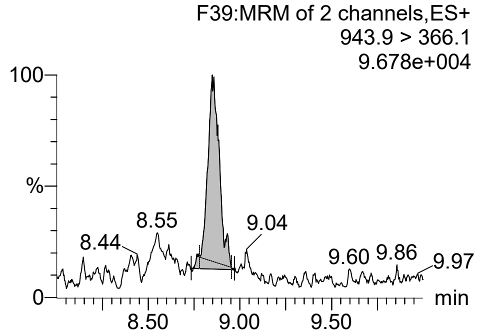

Sample Name: 20211111_H15_R2_IgM

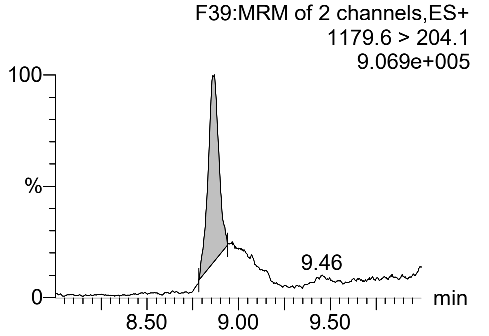

Sample Name: 20211111_A31_R2_IgM

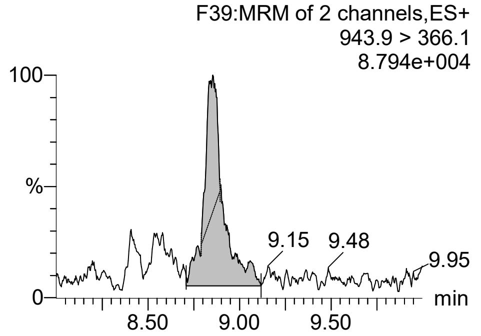

Sample Name: 20211111_A31_R2_IgM

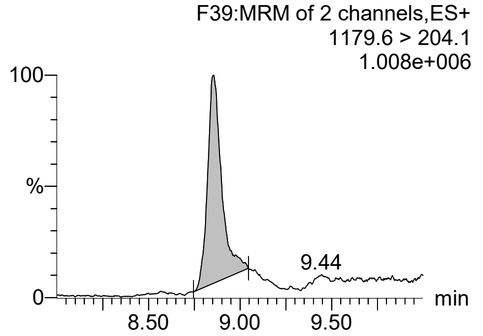

Sample Name: 20211111_A32_R2_IgM

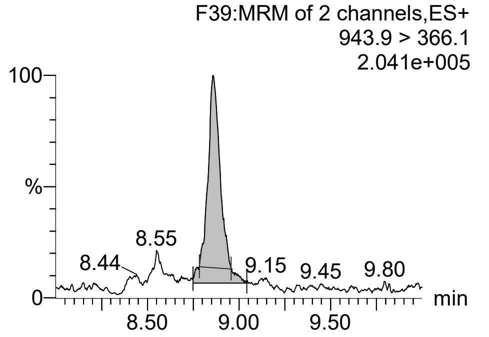

Sample Name: 20211111_A32_R2_IgM

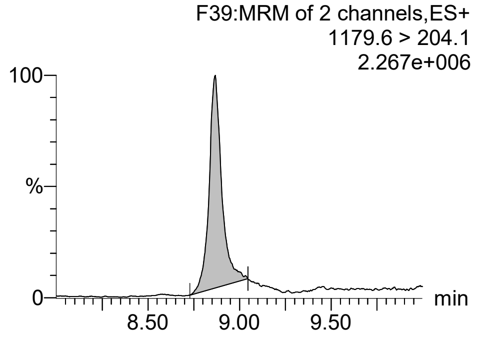

Sample Name: 20211111_L31_R2_IgM

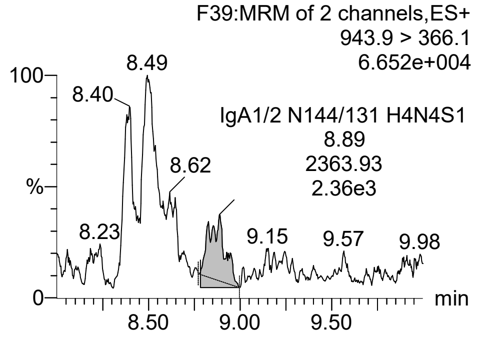

Dataset: C:\MassLynx\10.pro\20211111_clinicalsamples_IgM_IgA.qld

Last Altered: Thursday, March 09, 2023 19:58:25 台北標準時間
Printed: Friday, August 01, 2025 15:01:57 台北標準時間

Sample Name: 20211111_L31_R2_IgM
F39:MRM of 2 channels,ES+
1179.6 > 204.1
2.341e+005

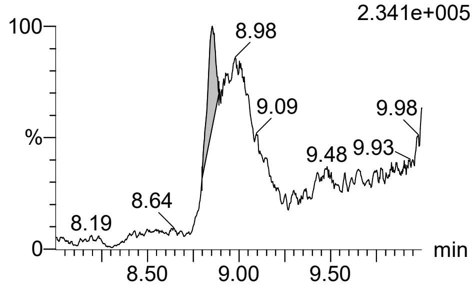

Sample Name: 20211111_L32_R2_IgM
F39:MRM of 2 channels,ES+
943.9 > 366.1
9.666e+004

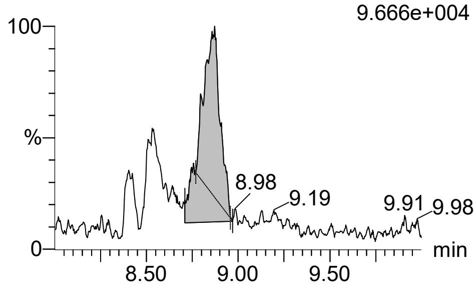

Sample Name: 20211111_L32_R2_IgM
F39:MRM of 2 channels,ES+
1179.6 > 204.1
1.058e+006

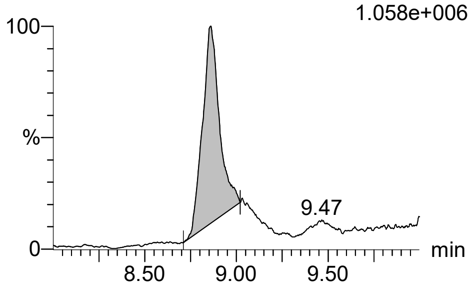

Sample Name: 20211111_H16_R2_IgM
F39:MRM of 2 channels,ES+
943.9 > 366.1
1.759e+005

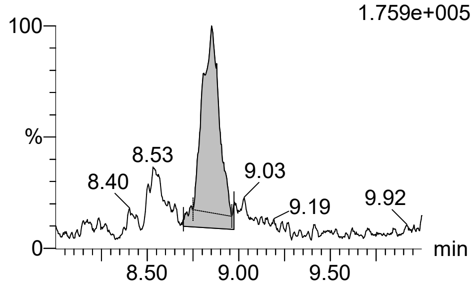

Sample Name: 20211111_H16_R2_IgM
F39:MRM of 2 channels,ES+
1179.6 > 204.1
1.983e+006

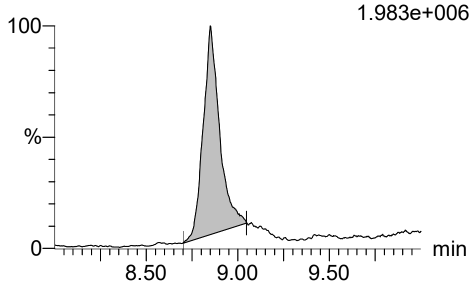

Sample Name: 20211111_A33_R2_IgM
F39:MRM of 2 channels,ES+
943.9 > 366.1
1.768e+005

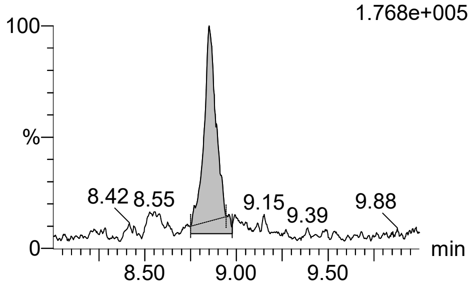

Sample Name: 20211111_A33_R2_IgM
F39:MRM of 2 channels,ES+
1179.6 > 204.1
1.676e+006

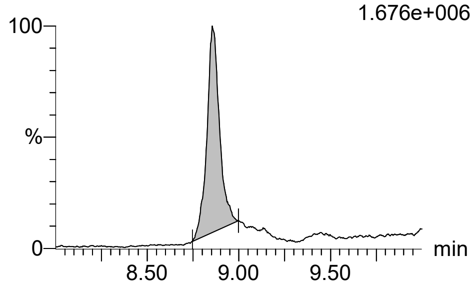

Sample Name: 20211111_A34_R2_IgM
F39:MRM of 2 channels,ES+
943.9 > 366.1
9.786e+004

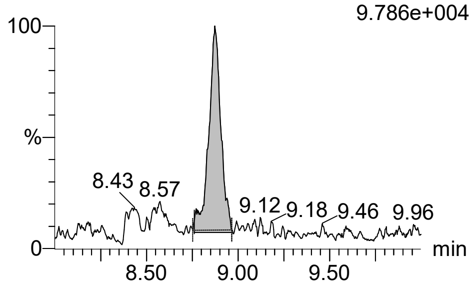

Sample Name: 20211111_A34_R2_IgM
F39:MRM of 2 channels,ES+
1179.6 > 204.1
9.795e+005

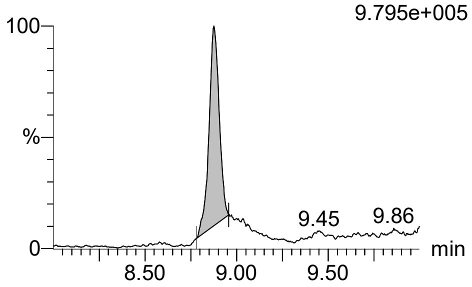

Sample Name: 20211111_H17_R2_IgM
F39:MRM of 2 channels,ES+
943.9 > 366.1
3.816e+005

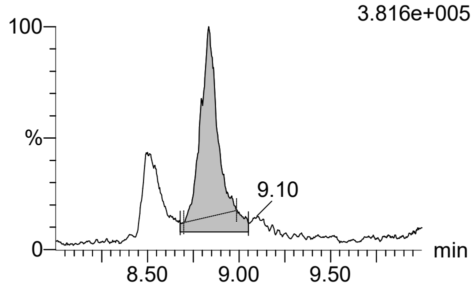

Sample Name: 20211111_H17_R2_IgM
F39:MRM of 2 channels,ES+
1179.6 > 204.1
3.823e+006

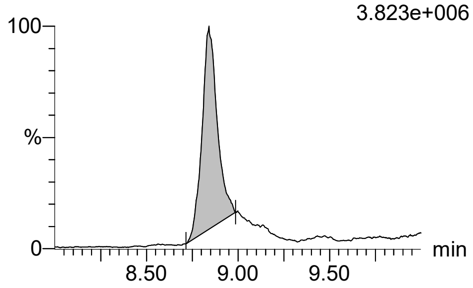

Sample Name: 20211111_A35_R2_IgM
F39:MRM of 2 channels,ES+
943.9 > 366.1
8.124e+004

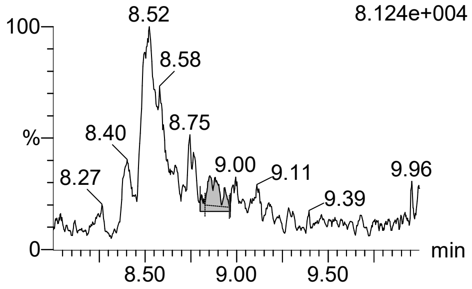

Sample Name: 20211111_A35_R2_IgM
F39:MRM of 2 channels,ES+
1179.6 > 204.1
2.857e+005

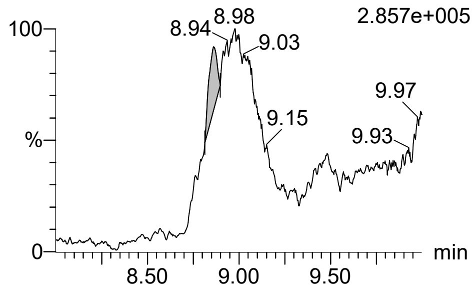

Sample Name: 20211111_QC_R16_IgM
F39:MRM of 2 channels,ES+
943.9 > 366.1
1.989e+005

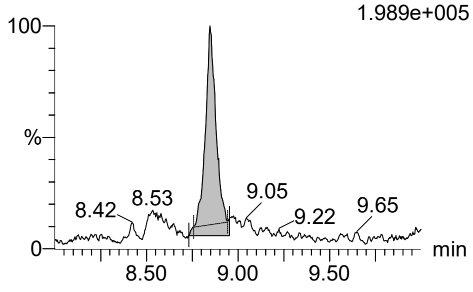

Sample Name: 20211111_QC_R16_IgM
F39:MRM of 2 channels,ES+
1179.6 > 204.1
1.748e+006

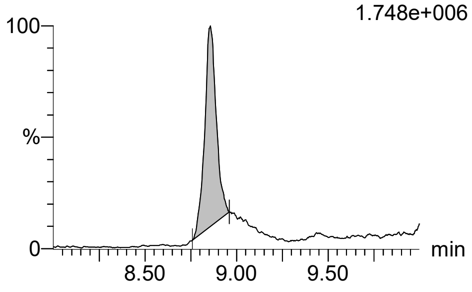

Dataset: C:\MassLynx\10.pro\20211111_clinicalsamples_IgM_IgA.qld

Last Altered: Thursday, March 09, 2023 19:58:25 台北標準時間
Printed: Friday, August 01, 2025 15:01:57 台北標準時間

Sample Name: 20211111_A36_R2_IgM
F39:MRM of 2 channels,ES+
943.9 > 366.1
8.450e+004

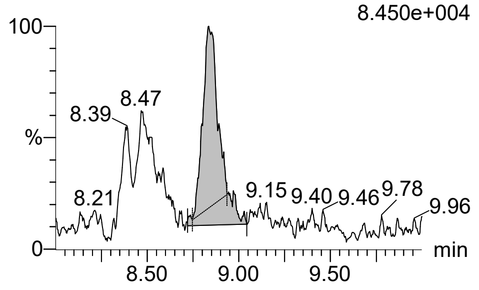

Sample Name: 20211111_A36_R2_IgM
F39:MRM of 2 channels,ES+
1179.6 > 204.1
1.144e+006

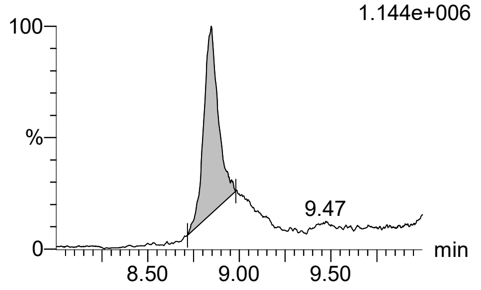

Sample Name: 20211111_H18_R2_IgM
F39:MRM of 2 channels,ES+
943.9 > 366.1
3.992e+005

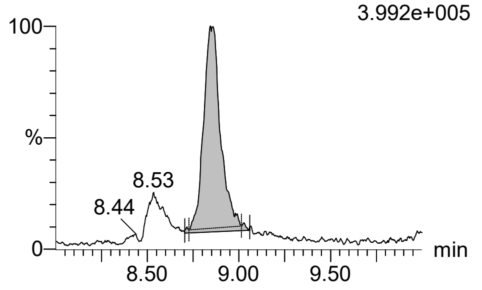

Sample Name: 20211111_H18_R2_IgM
F39:MRM of 2 channels,ES+
1179.6 > 204.1
4.758e+006

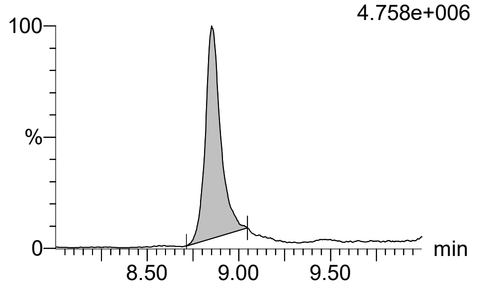

Sample Name: 20211111_A37_R2_IgM
F39:MRM of 2 channels,ES+
943.9 > 366.1
1.828e+005

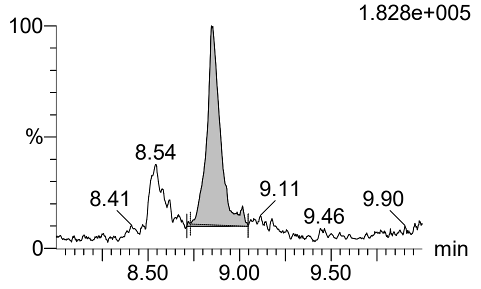

Sample Name: 20211111_A37_R2_IgM
F39:MRM of 2 channels,ES+
1179.6 > 204.1
1.833e+006

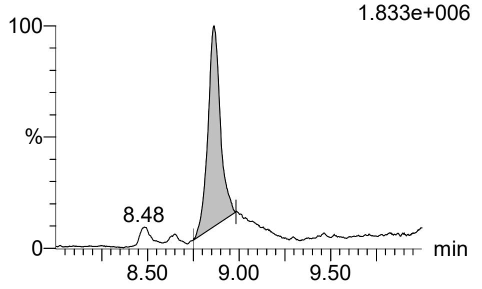

Sample Name: 20211111_A38_R2_IgM
F39:MRM of 2 channels,ES+
943.9 > 366.1
2.927e+005

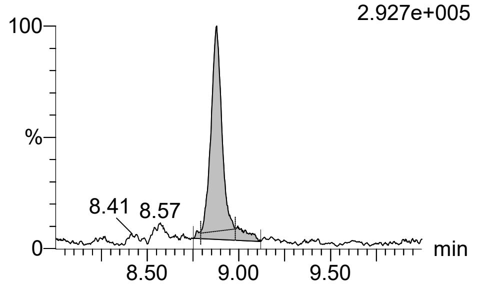

Sample Name: 20211111_A38_R2_IgM
F39:MRM of 2 channels,ES+
1179.6 > 204.1
2.915e+006

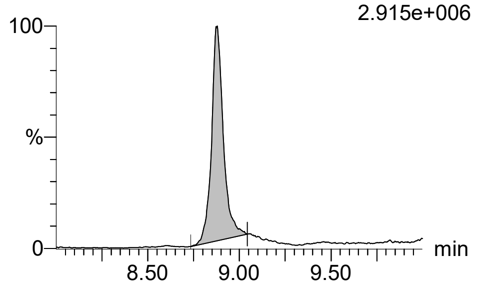

Sample Name: 20211111_H19_R2_IgM
F39:MRM of 2 channels,ES+
943.9 > 366.1
1.223e+005

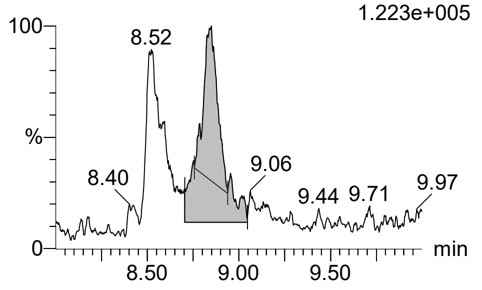

Sample Name: 20211111_H19_R2_IgM
F39:MRM of 2 channels,ES+
1179.6 > 204.1
1.242e+006

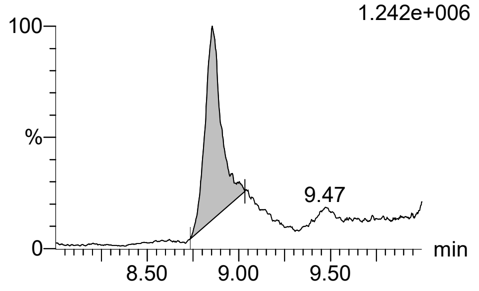

Sample Name: 20211111_A39_R2_IgM
F39:MRM of 2 channels,ES+
943.9 > 366.1
2.445e+005

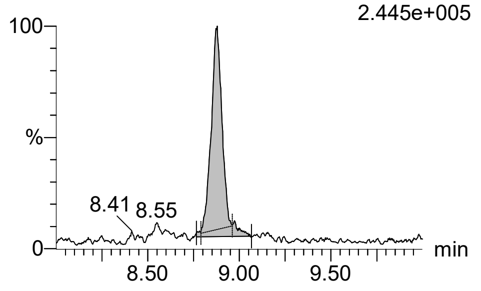

Sample Name: 20211111_A39_R2_IgM
F39:MRM of 2 channels,ES+
1179.6 > 204.1
2.249e+006

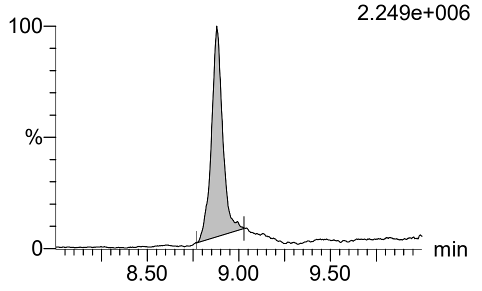

Sample Name: 20211111_A40_R2_IgM
F39:MRM of 2 channels,ES+
943.9 > 366.1
7.830e+004

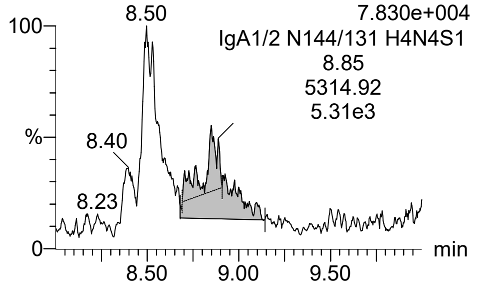

Sample Name: 20211111_A40_R2_IgM
F39:MRM of 2 channels,ES+
1179.6 > 204.1
3.688e+005

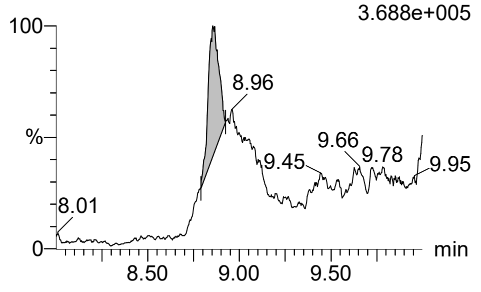

Sample Name: 20211111_H20_R2_IgM
F39:MRM of 2 channels,ES+
943.9 > 366.1
3.287e+005

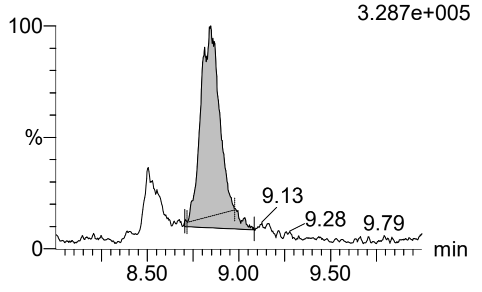

Dataset: C:\MassLynx\10.pro\20211111_clinicalsamples_IgM_IgA.qld

Last Altered: Thursday, March 09, 2023 19:58:25 台北標準時間
Printed: Friday, August 01, 2025 15:01:57 台北標準時間

Sample Name: 20211111_H20_R2_IgM
F39:MRM of 2 channels,ES+
1179.6 > 204.1
3.755e+006

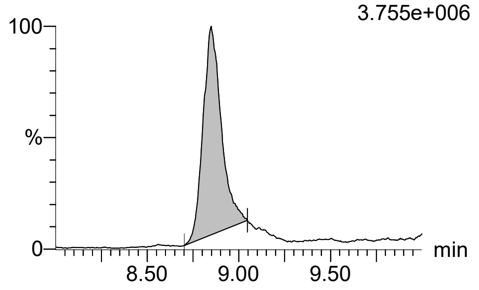

Sample Name: 20211111_A41_R2_IgM
F39:MRM of 2 channels,ES+
943.9 > 366.1
1.354e+005

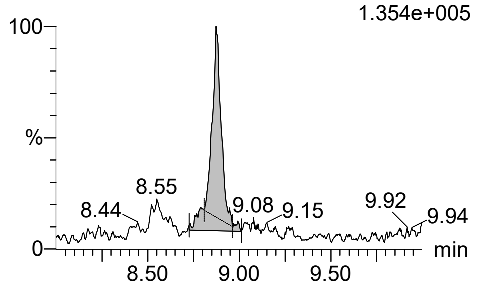

Sample Name: 20211111_A41_R2_IgM
F39:MRM of 2 channels,ES+
1179.6 > 204.1
1.037e+006

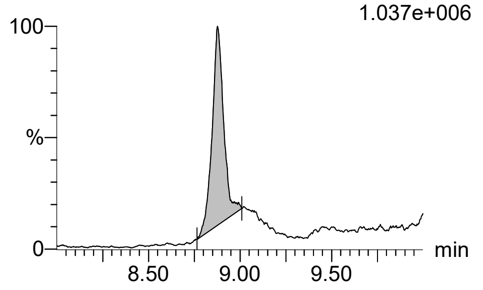

Sample Name: 20211111_A42_R2_IgM
F39:MRM of 2 channels,ES+
943.9 > 366.1
6.850e+004

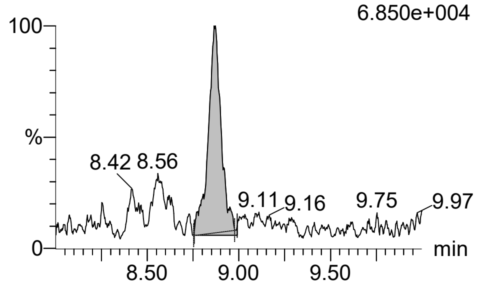

Sample Name: 20211111_A42_R2_IgM
F39:MRM of 2 channels,ES+
1179.6 > 204.1
6.738e+005

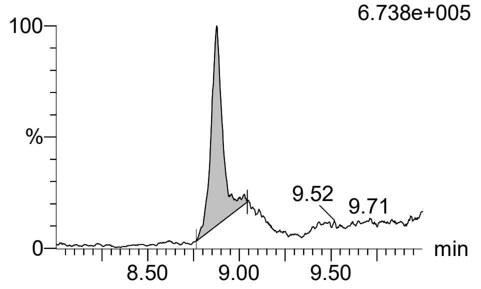

Sample Name: 20211111_A43_R2_IgM
F39:MRM of 2 channels,ES+
943.9 > 366.1
7.921e+004

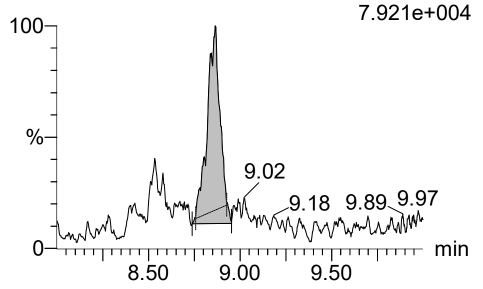

Sample Name: 20211111_A43_R2_IgM
F39:MRM of 2 channels,ES+
1179.6 > 204.1
7.779e+005

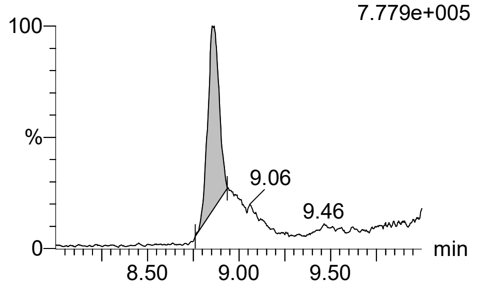

Sample Name: 20211111_A44_R2_IgM
F39:MRM of 2 channels,ES+
943.9 > 366.1
9.275e+004

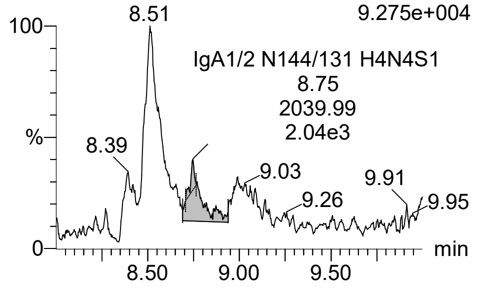

Sample Name: 20211111_A44_R2_IgM
F39:MRM of 2 channels,ES+
1179.6 > 204.1
2.565e+005

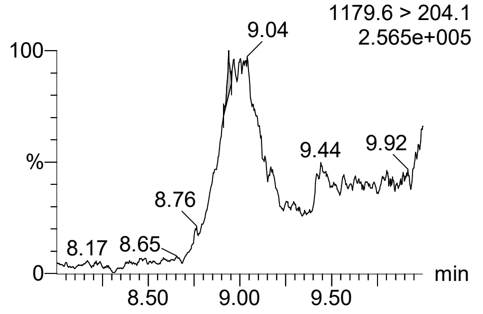

Sample Name: 20211111_QC_R17_IgM
F39:MRM of 2 channels,ES+
943.9 > 366.1
1.281e+005

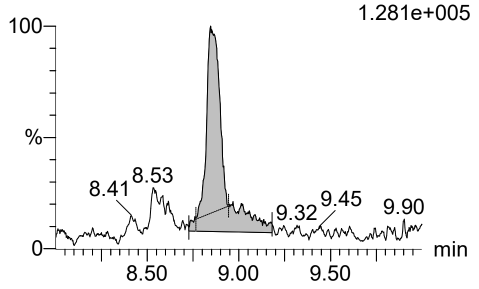

Sample Name: 20211111_QC_R17_IgM
F39:MRM of 2 channels,ES+
1179.6 > 204.1
1.471e+006

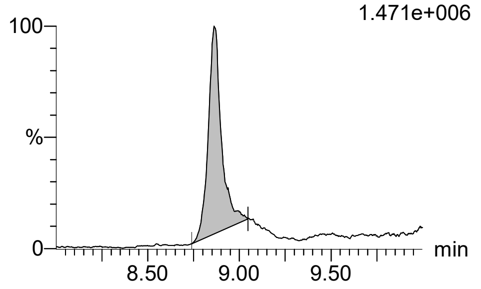

Sample Name: 20211111_L2_R3_IgM
F39:MRM of 2 channels,ES+
943.9 > 366.1
1.476e+005

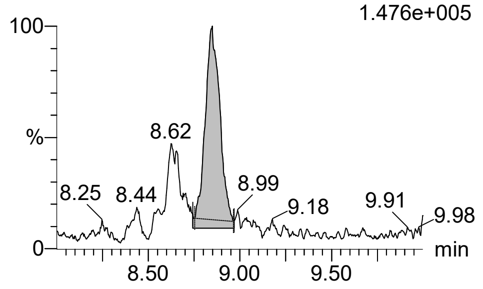

Sample Name: 20211111_L2_R3_IgM
F39:MRM of 2 channels,ES+
1179.6 > 204.1
1.409e+006

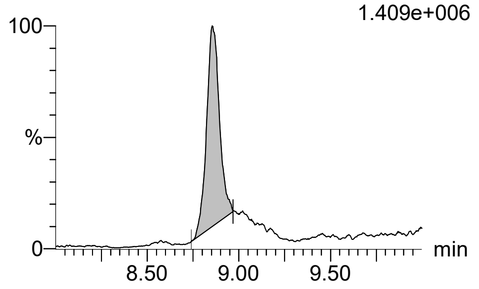

Sample Name: 20211111_H1_R3_IgM
F39:MRM of 2 channels,ES+
943.9 > 366.1
4.729e+005

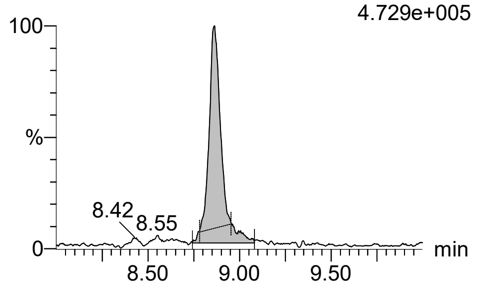

Sample Name: 20211111_H1_R3_IgM
F39:MRM of 2 channels,ES+
1179.6 > 204.1
4.975e+006

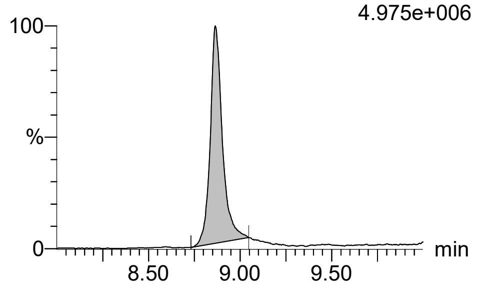

Sample Name: 20211111_A3_R3_IgM
F39:MRM of 2 channels,ES+
943.9 > 366.1
4.738e+005

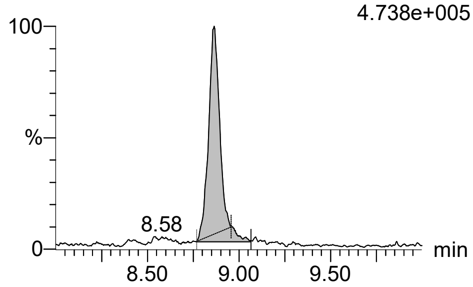

Sample Name: 20211111_A3_R3_IgM
F39:MRM of 2 channels,ES+
1179.6 > 204.1
4.597e+006

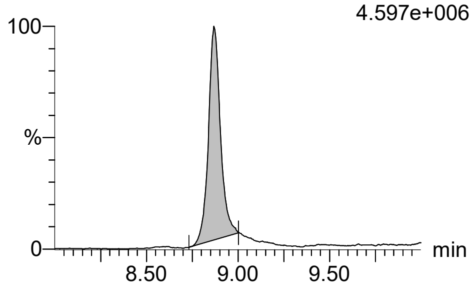

Sample Name: 20211111_A4_R3_IgM
F39:MRM of 2 channels,ES+
943.9 > 366.1
2.280e+005

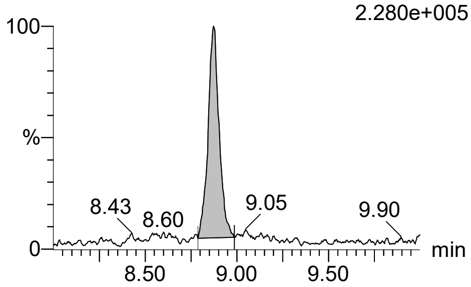

Sample Name: 20211111_A4_R3_IgM
F39:MRM of 2 channels,ES+
1179.6 > 204.1
2.054e+006

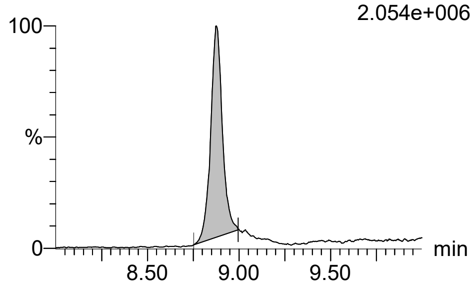

Sample Name: 20211111_L3_R3_IgM
F39:MRM of 2 channels,ES+
943.9 > 366.1
4.166e+004

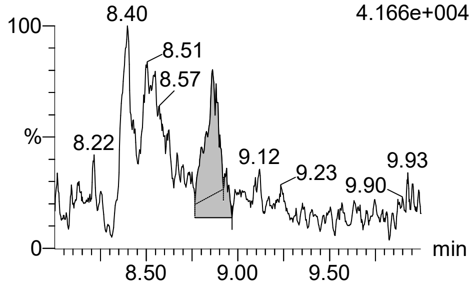

Sample Name: 20211111_L3_R3_IgM
F39:MRM of 2 channels,ES+
1179.6 > 204.1
3.562e+005

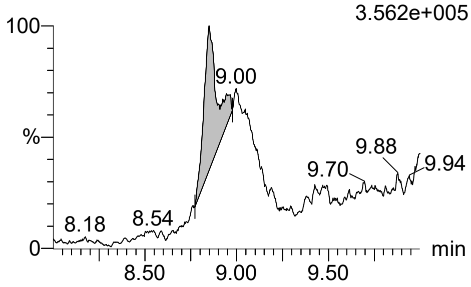

Sample Name: 20211111_L4_R3_IgM
F39:MRM of 2 channels,ES+
943.9 > 366.1
1.503e+005

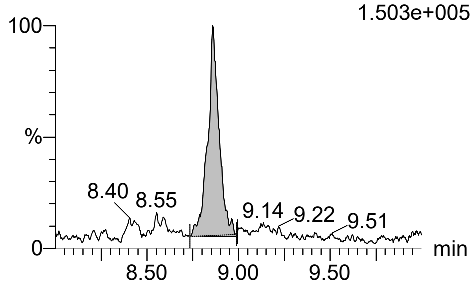

Sample Name: 20211111_L4_R3_IgM
F39:MRM of 2 channels,ES+
1179.6 > 204.1
1.335e+006

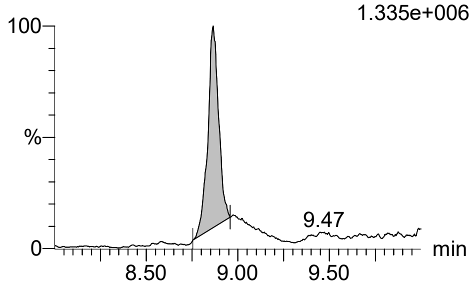

Sample Name: 20211111_H2_R3_IgM
F39:MRM of 2 channels,ES+
943.9 > 366.1
6.787e+005

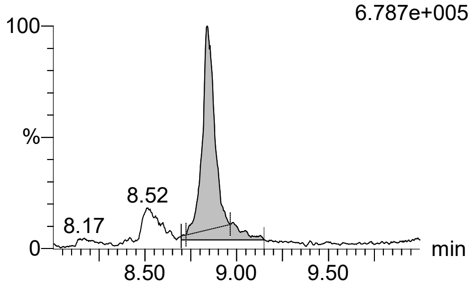

Sample Name: 20211111_H2_R3_IgM
F39:MRM of 2 channels,ES+
1179.6 > 204.1
7.056e+006

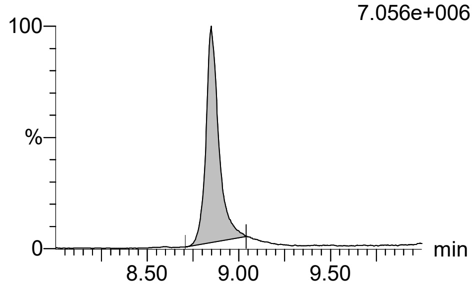

Sample Name: 20211111_A5_R3_IgM
F39:MRM of 2 channels,ES+
943.9 > 366.1
7.684e+005

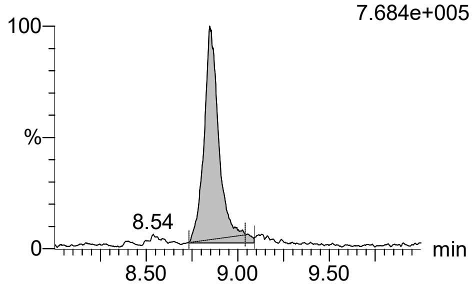

Sample Name: 20211111_A5_R3_IgM
F39:MRM of 2 channels,ES+
1179.6 > 204.1
8.121e+006

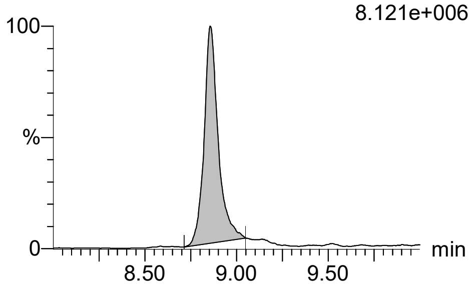

Sample Name: 20211111_A6_R3_IgM
F39:MRM of 2 channels,ES+
943.9 > 366.1
1.366e+005

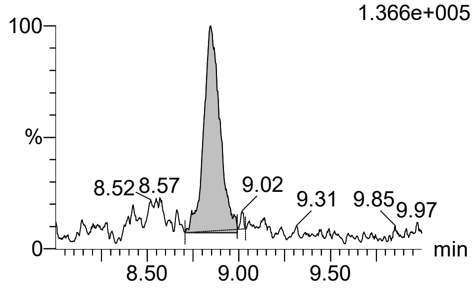

Sample Name: 20211111_A6_R3_IgM
F39:MRM of 2 channels,ES+
1179.6 > 204.1
1.526e+006

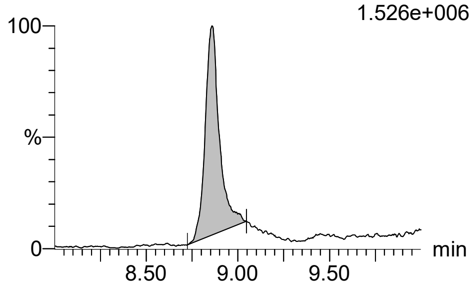

Sample Name: 20211111_QC_R18_IgM
F39:MRM of 2 channels,ES+
943.9 > 366.1
1.552e+005

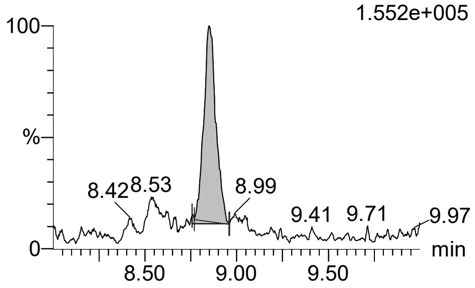

Sample Name: 20211111_QC_R18_IgM
F39:MRM of 2 channels,ES+
1179.6 > 204.1
1.601e+006

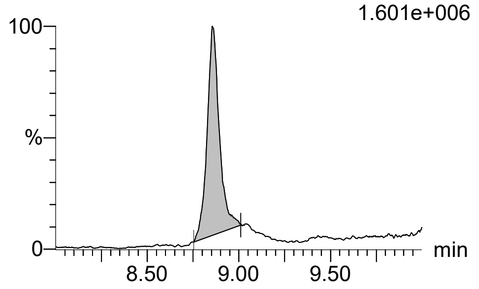

Sample Name: 20211111_L5_R3_IgM
F39:MRM of 2 channels,ES+
943.9 > 366.1
2.712e+005

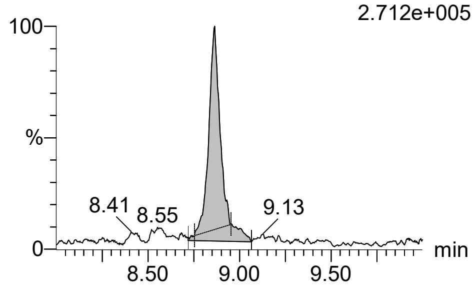

Sample Name: 20211111_L5_R3_IgM
F39:MRM of 2 channels,ES+
1179.6 > 204.1
2.651e+006

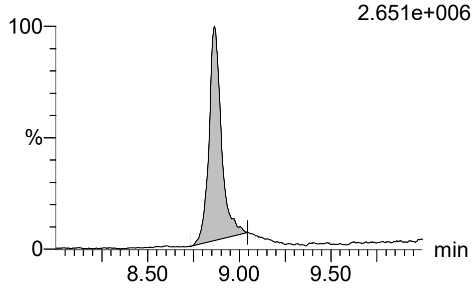

Sample Name: 20211111_L6_R3_IgM
F39:MRM of 2 channels,ES+
943.9 > 366.1
1.444e+005

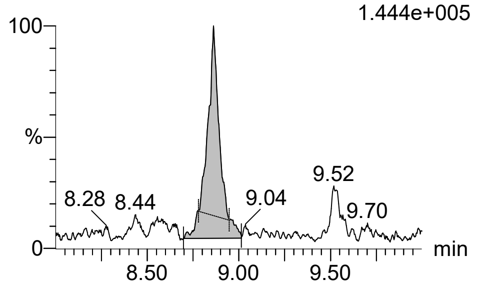

Sample Name: 20211111_L6_R3_IgM
F39:MRM of 2 channels,ES+
1179.6 > 204.1
1.339e+006

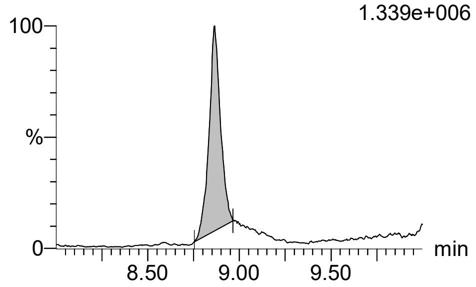

Sample Name: 20211111_H3_R3_IgM
F39:MRM of 2 channels,ES+
943.9 > 366.1
1.060e+005

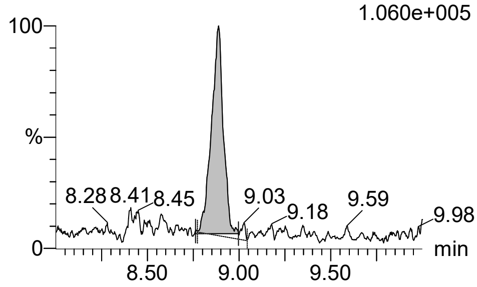

Sample Name: 20211111_H3_R3_IgM
F39:MRM of 2 channels,ES+
1179.6 > 204.1
8.641e+005

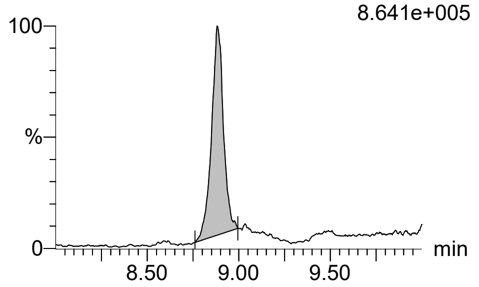

Sample Name: 20211111_A7_R3_IgM
F39:MRM of 2 channels,ES+
943.9 > 366.1
6.004e+005

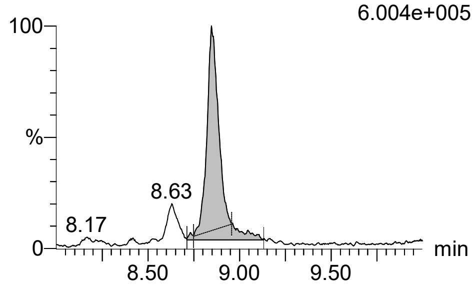

Sample Name: 20211111_A7_R3_IgM
F39:MRM of 2 channels,ES+
1179.6 > 204.1
6.178e+006

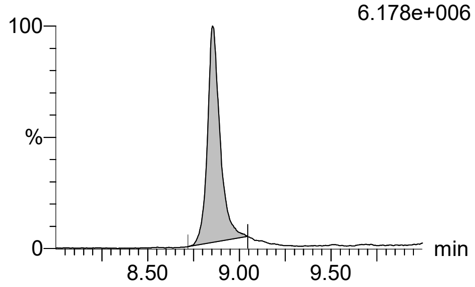

Sample Name: 20211111_A8_R3_IgM
F39:MRM of 2 channels,ES+
943.9 > 366.1
5.246e+005

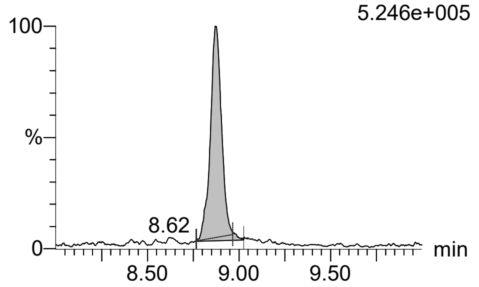

Sample Name: 20211111_A8_R3_IgM
F39:MRM of 2 channels,ES+
1179.6 > 204.1
4.799e+006

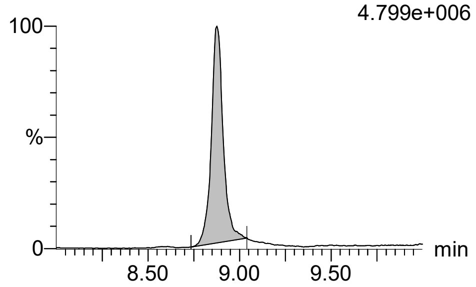

Sample Name: 20211111_L7_R3_IgM
F39:MRM of 2 channels,ES+
943.9 > 366.1
9.380e+004

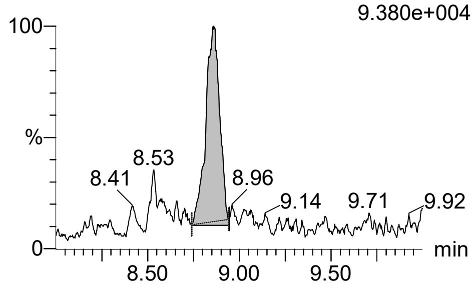

Sample Name: 20211111_L7_R3_IgM
F39:MRM of 2 channels,ES+
1179.6 > 204.1
1.127e+006

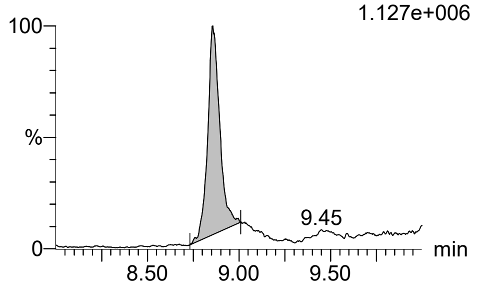

Sample Name: 20211111_L8_R3_IgM
F39:MRM of 2 channels,ES+
943.9 > 366.1
3.320e+004

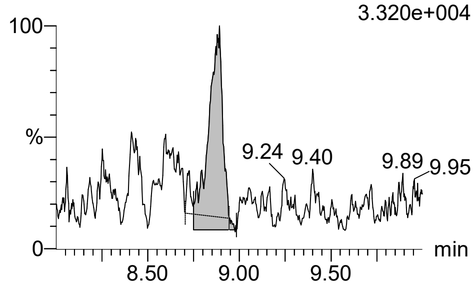

Sample Name: 20211111_L8_R3_IgM
F39:MRM of 2 channels,ES+
1179.6 > 204.1
2.269e+005

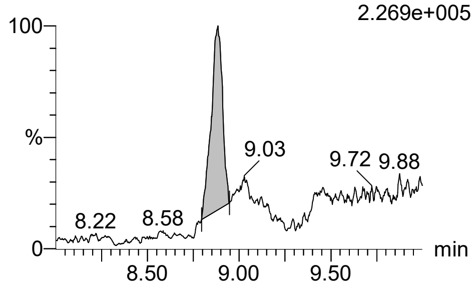

Sample Name: 20211111_H4_R3_IgM
F39:MRM of 2 channels,ES+
943.9 > 366.1
8.269e+004

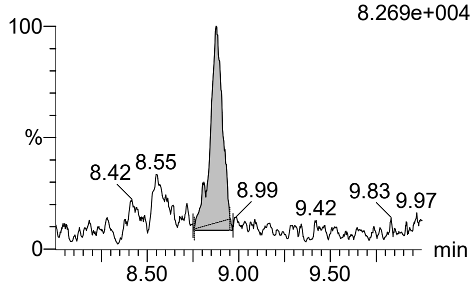

Sample Name: 20211111_H4_R3_IgM
F39:MRM of 2 channels,ES+
1179.6 > 204.1
7.289e+005

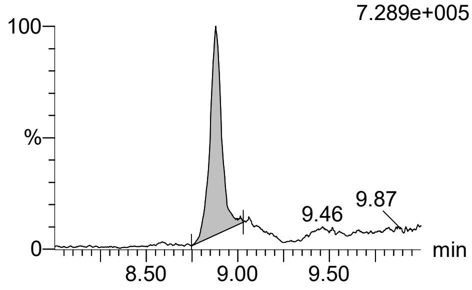

Sample Name: 20211111_A9_R3_IgM
F39:MRM of 2 channels,ES+
943.9 > 366.1
2.609e+005

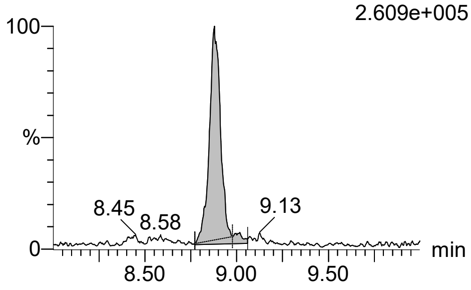

Sample Name: 20211111_A9_R3_IgM
F39:MRM of 2 channels,ES+
1179.6 > 204.1
2.250e+006

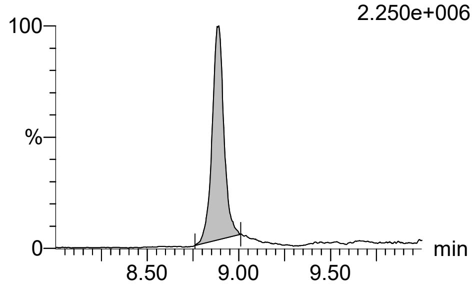

Sample Name: 20211111_A10_R3_IgM
F39:MRM of 2 channels,ES+
943.9 > 366.1
7.809e+005

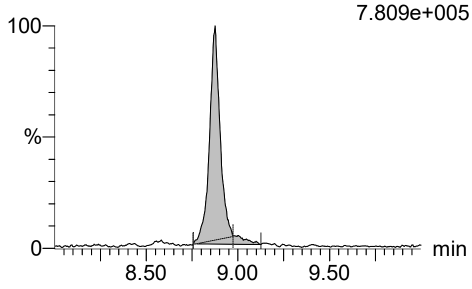

Sample Name: 20211111_A10_R3_IgM
F39:MRM of 2 channels,ES+
1179.6 > 204.1
7.123e+006

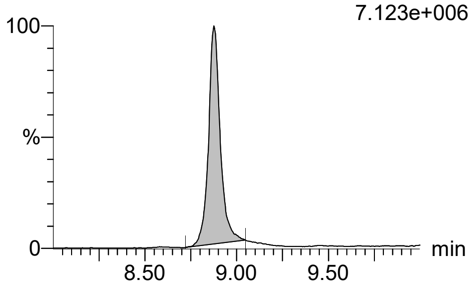

Sample Name: 20211111_L9_R3_IgM
F39:MRM of 2 channels,ES+
943.9 > 366.1
2.012e+005

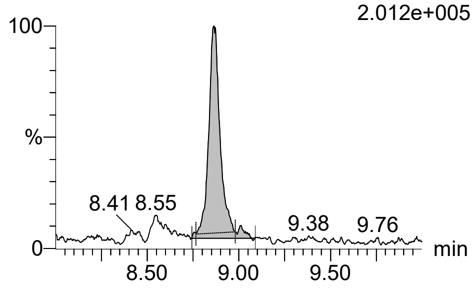

Sample Name: 20211111_L9_R3_IgM
F39:MRM of 2 channels,ES+
1179.6 > 204.1
1.995e+006

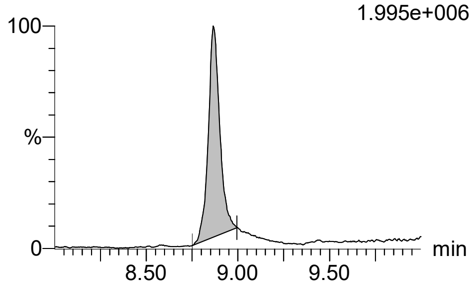

Sample Name: 20211111_L10_R3_IgM
F39:MRM of 2 channels,ES+
943.9 > 366.1
1.114e+005

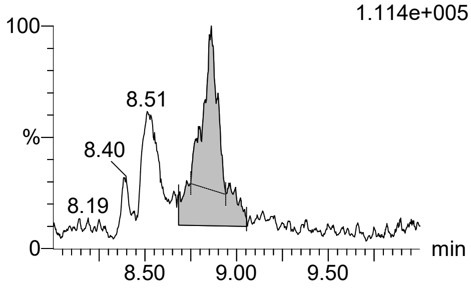

Sample Name: 20211111_L10_R3_IgM
F39:MRM of 2 channels,ES+
1179.6 > 204.1
1.260e+006

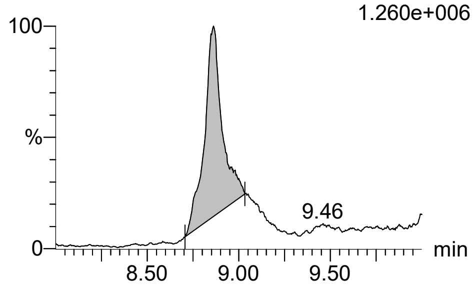

Sample Name: 20211111_QC_R19_IgM
F39:MRM of 2 channels,ES+
943.9 > 366.1
1.480e+005

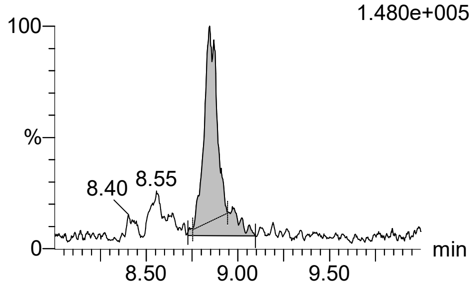

Sample Name: 20211111_QC_R19_IgM
F39:MRM of 2 channels,ES+
1179.6 > 204.1
1.455e+006

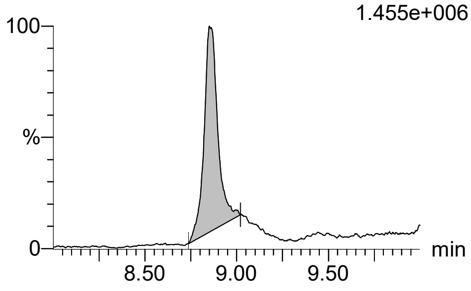

Sample Name: 20211111_H5_R3_IgM
F39:MRM of 2 channels,ES+
943.9 > 366.1
1.097e+005

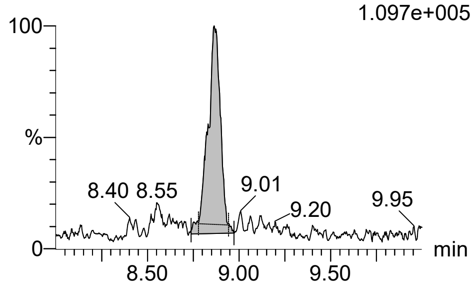

Sample Name: 20211111_H5_R3_IgM
F39:MRM of 2 channels,ES+
1179.6 > 204.1
9.227e+005

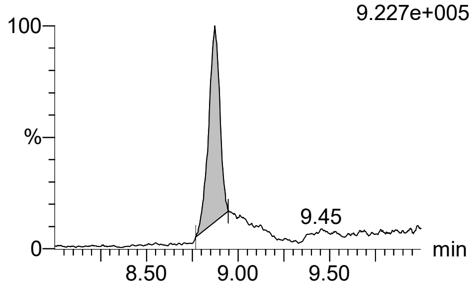

Sample Name: 20211111_A11_R3_IgM
F39:MRM of 2 channels,ES+
943.9 > 366.1
3.007e+005

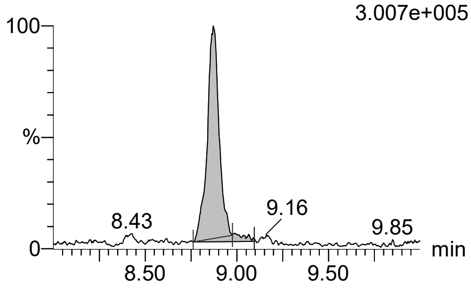

Dataset: C:\MassLynx\10.pro\20211111_clinicalsamples_IgM_IgA.qld

Last Altered: Thursday, March 09, 2023 19:58:25 台北標準時間
Printed: Friday, August 01, 2025 15:01:57 台北標準時間

Sample Name: 20211111_A11_R3_IgM
F39:MRM of 2 channels,ES+
1179.6 > 204.1
2.848e+006

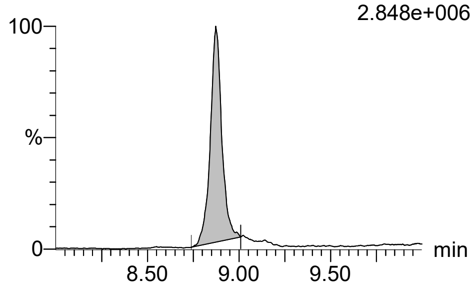

Sample Name: 20211111_A12_R3_IgM
F39:MRM of 2 channels,ES+
943.9 > 366.1
8.763e+004

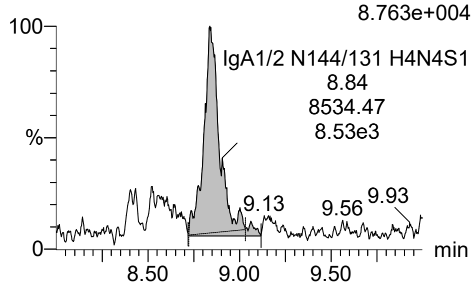

Sample Name: 20211111_A12_R3_IgM
F39:MRM of 2 channels,ES+
1179.6 > 204.1
9.371e+005

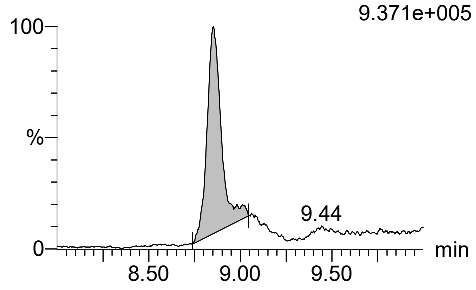

Sample Name: 20211111_L11_R3_IgM
F39:MRM of 2 channels,ES+
943.9 > 366.1
1.462e+005

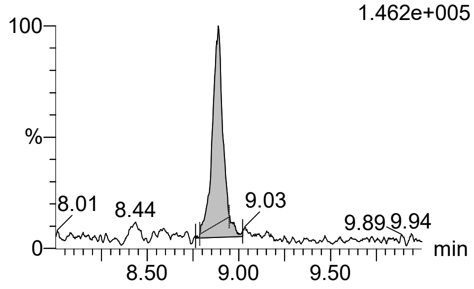

Sample Name: 20211111_L11_R3_IgM
F39:MRM of 2 channels,ES+
1179.6 > 204.1
1.211e+006

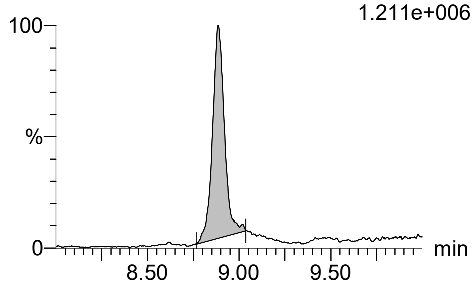

Sample Name: 20211111_L12_R3_IgM
F39:MRM of 2 channels,ES+
943.9 > 366.1
6.086e+004

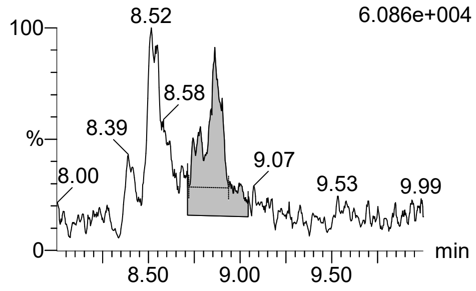

Sample Name: 20211111_L12_R3_IgM
F39:MRM of 2 channels,ES+
1179.6 > 204.1
5.738e+005

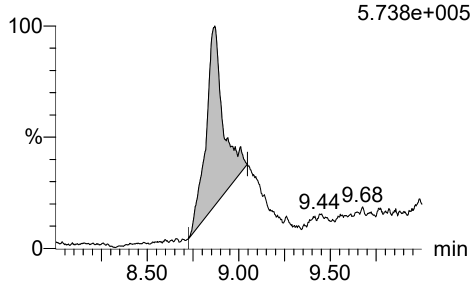

Sample Name: 20211111_H6_R3_IgM
F39:MRM of 2 channels,ES+
943.9 > 366.1
4.456e+004

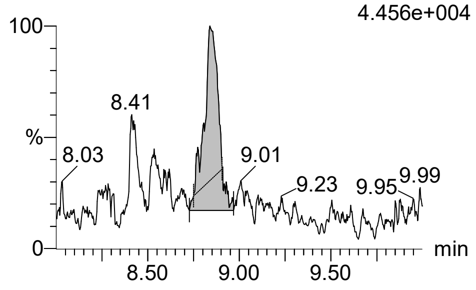

Sample Name: 20211111_H6_R3_IgM
F39:MRM of 2 channels,ES+
1179.6 > 204.1
4.762e+005

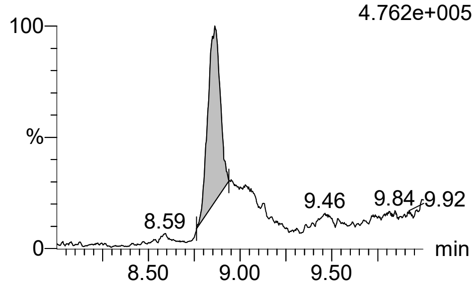

Sample Name: 20211111_A13_R3_IgM
F39:MRM of 2 channels,ES+
943.9 > 366.1
5.314e+004

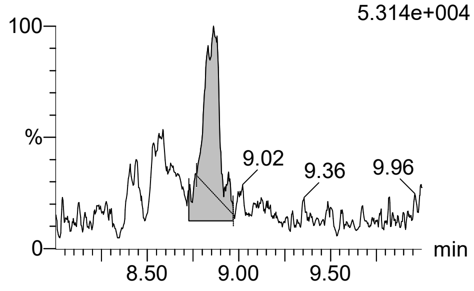

Sample Name: 20211111_A13_R3_IgM
F39:MRM of 2 channels,ES+
1179.6 > 204.1
5.591e+005

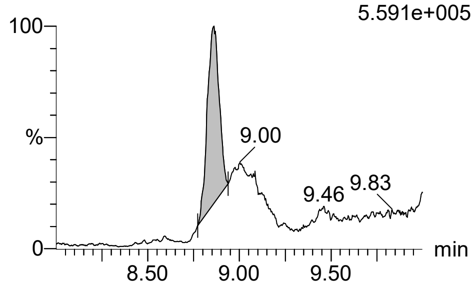

Sample Name: 20211111_A14_R3_IgM
F39:MRM of 2 channels,ES+
943.9 > 366.1
6.558e+005

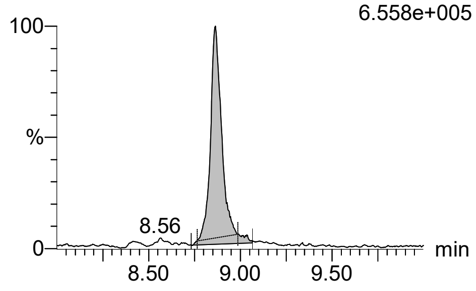

Sample Name: 20211111_A14_R3_IgM
F39:MRM of 2 channels,ES+
1179.6 > 204.1
6.885e+006

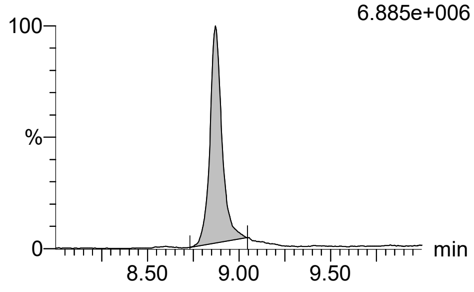

Sample Name: 20211111_L13_R3_IgM
F39:MRM of 2 channels,ES+
943.9 > 366.1
4.974e+004

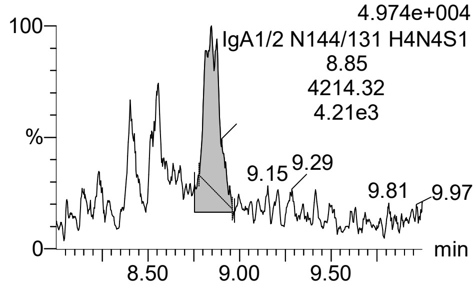

Sample Name: 20211111_L13_R3_IgM
F39:MRM of 2 channels,ES+
1179.6 > 204.1
5.946e+005

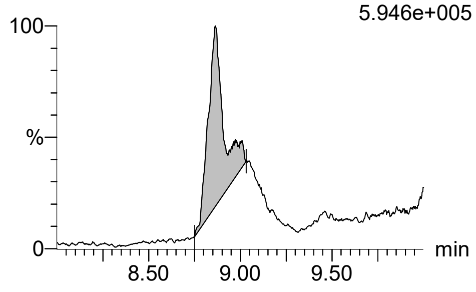

Dataset: C:\MassLynx\10.pro\20211111_clinicalsamples_IgM_IgA.qld

Last Altered: Thursday, March 09, 2023 19:58:25 台北標準時間
Printed: Friday, August 01, 2025 15:01:57 台北標準時間

Sample Name: 20211111_L14_R3_IgM
F39:MRM of 2 channels,ES+
943.9 > 366.1
1.402e+005

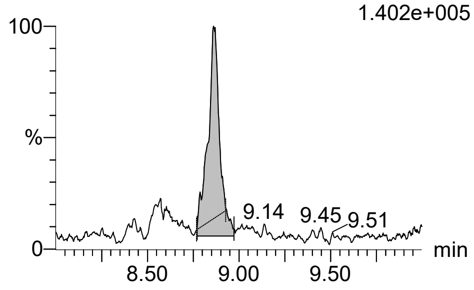

Sample Name: 20211111_L14_R3_IgM
F39:MRM of 2 channels,ES+
1179.6 > 204.1
1.308e+006

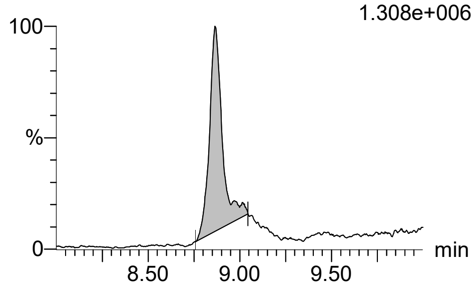

Sample Name: 20211111_H7_R3_IgM
F39:MRM of 2 channels,ES+
943.9 > 366.1
8.884e+004

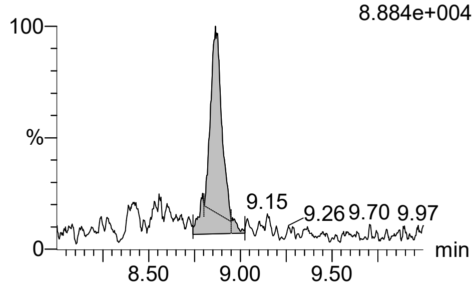

Sample Name: 20211111_H7_R3_IgM
F39:MRM of 2 channels,ES+
1179.6 > 204.1
8.382e+005

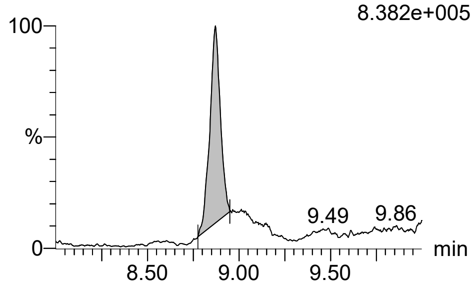

Sample Name: 20211111_A15_R3_IgM
F39:MRM of 2 channels,ES+
943.9 > 366.1
4.392e+005

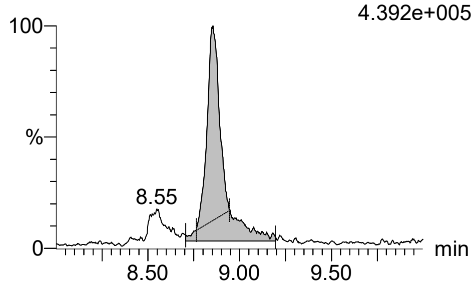

Sample Name: 20211111_A15_R3_IgM
F39:MRM of 2 channels,ES+
1179.6 > 204.1
4.910e+006

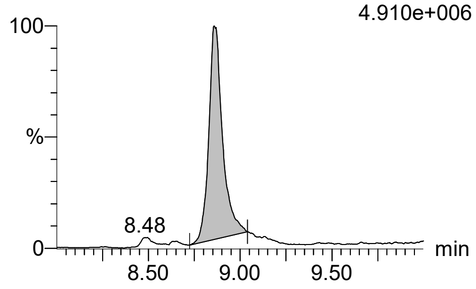

Sample Name: 20211111_QC_R20_IgM
F39:MRM of 2 channels,ES+
943.9 > 366.1
1.817e+005

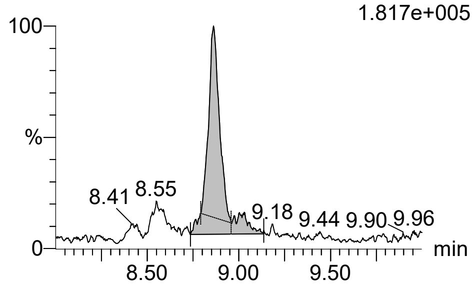

Sample Name: 20211111_QC_R20_IgM
F39:MRM of 2 channels,ES+
1179.6 > 204.1
1.912e+006

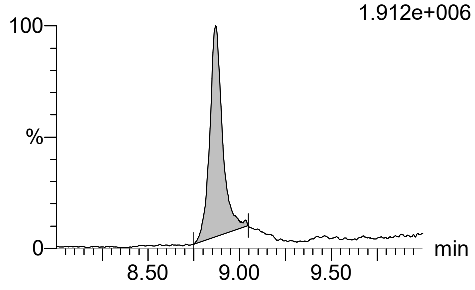

Sample Name: 20211111_A16_R3_IgM
F39:MRM of 2 channels,ES+
943.9 > 366.1
3.765e+005

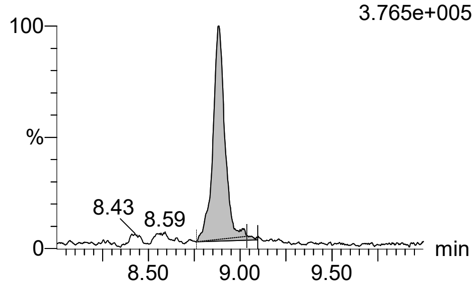

Sample Name: 20211111_A16_R3_IgM
F39:MRM of 2 channels,ES+
1179.6 > 204.1
3.815e+006

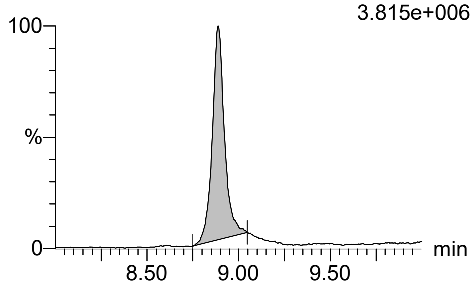

Sample Name: 20211111_L15_R3_IgM
F39:MRM of 2 channels,ES+
943.9 > 366.1
1.678e+005

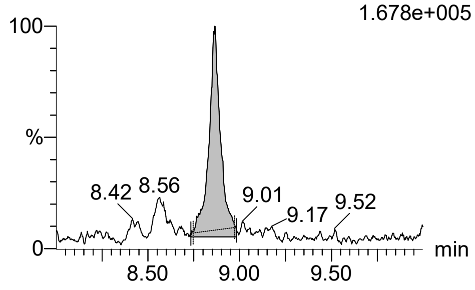

Sample Name: 20211111_L15_R3_IgM
F39:MRM of 2 channels,ES+
1179.6 > 204.1
1.543e+006

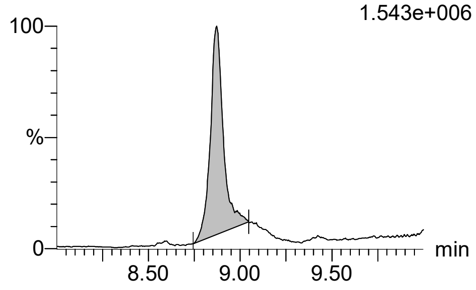

Sample Name: 20211111_L16_R3_IgM
F39:MRM of 2 channels,ES+
943.9 > 366.1
5.933e+004

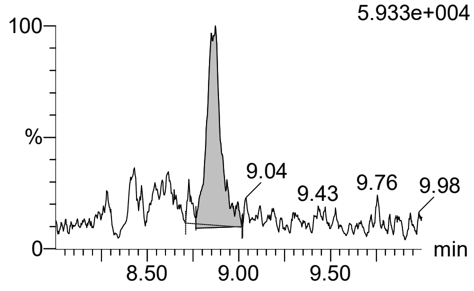

Sample Name: 20211111_L16_R3_IgM
F39:MRM of 2 channels,ES+
1179.6 > 204.1
7.087e+005

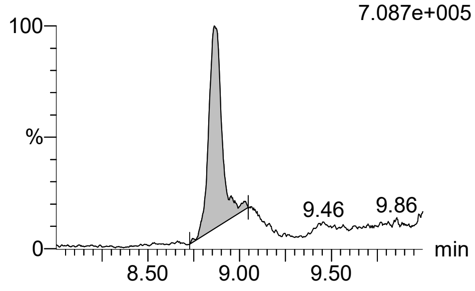

Sample Name: 20211111_H8_R3_IgM
F39:MRM of 2 channels,ES+
943.9 > 366.1
1.502e+005

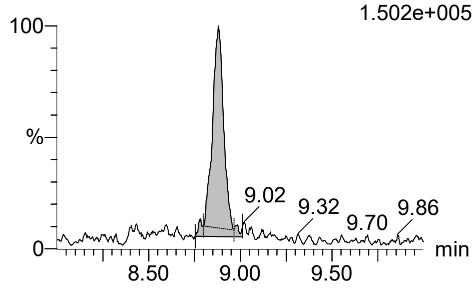

Sample Name: 20211111_H8_R3_IgM
F39:MRM of 2 channels,ES+
1179.6 > 204.1
1.555e+006

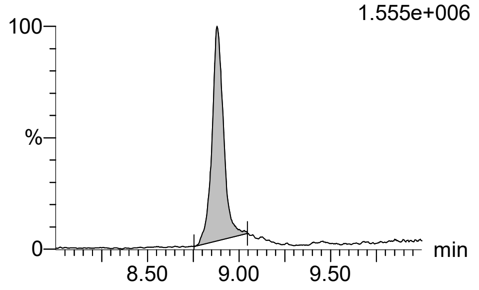

Sample Name: 20211111_A17_R3_IgM
F39:MRM of 2 channels,ES+
943.9 > 366.1
3.237e+005

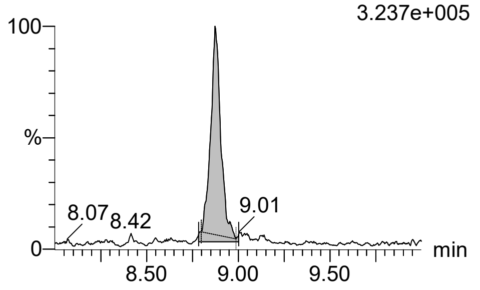

Sample Name: 20211111_A17_R3_IgM
F39:MRM of 2 channels,ES+
1179.6 > 204.1
2.864e+006

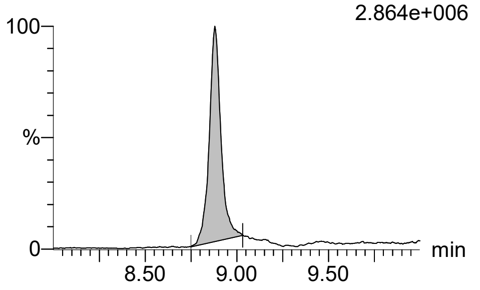

Sample Name: 20211111_A18_R3_IgM
F39:MRM of 2 channels,ES+
943.9 > 366.1
6.419e+004

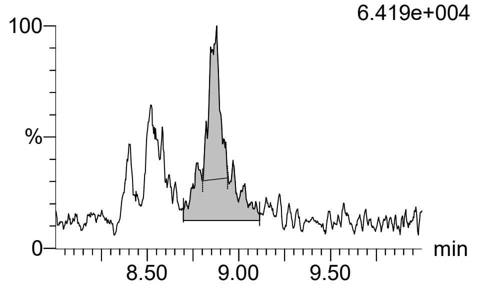

Sample Name: 20211111_A18_R3_IgM
F39:MRM of 2 channels,ES+
1179.6 > 204.1
8.094e+005

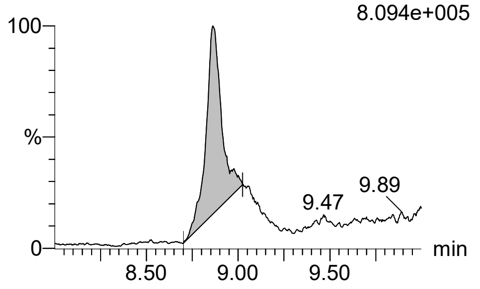

Sample Name: 20211111_L17_R3_IgM
F39:MRM of 2 channels,ES+
943.9 > 366.1
1.757e+005

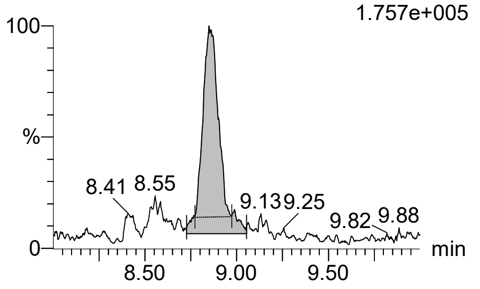

Sample Name: 20211111_L17_R3_IgM
F39:MRM of 2 channels,ES+
1179.6 > 204.1
2.205e+006

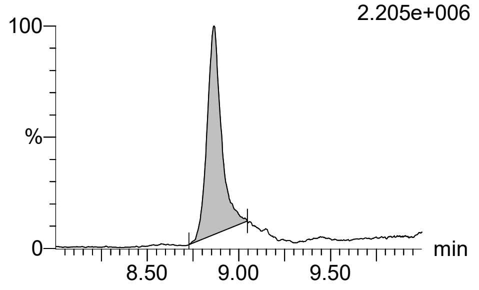

Sample Name: 20211111_L18_R3_IgM
F39:MRM of 2 channels,ES+
943.9 > 366.1
3.251e+004

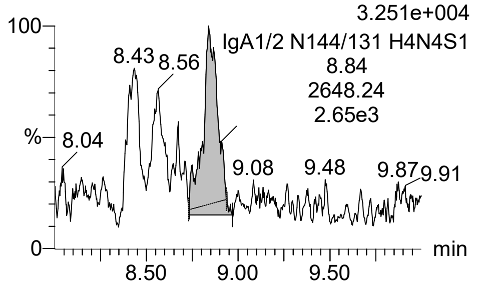

Sample Name: 20211111_L18_R3_IgM
F39:MRM of 2 channels,ES+
1179.6 > 204.1
3.580e+005

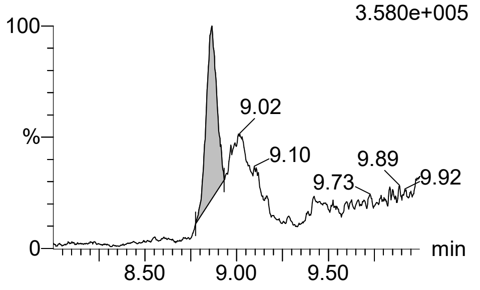

Sample Name: 20211111_H9_R3_IgM
F39:MRM of 2 channels,ES+
943.9 > 366.1
6.768e+004

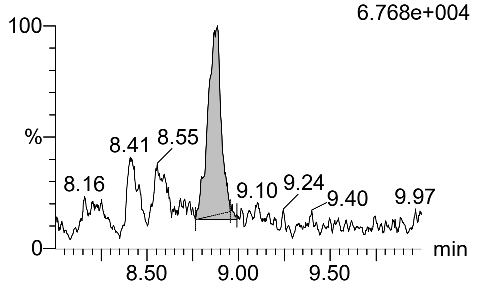

Sample Name: 20211111_H9_R3_IgM
F39:MRM of 2 channels,ES+
1179.6 > 204.1
7.907e+005

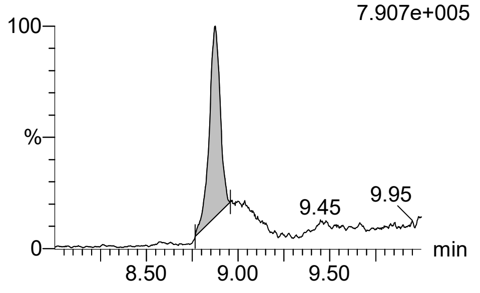

Sample Name: 20211111_A19_R3_IgM
F39:MRM of 2 channels,ES+
943.9 > 366.1
1.364e+005

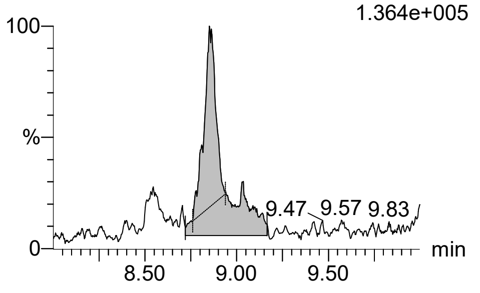

Sample Name: 20211111_A19_R3_IgM
F39:MRM of 2 channels,ES+
1179.6 > 204.1
1.437e+006

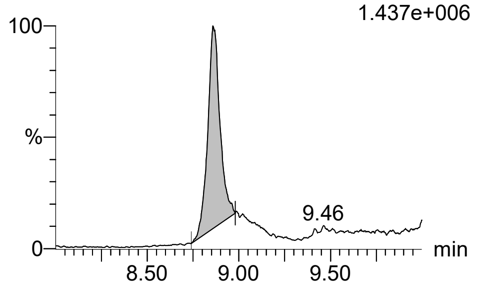

Sample Name: 20211111_A20_R3_IgM
F39:MRM of 2 channels,ES+
943.9 > 366.1
3.829e+004

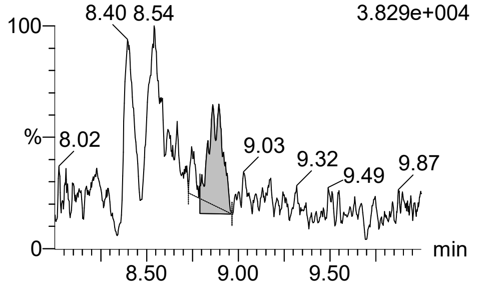

Sample Name: 20211111_A20_R3_IgM
F39:MRM of 2 channels,ES+
1179.6 > 204.1
2.938e+005

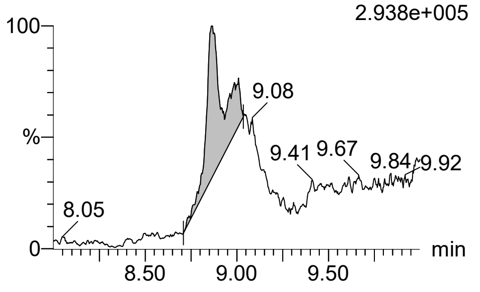

Dataset: C:\MassLynx\10.pro\20211111_clinicalsamples_IgM_IgA.qld

Last Altered: Thursday, March 09, 2023 19:58:25 台北標準時間
Printed: Friday, August 01, 2025 15:01:57 台北標準時間

Sample Name: 20211111_L19_R3_IgM
F39:MRM of 2 channels,ES+
943.9 > 366.1
8.077e+004

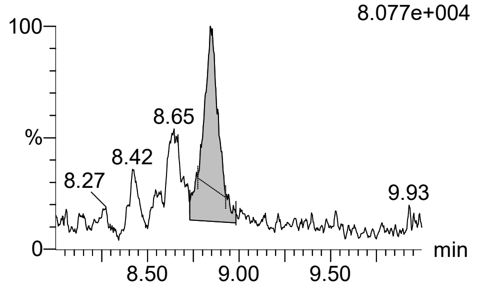

Sample Name: 20211111_L19_R3_IgM
F39:MRM of 2 channels,ES+
1179.6 > 204.1
7.724e+005

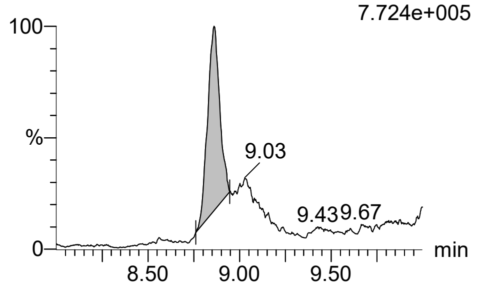

Sample Name: 20211111_QC_R21_IgM
F39:MRM of 2 channels,ES+
943.9 > 366.1
1.858e+005

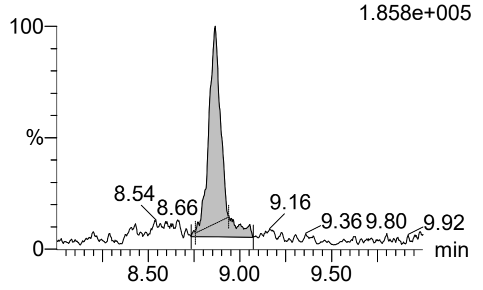

Sample Name: 20211111_QC_R21_IgM
F39:MRM of 2 channels,ES+
1179.6 > 204.1
1.891e+006

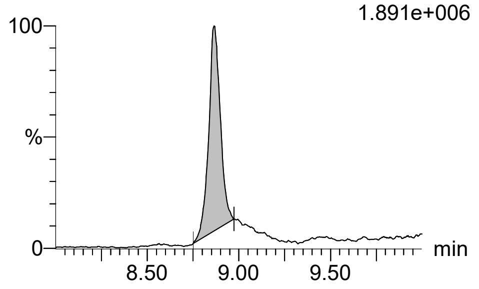

Sample Name: 20211111_L20_R3_IgM
F39:MRM of 2 channels,ES+
943.9 > 366.1
7.568e+004

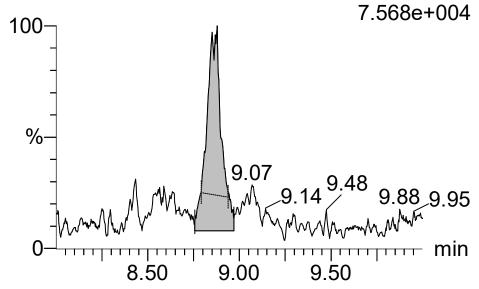

Sample Name: 20211111_L20_R3_IgM
F39:MRM of 2 channels,ES+
1179.6 > 204.1
7.755e+005

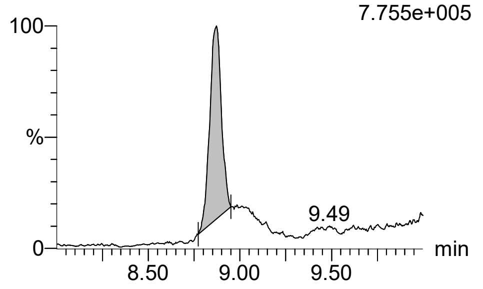

Sample Name: 20211111_H10_R3_IgM
F39:MRM of 2 channels,ES+
943.9 > 366.1
9.842e+004

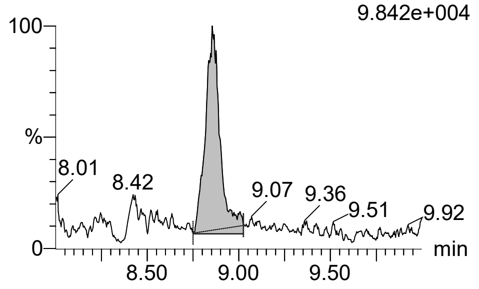

Sample Name: 20211111_H10_R3_IgM
F39:MRM of 2 channels,ES+
1179.6 > 204.1
8.651e+005

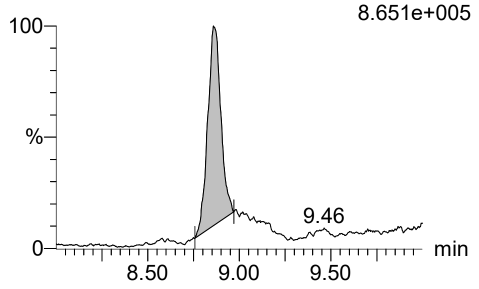

Sample Name: 20211111_A21_R3_IgM
F39:MRM of 2 channels,ES+
943.9 > 366.1
1.735e+005

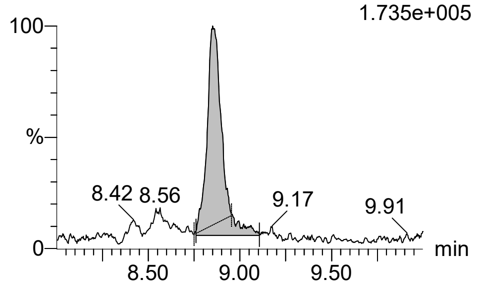

Sample Name: 20211111_A21_R3_IgM
F39:MRM of 2 channels,ES+
1179.6 > 204.1
2.168e+006

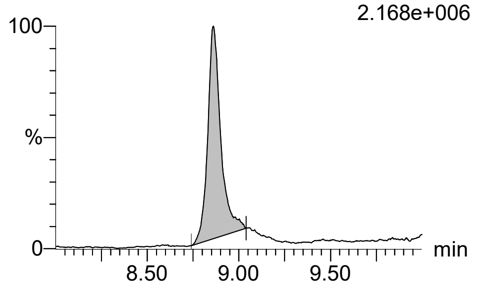

Sample Name: 20211111_A22_R3_IgM
F39:MRM of 2 channels,ES+
943.9 > 366.1
2.972e+004

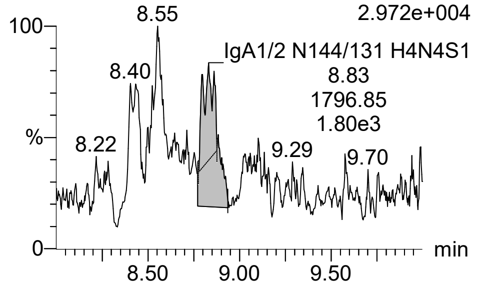

Sample Name: 20211111_A22_R3_IgM
F39:MRM of 2 channels,ES+
1179.6 > 204.1
1.647e+005

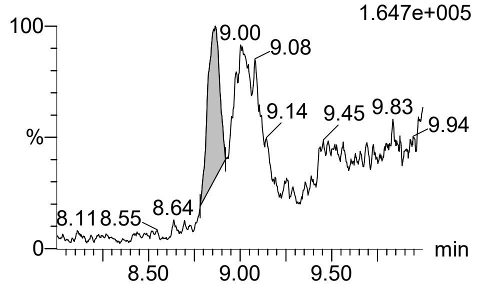

Sample Name: 20211111_L21_R3_IgM
F39:MRM of 2 channels,ES+
943.9 > 366.1
9.671e+004

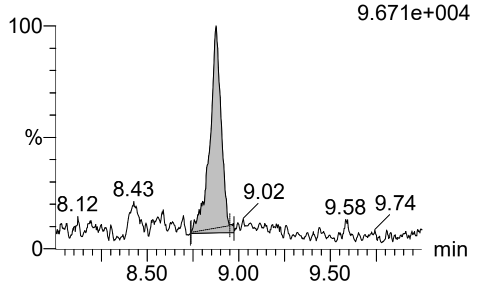

Sample Name: 20211111_L21_R3_IgM
F39:MRM of 2 channels,ES+
1179.6 > 204.1
8.221e+005

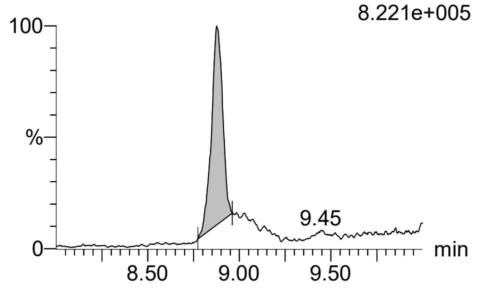

Sample Name: 20211111_L22_R3_IgM
F39:MRM of 2 channels,ES+
943.9 > 366.1
1.841e+005

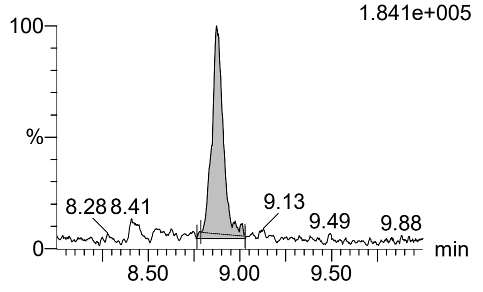

Sample Name: 20211111_L22_R3_IgM
F39:MRM of 2 channels,ES+
1179.6 > 204.1
1.807e+006

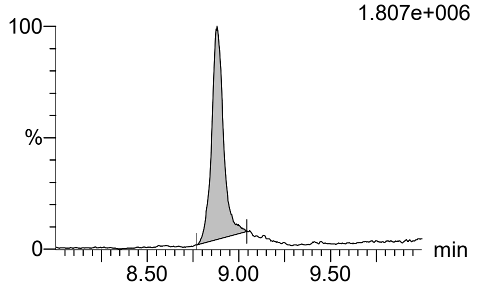

Sample Name: 20211111_H11_R3_IgM
F39:MRM of 2 channels,ES+
943.9 > 366.1
3.502e+005

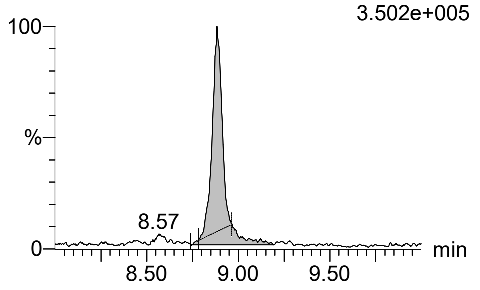

Sample Name: 20211111_H11_R3_IgM
F39:MRM of 2 channels,ES+
1179.6 > 204.1
3.151e+006

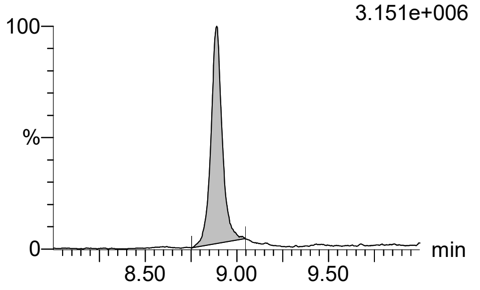

Sample Name: 20211111_A23_R3_IgM
F39:MRM of 2 channels,ES+
943.9 > 366.1
6.822e+004

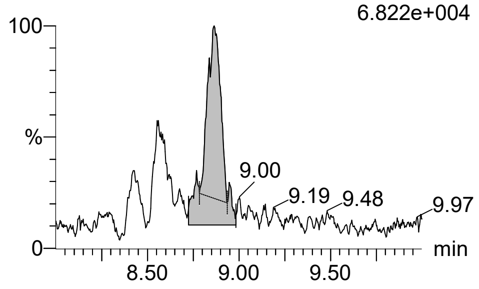

Sample Name: 20211111_A23_R3_IgM
F39:MRM of 2 channels,ES+
1179.6 > 204.1
7.033e+005

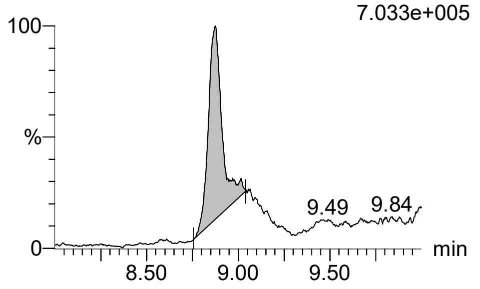

Sample Name: 20211111_A24_R3_IgM
F39:MRM of 2 channels,ES+
943.9 > 366.1
1.067e+005

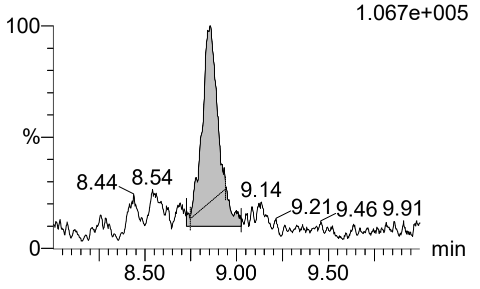

Sample Name: 20211111_A24_R3_IgM
F39:MRM of 2 channels,ES+
1179.6 > 204.1
1.261e+006

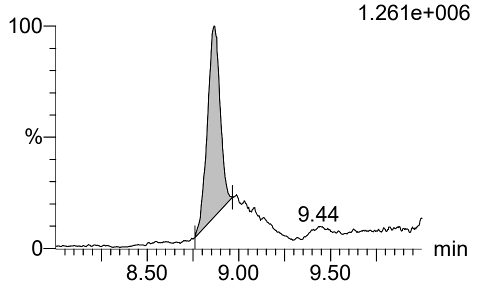

Sample Name: 20211111_L23_R3_IgM
F39:MRM of 2 channels,ES+
943.9 > 366.1
1.600e+005

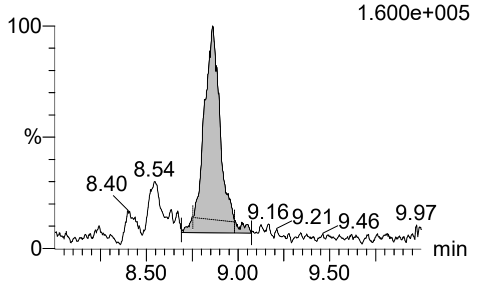

Sample Name: 20211111_L23_R3_IgM
F39:MRM of 2 channels,ES+
1179.6 > 204.1
1.866e+006

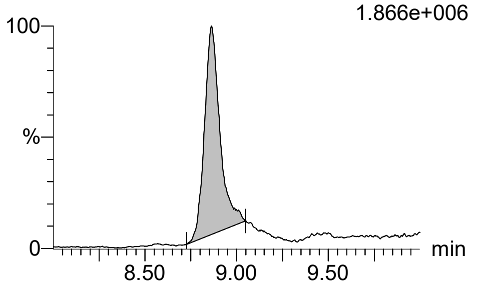

Sample Name: 20211111_L24_R3_IgM
F39:MRM of 2 channels,ES+
943.9 > 366.1
3.528e+004

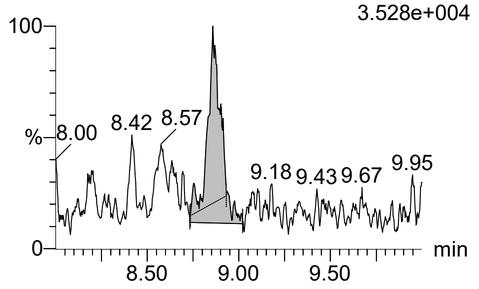

Sample Name: 20211111_L24_R3_IgM
F39:MRM of 2 channels,ES+
1179.6 > 204.1
2.701e+005

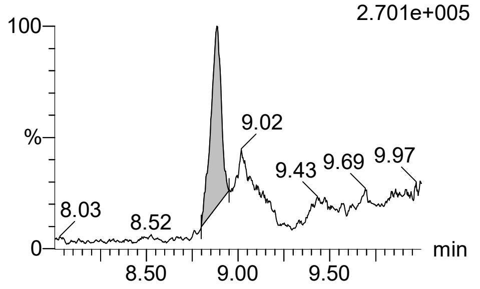

Sample Name: 20211111_H12_R3_IgM
F39:MRM of 2 channels,ES+
943.9 > 366.1
6.771e+004

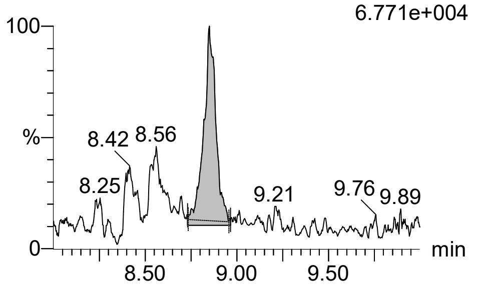

Sample Name: 20211111_H12_R3_IgM
F39:MRM of 2 channels,ES+
1179.6 > 204.1
5.976e+005

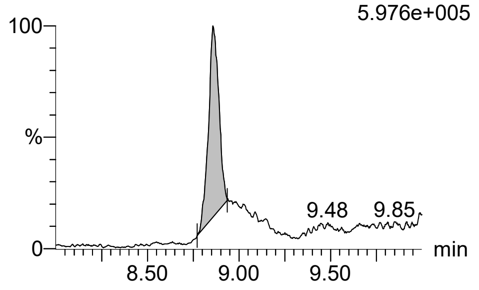

Sample Name: 20211111_QC_R22_IgM
F39:MRM of 2 channels,ES+
943.9 > 366.1
1.945e+005

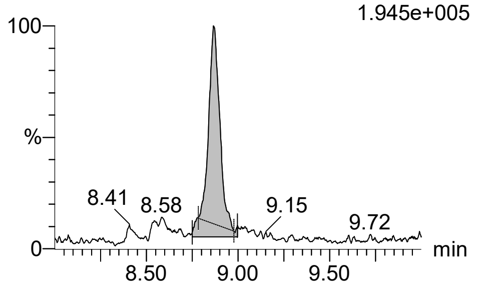

Sample Name: 20211111_QC_R22_IgM
F39:MRM of 2 channels,ES+
1179.6 > 204.1
2.059e+006

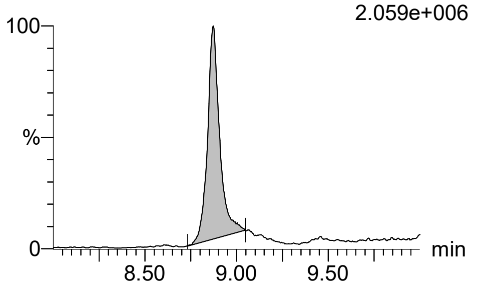

Sample Name: 20211111_A25_R3_IgM
F39:MRM of 2 channels,ES+
943.9 > 366.1
5.247e+005

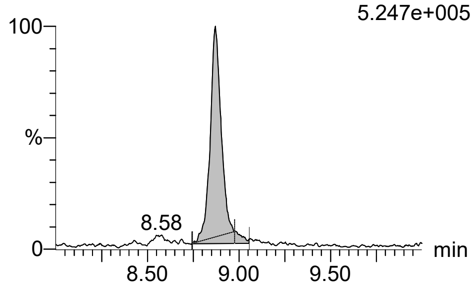

Sample Name: 20211111_A25_R3_IgM
F39:MRM of 2 channels,ES+
1179.6 > 204.1
5.444e+006

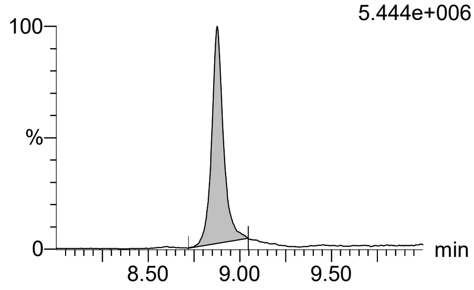

Sample Name: 20211111_A26_R3_IgM
F39:MRM of 2 channels,ES+
943.9 > 366.1
2.305e+005

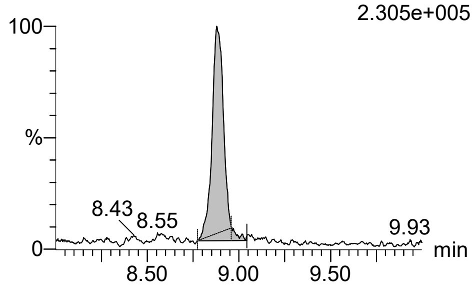

Sample Name: 20211111_A26_R3_IgM
F39:MRM of 2 channels,ES+
1179.6 > 204.1
2.368e+006

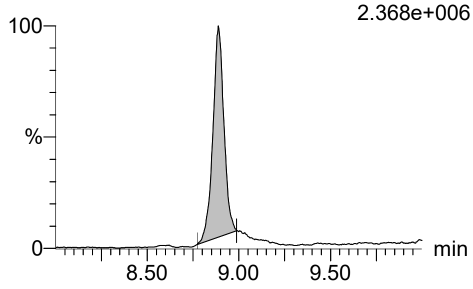

Sample Name: 20211111_L25_R3_IgM
F39:MRM of 2 channels,ES+
943.9 > 366.1
1.987e+005

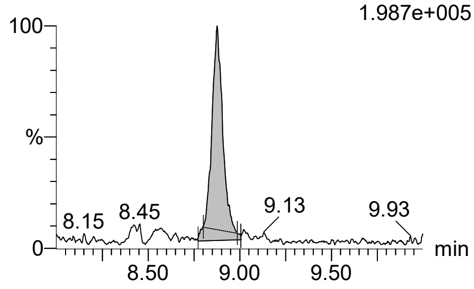

Sample Name: 20211111_L25_R3_IgM
F39:MRM of 2 channels,ES+
1179.6 > 204.1
2.030e+006

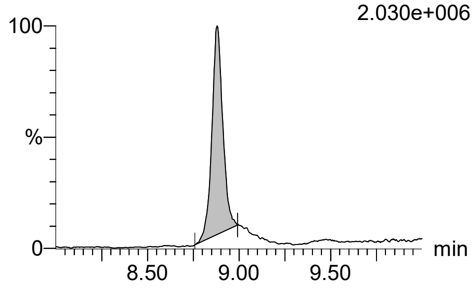

Sample Name: 20211111_L26_R3_IgM
F39:MRM of 2 channels,ES+
943.9 > 366.1
1.381e+005

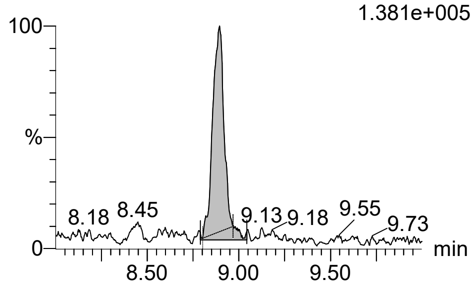

Sample Name: 20211111_L26_R3_IgM
F39:MRM of 2 channels,ES+
1179.6 > 204.1
1.329e+006

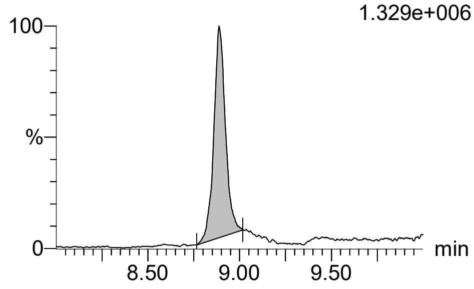

Sample Name: 20211111_H13_R3_IgM
F39:MRM of 2 channels,ES+
943.9 > 366.1
1.843e+005

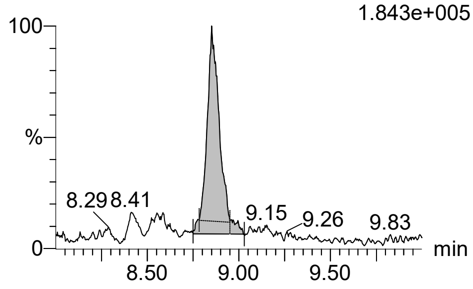

Sample Name: 20211111_H13_R3_IgM
F39:MRM of 2 channels,ES+
1179.6 > 204.1
2.055e+006

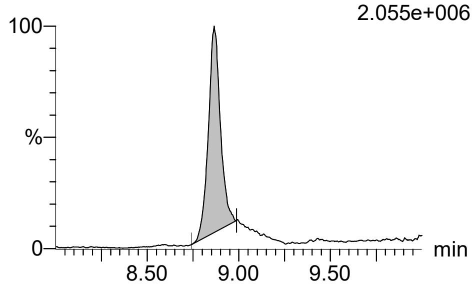

Sample Name: 20211111_A27_R3_IgM
F39:MRM of 2 channels,ES+
943.9 > 366.1
2.116e+005

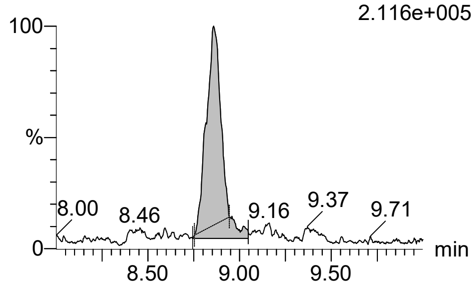

Sample Name: 20211111_A27_R3_IgM
F39:MRM of 2 channels,ES+
1179.6 > 204.1
2.179e+006

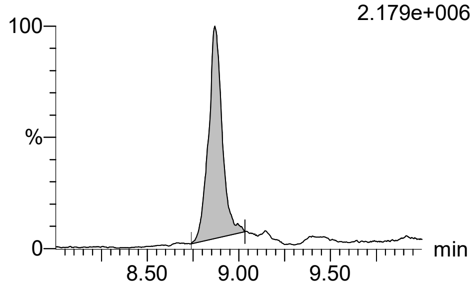

Sample Name: 20211111_A28_R3_IgM
F39:MRM of 2 channels,ES+
943.9 > 366.1
7.134e+004

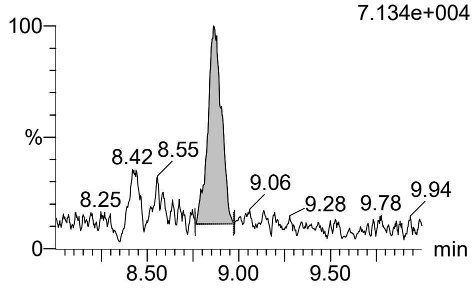

Sample Name: 20211111_A28_R3_IgM
F39:MRM of 2 channels,ES+
1179.6 > 204.1
8.725e+005

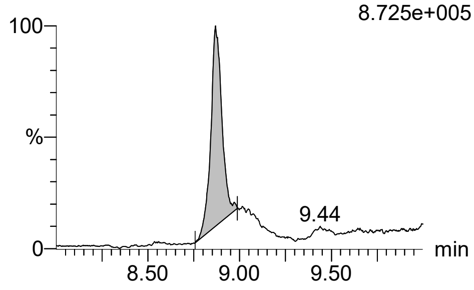

Sample Name: 20211111_L27_R3_IgM
F39:MRM of 2 channels,ES+
943.9 > 366.1
2.468e+005

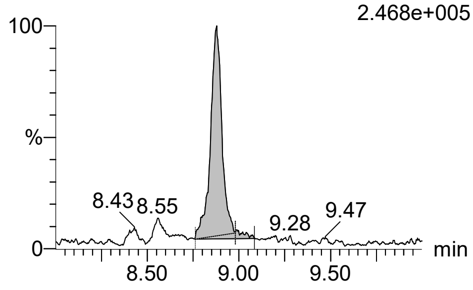

Dataset: C:\MassLynx\10.pro\20211111_clinicalsamples_IgM_IgA.qld

Last Altered: Thursday, March 09, 2023 19:58:25 台北標準時間
Printed: Friday, August 01, 2025 15:01:57 台北標準時間

Sample Name: 20211111_L27_R3_IgM
F39:MRM of 2 channels,ES+
1179.6 > 204.1
2.523e+006

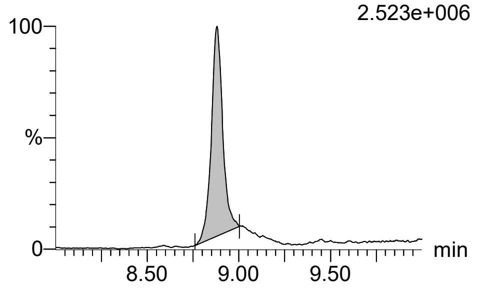

Sample Name: 20211111_L28_R3_IgM
F39:MRM of 2 channels,ES+
943.9 > 366.1
7.227e+004

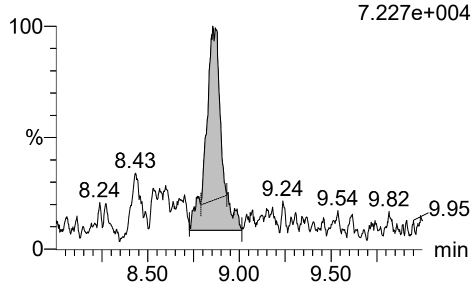

Sample Name: 20211111_L28_R3_IgM
F39:MRM of 2 channels,ES+
1179.6 > 204.1
7.311e+005

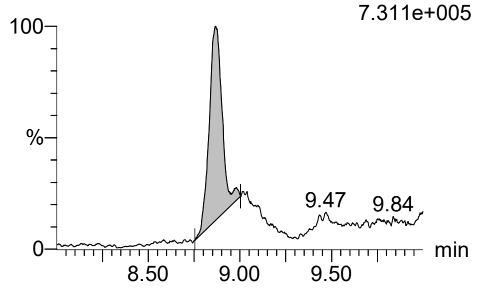

Sample Name: 20211111_H14_R3_IgM
F39:MRM of 2 channels,ES+
943.9 > 366.1
1.926e+005

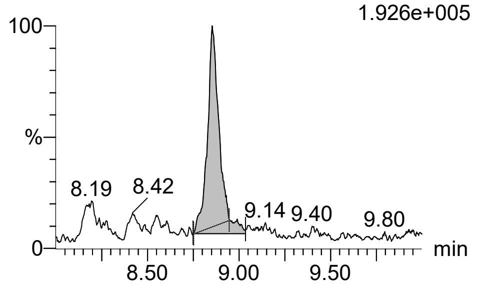

Sample Name: 20211111_H14_R3_IgM
F39:MRM of 2 channels,ES+
1179.6 > 204.1
1.901e+006

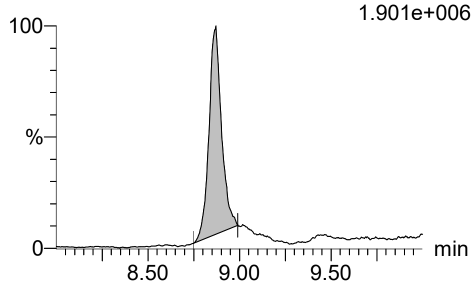

Sample Name: 20211111_A29_R3_IgM
F39:MRM of 2 channels,ES+
943.9 > 366.1
5.360e+004

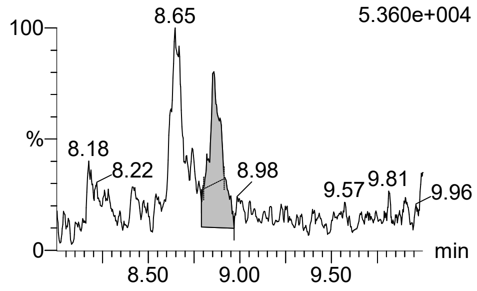

Sample Name: 20211111_A29_R3_IgM
F39:MRM of 2 channels,ES+
1179.6 > 204.1
3.055e+005

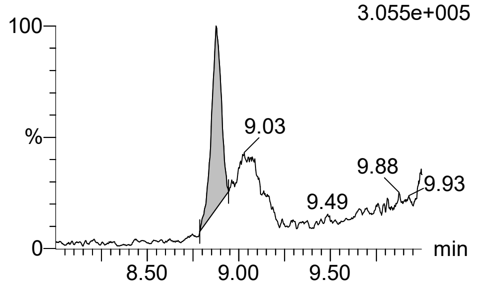

Sample Name: 20211111_A30_R3_IgM
F39:MRM of 2 channels,ES+
943.9 > 366.1
3.362e+005

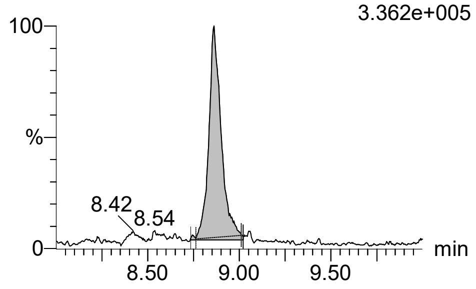

Sample Name: 20211111_A30_R3_IgM
F39:MRM of 2 channels,ES+
1179.6 > 204.1
3.620e+006

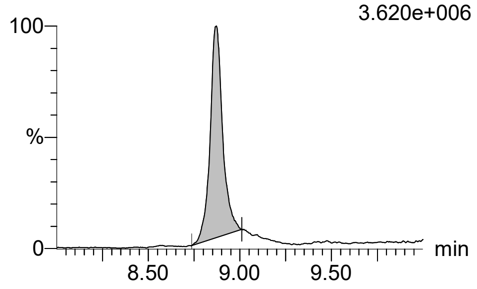

Sample Name: 20211111_QC_R23_IgM
F39:MRM of 2 channels,ES+
943.9 > 366.1
2.131e+005

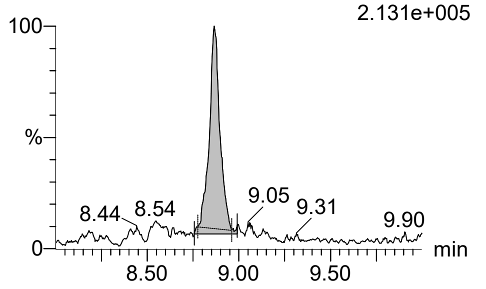

Sample Name: 20211111_QC_R23_IgM
F39:MRM of 2 channels,ES+
1179.6 > 204.1
2.018e+006

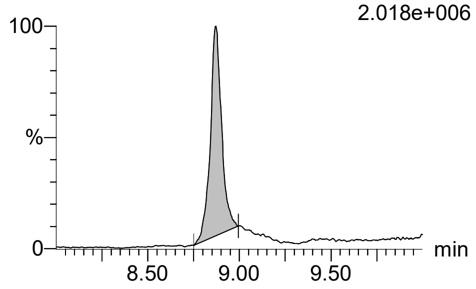

Sample Name: 20211111_L29_R3_IgM
F39:MRM of 2 channels,ES+
943.9 > 366.1
5.687e+004

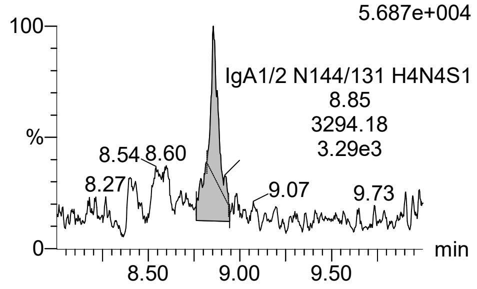

Sample Name: 20211111_L29_R3_IgM
F39:MRM of 2 channels,ES+
1179.6 > 204.1
4.618e+005

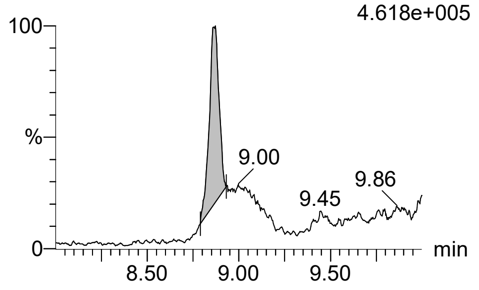

Sample Name: 20211111_L30_R3_IgM
F39:MRM of 2 channels,ES+
943.9 > 366.1
3.406e+004

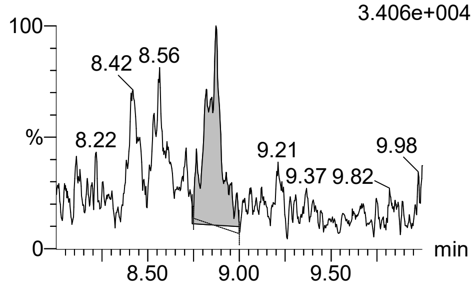

Sample Name: 20211111_L30_R3_IgM
F39:MRM of 2 channels,ES+
1179.6 > 204.1
2.562e+005

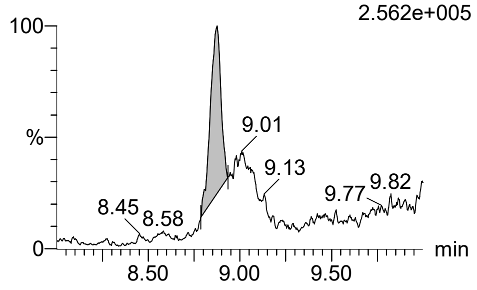

Dataset: C:\MassLynx\10.pro\20211111_clinicalsamples_IgM_IgA.qld

Last Altered: Thursday, March 09, 2023 19:58:25 台北標準時間
Printed: Friday, August 01, 2025 15:01:57 台北標準時間

Sample Name: 20211111_H15_R3_IgM
F39:MRM of 2 channels,ES+
943.9 > 366.1
9.337e+004

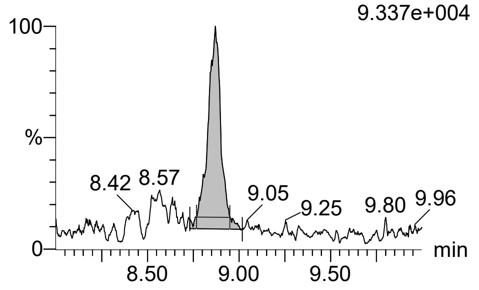

Sample Name: 20211111_H15_R3_IgM
F39:MRM of 2 channels,ES+
1179.6 > 204.1
8.805e+005

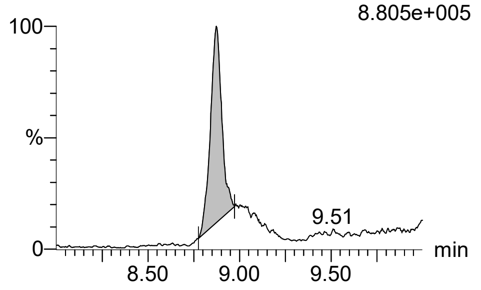

Sample Name: 20211111_A31_R3_IgM
F39:MRM of 2 channels,ES+
943.9 > 366.1
8.623e+004

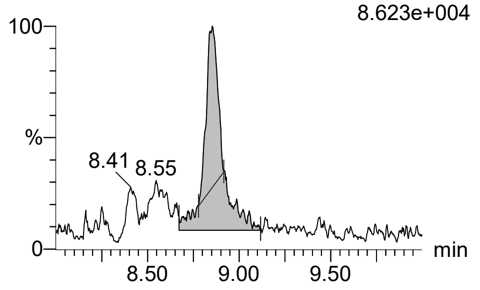

Sample Name: 20211111_A31_R3_IgM
F39:MRM of 2 channels,ES+
1179.6 > 204.1
1.039e+006

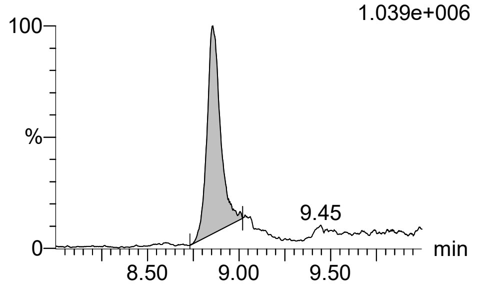

Sample Name: 20211111_A32_R3_IgM
F39:MRM of 2 channels,ES+
943.9 > 366.1
1.815e+005

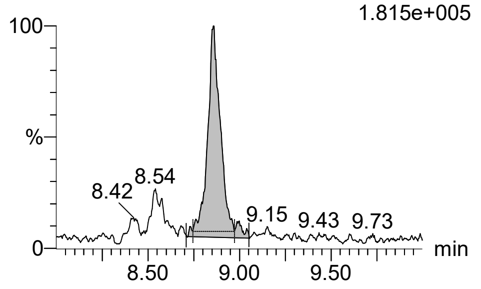

Sample Name: 20211111_A32_R3_IgM
F39:MRM of 2 channels,ES+
1179.6 > 204.1
1.985e+006

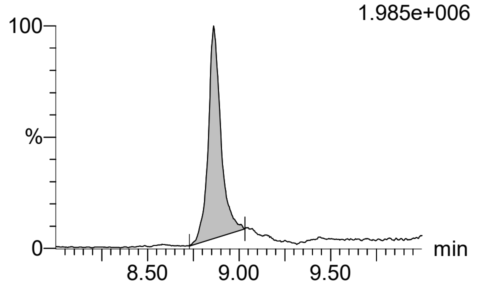

Sample Name: 20211111_L31_R3_IgM
F39:MRM of 2 channels,ES+
943.9 > 366.1
3.978e+004

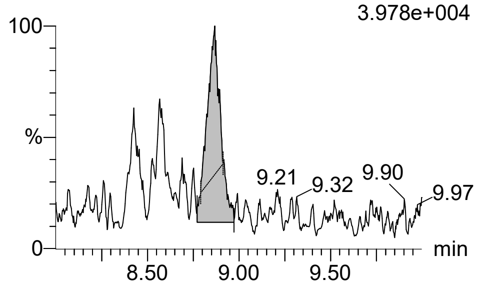

Sample Name: 20211111_L31_R3_IgM
F39:MRM of 2 channels,ES+
1179.6 > 204.1
4.199e+005

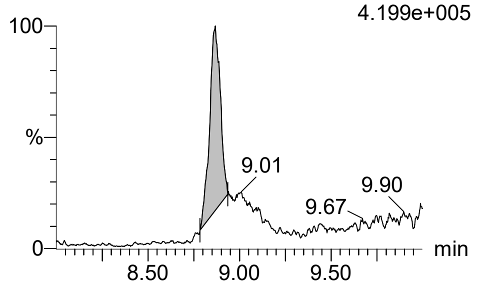

Sample Name: 20211111_L32_R3_IgM
F39:MRM of 2 channels,ES+
943.9 > 366.1
1.987e+005

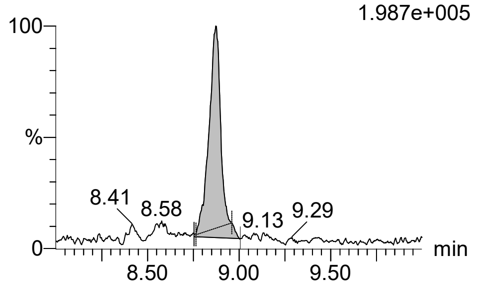

Sample Name: 20211111_L32_R3_IgM
F39:MRM of 2 channels,ES+
1179.6 > 204.1
2.027e+006

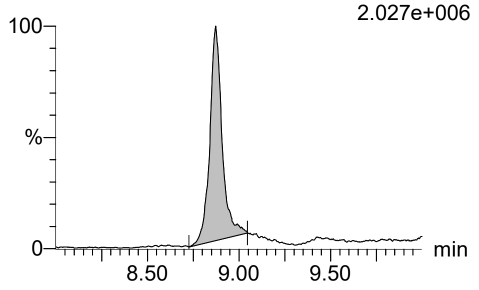

Sample Name: 20211111_H16_R3_IgM
F39:MRM of 2 channels,ES+
943.9 > 366.1
2.285e+005

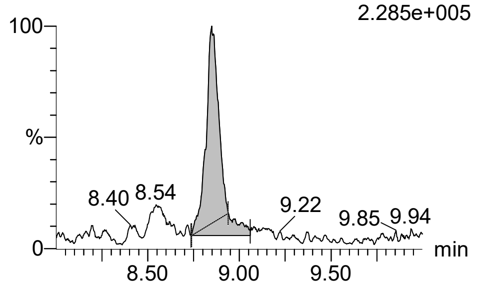

Sample Name: 20211111_H16_R3_IgM
F39:MRM of 2 channels,ES+
1179.6 > 204.1
2.387e+006

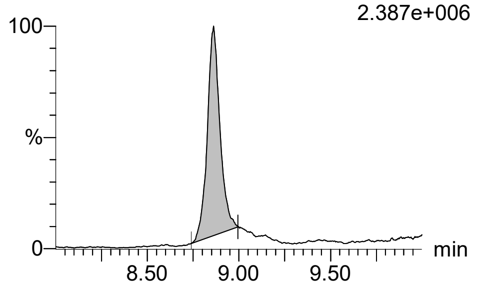

Sample Name: 20211111_A33_R3_IgM
F39:MRM of 2 channels,ES+
943.9 > 366.1
1.765e+005

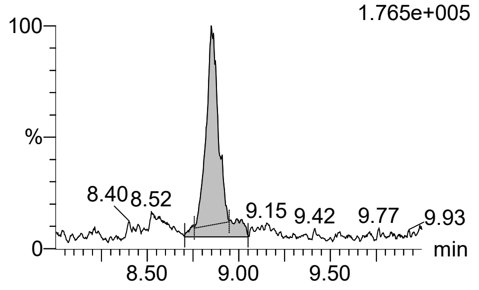

Sample Name: 20211111_A33_R3_IgM
F39:MRM of 2 channels,ES+
1179.6 > 204.1
1.690e+006

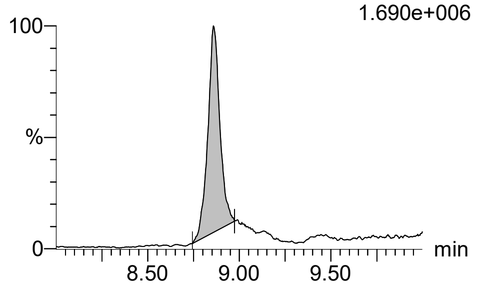

Sample Name: 20211111_A34_R3_IgM
F39:MRM of 2 channels,ES+
943.9 > 366.1
8.807e+004

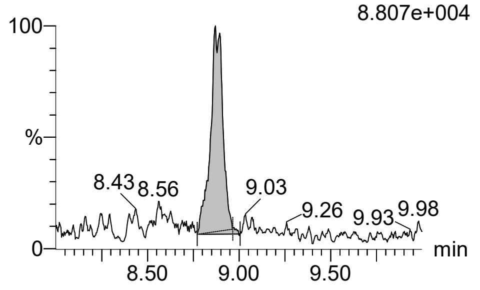

Sample Name: 20211111_A34_R3_IgM
F39:MRM of 2 channels,ES+
1179.6 > 204.1
8.695e+005

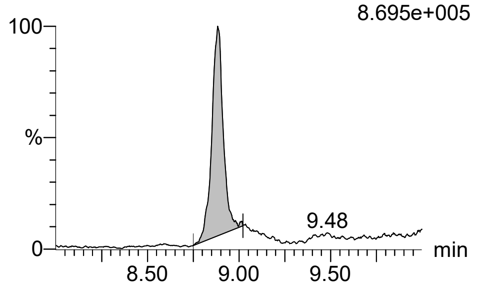

Sample Name: 20211111_H17_R3_IgM
F39:MRM of 2 channels,ES+
943.9 > 366.1
3.626e+005

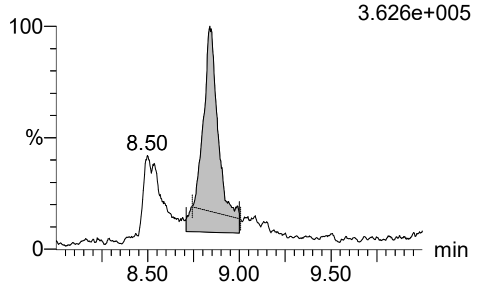

Sample Name: 20211111_H17_R3_IgM
F39:MRM of 2 channels,ES+
1179.6 > 204.1
3.708e+006

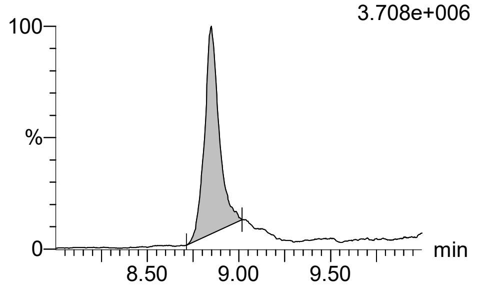

Sample Name: 20211111_A35_R3_IgM
F39:MRM of 2 channels,ES+
943.9 > 366.1
5.592e+004

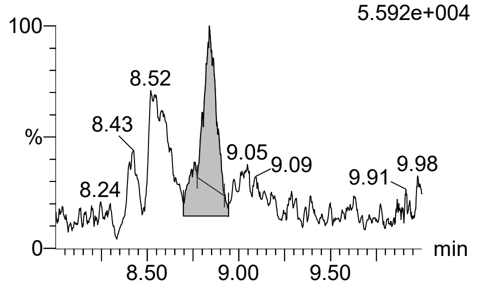

Sample Name: 20211111_A35_R3_IgM
F39:MRM of 2 channels,ES+
1179.6 > 204.1
4.315e+005

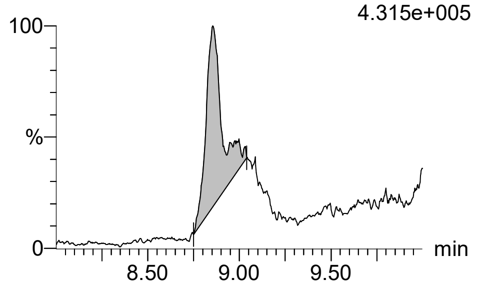

Sample Name: 20211111_QC_R24_IgM
F39:MRM of 2 channels,ES+
943.9 > 366.1
1.597e+005

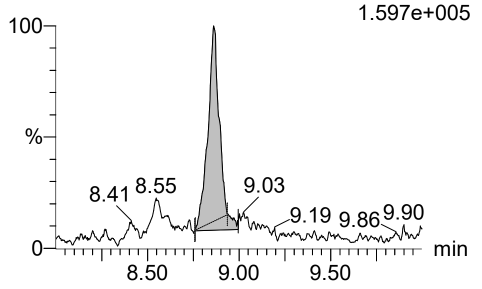

Sample Name: 20211111_QC_R24_IgM
F39:MRM of 2 channels,ES+
1179.6 > 204.1
1.825e+006

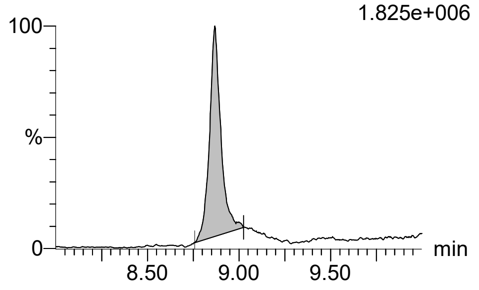

Sample Name: 20211111_A36_R3_IgM
F39:MRM of 2 channels,ES+
943.9 > 366.1
2.089e+005

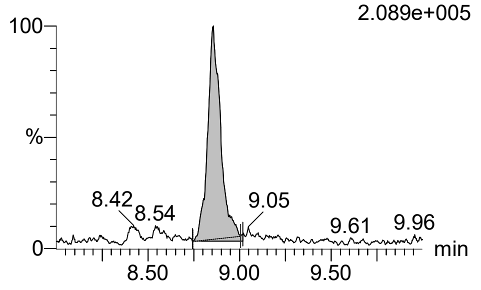

Sample Name: 20211111_A36_R3_IgM
F39:MRM of 2 channels,ES+
1179.6 > 204.1
2.227e+006

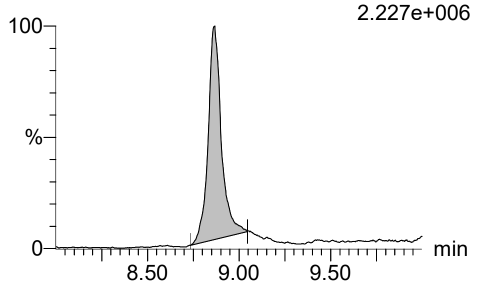

Sample Name: 20211111_H18_R3_IgM
F39:MRM of 2 channels,ES+
943.9 > 366.1
5.456e+005

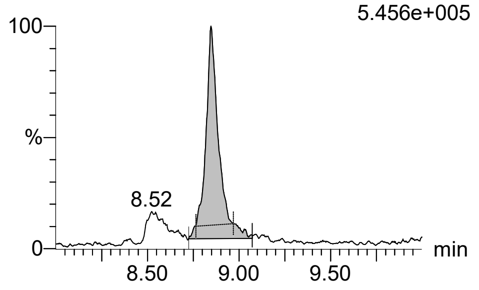

Sample Name: 20211111_H18_R3_IgM
F39:MRM of 2 channels,ES+
1179.6 > 204.1
5.268e+006

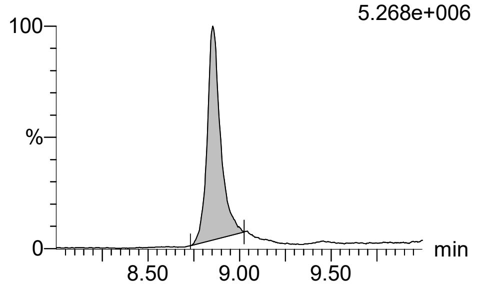

Sample Name: 20211111_A37_R3_IgM
F39:MRM of 2 channels,ES+
943.9 > 366.1
1.569e+005

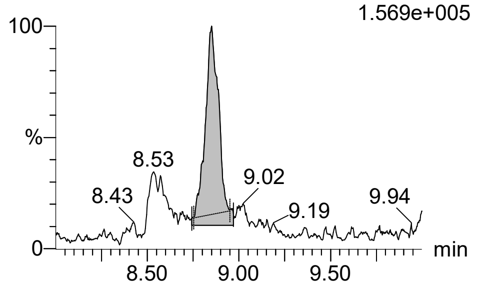

Sample Name: 20211111_A37_R3_IgM
F39:MRM of 2 channels,ES+
1179.6 > 204.1
1.604e+006

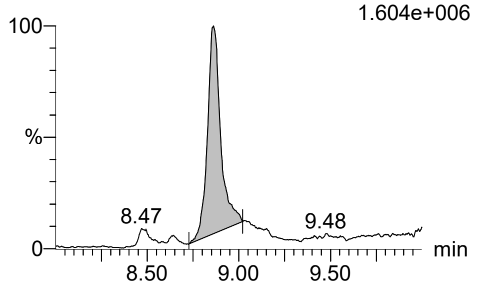

Sample Name: 20211111_A38_R3_IgM
F39:MRM of 2 channels,ES+
943.9 > 366.1
3.094e+005

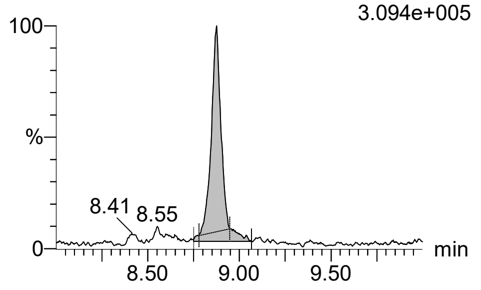

Sample Name: 20211111_A38_R3_IgM
F39:MRM of 2 channels,ES+
1179.6 > 204.1
2.489e+006

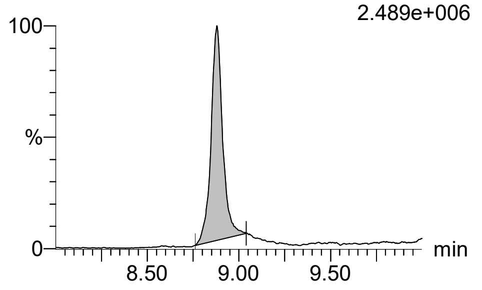

Dataset: C:\MassLynx\10.pro\20211111_clinicalsamples_IgM_IgA.qld

Last Altered: Thursday, March 09, 2023 19:58:25 台北標準時間
Printed: Friday, August 01, 2025 15:01:57 台北標準時間

Sample Name: 20211111_H19_R3_IgM
F39:MRM of 2 channels,ES+
943.9 > 366.1
1.307e+005

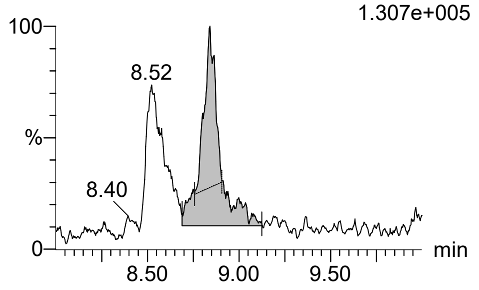

Sample Name: 20211111_H19_R3_IgM
F39:MRM of 2 channels,ES+
1179.6 > 204.1
1.175e+006

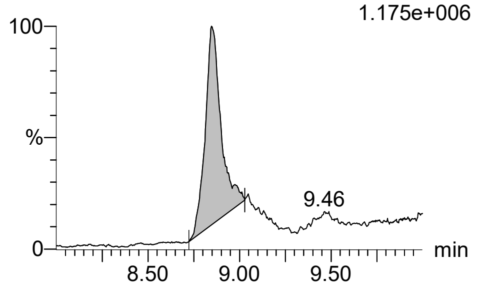

Sample Name: 20211111_A39_R3_IgM
F39:MRM of 2 channels,ES+
943.9 > 366.1
2.323e+005

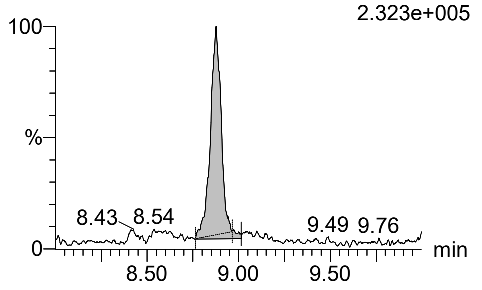

Sample Name: 20211111_A39_R3_IgM
F39:MRM of 2 channels,ES+
1179.6 > 204.1
1.999e+006

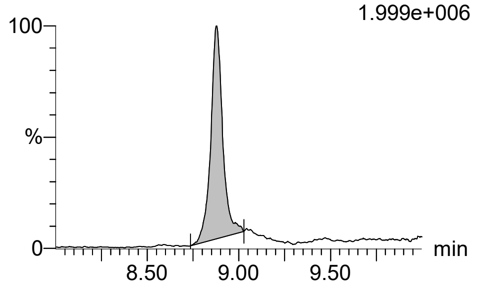

Sample Name: 20211111_A40_R3_IgM
F39:MRM of 2 channels,ES+
943.9 > 366.1
7.716e+004

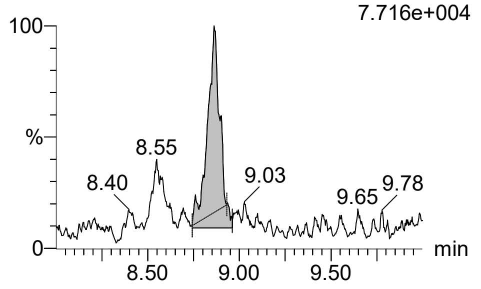

Sample Name: 20211111_A40_R3_IgM
F39:MRM of 2 channels,ES+
1179.6 > 204.1
7.287e+005

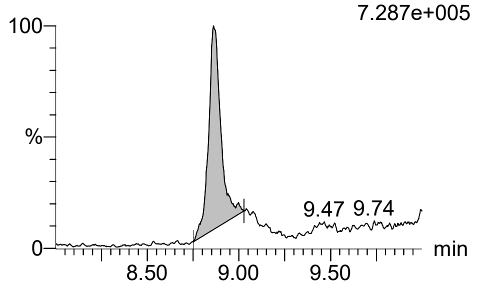

Sample Name: 20211111_H20_R3_IgM
F39:MRM of 2 channels,ES+
943.9 > 366.1
3.524e+005

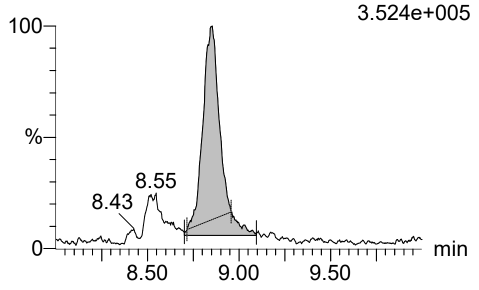

Sample Name: 20211111_H20_R3_IgM
F39:MRM of 2 channels,ES+
1179.6 > 204.1
4.081e+006

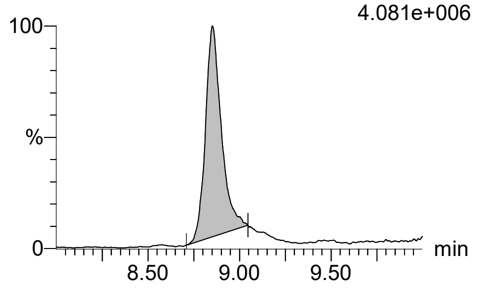

Sample Name: 20211111_A41_R3_IgM
F39:MRM of 2 channels,ES+
943.9 > 366.1
6.586e+004

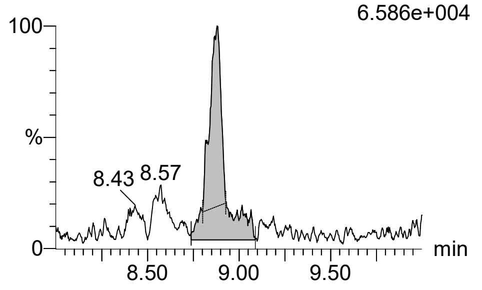

Sample Name: 20211111_A41_R3_IgM
F39:MRM of 2 channels,ES+
1179.6 > 204.1
6.576e+005

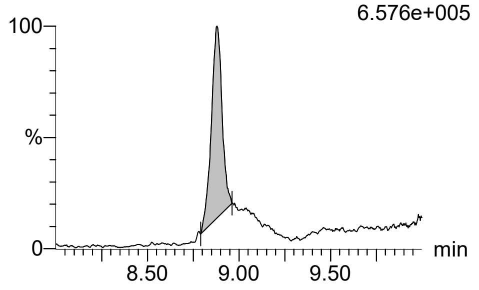

Sample Name: 20211111_A42_R3_IgM
F39:MRM of 2 channels,ES+
943.9 > 366.1
5.237e+004

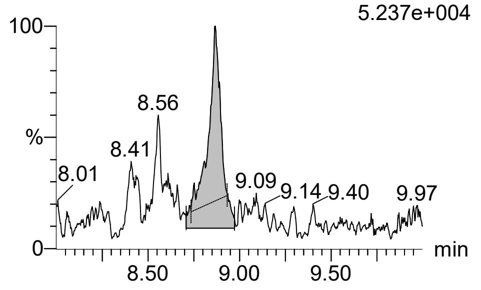

Sample Name: 20211111_A42_R3_IgM
F39:MRM of 2 channels,ES+
1179.6 > 204.1
4.993e+005

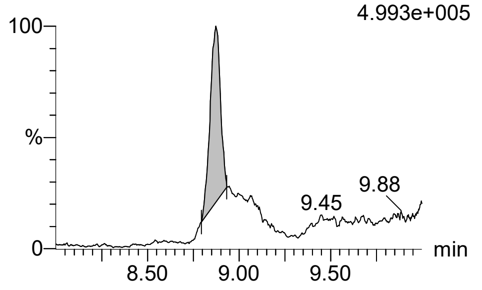

Sample Name: 20211111_A43_R3_IgM
F39:MRM of 2 channels,ES+
943.9 > 366.1
6.982e+004

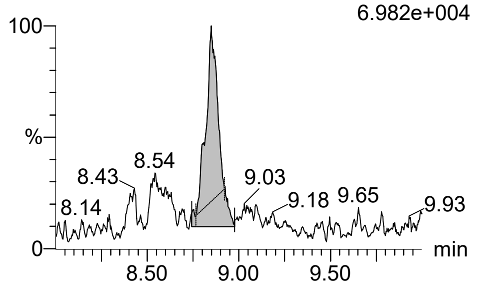

Sample Name: 20211111_A43_R3_IgM
F39:MRM of 2 channels,ES+
1179.6 > 204.1
7.585e+005

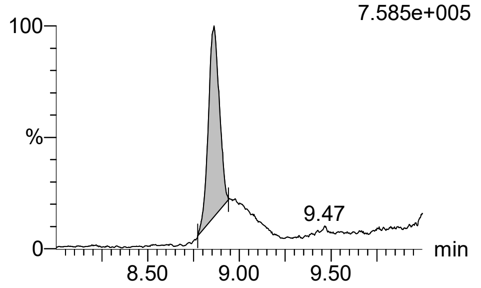

Sample Name: 20211111_A44_R3_IgM
F39:MRM of 2 channels,ES+
943.9 > 366.1
3.753e+004

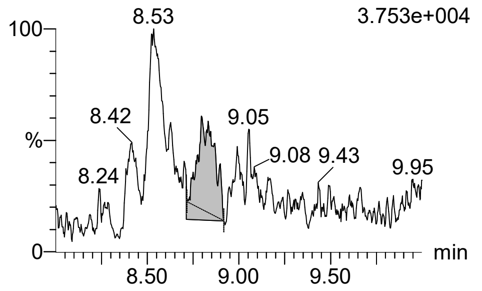

Dataset: C:\MassLynx\10.pro\20211111_clinicalsamples_IgM_IgA.qld

Last Altered: Thursday, March 09, 2023 19:58:25 台北標準時間
Printed: Friday, August 01, 2025 15:01:57 台北標準時間

Sample Name: 20211111_A44_R3_IgM

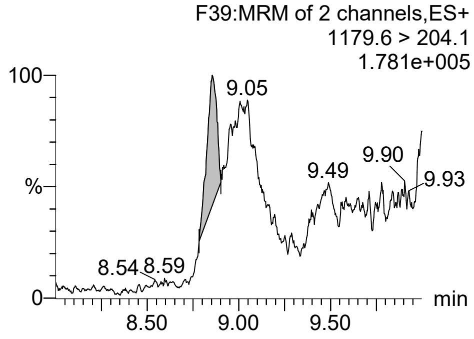

Sample Name: 20211111_QC_R25_IgM

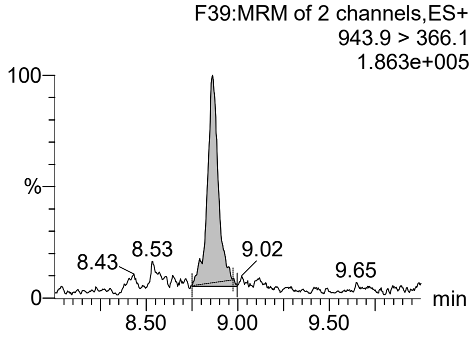

Sample Name: 20211111_QC_R25_IgM

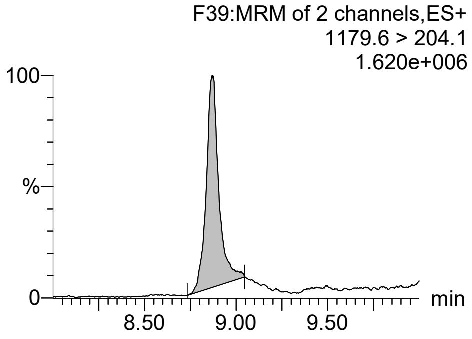

Dataset: C:\MassLynx\10.pro\20211111_clinicalsamples_IgM_IgA.qld

Last Altered: Thursday, March 09, 2023 19:58:25 台北標準時間
Printed: Friday, August 01, 2025 15:01:57 台北標準時間

	#	Name	RT	Area	IS Area	Response	Prima...
1	1	20211112_QC_R001_IgM	8.85	17906.3	15421.4	1.161	MM
2	2	20211112_QC_R002_IgM	8.85	18663.9	13188.8	1.415	MM
3	3	20211111_A1_R2_IgM	8.87	13367.3	11871.0	1.126	MM
4	4	20211111_A1_R3_IgM	8.85	13509.3	12030.5	1.123	MM
5	5	20211111_A2_R2_IgM	8.84	16657.0	11446.8	1.455	MM
6	6	20211111_A2_R3_IgM	8.85	18031.4	11098.4	1.625	MM
7	7	20211111_L1_R2_IgM	8.87	5691.6	11779.4	0.483	MM
8	8	20211111_L1_R3_IgM	8.86	4467.9	11176.2	0.400	MM
9	9	20211111_L2_R2_IgM	8.84	12878.8	11375.0	1.132	MM
10	10	20211111_H1_R2_IgM	8.85	28187.2	13595.2	2.073	MM
11	11	20211111_A3_R2_IgM	8.86	25258.9	14112.3	1.790	MM
12	12	20211111_A4_R2_IgM	8.85	12955.5	11977.5	1.082	MM
13	13	20211111_L3_R2_IgM	8.86	3495.4	12242.1	0.286	MM
14	14	20211111_L4_R2_IgM	8.86	9917.1	12797.0	0.775	MM
15	15	20211111_H2_R2_IgM	8.85	54041.5	10006.0	5.401	MM
16	16	20211111_A5_R2_IgM	8.86	56643.5	11434.6	4.954	MM
17	17	20211111_A6_R2_IgM	8.85	15782.3	13193.7	1.196	MM
18	18	20211111_QC_R10_IgM	8.86	16122.8	12566.1	1.283	MM
19	19	20211111_L5_R2_IgM	8.84	14415.7	13616.4	1.059	MM
20	20	20211111_L6_R2_IgM	8.84	13916.6	13698.5	1.016	MM
21	21	20211111_H3_R2_IgM	8.85	5862.1	15793.1	0.371	MM
22	22	20211111_A7_R2_IgM	8.84	50603.7	12936.9	3.912	MM
23	23	20211111_A8_R2_IgM	8.86	28491.3	13108.0	2.174	MM
24	24	20211111_L7_R2_IgM	8.85	8476.8	13278.5	0.638	MM
25	25	20211111_L8_R2_IgM	8.85	2941.1	15233.6	0.193	MM
26	26	20211111_H4_R2_IgM	8.85	5186.4	16521.9	0.314	MM
27	27	20211111_A9_R2_IgM	8.86	10630.2	16451.6	0.646	MM
28	28	20211111_A10_R2_IgM	8.86	38547.6	13036.0	2.957	MM
29	29	20211111_L9_R2_IgM	8.88	10386.3	14554.1	0.714	MM
30	30	20211111_L10_R2_IgM	8.87	15790.9	12250.4	1.289	MM
31	31	20211111_QC_R11_IgM	8.85	14631.4	11459.2	1.277	MM
32	32	20211111_H5_R2_IgM	8.82	8556.3	14358.2	0.596	MM
33	33	20211111_A11_R2_IgM	8.84	9344.1	16184.1	0.577	MM
34	34	20211111_A12_R2_IgM	8.83	7295.8	13465.4	0.542	MM
35	35	20211111_L11_R2_IgM	8.88	12101.9	13277.9	0.911	MM
36	36	20211111_L12_R2_IgM	8.87	5675.6	15958.8	0.356	MM
37	37	20211111_H6_R2_IgM	8.84	4272.3	14003.5	0.305	MM
38	38	20211111_A13_R2_IgM	8.84	5414.2	13656.1	0.396	MM
39	39	20211111_A14_R2_IgM	8.85	48820.0	14071.3	3.469	MM
40	40	20211111_L13_R2_IgM	8.86	7835.3	14785.6	0.530	MM
41	41	20211111_L14_R2_IgM	8.86	9862.9	14772.2	0.668	MM
42	42	20211111_H7_R2_IgM	8.85	3580.2	15164.4	0.236	MM
43	43	20211111_A15_R2_IgM	8.85	47730.5	15721.5	3.036	MM
44	44	20211111_QC_R12_IgM	8.86	20167.2	13711.5	1.471	MM
45	45	20211111_A16_R2_IgM	8.87	34116.3	14275.6	2.390	MM
46	46	20211111_L15_R2_IgM	8.86	10489.4	12935.8	0.811	MM
47	47	20211111_L16_R2_IgM	8.86	6437.7	13342.8	0.482	MM
48	48	20211111_H8_R2_IgM	8.88	9975.5	15835.1	0.630	MM
49	49	20211111_A17_R2_IgM	8.88	23978.1	15786.7	1.519	MM
50	50	20211111_A18_R2_IgM	8.88	9434.7	16986.8	0.555	MM
51	51	20211111_L17_R2_IgM	8.85	18710.4	13342.7	1.402	MM
52	52	20211111_L18_R2_IgM	8.87	3069.3	14692.0	0.209	MM
53	53	20211111_H9_R2_IgM	8.88	4734.5	15639.7	0.303	MM
54	54	20211111_A19_R2_IgM	8.84	14063.1	14198.7	0.990	MM
55	55	20211111_A20_R2_IgM	8.87	2112.1	14162.7	0.149	MM
56	56	20211111_L19_R2_IgM	8.83	5946.8	9293.4	0.640	MM

	#	Name	RT	Area	IS Area	Response	Prima...
57	57	20211111_QC_R13_IgM	8.87	15203.6	13903.0	1.094	bb
58	58	20211111_L20_R2_IgM	8.99	8160.0	12781.6	0.638	MM
59	59	20211111_H10_R2_IgM	8.88	5362.5	14796.4	0.362	MM
60	60	20211111_A21_R2_IgM	8.87	15223.8	13669.0	1.114	MM
61	61	20211111_A22_R2_IgM	8.80	2316.3	14098.3	0.164	MM
62	62	20211111_L21_R2_IgM	8.88	7344.9	15678.2	0.468	MM
63	63	20211111_L22_R2_IgM	8.87	14078.3	15724.5	0.895	MM
64	64	20211111_H11_R2_IgM	8.87	23712.9	15890.2	1.492	MM
65	65	20211111_A23_R2_IgM	8.83	6344.0	13423.2	0.473	MM
66	66	20211111_A24_R2_IgM	8.85	9983.6	12438.0	0.803	MM
67	67	20211111_L23_R2_IgM	8.86	17989.7	14075.0	1.278	MM
68	68	20211111_L24_R2_IgM	8.86	1606.7	15972.1	0.101	MM
69	69	20211111_H12_R2_IgM	8.85	4016.3	16270.9	0.247	MM
70	70	20211111_QC_R14_IgM	8.86	16810.2	15806.8	1.063	MM
71	71	20211111_A25_R2_IgM	8.87	50405.6	14552.1	3.464	MM
72	72	20211111_A26_R2_IgM	8.89	19494.4	11492.7	1.696	MM
73	73	20211111_L25_R2_IgM	8.87	16121.2	14403.3	1.119	MM
74	74	20211111_L26_R2_IgM	8.88	11680.3	16533.9	0.706	MM
75	75	20211111_H13_R2_IgM	8.86	15777.8	15520.1	1.017	MM
76	76	20211111_A27_R2_IgM	8.86	18560.8	13337.4	1.392	MM
77	77	20211111_A28_R2_IgM	8.84	3194.6	11895.6	0.269	MM
78	78	20211111_L27_R2_IgM	8.86	15663.7	14125.7	1.109	MM
79	79	20211111_L28_R2_IgM	8.85	6179.1	11807.5	0.523	MM
80	80	20211111_H14_R2_IgM	8.84	14311.5	15035.7	0.952	MM
81	81	20211111_A29_R2_IgM	8.84	3162.9	13554.4	0.233	MM
82	82	20211111_A30_R2_IgM	8.87	26682.9	12984.1	2.055	MM
83	83	20211111_QC_R15_IgM	8.86	14622.8	12299.5	1.189	MM
84	84	20211111_L29_R2_IgM	8.86	3504.7	13590.7	0.258	MM
85	85	20211111_L30_R2_IgM	8.87	1863.7	12511.4	0.149	MM
86	86	20211111_H15_R2_IgM	8.85	6313.0	11885.0	0.531	MM
87	87	20211111_A31_R2_IgM	8.85	9956.6	13422.1	0.742	MM
88	88	20211111_A32_R2_IgM	8.86	16029.5	11461.3	1.399	MM
89	89	20211111_L31_R2_IgM	8.89	2363.9	13753.4	0.172	MM
90	90	20211111_L32_R2_IgM	8.87	10358.3	12949.0	0.800	MM
91	91	20211111_H16_R2_IgM	8.85	18590.8	11465.4	1.621	MM
92	92	20211111_A33_R2_IgM	8.85	13734.7	10551.5	1.302	MM
93	93	20211111_A34_R2_IgM	8.87	6894.5	12409.9	0.556	MM
94	94	20211111_H17_R2_IgM	8.83	42269.7	10160.9	4.160	MM
95	95	20211111_A35_R2_IgM	8.85	1169.0	10460.6	0.112	MM
96	96	20211111_QC_R16_IgM	8.85	13460.9	10433.5	1.290	MM
97	97	20211111_A36_R2_IgM	8.84	8239.4	12504.8	0.659	MM
98	98	20211111_H18_R2_IgM	8.84	40309.0	9140.4	4.410	MM
99	99	20211111_A37_R2_IgM	8.85	14323.2	9796.9	1.462	MM
100	1...	20211111_A38_R2_IgM	8.88	21598.3	11224.3	1.924	MM
101	1...	20211111_H19_R2_IgM	8.85	14271.3	11739.2	1.216	MM
102	1...	20211111_A39_R2_IgM	8.88	16834.6	12718.9	1.324	MM
103	1...	20211111_A40_R2_IgM	8.85	5314.9	10006.1	0.531	MM
104	1...	20211111_H20_R2_IgM	8.85	38238.7	7988.2	4.787	MM
105	1...	20211111_A41_R2_IgM	8.87	8348.1	11513.3	0.725	MM
106	1...	20211111_A42_R2_IgM	8.86	5286.2	10238.5	0.516	MM
107	1...	20211111_A43_R2_IgM	8.86	5806.9	11098.9	0.523	MM
108	1...	20211111_A44_R2_IgM	8.75	2040.0	10969.9	0.186	MM
109	1...	20211111_QC_R17_IgM	8.85	13017.8	9700.8	1.342	MM
110	1...	20211111_L2_R3_IgM	8.85	12687.7	8528.0	1.488	MM
111	1...	20211111_H1_R3_IgM	8.86	36904.8	9438.2	3.910	MM
112	1...	20211111_A3_R3_IgM	8.86	34635.6	11598.6	2.986	MM

	#	Name	RT	Area	IS Area	Response	Prima...
113	1...	20211111_A4_R3_IgM	8.87	14961.7	11364.1	1.317	bb
114	1...	20211111_L3_R3_IgM	8.86	2640.0	10753.4	0.246	MM
115	1...	20211111_L4_R3_IgM	8.86	10293.0	10291.6	1.000	MM
116	1...	20211111_H2_R3_IgM	8.84	58983.5	9315.8	6.332	MM
117	1...	20211111_A5_R3_IgM	8.84	71695.7	9102.2	7.877	MM
118	1...	20211111_A6_R3_IgM	8.85	12985.4	10356.4	1.254	MM
119	1...	20211111_QC_R18_IgM	8.85	10684.8	8398.3	1.272	MM
120	1...	20211111_L5_R3_IgM	8.86	20955.6	9786.7	2.141	MM
121	1...	20211111_L6_R3_IgM	8.86	11132.0	9911.3	1.123	MM
122	1...	20211111_H3_R3_IgM	8.89	7378.0	12155.5	0.607	MM
123	1...	20211111_A7_R3_IgM	8.85	48344.5	11804.8	4.095	MM
124	1...	20211111_A8_R3_IgM	8.87	36592.0	10510.6	3.481	MM
125	1...	20211111_L7_R3_IgM	8.86	7383.3	12177.9	0.606	MM
126	1...	20211111_L8_R3_IgM	8.89	2738.3	11668.6	0.235	MM
127	1...	20211111_H4_R3_IgM	8.87	5956.9	11192.0	0.532	MM
128	1...	20211111_A9_R3_IgM	8.88	18890.0	14077.4	1.342	MM
129	1...	20211111_A10_R3_IgM	8.87	54398.3	11190.4	4.861	MM
130	1...	20211111_L9_R3_IgM	8.86	14547.7	14649.3	0.993	MM
131	1...	20211111_L10_R3_IgM	8.86	13237.6	9361.3	1.414	MM
132	1...	20211111_QC_R19_IgM	8.85	13556.9	10125.5	1.339	MM
133	1...	20211111_H5_R3_IgM	8.86	8129.1	10010.9	0.812	MM
134	1...	20211111_A11_R3_IgM	8.87	22803.3	12697.2	1.796	MM
135	1...	20211111_A12_R3_IgM	8.84	8534.5	13148.5	0.649	MM
136	1...	20211111_L11_R3_IgM	8.89	9522.8	10786.6	0.883	MM
137	1...	20211111_L12_R3_IgM	8.86	5825.8	11681.8	0.499	MM
138	1...	20211111_H6_R3_IgM	8.84	3448.6	12095.5	0.285	MM
139	1...	20211111_A13_R3_IgM	8.86	5075.0	12908.3	0.393	MM
140	1...	20211111_A14_R3_IgM	8.86	47740.6	11322.6	4.216	MM
141	1...	20211111_L13_R3_IgM	8.85	4214.3	11516.1	0.366	MM
142	1...	20211111_L14_R3_IgM	8.86	9829.7	10790.7	0.911	MM
143	1...	20211111_H7_R3_IgM	8.86	6960.7	11572.1	0.602	MM
144	1...	20211111_A15_R3_IgM	8.85	43950.6	12434.4	3.535	MM
145	1...	20211111_QC_R20_IgM	8.86	14655.7	10701.0	1.370	MM
146	1...	20211111_A16_R3_IgM	8.88	27773.9	11452.1	2.425	MM
147	1...	20211111_L15_R3_IgM	8.86	11876.0	10531.1	1.128	MM
148	1...	20211111_L16_R3_IgM	8.87	4876.7	12049.4	0.405	MM
149	1...	20211111_H8_R3_IgM	8.88	10721.0	13434.9	0.798	MM
150	1...	20211111_A17_R3_IgM	8.87	21000.3	12452.0	1.686	MM
151	1...	20211111_A18_R3_IgM	8.88	6909.6	12506.5	0.552	MM
152	1...	20211111_L17_R3_IgM	8.85	17770.5	9948.9	1.786	MM
153	1...	20211111_L18_R3_IgM	8.84	2648.2	13462.1	0.197	MM
154	1...	20211111_H9_R3_IgM	8.88	4999.6	13178.7	0.379	MM
155	1...	20211111_A19_R3_IgM	8.85	15619.4	11298.6	1.382	MM
156	1...	20211111_A20_R3_IgM	8.89	1725.6	11175.6	0.154	MM
157	1...	20211111_L19_R3_IgM	8.84	6866.7	11558.2	0.594	MM
158	1...	20211111_QC_R21_IgM	8.86	14431.2	9977.0	1.446	MM
159	1...	20211111_L20_R3_IgM	8.88	6456.3	13508.1	0.478	MM
160	1...	20211111_H10_R3_IgM	8.85	8512.0	12374.8	0.688	MM
161	1...	20211111_A21_R3_IgM	8.85	15592.7	12443.6	1.253	MM
162	1...	20211111_A22_R3_IgM	8.83	1796.8	11589.3	0.155	MM
163	1...	20211111_L21_R3_IgM	8.87	6440.7	13107.4	0.491	MM
164	1...	20211111_L22_R3_IgM	8.87	12860.8	12021.5	1.070	MM
165	1...	20211111_H11_R3_IgM	8.88	25074.8	12687.8	1.976	MM
166	1...	20211111_A23_R3_IgM	8.86	6450.8	11611.8	0.556	MM
167	1...	20211111_A24_R3_IgM	8.85	10268.4	10476.8	0.980	MM
168	1...	20211111_L23_R3_IgM	8.86	15689.5	11873.1	1.321	MM

Dataset: C:\MassLynx\10.pro\20211111_clinicalsamples_IgM_IgA.qld

Last Altered: Thursday, March 09, 2023 19:58:25 台北標準時間
Printed: Friday, August 01, 2025 15:01:57 台北標準時間

	# Name	RT	Area	IS Area	Response	Prima...
169	1... 20211111_L24_R3_IgM	8.86	2703.2	13468.0	0.201	MM
170	1... 20211111_H12_R3_IgM	8.85	4730.5	14229.1	0.332	MM
171	1... 20211111_QC_R22_IgM	8.86	14407.0	11264.4	1.279	MM
172	1... 20211111_A25_R3_IgM	8.87	38055.2	11775.7	3.232	MM
173	1... 20211111_A26_R3_IgM	8.88	16573.8	10473.9	1.582	MM
174	1... 20211111_L25_R3_IgM	8.88	13869.5	13256.8	1.046	MM
175	1... 20211111_L26_R3_IgM	8.89	10001.8	13565.0	0.737	MM
176	1... 20211111_H13_R3_IgM	8.85	14036.4	13146.0	1.068	MM
177	1... 20211111_A27_R3_IgM	8.86	20168.7	12025.6	1.677	MM
178	1... 20211111_A28_R3_IgM	8.86	5399.4	11229.1	0.481	MM
179	1... 20211111_L27_R3_IgM	8.88	17605.1	14960.0	1.177	MM
180	1... 20211111_L28_R3_IgM	8.85	6354.1	11359.0	0.559	MM
181	1... 20211111_H14_R3_IgM	8.85	13844.8	12830.8	1.079	MM
182	1... 20211111_A29_R3_IgM	8.86	3085.8	13434.3	0.230	MM
183	1... 20211111_A30_R3_IgM	8.86	25492.4	11272.2	2.262	MM
184	1... 20211111_QC_R23_IgM	8.86	13906.3	11872.2	1.171	MM
185	1... 20211111_L29_R3_IgM	8.85	3294.2	12948.7	0.254	MM
186	1... 20211111_L30_R3_IgM	8.87	2878.8	11026.6	0.261	MM
187	1... 20211111_H15_R3_IgM	8.87	6987.6	10786.3	0.648	MM
188	1... 20211111_A31_R3_IgM	8.85	8664.6	12010.5	0.721	MM
189	1... 20211111_A32_R3_IgM	8.86	14749.8	11107.7	1.328	MM
190	1... 20211111_L31_R3_IgM	8.87	2973.0	11021.8	0.270	MM
191	1... 20211111_L32_R3_IgM	8.87	14540.9	11683.8	1.245	MM
192	1... 20211111_H16_R3_IgM	8.85	19380.5	9831.7	1.971	MM
193	1... 20211111_A33_R3_IgM	8.85	14952.2	11213.1	1.333	MM
194	1... 20211111_A34_R3_IgM	8.87	6528.4	11550.1	0.565	MM
195	1... 20211111_H17_R3_IgM	8.84	35038.7	8874.4	3.948	MM
196	1... 20211111_A35_R3_IgM	8.84	4720.1	9856.3	0.479	MM
197	1... 20211111_QC_R24_IgM	8.86	10994.6	7561.8	1.454	MM
198	1... 20211111_A36_R3_IgM	8.86	16785.5	9562.8	1.755	MM
199	1... 20211111_H18_R3_IgM	8.85	43192.6	8165.3	5.290	MM
200	2... 20211111_A37_R3_IgM	8.85	12401.9	9278.8	1.337	MM
201	2... 20211111_A38_R3_IgM	8.87	20212.9	10920.3	1.851	MM
202	2... 20211111_H19_R3_IgM	8.84	12870.7	9285.3	1.386	MM
203	2... 20211111_A39_R3_IgM	8.88	15627.8	9083.4	1.720	MM
204	2... 20211111_A40_R3_IgM	8.86	5610.2	8382.4	0.669	MM
205	2... 20211111_H20_R3_IgM	8.85	37742.3	7807.9	4.834	MM
206	2... 20211111_A41_R3_IgM	8.88	6310.2	5925.2	1.065	MM
207	2... 20211111_A42_R3_IgM	8.87	4265.6	9352.6	0.456	MM
208	2... 20211111_A43_R3_IgM	8.85	5314.5	10216.3	0.520	MM
209	2... 20211111_A44_R3_IgM	8.80	2018.5	10262.7	0.197	MM
210	2... 20211111_QC_R25_IgM	8.86	12468.6	8575.3	1.454	MM

Dataset: C:\MassLynx\10.pro\20211111_clinicalsamples_IgM_IgA.qld

Last Altered: Thursday, March 09, 2023 19:58:25 台北標準時間
Printed: Friday, August 01, 2025 15:03:19 台北標準時間

Method: C:\MassLynx\10_Pro_IgA.PRO\MethDB\20211111_IgM_IgA_DMRM_new.mdb 24 Feb 2022 13:44:14
Calibration: 15 Nov 2021 14:42:11

Sample Name: 20211112_QC_R001_IgM

F82:MRM of 1 channel,ES+
1157.6 > 204.1
3.206e+006

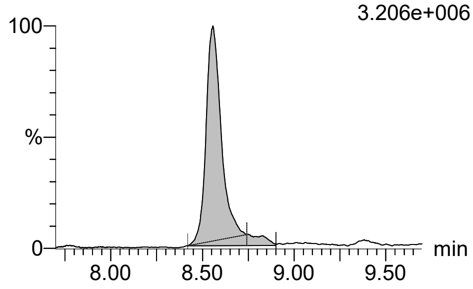

Sample Name: 20211112_QC_R002_IgM

F82:MRM of 1 channel,ES+
1157.6 > 204.1
2.951e+006

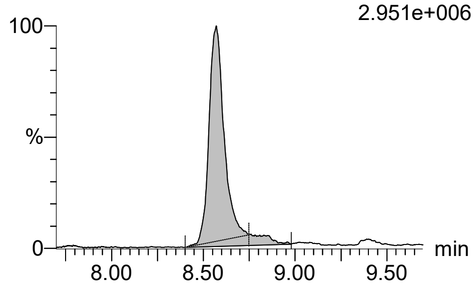

Sample Name: 20211111_A1_R2_IgM

F82:MRM of 1 channel,ES+
1157.6 > 204.1
2.282e+006

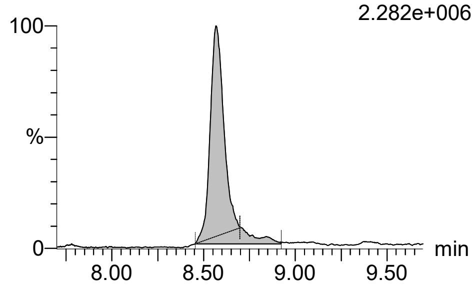

Sample Name: 20211111_A1_R3_IgM

F82:MRM of 1 channel,ES+
1157.6 > 204.1
2.222e+006

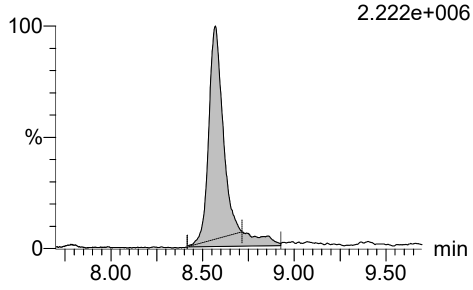

Sample Name: 20211111_A2_R2_IgM

F82:MRM of 1 channel,ES+
1157.6 > 204.1
7.666e+006

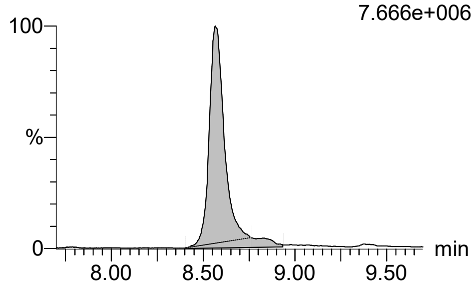

Sample Name: 20211111_A2_R3_IgM

F82:MRM of 1 channel,ES+
1157.6 > 204.1
7.362e+006

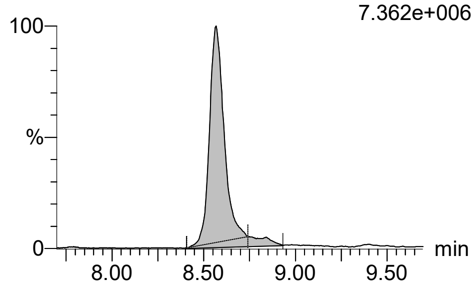

Sample Name: 20211111_L1_R2_IgM

F82:MRM of 1 channel,ES+
1157.6 > 204.1
1.097e+006

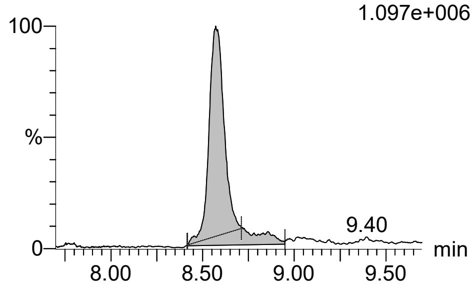

Sample Name: 20211111_L1_R3_IgM

F82:MRM of 1 channel,ES+
1157.6 > 204.1
1.070e+006

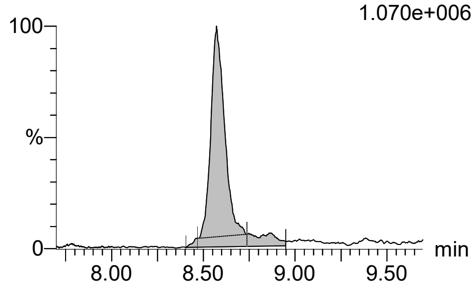

Sample Name: 20211111_L2_R2_IgM

F82:MRM of 1 channel,ES+
1157.6 > 204.1
4.405e+006

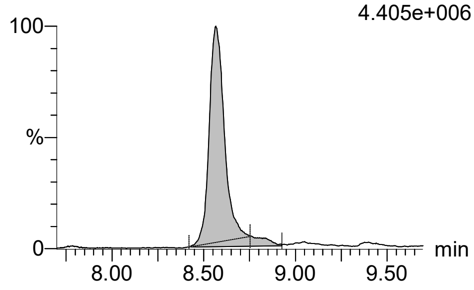

Sample Name: 20211111_H1_R2_IgM

F82:MRM of 1 channel,ES+
1157.6 > 204.1
4.757e+006

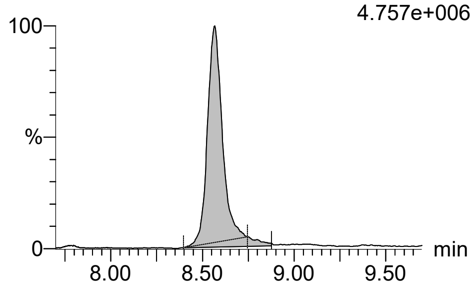

Sample Name: 20211111_A3_R2_IgM

F82:MRM of 1 channel,ES+
1157.6 > 204.1
6.380e+006

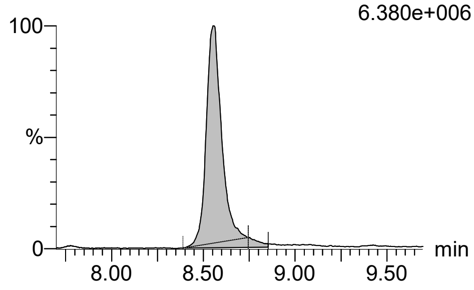

Sample Name: 20211111_A4_R2_IgM

F82:MRM of 1 channel,ES+
1157.6 > 204.1
3.176e+006

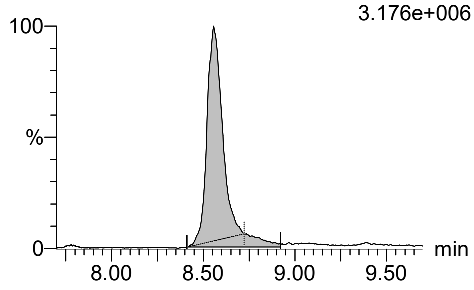

Dataset: C:\MassLynx\10.pro\20211111_clinicalsamples_IgM_IgA.qld

Last Altered: Thursday, March 09, 2023 19:58:25 台北標準時間
Printed: Friday, August 01, 2025 15:03:19 台北標準時間

Sample Name: 20211111_L3_R2_IgM
F82:MRM of 1 channel,ES+
1157.6 > 204.1
1.917e+006

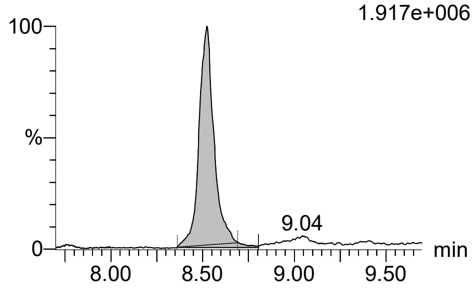

Sample Name: 20211111_L4_R2_IgM
F82:MRM of 1 channel,ES+
1157.6 > 204.1
4.388e+006

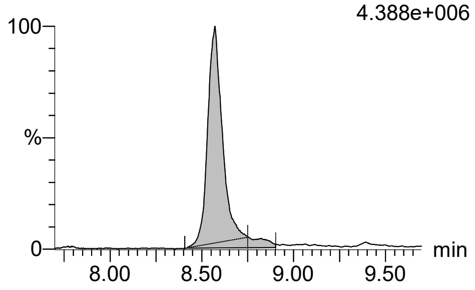

Sample Name: 20211111_H2_R2_IgM
F82:MRM of 1 channel,ES+
1157.6 > 204.1
1.025e+007

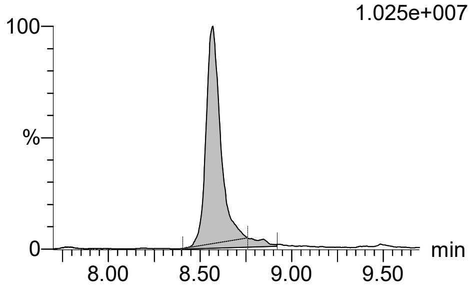

Sample Name: 20211111_A5_R2_IgM
F82:MRM of 1 channel,ES+
1157.6 > 204.1
1.139e+007

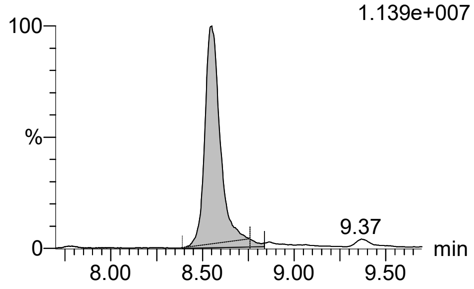

Sample Name: 20211111_A6_R2_IgM
F82:MRM of 1 channel,ES+
1157.6 > 204.1
3.333e+006

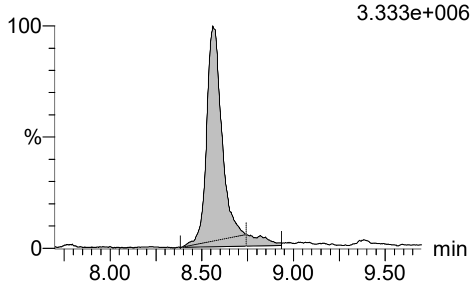

Sample Name: 20211111_QC_R10_IgM
F82:MRM of 1 channel,ES+
1157.6 > 204.1
3.107e+006

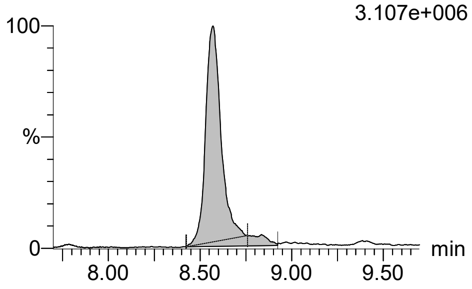

Sample Name: 20211111_L5_R2_IgM
F82:MRM of 1 channel,ES+
1157.6 > 204.1
3.851e+006

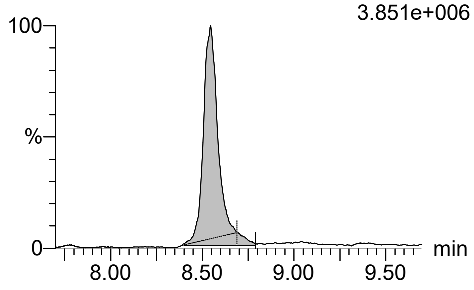

Sample Name: 20211111_L6_R2_IgM
F82:MRM of 1 channel,ES+
1157.6 > 204.1
3.707e+006

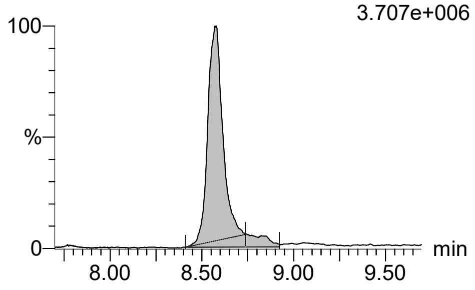

Sample Name: 20211111_H3_R2_IgM
F82:MRM of 1 channel,ES+
1157.6 > 204.1
2.786e+006

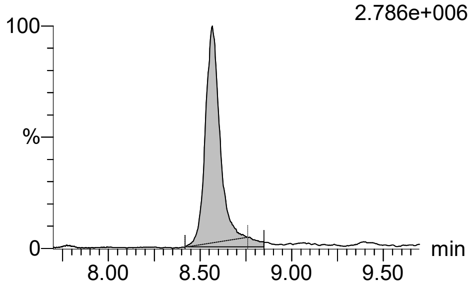

Sample Name: 20211111_A7_R2_IgM
F82:MRM of 1 channel,ES+
1157.6 > 204.1
3.936e+006

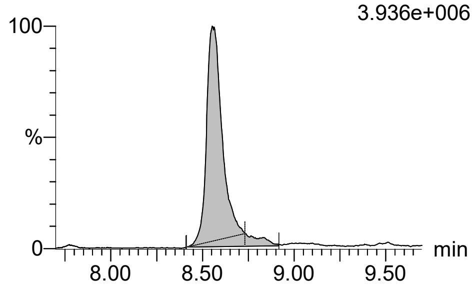

Sample Name: 20211111_A8_R2_IgM
F82:MRM of 1 channel,ES+
1157.6 > 204.1
5.658e+006

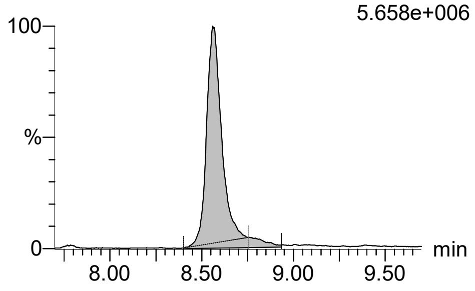

Sample Name: 20211111_L7_R2_IgM
F82:MRM of 1 channel,ES+
1157.6 > 204.1
1.974e+006

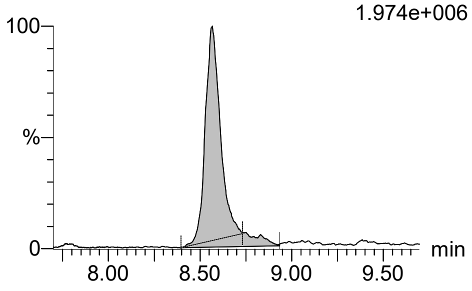

Sample Name: 20211111_L8_R2_IgM
F82:MRM of 1 channel,ES+
1157.6 > 204.1
7.697e+005

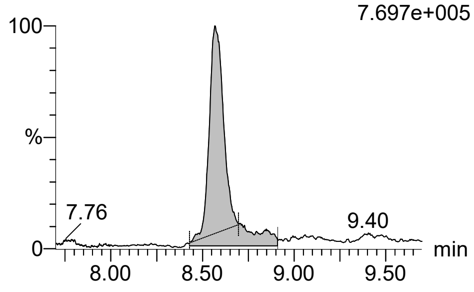

Sample Name: 20211111_H4_R2_IgM
F82:MRM of 1 channel,ES+
1157.6 > 204.1
1.975e+006

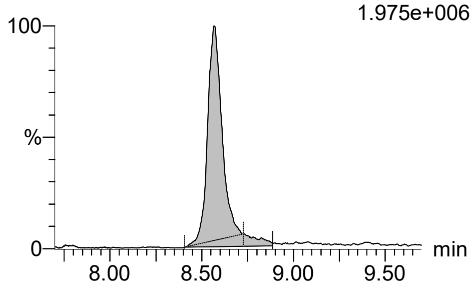

Sample Name: 20211111_A9_R2_IgM
F82:MRM of 1 channel,ES+
1157.6 > 204.1
2.728e+006

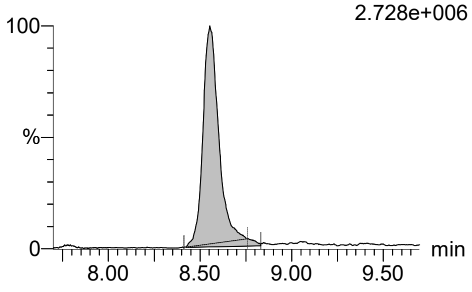

Dataset: C:\MassLynx\10.pro\20211111_clinicalsamples_IgM_IgA.qld

Last Altered: Thursday, March 09, 2023 19:58:25 台北標準時間
Printed: Friday, August 01, 2025 15:03:19 台北標準時間

Sample Name: 20211111_A10_R2_IgM
F82:MRM of 1 channel,ES+
1157.6 > 204.1
5.443e+006

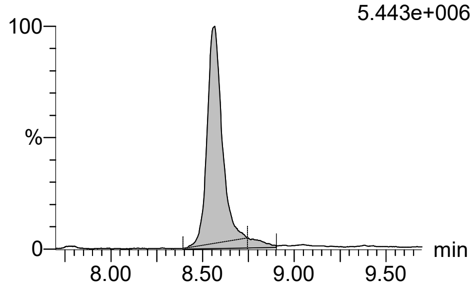

Sample Name: 20211111_L9_R2_IgM
F82:MRM of 1 channel,ES+
1157.6 > 204.1
2.055e+006

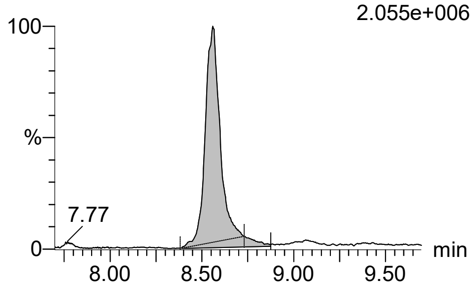

Sample Name: 20211111_L10_R2_IgM
F82:MRM of 1 channel,ES+
1157.6 > 204.1
5.671e+006

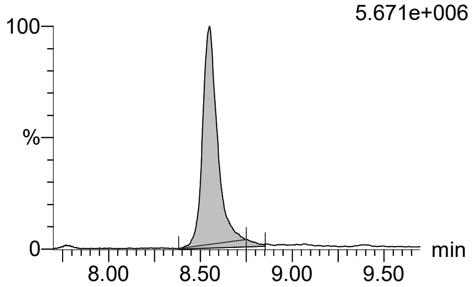

Sample Name: 20211111_QC_R11_IgM
F82:MRM of 1 channel,ES+
1157.6 > 204.1
3.748e+006

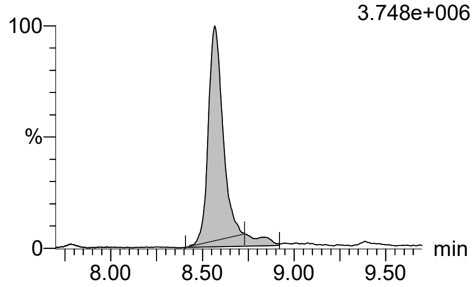

Sample Name: 20211111_H5_R2_IgM
F82:MRM of 1 channel,ES+
1157.6 > 204.1
2.824e+006

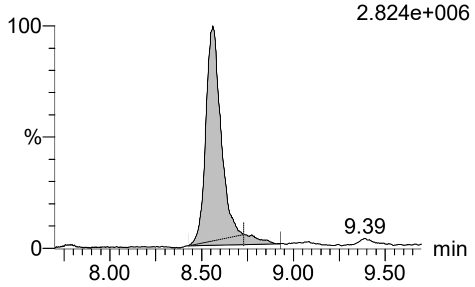

Sample Name: 20211111_A11_R2_IgM
F82:MRM of 1 channel,ES+
1157.6 > 204.1
7.268e+006

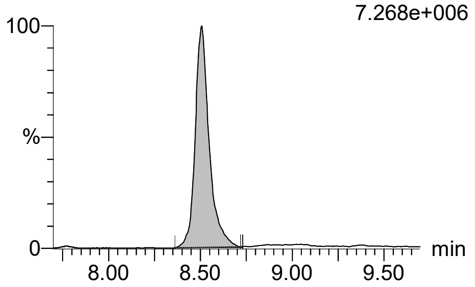

Sample Name: 20211111_A12_R2_IgM
F82:MRM of 1 channel,ES+
1157.6 > 204.1
2.226e+006

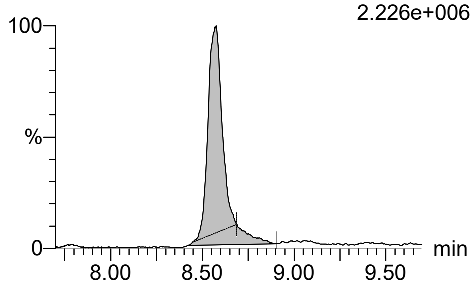

Sample Name: 20211111_L11_R2_IgM
F82:MRM of 1 channel,ES+
1157.6 > 204.1
2.685e+006

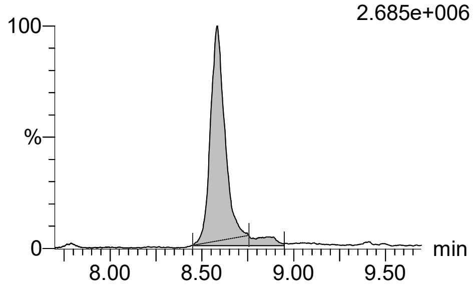

Sample Name: 20211111_L12_R2_IgM
F82:MRM of 1 channel,ES+
1157.6 > 204.1
2.133e+006

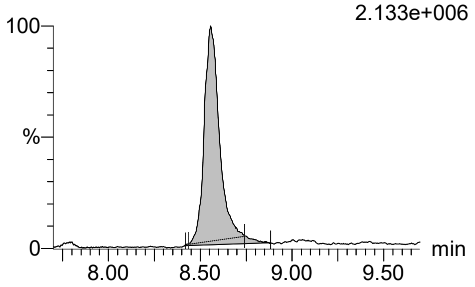

Sample Name: 20211111_H6_R2_IgM
F82:MRM of 1 channel,ES+
1157.6 > 204.1
2.415e+006

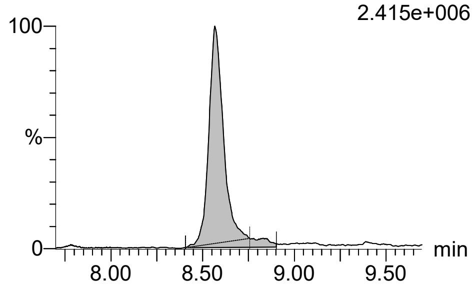

Sample Name: 20211111_A13_R2_IgM
F82:MRM of 1 channel,ES+
1157.6 > 204.1
2.731e+006

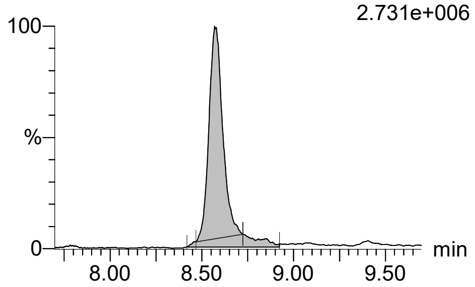

Sample Name: 20211111_A14_R2_IgM
F82:MRM of 1 channel,ES+
1157.6 > 204.1
1.129e+007

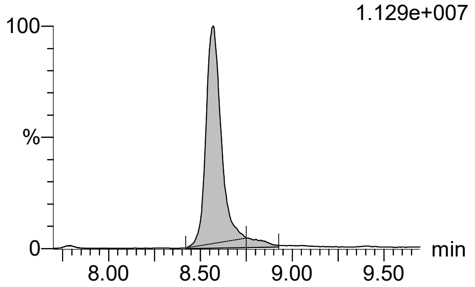

Sample Name: 20211111_L13_R2_IgM
F82:MRM of 1 channel,ES+
1157.6 > 204.1
1.359e+006

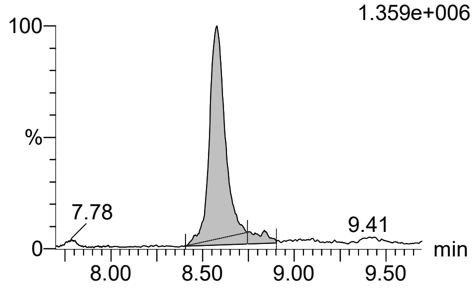

Sample Name: 20211111_L14_R2_IgM
F82:MRM of 1 channel,ES+
1157.6 > 204.1
2.852e+006

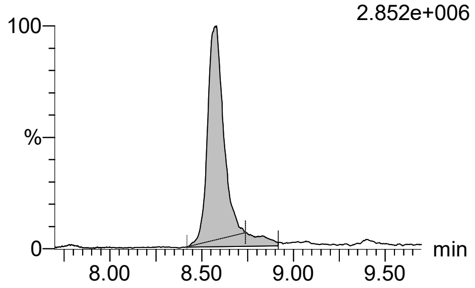

Sample Name: 20211111_H7_R2_IgM
F82:MRM of 1 channel,ES+
1157.6 > 204.1
3.432e+006

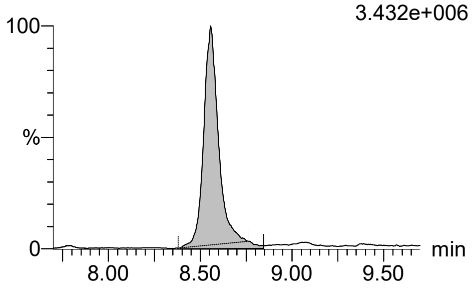

Dataset: C:\MassLynx\10.pro\20211111_clinicalsamples_IgM_IgA.qld

Last Altered: Thursday, March 09, 2023 19:58:25 台北標準時間
Printed: Friday, August 01, 2025 15:03:19 台北標準時間

Sample Name: 20211111_A15_R2_IgM

F82:MRM of 1 channel,ES+
1157.6 > 204.1
1.423e+007

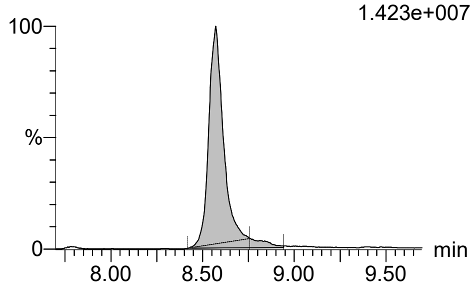

Sample Name: 20211111_QC_R12_IgM

F82:MRM of 1 channel,ES+
1157.6 > 204.1
3.945e+006

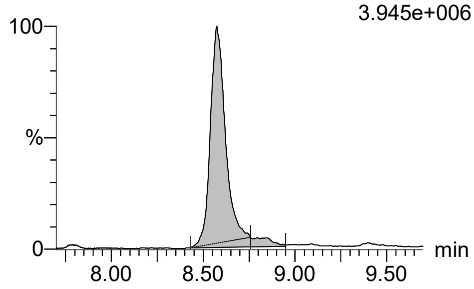

Sample Name: 20211111_A16_R2_IgM

F82:MRM of 1 channel,ES+
1157.6 > 204.1
5.742e+006

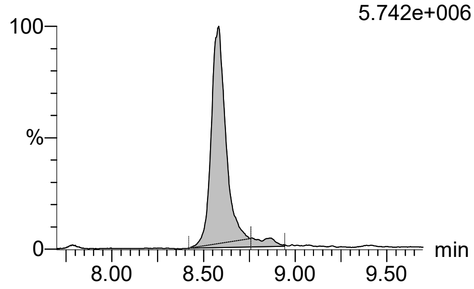

Sample Name: 20211111_L15_R2_IgM

F82:MRM of 1 channel,ES+
1157.6 > 204.1
5.690e+006

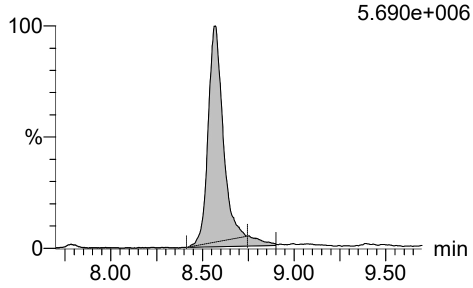

Sample Name: 20211111_L16_R2_IgM

F82:MRM of 1 channel,ES+
1157.6 > 204.1
9.314e+005

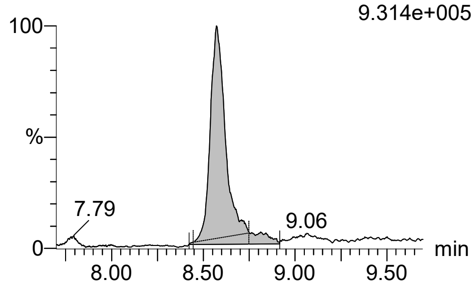

Sample Name: 20211111_H8_R2_IgM

F82:MRM of 1 channel,ES+
1157.6 > 204.1
1.678e+006

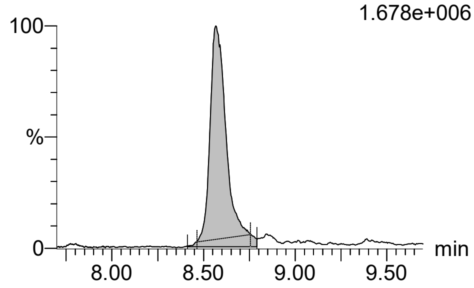

Sample Name: 20211111_A17_R2_IgM

F82:MRM of 1 channel,ES+
1157.6 > 204.1
5.195e+006

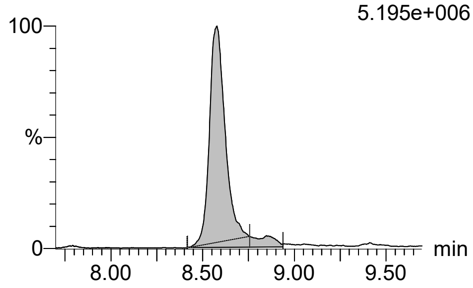

Sample Name: 20211111_A18_R2_IgM

F82:MRM of 1 channel,ES+
1157.6 > 204.1
2.253e+006

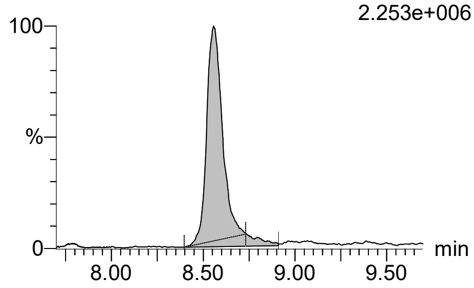

Sample Name: 20211111_L17_R2_IgM

F82:MRM of 1 channel,ES+
1157.6 > 204.1
4.300e+006

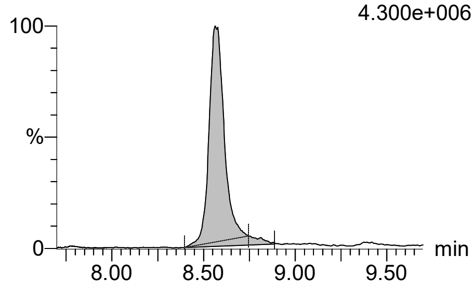

Sample Name: 20211111_L18_R2_IgM

F82:MRM of 1 channel,ES+
1157.6 > 204.1
8.004e+005

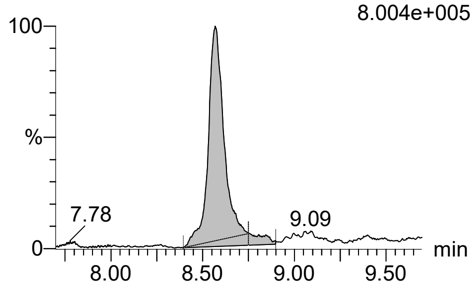

Sample Name: 20211111_H9_R2_IgM

F82:MRM of 1 channel,ES+
1157.6 > 204.1
2.071e+006

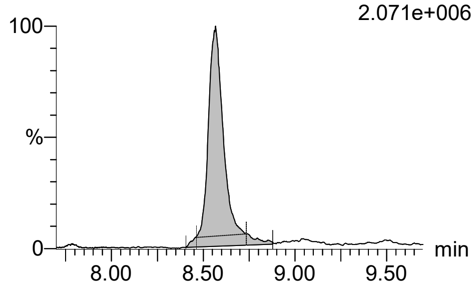

Sample Name: 20211111_A19_R2_IgM

F82:MRM of 1 channel,ES+
1157.6 > 204.1
3.525e+006

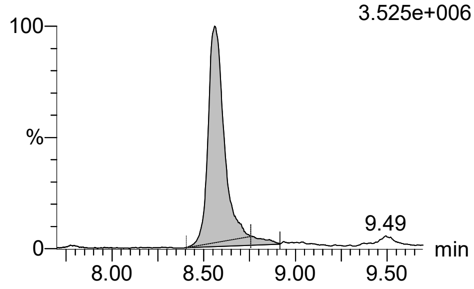

Sample Name: 20211111_A20_R2_IgM

F82:MRM of 1 channel,ES+
1157.6 > 204.1
1.902e+006

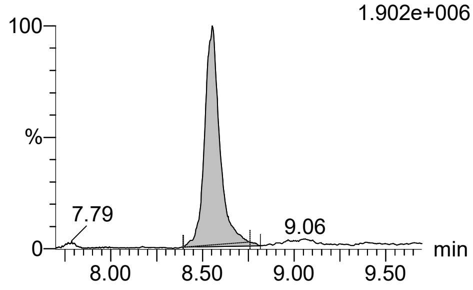

Sample Name: 20211111_L19_R2_IgM

F82:MRM of 1 channel,ES+
1157.6 > 204.1
3.369e+006

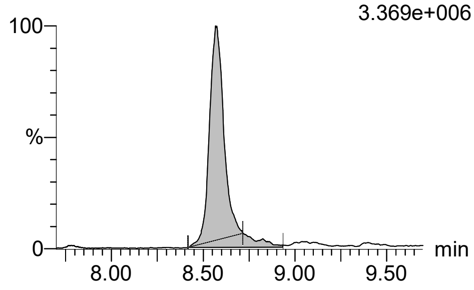

Sample Name: 20211111_QC_R13_IgM

F82:MRM of 1 channel,ES+
1157.6 > 204.1
3.854e+006

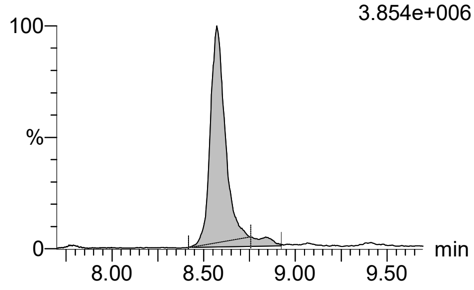

Dataset: C:\MassLynx\10.pro\20211111_clinicalsamples_IgM_IgA.qld

Last Altered: Thursday, March 09, 2023 19:58:25 台北標準時間
Printed: Friday, August 01, 2025 15:03:19 台北標準時間

Sample Name: 20211111_L20_R2_IgM
F82:MRM of 1 channel,ES+
1157.6 > 204.1
2.059e+006

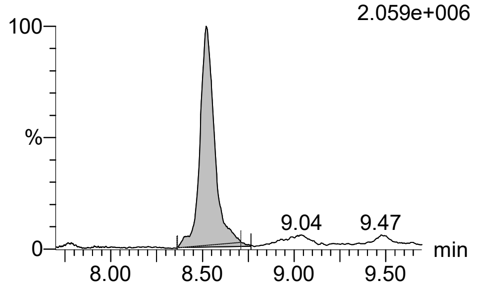

Sample Name: 20211111_H10_R2_IgM
F82:MRM of 1 channel,ES+
1157.6 > 204.1
4.217e+006

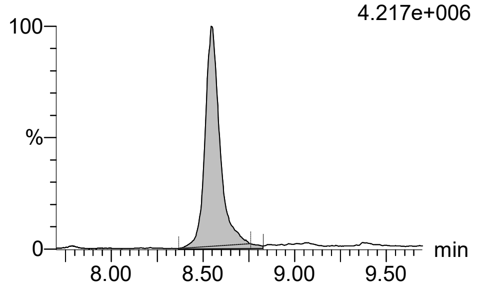

Sample Name: 20211111_A21_R2_IgM
F82:MRM of 1 channel,ES+
1157.6 > 204.1
3.194e+006

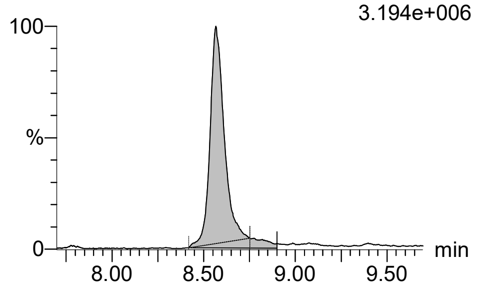

Sample Name: 20211111_A22_R2_IgM
F82:MRM of 1 channel,ES+
1157.6 > 204.1
5.785e+005

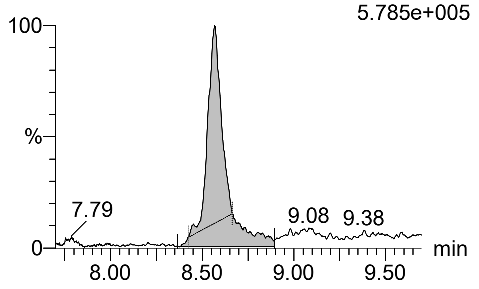

Sample Name: 20211111_L21_R2_IgM
F82:MRM of 1 channel,ES+
1157.6 > 204.1
2.637e+006

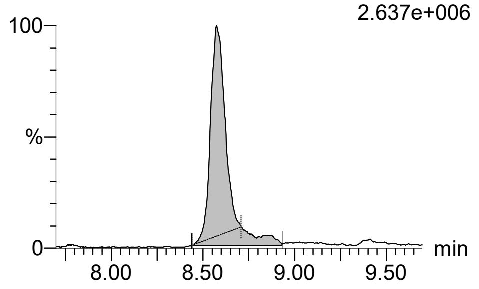

Sample Name: 20211111_L22_R2_IgM
F82:MRM of 1 channel,ES+
1157.6 > 204.1
2.124e+006

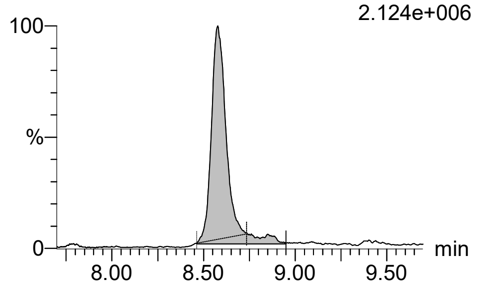

Sample Name: 20211111_H11_R2_IgM
F82:MRM of 1 channel,ES+
1157.6 > 204.1
3.930e+006

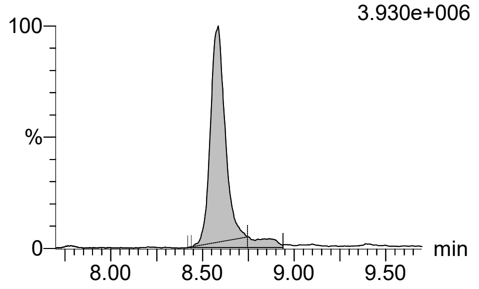

Sample Name: 20211111_A23_R2_IgM
F82:MRM of 1 channel,ES+
1157.6 > 204.1
2.504e+006

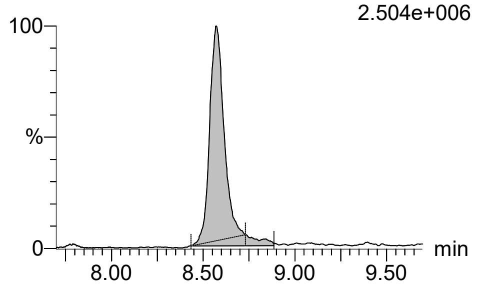

Sample Name: 20211111_A24_R2_IgM
F82:MRM of 1 channel,ES+
1157.6 > 204.1
5.187e+006

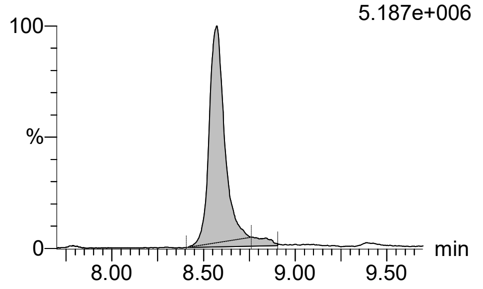

Sample Name: 20211111_L23_R2_IgM
F82:MRM of 1 channel,ES+
1157.6 > 204.1
5.819e+006

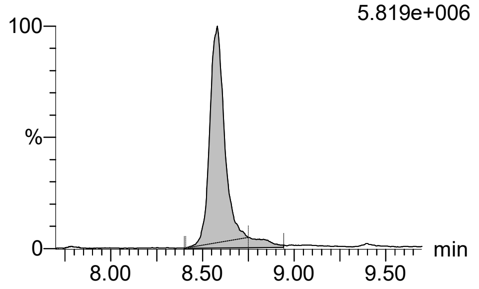

Sample Name: 20211111_L24_R2_IgM
F82:MRM of 1 channel,ES+
1157.6 > 204.1
8.167e+005

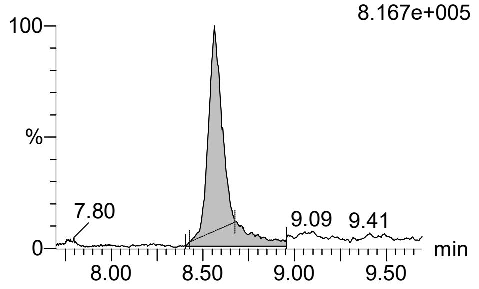

Sample Name: 20211111_H12_R2_IgM
F82:MRM of 1 channel,ES+
1157.6 > 204.1
1.146e+006

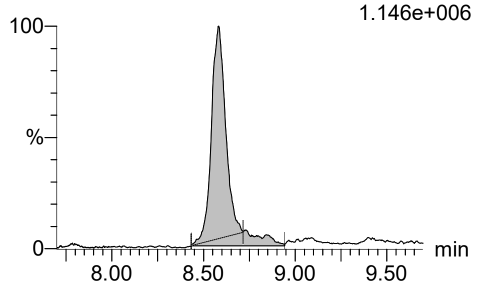

Sample Name: 20211111_QC_R14_IgM
F82:MRM of 1 channel,ES+
1157.6 > 204.1
3.998e+006

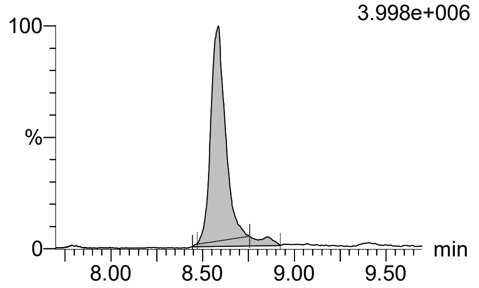

Sample Name: 20211111_A25_R2_IgM
F82:MRM of 1 channel,ES+
1157.6 > 204.1
8.012e+006

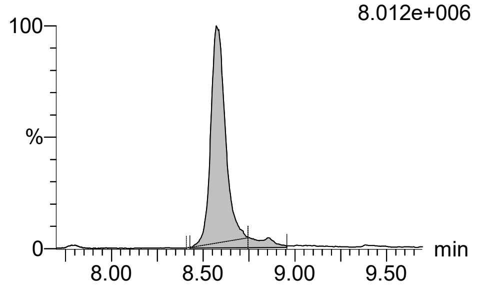

Sample Name: 20211111_A26_R2_IgM
F82:MRM of 1 channel,ES+
1157.6 > 204.1
3.265e+006

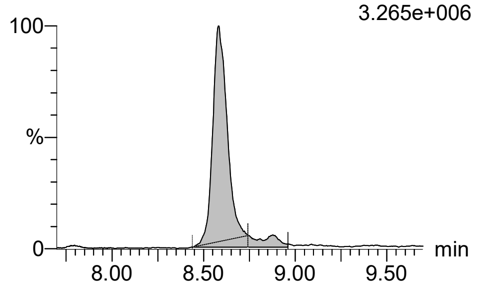

Dataset: C:\MassLynx\10.pro\20211111_clinicalsamples_IgM_IgA.qld

Last Altered: Thursday, March 09, 2023 19:58:25 台北標準時間
Printed: Friday, August 01, 2025 15:03:19 台北標準時間

Sample Name: 20211111_L25_R2_IgM
F82:MRM of 1 channel,ES+
1157.6 > 204.1
1.725e+006

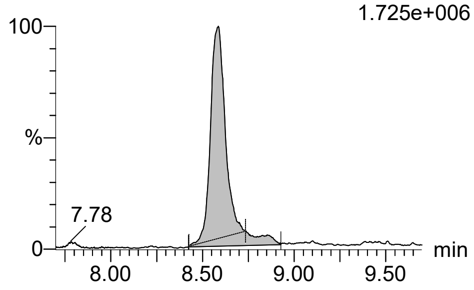

Sample Name: 20211111_L26_R2_IgM
F82:MRM of 1 channel,ES+
1157.6 > 204.1
2.448e+006

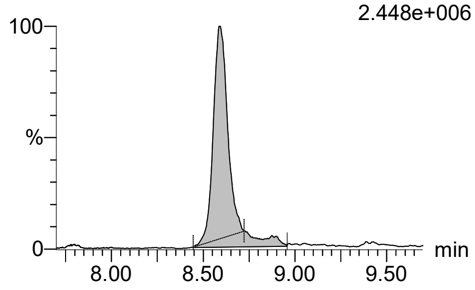

Sample Name: 20211111_H13_R2_IgM
F82:MRM of 1 channel,ES+
1157.6 > 204.1
4.554e+006

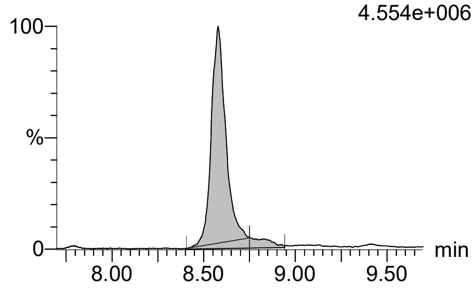

Sample Name: 20211111_A27_R2_IgM
F82:MRM of 1 channel,ES+
1157.6 > 204.1
3.665e+006

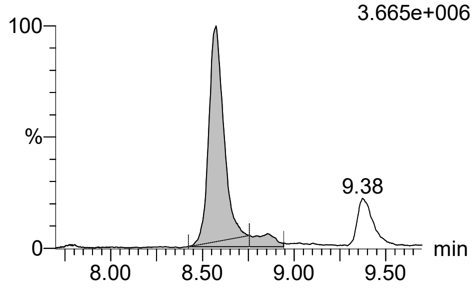

Sample Name: 20211111_A28_R2_IgM
F82:MRM of 1 channel,ES+
1157.6 > 204.1
1.644e+006

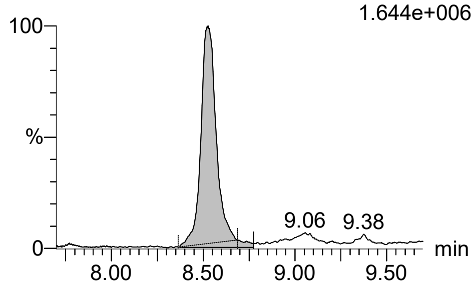

Sample Name: 20211111_L27_R2_IgM
F82:MRM of 1 channel,ES+
1157.6 > 204.1
3.703e+006

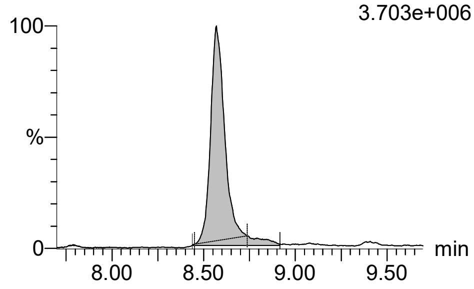

Sample Name: 20211111_L28_R2_IgM
F82:MRM of 1 channel,ES+
1157.6 > 204.1
1.233e+006

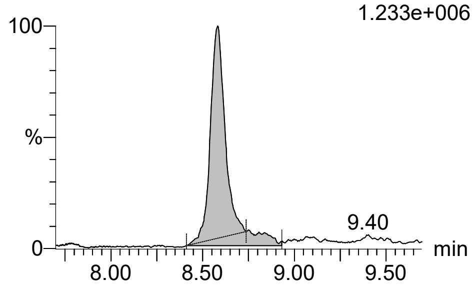

Sample Name: 20211111_H14_R2_IgM
F82:MRM of 1 channel,ES+
1157.6 > 204.1
3.143e+006

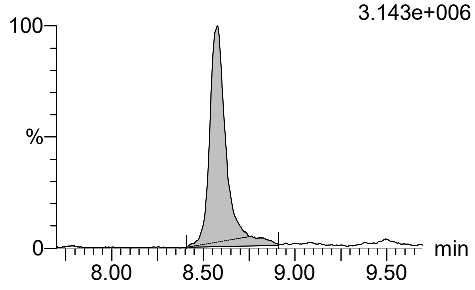

Sample Name: 20211111_A29_R2_IgM
F82:MRM of 1 channel,ES+
1157.6 > 204.1
5.425e+005

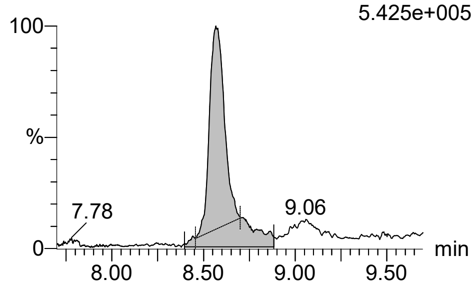

Sample Name: 20211111_A30_R2_IgM
F82:MRM of 1 channel,ES+
1157.6 > 204.1
6.593e+006

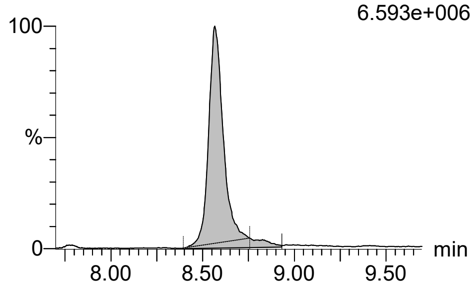

Sample Name: 20211111_QC_R15_IgM
F82:MRM of 1 channel,ES+
1157.6 > 204.1
3.364e+006

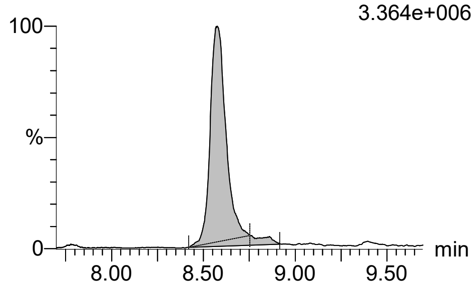

Sample Name: 20211111_L29_R2_IgM
F82:MRM of 1 channel,ES+
1157.6 > 204.1
9.381e+005

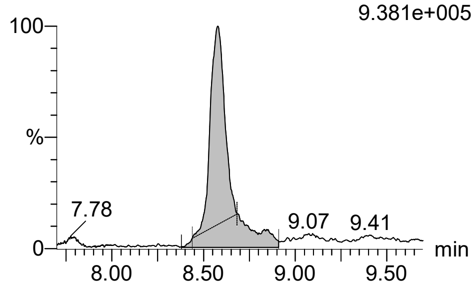

Sample Name: 20211111_L30_R2_IgM
F82:MRM of 1 channel,ES+
1157.6 > 204.1
9.891e+005

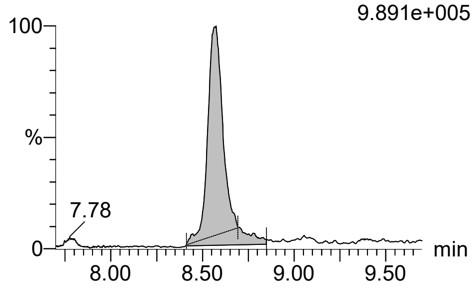

Sample Name: 20211111_H15_R2_IgM
F82:MRM of 1 channel,ES+
1157.6 > 204.1
2.627e+006

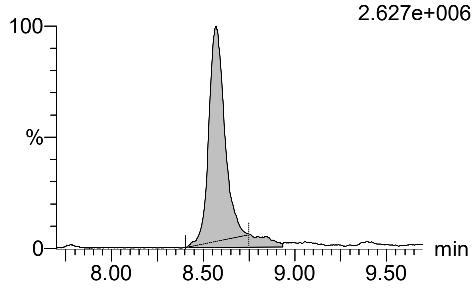

Sample Name: 20211111_A31_R2_IgM
F82:MRM of 1 channel,ES+
1157.6 > 204.1
2.332e+006

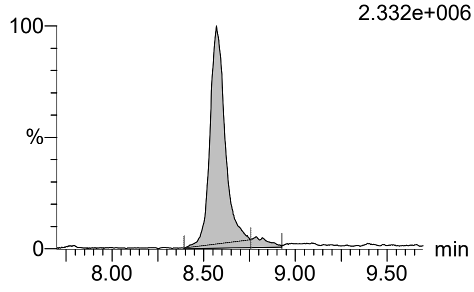

Dataset: C:\MassLynx\10.pro\20211111_clinicalsamples_IgM_IgA.qld

Last Altered: Thursday, March 09, 2023 19:58:25 台北標準時間
Printed: Friday, August 01, 2025 15:03:19 台北標準時間

Sample Name: 20211111_A32_R2_IgM
F82:MRM of 1 channel,ES+
1157.6 > 204.1
3.994e+006

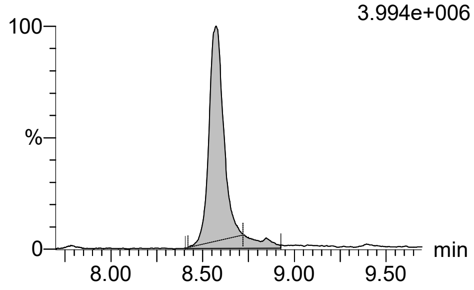

Sample Name: 20211111_L31_R2_IgM
F82:MRM of 1 channel,ES+
1157.6 > 204.1
6.635e+005

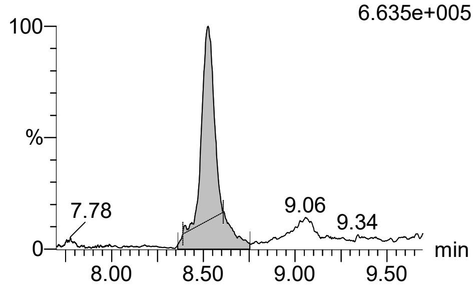

Sample Name: 20211111_L32_R2_IgM
F82:MRM of 1 channel,ES+
1157.6 > 204.1
2.981e+006

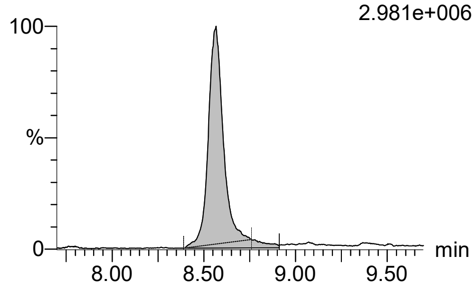

Sample Name: 20211111_H16_R2_IgM
F82:MRM of 1 channel,ES+
1157.6 > 204.1
4.893e+006

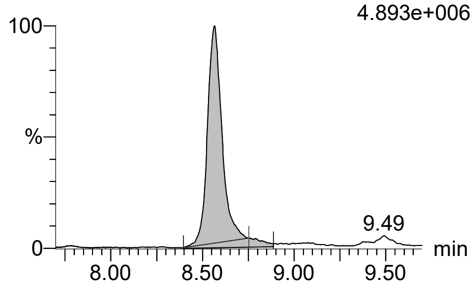

Sample Name: 20211111_A33_R2_IgM
F82:MRM of 1 channel,ES+
1157.6 > 204.1
5.349e+006

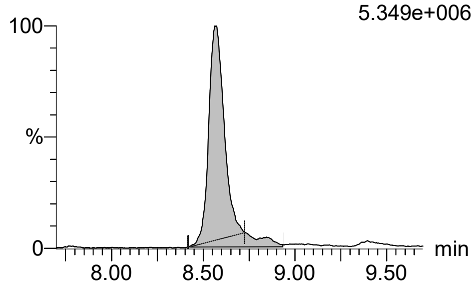

Sample Name: 20211111_A34_R2_IgM
F82:MRM of 1 channel,ES+
1157.6 > 204.1
1.499e+006

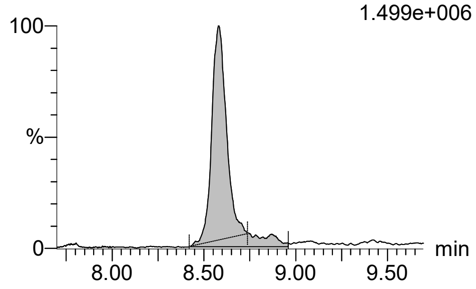

Sample Name: 20211111_H17_R2_IgM
F82:MRM of 1 channel,ES+
1157.6 > 204.1
1.643e+007

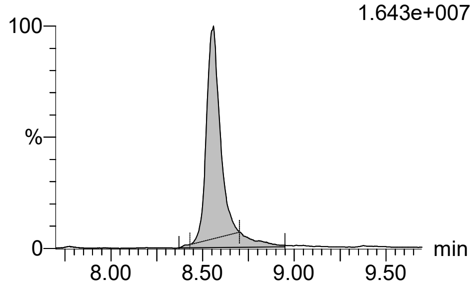

Sample Name: 20211111_A35_R2_IgM
F82:MRM of 1 channel,ES+
1157.6 > 204.1
1.980e+006

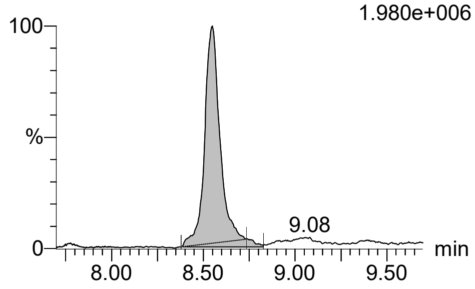

Sample Name: 20211111_QC_R16_IgM
F82:MRM of 1 channel,ES+
1157.6 > 204.1
3.246e+006

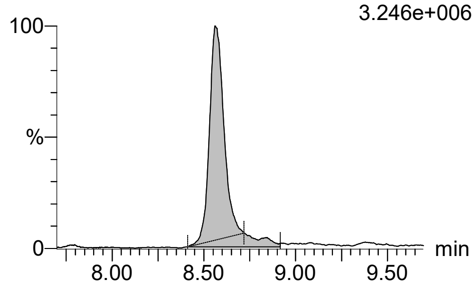

Sample Name: 20211111_A36_R2_IgM
F82:MRM of 1 channel,ES+
1157.6 > 204.1
3.129e+006

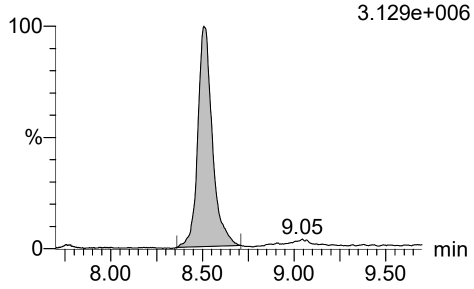

Sample Name: 20211111_H18_R2_IgM
F82:MRM of 1 channel,ES+
1157.6 > 204.1
7.904e+006

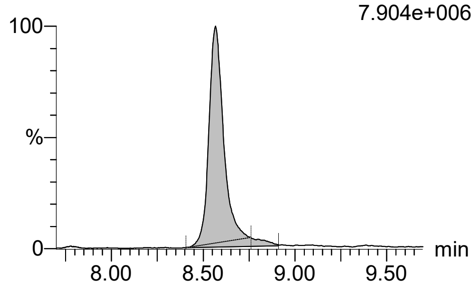

Sample Name: 20211111_A37_R2_IgM
F82:MRM of 1 channel,ES+
1157.6 > 204.1
5.017e+006

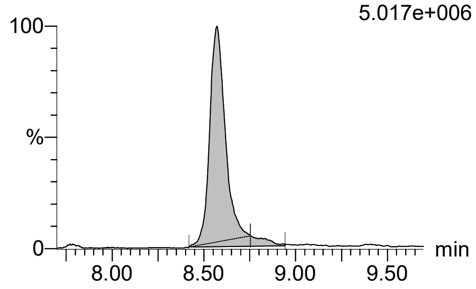

Sample Name: 20211111_A38_R2_IgM
F82:MRM of 1 channel,ES+
1157.6 > 204.1
3.407e+006

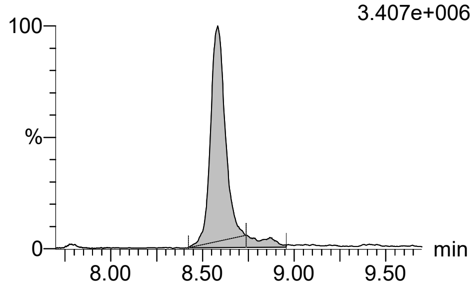

Sample Name: 20211111_H19_R2_IgM
F82:MRM of 1 channel,ES+
1157.6 > 204.1
4.872e+006

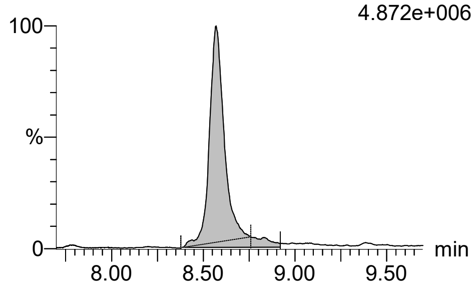

Sample Name: 20211111_A39_R2_IgM
F82:MRM of 1 channel,ES+
1157.6 > 204.1
3.527e+006

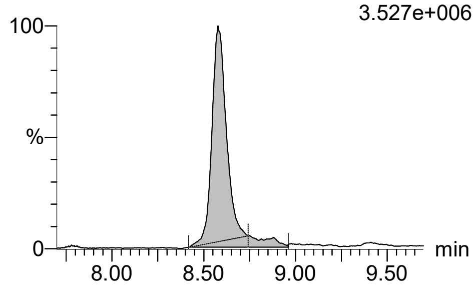

Dataset: C:\MassLynx\10.pro\20211111_clinicalsamples_IgM_IgA.qld

Last Altered: Thursday, March 09, 2023 19:58:25 台北標準時間
Printed: Friday, August 01, 2025 15:03:19 台北標準時間

Sample Name: 20211111_A40_R2_IgM
F82:MRM of 1 channel,ES+
1157.6 > 204.1
1.899e+006

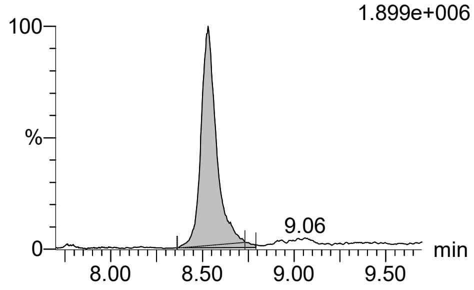

Sample Name: 20211111_H20_R2_IgM
F82:MRM of 1 channel,ES+
1157.6 > 204.1
1.579e+007

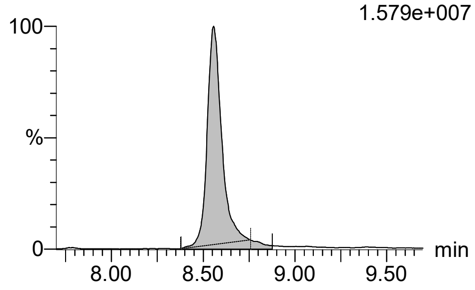

Sample Name: 20211111_A41_R2_IgM
F82:MRM of 1 channel,ES+
1157.6 > 204.1
1.981e+006

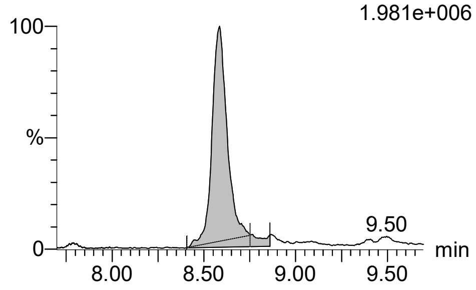

Sample Name: 20211111_A42_R2_IgM
F82:MRM of 1 channel,ES+
1157.6 > 204.1
1.229e+006

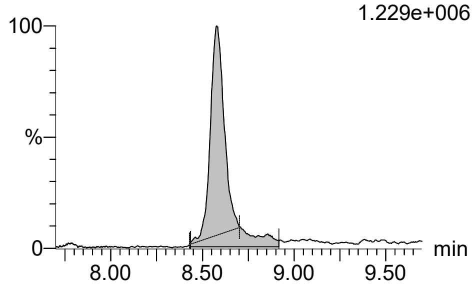

Sample Name: 20211111_A43_R2_IgM
F82:MRM of 1 channel,ES+
1157.6 > 204.1
1.204e+006

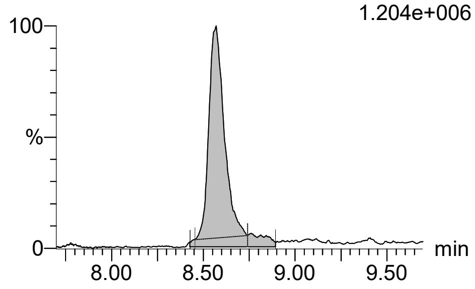

Sample Name: 20211111_A44_R2_IgM
F82:MRM of 1 channel,ES+
1157.6 > 204.1
7.474e+005

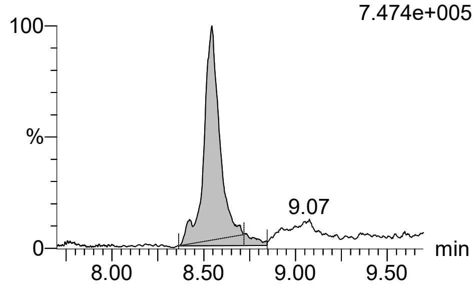

Sample Name: 20211111_QC_R17_IgM
F82:MRM of 1 channel,ES+
1157.6 > 204.1
2.880e+006

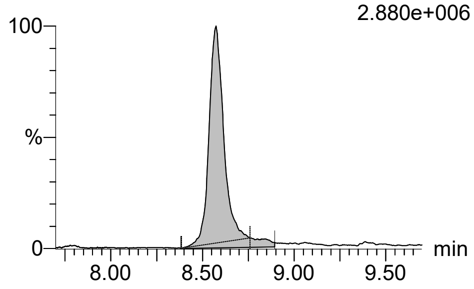

Sample Name: 20211111_L2_R3_IgM
F82:MRM of 1 channel,ES+
1157.6 > 204.1
4.700e+006

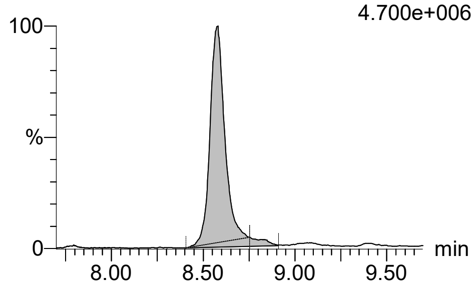

Sample Name: 20211111_H1_R3_IgM
F82:MRM of 1 channel,ES+
1157.6 > 204.1
4.448e+006

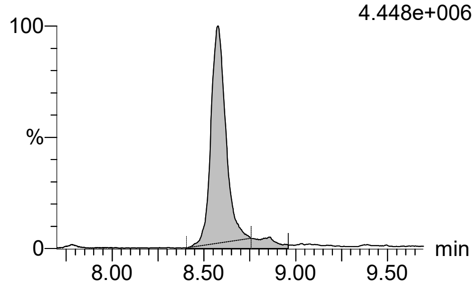

Sample Name: 20211111_A3_R3_IgM
F82:MRM of 1 channel,ES+
1157.6 > 204.1
5.940e+006

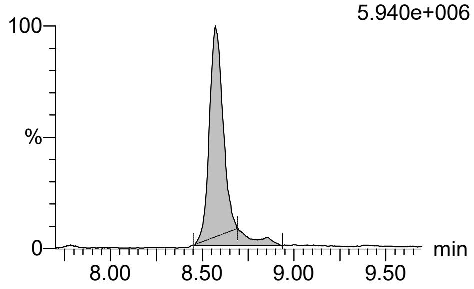

Sample Name: 20211111_A4_R3_IgM
F82:MRM of 1 channel,ES+
1157.6 > 204.1
2.837e+006

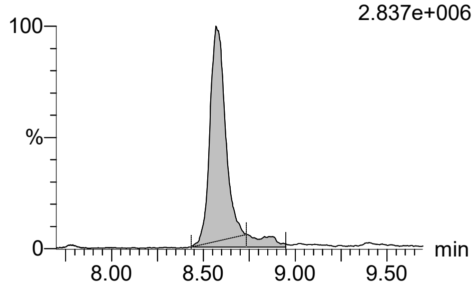

Sample Name: 20211111_L3_R3_IgM
F82:MRM of 1 channel,ES+
1157.6 > 204.1
1.675e+006

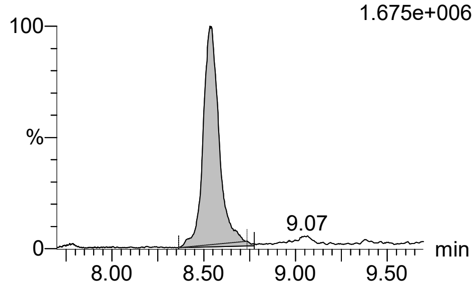

Sample Name: 20211111_L4_R3_IgM
F82:MRM of 1 channel,ES+
1157.6 > 204.1
3.933e+006

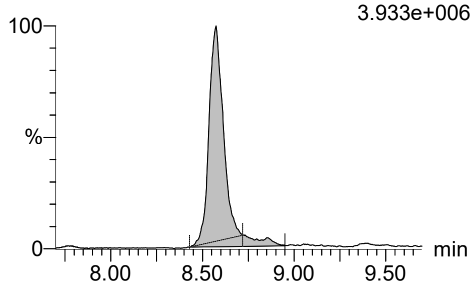

Sample Name: 20211111_H2_R3_IgM
F82:MRM of 1 channel,ES+
1157.6 > 204.1
1.060e+007

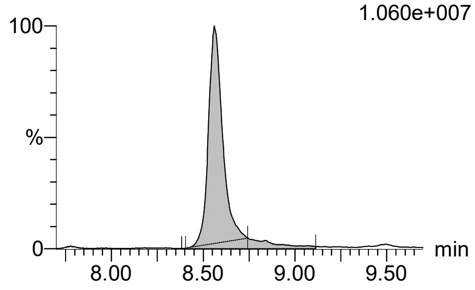

Sample Name: 20211111_A5_R3_IgM
F82:MRM of 1 channel,ES+
1157.6 > 204.1
1.046e+007

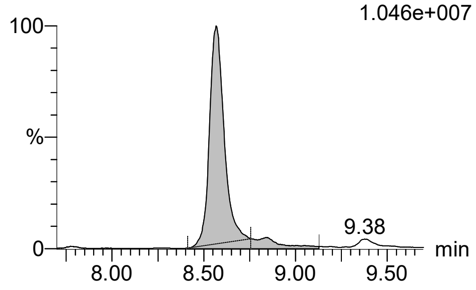

Sample Name: 20211111_A6_R3_IgM
F82:MRM of 1 channel,ES+
1157.6 > 204.1
2.791e+006

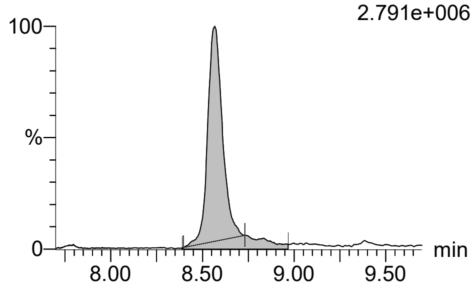

Sample Name: 20211111_QC_R18_IgM
F82:MRM of 1 channel,ES+
1157.6 > 204.1
2.925e+006

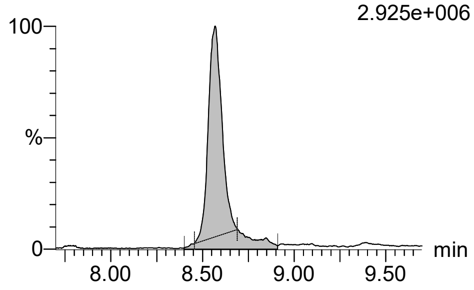

Sample Name: 20211111_L5_R3_IgM
F82:MRM of 1 channel,ES+
1157.6 > 204.1
3.068e+006

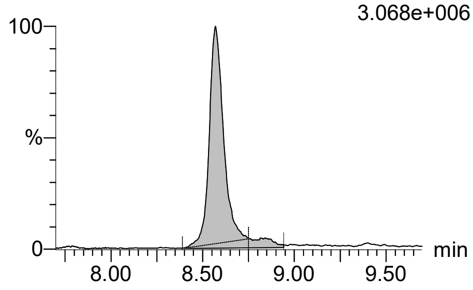

Sample Name: 20211111_L6_R3_IgM
F82:MRM of 1 channel,ES+
1157.6 > 204.1
3.277e+006

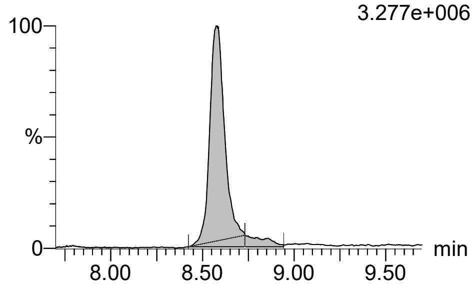

Sample Name: 20211111_H3_R3_IgM
F82:MRM of 1 channel,ES+
1157.6 > 204.1
2.255e+006

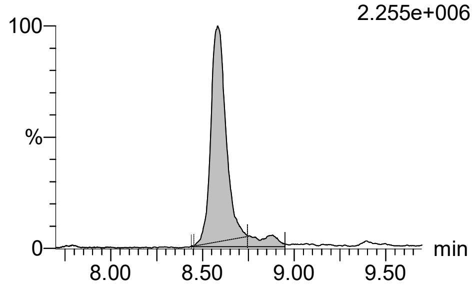

Sample Name: 20211111_A7_R3_IgM
F82:MRM of 1 channel,ES+
1157.6 > 204.1
4.159e+006

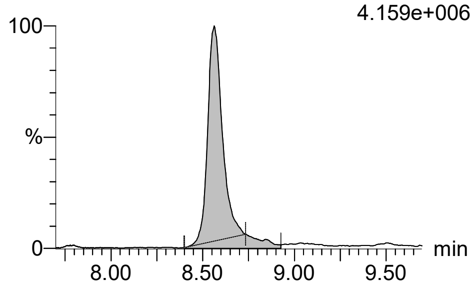

Sample Name: 20211111_A8_R3_IgM
F82:MRM of 1 channel,ES+
1157.6 > 204.1
4.783e+006

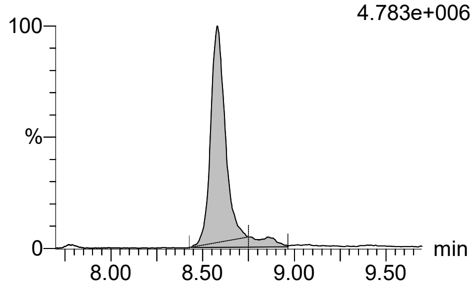

Sample Name: 20211111_L7_R3_IgM
F82:MRM of 1 channel,ES+
1157.6 > 204.1
1.847e+006

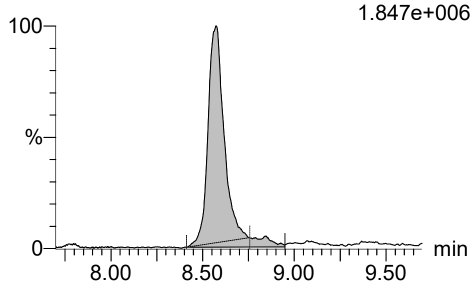

Sample Name: 20211111_L8_R3_IgM
F82:MRM of 1 channel,ES+
1157.6 > 204.1
6.789e+005

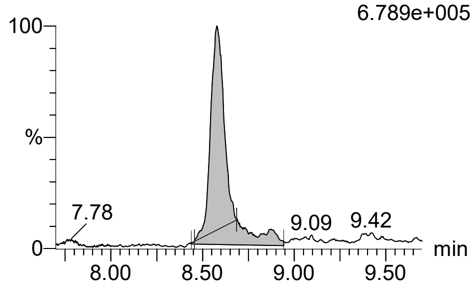

Sample Name: 20211111_H4_R3_IgM
F82:MRM of 1 channel,ES+
1157.6 > 204.1
1.586e+006

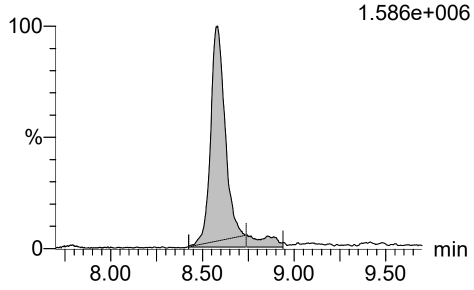

Sample Name: 20211111_A9_R3_IgM
F82:MRM of 1 channel,ES+
1157.6 > 204.1
2.199e+006

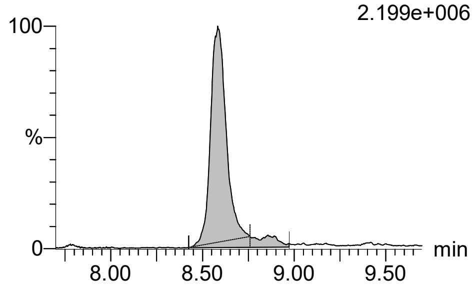

Sample Name: 20211111_A10_R3_IgM
F82:MRM of 1 channel,ES+
1157.6 > 204.1
5.021e+006

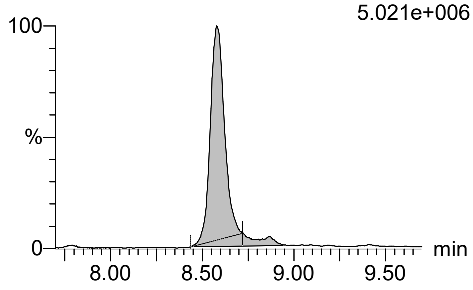

Sample Name: 20211111_L9_R3_IgM
F82:MRM of 1 channel,ES+
1157.6 > 204.1
1.899e+006

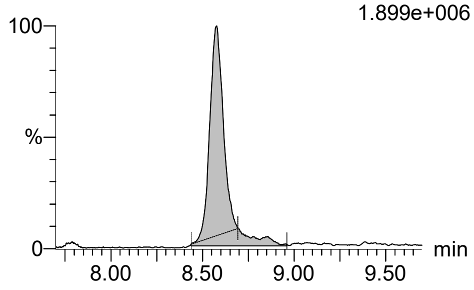

Sample Name: 20211111_L10_R3_IgM
F82:MRM of 1 channel,ES+
1157.6 > 204.1
4.715e+006

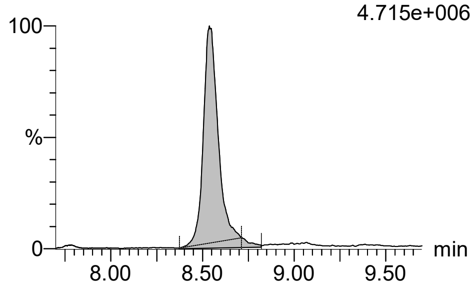

Sample Name: 20211111_QC_R19_IgM
F82:MRM of 1 channel,ES+
1157.6 > 204.1
3.020e+006

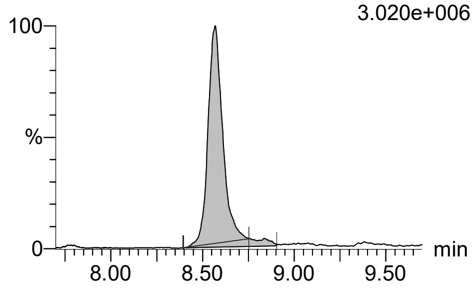

Dataset: C:\MassLynx\10.pro\20211111_clinicalsamples_IgM_IgA.qld

Last Altered: Thursday, March 09, 2023 19:58:25 台北標準時間
Printed: Friday, August 01, 2025 15:03:19 台北標準時間

Sample Name: 20211111_H5_R3_IgM
F82:MRM of 1 channel,ES+
1157.6 > 204.1
2.150e+006

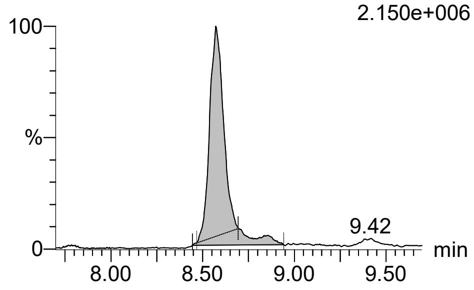

Sample Name: 20211111_A11_R3_IgM
F82:MRM of 1 channel,ES+
1157.6 > 204.1
4.797e+006

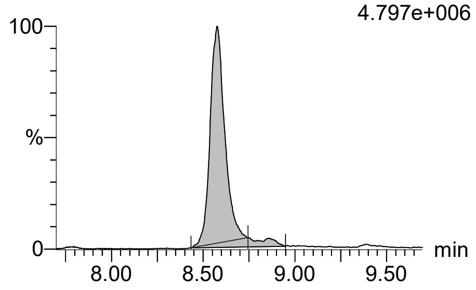

Sample Name: 20211111_A12_R3_IgM
F82:MRM of 1 channel,ES+
1157.6 > 204.1
2.329e+006

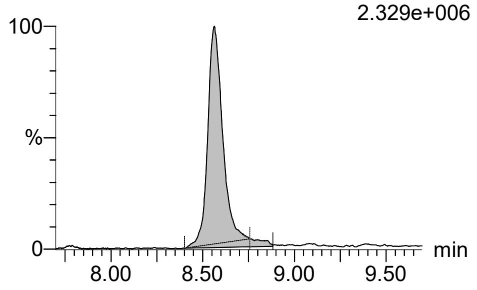

Sample Name: 20211111_L11_R3_IgM
F82:MRM of 1 channel,ES+
1157.6 > 204.1
1.927e+006

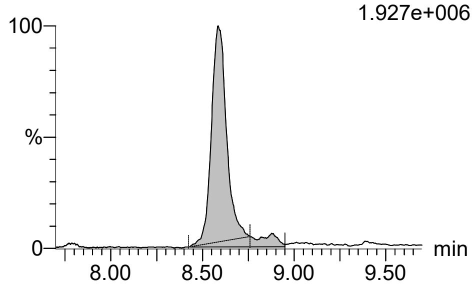

Sample Name: 20211111_L12_R3_IgM
F82:MRM of 1 channel,ES+
1157.6 > 204.1
1.940e+006

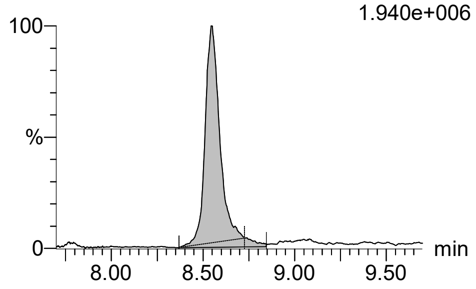

Sample Name: 20211111_H6_R3_IgM
F82:MRM of 1 channel,ES+
1157.6 > 204.1
2.160e+006

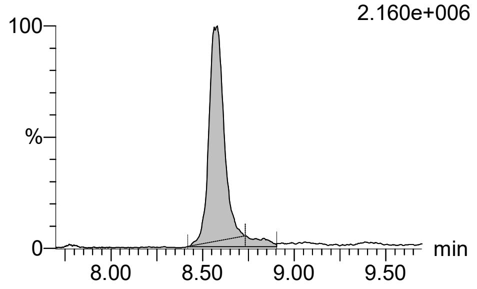

Sample Name: 20211111_A13_R3_IgM
F82:MRM of 1 channel,ES+
1157.6 > 204.1
2.666e+006

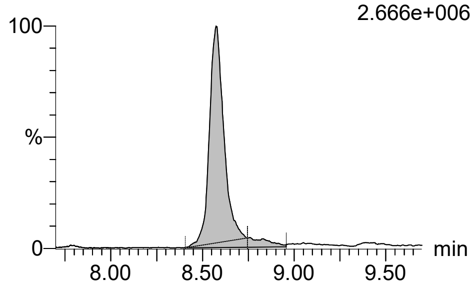

Sample Name: 20211111_A14_R3_IgM
F82:MRM of 1 channel,ES+
1157.6 > 204.1
9.376e+006

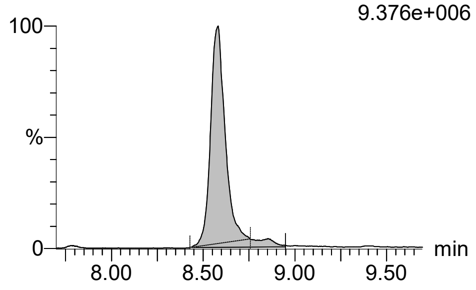

Sample Name: 20211111_L13_R3_IgM
F82:MRM of 1 channel,ES+
1157.6 > 204.1
1.227e+006

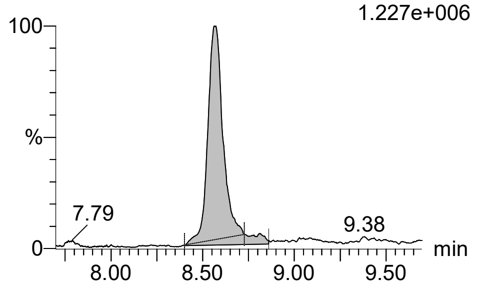

Sample Name: 20211111_L14_R3_IgM
F82:MRM of 1 channel,ES+
1157.6 > 204.1
2.489e+006

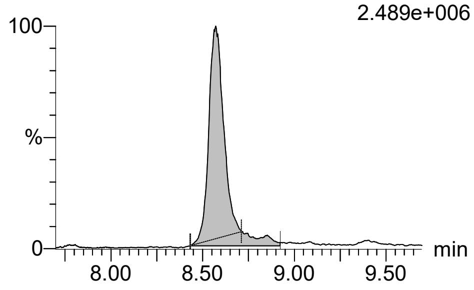

Sample Name: 20211111_H7_R3_IgM
F82:MRM of 1 channel,ES+
1157.6 > 204.1
2.753e+006

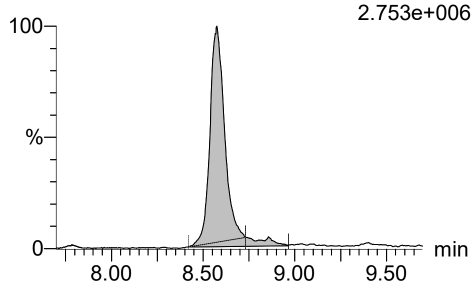

Sample Name: 20211111_A15_R3_IgM
F82:MRM of 1 channel,ES+
1157.6 > 204.1
1.181e+007

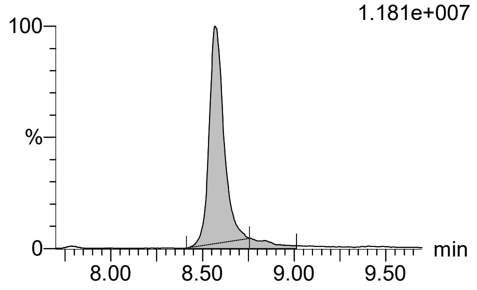

Sample Name: 20211111_QC_R20_IgM
F82:MRM of 1 channel,ES+
1157.6 > 204.1
3.464e+006

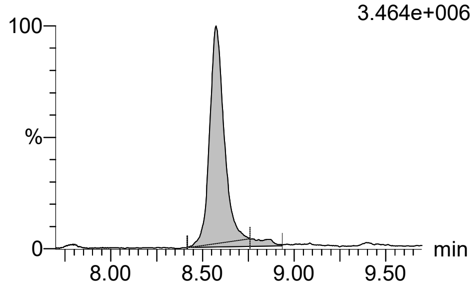

Sample Name: 20211111_A16_R3_IgM
F82:MRM of 1 channel,ES+
1157.6 > 204.1
4.962e+006

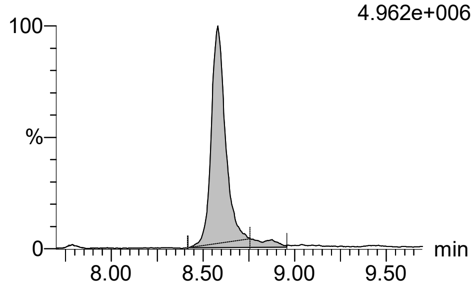

Sample Name: 20211111_L15_R3_IgM
F82:MRM of 1 channel,ES+
1157.6 > 204.1
4.889e+006

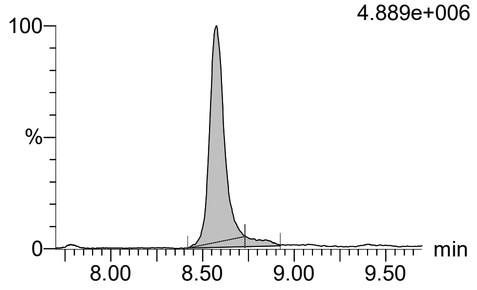

Dataset: C:\MassLynx\10.pro\20211111_clinicalsamples_IgM_IgA.qld

Last Altered: Thursday, March 09, 2023 19:58:25 台北標準時間
Printed: Friday, August 01, 2025 15:03:19 台北標準時間

Sample Name: 20211111_L16_R3_IgM
F82:MRM of 1 channel,ES+
1157.6 > 204.1
8.570e+005

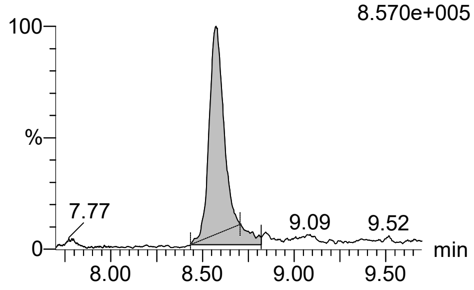

Sample Name: 20211111_H8_R3_IgM
F82:MRM of 1 channel,ES+
1157.6 > 204.1
1.533e+006

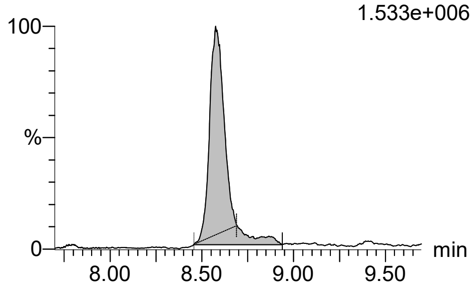

Sample Name: 20211111_A17_R3_IgM
F82:MRM of 1 channel,ES+
1157.6 > 204.1
4.429e+006

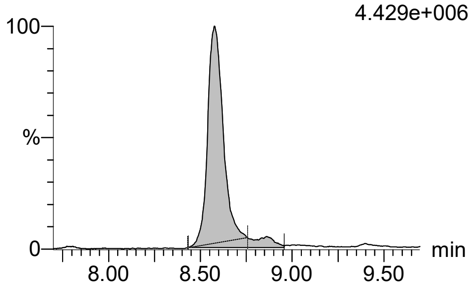

Sample Name: 20211111_A18_R3_IgM
F82:MRM of 1 channel,ES+
1157.6 > 204.1
2.174e+006

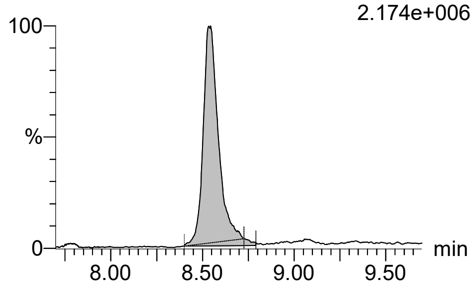

Sample Name: 20211111_L17_R3_IgM
F82:MRM of 1 channel,ES+
1157.6 > 204.1
3.967e+006

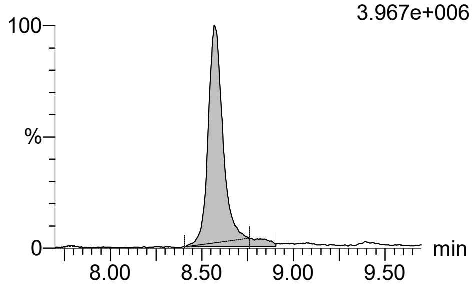

Sample Name: 20211111_L18_R3_IgM
F82:MRM of 1 channel,ES+
1157.6 > 204.1
7.758e+005

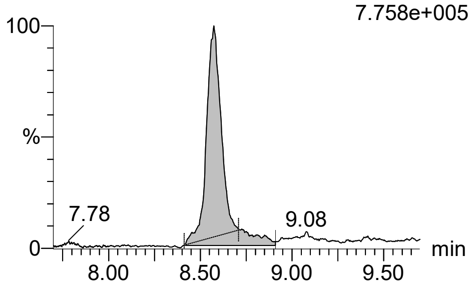

Sample Name: 20211111_H9_R3_IgM
F82:MRM of 1 channel,ES+
1157.6 > 204.1
1.784e+006

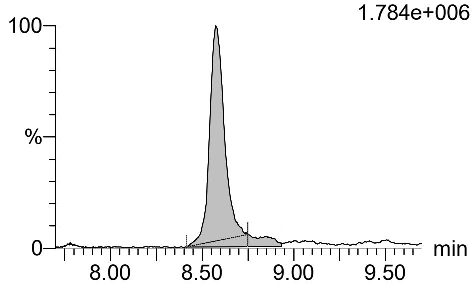

Sample Name: 20211111_A19_R3_IgM
F82:MRM of 1 channel,ES+
1157.6 > 204.1
3.406e+006

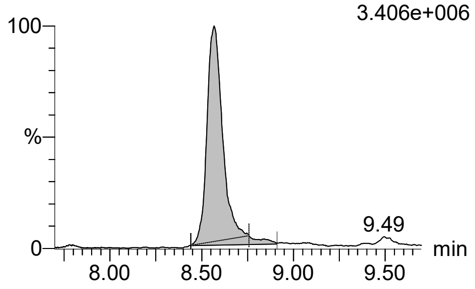

Sample Name: 20211111_A20_R3_IgM
F82:MRM of 1 channel,ES+
1157.6 > 204.1
1.758e+006

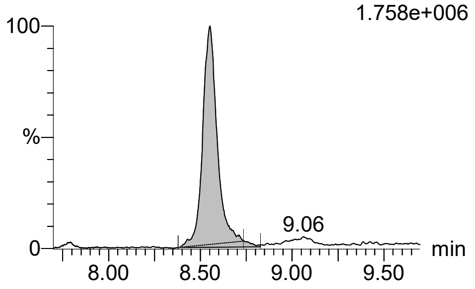

Sample Name: 20211111_L19_R3_IgM
F82:MRM of 1 channel,ES+
1157.6 > 204.1
3.745e+006

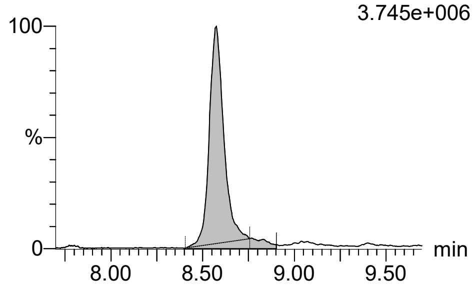

Sample Name: 20211111_QC_R21_IgM
F82:MRM of 1 channel,ES+
1157.6 > 204.1
3.379e+006

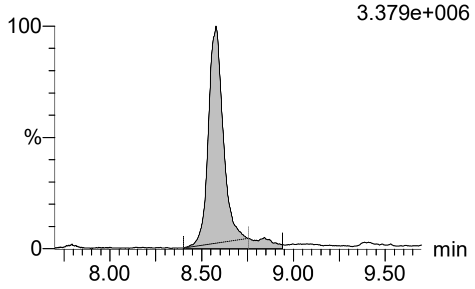

Sample Name: 20211111_L20_R3_IgM
F82:MRM of 1 channel,ES+
1157.6 > 204.1
1.566e+006

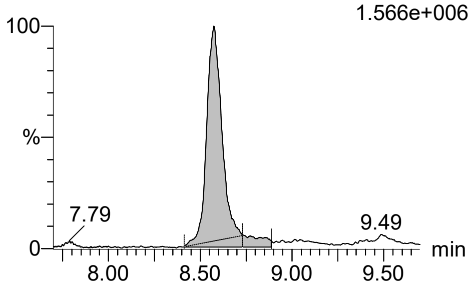

Sample Name: 20211111_H10_R3_IgM
F82:MRM of 1 channel,ES+
1157.6 > 204.1
3.590e+006

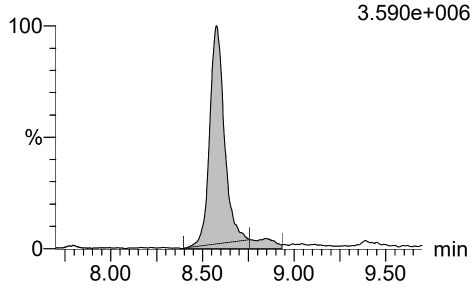

Sample Name: 20211111_A21_R3_IgM
F82:MRM of 1 channel,ES+
1157.6 > 204.1
2.982e+006

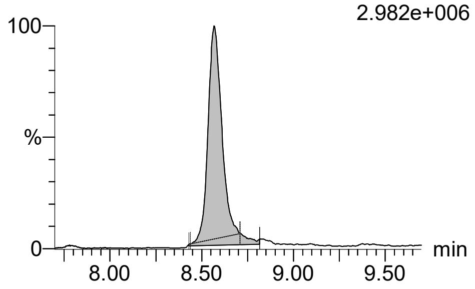

Sample Name: 20211111_A22_R3_IgM
F82:MRM of 1 channel,ES+
1157.6 > 204.1
5.430e+005

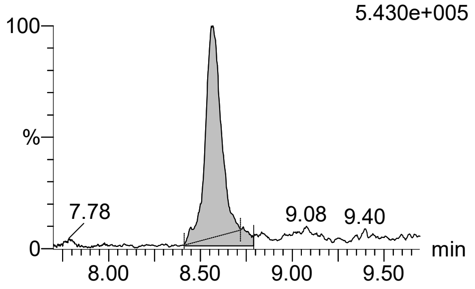

Dataset: C:\MassLynx\10.pro\20211111_clinicalsamples_IgM_IgA.qld

Last Altered: Thursday, March 09, 2023 19:58:25 台北標準時間
Printed: Friday, August 01, 2025 15:03:19 台北標準時間

Sample Name: 20211111_L21_R3_IgM
F82:MRM of 1 channel,ES+
1157.6 > 204.1
2.343e+006

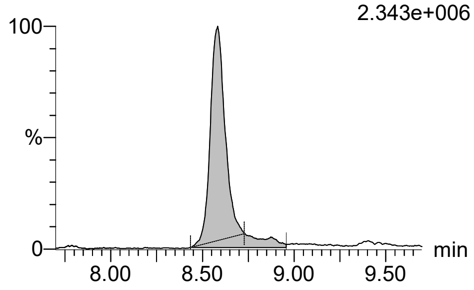

Sample Name: 20211111_L22_R3_IgM
F82:MRM of 1 channel,ES+
1157.6 > 204.1
2.000e+006

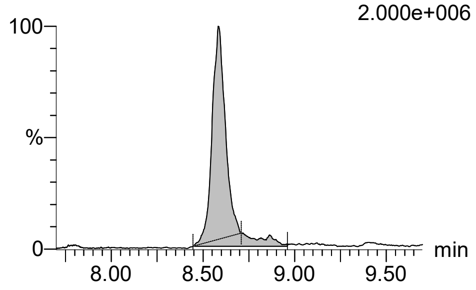

Sample Name: 20211111_H11_R3_IgM
F82:MRM of 1 channel,ES+
1157.6 > 204.1
3.532e+006

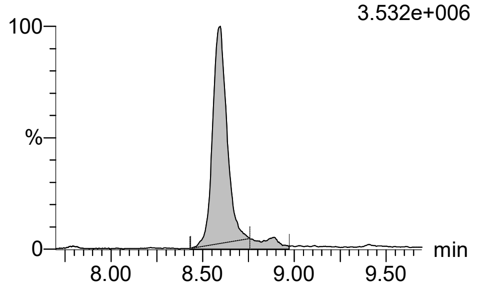

Sample Name: 20211111_A23_R3_IgM
F82:MRM of 1 channel,ES+
1157.6 > 204.1
2.449e+006

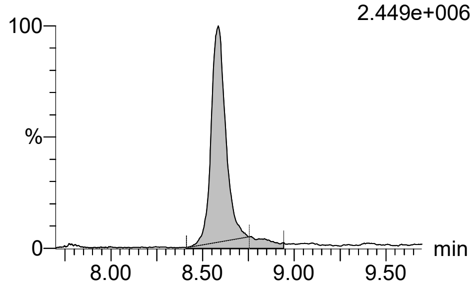

Sample Name: 20211111_A24_R3_IgM
F82:MRM of 1 channel,ES+
1157.6 > 204.1
5.374e+006

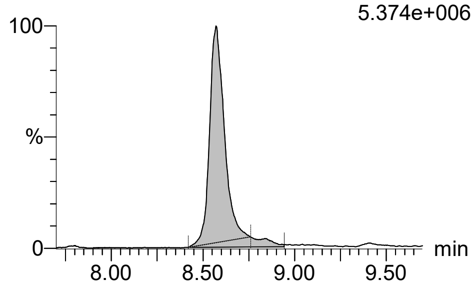

Sample Name: 20211111_L23_R3_IgM
F82:MRM of 1 channel,ES+
1157.6 > 204.1
5.261e+006

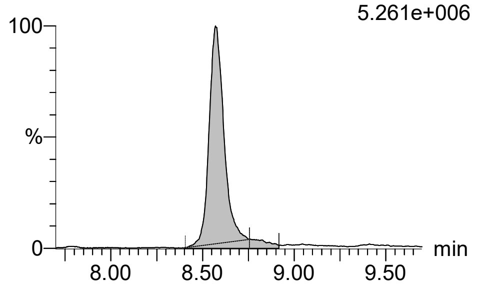

Sample Name: 20211111_L24_R3_IgM
F82:MRM of 1 channel,ES+
1157.6 > 204.1
6.265e+005

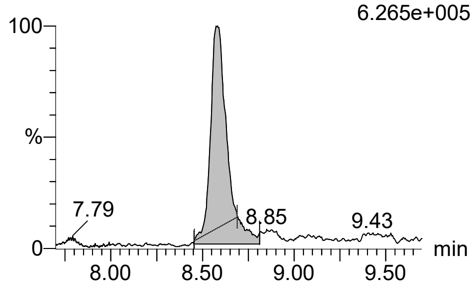

Sample Name: 20211111_H12_R3_IgM
F82:MRM of 1 channel,ES+
1157.6 > 204.1
1.119e+006

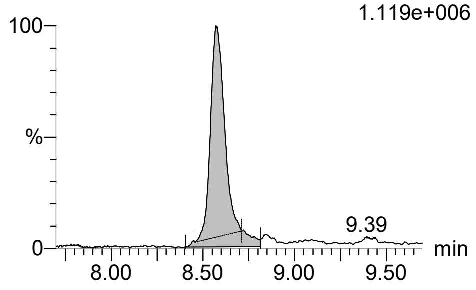

Sample Name: 20211111_QC_R22_IgM
F82:MRM of 1 channel,ES+
1157.6 > 204.1
3.418e+006

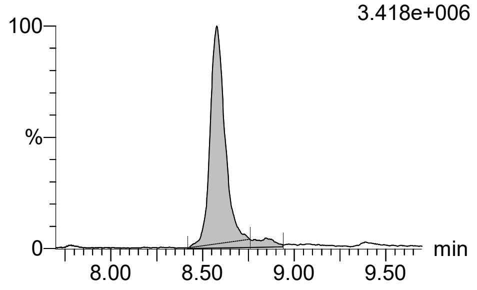

Sample Name: 20211111_A25_R3_IgM
F82:MRM of 1 channel,ES+
1157.6 > 204.1
6.581e+006

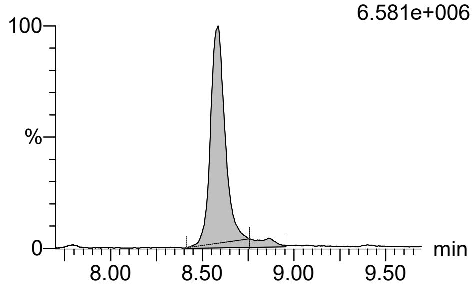

Sample Name: 20211111_A26_R3_IgM
F82:MRM of 1 channel,ES+
1157.6 > 204.1
3.051e+006

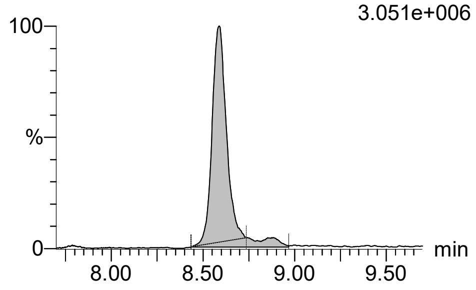

Sample Name: 20211111_L25_R3_IgM
F82:MRM of 1 channel,ES+
1157.6 > 204.1
1.548e+006

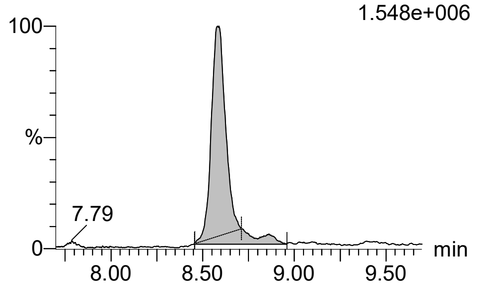

Sample Name: 20211111_L26_R3_IgM
F82:MRM of 1 channel,ES+
1157.6 > 204.1
2.065e+006

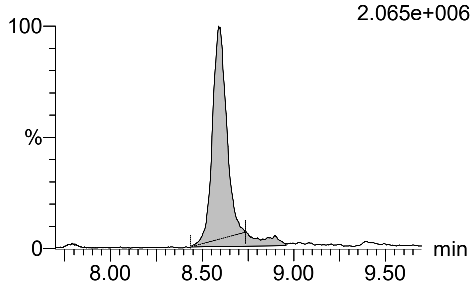

Sample Name: 20211111_H13_R3_IgM
F82:MRM of 1 channel,ES+
1157.6 > 204.1
4.588e+006

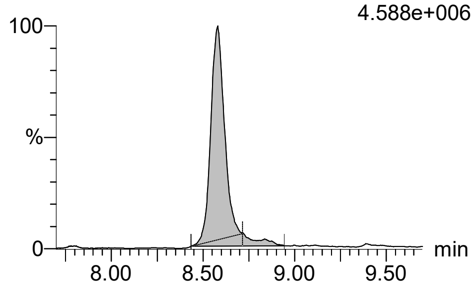

Sample Name: 20211111_A27_R3_IgM
F82:MRM of 1 channel,ES+
1157.6 > 204.1
3.226e+006

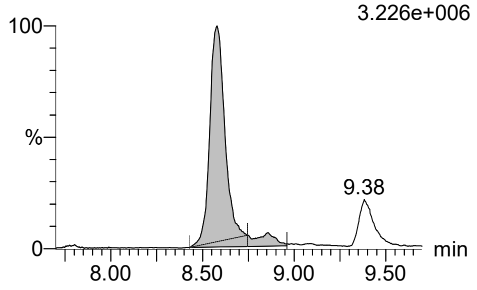

Sample Name: 20211111_A28_R3_IgM
F82:MRM of 1 channel,ES+
1157.6 > 204.1
1.456e+006

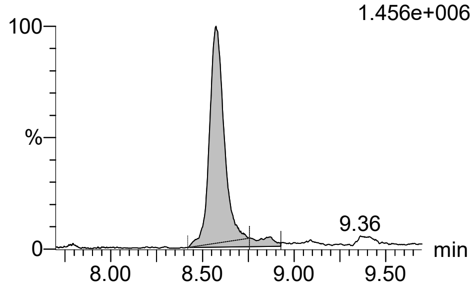

Sample Name: 20211111_L27_R3_IgM
F82:MRM of 1 channel,ES+
1157.6 > 204.1
3.427e+006

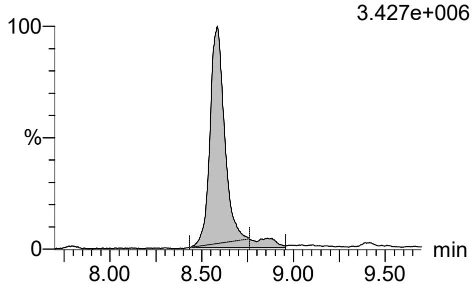

Sample Name: 20211111_L28_R3_IgM
F82:MRM of 1 channel,ES+
1157.6 > 204.1
1.318e+006

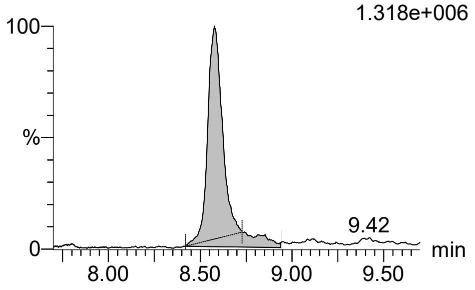

Sample Name: 20211111_H14_R3_IgM
F82:MRM of 1 channel,ES+
1157.6 > 204.1
3.237e+006

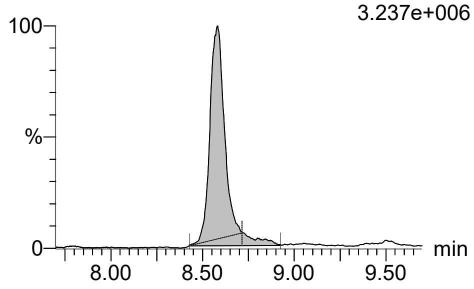

Sample Name: 20211111_A29_R3_IgM
F82:MRM of 1 channel,ES+
1157.6 > 204.1
5.434e+005

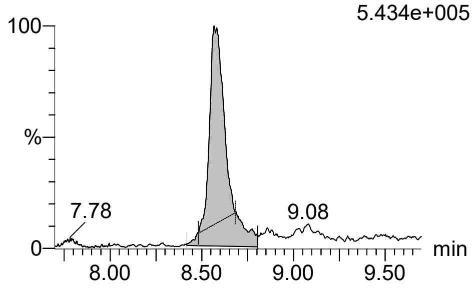

Sample Name: 20211111_A30_R3_IgM
F82:MRM of 1 channel,ES+
1157.6 > 204.1
5.964e+006

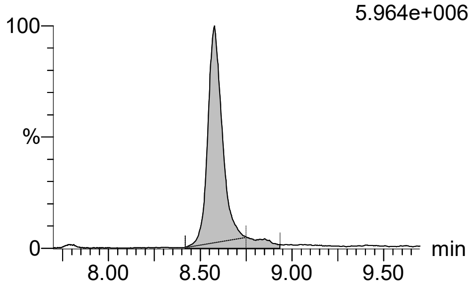

Sample Name: 20211111_QC_R23_IgM
F82:MRM of 1 channel,ES+
1157.6 > 204.1
3.202e+006

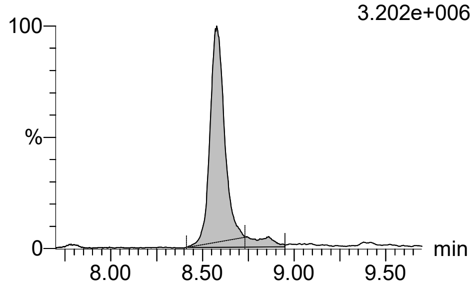

Sample Name: 20211111_L29_R3_IgM
F82:MRM of 1 channel,ES+
1157.6 > 204.1
9.650e+005

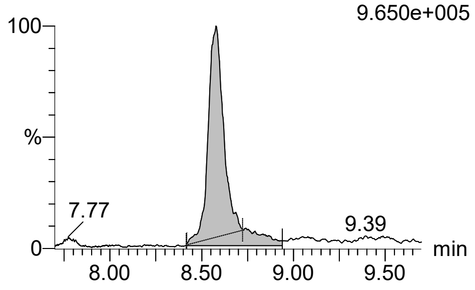

Sample Name: 20211111_L30_R3_IgM
F82:MRM of 1 channel,ES+
1157.6 > 204.1
8.860e+005

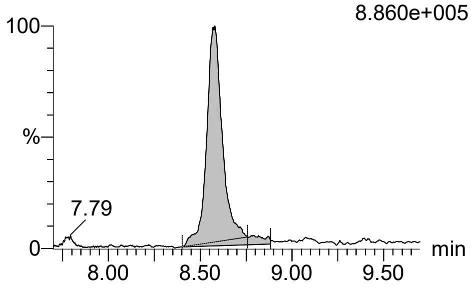

Sample Name: 20211111_H15_R3_IgM
F82:MRM of 1 channel,ES+
1157.6 > 204.1
2.450e+006

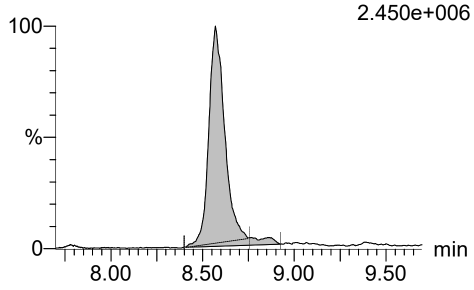

Sample Name: 20211111_A31_R3_IgM
F82:MRM of 1 channel,ES+
1157.6 > 204.1
2.279e+006

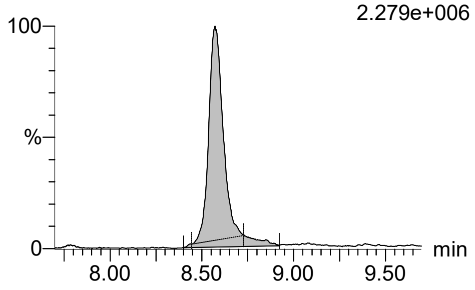

Sample Name: 20211111_A32_R3_IgM
F82:MRM of 1 channel,ES+
1157.6 > 204.1
3.606e+006

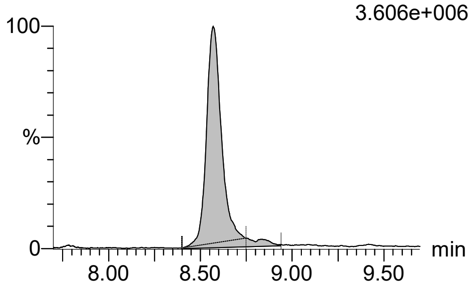

Sample Name: 20211111_L31_R3_IgM
F82:MRM of 1 channel,ES+
1157.6 > 204.1
4.990e+005

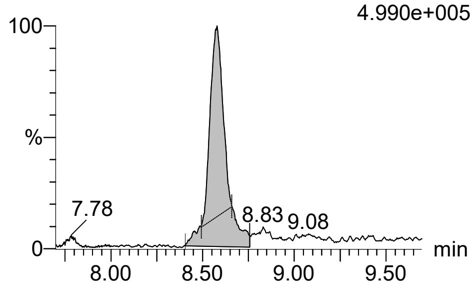

Sample Name: 20211111_L32_R3_IgM
F82:MRM of 1 channel,ES+
1157.6 > 204.1
2.584e+006

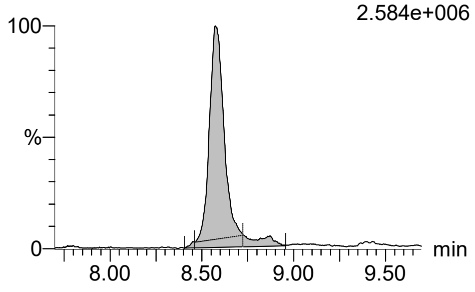

Sample Name: 20211111_H16_R3_IgM
F82:MRM of 1 channel,ES+
1157.6 > 204.1
3.931e+006

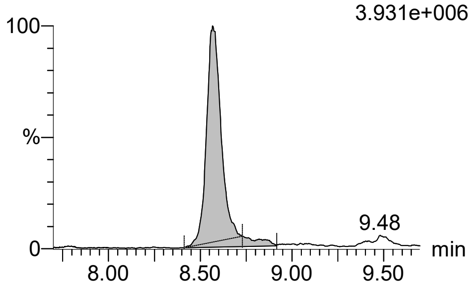

Sample Name: 20211111_A33_R3_IgM
F82:MRM of 1 channel,ES+
1157.6 > 204.1
5.213e+006

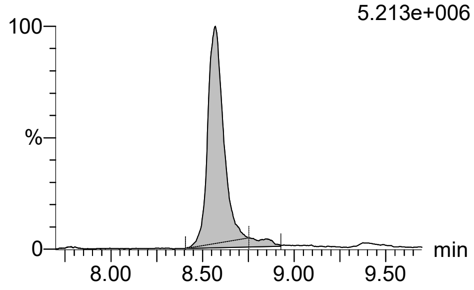

Sample Name: 20211111_A34_R3_IgM
F82:MRM of 1 channel,ES+
1157.6 > 204.1
1.261e+006

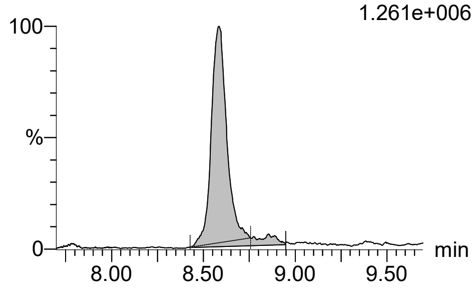

Sample Name: 20211111_H17_R3_IgM
F82:MRM of 1 channel,ES+
1157.6 > 204.1
1.342e+007

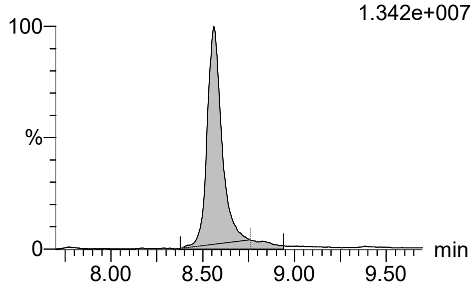

Sample Name: 20211111_A35_R3_IgM
F82:MRM of 1 channel,ES+
1157.6 > 204.1
1.751e+006

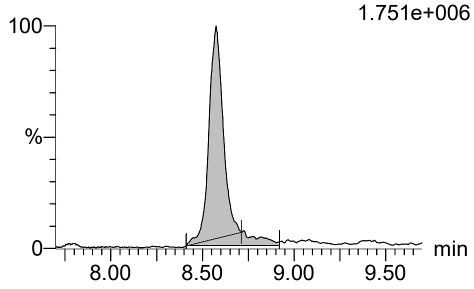

Sample Name: 20211111_QC_R24_IgM
F82:MRM of 1 channel,ES+
1157.6 > 204.1
2.770e+006

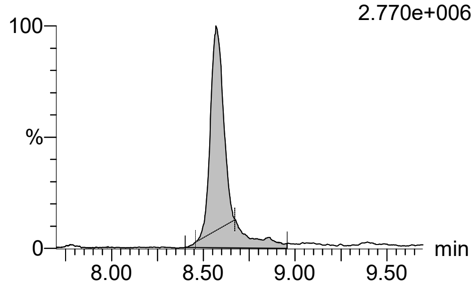

Sample Name: 20211111_A36_R3_IgM
F82:MRM of 1 channel,ES+
1157.6 > 204.1
2.452e+006

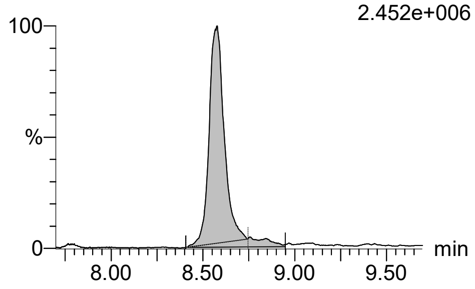

Sample Name: 20211111_H18_R3_IgM
F82:MRM of 1 channel,ES+
1157.6 > 204.1
7.010e+006

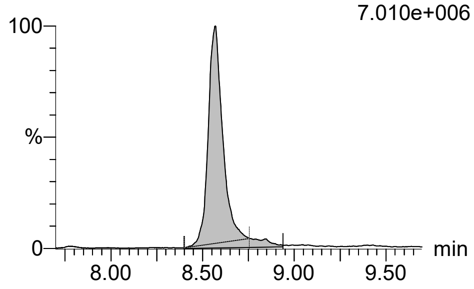

Sample Name: 20211111_A37_R3_IgM
F82:MRM of 1 channel,ES+
1157.6 > 204.1
4.027e+006

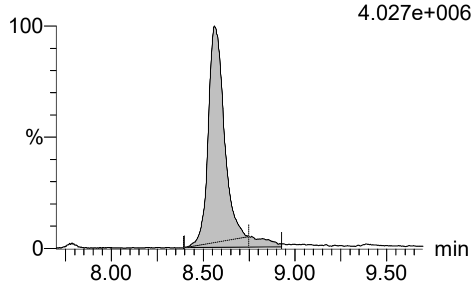

Sample Name: 20211111_A38_R3_IgM
F82:MRM of 1 channel,ES+
1157.6 > 204.1
3.014e+006

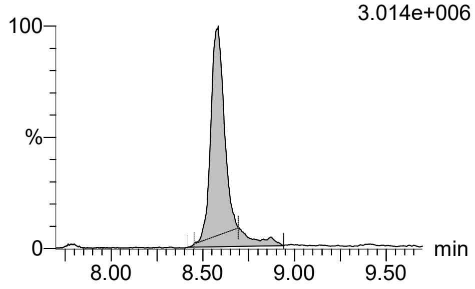

Sample Name: 20211111_H19_R3_IgM
F82:MRM of 1 channel,ES+
1157.6 > 204.1
4.125e+006

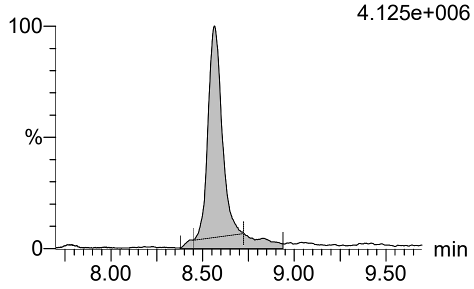

Sample Name: 20211111_A39_R3_IgM
F82:MRM of 1 channel,ES+
1157.6 > 204.1
3.111e+006

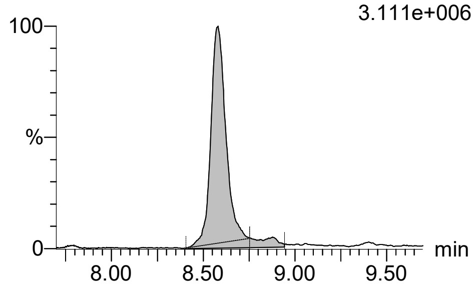

Sample Name: 20211111_A40_R3_IgM
F82:MRM of 1 channel,ES+
1157.6 > 204.1
1.556e+006

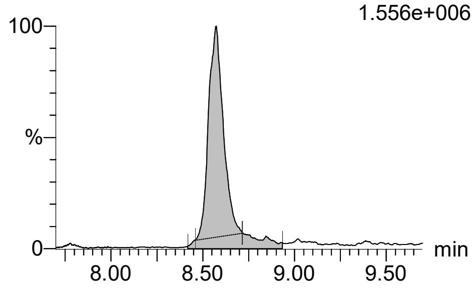

Sample Name: 20211111_H20_R3_IgM
F82:MRM of 1 channel,ES+
1157.6 > 204.1
1.298e+007

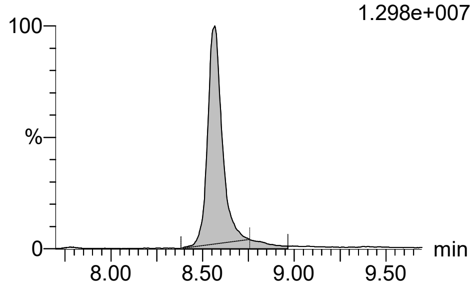

Sample Name: 20211111_A41_R3_IgM
F82:MRM of 1 channel,ES+
1157.6 > 204.1
1.233e+006

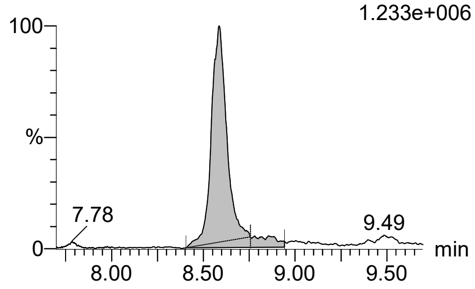

Sample Name: 20211111_A42_R3_IgM
F82:MRM of 1 channel,ES+
1157.6 > 204.1
1.222e+006

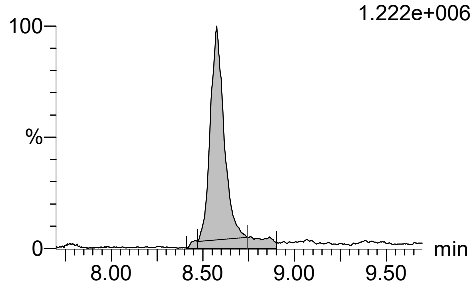

Dataset: C:\MassLynx\10.pro\20211111_clinicalsamples_IgM_IgA.qld

Last Altered: Thursday, March 09, 2023 19:58:25 台北標準時間
Printed: Friday, August 01, 2025 15:03:19 台北標準時間

Sample Name: 20211111_A43_R3_IgM

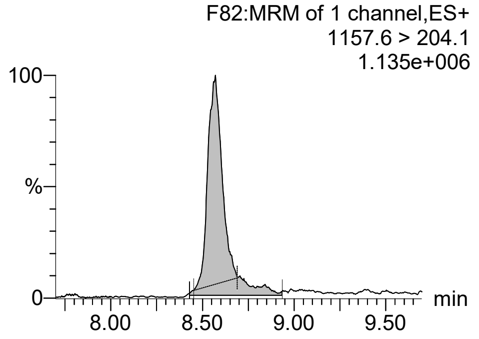

Sample Name: 20211111_A44_R3_IgM

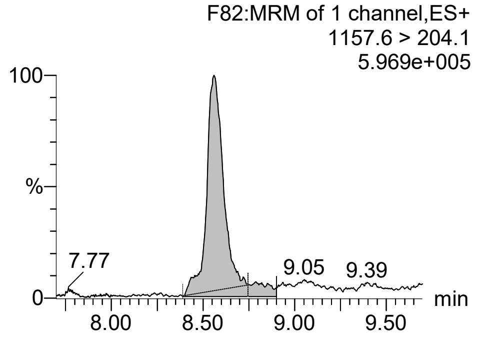

Sample Name: 20211111_QC_R25_IgM

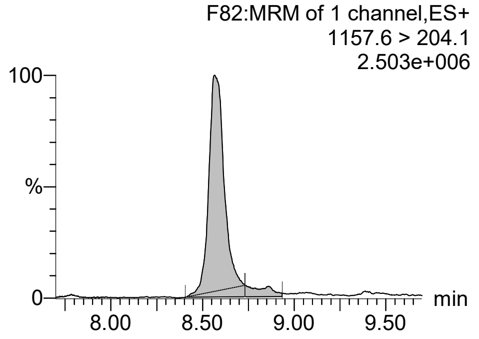

Dataset: C:\MassLynx\10.pro\20211111_clinicalsamples_IgM_IgA.qld

Last Altered: Thursday, March 09, 2023 19:58:25 台北標準時間
Printed: Friday, August 01, 2025 15:03:19 台北標準時間

	#	Name	RT	Area	IS Area	Response	Prima...
1	1	20211112_QC_R001_IgM	8.56	335085.2	15421.4	21.729	MM
2	2	20211112_QC_R002_IgM	8.57	312069.0	13188.8	23.662	MM
3	3	20211111_A1_R2_IgM	8.57	220730.1	11871.0	18.594	MM
4	4	20211111_A1_R3_IgM	8.57	227791.7	12030.5	18.935	MM
5	5	20211111_A2_R2_IgM	8.57	806384.6	11446.8	70.446	MM
6	6	20211111_A2_R3_IgM	8.57	754109.4	11098.4	67.947	MM
7	7	20211111_L1_R2_IgM	8.57	120970.8	11779.4	10.270	MM
8	8	20211111_L1_R3_IgM	8.57	111005.4	11176.2	9.932	MM
9	9	20211111_L2_R2_IgM	8.57	459544.2	11375.0	40.399	MM
10	10	20211111_H1_R2_IgM	8.57	476497.0	13595.2	35.049	MM
11	11	20211111_A3_R2_IgM	8.55	660131.7	14112.3	46.777	MM
12	12	20211111_A4_R2_IgM	8.56	345683.0	11977.5	28.861	MM
13	13	20211111_L3_R2_IgM	8.52	184890.9	12242.1	15.103	MM
14	14	20211111_L4_R2_IgM	8.57	446076.0	12797.0	34.858	MM
15	15	20211111_H2_R2_IgM	8.57	1032444.0	10006.0	103.182	MM
16	16	20211111_A5_R2_IgM	8.55	1165688.4	11434.6	101.944	MM
17	17	20211111_A6_R2_IgM	8.56	353170.8	13193.7	26.768	MM
18	18	20211111_QC_R10_IgM	8.57	334273.0	12566.1	26.601	MM
19	19	20211111_L5_R2_IgM	8.55	370662.2	13616.4	27.222	MM
20	20	20211111_L6_R2_IgM	8.57	398196.6	13698.5	29.069	MM
21	21	20211111_H3_R2_IgM	8.57	274234.6	15793.1	17.364	MM
22	22	20211111_A7_R2_IgM	8.55	424904.9	12936.9	32.844	MM
23	23	20211111_A8_R2_IgM	8.56	591758.3	13108.0	45.145	MM
24	24	20211111_L7_R2_IgM	8.57	211630.3	13278.5	15.938	MM
25	25	20211111_L8_R2_IgM	8.57	85331.1	15233.6	5.602	MM
26	26	20211111_H4_R2_IgM	8.57	199314.5	16521.9	12.064	MM
27	27	20211111_A9_R2_IgM	8.55	281604.4	16451.6	17.117	MM
28	28	20211111_A10_R2_IgM	8.57	565245.9	13036.0	43.360	MM
29	29	20211111_L9_R2_IgM	8.56	206263.5	14554.1	14.172	MM
30	30	20211111_L10_R2_IgM	8.55	559046.8	12250.4	45.635	MM
31	31	20211111_QC_R11_IgM	8.57	383151.6	11459.2	33.436	MM
32	32	20211111_H5_R2_IgM	8.56	286877.3	14358.2	19.980	MM
33	33	20211111_A11_R2_IgM	8.51	636808.7	16184.1	39.348	MM
34	34	20211111_A12_R2_IgM	8.58	224958.6	13465.4	16.706	MM
35	35	20211111_L11_R2_IgM	8.58	266120.0	13277.9	20.042	MM
36	36	20211111_L12_R2_IgM	8.56	210471.4	15958.8	13.188	MM
37	37	20211111_H6_R2_IgM	8.57	241935.8	14003.5	17.277	MM
38	38	20211111_A13_R2_IgM	8.57	274045.3	13656.1	20.068	MM
39	39	20211111_A14_R2_IgM	8.57	1181506.5	14071.3	83.966	MM
40	40	20211111_L13_R2_IgM	8.58	145931.2	14785.6	9.870	MM
41	41	20211111_L14_R2_IgM	8.58	322147.0	14772.2	21.808	MM
42	42	20211111_H7_R2_IgM	8.56	338963.1	15164.4	22.353	MM
43	43	20211111_A15_R2_IgM	8.57	1413926.3	15721.5	89.936	MM
44	44	20211111_QC_R12_IgM	8.57	407232.8	13711.5	29.700	MM
45	45	20211111_A16_R2_IgM	8.58	570208.3	14275.6	39.943	MM
46	46	20211111_L15_R2_IgM	8.57	573607.4	12935.8	44.343	MM
47	47	20211111_L16_R2_IgM	8.57	96688.5	13342.8	7.246	MM
48	48	20211111_H8_R2_IgM	8.57	178641.3	15835.1	11.281	MM
49	49	20211111_A17_R2_IgM	8.58	570819.9	15786.7	36.158	MM
50	50	20211111_A18_R2_IgM	8.56	244744.5	16986.8	14.408	MM
51	51	20211111_L17_R2_IgM	8.56	437945.3	13342.7	32.823	MM
52	52	20211111_L18_R2_IgM	8.57	86255.4	14692.0	5.871	MM
53	53	20211111_H9_R2_IgM	8.57	209565.7	15639.7	13.400	MM
54	54	20211111_A19_R2_IgM	8.56	376476.6	14198.7	26.515	MM
55	55	20211111_A20_R2_IgM	8.55	182772.6	14162.7	12.905	MM
56	56	20211111_L19_R2_IgM	8.57	341801.6	9293.4	36.779	MM

Dataset: C:\MassLynx\10.pro\20211111_clinicalsamples_IgM_IgA.qld

Last Altered: Thursday, March 09, 2023 19:58:25 台北標準時間
Printed: Friday, August 01, 2025 15:03:19 台北標準時間

	#	Name	RT	Area	IS Area	Response	Prima...
57	57	20211111_QC_R13_IgM	8.57	391785.9	13903.0	28.180	MM
58	58	20211111_L20_R2_IgM	8.52	190318.9	12781.6	14.890	MM
59	59	20211111_H10_R2_IgM	8.54	411953.2	14796.4	27.841	MM
60	60	20211111_A21_R2_IgM	8.57	317206.3	13669.0	23.206	MM
61	61	20211111_A22_R2_IgM	8.57	65268.3	14098.3	4.630	MM
62	62	20211111_L21_R2_IgM	8.58	278331.0	15678.2	17.753	MM
63	63	20211111_L22_R2_IgM	8.58	209359.3	15724.5	13.314	MM
64	64	20211111_H11_R2_IgM	8.59	409674.7	15890.2	25.782	MM
65	65	20211111_A23_R2_IgM	8.57	245195.6	13423.2	18.267	MM
66	66	20211111_A24_R2_IgM	8.57	541826.7	12438.0	43.562	MM
67	67	20211111_L23_R2_IgM	8.58	600145.8	14075.0	42.639	MM
68	68	20211111_L24_R2_IgM	8.56	86187.2	15972.1	5.396	MM
69	69	20211111_H12_R2_IgM	8.58	117061.5	16270.9	7.195	MM
70	70	20211111_QC_R14_IgM	8.59	403906.9	15806.8	25.553	MM
71	71	20211111_A25_R2_IgM	8.57	824453.4	14552.1	56.655	MM
72	72	20211111_A26_R2_IgM	8.58	337403.6	11492.7	29.358	MM
73	73	20211111_L25_R2_IgM	8.59	180276.5	14403.3	12.516	MM
74	74	20211111_L26_R2_IgM	8.59	256141.5	16533.9	15.492	MM
75	75	20211111_H13_R2_IgM	8.58	463957.0	15520.1	29.894	MM
76	76	20211111_A27_R2_IgM	8.57	394278.6	13337.4	29.562	MM
77	77	20211111_A28_R2_IgM	8.53	165728.2	11895.6	13.932	MM
78	78	20211111_L27_R2_IgM	8.57	351934.9	14125.7	24.915	MM
79	79	20211111_L28_R2_IgM	8.58	131368.5	11807.5	11.126	MM
80	80	20211111_H14_R2_IgM	8.58	327756.3	15035.7	21.799	MM
81	81	20211111_A29_R2_IgM	8.57	66925.5	13554.4	4.938	MM
82	82	20211111_A30_R2_IgM	8.57	669901.3	12984.1	51.594	MM
83	83	20211111_QC_R15_IgM	8.58	358689.4	12299.5	29.163	MM
84	84	20211111_L29_R2_IgM	8.58	114528.7	13590.7	8.427	MM
85	85	20211111_L30_R2_IgM	8.57	104810.7	12511.4	8.377	MM
86	86	20211111_H15_R2_IgM	8.57	296415.7	11885.0	24.940	MM
87	87	20211111_A31_R2_IgM	8.57	241754.1	13422.1	18.012	MM
88	88	20211111_A32_R2_IgM	8.57	418718.6	11461.3	36.533	MM
89	89	20211111_L31_R2_IgM	8.53	68591.1	13753.4	4.987	MM
90	90	20211111_L32_R2_IgM	8.57	301570.4	12949.0	23.289	MM
91	91	20211111_H16_R2_IgM	8.57	492244.7	11465.4	42.933	MM
92	92	20211111_A33_R2_IgM	8.56	589325.8	10551.5	55.852	MM
93	93	20211111_A34_R2_IgM	8.58	161389.6	12409.9	13.005	MM
94	94	20211111_H17_R2_IgM	8.56	1619778.4	10160.9	159.413	MM
95	95	20211111_A35_R2_IgM	8.55	200837.3	10460.6	19.199	MM
96	96	20211111_QC_R16_IgM	8.56	338221.1	10433.5	32.417	MM
97	97	20211111_A36_R2_IgM	8.51	282213.3	12504.8	22.568	bb
98	98	20211111_H18_R2_IgM	8.57	779547.4	9140.4	85.286	MM
99	99	20211111_A37_R2_IgM	8.57	534463.9	9796.9	54.555	MM
100	1...	20211111_A38_R2_IgM	8.58	354554.5	11224.3	31.588	MM
101	1...	20211111_H19_R2_IgM	8.57	501775.5	11739.2	42.744	MM
102	1...	20211111_A39_R2_IgM	8.58	361537.3	12718.9	28.425	MM
103	1...	20211111_A40_R2_IgM	8.53	187586.0	10006.1	18.747	MM
104	1...	20211111_H20_R2_IgM	8.56	1589508.3	7988.2	198.982	MM
105	1...	20211111_A41_R2_IgM	8.59	206098.6	11513.3	17.901	MM
106	1...	20211111_A42_R2_IgM	8.58	130200.6	10238.5	12.717	MM
107	1...	20211111_A43_R2_IgM	8.57	134273.4	11098.9	12.098	MM
108	1...	20211111_A44_R2_IgM	8.54	79604.0	10969.9	7.257	MM
109	1...	20211111_QC_R17_IgM	8.57	294935.1	9700.8	30.403	MM
110	1...	20211111_L2_R3_IgM	8.58	466033.3	8528.0	54.647	MM
111	1...	20211111_H1_R3_IgM	8.58	475319.8	9438.2	50.361	MM
112	1...	20211111_A3_R3_IgM	8.57	577563.0	11598.6	49.796	MM

Dataset: C:\MassLynx\10.pro\20211111_clinicalsamples_IgM_IgA.qld

Last Altered: Thursday, March 09, 2023 19:58:25 台北標準時間
Printed: Friday, August 01, 2025 15:03:19 台北標準時間

	#	Name	RT	Area	IS Area	Response	Prima...
113	1...	20211111_A4_R3_IgM	8.57	312367.2	11364.1	27.487	MM
114	1...	20211111_L3_R3_IgM	8.53	169356.6	10753.4	15.749	MM
115	1...	20211111_L4_R3_IgM	8.57	391970.2	10291.6	38.087	MM
116	1...	20211111_H2_R3_IgM	8.56	1094916.0	9315.8	117.534	MM
117	1...	20211111_A5_R3_IgM	8.57	1120012.6	9102.2	123.049	MM
118	1...	20211111_A6_R3_IgM	8.57	307066.2	10356.4	29.650	MM
119	1...	20211111_QC_R18_IgM	8.57	307715.7	8398.3	36.640	MM
120	1...	20211111_L5_R3_IgM	8.57	306776.0	9786.7	31.346	MM
121	1...	20211111_L6_R3_IgM	8.57	342381.4	9911.3	34.545	MM
122	1...	20211111_H3_R3_IgM	8.58	241437.8	12155.5	19.862	MM
123	1...	20211111_A7_R3_IgM	8.56	446458.6	11804.8	37.820	MM
124	1...	20211111_A8_R3_IgM	8.58	487611.6	10510.6	46.392	MM
125	1...	20211111_L7_R3_IgM	8.58	204713.7	12177.9	16.810	MM
126	1...	20211111_L8_R3_IgM	8.58	71136.0	11668.6	6.096	MM
127	1...	20211111_H4_R3_IgM	8.58	164187.7	11192.0	14.670	MM
128	1...	20211111_A9_R3_IgM	8.58	242858.7	14077.4	17.252	MM
129	1...	20211111_A10_R3_IgM	8.58	503495.6	11190.4	44.994	MM
130	1...	20211111_L9_R3_IgM	8.58	187569.3	14649.3	12.804	MM
131	1...	20211111_L10_R3_IgM	8.54	470199.6	9361.3	50.228	MM
132	1...	20211111_QC_R19_IgM	8.57	299307.5	10125.5	29.560	MM
133	1...	20211111_H5_R3_IgM	8.57	212455.2	10010.9	21.222	MM
134	1...	20211111_A11_R3_IgM	8.58	482907.3	12697.2	38.032	MM
135	1...	20211111_A12_R3_IgM	8.56	233114.8	13148.5	17.729	MM
136	1...	20211111_L11_R3_IgM	8.59	207076.6	10786.6	19.198	MM
137	1...	20211111_L12_R3_IgM	8.54	191644.9	11681.8	16.405	MM
138	1...	20211111_H6_R3_IgM	8.58	227239.5	12095.5	18.787	MM
139	1...	20211111_A13_R3_IgM	8.57	266296.5	12908.3	20.630	MM
140	1...	20211111_A14_R3_IgM	8.58	934711.0	11322.6	82.553	MM
141	1...	20211111_L13_R3_IgM	8.57	126842.4	11516.1	11.014	MM
142	1...	20211111_L14_R3_IgM	8.57	261118.6	10790.7	24.199	MM
143	1...	20211111_H7_R3_IgM	8.58	276000.6	11572.1	23.851	MM
144	1...	20211111_A15_R3_IgM	8.57	1246082.4	12434.4	100.212	MM
145	1...	20211111_QC_R20_IgM	8.57	339406.2	10701.0	31.717	MM
146	1...	20211111_A16_R3_IgM	8.58	483235.6	11452.1	42.196	MM
147	1...	20211111_L15_R3_IgM	8.57	485611.2	10531.1	46.112	MM
148	1...	20211111_L16_R3_IgM	8.57	88143.3	12049.4	7.315	MM
149	1...	20211111_H8_R3_IgM	8.58	156387.4	13434.9	11.640	MM
150	1...	20211111_A17_R3_IgM	8.58	480354.4	12452.0	38.576	MM
151	1...	20211111_A18_R3_IgM	8.54	213584.6	12506.5	17.078	MM
152	1...	20211111_L17_R3_IgM	8.57	397452.5	9948.9	39.949	MM
153	1...	20211111_L18_R3_IgM	8.57	81397.3	13462.1	6.046	MM
154	1...	20211111_H9_R3_IgM	8.57	189062.9	13178.7	14.346	MM
155	1...	20211111_A19_R3_IgM	8.57	351603.3	11298.6	31.119	MM
156	1...	20211111_A20_R3_IgM	8.55	165864.7	11175.6	14.842	MM
157	1...	20211111_L19_R3_IgM	8.58	381928.3	11558.2	33.044	MM
158	1...	20211111_QC_R21_IgM	8.58	355532.4	9977.0	35.635	MM
159	1...	20211111_L20_R3_IgM	8.57	167017.2	13508.1	12.364	MM
160	1...	20211111_H10_R3_IgM	8.58	374275.9	12374.8	30.245	MM
161	1...	20211111_A21_R3_IgM	8.57	274640.2	12443.6	22.071	MM
162	1...	20211111_A22_R3_IgM	8.57	59916.4	11589.3	5.170	MM
163	1...	20211111_L21_R3_IgM	8.58	251178.5	13107.4	19.163	MM
164	1...	20211111_L22_R3_IgM	8.58	189902.3	12021.5	15.797	MM
165	1...	20211111_H11_R3_IgM	8.60	366765.0	12687.8	28.907	MM
166	1...	20211111_A23_R3_IgM	8.59	257329.9	11611.8	22.161	MM
167	1...	20211111_A24_R3_IgM	8.57	552462.4	10476.8	52.732	MM
168	1...	20211111_L23_R3_IgM	8.57	541792.3	11873.1	45.632	MM

Dataset: C:\MassLynx\10.pro\20211111_clinicalsamples_IgM_IgA.qld

Last Altered: Thursday, March 09, 2023 19:58:25 台北標準時間
Printed: Friday, August 01, 2025 15:03:19 台北標準時間

	# Name	RT	Area	IS Area	Response	Prima...
169	1... 20211111_L24_R3_IgM	8.58	65469.9	13468.0	4.861	MM
170	1... 20211111_H12_R3_IgM	8.57	112552.8	14229.1	7.910	MM
171	1... 20211111_QC_R22_IgM	8.58	350250.3	11264.4	31.094	MM
172	1... 20211111_A25_R3_IgM	8.59	667320.2	11775.7	56.669	MM
173	1... 20211111_A26_R3_IgM	8.59	307003.6	10473.9	29.311	MM
174	1... 20211111_L25_R3_IgM	8.59	155821.3	13256.8	11.754	MM
175	1... 20211111_L26_R3_IgM	8.59	210193.3	13565.0	15.495	MM
176	1... 20211111_H13_R3_IgM	8.58	433103.6	13146.0	32.946	MM
177	1... 20211111_A27_R3_IgM	8.58	343259.1	12025.6	28.544	MM
178	1... 20211111_A28_R3_IgM	8.57	146981.3	11229.1	13.089	MM
179	1... 20211111_L27_R3_IgM	8.59	346433.9	14960.0	23.157	MM
180	1... 20211111_L28_R3_IgM	8.58	141636.0	11359.0	12.469	MM
181	1... 20211111_H14_R3_IgM	8.58	322312.8	12830.8	25.120	MM
182	1... 20211111_A29_R3_IgM	8.57	59517.9	13434.3	4.430	MM
183	1... 20211111_A30_R3_IgM	8.58	613417.8	11272.2	54.419	MM
184	1... 20211111_QC_R23_IgM	8.58	324704.6	11872.2	27.350	MM
185	1... 20211111_L29_R3_IgM	8.58	108462.0	12948.7	8.376	MM
186	1... 20211111_L30_R3_IgM	8.58	93302.6	11026.6	8.462	MM
187	1... 20211111_H15_R3_IgM	8.57	259536.9	10786.3	24.062	MM
188	1... 20211111_A31_R3_IgM	8.57	227095.5	12010.5	18.908	MM
189	1... 20211111_A32_R3_IgM	8.57	368287.1	11107.7	33.156	MM
190	1... 20211111_L31_R3_IgM	8.58	51202.6	11021.8	4.646	MM
191	1... 20211111_L32_R3_IgM	8.58	264537.8	11683.8	22.641	MM
192	1... 20211111_H16_R3_IgM	8.57	392490.0	9831.7	39.921	MM
193	1... 20211111_A33_R3_IgM	8.57	555859.6	11213.1	49.572	MM
194	1... 20211111_A34_R3_IgM	8.59	133675.9	11550.1	11.574	MM
195	1... 20211111_H17_R3_IgM	8.56	1384455.5	8874.4	156.005	MM
196	1... 20211111_A35_R3_IgM	8.57	175508.3	9856.3	17.807	MM
197	1... 20211111_QC_R24_IgM	8.57	285354.9	7561.8	37.736	MM
198	1... 20211111_A36_R3_IgM	8.58	253403.5	9562.8	26.499	MM
199	1... 20211111_H18_R3_IgM	8.57	710023.8	8165.3	86.956	MM
200	2... 20211111_A37_R3_IgM	8.56	444696.8	9278.8	47.926	MM
201	2... 20211111_A38_R3_IgM	8.58	295340.6	10920.3	27.045	MM
202	2... 20211111_H19_R3_IgM	8.57	445387.3	9285.3	47.967	MM
203	2... 20211111_A39_R3_IgM	8.58	329281.4	9083.4	36.251	MM
204	2... 20211111_A40_R3_IgM	8.57	174300.7	8382.4	20.794	MM
205	2... 20211111_H20_R3_IgM	8.57	1325255.6	7807.9	169.732	MM
206	2... 20211111_A41_R3_IgM	8.59	133987.6	5925.2	22.613	MM
207	2... 20211111_A42_R3_IgM	8.58	124632.3	9352.6	13.326	MM
208	2... 20211111_A43_R3_IgM	8.57	119421.7	10216.3	11.689	MM
209	2... 20211111_A44_R3_IgM	8.56	70823.6	10262.7	6.901	MM
210	2... 20211111_QC_R25_IgM	8.56	269861.6	8575.3	31.469	MM

Dataset: C:\MassLynx\10.pro\20211111_clinicalsamples_IgM_IgA.qld

Last Altered: Thursday, March 09, 2023 19:58:25 台北標準時間
Printed: Friday, August 01, 2025 15:04:17 台北標準時間

Method: C:\MassLynx\10_Pro_IgA.PRO\MethDB\20211111_IgM_IgA_DMRM_new.mdb 24 Feb 2022 13:44:14
Calibration: 15 Nov 2021 14:42:11

Sample Name: 20211112_QC_R001_IgM
F48:MRM of 2 channels,ES+
984.5 > 204.1
5.752e+006

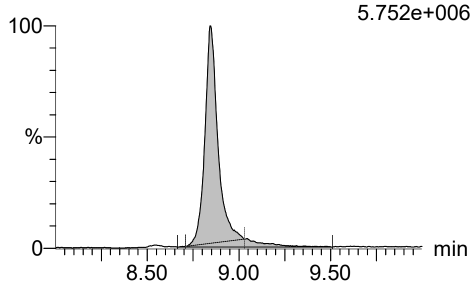

Sample Name: 20211112_QC_R001_IgM
F48:MRM of 2 channels,ES+
1230.4 > 366.1
1.532e+006

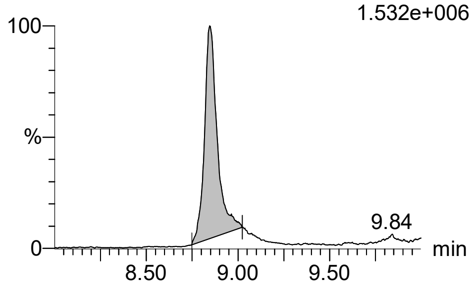

Sample Name: 20211112_QC_R002_IgM
F48:MRM of 2 channels,ES+
984.5 > 204.1
5.760e+006

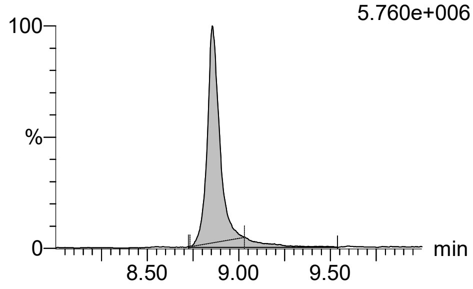

Sample Name: 20211112_QC_R002_IgM
F48:MRM of 2 channels,ES+
1230.4 > 366.1
1.559e+006

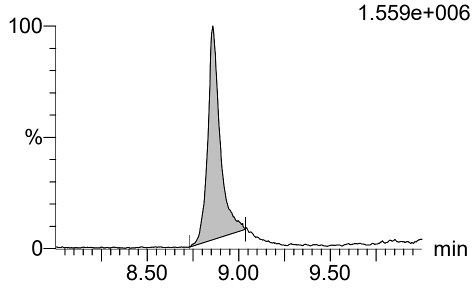

Sample Name: 20211111_A1_R2_IgM
F48:MRM of 2 channels,ES+
984.5 > 204.1
3.483e+006

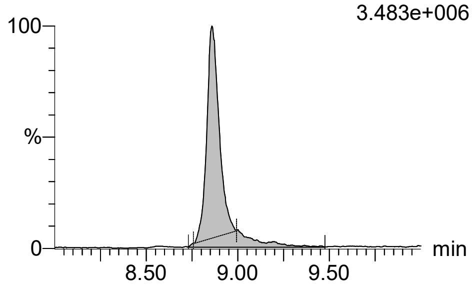

Sample Name: 20211111_A1_R2_IgM
F48:MRM of 2 channels,ES+
1230.4 > 366.1
9.626e+005

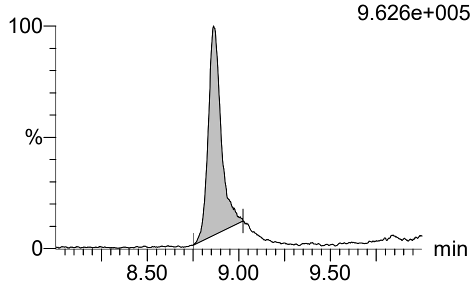

Sample Name: 20211111_A1_R3_IgM
F48:MRM of 2 channels,ES+
984.5 > 204.1
3.487e+006

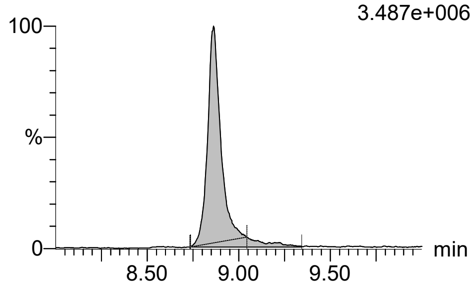

Sample Name: 20211111_A1_R3_IgM
F48:MRM of 2 channels,ES+
1230.4 > 366.1
9.635e+005

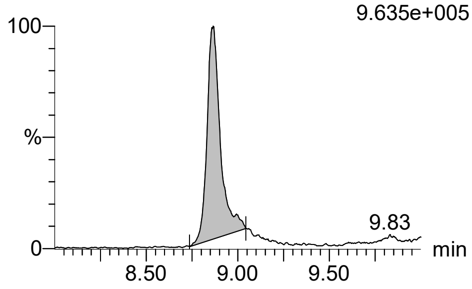

Sample Name: 20211111_A2_R2_IgM
F48:MRM of 2 channels,ES+
984.5 > 204.1
1.187e+007

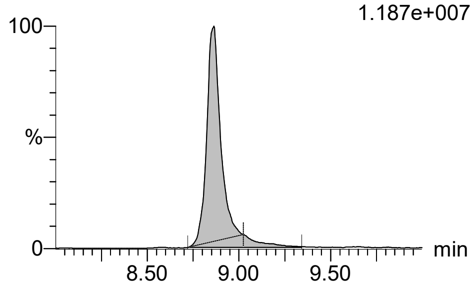

Sample Name: 20211111_A2_R2_IgM
F48:MRM of 2 channels,ES+
1230.4 > 366.1
3.508e+006

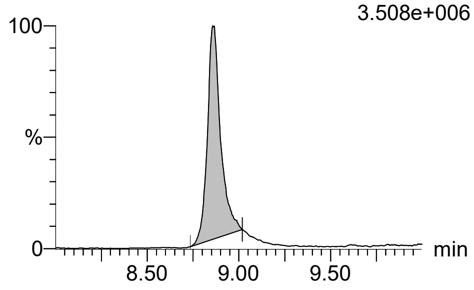

Sample Name: 20211111_A2_R3_IgM
F48:MRM of 2 channels,ES+
984.5 > 204.1
1.207e+007

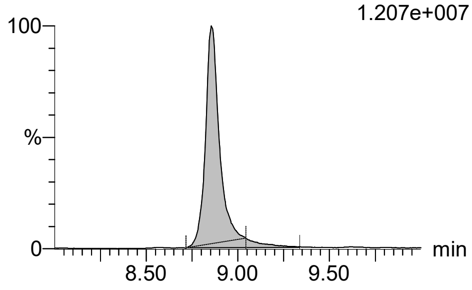

Sample Name: 20211111_A2_R3_IgM
F48:MRM of 2 channels,ES+
1230.4 > 366.1
3.485e+006

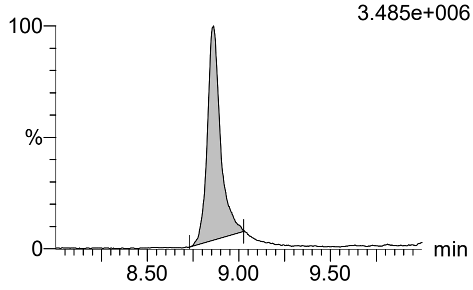

Dataset: C:\MassLynx\10.pro\20211111_clinicalsamples_IgM_IgA.qld

Last Altered: Thursday, March 09, 2023 19:58:25 台北標準時間
Printed: Friday, August 01, 2025 15:04:17 台北標準時間

Sample Name: 20211111_L1_R2_IgM
F48:MRM of 2 channels,ES+
984.5 > 204.1
2.226e+006

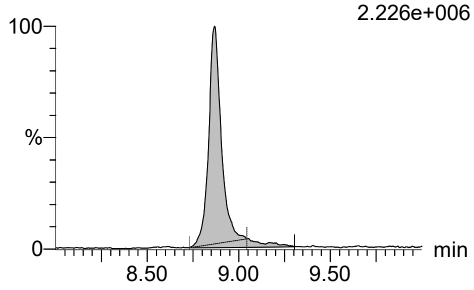

Sample Name: 20211111_L1_R2_IgM
F48:MRM of 2 channels,ES+
1230.4 > 366.1
6.219e+005

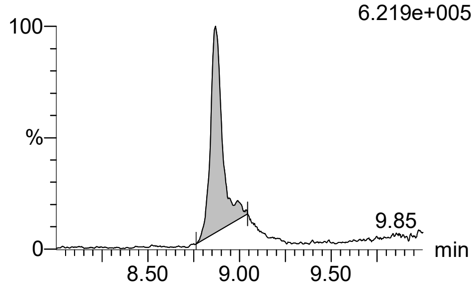

Sample Name: 20211111_L1_R3_IgM
F48:MRM of 2 channels,ES+
984.5 > 204.1
2.140e+006

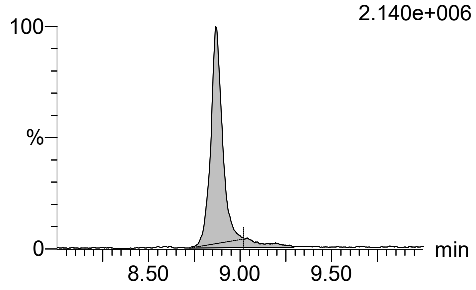

Sample Name: 20211111_L1_R3_IgM
F48:MRM of 2 channels,ES+
1230.4 > 366.1
5.518e+005

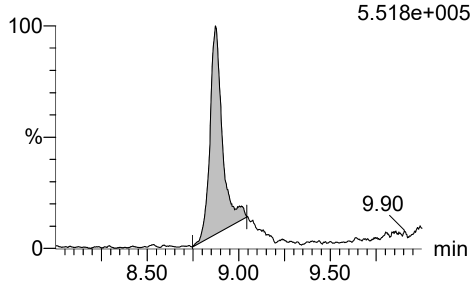

Sample Name: 20211111_L2_R2_IgM
F48:MRM of 2 channels,ES+
984.5 > 204.1
4.937e+006

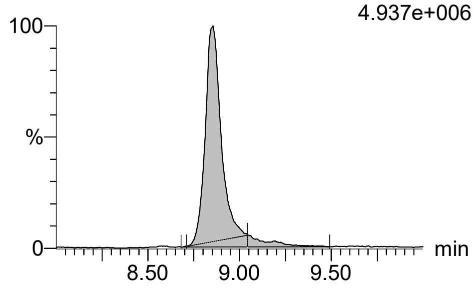

Sample Name: 20211111_L2_R2_IgM
F48:MRM of 2 channels,ES+
1230.4 > 366.1
1.426e+006

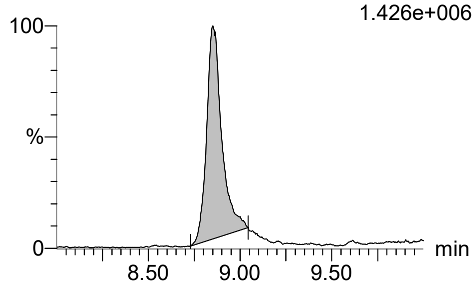

Sample Name: 20211111_H1_R2_IgM
F48:MRM of 2 channels,ES+
984.5 > 204.1
3.751e+006

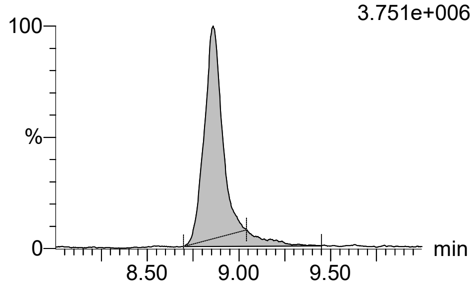

Sample Name: 20211111_H1_R2_IgM
F48:MRM of 2 channels,ES+
1230.4 > 366.1
1.104e+006

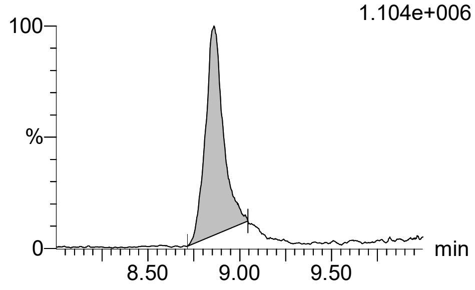

Sample Name: 20211111_A3_R2_IgM
F48:MRM of 2 channels,ES+
984.5 > 204.1
4.966e+006

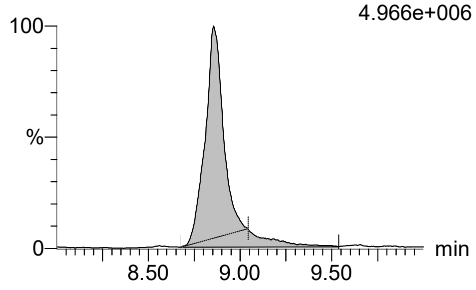

Sample Name: 20211111_A3_R2_IgM
F48:MRM of 2 channels,ES+
1230.4 > 366.1
1.465e+006

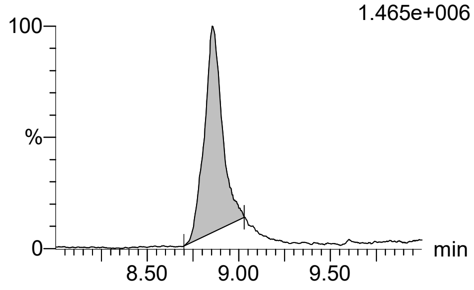

Sample Name: 20211111_A4_R2_IgM
F48:MRM of 2 channels,ES+
984.5 > 204.1
3.444e+006

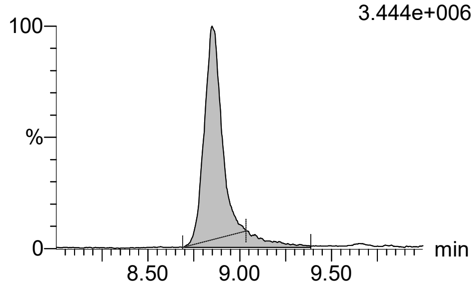

Sample Name: 20211111_A4_R2_IgM
F48:MRM of 2 channels,ES+
1230.4 > 366.1
1.038e+006

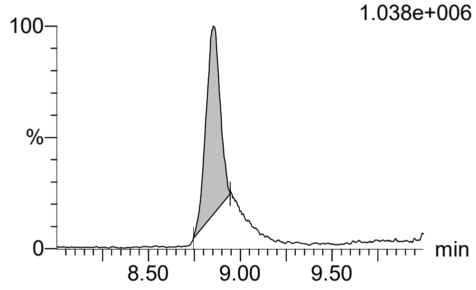

Sample Name: 20211111_L3_R2_IgM
F48:MRM of 2 channels,ES+
984.5 > 204.1
1.156e+006

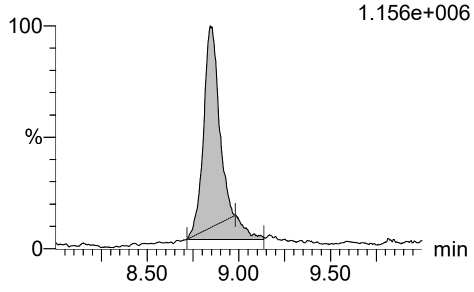

Sample Name: 20211111_L3_R2_IgM
F48:MRM of 2 channels,ES+
1230.4 > 366.1
3.563e+005

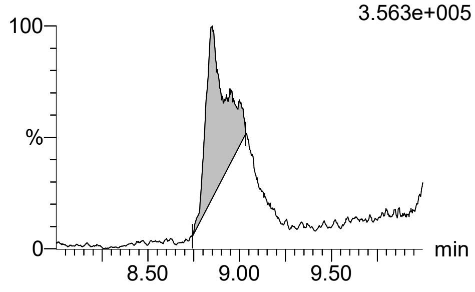

Sample Name: 20211111_L4_R2_IgM
F48:MRM of 2 channels,ES+
984.5 > 204.1
5.237e+006

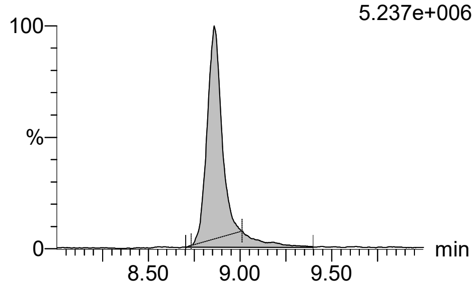

Dataset: C:\MassLynx\10.pro\20211111_clinicalsamples_IgM_IgA.qld

Last Altered: Thursday, March 09, 2023 19:58:25 台北標準時間
Printed: Friday, August 01, 2025 15:04:17 台北標準時間

Sample Name: 20211111_L4_R2_IgM
F48:MRM of 2 channels,ES+
1230.4 > 366.1
1.549e+006

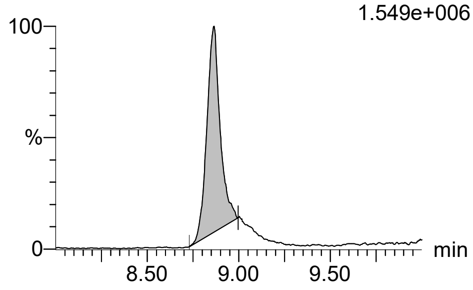

Sample Name: 20211111_H2_R2_IgM
F48:MRM of 2 channels,ES+
984.5 > 204.1
1.341e+007

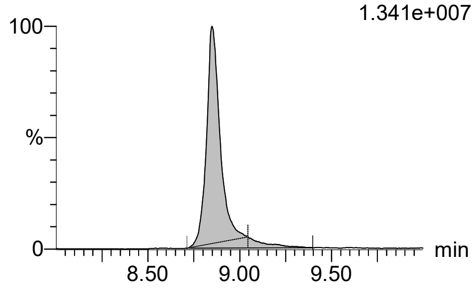

Sample Name: 20211111_H2_R2_IgM
F48:MRM of 2 channels,ES+
1230.4 > 366.1
3.920e+006

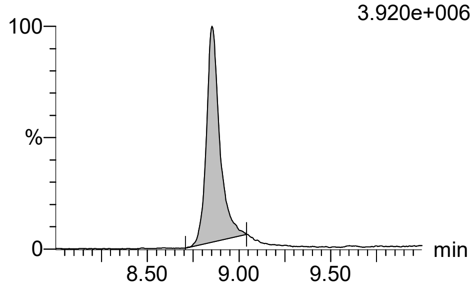

Sample Name: 20211111_A5_R2_IgM
F48:MRM of 2 channels,ES+
984.5 > 204.1
1.699e+007

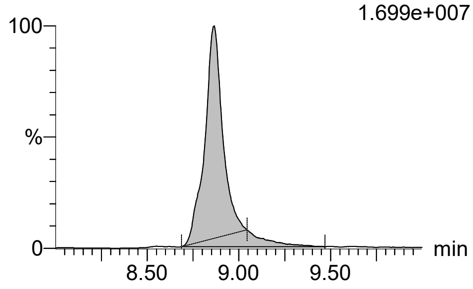

Sample Name: 20211111_A5_R2_IgM
F48:MRM of 2 channels,ES+
1230.4 > 366.1
5.041e+006

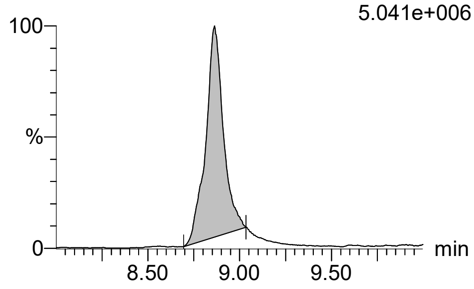

Sample Name: 20211111_A6_R2_IgM
F48:MRM of 2 channels,ES+
984.5 > 204.1
4.987e+006

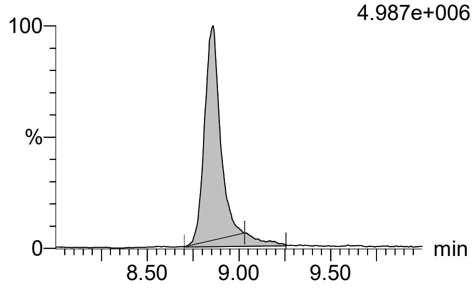

Sample Name: 20211111_A6_R2_IgM
F48:MRM of 2 channels,ES+
1230.4 > 366.1
1.508e+006

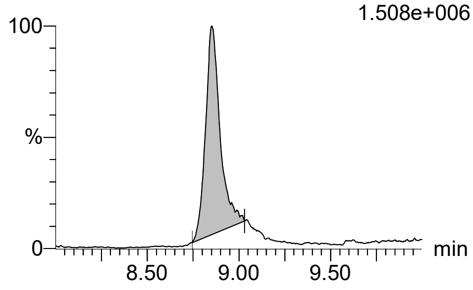

Sample Name: 20211111_QC_R10_IgM
F48:MRM of 2 channels,ES+
984.5 > 204.1
5.530e+006

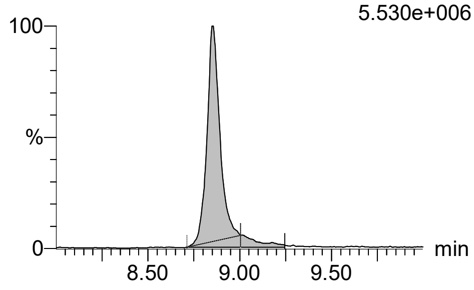

Sample Name: 20211111_QC_R10_IgM
F48:MRM of 2 channels,ES+
1230.4 > 366.1
1.529e+006

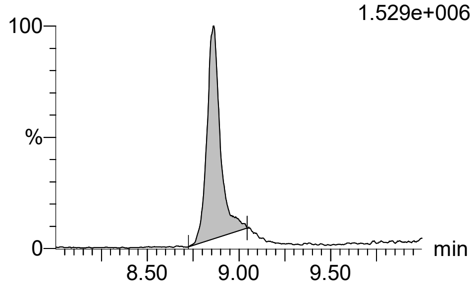

Sample Name: 20211111_L5_R2_IgM
F48:MRM of 2 channels,ES+
984.5 > 204.1
2.532e+006

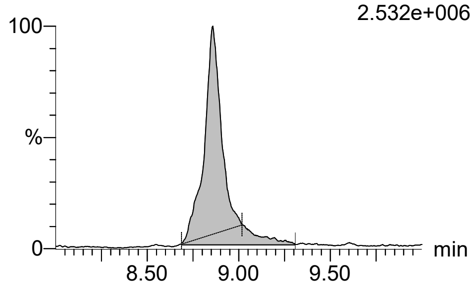

Sample Name: 20211111_L5_R2_IgM
F48:MRM of 2 channels,ES+
1230.4 > 366.1
6.769e+005

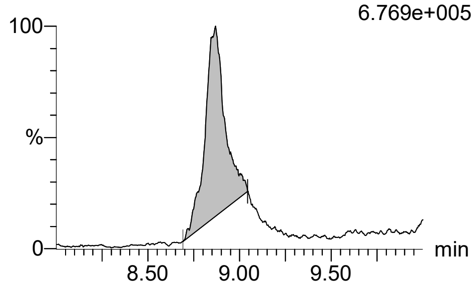

Sample Name: 20211111_L6_R2_IgM
F48:MRM of 2 channels,ES+
984.5 > 204.1
5.785e+006

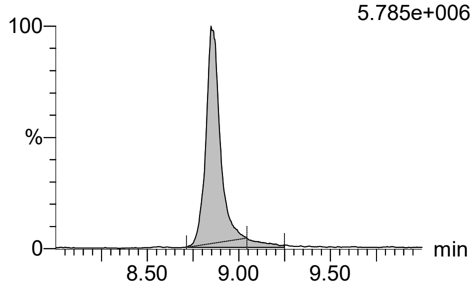

Sample Name: 20211111_L6_R2_IgM
F48:MRM of 2 channels,ES+
1230.4 > 366.1
1.675e+006

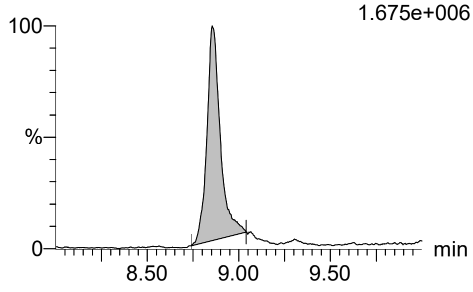

Sample Name: 20211111_H3_R2_IgM
F48:MRM of 2 channels,ES+
984.5 > 204.1
2.765e+006

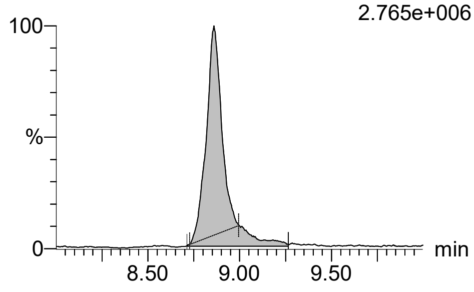

Sample Name: 20211111_H3_R2_IgM
F48:MRM of 2 channels,ES+
1230.4 > 366.1
8.312e+005

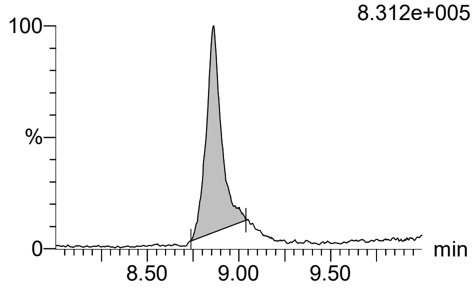

Sample Name: 20211111_A7_R2_IgM

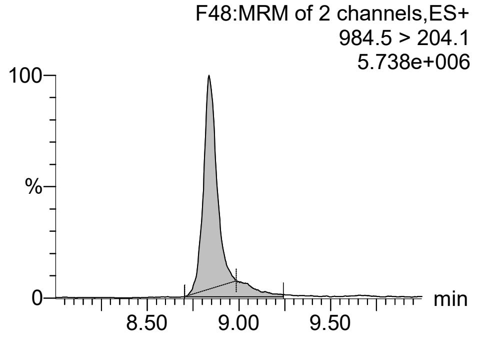

Sample Name: 20211111_A7_R2_IgM

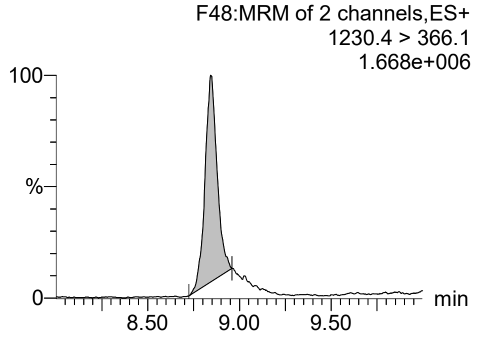

Sample Name: 20211111_A8_R2_IgM

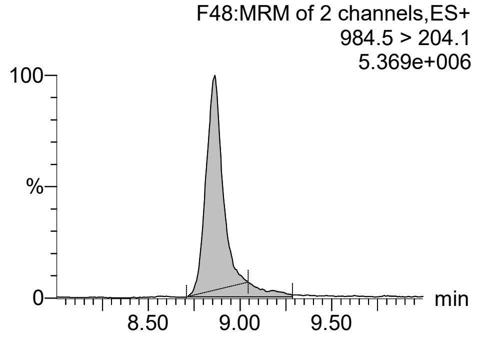

Sample Name: 20211111_A8_R2_IgM

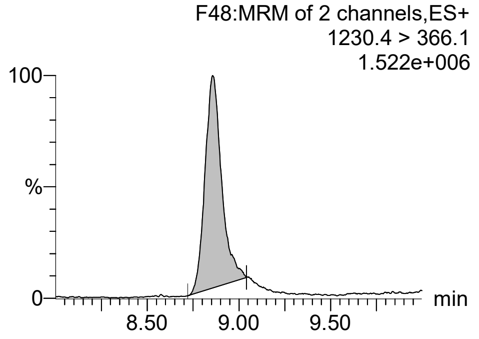

Sample Name: 20211111_L7_R2_IgM

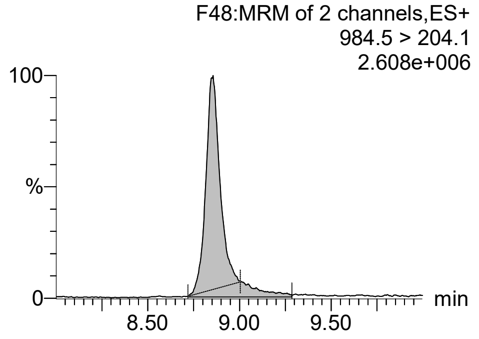

Sample Name: 20211111_L7_R2_IgM

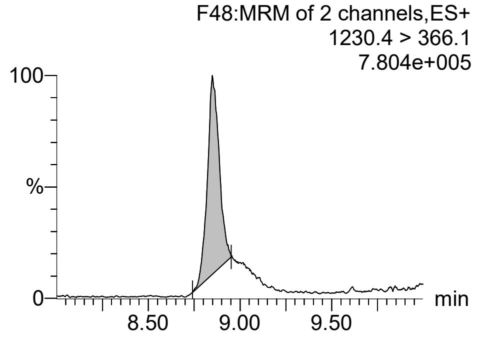

Sample Name: 20211111_L8_R2_IgM

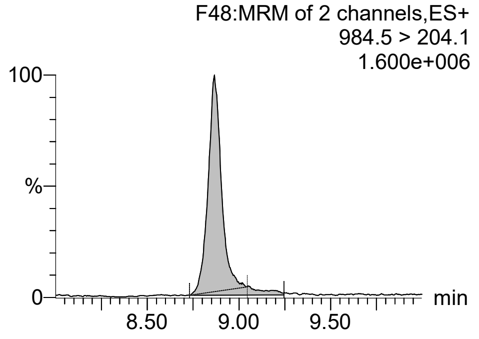

Sample Name: 20211111_L8_R2_IgM

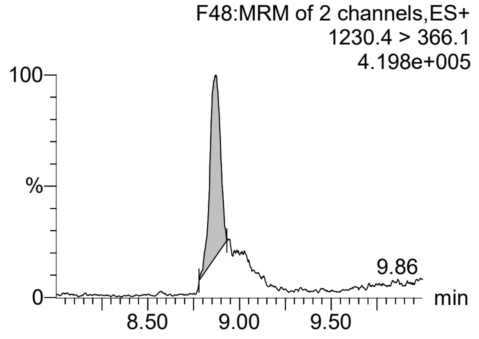

Sample Name: 20211111_H4_R2_IgM

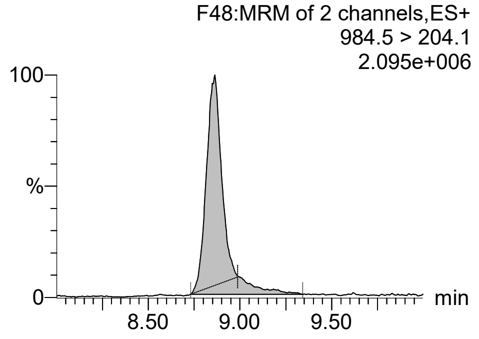

Sample Name: 20211111_H4_R2_IgM

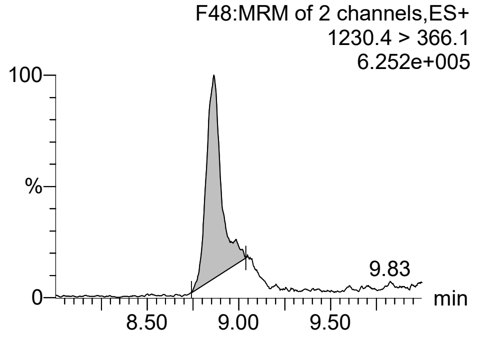

Sample Name: 20211111_A9_R2_IgM

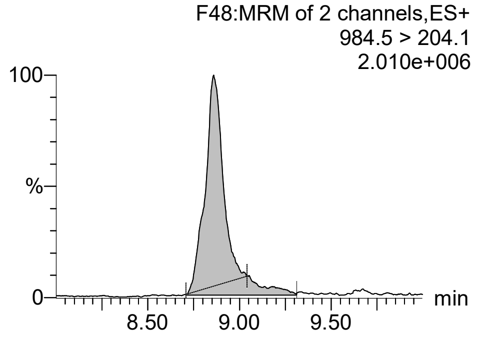

Sample Name: 20211111_A9_R2_IgM

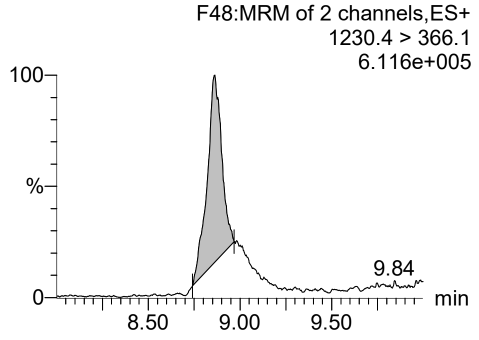

Sample Name: 20211111_A10_R2_IgM

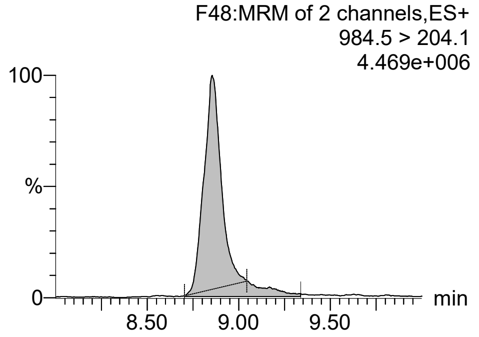

Sample Name: 20211111_A10_R2_IgM

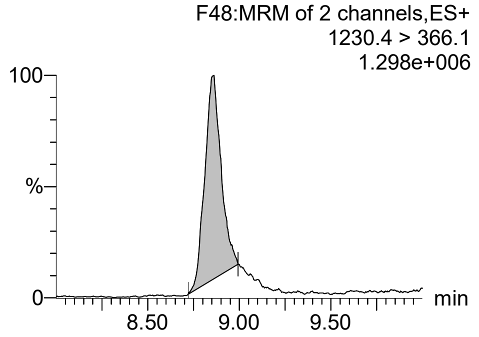

Sample Name: 20211111_L9_R2_IgM

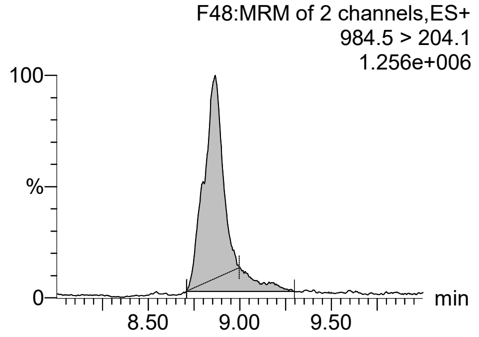

Sample Name: 20211111_L9_R2_IgM
F48:MRM of 2 channels,ES+
1230.4 > 366.1
3.798e+005

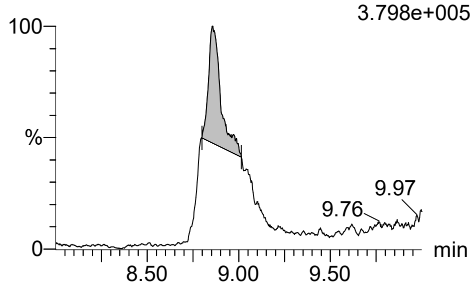

Sample Name: 20211111_L10_R2_IgM
F48:MRM of 2 channels,ES+
984.5 > 204.1
4.104e+006

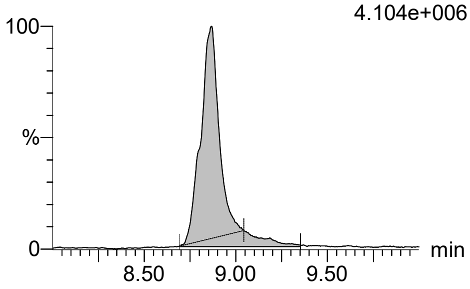

Sample Name: 20211111_L10_R2_IgM
F48:MRM of 2 channels,ES+
1230.4 > 366.1
1.273e+006

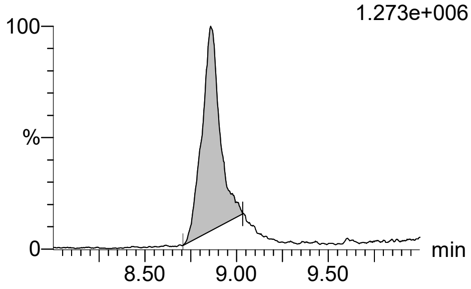

Sample Name: 20211111_QC_R11_IgM
F48:MRM of 2 channels,ES+
984.5 > 204.1
5.747e+006

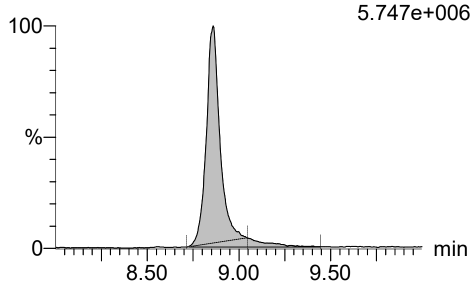

Sample Name: 20211111_QC_R11_IgM
F48:MRM of 2 channels,ES+
1230.4 > 366.1
1.706e+006

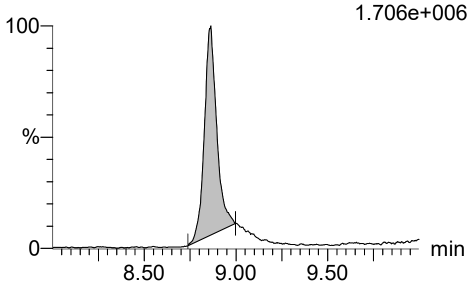

Sample Name: 20211111_H5_R2_IgM
F48:MRM of 2 channels,ES+
984.5 > 204.1
3.535e+006

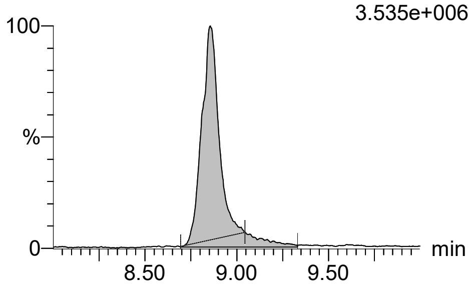

Sample Name: 20211111_H5_R2_IgM
F48:MRM of 2 channels,ES+
1230.4 > 366.1
1.107e+006

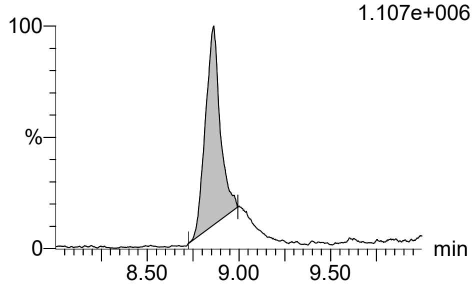

Sample Name: 20211111_A11_R2_IgM
F48:MRM of 2 channels,ES+
984.5 > 204.1
4.547e+006

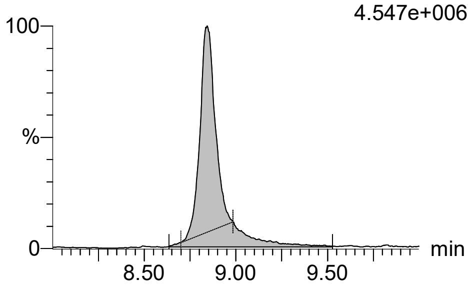

Sample Name: 20211111_A11_R2_IgM
F48:MRM of 2 channels,ES+
1230.4 > 366.1
1.376e+006

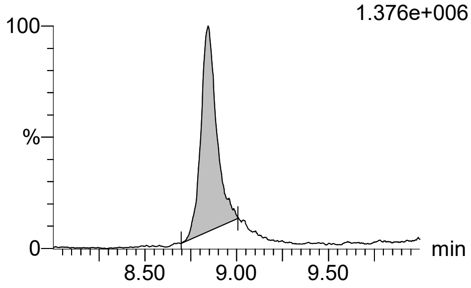

Sample Name: 20211111_A12_R2_IgM
F48:MRM of 2 channels,ES+
984.5 > 204.1
1.912e+006

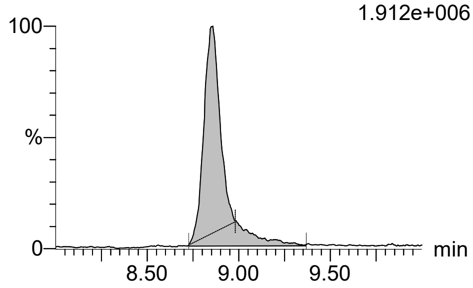

Sample Name: 20211111_A12_R2_IgM
F48:MRM of 2 channels,ES+
1230.4 > 366.1
5.876e+005

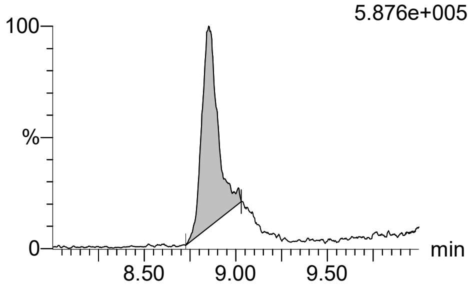

Sample Name: 20211111_L11_R2_IgM
F48:MRM of 2 channels,ES+
984.5 > 204.1
4.864e+006

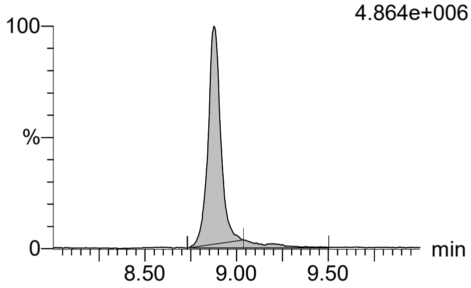

Sample Name: 20211111_L11_R2_IgM
F48:MRM of 2 channels,ES+
1230.4 > 366.1
1.426e+006

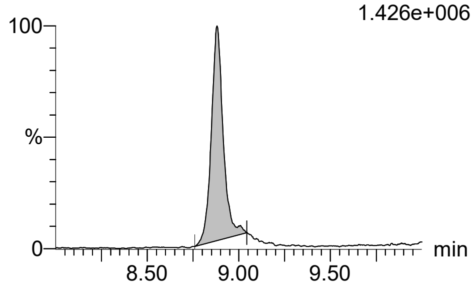

Sample Name: 20211111_L12_R2_IgM
F48:MRM of 2 channels,ES+
984.5 > 204.1
1.669e+006

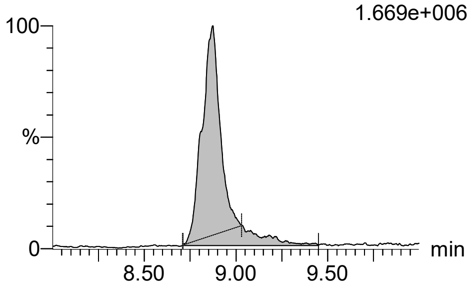

Sample Name: 20211111_L12_R2_IgM
F48:MRM of 2 channels,ES+
1230.4 > 366.1
5.031e+005

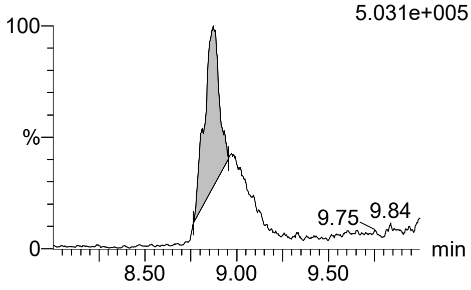

Dataset: C:\MassLynx\10.pro\20211111_clinicalsamples_IgM_IgA.qld

Last Altered: Thursday, March 09, 2023 19:58:25 台北標準時間
Printed: Friday, August 01, 2025 15:04:17 台北標準時間

Sample Name: 20211111_H6_R2_IgM
F48:MRM of 2 channels,ES+
984.5 > 204.1
2.450e+006

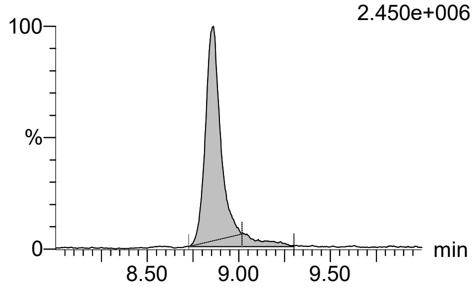

Sample Name: 20211111_H6_R2_IgM
F48:MRM of 2 channels,ES+
1230.4 > 366.1
8.042e+005

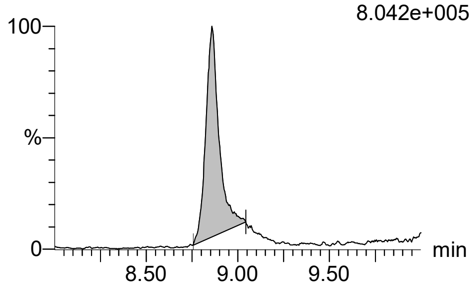

Sample Name: 20211111_A13_R2_IgM
F48:MRM of 2 channels,ES+
984.5 > 204.1
3.072e+006

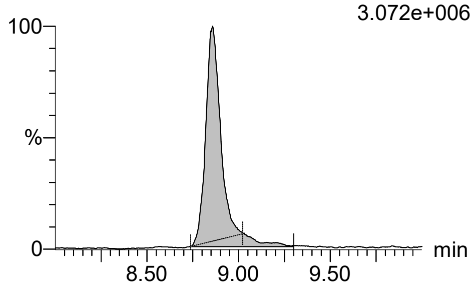

Sample Name: 20211111_A13_R2_IgM
F48:MRM of 2 channels,ES+
1230.4 > 366.1
9.192e+005

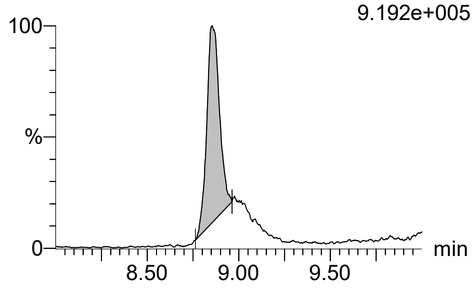

Sample Name: 20211111_A14_R2_IgM
F48:MRM of 2 channels,ES+
984.5 > 204.1
1.073e+007

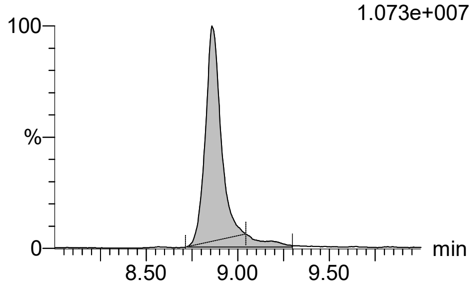

Sample Name: 20211111_A14_R2_IgM
F48:MRM of 2 channels,ES+
1230.4 > 366.1
3.328e+006

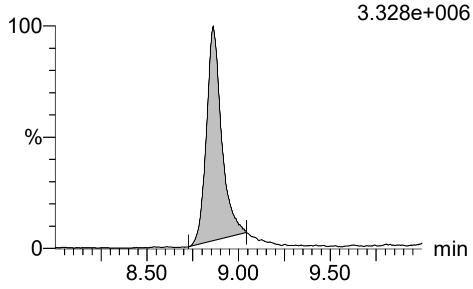

Sample Name: 20211111_L13_R2_IgM
F48:MRM of 2 channels,ES+
984.5 > 204.1
2.143e+006

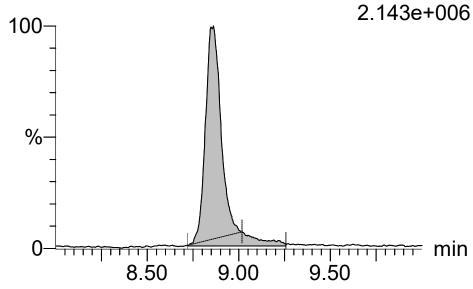

Sample Name: 20211111_L13_R2_IgM
F48:MRM of 2 channels,ES+
1230.4 > 366.1
6.533e+005

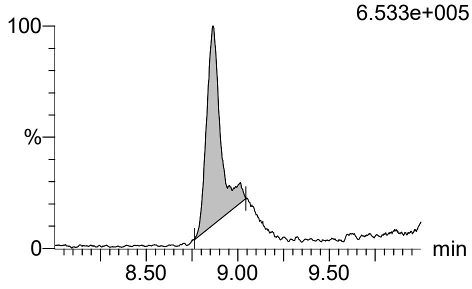

Sample Name: 20211111_L14_R2_IgM
F48:MRM of 2 channels,ES+
984.5 > 204.1
3.855e+006

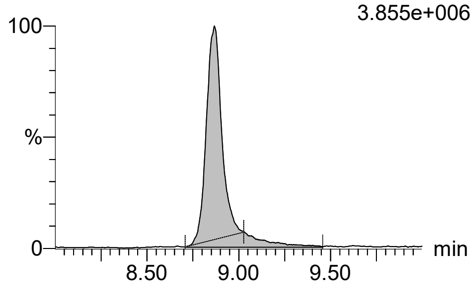

Sample Name: 20211111_L14_R2_IgM
F48:MRM of 2 channels,ES+
1230.4 > 366.1
1.260e+006

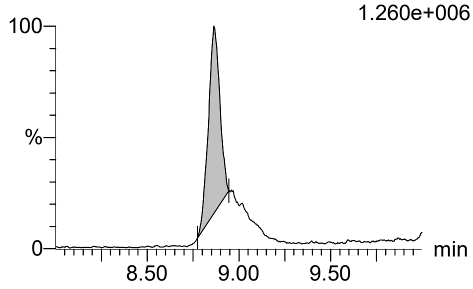

Sample Name: 20211111_H7_R2_IgM
F48:MRM of 2 channels,ES+
984.5 > 204.1
1.692e+006

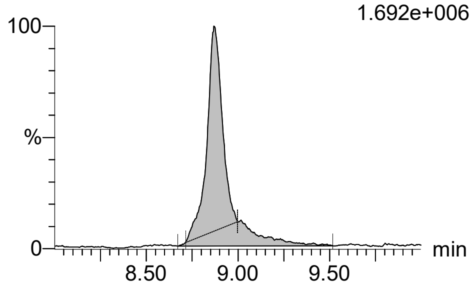

Sample Name: 20211111_H7_R2_IgM
F48:MRM of 2 channels,ES+
1230.4 > 366.1
4.841e+005

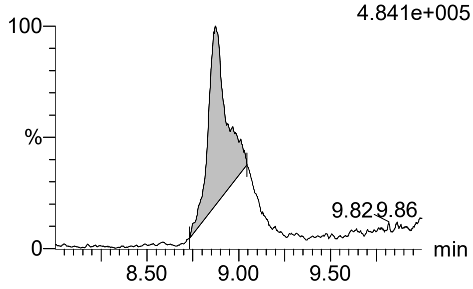

Sample Name: 20211111_A15_R2_IgM
F48:MRM of 2 channels,ES+
984.5 > 204.1
1.405e+007

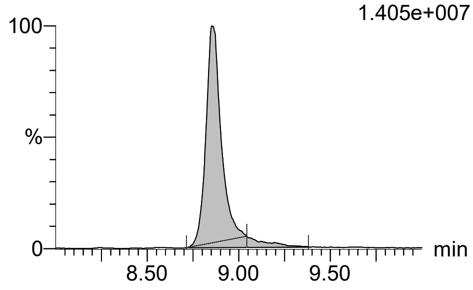

Sample Name: 20211111_A15_R2_IgM
F48:MRM of 2 channels,ES+
1230.4 > 366.1
4.485e+006

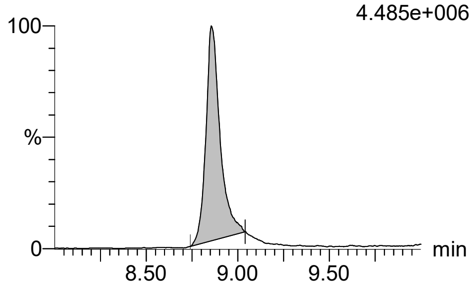

Sample Name: 20211111_QC_R12_IgM
F48:MRM of 2 channels,ES+
984.5 > 204.1
6.249e+006

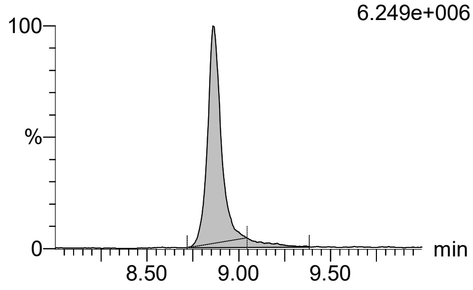

Sample Name: 20211111_QC_R12_IgM
F48:MRM of 2 channels,ES+
1230.4 > 366.1
1.824e+006

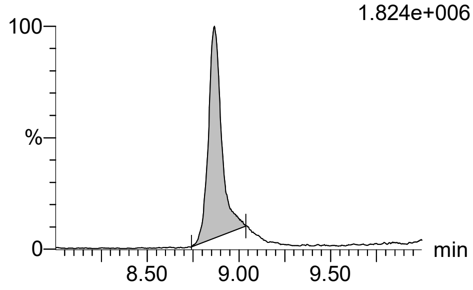

Sample Name: 20211111_A16_R2_IgM
F48:MRM of 2 channels,ES+
984.5 > 204.1
8.579e+006

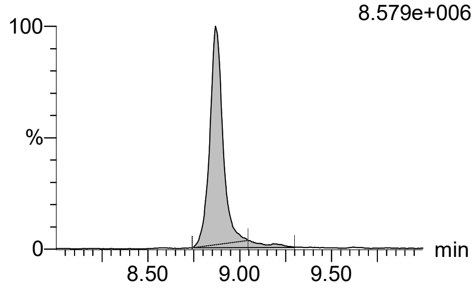

Sample Name: 20211111_A16_R2_IgM
F48:MRM of 2 channels,ES+
1230.4 > 366.1
2.547e+006

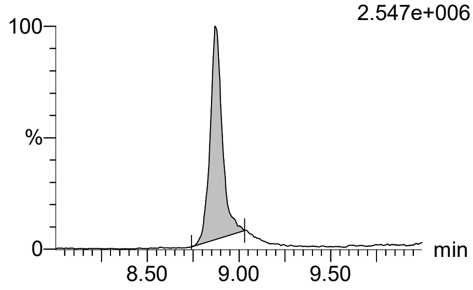

Sample Name: 20211111_L15_R2_IgM
F48:MRM of 2 channels,ES+
984.5 > 204.1
4.670e+006

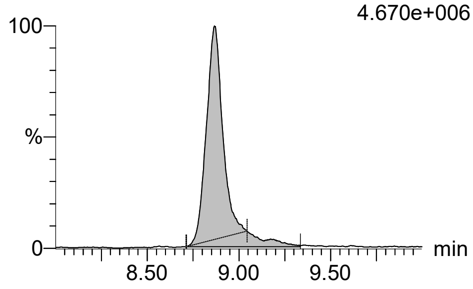

Sample Name: 20211111_L15_R2_IgM
F48:MRM of 2 channels,ES+
1230.4 > 366.1
1.422e+006

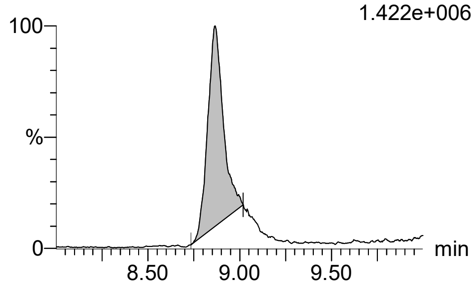

Sample Name: 20211111_L16_R2_IgM
F48:MRM of 2 channels,ES+
984.5 > 204.1
1.135e+006

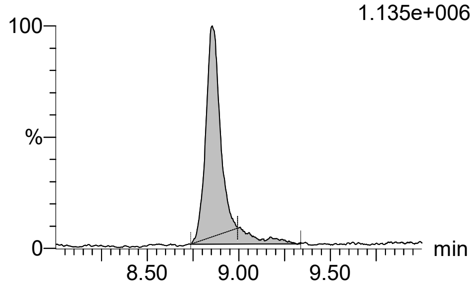

Sample Name: 20211111_L16_R2_IgM
F48:MRM of 2 channels,ES+
1230.4 > 366.1
3.286e+005

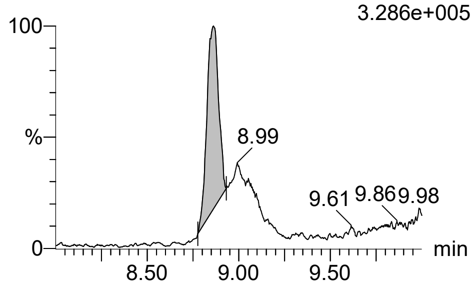

Sample Name: 20211111_H8_R2_IgM
F48:MRM of 2 channels,ES+
984.5 > 204.1
3.215e+006

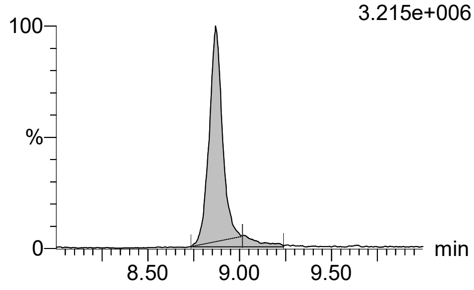

Sample Name: 20211111_H8_R2_IgM
F48:MRM of 2 channels,ES+
1230.4 > 366.1
9.377e+005

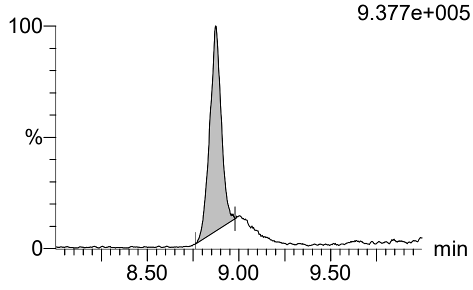

Sample Name: 20211111_A17_R2_IgM
F48:MRM of 2 channels,ES+
984.5 > 204.1
1.094e+007

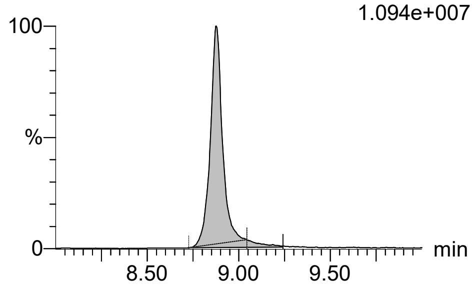

Sample Name: 20211111_A17_R2_IgM
F48:MRM of 2 channels,ES+
1230.4 > 366.1
3.123e+006

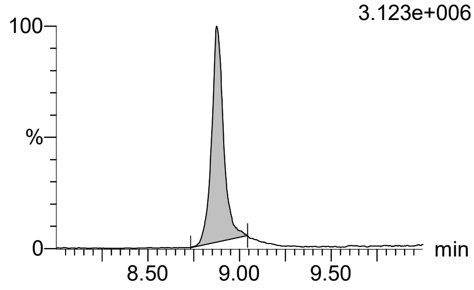

Sample Name: 20211111_A18_R2_IgM
F48:MRM of 2 channels,ES+
984.5 > 204.1
1.795e+006

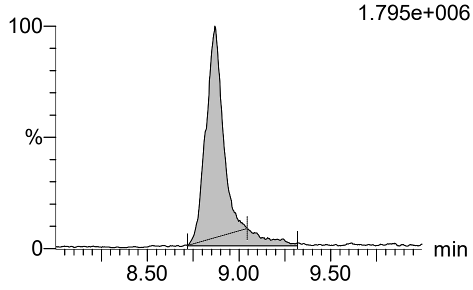

Sample Name: 20211111_A18_R2_IgM
F48:MRM of 2 channels,ES+
1230.4 > 366.1
5.352e+005

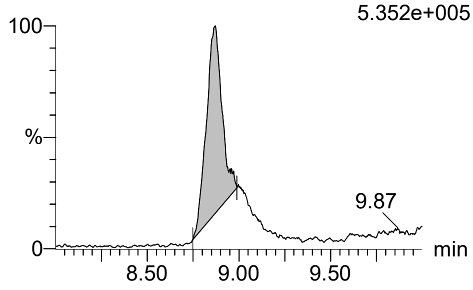

Sample Name: 20211111_L17_R2_IgM
F48:MRM of 2 channels,ES+
984.5 > 204.1
4.832e+006

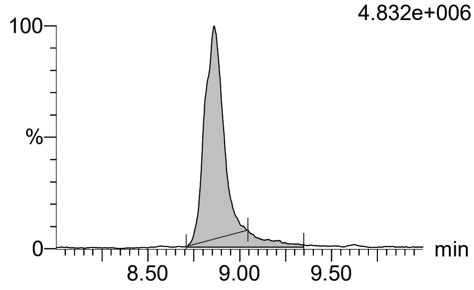

Sample Name: 20211111_L17_R2_IgM
F48:MRM of 2 channels,ES+
1230.4 > 366.1
1.458e+006

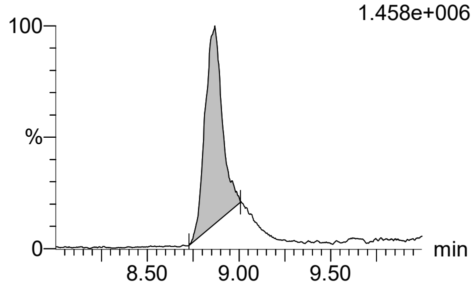

Sample Name: 20211111_L18_R2_IgM
F48:MRM of 2 channels,ES+
984.5 > 204.1
8.485e+005

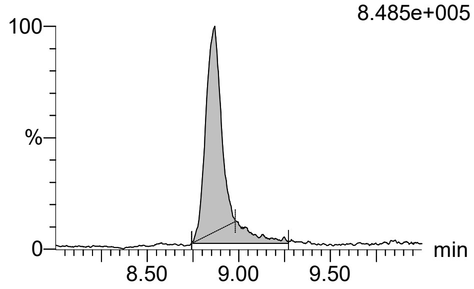

Sample Name: 20211111_L18_R2_IgM
F48:MRM of 2 channels,ES+
1230.4 > 366.1
2.692e+005

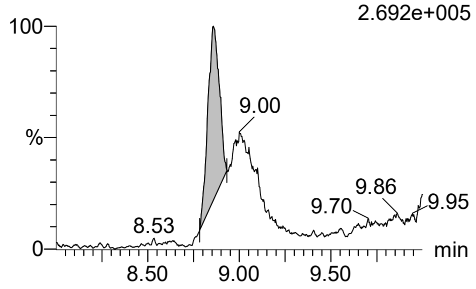

Sample Name: 20211111_H9_R2_IgM
F48:MRM of 2 channels,ES+
984.5 > 204.1
1.691e+006

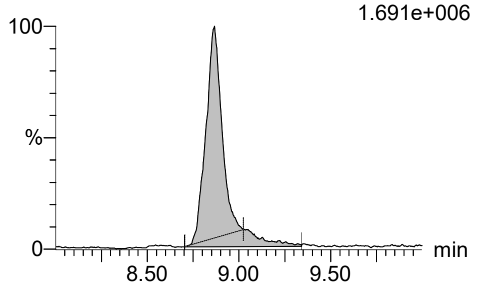

Sample Name: 20211111_H9_R2_IgM
F48:MRM of 2 channels,ES+
1230.4 > 366.1
5.168e+005

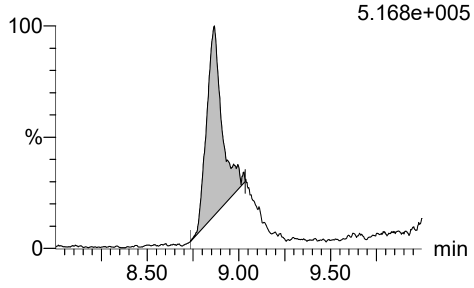

Sample Name: 20211111_A19_R2_IgM
F48:MRM of 2 channels,ES+
984.5 > 204.1
5.251e+006

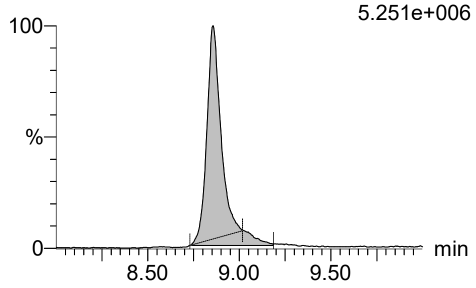

Sample Name: 20211111_A19_R2_IgM
F48:MRM of 2 channels,ES+
1230.4 > 366.1
1.617e+006

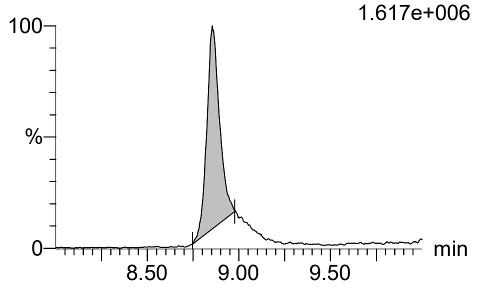

Sample Name: 20211111_A20_R2_IgM
F48:MRM of 2 channels,ES+
984.5 > 204.1
1.047e+006

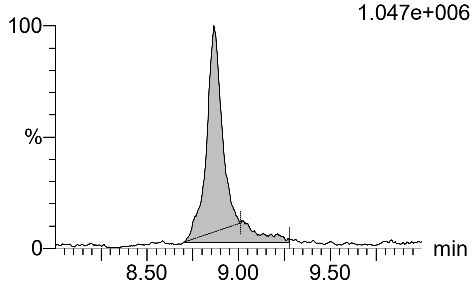

Sample Name: 20211111_A20_R2_IgM
F48:MRM of 2 channels,ES+
1230.4 > 366.1
2.944e+005

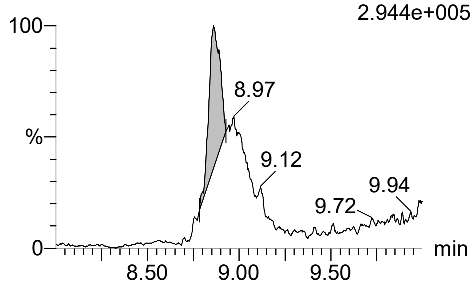

Sample Name: 20211111_L19_R2_IgM
F48:MRM of 2 channels,ES+
984.5 > 204.1
2.818e+006

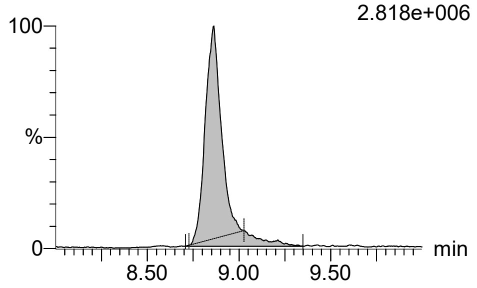

Sample Name: 20211111_L19_R2_IgM
F48:MRM of 2 channels,ES+
1230.4 > 366.1
8.458e+005

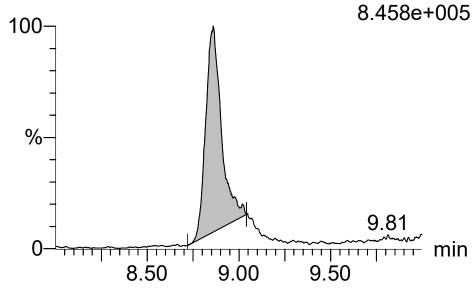

Sample Name: 20211111_QC_R13_IgM
F48:MRM of 2 channels,ES+
984.5 > 204.1
6.140e+006

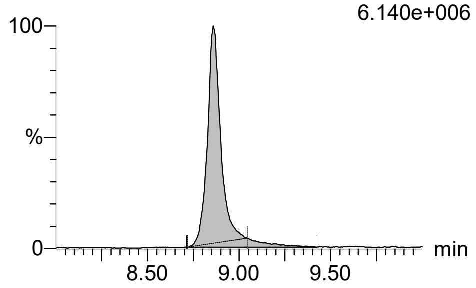

Sample Name: 20211111_QC_R13_IgM
F48:MRM of 2 channels,ES+
1230.4 > 366.1
1.806e+006

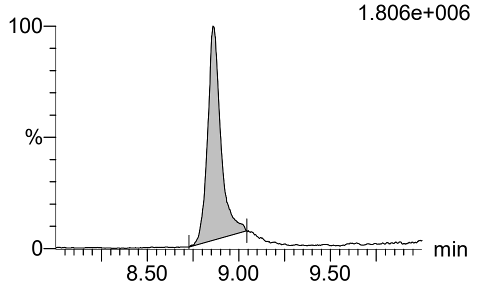

Sample Name: 20211111_L20_R2_IgM
F48:MRM of 2 channels,ES+
984.5 > 204.1
1.277e+006

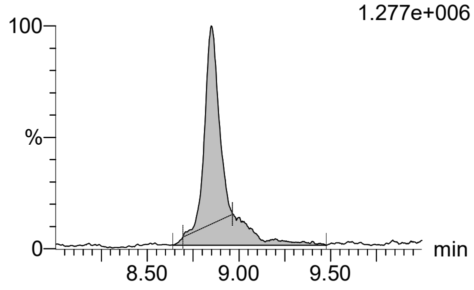

Sample Name: 20211111_L20_R2_IgM
F48:MRM of 2 channels,ES+
1230.4 > 366.1
3.853e+005

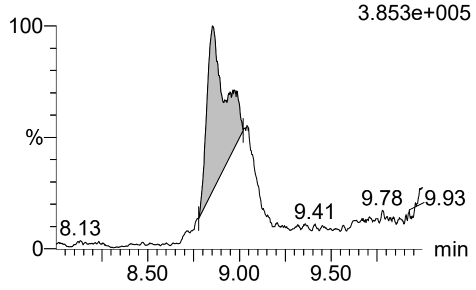

Sample Name: 20211111_H10_R2_IgM
F48:MRM of 2 channels,ES+
984.5 > 204.1
3.371e+006

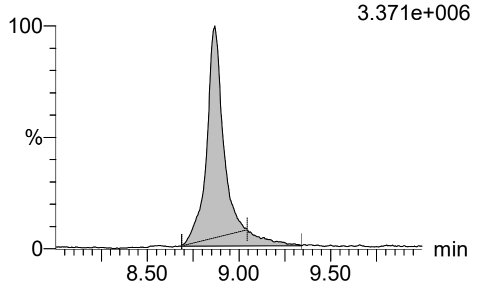

Sample Name: 20211111_H10_R2_IgM
F48:MRM of 2 channels,ES+
1230.4 > 366.1
9.743e+005

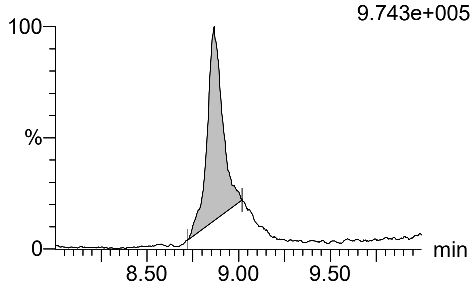

Sample Name: 20211111_A21_R2_IgM
F48:MRM of 2 channels,ES+
984.5 > 204.1
3.211e+006

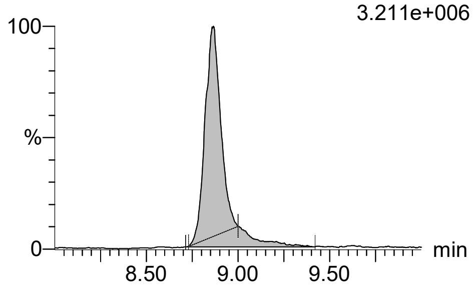

Sample Name: 20211111_A21_R2_IgM
F48:MRM of 2 channels,ES+
1230.4 > 366.1
9.248e+005

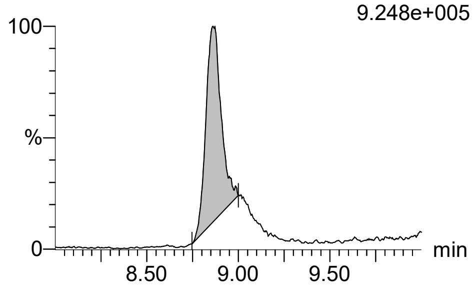

Sample Name: 20211111_A22_R2_IgM
F48:MRM of 2 channels,ES+
984.5 > 204.1
6.601e+005

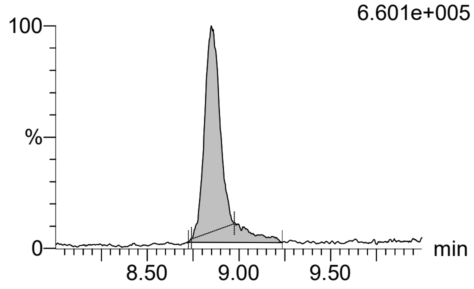

Sample Name: 20211111_A22_R2_IgM
F48:MRM of 2 channels,ES+
1230.4 > 366.1
1.886e+005

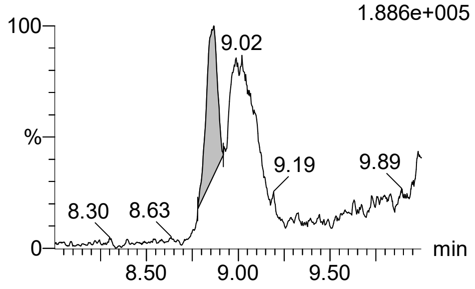

Sample Name: 20211111_L21_R2_IgM
F48:MRM of 2 channels,ES+
984.5 > 204.1
5.349e+006

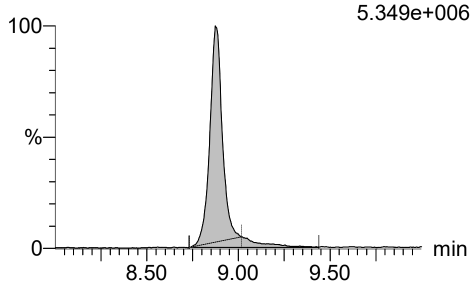

Sample Name: 20211111_L21_R2_IgM
F48:MRM of 2 channels,ES+
1230.4 > 366.1
1.607e+006

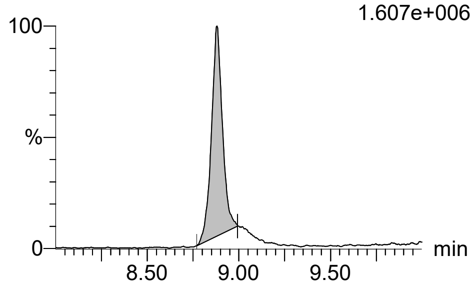

Sample Name: 20211111_L22_R2_IgM
F48:MRM of 2 channels,ES+
984.5 > 204.1
4.366e+006

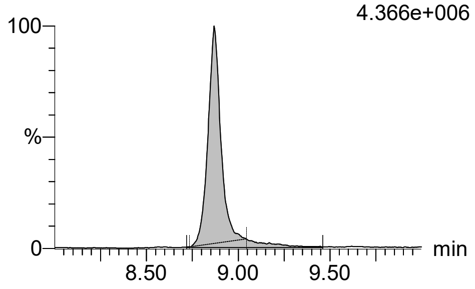

Sample Name: 20211111_L22_R2_IgM
F48:MRM of 2 channels,ES+
1230.4 > 366.1
1.125e+006

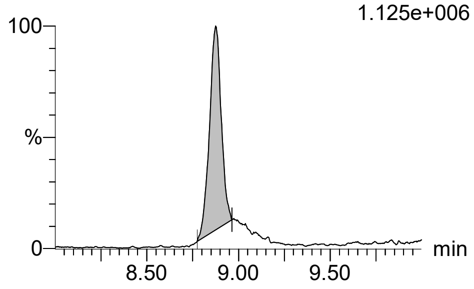

Sample Name: 20211111_H11_R2_IgM
F48:MRM of 2 channels,ES+
984.5 > 204.1
7.148e+006

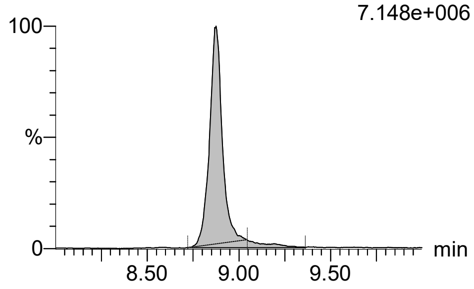

Sample Name: 20211111_H11_R2_IgM
F48:MRM of 2 channels,ES+
1230.4 > 366.1
1.983e+006

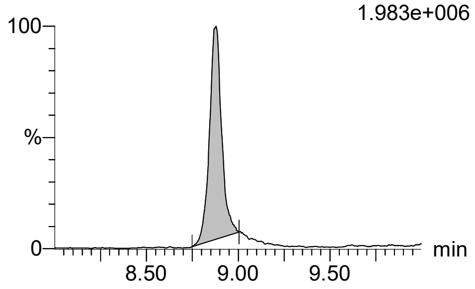

Sample Name: 20211111_A23_R2_IgM
F48:MRM of 2 channels,ES+
984.5 > 204.1
2.909e+006

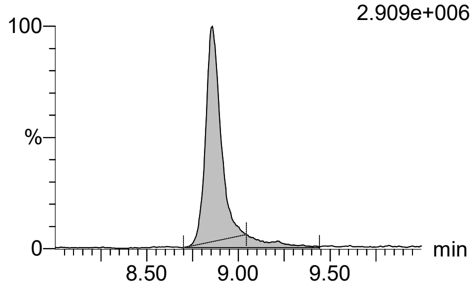

Sample Name: 20211111_A23_R2_IgM
F48:MRM of 2 channels,ES+
1230.4 > 366.1
8.788e+005

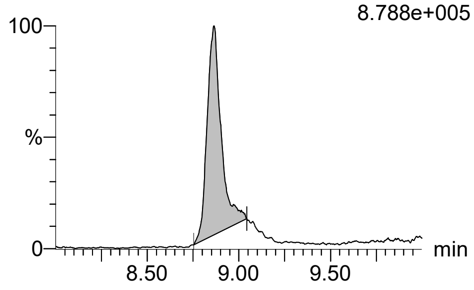

Sample Name: 20211111_A24_R2_IgM
F48:MRM of 2 channels,ES+
984.5 > 204.1
7.415e+006

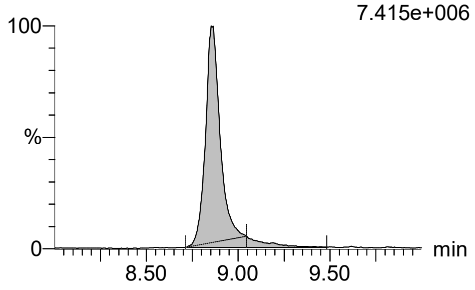

Sample Name: 20211111_A24_R2_IgM
F48:MRM of 2 channels,ES+
1230.4 > 366.1
2.268e+006

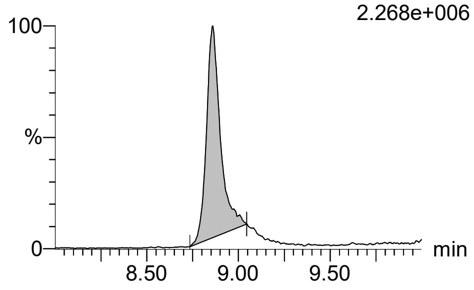

Dataset: C:\MassLynx\10.pro\20211111_clinicalsamples_IgM_IgA.qld

Last Altered: Thursday, March 09, 2023 19:58:25 台北標準時間
Printed: Friday, August 01, 2025 15:04:17 台北標準時間

Sample Name: 20211111_L23_R2_IgM
F48:MRM of 2 channels,ES+
984.5 > 204.1
6.348e+006

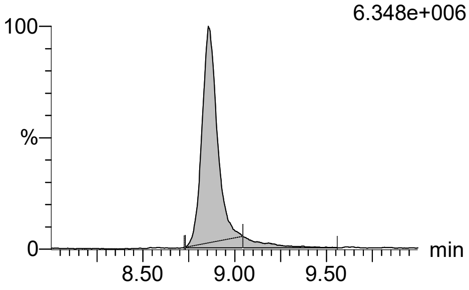

Sample Name: 20211111_L23_R2_IgM
F48:MRM of 2 channels,ES+
1230.4 > 366.1
1.832e+006

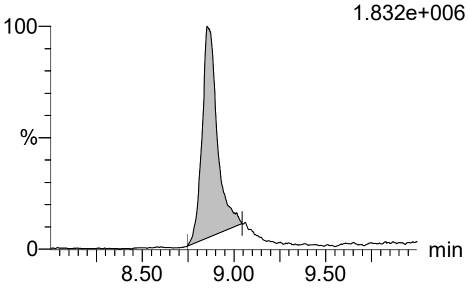

Sample Name: 20211111_L24_R2_IgM
F48:MRM of 2 channels,ES+
984.5 > 204.1
7.775e+005

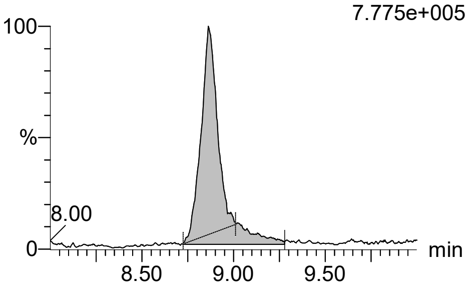

Sample Name: 20211111_L24_R2_IgM
F48:MRM of 2 channels,ES+
1230.4 > 366.1
2.300e+005

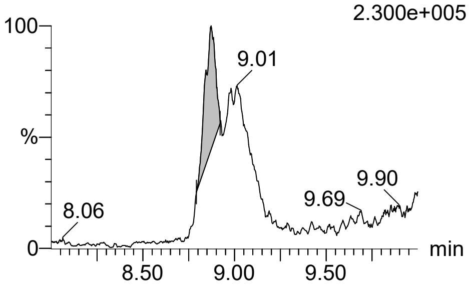

Sample Name: 20211111_H12_R2_IgM
F48:MRM of 2 channels,ES+
984.5 > 204.1
1.870e+006

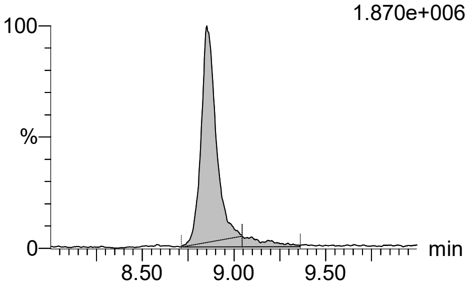

Sample Name: 20211111_H12_R2_IgM
F48:MRM of 2 channels,ES+
1230.4 > 366.1
5.200e+005

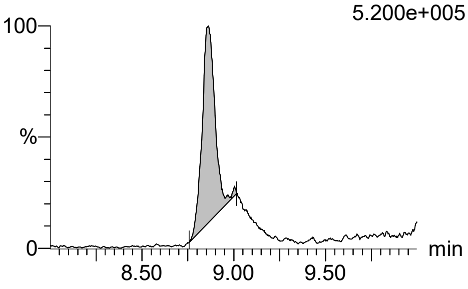

Sample Name: 20211111_QC_R14_IgM
F48:MRM of 2 channels,ES+
984.5 > 204.1
6.761e+006

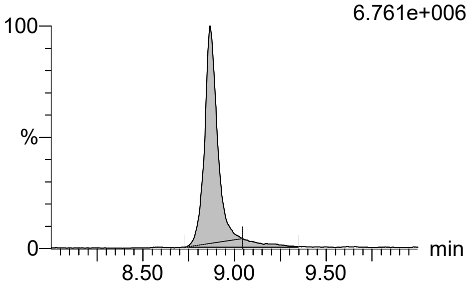

Sample Name: 20211111_QC_R14_IgM
F48:MRM of 2 channels,ES+
1230.4 > 366.1
1.987e+006

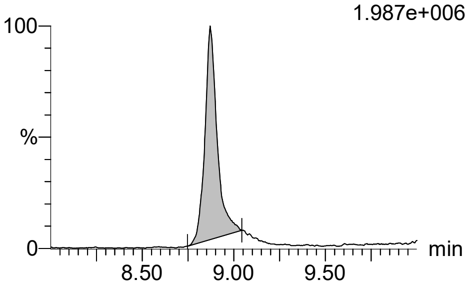

Sample Name: 20211111_A25_R2_IgM
F48:MRM of 2 channels,ES+
984.5 > 204.1
1.361e+007

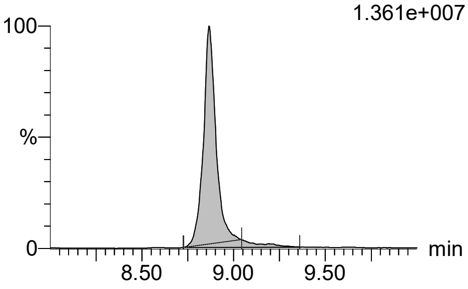

Sample Name: 20211111_A25_R2_IgM
F48:MRM of 2 channels,ES+
1230.4 > 366.1
4.028e+006

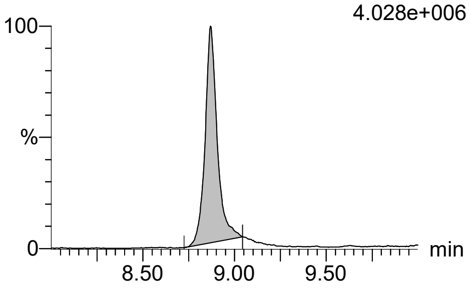

Sample Name: 20211111_A26_R2_IgM
F48:MRM of 2 channels,ES+
984.5 > 204.1
7.816e+006

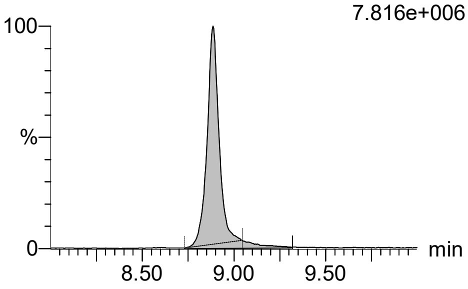

Sample Name: 20211111_A26_R2_IgM
F48:MRM of 2 channels,ES+
1230.4 > 366.1
2.106e+006

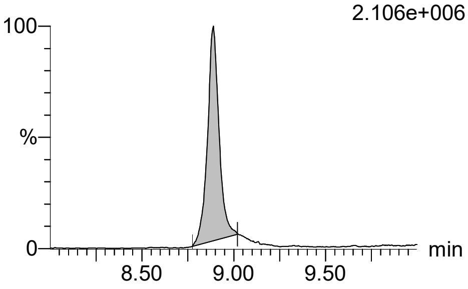

Sample Name: 20211111_L25_R2_IgM
F48:MRM of 2 channels,ES+
984.5 > 204.1
2.916e+006

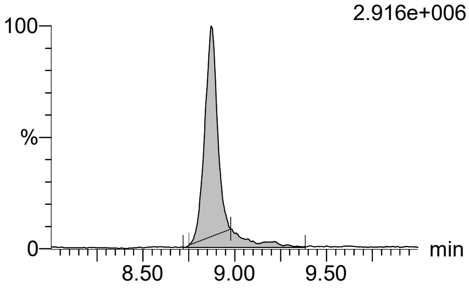

Sample Name: 20211111_L25_R2_IgM
F48:MRM of 2 channels,ES+
1230.4 > 366.1
8.461e+005

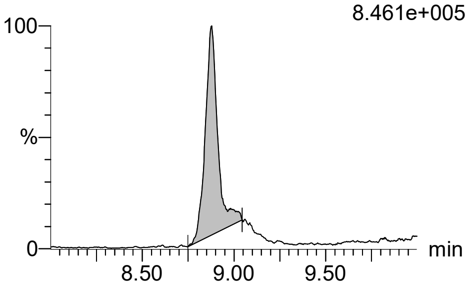

Sample Name: 20211111_L26_R2_IgM
F48:MRM of 2 channels,ES+
984.5 > 204.1
4.932e+006

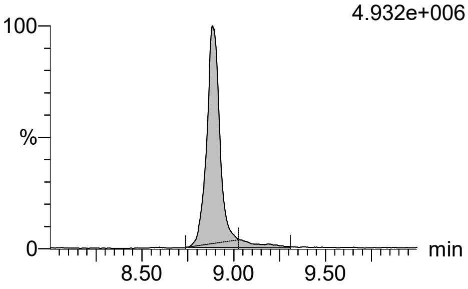

Sample Name: 20211111_L26_R2_IgM
F48:MRM of 2 channels,ES+
1230.4 > 366.1
1.365e+006

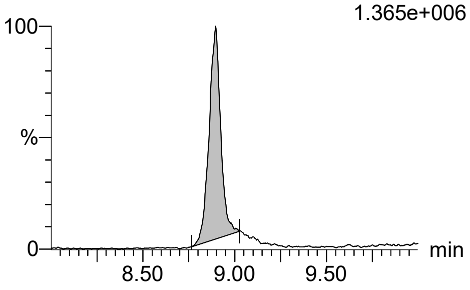

Sample Name: 20211111_H13_R2_IgM
F48:MRM of 2 channels,ES+
984.5 > 204.1
5.896e+006

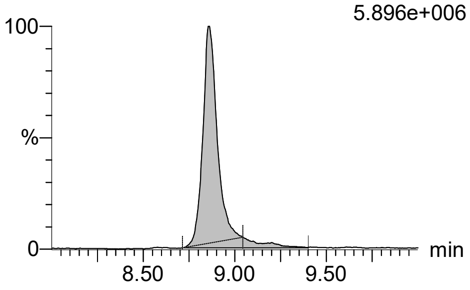

Sample Name: 20211111_H13_R2_IgM
F48:MRM of 2 channels,ES+
1230.4 > 366.1
1.776e+006

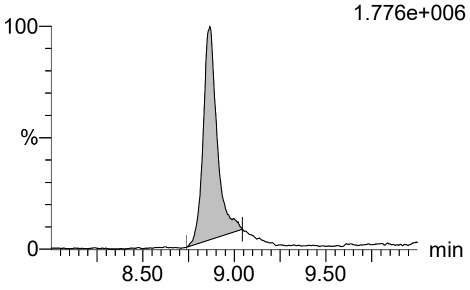

Sample Name: 20211111_A27_R2_IgM
F48:MRM of 2 channels,ES+
984.5 > 204.1
1.148e+007

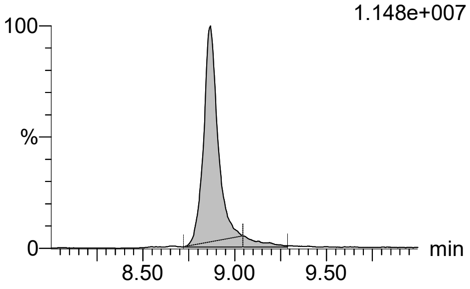

Sample Name: 20211111_A27_R2_IgM
F48:MRM of 2 channels,ES+
1230.4 > 366.1
3.517e+006

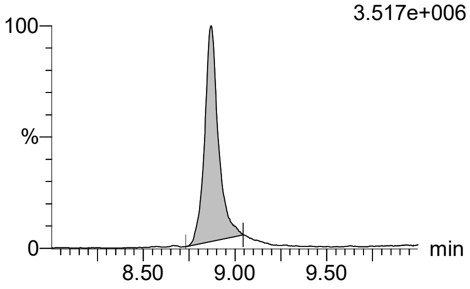

Sample Name: 20211111_A28_R2_IgM
F48:MRM of 2 channels,ES+
984.5 > 204.1
1.245e+006

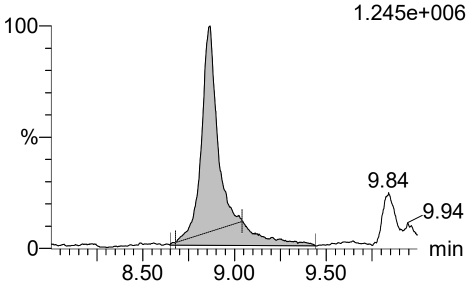

Sample Name: 20211111_A28_R2_IgM
F48:MRM of 2 channels,ES+
1230.4 > 366.1
3.591e+005

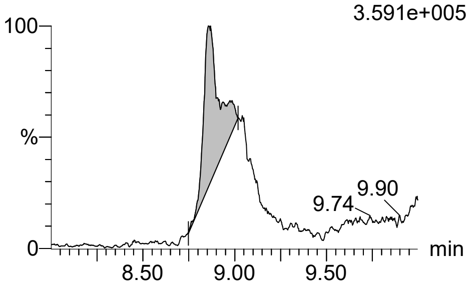

Sample Name: 20211111_L27_R2_IgM
F48:MRM of 2 channels,ES+
984.5 > 204.1
4.562e+006

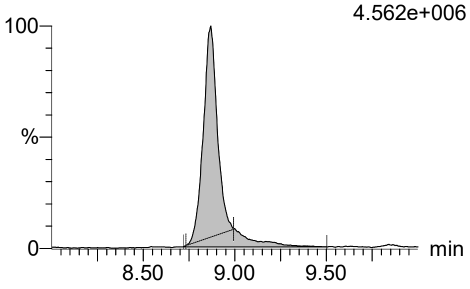

Sample Name: 20211111_L27_R2_IgM
F48:MRM of 2 channels,ES+
1230.4 > 366.1
1.300e+006

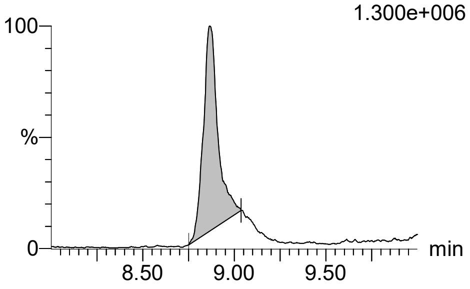

Sample Name: 20211111_L28_R2_IgM
F48:MRM of 2 channels,ES+
984.5 > 204.1
2.016e+006

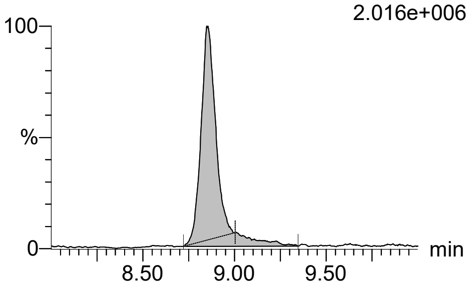

Sample Name: 20211111_L28_R2_IgM
F48:MRM of 2 channels,ES+
1230.4 > 366.1
5.457e+005

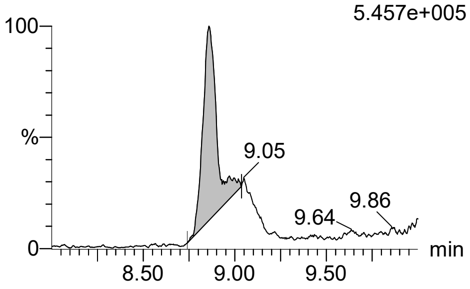

Sample Name: 20211111_H14_R2_IgM
F48:MRM of 2 channels,ES+
984.5 > 204.1
3.964e+006

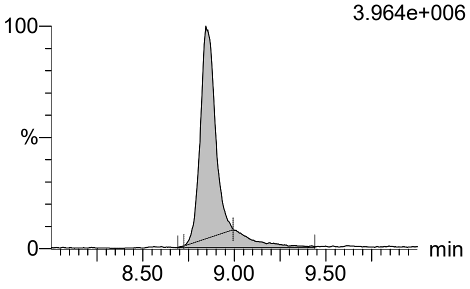

Sample Name: 20211111_H14_R2_IgM
F48:MRM of 2 channels,ES+
1230.4 > 366.1
1.144e+006

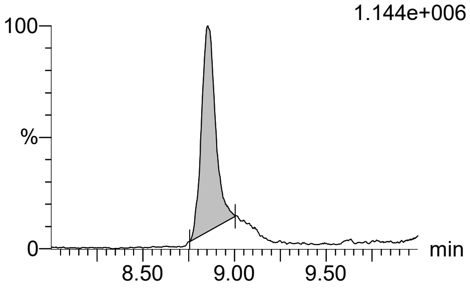

Sample Name: 20211111_A29_R2_IgM
F48:MRM of 2 channels,ES+
984.5 > 204.1
8.410e+005

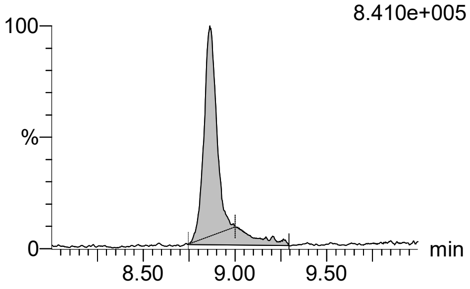

Sample Name: 20211111_A29_R2_IgM
F48:MRM of 2 channels,ES+
1230.4 > 366.1
2.371e+005

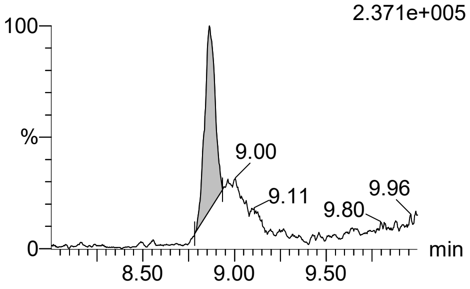

Sample Name: 20211111_A30_R2_IgM
F48:MRM of 2 channels,ES+
984.5 > 204.1
7.135e+006

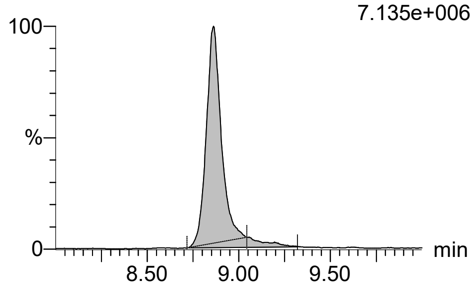

Sample Name: 20211111_A30_R2_IgM
F48:MRM of 2 channels,ES+
1230.4 > 366.1
2.083e+006

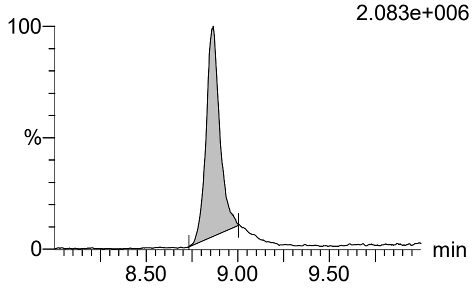

Sample Name: 20211111_QC_R15_IgM
F48:MRM of 2 channels,ES+
984.5 > 204.1
6.315e+006

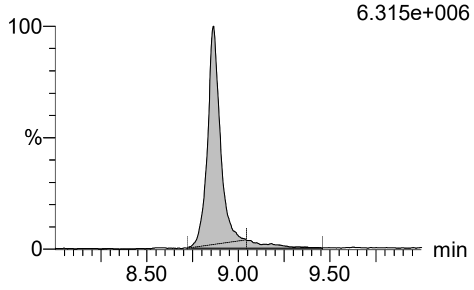

Sample Name: 20211111_QC_R15_IgM
F48:MRM of 2 channels,ES+
1230.4 > 366.1
1.720e+006

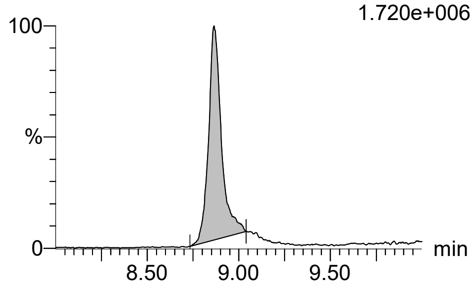

Sample Name: 20211111_L29_R2_IgM
F48:MRM of 2 channels,ES+
984.5 > 204.1
1.526e+006

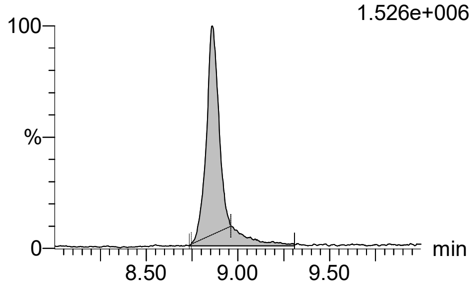

Sample Name: 20211111_L29_R2_IgM
F48:MRM of 2 channels,ES+
1230.4 > 366.1
4.292e+005

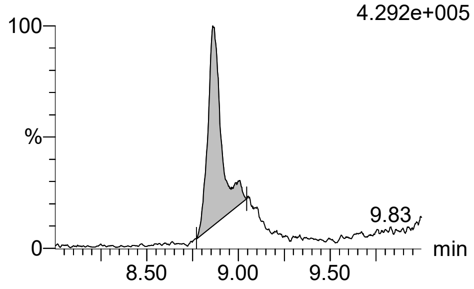

Sample Name: 20211111_L30_R2_IgM
F48:MRM of 2 channels,ES+
984.5 > 204.1
1.020e+006

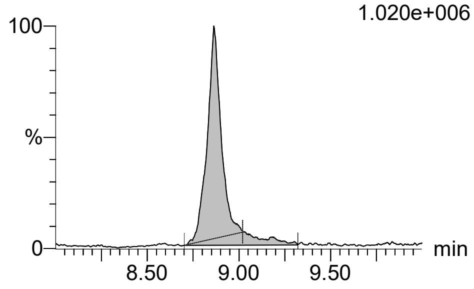

Sample Name: 20211111_L30_R2_IgM
F48:MRM of 2 channels,ES+
1230.4 > 366.1
2.941e+005

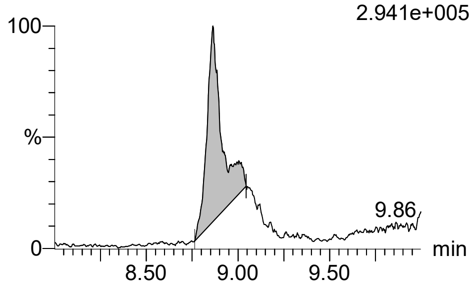

Sample Name: 20211111_H15_R2_IgM
F48:MRM of 2 channels,ES+
984.5 > 204.1
4.085e+006

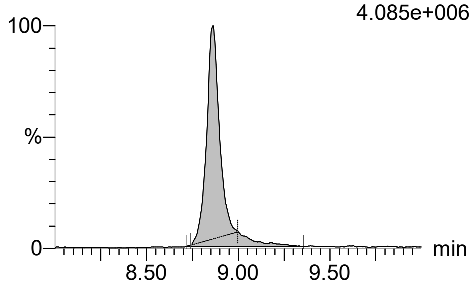

Sample Name: 20211111_H15_R2_IgM
F48:MRM of 2 channels,ES+
1230.4 > 366.1
1.169e+006

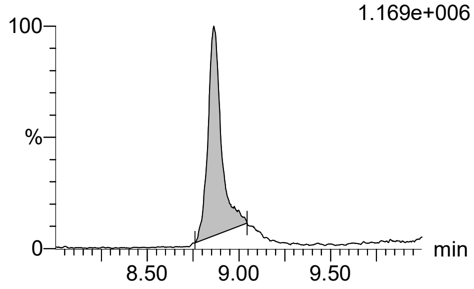

Sample Name: 20211111_A31_R2_IgM
F48:MRM of 2 channels,ES+
984.5 > 204.1
2.380e+006

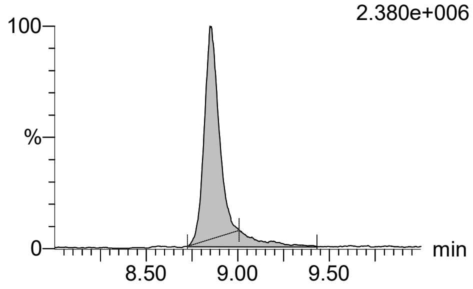

Sample Name: 20211111_A31_R2_IgM
F48:MRM of 2 channels,ES+
1230.4 > 366.1
7.191e+005

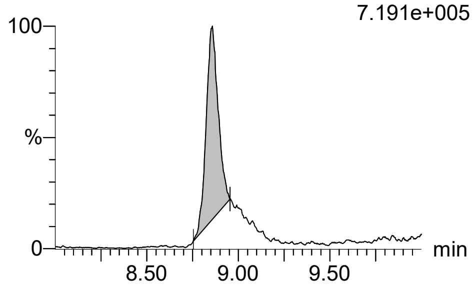

Sample Name: 20211111_A32_R2_IgM
F48:MRM of 2 channels,ES+
984.5 > 204.1
6.010e+006

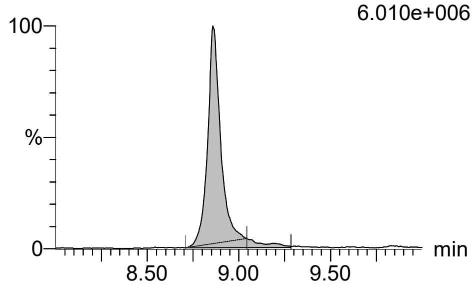

Sample Name: 20211111_A32_R2_IgM
F48:MRM of 2 channels,ES+
1230.4 > 366.1
1.712e+006

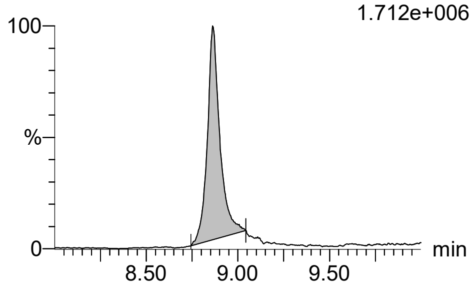

Sample Name: 20211111_L31_R2_IgM
F48:MRM of 2 channels,ES+
984.5 > 204.1
3.001e+005

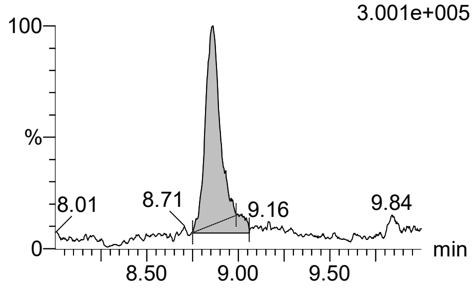

Sample Name: 20211111_L31_R2_IgM
F48:MRM of 2 channels,ES+
1230.4 > 366.1
1.215e+005

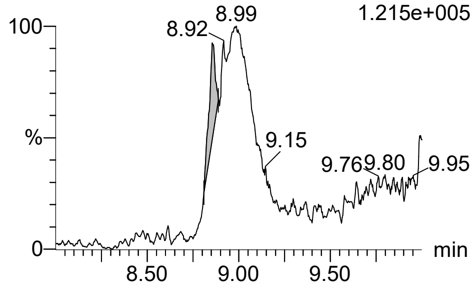

Sample Name: 20211111_L32_R2_IgM
F48:MRM of 2 channels,ES+
984.5 > 204.1
2.843e+006

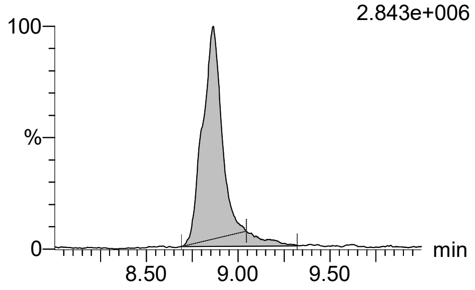

Sample Name: 20211111_L32_R2_IgM
F48:MRM of 2 channels,ES+
1230.4 > 366.1
8.527e+005

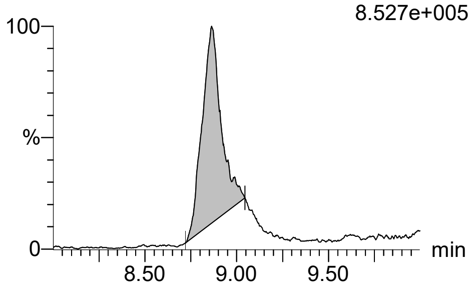

Sample Name: 20211111_H16_R2_IgM
F48:MRM of 2 channels,ES+
984.5 > 204.1
4.765e+006

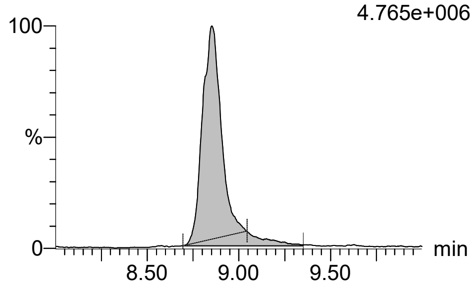

Sample Name: 20211111_H16_R2_IgM
F48:MRM of 2 channels,ES+
1230.4 > 366.1
1.450e+006

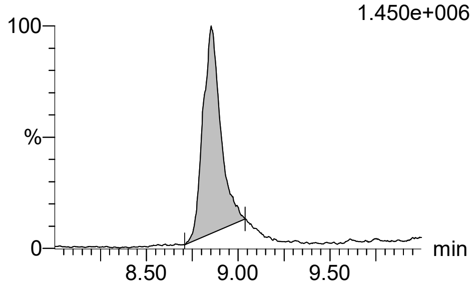

Sample Name: 20211111_A33_R2_IgM
F48:MRM of 2 channels,ES+
984.5 > 204.1
1.067e+007

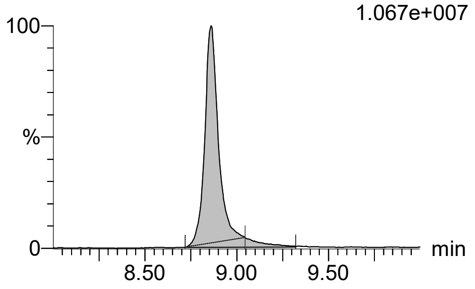

Sample Name: 20211111_A33_R2_IgM
F48:MRM of 2 channels,ES+
1230.4 > 366.1
3.302e+006

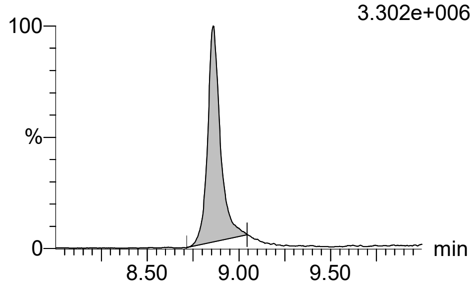

Sample Name: 20211111_A34_R2_IgM
F48:MRM of 2 channels,ES+
984.5 > 204.1
3.306e+006

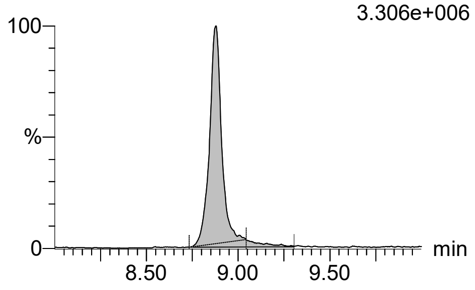

Sample Name: 20211111_A34_R2_IgM
F48:MRM of 2 channels,ES+
1230.4 > 366.1
8.649e+005

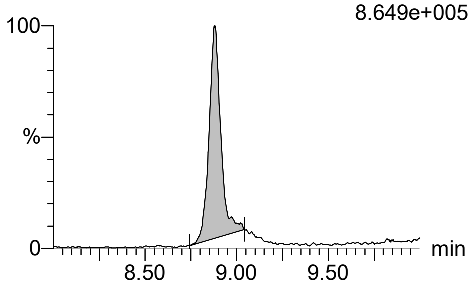

Sample Name: 20211111_H17_R2_IgM
F48:MRM of 2 channels,ES+
984.5 > 204.1
1.635e+007

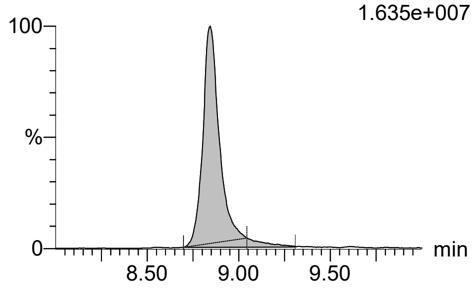

Sample Name: 20211111_H17_R2_IgM
F48:MRM of 2 channels,ES+
1230.4 > 366.1
4.841e+006

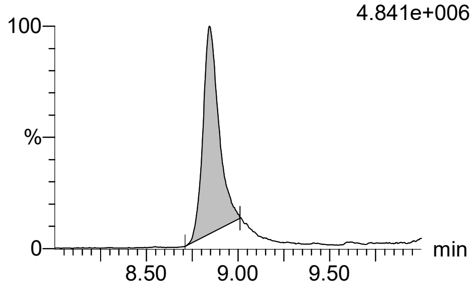

Sample Name: 20211111_A35_R2_IgM
F48:MRM of 2 channels,ES+
984.5 > 204.1
1.189e+006

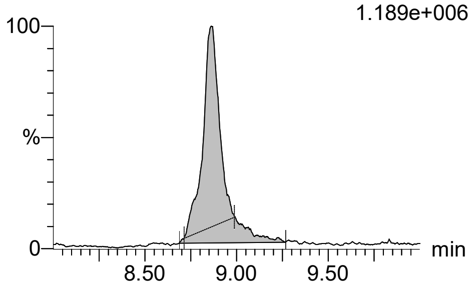

Sample Name: 20211111_A35_R2_IgM
F48:MRM of 2 channels,ES+
1230.4 > 366.1
3.849e+005

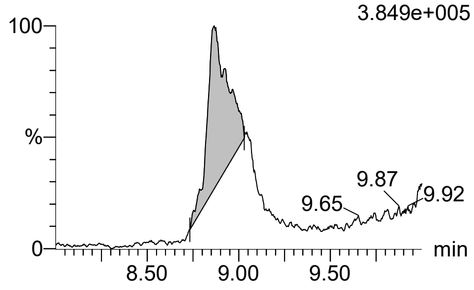

Sample Name: 20211111_QC_R16_IgM
F48:MRM of 2 channels,ES+
984.5 > 204.1
5.258e+006

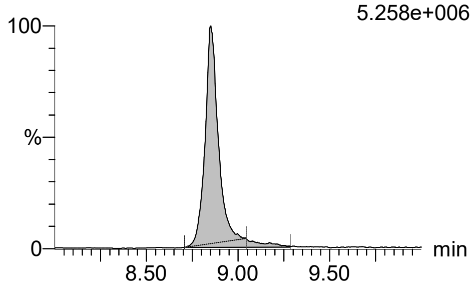

Sample Name: 20211111_QC_R16_IgM
F48:MRM of 2 channels,ES+
1230.4 > 366.1
1.509e+006

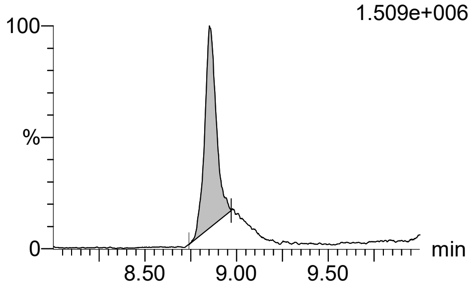

Dataset: C:\MassLynx\10.pro\20211111_clinicalsamples_IgM_IgA.qld

Last Altered: Thursday, March 09, 2023 19:58:25 台北標準時間
Printed: Friday, August 01, 2025 15:04:17 台北標準時間

Sample Name: 20211111_A36_R2_IgM
F48:MRM of 2 channels,ES+
984.5 > 204.1
1.446e+006

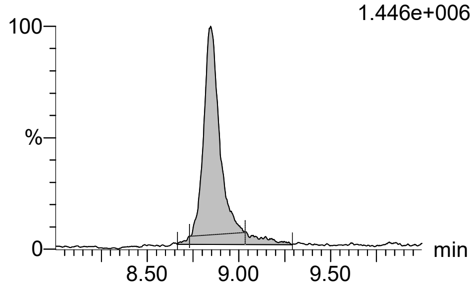

Sample Name: 20211111_A36_R2_IgM
F48:MRM of 2 channels,ES+
1230.4 > 366.1
4.172e+005

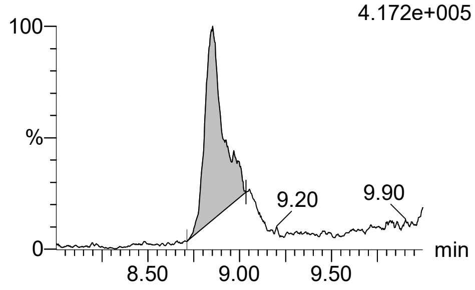

Sample Name: 20211111_H18_R2_IgM
F48:MRM of 2 channels,ES+
984.5 > 204.1
7.644e+006

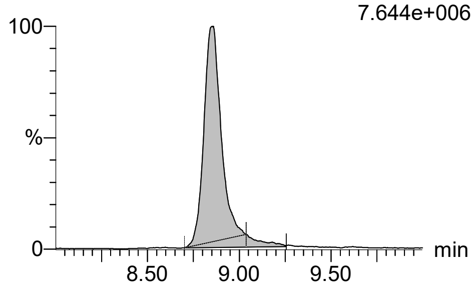

Sample Name: 20211111_H18_R2_IgM
F48:MRM of 2 channels,ES+
1230.4 > 366.1
2.415e+006

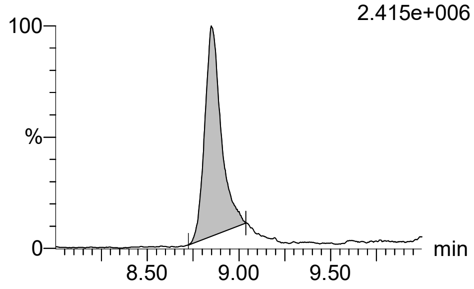

Sample Name: 20211111_A37_R2_IgM
F48:MRM of 2 channels,ES+
984.5 > 204.1
6.362e+006

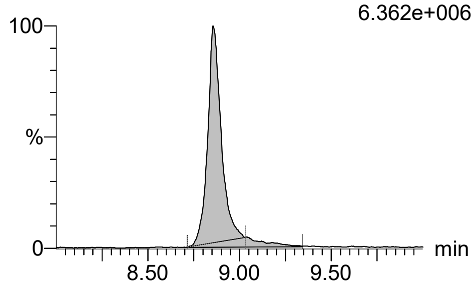

Sample Name: 20211111_A37_R2_IgM
F48:MRM of 2 channels,ES+
1230.4 > 366.1
1.871e+006

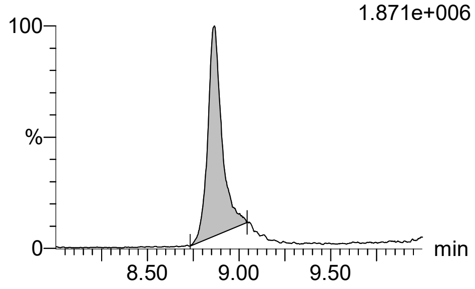

Sample Name: 20211111_A38_R2_IgM
F48:MRM of 2 channels,ES+
984.5 > 204.1
6.795e+006

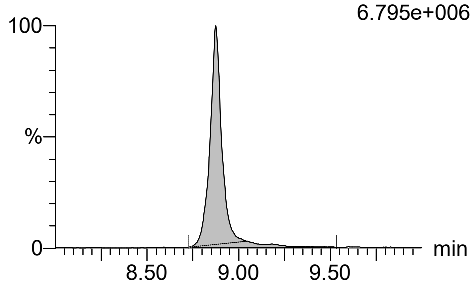

Sample Name: 20211111_A38_R2_IgM
F48:MRM of 2 channels,ES+
1230.4 > 366.1
1.757e+006

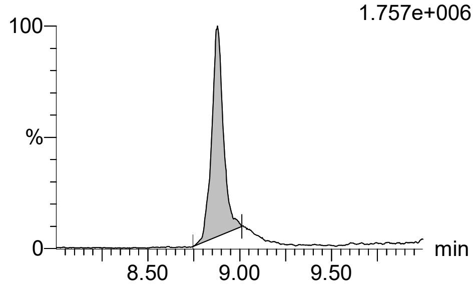

Sample Name: 20211111_H19_R2_IgM
F48:MRM of 2 channels,ES+
984.5 > 204.1
6.207e+006

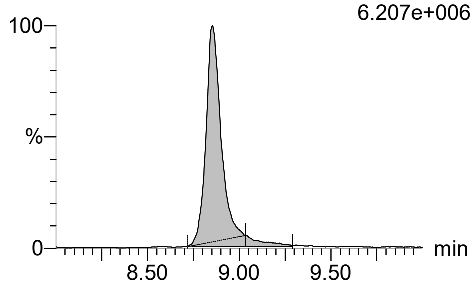

Sample Name: 20211111_H19_R2_IgM
F48:MRM of 2 channels,ES+
1230.4 > 366.1
1.848e+006

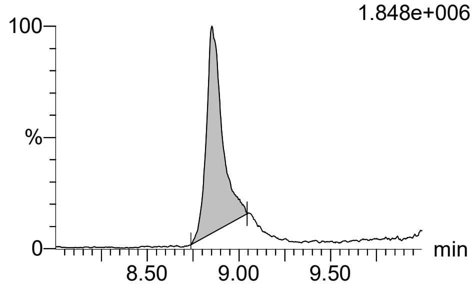

Sample Name: 20211111_A39_R2_IgM
F48:MRM of 2 channels,ES+
984.5 > 204.1
7.584e+006

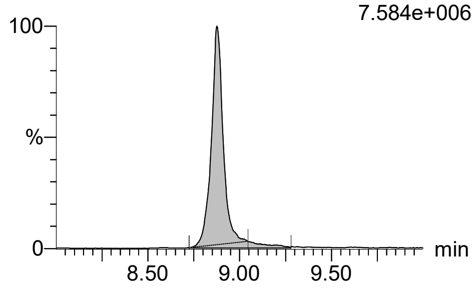

Sample Name: 20211111_A39_R2_IgM
F48:MRM of 2 channels,ES+
1230.4 > 366.1
2.016e+006

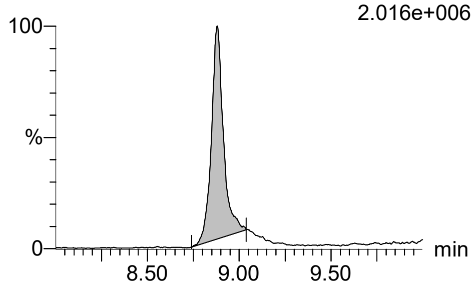

Sample Name: 20211111_A40_R2_IgM
F48:MRM of 2 channels,ES+
984.5 > 204.1
1.114e+006

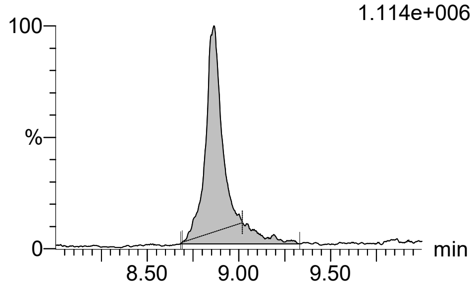

Sample Name: 20211111_A40_R2_IgM
F48:MRM of 2 channels,ES+
1230.4 > 366.1
3.642e+005

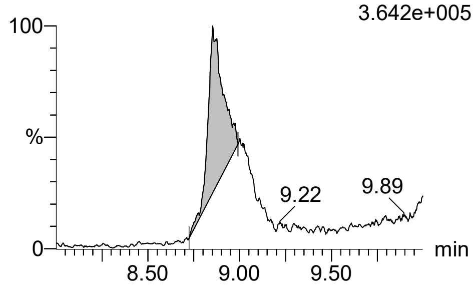

Sample Name: 20211111_H20_R2_IgM
F48:MRM of 2 channels,ES+
984.5 > 204.1
1.051e+007

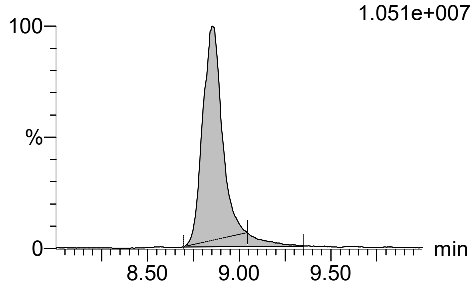

Dataset: C:\MassLynx\10.pro\20211111_clinicalsamples_IgM_IgA.qld

Last Altered: Thursday, March 09, 2023 19:58:25 台北標準時間
Printed: Friday, August 01, 2025 15:04:17 台北標準時間

Sample Name: 20211111_H20_R2_IgM
F48:MRM of 2 channels,ES+
1230.4 > 366.1
3.348e+006

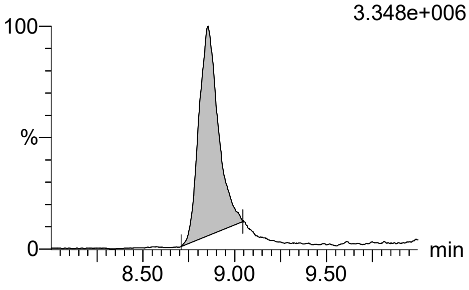

Sample Name: 20211111_A41_R2_IgM
F48:MRM of 2 channels,ES+
984.5 > 204.1
5.019e+006

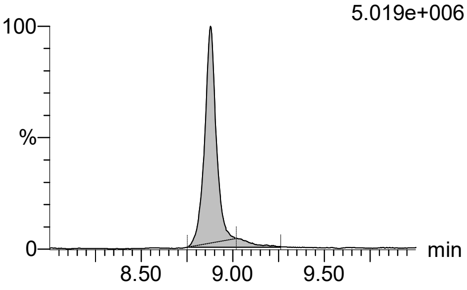

Sample Name: 20211111_A41_R2_IgM
F48:MRM of 2 channels,ES+
1230.4 > 366.1
1.419e+006

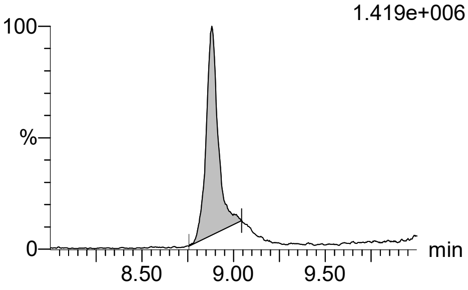

Sample Name: 20211111_A42_R2_IgM
F48:MRM of 2 channels,ES+
984.5 > 204.1
2.414e+006

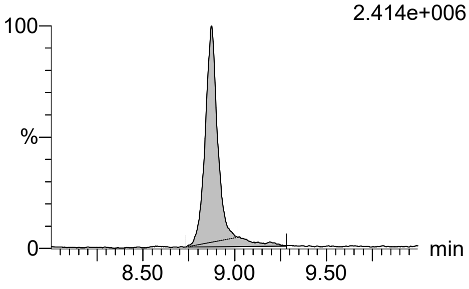

Sample Name: 20211111_A42_R2_IgM
F48:MRM of 2 channels,ES+
1230.4 > 366.1
6.871e+005

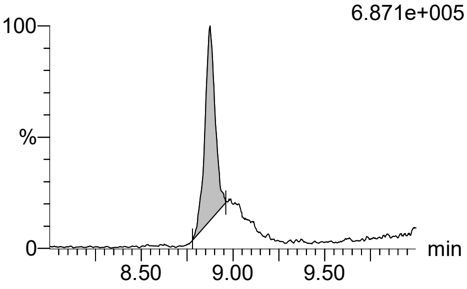

Sample Name: 20211111_A43_R2_IgM
F48:MRM of 2 channels,ES+
984.5 > 204.1
1.731e+006

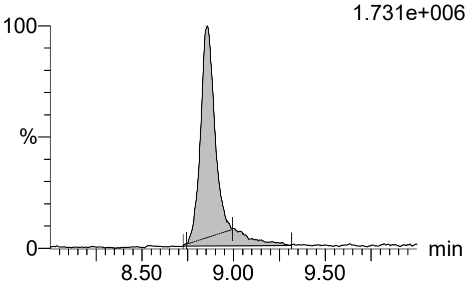

Sample Name: 20211111_A43_R2_IgM
F48:MRM of 2 channels,ES+
1230.4 > 366.1
5.171e+005

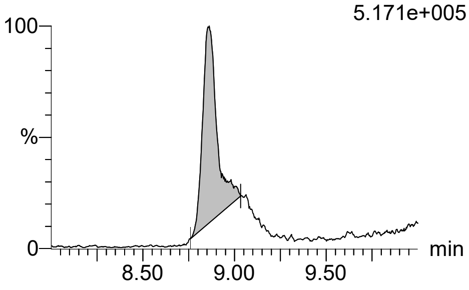

Sample Name: 20211111_A44_R2_IgM
F48:MRM of 2 channels,ES+
984.5 > 204.1
3.989e+005

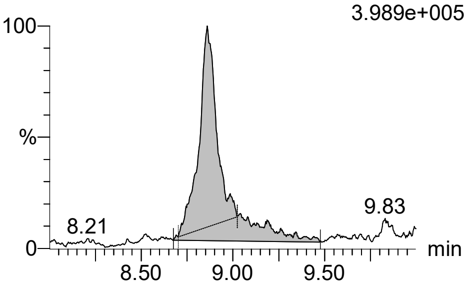

Sample Name: 20211111_A44_R2_IgM
F48:MRM of 2 channels,ES+
1230.4 > 366.1
3.116e+005

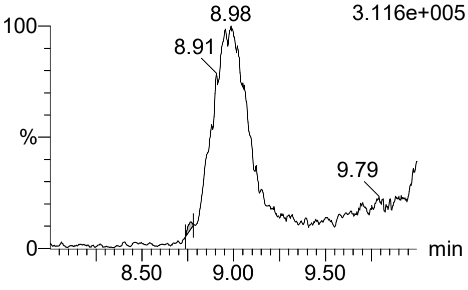

Sample Name: 20211111_QC_R17_IgM
F48:MRM of 2 channels,ES+
984.5 > 204.1
4.010e+006

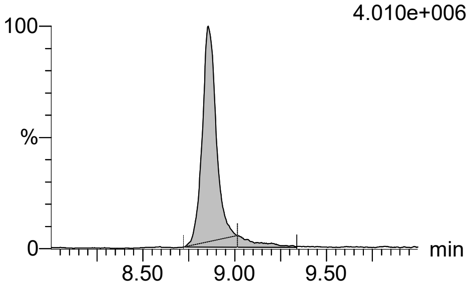

Sample Name: 20211111_QC_R17_IgM
F48:MRM of 2 channels,ES+
1230.4 > 366.1
1.232e+006

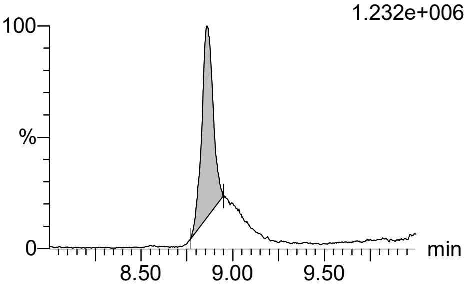

Sample Name: 20211111_L2_R3_IgM
F48:MRM of 2 channels,ES+
984.5 > 204.1
4.739e+006

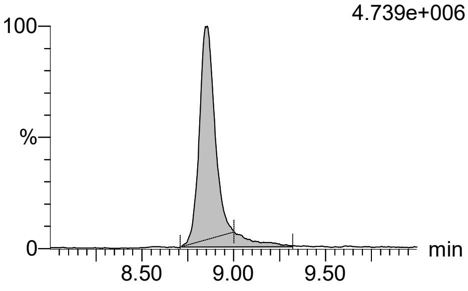

Sample Name: 20211111_L2_R3_IgM
F48:MRM of 2 channels,ES+
1230.4 > 366.1
1.427e+006

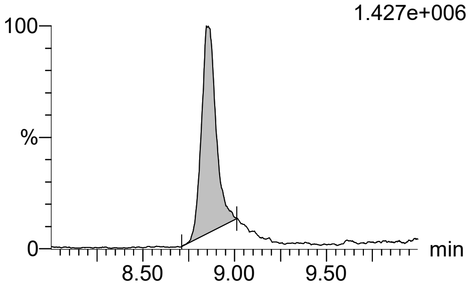

Sample Name: 20211111_H1_R3_IgM
F48:MRM of 2 channels,ES+
984.5 > 204.1
7.277e+006

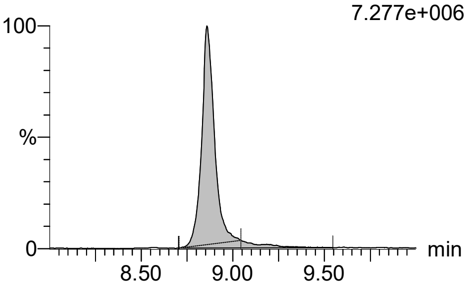

Sample Name: 20211111_H1_R3_IgM
F48:MRM of 2 channels,ES+
1230.4 > 366.1
2.018e+006

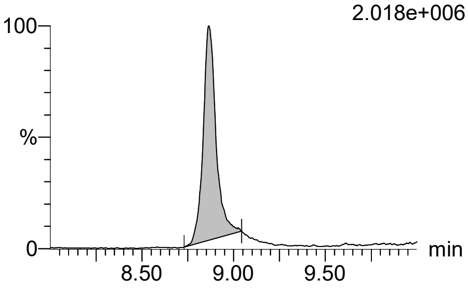

Dataset: C:\MassLynx\10.pro\20211111_clinicalsamples_IgM_IgA.qld

Last Altered: Thursday, March 09, 2023 19:58:25 台北標準時間
Printed: Friday, August 01, 2025 15:04:17 台北標準時間

Sample Name: 20211111_A3_R3_IgM
F48:MRM of 2 channels,ES+
984.5 > 204.1
1.040e+007

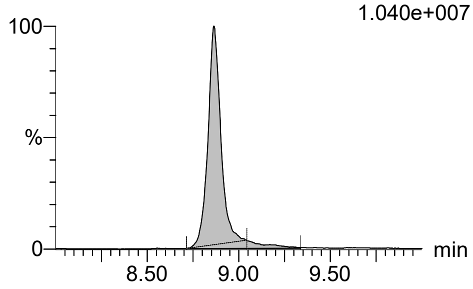

Sample Name: 20211111_A3_R3_IgM
F48:MRM of 2 channels,ES+
1230.4 > 366.1
2.980e+006

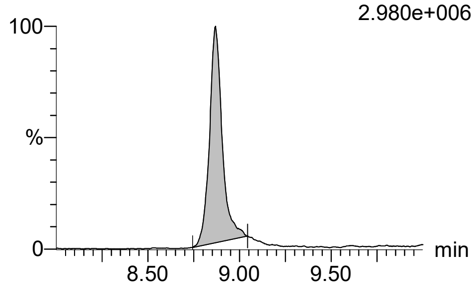

Sample Name: 20211111_A4_R3_IgM
F48:MRM of 2 channels,ES+
984.5 > 204.1
6.980e+006

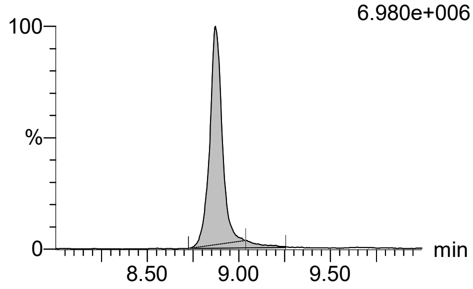

Sample Name: 20211111_A4_R3_IgM
F48:MRM of 2 channels,ES+
1230.4 > 366.1
1.951e+006

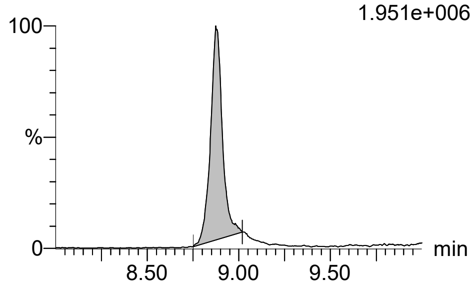

Sample Name: 20211111_L3_R3_IgM
F48:MRM of 2 channels,ES+
984.5 > 204.1
1.158e+006

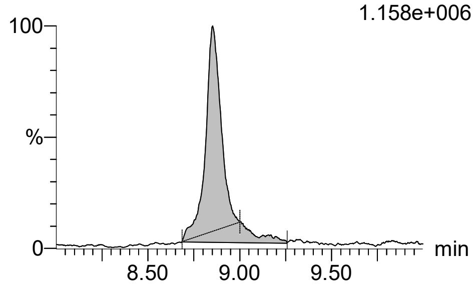

Sample Name: 20211111_L3_R3_IgM
F48:MRM of 2 channels,ES+
1230.4 > 366.1
3.381e+005

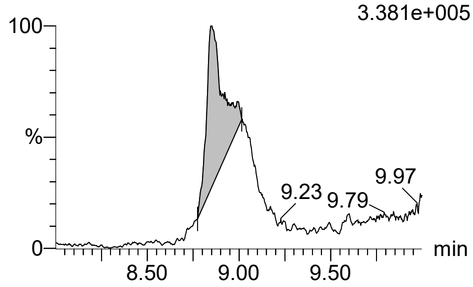

Sample Name: 20211111_L4_R3_IgM
F48:MRM of 2 channels,ES+
984.5 > 204.1
7.430e+006

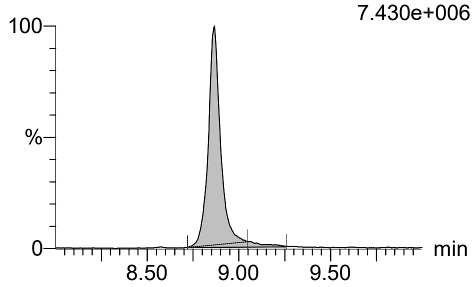

Sample Name: 20211111_L4_R3_IgM
F48:MRM of 2 channels,ES+
1230.4 > 366.1
2.027e+006

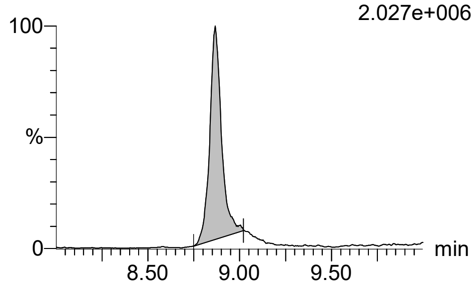

Sample Name: 20211111_H2_R3_IgM
F48:MRM of 2 channels,ES+
984.5 > 204.1
1.372e+007

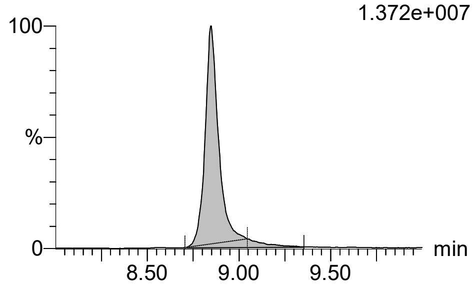

Sample Name: 20211111_H2_R3_IgM
F48:MRM of 2 channels,ES+
1230.4 > 366.1
4.077e+006

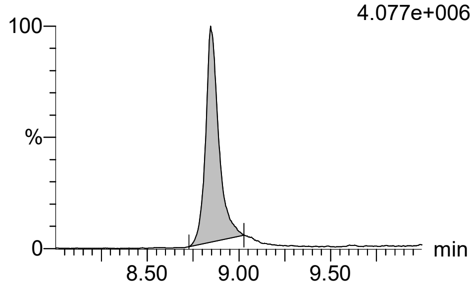

Sample Name: 20211111_A5_R3_IgM
F48:MRM of 2 channels,ES+
984.5 > 204.1
2.715e+007

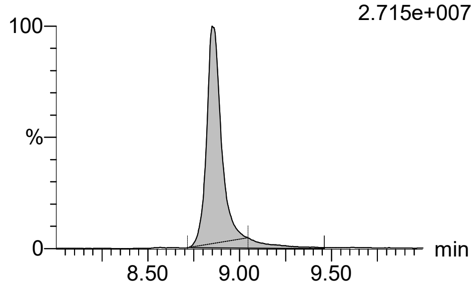

Sample Name: 20211111_A5_R3_IgM
F48:MRM of 2 channels,ES+
1230.4 > 366.1
8.540e+006

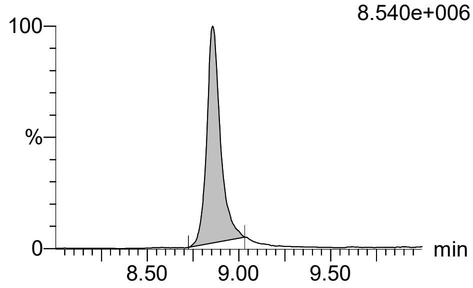

Sample Name: 20211111_A6_R3_IgM
F48:MRM of 2 channels,ES+
984.5 > 204.1
4.259e+006

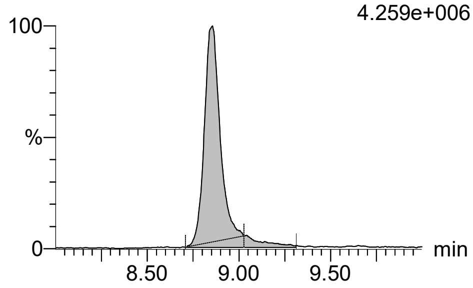

Sample Name: 20211111_A6_R3_IgM
F48:MRM of 2 channels,ES+
1230.4 > 366.1
1.367e+006

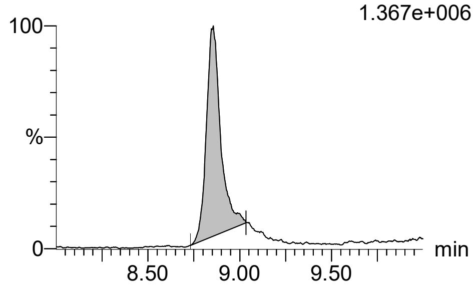

Sample Name: 20211111_QC_R18_IgM
F48:MRM of 2 channels,ES+
984.5 > 204.1
4.288e+006

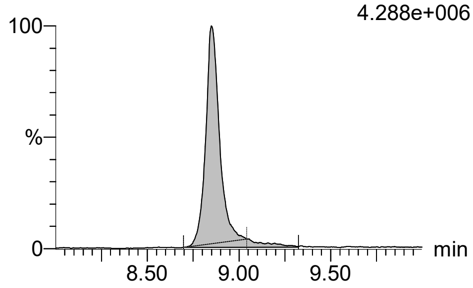

Sample Name: 20211111_QC_R18_IgM
F48:MRM of 2 channels,ES+
1230.4 > 366.1
1.280e+006

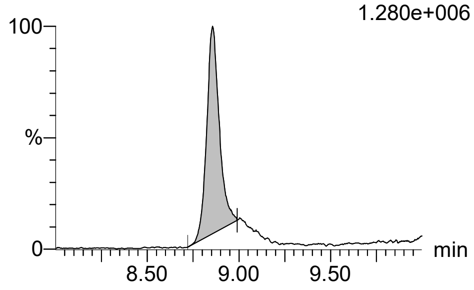

Sample Name: 20211111_L5_R3_IgM
F48:MRM of 2 channels,ES+
984.5 > 204.1
5.362e+006

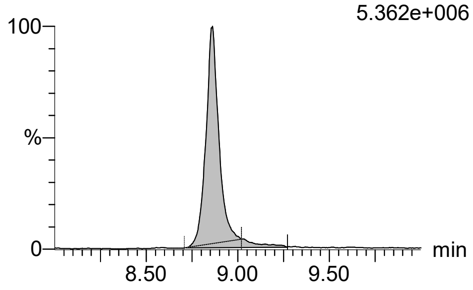

Sample Name: 20211111_L5_R3_IgM
F48:MRM of 2 channels,ES+
1230.4 > 366.1
1.523e+006

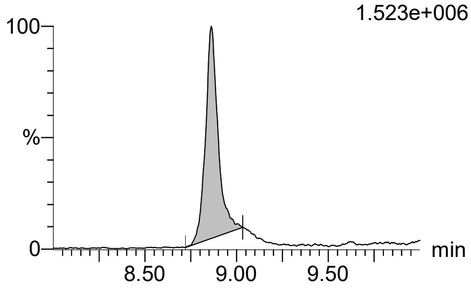

Sample Name: 20211111_L6_R3_IgM
F48:MRM of 2 channels,ES+
984.5 > 204.1
5.354e+006

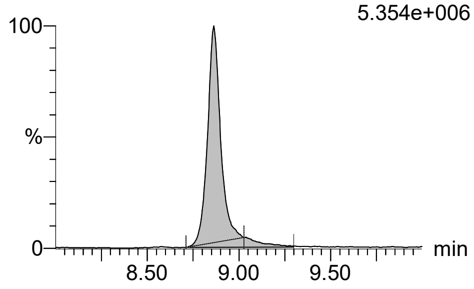

Sample Name: 20211111_L6_R3_IgM
F48:MRM of 2 channels,ES+
1230.4 > 366.1
1.495e+006

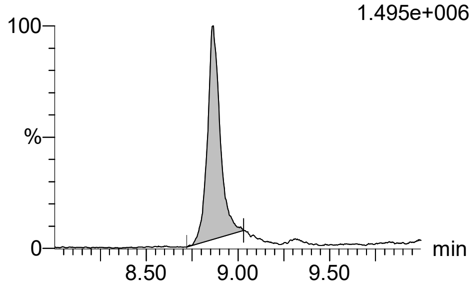

Sample Name: 20211111_H3_R3_IgM
F48:MRM of 2 channels,ES+
984.5 > 204.1
5.543e+006

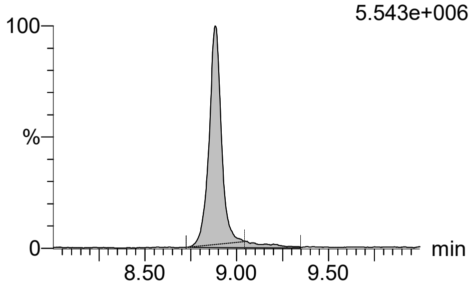

Sample Name: 20211111_H3_R3_IgM
F48:MRM of 2 channels,ES+
1230.4 > 366.1
1.372e+006

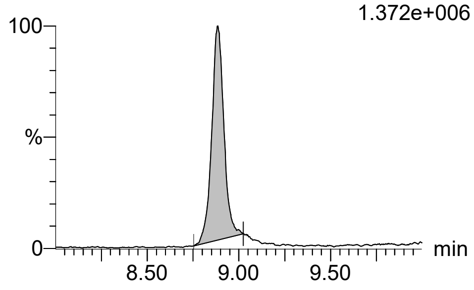

Sample Name: 20211111_A7_R3_IgM
F48:MRM of 2 channels,ES+
984.5 > 204.1
5.925e+006

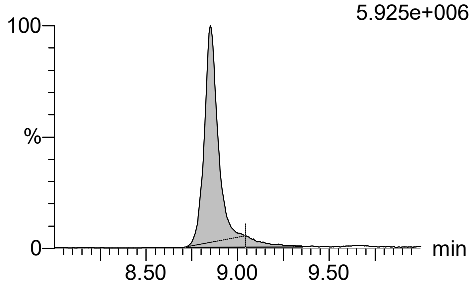

Sample Name: 20211111_A7_R3_IgM
F48:MRM of 2 channels,ES+
1230.4 > 366.1
1.749e+006

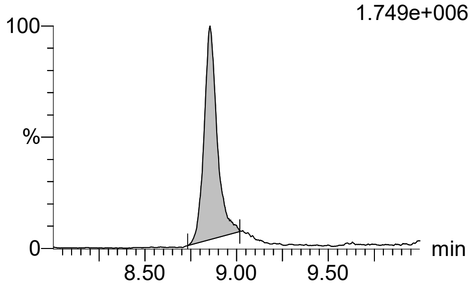

Sample Name: 20211111_A8_R3_IgM
F48:MRM of 2 channels,ES+
984.5 > 204.1
9.818e+006

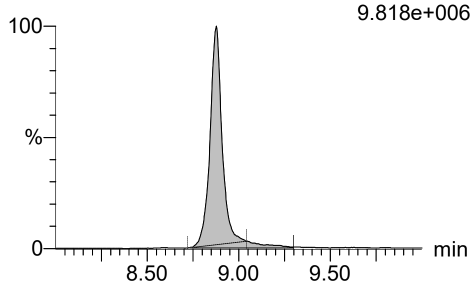

Sample Name: 20211111_A8_R3_IgM
F48:MRM of 2 channels,ES+
1230.4 > 366.1
2.677e+006

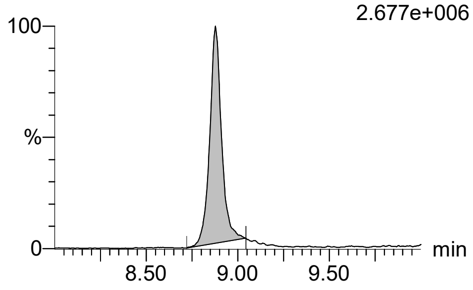

Sample Name: 20211111_L7_R3_IgM
F48:MRM of 2 channels,ES+
984.5 > 204.1
2.742e+006

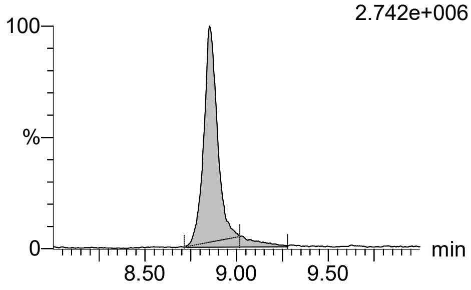

Sample Name: 20211111_L7_R3_IgM
F48:MRM of 2 channels,ES+
1230.4 > 366.1
7.948e+005

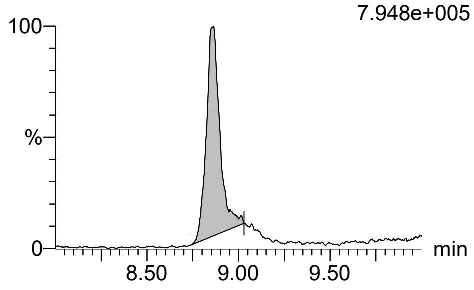

Sample Name: 20211111_L8_R3_IgM
F48:MRM of 2 channels,ES+
984.5 > 204.1
1.754e+006

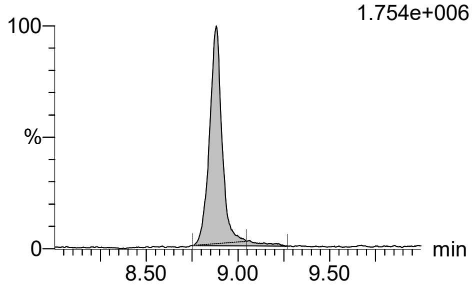

Sample Name: 20211111_L8_R3_IgM
F48:MRM of 2 channels,ES+
1230.4 > 366.1
4.274e+005

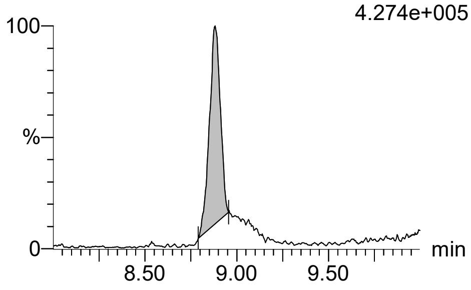

Sample Name: 20211111_H4_R3_IgM
F48:MRM of 2 channels,ES+
984.5 > 204.1
3.269e+006

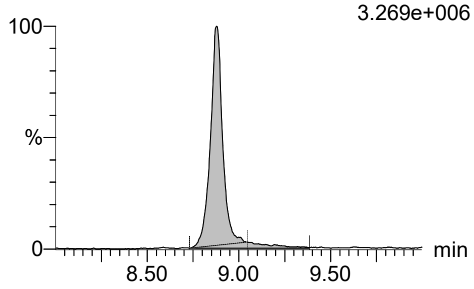

Sample Name: 20211111_H4_R3_IgM
F48:MRM of 2 channels,ES+
1230.4 > 366.1
8.215e+005

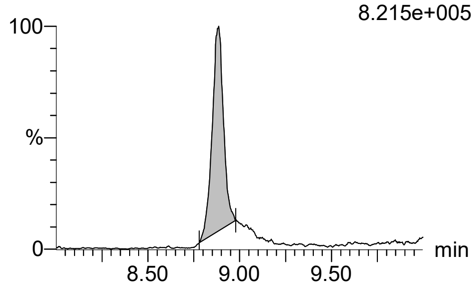

Sample Name: 20211111_A9_R3_IgM
F48:MRM of 2 channels,ES+
984.5 > 204.1
5.466e+006

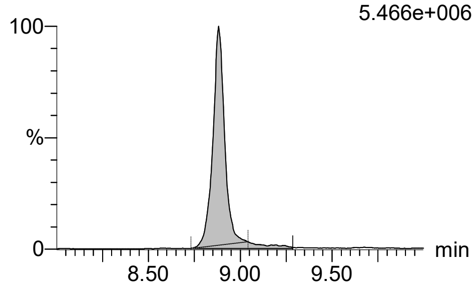

Sample Name: 20211111_A9_R3_IgM
F48:MRM of 2 channels,ES+
1230.4 > 366.1
1.330e+006

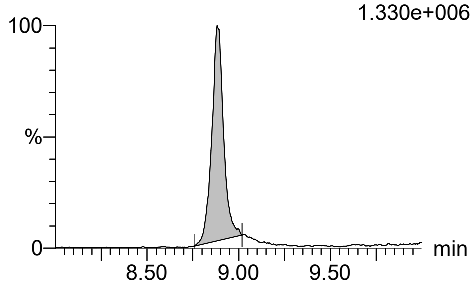

Sample Name: 20211111_A10_R3_IgM
F48:MRM of 2 channels,ES+
984.5 > 204.1
9.637e+006

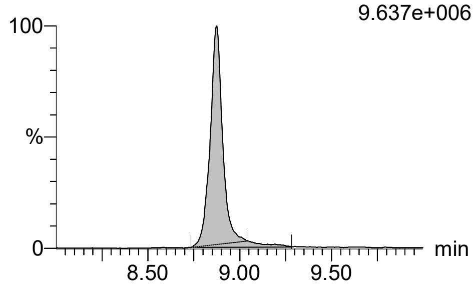

Sample Name: 20211111_A10_R3_IgM
F48:MRM of 2 channels,ES+
1230.4 > 366.1
2.628e+006

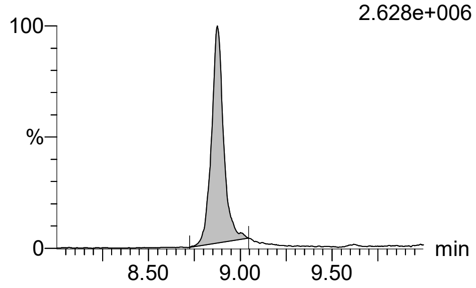

Sample Name: 20211111_L9_R3_IgM
F48:MRM of 2 channels,ES+
984.5 > 204.1
3.224e+006

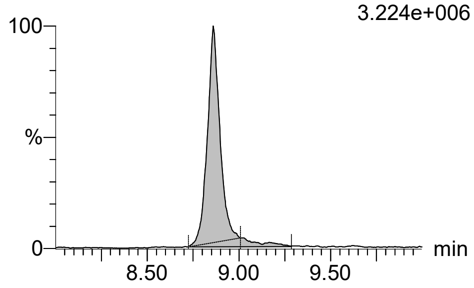

Sample Name: 20211111_L9_R3_IgM
F48:MRM of 2 channels,ES+
1230.4 > 366.1
8.485e+005

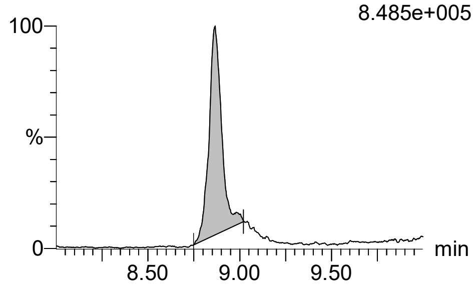

Sample Name: 20211111_L10_R3_IgM
F48:MRM of 2 channels,ES+
984.5 > 204.1
3.301e+006

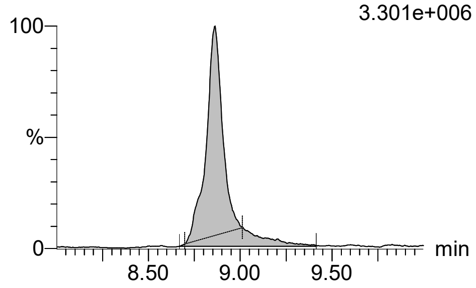

Sample Name: 20211111_L10_R3_IgM
F48:MRM of 2 channels,ES+
1230.4 > 366.1
9.470e+005

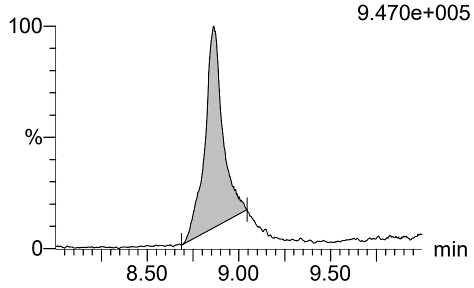

Sample Name: 20211111_QC_R19_IgM
F48:MRM of 2 channels,ES+
984.5 > 204.1
4.058e+006

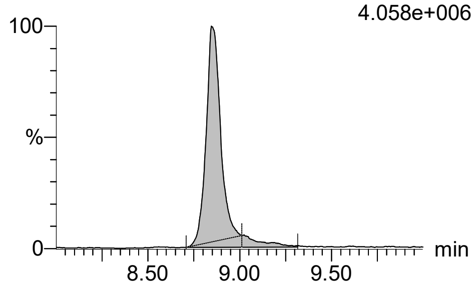

Sample Name: 20211111_QC_R19_IgM
F48:MRM of 2 channels,ES+
1230.4 > 366.1
1.256e+006

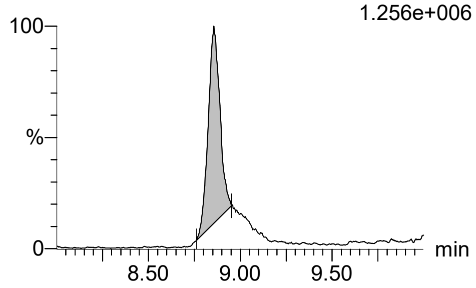

Sample Name: 20211111_H5_R3_IgM
F48:MRM of 2 channels,ES+
984.5 > 204.1
6.049e+006

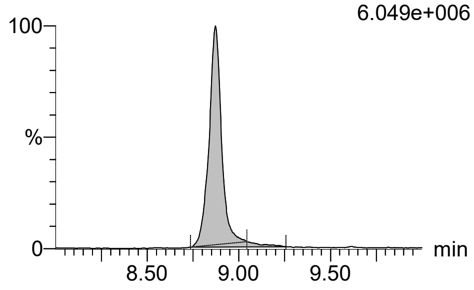

Sample Name: 20211111_H5_R3_IgM
F48:MRM of 2 channels,ES+
1230.4 > 366.1
1.654e+006

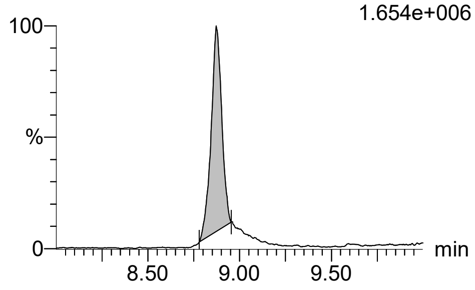

Sample Name: 20211111_A11_R3_IgM
F48:MRM of 2 channels,ES+
984.5 > 204.1
1.333e+007

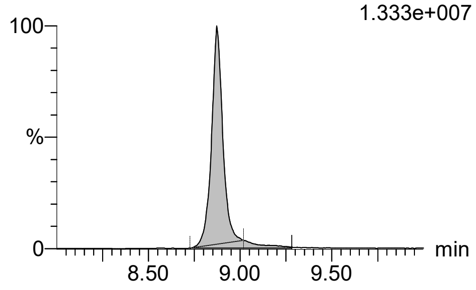

Dataset: C:\MassLynx\10.pro\20211111_clinicalsamples_IgM_IgA.qld

Last Altered: Thursday, March 09, 2023 19:58:25 台北標準時間
Printed: Friday, August 01, 2025 15:04:17 台北標準時間

Sample Name: 20211111_A11_R3_IgM
F48:MRM of 2 channels,ES+
1230.4 > 366.1
3.421e+006

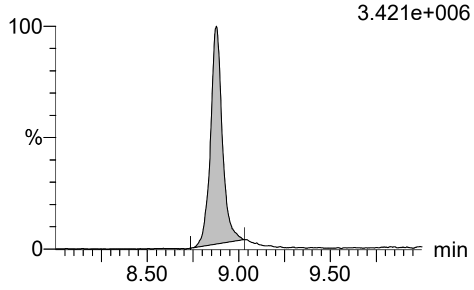

Sample Name: 20211111_A12_R3_IgM
F48:MRM of 2 channels,ES+
984.5 > 204.1
2.494e+006

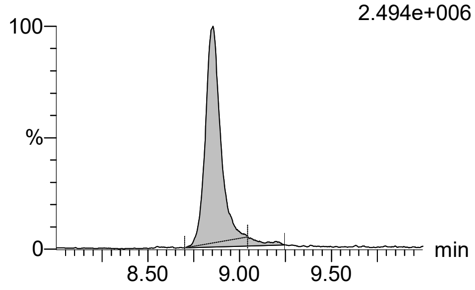

Sample Name: 20211111_A12_R3_IgM
F48:MRM of 2 channels,ES+
1230.4 > 366.1
7.626e+005

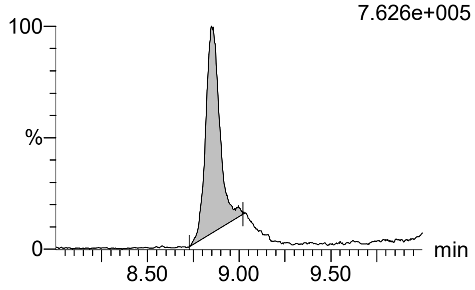

Sample Name: 20211111_L11_R3_IgM
F48:MRM of 2 channels,ES+
984.5 > 204.1
4.379e+006

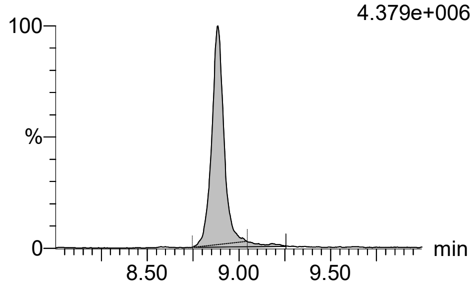

Sample Name: 20211111_L11_R3_IgM
F48:MRM of 2 channels,ES+
1230.4 > 366.1
1.074e+006

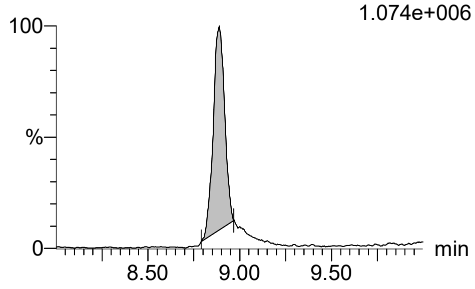

Sample Name: 20211111_L12_R3_IgM
F48:MRM of 2 channels,ES+
984.5 > 204.1
1.314e+006

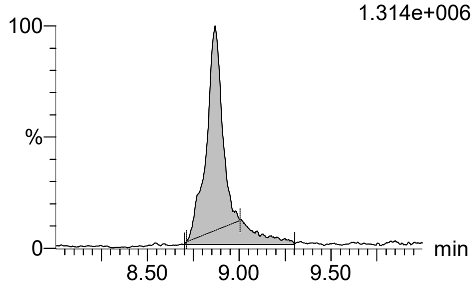

Sample Name: 20211111_L12_R3_IgM
F48:MRM of 2 channels,ES+
1230.4 > 366.1
3.746e+005

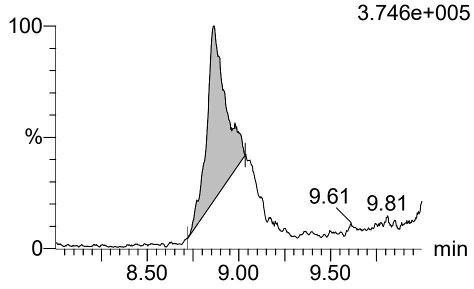

Sample Name: 20211111_H6_R3_IgM
F48:MRM of 2 channels,ES+
984.5 > 204.1
2.042e+006

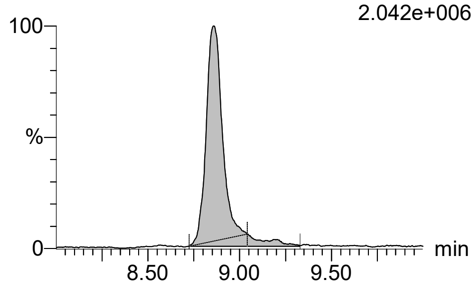

Sample Name: 20211111_H6_R3_IgM
F48:MRM of 2 channels,ES+
1230.4 > 366.1
6.667e+005

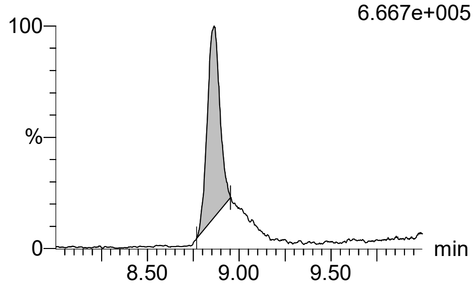

Sample Name: 20211111_A13_R3_IgM
F48:MRM of 2 channels,ES+
984.5 > 204.1
2.910e+006

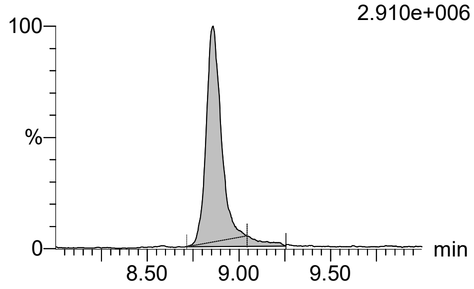

Sample Name: 20211111_A13_R3_IgM
F48:MRM of 2 channels,ES+
1230.4 > 366.1
9.191e+005

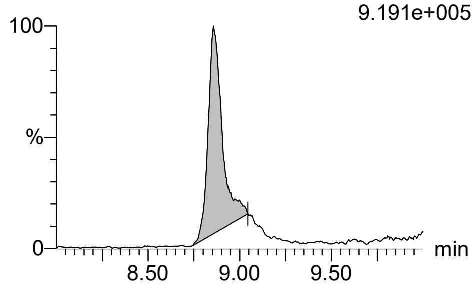

Sample Name: 20211111_A14_R3_IgM
F48:MRM of 2 channels,ES+
984.5 > 204.1
1.434e+007

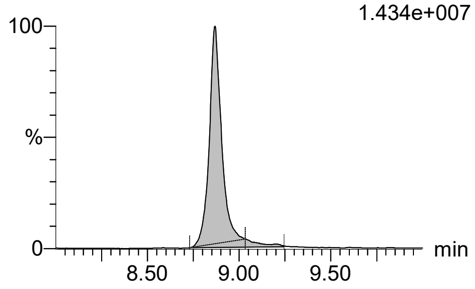

Sample Name: 20211111_A14_R3_IgM
F48:MRM of 2 channels,ES+
1230.4 > 366.1
4.325e+006

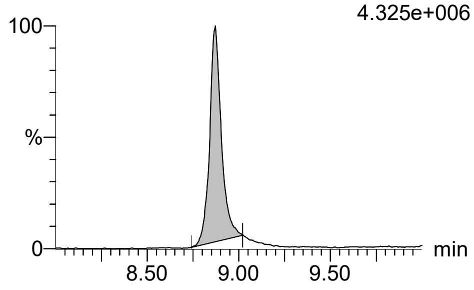

Sample Name: 20211111_L13_R3_IgM
F48:MRM of 2 channels,ES+
984.5 > 204.1
1.138e+006

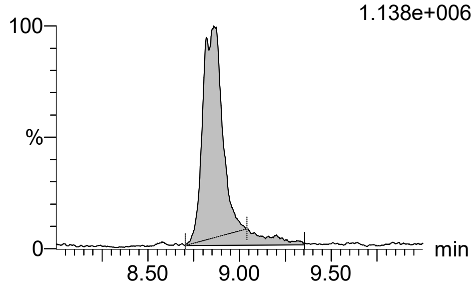

Sample Name: 20211111_L13_R3_IgM
F48:MRM of 2 channels,ES+
1230.4 > 366.1
3.823e+005

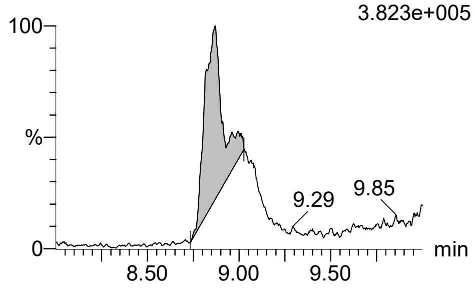

Dataset: C:\MassLynx\10.pro\20211111_clinicalsamples_IgM_IgA.qld

Last Altered: Thursday, March 09, 2023 19:58:25 台北標準時間
Printed: Friday, August 01, 2025 15:04:17 台北標準時間

Sample Name: 20211111_L14_R3_IgM
F48:MRM of 2 channels,ES+
984.5 > 204.1
4.287e+006

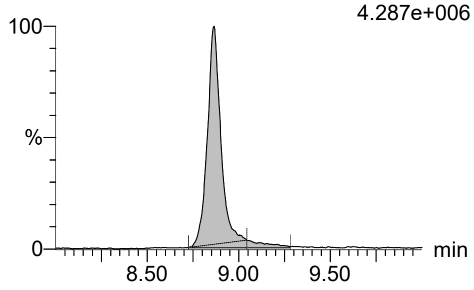

Sample Name: 20211111_L14_R3_IgM
F48:MRM of 2 channels,ES+
1230.4 > 366.1
1.246e+006

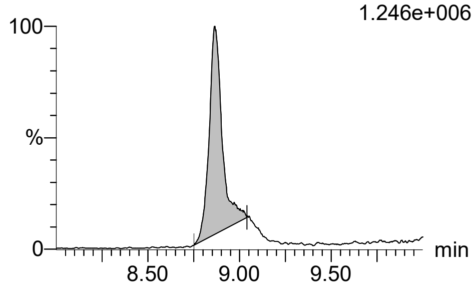

Sample Name: 20211111_H7_R3_IgM
F48:MRM of 2 channels,ES+
984.5 > 204.1
4.171e+006

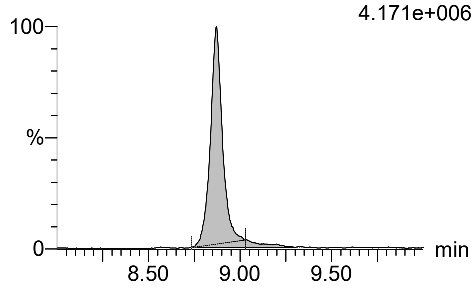

Sample Name: 20211111_H7_R3_IgM
F48:MRM of 2 channels,ES+
1230.4 > 366.1
1.152e+006

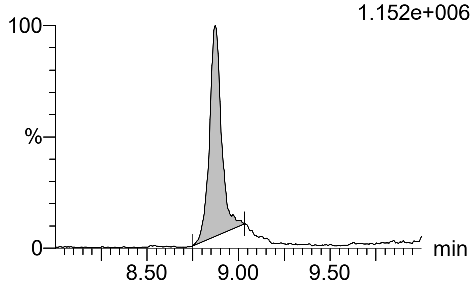

Sample Name: 20211111_A15_R3_IgM
F48:MRM of 2 channels,ES+
984.5 > 204.1
1.326e+007

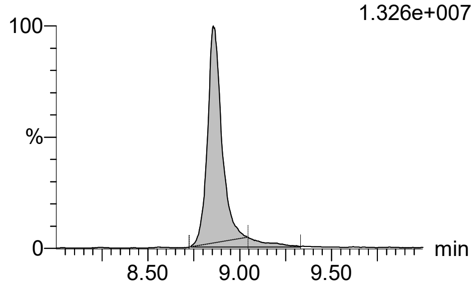

Sample Name: 20211111_A15_R3_IgM
F48:MRM of 2 channels,ES+
1230.4 > 366.1
4.062e+006

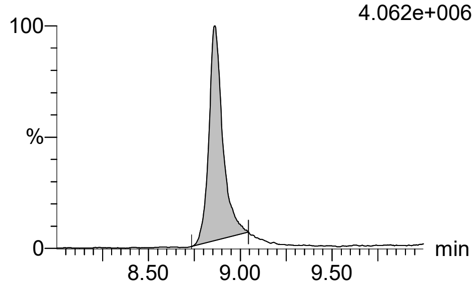

Sample Name: 20211111_QC_R20_IgM
F48:MRM of 2 channels,ES+
984.5 > 204.1
5.281e+006

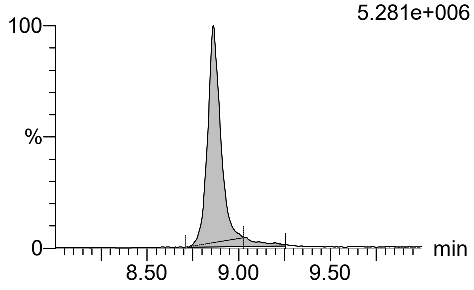

Sample Name: 20211111_QC_R20_IgM
F48:MRM of 2 channels,ES+
1230.4 > 366.1
1.502e+006

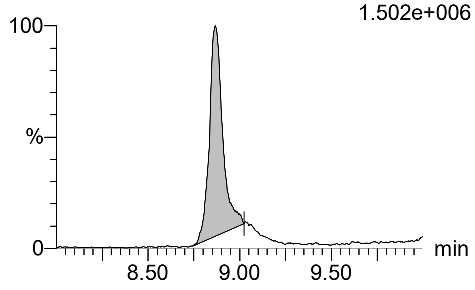

Sample Name: 20211111_A16_R3_IgM
F48:MRM of 2 channels,ES+
984.5 > 204.1
7.213e+006

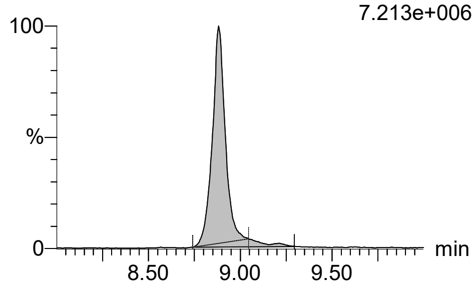

Sample Name: 20211111_A16_R3_IgM
F48:MRM of 2 channels,ES+
1230.4 > 366.1
2.130e+006

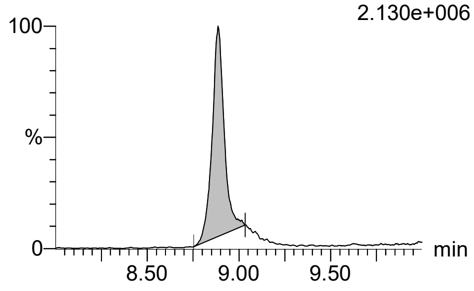

Sample Name: 20211111_L15_R3_IgM
F48:MRM of 2 channels,ES+
984.5 > 204.1
6.481e+006

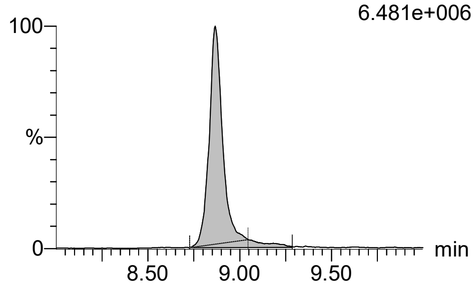

Sample Name: 20211111_L15_R3_IgM
F48:MRM of 2 channels,ES+
1230.4 > 366.1
1.957e+006

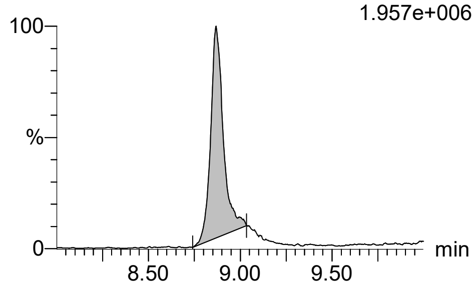

Sample Name: 20211111_L16_R3_IgM
F48:MRM of 2 channels,ES+
984.5 > 204.1
1.141e+006

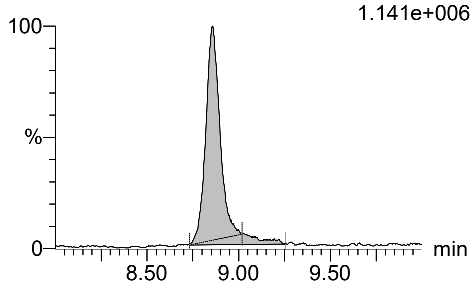

Sample Name: 20211111_L16_R3_IgM
F48:MRM of 2 channels,ES+
1230.4 > 366.1
3.462e+005

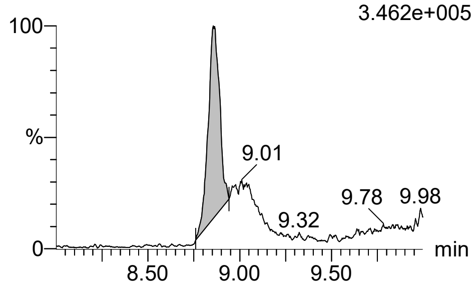

Sample Name: 20211111_H8_R3_IgM
F48:MRM of 2 channels,ES+
984.5 > 204.1
3.680e+006

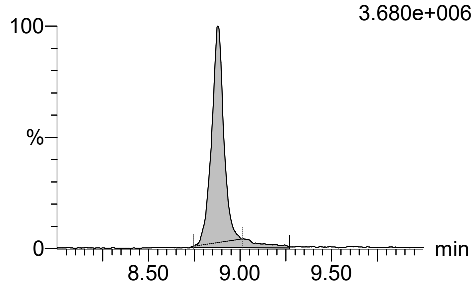

Dataset: C:\MassLynx\10.pro\20211111_clinicalsamples_IgM_IgA.qld

Last Altered: Thursday, March 09, 2023 19:58:25 台北標準時間
Printed: Friday, August 01, 2025 15:04:17 台北標準時間

Sample Name: 20211111_H8_R3_IgM
F48:MRM of 2 channels,ES+
1230.4 > 366.1
9.946e+005

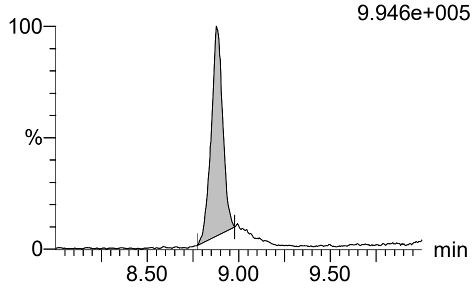

Sample Name: 20211111_A17_R3_IgM
F48:MRM of 2 channels,ES+
984.5 > 204.1
9.392e+006

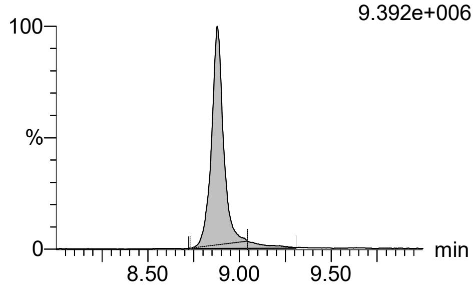

Sample Name: 20211111_A17_R3_IgM
F48:MRM of 2 channels,ES+
1230.4 > 366.1
2.804e+006

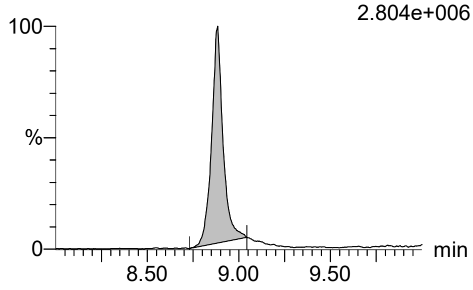

Sample Name: 20211111_A18_R3_IgM
F48:MRM of 2 channels,ES+
984.5 > 204.1
1.269e+006

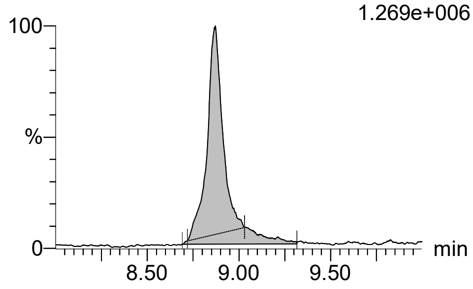

Sample Name: 20211111_A18_R3_IgM
F48:MRM of 2 channels,ES+
1230.4 > 366.1
3.995e+005

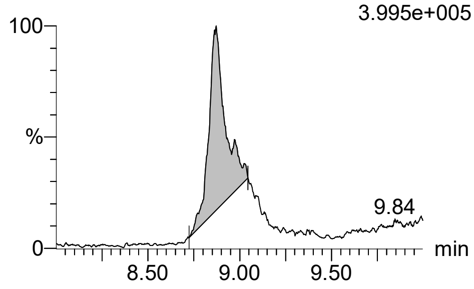

Sample Name: 20211111_L17_R3_IgM
F48:MRM of 2 channels,ES+
984.5 > 204.1
5.225e+006

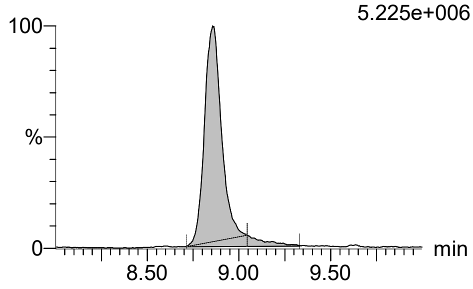

Sample Name: 20211111_L17_R3_IgM
F48:MRM of 2 channels,ES+
1230.4 > 366.1
1.665e+006

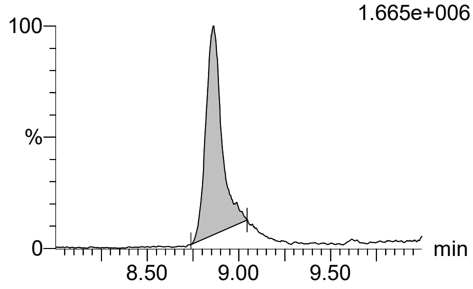

Sample Name: 20211111_L18_R3_IgM
F48:MRM of 2 channels,ES+
984.5 > 204.1
8.985e+005

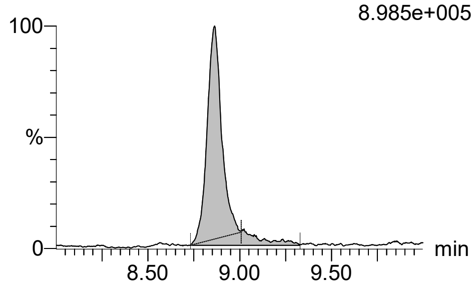

Sample Name: 20211111_L18_R3_IgM
F48:MRM of 2 channels,ES+
1230.4 > 366.1
2.742e+005

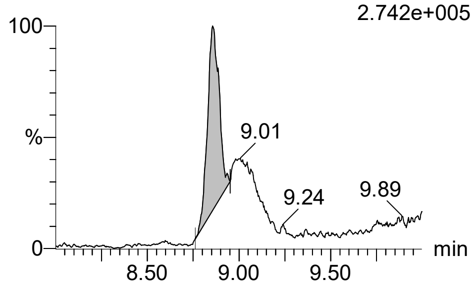

Sample Name: 20211111_H9_R3_IgM
F48:MRM of 2 channels,ES+
984.5 > 204.1
3.054e+006

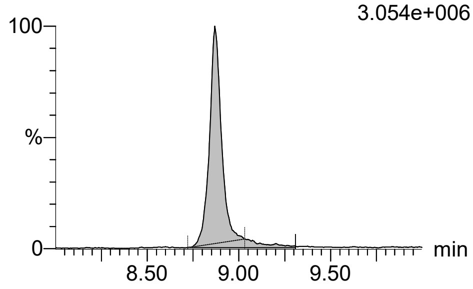

Sample Name: 20211111_H9_R3_IgM
F48:MRM of 2 channels,ES+
1230.4 > 366.1
8.831e+005

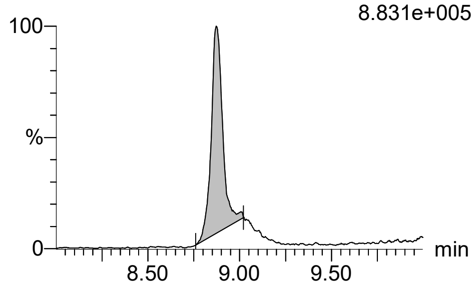

Sample Name: 20211111_A19_R3_IgM
F48:MRM of 2 channels,ES+
984.5 > 204.1
5.364e+006

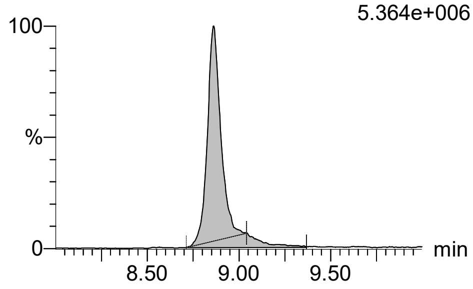

Sample Name: 20211111_A19_R3_IgM
F48:MRM of 2 channels,ES+
1230.4 > 366.1
1.653e+006

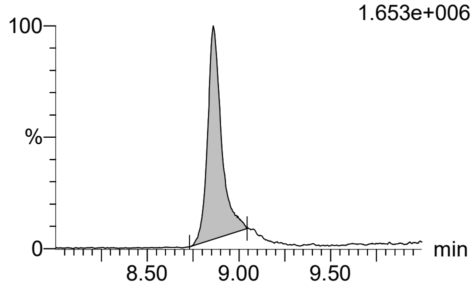

Sample Name: 20211111_A20_R3_IgM
F48:MRM of 2 channels,ES+
984.5 > 204.1
8.882e+005

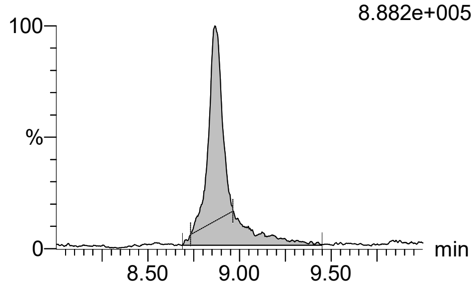

Sample Name: 20211111_A20_R3_IgM
F48:MRM of 2 channels,ES+
1230.4 > 366.1
2.616e+005

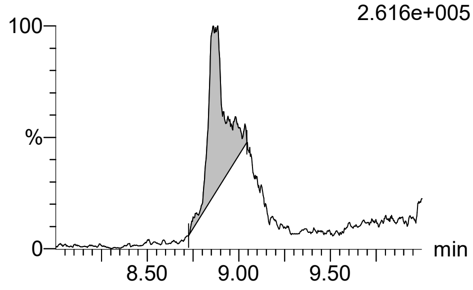

Sample Name: 20211111_L19_R3_IgM
F48:MRM of 2 channels,ES+
984.5 > 204.1
2.989e+006

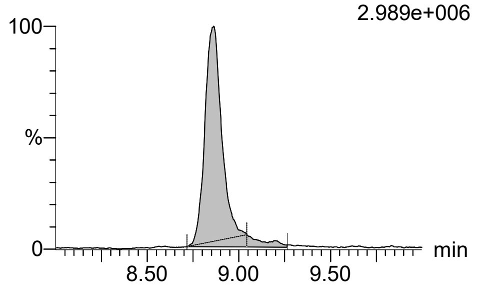

Sample Name: 20211111_L19_R3_IgM
F48:MRM of 2 channels,ES+
1230.4 > 366.1
1.012e+006

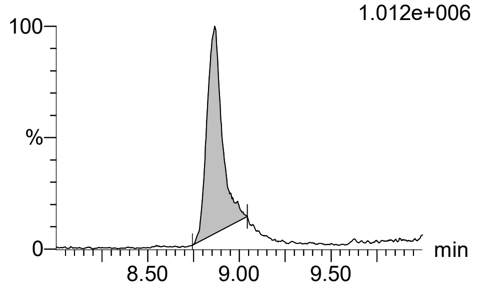

Sample Name: 20211111_QC_R21_IgM
F48:MRM of 2 channels,ES+
984.5 > 204.1
5.217e+006

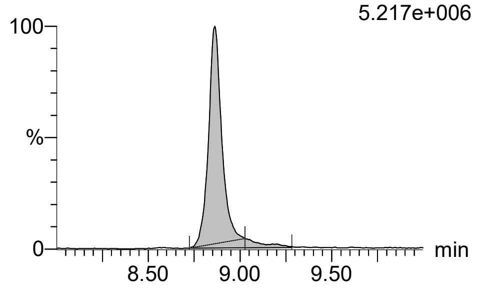

Sample Name: 20211111_QC_R21_IgM
F48:MRM of 2 channels,ES+
1230.4 > 366.1
1.593e+006

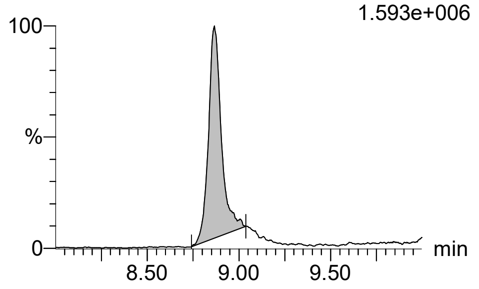

Sample Name: 20211111_L20_R3_IgM
F48:MRM of 2 channels,ES+
984.5 > 204.1
2.716e+006

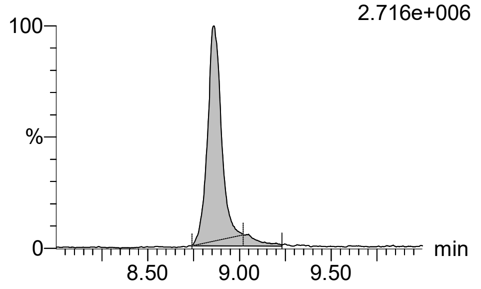

Sample Name: 20211111_L20_R3_IgM
F48:MRM of 2 channels,ES+
1230.4 > 366.1
8.825e+005

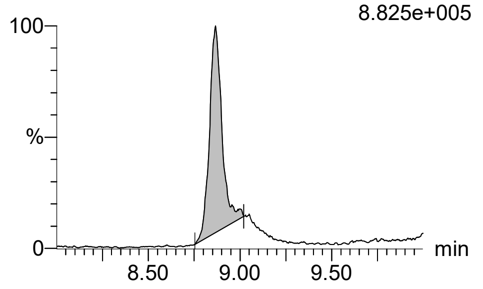

Sample Name: 20211111_H10_R3_IgM
F48:MRM of 2 channels,ES+
984.5 > 204.1
6.391e+006

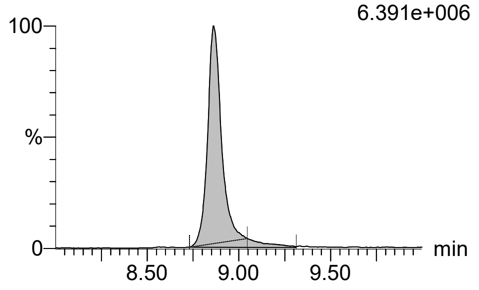

Sample Name: 20211111_H10_R3_IgM
F48:MRM of 2 channels,ES+
1230.4 > 366.1
1.987e+006

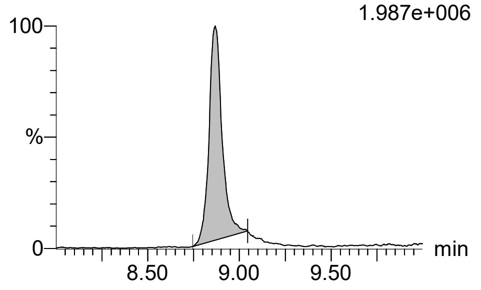

Sample Name: 20211111_A21_R3_IgM
F48:MRM of 2 channels,ES+
984.5 > 204.1
3.983e+006

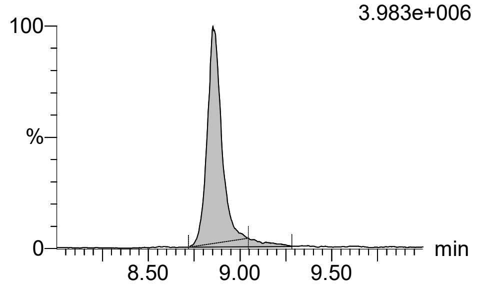

Sample Name: 20211111_A21_R3_IgM
F48:MRM of 2 channels,ES+
1230.4 > 366.1
1.242e+006

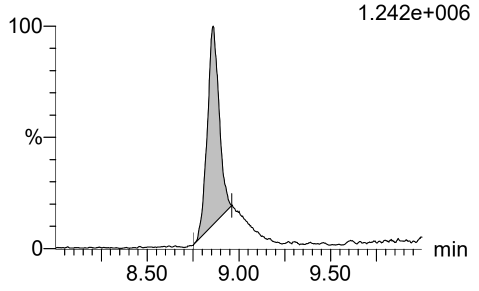

Sample Name: 20211111_A22_R3_IgM
F48:MRM of 2 channels,ES+
984.5 > 204.1
6.138e+005

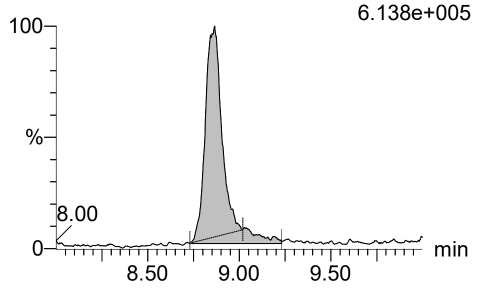

Sample Name: 20211111_A22_R3_IgM
F48:MRM of 2 channels,ES+
1230.4 > 366.1
1.968e+005

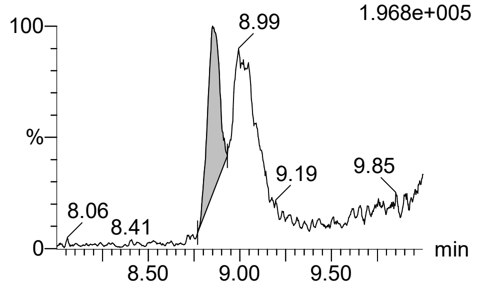

Sample Name: 20211111_L21_R3_IgM
F48:MRM of 2 channels,ES+
984.5 > 204.1
4.983e+006

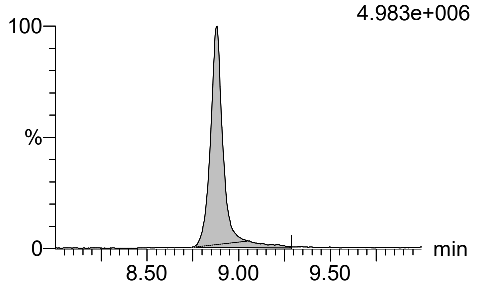

Sample Name: 20211111_L21_R3_IgM
F48:MRM of 2 channels,ES+
1230.4 > 366.1
1.387e+006

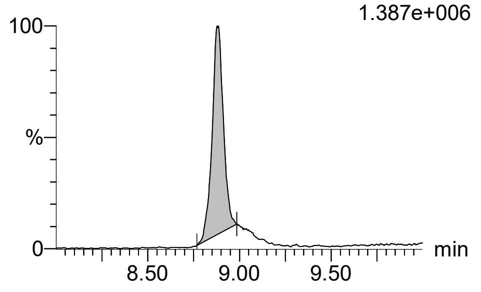

Sample Name: 20211111_L22_R3_IgM
F48:MRM of 2 channels,ES+
984.5 > 204.1
4.031e+006

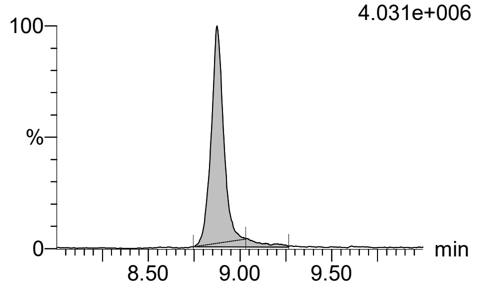

Dataset: C:\MassLynx\10.pro\20211111_clinicalsamples_IgM_IgA.qld

Last Altered: Thursday, March 09, 2023 19:58:25 台北標準時間
Printed: Friday, August 01, 2025 15:04:17 台北標準時間

Sample Name: 20211111_L22_R3_IgM
F48:MRM of 2 channels,ES+
1230.4 > 366.1
1.110e+006

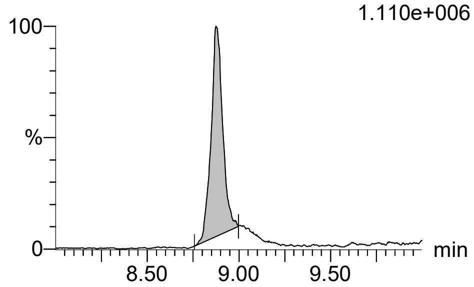

Sample Name: 20211111_H11_R3_IgM
F48:MRM of 2 channels,ES+
984.5 > 204.1
8.286e+006

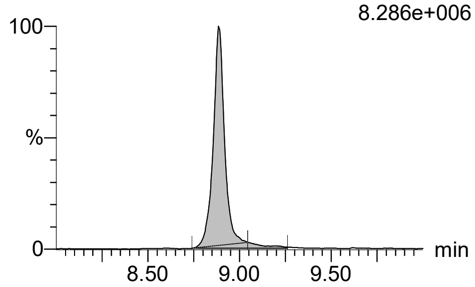

Sample Name: 20211111_H11_R3_IgM
F48:MRM of 2 channels,ES+
1230.4 > 366.1
2.076e+006

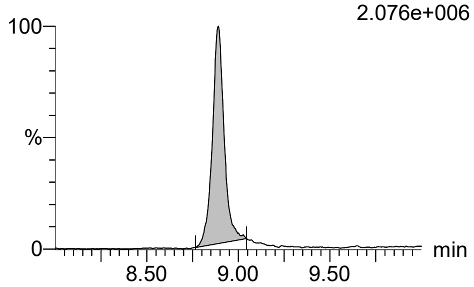

Sample Name: 20211111_A23_R3_IgM
F48:MRM of 2 channels,ES+
984.5 > 204.1
2.819e+006

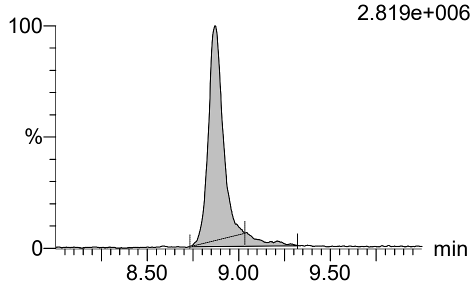

Sample Name: 20211111_A23_R3_IgM
F48:MRM of 2 channels,ES+
1230.4 > 366.1
9.020e+005

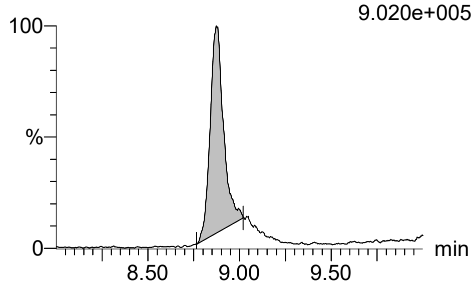

Sample Name: 20211111_A24_R3_IgM
F48:MRM of 2 channels,ES+
984.5 > 204.1
7.062e+006

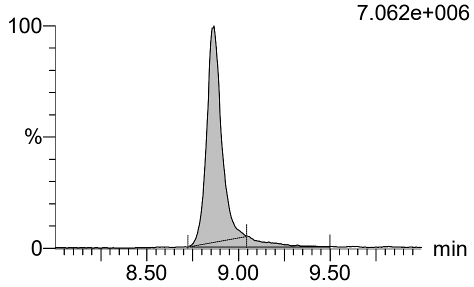

Sample Name: 20211111_A24_R3_IgM
F48:MRM of 2 channels,ES+
1230.4 > 366.1
2.173e+006

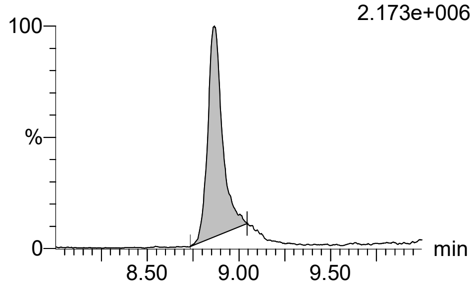

Sample Name: 20211111_L23_R3_IgM
F48:MRM of 2 channels,ES+
984.5 > 204.1
4.390e+006

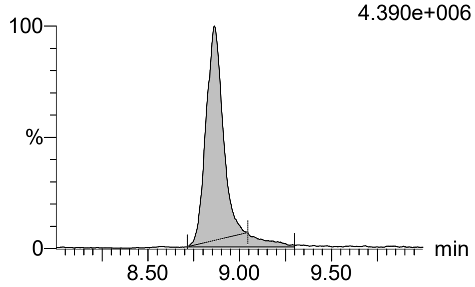

Sample Name: 20211111_L23_R3_IgM
F48:MRM of 2 channels,ES+
1230.4 > 366.1
1.399e+006

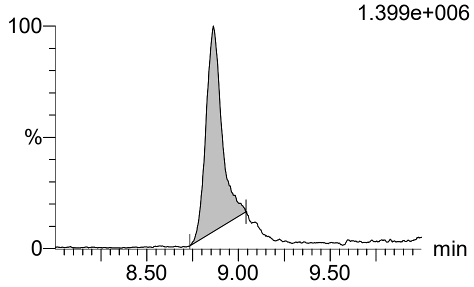

Sample Name: 20211111_L24_R3_IgM
F48:MRM of 2 channels,ES+
984.5 > 204.1
1.643e+006

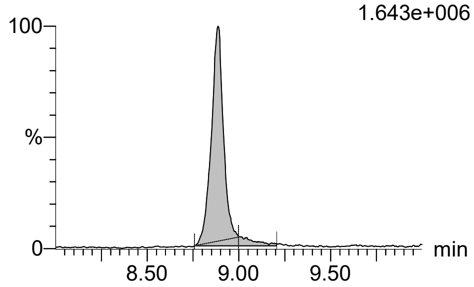

Sample Name: 20211111_L24_R3_IgM
F48:MRM of 2 channels,ES+
1230.4 > 366.1
4.504e+005

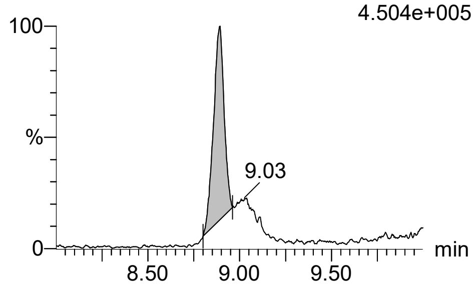

Sample Name: 20211111_H12_R3_IgM
F48:MRM of 2 channels,ES+
984.5 > 204.1
1.969e+006

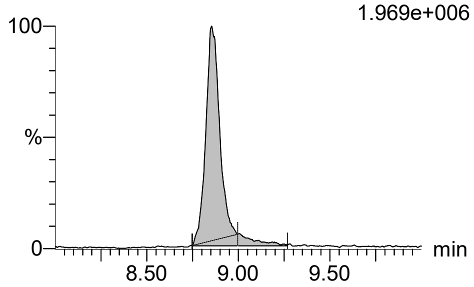

Sample Name: 20211111_H12_R3_IgM
F48:MRM of 2 channels,ES+
1230.4 > 366.1
5.840e+005

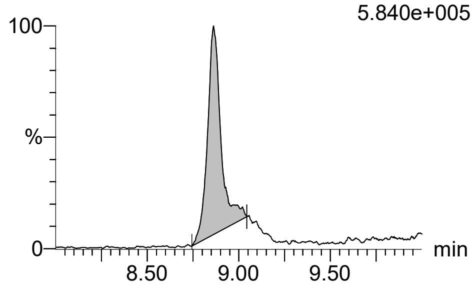

Sample Name: 20211111_QC_R22_IgM
F48:MRM of 2 channels,ES+
984.5 > 204.1
5.932e+006

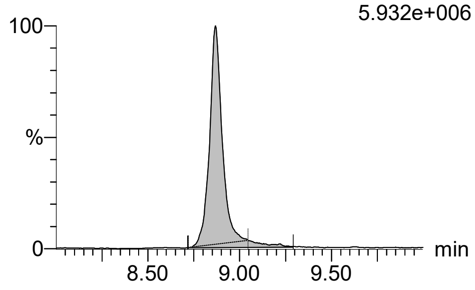

Sample Name: 20211111_QC_R22_IgM
F48:MRM of 2 channels,ES+
1230.4 > 366.1
1.726e+006

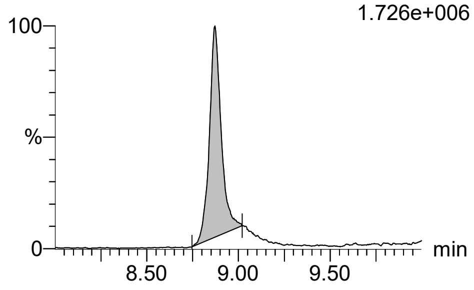

Sample Name: 20211111_A25_R3_IgM
F48:MRM of 2 channels,ES+
984.5 > 204.1
1.173e+007

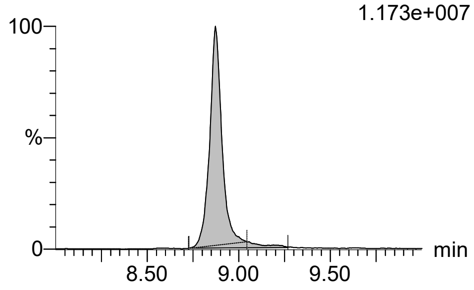

Sample Name: 20211111_A25_R3_IgM
F48:MRM of 2 channels,ES+
1230.4 > 366.1
3.299e+006

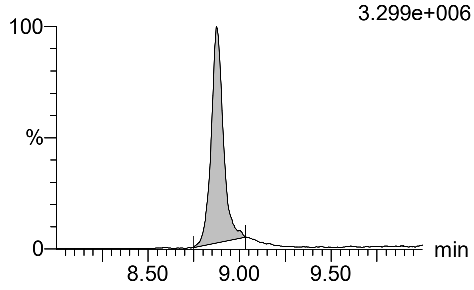

Sample Name: 20211111_A26_R3_IgM
F48:MRM of 2 channels,ES+
984.5 > 204.1
7.708e+006

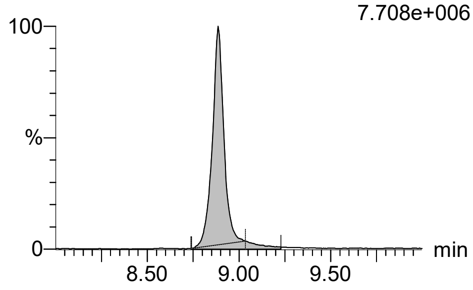

Sample Name: 20211111_A26_R3_IgM
F48:MRM of 2 channels,ES+
1230.4 > 366.1
1.859e+006

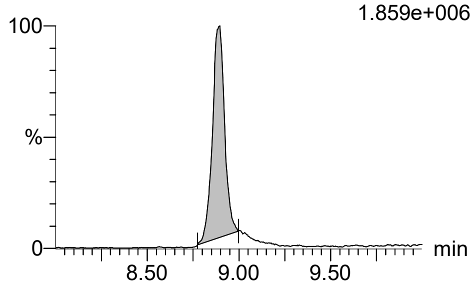

Sample Name: 20211111_L25_R3_IgM
F48:MRM of 2 channels,ES+
984.5 > 204.1
2.859e+006

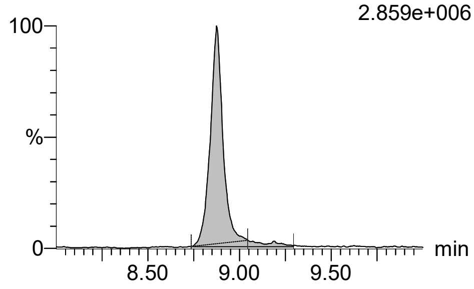

Sample Name: 20211111_L25_R3_IgM
F48:MRM of 2 channels,ES+
1230.4 > 366.1
7.915e+005

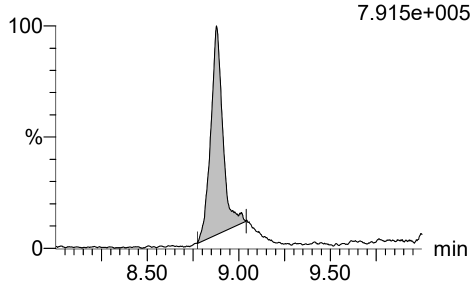

Sample Name: 20211111_L26_R3_IgM
F48:MRM of 2 channels,ES+
984.5 > 204.1
4.415e+006

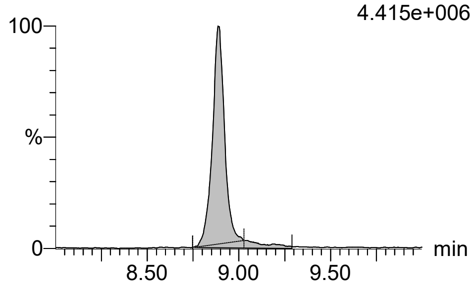

Sample Name: 20211111_L26_R3_IgM
F48:MRM of 2 channels,ES+
1230.4 > 366.1
1.116e+006

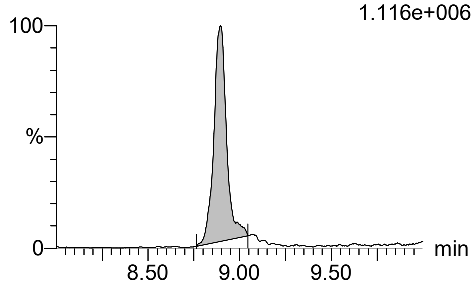

Sample Name: 20211111_H13_R3_IgM
F48:MRM of 2 channels,ES+
984.5 > 204.1
5.784e+006

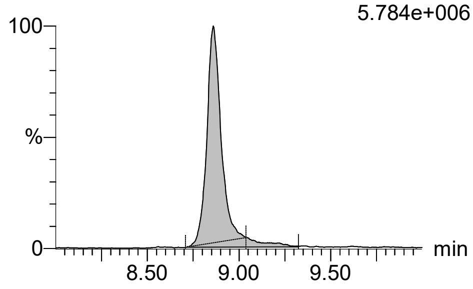

Sample Name: 20211111_H13_R3_IgM
F48:MRM of 2 channels,ES+
1230.4 > 366.1
1.717e+006

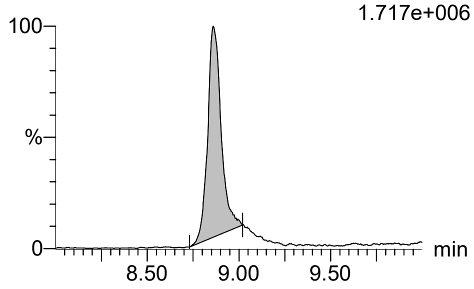

Sample Name: 20211111_A27_R3_IgM
F48:MRM of 2 channels,ES+
984.5 > 204.1
1.214e+007

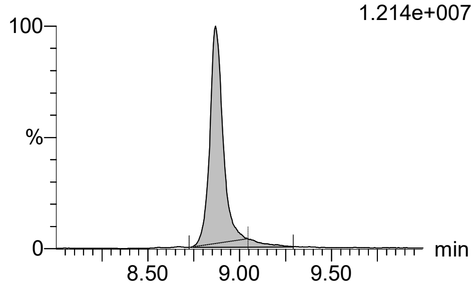

Sample Name: 20211111_A27_R3_IgM
F48:MRM of 2 channels,ES+
1230.4 > 366.1
3.802e+006

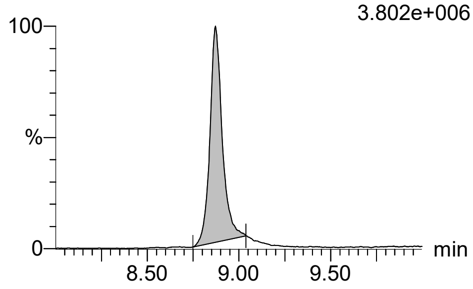

Sample Name: 20211111_A28_R3_IgM
F48:MRM of 2 channels,ES+
984.5 > 204.1
2.563e+006

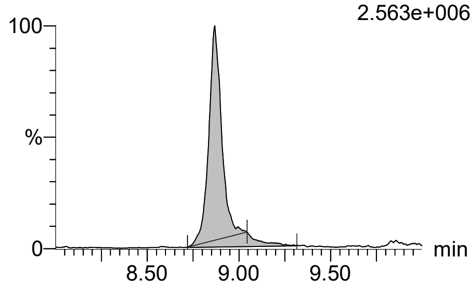

Sample Name: 20211111_A28_R3_IgM
F48:MRM of 2 channels,ES+
1230.4 > 366.1
7.758e+005

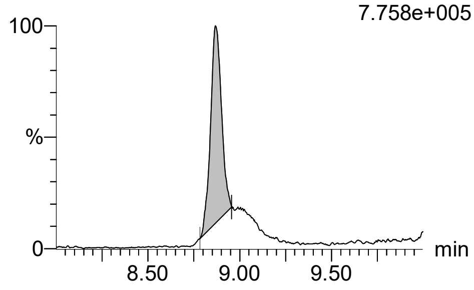

Sample Name: 20211111_L27_R3_IgM
F48:MRM of 2 channels,ES+
984.5 > 204.1
7.014e+006

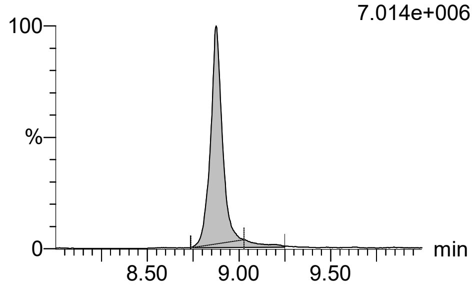

Sample Name: 20211111_L27_R3_IgM
F48:MRM of 2 channels,ES+
1230.4 > 366.1
1.942e+006

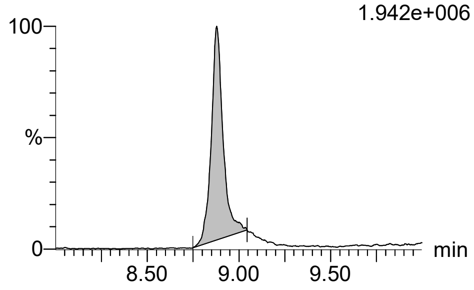

Sample Name: 20211111_L28_R3_IgM
F48:MRM of 2 channels,ES+
984.5 > 204.1
2.089e+006

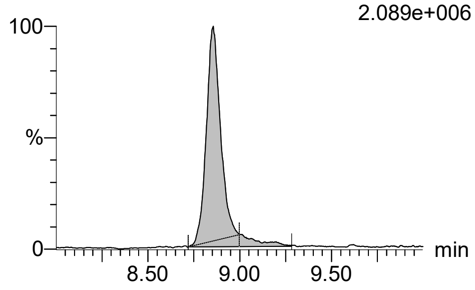

Sample Name: 20211111_L28_R3_IgM
F48:MRM of 2 channels,ES+
1230.4 > 366.1
5.967e+005

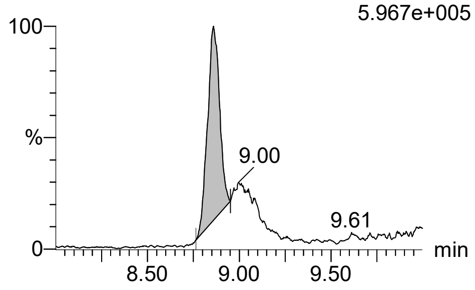

Sample Name: 20211111_H14_R3_IgM
F48:MRM of 2 channels,ES+
984.5 > 204.1
4.056e+006

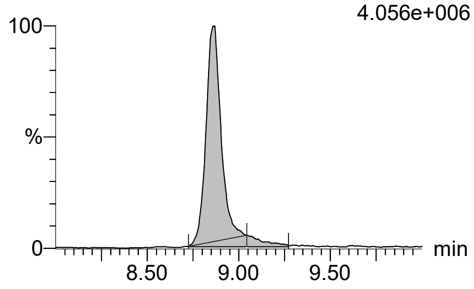

Sample Name: 20211111_H14_R3_IgM
F48:MRM of 2 channels,ES+
1230.4 > 366.1
1.268e+006

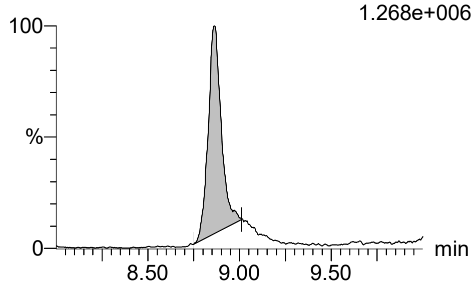

Sample Name: 20211111_A29_R3_IgM
F48:MRM of 2 channels,ES+
984.5 > 204.1
1.051e+006

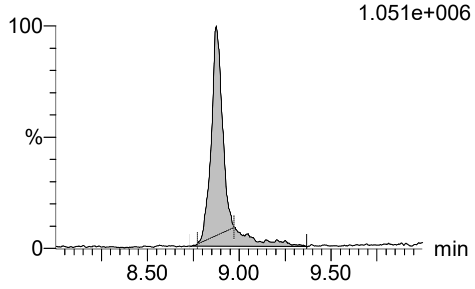

Sample Name: 20211111_A29_R3_IgM
F48:MRM of 2 channels,ES+
1230.4 > 366.1
2.873e+005

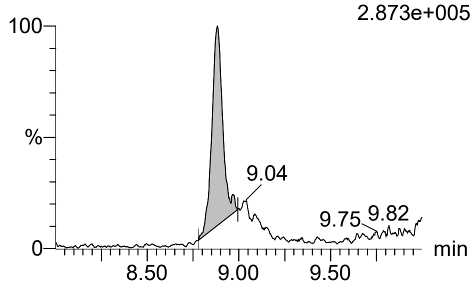

Sample Name: 20211111_A30_R3_IgM
F48:MRM of 2 channels,ES+
984.5 > 204.1
7.454e+006

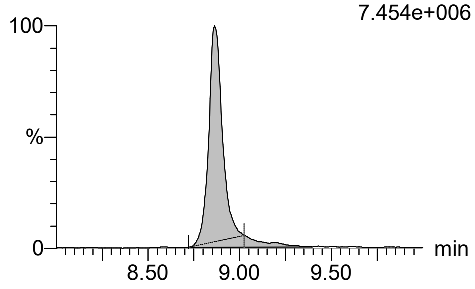

Sample Name: 20211111_A30_R3_IgM
F48:MRM of 2 channels,ES+
1230.4 > 366.1
2.260e+006

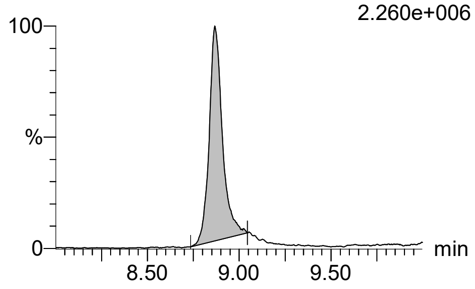

Sample Name: 20211111_QC_R23_IgM
F48:MRM of 2 channels,ES+
984.5 > 204.1
5.932e+006

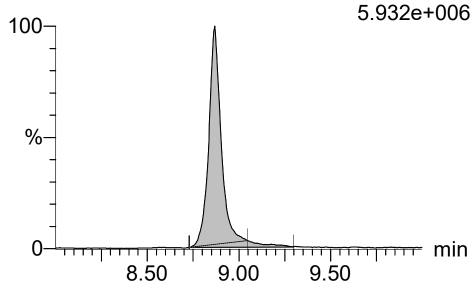

Sample Name: 20211111_QC_R23_IgM
F48:MRM of 2 channels,ES+
1230.4 > 366.1
1.690e+006

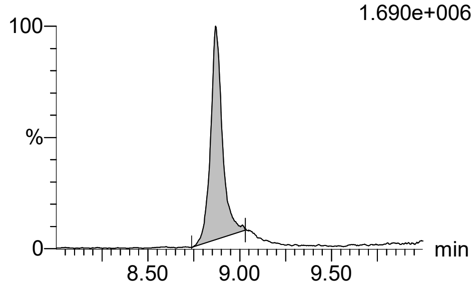

Sample Name: 20211111_L29_R3_IgM
F48:MRM of 2 channels,ES+
984.5 > 204.1
1.504e+006

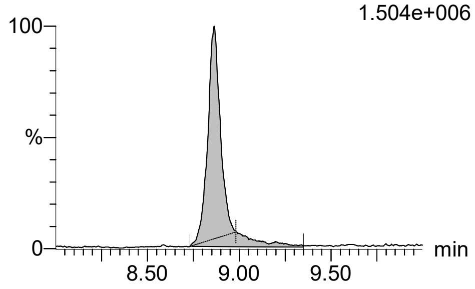

Sample Name: 20211111_L29_R3_IgM
F48:MRM of 2 channels,ES+
1230.4 > 366.1
4.391e+005

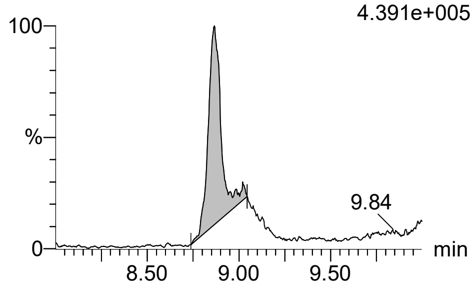

Sample Name: 20211111_L30_R3_IgM
F48:MRM of 2 channels,ES+
984.5 > 204.1
1.104e+006

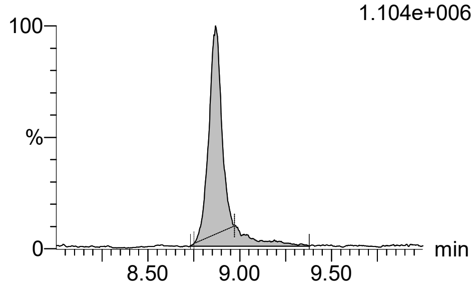

Sample Name: 20211111_L30_R3_IgM
F48:MRM of 2 channels,ES+
1230.4 > 366.1
3.267e+005

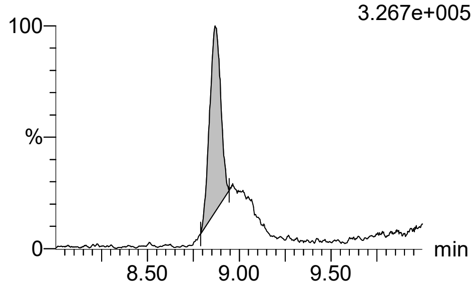

Sample Name: 20211111_H15_R3_IgM
F48:MRM of 2 channels,ES+
984.5 > 204.1
4.181e+006

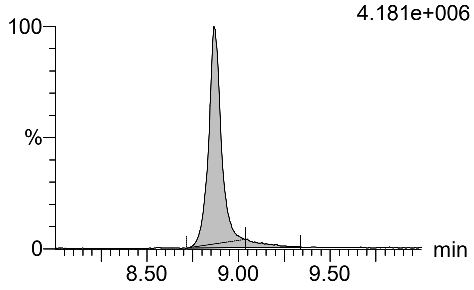

Sample Name: 20211111_H15_R3_IgM
F48:MRM of 2 channels,ES+
1230.4 > 366.1
1.223e+006

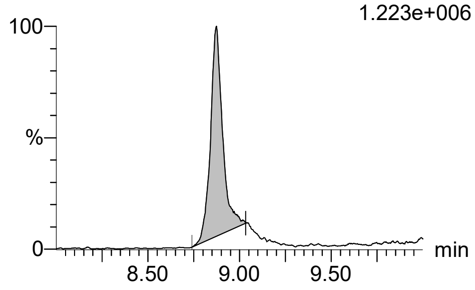

Sample Name: 20211111_A31_R3_IgM
F48:MRM of 2 channels,ES+
984.5 > 204.1
2.295e+006

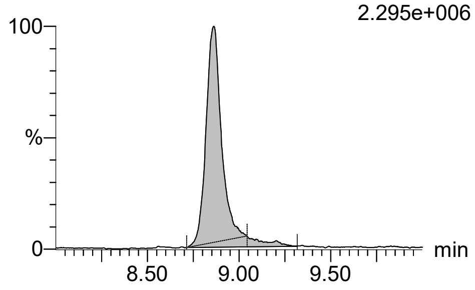

Sample Name: 20211111_A31_R3_IgM
F48:MRM of 2 channels,ES+
1230.4 > 366.1
7.321e+005

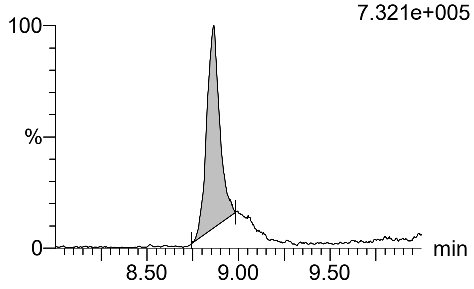

Sample Name: 20211111_A32_R3_IgM
F48:MRM of 2 channels,ES+
984.5 > 204.1
5.007e+006

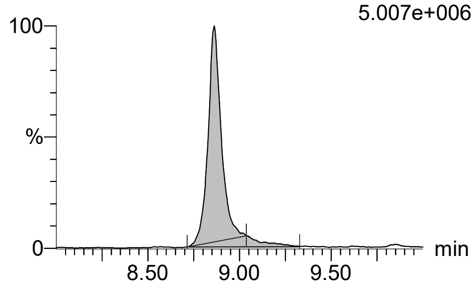

Sample Name: 20211111_A32_R3_IgM
F48:MRM of 2 channels,ES+
1230.4 > 366.1
1.428e+006

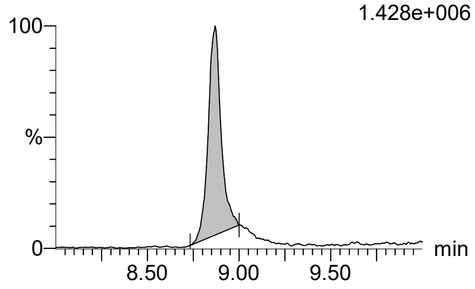

Sample Name: 20211111_L31_R3_IgM
F48:MRM of 2 channels,ES+
984.5 > 204.1
6.468e+005

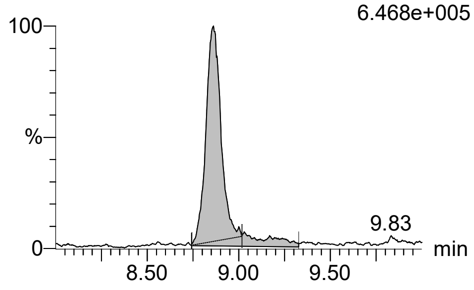

Sample Name: 20211111_L31_R3_IgM
F48:MRM of 2 channels,ES+
1230.4 > 366.1
1.963e+005

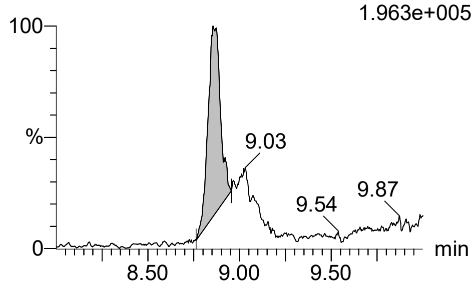

Sample Name: 20211111_L32_R3_IgM
F48:MRM of 2 channels,ES+
984.5 > 204.1
6.717e+006

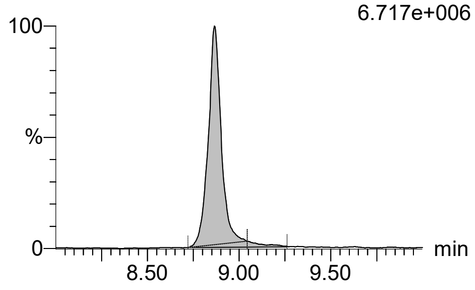

Sample Name: 20211111_L32_R3_IgM
F48:MRM of 2 channels,ES+
1230.4 > 366.1
1.897e+006

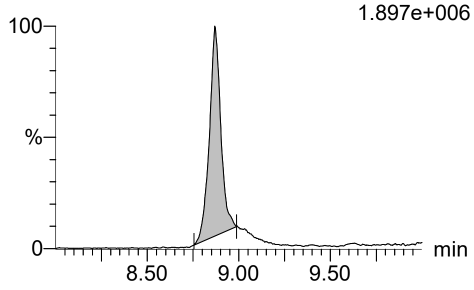

Sample Name: 20211111_H16_R3_IgM
F48:MRM of 2 channels,ES+
984.5 > 204.1
6.529e+006

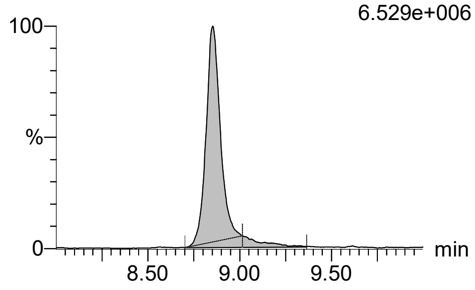

Sample Name: 20211111_H16_R3_IgM
F48:MRM of 2 channels,ES+
1230.4 > 366.1
1.950e+006

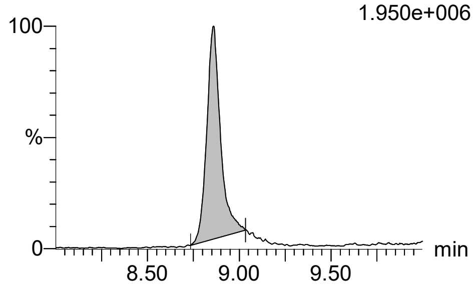

Sample Name: 20211111_A33_R3_IgM
F48:MRM of 2 channels,ES+
984.5 > 204.1
1.130e+007

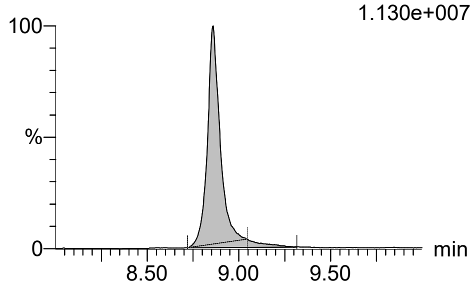

Sample Name: 20211111_A33_R3_IgM
F48:MRM of 2 channels,ES+
1230.4 > 366.1
3.350e+006

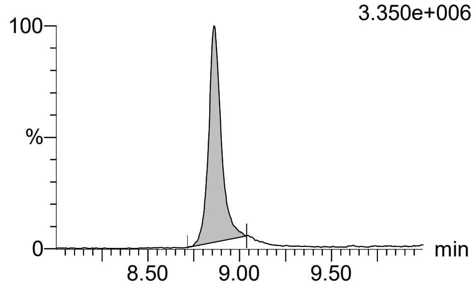

Sample Name: 20211111_A34_R3_IgM
F48:MRM of 2 channels,ES+
984.5 > 204.1
3.078e+006

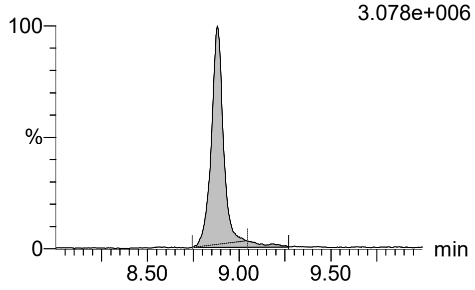

Dataset: C:\MassLynx\10.pro\20211111_clinicalsamples_IgM_IgA.qld

Last Altered: Thursday, March 09, 2023 19:58:25 台北標準時間
Printed: Friday, August 01, 2025 15:04:17 台北標準時間

Sample Name: 20211111_A34_R3_IgM
F48:MRM of 2 channels,ES+
1230.4 > 366.1
8.298e+005

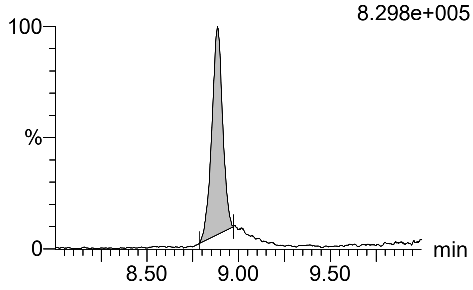

Sample Name: 20211111_H17_R3_IgM
F48:MRM of 2 channels,ES+
984.5 > 204.1
1.599e+007

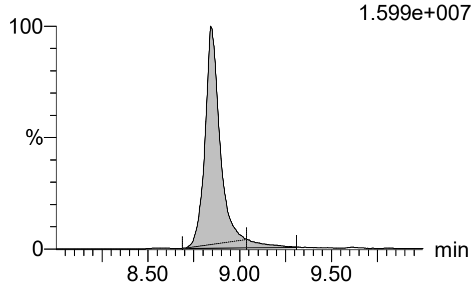

Sample Name: 20211111_H17_R3_IgM
F48:MRM of 2 channels,ES+
1230.4 > 366.1
4.918e+006

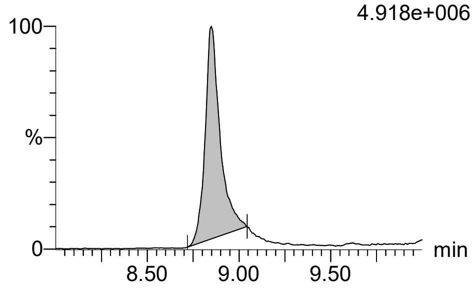

Sample Name: 20211111_A35_R3_IgM
F48:MRM of 2 channels,ES+
984.5 > 204.1
2.169e+006

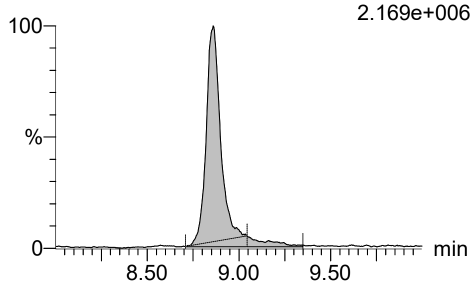

Sample Name: 20211111_A35_R3_IgM
F48:MRM of 2 channels,ES+
1230.4 > 366.1
7.004e+005

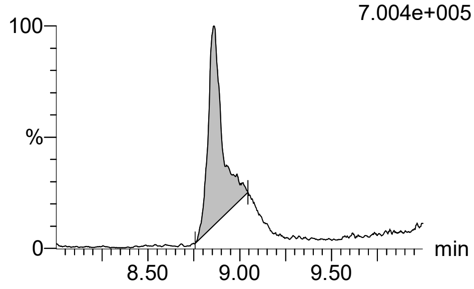

Sample Name: 20211111_QC_R24_IgM
F48:MRM of 2 channels,ES+
984.5 > 204.1
5.022e+006

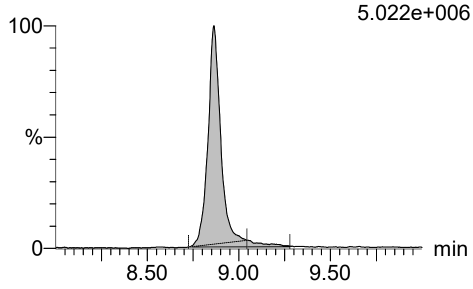

Sample Name: 20211111_QC_R24_IgM
F48:MRM of 2 channels,ES+
1230.4 > 366.1
1.492e+006

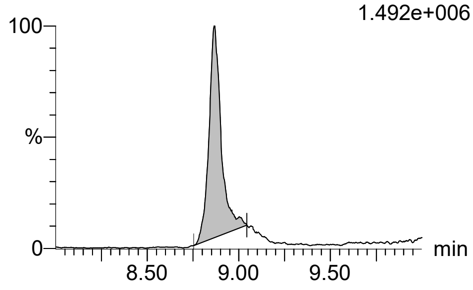

Sample Name: 20211111_A36_R3_IgM
F48:MRM of 2 channels,ES+
984.5 > 204.1
3.105e+006

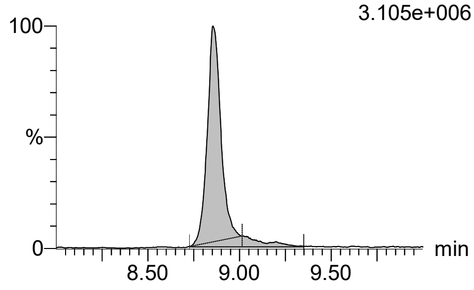

Sample Name: 20211111_A36_R3_IgM
F48:MRM of 2 channels,ES+
1230.4 > 366.1
9.818e+005

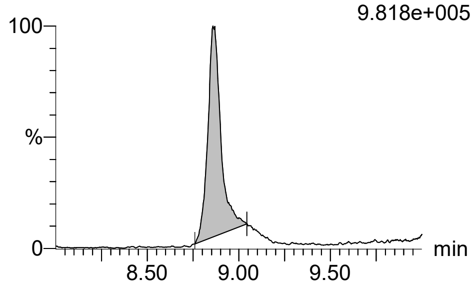

Sample Name: 20211111_H18_R3_IgM
F48:MRM of 2 channels,ES+
984.5 > 204.1
9.149e+006

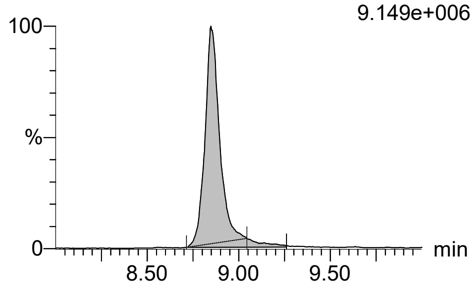

Sample Name: 20211111_H18_R3_IgM
F48:MRM of 2 channels,ES+
1230.4 > 366.1
2.856e+006

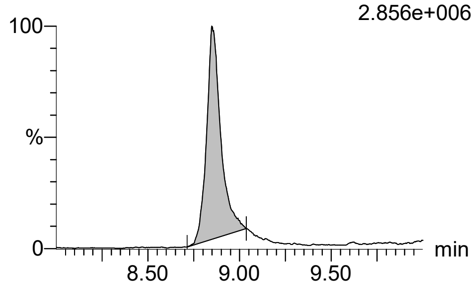

Sample Name: 20211111_A37_R3_IgM
F48:MRM of 2 channels,ES+
984.5 > 204.1
5.422e+006

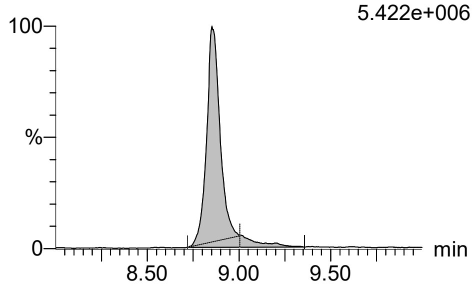

Sample Name: 20211111_A37_R3_IgM
F48:MRM of 2 channels,ES+
1230.4 > 366.1
1.702e+006

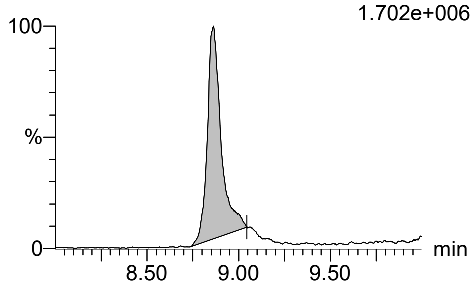

Sample Name: 20211111_A38_R3_IgM
F48:MRM of 2 channels,ES+
984.5 > 204.1
5.782e+006

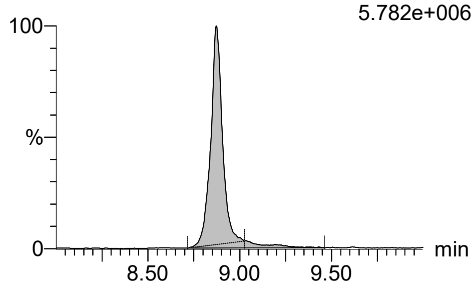

Sample Name: 20211111_A38_R3_IgM
F48:MRM of 2 channels,ES+
1230.4 > 366.1
1.447e+006

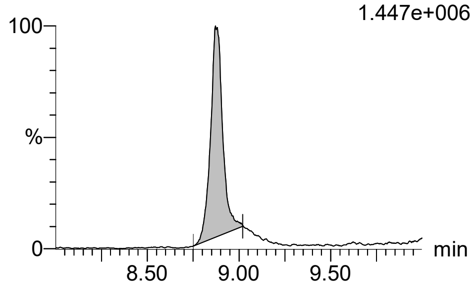

Sample Name: 20211111_H19_R3_IgM
F48:MRM of 2 channels,ES+
984.5 > 204.1
5.589e+006

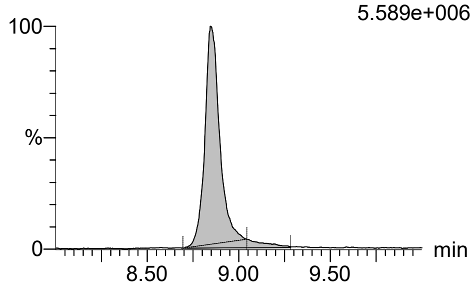

Sample Name: 20211111_H19_R3_IgM
F48:MRM of 2 channels,ES+
1230.4 > 366.1
1.801e+006

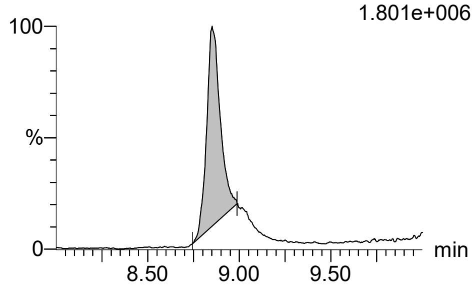

Sample Name: 20211111_A39_R3_IgM
F48:MRM of 2 channels,ES+
984.5 > 204.1
6.762e+006

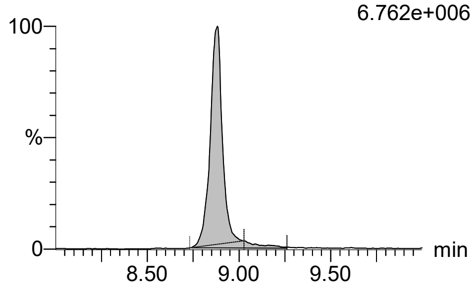

Sample Name: 20211111_A39_R3_IgM
F48:MRM of 2 channels,ES+
1230.4 > 366.1
1.860e+006

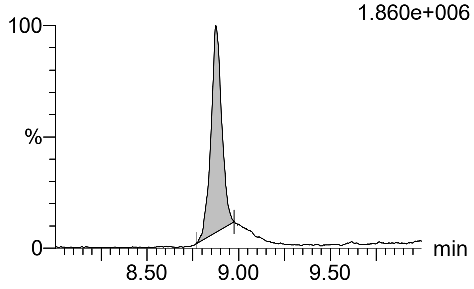

Sample Name: 20211111_A40_R3_IgM
F48:MRM of 2 channels,ES+
984.5 > 204.1
2.438e+006

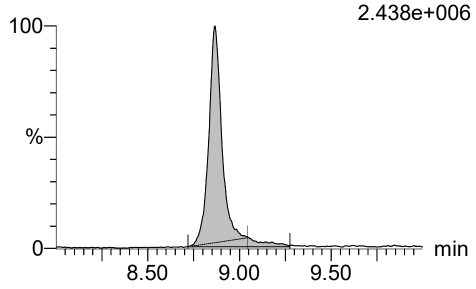

Sample Name: 20211111_A40_R3_IgM
F48:MRM of 2 channels,ES+
1230.4 > 366.1
7.418e+005

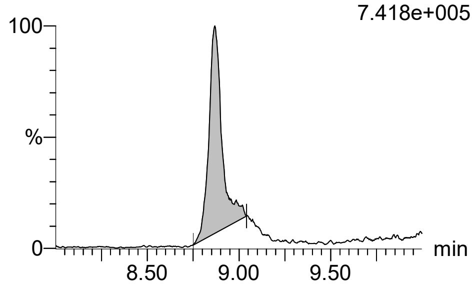

Sample Name: 20211111_H20_R3_IgM
F48:MRM of 2 channels,ES+
984.5 > 204.1
1.117e+007

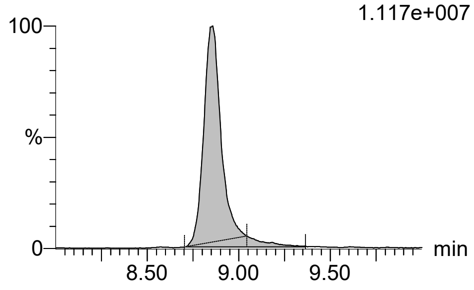

Sample Name: 20211111_H20_R3_IgM
F48:MRM of 2 channels,ES+
1230.4 > 366.1
3.592e+006

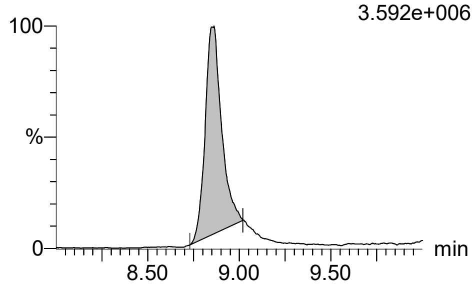

Sample Name: 20211111_A41_R3_IgM
F48:MRM of 2 channels,ES+
984.5 > 204.1
3.380e+006

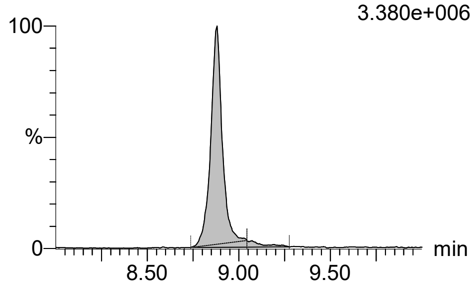

Sample Name: 20211111_A41_R3_IgM
F48:MRM of 2 channels,ES+
1230.4 > 366.1
8.611e+005

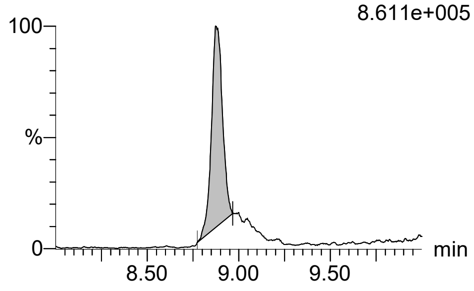

Sample Name: 20211111_A42_R3_IgM
F48:MRM of 2 channels,ES+
984.5 > 204.1
1.731e+006

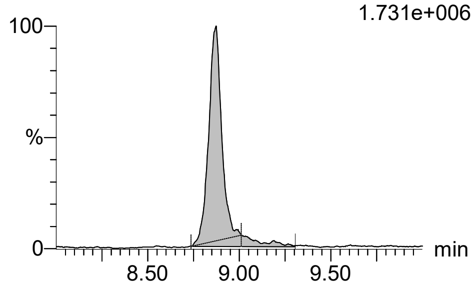

Sample Name: 20211111_A42_R3_IgM
F48:MRM of 2 channels,ES+
1230.4 > 366.1
5.424e+005

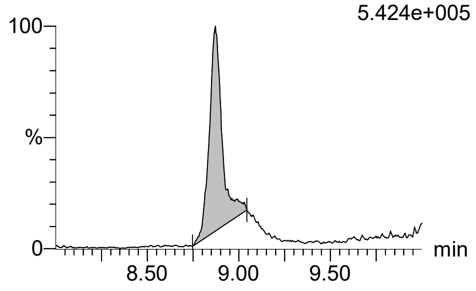

Sample Name: 20211111_A43_R3_IgM
F48:MRM of 2 channels,ES+
984.5 > 204.1
1.643e+006

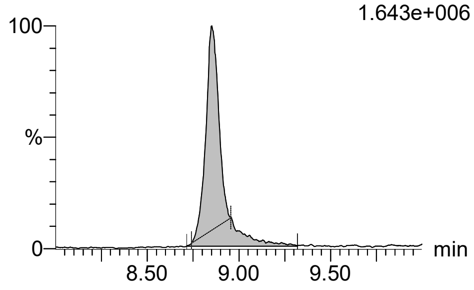

Sample Name: 20211111_A43_R3_IgM
F48:MRM of 2 channels,ES+
1230.4 > 366.1
5.160e+005

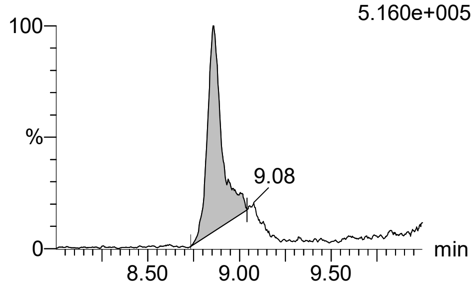

Sample Name: 20211111_A44_R3_IgM
F48:MRM of 2 channels,ES+
984.5 > 204.1
6.343e+005

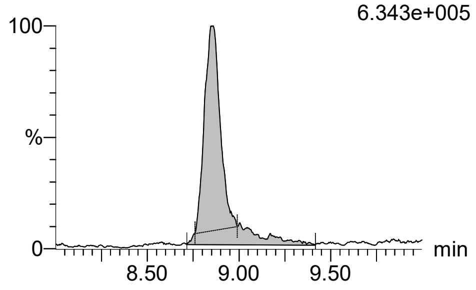

Dataset: C:\MassLynx\10.pro\20211111_clinicalsamples_IgM_IgA.qld

Last Altered: Thursday, March 09, 2023 19:58:25 台北標準時間
Printed: Friday, August 01, 2025 15:04:17 台北標準時間

Sample Name: 20211111_A44_R3_IgM

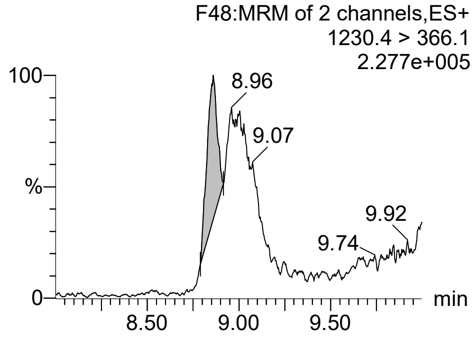

Sample Name: 20211111_QC_R25_IgM

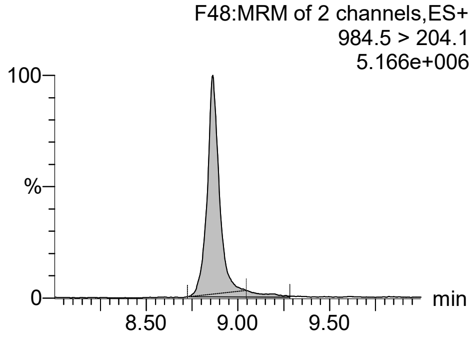

Sample Name: 20211111_QC_R25_IgM

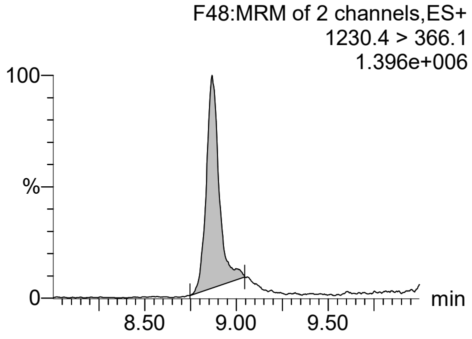

Dataset: C:\MassLynx\10.pro\20211111_clinicalsamples_IgM_IgA.qld

Last Altered: Thursday, March 09, 2023 19:58:25 台北標準時間
Printed: Friday, August 01, 2025 15:04:17 台北標準時間

	#	Name	RT	Area	IS Area	Response	Prima...
1	1	20211112_QC_R001_IgM	8.84	524242.7	15421.4	33.994	MM
2	2	20211112_QC_R002_IgM	8.85	505605.3	13188.8	38.336	MM
3	3	20211111_A1_R2_IgM	8.86	329388.1	11871.0	27.747	MM
4	4	20211111_A1_R3_IgM	8.86	321536.4	12030.5	26.727	MM
5	5	20211111_A2_R2_IgM	8.86	1163523.5	11446.8	101.646	MM
6	6	20211111_A2_R3_IgM	8.85	1145825.5	11098.4	103.242	MM
7	7	20211111_L1_R2_IgM	8.87	195527.8	11779.4	16.599	MM
8	8	20211111_L1_R3_IgM	8.86	176095.4	11176.2	15.756	MM
9	9	20211111_L2_R2_IgM	8.85	557104.2	11375.0	48.976	MM
10	10	20211111_H1_R2_IgM	8.86	480774.0	13595.2	35.364	MM
11	11	20211111_A3_R2_IgM	8.85	668551.5	14112.3	47.374	MM
12	12	20211111_A4_R2_IgM	8.85	436772.8	11977.5	36.466	MM
13	13	20211111_L3_R2_IgM	8.84	125717.7	12242.1	10.269	MM
14	14	20211111_L4_R2_IgM	8.86	555100.9	12797.0	43.377	MM
15	15	20211111_H2_R2_IgM	8.85	1286364.4	10006.0	128.559	MM
16	16	20211111_A5_R2_IgM	8.87	2203477.5	11434.6	192.702	MM
17	17	20211111_A6_R2_IgM	8.86	552449.7	13193.7	41.872	MM
18	18	20211111_QC_R10_IgM	8.85	510030.3	12566.1	40.588	MM
19	19	20211111_L5_R2_IgM	8.86	312858.5	13616.4	22.977	MM
20	20	20211111_L6_R2_IgM	8.85	564632.1	13698.5	41.219	MM
21	21	20211111_H3_R2_IgM	8.86	313110.7	15793.1	19.826	MM
22	22	20211111_A7_R2_IgM	8.84	570259.8	12936.9	44.080	MM
23	23	20211111_A8_R2_IgM	8.86	633798.7	13108.0	48.352	MM
24	24	20211111_L7_R2_IgM	8.86	271965.2	13278.5	20.482	MM
25	25	20211111_L8_R2_IgM	8.87	145061.9	15233.6	9.523	MM
26	26	20211111_H4_R2_IgM	8.86	228306.7	16521.9	13.818	MM
27	27	20211111_A9_R2_IgM	8.86	261360.6	16451.6	15.887	MM
28	28	20211111_A10_R2_IgM	8.85	564679.2	13036.0	43.317	MM
29	29	20211111_L9_R2_IgM	8.86	169577.3	14554.1	11.652	MM
30	30	20211111_L10_R2_IgM	8.87	541540.7	12250.4	44.206	MM
31	31	20211111_QC_R11_IgM	8.86	550926.6	11459.2	48.077	MM
32	32	20211111_H5_R2_IgM	8.86	436592.3	14358.2	30.407	MM
33	33	20211111_A11_R2_IgM	8.84	556383.7	16184.1	34.378	MM
34	34	20211111_A12_R2_IgM	8.86	232985.4	13465.4	17.303	MM
35	35	20211111_L11_R2_IgM	8.88	430583.8	13277.9	32.429	MM
36	36	20211111_L12_R2_IgM	8.88	223063.9	15958.8	13.978	MM
37	37	20211111_H6_R2_IgM	8.86	257921.3	14003.5	18.418	MM
38	38	20211111_A13_R2_IgM	8.86	314605.9	13656.1	23.038	MM
39	39	20211111_A14_R2_IgM	8.86	1186815.8	14071.3	84.343	MM
40	40	20211111_L13_R2_IgM	8.86	235043.7	14785.6	15.897	MM
41	41	20211111_L14_R2_IgM	8.87	433000.1	14772.2	29.312	MM
42	42	20211111_H7_R2_IgM	8.87	208465.3	15164.4	13.747	MM
43	43	20211111_A15_R2_IgM	8.85	1495529.6	15721.5	95.127	MM
44	44	20211111_QC_R12_IgM	8.86	588789.4	13711.5	42.941	MM
45	45	20211111_A16_R2_IgM	8.87	762715.4	14275.6	53.428	MM
46	46	20211111_L15_R2_IgM	8.87	555156.9	12935.8	42.916	MM
47	47	20211111_L16_R2_IgM	8.85	116815.0	13342.8	8.755	MM
48	48	20211111_H8_R2_IgM	8.87	296582.7	15835.1	18.729	MM
49	49	20211111_A17_R2_IgM	8.87	897079.3	15786.7	56.825	MM
50	50	20211111_A18_R2_IgM	8.87	220209.3	16986.8	12.964	MM
51	51	20211111_L17_R2_IgM	8.86	645501.3	13342.7	48.379	MM
52	52	20211111_L18_R2_IgM	8.87	94267.7	14692.0	6.416	MM
53	53	20211111_H9_R2_IgM	8.87	196048.8	15639.7	12.535	MM
54	54	20211111_A19_R2_IgM	8.86	518982.4	14198.7	36.551	MM
55	55	20211111_A20_R2_IgM	8.87	118644.2	14162.7	8.377	MM
56	56	20211111_L19_R2_IgM	8.86	323259.7	9293.4	34.784	MM

Dataset: C:\MassLynx\10.pro\20211111_clinicalsamples_IgM_IgA.qld

Last Altered: Thursday, March 09, 2023 19:58:25 台北標準時間
Printed: Friday, August 01, 2025 15:04:17 台北標準時間

	# Name	RT	Area	IS Area	Response	Prima...
57	57 20211111_QC_R13_IgM	8.86	566554.8	13903.0	40.751	MM
58	58 20211111_L20_R2_IgM	8.85	155278.4	12781.6	12.149	MM
59	59 20211111_H10_R2_IgM	8.87	393085.8	14796.4	26.566	MM
60	60 20211111_A21_R2_IgM	8.86	373816.4	13669.0	27.348	MM
61	61 20211111_A22_R2_IgM	8.85	72045.6	14098.3	5.110	MM
62	62 20211111_L21_R2_IgM	8.87	472007.2	15678.2	30.106	MM
63	63 20211111_L22_R2_IgM	8.87	391471.7	15724.5	24.896	MM
64	64 20211111_H11_R2_IgM	8.88	631259.9	15890.2	39.726	MM
65	65 20211111_A23_R2_IgM	8.86	320216.9	13423.2	23.856	MM
66	66 20211111_A24_R2_IgM	8.85	767758.3	12438.0	61.727	MM
67	67 20211111_L23_R2_IgM	8.86	683448.0	14075.0	48.558	MM
68	68 20211111_L24_R2_IgM	8.86	93168.0	15972.1	5.833	MM
69	69 20211111_H12_R2_IgM	8.85	199266.0	16270.9	12.247	MM
70	70 20211111_QC_R14_IgM	8.86	603617.8	15806.8	38.187	MM
71	71 20211111_A25_R2_IgM	8.86	1173725.1	14552.1	80.657	MM
72	72 20211111_A26_R2_IgM	8.89	649714.8	11492.7	56.533	MM
73	73 20211111_L25_R2_IgM	8.87	277155.5	14403.3	19.242	MM
74	74 20211111_L26_R2_IgM	8.89	435309.2	16533.9	26.328	MM
75	75 20211111_H13_R2_IgM	8.85	600198.7	15520.1	38.672	MM
76	76 20211111_A27_R2_IgM	8.87	1145451.4	13337.4	85.882	MM
77	77 20211111_A28_R2_IgM	8.87	155920.2	11895.6	13.107	MM
78	78 20211111_L27_R2_IgM	8.87	475195.0	14125.7	33.640	MM
79	79 20211111_L28_R2_IgM	8.85	210385.6	11807.5	17.818	MM
80	80 20211111_H14_R2_IgM	8.84	441775.0	15035.7	29.382	MM
81	81 20211111_A29_R2_IgM	8.86	80891.8	13554.4	5.968	MM
82	82 20211111_A30_R2_IgM	8.86	741755.8	12984.1	57.128	MM
83	83 20211111_QC_R15_IgM	8.86	560316.2	12299.5	45.556	MM
84	84 20211111_L29_R2_IgM	8.86	141562.3	13590.7	10.416	MM
85	85 20211111_L30_R2_IgM	8.86	102146.3	12511.4	8.164	MM
86	86 20211111_H15_R2_IgM	8.86	382719.3	11885.0	32.202	MM
87	87 20211111_A31_R2_IgM	8.85	256005.0	13422.1	19.073	MM
88	88 20211111_A32_R2_IgM	8.86	539249.0	11461.3	47.049	MM
89	89 20211111_L31_R2_IgM	8.86	28672.1	13753.4	2.085	MM
90	90 20211111_L32_R2_IgM	8.86	378520.0	12949.0	29.232	MM
91	91 20211111_H16_R2_IgM	8.85	623021.6	11465.4	54.339	MM
92	92 20211111_A33_R2_IgM	8.86	1004831.3	10551.5	95.231	MM
93	93 20211111_A34_R2_IgM	8.88	272664.5	12409.9	21.972	MM
94	94 20211111_H17_R2_IgM	8.84	1819337.9	10160.9	179.052	MM
95	95 20211111_A35_R2_IgM	8.86	145831.8	10460.6	13.941	MM
96	96 20211111_QC_R16_IgM	8.85	477943.2	10433.5	45.809	MM
97	97 20211111_A36_R2_IgM	8.85	161997.3	12504.8	12.955	MM
98	98 20211111_H18_R2_IgM	8.85	906355.3	9140.4	99.159	MM
99	99 20211111_A37_R2_IgM	8.85	608771.4	9796.9	62.139	MM
100	1... 20211111_A38_R2_IgM	8.88	541640.7	11224.3	48.256	MM
101	1... 20211111_H19_R2_IgM	8.85	647997.1	11739.2	55.200	MM
102	1... 20211111_A39_R2_IgM	8.88	609764.3	12718.9	47.941	MM
103	1... 20211111_A40_R2_IgM	8.86	133697.8	10006.1	13.362	MM
104	1... 20211111_H20_R2_IgM	8.85	1408485.6	7988.2	176.321	MM
105	1... 20211111_A41_R2_IgM	8.88	400855.9	11513.3	34.817	MM
106	1... 20211111_A42_R2_IgM	8.88	204614.7	10238.5	19.985	MM
107	1... 20211111_A43_R2_IgM	8.86	178927.5	11098.9	16.121	MM
108	1... 20211111_A44_R2_IgM	8.86	57073.8	10969.9	5.203	MM
109	1... 20211111_QC_R17_IgM	8.86	394604.8	9700.8	40.677	MM
110	1... 20211111_L2_R3_IgM	8.86	522688.5	8528.0	61.291	MM
111	1... 20211111_H1_R3_IgM	8.86	648032.4	9438.2	68.661	MM
112	1... 20211111_A3_R3_IgM	8.86	932118.6	11598.6	80.364	MM

Dataset: C:\MassLynx\10.pro\20211111_clinicalsamples_IgM_IgA.qld

Last Altered: Thursday, March 09, 2023 19:58:25 台北標準時間
Printed: Friday, August 01, 2025 15:04:17 台北標準時間

	#	Name	RT	Area	IS Area	Response	Prima...
113	1...	20211111_A4_R3_IgM	8.87	588089.9	11364.1	51.750	MM
114	1...	20211111_L3_R3_IgM	8.85	131190.7	10753.4	12.200	MM
115	1...	20211111_L4_R3_IgM	8.87	606177.8	10291.6	58.901	MM
116	1...	20211111_H2_R3_IgM	8.85	1279491.0	9315.8	137.347	MM
117	1...	20211111_A5_R3_IgM	8.85	2804928.3	9102.2	308.161	MM
118	1...	20211111_A6_R3_IgM	8.86	469231.3	10356.4	45.308	MM
119	1...	20211111_QC_R18_IgM	8.85	409880.7	8398.3	48.805	MM
120	1...	20211111_L5_R3_IgM	8.86	474518.8	9786.7	48.486	MM
121	1...	20211111_L6_R3_IgM	8.86	490340.2	9911.3	49.473	MM
122	1...	20211111_H3_R3_IgM	8.88	465256.3	12155.5	38.275	MM
123	1...	20211111_A7_R3_IgM	8.85	571318.6	11804.8	48.397	MM
124	1...	20211111_A8_R3_IgM	8.88	809760.5	10510.6	77.042	MM
125	1...	20211111_L7_R3_IgM	8.85	261862.8	12177.9	21.503	MM
126	1...	20211111_L8_R3_IgM	8.88	141655.2	11668.6	12.140	MM
127	1...	20211111_H4_R3_IgM	8.88	272987.5	11192.0	24.391	MM
128	1...	20211111_A9_R3_IgM	8.88	437249.6	14077.4	31.060	MM
129	1...	20211111_A10_R3_IgM	8.88	806891.7	11190.4	72.106	MM
130	1...	20211111_L9_R3_IgM	8.86	278414.2	14649.3	19.005	MM
131	1...	20211111_L10_R3_IgM	8.86	402800.0	9361.3	43.028	MM
132	1...	20211111_QC_R19_IgM	8.85	413456.6	10125.5	40.833	MM
133	1...	20211111_H5_R3_IgM	8.87	497398.6	10010.9	49.686	MM
134	1...	20211111_A11_R3_IgM	8.87	1034132.1	12697.2	81.445	MM
135	1...	20211111_A12_R3_IgM	8.85	256687.8	13148.5	19.522	MM
136	1...	20211111_L11_R3_IgM	8.89	354066.0	10786.6	32.825	MM
137	1...	20211111_L12_R3_IgM	8.87	159699.7	11681.8	13.671	MM
138	1...	20211111_H6_R3_IgM	8.86	229425.5	12095.5	18.968	MM
139	1...	20211111_A13_R3_IgM	8.86	300292.0	12908.3	23.263	MM
140	1...	20211111_A14_R3_IgM	8.87	1194073.1	11322.6	105.459	MM
141	1...	20211111_L13_R3_IgM	8.86	159667.9	11516.1	13.865	MM
142	1...	20211111_L14_R3_IgM	8.86	392776.0	10790.7	36.400	MM
143	1...	20211111_H7_R3_IgM	8.87	344889.6	11572.1	29.804	MM
144	1...	20211111_A15_R3_IgM	8.86	1309999.8	12434.4	105.353	MM
145	1...	20211111_QC_R20_IgM	8.86	472294.5	10701.0	44.136	MM
146	1...	20211111_A16_R3_IgM	8.88	637199.0	11452.1	55.640	MM
147	1...	20211111_L15_R3_IgM	8.87	593463.2	10531.1	56.353	MM
148	1...	20211111_L16_R3_IgM	8.86	111338.3	12049.4	9.240	MM
149	1...	20211111_H8_R3_IgM	8.88	313388.9	13434.9	23.326	MM
150	1...	20211111_A17_R3_IgM	8.88	771073.2	12452.0	61.923	MM
151	1...	20211111_A18_R3_IgM	8.87	145198.0	12506.5	11.610	MM
152	1...	20211111_L17_R3_IgM	8.86	611621.9	9948.9	61.476	MM
153	1...	20211111_L18_R3_IgM	8.86	94107.3	13462.1	6.991	MM
154	1...	20211111_H9_R3_IgM	8.87	255004.3	13178.7	19.350	MM
155	1...	20211111_A19_R3_IgM	8.86	529924.2	11298.6	46.902	MM
156	1...	20211111_A20_R3_IgM	8.86	105382.3	11175.6	9.430	MM
157	1...	20211111_L19_R3_IgM	8.86	347271.6	11558.2	30.046	MM
158	1...	20211111_QC_R21_IgM	8.86	476862.5	9977.0	47.796	MM
159	1...	20211111_L20_R3_IgM	8.86	258946.7	13508.1	19.170	MM
160	1...	20211111_H10_R3_IgM	8.86	607965.8	12374.8	49.129	MM
161	1...	20211111_A21_R3_IgM	8.85	389768.4	12443.6	31.323	MM
162	1...	20211111_A22_R3_IgM	8.86	69831.1	11589.3	6.025	MM
163	1...	20211111_L21_R3_IgM	8.88	409774.8	13107.4	31.263	MM
164	1...	20211111_L22_R3_IgM	8.88	342931.8	12021.5	28.527	MM
165	1...	20211111_H11_R3_IgM	8.88	629976.3	12687.8	49.652	MM
166	1...	20211111_A23_R3_IgM	8.87	291694.4	11611.8	25.120	MM
167	1...	20211111_A24_R3_IgM	8.87	736933.8	10476.8	70.339	MM
168	1...	20211111_L23_R3_IgM	8.86	528041.6	11873.1	44.474	MM

Dataset: C:\MassLynx\10.pro\20211111_clinicalsamples_IgM_IgA.qld

Last Altered: Thursday, March 09, 2023 19:58:25 台北標準時間
Printed: Friday, August 01, 2025 15:04:17 台北標準時間

	#	Name	RT	Area	IS Area	Response	Prima...
169	1...	20211111_L24_R3_IgM	8.89	134051.0	13468.0	9.953	MM
170	1...	20211111_H12_R3_IgM	8.85	188522.1	14229.1	13.249	MM
171	1...	20211111_QC_R22_IgM	8.87	506306.8	11264.4	44.948	MM
172	1...	20211111_A25_R3_IgM	8.87	953152.1	11775.7	80.942	MM
173	1...	20211111_A26_R3_IgM	8.89	615129.4	10473.9	58.730	MM
174	1...	20211111_L25_R3_IgM	8.87	245802.2	13256.8	18.542	MM
175	1...	20211111_L26_R3_IgM	8.89	369903.9	13565.0	27.269	MM
176	1...	20211111_H13_R3_IgM	8.86	566779.5	13146.0	43.114	MM
177	1...	20211111_A27_R3_IgM	8.87	1091687.8	12025.6	90.780	MM
178	1...	20211111_A28_R3_IgM	8.87	237557.7	11229.1	21.156	MM
179	1...	20211111_L27_R3_IgM	8.88	559656.2	14960.0	37.410	MM
180	1...	20211111_L28_R3_IgM	8.86	204853.5	11359.0	18.034	MM
181	1...	20211111_H14_R3_IgM	8.86	429833.6	12830.8	33.500	MM
182	1...	20211111_A29_R3_IgM	8.88	89318.4	13434.3	6.649	MM
183	1...	20211111_A30_R3_IgM	8.86	728252.8	11272.2	64.606	MM
184	1...	20211111_QC_R23_IgM	8.87	491271.2	11872.2	41.380	MM
185	1...	20211111_L29_R3_IgM	8.86	138469.2	12948.7	10.694	MM
186	1...	20211111_L30_R3_IgM	8.87	106815.4	11026.6	9.687	MM
187	1...	20211111_H15_R3_IgM	8.86	362291.1	10786.3	33.588	MM
188	1...	20211111_A31_R3_IgM	8.86	244581.2	12010.5	20.364	MM
189	1...	20211111_A32_R3_IgM	8.86	470633.3	11107.7	42.370	MM
190	1...	20211111_L31_R3_IgM	8.86	70213.2	11021.8	6.370	MM
191	1...	20211111_L32_R3_IgM	8.86	578515.5	11683.8	49.514	MM
192	1...	20211111_H16_R3_IgM	8.85	656648.8	9831.7	66.789	MM
193	1...	20211111_A33_R3_IgM	8.86	976116.0	11213.1	87.051	MM
194	1...	20211111_A34_R3_IgM	8.88	246563.2	11550.1	21.347	MM
195	1...	20211111_H17_R3_IgM	8.84	1586467.8	8874.4	178.768	MM
196	1...	20211111_A35_R3_IgM	8.86	220030.3	9856.3	22.324	MM
197	1...	20211111_QC_R24_IgM	8.86	426191.1	7561.8	56.361	MM
198	1...	20211111_A36_R3_IgM	8.85	301883.7	9562.8	31.568	MM
199	1...	20211111_H18_R3_IgM	8.85	907773.4	8165.3	111.175	MM
200	2...	20211111_A37_R3_IgM	8.85	511629.5	9278.8	55.140	MM
201	2...	20211111_A38_R3_IgM	8.87	470789.4	10920.3	43.111	MM
202	2...	20211111_H19_R3_IgM	8.84	569732.6	9285.3	61.359	MM
203	2...	20211111_A39_R3_IgM	8.88	557027.5	9083.4	61.324	MM
204	2...	20211111_A40_R3_IgM	8.87	220453.6	8382.4	26.300	MM
205	2...	20211111_H20_R3_IgM	8.85	1328140.8	7807.9	170.102	MM
206	2...	20211111_A41_R3_IgM	8.88	264161.6	5925.2	44.582	MM
207	2...	20211111_A42_R3_IgM	8.87	158961.4	9352.6	16.996	MM
208	2...	20211111_A43_R3_IgM	8.85	164280.4	10216.3	16.080	MM
209	2...	20211111_A44_R3_IgM	8.85	77483.6	10262.7	7.550	MM
210	2...	20211111_QC_R25_IgM	8.86	419916.4	8575.3	48.968	MM

Dataset: C:\MassLynx\10.pro\20211111_clinicalsamples_IgM_IgA.qld

Last Altered: Thursday, March 09, 2023 19:58:25 台北標準時間
Printed: Friday, August 01, 2025 15:05:35 台北標準時間

Method: C:\MassLynx\10_Pro_IgA.PRO\MethDB\20211111_IgM_IgA_DMRM_new.mdb 24 Feb 2022 13:44:14
Calibration: 15 Nov 2021 14:42:11

Sample Name: 20211112_QC_R001_IgM

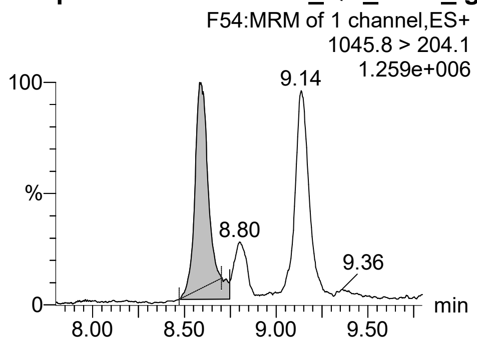

Sample Name: 20211112_QC_R002_IgM

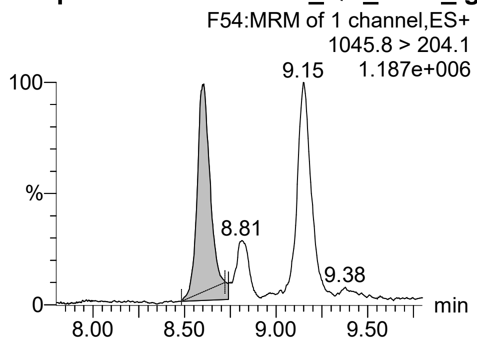

Sample Name: 20211111_A1_R2_IgM

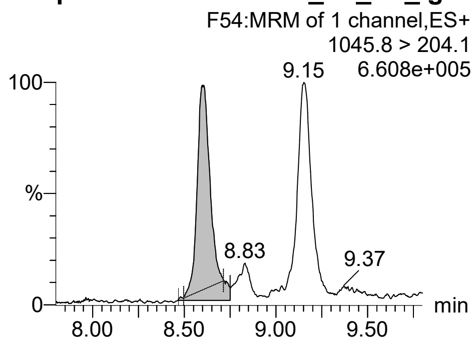

Sample Name: 20211111_A1_R3_IgM

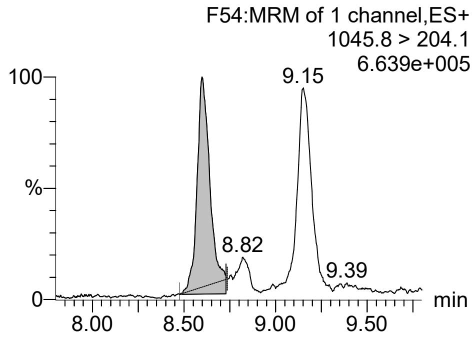

Sample Name: 20211111_A2_R2_IgM

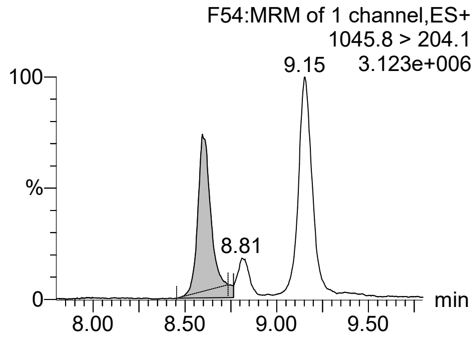

Sample Name: 20211111_A2_R3_IgM

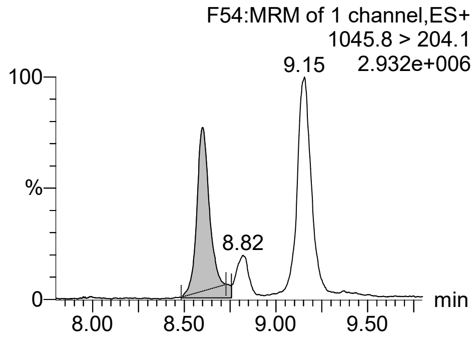

Sample Name: 20211111_L1_R2_IgM

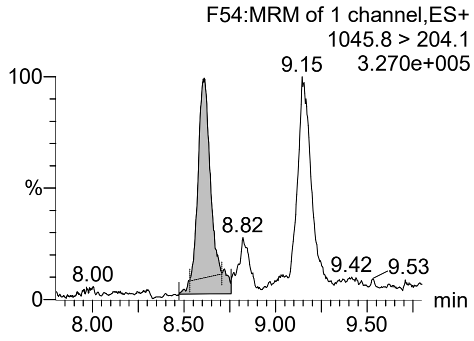

Sample Name: 20211111_L1_R3_IgM

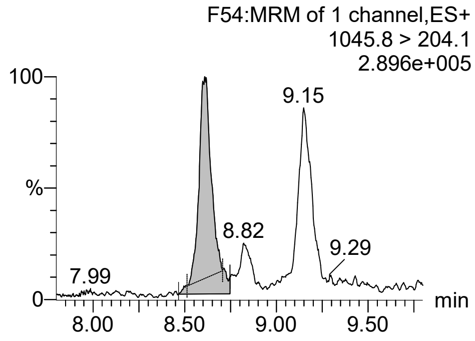

Sample Name: 20211111_L2_R2_IgM

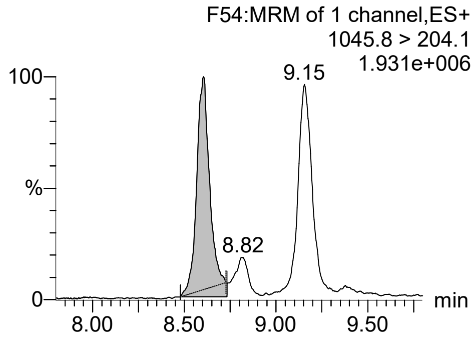

Sample Name: 20211111_H1_R2_IgM

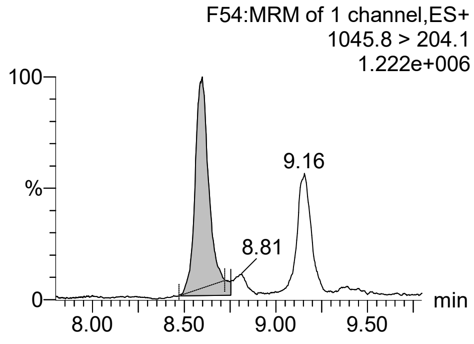

Sample Name: 20211111_A3_R2_IgM

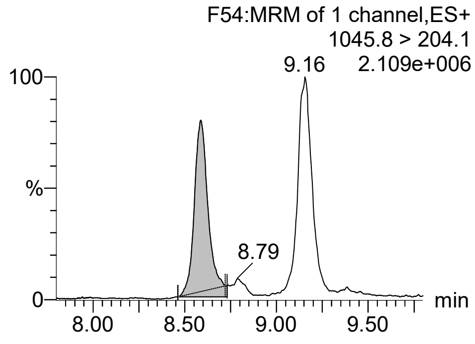

Sample Name: 20211111_A4_R2_IgM

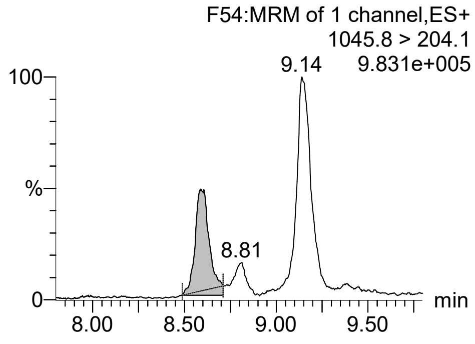

Sample Name: 20211111_L3_R2_IgM
F54:MRM of 1 channel,ES+
1045.8 > 204.1
5.493e+005

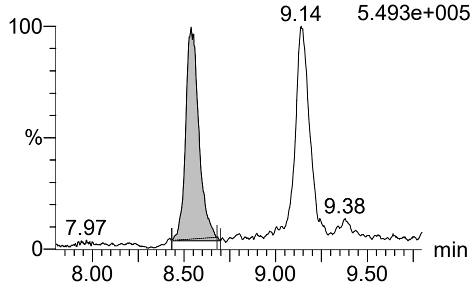

Sample Name: 20211111_L4_R2_IgM
F54:MRM of 1 channel,ES+
1045.8 > 204.1
1.281e+006

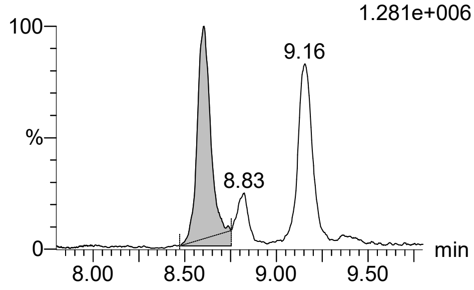

Sample Name: 20211111_H2_R2_IgM
F54:MRM of 1 channel,ES+
1045.8 > 204.1
2.070e+006

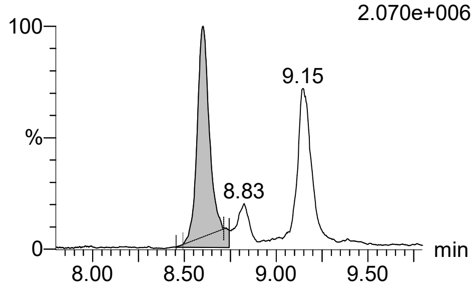

Sample Name: 20211111_A5_R2_IgM
F54:MRM of 1 channel,ES+
1045.8 > 204.1
5.314e+006

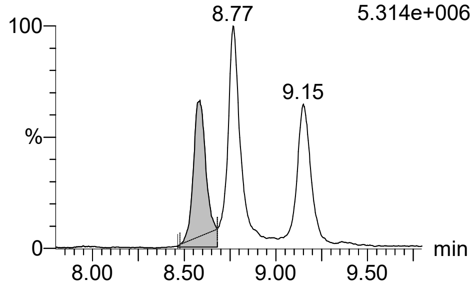

Sample Name: 20211111_A6_R2_IgM
F54:MRM of 1 channel,ES+
1045.8 > 204.1
2.078e+006

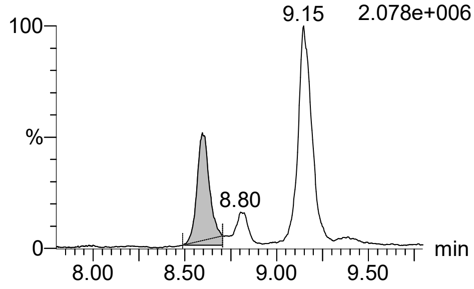

Sample Name: 20211111_QC_R10_IgM
F54:MRM of 1 channel,ES+
1045.8 > 204.1
1.278e+006

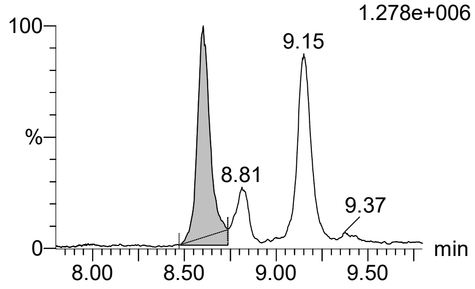

Sample Name: 20211111_L5_R2_IgM
F54:MRM of 1 channel,ES+
1045.8 > 204.1
1.931e+006

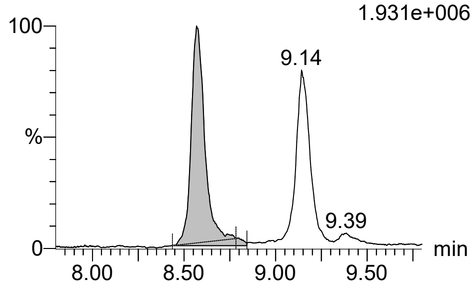

Sample Name: 20211111_L6_R2_IgM
F54:MRM of 1 channel,ES+
1045.8 > 204.1
1.672e+006

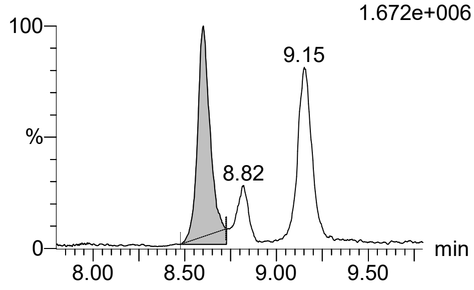

Sample Name: 20211111_H3_R2_IgM
F54:MRM of 1 channel,ES+
1045.8 > 204.1
1.341e+006

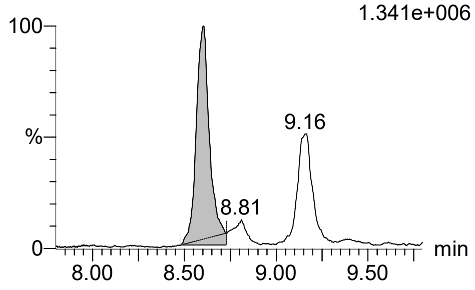

Sample Name: 20211111_A7_R2_IgM
F54:MRM of 1 channel,ES+
1045.8 > 204.1
2.420e+006

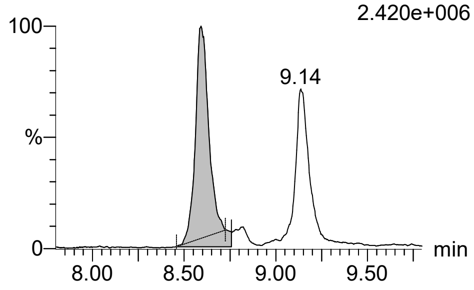

Sample Name: 20211111_A8_R2_IgM
F54:MRM of 1 channel,ES+
1045.8 > 204.1
2.156e+006

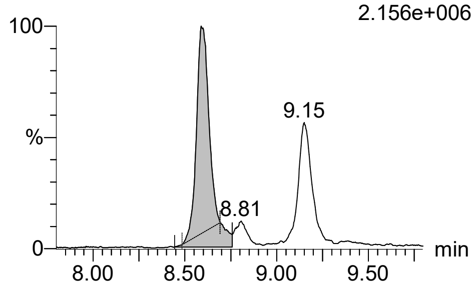

Sample Name: 20211111_L7_R2_IgM
F54:MRM of 1 channel,ES+
1045.8 > 204.1
5.713e+005

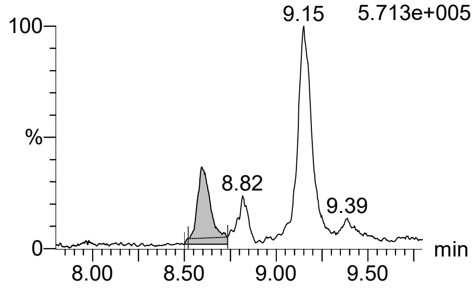

Sample Name: 20211111_L8_R2_IgM
F54:MRM of 1 channel,ES+
1045.8 > 204.1
5.604e+005

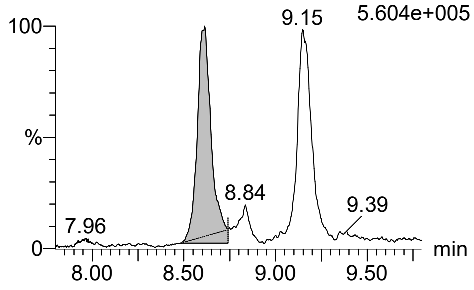

Sample Name: 20211111_H4_R2_IgM
F54:MRM of 1 channel,ES+
1045.8 > 204.1
1.089e+006

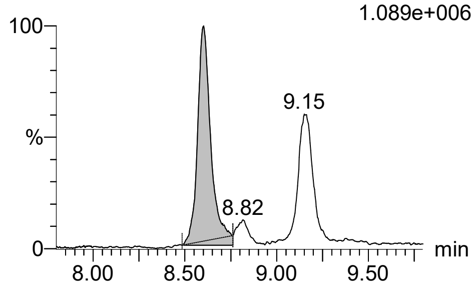

Sample Name: 20211111_A9_R2_IgM
F54:MRM of 1 channel,ES+
1045.8 > 204.1
1.097e+006

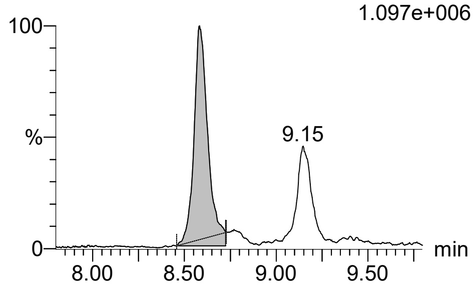

Sample Name: 20211111_A10_R2_IgM

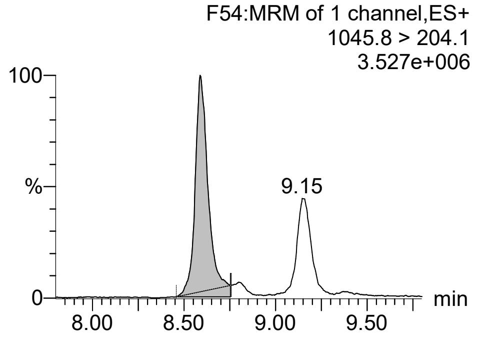

Sample Name: 20211111_L9_R2_IgM

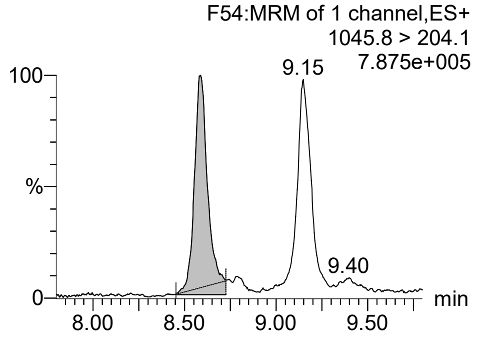

Sample Name: 20211111_L10_R2_IgM

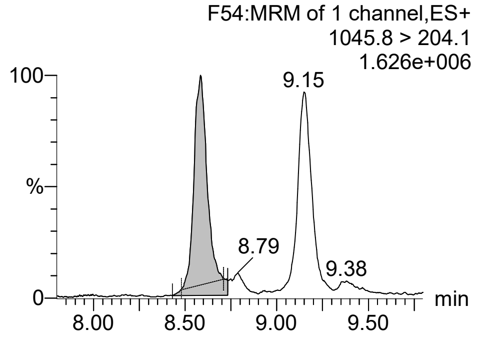

Sample Name: 20211111_QC_R11_IgM

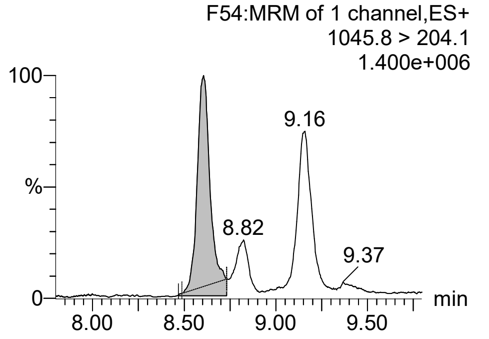

Sample Name: 20211111_H5_R2_IgM

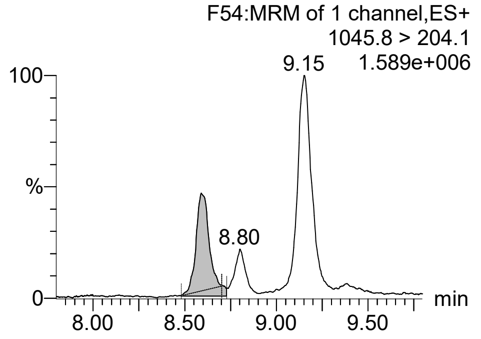

Sample Name: 20211111_A11_R2_IgM

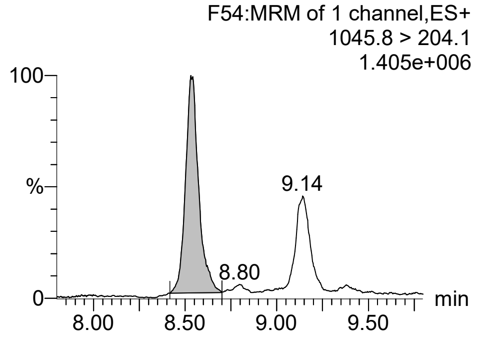

Sample Name: 20211111_A12_R2_IgM

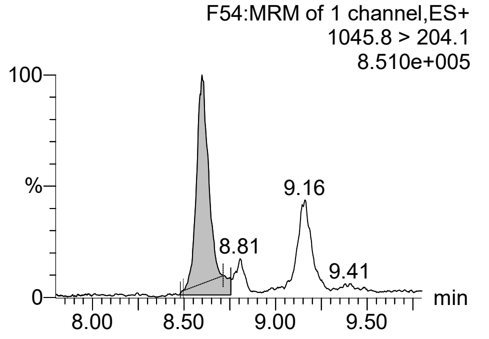

Sample Name: 20211111_L11_R2_IgM

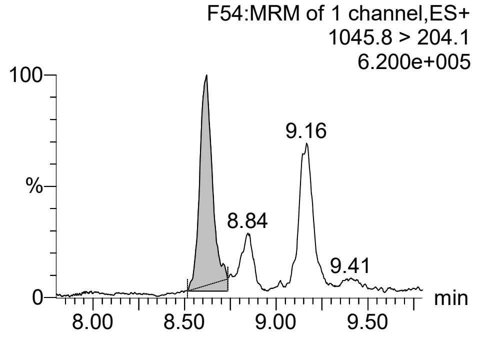

Sample Name: 20211111_L12_R2_IgM

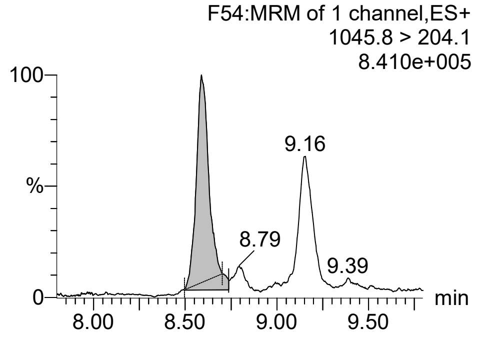

Sample Name: 20211111_H6_R2_IgM

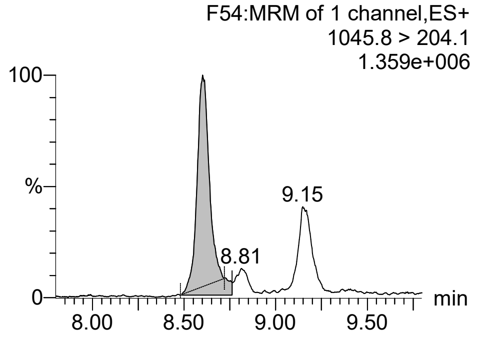

Sample Name: 20211111_A13_R2_IgM

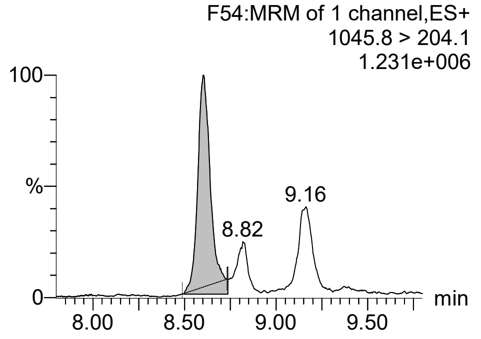

Sample Name: 20211111_A14_R2_IgM

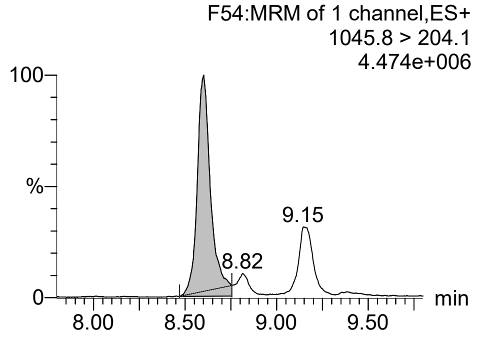

Sample Name: 20211111_L13_R2_IgM

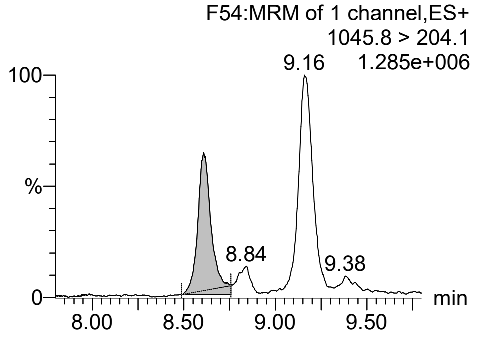

Sample Name: 20211111_L14_R2_IgM

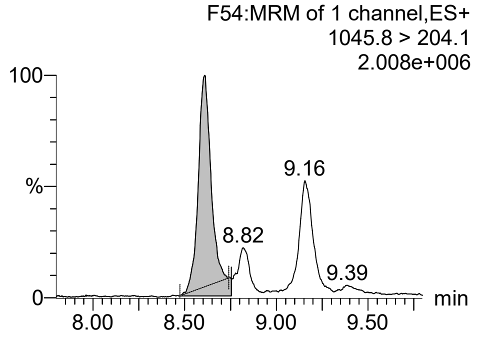

Sample Name: 20211111_H7_R2_IgM

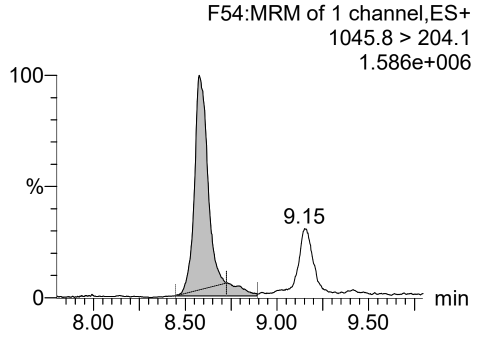

Dataset: C:\MassLynx\10.pro\20211111_clinicalsamples_IgM_IgA.qld

Last Altered: Thursday, March 09, 2023 19:58:25 台北標準時間
Printed: Friday, August 01, 2025 15:05:35 台北標準時間

Sample Name: 20211111_A15_R2_IgM
F54:MRM of 1 channel,ES+
1045.8 > 204.1
5.009e+006

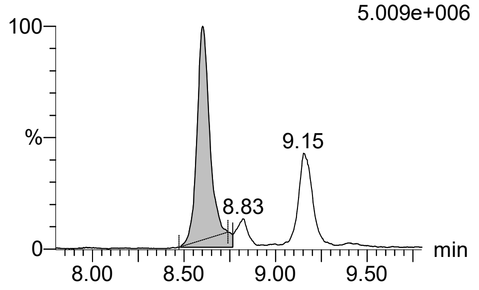

Sample Name: 20211111_QC_R12_IgM
F54:MRM of 1 channel,ES+
1045.8 > 204.1
1.539e+006

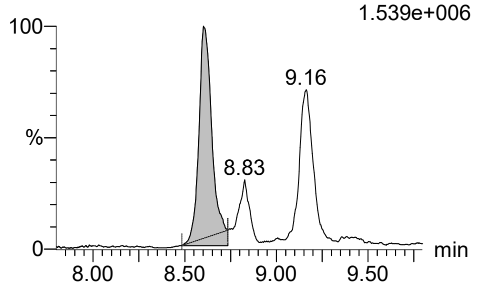

Sample Name: 20211111_A16_R2_IgM
F54:MRM of 1 channel,ES+
1045.8 > 204.1
2.544e+006

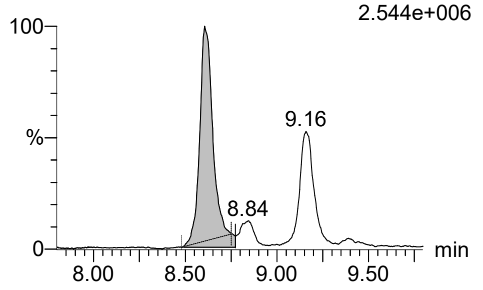

Sample Name: 20211111_L15_R2_IgM
F54:MRM of 1 channel,ES+
1045.8 > 204.1
2.626e+006

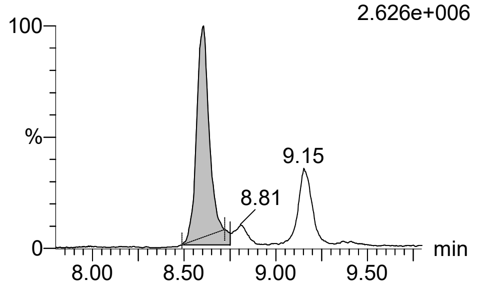

Sample Name: 20211111_L16_R2_IgM
F54:MRM of 1 channel,ES+
1045.8 > 204.1
4.533e+005

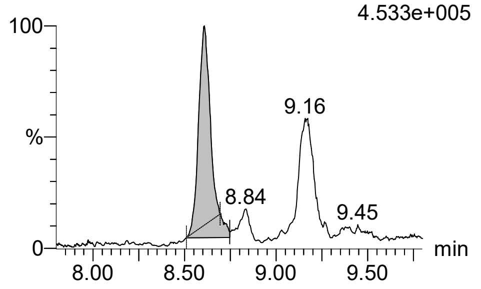

Sample Name: 20211111_H8_R2_IgM
F54:MRM of 1 channel,ES+
1045.8 > 204.1
9.493e+005

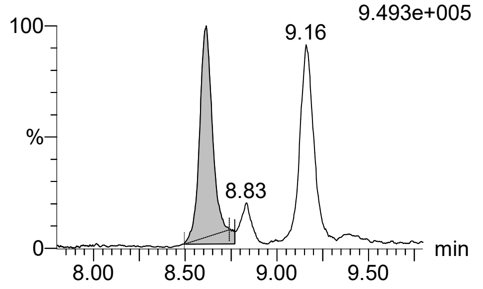

Sample Name: 20211111_A17_R2_IgM
F54:MRM of 1 channel,ES+
1045.8 > 204.1
1.829e+006

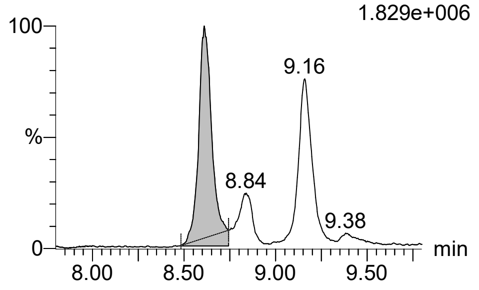

Sample Name: 20211111_A18_R2_IgM
F54:MRM of 1 channel,ES+
1045.8 > 204.1
2.050e+006

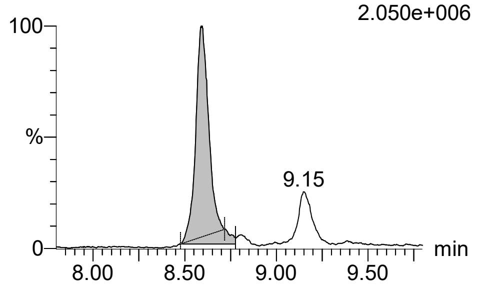

Sample Name: 20211111_L17_R2_IgM
F54:MRM of 1 channel,ES+
1045.8 > 204.1
2.152e+006

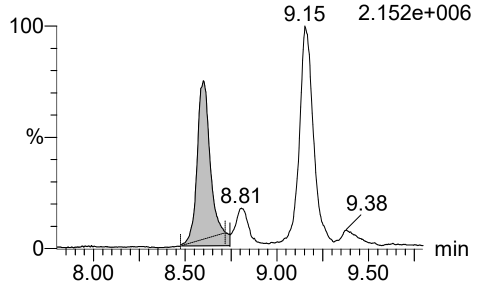

Sample Name: 20211111_L18_R2_IgM
F54:MRM of 1 channel,ES+
1045.8 > 204.1
2.205e+005

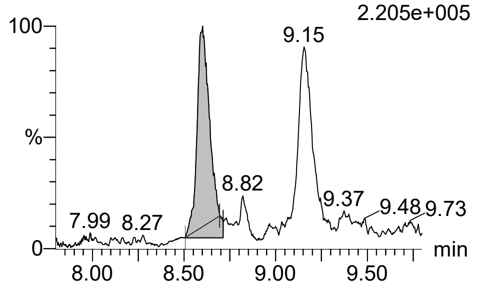

Sample Name: 20211111_H9_R2_IgM
F54:MRM of 1 channel,ES+
1045.8 > 204.1
7.220e+005

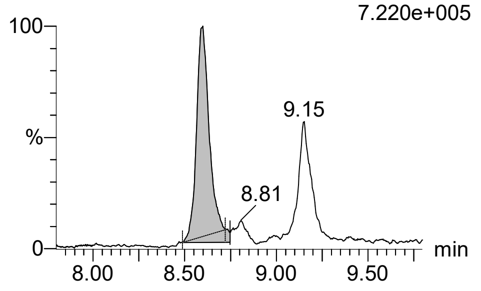

Sample Name: 20211111_A19_R2_IgM
F54:MRM of 1 channel,ES+
1045.8 > 204.1
1.352e+006

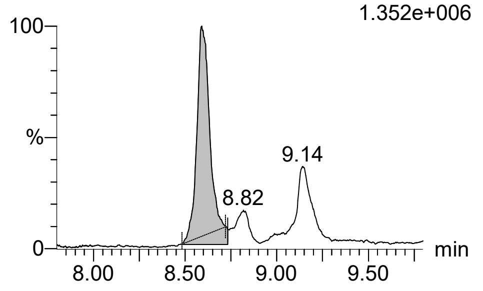

Sample Name: 20211111_A20_R2_IgM
F54:MRM of 1 channel,ES+
1045.8 > 204.1
8.978e+005

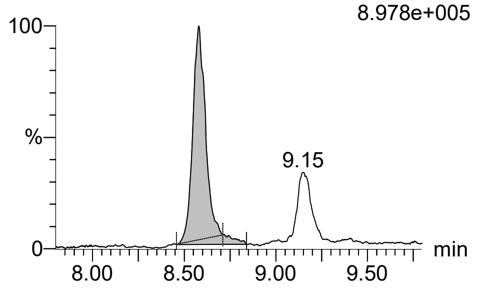

Sample Name: 20211111_L19_R2_IgM
F54:MRM of 1 channel,ES+
1045.8 > 204.1
1.257e+006

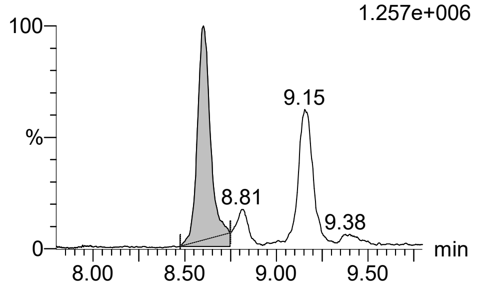

Sample Name: 20211111_QC_R13_IgM
F54:MRM of 1 channel,ES+
1045.8 > 204.1
1.418e+006

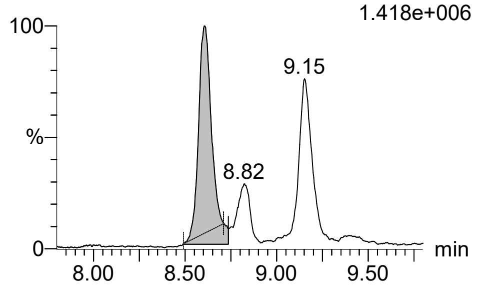

Dataset: C:\MassLynx\10.pro\20211111_clinicalsamples_IgM_IgA.qld

Last Altered: Thursday, March 09, 2023 19:58:25 台北標準時間
Printed: Friday, August 01, 2025 15:05:35 台北標準時間

Sample Name: 20211111_L20_R2_IgM
F54:MRM of 1 channel,ES+
1045.8 > 204.1
5.804e+005

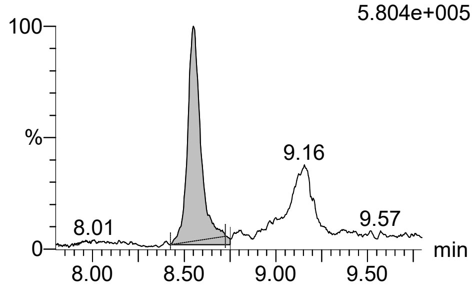

Sample Name: 20211111_H10_R2_IgM
F54:MRM of 1 channel,ES+
1045.8 > 204.1
1.320e+006

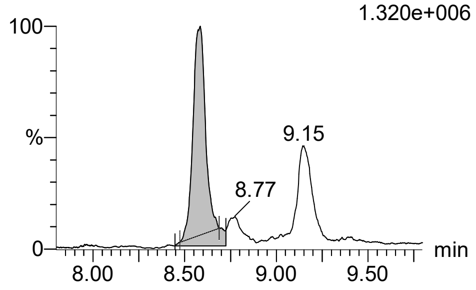

Sample Name: 20211111_A21_R2_IgM
F54:MRM of 1 channel,ES+
1045.8 > 204.1
1.566e+006

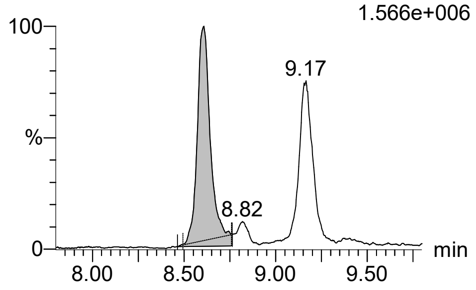

Sample Name: 20211111_A22_R2_IgM
F54:MRM of 1 channel,ES+
1045.8 > 204.1
4.197e+005

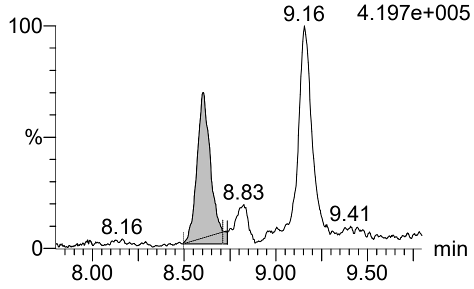

Sample Name: 20211111_L21_R2_IgM
F54:MRM of 1 channel,ES+
1045.8 > 204.1
8.223e+005

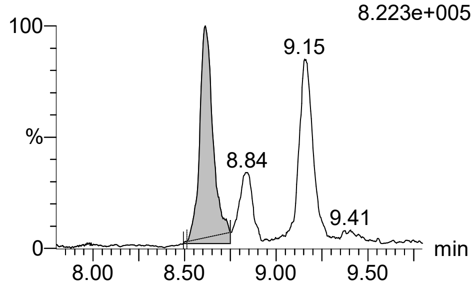

Sample Name: 20211111_L22_R2_IgM
F54:MRM of 1 channel,ES+
1045.8 > 204.1
1.138e+006

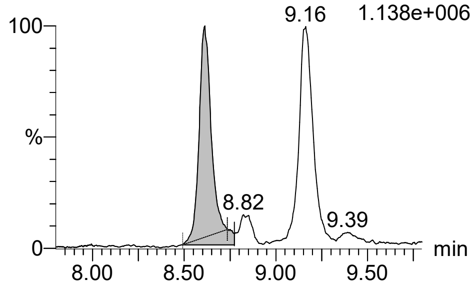

Sample Name: 20211111_H11_R2_IgM
F54:MRM of 1 channel,ES+
1045.8 > 204.1
1.081e+006

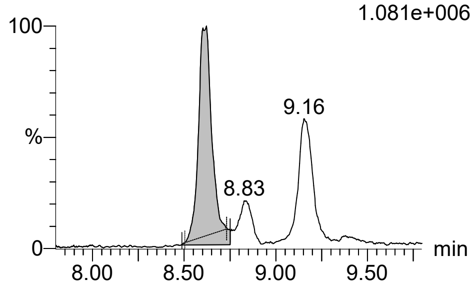

Sample Name: 20211111_A23_R2_IgM
F54:MRM of 1 channel,ES+
1045.8 > 204.1
7.219e+005

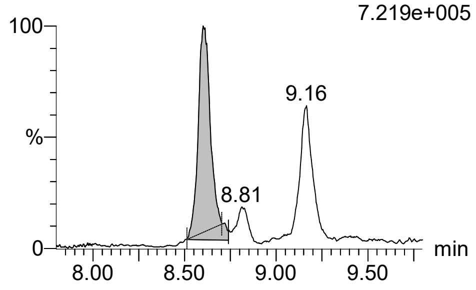

Sample Name: 20211111_A24_R2_IgM
F54:MRM of 1 channel,ES+
1045.8 > 204.1
1.891e+006

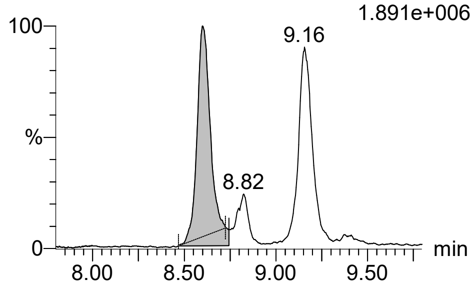

Sample Name: 20211111_L23_R2_IgM
F54:MRM of 1 channel,ES+
1045.8 > 204.1
1.124e+006

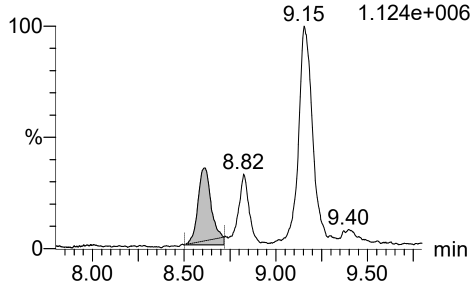

Sample Name: 20211111_L24_R2_IgM
F54:MRM of 1 channel,ES+
1045.8 > 204.1
5.703e+005

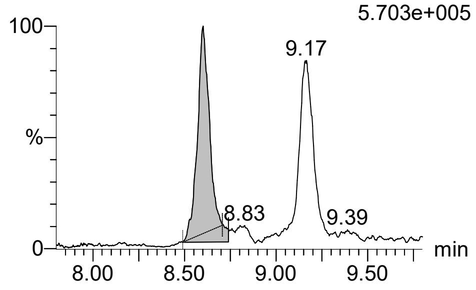

Sample Name: 20211111_H12_R2_IgM
F54:MRM of 1 channel,ES+
1045.8 > 204.1
6.128e+005

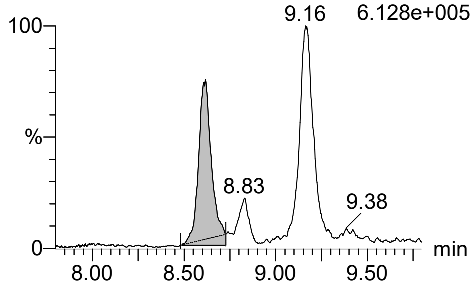

Sample Name: 20211111_QC_R14_IgM
F54:MRM of 1 channel,ES+
1045.8 > 204.1
1.629e+006

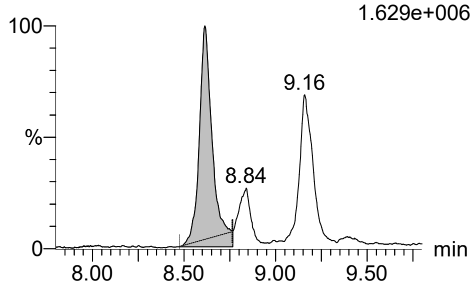

Sample Name: 20211111_A25_R2_IgM
F54:MRM of 1 channel,ES+
1045.8 > 204.1
2.293e+006

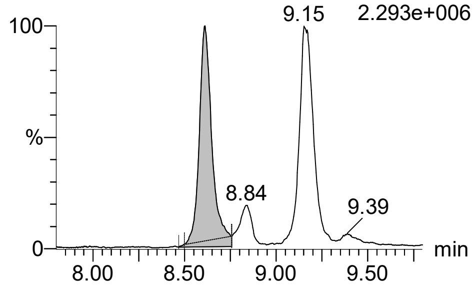

Sample Name: 20211111_A26_R2_IgM
F54:MRM of 1 channel,ES+
1045.8 > 204.1
8.418e+005

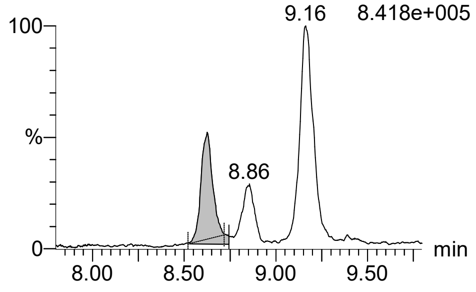

Dataset: C:\MassLynx\10.pro\20211111_clinicalsamples_IgM_IgA.qld

Last Altered: Thursday, March 09, 2023 19:58:25 台北標準時間
Printed: Friday, August 01, 2025 15:05:35 台北標準時間

Sample Name: 20211111_L25_R2_IgM

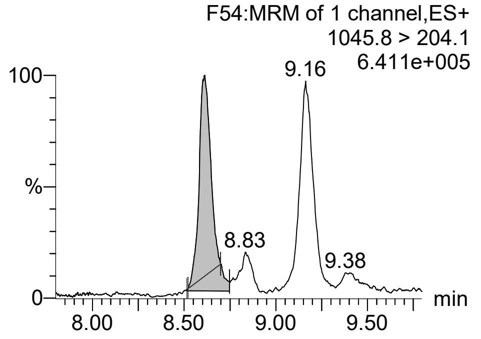

Sample Name: 20211111_L26_R2_IgM

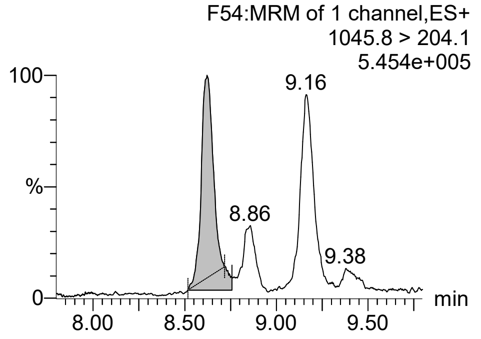

Sample Name: 20211111_H13_R2_IgM

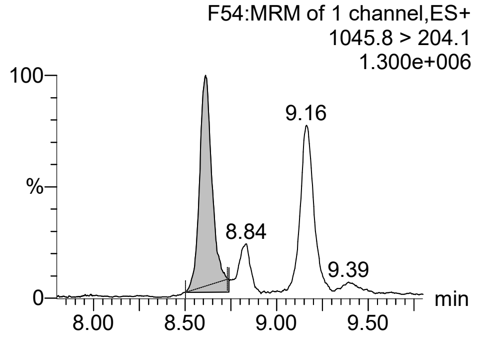

Sample Name: 20211111_A27_R2_IgM

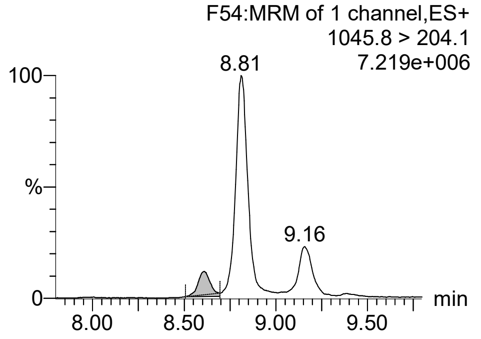

Sample Name: 20211111_A28_R2_IgM

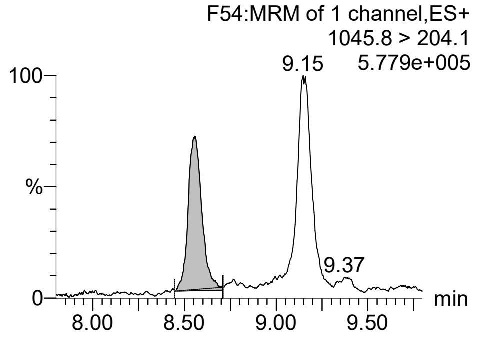

Sample Name: 20211111_L27_R2_IgM

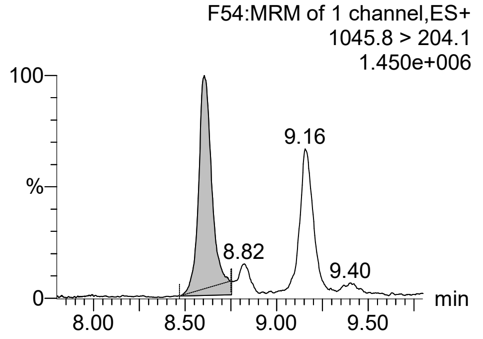

Sample Name: 20211111_L28_R2_IgM

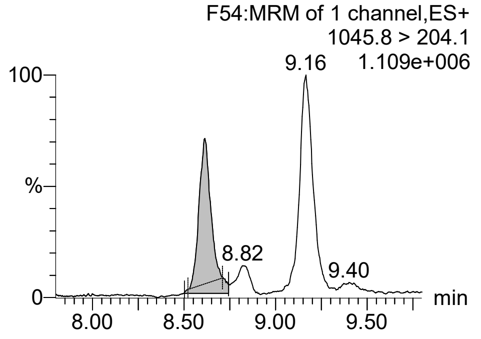

Sample Name: 20211111_H14_R2_IgM

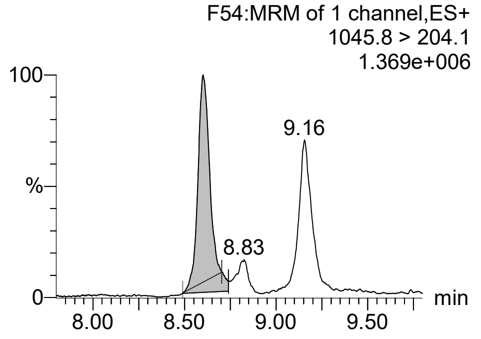

Sample Name: 20211111_A29_R2_IgM

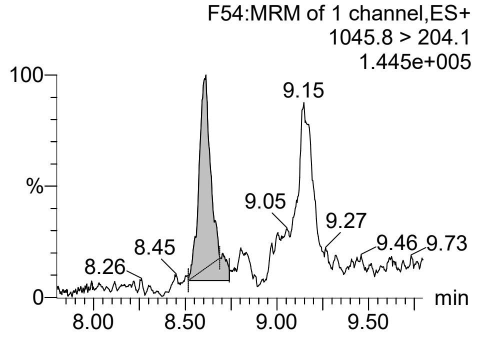

Sample Name: 20211111_A30_R2_IgM

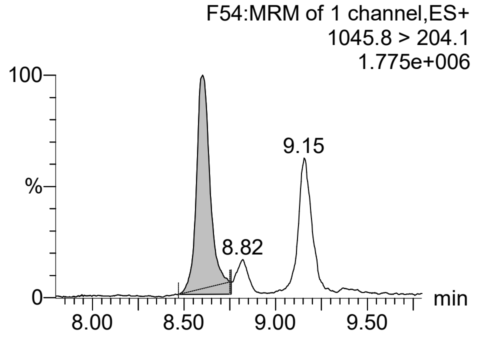

Sample Name: 20211111_QC_R15_IgM

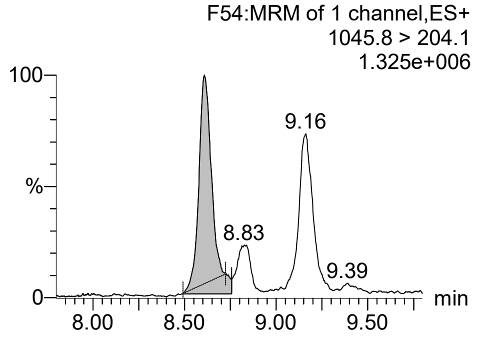

Sample Name: 20211111_L29_R2_IgM

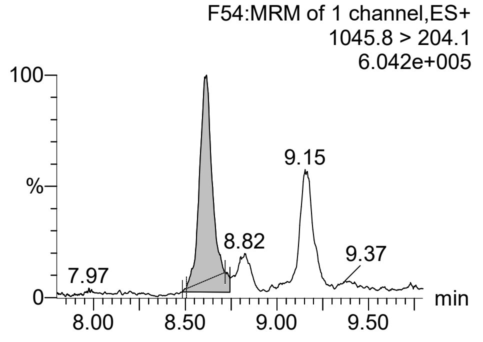

Sample Name: 20211111_L30_R2_IgM

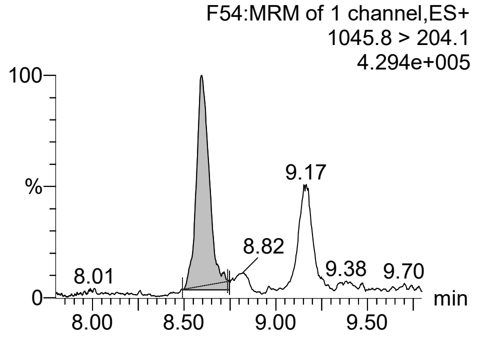

Sample Name: 20211111_H15_R2_IgM

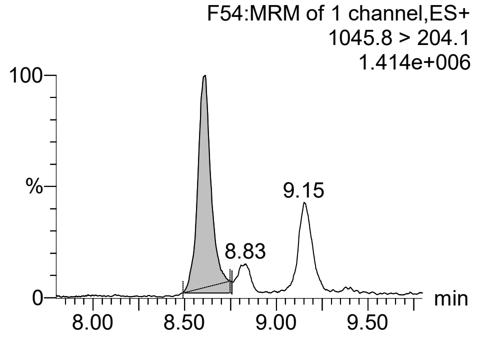

Sample Name: 20211111_A31_R2_IgM

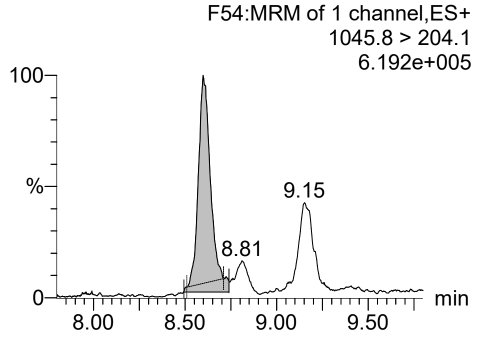

Sample Name: 20211111_A32_R2_IgM

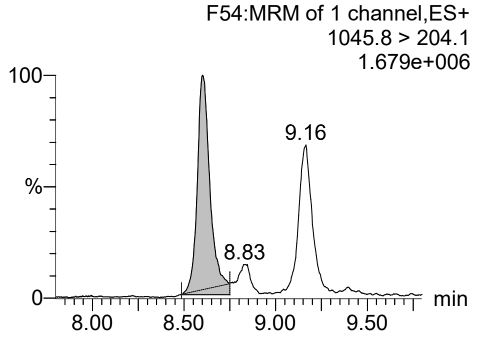

Sample Name: 20211111_L31_R2_IgM

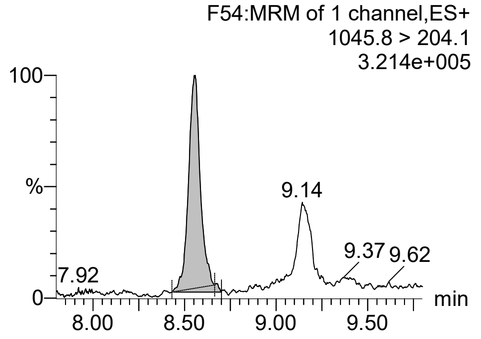

Sample Name: 20211111_L32_R2_IgM

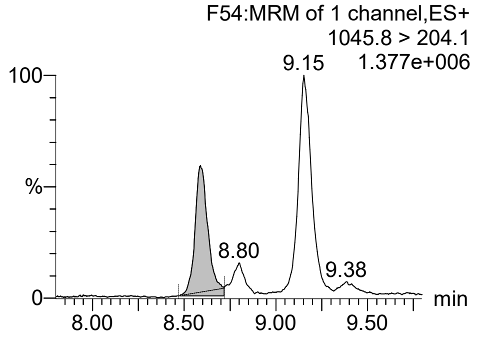

Sample Name: 20211111_H16_R2_IgM

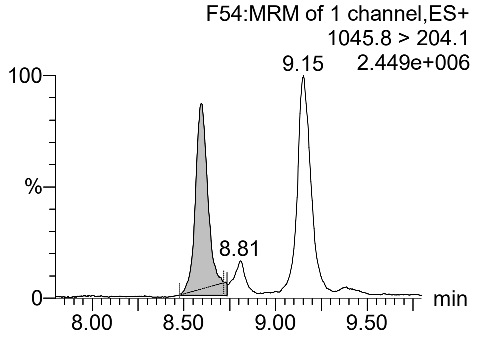

Sample Name: 20211111_A33_R2_IgM

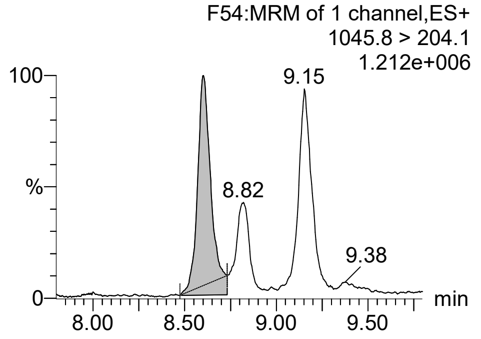

Sample Name: 20211111_A34_R2_IgM

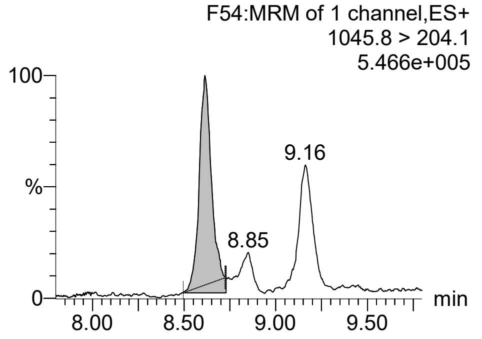

Sample Name: 20211111_H17_R2_IgM

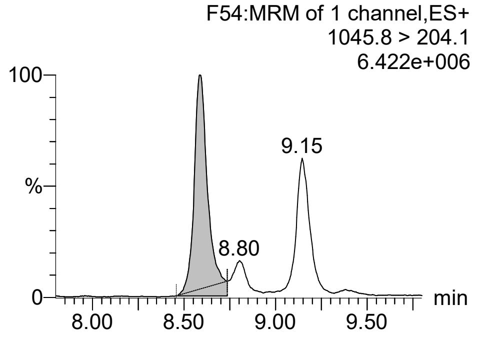

Sample Name: 20211111_A35_R2_IgM

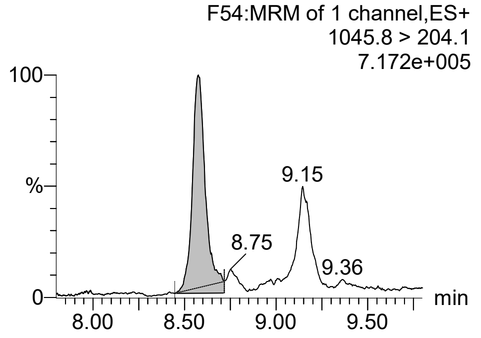

Sample Name: 20211111_QC_R16_IgM

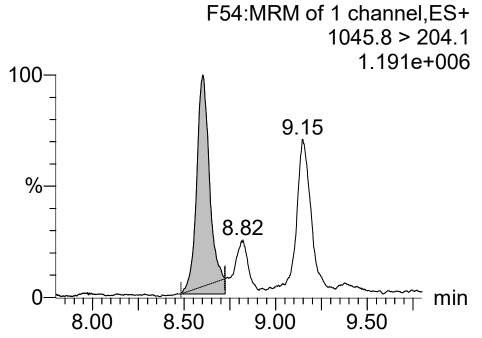

Sample Name: 20211111_A36_R2_IgM

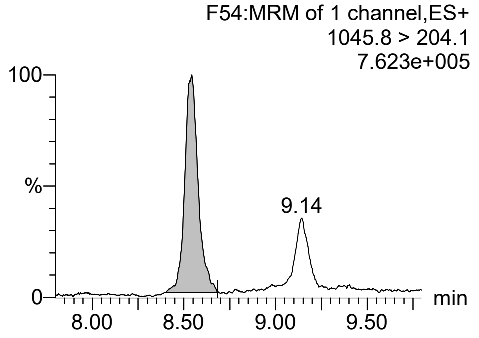

Sample Name: 20211111_H18_R2_IgM

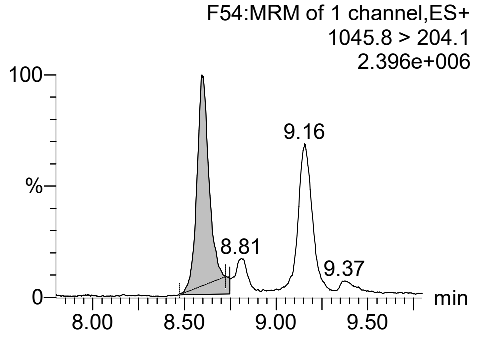

Sample Name: 20211111_A37_R2_IgM

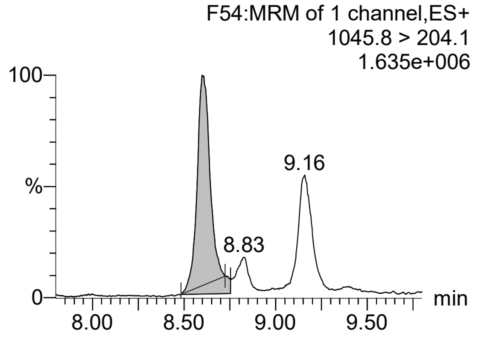

Sample Name: 20211111_A38_R2_IgM

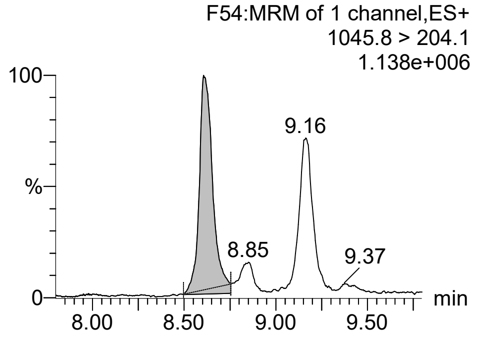

Sample Name: 20211111_H19_R2_IgM

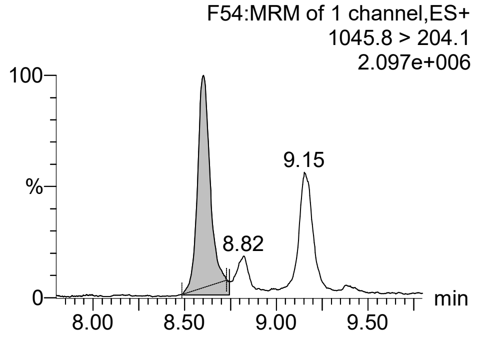

Sample Name: 20211111_A39_R2_IgM

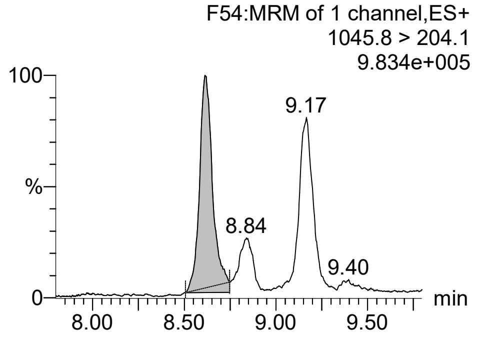

Dataset: C:\MassLynx\10.pro\20211111_clinicalsamples_IgM_IgA.qld

Last Altered: Thursday, March 09, 2023 19:58:25 台北標準時間
Printed: Friday, August 01, 2025 15:05:35 台北標準時間

Sample Name: 20211111_A40_R2_IgM
F54:MRM of 1 channel,ES+
1045.8 > 204.1
1.127e+006

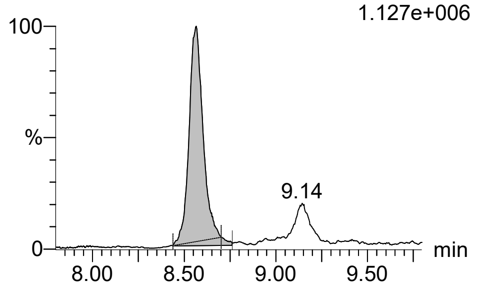

Sample Name: 20211111_H20_R2_IgM
F54:MRM of 1 channel,ES+
1045.8 > 204.1
4.648e+006

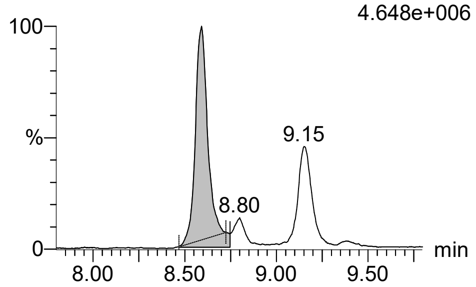

Sample Name: 20211111_A41_R2_IgM
F54:MRM of 1 channel,ES+
1045.8 > 204.1
6.090e+005

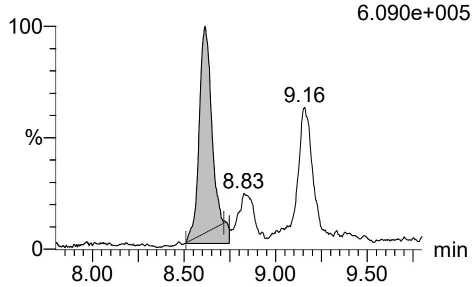

Sample Name: 20211111_A42_R2_IgM
F54:MRM of 1 channel,ES+
1045.8 > 204.1
6.405e+005

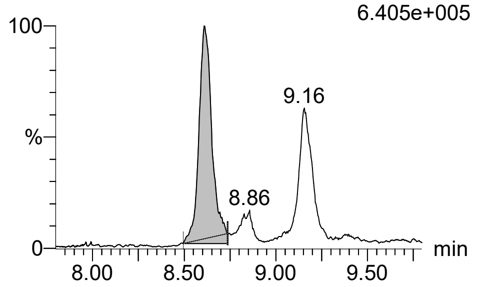

Sample Name: 20211111_A43_R2_IgM
F54:MRM of 1 channel,ES+
1045.8 > 204.1
5.614e+005

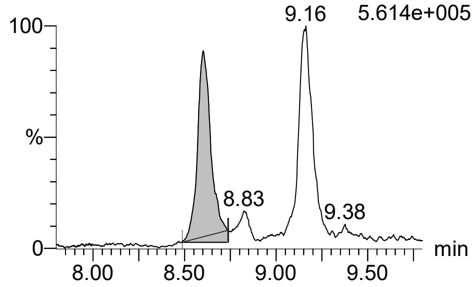

Sample Name: 20211111_A44_R2_IgM
F54:MRM of 1 channel,ES+
1045.8 > 204.1
3.284e+005

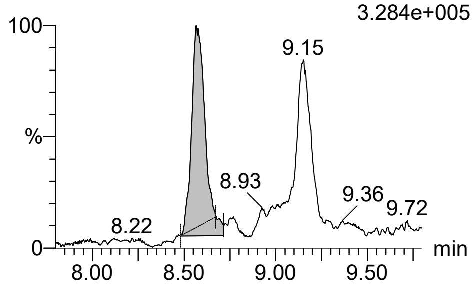

Sample Name: 20211111_QC_R17_IgM
F54:MRM of 1 channel,ES+
1045.8 > 204.1
1.089e+006

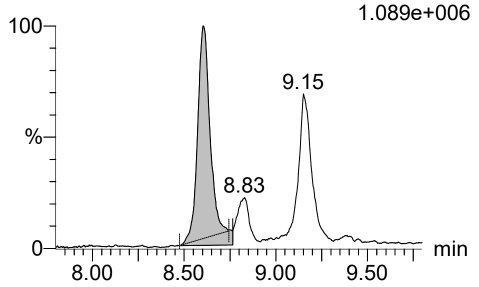

Sample Name: 20211111_L2_R3_IgM
F54:MRM of 1 channel,ES+
1045.8 > 204.1
2.018e+006

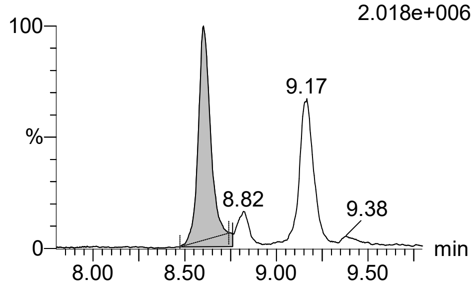

Sample Name: 20211111_H1_R3_IgM
F54:MRM of 1 channel,ES+
1045.8 > 204.1
1.154e+006

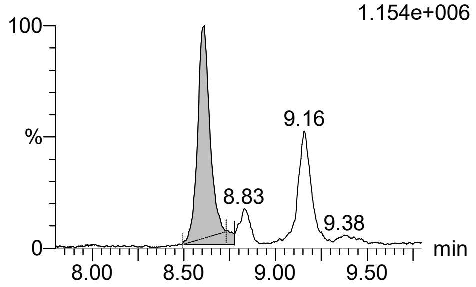

Sample Name: 20211111_A3_R3_IgM
F54:MRM of 1 channel,ES+
1045.8 > 204.1
1.755e+006

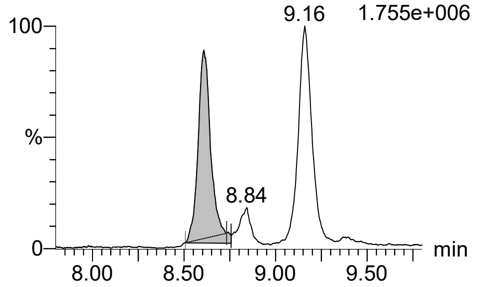

Sample Name: 20211111_A4_R3_IgM
F54:MRM of 1 channel,ES+
1045.8 > 204.1
8.528e+005

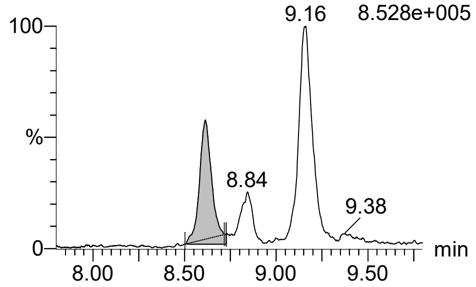

Sample Name: 20211111_L3_R3_IgM
F54:MRM of 1 channel,ES+
1045.8 > 204.1
4.869e+005

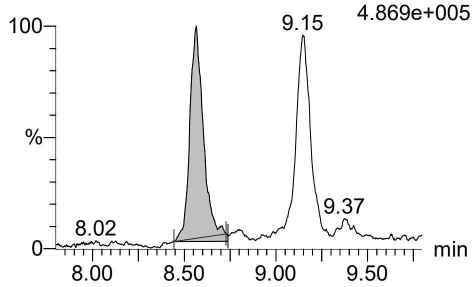

Sample Name: 20211111_L4_R3_IgM
F54:MRM of 1 channel,ES+
1045.8 > 204.1
1.203e+006

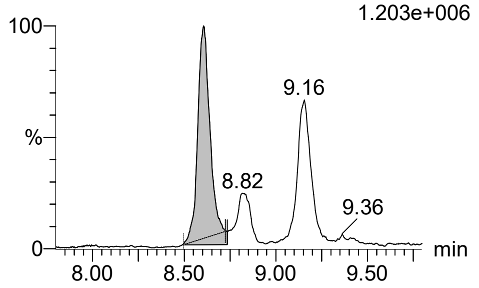

Sample Name: 20211111_H2_R3_IgM
F54:MRM of 1 channel,ES+
1045.8 > 204.1
2.057e+006

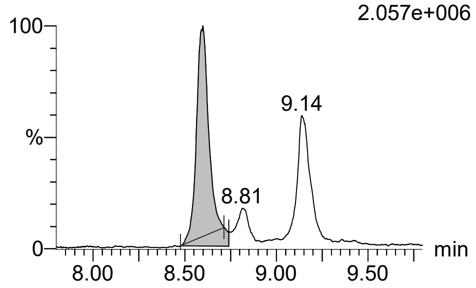

Sample Name: 20211111_A5_R3_IgM
F54:MRM of 1 channel,ES+
1045.8 > 204.1
6.591e+006

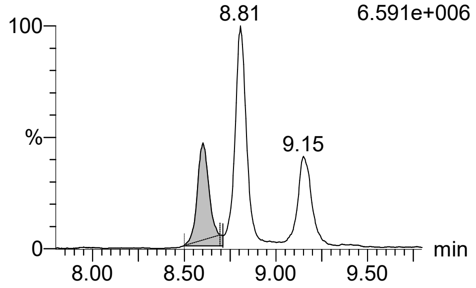

Dataset: C:\MassLynx\10.pro\20211111_clinicalsamples_IgM_IgA.qld

Last Altered: Thursday, March 09, 2023 19:58:25 台北標準時間
Printed: Friday, August 01, 2025 15:05:35 台北標準時間

Sample Name: 20211111_A6_R3_IgM
F54:MRM of 1 channel,ES+
1045.8 > 204.1
1.423e+006

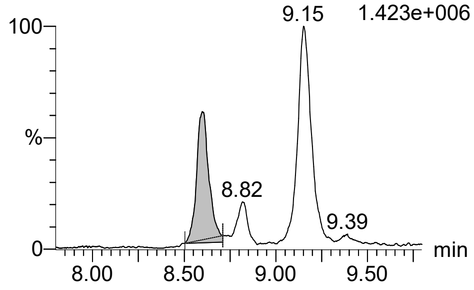

Sample Name: 20211111_QC_R18_IgM
F54:MRM of 1 channel,ES+
1045.8 > 204.1
1.032e+006

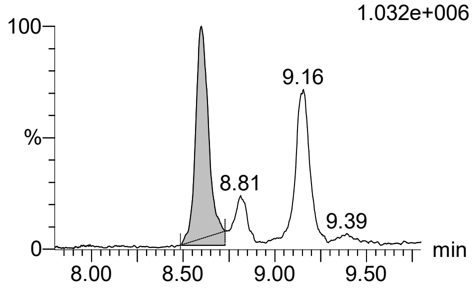

Sample Name: 20211111_L5_R3_IgM
F54:MRM of 1 channel,ES+
1045.8 > 204.1
1.549e+006

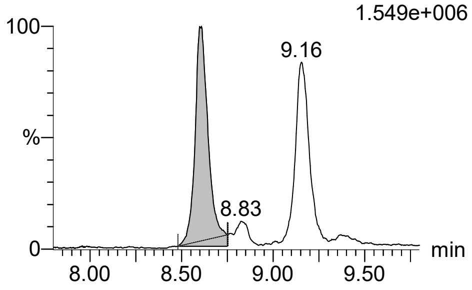

Sample Name: 20211111_L6_R3_IgM
F54:MRM of 1 channel,ES+
1045.8 > 204.1
1.291e+006

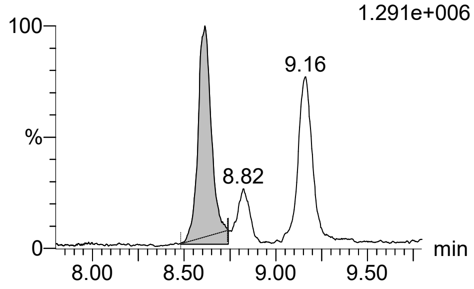

Sample Name: 20211111_H3_R3_IgM
F54:MRM of 1 channel,ES+
1045.8 > 204.1
1.057e+006

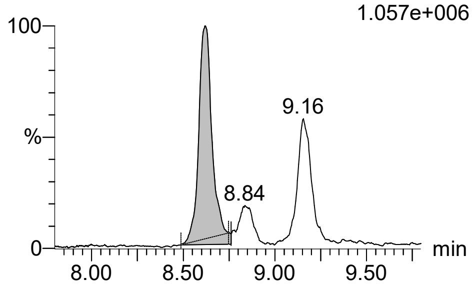

Sample Name: 20211111_A7_R3_IgM
F54:MRM of 1 channel,ES+
1045.8 > 204.1
2.507e+006

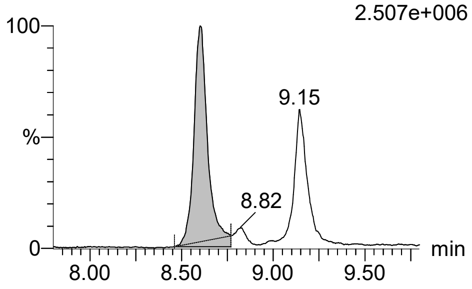

Sample Name: 20211111_A8_R3_IgM
F54:MRM of 1 channel,ES+
1045.8 > 204.1
1.816e+006

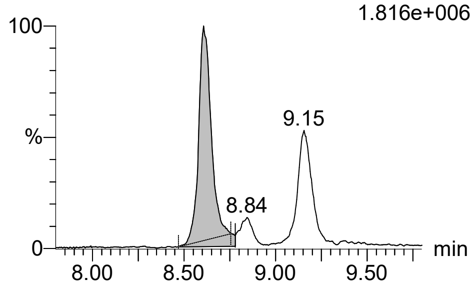

Sample Name: 20211111_L7_R3_IgM
F54:MRM of 1 channel,ES+
1045.8 > 204.1
4.919e+005

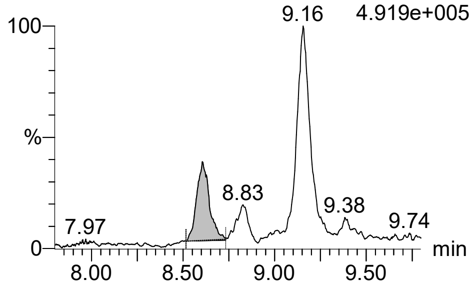

Sample Name: 20211111_L8_R3_IgM
F54:MRM of 1 channel,ES+
1045.8 > 204.1
4.911e+005

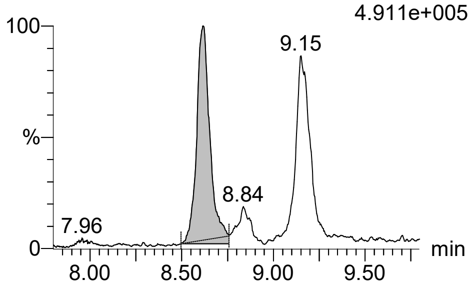

Sample Name: 20211111_H4_R3_IgM
F54:MRM of 1 channel,ES+
1045.8 > 204.1
8.578e+005

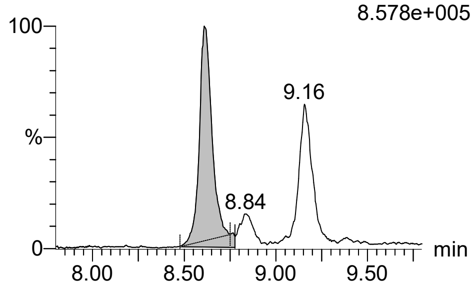

Sample Name: 20211111_A9_R3_IgM
F54:MRM of 1 channel,ES+
1045.8 > 204.1
9.424e+005

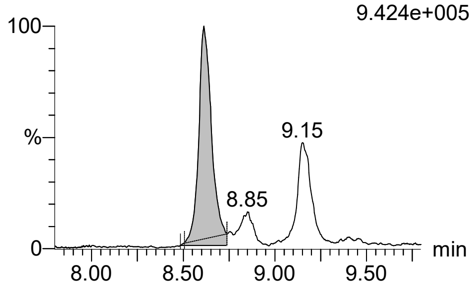

Sample Name: 20211111_A10_R3_IgM
F54:MRM of 1 channel,ES+
1045.8 > 204.1
3.164e+006

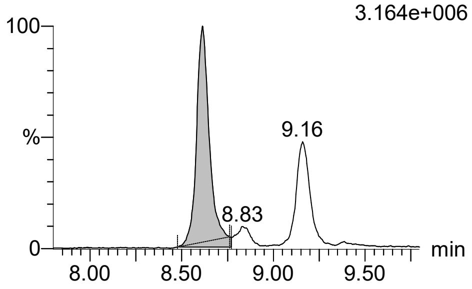

Sample Name: 20211111_L9_R3_IgM
F54:MRM of 1 channel,ES+
1045.8 > 204.1
7.139e+005

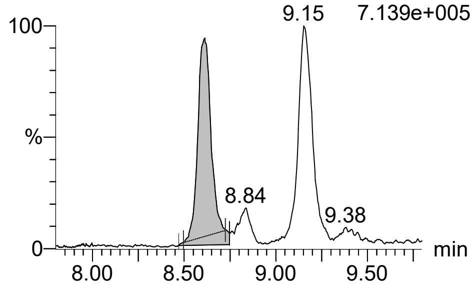

Sample Name: 20211111_L10_R3_IgM
F54:MRM of 1 channel,ES+
1045.8 > 204.1
1.395e+006

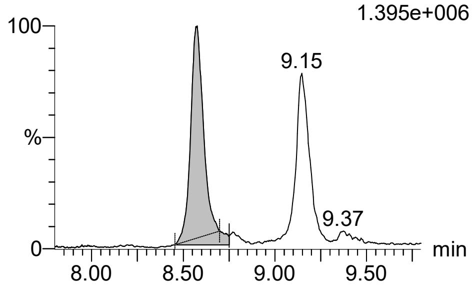

Sample Name: 20211111_QC_R19_IgM
F54:MRM of 1 channel,ES+
1045.8 > 204.1
1.094e+006

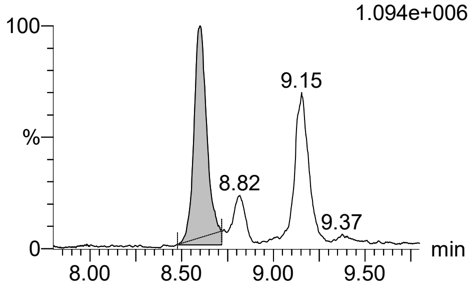

Dataset: C:\MassLynx\10.pro\20211111_clinicalsamples_IgM_IgA.qld

Last Altered: Thursday, March 09, 2023 19:58:25 台北標準時間
Printed: Friday, August 01, 2025 15:05:35 台北標準時間

Sample Name: 20211111_H5_R3_IgM

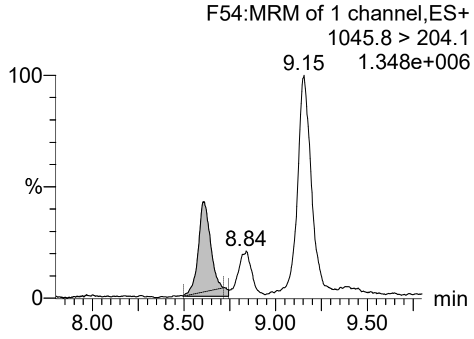

Sample Name: 20211111_A11_R3_IgM

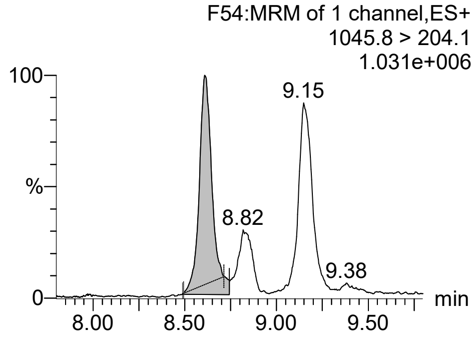

Sample Name: 20211111_A12_R3_IgM

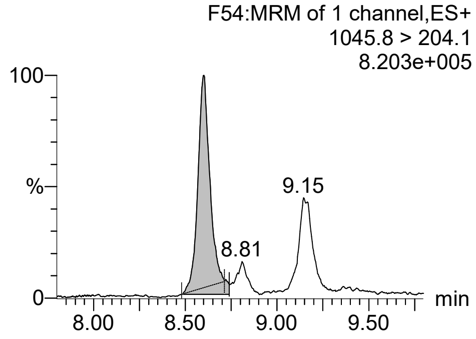

Sample Name: 20211111_L11_R3_IgM

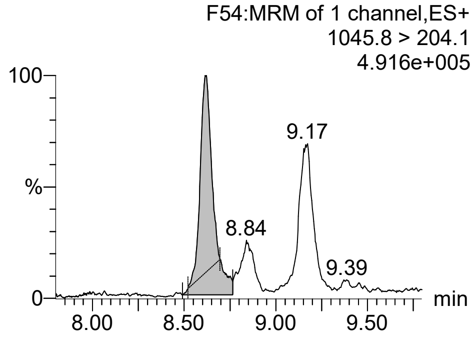

Sample Name: 20211111_L12_R3_IgM

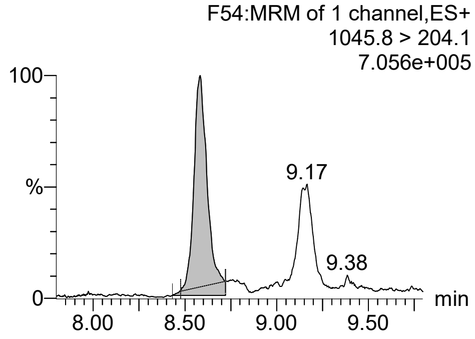

Sample Name: 20211111_H6_R3_IgM

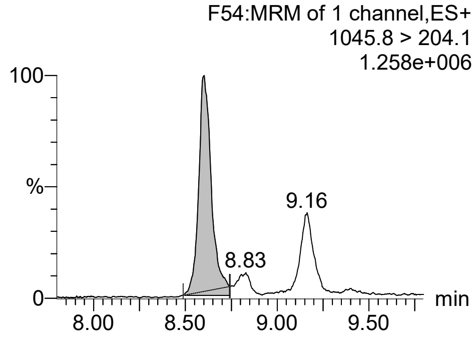

Sample Name: 20211111_A13_R3_IgM

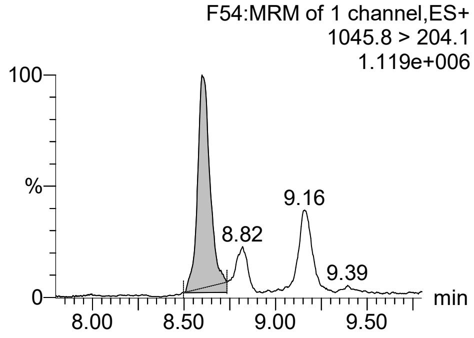

Sample Name: 20211111_A14_R3_IgM

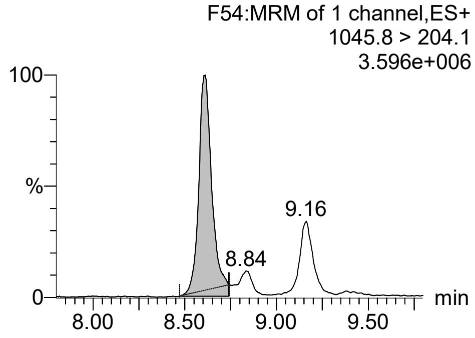

Sample Name: 20211111_L13_R3_IgM

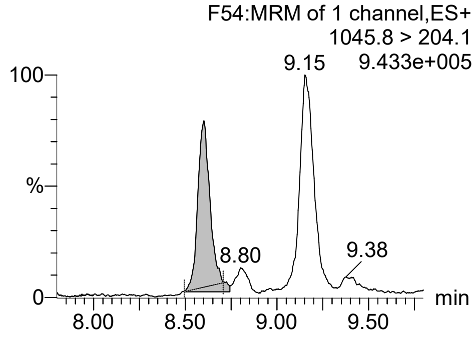

Sample Name: 20211111_L14_R3_IgM

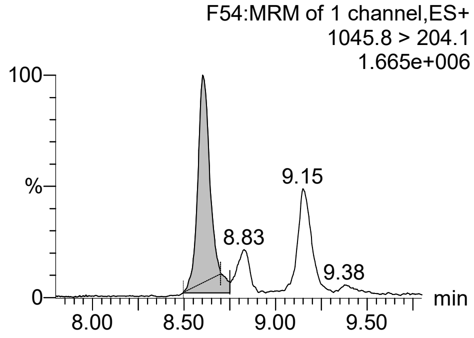

Sample Name: 20211111_H7_R3_IgM

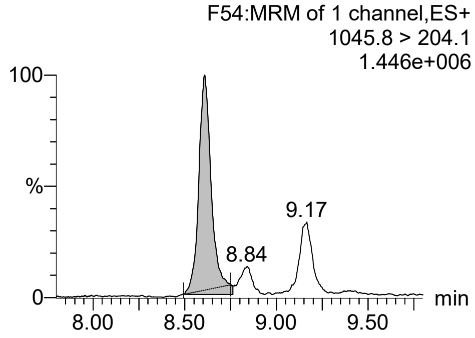

Sample Name: 20211111_A15_R3_IgM

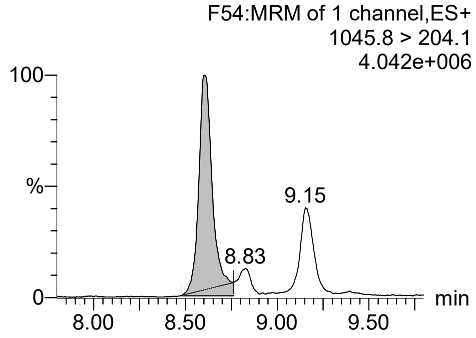

Sample Name: 20211111_QC_R20_IgM

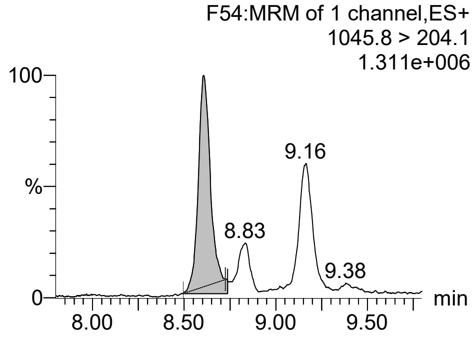

Sample Name: 20211111_A16_R3_IgM

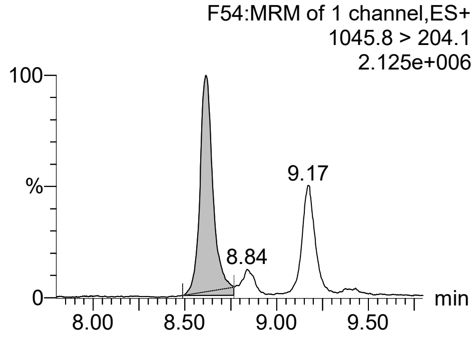

Sample Name: 20211111_L15_R3_IgM

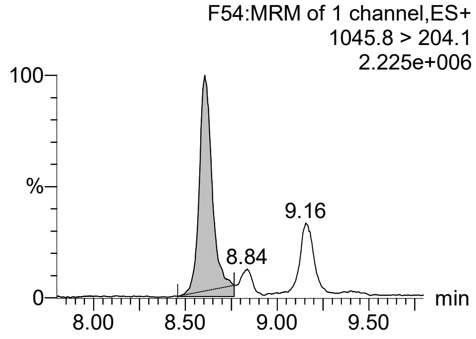

Dataset: C:\MassLynx\10.pro\20211111_clinicalsamples_IgM_IgA.qld

Last Altered: Thursday, March 09, 2023 19:58:25 台北標準時間
Printed: Friday, August 01, 2025 15:05:35 台北標準時間

Sample Name: 20211111_L16_R3_IgM
F54:MRM of 1 channel,ES+
1045.8 > 204.1
4.238e+005

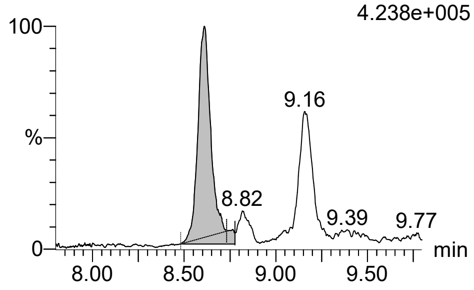

Sample Name: 20211111_H8_R3_IgM
F54:MRM of 1 channel,ES+
1045.8 > 204.1
8.093e+005

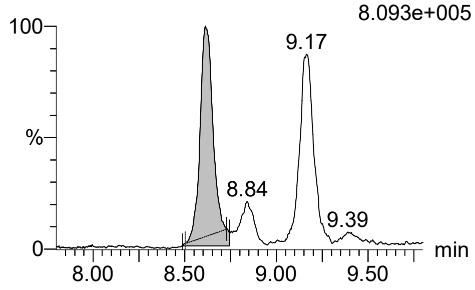

Sample Name: 20211111_A17_R3_IgM
F54:MRM of 1 channel,ES+
1045.8 > 204.1
1.534e+006

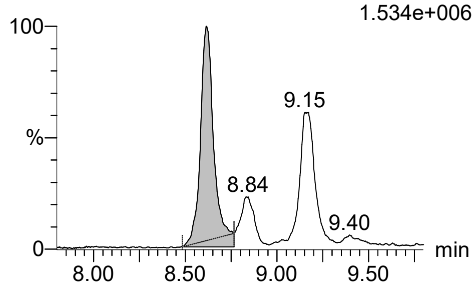

Sample Name: 20211111_A18_R3_IgM
F54:MRM of 1 channel,ES+
1045.8 > 204.1
1.781e+006

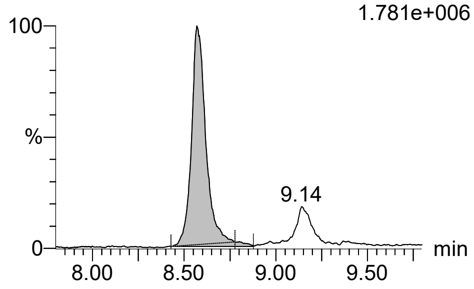

Sample Name: 20211111_L17_R3_IgM
F54:MRM of 1 channel,ES+
1045.8 > 204.1
1.709e+006

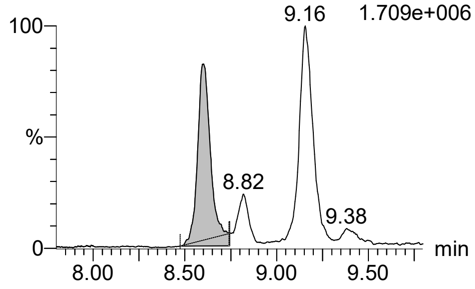

Sample Name: 20211111_L18_R3_IgM
F54:MRM of 1 channel,ES+
1045.8 > 204.1
1.845e+005

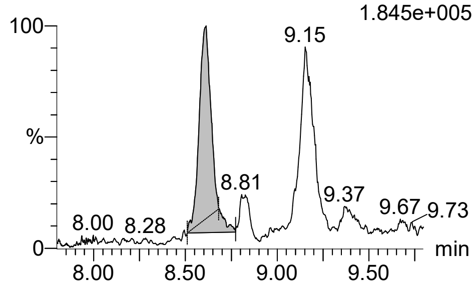

Sample Name: 20211111_H9_R3_IgM
F54:MRM of 1 channel,ES+
1045.8 > 204.1
6.507e+005

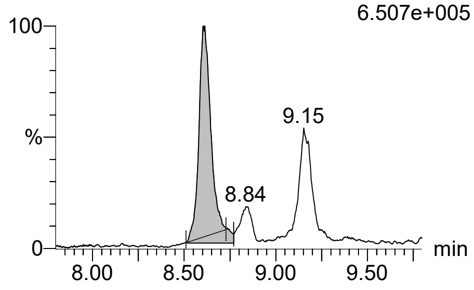

Sample Name: 20211111_A19_R3_IgM
F54:MRM of 1 channel,ES+
1045.8 > 204.1
1.272e+006

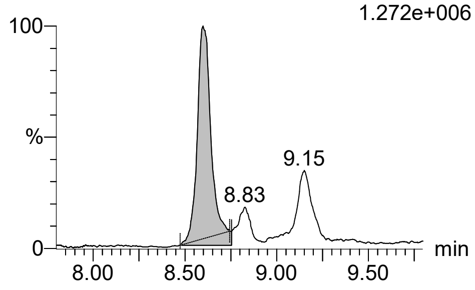

Sample Name: 20211111_A20_R3_IgM
F54:MRM of 1 channel,ES+
1045.8 > 204.1
8.099e+005

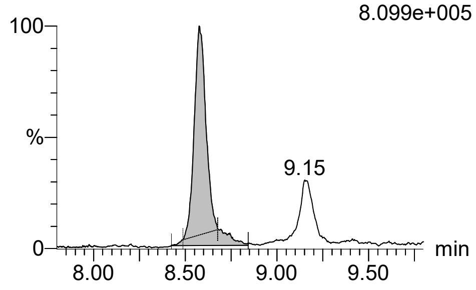

Sample Name: 20211111_L19_R3_IgM
F54:MRM of 1 channel,ES+
1045.8 > 204.1
1.399e+006

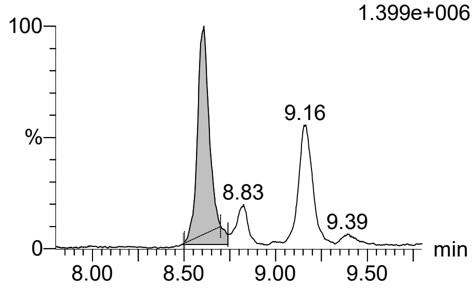

Sample Name: 20211111_QC_R21_IgM
F54:MRM of 1 channel,ES+
1045.8 > 204.1
1.292e+006

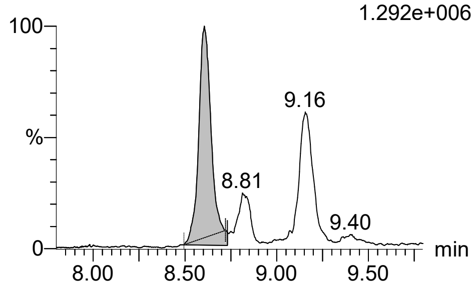

Sample Name: 20211111_L20_R3_IgM
F54:MRM of 1 channel,ES+
1045.8 > 204.1
4.877e+005

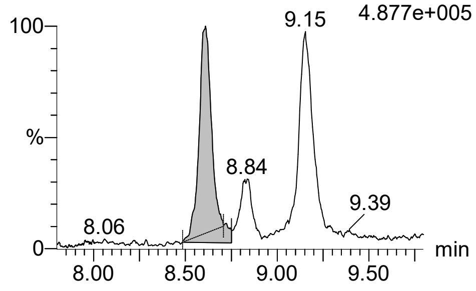

Sample Name: 20211111_H10_R3_IgM
F54:MRM of 1 channel,ES+
1045.8 > 204.1
1.214e+006

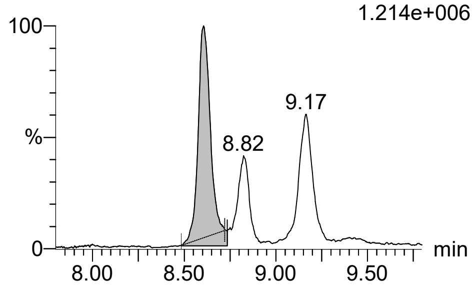

Sample Name: 20211111_A21_R3_IgM
F54:MRM of 1 channel,ES+
1045.8 > 204.1
1.479e+006

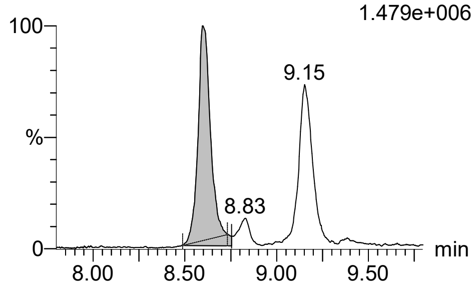

Sample Name: 20211111_A22_R3_IgM
F54:MRM of 1 channel,ES+
1045.8 > 204.1
3.643e+005

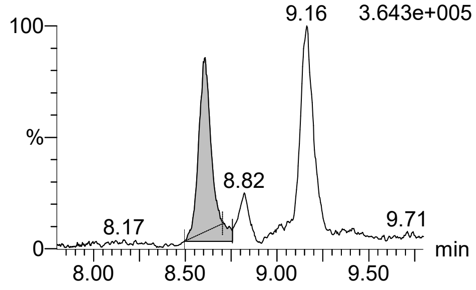

Dataset: C:\MassLynx\10.pro\20211111_clinicalsamples_IgM_IgA.qld

Last Altered: Thursday, March 09, 2023 19:58:25 台北標準時間
Printed: Friday, August 01, 2025 15:05:35 台北標準時間

Sample Name: 20211111_L21_R3_IgM

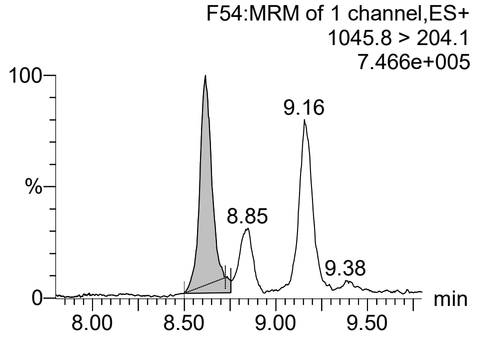

Sample Name: 20211111_L22_R3_IgM

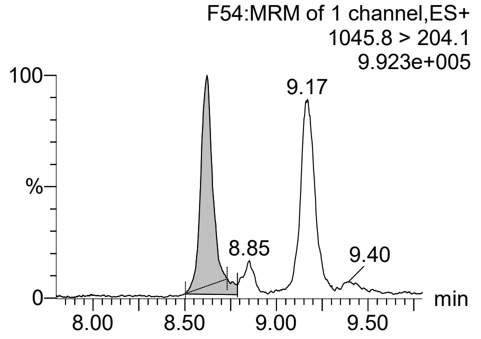

Sample Name: 20211111_H11_R3_IgM

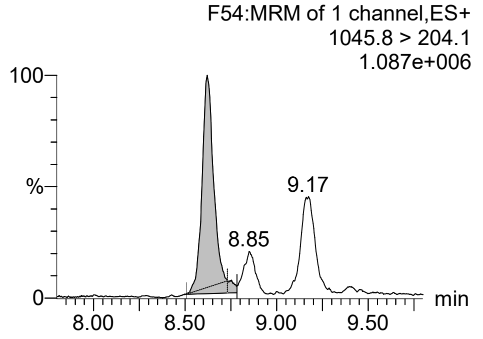

Sample Name: 20211111_A23_R3_IgM

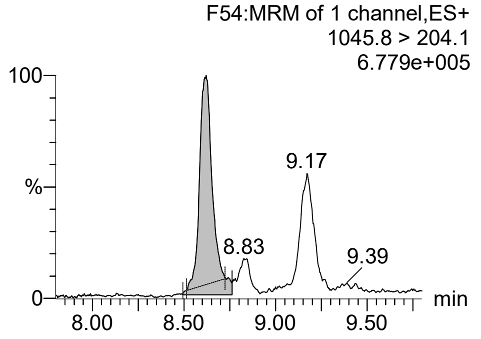

Sample Name: 20211111_A24_R3_IgM

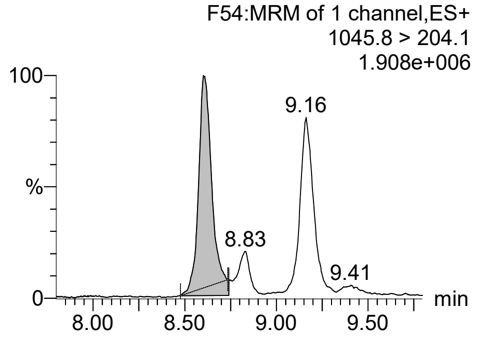

Sample Name: 20211111_L23_R3_IgM

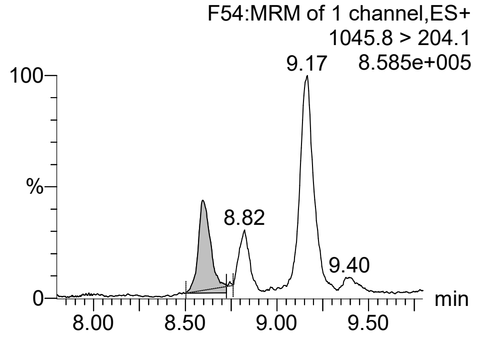

Sample Name: 20211111_L24_R3_IgM

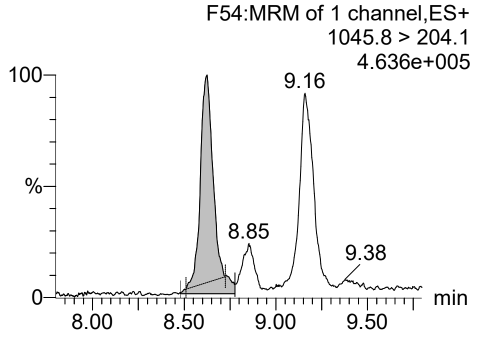

Sample Name: 20211111_H12_R3_IgM

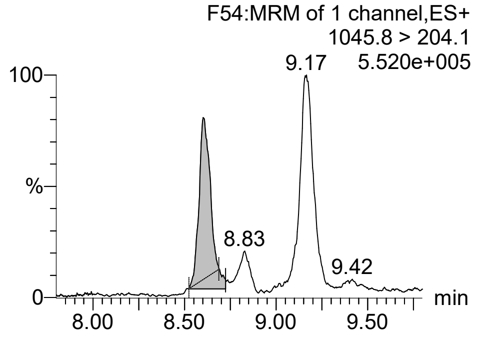

Sample Name: 20211111_QC_R22_IgM

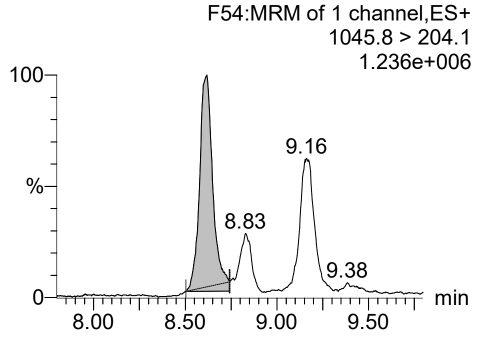

Sample Name: 20211111_A25_R3_IgM

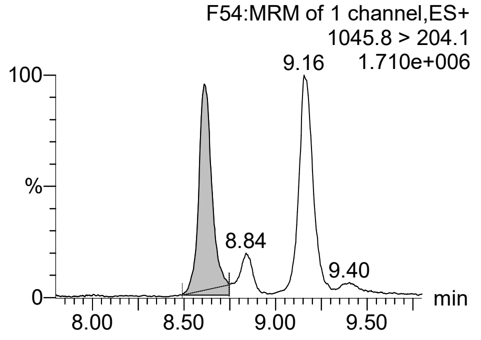

Sample Name: 20211111_A26_R3_IgM

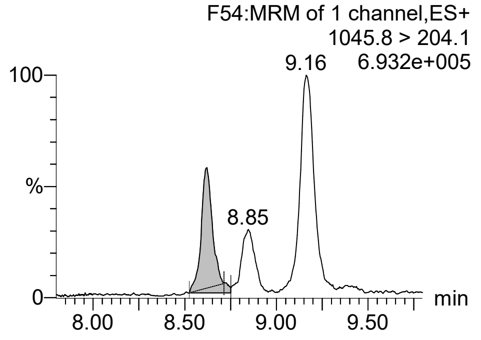

Sample Name: 20211111_L25_R3_IgM

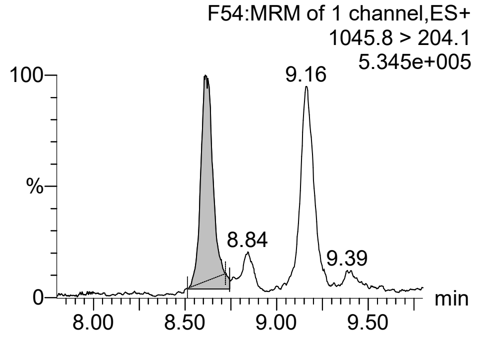

Sample Name: 20211111_L26_R3_IgM

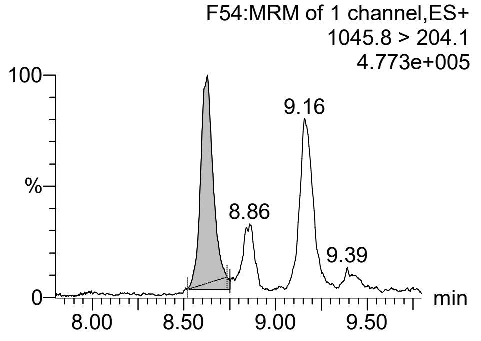

Sample Name: 20211111_H13_R3_IgM

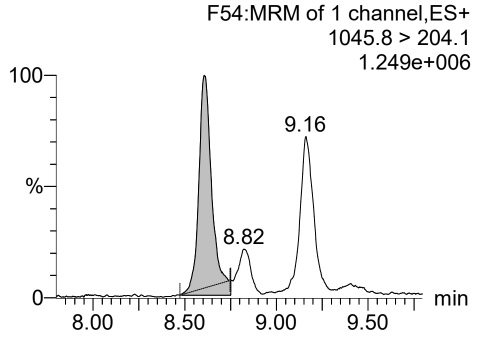

Sample Name: 20211111_A27_R3_IgM

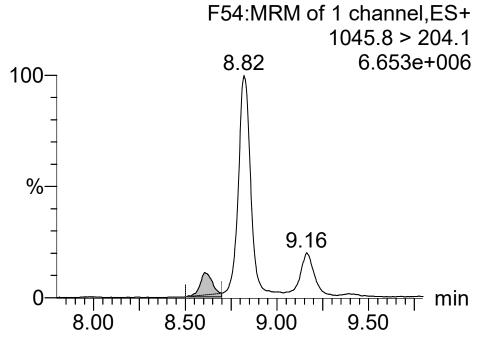

Dataset: C:\MassLynx\10.pro\20211111_clinicalsamples_IgM_IgA.qld

Last Altered: Thursday, March 09, 2023 19:58:25 台北標準時間
Printed: Friday, August 01, 2025 15:05:35 台北標準時間

Sample Name: 20211111_A28_R3_IgM
F54:MRM of 1 channel,ES+
1045.8 > 204.1
7.067e+005

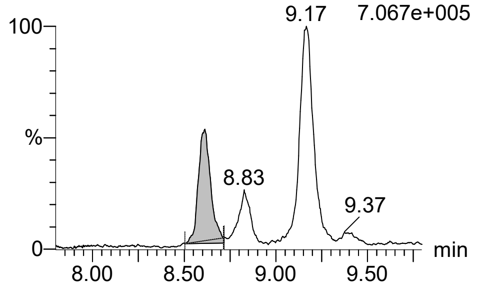

Sample Name: 20211111_L27_R3_IgM
F54:MRM of 1 channel,ES+
1045.8 > 204.1
1.392e+006

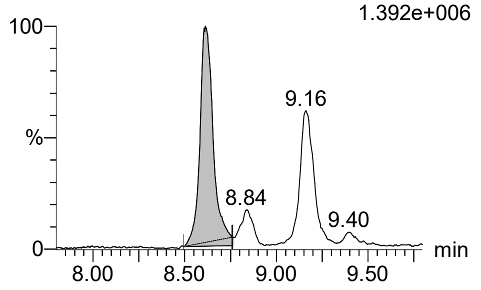

Sample Name: 20211111_L28_R3_IgM
F54:MRM of 1 channel,ES+
1045.8 > 204.1
1.006e+006

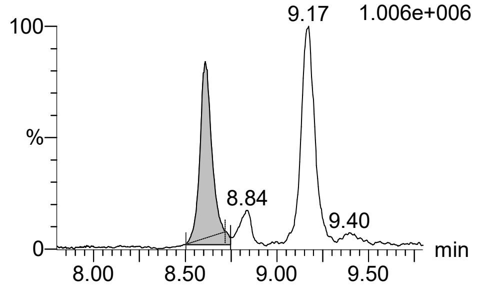

Sample Name: 20211111_H14_R3_IgM
F54:MRM of 1 channel,ES+
1045.8 > 204.1
1.275e+006

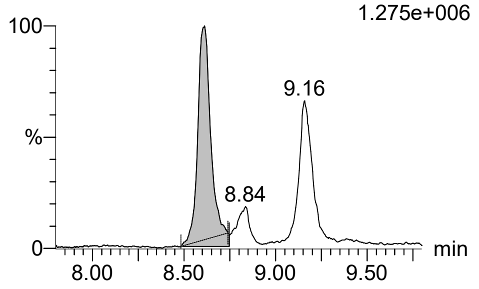

Sample Name: 20211111_A29_R3_IgM
F54:MRM of 1 channel,ES+
1045.8 > 204.1
1.238e+005

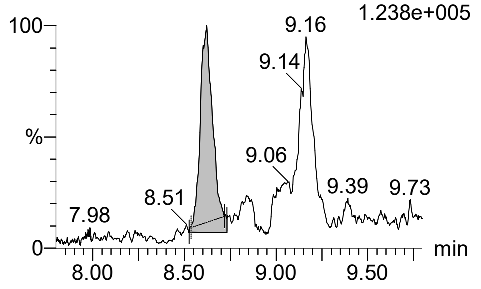

Sample Name: 20211111_A30_R3_IgM
F54:MRM of 1 channel,ES+
1045.8 > 204.1
1.668e+006

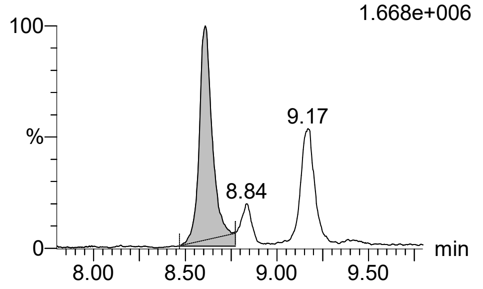

Sample Name: 20211111_QC_R23_IgM
F54:MRM of 1 channel,ES+
1045.8 > 204.1
1.192e+006

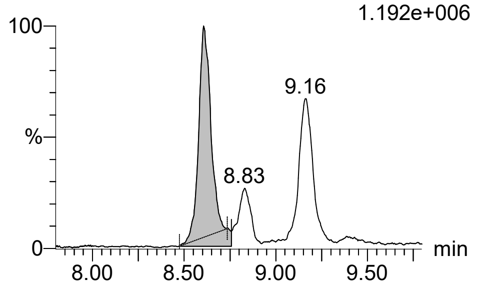

Sample Name: 20211111_L29_R3_IgM
F54:MRM of 1 channel,ES+
1045.8 > 204.1
6.131e+005

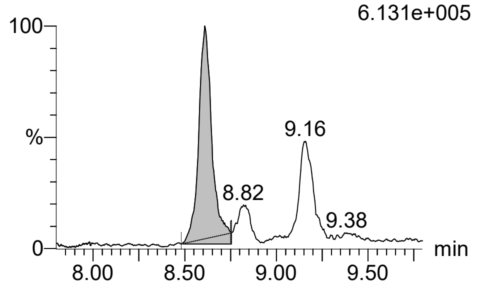

Sample Name: 20211111_L30_R3_IgM
F54:MRM of 1 channel,ES+
1045.8 > 204.1
3.715e+005

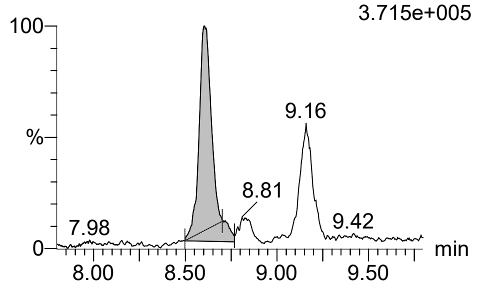

Sample Name: 20211111_H15_R3_IgM
F54:MRM of 1 channel,ES+
1045.8 > 204.1
1.223e+006

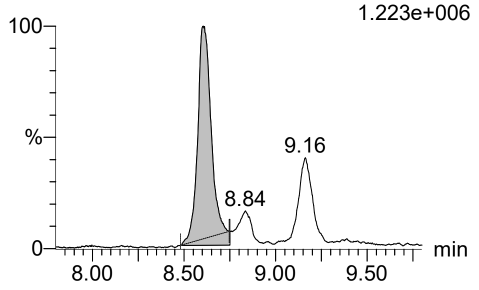

Sample Name: 20211111_A31_R3_IgM
F54:MRM of 1 channel,ES+
1045.8 > 204.1
5.694e+005

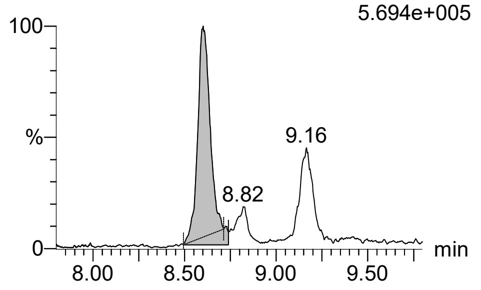

Sample Name: 20211111_A32_R3_IgM
F54:MRM of 1 channel,ES+
1045.8 > 204.1
1.462e+006

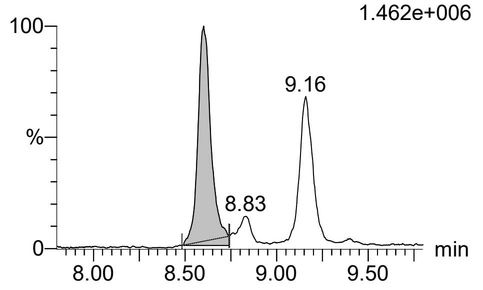

Sample Name: 20211111_L31_R3_IgM
F54:MRM of 1 channel,ES+
1045.8 > 204.1
2.334e+005

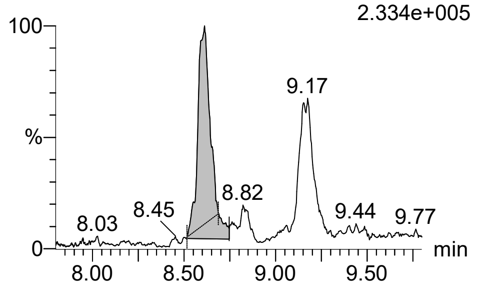

Sample Name: 20211111_L32_R3_IgM
F54:MRM of 1 channel,ES+
1045.8 > 204.1
1.347e+006

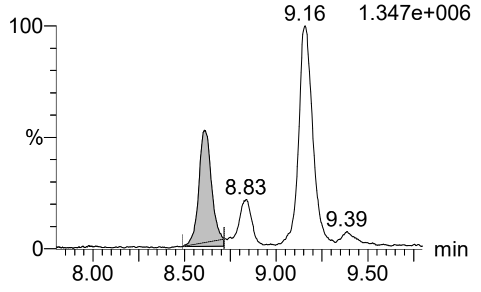

Sample Name: 20211111_H16_R3_IgM
F54:MRM of 1 channel,ES+
1045.8 > 204.1
2.147e+006

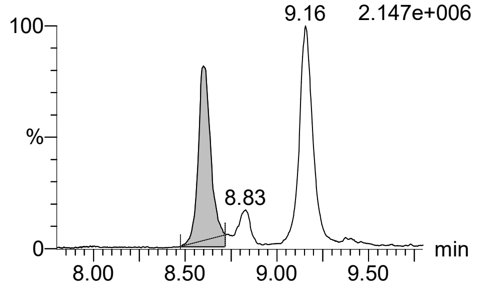

Sample Name: 20211111_A33_R3_IgM
F54:MRM of 1 channel,ES+
1045.8 > 204.1
1.167e+006

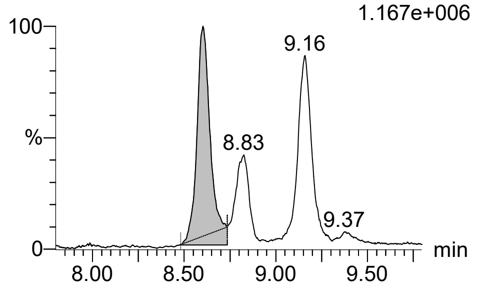

Sample Name: 20211111_A34_R3_IgM
F54:MRM of 1 channel,ES+
1045.8 > 204.1
4.432e+005

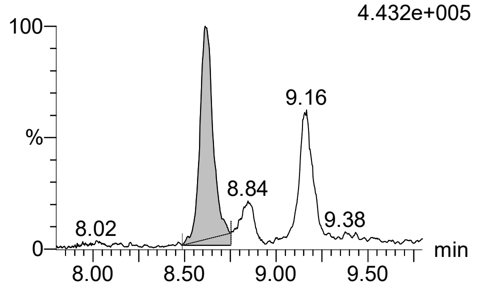

Sample Name: 20211111_H17_R3_IgM
F54:MRM of 1 channel,ES+
1045.8 > 204.1
5.643e+006

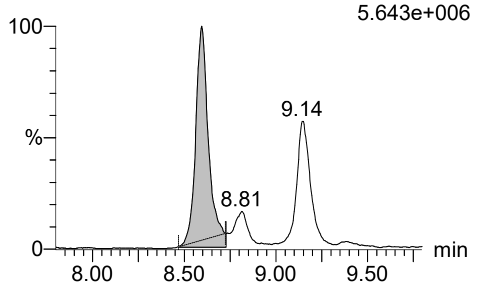

Sample Name: 20211111_A35_R3_IgM
F54:MRM of 1 channel,ES+
1045.8 > 204.1
6.592e+005

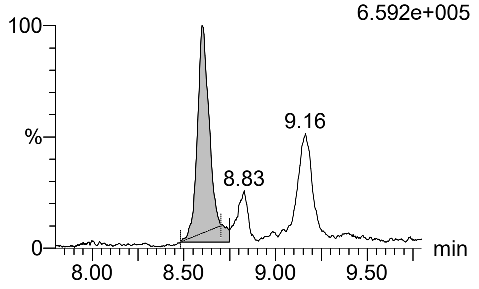

Sample Name: 20211111_QC_R24_IgM
F54:MRM of 1 channel,ES+
1045.8 > 204.1
1.026e+006

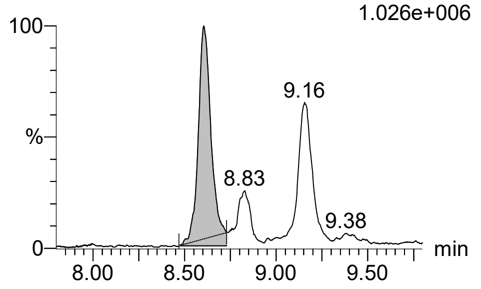

Sample Name: 20211111_A36_R3_IgM
F54:MRM of 1 channel,ES+
1045.8 > 204.1
6.927e+005

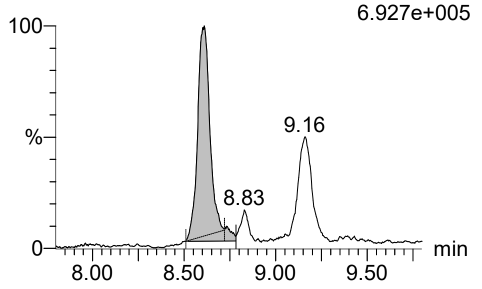

Sample Name: 20211111_H18_R3_IgM
F54:MRM of 1 channel,ES+
1045.8 > 204.1
2.218e+006

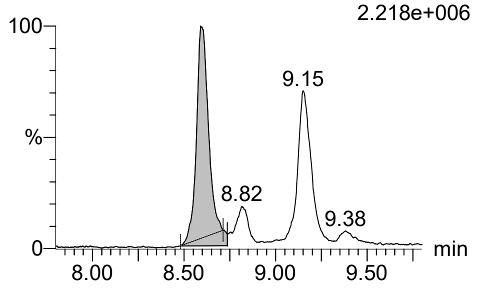

Sample Name: 20211111_A37_R3_IgM
F54:MRM of 1 channel,ES+
1045.8 > 204.1
1.381e+006

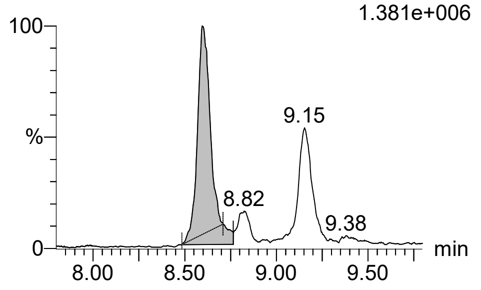

Sample Name: 20211111_A38_R3_IgM
F54:MRM of 1 channel,ES+
1045.8 > 204.1
1.034e+006

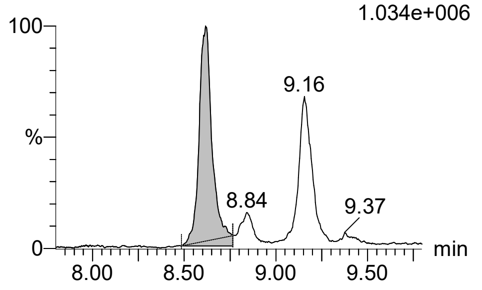

Sample Name: 20211111_H19_R3_IgM
F54:MRM of 1 channel,ES+
1045.8 > 204.1
1.818e+006

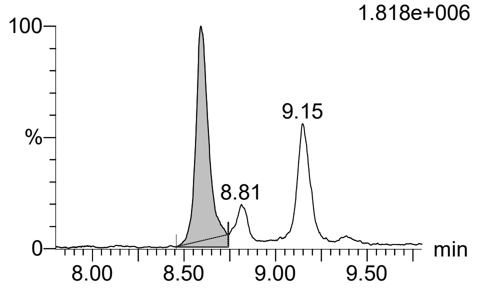

Sample Name: 20211111_A39_R3_IgM
F54:MRM of 1 channel,ES+
1045.8 > 204.1
8.726e+005

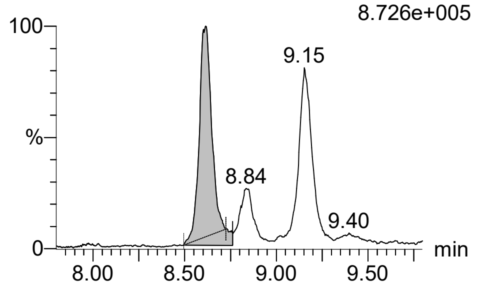

Sample Name: 20211111_A40_R3_IgM
F54:MRM of 1 channel,ES+
1045.8 > 204.1
1.040e+006

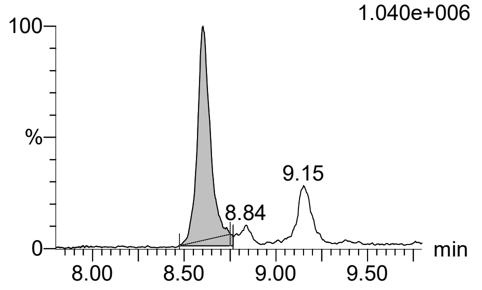

Sample Name: 20211111_H20_R3_IgM
F54:MRM of 1 channel,ES+
1045.8 > 204.1
3.832e+006

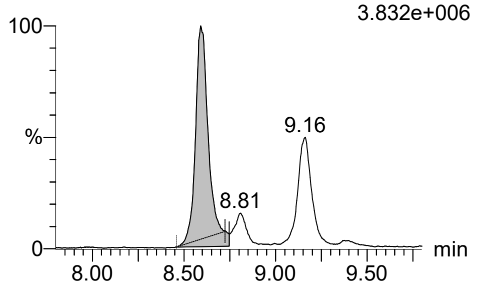

Sample Name: 20211111_A41_R3_IgM
F54:MRM of 1 channel,ES+
1045.8 > 204.1
4.153e+005

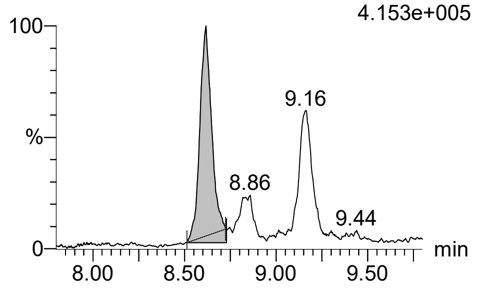

Sample Name: 20211111_A42_R3_IgM
F54:MRM of 1 channel,ES+
1045.8 > 204.1
6.298e+005

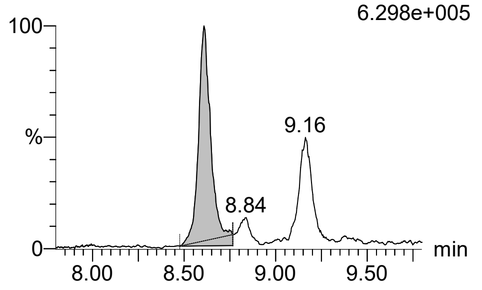

Sample Name: 20211111_A43_R3_IgM

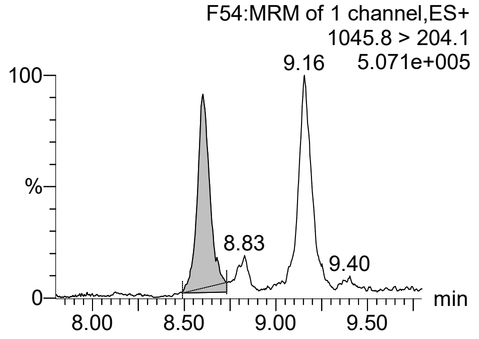

Sample Name: 20211111_A44_R3_IgM

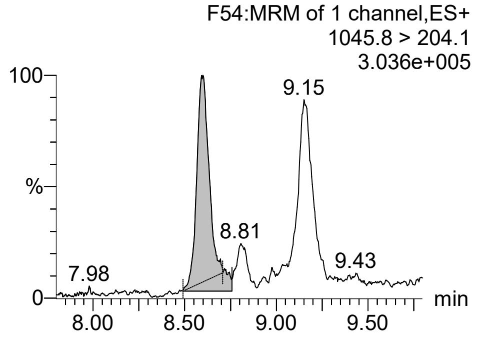

Sample Name: 20211111_QC_R25_IgM

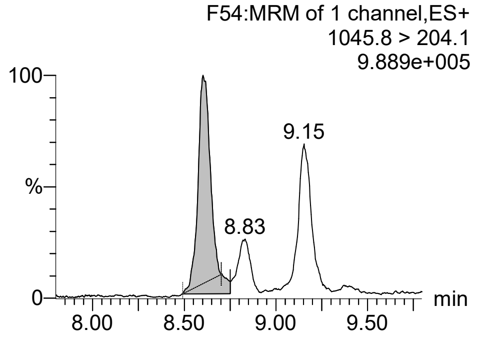

Dataset: C:\MassLynx\10.pro\20211111_clinicalsamples_IgM_IgA.qld

Last Altered: Thursday, March 09, 2023 19:58:25 台北標準時間
Printed: Friday, August 01, 2025 15:05:35 台北標準時間

	#	Name	RT	Area	IS Area	Response	Prima...
1	1	20211112_QC_R001_IgM	8.58	110774.6	15421.4	7.183	MM
2	2	20211112_QC_R002_IgM	8.60	99875.8	13188.8	7.573	MM
3	3	20211111_A1_R2_IgM	8.60	57012.4	11871.0	4.803	MM
4	4	20211111_A1_R3_IgM	8.60	53644.4	12030.5	4.459	MM
5	5	20211111_A2_R2_IgM	8.60	202761.7	11446.8	17.713	MM
6	6	20211111_A2_R3_IgM	8.60	195290.5	11098.4	17.596	MM
7	7	20211111_L1_R2_IgM	8.61	27284.6	11779.4	2.316	MM
8	8	20211111_L1_R3_IgM	8.61	24625.5	11176.2	2.203	MM
9	9	20211111_L2_R2_IgM	8.60	162218.5	11375.0	14.261	MM
10	10	20211111_H1_R2_IgM	8.60	107997.8	13595.2	7.944	MM
11	11	20211111_A3_R2_IgM	8.59	148781.1	14112.3	10.543	MM
12	12	20211111_A4_R2_IgM	8.59	42590.3	11977.5	3.556	MM
13	13	20211111_L3_R2_IgM	8.54	44264.0	12242.1	3.616	MM
14	14	20211111_L4_R2_IgM	8.60	113828.2	12797.0	8.895	MM
15	15	20211111_H2_R2_IgM	8.60	173705.5	10006.0	17.360	MM
16	16	20211111_A5_R2_IgM	8.59	296026.5	11434.6	25.889	MM
17	17	20211111_A6_R2_IgM	8.60	86239.1	13193.7	6.536	MM
18	18	20211111_QC_R10_IgM	8.60	105320.5	12566.1	8.381	MM
19	19	20211111_L5_R2_IgM	8.57	172460.6	13616.4	12.666	MM
20	20	20211111_L6_R2_IgM	8.60	135582.7	13698.5	9.898	MM
21	21	20211111_H3_R2_IgM	8.61	111716.0	15793.1	7.074	MM
22	22	20211111_A7_R2_IgM	8.59	221726.7	12936.9	17.139	MM
23	23	20211111_A8_R2_IgM	8.59	192761.1	13108.0	14.706	MM
24	24	20211111_L7_R2_IgM	8.60	17423.8	13278.5	1.312	MM
25	25	20211111_L8_R2_IgM	8.61	49207.7	15233.6	3.230	MM
26	26	20211111_H4_R2_IgM	8.60	90253.2	16521.9	5.463	MM
27	27	20211111_A9_R2_IgM	8.58	95378.0	16451.6	5.797	MM
28	28	20211111_A10_R2_IgM	8.59	295116.7	13036.0	22.639	MM
29	29	20211111_L9_R2_IgM	8.59	66819.5	14554.1	4.591	MM
30	30	20211111_L10_R2_IgM	8.58	145307.7	12250.4	11.861	MM
31	31	20211111_QC_R11_IgM	8.61	117463.7	11459.2	10.251	MM
32	32	20211111_H5_R2_IgM	8.59	65463.0	14358.2	4.559	MM
33	33	20211111_A11_R2_IgM	8.53	113351.0	16184.1	7.004	bb
34	34	20211111_A12_R2_IgM	8.60	71111.8	13465.4	5.281	MM
35	35	20211111_L11_R2_IgM	8.62	49337.0	13277.9	3.716	MM
36	36	20211111_L12_R2_IgM	8.59	68288.2	15958.8	4.279	MM
37	37	20211111_H6_R2_IgM	8.60	114413.3	14003.5	8.170	MM
38	38	20211111_A13_R2_IgM	8.60	99566.6	13656.1	7.291	MM
39	39	20211111_A14_R2_IgM	8.60	381764.0	14071.3	27.131	MM
40	40	20211111_L13_R2_IgM	8.61	71294.7	14785.6	4.822	MM
41	41	20211111_L14_R2_IgM	8.61	174320.6	14772.2	11.801	MM
42	42	20211111_H7_R2_IgM	8.58	148292.9	15164.4	9.779	MM
43	43	20211111_A15_R2_IgM	8.60	429577.1	15721.5	27.324	MM
44	44	20211111_QC_R12_IgM	8.60	127755.2	13711.5	9.317	MM
45	45	20211111_A16_R2_IgM	8.61	221667.2	14275.6	15.528	MM
46	46	20211111_L15_R2_IgM	8.61	218638.5	12935.8	16.902	MM
47	47	20211111_L16_R2_IgM	8.61	34204.8	13342.8	2.564	MM
48	48	20211111_H8_R2_IgM	8.62	80109.2	15835.1	5.059	MM
49	49	20211111_A17_R2_IgM	8.61	154897.1	15786.7	9.812	MM
50	50	20211111_A18_R2_IgM	8.59	178764.4	16986.8	10.524	MM
51	51	20211111_L17_R2_IgM	8.60	138849.8	13342.7	10.406	MM
52	52	20211111_L18_R2_IgM	8.60	17295.0	14692.0	1.177	MM
53	53	20211111_H9_R2_IgM	8.60	59195.3	15639.7	3.785	MM
54	54	20211111_A19_R2_IgM	8.59	114271.8	14198.7	8.048	MM
55	55	20211111_A20_R2_IgM	8.58	76418.8	14162.7	5.396	MM
56	56	20211111_L19_R2_IgM	8.60	107493.4	9293.4	11.567	MM

Dataset: C:\MassLynx\10.pro\20211111_clinicalsamples_IgM_IgA.qld

Last Altered: Thursday, March 09, 2023 19:58:25 台北標準時間
Printed: Friday, August 01, 2025 15:05:35 台北標準時間

	# Name	RT	Area	IS Area	Response	Prima...
57	57 20211111_QC_R13_IgM	8.60	120107.6	13903.0	8.639	MM
58	58 20211111_L20_R2_IgM	8.55	48882.0	12781.6	3.824	MM
59	59 20211111_H10_R2_IgM	8.59	114012.9	14796.4	7.705	MM
60	60 20211111_A21_R2_IgM	8.61	132431.0	13669.0	9.688	MM
61	61 20211111_A22_R2_IgM	8.60	25091.9	14098.3	1.780	MM
62	62 20211111_L21_R2_IgM	8.61	69407.1	15678.2	4.427	MM
63	63 20211111_L22_R2_IgM	8.61	92557.0	15724.5	5.886	MM
64	64 20211111_H11_R2_IgM	8.62	93886.7	15890.2	5.908	MM
65	65 20211111_A23_R2_IgM	8.60	56614.5	13423.2	4.218	MM
66	66 20211111_A24_R2_IgM	8.60	166139.5	12438.0	13.357	MM
67	67 20211111_L23_R2_IgM	8.61	33664.5	14075.0	2.392	MM
68	68 20211111_L24_R2_IgM	8.60	44808.9	15972.1	2.805	MM
69	69 20211111_H12_R2_IgM	8.62	38077.8	16270.9	2.340	MM
70	70 20211111_QC_R14_IgM	8.61	134670.1	15806.8	8.520	MM
71	71 20211111_A25_R2_IgM	8.61	184512.8	14552.1	12.679	MM
72	72 20211111_A26_R2_IgM	8.63	33744.4	11492.7	2.936	MM
73	73 20211111_L25_R2_IgM	8.61	50848.0	14403.3	3.530	MM
74	74 20211111_L26_R2_IgM	8.62	44206.4	16533.9	2.674	MM
75	75 20211111_H13_R2_IgM	8.61	102718.8	15520.1	6.618	MM
76	76 20211111_A27_R2_IgM	8.61	64122.8	13337.4	4.808	MM
77	77 20211111_A28_R2_IgM	8.56	34937.7	11895.6	2.937	MM
78	78 20211111_L27_R2_IgM	8.60	123826.6	14125.7	8.766	MM
79	79 20211111_L28_R2_IgM	8.61	63152.2	11807.5	5.348	MM
80	80 20211111_H14_R2_IgM	8.60	106895.2	15035.7	7.109	MM
81	81 20211111_A29_R2_IgM	8.61	10743.6	13554.4	0.793	MM
82	82 20211111_A30_R2_IgM	8.60	156103.5	12984.1	12.023	MM
83	83 20211111_QC_R15_IgM	8.61	112175.8	12299.5	9.120	MM
84	84 20211111_L29_R2_IgM	8.62	50298.4	13590.7	3.701	MM
85	85 20211111_L30_R2_IgM	8.60	34413.4	12511.4	2.751	MM
86	86 20211111_H15_R2_IgM	8.61	120347.1	11885.0	10.126	MM
87	87 20211111_A31_R2_IgM	8.60	48313.6	13422.1	3.600	MM
88	88 20211111_A32_R2_IgM	8.60	135780.7	11461.3	11.847	MM
89	89 20211111_L31_R2_IgM	8.56	24047.2	13753.4	1.748	MM
90	90 20211111_L32_R2_IgM	8.59	66896.9	12949.0	5.166	MM
91	91 20211111_H16_R2_IgM	8.60	174233.8	11465.4	15.196	MM
92	92 20211111_A33_R2_IgM	8.60	102586.7	10551.5	9.722	MM
93	93 20211111_A34_R2_IgM	8.61	42866.4	12409.9	3.454	MM
94	94 20211111_H17_R2_IgM	8.58	558526.0	10160.9	54.968	MM
95	95 20211111_A35_R2_IgM	8.57	59094.4	10460.6	5.649	MM
96	96 20211111_QC_R16_IgM	8.60	98789.5	10433.5	9.468	MM
97	97 20211111_A36_R2_IgM	8.54	62822.6	12504.8	5.024	bb
98	98 20211111_H18_R2_IgM	8.60	196906.4	9140.4	21.542	MM
99	99 20211111_A37_R2_IgM	8.60	142593.1	9796.9	14.555	MM
100	1... 20211111_A38_R2_IgM	8.61	95413.4	11224.3	8.501	MM
101	1... 20211111_H19_R2_IgM	8.60	174821.3	11739.2	14.892	MM
102	1... 20211111_A39_R2_IgM	8.61	80066.3	12718.9	6.295	MM
103	1... 20211111_A40_R2_IgM	8.57	101742.4	10006.1	10.168	MM
104	1... 20211111_H20_R2_IgM	8.59	386431.9	7988.2	48.375	MM
105	1... 20211111_A41_R2_IgM	8.61	50678.7	11513.3	4.402	MM
106	1... 20211111_A42_R2_IgM	8.61	51739.2	10238.5	5.053	MM
107	1... 20211111_A43_R2_IgM	8.60	42302.6	11098.9	3.811	MM
108	1... 20211111_A44_R2_IgM	8.57	27144.3	10969.9	2.474	MM
109	1... 20211111_QC_R17_IgM	8.61	90863.0	9700.8	9.367	MM
110	1... 20211111_L2_R3_IgM	8.60	165783.0	8528.0	19.440	MM
111	1... 20211111_H1_R3_IgM	8.61	96065.6	9438.2	10.178	MM
112	1... 20211111_A3_R3_IgM	8.61	124587.9	11598.6	10.742	MM

Dataset: C:\MassLynx\10.pro\20211111_clinicalsamples_IgM_IgA.qld

Last Altered: Thursday, March 09, 2023 19:58:25 台北標準時間
Printed: Friday, August 01, 2025 15:05:35 台北標準時間

	#	Name	RT	Area	IS Area	Response	Prima...
113	1...	20211111_A4_R3_IgM	8.61	37982.0	11364.1	3.342	MM
114	1...	20211111_L3_R3_IgM	8.57	41012.0	10753.4	3.814	MM
115	1...	20211111_L4_R3_IgM	8.61	94897.2	10291.6	9.221	MM
116	1...	20211111_H2_R3_IgM	8.60	171013.2	9315.8	18.357	MM
117	1...	20211111_A5_R3_IgM	8.60	239564.2	9102.2	26.319	MM
118	1...	20211111_A6_R3_IgM	8.60	66246.8	10356.4	6.397	MM
119	1...	20211111_QC_R18_IgM	8.60	85626.0	8398.3	10.196	MM
120	1...	20211111_L5_R3_IgM	8.61	129500.7	9786.7	13.232	MM
121	1...	20211111_L6_R3_IgM	8.61	110837.6	9911.3	11.183	MM
122	1...	20211111_H3_R3_IgM	8.62	89466.6	12155.5	7.360	MM
123	1...	20211111_A7_R3_IgM	8.60	220981.2	11804.8	18.720	MM
124	1...	20211111_A8_R3_IgM	8.61	153953.5	10510.6	14.647	MM
125	1...	20211111_L7_R3_IgM	8.60	13941.7	12177.9	1.145	MM
126	1...	20211111_L8_R3_IgM	8.62	40325.4	11668.6	3.456	MM
127	1...	20211111_H4_R3_IgM	8.61	72988.8	11192.0	6.522	MM
128	1...	20211111_A9_R3_IgM	8.61	77070.2	14077.4	5.475	MM
129	1...	20211111_A10_R3_IgM	8.61	261199.4	11190.4	23.341	MM
130	1...	20211111_L9_R3_IgM	8.61	58978.0	14649.3	4.026	MM
131	1...	20211111_L10_R3_IgM	8.58	120454.6	9361.3	12.867	MM
132	1...	20211111_QC_R19_IgM	8.60	89929.6	10125.5	8.881	MM
133	1...	20211111_H5_R3_IgM	8.60	48244.5	10010.9	4.819	MM
134	1...	20211111_A11_R3_IgM	8.61	83691.3	12697.2	6.591	MM
135	1...	20211111_A12_R3_IgM	8.60	64679.8	13148.5	4.919	MM
136	1...	20211111_L11_R3_IgM	8.62	41102.2	10786.6	3.810	MM
137	1...	20211111_L12_R3_IgM	8.58	61112.1	11681.8	5.231	MM
138	1...	20211111_H6_R3_IgM	8.60	100496.7	12095.5	8.309	MM
139	1...	20211111_A13_R3_IgM	8.60	88899.1	12908.3	6.887	MM
140	1...	20211111_A14_R3_IgM	8.61	299376.5	11322.6	26.441	MM
141	1...	20211111_L13_R3_IgM	8.60	58325.0	11516.1	5.065	MM
142	1...	20211111_L14_R3_IgM	8.60	133642.0	10790.7	12.385	MM
143	1...	20211111_H7_R3_IgM	8.61	114592.7	11572.1	9.903	MM
144	1...	20211111_A15_R3_IgM	8.61	352668.7	12434.4	28.362	MM
145	1...	20211111_QC_R20_IgM	8.61	102917.2	10701.0	9.618	MM
146	1...	20211111_A16_R3_IgM	8.62	175009.6	11452.1	15.282	MM
147	1...	20211111_L15_R3_IgM	8.61	187912.0	10531.1	17.844	MM
148	1...	20211111_L16_R3_IgM	8.61	35825.0	12049.4	2.973	MM
149	1...	20211111_H8_R3_IgM	8.61	68843.2	13434.9	5.124	MM
150	1...	20211111_A17_R3_IgM	8.61	129728.6	12452.0	10.418	MM
151	1...	20211111_A18_R3_IgM	8.57	164745.3	12506.5	13.173	MM
152	1...	20211111_L17_R3_IgM	8.60	118498.4	9948.9	11.911	MM
153	1...	20211111_L18_R3_IgM	8.61	13879.5	13462.1	1.031	MM
154	1...	20211111_H9_R3_IgM	8.61	51087.5	13178.7	3.877	MM
155	1...	20211111_A19_R3_IgM	8.60	113254.9	11298.6	10.024	MM
156	1...	20211111_A20_R3_IgM	8.57	68266.2	11175.6	6.108	MM
157	1...	20211111_L19_R3_IgM	8.61	109299.0	11558.2	9.456	MM
158	1...	20211111_QC_R21_IgM	8.61	102545.8	9977.0	10.278	MM
159	1...	20211111_L20_R3_IgM	8.61	40105.4	13508.1	2.969	MM
160	1...	20211111_H10_R3_IgM	8.61	97554.9	12374.8	7.883	MM
161	1...	20211111_A21_R3_IgM	8.60	120225.5	12443.6	9.662	MM
162	1...	20211111_A22_R3_IgM	8.61	25467.6	11589.3	2.198	MM
163	1...	20211111_L21_R3_IgM	8.62	58588.8	13107.4	4.470	MM
164	1...	20211111_L22_R3_IgM	8.62	79023.7	12021.5	6.574	MM
165	1...	20211111_H11_R3_IgM	8.62	82556.9	12687.8	6.507	MM
166	1...	20211111_A23_R3_IgM	8.62	57003.3	11611.8	4.909	MM
167	1...	20211111_A24_R3_IgM	8.60	161583.7	10476.8	15.423	MM
168	1...	20211111_L23_R3_IgM	8.60	28950.4	11873.1	2.438	MM

Dataset: C:\MassLynx\10.pro\20211111_clinicalsamples_IgM_IgA.qld

Last Altered: Thursday, March 09, 2023 19:58:25 台北標準時間
Printed: Friday, August 01, 2025 15:05:35 台北標準時間

	# Name	RT	Area	IS Area	Response	Prima...
169	1... 20211111_L24_R3_IgM	8.63	39656.4	13468.0	2.944	MM
170	1... 20211111_H12_R3_IgM	8.60	32372.8	14229.1	2.275	MM
171	1... 20211111_QC_R22_IgM	8.62	100805.9	11264.4	8.949	MM
172	1... 20211111_A25_R3_IgM	8.61	138686.5	11775.7	11.777	MM
173	1... 20211111_A26_R3_IgM	8.62	29754.3	10473.9	2.841	MM
174	1... 20211111_L25_R3_IgM	8.61	41434.2	13256.8	3.126	MM
175	1... 20211111_L26_R3_IgM	8.63	37290.4	13565.0	2.749	MM
176	1... 20211111_H13_R3_IgM	8.61	103172.1	13146.0	7.848	MM
177	1... 20211111_A27_R3_IgM	8.61	58925.3	12025.6	4.900	MM
178	1... 20211111_A28_R3_IgM	8.61	28311.6	11229.1	2.521	MM
179	1... 20211111_L27_R3_IgM	8.61	116550.7	14960.0	7.791	MM
180	1... 20211111_L28_R3_IgM	8.61	65735.7	11359.0	5.787	MM
181	1... 20211111_H14_R3_IgM	8.61	108164.6	12830.8	8.430	MM
182	1... 20211111_A29_R3_IgM	8.62	9253.7	13434.3	0.689	MM
183	1... 20211111_A30_R3_IgM	8.61	143759.1	11272.2	12.753	MM
184	1... 20211111_QC_R23_IgM	8.61	100135.3	11872.2	8.434	MM
185	1... 20211111_L29_R3_IgM	8.61	51122.0	12948.7	3.948	MM
186	1... 20211111_L30_R3_IgM	8.60	30722.4	11026.6	2.786	MM
187	1... 20211111_H15_R3_IgM	8.60	107629.5	10786.3	9.978	MM
188	1... 20211111_A31_R3_IgM	8.60	46979.8	12010.5	3.912	MM
189	1... 20211111_A32_R3_IgM	8.60	120906.5	11107.7	10.885	MM
190	1... 20211111_L31_R3_IgM	8.61	18595.9	11021.8	1.687	MM
191	1... 20211111_L32_R3_IgM	8.61	57499.7	11683.8	4.921	MM
192	1... 20211111_H16_R3_IgM	8.60	143640.7	9831.7	14.610	MM
193	1... 20211111_A33_R3_IgM	8.60	98311.4	11213.1	8.768	MM
194	1... 20211111_A34_R3_IgM	8.61	38020.4	11550.1	3.292	MM
195	1... 20211111_H17_R3_IgM	8.60	458956.0	8874.4	51.717	MM
196	1... 20211111_A35_R3_IgM	8.60	50889.2	9856.3	5.163	MM
197	1... 20211111_QC_R24_IgM	8.60	83749.3	7561.8	11.075	MM
198	1... 20211111_A36_R3_IgM	8.61	55207.5	9562.8	5.773	MM
199	1... 20211111_H18_R3_IgM	8.59	178597.8	8165.3	21.873	MM
200	2... 20211111_A37_R3_IgM	8.60	119628.9	9278.8	12.893	MM
201	2... 20211111_A38_R3_IgM	8.62	85474.1	10920.3	7.827	MM
202	2... 20211111_H19_R3_IgM	8.59	152415.6	9285.3	16.415	MM
203	2... 20211111_A39_R3_IgM	8.62	73462.1	9083.4	8.088	MM
204	2... 20211111_A40_R3_IgM	8.60	87777.0	8382.4	10.472	MM
205	2... 20211111_H20_R3_IgM	8.59	319571.9	7807.9	40.929	MM
206	2... 20211111_A41_R3_IgM	8.62	31370.1	5925.2	5.294	MM
207	2... 20211111_A42_R3_IgM	8.61	50003.9	9352.6	5.347	MM
208	2... 20211111_A43_R3_IgM	8.60	36744.9	10216.3	3.597	MM
209	2... 20211111_A44_R3_IgM	8.60	25468.7	10262.7	2.482	MM
210	2... 20211111_QC_R25_IgM	8.60	82036.4	8575.3	9.567	MM

Dataset: C:\MassLynx\10.pro\20211111_clinicalsamples_IgM_IgA.qld

Last Altered: Thursday, March 09, 2023 19:58:25 台北標準時間
Printed: Friday, August 01, 2025 15:06:51 台北標準時間

Method: C:\MassLynx\10_Pro_IgA.PRO\MethDB\20211111_IgM_IgA_DMRM_new.mdb 24 Feb 2022 13:44:14
Calibration: 15 Nov 2021 14:42:11

Sample Name: 20211112_QC_R001_IgM

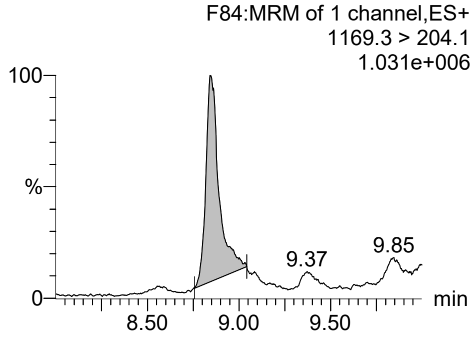

Sample Name: 20211112_QC_R002_IgM

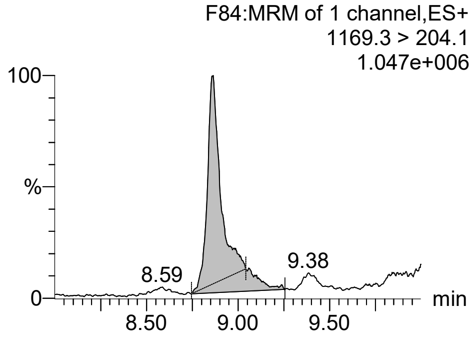

Sample Name: 20211111_A1_R2_IgM

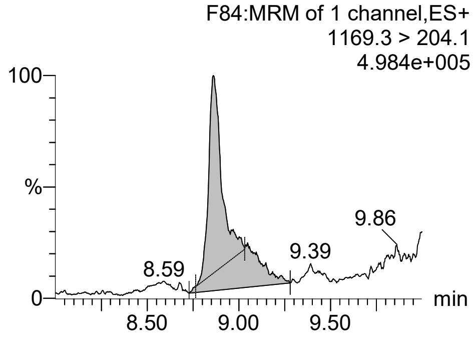

Sample Name: 20211111_A1_R3_IgM

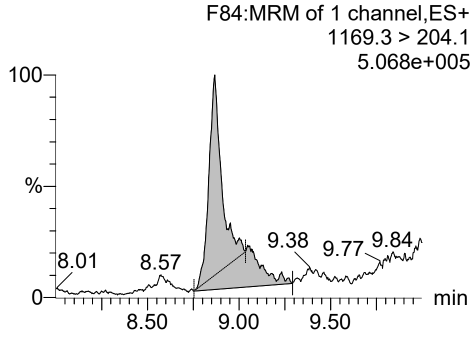

Sample Name: 20211111_A2_R2_IgM

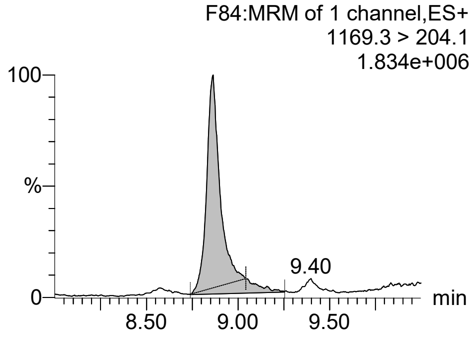

Sample Name: 20211111_A2_R3_IgM

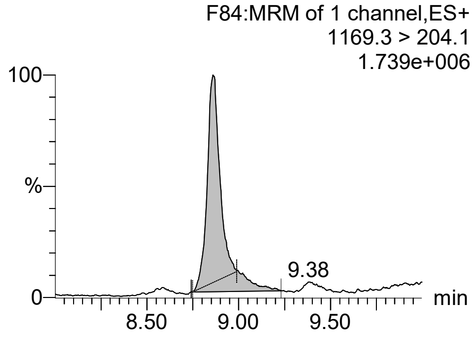

Sample Name: 20211111_L1_R2_IgM

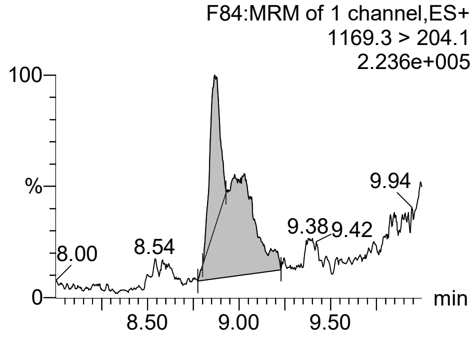

Sample Name: 20211111_L1_R3_IgM

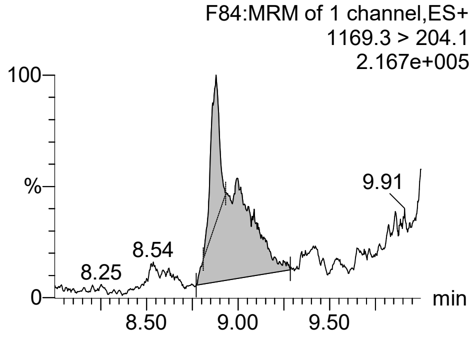

Sample Name: 20211111_L2_R2_IgM

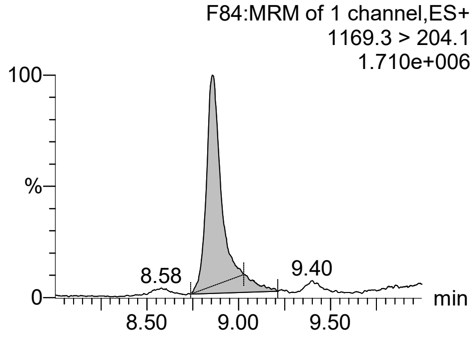

Sample Name: 20211111_H1_R2_IgM

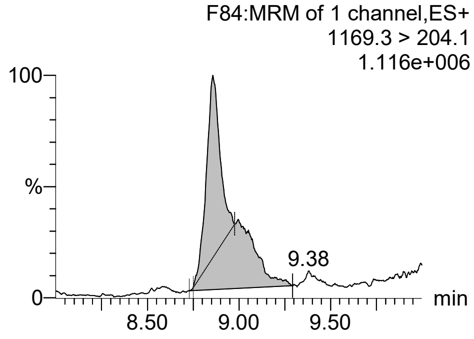

Sample Name: 20211111_A3_R2_IgM

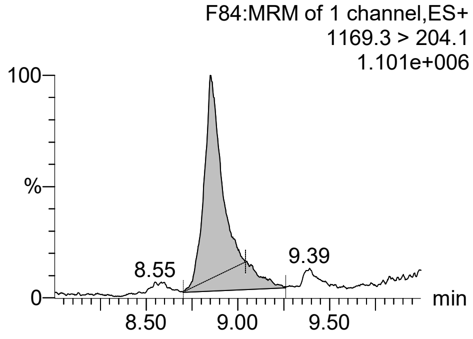

Sample Name: 20211111_A4_R2_IgM

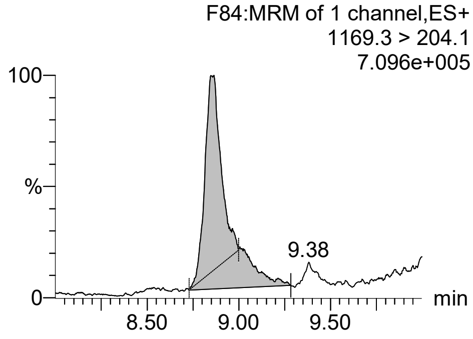

Dataset: C:\MassLynx\10.pro\20211111_clinicalsamples_IgM_IgA.qld

Last Altered: Thursday, March 09, 2023 19:58:25 台北標準時間
Printed: Friday, August 01, 2025 15:06:51 台北標準時間

Sample Name: 20211111_L3_R2_IgM
F84:MRM of 1 channel,ES+
1169.3 > 204.1
5.033e+005

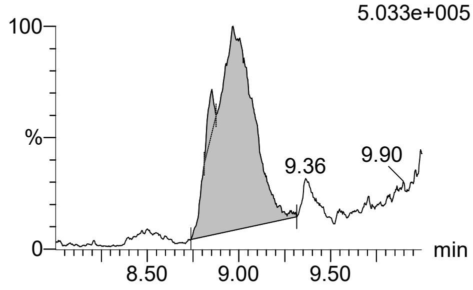

Sample Name: 20211111_L4_R2_IgM
F84:MRM of 1 channel,ES+
1169.3 > 204.1
7.491e+005

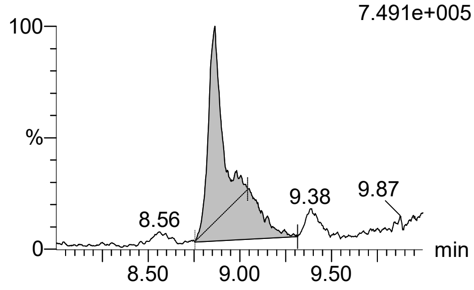

Sample Name: 20211111_H2_R2_IgM
F84:MRM of 1 channel,ES+
1169.3 > 204.1
1.592e+006

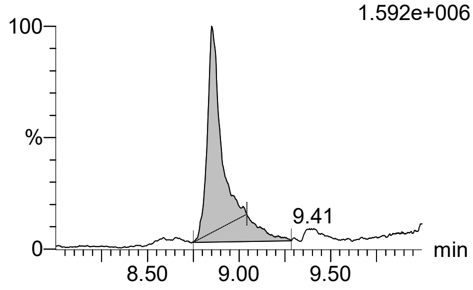

Sample Name: 20211111_A5_R2_IgM
F84:MRM of 1 channel,ES+
1169.3 > 204.1
1.645e+006

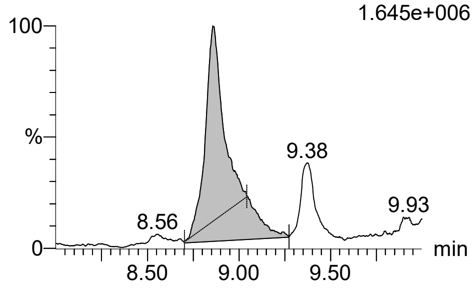

Sample Name: 20211111_A6_R2_IgM
F84:MRM of 1 channel,ES+
1169.3 > 204.1
1.161e+006

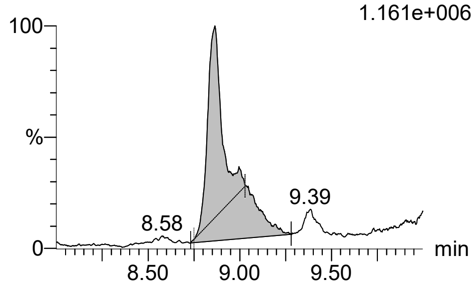

Sample Name: 20211111_QC_R10_IgM
F84:MRM of 1 channel,ES+
1169.3 > 204.1
1.043e+006

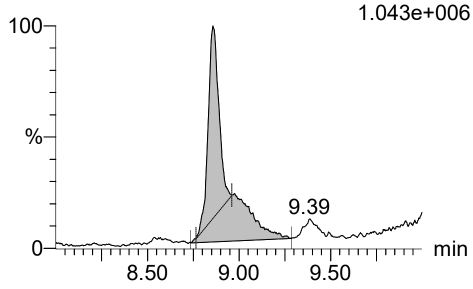

Sample Name: 20211111_L5_R2_IgM
F84:MRM of 1 channel,ES+
1169.3 > 204.1
7.475e+005

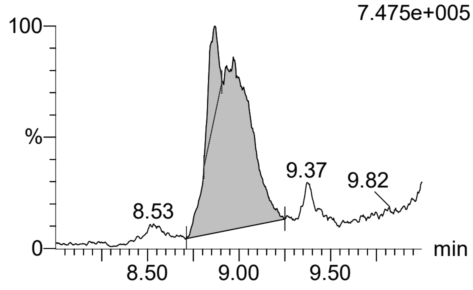

Sample Name: 20211111_L6_R2_IgM
F84:MRM of 1 channel,ES+
1169.3 > 204.1
1.666e+006

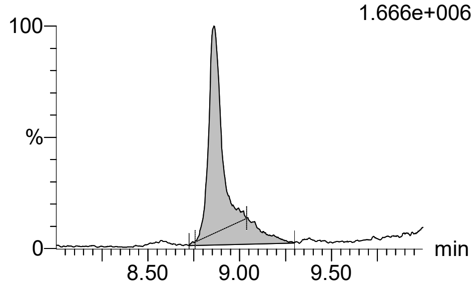

Sample Name: 20211111_H3_R2_IgM
F84:MRM of 1 channel,ES+
1169.3 > 204.1
4.997e+005

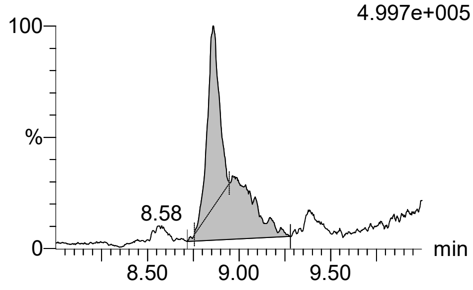

Sample Name: 20211111_A7_R2_IgM
F84:MRM of 1 channel,ES+
1169.3 > 204.1
1.485e+006

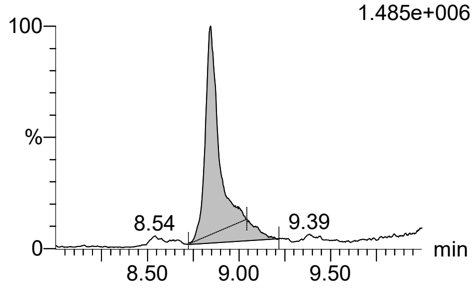

Sample Name: 20211111_A8_R2_IgM
F84:MRM of 1 channel,ES+
1169.3 > 204.1
9.802e+005

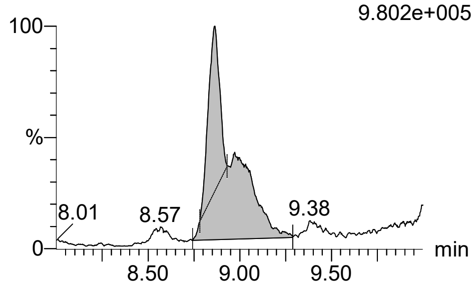

Sample Name: 20211111_L7_R2_IgM
F84:MRM of 1 channel,ES+
1169.3 > 204.1
2.418e+005

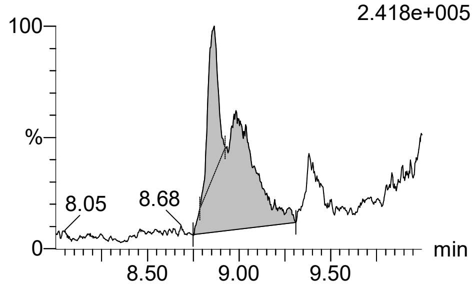

Sample Name: 20211111_L8_R2_IgM
F84:MRM of 1 channel,ES+
1169.3 > 204.1
4.145e+005

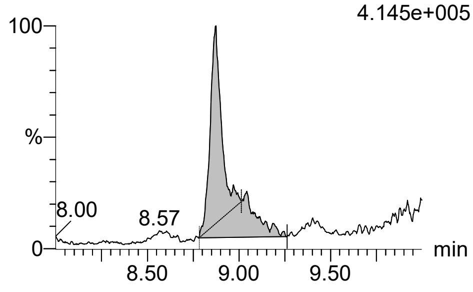

Sample Name: 20211111_H4_R2_IgM
F84:MRM of 1 channel,ES+
1169.3 > 204.1
6.798e+005

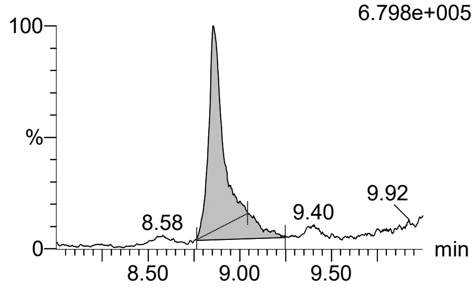

Sample Name: 20211111_A9_R2_IgM
F84:MRM of 1 channel,ES+
1169.3 > 204.1
6.028e+005

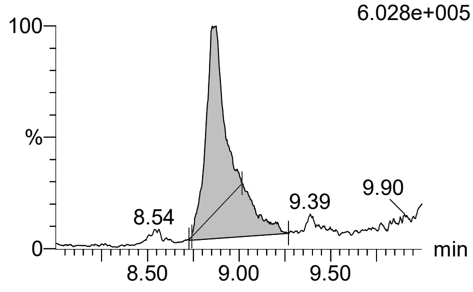

Sample Name: 20211111_A10_R2_IgM

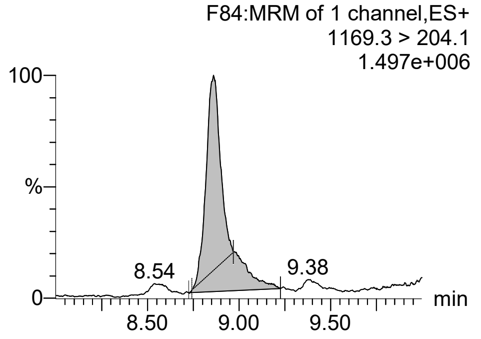

Sample Name: 20211111_L9_R2_IgM

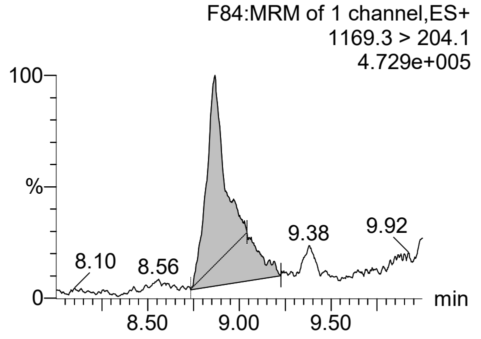

Sample Name: 20211111_L10_R2_IgM

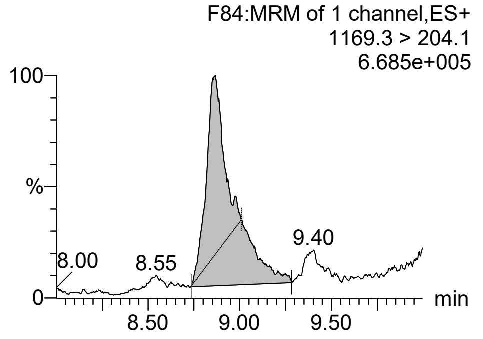

Sample Name: 20211111_QC_R11_IgM

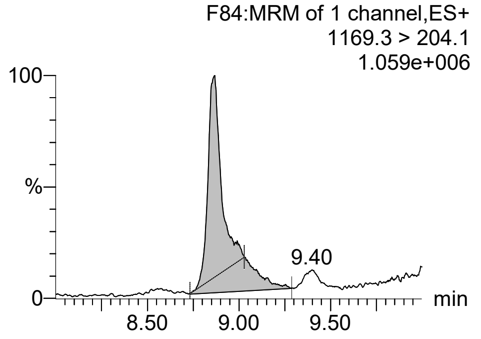

Sample Name: 20211111_H5_R2_IgM

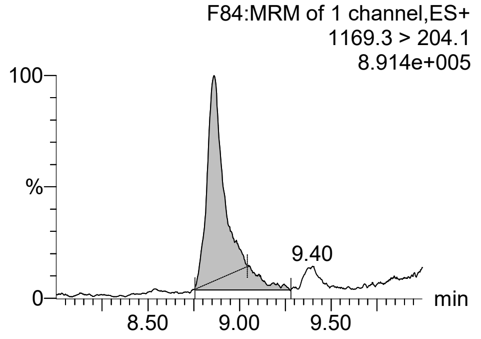

Sample Name: 20211111_A11_R2_IgM

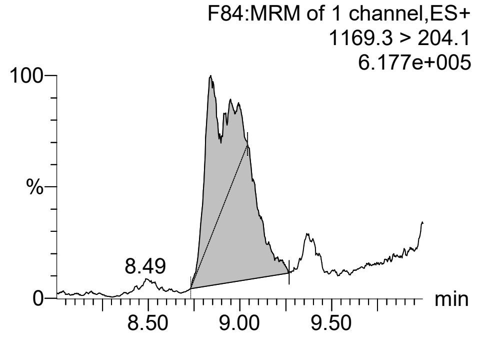

Sample Name: 20211111_A12_R2_IgM

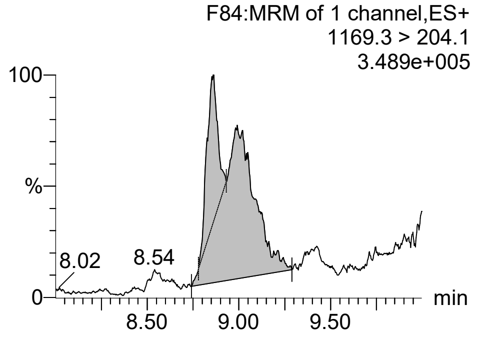

Sample Name: 20211111_L11_R2_IgM

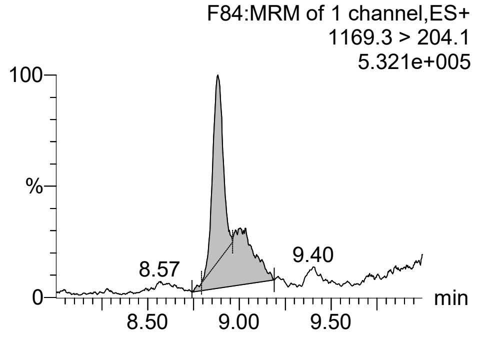

Sample Name: 20211111_L12_R2_IgM

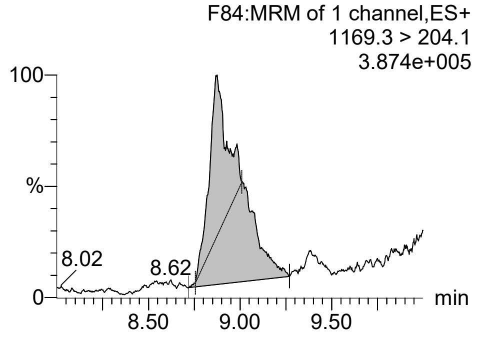

Sample Name: 20211111_H6_R2_IgM

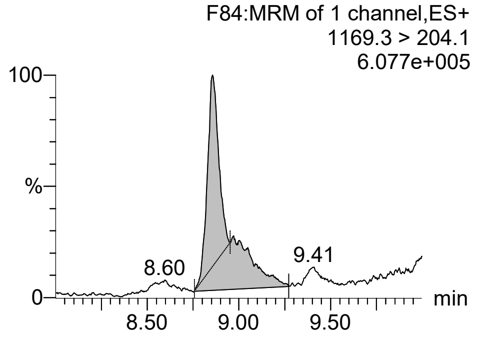

Sample Name: 20211111_A13_R2_IgM

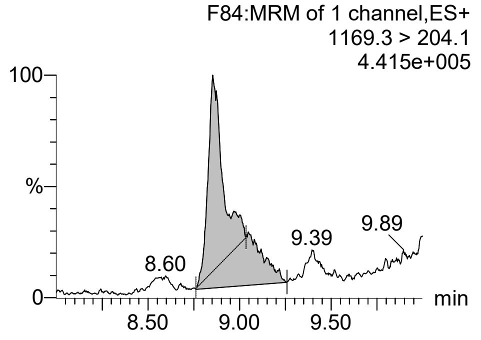

Sample Name: 20211111_A14_R2_IgM

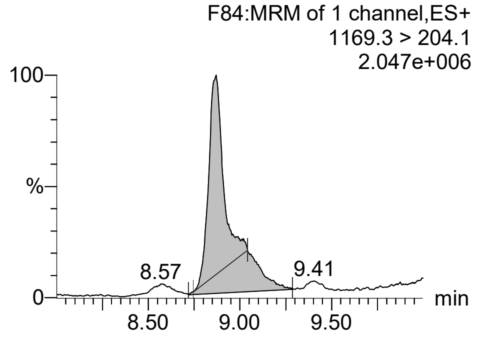

Sample Name: 20211111_L13_R2_IgM

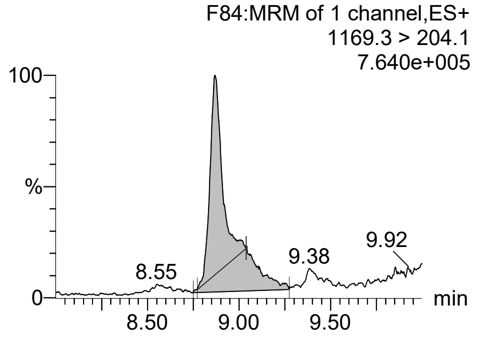

Sample Name: 20211111_L14_R2_IgM

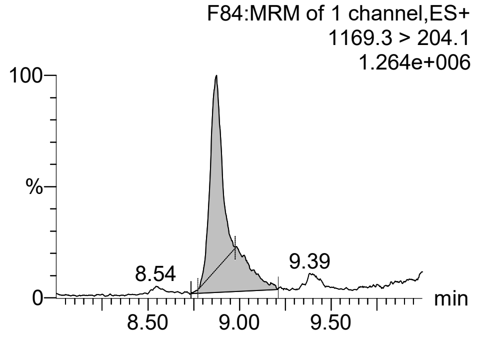

Sample Name: 20211111_H7_R2_IgM

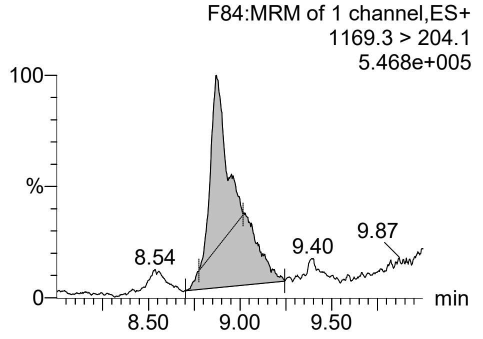

Dataset: C:\MassLynx\10.pro\20211111_clinicalsamples_IgM_IgA.qld

Last Altered: Thursday, March 09, 2023 19:58:25 台北標準時間
Printed: Friday, August 01, 2025 15:06:51 台北標準時間

Sample Name: 20211111_A15_R2_IgM
F84:MRM of 1 channel,ES+
1169.3 > 204.1
2.898e+006

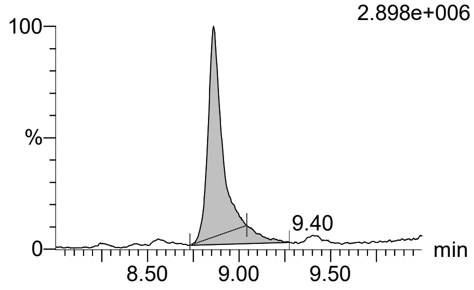

Sample Name: 20211111_QC_R12_IgM
F84:MRM of 1 channel,ES+
1169.3 > 204.1
1.246e+006

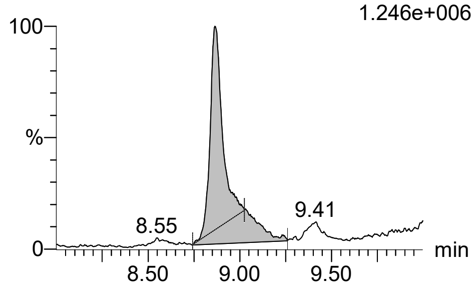

Sample Name: 20211111_A16_R2_IgM
F84:MRM of 1 channel,ES+
1169.3 > 204.1
1.749e+006

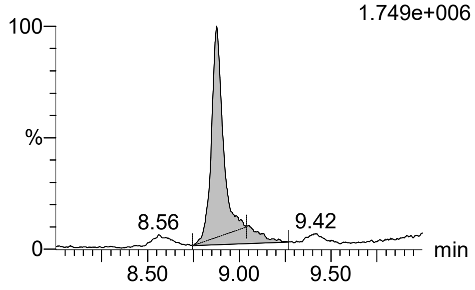

Sample Name: 20211111_L15_R2_IgM
F84:MRM of 1 channel,ES+
1169.3 > 204.1
1.075e+006

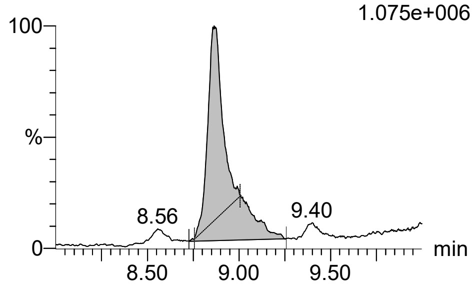

Sample Name: 20211111_L16_R2_IgM
F84:MRM of 1 channel,ES+
1169.3 > 204.1
4.819e+005

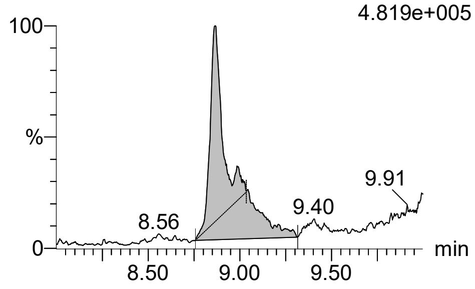

Sample Name: 20211111_H8_R2_IgM
F84:MRM of 1 channel,ES+
1169.3 > 204.1
7.461e+005

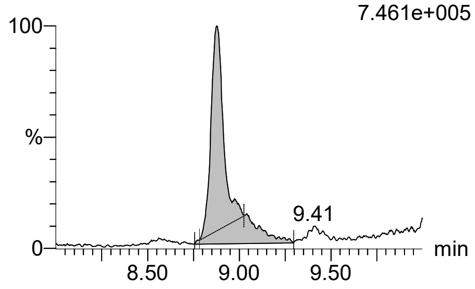

Sample Name: 20211111_A17_R2_IgM
F84:MRM of 1 channel,ES+
1169.3 > 204.1
2.482e+006

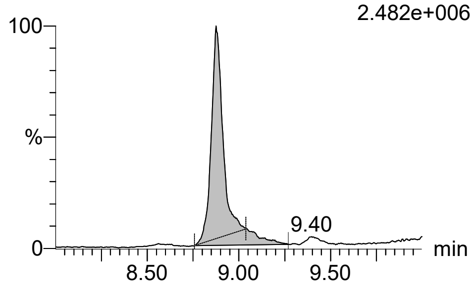

Sample Name: 20211111_A18_R2_IgM
F84:MRM of 1 channel,ES+
1169.3 > 204.1
5.289e+005

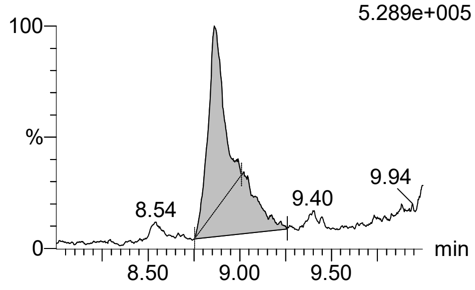

Sample Name: 20211111_L17_R2_IgM
F84:MRM of 1 channel,ES+
1169.3 > 204.1
1.033e+006

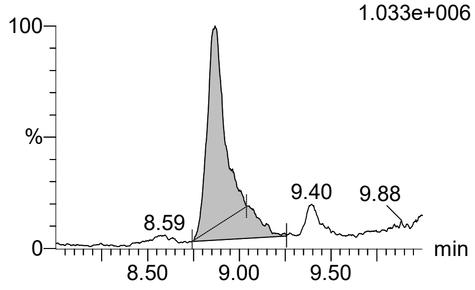

Sample Name: 20211111_L18_R2_IgM
F84:MRM of 1 channel,ES+
1169.3 > 204.1
1.446e+005

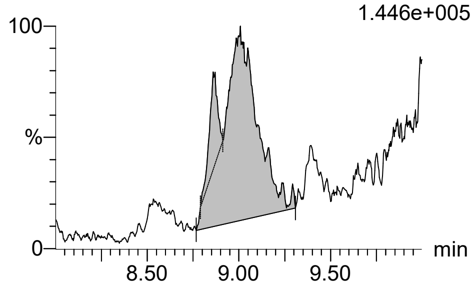

Sample Name: 20211111_H9_R2_IgM
F84:MRM of 1 channel,ES+
1169.3 > 204.1
2.468e+005

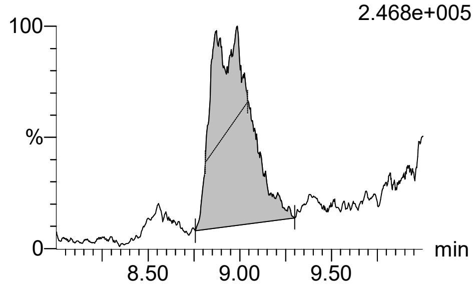

Sample Name: 20211111_A19_R2_IgM
F84:MRM of 1 channel,ES+
1169.3 > 204.1
6.779e+005

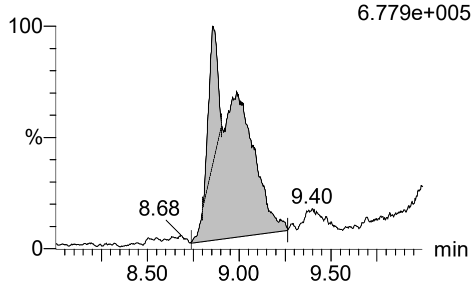

Sample Name: 20211111_A20_R2_IgM
F84:MRM of 1 channel,ES+
1169.3 > 204.1
2.397e+005

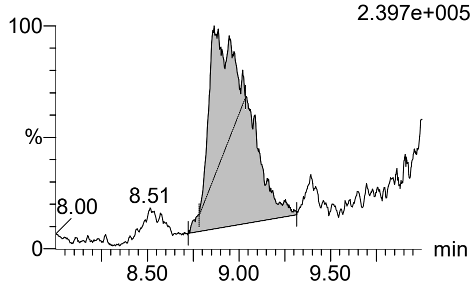

Sample Name: 20211111_L19_R2_IgM
F84:MRM of 1 channel,ES+
1169.3 > 204.1
7.081e+005

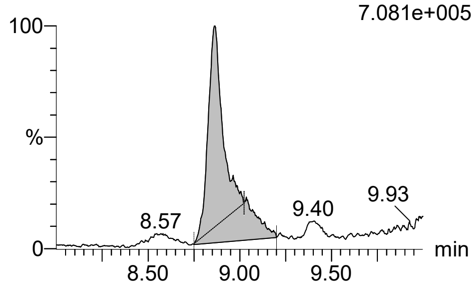

Sample Name: 20211111_QC_R13_IgM
F84:MRM of 1 channel,ES+
1169.3 > 204.1
1.179e+006

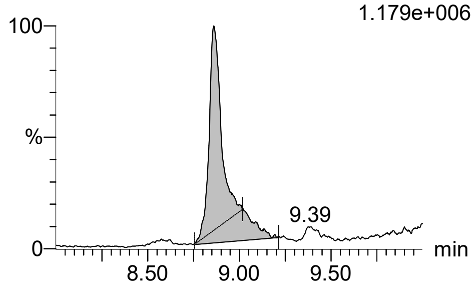

Sample Name: 20211111_L20_R2_IgM
F84:MRM of 1 channel,ES+
1169.3 > 204.1
5.989e+005

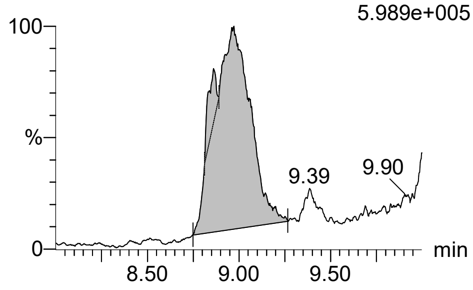

Sample Name: 20211111_H10_R2_IgM
F84:MRM of 1 channel,ES+
1169.3 > 204.1
6.226e+005

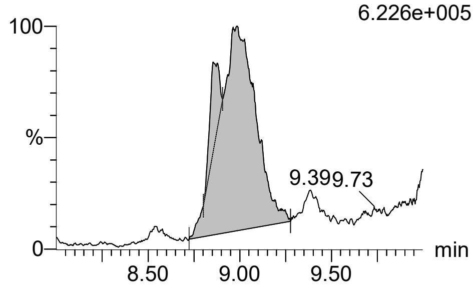

Sample Name: 20211111_A21_R2_IgM
F84:MRM of 1 channel,ES+
1169.3 > 204.1
8.372e+005

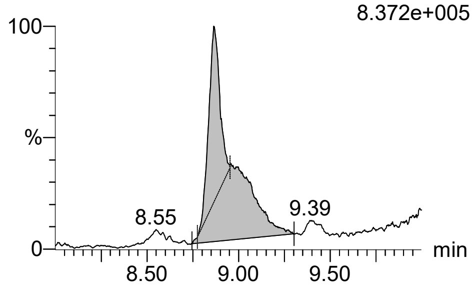

Sample Name: 20211111_A22_R2_IgM
F84:MRM of 1 channel,ES+
1169.3 > 204.1
1.942e+005

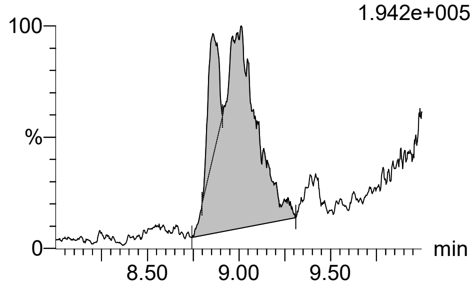

Sample Name: 20211111_L21_R2_IgM
F84:MRM of 1 channel,ES+
1169.3 > 204.1
6.535e+005

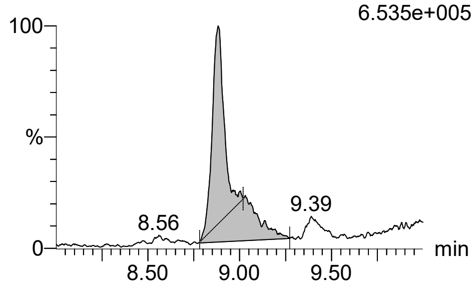

Sample Name: 20211111_L22_R2_IgM
F84:MRM of 1 channel,ES+
1169.3 > 204.1
8.881e+005

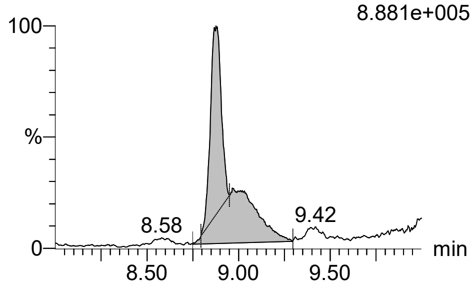

Sample Name: 20211111_H11_R2_IgM
F84:MRM of 1 channel,ES+
1169.3 > 204.1
1.272e+006

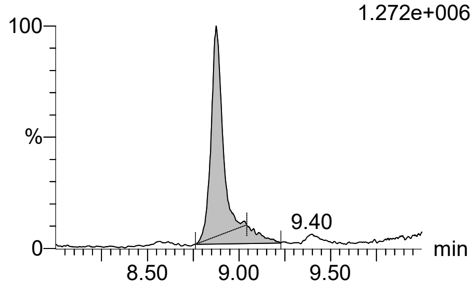

Sample Name: 20211111_A23_R2_IgM
F84:MRM of 1 channel,ES+
1169.3 > 204.1
3.308e+005

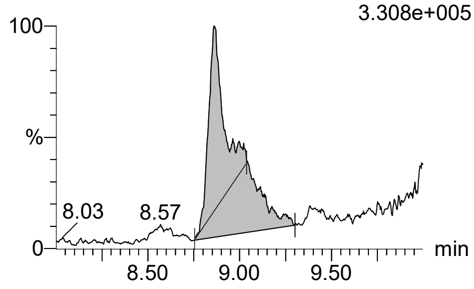

Sample Name: 20211111_A24_R2_IgM
F84:MRM of 1 channel,ES+
1169.3 > 204.1
1.210e+006

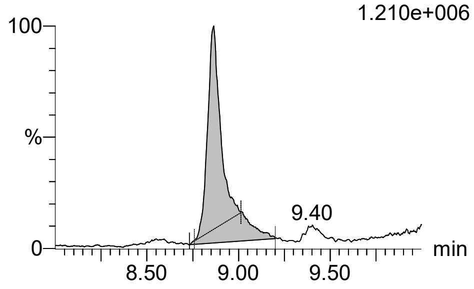

Sample Name: 20211111_L23_R2_IgM
F84:MRM of 1 channel,ES+
1169.3 > 204.1
5.780e+005

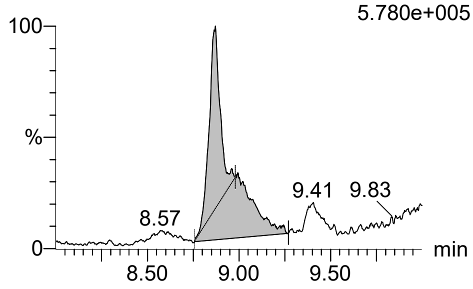

Sample Name: 20211111_L24_R2_IgM
F84:MRM of 1 channel,ES+
1169.3 > 204.1
2.604e+005

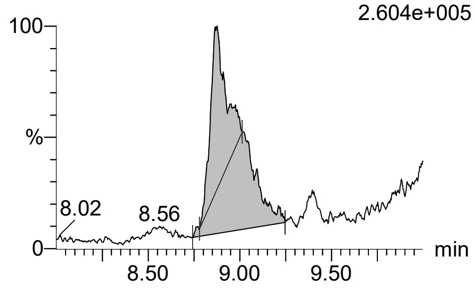

Sample Name: 20211111_H12_R2_IgM
F84:MRM of 1 channel,ES+
1169.3 > 204.1
4.258e+005

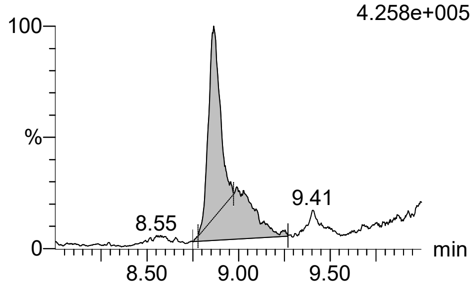

Sample Name: 20211111_QC_R14_IgM
F84:MRM of 1 channel,ES+
1169.3 > 204.1
1.323e+006

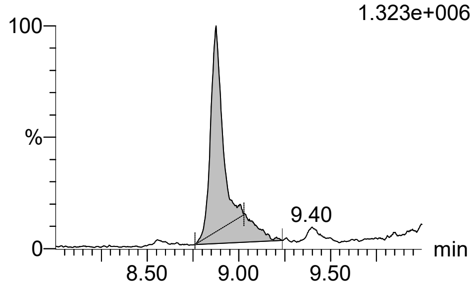

Sample Name: 20211111_A25_R2_IgM
F84:MRM of 1 channel,ES+
1169.3 > 204.1
2.407e+006

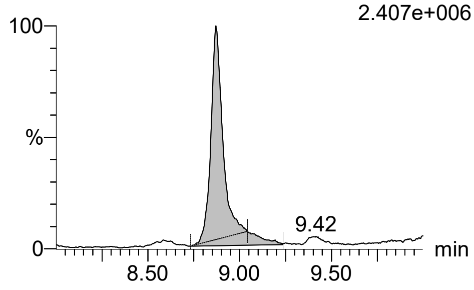

Sample Name: 20211111_A26_R2_IgM
F84:MRM of 1 channel,ES+
1169.3 > 204.1
1.328e+006

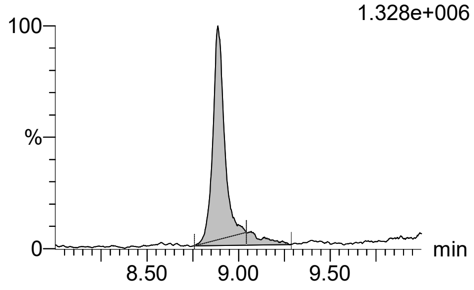

Dataset: C:\MassLynx\10.pro\20211111_clinicalsamples_IgM_IgA.qld

Last Altered: Thursday, March 09, 2023 19:58:25 台北標準時間
Printed: Friday, August 01, 2025 15:06:51 台北標準時間

Sample Name: 20211111_L25_R2_IgM
F84:MRM of 1 channel,ES+
1169.3 > 204.1
8.475e+005

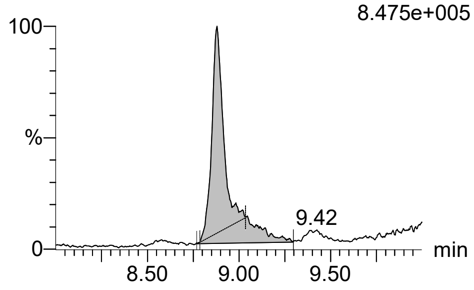

Sample Name: 20211111_L26_R2_IgM
F84:MRM of 1 channel,ES+
1169.3 > 204.1
5.230e+005

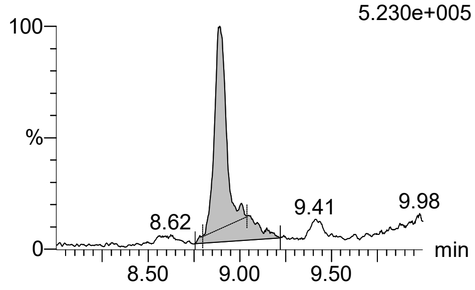

Sample Name: 20211111_H13_R2_IgM
F84:MRM of 1 channel,ES+
1169.3 > 204.1
1.054e+006

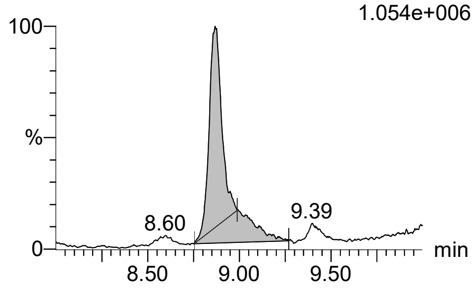

Sample Name: 20211111_A27_R2_IgM
F84:MRM of 1 channel,ES+
1169.3 > 204.1
1.262e+006

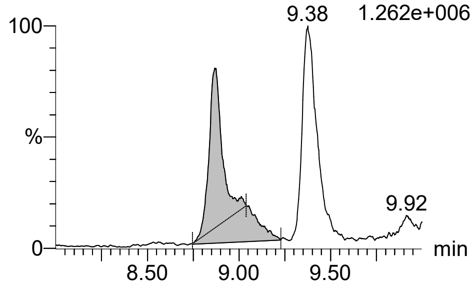

Sample Name: 20211111_A28_R2_IgM
F84:MRM of 1 channel,ES+
1169.3 > 204.1
5.722e+005

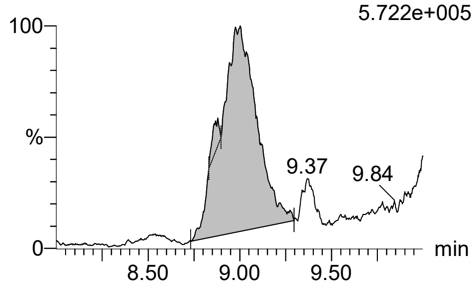

Sample Name: 20211111_L27_R2_IgM
F84:MRM of 1 channel,ES+
1169.3 > 204.1
8.584e+005

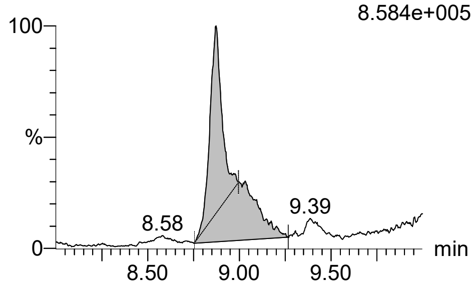

Sample Name: 20211111_L28_R2_IgM
F84:MRM of 1 channel,ES+
1169.3 > 204.1
6.253e+005

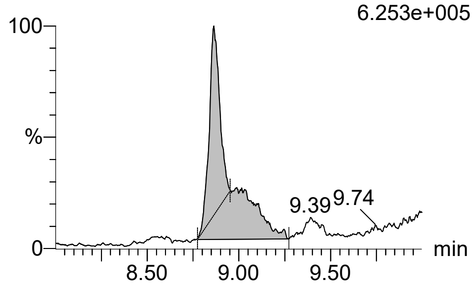

Sample Name: 20211111_H14_R2_IgM
F84:MRM of 1 channel,ES+
1169.3 > 204.1
8.185e+005

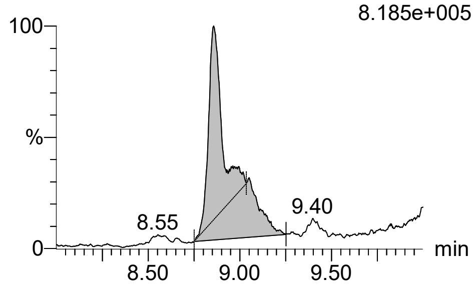

Sample Name: 20211111_A29_R2_IgM
F84:MRM of 1 channel,ES+
1169.3 > 204.1
1.744e+005

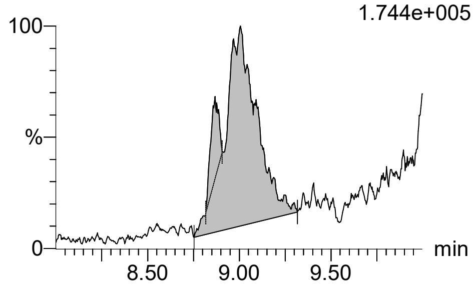

Sample Name: 20211111_A30_R2_IgM
F84:MRM of 1 channel,ES+
1169.3 > 204.1
1.367e+006

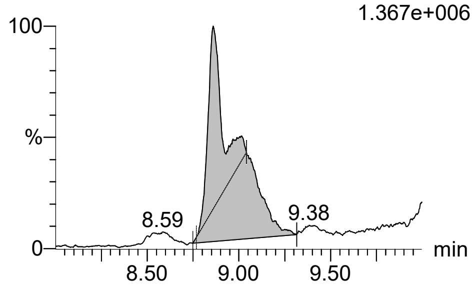

Sample Name: 20211111_QC_R15_IgM
F84:MRM of 1 channel,ES+
1169.3 > 204.1
1.108e+006

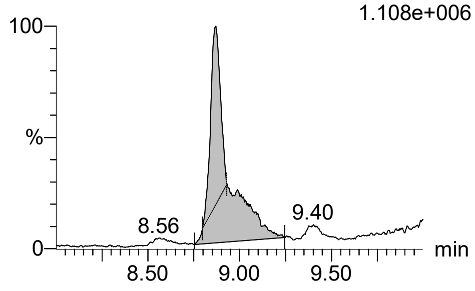

Sample Name: 20211111_L29_R2_IgM
F84:MRM of 1 channel,ES+
1169.3 > 204.1
4.337e+005

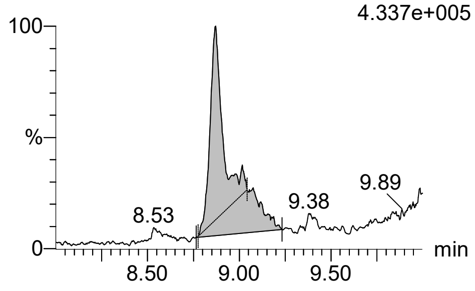

Sample Name: 20211111_L30_R2_IgM
F84:MRM of 1 channel,ES+
1169.3 > 204.1
2.443e+005

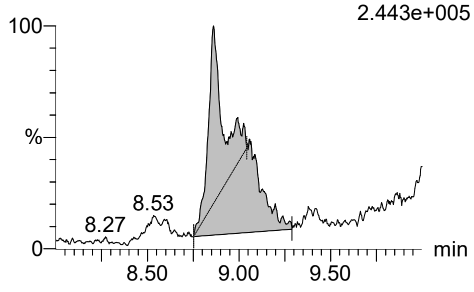

Sample Name: 20211111_H15_R2_IgM
F84:MRM of 1 channel,ES+
1169.3 > 204.1
1.046e+006

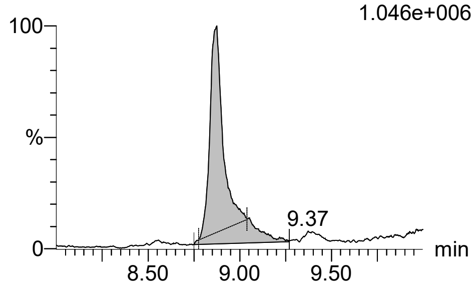

Sample Name: 20211111_A31_R2_IgM
F84:MRM of 1 channel,ES+
1169.3 > 204.1
4.377e+005

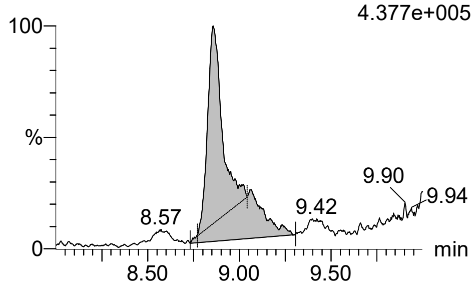

Dataset: C:\MassLynx\10.pro\20211111_clinicalsamples_IgM_IgA.qld

Last Altered: Thursday, March 09, 2023 19:58:25 台北標準時間
Printed: Friday, August 01, 2025 15:06:51 台北標準時間

Sample Name: 20211111_A32_R2_IgM
F84:MRM of 1 channel,ES+
1169.3 > 204.1
1.361e+006

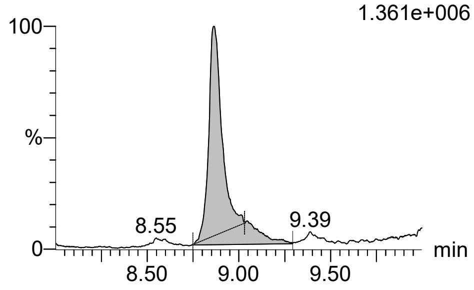

Sample Name: 20211111_L31_R2_IgM
F84:MRM of 1 channel,ES+
1169.3 > 204.1
2.026e+005

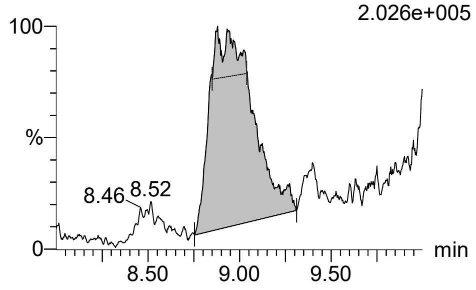

Sample Name: 20211111_L32_R2_IgM
F84:MRM of 1 channel,ES+
1169.3 > 204.1
5.885e+005

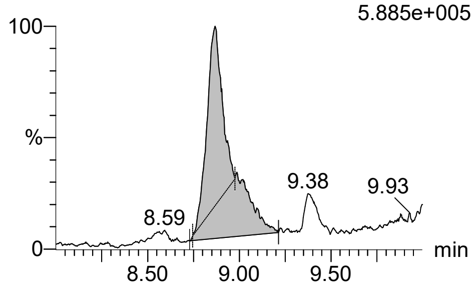

Sample Name: 20211111_H16_R2_IgM
F84:MRM of 1 channel,ES+
1169.3 > 204.1
1.713e+006

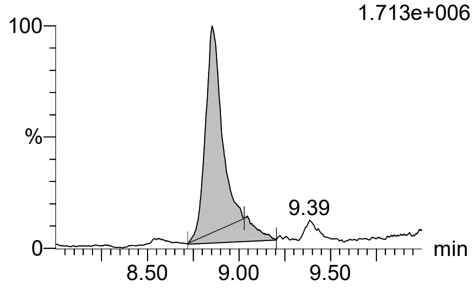

Sample Name: 20211111_A33_R2_IgM
F84:MRM of 1 channel,ES+
1169.3 > 204.1
1.251e+006

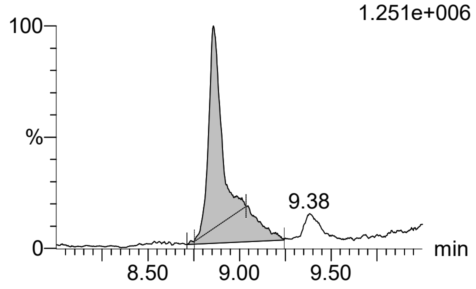

Sample Name: 20211111_A34_R2_IgM
F84:MRM of 1 channel,ES+
1169.3 > 204.1
4.693e+005

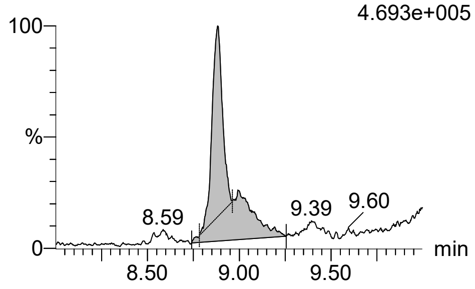

Sample Name: 20211111_H17_R2_IgM
F84:MRM of 1 channel,ES+
1169.3 > 204.1
3.988e+006

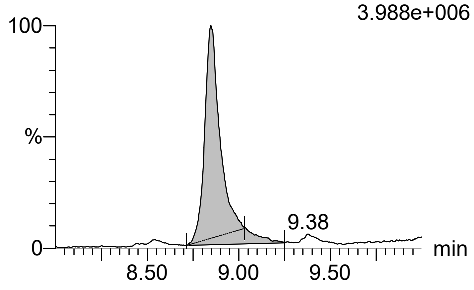

Sample Name: 20211111_A35_R2_IgM
F84:MRM of 1 channel,ES+
1169.3 > 204.1
2.403e+005

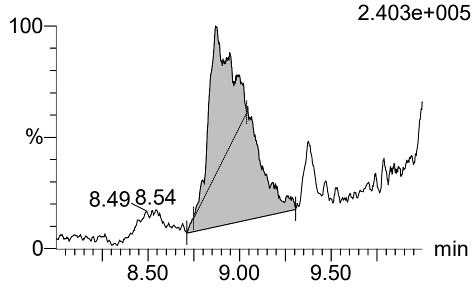

Sample Name: 20211111_QC_R16_IgM
F84:MRM of 1 channel,ES+
1169.3 > 204.1
9.641e+005

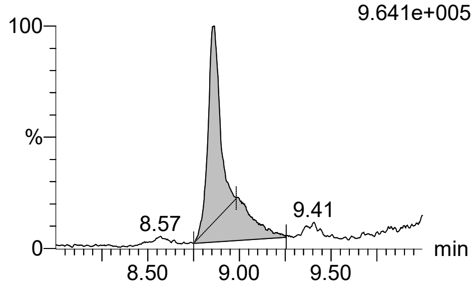

Sample Name: 20211111_A36_R2_IgM
F84:MRM of 1 channel,ES+
1169.3 > 204.1
4.802e+005

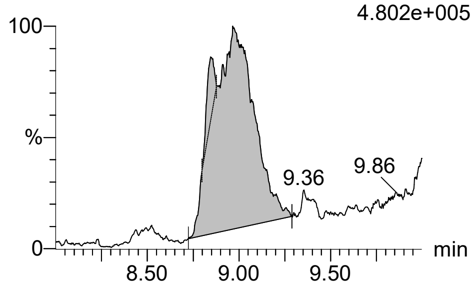

Sample Name: 20211111_H18_R2_IgM
F84:MRM of 1 channel,ES+
1169.3 > 204.1
1.515e+006

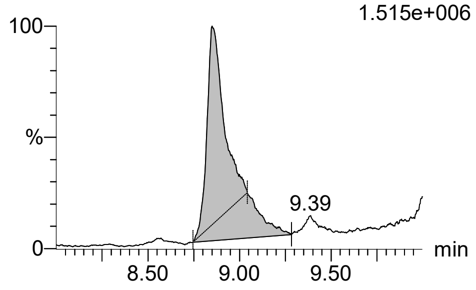

Sample Name: 20211111_A37_R2_IgM
F84:MRM of 1 channel,ES+
1169.3 > 204.1
1.205e+006

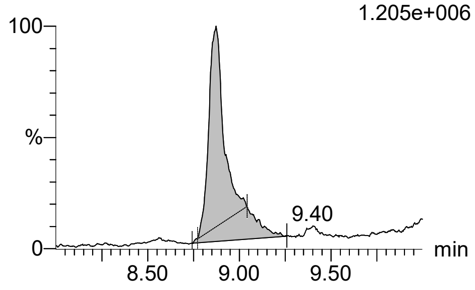

Sample Name: 20211111_A38_R2_IgM
F84:MRM of 1 channel,ES+
1169.3 > 204.1
1.064e+006

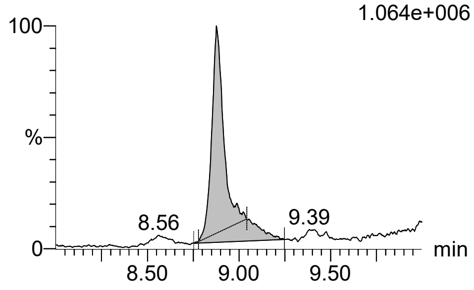

Sample Name: 20211111_H19_R2_IgM
F84:MRM of 1 channel,ES+
1169.3 > 204.1
1.191e+006

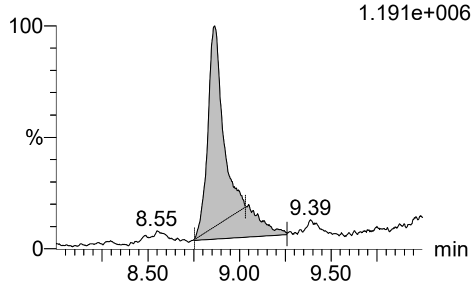

Sample Name: 20211111_A39_R2_IgM
F84:MRM of 1 channel,ES+
1169.3 > 204.1
1.536e+006

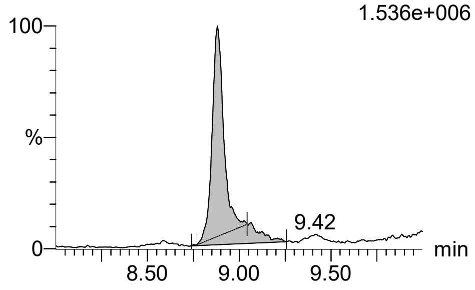

Dataset: C:\MassLynx\10.pro\20211111_clinicalsamples_IgM_IgA.qld

Last Altered: Thursday, March 09, 2023 19:58:25 台北標準時間
Printed: Friday, August 01, 2025 15:06:51 台北標準時間

Sample Name: 20211111_A40_R2_IgM
F84:MRM of 1 channel,ES+
1169.3 > 204.1
3.785e+005

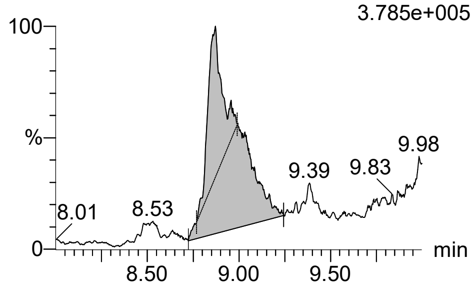

Sample Name: 20211111_H20_R2_IgM
F84:MRM of 1 channel,ES+
1169.3 > 204.1
1.458e+006

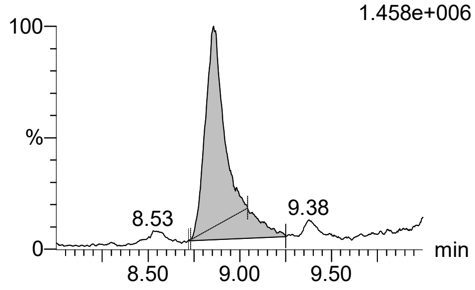

Sample Name: 20211111_A41_R2_IgM
F84:MRM of 1 channel,ES+
1169.3 > 204.1
4.881e+005

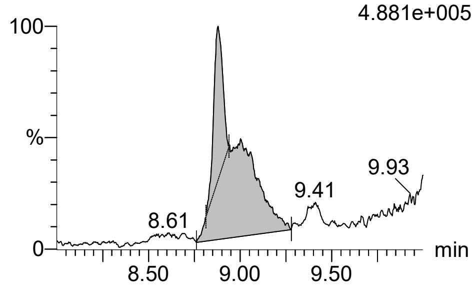

Sample Name: 20211111_A42_R2_IgM
F84:MRM of 1 channel,ES+
1169.3 > 204.1
4.903e+005

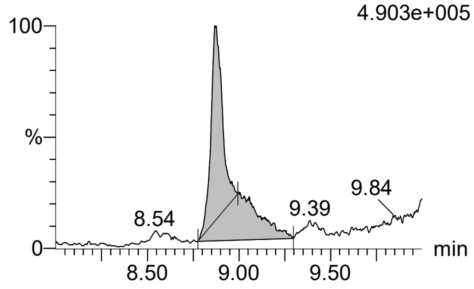

Sample Name: 20211111_A43_R2_IgM
F84:MRM of 1 channel,ES+
1169.3 > 204.1
3.978e+005

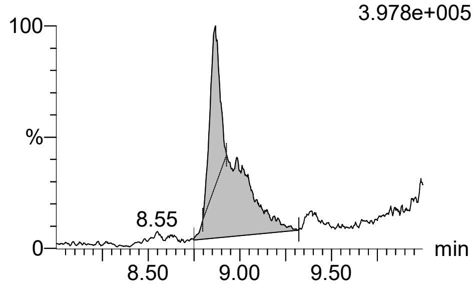

Sample Name: 20211111_A44_R2_IgM
F84:MRM of 1 channel,ES+
1169.3 > 204.1
2.323e+005

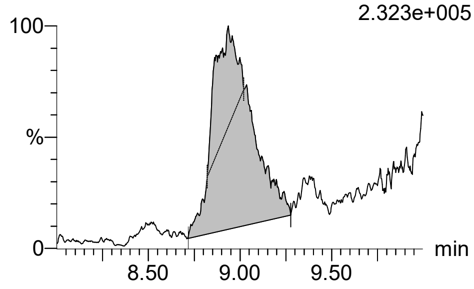

Sample Name: 20211111_QC_R17_IgM
F84:MRM of 1 channel,ES+
1169.3 > 204.1
7.822e+005

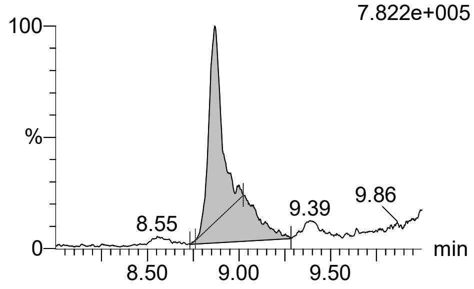

Sample Name: 20211111_L2_R3_IgM
F84:MRM of 1 channel,ES+
1169.3 > 204.1
1.665e+006

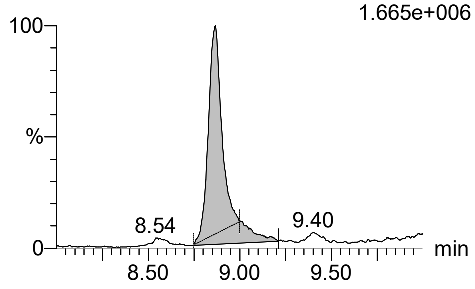

Sample Name: 20211111_H1_R3_IgM
F84:MRM of 1 channel,ES+
1169.3 > 204.1
1.677e+006

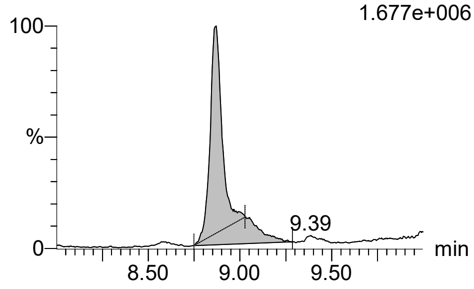

Sample Name: 20211111_A3_R3_IgM
F84:MRM of 1 channel,ES+
1169.3 > 204.1
1.839e+006

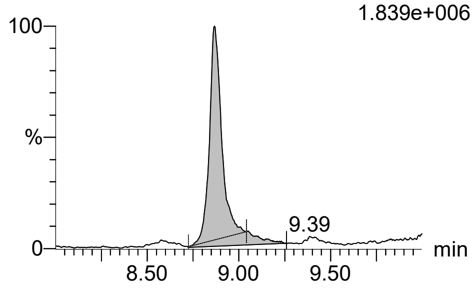

Sample Name: 20211111_A4_R3_IgM
F84:MRM of 1 channel,ES+
1169.3 > 204.1
1.125e+006

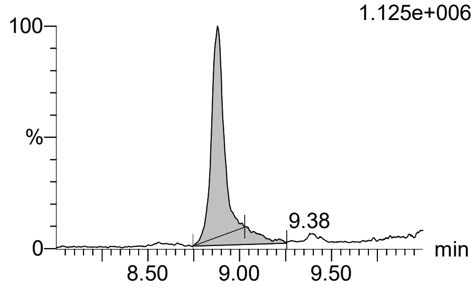

Sample Name: 20211111_L3_R3_IgM
F84:MRM of 1 channel,ES+
1169.3 > 204.1
4.046e+005

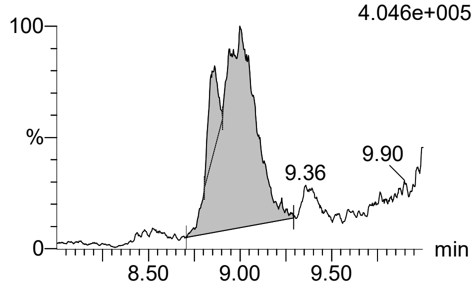

Sample Name: 20211111_L4_R3_IgM
F84:MRM of 1 channel,ES+
1169.3 > 204.1
8.367e+005

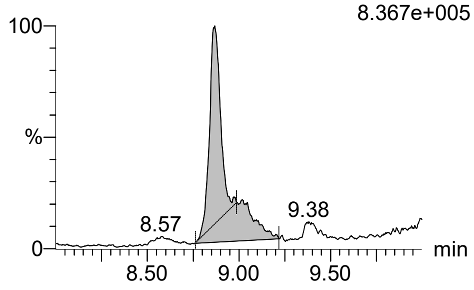

Sample Name: 20211111_H2_R3_IgM
F84:MRM of 1 channel,ES+
1169.3 > 204.1
1.514e+006

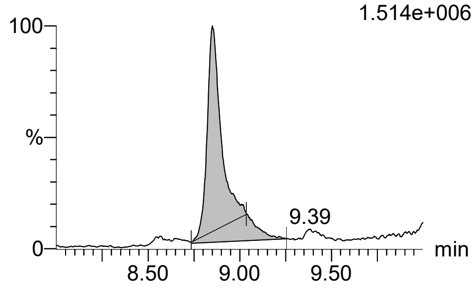

Sample Name: 20211111_A5_R3_IgM
F84:MRM of 1 channel,ES+
1169.3 > 204.1
2.413e+006

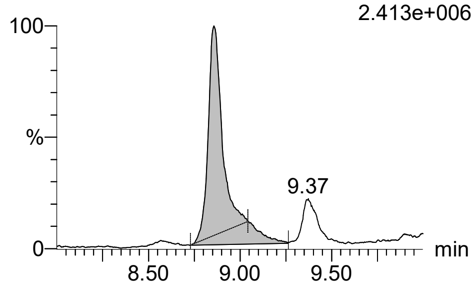

Sample Name: 20211111_A6_R3_IgM
F84:MRM of 1 channel,ES+
1169.3 > 204.1
1.060e+006

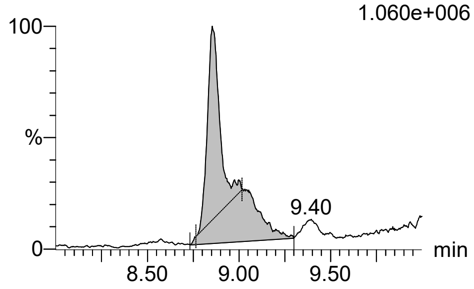

Sample Name: 20211111_QC_R18_IgM
F84:MRM of 1 channel,ES+
1169.3 > 204.1
8.084e+005

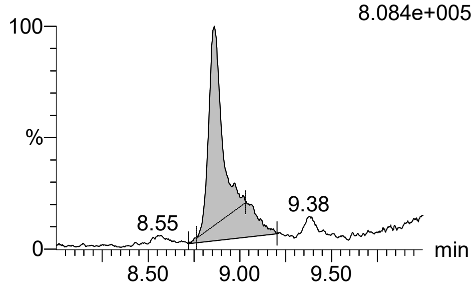

Sample Name: 20211111_L5_R3_IgM
F84:MRM of 1 channel,ES+
1169.3 > 204.1
1.240e+006

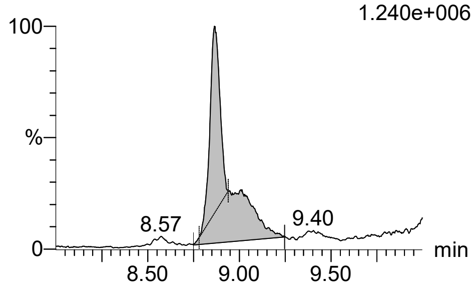

Sample Name: 20211111_L6_R3_IgM
F84:MRM of 1 channel,ES+
1169.3 > 204.1
1.384e+006

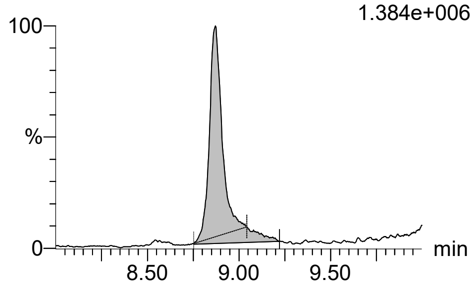

Sample Name: 20211111_H3_R3_IgM
F84:MRM of 1 channel,ES+
1169.3 > 204.1
7.544e+005

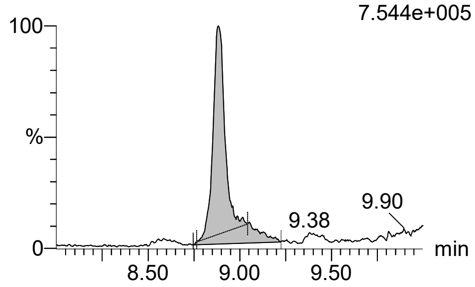

Sample Name: 20211111_A7_R3_IgM
F84:MRM of 1 channel,ES+
1169.3 > 204.1
1.391e+006

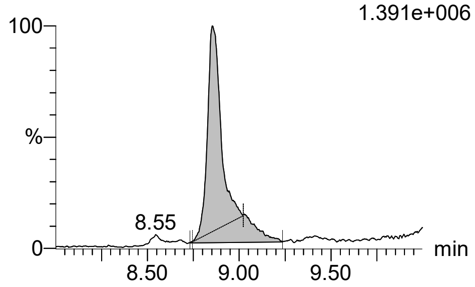

Sample Name: 20211111_A8_R3_IgM
F84:MRM of 1 channel,ES+
1169.3 > 204.1
1.495e+006

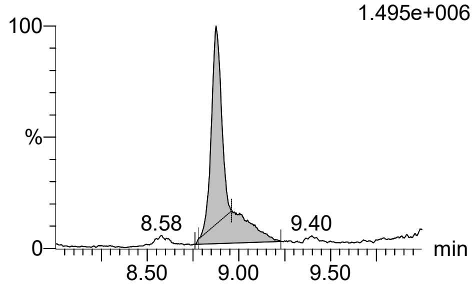

Sample Name: 20211111_L7_R3_IgM
F84:MRM of 1 channel,ES+
1169.3 > 204.1
2.502e+005

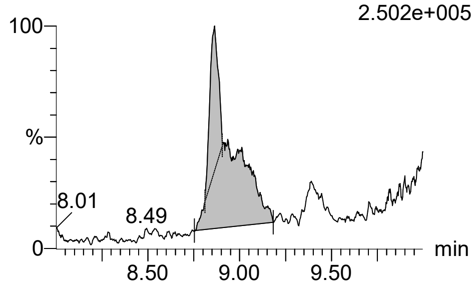

Sample Name: 20211111_L8_R3_IgM
F84:MRM of 1 channel,ES+
1169.3 > 204.1
4.158e+005

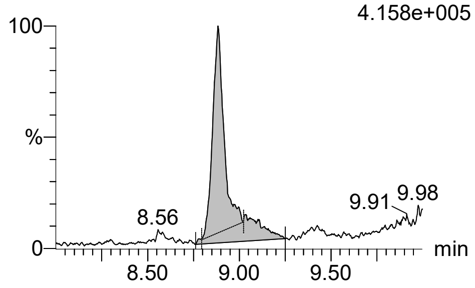

Sample Name: 20211111_H4_R3_IgM
F84:MRM of 1 channel,ES+
1169.3 > 204.1
8.304e+005

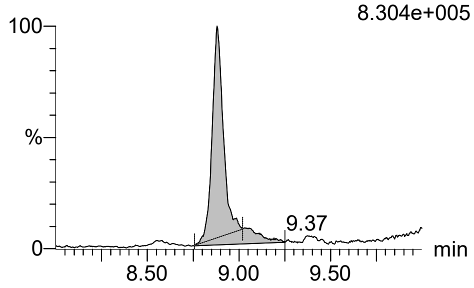

Sample Name: 20211111_A9_R3_IgM
F84:MRM of 1 channel,ES+
1169.3 > 204.1
1.174e+006

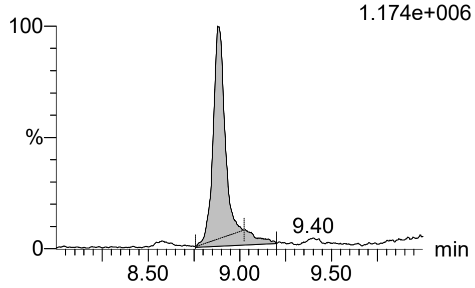

Sample Name: 20211111_A10_R3_IgM
F84:MRM of 1 channel,ES+
1169.3 > 204.1
2.565e+006

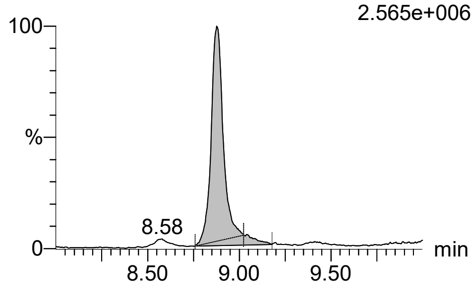

Sample Name: 20211111_L9_R3_IgM
F84:MRM of 1 channel,ES+
1169.3 > 204.1
8.367e+005

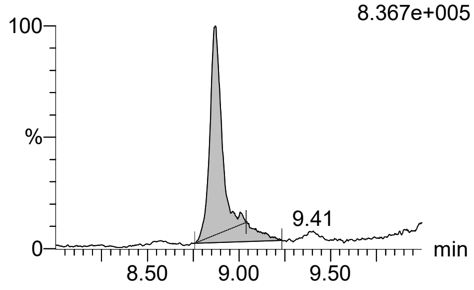

Sample Name: 20211111_L10_R3_IgM
F84:MRM of 1 channel,ES+
1169.3 > 204.1
5.596e+005

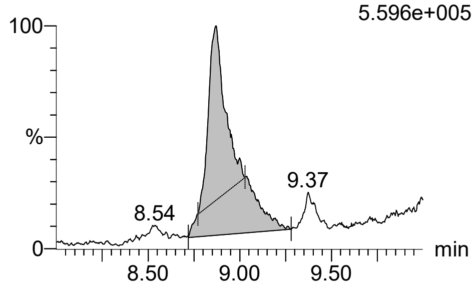

Sample Name: 20211111_QC_R19_IgM
F84:MRM of 1 channel,ES+
1169.3 > 204.1
7.949e+005

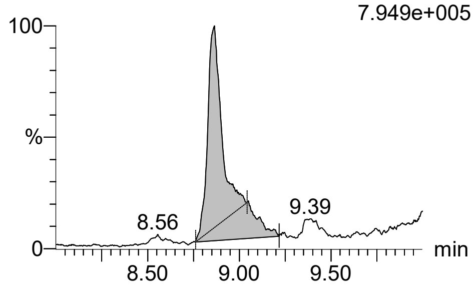

Dataset: C:\MassLynx\10.pro\20211111_clinicalsamples_IgM_IgA.qld

Last Altered: Thursday, March 09, 2023 19:58:25 台北標準時間
Printed: Friday, August 01, 2025 15:06:51 台北標準時間

Sample Name: 20211111_H5_R3_IgM

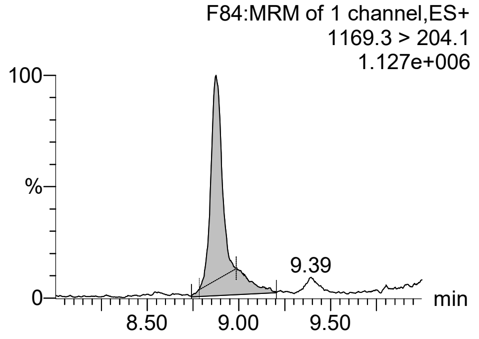

Sample Name: 20211111_A11_R3_IgM

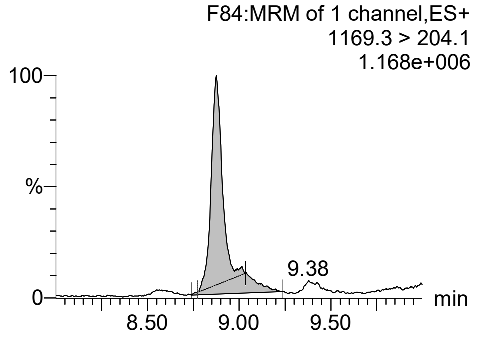

Sample Name: 20211111_A12_R3_IgM

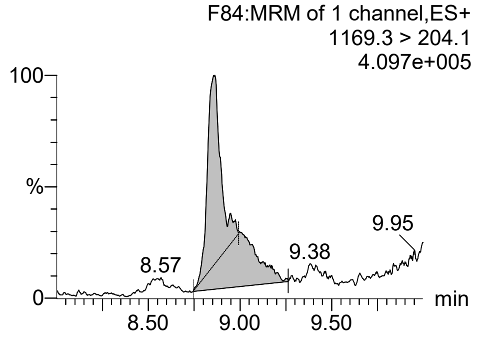

Sample Name: 20211111_L11_R3_IgM

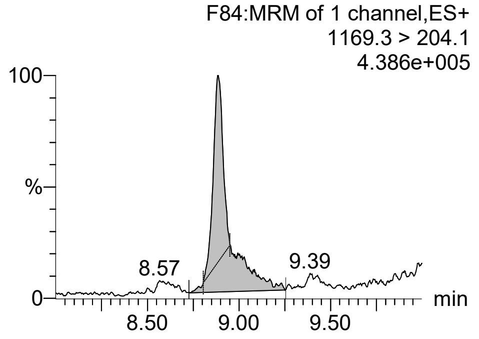

Sample Name: 20211111_L12_R3_IgM

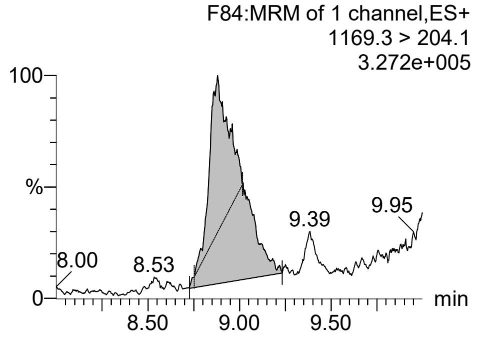

Sample Name: 20211111_H6_R3_IgM

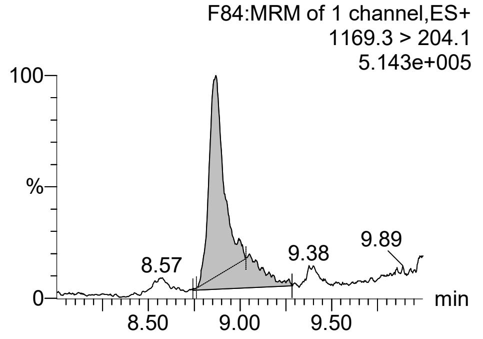

Sample Name: 20211111_A13_R3_IgM

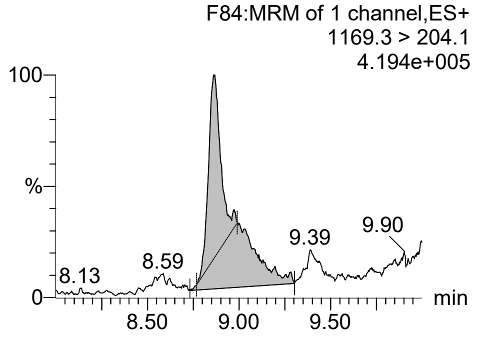

Sample Name: 20211111_A14_R3_IgM

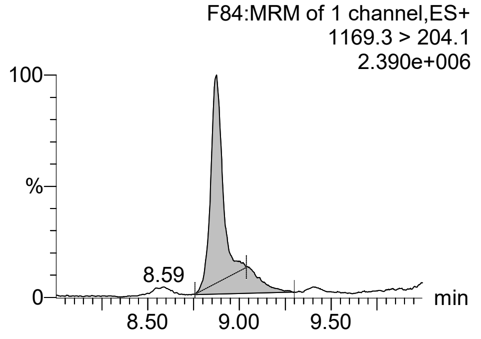

Sample Name: 20211111_L13_R3_IgM

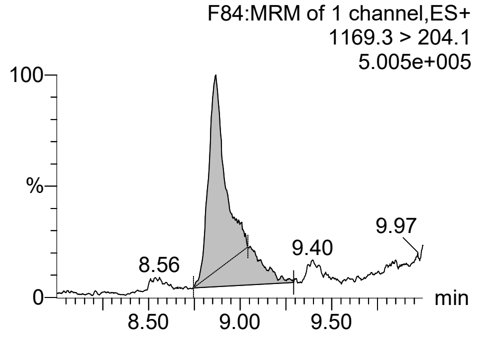

Sample Name: 20211111_L14_R3_IgM

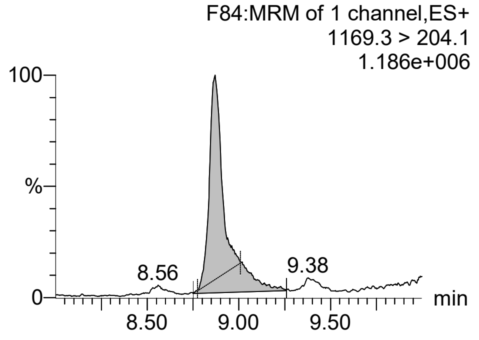

Sample Name: 20211111_H7_R3_IgM

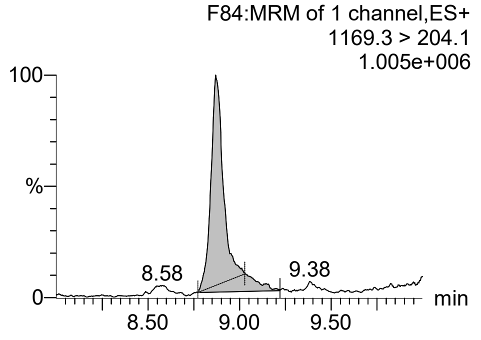

Sample Name: 20211111_A15_R3_IgM

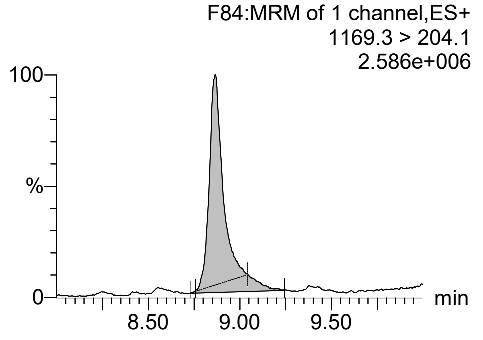

Sample Name: 20211111_QC_R20_IgM

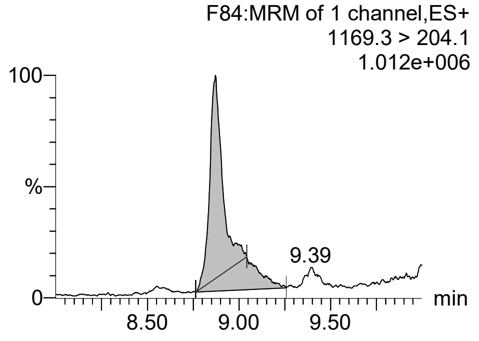

Sample Name: 20211111_A16_R3_IgM

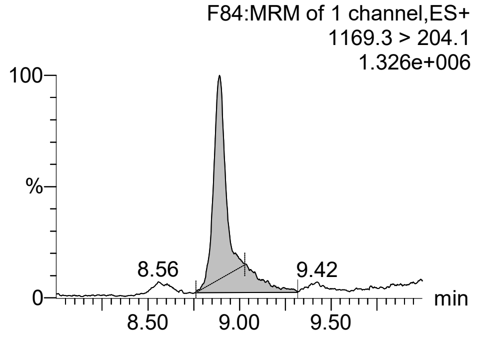

Sample Name: 20211111_L15_R3_IgM

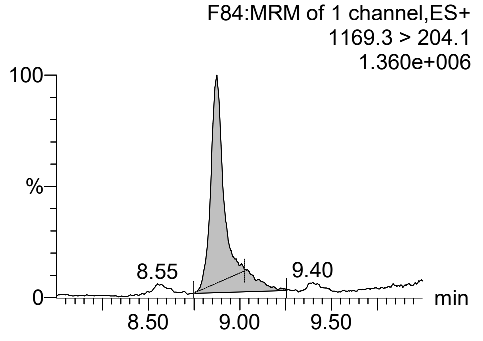

Sample Name: 20211111_L16_R3_IgM
F84:MRM of 1 channel,ES+
1169.3 > 204.1
4.294e+005

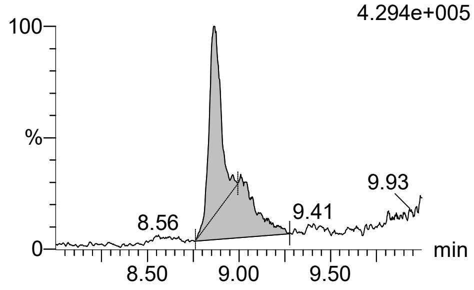

Sample Name: 20211111_H8_R3_IgM
F84:MRM of 1 channel,ES+
1169.3 > 204.1
7.880e+005

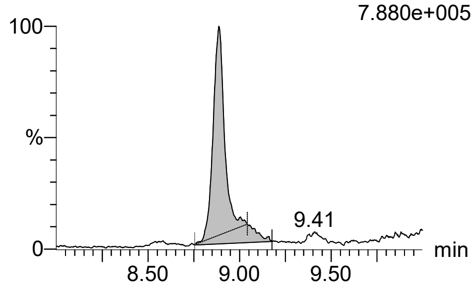

Sample Name: 20211111_A17_R3_IgM
F84:MRM of 1 channel,ES+
1169.3 > 204.1
2.117e+006

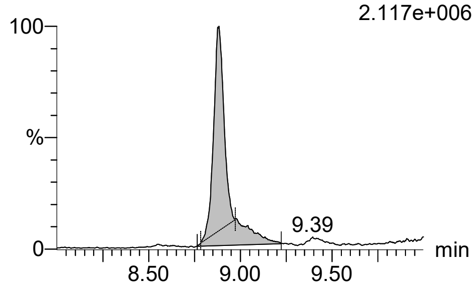

Sample Name: 20211111_A18_R3_IgM
F84:MRM of 1 channel,ES+
1169.3 > 204.1
4.178e+005

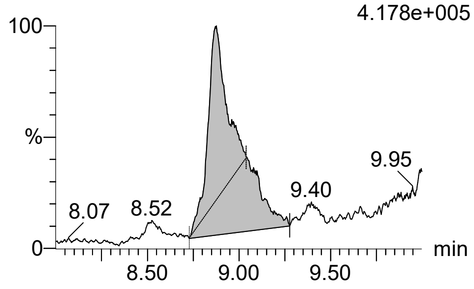

Sample Name: 20211111_L17_R3_IgM
F84:MRM of 1 channel,ES+
1169.3 > 204.1
1.128e+006

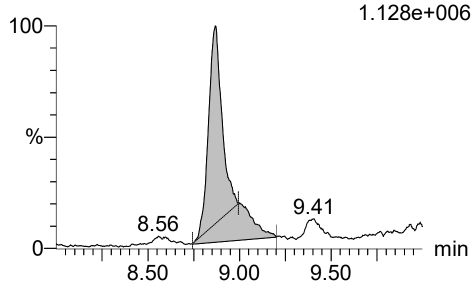

Sample Name: 20211111_L18_R3_IgM
F84:MRM of 1 channel,ES+
1169.3 > 204.1
1.319e+005

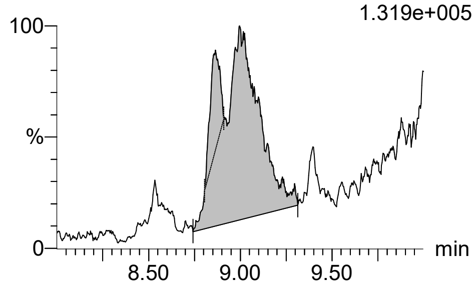

Sample Name: 20211111_H9_R3_IgM
F84:MRM of 1 channel,ES+
1169.3 > 204.1
3.597e+005

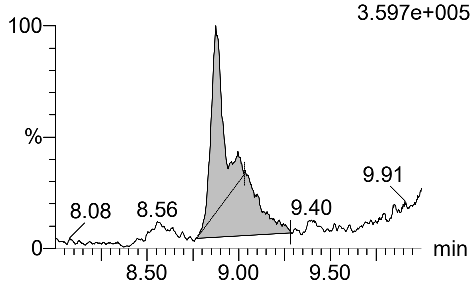

Sample Name: 20211111_A19_R3_IgM
F84:MRM of 1 channel,ES+
1169.3 > 204.1
6.979e+005

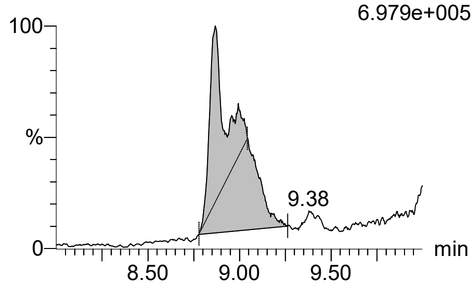

Sample Name: 20211111_A20_R3_IgM
F84:MRM of 1 channel,ES+
1169.3 > 204.1
2.264e+005

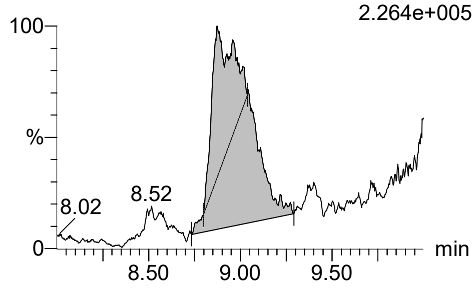

Sample Name: 20211111_L19_R3_IgM
F84:MRM of 1 channel,ES+
1169.3 > 204.1
8.361e+005

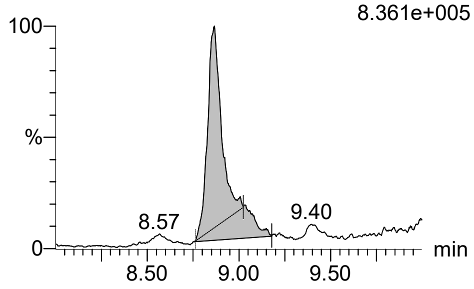

Sample Name: 20211111_QC_R21_IgM
F84:MRM of 1 channel,ES+
1169.3 > 204.1
9.463e+005

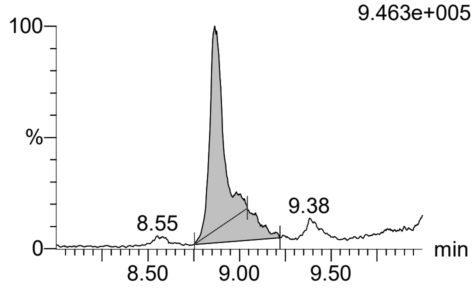

Sample Name: 20211111_L20_R3_IgM
F84:MRM of 1 channel,ES+
1169.3 > 204.1
7.381e+005

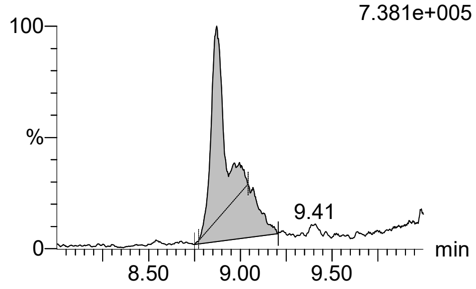

Sample Name: 20211111_H10_R3_IgM
F84:MRM of 1 channel,ES+
1169.3 > 204.1
9.581e+005

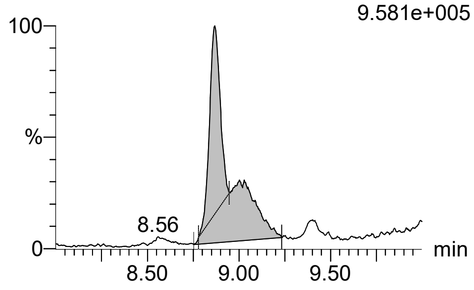

Sample Name: 20211111_A21_R3_IgM
F84:MRM of 1 channel,ES+
1169.3 > 204.1
9.355e+005

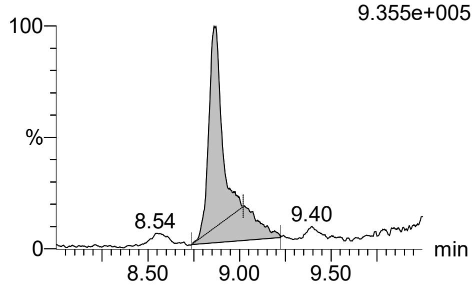

Sample Name: 20211111_A22_R3_IgM
F84:MRM of 1 channel,ES+
1169.3 > 204.1
2.083e+005

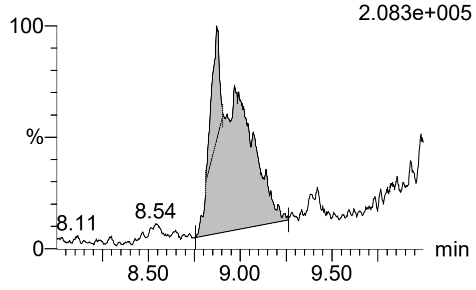

Dataset: C:\MassLynx\10.pro\20211111_clinicalsamples_IgM_IgA.qld

Last Altered: Thursday, March 09, 2023 19:58:25 台北標準時間
Printed: Friday, August 01, 2025 15:06:51 台北標準時間

Sample Name: 20211111_L21_R3_IgM
F84:MRM of 1 channel,ES+
1169.3 > 204.1
5.785e+005

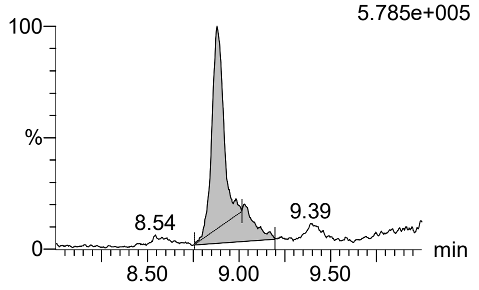

Sample Name: 20211111_L22_R3_IgM
F84:MRM of 1 channel,ES+
1169.3 > 204.1
9.042e+005

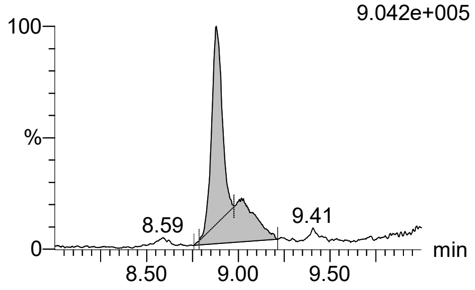

Sample Name: 20211111_H11_R3_IgM
F84:MRM of 1 channel,ES+
1169.3 > 204.1
1.178e+006

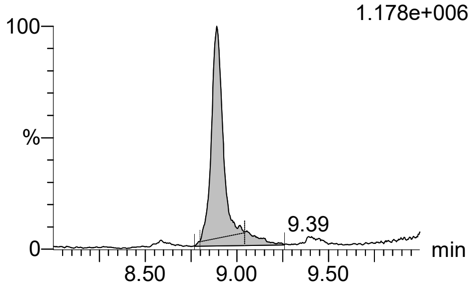

Sample Name: 20211111_A23_R3_IgM
F84:MRM of 1 channel,ES+
1169.3 > 204.1
3.238e+005

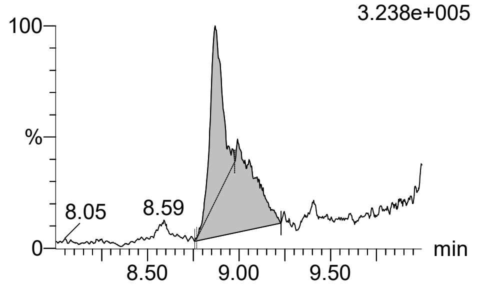

Sample Name: 20211111_A24_R3_IgM
F84:MRM of 1 channel,ES+
1169.3 > 204.1
1.200e+006

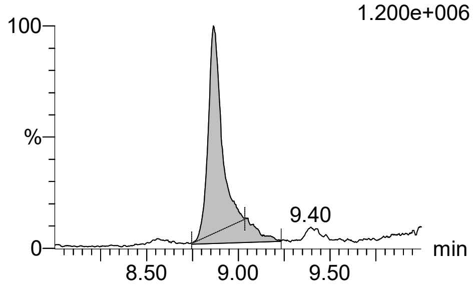

Sample Name: 20211111_L23_R3_IgM
F84:MRM of 1 channel,ES+
1169.3 > 204.1
3.983e+005

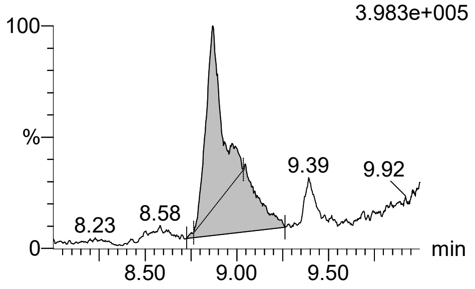

Sample Name: 20211111_L24_R3_IgM
F84:MRM of 1 channel,ES+
1169.3 > 204.1
3.811e+005

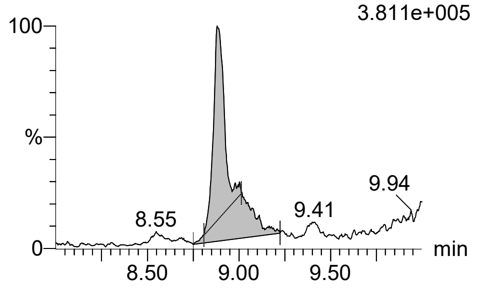

Sample Name: 20211111_H12_R3_IgM
F84:MRM of 1 channel,ES+
1169.3 > 204.1
4.653e+005

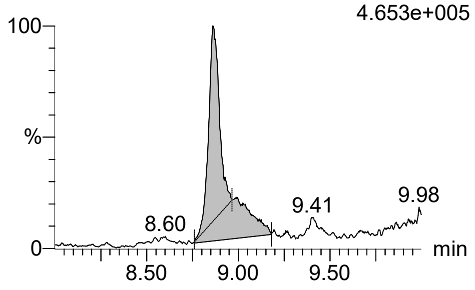

Sample Name: 20211111_QC_R22_IgM
F84:MRM of 1 channel,ES+
1169.3 > 204.1
1.110e+006

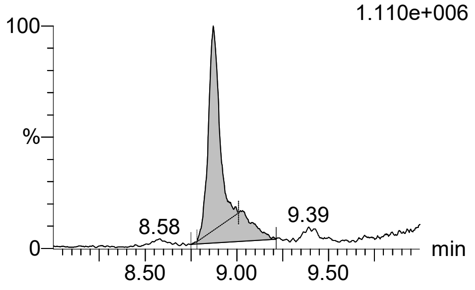

Sample Name: 20211111_A25_R3_IgM
F84:MRM of 1 channel,ES+
1169.3 > 204.1
1.987e+006

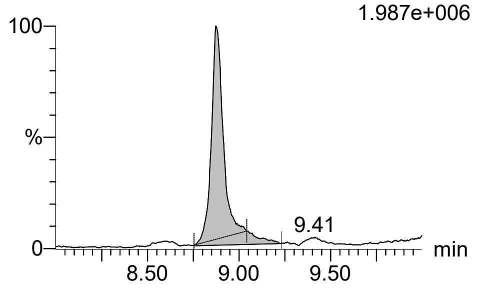

Sample Name: 20211111_A26_R3_IgM
F84:MRM of 1 channel,ES+
1169.3 > 204.1
1.189e+006

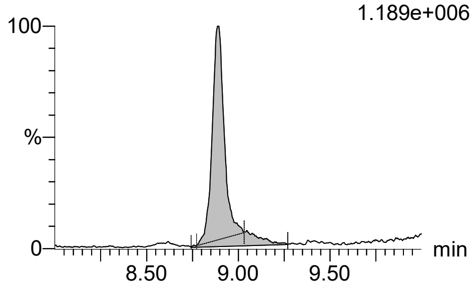

Sample Name: 20211111_L25_R3_IgM
F84:MRM of 1 channel,ES+
1169.3 > 204.1
7.352e+005

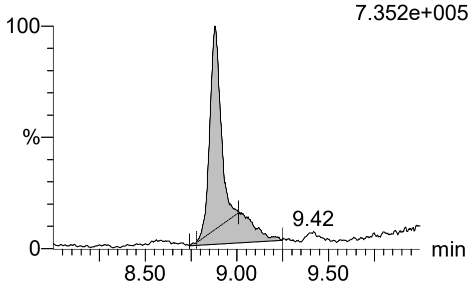

Sample Name: 20211111_L26_R3_IgM
F84:MRM of 1 channel,ES+
1169.3 > 204.1
4.266e+005

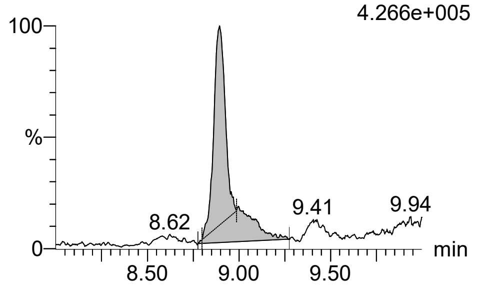

Sample Name: 20211111_H13_R3_IgM
F84:MRM of 1 channel,ES+
1169.3 > 204.1
1.056e+006

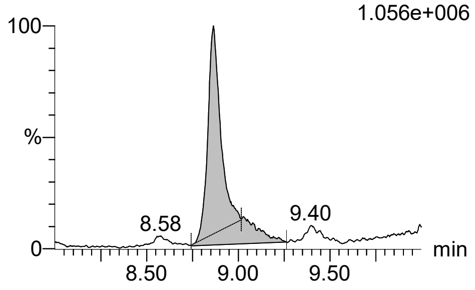

Sample Name: 20211111_A27_R3_IgM
F84:MRM of 1 channel,ES+
1169.3 > 204.1
1.039e+006

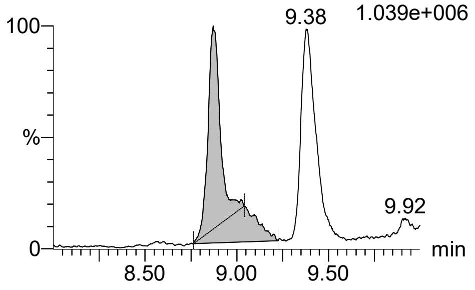

Dataset: C:\MassLynx\10.pro\20211111_clinicalsamples_IgM_IgA.qld

Last Altered: Thursday, March 09, 2023 19:58:25 台北標準時間
Printed: Friday, August 01, 2025 15:06:51 台北標準時間

Sample Name: 20211111_A28_R3_IgM
F84:MRM of 1 channel,ES+
1169.3 > 204.1
5.548e+005

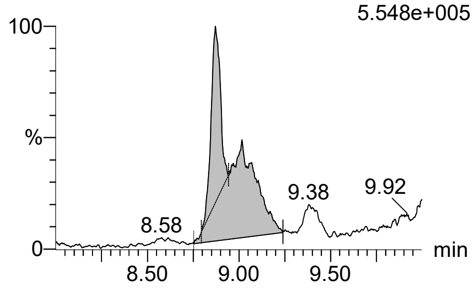

Sample Name: 20211111_L27_R3_IgM
F84:MRM of 1 channel,ES+
1169.3 > 204.1
1.127e+006

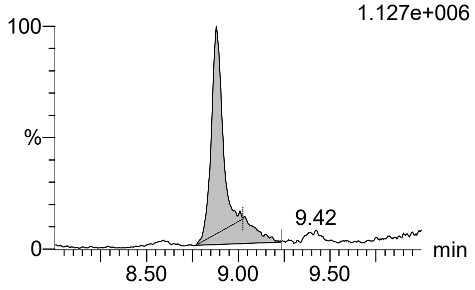

Sample Name: 20211111_L28_R3_IgM
F84:MRM of 1 channel,ES+
1169.3 > 204.1
6.708e+005

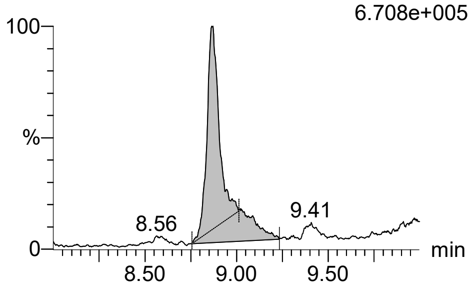

Sample Name: 20211111_H14_R3_IgM
F84:MRM of 1 channel,ES+
1169.3 > 204.1
8.780e+005

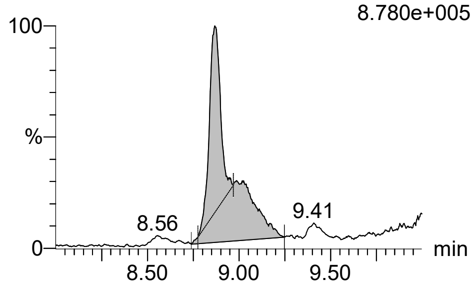

Sample Name: 20211111_A29_R3_IgM
F84:MRM of 1 channel,ES+
1169.3 > 204.1
1.387e+005

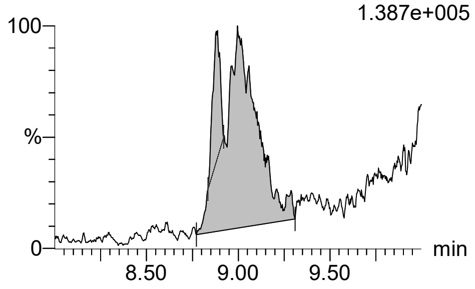

Sample Name: 20211111_A30_R3_IgM
F84:MRM of 1 channel,ES+
1169.3 > 204.1
1.472e+006

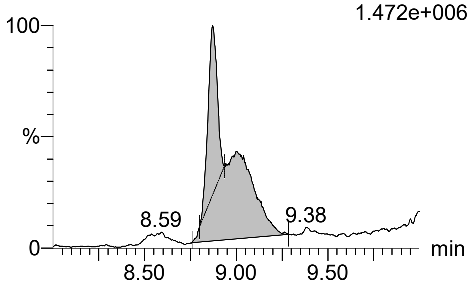

Sample Name: 20211111_QC_R23_IgM
F84:MRM of 1 channel,ES+
1169.3 > 204.1
1.028e+006

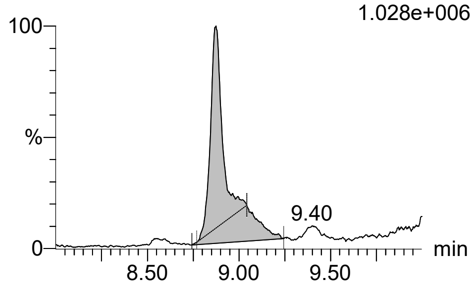

Sample Name: 20211111_L29_R3_IgM
F84:MRM of 1 channel,ES+
1169.3 > 204.1
4.261e+005

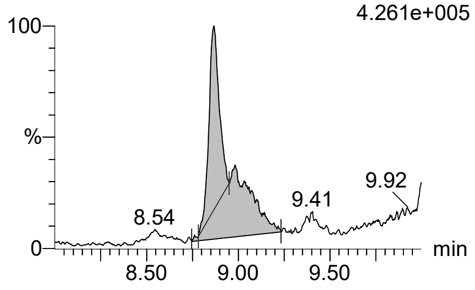

Sample Name: 20211111_L30_R3_IgM
F84:MRM of 1 channel,ES+
1169.3 > 204.1
2.820e+005

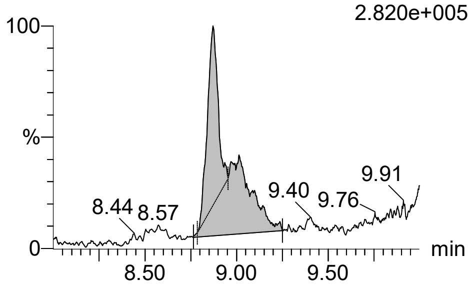

Sample Name: 20211111_H15_R3_IgM
F84:MRM of 1 channel,ES+
1169.3 > 204.1
1.035e+006

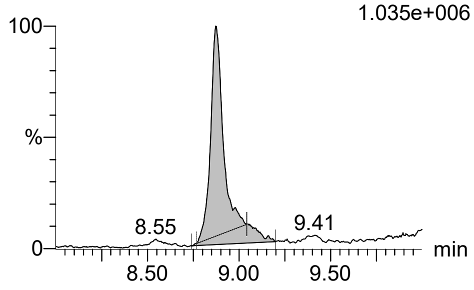

Sample Name: 20211111_A31_R3_IgM
F84:MRM of 1 channel,ES+
1169.3 > 204.1
4.536e+005

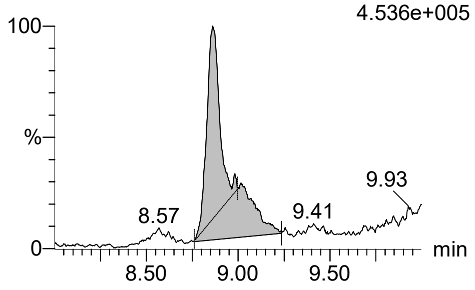

Sample Name: 20211111_A32_R3_IgM
F84:MRM of 1 channel,ES+
1169.3 > 204.1
1.279e+006

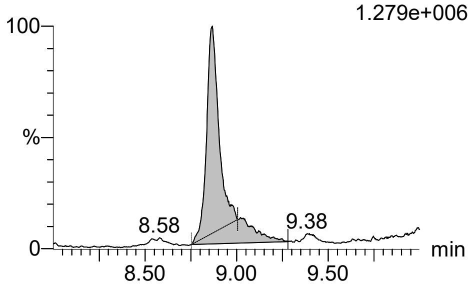

Sample Name: 20211111_L31_R3_IgM
F84:MRM of 1 channel,ES+
1169.3 > 204.1
2.533e+005

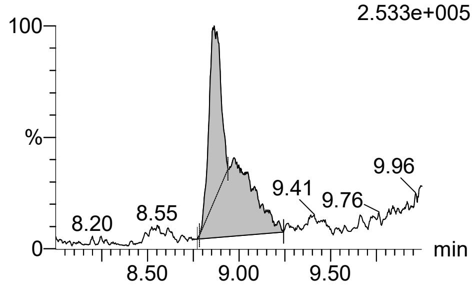

Sample Name: 20211111_L32_R3_IgM
F84:MRM of 1 channel,ES+
1169.3 > 204.1
1.017e+006

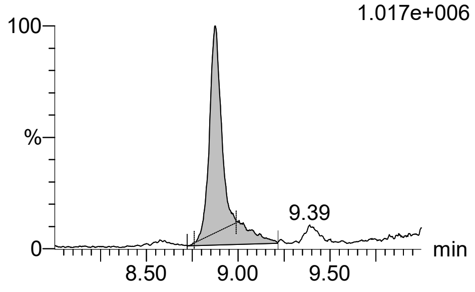

Sample Name: 20211111_H16_R3_IgM
F84:MRM of 1 channel,ES+
1169.3 > 204.1
2.036e+006

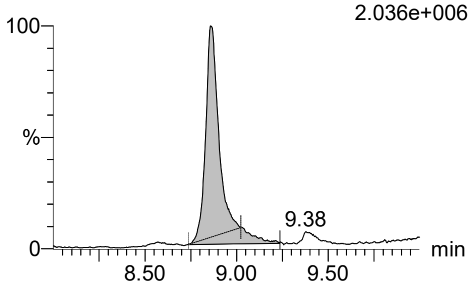

Dataset: C:\MassLynx\10.pro\20211111_clinicalsamples_IgM_IgA.qld

Last Altered: Thursday, March 09, 2023 19:58:25 台北標準時間
Printed: Friday, August 01, 2025 15:06:51 台北標準時間

Sample Name: 20211111_A33_R3_IgM
F84:MRM of 1 channel,ES+
1169.3 > 204.1
1.199e+006

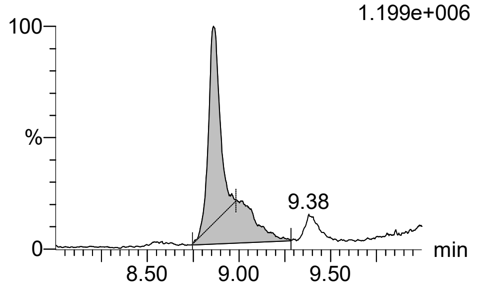

Sample Name: 20211111_A34_R3_IgM
F84:MRM of 1 channel,ES+
1169.3 > 204.1
4.299e+005

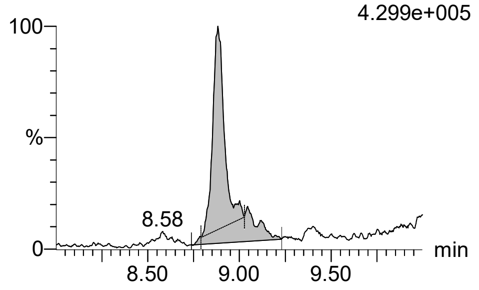

Sample Name: 20211111_H17_R3_IgM
F84:MRM of 1 channel,ES+
1169.3 > 204.1
3.883e+006

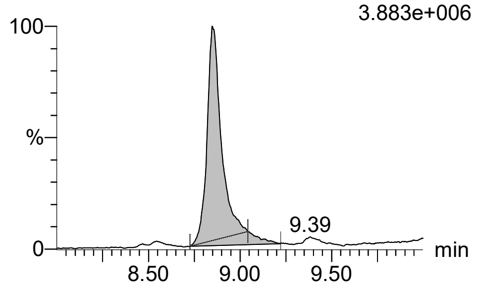

Sample Name: 20211111_A35_R3_IgM
F84:MRM of 1 channel,ES+
1169.3 > 204.1
3.045e+005

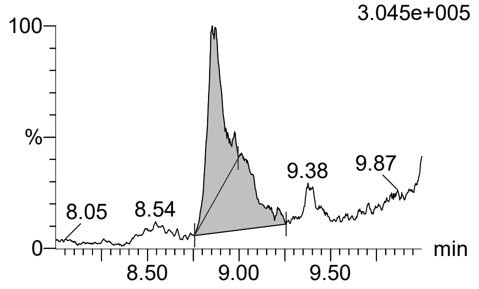

Sample Name: 20211111_QC_R24_IgM
F84:MRM of 1 channel,ES+
1169.3 > 204.1
9.025e+005

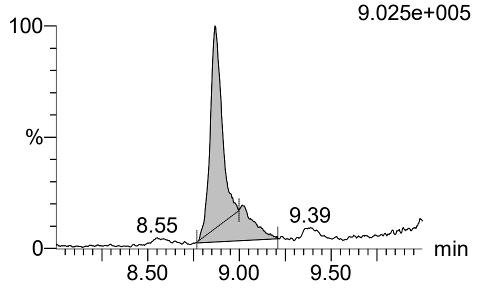

Sample Name: 20211111_A36_R3_IgM
F84:MRM of 1 channel,ES+
1169.3 > 204.1
7.147e+005

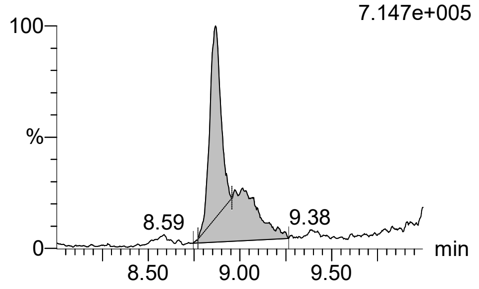

Sample Name: 20211111_H18_R3_IgM
F84:MRM of 1 channel,ES+
1169.3 > 204.1
1.655e+006

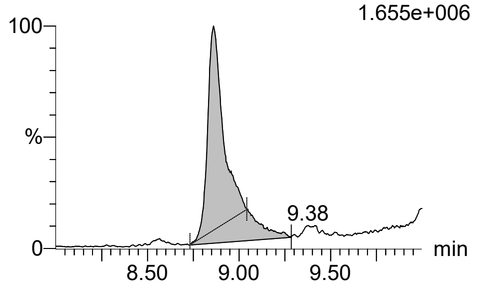

Sample Name: 20211111_A37_R3_IgM
F84:MRM of 1 channel,ES+
1169.3 > 204.1
1.107e+006

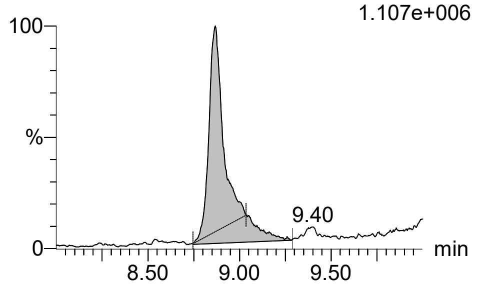

Sample Name: 20211111_A38_R3_IgM
F84:MRM of 1 channel,ES+
1169.3 > 204.1
9.230e+005

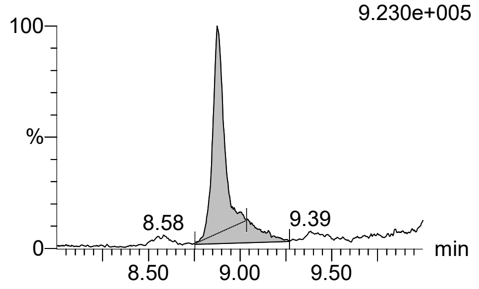

Sample Name: 20211111_H19_R3_IgM
F84:MRM of 1 channel,ES+
1169.3 > 204.1
1.116e+006

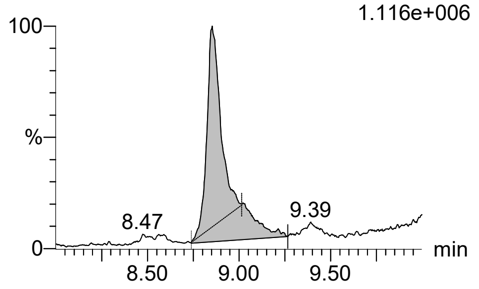

Sample Name: 20211111_A39_R3_IgM
F84:MRM of 1 channel,ES+
1169.3 > 204.1
1.392e+006

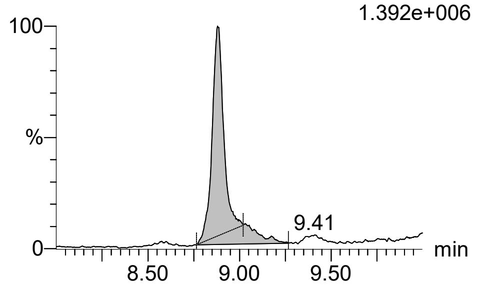

Sample Name: 20211111_A40_R3_IgM
F84:MRM of 1 channel,ES+
1169.3 > 204.1
6.333e+005

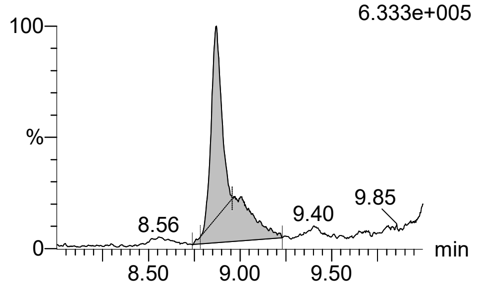

Sample Name: 20211111_H20_R3_IgM
F84:MRM of 1 channel,ES+
1169.3 > 204.1
1.458e+006

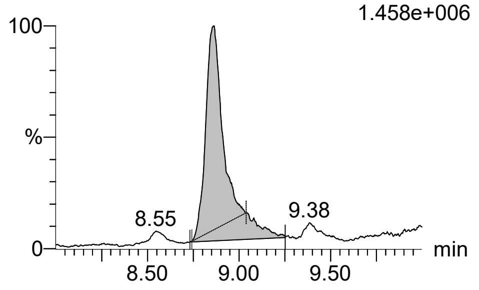

Sample Name: 20211111_A41_R3_IgM
F84:MRM of 1 channel,ES+
1169.3 > 204.1
3.324e+005

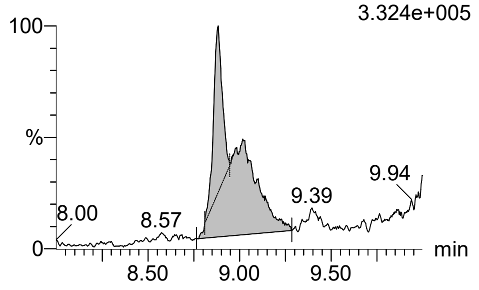

Sample Name: 20211111_A42_R3_IgM
F84:MRM of 1 channel,ES+
1169.3 > 204.1
4.141e+005

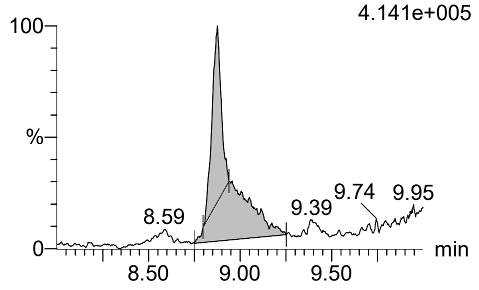

Dataset: C:\MassLynx\10.pro\20211111_clinicalsamples_IgM_IgA.qld

Last Altered: Thursday, March 09, 2023 19:58:25 台北標準時間
Printed: Friday, August 01, 2025 15:06:51 台北標準時間

Sample Name: 20211111_A43_R3_IgM

F84:MRM of 1 channel,ES+
1169.3 > 204.1
3.865e+005

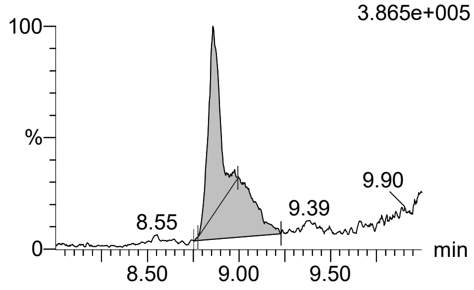

Sample Name: 20211111_A44_R3_IgM

F84:MRM of 1 channel,ES+
1169.3 > 204.1
2.139e+005

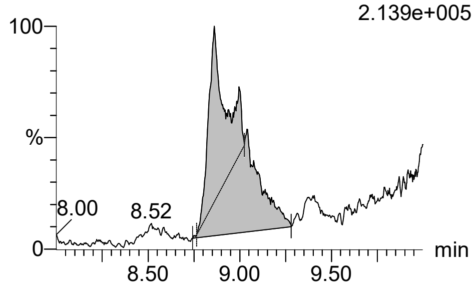

Sample Name: 20211111_QC_R25_IgM

F84:MRM of 1 channel,ES+
1169.3 > 204.1
8.975e+005

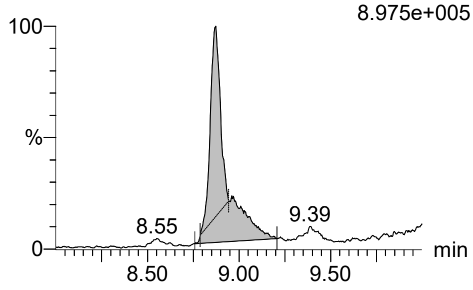

Dataset: C:\MassLynx\10.pro\20211111_clinicalsamples_IgM_IgA.qld

Last Altered: Thursday, March 09, 2023 19:58:25 台北標準時間
Printed: Friday, August 01, 2025 15:06:51 台北標準時間

	#	Name	RT	Area	IS Area	Response	Prima...
1	1	20211112_QC_R001_IgM	8.84	78904.2	15421.4	5.117	bb
2	2	20211112_QC_R002_IgM	8.86	105638.9	13188.8	8.010	MM
3	3	20211111_A1_R2_IgM	8.86	60067.1	11871.0	5.060	MM
4	4	20211111_A1_R3_IgM	8.87	57334.7	12030.5	4.766	MM
5	5	20211111_A2_R2_IgM	8.86	173218.4	11446.8	15.132	MM
6	6	20211111_A2_R3_IgM	8.86	160641.1	11098.4	14.474	MM
7	7	20211111_L1_R2_IgM	8.87	33586.6	11779.4	2.851	MM
8	8	20211111_L1_R3_IgM	8.88	30902.3	11176.2	2.765	MM
9	9	20211111_L2_R2_IgM	8.86	171197.6	11375.0	15.050	MM
10	10	20211111_H1_R2_IgM	8.86	161103.3	13595.2	11.850	MM
11	11	20211111_A3_R2_IgM	8.85	143139.8	14112.3	10.143	MM
12	12	20211111_A4_R2_IgM	8.86	93209.2	11977.5	7.782	MM
13	13	20211111_L3_R2_IgM	8.97	114642.1	12242.1	9.365	MM
14	14	20211111_L4_R2_IgM	8.86	96666.0	12797.0	7.554	MM
15	15	20211111_H2_R2_IgM	8.85	167789.2	10006.0	16.769	MM
16	16	20211111_A5_R2_IgM	8.86	242969.0	11434.6	21.249	MM
17	17	20211111_A6_R2_IgM	8.86	157769.8	13193.7	11.958	MM
18	18	20211111_QC_R10_IgM	8.86	112954.4	12566.1	8.989	MM
19	19	20211111_L5_R2_IgM	8.87	163161.5	13616.4	11.983	MM
20	20	20211111_L6_R2_IgM	8.86	176594.0	13698.5	12.891	MM
21	21	20211111_H3_R2_IgM	8.86	65990.7	15793.1	4.178	MM
22	22	20211111_A7_R2_IgM	8.84	148325.0	12936.9	11.465	MM
23	23	20211111_A8_R2_IgM	8.86	145664.4	13108.0	11.113	MM
24	24	20211111_L7_R2_IgM	8.86	40488.4	13278.5	3.049	MM
25	25	20211111_L8_R2_IgM	8.87	41845.8	15233.6	2.747	MM
26	26	20211111_H4_R2_IgM	8.85	71072.6	16521.9	4.302	MM
27	27	20211111_A9_R2_IgM	8.87	89430.7	16451.6	5.436	MM
28	28	20211111_A10_R2_IgM	8.86	173835.3	13036.0	13.335	MM
29	29	20211111_L9_R2_IgM	8.87	68768.0	14554.1	4.725	MM
30	30	20211111_L10_R2_IgM	8.87	105235.3	12250.4	8.590	MM
31	31	20211111_QC_R11_IgM	8.87	120471.6	11459.2	10.513	MM
32	32	20211111_H5_R2_IgM	8.86	105815.4	14358.2	7.370	MM
33	33	20211111_A11_R2_IgM	8.84	146876.2	16184.1	9.075	MM
34	34	20211111_A12_R2_IgM	8.86	68912.4	13465.4	5.118	MM
35	35	20211111_L11_R2_IgM	8.88	56901.1	13277.9	4.285	MM
36	36	20211111_L12_R2_IgM	8.87	69761.4	15958.8	4.371	MM
37	37	20211111_H6_R2_IgM	8.86	69738.7	14003.5	4.980	MM
38	38	20211111_A13_R2_IgM	8.85	60253.3	13656.1	4.412	MM
39	39	20211111_A14_R2_IgM	8.87	264853.4	14071.3	18.822	MM
40	40	20211111_L13_R2_IgM	8.87	87507.1	14785.6	5.918	MM
41	41	20211111_L14_R2_IgM	8.87	134037.1	14772.2	9.074	MM
42	42	20211111_H7_R2_IgM	8.87	85257.2	15164.4	5.622	MM
43	43	20211111_A15_R2_IgM	8.86	288891.8	15721.5	18.376	MM
44	44	20211111_QC_R12_IgM	8.86	132617.9	13711.5	9.672	MM
45	45	20211111_A16_R2_IgM	8.88	149881.0	14275.6	10.499	MM
46	46	20211111_L15_R2_IgM	8.86	135212.9	12935.8	10.453	MM
47	47	20211111_L16_R2_IgM	8.86	58184.3	13342.8	4.361	MM
48	48	20211111_H8_R2_IgM	8.88	77572.0	15835.1	4.899	MM
49	49	20211111_A17_R2_IgM	8.88	212307.2	15786.7	13.449	MM
50	50	20211111_A18_R2_IgM	8.86	75321.0	16986.8	4.434	MM
51	51	20211111_L17_R2_IgM	8.87	135746.8	13342.7	10.174	MM
52	52	20211111_L18_R2_IgM	9.01	30086.3	14692.0	2.048	MM
53	53	20211111_H9_R2_IgM	8.99	54936.7	15639.7	3.513	MM
54	54	20211111_A19_R2_IgM	8.86	129099.9	14198.7	9.092	MM
55	55	20211111_A20_R2_IgM	8.86	53399.4	14162.7	3.770	MM
56	56	20211111_L19_R2_IgM	8.86	86383.9	9293.4	9.295	MM

Dataset: C:\MassLynx\10.pro\20211111_clinicalsamples_IgM_IgA.qld

Last Altered: Thursday, March 09, 2023 19:58:25 台北標準時間
Printed: Friday, August 01, 2025 15:06:51 台北標準時間

	# Name	RT	Area	IS Area	Response	Prima...
57	57 20211111_QC_R13_IgM	8.86	117104.1	13903.0	8.423	MM
58	58 20211111_L20_R2_IgM	8.97	130110.9	12781.6	10.180	MM
59	59 20211111_H10_R2_IgM	8.99	146284.6	14796.4	9.886	MM
60	60 20211111_A21_R2_IgM	8.86	115922.4	13669.0	8.481	MM
61	61 20211111_A22_R2_IgM	9.01	47360.4	14098.3	3.359	MM
62	62 20211111_L21_R2_IgM	8.88	71396.7	15678.2	4.554	MM
63	63 20211111_L22_R2_IgM	8.88	106353.4	15724.5	6.764	MM
64	64 20211111_H11_R2_IgM	8.87	109290.6	15890.2	6.878	MM
65	65 20211111_A23_R2_IgM	8.86	50769.8	13423.2	3.782	MM
66	66 20211111_A24_R2_IgM	8.86	123025.8	12438.0	9.891	MM
67	67 20211111_L23_R2_IgM	8.87	69984.7	14075.0	4.972	MM
68	68 20211111_L24_R2_IgM	8.88	43504.7	15972.1	2.724	MM
69	69 20211111_H12_R2_IgM	8.86	49316.9	16270.9	3.031	MM
70	70 20211111_QC_R14_IgM	8.88	125753.0	15806.8	7.956	MM
71	71 20211111_A25_R2_IgM	8.87	207246.7	14552.1	14.242	MM
72	72 20211111_A26_R2_IgM	8.89	111511.1	11492.7	9.703	MM
73	73 20211111_L25_R2_IgM	8.88	80284.4	14403.3	5.574	MM
74	74 20211111_L26_R2_IgM	8.89	48856.5	16533.9	2.955	MM
75	75 20211111_H13_R2_IgM	8.87	110603.7	15520.1	7.126	MM
76	76 20211111_A27_R2_IgM	8.87	123444.2	13337.4	9.255	MM
77	77 20211111_A28_R2_IgM	9.00	123722.8	11895.6	10.401	MM
78	78 20211111_L27_R2_IgM	8.87	109427.4	14125.7	7.747	MM
79	79 20211111_L28_R2_IgM	8.86	71325.8	11807.5	6.041	MM
80	80 20211111_H14_R2_IgM	8.86	107038.8	15035.7	7.119	MM
81	81 20211111_A29_R2_IgM	9.01	35061.4	13554.4	2.587	MM
82	82 20211111_A30_R2_IgM	8.86	226734.9	12984.1	17.463	MM
83	83 20211111_QC_R15_IgM	8.87	120700.3	12299.5	9.813	MM
84	84 20211111_L29_R2_IgM	8.87	49314.7	13590.7	3.629	MM
85	85 20211111_L30_R2_IgM	8.86	40379.1	12511.4	3.227	MM
86	86 20211111_H15_R2_IgM	8.87	107063.6	11885.0	9.008	MM
87	87 20211111_A31_R2_IgM	8.86	59144.4	13422.1	4.406	MM
88	88 20211111_A32_R2_IgM	8.86	139358.0	11461.3	12.159	MM
89	89 20211111_L31_R2_IgM	8.88	49272.2	13753.4	3.583	MM
90	90 20211111_L32_R2_IgM	8.87	81579.1	12949.0	6.300	MM
91	91 20211111_H16_R2_IgM	8.85	205390.5	11465.4	17.914	MM
92	92 20211111_A33_R2_IgM	8.86	138516.9	10551.5	13.128	MM
93	93 20211111_A34_R2_IgM	8.88	48165.1	12409.9	3.881	MM
94	94 20211111_H17_R2_IgM	8.85	430473.2	10160.9	42.366	MM
95	95 20211111_A35_R2_IgM	8.87	51387.6	10460.6	4.912	MM
96	96 20211111_QC_R16_IgM	8.86	104766.8	10433.5	10.041	MM
97	97 20211111_A36_R2_IgM	8.97	117836.9	12504.8	9.423	MM
98	98 20211111_H18_R2_IgM	8.85	216726.4	9140.4	23.711	MM
99	99 20211111_A37_R2_IgM	8.87	141259.3	9796.9	14.419	MM
100	1... 20211111_A38_R2_IgM	8.88	97034.1	11224.3	8.645	MM
101	1... 20211111_H19_R2_IgM	8.86	142629.2	11739.2	12.150	MM
102	1... 20211111_A39_R2_IgM	8.88	136981.6	12718.9	10.770	MM
103	1... 20211111_A40_R2_IgM	8.87	65173.0	10006.1	6.513	MM
104	1... 20211111_H20_R2_IgM	8.86	198165.9	7988.2	24.807	MM
105	1... 20211111_A41_R2_IgM	8.88	73262.0	11513.3	6.363	MM
106	1... 20211111_A42_R2_IgM	8.87	56422.1	10238.5	5.511	MM
107	1... 20211111_A43_R2_IgM	8.87	52981.3	11098.9	4.774	MM
108	1... 20211111_A44_R2_IgM	8.94	52424.4	10969.9	4.779	MM
109	1... 20211111_QC_R17_IgM	8.87	95622.2	9700.8	9.857	MM
110	1... 20211111_L2_R3_IgM	8.87	166481.9	8528.0	19.522	MM
111	1... 20211111_H1_R3_IgM	8.87	163885.2	9438.2	17.364	MM
112	1... 20211111_A3_R3_IgM	8.87	162242.7	11598.6	13.988	MM

Dataset: C:\MassLynx\10.pro\20211111_clinicalsamples_IgM_IgA.qld

Last Altered: Thursday, March 09, 2023 19:58:25 台北標準時間
Printed: Friday, August 01, 2025 15:06:51 台北標準時間

	#	Name	RT	Area	IS Area	Response	Prima...
113	1...	20211111_A4_R3_IgM	8.88	103137.4	11364.1	9.076	MM
114	1...	20211111_L3_R3_IgM	9.00	92579.6	10753.4	8.609	MM
115	1...	20211111_L4_R3_IgM	8.87	85168.7	10291.6	8.276	MM
116	1...	20211111_H2_R3_IgM	8.85	162621.8	9315.8	17.457	MM
117	1...	20211111_A5_R3_IgM	8.86	255866.7	9102.2	28.111	MM
118	1...	20211111_A6_R3_IgM	8.85	136841.3	10356.4	13.213	MM
119	1...	20211111_QC_R18_IgM	8.86	89472.5	8398.3	10.654	MM
120	1...	20211111_L5_R3_IgM	8.86	135860.1	9786.7	13.882	MM
121	1...	20211111_L6_R3_IgM	8.87	126795.5	9911.3	12.793	MM
122	1...	20211111_H3_R3_IgM	8.88	67561.6	12155.5	5.558	MM
123	1...	20211111_A7_R3_IgM	8.85	147323.1	11804.8	12.480	MM
124	1...	20211111_A8_R3_IgM	8.88	133818.3	10510.6	12.732	MM
125	1...	20211111_L7_R3_IgM	8.86	31167.5	12177.9	2.559	MM
126	1...	20211111_L8_R3_IgM	8.88	37957.6	11668.6	3.253	MM
127	1...	20211111_H4_R3_IgM	8.88	68458.5	11192.0	6.117	MM
128	1...	20211111_A9_R3_IgM	8.88	98766.6	14077.4	7.016	MM
129	1...	20211111_A10_R3_IgM	8.88	210542.8	11190.4	18.815	MM
130	1...	20211111_L9_R3_IgM	8.87	74645.5	14649.3	5.096	MM
131	1...	20211111_L10_R3_IgM	8.87	85674.6	9361.3	9.152	MM
132	1...	20211111_QC_R19_IgM	8.86	90797.6	10125.5	8.967	MM
133	1...	20211111_H5_R3_IgM	8.88	100510.6	10010.9	10.040	MM
134	1...	20211111_A11_R3_IgM	8.88	99686.4	12697.2	7.851	MM
135	1...	20211111_A12_R3_IgM	8.86	53533.8	13148.5	4.071	MM
136	1...	20211111_L11_R3_IgM	8.88	41950.8	10786.6	3.889	MM
137	1...	20211111_L12_R3_IgM	8.88	59585.6	11681.8	5.101	MM
138	1...	20211111_H6_R3_IgM	8.87	61187.3	12095.5	5.059	MM
139	1...	20211111_A13_R3_IgM	8.87	57023.7	12908.3	4.418	MM
140	1...	20211111_A14_R3_IgM	8.88	233056.7	11322.6	20.583	MM
141	1...	20211111_L13_R3_IgM	8.87	66469.1	11516.1	5.772	MM
142	1...	20211111_L14_R3_IgM	8.87	118323.7	10790.7	10.965	MM
143	1...	20211111_H7_R3_IgM	8.87	89995.2	11572.1	7.777	MM
144	1...	20211111_A15_R3_IgM	8.87	252905.4	12434.4	20.339	MM
145	1...	20211111_QC_R20_IgM	8.87	108594.3	10701.0	10.148	MM
146	1...	20211111_A16_R3_IgM	8.89	128870.6	11452.1	11.253	MM
147	1...	20211111_L15_R3_IgM	8.87	127806.9	10531.1	12.136	MM
148	1...	20211111_L16_R3_IgM	8.86	52211.8	12049.4	4.333	MM
149	1...	20211111_H8_R3_IgM	8.89	66643.1	13434.9	4.960	MM
150	1...	20211111_A17_R3_IgM	8.88	177973.4	12452.0	14.293	MM
151	1...	20211111_A18_R3_IgM	8.88	72964.3	12506.5	5.834	MM
152	1...	20211111_L17_R3_IgM	8.87	126141.6	9948.9	12.679	MM
153	1...	20211111_L18_R3_IgM	9.00	29706.3	13462.1	2.207	MM
154	1...	20211111_H9_R3_IgM	8.87	46382.7	13178.7	3.520	MM
155	1...	20211111_A19_R3_IgM	8.87	112104.2	11298.6	9.922	MM
156	1...	20211111_A20_R3_IgM	8.87	48836.3	11175.6	4.370	MM
157	1...	20211111_L19_R3_IgM	8.87	90405.9	11558.2	7.822	MM
158	1...	20211111_QC_R21_IgM	8.86	104005.2	9977.0	10.424	MM
159	1...	20211111_L20_R3_IgM	8.87	91689.2	13508.1	6.788	MM
160	1...	20211111_H10_R3_IgM	8.87	115463.3	12374.8	9.331	MM
161	1...	20211111_A21_R3_IgM	8.86	103363.8	12443.6	8.307	MM
162	1...	20211111_A22_R3_IgM	8.87	36998.0	11589.3	3.192	MM
163	1...	20211111_L21_R3_IgM	8.88	56778.8	13107.4	4.332	MM
164	1...	20211111_L22_R3_IgM	8.88	90456.7	12021.5	7.525	MM
165	1...	20211111_H11_R3_IgM	8.89	98184.9	12687.8	7.739	MM
166	1...	20211111_A23_R3_IgM	8.87	46898.1	11611.8	4.039	MM
167	1...	20211111_A24_R3_IgM	8.86	123253.1	10476.8	11.764	MM
168	1...	20211111_L23_R3_IgM	8.87	59568.7	11873.1	5.017	MM

Dataset: C:\MassLynx\10.pro\20211111_clinicalsamples_IgM_IgA.qld

Last Altered: Thursday, March 09, 2023 19:58:25 台北標準時間
Printed: Friday, August 01, 2025 15:06:51 台北標準時間

	# Name	RT	Area	IS Area	Response	Prima...
169	1... 20211111_L24_R3_IgM	8.88	38505.4	13468.0	2.859	MM
170	1... 20211111_H12_R3_IgM	8.86	46418.7	14229.1	3.262	MM
171	1... 20211111_QC_R22_IgM	8.87	103077.8	11264.4	9.151	MM
172	1... 20211111_A25_R3_IgM	8.87	166887.0	11775.7	14.172	MM
173	1... 20211111_A26_R3_IgM	8.89	104272.7	10473.9	9.956	MM
174	1... 20211111_L25_R3_IgM	8.88	69595.1	13256.8	5.250	MM
175	1... 20211111_L26_R3_IgM	8.89	41344.6	13565.0	3.048	MM
176	1... 20211111_H13_R3_IgM	8.86	108751.4	13146.0	8.273	MM
177	1... 20211111_A27_R3_IgM	8.87	110320.8	12025.6	9.174	MM
178	1... 20211111_A28_R3_IgM	8.87	76012.0	11229.1	6.769	MM
179	1... 20211111_L27_R3_IgM	8.88	101175.7	14960.0	6.763	MM
180	1... 20211111_L28_R3_IgM	8.87	69055.3	11359.0	6.079	MM
181	1... 20211111_H14_R3_IgM	8.87	109498.4	12830.8	8.534	MM
182	1... 20211111_A29_R3_IgM	9.00	29548.6	13434.3	2.199	MM
183	1... 20211111_A30_R3_IgM	8.87	206904.0	11272.2	18.355	MM
184	1... 20211111_QC_R23_IgM	8.87	106546.2	11872.2	8.974	MM
185	1... 20211111_L29_R3_IgM	8.87	50984.2	12948.7	3.937	MM
186	1... 20211111_L30_R3_IgM	8.87	35436.1	11026.6	3.214	MM
187	1... 20211111_H15_R3_IgM	8.87	96916.7	10786.3	8.985	MM
188	1... 20211111_A31_R3_IgM	8.86	55880.9	12010.5	4.653	MM
189	1... 20211111_A32_R3_IgM	8.87	125571.3	11107.7	11.305	MM
190	1... 20211111_L31_R3_IgM	8.86	34884.1	11021.8	3.165	MM
191	1... 20211111_L32_R3_IgM	8.88	93188.6	11683.8	7.976	MM
192	1... 20211111_H16_R3_IgM	8.86	190112.7	9831.7	19.337	MM
193	1... 20211111_A33_R3_IgM	8.86	132867.6	11213.1	11.849	MM
194	1... 20211111_A34_R3_IgM	8.88	41710.6	11550.1	3.611	MM
195	1... 20211111_H17_R3_IgM	8.85	384181.1	8874.4	43.291	MM
196	1... 20211111_A35_R3_IgM	8.85	46124.2	9856.3	4.680	MM
197	1... 20211111_QC_R24_IgM	8.87	87925.0	7561.8	11.628	MM
198	1... 20211111_A36_R3_IgM	8.87	84462.3	9562.8	8.832	MM
199	1... 20211111_H18_R3_IgM	8.86	204615.6	8165.3	25.059	MM
200	2... 20211111_A37_R3_IgM	8.87	122357.5	9278.8	13.187	MM
201	2... 20211111_A38_R3_IgM	8.87	84218.5	10920.3	7.712	MM
202	2... 20211111_H19_R3_IgM	8.85	129401.0	9285.3	13.936	MM
203	2... 20211111_A39_R3_IgM	8.88	121997.5	9083.4	13.431	MM
204	2... 20211111_A40_R3_IgM	8.87	65101.5	8382.4	7.766	MM
205	2... 20211111_H20_R3_IgM	8.86	178738.0	7807.9	22.892	MM
206	2... 20211111_A41_R3_IgM	8.88	45539.1	5925.2	7.686	MM
207	2... 20211111_A42_R3_IgM	8.88	44666.4	9352.6	4.776	MM
208	2... 20211111_A43_R3_IgM	8.86	46379.8	10216.3	4.540	MM
209	2... 20211111_A44_R3_IgM	8.86	38117.9	10262.7	3.714	MM
210	2... 20211111_QC_R25_IgM	8.87	83810.2	8575.3	9.773	MM

Dataset: C:\MassLynx\10.pro\20211111_clinicalsamples_IgM_IgA.qld

Last Altered: Thursday, March 09, 2023 19:58:25 台北標準時間
Printed: Friday, August 01, 2025 15:08:19 台北標準時間

Method: C:\MassLynx\10_Pro_IgA.PRO\MethDB\20211111_IgM_IgA_DMRM_new.mdb 24 Feb 2022 13:44:14
Calibration: 15 Nov 2021 14:42:11

Sample Name: 20211112_QC_R001_IgM
F47:MRM of 3 channels,ES+
976.3 > 204.1
1.958e+007

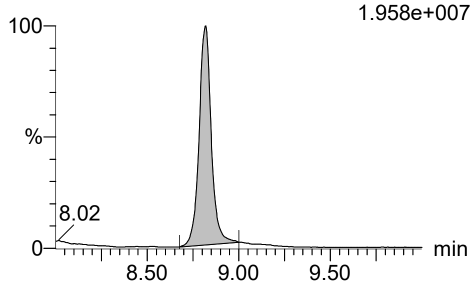

Sample Name: 20211112_QC_R001_IgM
F47:MRM of 3 channels,ES+
976.3 > 366.1
7.156e+006

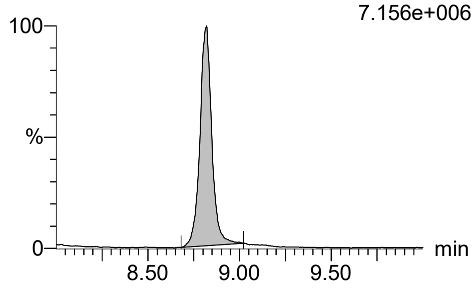

Sample Name: 20211112_QC_R001_IgM
F47:MRM of 3 channels,ES+
1220.1 > 366.1
1.335e+007

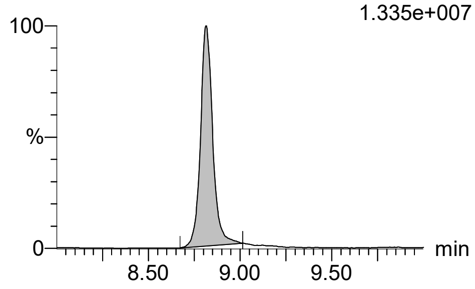

Sample Name: 20211112_QC_R002_IgM
F47:MRM of 3 channels,ES+
976.3 > 204.1
1.816e+007

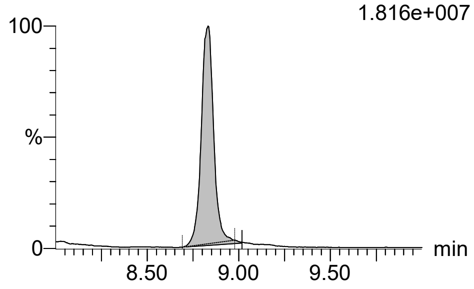

Sample Name: 20211112_QC_R002_IgM
F47:MRM of 3 channels,ES+
976.3 > 366.1
6.476e+006

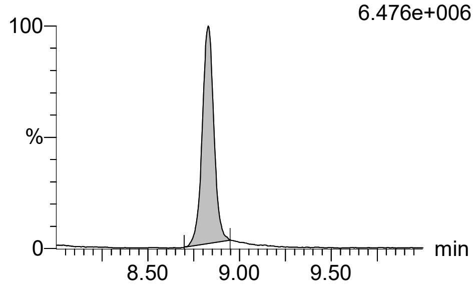

Sample Name: 20211112_QC_R002_IgM
F47:MRM of 3 channels,ES+
1220.1 > 366.1
1.298e+007

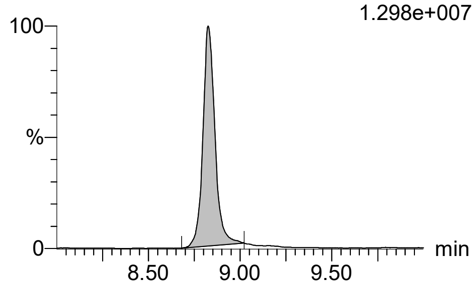

Sample Name: 20211111_A1_R2_IgM
F47:MRM of 3 channels,ES+
976.3 > 204.1
8.326e+006

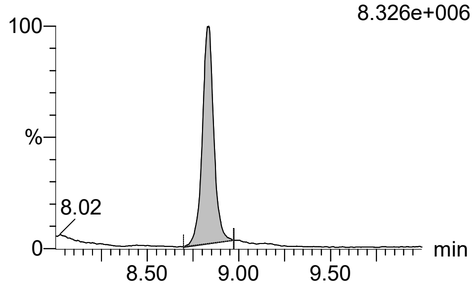

Sample Name: 20211111_A1_R2_IgM
F47:MRM of 3 channels,ES+
976.3 > 366.1
2.968e+006

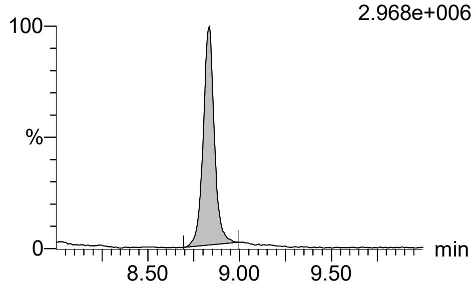

Sample Name: 20211111_A1_R2_IgM
F47:MRM of 3 channels,ES+
1220.1 > 366.1
5.627e+006

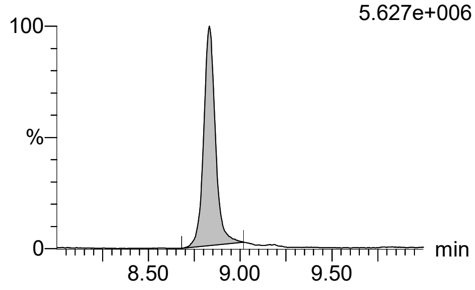

Sample Name: 20211111_A1_R3_IgM
F47:MRM of 3 channels,ES+
976.3 > 204.1
8.564e+006

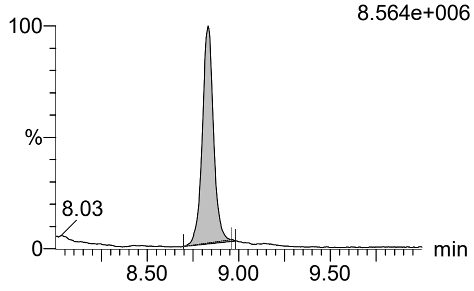

Sample Name: 20211111_A1_R3_IgM
F47:MRM of 3 channels,ES+
976.3 > 366.1
3.012e+006

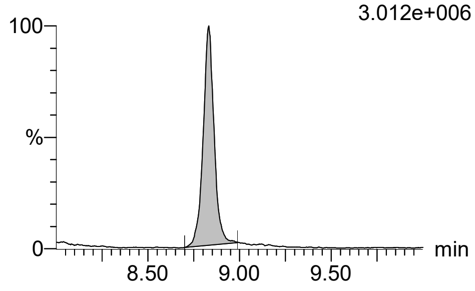

Sample Name: 20211111_A1_R3_IgM
F47:MRM of 3 channels,ES+
1220.1 > 366.1
5.788e+006

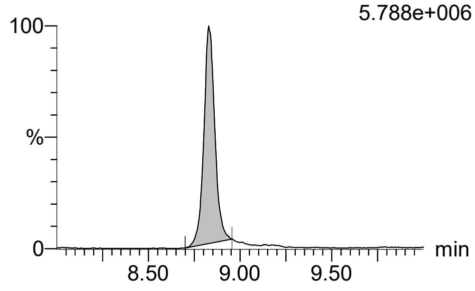

Sample Name: 20211111_A2_R2_IgM
F47:MRM of 3 channels,ES+
976.3 > 204.1
3.944e+007

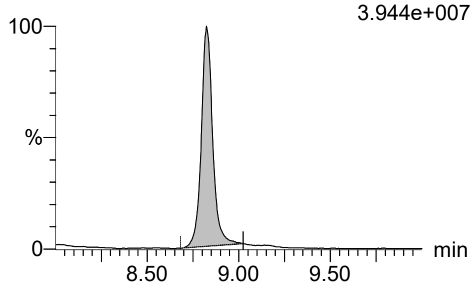

Sample Name: 20211111_A2_R2_IgM
F47:MRM of 3 channels,ES+
976.3 > 366.1
1.402e+007

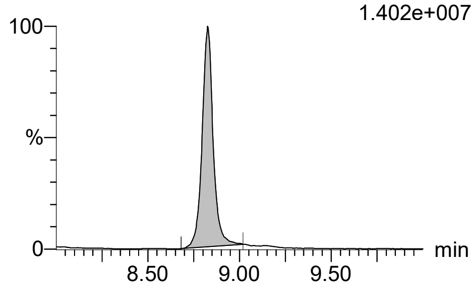

Sample Name: 20211111_A2_R2_IgM
F47:MRM of 3 channels,ES+
1220.1 > 366.1
2.741e+007

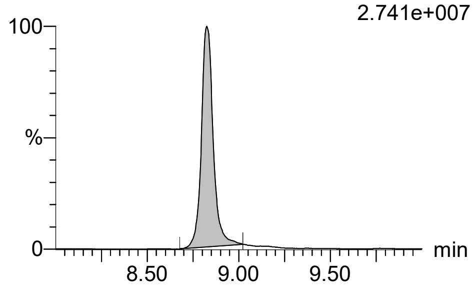

Sample Name: 20211111_A2_R3_IgM
F47:MRM of 3 channels,ES+
976.3 > 204.1
3.857e+007

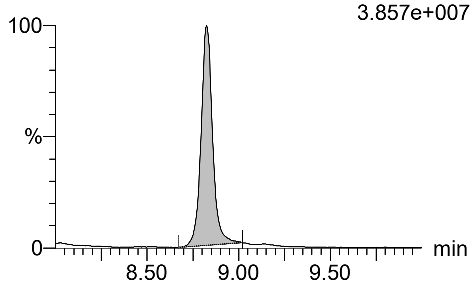

Sample Name: 20211111_A2_R3_IgM
F47:MRM of 3 channels,ES+
976.3 > 366.1
1.369e+007

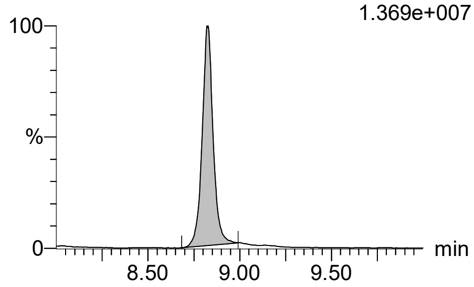

Sample Name: 20211111_A2_R3_IgM
F47:MRM of 3 channels,ES+
1220.1 > 366.1
2.644e+007

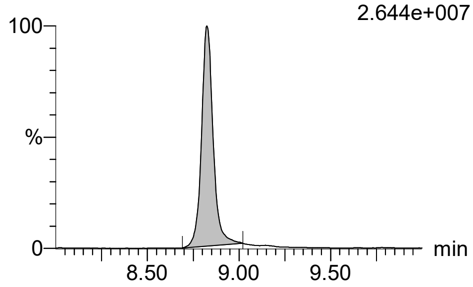

Sample Name: 20211111_L1_R2_IgM
F47:MRM of 3 channels,ES+
976.3 > 204.1
5.118e+006

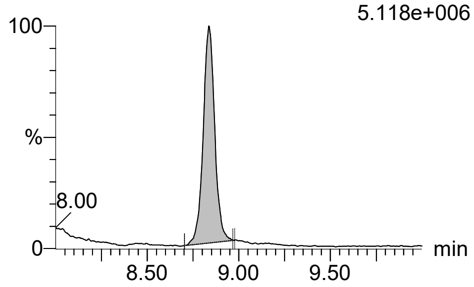

Sample Name: 20211111_L1_R2_IgM
F47:MRM of 3 channels,ES+
976.3 > 366.1
1.669e+006

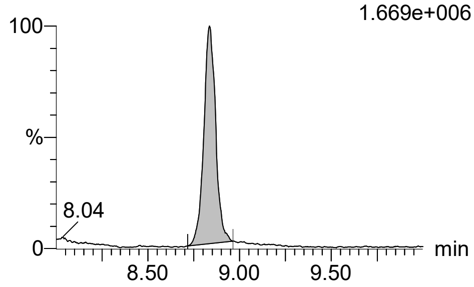

Sample Name: 20211111_L1_R2_IgM
F47:MRM of 3 channels,ES+
1220.1 > 366.1
3.297e+006

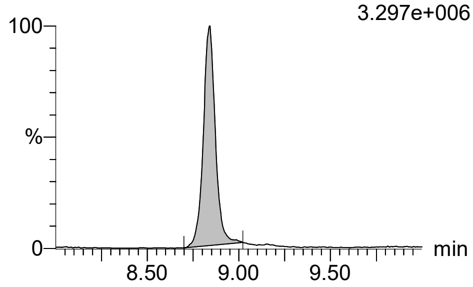

Sample Name: 20211111_L1_R3_IgM
F47:MRM of 3 channels,ES+
976.3 > 204.1
4.269e+006

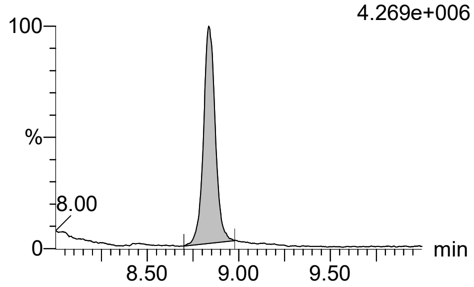

Sample Name: 20211111_L1_R3_IgM
F47:MRM of 3 channels,ES+
976.3 > 366.1
1.325e+006

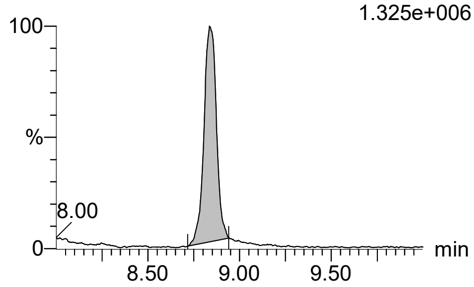

Sample Name: 20211111_L1_R3_IgM
F47:MRM of 3 channels,ES+
1220.1 > 366.1
2.840e+006

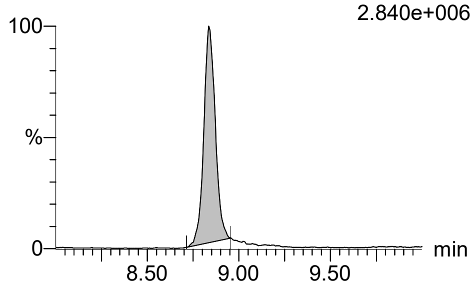

Sample Name: 20211111_L2_R2_IgM
F47:MRM of 3 channels,ES+
976.3 > 204.1
3.272e+007

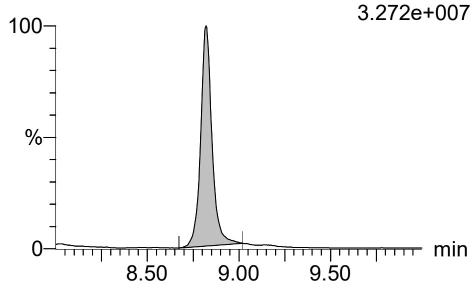

Sample Name: 20211111_L2_R2_IgM
F47:MRM of 3 channels,ES+
976.3 > 366.1
1.154e+007

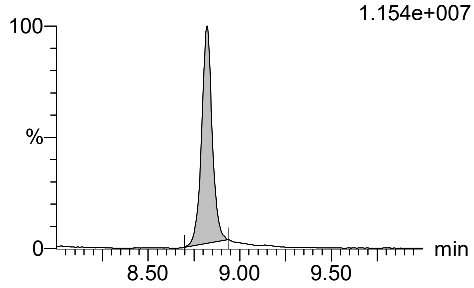

Sample Name: 20211111_L2_R2_IgM
F47:MRM of 3 channels,ES+
1220.1 > 366.1
2.290e+007

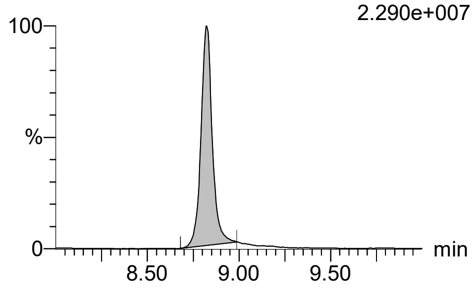

Dataset: C:\MassLynx\10.pro\20211111_clinicalsamples_IgM_IgA.qld

Last Altered: Thursday, March 09, 2023 19:58:25 台北標準時間
Printed: Friday, August 01, 2025 15:08:19 台北標準時間

Sample Name: 20211111_H1_R2_IgM
F47:MRM of 3 channels,ES+
976.3 > 204.1
1.788e+007

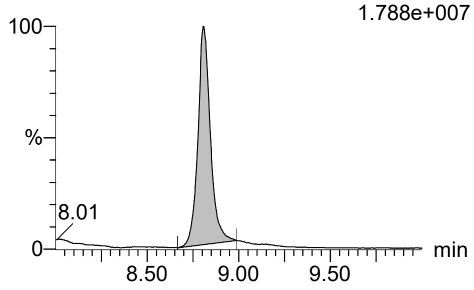

Sample Name: 20211111_H1_R2_IgM
F47:MRM of 3 channels,ES+
976.3 > 366.1
6.011e+006

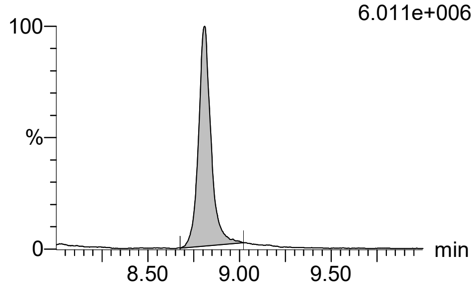

Sample Name: 20211111_H1_R2_IgM
F47:MRM of 3 channels,ES+
1220.1 > 366.1
1.359e+007

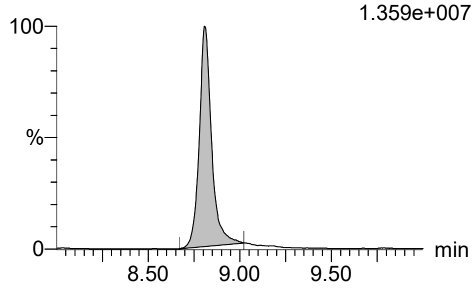

Sample Name: 20211111_A3_R2_IgM
F47:MRM of 3 channels,ES+
976.3 > 204.1
2.282e+007

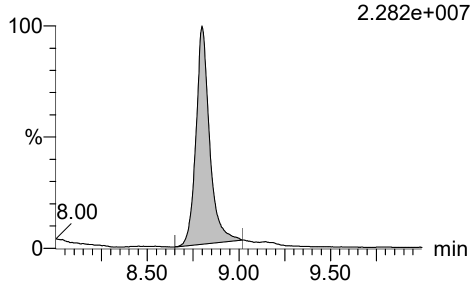

Sample Name: 20211111_A3_R2_IgM
F47:MRM of 3 channels,ES+
976.3 > 366.1
7.560e+006

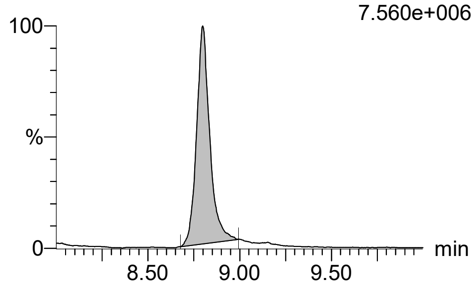

Sample Name: 20211111_A3_R2_IgM
F47:MRM of 3 channels,ES+
1220.1 > 366.1
1.712e+007

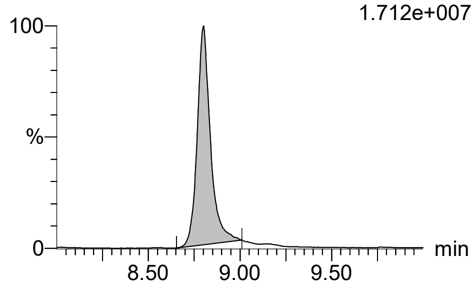

Sample Name: 20211111_A4_R2_IgM
F47:MRM of 3 channels,ES+
976.3 > 204.1
1.663e+007

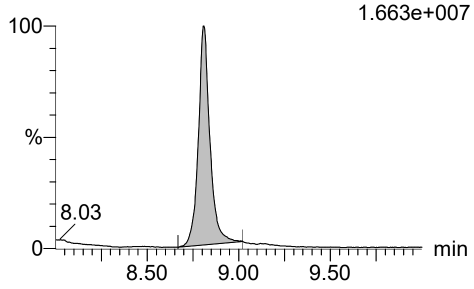

Sample Name: 20211111_A4_R2_IgM
F47:MRM of 3 channels,ES+
976.3 > 366.1
5.482e+006

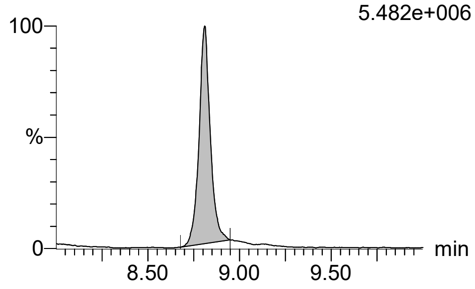

Sample Name: 20211111_A4_R2_IgM
F47:MRM of 3 channels,ES+
1220.1 > 366.1
1.196e+007

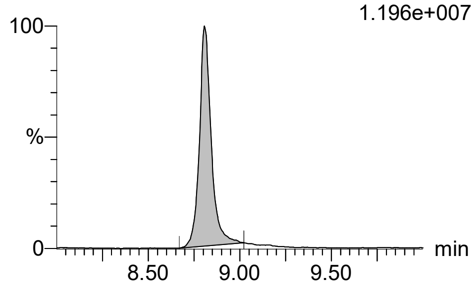

Sample Name: 20211111_L3_R2_IgM
F47:MRM of 3 channels,ES+
976.3 > 204.1
3.151e+006

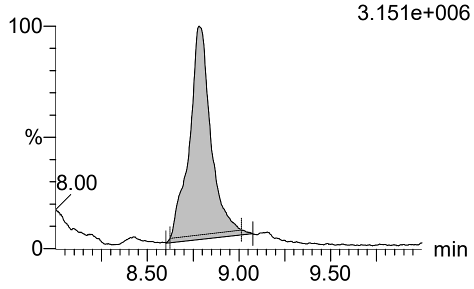

Sample Name: 20211111_L3_R2_IgM
F47:MRM of 3 channels,ES+
976.3 > 366.1
9.565e+005

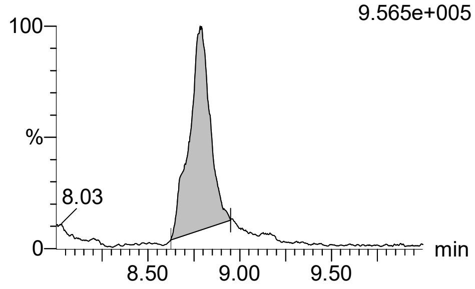

Sample Name: 20211111_L3_R2_IgM
F47:MRM of 3 channels,ES+
1220.1 > 366.1
2.299e+006

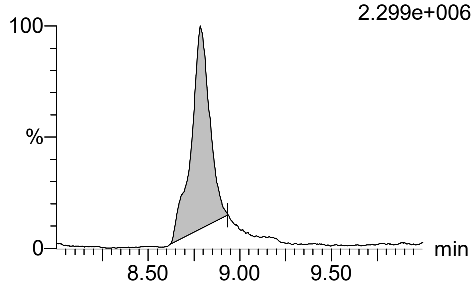

Sample Name: 20211111_L4_R2_IgM
F47:MRM of 3 channels,ES+
976.3 > 204.1
1.794e+007

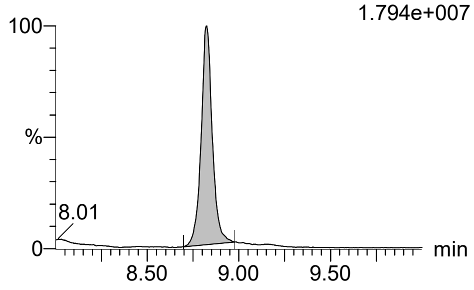

Sample Name: 20211111_L4_R2_IgM
F47:MRM of 3 channels,ES+
976.3 > 366.1
6.193e+006

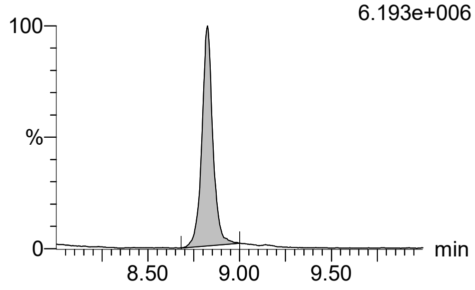

Sample Name: 20211111_L4_R2_IgM
F47:MRM of 3 channels,ES+
1220.1 > 366.1
1.250e+007

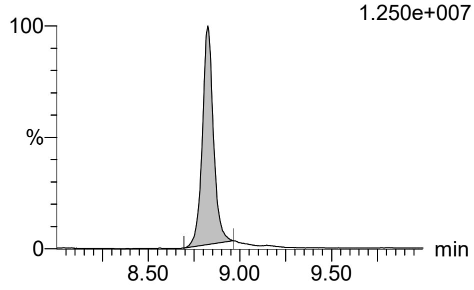

Sample Name: 20211111_H2_R2_IgM
F47:MRM of 3 channels,ES+
976.3 > 204.1
4.233e+007

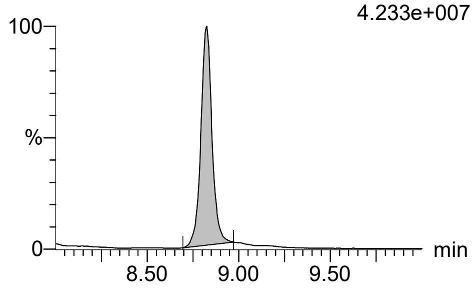

Sample Name: 20211111_H2_R2_IgM
F47:MRM of 3 channels,ES+
976.3 > 366.1
1.480e+007

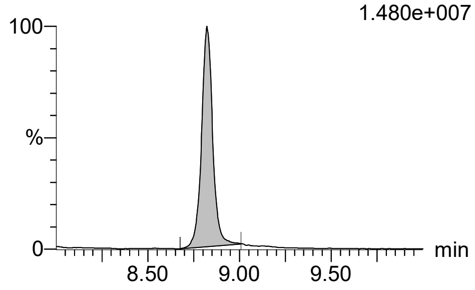

Sample Name: 20211111_H2_R2_IgM
F47:MRM of 3 channels,ES+
1220.1 > 366.1
2.986e+007

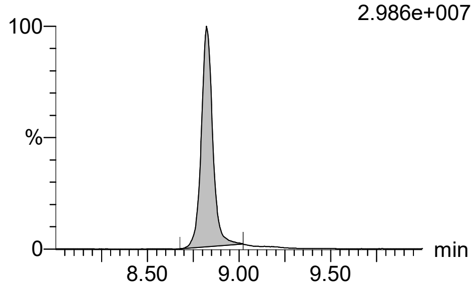

Sample Name: 20211111_A5_R2_IgM
F47:MRM of 3 channels,ES+
976.3 > 204.1
2.849e+007

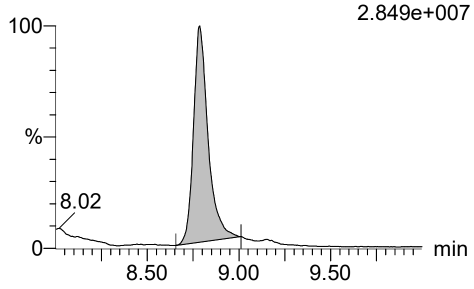

Sample Name: 20211111_A5_R2_IgM
F47:MRM of 3 channels,ES+
976.3 > 366.1
9.241e+006

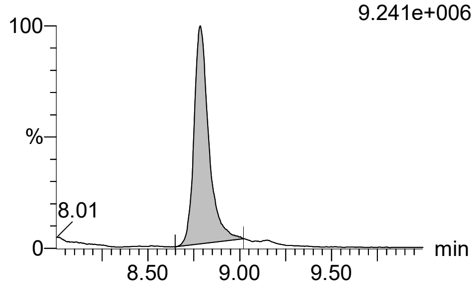

Sample Name: 20211111_A5_R2_IgM
F47:MRM of 3 channels,ES+
1220.1 > 366.1
2.114e+007

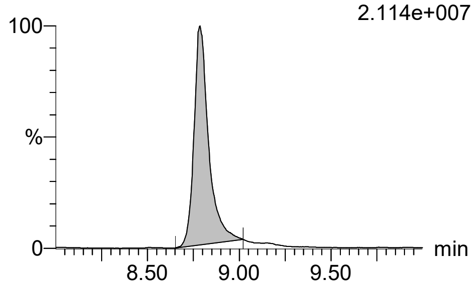

Sample Name: 20211111_A6_R2_IgM
F47:MRM of 3 channels,ES+
976.3 > 204.1
3.020e+007

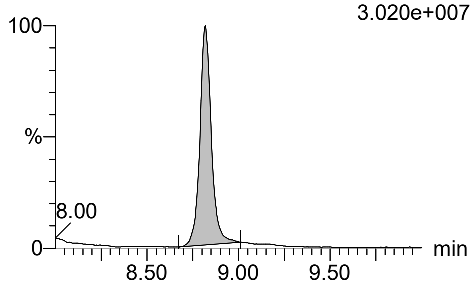

Sample Name: 20211111_A6_R2_IgM
F47:MRM of 3 channels,ES+
976.3 > 366.1
1.047e+007

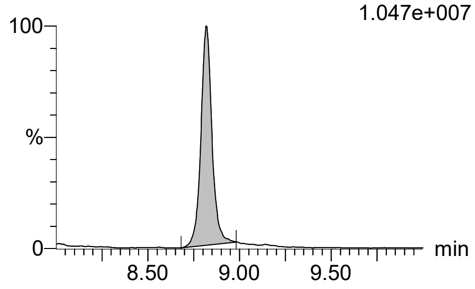

Sample Name: 20211111_A6_R2_IgM
F47:MRM of 3 channels,ES+
1220.1 > 366.1
2.216e+007

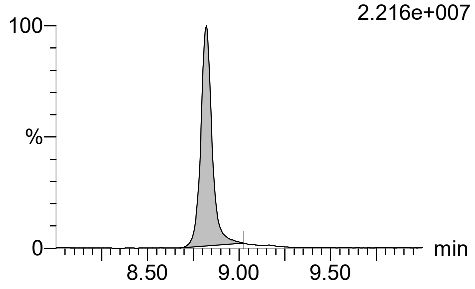

Sample Name: 20211111_QC_R10_IgM
F47:MRM of 3 channels,ES+
976.3 > 204.1
2.027e+007

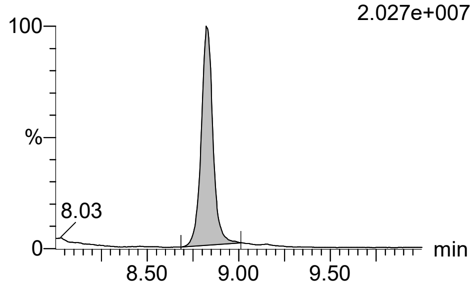

Sample Name: 20211111_QC_R10_IgM
F47:MRM of 3 channels,ES+
976.3 > 366.1
7.112e+006

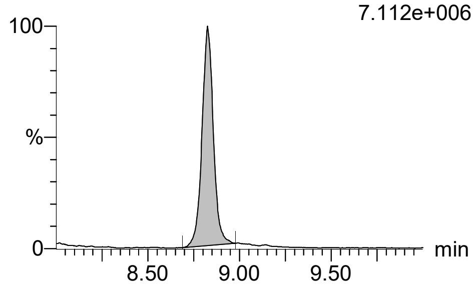

Sample Name: 20211111_QC_R10_IgM
F47:MRM of 3 channels,ES+
1220.1 > 366.1
1.426e+007

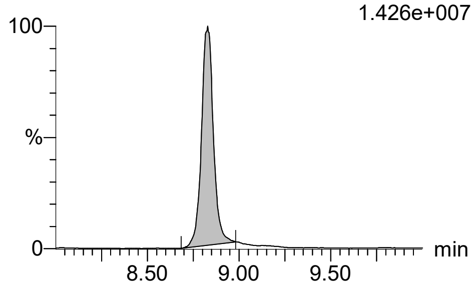

Sample Name: 20211111_L5_R2_IgM
F47:MRM of 3 channels,ES+
976.3 > 204.1
1.373e+007

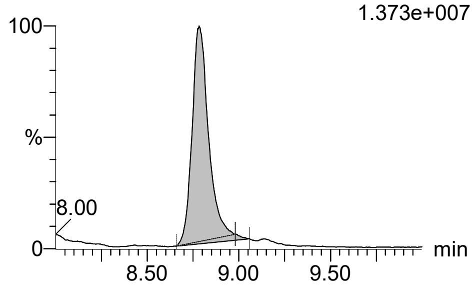

Sample Name: 20211111_L5_R2_IgM
F47:MRM of 3 channels,ES+
976.3 > 366.1
4.551e+006

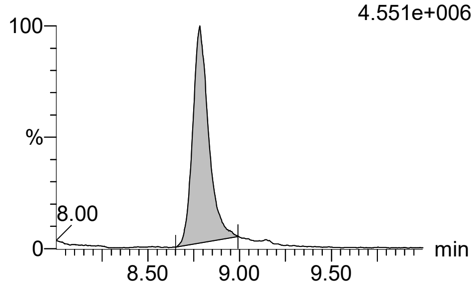

Sample Name: 20211111_L5_R2_IgM
F47:MRM of 3 channels,ES+
1220.1 > 366.1
1.018e+007

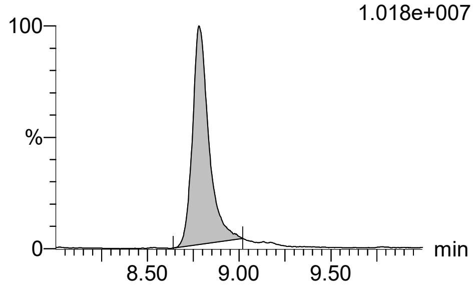

Dataset: C:\MassLynx\10.pro\20211111_clinicalsamples_IgM_IgA.qld

Last Altered: Thursday, March 09, 2023 19:58:25 台北標準時間
Printed: Friday, August 01, 2025 15:08:19 台北標準時間

Sample Name: 20211111_L6_R2_IgM
F47:MRM of 3 channels,ES+
976.3 > 204.1
2.849e+007

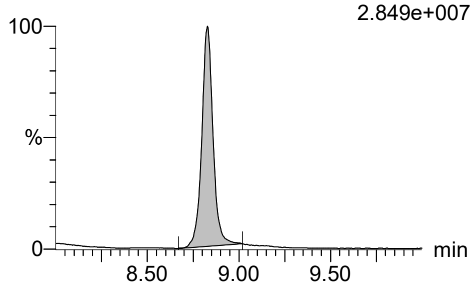

Sample Name: 20211111_L6_R2_IgM
F47:MRM of 3 channels,ES+
976.3 > 366.1
9.770e+006

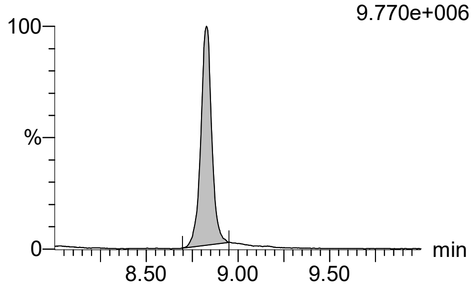

Sample Name: 20211111_L6_R2_IgM
F47:MRM of 3 channels,ES+
1220.1 > 366.1
2.012e+007

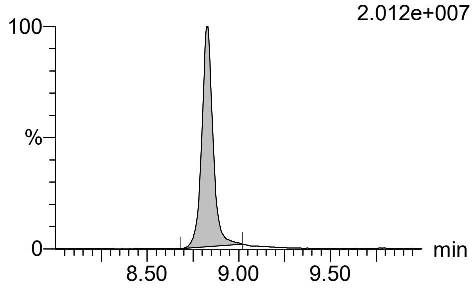

Sample Name: 20211111_H3_R2_IgM
F47:MRM of 3 channels,ES+
976.3 > 204.1
1.012e+007

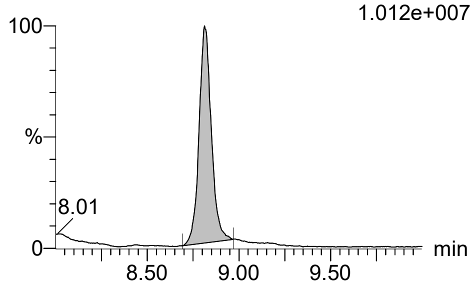

Sample Name: 20211111_H3_R2_IgM
F47:MRM of 3 channels,ES+
976.3 > 366.1
3.380e+006

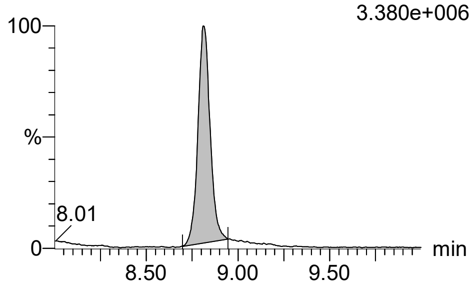

Sample Name: 20211111_H3_R2_IgM
F47:MRM of 3 channels,ES+
1220.1 > 366.1
7.312e+006

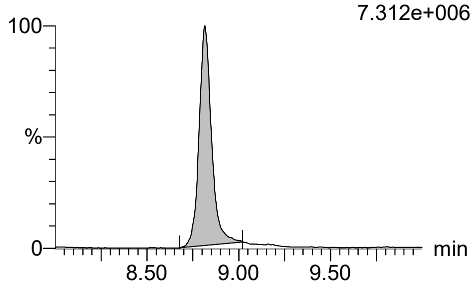

Sample Name: 20211111_A7_R2_IgM
F47:MRM of 3 channels,ES+
976.3 > 204.1
2.440e+007

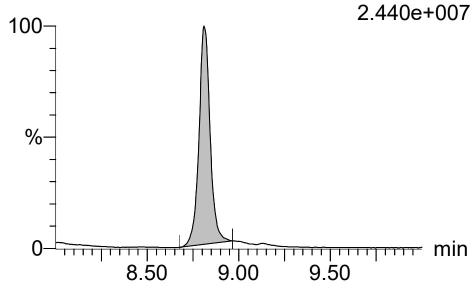

Sample Name: 20211111_A7_R2_IgM
F47:MRM of 3 channels,ES+
976.3 > 366.1
8.623e+006

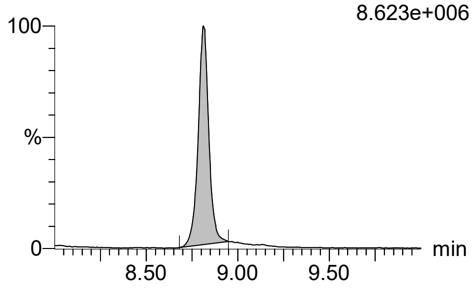

Sample Name: 20211111_A7_R2_IgM
F47:MRM of 3 channels,ES+
1220.1 > 366.1
1.699e+007

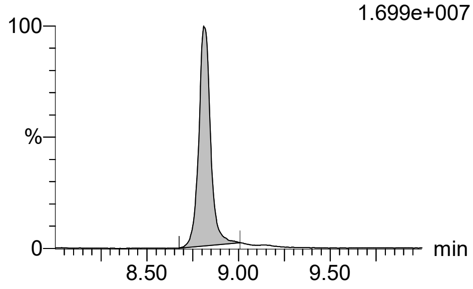

Sample Name: 20211111_A8_R2_IgM
F47:MRM of 3 channels,ES+
976.3 > 204.1
1.845e+007

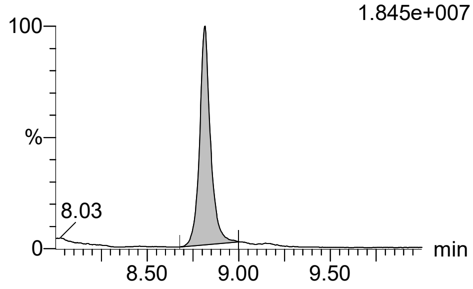

Sample Name: 20211111_A8_R2_IgM
F47:MRM of 3 channels,ES+
976.3 > 366.1
5.998e+006

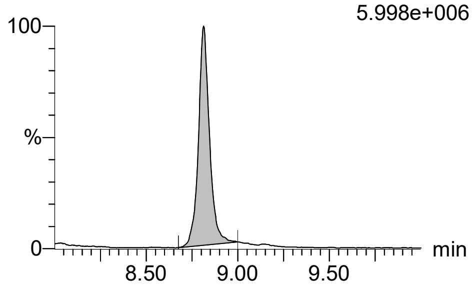

Sample Name: 20211111_A8_R2_IgM
F47:MRM of 3 channels,ES+
1220.1 > 366.1
1.331e+007

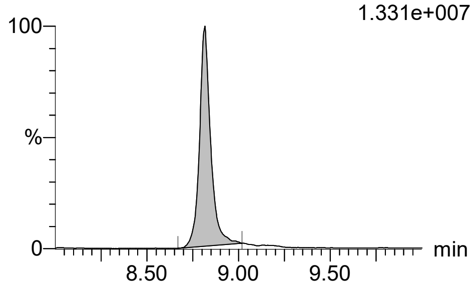

Sample Name: 20211111_L7_R2_IgM
F47:MRM of 3 channels,ES+
976.3 > 204.1
1.450e+007

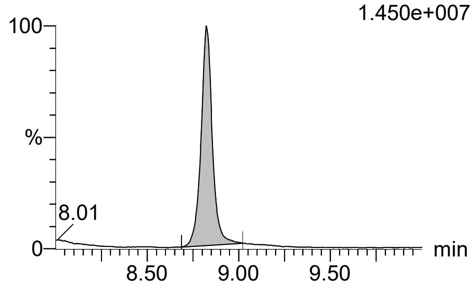

Sample Name: 20211111_L7_R2_IgM
F47:MRM of 3 channels,ES+
976.3 > 366.1
4.868e+006

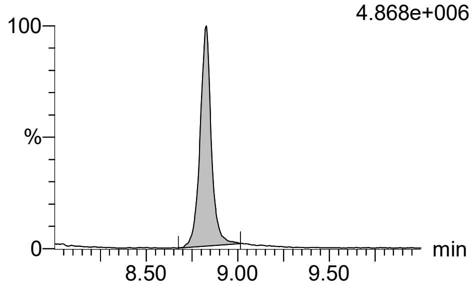

Sample Name: 20211111_L7_R2_IgM
F47:MRM of 3 channels,ES+
1220.1 > 366.1
1.049e+007

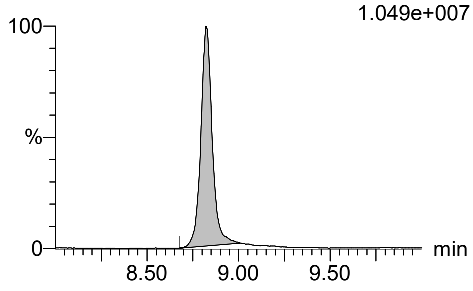

Sample Name: 20211111_L8_R2_IgM
F47:MRM of 3 channels,ES+
976.3 > 204.1
6.364e+006

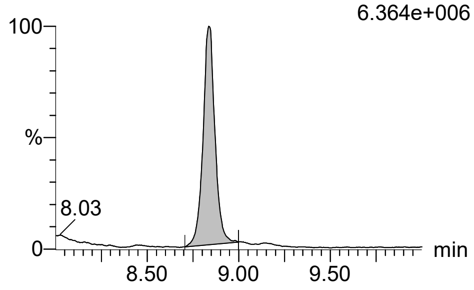

Sample Name: 20211111_L8_R2_IgM
F47:MRM of 3 channels,ES+
976.3 > 366.1
2.212e+006

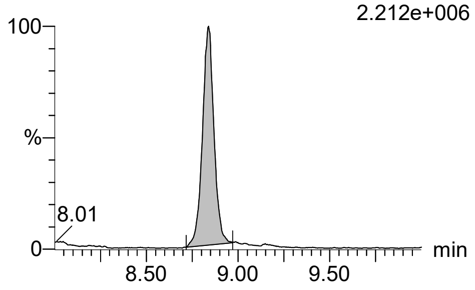

Sample Name: 20211111_L8_R2_IgM
F47:MRM of 3 channels,ES+
1220.1 > 366.1
4.284e+006

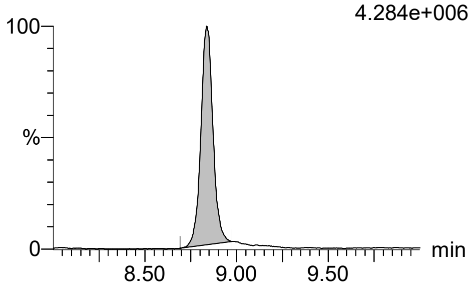

Sample Name: 20211111_H4_R2_IgM
F47:MRM of 3 channels,ES+
976.3 > 204.1
1.214e+007

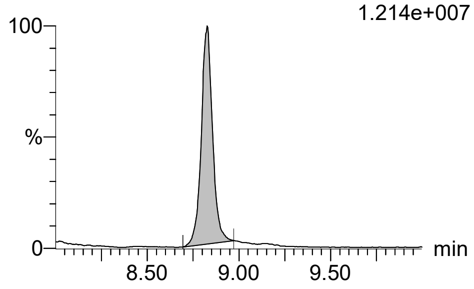

Sample Name: 20211111_H4_R2_IgM
F47:MRM of 3 channels,ES+
976.3 > 366.1
4.299e+006

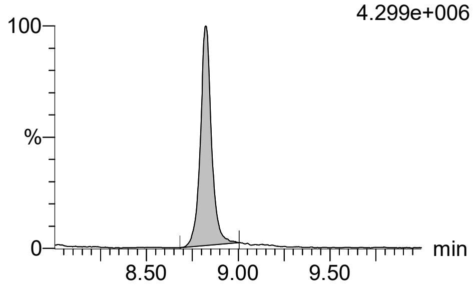

Sample Name: 20211111_H4_R2_IgM
F47:MRM of 3 channels,ES+
1220.1 > 366.1
8.563e+006

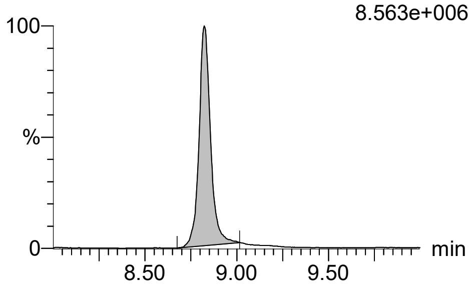

Sample Name: 20211111_A9_R2_IgM
F47:MRM of 3 channels,ES+
976.3 > 204.1
7.035e+006

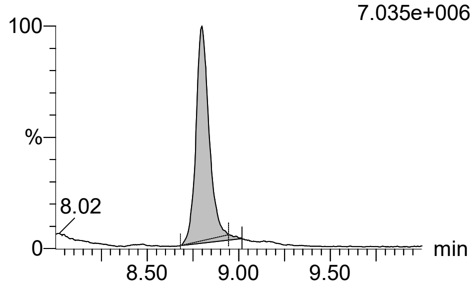

Sample Name: 20211111_A9_R2_IgM
F47:MRM of 3 channels,ES+
976.3 > 366.1
2.240e+006

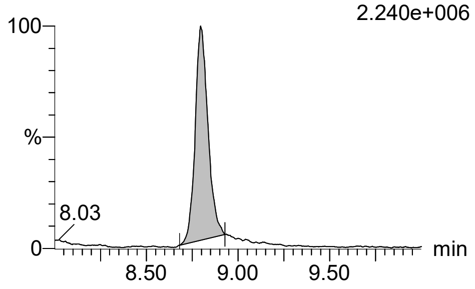

Sample Name: 20211111_A9_R2_IgM
F47:MRM of 3 channels,ES+
1220.1 > 366.1
5.120e+006

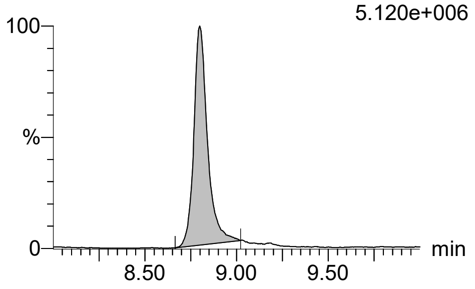

Sample Name: 20211111_A10_R2_IgM
F47:MRM of 3 channels,ES+
976.3 > 204.1
2.650e+007

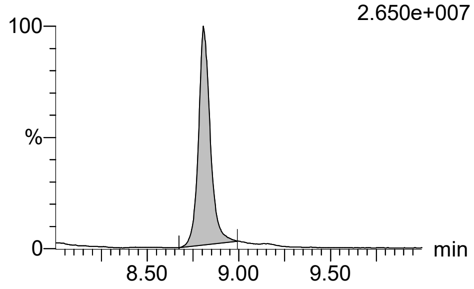

Sample Name: 20211111_A10_R2_IgM
F47:MRM of 3 channels,ES+
976.3 > 366.1
8.710e+006

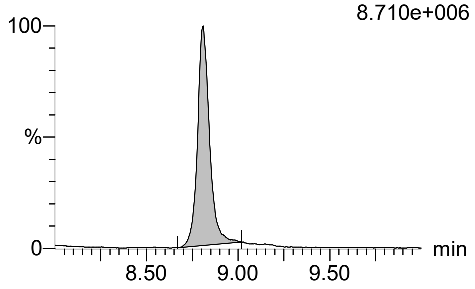

Sample Name: 20211111_A10_R2_IgM
F47:MRM of 3 channels,ES+
1220.1 > 366.1
2.015e+007

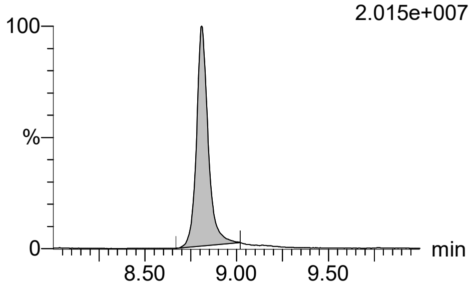

Sample Name: 20211111_L9_R2_IgM
F47:MRM of 3 channels,ES+
976.3 > 204.1
1.188e+007

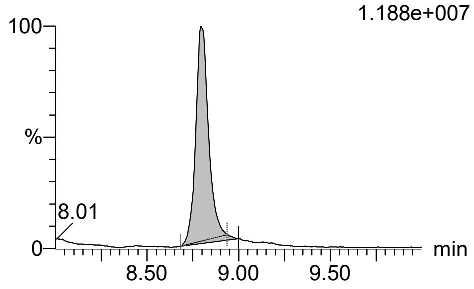

Sample Name: 20211111_L9_R2_IgM
F47:MRM of 3 channels,ES+
976.3 > 366.1
3.979e+006

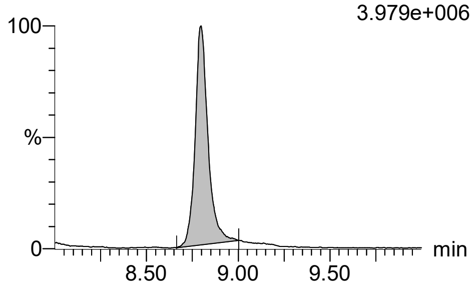

Sample Name: 20211111_L9_R2_IgM
F47:MRM of 3 channels,ES+
1220.1 > 366.1
9.145e+006

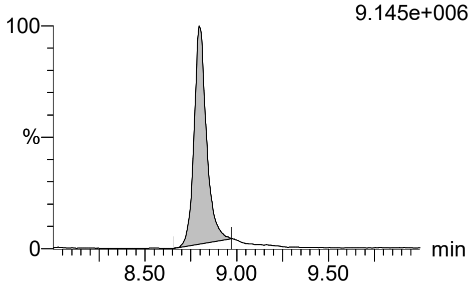

Dataset: C:\MassLynx\10.pro\20211111_clinicalsamples_IgM_IgA.qld

Last Altered: Thursday, March 09, 2023 19:58:25 台北標準時間
Printed: Friday, August 01, 2025 15:08:19 台北標準時間

Sample Name: 20211111_L10_R2_IgM
F47:MRM of 3 channels,ES+
976.3 > 204.1
1.742e+007

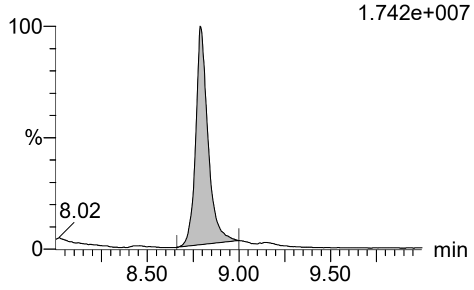

Sample Name: 20211111_L10_R2_IgM
F47:MRM of 3 channels,ES+
976.3 > 366.1
5.765e+006

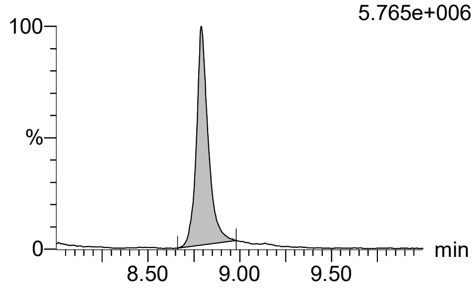

Sample Name: 20211111_L10_R2_IgM
F47:MRM of 3 channels,ES+
1220.1 > 366.1
1.316e+007

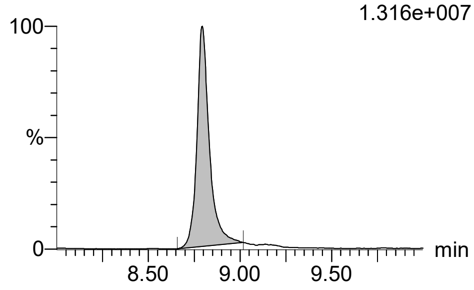

Sample Name: 20211111_QC_R11_IgM
F47:MRM of 3 channels,ES+
976.3 > 204.1
2.204e+007

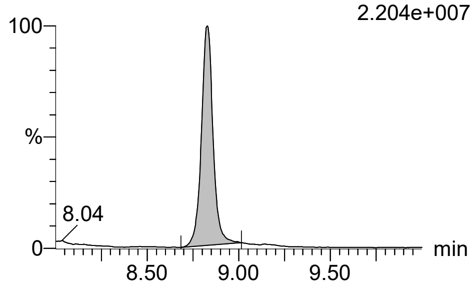

Sample Name: 20211111_QC_R11_IgM
F47:MRM of 3 channels,ES+
976.3 > 366.1
7.748e+006

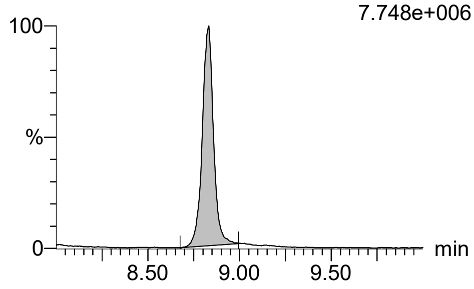

Sample Name: 20211111_QC_R11_IgM
F47:MRM of 3 channels,ES+
1220.1 > 366.1
1.642e+007

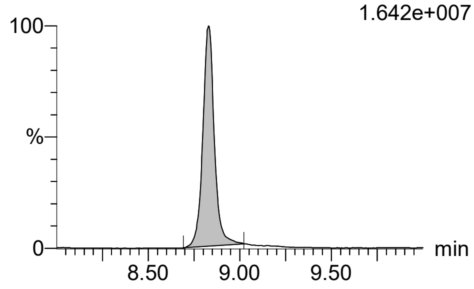

Sample Name: 20211111_H5_R2_IgM
F47:MRM of 3 channels,ES+
976.3 > 204.1
2.517e+007

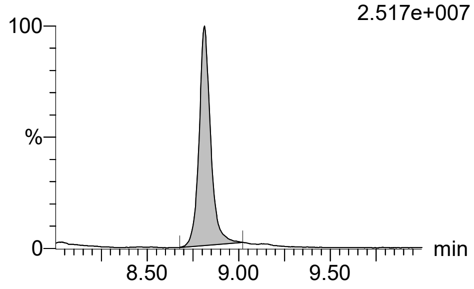

Sample Name: 20211111_H5_R2_IgM
F47:MRM of 3 channels,ES+
976.3 > 366.1
8.314e+006

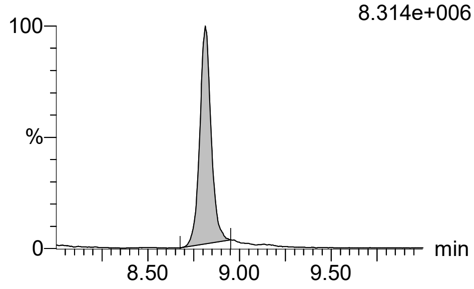

Sample Name: 20211111_H5_R2_IgM
F47:MRM of 3 channels,ES+
1220.1 > 366.1
1.928e+007

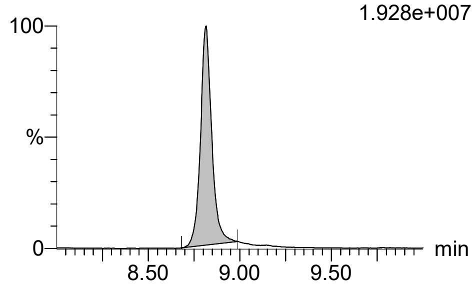

Sample Name: 20211111_A11_R2_IgM
F47:MRM of 3 channels,ES+
976.3 > 204.1
3.177e+006

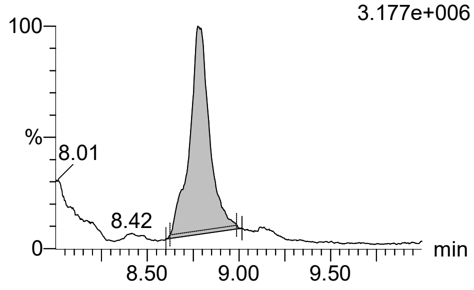

Sample Name: 20211111_A11_R2_IgM
F47:MRM of 3 channels,ES+
976.3 > 366.1
9.958e+005

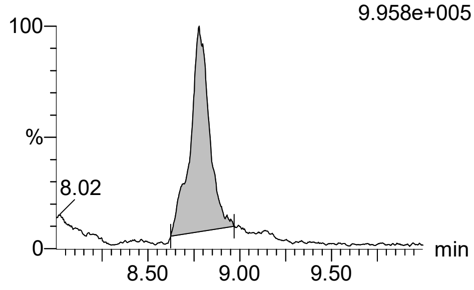

Sample Name: 20211111_A11_R2_IgM
F47:MRM of 3 channels,ES+
1220.1 > 366.1
2.356e+006

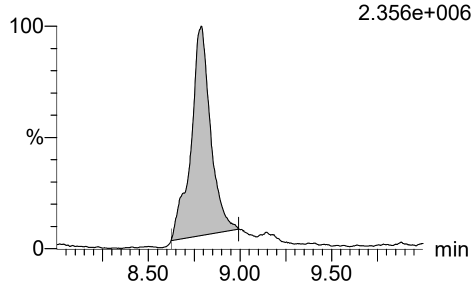

Sample Name: 20211111_A12_R2_IgM
F47:MRM of 3 channels,ES+
976.3 > 204.1
8.135e+006

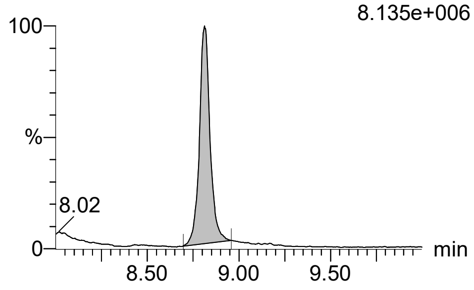

Sample Name: 20211111_A12_R2_IgM
F47:MRM of 3 channels,ES+
976.3 > 366.1
2.700e+006

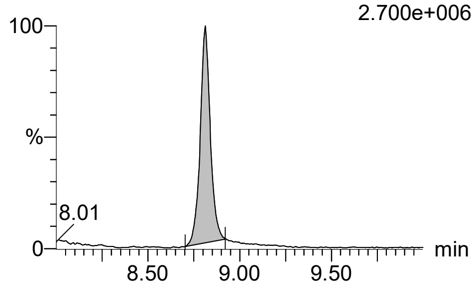

Sample Name: 20211111_A12_R2_IgM
F47:MRM of 3 channels,ES+
1220.1 > 366.1
6.134e+006

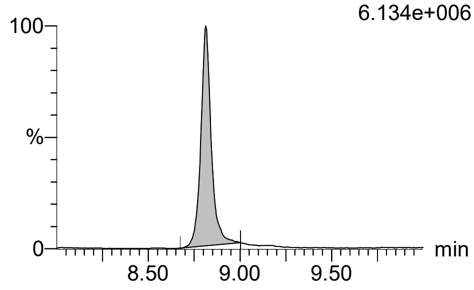

Sample Name: 20211111_L11_R2_IgM
F47:MRM of 3 channels,ES+
976.3 > 204.1
1.153e+007

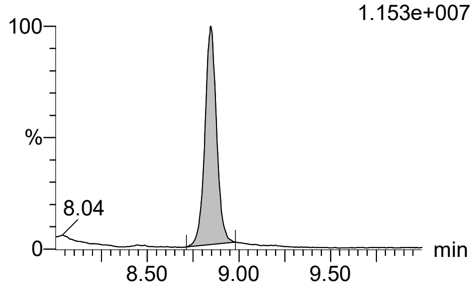

Sample Name: 20211111_L11_R2_IgM
F47:MRM of 3 channels,ES+
976.3 > 366.1
3.928e+006

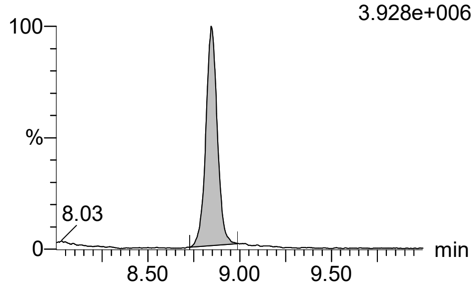

Sample Name: 20211111_L11_R2_IgM
F47:MRM of 3 channels,ES+
1220.1 > 366.1
7.868e+006

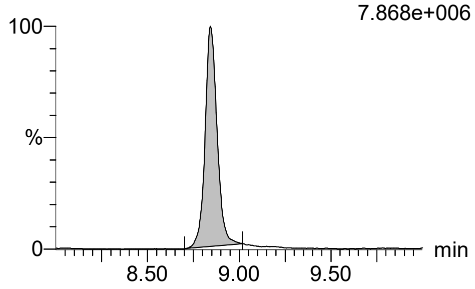

Sample Name: 20211111_L12_R2_IgM
F47:MRM of 3 channels,ES+
976.3 > 204.1
8.900e+006

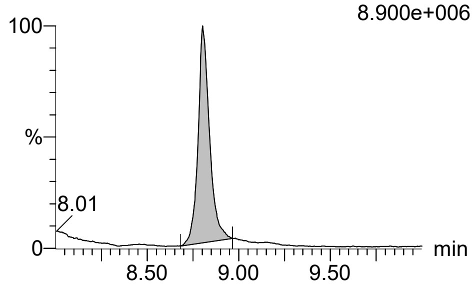

Sample Name: 20211111_L12_R2_IgM
F47:MRM of 3 channels,ES+
976.3 > 366.1
2.999e+006

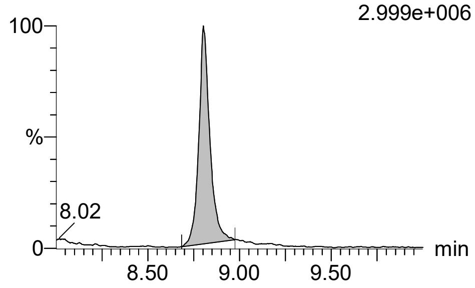

Sample Name: 20211111_L12_R2_IgM
F47:MRM of 3 channels,ES+
1220.1 > 366.1
7.013e+006

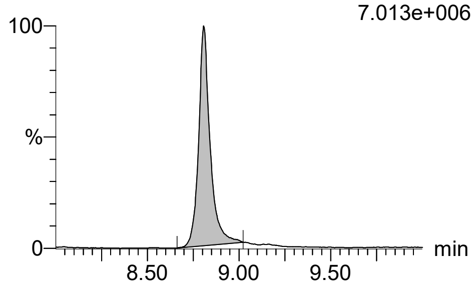

Sample Name: 20211111_H6_R2_IgM
F47:MRM of 3 channels,ES+
976.3 > 204.1
8.819e+006

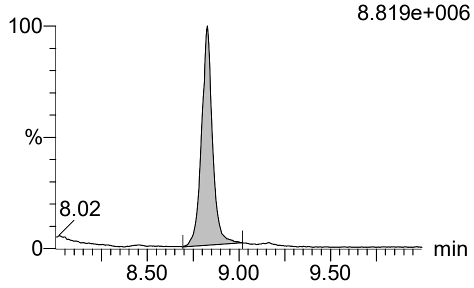

Sample Name: 20211111_H6_R2_IgM
F47:MRM of 3 channels,ES+
976.3 > 366.1
2.972e+006

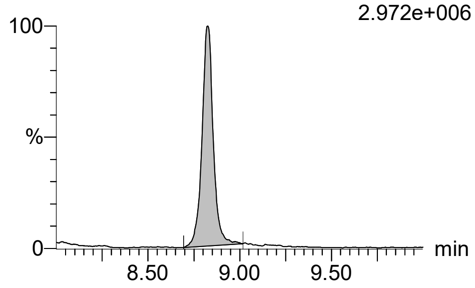

Sample Name: 20211111_H6_R2_IgM
F47:MRM of 3 channels,ES+
1220.1 > 366.1
6.402e+006

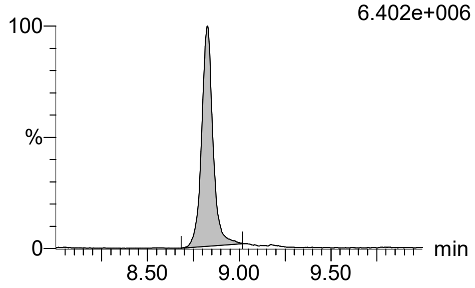

Sample Name: 20211111_A13_R2_IgM
F47:MRM of 3 channels,ES+
976.3 > 204.1
1.001e+007

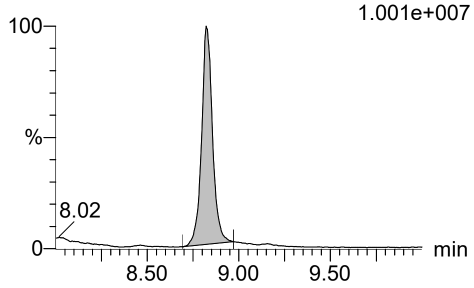

Sample Name: 20211111_A13_R2_IgM
F47:MRM of 3 channels,ES+
976.3 > 366.1
3.435e+006

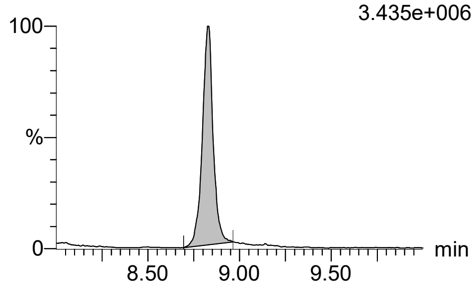

Sample Name: 20211111_A13_R2_IgM
F47:MRM of 3 channels,ES+
1220.1 > 366.1
7.122e+006

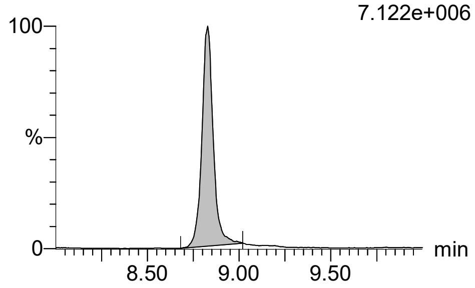

Sample Name: 20211111_A14_R2_IgM
F47:MRM of 3 channels,ES+
976.3 > 204.1
2.953e+007

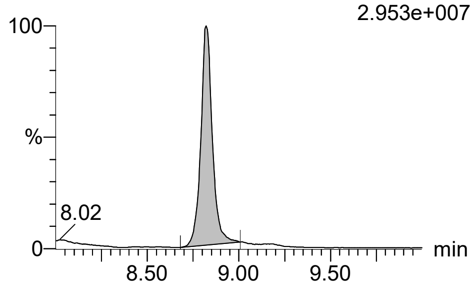

Sample Name: 20211111_A14_R2_IgM
F47:MRM of 3 channels,ES+
976.3 > 366.1
1.023e+007

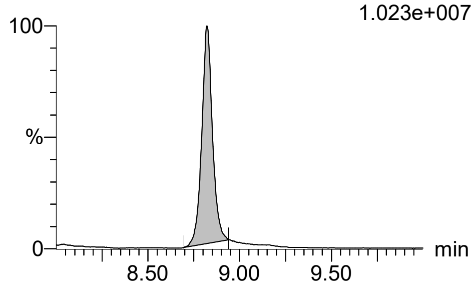

Sample Name: 20211111_A14_R2_IgM
F47:MRM of 3 channels,ES+
1220.1 > 366.1
2.278e+007

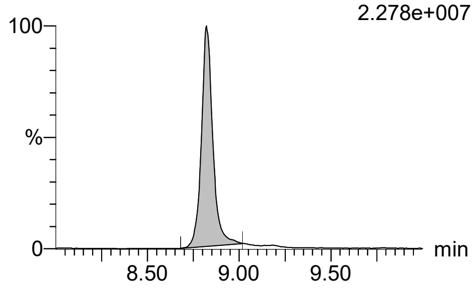

Dataset: C:\MassLynx\10.pro\20211111_clinicalsamples_IgM_IgA.qld

Last Altered: Thursday, March 09, 2023 19:58:25 台北標準時間
Printed: Friday, August 01, 2025 15:08:19 台北標準時間

Sample Name: 20211111_L13_R2_IgM
F47:MRM of 3 channels,ES+
976.3 > 204.1
2.306e+007

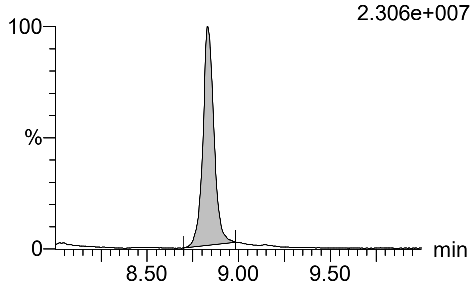

Sample Name: 20211111_L13_R2_IgM
F47:MRM of 3 channels,ES+
976.3 > 366.1
8.484e+006

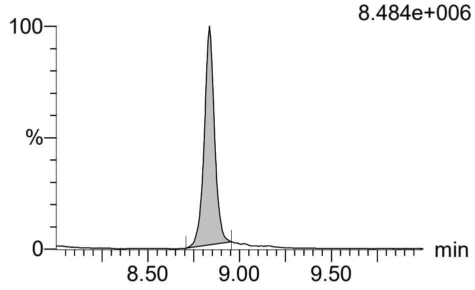

Sample Name: 20211111_L13_R2_IgM
F47:MRM of 3 channels,ES+
1220.1 > 366.1
1.651e+007

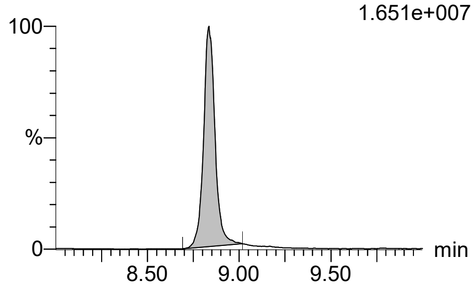

Sample Name: 20211111_L14_R2_IgM
F47:MRM of 3 channels,ES+
976.3 > 204.1
2.569e+007

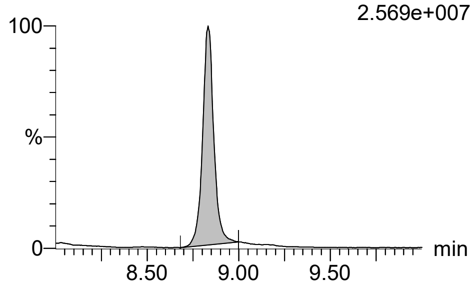

Sample Name: 20211111_L14_R2_IgM
F47:MRM of 3 channels,ES+
976.3 > 366.1
8.814e+006

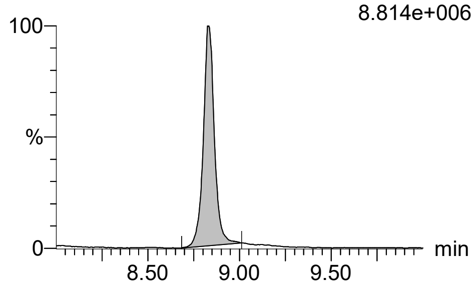

Sample Name: 20211111_L14_R2_IgM
F47:MRM of 3 channels,ES+
1220.1 > 366.1
1.935e+007

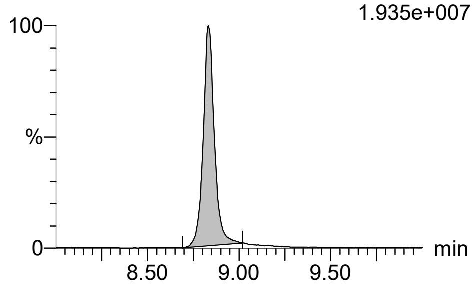

Sample Name: 20211111_H7_R2_IgM
F47:MRM of 3 channels,ES+
976.3 > 204.1
4.471e+006

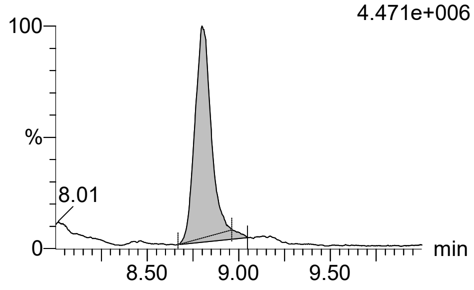

Sample Name: 20211111_H7_R2_IgM
F47:MRM of 3 channels,ES+
976.3 > 366.1
1.418e+006

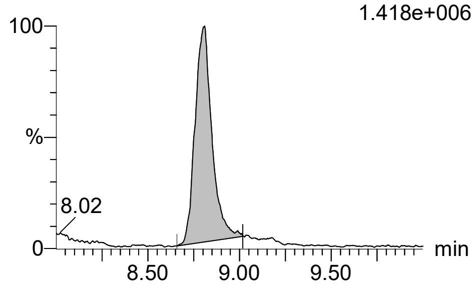

Sample Name: 20211111_H7_R2_IgM
F47:MRM of 3 channels,ES+
1220.1 > 366.1
3.316e+006

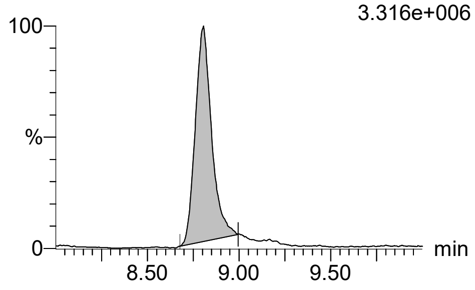

Sample Name: 20211111_A15_R2_IgM
F47:MRM of 3 channels,ES+
976.3 > 204.1
4.728e+007

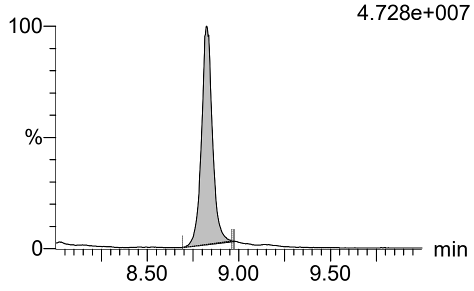

Sample Name: 20211111_A15_R2_IgM
F47:MRM of 3 channels,ES+
976.3 > 366.1
1.670e+007

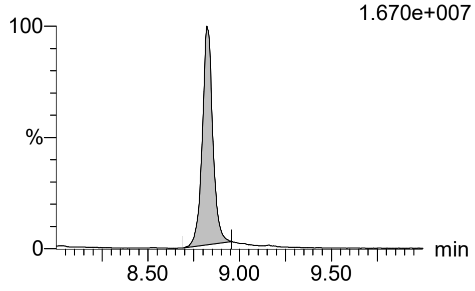

Sample Name: 20211111_A15_R2_IgM
F47:MRM of 3 channels,ES+
1220.1 > 366.1
3.750e+007

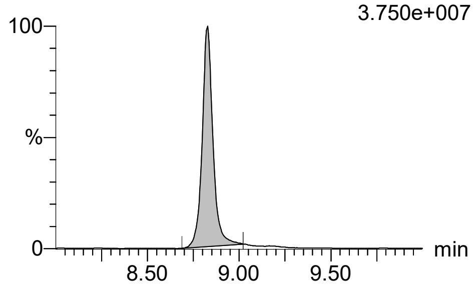

Sample Name: 20211111_QC_R12_IgM
F47:MRM of 3 channels,ES+
976.3 > 204.1
2.418e+007

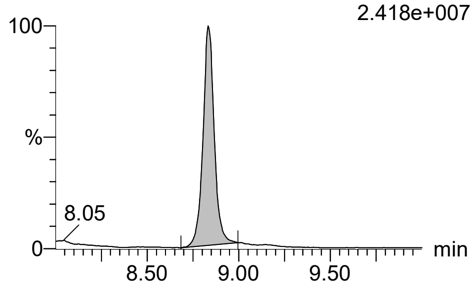

Sample Name: 20211111_QC_R12_IgM
F47:MRM of 3 channels,ES+
976.3 > 366.1
8.324e+006

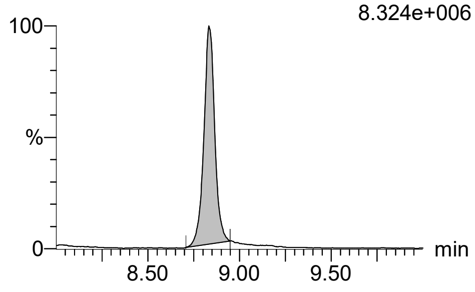

Sample Name: 20211111_QC_R12_IgM
F47:MRM of 3 channels,ES+
1220.1 > 366.1
1.787e+007

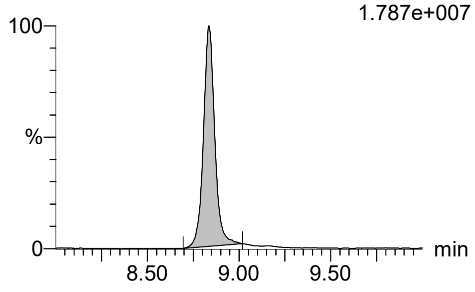

Dataset: C:\MassLynx\10.pro\20211111_clinicalsamples_IgM_IgA.qld

Last Altered: Thursday, March 09, 2023 19:58:25 台北標準時間
Printed: Friday, August 01, 2025 15:08:19 台北標準時間

Sample Name: 20211111_A16_R2_IgM
F47:MRM of 3 channels,ES+
976.3 > 204.1
3.793e+007

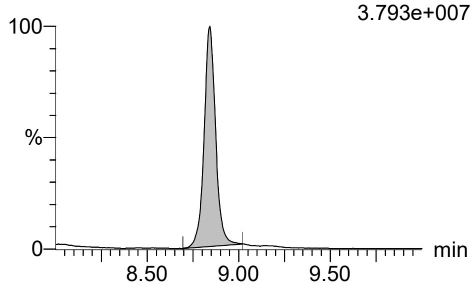

Sample Name: 20211111_A16_R2_IgM
F47:MRM of 3 channels,ES+
976.3 > 366.1
1.309e+007

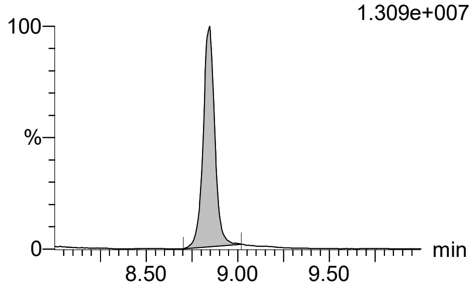

Sample Name: 20211111_A16_R2_IgM
F47:MRM of 3 channels,ES+
1220.1 > 366.1
2.720e+007

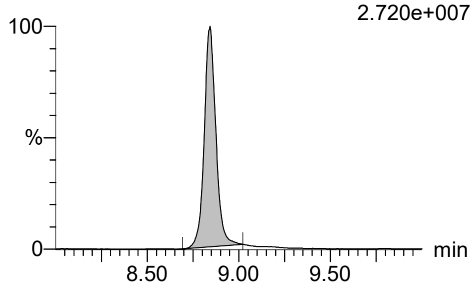

Sample Name: 20211111_L15_R2_IgM
F47:MRM of 3 channels,ES+
976.3 > 204.1
1.724e+007

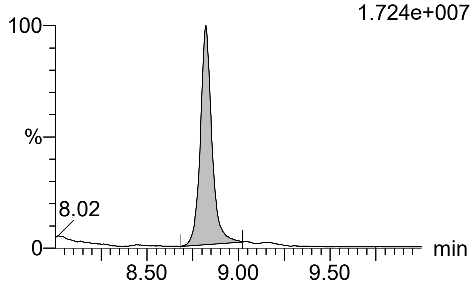

Sample Name: 20211111_L15_R2_IgM
F47:MRM of 3 channels,ES+
976.3 > 366.1
5.838e+006

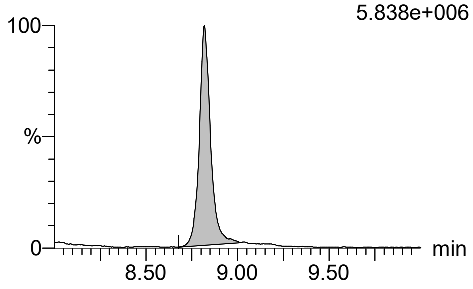

Sample Name: 20211111_L15_R2_IgM
F47:MRM of 3 channels,ES+
1220.1 > 366.1
1.269e+007

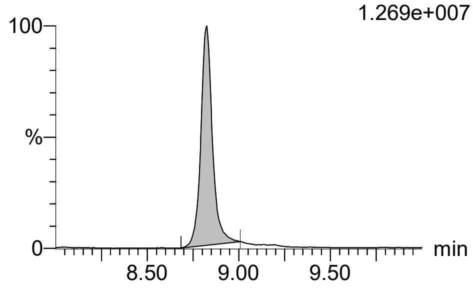

Sample Name: 20211111_L16_R2_IgM
F47:MRM of 3 channels,ES+
976.3 > 204.1
8.209e+006

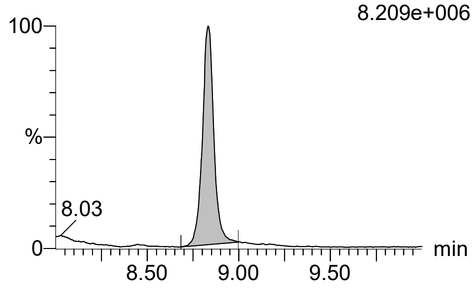

Sample Name: 20211111_L16_R2_IgM
F47:MRM of 3 channels,ES+
976.3 > 366.1
2.901e+006

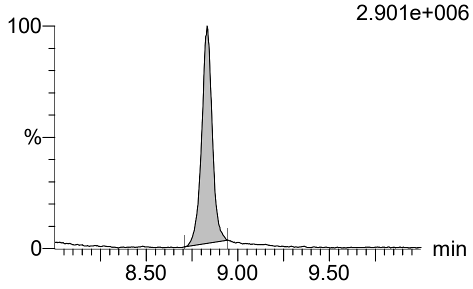

Sample Name: 20211111_L16_R2_IgM
F47:MRM of 3 channels,ES+
1220.1 > 366.1
5.998e+006

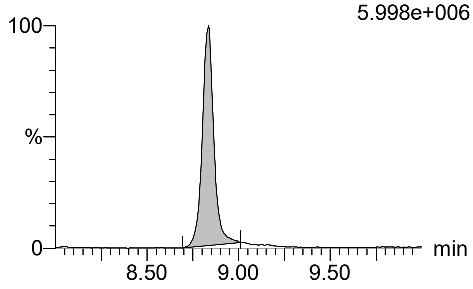

Sample Name: 20211111_H8_R2_IgM
F47:MRM of 3 channels,ES+
976.3 > 204.1
1.778e+007

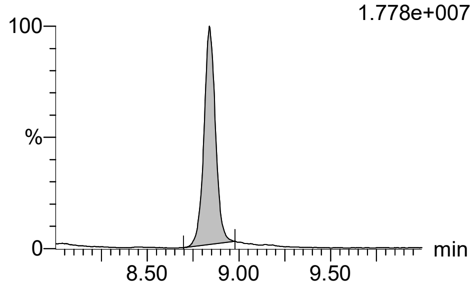

Sample Name: 20211111_H8_R2_IgM
F47:MRM of 3 channels,ES+
976.3 > 366.1
6.083e+006

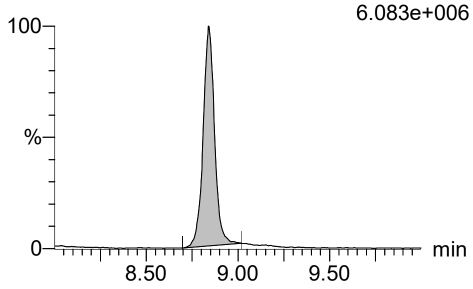

Sample Name: 20211111_H8_R2_IgM
F47:MRM of 3 channels,ES+
1220.1 > 366.1
1.236e+007

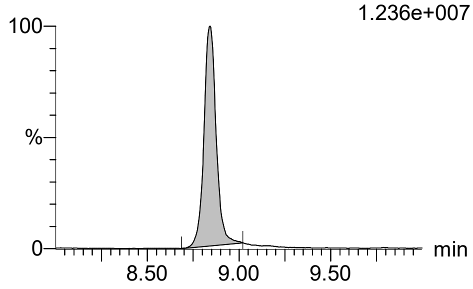

Sample Name: 20211111_A17_R2_IgM
F47:MRM of 3 channels,ES+
976.3 > 204.1
3.166e+007

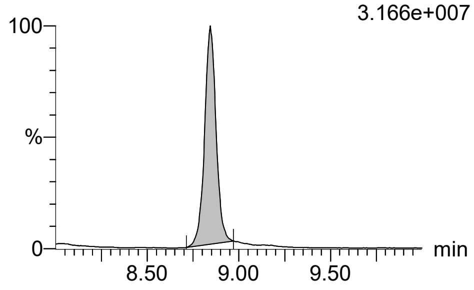

Sample Name: 20211111_A17_R2_IgM
F47:MRM of 3 channels,ES+
976.3 > 366.1
1.018e+007

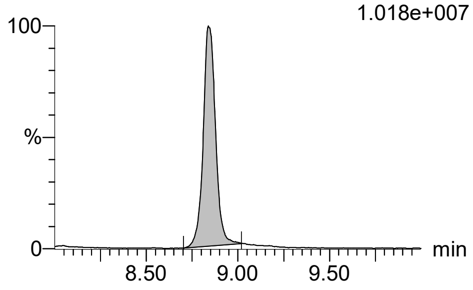

Sample Name: 20211111_A17_R2_IgM
F47:MRM of 3 channels,ES+
1220.1 > 366.1
2.318e+007

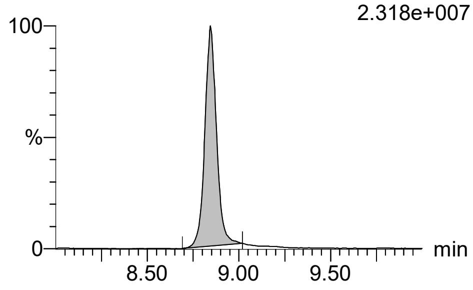

Sample Name: 20211111_A18_R2_IgM
F47:MRM of 3 channels,ES+
976.3 > 204.1
8.155e+006

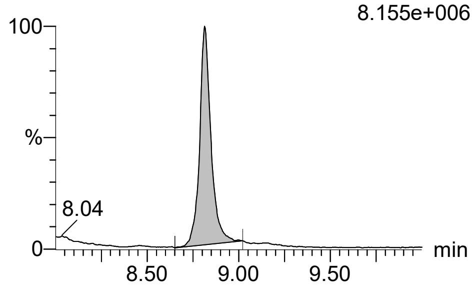

Sample Name: 20211111_A18_R2_IgM
F47:MRM of 3 channels,ES+
976.3 > 366.1
2.656e+006

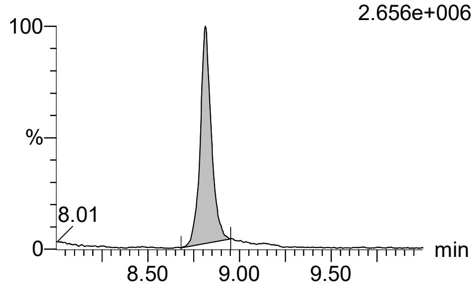

Sample Name: 20211111_A18_R2_IgM
F47:MRM of 3 channels,ES+
1220.1 > 366.1
5.943e+006

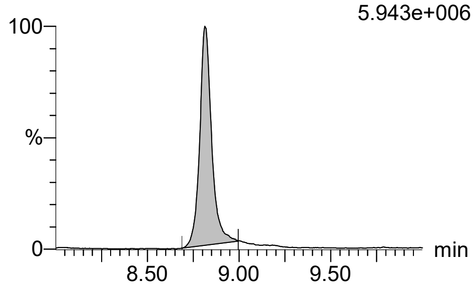

Sample Name: 20211111_L17_R2_IgM
F47:MRM of 3 channels,ES+
976.3 > 204.1
5.298e+007

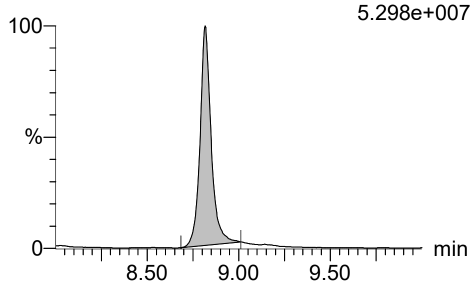

Sample Name: 20211111_L17_R2_IgM
F47:MRM of 3 channels,ES+
976.3 > 366.1
1.803e+007

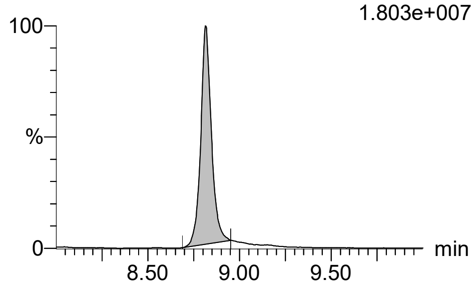

Sample Name: 20211111_L17_R2_IgM
F47:MRM of 3 channels,ES+
1220.1 > 366.1
4.059e+007

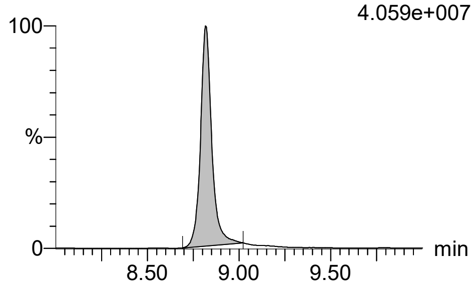

Sample Name: 20211111_L18_R2_IgM
F47:MRM of 3 channels,ES+
976.3 > 204.1
3.531e+006

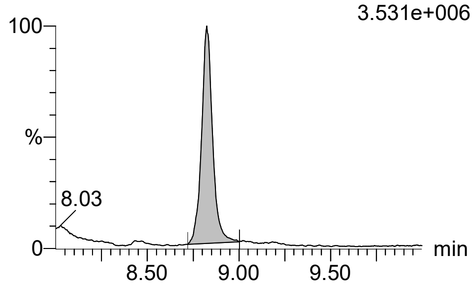

Sample Name: 20211111_L18_R2_IgM
F47:MRM of 3 channels,ES+
976.3 > 366.1
1.162e+006

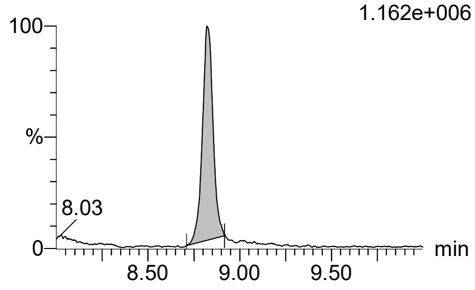

Sample Name: 20211111_L18_R2_IgM
F47:MRM of 3 channels,ES+
1220.1 > 366.1
2.522e+006

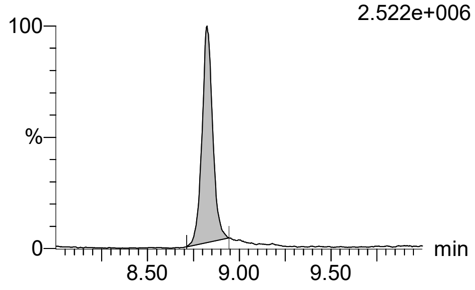

Sample Name: 20211111_H9_R2_IgM
F47:MRM of 3 channels,ES+
976.3 > 204.1
7.186e+006

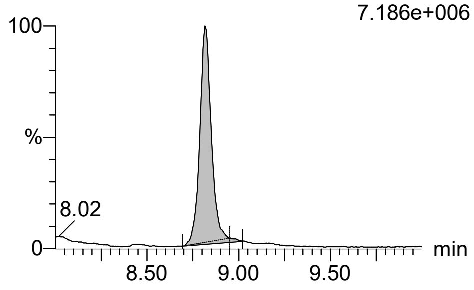

Sample Name: 20211111_H9_R2_IgM
F47:MRM of 3 channels,ES+
976.3 > 366.1
2.464e+006

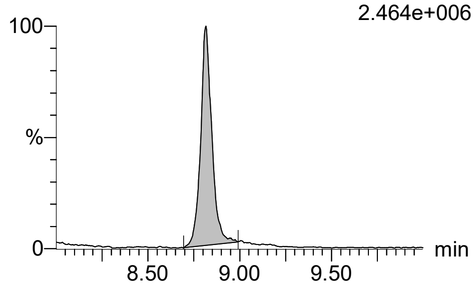

Sample Name: 20211111_H9_R2_IgM
F47:MRM of 3 channels,ES+
1220.1 > 366.1
5.382e+006

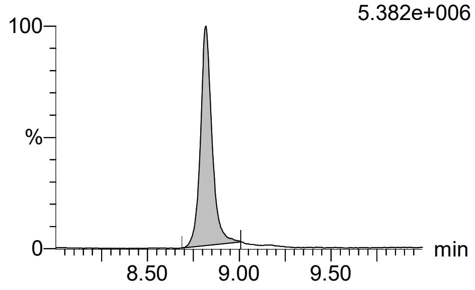

Sample Name: 20211111_A19_R2_IgM
F47:MRM of 3 channels,ES+
976.3 > 204.1
1.570e+007

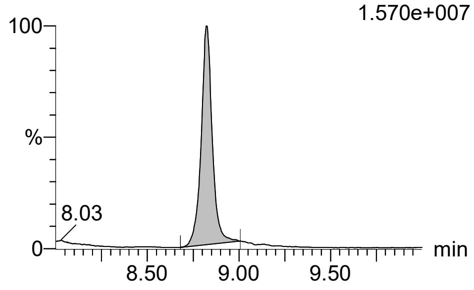

Sample Name: 20211111_A19_R2_IgM
F47:MRM of 3 channels,ES+
976.3 > 366.1
5.088e+006

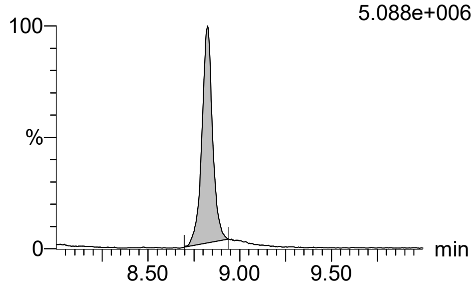

Sample Name: 20211111_A19_R2_IgM
F47:MRM of 3 channels,ES+
1220.1 > 366.1
1.251e+007

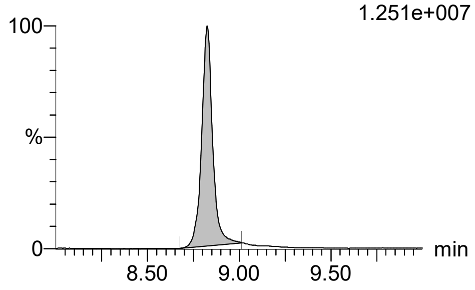

Dataset: C:\MassLynx\10.pro\20211111_clinicalsamples_IgM_IgA.qld

Last Altered: Thursday, March 09, 2023 19:58:25 台北標準時間
Printed: Friday, August 01, 2025 15:08:19 台北標準時間

Sample Name: 20211111_A20_R2_IgM
F47:MRM of 3 channels,ES+
976.3 > 204.1
2.187e+006

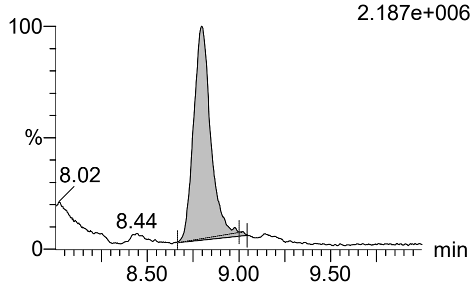

Sample Name: 20211111_A20_R2_IgM
F47:MRM of 3 channels,ES+
976.3 > 366.1
6.945e+005

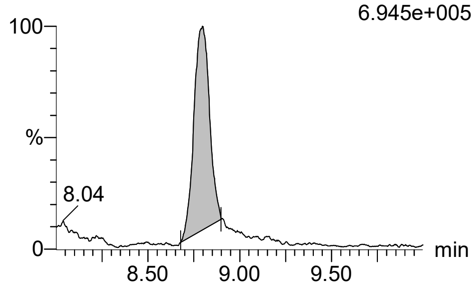

Sample Name: 20211111_A20_R2_IgM
F47:MRM of 3 channels,ES+
1220.1 > 366.1
1.653e+006

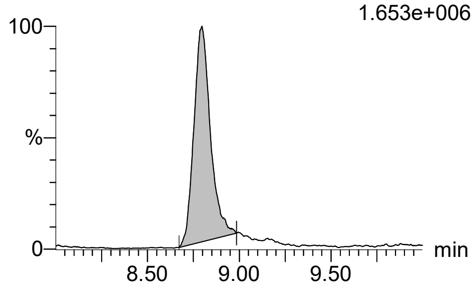

Sample Name: 20211111_L19_R2_IgM
F47:MRM of 3 channels,ES+
976.3 > 204.1
1.820e+007

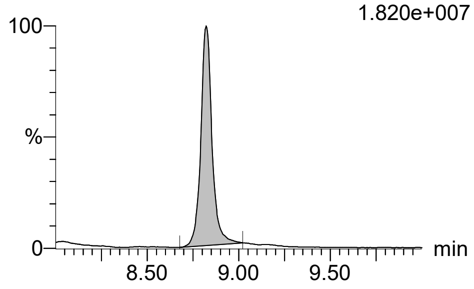

Sample Name: 20211111_L19_R2_IgM
F47:MRM of 3 channels,ES+
976.3 > 366.1
6.062e+006

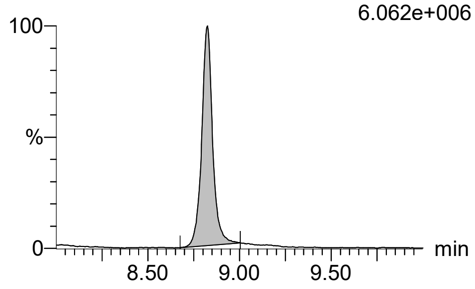

Sample Name: 20211111_L19_R2_IgM
F47:MRM of 3 channels,ES+
1220.1 > 366.1
1.385e+007

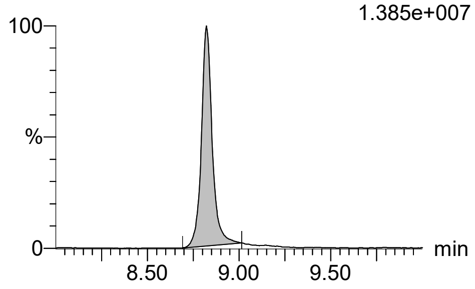

Sample Name: 20211111_QC_R13_IgM
F47:MRM of 3 channels,ES+
976.3 > 204.1
2.323e+007

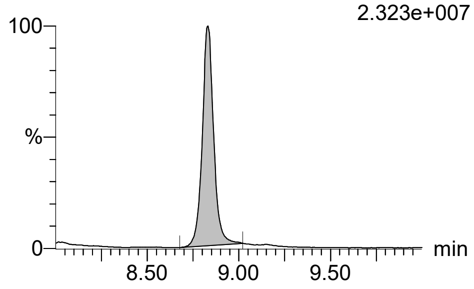

Sample Name: 20211111_QC_R13_IgM
F47:MRM of 3 channels,ES+
976.3 > 366.1
8.315e+006

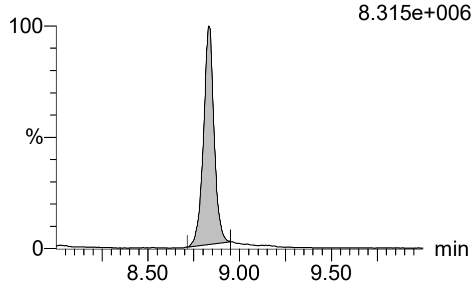

Sample Name: 20211111_QC_R13_IgM
F47:MRM of 3 channels,ES+
1220.1 > 366.1
1.748e+007

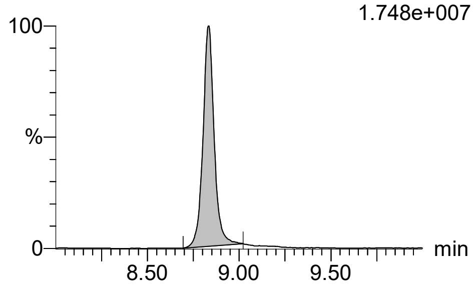

Sample Name: 20211111_L20_R2_IgM
F47:MRM of 3 channels,ES+
976.3 > 204.1
3.511e+006

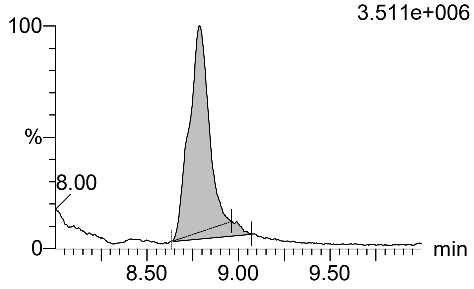

Sample Name: 20211111_L20_R2_IgM
F47:MRM of 3 channels,ES+
976.3 > 366.1
1.035e+006

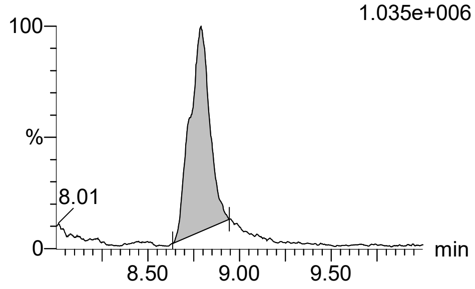

Sample Name: 20211111_L20_R2_IgM
F47:MRM of 3 channels,ES+
1220.1 > 366.1
2.523e+006

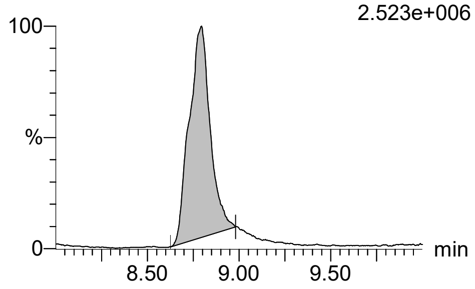

Sample Name: 20211111_H10_R2_IgM
F47:MRM of 3 channels,ES+
976.3 > 204.1
4.926e+006

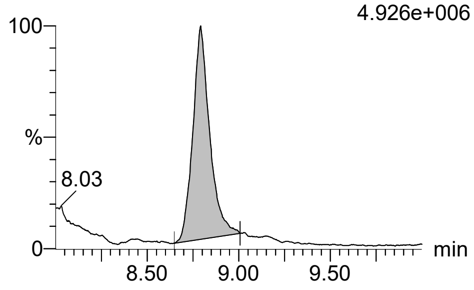

Sample Name: 20211111_H10_R2_IgM
F47:MRM of 3 channels,ES+
976.3 > 366.1
1.510e+006

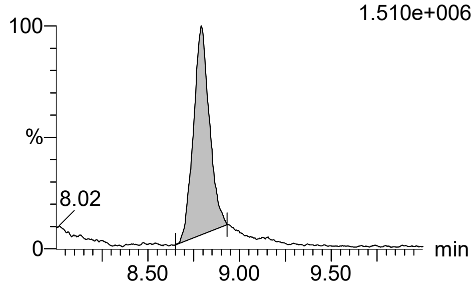

Sample Name: 20211111_H10_R2_IgM
F47:MRM of 3 channels,ES+
1220.1 > 366.1
3.516e+006

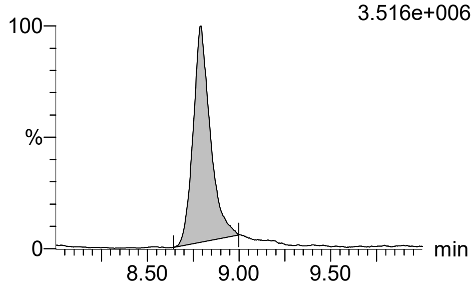

Dataset: C:\MassLynx\10.pro\20211111_clinicalsamples_IgM_IgA.qld

Last Altered: Thursday, March 09, 2023 19:58:25 台北標準時間
Printed: Friday, August 01, 2025 15:08:19 台北標準時間

Sample Name: 20211111_A21_R2_IgM
F47:MRM of 3 channels,ES+
976.3 > 204.1
2.010e+007

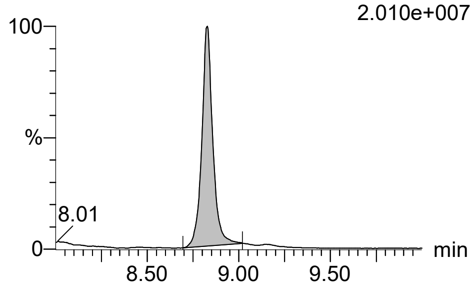

Sample Name: 20211111_A21_R2_IgM
F47:MRM of 3 channels,ES+
976.3 > 366.1
7.104e+006

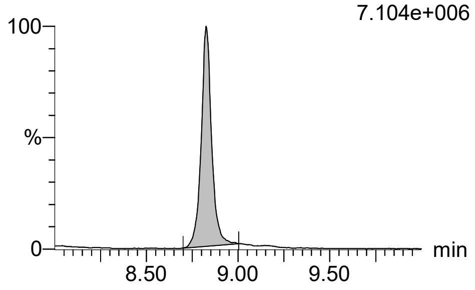

Sample Name: 20211111_A21_R2_IgM
F47:MRM of 3 channels,ES+
1220.1 > 366.1
1.455e+007

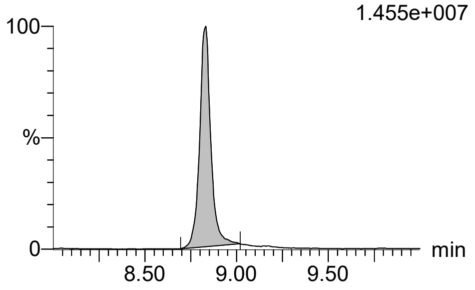

Sample Name: 20211111_A22_R2_IgM
F47:MRM of 3 channels,ES+
976.3 > 204.1
5.613e+006

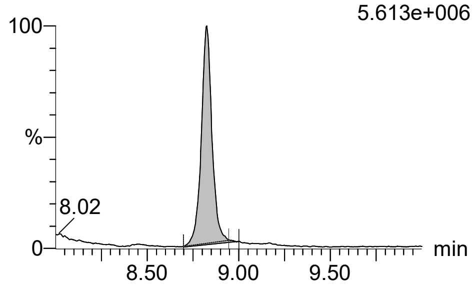

Sample Name: 20211111_A22_R2_IgM
F47:MRM of 3 channels,ES+
976.3 > 366.1
1.835e+006

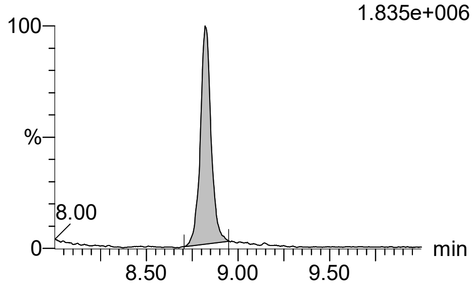

Sample Name: 20211111_A22_R2_IgM
F47:MRM of 3 channels,ES+
1220.1 > 366.1
4.087e+006

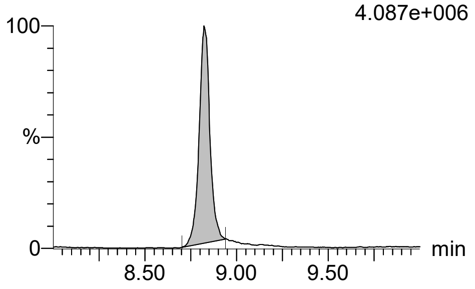

Sample Name: 20211111_L21_R2_IgM
F47:MRM of 3 channels,ES+
976.3 > 204.1
1.342e+007

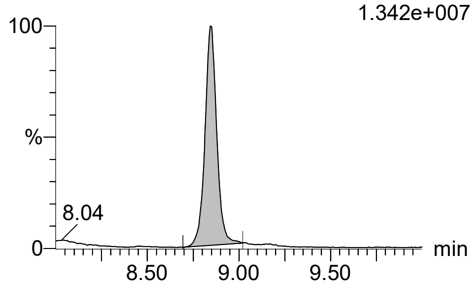

Sample Name: 20211111_L21_R2_IgM
F47:MRM of 3 channels,ES+
976.3 > 366.1
4.722e+006

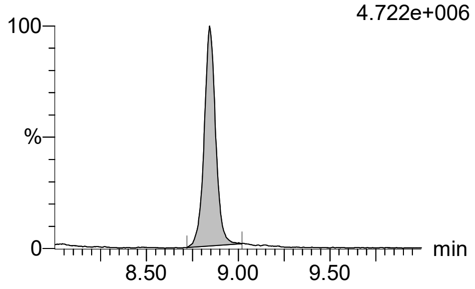

Sample Name: 20211111_L21_R2_IgM
F47:MRM of 3 channels,ES+
1220.1 > 366.1
9.231e+006

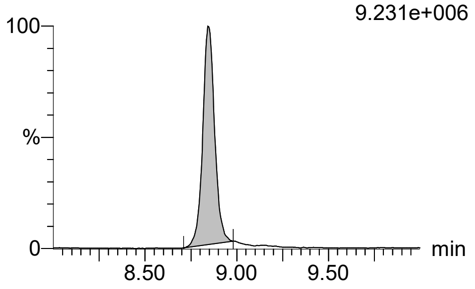

Sample Name: 20211111_L22_R2_IgM
F47:MRM of 3 channels,ES+
976.3 > 204.1
2.293e+007

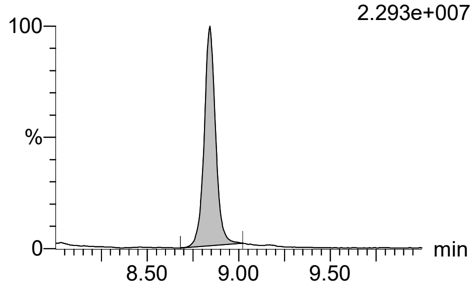

Sample Name: 20211111_L22_R2_IgM
F47:MRM of 3 channels,ES+
976.3 > 366.1
7.910e+006

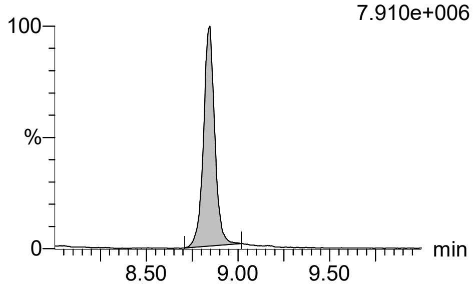

Sample Name: 20211111_L22_R2_IgM
F47:MRM of 3 channels,ES+
1220.1 > 366.1
1.559e+007

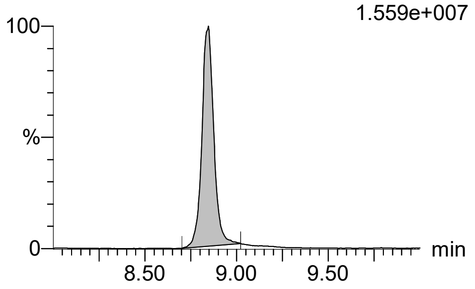

Sample Name: 20211111_H11_R2_IgM
F47:MRM of 3 channels,ES+
976.3 > 204.1
1.819e+007

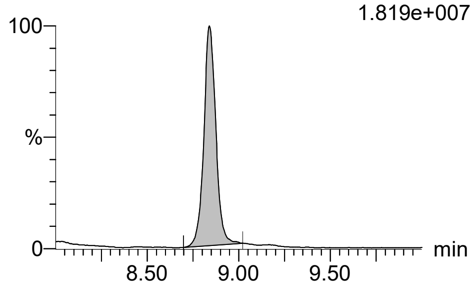

Sample Name: 20211111_H11_R2_IgM
F47:MRM of 3 channels,ES+
976.3 > 366.1
6.371e+006

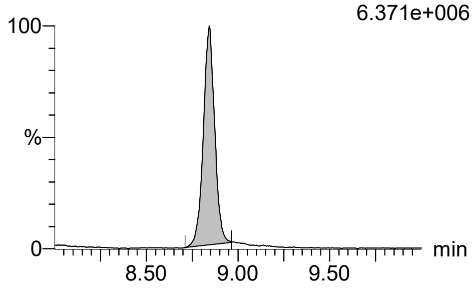

Sample Name: 20211111_H11_R2_IgM
F47:MRM of 3 channels,ES+
1220.1 > 366.1
1.327e+007

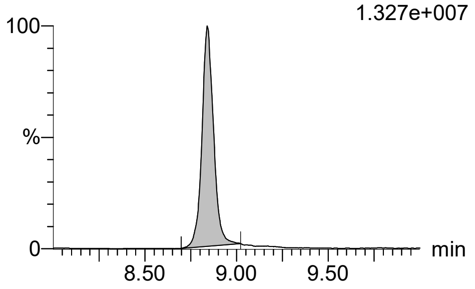

Sample Name: 20211111_A23_R2_IgM
F47:MRM of 3 channels,ES+
976.3 > 204.1
7.987e+006

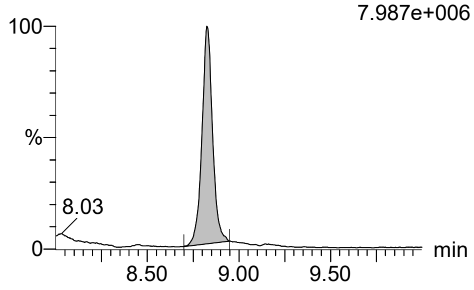

Sample Name: 20211111_A23_R2_IgM
F47:MRM of 3 channels,ES+
976.3 > 366.1
2.751e+006

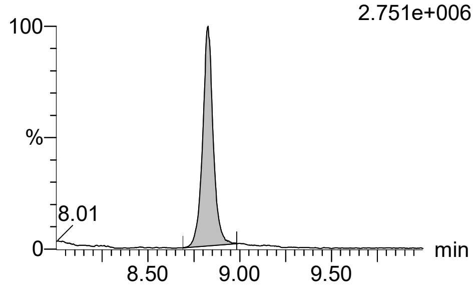

Sample Name: 20211111_A23_R2_IgM
F47:MRM of 3 channels,ES+
1220.1 > 366.1
5.712e+006

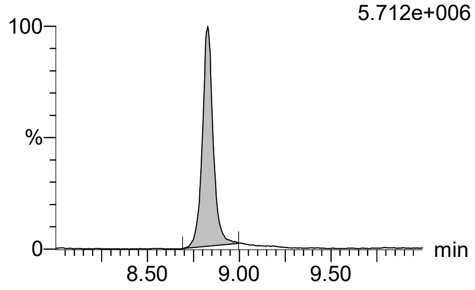

Sample Name: 20211111_A24_R2_IgM
F47:MRM of 3 channels,ES+
976.3 > 204.1
2.709e+007

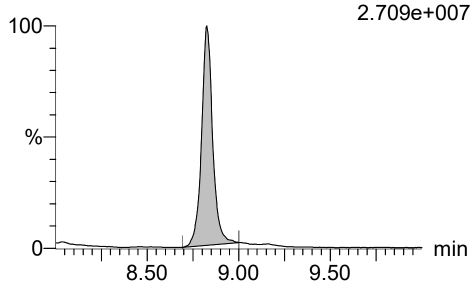

Sample Name: 20211111_A24_R2_IgM
F47:MRM of 3 channels,ES+
976.3 > 366.1
9.266e+006

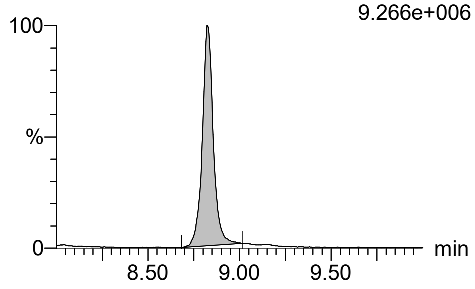

Sample Name: 20211111_A24_R2_IgM
F47:MRM of 3 channels,ES+
1220.1 > 366.1
1.862e+007

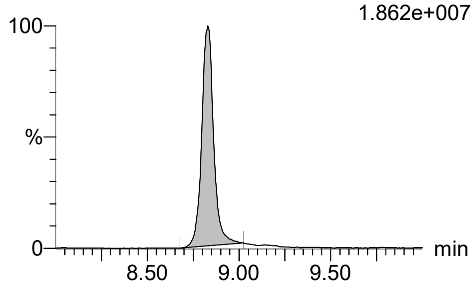

Sample Name: 20211111_L23_R2_IgM
F47:MRM of 3 channels,ES+
976.3 > 204.1
3.763e+007

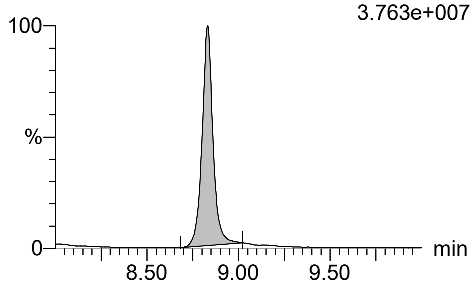

Sample Name: 20211111_L23_R2_IgM
F47:MRM of 3 channels,ES+
976.3 > 366.1
1.286e+007

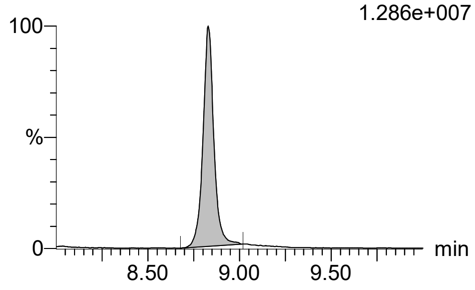

Sample Name: 20211111_L23_R2_IgM
F47:MRM of 3 channels,ES+
1220.1 > 366.1
2.786e+007

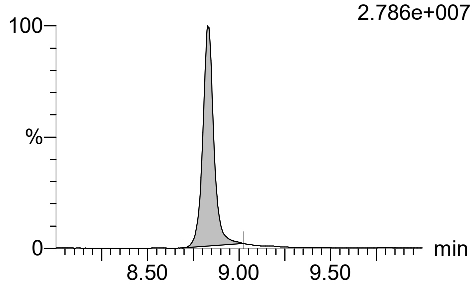

Sample Name: 20211111_L24_R2_IgM
F47:MRM of 3 channels,ES+
976.3 > 204.1
4.542e+006

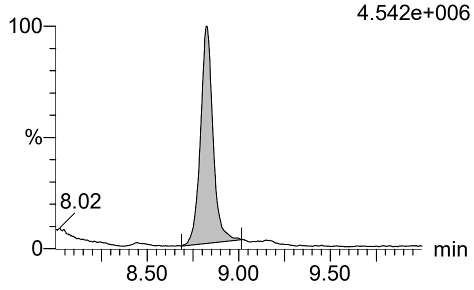

Sample Name: 20211111_L24_R2_IgM
F47:MRM of 3 channels,ES+
976.3 > 366.1
1.506e+006

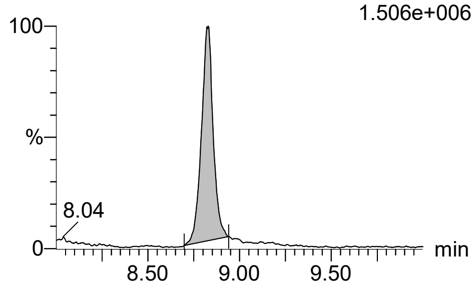

Sample Name: 20211111_L24_R2_IgM
F47:MRM of 3 channels,ES+
1220.1 > 366.1
3.544e+006

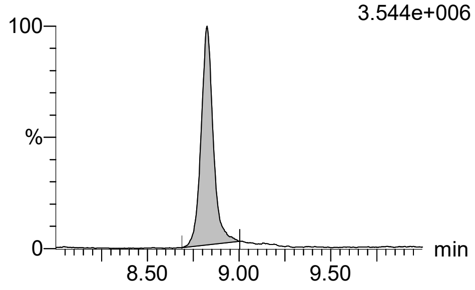

Sample Name: 20211111_H12_R2_IgM
F47:MRM of 3 channels,ES+
976.3 > 204.1
1.174e+007

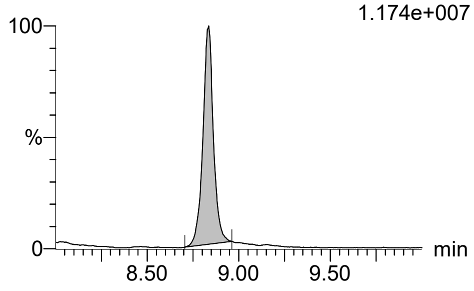

Sample Name: 20211111_H12_R2_IgM
F47:MRM of 3 channels,ES+
976.3 > 366.1
4.010e+006

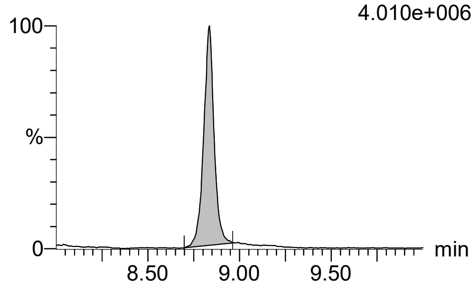

Sample Name: 20211111_H12_R2_IgM
F47:MRM of 3 channels,ES+
1220.1 > 366.1
8.237e+006

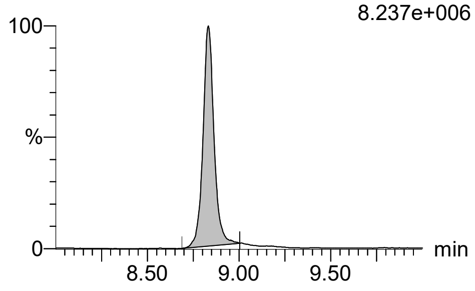

Sample Name: 20211111_QC_R14_IgM
F47:MRM of 3 channels,ES+
976.3 > 204.1
2.423e+007

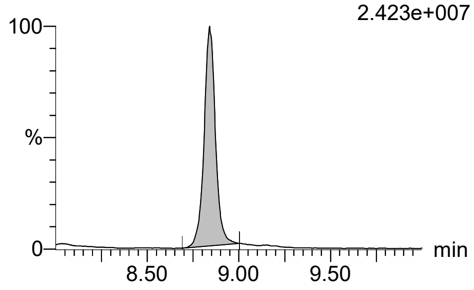

Sample Name: 20211111_QC_R14_IgM
F47:MRM of 3 channels,ES+
976.3 > 366.1
8.122e+006

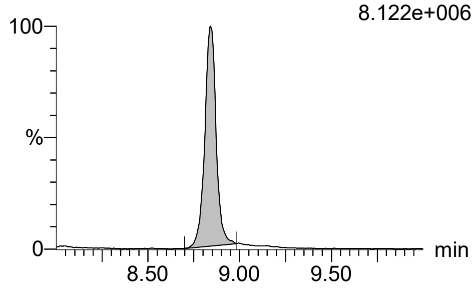

Sample Name: 20211111_QC_R14_IgM
F47:MRM of 3 channels,ES+
1220.1 > 366.1
1.760e+007

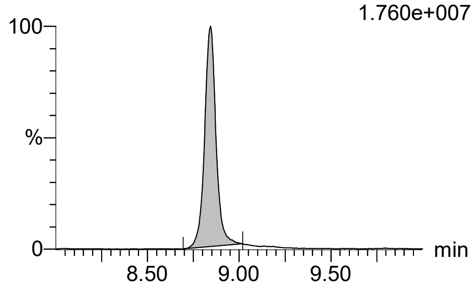

Sample Name: 20211111_A25_R2_IgM
F47:MRM of 3 channels,ES+
976.3 > 204.1
4.764e+007

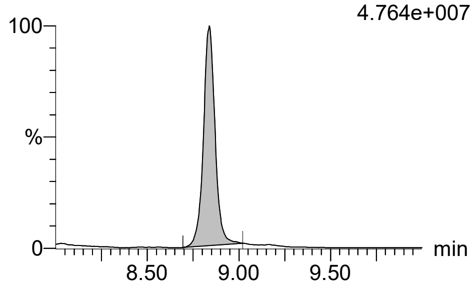

Sample Name: 20211111_A25_R2_IgM
F47:MRM of 3 channels,ES+
976.3 > 366.1
1.712e+007

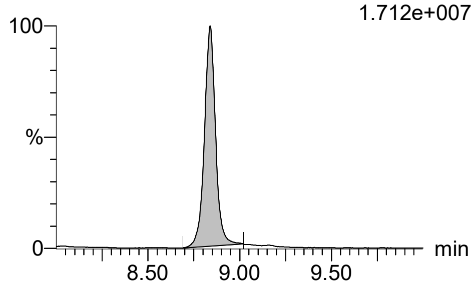

Sample Name: 20211111_A25_R2_IgM
F47:MRM of 3 channels,ES+
1220.1 > 366.1
3.368e+007

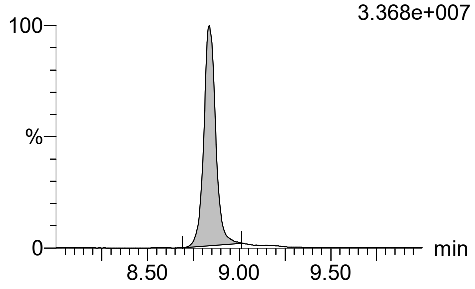

Sample Name: 20211111_A26_R2_IgM
F47:MRM of 3 channels,ES+
976.3 > 204.1
1.942e+007

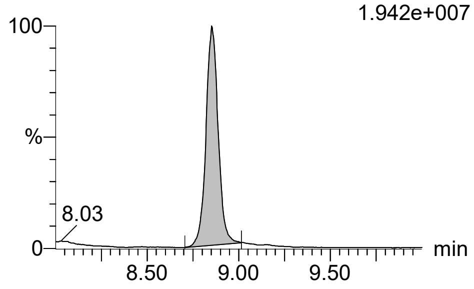

Sample Name: 20211111_A26_R2_IgM
F47:MRM of 3 channels,ES+
976.3 > 366.1
6.450e+006

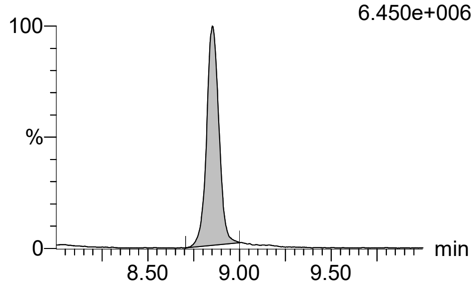

Sample Name: 20211111_A26_R2_IgM
F47:MRM of 3 channels,ES+
1220.1 > 366.1
1.351e+007

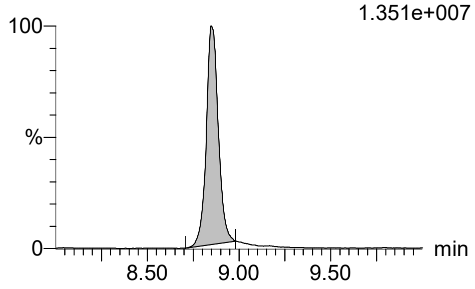

Sample Name: 20211111_L25_R2_IgM
F47:MRM of 3 channels,ES+
976.3 > 204.1
1.818e+007

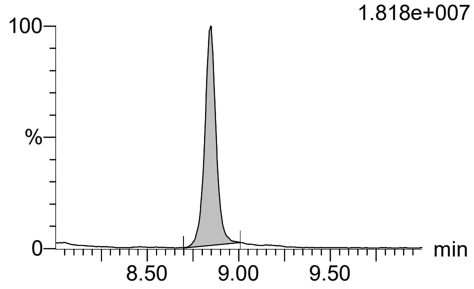

Sample Name: 20211111_L25_R2_IgM
F47:MRM of 3 channels,ES+
976.3 > 366.1
6.121e+006

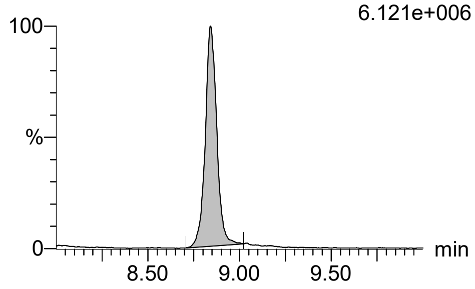

Sample Name: 20211111_L25_R2_IgM
F47:MRM of 3 channels,ES+
1220.1 > 366.1
1.278e+007

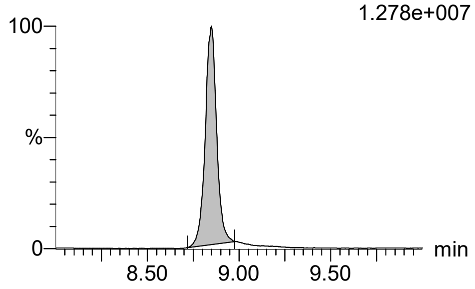

Sample Name: 20211111_L26_R2_IgM
F47:MRM of 3 channels,ES+
976.3 > 204.1
1.351e+007

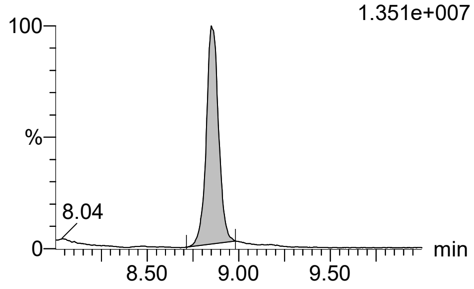

Sample Name: 20211111_L26_R2_IgM
F47:MRM of 3 channels,ES+
976.3 > 366.1
4.804e+006

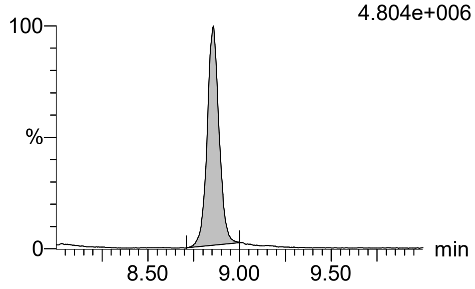

Sample Name: 20211111_L26_R2_IgM
F47:MRM of 3 channels,ES+
1220.1 > 366.1
9.700e+006

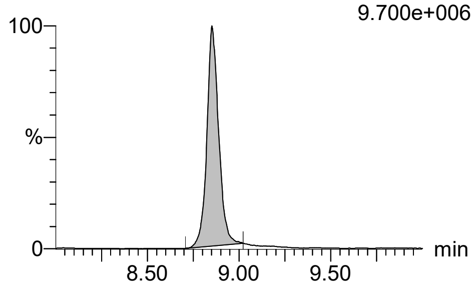

Sample Name: 20211111_H13_R2_IgM
F47:MRM of 3 channels,ES+
976.3 > 204.1
2.608e+007

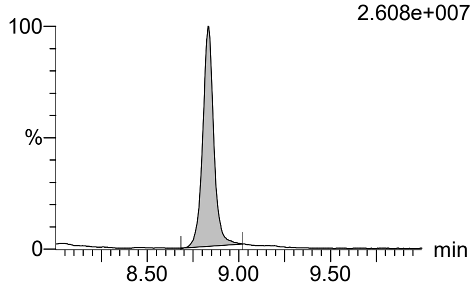

Sample Name: 20211111_H13_R2_IgM
F47:MRM of 3 channels,ES+
976.3 > 366.1
8.904e+006

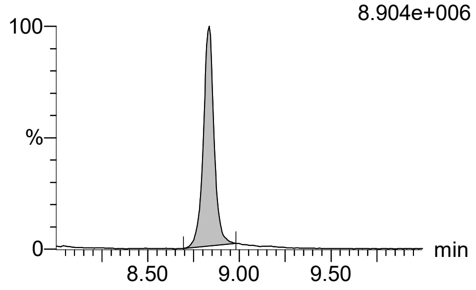

Sample Name: 20211111_H13_R2_IgM
F47:MRM of 3 channels,ES+
1220.1 > 366.1
1.887e+007

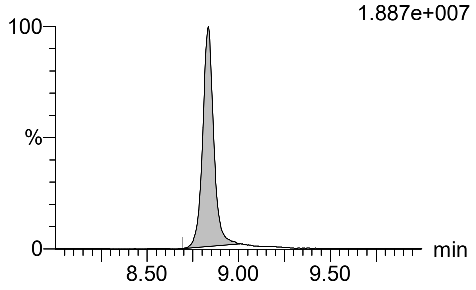

Sample Name: 20211111_A27_R2_IgM
F47:MRM of 3 channels,ES+
976.3 > 204.1
2.016e+007

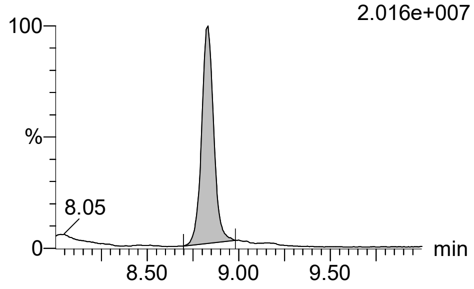

Sample Name: 20211111_A27_R2_IgM
F47:MRM of 3 channels,ES+
976.3 > 366.1
6.967e+006

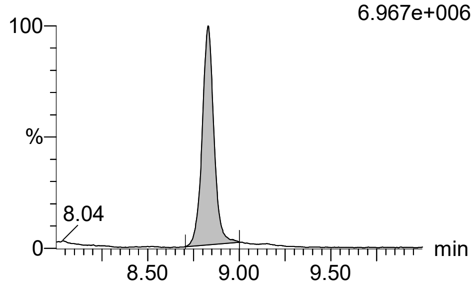

Sample Name: 20211111_A27_R2_IgM
F47:MRM of 3 channels,ES+
1220.1 > 366.1
1.593e+007

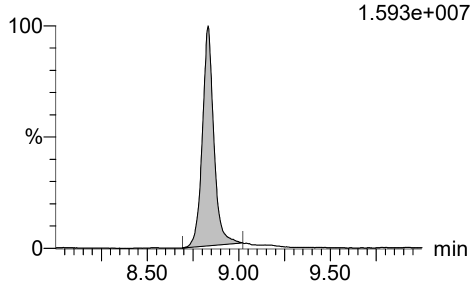

Sample Name: 20211111_A28_R2_IgM
F47:MRM of 3 channels,ES+
976.3 > 204.1
3.599e+006

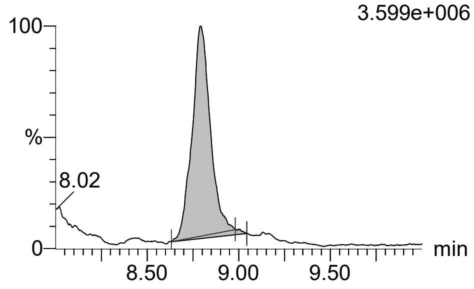

Sample Name: 20211111_A28_R2_IgM
F47:MRM of 3 channels,ES+
976.3 > 366.1
1.133e+006

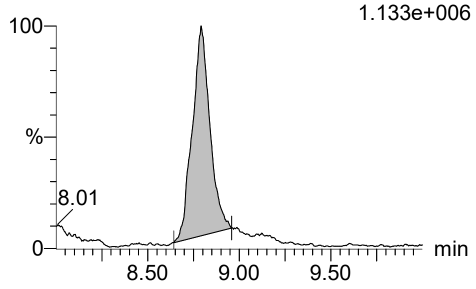

Sample Name: 20211111_A28_R2_IgM
F47:MRM of 3 channels,ES+
1220.1 > 366.1
2.533e+006

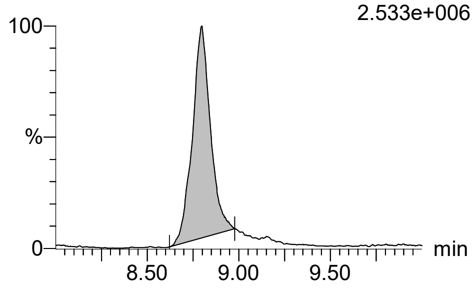

Sample Name: 20211111_L27_R2_IgM
F47:MRM of 3 channels,ES+
976.3 > 204.1
1.943e+007

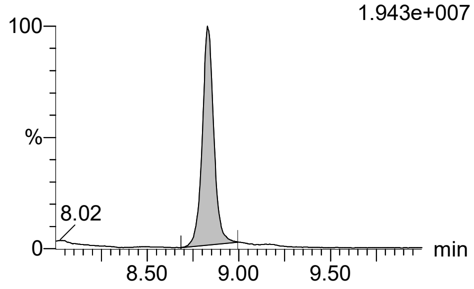

Sample Name: 20211111_L27_R2_IgM
F47:MRM of 3 channels,ES+
976.3 > 366.1
6.562e+006

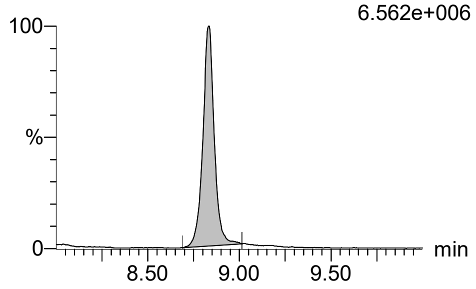

Sample Name: 20211111_L27_R2_IgM
F47:MRM of 3 channels,ES+
1220.1 > 366.1
1.431e+007

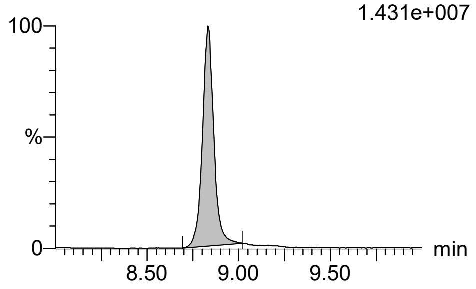

Sample Name: 20211111_L28_R2_IgM
F47:MRM of 3 channels,ES+
976.3 > 204.1
2.091e+007

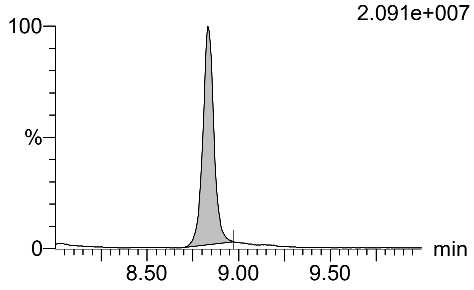

Sample Name: 20211111_L28_R2_IgM
F47:MRM of 3 channels,ES+
976.3 > 366.1
7.035e+006

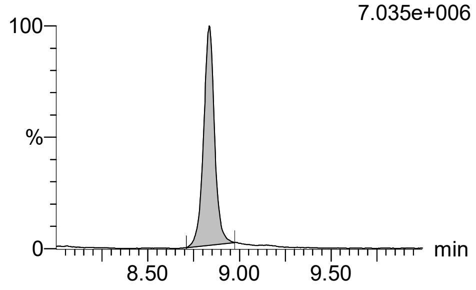

Sample Name: 20211111_L28_R2_IgM
F47:MRM of 3 channels,ES+
1220.1 > 366.1
1.459e+007

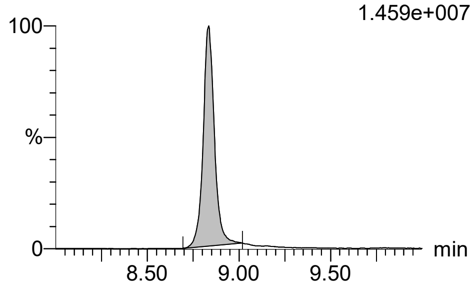

Dataset: C:\MassLynx\10.pro\20211111_clinicalsamples_IgM_IgA.qld

Last Altered: Thursday, March 09, 2023 19:58:25 台北標準時間
Printed: Friday, August 01, 2025 15:08:19 台北標準時間

Sample Name: 20211111_H14_R2_IgM
F47:MRM of 3 channels,ES+
976.3 > 204.1
2.815e+007

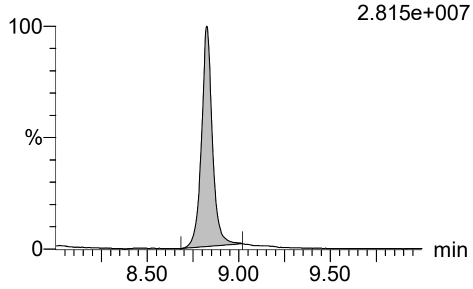

Sample Name: 20211111_H14_R2_IgM
F47:MRM of 3 channels,ES+
976.3 > 366.1
9.353e+006

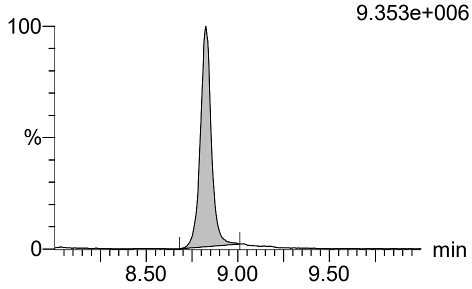

Sample Name: 20211111_H14_R2_IgM
F47:MRM of 3 channels,ES+
1220.1 > 366.1
1.969e+007

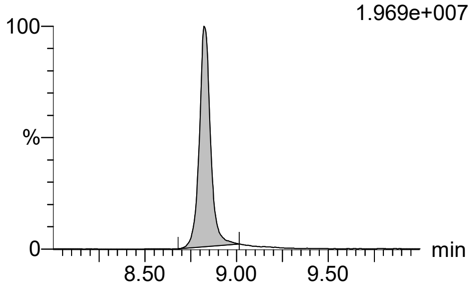

Sample Name: 20211111_A29_R2_IgM
F47:MRM of 3 channels,ES+
976.3 > 204.1
1.778e+006

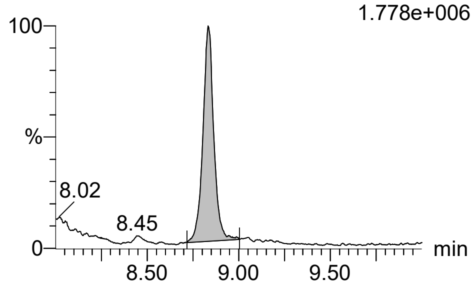

Sample Name: 20211111_A29_R2_IgM
F47:MRM of 3 channels,ES+
976.3 > 366.1
5.375e+005

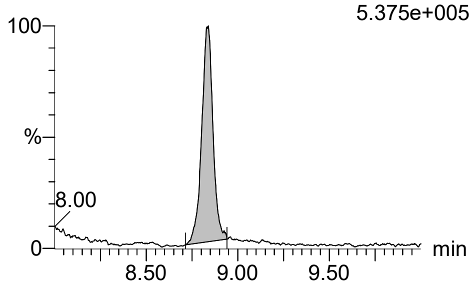

Sample Name: 20211111_A29_R2_IgM
F47:MRM of 3 channels,ES+
1220.1 > 366.1
1.232e+006

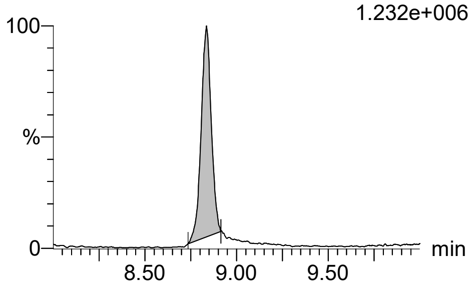

Sample Name: 20211111_A30_R2_IgM
F47:MRM of 3 channels,ES+
976.3 > 204.1
2.111e+007

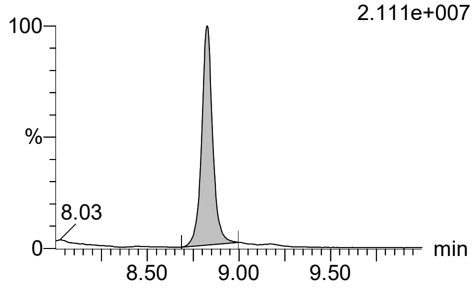

Sample Name: 20211111_A30_R2_IgM
F47:MRM of 3 channels,ES+
976.3 > 366.1
7.097e+006

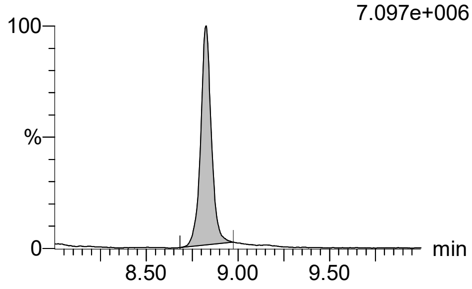

Sample Name: 20211111_A30_R2_IgM
F47:MRM of 3 channels,ES+
1220.1 > 366.1
1.496e+007

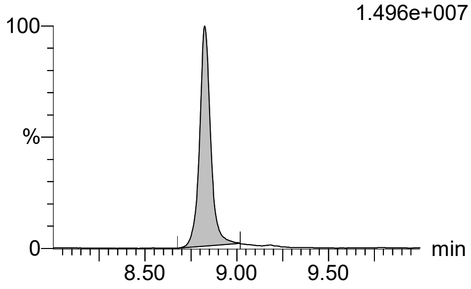

Sample Name: 20211111_QC_R15_IgM
F47:MRM of 3 channels,ES+
976.3 > 204.1
2.224e+007

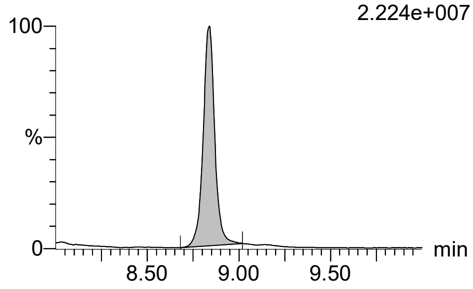

Sample Name: 20211111_QC_R15_IgM
F47:MRM of 3 channels,ES+
976.3 > 366.1
7.595e+006

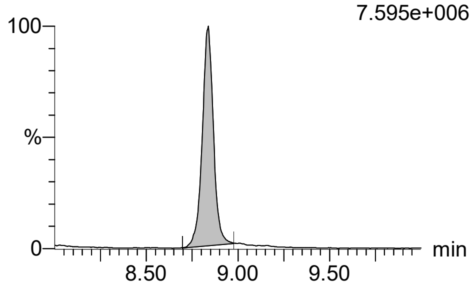

Sample Name: 20211111_QC_R15_IgM
F47:MRM of 3 channels,ES+
1220.1 > 366.1
1.499e+007

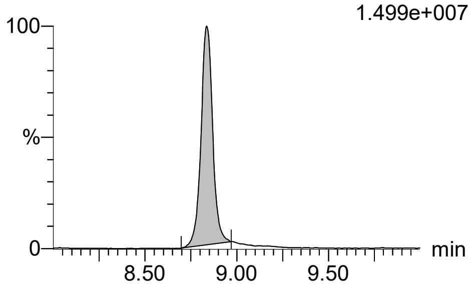

Sample Name: 20211111_L29_R2_IgM
F47:MRM of 3 channels,ES+
976.3 > 204.1
7.347e+006

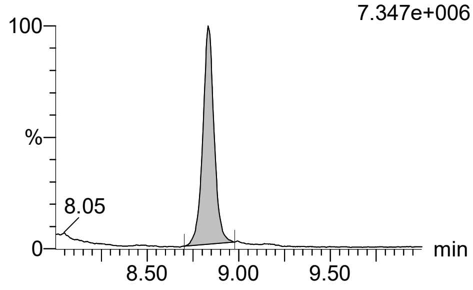

Sample Name: 20211111_L29_R2_IgM
F47:MRM of 3 channels,ES+
976.3 > 366.1
2.404e+006

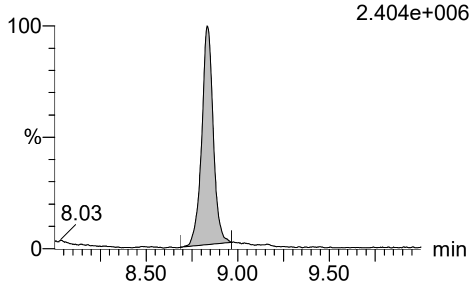

Sample Name: 20211111_L29_R2_IgM
F47:MRM of 3 channels,ES+
1220.1 > 366.1
4.959e+006

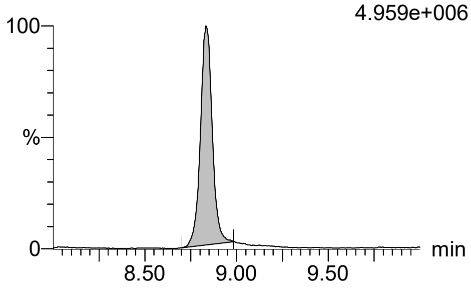

Sample Name: 20211111_L30_R2_IgM
F47:MRM of 3 channels,ES+
976.3 > 204.1
3.252e+006

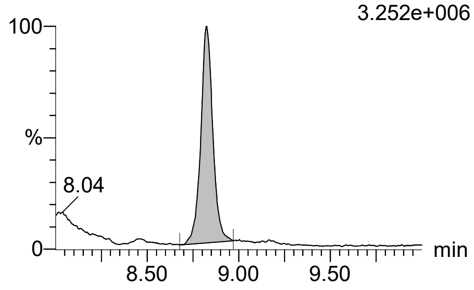

Sample Name: 20211111_L30_R2_IgM
F47:MRM of 3 channels,ES+
976.3 > 366.1
1.053e+006

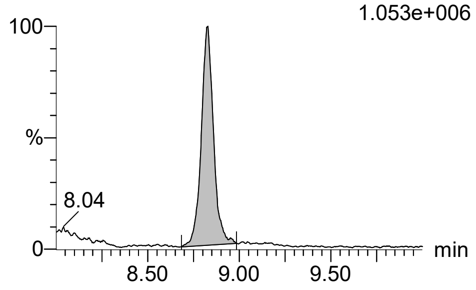

Sample Name: 20211111_L30_R2_IgM
F47:MRM of 3 channels,ES+
1220.1 > 366.1
2.355e+006

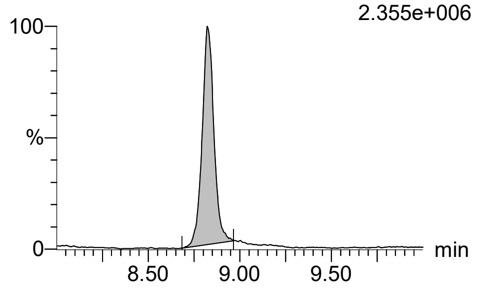

Sample Name: 20211111_H15_R2_IgM
F47:MRM of 3 channels,ES+
976.3 > 204.1
1.558e+007

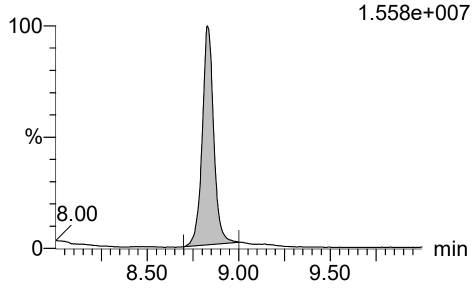

Sample Name: 20211111_H15_R2_IgM
F47:MRM of 3 channels,ES+
976.3 > 366.1
5.255e+006

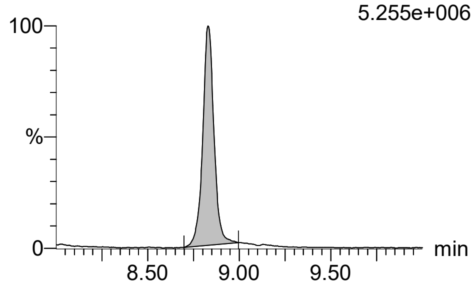

Sample Name: 20211111_H15_R2_IgM
F47:MRM of 3 channels,ES+
1220.1 > 366.1
1.111e+007

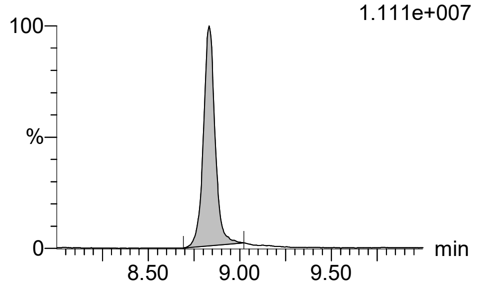

Sample Name: 20211111_A31_R2_IgM
F47:MRM of 3 channels,ES+
976.3 > 204.1
7.987e+006

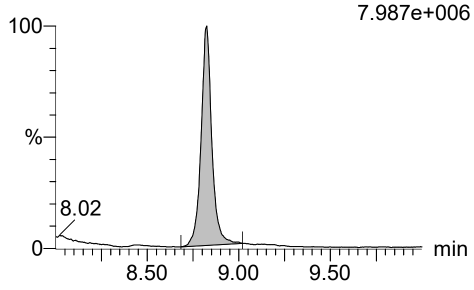

Sample Name: 20211111_A31_R2_IgM
F47:MRM of 3 channels,ES+
976.3 > 366.1
2.667e+006

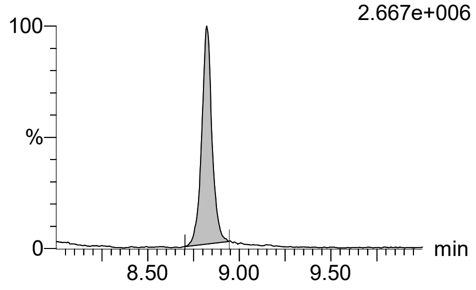

Sample Name: 20211111_A31_R2_IgM
F47:MRM of 3 channels,ES+
1220.1 > 366.1
5.640e+006

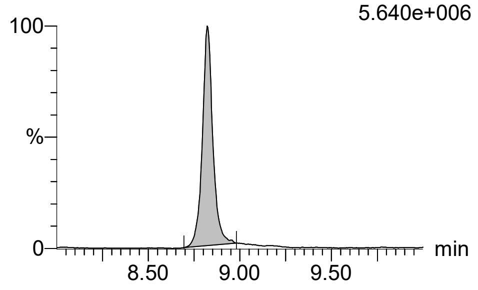

Sample Name: 20211111_A32_R2_IgM
F47:MRM of 3 channels,ES+
976.3 > 204.1
2.386e+007

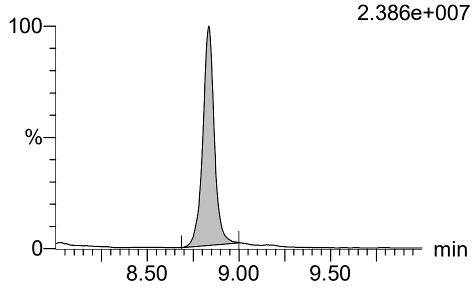

Sample Name: 20211111_A32_R2_IgM
F47:MRM of 3 channels,ES+
976.3 > 366.1
8.031e+006

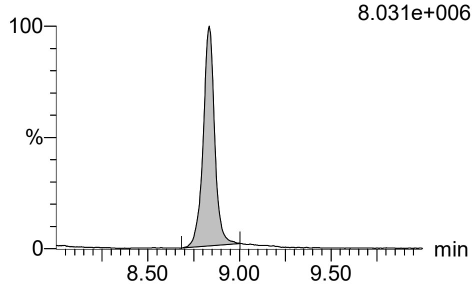

Sample Name: 20211111_A32_R2_IgM
F47:MRM of 3 channels,ES+
1220.1 > 366.1
1.752e+007

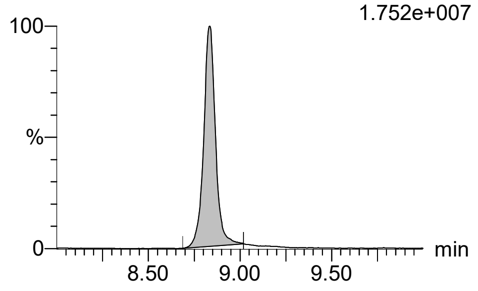

Sample Name: 20211111_L31_R2_IgM
F47:MRM of 3 channels,ES+
976.3 > 204.1
1.132e+006

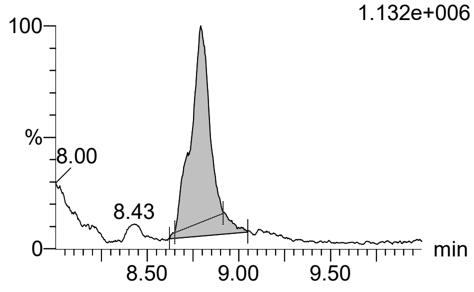

Sample Name: 20211111_L31_R2_IgM
F47:MRM of 3 channels,ES+
976.3 > 366.1
3.368e+005

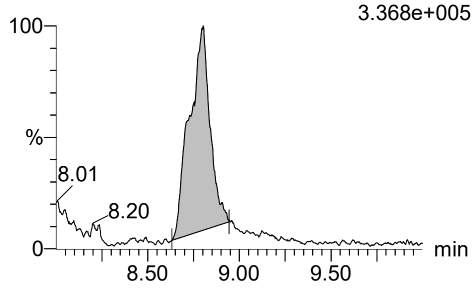

Sample Name: 20211111_L31_R2_IgM
F47:MRM of 3 channels,ES+
1220.1 > 366.1
7.909e+005

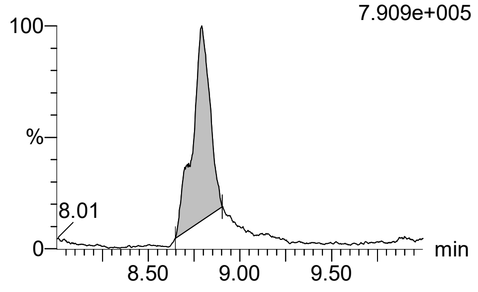

Dataset: C:\MassLynx\10.pro\20211111_clinicalsamples_IgM_IgA.qld

Last Altered: Thursday, March 09, 2023 19:58:25 台北標準時間
Printed: Friday, August 01, 2025 15:08:19 台北標準時間

Sample Name: 20211111_L32_R2_IgM
F47:MRM of 3 channels,ES+
976.3 > 204.1
2.807e+007

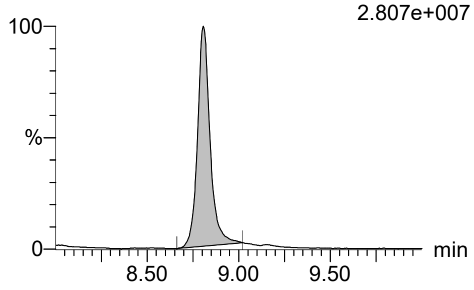

Sample Name: 20211111_L32_R2_IgM
F47:MRM of 3 channels,ES+
976.3 > 366.1
9.271e+006

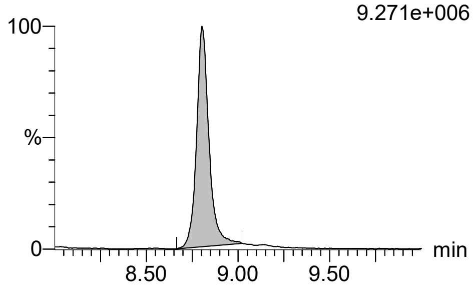

Sample Name: 20211111_L32_R2_IgM
F47:MRM of 3 channels,ES+
1220.1 > 366.1
2.180e+007

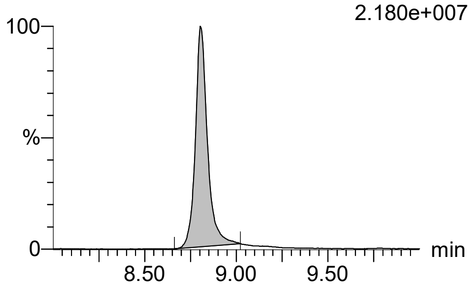

Sample Name: 20211111_H16_R2_IgM
F47:MRM of 3 channels,ES+
976.3 > 204.1
4.479e+007

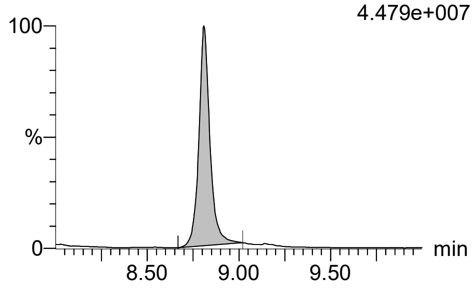

Sample Name: 20211111_H16_R2_IgM
F47:MRM of 3 channels,ES+
976.3 > 366.1
1.499e+007

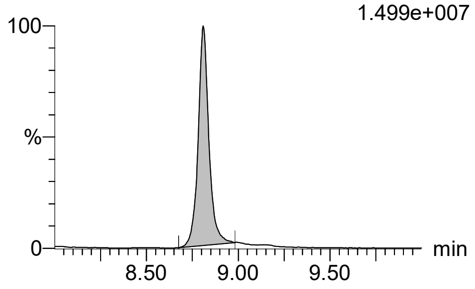

Sample Name: 20211111_H16_R2_IgM
F47:MRM of 3 channels,ES+
1220.1 > 366.1
3.376e+007

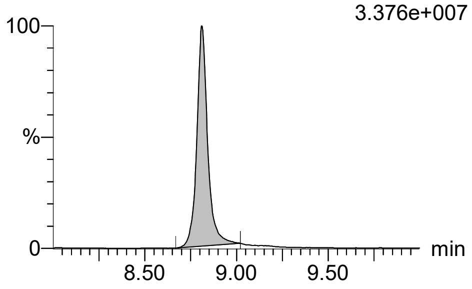

Sample Name: 20211111_A33_R2_IgM
F47:MRM of 3 channels,ES+
976.3 > 204.1
2.045e+007

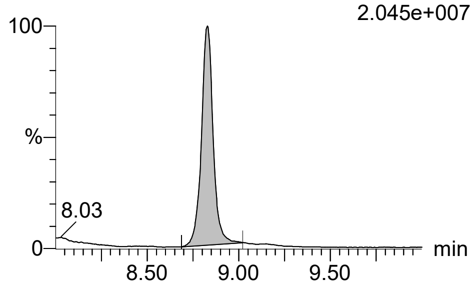

Sample Name: 20211111_A33_R2_IgM
F47:MRM of 3 channels,ES+
976.3 > 366.1
7.031e+006

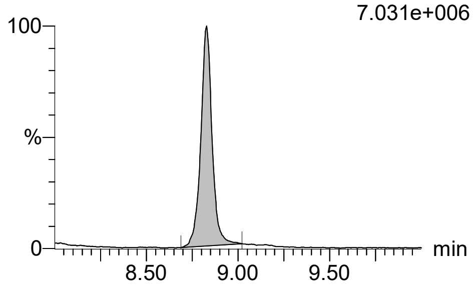

Sample Name: 20211111_A33_R2_IgM
F47:MRM of 3 channels,ES+
1220.1 > 366.1
1.508e+007

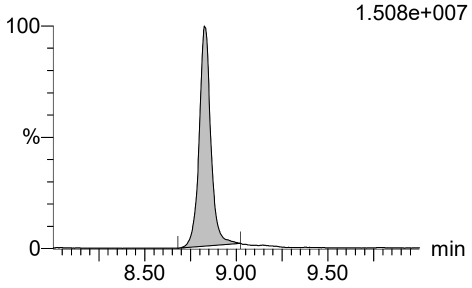

Sample Name: 20211111_A34_R2_IgM
F47:MRM of 3 channels,ES+
976.3 > 204.1
7.125e+006

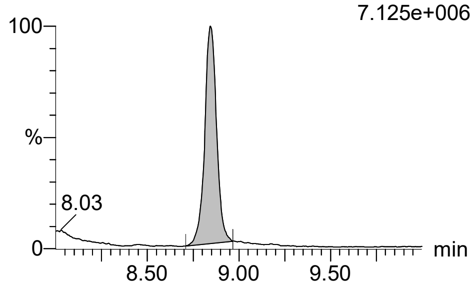

Sample Name: 20211111_A34_R2_IgM
F47:MRM of 3 channels,ES+
976.3 > 366.1
2.440e+006

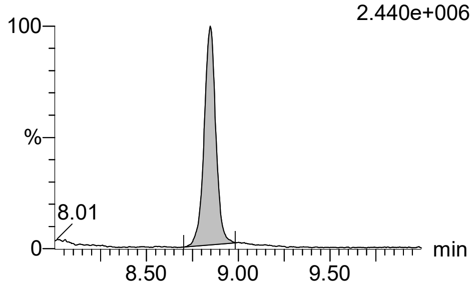

Sample Name: 20211111_A34_R2_IgM
F47:MRM of 3 channels,ES+
1220.1 > 366.1
4.654e+006

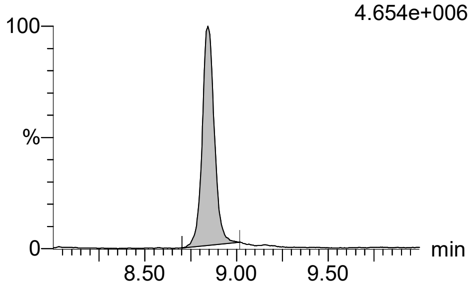

Sample Name: 20211111_H17_R2_IgM
F47:MRM of 3 channels,ES+
976.3 > 204.1
1.110e+008

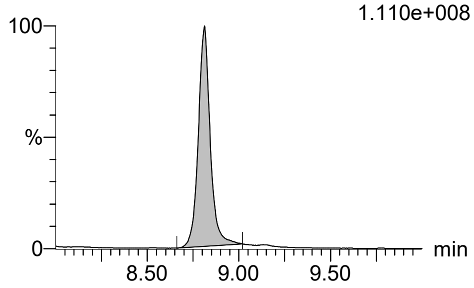

Sample Name: 20211111_H17_R2_IgM
F47:MRM of 3 channels,ES+
976.3 > 366.1
3.533e+007

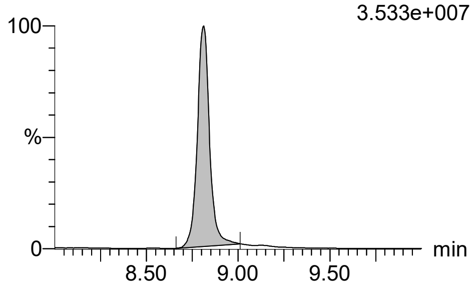

Sample Name: 20211111_H17_R2_IgM
F47:MRM of 3 channels,ES+
1220.1 > 366.1
7.981e+007

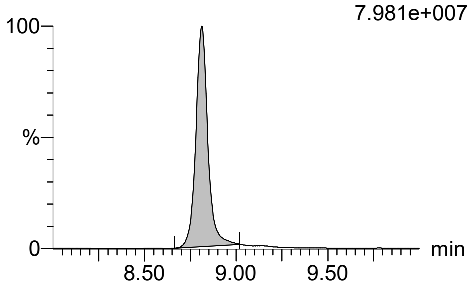

Dataset: C:\MassLynx\10.pro\20211111_clinicalsamples_IgM_IgA.qld

Last Altered: Thursday, March 09, 2023 19:58:25 台北標準時間
Printed: Friday, August 01, 2025 15:08:19 台北標準時間

Sample Name: 20211111_A35_R2_IgM
F47:MRM of 3 channels,ES+
976.3 > 204.1
2.980e+006

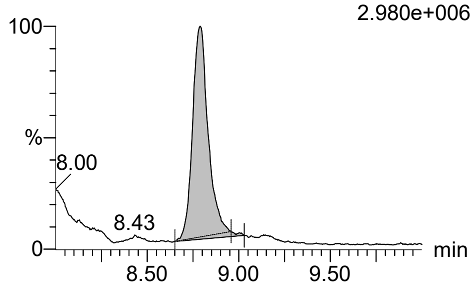

Sample Name: 20211111_A35_R2_IgM
F47:MRM of 3 channels,ES+
976.3 > 366.1
9.251e+005

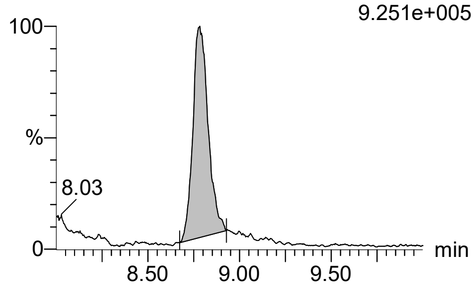

Sample Name: 20211111_A35_R2_IgM
F47:MRM of 3 channels,ES+
1220.1 > 366.1
2.006e+006

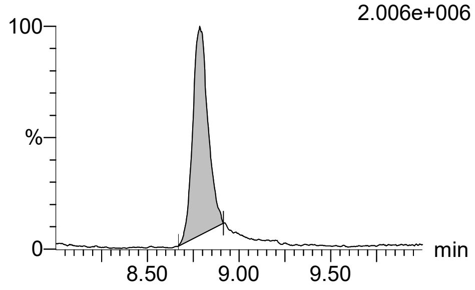

Sample Name: 20211111_QC_R16_IgM
F47:MRM of 3 channels,ES+
976.3 > 204.1
2.038e+007

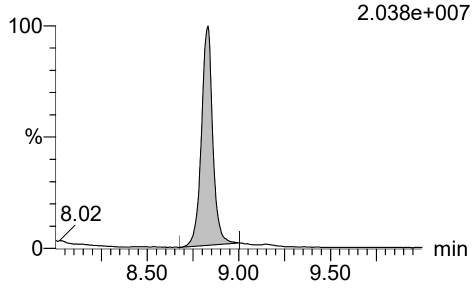

Sample Name: 20211111_QC_R16_IgM
F47:MRM of 3 channels,ES+
976.3 > 366.1
7.093e+006

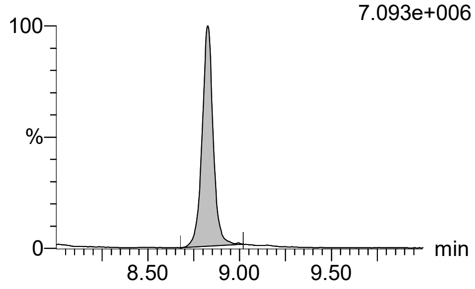

Sample Name: 20211111_QC_R16_IgM
F47:MRM of 3 channels,ES+
1220.1 > 366.1
1.417e+007

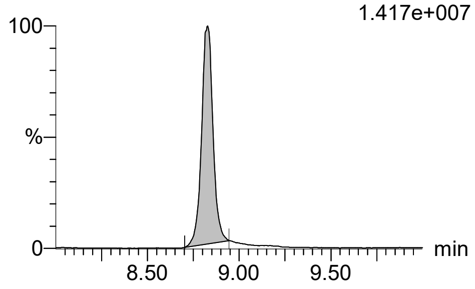

Sample Name: 20211111_A36_R2_IgM
F47:MRM of 3 channels,ES+
976.3 > 204.1
2.475e+006

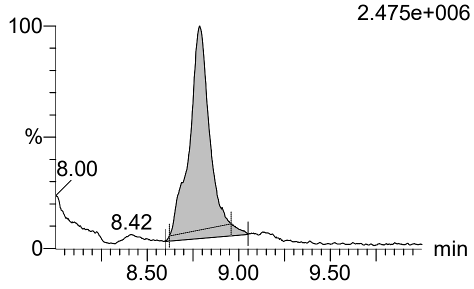

Sample Name: 20211111_A36_R2_IgM
F47:MRM of 3 channels,ES+
976.3 > 366.1
7.172e+005

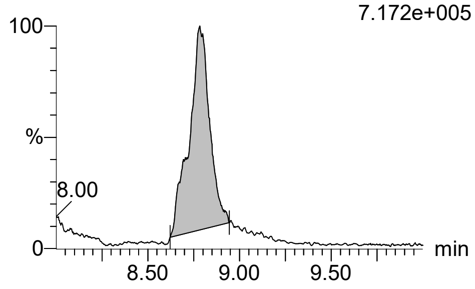

Sample Name: 20211111_A36_R2_IgM
F47:MRM of 3 channels,ES+
1220.1 > 366.1
1.675e+006

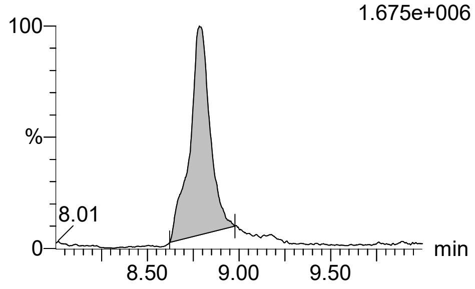

Sample Name: 20211111_H18_R2_IgM
F47:MRM of 3 channels,ES+
976.3 > 204.1
5.683e+007

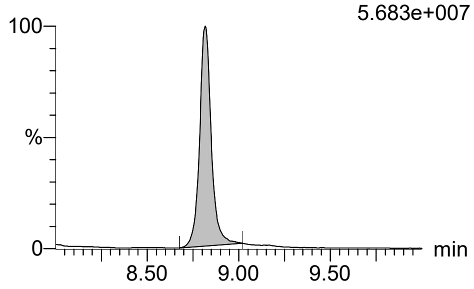

Sample Name: 20211111_H18_R2_IgM
F47:MRM of 3 channels,ES+
976.3 > 366.1
2.010e+007

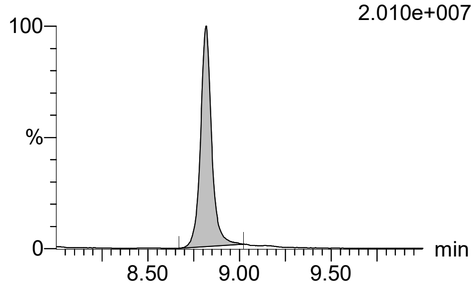

Sample Name: 20211111_H18_R2_IgM
F47:MRM of 3 channels,ES+
1220.1 > 366.1
4.447e+007

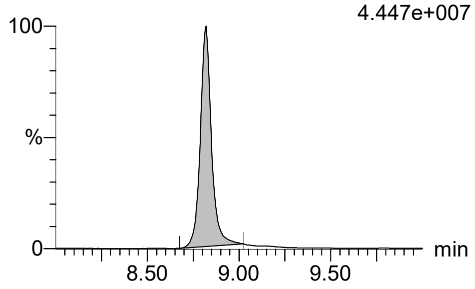

Sample Name: 20211111_A37_R2_IgM
F47:MRM of 3 channels,ES+
976.3 > 204.1
2.026e+007

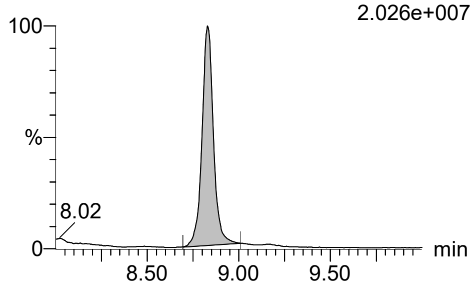

Sample Name: 20211111_A37_R2_IgM
F47:MRM of 3 channels,ES+
976.3 > 366.1
6.685e+006

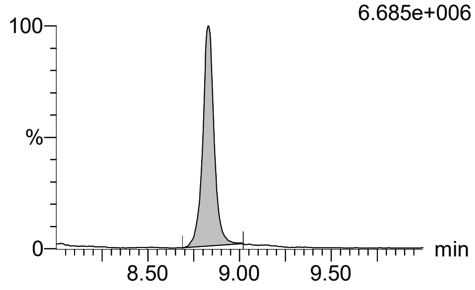

Sample Name: 20211111_A37_R2_IgM
F47:MRM of 3 channels,ES+
1220.1 > 366.1
1.525e+007

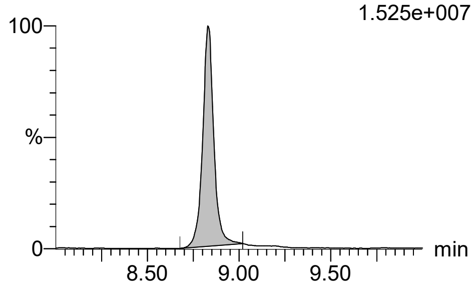

Dataset: C:\MassLynx\10.pro\20211111_clinicalsamples_IgM_IgA.qld

Last Altered: Thursday, March 09, 2023 19:58:25 台北標準時間
Printed: Friday, August 01, 2025 15:08:19 台北標準時間

Sample Name: 20211111_A38_R2_IgM
F47:MRM of 3 channels,ES+
976.3 > 204.1
2.318e+007

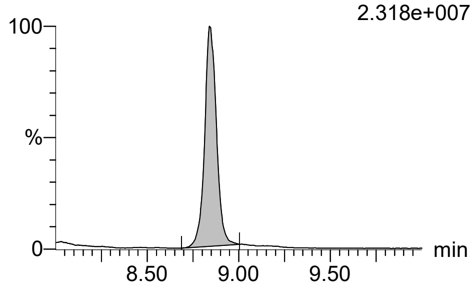

Sample Name: 20211111_A38_R2_IgM
F47:MRM of 3 channels,ES+
976.3 > 366.1
7.853e+006

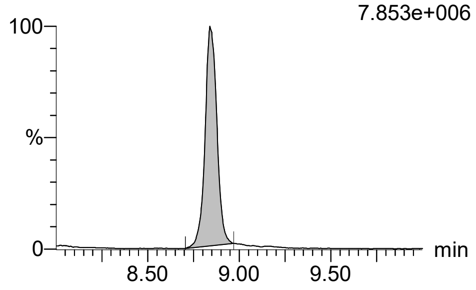

Sample Name: 20211111_A38_R2_IgM
F47:MRM of 3 channels,ES+
1220.1 > 366.1
1.586e+007

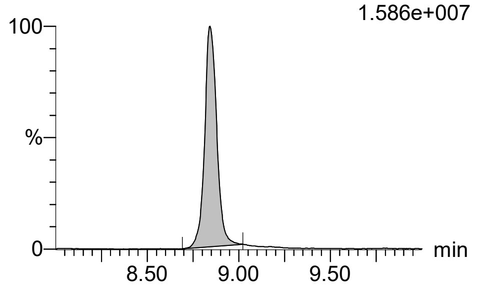

Sample Name: 20211111_H19_R2_IgM
F47:MRM of 3 channels,ES+
976.3 > 204.1
2.978e+007

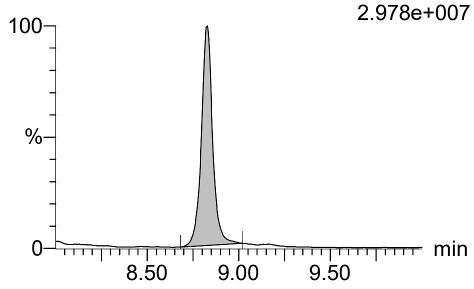

Sample Name: 20211111_H19_R2_IgM
F47:MRM of 3 channels,ES+
976.3 > 366.1
1.034e+007

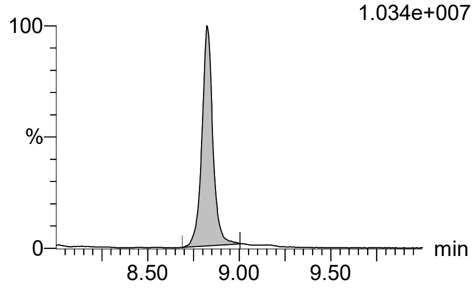

Sample Name: 20211111_H19_R2_IgM
F47:MRM of 3 channels,ES+
1220.1 > 366.1
2.158e+007

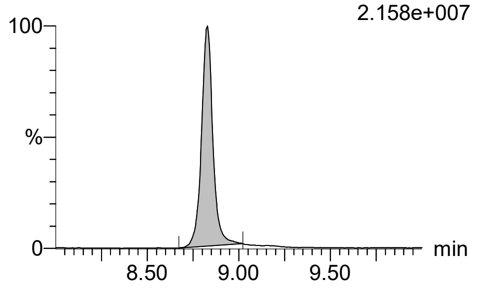

Sample Name: 20211111_A39_R2_IgM
F47:MRM of 3 channels,ES+
976.3 > 204.1
2.242e+007

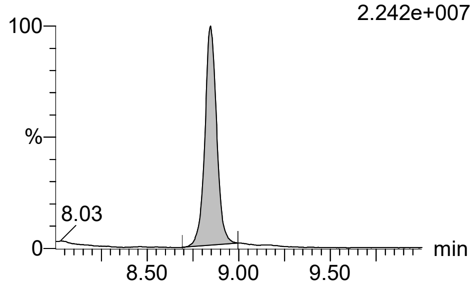

Sample Name: 20211111_A39_R2_IgM
F47:MRM of 3 channels,ES+
976.3 > 366.1
7.680e+006

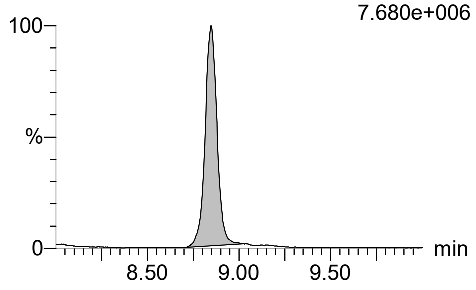

Sample Name: 20211111_A39_R2_IgM
F47:MRM of 3 channels,ES+
1220.1 > 366.1
1.572e+007

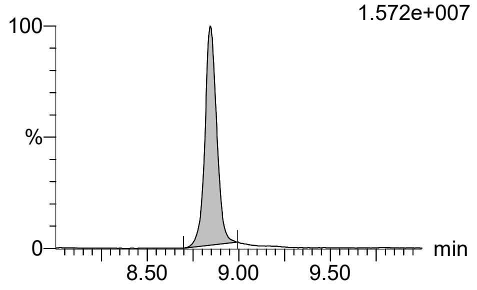

Sample Name: 20211111_A40_R2_IgM
F47:MRM of 3 channels,ES+
976.3 > 204.1
2.549e+006

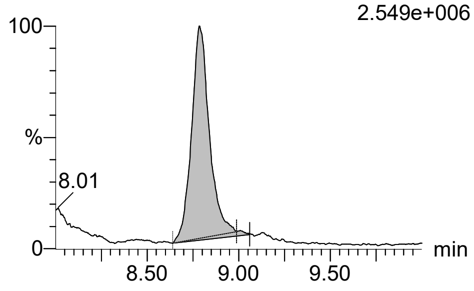

Sample Name: 20211111_A40_R2_IgM
F47:MRM of 3 channels,ES+
976.3 > 366.1
8.169e+005

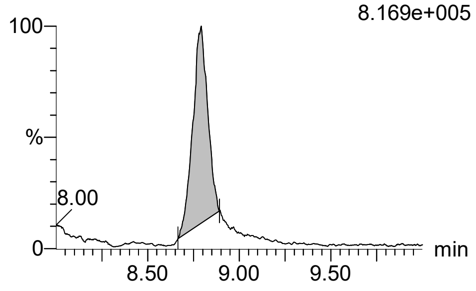

Sample Name: 20211111_A40_R2_IgM
F47:MRM of 3 channels,ES+
1220.1 > 366.1
1.854e+006

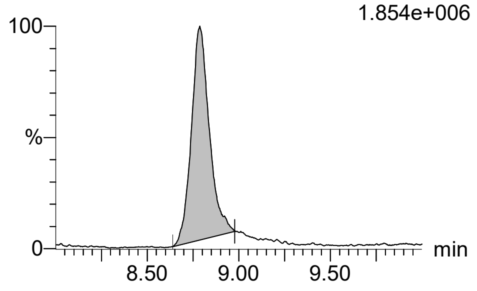

Sample Name: 20211111_H20_R2_IgM
F47:MRM of 3 channels,ES+
976.3 > 204.1
7.431e+007

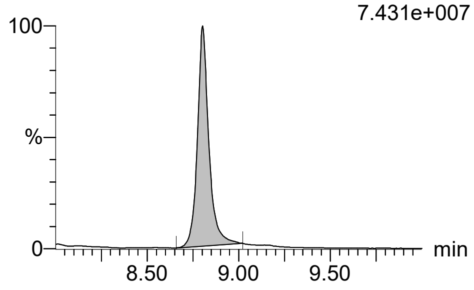

Sample Name: 20211111_H20_R2_IgM
F47:MRM of 3 channels,ES+
976.3 > 366.1
2.523e+007

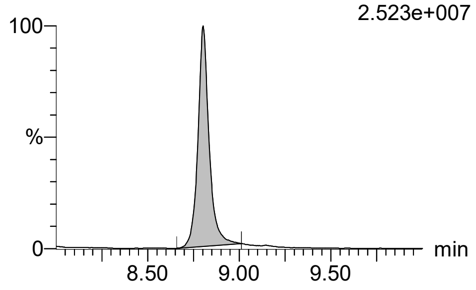

Sample Name: 20211111_H20_R2_IgM
F47:MRM of 3 channels,ES+
1220.1 > 366.1
5.640e+007

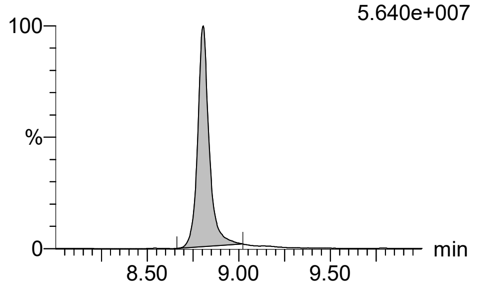

Dataset: C:\MassLynx\10.pro\20211111_clinicalsamples_IgM_IgA.qld

Last Altered: Thursday, March 09, 2023 19:58:25 台北標準時間
Printed: Friday, August 01, 2025 15:08:19 台北標準時間

Sample Name: 20211111_A41_R2_IgM
F47:MRM of 3 channels,ES+
976.3 > 204.1
1.155e+007

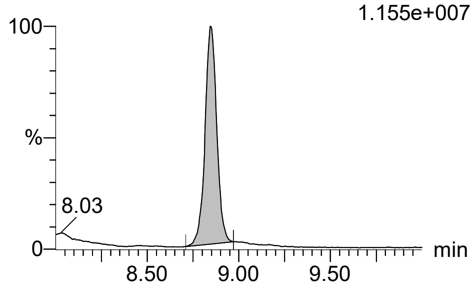

Sample Name: 20211111_A41_R2_IgM
F47:MRM of 3 channels,ES+
976.3 > 366.1
4.052e+006

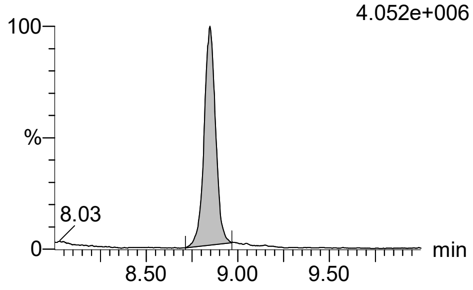

Sample Name: 20211111_A41_R2_IgM
F47:MRM of 3 channels,ES+
1220.1 > 366.1
7.904e+006

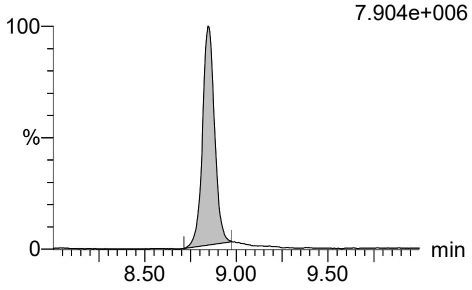

Sample Name: 20211111_A42_R2_IgM
F47:MRM of 3 channels,ES+
976.3 > 204.1
7.083e+006

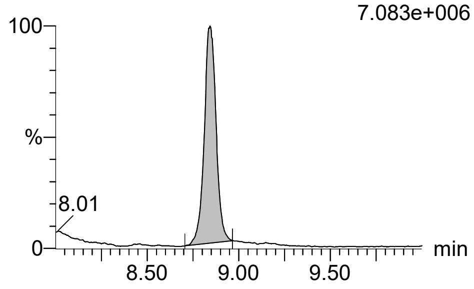

Sample Name: 20211111_A42_R2_IgM
F47:MRM of 3 channels,ES+
976.3 > 366.1
2.486e+006

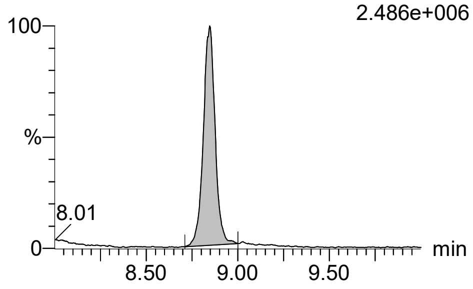

Sample Name: 20211111_A42_R2_IgM
F47:MRM of 3 channels,ES+
1220.1 > 366.1
5.156e+006

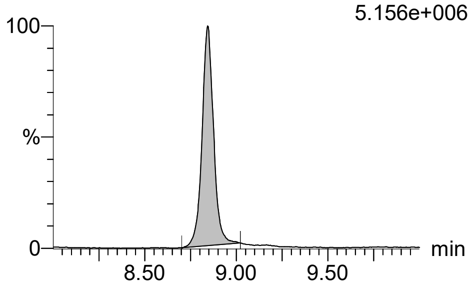

Sample Name: 20211111_A43_R2_IgM
F47:MRM of 3 channels,ES+
976.3 > 204.1
9.877e+006

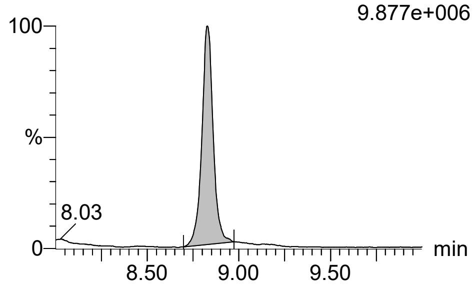

Sample Name: 20211111_A43_R2_IgM
F47:MRM of 3 channels,ES+
976.3 > 366.1
3.464e+006

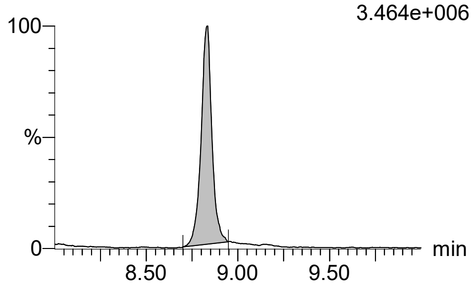

Sample Name: 20211111_A43_R2_IgM
F47:MRM of 3 channels,ES+
1220.1 > 366.1
7.393e+006

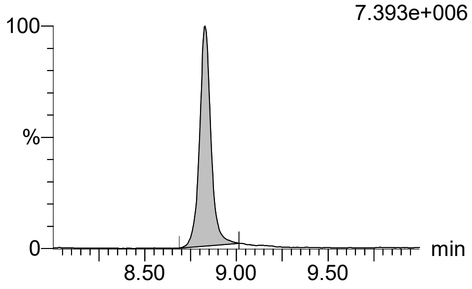

Sample Name: 20211111_A44_R2_IgM
F47:MRM of 3 channels,ES+
976.3 > 204.1
2.157e+006

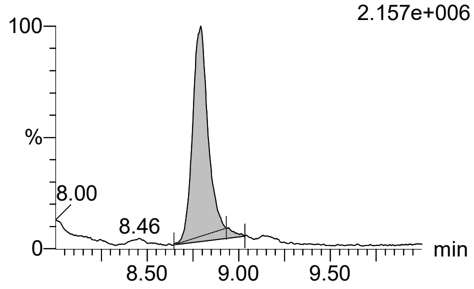

Sample Name: 20211111_A44_R2_IgM
F47:MRM of 3 channels,ES+
976.3 > 366.1
7.035e+005

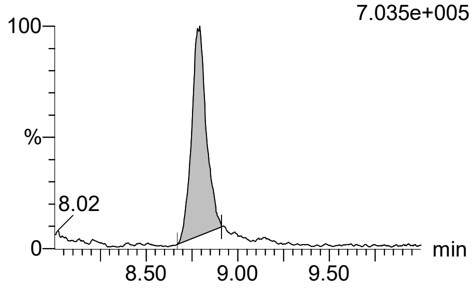

Sample Name: 20211111_A44_R2_IgM
F47:MRM of 3 channels,ES+
1220.1 > 366.1
1.514e+006

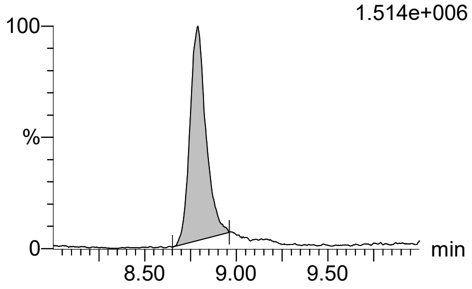

Sample Name: 20211111_QC_R17_IgM
F47:MRM of 3 channels,ES+
976.3 > 204.1
1.789e+007

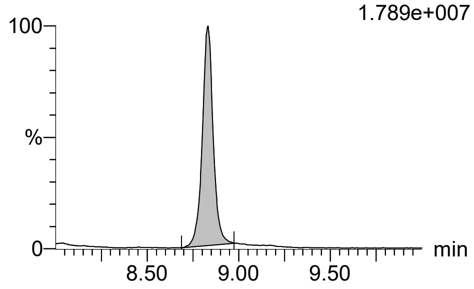

Sample Name: 20211111_QC_R17_IgM
F47:MRM of 3 channels,ES+
976.3 > 366.1
6.284e+006

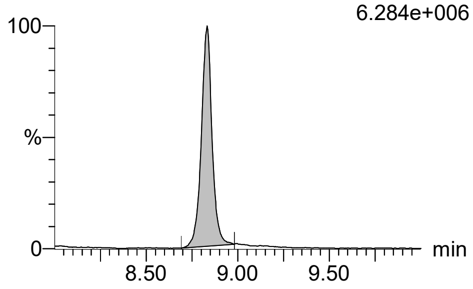

Sample Name: 20211111_QC_R17_IgM
F47:MRM of 3 channels,ES+
1220.1 > 366.1
1.288e+007

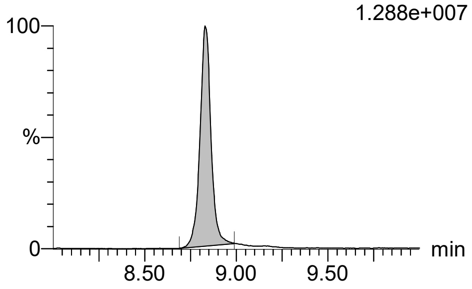

Sample Name: 20211111_L2_R3_IgM
F47:MRM of 3 channels,ES+
976.3 > 204.1
3.331e+007

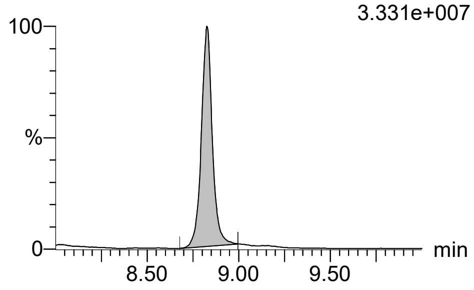

Sample Name: 20211111_L2_R3_IgM
F47:MRM of 3 channels,ES+
976.3 > 366.1
1.147e+007

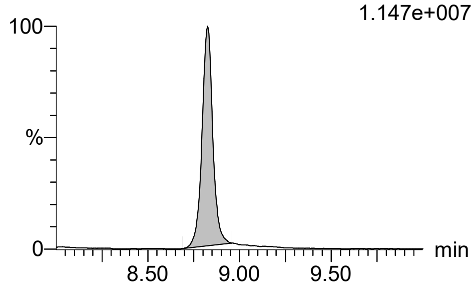

Sample Name: 20211111_L2_R3_IgM
F47:MRM of 3 channels,ES+
1220.1 > 366.1
2.569e+007

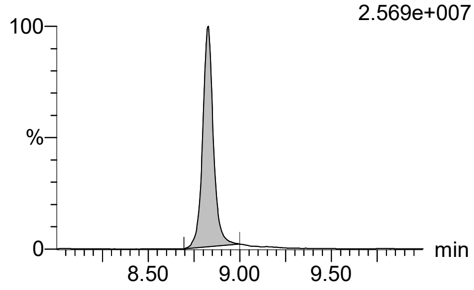

Sample Name: 20211111_H1_R3_IgM
F47:MRM of 3 channels,ES+
976.3 > 204.1
2.568e+007

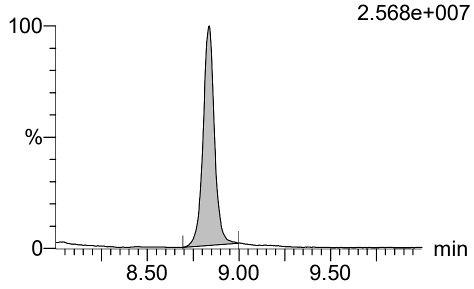

Sample Name: 20211111_H1_R3_IgM
F47:MRM of 3 channels,ES+
976.3 > 366.1
8.792e+006

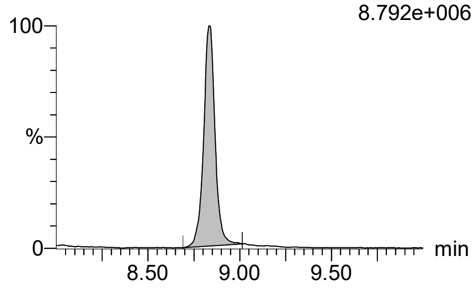

Sample Name: 20211111_H1_R3_IgM
F47:MRM of 3 channels,ES+
1220.1 > 366.1
1.873e+007

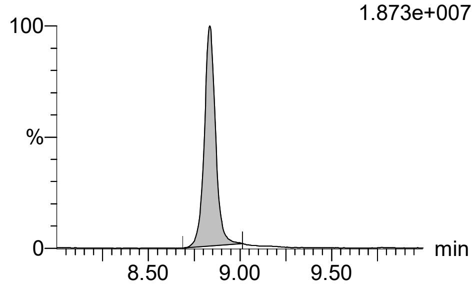

Sample Name: 20211111_A3_R3_IgM
F47:MRM of 3 channels,ES+
976.3 > 204.1
3.803e+007

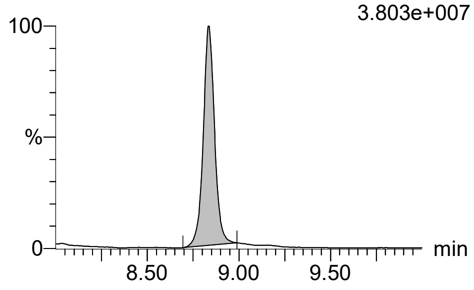

Sample Name: 20211111_A3_R3_IgM
F47:MRM of 3 channels,ES+
976.3 > 366.1
1.313e+007

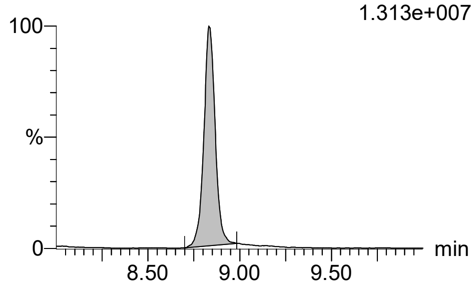

Sample Name: 20211111_A3_R3_IgM
F47:MRM of 3 channels,ES+
1220.1 > 366.1
2.641e+007

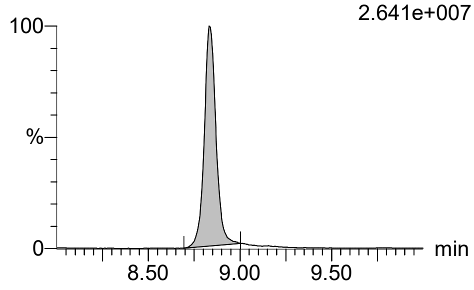

Sample Name: 20211111_A4_R3_IgM
F47:MRM of 3 channels,ES+
976.3 > 204.1
2.025e+007

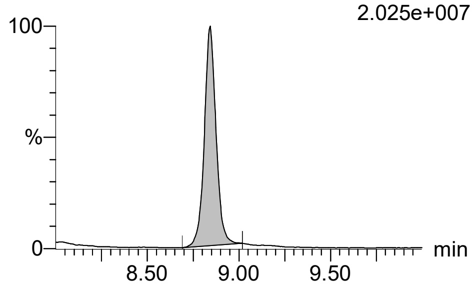

Sample Name: 20211111_A4_R3_IgM
F47:MRM of 3 channels,ES+
976.3 > 366.1
6.901e+006

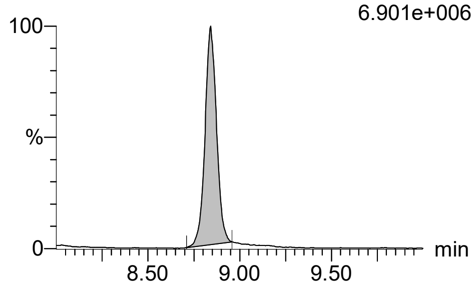

Sample Name: 20211111_A4_R3_IgM
F47:MRM of 3 channels,ES+
1220.1 > 366.1
1.393e+007

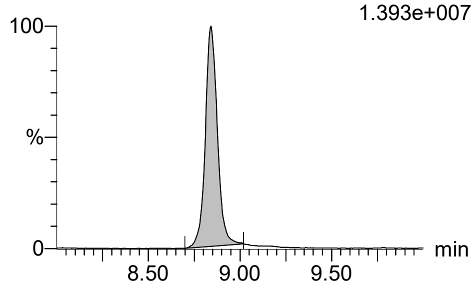

Sample Name: 20211111_L3_R3_IgM
F47:MRM of 3 channels,ES+
976.3 > 204.1
4.215e+006

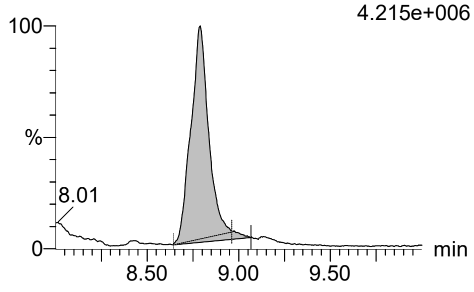

Sample Name: 20211111_L3_R3_IgM
F47:MRM of 3 channels,ES+
976.3 > 366.1
1.277e+006

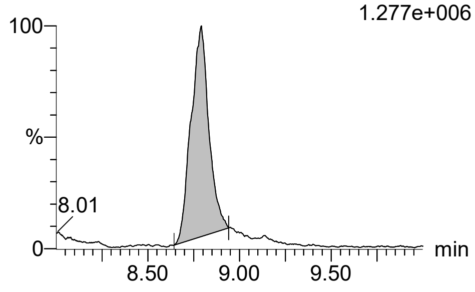

Sample Name: 20211111_L3_R3_IgM
F47:MRM of 3 channels,ES+
1220.1 > 366.1
2.863e+006

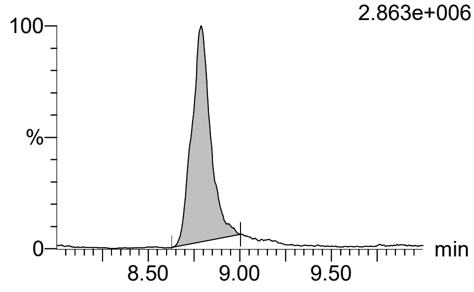

Dataset: C:\MassLynx\10.pro\20211111_clinicalsamples_IgM_IgA.qld

Last Altered: Thursday, March 09, 2023 19:58:25 台北標準時間
Printed: Friday, August 01, 2025 15:08:19 台北標準時間

Sample Name: 20211111_L4_R3_IgM
F47:MRM of 3 channels,ES+
976.3 > 204.1
1.721e+007

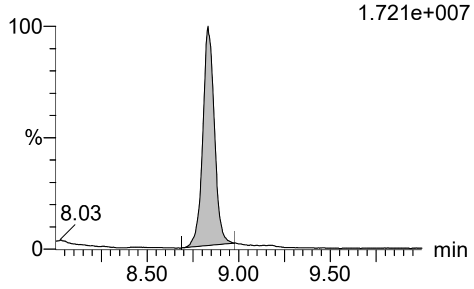

Sample Name: 20211111_L4_R3_IgM
F47:MRM of 3 channels,ES+
976.3 > 366.1
5.638e+006

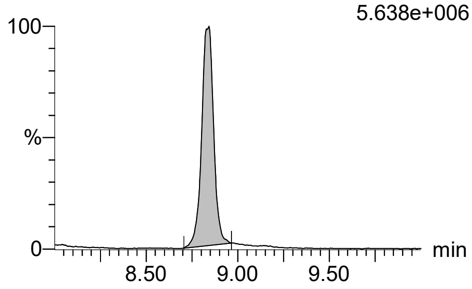

Sample Name: 20211111_L4_R3_IgM
F47:MRM of 3 channels,ES+
1220.1 > 366.1
1.227e+007

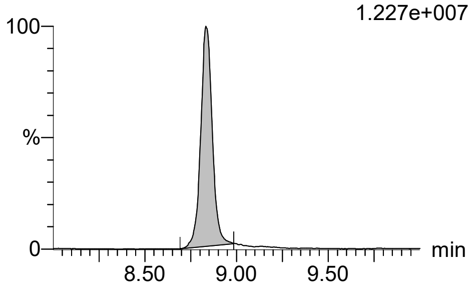

Sample Name: 20211111_H2_R3_IgM
F47:MRM of 3 channels,ES+
976.3 > 204.1
4.226e+007

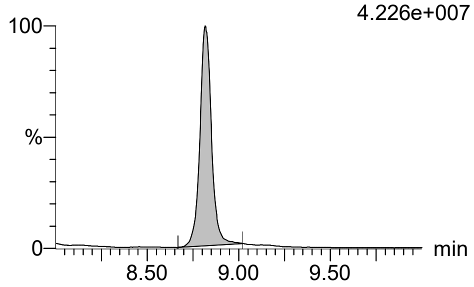

Sample Name: 20211111_H2_R3_IgM
F47:MRM of 3 channels,ES+
976.3 > 366.1
1.508e+007

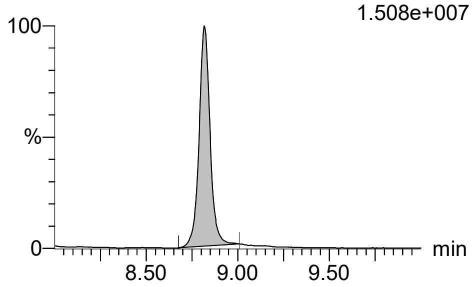

Sample Name: 20211111_H2_R3_IgM
F47:MRM of 3 channels,ES+
1220.1 > 366.1
3.091e+007

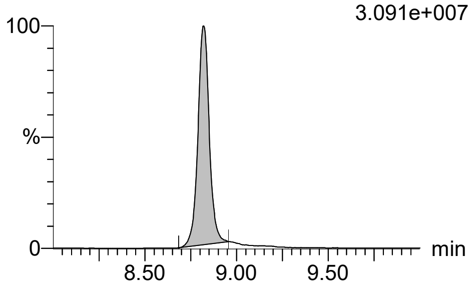

Sample Name: 20211111_A5_R3_IgM
F47:MRM of 3 channels,ES+
976.3 > 204.1
5.374e+007

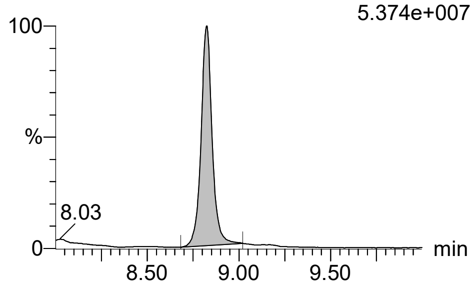

Sample Name: 20211111_A5_R3_IgM
F47:MRM of 3 channels,ES+
976.3 > 366.1
1.898e+007

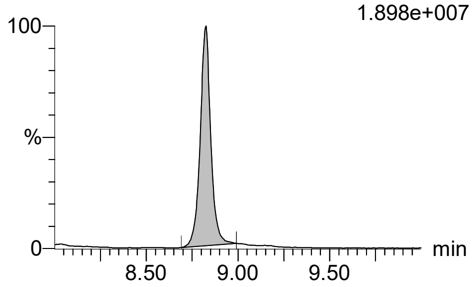

Sample Name: 20211111_A5_R3_IgM
F47:MRM of 3 channels,ES+
1220.1 > 366.1
3.943e+007

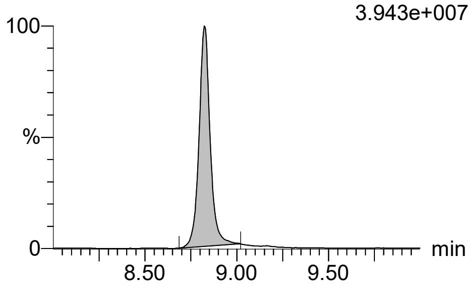

Sample Name: 20211111_A6_R3_IgM
F47:MRM of 3 channels,ES+
976.3 > 204.1
2.772e+007

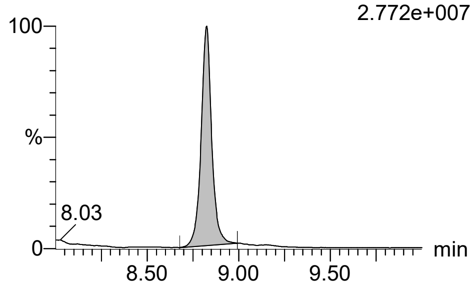

Sample Name: 20211111_A6_R3_IgM
F47:MRM of 3 channels,ES+
976.3 > 366.1
9.540e+006

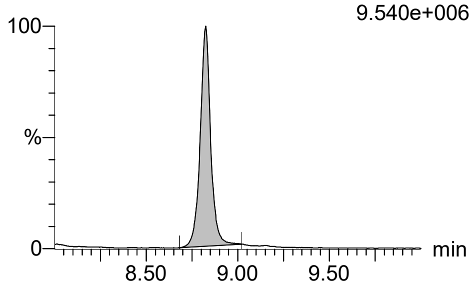

Sample Name: 20211111_A6_R3_IgM
F47:MRM of 3 channels,ES+
1220.1 > 366.1
1.925e+007

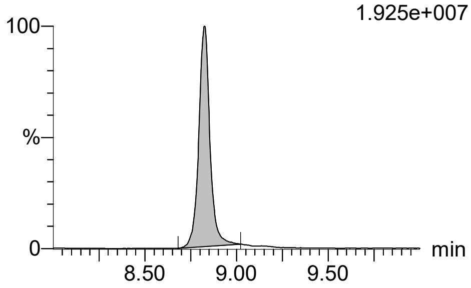

Sample Name: 20211111_QC_R18_IgM
F47:MRM of 3 channels,ES+
976.3 > 204.1
1.779e+007

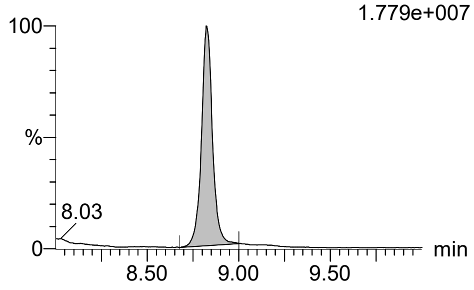

Sample Name: 20211111_QC_R18_IgM
F47:MRM of 3 channels,ES+
976.3 > 366.1
6.390e+006

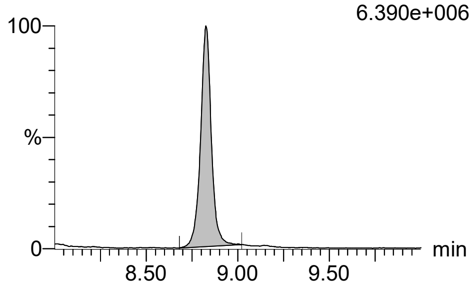

Sample Name: 20211111_QC_R18_IgM
F47:MRM of 3 channels,ES+
1220.1 > 366.1
1.308e+007

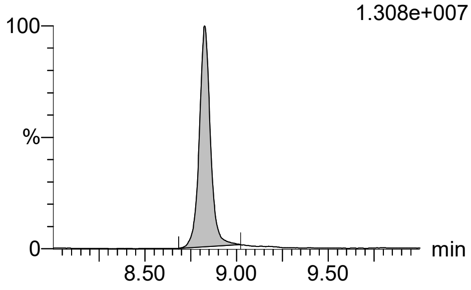

Dataset: C:\MassLynx\10.pro\20211111_clinicalsamples_IgM_IgA.qld

Last Altered: Thursday, March 09, 2023 19:58:25 台北標準時間
Printed: Friday, August 01, 2025 15:08:19 台北標準時間

Sample Name: 20211111_L5_R3_IgM
F47:MRM of 3 channels,ES+
976.3 > 204.1
3.107e+007

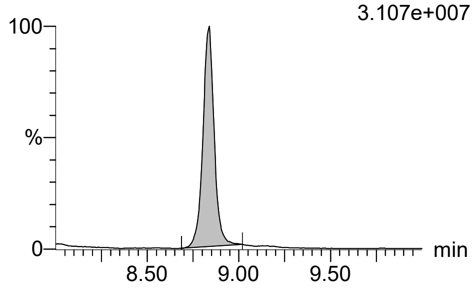

Sample Name: 20211111_L5_R3_IgM
F47:MRM of 3 channels,ES+
976.3 > 366.1
1.086e+007

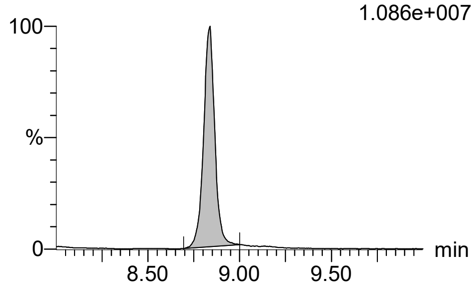

Sample Name: 20211111_L5_R3_IgM
F47:MRM of 3 channels,ES+
1220.1 > 366.1
2.240e+007

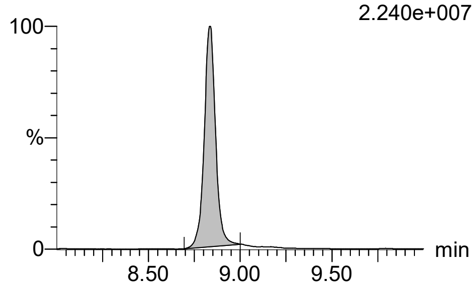

Sample Name: 20211111_L6_R3_IgM
F47:MRM of 3 channels,ES+
976.3 > 204.1
2.411e+007

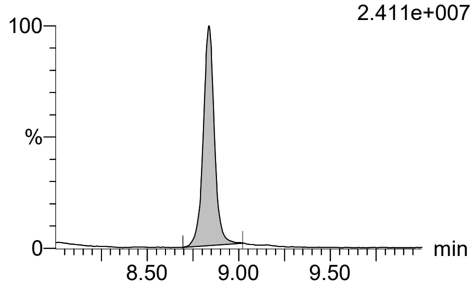

Sample Name: 20211111_L6_R3_IgM
F47:MRM of 3 channels,ES+
976.3 > 366.1
8.248e+006

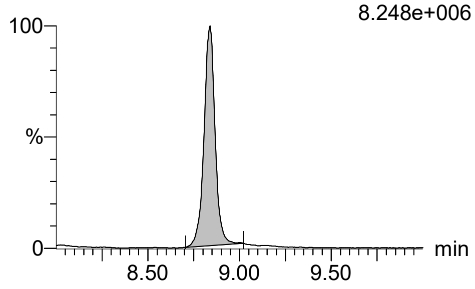

Sample Name: 20211111_L6_R3_IgM
F47:MRM of 3 channels,ES+
1220.1 > 366.1
1.644e+007

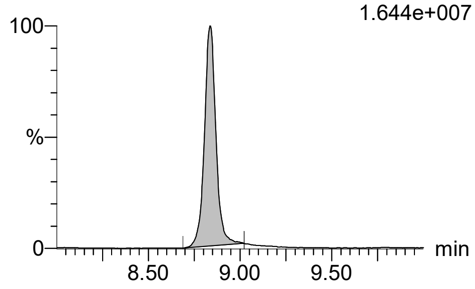

Sample Name: 20211111_H3_R3_IgM
F47:MRM of 3 channels,ES+
976.3 > 204.1
1.237e+007

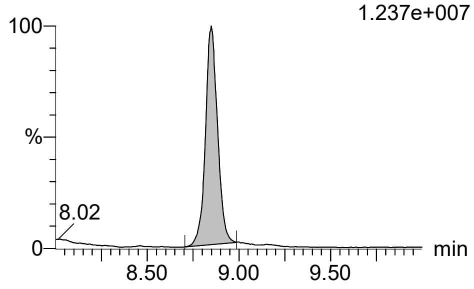

Sample Name: 20211111_H3_R3_IgM
F47:MRM of 3 channels,ES+
976.3 > 366.1
4.275e+006

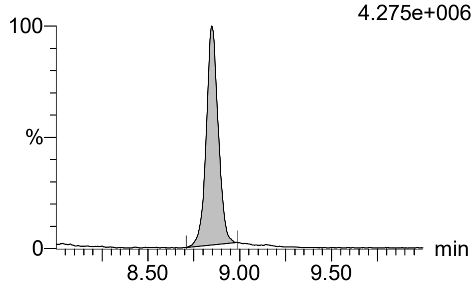

Sample Name: 20211111_H3_R3_IgM
F47:MRM of 3 channels,ES+
1220.1 > 366.1
8.314e+006

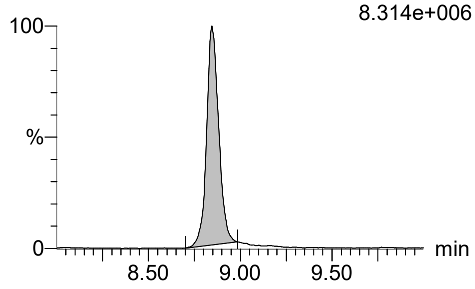

Sample Name: 20211111_A7_R3_IgM
F47:MRM of 3 channels,ES+
976.3 > 204.1
2.452e+007

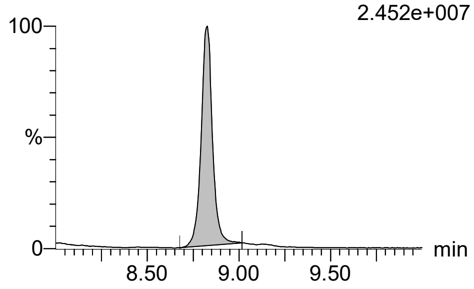

Sample Name: 20211111_A7_R3_IgM
F47:MRM of 3 channels,ES+
976.3 > 366.1
8.659e+006

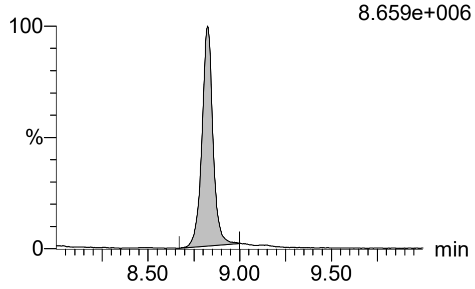

Sample Name: 20211111_A7_R3_IgM
F47:MRM of 3 channels,ES+
1220.1 > 366.1
1.868e+007

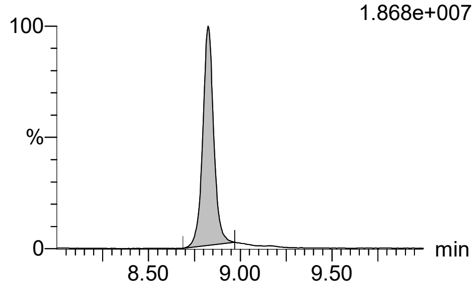

Sample Name: 20211111_A8_R3_IgM
F47:MRM of 3 channels,ES+
976.3 > 204.1
1.905e+007

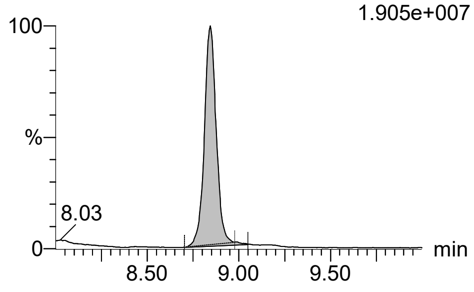

Sample Name: 20211111_A8_R3_IgM
F47:MRM of 3 channels,ES+
976.3 > 366.1
6.484e+006

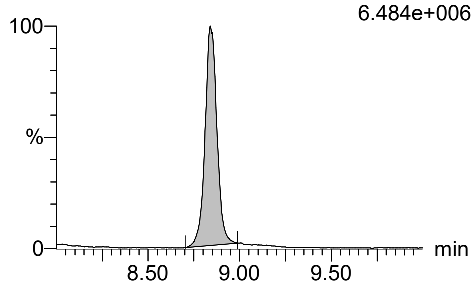

Sample Name: 20211111_A8_R3_IgM
F47:MRM of 3 channels,ES+
1220.1 > 366.1
1.255e+007

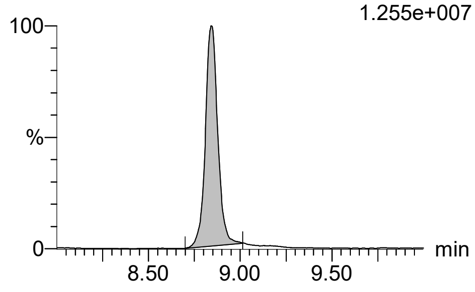

Dataset: C:\MassLynx\10.pro\20211111_clinicalsamples_IgM_IgA.qld

Last Altered: Thursday, March 09, 2023 19:58:25 台北標準時間
Printed: Friday, August 01, 2025 15:08:19 台北標準時間

Sample Name: 20211111_L7_R3_IgM
F47:MRM of 3 channels,ES+
976.3 > 204.1
1.395e+007

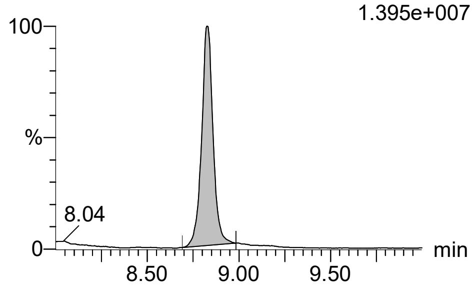

Sample Name: 20211111_L7_R3_IgM
F47:MRM of 3 channels,ES+
976.3 > 366.1
4.759e+006

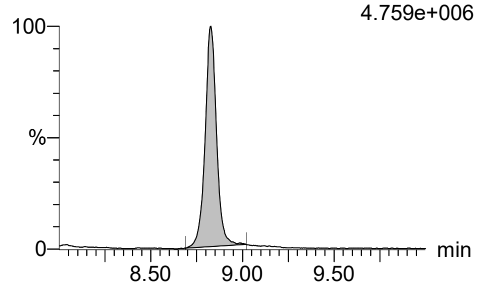

Sample Name: 20211111_L7_R3_IgM
F47:MRM of 3 channels,ES+
1220.1 > 366.1
1.045e+007

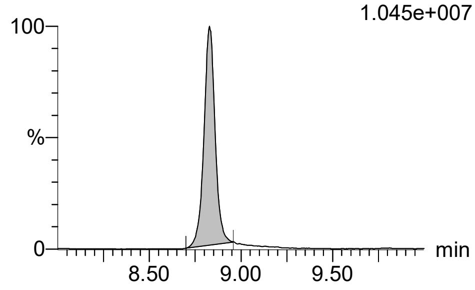

Sample Name: 20211111_L8_R3_IgM
F47:MRM of 3 channels,ES+
976.3 > 204.1
5.666e+006

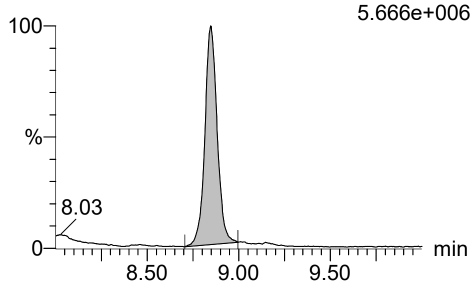

Sample Name: 20211111_L8_R3_IgM
F47:MRM of 3 channels,ES+
976.3 > 366.1
1.912e+006

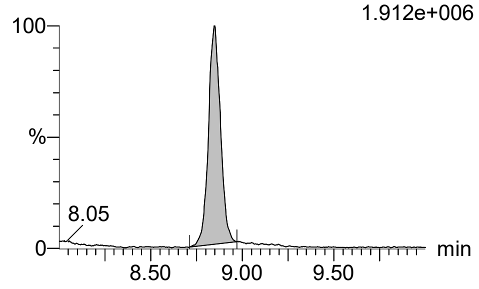

Sample Name: 20211111_L8_R3_IgM
F47:MRM of 3 channels,ES+
1220.1 > 366.1
3.831e+006

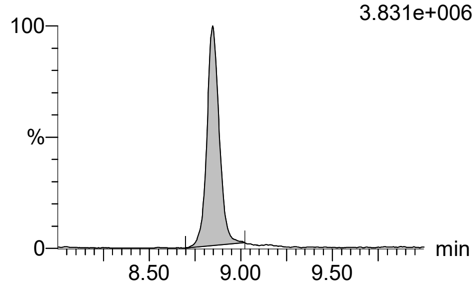

Sample Name: 20211111_H4_R3_IgM
F47:MRM of 3 channels,ES+
976.3 > 204.1
1.143e+007

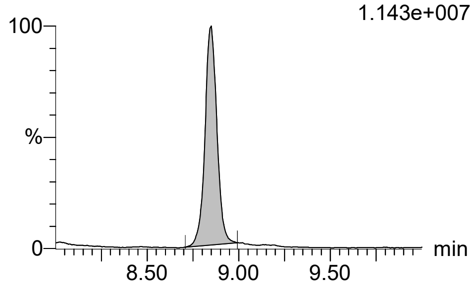

Sample Name: 20211111_H4_R3_IgM
F47:MRM of 3 channels,ES+
976.3 > 366.1
3.770e+006

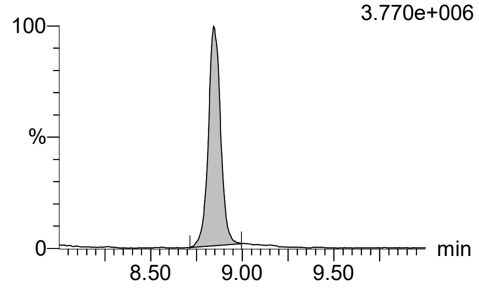

Sample Name: 20211111_H4_R3_IgM
F47:MRM of 3 channels,ES+
1220.1 > 366.1
7.563e+006

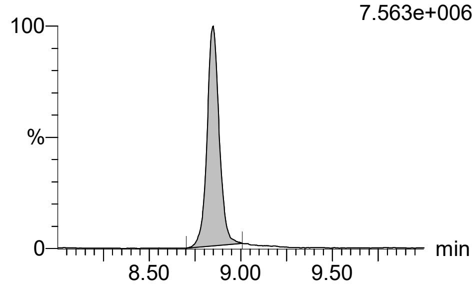

Sample Name: 20211111_A9_R3_IgM
F47:MRM of 3 channels,ES+
976.3 > 204.1
1.117e+007

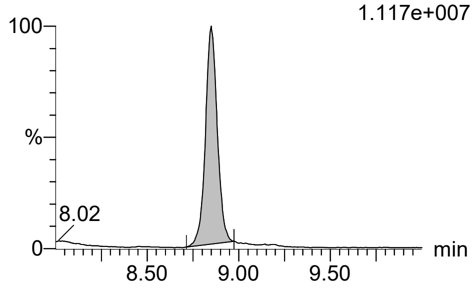

Sample Name: 20211111_A9_R3_IgM
F47:MRM of 3 channels,ES+
976.3 > 366.1
3.791e+006

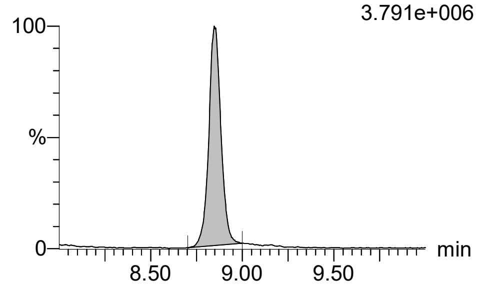

Sample Name: 20211111_A9_R3_IgM
F47:MRM of 3 channels,ES+
1220.1 > 366.1
7.558e+006

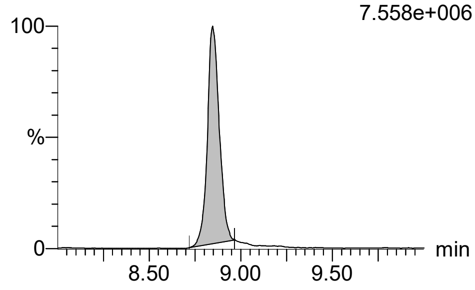

Sample Name: 20211111_A10_R3_IgM
F47:MRM of 3 channels,ES+
976.3 > 204.1
3.494e+007

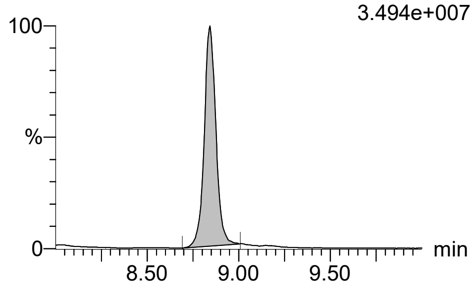

Sample Name: 20211111_A10_R3_IgM
F47:MRM of 3 channels,ES+
976.3 > 366.1
1.172e+007

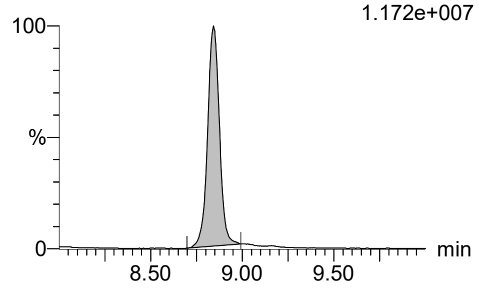

Sample Name: 20211111_A10_R3_IgM
F47:MRM of 3 channels,ES+
1220.1 > 366.1
2.433e+007

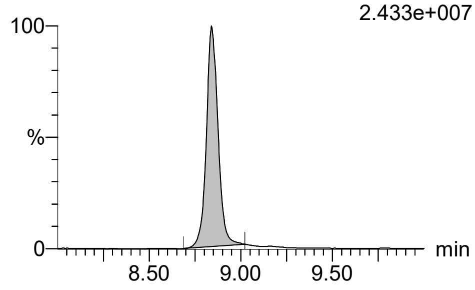

Sample Name: 20211111_L9_R3_IgM
F47:MRM of 3 channels,ES+
976.3 > 204.1
2.049e+007

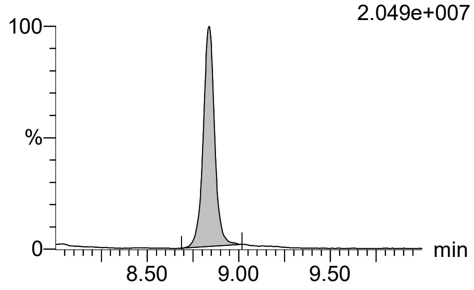

Sample Name: 20211111_L9_R3_IgM
F47:MRM of 3 channels,ES+
976.3 > 366.1
7.263e+006

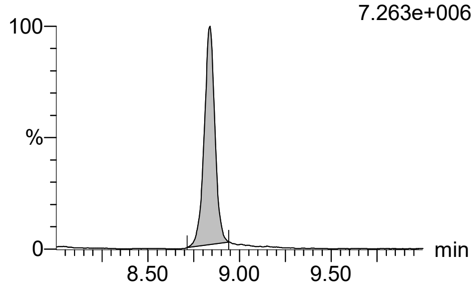

Sample Name: 20211111_L9_R3_IgM
F47:MRM of 3 channels,ES+
1220.1 > 366.1
1.439e+007

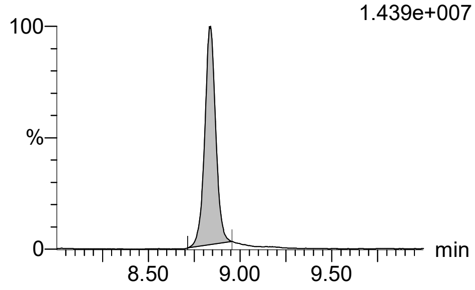

Sample Name: 20211111_L10_R3_IgM
F47:MRM of 3 channels,ES+
976.3 > 204.1
9.227e+006

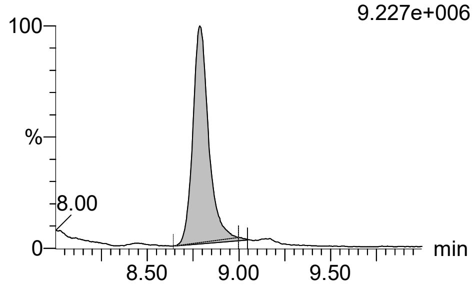

Sample Name: 20211111_L10_R3_IgM
F47:MRM of 3 channels,ES+
976.3 > 366.1
2.931e+006

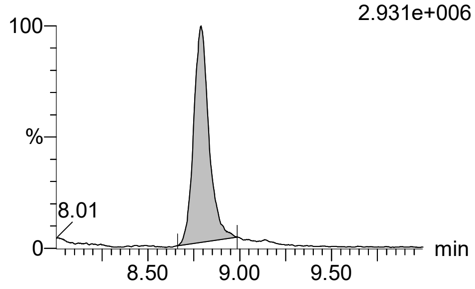

Sample Name: 20211111_L10_R3_IgM
F47:MRM of 3 channels,ES+
1220.1 > 366.1
6.854e+006

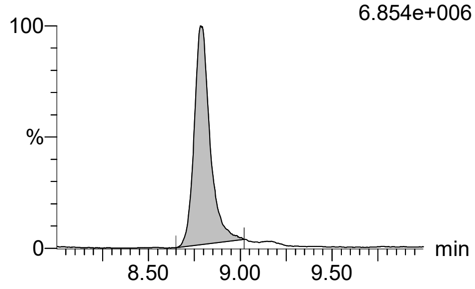

Sample Name: 20211111_QC_R19_IgM
F47:MRM of 3 channels,ES+
976.3 > 204.1
1.843e+007

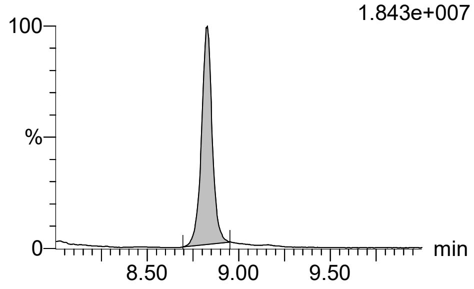

Sample Name: 20211111_QC_R19_IgM
F47:MRM of 3 channels,ES+
976.3 > 366.1
6.299e+006

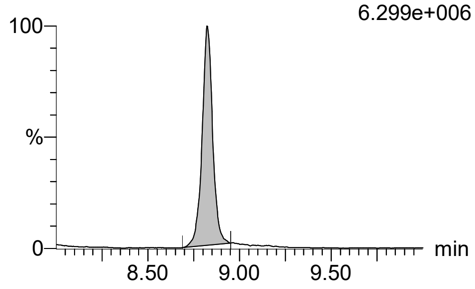

Sample Name: 20211111_QC_R19_IgM
F47:MRM of 3 channels,ES+
1220.1 > 366.1
1.325e+007

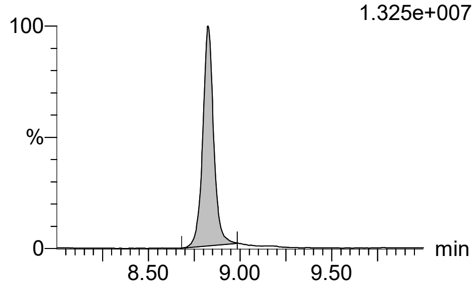

Sample Name: 20211111_H5_R3_IgM
F47:MRM of 3 channels,ES+
976.3 > 204.1
2.468e+007

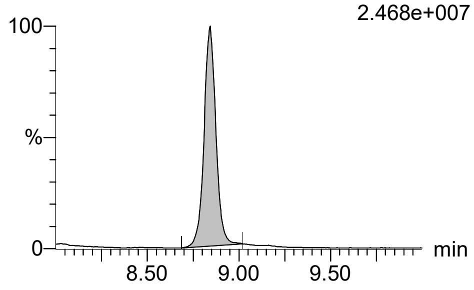

Sample Name: 20211111_H5_R3_IgM
F47:MRM of 3 channels,ES+
976.3 > 366.1
8.532e+006

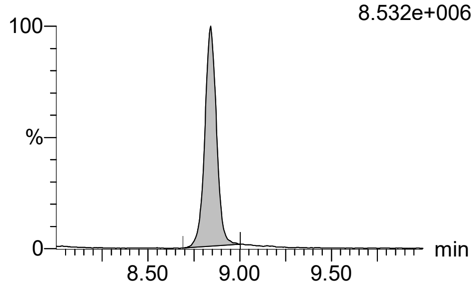

Sample Name: 20211111_H5_R3_IgM
F47:MRM of 3 channels,ES+
1220.1 > 366.1
1.615e+007

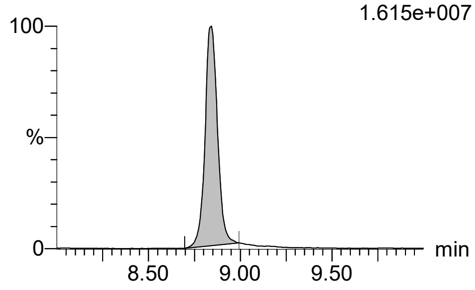

Sample Name: 20211111_A11_R3_IgM
F47:MRM of 3 channels,ES+
976.3 > 204.1
1.403e+007

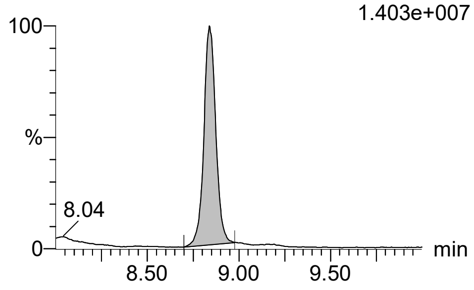

Sample Name: 20211111_A11_R3_IgM
F47:MRM of 3 channels,ES+
976.3 > 366.1
4.903e+006

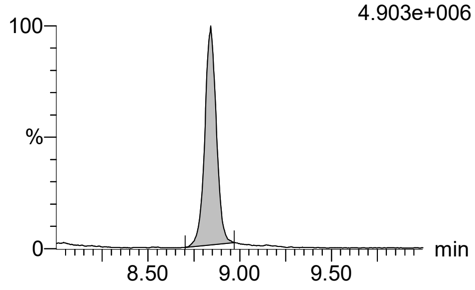

Sample Name: 20211111_A11_R3_IgM
F47:MRM of 3 channels,ES+
1220.1 > 366.1
9.801e+006

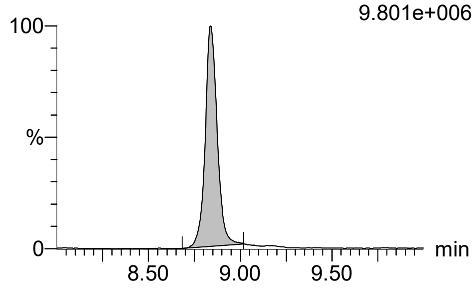

Dataset: C:\MassLynx\10.pro\20211111_clinicalsamples_IgM_IgA.qld

Last Altered: Thursday, March 09, 2023 19:58:25 台北標準時間
Printed: Friday, August 01, 2025 15:08:19 台北標準時間

Sample Name: 20211111_A12_R3_IgM
F47:MRM of 3 channels,ES+
976.3 > 204.1
9.161e+006

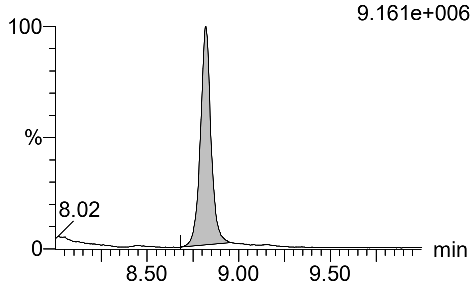

Sample Name: 20211111_A12_R3_IgM
F47:MRM of 3 channels,ES+
976.3 > 366.1
3.117e+006

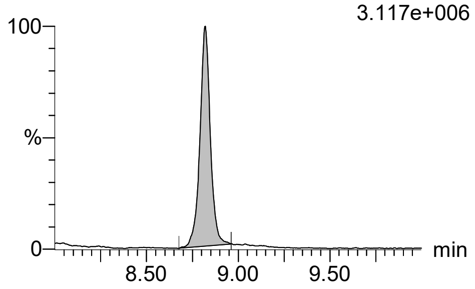

Sample Name: 20211111_A12_R3_IgM
F47:MRM of 3 channels,ES+
1220.1 > 366.1
6.476e+006

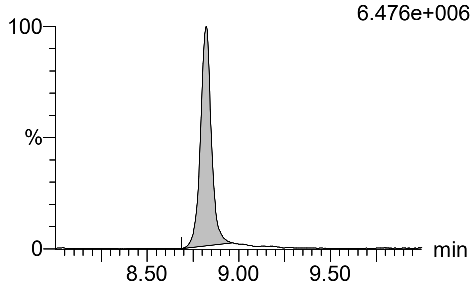

Sample Name: 20211111_L11_R3_IgM
F47:MRM of 3 channels,ES+
976.3 > 204.1
9.043e+006

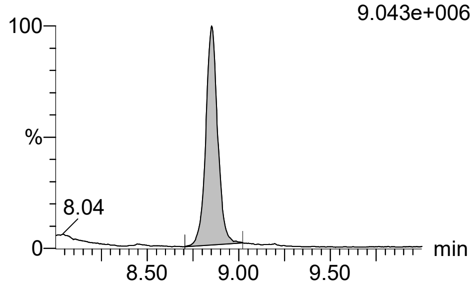

Sample Name: 20211111_L11_R3_IgM
F47:MRM of 3 channels,ES+
976.3 > 366.1
3.040e+006

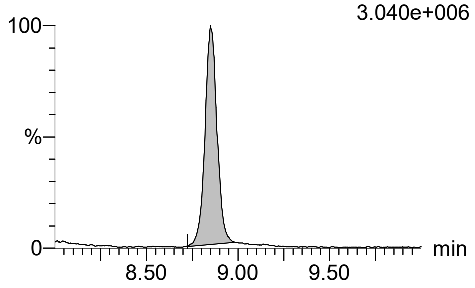

Sample Name: 20211111_L11_R3_IgM
F47:MRM of 3 channels,ES+
1220.1 > 366.1
5.885e+006

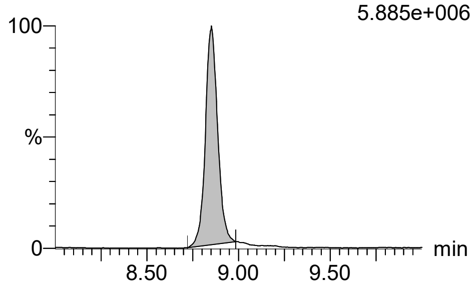

Sample Name: 20211111_L12_R3_IgM
F47:MRM of 3 channels,ES+
976.3 > 204.1
4.783e+006

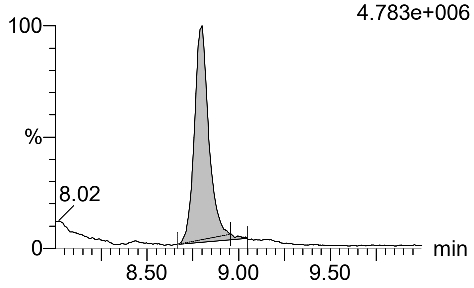

Sample Name: 20211111_L12_R3_IgM
F47:MRM of 3 channels,ES+
976.3 > 366.1
1.524e+006

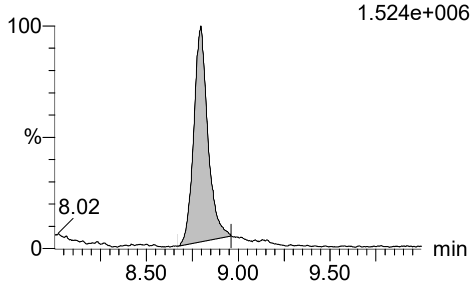

Sample Name: 20211111_L12_R3_IgM
F47:MRM of 3 channels,ES+
1220.1 > 366.1
3.634e+006

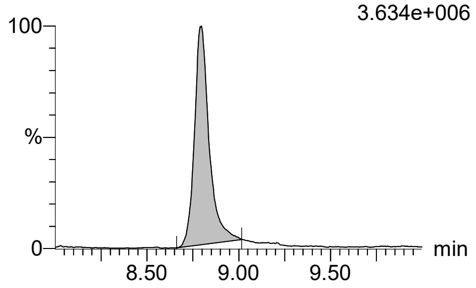

Sample Name: 20211111_H6_R3_IgM
F47:MRM of 3 channels,ES+
976.3 > 204.1
8.418e+006

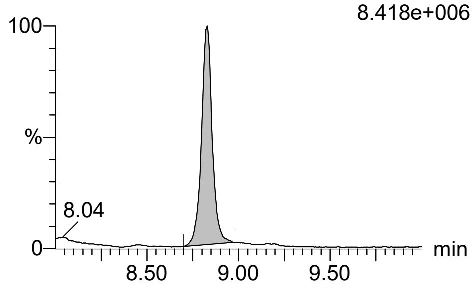

Sample Name: 20211111_H6_R3_IgM
F47:MRM of 3 channels,ES+
976.3 > 366.1
3.004e+006

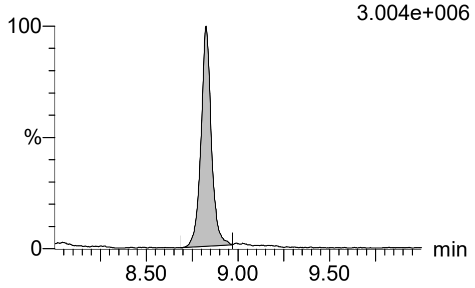

Sample Name: 20211111_H6_R3_IgM
F47:MRM of 3 channels,ES+
1220.1 > 366.1
6.175e+006

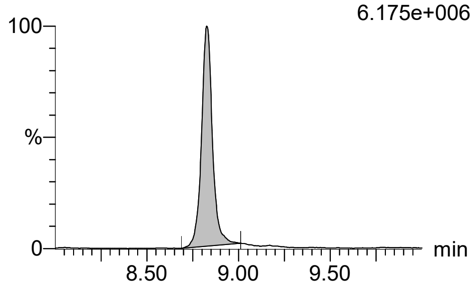

Sample Name: 20211111_A13_R3_IgM
F47:MRM of 3 channels,ES+
976.3 > 204.1
9.826e+006

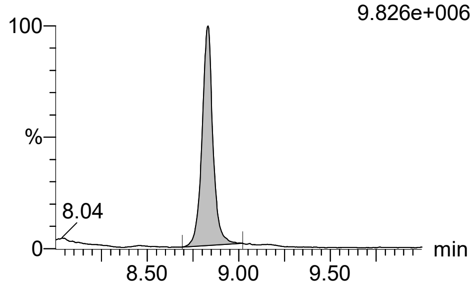

Sample Name: 20211111_A13_R3_IgM
F47:MRM of 3 channels,ES+
976.3 > 366.1
3.506e+006

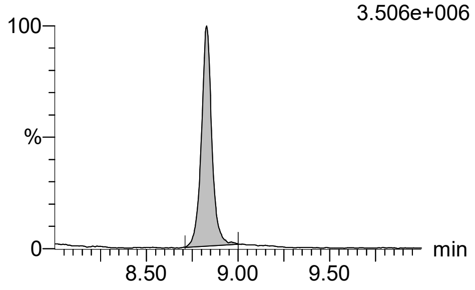

Sample Name: 20211111_A13_R3_IgM
F47:MRM of 3 channels,ES+
1220.1 > 366.1
7.358e+006

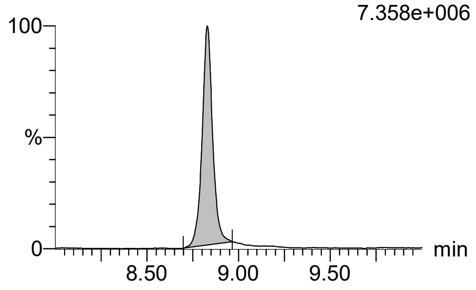

Dataset: C:\MassLynx\10.pro\20211111_clinicalsamples_IgM_IgA.qld

Last Altered: Thursday, March 09, 2023 19:58:25 台北標準時間
Printed: Friday, August 01, 2025 15:08:19 台北標準時間

Sample Name: 20211111_A14_R3_IgM
F47:MRM of 3 channels,ES+
976.3 > 204.1
2.917e+007

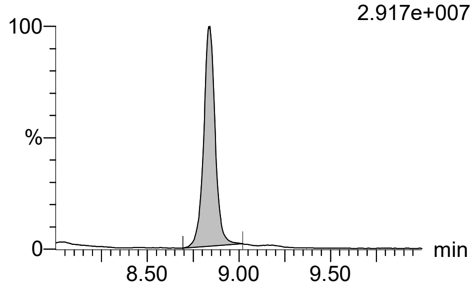

Sample Name: 20211111_A14_R3_IgM
F47:MRM of 3 channels,ES+
976.3 > 366.1
1.006e+007

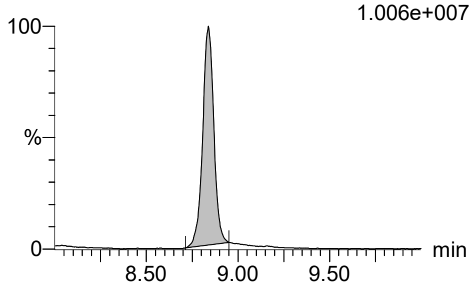

Sample Name: 20211111_A14_R3_IgM
F47:MRM of 3 channels,ES+
1220.1 > 366.1
2.151e+007

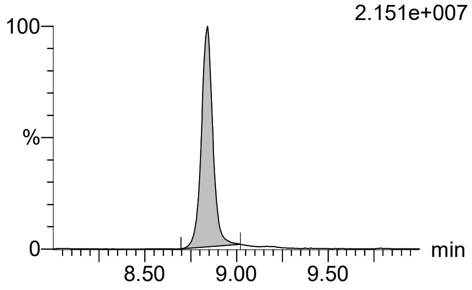

Sample Name: 20211111_L13_R3_IgM
F47:MRM of 3 channels,ES+
976.3 > 204.1
1.730e+007

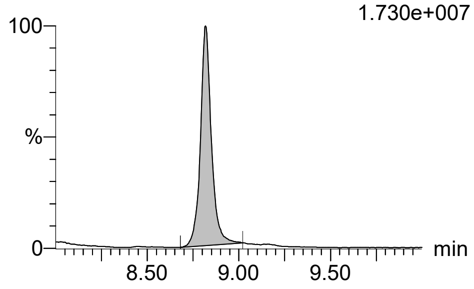

Sample Name: 20211111_L13_R3_IgM
F47:MRM of 3 channels,ES+
976.3 > 366.1
5.877e+006

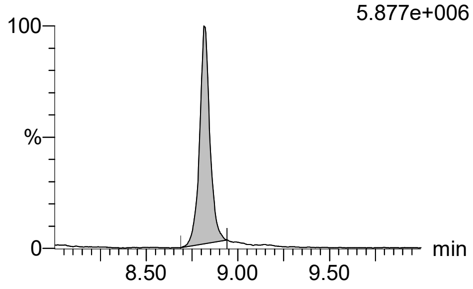

Sample Name: 20211111_L13_R3_IgM
F47:MRM of 3 channels,ES+
1220.1 > 366.1
1.301e+007

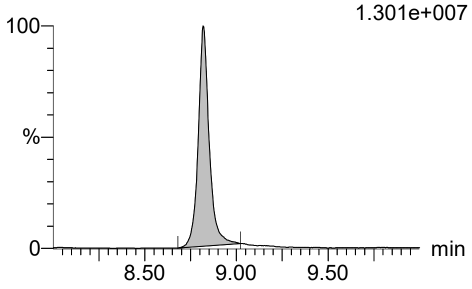

Sample Name: 20211111_L14_R3_IgM
F47:MRM of 3 channels,ES+
976.3 > 204.1
2.299e+007

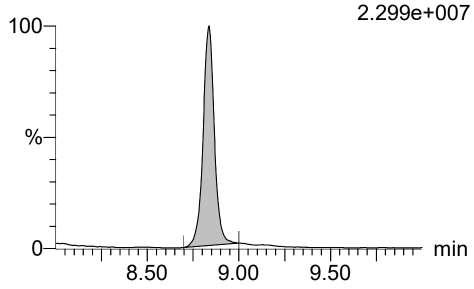

Sample Name: 20211111_L14_R3_IgM
F47:MRM of 3 channels,ES+
976.3 > 366.1
7.813e+006

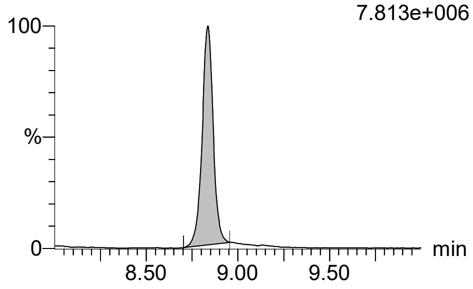

Sample Name: 20211111_L14_R3_IgM
F47:MRM of 3 channels,ES+
1220.1 > 366.1
1.678e+007

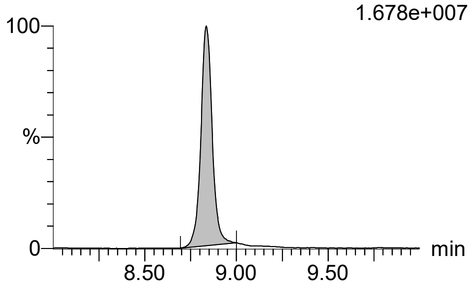

Sample Name: 20211111_H7_R3_IgM
F47:MRM of 3 channels,ES+
976.3 > 204.1
1.191e+007

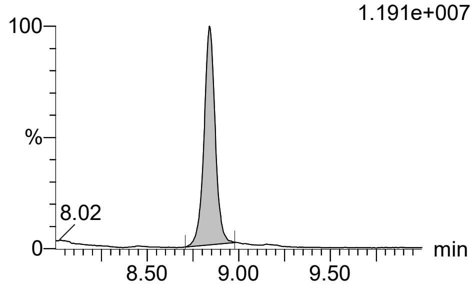

Sample Name: 20211111_H7_R3_IgM
F47:MRM of 3 channels,ES+
976.3 > 366.1
4.137e+006

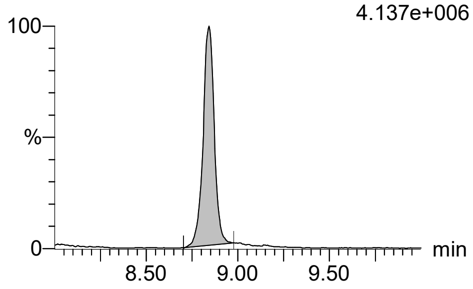

Sample Name: 20211111_H7_R3_IgM
F47:MRM of 3 channels,ES+
1220.1 > 366.1
8.161e+006

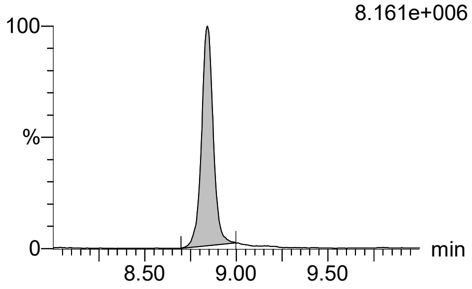

Sample Name: 20211111_A15_R3_IgM
F47:MRM of 3 channels,ES+
976.3 > 204.1
4.308e+007

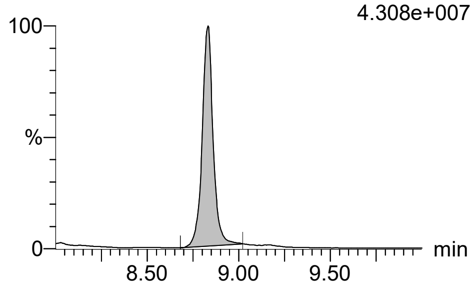

Sample Name: 20211111_A15_R3_IgM
F47:MRM of 3 channels,ES+
976.3 > 366.1
1.479e+007

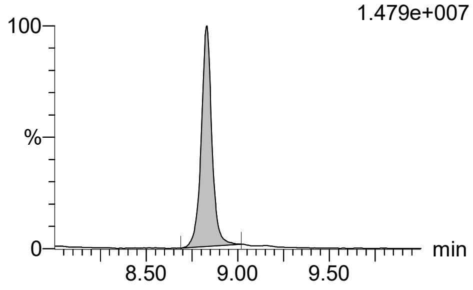

Sample Name: 20211111_A15_R3_IgM
F47:MRM of 3 channels,ES+
1220.1 > 366.1
3.120e+007

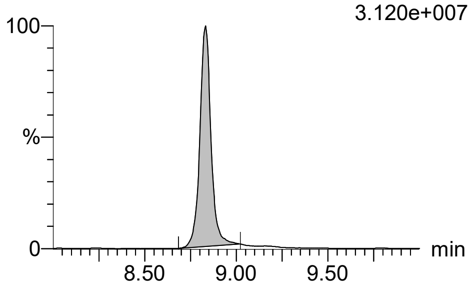

Dataset: C:\MassLynx\10.pro\20211111_clinicalsamples_IgM_IgA.qld

Last Altered: Thursday, March 09, 2023 19:58:25 台北標準時間
Printed: Friday, August 01, 2025 15:08:19 台北標準時間

Sample Name: 20211111_QC_R20_IgM
F47:MRM of 3 channels,ES+
976.3 > 204.1
2.029e+007

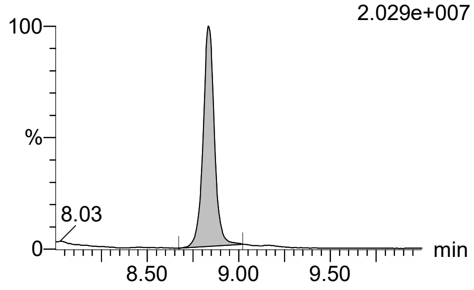

Sample Name: 20211111_QC_R20_IgM
F47:MRM of 3 channels,ES+
976.3 > 366.1
7.219e+006

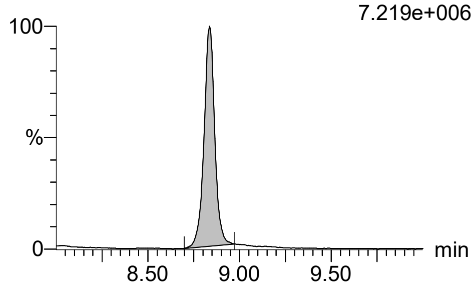

Sample Name: 20211111_QC_R20_IgM
F47:MRM of 3 channels,ES+
1220.1 > 366.1
1.462e+007

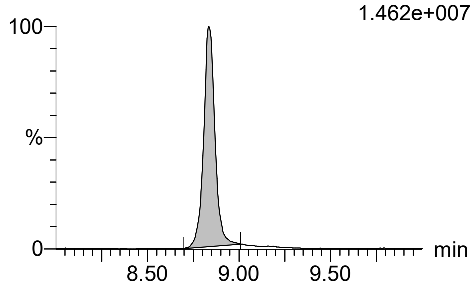

Sample Name: 20211111_A16_R3_IgM
F47:MRM of 3 channels,ES+
976.3 > 204.1
2.986e+007

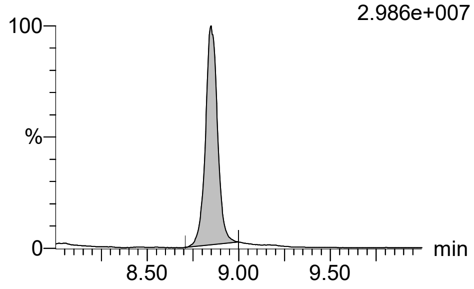

Sample Name: 20211111_A16_R3_IgM
F47:MRM of 3 channels,ES+
976.3 > 366.1
1.044e+007

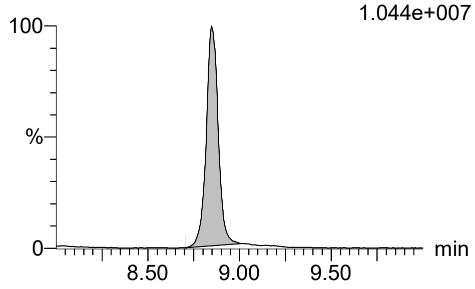

Sample Name: 20211111_A16_R3_IgM
F47:MRM of 3 channels,ES+
1220.1 > 366.1
2.186e+007

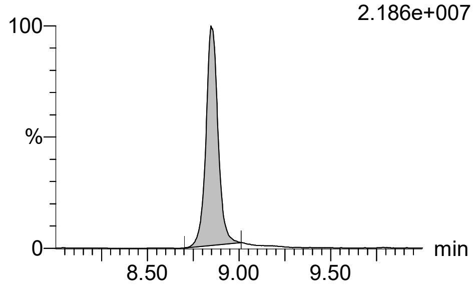

Sample Name: 20211111_L15_R3_IgM
F47:MRM of 3 channels,ES+
976.3 > 204.1
1.803e+007

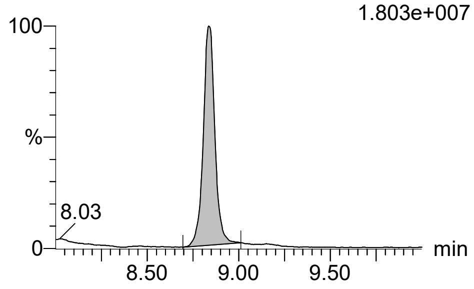

Sample Name: 20211111_L15_R3_IgM
F47:MRM of 3 channels,ES+
976.3 > 366.1
6.300e+006

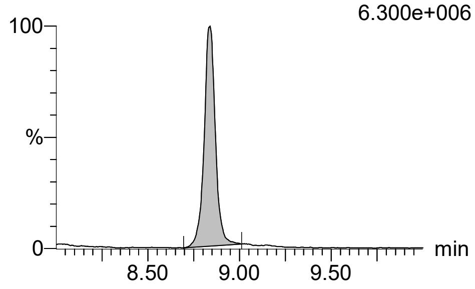

Sample Name: 20211111_L15_R3_IgM
F47:MRM of 3 channels,ES+
1220.1 > 366.1
1.356e+007

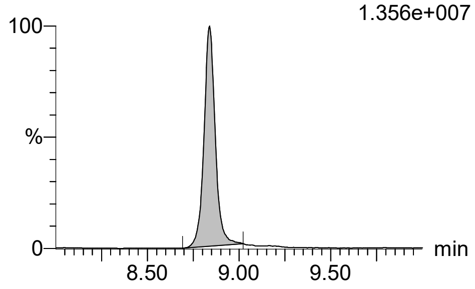

Sample Name: 20211111_L16_R3_IgM
F47:MRM of 3 channels,ES+
976.3 > 204.1
8.532e+006

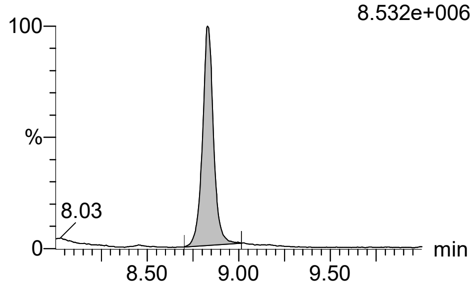

Sample Name: 20211111_L16_R3_IgM
F47:MRM of 3 channels,ES+
976.3 > 366.1
2.781e+006

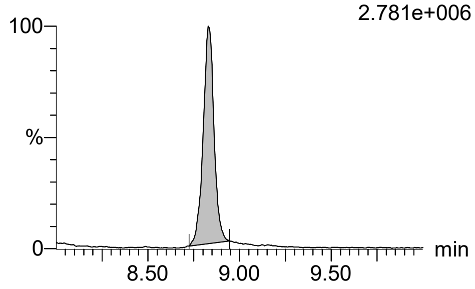

Sample Name: 20211111_L16_R3_IgM
F47:MRM of 3 channels,ES+
1220.1 > 366.1
6.190e+006

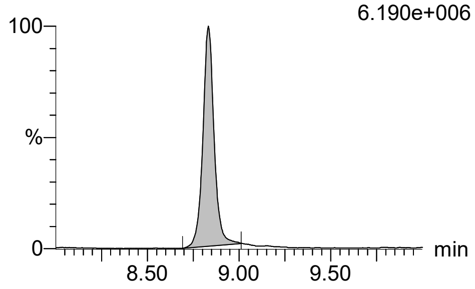

Sample Name: 20211111_H8_R3_IgM
F47:MRM of 3 channels,ES+
976.3 > 204.1
1.755e+007

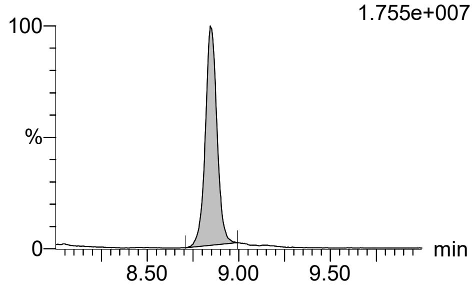

Sample Name: 20211111_H8_R3_IgM
F47:MRM of 3 channels,ES+
976.3 > 366.1
5.987e+006

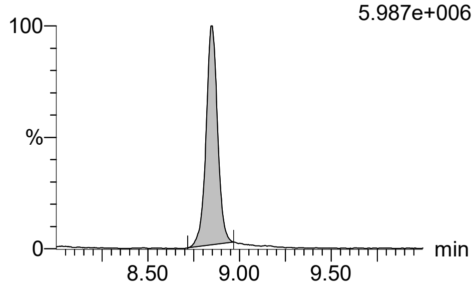

Sample Name: 20211111_H8_R3_IgM
F47:MRM of 3 channels,ES+
1220.1 > 366.1
1.170e+007

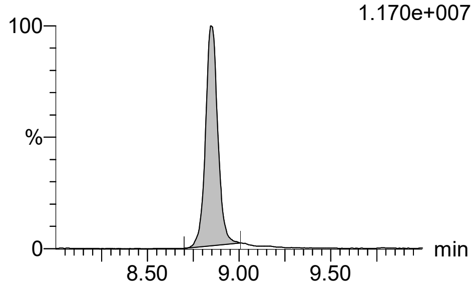

Sample Name: 20211111_A17_R3_IgM
F47:MRM of 3 channels,ES+
976.3 > 204.1
2.692e+007

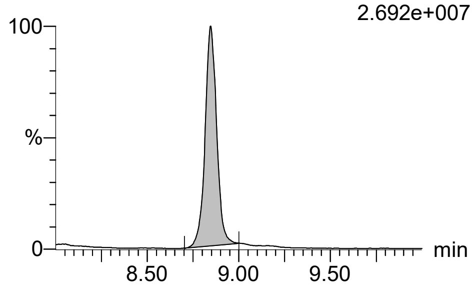

Sample Name: 20211111_A17_R3_IgM
F47:MRM of 3 channels,ES+
976.3 > 366.1
8.858e+006

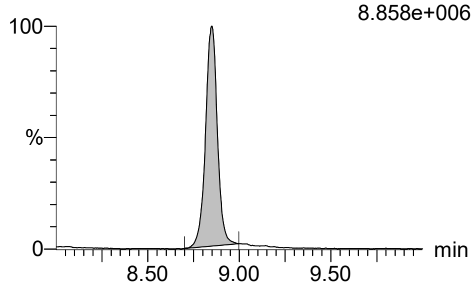

Sample Name: 20211111_A17_R3_IgM
F47:MRM of 3 channels,ES+
1220.1 > 366.1
1.965e+007

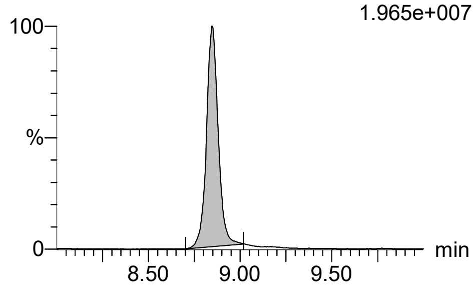

Sample Name: 20211111_A18_R3_IgM
F47:MRM of 3 channels,ES+
976.3 > 204.1
3.444e+006

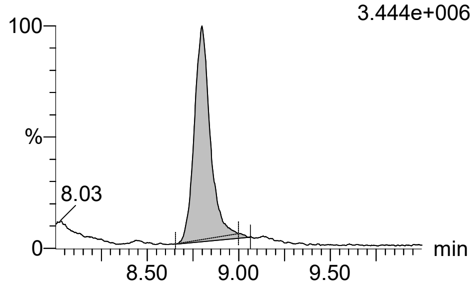

Sample Name: 20211111_A18_R3_IgM
F47:MRM of 3 channels,ES+
976.3 > 366.1
1.061e+006

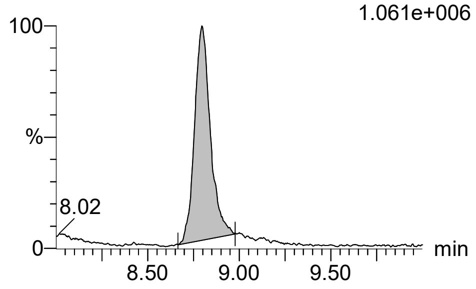

Sample Name: 20211111_A18_R3_IgM
F47:MRM of 3 channels,ES+
1220.1 > 366.1
2.692e+006

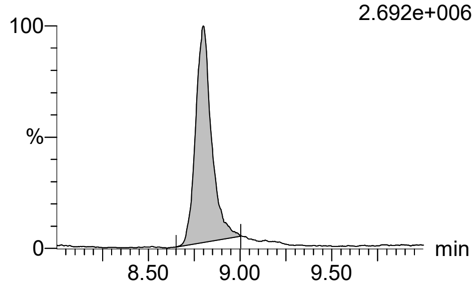

Sample Name: 20211111_L17_R3_IgM
F47:MRM of 3 channels,ES+
976.3 > 204.1
5.363e+007

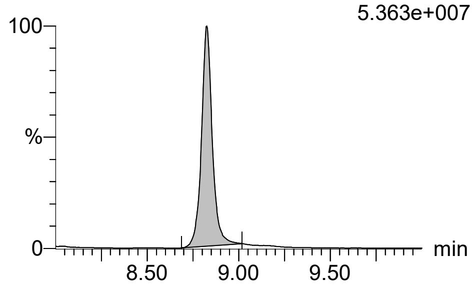

Sample Name: 20211111_L17_R3_IgM
F47:MRM of 3 channels,ES+
976.3 > 366.1
1.837e+007

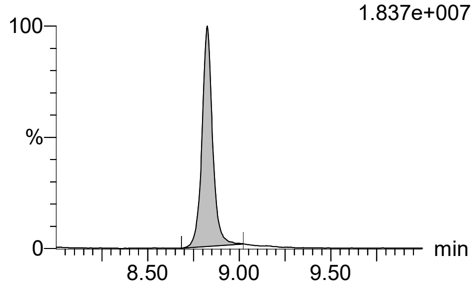

Sample Name: 20211111_L17_R3_IgM
F47:MRM of 3 channels,ES+
1220.1 > 366.1
4.091e+007

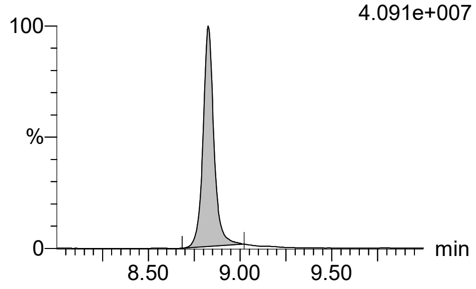

Sample Name: 20211111_L18_R3_IgM
F47:MRM of 3 channels,ES+
976.3 > 204.1
3.545e+006

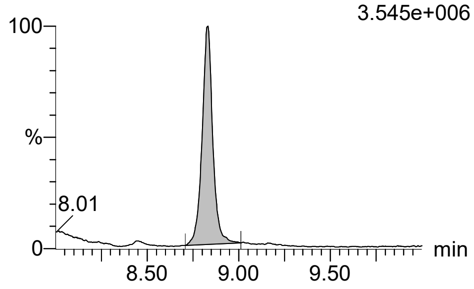

Sample Name: 20211111_L18_R3_IgM
F47:MRM of 3 channels,ES+
976.3 > 366.1
1.190e+006

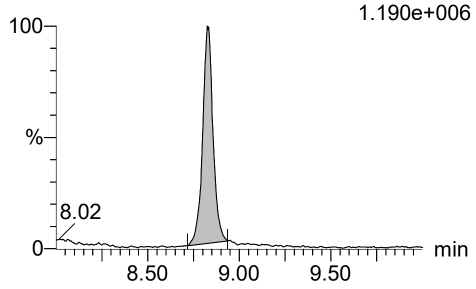

Sample Name: 20211111_L18_R3_IgM
F47:MRM of 3 channels,ES+
1220.1 > 366.1
2.504e+006

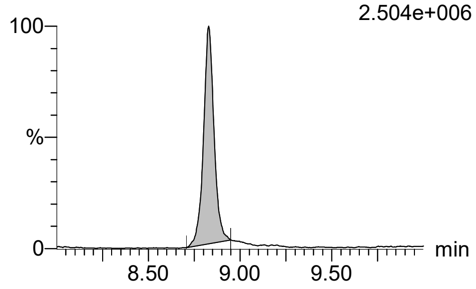

Sample Name: 20211111_H9_R3_IgM
F47:MRM of 3 channels,ES+
976.3 > 204.1
8.577e+006

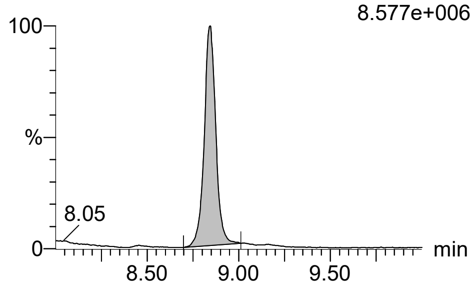

Sample Name: 20211111_H9_R3_IgM
F47:MRM of 3 channels,ES+
976.3 > 366.1
2.860e+006

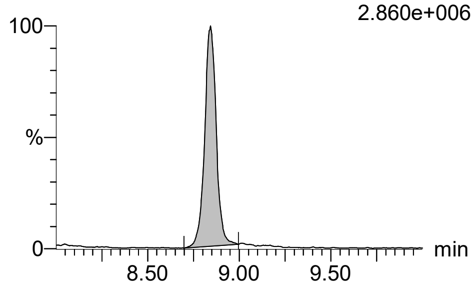

Sample Name: 20211111_H9_R3_IgM
F47:MRM of 3 channels,ES+
1220.1 > 366.1
5.995e+006

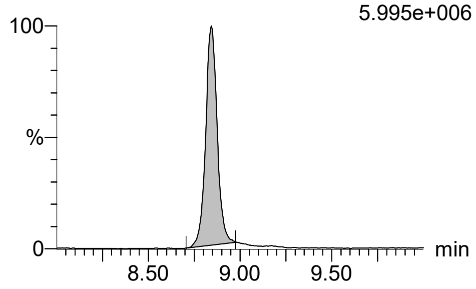

Dataset: C:\MassLynx\10.pro\20211111_clinicalsamples_IgM_IgA.qld

Last Altered: Thursday, March 09, 2023 19:58:25 台北標準時間
Printed: Friday, August 01, 2025 15:08:19 台北標準時間

Sample Name: 20211111_A19_R3_IgM
F47:MRM of 3 channels,ES+
976.3 > 204.1
1.481e+007

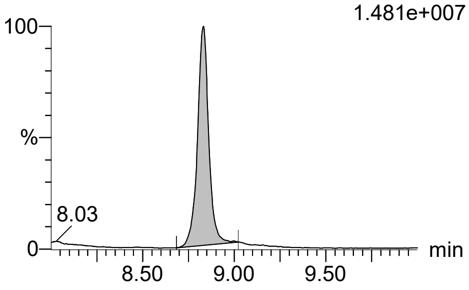

Sample Name: 20211111_A19_R3_IgM
F47:MRM of 3 channels,ES+
976.3 > 366.1
4.870e+006

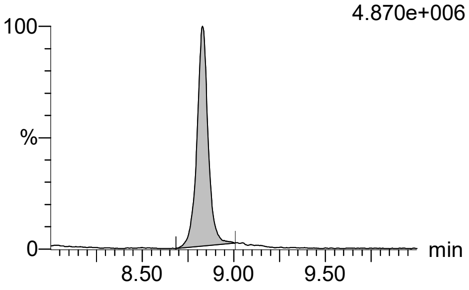

Sample Name: 20211111_A19_R3_IgM
F47:MRM of 3 channels,ES+
1220.1 > 366.1
1.200e+007

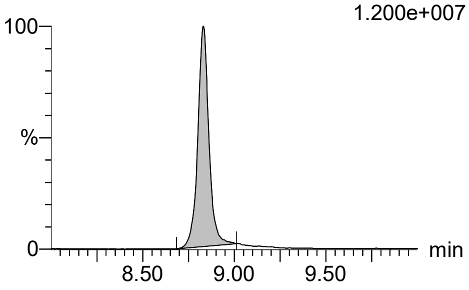

Sample Name: 20211111_A20_R3_IgM
F47:MRM of 3 channels,ES+
976.3 > 204.1
1.822e+006

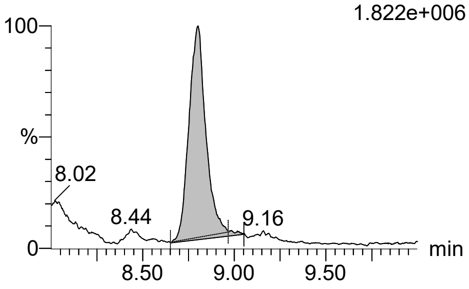

Sample Name: 20211111_A20_R3_IgM
F47:MRM of 3 channels,ES+
976.3 > 366.1
6.041e+005

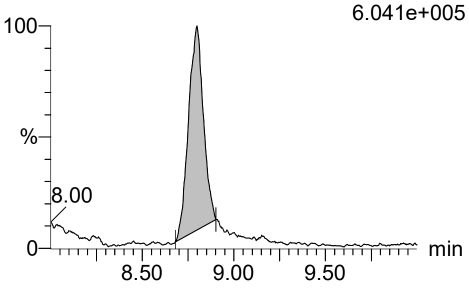

Sample Name: 20211111_A20_R3_IgM
F47:MRM of 3 channels,ES+
1220.1 > 366.1
1.311e+006

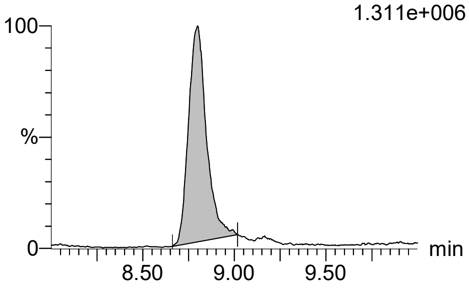

Sample Name: 20211111_L19_R3_IgM
F47:MRM of 3 channels,ES+
976.3 > 204.1
2.120e+007

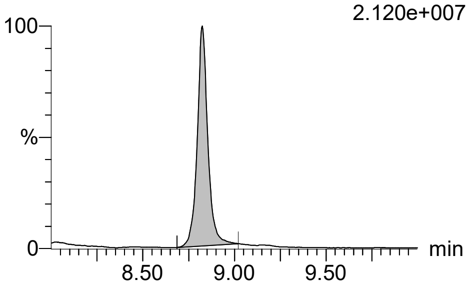

Sample Name: 20211111_L19_R3_IgM
F47:MRM of 3 channels,ES+
976.3 > 366.1
7.264e+006

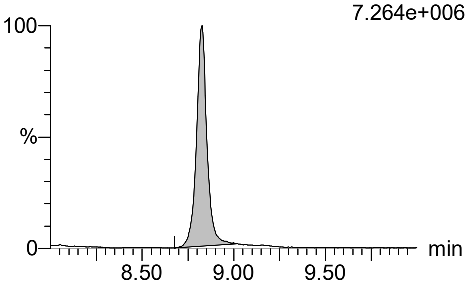

Sample Name: 20211111_L19_R3_IgM
F47:MRM of 3 channels,ES+
1220.1 > 366.1
1.610e+007

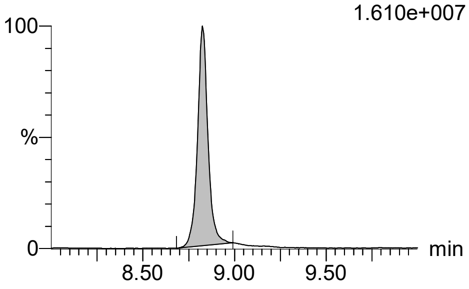

Sample Name: 20211111_QC_R21_IgM
F47:MRM of 3 channels,ES+
976.3 > 204.1
2.071e+007

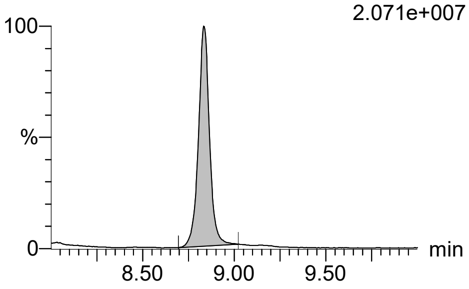

Sample Name: 20211111_QC_R21_IgM
F47:MRM of 3 channels,ES+
976.3 > 366.1
7.212e+006

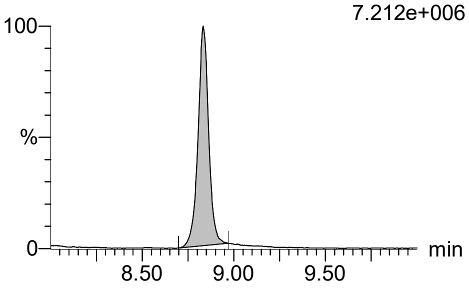

Sample Name: 20211111_QC_R21_IgM
F47:MRM of 3 channels,ES+
1220.1 > 366.1
1.527e+007

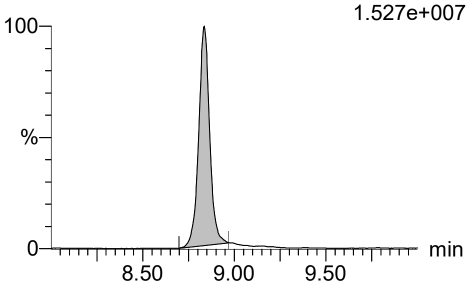

Sample Name: 20211111_L20_R3_IgM
F47:MRM of 3 channels,ES+
976.3 > 204.1
1.465e+007

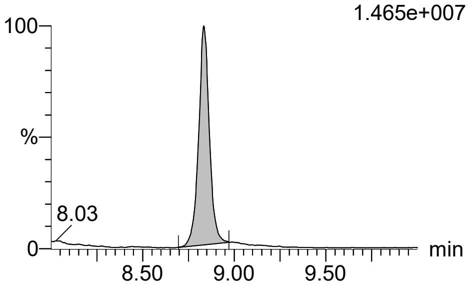

Sample Name: 20211111_L20_R3_IgM
F47:MRM of 3 channels,ES+
976.3 > 366.1
4.982e+006

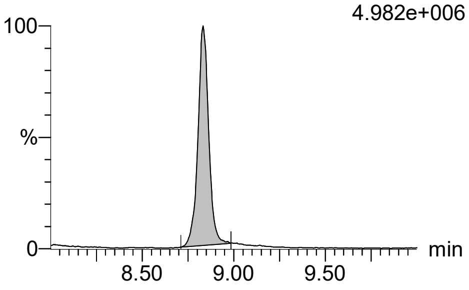

Sample Name: 20211111_L20_R3_IgM
F47:MRM of 3 channels,ES+
1220.1 > 366.1
1.058e+007

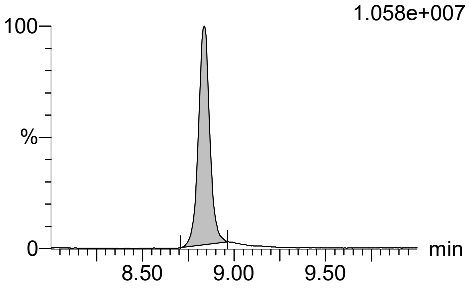

Sample Name: 20211111_H10_R3_IgM
F47:MRM of 3 channels,ES+
976.3 > 204.1
1.492e+007

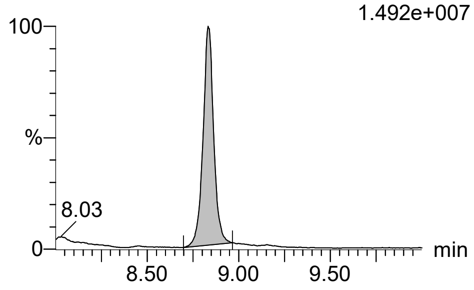

Sample Name: 20211111_H10_R3_IgM
F47:MRM of 3 channels,ES+
976.3 > 366.1
5.054e+006

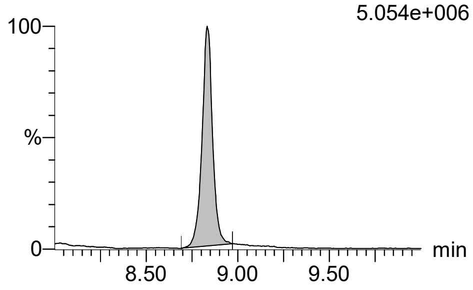

Sample Name: 20211111_H10_R3_IgM
F47:MRM of 3 channels,ES+
1220.1 > 366.1
1.066e+007

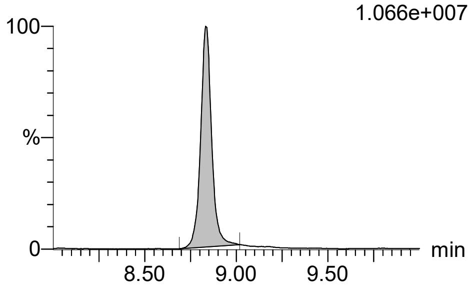

Sample Name: 20211111_A21_R3_IgM
F47:MRM of 3 channels,ES+
976.3 > 204.1
2.116e+007

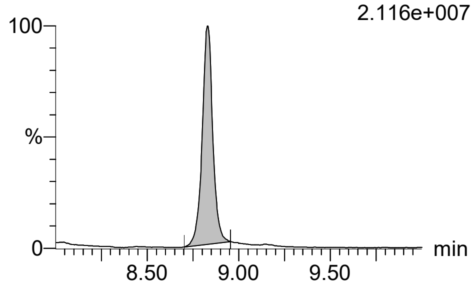

Sample Name: 20211111_A21_R3_IgM
F47:MRM of 3 channels,ES+
976.3 > 366.1
7.356e+006

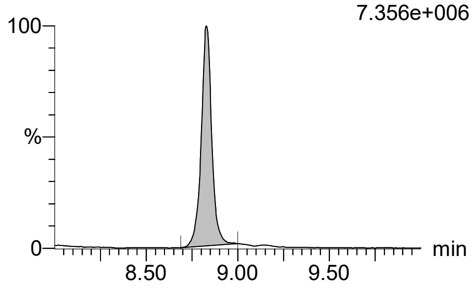

Sample Name: 20211111_A21_R3_IgM
F47:MRM of 3 channels,ES+
1220.1 > 366.1
1.573e+007

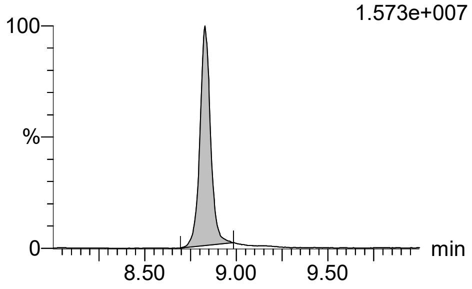

Sample Name: 20211111_A22_R3_IgM
F47:MRM of 3 channels,ES+
976.3 > 204.1
5.634e+006

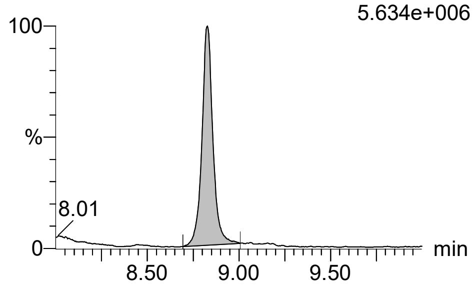

Sample Name: 20211111_A22_R3_IgM
F47:MRM of 3 channels,ES+
976.3 > 366.1
1.823e+006

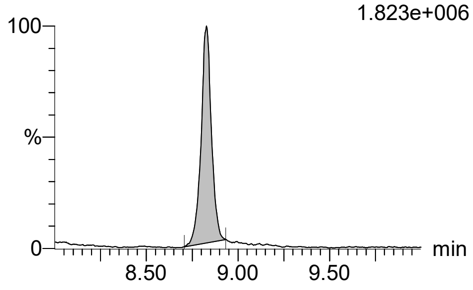

Sample Name: 20211111_A22_R3_IgM
F47:MRM of 3 channels,ES+
1220.1 > 366.1
4.088e+006

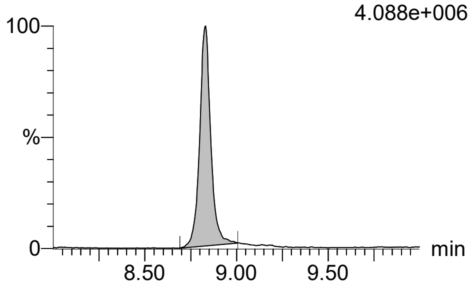

Sample Name: 20211111_L21_R3_IgM
F47:MRM of 3 channels,ES+
976.3 > 204.1
1.165e+007

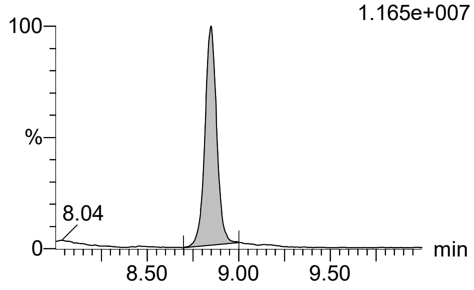

Sample Name: 20211111_L21_R3_IgM
F47:MRM of 3 channels,ES+
976.3 > 366.1
4.096e+006

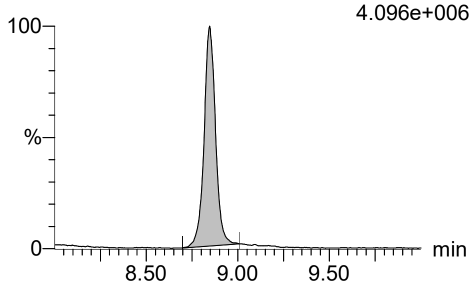

Sample Name: 20211111_L21_R3_IgM
F47:MRM of 3 channels,ES+
1220.1 > 366.1
8.068e+006

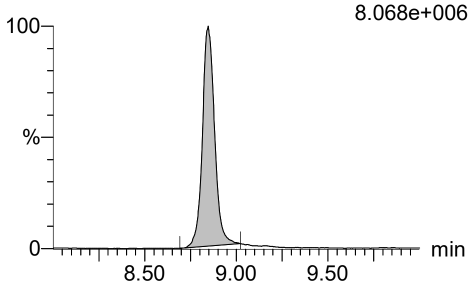

Sample Name: 20211111_L22_R3_IgM
F47:MRM of 3 channels,ES+
976.3 > 204.1
2.060e+007

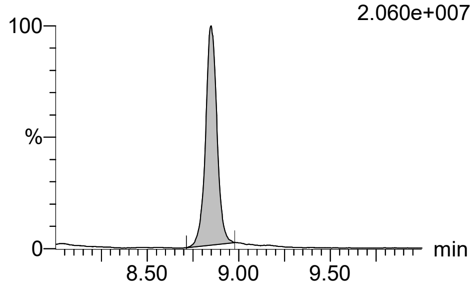

Sample Name: 20211111_L22_R3_IgM
F47:MRM of 3 channels,ES+
976.3 > 366.1
7.108e+006

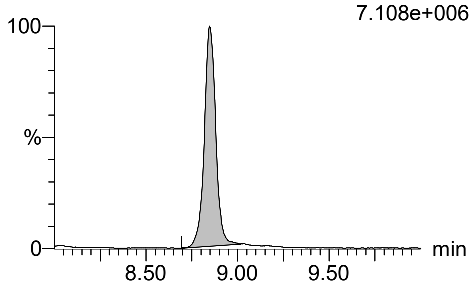

Sample Name: 20211111_L22_R3_IgM
F47:MRM of 3 channels,ES+
1220.1 > 366.1
1.510e+007

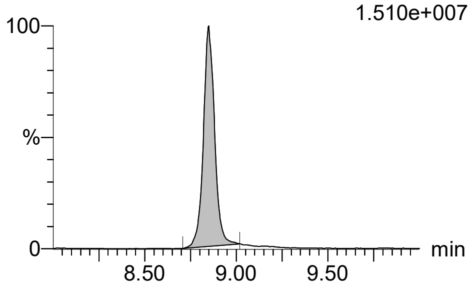

Dataset: C:\MassLynx\10.pro\20211111_clinicalsamples_IgM_IgA.qld

Last Altered: Thursday, March 09, 2023 19:58:25 台北標準時間
Printed: Friday, August 01, 2025 15:08:19 台北標準時間

Sample Name: 20211111_H11_R3_IgM
F47:MRM of 3 channels,ES+
976.3 > 204.1
1.681e+007

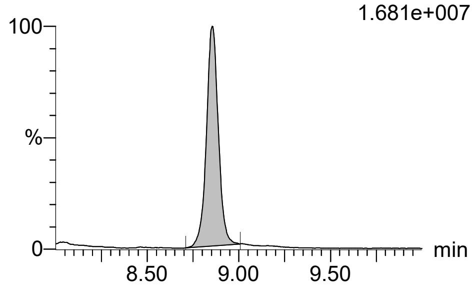

Sample Name: 20211111_H11_R3_IgM
F47:MRM of 3 channels,ES+
976.3 > 366.1
6.120e+006

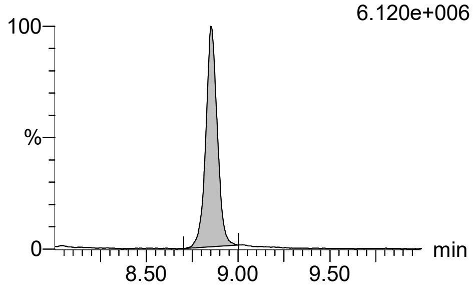

Sample Name: 20211111_H11_R3_IgM
F47:MRM of 3 channels,ES+
1220.1 > 366.1
1.202e+007

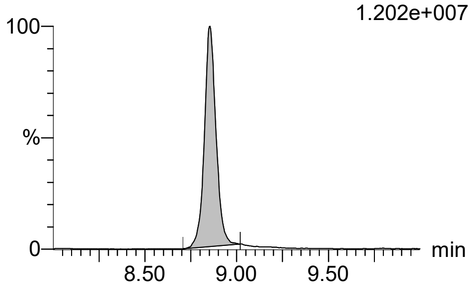

Sample Name: 20211111_A23_R3_IgM
F47:MRM of 3 channels,ES+
976.3 > 204.1
7.863e+006

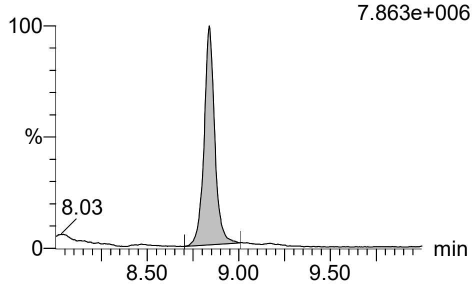

Sample Name: 20211111_A23_R3_IgM
F47:MRM of 3 channels,ES+
976.3 > 366.1
2.614e+006

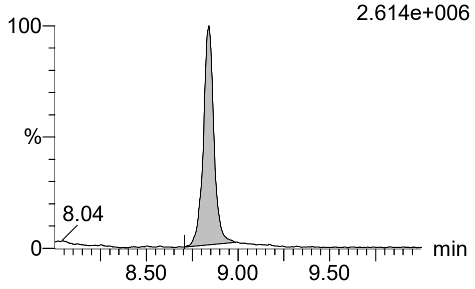

Sample Name: 20211111_A23_R3_IgM
F47:MRM of 3 channels,ES+
1220.1 > 366.1
5.705e+006

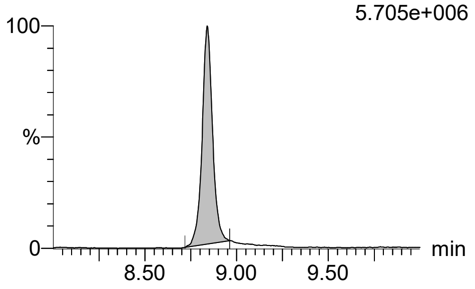

Sample Name: 20211111_A24_R3_IgM
F47:MRM of 3 channels,ES+
976.3 > 204.1
2.724e+007

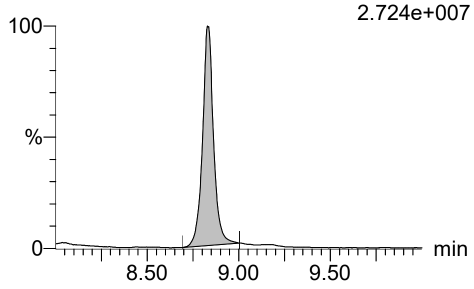

Sample Name: 20211111_A24_R3_IgM
F47:MRM of 3 channels,ES+
976.3 > 366.1
9.112e+006

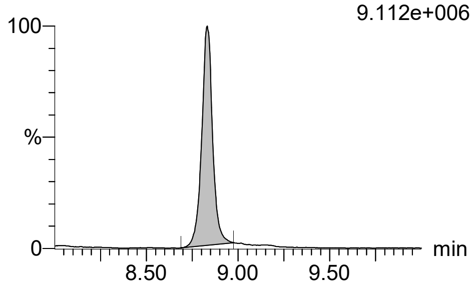

Sample Name: 20211111_A24_R3_IgM
F47:MRM of 3 channels,ES+
1220.1 > 366.1
2.065e+007

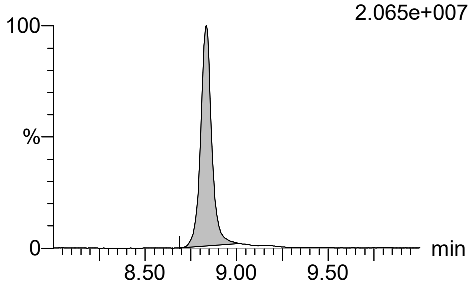

Sample Name: 20211111_L23_R3_IgM
F47:MRM of 3 channels,ES+
976.3 > 204.1
3.034e+007

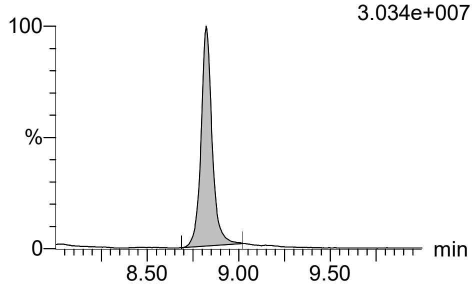

Sample Name: 20211111_L23_R3_IgM
F47:MRM of 3 channels,ES+
976.3 > 366.1
1.021e+007

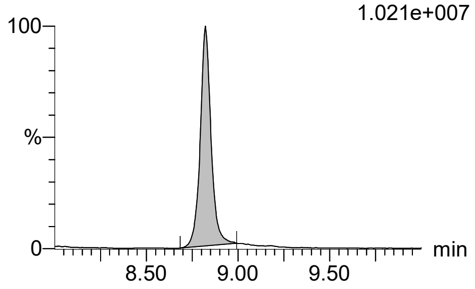

Sample Name: 20211111_L23_R3_IgM
F47:MRM of 3 channels,ES+
1220.1 > 366.1
2.376e+007

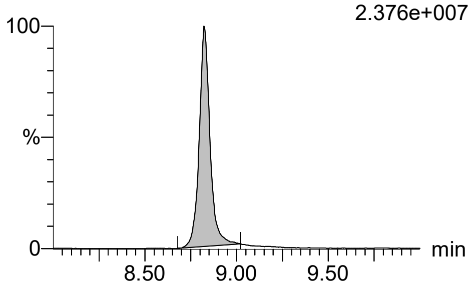

Sample Name: 20211111_L24_R3_IgM
F47:MRM of 3 channels,ES+
976.3 > 204.1
5.973e+006

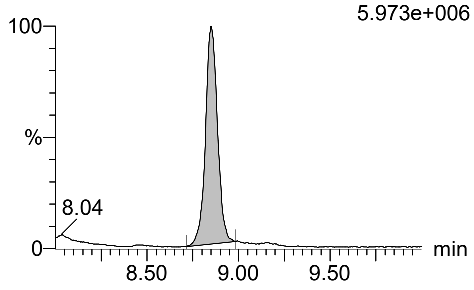

Sample Name: 20211111_L24_R3_IgM
F47:MRM of 3 channels,ES+
976.3 > 366.1
2.003e+006

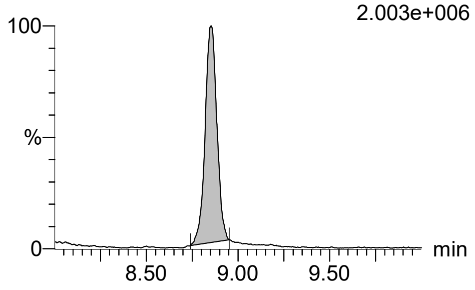

Sample Name: 20211111_L24_R3_IgM
F47:MRM of 3 channels,ES+
1220.1 > 366.1
3.947e+006

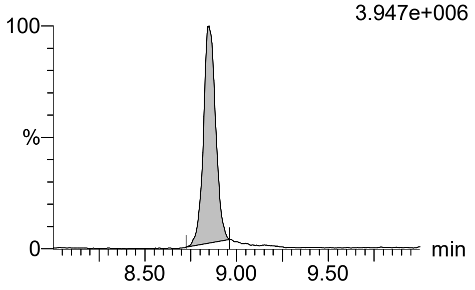

Sample Name: 20211111_H12_R3_IgM
F47:MRM of 3 channels,ES+
976.3 > 204.1
1.151e+007

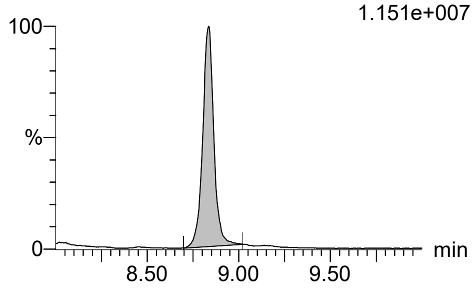

Sample Name: 20211111_H12_R3_IgM
F47:MRM of 3 channels,ES+
976.3 > 366.1
4.043e+006

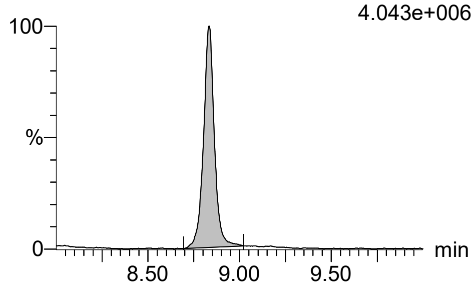

Sample Name: 20211111_H12_R3_IgM
F47:MRM of 3 channels,ES+
1220.1 > 366.1
8.133e+006

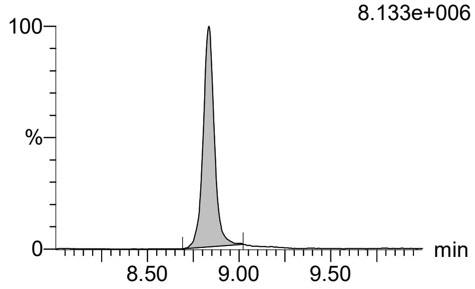

Sample Name: 20211111_QC_R22_IgM
F47:MRM of 3 channels,ES+
976.3 > 204.1
2.163e+007

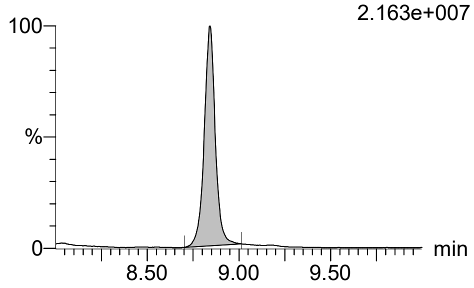

Sample Name: 20211111_QC_R22_IgM
F47:MRM of 3 channels,ES+
976.3 > 366.1
7.306e+006

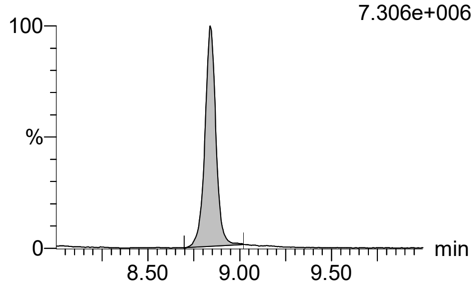

Sample Name: 20211111_QC_R22_IgM
F47:MRM of 3 channels,ES+
1220.1 > 366.1
1.491e+007

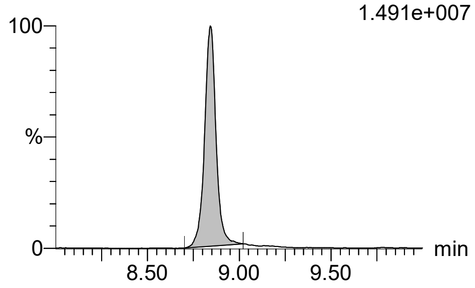

Sample Name: 20211111_A25_R3_IgM
F47:MRM of 3 channels,ES+
976.3 > 204.1
3.950e+007

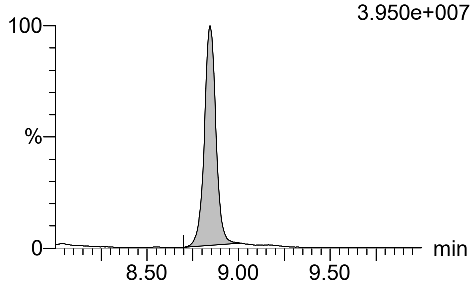

Sample Name: 20211111_A25_R3_IgM
F47:MRM of 3 channels,ES+
976.3 > 366.1
1.310e+007

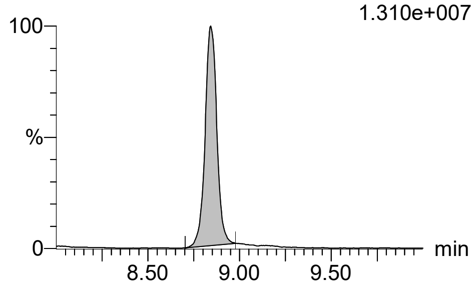

Sample Name: 20211111_A25_R3_IgM
F47:MRM of 3 channels,ES+
1220.1 > 366.1
2.814e+007

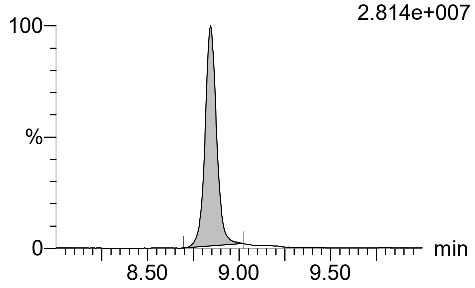

Sample Name: 20211111_A26_R3_IgM
F47:MRM of 3 channels,ES+
976.3 > 204.1
1.872e+007

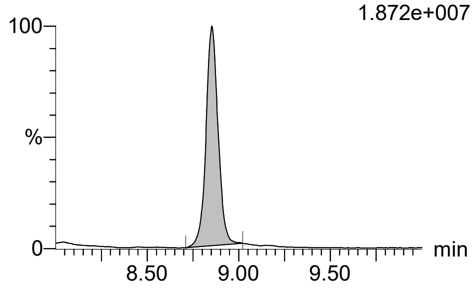

Sample Name: 20211111_A26_R3_IgM
F47:MRM of 3 channels,ES+
976.3 > 366.1
6.396e+006

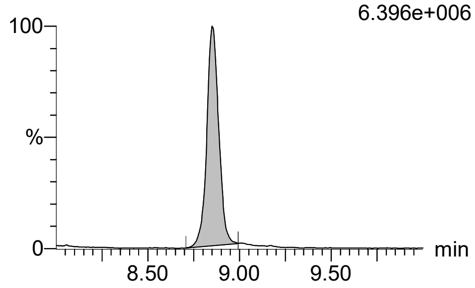

Sample Name: 20211111_A26_R3_IgM
F47:MRM of 3 channels,ES+
1220.1 > 366.1
1.282e+007

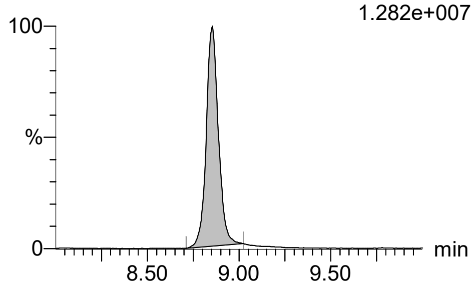

Sample Name: 20211111_L25_R3_IgM
F47:MRM of 3 channels,ES+
976.3 > 204.1
1.651e+007

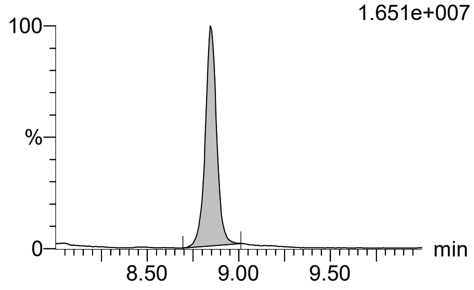

Sample Name: 20211111_L25_R3_IgM
F47:MRM of 3 channels,ES+
976.3 > 366.1
5.452e+006

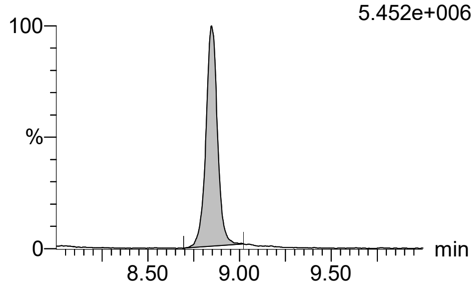

Sample Name: 20211111_L25_R3_IgM
F47:MRM of 3 channels,ES+
1220.1 > 366.1
1.133e+007

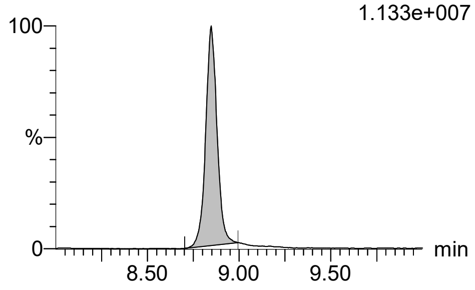

Dataset: C:\MassLynx\10.pro\20211111_clinicalsamples_IgM_IgA.qld

Last Altered: Thursday, March 09, 2023 19:58:25 台北標準時間
Printed: Friday, August 01, 2025 15:08:19 台北標準時間

Sample Name: 20211111_L26_R3_IgM
F47:MRM of 3 channels,ES+
976.3 > 204.1
1.182e+007

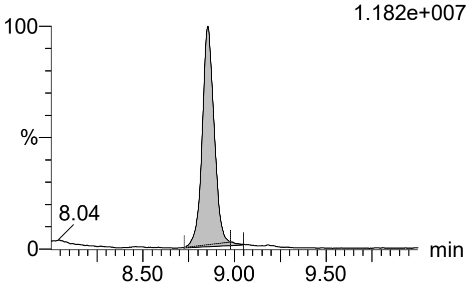

Sample Name: 20211111_L26_R3_IgM
F47:MRM of 3 channels,ES+
976.3 > 366.1
3.889e+006

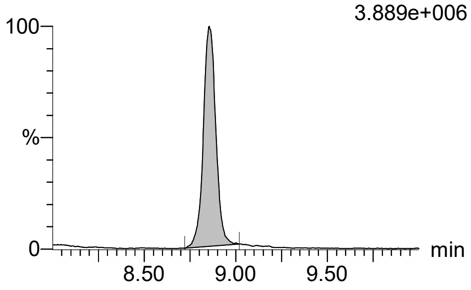

Sample Name: 20211111_L26_R3_IgM
F47:MRM of 3 channels,ES+
1220.1 > 366.1
8.122e+006

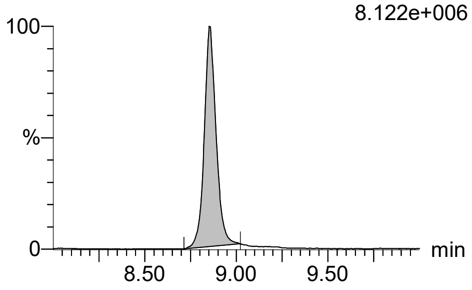

Sample Name: 20211111_H13_R3_IgM
F47:MRM of 3 channels,ES+
976.3 > 204.1
2.637e+007

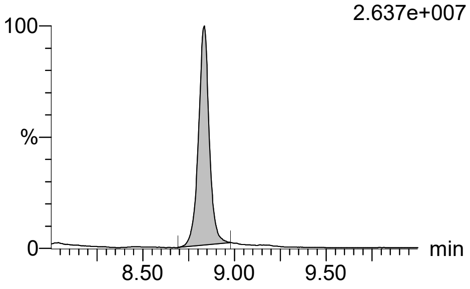

Sample Name: 20211111_H13_R3_IgM
F47:MRM of 3 channels,ES+
976.3 > 366.1
9.081e+006

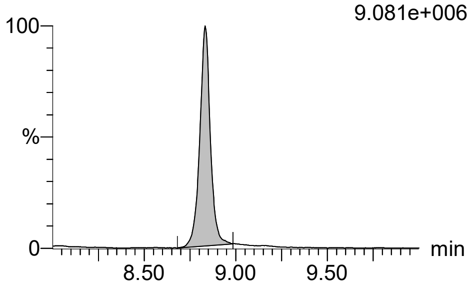

Sample Name: 20211111_H13_R3_IgM
F47:MRM of 3 channels,ES+
1220.1 > 366.1
1.897e+007

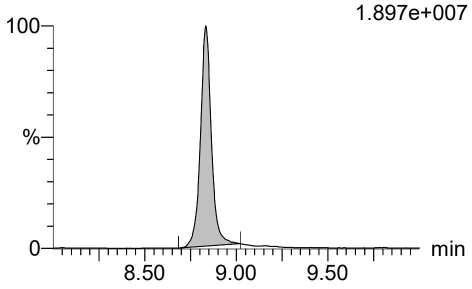

Sample Name: 20211111_A27_R3_IgM
F47:MRM of 3 channels,ES+
976.3 > 204.1
2.052e+007

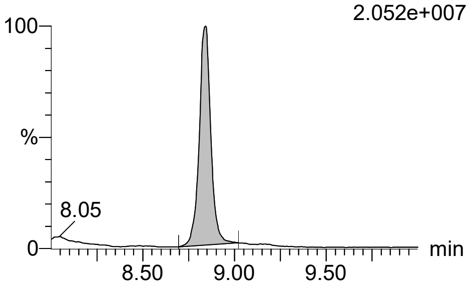

Sample Name: 20211111_A27_R3_IgM
F47:MRM of 3 channels,ES+
976.3 > 366.1
7.225e+006

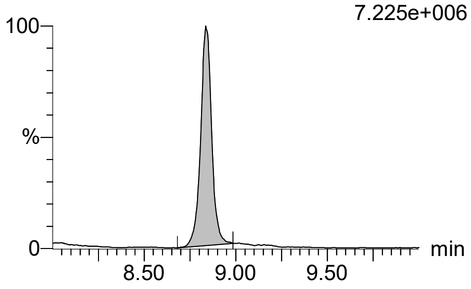

Sample Name: 20211111_A27_R3_IgM
F47:MRM of 3 channels,ES+
1220.1 > 366.1
1.468e+007

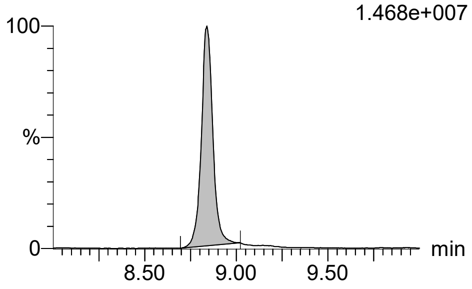

Sample Name: 20211111_A28_R3_IgM
F47:MRM of 3 channels,ES+
976.3 > 204.1
1.231e+007

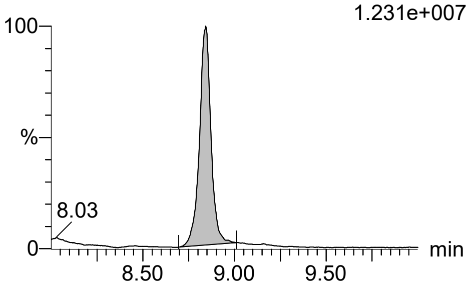

Sample Name: 20211111_A28_R3_IgM
F47:MRM of 3 channels,ES+
976.3 > 366.1
4.108e+006

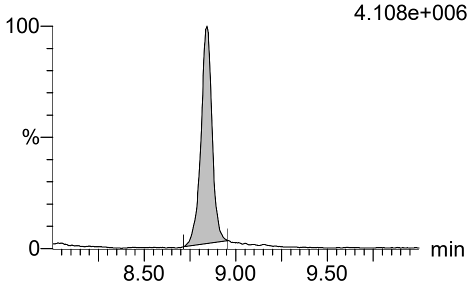

Sample Name: 20211111_A28_R3_IgM
F47:MRM of 3 channels,ES+
1220.1 > 366.1
8.820e+006

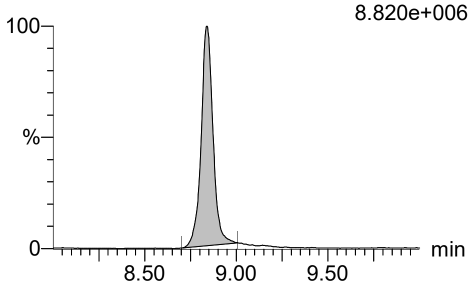

Sample Name: 20211111_L27_R3_IgM
F47:MRM of 3 channels,ES+
976.3 > 204.1
2.159e+007

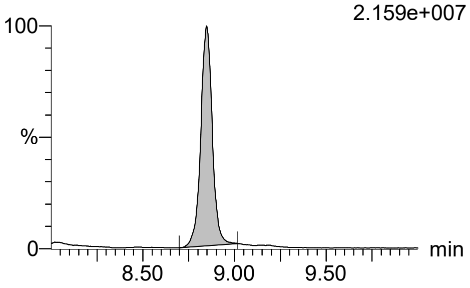

Sample Name: 20211111_L27_R3_IgM
F47:MRM of 3 channels,ES+
976.3 > 366.1
7.390e+006

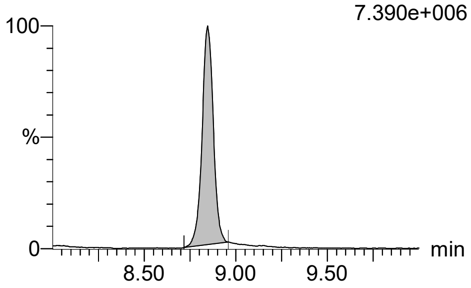

Sample Name: 20211111_L27_R3_IgM
F47:MRM of 3 channels,ES+
1220.1 > 366.1
1.496e+007

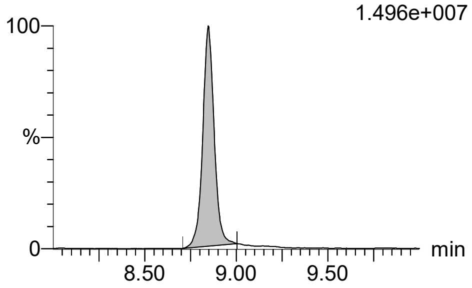

Dataset: C:\MassLynx\10.pro\20211111_clinicalsamples_IgM_IgA.qld

Last Altered: Thursday, March 09, 2023 19:58:25 台北標準時間
Printed: Friday, August 01, 2025 15:08:19 台北標準時間

Sample Name: 20211111_L28_R3_IgM
F47:MRM of 3 channels,ES+
976.3 > 204.1
2.272e+007

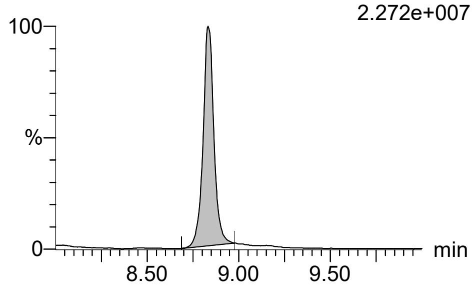

Sample Name: 20211111_L28_R3_IgM
F47:MRM of 3 channels,ES+
976.3 > 366.1
7.595e+006

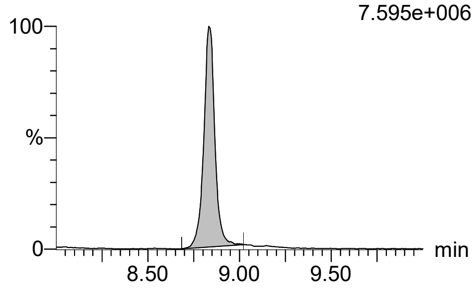

Sample Name: 20211111_L28_R3_IgM
F47:MRM of 3 channels,ES+
1220.1 > 366.1
1.611e+007

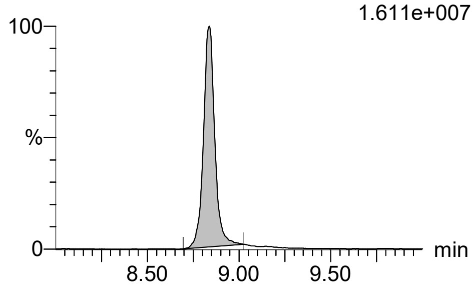

Sample Name: 20211111_H14_R3_IgM
F47:MRM of 3 channels,ES+
976.3 > 204.1
2.872e+007

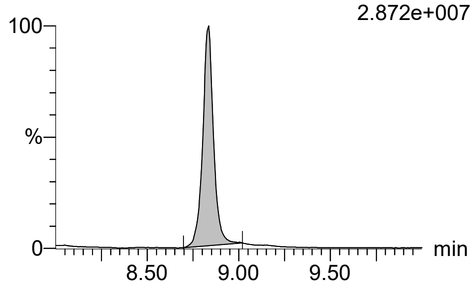

Sample Name: 20211111_H14_R3_IgM
F47:MRM of 3 channels,ES+
976.3 > 366.1
9.409e+006

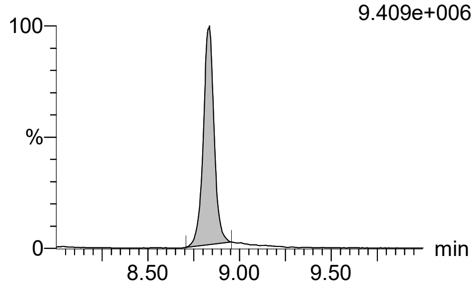

Sample Name: 20211111_H14_R3_IgM
F47:MRM of 3 channels,ES+
1220.1 > 366.1
2.038e+007

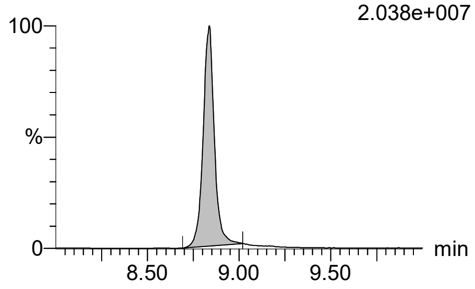

Sample Name: 20211111_A29_R3_IgM
F47:MRM of 3 channels,ES+
976.3 > 204.1
1.671e+006

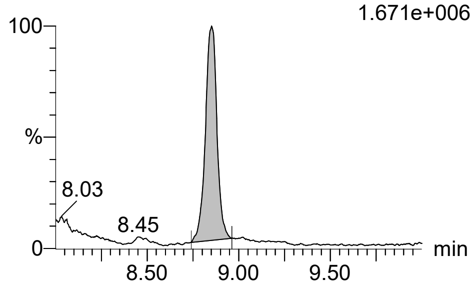

Sample Name: 20211111_A29_R3_IgM
F47:MRM of 3 channels,ES+
976.3 > 366.1
5.779e+005

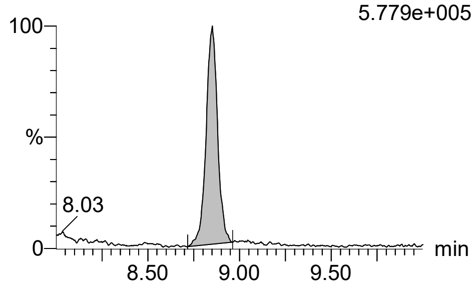

Sample Name: 20211111_A29_R3_IgM
F47:MRM of 3 channels,ES+
1220.1 > 366.1
1.202e+006

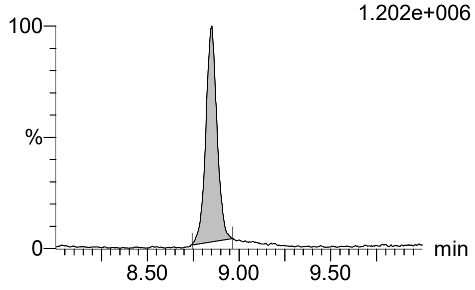

Sample Name: 20211111_A30_R3_IgM
F47:MRM of 3 channels,ES+
976.3 > 204.1
2.101e+007

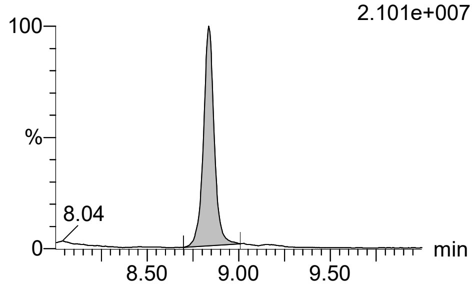

Sample Name: 20211111_A30_R3_IgM
F47:MRM of 3 channels,ES+
976.3 > 366.1
6.884e+006

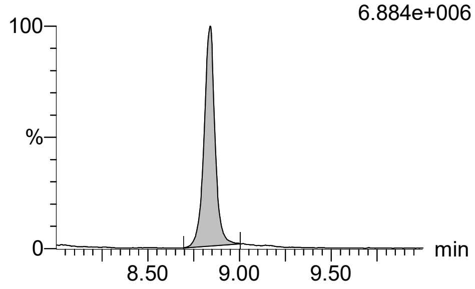

Sample Name: 20211111_A30_R3_IgM
F47:MRM of 3 channels,ES+
1220.1 > 366.1
1.494e+007

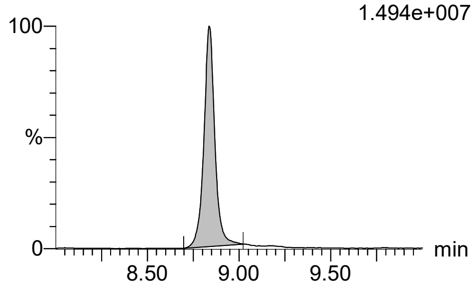

Sample Name: 20211111_QC_R23_IgM
F47:MRM of 3 channels,ES+
976.3 > 204.1
2.019e+007

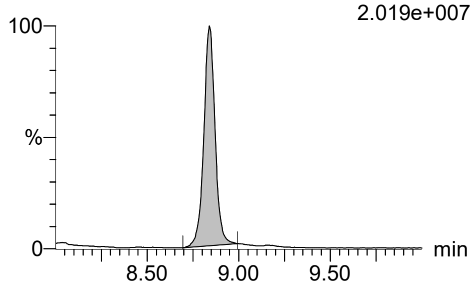

Sample Name: 20211111_QC_R23_IgM
F47:MRM of 3 channels,ES+
976.3 > 366.1
7.037e+006

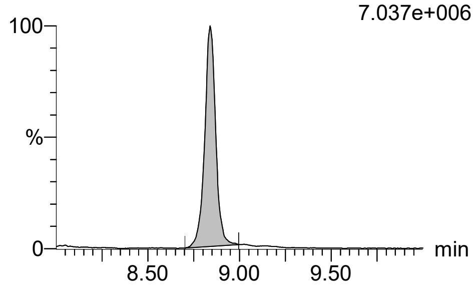

Sample Name: 20211111_QC_R23_IgM
F47:MRM of 3 channels,ES+
1220.1 > 366.1
1.405e+007

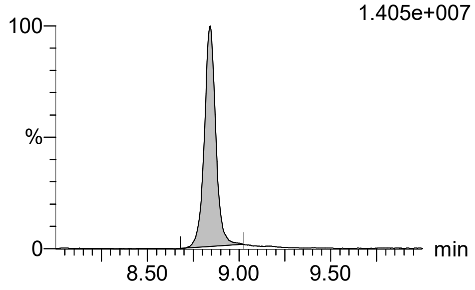

Dataset: C:\MassLynx\10.pro\20211111_clinicalsamples_IgM_IgA.qld

Last Altered: Thursday, March 09, 2023 19:58:25 台北標準時間
Printed: Friday, August 01, 2025 15:08:19 台北標準時間

Sample Name: 20211111_L29_R3_IgM
F47:MRM of 3 channels,ES+
976.3 > 204.1
7.019e+006

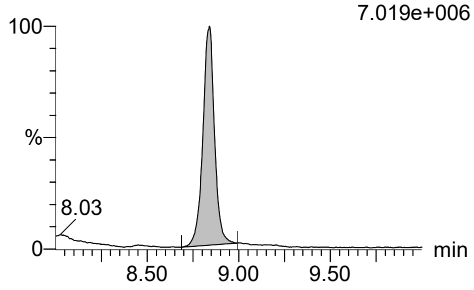

Sample Name: 20211111_L29_R3_IgM
F47:MRM of 3 channels,ES+
976.3 > 366.1
2.460e+006

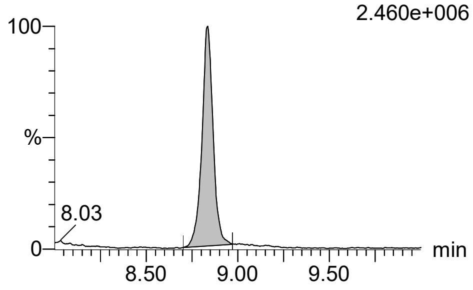

Sample Name: 20211111_L29_R3_IgM
F47:MRM of 3 channels,ES+
1220.1 > 366.1
5.232e+006

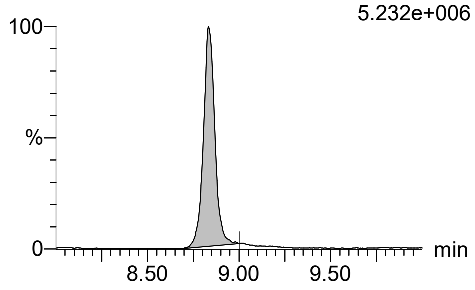

Sample Name: 20211111_L30_R3_IgM
F47:MRM of 3 channels,ES+
976.3 > 204.1
3.542e+006

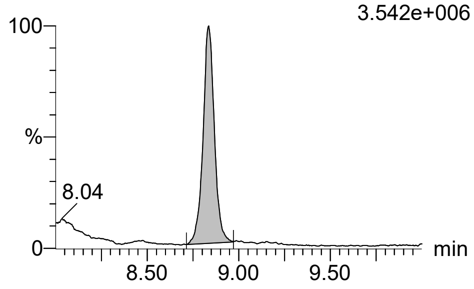

Sample Name: 20211111_L30_R3_IgM
F47:MRM of 3 channels,ES+
976.3 > 366.1
1.145e+006

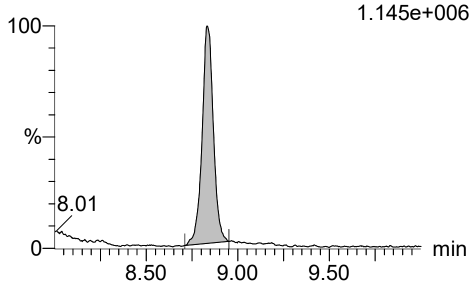

Sample Name: 20211111_L30_R3_IgM
F47:MRM of 3 channels,ES+
1220.1 > 366.1
2.462e+006

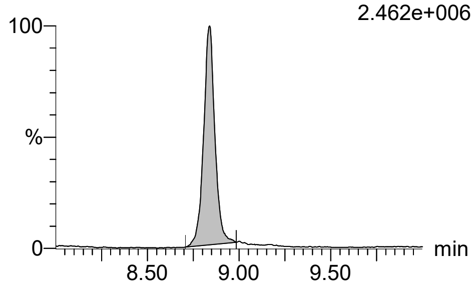

Sample Name: 20211111_H15_R3_IgM
F47:MRM of 3 channels,ES+
976.3 > 204.1
1.468e+007

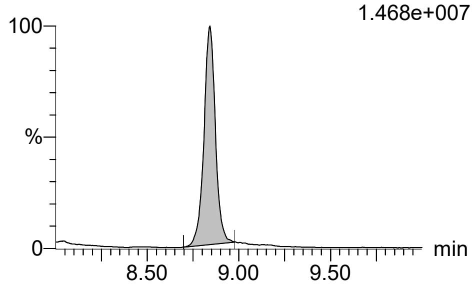

Sample Name: 20211111_H15_R3_IgM
F47:MRM of 3 channels,ES+
976.3 > 366.1
4.871e+006

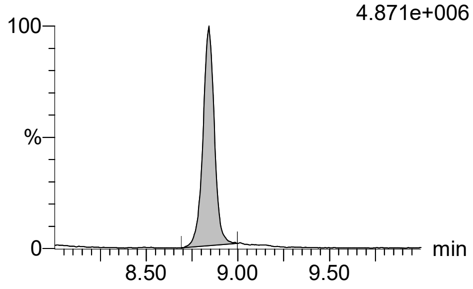

Sample Name: 20211111_H15_R3_IgM
F47:MRM of 3 channels,ES+
1220.1 > 366.1
1.017e+007

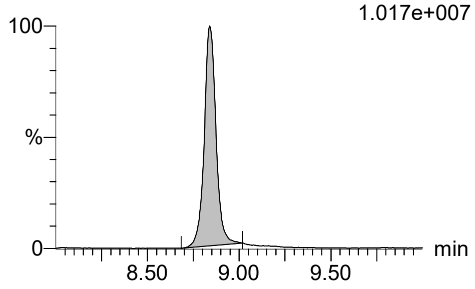

Sample Name: 20211111_A31_R3_IgM
F47:MRM of 3 channels,ES+
976.3 > 204.1
8.050e+006

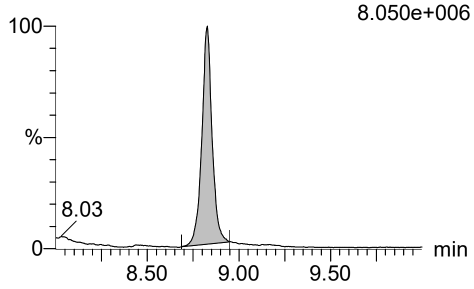

Sample Name: 20211111_A31_R3_IgM
F47:MRM of 3 channels,ES+
976.3 > 366.1
2.718e+006

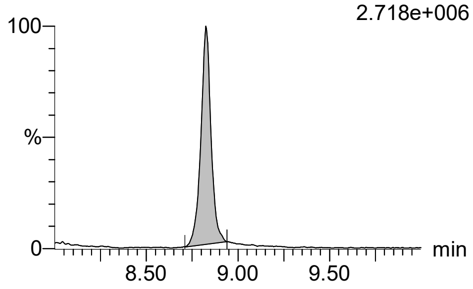

Sample Name: 20211111_A31_R3_IgM
F47:MRM of 3 channels,ES+
1220.1 > 366.1
5.494e+006

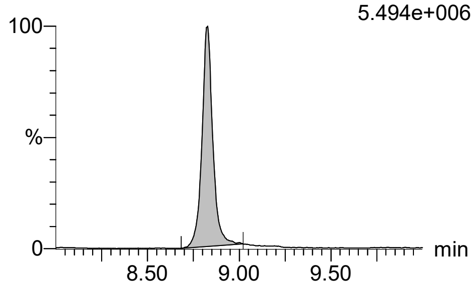

Sample Name: 20211111_A32_R3_IgM
F47:MRM of 3 channels,ES+
976.3 > 204.1
2.132e+007

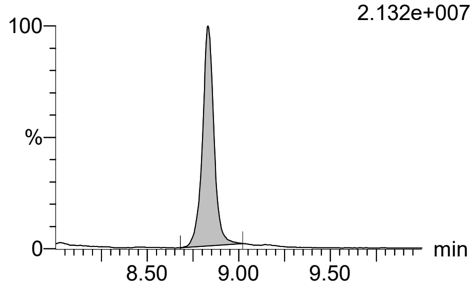

Sample Name: 20211111_A32_R3_IgM
F47:MRM of 3 channels,ES+
976.3 > 366.1
7.244e+006

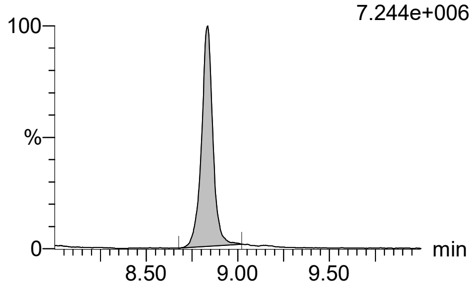

Sample Name: 20211111_A32_R3_IgM
F47:MRM of 3 channels,ES+
1220.1 > 366.1
1.563e+007

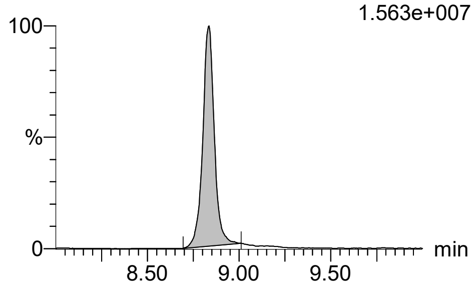

Sample Name: 20211111_L31_R3_IgM
F47:MRM of 3 channels,ES+
976.3 > 204.1
5.161e+006

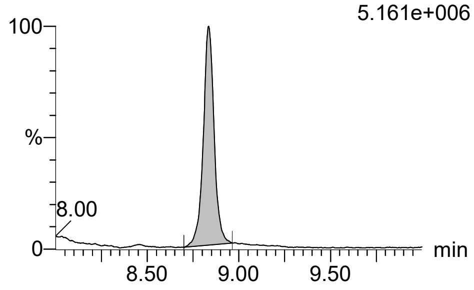

Sample Name: 20211111_L31_R3_IgM
F47:MRM of 3 channels,ES+
976.3 > 366.1
1.706e+006

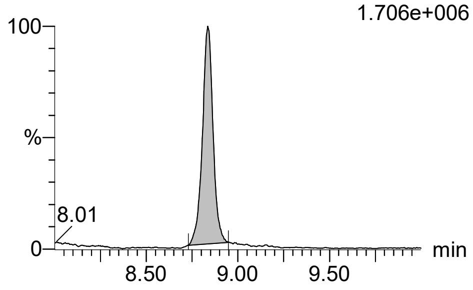

Sample Name: 20211111_L31_R3_IgM
F47:MRM of 3 channels,ES+
1220.1 > 366.1
3.325e+006

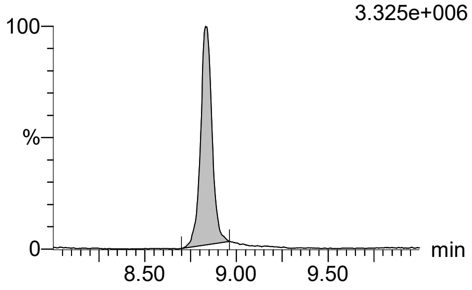

Sample Name: 20211111_L32_R3_IgM
F47:MRM of 3 channels,ES+
976.3 > 204.1
4.163e+007

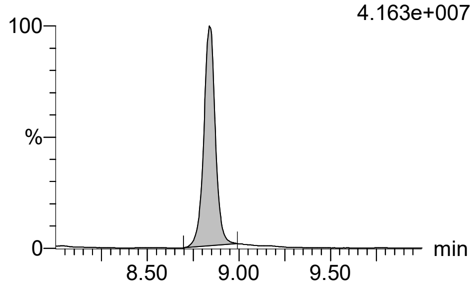

Sample Name: 20211111_L32_R3_IgM
F47:MRM of 3 channels,ES+
976.3 > 366.1
1.446e+007

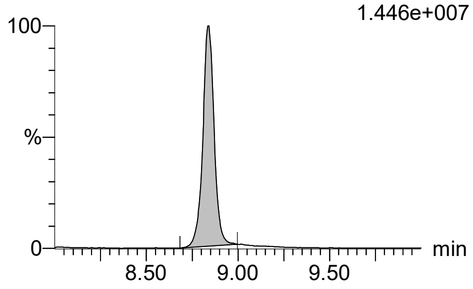

Sample Name: 20211111_L32_R3_IgM
F47:MRM of 3 channels,ES+
1220.1 > 366.1
2.893e+007

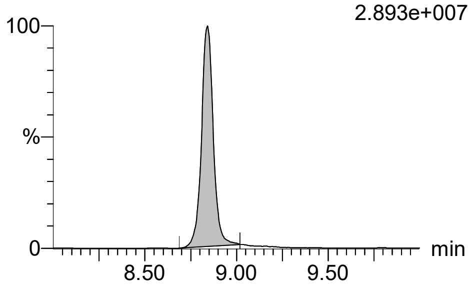

Sample Name: 20211111_H16_R3_IgM
F47:MRM of 3 channels,ES+
976.3 > 204.1
4.621e+007

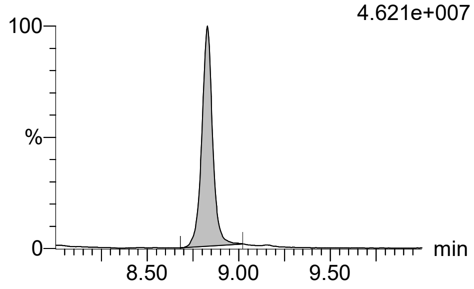

Sample Name: 20211111_H16_R3_IgM
F47:MRM of 3 channels,ES+
976.3 > 366.1
1.530e+007

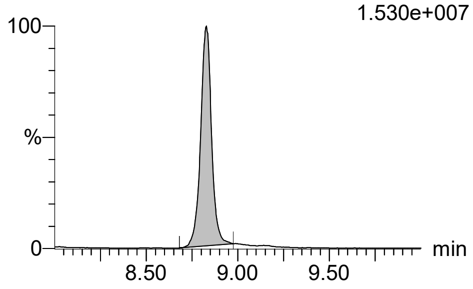

Sample Name: 20211111_H16_R3_IgM
F47:MRM of 3 channels,ES+
1220.1 > 366.1
3.195e+007

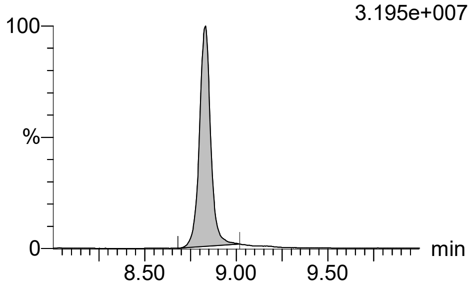

Sample Name: 20211111_A33_R3_IgM
F47:MRM of 3 channels,ES+
976.3 > 204.1
2.019e+007

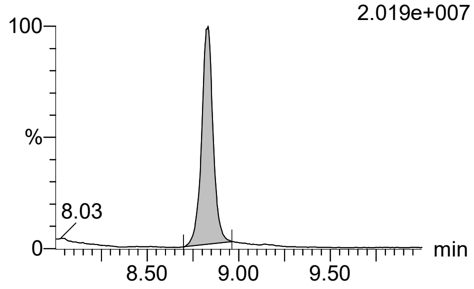

Sample Name: 20211111_A33_R3_IgM
F47:MRM of 3 channels,ES+
976.3 > 366.1
6.848e+006

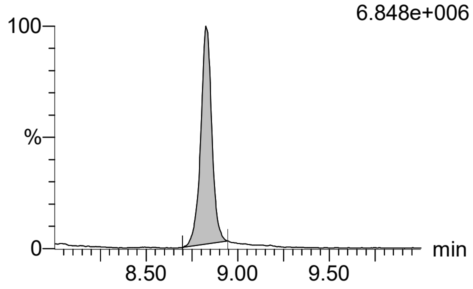

Sample Name: 20211111_A33_R3_IgM
F47:MRM of 3 channels,ES+
1220.1 > 366.1
1.536e+007

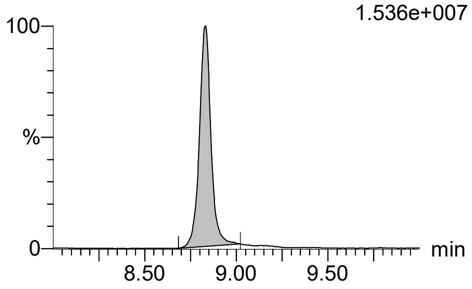

Sample Name: 20211111_A34_R3_IgM
F47:MRM of 3 channels,ES+
976.3 > 204.1
6.483e+006

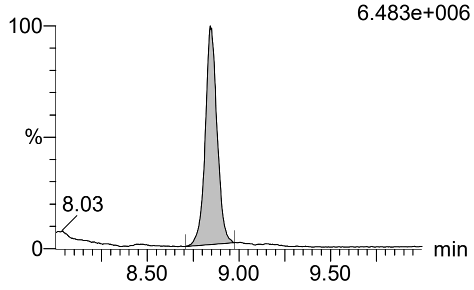

Sample Name: 20211111_A34_R3_IgM
F47:MRM of 3 channels,ES+
976.3 > 366.1
2.049e+006

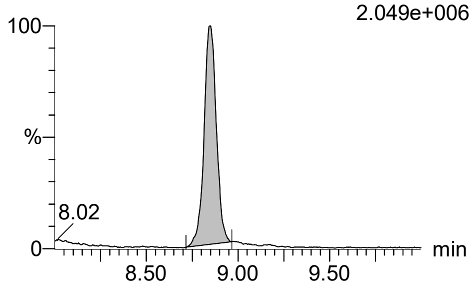

Sample Name: 20211111_A34_R3_IgM
F47:MRM of 3 channels,ES+
1220.1 > 366.1
4.227e+006

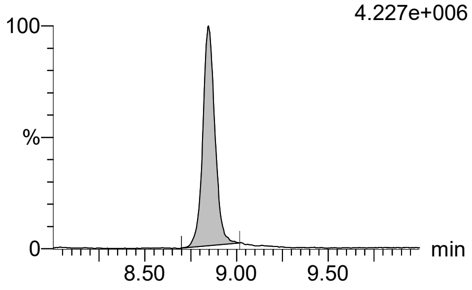

Sample Name: 20211111_H17_R3_IgM
F47:MRM of 3 channels,ES+
976.3 > 204.1
9.443e+007

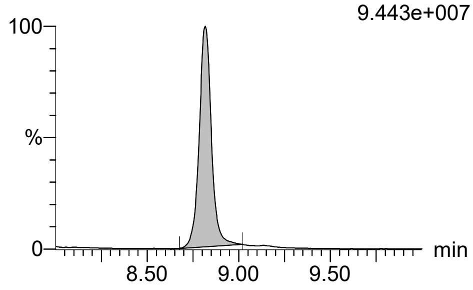

Sample Name: 20211111_H17_R3_IgM
F47:MRM of 3 channels,ES+
976.3 > 366.1
3.194e+007

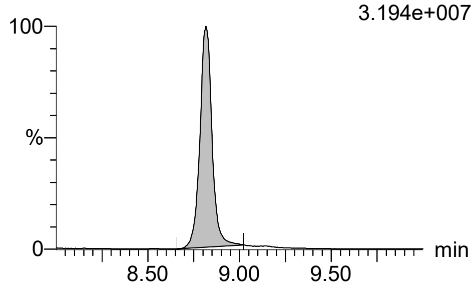

Sample Name: 20211111_H17_R3_IgM
F47:MRM of 3 channels,ES+
1220.1 > 366.1
7.294e+007

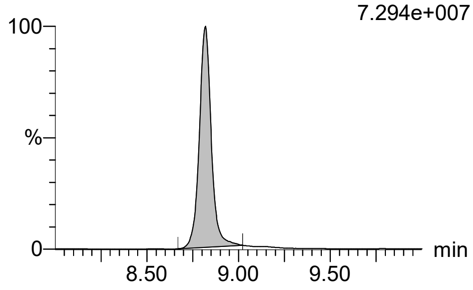

Sample Name: 20211111_A35_R3_IgM
F47:MRM of 3 channels,ES+
976.3 > 204.1
7.275e+006

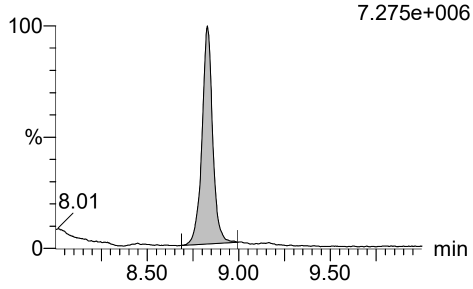

Sample Name: 20211111_A35_R3_IgM
F47:MRM of 3 channels,ES+
976.3 > 366.1
2.317e+006

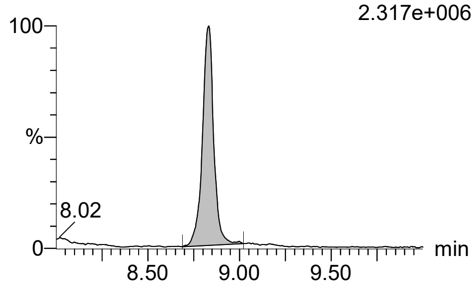

Sample Name: 20211111_A35_R3_IgM
F47:MRM of 3 channels,ES+
1220.1 > 366.1
5.019e+006

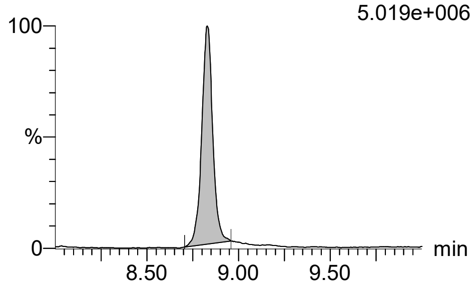

Sample Name: 20211111_QC_R24_IgM
F47:MRM of 3 channels,ES+
976.3 > 204.1
1.820e+007

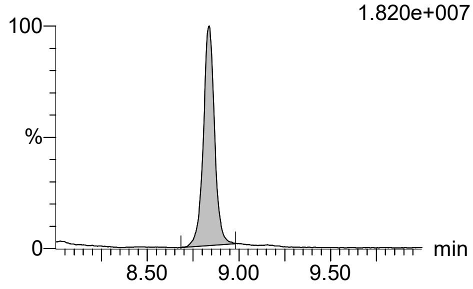

Sample Name: 20211111_QC_R24_IgM
F47:MRM of 3 channels,ES+
976.3 > 366.1
6.537e+006

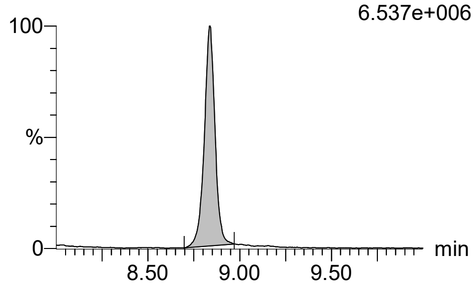

Sample Name: 20211111_QC_R24_IgM
F47:MRM of 3 channels,ES+
1220.1 > 366.1
1.245e+007

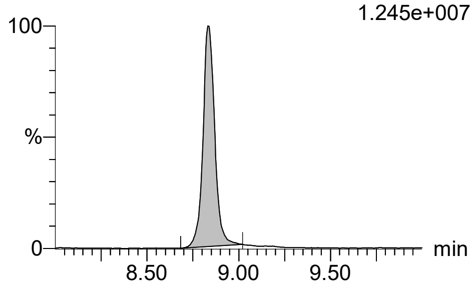

Sample Name: 20211111_A36_R3_IgM
F47:MRM of 3 channels,ES+
976.3 > 204.1
1.196e+007

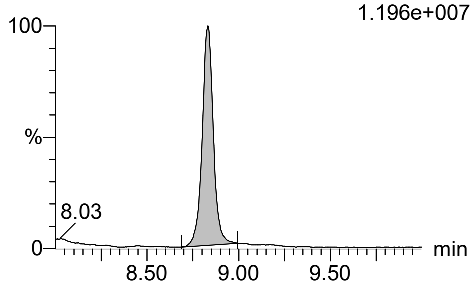

Sample Name: 20211111_A36_R3_IgM
F47:MRM of 3 channels,ES+
976.3 > 366.1
4.174e+006

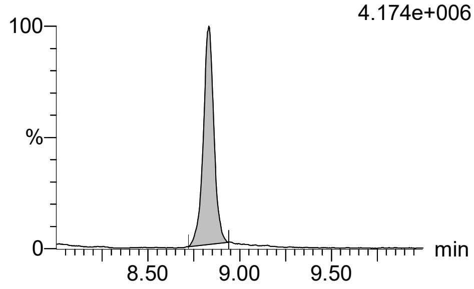

Sample Name: 20211111_A36_R3_IgM
F47:MRM of 3 channels,ES+
1220.1 > 366.1
8.669e+006

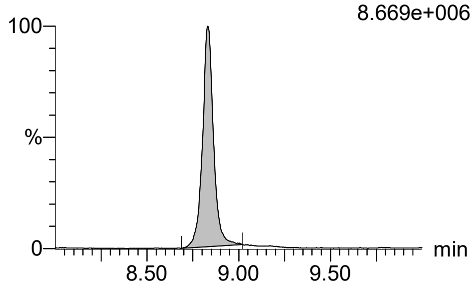

Sample Name: 20211111_H18_R3_IgM
F47:MRM of 3 channels,ES+
976.3 > 204.1
5.815e+007

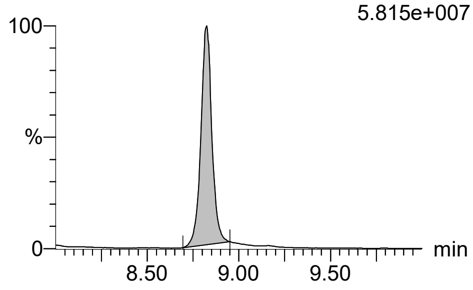

Sample Name: 20211111_H18_R3_IgM
F47:MRM of 3 channels,ES+
976.3 > 366.1
1.981e+007

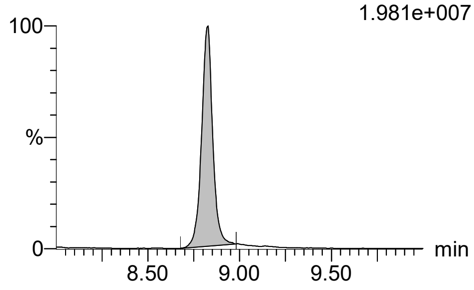

Sample Name: 20211111_H18_R3_IgM
F47:MRM of 3 channels,ES+
1220.1 > 366.1
4.201e+007

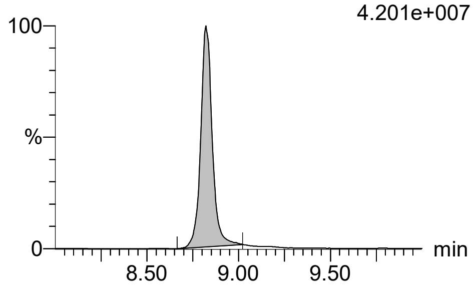

Sample Name: 20211111_A37_R3_IgM
F47:MRM of 3 channels,ES+
976.3 > 204.1
1.735e+007

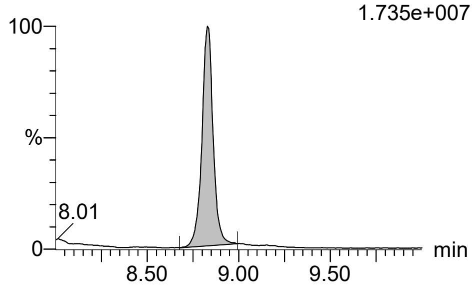

Sample Name: 20211111_A37_R3_IgM
F47:MRM of 3 channels,ES+
976.3 > 366.1
5.867e+006

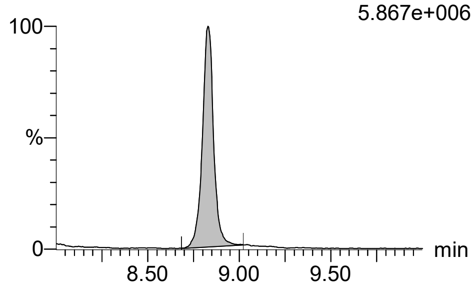

Sample Name: 20211111_A37_R3_IgM
F47:MRM of 3 channels,ES+
1220.1 > 366.1
1.248e+007

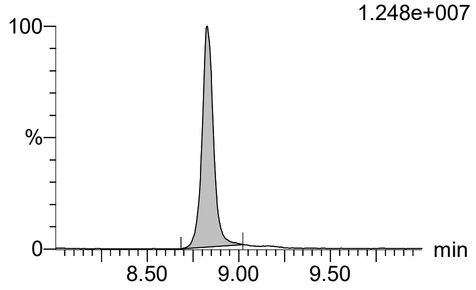

Sample Name: 20211111_A38_R3_IgM
F47:MRM of 3 channels,ES+
976.3 > 204.1
2.105e+007

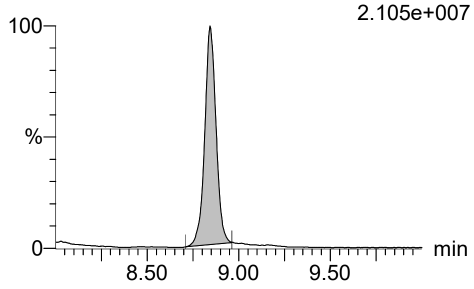

Sample Name: 20211111_A38_R3_IgM
F47:MRM of 3 channels,ES+
976.3 > 366.1
7.112e+006

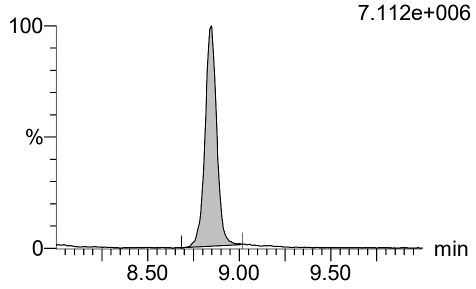

Sample Name: 20211111_A38_R3_IgM
F47:MRM of 3 channels,ES+
1220.1 > 366.1
1.432e+007

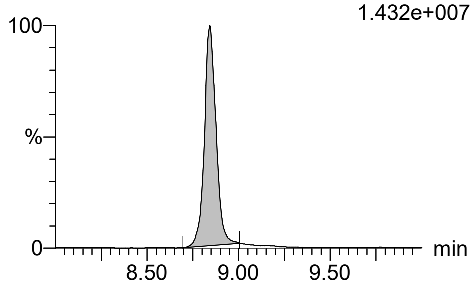

Sample Name: 20211111_H19_R3_IgM
F47:MRM of 3 channels,ES+
976.3 > 204.1
2.640e+007

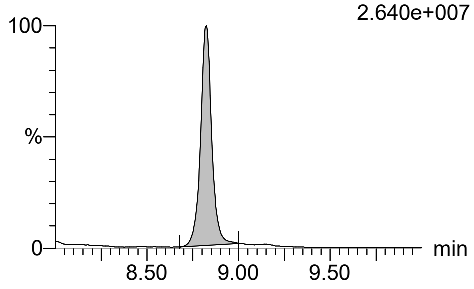

Sample Name: 20211111_H19_R3_IgM
F47:MRM of 3 channels,ES+
976.3 > 366.1
8.788e+006

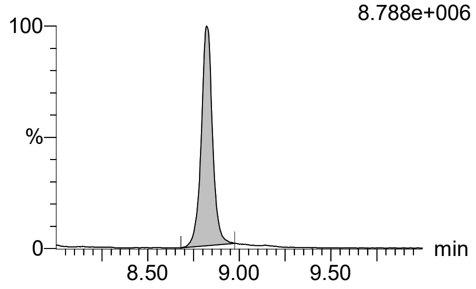

Sample Name: 20211111_H19_R3_IgM
F47:MRM of 3 channels,ES+
1220.1 > 366.1
1.928e+007

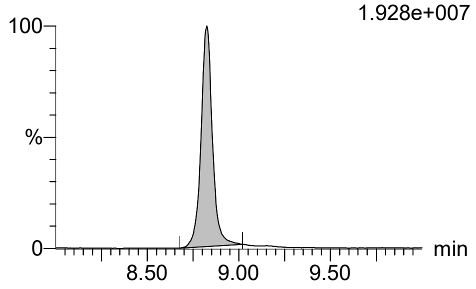

Sample Name: 20211111_A39_R3_IgM
F47:MRM of 3 channels,ES+
976.3 > 204.1
1.971e+007

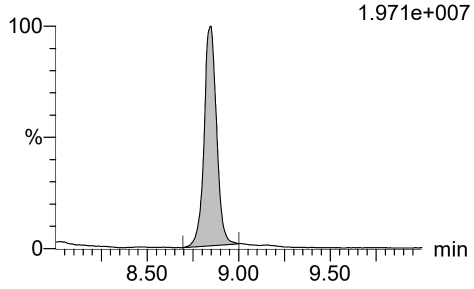

Sample Name: 20211111_A39_R3_IgM
F47:MRM of 3 channels,ES+
976.3 > 366.1
7.213e+006

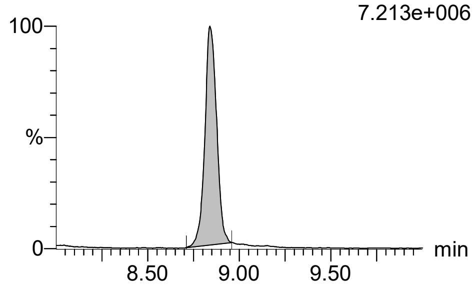

Sample Name: 20211111_A39_R3_IgM
F47:MRM of 3 channels,ES+
1220.1 > 366.1
1.439e+007

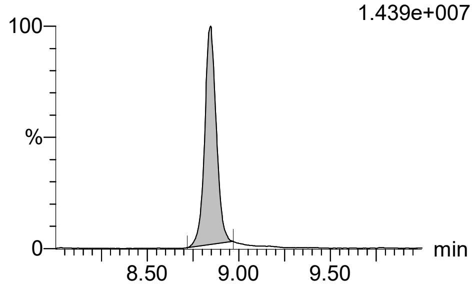

Sample Name: 20211111_A40_R3_IgM
F47:MRM of 3 channels,ES+
976.3 > 204.1
7.618e+006

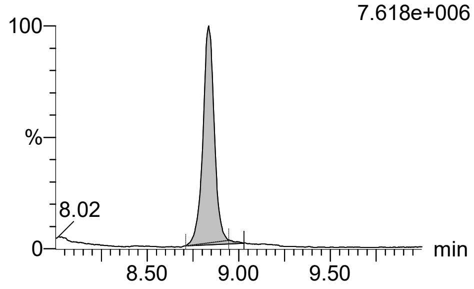

Sample Name: 20211111_A40_R3_IgM
F47:MRM of 3 channels,ES+
976.3 > 366.1
2.661e+006

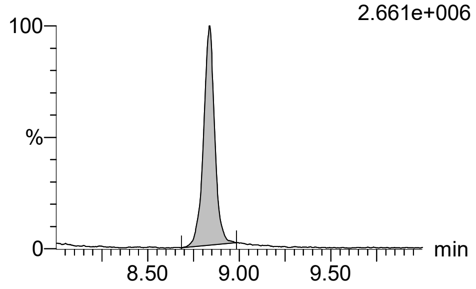

Sample Name: 20211111_A40_R3_IgM
F47:MRM of 3 channels,ES+
1220.1 > 366.1
5.745e+006

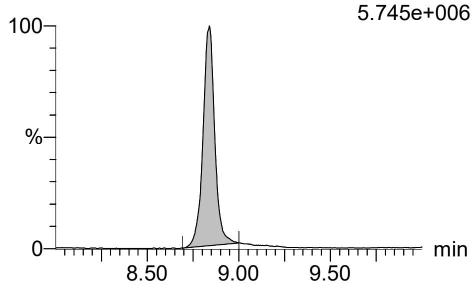

Sample Name: 20211111_H20_R3_IgM
F47:MRM of 3 channels,ES+
976.3 > 204.1
7.215e+007

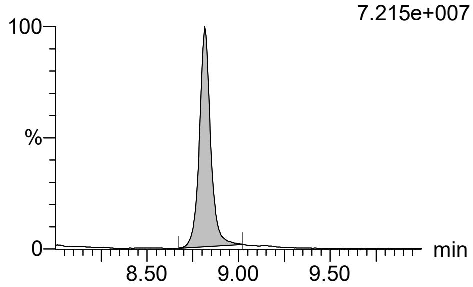

Sample Name: 20211111_H20_R3_IgM
F47:MRM of 3 channels,ES+
976.3 > 366.1
2.386e+007

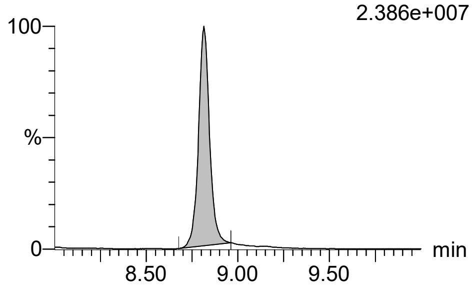

Sample Name: 20211111_H20_R3_IgM
F47:MRM of 3 channels,ES+
1220.1 > 366.1
5.437e+007

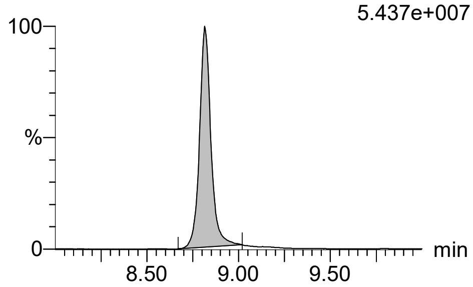

Sample Name: 20211111_A41_R3_IgM
F47:MRM of 3 channels,ES+
976.3 > 204.1
7.395e+006

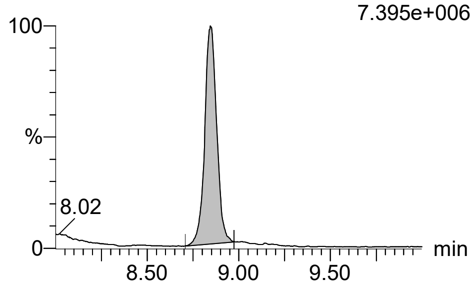

Sample Name: 20211111_A41_R3_IgM
F47:MRM of 3 channels,ES+
976.3 > 366.1
2.520e+006

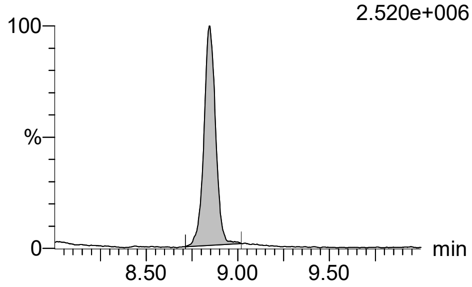

Sample Name: 20211111_A41_R3_IgM
F47:MRM of 3 channels,ES+
1220.1 > 366.1
5.135e+006

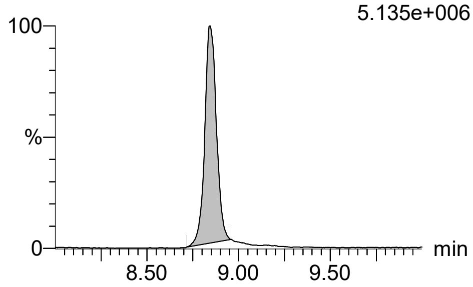

Sample Name: 20211111_A42_R3_IgM
F47:MRM of 3 channels,ES+
976.3 > 204.1
6.147e+006

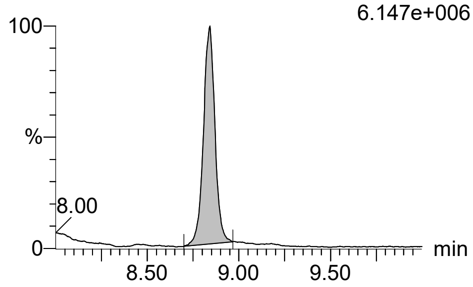

Sample Name: 20211111_A42_R3_IgM
F47:MRM of 3 channels,ES+
976.3 > 366.1
2.254e+006

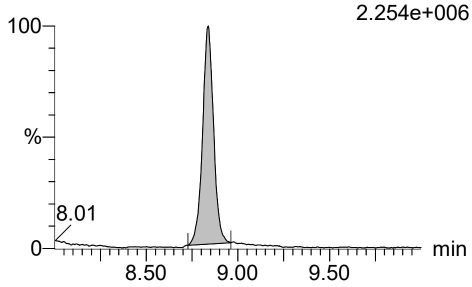

Sample Name: 20211111_A42_R3_IgM
F47:MRM of 3 channels,ES+
1220.1 > 366.1
4.590e+006

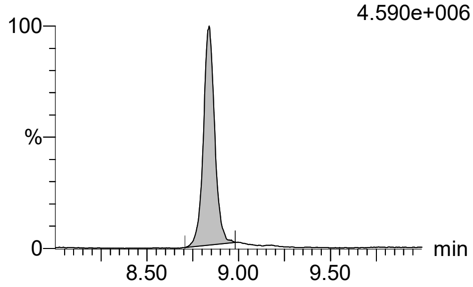

Sample Name: 20211111_A43_R3_IgM
F47:MRM of 3 channels,ES+
976.3 > 204.1
9.293e+006

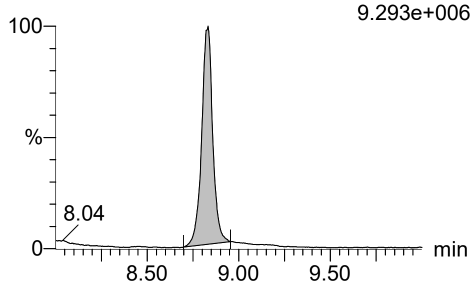

Sample Name: 20211111_A43_R3_IgM
F47:MRM of 3 channels,ES+
976.3 > 366.1
3.146e+006

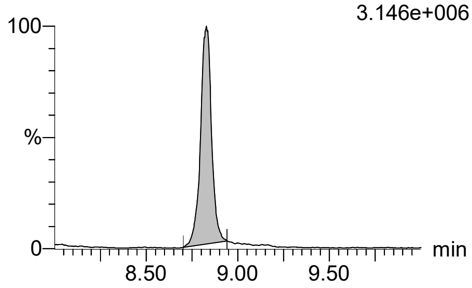

Sample Name: 20211111_A43_R3_IgM
F47:MRM of 3 channels,ES+
1220.1 > 366.1
6.735e+006

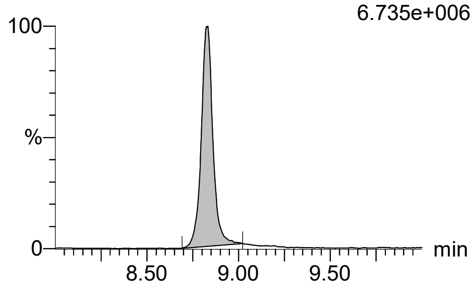

Sample Name: 20211111_A44_R3_IgM
F47:MRM of 3 channels,ES+
976.3 > 204.1
4.899e+006

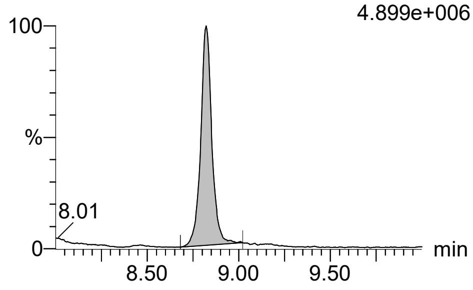

Sample Name: 20211111_A44_R3_IgM
F47:MRM of 3 channels,ES+
976.3 > 366.1
1.722e+006

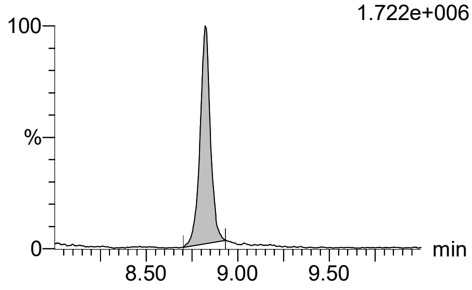

Sample Name: 20211111_A44_R3_IgM
F47:MRM of 3 channels,ES+
1220.1 > 366.1
3.670e+006

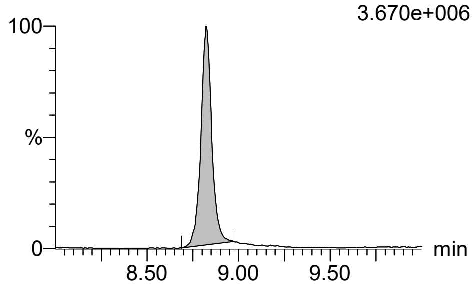

Sample Name: 20211111_QC_R25_IgM

F47:MRM of 3 channels,ES+
976.3 > 204.1
1.732e+007

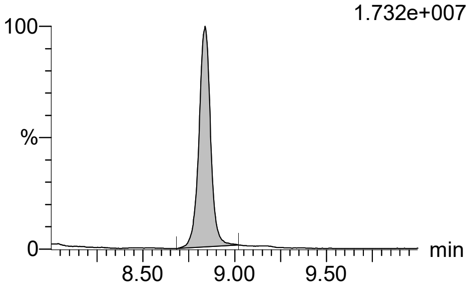

Sample Name: 20211111_QC_R25_IgM

F47:MRM of 3 channels,ES+
976.3 > 366.1
6.114e+006

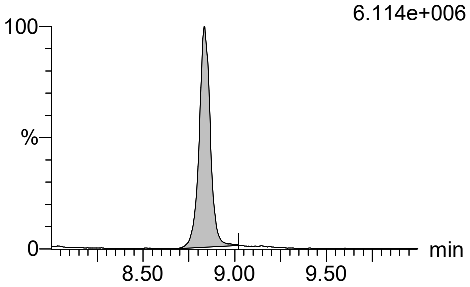

Sample Name: 20211111_QC_R25_IgM

F47:MRM of 3 channels,ES+
1220.1 > 366.1
1.266e+007

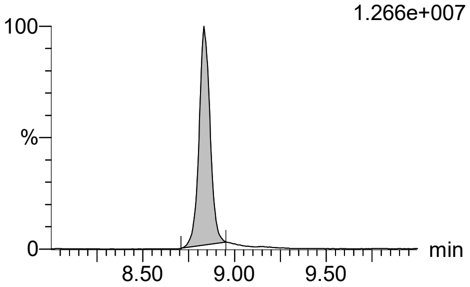

Dataset: C:\MassLynx\10.pro\20211111_clinicalsamples_IgM_IgA.qld

Last Altered: Thursday, March 09, 2023 19:58:25 台北標準時間
Printed: Friday, August 01, 2025 15:08:19 台北標準時間

	#	Name	RT	Area	IS Area	Response	Prima...
1	1	20211112_QC_R001_IgM	8.82	1475837.6	15421.4	95.700	bb
2	2	20211112_QC_R002_IgM	8.83	1420265.3	13188.8	107.687	MM
3	3	20211111_A1_R2_IgM	8.83	587843.5	11871.0	49.519	MM
4	4	20211111_A1_R3_IgM	8.83	586305.7	12030.5	48.735	MM
5	5	20211111_A2_R2_IgM	8.82	2773305.5	11446.8	242.277	MM
6	6	20211111_A2_R3_IgM	8.82	2731430.3	11098.4	246.110	MM
7	7	20211111_L1_R2_IgM	8.83	361763.9	11779.4	30.712	MM
8	8	20211111_L1_R3_IgM	8.83	309039.7	11176.2	27.652	bb
9	9	20211111_L2_R2_IgM	8.82	2301402.8	11375.0	202.321	bb
10	10	20211111_H1_R2_IgM	8.81	1373134.6	13595.2	101.002	bb
11	11	20211111_A3_R2_IgM	8.80	1908558.8	14112.3	135.241	bb
12	12	20211111_A4_R2_IgM	8.81	1189725.6	11977.5	99.330	bb
13	13	20211111_L3_R2_IgM	8.78	416312.3	12242.1	34.007	MM
14	14	20211111_L4_R2_IgM	8.82	1233542.1	12797.0	96.393	bb
15	15	20211111_H2_R2_IgM	8.83	2904857.3	10006.0	290.310	bb
16	16	20211111_A5_R2_IgM	8.79	2535870.3	11434.6	221.771	bb
17	17	20211111_A6_R2_IgM	8.82	2140318.5	13193.7	162.223	bb
18	18	20211111_QC_R10_IgM	8.82	1464243.3	12566.1	116.523	bb
19	19	20211111_L5_R2_IgM	8.78	1427898.6	13616.4	104.866	MM
20	20	20211111_L6_R2_IgM	8.83	1982513.6	13698.5	144.725	bb
21	21	20211111_H3_R2_IgM	8.81	755649.1	15793.1	47.847	bb
22	22	20211111_A7_R2_IgM	8.81	1673571.5	12936.9	129.364	bb
23	23	20211111_A8_R2_IgM	8.82	1242989.8	13108.0	94.827	bb
24	24	20211111_L7_R2_IgM	8.82	1014563.9	13278.5	76.407	bb
25	25	20211111_L8_R2_IgM	8.84	466620.2	15233.6	30.631	bb
26	26	20211111_H4_R2_IgM	8.83	837482.1	16521.9	50.689	bb
27	27	20211111_A9_R2_IgM	8.80	583644.3	16451.6	35.476	MM
28	28	20211111_A10_R2_IgM	8.80	1924228.1	13036.0	147.608	bb
29	29	20211111_L9_R2_IgM	8.79	959418.8	14554.1	65.921	MM
30	30	20211111_L10_R2_IgM	8.79	1318770.4	12250.4	107.651	bb
31	31	20211111_QC_R11_IgM	8.83	1593834.1	11459.2	139.088	bb
32	32	20211111_H5_R2_IgM	8.81	1805928.8	14358.2	125.777	bb
33	33	20211111_A11_R2_IgM	8.77	374515.0	16184.1	23.141	MM
34	34	20211111_A12_R2_IgM	8.81	529552.6	13465.4	39.327	bb
35	35	20211111_L11_R2_IgM	8.85	827630.0	13277.9	62.332	bb
36	36	20211111_L12_R2_IgM	8.80	624333.0	15958.8	39.122	bb
37	37	20211111_H6_R2_IgM	8.83	592234.6	14003.5	42.292	bb
38	38	20211111_A13_R2_IgM	8.82	670986.6	13656.1	49.135	bb
39	39	20211111_A14_R2_IgM	8.82	2107036.5	14071.3	149.740	bb
40	40	20211111_L13_R2_IgM	8.83	1581781.1	14785.6	106.981	bb
41	41	20211111_L14_R2_IgM	8.83	1773089.3	14772.2	120.029	bb
42	42	20211111_H7_R2_IgM	8.80	463874.3	15164.4	30.590	MM
43	43	20211111_A15_R2_IgM	8.82	3185565.0	15721.5	202.625	MM
44	44	20211111_QC_R12_IgM	8.83	1680923.3	13711.5	122.592	bb
45	45	20211111_A16_R2_IgM	8.84	2700344.3	14275.6	189.159	bb
46	46	20211111_L15_R2_IgM	8.82	1226128.6	12935.8	94.785	bb
47	47	20211111_L16_R2_IgM	8.83	580159.8	13342.8	43.481	bb
48	48	20211111_H8_R2_IgM	8.84	1258237.9	15835.1	79.459	bb
49	49	20211111_A17_R2_IgM	8.84	2228391.0	15786.7	141.156	bb
50	50	20211111_A18_R2_IgM	8.81	571295.3	16986.8	33.632	bb
51	51	20211111_L17_R2_IgM	8.82	3642201.0	13342.7	272.973	bb
52	52	20211111_L18_R2_IgM	8.82	237702.1	14692.0	16.179	bb
53	53	20211111_H9_R2_IgM	8.82	518448.0	15639.7	33.150	MM
54	54	20211111_A19_R2_IgM	8.82	1070588.8	14198.7	75.400	bb
55	55	20211111_A20_R2_IgM	8.80	227730.8	14162.7	16.080	MM
56	56	20211111_L19_R2_IgM	8.82	1265920.5	9293.4	136.217	bb

Dataset: C:\MassLynx\10.pro\20211111_clinicalsamples_IgM_IgA.qld

Last Altered: Thursday, March 09, 2023 19:58:25 台北標準時間
Printed: Friday, August 01, 2025 15:08:19 台北標準時間

	# Name	RT	Area	IS Area	Response	Prima...
57	57 20211111_QC_R13_IgM	8.83	1640229.3	13903.0	117.977	bb
58	58 20211111_L20_R2_IgM	8.79	453494.6	12781.6	35.480	MM
59	59 20211111_H10_R2_IgM	8.79	499070.8	14796.4	33.729	bb
60	60 20211111_A21_R2_IgM	8.83	1361441.9	13669.0	99.600	bb
61	61 20211111_A22_R2_IgM	8.83	374524.7	14098.3	26.565	MM
62	62 20211111_L21_R2_IgM	8.84	977498.6	15678.2	62.348	bb
63	63 20211111_L22_R2_IgM	8.84	1621677.8	15724.5	103.131	bb
64	64 20211111_H11_R2_IgM	8.84	1352104.9	15890.2	85.091	bb
65	65 20211111_A23_R2_IgM	8.82	517578.8	13423.2	38.559	bb
66	66 20211111_A24_R2_IgM	8.83	1860606.0	12438.0	149.591	bb
67	67 20211111_L23_R2_IgM	8.83	2535407.3	14075.0	180.136	bb
68	68 20211111_L24_R2_IgM	8.82	348236.9	15972.1	21.803	bb
69	69 20211111_H12_R2_IgM	8.84	756221.5	16270.9	46.477	bb
70	70 20211111_QC_R14_IgM	8.84	1706612.3	15806.8	107.967	bb
71	71 20211111_A25_R2_IgM	8.84	3440248.5	14552.1	236.409	bb
72	72 20211111_A26_R2_IgM	8.85	1461630.1	11492.7	127.179	bb
73	73 20211111_L25_R2_IgM	8.85	1272744.1	14403.3	88.364	bb
74	74 20211111_L26_R2_IgM	8.85	1016653.2	16533.9	61.489	bb
75	75 20211111_H13_R2_IgM	8.83	1786048.9	15520.1	115.079	bb
76	76 20211111_A27_R2_IgM	8.83	1495725.5	13337.4	112.145	bb
77	77 20211111_A28_R2_IgM	8.79	403661.8	11895.6	33.934	MM
78	78 20211111_L27_R2_IgM	8.83	1379249.4	14125.7	97.641	bb
79	79 20211111_L28_R2_IgM	8.83	1431123.8	11807.5	121.205	bb
80	80 20211111_H14_R2_IgM	8.82	1900283.5	15035.7	126.385	bb
81	81 20211111_A29_R2_IgM	8.83	117138.5	13554.4	8.642	bb
82	82 20211111_A30_R2_IgM	8.83	1435763.0	12984.1	110.579	bb
83	83 20211111_QC_R15_IgM	8.84	1598498.4	12299.5	129.964	bb
84	84 20211111_L29_R2_IgM	8.83	509453.0	13590.7	37.485	bb
85	85 20211111_L30_R2_IgM	8.83	238664.5	12511.4	19.076	bb
86	86 20211111_H15_R2_IgM	8.83	1104358.9	11885.0	92.920	bb
87	87 20211111_A31_R2_IgM	8.82	530247.9	13422.1	39.506	bb
88	88 20211111_A32_R2_IgM	8.83	1695752.0	11461.3	147.954	bb
89	89 20211111_L31_R2_IgM	8.79	136938.4	13753.4	9.957	MM
90	90 20211111_L32_R2_IgM	8.81	2184606.8	12949.0	168.709	bb
91	91 20211111_H16_R2_IgM	8.81	3062444.3	11465.4	267.102	bb
92	92 20211111_A33_R2_IgM	8.83	1479016.6	10551.5	140.171	bb
93	93 20211111_A34_R2_IgM	8.84	518604.8	12409.9	41.790	bb
94	94 20211111_H17_R2_IgM	8.81	8189502.5	10160.9	805.980	bb
95	95 20211111_A35_R2_IgM	8.79	284284.8	10460.6	27.177	MM
96	96 20211111_QC_R16_IgM	8.83	1430212.5	10433.5	137.079	bb
97	97 20211111_A36_R2_IgM	8.79	312989.3	12504.8	25.029	MM
98	98 20211111_H18_R2_IgM	8.82	4112263.3	9140.4	449.900	bb
99	99 20211111_A37_R2_IgM	8.83	1426775.0	9796.9	145.636	bb
100	1... 20211111_A38_R2_IgM	8.84	1699948.3	11224.3	151.453	bb
101	1... 20211111_H19_R2_IgM	8.83	2086402.4	11739.2	177.730	bb
102	1... 20211111_A39_R2_IgM	8.85	1634196.8	12718.9	128.485	bb
103	1... 20211111_A40_R2_IgM	8.79	282400.3	10006.1	28.223	MM
104	1... 20211111_H20_R2_IgM	8.80	5280488.0	7988.2	661.036	bb
105	1... 20211111_A41_R2_IgM	8.84	828717.1	11513.3	71.979	bb
106	1... 20211111_A42_R2_IgM	8.84	513097.5	10238.5	50.114	bb
107	1... 20211111_A43_R2_IgM	8.83	690409.3	11098.9	62.205	bb
108	1... 20211111_A44_R2_IgM	8.79	212464.0	10969.9	19.368	MM
109	1... 20211111_QC_R17_IgM	8.83	1203715.9	9700.8	124.084	bb
110	1... 20211111_L2_R3_IgM	8.83	2258265.5	8528.0	264.805	bb
111	1... 20211111_H1_R3_IgM	8.84	1809262.6	9438.2	191.696	bb
112	1... 20211111_A3_R3_IgM	8.83	2671486.8	11598.6	230.327	bb

Dataset: C:\MassLynx\10.pro\20211111_clinicalsamples_IgM_IgA.qld

Last Altered: Thursday, March 09, 2023 19:58:25 台北標準時間
Printed: Friday, August 01, 2025 15:08:19 台北標準時間

	# Name	RT	Area	IS Area	Response	Prima...
113	1... 20211111_A4_R3_IgM	8.84	1468143.1	11364.1	129.192	bb
114	1... 20211111_L3_R3_IgM	8.79	474824.9	10753.4	44.156	MM
115	1... 20211111_L4_R3_IgM	8.83	1229958.0	10291.6	119.511	bb
116	1... 20211111_H2_R3_IgM	8.82	3038235.8	9315.8	326.139	bb
117	1... 20211111_A5_R3_IgM	8.82	3681181.5	9102.2	404.429	bb
118	1... 20211111_A6_R3_IgM	8.83	1824461.9	10356.4	176.168	bb
119	1... 20211111_QC_R18_IgM	8.82	1210702.3	8398.3	144.160	bb
120	1... 20211111_L5_R3_IgM	8.84	2170765.0	9786.7	221.807	bb
121	1... 20211111_L6_R3_IgM	8.84	1669154.9	9911.3	168.410	bb
122	1... 20211111_H3_R3_IgM	8.85	918713.2	12155.5	75.580	bb
123	1... 20211111_A7_R3_IgM	8.83	1719993.5	11804.8	145.702	bb
124	1... 20211111_A8_R3_IgM	8.84	1426160.6	10510.6	135.688	MM
125	1... 20211111_L7_R3_IgM	8.82	982372.4	12177.9	80.668	bb
126	1... 20211111_L8_R3_IgM	8.85	428784.8	11668.6	36.747	bb
127	1... 20211111_H4_R3_IgM	8.85	844817.0	11192.0	75.484	bb
128	1... 20211111_A9_R3_IgM	8.85	812508.2	14077.4	57.717	bb
129	1... 20211111_A10_R3_IgM	8.84	2522339.3	11190.4	225.403	bb
130	1... 20211111_L9_R3_IgM	8.84	1452898.8	14649.3	99.179	bb
131	1... 20211111_L10_R3_IgM	8.79	911020.1	9361.3	97.318	MM
132	1... 20211111_QC_R19_IgM	8.83	1212578.9	10125.5	119.755	bb
133	1... 20211111_H5_R3_IgM	8.84	1813418.4	10010.9	181.144	bb
134	1... 20211111_A11_R3_IgM	8.84	1027165.4	12697.2	80.897	bb
135	1... 20211111_A12_R3_IgM	8.82	597553.1	13148.5	45.446	bb
136	1... 20211111_L11_R3_IgM	8.85	664732.4	10786.6	61.626	bb
137	1... 20211111_L12_R3_IgM	8.80	432111.6	11681.8	36.990	MM
138	1... 20211111_H6_R3_IgM	8.83	555353.7	12095.5	45.914	bb
139	1... 20211111_A13_R3_IgM	8.83	661191.7	12908.3	51.222	bb
140	1... 20211111_A14_R3_IgM	8.84	2081820.5	11322.6	183.864	bb
141	1... 20211111_L13_R3_IgM	8.82	1176177.4	11516.1	102.133	bb
142	1... 20211111_L14_R3_IgM	8.84	1611940.3	10790.7	149.383	bb
143	1... 20211111_H7_R3_IgM	8.84	819808.8	11572.1	70.844	bb
144	1... 20211111_A15_R3_IgM	8.83	2922240.3	12434.4	235.012	bb
145	1... 20211111_QC_R20_IgM	8.84	1425436.8	10701.0	133.206	bb
146	1... 20211111_A16_R3_IgM	8.85	2226449.0	11452.1	194.414	bb
147	1... 20211111_L15_R3_IgM	8.84	1290930.3	10531.1	122.583	bb
148	1... 20211111_L16_R3_IgM	8.83	583969.0	12049.4	48.464	bb
149	1... 20211111_H8_R3_IgM	8.84	1263512.8	13434.9	94.047	bb
150	1... 20211111_A17_R3_IgM	8.85	1933130.6	12452.0	155.246	bb
151	1... 20211111_A18_R3_IgM	8.80	342094.5	12506.5	27.353	MM
152	1... 20211111_L17_R3_IgM	8.83	3604149.3	9948.9	362.266	bb
153	1... 20211111_L18_R3_IgM	8.83	232910.3	13462.1	17.301	bb
154	1... 20211111_H9_R3_IgM	8.84	613658.9	13178.7	46.564	bb
155	1... 20211111_A19_R3_IgM	8.83	1029379.0	11298.6	91.107	bb
156	1... 20211111_A20_R3_IgM	8.80	190407.5	11175.6	17.038	MM
157	1... 20211111_L19_R3_IgM	8.82	1391483.9	11558.2	120.390	bb
158	1... 20211111_QC_R21_IgM	8.83	1421292.5	9977.0	142.457	bb
159	1... 20211111_L20_R3_IgM	8.83	970751.9	13508.1	71.864	bb
160	1... 20211111_H10_R3_IgM	8.83	976176.9	12374.8	78.884	bb
161	1... 20211111_A21_R3_IgM	8.83	1384198.6	12443.6	111.238	bb
162	1... 20211111_A22_R3_IgM	8.83	381264.2	11589.3	32.898	bb
163	1... 20211111_L21_R3_IgM	8.85	850846.6	13107.4	64.914	bb
164	1... 20211111_L22_R3_IgM	8.85	1454142.8	12021.5	120.962	bb
165	1... 20211111_H11_R3_IgM	8.85	1239916.3	12687.8	97.725	bb
166	1... 20211111_A23_R3_IgM	8.84	515433.7	11611.8	44.389	bb
167	1... 20211111_A24_R3_IgM	8.83	1854363.1	10476.8	176.997	bb
168	1... 20211111_L23_R3_IgM	8.82	2078996.6	11873.1	175.102	bb

Dataset: C:\MassLynx\10.pro\20211111_clinicalsamples_IgM_IgA.qld

Last Altered: Thursday, March 09, 2023 19:58:25 台北標準時間
Printed: Friday, August 01, 2025 15:08:19 台北標準時間

	# Name	RT	Area	IS Area	Response	Prima...
169	1... 20211111_L24_R3_IgM	8.85	440611.8	13468.0	32.716	bb
170	1... 20211111_H12_R3_IgM	8.83	793243.1	14229.1	55.748	bb
171	1... 20211111_QC_R22_IgM	8.84	1486685.6	11264.4	131.981	bb
172	1... 20211111_A25_R3_IgM	8.84	2844887.3	11775.7	241.589	bb
173	1... 20211111_A26_R3_IgM	8.85	1403842.1	10473.9	134.033	bb
174	1... 20211111_L25_R3_IgM	8.84	1162587.1	13256.8	87.697	bb
175	1... 20211111_L26_R3_IgM	8.86	892975.5	13565.0	65.829	MM
176	1... 20211111_H13_R3_IgM	8.84	1752722.8	13146.0	133.327	bb
177	1... 20211111_A27_R3_IgM	8.84	1468911.0	12025.6	122.148	bb
178	1... 20211111_A28_R3_IgM	8.84	872038.4	11229.1	77.659	bb
179	1... 20211111_L27_R3_IgM	8.85	1540081.4	14960.0	102.946	bb
180	1... 20211111_L28_R3_IgM	8.83	1523629.8	11359.0	134.134	bb
181	1... 20211111_H14_R3_IgM	8.83	1920251.8	12830.8	149.660	bb
182	1... 20211111_A29_R3_IgM	8.85	116714.4	13434.3	8.688	bb
183	1... 20211111_A30_R3_IgM	8.84	1403481.1	11272.2	124.508	bb
184	1... 20211111_QC_R23_IgM	8.84	1407720.5	11872.2	118.573	bb
185	1... 20211111_L29_R3_IgM	8.84	504127.2	12948.7	38.933	bb
186	1... 20211111_L30_R3_IgM	8.84	245665.6	11026.6	22.279	bb
187	1... 20211111_H15_R3_IgM	8.84	1035869.4	10786.3	96.036	bb
188	1... 20211111_A31_R3_IgM	8.83	505609.3	12010.5	42.097	bb
189	1... 20211111_A32_R3_IgM	8.83	1528844.8	11107.7	137.639	bb
190	1... 20211111_L31_R3_IgM	8.83	345107.0	11021.8	31.311	bb
191	1... 20211111_L32_R3_IgM	8.84	2988130.0	11683.8	255.750	bb
192	1... 20211111_H16_R3_IgM	8.83	3102789.5	9831.7	315.592	bb
193	1... 20211111_A33_R3_IgM	8.83	1421568.4	11213.1	126.777	bb
194	1... 20211111_A34_R3_IgM	8.84	470148.1	11550.1	40.705	bb
195	1... 20211111_H17_R3_IgM	8.82	7190227.0	8874.4	810.219	bb
196	1... 20211111_A35_R3_IgM	8.83	477007.6	9856.3	48.396	bb
197	1... 20211111_QC_R24_IgM	8.84	1264513.0	7561.8	167.224	bb
198	1... 20211111_A36_R3_IgM	8.83	824345.3	9562.8	86.203	bb
199	1... 20211111_H18_R3_IgM	8.82	3936452.8	8165.3	482.096	bb
200	2... 20211111_A37_R3_IgM	8.83	1197635.1	9278.8	129.072	bb
201	2... 20211111_A38_R3_IgM	8.84	1461838.6	10920.3	133.864	bb
202	2... 20211111_H19_R3_IgM	8.82	1832325.4	9285.3	197.336	bb
203	2... 20211111_A39_R3_IgM	8.85	1519224.1	9083.4	167.253	bb
204	2... 20211111_A40_R3_IgM	8.84	545825.5	8382.4	65.116	MM
205	2... 20211111_H20_R3_IgM	8.81	5022055.0	7807.9	643.200	bb
206	2... 20211111_A41_R3_IgM	8.84	544430.6	5925.2	91.883	bb
207	2... 20211111_A42_R3_IgM	8.84	434023.9	9352.6	46.407	bb
208	2... 20211111_A43_R3_IgM	8.83	628980.1	10216.3	61.566	bb
209	2... 20211111_A44_R3_IgM	8.82	335828.4	10262.7	32.723	bb
210	2... 20211111_QC_R25_IgM	8.84	1248454.5	8575.3	145.586	bb

Dataset: C:\MassLynx\10.pro\20211111_clinicalsamples_IgM_IgA.qld

Last Altered: Thursday, March 09, 2023 19:58:25 台北標準時間
Printed: Friday, August 01, 2025 15:09:56 台北標準時間

Method: C:\MassLynx\10_Pro_IgA.PRO\MethDB\20211111_IgM_IgA_DMRM_new.mdb 24 Feb 2022 13:44:14
Calibration: 15 Nov 2021 14:42:11

Sample Name: 20211112_QC_R001_IgM

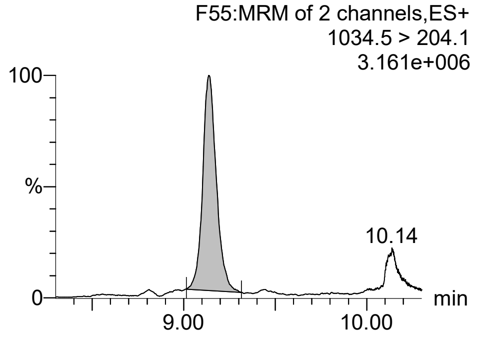

Sample Name: 20211112_QC_R001_IgM

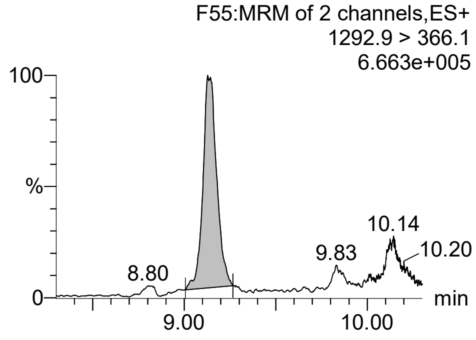

Sample Name: 20211112_QC_R002_IgM

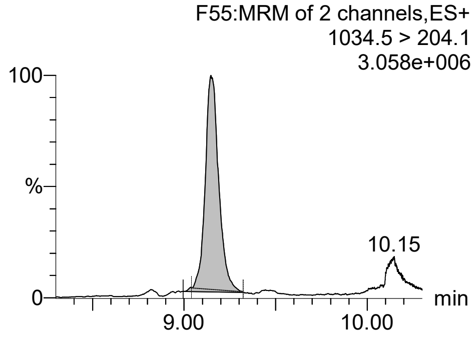

Sample Name: 20211112_QC_R002_IgM

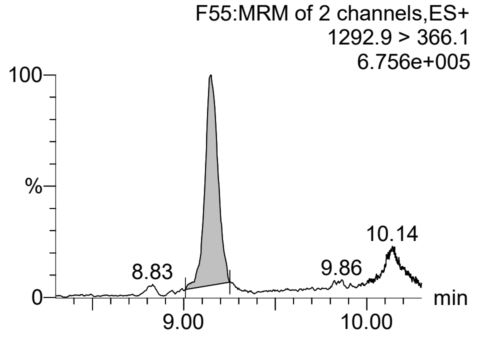

Sample Name: 20211111_A1_R2_IgM

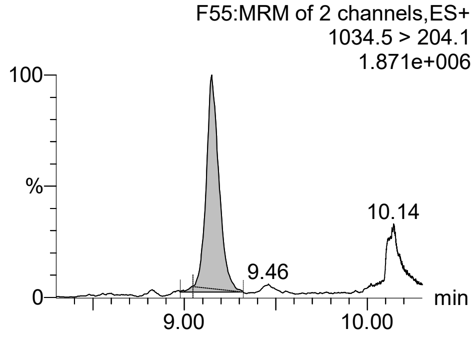

Sample Name: 20211111_A1_R2_IgM

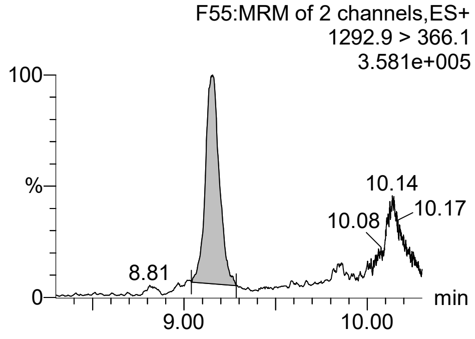

Sample Name: 20211111_A1_R3_IgM

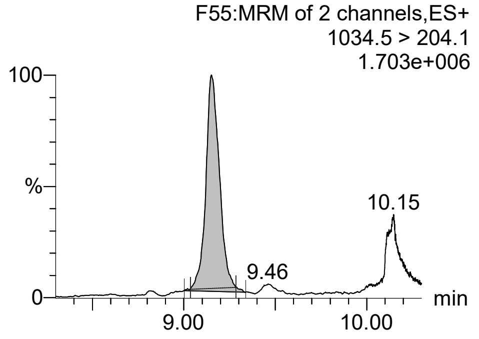

Sample Name: 20211111_A1_R3_IgM

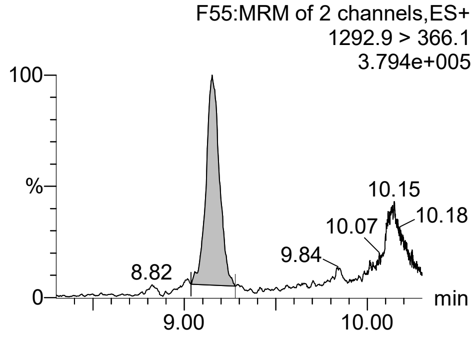

Sample Name: 20211111_A2_R2_IgM

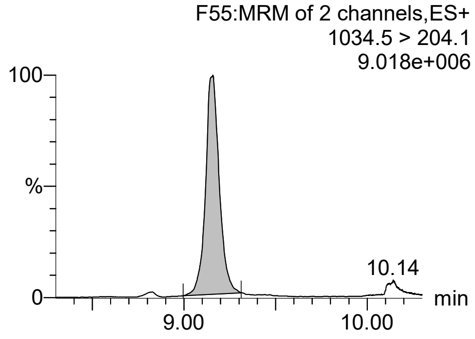

Sample Name: 20211111_A2_R2_IgM

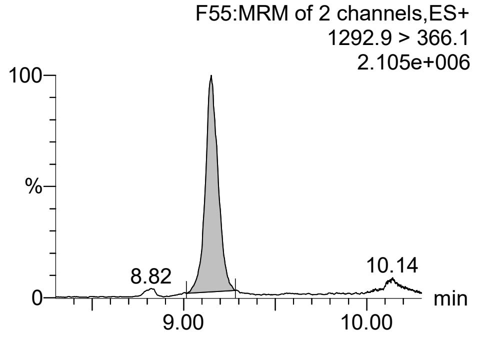

Sample Name: 20211111_A2_R3_IgM

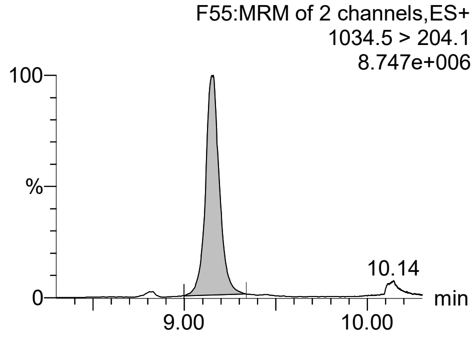

Sample Name: 20211111_A2_R3_IgM

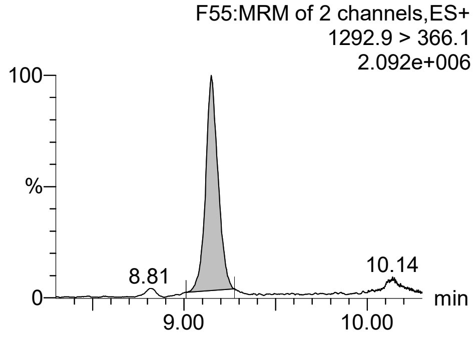

Sample Name: 20211111_L1_R2_IgM
F55:MRM of 2 channels,ES+
1034.5 > 204.1
8.696e+005

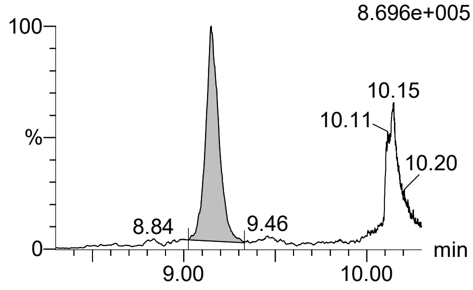

Sample Name: 20211111_L1_R2_IgM
F55:MRM of 2 channels,ES+
1292.9 > 366.1
1.834e+005

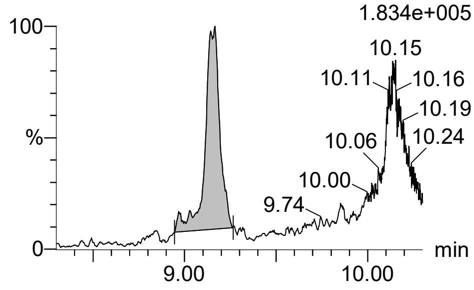

Sample Name: 20211111_L1_R3_IgM
F55:MRM of 2 channels,ES+
1034.5 > 204.1
6.338e+005

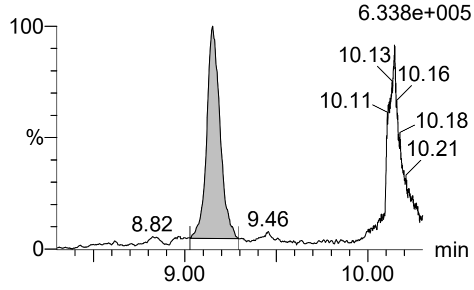

Sample Name: 20211111_L1_R3_IgM
F55:MRM of 2 channels,ES+
1292.9 > 366.1
1.544e+005

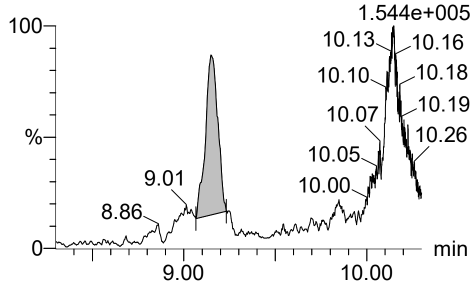

Sample Name: 20211111_L2_R2_IgM
F55:MRM of 2 channels,ES+
1034.5 > 204.1
5.392e+006

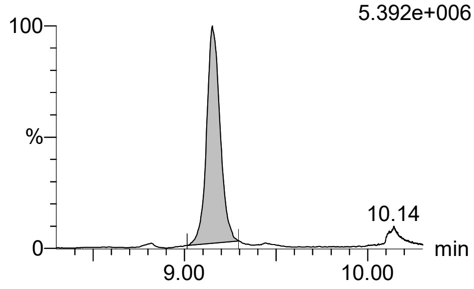

Sample Name: 20211111_L2_R2_IgM
F55:MRM of 2 channels,ES+
1292.9 > 366.1
1.226e+006

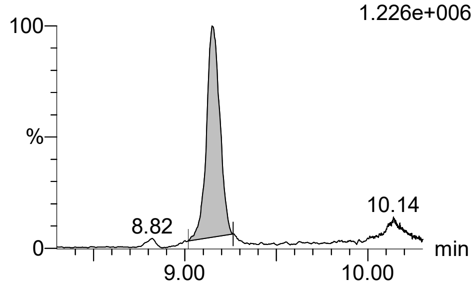

Sample Name: 20211111_H1_R2_IgM
F55:MRM of 2 channels,ES+
1034.5 > 204.1
2.289e+006

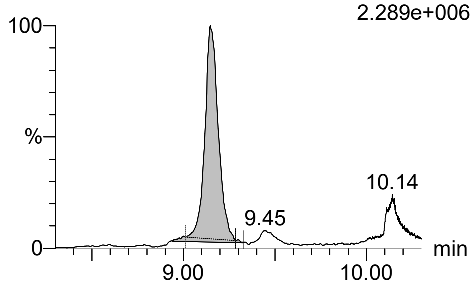

Sample Name: 20211111_H1_R2_IgM
F55:MRM of 2 channels,ES+
1292.9 > 366.1
5.262e+005

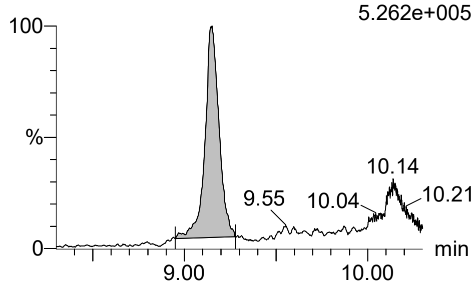

Sample Name: 20211111_A3_R2_IgM
F55:MRM of 2 channels,ES+
1034.5 > 204.1
6.933e+006

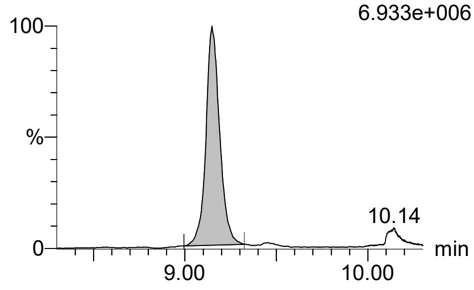

Sample Name: 20211111_A3_R2_IgM
F55:MRM of 2 channels,ES+
1292.9 > 366.1
1.644e+006

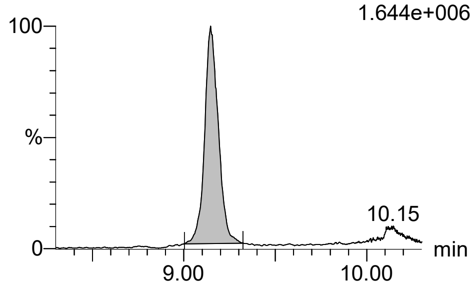

Sample Name: 20211111_A4_R2_IgM
F55:MRM of 2 channels,ES+
1034.5 > 204.1
3.054e+006

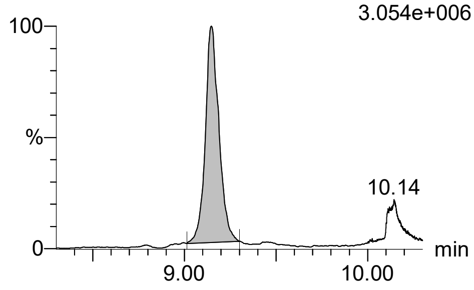

Sample Name: 20211111_A4_R2_IgM
F55:MRM of 2 channels,ES+
1292.9 > 366.1
7.709e+005

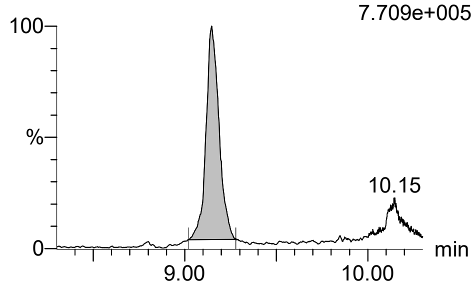

Sample Name: 20211111_L3_R2_IgM
F55:MRM of 2 channels,ES+
1034.5 > 204.1
2.217e+006

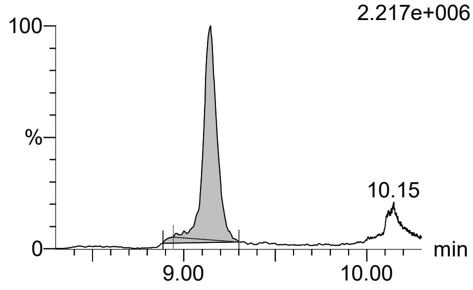

Sample Name: 20211111_L3_R2_IgM
F55:MRM of 2 channels,ES+
1292.9 > 366.1
6.074e+005

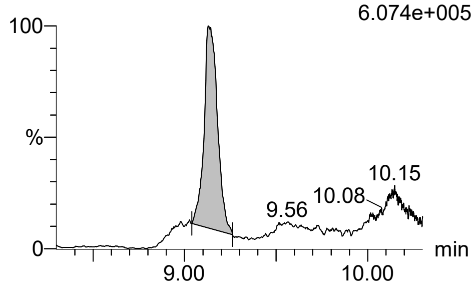

Sample Name: 20211111_L4_R2_IgM
F55:MRM of 2 channels,ES+
1034.5 > 204.1
3.149e+006

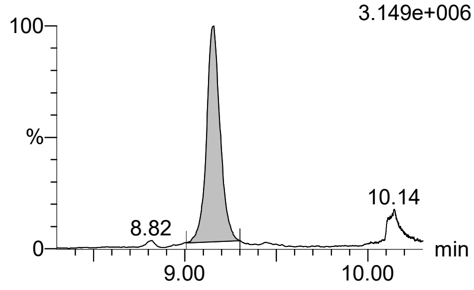

Dataset: C:\MassLynx\10.pro\20211111_clinicalsamples_IgM_IgA.qld

Last Altered: Thursday, March 09, 2023 19:58:25 台北標準時間
Printed: Friday, August 01, 2025 15:09:56 台北標準時間

Sample Name: 20211111_L4_R2_IgM
F55:MRM of 2 channels,ES+
1292.9 > 366.1
7.549e+005

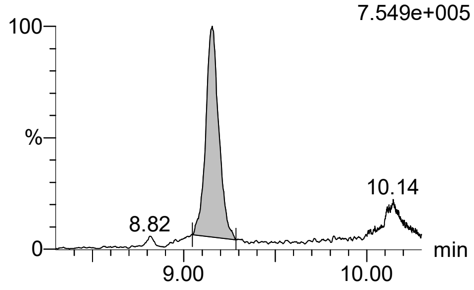

Sample Name: 20211111_H2_R2_IgM
F55:MRM of 2 channels,ES+
1034.5 > 204.1
5.696e+006

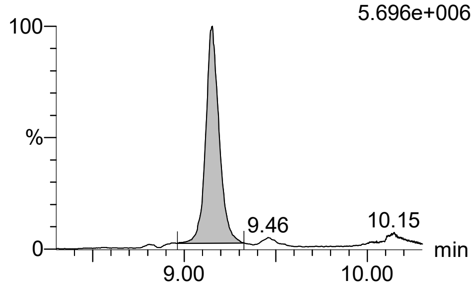

Sample Name: 20211111_H2_R2_IgM
F55:MRM of 2 channels,ES+
1292.9 > 366.1
1.478e+006

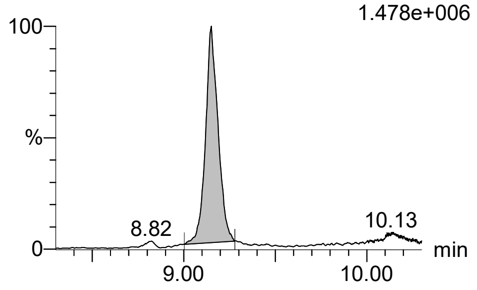

Sample Name: 20211111_A5_R2_IgM
F55:MRM of 2 channels,ES+
1034.5 > 204.1
1.429e+007

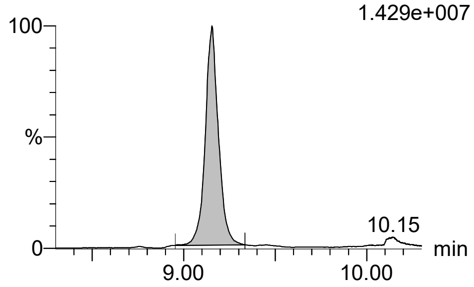

Sample Name: 20211111_A5_R2_IgM
F55:MRM of 2 channels,ES+
1292.9 > 366.1
3.535e+006

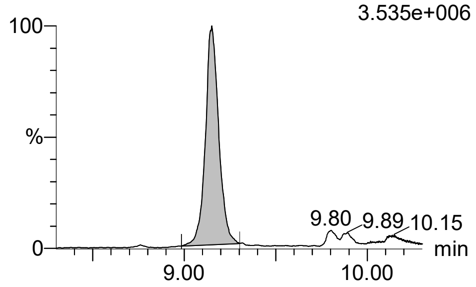

Sample Name: 20211111_A6_R2_IgM
F55:MRM of 2 channels,ES+
1034.5 > 204.1
6.939e+006

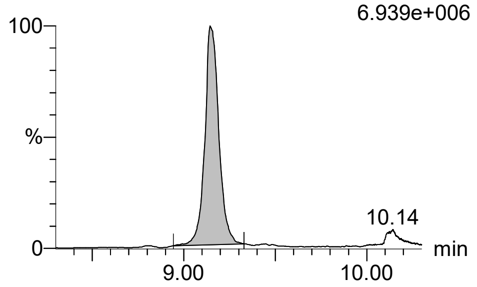

Sample Name: 20211111_A6_R2_IgM
F55:MRM of 2 channels,ES+
1292.9 > 366.1
1.698e+006

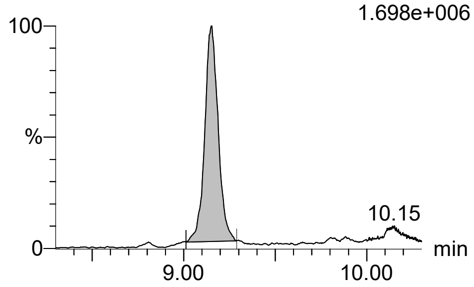

Sample Name: 20211111_QC_R10_IgM
F55:MRM of 2 channels,ES+
1034.5 > 204.1
3.463e+006

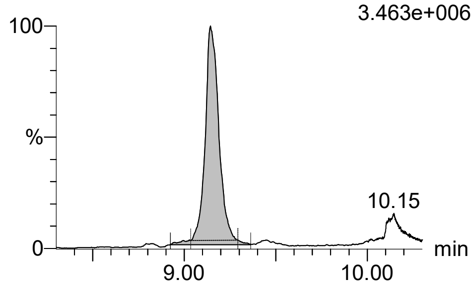

Sample Name: 20211111_QC_R10_IgM
F55:MRM of 2 channels,ES+
1292.9 > 366.1
8.620e+005

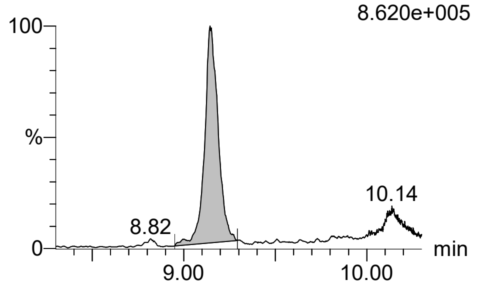

Sample Name: 20211111_L5_R2_IgM
F55:MRM of 2 channels,ES+
1034.5 > 204.1
6.213e+006

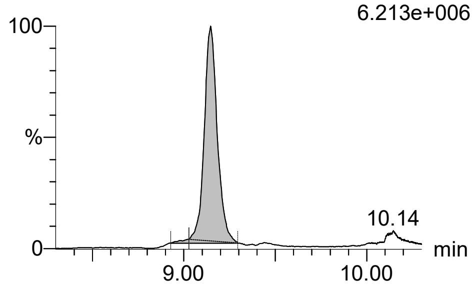

Sample Name: 20211111_L5_R2_IgM
F55:MRM of 2 channels,ES+
1292.9 > 366.1
1.600e+006

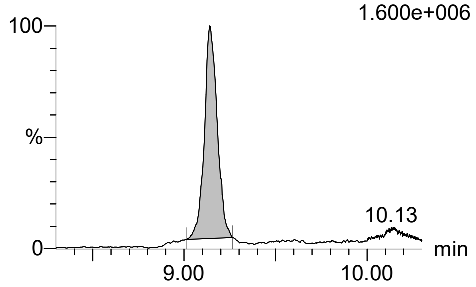

Sample Name: 20211111_L6_R2_IgM
F55:MRM of 2 channels,ES+
1034.5 > 204.1
4.559e+006

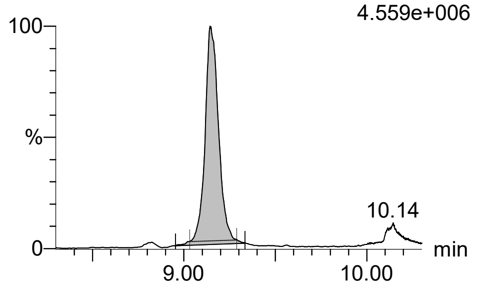

Sample Name: 20211111_L6_R2_IgM
F55:MRM of 2 channels,ES+
1292.9 > 366.1
1.050e+006

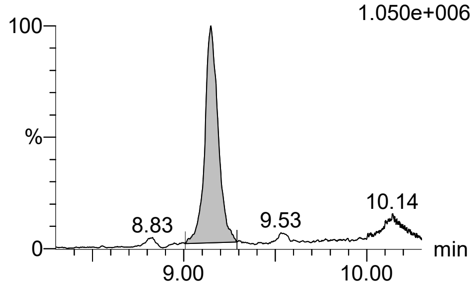

Sample Name: 20211111_H3_R2_IgM
F55:MRM of 2 channels,ES+
1034.5 > 204.1
2.383e+006

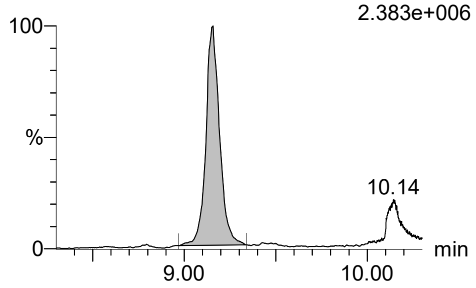

Sample Name: 20211111_H3_R2_IgM
F55:MRM of 2 channels,ES+
1292.9 > 366.1
5.274e+005

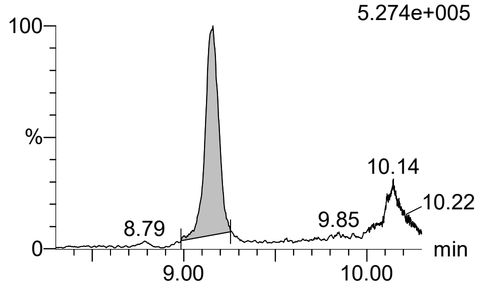

Dataset: C:\MassLynx\10.pro\20211111_clinicalsamples_IgM_IgA.qld

Last Altered: Thursday, March 09, 2023 19:58:25 台北標準時間
Printed: Friday, August 01, 2025 15:09:56 台北標準時間

Sample Name: 20211111_A7_R2_IgM
F55:MRM of 2 channels,ES+
1034.5 > 204.1
6.523e+006

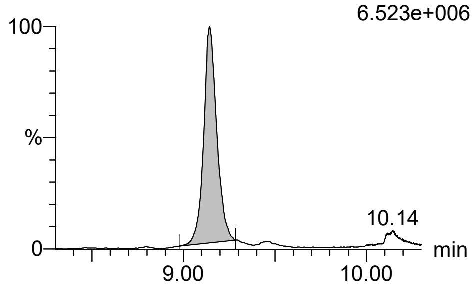

Sample Name: 20211111_A7_R2_IgM
F55:MRM of 2 channels,ES+
1292.9 > 366.1
1.615e+006

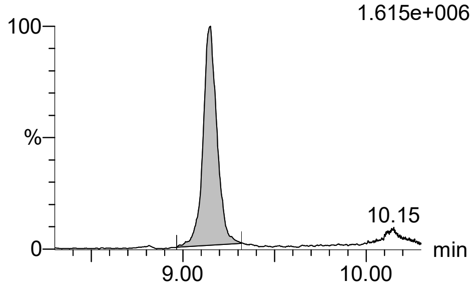

Sample Name: 20211111_A8_R2_IgM
F55:MRM of 2 channels,ES+
1034.5 > 204.1
4.030e+006

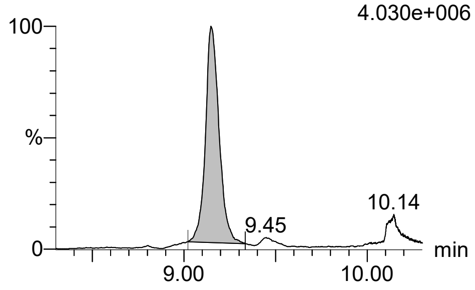

Sample Name: 20211111_A8_R2_IgM
F55:MRM of 2 channels,ES+
1292.9 > 366.1
9.790e+005

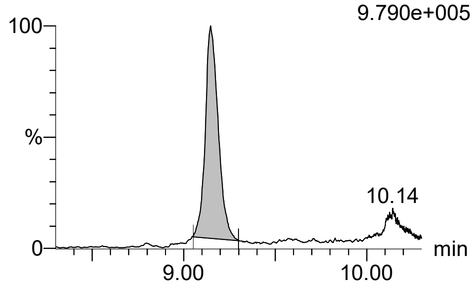

Sample Name: 20211111_L7_R2_IgM
F55:MRM of 2 channels,ES+
1034.5 > 204.1
1.759e+006

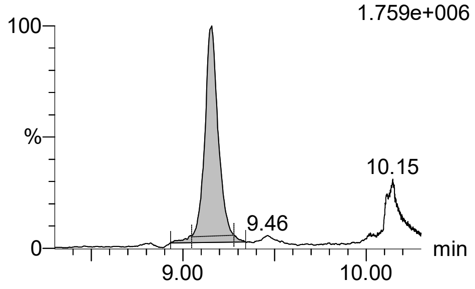

Sample Name: 20211111_L7_R2_IgM
F55:MRM of 2 channels,ES+
1292.9 > 366.1
3.974e+005

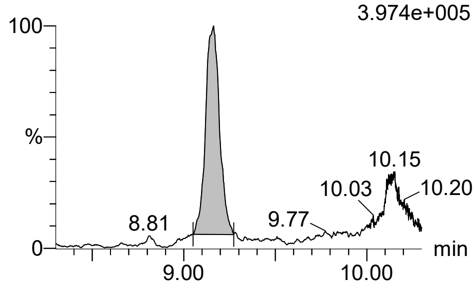

Sample Name: 20211111_L8_R2_IgM
F55:MRM of 2 channels,ES+
1034.5 > 204.1
1.501e+006

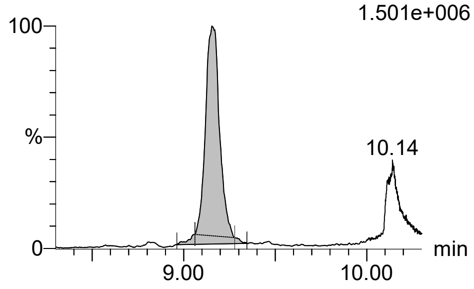

Sample Name: 20211111_L8_R2_IgM
F55:MRM of 2 channels,ES+
1292.9 > 366.1
3.407e+005

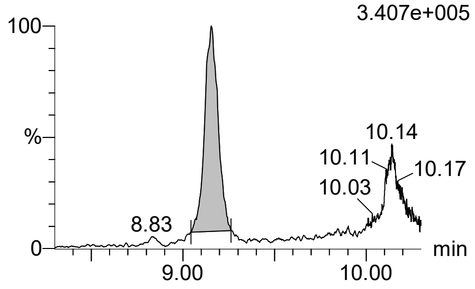

Sample Name: 20211111_H4_R2_IgM
F55:MRM of 2 channels,ES+
1034.5 > 204.1
2.120e+006

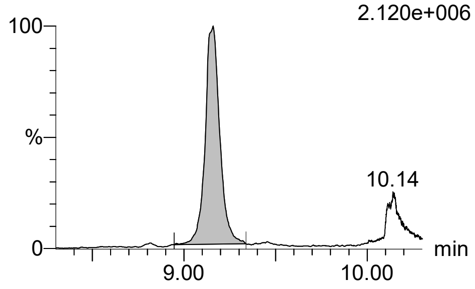

Sample Name: 20211111_H4_R2_IgM
F55:MRM of 2 channels,ES+
1292.9 > 366.1
4.972e+005

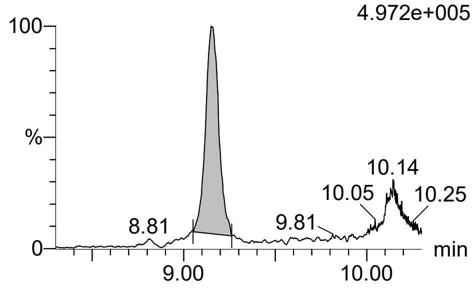

Sample Name: 20211111_A9_R2_IgM
F55:MRM of 2 channels,ES+
1034.5 > 204.1
1.691e+006

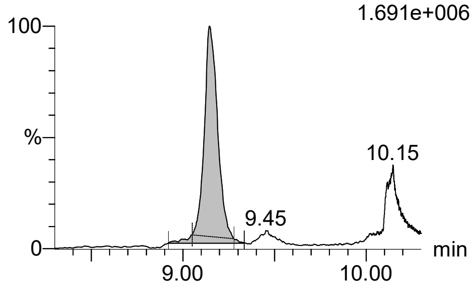

Sample Name: 20211111_A9_R2_IgM
F55:MRM of 2 channels,ES+
1292.9 > 366.1
3.826e+005

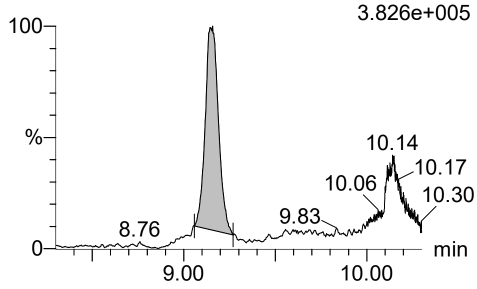

Sample Name: 20211111_A10_R2_IgM
F55:MRM of 2 channels,ES+
1034.5 > 204.1
5.885e+006

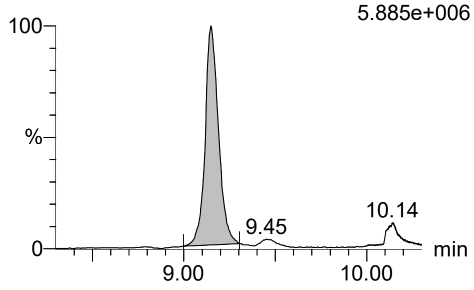

Sample Name: 20211111_A10_R2_IgM
F55:MRM of 2 channels,ES+
1292.9 > 366.1
1.353e+006

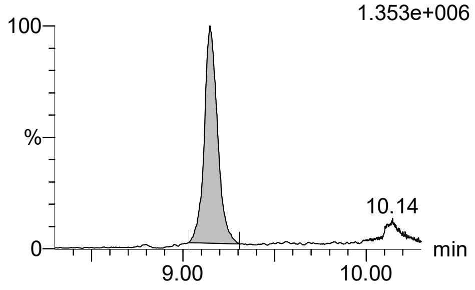

Sample Name: 20211111_L9_R2_IgM
F55:MRM of 2 channels,ES+
1034.5 > 204.1
2.807e+006

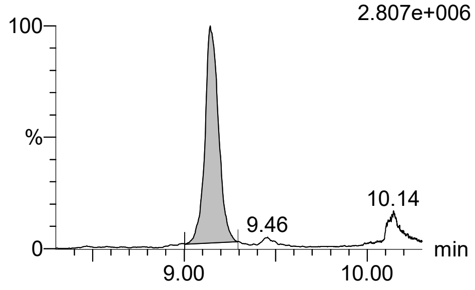

Sample Name: 20211111_L9_R2_IgM
F55:MRM of 2 channels,ES+
1292.9 > 366.1
6.774e+005

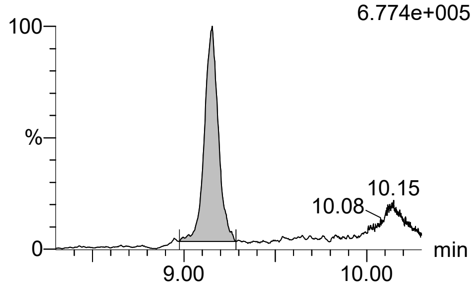

Sample Name: 20211111_L10_R2_IgM
F55:MRM of 2 channels,ES+
1034.5 > 204.1
6.310e+006

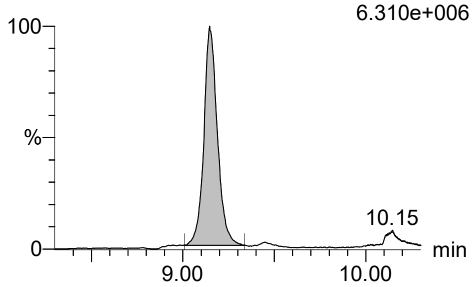

Sample Name: 20211111_L10_R2_IgM
F55:MRM of 2 channels,ES+
1292.9 > 366.1
1.597e+006

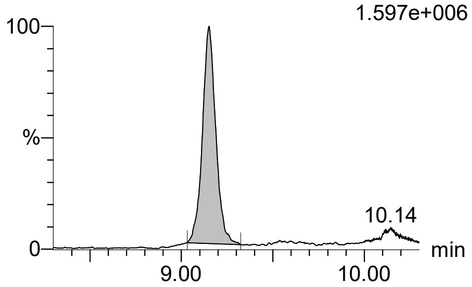

Sample Name: 20211111_QC_R11_IgM
F55:MRM of 2 channels,ES+
1034.5 > 204.1
3.925e+006

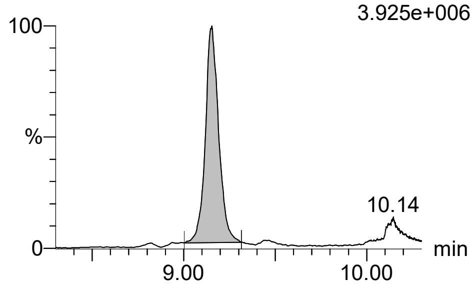

Sample Name: 20211111_QC_R11_IgM
F55:MRM of 2 channels,ES+
1292.9 > 366.1
9.864e+005

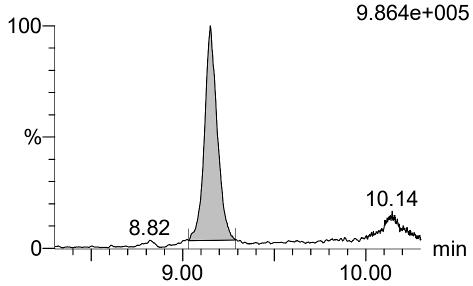

Sample Name: 20211111_H5_R2_IgM
F55:MRM of 2 channels,ES+
1034.5 > 204.1
6.053e+006

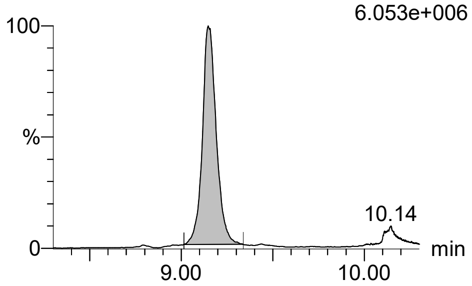

Sample Name: 20211111_H5_R2_IgM
F55:MRM of 2 channels,ES+
1292.9 > 366.1
1.585e+006

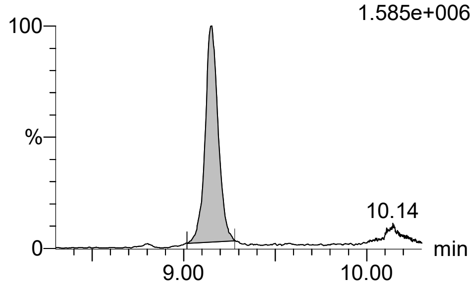

Sample Name: 20211111_A11_R2_IgM
F55:MRM of 2 channels,ES+
1034.5 > 204.1
3.584e+006

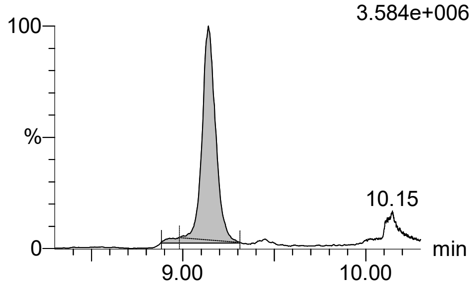

Sample Name: 20211111_A11_R2_IgM
F55:MRM of 2 channels,ES+
1292.9 > 366.1
1.015e+006

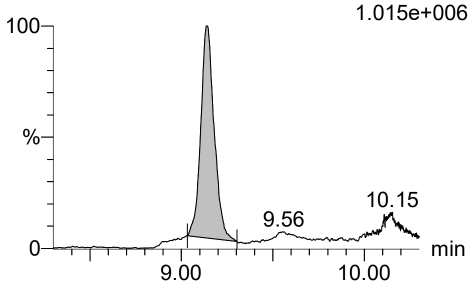

Sample Name: 20211111_A12_R2_IgM
F55:MRM of 2 channels,ES+
1034.5 > 204.1
1.384e+006

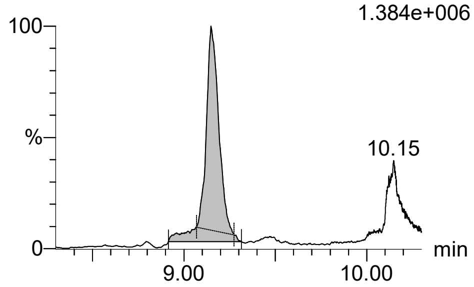

Sample Name: 20211111_A12_R2_IgM
F55:MRM of 2 channels,ES+
1292.9 > 366.1
3.205e+005

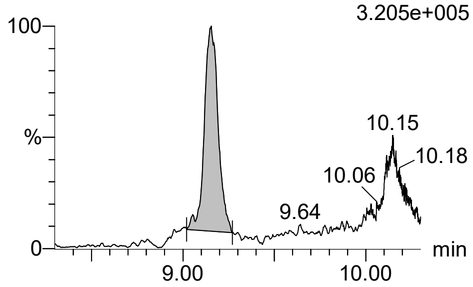

Sample Name: 20211111_L11_R2_IgM
F55:MRM of 2 channels,ES+
1034.5 > 204.1
1.459e+006

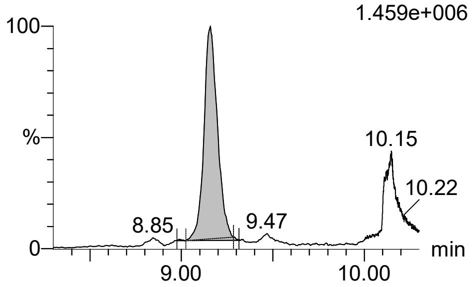

Sample Name: 20211111_L11_R2_IgM
F55:MRM of 2 channels,ES+
1292.9 > 366.1
3.233e+005

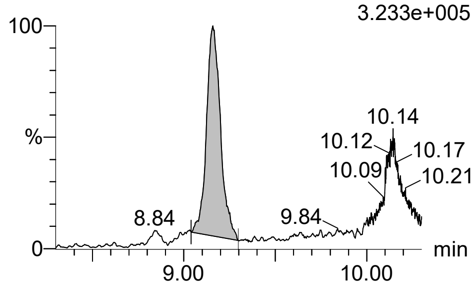

Sample Name: 20211111_L12_R2_IgM
F55:MRM of 2 channels,ES+
1034.5 > 204.1
2.098e+006

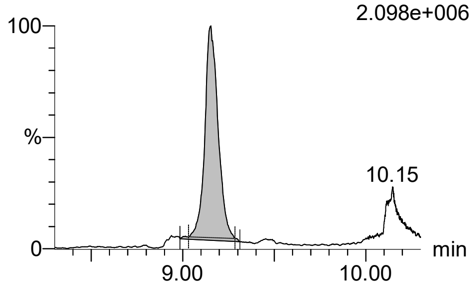

Sample Name: 20211111_L12_R2_IgM
F55:MRM of 2 channels,ES+
1292.9 > 366.1
5.160e+005

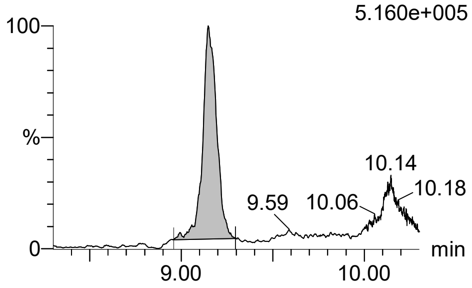

Sample Name: 20211111_H6_R2_IgM
F55:MRM of 2 channels,ES+
1034.5 > 204.1
1.795e+006

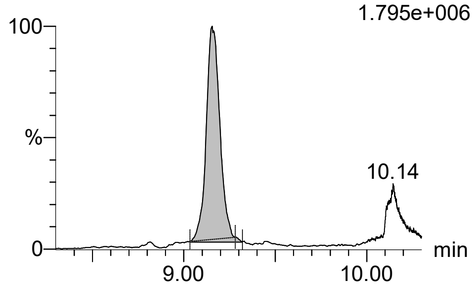

Sample Name: 20211111_H6_R2_IgM
F55:MRM of 2 channels,ES+
1292.9 > 366.1
4.518e+005

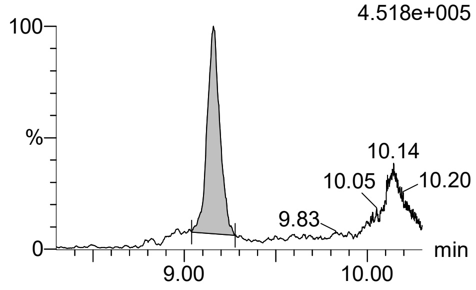

Sample Name: 20211111_A13_R2_IgM
F55:MRM of 2 channels,ES+
1034.5 > 204.1
1.801e+006

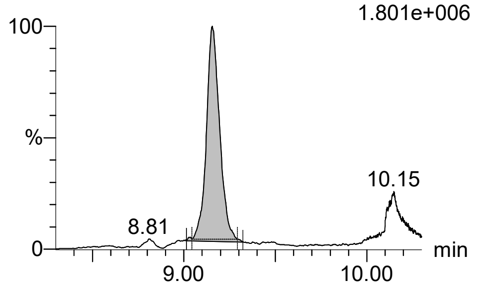

Sample Name: 20211111_A13_R2_IgM
F55:MRM of 2 channels,ES+
1292.9 > 366.1
4.407e+005

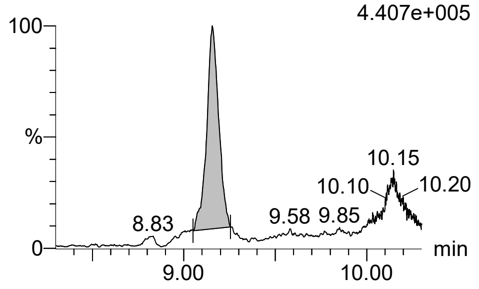

Sample Name: 20211111_A14_R2_IgM
F55:MRM of 2 channels,ES+
1034.5 > 204.1
5.807e+006

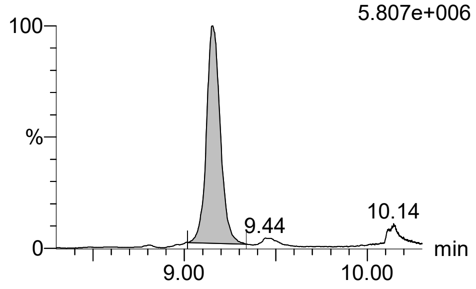

Sample Name: 20211111_A14_R2_IgM
F55:MRM of 2 channels,ES+
1292.9 > 366.1
1.443e+006

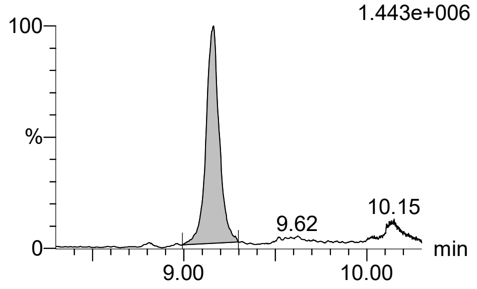

Sample Name: 20211111_L13_R2_IgM
F55:MRM of 2 channels,ES+
1034.5 > 204.1
4.373e+006

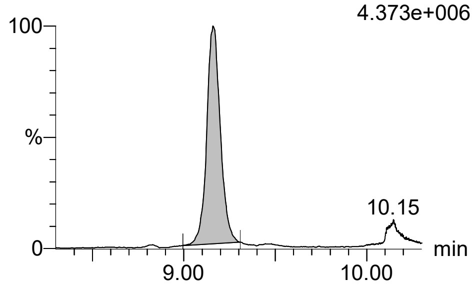

Sample Name: 20211111_L13_R2_IgM
F55:MRM of 2 channels,ES+
1292.9 > 366.1
1.027e+006

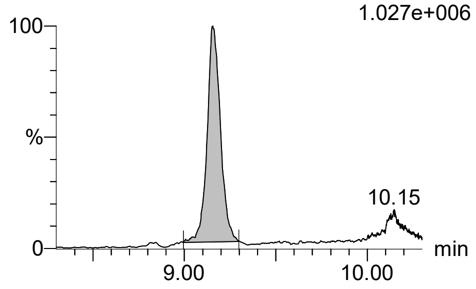

Sample Name: 20211111_L14_R2_IgM
F55:MRM of 2 channels,ES+
1034.5 > 204.1
4.017e+006

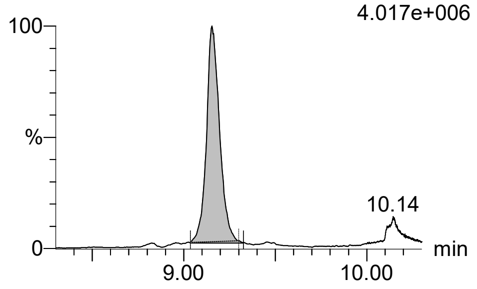

Sample Name: 20211111_L14_R2_IgM
F55:MRM of 2 channels,ES+
1292.9 > 366.1
9.374e+005

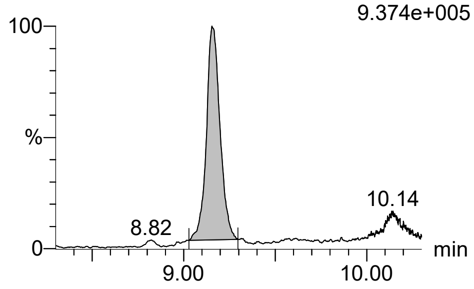

Sample Name: 20211111_H7_R2_IgM
F55:MRM of 2 channels,ES+
1034.5 > 204.1
1.950e+006

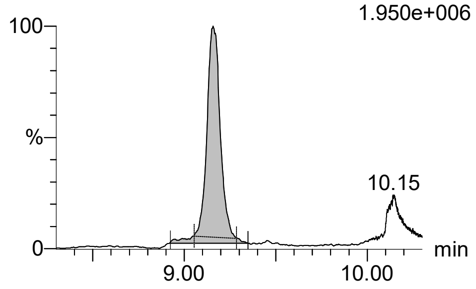

Sample Name: 20211111_H7_R2_IgM
F55:MRM of 2 channels,ES+
1292.9 > 366.1
5.280e+005

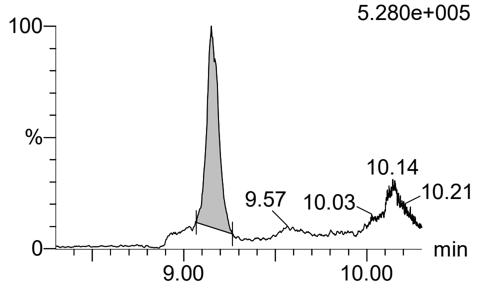

Sample Name: 20211111_A15_R2_IgM
F55:MRM of 2 channels,ES+
1034.5 > 204.1
9.217e+006

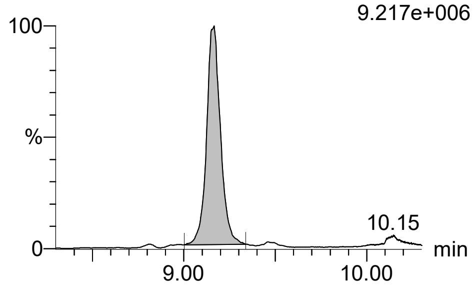

Sample Name: 20211111_A15_R2_IgM
F55:MRM of 2 channels,ES+
1292.9 > 366.1
2.255e+006

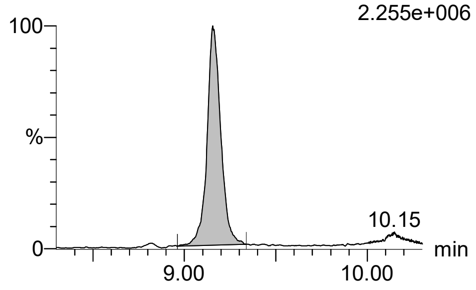

Sample Name: 20211111_QC_R12_IgM
F55:MRM of 2 channels,ES+
1034.5 > 204.1
4.241e+006

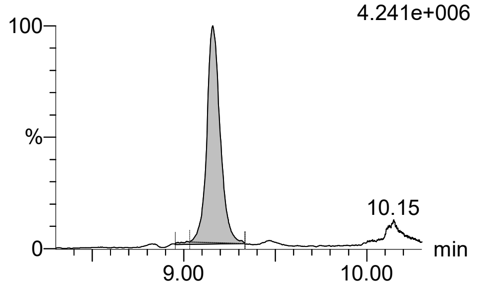

Dataset: C:\MassLynx\10.pro\20211111_clinicalsamples_IgM_IgA.qld

Last Altered: Thursday, March 09, 2023 19:58:25 台北標準時間
Printed: Friday, August 01, 2025 15:09:56 台北標準時間

Sample Name: 20211111_QC_R12_IgM
F55:MRM of 2 channels,ES+
1292.9 > 366.1
1.020e+006

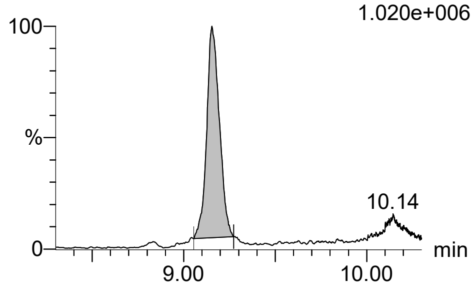

Sample Name: 20211111_A16_R2_IgM
F55:MRM of 2 channels,ES+
1034.5 > 204.1
5.353e+006

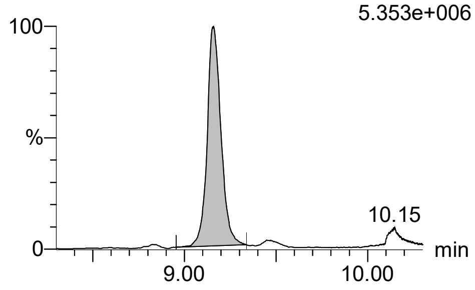

Sample Name: 20211111_A16_R2_IgM
F55:MRM of 2 channels,ES+
1292.9 > 366.1
1.250e+006

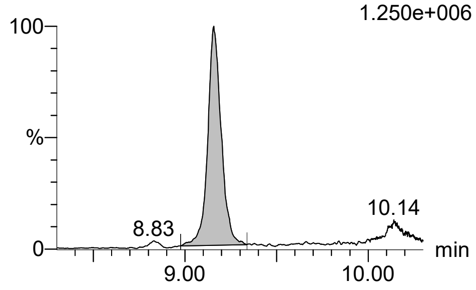

Sample Name: 20211111_L15_R2_IgM
F55:MRM of 2 channels,ES+
1034.5 > 204.1
3.818e+006

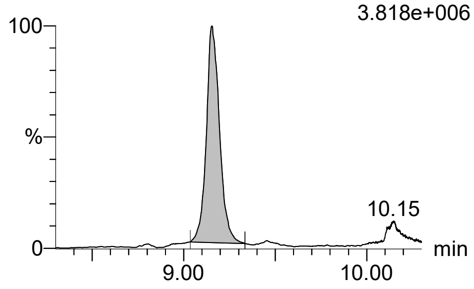

Sample Name: 20211111_L15_R2_IgM
F55:MRM of 2 channels,ES+
1292.9 > 366.1
9.036e+005

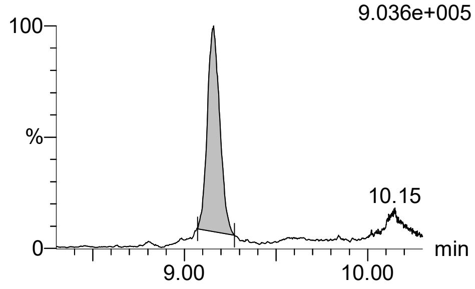

Sample Name: 20211111_L16_R2_IgM
F55:MRM of 2 channels,ES+
1034.5 > 204.1
9.917e+005

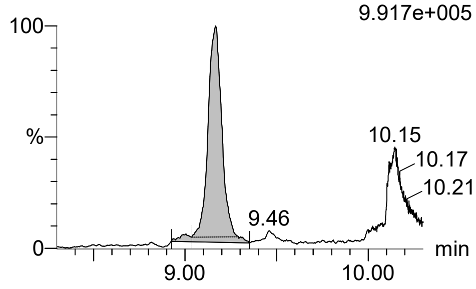

Sample Name: 20211111_L16_R2_IgM
F55:MRM of 2 channels,ES+
1292.9 > 366.1
2.394e+005

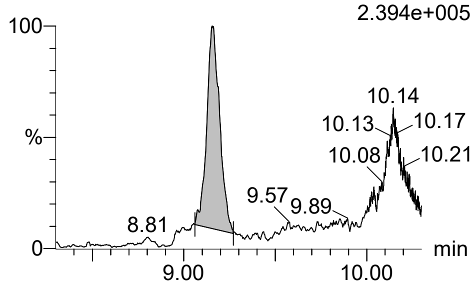

Sample Name: 20211111_H8_R2_IgM
F55:MRM of 2 channels,ES+
1034.5 > 204.1
3.108e+006

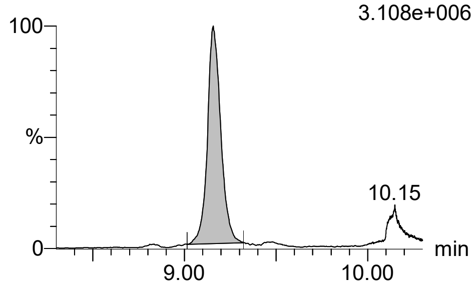

Sample Name: 20211111_H8_R2_IgM
F55:MRM of 2 channels,ES+
1292.9 > 366.1
6.990e+005

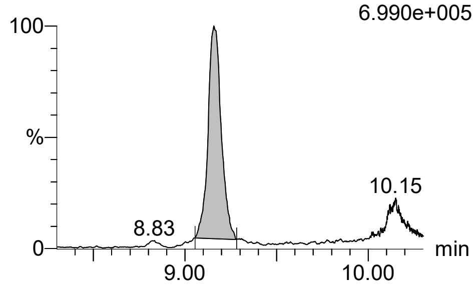

Sample Name: 20211111_A17_R2_IgM
F55:MRM of 2 channels,ES+
1034.5 > 204.1
4.825e+006

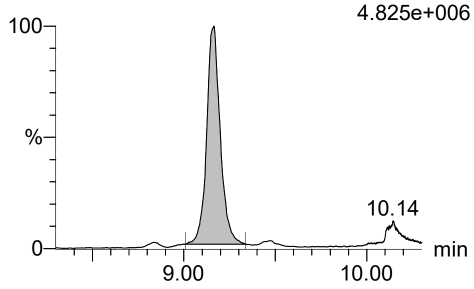

Sample Name: 20211111_A17_R2_IgM
F55:MRM of 2 channels,ES+
1292.9 > 366.1
1.084e+006

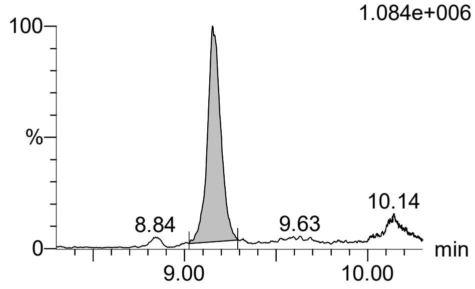

Sample Name: 20211111_A18_R2_IgM
F55:MRM of 2 channels,ES+
1034.5 > 204.1
1.856e+006

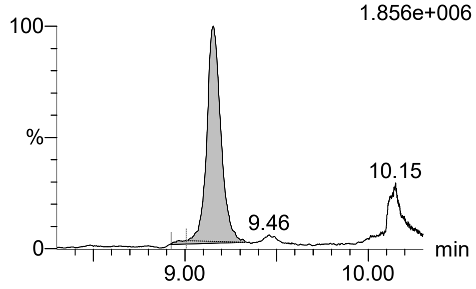

Sample Name: 20211111_A18_R2_IgM
F55:MRM of 2 channels,ES+
1292.9 > 366.1
4.845e+005

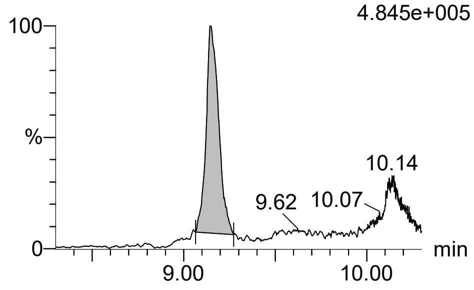

Sample Name: 20211111_L17_R2_IgM
F55:MRM of 2 channels,ES+
1034.5 > 204.1
9.375e+006

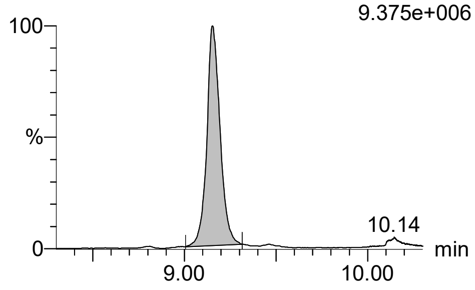

Sample Name: 20211111_L17_R2_IgM
F55:MRM of 2 channels,ES+
1292.9 > 366.1
2.220e+006

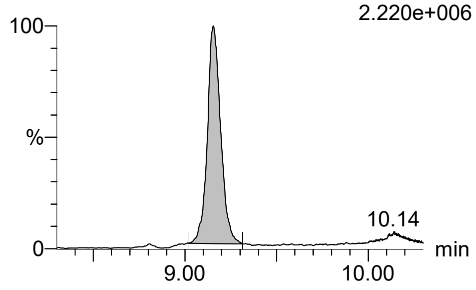

Sample Name: 20211111_L18_R2_IgM
F55:MRM of 2 channels,ES+
1034.5 > 204.1
6.738e+005

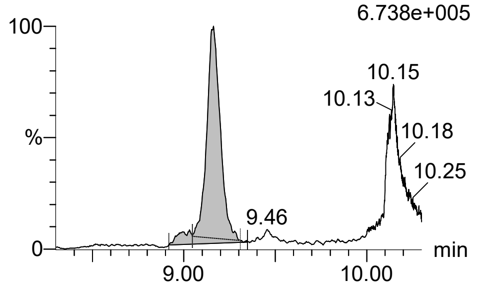

Sample Name: 20211111_L18_R2_IgM
F55:MRM of 2 channels,ES+
1292.9 > 366.1
1.697e+005

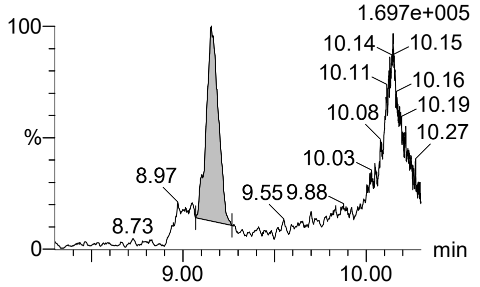

Sample Name: 20211111_H9_R2_IgM
F55:MRM of 2 channels,ES+
1034.5 > 204.1
1.556e+006

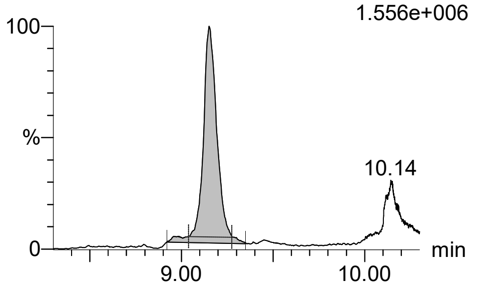

Sample Name: 20211111_H9_R2_IgM
F55:MRM of 2 channels,ES+
1292.9 > 366.1
3.938e+005

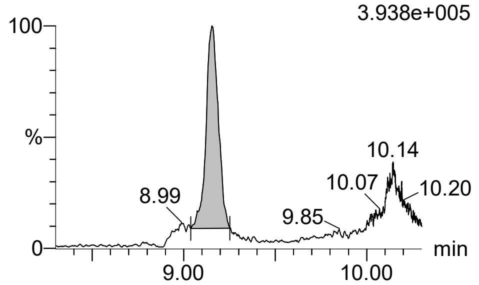

Sample Name: 20211111_A19_R2_IgM
F55:MRM of 2 channels,ES+
1034.5 > 204.1
2.240e+006

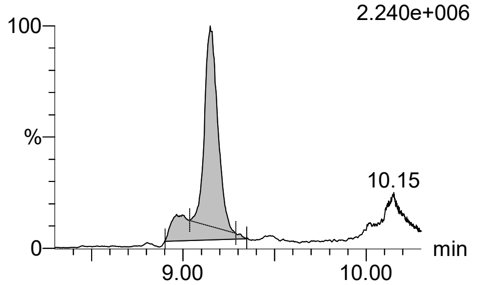

Sample Name: 20211111_A19_R2_IgM
F55:MRM of 2 channels,ES+
1292.9 > 366.1
6.112e+005

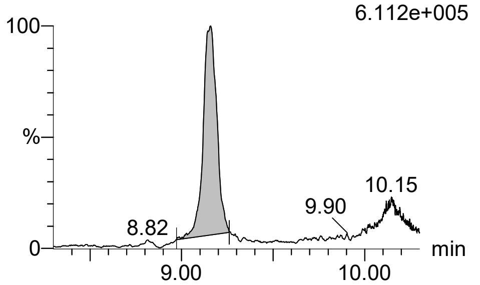

Sample Name: 20211111_A20_R2_IgM
F55:MRM of 2 channels,ES+
1034.5 > 204.1
1.256e+006

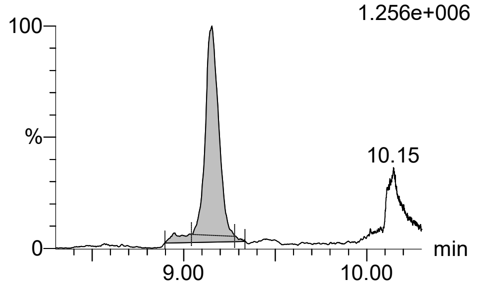

Sample Name: 20211111_A20_R2_IgM
F55:MRM of 2 channels,ES+
1292.9 > 366.1
3.336e+005

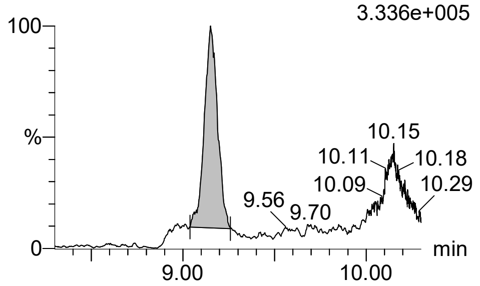

Sample Name: 20211111_L19_R2_IgM
F55:MRM of 2 channels,ES+
1034.5 > 204.1
3.307e+006

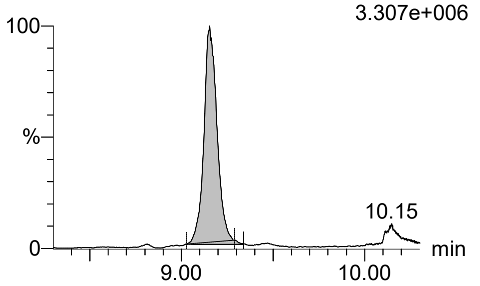

Sample Name: 20211111_L19_R2_IgM
F55:MRM of 2 channels,ES+
1292.9 > 366.1
7.785e+005

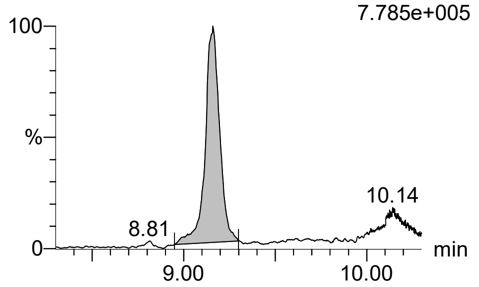

Sample Name: 20211111_QC_R13_IgM
F55:MRM of 2 channels,ES+
1034.5 > 204.1
4.065e+006

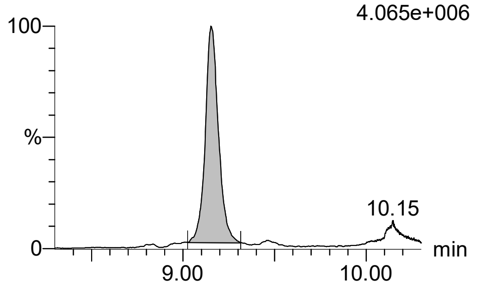

Sample Name: 20211111_QC_R13_IgM
F55:MRM of 2 channels,ES+
1292.9 > 366.1
9.403e+005

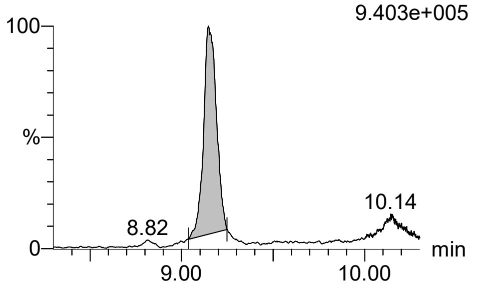

Sample Name: 20211111_L20_R2_IgM
F55:MRM of 2 channels,ES+
1034.5 > 204.1
1.861e+006

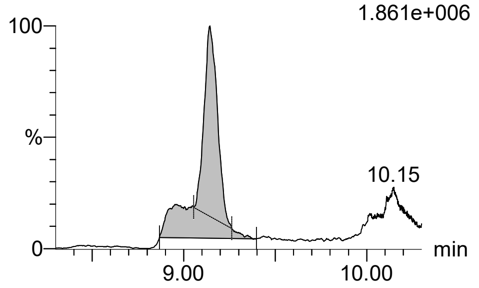

Sample Name: 20211111_L20_R2_IgM
F55:MRM of 2 channels,ES+
1292.9 > 366.1
6.381e+005

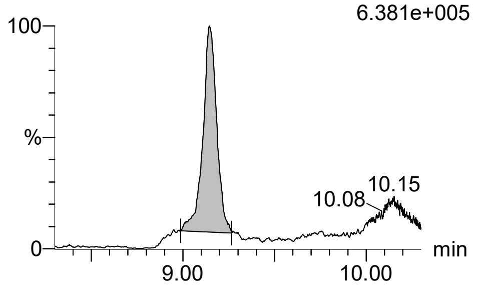

Sample Name: 20211111_H10_R2_IgM
F55:MRM of 2 channels,ES+
1034.5 > 204.1
2.912e+006

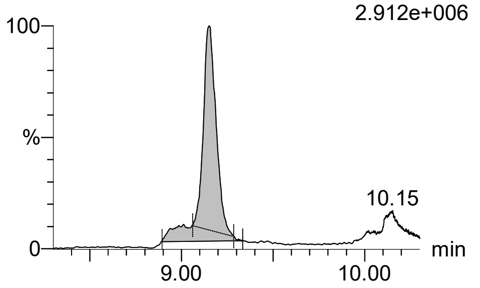

Sample Name: 20211111_H10_R2_IgM
F55:MRM of 2 channels,ES+
1292.9 > 366.1
8.192e+005

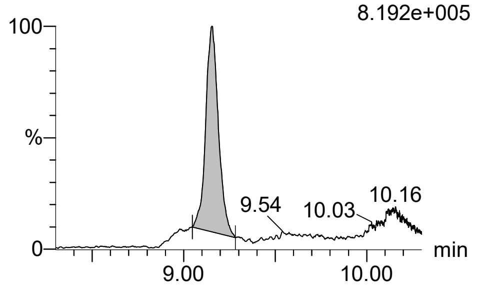

Sample Name: 20211111_A21_R2_IgM
F55:MRM of 2 channels,ES+
1034.5 > 204.1
5.059e+006

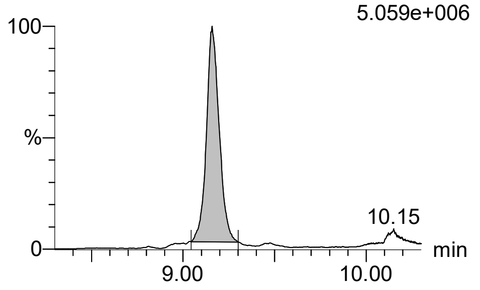

Sample Name: 20211111_A21_R2_IgM
F55:MRM of 2 channels,ES+
1292.9 > 366.1
1.231e+006

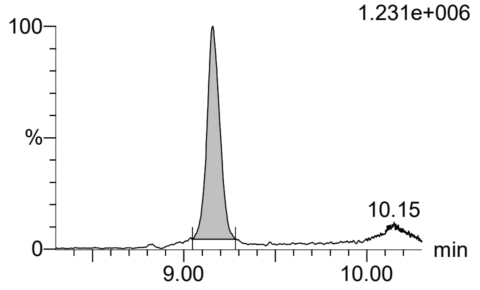

Sample Name: 20211111_A22_R2_IgM
F55:MRM of 2 channels,ES+
1034.5 > 204.1
1.547e+006

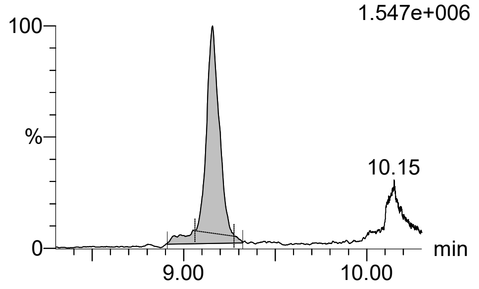

Sample Name: 20211111_A22_R2_IgM
F55:MRM of 2 channels,ES+
1292.9 > 366.1
4.015e+005

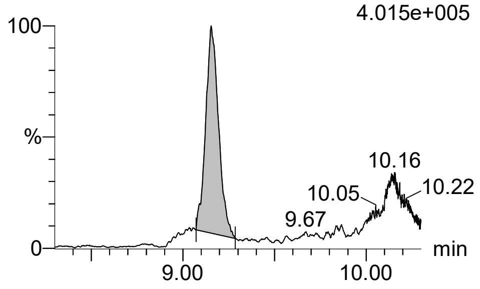

Sample Name: 20211111_L21_R2_IgM
F55:MRM of 2 channels,ES+
1034.5 > 204.1
2.578e+006

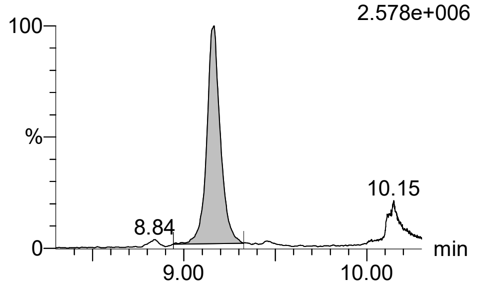

Sample Name: 20211111_L21_R2_IgM
F55:MRM of 2 channels,ES+
1292.9 > 366.1
5.464e+005

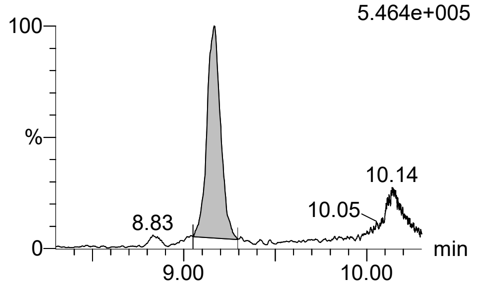

Sample Name: 20211111_L22_R2_IgM
F55:MRM of 2 channels,ES+
1034.5 > 204.1
4.067e+006

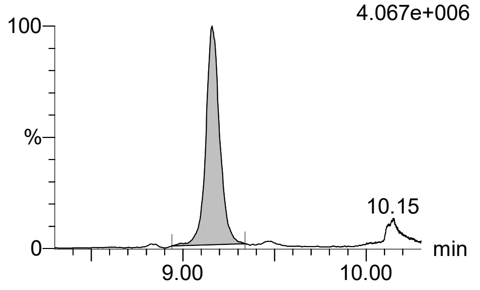

Sample Name: 20211111_L22_R2_IgM
F55:MRM of 2 channels,ES+
1292.9 > 366.1
9.141e+005

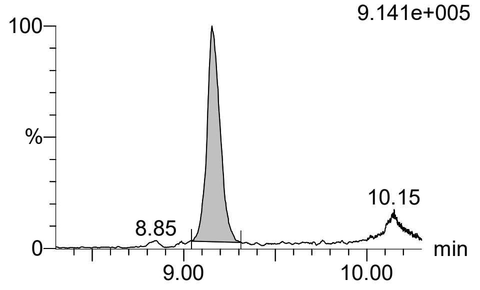

Sample Name: 20211111_H11_R2_IgM
F55:MRM of 2 channels,ES+
1034.5 > 204.1
2.221e+006

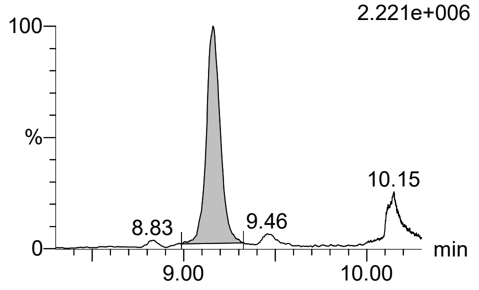

Sample Name: 20211111_H11_R2_IgM
F55:MRM of 2 channels,ES+
1292.9 > 366.1
4.973e+005

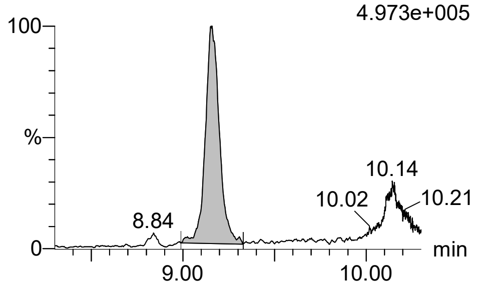

Sample Name: 20211111_A23_R2_IgM
F55:MRM of 2 channels,ES+
1034.5 > 204.1
1.610e+006

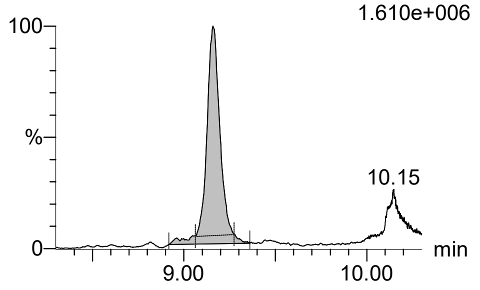

Sample Name: 20211111_A23_R2_IgM
F55:MRM of 2 channels,ES+
1292.9 > 366.1
3.678e+005

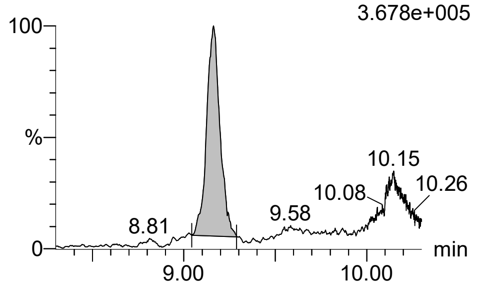

Sample Name: 20211111_A24_R2_IgM
F55:MRM of 2 channels,ES+
1034.5 > 204.1
6.610e+006

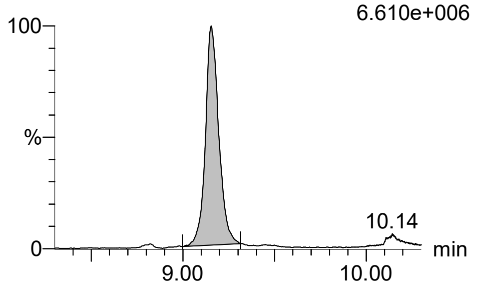

Sample Name: 20211111_A24_R2_IgM
F55:MRM of 2 channels,ES+
1292.9 > 366.1
1.552e+006

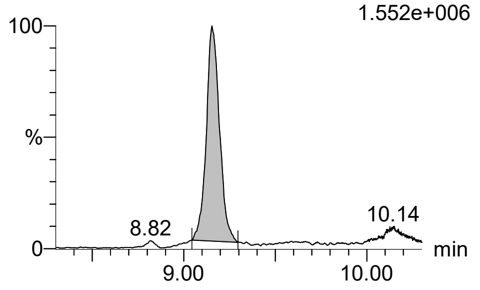

Dataset: C:\MassLynx\10.pro\20211111_clinicalsamples_IgM_IgA.qld

Last Altered: Thursday, March 09, 2023 19:58:25 台北標準時間
Printed: Friday, August 01, 2025 15:09:56 台北標準時間

Sample Name: 20211111_L23_R2_IgM
F55:MRM of 2 channels,ES+
1034.5 > 204.1
4.257e+006

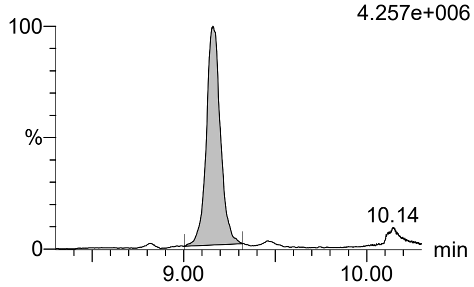

Sample Name: 20211111_L23_R2_IgM
F55:MRM of 2 channels,ES+
1292.9 > 366.1
1.052e+006

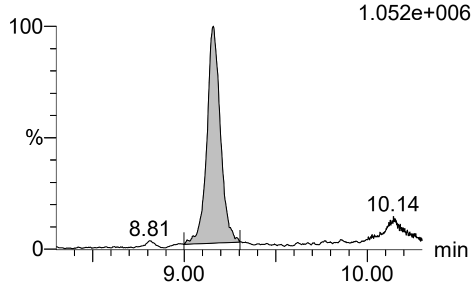

Sample Name: 20211111_L24_R2_IgM
F55:MRM of 2 channels,ES+
1034.5 > 204.1
1.905e+006

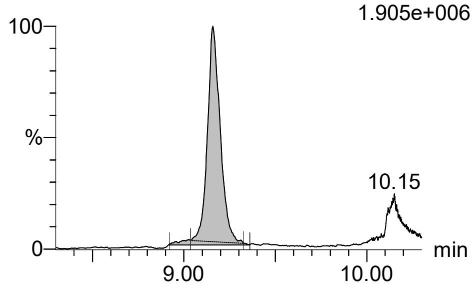

Sample Name: 20211111_L24_R2_IgM
F55:MRM of 2 channels,ES+
1292.9 > 366.1
4.547e+005

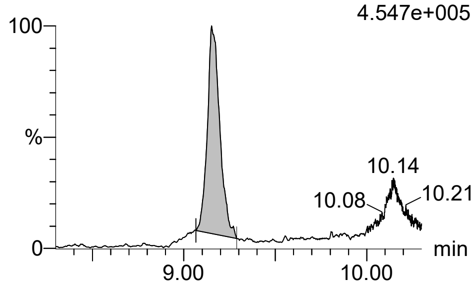

Sample Name: 20211111_H12_R2_IgM
F55:MRM of 2 channels,ES+
1034.5 > 204.1
2.288e+006

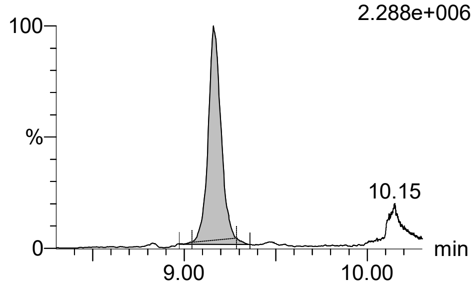

Sample Name: 20211111_H12_R2_IgM
F55:MRM of 2 channels,ES+
1292.9 > 366.1
5.221e+005

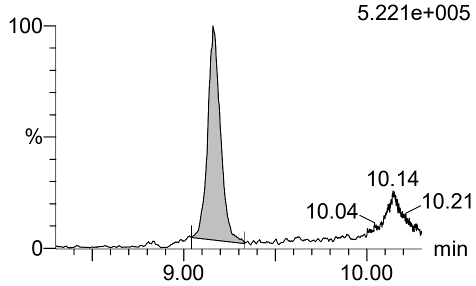

Sample Name: 20211111_QC_R14_IgM
F55:MRM of 2 channels,ES+
1034.5 > 204.1
4.208e+006

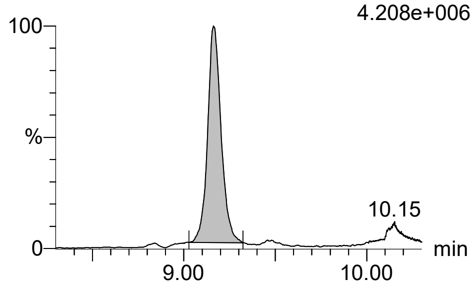

Sample Name: 20211111_QC_R14_IgM
F55:MRM of 2 channels,ES+
1292.9 > 366.1
9.218e+005

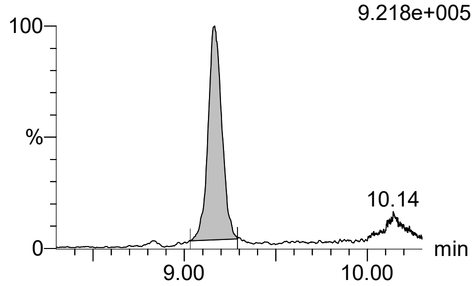

Sample Name: 20211111_A25_R2_IgM
F55:MRM of 2 channels,ES+
1034.5 > 204.1
9.243e+006

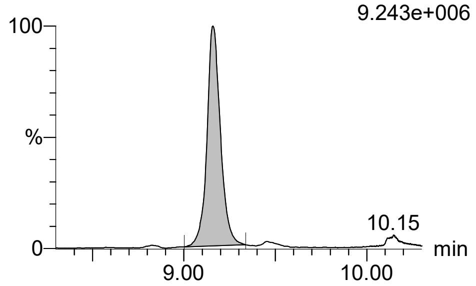

Sample Name: 20211111_A25_R2_IgM
F55:MRM of 2 channels,ES+
1292.9 > 366.1
2.023e+006

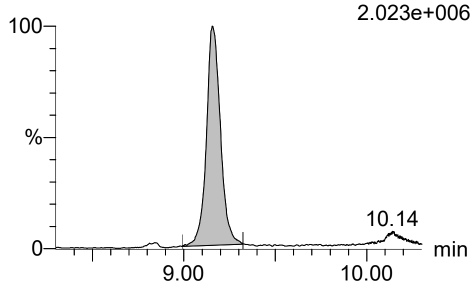

Sample Name: 20211111_A26_R2_IgM
F55:MRM of 2 channels,ES+
1034.5 > 204.1
3.000e+006

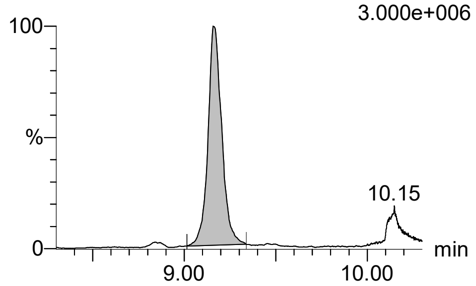

Sample Name: 20211111_A26_R2_IgM
F55:MRM of 2 channels,ES+
1292.9 > 366.1
6.687e+005

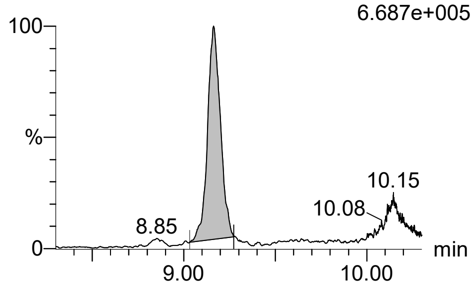

Sample Name: 20211111_L25_R2_IgM
F55:MRM of 2 channels,ES+
1034.5 > 204.1
2.408e+006

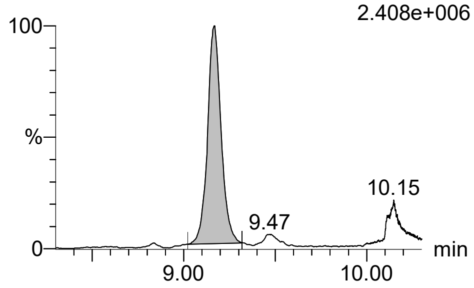

Sample Name: 20211111_L25_R2_IgM
F55:MRM of 2 channels,ES+
1292.9 > 366.1
5.422e+005

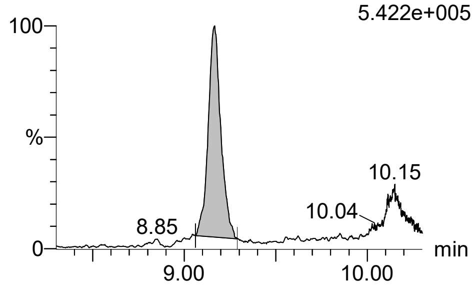

Sample Name: 20211111_L26_R2_IgM
F55:MRM of 2 channels,ES+
1034.5 > 204.1
1.524e+006

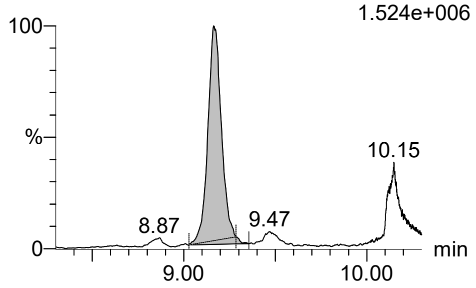

Sample Name: 20211111_L26_R2_IgM
F55:MRM of 2 channels,ES+
1292.9 > 366.1
3.305e+005

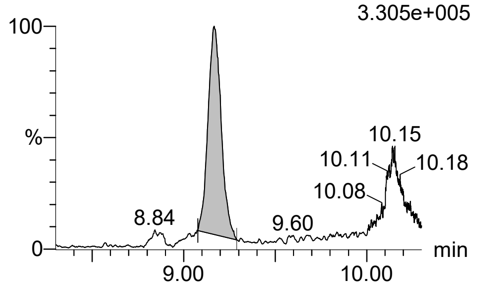

Sample Name: 20211111_H13_R2_IgM
F55:MRM of 2 channels,ES+
1034.5 > 204.1
3.886e+006

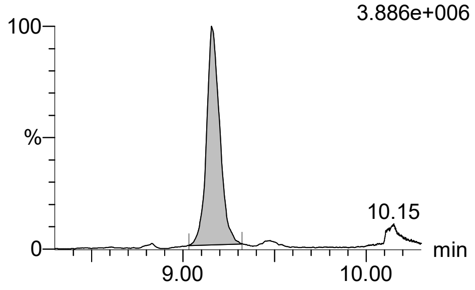

Sample Name: 20211111_H13_R2_IgM
F55:MRM of 2 channels,ES+
1292.9 > 366.1
8.875e+005

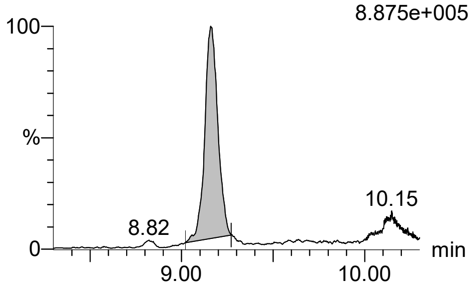

Sample Name: 20211111_A27_R2_IgM
F55:MRM of 2 channels,ES+
1034.5 > 204.1
6.205e+006

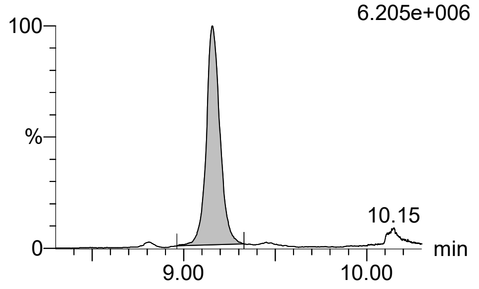

Sample Name: 20211111_A27_R2_IgM
F55:MRM of 2 channels,ES+
1292.9 > 366.1
1.452e+006

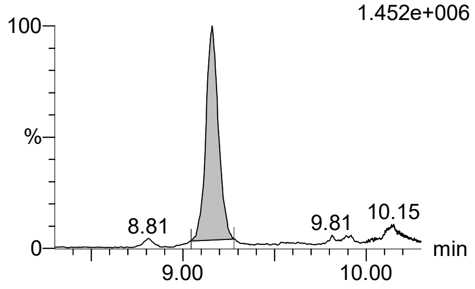

Sample Name: 20211111_A28_R2_IgM
F55:MRM of 2 channels,ES+
1034.5 > 204.1
2.806e+006

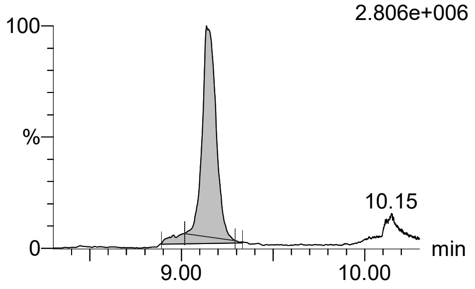

Sample Name: 20211111_A28_R2_IgM
F55:MRM of 2 channels,ES+
1292.9 > 366.1
8.133e+005

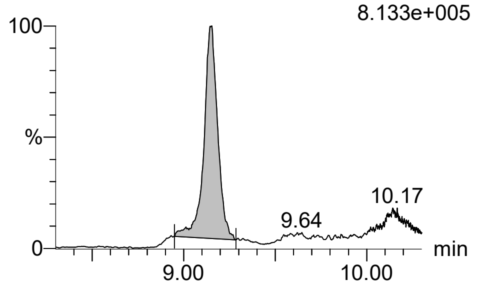

Sample Name: 20211111_L27_R2_IgM
F55:MRM of 2 channels,ES+
1034.5 > 204.1
3.796e+006

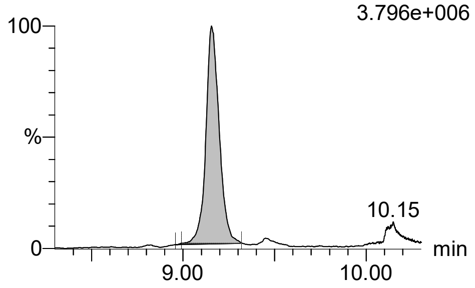

Sample Name: 20211111_L27_R2_IgM
F55:MRM of 2 channels,ES+
1292.9 > 366.1
8.509e+005

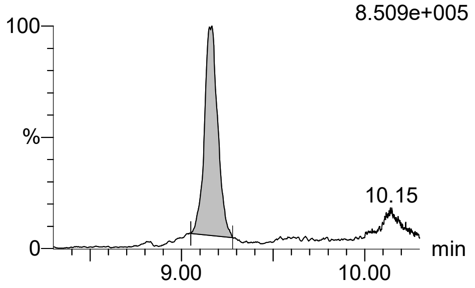

Sample Name: 20211111_L28_R2_IgM
F55:MRM of 2 channels,ES+
1034.5 > 204.1
3.921e+006

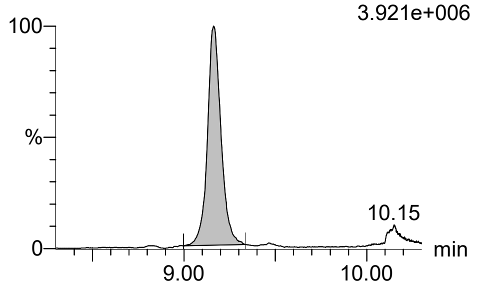

Sample Name: 20211111_L28_R2_IgM
F55:MRM of 2 channels,ES+
1292.9 > 366.1
9.140e+005

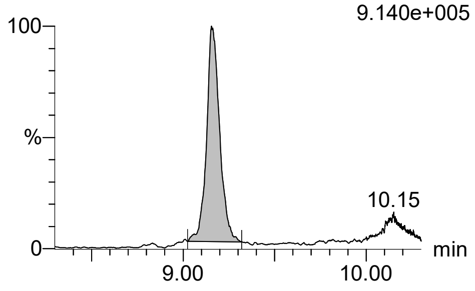

Sample Name: 20211111_H14_R2_IgM
F55:MRM of 2 channels,ES+
1034.5 > 204.1
3.687e+006

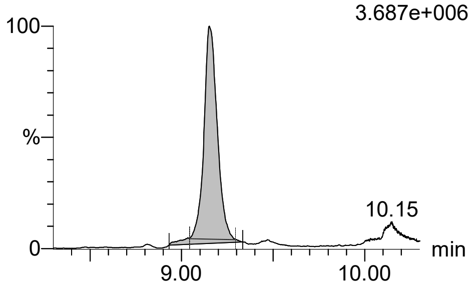

Sample Name: 20211111_H14_R2_IgM
F55:MRM of 2 channels,ES+
1292.9 > 366.1
8.839e+005

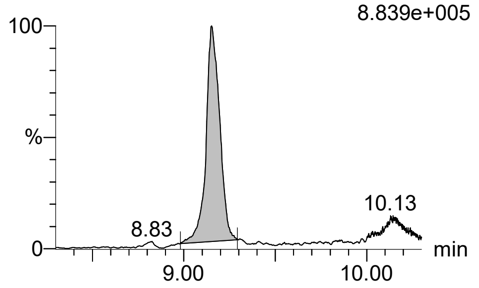

Sample Name: 20211111_A29_R2_IgM
F55:MRM of 2 channels,ES+
1034.5 > 204.1
4.095e+005

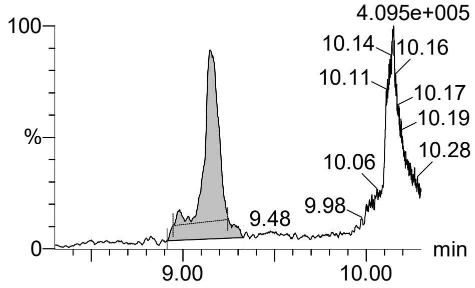

Sample Name: 20211111_A29_R2_IgM
F55:MRM of 2 channels,ES+
1292.9 > 366.1
1.300e+005

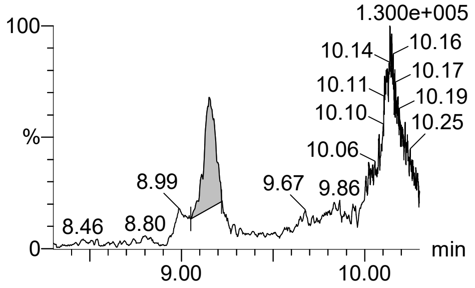

Dataset: C:\MassLynx\10.pro\20211111_clinicalsamples_IgM_IgA.qld

Last Altered: Thursday, March 09, 2023 19:58:25 台北標準時間
Printed: Friday, August 01, 2025 15:09:56 台北標準時間

Sample Name: 20211111_A30_R2_IgM
F55:MRM of 2 channels,ES+
1034.5 > 204.1
4.387e+006

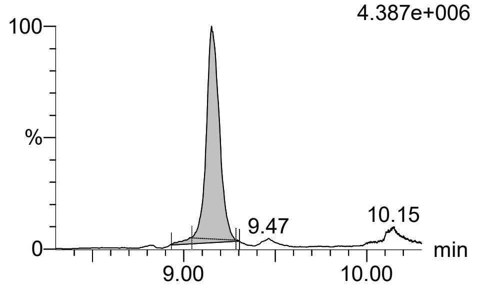

Sample Name: 20211111_A30_R2_IgM
F55:MRM of 2 channels,ES+
1292.9 > 366.1
9.766e+005

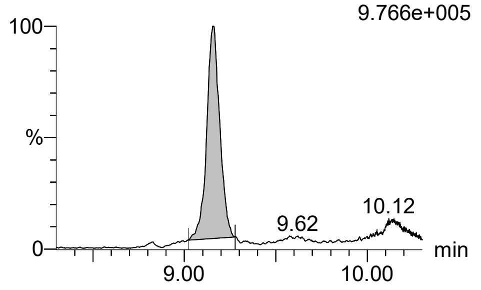

Sample Name: 20211111_QC_R15_IgM
F55:MRM of 2 channels,ES+
1034.5 > 204.1
3.546e+006

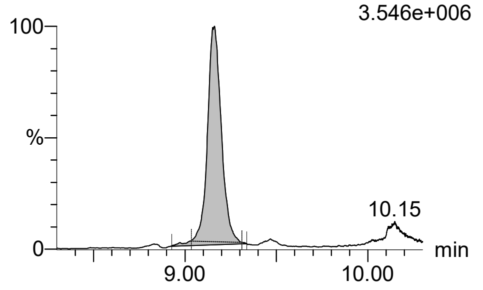

Sample Name: 20211111_QC_R15_IgM
F55:MRM of 2 channels,ES+
1292.9 > 366.1
8.741e+005

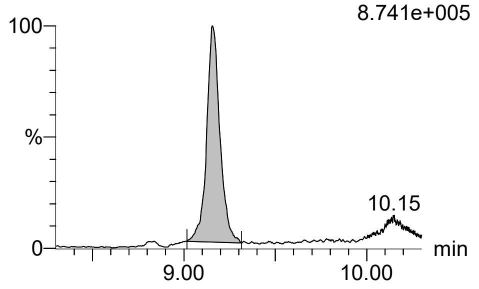

Sample Name: 20211111_L29_R2_IgM
F55:MRM of 2 channels,ES+
1034.5 > 204.1
1.123e+006

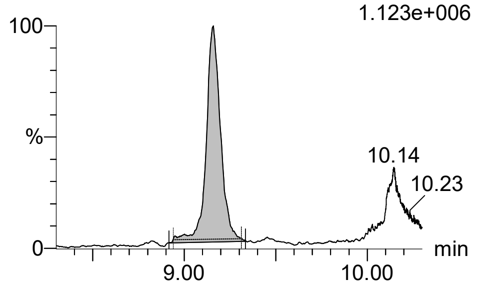

Sample Name: 20211111_L29_R2_IgM
F55:MRM of 2 channels,ES+
1292.9 > 366.1
2.681e+005

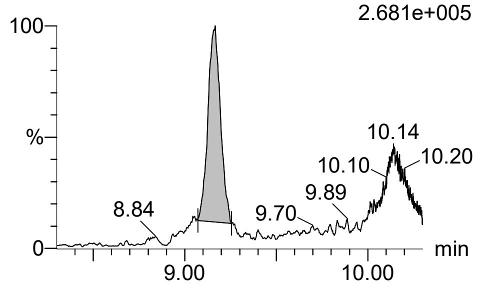

Sample Name: 20211111_L30_R2_IgM
F55:MRM of 2 channels,ES+
1034.5 > 204.1
7.448e+005

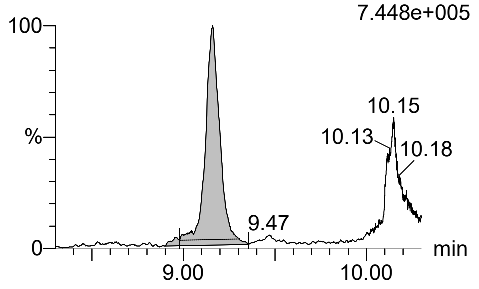

Sample Name: 20211111_L30_R2_IgM
F55:MRM of 2 channels,ES+
1292.9 > 366.1
1.878e+005

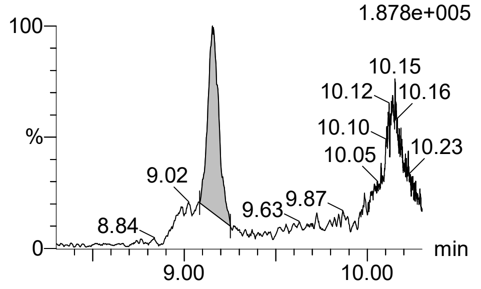

Sample Name: 20211111_H15_R2_IgM
F55:MRM of 2 channels,ES+
1034.5 > 204.1
2.240e+006

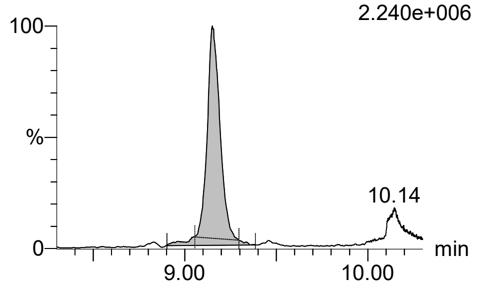

Sample Name: 20211111_H15_R2_IgM
F55:MRM of 2 channels,ES+
1292.9 > 366.1
5.276e+005

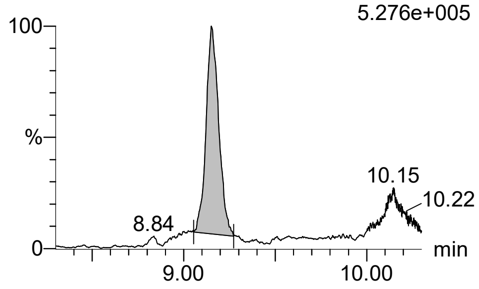

Sample Name: 20211111_A31_R2_IgM
F55:MRM of 2 channels,ES+
1034.5 > 204.1
9.659e+005

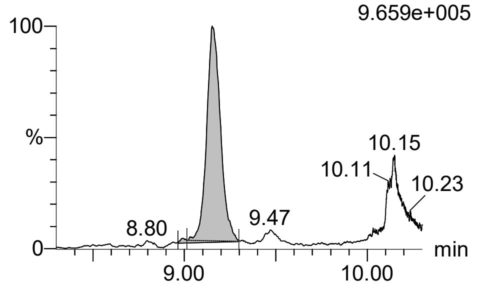

Sample Name: 20211111_A31_R2_IgM
F55:MRM of 2 channels,ES+
1292.9 > 366.1
2.479e+005

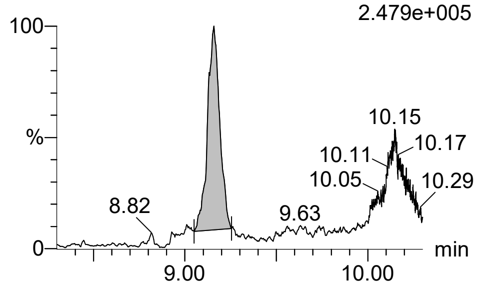

Sample Name: 20211111_A32_R2_IgM
F55:MRM of 2 channels,ES+
1034.5 > 204.1
4.312e+006

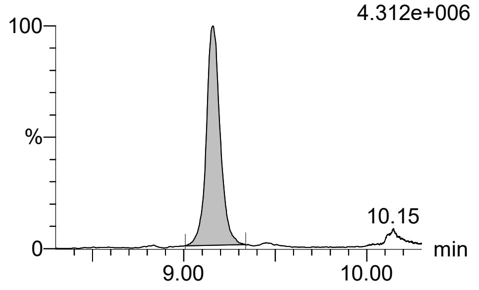

Sample Name: 20211111_A32_R2_IgM
F55:MRM of 2 channels,ES+
1292.9 > 366.1
1.076e+006

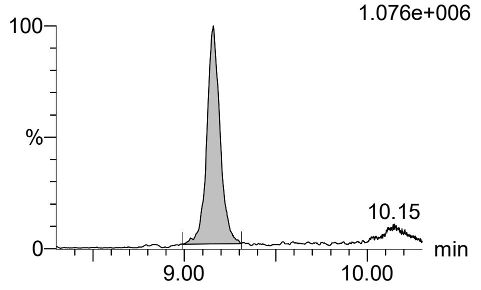

Sample Name: 20211111_L31_R2_IgM
F55:MRM of 2 channels,ES+
1034.5 > 204.1
6.176e+005

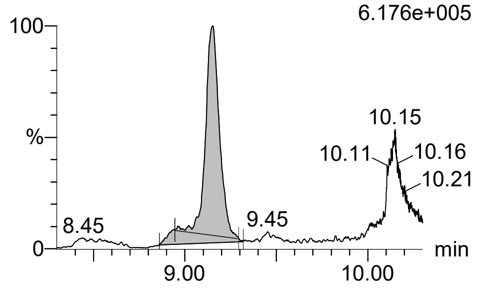

Dataset: C:\MassLynx\10.pro\20211111_clinicalsamples_IgM_IgA.qld

Last Altered: Thursday, March 09, 2023 19:58:25 台北標準時間
Printed: Friday, August 01, 2025 15:09:56 台北標準時間

Sample Name: 20211111_L31_R2_IgM
F55:MRM of 2 channels,ES+
1292.9 > 366.1
1.927e+005

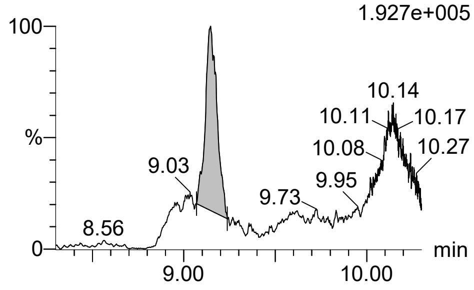

Sample Name: 20211111_L32_R2_IgM
F55:MRM of 2 channels,ES+
1034.5 > 204.1
6.102e+006

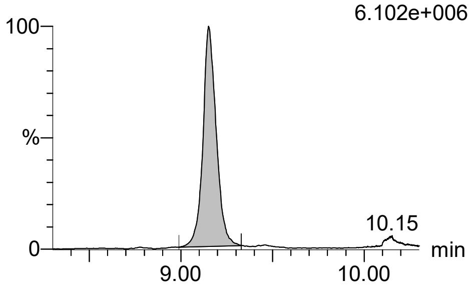

Sample Name: 20211111_L32_R2_IgM
F55:MRM of 2 channels,ES+
1292.9 > 366.1
1.448e+006

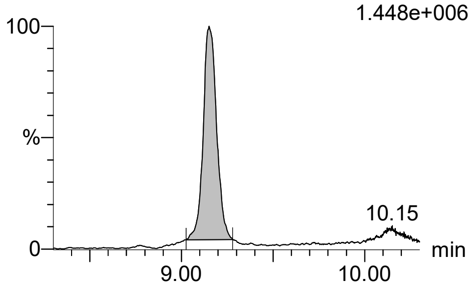

Sample Name: 20211111_H16_R2_IgM
F55:MRM of 2 channels,ES+
1034.5 > 204.1
1.123e+007

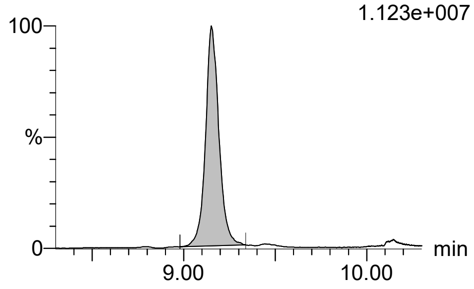

Sample Name: 20211111_H16_R2_IgM
F55:MRM of 2 channels,ES+
1292.9 > 366.1
2.860e+006

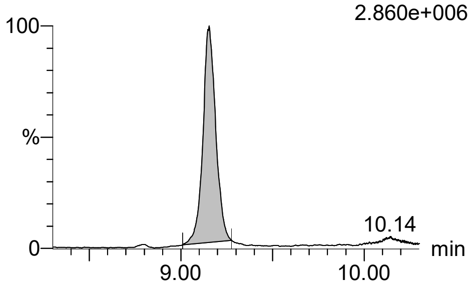

Sample Name: 20211111_A33_R2_IgM
F55:MRM of 2 channels,ES+
1034.5 > 204.1
4.241e+006

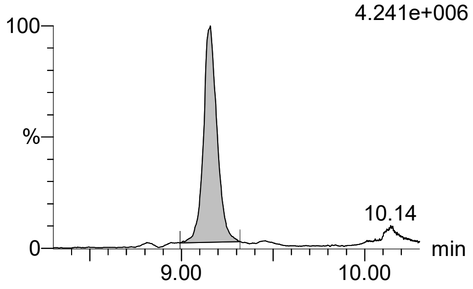

Sample Name: 20211111_A33_R2_IgM
F55:MRM of 2 channels,ES+
1292.9 > 366.1
1.064e+006

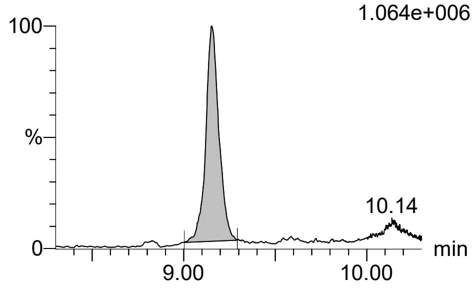

Sample Name: 20211111_A34_R2_IgM
F55:MRM of 2 channels,ES+
1034.5 > 204.1
1.081e+006

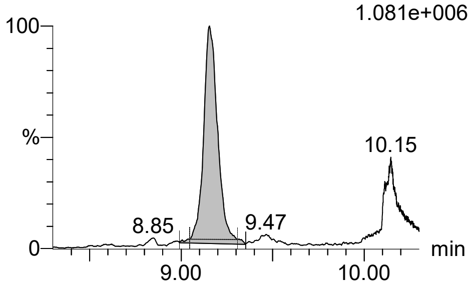

Sample Name: 20211111_A34_R2_IgM
F55:MRM of 2 channels,ES+
1292.9 > 366.1
2.493e+005

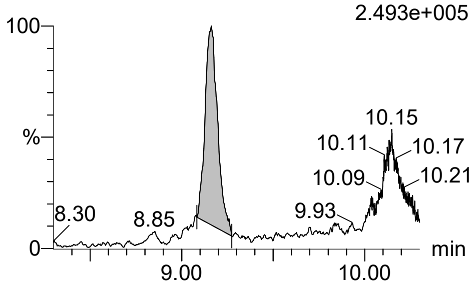

Sample Name: 20211111_H17_R2_IgM
F55:MRM of 2 channels,ES+
1034.5 > 204.1
2.623e+007

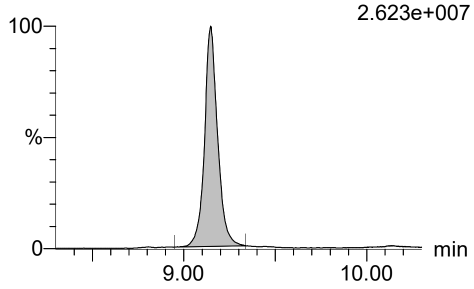

Sample Name: 20211111_H17_R2_IgM
F55:MRM of 2 channels,ES+
1292.9 > 366.1
7.047e+006

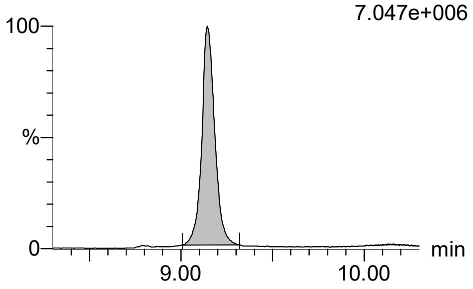

Sample Name: 20211111_A35_R2_IgM
F55:MRM of 2 channels,ES+
1034.5 > 204.1
1.436e+006

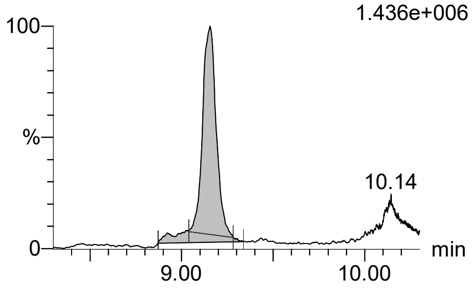

Sample Name: 20211111_A35_R2_IgM
F55:MRM of 2 channels,ES+
1292.9 > 366.1
4.232e+005

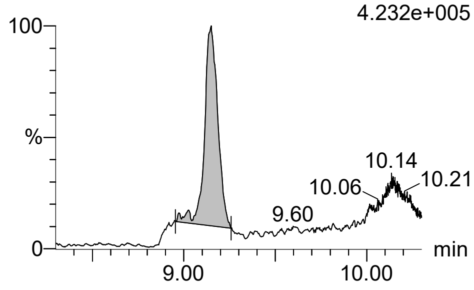

Sample Name: 20211111_QC_R16_IgM
F55:MRM of 2 channels,ES+
1034.5 > 204.1
3.300e+006

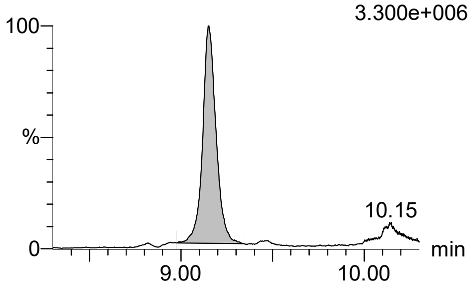

Sample Name: 20211111_QC_R16_IgM
F55:MRM of 2 channels,ES+
1292.9 > 366.1
8.171e+005

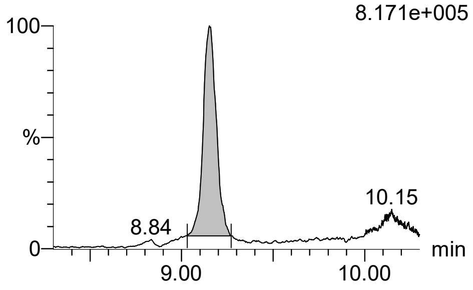

Sample Name: 20211111_A36_R2_IgM
F55:MRM of 2 channels,ES+
1034.5 > 204.1
1.497e+006

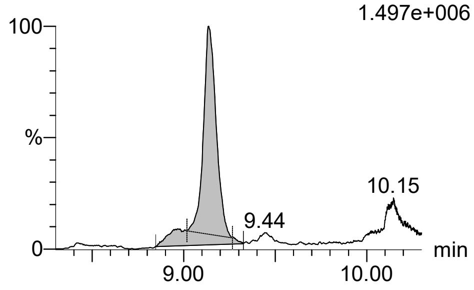

Sample Name: 20211111_A36_R2_IgM
F55:MRM of 2 channels,ES+
1292.9 > 366.1
4.584e+005

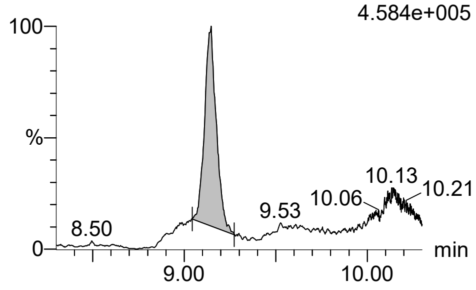

Sample Name: 20211111_H18_R2_IgM
F55:MRM of 2 channels,ES+
1034.5 > 204.1
8.924e+006

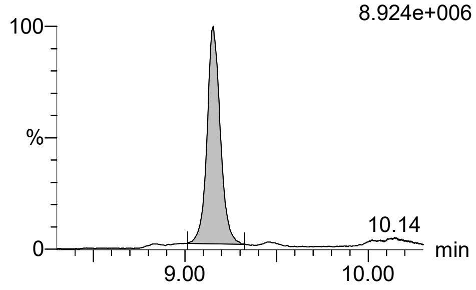

Sample Name: 20211111_H18_R2_IgM
F55:MRM of 2 channels,ES+
1292.9 > 366.1
2.303e+006

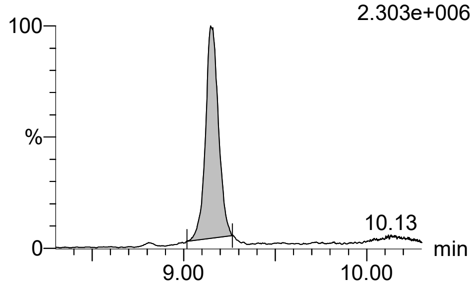

Sample Name: 20211111_A37_R2_IgM
F55:MRM of 2 channels,ES+
1034.5 > 204.1
4.067e+006

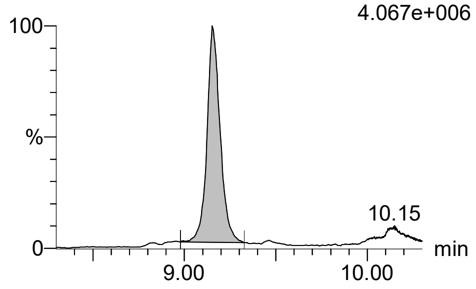

Sample Name: 20211111_A37_R2_IgM
F55:MRM of 2 channels,ES+
1292.9 > 366.1
1.011e+006

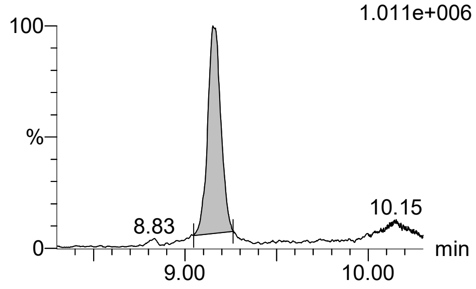

Sample Name: 20211111_A38_R2_IgM
F55:MRM of 2 channels,ES+
1034.5 > 204.1
2.987e+006

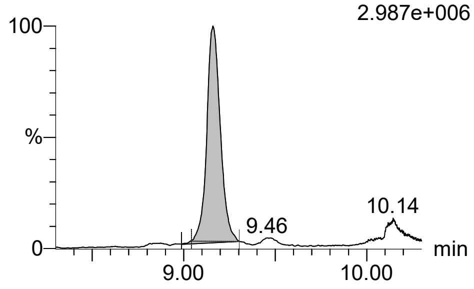

Sample Name: 20211111_A38_R2_IgM
F55:MRM of 2 channels,ES+
1292.9 > 366.1
7.530e+005

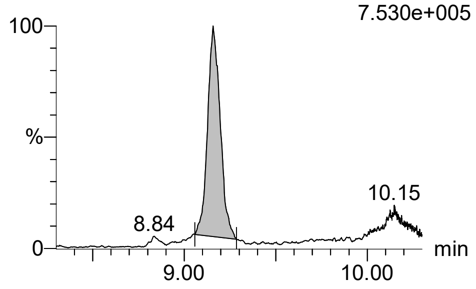

Sample Name: 20211111_H19_R2_IgM
F55:MRM of 2 channels,ES+
1034.5 > 204.1
6.120e+006

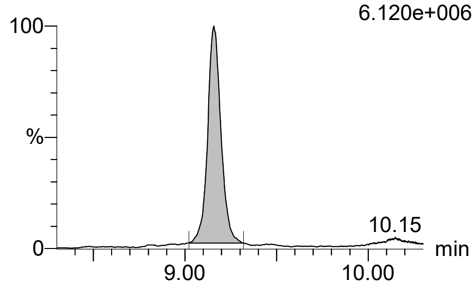

Sample Name: 20211111_H19_R2_IgM
F55:MRM of 2 channels,ES+
1292.9 > 366.1
1.582e+006

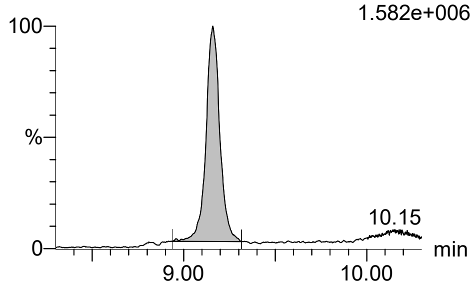

Sample Name: 20211111_A39_R2_IgM
F55:MRM of 2 channels,ES+
1034.5 > 204.1
3.054e+006

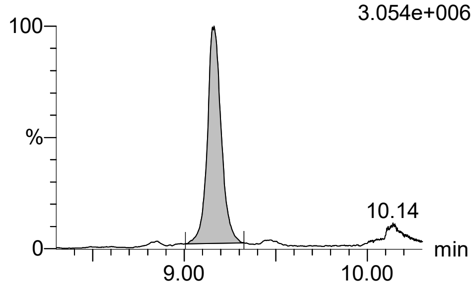

Sample Name: 20211111_A39_R2_IgM
F55:MRM of 2 channels,ES+
1292.9 > 366.1
7.566e+005

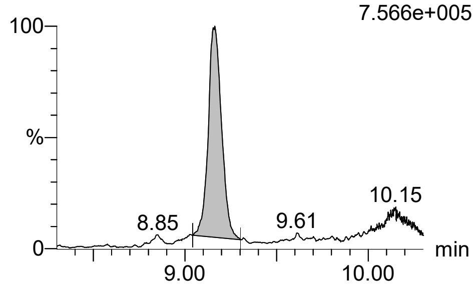

Sample Name: 20211111_A40_R2_IgM
F55:MRM of 2 channels,ES+
1034.5 > 204.1
1.259e+006

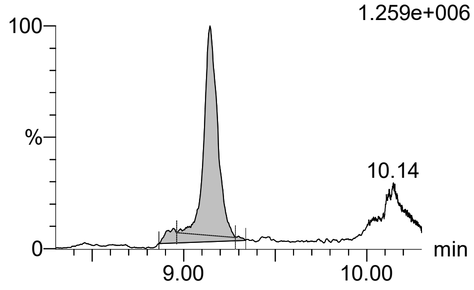

Sample Name: 20211111_A40_R2_IgM
F55:MRM of 2 channels,ES+
1292.9 > 366.1
3.806e+005

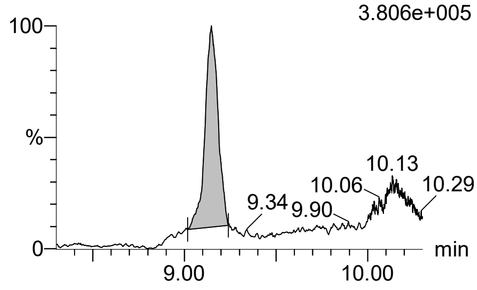

Sample Name: 20211111_H20_R2_IgM
F55:MRM of 2 channels,ES+
1034.5 > 204.1
1.343e+007

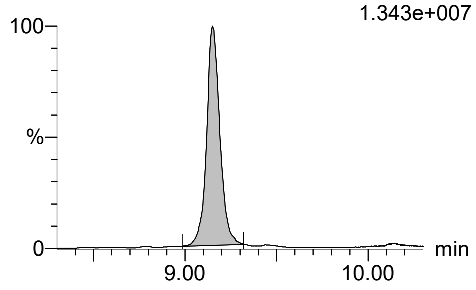

Sample Name: 20211111_H20_R2_IgM
F55:MRM of 2 channels,ES+
1292.9 > 366.1
3.848e+006

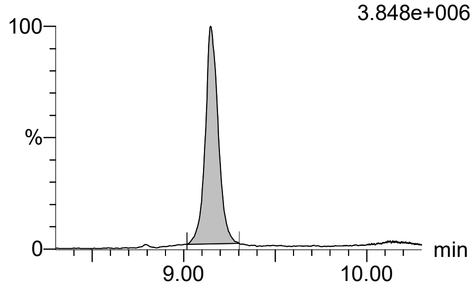

Sample Name: 20211111_A41_R2_IgM
F55:MRM of 2 channels,ES+
1034.5 > 204.1
1.570e+006

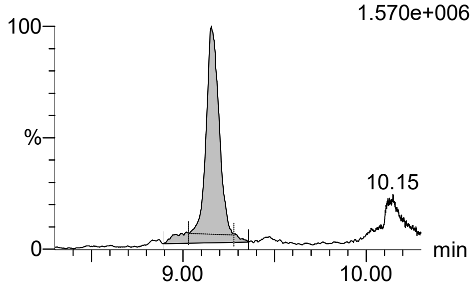

Sample Name: 20211111_A41_R2_IgM
F55:MRM of 2 channels,ES+
1292.9 > 366.1
3.877e+005

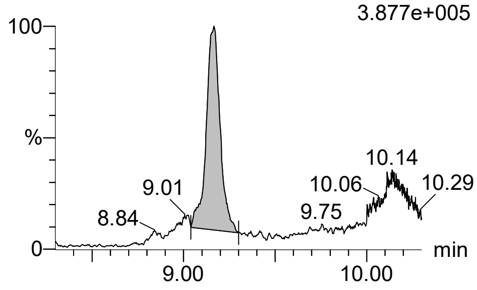

Sample Name: 20211111_A42_R2_IgM
F55:MRM of 2 channels,ES+
1034.5 > 204.1
1.355e+006

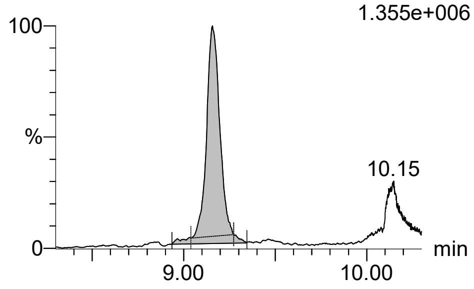

Sample Name: 20211111_A42_R2_IgM
F55:MRM of 2 channels,ES+
1292.9 > 366.1
3.118e+005

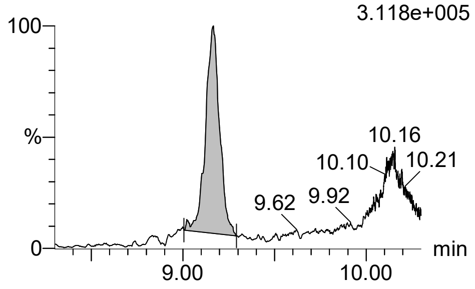

Sample Name: 20211111_A43_R2_IgM
F55:MRM of 2 channels,ES+
1034.5 > 204.1
2.171e+006

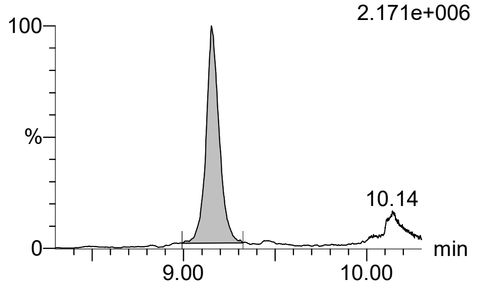

Sample Name: 20211111_A43_R2_IgM
F55:MRM of 2 channels,ES+
1292.9 > 366.1
5.484e+005

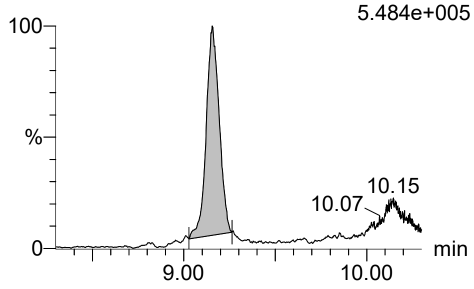

Sample Name: 20211111_A44_R2_IgM
F55:MRM of 2 channels,ES+
1034.5 > 204.1
1.292e+006

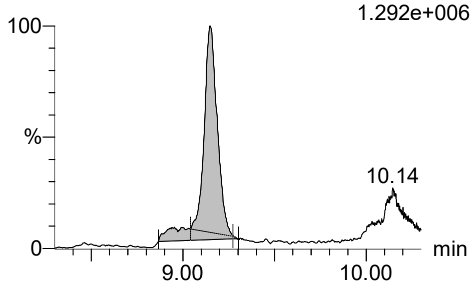

Sample Name: 20211111_A44_R2_IgM
F55:MRM of 2 channels,ES+
1292.9 > 366.1
3.674e+005

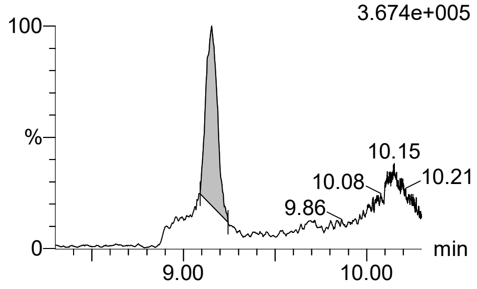

Sample Name: 20211111_QC_R17_IgM
F55:MRM of 2 channels,ES+
1034.5 > 204.1
2.927e+006

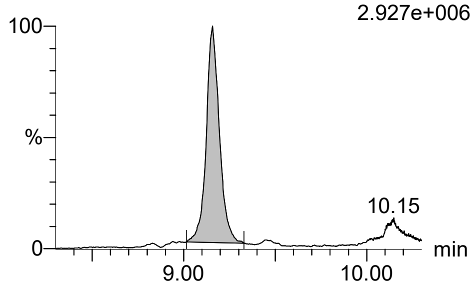

Sample Name: 20211111_QC_R17_IgM
F55:MRM of 2 channels,ES+
1292.9 > 366.1
7.177e+005

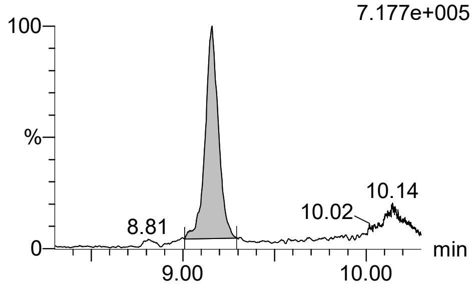

Sample Name: 20211111_L2_R3_IgM
F55:MRM of 2 channels,ES+
1034.5 > 204.1
5.553e+006

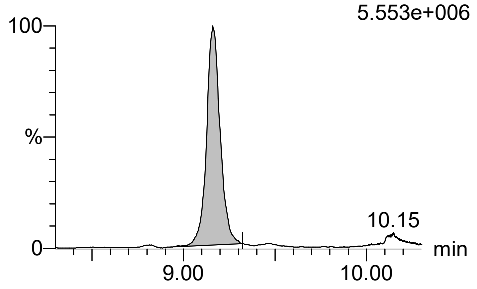

Sample Name: 20211111_L2_R3_IgM
F55:MRM of 2 channels,ES+
1292.9 > 366.1
1.413e+006

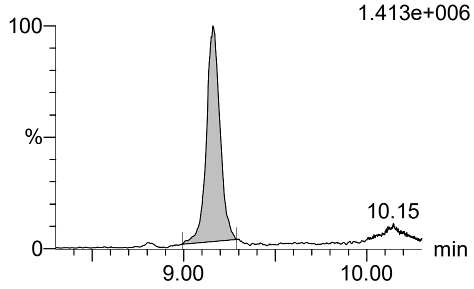

Sample Name: 20211111_H1_R3_IgM
F55:MRM of 2 channels,ES+
1034.5 > 204.1
2.140e+006

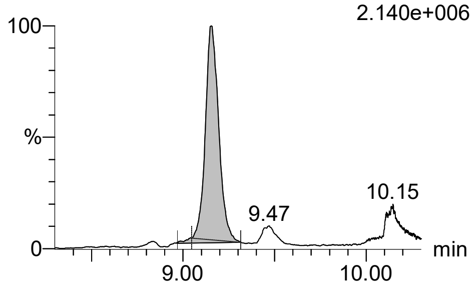

Sample Name: 20211111_H1_R3_IgM
F55:MRM of 2 channels,ES+
1292.9 > 366.1
5.169e+005

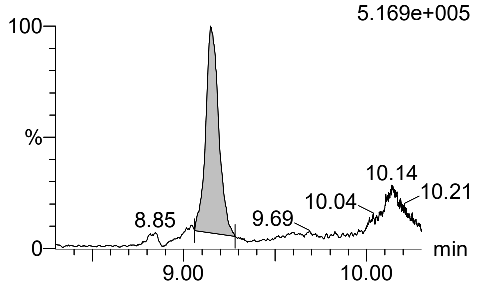

Dataset: C:\MassLynx\10.pro\20211111_clinicalsamples_IgM_IgA.qld

Last Altered: Thursday, March 09, 2023 19:58:25 台北標準時間
Printed: Friday, August 01, 2025 15:09:56 台北標準時間

Sample Name: 20211111_A3_R3_IgM
F55:MRM of 2 channels,ES+
1034.5 > 204.1
6.774e+006

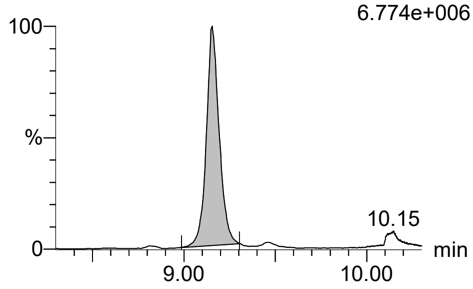

Sample Name: 20211111_A3_R3_IgM
F55:MRM of 2 channels,ES+
1292.9 > 366.1
1.508e+006

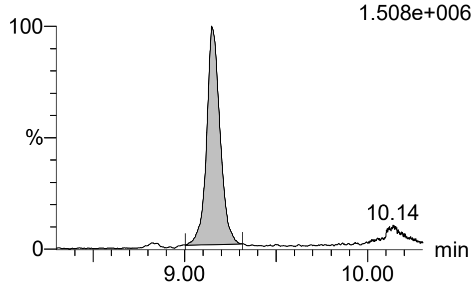

Sample Name: 20211111_A4_R3_IgM
F55:MRM of 2 channels,ES+
1034.5 > 204.1
2.999e+006

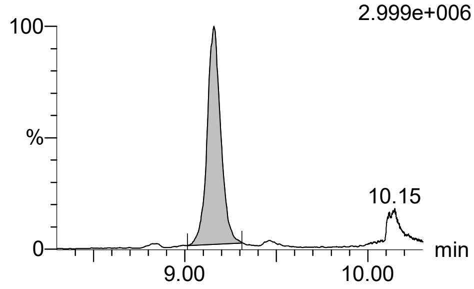

Sample Name: 20211111_A4_R3_IgM
F55:MRM of 2 channels,ES+
1292.9 > 366.1
7.242e+005

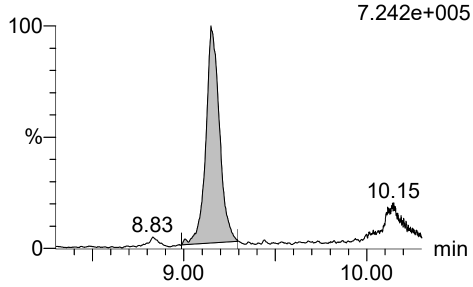

Sample Name: 20211111_L3_R3_IgM
F55:MRM of 2 channels,ES+
1034.5 > 204.1
2.165e+006

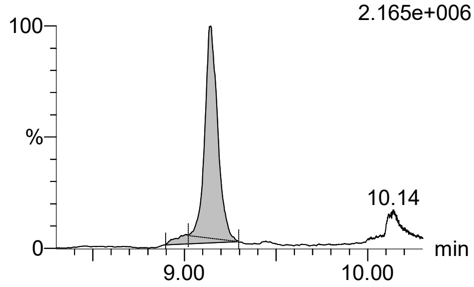

Sample Name: 20211111_L3_R3_IgM
F55:MRM of 2 channels,ES+
1292.9 > 366.1
5.932e+005

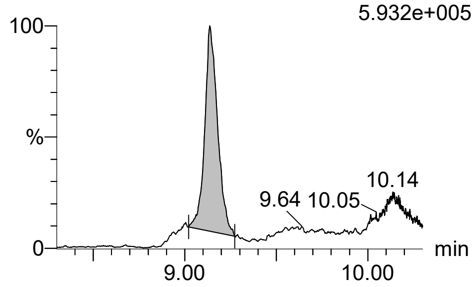

Sample Name: 20211111_L4_R3_IgM
F55:MRM of 2 channels,ES+
1034.5 > 204.1
2.945e+006

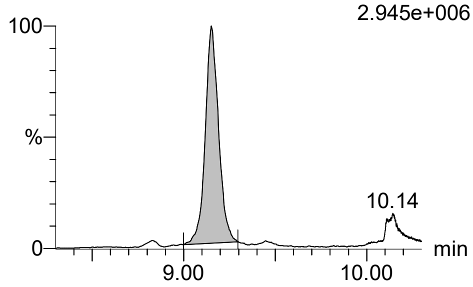

Sample Name: 20211111_L4_R3_IgM
F55:MRM of 2 channels,ES+
1292.9 > 366.1
6.903e+005

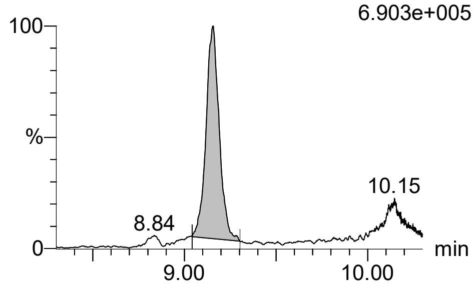

Sample Name: 20211111_H2_R3_IgM
F55:MRM of 2 channels,ES+
1034.5 > 204.1
5.787e+006

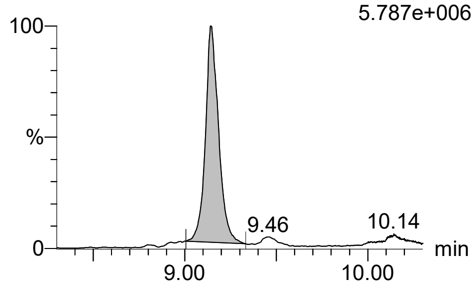

Sample Name: 20211111_H2_R3_IgM
F55:MRM of 2 channels,ES+
1292.9 > 366.1
1.610e+006

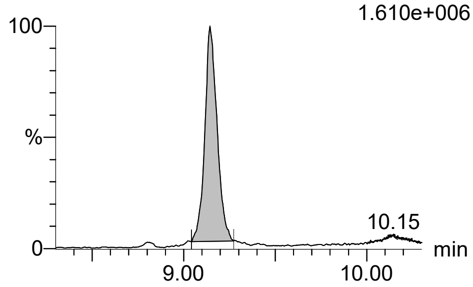

Sample Name: 20211111_A5_R3_IgM
F55:MRM of 2 channels,ES+
1034.5 > 204.1
1.221e+007

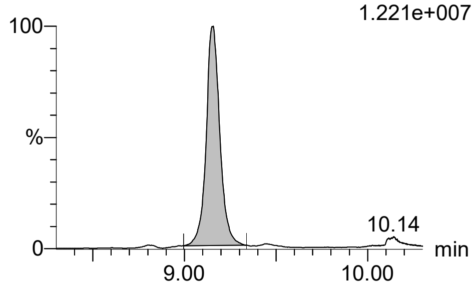

Sample Name: 20211111_A5_R3_IgM
F55:MRM of 2 channels,ES+
1292.9 > 366.1
3.139e+006

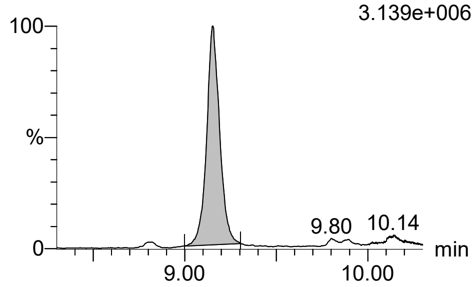

Sample Name: 20211111_A6_R3_IgM
F55:MRM of 2 channels,ES+
1034.5 > 204.1
5.809e+006

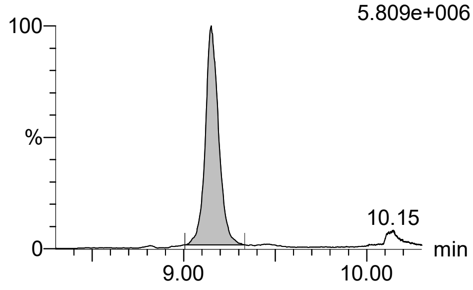

Sample Name: 20211111_A6_R3_IgM
F55:MRM of 2 channels,ES+
1292.9 > 366.1
1.468e+006

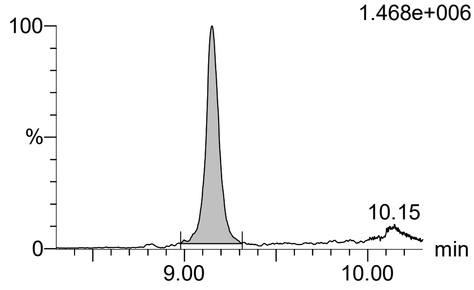

Sample Name: 20211111_QC_R18_IgM
F55:MRM of 2 channels,ES+
1034.5 > 204.1
3.107e+006

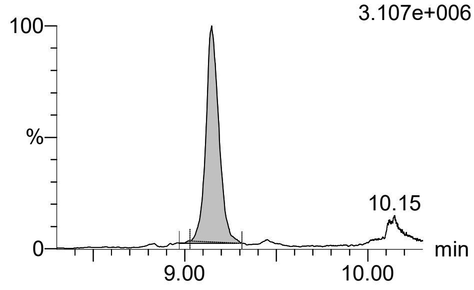

Dataset: C:\MassLynx\10.pro\20211111_clinicalsamples_IgM_IgA.qld

Last Altered: Thursday, March 09, 2023 19:58:25 台北標準時間
Printed: Friday, August 01, 2025 15:09:56 台北標準時間

Sample Name: 20211111_QC_R18_IgM
F55:MRM of 2 channels,ES+
1292.9 > 366.1
7.326e+005

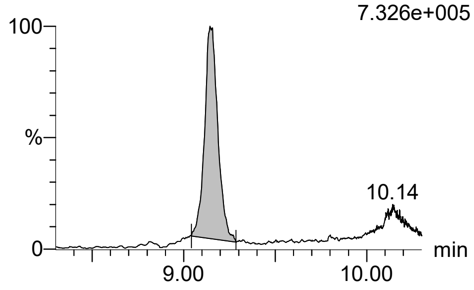

Sample Name: 20211111_L5_R3_IgM
F55:MRM of 2 channels,ES+
1034.5 > 204.1
4.830e+006

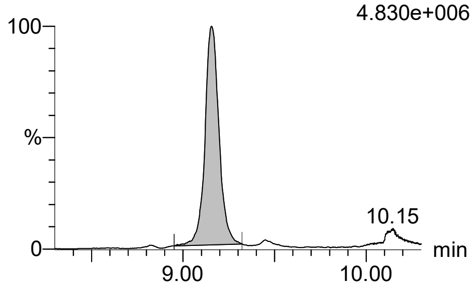

Sample Name: 20211111_L5_R3_IgM
F55:MRM of 2 channels,ES+
1292.9 > 366.1
1.253e+006

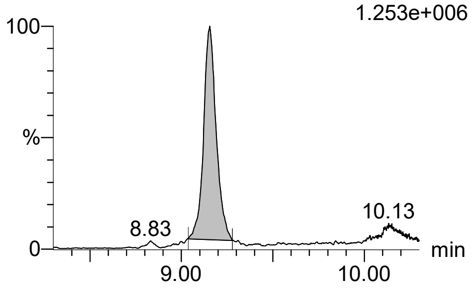

Sample Name: 20211111_L6_R3_IgM
F55:MRM of 2 channels,ES+
1034.5 > 204.1
3.826e+006

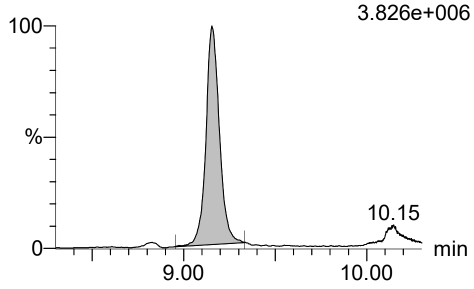

Sample Name: 20211111_L6_R3_IgM
F55:MRM of 2 channels,ES+
1292.9 > 366.1
8.964e+005

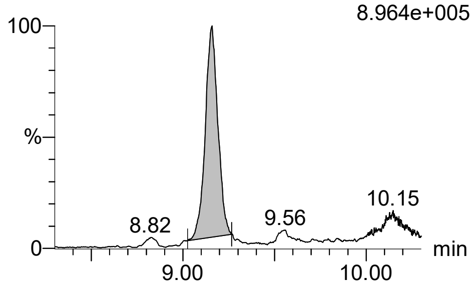

Sample Name: 20211111_H3_R3_IgM
F55:MRM of 2 channels,ES+
1034.5 > 204.1
2.092e+006

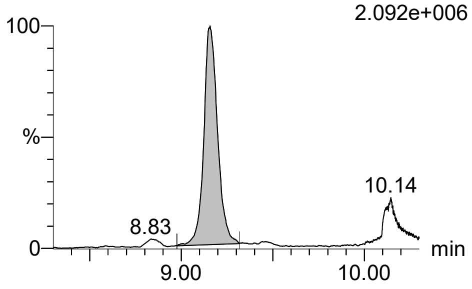

Sample Name: 20211111_H3_R3_IgM
F55:MRM of 2 channels,ES+
1292.9 > 366.1
4.671e+005

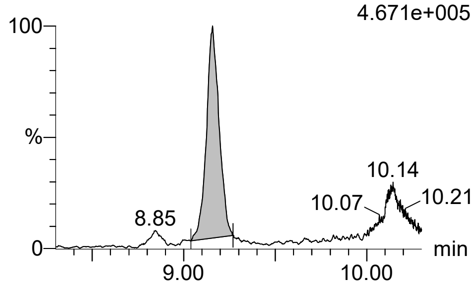

Sample Name: 20211111_A7_R3_IgM
F55:MRM of 2 channels,ES+
1034.5 > 204.1
6.756e+006

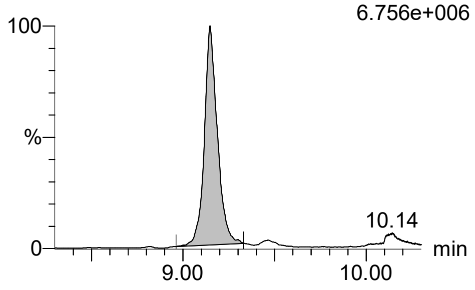

Sample Name: 20211111_A7_R3_IgM
F55:MRM of 2 channels,ES+
1292.9 > 366.1
1.717e+006

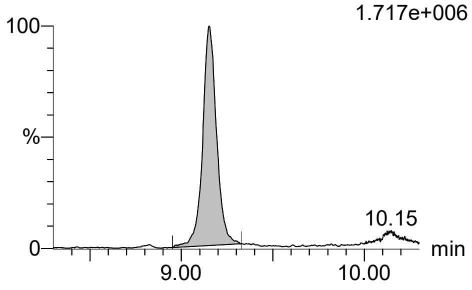

Sample Name: 20211111_A8_R3_IgM
F55:MRM of 2 channels,ES+
1034.5 > 204.1
3.546e+006

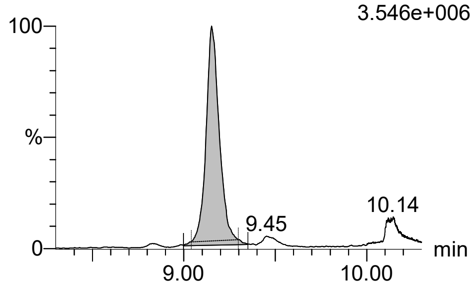

Sample Name: 20211111_A8_R3_IgM
F55:MRM of 2 channels,ES+
1292.9 > 366.1
8.374e+005

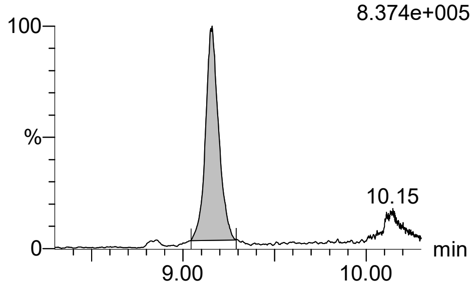

Sample Name: 20211111_L7_R3_IgM
F55:MRM of 2 channels,ES+
1034.5 > 204.1
1.665e+006

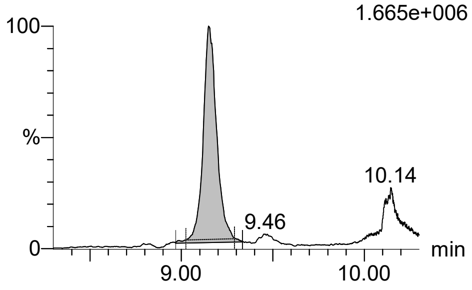

Sample Name: 20211111_L7_R3_IgM
F55:MRM of 2 channels,ES+
1292.9 > 366.1
4.184e+005

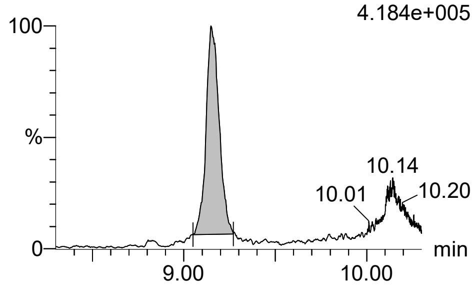

Sample Name: 20211111_L8_R3_IgM
F55:MRM of 2 channels,ES+
1034.5 > 204.1
1.357e+006

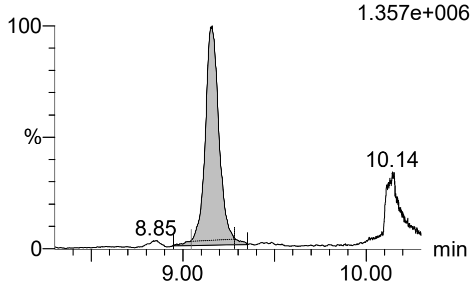

Sample Name: 20211111_L8_R3_IgM
F55:MRM of 2 channels,ES+
1292.9 > 366.1
3.064e+005

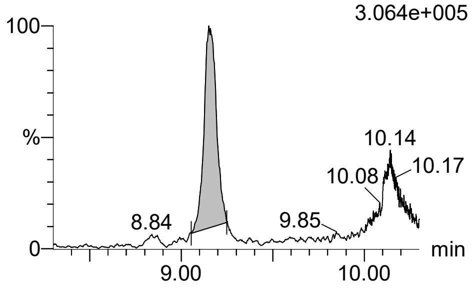

Sample Name: 20211111_H4_R3_IgM
F55:MRM of 2 channels,ES+
1034.5 > 204.1
1.884e+006

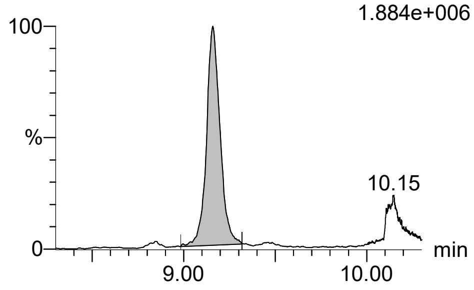

Sample Name: 20211111_H4_R3_IgM
F55:MRM of 2 channels,ES+
1292.9 > 366.1
4.150e+005

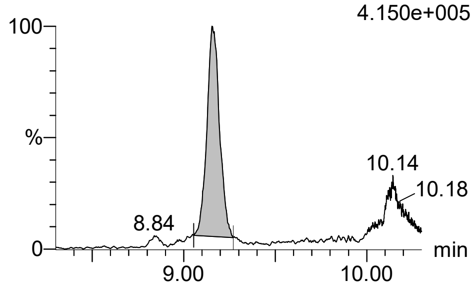

Sample Name: 20211111_A9_R3_IgM
F55:MRM of 2 channels,ES+
1034.5 > 204.1
1.541e+006

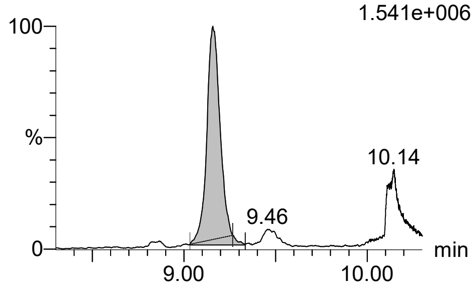

Sample Name: 20211111_A9_R3_IgM
F55:MRM of 2 channels,ES+
1292.9 > 366.1
3.424e+005

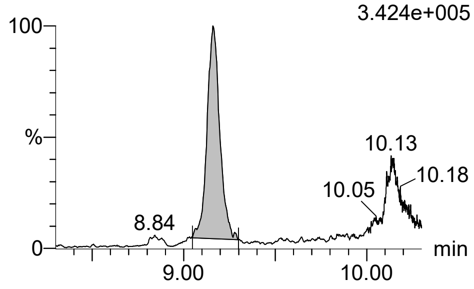

Sample Name: 20211111_A10_R3_IgM
F55:MRM of 2 channels,ES+
1034.5 > 204.1
5.475e+006

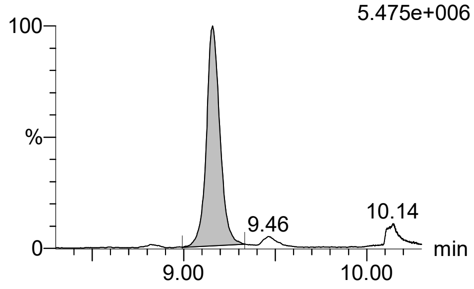

Sample Name: 20211111_A10_R3_IgM
F55:MRM of 2 channels,ES+
1292.9 > 366.1
1.205e+006

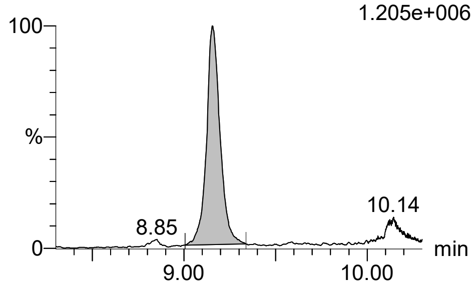

Sample Name: 20211111_L9_R3_IgM
F55:MRM of 2 channels,ES+
1034.5 > 204.1
2.693e+006

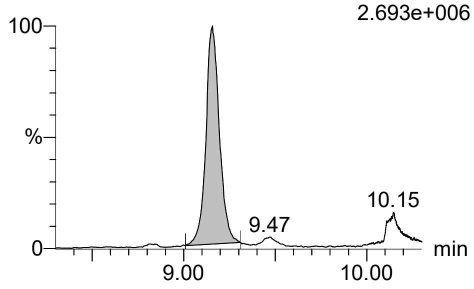

Sample Name: 20211111_L9_R3_IgM
F55:MRM of 2 channels,ES+
1292.9 > 366.1
5.998e+005

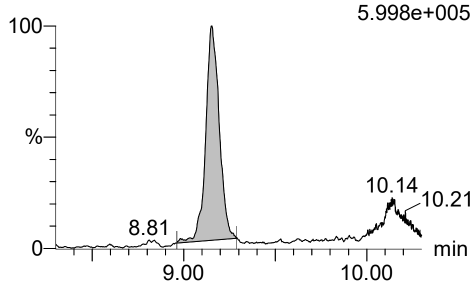

Sample Name: 20211111_L10_R3_IgM
F55:MRM of 2 channels,ES+
1034.5 > 204.1
5.055e+006

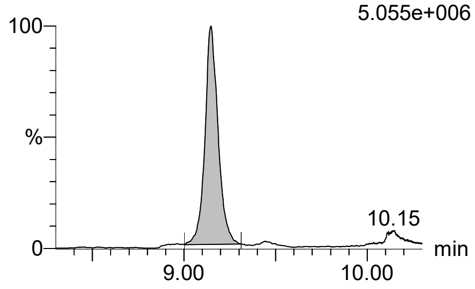

Sample Name: 20211111_L10_R3_IgM
F55:MRM of 2 channels,ES+
1292.9 > 366.1
1.372e+006

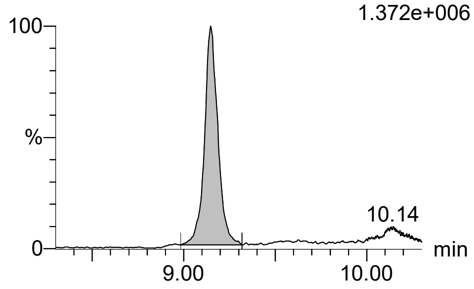

Sample Name: 20211111_QC_R19_IgM
F55:MRM of 2 channels,ES+
1034.5 > 204.1
3.008e+006

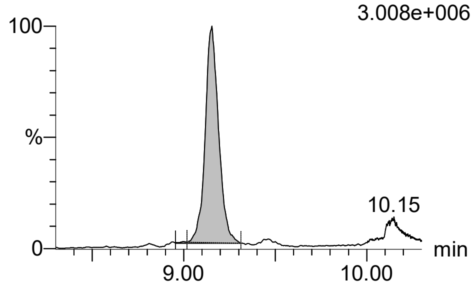

Sample Name: 20211111_QC_R19_IgM
F55:MRM of 2 channels,ES+
1292.9 > 366.1
7.245e+005

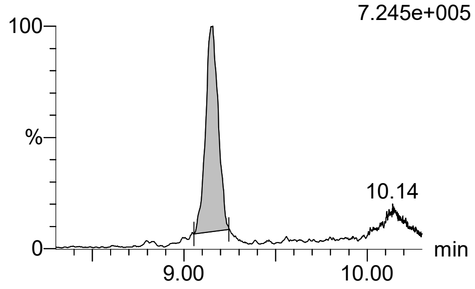

Sample Name: 20211111_H5_R3_IgM
F55:MRM of 2 channels,ES+
1034.5 > 204.1
4.748e+006

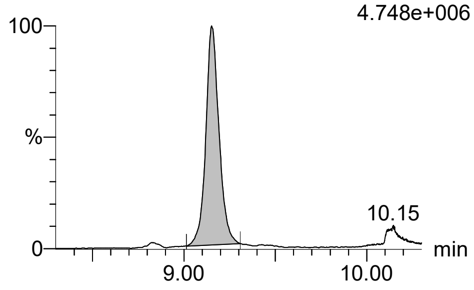

Sample Name: 20211111_H5_R3_IgM
F55:MRM of 2 channels,ES+
1292.9 > 366.1
1.036e+006

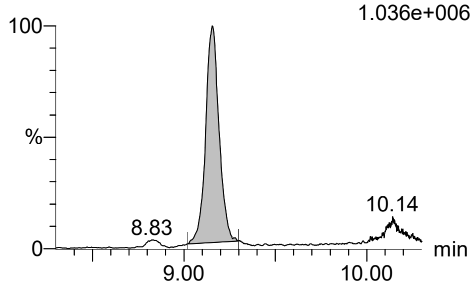

Sample Name: 20211111_A11_R3_IgM
F55:MRM of 2 channels,ES+
1034.5 > 204.1
3.043e+006

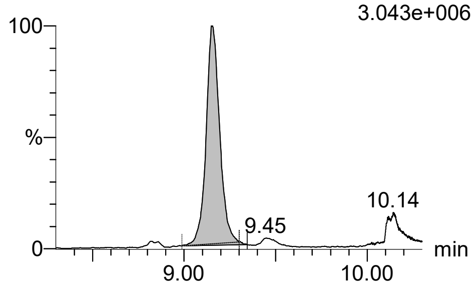

Sample Name: 20211111_A11_R3_IgM
F55:MRM of 2 channels,ES+
1292.9 > 366.1
6.745e+005

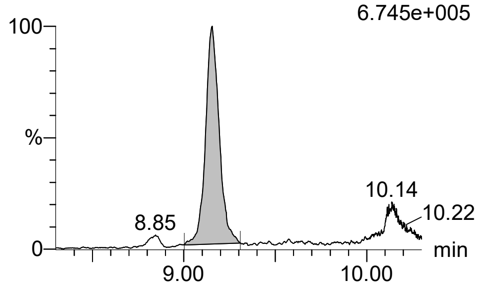

Sample Name: 20211111_A12_R3_IgM
F55:MRM of 2 channels,ES+
1034.5 > 204.1
1.297e+006

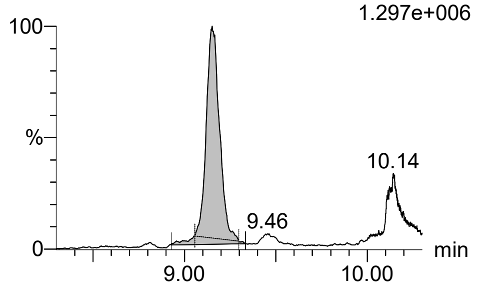

Sample Name: 20211111_A12_R3_IgM
F55:MRM of 2 channels,ES+
1292.9 > 366.1
3.213e+005

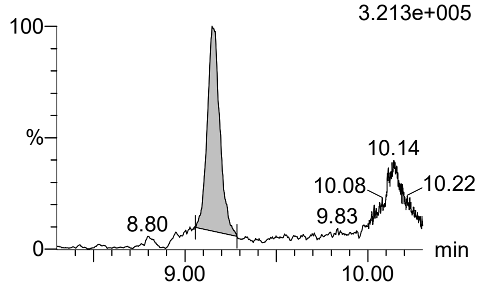

Sample Name: 20211111_L11_R3_IgM
F55:MRM of 2 channels,ES+
1034.5 > 204.1
1.183e+006

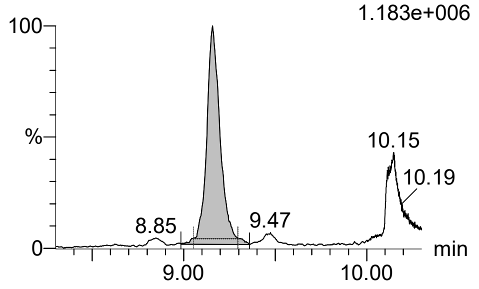

Sample Name: 20211111_L11_R3_IgM
F55:MRM of 2 channels,ES+
1292.9 > 366.1
2.469e+005

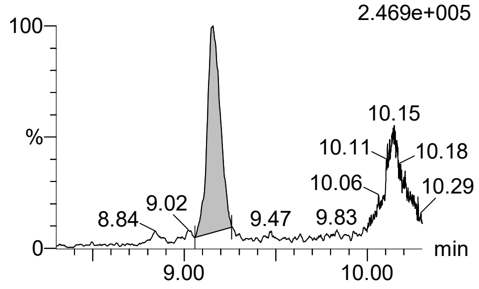

Sample Name: 20211111_L12_R3_IgM
F55:MRM of 2 channels,ES+
1034.5 > 204.1
1.760e+006

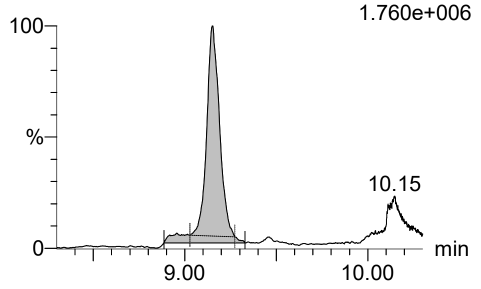

Sample Name: 20211111_L12_R3_IgM
F55:MRM of 2 channels,ES+
1292.9 > 366.1
4.769e+005

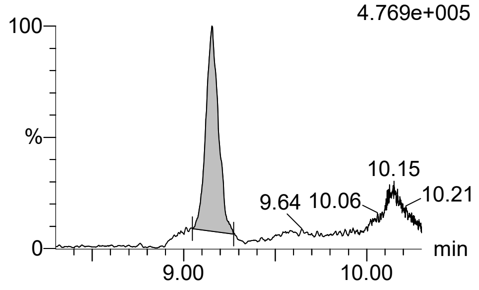

Sample Name: 20211111_H6_R3_IgM
F55:MRM of 2 channels,ES+
1034.5 > 204.1
1.691e+006

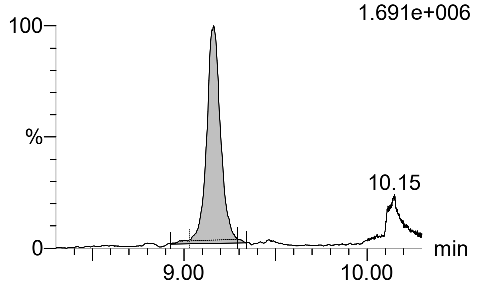

Sample Name: 20211111_H6_R3_IgM
F55:MRM of 2 channels,ES+
1292.9 > 366.1
4.160e+005

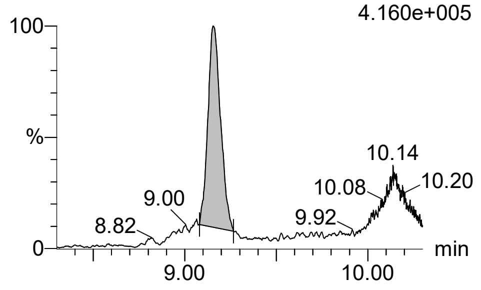

Sample Name: 20211111_A13_R3_IgM
F55:MRM of 2 channels,ES+
1034.5 > 204.1
1.687e+006

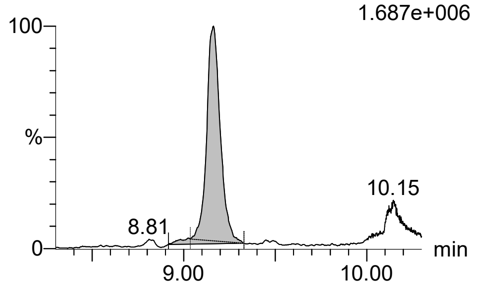

Sample Name: 20211111_A13_R3_IgM
F55:MRM of 2 channels,ES+
1292.9 > 366.1
4.199e+005

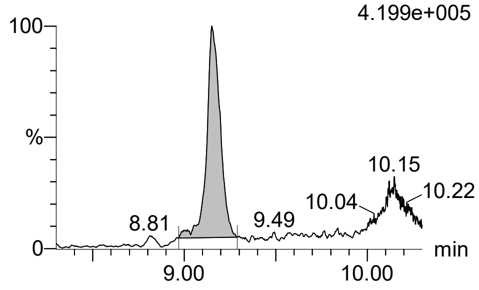

Sample Name: 20211111_A14_R3_IgM
F55:MRM of 2 channels,ES+
1034.5 > 204.1
4.995e+006

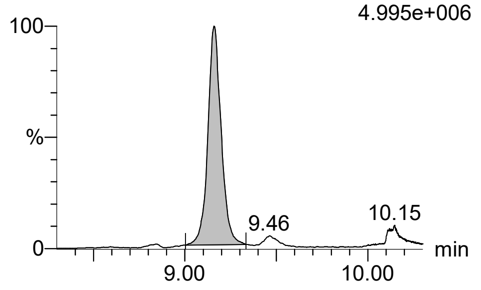

Sample Name: 20211111_A14_R3_IgM
F55:MRM of 2 channels,ES+
1292.9 > 366.1
1.145e+006

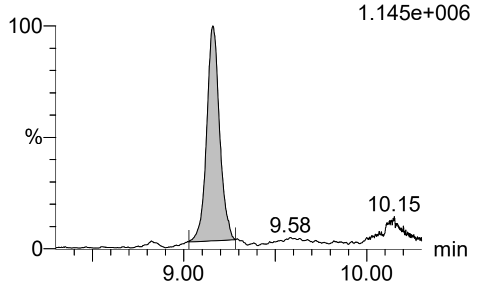

Sample Name: 20211111_L13_R3_IgM
F55:MRM of 2 channels,ES+
1034.5 > 204.1
3.694e+006

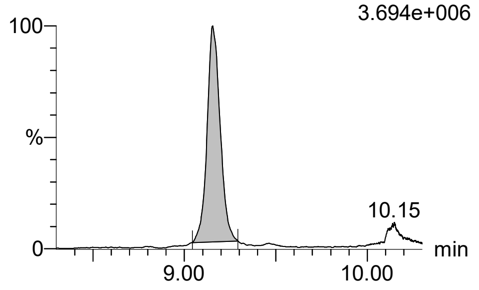

Sample Name: 20211111_L13_R3_IgM
F55:MRM of 2 channels,ES+
1292.9 > 366.1
9.190e+005

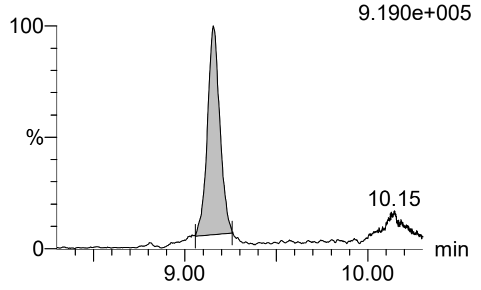

Sample Name: 20211111_L14_R3_IgM
F55:MRM of 2 channels,ES+
1034.5 > 204.1
3.440e+006

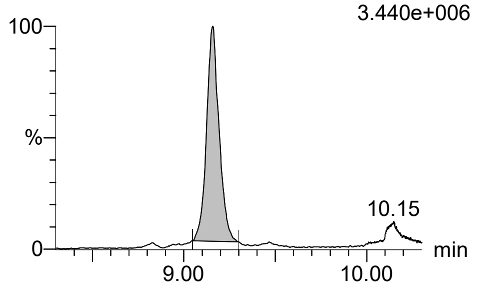

Sample Name: 20211111_L14_R3_IgM
F55:MRM of 2 channels,ES+
1292.9 > 366.1
8.397e+005

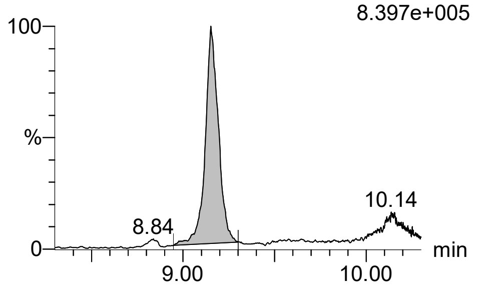

Sample Name: 20211111_H7_R3_IgM
F55:MRM of 2 channels,ES+
1034.5 > 204.1
1.879e+006

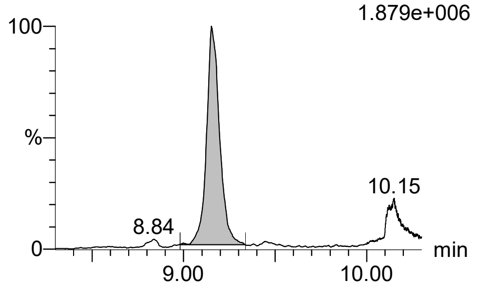

Sample Name: 20211111_H7_R3_IgM
F55:MRM of 2 channels,ES+
1292.9 > 366.1
4.489e+005

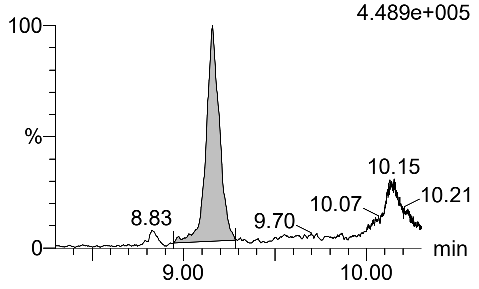

Sample Name: 20211111_A15_R3_IgM
F55:MRM of 2 channels,ES+
1034.5 > 204.1
8.339e+006

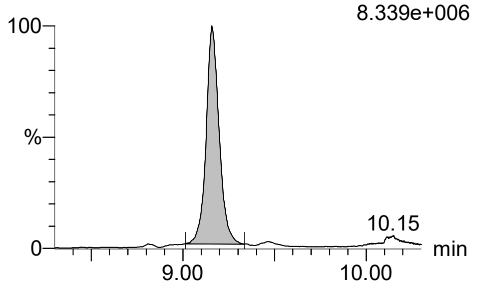

Sample Name: 20211111_A15_R3_IgM
F55:MRM of 2 channels,ES+
1292.9 > 366.1
2.019e+006

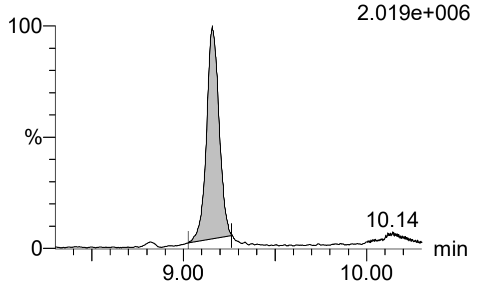

Sample Name: 20211111_QC_R20_IgM
F55:MRM of 2 channels,ES+
1034.5 > 204.1
3.523e+006

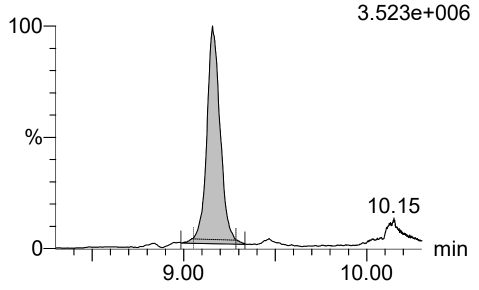

Sample Name: 20211111_QC_R20_IgM
F55:MRM of 2 channels,ES+
1292.9 > 366.1
8.383e+005

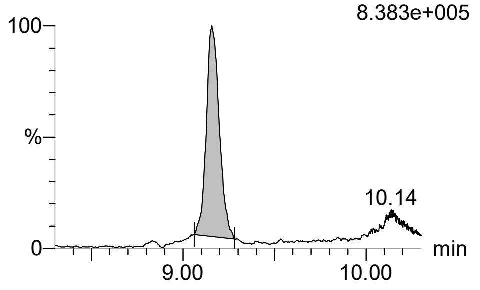

Sample Name: 20211111_A16_R3_IgM
F55:MRM of 2 channels,ES+
1034.5 > 204.1
4.519e+006

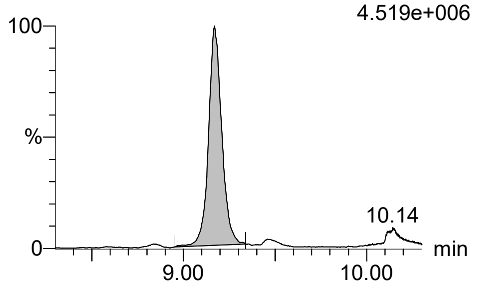

Sample Name: 20211111_A16_R3_IgM
F55:MRM of 2 channels,ES+
1292.9 > 366.1
1.049e+006

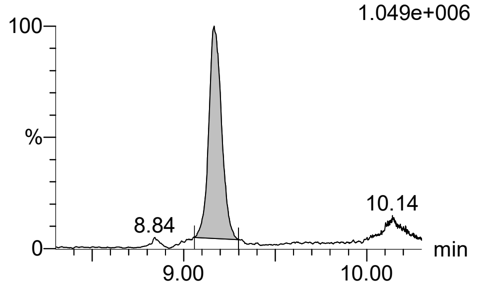

Sample Name: 20211111_L15_R3_IgM
F55:MRM of 2 channels,ES+
1034.5 > 204.1
3.394e+006

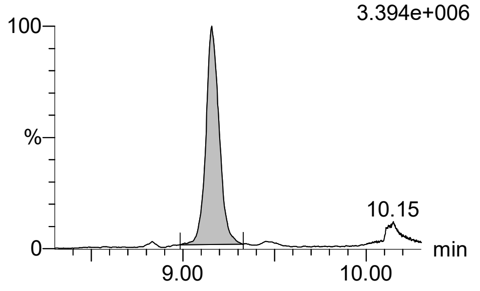

Sample Name: 20211111_L15_R3_IgM
F55:MRM of 2 channels,ES+
1292.9 > 366.1
7.638e+005

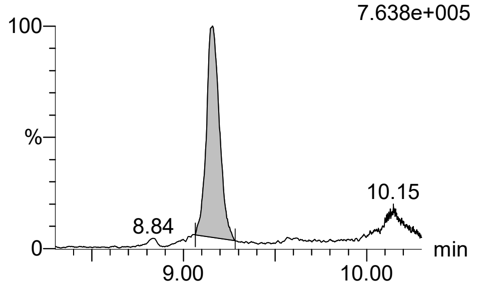

Sample Name: 20211111_L16_R3_IgM
F55:MRM of 2 channels,ES+
1034.5 > 204.1
9.887e+005

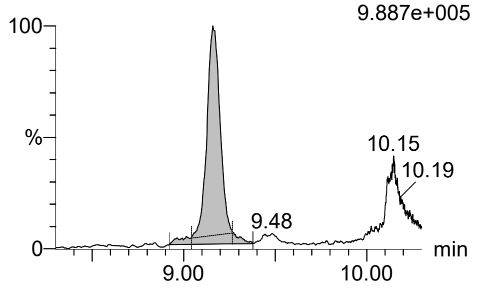

Sample Name: 20211111_L16_R3_IgM
F55:MRM of 2 channels,ES+
1292.9 > 366.1
2.709e+005

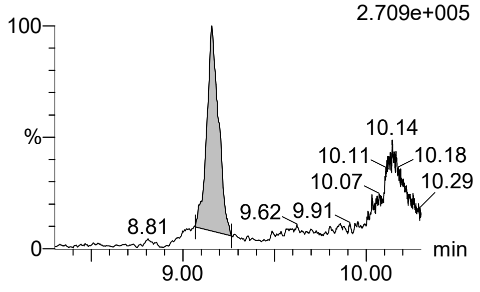

Sample Name: 20211111_H8_R3_IgM
F55:MRM of 2 channels,ES+
1034.5 > 204.1
2.876e+006

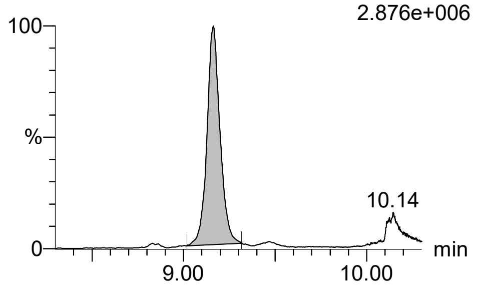

Sample Name: 20211111_H8_R3_IgM
F55:MRM of 2 channels,ES+
1292.9 > 366.1
6.467e+005

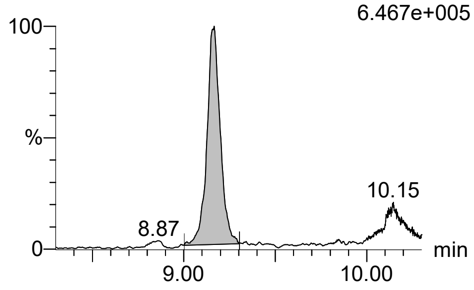

Sample Name: 20211111_A17_R3_IgM
F55:MRM of 2 channels,ES+
1034.5 > 204.1
4.145e+006

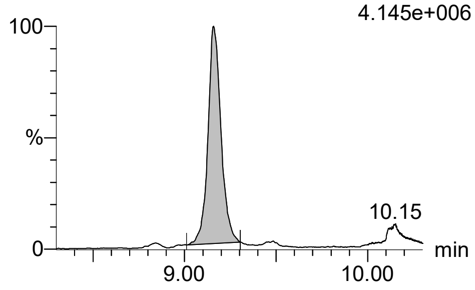

Sample Name: 20211111_A17_R3_IgM
F55:MRM of 2 channels,ES+
1292.9 > 366.1
9.321e+005

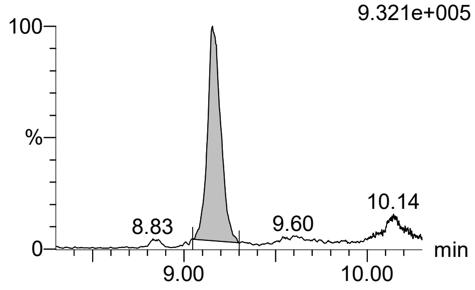

Sample Name: 20211111_A18_R3_IgM
F55:MRM of 2 channels,ES+
1034.5 > 204.1
1.580e+006

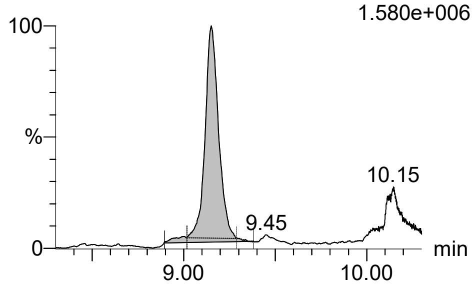

Sample Name: 20211111_A18_R3_IgM
F55:MRM of 2 channels,ES+
1292.9 > 366.1
4.483e+005

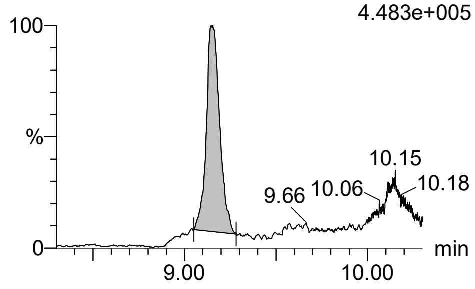

Sample Name: 20211111_L17_R3_IgM
F55:MRM of 2 channels,ES+
1034.5 > 204.1
7.800e+006

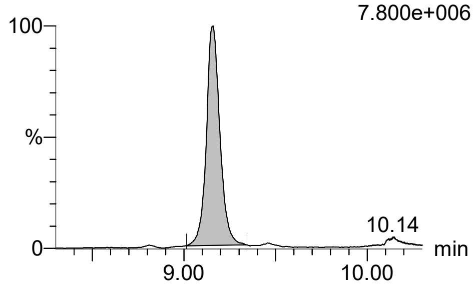

Sample Name: 20211111_L17_R3_IgM
F55:MRM of 2 channels,ES+
1292.9 > 366.1
1.940e+006

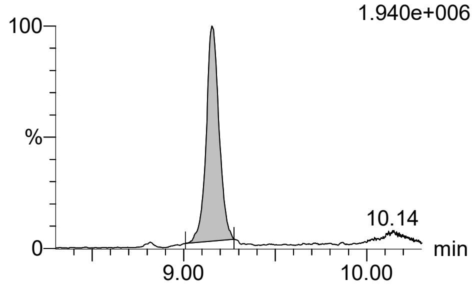

Sample Name: 20211111_L18_R3_IgM
F55:MRM of 2 channels,ES+
1034.5 > 204.1
6.127e+005

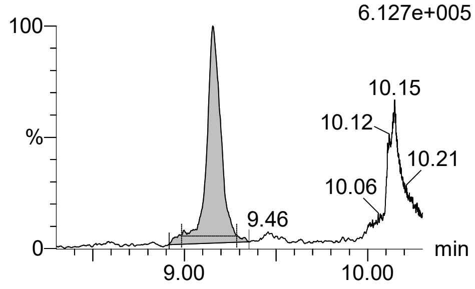

Sample Name: 20211111_L18_R3_IgM
F55:MRM of 2 channels,ES+
1292.9 > 366.1
1.498e+005

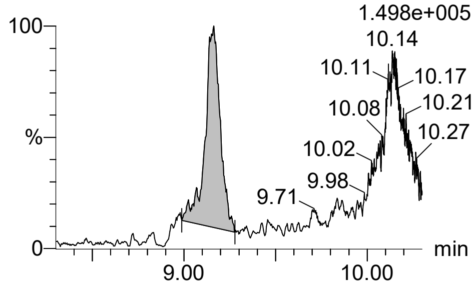

Sample Name: 20211111_H9_R3_IgM
F55:MRM of 2 channels,ES+
1034.5 > 204.1
1.459e+006

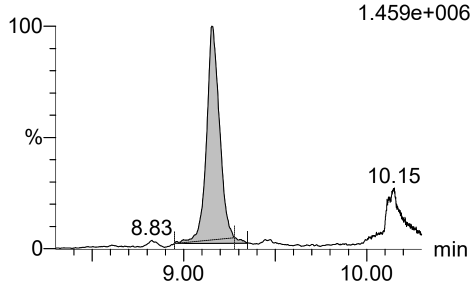

Sample Name: 20211111_H9_R3_IgM
F55:MRM of 2 channels,ES+
1292.9 > 366.1
3.251e+005

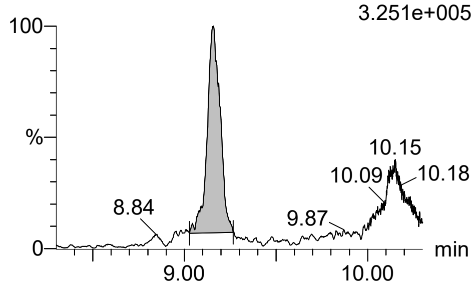

Sample Name: 20211111_A19_R3_IgM
F55:MRM of 2 channels,ES+
1034.5 > 204.1
2.257e+006

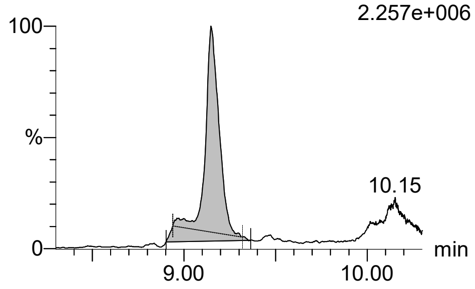

Sample Name: 20211111_A19_R3_IgM
F55:MRM of 2 channels,ES+
1292.9 > 366.1
5.990e+005

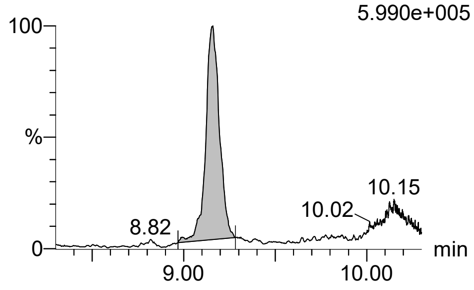

Sample Name: 20211111_A20_R3_IgM
F55:MRM of 2 channels,ES+
1034.5 > 204.1
1.131e+006

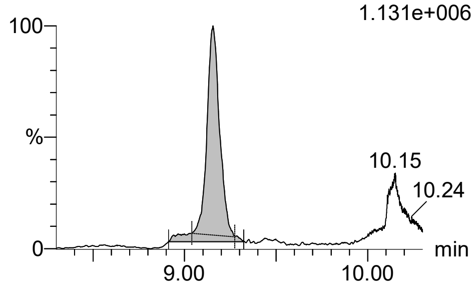

Sample Name: 20211111_A20_R3_IgM
F55:MRM of 2 channels,ES+
1292.9 > 366.1
3.105e+005

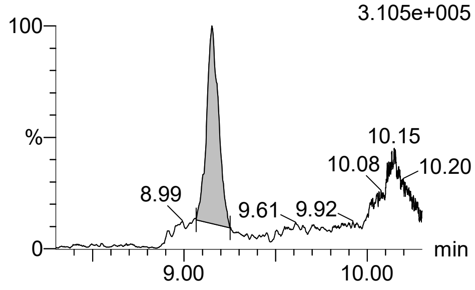

Dataset: C:\MassLynx\10.pro\20211111_clinicalsamples_IgM_IgA.qld

Last Altered: Thursday, March 09, 2023 19:58:25 台北標準時間
Printed: Friday, August 01, 2025 15:09:56 台北標準時間

Sample Name: 20211111_L19_R3_IgM
F55:MRM of 2 channels,ES+
1034.5 > 204.1
3.437e+006

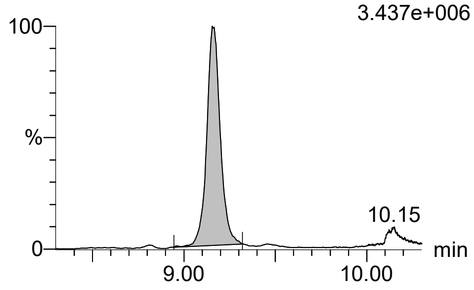

Sample Name: 20211111_L19_R3_IgM
F55:MRM of 2 channels,ES+
1292.9 > 366.1
8.846e+005

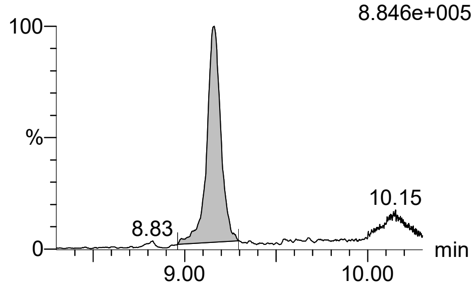

Sample Name: 20211111_QC_R21_IgM
F55:MRM of 2 channels,ES+
1034.5 > 204.1
3.605e+006

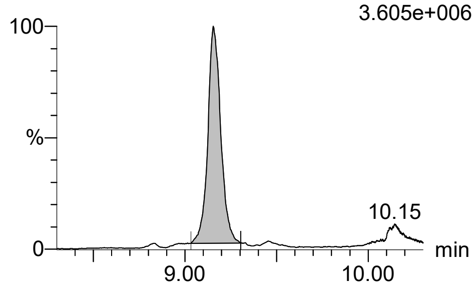

Sample Name: 20211111_QC_R21_IgM
F55:MRM of 2 channels,ES+
1292.9 > 366.1
8.603e+005

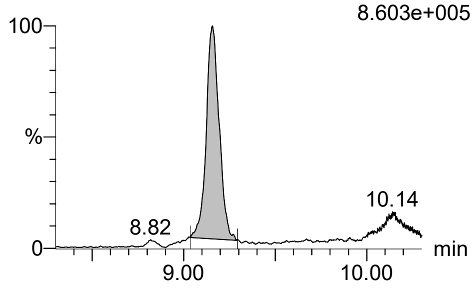

Sample Name: 20211111_L20_R3_IgM
F55:MRM of 2 channels,ES+
1034.5 > 204.1
2.182e+006

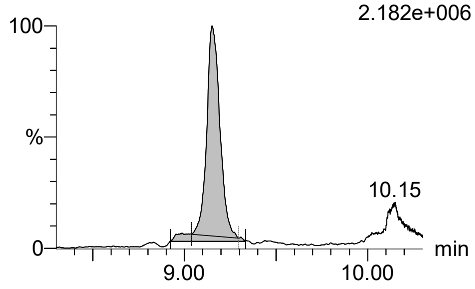

Sample Name: 20211111_L20_R3_IgM
F55:MRM of 2 channels,ES+
1292.9 > 366.1
5.865e+005

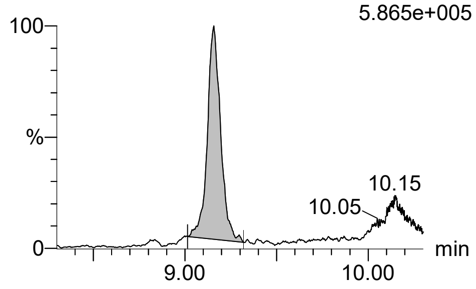

Sample Name: 20211111_H10_R3_IgM
F55:MRM of 2 channels,ES+
1034.5 > 204.1
2.912e+006

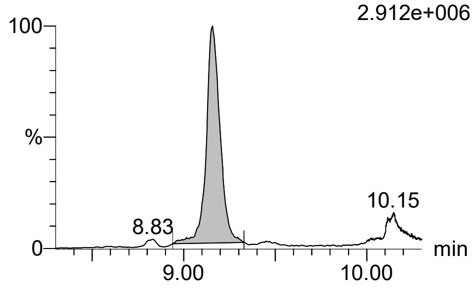

Sample Name: 20211111_H10_R3_IgM
F55:MRM of 2 channels,ES+
1292.9 > 366.1
7.199e+005

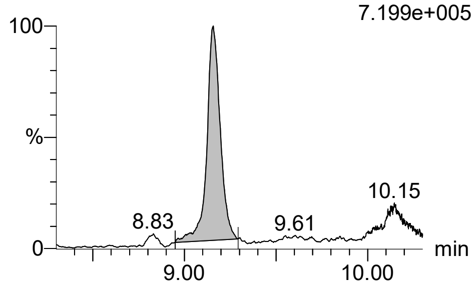

Sample Name: 20211111_A21_R3_IgM
F55:MRM of 2 channels,ES+
1034.5 > 204.1
4.878e+006

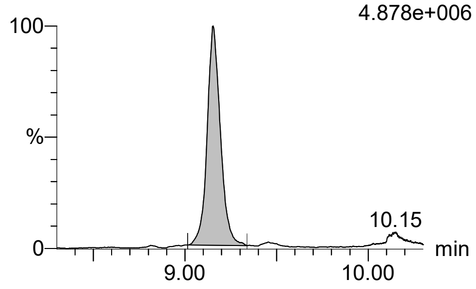

Sample Name: 20211111_A21_R3_IgM
F55:MRM of 2 channels,ES+
1292.9 > 366.1
1.145e+006

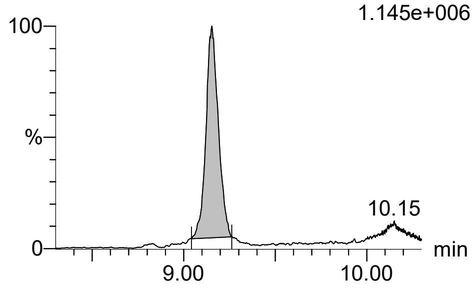

Sample Name: 20211111_A22_R3_IgM
F55:MRM of 2 channels,ES+
1034.5 > 204.1
1.573e+006

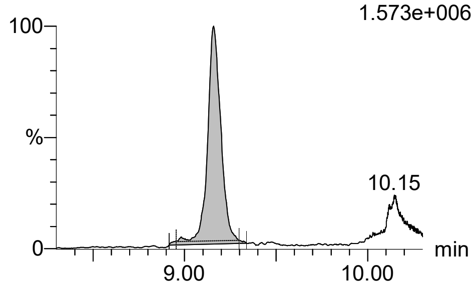

Sample Name: 20211111_A22_R3_IgM
F55:MRM of 2 channels,ES+
1292.9 > 366.1
4.000e+005

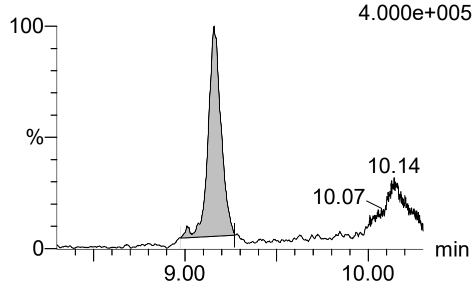

Sample Name: 20211111_L21_R3_IgM
F55:MRM of 2 channels,ES+
1034.5 > 204.1
2.264e+006

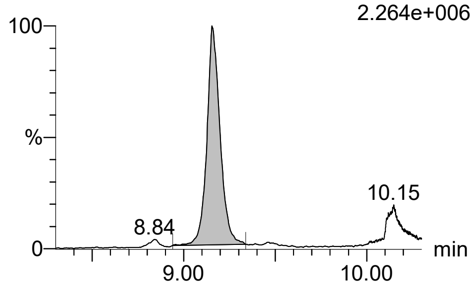

Sample Name: 20211111_L21_R3_IgM
F55:MRM of 2 channels,ES+
1292.9 > 366.1
5.187e+005

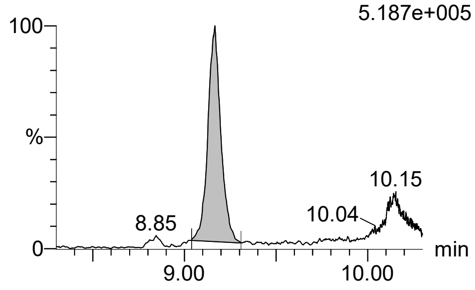

Sample Name: 20211111_L22_R3_IgM
F55:MRM of 2 channels,ES+
1034.5 > 204.1
3.460e+006

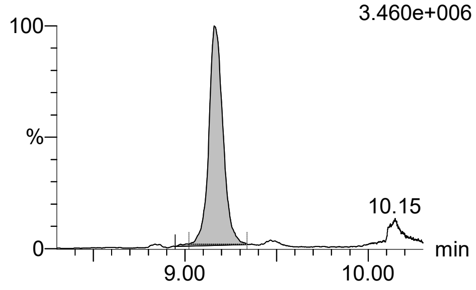

Sample Name: 20211111_L22_R3_IgM

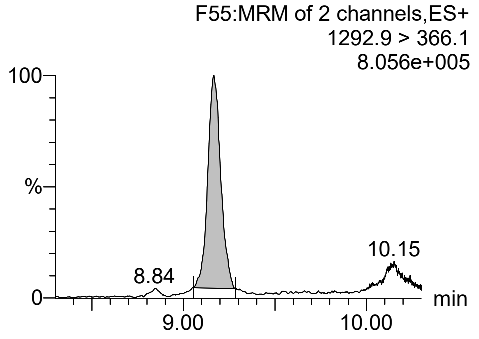

Sample Name: 20211111_H11_R3_IgM

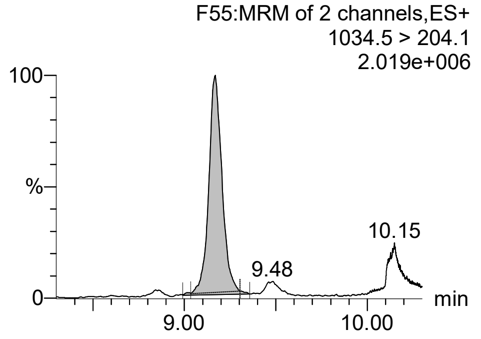

Sample Name: 20211111_H11_R3_IgM

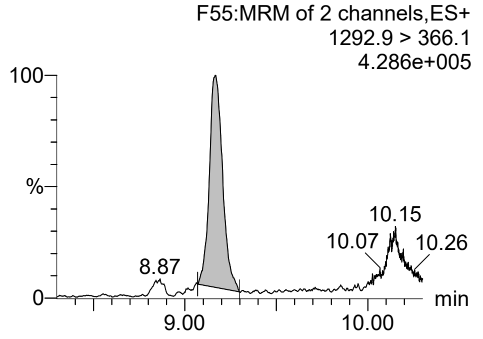

Sample Name: 20211111_A23_R3_IgM

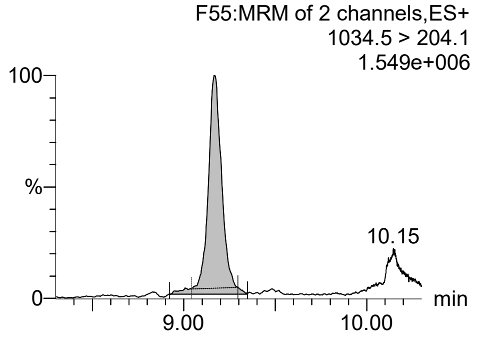

Sample Name: 20211111_A23_R3_IgM

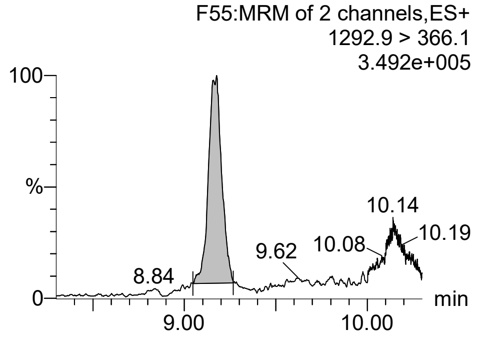

Sample Name: 20211111_A24_R3_IgM

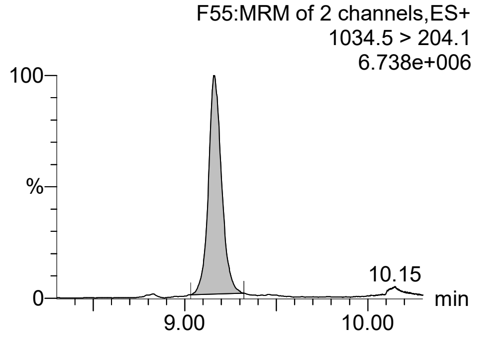

Sample Name: 20211111_A24_R3_IgM

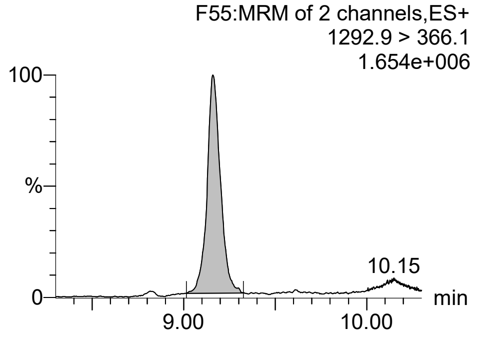

Sample Name: 20211111_L23_R3_IgM

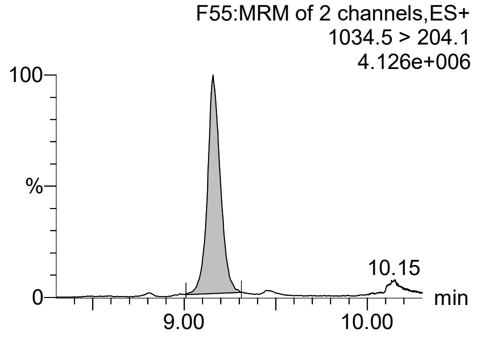

Sample Name: 20211111_L23_R3_IgM

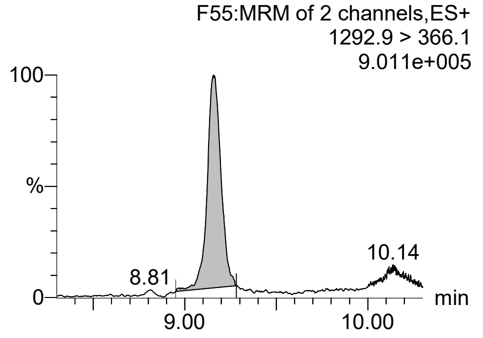

Sample Name: 20211111_L24_R3_IgM

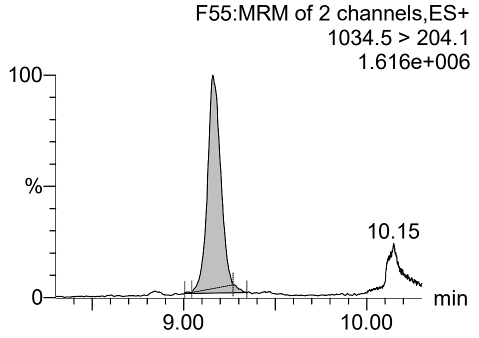

Sample Name: 20211111_L24_R3_IgM

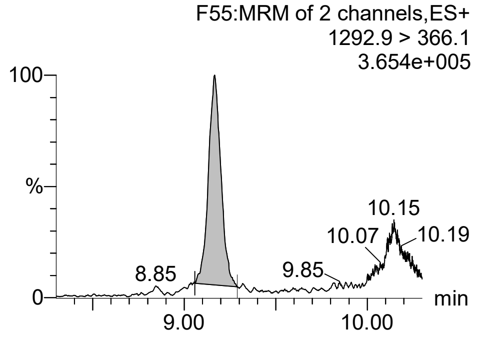

Sample Name: 20211111_H12_R3_IgM

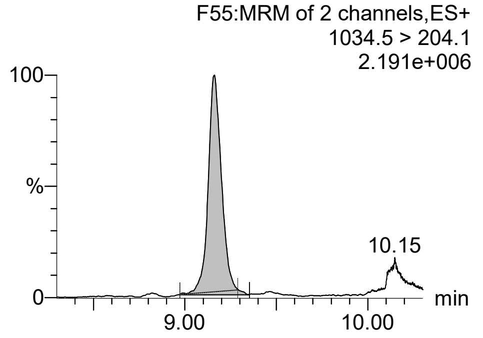

Sample Name: 20211111_H12_R3_IgM

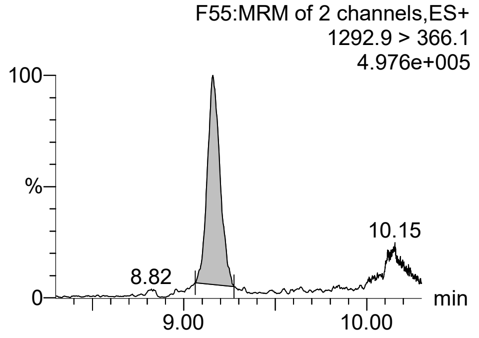

Sample Name: 20211111_QC_R22_IgM

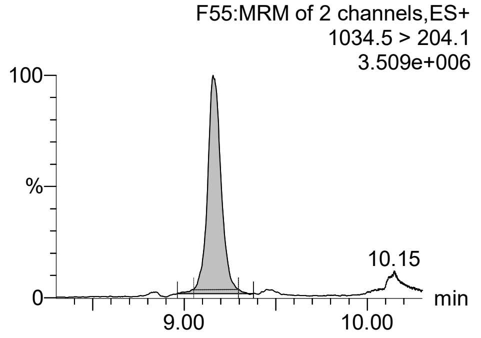

Sample Name: 20211111_QC_R22_IgM

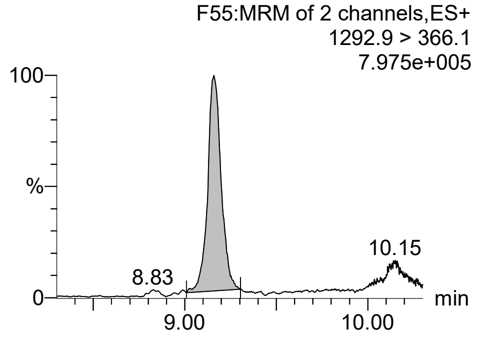

Sample Name: 20211111_A25_R3_IgM
F55:MRM of 2 channels,ES+
1034.5 > 204.1
7.369e+006

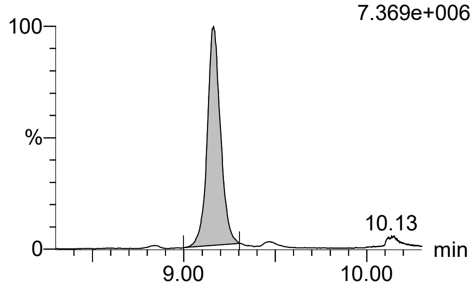

Sample Name: 20211111_A25_R3_IgM
F55:MRM of 2 channels,ES+
1292.9 > 366.1
1.676e+006

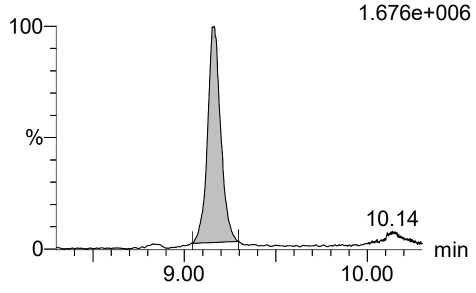

Sample Name: 20211111_A26_R3_IgM
F55:MRM of 2 channels,ES+
1034.5 > 204.1
2.872e+006

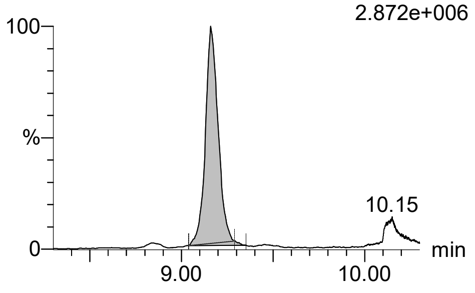

Sample Name: 20211111_A26_R3_IgM
F55:MRM of 2 channels,ES+
1292.9 > 366.1
6.359e+005

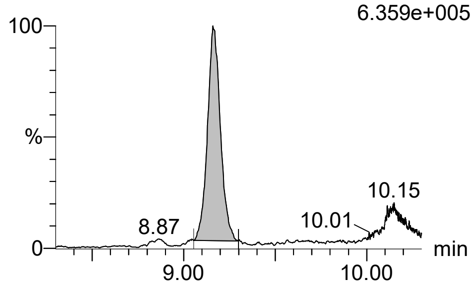

Sample Name: 20211111_L25_R3_IgM
F55:MRM of 2 channels,ES+
1034.5 > 204.1
2.074e+006

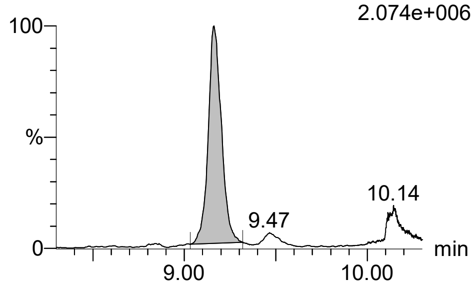

Sample Name: 20211111_L25_R3_IgM
F55:MRM of 2 channels,ES+
1292.9 > 366.1
4.581e+005

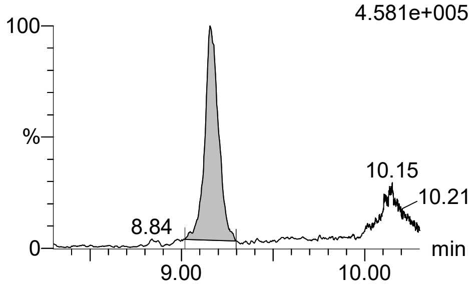

Sample Name: 20211111_L26_R3_IgM
F55:MRM of 2 channels,ES+
1034.5 > 204.1
1.298e+006

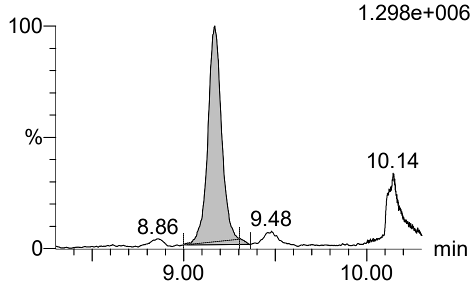

Sample Name: 20211111_L26_R3_IgM
F55:MRM of 2 channels,ES+
1292.9 > 366.1
2.736e+005

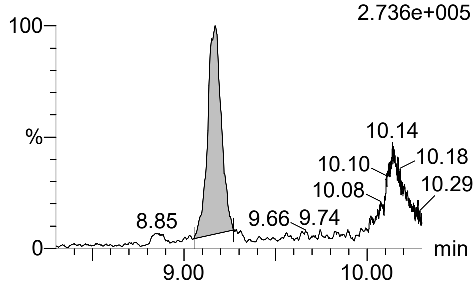

Sample Name: 20211111_H13_R3_IgM
F55:MRM of 2 channels,ES+
1034.5 > 204.1
3.752e+006

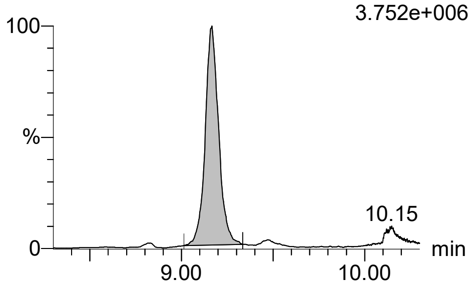

Sample Name: 20211111_H13_R3_IgM
F55:MRM of 2 channels,ES+
1292.9 > 366.1
8.902e+005

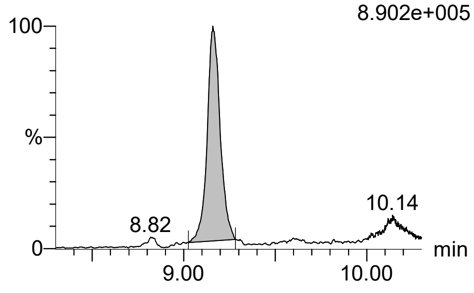

Sample Name: 20211111_A27_R3_IgM
F55:MRM of 2 channels,ES+
1034.5 > 204.1
5.365e+006

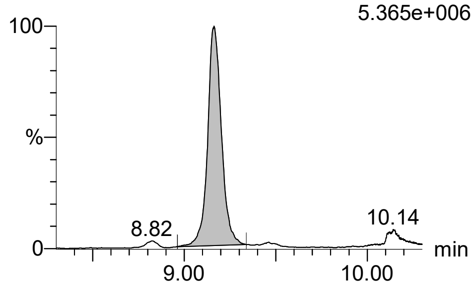

Sample Name: 20211111_A27_R3_IgM
F55:MRM of 2 channels,ES+
1292.9 > 366.1
1.205e+006

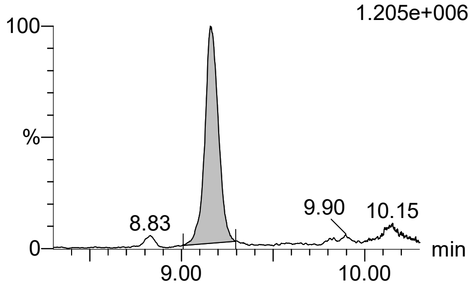

Sample Name: 20211111_A28_R3_IgM
F55:MRM of 2 channels,ES+
1034.5 > 204.1
2.835e+006

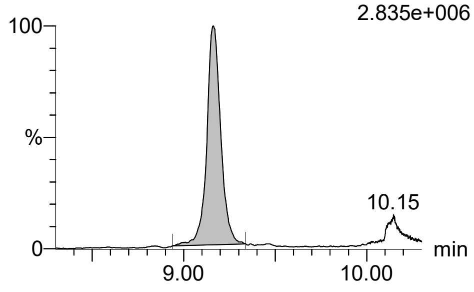

Sample Name: 20211111_A28_R3_IgM
F55:MRM of 2 channels,ES+
1292.9 > 366.1
6.997e+005

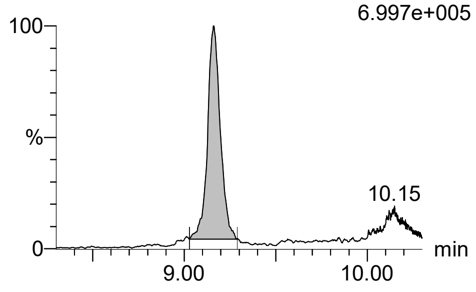

Sample Name: 20211111_L27_R3_IgM
F55:MRM of 2 channels,ES+
1034.5 > 204.1
3.717e+006

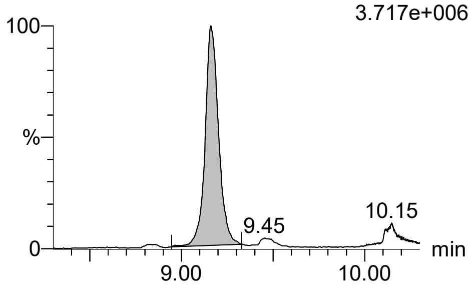

Sample Name: 20211111_L27_R3_IgM
F55:MRM of 2 channels,ES+
1292.9 > 366.1
8.111e+005

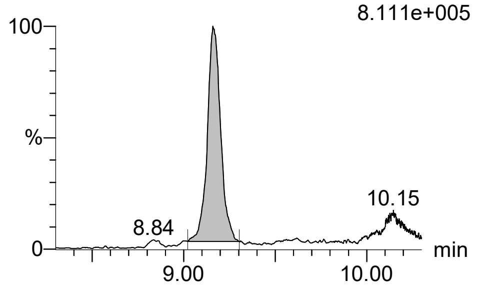

Sample Name: 20211111_L28_R3_IgM
F55:MRM of 2 channels,ES+
1034.5 > 204.1
4.180e+006

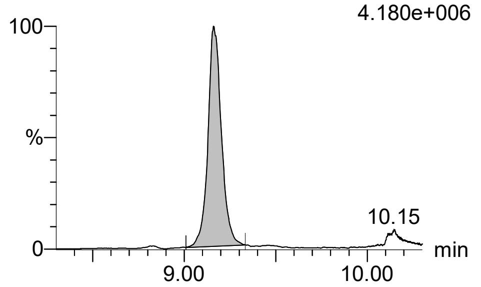

Sample Name: 20211111_L28_R3_IgM
F55:MRM of 2 channels,ES+
1292.9 > 366.1
9.972e+005

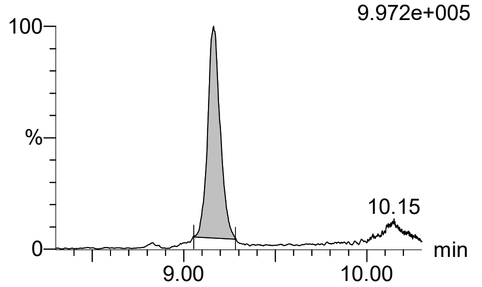

Sample Name: 20211111_H14_R3_IgM
F55:MRM of 2 channels,ES+
1034.5 > 204.1
3.672e+006

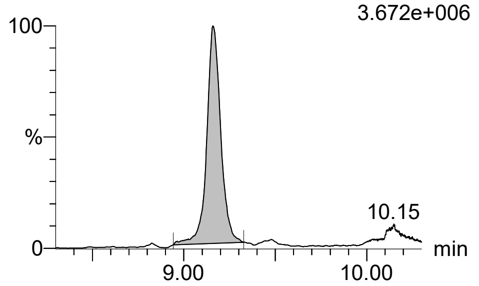

Sample Name: 20211111_H14_R3_IgM
F55:MRM of 2 channels,ES+
1292.9 > 366.1
9.791e+005

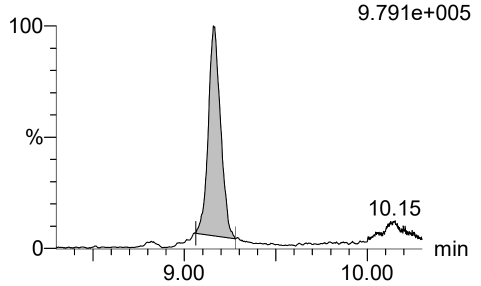

Sample Name: 20211111_A29_R3_IgM
F55:MRM of 2 channels,ES+
1034.5 > 204.1
3.616e+005

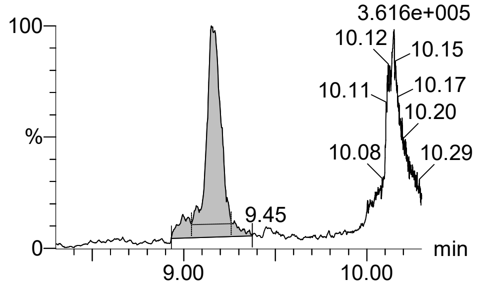

Sample Name: 20211111_A29_R3_IgM
F55:MRM of 2 channels,ES+
1292.9 > 366.1
1.183e+005

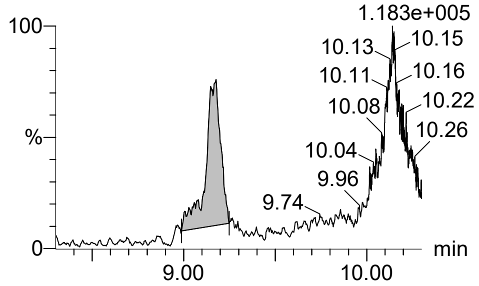

Sample Name: 20211111_A30_R3_IgM
F55:MRM of 2 channels,ES+
1034.5 > 204.1
3.960e+006

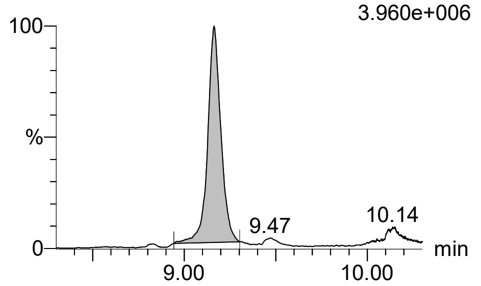

Sample Name: 20211111_A30_R3_IgM
F55:MRM of 2 channels,ES+
1292.9 > 366.1
8.566e+005

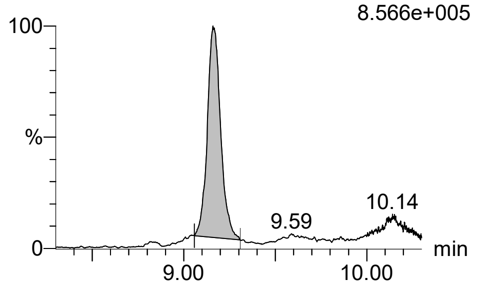

Sample Name: 20211111_QC_R23_IgM
F55:MRM of 2 channels,ES+
1034.5 > 204.1
3.385e+006

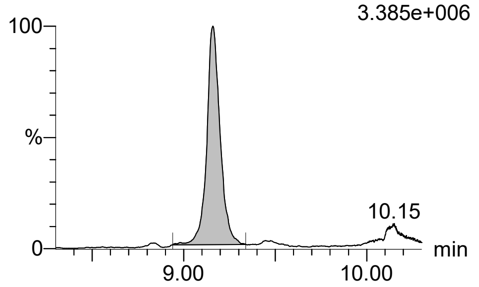

Sample Name: 20211111_QC_R23_IgM
F55:MRM of 2 channels,ES+
1292.9 > 366.1
7.508e+005

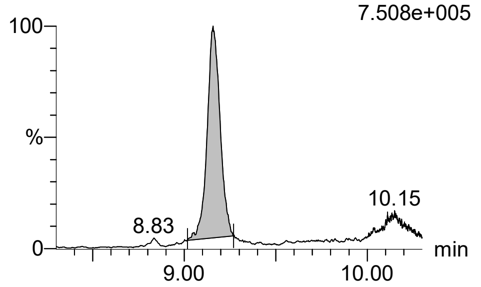

Sample Name: 20211111_L29_R3_IgM
F55:MRM of 2 channels,ES+
1034.5 > 204.1
1.109e+006

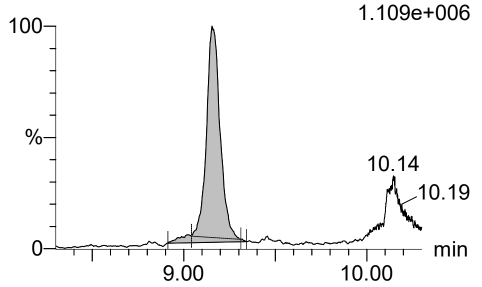

Sample Name: 20211111_L29_R3_IgM
F55:MRM of 2 channels,ES+
1292.9 > 366.1
2.712e+005

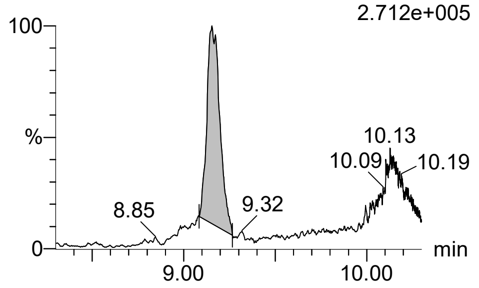

Sample Name: 20211111_L30_R3_IgM
F55:MRM of 2 channels,ES+
1034.5 > 204.1
6.605e+005

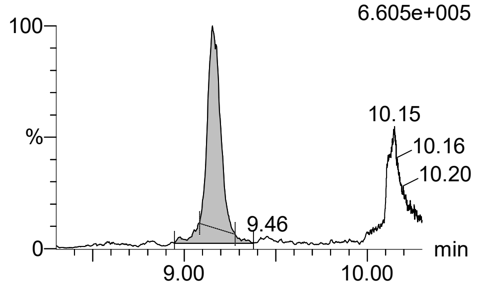

Sample Name: 20211111_L30_R3_IgM
F55:MRM of 2 channels,ES+
1292.9 > 366.1
1.543e+005

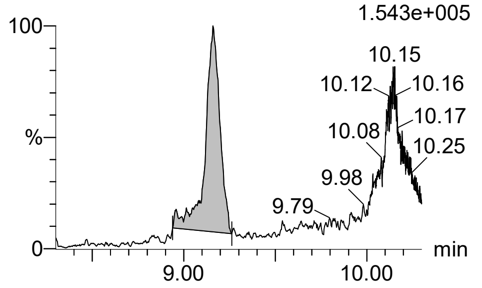

Sample Name: 20211111_H15_R3_IgM
F55:MRM of 2 channels,ES+
1034.5 > 204.1
1.947e+006

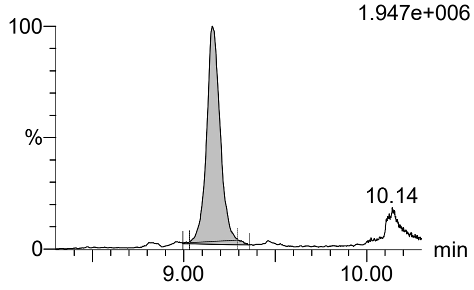

Sample Name: 20211111_H15_R3_IgM
F55:MRM of 2 channels,ES+
1292.9 > 366.1
4.344e+005

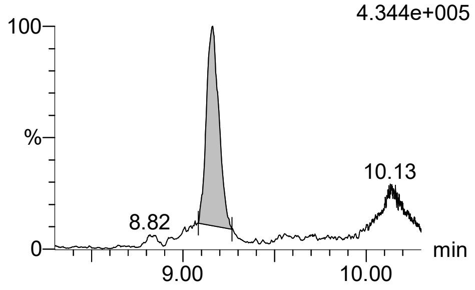

Sample Name: 20211111_A31_R3_IgM
F55:MRM of 2 channels,ES+
1034.5 > 204.1
1.033e+006

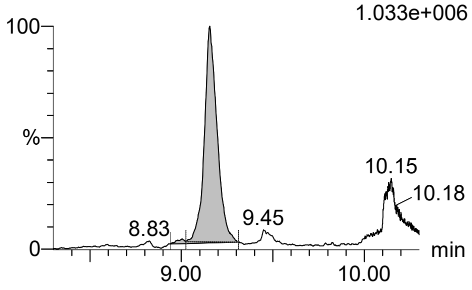

Sample Name: 20211111_A31_R3_IgM
F55:MRM of 2 channels,ES+
1292.9 > 366.1
2.181e+005

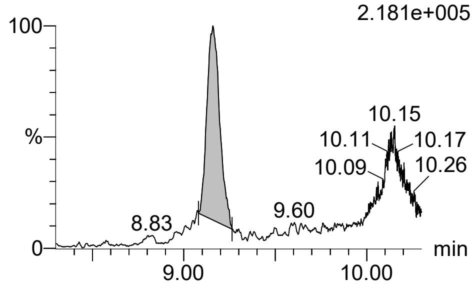

Sample Name: 20211111_A32_R3_IgM
F55:MRM of 2 channels,ES+
1034.5 > 204.1
3.955e+006

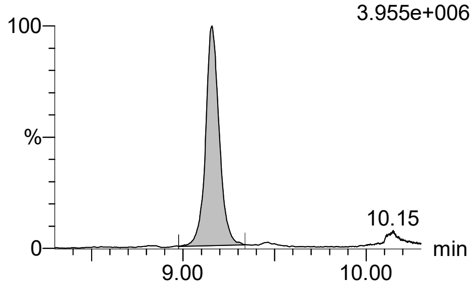

Sample Name: 20211111_A32_R3_IgM
F55:MRM of 2 channels,ES+
1292.9 > 366.1
9.676e+005

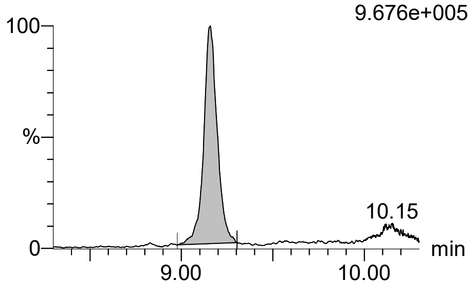

Sample Name: 20211111_L31_R3_IgM
F55:MRM of 2 channels,ES+
1034.5 > 204.1
5.982e+005

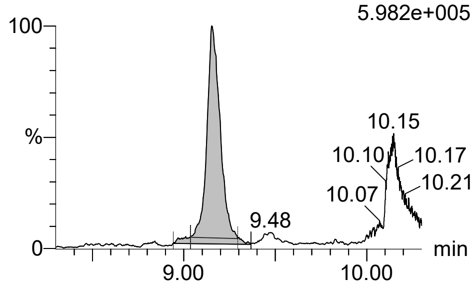

Sample Name: 20211111_L31_R3_IgM
F55:MRM of 2 channels,ES+
1292.9 > 366.1
1.418e+005

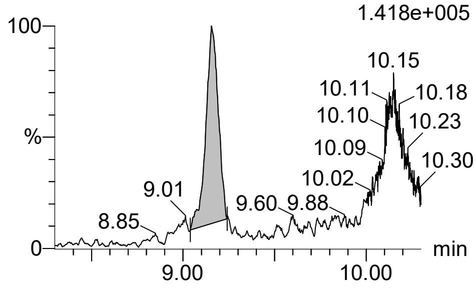

Sample Name: 20211111_L32_R3_IgM
F55:MRM of 2 channels,ES+
1034.5 > 204.1
5.480e+006

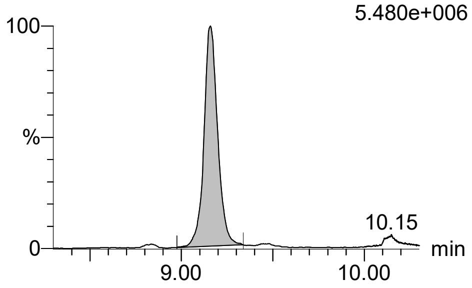

Sample Name: 20211111_L32_R3_IgM
F55:MRM of 2 channels,ES+
1292.9 > 366.1
1.301e+006

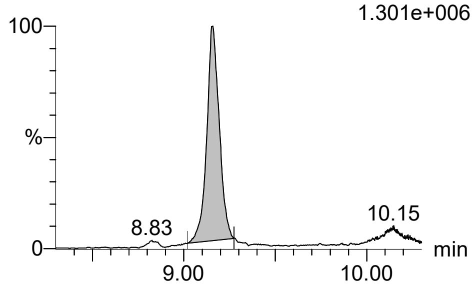

Sample Name: 20211111_H16_R3_IgM
F55:MRM of 2 channels,ES+
1034.5 > 204.1
9.182e+006

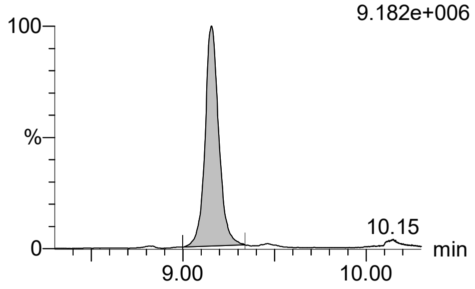

Sample Name: 20211111_H16_R3_IgM
F55:MRM of 2 channels,ES+
1292.9 > 366.1
2.349e+006

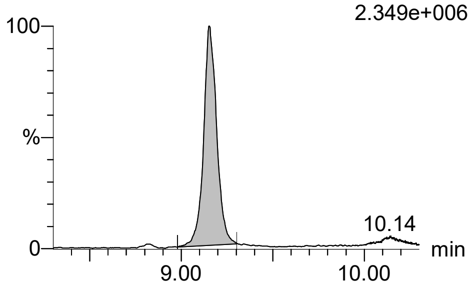

Sample Name: 20211111_A33_R3_IgM
F55:MRM of 2 channels,ES+
1034.5 > 204.1
4.264e+006

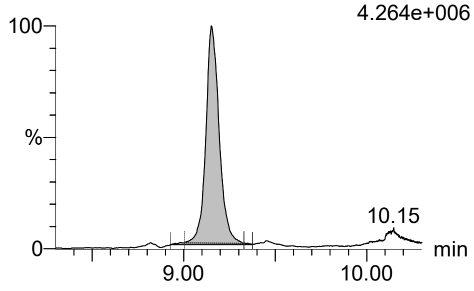

Sample Name: 20211111_A33_R3_IgM
F55:MRM of 2 channels,ES+
1292.9 > 366.1
1.117e+006

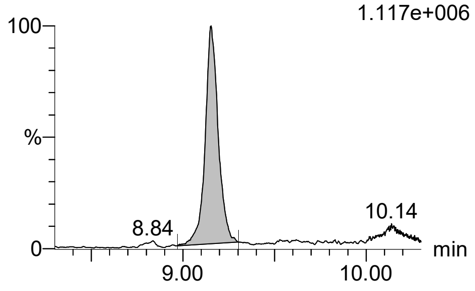

Sample Name: 20211111_A34_R3_IgM
F55:MRM of 2 channels,ES+
1034.5 > 204.1
9.340e+005

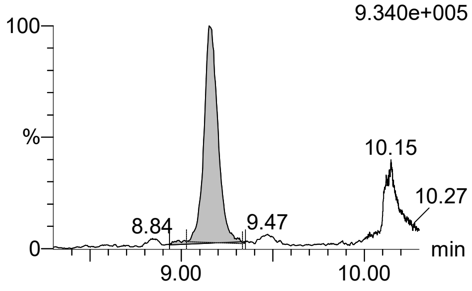

Sample Name: 20211111_A34_R3_IgM
F55:MRM of 2 channels,ES+
1292.9 > 366.1
2.234e+005

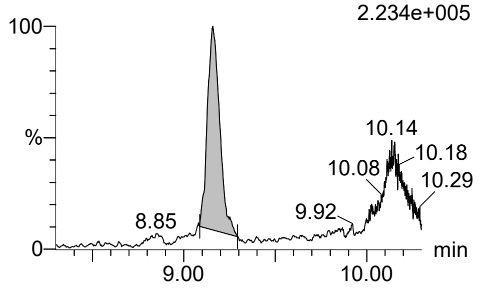

Sample Name: 20211111_H17_R3_IgM
F55:MRM of 2 channels,ES+
1034.5 > 204.1
2.128e+007

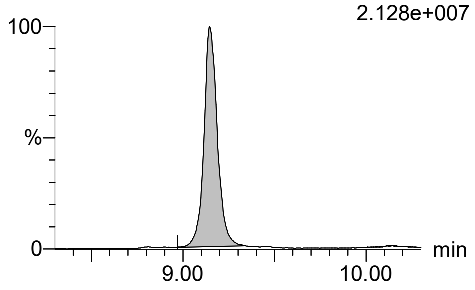

Sample Name: 20211111_H17_R3_IgM
F55:MRM of 2 channels,ES+
1292.9 > 366.1
5.914e+006

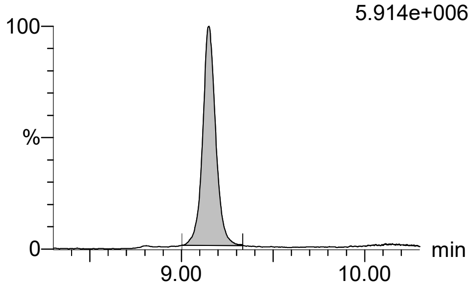

Sample Name: 20211111_A35_R3_IgM
F55:MRM of 2 channels,ES+
1034.5 > 204.1
1.414e+006

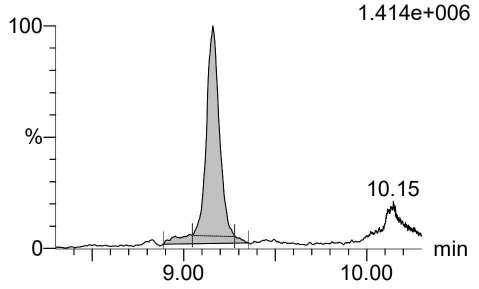

Sample Name: 20211111_A35_R3_IgM
F55:MRM of 2 channels,ES+
1292.9 > 366.1
3.684e+005

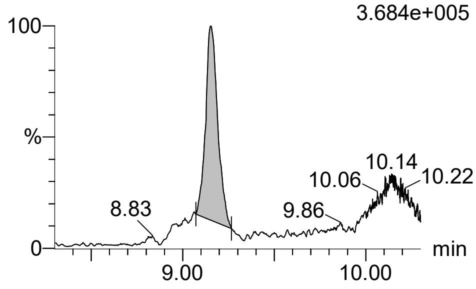

Sample Name: 20211111_QC_R24_IgM
F55:MRM of 2 channels,ES+
1034.5 > 204.1
2.925e+006

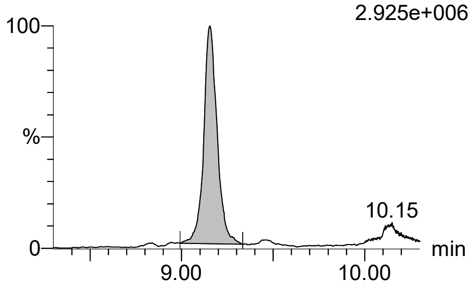

Sample Name: 20211111_QC_R24_IgM
F55:MRM of 2 channels,ES+
1292.9 > 366.1
7.162e+005

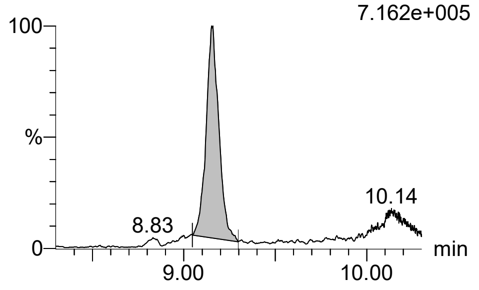

Sample Name: 20211111_A36_R3_IgM
F55:MRM of 2 channels,ES+
1034.5 > 204.1
1.416e+006

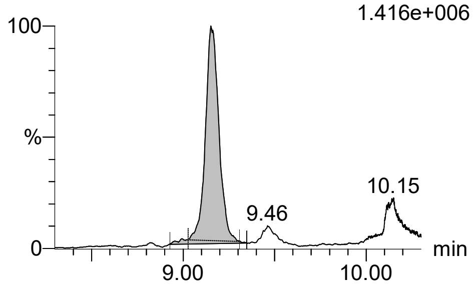

Sample Name: 20211111_A36_R3_IgM
F55:MRM of 2 channels,ES+
1292.9 > 366.1
3.493e+005

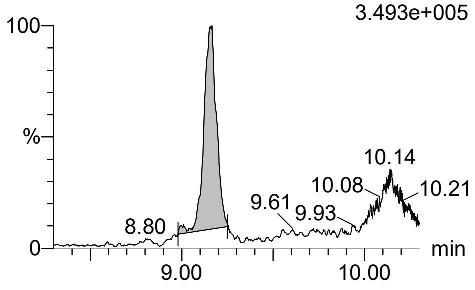

Sample Name: 20211111_H18_R3_IgM
F55:MRM of 2 channels,ES+
1034.5 > 204.1
8.141e+006

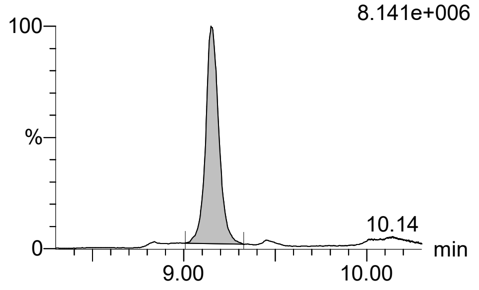

Sample Name: 20211111_H18_R3_IgM
F55:MRM of 2 channels,ES+
1292.9 > 366.1
2.077e+006

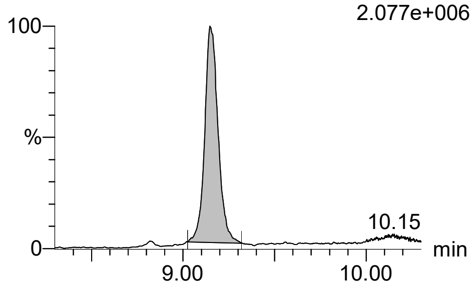

Sample Name: 20211111_A37_R3_IgM
F55:MRM of 2 channels,ES+
1034.5 > 204.1
3.011e+006

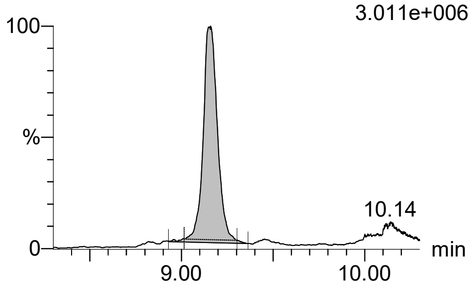

Sample Name: 20211111_A37_R3_IgM
F55:MRM of 2 channels,ES+
1292.9 > 366.1
7.766e+005

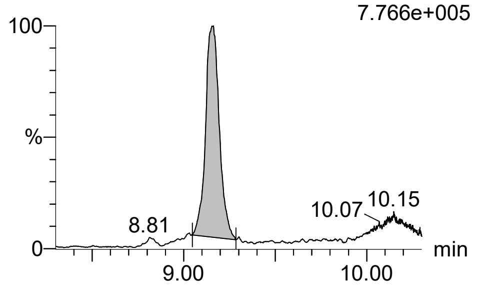

Sample Name: 20211111_A38_R3_IgM
F55:MRM of 2 channels,ES+
1034.5 > 204.1
2.596e+006

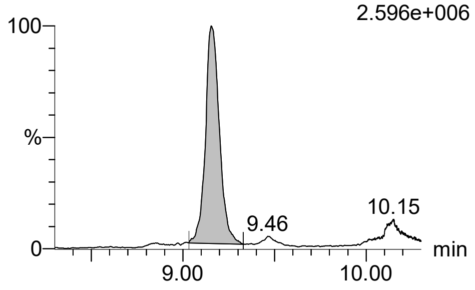

Sample Name: 20211111_A38_R3_IgM
F55:MRM of 2 channels,ES+
1292.9 > 366.1
6.040e+005

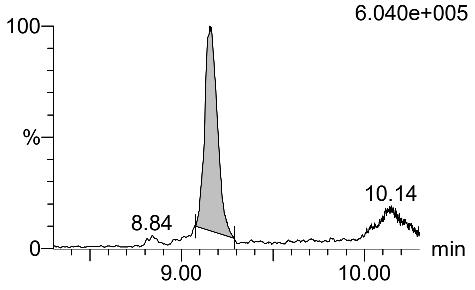

Sample Name: 20211111_H19_R3_IgM
F55:MRM of 2 channels,ES+
1034.5 > 204.1
5.196e+006

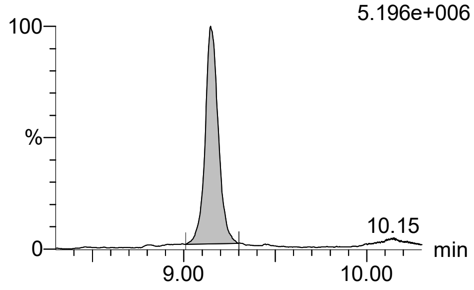

Sample Name: 20211111_H19_R3_IgM
F55:MRM of 2 channels,ES+
1292.9 > 366.1
1.428e+006

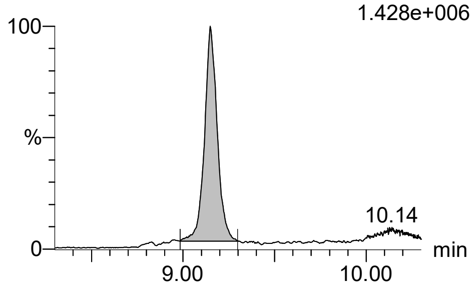

Sample Name: 20211111_A39_R3_IgM
F55:MRM of 2 channels,ES+
1034.5 > 204.1
2.698e+006

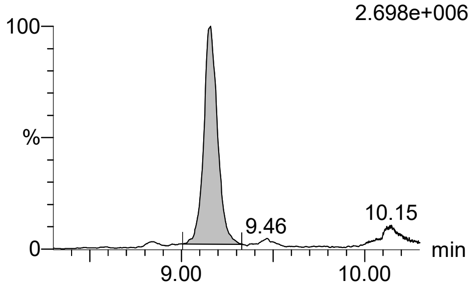

Sample Name: 20211111_A39_R3_IgM
F55:MRM of 2 channels,ES+
1292.9 > 366.1
6.979e+005

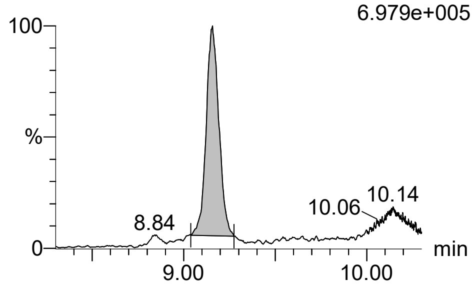

Sample Name: 20211111_A40_R3_IgM
F55:MRM of 2 channels,ES+
1034.5 > 204.1
1.195e+006

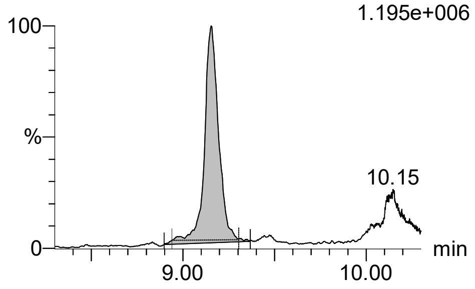

Sample Name: 20211111_A40_R3_IgM
F55:MRM of 2 channels,ES+
1292.9 > 366.1
2.949e+005

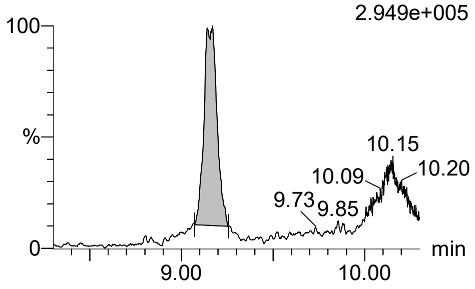

Sample Name: 20211111_H20_R3_IgM
F55:MRM of 2 channels,ES+
1034.5 > 204.1
1.083e+007

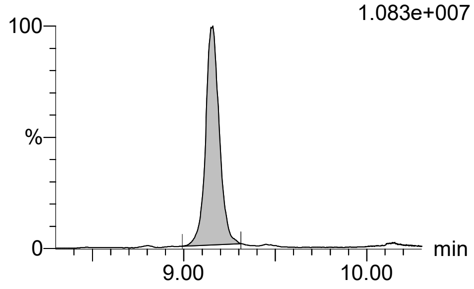

Sample Name: 20211111_H20_R3_IgM
F55:MRM of 2 channels,ES+
1292.9 > 366.1
2.957e+006

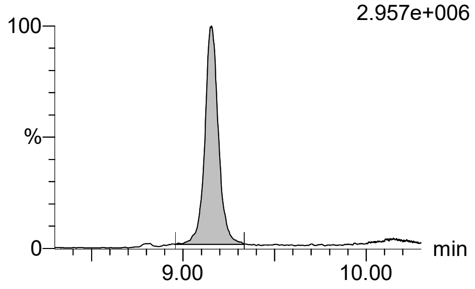

Sample Name: 20211111_A41_R3_IgM
F55:MRM of 2 channels,ES+
1034.5 > 204.1
1.031e+006

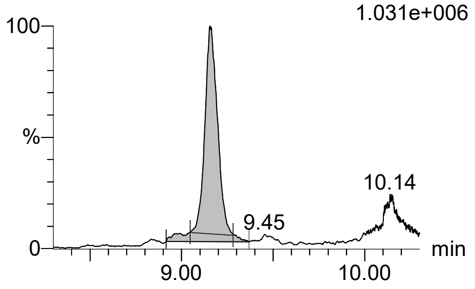

Sample Name: 20211111_A41_R3_IgM
F55:MRM of 2 channels,ES+
1292.9 > 366.1
2.528e+005

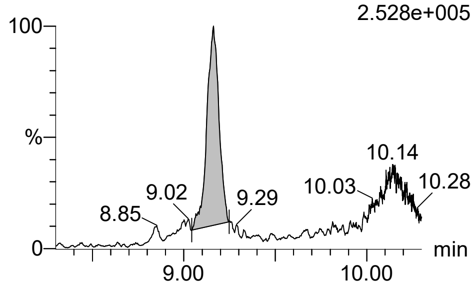

Sample Name: 20211111_A42_R3_IgM
F55:MRM of 2 channels,ES+
1034.5 > 204.1
1.211e+006

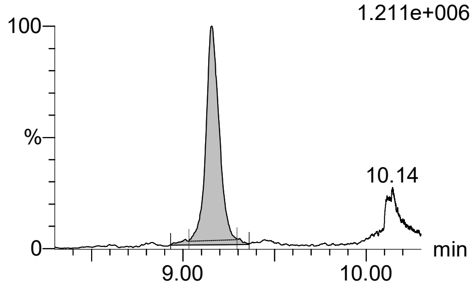

Sample Name: 20211111_A42_R3_IgM
F55:MRM of 2 channels,ES+
1292.9 > 366.1
2.946e+005

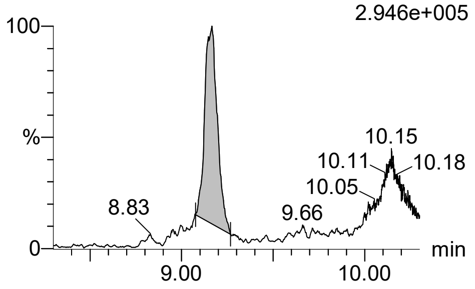

Sample Name: 20211111_A43_R3_IgM
F55:MRM of 2 channels,ES+
1034.5 > 204.1
1.927e+006

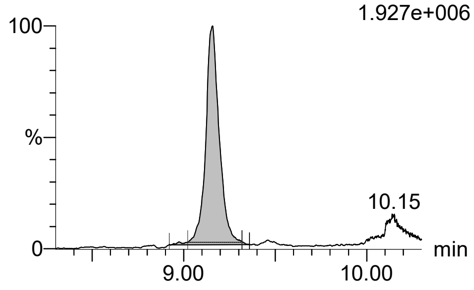

Sample Name: 20211111_A43_R3_IgM
F55:MRM of 2 channels,ES+
1292.9 > 366.1
5.278e+005

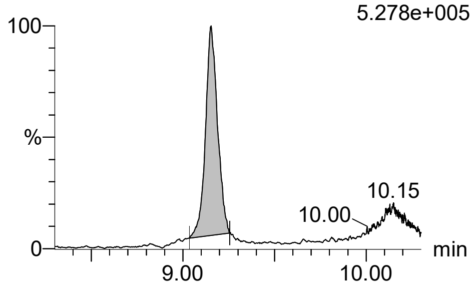

Sample Name: 20211111_A44_R3_IgM
F55:MRM of 2 channels,ES+
1034.5 > 204.1
1.134e+006

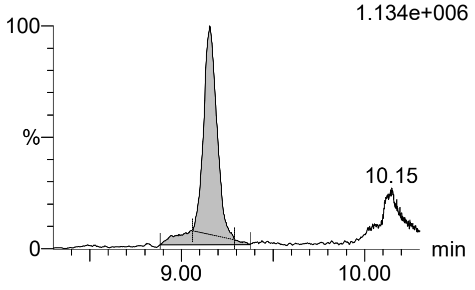

Dataset: C:\MassLynx\10.pro\20211111_clinicalsamples_IgM_IgA.qld

Last Altered: Thursday, March 09, 2023 19:58:25 台北標準時間
Printed: Friday, August 01, 2025 15:09:56 台北標準時間

Sample Name: 20211111_A44_R3_IgM

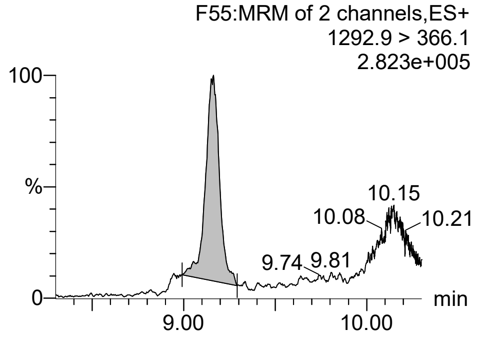

Sample Name: 20211111_QC_R25_IgM

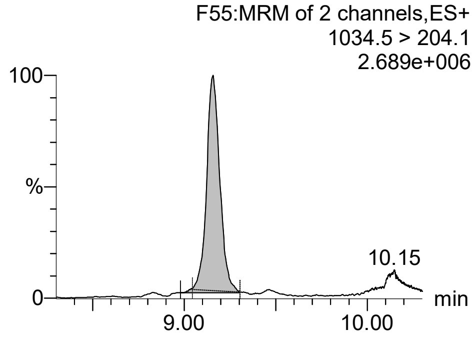

Sample Name: 20211111_QC_R25_IgM

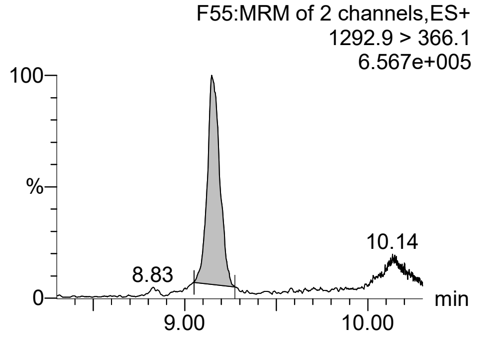

Dataset: C:\MassLynx\10.pro\20211111_clinicalsamples_IgM_IgA.qld

Last Altered: Thursday, March 09, 2023 19:58:25 台北標準時間
Printed: Friday, August 01, 2025 15:09:56 台北標準時間

	#	Name	RT	Area	IS Area	Response	Prima...
1	1	20211112_QC_R001_IgM	9.14	263117.3	15421.4	17.062	bb
2	2	20211112_QC_R002_IgM	9.15	254945.8	13188.8	19.330	MM
3	3	20211111_A1_R2_IgM	9.15	150375.1	11871.0	12.667	MM
4	4	20211111_A1_R3_IgM	9.15	144609.2	12030.5	12.020	MM
5	5	20211111_A2_R2_IgM	9.16	766445.1	11446.8	66.957	bb
6	6	20211111_A2_R3_IgM	9.15	752251.1	11098.4	67.780	bb
7	7	20211111_L1_R2_IgM	9.15	69786.3	11779.4	5.924	bb
8	8	20211111_L1_R3_IgM	9.15	50384.1	11176.2	4.508	bb
9	9	20211111_L2_R2_IgM	9.15	445960.2	11375.0	39.205	bb
10	10	20211111_H1_R2_IgM	9.15	195611.3	13595.2	14.388	MM
11	11	20211111_A3_R2_IgM	9.15	589421.4	14112.3	41.766	bb
12	12	20211111_A4_R2_IgM	9.15	253633.3	11977.5	21.176	bb
13	13	20211111_L3_R2_IgM	9.15	194384.2	12242.1	15.878	MM
14	14	20211111_L4_R2_IgM	9.16	261293.1	12797.0	20.418	bb
15	15	20211111_H2_R2_IgM	9.15	468332.7	10006.0	46.805	bb
16	16	20211111_A5_R2_IgM	9.15	1165428.4	11434.6	101.921	bb
17	17	20211111_A6_R2_IgM	9.14	600256.9	13193.7	45.496	bb
18	18	20211111_QC_R10_IgM	9.14	301253.3	12566.1	23.973	MM
19	19	20211111_L5_R2_IgM	9.15	508203.0	13616.4	37.323	MM
20	20	20211111_L6_R2_IgM	9.14	387708.5	13698.5	28.303	MM
21	21	20211111_H3_R2_IgM	9.16	201774.9	15793.1	12.776	bb
22	22	20211111_A7_R2_IgM	9.14	522582.1	12936.9	40.395	bb
23	23	20211111_A8_R2_IgM	9.15	335077.7	13108.0	25.563	bb
24	24	20211111_L7_R2_IgM	9.16	148871.7	13278.5	11.212	MM
25	25	20211111_L8_R2_IgM	9.15	138541.3	15233.6	9.094	MM
26	26	20211111_H4_R2_IgM	9.16	191394.5	16521.9	11.584	bb
27	27	20211111_A9_R2_IgM	9.14	146414.5	16451.6	8.900	MM
28	28	20211111_A10_R2_IgM	9.15	485934.1	13036.0	37.276	bb
29	29	20211111_L9_R2_IgM	9.14	233165.2	14554.1	16.021	bb
30	30	20211111_L10_R2_IgM	9.15	527651.2	12250.4	43.072	bb
31	31	20211111_QC_R11_IgM	9.15	319621.6	11459.2	27.892	bb
32	32	20211111_H5_R2_IgM	9.15	518266.1	14358.2	36.095	bb
33	33	20211111_A11_R2_IgM	9.14	311141.0	16184.1	19.225	MM
34	34	20211111_A12_R2_IgM	9.15	119629.2	13465.4	8.884	MM
35	35	20211111_L11_R2_IgM	9.16	119909.7	13277.9	9.031	MM
36	36	20211111_L12_R2_IgM	9.15	172494.6	15958.8	10.809	MM
37	37	20211111_H6_R2_IgM	9.16	152193.7	14003.5	10.868	MM
38	38	20211111_A13_R2_IgM	9.16	146805.1	13656.1	10.750	MM
39	39	20211111_A14_R2_IgM	9.16	495461.2	14071.3	35.211	bb
40	40	20211111_L13_R2_IgM	9.16	365853.6	14785.6	24.744	bb
41	41	20211111_L14_R2_IgM	9.15	324578.3	14772.2	21.972	MM
42	42	20211111_H7_R2_IgM	9.16	169575.5	15164.4	11.183	MM
43	43	20211111_A15_R2_IgM	9.17	789569.6	15721.5	50.222	bb
44	44	20211111_QC_R12_IgM	9.16	354104.7	13711.5	25.825	MM
45	45	20211111_A16_R2_IgM	9.16	456292.5	14275.6	31.963	bb
46	46	20211111_L15_R2_IgM	9.15	309502.7	12935.8	23.926	bb
47	47	20211111_L16_R2_IgM	9.17	88899.7	13342.8	6.663	MM
48	48	20211111_H8_R2_IgM	9.16	254825.0	15835.1	16.092	bb
49	49	20211111_A17_R2_IgM	9.17	404423.9	15786.7	25.618	bb
50	50	20211111_A18_R2_IgM	9.15	163061.6	16986.8	9.599	MM
51	51	20211111_L17_R2_IgM	9.15	768239.1	13342.7	57.578	bb
52	52	20211111_L18_R2_IgM	9.16	61551.9	14692.0	4.189	MM
53	53	20211111_H9_R2_IgM	9.15	134347.3	15639.7	8.590	MM
54	54	20211111_A19_R2_IgM	9.15	227291.9	14198.7	16.008	MM
55	55	20211111_A20_R2_IgM	9.15	112644.0	14162.7	7.954	MM
56	56	20211111_L19_R2_IgM	9.15	270559.8	9293.4	29.113	MM

Dataset: C:\MassLynx\10.pro\20211111_clinicalsamples_IgM_IgA.qld

Last Altered: Thursday, March 09, 2023 19:58:25 台北標準時間
Printed: Friday, August 01, 2025 15:09:56 台北標準時間

	# Name	RT	Area	IS Area	Response	Prima...
57	57 20211111_QC_R13_IgM	9.15	328344.1	13903.0	23.617	bb
58	58 20211111_L20_R2_IgM	9.14	213694.9	12781.6	16.719	MM
59	59 20211111_H10_R2_IgM	9.15	268036.4	14796.4	18.115	MM
60	60 20211111_A21_R2_IgM	9.16	390895.8	13669.0	28.597	bb
61	61 20211111_A22_R2_IgM	9.16	136322.3	14098.3	9.669	MM
62	62 20211111_L21_R2_IgM	9.17	215920.6	15678.2	13.772	bb
63	63 20211111_L22_R2_IgM	9.16	347524.8	15724.5	22.101	bb
64	64 20211111_H11_R2_IgM	9.16	189337.5	15890.2	11.915	bb
65	65 20211111_A23_R2_IgM	9.16	136965.1	13423.2	10.204	MM
66	66 20211111_A24_R2_IgM	9.15	544915.4	12438.0	43.811	bb
67	67 20211111_L23_R2_IgM	9.16	369026.8	14075.0	26.219	bb
68	68 20211111_L24_R2_IgM	9.16	158100.4	15972.1	9.899	MM
69	69 20211111_H12_R2_IgM	9.16	185666.3	16270.9	11.411	MM
70	70 20211111_QC_R14_IgM	9.16	348160.7	15806.8	22.026	bb
71	71 20211111_A25_R2_IgM	9.16	774649.3	14552.1	53.233	bb
72	72 20211111_A26_R2_IgM	9.16	255113.1	11492.7	22.198	bb
73	73 20211111_L25_R2_IgM	9.17	200660.5	14403.3	13.932	bb
74	74 20211111_L26_R2_IgM	9.16	129968.9	16533.9	7.861	MM
75	75 20211111_H13_R2_IgM	9.16	315991.9	15520.1	20.360	bb
76	76 20211111_A27_R2_IgM	9.16	524158.6	13337.4	39.300	bb
77	77 20211111_A28_R2_IgM	9.14	263756.2	11895.6	22.172	MM
78	78 20211111_L27_R2_IgM	9.16	310954.1	14125.7	22.013	MM
79	79 20211111_L28_R2_IgM	9.16	329154.4	11807.5	27.877	bb
80	80 20211111_H14_R2_IgM	9.15	314727.6	15035.7	20.932	MM
81	81 20211111_A29_R2_IgM	9.15	38214.5	13554.4	2.819	MM
82	82 20211111_A30_R2_IgM	9.15	358946.1	12984.1	27.645	MM
83	83 20211111_QC_R15_IgM	9.16	312090.1	12299.5	25.374	MM
84	84 20211111_L29_R2_IgM	9.16	99399.5	13590.7	7.314	MM
85	85 20211111_L30_R2_IgM	9.16	68839.3	12511.4	5.502	MM
86	86 20211111_H15_R2_IgM	9.15	192107.4	11885.0	16.164	MM
87	87 20211111_A31_R2_IgM	9.15	83457.4	13422.1	6.218	MM
88	88 20211111_A32_R2_IgM	9.16	370388.4	11461.3	32.316	bb
89	89 20211111_L31_R2_IgM	9.15	60587.9	13753.4	4.405	MM
90	90 20211111_L32_R2_IgM	9.15	503363.6	12949.0	38.873	bb
91	91 20211111_H16_R2_IgM	9.15	934514.4	11465.4	81.507	bb
92	92 20211111_A33_R2_IgM	9.16	351786.2	10551.5	33.340	bb
93	93 20211111_A34_R2_IgM	9.16	95203.7	12409.9	7.672	MM
94	94 20211111_H17_R2_IgM	9.15	2130189.8	10160.9	209.645	bb
95	95 20211111_A35_R2_IgM	9.16	135804.9	10460.6	12.983	MM
96	96 20211111_QC_R16_IgM	9.15	271711.9	10433.5	26.042	bb
97	97 20211111_A36_R2_IgM	9.14	143103.2	12504.8	11.444	MM
98	98 20211111_H18_R2_IgM	9.15	723777.0	9140.4	79.184	bb
99	99 20211111_A37_R2_IgM	9.15	325767.4	9796.9	33.252	bb
100	1... 20211111_A38_R2_IgM	9.16	250201.3	11224.3	22.291	MM
101	1... 20211111_H19_R2_IgM	9.16	498290.3	11739.2	42.447	bb
102	1... 20211111_A39_R2_IgM	9.16	259853.2	12718.9	20.430	bb
103	1... 20211111_A40_R2_IgM	9.14	118645.8	10006.1	11.857	MM
104	1... 20211111_H20_R2_IgM	9.15	1100311.1	7988.2	137.742	bb
105	1... 20211111_A41_R2_IgM	9.16	141165.0	11513.3	12.261	MM
106	1... 20211111_A42_R2_IgM	9.16	118774.0	10238.5	11.601	MM
107	1... 20211111_A43_R2_IgM	9.15	173879.0	11098.9	15.666	bb
108	1... 20211111_A44_R2_IgM	9.15	117267.7	10969.9	10.690	MM
109	1... 20211111_QC_R17_IgM	9.16	235138.0	9700.8	24.239	bb
110	1... 20211111_L2_R3_IgM	9.16	467555.2	8528.0	54.826	bb
111	1... 20211111_H1_R3_IgM	9.16	179396.2	9438.2	19.007	MM
112	1... 20211111_A3_R3_IgM	9.16	537323.7	11598.6	46.326	bb

Dataset: C:\MassLynx\10.pro\20211111_clinicalsamples_IgM_IgA.qld

Last Altered: Thursday, March 09, 2023 19:58:25 台北標準時間
Printed: Friday, August 01, 2025 15:09:56 台北標準時間

	#	Name	RT	Area	IS Area	Response	Prima...
113	1...	20211111_A4_R3_IgM	9.16	250768.1	11364.1	22.067	bb
114	1...	20211111_L3_R3_IgM	9.14	183828.3	10753.4	17.095	MM
115	1...	20211111_L4_R3_IgM	9.15	240564.1	10291.6	23.375	bb
116	1...	20211111_H2_R3_IgM	9.14	466198.0	9315.8	50.044	bb
117	1...	20211111_A5_R3_IgM	9.16	1031666.9	9102.2	113.343	bb
118	1...	20211111_A6_R3_IgM	9.15	477829.2	10356.4	46.139	bb
119	1...	20211111_QC_R18_IgM	9.15	250181.3	8398.3	29.790	MM
120	1...	20211111_L5_R3_IgM	9.16	419256.1	9786.7	42.839	bb
121	1...	20211111_L6_R3_IgM	9.15	317358.9	9911.3	32.020	bb
122	1...	20211111_H3_R3_IgM	9.16	177573.4	12155.5	14.608	bb
123	1...	20211111_A7_R3_IgM	9.15	544197.3	11804.8	46.099	bb
124	1...	20211111_A8_R3_IgM	9.15	299760.2	10510.6	28.520	MM
125	1...	20211111_L7_R3_IgM	9.15	139390.9	12177.9	11.446	MM
126	1...	20211111_L8_R3_IgM	9.16	118729.8	11668.6	10.175	MM
127	1...	20211111_H4_R3_IgM	9.16	157761.1	11192.0	14.096	bb
128	1...	20211111_A9_R3_IgM	9.16	130416.9	14077.4	9.264	MM
129	1...	20211111_A10_R3_IgM	9.16	459400.0	11190.4	41.053	bb
130	1...	20211111_L9_R3_IgM	9.16	223142.9	14649.3	15.232	bb
131	1...	20211111_L10_R3_IgM	9.15	409503.2	9361.3	43.744	bb
132	1...	20211111_QC_R19_IgM	9.15	248639.4	10125.5	24.556	MM
133	1...	20211111_H5_R3_IgM	9.15	387433.0	10010.9	38.701	bb
134	1...	20211111_A11_R3_IgM	9.15	259291.3	12697.2	20.421	MM
135	1...	20211111_A12_R3_IgM	9.15	112516.8	13148.5	8.557	MM
136	1...	20211111_L11_R3_IgM	9.16	99165.9	10786.6	9.193	MM
137	1...	20211111_L12_R3_IgM	9.15	155581.4	11681.8	13.318	MM
138	1...	20211111_H6_R3_IgM	9.16	147291.7	12095.5	12.177	MM
139	1...	20211111_A13_R3_IgM	9.16	147915.8	12908.3	11.459	MM
140	1...	20211111_A14_R3_IgM	9.16	412174.6	11322.6	36.403	bb
141	1...	20211111_L13_R3_IgM	9.16	295136.1	11516.1	25.628	bb
142	1...	20211111_L14_R3_IgM	9.16	261386.2	10790.7	24.223	bb
143	1...	20211111_H7_R3_IgM	9.15	150884.9	11572.1	13.039	bb
144	1...	20211111_A15_R3_IgM	9.16	666499.7	12434.4	53.601	bb
145	1...	20211111_QC_R20_IgM	9.16	291755.9	10701.0	27.264	MM
146	1...	20211111_A16_R3_IgM	9.17	369495.9	11452.1	32.265	bb
147	1...	20211111_L15_R3_IgM	9.16	277808.7	10531.1	26.380	bb
148	1...	20211111_L16_R3_IgM	9.16	88899.7	12049.4	7.378	MM
149	1...	20211111_H8_R3_IgM	9.16	230697.4	13434.9	17.171	bb
150	1...	20211111_A17_R3_IgM	9.16	331027.7	12452.0	26.584	bb
151	1...	20211111_A18_R3_IgM	9.15	138526.1	12506.5	11.076	MM
152	1...	20211111_L17_R3_IgM	9.16	653360.1	9948.9	65.672	bb
153	1...	20211111_L18_R3_IgM	9.15	54640.1	13462.1	4.059	MM
154	1...	20211111_H9_R3_IgM	9.15	119802.5	13178.7	9.091	MM
155	1...	20211111_A19_R3_IgM	9.15	217978.6	11298.6	19.293	MM
156	1...	20211111_A20_R3_IgM	9.16	96244.0	11175.6	8.612	MM
157	1...	20211111_L19_R3_IgM	9.16	287067.2	11558.2	24.837	bb
158	1...	20211111_QC_R21_IgM	9.15	284412.2	9977.0	28.507	bb
159	1...	20211111_L20_R3_IgM	9.15	190904.8	13508.1	14.133	MM
160	1...	20211111_H10_R3_IgM	9.16	246561.4	12374.8	19.925	bb
161	1...	20211111_A21_R3_IgM	9.15	400327.7	12443.6	32.171	bb
162	1...	20211111_A22_R3_IgM	9.16	131127.5	11589.3	11.315	MM
163	1...	20211111_L21_R3_IgM	9.15	184541.0	13107.4	14.079	bb
164	1...	20211111_L22_R3_IgM	9.16	303935.3	12021.5	25.283	MM
165	1...	20211111_H11_R3_IgM	9.17	168818.5	12687.8	13.306	MM
166	1...	20211111_A23_R3_IgM	9.17	134179.8	11611.8	11.555	MM
167	1...	20211111_A24_R3_IgM	9.16	546823.3	10476.8	52.194	bb
168	1...	20211111_L23_R3_IgM	9.16	327433.8	11873.1	27.578	bb

Dataset: C:\MassLynx\10.pro\20211111_clinicalsamples_IgM_IgA.qld

Last Altered: Thursday, March 09, 2023 19:58:25 台北標準時間
Printed: Friday, August 01, 2025 15:09:56 台北標準時間

	#	Name	RT	Area	IS Area	Response	Prima...
169	1...	20211111_L24_R3_IgM	9.16	135223.5	13468.0	10.040	MM
170	1...	20211111_H12_R3_IgM	9.16	185807.5	14229.1	13.058	MM
171	1...	20211111_QC_R22_IgM	9.16	297041.4	11264.4	26.370	MM
172	1...	20211111_A25_R3_IgM	9.16	601549.1	11775.7	51.084	bb
173	1...	20211111_A26_R3_IgM	9.16	227775.7	10473.9	21.747	MM
174	1...	20211111_L25_R3_IgM	9.16	168241.3	13256.8	12.691	bb
175	1...	20211111_L26_R3_IgM	9.17	112256.5	13565.0	8.275	MM
176	1...	20211111_H13_R3_IgM	9.17	312490.8	13146.0	23.771	bb
177	1...	20211111_A27_R3_IgM	9.16	457929.3	12025.6	38.079	bb
178	1...	20211111_A28_R3_IgM	9.16	244599.9	11229.1	21.783	bb
179	1...	20211111_L27_R3_IgM	9.16	308310.8	14960.0	20.609	bb
180	1...	20211111_L28_R3_IgM	9.16	348942.4	11359.0	30.719	bb
181	1...	20211111_H14_R3_IgM	9.16	316140.4	12830.8	24.639	bb
182	1...	20211111_A29_R3_IgM	9.15	35934.2	13434.3	2.675	MM
183	1...	20211111_A30_R3_IgM	9.16	318037.5	11272.2	28.214	bb
184	1...	20211111_QC_R23_IgM	9.16	279315.8	11872.2	23.527	bb
185	1...	20211111_L29_R3_IgM	9.15	98756.0	12948.7	7.627	MM
186	1...	20211111_L30_R3_IgM	9.15	58903.1	11026.6	5.342	MM
187	1...	20211111_H15_R3_IgM	9.16	166102.1	10786.3	15.399	MM
188	1...	20211111_A31_R3_IgM	9.16	81884.2	12010.5	6.818	MM
189	1...	20211111_A32_R3_IgM	9.16	333832.4	11107.7	30.054	bb
190	1...	20211111_L31_R3_IgM	9.15	52234.1	11021.8	4.739	MM
191	1...	20211111_L32_R3_IgM	9.16	467262.7	11683.8	39.992	bb
192	1...	20211111_H16_R3_IgM	9.16	782278.6	9831.7	79.567	bb
193	1...	20211111_A33_R3_IgM	9.15	358728.2	11213.1	31.992	MM
194	1...	20211111_A34_R3_IgM	9.15	81199.1	11550.1	7.030	MM
195	1...	20211111_H17_R3_IgM	9.14	1771036.8	8874.4	199.566	bb
196	1...	20211111_A35_R3_IgM	9.16	124580.4	9856.3	12.640	MM
197	1...	20211111_QC_R24_IgM	9.16	241206.3	7561.8	31.898	bb
198	1...	20211111_A36_R3_IgM	9.15	122151.5	9562.8	12.774	MM
199	1...	20211111_H18_R3_IgM	9.15	661620.0	8165.3	81.028	bb
200	2...	20211111_A37_R3_IgM	9.16	266654.9	9278.8	28.738	MM
201	2...	20211111_A38_R3_IgM	9.15	222000.1	10920.3	20.329	bb
202	2...	20211111_H19_R3_IgM	9.15	419726.7	9285.3	45.203	bb
203	2...	20211111_A39_R3_IgM	9.16	229392.7	9083.4	25.254	bb
204	2...	20211111_A40_R3_IgM	9.15	105194.3	8382.4	12.549	MM
205	2...	20211111_H20_R3_IgM	9.16	916806.3	7807.9	117.420	bb
206	2...	20211111_A41_R3_IgM	9.16	89547.0	5925.2	15.113	MM
207	2...	20211111_A42_R3_IgM	9.16	104221.9	9352.6	11.144	MM
208	2...	20211111_A43_R3_IgM	9.16	163193.5	10216.3	15.974	MM
209	2...	20211111_A44_R3_IgM	9.15	107135.7	10262.7	10.439	MM
210	2...	20211111_QC_R25_IgM	9.16	220629.2	8575.3	25.728	MM

Dataset: C:\MassLynx\10.pro\20211111_clinicalsamples_IgM_IgA.qld

Last Altered: Thursday, March 09, 2023 19:58:25 台北標準時間
Printed: Friday, August 01, 2025 15:11:07 台北標準時間

Method: C:\MassLynx\10_Pro_IgA.PRO\MethDB\20211111_IgM_IgA_DMRM_new.mdb 24 Feb 2022 13:44:14
Calibration: 15 Nov 2021 14:42:11

Sample Name: 20211112_QC_R001_IgM

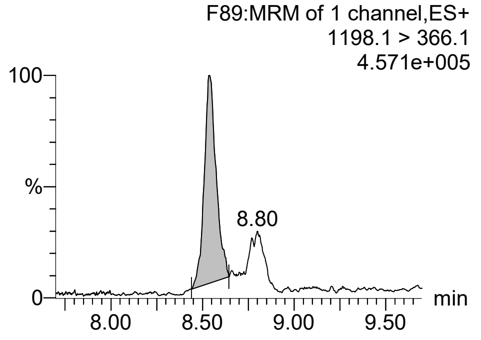

Sample Name: 20211112_QC_R002_IgM

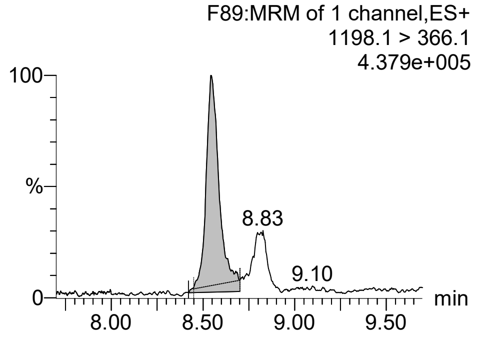

Sample Name: 20211111_A1_R2_IgM

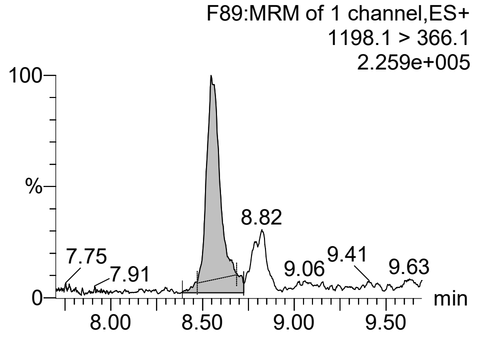

Sample Name: 20211111_A1_R3_IgM

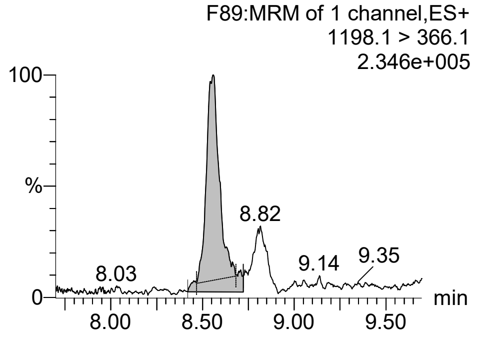

Sample Name: 20211111_A2_R2_IgM

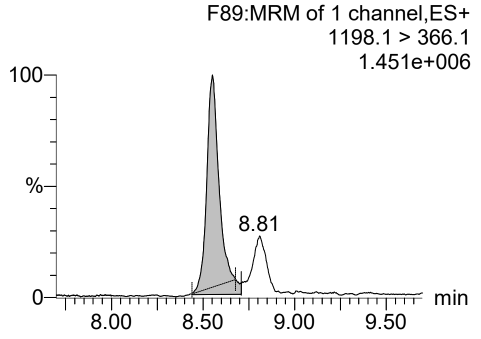

Sample Name: 20211111_A2_R3_IgM

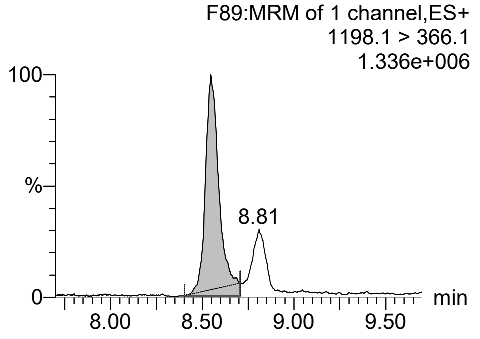

Sample Name: 20211111_L1_R2_IgM

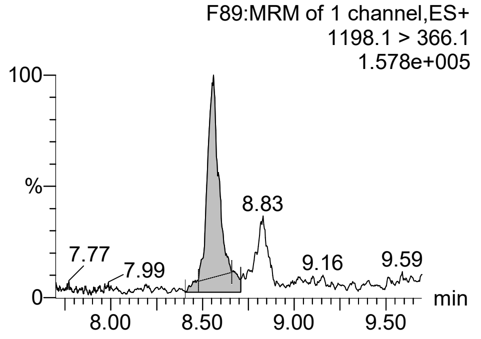

Sample Name: 20211111_L1_R3_IgM

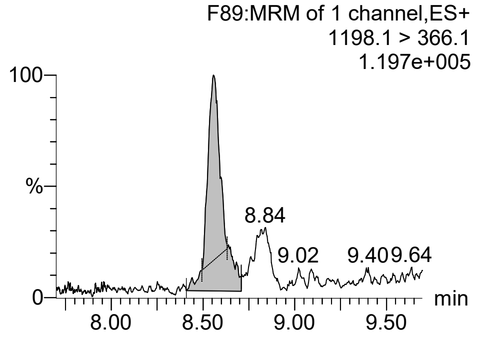

Sample Name: 20211111_L2_R2_IgM

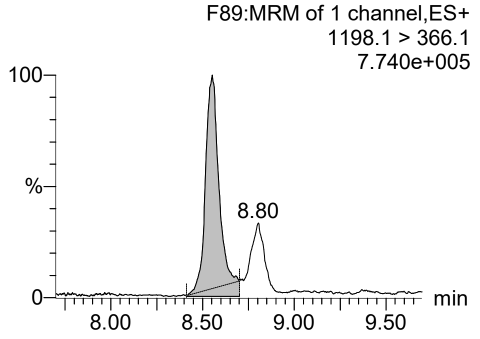

Sample Name: 20211111_H1_R2_IgM

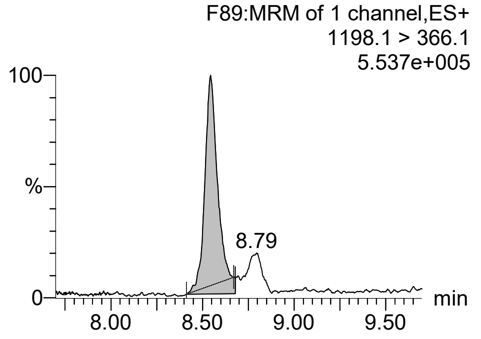

Sample Name: 20211111_A3_R2_IgM

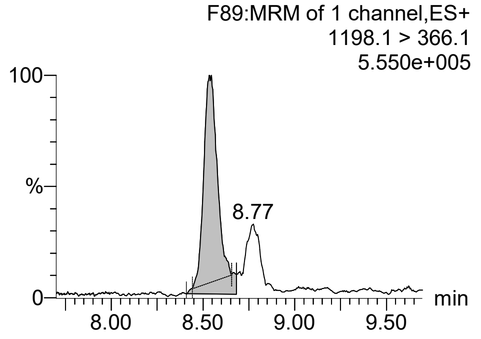

Sample Name: 20211111_A4_R2_IgM

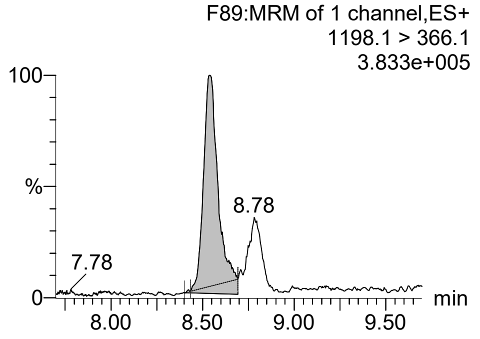

Sample Name: 20211111_L3_R2_IgM
F89:MRM of 1 channel,ES+
1198.1 > 366.1
3.479e+005

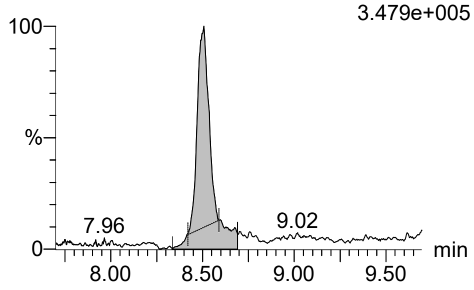

Sample Name: 20211111_L4_R2_IgM
F89:MRM of 1 channel,ES+
1198.1 > 366.1
6.863e+005

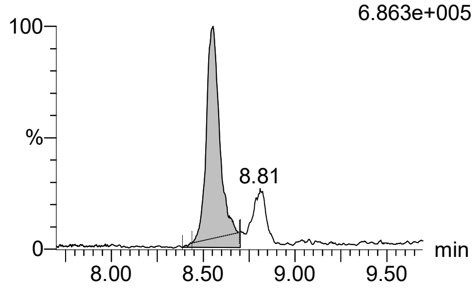

Sample Name: 20211111_H2_R2_IgM
F89:MRM of 1 channel,ES+
1198.1 > 366.1
8.955e+005

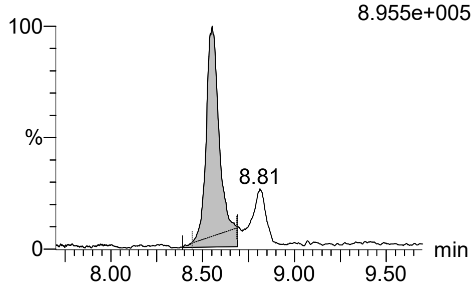

Sample Name: 20211111_A5_R2_IgM
F89:MRM of 1 channel,ES+
1198.1 > 366.1
1.008e+006

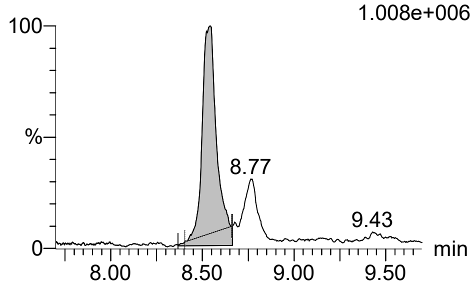

Sample Name: 20211111_A6_R2_IgM
F89:MRM of 1 channel,ES+
1198.1 > 366.1
4.489e+005

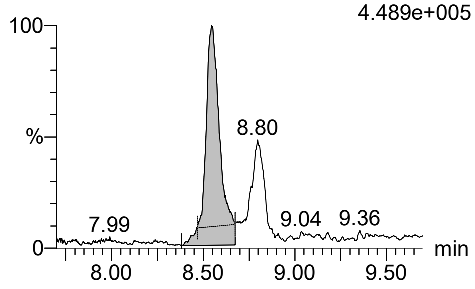

Sample Name: 20211111_QC_R10_IgM
F89:MRM of 1 channel,ES+
1198.1 > 366.1
4.686e+005

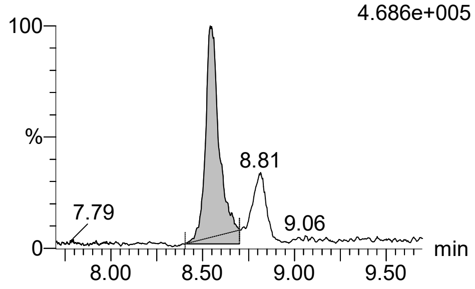

Sample Name: 20211111_L5_R2_IgM
F89:MRM of 1 channel,ES+
1198.1 > 366.1
4.711e+005

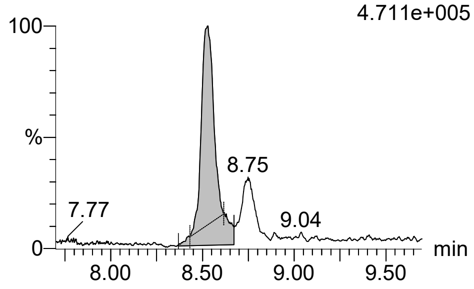

Sample Name: 20211111_L6_R2_IgM
F89:MRM of 1 channel,ES+
1198.1 > 366.1
7.438e+005

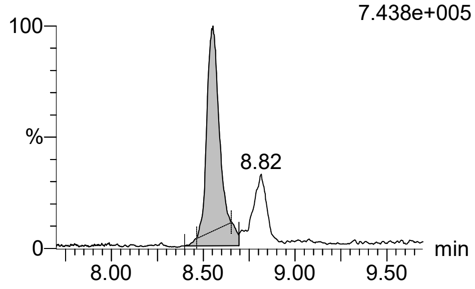

Sample Name: 20211111_H3_R2_IgM
F89:MRM of 1 channel,ES+
1198.1 > 366.1
4.589e+005

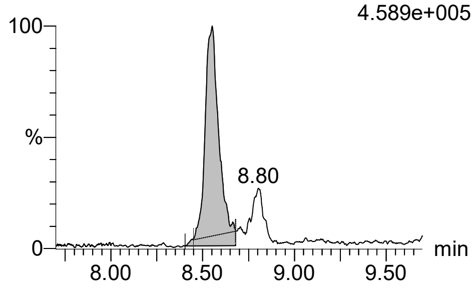

Sample Name: 20211111_A7_R2_IgM
F89:MRM of 1 channel,ES+
1198.1 > 366.1
3.135e+005

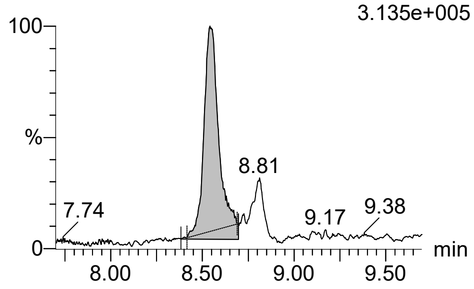

Sample Name: 20211111_A8_R2_IgM
F89:MRM of 1 channel,ES+
1198.1 > 366.1
5.398e+005

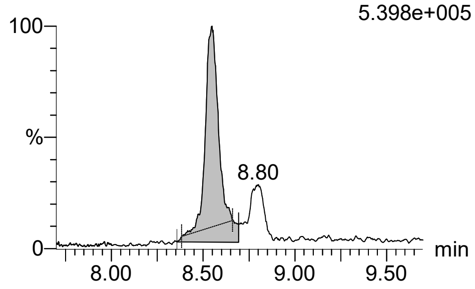

Sample Name: 20211111_L7_R2_IgM
F89:MRM of 1 channel,ES+
1198.1 > 366.1
3.400e+005

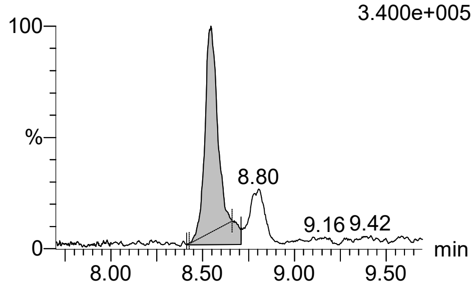

Sample Name: 20211111_L8_R2_IgM
F89:MRM of 1 channel,ES+
1198.1 > 366.1
1.688e+005

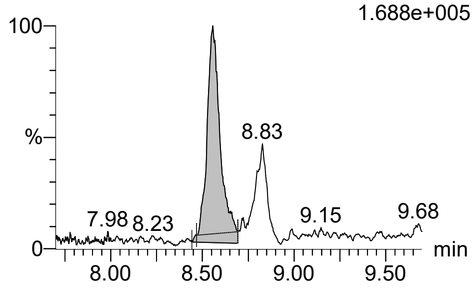

Sample Name: 20211111_H4_R2_IgM
F89:MRM of 1 channel,ES+
1198.1 > 366.1
3.272e+005

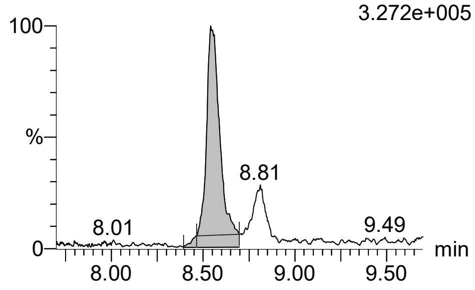

Sample Name: 20211111_A9_R2_IgM
F89:MRM of 1 channel,ES+
1198.1 > 366.1
3.310e+005

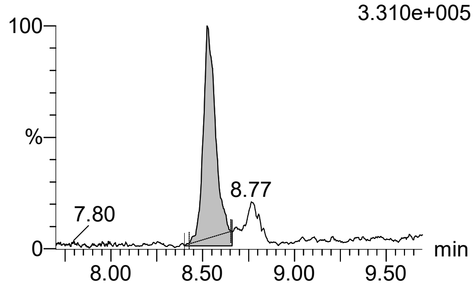

Sample Name: 20211111_A10_R2_IgM

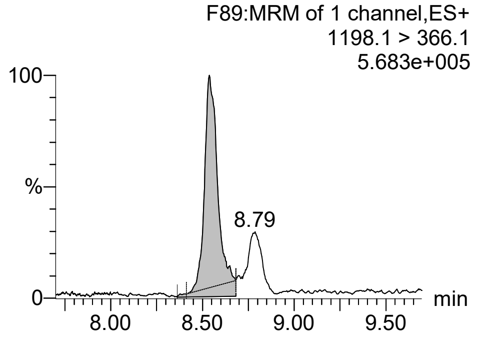

Sample Name: 20211111_L9_R2_IgM

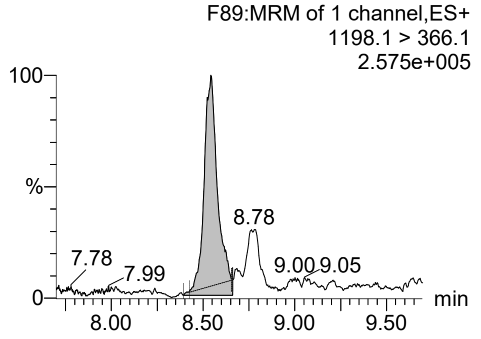

Sample Name: 20211111_L10_R2_IgM

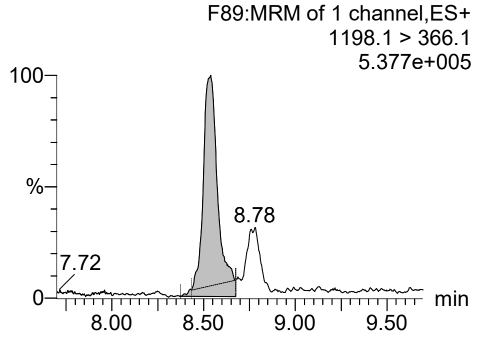

Sample Name: 20211111_QC_R11_IgM

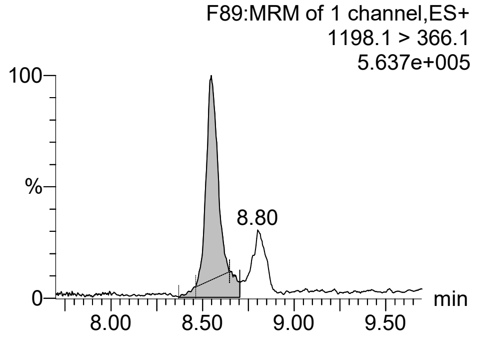

Sample Name: 20211111_H5_R2_IgM

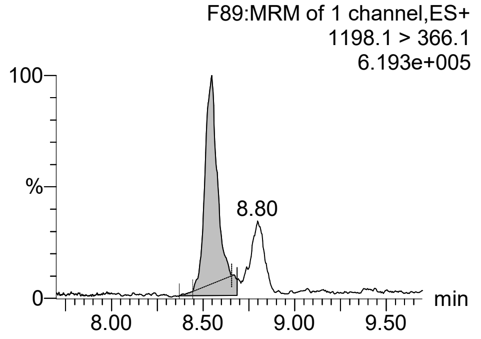

Sample Name: 20211111_A11_R2_IgM

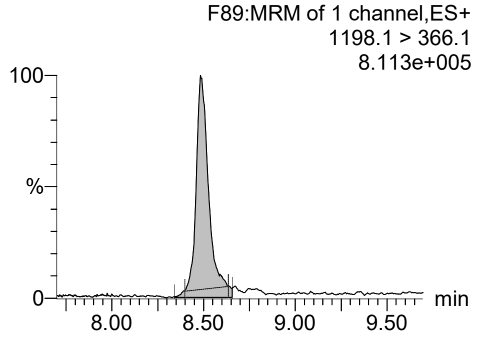

Sample Name: 20211111_A12_R2_IgM

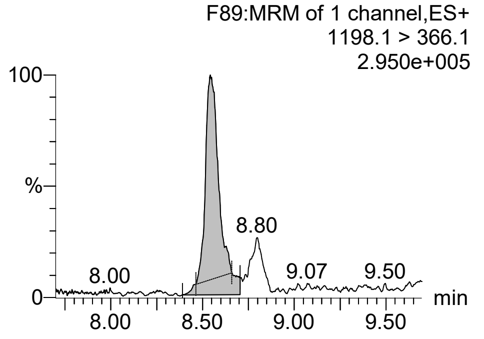

Sample Name: 20211111_L11_R2_IgM

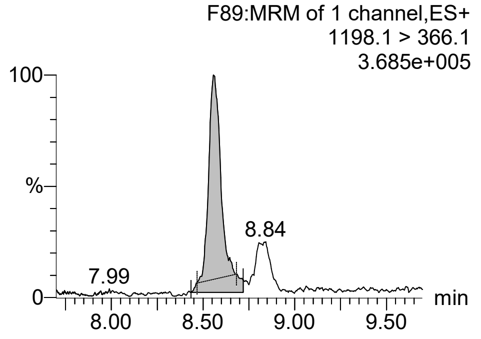

Sample Name: 20211111_L12_R2_IgM

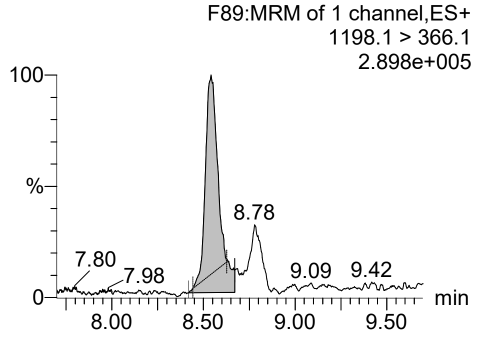

Sample Name: 20211111_H6_R2_IgM

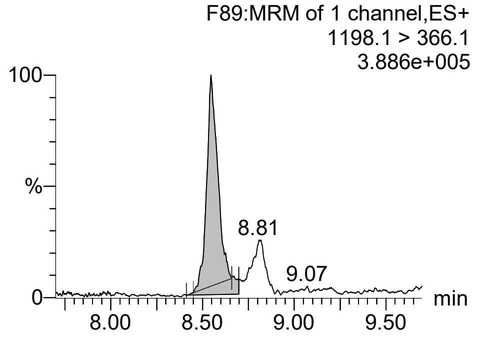

Sample Name: 20211111_A13_R2_IgM

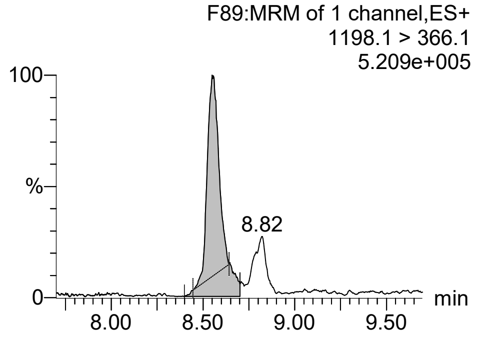

Sample Name: 20211111_A14_R2_IgM

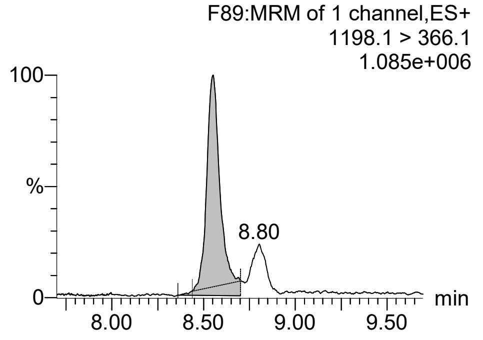

Sample Name: 20211111_L13_R2_IgM

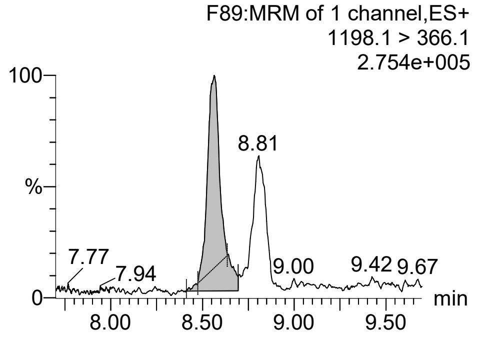

Sample Name: 20211111_L14_R2_IgM

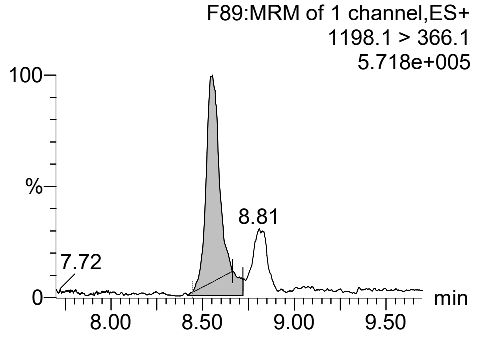

Sample Name: 20211111_H7_R2_IgM

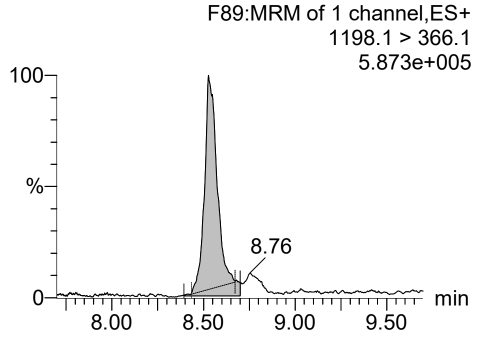

Sample Name: 20211111_A15_R2_IgM
F89:MRM of 1 channel,ES+
1198.1 > 366.1
1.680e+006

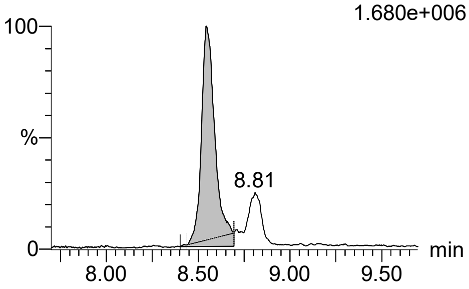

Sample Name: 20211111_QC_R12_IgM
F89:MRM of 1 channel,ES+
1198.1 > 366.1
5.789e+005

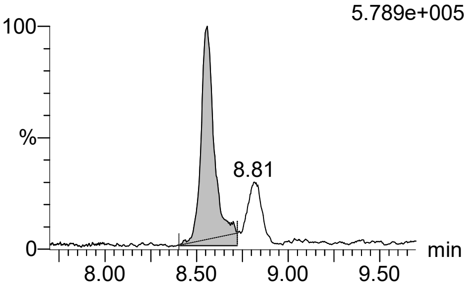

Sample Name: 20211111_A16_R2_IgM
F89:MRM of 1 channel,ES+
1198.1 > 366.1
6.154e+005

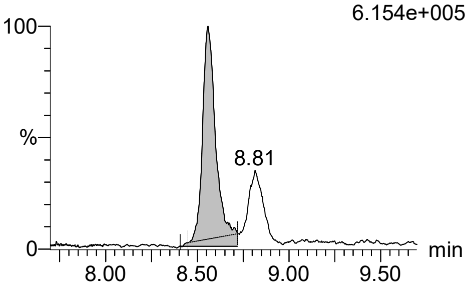

Sample Name: 20211111_L15_R2_IgM
F89:MRM of 1 channel,ES+
1198.1 > 366.1
8.217e+005

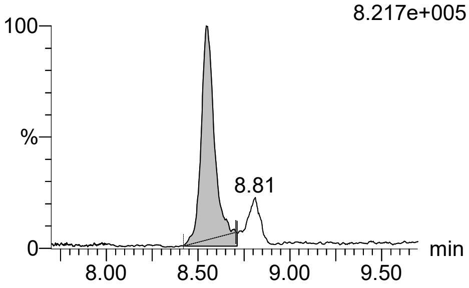

Sample Name: 20211111_L16_R2_IgM
F89:MRM of 1 channel,ES+
1198.1 > 366.1
1.292e+005

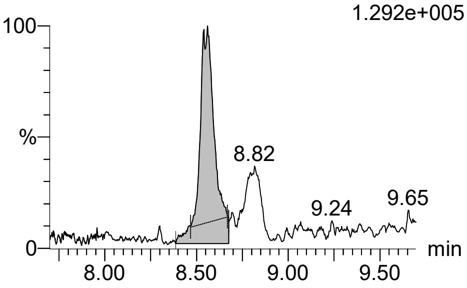

Sample Name: 20211111_H8_R2_IgM
F89:MRM of 1 channel,ES+
1198.1 > 366.1
2.693e+005

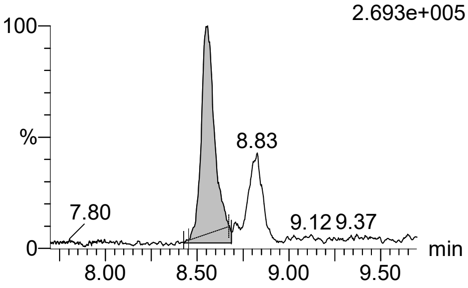

Sample Name: 20211111_A17_R2_IgM
F89:MRM of 1 channel,ES+
1198.1 > 366.1
8.225e+005

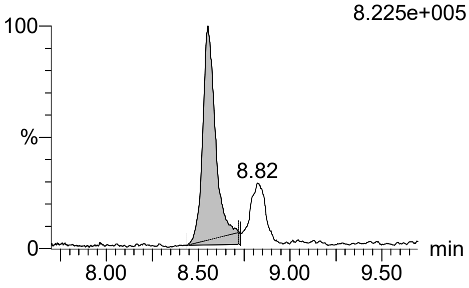

Sample Name: 20211111_A18_R2_IgM
F89:MRM of 1 channel,ES+
1198.1 > 366.1
2.692e+005

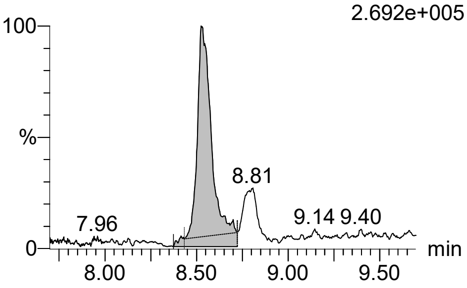

Sample Name: 20211111_L17_R2_IgM
F89:MRM of 1 channel,ES+
1198.1 > 366.1
8.115e+005

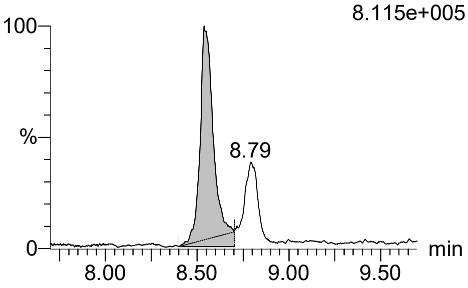

Sample Name: 20211111_L18_R2_IgM
F89:MRM of 1 channel,ES+
1198.1 > 366.1
1.093e+005

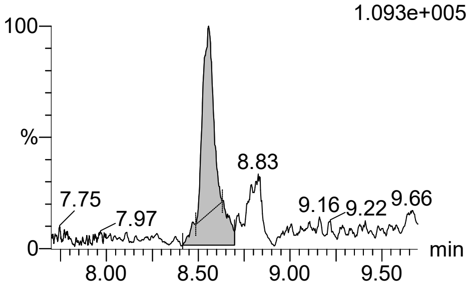

Sample Name: 20211111_H9_R2_IgM
F89:MRM of 1 channel,ES+
1198.1 > 366.1
2.802e+005

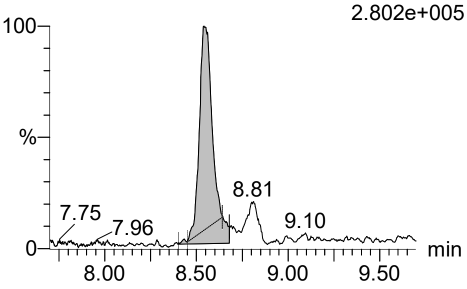

Sample Name: 20211111_A19_R2_IgM
F89:MRM of 1 channel,ES+
1198.1 > 366.1
6.001e+005

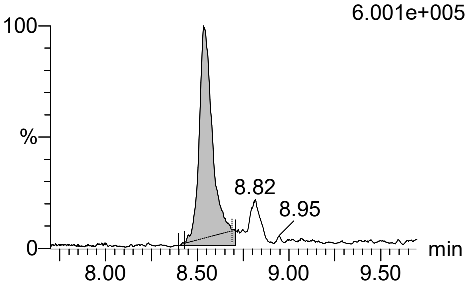

Sample Name: 20211111_A20_R2_IgM
F89:MRM of 1 channel,ES+
1198.1 > 366.1
2.078e+005

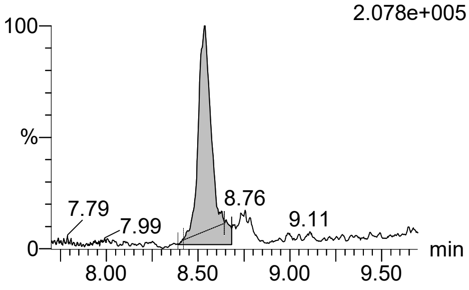

Sample Name: 20211111_L19_R2_IgM
F89:MRM of 1 channel,ES+
1198.1 > 366.1
7.179e+005

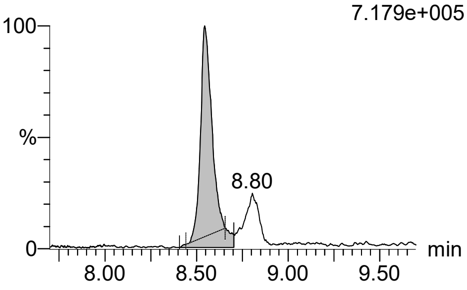

Sample Name: 20211111_QC_R13_IgM
F89:MRM of 1 channel,ES+
1198.1 > 366.1
5.753e+005

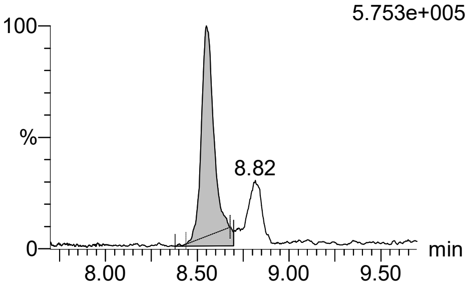

Dataset: C:\MassLynx\10.pro\20211111_clinicalsamples_IgM_IgA.qld

Last Altered: Thursday, March 09, 2023 19:58:25 台北標準時間
Printed: Friday, August 01, 2025 15:11:07 台北標準時間

Sample Name: 20211111_L20_R2_IgM

F89:MRM of 1 channel,ES+
1198.1 > 366.1
3.753e+005

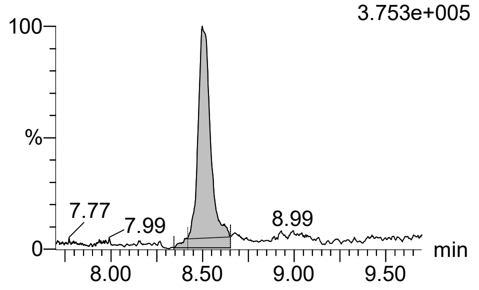

Sample Name: 20211111_H10_R2_IgM

F89:MRM of 1 channel,ES+
1198.1 > 366.1
7.863e+005

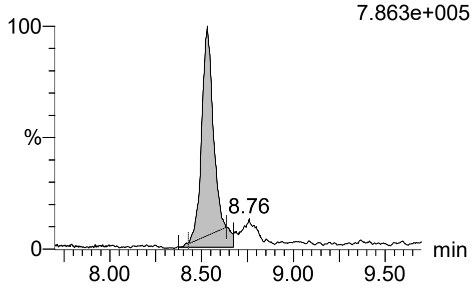

Sample Name: 20211111_A21_R2_IgM

F89:MRM of 1 channel,ES+
1198.1 > 366.1
3.243e+005

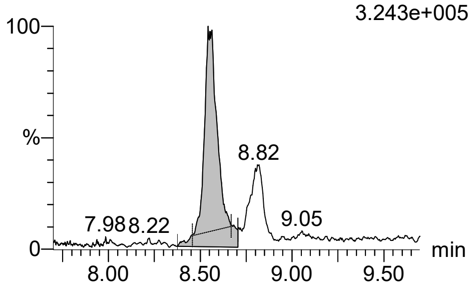

Sample Name: 20211111_A22_R2_IgM

F89:MRM of 1 channel,ES+
1198.1 > 366.1
1.138e+005

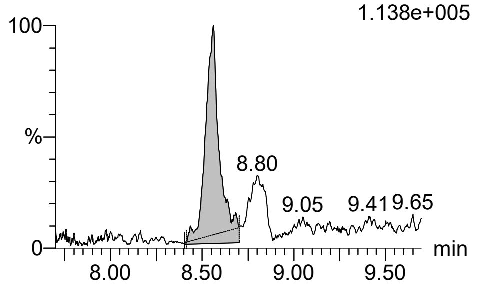

Sample Name: 20211111_L21_R2_IgM

F89:MRM of 1 channel,ES+
1198.1 > 366.1
5.017e+005

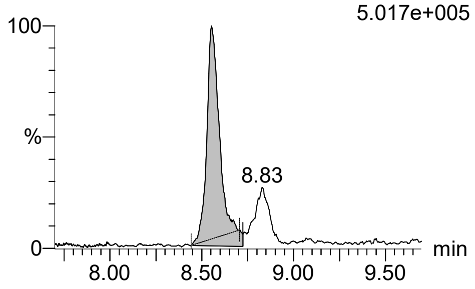

Sample Name: 20211111_L22_R2_IgM

F89:MRM of 1 channel,ES+
1198.1 > 366.1
3.029e+005

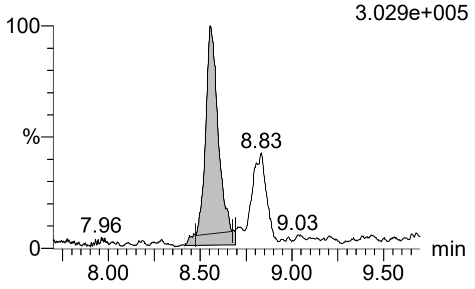

Sample Name: 20211111_H11_R2_IgM

F89:MRM of 1 channel,ES+
1198.1 > 366.1
4.453e+005

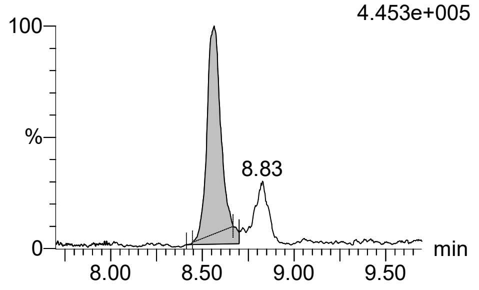

Sample Name: 20211111_A23_R2_IgM

F89:MRM of 1 channel,ES+
1198.1 > 366.1
3.482e+005

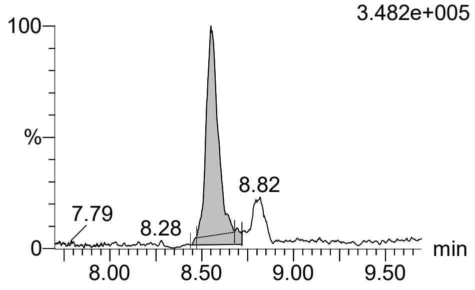

Sample Name: 20211111_A24_R2_IgM

F89:MRM of 1 channel,ES+
1198.1 > 366.1
9.157e+005

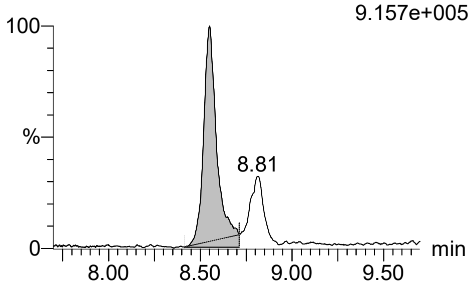

Sample Name: 20211111_L23_R2_IgM

F89:MRM of 1 channel,ES+
1198.1 > 366.1
1.026e+006

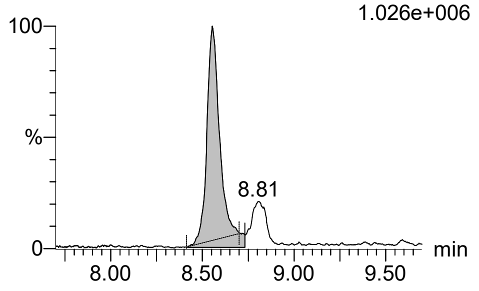

Sample Name: 20211111_L24_R2_IgM

F89:MRM of 1 channel,ES+
1198.1 > 366.1
1.564e+005

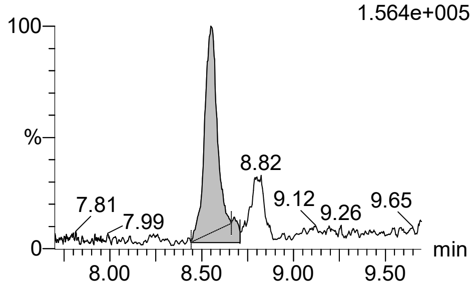

Sample Name: 20211111_H12_R2_IgM

F89:MRM of 1 channel,ES+
1198.1 > 366.1
1.877e+005

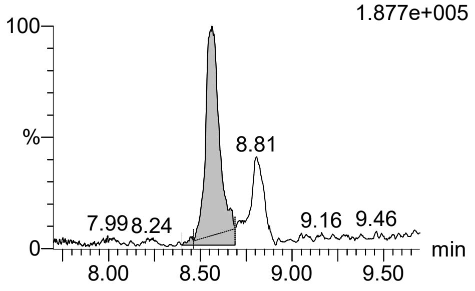

Sample Name: 20211111_QC_R14_IgM

F89:MRM of 1 channel,ES+
1198.1 > 366.1
5.945e+005

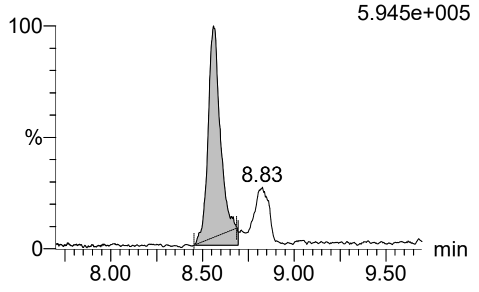

Sample Name: 20211111_A25_R2_IgM

F89:MRM of 1 channel,ES+
1198.1 > 366.1
8.540e+005

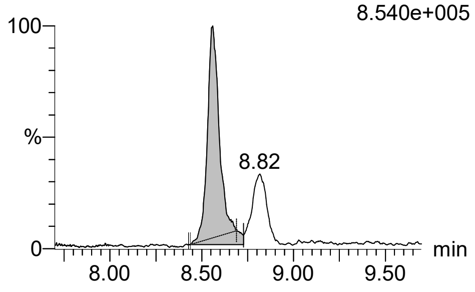

Sample Name: 20211111_A26_R2_IgM

F89:MRM of 1 channel,ES+
1198.1 > 366.1
4.366e+005

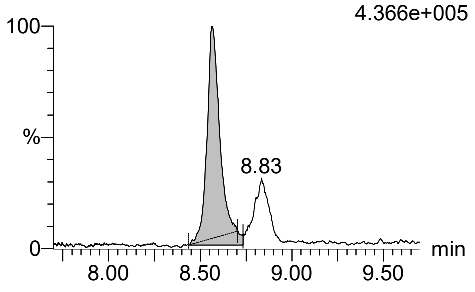

Dataset: C:\MassLynx\10.pro\20211111_clinicalsamples_IgM_IgA.qld

Last Altered: Thursday, March 09, 2023 19:58:25 台北標準時間
Printed: Friday, August 01, 2025 15:11:07 台北標準時間

Sample Name: 20211111_L25_R2_IgM
F89:MRM of 1 channel,ES+
1198.1 > 366.1
2.193e+005

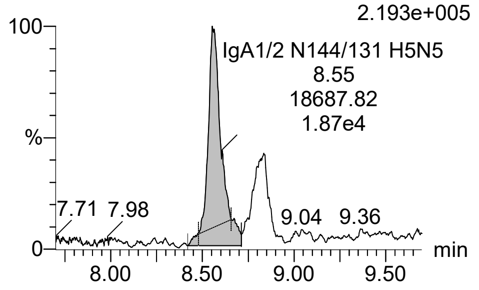

Sample Name: 20211111_L26_R2_IgM
F89:MRM of 1 channel,ES+
1198.1 > 366.1
3.921e+005

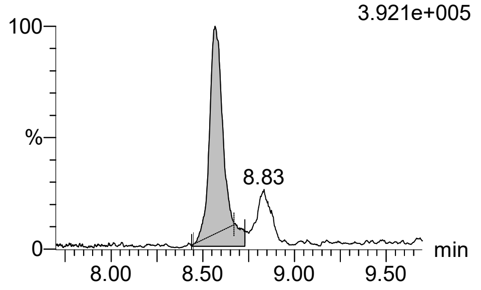

Sample Name: 20211111_H13_R2_IgM
F89:MRM of 1 channel,ES+
1198.1 > 366.1
6.498e+005

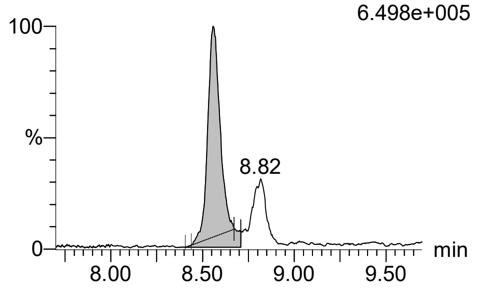

Sample Name: 20211111_A27_R2_IgM
F89:MRM of 1 channel,ES+
1198.1 > 366.1
5.220e+005

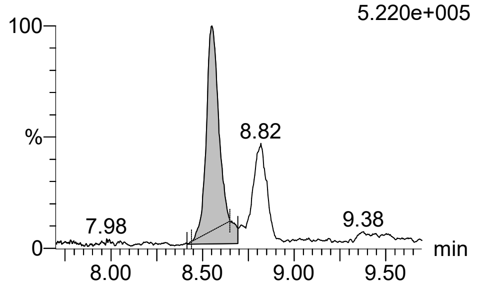

Sample Name: 20211111_A28_R2_IgM
F89:MRM of 1 channel,ES+
1198.1 > 366.1
2.257e+005

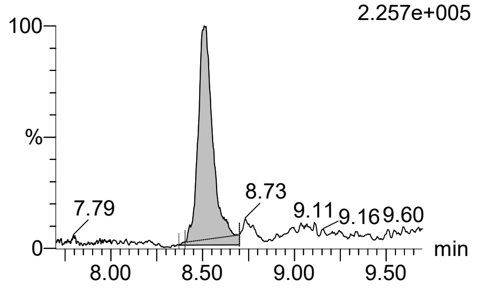

Sample Name: 20211111_L27_R2_IgM
F89:MRM of 1 channel,ES+
1198.1 > 366.1
4.712e+005

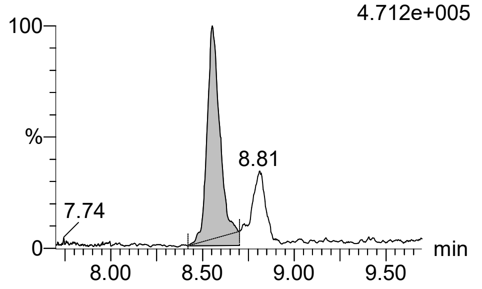

Sample Name: 20211111_L28_R2_IgM
F89:MRM of 1 channel,ES+
1198.1 > 366.1
2.675e+005

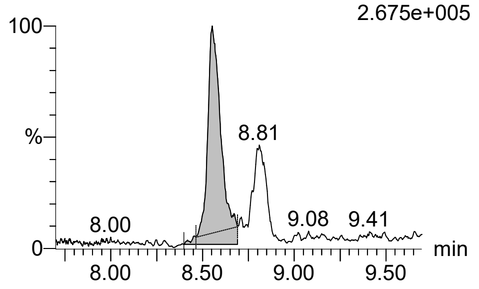

Sample Name: 20211111_H14_R2_IgM
F89:MRM of 1 channel,ES+
1198.1 > 366.1
5.006e+005

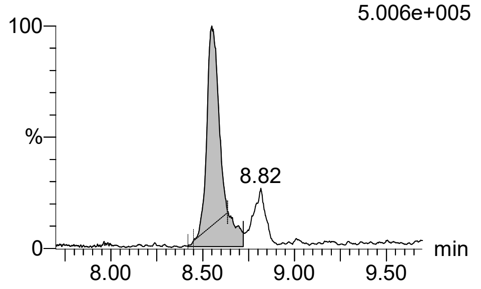

Sample Name: 20211111_A29_R2_IgM
F89:MRM of 1 channel,ES+
1198.1 > 366.1
5.712e+004

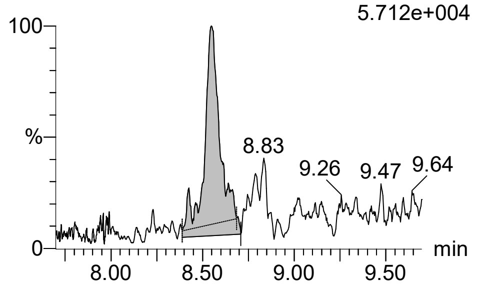

Sample Name: 20211111_A30_R2_IgM
F89:MRM of 1 channel,ES+
1198.1 > 366.1
6.453e+005

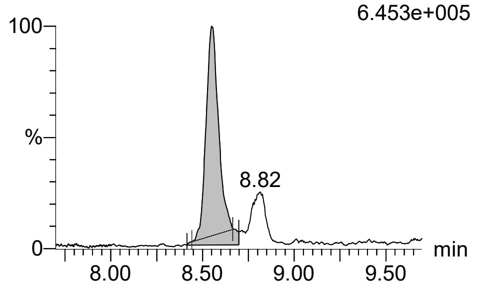

Sample Name: 20211111_QC_R15_IgM
F89:MRM of 1 channel,ES+
1198.1 > 366.1
5.357e+005

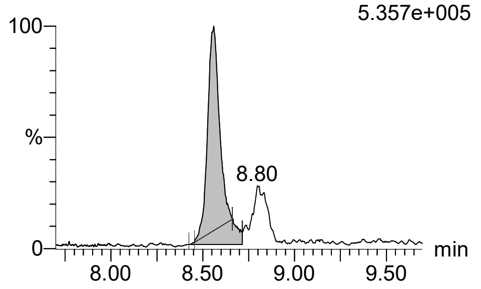

Sample Name: 20211111_L29_R2_IgM
F89:MRM of 1 channel,ES+
1198.1 > 366.1
1.846e+005

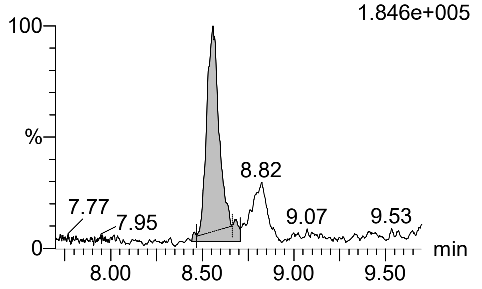

Sample Name: 20211111_L30_R2_IgM
F89:MRM of 1 channel,ES+
1198.1 > 366.1
1.392e+005

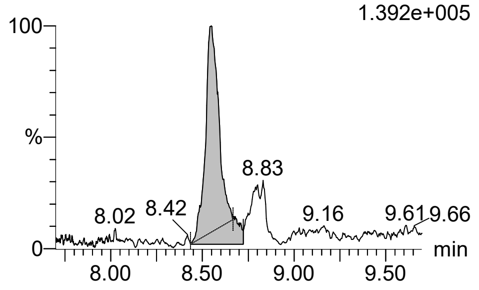

Sample Name: 20211111_H15_R2_IgM
F89:MRM of 1 channel,ES+
1198.1 > 366.1
6.266e+005

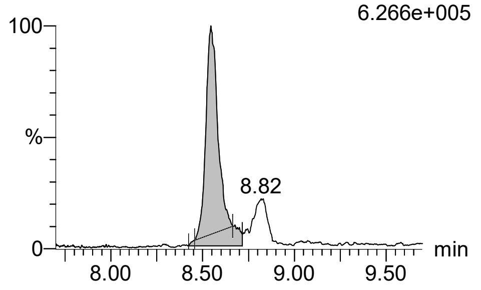

Sample Name: 20211111_A31_R2_IgM
F89:MRM of 1 channel,ES+
1198.1 > 366.1
2.768e+005

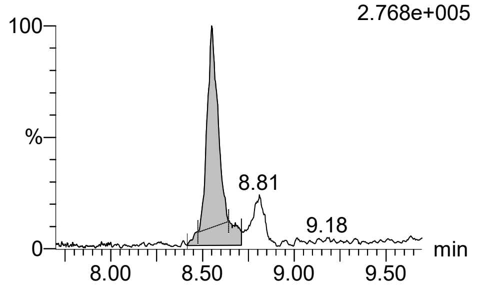

Sample Name: 20211111_A32_R2_IgM
F89:MRM of 1 channel,ES+
1198.1 > 366.1
5.565e+005

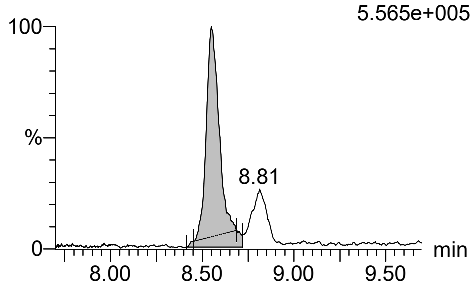

Sample Name: 20211111_L31_R2_IgM
F89:MRM of 1 channel,ES+
1198.1 > 366.1
9.381e+004

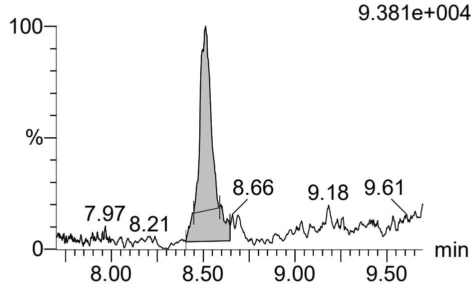

Sample Name: 20211111_L32_R2_IgM
F89:MRM of 1 channel,ES+
1198.1 > 366.1
5.556e+005

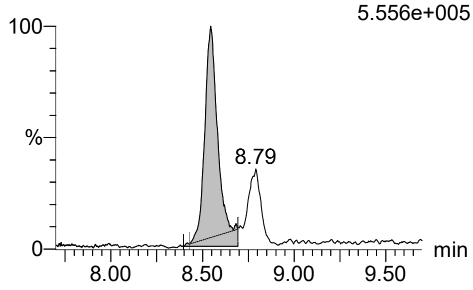

Sample Name: 20211111_H16_R2_IgM
F89:MRM of 1 channel,ES+
1198.1 > 366.1
7.979e+005

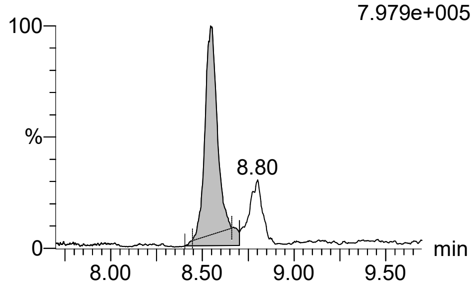

Sample Name: 20211111_A33_R2_IgM
F89:MRM of 1 channel,ES+
1198.1 > 366.1
1.025e+006

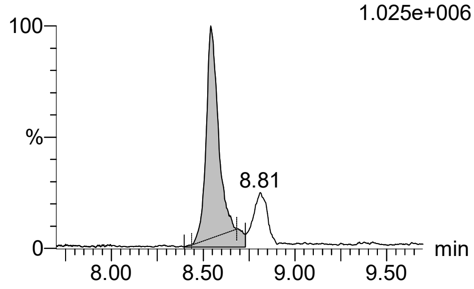

Sample Name: 20211111_A34_R2_IgM
F89:MRM of 1 channel,ES+
1198.1 > 366.1
2.010e+005

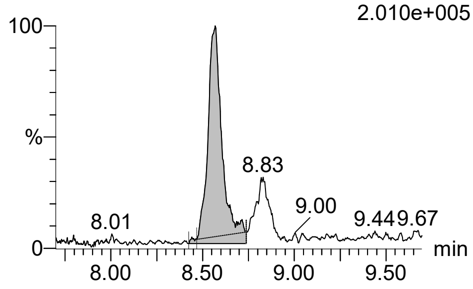

Sample Name: 20211111_H17_R2_IgM
F89:MRM of 1 channel,ES+
1198.1 > 366.1
3.040e+006

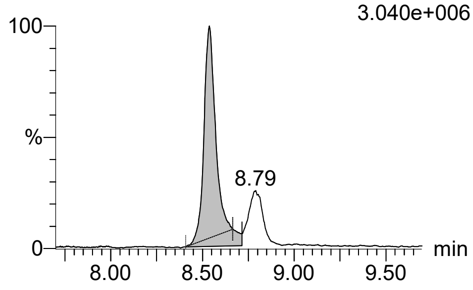

Sample Name: 20211111_A35_R2_IgM
F89:MRM of 1 channel,ES+
1198.1 > 366.1
3.636e+005

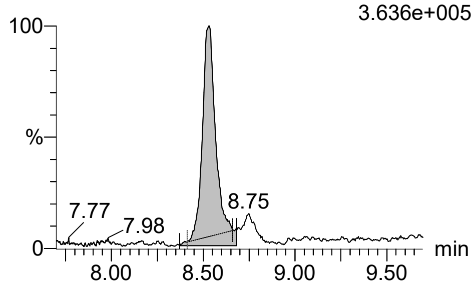

Sample Name: 20211111_QC_R16_IgM
F89:MRM of 1 channel,ES+
1198.1 > 366.1
4.973e+005

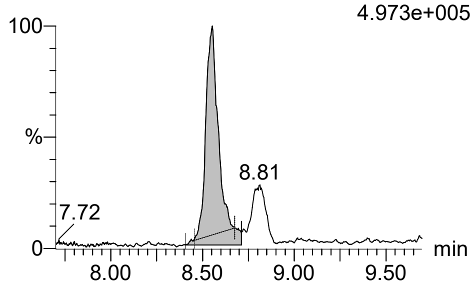

Sample Name: 20211111_A36_R2_IgM
F89:MRM of 1 channel,ES+
1198.1 > 366.1
3.011e+005

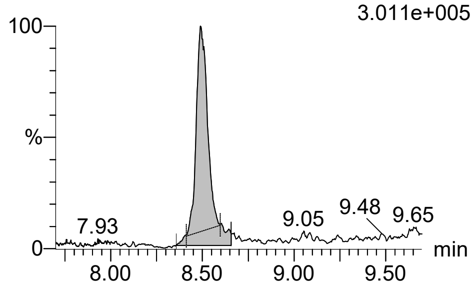

Sample Name: 20211111_H18_R2_IgM
F89:MRM of 1 channel,ES+
1198.1 > 366.1
1.010e+006

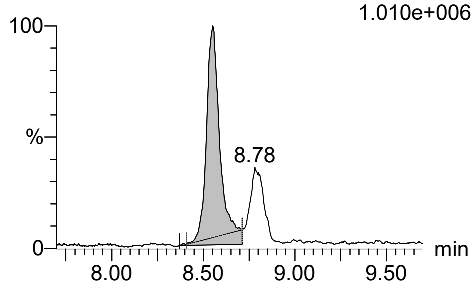

Sample Name: 20211111_A37_R2_IgM
F89:MRM of 1 channel,ES+
1198.1 > 366.1
7.717e+005

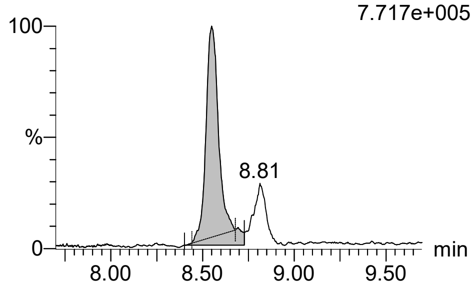

Sample Name: 20211111_A38_R2_IgM
F89:MRM of 1 channel,ES+
1198.1 > 366.1
4.177e+005

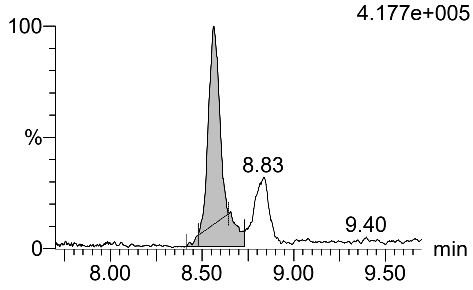

Sample Name: 20211111_H19_R2_IgM
F89:MRM of 1 channel,ES+
1198.1 > 366.1
7.942e+005

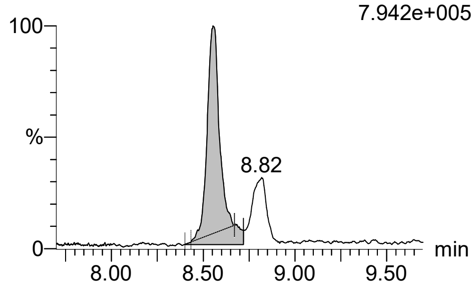

Sample Name: 20211111_A39_R2_IgM
F89:MRM of 1 channel,ES+
1198.1 > 366.1
5.467e+005

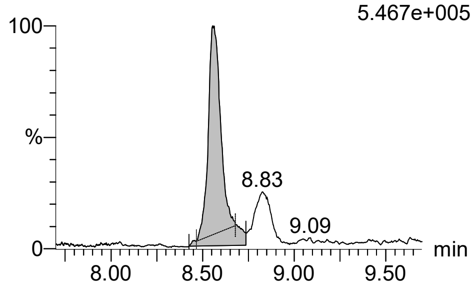

Sample Name: 20211111_A40_R2_IgM
F89:MRM of 1 channel,ES+
1198.1 > 366.1
2.984e+005

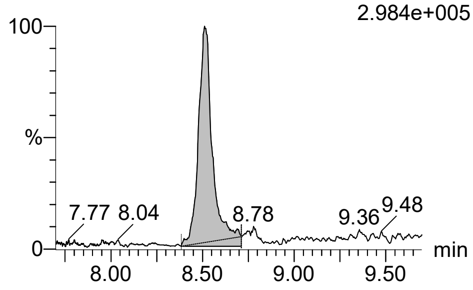

Sample Name: 20211111_H20_R2_IgM
F89:MRM of 1 channel,ES+
1198.1 > 366.1
2.518e+006

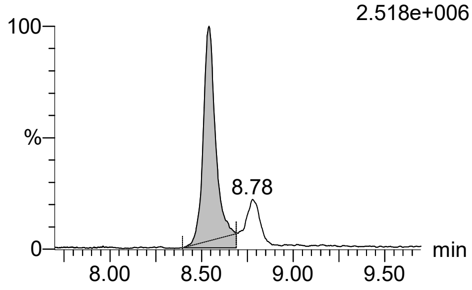

Sample Name: 20211111_A41_R2_IgM
F89:MRM of 1 channel,ES+
1198.1 > 366.1
3.546e+005

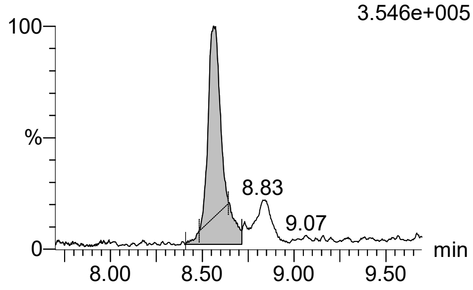

Sample Name: 20211111_A42_R2_IgM
F89:MRM of 1 channel,ES+
1198.1 > 366.1
1.699e+005

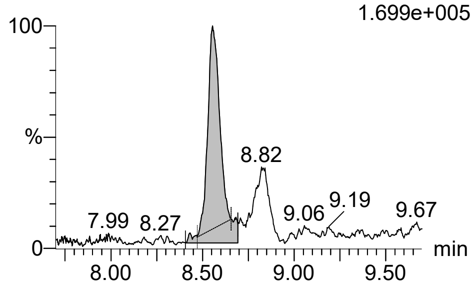

Sample Name: 20211111_A43_R2_IgM
F89:MRM of 1 channel,ES+
1198.1 > 366.1
1.591e+005

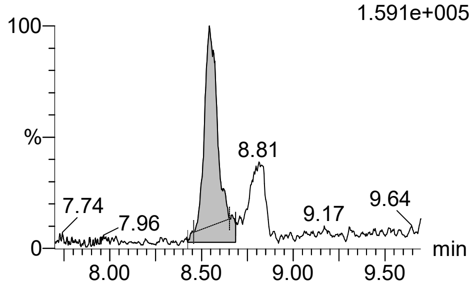

Sample Name: 20211111_A44_R2_IgM
F89:MRM of 1 channel,ES+
1198.1 > 366.1
1.491e+005

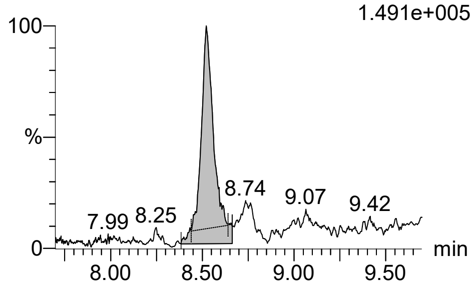

Sample Name: 20211111_QC_R17_IgM
F89:MRM of 1 channel,ES+
1198.1 > 366.1
3.984e+005

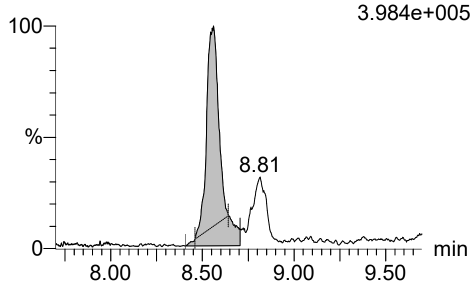

Sample Name: 20211111_L2_R3_IgM
F89:MRM of 1 channel,ES+
1198.1 > 366.1
8.667e+005

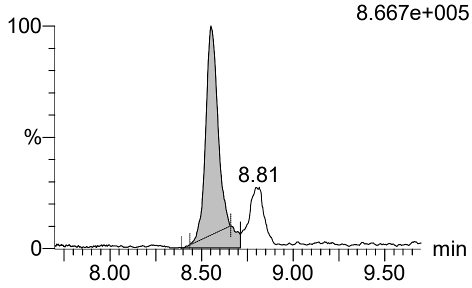

Sample Name: 20211111_H1_R3_IgM
F89:MRM of 1 channel,ES+
1198.1 > 366.1
5.304e+005

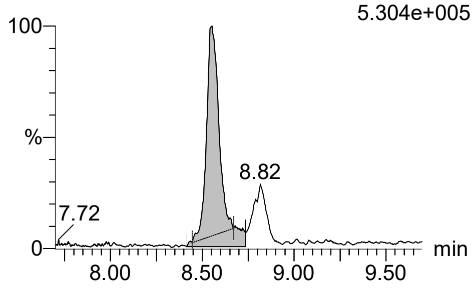

Sample Name: 20211111_A3_R3_IgM
F89:MRM of 1 channel,ES+
1198.1 > 366.1
5.266e+005

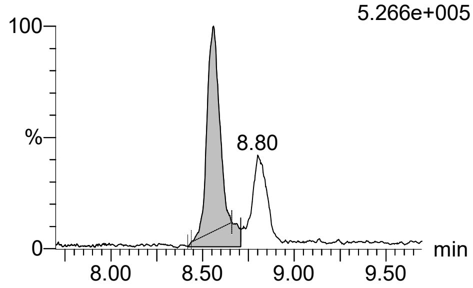

Sample Name: 20211111_A4_R3_IgM
F89:MRM of 1 channel,ES+
1198.1 > 366.1
4.037e+005

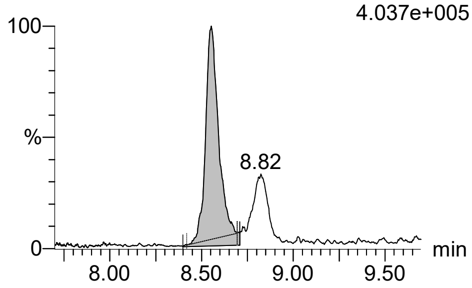

Sample Name: 20211111_L3_R3_IgM
F89:MRM of 1 channel,ES+
1198.1 > 366.1
2.993e+005

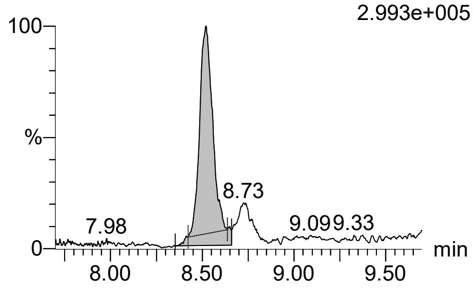

Sample Name: 20211111_L4_R3_IgM
F89:MRM of 1 channel,ES+
1198.1 > 366.1
6.443e+005

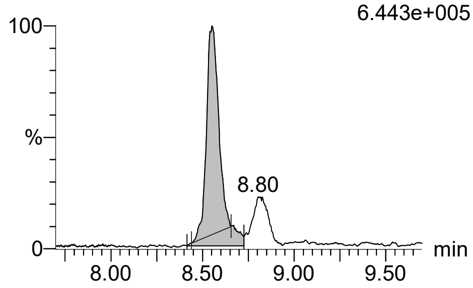

Sample Name: 20211111_H2_R3_IgM
F89:MRM of 1 channel,ES+
1198.1 > 366.1
9.161e+005

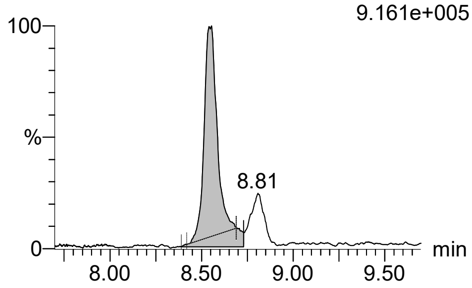

Sample Name: 20211111_A5_R3_IgM
F89:MRM of 1 channel,ES+
1198.1 > 366.1
9.695e+005

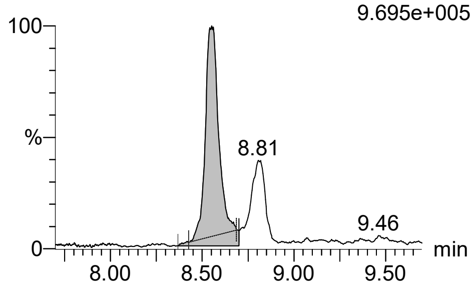

Sample Name: 20211111_A6_R3_IgM
F89:MRM of 1 channel,ES+
1198.1 > 366.1
4.159e+005

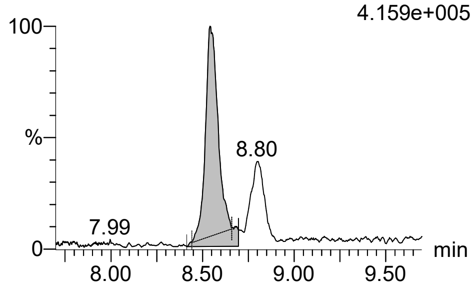

Sample Name: 20211111_QC_R18_IgM
F89:MRM of 1 channel,ES+
1198.1 > 366.1
4.247e+005

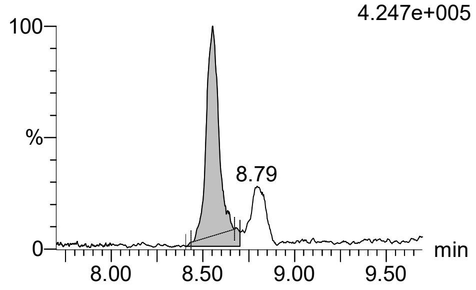

Sample Name: 20211111_L5_R3_IgM
F89:MRM of 1 channel,ES+
1198.1 > 366.1
4.310e+005

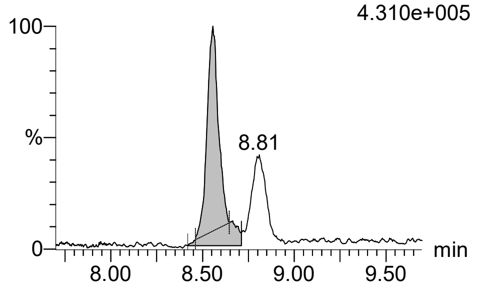

Sample Name: 20211111_L6_R3_IgM
F89:MRM of 1 channel,ES+
1198.1 > 366.1
6.656e+005

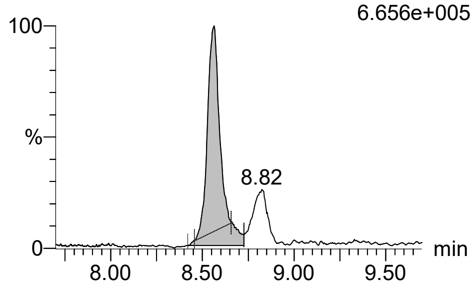

Sample Name: 20211111_H3_R3_IgM
F89:MRM of 1 channel,ES+
1198.1 > 366.1
4.018e+005

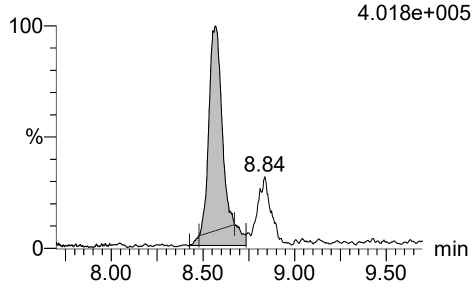

Sample Name: 20211111_A7_R3_IgM
F89:MRM of 1 channel,ES+
1198.1 > 366.1
3.728e+005

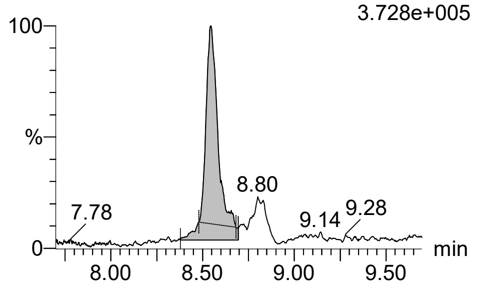

Sample Name: 20211111_A8_R3_IgM
F89:MRM of 1 channel,ES+
1198.1 > 366.1
4.657e+005

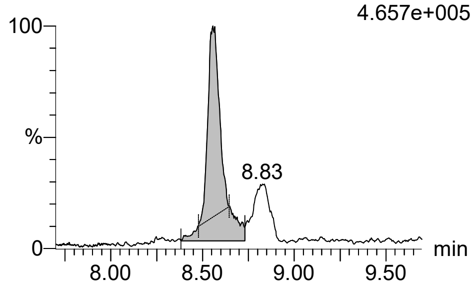

Sample Name: 20211111_L7_R3_IgM
F89:MRM of 1 channel,ES+
1198.1 > 366.1
3.520e+005

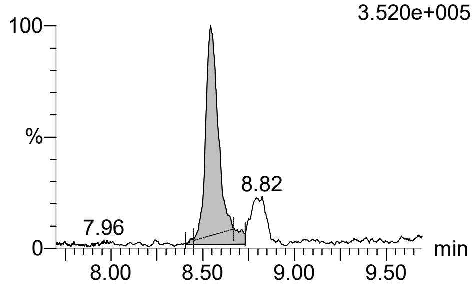

Sample Name: 20211111_L8_R3_IgM
F89:MRM of 1 channel,ES+
1198.1 > 366.1
1.381e+005

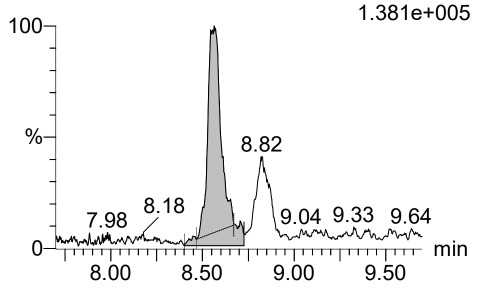

Sample Name: 20211111_H4_R3_IgM
F89:MRM of 1 channel,ES+
1198.1 > 366.1
2.754e+005

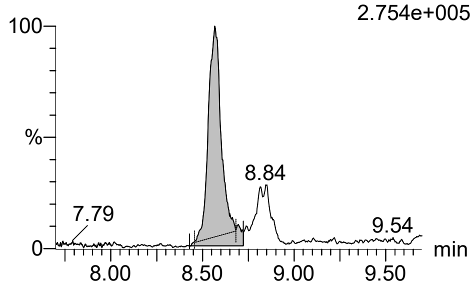

Sample Name: 20211111_A9_R3_IgM
F89:MRM of 1 channel,ES+
1198.1 > 366.1
2.680e+005

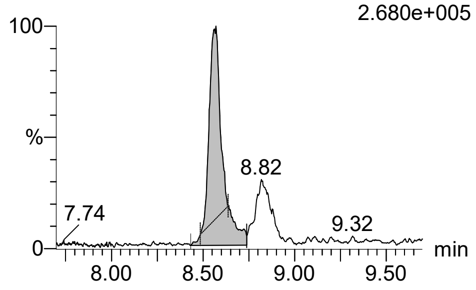

Sample Name: 20211111_A10_R3_IgM
F89:MRM of 1 channel,ES+
1198.1 > 366.1
5.342e+005

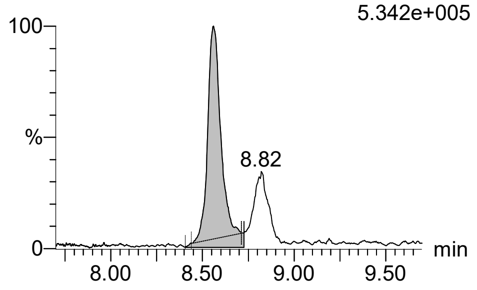

Sample Name: 20211111_L9_R3_IgM
F89:MRM of 1 channel,ES+
1198.1 > 366.1
2.423e+005

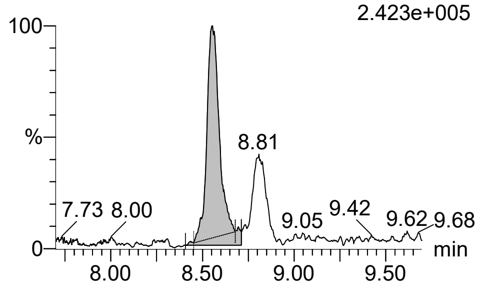

Sample Name: 20211111_L10_R3_IgM
F89:MRM of 1 channel,ES+
1198.1 > 366.1
4.830e+005

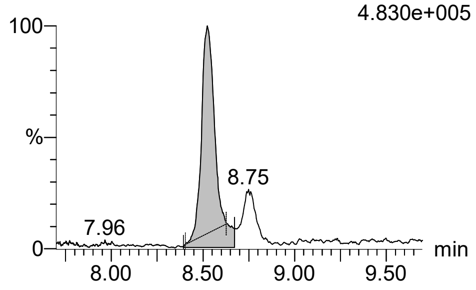

Sample Name: 20211111_QC_R19_IgM
F89:MRM of 1 channel,ES+
1198.1 > 366.1
4.228e+005

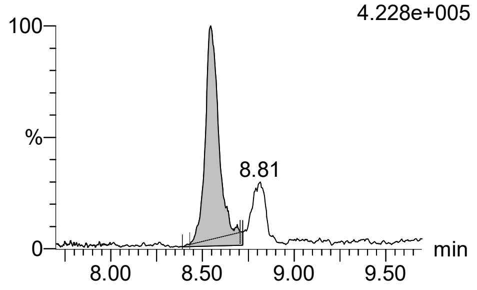

Sample Name: 20211111_H5_R3_IgM
F89:MRM of 1 channel,ES+
1198.1 > 366.1
4.745e+005

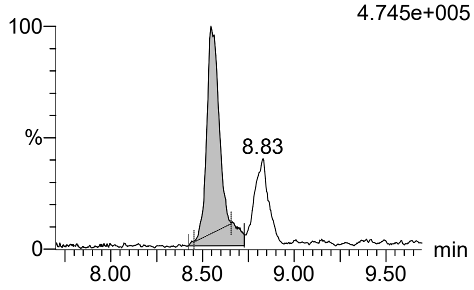

Sample Name: 20211111_A11_R3_IgM
F89:MRM of 1 channel,ES+
1198.1 > 366.1
5.664e+005

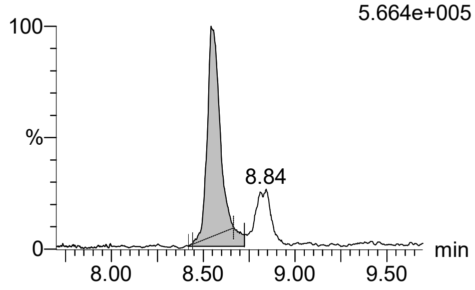

Sample Name: 20211111_A12_R3_IgM
F89:MRM of 1 channel,ES+
1198.1 > 366.1
3.330e+005

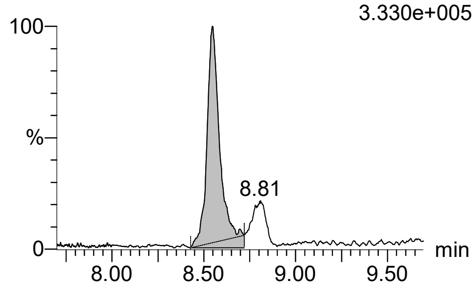

Sample Name: 20211111_L11_R3_IgM
F89:MRM of 1 channel,ES+
1198.1 > 366.1
2.980e+005

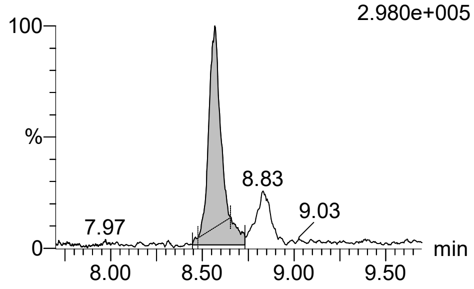

Sample Name: 20211111_L12_R3_IgM
F89:MRM of 1 channel,ES+
1198.1 > 366.1
2.661e+005

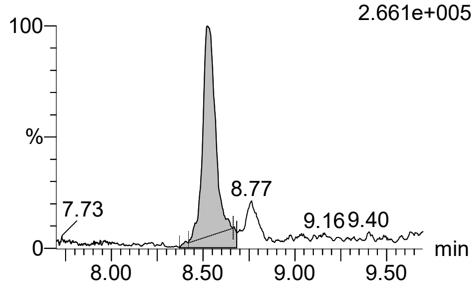

Sample Name: 20211111_H6_R3_IgM
F89:MRM of 1 channel,ES+
1198.1 > 366.1
3.426e+005

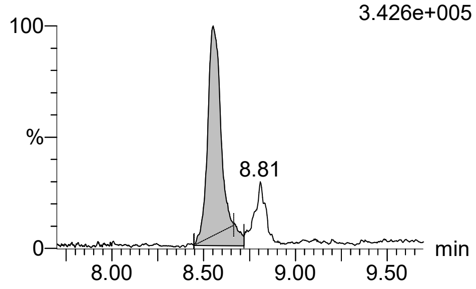

Sample Name: 20211111_A13_R3_IgM
F89:MRM of 1 channel,ES+
1198.1 > 366.1
5.036e+005

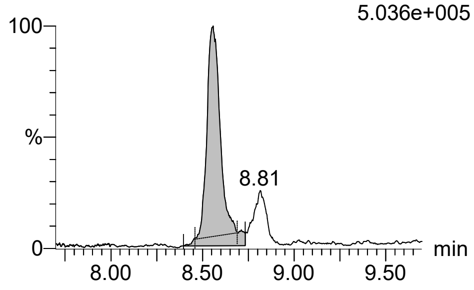

Sample Name: 20211111_A14_R3_IgM
F89:MRM of 1 channel,ES+
1198.1 > 366.1
9.069e+005

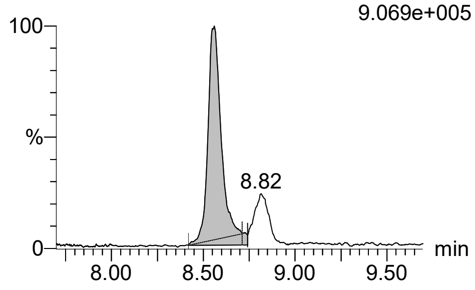

Sample Name: 20211111_L13_R3_IgM
F89:MRM of 1 channel,ES+
1198.1 > 366.1
2.465e+005

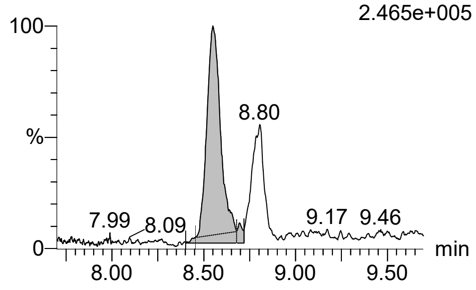

Sample Name: 20211111_L14_R3_IgM
F89:MRM of 1 channel,ES+
1198.1 > 366.1
5.720e+005

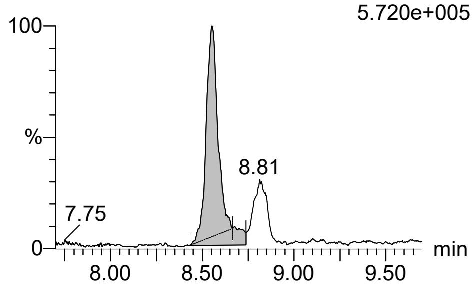

Sample Name: 20211111_H7_R3_IgM
F89:MRM of 1 channel,ES+
1198.1 > 366.1
5.119e+005

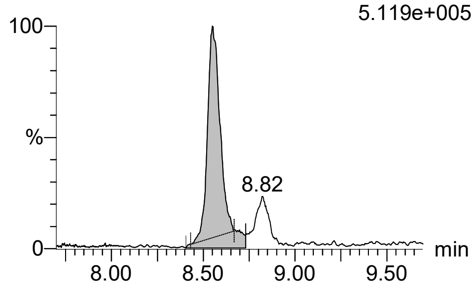

Sample Name: 20211111_A15_R3_IgM
F89:MRM of 1 channel,ES+
1198.1 > 366.1
1.585e+006

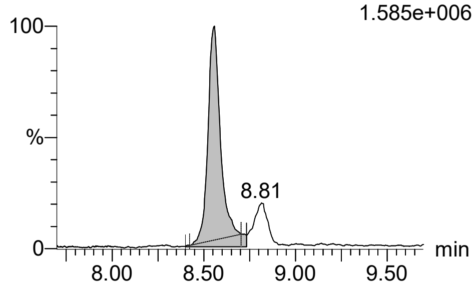

Sample Name: 20211111_QC_R20_IgM
F89:MRM of 1 channel,ES+
1198.1 > 366.1
4.572e+005

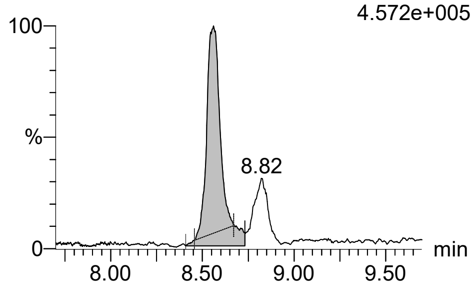

Sample Name: 20211111_A16_R3_IgM
F89:MRM of 1 channel,ES+
1198.1 > 366.1
5.426e+005

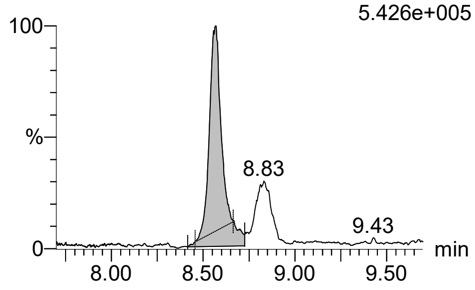

Sample Name: 20211111_L15_R3_IgM
F89:MRM of 1 channel,ES+
1198.1 > 366.1
7.470e+005

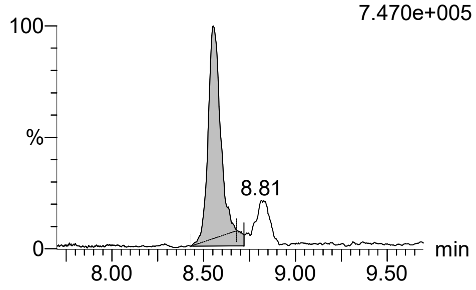

Sample Name: 20211111_L16_R3_IgM

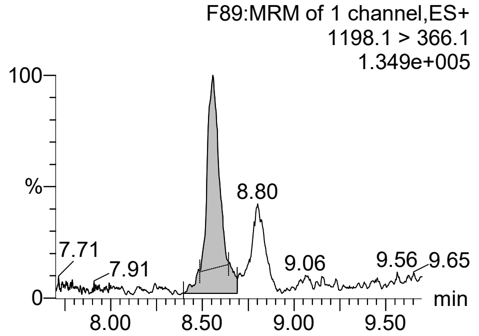

Sample Name: 20211111_H8_R3_IgM

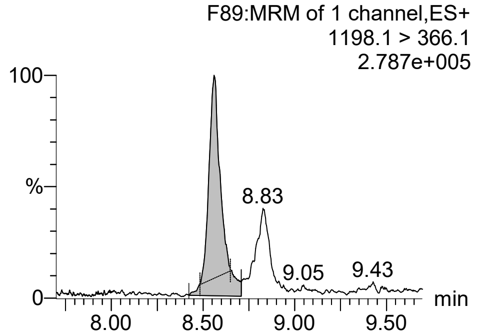

Sample Name: 20211111_A17_R3_IgM

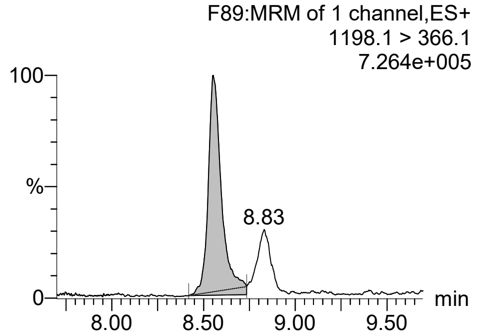

Sample Name: 20211111_A18_R3_IgM

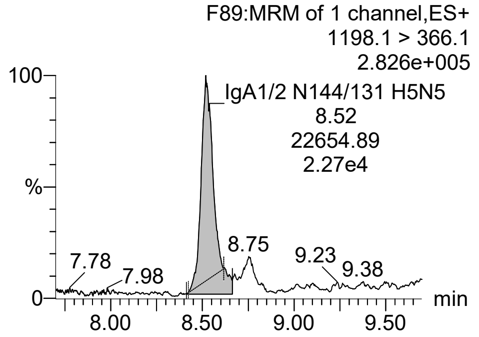

Sample Name: 20211111_L17_R3_IgM

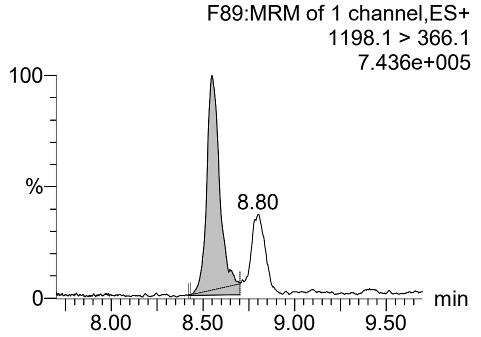

Sample Name: 20211111_L18_R3_IgM

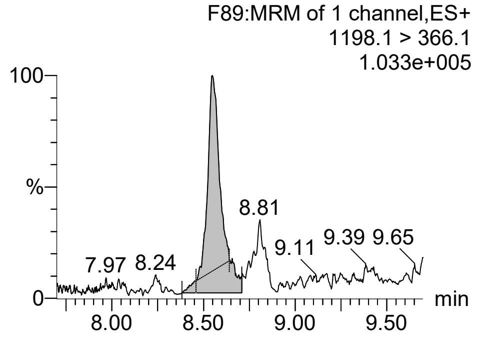

Sample Name: 20211111_H9_R3_IgM

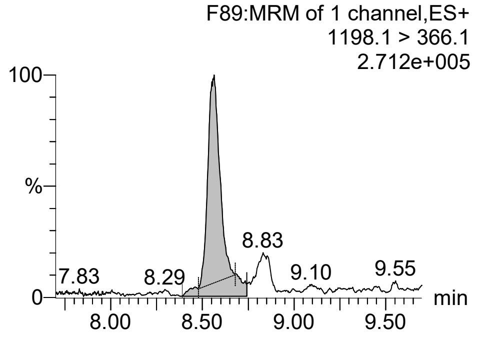

Sample Name: 20211111_A19_R3_IgM

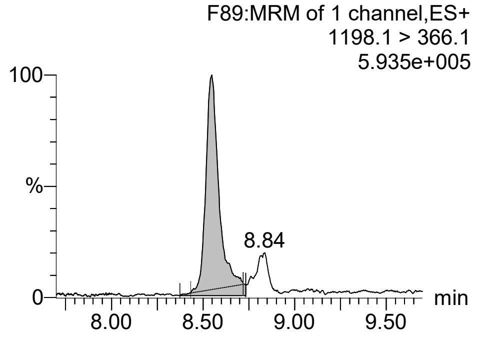

Sample Name: 20211111_A20_R3_IgM

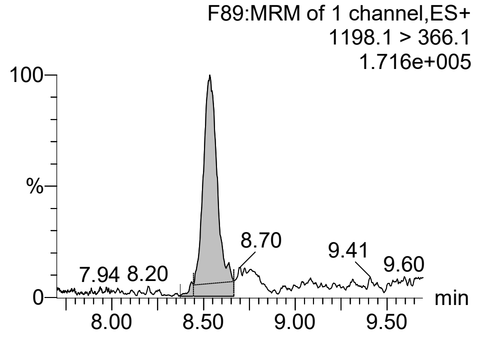

Sample Name: 20211111_L19_R3_IgM

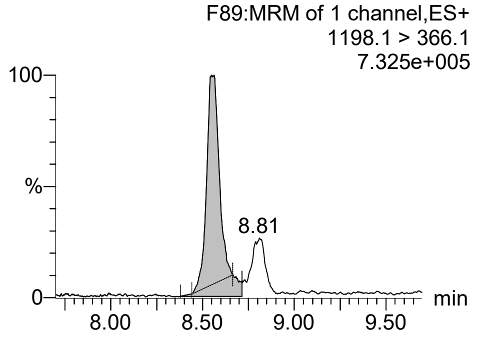

Sample Name: 20211111_QC_R21_IgM

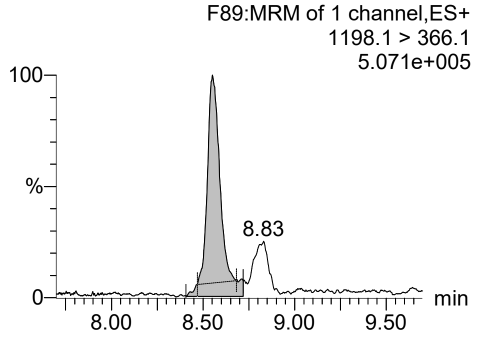

Sample Name: 20211111_L20_R3_IgM

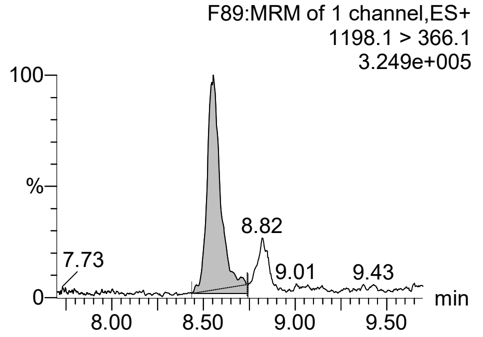

Sample Name: 20211111_H10_R3_IgM

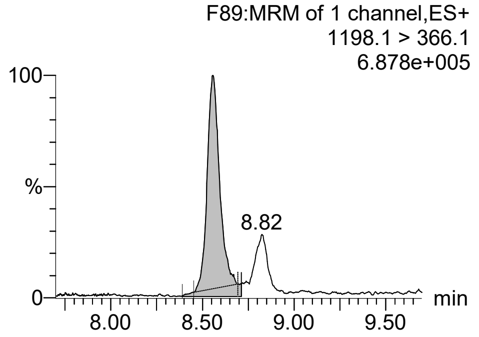

Sample Name: 20211111_A21_R3_IgM

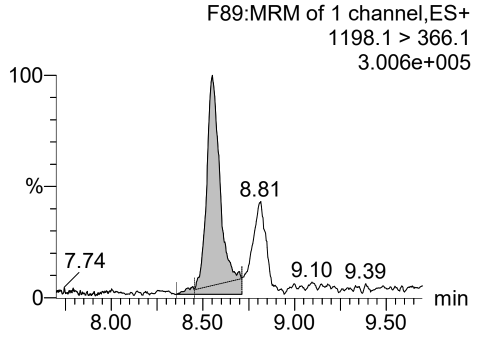

Sample Name: 20211111_A22_R3_IgM

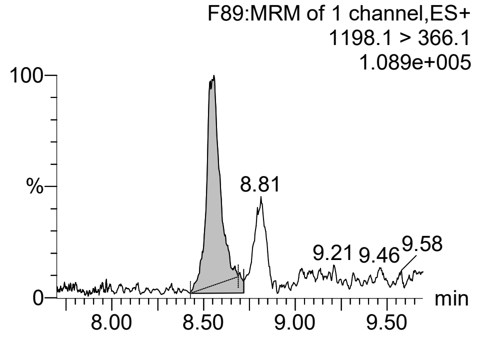

Dataset: C:\MassLynx\10.pro\20211111_clinicalsamples_IgM_IgA.qld

Last Altered: Thursday, March 09, 2023 19:58:25 台北標準時間
Printed: Friday, August 01, 2025 15:11:07 台北標準時間

Sample Name: 20211111_L21_R3_IgM
F89:MRM of 1 channel,ES+
1198.1 > 366.1
4.498e+005

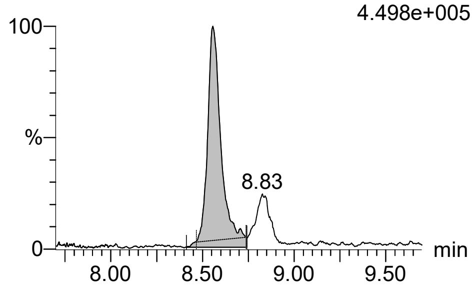

Sample Name: 20211111_L22_R3_IgM
F89:MRM of 1 channel,ES+
1198.1 > 366.1
2.704e+005

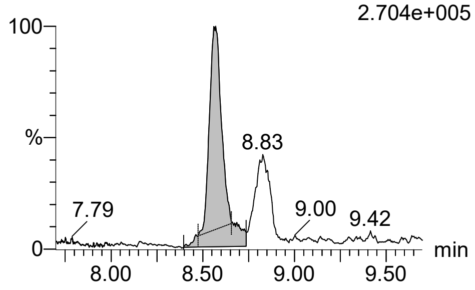

Sample Name: 20211111_H11_R3_IgM
F89:MRM of 1 channel,ES+
1198.1 > 366.1
4.648e+005

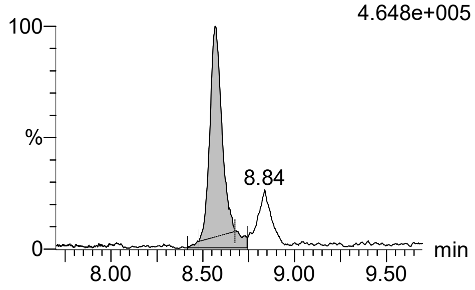

Sample Name: 20211111_A23_R3_IgM
F89:MRM of 1 channel,ES+
1198.1 > 366.1
3.012e+005

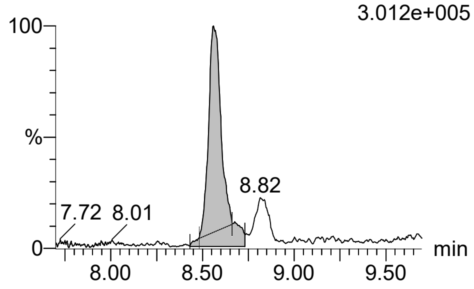

Sample Name: 20211111_A24_R3_IgM
F89:MRM of 1 channel,ES+
1198.1 > 366.1
9.719e+005

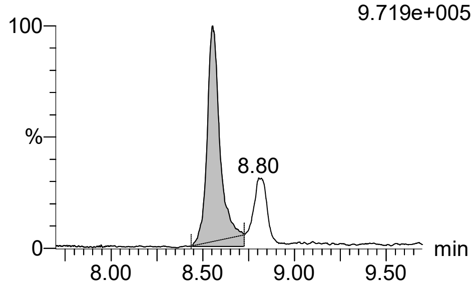

Sample Name: 20211111_L23_R3_IgM
F89:MRM of 1 channel,ES+
1198.1 > 366.1
9.272e+005

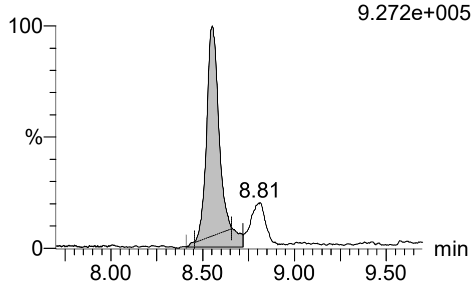

Sample Name: 20211111_L24_R3_IgM
F89:MRM of 1 channel,ES+
1198.1 > 366.1
1.375e+005

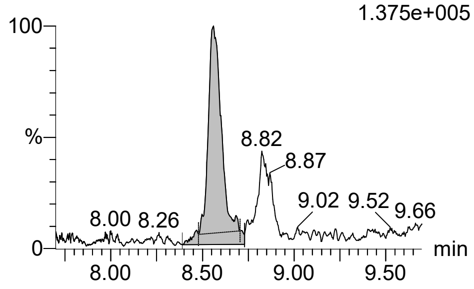

Sample Name: 20211111_H12_R3_IgM
F89:MRM of 1 channel,ES+
1198.1 > 366.1
2.077e+005

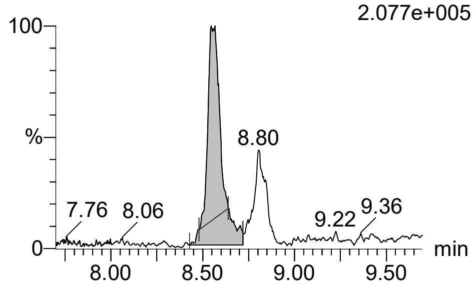

Sample Name: 20211111_QC_R22_IgM
F89:MRM of 1 channel,ES+
1198.1 > 366.1
4.988e+005

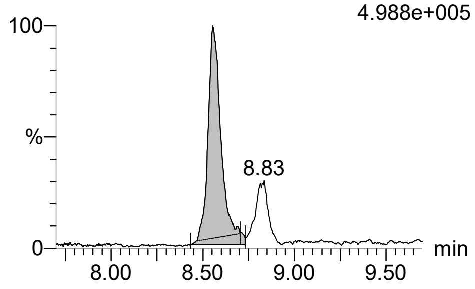

Sample Name: 20211111_A25_R3_IgM
F89:MRM of 1 channel,ES+
1198.1 > 366.1
6.665e+005

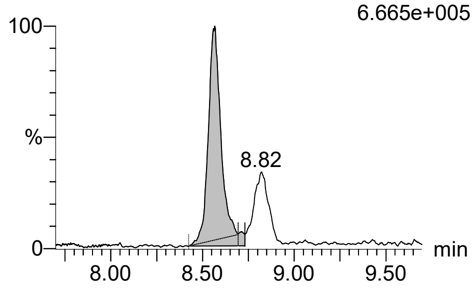

Sample Name: 20211111_A26_R3_IgM
F89:MRM of 1 channel,ES+
1198.1 > 366.1
4.058e+005

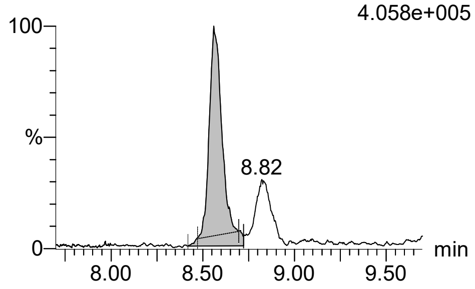

Sample Name: 20211111_L25_R3_IgM
F89:MRM of 1 channel,ES+
1198.1 > 366.1
1.996e+005

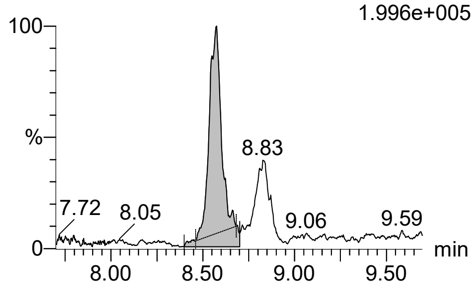

Sample Name: 20211111_L26_R3_IgM
F89:MRM of 1 channel,ES+
1198.1 > 366.1
3.588e+005

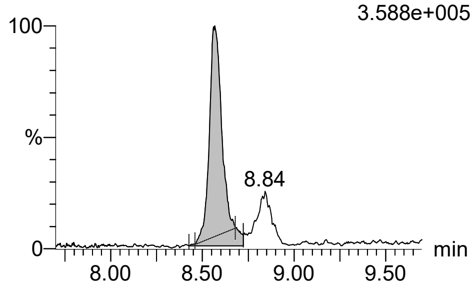

Sample Name: 20211111_H13_R3_IgM
F89:MRM of 1 channel,ES+
1198.1 > 366.1
6.626e+005

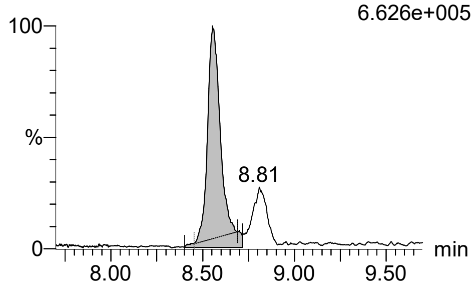

Sample Name: 20211111_A27_R3_IgM
F89:MRM of 1 channel,ES+
1198.1 > 366.1
5.144e+005

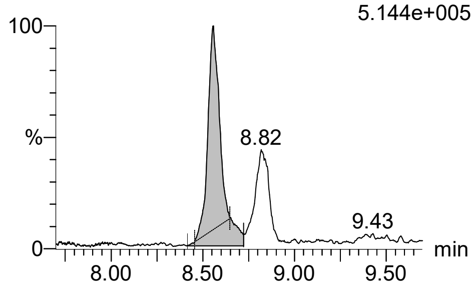

Dataset: C:\MassLynx\10.pro\20211111_clinicalsamples_IgM_IgA.qld

Last Altered: Thursday, March 09, 2023 19:58:25 台北標準時間
Printed: Friday, August 01, 2025 15:11:07 台北標準時間

Sample Name: 20211111_A28_R3_IgM

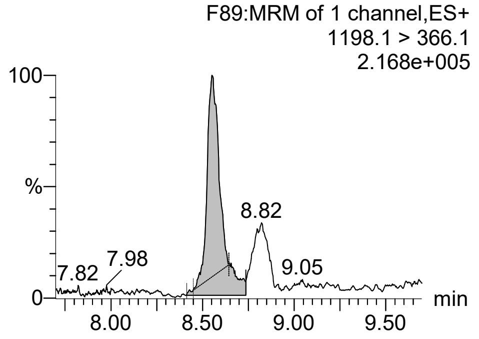

Sample Name: 20211111_L27_R3_IgM

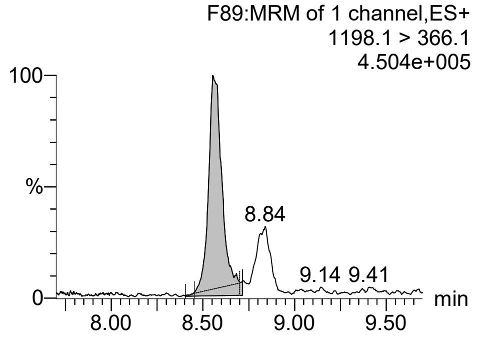

Sample Name: 20211111_L28_R3_IgM

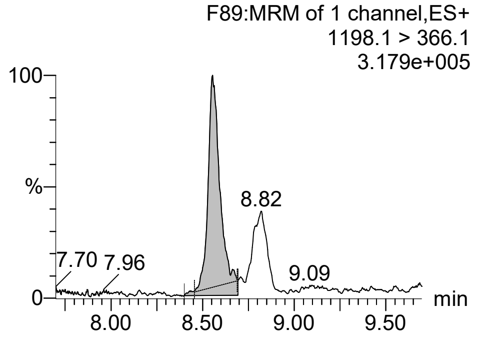

Sample Name: 20211111_H14_R3_IgM

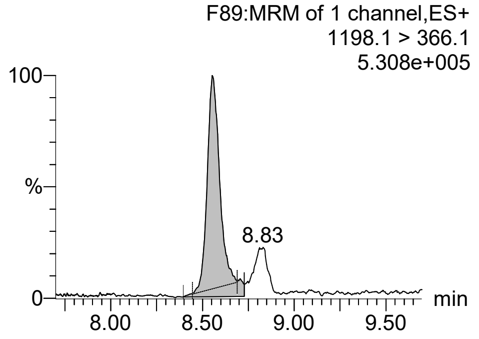

Sample Name: 20211111_A29_R3_IgM

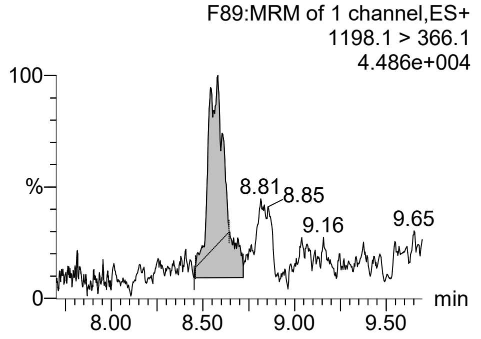

Sample Name: 20211111_A30_R3_IgM

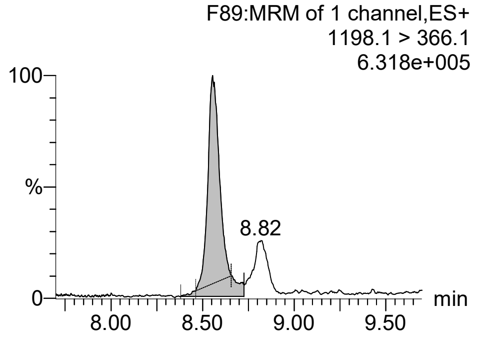

Sample Name: 20211111_QC_R23_IgM

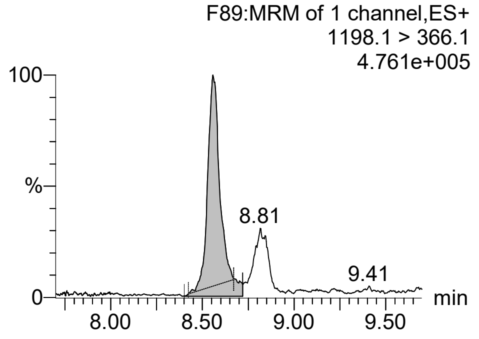

Sample Name: 20211111_L29_R3_IgM

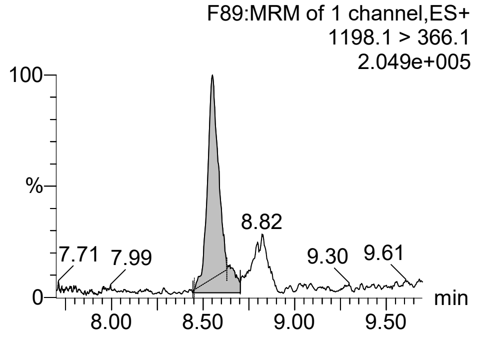

Sample Name: 20211111_L30_R3_IgM

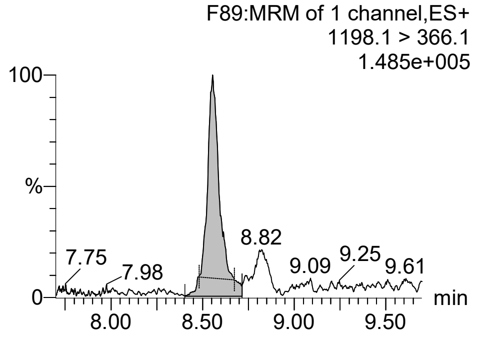

Sample Name: 20211111_H15_R3_IgM

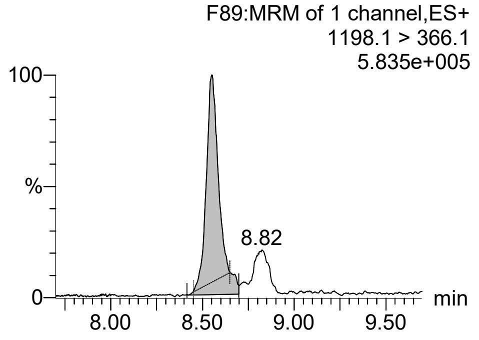

Sample Name: 20211111_A31_R3_IgM

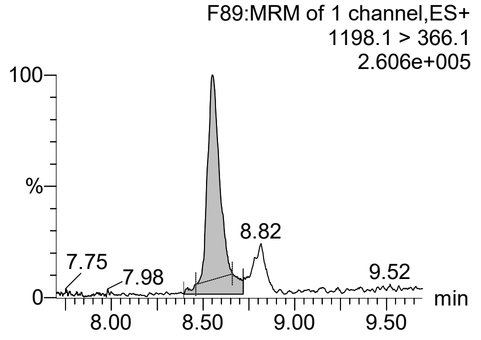

Sample Name: 20211111_A32_R3_IgM

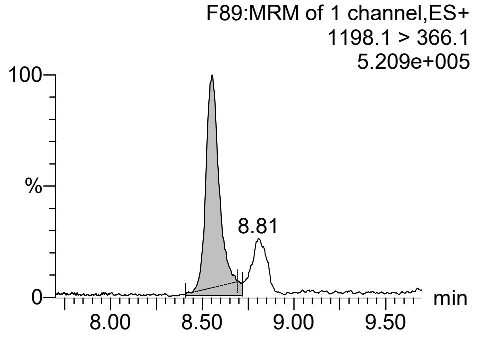

Sample Name: 20211111_L31_R3_IgM

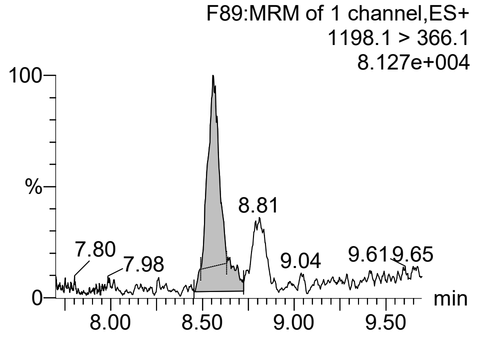

Sample Name: 20211111_L32_R3_IgM

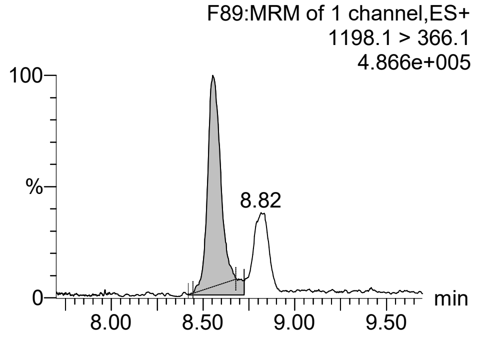

Sample Name: 20211111_H16_R3_IgM

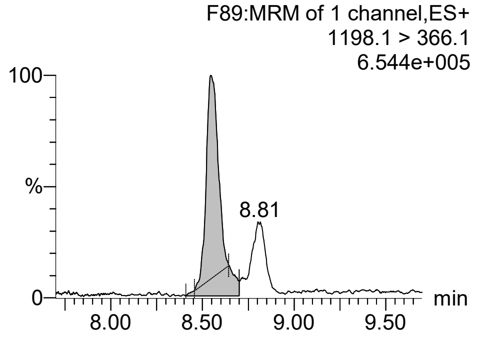

Sample Name: 20211111_A33_R3_IgM
F89:MRM of 1 channel,ES+
1198.1 > 366.1
1.007e+006

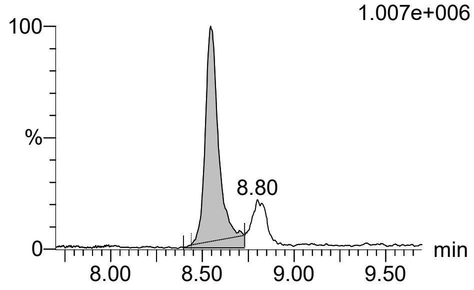

Sample Name: 20211111_A34_R3_IgM
F89:MRM of 1 channel,ES+
1198.1 > 366.1
1.976e+005

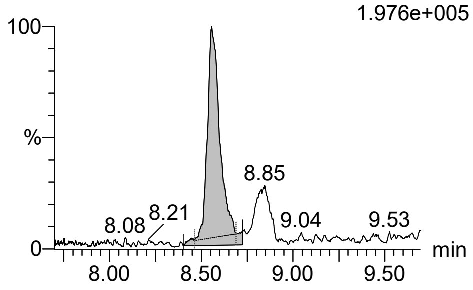

Sample Name: 20211111_H17_R3_IgM
F89:MRM of 1 channel,ES+
1198.1 > 366.1
2.570e+006

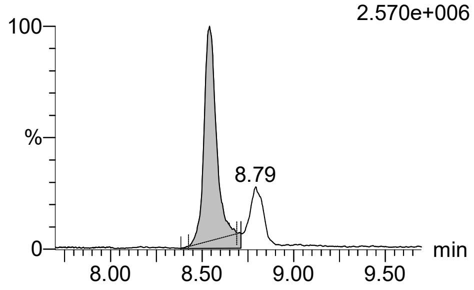

Sample Name: 20211111_A35_R3_IgM
F89:MRM of 1 channel,ES+
1198.1 > 366.1
3.469e+005

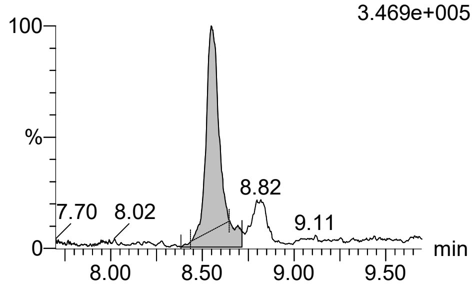

Sample Name: 20211111_QC_R24_IgM
F89:MRM of 1 channel,ES+
1198.1 > 366.1
4.312e+005

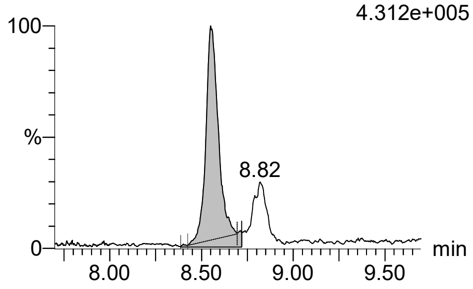

Sample Name: 20211111_A36_R3_IgM
F89:MRM of 1 channel,ES+
1198.1 > 366.1
2.495e+005

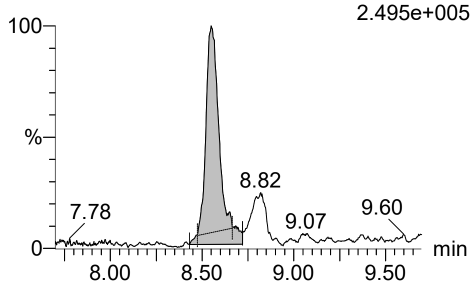

Sample Name: 20211111_H18_R3_IgM
F89:MRM of 1 channel,ES+
1198.1 > 366.1
9.499e+005

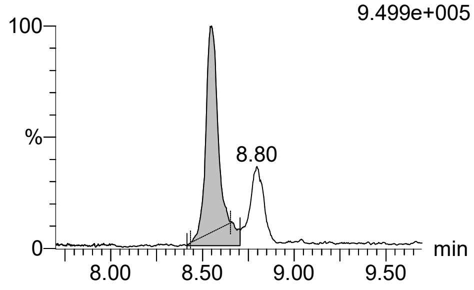

Sample Name: 20211111_A37_R3_IgM
F89:MRM of 1 channel,ES+
1198.1 > 366.1
6.437e+005

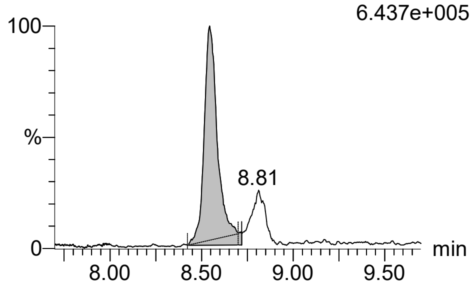

Sample Name: 20211111_A38_R3_IgM
F89:MRM of 1 channel,ES+
1198.1 > 366.1
3.672e+005

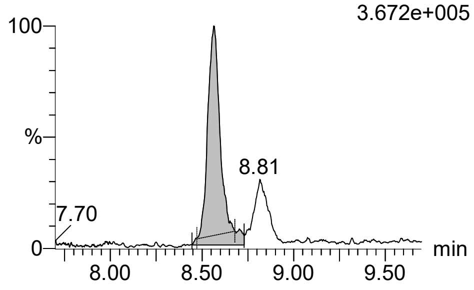

Sample Name: 20211111_H19_R3_IgM
F89:MRM of 1 channel,ES+
1198.1 > 366.1
6.983e+005

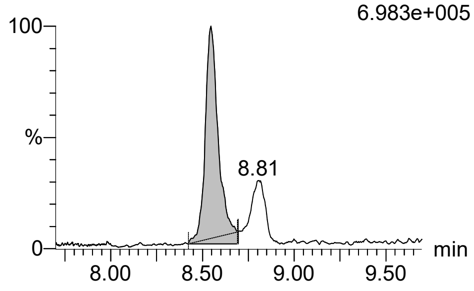

Sample Name: 20211111_A39_R3_IgM
F89:MRM of 1 channel,ES+
1198.1 > 366.1
5.071e+005

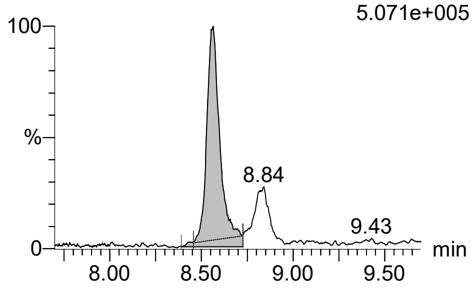

Sample Name: 20211111_A40_R3_IgM
F89:MRM of 1 channel,ES+
1198.1 > 366.1
2.651e+005

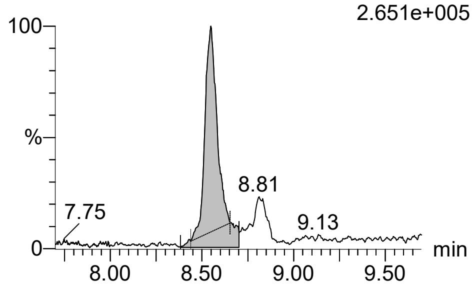

Sample Name: 20211111_H20_R3_IgM
F89:MRM of 1 channel,ES+
1198.1 > 366.1
2.128e+006

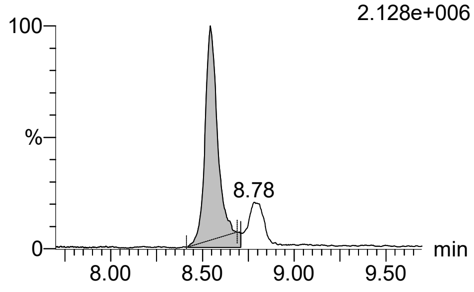

Sample Name: 20211111_A41_R3_IgM
F89:MRM of 1 channel,ES+
1198.1 > 366.1
2.392e+005

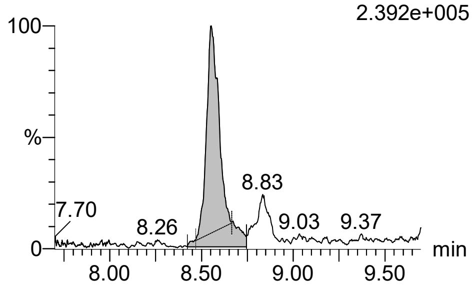

Sample Name: 20211111_A42_R3_IgM
F89:MRM of 1 channel,ES+
1198.1 > 366.1
1.697e+005

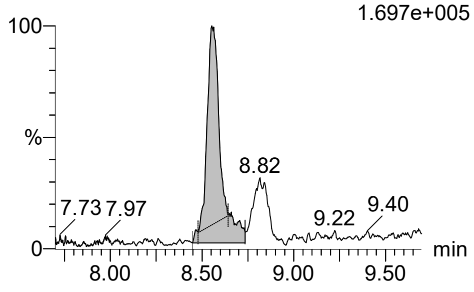

Dataset: C:\MassLynx\10.pro\20211111_clinicalsamples_IgM_IgA.qld

Last Altered: Thursday, March 09, 2023 19:58:25 台北標準時間
Printed: Friday, August 01, 2025 15:11:07 台北標準時間

Sample Name: 20211111_A43_R3_IgM

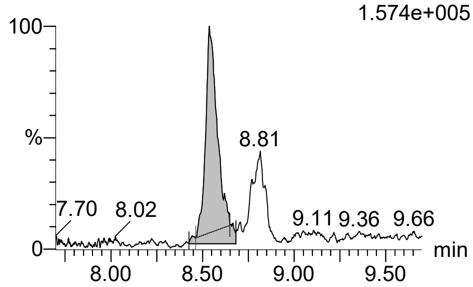

Sample Name: 20211111_A44_R3_IgM

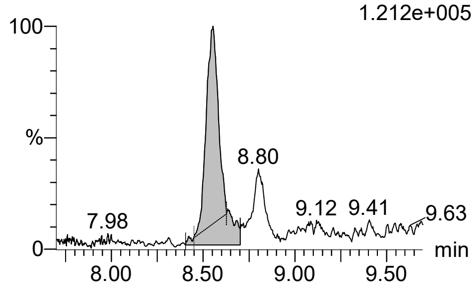

Sample Name: 20211111_QC_R25_IgM

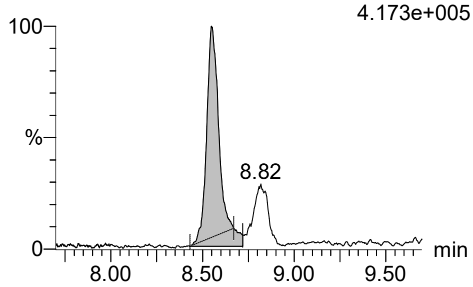

Dataset: C:\MassLynx\10.pro\20211111_clinicalsamples_IgM_IgA.qld

Last Altered: Thursday, March 09, 2023 19:58:25 台北標準時間
Printed: Friday, August 01, 2025 15:11:07 台北標準時間

	#	Name	RT	Area	IS Area	Response	Prima...
1	1	20211112_QC_R001_IgM	8.54	32046.0	15421.4	2.078	bb
2	2	20211112_QC_R002_IgM	8.54	36154.1	13188.8	2.741	MM
3	3	20211111_A1_R2_IgM	8.55	20577.1	11871.0	1.733	MM
4	4	20211111_A1_R3_IgM	8.56	21464.0	12030.5	1.784	MM
5	5	20211111_A2_R2_IgM	8.55	118213.9	11446.8	10.327	MM
6	6	20211111_A2_R3_IgM	8.55	116551.1	11098.4	10.502	MM
7	7	20211111_L1_R2_IgM	8.56	12591.6	11779.4	1.069	MM
8	8	20211111_L1_R3_IgM	8.56	10947.3	11176.2	0.980	MM
9	9	20211111_L2_R2_IgM	8.55	68865.4	11375.0	6.054	MM
10	10	20211111_H1_R2_IgM	8.54	45255.0	13595.2	3.329	MM
11	11	20211111_A3_R2_IgM	8.55	51473.9	14112.3	3.647	MM
12	12	20211111_A4_R2_IgM	8.54	35658.4	11977.5	2.977	MM
13	13	20211111_L3_R2_IgM	8.51	32555.1	12242.1	2.659	MM
14	14	20211111_L4_R2_IgM	8.55	61613.6	12797.0	4.815	MM
15	15	20211111_H2_R2_IgM	8.55	77488.1	10006.0	7.744	MM
16	16	20211111_A5_R2_IgM	8.54	93865.6	11434.6	8.209	MM
17	17	20211111_A6_R2_IgM	8.54	40010.5	13193.7	3.033	MM
18	18	20211111_QC_R10_IgM	8.54	41261.8	12566.1	3.284	MM
19	19	20211111_L5_R2_IgM	8.53	43221.8	13616.4	3.174	MM
20	20	20211111_L6_R2_IgM	8.55	63180.9	13698.5	4.612	MM
21	21	20211111_H3_R2_IgM	8.55	40924.3	15793.1	2.591	MM
22	22	20211111_A7_R2_IgM	8.54	29203.1	12936.9	2.257	MM
23	23	20211111_A8_R2_IgM	8.55	49383.5	13108.0	3.767	MM
24	24	20211111_L7_R2_IgM	8.54	30289.8	13278.5	2.281	MM
25	25	20211111_L8_R2_IgM	8.56	13815.6	15233.6	0.907	MM
26	26	20211111_H4_R2_IgM	8.54	29060.5	16521.9	1.759	MM
27	27	20211111_A9_R2_IgM	8.52	26628.1	16451.6	1.619	MM
28	28	20211111_A10_R2_IgM	8.54	49870.4	13036.0	3.826	MM
29	29	20211111_L9_R2_IgM	8.54	22130.0	14554.1	1.521	MM
30	30	20211111_L10_R2_IgM	8.54	50373.8	12250.4	4.112	MM
31	31	20211111_QC_R11_IgM	8.55	49311.5	11459.2	4.303	MM
32	32	20211111_H5_R2_IgM	8.55	52442.6	14358.2	3.652	MM
33	33	20211111_A11_R2_IgM	8.48	65902.7	16184.1	4.072	MM
34	34	20211111_A12_R2_IgM	8.54	26938.7	13465.4	2.001	MM
35	35	20211111_L11_R2_IgM	8.56	30888.0	13277.9	2.326	MM
36	36	20211111_L12_R2_IgM	8.54	24474.6	15958.8	1.534	MM
37	37	20211111_H6_R2_IgM	8.55	29750.6	14003.5	2.125	MM
38	38	20211111_A13_R2_IgM	8.55	46143.8	13656.1	3.379	MM
39	39	20211111_A14_R2_IgM	8.55	95020.9	14071.3	6.753	MM
40	40	20211111_L13_R2_IgM	8.56	23562.0	14785.6	1.594	MM
41	41	20211111_L14_R2_IgM	8.55	53931.3	14772.2	3.651	MM
42	42	20211111_H7_R2_IgM	8.53	49945.7	15164.4	3.294	MM
43	43	20211111_A15_R2_IgM	8.54	144937.3	15721.5	9.219	MM
44	44	20211111_QC_R12_IgM	8.56	49647.0	13711.5	3.621	MM
45	45	20211111_A16_R2_IgM	8.56	52901.4	14275.6	3.706	MM
46	46	20211111_L15_R2_IgM	8.54	71394.7	12935.8	5.519	MM
47	47	20211111_L16_R2_IgM	8.56	12052.0	13342.8	0.903	MM
48	48	20211111_H8_R2_IgM	8.56	22938.9	15835.1	1.449	MM
49	49	20211111_A17_R2_IgM	8.55	68545.3	15786.7	4.342	MM
50	50	20211111_A18_R2_IgM	8.52	25940.6	16986.8	1.527	MM
51	51	20211111_L17_R2_IgM	8.54	70660.6	13342.7	5.296	MM
52	52	20211111_L18_R2_IgM	8.56	9716.5	14692.0	0.661	MM
53	53	20211111_H9_R2_IgM	8.54	24123.4	15639.7	1.542	MM
54	54	20211111_A19_R2_IgM	8.53	52646.1	14198.7	3.708	MM
55	55	20211111_A20_R2_IgM	8.53	18071.9	14162.7	1.276	MM
56	56	20211111_L19_R2_IgM	8.54	57742.7	9293.4	6.213	MM

Dataset: C:\MassLynx\10.pro\20211111_clinicalsamples_IgM_IgA.qld

Last Altered: Thursday, March 09, 2023 19:58:25 台北標準時間
Printed: Friday, August 01, 2025 15:11:07 台北標準時間

	# Name	RT	Area	IS Area	Response	Prima...
57	57 20211111_QC_R13_IgM	8.55	48393.8	13903.0	3.481	MM
58	58 20211111_L20_R2_IgM	8.50	31632.9	12781.6	2.475	MM
59	59 20211111_H10_R2_IgM	8.53	63191.5	14796.4	4.271	MM
60	60 20211111_A21_R2_IgM	8.54	29137.3	13669.0	2.132	MM
61	61 20211111_A22_R2_IgM	8.56	9932.5	14098.3	0.705	MM
62	62 20211111_L21_R2_IgM	8.55	42232.9	15678.2	2.694	MM
63	63 20211111_L22_R2_IgM	8.55	25528.7	15724.5	1.623	MM
64	64 20211111_H11_R2_IgM	8.56	40108.0	15890.2	2.524	MM
65	65 20211111_A23_R2_IgM	8.55	28081.5	13423.2	2.092	MM
66	66 20211111_A24_R2_IgM	8.55	78105.7	12438.0	6.280	MM
67	67 20211111_L23_R2_IgM	8.55	87881.9	14075.0	6.244	MM
68	68 20211111_L24_R2_IgM	8.55	13514.3	15972.1	0.846	MM
69	69 20211111_H12_R2_IgM	8.56	17120.4	16270.9	1.052	MM
70	70 20211111_QC_R14_IgM	8.56	48168.1	15806.8	3.047	MM
71	71 20211111_A25_R2_IgM	8.56	68160.1	14552.1	4.684	MM
72	72 20211111_A26_R2_IgM	8.56	36875.7	11492.7	3.209	MM
73	73 20211111_L25_R2_IgM	8.55	18687.8	14403.3	1.297	MM
74	74 20211111_L26_R2_IgM	8.57	35424.9	16533.9	2.143	MM
75	75 20211111_H13_R2_IgM	8.56	55793.6	15520.1	3.595	MM
76	76 20211111_A27_R2_IgM	8.55	44869.4	13337.4	3.364	MM
77	77 20211111_A28_R2_IgM	8.51	21283.0	11895.6	1.789	MM
78	78 20211111_L27_R2_IgM	8.55	39270.9	14125.7	2.780	MM
79	79 20211111_L28_R2_IgM	8.55	22954.0	11807.5	1.944	MM
80	80 20211111_H14_R2_IgM	8.55	45060.3	15035.7	2.997	MM
81	81 20211111_A29_R2_IgM	8.55	5556.6	13554.4	0.410	MM
82	82 20211111_A30_R2_IgM	8.55	55221.6	12984.1	4.253	MM
83	83 20211111_QC_R15_IgM	8.56	45193.5	12299.5	3.674	MM
84	84 20211111_L29_R2_IgM	8.56	15431.7	13590.7	1.135	MM
85	85 20211111_L30_R2_IgM	8.55	13210.2	12511.4	1.056	MM
86	86 20211111_H15_R2_IgM	8.54	53646.3	11885.0	4.514	MM
87	87 20211111_A31_R2_IgM	8.55	22544.7	13422.1	1.680	MM
88	88 20211111_A32_R2_IgM	8.55	49420.5	11461.3	4.312	MM
89	89 20211111_L31_R2_IgM	8.51	7635.2	13753.4	0.555	MM
90	90 20211111_L32_R2_IgM	8.54	47397.2	12949.0	3.660	MM
91	91 20211111_H16_R2_IgM	8.54	68836.1	11465.4	6.004	MM
92	92 20211111_A33_R2_IgM	8.54	89062.6	10551.5	8.441	MM
93	93 20211111_A34_R2_IgM	8.57	18096.2	12409.9	1.458	MM
94	94 20211111_H17_R2_IgM	8.54	246157.5	10160.9	24.226	MM
95	95 20211111_A35_R2_IgM	8.53	32258.5	10460.6	3.084	MM
96	96 20211111_QC_R16_IgM	8.55	41891.4	10433.5	4.015	MM
97	97 20211111_A36_R2_IgM	8.49	24754.7	12504.8	1.980	MM
98	98 20211111_H18_R2_IgM	8.55	86616.3	9140.4	9.476	MM
99	99 20211111_A37_R2_IgM	8.55	66827.0	9796.9	6.821	MM
100	1... 20211111_A38_R2_IgM	8.56	36435.8	11224.3	3.246	MM
101	1... 20211111_H19_R2_IgM	8.55	68308.0	11739.2	5.819	MM
102	1... 20211111_A39_R2_IgM	8.55	48579.9	12718.9	3.819	MM
103	1... 20211111_A40_R2_IgM	8.51	26771.1	10006.1	2.675	MM
104	1... 20211111_H20_R2_IgM	8.54	207866.4	7988.2	26.022	MM
105	1... 20211111_A41_R2_IgM	8.56	32361.4	11513.3	2.811	MM
106	1... 20211111_A42_R2_IgM	8.55	14318.6	10238.5	1.399	MM
107	1... 20211111_A43_R2_IgM	8.54	14077.2	11098.9	1.268	MM
108	1... 20211111_A44_R2_IgM	8.52	12214.9	10969.9	1.113	MM
109	1... 20211111_QC_R17_IgM	8.56	35287.3	9700.8	3.638	MM
110	1... 20211111_L2_R3_IgM	8.55	76370.7	8528.0	8.955	MM
111	1... 20211111_H1_R3_IgM	8.55	47260.7	9438.2	5.007	MM
112	1... 20211111_A3_R3_IgM	8.56	47601.6	11598.6	4.104	MM

Dataset: C:\MassLynx\10.pro\20211111_clinicalsamples_IgM_IgA.qld

Last Altered: Thursday, March 09, 2023 19:58:25 台北標準時間
Printed: Friday, August 01, 2025 15:11:07 台北標準時間

	#	Name	RT	Area	IS Area	Response	Prima...
113	1...	20211111_A4_R3_IgM	8.55	34669.4	11364.1	3.051	MM
114	1...	20211111_L3_R3_IgM	8.52	26863.7	10753.4	2.498	MM
115	1...	20211111_L4_R3_IgM	8.55	56271.3	10291.6	5.468	MM
116	1...	20211111_H2_R3_IgM	8.55	84790.7	9315.8	9.102	MM
117	1...	20211111_A5_R3_IgM	8.55	88341.1	9102.2	9.705	MM
118	1...	20211111_A6_R3_IgM	8.54	36432.1	10356.4	3.518	MM
119	1...	20211111_QC_R18_IgM	8.55	36720.2	8398.3	4.372	MM
120	1...	20211111_L5_R3_IgM	8.56	36037.2	9786.7	3.682	MM
121	1...	20211111_L6_R3_IgM	8.56	56870.4	9911.3	5.738	MM
122	1...	20211111_H3_R3_IgM	8.57	37010.5	12155.5	3.045	MM
123	1...	20211111_A7_R3_IgM	8.55	30101.9	11804.8	2.550	MM
124	1...	20211111_A8_R3_IgM	8.56	42307.7	10510.6	4.025	MM
125	1...	20211111_L7_R3_IgM	8.54	30263.7	12177.9	2.485	MM
126	1...	20211111_L8_R3_IgM	8.56	13071.0	11668.6	1.120	MM
127	1...	20211111_H4_R3_IgM	8.57	24091.3	11192.0	2.153	MM
128	1...	20211111_A9_R3_IgM	8.57	22644.0	14077.4	1.609	MM
129	1...	20211111_A10_R3_IgM	8.56	47790.0	11190.4	4.271	MM
130	1...	20211111_L9_R3_IgM	8.55	20500.9	14649.3	1.399	MM
131	1...	20211111_L10_R3_IgM	8.52	43501.0	9361.3	4.647	MM
132	1...	20211111_QC_R19_IgM	8.54	37819.5	10125.5	3.735	MM
133	1...	20211111_H5_R3_IgM	8.55	41633.0	10010.9	4.159	MM
134	1...	20211111_A11_R3_IgM	8.54	49430.2	12697.2	3.893	MM
135	1...	20211111_A12_R3_IgM	8.55	27584.0	13148.5	2.098	MM
136	1...	20211111_L11_R3_IgM	8.57	24563.2	10786.6	2.277	MM
137	1...	20211111_L12_R3_IgM	8.52	24123.6	11681.8	2.065	MM
138	1...	20211111_H6_R3_IgM	8.55	30358.2	12095.5	2.510	MM
139	1...	20211111_A13_R3_IgM	8.56	45647.6	12908.3	3.536	MM
140	1...	20211111_A14_R3_IgM	8.56	78945.8	11322.6	6.972	MM
141	1...	20211111_L13_R3_IgM	8.55	22510.0	11516.1	1.955	MM
142	1...	20211111_L14_R3_IgM	8.55	47590.8	10790.7	4.410	MM
143	1...	20211111_H7_R3_IgM	8.55	45861.5	11572.1	3.963	MM
144	1...	20211111_A15_R3_IgM	8.56	133779.8	12434.4	10.759	MM
145	1...	20211111_QC_R20_IgM	8.56	42857.4	10701.0	4.005	MM
146	1...	20211111_A16_R3_IgM	8.57	45656.5	11452.1	3.987	MM
147	1...	20211111_L15_R3_IgM	8.55	62570.3	10531.1	5.941	MM
148	1...	20211111_L16_R3_IgM	8.56	11565.2	12049.4	0.960	MM
149	1...	20211111_H8_R3_IgM	8.56	22307.6	13434.9	1.660	MM
150	1...	20211111_A17_R3_IgM	8.55	60810.9	12452.0	4.884	MM
151	1...	20211111_A18_R3_IgM	8.52	22654.9	12506.5	1.811	MM
152	1...	20211111_L17_R3_IgM	8.55	62195.5	9948.9	6.251	MM
153	1...	20211111_L18_R3_IgM	8.55	9075.8	13462.1	0.674	MM
154	1...	20211111_H9_R3_IgM	8.56	24297.0	13178.7	1.844	MM
155	1...	20211111_A19_R3_IgM	8.55	51571.1	11298.6	4.564	MM
156	1...	20211111_A20_R3_IgM	8.53	15995.3	11175.6	1.431	MM
157	1...	20211111_L19_R3_IgM	8.55	66073.9	11558.2	5.717	MM
158	1...	20211111_QC_R21_IgM	8.55	44172.8	9977.0	4.427	MM
159	1...	20211111_L20_R3_IgM	8.55	27911.1	13508.1	2.066	MM
160	1...	20211111_H10_R3_IgM	8.56	58753.1	12374.8	4.748	MM
161	1...	20211111_A21_R3_IgM	8.55	26912.0	12443.6	2.163	MM
162	1...	20211111_A22_R3_IgM	8.56	9895.2	11589.3	0.854	MM
163	1...	20211111_L21_R3_IgM	8.56	38988.6	13107.4	2.975	MM
164	1...	20211111_L22_R3_IgM	8.57	24454.9	12021.5	2.034	MM
165	1...	20211111_H11_R3_IgM	8.57	39568.4	12687.8	3.119	MM
166	1...	20211111_A23_R3_IgM	8.56	27291.5	11611.8	2.350	MM
167	1...	20211111_A24_R3_IgM	8.55	81363.2	10476.8	7.766	MM
168	1...	20211111_L23_R3_IgM	8.55	79543.3	11873.1	6.699	MM

Dataset: C:\MassLynx\10.pro\20211111_clinicalsamples_IgM_IgA.qld

Last Altered: Thursday, March 09, 2023 19:58:25 台北標準時間
Printed: Friday, August 01, 2025 15:11:07 台北標準時間

	# Name	RT	Area	IS Area	Response	Prima...
169	1... 20211111_L24_R3_IgM	8.56	12434.1	13468.0	0.923	MM
170	1... 20211111_H12_R3_IgM	8.55	18225.0	14229.1	1.281	MM
171	1... 20211111_QC_R22_IgM	8.55	41483.3	11264.4	3.683	MM
172	1... 20211111_A25_R3_IgM	8.57	55649.0	11775.7	4.726	MM
173	1... 20211111_A26_R3_IgM	8.56	34349.8	10473.9	3.280	MM
174	1... 20211111_L25_R3_IgM	8.58	17170.4	13256.8	1.295	MM
175	1... 20211111_L26_R3_IgM	8.57	30244.9	13565.0	2.230	MM
176	1... 20211111_H13_R3_IgM	8.55	57170.7	13146.0	4.349	MM
177	1... 20211111_A27_R3_IgM	8.56	43549.8	12025.6	3.621	MM
178	1... 20211111_A28_R3_IgM	8.55	19487.5	11229.1	1.735	MM
179	1... 20211111_L27_R3_IgM	8.55	39379.4	14960.0	2.632	MM
180	1... 20211111_L28_R3_IgM	8.55	24919.7	11359.0	2.194	MM
181	1... 20211111_H14_R3_IgM	8.55	45927.4	12830.8	3.579	MM
182	1... 20211111_A29_R3_IgM	8.58	4432.8	13434.3	0.330	MM
183	1... 20211111_A30_R3_IgM	8.55	52812.1	11272.2	4.685	MM
184	1... 20211111_QC_R23_IgM	8.56	39814.8	11872.2	3.354	MM
185	1... 20211111_L29_R3_IgM	8.55	16001.3	12948.7	1.236	MM
186	1... 20211111_L30_R3_IgM	8.55	12019.4	11026.6	1.090	MM
187	1... 20211111_H15_R3_IgM	8.55	48078.0	10786.3	4.457	MM
188	1... 20211111_A31_R3_IgM	8.55	22421.8	12010.5	1.867	MM
189	1... 20211111_A32_R3_IgM	8.55	46118.6	11107.7	4.152	MM
190	1... 20211111_L31_R3_IgM	8.56	6895.3	11021.8	0.626	MM
191	1... 20211111_L32_R3_IgM	8.55	43469.1	11683.8	3.720	MM
192	1... 20211111_H16_R3_IgM	8.54	58001.4	9831.7	5.899	MM
193	1... 20211111_A33_R3_IgM	8.54	88950.4	11213.1	7.933	MM
194	1... 20211111_A34_R3_IgM	8.56	16423.8	11550.1	1.422	MM
195	1... 20211111_H17_R3_IgM	8.54	220213.8	8874.4	24.814	MM
196	1... 20211111_A35_R3_IgM	8.55	31497.5	9856.3	3.196	MM
197	1... 20211111_QC_R24_IgM	8.55	36911.0	7561.8	4.881	MM
198	1... 20211111_A36_R3_IgM	8.55	21703.1	9562.8	2.270	MM
199	1... 20211111_H18_R3_IgM	8.55	80836.5	8165.3	9.900	MM
200	2... 20211111_A37_R3_IgM	8.54	55137.9	9278.8	5.942	MM
201	2... 20211111_A38_R3_IgM	8.56	30317.9	10920.3	2.776	MM
202	2... 20211111_H19_R3_IgM	8.55	57997.3	9285.3	6.246	MM
203	2... 20211111_A39_R3_IgM	8.56	43305.7	9083.4	4.768	MM
204	2... 20211111_A40_R3_IgM	8.55	23795.9	8382.4	2.839	MM
205	2... 20211111_H20_R3_IgM	8.54	179381.5	7807.9	22.974	MM
206	2... 20211111_A41_R3_IgM	8.55	21672.2	5925.2	3.658	MM
207	2... 20211111_A42_R3_IgM	8.55	14587.6	9352.6	1.560	MM
208	2... 20211111_A43_R3_IgM	8.54	12422.3	10216.3	1.216	MM
209	2... 20211111_A44_R3_IgM	8.55	11121.C	10262.7	1.084	MM
210	2... 20211111_QC_R25_IgM	8.55	34407.9	8575.3	4.012	MM

Dataset: C:\MassLynx\10.pro\20211111_clinicalsamples_IgM_IgA.qld

Last Altered: Thursday, March 09, 2023 19:58:25 台北標準時間
Printed: Friday, August 01, 2025 15:12:14 台北標準時間

Method: C:\MassLynx\10_Pro_IgA.PRO\MethDB\20211111_IgM_IgA_DMRM_new.mdb 24 Feb 2022 13:44:14
Calibration: 15 Nov 2021 14:42:11

Sample Name: 20211112_QC_R001_IgM
F50:MRM of 1 channel,ES+
1016.9 > 366.1
8.425e+006

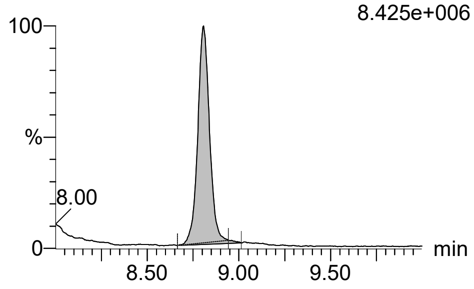

Sample Name: 20211112_QC_R002_IgM
F50:MRM of 1 channel,ES+
1016.9 > 366.1
7.801e+006

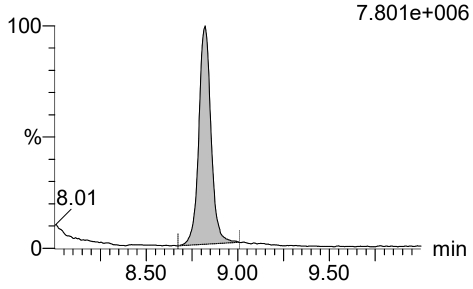

Sample Name: 20211111_A1_R2_IgM
F50:MRM of 1 channel,ES+
1016.9 > 366.1
3.034e+006

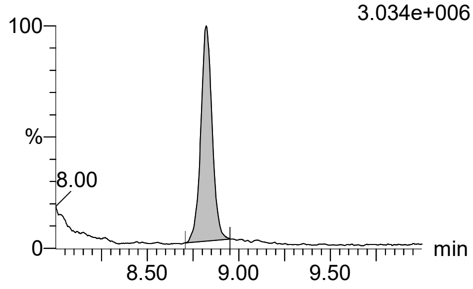

Sample Name: 20211111_A1_R3_IgM
F50:MRM of 1 channel,ES+
1016.9 > 366.1
2.994e+006

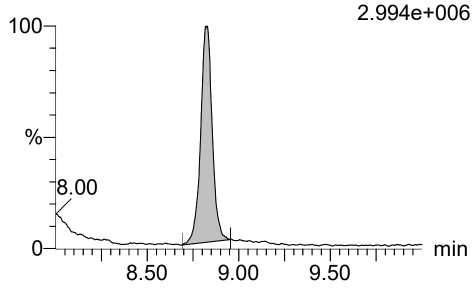

Sample Name: 20211111_A2_R2_IgM
F50:MRM of 1 channel,ES+
1016.9 > 366.1
2.957e+007

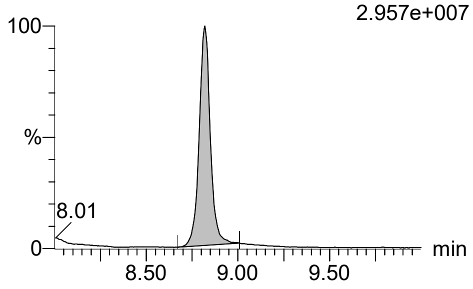

Sample Name: 20211111_A2_R3_IgM
F50:MRM of 1 channel,ES+
1016.9 > 366.1
2.862e+007

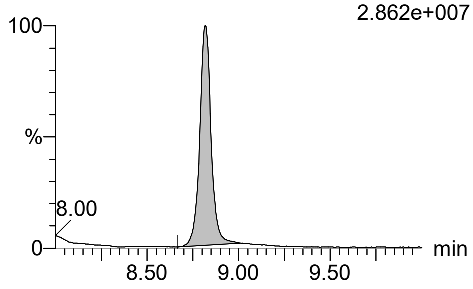

Sample Name: 20211111_L1_R2_IgM
F50:MRM of 1 channel,ES+
1016.9 > 366.1
2.526e+006

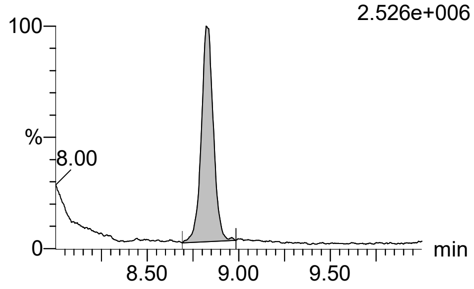

Sample Name: 20211111_L1_R3_IgM
F50:MRM of 1 channel,ES+
1016.9 > 366.1
2.131e+006

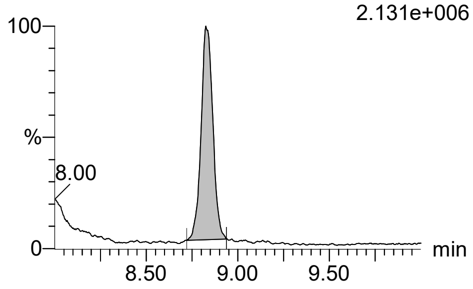

Sample Name: 20211111_L2_R2_IgM
F50:MRM of 1 channel,ES+
1016.9 > 366.1
1.193e+007

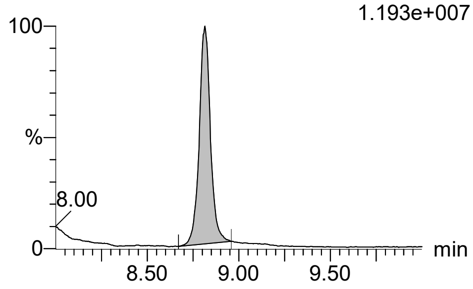

Sample Name: 20211111_H1_R2_IgM
F50:MRM of 1 channel,ES+
1016.9 > 366.1
4.579e+006

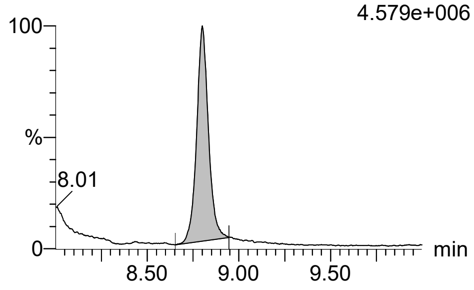

Sample Name: 20211111_A3_R2_IgM
F50:MRM of 1 channel,ES+
1016.9 > 366.1
6.138e+006

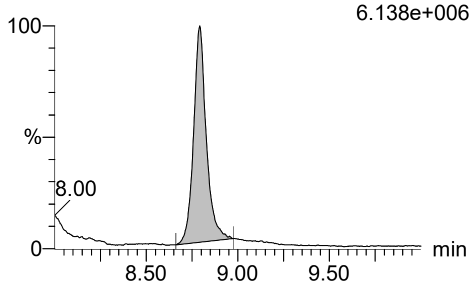

Sample Name: 20211111_A4_R2_IgM
F50:MRM of 1 channel,ES+
1016.9 > 366.1
6.368e+006

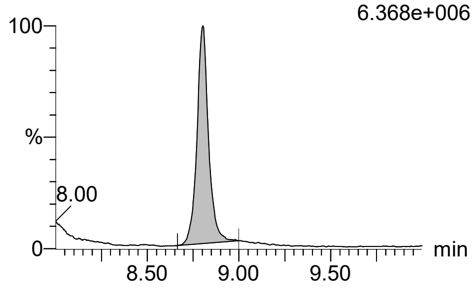

Sample Name: 20211111_L3_R2_IgM
F50:MRM of 1 channel,ES+
1016.9 > 366.1
1.178e+006

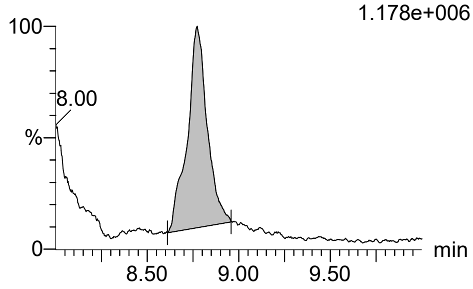

Sample Name: 20211111_L4_R2_IgM
F50:MRM of 1 channel,ES+
1016.9 > 366.1
1.107e+007

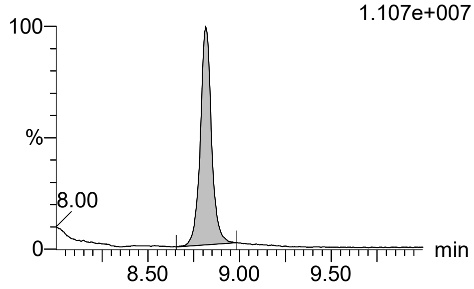

Sample Name: 20211111_H2_R2_IgM
F50:MRM of 1 channel,ES+
1016.9 > 366.1
1.453e+007

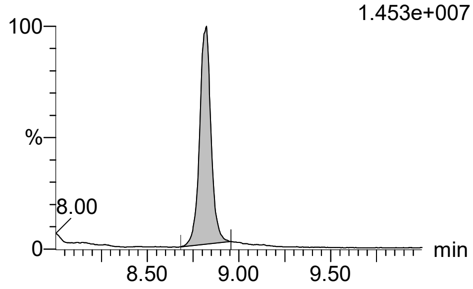

Sample Name: 20211111_A5_R2_IgM
F50:MRM of 1 channel,ES+
1016.9 > 366.1
1.653e+007

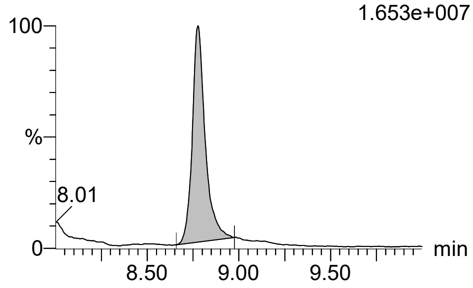

Sample Name: 20211111_A6_R2_IgM
F50:MRM of 1 channel,ES+
1016.9 > 366.1
9.636e+006

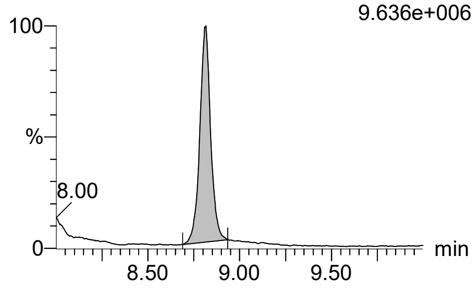

Sample Name: 20211111_QC_R10_IgM
F50:MRM of 1 channel,ES+
1016.9 > 366.1
8.363e+006

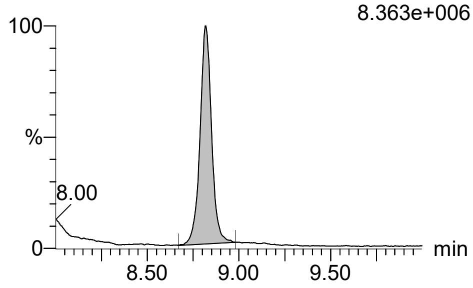

Sample Name: 20211111_L5_R2_IgM
F50:MRM of 1 channel,ES+
1016.9 > 366.1
3.435e+006

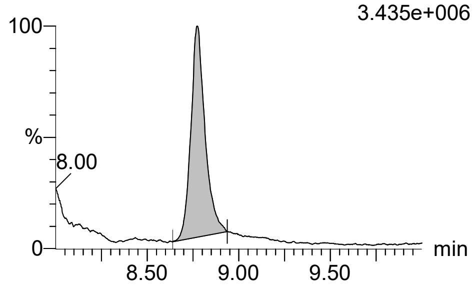

Sample Name: 20211111_L6_R2_IgM
F50:MRM of 1 channel,ES+
1016.9 > 366.1
1.331e+007

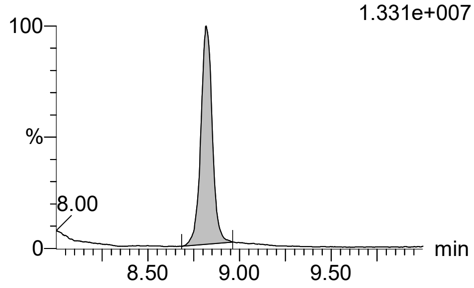

Sample Name: 20211111_H3_R2_IgM
F50:MRM of 1 channel,ES+
1016.9 > 366.1
6.055e+006

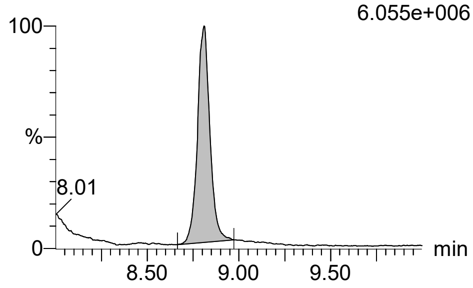

Sample Name: 20211111_A7_R2_IgM
F50:MRM of 1 channel,ES+
1016.9 > 366.1
5.166e+006

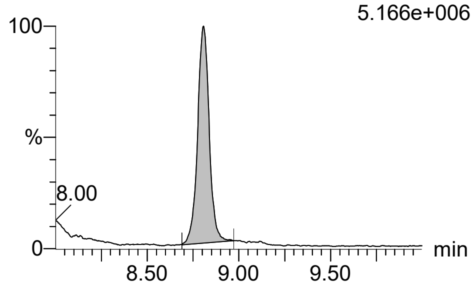

Sample Name: 20211111_A8_R2_IgM
F50:MRM of 1 channel,ES+
1016.9 > 366.1
6.891e+006

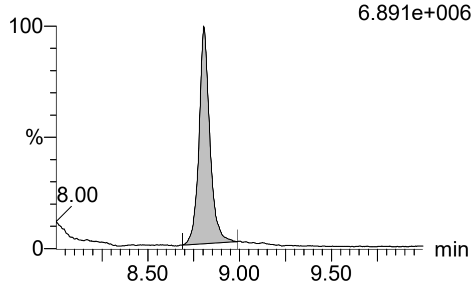

Sample Name: 20211111_L7_R2_IgM
F50:MRM of 1 channel,ES+
1016.9 > 366.1
4.166e+006

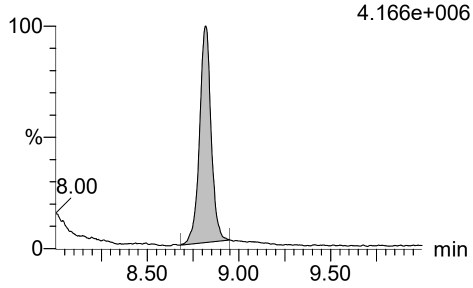

Sample Name: 20211111_L8_R2_IgM
F50:MRM of 1 channel,ES+
1016.9 > 366.1
3.866e+006

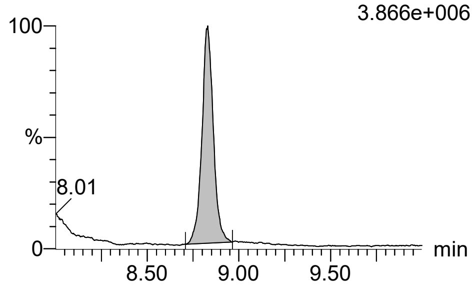

Sample Name: 20211111_H4_R2_IgM
F50:MRM of 1 channel,ES+
1016.9 > 366.1
5.149e+006

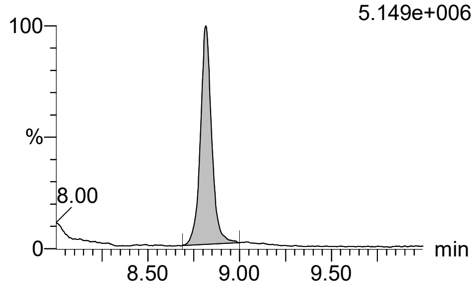

Sample Name: 20211111_A9_R2_IgM
F50:MRM of 1 channel,ES+
1016.9 > 366.1
2.468e+006

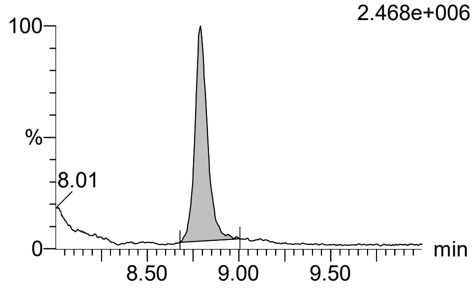

Dataset: C:\MassLynx\10.pro\20211111_clinicalsamples_IgM_IgA.qld

Last Altered: Thursday, March 09, 2023 19:58:25 台北標準時間
Printed: Friday, August 01, 2025 15:12:14 台北標準時間

Sample Name: 20211111_A10_R2_IgM
F50:MRM of 1 channel,ES+
1016.9 > 366.1
6.753e+006

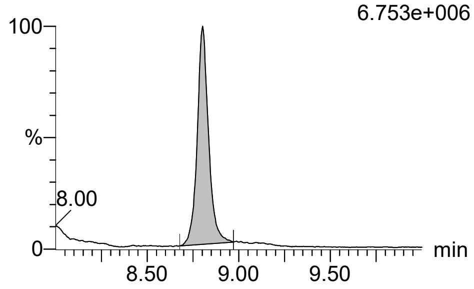

Sample Name: 20211111_L9_R2_IgM
F50:MRM of 1 channel,ES+
1016.9 > 366.1
2.533e+006

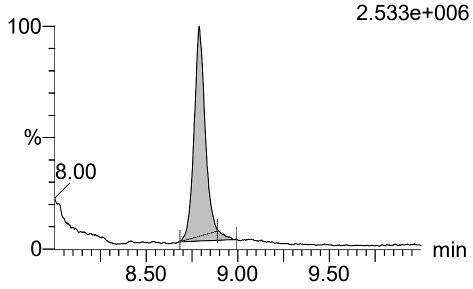

Sample Name: 20211111_L10_R2_IgM
F50:MRM of 1 channel,ES+
1016.9 > 366.1
6.824e+006

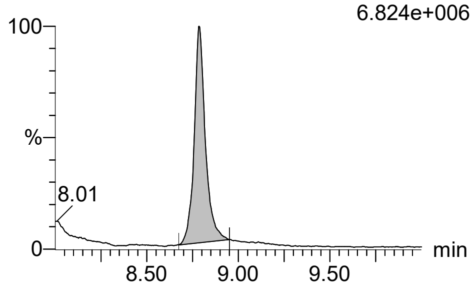

Sample Name: 20211111_QC_R11_IgM
F50:MRM of 1 channel,ES+
1016.9 > 366.1
9.036e+006

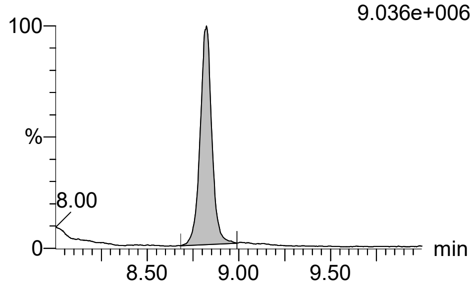

Sample Name: 20211111_H5_R2_IgM
F50:MRM of 1 channel,ES+
1016.9 > 366.1
1.256e+007

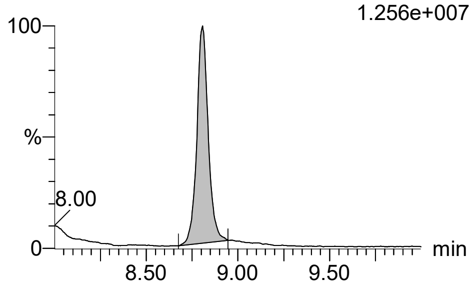

Sample Name: 20211111_A11_R2_IgM
F50:MRM of 1 channel,ES+
1016.9 > 366.1
2.244e+006

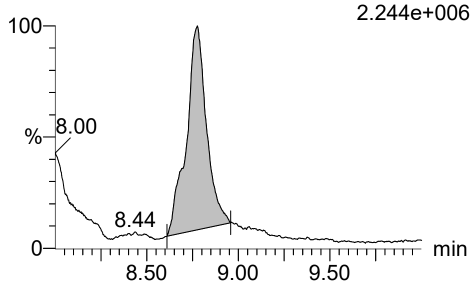

Sample Name: 20211111_A12_R2_IgM
F50:MRM of 1 channel,ES+
1016.9 > 366.1
3.813e+006

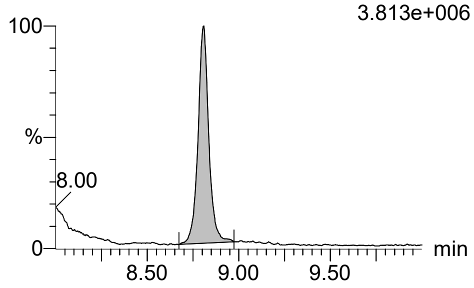

Sample Name: 20211111_L11_R2_IgM
F50:MRM of 1 channel,ES+
1016.9 > 366.1
4.931e+006

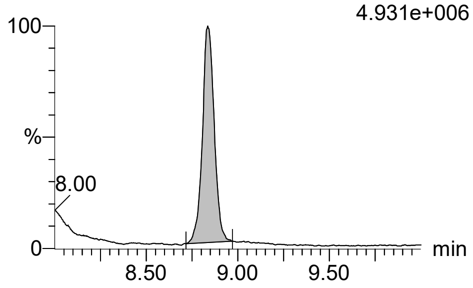

Sample Name: 20211111_L12_R2_IgM
F50:MRM of 1 channel,ES+
1016.9 > 366.1
3.663e+006

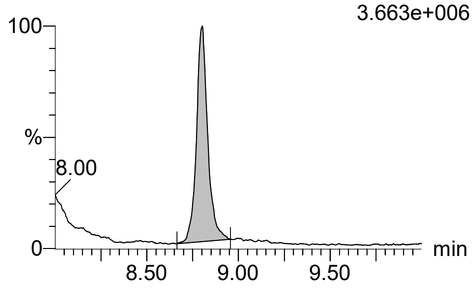

Sample Name: 20211111_H6_R2_IgM
F50:MRM of 1 channel,ES+
1016.9 > 366.1
5.412e+006

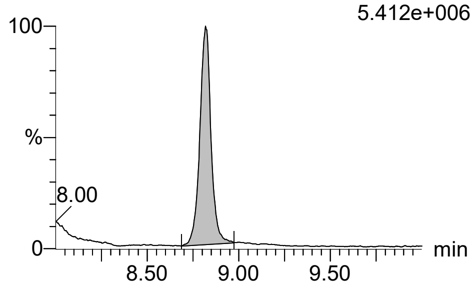

Sample Name: 20211111_A13_R2_IgM
F50:MRM of 1 channel,ES+
1016.9 > 366.1
8.936e+006

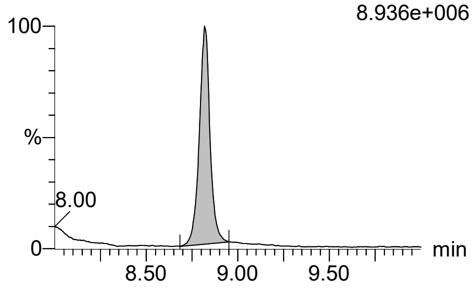

Sample Name: 20211111_A14_R2_IgM
F50:MRM of 1 channel,ES+
1016.9 > 366.1
1.225e+007

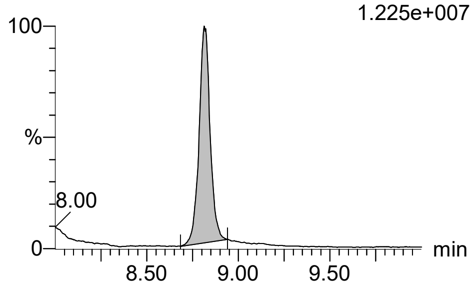

Sample Name: 20211111_L13_R2_IgM
F50:MRM of 1 channel,ES+
1016.9 > 366.1
5.466e+006

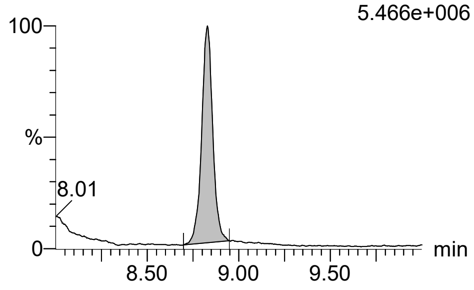

Sample Name: 20211111_L14_R2_IgM
F50:MRM of 1 channel,ES+
1016.9 > 366.1
1.046e+007

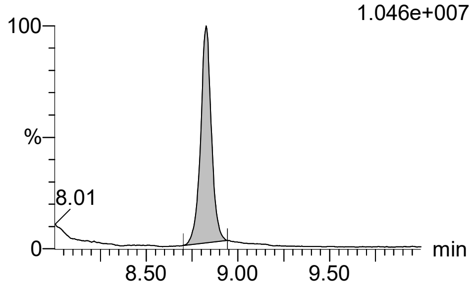

Sample Name: 20211111_H7_R2_IgM
F50:MRM of 1 channel,ES+
1016.9 > 366.1
3.022e+006

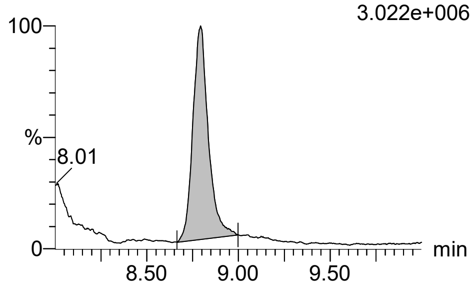

Sample Name: 20211111_A15_R2_IgM
F50:MRM of 1 channel,ES+
1016.9 > 366.1
2.530e+007

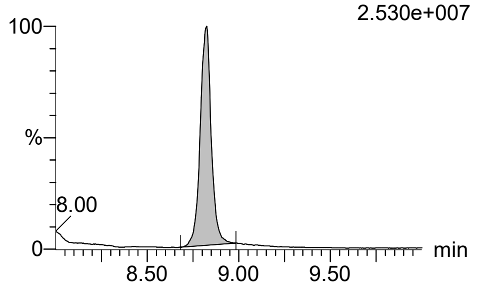

Sample Name: 20211111_QC_R12_IgM
F50:MRM of 1 channel,ES+
1016.9 > 366.1
1.003e+007

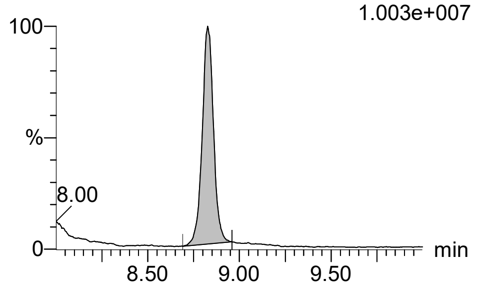

Sample Name: 20211111_A16_R2_IgM
F50:MRM of 1 channel,ES+
1016.9 > 366.1
1.020e+007

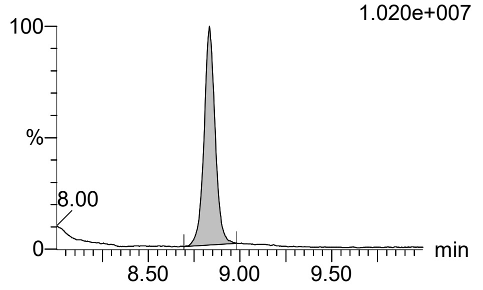

Sample Name: 20211111_L15_R2_IgM
F50:MRM of 1 channel,ES+
1016.9 > 366.1
1.042e+007

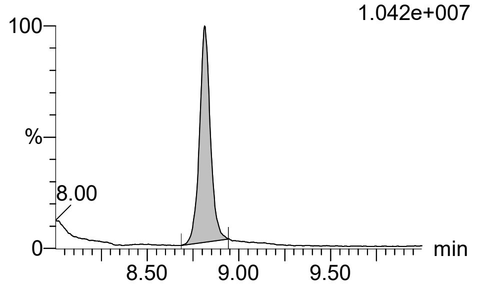

Sample Name: 20211111_L16_R2_IgM
F50:MRM of 1 channel,ES+
1016.9 > 366.1
1.693e+006

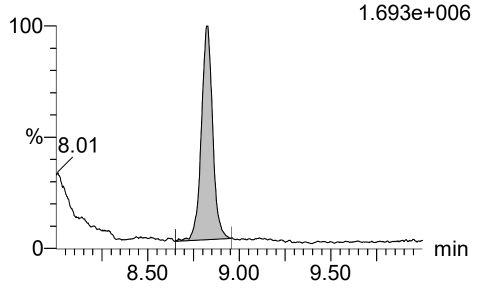

Sample Name: 20211111_H8_R2_IgM
F50:MRM of 1 channel,ES+
1016.9 > 366.1
4.981e+006

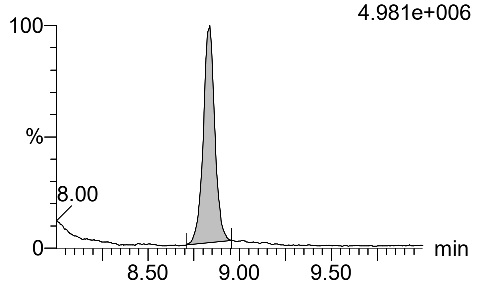

Sample Name: 20211111_A17_R2_IgM
F50:MRM of 1 channel,ES+
1016.9 > 366.1
1.483e+007

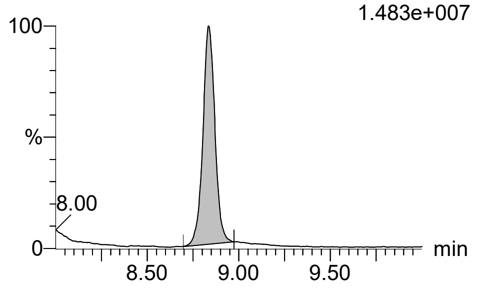

Sample Name: 20211111_A18_R2_IgM
F50:MRM of 1 channel,ES+
1016.9 > 366.1
2.986e+006

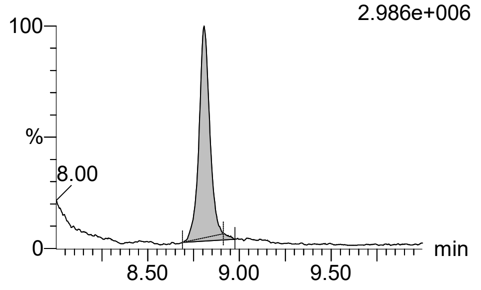

Sample Name: 20211111_L17_R2_IgM
F50:MRM of 1 channel,ES+
1016.9 > 366.1
1.365e+007

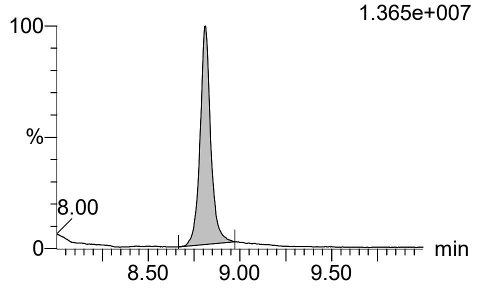

Sample Name: 20211111_L18_R2_IgM
F50:MRM of 1 channel,ES+
1016.9 > 366.1
1.474e+006

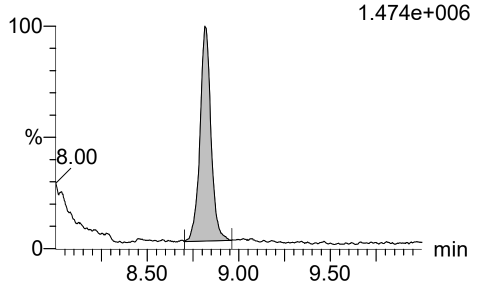

Sample Name: 20211111_H9_R2_IgM
F50:MRM of 1 channel,ES+
1016.9 > 366.1
3.370e+006

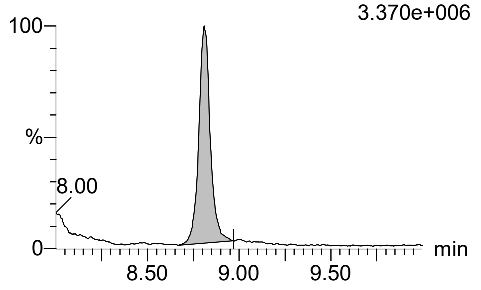

Sample Name: 20211111_A19_R2_IgM
F50:MRM of 1 channel,ES+
1016.9 > 366.1
9.340e+006

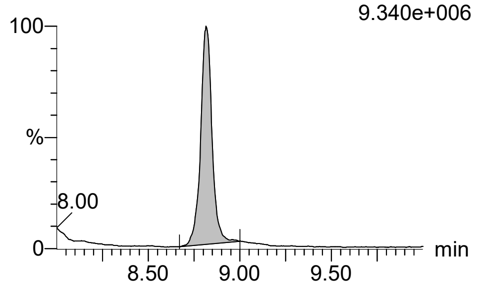

Sample Name: 20211111_A20_R2_IgM
F50:MRM of 1 channel,ES+
1016.9 > 366.1
1.153e+006

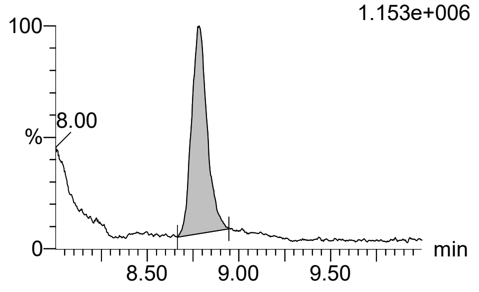

Sample Name: 20211111_L19_R2_IgM
F50:MRM of 1 channel,ES+
1016.9 > 366.1
8.347e+006

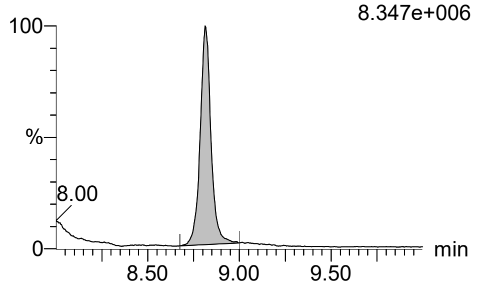

Sample Name: 20211111_QC_R13_IgM
F50:MRM of 1 channel,ES+
1016.9 > 366.1
9.732e+006

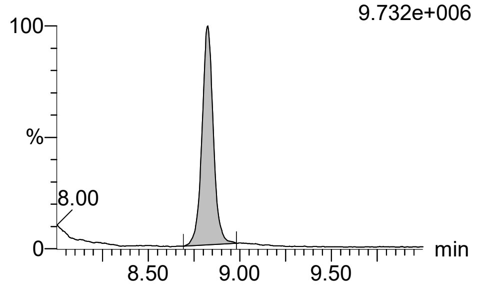

Sample Name: 20211111_L20_R2_IgM
F50:MRM of 1 channel,ES+
1016.9 > 366.1
1.336e+006

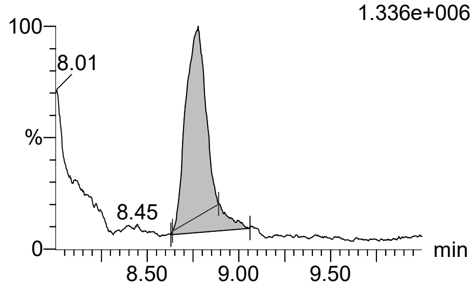

Sample Name: 20211111_H10_R2_IgM
F50:MRM of 1 channel,ES+
1016.9 > 366.1
4.999e+006

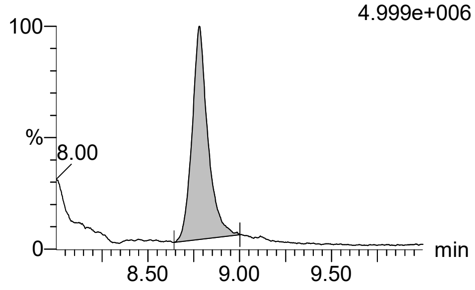

Sample Name: 20211111_A21_R2_IgM
F50:MRM of 1 channel,ES+
1016.9 > 366.1
6.052e+006

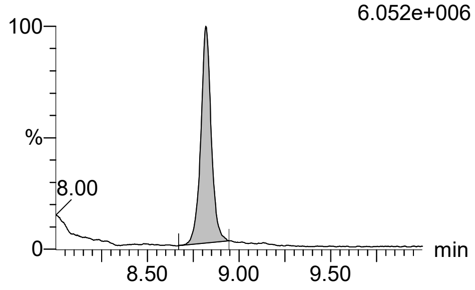

Sample Name: 20211111_A22_R2_IgM
F50:MRM of 1 channel,ES+
1016.9 > 366.1
2.374e+006

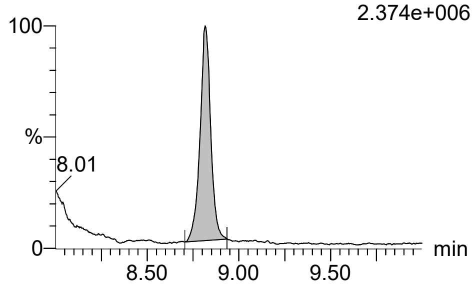

Sample Name: 20211111_L21_R2_IgM
F50:MRM of 1 channel,ES+
1016.9 > 366.1
8.188e+006

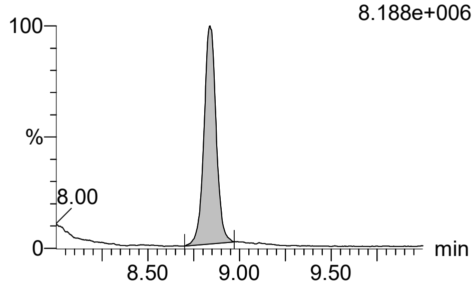

Sample Name: 20211111_L22_R2_IgM
F50:MRM of 1 channel,ES+
1016.9 > 366.1
5.490e+006

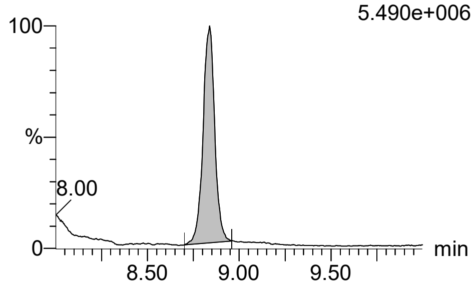

Sample Name: 20211111_H11_R2_IgM
F50:MRM of 1 channel,ES+
1016.9 > 366.1
6.428e+006

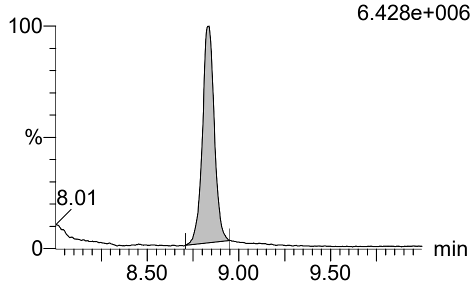

Sample Name: 20211111_A23_R2_IgM
F50:MRM of 1 channel,ES+
1016.9 > 366.1
4.887e+006

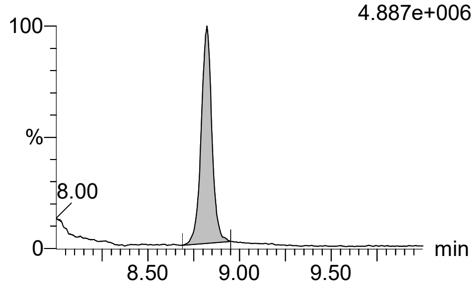

Sample Name: 20211111_A24_R2_IgM
F50:MRM of 1 channel,ES+
1016.9 > 366.1
1.892e+007

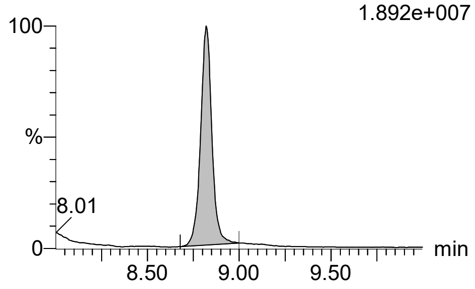

Sample Name: 20211111_L23_R2_IgM
F50:MRM of 1 channel,ES+
1016.9 > 366.1
1.282e+007

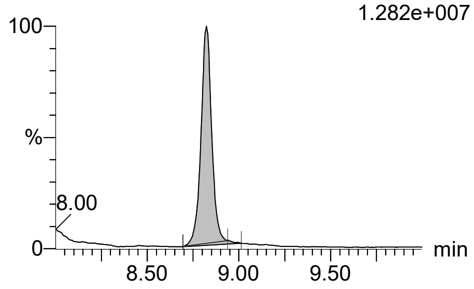

Sample Name: 20211111_L24_R2_IgM
F50:MRM of 1 channel,ES+
1016.9 > 366.1
2.628e+006

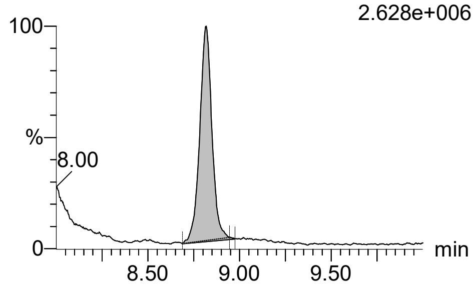

Sample Name: 20211111_H12_R2_IgM
F50:MRM of 1 channel,ES+
1016.9 > 366.1
4.298e+006

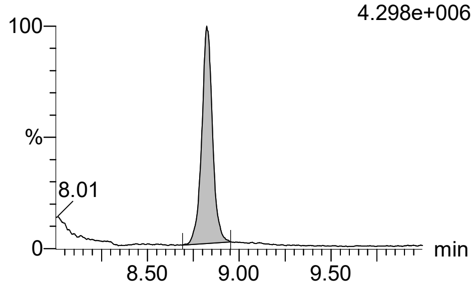

Sample Name: 20211111_QC_R14_IgM
F50:MRM of 1 channel,ES+
1016.9 > 366.1
1.015e+007

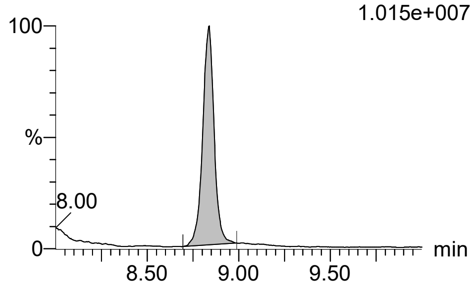

Sample Name: 20211111_A25_R2_IgM
F50:MRM of 1 channel,ES+
1016.9 > 366.1
1.320e+007

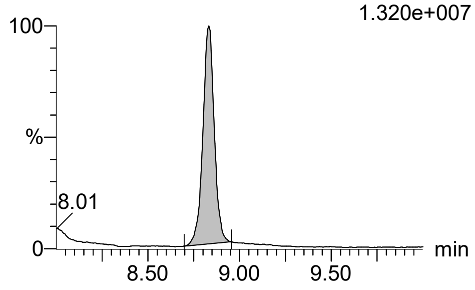

Sample Name: 20211111_A26_R2_IgM
F50:MRM of 1 channel,ES+
1016.9 > 366.1
7.612e+006

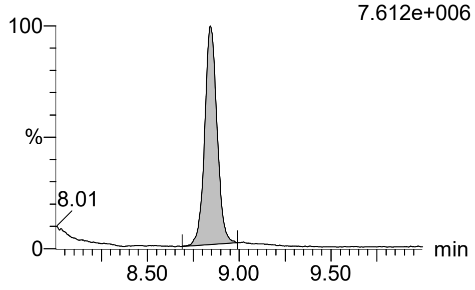

Sample Name: 20211111_L25_R2_IgM
F50:MRM of 1 channel,ES+
1016.9 > 366.1
3.219e+006

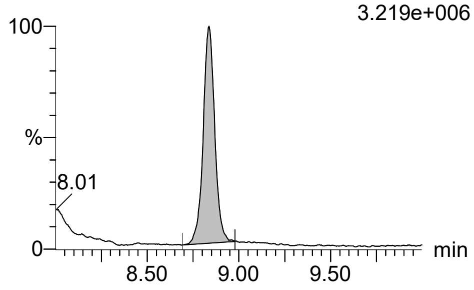

Sample Name: 20211111_L26_R2_IgM
F50:MRM of 1 channel,ES+
1016.9 > 366.1
5.197e+006

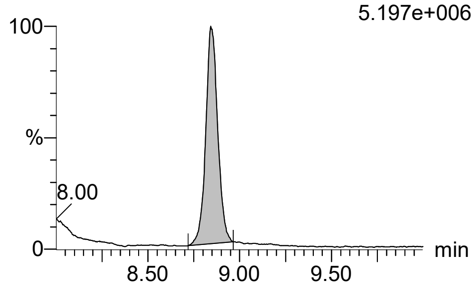

Sample Name: 20211111_H13_R2_IgM
F50:MRM of 1 channel,ES+
1016.9 > 366.1
1.051e+007

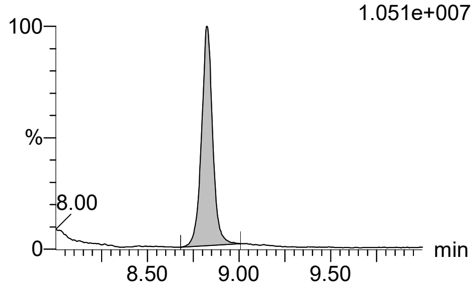

Sample Name: 20211111_A27_R2_IgM
F50:MRM of 1 channel,ES+
1016.9 > 366.1
1.647e+007

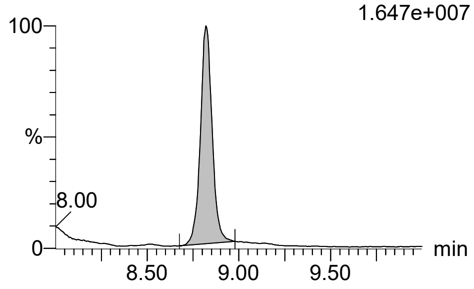

Sample Name: 20211111_A28_R2_IgM
F50:MRM of 1 channel,ES+
1016.9 > 366.1
1.081e+006

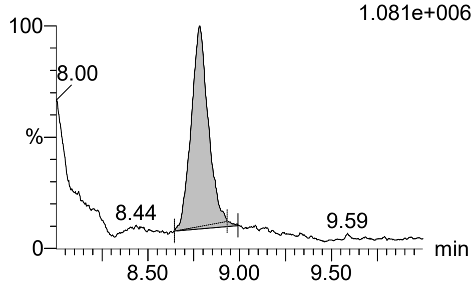

Sample Name: 20211111_L27_R2_IgM
F50:MRM of 1 channel,ES+
1016.9 > 366.1
7.564e+006

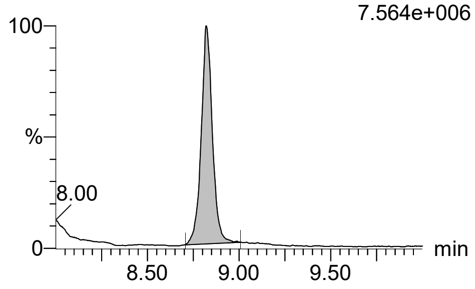

Sample Name: 20211111_L28_R2_IgM
F50:MRM of 1 channel,ES+
1016.9 > 366.1
4.820e+006

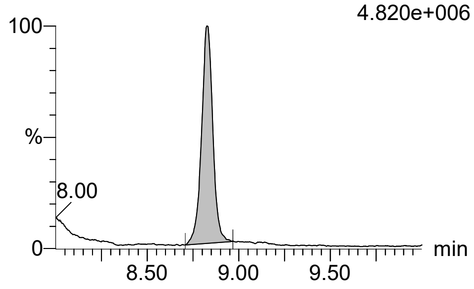

Sample Name: 20211111_H14_R2_IgM
F50:MRM of 1 channel,ES+
1016.9 > 366.1
8.287e+006

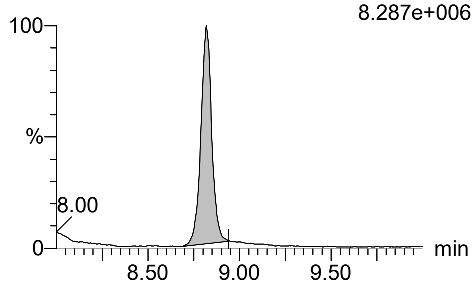

Sample Name: 20211111_A29_R2_IgM
F50:MRM of 1 channel,ES+
1016.9 > 366.1
7.693e+005

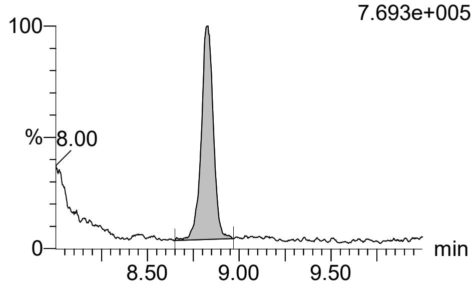

Sample Name: 20211111_A30_R2_IgM
F50:MRM of 1 channel,ES+
1016.9 > 366.1
9.077e+006

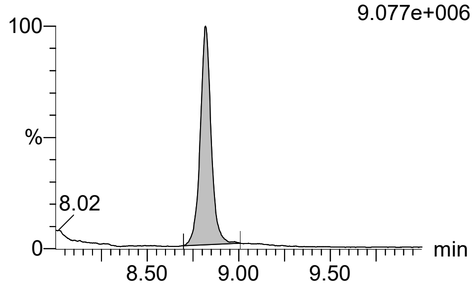

Sample Name: 20211111_QC_R15_IgM
F50:MRM of 1 channel,ES+
1016.9 > 366.1
9.122e+006

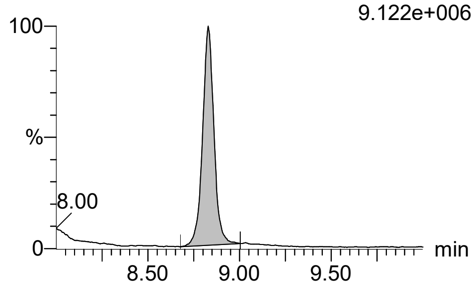

Sample Name: 20211111_L29_R2_IgM
F50:MRM of 1 channel,ES+
1016.9 > 366.1
2.798e+006

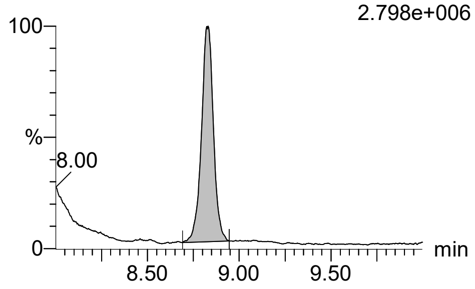

Sample Name: 20211111_L30_R2_IgM
F50:MRM of 1 channel,ES+
1016.9 > 366.1
1.786e+006

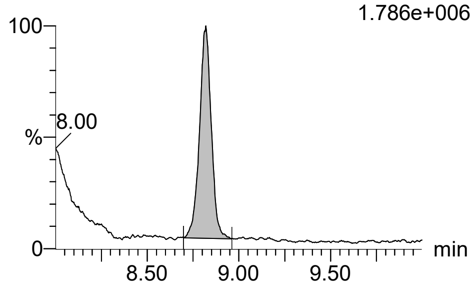

Sample Name: 20211111_H15_R2_IgM
F50:MRM of 1 channel,ES+
1016.9 > 366.1
8.259e+006

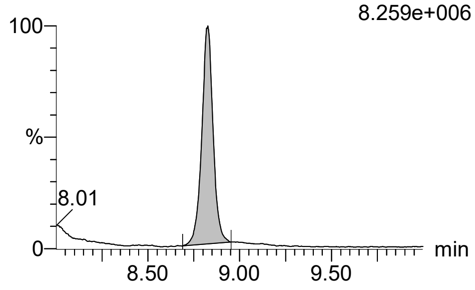

Sample Name: 20211111_A31_R2_IgM
F50:MRM of 1 channel,ES+
1016.9 > 366.1
3.093e+006

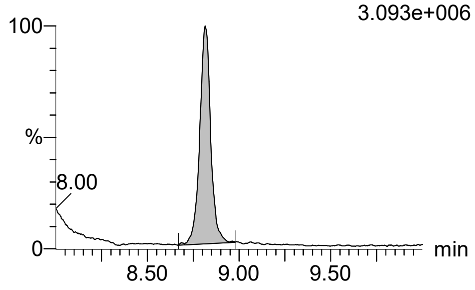

Dataset: C:\MassLynx\10.pro\20211111_clinicalsamples_IgM_IgA.qld

Last Altered: Thursday, March 09, 2023 19:58:25 台北標準時間
Printed: Friday, August 01, 2025 15:12:14 台北標準時間

Sample Name: 20211111_A32_R2_IgM
F50:MRM of 1 channel,ES+
1016.9 > 366.1
8.815e+006

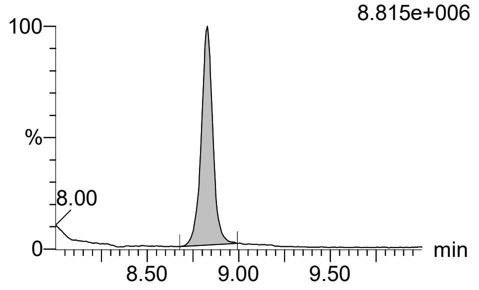

Sample Name: 20211111_L31_R2_IgM
F50:MRM of 1 channel,ES+
1016.9 > 366.1
3.623e+005

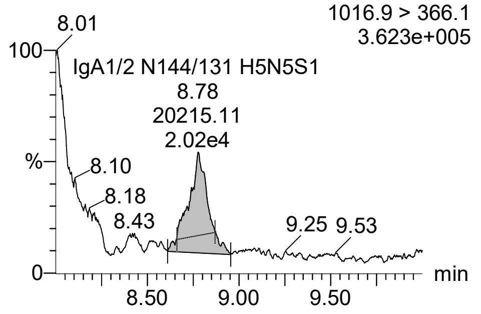

Sample Name: 20211111_L32_R2_IgM
F50:MRM of 1 channel,ES+
1016.9 > 366.1
7.725e+006

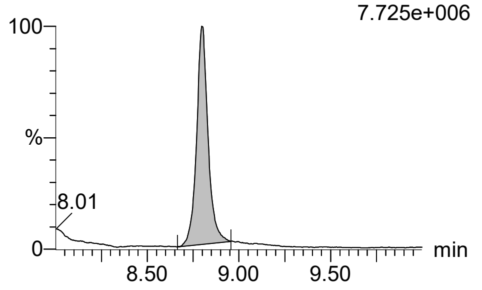

Sample Name: 20211111_H16_R2_IgM
F50:MRM of 1 channel,ES+
1016.9 > 366.1
1.500e+007

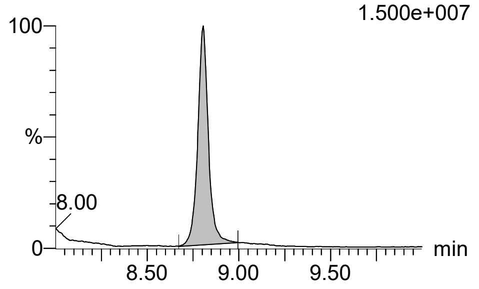

Sample Name: 20211111_A33_R2_IgM
F50:MRM of 1 channel,ES+
1016.9 > 366.1
1.822e+007

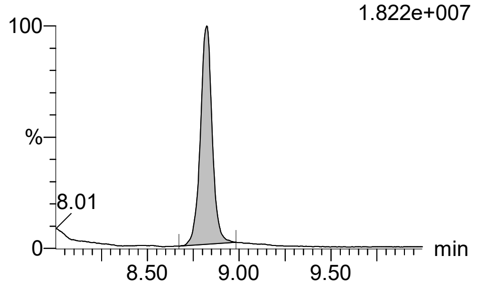

Sample Name: 20211111_A34_R2_IgM
F50:MRM of 1 channel,ES+
1016.9 > 366.1
3.389e+006

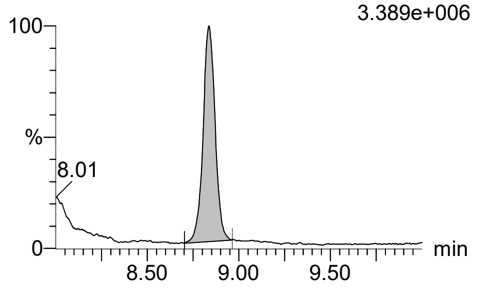

Sample Name: 20211111_H17_R2_IgM
F50:MRM of 1 channel,ES+
1016.9 > 366.1
4.980e+007

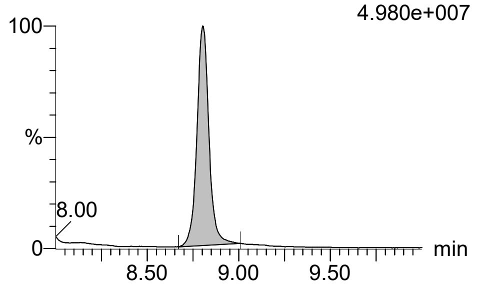

Sample Name: 20211111_A35_R2_IgM
F50:MRM of 1 channel,ES+
1016.9 > 366.1
2.443e+006

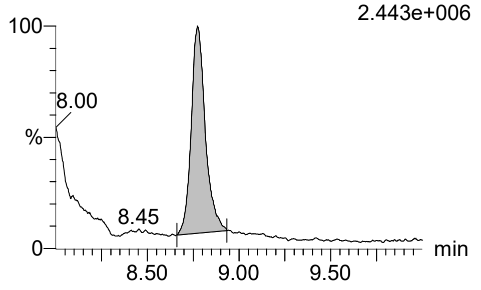

Sample Name: 20211111_QC_R16_IgM
F50:MRM of 1 channel,ES+
1016.9 > 366.1
7.930e+006

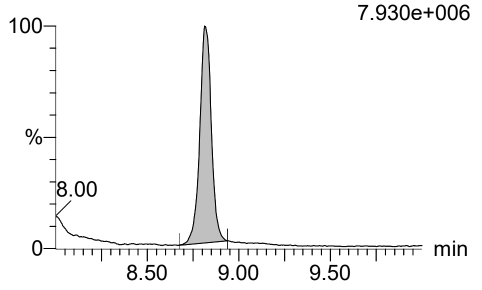

Sample Name: 20211111_A36_R2_IgM
F50:MRM of 1 channel,ES+
1016.9 > 366.1
6.114e+005

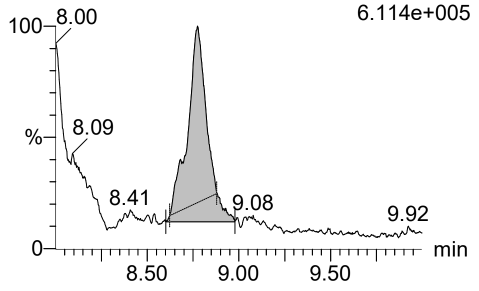

Sample Name: 20211111_H18_R2_IgM
F50:MRM of 1 channel,ES+
1016.9 > 366.1
1.453e+007

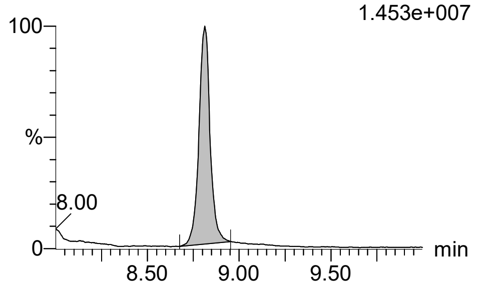

Sample Name: 20211111_A37_R2_IgM
F50:MRM of 1 channel,ES+
1016.9 > 366.1
1.306e+007

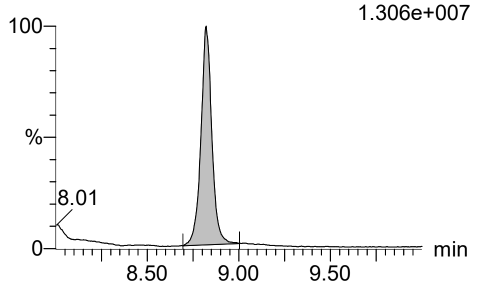

Sample Name: 20211111_A38_R2_IgM
F50:MRM of 1 channel,ES+
1016.9 > 366.1
5.927e+006

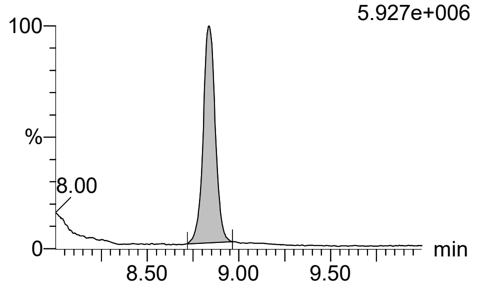

Sample Name: 20211111_H19_R2_IgM
F50:MRM of 1 channel,ES+
1016.9 > 366.1
1.644e+007

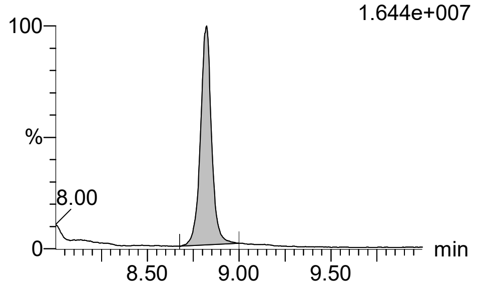

Sample Name: 20211111_A39_R2_IgM
F50:MRM of 1 channel,ES+
1016.9 > 366.1
9.802e+006

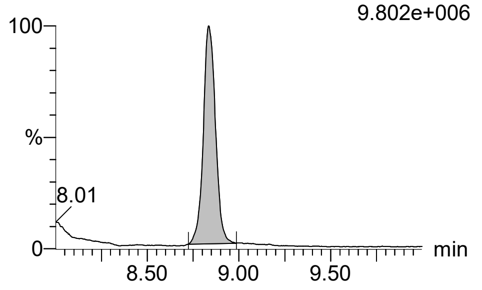

Dataset: C:\MassLynx\10.pro\20211111_clinicalsamples_IgM_IgA.qld

Last Altered: Thursday, March 09, 2023 19:58:25 台北標準時間
Printed: Friday, August 01, 2025 15:12:14 台北標準時間

Sample Name: 20211111_A40_R2_IgM
F50:MRM of 1 channel,ES+
1016.9 > 366.1
1.362e+006

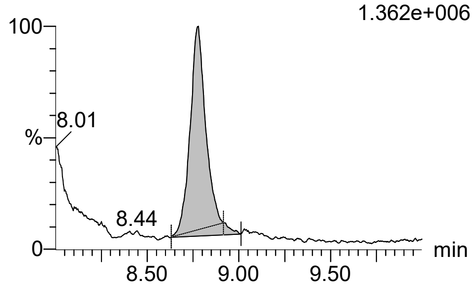

Sample Name: 20211111_H20_R2_IgM
F50:MRM of 1 channel,ES+
1016.9 > 366.1
3.222e+007

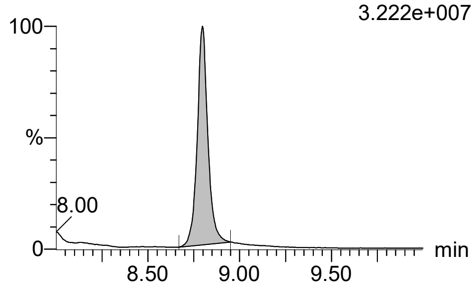

Sample Name: 20211111_A41_R2_IgM
F50:MRM of 1 channel,ES+
1016.9 > 366.1
6.723e+006

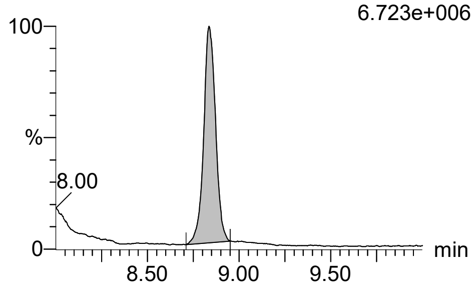

Sample Name: 20211111_A42_R2_IgM
F50:MRM of 1 channel,ES+
1016.9 > 366.1
3.490e+006

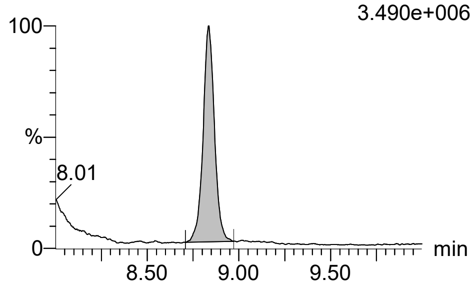

Sample Name: 20211111_A43_R2_IgM
F50:MRM of 1 channel,ES+
1016.9 > 366.1
3.119e+006

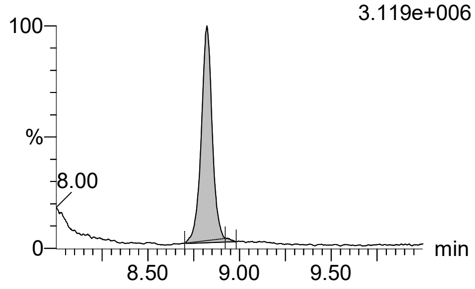

Sample Name: 20211111_A44_R2_IgM
F50:MRM of 1 channel,ES+
1016.9 > 366.1
1.056e+006

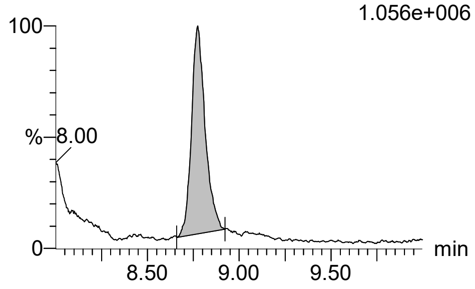

Sample Name: 20211111_QC_R17_IgM
F50:MRM of 1 channel,ES+
1016.9 > 366.1
7.106e+006

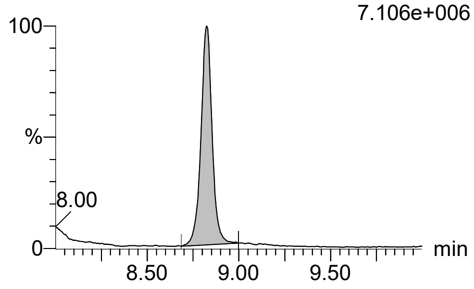

Sample Name: 20211111_L2_R3_IgM
F50:MRM of 1 channel,ES+
1016.9 > 366.1
1.269e+007

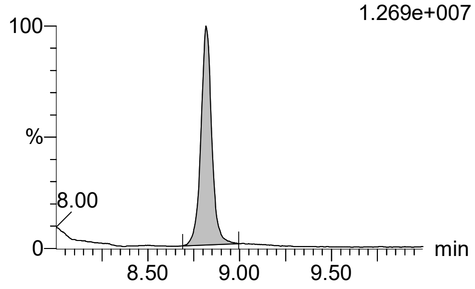

Sample Name: 20211111_H1_R3_IgM
F50:MRM of 1 channel,ES+
1016.9 > 366.1
6.452e+006

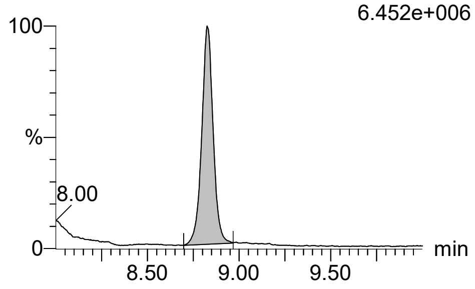

Sample Name: 20211111_A3_R3_IgM
F50:MRM of 1 channel,ES+
1016.9 > 366.1
9.559e+006

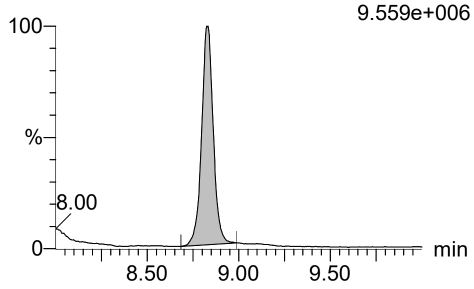

Sample Name: 20211111_A4_R3_IgM
F50:MRM of 1 channel,ES+
1016.9 > 366.1
7.659e+006

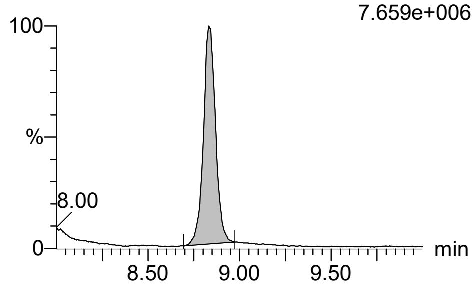

Sample Name: 20211111_L3_R3_IgM
F50:MRM of 1 channel,ES+
1016.9 > 366.1
1.613e+006

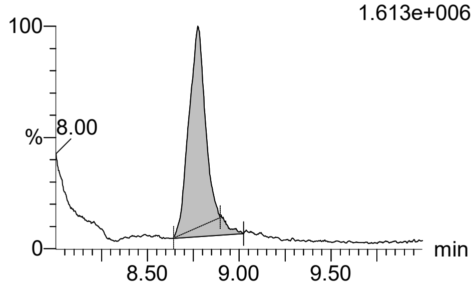

Sample Name: 20211111_L4_R3_IgM
F50:MRM of 1 channel,ES+
1016.9 > 366.1
1.019e+007

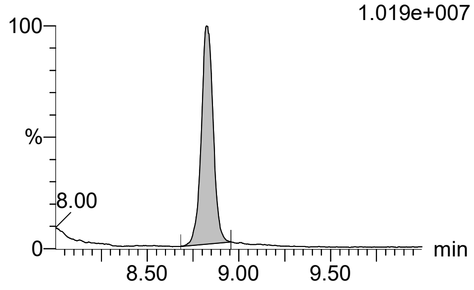

Sample Name: 20211111_H2_R3_IgM
F50:MRM of 1 channel,ES+
1016.9 > 366.1
1.457e+007

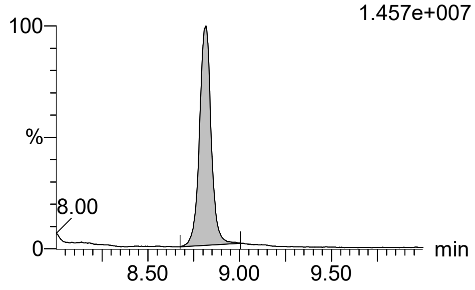

Sample Name: 20211111_A5_R3_IgM
F50:MRM of 1 channel,ES+
1016.9 > 366.1
2.758e+007

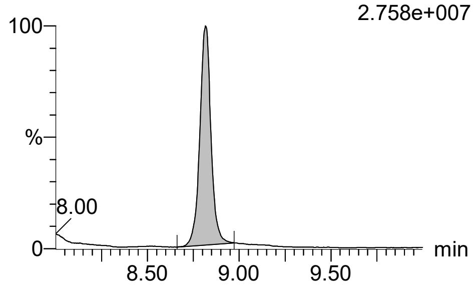

Sample Name: 20211111_A6_R3_IgM
F50:MRM of 1 channel,ES+
1016.9 > 366.1
8.222e+006

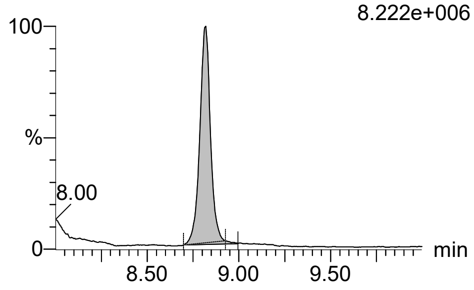

Sample Name: 20211111_QC_R18_IgM
F50:MRM of 1 channel,ES+
1016.9 > 366.1
7.481e+006

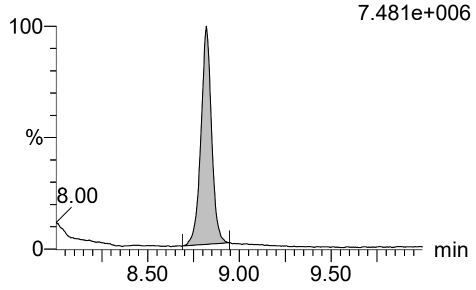

Sample Name: 20211111_L5_R3_IgM
F50:MRM of 1 channel,ES+
1016.9 > 366.1
7.013e+006

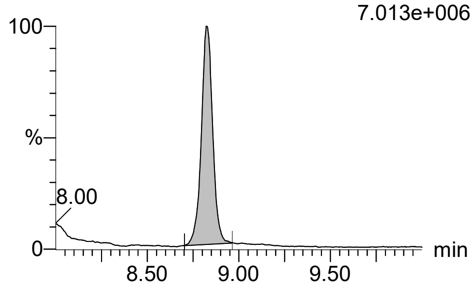

Sample Name: 20211111_L6_R3_IgM
F50:MRM of 1 channel,ES+
1016.9 > 366.1
1.131e+007

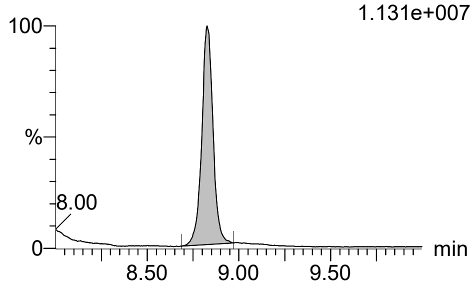

Sample Name: 20211111_H3_R3_IgM
F50:MRM of 1 channel,ES+
1016.9 > 366.1
7.802e+006

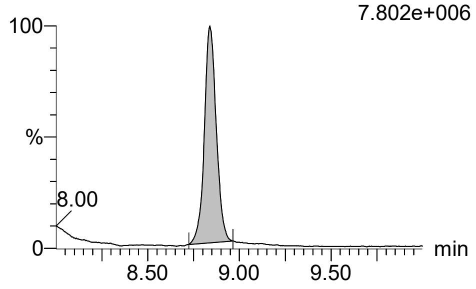

Sample Name: 20211111_A7_R3_IgM
F50:MRM of 1 channel,ES+
1016.9 > 366.1
5.454e+006

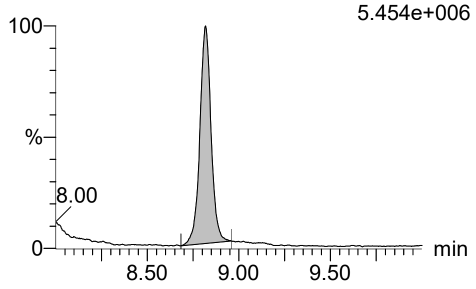

Sample Name: 20211111_A8_R3_IgM
F50:MRM of 1 channel,ES+
1016.9 > 366.1
6.692e+006

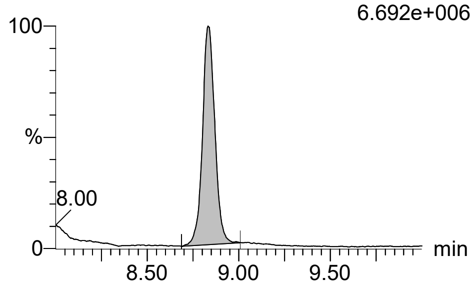

Sample Name: 20211111_L7_R3_IgM
F50:MRM of 1 channel,ES+
1016.9 > 366.1
4.209e+006

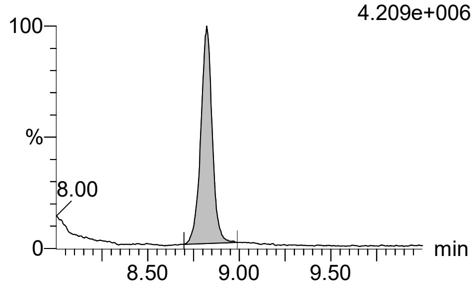

Sample Name: 20211111_L8_R3_IgM
F50:MRM of 1 channel,ES+
1016.9 > 366.1
3.301e+006

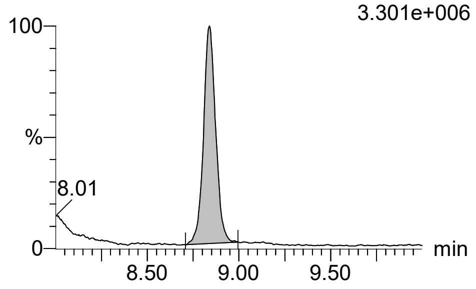

Sample Name: 20211111_H4_R3_IgM
F50:MRM of 1 channel,ES+
1016.9 > 366.1
4.759e+006

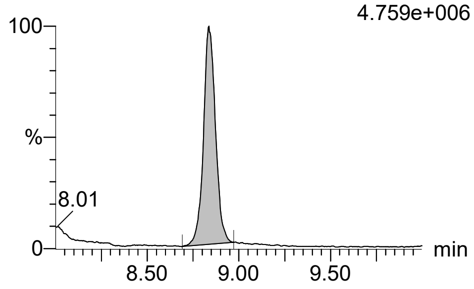

Sample Name: 20211111_A9_R3_IgM
F50:MRM of 1 channel,ES+
1016.9 > 366.1
3.881e+006

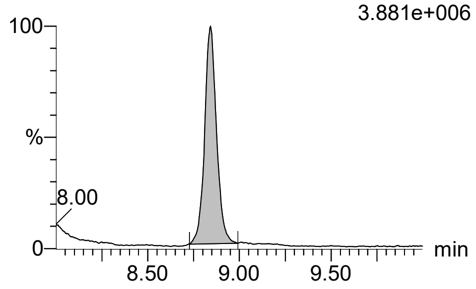

Sample Name: 20211111_A10_R3_IgM
F50:MRM of 1 channel,ES+
1016.9 > 366.1
8.340e+006

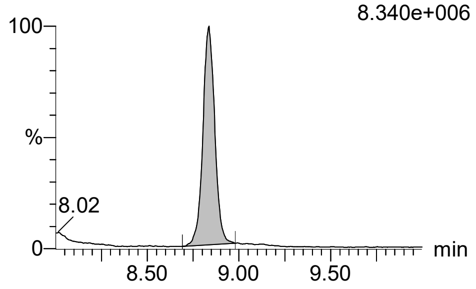

Sample Name: 20211111_L9_R3_IgM
F50:MRM of 1 channel,ES+
1016.9 > 366.1
3.831e+006

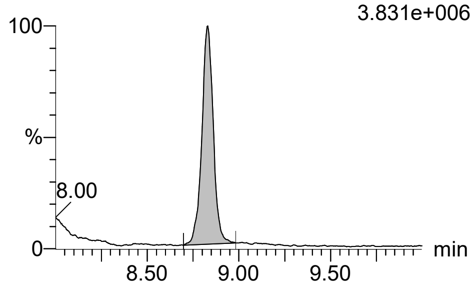

Sample Name: 20211111_L10_R3_IgM
F50:MRM of 1 channel,ES+
1016.9 > 366.1
3.567e+006

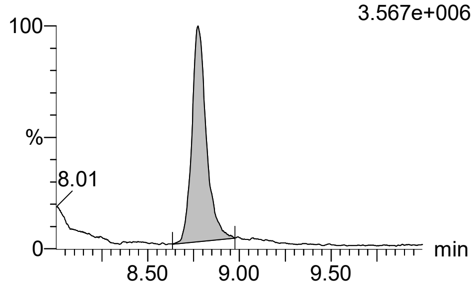

Sample Name: 20211111_QC_R19_IgM
F50:MRM of 1 channel,ES+
1016.9 > 366.1
7.063e+006

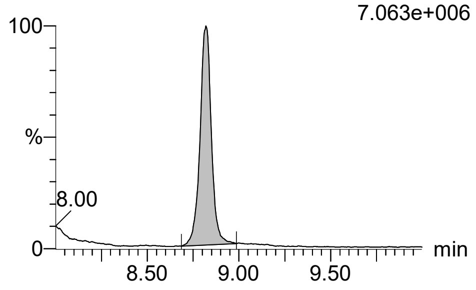

Sample Name: 20211111_H5_R3_IgM
F50:MRM of 1 channel,ES+
1016.9 > 366.1
1.220e+007

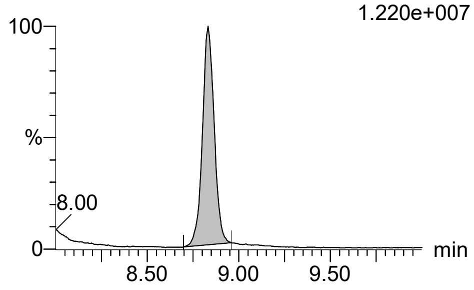

Sample Name: 20211111_A11_R3_IgM
F50:MRM of 1 channel,ES+
1016.9 > 366.1
1.119e+007

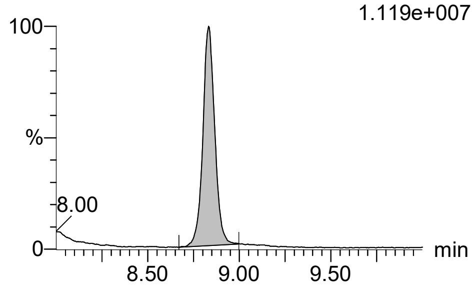

Sample Name: 20211111_A12_R3_IgM
F50:MRM of 1 channel,ES+
1016.9 > 366.1
4.161e+006

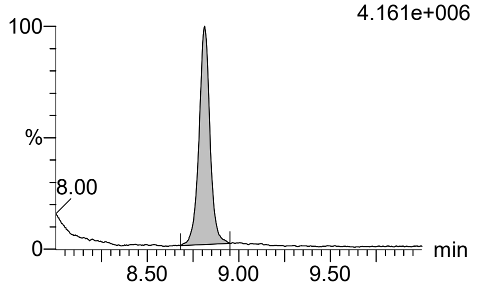

Sample Name: 20211111_L11_R3_IgM
F50:MRM of 1 channel,ES+
1016.9 > 366.1
3.840e+006

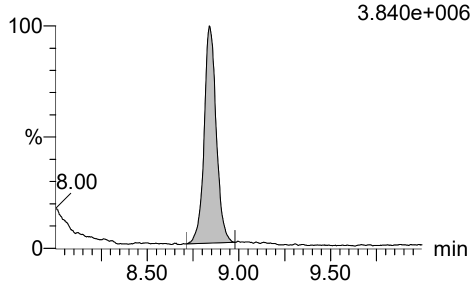

Sample Name: 20211111_L12_R3_IgM
F50:MRM of 1 channel,ES+
1016.9 > 366.1
1.996e+006

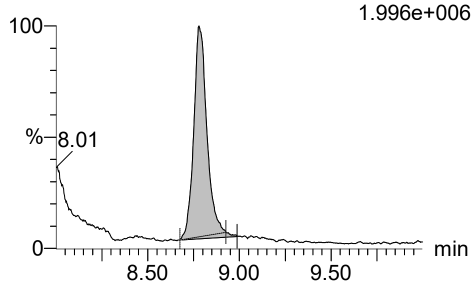

Sample Name: 20211111_H6_R3_IgM
F50:MRM of 1 channel,ES+
1016.9 > 366.1
5.188e+006

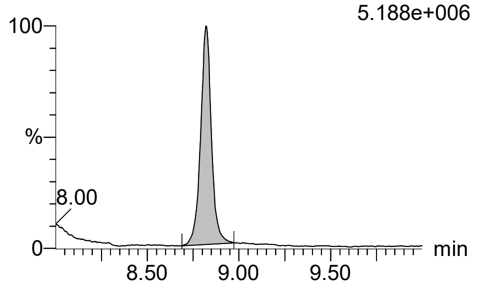

Sample Name: 20211111_A13_R3_IgM
F50:MRM of 1 channel,ES+
1016.9 > 366.1
8.684e+006

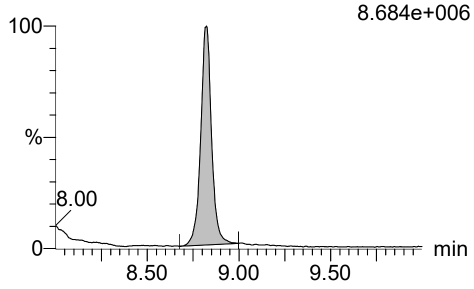

Sample Name: 20211111_A14_R3_IgM
F50:MRM of 1 channel,ES+
1016.9 > 366.1
1.181e+007

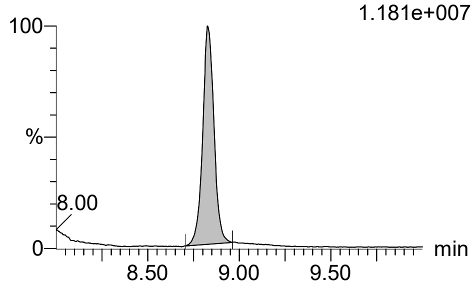

Sample Name: 20211111_L13_R3_IgM
F50:MRM of 1 channel,ES+
1016.9 > 366.1
4.084e+006

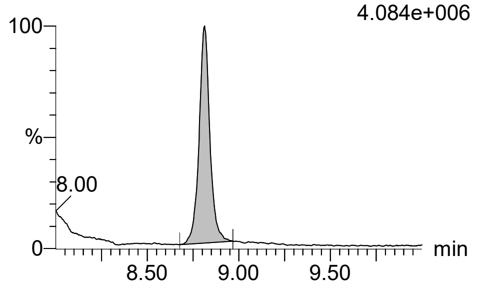

Sample Name: 20211111_L14_R3_IgM
F50:MRM of 1 channel,ES+
1016.9 > 366.1
9.235e+006

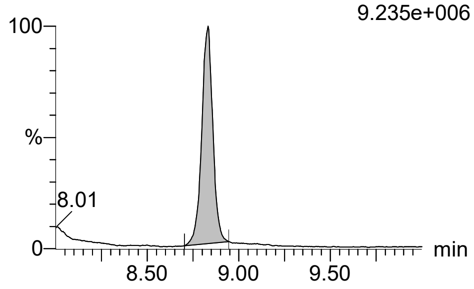

Sample Name: 20211111_H7_R3_IgM
F50:MRM of 1 channel,ES+
1016.9 > 366.1
7.676e+006

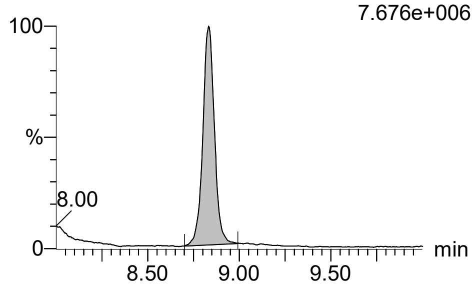

Sample Name: 20211111_A15_R3_IgM
F50:MRM of 1 channel,ES+
1016.9 > 366.1
2.241e+007

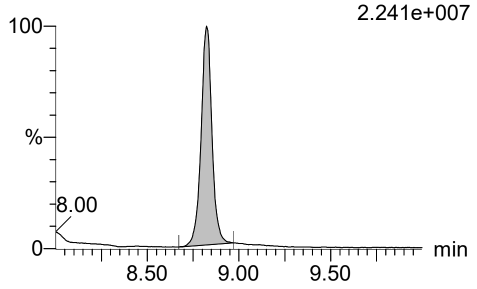

Sample Name: 20211111_QC_R20_IgM
F50:MRM of 1 channel,ES+
1016.9 > 366.1
8.491e+006

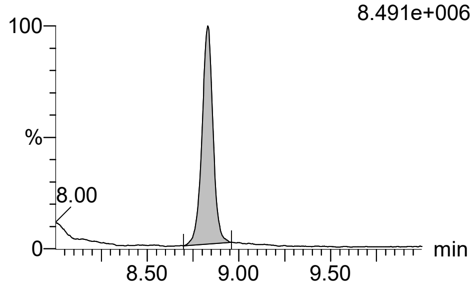

Sample Name: 20211111_A16_R3_IgM
F50:MRM of 1 channel,ES+
1016.9 > 366.1
7.751e+006

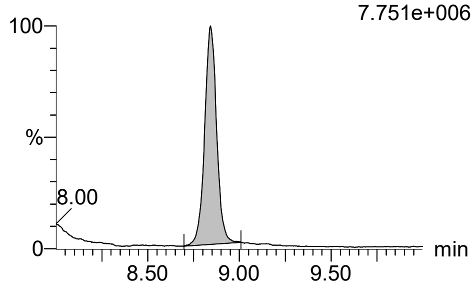

Sample Name: 20211111_L15_R3_IgM
F50:MRM of 1 channel,ES+
1016.9 > 366.1
1.117e+007

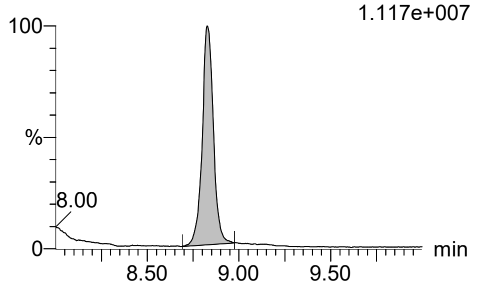

Sample Name: 20211111_L16_R3_IgM
F50:MRM of 1 channel,ES+
1016.9 > 366.1
1.718e+006

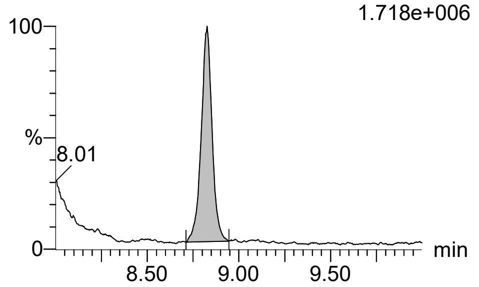

Sample Name: 20211111_H8_R3_IgM
F50:MRM of 1 channel,ES+
1016.9 > 366.1
4.766e+006

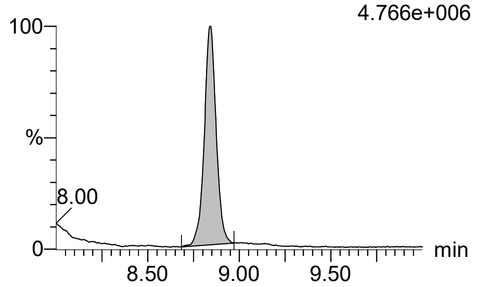

Sample Name: 20211111_A17_R3_IgM
F50:MRM of 1 channel,ES+
1016.9 > 366.1
1.278e+007

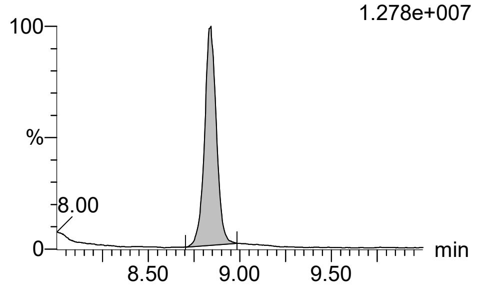

Sample Name: 20211111_A18_R3_IgM
F50:MRM of 1 channel,ES+
1016.9 > 366.1
1.266e+006

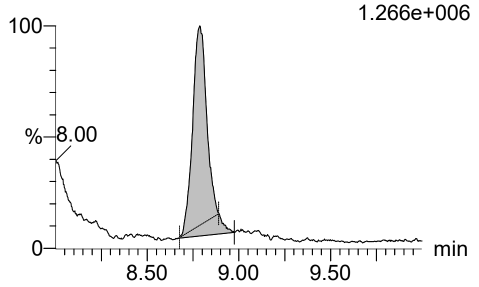

Sample Name: 20211111_L17_R3_IgM
F50:MRM of 1 channel,ES+
1016.9 > 366.1
1.282e+007

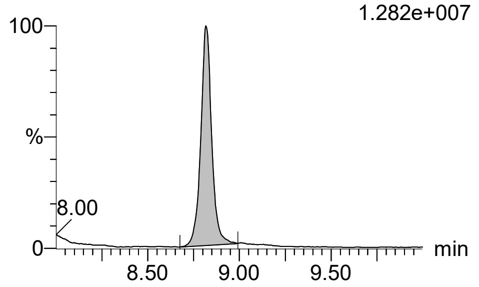

Sample Name: 20211111_L18_R3_IgM
F50:MRM of 1 channel,ES+
1016.9 > 366.1
1.454e+006

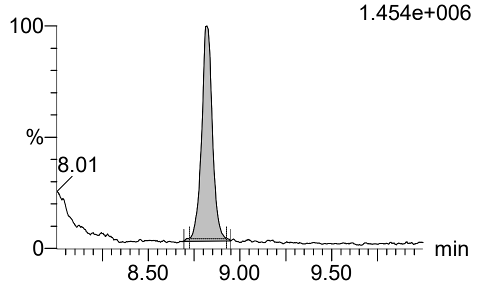

Sample Name: 20211111_H9_R3_IgM
F50:MRM of 1 channel,ES+
1016.9 > 366.1
4.044e+006

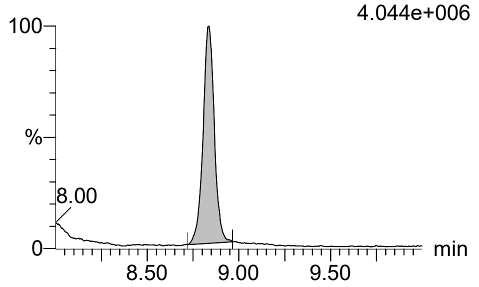

Sample Name: 20211111_A19_R3_IgM
F50:MRM of 1 channel,ES+
1016.9 > 366.1
8.613e+006

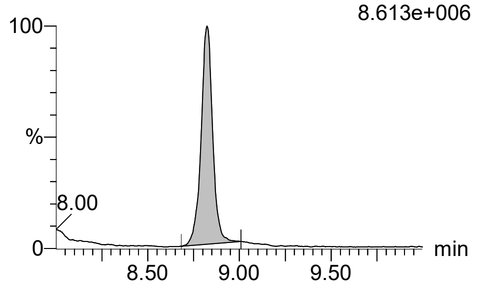

Sample Name: 20211111_A20_R3_IgM
F50:MRM of 1 channel,ES+
1016.9 > 366.1
9.780e+005

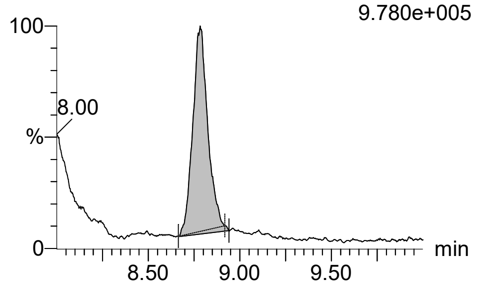

Sample Name: 20211111_L19_R3_IgM
F50:MRM of 1 channel,ES+
1016.9 > 366.1
9.690e+006

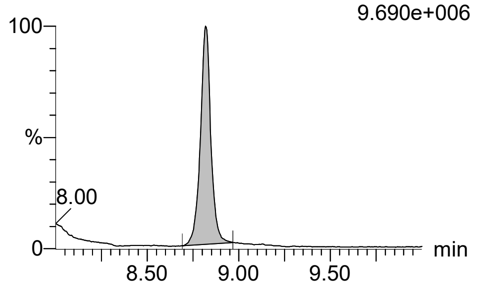

Sample Name: 20211111_QC_R21_IgM
F50:MRM of 1 channel,ES+
1016.9 > 366.1
8.626e+006

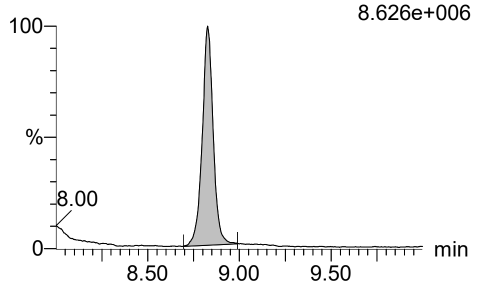

Sample Name: 20211111_L20_R3_IgM
F50:MRM of 1 channel,ES+
1016.9 > 366.1
6.119e+006

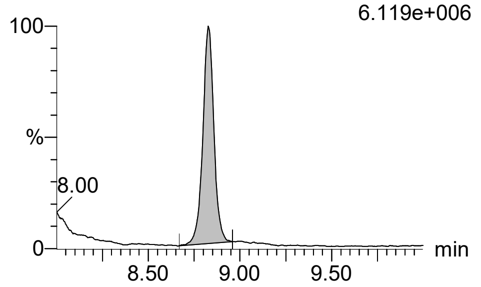

Sample Name: 20211111_H10_R3_IgM
F50:MRM of 1 channel,ES+
1016.9 > 366.1
1.475e+007

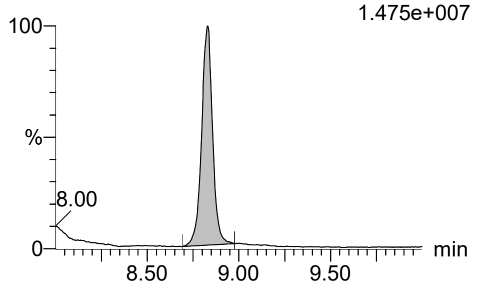

Sample Name: 20211111_A21_R3_IgM
F50:MRM of 1 channel,ES+
1016.9 > 366.1
6.215e+006

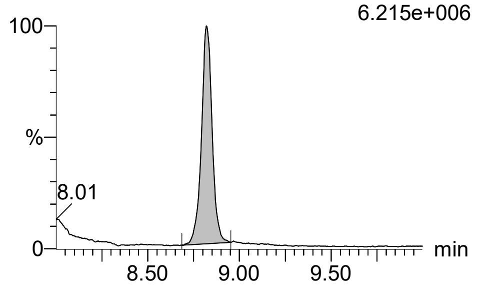

Sample Name: 20211111_A22_R3_IgM
F50:MRM of 1 channel,ES+
1016.9 > 366.1
2.407e+006

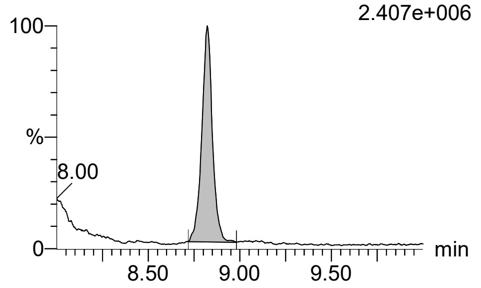

Sample Name: 20211111_L21_R3_IgM
F50:MRM of 1 channel,ES+
1016.9 > 366.1
7.329e+006

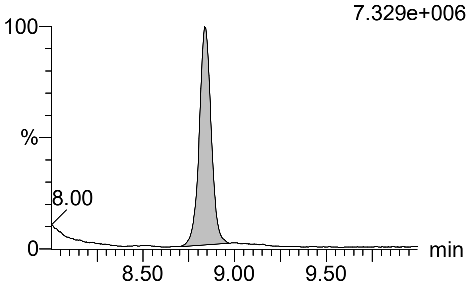

Sample Name: 20211111_L22_R3_IgM
F50:MRM of 1 channel,ES+
1016.9 > 366.1
5.150e+006

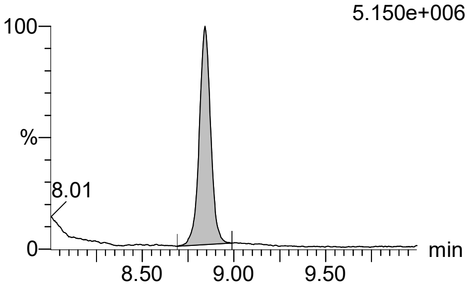

Sample Name: 20211111_H11_R3_IgM
F50:MRM of 1 channel,ES+
1016.9 > 366.1
6.002e+006

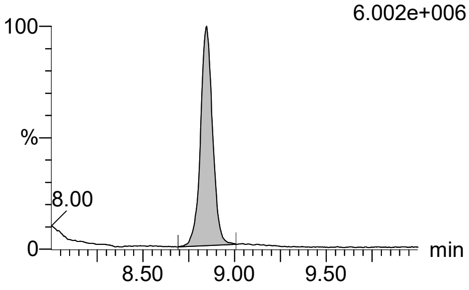

Sample Name: 20211111_A23_R3_IgM
F50:MRM of 1 channel,ES+
1016.9 > 366.1
4.795e+006

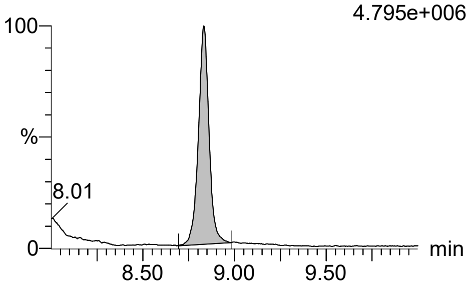

Sample Name: 20211111_A24_R3_IgM
F50:MRM of 1 channel,ES+
1016.9 > 366.1
2.029e+007

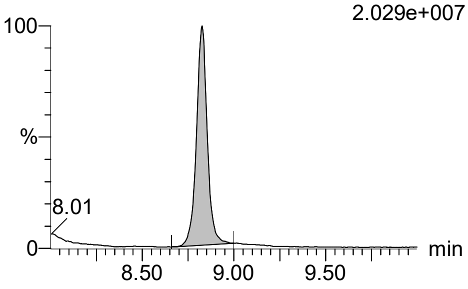

Sample Name: 20211111_L23_R3_IgM
F50:MRM of 1 channel,ES+
1016.9 > 366.1
1.012e+007

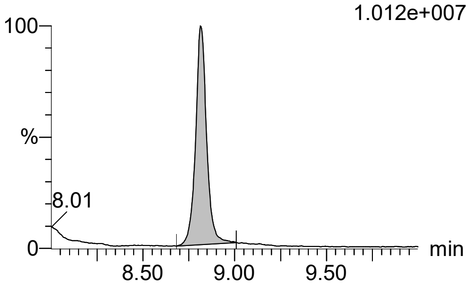

Sample Name: 20211111_L24_R3_IgM
F50:MRM of 1 channel,ES+
1016.9 > 366.1
3.490e+006

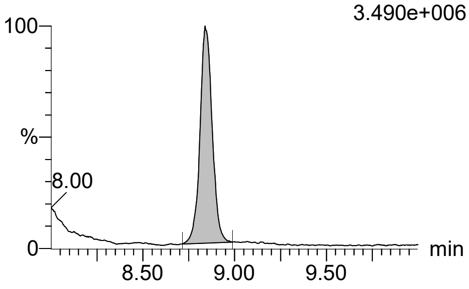

Sample Name: 20211111_H12_R3_IgM
F50:MRM of 1 channel,ES+
1016.9 > 366.1
4.063e+006

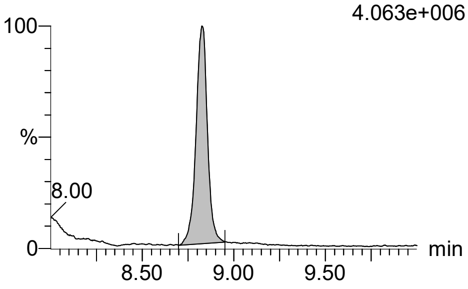

Sample Name: 20211111_QC_R22_IgM
F50:MRM of 1 channel,ES+
1016.9 > 366.1
8.163e+006

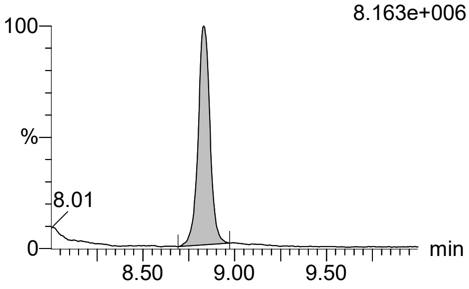

Sample Name: 20211111_A25_R3_IgM
F50:MRM of 1 channel,ES+
1016.9 > 366.1
1.078e+007

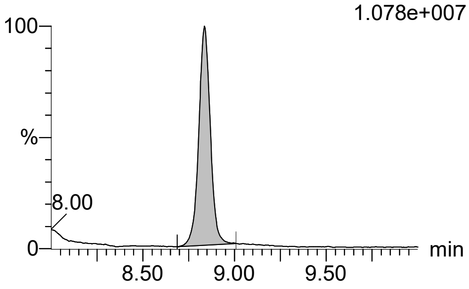

Sample Name: 20211111_A26_R3_IgM
F50:MRM of 1 channel,ES+
1016.9 > 366.1
7.213e+006

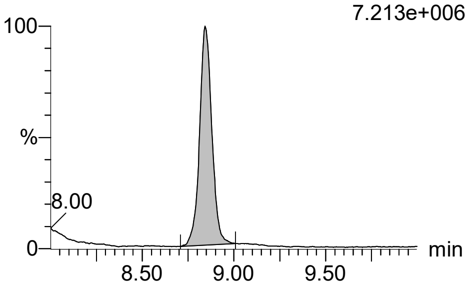

Sample Name: 20211111_L25_R3_IgM
F50:MRM of 1 channel,ES+
1016.9 > 366.1
2.883e+006

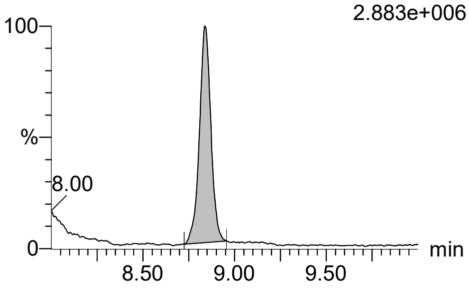

Sample Name: 20211111_L26_R3_IgM
F50:MRM of 1 channel,ES+
1016.9 > 366.1
4.337e+006

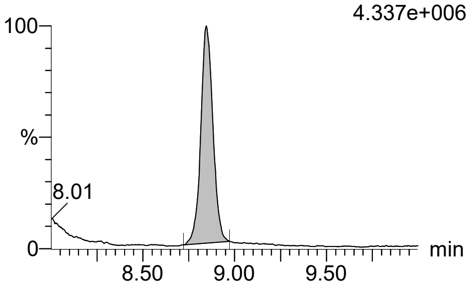

Sample Name: 20211111_H13_R3_IgM
F50:MRM of 1 channel,ES+
1016.9 > 366.1
1.016e+007

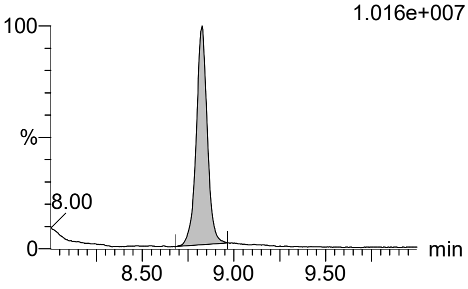

Sample Name: 20211111_A27_R3_IgM
F50:MRM of 1 channel,ES+
1016.9 > 366.1
1.610e+007

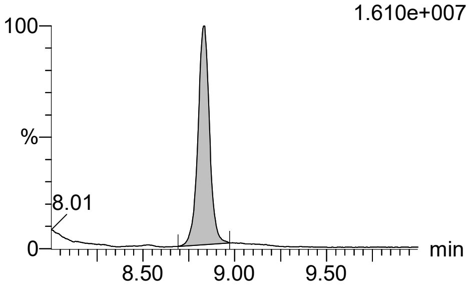

Quantify Compound Report **MassLynx V4.2 SCN943**

Dataset: C:\MassLynx\10.pro\20211111_clinicalsamples_IgM_IgA.qld

Last Altered: Thursday, March 09, 2023 19:58:25 台北標準時間
Printed: Friday, August 01, 2025 15:12:14 台北標準時間

Sample Name: 20211111_A28_R3_IgM

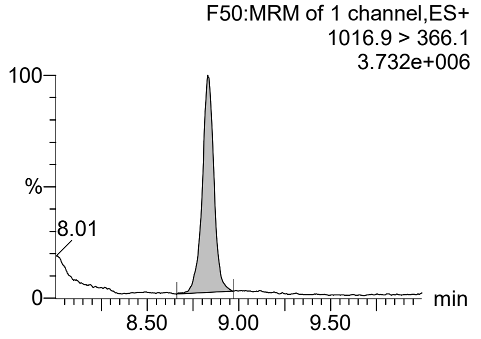

Sample Name: 20211111_L27_R3_IgM

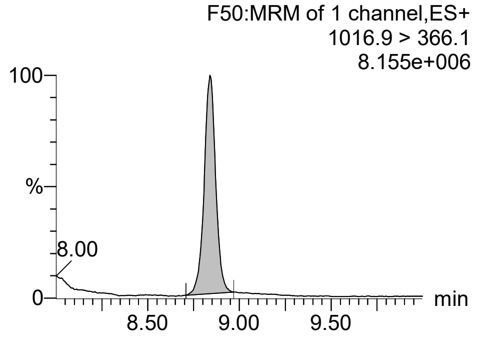

Sample Name: 20211111_L28_R3_IgM

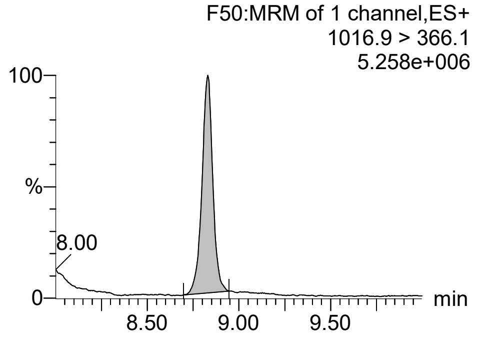

Sample Name: 20211111_H14_R3_IgM

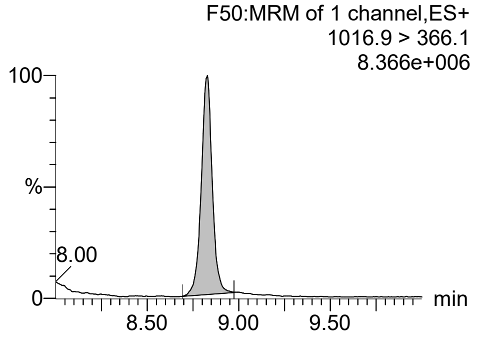

Sample Name: 20211111_A29_R3_IgM

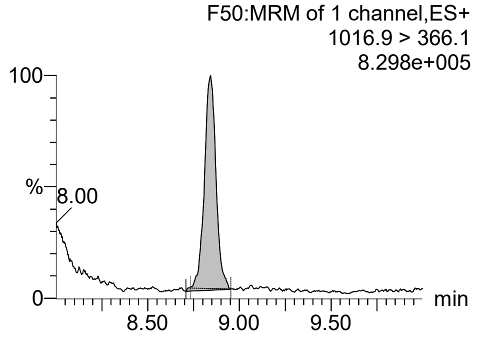

Sample Name: 20211111_A30_R3_IgM

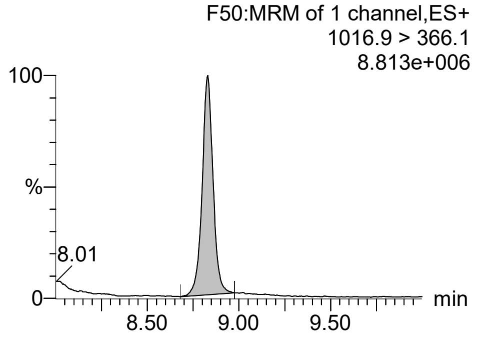

Sample Name: 20211111_QC_R23_IgM

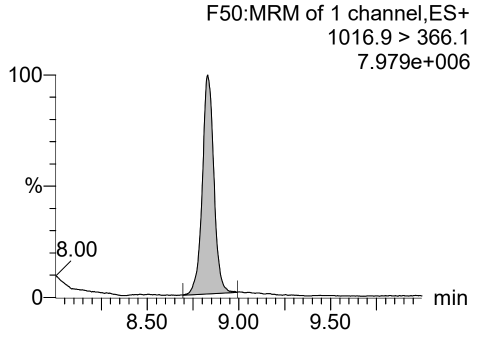

Sample Name: 20211111_L29_R3_IgM

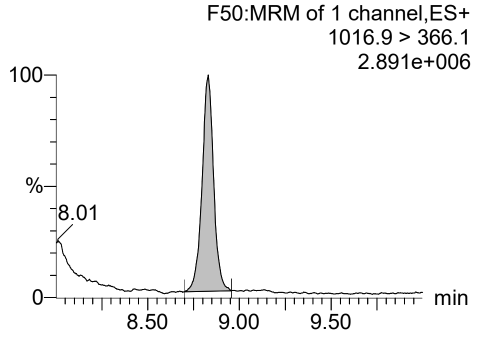

Sample Name: 20211111_L30_R3_IgM

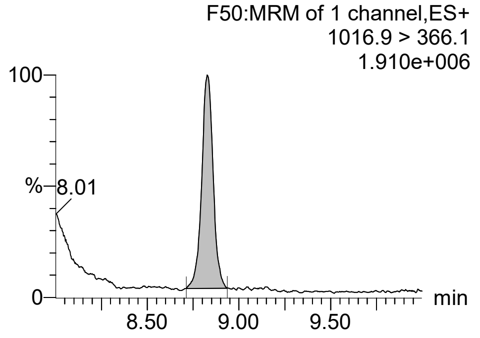

Sample Name: 20211111_H15_R3_IgM

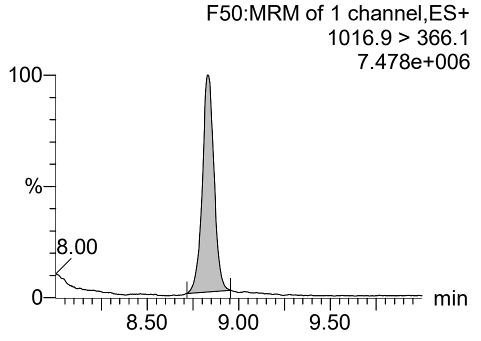

Sample Name: 20211111_A31_R3_IgM

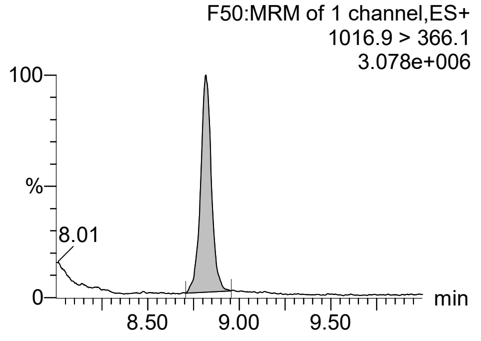

Sample Name: 20211111_A32_R3_IgM

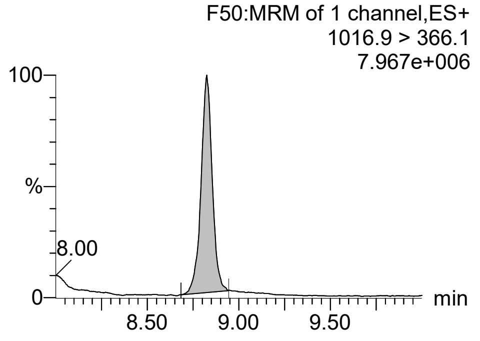

Sample Name: 20211111_L31_R3_IgM

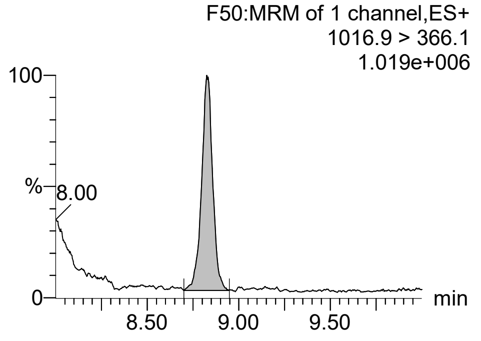

Sample Name: 20211111_L32_R3_IgM

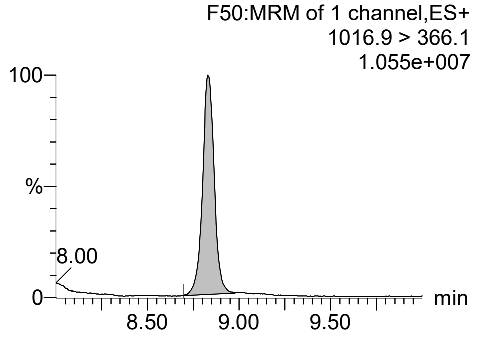

Sample Name: 20211111_H16_R3_IgM

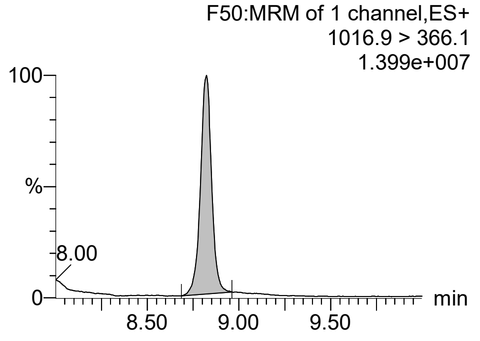

Dataset: C:\MassLynx\10.pro\20211111_clinicalsamples_IgM_IgA.qld

Last Altered: Thursday, March 09, 2023 19:58:25 台北標準時間
Printed: Friday, August 01, 2025 15:12:14 台北標準時間

Sample Name: 20211111_A33_R3_IgM

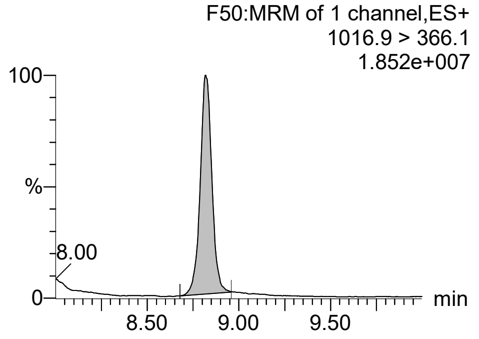

Sample Name: 20211111_A34_R3_IgM

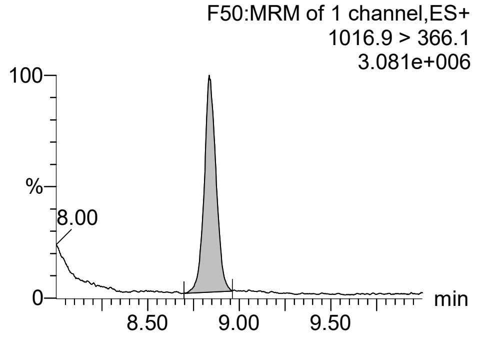

Sample Name: 20211111_H17_R3_IgM

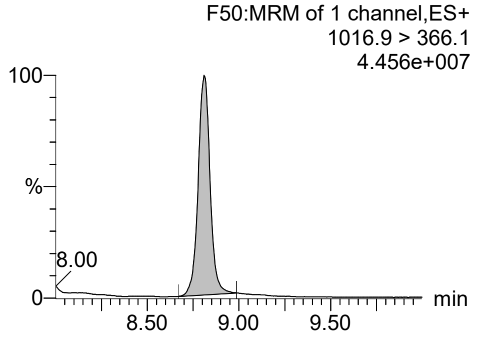

Sample Name: 20211111_A35_R3_IgM

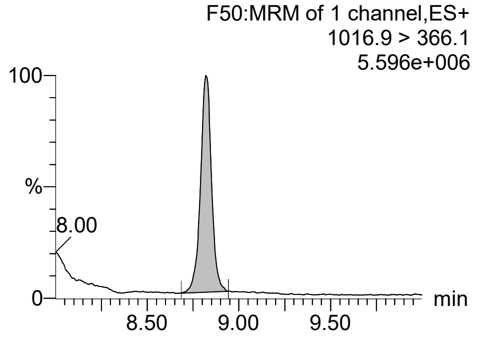

Sample Name: 20211111_QC_R24_IgM

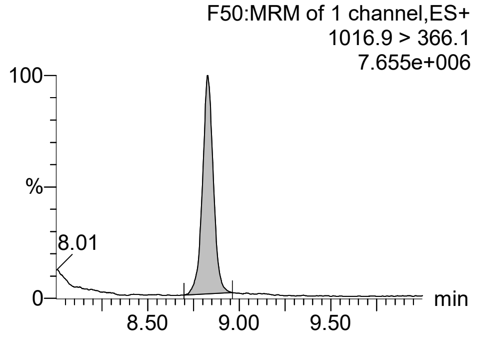

Sample Name: 20211111_A36_R3_IgM

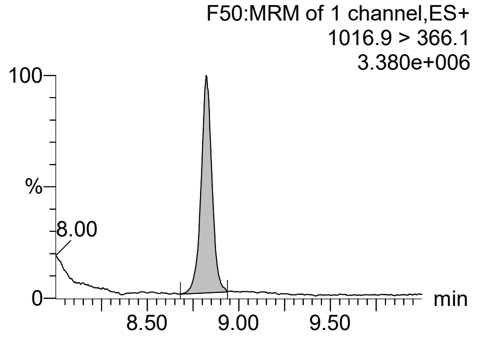

Sample Name: 20211111_H18_R3_IgM

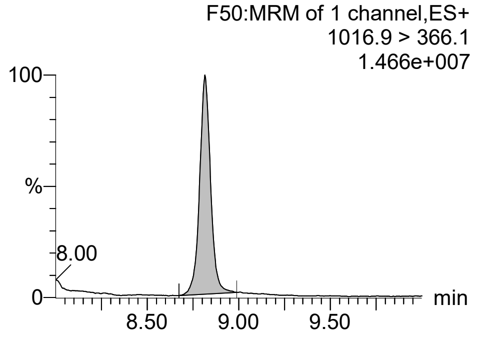

Sample Name: 20211111_A37_R3_IgM

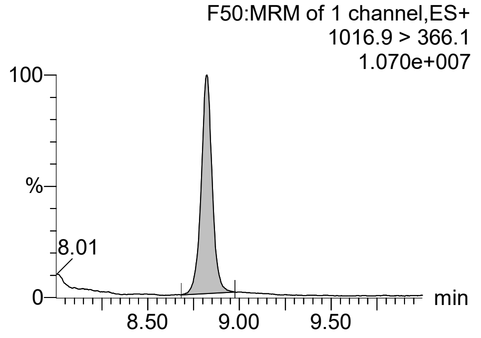

Sample Name: 20211111_A38_R3_IgM

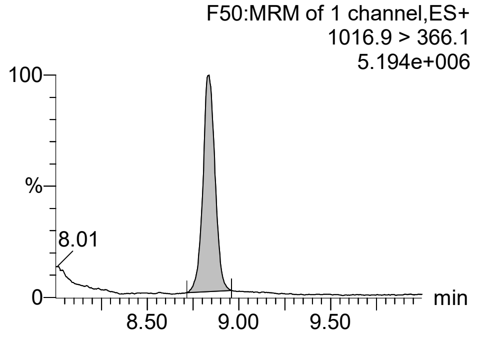

Sample Name: 20211111_H19_R3_IgM

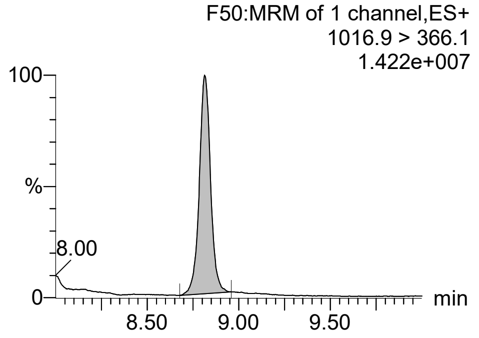

Sample Name: 20211111_A39_R3_IgM

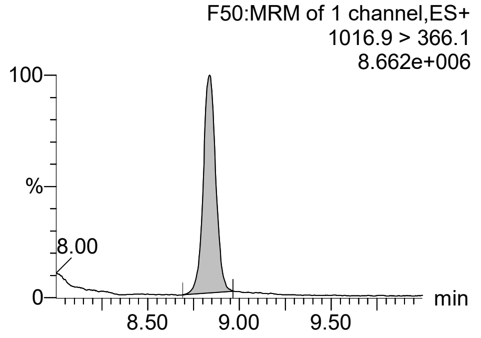

Sample Name: 20211111_A40_R3_IgM

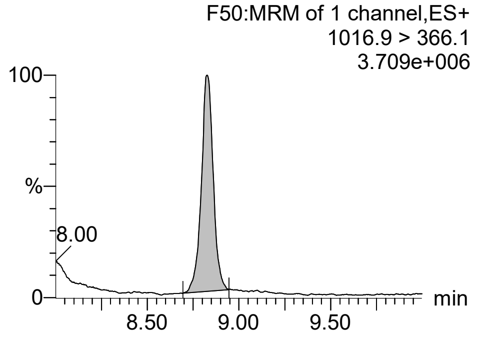

Sample Name: 20211111_H20_R3_IgM

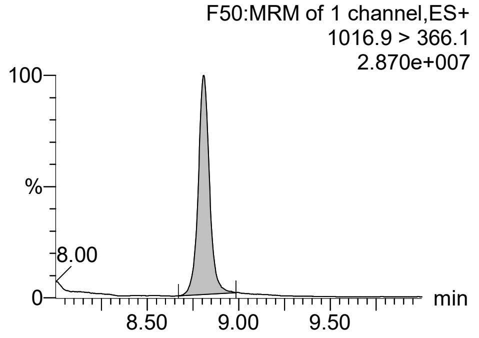

Sample Name: 20211111_A41_R3_IgM

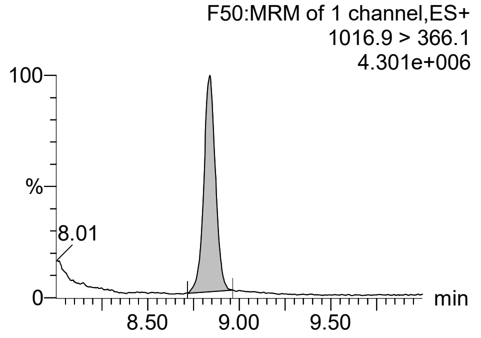

Sample Name: 20211111_A42_R3_IgM

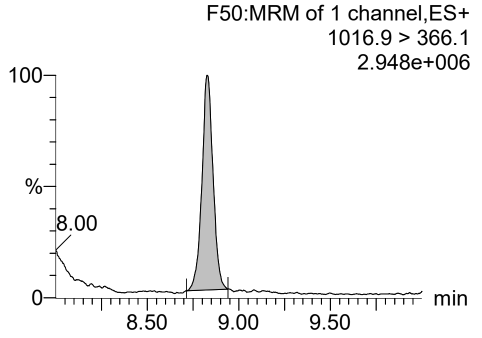

Dataset: C:\MassLynx\10.pro\20211111_clinicalsamples_IgM_IgA.qld

Last Altered: Thursday, March 09, 2023 19:58:25 台北標準時間
Printed: Friday, August 01, 2025 15:12:14 台北標準時間

Sample Name: 20211111_A43_R3_IgM

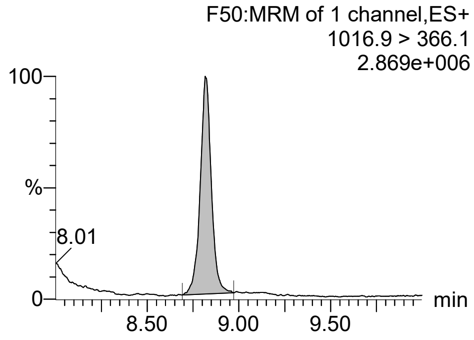

Sample Name: 20211111_A44_R3_IgM

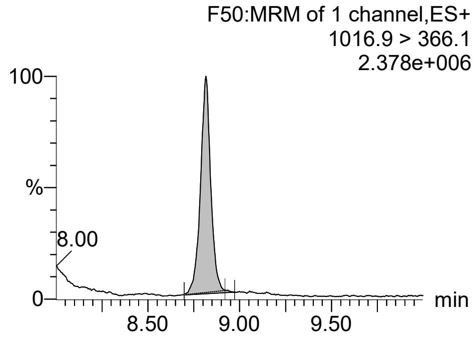

Sample Name: 20211111_QC_R25_IgM

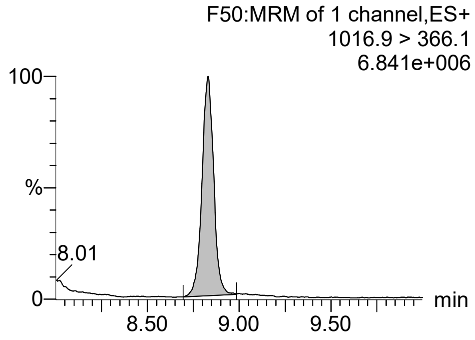

Dataset: C:\MassLynx\10.pro\20211111_clinicalsamples_IgM_IgA.qld

Last Altered: Thursday, March 09, 2023 19:58:25 台北標準時間
Printed: Friday, August 01, 2025 15:12:14 台北標準時間

	#	Name	RT	Area	IS Area	Response	Prima...
1	1	20211112_QC_R001_IgM	8.81	623277.3	15421.4	40.416	MM
2	2	20211112_QC_R002_IgM	8.82	588857.9	13188.8	44.648	MM
3	3	20211111_A1_R2_IgM	8.82	209936.0	11871.0	17.685	bb
4	4	20211111_A1_R3_IgM	8.83	211643.2	12030.5	17.592	bb
5	5	20211111_A2_R2_IgM	8.82	2033128.9	11446.8	177.615	bb
6	6	20211111_A2_R3_IgM	8.82	2022582.6	11098.4	182.241	bb
7	7	20211111_L1_R2_IgM	8.82	185392.3	11779.4	15.739	bb
8	8	20211111_L1_R3_IgM	8.83	150410.2	11176.2	13.458	bb
9	9	20211111_L2_R2_IgM	8.81	817295.9	11375.0	71.850	bb
10	10	20211111_H1_R2_IgM	8.80	328237.3	13595.2	24.144	bb
11	11	20211111_A3_R2_IgM	8.79	475410.5	14112.3	33.688	bb
12	12	20211111_A4_R2_IgM	8.80	452996.2	11977.5	37.821	bb
13	13	20211111_L3_R2_IgM	8.77	133164.3	12242.1	10.878	bb
14	14	20211111_L4_R2_IgM	8.82	760541.8	12797.0	59.431	bb
15	15	20211111_H2_R2_IgM	8.82	1010695.8	10006.0	101.009	bb
16	16	20211111_A5_R2_IgM	8.78	1303317.6	11434.6	113.980	bb
17	17	20211111_A6_R2_IgM	8.82	632736.7	13193.7	47.958	bb
18	18	20211111_QC_R10_IgM	8.82	595096.7	12566.1	47.357	bb
19	19	20211111_L5_R2_IgM	8.77	291472.7	13616.4	21.406	bb
20	20	20211111_L6_R2_IgM	8.82	937599.1	13698.5	68.445	bb
21	21	20211111_H3_R2_IgM	8.81	458207.4	15793.1	29.013	bb
22	22	20211111_A7_R2_IgM	8.81	367642.6	12936.9	28.418	bb
23	23	20211111_A8_R2_IgM	8.80	456378.9	13108.0	34.817	bb
24	24	20211111_L7_R2_IgM	8.82	294414.0	13278.5	22.172	bb
25	25	20211111_L8_R2_IgM	8.83	269405.8	15233.6	17.685	bb
26	26	20211111_H4_R2_IgM	8.82	355577.0	16521.9	21.522	bb
27	27	20211111_A9_R2_IgM	8.79	188229.3	16451.6	11.441	bb
28	28	20211111_A10_R2_IgM	8.80	469490.8	13036.0	36.015	bb
29	29	20211111_L9_R2_IgM	8.79	177257.5	14554.1	12.179	MM
30	30	20211111_L10_R2_IgM	8.79	464764.0	12250.4	37.939	bb
31	31	20211111_QC_R11_IgM	8.82	648178.7	11459.2	56.564	bb
32	32	20211111_H5_R2_IgM	8.81	874186.9	14358.2	60.884	bb
33	33	20211111_A11_R2_IgM	8.78	260258.3	16184.1	16.081	bb
34	34	20211111_A12_R2_IgM	8.81	251991.4	13465.4	18.714	bb
35	35	20211111_L11_R2_IgM	8.83	356418.2	13277.9	26.843	bb
36	36	20211111_L12_R2_IgM	8.80	251101.5	15958.8	15.734	bb
37	37	20211111_H6_R2_IgM	8.82	371877.7	14003.5	26.556	bb
38	38	20211111_A13_R2_IgM	8.82	588213.9	13656.1	43.073	bb
39	39	20211111_A14_R2_IgM	8.81	827627.2	14071.3	58.817	bb
40	40	20211111_L13_R2_IgM	8.83	369829.6	14785.6	25.013	bb
41	41	20211111_L14_R2_IgM	8.83	686739.6	14772.2	46.489	bb
42	42	20211111_H7_R2_IgM	8.79	281922.2	15164.4	18.591	bb
43	43	20211111_A15_R2_IgM	8.83	1720009.1	15721.5	109.405	bb
44	44	20211111_QC_R12_IgM	8.83	694291.1	13711.5	50.636	bb
45	45	20211111_A16_R2_IgM	8.83	694871.6	14275.6	48.676	bb
46	46	20211111_L15_R2_IgM	8.81	689509.6	12935.8	53.302	bb
47	47	20211111_L16_R2_IgM	8.82	115209.2	13342.8	8.635	bb
48	48	20211111_H8_R2_IgM	8.84	350690.8	15835.1	22.146	bb
49	49	20211111_A17_R2_IgM	8.83	1099848.4	15786.7	69.669	bb
50	50	20211111_A18_R2_IgM	8.81	201313.7	16986.8	11.851	MM
51	51	20211111_L17_R2_IgM	8.81	895187.8	13342.7	67.092	bb
52	52	20211111_L18_R2_IgM	8.81	97917.2	14692.0	6.665	bb
53	53	20211111_H9_R2_IgM	8.81	236740.0	15639.7	15.137	bb
54	54	20211111_A19_R2_IgM	8.82	656380.5	14198.7	46.228	bb
55	55	20211111_A20_R2_IgM	8.78	104805.3	14162.7	7.400	bb
56	56	20211111_L19_R2_IgM	8.81	556650.8	9293.4	59.897	bb

Dataset: C:\MassLynx\10.pro\20211111_clinicalsamples_IgM_IgA.qld

Last Altered: Thursday, March 09, 2023 19:58:25 台北標準時間
Printed: Friday, August 01, 2025 15:12:14 台北標準時間

	# Name	RT	Area	IS Area	Response	Prima...
57	57 20211111_QC_R13_IgM	8.83	680275.5	13903.0	48.930	bb
58	58 20211111_L20_R2_IgM	8.78	173377.6	12781.6	13.565	MM
59	59 20211111_H10_R2_IgM	8.78	473885.4	14796.4	32.027	bb
60	60 20211111_A21_R2_IgM	8.82	385086.1	13669.0	28.172	bb
61	61 20211111_A22_R2_IgM	8.82	153090.8	14098.3	10.859	bb
62	62 20211111_L21_R2_IgM	8.84	612033.1	15678.2	39.037	bb
63	63 20211111_L22_R2_IgM	8.84	390956.7	15724.5	24.863	bb
64	64 20211111_H11_R2_IgM	8.84	467847.2	15890.2	29.443	bb
65	65 20211111_A23_R2_IgM	8.82	320617.1	13423.2	23.885	bb
66	66 20211111_A24_R2_IgM	8.82	1318537.6	12438.0	106.009	bb
67	67 20211111_L23_R2_IgM	8.82	862398.5	14075.0	61.272	MM
68	68 20211111_L24_R2_IgM	8.82	195929.3	15972.1	12.267	MM
69	69 20211111_H12_R2_IgM	8.82	283772.8	16270.9	17.441	bb
70	70 20211111_QC_R14_IgM	8.84	718073.1	15806.8	45.428	bb
71	71 20211111_A25_R2_IgM	8.83	920238.4	14552.1	63.238	bb
72	72 20211111_A26_R2_IgM	8.84	579529.9	11492.7	50.426	bb
73	73 20211111_L25_R2_IgM	8.84	227340.3	14403.3	15.784	bb
74	74 20211111_L26_R2_IgM	8.84	380719.0	16533.9	23.027	bb
75	75 20211111_H13_R2_IgM	8.83	723195.8	15520.1	46.597	bb
76	76 20211111_A27_R2_IgM	8.82	1189931.0	13337.4	89.217	bb
77	77 20211111_A28_R2_IgM	8.78	107515.6	11895.6	9.038	MM
78	78 20211111_L27_R2_IgM	8.82	518746.5	14125.7	36.724	bb
79	79 20211111_L28_R2_IgM	8.82	335283.8	11807.5	28.396	bb
80	80 20211111_H14_R2_IgM	8.82	550893.0	15035.7	36.639	bb
81	81 20211111_A29_R2_IgM	8.83	55991.2	13554.4	4.131	bb
82	82 20211111_A30_R2_IgM	8.82	610778.9	12984.1	47.041	bb
83	83 20211111_QC_R15_IgM	8.83	638012.2	12299.5	51.873	bb
84	84 20211111_L29_R2_IgM	8.83	203587.6	13590.7	14.980	bb
85	85 20211111_L30_R2_IgM	8.82	125280.5	12511.4	10.013	bb
86	86 20211111_H15_R2_IgM	8.83	572045.7	11885.0	48.132	bb
87	87 20211111_A31_R2_IgM	8.82	208337.8	13422.1	15.522	bb
88	88 20211111_A32_R2_IgM	8.83	628891.9	11461.3	54.871	bb
89	89 20211111_L31_R2_IgM	8.78	20215.1	13753.4	1.470	MM
90	90 20211111_L32_R2_IgM	8.80	542940.3	12949.0	41.929	bb
91	91 20211111_H16_R2_IgM	8.81	983434.9	11465.4	85.774	bb
92	92 20211111_A33_R2_IgM	8.82	1326329.6	10551.5	125.700	bb
93	93 20211111_A34_R2_IgM	8.83	248675.6	12409.9	20.039	bb
94	94 20211111_H17_R2_IgM	8.80	3711065.3	10160.9	365.229	bb
95	95 20211111_A35_R2_IgM	8.77	202054.7	10460.6	19.316	bb
96	96 20211111_QC_R16_IgM	8.81	563185.0	10433.5	53.979	bb
97	97 20211111_A36_R2_IgM	8.77	67902.8	12504.8	5.430	MM
98	98 20211111_H18_R2_IgM	8.81	994867.9	9140.4	108.843	bb
99	99 20211111_A37_R2_IgM	8.82	880562.9	9796.9	89.882	bb
100	1... 20211111_A38_R2_IgM	8.84	440364.2	11224.3	39.233	bb
101	1... 20211111_H19_R2_IgM	8.82	1122876.3	11739.2	95.652	bb
102	1... 20211111_A39_R2_IgM	8.83	733122.9	12718.9	57.640	bb
103	1... 20211111_A40_R2_IgM	8.78	133944.2	10006.1	13.386	MM
104	1... 20211111_H20_R2_IgM	8.80	2111966.5	7988.2	264.386	bb
105	1... 20211111_A41_R2_IgM	8.84	486943.1	11513.3	42.294	bb
106	1... 20211111_A42_R2_IgM	8.83	241640.3	10238.5	23.601	bb
107	1... 20211111_A43_R2_IgM	8.82	212083.6	11098.9	19.109	MM
108	1... 20211111_A44_R2_IgM	8.77	84881.3	10969.9	7.738	bb
109	1... 20211111_QC_R17_IgM	8.82	489457.7	9700.8	50.455	bb
110	1... 20211111_L2_R3_IgM	8.82	849338.1	8528.0	99.594	bb
111	1... 20211111_H1_R3_IgM	8.82	450259.7	9438.2	47.706	bb
112	1... 20211111_A3_R3_IgM	8.82	689059.2	11598.6	59.409	bb

Dataset: C:\MassLynx\10.pro\20211111_clinicalsamples_IgM_IgA.qld

Last Altered: Thursday, March 09, 2023 19:58:25 台北標準時間
Printed: Friday, August 01, 2025 15:12:14 台北標準時間

	# Name	RT	Area	IS Area	Response	Prima...
113	1... 20211111_A4_R3_IgM	8.83	570899.4	11364.1	50.237	bb
114	1... 20211111_L3_R3_IgM	8.77	171566.6	10753.4	15.955	MM
115	1... 20211111_L4_R3_IgM	8.82	749586.7	10291.6	72.835	bb
116	1... 20211111_H2_R3_IgM	8.82	1049619.4	9315.8	112.671	bb
117	1... 20211111_A5_R3_IgM	8.82	1888274.1	9102.2	207.453	bb
118	1... 20211111_A6_R3_IgM	8.82	547533.8	10356.4	52.869	MM
119	1... 20211111_QC_R18_IgM	8.82	485977.4	8398.3	57.866	bb
120	1... 20211111_L5_R3_IgM	8.83	487286.8	9786.7	49.790	bb
121	1... 20211111_L6_R3_IgM	8.83	784917.8	9911.3	79.194	bb
122	1... 20211111_H3_R3_IgM	8.84	567919.6	12155.5	46.721	bb
123	1... 20211111_A7_R3_IgM	8.82	373329.2	11804.8	31.625	bb
124	1... 20211111_A8_R3_IgM	8.83	511651.2	10510.6	48.679	bb
125	1... 20211111_L7_R3_IgM	8.82	294076.5	12177.9	24.148	bb
126	1... 20211111_L8_R3_IgM	8.84	246783.8	11668.6	21.149	bb
127	1... 20211111_H4_R3_IgM	8.84	352485.8	11192.0	31.495	bb
128	1... 20211111_A9_R3_IgM	8.84	282854.6	14077.4	20.093	bb
129	1... 20211111_A10_R3_IgM	8.84	611079.9	11190.4	54.608	bb
130	1... 20211111_L9_R3_IgM	8.83	269682.7	14649.3	18.409	bb
131	1... 20211111_L10_R3_IgM	8.77	314463.0	9361.3	33.592	bb
132	1... 20211111_QC_R19_IgM	8.82	494596.5	10125.5	48.847	bb
133	1... 20211111_H5_R3_IgM	8.83	881429.6	10010.9	88.047	bb
134	1... 20211111_A11_R3_IgM	8.83	823208.9	12697.2	64.834	bb
135	1... 20211111_A12_R3_IgM	8.81	279399.1	13148.5	21.249	bb
136	1... 20211111_L11_R3_IgM	8.84	284311.0	10786.6	26.358	bb
137	1... 20211111_L12_R3_IgM	8.78	162014.0	11681.8	13.869	MM
138	1... 20211111_H6_R3_IgM	8.82	344364.0	12095.5	28.471	bb
139	1... 20211111_A13_R3_IgM	8.82	583417.1	12908.3	45.197	bb
140	1... 20211111_A14_R3_IgM	8.82	821909.2	11322.6	72.590	bb
141	1... 20211111_L13_R3_IgM	8.81	269608.1	11516.1	23.411	bb
142	1... 20211111_L14_R3_IgM	8.83	628984.5	10790.7	58.290	bb
143	1... 20211111_H7_R3_IgM	8.83	549271.4	11572.1	47.465	bb
144	1... 20211111_A15_R3_IgM	8.82	1504719.6	12434.4	121.012	bb
145	1... 20211111_QC_R20_IgM	8.83	576045.8	10701.0	53.831	bb
146	1... 20211111_A16_R3_IgM	8.84	571485.3	11452.1	49.902	bb
147	1... 20211111_L15_R3_IgM	8.83	768835.1	10531.1	73.006	bb
148	1... 20211111_L16_R3_IgM	8.83	112610.7	12049.4	9.346	bb
149	1... 20211111_H8_R3_IgM	8.84	348464.8	13434.9	25.937	bb
150	1... 20211111_A17_R3_IgM	8.84	932409.4	12452.0	74.880	bb
151	1... 20211111_A18_R3_IgM	8.79	115120.3	12506.5	9.205	MM
152	1... 20211111_L17_R3_IgM	8.82	866309.7	9948.9	87.076	bb
153	1... 20211111_L18_R3_IgM	8.82	96184.9	13462.1	7.145	MM
154	1... 20211111_H9_R3_IgM	8.84	279356.3	13178.7	21.197	bb
155	1... 20211111_A19_R3_IgM	8.82	615220.3	11298.6	54.451	bb
156	1... 20211111_A20_R3_IgM	8.78	87369.6	11175.6	7.818	MM
157	1... 20211111_L19_R3_IgM	8.82	622881.5	11558.2	53.891	bb
158	1... 20211111_QC_R21_IgM	8.83	584458.1	9977.0	58.580	bb
159	1... 20211111_L20_R3_IgM	8.83	415498.2	13508.1	30.759	bb
160	1... 20211111_H10_R3_IgM	8.83	995187.4	12374.8	80.421	bb
161	1... 20211111_A21_R3_IgM	8.82	407645.3	12443.6	32.760	bb
162	1... 20211111_A22_R3_IgM	8.82	155487.7	11589.3	13.417	bb
163	1... 20211111_L21_R3_IgM	8.84	537065.6	13107.4	40.974	bb
164	1... 20211111_L22_R3_IgM	8.84	359664.2	12021.5	29.919	bb
165	1... 20211111_H11_R3_IgM	8.85	446487.8	12687.8	35.190	bb
166	1... 20211111_A23_R3_IgM	8.83	313570.6	11611.8	27.004	bb
167	1... 20211111_A24_R3_IgM	8.83	1357393.0	10476.8	129.562	bb
168	1... 20211111_L23_R3_IgM	8.81	683773.8	11873.1	57.590	bb

Dataset: C:\MassLynx\10.pro\20211111_clinicalsamples_IgM_IgA.qld

Last Altered: Thursday, March 09, 2023 19:58:25 台北標準時間
Printed: Friday, August 01, 2025 15:12:14 台北標準時間

	# Name	RT	Area	IS Area	Response	Prima...
169	1... 20211111_L24_R3_IgM	8.84	259639.6	13468.0	19.278	bb
170	1... 20211111_H12_R3_IgM	8.83	284940.0	14229.1	20.025	bb
171	1... 20211111_QC_R22_IgM	8.83	597557.1	11264.4	53.048	bb
172	1... 20211111_A25_R3_IgM	8.84	769870.4	11775.7	65.378	bb
173	1... 20211111_A26_R3_IgM	8.84	546107.3	10473.9	52.140	bb
174	1... 20211111_L25_R3_IgM	8.84	201820.0	13256.8	15.224	bb
175	1... 20211111_L26_R3_IgM	8.85	322138.8	13565.0	23.748	bb
176	1... 20211111_H13_R3_IgM	8.83	687630.9	13146.0	52.307	bb
177	1... 20211111_A27_R3_IgM	8.83	1144911.0	12025.6	95.206	bb
178	1... 20211111_A28_R3_IgM	8.83	266467.9	11229.1	23.730	bb
179	1... 20211111_L27_R3_IgM	8.84	577269.6	14960.0	38.587	bb
180	1... 20211111_L28_R3_IgM	8.83	348961.9	11359.0	30.721	bb
181	1... 20211111_H14_R3_IgM	8.83	565907.9	12830.8	44.106	bb
182	1... 20211111_A29_R3_IgM	8.84	59082.9	13434.3	4.398	MM
183	1... 20211111_A30_R3_IgM	8.83	592409.5	11272.2	52.555	bb
184	1... 20211111_QC_R23_IgM	8.83	570193.3	11872.2	48.028	bb
185	1... 20211111_L29_R3_IgM	8.83	203505.4	12948.7	15.716	bb
186	1... 20211111_L30_R3_IgM	8.83	131476.1	11026.6	11.924	bb
187	1... 20211111_H15_R3_IgM	8.83	535923.2	10786.3	49.686	bb
188	1... 20211111_A31_R3_IgM	8.82	203727.7	12010.5	16.963	bb
189	1... 20211111_A32_R3_IgM	8.83	545515.4	11107.7	49.112	bb
190	1... 20211111_L31_R3_IgM	8.83	68593.6	11021.8	6.223	bb
191	1... 20211111_L32_R3_IgM	8.83	761918.7	11683.8	65.212	bb
192	1... 20211111_H16_R3_IgM	8.82	963865.9	9831.7	98.037	bb
193	1... 20211111_A33_R3_IgM	8.82	1333131.8	11213.1	118.890	bb
194	1... 20211111_A34_R3_IgM	8.83	222416.0	11550.1	19.257	bb
195	1... 20211111_H17_R3_IgM	8.81	3286806.3	8874.4	370.368	bb
196	1... 20211111_A35_R3_IgM	8.82	376337.6	9856.3	38.183	bb
197	1... 20211111_QC_R24_IgM	8.83	512403.4	7561.8	67.762	bb
198	1... 20211111_A36_R3_IgM	8.82	224734.9	9562.8	23.501	bb
199	1... 20211111_H18_R3_IgM	8.82	994094.6	8165.3	121.746	bb
200	2... 20211111_A37_R3_IgM	8.82	726556.9	9278.8	78.303	bb
201	2... 20211111_A38_R3_IgM	8.84	385002.0	10920.3	35.256	bb
202	2... 20211111_H19_R3_IgM	8.81	986873.5	9285.3	106.283	bb
203	2... 20211111_A39_R3_IgM	8.84	667238.9	9083.4	73.457	bb
204	2... 20211111_A40_R3_IgM	8.83	269294.6	8382.4	32.126	bb
205	2... 20211111_H20_R3_IgM	8.81	1997600.9	7807.9	255.843	bb
206	2... 20211111_A41_R3_IgM	8.84	306569.8	5925.2	51.740	bb
207	2... 20211111_A42_R3_IgM	8.83	199142.8	9352.6	21.293	bb
208	2... 20211111_A43_R3_IgM	8.82	194898.7	10216.3	19.077	bb
209	2... 20211111_A44_R3_IgM	8.82	150387.4	10262.7	14.654	MM
210	2... 20211111_QC_R25_IgM	8.83	493481.2	8575.3	57.547	bb

Dataset: C:\MassLynx\10.pro\20211111_clinicalsamples_IgM_IgA.qld

Last Altered: Thursday, March 09, 2023 19:58:25 台北標準時間
Printed: Friday, August 01, 2025 15:13:08 台北標準時間

Method: C:\MassLynx\10_Pro_IgA.PRO\MethDB\20211111_IgM_IgA_DMRM_new.mdb 24 Feb 2022 13:44:14
Calibration: 15 Nov 2021 14:42:11

Sample Name: 20211112_QC_R001_IgM
F65:MRM of 1 channel,ES+
1075.1 > 366.1
6.625e+005

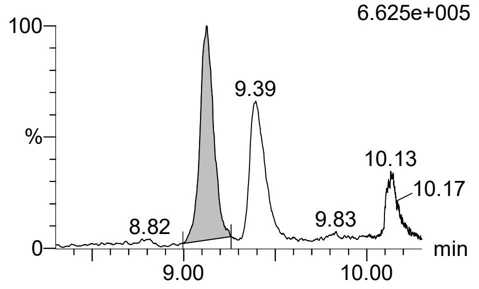

Sample Name: 20211112_QC_R002_IgM
F65:MRM of 1 channel,ES+
1075.1 > 366.1
6.363e+005

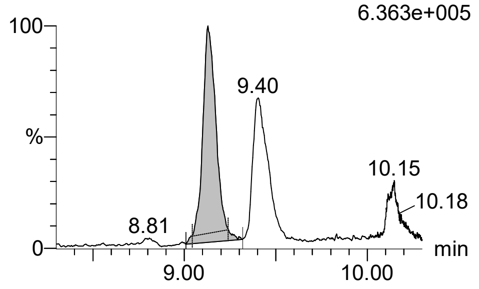

Sample Name: 20211111_A1_R2_IgM
F65:MRM of 1 channel,ES+
1075.1 > 366.1
3.080e+005

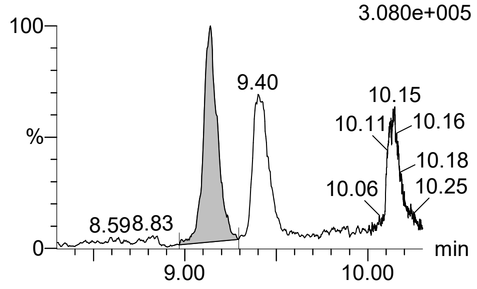

Sample Name: 20211111_A1_R3_IgM
F65:MRM of 1 channel,ES+
1075.1 > 366.1
3.044e+005

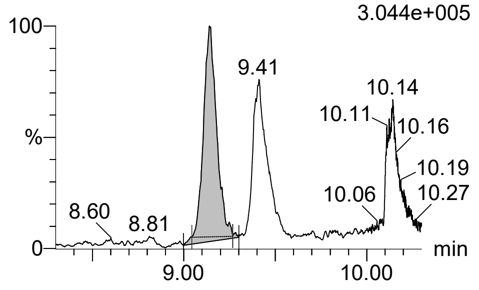

Sample Name: 20211111_A2_R2_IgM
F65:MRM of 1 channel,ES+
1075.1 > 366.1
2.425e+006

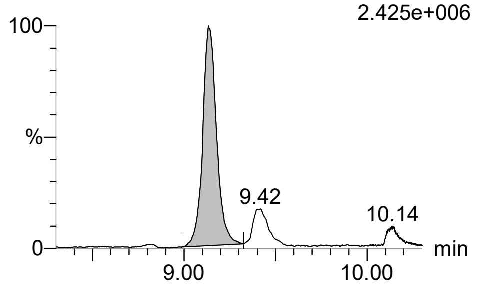

Sample Name: 20211111_A2_R3_IgM
F65:MRM of 1 channel,ES+
1075.1 > 366.1
2.309e+006

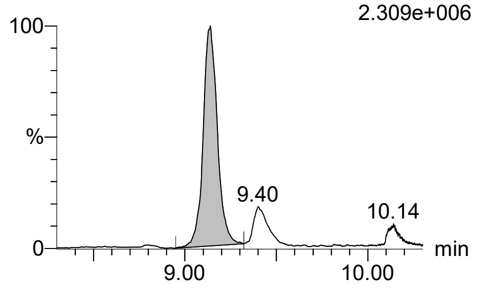

Sample Name: 20211111_L1_R2_IgM
F65:MRM of 1 channel,ES+
1075.1 > 366.1
2.194e+005

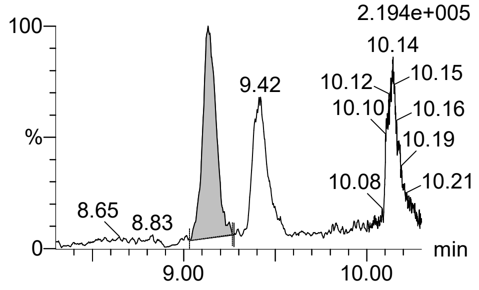

Sample Name: 20211111_L1_R3_IgM
F65:MRM of 1 channel,ES+
1075.1 > 366.1
1.835e+005

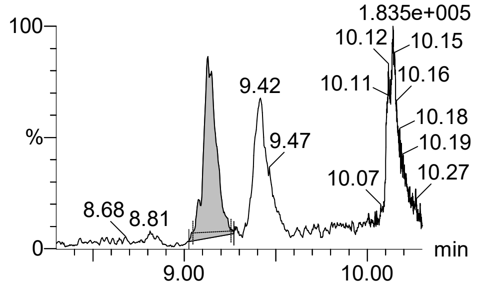

Sample Name: 20211111_L2_R2_IgM
F65:MRM of 1 channel,ES+
1075.1 > 366.1
8.468e+005

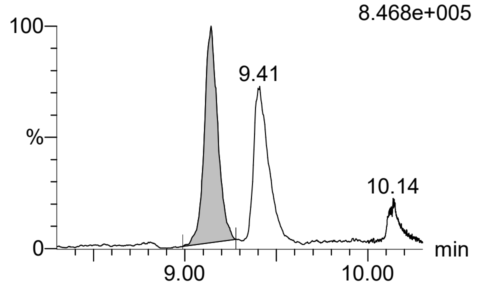

Sample Name: 20211111_H1_R2_IgM
F65:MRM of 1 channel,ES+
1075.1 > 366.1
4.407e+005

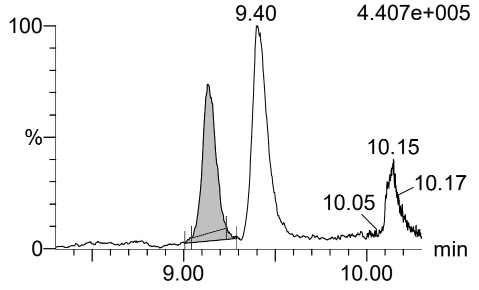

Sample Name: 20211111_A3_R2_IgM
F65:MRM of 1 channel,ES+
1075.1 > 366.1
8.536e+005

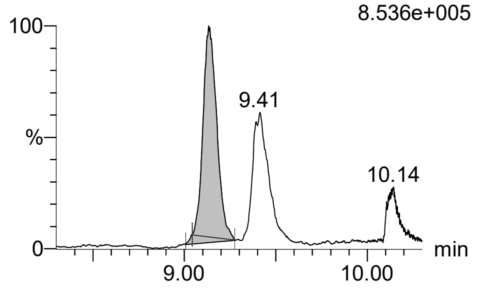

Sample Name: 20211111_A4_R2_IgM
F65:MRM of 1 channel,ES+
1075.1 > 366.1
5.849e+005

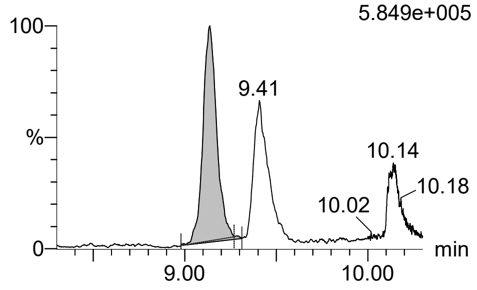

Dataset: C:\MassLynx\10.pro\20211111_clinicalsamples_IgM_IgA.qld

Last Altered: Thursday, March 09, 2023 19:58:25 台北標準時間
Printed: Friday, August 01, 2025 15:13:08 台北標準時間

Sample Name: 20211111_L3_R2_IgM
F65:MRM of 1 channel,ES+
1075.1 > 366.1
3.570e+005

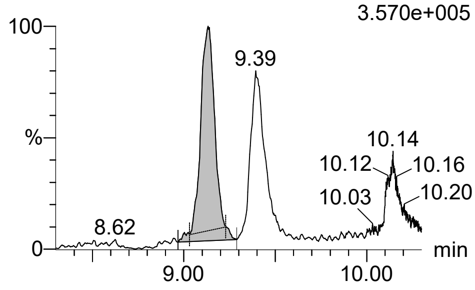

Sample Name: 20211111_L4_R2_IgM
F65:MRM of 1 channel,ES+
1075.1 > 366.1
8.490e+005

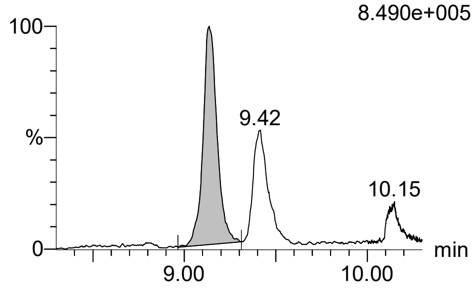

Sample Name: 20211111_H2_R2_IgM
F65:MRM of 1 channel,ES+
1075.1 > 366.1
1.074e+006

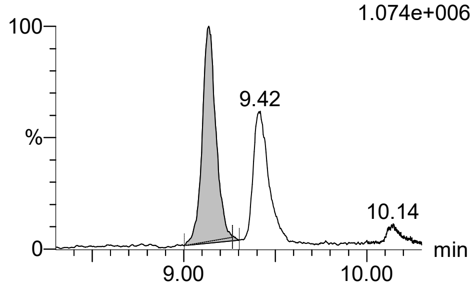

Sample Name: 20211111_A5_R2_IgM
F65:MRM of 1 channel,ES+
1075.1 > 366.1
5.587e+006

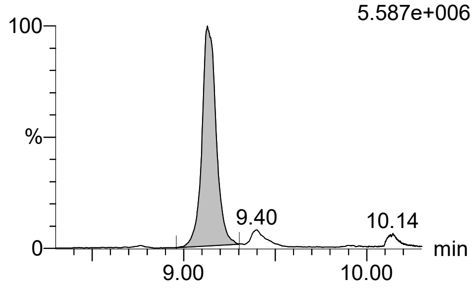

Sample Name: 20211111_A6_R2_IgM
F65:MRM of 1 channel,ES+
1075.1 > 366.1
1.074e+006

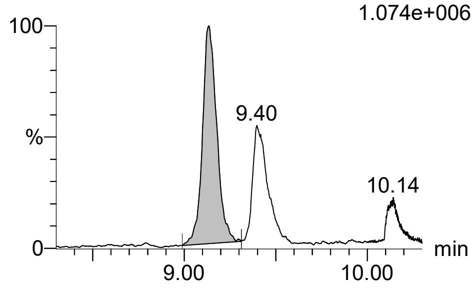

Sample Name: 20211111_QC_R10_IgM
F65:MRM of 1 channel,ES+
1075.1 > 366.1
6.661e+005

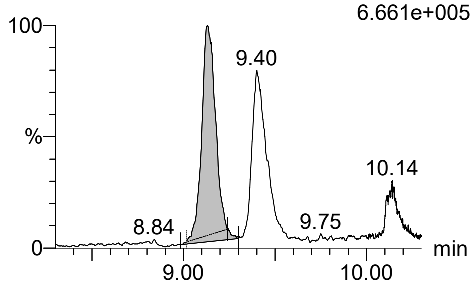

Sample Name: 20211111_L5_R2_IgM
F65:MRM of 1 channel,ES+
1075.1 > 366.1
6.686e+005

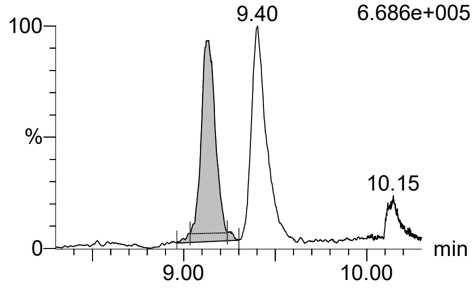

Sample Name: 20211111_L6_R2_IgM
F65:MRM of 1 channel,ES+
1075.1 > 366.1
9.241e+005

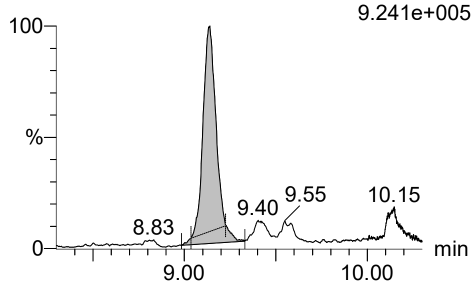

Sample Name: 20211111_H3_R2_IgM
F65:MRM of 1 channel,ES+
1075.1 > 366.1
6.046e+005

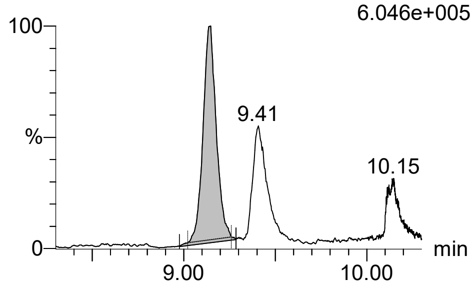

Sample Name: 20211111_A7_R2_IgM
F65:MRM of 1 channel,ES+
1075.1 > 366.1
6.736e+005

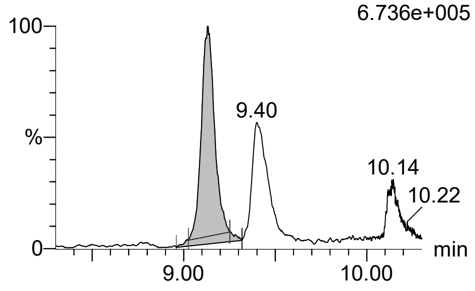

Sample Name: 20211111_A8_R2_IgM
F65:MRM of 1 channel,ES+
1075.1 > 366.1
7.352e+005

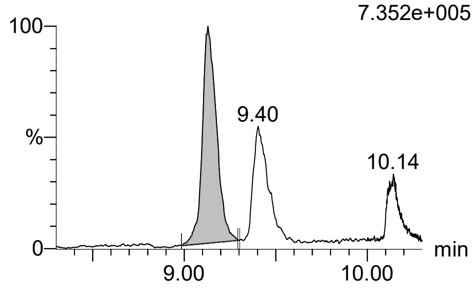

Sample Name: 20211111_L7_R2_IgM
F65:MRM of 1 channel,ES+
1075.1 > 366.1
4.488e+005

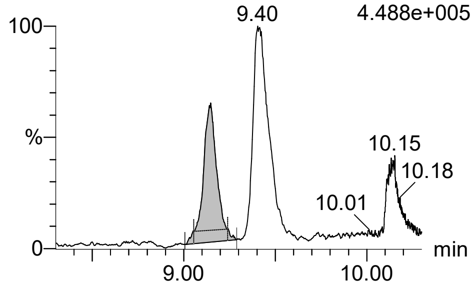

Sample Name: 20211111_L8_R2_IgM
F65:MRM of 1 channel,ES+
1075.1 > 366.1
4.007e+005

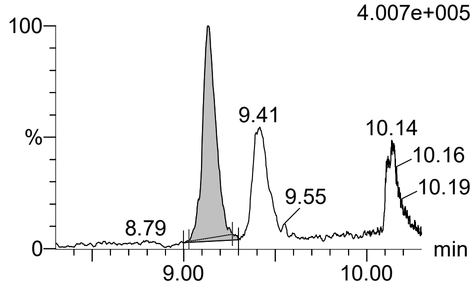

Sample Name: 20211111_H4_R2_IgM
F65:MRM of 1 channel,ES+
1075.1 > 366.1
3.974e+005

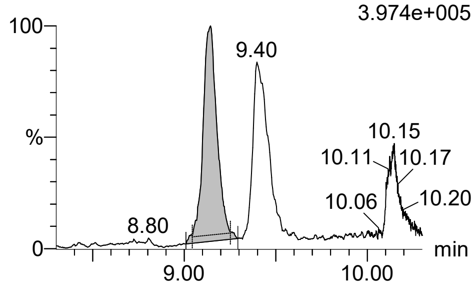

Sample Name: 20211111_A9_R2_IgM
F65:MRM of 1 channel,ES+
1075.1 > 366.1
3.720e+005

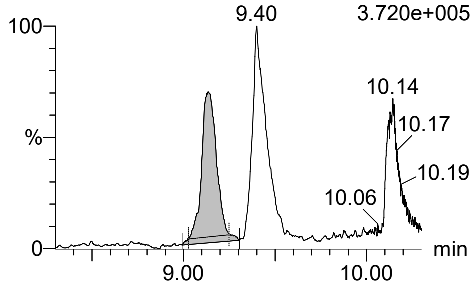

Dataset: C:\MassLynx\10.pro\20211111_clinicalsamples_IgM_IgA.qld

Last Altered: Thursday, March 09, 2023 19:58:25 台北標準時間
Printed: Friday, August 01, 2025 15:13:08 台北標準時間

Sample Name: 20211111_A10_R2_IgM
F65:MRM of 1 channel,ES+
1075.1 > 366.1
7.335e+005

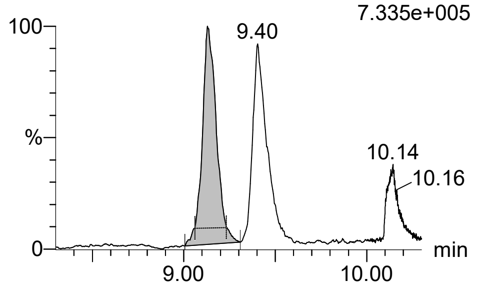

Sample Name: 20211111_L9_R2_IgM
F65:MRM of 1 channel,ES+
1075.1 > 366.1
4.843e+005

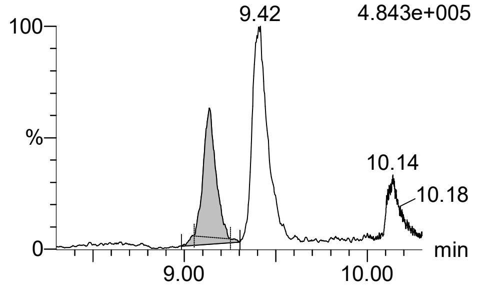

Sample Name: 20211111_L10_R2_IgM
F65:MRM of 1 channel,ES+
1075.1 > 366.1
9.787e+005

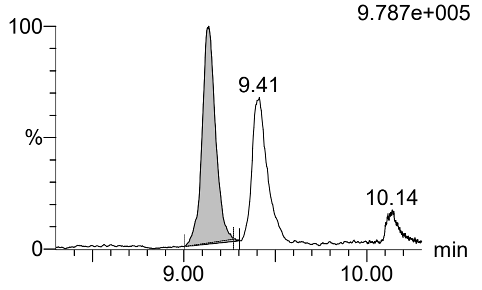

Sample Name: 20211111_QC_R11_IgM
F65:MRM of 1 channel,ES+
1075.1 > 366.1
7.513e+005

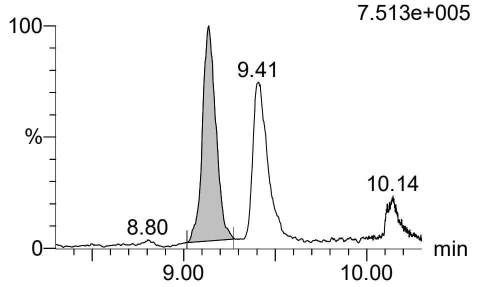

Sample Name: 20211111_H5_R2_IgM
F65:MRM of 1 channel,ES+
1075.1 > 366.1
1.298e+006

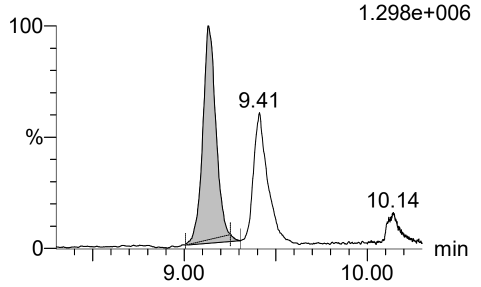

Sample Name: 20211111_A11_R2_IgM
F65:MRM of 1 channel,ES+
1075.1 > 366.1
1.166e+006

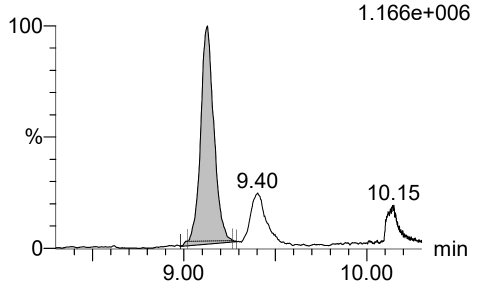

Sample Name: 20211111_A12_R2_IgM
F65:MRM of 1 channel,ES+
1075.1 > 366.1
3.455e+005

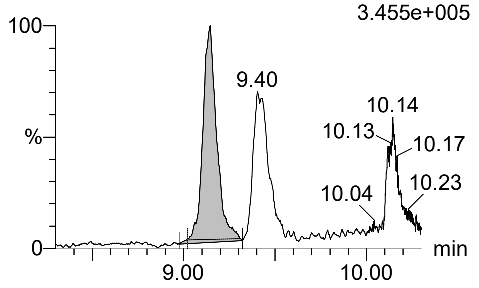

Sample Name: 20211111_L11_R2_IgM
F65:MRM of 1 channel,ES+
1075.1 > 366.1
3.359e+005

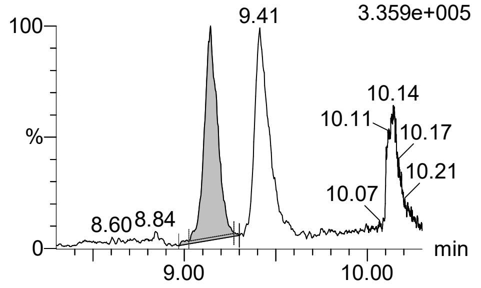

Sample Name: 20211111_L12_R2_IgM
F65:MRM of 1 channel,ES+
1075.1 > 366.1
4.217e+005

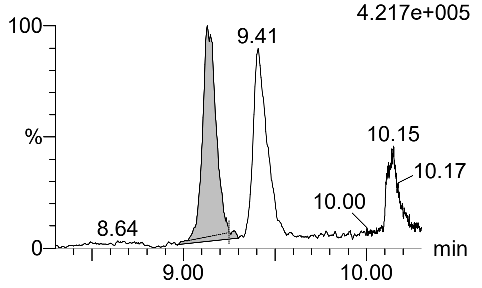

Sample Name: 20211111_H6_R2_IgM
F65:MRM of 1 channel,ES+
1075.1 > 366.1
4.707e+005

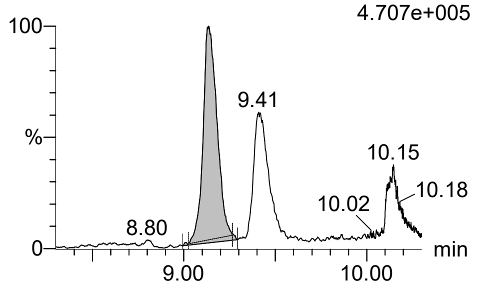

Sample Name: 20211111_A13_R2_IgM
F65:MRM of 1 channel,ES+
1075.1 > 366.1
6.959e+005

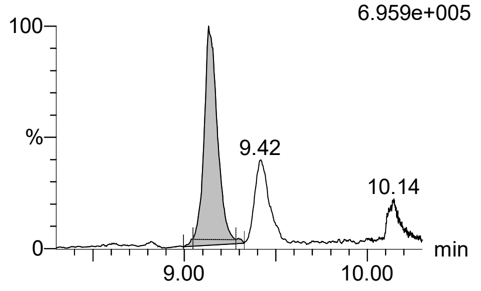

Sample Name: 20211111_A14_R2_IgM
F65:MRM of 1 channel,ES+
1075.1 > 366.1
1.287e+006

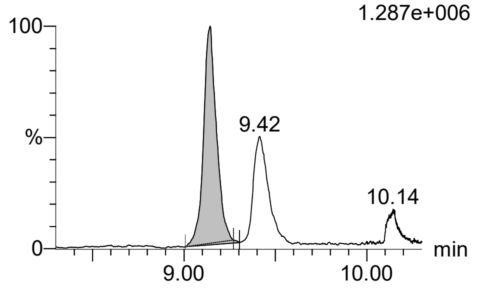

Sample Name: 20211111_L13_R2_IgM
F65:MRM of 1 channel,ES+
1075.1 > 366.1
7.414e+005

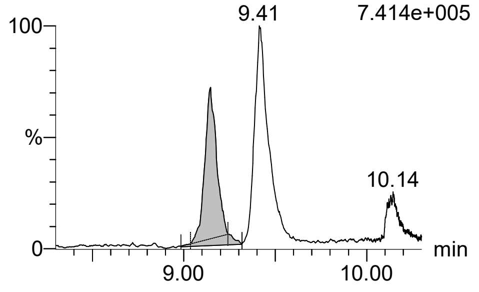

Sample Name: 20211111_L14_R2_IgM
F65:MRM of 1 channel,ES+
1075.1 > 366.1
7.520e+005

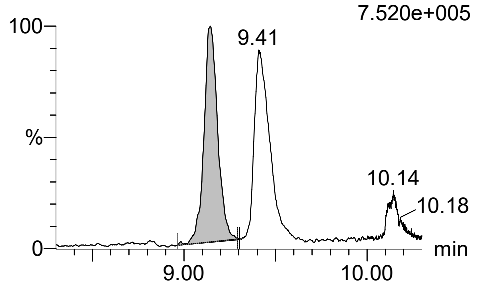

Sample Name: 20211111_H7_R2_IgM
F65:MRM of 1 channel,ES+
1075.1 > 366.1
6.159e+005

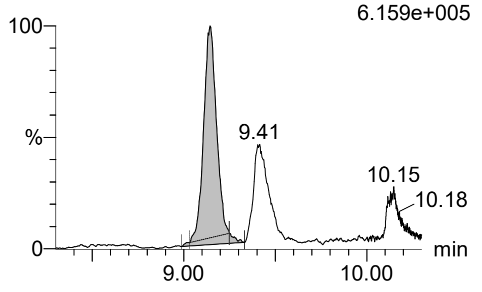

Sample Name: 20211111_A15_R2_IgM
F65:MRM of 1 channel,ES+
1075.1 > 366.1
2.019e+006

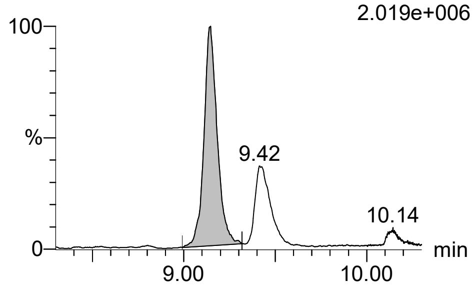

Sample Name: 20211111_QC_R12_IgM
F65:MRM of 1 channel,ES+
1075.1 > 366.1
8.269e+005

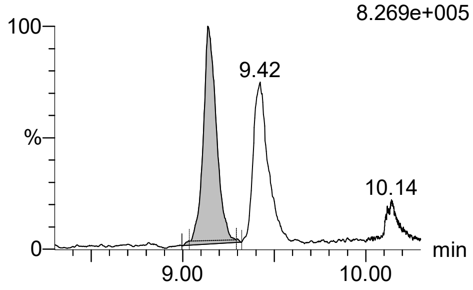

Sample Name: 20211111_A16_R2_IgM
F65:MRM of 1 channel,ES+
1075.1 > 366.1
8.685e+005

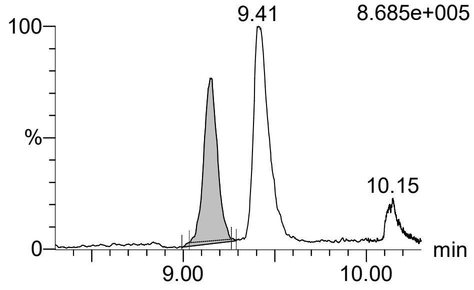

Sample Name: 20211111_L15_R2_IgM
F65:MRM of 1 channel,ES+
1075.1 > 366.1
9.303e+005

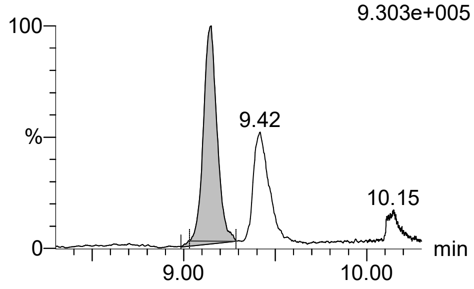

Sample Name: 20211111_L16_R2_IgM
F65:MRM of 1 channel,ES+
1075.1 > 366.1
3.016e+005

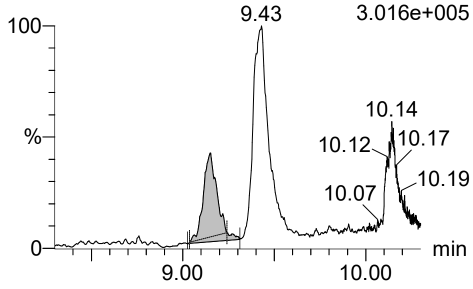

Sample Name: 20211111_H8_R2_IgM
F65:MRM of 1 channel,ES+
1075.1 > 366.1
5.050e+005

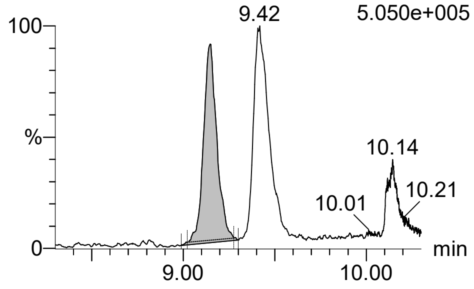

Sample Name: 20211111_A17_R2_IgM
F65:MRM of 1 channel,ES+
1075.1 > 366.1
1.195e+006

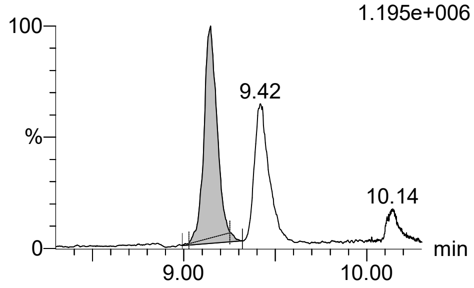

Sample Name: 20211111_A18_R2_IgM
F65:MRM of 1 channel,ES+
1075.1 > 366.1
3.197e+005

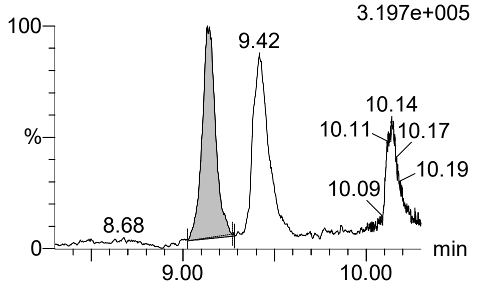

Sample Name: 20211111_L17_R2_IgM
F65:MRM of 1 channel,ES+
1075.1 > 366.1
1.246e+006

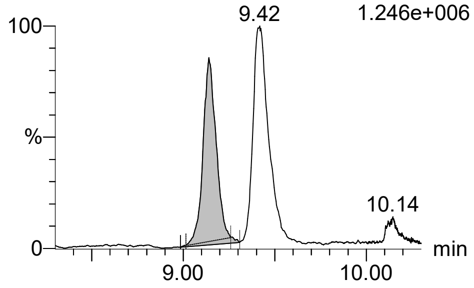

Sample Name: 20211111_L18_R2_IgM
F65:MRM of 1 channel,ES+
1075.1 > 366.1
1.958e+005

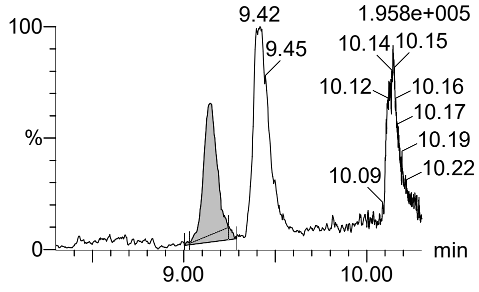

Sample Name: 20211111_H9_R2_IgM
F65:MRM of 1 channel,ES+
1075.1 > 366.1
3.323e+005

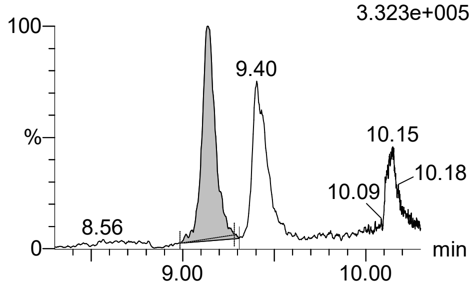

Sample Name: 20211111_A19_R2_IgM
F65:MRM of 1 channel,ES+
1075.1 > 366.1
6.767e+005

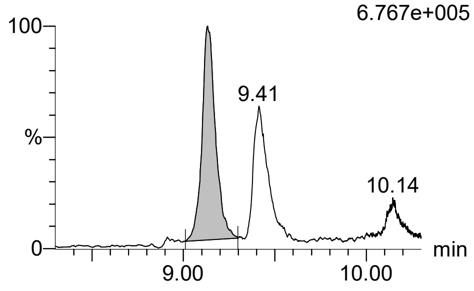

Sample Name: 20211111_A20_R2_IgM
F65:MRM of 1 channel,ES+
1075.1 > 366.1
2.772e+005

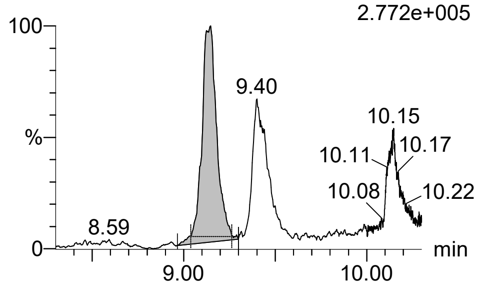

Sample Name: 20211111_L19_R2_IgM
F65:MRM of 1 channel,ES+
1075.1 > 366.1
5.967e+005

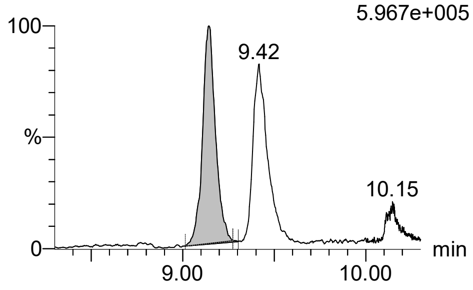

Sample Name: 20211111_QC_R13_IgM
F65:MRM of 1 channel,ES+
1075.1 > 366.1
7.847e+005

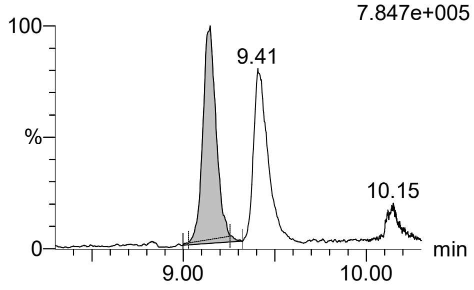

Dataset: C:\MassLynx\10.pro\20211111_clinicalsamples_IgM_IgA.qld

Last Altered: Thursday, March 09, 2023 19:58:25 台北標準時間
Printed: Friday, August 01, 2025 15:13:08 台北標準時間

Sample Name: 20211111_L20_R2_IgM
F65:MRM of 1 channel,ES+
1075.1 > 366.1
3.154e+005

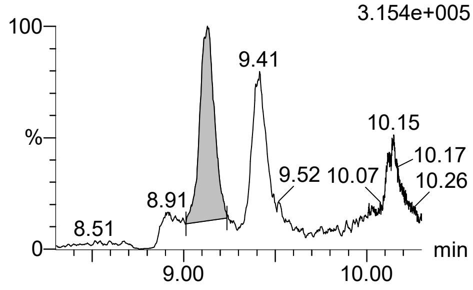

Sample Name: 20211111_H10_R2_IgM
F65:MRM of 1 channel,ES+
1075.1 > 366.1
1.212e+006

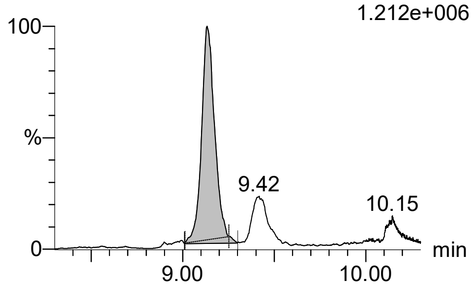

Sample Name: 20211111_A21_R2_IgM
F65:MRM of 1 channel,ES+
1075.1 > 366.1
7.157e+005

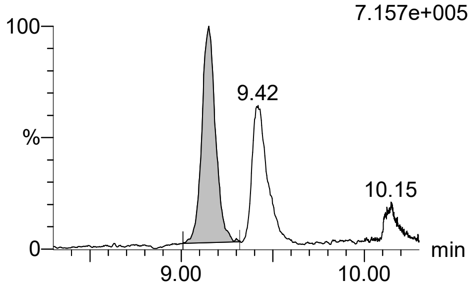

Sample Name: 20211111_A22_R2_IgM
F65:MRM of 1 channel,ES+
1075.1 > 366.1
2.564e+005

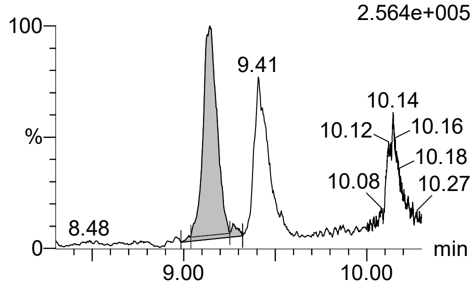

Sample Name: 20211111_L21_R2_IgM
F65:MRM of 1 channel,ES+
1075.1 > 366.1
6.969e+005

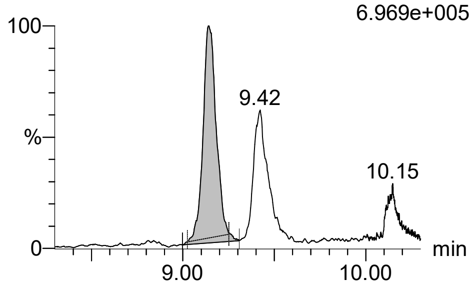

Sample Name: 20211111_L22_R2_IgM
F65:MRM of 1 channel,ES+
1075.1 > 366.1
7.135e+005

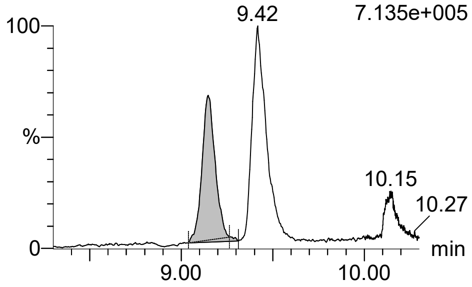

Sample Name: 20211111_H11_R2_IgM
F65:MRM of 1 channel,ES+
1075.1 > 366.1
4.846e+005

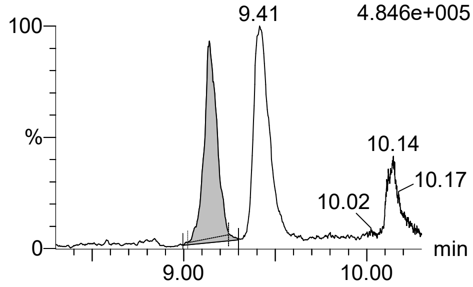

Sample Name: 20211111_A23_R2_IgM
F65:MRM of 1 channel,ES+
1075.1 > 366.1
4.617e+005

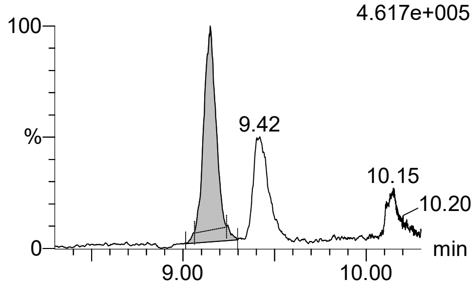

Sample Name: 20211111_A24_R2_IgM
F65:MRM of 1 channel,ES+
1075.1 > 366.1
1.908e+006

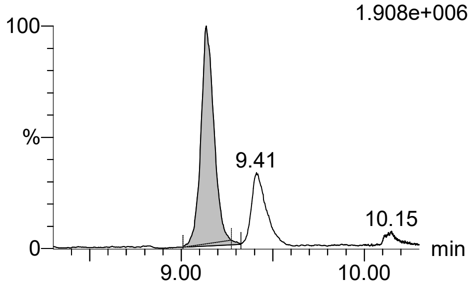

Sample Name: 20211111_L23_R2_IgM
F65:MRM of 1 channel,ES+
1075.1 > 366.1
8.435e+005

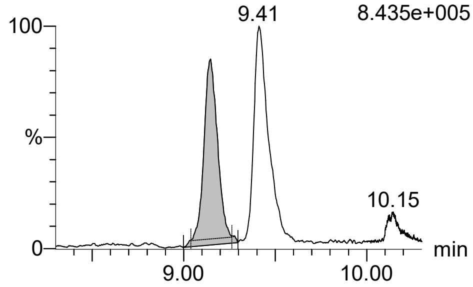

Sample Name: 20211111_L24_R2_IgM
F65:MRM of 1 channel,ES+
1075.1 > 366.1
4.404e+005

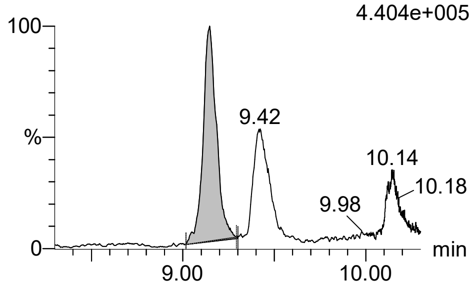

Sample Name: 20211111_H12_R2_IgM
F65:MRM of 1 channel,ES+
1075.1 > 366.1
3.845e+005

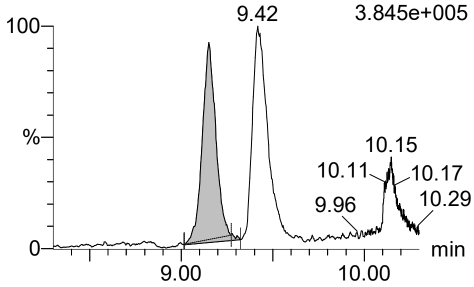

Sample Name: 20211111_QC_R14_IgM
F65:MRM of 1 channel,ES+
1075.1 > 366.1
8.135e+005

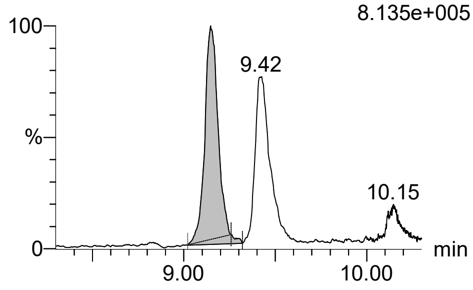

Sample Name: 20211111_A25_R2_IgM
F65:MRM of 1 channel,ES+
1075.1 > 366.1
1.218e+006

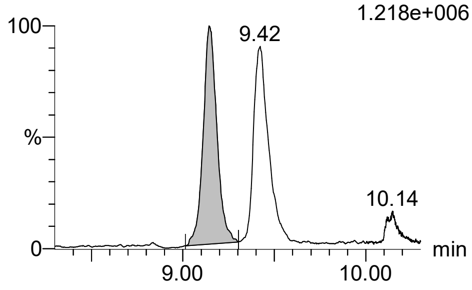

Sample Name: 20211111_A26_R2_IgM
F65:MRM of 1 channel,ES+
1075.1 > 366.1
5.669e+005

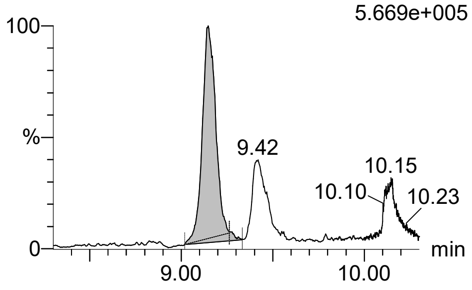

Sample Name: 20211111_L25_R2_IgM

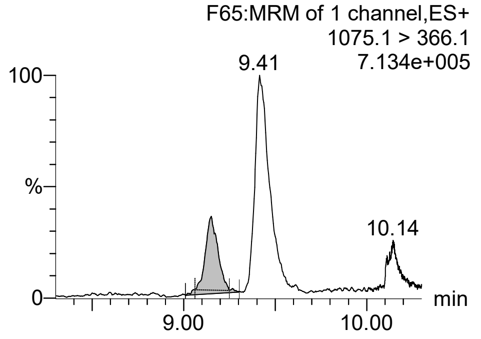

Sample Name: 20211111_L26_R2_IgM

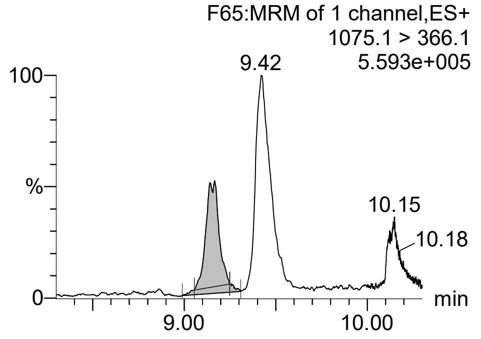

Sample Name: 20211111_H13_R2_IgM

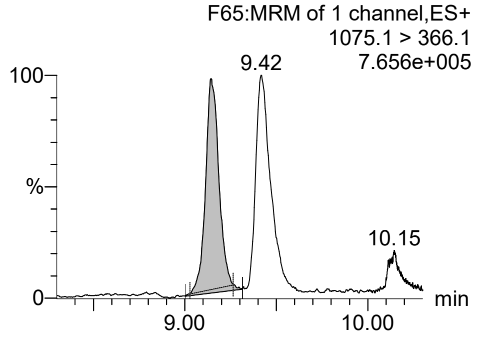

Sample Name: 20211111_A27_R2_IgM

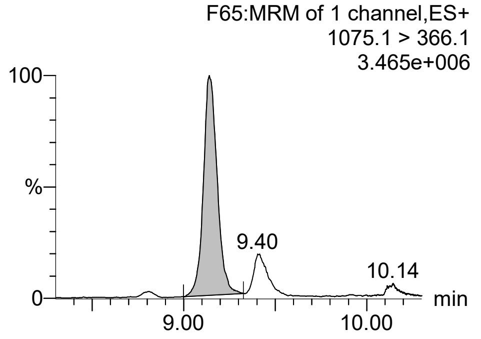

Sample Name: 20211111_A28_R2_IgM

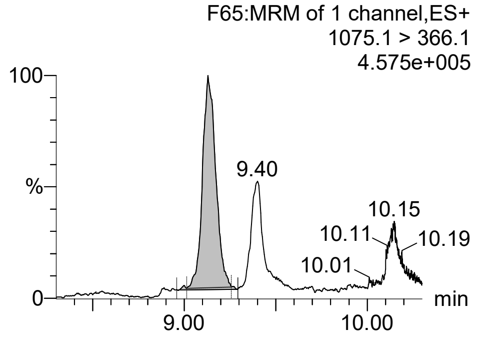

Sample Name: 20211111_L27_R2_IgM

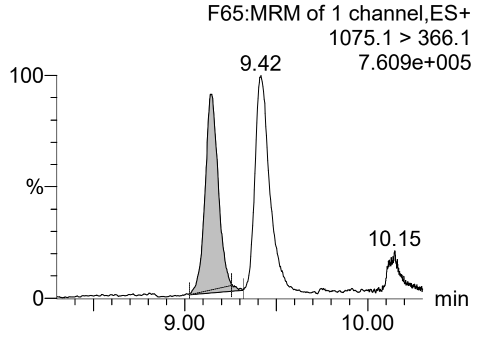

Sample Name: 20211111_L28_R2_IgM

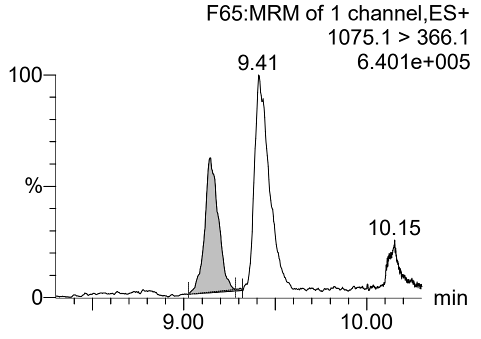

Sample Name: 20211111_H14_R2_IgM

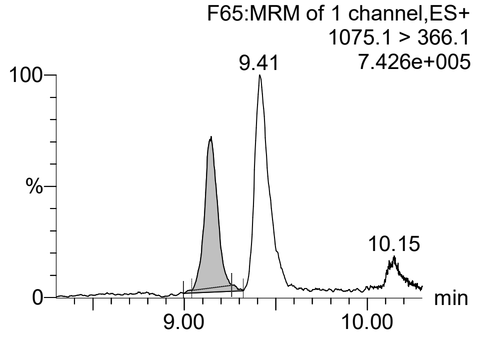

Sample Name: 20211111_A29_R2_IgM

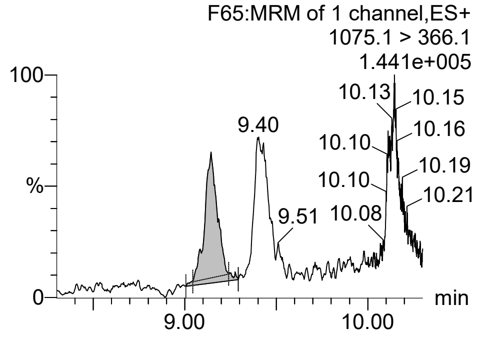

Sample Name: 20211111_A30_R2_IgM

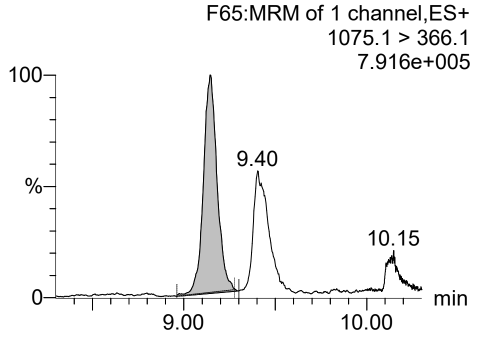

Sample Name: 20211111_QC_R15_IgM

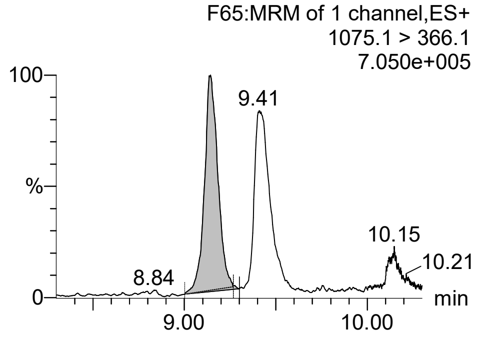

Sample Name: 20211111_L29_R2_IgM

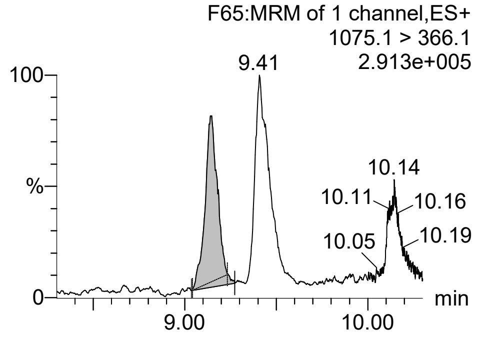

Sample Name: 20211111_L30_R2_IgM

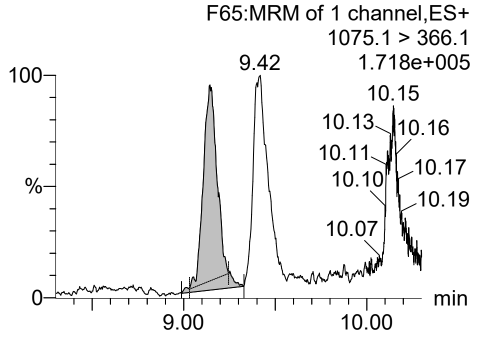

Sample Name: 20211111_H15_R2_IgM

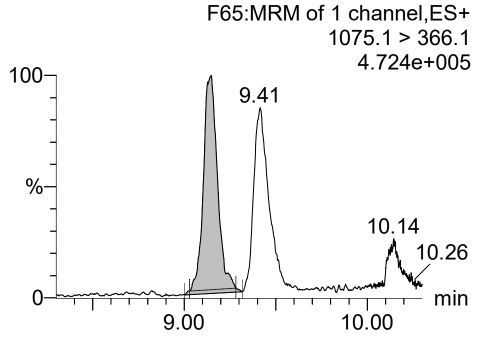

Sample Name: 20211111_A31_R2_IgM

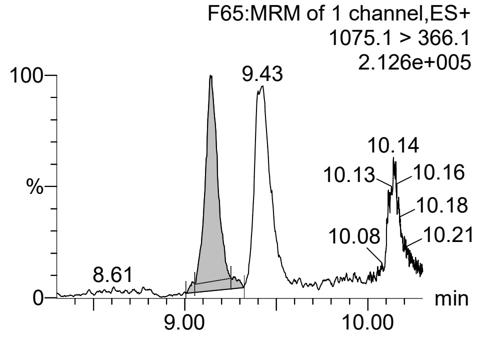

Sample Name: 20211111_A32_R2_IgM

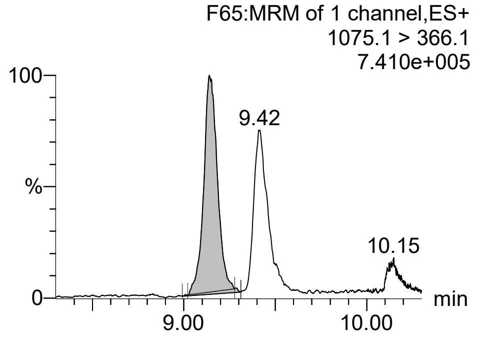

Sample Name: 20211111_L31_R2_IgM

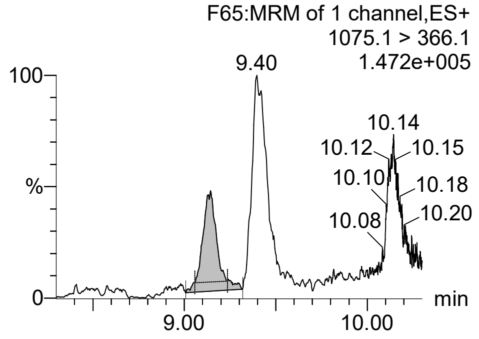

Sample Name: 20211111_L32_R2_IgM

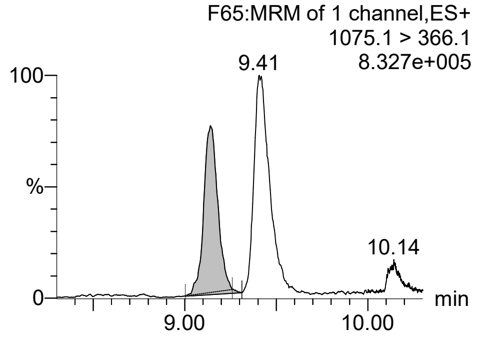

Sample Name: 20211111_H16_R2_IgM

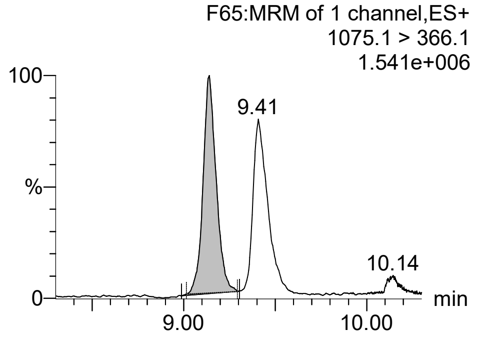

Sample Name: 20211111_A33_R2_IgM

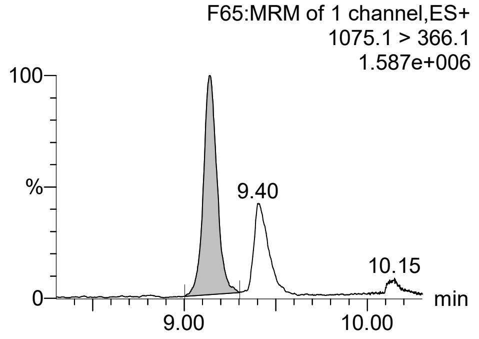

Sample Name: 20211111_A34_R2_IgM

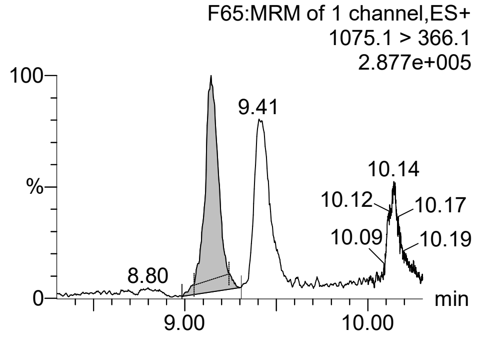

Sample Name: 20211111_H17_R2_IgM

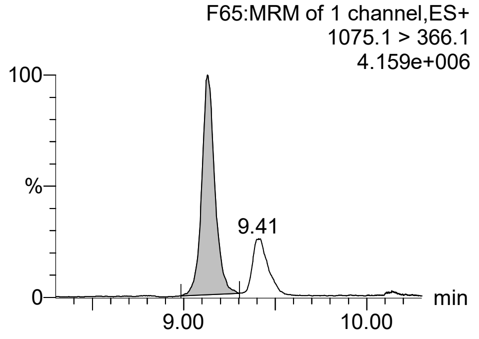

Sample Name: 20211111_A35_R2_IgM

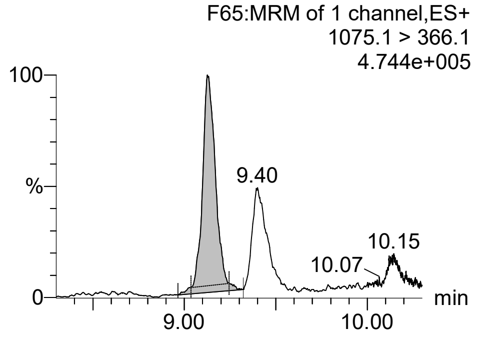

Sample Name: 20211111_QC_R16_IgM

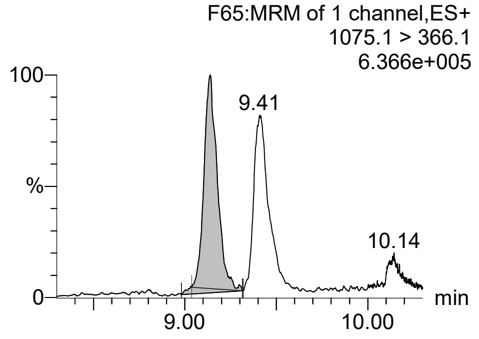

Sample Name: 20211111_A36_R2_IgM

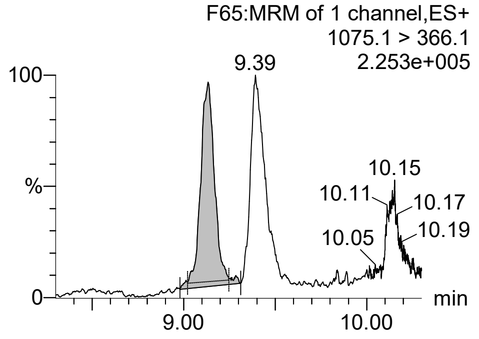

Sample Name: 20211111_H18_R2_IgM

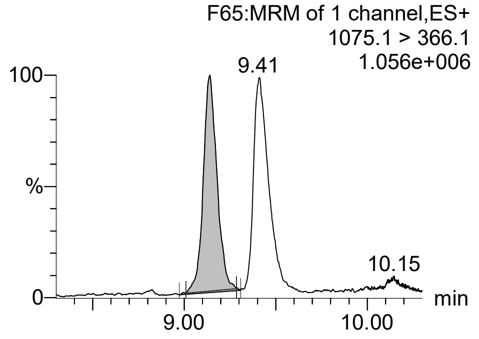

Sample Name: 20211111_A37_R2_IgM

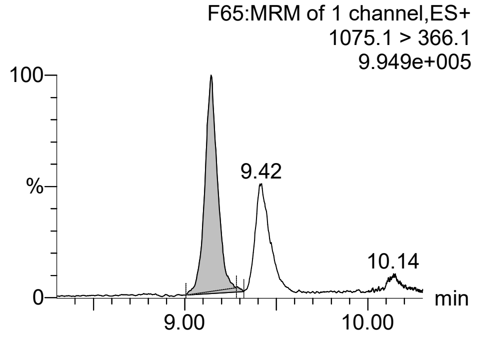

Sample Name: 20211111_A38_R2_IgM

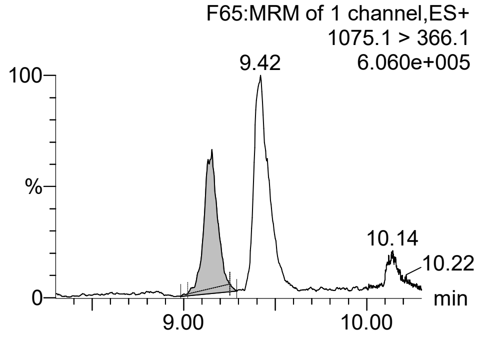

Sample Name: 20211111_H19_R2_IgM

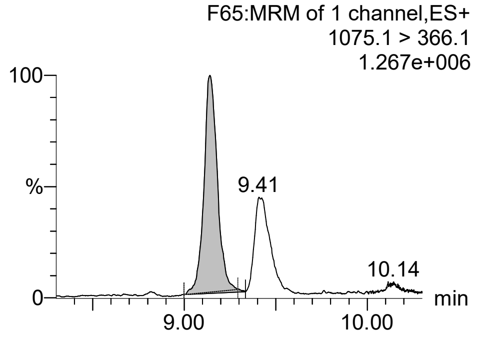

Sample Name: 20211111_A39_R2_IgM

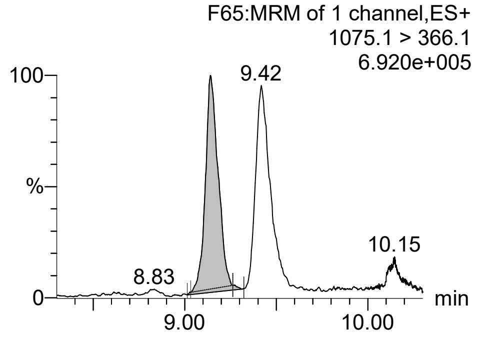

Dataset: C:\MassLynx\10.pro\20211111_clinicalsamples_IgM_IgA.qld

Last Altered: Thursday, March 09, 2023 19:58:25 台北標準時間
Printed: Friday, August 01, 2025 15:13:08 台北標準時間

Sample Name: 20211111_A40_R2_IgM
F65:MRM of 1 channel,ES+
1075.1 > 366.1
2.756e+005

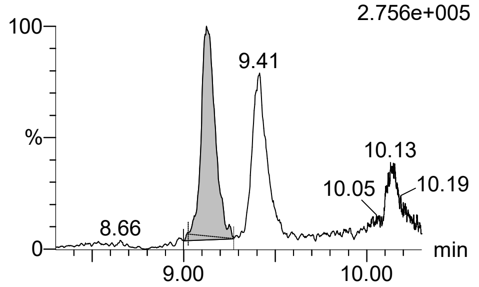

Sample Name: 20211111_H20_R2_IgM
F65:MRM of 1 channel,ES+
1075.1 > 366.1
2.297e+006

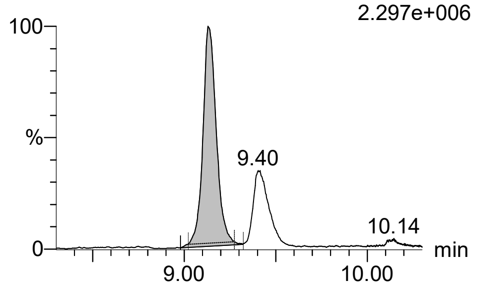

Sample Name: 20211111_A41_R2_IgM
F65:MRM of 1 channel,ES+
1075.1 > 366.1
4.413e+005

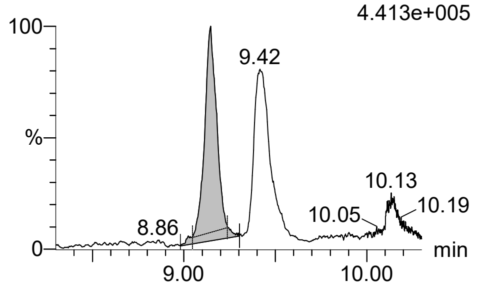

Sample Name: 20211111_A42_R2_IgM
F65:MRM of 1 channel,ES+
1075.1 > 366.1
2.944e+005

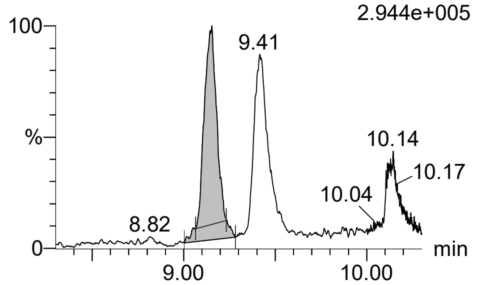

Sample Name: 20211111_A43_R2_IgM
F65:MRM of 1 channel,ES+
1075.1 > 366.1
3.238e+005

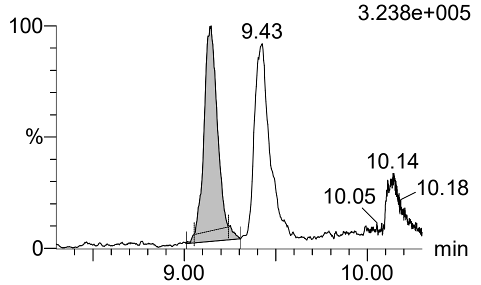

Sample Name: 20211111_A44_R2_IgM
F65:MRM of 1 channel,ES+
1075.1 > 366.1
2.254e+005

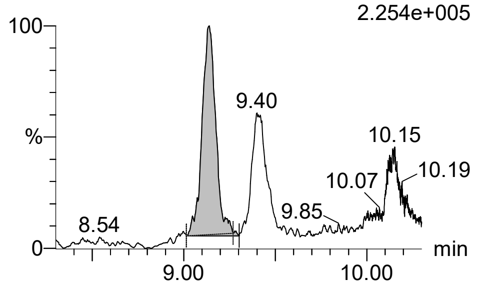

Sample Name: 20211111_QC_R17_IgM
F65:MRM of 1 channel,ES+
1075.1 > 366.1
5.509e+005

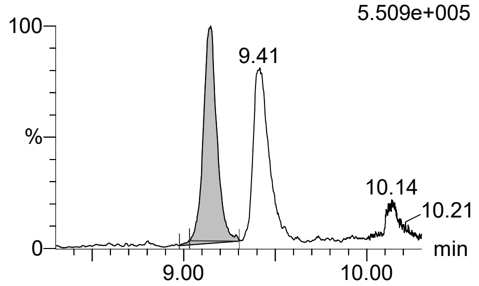

Sample Name: 20211111_L2_R3_IgM
F65:MRM of 1 channel,ES+
1075.1 > 366.1
7.998e+005

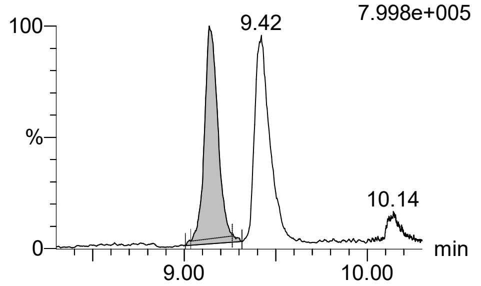

Sample Name: 20211111_H1_R3_IgM
F65:MRM of 1 channel,ES+
1075.1 > 366.1
6.145e+005

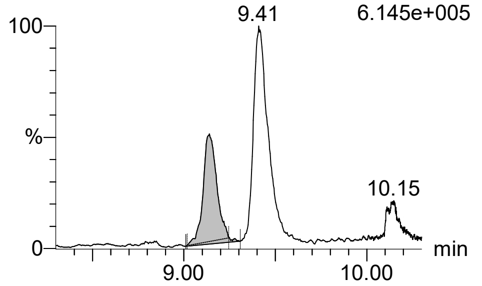

Sample Name: 20211111_A3_R3_IgM
F65:MRM of 1 channel,ES+
1075.1 > 366.1
8.623e+005

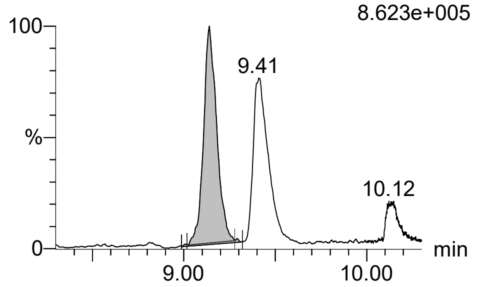

Sample Name: 20211111_A4_R3_IgM
F65:MRM of 1 channel,ES+
1075.1 > 366.1
5.628e+005

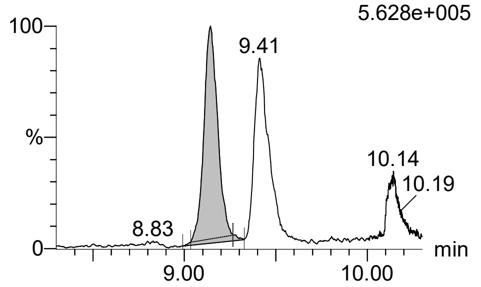

Sample Name: 20211111_L3_R3_IgM
F65:MRM of 1 channel,ES+
1075.1 > 366.1
3.693e+005

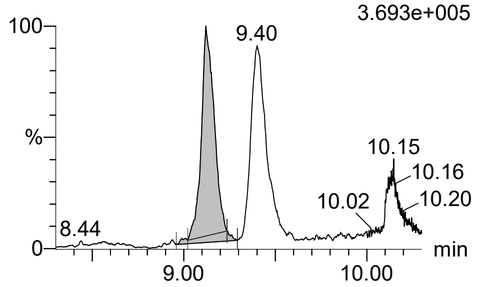

Sample Name: 20211111_L4_R3_IgM
F65:MRM of 1 channel,ES+
1075.1 > 366.1
7.831e+005

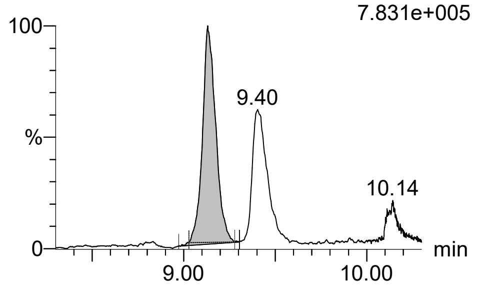

Sample Name: 20211111_H2_R3_IgM
F65:MRM of 1 channel,ES+
1075.1 > 366.1
1.058e+006

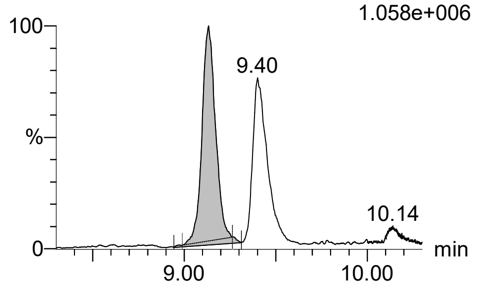

Sample Name: 20211111_A5_R3_IgM
F65:MRM of 1 channel,ES+
1075.1 > 366.1
5.401e+006

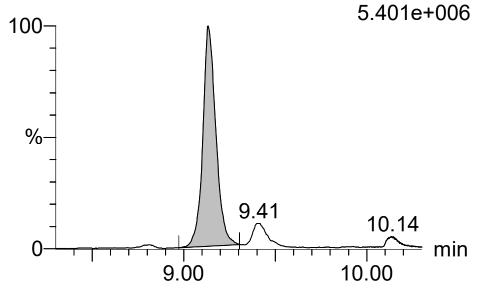

Dataset: C:\MassLynx\10.pro\20211111_clinicalsamples_IgM_IgA.qld

Last Altered: Thursday, March 09, 2023 19:58:25 台北標準時間
Printed: Friday, August 01, 2025 15:13:08 台北標準時間

Sample Name: 20211111_A6_R3_IgM
F65:MRM of 1 channel,ES+
1075.1 > 366.1
8.308e+005

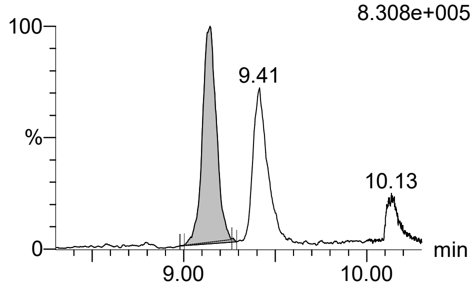

Sample Name: 20211111_QC_R18_IgM
F65:MRM of 1 channel,ES+
1075.1 > 366.1
5.891e+005

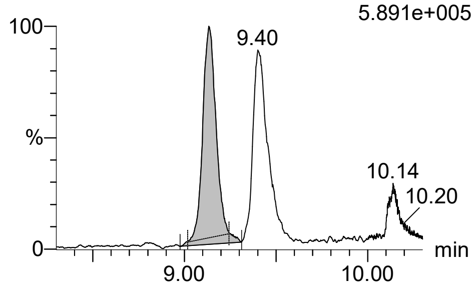

Sample Name: 20211111_L5_R3_IgM
F65:MRM of 1 channel,ES+
1075.1 > 366.1
8.803e+005

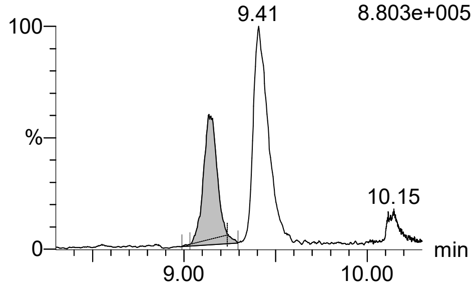

Sample Name: 20211111_L6_R3_IgM
F65:MRM of 1 channel,ES+
1075.1 > 366.1
7.705e+005

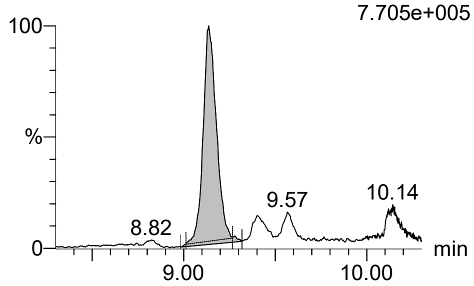

Sample Name: 20211111_H3_R3_IgM
F65:MRM of 1 channel,ES+
1075.1 > 366.1
5.133e+005

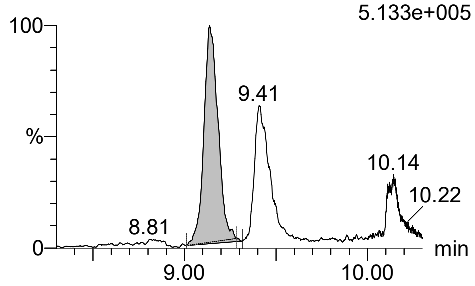

Sample Name: 20211111_A7_R3_IgM
F65:MRM of 1 channel,ES+
1075.1 > 366.1
6.823e+005

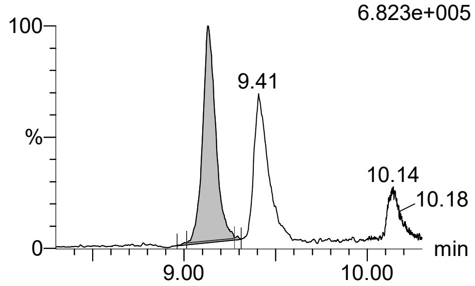

Sample Name: 20211111_A8_R3_IgM
F65:MRM of 1 channel,ES+
1075.1 > 366.1
6.258e+005

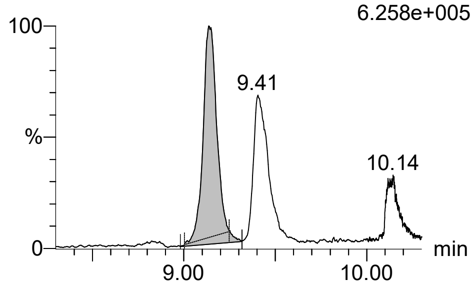

Sample Name: 20211111_L7_R3_IgM
F65:MRM of 1 channel,ES+
1075.1 > 366.1
5.442e+005

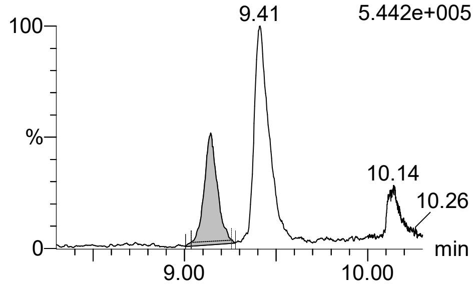

Sample Name: 20211111_L8_R3_IgM
F65:MRM of 1 channel,ES+
1075.1 > 366.1
3.186e+005

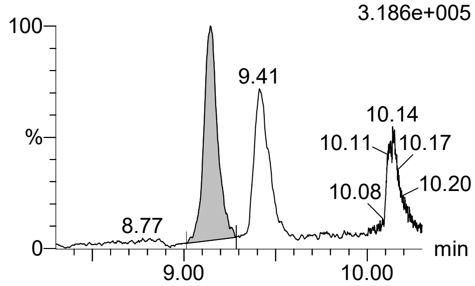

Sample Name: 20211111_H4_R3_IgM
F65:MRM of 1 channel,ES+
1075.1 > 366.1
3.550e+005

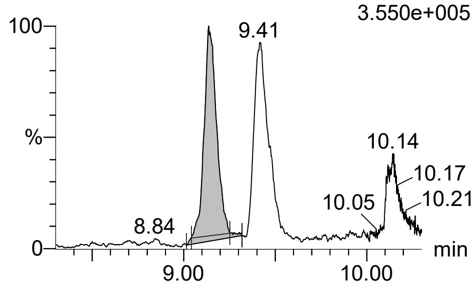

Sample Name: 20211111_A9_R3_IgM
F65:MRM of 1 channel,ES+
1075.1 > 366.1
3.792e+005

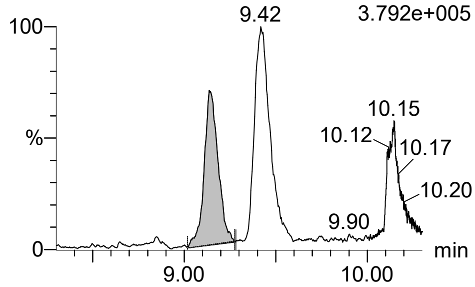

Sample Name: 20211111_A10_R3_IgM
F65:MRM of 1 channel,ES+
1075.1 > 366.1
7.339e+005

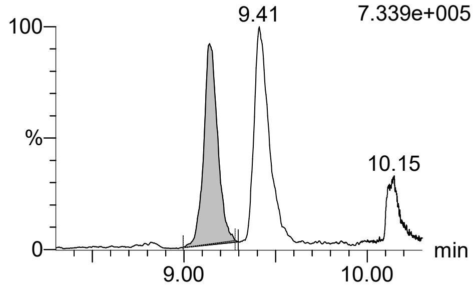

Sample Name: 20211111_L9_R3_IgM
F65:MRM of 1 channel,ES+
1075.1 > 366.1
6.329e+005

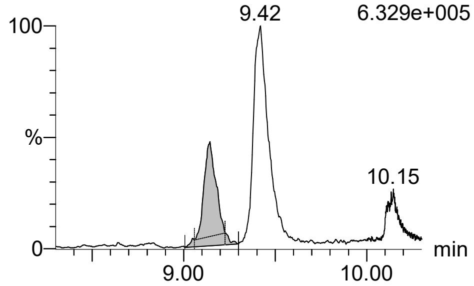

Sample Name: 20211111_L10_R3_IgM
F65:MRM of 1 channel,ES+
1075.1 > 366.1
7.874e+005

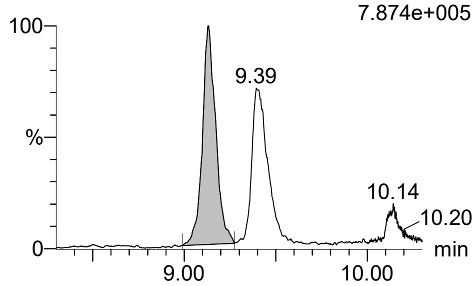

Sample Name: 20211111_QC_R19_IgM
F65:MRM of 1 channel,ES+
1075.1 > 366.1
5.568e+005

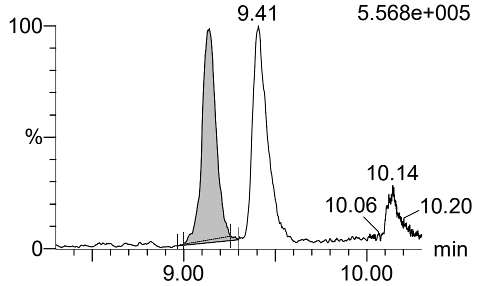

Sample Name: 20211111_H5_R3_IgM
F65:MRM of 1 channel,ES+
1075.1 > 366.1
9.754e+005

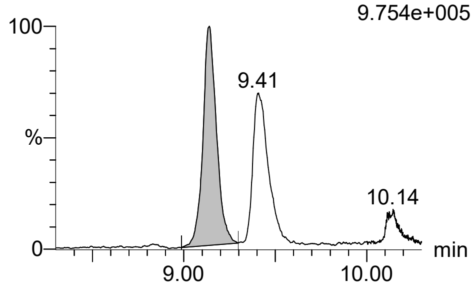

Sample Name: 20211111_A11_R3_IgM
F65:MRM of 1 channel,ES+
1075.1 > 366.1
1.031e+006

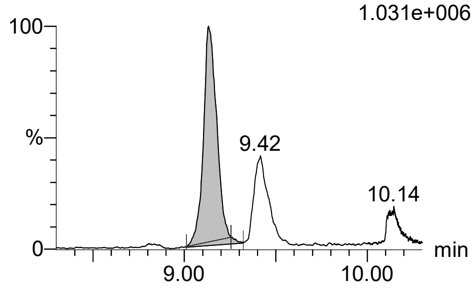

Sample Name: 20211111_A12_R3_IgM
F65:MRM of 1 channel,ES+
1075.1 > 366.1
3.288e+005

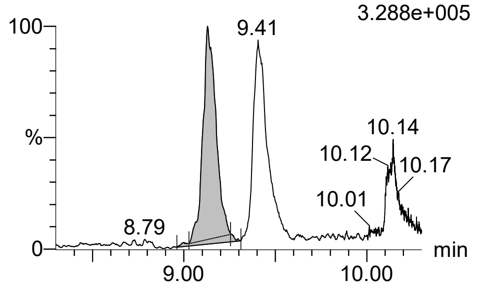

Sample Name: 20211111_L11_R3_IgM
F65:MRM of 1 channel,ES+
1075.1 > 366.1
3.186e+005

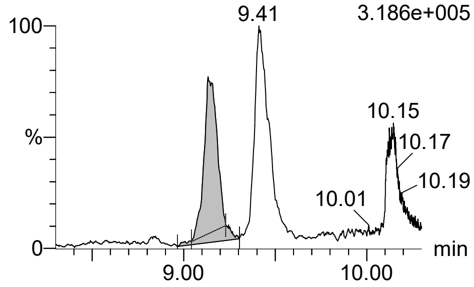

Sample Name: 20211111_L12_R3_IgM
F65:MRM of 1 channel,ES+
1075.1 > 366.1
3.452e+005

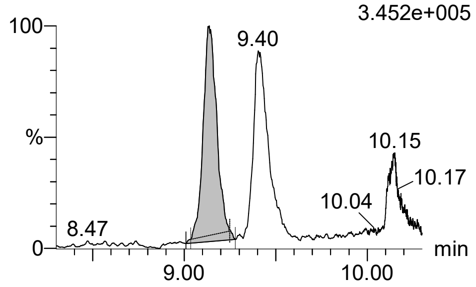

Sample Name: 20211111_H6_R3_IgM
F65:MRM of 1 channel,ES+
1075.1 > 366.1
4.340e+005

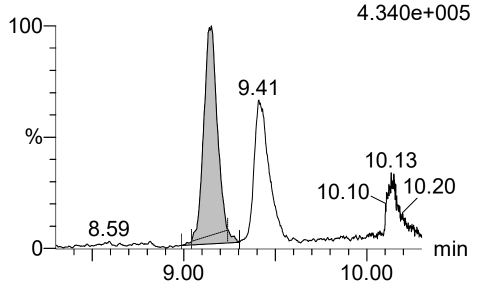

Sample Name: 20211111_A13_R3_IgM
F65:MRM of 1 channel,ES+
1075.1 > 366.1
6.430e+005

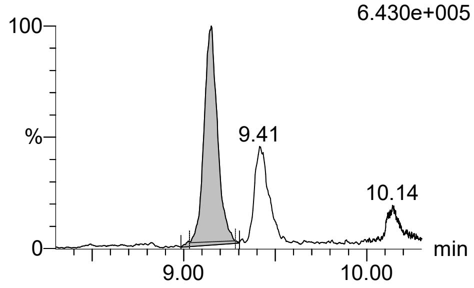

Sample Name: 20211111_A14_R3_IgM
F65:MRM of 1 channel,ES+
1075.1 > 366.1
9.625e+005

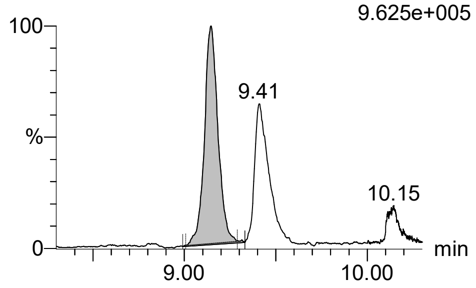

Sample Name: 20211111_L13_R3_IgM
F65:MRM of 1 channel,ES+
1075.1 > 366.1
5.828e+005

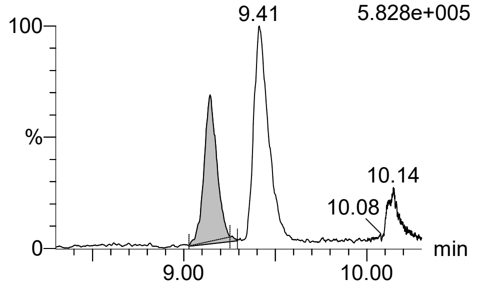

Sample Name: 20211111_L14_R3_IgM
F65:MRM of 1 channel,ES+
1075.1 > 366.1
5.908e+005

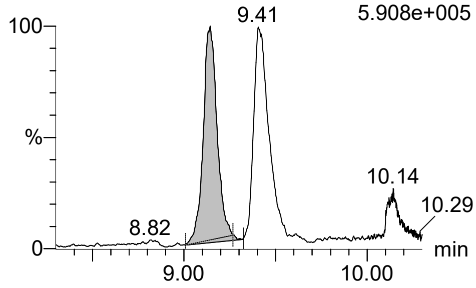

Sample Name: 20211111_H7_R3_IgM
F65:MRM of 1 channel,ES+
1075.1 > 366.1
5.530e+005

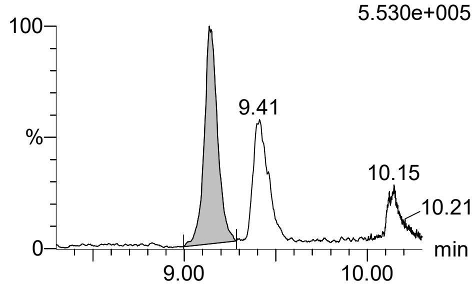

Sample Name: 20211111_A15_R3_IgM
F65:MRM of 1 channel,ES+
1075.1 > 366.1
1.623e+006

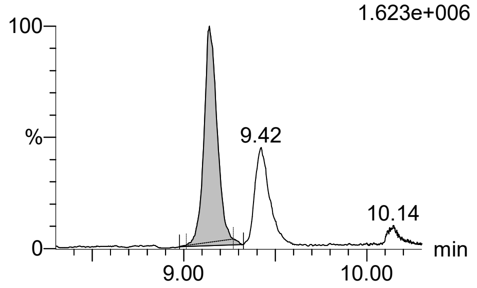

Sample Name: 20211111_QC_R20_IgM
F65:MRM of 1 channel,ES+
1075.1 > 366.1
6.617e+005

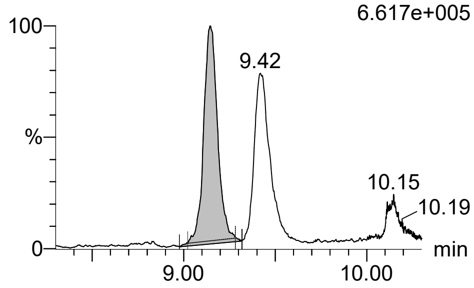

Sample Name: 20211111_A16_R3_IgM
F65:MRM of 1 channel,ES+
1075.1 > 366.1
7.244e+005

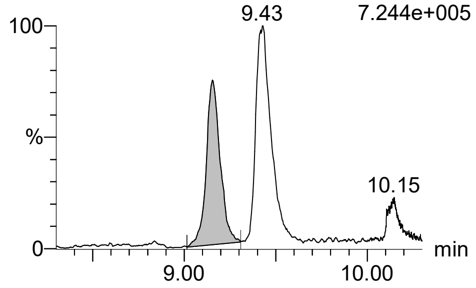

Sample Name: 20211111_L15_R3_IgM
F65:MRM of 1 channel,ES+
1075.1 > 366.1
8.264e+005

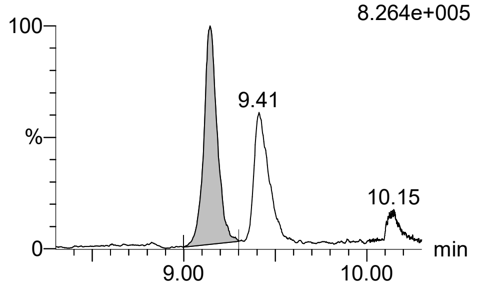

Dataset: C:\MassLynx\10.pro\20211111_clinicalsamples_IgM_IgA.qld

Last Altered: Thursday, March 09, 2023 19:58:25 台北標準時間
Printed: Friday, August 01, 2025 15:13:08 台北標準時間

Sample Name: 20211111_L16_R3_IgM
F65:MRM of 1 channel,ES+
1075.1 > 366.1
2.912e+005

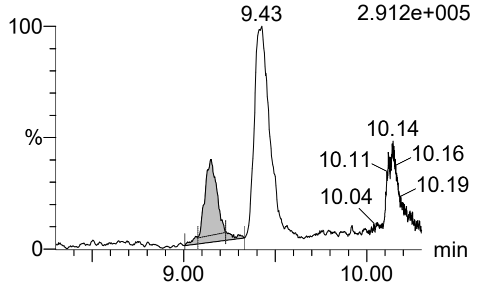

Sample Name: 20211111_H8_R3_IgM
F65:MRM of 1 channel,ES+
1075.1 > 366.1
4.537e+005

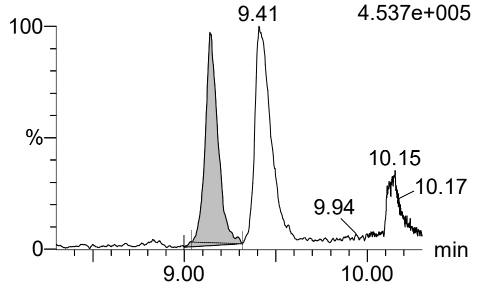

Sample Name: 20211111_A17_R3_IgM
F65:MRM of 1 channel,ES+
1075.1 > 366.1
9.018e+005

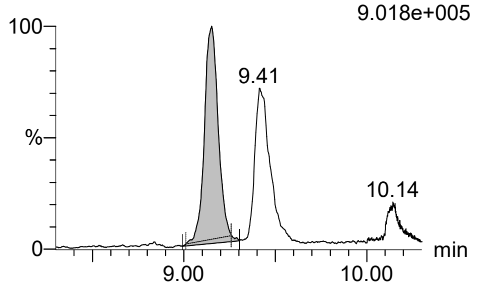

Sample Name: 20211111_A18_R3_IgM
F65:MRM of 1 channel,ES+
1075.1 > 366.1
2.736e+005

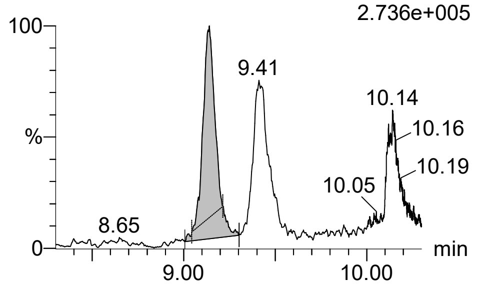

Sample Name: 20211111_L17_R3_IgM
F65:MRM of 1 channel,ES+
1075.1 > 366.1
1.202e+006

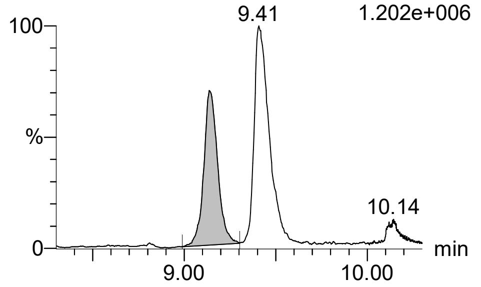

Sample Name: 20211111_L18_R3_IgM
F65:MRM of 1 channel,ES+
1075.1 > 366.1
1.952e+005

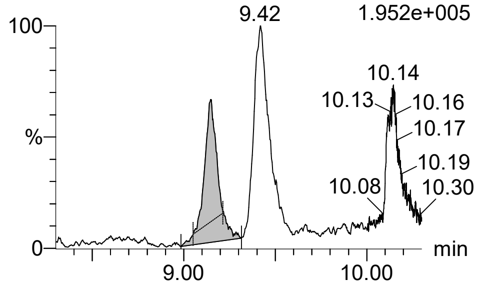

Sample Name: 20211111_H9_R3_IgM
F65:MRM of 1 channel,ES+
1075.1 > 366.1
2.985e+005

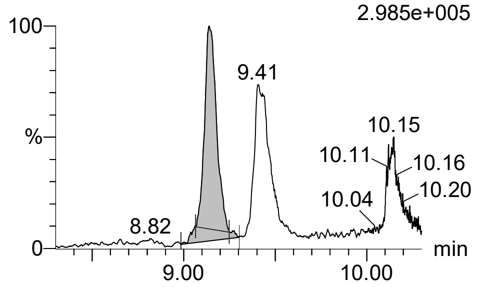

Sample Name: 20211111_A19_R3_IgM
F65:MRM of 1 channel,ES+
1075.1 > 366.1
6.630e+005

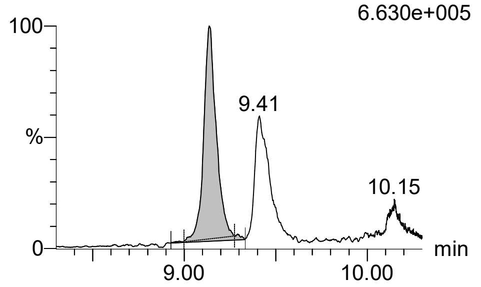

Sample Name: 20211111_A20_R3_IgM
F65:MRM of 1 channel,ES+
1075.1 > 366.1
2.532e+005

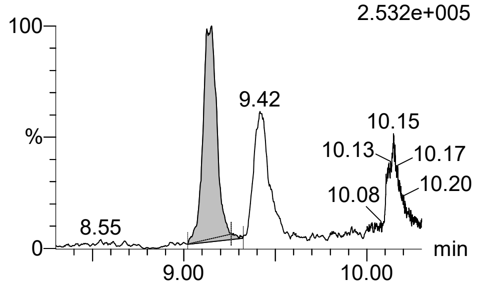

Sample Name: 20211111_L19_R3_IgM
F65:MRM of 1 channel,ES+
1075.1 > 366.1
5.969e+005

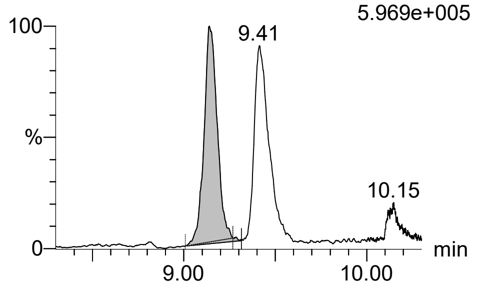

Sample Name: 20211111_QC_R21_IgM
F65:MRM of 1 channel,ES+
1075.1 > 366.1
6.868e+005

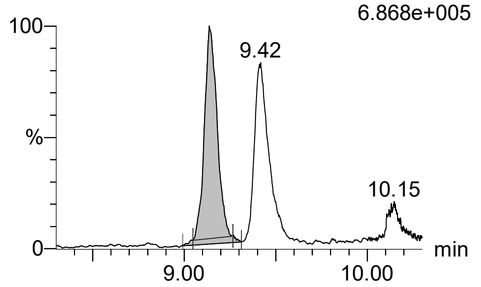

Sample Name: 20211111_L20_R3_IgM
F65:MRM of 1 channel,ES+
1075.1 > 366.1
4.262e+005

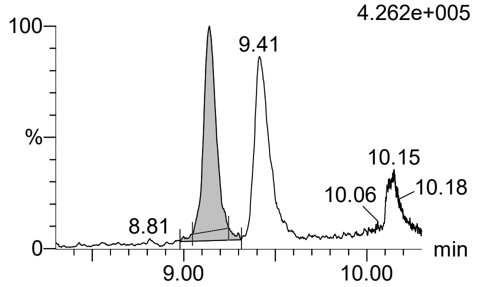

Sample Name: 20211111_H10_R3_IgM
F65:MRM of 1 channel,ES+
1075.1 > 366.1
1.135e+006

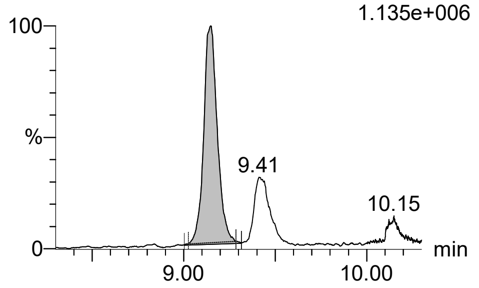

Sample Name: 20211111_A21_R3_IgM
F65:MRM of 1 channel,ES+
1075.1 > 366.1
6.815e+005

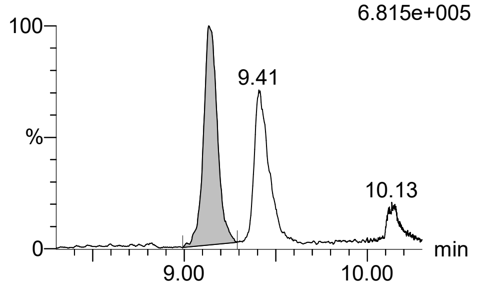

Sample Name: 20211111_A22_R3_IgM
F65:MRM of 1 channel,ES+
1075.1 > 366.1
2.705e+005

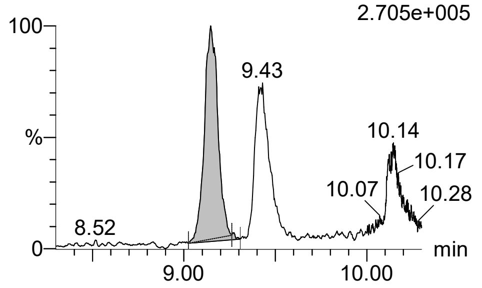

Sample Name: 20211111_L21_R3_IgM

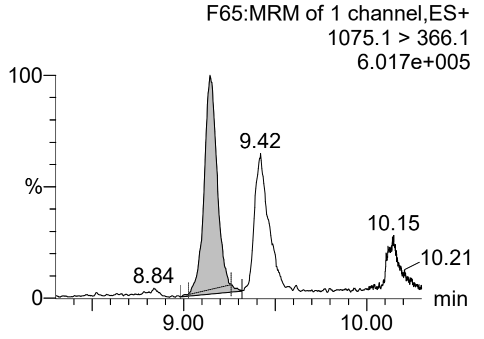

Sample Name: 20211111_L22_R3_IgM

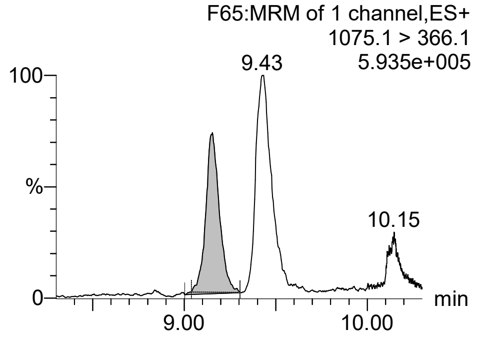

Sample Name: 20211111_H11_R3_IgM

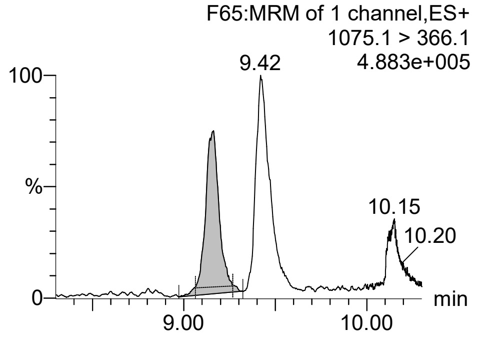

Sample Name: 20211111_A23_R3_IgM

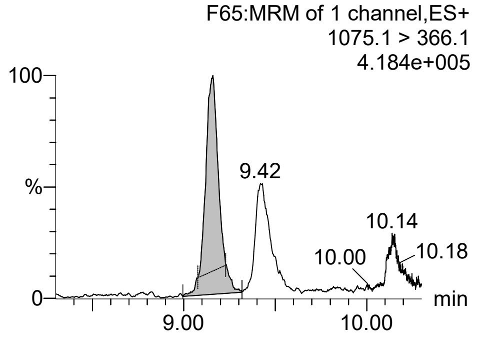

Sample Name: 20211111_A24_R3_IgM

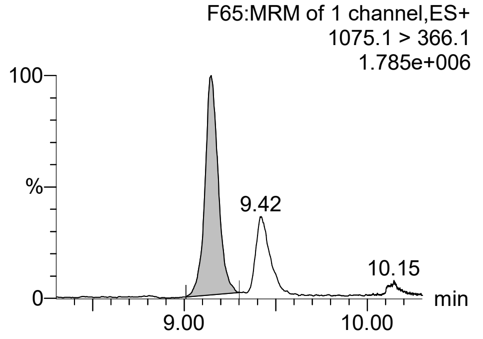

Sample Name: 20211111_L23_R3_IgM

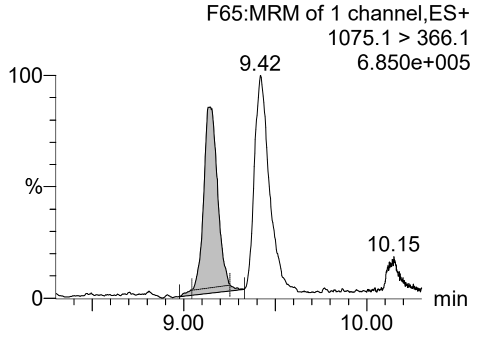

Sample Name: 20211111_L24_R3_IgM

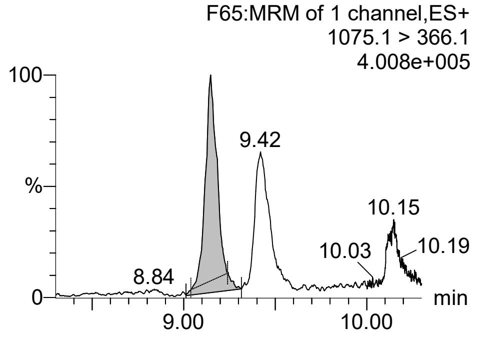

Sample Name: 20211111_H12_R3_IgM

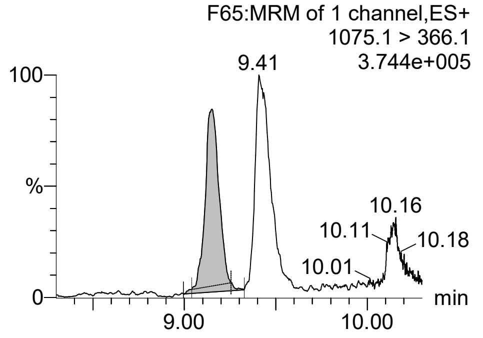

Sample Name: 20211111_QC_R22_IgM

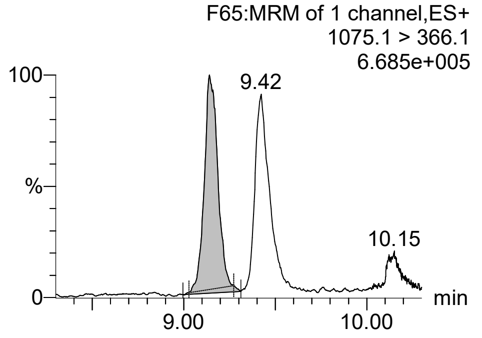

Sample Name: 20211111_A25_R3_IgM

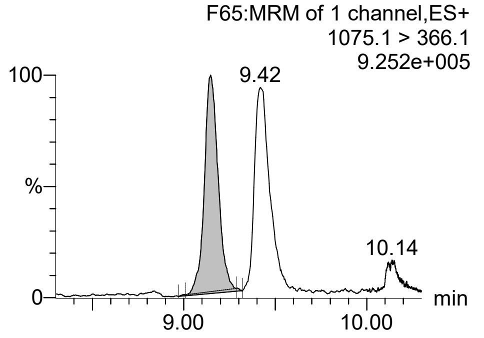

Sample Name: 20211111_A26_R3_IgM

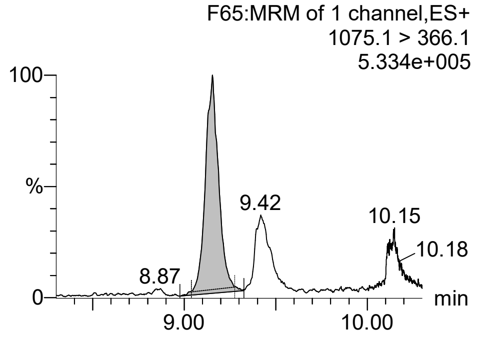

Sample Name: 20211111_L25_R3_IgM

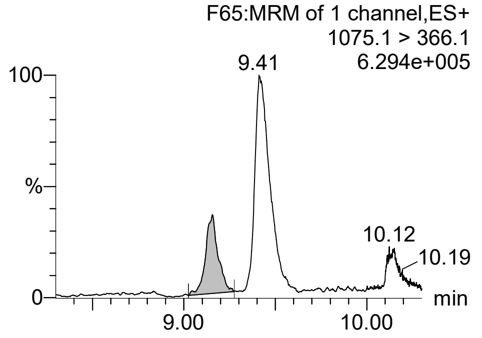

Sample Name: 20211111_L26_R3_IgM

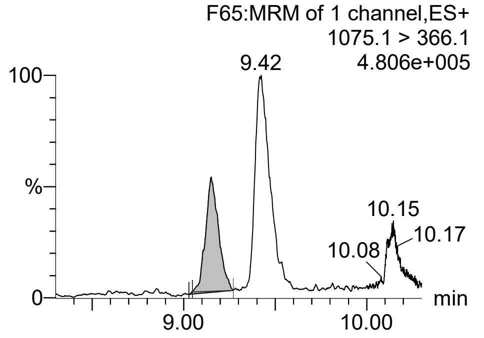

Sample Name: 20211111_H13_R3_IgM

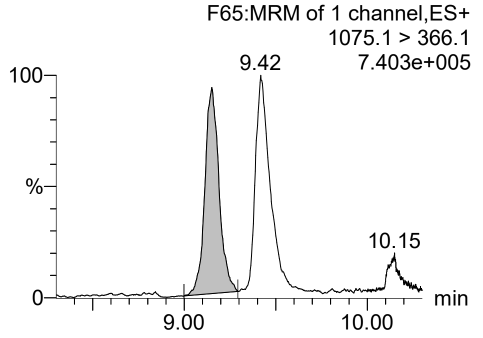

Sample Name: 20211111_A27_R3_IgM

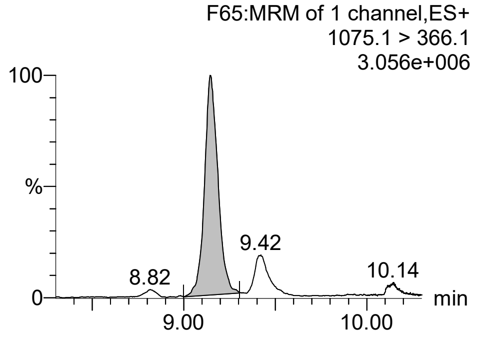

Dataset: C:\MassLynx\10.pro\20211111_clinicalsamples_IgM_IgA.qld

Last Altered: Thursday, March 09, 2023 19:58:25 台北標準時間
Printed: Friday, August 01, 2025 15:13:08 台北標準時間

Sample Name: 20211111_A28_R3_IgM
F65:MRM of 1 channel,ES+
1075.1 > 366.1
4.977e+005

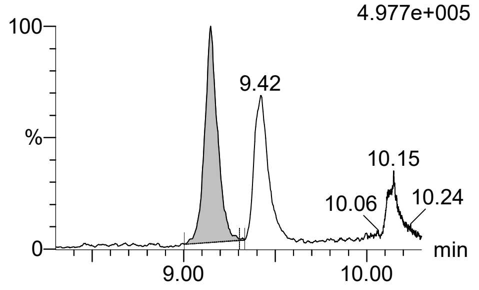

Sample Name: 20211111_L27_R3_IgM
F65:MRM of 1 channel,ES+
1075.1 > 366.1
7.267e+005

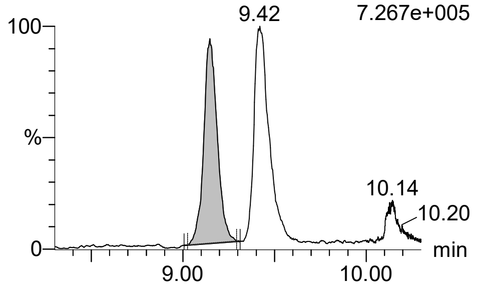

Sample Name: 20211111_L28_R3_IgM
F65:MRM of 1 channel,ES+
1075.1 > 366.1
6.492e+005

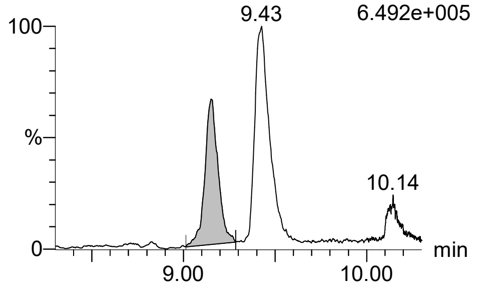

Sample Name: 20211111_H14_R3_IgM
F65:MRM of 1 channel,ES+
1075.1 > 366.1
6.595e+005

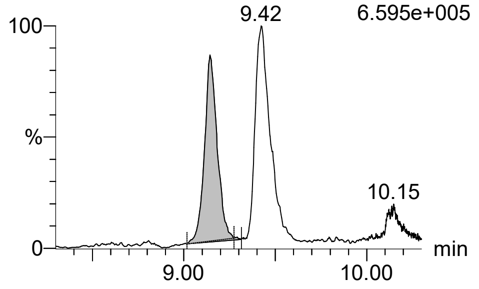

Sample Name: 20211111_A29_R3_IgM
F65:MRM of 1 channel,ES+
1075.1 > 366.1
1.262e+005

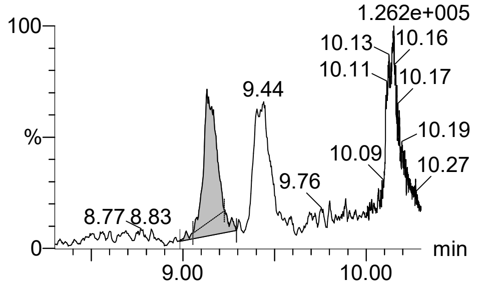

Sample Name: 20211111_A30_R3_IgM
F65:MRM of 1 channel,ES+
1075.1 > 366.1
7.541e+005

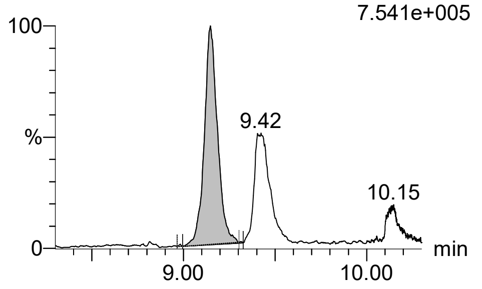

Sample Name: 20211111_QC_R23_IgM
F65:MRM of 1 channel,ES+
1075.1 > 366.1
6.241e+005

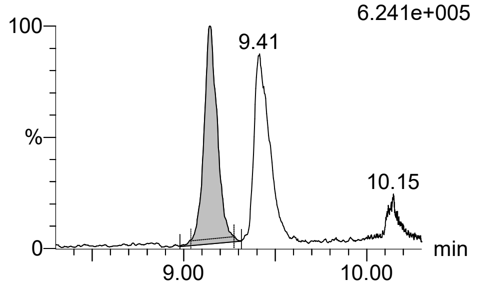

Sample Name: 20211111_L29_R3_IgM
F65:MRM of 1 channel,ES+
1075.1 > 366.1
2.723e+005

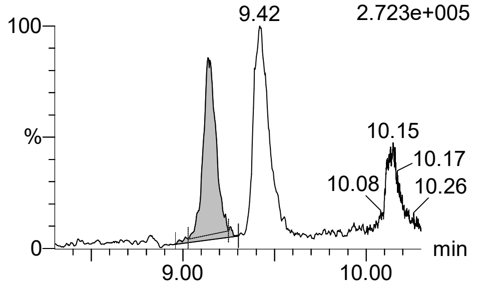

Sample Name: 20211111_L30_R3_IgM
F65:MRM of 1 channel,ES+
1075.1 > 366.1
1.702e+005

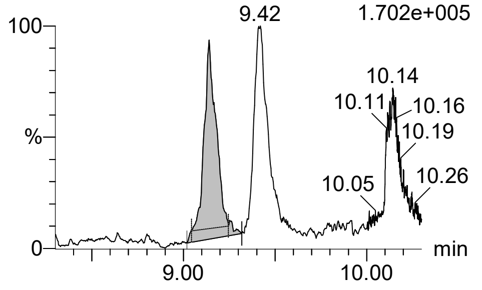

Sample Name: 20211111_H15_R3_IgM
F65:MRM of 1 channel,ES+
1075.1 > 366.1
4.282e+005

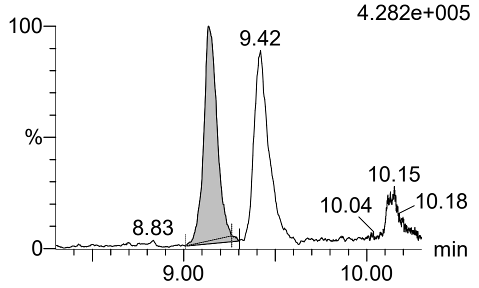

Sample Name: 20211111_A31_R3_IgM
F65:MRM of 1 channel,ES+
1075.1 > 366.1
2.119e+005

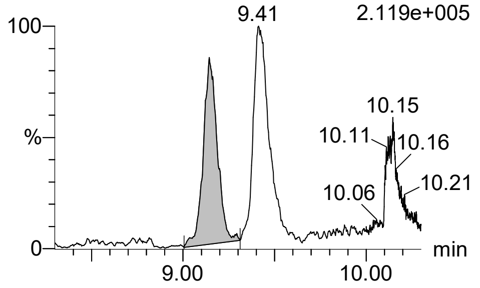

Sample Name: 20211111_A32_R3_IgM
F65:MRM of 1 channel,ES+
1075.1 > 366.1
6.544e+005

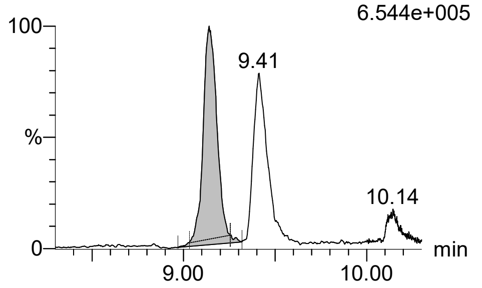

Sample Name: 20211111_L31_R3_IgM
F65:MRM of 1 channel,ES+
1075.1 > 366.1
2.232e+005

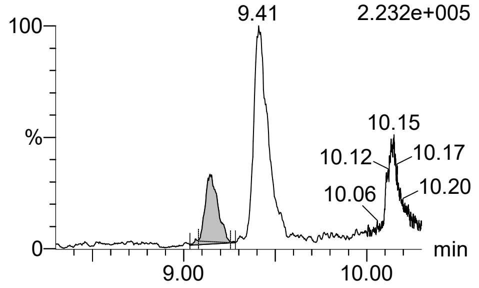

Sample Name: 20211111_L32_R3_IgM
F65:MRM of 1 channel,ES+
1075.1 > 366.1
9.966e+005

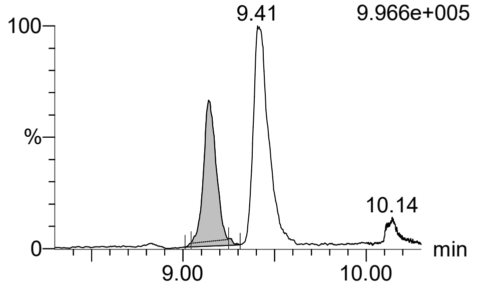

Sample Name: 20211111_H16_R3_IgM
F65:MRM of 1 channel,ES+
1075.1 > 366.1
1.261e+006

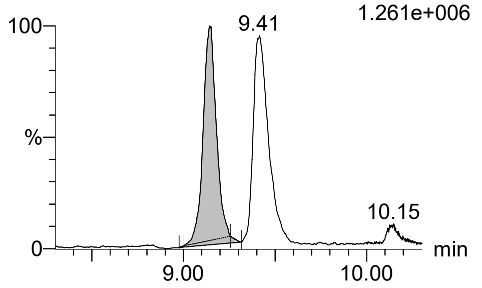

Dataset: C:\MassLynx\10.pro\20211111_clinicalsamples_IgM_IgA.qld

Last Altered: Thursday, March 09, 2023 19:58:25 台北標準時間
Printed: Friday, August 01, 2025 15:13:08 台北標準時間

Sample Name: 20211111_A33_R3_IgM
F65:MRM of 1 channel,ES+
1075.1 > 366.1
1.518e+006

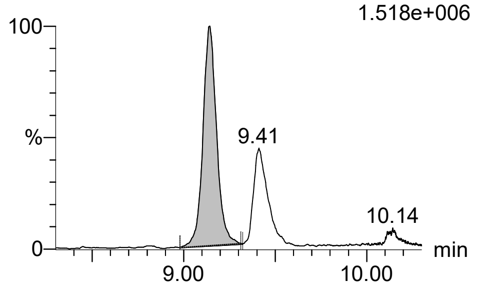

Sample Name: 20211111_A34_R3_IgM
F65:MRM of 1 channel,ES+
1075.1 > 366.1
2.365e+005

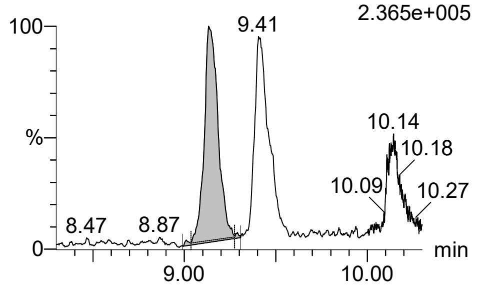

Sample Name: 20211111_H17_R3_IgM
F65:MRM of 1 channel,ES+
1075.1 > 366.1
3.329e+006

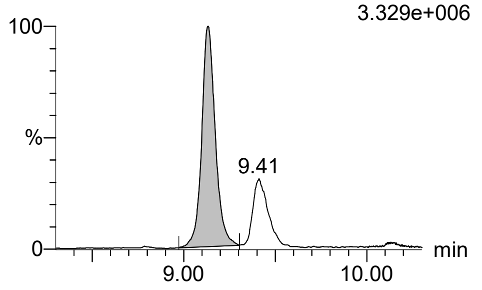

Sample Name: 20211111_A35_R3_IgM
F65:MRM of 1 channel,ES+
1075.1 > 366.1
4.058e+005

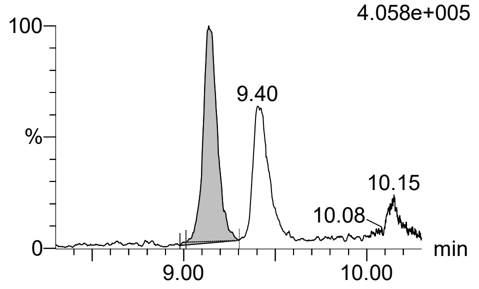

Sample Name: 20211111_QC_R24_IgM
F65:MRM of 1 channel,ES+
1075.1 > 366.1
5.325e+005

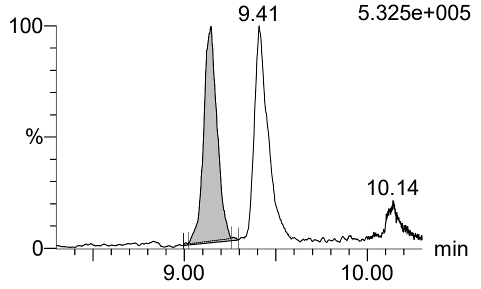

Sample Name: 20211111_A36_R3_IgM
F65:MRM of 1 channel,ES+
1075.1 > 366.1
3.295e+005

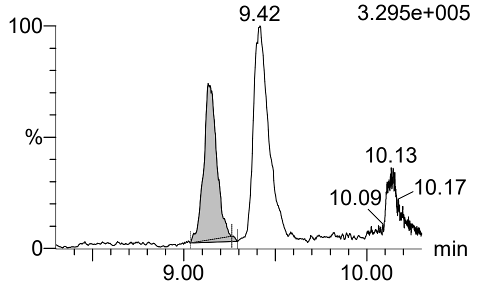

Sample Name: 20211111_H18_R3_IgM
F65:MRM of 1 channel,ES+
1075.1 > 366.1
1.036e+006

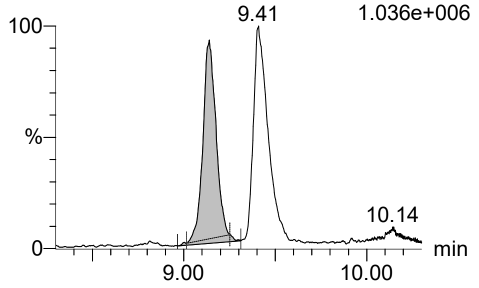

Sample Name: 20211111_A37_R3_IgM
F65:MRM of 1 channel,ES+
1075.1 > 366.1
7.169e+005

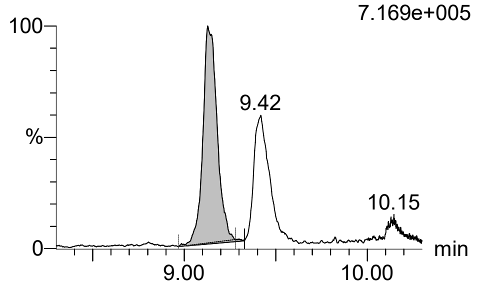

Sample Name: 20211111_A38_R3_IgM
F65:MRM of 1 channel,ES+
1075.1 > 366.1
5.417e+005

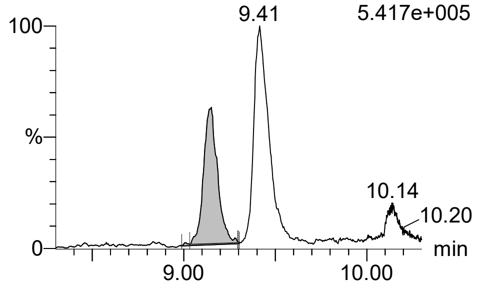

Sample Name: 20211111_H19_R3_IgM
F65:MRM of 1 channel,ES+
1075.1 > 366.1
1.058e+006

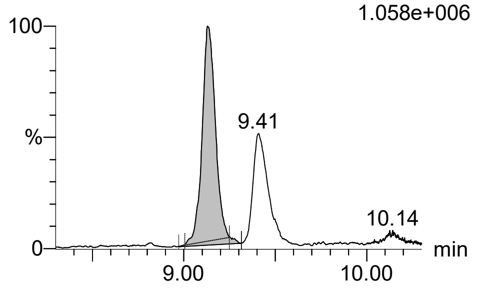

Sample Name: 20211111_A39_R3_IgM
F65:MRM of 1 channel,ES+
1075.1 > 366.1
6.177e+005

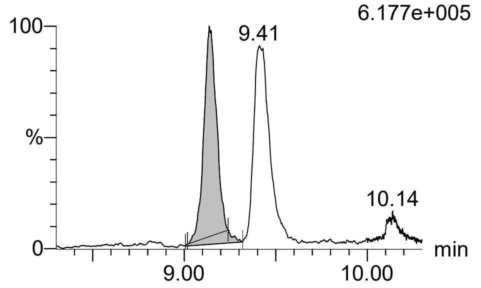

Sample Name: 20211111_A40_R3_IgM
F65:MRM of 1 channel,ES+
1075.1 > 366.1
2.962e+005

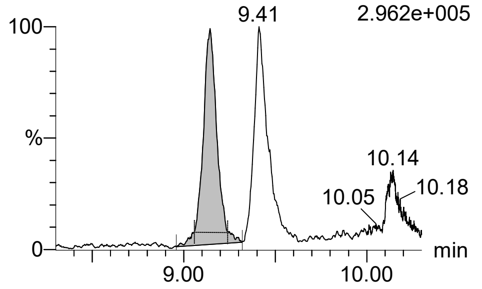

Sample Name: 20211111_H20_R3_IgM
F65:MRM of 1 channel,ES+
1075.1 > 366.1
2.022e+006

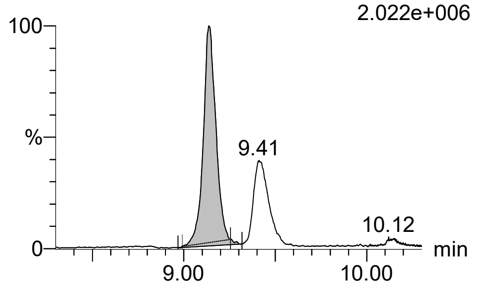

Sample Name: 20211111_A41_R3_IgM
F65:MRM of 1 channel,ES+
1075.1 > 366.1
2.728e+005

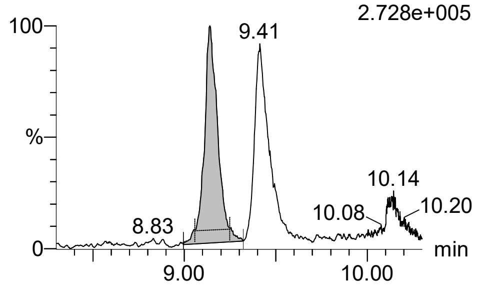

Sample Name: 20211111_A42_R3_IgM
F65:MRM of 1 channel,ES+
1075.1 > 366.1
2.511e+005

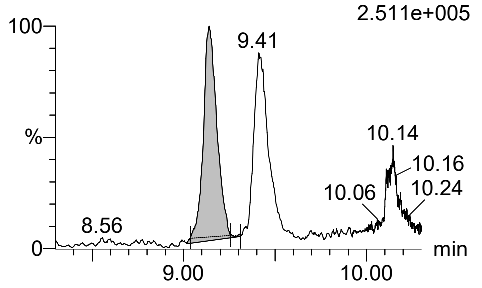

Dataset: C:\MassLynx\10.pro\20211111_clinicalsamples_IgM_IgA.qld

Last Altered: Thursday, March 09, 2023 19:58:25 台北標準時間
Printed: Friday, August 01, 2025 15:13:08 台北標準時間

Sample Name: 20211111_A43_R3_IgM

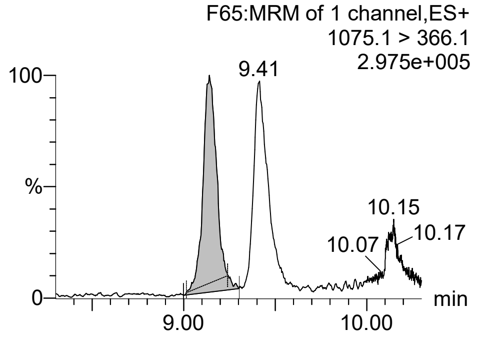

Sample Name: 20211111_A44_R3_IgM

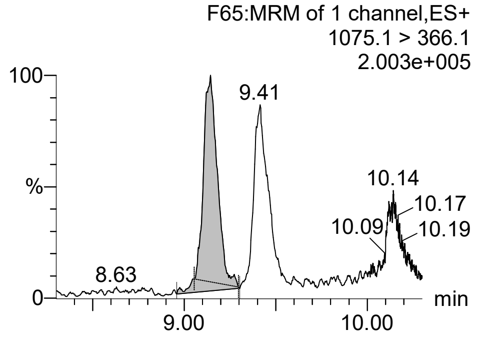

Sample Name: 20211111_QC_R25_IgM

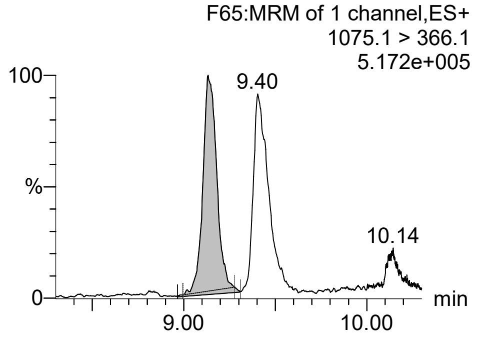

Dataset: C:\MassLynx\10.pro\20211111_clinicalsamples_IgM_IgA.qld

Last Altered: Thursday, March 09, 2023 19:58:25 台北標準時間
Printed: Friday, August 01, 2025 15:13:08 台北標準時間

	#	Name	RT	Area	IS Area	Response	Prima...
1	1	20211112_QC_R001_IgM	9.12	54927.6	15421.4	3.562	bb
2	2	20211112_QC_R002_IgM	9.13	53249.5	13188.8	4.037	MM
3	3	20211111_A1_R2_IgM	9.14	26477.2	11871.0	2.230	bb
4	4	20211111_A1_R3_IgM	9.14	25216.0	12030.5	2.096	MM
5	5	20211111_A2_R2_IgM	9.13	207889.4	11446.8	18.161	bb
6	6	20211111_A2_R3_IgM	9.14	203784.3	11098.4	18.362	bb
7	7	20211111_L1_R2_IgM	9.13	17695.5	11779.4	1.502	MM
8	8	20211111_L1_R3_IgM	9.13	12287.9	11176.2	1.099	MM
9	9	20211111_L2_R2_IgM	9.14	69739.4	11375.0	6.131	bb
10	10	20211111_H1_R2_IgM	9.13	27654.5	13595.2	2.034	MM
11	11	20211111_A3_R2_IgM	9.13	74226.9	14112.3	5.260	MM
12	12	20211111_A4_R2_IgM	9.14	49758.8	11977.5	4.154	MM
13	13	20211111_L3_R2_IgM	9.13	32024.4	12242.1	2.616	MM
14	14	20211111_L4_R2_IgM	9.14	74128.3	12797.0	5.793	bb
15	15	20211111_H2_R2_IgM	9.14	91148.9	10006.0	9.109	MM
16	16	20211111_A5_R2_IgM	9.13	491527.7	11434.6	42.986	bb
17	17	20211111_A6_R2_IgM	9.13	94362.8	13193.7	7.152	bb
18	18	20211111_QC_R10_IgM	9.13	58528.6	12566.1	4.658	MM
19	19	20211111_L5_R2_IgM	9.14	57151.0	13616.4	4.197	MM
20	20	20211111_L6_R2_IgM	9.14	78195.8	13698.5	5.708	MM
21	21	20211111_H3_R2_IgM	9.15	50063.8	15793.1	3.170	MM
22	22	20211111_A7_R2_IgM	9.13	59215.9	12936.9	4.577	MM
23	23	20211111_A8_R2_IgM	9.13	64173.2	13108.0	4.896	MM
24	24	20211111_L7_R2_IgM	9.15	24150.6	13278.5	1.819	MM
25	25	20211111_L8_R2_IgM	9.13	33378.6	15233.6	2.191	MM
26	26	20211111_H4_R2_IgM	9.14	34484.0	16521.9	2.087	MM
27	27	20211111_A9_R2_IgM	9.14	24954.2	16451.6	1.517	MM
28	28	20211111_A10_R2_IgM	9.13	63919.9	13036.0	4.903	MM
29	29	20211111_L9_R2_IgM	9.14	25901.6	14554.1	1.780	MM
30	30	20211111_L10_R2_IgM	9.14	81869.3	12250.4	6.683	MM
31	31	20211111_QC_R11_IgM	9.14	61283.9	11459.2	5.348	bb
32	32	20211111_H5_R2_IgM	9.13	107211.2	14358.2	7.467	MM
33	33	20211111_A11_R2_IgM	9.13	96735.5	16184.1	5.977	MM
34	34	20211111_A12_R2_IgM	9.15	29800.8	13465.4	2.213	MM
35	35	20211111_L11_R2_IgM	9.14	27398.4	13277.9	2.063	MM
36	36	20211111_L12_R2_IgM	9.13	36391.1	15958.8	2.280	MM
37	37	20211111_H6_R2_IgM	9.14	41204.7	14003.5	2.942	MM
38	38	20211111_A13_R2_IgM	9.13	58967.5	13656.1	4.318	MM
39	39	20211111_A14_R2_IgM	9.14	104118.9	14071.3	7.399	MM
40	40	20211111_L13_R2_IgM	9.15	43262.9	14785.6	2.926	MM
41	41	20211111_L14_R2_IgM	9.14	63178.6	14772.2	4.277	MM
42	42	20211111_H7_R2_IgM	9.14	52100.9	15164.4	3.436	MM
43	43	20211111_A15_R2_IgM	9.15	165639.7	15721.5	10.536	bb
44	44	20211111_QC_R12_IgM	9.14	68188.7	13711.5	4.973	MM
45	45	20211111_A16_R2_IgM	9.15	57014.3	14275.6	3.994	MM
46	46	20211111_L15_R2_IgM	9.15	79906.5	12935.8	6.177	MM
47	47	20211111_L16_R2_IgM	9.15	10979.8	13342.8	0.823	MM
48	48	20211111_H8_R2_IgM	9.15	38567.0	15835.1	2.436	MM
49	49	20211111_A17_R2_IgM	9.15	99626.2	15786.7	6.311	MM
50	50	20211111_A18_R2_IgM	9.13	25836.9	16986.8	1.521	MM
51	51	20211111_L17_R2_IgM	9.14	88929.7	13342.7	6.665	MM
52	52	20211111_L18_R2_IgM	9.14	11119.7	14692.0	0.757	MM
53	53	20211111_H9_R2_IgM	9.14	27873.3	15639.7	1.782	MM
54	54	20211111_A19_R2_IgM	9.13	55390.5	14198.7	3.901	bb
55	55	20211111_A20_R2_IgM	9.15	25378.5	14162.7	1.792	MM
56	56	20211111_L19_R2_IgM	9.14	48082.7	9293.4	5.174	MM

Dataset: C:\MassLynx\10.pro\20211111_clinicalsamples_IgM_IgA.qld

Last Altered: Thursday, March 09, 2023 19:58:25 台北標準時間
Printed: Friday, August 01, 2025 15:13:08 台北標準時間

	#	Name	RT	Area	IS Area	Response	Prima...
57	57	20211111_QC_R13_IgM	9.15	65544.0	13903.0	4.714	MM
58	58	20211111_L20_R2_IgM	9.13	25467.4	12781.6	1.992	bb
59	59	20211111_H10_R2_IgM	9.13	99105.9	14796.4	6.698	MM
60	60	20211111_A21_R2_IgM	9.15	60014.9	13669.0	4.391	bb
61	61	20211111_A22_R2_IgM	9.14	22663.1	14098.3	1.608	MM
62	62	20211111_L21_R2_IgM	9.14	58665.7	15678.2	3.742	MM
63	63	20211111_L22_R2_IgM	9.15	40114.1	15724.5	2.551	MM
64	64	20211111_H11_R2_IgM	9.14	36494.7	15890.2	2.297	MM
65	65	20211111_A23_R2_IgM	9.15	36404.3	13423.2	2.712	MM
66	66	20211111_A24_R2_IgM	9.14	158462.4	12438.0	12.740	MM
67	67	20211111_L23_R2_IgM	9.15	63153.1	14075.0	4.487	MM
68	68	20211111_L24_R2_IgM	9.15	36107.9	15972.1	2.261	MM
69	69	20211111_H12_R2_IgM	9.15	30137.7	16270.9	1.852	MM
70	70	20211111_QC_R14_IgM	9.15	69124.6	15806.8	4.373	MM
71	71	20211111_A25_R2_IgM	9.14	103473.9	14552.1	7.111	bb
72	72	20211111_A26_R2_IgM	9.15	48131.0	11492.7	4.188	MM
73	73	20211111_L25_R2_IgM	9.15	20871.1	14403.3	1.449	MM
74	74	20211111_L26_R2_IgM	9.17	25892.1	16533.9	1.566	MM
75	75	20211111_H13_R2_IgM	9.14	63641.1	15520.1	4.101	MM
76	76	20211111_A27_R2_IgM	9.14	296781.3	13337.4	22.252	bb
77	77	20211111_A28_R2_IgM	9.13	37520.1	11895.6	3.154	MM
78	78	20211111_L27_R2_IgM	9.14	58053.6	14125.7	4.110	MM
79	79	20211111_L28_R2_IgM	9.15	33802.9	11807.5	2.863	MM
80	80	20211111_H14_R2_IgM	9.15	44777.4	15035.7	2.978	MM
81	81	20211111_A29_R2_IgM	9.14	7170.7	13554.4	0.529	MM
82	82	20211111_A30_R2_IgM	9.15	67422.5	12984.1	5.193	MM
83	83	20211111_QC_R15_IgM	9.14	58731.8	12299.5	4.775	MM
84	84	20211111_L29_R2_IgM	9.14	18080.7	13590.7	1.330	MM
85	85	20211111_L30_R2_IgM	9.14	14828.7	12511.4	1.185	MM
86	86	20211111_H15_R2_IgM	9.15	42244.5	11885.0	3.554	MM
87	87	20211111_A31_R2_IgM	9.14	17545.5	13422.1	1.307	MM
88	88	20211111_A32_R2_IgM	9.14	63936.9	11461.3	5.578	MM
89	89	20211111_L31_R2_IgM	9.14	6199.9	13753.4	0.451	MM
90	90	20211111_L32_R2_IgM	9.14	57151.2	12949.0	4.414	MM
91	91	20211111_H16_R2_IgM	9.14	124890.9	11465.4	10.893	MM
92	92	20211111_A33_R2_IgM	9.14	133489.1	10551.5	12.651	bb
93	93	20211111_A34_R2_IgM	9.14	24416.5	12409.9	1.968	MM
94	94	20211111_H17_R2_IgM	9.13	337298.1	10160.9	33.196	bb
95	95	20211111_A35_R2_IgM	9.13	38885.0	10460.6	3.717	MM
96	96	20211111_QC_R16_IgM	9.14	54670.7	10433.5	5.240	MM
97	97	20211111_A36_R2_IgM	9.13	18659.9	12504.8	1.492	MM
98	98	20211111_H18_R2_IgM	9.14	90416.6	9140.4	9.892	MM
99	99	20211111_A37_R2_IgM	9.14	79173.8	9796.9	8.082	MM
100	1...	20211111_A38_R2_IgM	9.15	34322.6	11224.3	3.058	MM
101	1...	20211111_H19_R2_IgM	9.14	106096.7	11739.2	9.038	MM
102	1...	20211111_A39_R2_IgM	9.14	55862.3	12718.9	4.392	MM
103	1...	20211111_A40_R2_IgM	9.12	23849.3	10006.1	2.383	MM
104	1...	20211111_H20_R2_IgM	9.13	193170.5	7988.2	24.182	MM
105	1...	20211111_A41_R2_IgM	9.15	34292.1	11513.3	2.978	MM
106	1...	20211111_A42_R2_IgM	9.15	24506.5	10238.5	2.394	MM
107	1...	20211111_A43_R2_IgM	9.15	27415.8	11098.9	2.470	MM
108	1...	20211111_A44_R2_IgM	9.14	18545.4	10969.9	1.691	MM
109	1...	20211111_QC_R17_IgM	9.15	46641.8	9700.8	4.808	MM
110	1...	20211111_L2_R3_IgM	9.14	68222.1	8528.0	8.000	MM
111	1...	20211111_H1_R3_IgM	9.14	27086.7	9438.2	2.870	MM
112	1...	20211111_A3_R3_IgM	9.14	71147.1	11598.6	6.134	MM

Dataset: C:\MassLynx\10.pro\20211111_clinicalsamples_IgM_IgA.qld

Last Altered: Thursday, March 09, 2023 19:58:25 台北標準時間
Printed: Friday, August 01, 2025 15:13:08 台北標準時間

	#	Name	RT	Area	IS Area	Response	Prima...
113	1...	20211111_A4_R3_IgM	9.14	49309.2	11364.1	4.339	MM
114	1...	20211111_L3_R3_IgM	9.12	30871.8	10753.4	2.871	MM
115	1...	20211111_L4_R3_IgM	9.13	64418.7	10291.6	6.259	MM
116	1...	20211111_H2_R3_IgM	9.13	91943.1	9315.8	9.870	MM
117	1...	20211111_A5_R3_IgM	9.13	439765.2	9102.2	48.314	bb
118	1...	20211111_A6_R3_IgM	9.14	72130.2	10356.4	6.965	MM
119	1...	20211111_QC_R18_IgM	9.13	50825.7	8398.3	6.052	MM
120	1...	20211111_L5_R3_IgM	9.14	46607.5	9786.7	4.762	MM
121	1...	20211111_L6_R3_IgM	9.14	65025.2	9911.3	6.561	MM
122	1...	20211111_H3_R3_IgM	9.14	45229.9	12155.5	3.721	MM
123	1...	20211111_A7_R3_IgM	9.13	55638.1	11804.8	4.713	MM
124	1...	20211111_A8_R3_IgM	9.14	55414.7	10510.6	5.272	MM
125	1...	20211111_L7_R3_IgM	9.14	23137.0	12177.9	1.900	MM
126	1...	20211111_L8_R3_IgM	9.14	26573.5	11668.6	2.277	bb
127	1...	20211111_H4_R3_IgM	9.14	28224.3	11192.0	2.522	MM
128	1...	20211111_A9_R3_IgM	9.14	23087.2	14077.4	1.640	MM
129	1...	20211111_A10_R3_IgM	9.14	58499.2	11190.4	5.228	MM
130	1...	20211111_L9_R3_IgM	9.14	25453.9	14649.3	1.738	MM
131	1...	20211111_L10_R3_IgM	9.13	64842.8	9361.3	6.927	bb
132	1...	20211111_QC_R19_IgM	9.14	48358.8	10125.5	4.776	MM
133	1...	20211111_H5_R3_IgM	9.14	81981.4	10010.9	8.189	bb
134	1...	20211111_A11_R3_IgM	9.13	87138.7	12697.2	6.863	MM
135	1...	20211111_A12_R3_IgM	9.13	27814.5	13148.5	2.115	MM
136	1...	20211111_L11_R3_IgM	9.13	21702.6	10786.6	2.012	MM
137	1...	20211111_L12_R3_IgM	9.14	29476.9	11681.8	2.523	MM
138	1...	20211111_H6_R3_IgM	9.15	37925.5	12095.5	3.136	MM
139	1...	20211111_A13_R3_IgM	9.15	54777.9	12908.3	4.244	MM
140	1...	20211111_A14_R3_IgM	9.15	81500.0	11322.6	7.198	MM
141	1...	20211111_L13_R3_IgM	9.14	32631.6	11516.1	2.834	MM
142	1...	20211111_L14_R3_IgM	9.14	50878.8	10790.7	4.715	MM
143	1...	20211111_H7_R3_IgM	9.14	45334.3	11572.1	3.918	bb
144	1...	20211111_A15_R3_IgM	9.14	135573.8	12434.4	10.903	MM
145	1...	20211111_QC_R20_IgM	9.14	57790.5	10701.0	5.400	MM
146	1...	20211111_A16_R3_IgM	9.15	46754.8	11452.1	4.083	bb
147	1...	20211111_L15_R3_IgM	9.14	68004.0	10531.1	6.457	bb
148	1...	20211111_L16_R3_IgM	9.15	9528.1	12049.4	0.791	MM
149	1...	20211111_H8_R3_IgM	9.14	35641.9	13434.9	2.653	MM
150	1...	20211111_A17_R3_IgM	9.15	78569.0	12452.0	6.310	MM
151	1...	20211111_A18_R3_IgM	9.14	21842.6	12506.5	1.746	MM
152	1...	20211111_L17_R3_IgM	9.14	72842.9	9948.9	7.322	bb
153	1...	20211111_L18_R3_IgM	9.15	11037.1	13462.1	0.820	MM
154	1...	20211111_H9_R3_IgM	9.14	24296.2	13178.7	1.844	MM
155	1...	20211111_A19_R3_IgM	9.14	52349.4	11298.6	4.633	MM
156	1...	20211111_A20_R3_IgM	9.15	21902.6	11175.6	1.960	MM
157	1...	20211111_L19_R3_IgM	9.14	49238.4	11558.2	4.260	MM
158	1...	20211111_QC_R21_IgM	9.14	56259.0	9977.0	5.639	MM
159	1...	20211111_L20_R3_IgM	9.14	34796.9	13508.1	2.576	MM
160	1...	20211111_H10_R3_IgM	9.15	96380.1	12374.8	7.788	MM
161	1...	20211111_A21_R3_IgM	9.13	58896.6	12443.6	4.733	bb
162	1...	20211111_A22_R3_IgM	9.15	22719.1	11589.3	1.960	MM
163	1...	20211111_L21_R3_IgM	9.14	49657.6	13107.4	3.789	MM
164	1...	20211111_L22_R3_IgM	9.15	37262.9	12021.5	3.100	MM
165	1...	20211111_H11_R3_IgM	9.16	31635.4	12687.8	2.493	MM
166	1...	20211111_A23_R3_IgM	9.16	35036.3	11611.8	3.017	MM
167	1...	20211111_A24_R3_IgM	9.15	147428.9	10476.8	14.072	bb
168	1...	20211111_L23_R3_IgM	9.14	52289.3	11873.1	4.404	MM

Dataset: C:\MassLynx\10.pro\20211111_clinicalsamples_IgM_IgA.qld

Last Altered: Thursday, March 09, 2023 19:58:25 台北標準時間
Printed: Friday, August 01, 2025 15:13:08 台北標準時間

	#	Name	RT	Area	IS Area	Response	Prima...
169	1...	20211111_L24_R3_IgM	9.15	31018.3	13468.0	2.303	MM
170	1...	20211111_H12_R3_IgM	9.15	28071.7	14229.1	1.973	MM
171	1...	20211111_QC_R22_IgM	9.14	57335.7	11264.4	5.090	MM
172	1...	20211111_A25_R3_IgM	9.15	79685.6	11775.7	6.767	MM
173	1...	20211111_A26_R3_IgM	9.15	44082.8	10473.9	4.209	MM
174	1...	20211111_L25_R3_IgM	9.16	17618.2	13256.8	1.329	bb
175	1...	20211111_L26_R3_IgM	9.15	20650.7	13565.0	1.522	MM
176	1...	20211111_H13_R3_IgM	9.15	61206.8	13146.0	4.656	bb
177	1...	20211111_A27_R3_IgM	9.14	255225.4	12025.6	21.223	bb
178	1...	20211111_A28_R3_IgM	9.15	39314.4	11229.1	3.501	MM
179	1...	20211111_L27_R3_IgM	9.15	57037.2	14960.0	3.813	MM
180	1...	20211111_L28_R3_IgM	9.15	35176.1	11359.0	3.097	bb
181	1...	20211111_H14_R3_IgM	9.14	44558.7	12830.8	3.473	MM
182	1...	20211111_A29_R3_IgM	9.13	7521.5	13434.3	0.560	MM
183	1...	20211111_A30_R3_IgM	9.15	61612.7	11272.2	5.466	MM
184	1...	20211111_QC_R23_IgM	9.14	52062.1	11872.2	4.385	MM
185	1...	20211111_L29_R3_IgM	9.14	19109.6	12948.7	1.476	MM
186	1...	20211111_L30_R3_IgM	9.14	12459.2	11026.6	1.130	MM
187	1...	20211111_H15_R3_IgM	9.14	36146.6	10786.3	3.351	MM
188	1...	20211111_A31_R3_IgM	9.14	15692.5	12010.5	1.307	bb
189	1...	20211111_A32_R3_IgM	9.14	57215.2	11107.7	5.151	MM
190	1...	20211111_L31_R3_IgM	9.14	5828.5	11021.8	0.529	MM
191	1...	20211111_L32_R3_IgM	9.14	54713.4	11683.8	4.683	MM
192	1...	20211111_H16_R3_IgM	9.14	106754.8	9831.7	10.858	MM
193	1...	20211111_A33_R3_IgM	9.14	129346.9	11213.1	11.535	MM
194	1...	20211111_A34_R3_IgM	9.13	20414.7	11550.1	1.767	MM
195	1...	20211111_H17_R3_IgM	9.13	280634.5	8874.4	31.623	bb
196	1...	20211111_A35_R3_IgM	9.14	35866.8	9856.3	3.639	MM
197	1...	20211111_QC_R24_IgM	9.15	44575.8	7561.8	5.895	MM
198	1...	20211111_A36_R3_IgM	9.13	19915.7	9562.8	2.083	MM
199	1...	20211111_H18_R3_IgM	9.14	81174.8	8165.3	9.941	MM
200	2...	20211111_A37_R3_IgM	9.13	63742.1	9278.8	6.870	MM
201	2...	20211111_A38_R3_IgM	9.15	29852.3	10920.3	2.734	MM
202	2...	20211111_H19_R3_IgM	9.13	90191.1	9285.3	9.713	MM
203	2...	20211111_A39_R3_IgM	9.14	51365.2	9083.4	5.655	MM
204	2...	20211111_A40_R3_IgM	9.14	25251.7	8382.4	3.012	MM
205	2...	20211111_H20_R3_IgM	9.14	162737.6	7807.9	20.843	MM
206	2...	20211111_A41_R3_IgM	9.14	22582.2	5925.2	3.811	MM
207	2...	20211111_A42_R3_IgM	9.14	21087.9	9352.6	2.255	MM
208	2...	20211111_A43_R3_IgM	9.14	25598.7	10216.3	2.506	MM
209	2...	20211111_A44_R3_IgM	9.14	18161.4	10262.7	1.770	MM
210	2...	20211111_QC_R25_IgM	9.13	45532.5	8575.3	5.310	MM

Dataset: C:\MassLynx\10.pro\20211111_clinicalsamples_IgM_IgA.qld

Last Altered: Thursday, March 09, 2023 19:58:25 台北標準時間
Printed: Friday, August 01, 2025 15:13:55 台北標準時間

Method: C:\MassLynx\10_Pro_IgA.PRO\MethDB\20211111_IgM_IgA_DMRM_new.mdb 24 Feb 2022 13:44:14
Calibration: 15 Nov 2021 14:42:11

Sample Name: 20211112_QC_R001_IgM

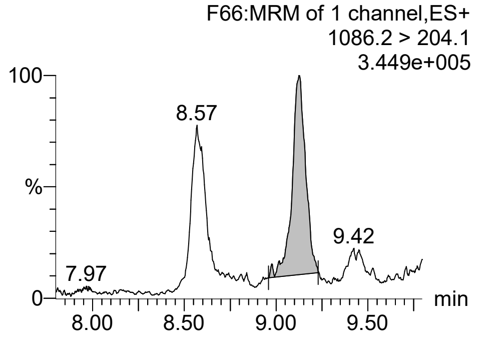

Sample Name: 20211112_QC_R002_IgM

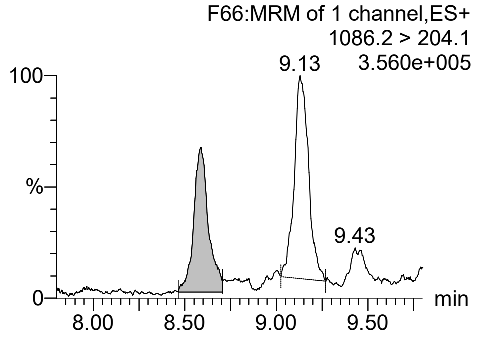

Sample Name: 20211111_A1_R2_IgM

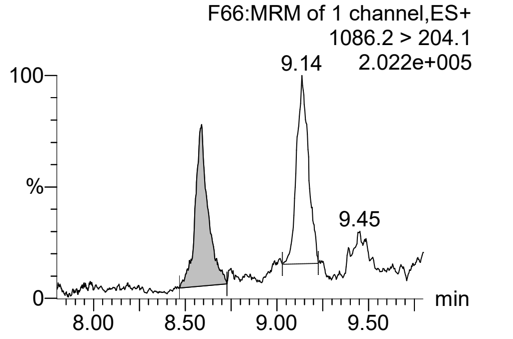

Sample Name: 20211111_A1_R3_IgM

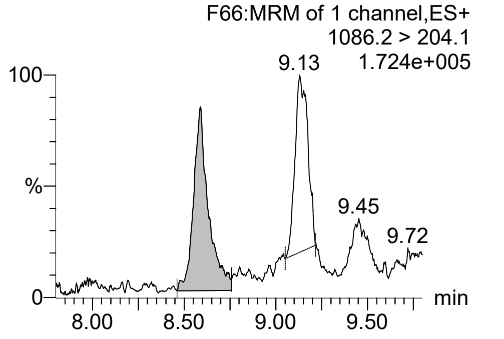

Sample Name: 20211111_A2_R2_IgM

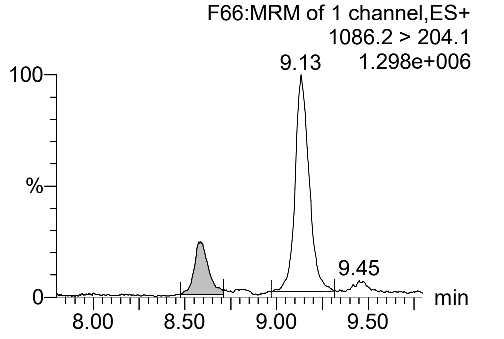

Sample Name: 20211111_A2_R3_IgM

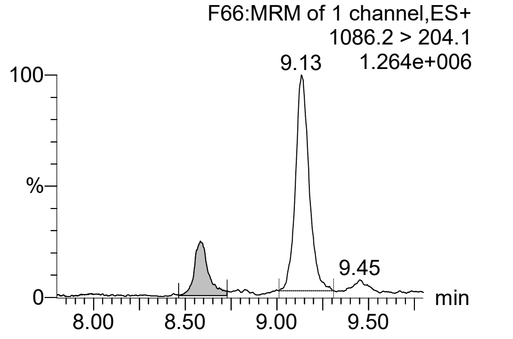

Sample Name: 20211111_L1_R2_IgM

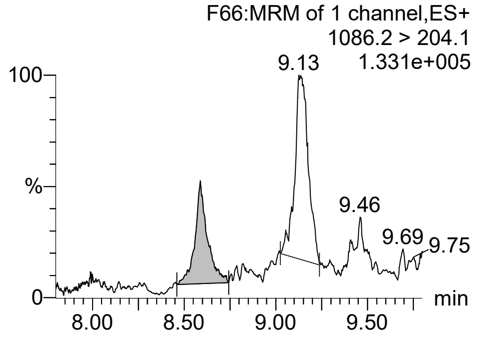

Sample Name: 20211111_L1_R3_IgM

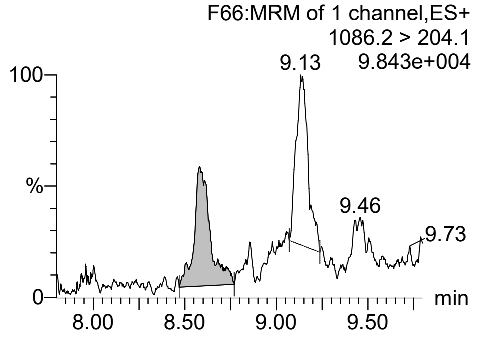

Sample Name: 20211111_L2_R2_IgM

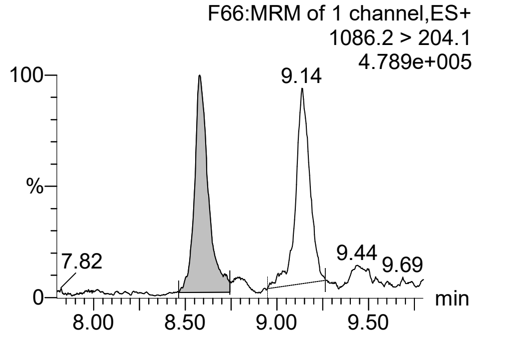

Sample Name: 20211111_H1_R2_IgM

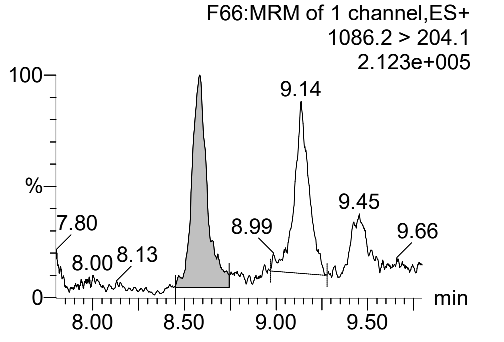

Sample Name: 20211111_A3_R2_IgM

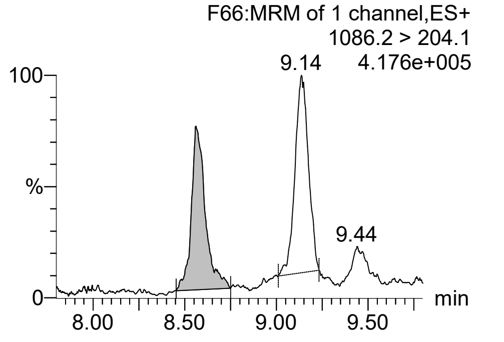

Sample Name: 20211111_A4_R2_IgM

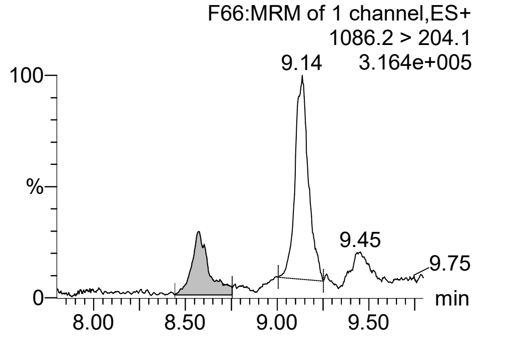

Dataset: C:\MassLynx\10.pro\20211111_clinicalsamples_IgM_IgA.qld

Last Altered: Thursday, March 09, 2023 19:58:25 台北標準時間
Printed: Friday, August 01, 2025 15:13:55 台北標準時間

Sample Name: 20211111_L3_R2_IgM
F66:MRM of 1 channel,ES+
1086.2 > 204.1
9.12 1.872e+005

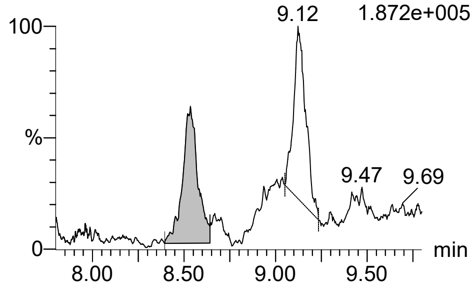

Sample Name: 20211111_L4_R2_IgM
F66:MRM of 1 channel,ES+
1086.2 > 204.1
9.14 4.375e+005

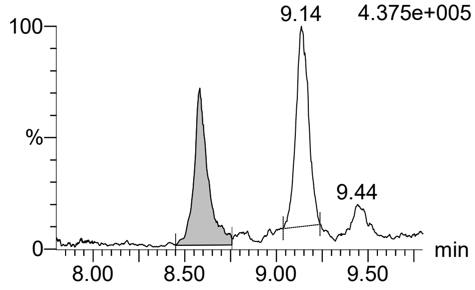

Sample Name: 20211111_H2_R2_IgM
F66:MRM of 1 channel,ES+
1086.2 > 204.1
5.872e+005

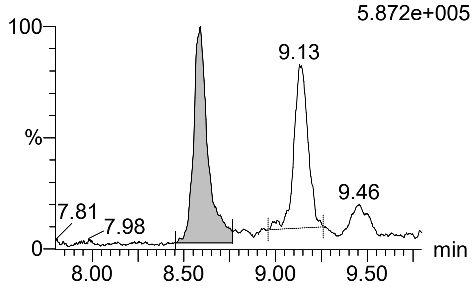

Sample Name: 20211111_A5_R2_IgM
F66:MRM of 1 channel,ES+
1086.2 > 204.1
9.14 2.241e+006

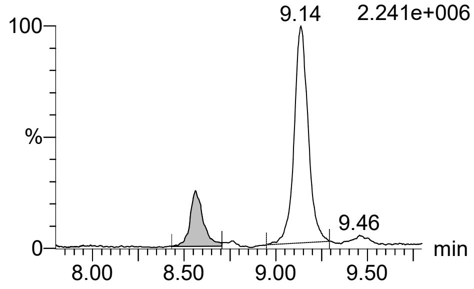

Sample Name: 20211111_A6_R2_IgM
F66:MRM of 1 channel,ES+
1086.2 > 204.1
9.13 5.280e+005

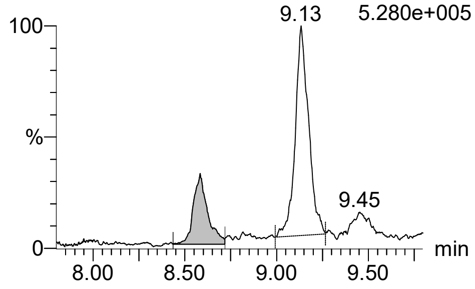

Sample Name: 20211111_QC_R10_IgM
F66:MRM of 1 channel,ES+
1086.2 > 204.1
9.13 3.429e+005

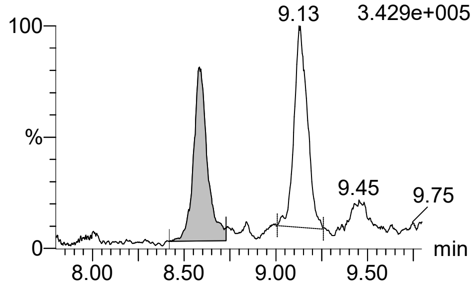

Sample Name: 20211111_L5_R2_IgM
F66:MRM of 1 channel,ES+
1086.2 > 204.1
5.032e+005

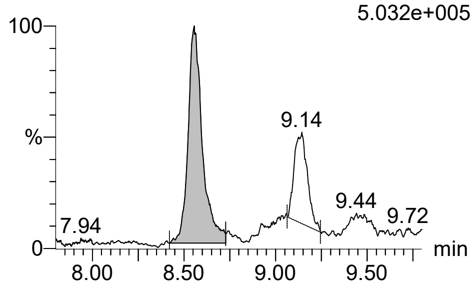

Sample Name: 20211111_L6_R2_IgM
F66:MRM of 1 channel,ES+
1086.2 > 204.1
9.13 4.615e+005

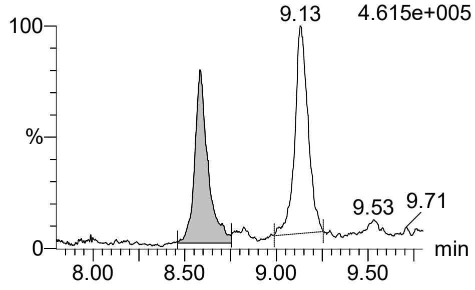

Sample Name: 20211111_H3_R2_IgM
F66:MRM of 1 channel,ES+
1086.2 > 204.1
9.13 3.235e+005

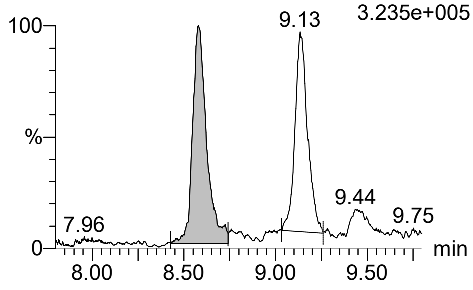

Sample Name: 20211111_A7_R2_IgM
F66:MRM of 1 channel,ES+
1086.2 > 204.1
6.955e+005

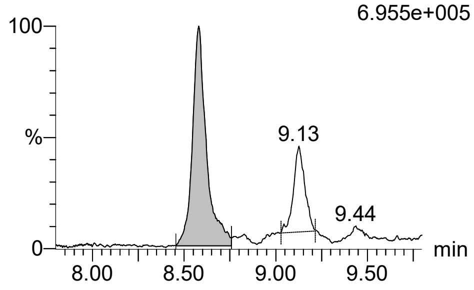

Sample Name: 20211111_A8_R2_IgM
F66:MRM of 1 channel,ES+
1086.2 > 204.1
5.181e+005

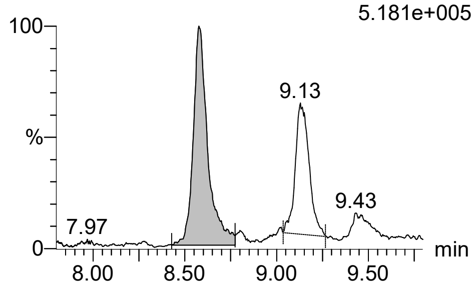

Sample Name: 20211111_L7_R2_IgM
F66:MRM of 1 channel,ES+
1086.2 > 204.1
9.14 1.553e+005

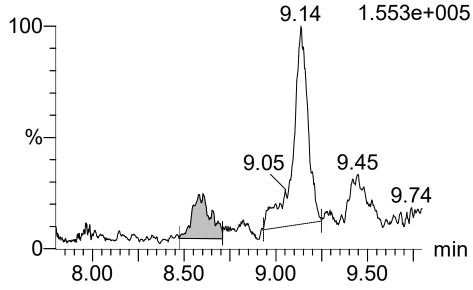

Sample Name: 20211111_L8_R2_IgM
F66:MRM of 1 channel,ES+
1086.2 > 204.1
9.13 2.097e+005

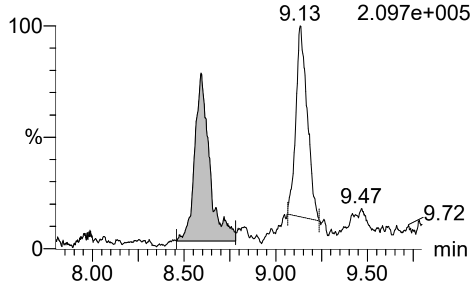

Sample Name: 20211111_H4_R2_IgM
F66:MRM of 1 channel,ES+
1086.2 > 204.1
2.656e+005

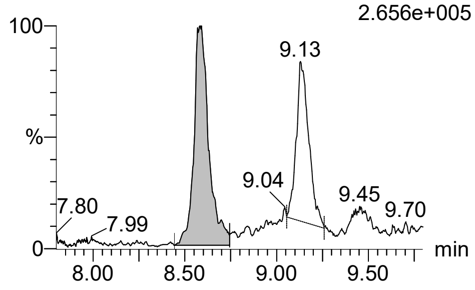

Sample Name: 20211111_A9_R2_IgM
F66:MRM of 1 channel,ES+
1086.2 > 204.1
3.332e+005

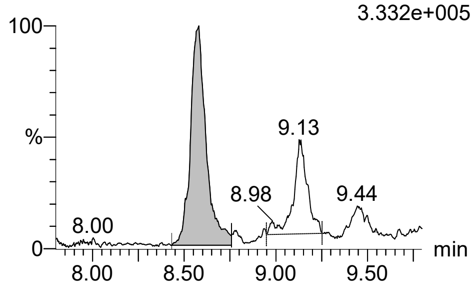

Dataset: C:\MassLynx\10.pro\20211111_clinicalsamples_IgM_IgA.qld

Last Altered: Thursday, March 09, 2023 19:58:25 台北標準時間
Printed: Friday, August 01, 2025 15:13:55 台北標準時間

Sample Name: 20211111_A10_R2_IgM
F66:MRM of 1 channel,ES+
1086.2 > 204.1
7.324e+005

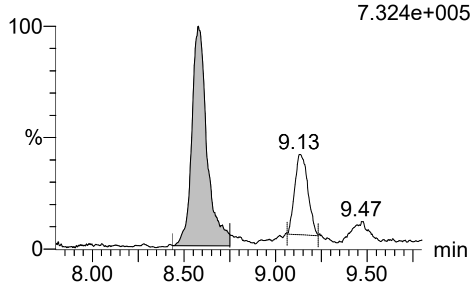

Sample Name: 20211111_L9_R2_IgM
F66:MRM of 1 channel,ES+
1086.2 > 204.1
1.509e+005

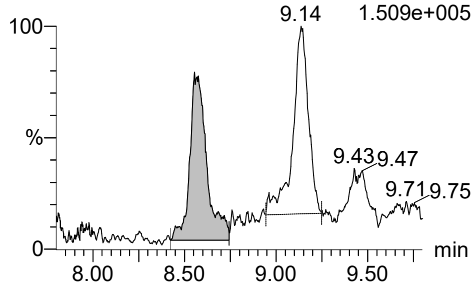

Sample Name: 20211111_L10_R2_IgM
F66:MRM of 1 channel,ES+
1086.2 > 204.1
4.007e+005

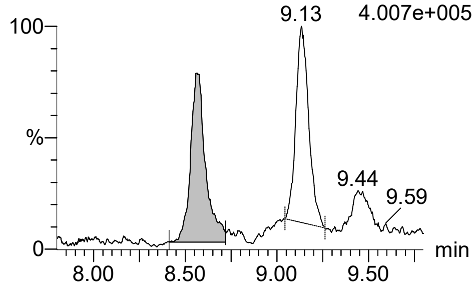

Sample Name: 20211111_QC_R11_IgM
F66:MRM of 1 channel,ES+
1086.2 > 204.1
3.320e+005

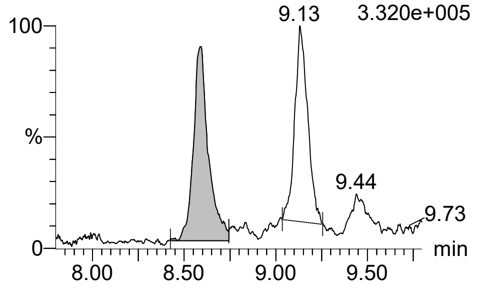

Sample Name: 20211111_H5_R2_IgM
F66:MRM of 1 channel,ES+
1086.2 > 204.1
5.374e+005

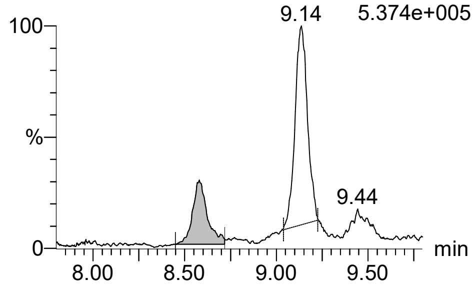

Sample Name: 20211111_A11_R2_IgM
F66:MRM of 1 channel,ES+
1086.2 > 204.1
3.745e+005

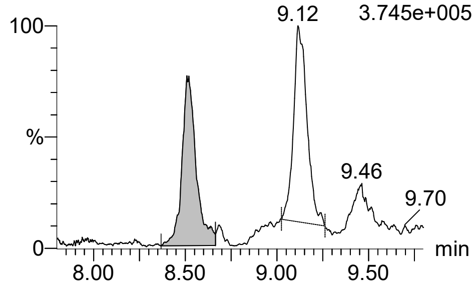

Sample Name: 20211111_A12_R2_IgM
F66:MRM of 1 channel,ES+
1086.2 > 204.1
2.107e+005

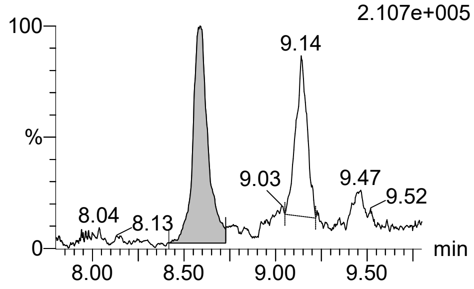

Sample Name: 20211111_L11_R2_IgM
F66:MRM of 1 channel,ES+
1086.2 > 204.1
1.501e+005

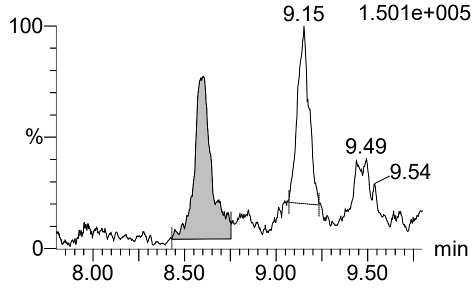

Sample Name: 20211111_L12_R2_IgM
F66:MRM of 1 channel,ES+
1086.2 > 204.1
1.841e+005

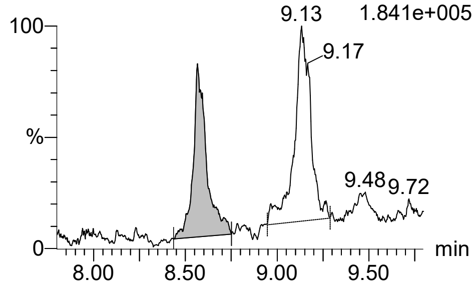

Sample Name: 20211111_H6_R2_IgM
F66:MRM of 1 channel,ES+
1086.2 > 204.1
2.749e+005

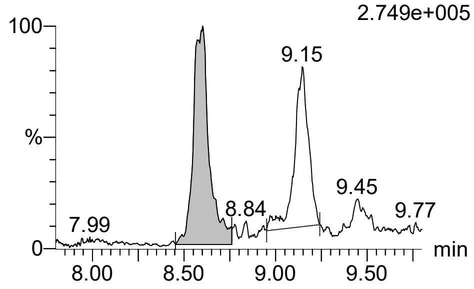

Sample Name: 20211111_A13_R2_IgM
F66:MRM of 1 channel,ES+
1086.2 > 204.1
3.182e+005

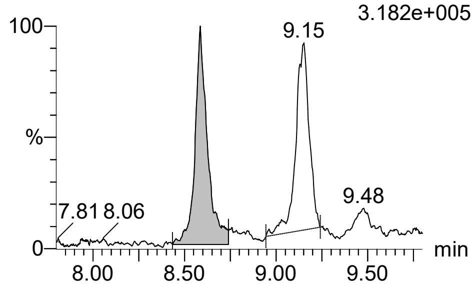

Sample Name: 20211111_A14_R2_IgM
F66:MRM of 1 channel,ES+
1086.2 > 204.1
9.627e+005

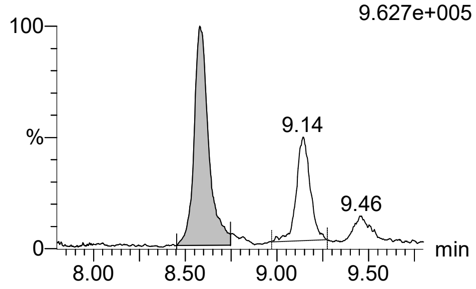

Sample Name: 20211111_L13_R2_IgM
F66:MRM of 1 channel,ES+
1086.2 > 204.1
2.563e+005

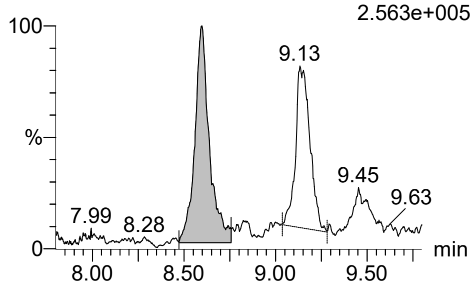

Sample Name: 20211111_L14_R2_IgM
F66:MRM of 1 channel,ES+
1086.2 > 204.1
6.361e+005

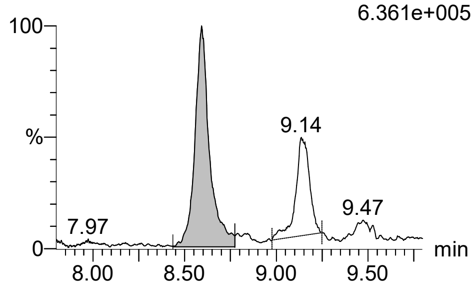

Sample Name: 20211111_H7_R2_IgM
F66:MRM of 1 channel,ES+
1086.2 > 204.1
4.452e+005

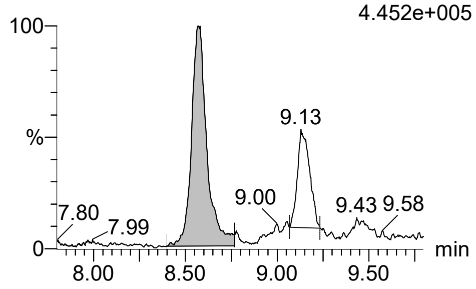

Sample Name: 20211111_A15_R2_IgM
F66:MRM of 1 channel,ES+
1086.2 > 204.1
1.015e+006

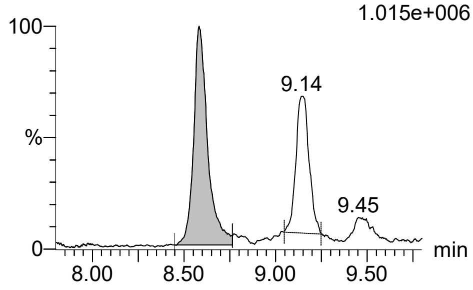

Sample Name: 20211111_QC_R12_IgM
F66:MRM of 1 channel,ES+
1086.2 > 204.1
3.419e+005

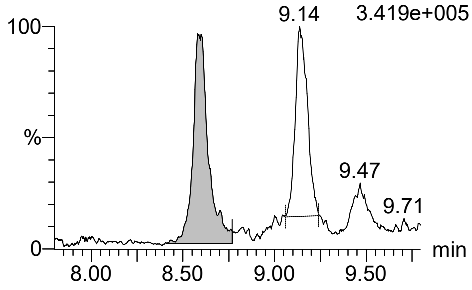

Sample Name: 20211111_A16_R2_IgM
F66:MRM of 1 channel,ES+
1086.2 > 204.1
5.220e+005

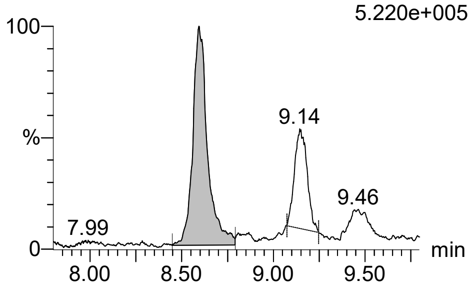

Sample Name: 20211111_L15_R2_IgM
F66:MRM of 1 channel,ES+
1086.2 > 204.1
6.770e+005

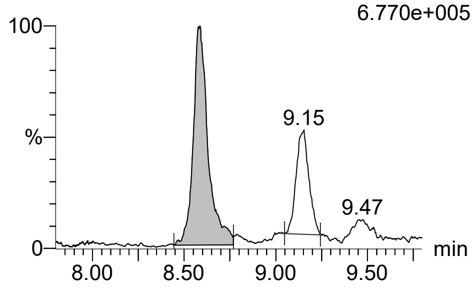

Sample Name: 20211111_L16_R2_IgM
F66:MRM of 1 channel,ES+
1086.2 > 204.1
1.048e+005

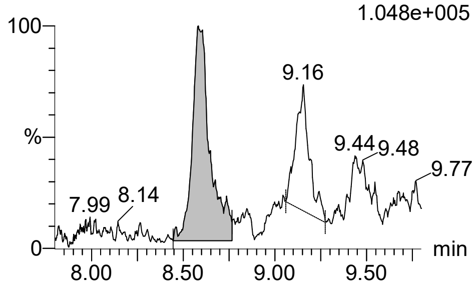

Sample Name: 20211111_H8_R2_IgM
F66:MRM of 1 channel,ES+
1086.2 > 204.1
2.120e+005

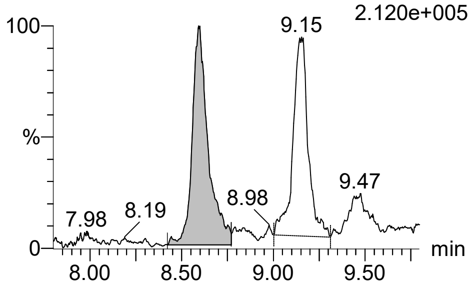

Sample Name: 20211111_A17_R2_IgM
F66:MRM of 1 channel,ES+
1086.2 > 204.1
4.599e+005

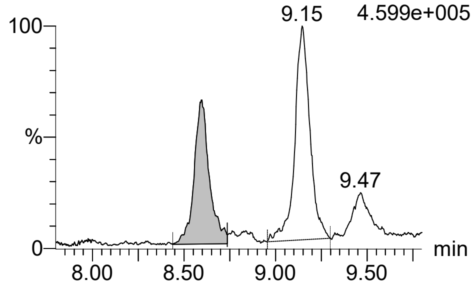

Sample Name: 20211111_A18_R2_IgM
F66:MRM of 1 channel,ES+
1086.2 > 204.1
5.432e+005

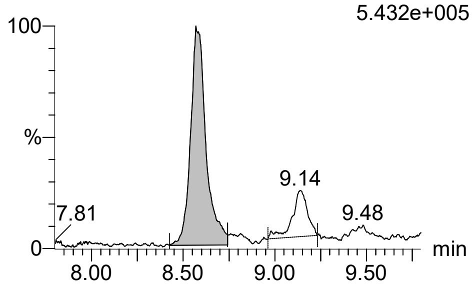

Sample Name: 20211111_L17_R2_IgM
F66:MRM of 1 channel,ES+
1086.2 > 204.1
3.743e+005

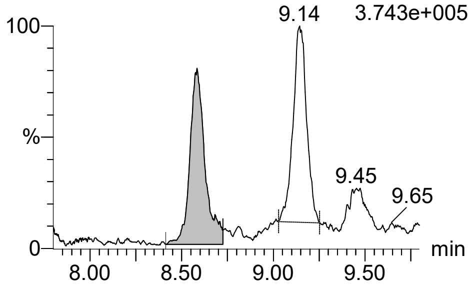

Sample Name: 20211111_L18_R2_IgM
F66:MRM of 1 channel,ES+
1086.2 > 204.1
7.631e+004

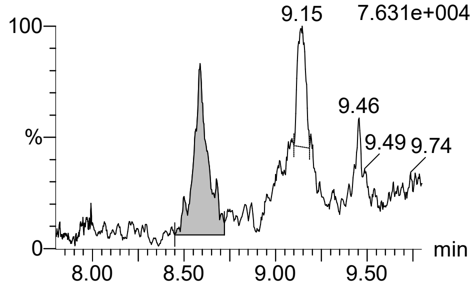

Sample Name: 20211111_H9_R2_IgM
F66:MRM of 1 channel,ES+
1086.2 > 204.1
1.470e+005

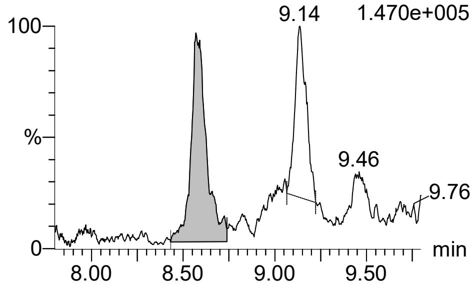

Sample Name: 20211111_A19_R2_IgM
F66:MRM of 1 channel,ES+
1086.2 > 204.1
2.874e+005

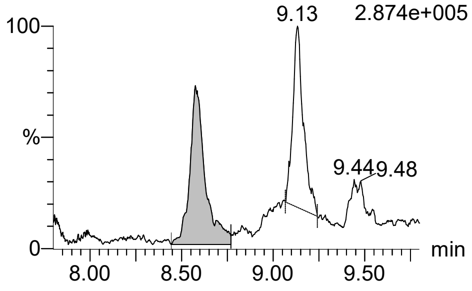

Sample Name: 20211111_A20_R2_IgM
F66:MRM of 1 channel,ES+
1086.2 > 204.1
1.937e+005

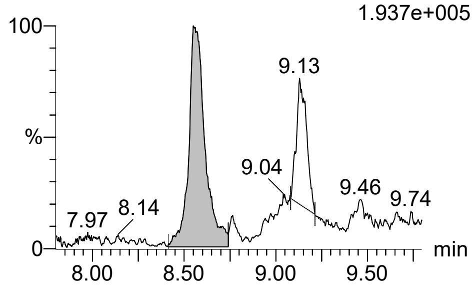

Sample Name: 20211111_L19_R2_IgM
F66:MRM of 1 channel,ES+
1086.2 > 204.1
2.917e+005

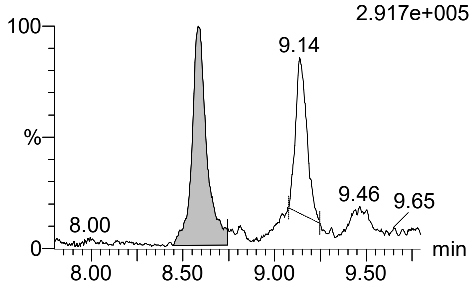

Sample Name: 20211111_QC_R13_IgM
F66:MRM of 1 channel,ES+
1086.2 > 204.1
3.373e+005

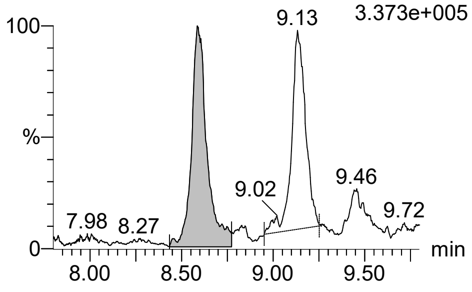

Dataset: C:\MassLynx\10.pro\20211111_clinicalsamples_IgM_IgA.qld

Last Altered: Thursday, March 09, 2023 19:58:25 台北標準時間
Printed: Friday, August 01, 2025 15:13:55 台北標準時間

Sample Name: 20211111_L20_R2_IgM

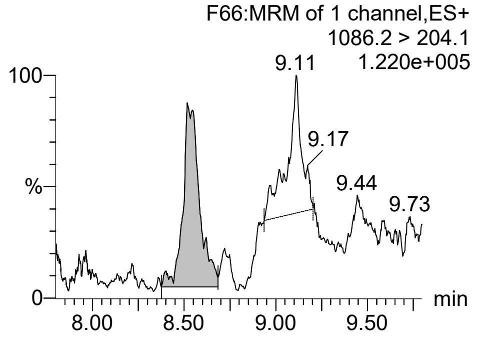

Sample Name: 20211111_H10_R2_IgM

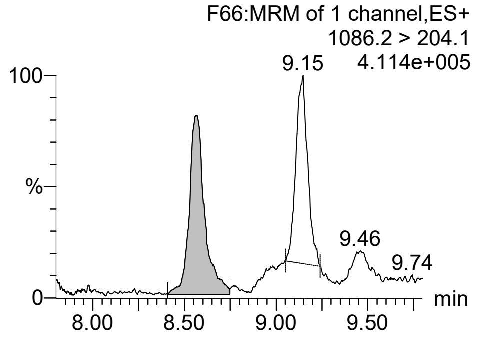

Sample Name: 20211111_A21_R2_IgM

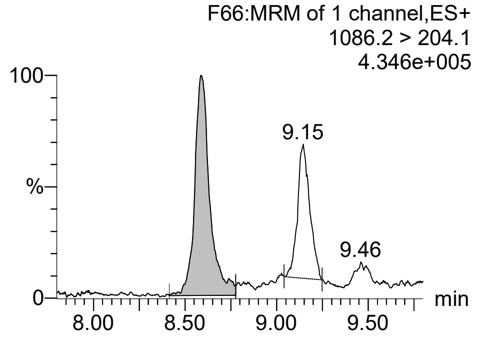

Sample Name: 20211111_A22_R2_IgM

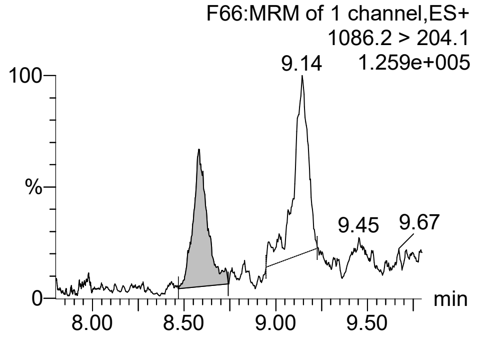

Sample Name: 20211111_L21_R2_IgM

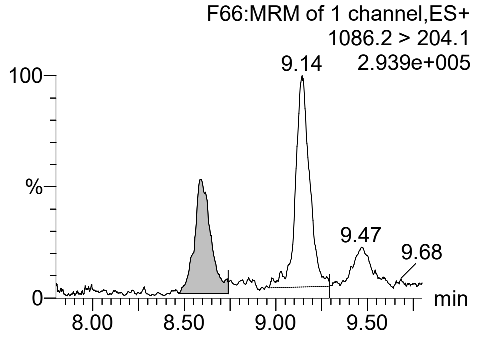

Sample Name: 20211111_L22_R2_IgM

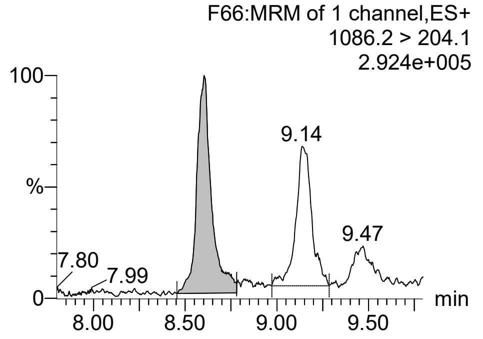

Sample Name: 20211111_H11_R2_IgM

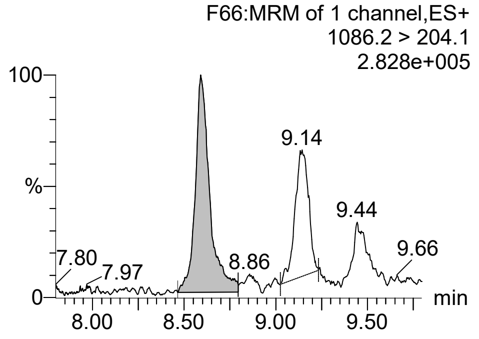

Sample Name: 20211111_A23_R2_IgM

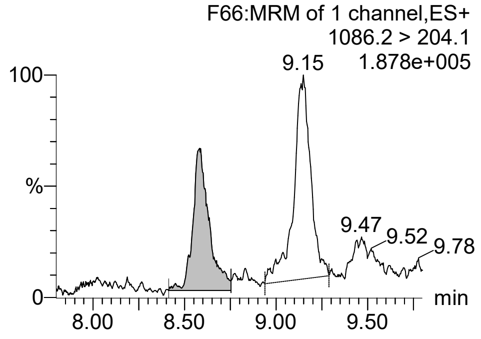

Sample Name: 20211111_A24_R2_IgM

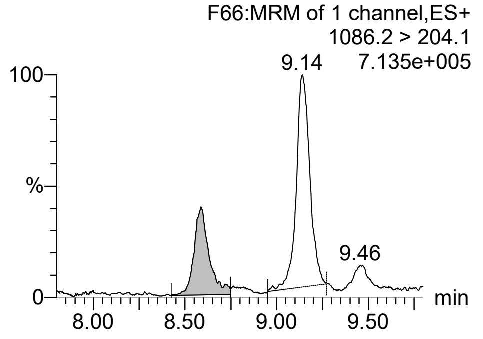

Sample Name: 20211111_L23_R2_IgM

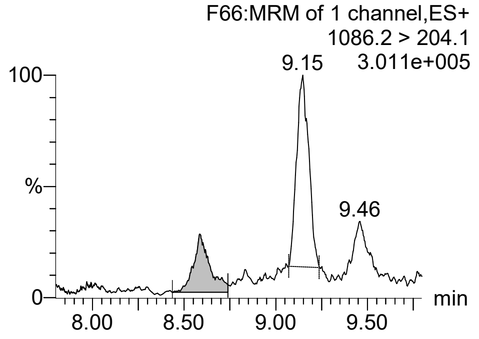

Sample Name: 20211111_L24_R2_IgM

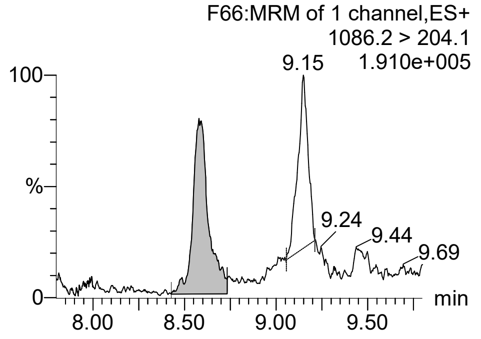

Sample Name: 20211111_H12_R2_IgM

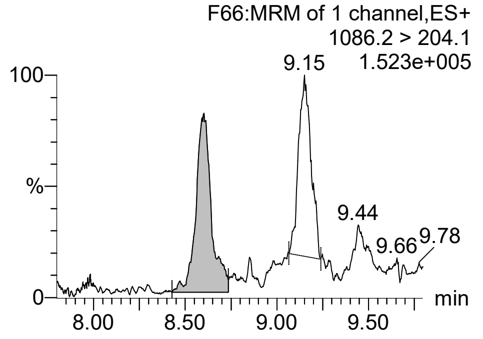

Sample Name: 20211111_QC_R14_IgM

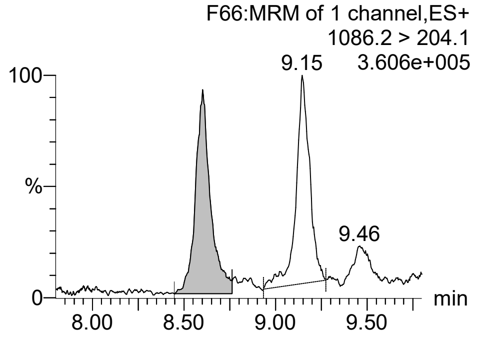

Sample Name: 20211111_A25_R2_IgM

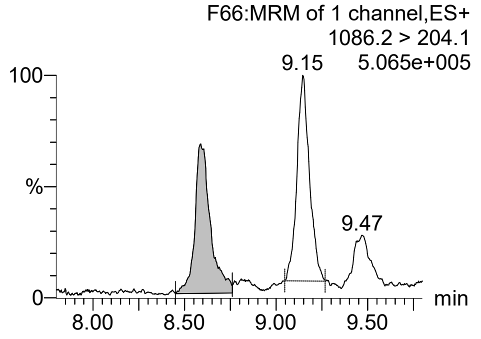

Sample Name: 20211111_A26_R2_IgM

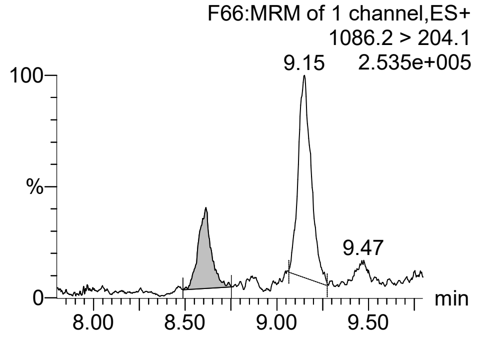

Dataset: C:\MassLynx\10.pro\20211111_clinicalsamples_IgM_IgA.qld

Last Altered: Thursday, March 09, 2023 19:58:25 台北標準時間
Printed: Friday, August 01, 2025 15:13:55 台北標準時間

Sample Name: 20211111_L25_R2_IgM

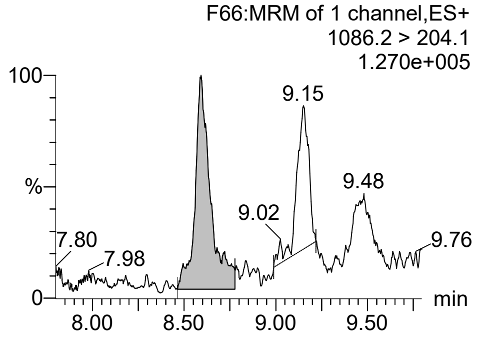

Sample Name: 20211111_L26_R2_IgM

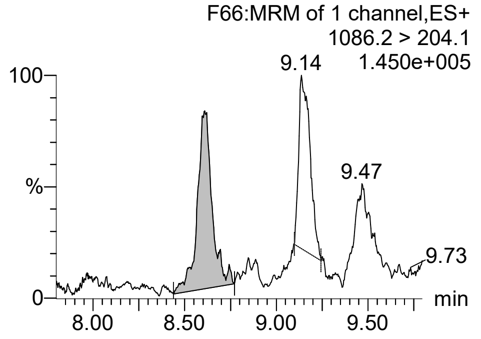

Sample Name: 20211111_H13_R2_IgM

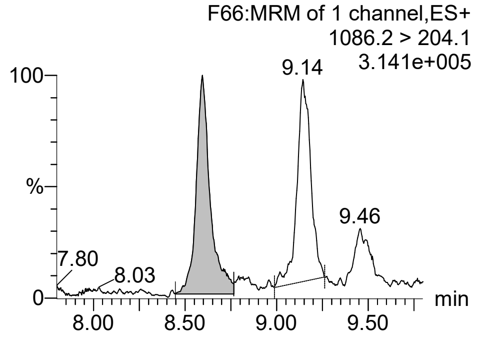

Sample Name: 20211111_A27_R2_IgM

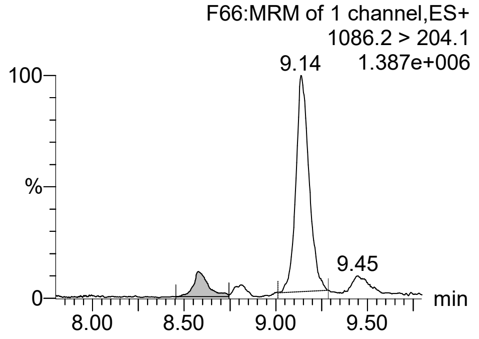

Sample Name: 20211111_A28_R2_IgM

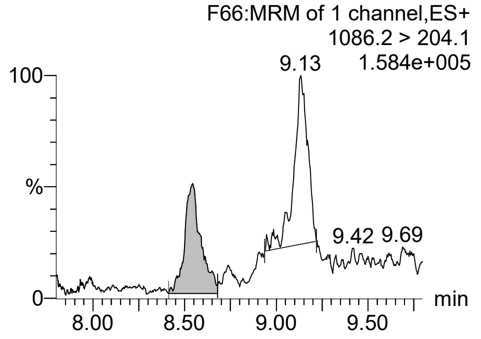

Sample Name: 20211111_L27_R2_IgM

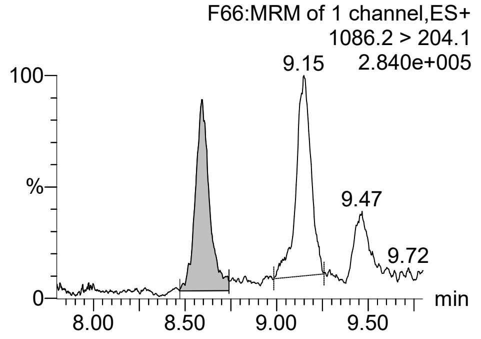

Sample Name: 20211111_L28_R2_IgM

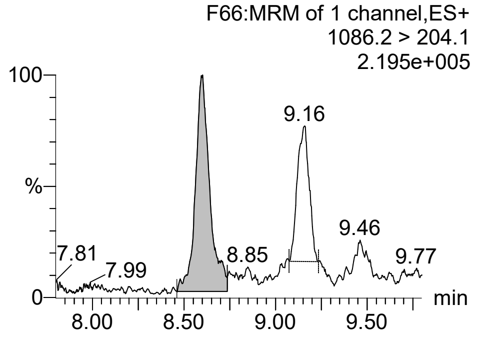

Sample Name: 20211111_H14_R2_IgM

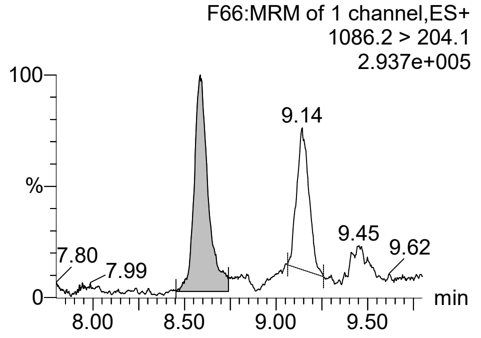

Sample Name: 20211111_A29_R2_IgM

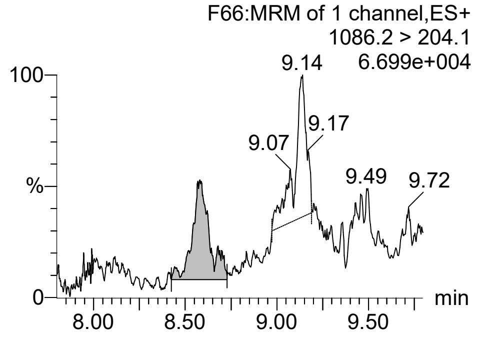

Sample Name: 20211111_A30_R2_IgM

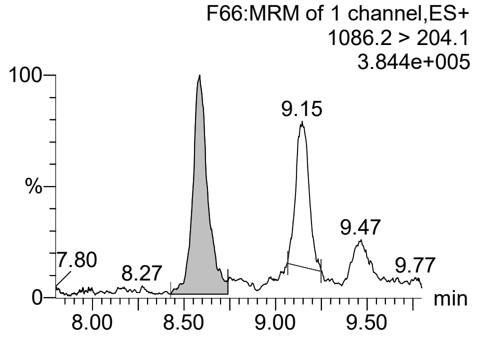

Sample Name: 20211111_QC_R15_IgM

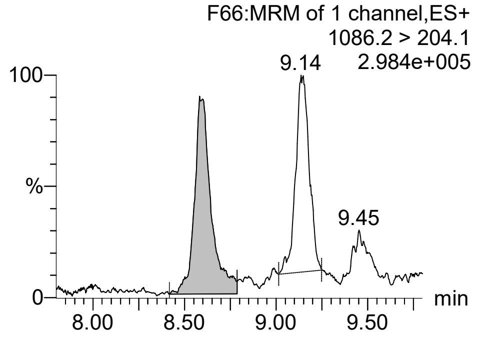

Sample Name: 20211111_L29_R2_IgM

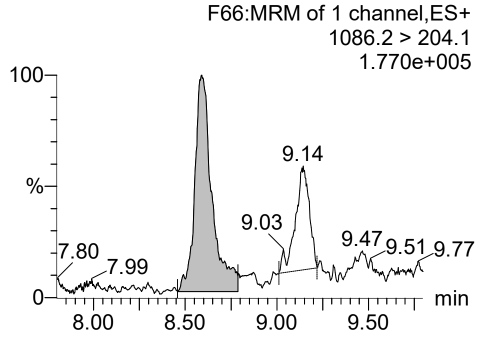

Sample Name: 20211111_L30_R2_IgM

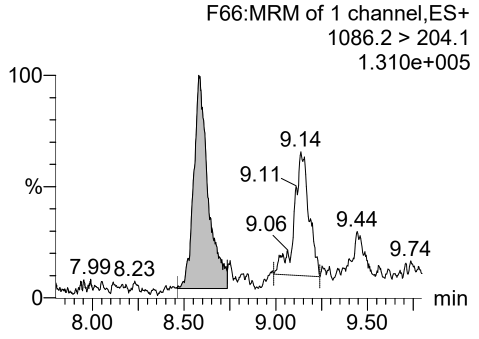

Sample Name: 20211111_H15_R2_IgM

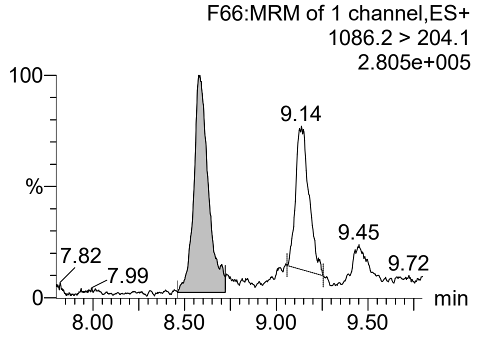

Sample Name: 20211111_A31_R2_IgM

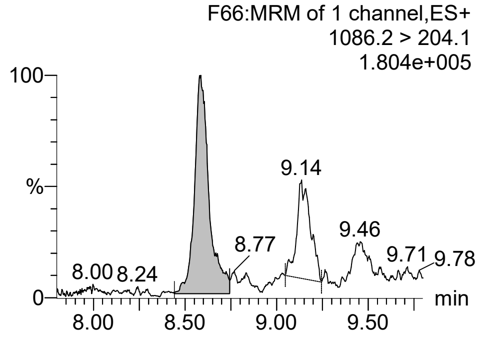

Sample Name: 20211111_A32_R2_IgM
F66:MRM of 1 channel,ES+
1086.2 > 204.1
3.406e+005

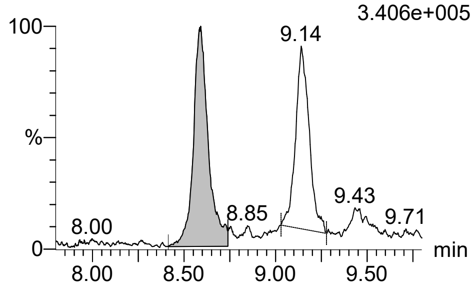

Sample Name: 20211111_L31_R2_IgM
F66:MRM of 1 channel,ES+
1086.2 > 204.1
8.921e+004

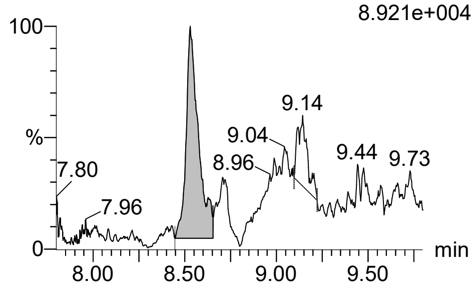

Sample Name: 20211111_L32_R2_IgM
F66:MRM of 1 channel,ES+
1086.2 > 204.1
9.13 2.622e+005

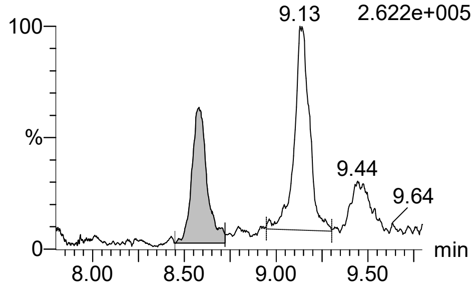

Sample Name: 20211111_H16_R2_IgM
F66:MRM of 1 channel,ES+
1086.2 > 204.1
9.14 5.782e+005

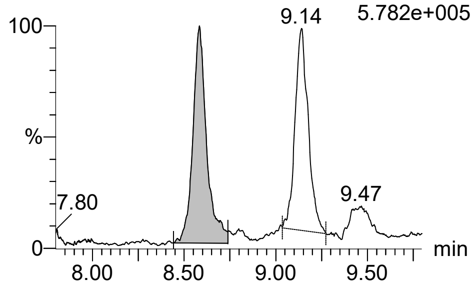

Sample Name: 20211111_A33_R2_IgM
F66:MRM of 1 channel,ES+
1086.2 > 204.1
9.14 6.480e+005

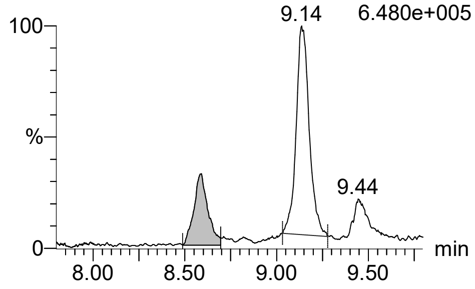

Sample Name: 20211111_A34_R2_IgM
F66:MRM of 1 channel,ES+
1086.2 > 204.1
1.698e+005

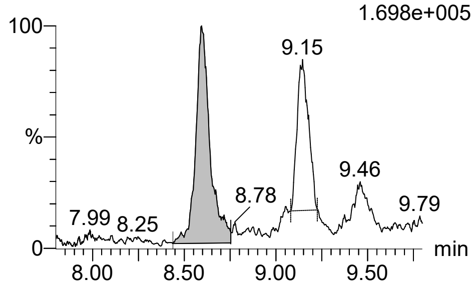

Sample Name: 20211111_H17_R2_IgM
F66:MRM of 1 channel,ES+
1086.2 > 204.1
1.308e+006

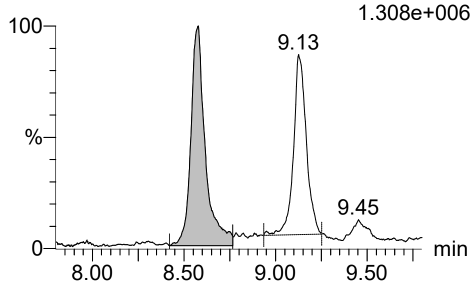

Sample Name: 20211111_A35_R2_IgM
F66:MRM of 1 channel,ES+
1086.2 > 204.1
9.13 1.736e+005

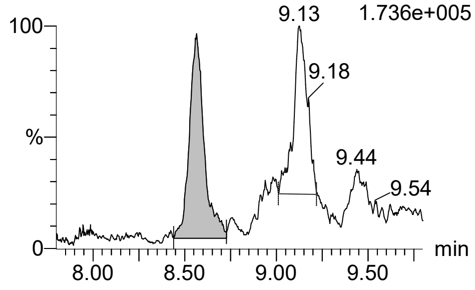

Sample Name: 20211111_QC_R16_IgM
F66:MRM of 1 channel,ES+
1086.2 > 204.1
9.14 2.735e+005

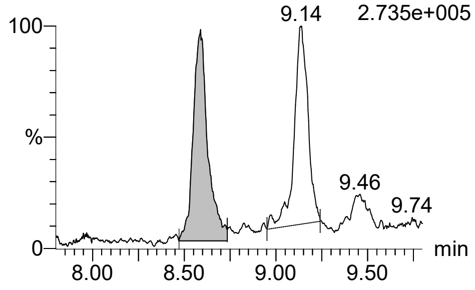

Sample Name: 20211111_A36_R2_IgM
F66:MRM of 1 channel,ES+
1086.2 > 204.1
1.503e+005

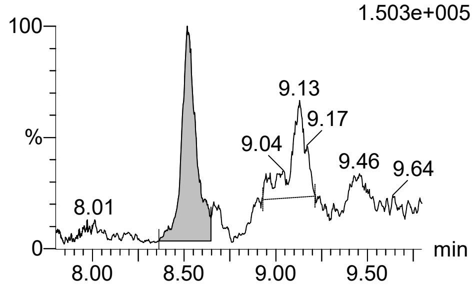

Sample Name: 20211111_H18_R2_IgM
F66:MRM of 1 channel,ES+
1086.2 > 204.1
5.013e+005

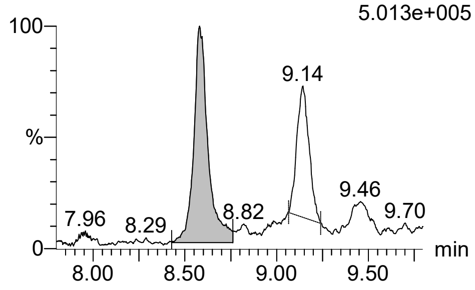

Sample Name: 20211111_A37_R2_IgM
F66:MRM of 1 channel,ES+
1086.2 > 204.1
9.14 3.647e+005

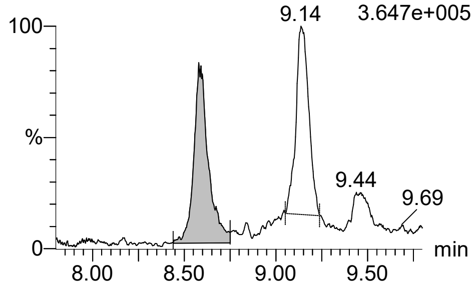

Sample Name: 20211111_A38_R2_IgM
F66:MRM of 1 channel,ES+
1086.2 > 204.1
2.544e+005

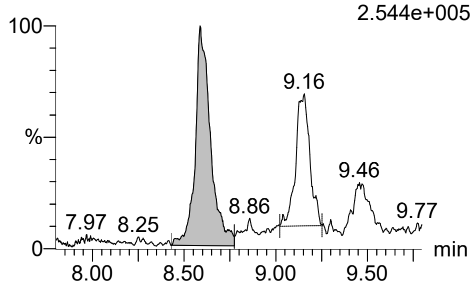

Sample Name: 20211111_H19_R2_IgM
F66:MRM of 1 channel,ES+
1086.2 > 204.1
9.14 4.565e+005

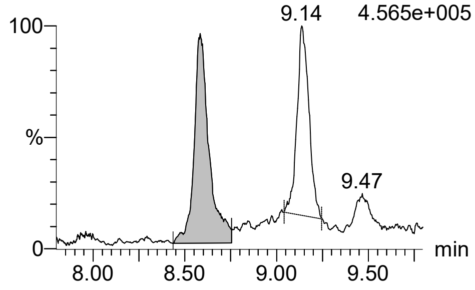

Sample Name: 20211111_A39_R2_IgM
F66:MRM of 1 channel,ES+
1086.2 > 204.1
9.14 2.923e+005

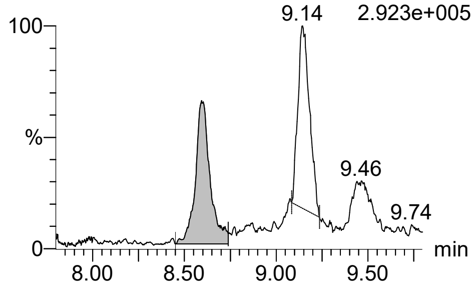

Dataset: C:\MassLynx\10.pro\20211111_clinicalsamples_IgM_IgA.qld

Last Altered: Thursday, March 09, 2023 19:58:25 台北標準時間
Printed: Friday, August 01, 2025 15:13:55 台北標準時間

Sample Name: 20211111_A40_R2_IgM

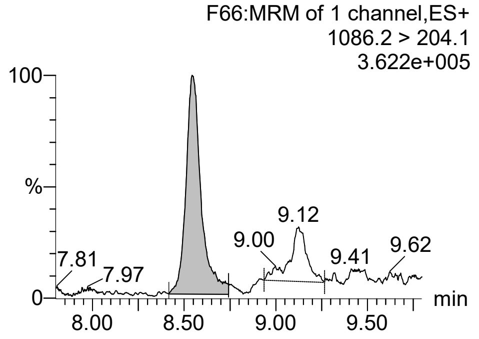

Sample Name: 20211111_H20_R2_IgM

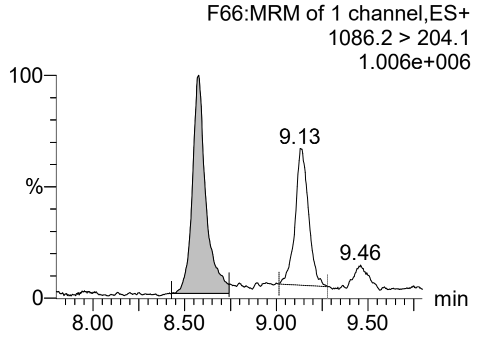

Sample Name: 20211111_A41_R2_IgM

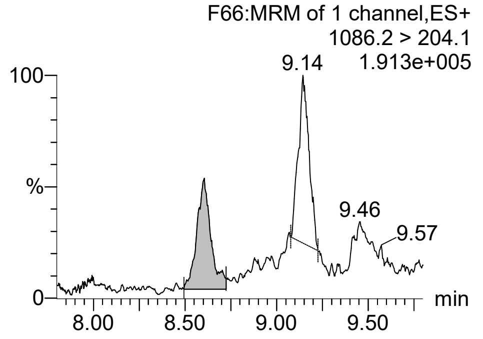

Sample Name: 20211111_A42_R2_IgM

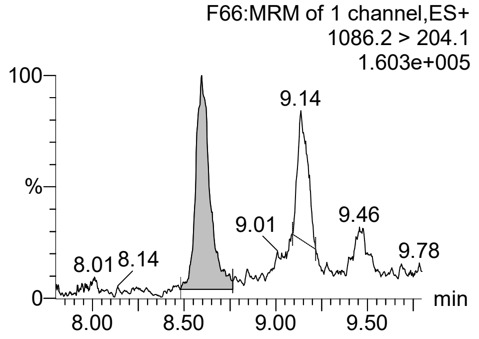

Sample Name: 20211111_A43_R2_IgM

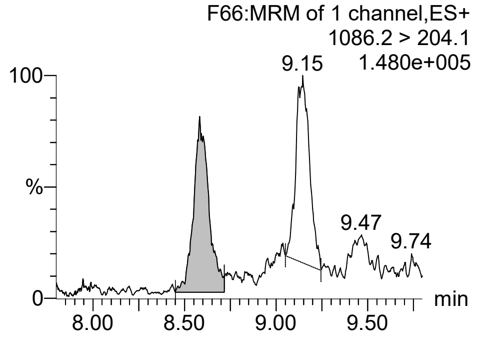

Sample Name: 20211111_A44_R2_IgM

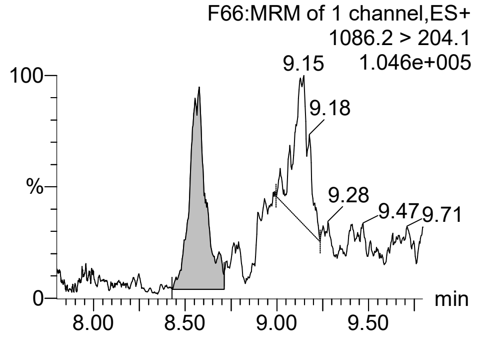

Sample Name: 20211111_QC_R17_IgM

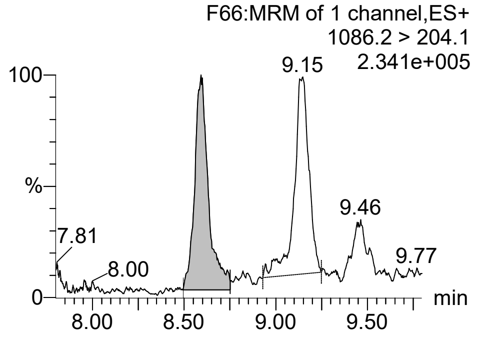

Sample Name: 20211111_L2_R3_IgM

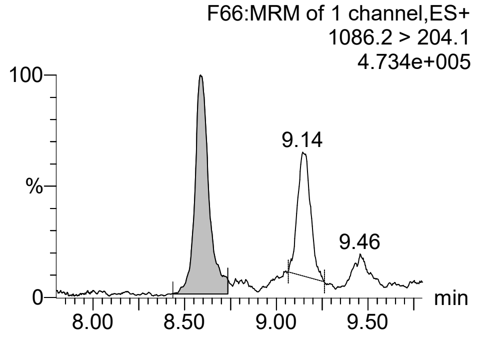

Sample Name: 20211111_H1_R3_IgM

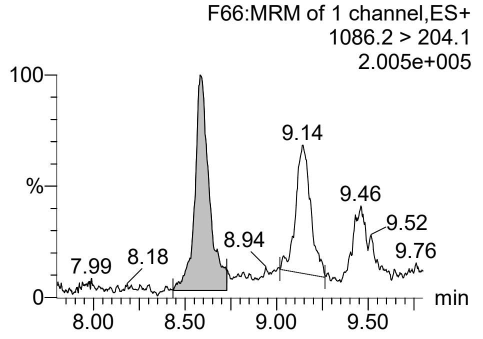

Sample Name: 20211111_A3_R3_IgM

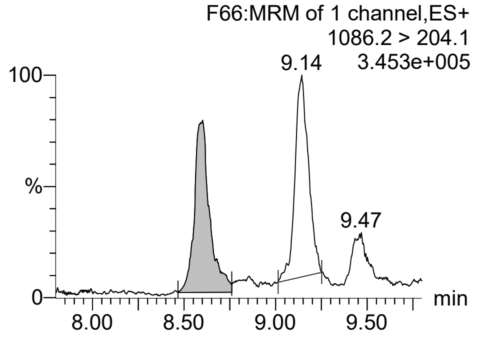

Sample Name: 20211111_A4_R3_IgM

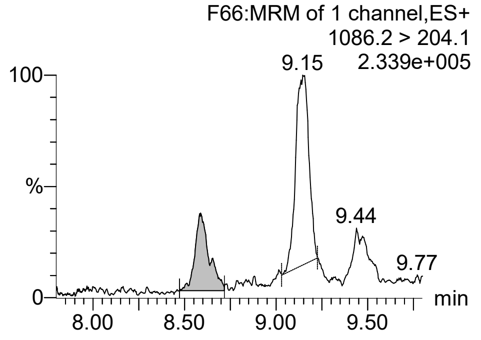

Sample Name: 20211111_L3_R3_IgM

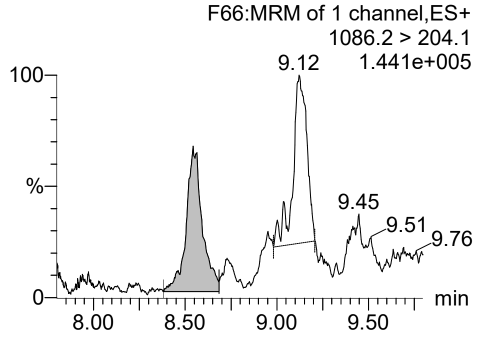

Sample Name: 20211111_L4_R3_IgM

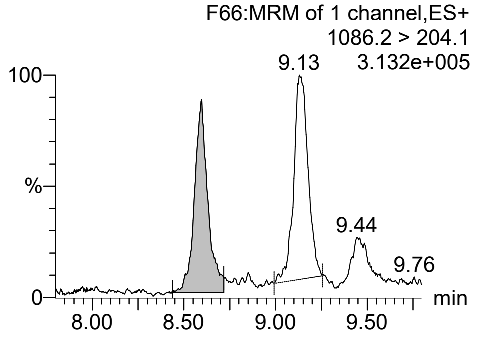

Sample Name: 20211111_H2_R3_IgM

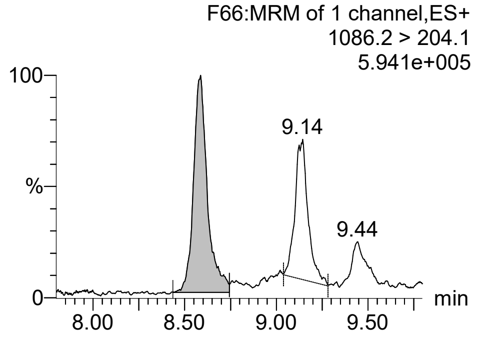

Sample Name: 20211111_A5_R3_IgM

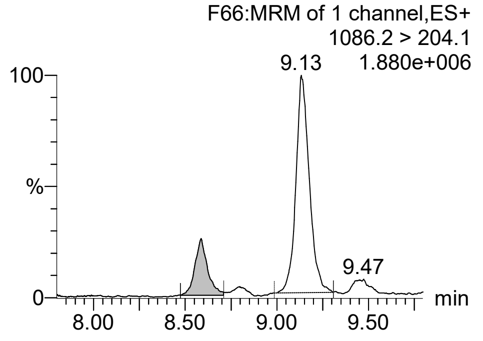

Sample Name: 20211111_A6_R3_IgM
F66:MRM of 1 channel,ES+
1086.2 > 204.1

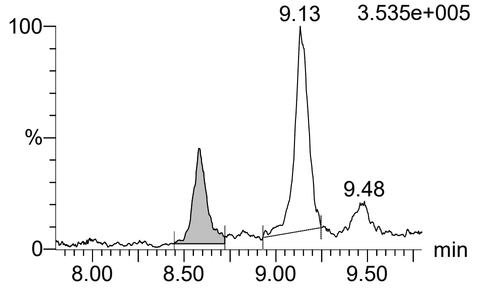

Sample Name: 20211111_QC_R18_IgM
F66:MRM of 1 channel,ES+
1086.2 > 204.1

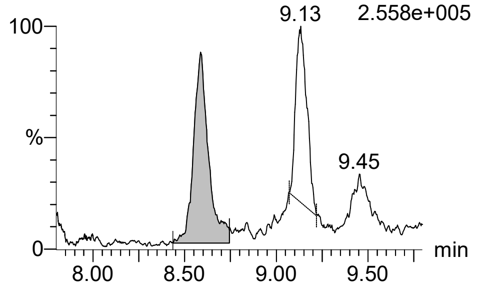

Sample Name: 20211111_L5_R3_IgM
F66:MRM of 1 channel,ES+
1086.2 > 204.1

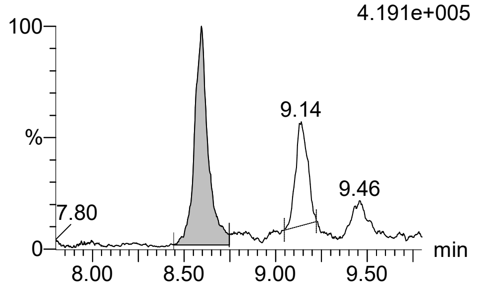

Sample Name: 20211111_L6_R3_IgM
F66:MRM of 1 channel,ES+
1086.2 > 204.1

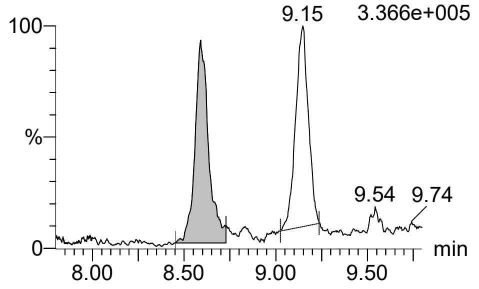

Sample Name: 20211111_H3_R3_IgM
F66:MRM of 1 channel,ES+
1086.2 > 204.1

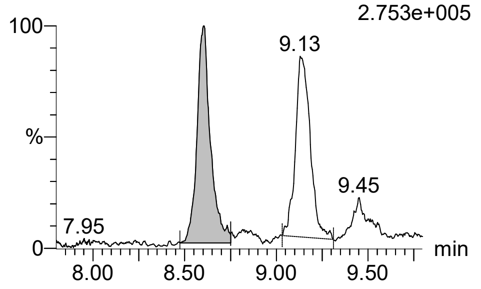

Sample Name: 20211111_A7_R3_IgM
F66:MRM of 1 channel,ES+
1086.2 > 204.1

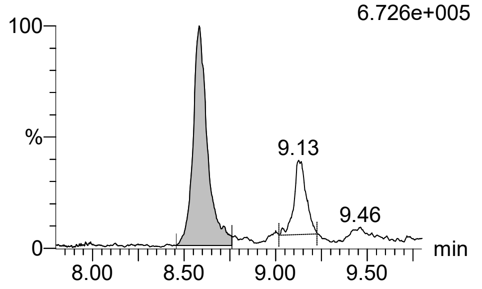

Sample Name: 20211111_A8_R3_IgM
F66:MRM of 1 channel,ES+
1086.2 > 204.1

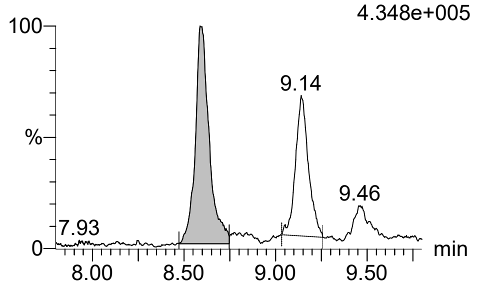

Sample Name: 20211111_L7_R3_IgM
F66:MRM of 1 channel,ES+
1086.2 > 204.1

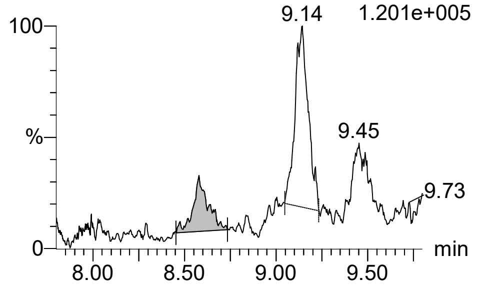

Sample Name: 20211111_L8_R3_IgM
F66:MRM of 1 channel,ES+
1086.2 > 204.1

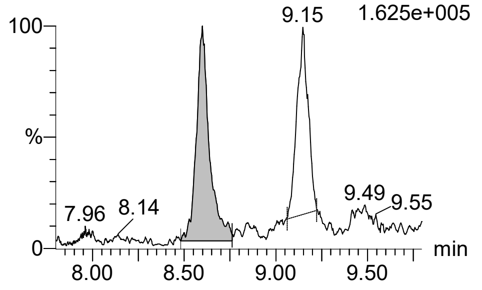

Sample Name: 20211111_H4_R3_IgM
F66:MRM of 1 channel,ES+
1086.2 > 204.1

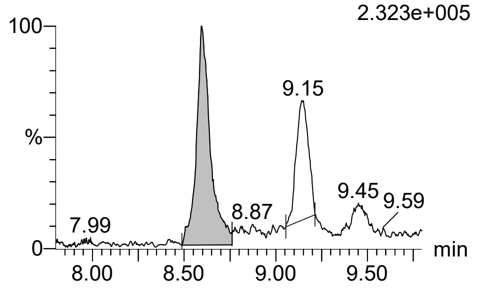

Sample Name: 20211111_A9_R3_IgM
F66:MRM of 1 channel,ES+
1086.2 > 204.1

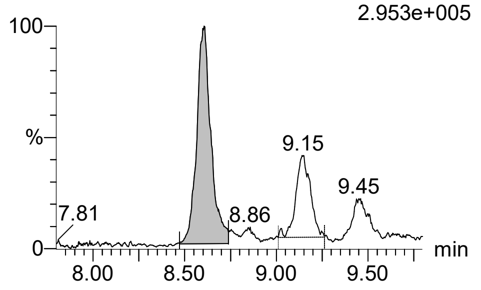

Sample Name: 20211111_A10_R3_IgM
F66:MRM of 1 channel,ES+
1086.2 > 204.1

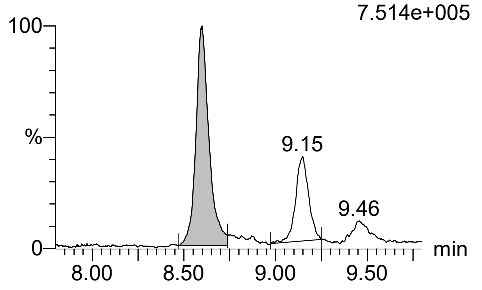

Sample Name: 20211111_L9_R3_IgM
F66:MRM of 1 channel,ES+
1086.2 > 204.1

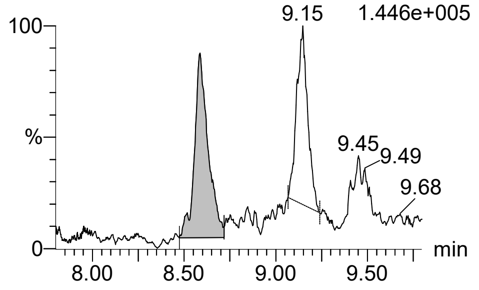

Sample Name: 20211111_L10_R3_IgM
F66:MRM of 1 channel,ES+
1086.2 > 204.1

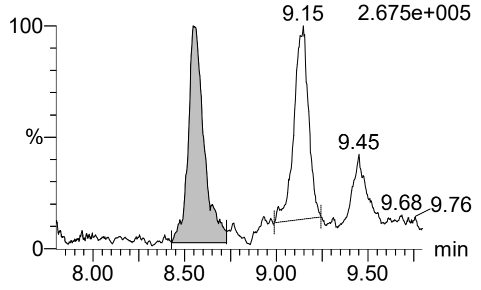

Sample Name: 20211111_QC_R19_IgM
F66:MRM of 1 channel,ES+
1086.2 > 204.1

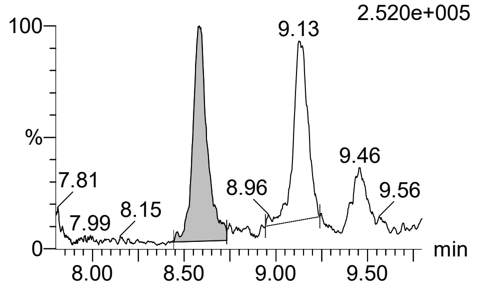

Dataset: C:\MassLynx\10.pro\20211111_clinicalsamples_IgM_IgA.qld

Last Altered: Thursday, March 09, 2023 19:58:25 台北標準時間
Printed: Friday, August 01, 2025 15:13:55 台北標準時間

Sample Name: 20211111_H5_R3_IgM

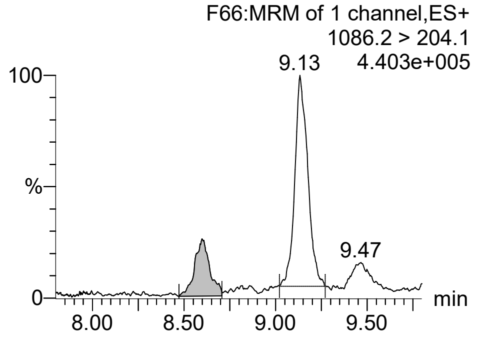

Sample Name: 20211111_A11_R3_IgM

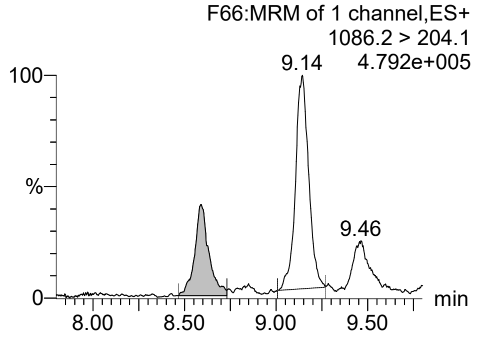

Sample Name: 20211111_A12_R3_IgM

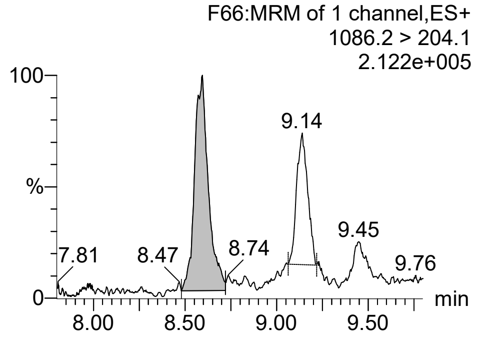

Sample Name: 20211111_L11_R3_IgM

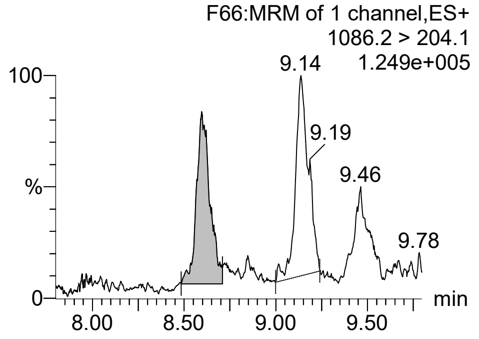

Sample Name: 20211111_L12_R3_IgM

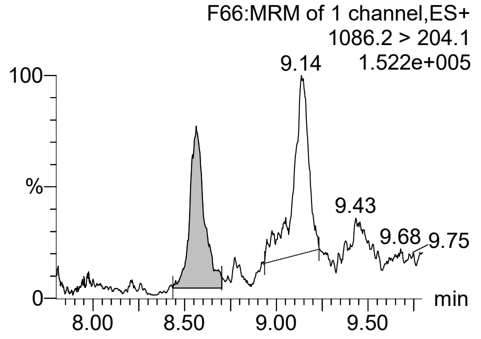

Sample Name: 20211111_H6_R3_IgM

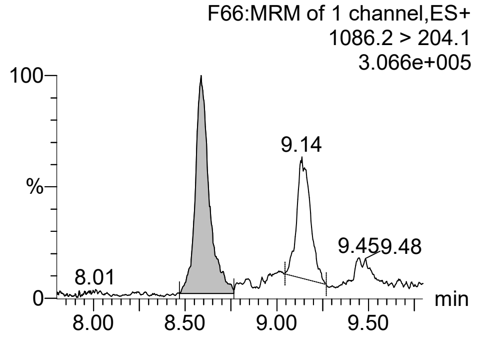

Sample Name: 20211111_A13_R3_IgM

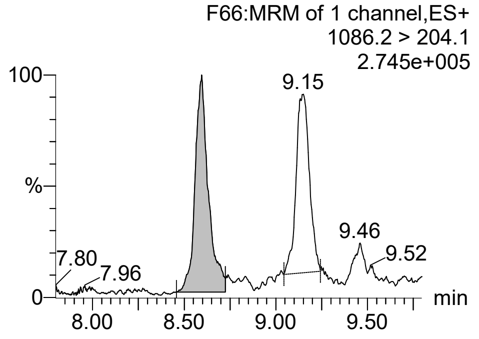

Sample Name: 20211111_A14_R3_IgM

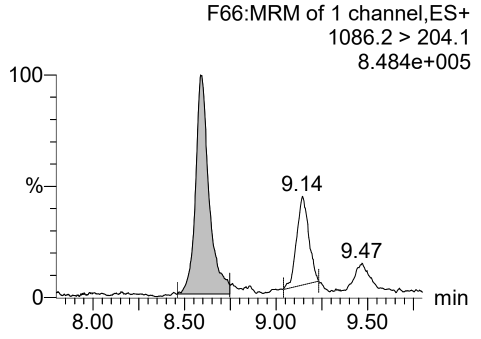

Sample Name: 20211111_L13_R3_IgM

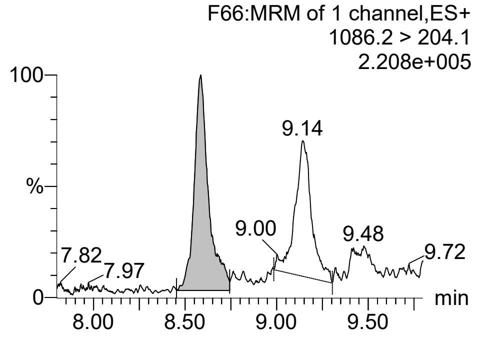

Sample Name: 20211111_L14_R3_IgM

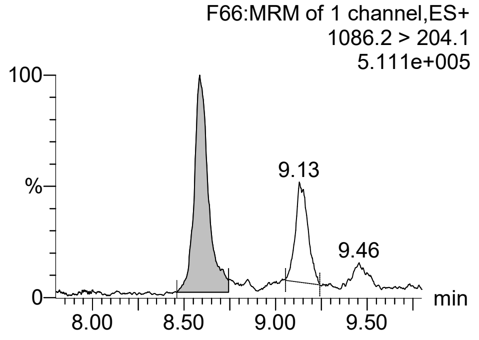

Sample Name: 20211111_H7_R3_IgM

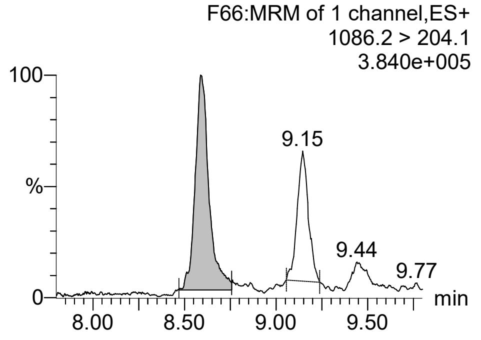

Sample Name: 20211111_A15_R3_IgM

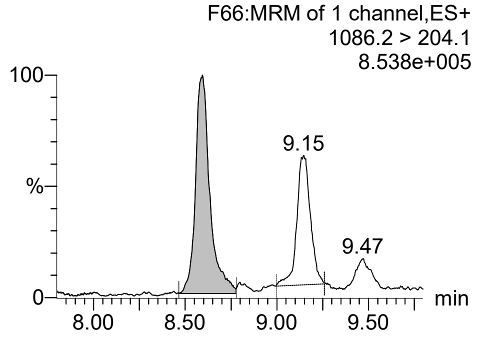

Sample Name: 20211111_QC_R20_IgM

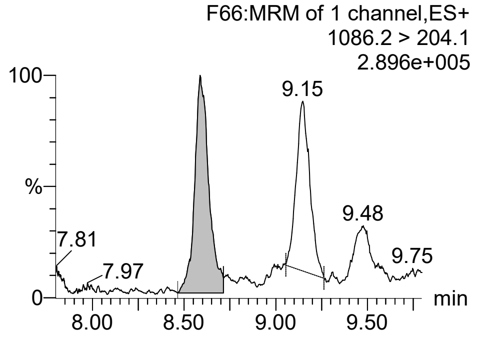

Sample Name: 20211111_A16_R3_IgM

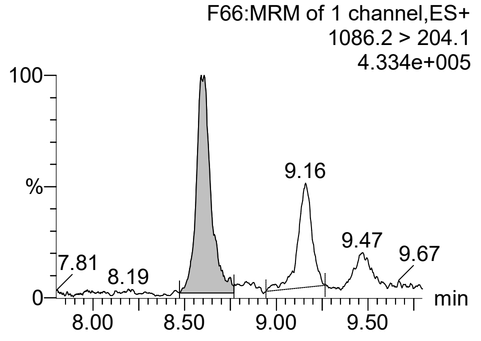

Sample Name: 20211111_L15_R3_IgM

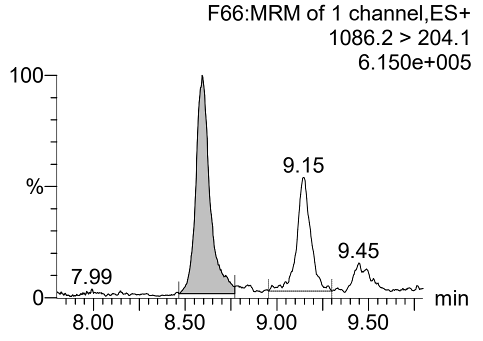

Dataset: C:\MassLynx\10.pro\20211111_clinicalsamples_IgM_IgA.qld

Last Altered: Thursday, March 09, 2023 19:58:25 台北標準時間
Printed: Friday, August 01, 2025 15:13:55 台北標準時間

Sample Name: 20211111_L16_R3_IgM

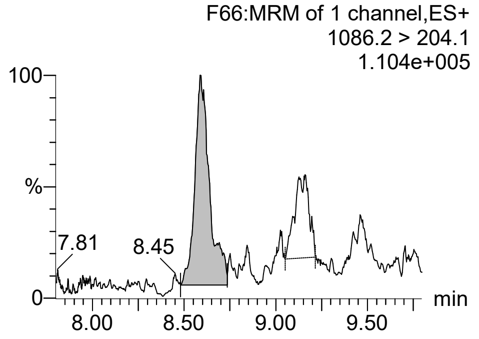

Sample Name: 20211111_H8_R3_IgM

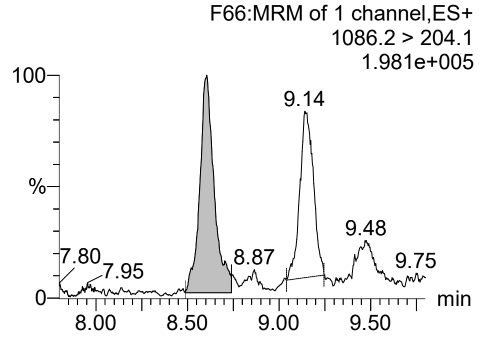

Sample Name: 20211111_A17_R3_IgM

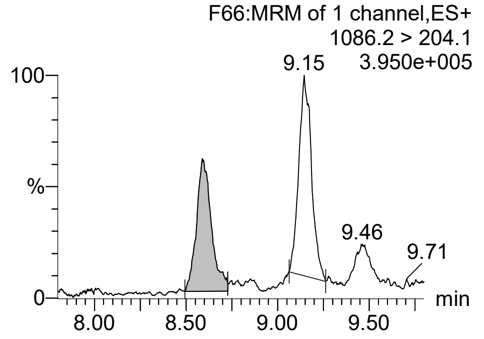

Sample Name: 20211111_A18_R3_IgM

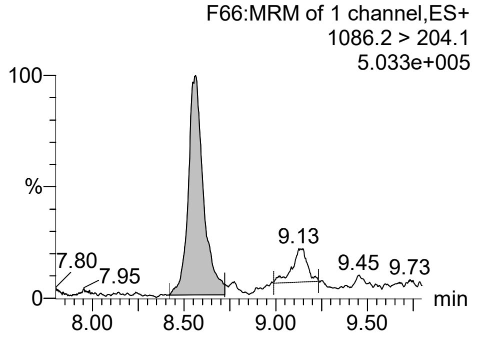

Sample Name: 20211111_L17_R3_IgM

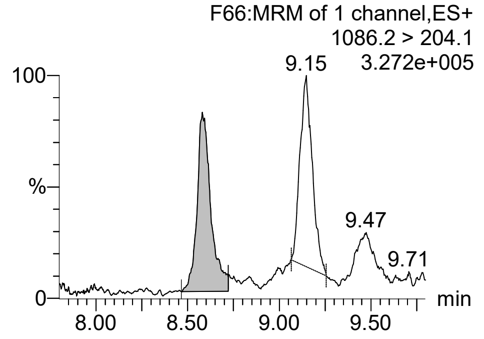

Sample Name: 20211111_L18_R3_IgM

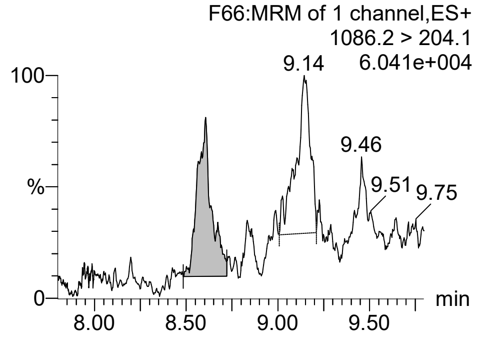

Sample Name: 20211111_H9_R3_IgM

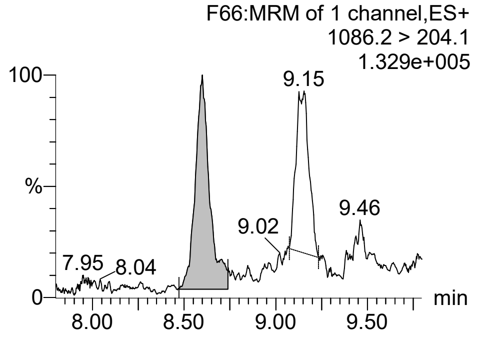

Sample Name: 20211111_A19_R3_IgM

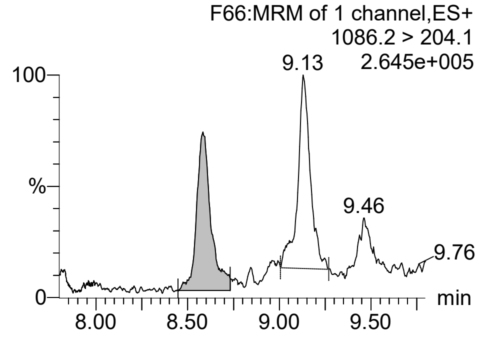

Sample Name: 20211111_A20_R3_IgM

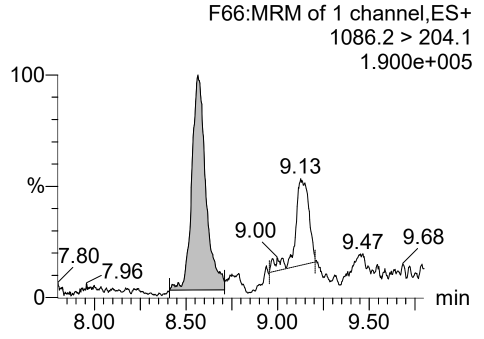

Sample Name: 20211111_L19_R3_IgM

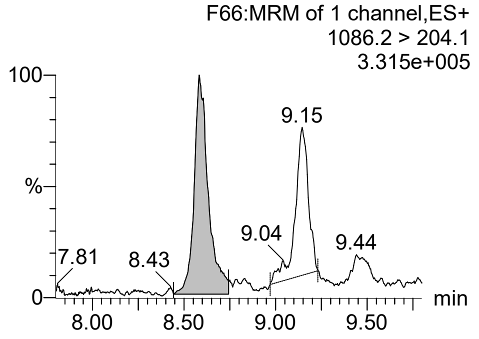

Sample Name: 20211111_QC_R21_IgM

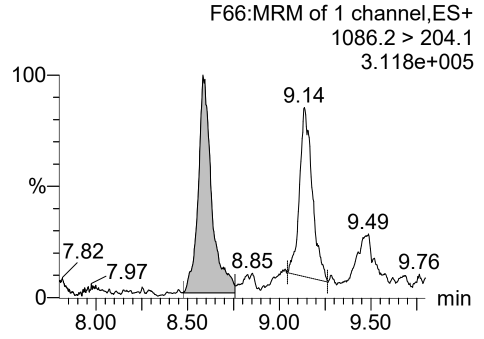

Sample Name: 20211111_L20_R3_IgM

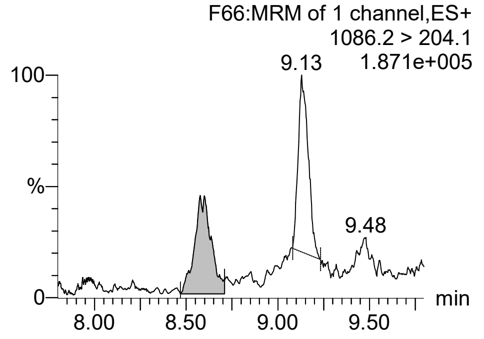

Sample Name: 20211111_H10_R3_IgM

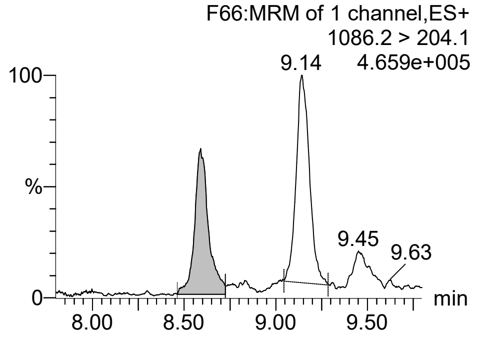

Sample Name: 20211111_A21_R3_IgM

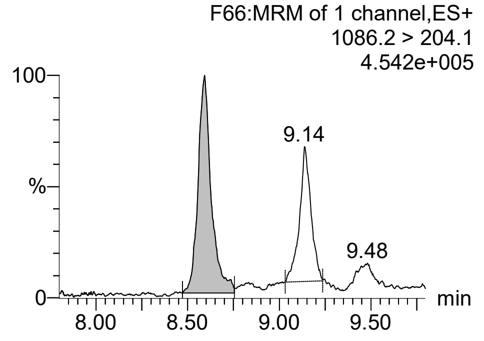

Sample Name: 20211111_A22_R3_IgM

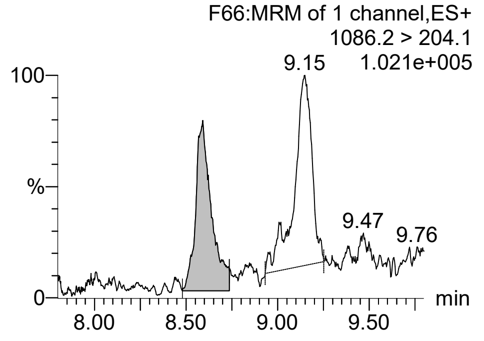

Dataset: C:\MassLynx\10.pro\20211111_clinicalsamples_IgM_IgA.qld

Last Altered: Thursday, March 09, 2023 19:58:25 台北標準時間
Printed: Friday, August 01, 2025 15:13:55 台北標準時間

Sample Name: 20211111_L21_R3_IgM
F66:MRM of 1 channel,ES+
1086.2 > 204.1

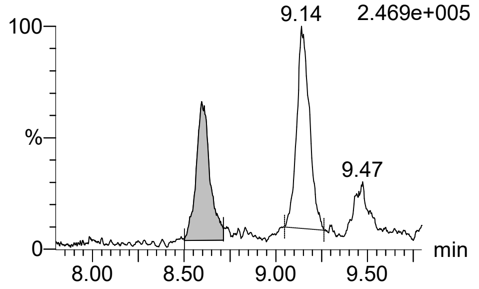

Sample Name: 20211111_L22_R3_IgM
F66:MRM of 1 channel,ES+
1086.2 > 204.1

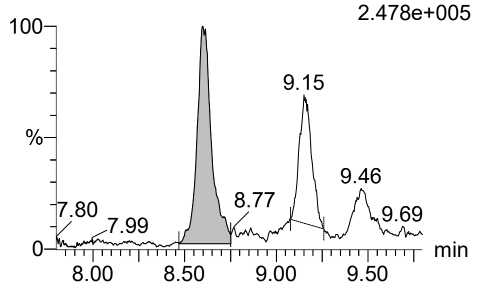

Sample Name: 20211111_H11_R3_IgM
F66:MRM of 1 channel,ES+
1086.2 > 204.1

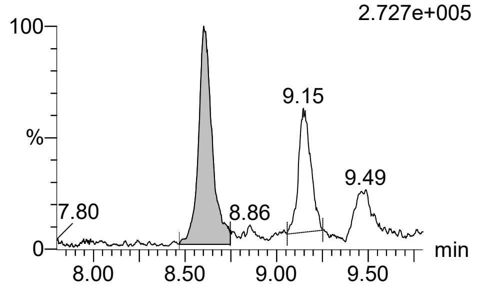

Sample Name: 20211111_A23_R3_IgM
F66:MRM of 1 channel,ES+
1086.2 > 204.1

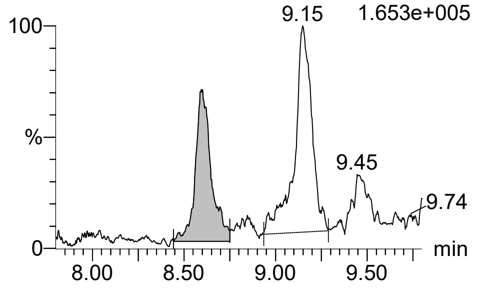

Sample Name: 20211111_A24_R3_IgM
F66:MRM of 1 channel,ES+
1086.2 > 204.1

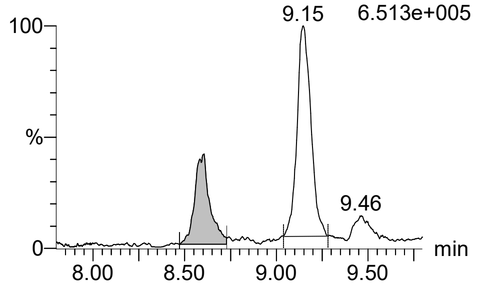

Sample Name: 20211111_L23_R3_IgM
F66:MRM of 1 channel,ES+
1086.2 > 204.1

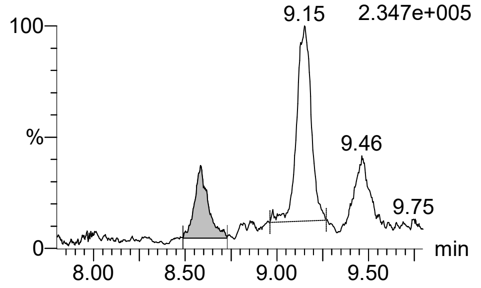

Sample Name: 20211111_L24_R3_IgM
F66:MRM of 1 channel,ES+
1086.2 > 204.1

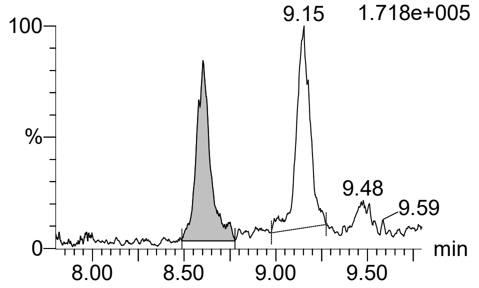

Sample Name: 20211111_H12_R3_IgM
F66:MRM of 1 channel,ES+
1086.2 > 204.1

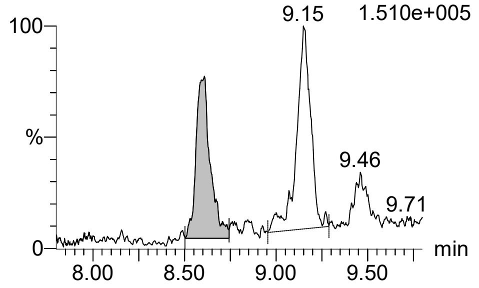

Sample Name: 20211111_QC_R22_IgM
F66:MRM of 1 channel,ES+
1086.2 > 204.1

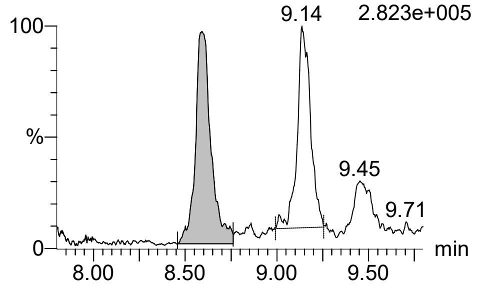

Sample Name: 20211111_A25_R3_IgM
F66:MRM of 1 channel,ES+
1086.2 > 204.1

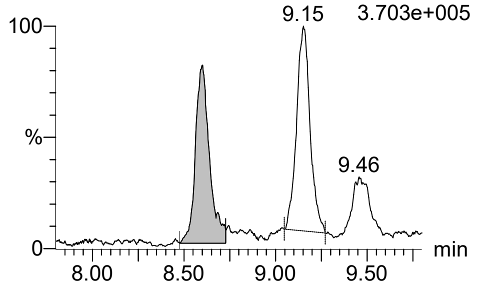

Sample Name: 20211111_A26_R3_IgM
F66:MRM of 1 channel,ES+
1086.2 > 204.1

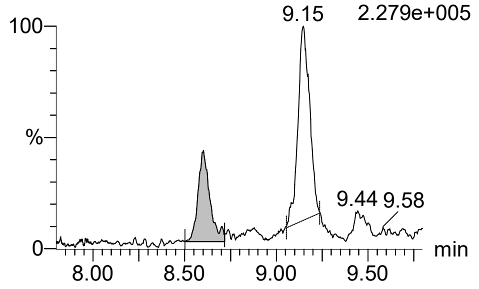

Sample Name: 20211111_L25_R3_IgM
F66:MRM of 1 channel,ES+
1086.2 > 204.1

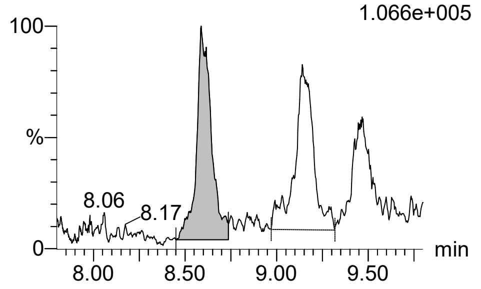

Sample Name: 20211111_L26_R3_IgM
F66:MRM of 1 channel,ES+
1086.2 > 204.1

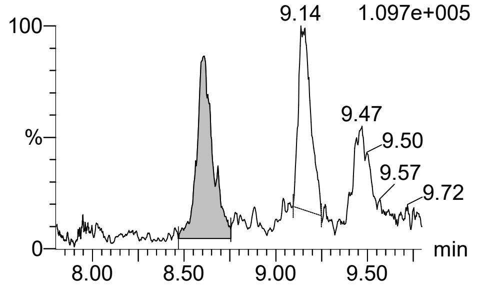

Sample Name: 20211111_H13_R3_IgM
F66:MRM of 1 channel,ES+
1086.2 > 204.1

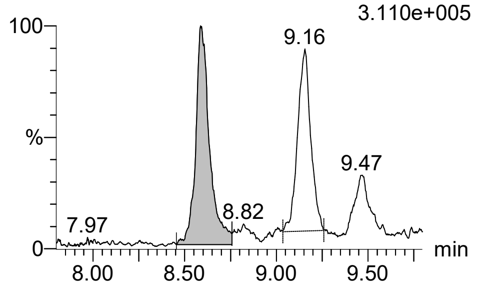

Sample Name: 20211111_A27_R3_IgM
F66:MRM of 1 channel,ES+
1086.2 > 204.1

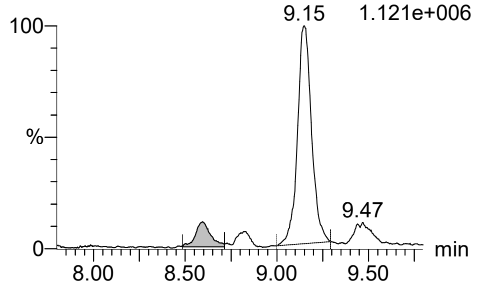

Dataset: C:\MassLynx\10.pro\20211111_clinicalsamples_IgM_IgA.qld

Last Altered: Thursday, March 09, 2023 19:58:25 台北標準時間
Printed: Friday, August 01, 2025 15:13:55 台北標準時間

Sample Name: 20211111_A28_R3_IgM
F66:MRM of 1 channel,ES+
1086.2 > 204.1
1.786e+005

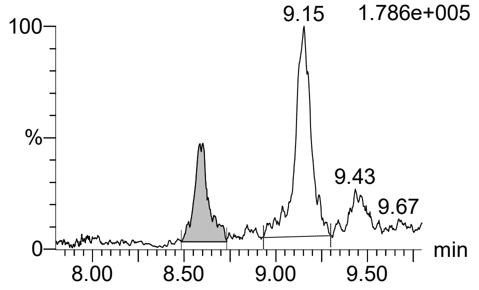

Sample Name: 20211111_L27_R3_IgM
F66:MRM of 1 channel,ES+
1086.2 > 204.1
2.525e+005

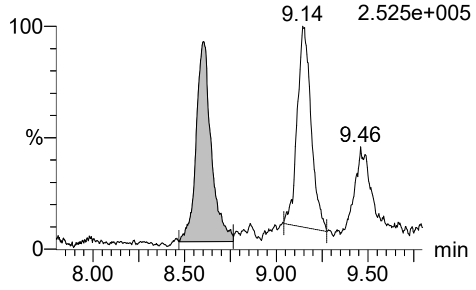

Sample Name: 20211111_L28_R3_IgM
F66:MRM of 1 channel,ES+
1086.2 > 204.1
2.370e+005

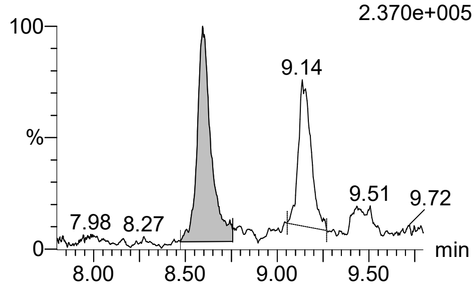

Sample Name: 20211111_H14_R3_IgM
F66:MRM of 1 channel,ES+
1086.2 > 204.1
2.933e+005

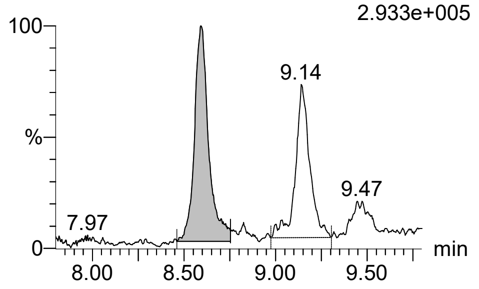

Sample Name: 20211111_A29_R3_IgM
F66:MRM of 1 channel,ES+
1086.2 > 204.1
5.752e+004

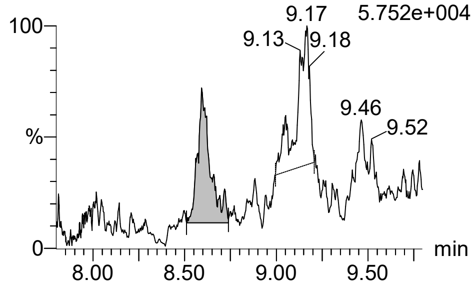

Sample Name: 20211111_A30_R3_IgM
F66:MRM of 1 channel,ES+
1086.2 > 204.1
3.716e+005

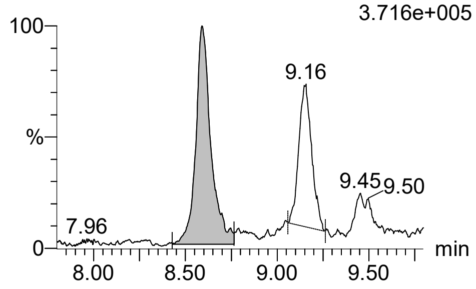

Sample Name: 20211111_QC_R23_IgM
F66:MRM of 1 channel,ES+
1086.2 > 204.1
2.843e+005

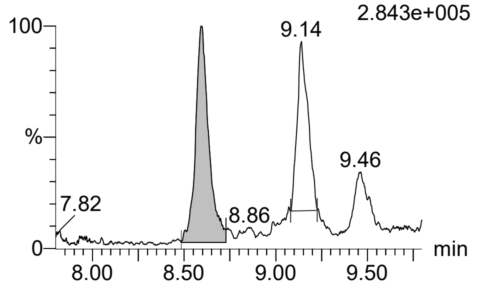

Sample Name: 20211111_L29_R3_IgM
F66:MRM of 1 channel,ES+
1086.2 > 204.1
1.864e+005

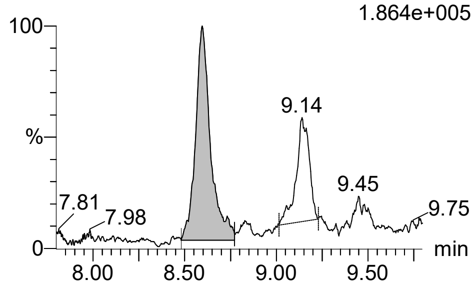

Sample Name: 20211111_L30_R3_IgM
F66:MRM of 1 channel,ES+
1086.2 > 204.1
1.319e+005

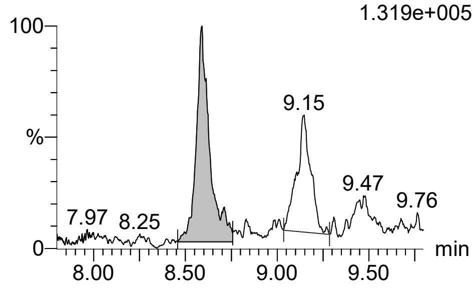

Sample Name: 20211111_H15_R3_IgM
F66:MRM of 1 channel,ES+
1086.2 > 204.1
2.700e+005

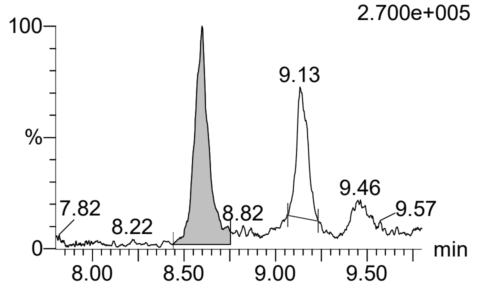

Sample Name: 20211111_A31_R3_IgM
F66:MRM of 1 channel,ES+
1086.2 > 204.1
1.904e+005

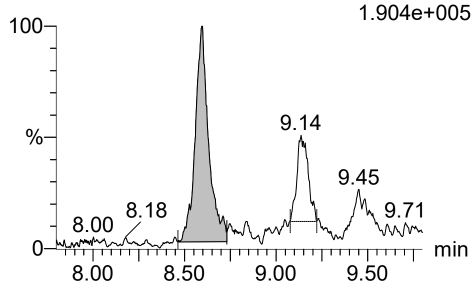

Sample Name: 20211111_A32_R3_IgM
F66:MRM of 1 channel,ES+
1086.2 > 204.1
2.995e+005

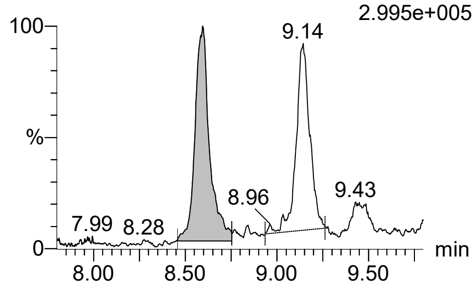

Sample Name: 20211111_L31_R3_IgM
F66:MRM of 1 channel,ES+
1086.2 > 204.1
6.959e+004

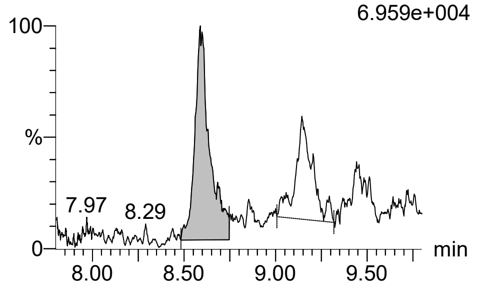

Sample Name: 20211111_L32_R3_IgM
F66:MRM of 1 channel,ES+
1086.2 > 204.1
2.721e+005

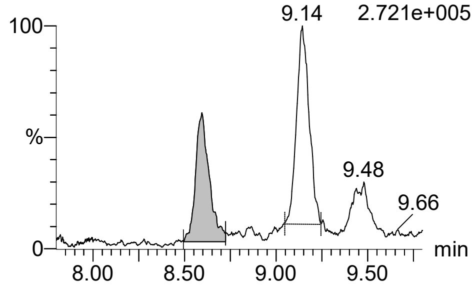

Sample Name: 20211111_H16_R3_IgM
F66:MRM of 1 channel,ES+
1086.2 > 204.1
4.925e+005

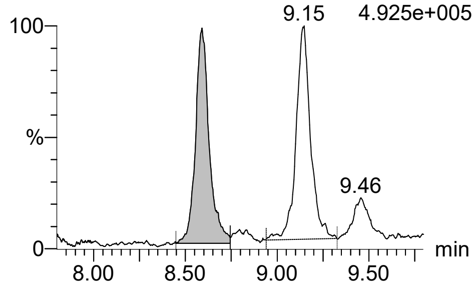

Dataset: C:\MassLynx\10.pro\20211111_clinicalsamples_IgM_IgA.qld

Last Altered: Thursday, March 09, 2023 19:58:25 台北標準時間
Printed: Friday, August 01, 2025 15:13:55 台北標準時間

Sample Name: 20211111_A33_R3_IgM
F66:MRM of 1 channel,ES+
1086.2 > 204.1
6.430e+005

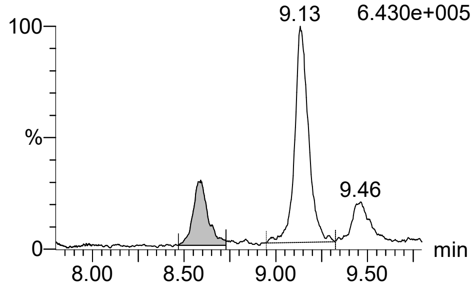

Sample Name: 20211111_A34_R3_IgM
F66:MRM of 1 channel,ES+
1086.2 > 204.1
1.382e+005

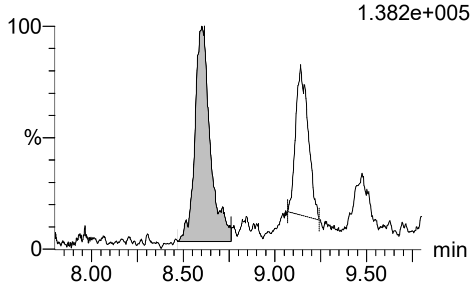

Sample Name: 20211111_H17_R3_IgM
F66:MRM of 1 channel,ES+
1086.2 > 204.1
1.071e+006

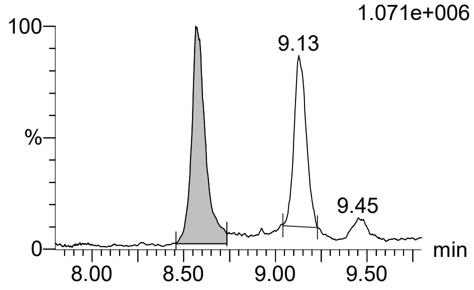

Sample Name: 20211111_A35_R3_IgM
F66:MRM of 1 channel,ES+
1086.2 > 204.1
1.864e+005

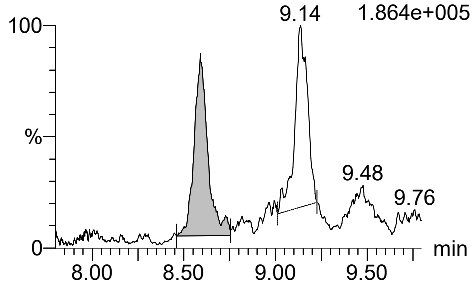

Sample Name: 20211111_QC_R24_IgM
F66:MRM of 1 channel,ES+
1086.2 > 204.1
2.376e+005

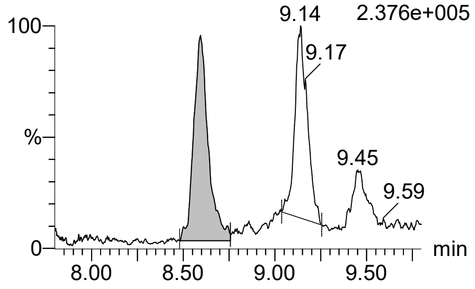

Sample Name: 20211111_A36_R3_IgM
F66:MRM of 1 channel,ES+
1086.2 > 204.1
1.124e+005

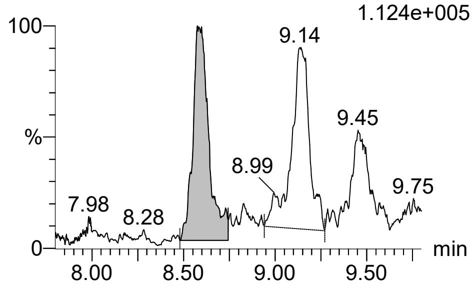

Sample Name: 20211111_H18_R3_IgM
F66:MRM of 1 channel,ES+
1086.2 > 204.1
4.447e+005

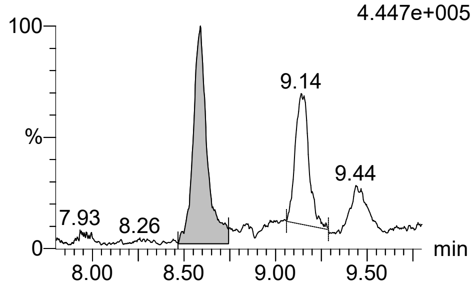

Sample Name: 20211111_A37_R3_IgM
F66:MRM of 1 channel,ES+
1086.2 > 204.1
3.202e+005

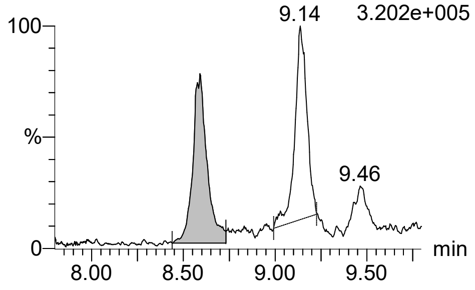

Sample Name: 20211111_A38_R3_IgM
F66:MRM of 1 channel,ES+
1086.2 > 204.1
2.242e+005

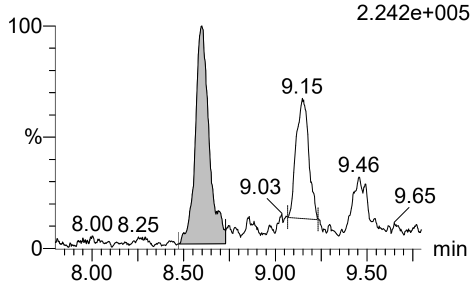

Sample Name: 20211111_H19_R3_IgM
F66:MRM of 1 channel,ES+
1086.2 > 204.1
3.746e+005

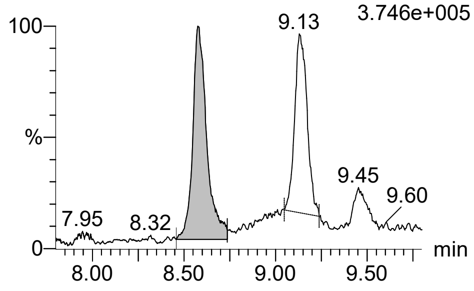

Sample Name: 20211111_A39_R3_IgM
F66:MRM of 1 channel,ES+
1086.2 > 204.1
2.430e+005

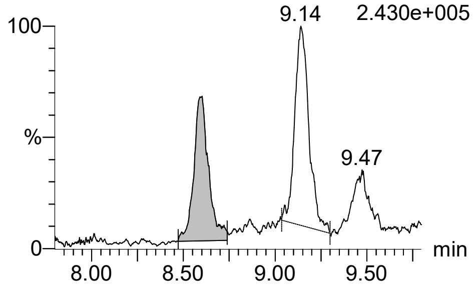

Sample Name: 20211111_A40_R3_IgM
F66:MRM of 1 channel,ES+
1086.2 > 204.1
2.876e+005

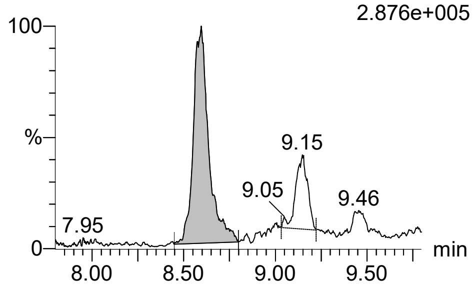

Sample Name: 20211111_H20_R3_IgM
F66:MRM of 1 channel,ES+
1086.2 > 204.1
7.635e+005

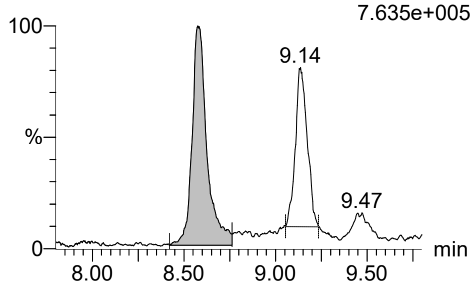

Sample Name: 20211111_A41_R3_IgM
F66:MRM of 1 channel,ES+
1086.2 > 204.1
1.211e+005

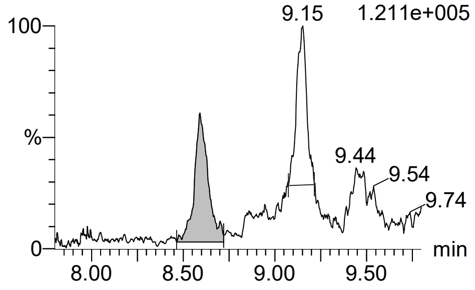

Sample Name: 20211111_A42_R3_IgM
F66:MRM of 1 channel,ES+
1086.2 > 204.1
1.597e+005

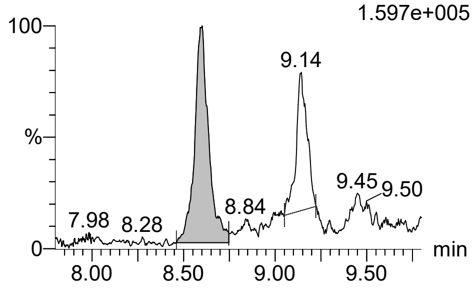

Dataset: C:\MassLynx\10.pro\20211111_clinicalsamples_IgM_IgA.qld

Last Altered: Thursday, March 09, 2023 19:58:25 台北標準時間
Printed: Friday, August 01, 2025 15:13:55 台北標準時間

Sample Name: 20211111_A43_R3_IgM

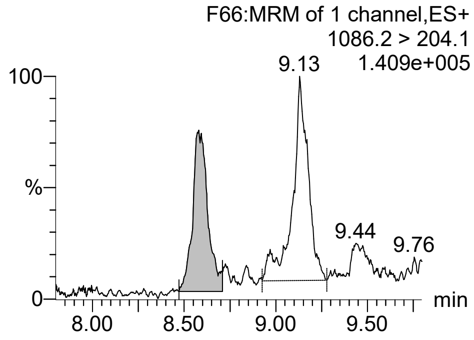

Sample Name: 20211111_A44_R3_IgM

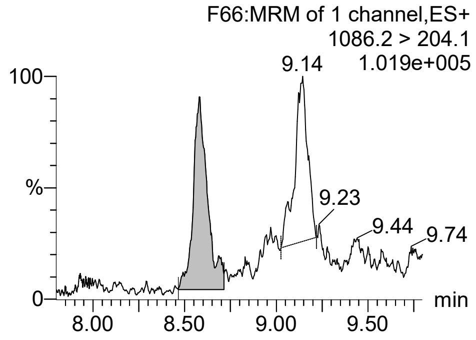

Sample Name: 20211111_QC_R25_IgM

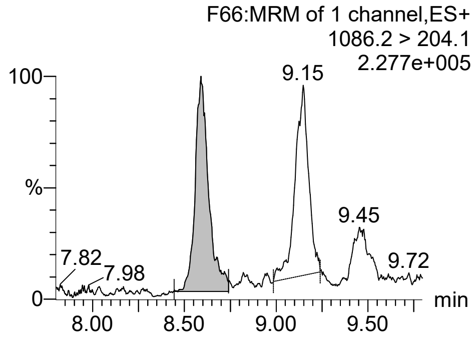

Dataset: C:\MassLynx\10.pro\20211111_clinicalsamples_IgM_IgA.qld

Last Altered: Thursday, March 09, 2023 19:58:25 台北標準時間
Printed: Friday, August 01, 2025 15:13:55 台北標準時間

	#	Name	RT	Area	IS Area	Response	Prima...
1	1	20211112_QC_R001_IgM	9.12	26307.8	15421.4	1.706	bb
2	2	20211112_QC_R002_IgM	8.59	21490.3	13188.8	1.629	MM
3	3	20211111_A1_R2_IgM	8.59	11719.4	11871.0	0.987	MM
4	4	20211111_A1_R3_IgM	8.59	13339.3	12030.5	1.109	MM
5	5	20211111_A2_R2_IgM	8.59	27669.7	11446.8	2.417	MM
6	6	20211111_A2_R3_IgM	8.58	27079.3	11098.4	2.440	MM
7	7	20211111_L1_R2_IgM	8.59	4801.6	11779.4	0.408	MM
8	8	20211111_L1_R3_IgM	8.58	5350.0	11176.2	0.479	MM
9	9	20211111_L2_R2_IgM	8.58	40578.2	11375.0	3.567	MM
10	10	20211111_H1_R2_IgM	8.58	19362.4	13595.2	1.424	MM
11	11	20211111_A3_R2_IgM	8.56	27079.1	14112.3	1.919	MM
12	12	20211111_A4_R2_IgM	8.57	9912.3	11977.5	0.828	MM
13	13	20211111_L3_R2_IgM	8.54	10948.0	12242.1	0.894	MM
14	14	20211111_L4_R2_IgM	8.58	26821.9	12797.0	2.096	MM
15	15	20211111_H2_R2_IgM	8.59	51544.3	10006.0	5.151	MM
16	16	20211111_A5_R2_IgM	8.56	45989.5	11434.6	4.022	MM
17	17	20211111_A6_R2_IgM	8.58	14886.7	13193.7	1.128	MM
18	18	20211111_QC_R10_IgM	8.58	23392.0	12566.1	1.862	MM
19	19	20211111_L5_R2_IgM	8.56	44014.6	13616.4	3.232	MM
20	20	20211111_L6_R2_IgM	8.58	29836.0	13698.5	2.178	MM
21	21	20211111_H3_R2_IgM	8.58	28062.8	15793.1	1.777	MM
22	22	20211111_A7_R2_IgM	8.58	60380.5	12936.9	4.667	MM
23	23	20211111_A8_R2_IgM	8.58	46784.0	13108.0	3.569	MM
24	24	20211111_L7_R2_IgM	8.60	3567.7	13278.5	0.269	MM
25	25	20211111_L8_R2_IgM	8.59	14907.3	15233.6	0.979	MM
26	26	20211111_H4_R2_IgM	8.58	24459.2	16521.9	1.480	MM
27	27	20211111_A9_R2_IgM	8.58	32227.7	16451.6	1.959	MM
28	28	20211111_A10_R2_IgM	8.58	68996.0	13036.0	5.293	MM
29	29	20211111_L9_R2_IgM	8.56	12135.4	14554.1	0.834	MM
30	30	20211111_L10_R2_IgM	8.57	28956.2	12250.4	2.364	MM
31	31	20211111_QC_R11_IgM	8.59	27482.2	11459.2	2.398	MM
32	32	20211111_H5_R2_IgM	8.58	14339.0	14358.2	0.999	MM
33	33	20211111_A11_R2_IgM	8.51	25598.7	16184.1	1.582	MM
34	34	20211111_A12_R2_IgM	8.59	19478.8	13465.4	1.447	MM
35	35	20211111_L11_R2_IgM	8.60	11862.4	13277.9	0.893	MM
36	36	20211111_L12_R2_IgM	8.57	12032.8	15958.8	0.754	MM
37	37	20211111_H6_R2_IgM	8.60	27028.0	14003.5	1.930	MM
38	38	20211111_A13_R2_IgM	8.59	24471.2	13656.1	1.792	MM
39	39	20211111_A14_R2_IgM	8.58	86958.3	14071.3	6.180	MM
40	40	20211111_L13_R2_IgM	8.60	22768.8	14785.6	1.540	MM
41	41	20211111_L14_R2_IgM	8.59	57587.0	14772.2	3.898	MM
42	42	20211111_H7_R2_IgM	8.57	42409.9	15164.4	2.797	MM
43	43	20211111_A15_R2_IgM	8.58	88128.8	15721.5	5.606	MM
44	44	20211111_QC_R12_IgM	8.58	31471.0	13711.5	2.295	MM
45	45	20211111_A16_R2_IgM	8.59	47257.2	14275.6	3.310	MM
46	46	20211111_L15_R2_IgM	8.59	62302.2	12935.8	4.816	MM
47	47	20211111_L16_R2_IgM	8.58	11287.9	13342.8	0.846	MM
48	48	20211111_H8_R2_IgM	8.59	20996.9	15835.1	1.326	MM
49	49	20211111_A17_R2_IgM	8.60	28670.3	15786.7	1.816	MM
50	50	20211111_A18_R2_IgM	8.57	51639.6	16986.8	3.040	MM
51	51	20211111_L17_R2_IgM	8.58	28141.2	13342.7	2.109	MM
52	52	20211111_L18_R2_IgM	8.59	5249.4	14692.0	0.357	MM
53	53	20211111_H9_R2_IgM	8.57	13342.4	15639.7	0.853	MM
54	54	20211111_A19_R2_IgM	8.57	19882.2	14198.7	1.400	MM
55	55	20211111_A20_R2_IgM	8.55	19675.3	14162.7	1.389	MM
56	56	20211111_L19_R2_IgM	8.58	26585.5	9293.4	2.861	MM

Dataset: C:\MassLynx\10.pro\20211111_clinicalsamples_IgM_IgA.qld

Last Altered: Thursday, March 09, 2023 19:58:25 台北標準時間
Printed: Friday, August 01, 2025 15:13:55 台北標準時間

	# Name	RT	Area	IS Area	Response	Prima...
57	57 20211111_QC_R13_IgM	8.59	32448.2	13903.0	2.334	MM
58	58 20211111_L20_R2_IgM	8.52	10483.1	12781.6	0.820	MM
59	59 20211111_H10_R2_IgM	8.56	32404.3	14796.4	2.190	MM
60	60 20211111_A21_R2_IgM	8.59	40695.9	13669.0	2.977	MM
61	61 20211111_A22_R2_IgM	8.58	6992.2	14098.3	0.496	MM
62	62 20211111_L21_R2_IgM	8.59	15604.6	15678.2	0.995	MM
63	63 20211111_L22_R2_IgM	8.60	26228.8	15724.5	1.668	MM
64	64 20211111_H11_R2_IgM	8.59	25108.9	15890.2	1.580	MM
65	65 20211111_A23_R2_IgM	8.58	12344.9	13423.2	0.920	MM
66	66 20211111_A24_R2_IgM	8.59	26802.8	12438.0	2.155	MM
67	67 20211111_L23_R2_IgM	8.58	8040.9	14075.0	0.571	MM
68	68 20211111_L24_R2_IgM	8.58	14869.3	15972.1	0.931	MM
69	69 20211111_H12_R2_IgM	8.60	11885.9	16270.9	0.731	MM
70	70 20211111_QC_R14_IgM	8.60	31192.9	15806.8	1.973	MM
71	71 20211111_A25_R2_IgM	8.58	33487.8	14552.1	2.301	MM
72	72 20211111_A26_R2_IgM	8.61	7388.3	11492.7	0.643	MM
73	73 20211111_L25_R2_IgM	8.59	11794.7	14403.3	0.819	MM
74	74 20211111_L26_R2_IgM	8.61	10832.0	16533.9	0.655	MM
75	75 20211111_H13_R2_IgM	8.60	27265.4	15520.1	1.757	MM
76	76 20211111_A27_R2_IgM	8.58	15062.8	13337.4	1.129	MM
77	77 20211111_A28_R2_IgM	8.54	8022.3	11895.6	0.674	MM
78	78 20211111_L27_R2_IgM	8.59	21481.8	14125.7	1.521	MM
79	79 20211111_L28_R2_IgM	8.60	19139.5	11807.5	1.621	MM
80	80 20211111_H14_R2_IgM	8.59	24947.1	15035.7	1.659	MM
81	81 20211111_A29_R2_IgM	8.58	3226.3	13554.4	0.238	MM
82	82 20211111_A30_R2_IgM	8.58	35828.2	12984.1	2.759	MM
83	83 20211111_QC_R15_IgM	8.58	27822.9	12299.5	2.262	MM
84	84 20211111_L29_R2_IgM	8.59	18542.3	13590.7	1.364	MM
85	85 20211111_L30_R2_IgM	8.58	11378.0	12511.4	0.909	MM
86	86 20211111_H15_R2_IgM	8.58	24979.0	11885.0	2.102	MM
87	87 20211111_A31_R2_IgM	8.59	16198.3	13422.1	1.207	MM
88	88 20211111_A32_R2_IgM	8.59	31591.7	11461.3	2.756	MM
89	89 20211111_L31_R2_IgM	8.53	6513.1	13753.4	0.474	MM
90	90 20211111_L32_R2_IgM	8.58	15164.3	12949.0	1.171	MM
91	91 20211111_H16_R2_IgM	8.58	48898.2	11465.4	4.265	MM
92	92 20211111_A33_R2_IgM	8.59	18300.7	10551.5	1.734	MM
93	93 20211111_A34_R2_IgM	8.59	14894.8	12409.9	1.200	MM
94	94 20211111_H17_R2_IgM	8.58	117612.4	10160.9	11.575	MM
95	95 20211111_A35_R2_IgM	8.57	14678.0	10460.6	1.403	MM
96	96 20211111_QC_R16_IgM	8.59	23774.1	10433.5	2.279	MM
97	97 20211111_A36_R2_IgM	8.52	13133.2	12504.8	1.050	MM
98	98 20211111_H18_R2_IgM	8.58	43612.7	9140.4	4.771	MM
99	99 20211111_A37_R2_IgM	8.58	26754.0	9796.9	2.731	MM
100	1... 20211111_A38_R2_IgM	8.59	24122.1	11224.3	2.149	MM
101	1... 20211111_H19_R2_IgM	8.58	40378.8	11739.2	3.440	MM
102	1... 20211111_A39_R2_IgM	8.59	17257.0	12718.9	1.357	MM
103	1... 20211111_A40_R2_IgM	8.54	33888.6	10006.1	3.387	MM
104	1... 20211111_H20_R2_IgM	8.58	83713.2	7988.2	10.480	MM
105	1... 20211111_A41_R2_IgM	8.60	8340.6	11513.3	0.724	MM
106	1... 20211111_A42_R2_IgM	8.60	13720.3	10238.5	1.340	MM
107	1... 20211111_A43_R2_IgM	8.58	11077.9	11098.9	0.998	MM
108	1... 20211111_A44_R2_IgM	8.58	9684.6	10969.9	0.883	MM
109	1... 20211111_QC_R17_IgM	8.59	19638.4	9700.8	2.024	MM
110	1... 20211111_L2_R3_IgM	8.59	41305.7	8528.0	4.844	MM
111	1... 20211111_H1_R3_IgM	8.58	17605.7	9438.2	1.865	MM
112	1... 20211111_A3_R3_IgM	8.60	25693.4	11598.6	2.215	MM

	# Name	RT	Area	IS Area	Response	Prima...
113	1... 20211111_A4_R3_IgM	8.59	7256.0	11364.1	0.638	MM
114	1... 20211111_L3_R3_IgM	8.54	9449.5	10753.4	0.879	MM
115	1... 20211111_L4_R3_IgM	8.60	22951.6	10291.6	2.230	MM
116	1... 20211111_H2_R3_IgM	8.59	50630.6	9315.8	5.435	MM
117	1... 20211111_A5_R3_IgM	8.59	37634.2	9102.2	4.135	MM
118	1... 20211111_A6_R3_IgM	8.58	13049.7	10356.4	1.260	MM
119	1... 20211111_QC_R18_IgM	8.59	20475.6	8398.3	2.438	MM
120	1... 20211111_L5_R3_IgM	8.60	34879.3	9786.7	3.564	MM
121	1... 20211111_L6_R3_IgM	8.59	26371.2	9911.3	2.661	MM
122	1... 20211111_H3_R3_IgM	8.60	22772.7	12155.5	1.873	MM
123	1... 20211111_A7_R3_IgM	8.58	60130.3	11804.8	5.094	MM
124	1... 20211111_A8_R3_IgM	8.59	37085.1	10510.6	3.528	MM
125	1... 20211111_L7_R3_IgM	8.58	2772.3	12177.9	0.228	MM
126	1... 20211111_L8_R3_IgM	8.60	12836.7	11668.6	1.100	MM
127	1... 20211111_H4_R3_IgM	8.60	20064.9	11192.0	1.793	MM
128	1... 20211111_A9_R3_IgM	8.61	25808.5	14077.4	1.833	MM
129	1... 20211111_A10_R3_IgM	8.60	61068.1	11190.4	5.457	MM
130	1... 20211111_L9_R3_IgM	8.59	10260.6	14649.3	0.700	MM
131	1... 20211111_L10_R3_IgM	8.55	25875.7	9361.3	2.764	MM
132	1... 20211111_QC_R19_IgM	8.58	22009.0	10125.5	2.174	MM
133	1... 20211111_H5_R3_IgM	8.60	10722.3	10010.9	1.071	MM
134	1... 20211111_A11_R3_IgM	8.59	17399.6	12697.2	1.370	MM
135	1... 20211111_A12_R3_IgM	8.59	17808.6	13148.5	1.354	MM
136	1... 20211111_L11_R3_IgM	8.60	8169.6	10786.6	0.757	MM
137	1... 20211111_L12_R3_IgM	8.56	9988.7	11681.8	0.855	MM
138	1... 20211111_H6_R3_IgM	8.59	26027.8	12095.5	2.152	MM
139	1... 20211111_A13_R3_IgM	8.60	22193.6	12908.3	1.719	MM
140	1... 20211111_A14_R3_IgM	8.59	70742.5	11322.6	6.248	MM
141	1... 20211111_L13_R3_IgM	8.59	19344.6	11516.1	1.680	MM
142	1... 20211111_L14_R3_IgM	8.58	44266.3	10790.7	4.102	MM
143	1... 20211111_H7_R3_IgM	8.59	32223.0	11572.1	2.785	MM
144	1... 20211111_A15_R3_IgM	8.59	75951.9	12434.4	6.108	MM
145	1... 20211111_QC_R20_IgM	8.59	25202.2	10701.0	2.355	MM
146	1... 20211111_A16_R3_IgM	8.61	39164.8	11452.1	3.420	MM
147	1... 20211111_L15_R3_IgM	8.59	53631.8	10531.1	5.093	MM
148	1... 20211111_L16_R3_IgM	8.59	9561.3	12049.4	0.794	MM
149	1... 20211111_H8_R3_IgM	8.60	17527.7	13434.9	1.305	MM
150	1... 20211111_A17_R3_IgM	8.59	22076.6	12452.0	1.773	MM
151	1... 20211111_A18_R3_IgM	8.56	47167.0	12506.5	3.771	MM
152	1... 20211111_L17_R3_IgM	8.58	22773.6	9948.9	2.289	MM
153	1... 20211111_L18_R3_IgM	8.61	3794.9	13462.1	0.282	MM
154	1... 20211111_H9_R3_IgM	8.60	11426.8	13178.7	0.867	MM
155	1... 20211111_A19_R3_IgM	8.58	16978.4	11298.6	1.503	MM
156	1... 20211111_A20_R3_IgM	8.57	16141.9	11175.6	1.444	MM
157	1... 20211111_L19_R3_IgM	8.58	29016.3	11558.2	2.510	MM
158	1... 20211111_QC_R21_IgM	8.58	25814.8	9977.0	2.587	MM
159	1... 20211111_L20_R3_IgM	8.58	8617.1	13508.1	0.638	MM
160	1... 20211111_H10_R3_IgM	8.59	26743.3	12374.8	2.161	MM
161	1... 20211111_A21_R3_IgM	8.59	36814.0	12443.6	2.958	MM
162	1... 20211111_A22_R3_IgM	8.59	7753.2	11589.3	0.669	MM
163	1... 20211111_L21_R3_IgM	8.60	13329.5	13107.4	1.017	MM
164	1... 20211111_L22_R3_IgM	8.60	22236.9	12021.5	1.850	MM
165	1... 20211111_H11_R3_IgM	8.60	22998.5	12687.8	1.813	MM
166	1... 20211111_A23_R3_IgM	8.60	11304.4	11611.8	0.974	MM
167	1... 20211111_A24_R3_IgM	8.61	25175.7	10476.8	2.403	MM
168	1... 20211111_L23_R3_IgM	8.58	6469.7	11873.1	0.545	MM

Dataset: C:\MassLynx\10.pro\20211111_clinicalsamples_IgM_IgA.qld

Last Altered: Thursday, March 09, 2023 19:58:25 台北標準時間
Printed: Friday, August 01, 2025 15:13:55 台北標準時間

	#	Name	RT	Area	IS Area	Response	Prima...
169	1...	20211111_L24_R3_IgM	8.60	12242.1	13468.0	0.909	MM
170	1...	20211111_H12_R3_IgM	8.61	9858.7	14229.1	0.693	MM
171	1...	20211111_QC_R22_IgM	8.59	26367.5	11264.4	2.341	MM
172	1...	20211111_A25_R3_IgM	8.60	26166.3	11775.7	2.222	MM
173	1...	20211111_A26_R3_IgM	8.60	6994.6	10473.9	0.668	MM
174	1...	20211111_L25_R3_IgM	8.59	9454.2	13256.8	0.713	MM
175	1...	20211111_L26_R3_IgM	8.61	9058.1	13565.0	0.668	MM
176	1...	20211111_H13_R3_IgM	8.59	26595.0	13146.0	2.023	MM
177	1...	20211111_A27_R3_IgM	8.59	11761.9	12025.6	0.978	MM
178	1...	20211111_A28_R3_IgM	8.60	7170.2	11229.1	0.639	MM
179	1...	20211111_L27_R3_IgM	8.60	21411.1	14960.0	1.431	MM
180	1...	20211111_L28_R3_IgM	8.59	19807.6	11359.0	1.744	MM
181	1...	20211111_H14_R3_IgM	8.59	25383.7	12830.8	1.978	MM
182	1...	20211111_A29_R3_IgM	8.59	2748.2	13434.3	0.205	MM
183	1...	20211111_A30_R3_IgM	8.59	34754.9	11272.2	3.083	MM
184	1...	20211111_QC_R23_IgM	8.60	22927.3	11872.2	1.931	MM
185	1...	20211111_L29_R3_IgM	8.60	16909.7	12948.7	1.306	MM
186	1...	20211111_L30_R3_IgM	8.59	10709.4	11026.6	0.971	MM
187	1...	20211111_H15_R3_IgM	8.60	23604.3	10786.3	2.188	MM
188	1...	20211111_A31_R3_IgM	8.59	15630.6	12010.5	1.301	MM
189	1...	20211111_A32_R3_IgM	8.60	26368.2	11107.7	2.374	MM
190	1...	20211111_L31_R3_IgM	8.59	6168.5	11021.8	0.560	MM
191	1...	20211111_L32_R3_IgM	8.59	13570.9	11683.8	1.162	MM
192	1...	20211111_H16_R3_IgM	8.59	40741.6	9831.7	4.144	MM
193	1...	20211111_A33_R3_IgM	8.59	17676.1	11213.1	1.576	MM
194	1...	20211111_A34_R3_IgM	8.60	12924.1	11550.1	1.119	MM
195	1...	20211111_H17_R3_IgM	8.57	91311.6	8874.4	10.289	MM
196	1...	20211111_A35_R3_IgM	8.59	13057.2	9856.3	1.325	MM
197	1...	20211111_QC_R24_IgM	8.59	20108.9	7561.8	2.659	MM
198	1...	20211111_A36_R3_IgM	8.57	10866.4	9562.8	1.136	MM
199	1...	20211111_H18_R3_IgM	8.59	37855.1	8165.3	4.636	MM
200	2...	20211111_A37_R3_IgM	8.59	21847.7	9278.8	2.355	MM
201	2...	20211111_A38_R3_IgM	8.60	19114.6	10920.3	1.750	MM
202	2...	20211111_H19_R3_IgM	8.58	31592.8	9285.3	3.402	MM
203	2...	20211111_A39_R3_IgM	8.60	14664.9	9083.4	1.614	MM
204	2...	20211111_A40_R3_IgM	8.60	26953.9	8382.4	3.216	MM
205	2...	20211111_H20_R3_IgM	8.58	70577.1	7807.9	9.039	MM
206	2...	20211111_A41_R3_IgM	8.59	6037.5	5925.2	1.019	MM
207	2...	20211111_A42_R3_IgM	8.60	13684.5	9352.6	1.463	MM
208	2...	20211111_A43_R3_IgM	8.58	9799.5	10216.3	0.959	MM
209	2...	20211111_A44_R3_IgM	8.58	7830.1	10262.7	0.763	MM
210	2...	20211111_QC_R25_IgM	8.59	18649.5	8575.3	2.175	MM

Dataset: C:\MassLynx\10.pro\20211111_clinicalsamples_IgM_IgA.qld

Last Altered: Thursday, March 09, 2023 19:58:25 台北標準時間
Printed: Friday, August 01, 2025 15:15:05 台北標準時間

Method: C:\MassLynx\10_Pro_IgA.PRO\MethDB\20211111_IgM_IgA_DMRM_new.mdb 24 Feb 2022 13:44:14
Calibration: 15 Nov 2021 14:42:11

Sample Name: 20211112_QC_R001_IgM

F43:MRM of 1 channel,ES+
968.1 > 366.1
2.802e+005

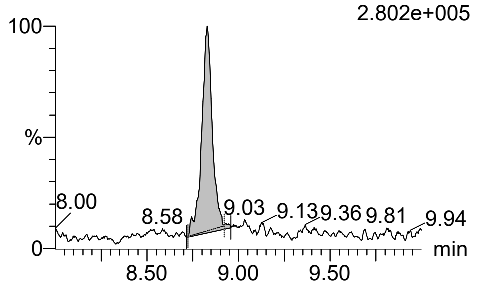

Sample Name: 20211112_QC_R002_IgM

F43:MRM of 1 channel,ES+
968.1 > 366.1
2.484e+005

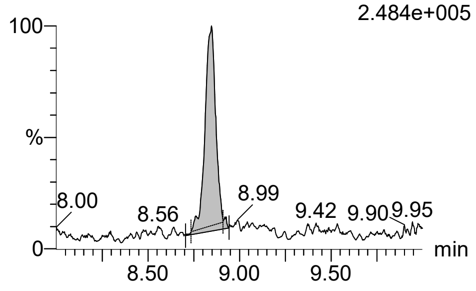

Sample Name: 20211111_A1_R2_IgM

F43:MRM of 1 channel,ES+
968.1 > 366.1
1.378e+005

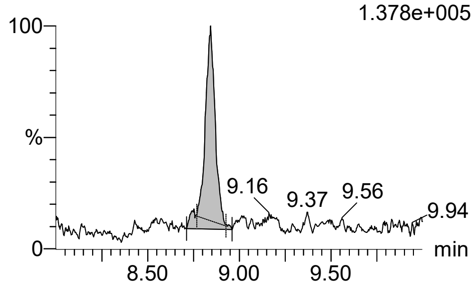

Sample Name: 20211111_A1_R3_IgM

F43:MRM of 1 channel,ES+
968.1 > 366.1
1.212e+005

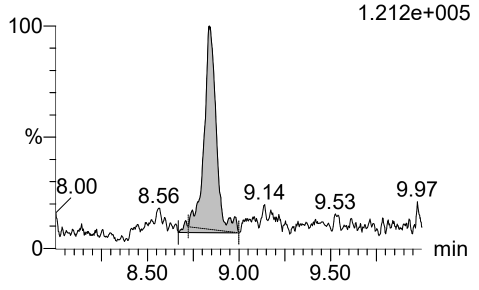

Sample Name: 20211111_A2_R2_IgM

F43:MRM of 1 channel,ES+
968.1 > 366.1
3.957e+005

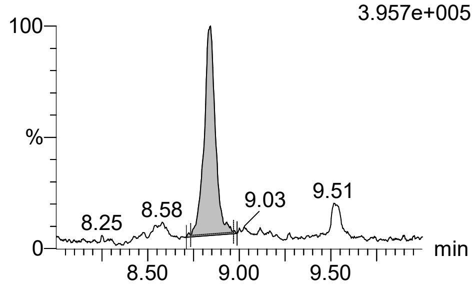

Sample Name: 20211111_A2_R3_IgM

F43:MRM of 1 channel,ES+
968.1 > 366.1
3.760e+005

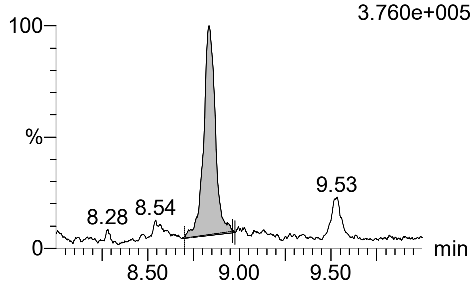

Sample Name: 20211111_L1_R2_IgM

F43:MRM of 1 channel,ES+
968.1 > 366.1
5.842e+004

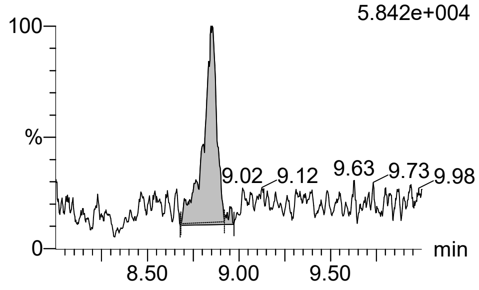

Sample Name: 20211111_L1_R3_IgM

F43:MRM of 1 channel,ES+
968.1 > 366.1
5.912e+004

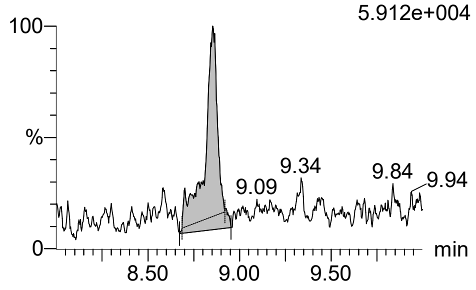

Sample Name: 20211111_L2_R2_IgM

F43:MRM of 1 channel,ES+
968.1 > 366.1
4.164e+005

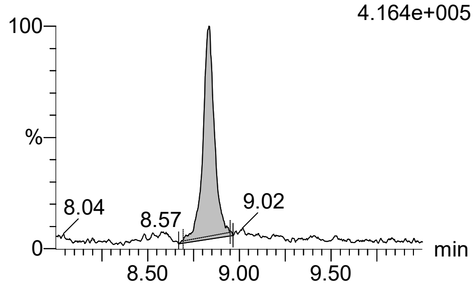

Sample Name: 20211111_H1_R2_IgM

F43:MRM of 1 channel,ES+
968.1 > 366.1
1.572e+005

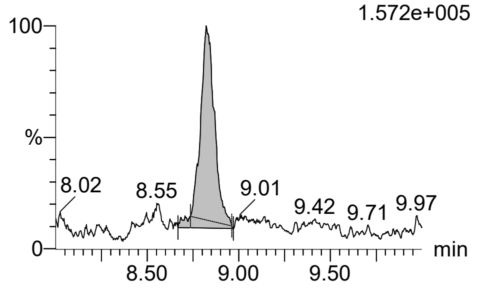

Sample Name: 20211111_A3_R2_IgM

F43:MRM of 1 channel,ES+
968.1 > 366.1
1.467e+005

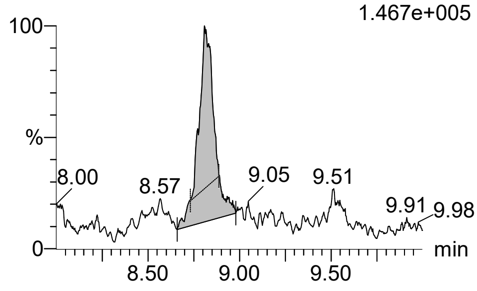

Sample Name: 20211111_A4_R2_IgM

F43:MRM of 1 channel,ES+
968.1 > 366.1
1.187e+005

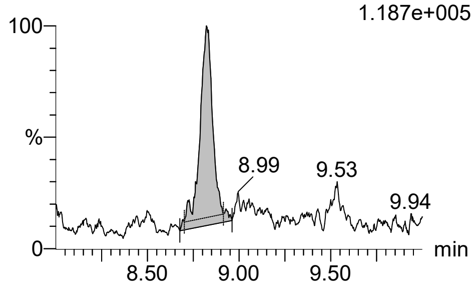

Dataset: C:\MassLynx\10.pro\20211111_clinicalsamples_IgM_IgA.qld

Last Altered: Thursday, March 09, 2023 19:58:25 台北標準時間
Printed: Friday, August 01, 2025 15:15:05 台北標準時間

Sample Name: 20211111_L3_R2_IgM
F43:MRM of 1 channel,ES+
968.1 > 366.1
3.969e+004

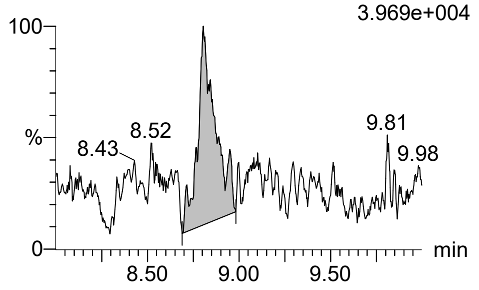

Sample Name: 20211111_L4_R2_IgM
F43:MRM of 1 channel,ES+
968.1 > 366.1
2.065e+005

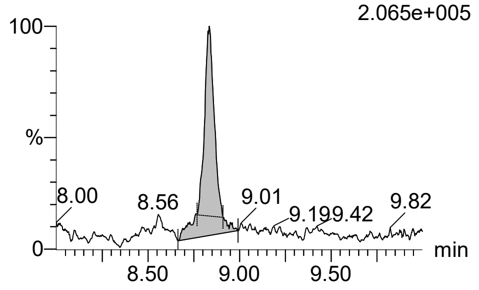

Sample Name: 20211111_H2_R2_IgM
F43:MRM of 1 channel,ES+
968.1 > 366.1
4.130e+005

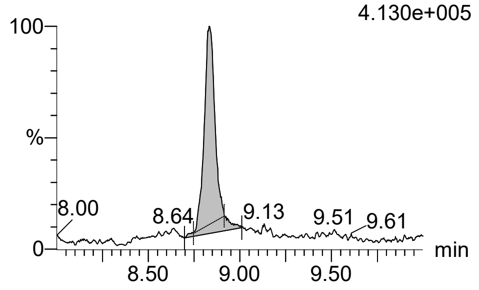

Sample Name: 20211111_A5_R2_IgM
F43:MRM of 1 channel,ES+
968.1 > 366.1
2.665e+005

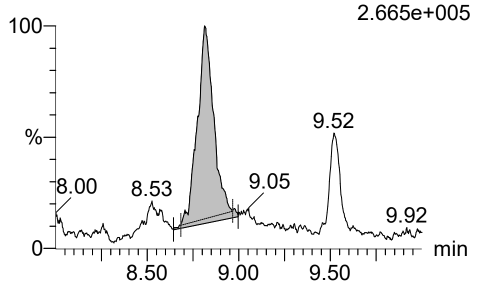

Sample Name: 20211111_A6_R2_IgM
F43:MRM of 1 channel,ES+
968.1 > 366.1
2.611e+005

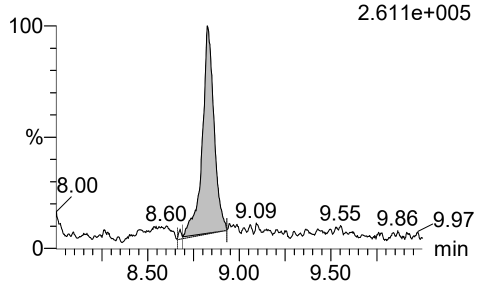

Sample Name: 20211111_QC_R10_IgM
F43:MRM of 1 channel,ES+
968.1 > 366.1
2.775e+005

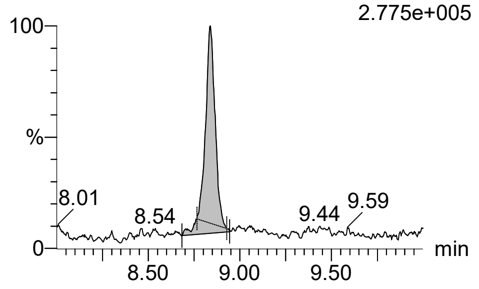

Sample Name: 20211111_L5_R2_IgM
F43:MRM of 1 channel,ES+
968.1 > 366.1
1.228e+005

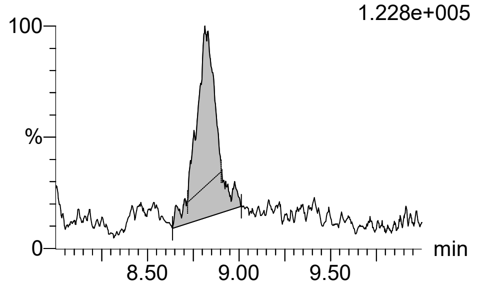

Sample Name: 20211111_L6_R2_IgM
F43:MRM of 1 channel,ES+
968.1 > 366.1
4.249e+005

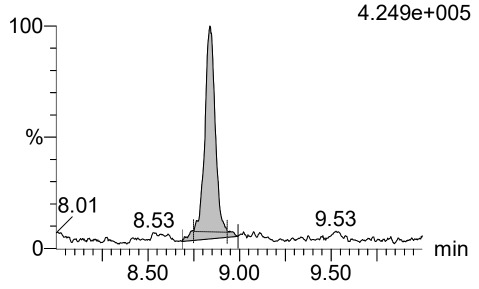

Sample Name: 20211111_H3_R2_IgM
F43:MRM of 1 channel,ES+
968.1 > 366.1
1.432e+005

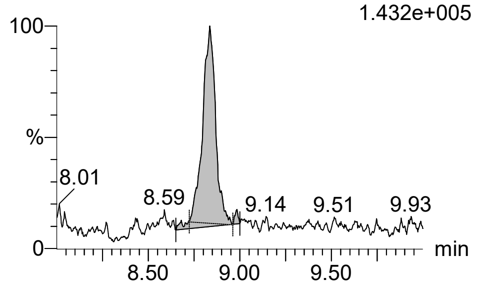

Sample Name: 20211111_A7_R2_IgM
F43:MRM of 1 channel,ES+
968.1 > 366.1
4.623e+005

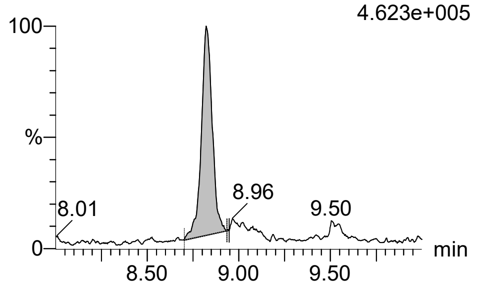

Sample Name: 20211111_A8_R2_IgM
F43:MRM of 1 channel,ES+
968.1 > 366.1
2.502e+005

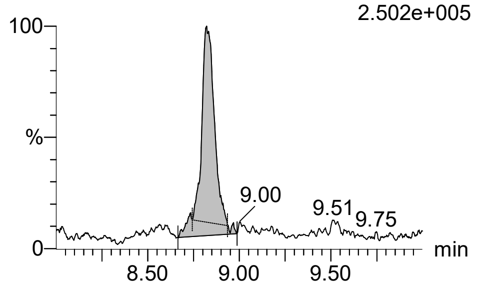

Sample Name: 20211111_L7_R2_IgM
F43:MRM of 1 channel,ES+
968.1 > 366.1
5.075e+004

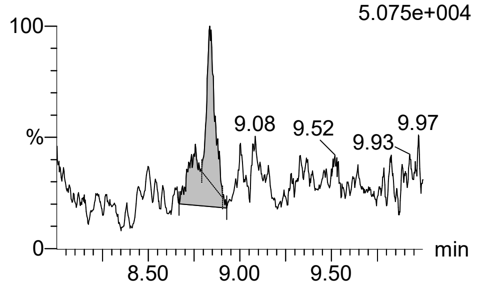

Sample Name: 20211111_L8_R2_IgM
F43:MRM of 1 channel,ES+
968.1 > 366.1
1.226e+005

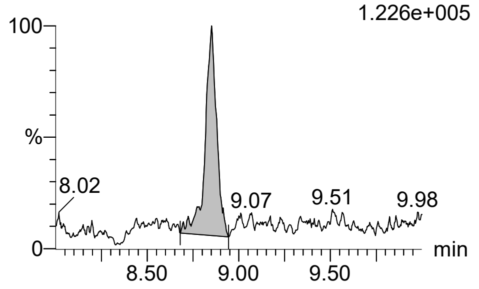

Sample Name: 20211111_H4_R2_IgM
F43:MRM of 1 channel,ES+
968.1 > 366.1
1.869e+005

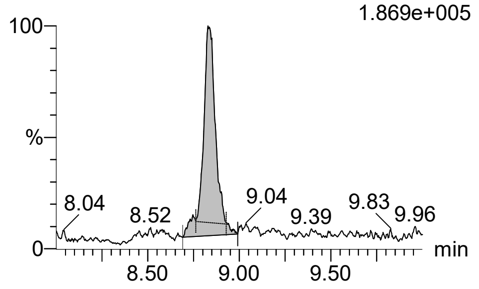

Sample Name: 20211111_A9_R2_IgM
F43:MRM of 1 channel,ES+
968.1 > 366.1
1.203e+005

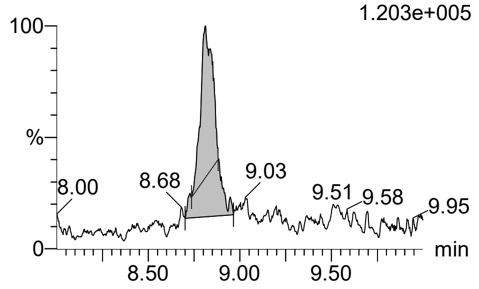

Sample Name: 20211111_A10_R2_IgM

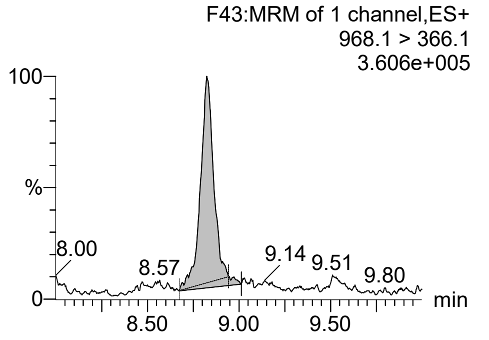

Sample Name: 20211111_L9_R2_IgM

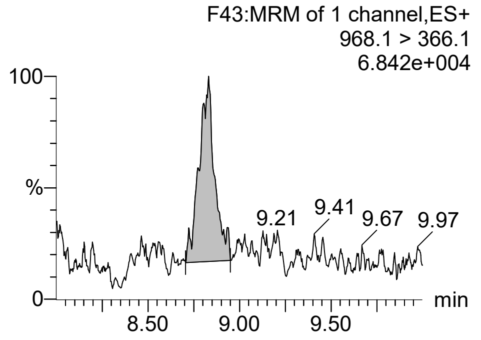

Sample Name: 20211111_L10_R2_IgM

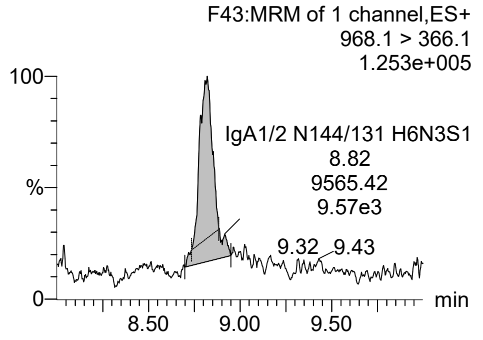

Sample Name: 20211111_QC_R11_IgM

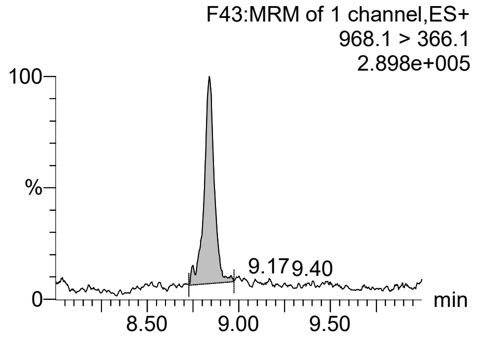

Sample Name: 20211111_H5_R2_IgM

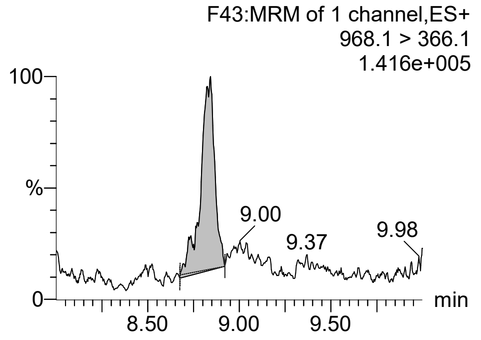

Sample Name: 20211111_A11_R2_IgM

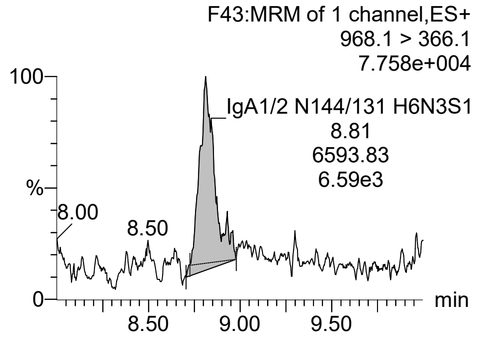

Sample Name: 20211111_A12_R2_IgM

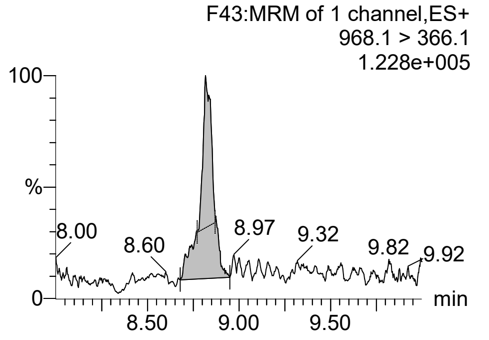

Sample Name: 20211111_L11_R2_IgM

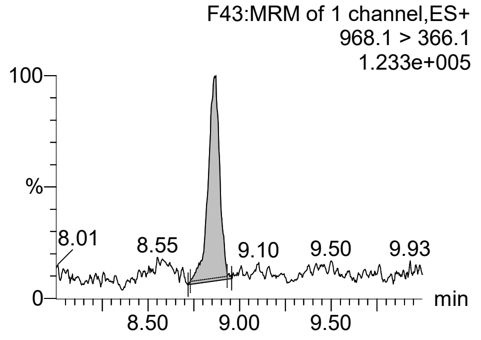

Sample Name: 20211111_L12_R2_IgM

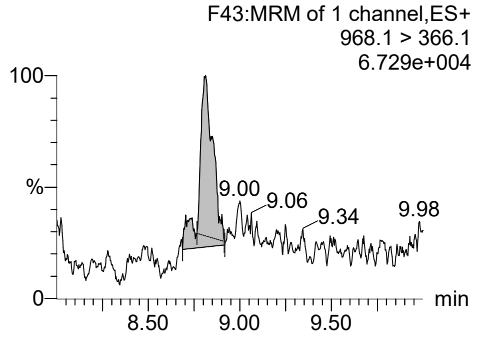

Sample Name: 20211111_H6_R2_IgM

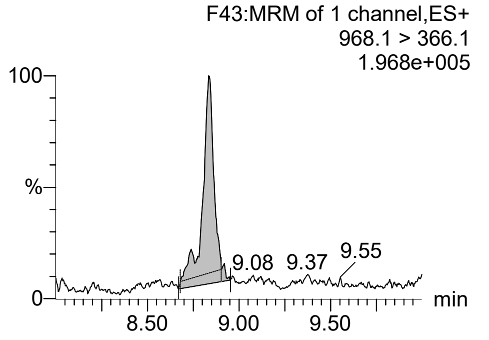

Sample Name: 20211111_A13_R2_IgM

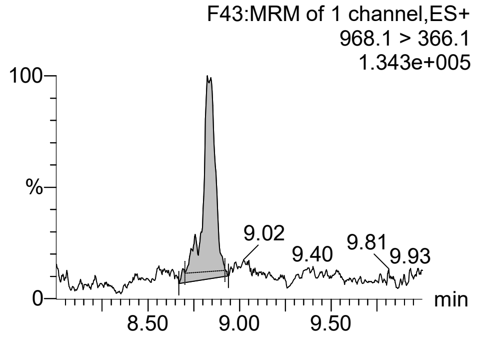

Sample Name: 20211111_A14_R2_IgM

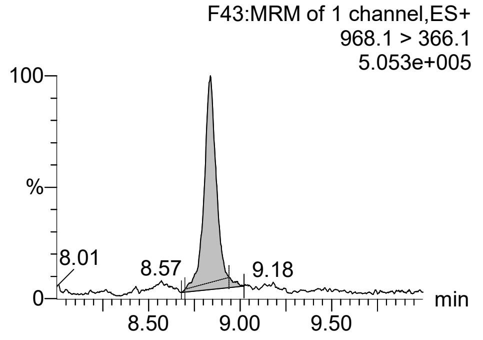

Sample Name: 20211111_L13_R2_IgM

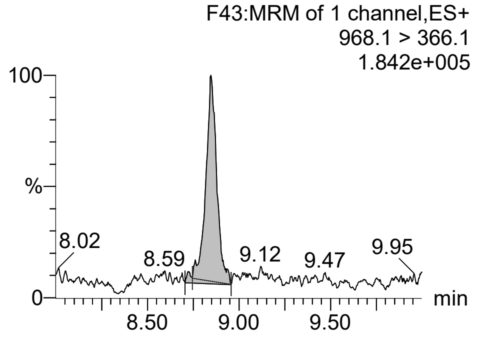

Sample Name: 20211111_L14_R2_IgM

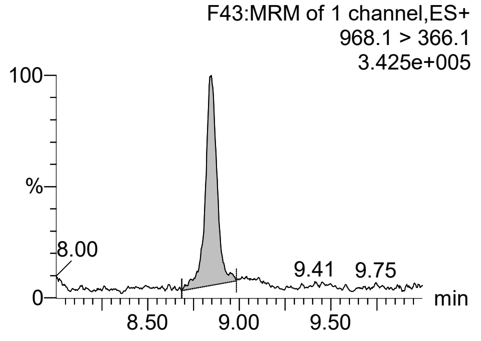

Sample Name: 20211111_H7_R2_IgM

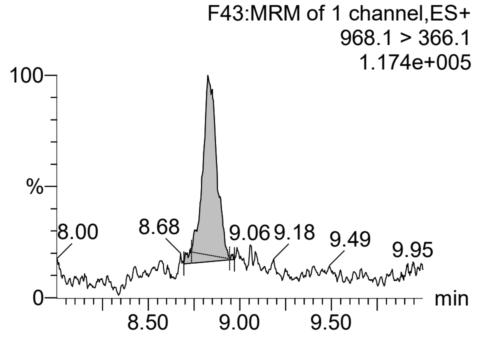

Dataset: C:\MassLynx\10.pro\20211111_clinicalsamples_IgM_IgA.qld

Last Altered: Thursday, March 09, 2023 19:58:25 台北標準時間
Printed: Friday, August 01, 2025 15:15:05 台北標準時間

Sample Name: 20211111_A15_R2_IgM
F43:MRM of 1 channel,ES+
968.1 > 366.1
6.447e+005

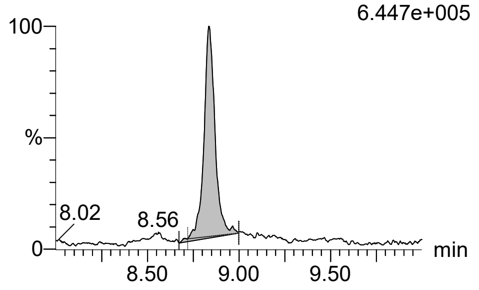

Sample Name: 20211111_QC_R12_IgM
F43:MRM of 1 channel,ES+
968.1 > 366.1
2.684e+005

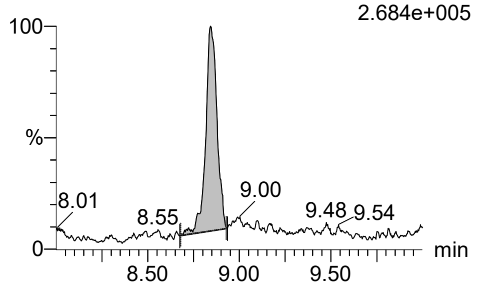

Sample Name: 20211111_A16_R2_IgM
F43:MRM of 1 channel,ES+
968.1 > 366.1
3.951e+005

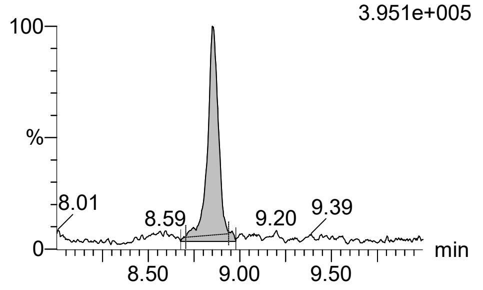

Sample Name: 20211111_L15_R2_IgM
F43:MRM of 1 channel,ES+
968.1 > 366.1
3.090e+005

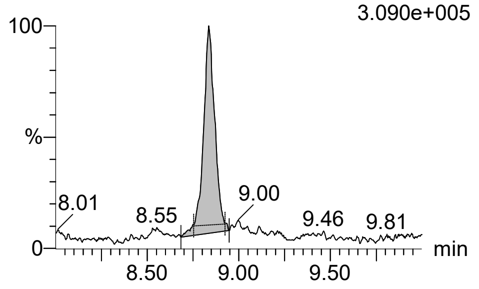

Sample Name: 20211111_L16_R2_IgM
F43:MRM of 1 channel,ES+
968.1 > 366.1
1.155e+005

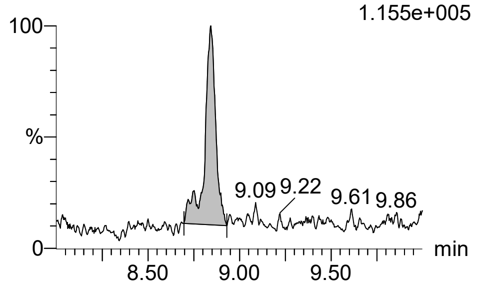

Sample Name: 20211111_H8_R2_IgM
F43:MRM of 1 channel,ES+
968.1 > 366.1
2.017e+005

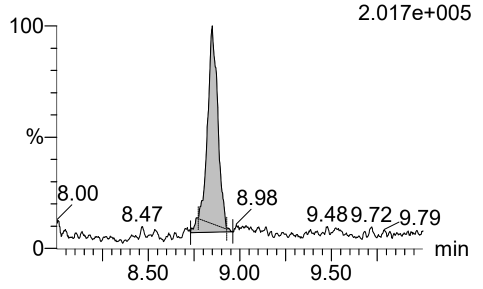

Sample Name: 20211111_A17_R2_IgM
F43:MRM of 1 channel,ES+
968.1 > 366.1
4.572e+005

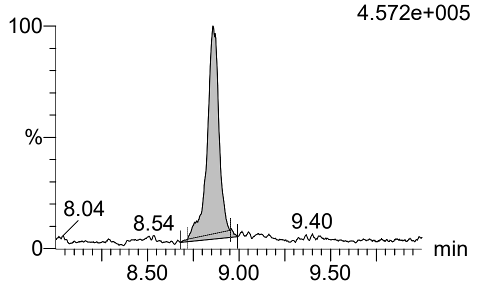

Sample Name: 20211111_A18_R2_IgM
F43:MRM of 1 channel,ES+
968.1 > 366.1
1.409e+005

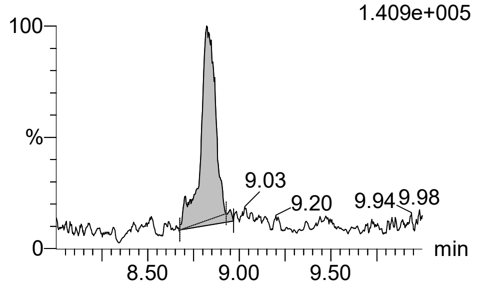

Sample Name: 20211111_L17_R2_IgM
F43:MRM of 1 channel,ES+
968.1 > 366.1
2.144e+005

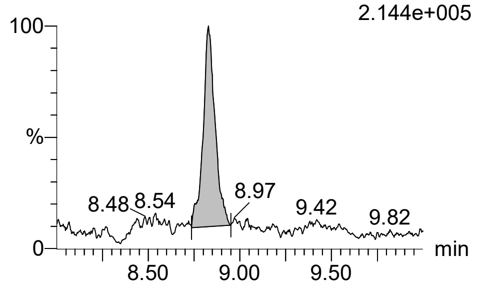

Sample Name: 20211111_L18_R2_IgM
F43:MRM of 1 channel,ES+
968.1 > 366.1
2.942e+004

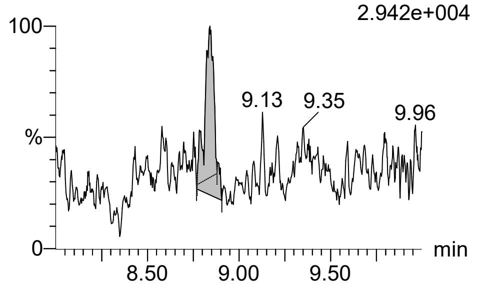

Sample Name: 20211111_H9_R2_IgM
F43:MRM of 1 channel,ES+
968.1 > 366.1
7.825e+004

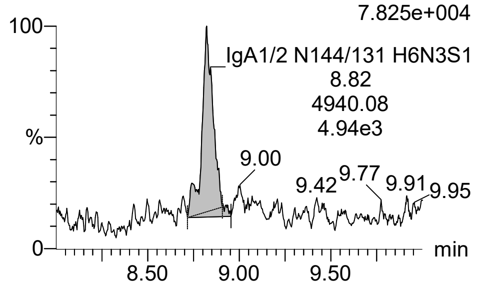

Sample Name: 20211111_A19_R2_IgM
F43:MRM of 1 channel,ES+
968.1 > 366.1
1.498e+005

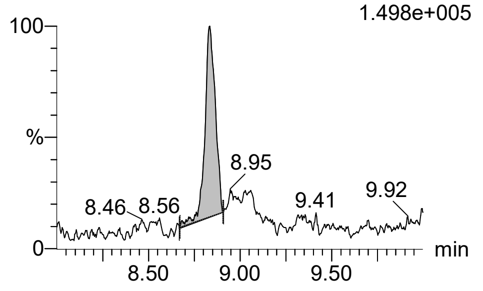

Sample Name: 20211111_A20_R2_IgM
F43:MRM of 1 channel,ES+
968.1 > 366.1
4.778e+004

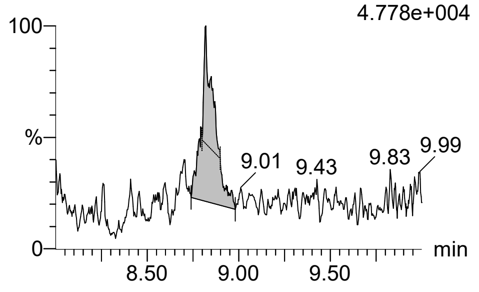

Sample Name: 20211111_L19_R2_IgM
F43:MRM of 1 channel,ES+
968.1 > 366.1
1.682e+005

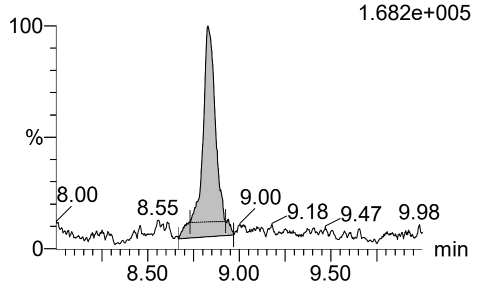

Sample Name: 20211111_QC_R13_IgM
F43:MRM of 1 channel,ES+
968.1 > 366.1
2.926e+005

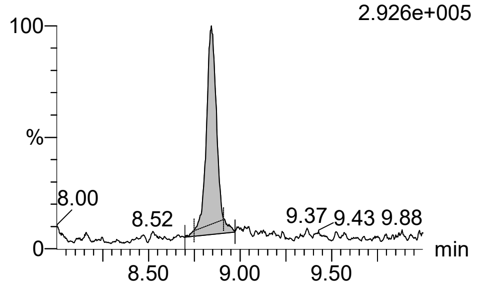

Dataset: C:\MassLynx\10.pro\20211111_clinicalsamples_IgM_IgA.qld

Last Altered: Thursday, March 09, 2023 19:58:25 台北標準時間
Printed: Friday, August 01, 2025 15:15:05 台北標準時間

Sample Name: 20211111_L20_R2_IgM
F43:MRM of 1 channel,ES+
968.1 > 366.1
4.476e+004

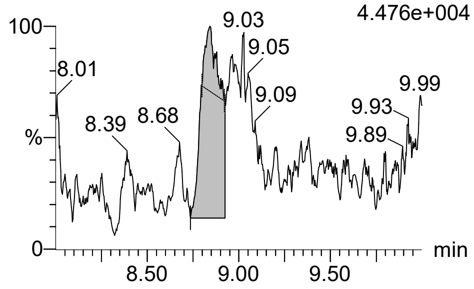

Sample Name: 20211111_H10_R2_IgM
F43:MRM of 1 channel,ES+
968.1 > 366.1
7.738e+004

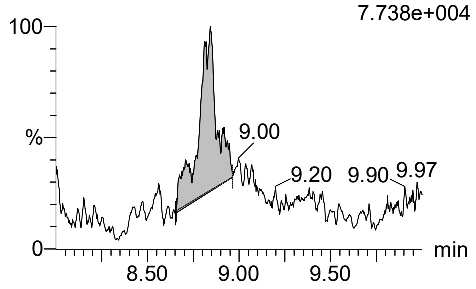

Sample Name: 20211111_A21_R2_IgM
F43:MRM of 1 channel,ES+
968.1 > 366.1
2.497e+005

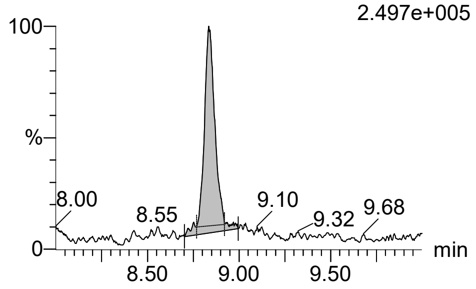

Sample Name: 20211111_A22_R2_IgM
F43:MRM of 1 channel,ES+
968.1 > 366.1
4.996e+004

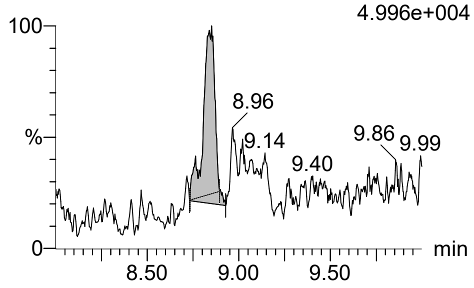

Sample Name: 20211111_L21_R2_IgM
F43:MRM of 1 channel,ES+
968.1 > 366.1
1.928e+005

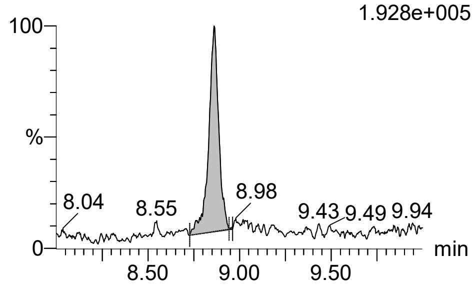

Sample Name: 20211111_L22_R2_IgM
F43:MRM of 1 channel,ES+
968.1 > 366.1
2.359e+005

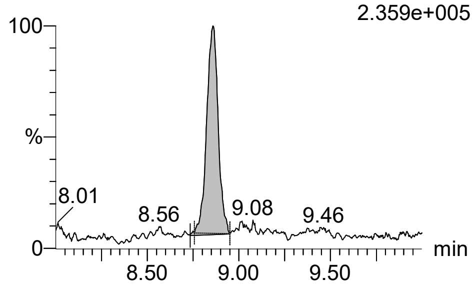

Sample Name: 20211111_H11_R2_IgM
F43:MRM of 1 channel,ES+
968.1 > 366.1
2.572e+005

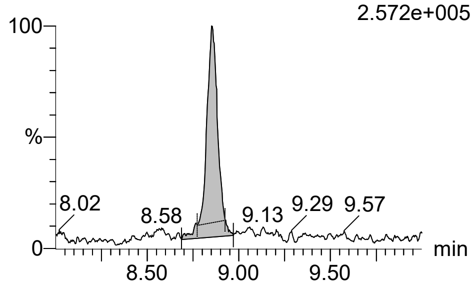

Sample Name: 20211111_A23_R2_IgM
F43:MRM of 1 channel,ES+
968.1 > 366.1
1.060e+005

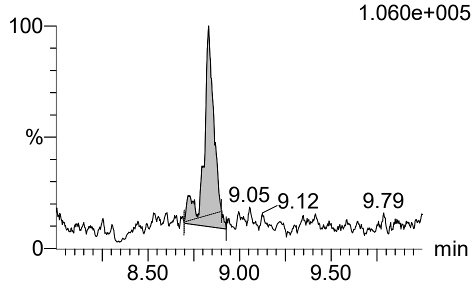

Sample Name: 20211111_A24_R2_IgM
F43:MRM of 1 channel,ES+
968.1 > 366.1
2.842e+005

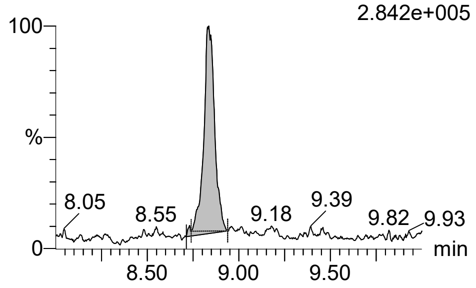

Sample Name: 20211111_L23_R2_IgM
F43:MRM of 1 channel,ES+
968.1 > 366.1
1.044e+005

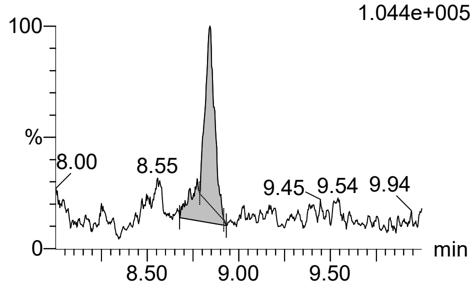

Sample Name: 20211111_L24_R2_IgM
F43:MRM of 1 channel,ES+
968.1 > 366.1
6.566e+004

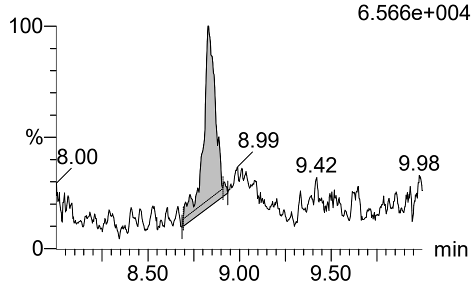

Sample Name: 20211111_H12_R2_IgM
F43:MRM of 1 channel,ES+
968.1 > 366.1
1.209e+005

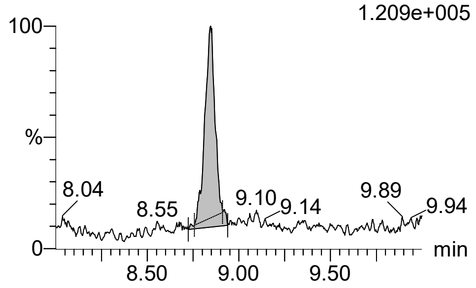

Sample Name: 20211111_QC_R14_IgM
F43:MRM of 1 channel,ES+
968.1 > 366.1
3.016e+005

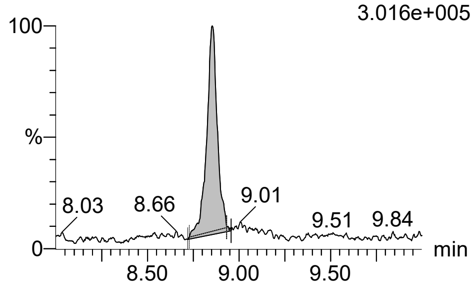

Sample Name: 20211111_A25_R2_IgM
F43:MRM of 1 channel,ES+
968.1 > 366.1
3.959e+005

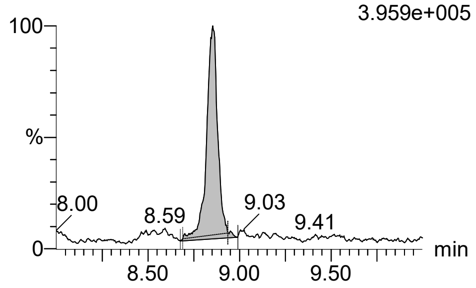

Sample Name: 20211111_A26_R2_IgM
F43:MRM of 1 channel,ES+
968.1 > 366.1
1.829e+005

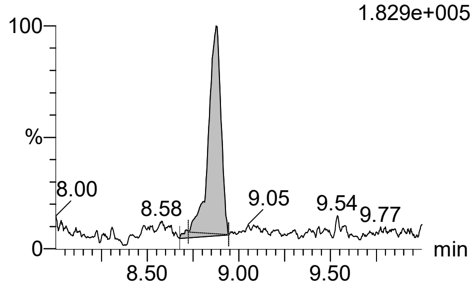

Sample Name: 20211111_L25_R2_IgM

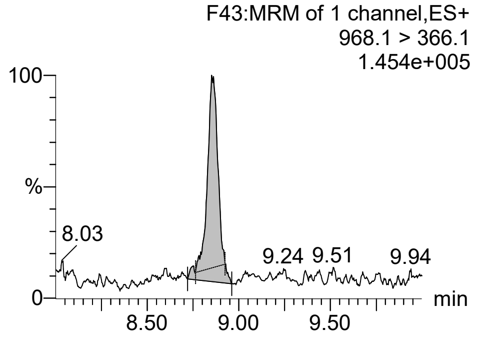

Sample Name: 20211111_L26_R2_IgM

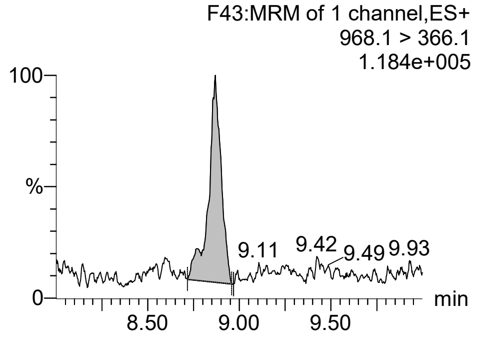

Sample Name: 20211111_H13_R2_IgM

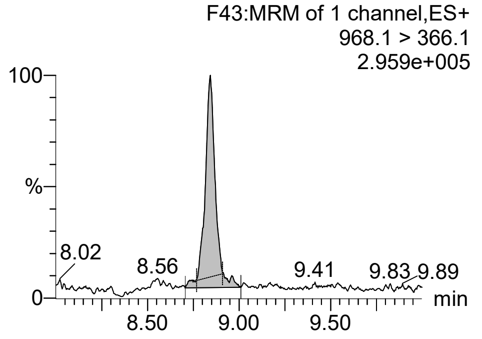

Sample Name: 20211111_A27_R2_IgM

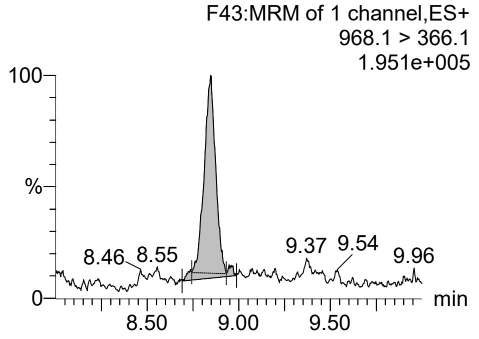

Sample Name: 20211111_A28_R2_IgM

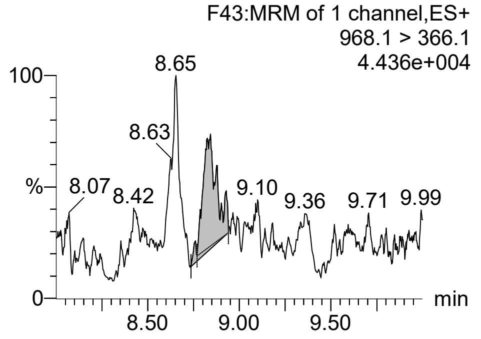

Sample Name: 20211111_L27_R2_IgM

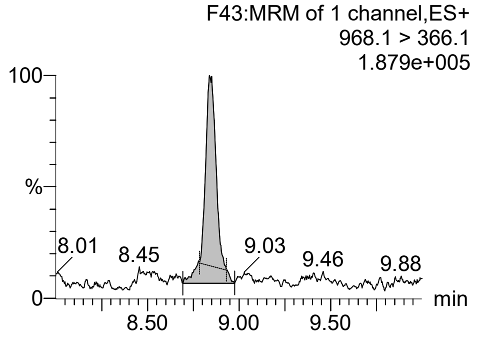

Sample Name: 20211111_L28_R2_IgM

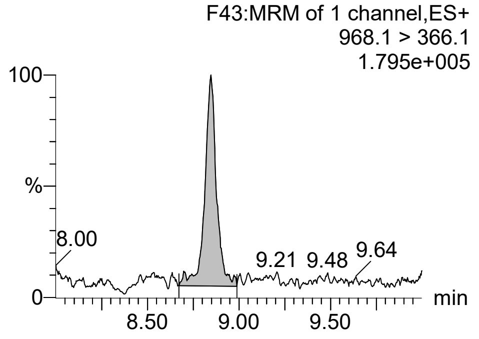

Sample Name: 20211111_H14_R2_IgM

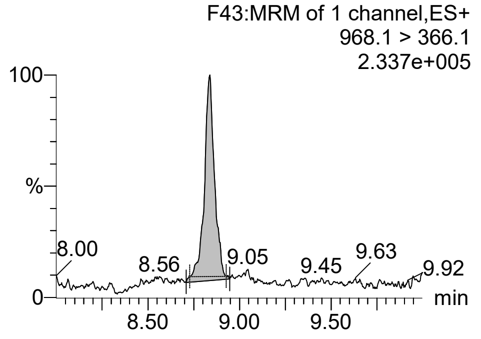

Sample Name: 20211111_A29_R2_IgM

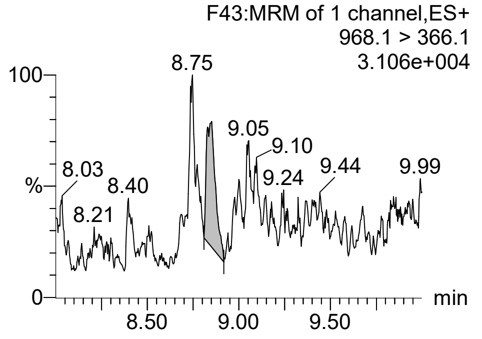

Sample Name: 20211111_A30_R2_IgM

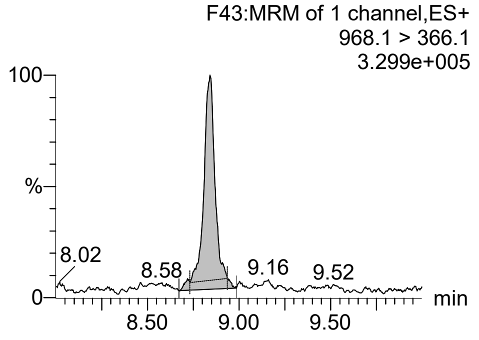

Sample Name: 20211111_QC_R15_IgM

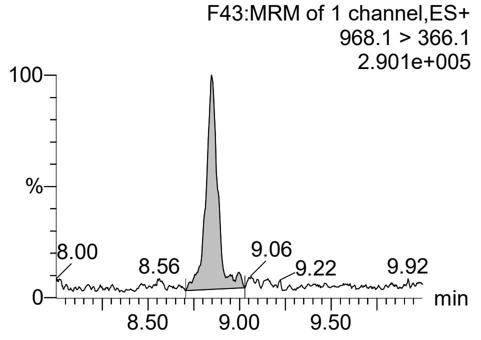

Sample Name: 20211111_L29_R2_IgM

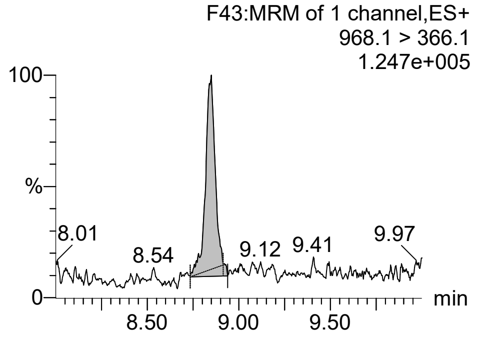

Sample Name: 20211111_L30_R2_IgM

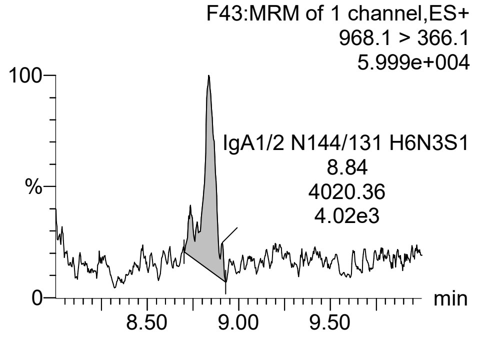

Sample Name: 20211111_H15_R2_IgM

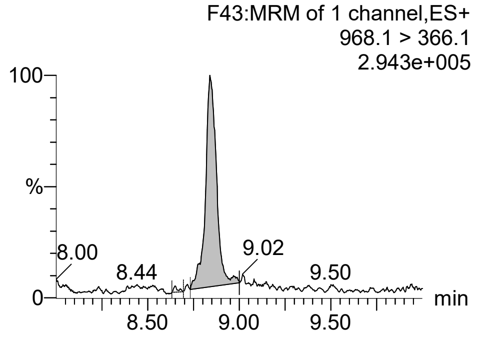

Sample Name: 20211111_A31_R2_IgM

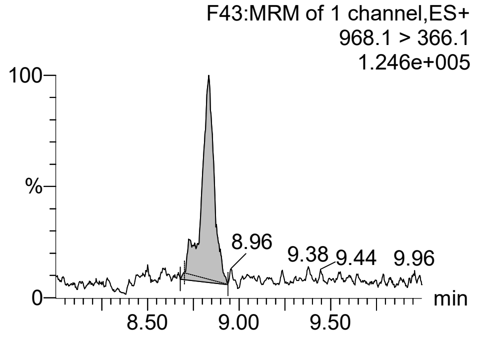

Dataset: C:\MassLynx\10.pro\20211111_clinicalsamples_IgM_IgA.qld

Last Altered: Thursday, March 09, 2023 19:58:25 台北標準時間
Printed: Friday, August 01, 2025 15:15:05 台北標準時間

Sample Name: 20211111_A32_R2_IgM
F43:MRM of 1 channel,ES+
968.1 > 366.1
3.374e+005

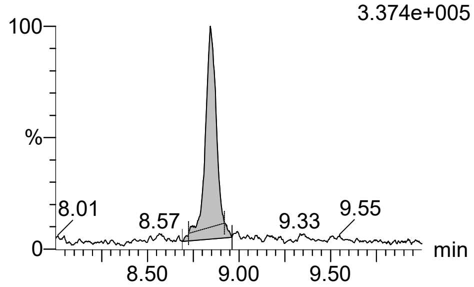

Sample Name: 20211111_L31_R2_IgM
F43:MRM of 1 channel,ES+
968.1 > 366.1
2.138e+004

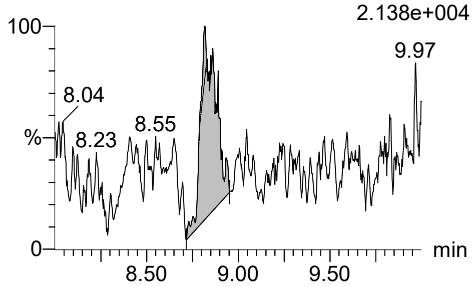

Sample Name: 20211111_L32_R2_IgM
F43:MRM of 1 channel,ES+
968.1 > 366.1
1.014e+005

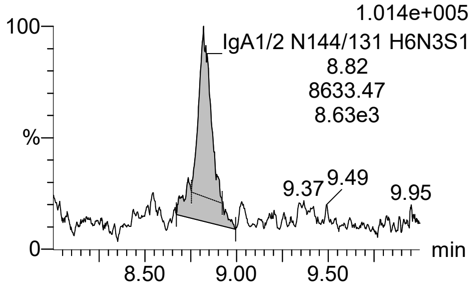

Sample Name: 20211111_H16_R2_IgM
F43:MRM of 1 channel,ES+
968.1 > 366.1
3.742e+005

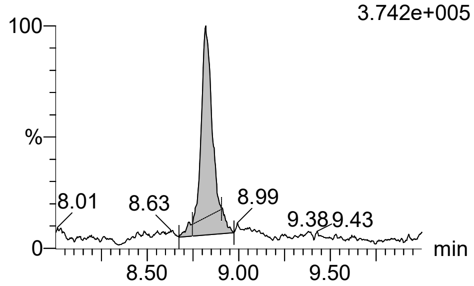

Sample Name: 20211111_A33_R2_IgM
F43:MRM of 1 channel,ES+
968.1 > 366.1
2.893e+005

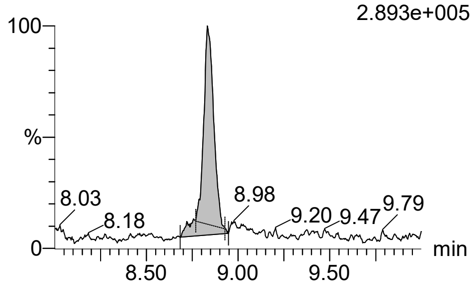

Sample Name: 20211111_A34_R2_IgM
F43:MRM of 1 channel,ES+
968.1 > 366.1
1.355e+005

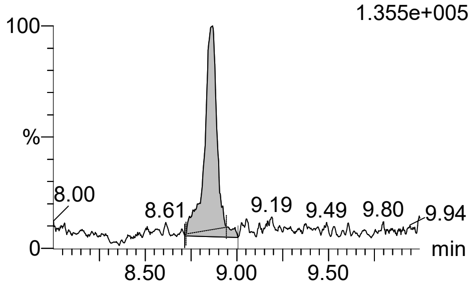

Sample Name: 20211111_H17_R2_IgM
F43:MRM of 1 channel,ES+
968.1 > 366.1
8.579e+005

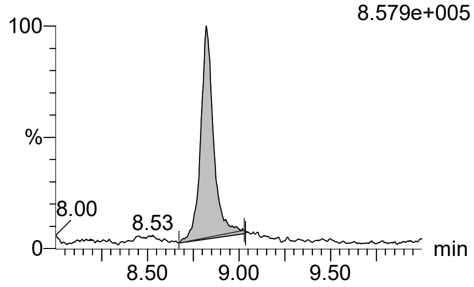

Sample Name: 20211111_A35_R2_IgM
F43:MRM of 1 channel,ES+
968.1 > 366.1
3.911e+004

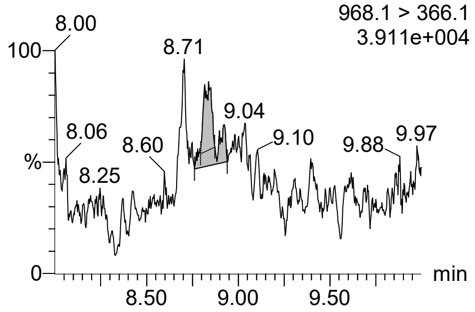

Sample Name: 20211111_QC_R16_IgM
F43:MRM of 1 channel,ES+
968.1 > 366.1
2.292e+005

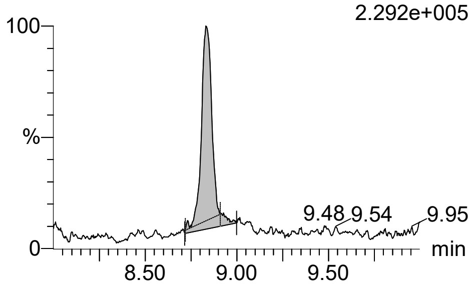

Sample Name: 20211111_A36_R2_IgM
F43:MRM of 1 channel,ES+
968.1 > 366.1
4.261e+004

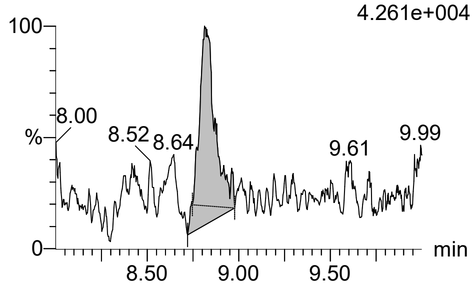

Sample Name: 20211111_H18_R2_IgM
F43:MRM of 1 channel,ES+
968.1 > 366.1
3.661e+005

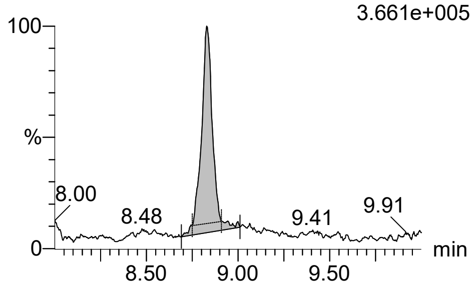

Sample Name: 20211111_A37_R2_IgM
F43:MRM of 1 channel,ES+
968.1 > 366.1
2.395e+005

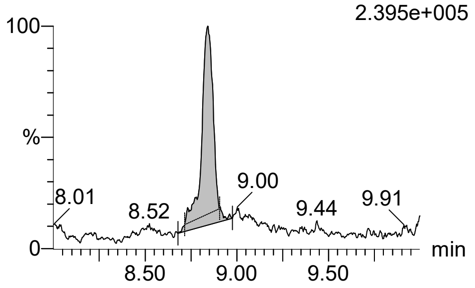

Sample Name: 20211111_A38_R2_IgM
F43:MRM of 1 channel,ES+
968.1 > 366.1
2.362e+005

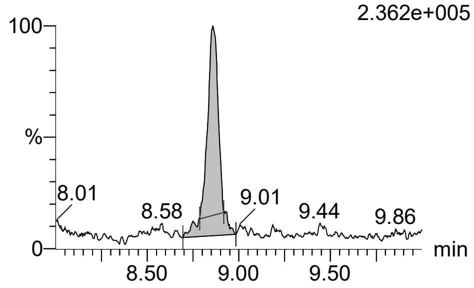

Sample Name: 20211111_H19_R2_IgM
F43:MRM of 1 channel,ES+
968.1 > 366.1
3.383e+005

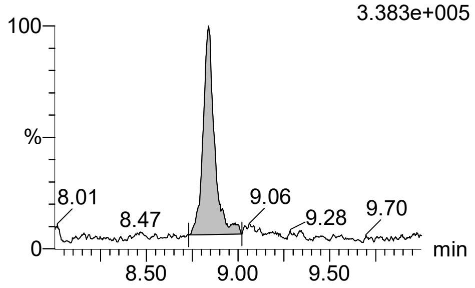

Sample Name: 20211111_A39_R2_IgM
F43:MRM of 1 channel,ES+
968.1 > 366.1
3.006e+005

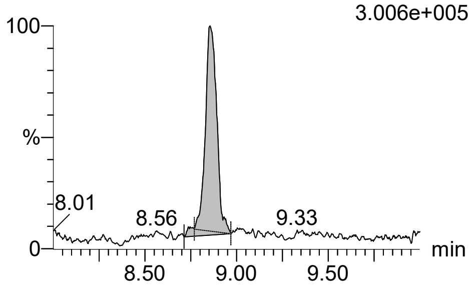

Sample Name: 20211111_A40_R2_IgM
F43:MRM of 1 channel,ES+
968.1 > 366.1
5.862e+004

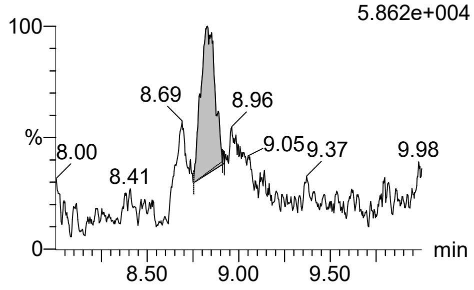

Sample Name: 20211111_H20_R2_IgM
F43:MRM of 1 channel,ES+
968.1 > 366.1
4.697e+005

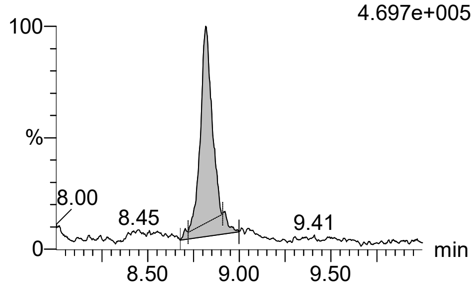

Sample Name: 20211111_A41_R2_IgM
F43:MRM of 1 channel,ES+
968.1 > 366.1
1.380e+005

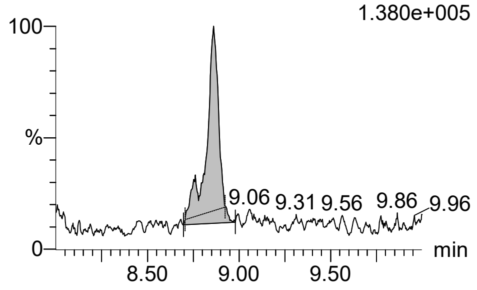

Sample Name: 20211111_A42_R2_IgM
F43:MRM of 1 channel,ES+
968.1 > 366.1
1.578e+005

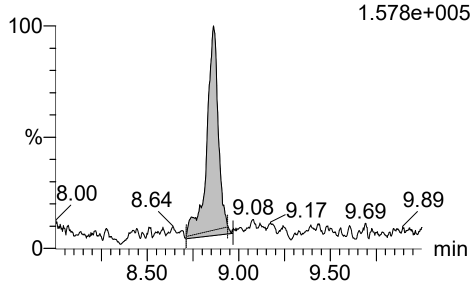

Sample Name: 20211111_A43_R2_IgM
F43:MRM of 1 channel,ES+
968.1 > 366.1
1.197e+005

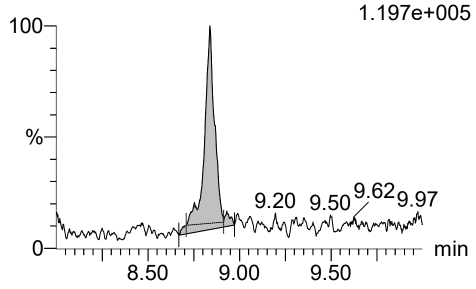

Sample Name: 20211111_A44_R2_IgM
F43:MRM of 1 channel,ES+
968.1 > 366.1
4.171e+004

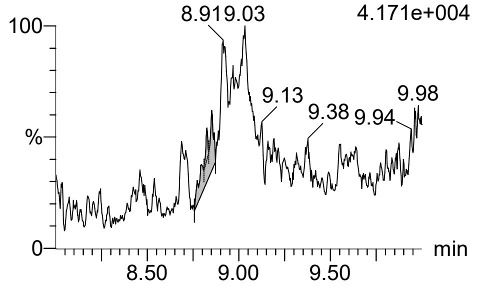

Sample Name: 20211111_QC_R17_IgM
F43:MRM of 1 channel,ES+
968.1 > 366.1
2.252e+005

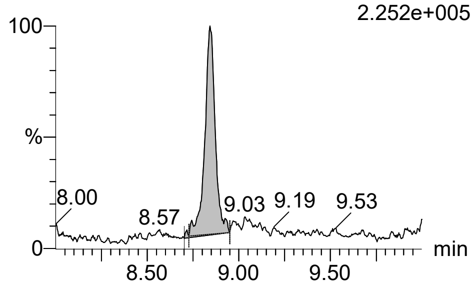

Sample Name: 20211111_L2_R3_IgM
F43:MRM of 1 channel,ES+
968.1 > 366.1
4.008e+005

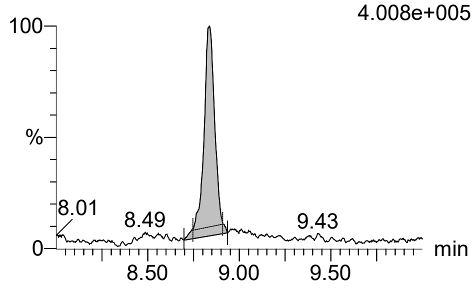

Sample Name: 20211111_H1_R3_IgM
F43:MRM of 1 channel,ES+
968.1 > 366.1
3.026e+005

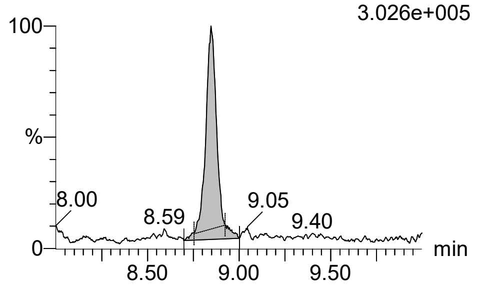

Sample Name: 20211111_A3_R3_IgM
F43:MRM of 1 channel,ES+
968.1 > 366.1
3.289e+005

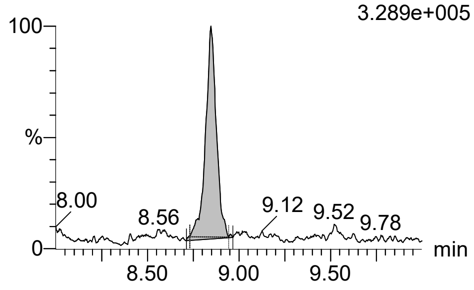

Sample Name: 20211111_A4_R3_IgM
F43:MRM of 1 channel,ES+
968.1 > 366.1
1.987e+005

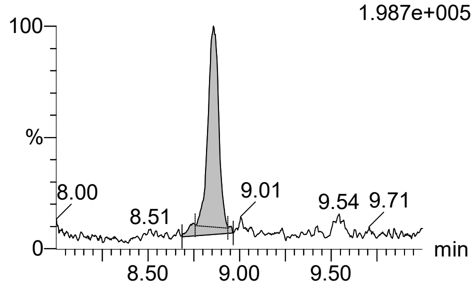

Sample Name: 20211111_L3_R3_IgM
F43:MRM of 1 channel,ES+
968.1 > 366.1
3.558e+004

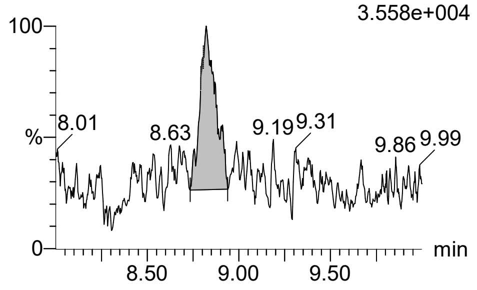

Sample Name: 20211111_L4_R3_IgM
F43:MRM of 1 channel,ES+
968.1 > 366.1
2.266e+005

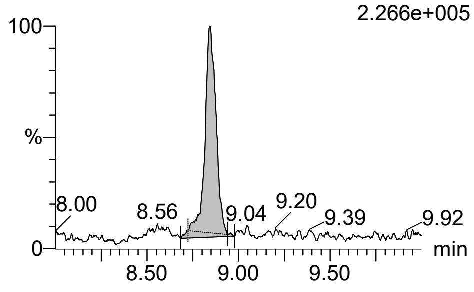

Sample Name: 20211111_H2_R3_IgM
F43:MRM of 1 channel,ES+
968.1 > 366.1
4.516e+005

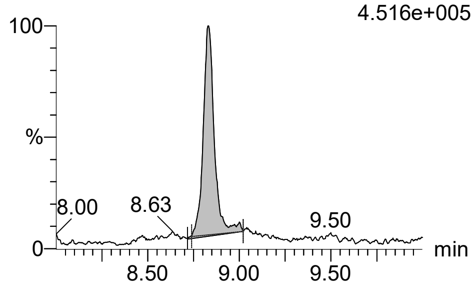

Sample Name: 20211111_A5_R3_IgM
F43:MRM of 1 channel,ES+
968.1 > 366.1
6.109e+005

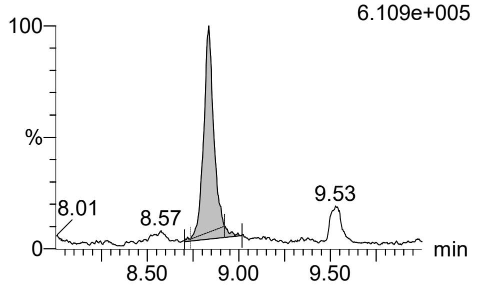

Dataset: C:\MassLynx\10.pro\20211111_clinicalsamples_IgM_IgA.qld

Last Altered: Thursday, March 09, 2023 19:58:25 台北標準時間
Printed: Friday, August 01, 2025 15:15:05 台北標準時間

Sample Name: 20211111_A6_R3_IgM
F43:MRM of 1 channel,ES+
968.1 > 366.1
2.274e+005

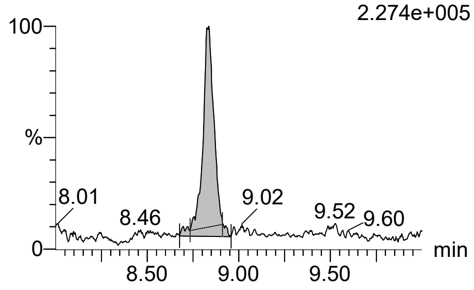

Sample Name: 20211111_QC_R18_IgM
F43:MRM of 1 channel,ES+
968.1 > 366.1
2.166e+005

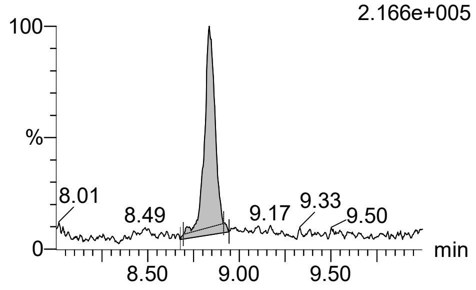

Sample Name: 20211111_L5_R3_IgM
F43:MRM of 1 channel,ES+
968.1 > 366.1
3.405e+005

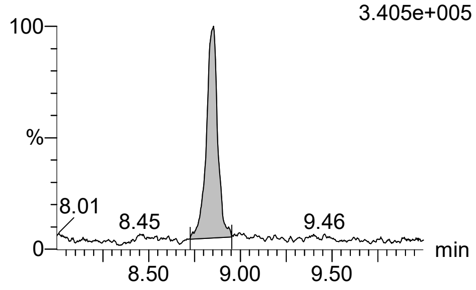

Sample Name: 20211111_L6_R3_IgM
F43:MRM of 1 channel,ES+
968.1 > 366.1
3.679e+005

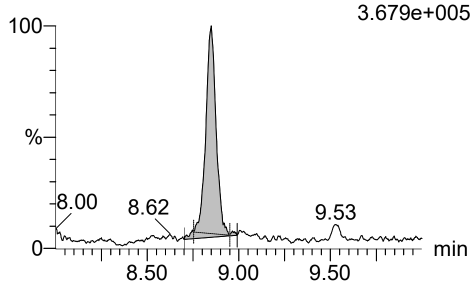

Sample Name: 20211111_H3_R3_IgM
F43:MRM of 1 channel,ES+
968.1 > 366.1
2.355e+005

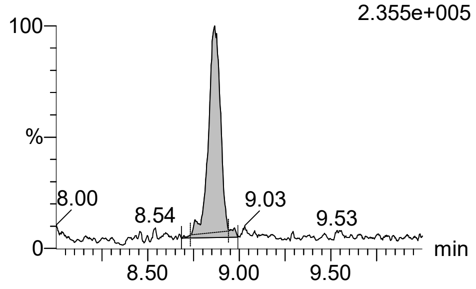

Sample Name: 20211111_A7_R3_IgM
F43:MRM of 1 channel,ES+
968.1 > 366.1
4.858e+005

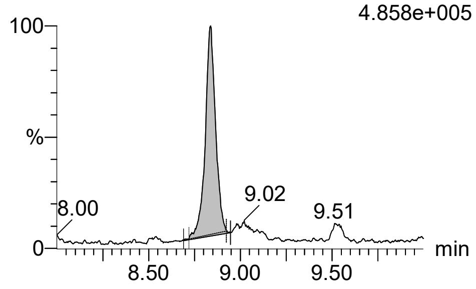

Sample Name: 20211111_A8_R3_IgM
F43:MRM of 1 channel,ES+
968.1 > 366.1
3.712e+005

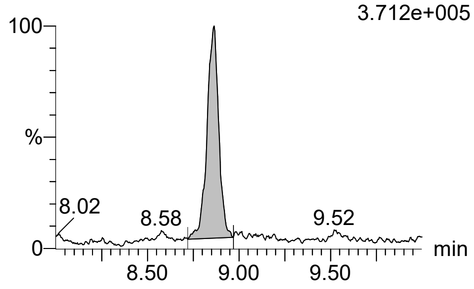

Sample Name: 20211111_L7_R3_IgM
F43:MRM of 1 channel,ES+
968.1 > 366.1
5.366e+004

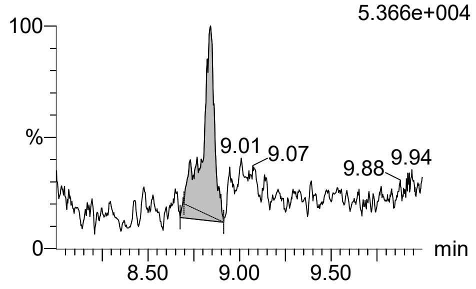

Sample Name: 20211111_L8_R3_IgM
F43:MRM of 1 channel,ES+
968.1 > 366.1
1.141e+005

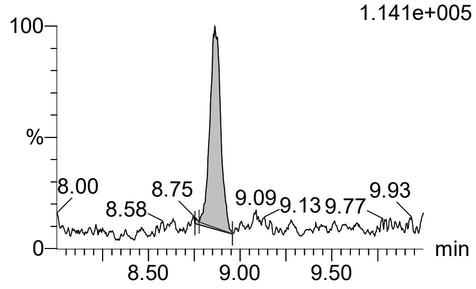

Sample Name: 20211111_H4_R3_IgM
F43:MRM of 1 channel,ES+
968.1 > 366.1
2.461e+005

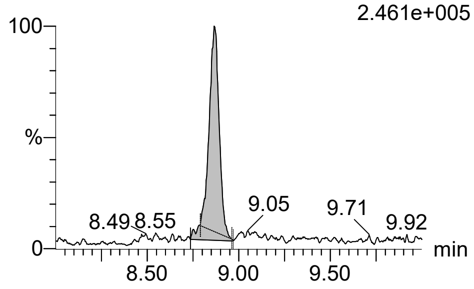

Sample Name: 20211111_A9_R3_IgM
F43:MRM of 1 channel,ES+
968.1 > 366.1
3.055e+005

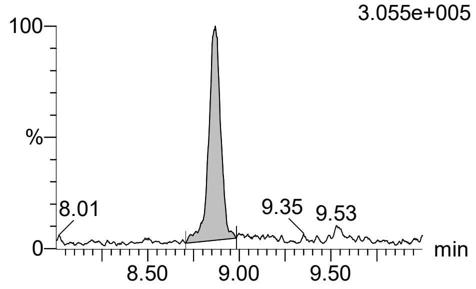

Sample Name: 20211111_A10_R3_IgM
F43:MRM of 1 channel,ES+
968.1 > 366.1
6.380e+005

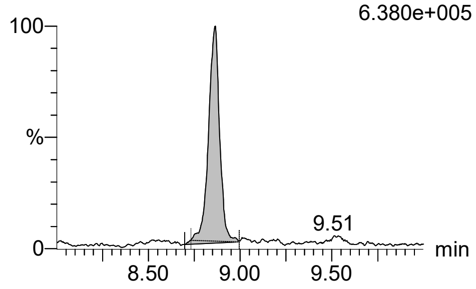

Sample Name: 20211111_L9_R3_IgM
F43:MRM of 1 channel,ES+
968.1 > 366.1
1.683e+005

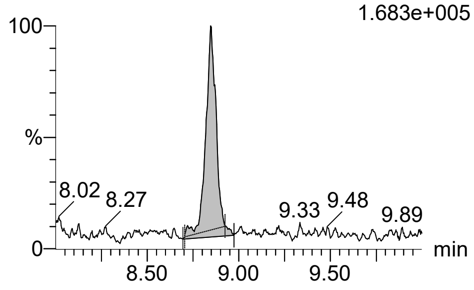

Sample Name: 20211111_L10_R3_IgM
F43:MRM of 1 channel,ES+
968.1 > 366.1
7.937e+004

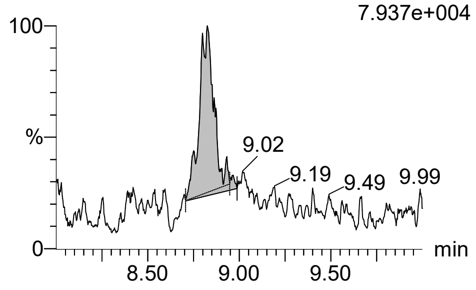

Sample Name: 20211111_QC_R19_IgM
F43:MRM of 1 channel,ES+
968.1 > 366.1
2.091e+005

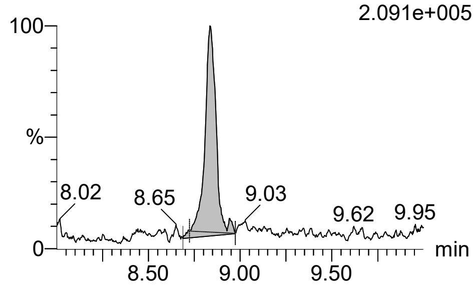

Dataset: C:\MassLynx\10.pro\20211111_clinicalsamples_IgM_IgA.qld

Last Altered: Thursday, March 09, 2023 19:58:25 台北標準時間
Printed: Friday, August 01, 2025 15:15:05 台北標準時間

Sample Name: 20211111_H5_R3_IgM
F43:MRM of 1 channel,ES+
968.1 > 366.1
2.071e+005

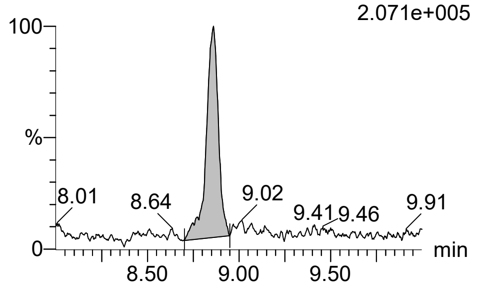

Sample Name: 20211111_A11_R3_IgM
F43:MRM of 1 channel,ES+
968.1 > 366.1
2.895e+005

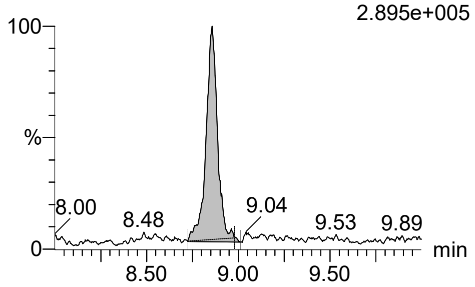

Sample Name: 20211111_A12_R3_IgM
F43:MRM of 1 channel,ES+
968.1 > 366.1
1.602e+005

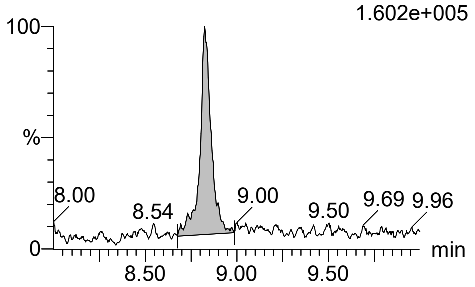

Sample Name: 20211111_L11_R3_IgM
F43:MRM of 1 channel,ES+
968.1 > 366.1
9.092e+004

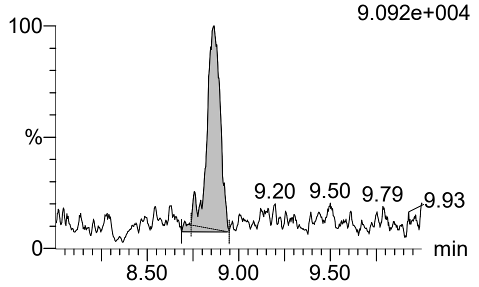

Sample Name: 20211111_L12_R3_IgM
F43:MRM of 1 channel,ES+
968.1 > 366.1
4.133e+004

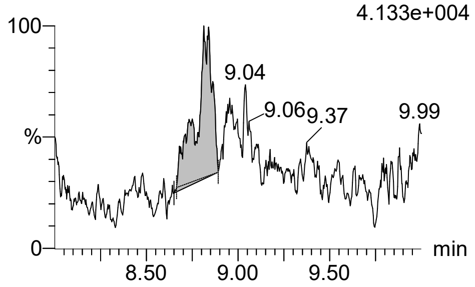

Sample Name: 20211111_H6_R3_IgM
F43:MRM of 1 channel,ES+
968.1 > 366.1
1.573e+005

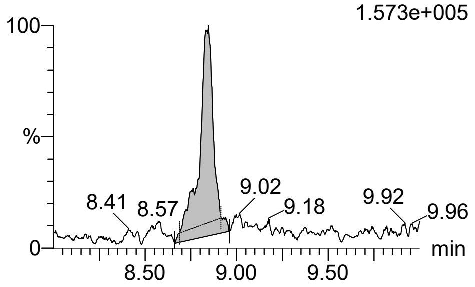

Sample Name: 20211111_A13_R3_IgM
F43:MRM of 1 channel,ES+
968.1 > 366.1
1.474e+005

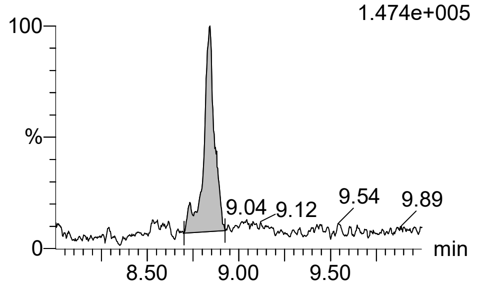

Sample Name: 20211111_A14_R3_IgM
F43:MRM of 1 channel,ES+
968.1 > 366.1
6.097e+005

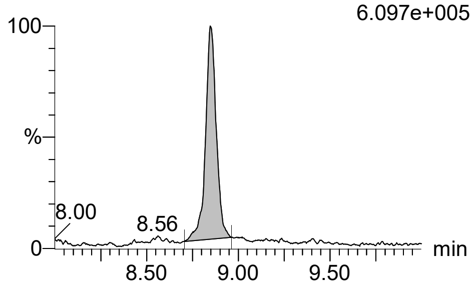

Sample Name: 20211111_L13_R3_IgM
F43:MRM of 1 channel,ES+
968.1 > 366.1
1.128e+005

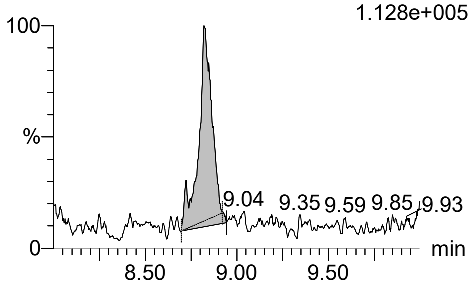

Sample Name: 20211111_L14_R3_IgM
F43:MRM of 1 channel,ES+
968.1 > 366.1
3.176e+005

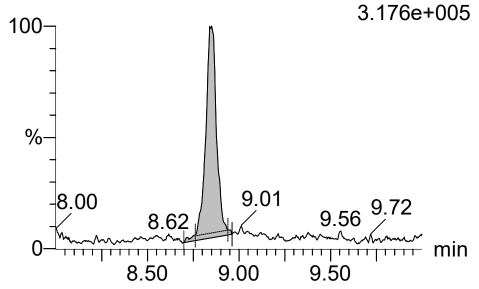

Sample Name: 20211111_H7_R3_IgM
F43:MRM of 1 channel,ES+
968.1 > 366.1
3.325e+005

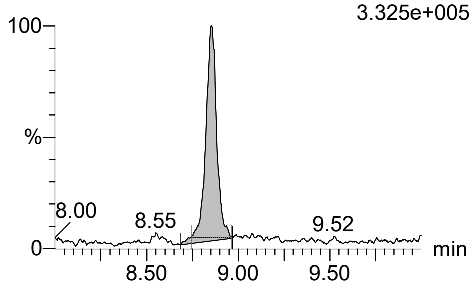

Sample Name: 20211111_A15_R3_IgM
F43:MRM of 1 channel,ES+
968.1 > 366.1
5.376e+005

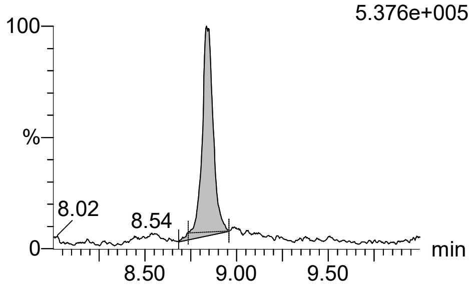

Sample Name: 20211111_QC_R20_IgM
F43:MRM of 1 channel,ES+
968.1 > 366.1
2.676e+005

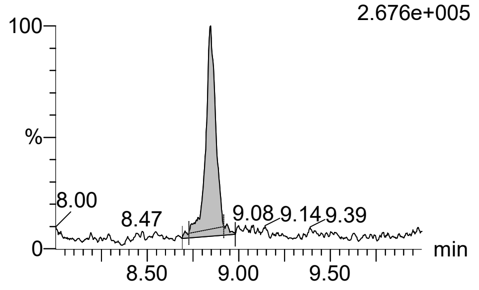

Sample Name: 20211111_A16_R3_IgM
F43:MRM of 1 channel,ES+
968.1 > 366.1
2.978e+005

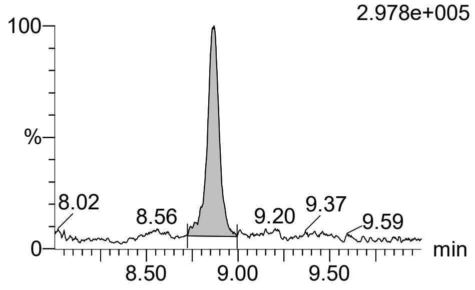

Sample Name: 20211111_L15_R3_IgM
F43:MRM of 1 channel,ES+
968.1 > 366.1
3.905e+005

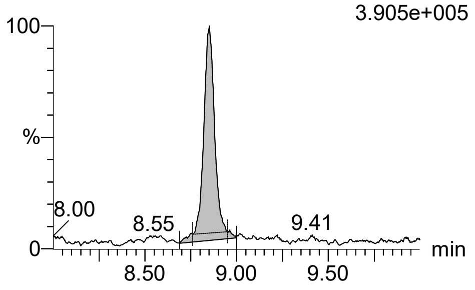

Dataset: C:\MassLynx\10.pro\20211111_clinicalsamples_IgM_IgA.qld

Last Altered: Thursday, March 09, 2023 19:58:25 台北標準時間
Printed: Friday, August 01, 2025 15:15:05 台北標準時間

Sample Name: 20211111_L16_R3_IgM
F43:MRM of 1 channel,ES+
968.1 > 366.1
1.130e+005

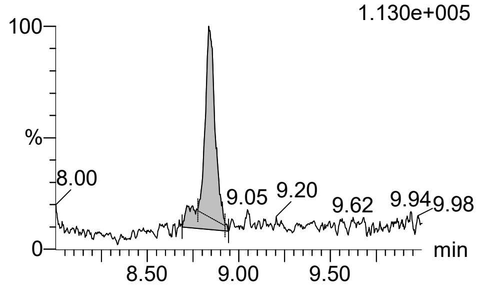

Sample Name: 20211111_H8_R3_IgM
F43:MRM of 1 channel,ES+
968.1 > 366.1
1.981e+005

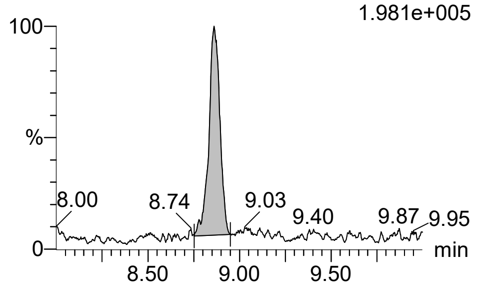

Sample Name: 20211111_A17_R3_IgM
F43:MRM of 1 channel,ES+
968.1 > 366.1
4.260e+005

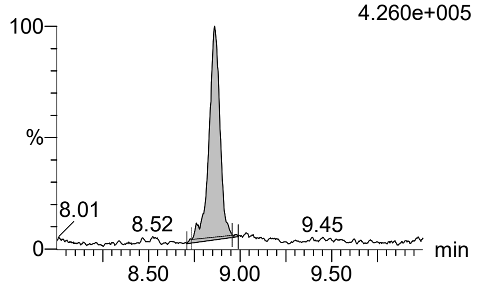

Sample Name: 20211111_A18_R3_IgM
F43:MRM of 1 channel,ES+
968.1 > 366.1
7.389e+004

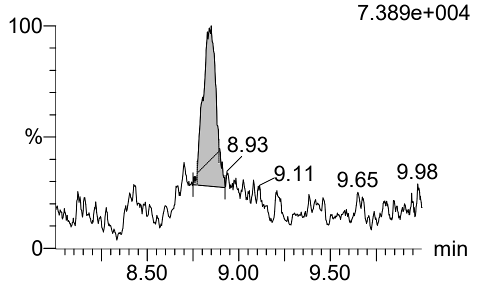

Sample Name: 20211111_L17_R3_IgM
F43:MRM of 1 channel,ES+
968.1 > 366.1
2.419e+005

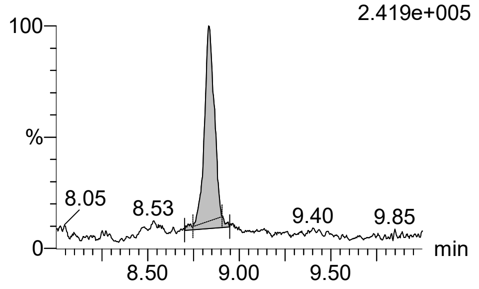

Sample Name: 20211111_L18_R3_IgM
F43:MRM of 1 channel,ES+
968.1 > 366.1
3.237e+004

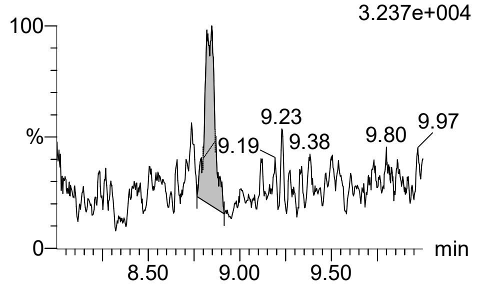

Sample Name: 20211111_H9_R3_IgM
F43:MRM of 1 channel,ES+
968.1 > 366.1
9.386e+004

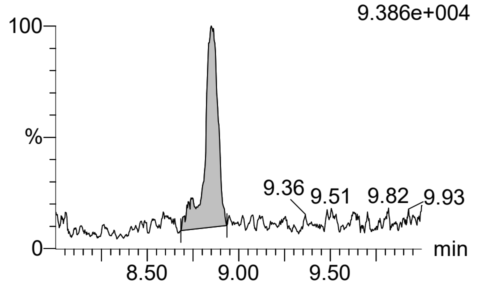

Sample Name: 20211111_A19_R3_IgM
F43:MRM of 1 channel,ES+
968.1 > 366.1
1.468e+005

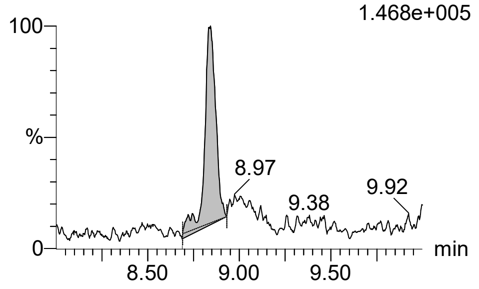

Sample Name: 20211111_A20_R3_IgM
F43:MRM of 1 channel,ES+
968.1 > 366.1
3.156e+004

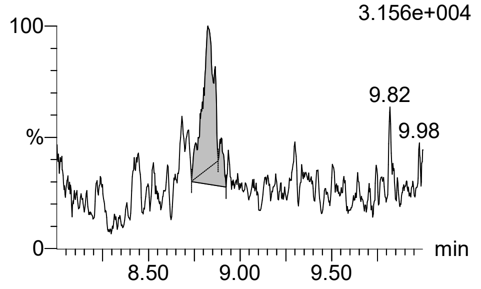

Sample Name: 20211111_L19_R3_IgM
F43:MRM of 1 channel,ES+
968.1 > 366.1
2.118e+005

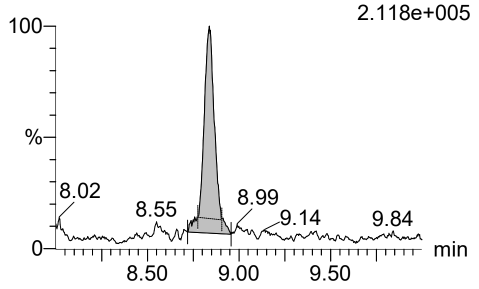

Sample Name: 20211111_QC_R21_IgM
F43:MRM of 1 channel,ES+
968.1 > 366.1
2.546e+005

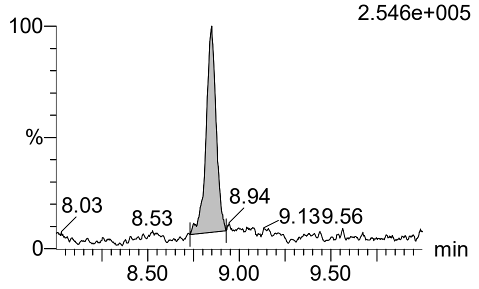

Sample Name: 20211111_L20_R3_IgM
F43:MRM of 1 channel,ES+
968.1 > 366.1
1.563e+005

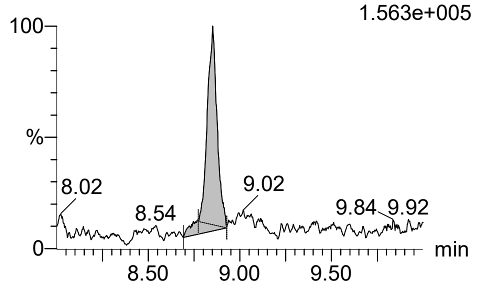

Sample Name: 20211111_H10_R3_IgM
F43:MRM of 1 channel,ES+
968.1 > 366.1
2.801e+005

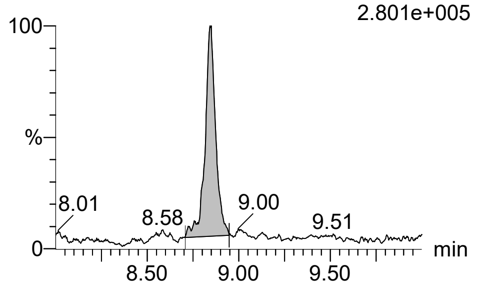

Sample Name: 20211111_A21_R3_IgM
F43:MRM of 1 channel,ES+
968.1 > 366.1
2.877e+005

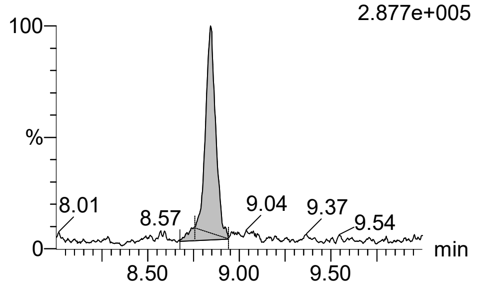

Sample Name: 20211111_A22_R3_IgM
F43:MRM of 1 channel,ES+
968.1 > 366.1
5.808e+004

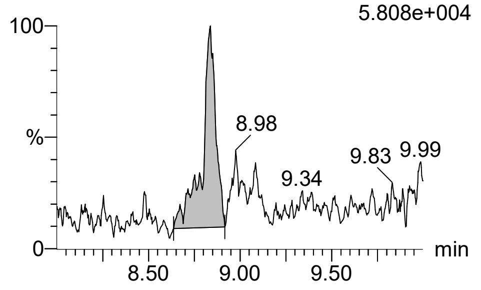

Sample Name: 20211111_L21_R3_IgM
F43:MRM of 1 channel,ES+
968.1 > 366.1
1.583e+005

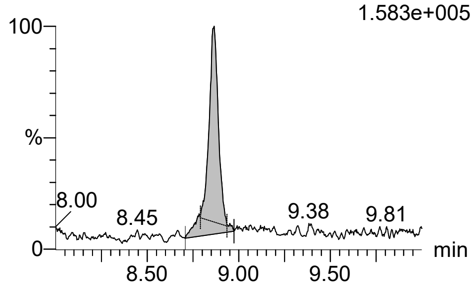

Sample Name: 20211111_L22_R3_IgM
F43:MRM of 1 channel,ES+
968.1 > 366.1
2.626e+005

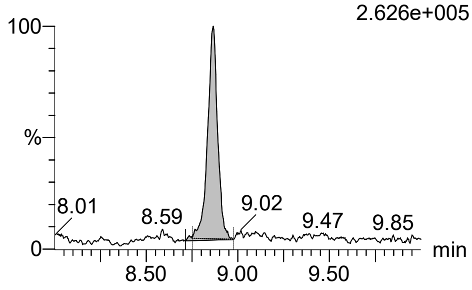

Sample Name: 20211111_H11_R3_IgM
F43:MRM of 1 channel,ES+
968.1 > 366.1
2.739e+005

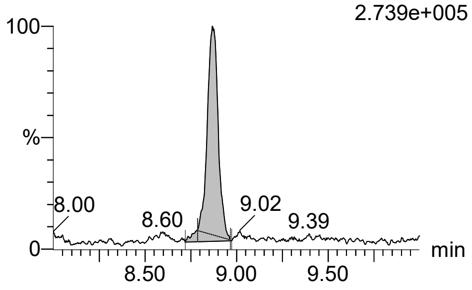

Sample Name: 20211111_A23_R3_IgM
F43:MRM of 1 channel,ES+
968.1 > 366.1
8.642e+004

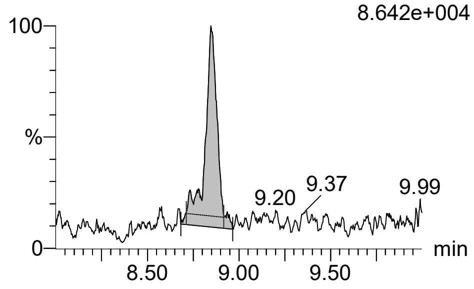

Sample Name: 20211111_A24_R3_IgM
F43:MRM of 1 channel,ES+
968.1 > 366.1
2.908e+005

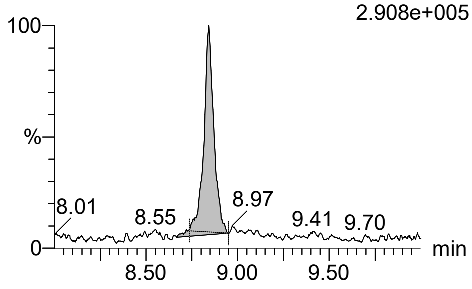

Sample Name: 20211111_L23_R3_IgM
F43:MRM of 1 channel,ES+
968.1 > 366.1
6.590e+004

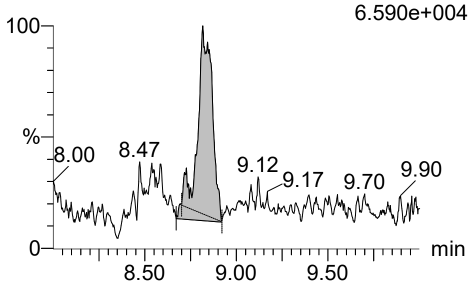

Sample Name: 20211111_L24_R3_IgM
F43:MRM of 1 channel,ES+
968.1 > 366.1
1.185e+005

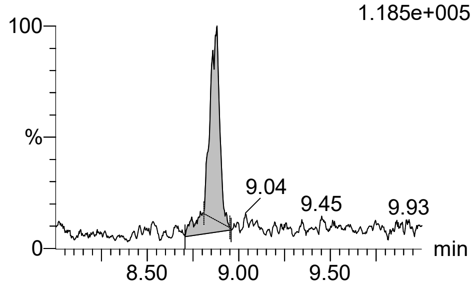

Sample Name: 20211111_H12_R3_IgM
F43:MRM of 1 channel,ES+
968.1 > 366.1
1.360e+005

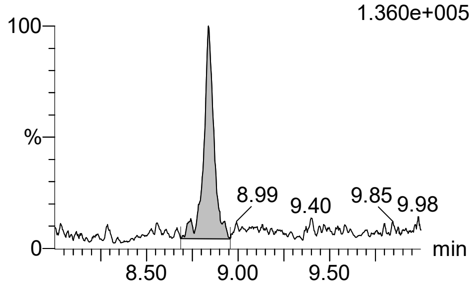

Sample Name: 20211111_QC_R22_IgM
F43:MRM of 1 channel,ES+
968.1 > 366.1
2.417e+005

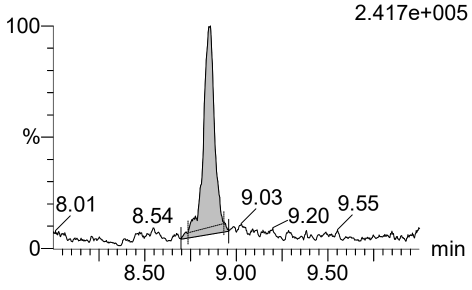

Sample Name: 20211111_A25_R3_IgM
F43:MRM of 1 channel,ES+
968.1 > 366.1
3.287e+005

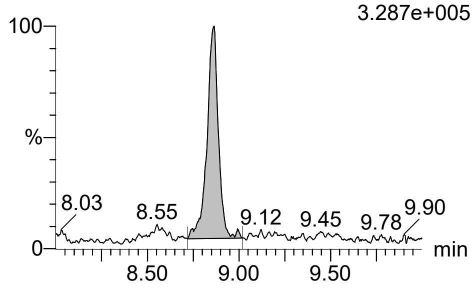

Sample Name: 20211111_A26_R3_IgM
F43:MRM of 1 channel,ES+
968.1 > 366.1
1.901e+005

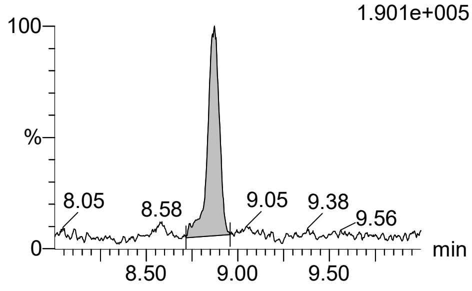

Sample Name: 20211111_L25_R3_IgM
F43:MRM of 1 channel,ES+
968.1 > 366.1
1.290e+005

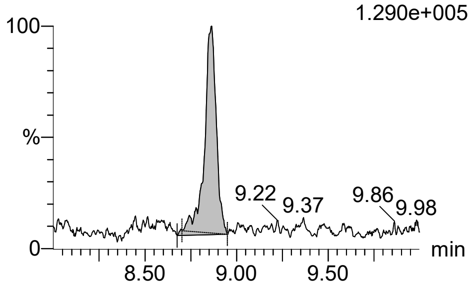

Sample Name: 20211111_L26_R3_IgM
F43:MRM of 1 channel,ES+
968.1 > 366.1
1.056e+005

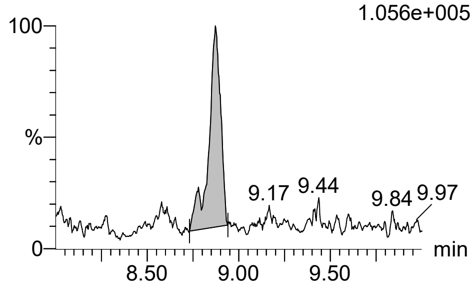

Sample Name: 20211111_H13_R3_IgM
F43:MRM of 1 channel,ES+
968.1 > 366.1
2.682e+005

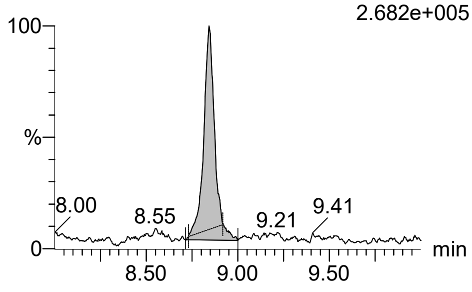

Sample Name: 20211111_A27_R3_IgM
F43:MRM of 1 channel,ES+
968.1 > 366.1
2.279e+005

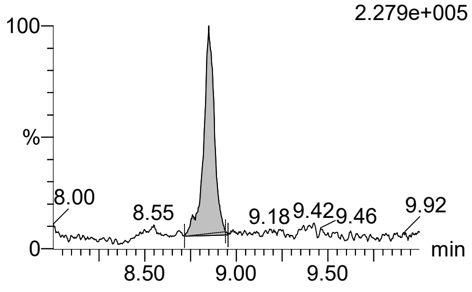

Dataset: C:\MassLynx\10.pro\20211111_clinicalsamples_IgM_IgA.qld

Last Altered: Thursday, March 09, 2023 19:58:25 台北標準時間
Printed: Friday, August 01, 2025 15:15:05 台北標準時間

Sample Name: 20211111_A28_R3_IgM

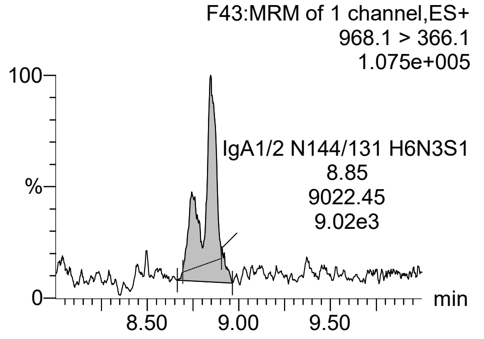

Sample Name: 20211111_L27_R3_IgM

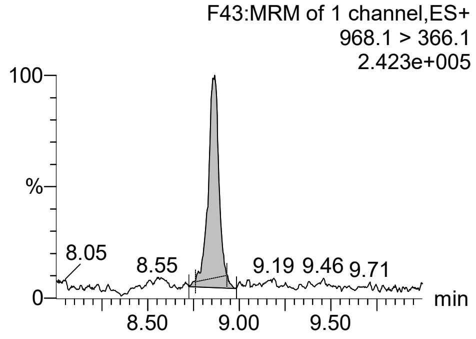

Sample Name: 20211111_L28_R3_IgM

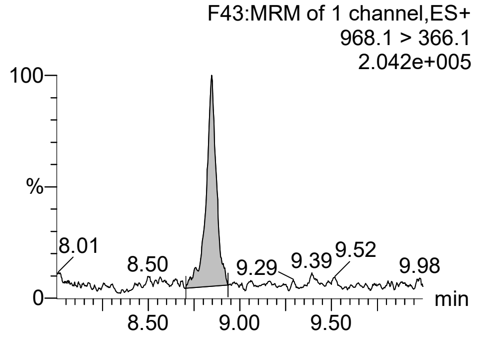

Sample Name: 20211111_H14_R3_IgM

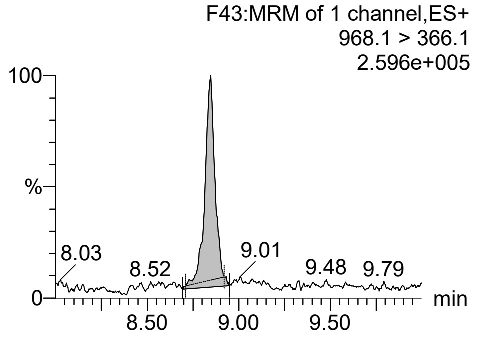

Sample Name: 20211111_A29_R3_IgM

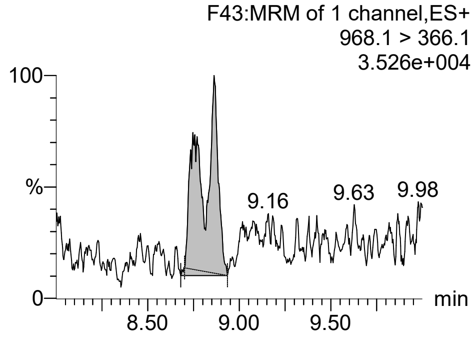

Sample Name: 20211111_A30_R3_IgM

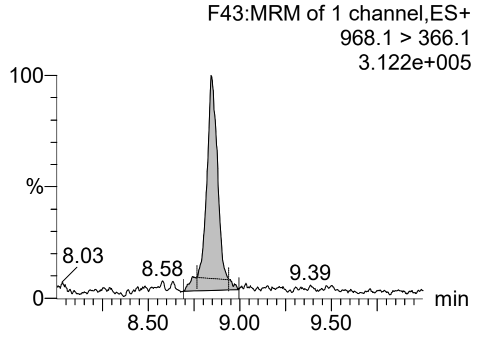

Sample Name: 20211111_QC_R23_IgM

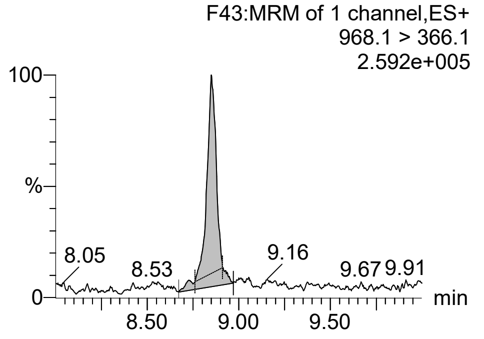

Sample Name: 20211111_L29_R3_IgM

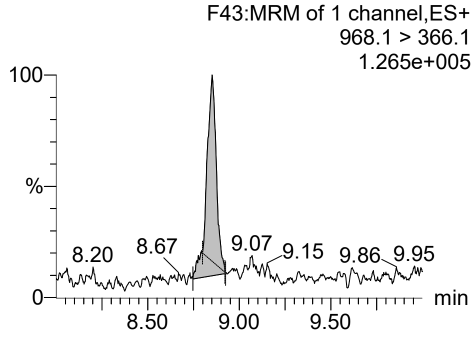

Sample Name: 20211111_L30_R3_IgM

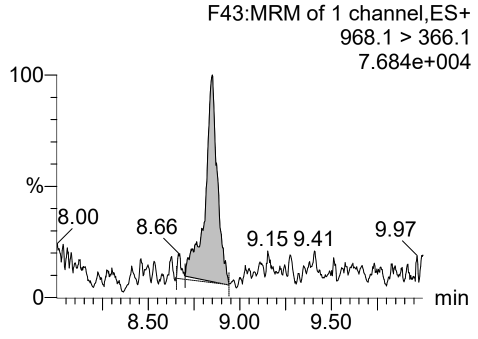

Sample Name: 20211111_H15_R3_IgM

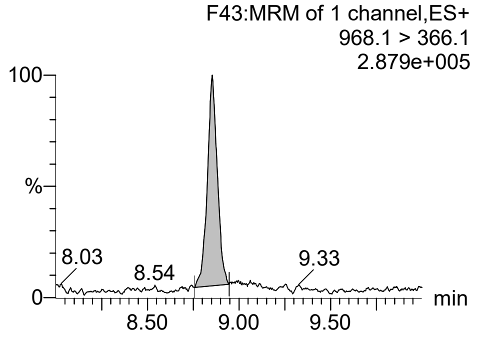

Sample Name: 20211111_A31_R3_IgM

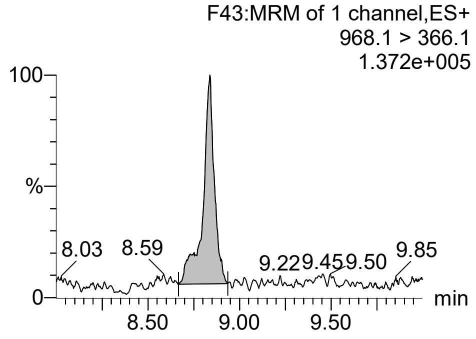

Sample Name: 20211111_A32_R3_IgM

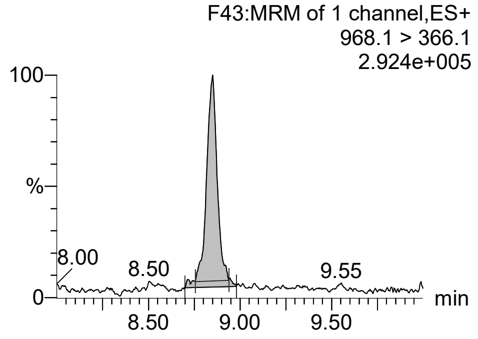

Sample Name: 20211111_L31_R3_IgM

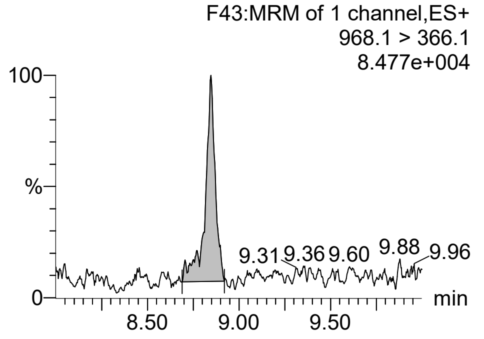

Sample Name: 20211111_L32_R3_IgM

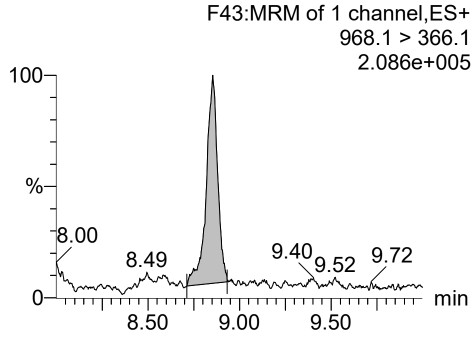

Sample Name: 20211111_H16_R3_IgM

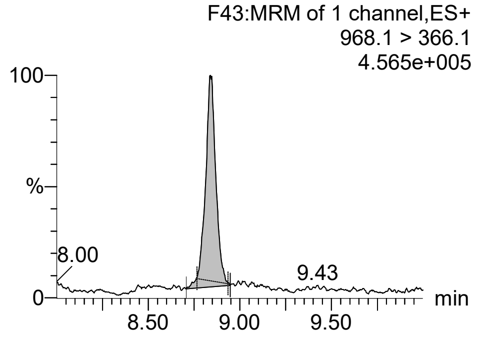

Dataset: C:\MassLynx\10.pro\20211111_clinicalsamples_IgM_IgA.qld

Last Altered: Thursday, March 09, 2023 19:58:25 台北標準時間
Printed: Friday, August 01, 2025 15:15:05 台北標準時間

Sample Name: 20211111_A33_R3_IgM
F43:MRM of 1 channel,ES+
968.1 > 366.1
2.837e+005

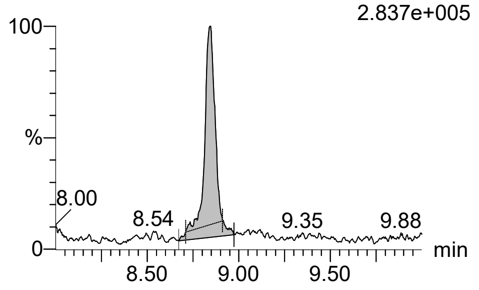

Sample Name: 20211111_A34_R3_IgM
F43:MRM of 1 channel,ES+
968.1 > 366.1
1.327e+005

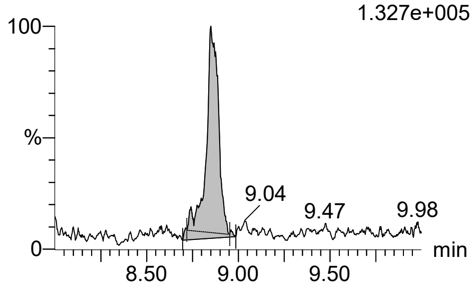

Sample Name: 20211111_H17_R3_IgM
F43:MRM of 1 channel,ES+
968.1 > 366.1
7.636e+005

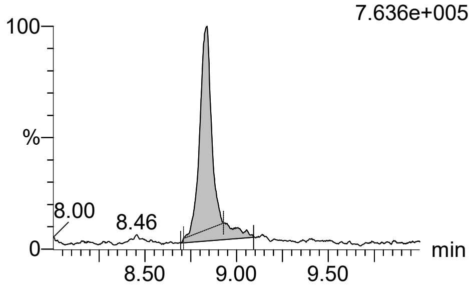

Sample Name: 20211111_A35_R3_IgM
F43:MRM of 1 channel,ES+
968.1 > 366.1
1.063e+005

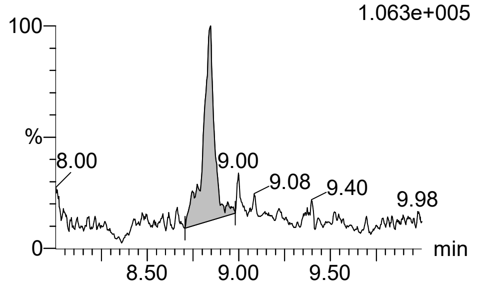

Sample Name: 20211111_QC_R24_IgM
F43:MRM of 1 channel,ES+
968.1 > 366.1
2.376e+005

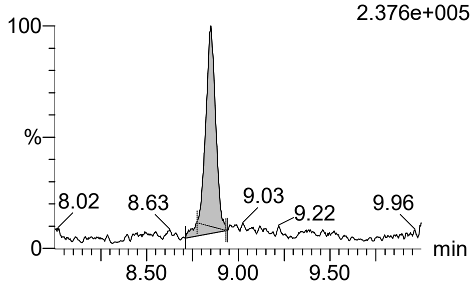

Sample Name: 20211111_A36_R3_IgM
F43:MRM of 1 channel,ES+
968.1 > 366.1
1.262e+005

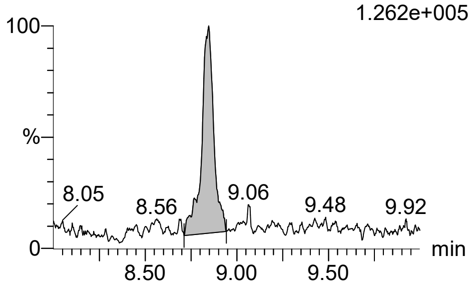

Sample Name: 20211111_H18_R3_IgM
F43:MRM of 1 channel,ES+
968.1 > 366.1
3.589e+005

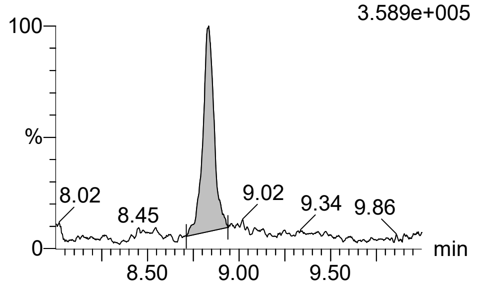

Sample Name: 20211111_A37_R3_IgM
F43:MRM of 1 channel,ES+
968.1 > 366.1
2.190e+005

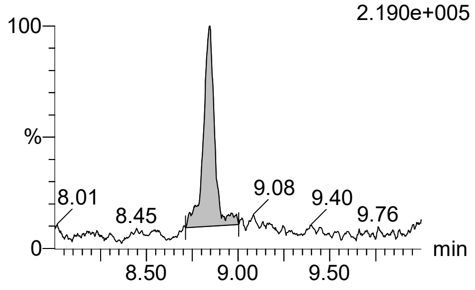

Sample Name: 20211111_A38_R3_IgM
F43:MRM of 1 channel,ES+
968.1 > 366.1
2.005e+005

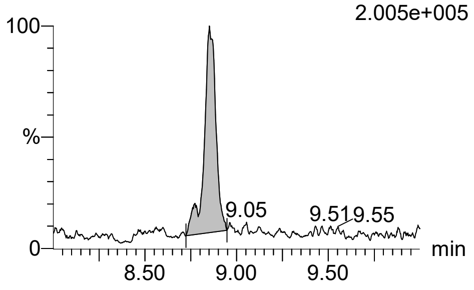

Sample Name: 20211111_H19_R3_IgM
F43:MRM of 1 channel,ES+
968.1 > 366.1
2.833e+005

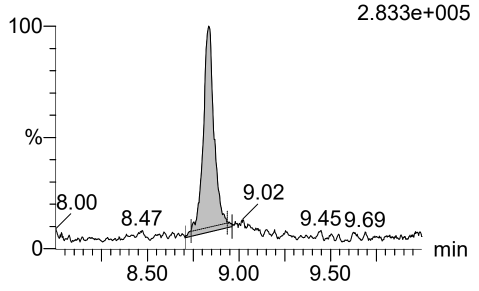

Sample Name: 20211111_A39_R3_IgM
F43:MRM of 1 channel,ES+
968.1 > 366.1
3.172e+005

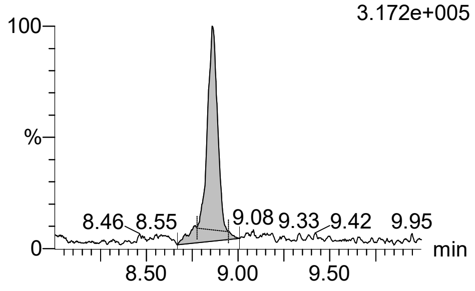

Sample Name: 20211111_A40_R3_IgM
F43:MRM of 1 channel,ES+
968.1 > 366.1
1.906e+005

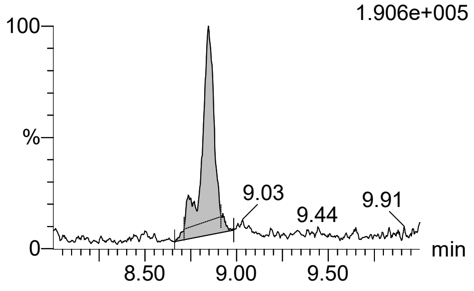

Sample Name: 20211111_H20_R3_IgM
F43:MRM of 1 channel,ES+
968.1 > 366.1
4.633e+005

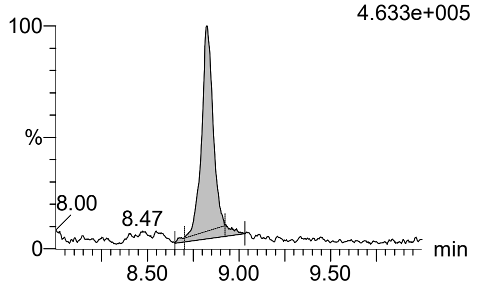

Sample Name: 20211111_A41_R3_IgM
F43:MRM of 1 channel,ES+
968.1 > 366.1
7.986e+004

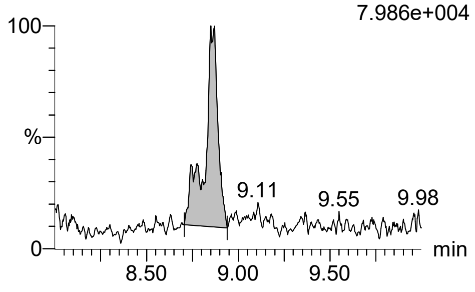

Sample Name: 20211111_A42_R3_IgM
F43:MRM of 1 channel,ES+
968.1 > 366.1
1.234e+005

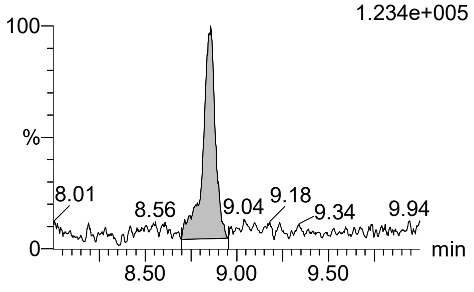

Dataset: C:\MassLynx\10.pro\20211111_clinicalsamples_IgM_IgA.qld

Last Altered: Thursday, March 09, 2023 19:58:25 台北標準時間
Printed: Friday, August 01, 2025 15:15:05 台北標準時間

Sample Name: 20211111_A43_R3_IgM

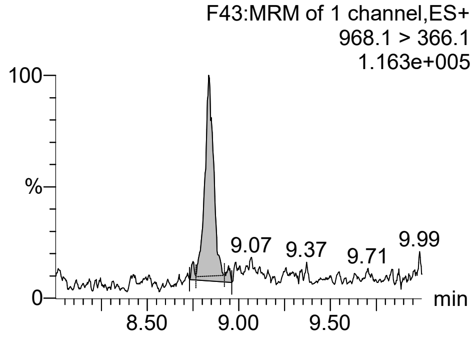

Sample Name: 20211111_A44_R3_IgM

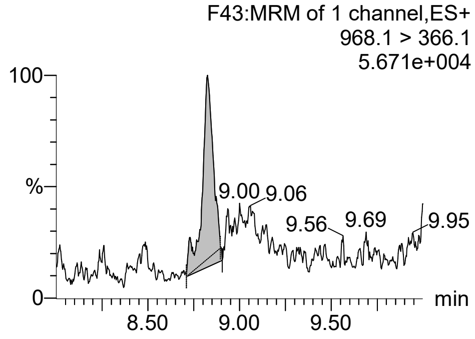

Sample Name: 20211111_QC_R25_IgM

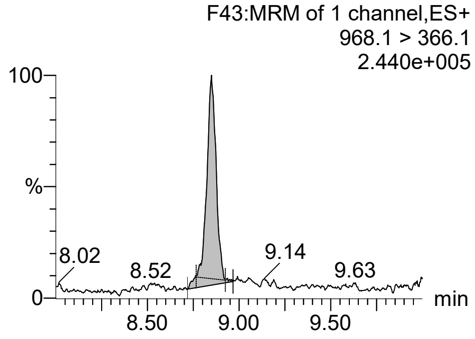

Dataset: C:\MassLynx\10.pro\20211111_clinicalsamples_IgM_IgA.qld

Last Altered: Thursday, March 09, 2023 19:58:25 台北標準時間
Printed: Friday, August 01, 2025 15:15:05 台北標準時間

	#	Name	RT	Area	IS Area	Response	Prima...
1	1	20211112_QC_R001_IgM	8.83	17577.9	15421.4	1.140	MM
2	2	20211112_QC_R002_IgM	8.85	15966.2	13188.8	1.211	MM
3	3	20211111_A1_R2_IgM	8.84	8429.1	11871.0	0.710	MM
4	4	20211111_A1_R3_IgM	8.84	8965.5	12030.5	0.745	MM
5	5	20211111_A2_R2_IgM	8.84	26630.2	11446.8	2.326	MM
6	6	20211111_A2_R3_IgM	8.83	26090.6	11098.4	2.351	MM
7	7	20211111_L1_R2_IgM	8.85	4549.8	11779.4	0.386	MM
8	8	20211111_L1_R3_IgM	8.85	4616.2	11176.2	0.413	MM
9	9	20211111_L2_R2_IgM	8.83	29407.5	11375.0	2.585	MM
10	10	20211111_H1_R2_IgM	8.82	13183.3	13595.2	0.970	MM
11	11	20211111_A3_R2_IgM	8.81	12829.8	14112.3	0.909	MM
12	12	20211111_A4_R2_IgM	8.82	8879.2	11977.5	0.741	MM
13	13	20211111_L3_R2_IgM	8.80	3910.0	12242.1	0.319	bb
14	14	20211111_L4_R2_IgM	8.83	14652.5	12797.0	1.145	MM
15	15	20211111_H2_R2_IgM	8.83	28356.6	10006.0	2.834	MM
16	16	20211111_A5_R2_IgM	8.81	25124.6	11434.6	2.197	MM
17	17	20211111_A6_R2_IgM	8.83	17886.6	13193.7	1.356	MM
18	18	20211111_QC_R10_IgM	8.84	16996.0	12566.1	1.353	MM
19	19	20211111_L5_R2_IgM	8.81	13571.5	13616.4	0.997	MM
20	20	20211111_L6_R2_IgM	8.84	28503.7	13698.5	2.081	MM
21	21	20211111_H3_R2_IgM	8.84	11206.0	15793.1	0.710	MM
22	22	20211111_A7_R2_IgM	8.82	29712.7	12936.9	2.297	MM
23	23	20211111_A8_R2_IgM	8.82	21175.4	13108.0	1.615	MM
24	24	20211111_L7_R2_IgM	8.84	3260.4	13278.5	0.246	MM
25	25	20211111_L8_R2_IgM	8.85	8347.6	15233.6	0.548	bb
26	26	20211111_H4_R2_IgM	8.83	14116.1	16521.9	0.854	MM
27	27	20211111_A9_R2_IgM	8.81	10318.4	16451.6	0.627	MM
28	28	20211111_A10_R2_IgM	8.82	29793.4	13036.0	2.285	MM
29	29	20211111_L9_R2_IgM	8.83	5550.2	14554.1	0.381	bb
30	30	20211111_L10_R2_IgM	8.82	9565.4	12250.4	0.781	MM
31	31	20211111_QC_R11_IgM	8.84	17249.3	11459.2	1.505	MM
32	32	20211111_H5_R2_IgM	8.84	10956.8	14358.2	0.763	MM
33	33	20211111_A11_R2_IgM	8.81	6593.8	16184.1	0.407	MM
34	34	20211111_A12_R2_IgM	8.82	9020.5	13465.4	0.670	MM
35	35	20211111_L11_R2_IgM	8.87	8296.0	13277.9	0.625	MM
36	36	20211111_L12_R2_IgM	8.81	4482.9	15958.8	0.281	MM
37	37	20211111_H6_R2_IgM	8.83	13715.9	14003.5	0.979	MM
38	38	20211111_A13_R2_IgM	8.82	10010.2	13656.1	0.733	MM
39	39	20211111_A14_R2_IgM	8.84	38254.0	14071.3	2.719	MM
40	40	20211111_L13_R2_IgM	8.85	12404.0	14785.6	0.839	MM
41	41	20211111_L14_R2_IgM	8.85	23699.7	14772.2	1.604	MM
42	42	20211111_H7_R2_IgM	8.83	9028.2	15164.4	0.595	MM
43	43	20211111_A15_R2_IgM	8.83	43529.7	15721.5	2.769	MM
44	44	20211111_QC_R12_IgM	8.84	17616.9	13711.5	1.285	MM
45	45	20211111_A16_R2_IgM	8.85	27603.6	14275.6	1.934	MM
46	46	20211111_L15_R2_IgM	8.84	20759.3	12935.8	1.605	MM
47	47	20211111_L16_R2_IgM	8.84	7126.1	13342.8	0.534	bb
48	48	20211111_H8_R2_IgM	8.85	12764.4	15835.1	0.806	MM
49	49	20211111_A17_R2_IgM	8.86	33666.2	15786.7	2.133	MM
50	50	20211111_A18_R2_IgM	8.82	12201.4	16986.8	0.718	MM
51	51	20211111_L17_R2_IgM	8.83	14510.9	13342.7	1.088	bb
52	52	20211111_L18_R2_IgM	8.84	1376.8	14692.0	0.094	MM
53	53	20211111_H9_R2_IgM	8.82	4940.1	15639.7	0.316	MM
54	54	20211111_A19_R2_IgM	8.83	8465.1	14198.7	0.596	MM
55	55	20211111_A20_R2_IgM	8.82	3030.3	14162.7	0.214	MM
56	56	20211111_L19_R2_IgM	8.83	13666.4	9293.4	1.471	MM

Dataset: C:\MassLynx\10.pro\20211111_clinicalsamples_IgM_IgA.qld

Last Altered: Thursday, March 09, 2023 19:58:25 台北標準時間
Printed: Friday, August 01, 2025 15:15:05 台北標準時間

	# Name	RT	Area	IS Area	Response	Prima...
57	57 20211111_QC_R13_IgM	8.84	18141.0	13903.0	1.305	MM
58	58 20211111_L20_R2_IgM	8.84	4601.8	12781.6	0.360	MM
59	59 20211111_H10_R2_IgM	8.84	6666.0	14796.4	0.451	MM
60	60 20211111_A21_R2_IgM	8.83	16277.6	13669.0	1.191	MM
61	61 20211111_A22_R2_IgM	8.85	2994.2	14098.3	0.212	MM
62	62 20211111_L21_R2_IgM	8.86	11150.1	15678.2	0.711	MM
63	63 20211111_L22_R2_IgM	8.86	16105.3	15724.5	1.024	MM
64	64 20211111_H11_R2_IgM	8.85	17613.9	15890.2	1.108	MM
65	65 20211111_A23_R2_IgM	8.83	6108.7	13423.2	0.455	MM
66	66 20211111_A24_R2_IgM	8.84	18539.5	12438.0	1.491	MM
67	67 20211111_L23_R2_IgM	8.84	6920.7	14075.0	0.492	MM
68	68 20211111_L24_R2_IgM	8.83	4103.5	15972.1	0.257	MM
69	69 20211111_H12_R2_IgM	8.85	7388.9	16270.9	0.454	MM
70	70 20211111_QC_R14_IgM	8.85	19133.1	15806.8	1.210	MM
71	71 20211111_A25_R2_IgM	8.85	26926.0	14552.1	1.850	MM
72	72 20211111_A26_R2_IgM	8.87	13977.7	11492.7	1.216	MM
73	73 20211111_L25_R2_IgM	8.85	9781.3	14403.3	0.679	MM
74	74 20211111_L26_R2_IgM	8.87	8694.4	16533.9	0.526	MM
75	75 20211111_H13_R2_IgM	8.84	18560.0	15520.1	1.196	MM
76	76 20211111_A27_R2_IgM	8.85	13773.4	13337.4	1.033	MM
77	77 20211111_A28_R2_IgM	8.84	2081.6	11895.6	0.175	MM
78	78 20211111_L27_R2_IgM	8.84	13013.7	14125.7	0.921	MM
79	79 20211111_L28_R2_IgM	8.85	12528.3	11807.5	1.061	bb
80	80 20211111_H14_R2_IgM	8.84	13943.3	15035.7	0.927	MM
81	81 20211111_A29_R2_IgM	8.85	988.2	13554.4	0.073	bb
82	82 20211111_A30_R2_IgM	8.84	23776.2	12984.1	1.831	MM
83	83 20211111_QC_R15_IgM	8.85	20792.0	12299.5	1.690	bb
84	84 20211111_L29_R2_IgM	8.85	7112.4	13590.7	0.523	MM
85	85 20211111_L30_R2_IgM	8.84	4020.4	12511.4	0.321	bb
86	86 20211111_H15_R2_IgM	8.84	19325.2	11885.0	1.626	MM
87	87 20211111_A31_R2_IgM	8.83	9214.8	13422.1	0.687	MM
88	88 20211111_A32_R2_IgM	8.84	23264.0	11461.3	2.030	MM
89	89 20211111_L31_R2_IgM	8.82	1760.3	13753.4	0.128	MM
90	90 20211111_L32_R2_IgM	8.82	8633.5	12949.0	0.667	MM
91	91 20211111_H16_R2_IgM	8.82	26956.4	11465.4	2.351	MM
92	92 20211111_A33_R2_IgM	8.83	19140.2	10551.5	1.814	MM
93	93 20211111_A34_R2_IgM	8.87	10359.8	12409.9	0.835	MM
94	94 20211111_H17_R2_IgM	8.82	67099.5	10160.9	6.604	MM
95	95 20211111_A35_R2_IgM	8.84	1095.1	10460.6	0.105	MM
96	96 20211111_QC_R16_IgM	8.83	14702.5	10433.5	1.409	MM
97	97 20211111_A36_R2_IgM	8.81	4018.7	12504.8	0.321	MM
98	98 20211111_H18_R2_IgM	8.83	24370.2	9140.4	2.666	MM
99	99 20211111_A37_R2_IgM	8.84	16282.8	9796.9	1.662	MM
100	1... 20211111_A38_R2_IgM	8.86	17183.6	11224.3	1.531	MM
101	1... 20211111_H19_R2_IgM	8.84	22499.9	11739.2	1.917	bb
102	1... 20211111_A39_R2_IgM	8.86	21207.0	12718.9	1.667	MM
103	1... 20211111_A40_R2_IgM	8.83	3490.7	10006.1	0.349	MM
104	1... 20211111_H20_R2_IgM	8.82	36917.4	7988.2	4.621	MM
105	1... 20211111_A41_R2_IgM	8.86	9770.1	11513.3	0.849	MM
106	1... 20211111_A42_R2_IgM	8.86	10922.0	10238.5	1.067	MM
107	1... 20211111_A43_R2_IgM	8.84	7602.5	11098.9	0.685	MM
108	1... 20211111_A44_R2_IgM	8.85	603.4	10969.9	0.055	MM
109	1... 20211111_QC_R17_IgM	8.84	14071.2	9700.8	1.451	MM
110	1... 20211111_L2_R3_IgM	8.84	26563.4	8528.0	3.115	MM
111	1... 20211111_H1_R3_IgM	8.85	20691.4	9438.2	2.192	MM
112	1... 20211111_A3_R3_IgM	8.85	21761.5	11598.6	1.876	MM

Dataset: C:\MassLynx\10.pro\20211111_clinicalsamples_IgM_IgA.qld

Last Altered: Thursday, March 09, 2023 19:58:25 台北標準時間
Printed: Friday, August 01, 2025 15:15:05 台北標準時間

	# Name	RT	Area	IS Area	Response	Prima...
113	1... 20211111_A4_R3_IgM	8.86	13901.0	11364.1	1.223	MM
114	1... 20211111_L3_R3_IgM	8.82	2581.4	10753.4	0.240	MM
115	1... 20211111_L4_R3_IgM	8.84	15635.0	10291.6	1.519	MM
116	1... 20211111_H2_R3_IgM	8.83	30049.1	9315.8	3.226	MM
117	1... 20211111_A5_R3_IgM	8.84	38998.6	9102.2	4.285	MM
118	1... 20211111_A6_R3_IgM	8.84	15833.0	10356.4	1.529	MM
119	1... 20211111_QC_R18_IgM	8.84	14147.1	8398.3	1.685	MM
120	1... 20211111_L5_R3_IgM	8.85	22894.5	9786.7	2.339	bb
121	1... 20211111_L6_R3_IgM	8.85	23751.8	9911.3	2.396	MM
122	1... 20211111_H3_R3_IgM	8.86	17675.0	12155.5	1.454	MM
123	1... 20211111_A7_R3_IgM	8.84	30001.0	11804.8	2.541	MM
124	1... 20211111_A8_R3_IgM	8.86	26095.5	10510.6	2.483	bb
125	1... 20211111_L7_R3_IgM	8.84	3763.5	12177.9	0.309	MM
126	1... 20211111_L8_R3_IgM	8.86	7142.1	11668.6	0.612	MM
127	1... 20211111_H4_R3_IgM	8.87	16030.5	11192.0	1.432	MM
128	1... 20211111_A9_R3_IgM	8.87	22153.4	14077.4	1.574	bb
129	1... 20211111_A10_R3_IgM	8.86	44298.1	11190.4	3.959	MM
130	1... 20211111_L9_R3_IgM	8.85	10882.7	14649.3	0.743	MM
131	1... 20211111_L10_R3_IgM	8.83	5827.5	9361.3	0.623	MM
132	1... 20211111_QC_R19_IgM	8.84	14269.4	10125.5	1.409	MM
133	1... 20211111_H5_R3_IgM	8.86	14970.6	10010.9	1.495	bb
134	1... 20211111_A11_R3_IgM	8.86	20497.0	12697.2	1.614	MM
135	1... 20211111_A12_R3_IgM	8.82	10611.5	13148.5	0.807	bb
136	1... 20211111_L11_R3_IgM	8.86	7328.3	10786.6	0.679	MM
137	1... 20211111_L12_R3_IgM	8.81	2873.9	11681.8	0.246	MM
138	1... 20211111_H6_R3_IgM	8.85	13522.2	12095.5	1.118	MM
139	1... 20211111_A13_R3_IgM	8.84	9146.3	12908.3	0.709	bb
140	1... 20211111_A14_R3_IgM	8.85	39307.0	11322.6	3.472	bb
141	1... 20211111_L13_R3_IgM	8.82	8733.9	11516.1	0.758	MM
142	1... 20211111_L14_R3_IgM	8.85	21052.3	10790.7	1.951	MM
143	1... 20211111_H7_R3_IgM	8.85	22596.8	11572.1	1.953	MM
144	1... 20211111_A15_R3_IgM	8.84	35925.2	12434.4	2.889	MM
145	1... 20211111_QC_R20_IgM	8.85	16731.3	10701.0	1.564	MM
146	1... 20211111_A16_R3_IgM	8.87	21341.2	11452.1	1.864	bb
147	1... 20211111_L15_R3_IgM	8.85	26922.0	10531.1	2.556	MM
148	1... 20211111_L16_R3_IgM	8.84	7419.1	12049.4	0.616	MM
149	1... 20211111_H8_R3_IgM	8.86	12848.8	13434.9	0.956	bb
150	1... 20211111_A17_R3_IgM	8.86	28564.5	12452.0	2.294	MM
151	1... 20211111_A18_R3_IgM	8.85	4380.5	12506.5	0.350	MM
152	1... 20211111_L17_R3_IgM	8.83	14525.8	9948.9	1.460	MM
153	1... 20211111_L18_R3_IgM	8.85	1664.9	13462.1	0.124	MM
154	1... 20211111_H9_R3_IgM	8.85	7032.0	13178.7	0.534	bb
155	1... 20211111_A19_R3_IgM	8.84	9322.6	11298.6	0.825	MM
156	1... 20211111_A20_R3_IgM	8.82	1903.5	11175.6	0.170	MM
157	1... 20211111_L19_R3_IgM	8.84	13611.7	11558.2	1.178	MM
158	1... 20211111_QC_R21_IgM	8.85	14842.1	9977.0	1.488	bb
159	1... 20211111_L20_R3_IgM	8.85	9689.8	13508.1	0.717	MM
160	1... 20211111_H10_R3_IgM	8.84	17610.3	12374.8	1.423	bb
161	1... 20211111_A21_R3_IgM	8.84	19082.2	12443.6	1.534	MM
162	1... 20211111_A22_R3_IgM	8.84	4497.1	11589.3	0.388	bb
163	1... 20211111_L21_R3_IgM	8.86	10017.2	13107.4	0.764	MM
164	1... 20211111_L22_R3_IgM	8.86	16194.5	12021.5	1.347	MM
165	1... 20211111_H11_R3_IgM	8.87	19265.2	12687.8	1.518	MM
166	1... 20211111_A23_R3_IgM	8.85	6111.4	11611.8	0.526	MM
167	1... 20211111_A24_R3_IgM	8.84	17780.3	10476.8	1.697	MM
168	1... 20211111_L23_R3_IgM	8.82	5552.7	11873.1	0.468	MM

Dataset: C:\MassLynx\10.pro\20211111_clinicalsamples_IgM_IgA.qld

Last Altered: Thursday, March 09, 2023 19:58:25 台北標準時間
Printed: Friday, August 01, 2025 15:15:05 台北標準時間

	# Name	RT	Area	IS Area	Response	Prima...
169	1... 20211111_L24_R3_IgM	8.88	7737.8	13468.0	0.575	MM
170	1... 20211111_H12_R3_IgM	8.84	8325.1	14229.1	0.585	bb
171	1... 20211111_QC_R22_IgM	8.86	16215.5	11264.4	1.440	MM
172	1... 20211111_A25_R3_IgM	8.86	22524.5	11775.7	1.913	bb
173	1... 20211111_A26_R3_IgM	8.87	13307.0	10473.9	1.270	bb
174	1... 20211111_L25_R3_IgM	8.86	9164.7	13256.8	0.691	MM
175	1... 20211111_L26_R3_IgM	8.87	7075.7	13565.0	0.522	bb
176	1... 20211111_H13_R3_IgM	8.84	18307.8	13146.0	1.393	MM
177	1... 20211111_A27_R3_IgM	8.85	14461.6	12025.6	1.203	MM
178	1... 20211111_A28_R3_IgM	8.85	9022.5	11229.1	0.803	MM
179	1... 20211111_L27_R3_IgM	8.86	15961.0	14960.0	1.067	MM
180	1... 20211111_L28_R3_IgM	8.85	12385.1	11359.0	1.090	bb
181	1... 20211111_H14_R3_IgM	8.85	16157.2	12830.8	1.259	MM
182	1... 20211111_A29_R3_IgM	8.86	3341.9	13434.3	0.249	MM
183	1... 20211111_A30_R3_IgM	8.84	22241.7	11272.2	1.973	MM
184	1... 20211111_QC_R23_IgM	8.85	16281.3	11872.2	1.371	MM
185	1... 20211111_L29_R3_IgM	8.85	6974.5	12948.7	0.539	MM
186	1... 20211111_L30_R3_IgM	8.85	5485.6	11026.6	0.497	MM
187	1... 20211111_H15_R3_IgM	8.85	16997.4	10786.3	1.576	bb
188	1... 20211111_A31_R3_IgM	8.84	9206.6	12010.5	0.767	bb
189	1... 20211111_A32_R3_IgM	8.85	19553.9	11107.7	1.760	MM
190	1... 20211111_L31_R3_IgM	8.85	4908.6	11021.8	0.445	bb
191	1... 20211111_L32_R3_IgM	8.85	13873.1	11683.8	1.187	bb
192	1... 20211111_H16_R3_IgM	8.84	28900.6	9831.7	2.940	MM
193	1... 20211111_A33_R3_IgM	8.84	19191.9	11213.1	1.712	MM
194	1... 20211111_A34_R3_IgM	8.85	10313.6	11550.1	0.893	MM
195	1... 20211111_H17_R3_IgM	8.84	62950.3	8874.4	7.093	MM
196	1... 20211111_A35_R3_IgM	8.85	6560.7	9856.3	0.666	bb
197	1... 20211111_QC_R24_IgM	8.85	14563.4	7561.8	1.926	MM
198	1... 20211111_A36_R3_IgM	8.85	8991.5	9562.8	0.940	bb
199	1... 20211111_H18_R3_IgM	8.83	23489.6	8165.3	2.877	bb
200	2... 20211111_A37_R3_IgM	8.85	13872.4	9278.8	1.495	bb
201	2... 20211111_A38_R3_IgM	8.85	13627.6	10920.3	1.248	bb
202	2... 20211111_H19_R3_IgM	8.83	18261.1	9285.3	1.967	MM
203	2... 20211111_A39_R3_IgM	8.86	22365.2	9083.4	2.462	MM
204	2... 20211111_A40_R3_IgM	8.85	14481.6	8382.4	1.728	MM
205	2... 20211111_H20_R3_IgM	8.82	35215.9	7807.9	4.510	MM
206	2... 20211111_A41_R3_IgM	8.85	6005.9	5925.2	1.014	bb
207	2... 20211111_A42_R3_IgM	8.86	9182.1	9352.6	0.982	bb
208	2... 20211111_A43_R3_IgM	8.84	6372.0	10216.3	0.624	MM
209	2... 20211111_A44_R3_IgM	8.83	3481.6	10262.7	0.339	MM
210	2... 20211111_QC_R25_IgM	8.85	14247.7	8575.3	1.661	MM

Dataset: C:\MassLynx\10.pro\20211111_clinicalsamples_IgM_IgA.qld

Last Altered: Thursday, March 09, 2023 19:58:25 台北標準時間
Printed: Friday, August 01, 2025 15:16:08 台北標準時間

Method: C:\MassLynx\10_Pro_IgA.PRO\MethDB\20211111_IgM_IgA_DMRM_new.mdb 24 Feb 2022 13:44:14
Calibration: 15 Nov 2021 14:42:11

Sample Name: 20211112_QC_R001_IgM

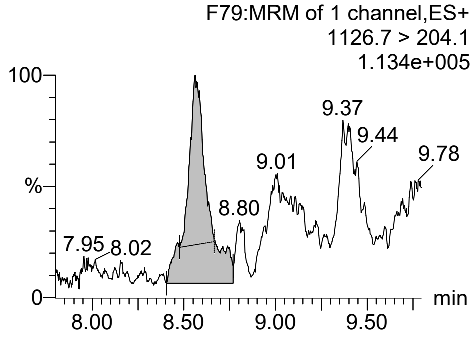

Sample Name: 20211112_QC_R002_IgM

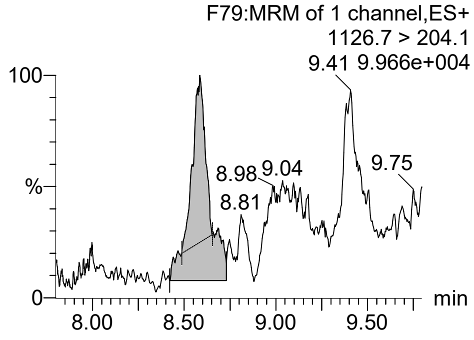

Sample Name: 20211111_A1_R2_IgM

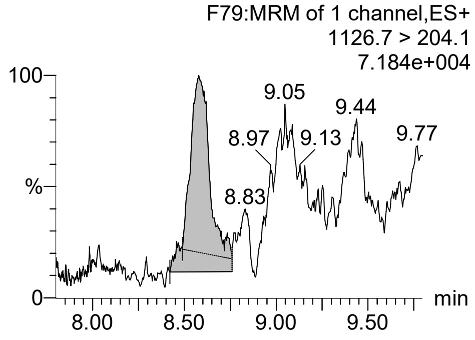

Sample Name: 20211111_A1_R3_IgM

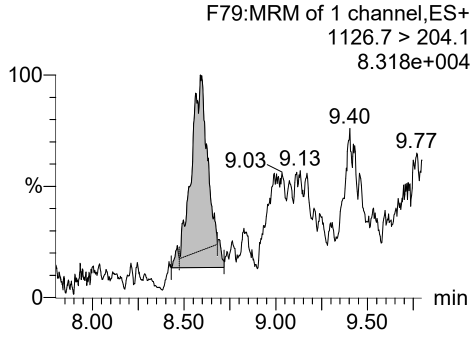

Sample Name: 20211111_A2_R2_IgM

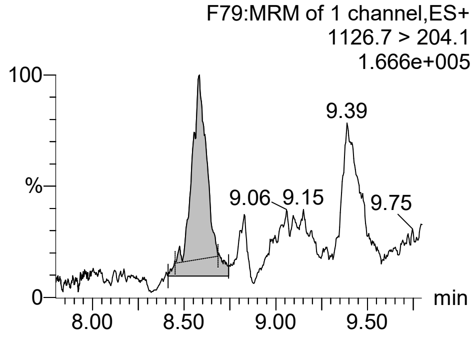

Sample Name: 20211111_A2_R3_IgM

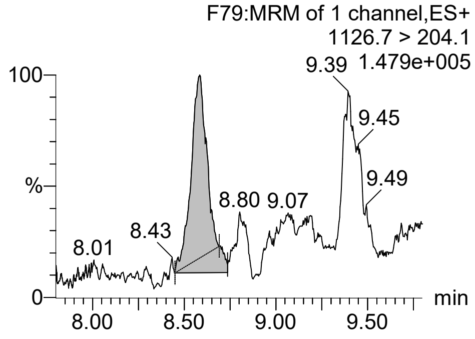

Sample Name: 20211111_L1_R2_IgM

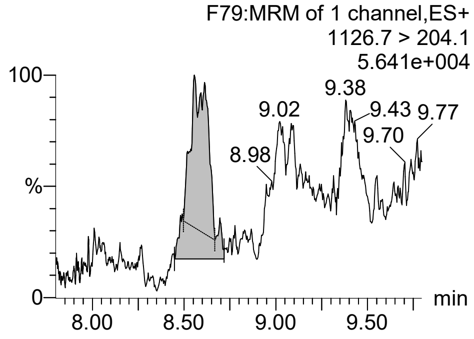

Sample Name: 20211111_L1_R3_IgM

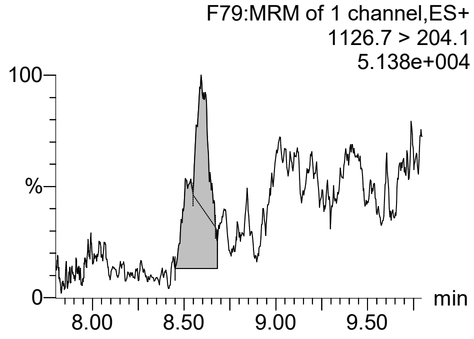

Sample Name: 20211111_L2_R2_IgM

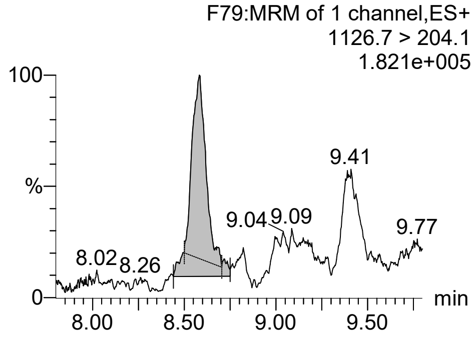

Sample Name: 20211111_H1_R2_IgM

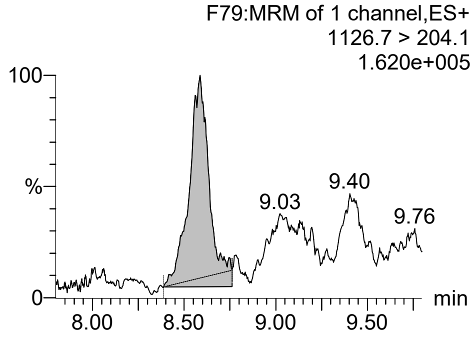

Sample Name: 20211111_A3_R2_IgM

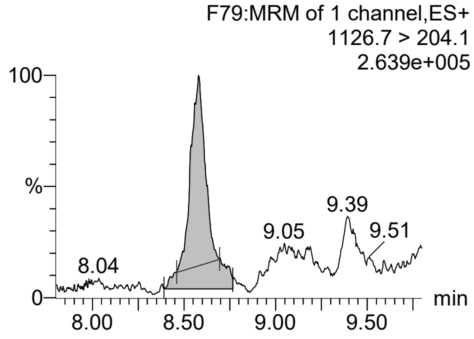

Sample Name: 20211111_A4_R2_IgM

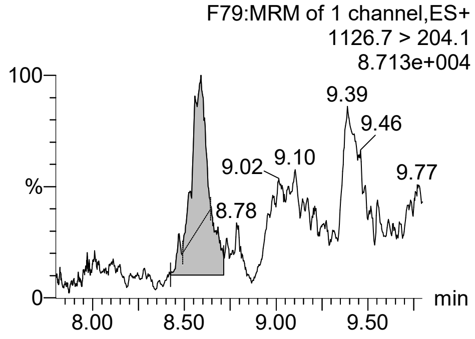

Sample Name: 20211111_L3_R2_IgM

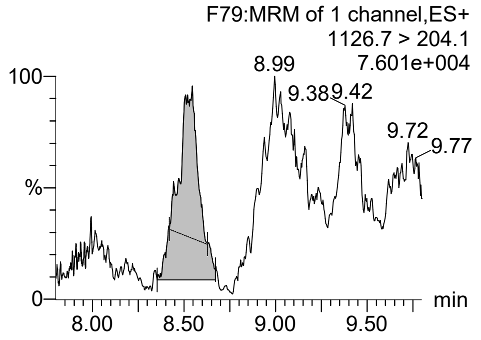

Sample Name: 20211111_L4_R2_IgM

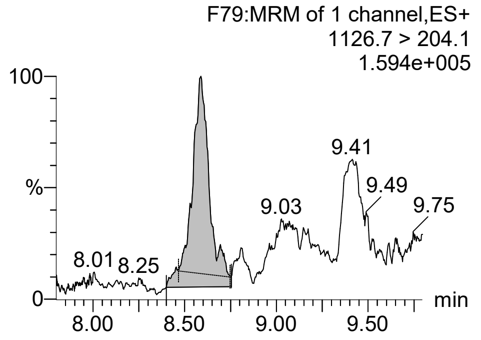

Sample Name: 20211111_H2_R2_IgM

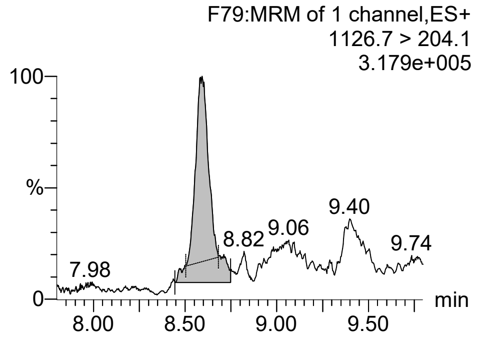

Sample Name: 20211111_A5_R2_IgM

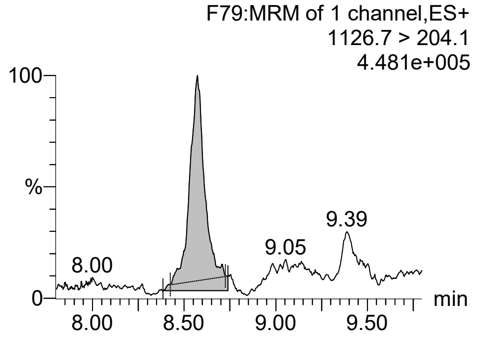

Sample Name: 20211111_A6_R2_IgM

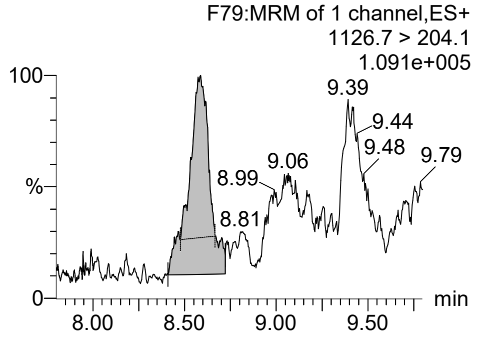

Sample Name: 20211111_QC_R10_IgM

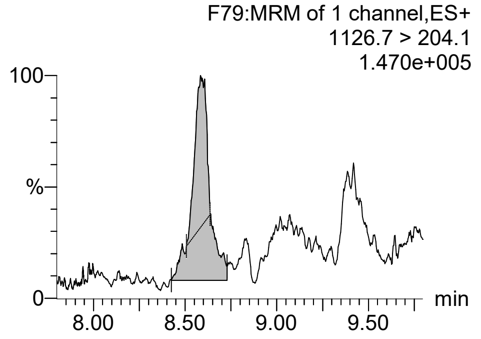

Sample Name: 20211111_L5_R2_IgM

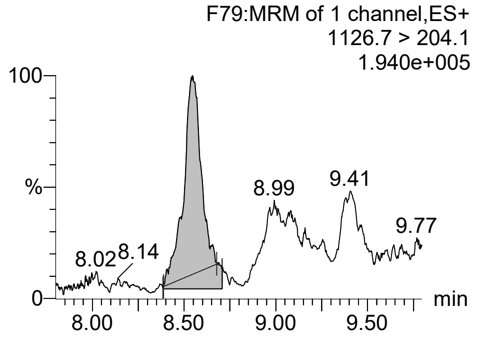

Sample Name: 20211111_L6_R2_IgM

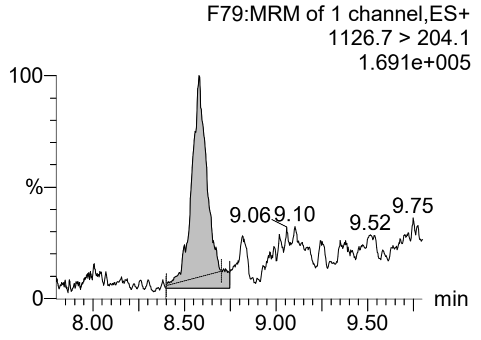

Sample Name: 20211111_H3_R2_IgM

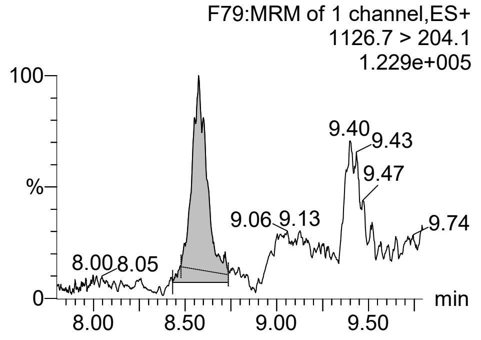

Sample Name: 20211111_A7_R2_IgM

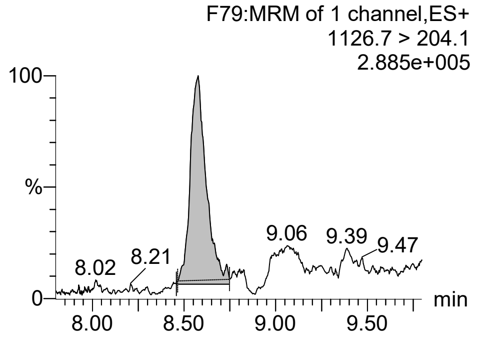

Sample Name: 20211111_A8_R2_IgM

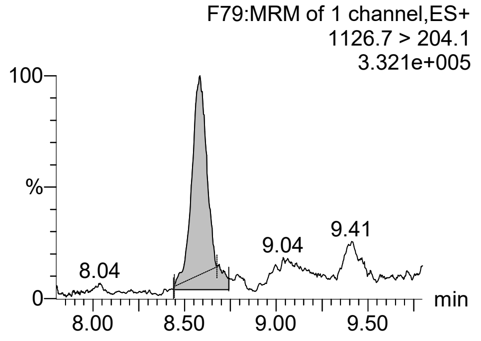

Sample Name: 20211111_L7_R2_IgM

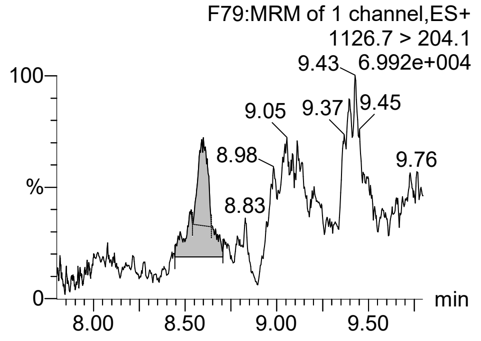

Sample Name: 20211111_L8_R2_IgM

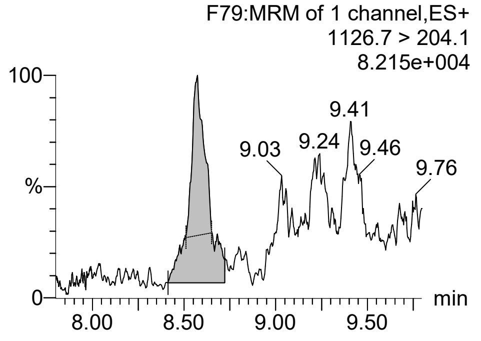

Sample Name: 20211111_H4_R2_IgM

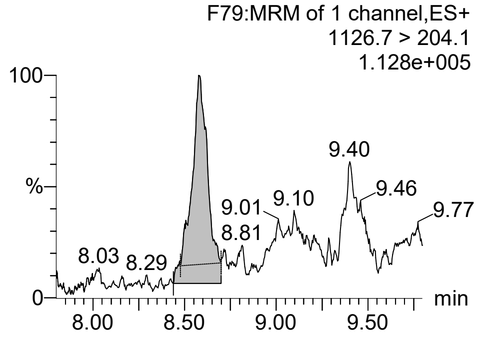

Sample Name: 20211111_A9_R2_IgM

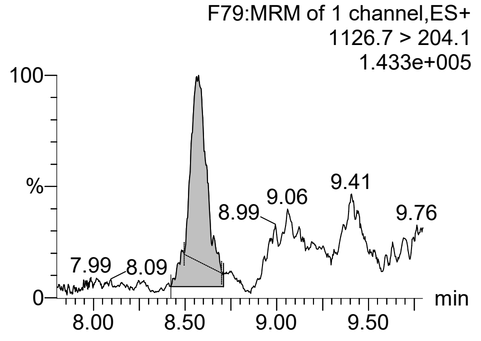

Dataset: C:\MassLynx\10.pro\20211111_clinicalsamples_IgM_IgA.qld

Last Altered: Thursday, March 09, 2023 19:58:25 台北標準時間
Printed: Friday, August 01, 2025 15:16:08 台北標準時間

Sample Name: 20211111_A10_R2_IgM
F79:MRM of 1 channel,ES+
1126.7 > 204.1
3.375e+005

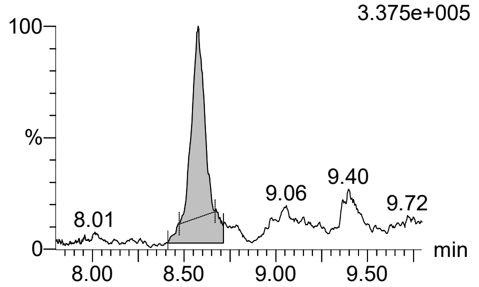

Sample Name: 20211111_L9_R2_IgM
F79:MRM of 1 channel,ES+
1126.7 > 204.1
1.153e+005

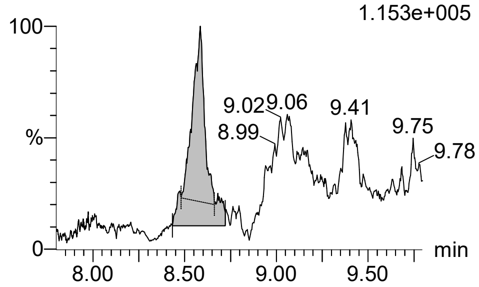

Sample Name: 20211111_L10_R2_IgM
F79:MRM of 1 channel,ES+
1126.7 > 204.1
2.539e+005

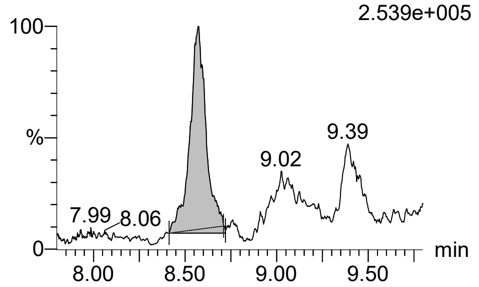

Sample Name: 20211111_QC_R11_IgM
F79:MRM of 1 channel,ES+
1126.7 > 204.1
1.727e+005

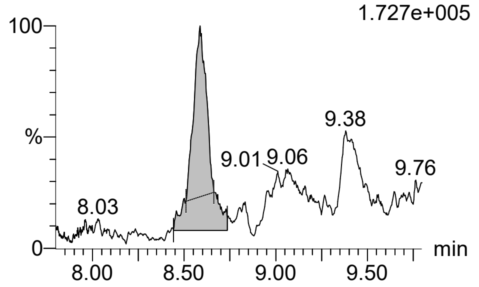

Sample Name: 20211111_H5_R2_IgM
F79:MRM of 1 channel,ES+
1126.7 > 204.1
1.443e+005

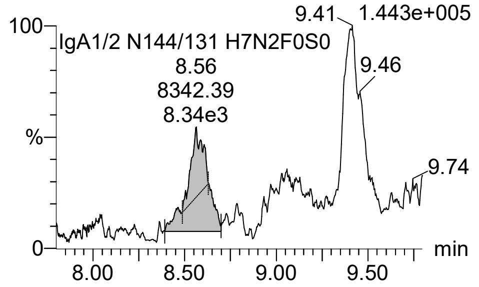

Sample Name: 20211111_A11_R2_IgM
F79:MRM of 1 channel,ES+
1126.7 > 204.1
2.250e+005

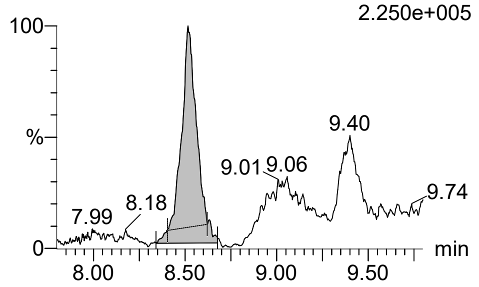

Sample Name: 20211111_A12_R2_IgM
F79:MRM of 1 channel,ES+
1126.7 > 204.1
1.112e+005

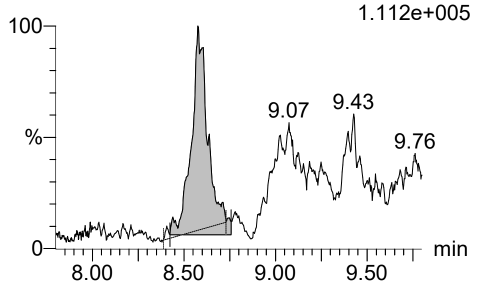

Sample Name: 20211111_L11_R2_IgM
F79:MRM of 1 channel,ES+
1126.7 > 204.1
1.052e+005

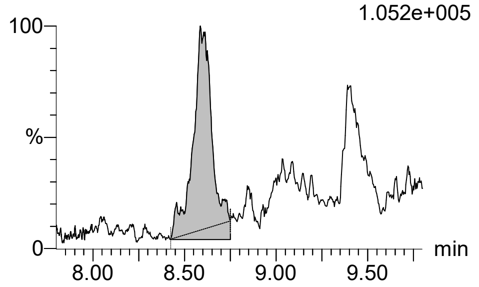

Sample Name: 20211111_L12_R2_IgM
F79:MRM of 1 channel,ES+
1126.7 > 204.1
9.172e+004

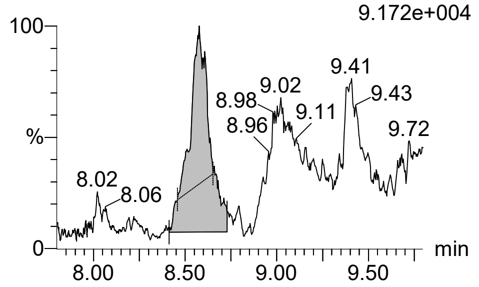

Sample Name: 20211111_H6_R2_IgM
F79:MRM of 1 channel,ES+
1126.7 > 204.1
1.395e+005

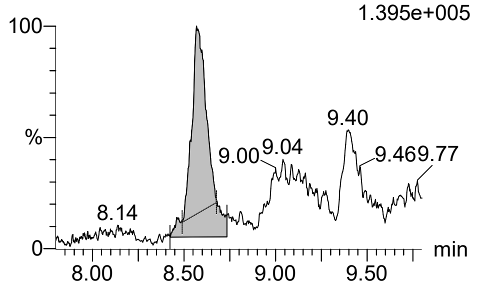

Sample Name: 20211111_A13_R2_IgM
F79:MRM of 1 channel,ES+
1126.7 > 204.1
1.204e+005

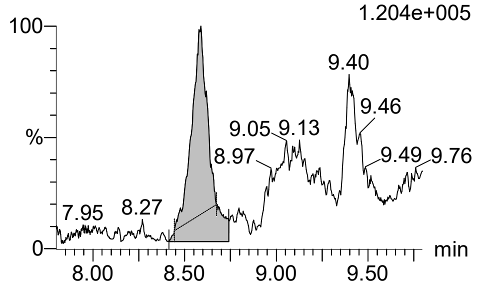

Sample Name: 20211111_A14_R2_IgM
F79:MRM of 1 channel,ES+
1126.7 > 204.1
6.092e+005

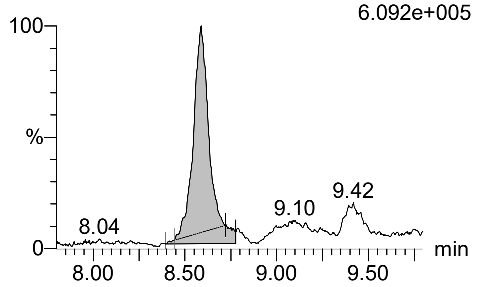

Sample Name: 20211111_L13_R2_IgM
F79:MRM of 1 channel,ES+
1126.7 > 204.1
1.016e+005

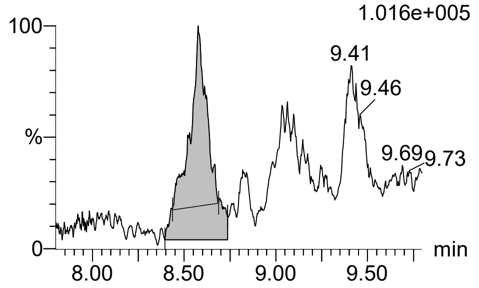

Sample Name: 20211111_L14_R2_IgM
F79:MRM of 1 channel,ES+
1126.7 > 204.1
1.683e+005

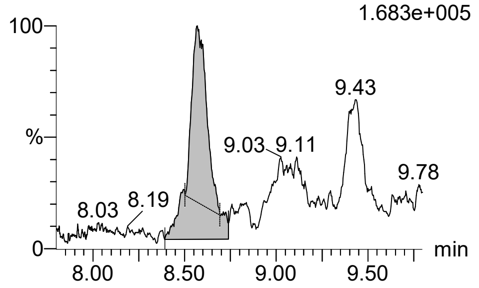

Sample Name: 20211111_H7_R2_IgM
F79:MRM of 1 channel,ES+
1126.7 > 204.1
1.808e+005

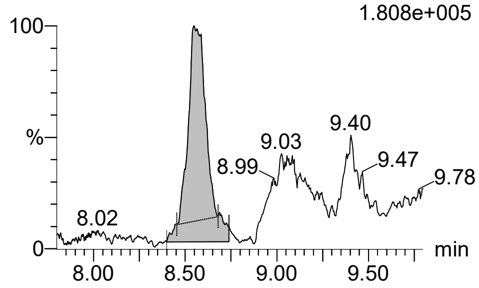

Dataset: C:\MassLynx\10.pro\20211111_clinicalsamples_IgM_IgA.qld

Last Altered: Thursday, March 09, 2023 19:58:25 台北標準時間
Printed: Friday, August 01, 2025 15:16:08 台北標準時間

Sample Name: 20211111_A15_R2_IgM
F79:MRM of 1 channel,ES+
1126.7 > 204.1
5.732e+005

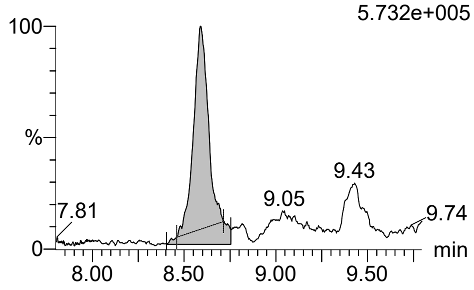

Sample Name: 20211111_QC_R12_IgM
F79:MRM of 1 channel,ES+
1126.7 > 204.1
1.826e+005

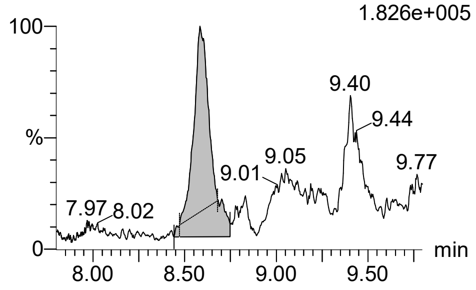

Sample Name: 20211111_A16_R2_IgM
F79:MRM of 1 channel,ES+
1126.7 > 204.1
3.518e+005

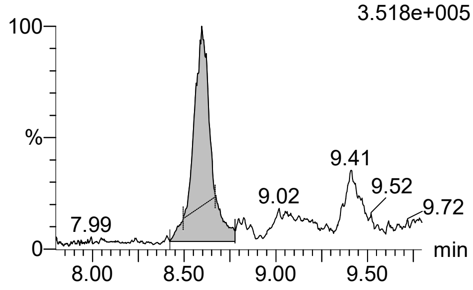

Sample Name: 20211111_L15_R2_IgM
F79:MRM of 1 channel,ES+
1126.7 > 204.1
2.995e+005

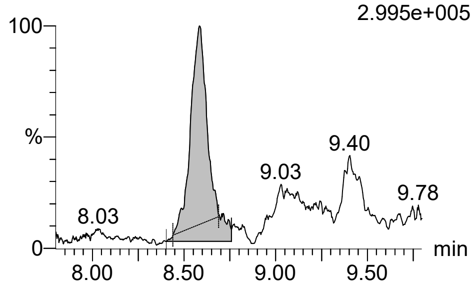

Sample Name: 20211111_L16_R2_IgM
F79:MRM of 1 channel,ES+
1126.7 > 204.1
6.708e+004

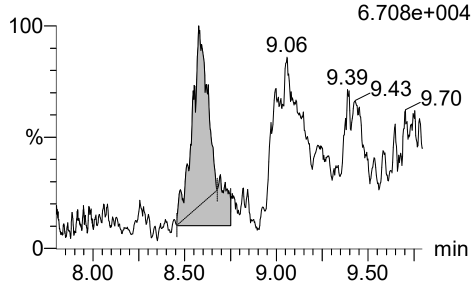

Sample Name: 20211111_H8_R2_IgM
F79:MRM of 1 channel,ES+
1126.7 > 204.1
7.443e+004

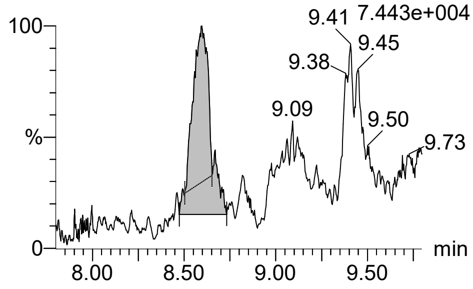

Sample Name: 20211111_A17_R2_IgM
F79:MRM of 1 channel,ES+
1126.7 > 204.1
1.921e+005

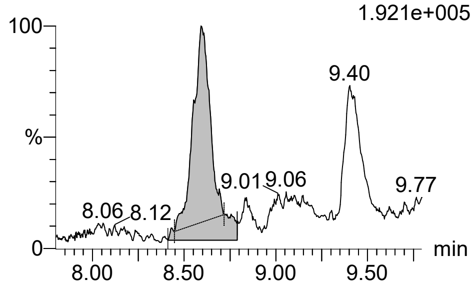

Sample Name: 20211111_A18_R2_IgM
F79:MRM of 1 channel,ES+
1126.7 > 204.1
1.779e+005

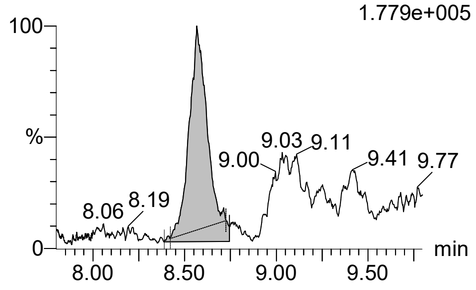

Sample Name: 20211111_L17_R2_IgM
F79:MRM of 1 channel,ES+
1126.7 > 204.1
2.045e+005

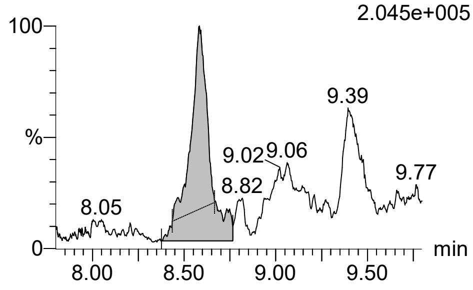

Sample Name: 20211111_L18_R2_IgM
F79:MRM of 1 channel,ES+
1126.7 > 204.1
5.486e+004

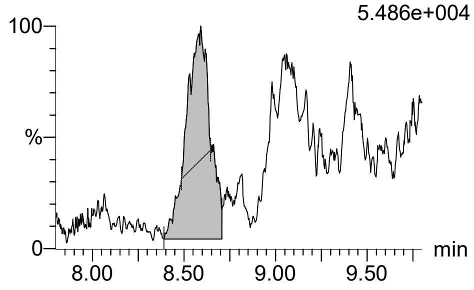

Sample Name: 20211111_H9_R2_IgM
F79:MRM of 1 channel,ES+
1126.7 > 204.1
1.023e+005

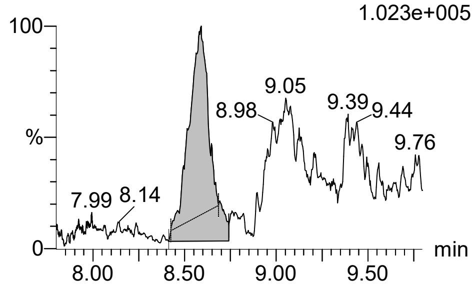

Sample Name: 20211111_A19_R2_IgM
F79:MRM of 1 channel,ES+
1126.7 > 204.1
1.203e+005

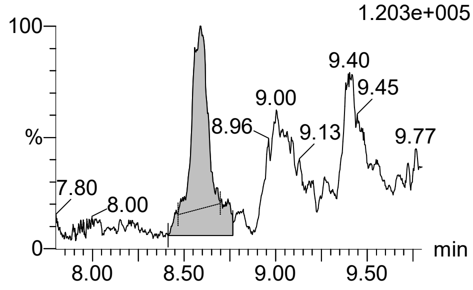

Sample Name: 20211111_A20_R2_IgM
F79:MRM of 1 channel,ES+
1126.7 > 204.1
1.213e+005

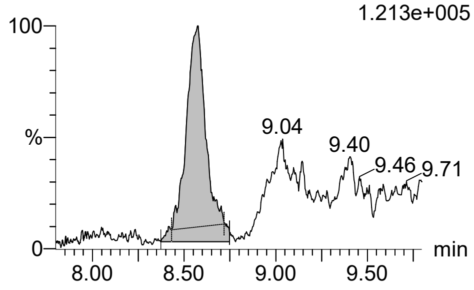

Sample Name: 20211111_L19_R2_IgM
F79:MRM of 1 channel,ES+
1126.7 > 204.1
1.367e+005

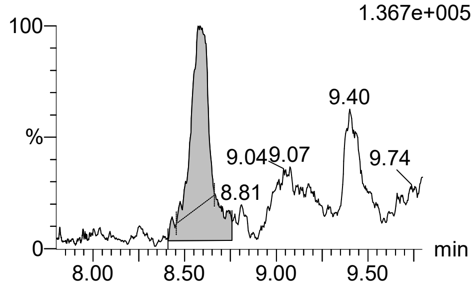

Sample Name: 20211111_QC_R13_IgM
F79:MRM of 1 channel,ES+
1126.7 > 204.1
1.770e+005

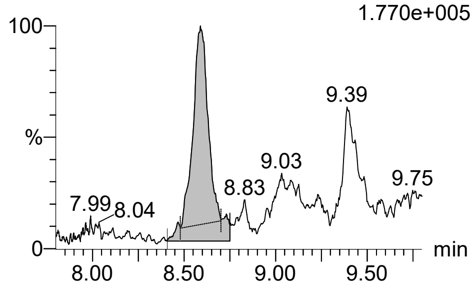

Sample Name: 20211111_L20_R2_IgM
F79:MRM of 1 channel,ES+
1126.7 > 204.1
8.299e+004

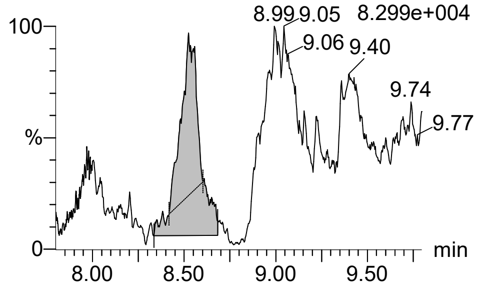

Sample Name: 20211111_H10_R2_IgM
F79:MRM of 1 channel,ES+
1126.7 > 204.1
1.442e+005

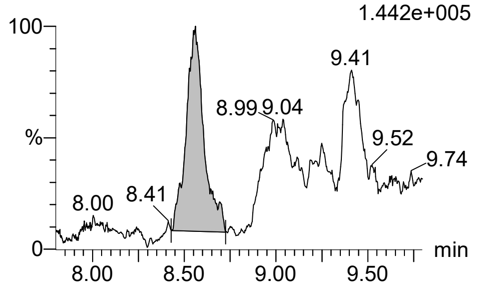

Sample Name: 20211111_A21_R2_IgM
F79:MRM of 1 channel,ES+
1126.7 > 204.1
2.019e+005

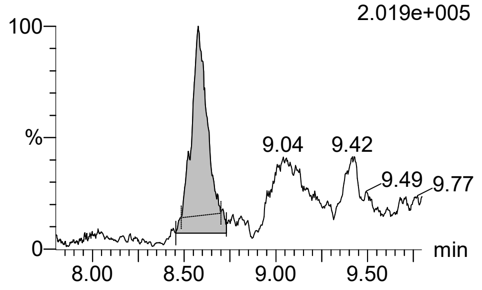

Sample Name: 20211111_A22_R2_IgM
F79:MRM of 1 channel,ES+
1126.7 > 204.1
6.143e+004

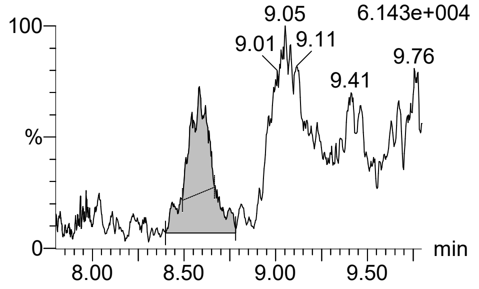

Sample Name: 20211111_L21_R2_IgM
F79:MRM of 1 channel,ES+
1126.7 > 204.1
1.052e+005

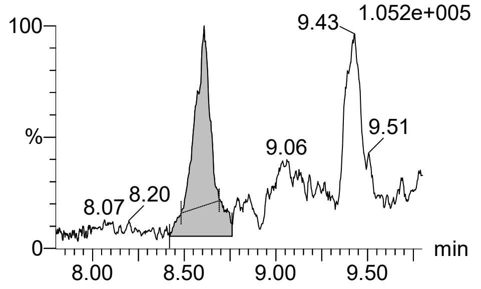

Sample Name: 20211111_L22_R2_IgM
F79:MRM of 1 channel,ES+
1126.7 > 204.1
1.262e+005

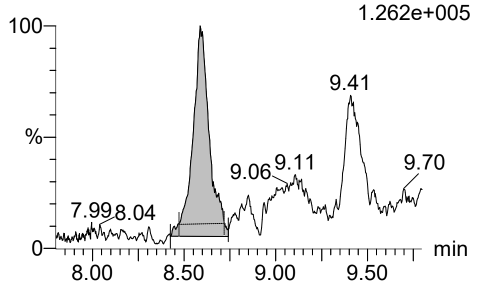

Sample Name: 20211111_H11_R2_IgM
F79:MRM of 1 channel,ES+
1126.7 > 204.1
1.709e+005

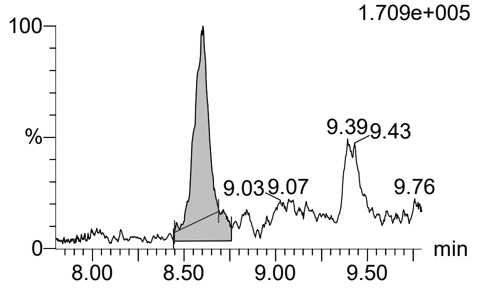

Sample Name: 20211111_A23_R2_IgM
F79:MRM of 1 channel,ES+
1126.7 > 204.1
1.178e+005

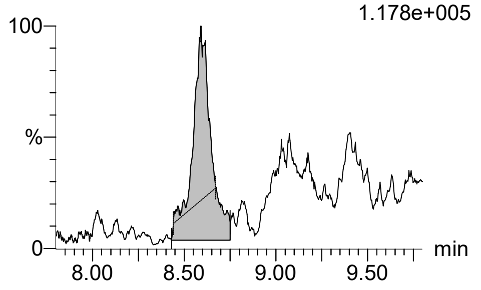

Sample Name: 20211111_A24_R2_IgM
F79:MRM of 1 channel,ES+
1126.7 > 204.1
1.970e+005

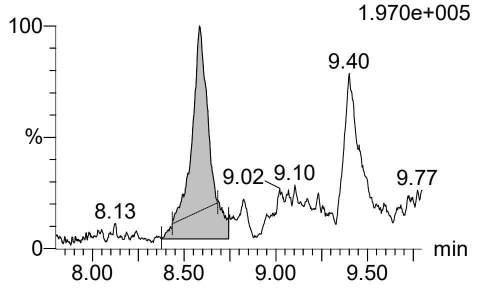

Sample Name: 20211111_L23_R2_IgM
F79:MRM of 1 channel,ES+
1126.7 > 204.1
1.465e+005

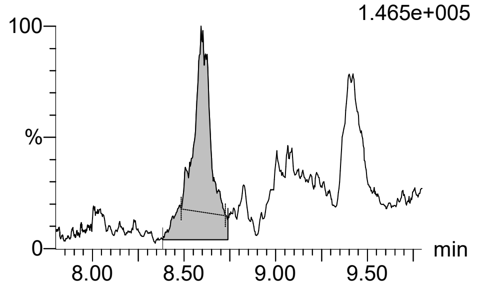

Sample Name: 20211111_L24_R2_IgM
F79:MRM of 1 channel,ES+
1126.7 > 204.1
8.494e+004

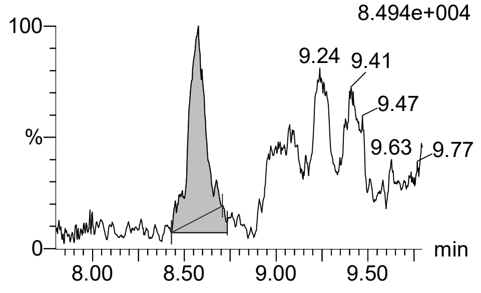

Sample Name: 20211111_H12_R2_IgM
F79:MRM of 1 channel,ES+
1126.7 > 204.1
6.104e+004

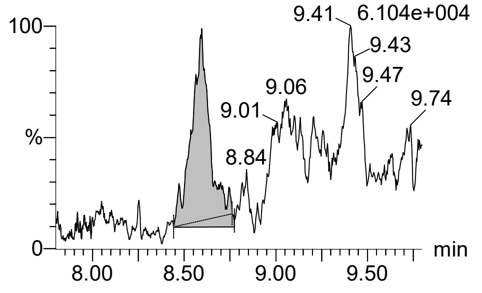

Sample Name: 20211111_QC_R14_IgM
F79:MRM of 1 channel,ES+
1126.7 > 204.1
1.820e+005

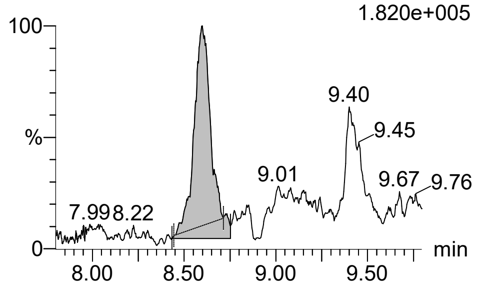

Sample Name: 20211111_A25_R2_IgM
F79:MRM of 1 channel,ES+
1126.7 > 204.1
4.211e+005

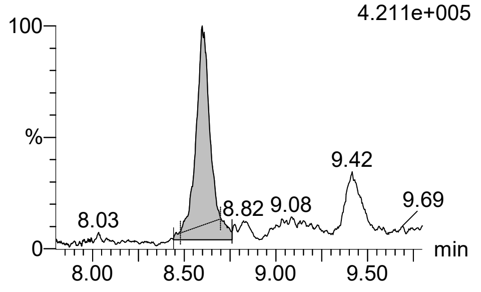

Sample Name: 20211111_A26_R2_IgM
F79:MRM of 1 channel,ES+
1126.7 > 204.1
1.113e+005

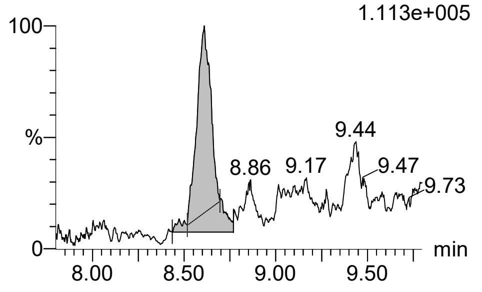

Dataset: C:\MassLynx\10.pro\20211111_clinicalsamples_IgM_IgA.qld

Last Altered: Thursday, March 09, 2023 19:58:25 台北標準時間
Printed: Friday, August 01, 2025 15:16:08 台北標準時間

Sample Name: 20211111_L25_R2_IgM

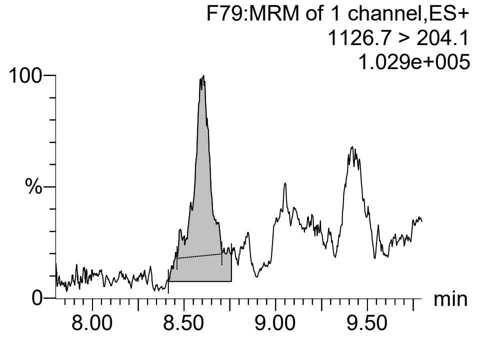

Sample Name: 20211111_L26_R2_IgM

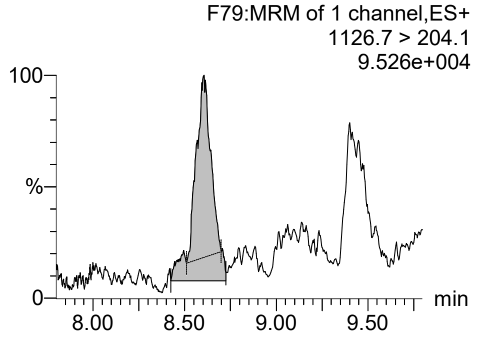

Sample Name: 20211111_H13_R2_IgM

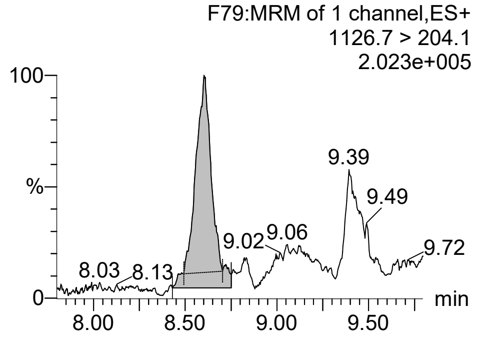

Sample Name: 20211111_A27_R2_IgM

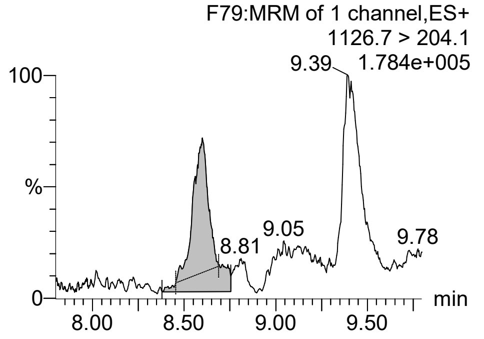

Sample Name: 20211111_A28_R2_IgM

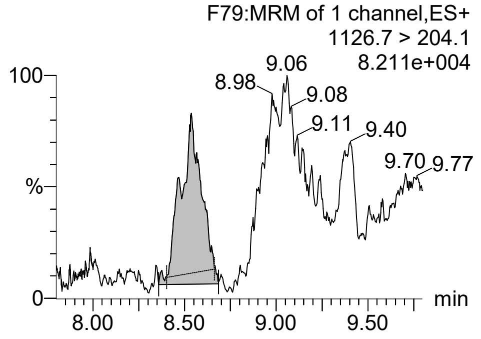

Sample Name: 20211111_L27_R2_IgM

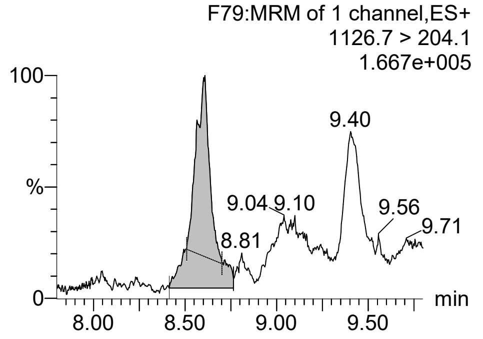

Sample Name: 20211111_L28_R2_IgM

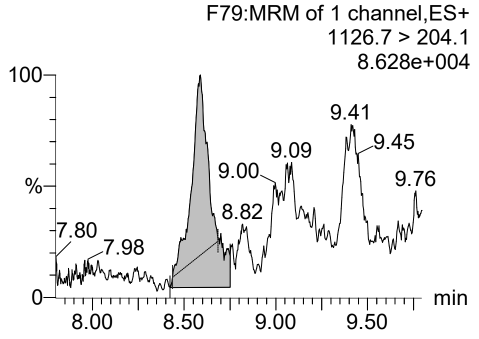

Sample Name: 20211111_H14_R2_IgM

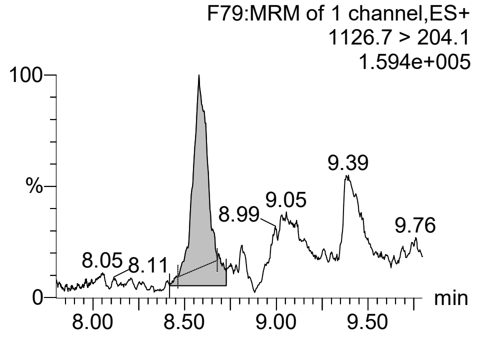

Sample Name: 20211111_A29_R2_IgM

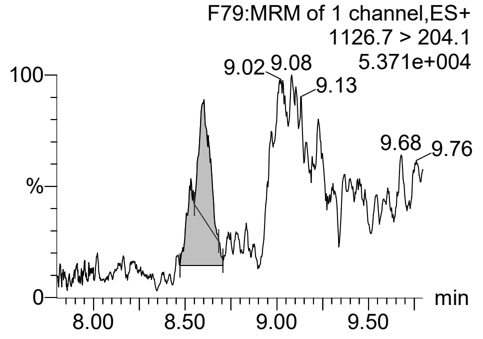

Sample Name: 20211111_A30_R2_IgM

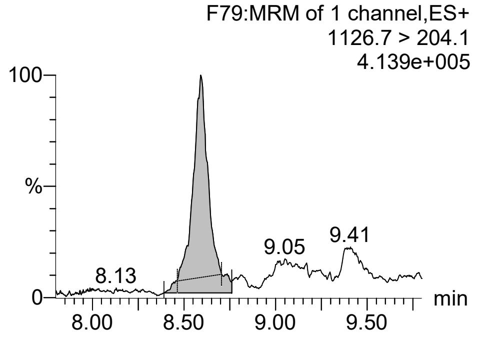

Sample Name: 20211111_QC_R15_IgM

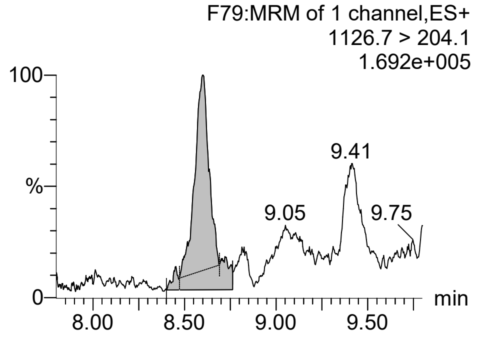

Sample Name: 20211111_L29_R2_IgM

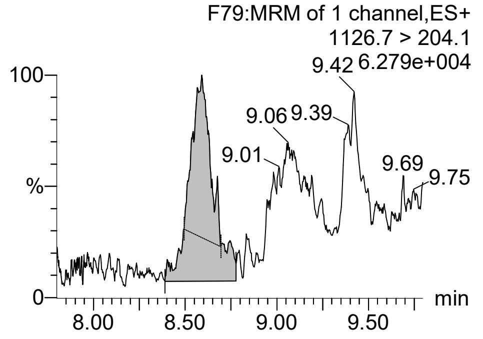

Sample Name: 20211111_L30_R2_IgM

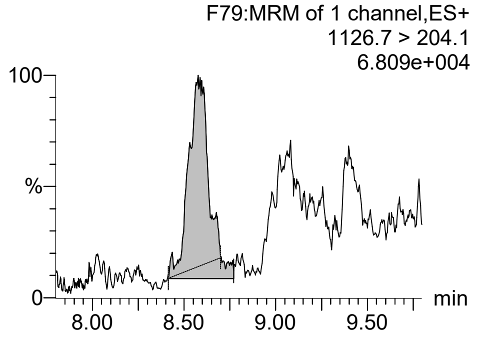

Sample Name: 20211111_H15_R2_IgM

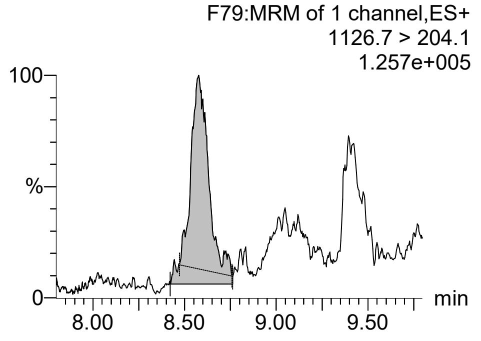

Sample Name: 20211111_A31_R2_IgM

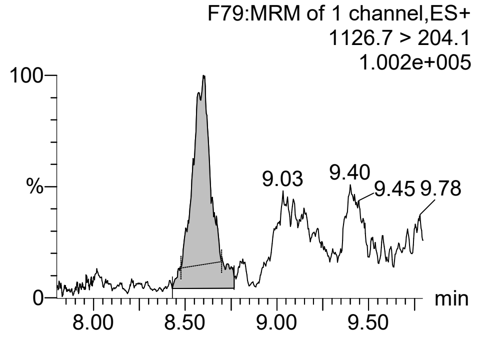

Sample Name: 20211111_A32_R2_IgM
F79:MRM of 1 channel,ES+
1126.7 > 204.1
2.079e+005

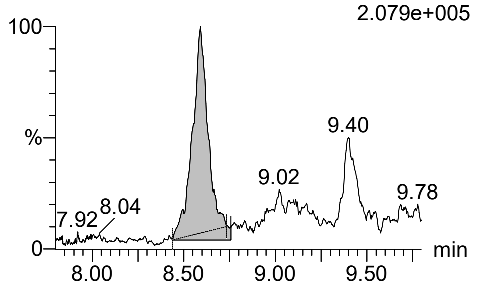

Sample Name: 20211111_L31_R2_IgM
F79:MRM of 1 channel,ES+
1126.7 > 204.1
7.455e+004

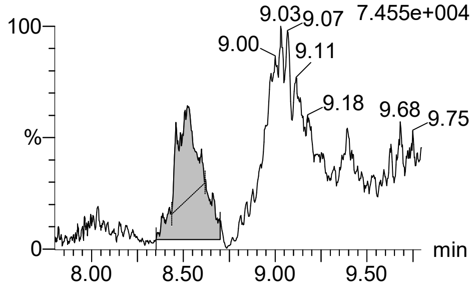

Sample Name: 20211111_L32_R2_IgM
F79:MRM of 1 channel,ES+
1126.7 > 204.1
1.377e+005

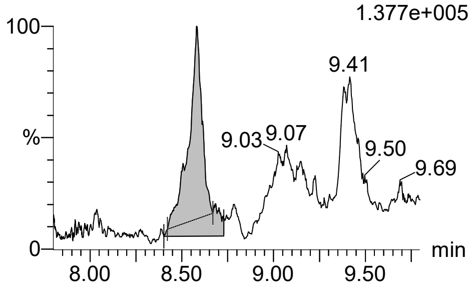

Sample Name: 20211111_H16_R2_IgM
F79:MRM of 1 channel,ES+
1126.7 > 204.1
1.935e+005

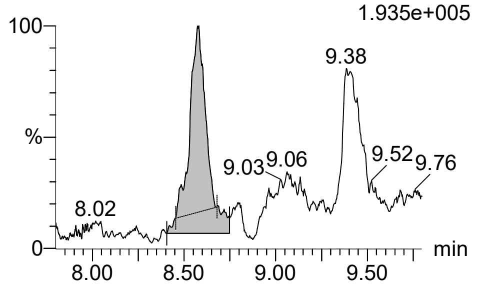

Sample Name: 20211111_A33_R2_IgM
F79:MRM of 1 channel,ES+
1126.7 > 204.1
1.819e+005

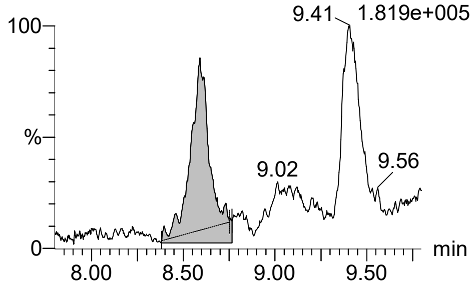

Sample Name: 20211111_A34_R2_IgM
F79:MRM of 1 channel,ES+
1126.7 > 204.1
7.658e+004

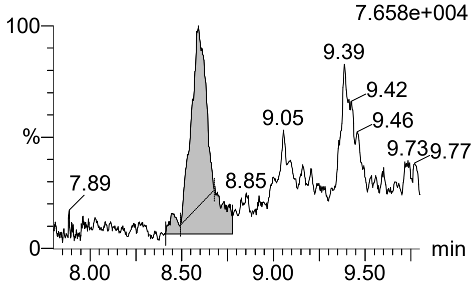

Sample Name: 20211111_H17_R2_IgM
F79:MRM of 1 channel,ES+
1126.7 > 204.1
6.127e+005

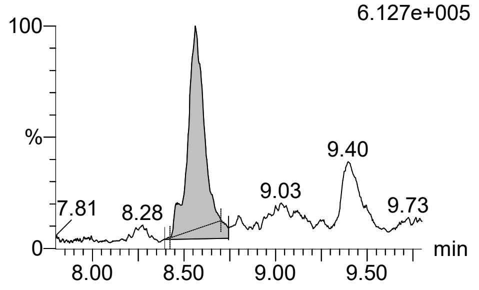

Sample Name: 20211111_A35_R2_IgM
F79:MRM of 1 channel,ES+
1126.7 > 204.1
9.028e+004

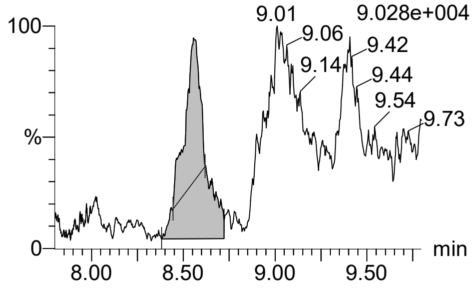

Sample Name: 20211111_QC_R16_IgM
F79:MRM of 1 channel,ES+
1126.7 > 204.1
1.502e+005

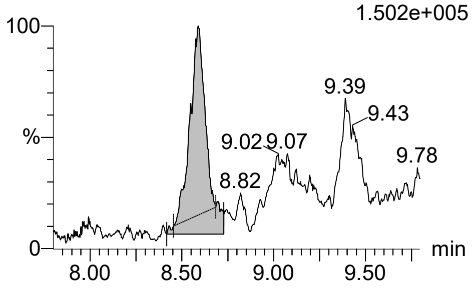

Sample Name: 20211111_A36_R2_IgM
F79:MRM of 1 channel,ES+
1126.7 > 204.1
1.335e+005

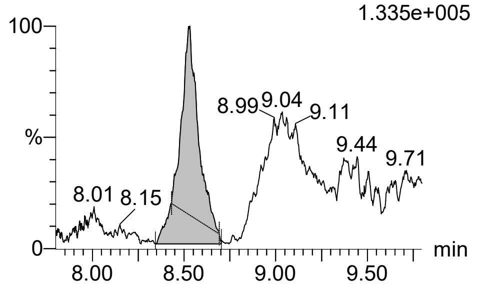

Sample Name: 20211111_H18_R2_IgM
F79:MRM of 1 channel,ES+
1126.7 > 204.1
2.939e+005

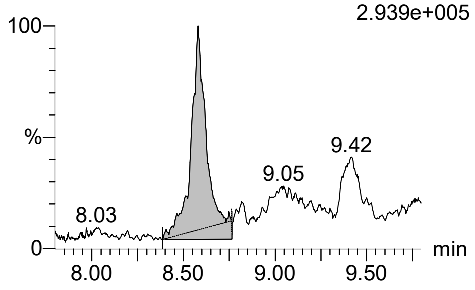

Sample Name: 20211111_A37_R2_IgM
F79:MRM of 1 channel,ES+
1126.7 > 204.1
2.127e+005

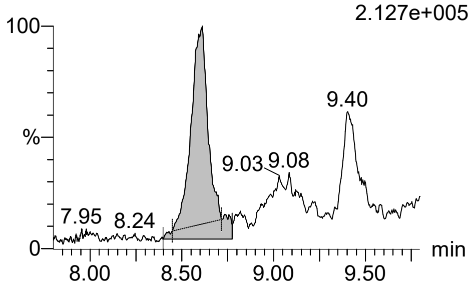

Sample Name: 20211111_A38_R2_IgM
F79:MRM of 1 channel,ES+
1126.7 > 204.1
1.734e+005

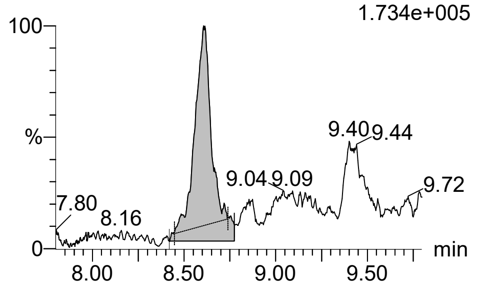

Sample Name: 20211111_H19_R2_IgM
F79:MRM of 1 channel,ES+
1126.7 > 204.1
2.395e+005

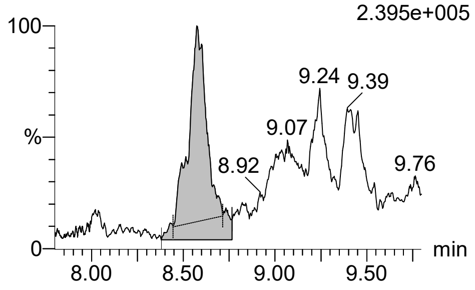

Sample Name: 20211111_A39_R2_IgM
F79:MRM of 1 channel,ES+
1126.7 > 204.1
1.458e+005

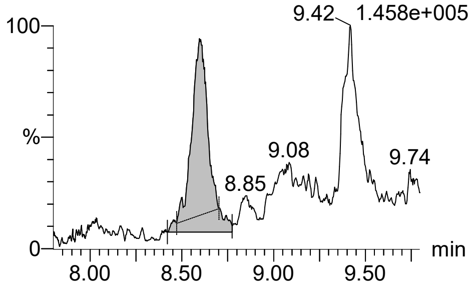

Sample Name: 20211111_A40_R2_IgM
F79:MRM of 1 channel,ES+
1126.7 > 204.1
1.175e+005

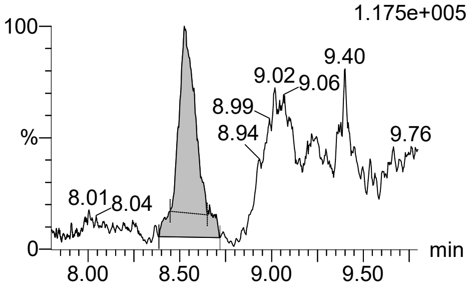

Sample Name: 20211111_H20_R2_IgM
F79:MRM of 1 channel,ES+
1126.7 > 204.1
4.713e+005

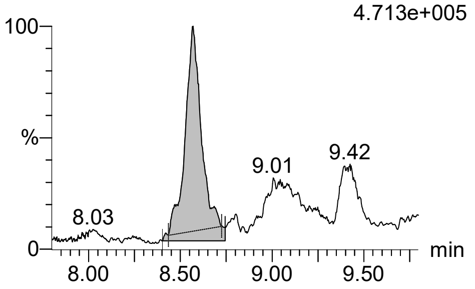

Sample Name: 20211111_A41_R2_IgM
F79:MRM of 1 channel,ES+
1126.7 > 204.1
9.145e+004

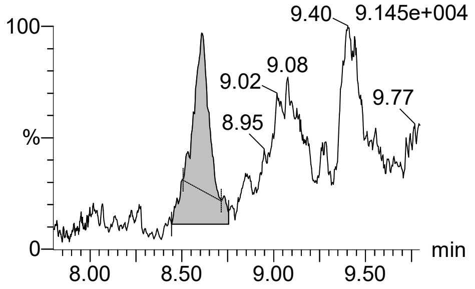

Sample Name: 20211111_A42_R2_IgM
F79:MRM of 1 channel,ES+
1126.7 > 204.1
8.102e+004

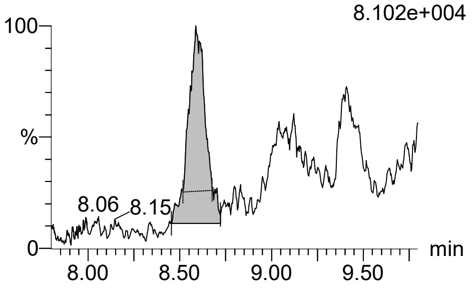

Sample Name: 20211111_A43_R2_IgM
F79:MRM of 1 channel,ES+
1126.7 > 204.1
7.002e+004

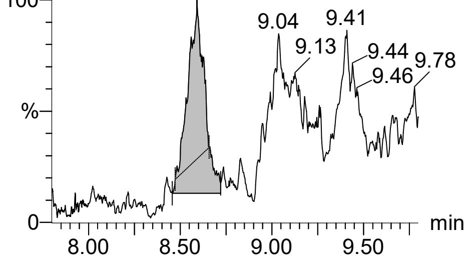

Sample Name: 20211111_A44_R2_IgM
F79:MRM of 1 channel,ES+
1126.7 > 204.1
9.161e+004

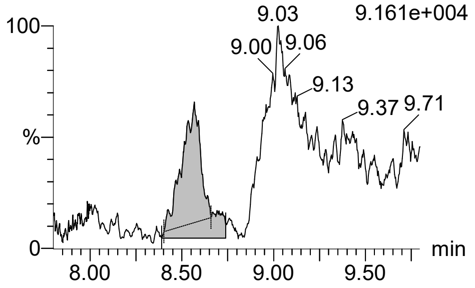

Sample Name: 20211111_QC_R17_IgM
F79:MRM of 1 channel,ES+
1126.7 > 204.1
1.257e+005

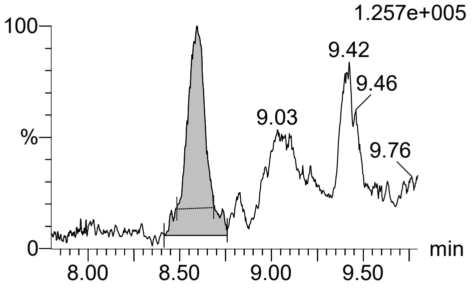

Sample Name: 20211111_L2_R3_IgM
F79:MRM of 1 channel,ES+
1126.7 > 204.1
2.268e+005

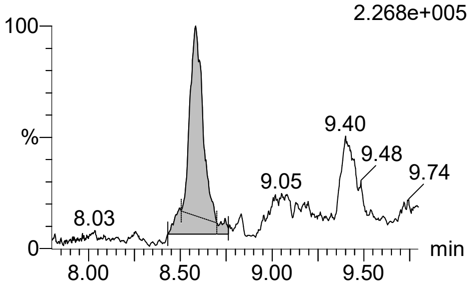

Sample Name: 20211111_H1_R3_IgM
F79:MRM of 1 channel,ES+
1126.7 > 204.1
2.002e+005

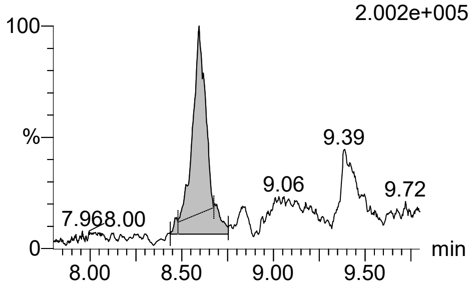

Sample Name: 20211111_A3_R3_IgM
F79:MRM of 1 channel,ES+
1126.7 > 204.1
3.001e+005

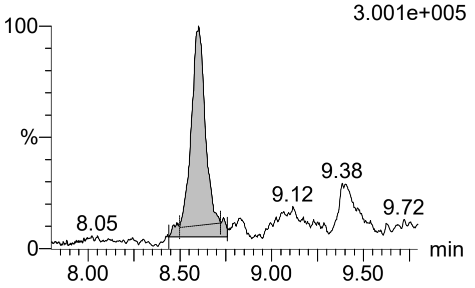

Sample Name: 20211111_A4_R3_IgM
F79:MRM of 1 channel,ES+
1126.7 > 204.1
9.710e+004

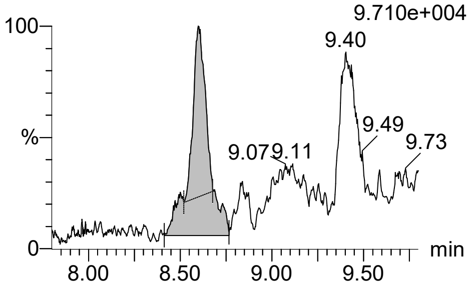

Sample Name: 20211111_L3_R3_IgM
F79:MRM of 1 channel,ES+
1126.7 > 204.1
7.637e+004

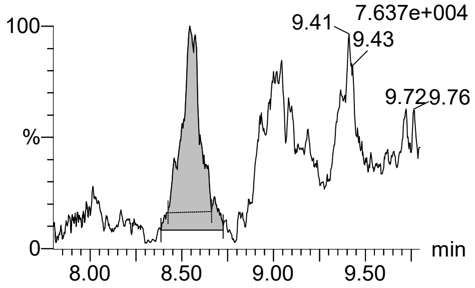

Sample Name: 20211111_L4_R3_IgM
F79:MRM of 1 channel,ES+
1126.7 > 204.1
1.564e+005

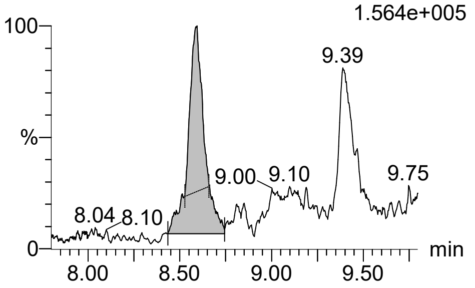

Sample Name: 20211111_H2_R3_IgM
F79:MRM of 1 channel,ES+
1126.7 > 204.1
3.486e+005

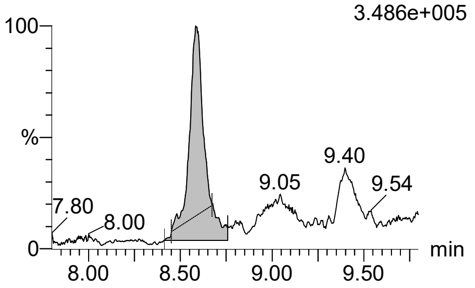

Sample Name: 20211111_A5_R3_IgM
F79:MRM of 1 channel,ES+
1126.7 > 204.1
3.821e+005

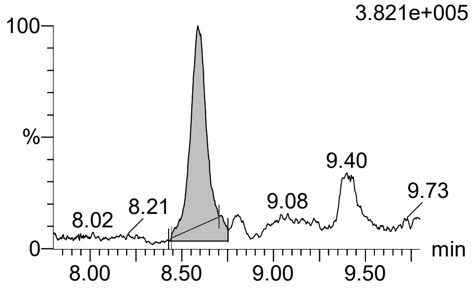

Dataset: C:\MassLynx\10.pro\20211111_clinicalsamples_IgM_IgA.qld

Last Altered: Thursday, March 09, 2023 19:58:25 台北標準時間
Printed: Friday, August 01, 2025 15:16:08 台北標準時間

Sample Name: 20211111_A6_R3_IgM
F79:MRM of 1 channel,ES+
1126.7 > 204.1
1.124e+005

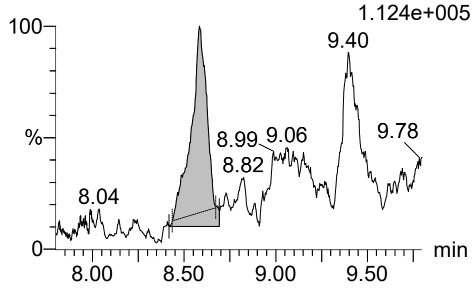

Sample Name: 20211111_QC_R18_IgM
F79:MRM of 1 channel,ES+
1126.7 > 204.1
1.385e+005

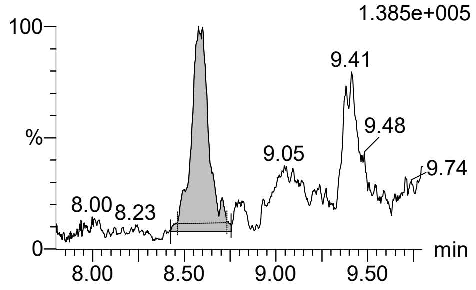

Sample Name: 20211111_L5_R3_IgM
F79:MRM of 1 channel,ES+
1126.7 > 204.1
1.501e+005

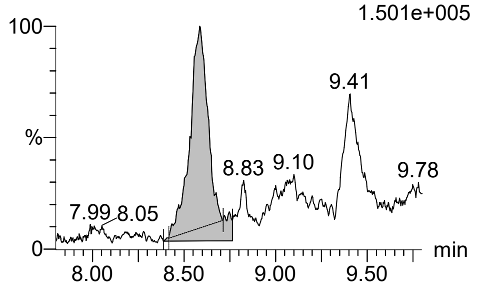

Sample Name: 20211111_L6_R3_IgM
F79:MRM of 1 channel,ES+
1126.7 > 204.1
1.339e+005

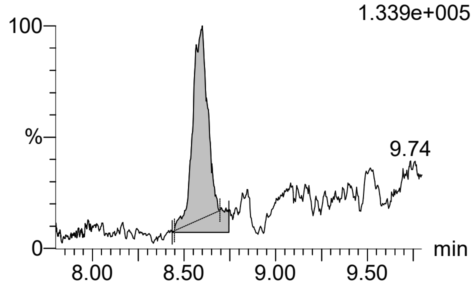

Sample Name: 20211111_H3_R3_IgM
F79:MRM of 1 channel,ES+
1126.7 > 204.1
1.066e+005

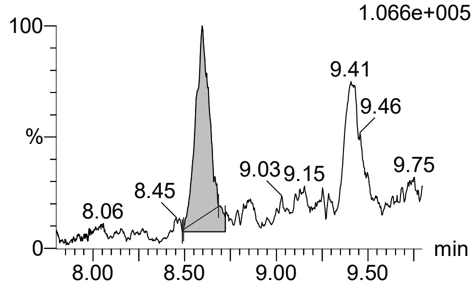

Sample Name: 20211111_A7_R3_IgM
F79:MRM of 1 channel,ES+
1126.7 > 204.1
2.849e+005

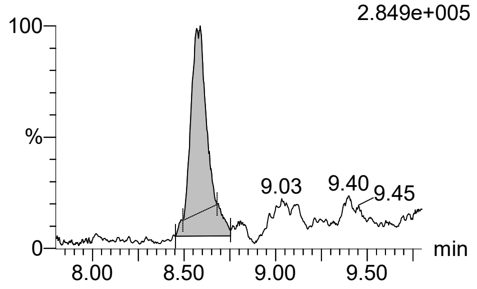

Sample Name: 20211111_A8_R3_IgM
F79:MRM of 1 channel,ES+
1126.7 > 204.1
2.988e+005

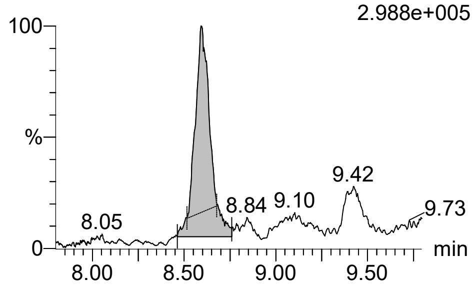

Sample Name: 20211111_L7_R3_IgM
F79:MRM of 1 channel,ES+
1126.7 > 204.1
9.41 7.130e+004

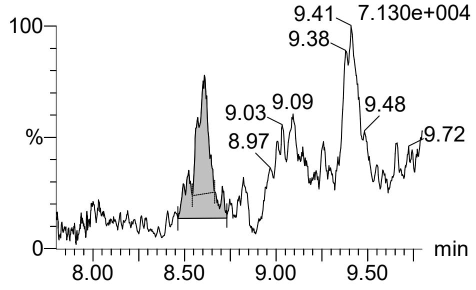

Sample Name: 20211111_L8_R3_IgM
F79:MRM of 1 channel,ES+
1126.7 > 204.1
7.268e+004

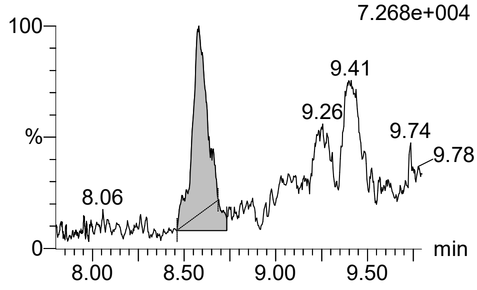

Sample Name: 20211111_H4_R3_IgM
F79:MRM of 1 channel,ES+
1126.7 > 204.1
9.532e+004

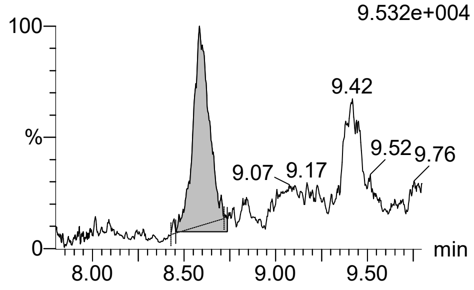

Sample Name: 20211111_A9_R3_IgM
F79:MRM of 1 channel,ES+
1126.7 > 204.1
1.374e+005

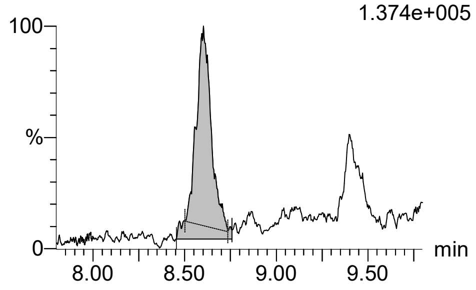

Sample Name: 20211111_A10_R3_IgM
F79:MRM of 1 channel,ES+
1126.7 > 204.1
3.273e+005

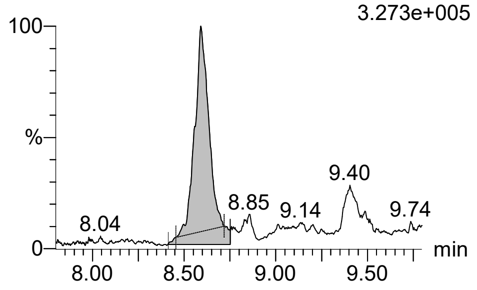

Sample Name: 20211111_L9_R3_IgM
F79:MRM of 1 channel,ES+
1126.7 > 204.1
9.514e+004

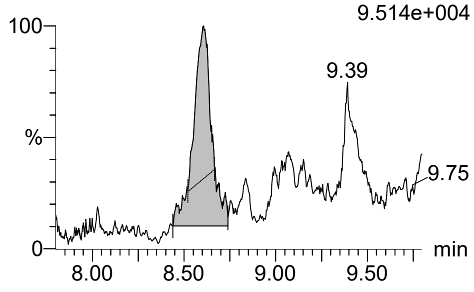

Sample Name: 20211111_L10_R3_IgM
F79:MRM of 1 channel,ES+
1126.7 > 204.1
2.317e+005

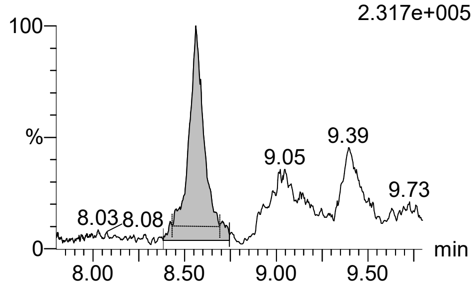

Sample Name: 20211111_QC_R19_IgM
F79:MRM of 1 channel,ES+
1126.7 > 204.1
1.357e+005

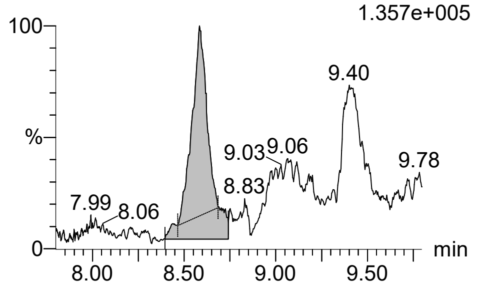

Sample Name: 20211111_H5_R3_IgM

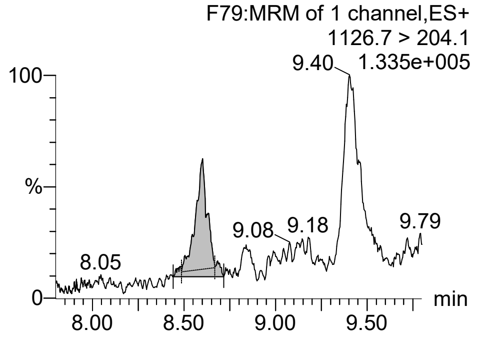

Sample Name: 20211111_A11_R3_IgM

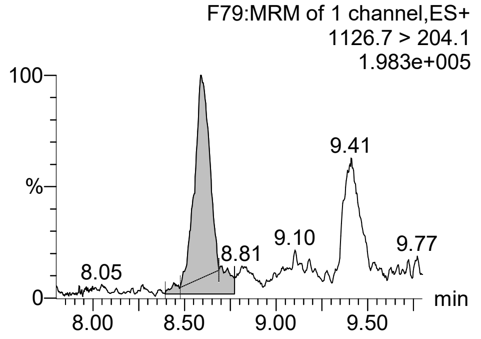

Sample Name: 20211111_A12_R3_IgM

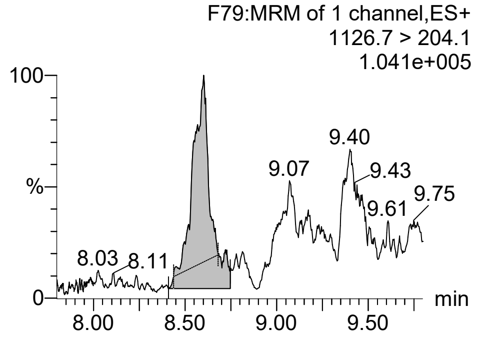

Sample Name: 20211111_L11_R3_IgM

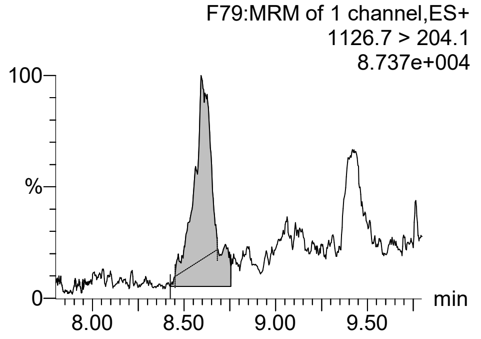

Sample Name: 20211111_L12_R3_IgM

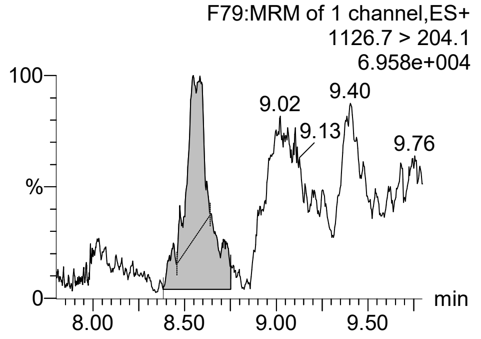

Sample Name: 20211111_H6_R3_IgM

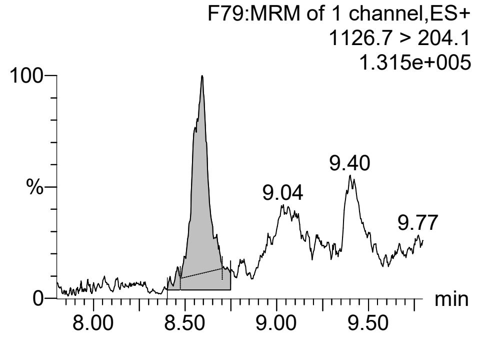

Sample Name: 20211111_A13_R3_IgM

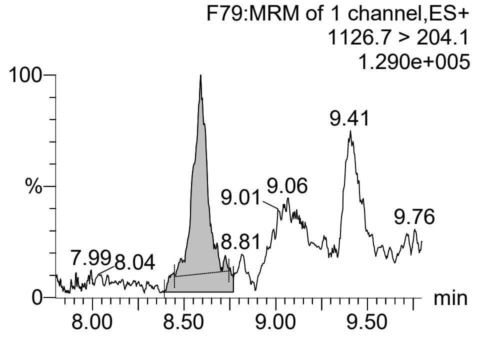

Sample Name: 20211111_A14_R3_IgM

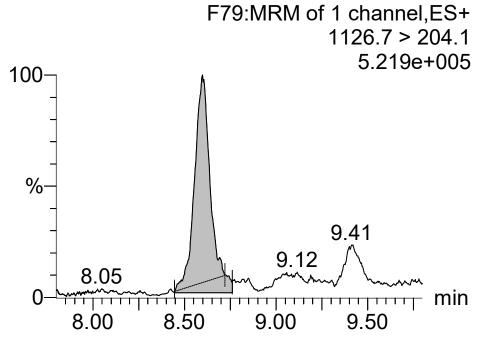

Sample Name: 20211111_L13_R3_IgM

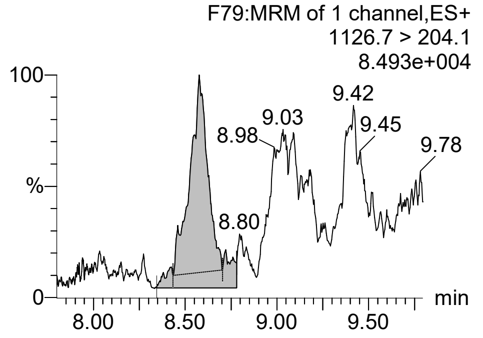

Sample Name: 20211111_L14_R3_IgM

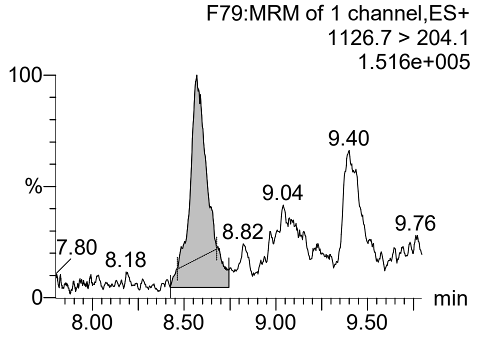

Sample Name: 20211111_H7_R3_IgM

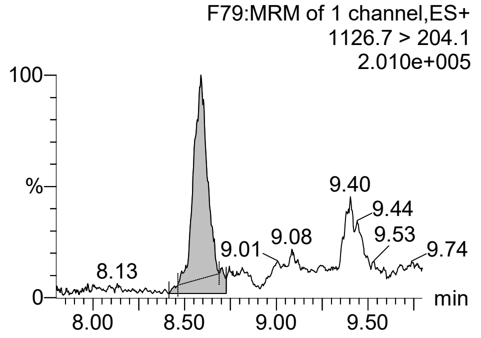

Sample Name: 20211111_A15_R3_IgM

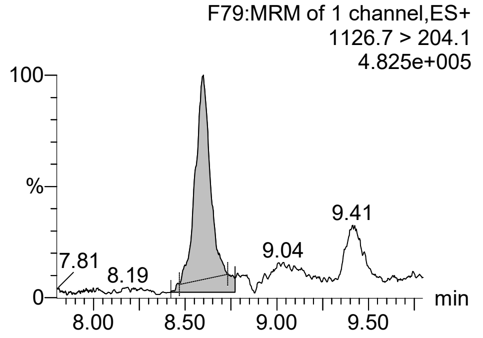

Sample Name: 20211111_QC_R20_IgM

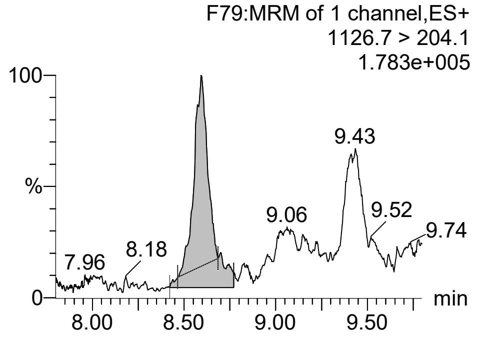

Sample Name: 20211111_A16_R3_IgM

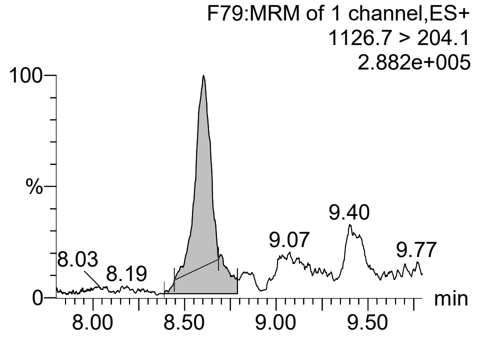

Sample Name: 20211111_L15_R3_IgM

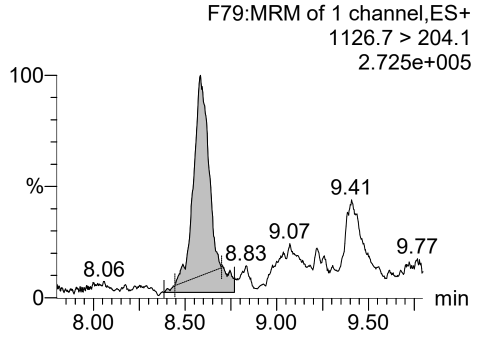

Sample Name: 20211111_L16_R3_IgM
F79:MRM of 1 channel,ES+
1126.7 > 204.1
5.813e+004

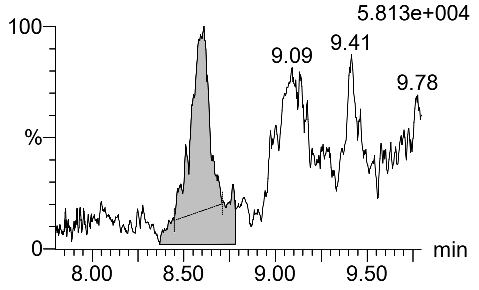

Sample Name: 20211111_H8_R3_IgM
F79:MRM of 1 channel,ES+
1126.7 > 204.1
7.788e+004

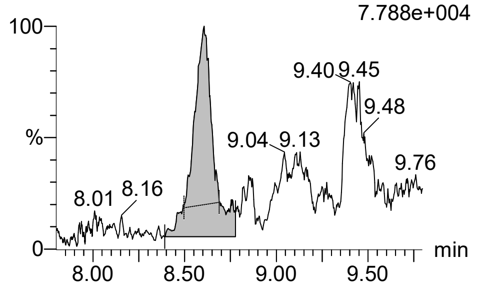

Sample Name: 20211111_A17_R3_IgM
F79:MRM of 1 channel,ES+
1126.7 > 204.1
1.546e+005

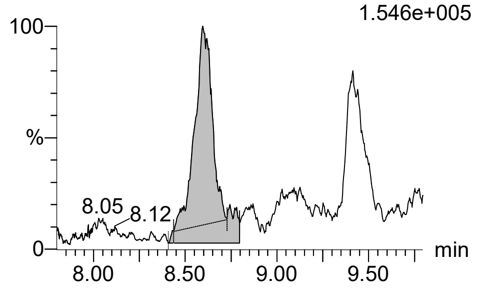

Sample Name: 20211111_A18_R3_IgM
F79:MRM of 1 channel,ES+
1126.7 > 204.1
1.441e+005

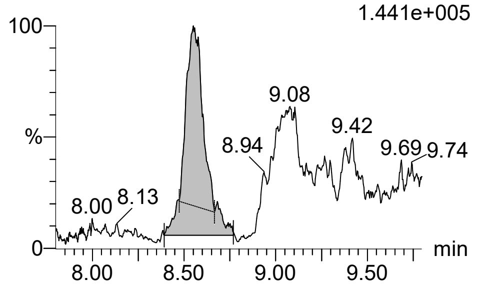

Sample Name: 20211111_L17_R3_IgM
F79:MRM of 1 channel,ES+
1126.7 > 204.1
1.525e+005

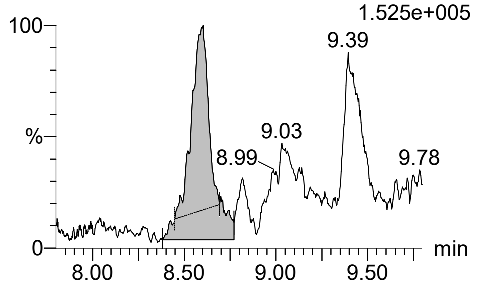

Sample Name: 20211111_L18_R3_IgM
F79:MRM of 1 channel,ES+
1126.7 > 204.1
5.545e+004

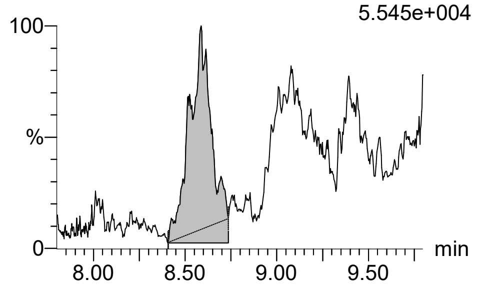

Sample Name: 20211111_H9_R3_IgM
F79:MRM of 1 channel,ES+
1126.7 > 204.1
1.066e+005

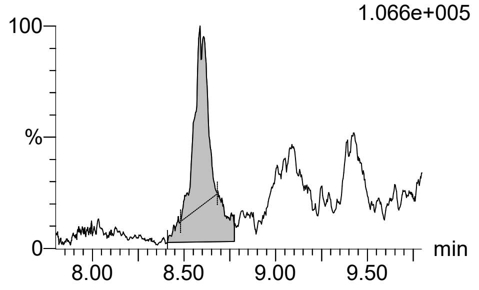

Sample Name: 20211111_A19_R3_IgM
F79:MRM of 1 channel,ES+
1126.7 > 204.1
1.242e+005

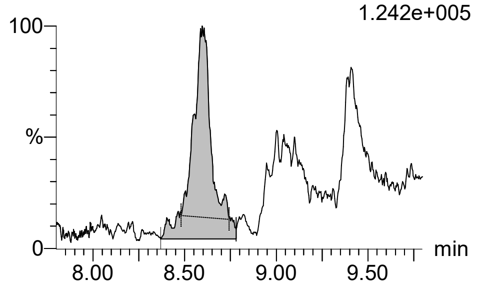

Sample Name: 20211111_A20_R3_IgM
F79:MRM of 1 channel,ES+
1126.7 > 204.1
1.339e+005

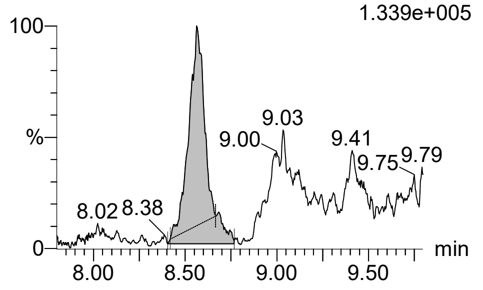

Sample Name: 20211111_L19_R3_IgM
F79:MRM of 1 channel,ES+
1126.7 > 204.1
1.615e+005

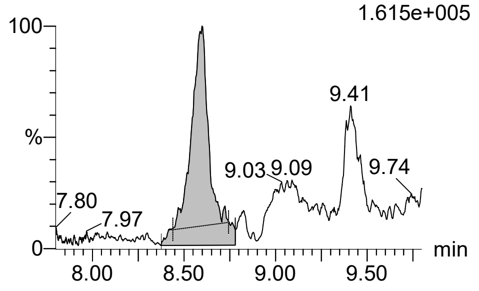

Sample Name: 20211111_QC_R21_IgM
F79:MRM of 1 channel,ES+
1126.7 > 204.1
1.700e+005

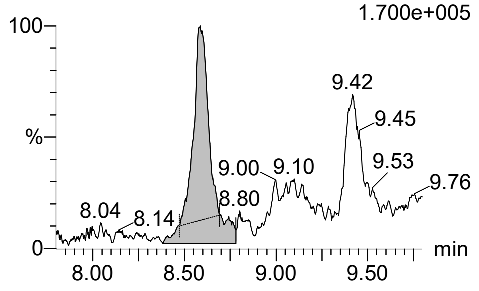

Sample Name: 20211111_L20_R3_IgM
F79:MRM of 1 channel,ES+
1126.7 > 204.1
7.326e+004

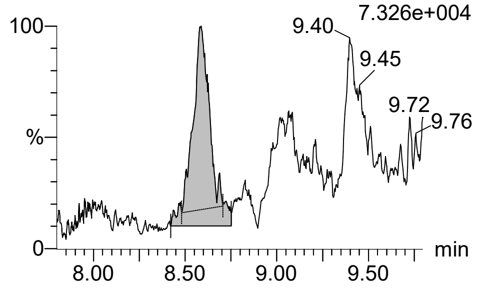

Sample Name: 20211111_H10_R3_IgM
F79:MRM of 1 channel,ES+
1126.7 > 204.1
1.400e+005

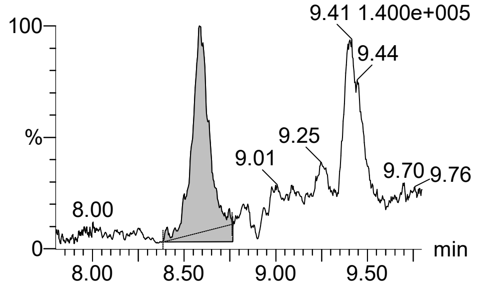

Sample Name: 20211111_A21_R3_IgM
F79:MRM of 1 channel,ES+
1126.7 > 204.1
2.083e+005

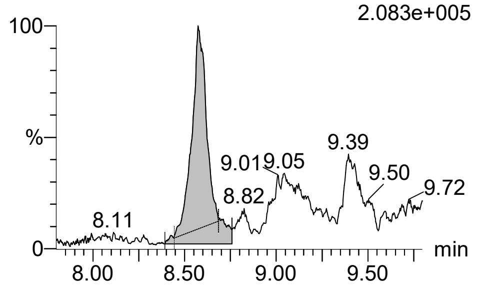

Sample Name: 20211111_A22_R3_IgM
F79:MRM of 1 channel,ES+
1126.7 > 204.1
5.313e+004

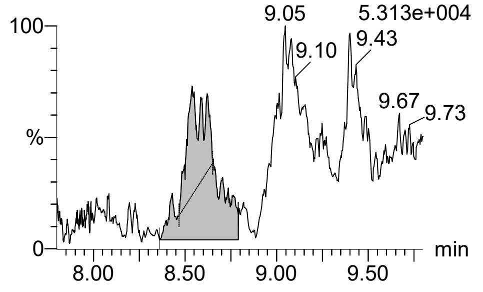

Dataset: C:\MassLynx\10.pro\20211111_clinicalsamples_IgM_IgA.qld

Last Altered: Thursday, March 09, 2023 19:58:25 台北標準時間
Printed: Friday, August 01, 2025 15:16:08 台北標準時間

Sample Name: 20211111_L21_R3_IgM

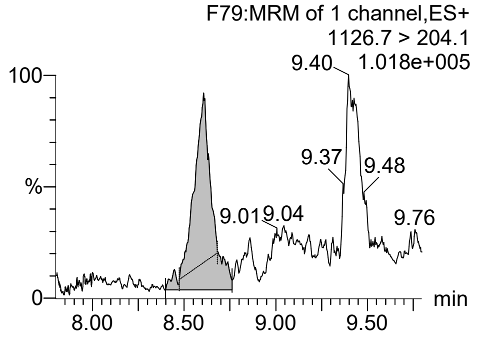

Sample Name: 20211111_L22_R3_IgM

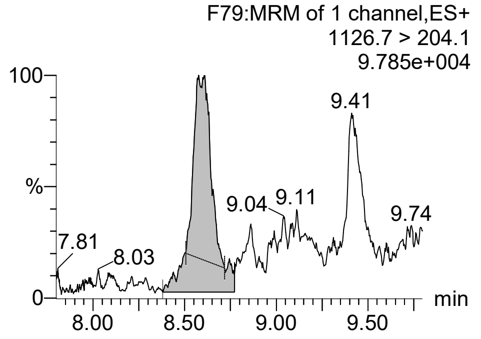

Sample Name: 20211111_H11_R3_IgM

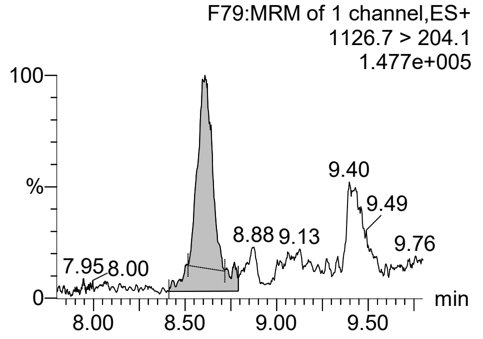

Sample Name: 20211111_A23_R3_IgM

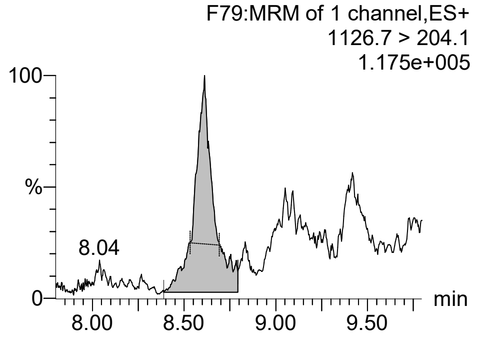

Sample Name: 20211111_A24_R3_IgM

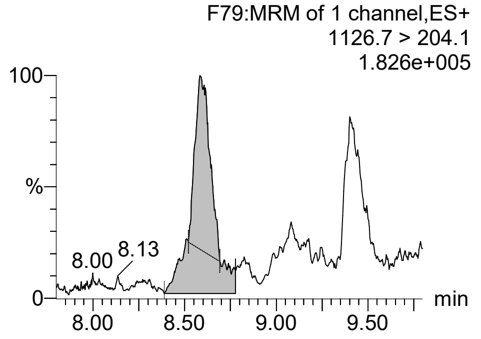

Sample Name: 20211111_L23_R3_IgM

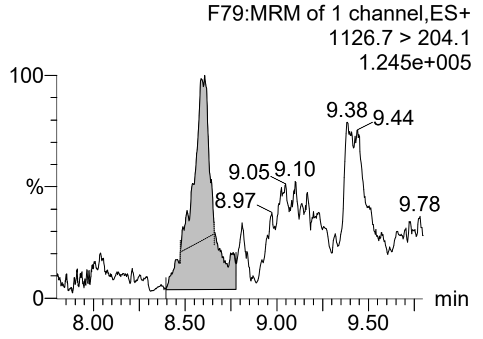

Sample Name: 20211111_L24_R3_IgM

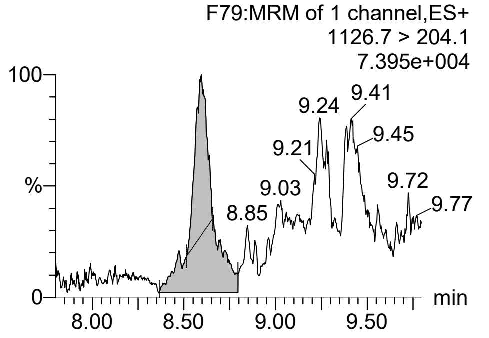

Sample Name: 20211111_H12_R3_IgM

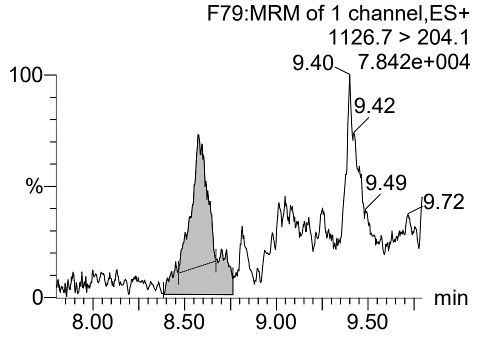

Sample Name: 20211111_QC_R22_IgM

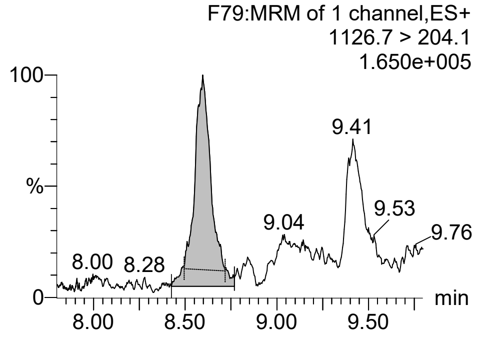

Sample Name: 20211111_A25_R3_IgM

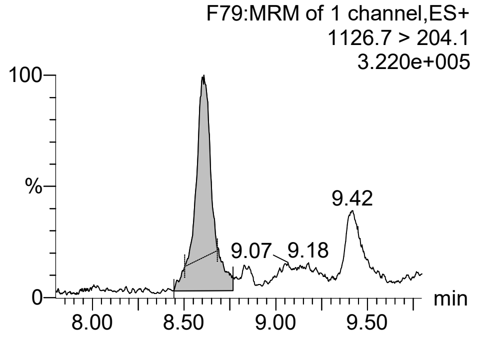

Sample Name: 20211111_A26_R3_IgM

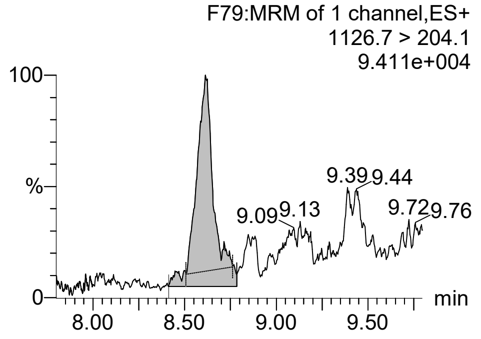

Sample Name: 20211111_L25_R3_IgM

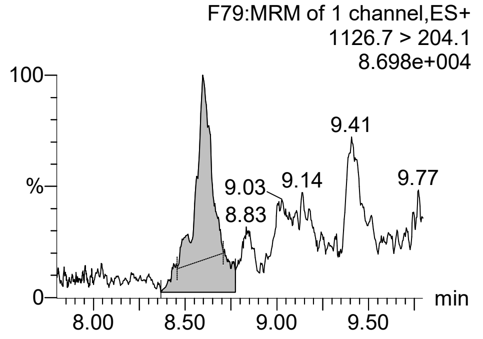

Sample Name: 20211111_L26_R3_IgM

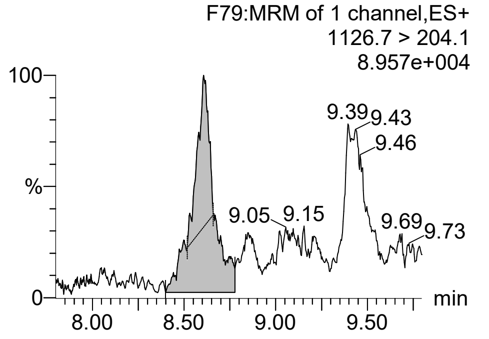

Sample Name: 20211111_H13_R3_IgM

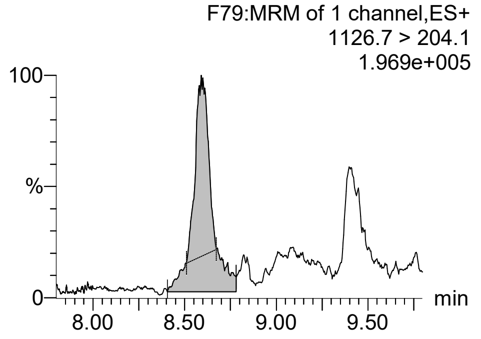

Sample Name: 20211111_A27_R3_IgM

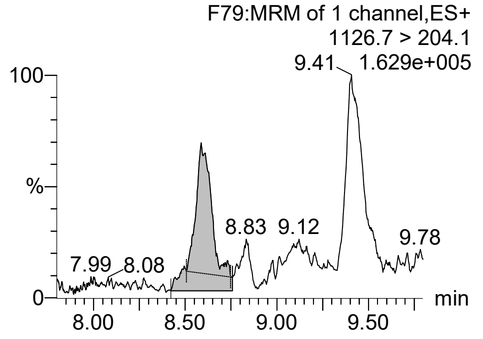

Dataset: C:\MassLynx\10.pro\20211111_clinicalsamples_IgM_IgA.qld

Last Altered: Thursday, March 09, 2023 19:58:25 台北標準時間
Printed: Friday, August 01, 2025 15:16:08 台北標準時間

Sample Name: 20211111_A28_R3_IgM
F79:MRM of 1 channel,ES+
1126.7 > 204.1
5.847e+004

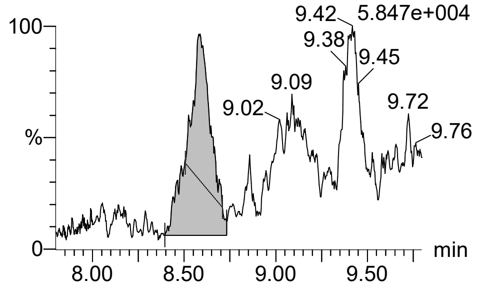

Sample Name: 20211111_L27_R3_IgM
F79:MRM of 1 channel,ES+
1126.7 > 204.1
1.534e+005

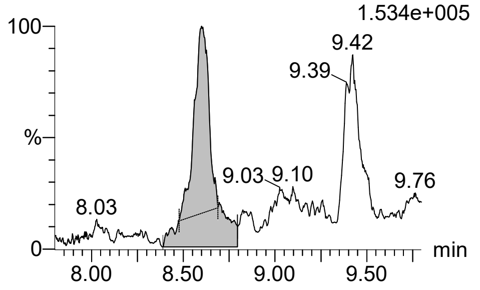

Sample Name: 20211111_L28_R3_IgM
F79:MRM of 1 channel,ES+
1126.7 > 204.1
8.359e+004

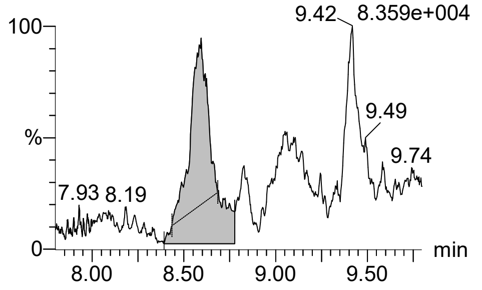

Sample Name: 20211111_H14_R3_IgM
F79:MRM of 1 channel,ES+
1126.7 > 204.1
1.637e+005

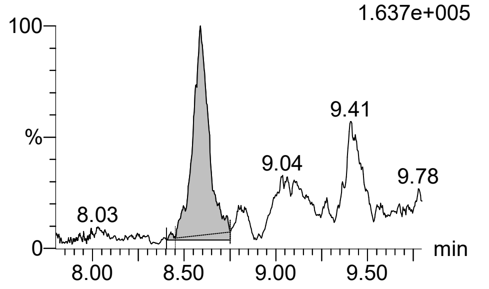

Sample Name: 20211111_A29_R3_IgM
F79:MRM of 1 channel,ES+
1126.7 > 204.1
4.433e+004

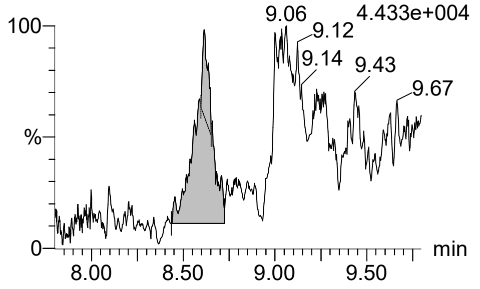

Sample Name: 20211111_A30_R3_IgM
F79:MRM of 1 channel,ES+
1126.7 > 204.1
4.022e+005

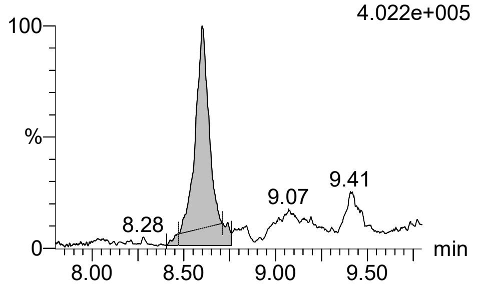

Sample Name: 20211111_QC_R23_IgM
F79:MRM of 1 channel,ES+
1126.7 > 204.1
1.501e+005

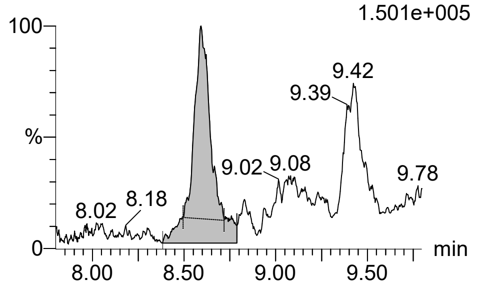

Sample Name: 20211111_L29_R3_IgM
F79:MRM of 1 channel,ES+
1126.7 > 204.1
6.720e+004

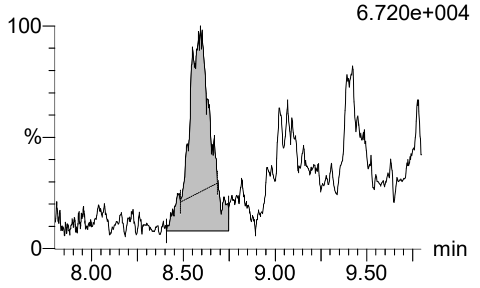

Sample Name: 20211111_L30_R3_IgM
F79:MRM of 1 channel,ES+
1126.7 > 204.1
7.140e+004

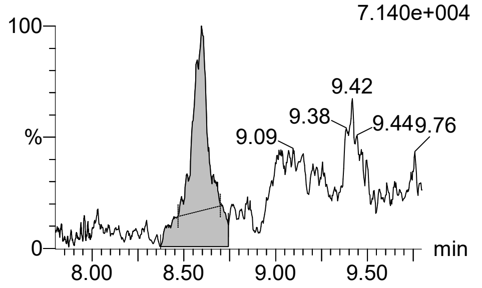

Sample Name: 20211111_H15_R3_IgM
F79:MRM of 1 channel,ES+
1126.7 > 204.1
1.106e+005

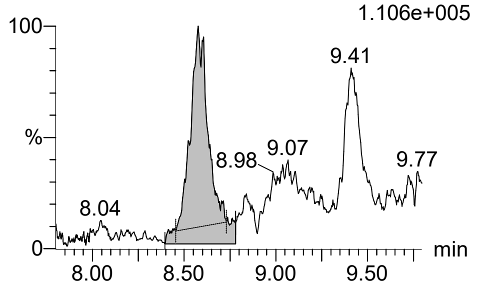

Sample Name: 20211111_A31_R3_IgM
F79:MRM of 1 channel,ES+
1126.7 > 204.1
1.330e+005

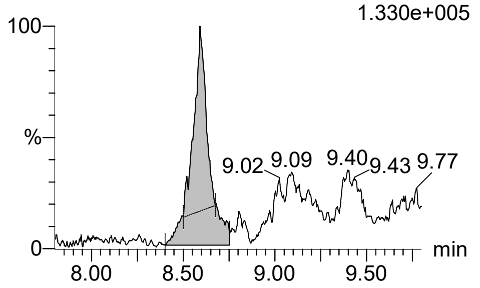

Sample Name: 20211111_A32_R3_IgM
F79:MRM of 1 channel,ES+
1126.7 > 204.1
1.666e+005

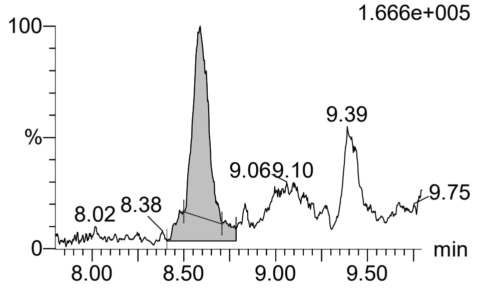

Sample Name: 20211111_L31_R3_IgM
F79:MRM of 1 channel,ES+
1126.7 > 204.1
3.823e+004

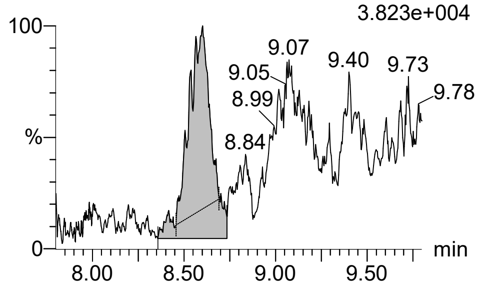

Sample Name: 20211111_L32_R3_IgM
F79:MRM of 1 channel,ES+
1126.7 > 204.1
1.185e+005

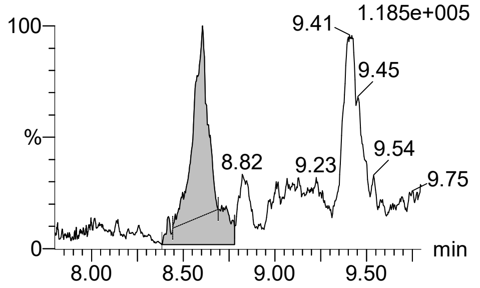

Sample Name: 20211111_H16_R3_IgM
F79:MRM of 1 channel,ES+
1126.7 > 204.1
1.747e+005

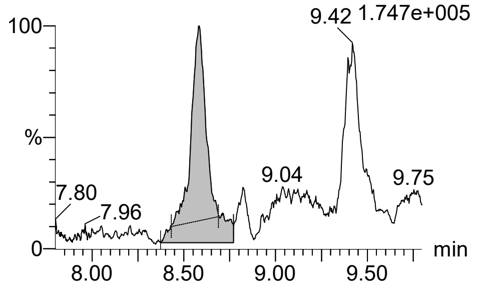

Dataset: C:\MassLynx\10.pro\20211111_clinicalsamples_IgM_IgA.qld

Last Altered: Thursday, March 09, 2023 19:58:25 台北標準時間
Printed: Friday, August 01, 2025 15:16:08 台北標準時間

Sample Name: 20211111_A33_R3_IgM
F79:MRM of 1 channel,ES+
1126.7 > 204.1
2.017e+005

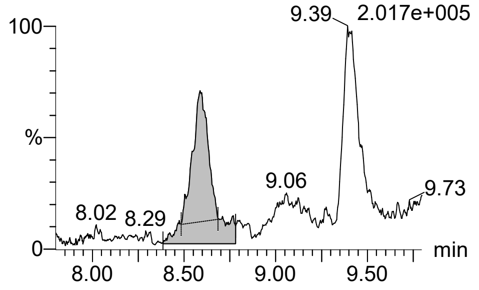

Sample Name: 20211111_A34_R3_IgM
F79:MRM of 1 channel,ES+
1126.7 > 204.1
6.485e+004

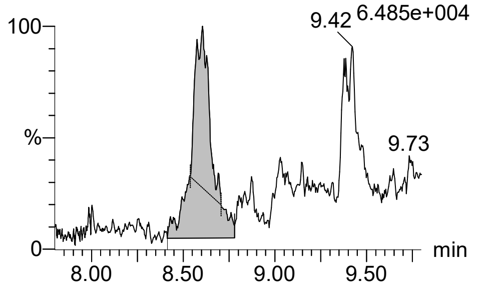

Sample Name: 20211111_H17_R3_IgM
F79:MRM of 1 channel,ES+
1126.7 > 204.1
4.868e+005

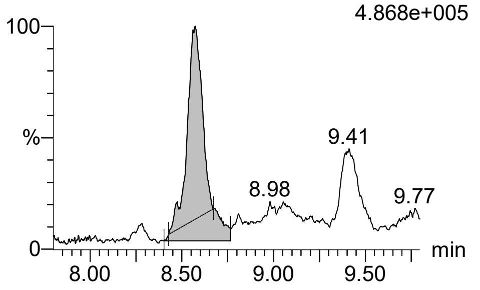

Sample Name: 20211111_A35_R3_IgM
F79:MRM of 1 channel,ES+
1126.7 > 204.1
9.314e+004

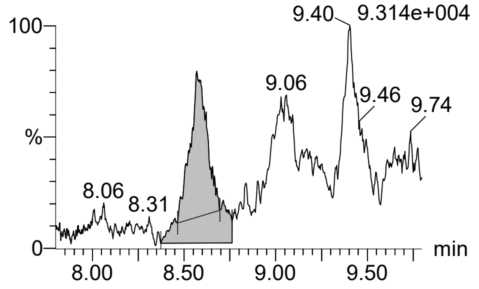

Sample Name: 20211111_QC_R24_IgM
F79:MRM of 1 channel,ES+
1126.7 > 204.1
1.444e+005

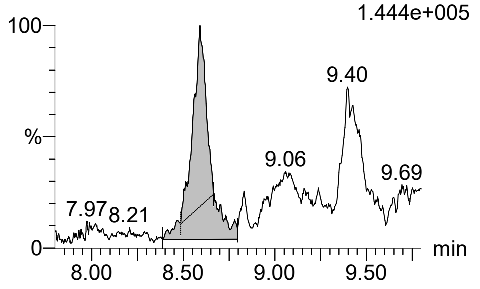

Sample Name: 20211111_A36_R3_IgM
F79:MRM of 1 channel,ES+
1126.7 > 204.1
1.495e+005

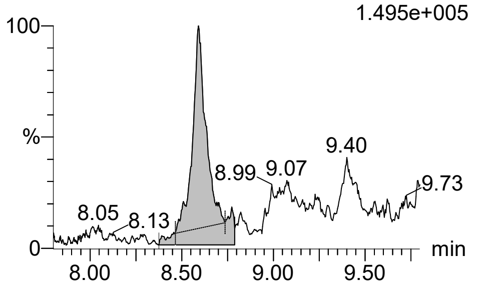

Sample Name: 20211111_H18_R3_IgM
F79:MRM of 1 channel,ES+
1126.7 > 204.1
2.276e+005

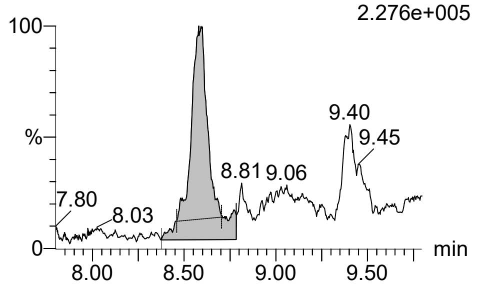

Sample Name: 20211111_A37_R3_IgM
F79:MRM of 1 channel,ES+
1126.7 > 204.1
2.000e+005

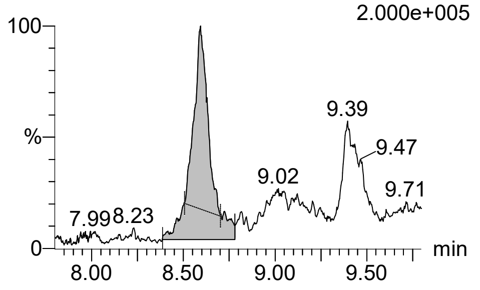

Sample Name: 20211111_A38_R3_IgM
F79:MRM of 1 channel,ES+
1126.7 > 204.1
1.598e+005

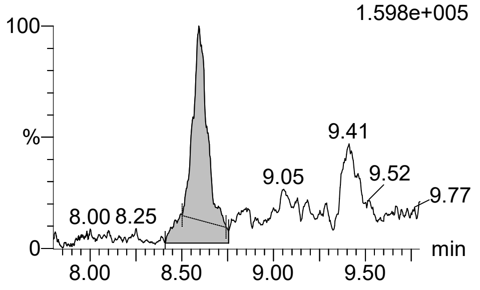

Sample Name: 20211111_H19_R3_IgM
F79:MRM of 1 channel,ES+
1126.7 > 204.1
2.175e+005

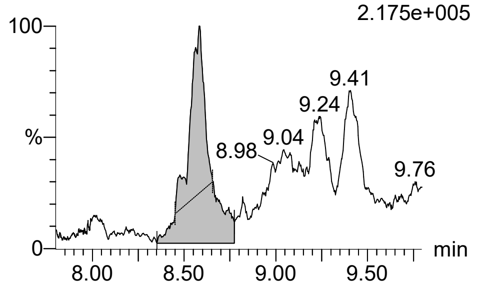

Sample Name: 20211111_A39_R3_IgM
F79:MRM of 1 channel,ES+
1126.7 > 204.1
1.257e+005

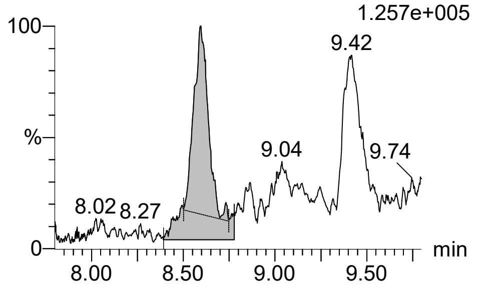

Sample Name: 20211111_A40_R3_IgM
F79:MRM of 1 channel,ES+
1126.7 > 204.1
9.882e+004

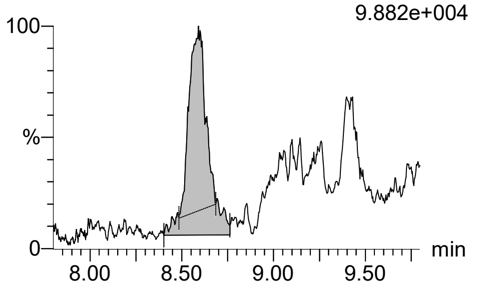

Sample Name: 20211111_H20_R3_IgM
F79:MRM of 1 channel,ES+
1126.7 > 204.1
3.567e+005

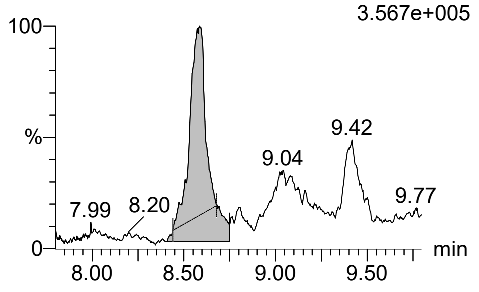

Sample Name: 20211111_A41_R3_IgM
F79:MRM of 1 channel,ES+
1126.7 > 204.1
7.546e+004

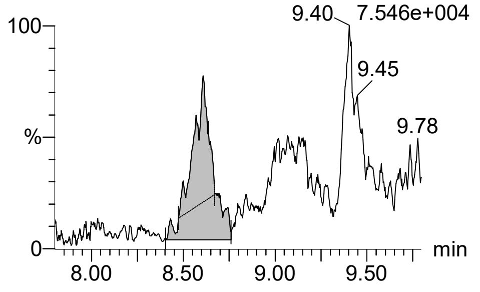

Sample Name: 20211111_A42_R3_IgM
F79:MRM of 1 channel,ES+
1126.7 > 204.1
6.490e+004

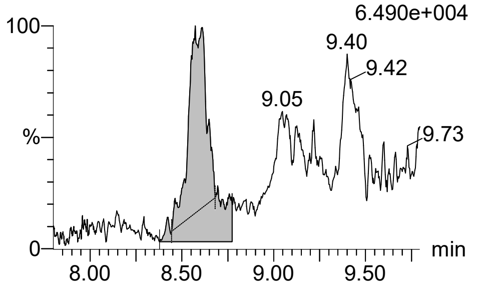

Sample Name: 20211111_A43_R3_IgM

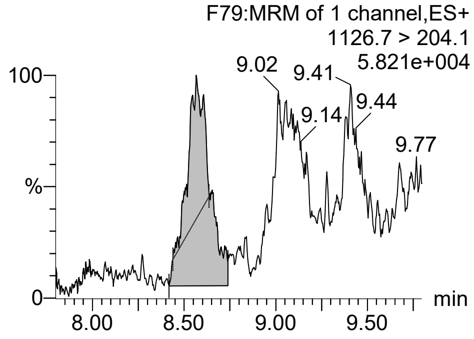

Sample Name: 20211111_A44_R3_IgM

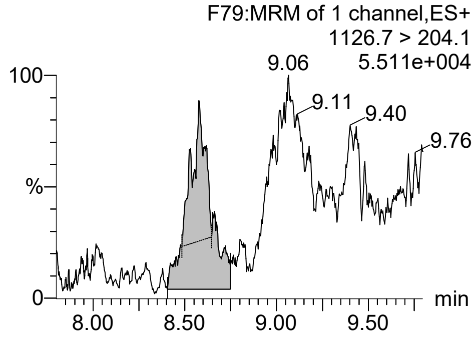

Sample Name: 20211111_QC_R25_IgM

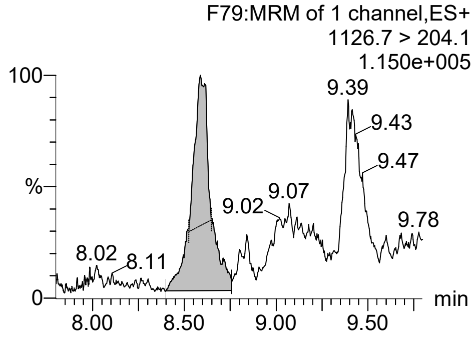

Dataset: C:\MassLynx\10.pro\20211111_clinicalsamples_IgM_IgA.qld

Last Altered: Thursday, March 09, 2023 19:58:25 台北標準時間
Printed: Friday, August 01, 2025 15:16:08 台北標準時間

	#	Name	RT	Area	IS Area	Response	Prima...
1	1	20211112_QC_R001_IgM	8.56	13512.0	15421.4	0.876	MM
2	2	20211112_QC_R002_IgM	8.59	10482.8	13188.8	0.795	MM
3	3	20211111_A1_R2_IgM	8.58	8556.7	11871.0	0.721	MM
4	4	20211111_A1_R3_IgM	8.59	7953.9	12030.5	0.661	MM
5	5	20211111_A2_R2_IgM	8.58	15698.8	11446.8	1.371	MM
6	6	20211111_A2_R3_IgM	8.59	14384.4	11098.4	1.296	MM
7	7	20211111_L1_R2_IgM	8.56	5984.0	11779.4	0.508	MM
8	8	20211111_L1_R3_IgM	8.59	4979.2	11176.2	0.446	MM
9	9	20211111_L2_R2_IgM	8.58	16869.8	11375.0	1.483	MM
10	10	20211111_H1_R2_IgM	8.59	19799.4	13595.2	1.456	MM
11	11	20211111_A3_R2_IgM	8.58	29071.8	14112.3	2.060	MM
12	12	20211111_A4_R2_IgM	8.59	8688.2	11977.5	0.725	MM
13	13	20211111_L3_R2_IgM	8.55	8863.3	12242.1	0.724	MM
14	14	20211111_L4_R2_IgM	8.59	16977.6	12797.0	1.327	MM
15	15	20211111_H2_R2_IgM	8.59	29106.4	10006.0	2.909	MM
16	16	20211111_A5_R2_IgM	8.57	44292.1	11434.6	3.874	MM
17	17	20211111_A6_R2_IgM	8.59	12872.1	13193.7	0.976	MM
18	18	20211111_QC_R10_IgM	8.58	14472.2	12566.1	1.152	MM
19	19	20211111_L5_R2_IgM	8.55	22830.9	13616.4	1.677	MM
20	20	20211111_L6_R2_IgM	8.58	16618.0	13698.5	1.213	MM
21	21	20211111_H3_R2_IgM	8.57	11532.3	15793.1	0.730	MM
22	22	20211111_A7_R2_IgM	8.58	27451.7	12936.9	2.122	MM
23	23	20211111_A8_R2_IgM	8.58	33174.2	13108.0	2.531	MM
24	24	20211111_L7_R2_IgM	8.60	3767.1	13278.5	0.284	MM
25	25	20211111_L8_R2_IgM	8.57	8575.8	15233.6	0.563	MM
26	26	20211111_H4_R2_IgM	8.58	10979.6	16521.9	0.665	MM
27	27	20211111_A9_R2_IgM	8.57	15179.6	16451.6	0.923	MM
28	28	20211111_A10_R2_IgM	8.58	32268.2	13036.0	2.475	MM
29	29	20211111_L9_R2_IgM	8.59	10099.6	14554.1	0.694	MM
30	30	20211111_L10_R2_IgM	8.57	24357.1	12250.4	1.988	MM
31	31	20211111_QC_R11_IgM	8.59	16529.2	11459.2	1.442	MM
32	32	20211111_H5_R2_IgM	8.56	8342.4	14358.2	0.581	MM
33	33	20211111_A11_R2_IgM	8.52	22164.3	16184.1	1.370	MM
34	34	20211111_A12_R2_IgM	8.58	11098.5	13465.4	0.824	MM
35	35	20211111_L11_R2_IgM	8.59	12414.0	13277.9	0.935	MM
36	36	20211111_L12_R2_IgM	8.58	10858.0	15958.8	0.680	MM
37	37	20211111_H6_R2_IgM	8.57	14369.2	14003.5	1.026	MM
38	38	20211111_A13_R2_IgM	8.59	13402.7	13656.1	0.981	MM
39	39	20211111_A14_R2_IgM	8.59	62585.2	14071.3	4.448	MM
40	40	20211111_L13_R2_IgM	8.58	12830.9	14785.6	0.868	MM
41	41	20211111_L14_R2_IgM	8.57	19992.2	14772.2	1.353	MM
42	42	20211111_H7_R2_IgM	8.55	21952.9	15164.4	1.448	MM
43	43	20211111_A15_R2_IgM	8.59	59773.3	15721.5	3.802	MM
44	44	20211111_QC_R12_IgM	8.58	19104.6	13711.5	1.393	MM
45	45	20211111_A16_R2_IgM	8.60	37275.1	14275.6	2.611	MM
46	46	20211111_L15_R2_IgM	8.58	33764.8	12935.8	2.610	MM
47	47	20211111_L16_R2_IgM	8.58	7142.8	13342.8	0.535	MM
48	48	20211111_H8_R2_IgM	8.60	7235.4	15835.1	0.457	MM
49	49	20211111_A17_R2_IgM	8.59	23395.5	15786.7	1.482	MM
50	50	20211111_A18_R2_IgM	8.57	20733.5	16986.8	1.221	MM
51	51	20211111_L17_R2_IgM	8.58	24297.7	13342.7	1.821	MM
52	52	20211111_L18_R2_IgM	8.59	7838.5	14692.0	0.534	MM
53	53	20211111_H9_R2_IgM	8.59	13370.4	15639.7	0.855	MM
54	54	20211111_A19_R2_IgM	8.59	14039.4	14198.7	0.989	MM
55	55	20211111_A20_R2_IgM	8.58	15449.9	14162.7	1.091	MM
56	56	20211111_L19_R2_IgM	8.58	17155.7	9293.4	1.846	MM

Dataset: C:\MassLynx\10.pro\20211111_clinicalsamples_IgM_IgA.qld

Last Altered: Thursday, March 09, 2023 19:58:25 台北標準時間
Printed: Friday, August 01, 2025 15:16:08 台北標準時間

	#	Name	RT	Area	IS Area	Response	Prima...
57	57	20211111_QC_R13_IgM	8.59	18968.6	13903.0	1.364	MM
58	58	20211111_L20_R2_IgM	8.52	9610.4	12781.6	0.752	MM
59	59	20211111_H10_R2_IgM	8.56	14705.7	14796.4	0.994	bb
60	60	20211111_A21_R2_IgM	8.58	19760.4	13669.0	1.446	MM
61	61	20211111_A22_R2_IgM	8.58	6038.5	14098.3	0.428	MM
62	62	20211111_L21_R2_IgM	8.61	11300.3	15678.2	0.721	MM
63	63	20211111_L22_R2_IgM	8.59	12340.4	15724.5	0.785	MM
64	64	20211111_H11_R2_IgM	8.60	17238.0	15890.2	1.085	MM
65	65	20211111_A23_R2_IgM	8.59	13133.8	13423.2	0.978	MM
66	66	20211111_A24_R2_IgM	8.58	21805.0	12438.0	1.753	MM
67	67	20211111_L23_R2_IgM	8.59	16809.8	14075.0	1.194	MM
68	68	20211111_L24_R2_IgM	8.58	9031.6	15972.1	0.565	MM
69	69	20211111_H12_R2_IgM	8.60	6448.5	16270.9	0.396	MM
70	70	20211111_QC_R14_IgM	8.60	19253.2	15806.8	1.218	MM
71	71	20211111_A25_R2_IgM	8.60	37761.5	14552.1	2.595	MM
72	72	20211111_A26_R2_IgM	8.61	10679.4	11492.7	0.929	MM
73	73	20211111_L25_R2_IgM	8.61	11759.7	14403.3	0.816	MM
74	74	20211111_L26_R2_IgM	8.61	9789.9	16533.9	0.592	MM
75	75	20211111_H13_R2_IgM	8.60	20735.3	15520.1	1.336	MM
76	76	20211111_A27_R2_IgM	8.60	14908.4	13337.4	1.118	MM
77	77	20211111_A28_R2_IgM	8.54	8442.8	11895.6	0.710	MM
78	78	20211111_L27_R2_IgM	8.61	18125.3	14125.7	1.283	MM
79	79	20211111_L28_R2_IgM	8.59	10295.6	11807.5	0.872	MM
80	80	20211111_H14_R2_IgM	8.58	15223.0	15035.7	1.012	MM
81	81	20211111_A29_R2_IgM	8.60	4207.8	13554.4	0.310	MM
82	82	20211111_A30_R2_IgM	8.59	42647.3	12984.1	3.285	MM
83	83	20211111_QC_R15_IgM	8.60	18370.6	12299.5	1.494	MM
84	84	20211111_L29_R2_IgM	8.59	8702.7	13590.7	0.640	MM
85	85	20211111_L30_R2_IgM	8.58	8192.4	12511.4	0.655	MM
86	86	20211111_H15_R2_IgM	8.58	14447.6	11885.0	1.216	MM
87	87	20211111_A31_R2_IgM	8.60	11893.4	13422.1	0.886	MM
88	88	20211111_A32_R2_IgM	8.59	20705.3	11461.3	1.807	MM
89	89	20211111_L31_R2_IgM	8.52	7240.6	13753.4	0.526	MM
90	90	20211111_L32_R2_IgM	8.58	12902.7	12949.0	0.996	MM
91	91	20211111_H16_R2_IgM	8.58	20620.9	11465.4	1.799	MM
92	92	20211111_A33_R2_IgM	8.59	19452.2	10551.5	1.844	MM
93	93	20211111_A34_R2_IgM	8.59	8648.1	12409.9	0.697	MM
94	94	20211111_H17_R2_IgM	8.56	64121.8	10160.9	6.311	MM
95	95	20211111_A35_R2_IgM	8.56	11123.7	10460.6	1.063	MM
96	96	20211111_QC_R16_IgM	8.59	15171.6	10433.5	1.454	MM
97	97	20211111_A36_R2_IgM	8.53	16037.0	12504.8	1.282	MM
98	98	20211111_H18_R2_IgM	8.58	28843.6	9140.4	3.156	MM
99	99	20211111_A37_R2_IgM	8.61	24608.8	9796.9	2.512	MM
100	1...	20211111_A38_R2_IgM	8.61	19358.2	11224.3	1.725	MM
101	1...	20211111_H19_R2_IgM	8.57	30276.3	11739.2	2.579	MM
102	1...	20211111_A39_R2_IgM	8.60	14374.2	12718.9	1.130	MM
103	1...	20211111_A40_R2_IgM	8.52	13073.7	10006.1	1.307	MM
104	1...	20211111_H20_R2_IgM	8.57	50010.4	7988.2	6.261	MM
105	1...	20211111_A41_R2_IgM	8.61	9314.8	11513.3	0.809	MM
106	1...	20211111_A42_R2_IgM	8.59	7852.9	10238.5	0.767	MM
107	1...	20211111_A43_R2_IgM	8.59	6539.1	11098.9	0.589	MM
108	1...	20211111_A44_R2_IgM	8.57	7961.9	10969.9	0.726	MM
109	1...	20211111_QC_R17_IgM	8.59	14412.6	9700.8	1.486	MM
110	1...	20211111_L2_R3_IgM	8.58	20591.2	8528.0	2.415	MM
111	1...	20211111_H1_R3_IgM	8.60	17739.0	9438.2	1.879	MM
112	1...	20211111_A3_R3_IgM	8.60	27747.9	11598.6	2.392	MM

	# Name	RT	Area	IS Area	Response	Prima...
113	1... 20211111_A4_R3_IgM	8.60	10333.4	11364.1	0.909	MM
114	1... 20211111_L3_R3_IgM	8.54	8814.2	10753.4	0.820	MM
115	1... 20211111_L4_R3_IgM	8.59	14629.7	10291.6	1.422	MM
116	1... 20211111_H2_R3_IgM	8.58	33432.4	9315.8	3.589	MM
117	1... 20211111_A5_R3_IgM	8.59	40184.6	9102.2	4.415	MM
118	1... 20211111_A6_R3_IgM	8.58	10611.1	10356.4	1.025	MM
119	1... 20211111_QC_R18_IgM	8.58	14363.9	8398.3	1.710	MM
120	1... 20211111_L5_R3_IgM	8.59	18232.1	9786.7	1.863	MM
121	1... 20211111_L6_R3_IgM	8.60	13204.0	9911.3	1.332	MM
122	1... 20211111_H3_R3_IgM	8.60	9175.5	12155.5	0.755	MM
123	1... 20211111_A7_R3_IgM	8.59	29133.2	11804.8	2.468	MM
124	1... 20211111_A8_R3_IgM	8.59	27589.8	10510.6	2.625	MM
125	1... 20211111_L7_R3_IgM	8.61	4333.7	12177.9	0.356	MM
126	1... 20211111_L8_R3_IgM	8.58	6991.9	11668.6	0.599	MM
127	1... 20211111_H4_R3_IgM	8.58	9239.6	11192.0	0.826	MM
128	1... 20211111_A9_R3_IgM	8.60	13572.5	14077.4	0.964	MM
129	1... 20211111_A10_R3_IgM	8.59	32089.0	11190.4	2.868	MM
130	1... 20211111_L9_R3_IgM	8.61	9756.7	14649.3	0.666	MM
131	1... 20211111_L10_R3_IgM	8.56	22875.9	9361.3	2.444	MM
132	1... 20211111_QC_R19_IgM	8.58	15001.6	10125.5	1.482	MM
133	1... 20211111_H5_R3_IgM	8.60	5937.9	10010.9	0.593	MM
134	1... 20211111_A11_R3_IgM	8.59	21836.0	12697.2	1.720	MM
135	1... 20211111_A12_R3_IgM	8.60	11361.9	13148.5	0.864	MM
136	1... 20211111_L11_R3_IgM	8.59	9945.4	10786.6	0.922	MM
137	1... 20211111_L12_R3_IgM	8.55	10030.8	11681.8	0.859	MM
138	1... 20211111_H6_R3_IgM	8.59	14102.4	12095.5	1.166	MM
139	1... 20211111_A13_R3_IgM	8.59	13736.4	12908.3	1.064	MM
140	1... 20211111_A14_R3_IgM	8.60	52599.7	11322.6	4.646	MM
141	1... 20211111_L13_R3_IgM	8.58	11195.0	11516.1	0.972	MM
142	1... 20211111_L14_R3_IgM	8.57	16474.0	10790.7	1.527	MM
143	1... 20211111_H7_R3_IgM	8.59	19152.0	11572.1	1.655	MM
144	1... 20211111_A15_R3_IgM	8.60	49943.1	12434.4	4.017	MM
145	1... 20211111_QC_R20_IgM	8.59	18164.0	10701.0	1.697	MM
146	1... 20211111_A16_R3_IgM	8.60	33945.8	11452.1	2.964	MM
147	1... 20211111_L15_R3_IgM	8.58	29452.6	10531.1	2.797	MM
148	1... 20211111_L16_R3_IgM	8.61	8754.2	12049.4	0.727	MM
149	1... 20211111_H8_R3_IgM	8.61	9218.2	13434.9	0.686	MM
150	1... 20211111_A17_R3_IgM	8.60	20197.7	12452.0	1.622	MM
151	1... 20211111_A18_R3_IgM	8.55	17183.9	12506.5	1.374	MM
152	1... 20211111_L17_R3_IgM	8.60	19832.7	9948.9	1.993	MM
153	1... 20211111_L18_R3_IgM	8.59	7671.9	13462.1	0.570	MM
154	1... 20211111_H9_R3_IgM	8.59	12064.4	13178.7	0.915	MM
155	1... 20211111_A19_R3_IgM	8.60	14907.1	11298.6	1.319	MM
156	1... 20211111_A20_R3_IgM	8.56	14743.4	11175.6	1.319	MM
157	1... 20211111_L19_R3_IgM	8.60	19871.5	11558.2	1.719	MM
158	1... 20211111_QC_R21_IgM	8.59	19310.9	9977.0	1.936	MM
159	1... 20211111_L20_R3_IgM	8.59	7644.5	13508.1	0.566	MM
160	1... 20211111_H10_R3_IgM	8.58	15815.0	12374.8	1.278	MM
161	1... 20211111_A21_R3_IgM	8.57	21902.6	12443.6	1.760	MM
162	1... 20211111_A22_R3_IgM	8.54	6878.6	11589.3	0.594	MM
163	1... 20211111_L21_R3_IgM	8.61	10519.6	13107.4	0.803	MM
164	1... 20211111_L22_R3_IgM	8.58	12880.0	12021.5	1.071	MM
165	1... 20211111_H11_R3_IgM	8.61	16348.2	12687.8	1.288	MM
166	1... 20211111_A23_R3_IgM	8.61	13234.7	11611.8	1.140	MM
167	1... 20211111_A24_R3_IgM	8.58	23830.0	10476.8	2.275	MM
168	1... 20211111_L23_R3_IgM	8.61	15729.3	11873.1	1.325	MM

Dataset: C:\MassLynx\10.pro\20211111_clinicalsamples_IgM_IgA.qld

Last Altered: Thursday, March 09, 2023 19:58:25 台北標準時間
Printed: Friday, August 01, 2025 15:16:08 台北標準時間

	# Name	RT	Area	IS Area	Response	Prima...
169	1... 20211111_L24_R3_IgM	8.60	9298.0	13468.0	0.690	MM
170	1... 20211111_H12_R3_IgM	8.57	7962.4	14229.1	0.560	MM
171	1... 20211111_QC_R22_IgM	8.60	17059.0	11264.4	1.514	MM
172	1... 20211111_A25_R3_IgM	8.61	32797.8	11775.7	2.785	MM
173	1... 20211111_A26_R3_IgM	8.61	10073.5	10473.9	0.962	MM
174	1... 20211111_L25_R3_IgM	8.60	10777.4	13256.8	0.813	MM
175	1... 20211111_L26_R3_IgM	8.61	11108.7	13565.0	0.819	MM
176	1... 20211111_H13_R3_IgM	8.59	21734.9	13146.0	1.653	MM
177	1... 20211111_A27_R3_IgM	8.59	12967.2	12025.6	1.078	MM
178	1... 20211111_A28_R3_IgM	8.59	7611.0	11229.1	0.678	MM
179	1... 20211111_L27_R3_IgM	8.60	19836.0	14960.0	1.326	MM
180	1... 20211111_L28_R3_IgM	8.59	11318.5	11359.0	0.996	MM
181	1... 20211111_H14_R3_IgM	8.59	16522.3	12830.8	1.288	MM
182	1... 20211111_A29_R3_IgM	8.61	4137.5	13434.3	0.308	MM
183	1... 20211111_A30_R3_IgM	8.60	39829.1	11272.2	3.533	MM
184	1... 20211111_QC_R23_IgM	8.59	17610.8	11872.2	1.483	MM
185	1... 20211111_L29_R3_IgM	8.59	8425.5	12948.7	0.651	MM
186	1... 20211111_L30_R3_IgM	8.60	8727.2	11026.6	0.791	MM
187	1... 20211111_H15_R3_IgM	8.58	13887.5	10786.3	1.288	MM
188	1... 20211111_A31_R3_IgM	8.59	13643.5	12010.5	1.136	MM
189	1... 20211111_A32_R3_IgM	8.59	18960.8	11107.7	1.707	MM
190	1... 20211111_L31_R3_IgM	8.60	5385.5	11021.8	0.489	MM
191	1... 20211111_L32_R3_IgM	8.60	14376.3	11683.8	1.230	MM
192	1... 20211111_H16_R3_IgM	8.58	19645.9	9831.7	1.998	MM
193	1... 20211111_A33_R3_IgM	8.59	18375.4	11213.1	1.639	MM
194	1... 20211111_A34_R3_IgM	8.60	7712.1	11550.1	0.668	MM
195	1... 20211111_H17_R3_IgM	8.57	54125.0	8874.4	6.099	MM
196	1... 20211111_A35_R3_IgM	8.57	10447.2	9856.3	1.060	MM
197	1... 20211111_QC_R24_IgM	8.59	15400.7	7561.8	2.037	MM
198	1... 20211111_A36_R3_IgM	8.59	15951.2	9562.8	1.668	MM
199	1... 20211111_H18_R3_IgM	8.58	26538.2	8165.3	3.250	MM
200	2... 20211111_A37_R3_IgM	8.59	21767.8	9278.8	2.346	MM
201	2... 20211111_A38_R3_IgM	8.59	17032.3	10920.3	1.560	MM
202	2... 20211111_H19_R3_IgM	8.59	28528.4	9285.3	3.072	MM
203	2... 20211111_A39_R3_IgM	8.60	14558.6	9083.4	1.603	MM
204	2... 20211111_A40_R3_IgM	8.59	11541.0	8382.4	1.377	MM
205	2... 20211111_H20_R3_IgM	8.59	41172.4	7807.9	5.273	MM
206	2... 20211111_A41_R3_IgM	8.61	7221.7	5925.2	1.219	MM
207	2... 20211111_A42_R3_IgM	8.57	9281.1	9352.6	0.992	MM
208	2... 20211111_A43_R3_IgM	8.57	7962.5	10216.3	0.779	MM
209	2... 20211111_A44_R3_IgM	8.58	6427.1	10262.7	0.626	MM
210	2... 20211111_QC_R25_IgM	8.59	13092.6	8575.3	1.527	MM

Dataset: C:\MassLynx\10.pro\20211111_clinicalsamples_IgM_IgA.qld

Last Altered: Thursday, March 09, 2023 19:58:25 台北標準時間
Printed: Friday, August 01, 2025 15:17:02 台北標準時間

Method: C:\MassLynx\10_Pro_IgA.PRO\MethDB\20211111_IgM_IgA_DMRM_new.mdb 24 Feb 2022 13:44:14
Calibration: 15 Nov 2021 14:42:11

Sample Name: 20211112_QC_R001_IgM

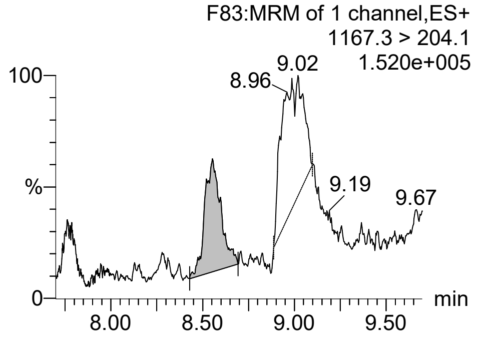

Sample Name: 20211112_QC_R002_IgM

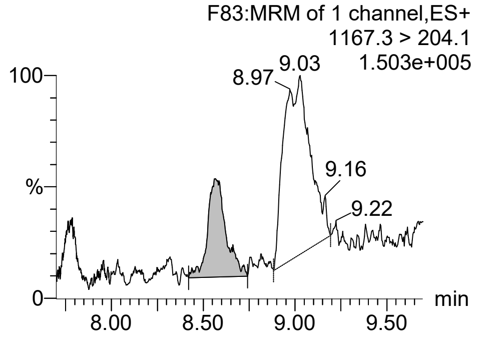

Sample Name: 20211111_A1_R2_IgM

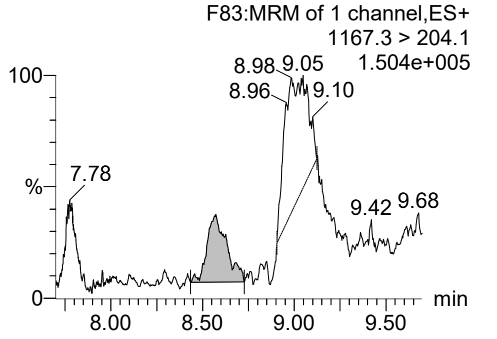

Sample Name: 20211111_A1_R3_IgM

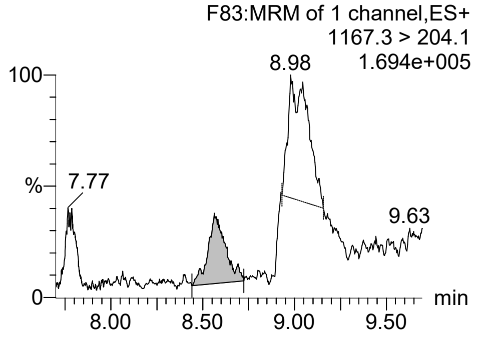

Sample Name: 20211111_A2_R2_IgM

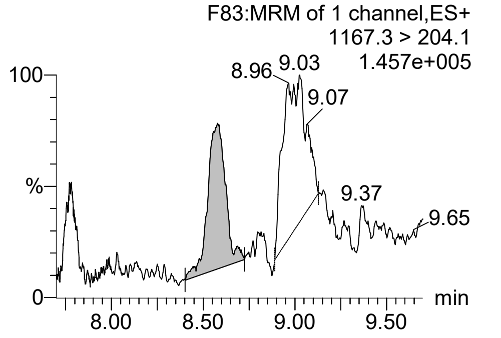

Sample Name: 20211111_A2_R3_IgM

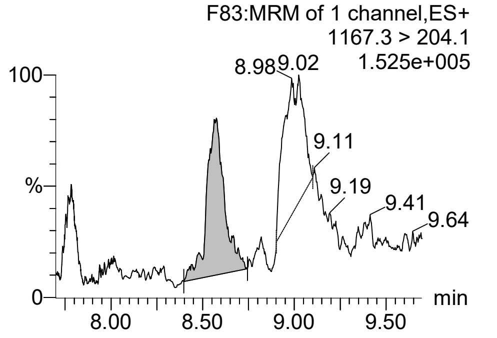

Sample Name: 20211111_L1_R2_IgM

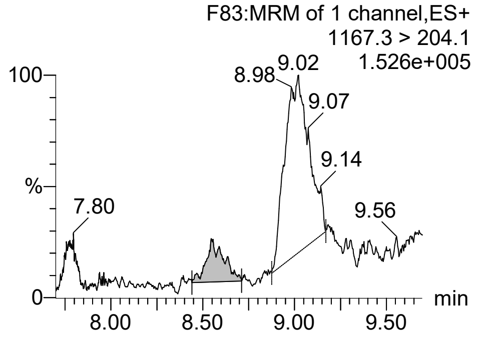

Sample Name: 20211111_L1_R3_IgM

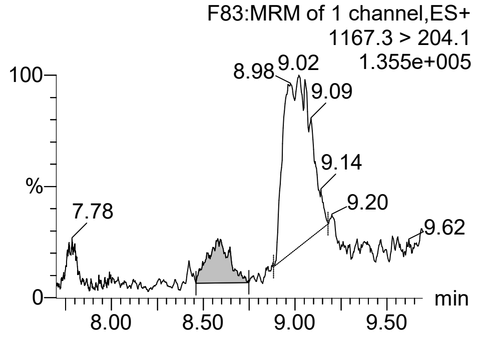

Sample Name: 20211111_L2_R2_IgM

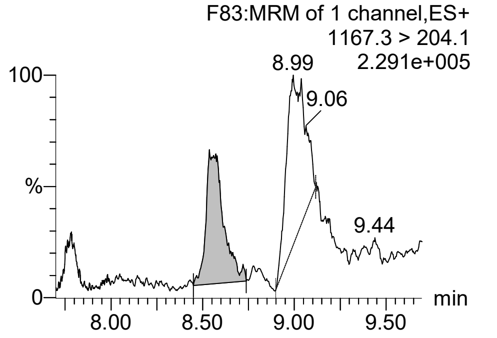

Sample Name: 20211111_H1_R2_IgM

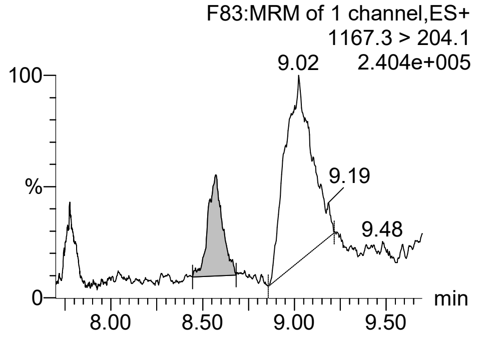

Sample Name: 20211111_A3_R2_IgM

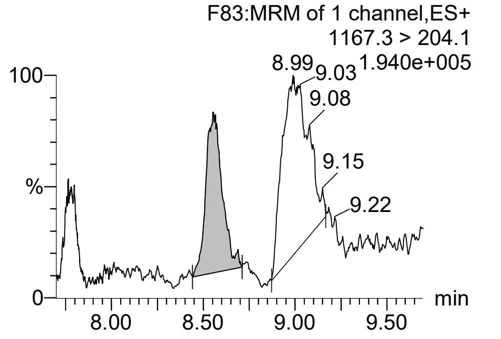

Sample Name: 20211111_A4_R2_IgM

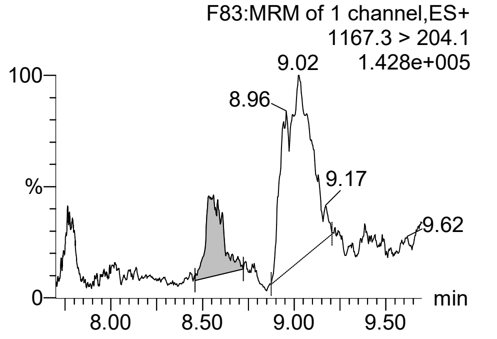

Sample Name: 20211111_L3_R2_IgM

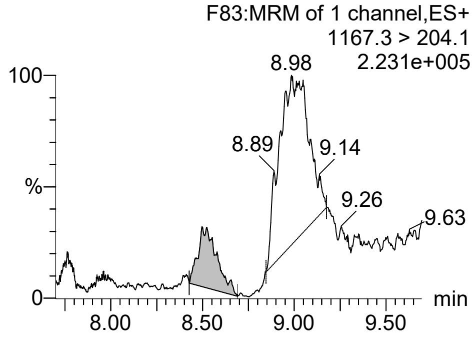

Sample Name: 20211111_L4_R2_IgM

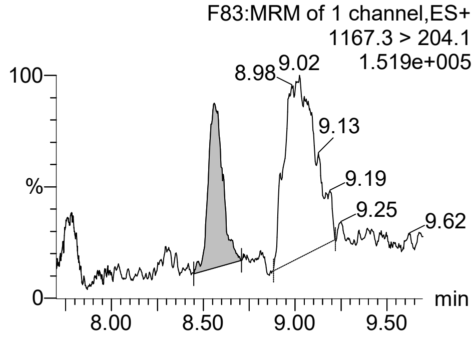

Sample Name: 20211111_H2_R2_IgM

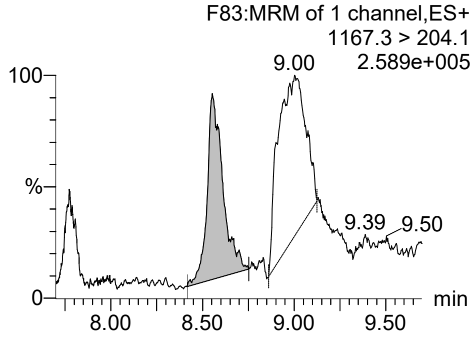

Sample Name: 20211111_A5_R2_IgM

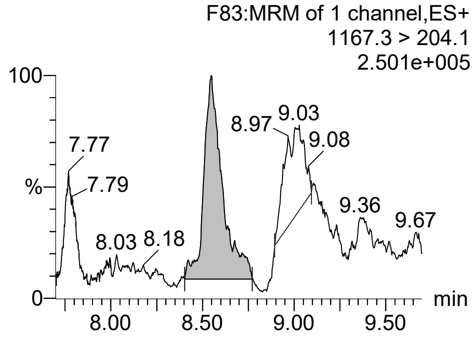

Sample Name: 20211111_A6_R2_IgM

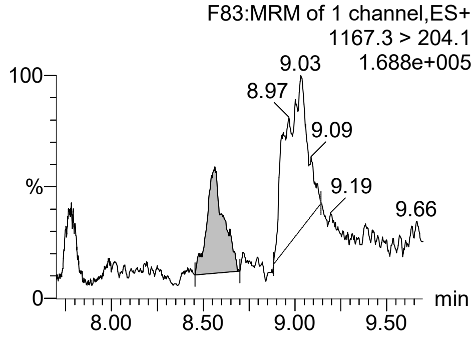

Sample Name: 20211111_QC_R10_IgM

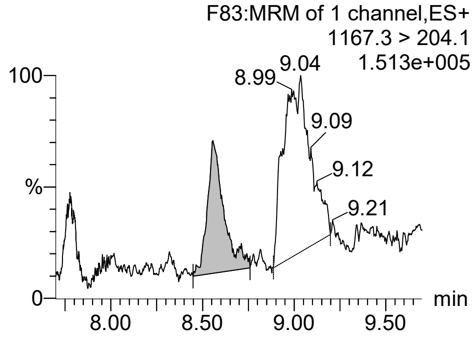

Sample Name: 20211111_L5_R2_IgM

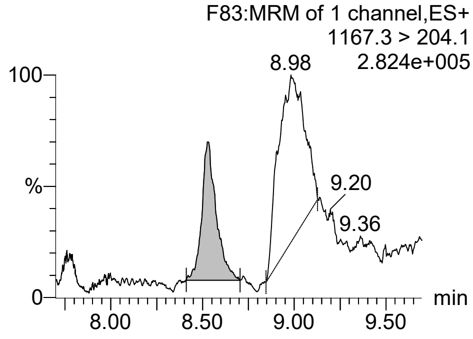

Sample Name: 20211111_L6_R2_IgM

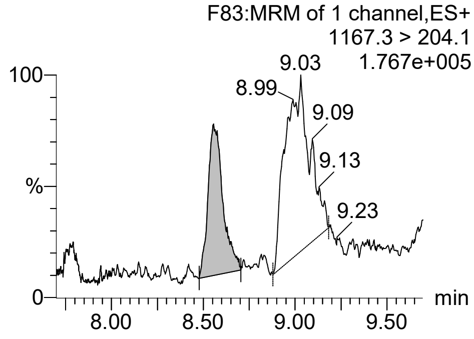

Sample Name: 20211111_H3_R2_IgM

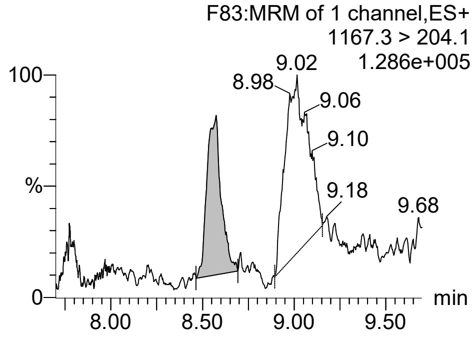

Sample Name: 20211111_A7_R2_IgM

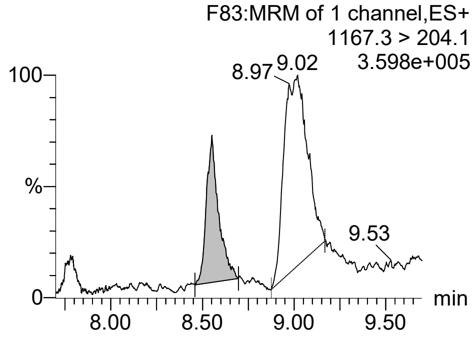

Sample Name: 20211111_A8_R2_IgM

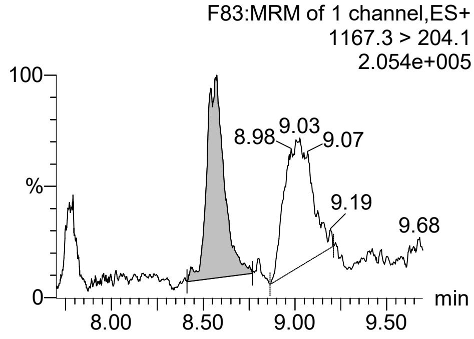

Sample Name: 20211111_L7_R2_IgM

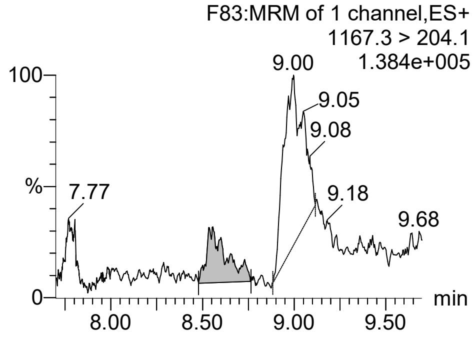

Sample Name: 20211111_L8_R2_IgM

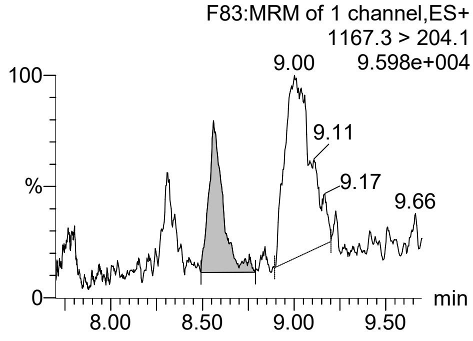

Sample Name: 20211111_H4_R2_IgM

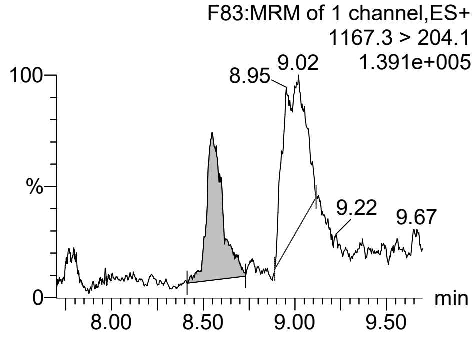

Sample Name: 20211111_A9_R2_IgM

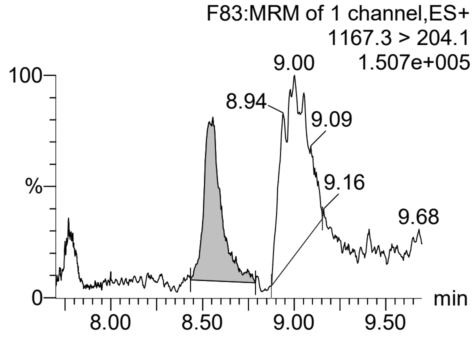

Dataset: C:\MassLynx\10.pro\20211111_clinicalsamples_IgM_IgA.qld

Last Altered: Thursday, March 09, 2023 19:58:25 台北標準時間
Printed: Friday, August 01, 2025 15:17:02 台北標準時間

Sample Name: 20211111_A10_R2_IgM

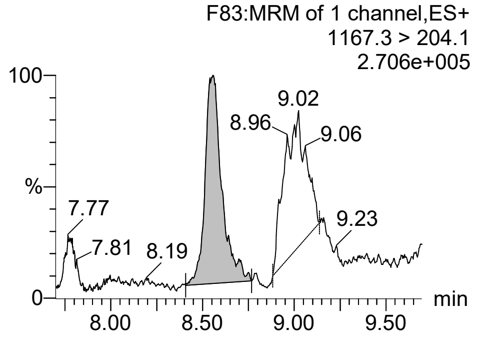

Sample Name: 20211111_L9_R2_IgM

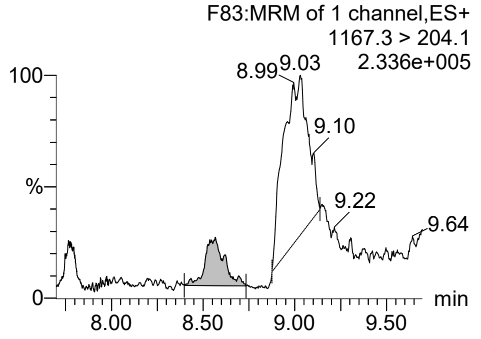

Sample Name: 20211111_L10_R2_IgM

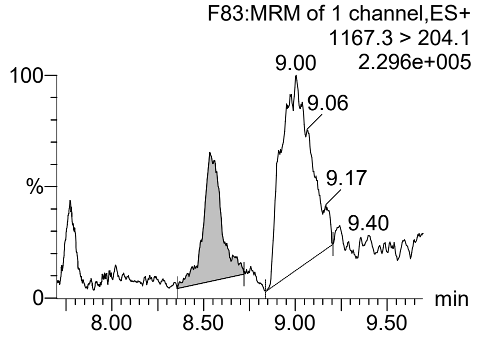

Sample Name: 20211111_QC_R11_IgM

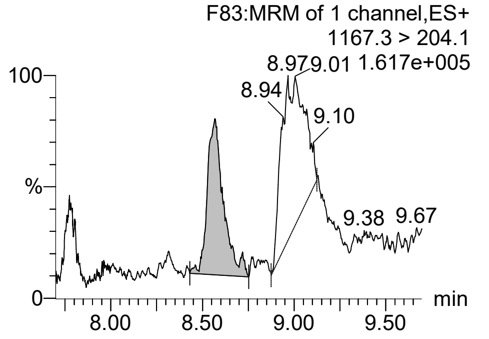

Sample Name: 20211111_H5_R2_IgM

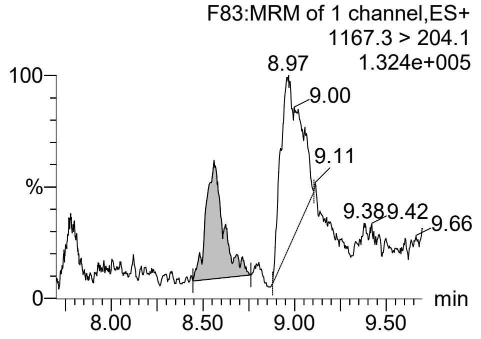

Sample Name: 20211111_A11_R2_IgM

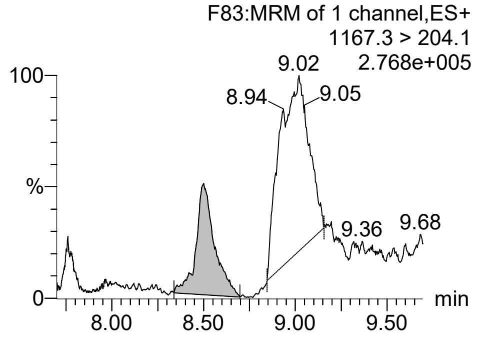

Sample Name: 20211111_A12_R2_IgM

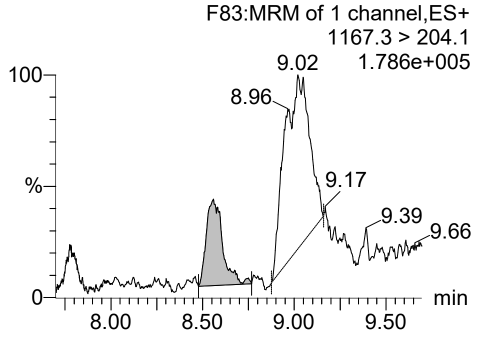

Sample Name: 20211111_L11_R2_IgM

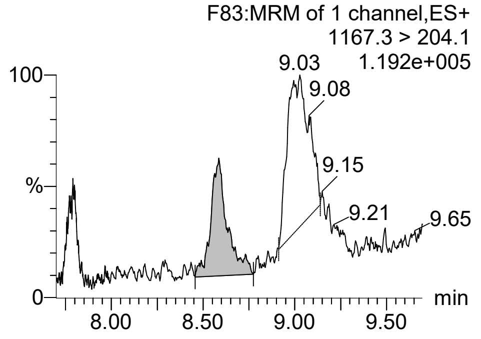

Sample Name: 20211111_L12_R2_IgM

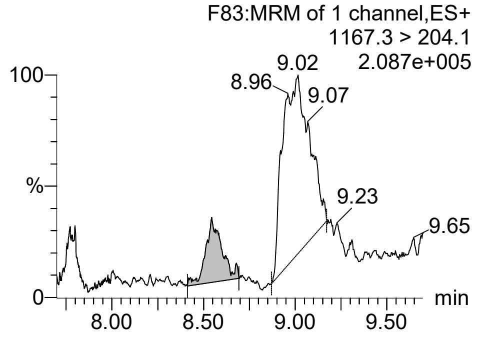

Sample Name: 20211111_H6_R2_IgM

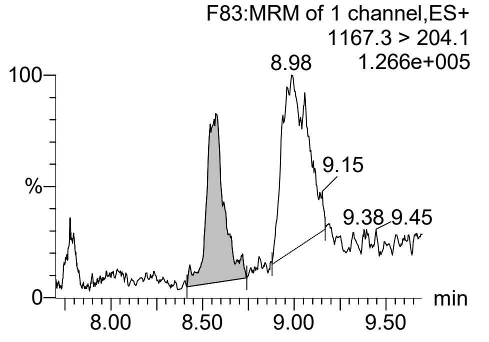

Sample Name: 20211111_A13_R2_IgM

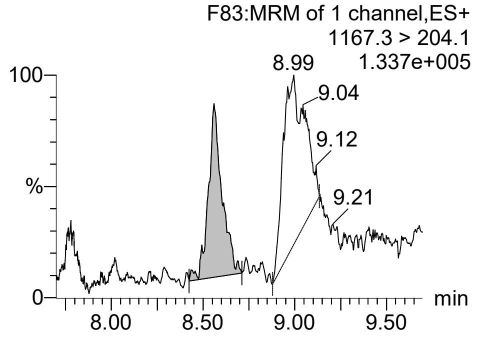

Sample Name: 20211111_A14_R2_IgM

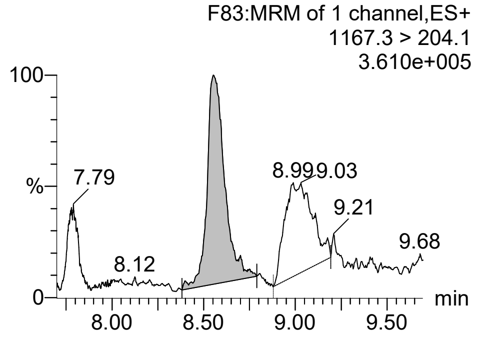

Sample Name: 20211111_L13_R2_IgM

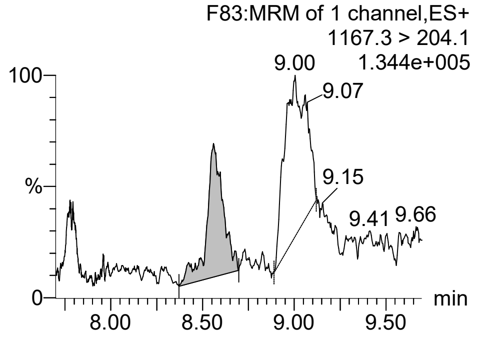

Sample Name: 20211111_L14_R2_IgM

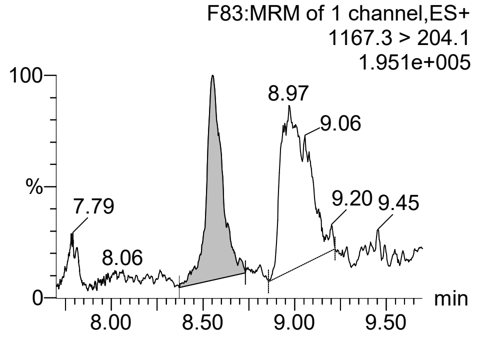

Sample Name: 20211111_H7_R2_IgM

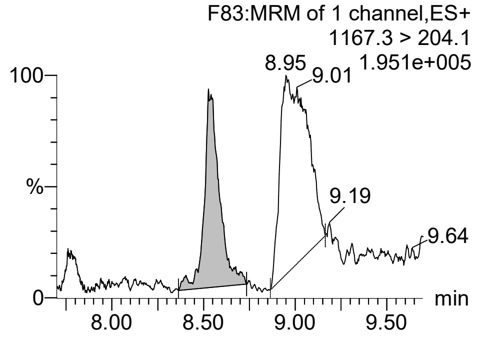

Sample Name: 20211111_A15_R2_IgM
F83:MRM of 1 channel,ES+
1167.3 > 204.1
3.546e+005

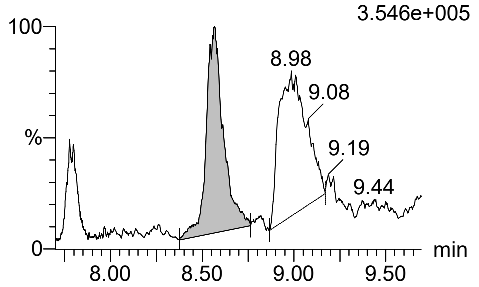

Sample Name: 20211111_QC_R12_IgM
F83:MRM of 1 channel,ES+
1167.3 > 204.1
1.629e+005

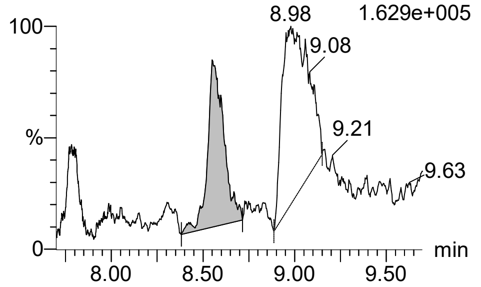

Sample Name: 20211111_A16_R2_IgM
F83:MRM of 1 channel,ES+
1167.3 > 204.1
2.059e+005

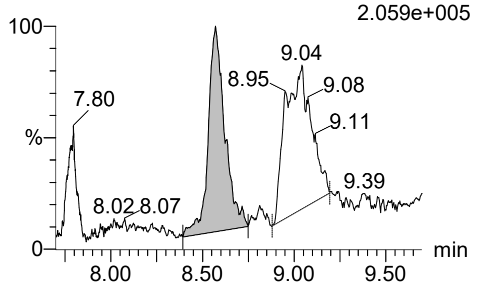

Sample Name: 20211111_L15_R2_IgM
F83:MRM of 1 channel,ES+
1167.3 > 204.1
2.657e+005

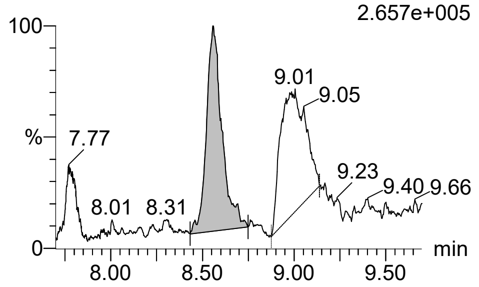

Sample Name: 20211111_L16_R2_IgM
F83:MRM of 1 channel,ES+
1167.3 > 204.1
1.349e+005

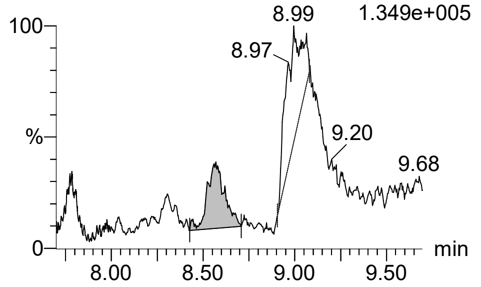

Sample Name: 20211111_H8_R2_IgM
F83:MRM of 1 channel,ES+
1167.3 > 204.1
9.962e+004

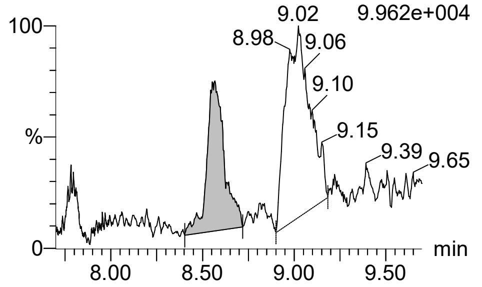

Sample Name: 20211111_A17_R2_IgM
F83:MRM of 1 channel,ES+
1167.3 > 204.1
1.412e+005

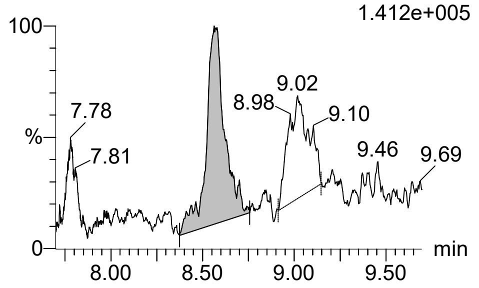

Sample Name: 20211111_A18_R2_IgM
F83:MRM of 1 channel,ES+
1167.3 > 204.1
1.555e+005

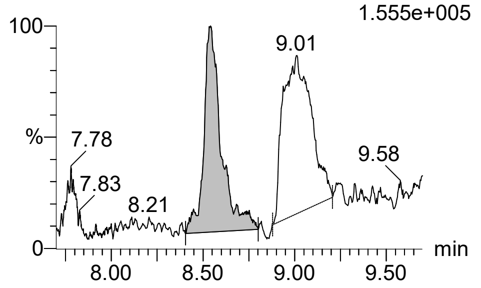

Sample Name: 20211111_L17_R2_IgM
F83:MRM of 1 channel,ES+
1167.3 > 204.1
2.131e+005

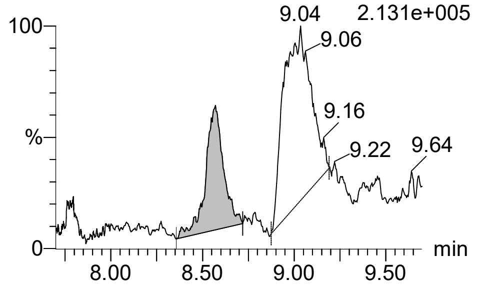

Sample Name: 20211111_L18_R2_IgM
F83:MRM of 1 channel,ES+
1167.3 > 204.1
1.623e+005

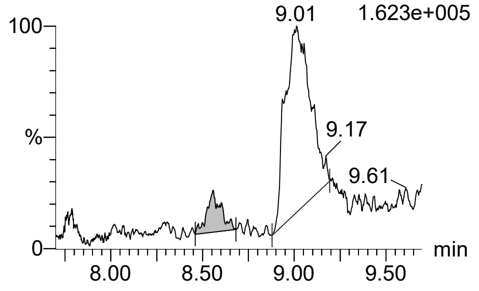

Sample Name: 20211111_H9_R2_IgM
F83:MRM of 1 channel,ES+
1167.3 > 204.1
2.249e+005

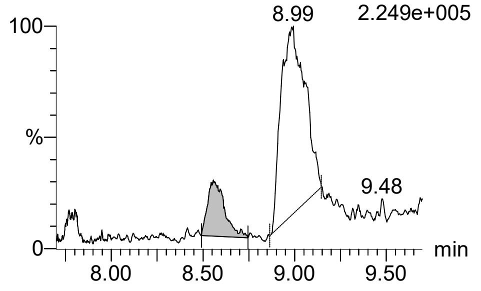

Sample Name: 20211111_A19_R2_IgM
F83:MRM of 1 channel,ES+
1167.3 > 204.1
1.856e+005

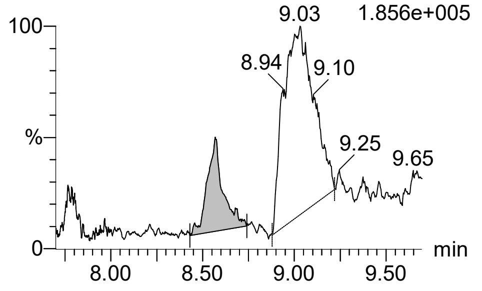

Sample Name: 20211111_A20_R2_IgM
F83:MRM of 1 channel,ES+
1167.3 > 204.1
2.155e+005

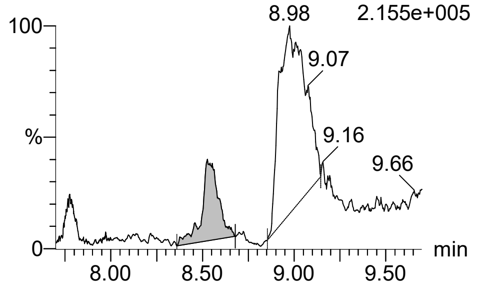

Sample Name: 20211111_L19_R2_IgM
F83:MRM of 1 channel,ES+
1167.3 > 204.1
2.743e+005

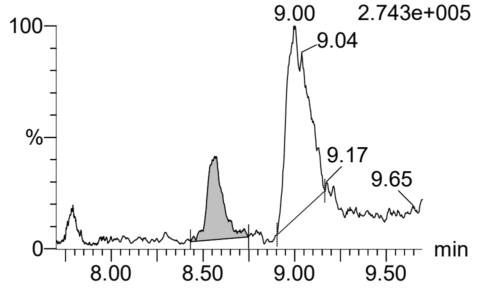

Sample Name: 20211111_QC_R13_IgM
F83:MRM of 1 channel,ES+
1167.3 > 204.1
1.709e+005

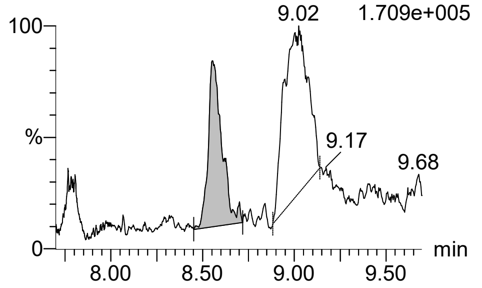

Sample Name: 20211111_L20_R2_IgM
F83:MRM of 1 channel,ES+
1167.3 > 204.1
2.630e+005

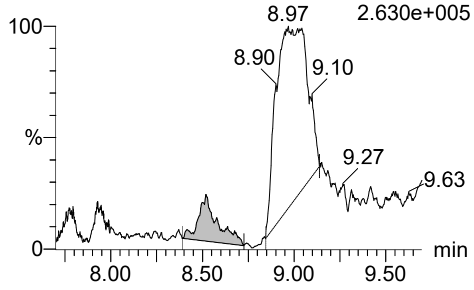

Sample Name: 20211111_H10_R2_IgM
F83:MRM of 1 channel,ES+
1167.3 > 204.1
2.088e+005

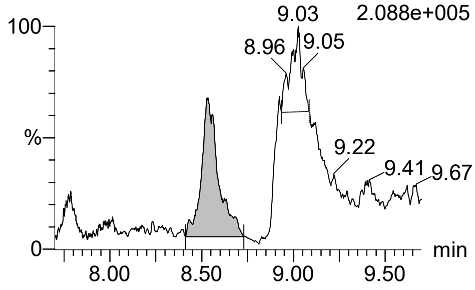

Sample Name: 20211111_A21_R2_IgM
F83:MRM of 1 channel,ES+
1167.3 > 204.1
2.296e+005

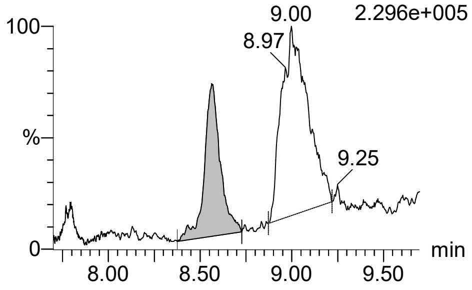

Sample Name: 20211111_A22_R2_IgM
F83:MRM of 1 channel,ES+
1167.3 > 204.1
1.316e+005

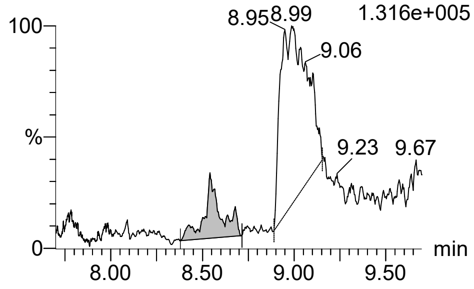

Sample Name: 20211111_L21_R2_IgM
F83:MRM of 1 channel,ES+
1167.3 > 204.1
1.029e+005

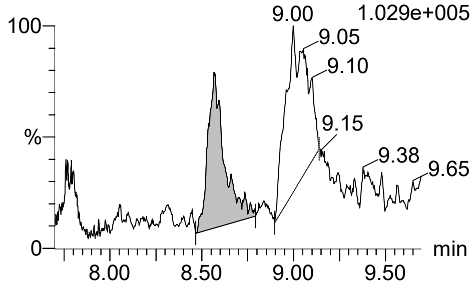

Sample Name: 20211111_L22_R2_IgM
F83:MRM of 1 channel,ES+
1167.3 > 204.1
1.096e+005

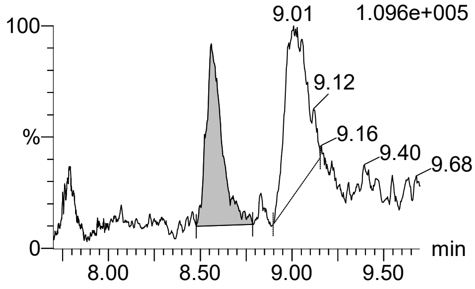

Sample Name: 20211111_H11_R2_IgM
F83:MRM of 1 channel,ES+
1167.3 > 204.1
1.209e+005

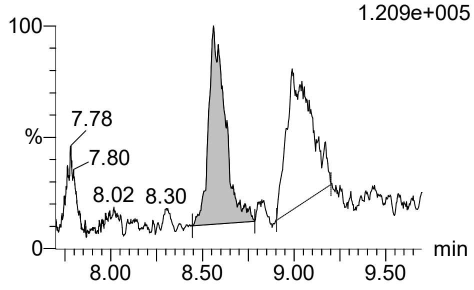

Sample Name: 20211111_A23_R2_IgM
F83:MRM of 1 channel,ES+
1167.3 > 204.1
1.496e+005

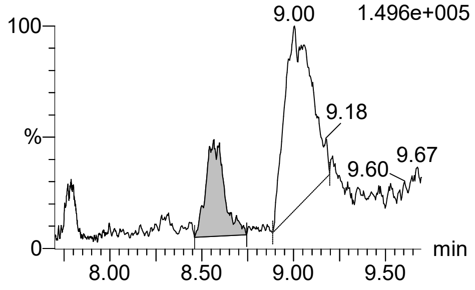

Sample Name: 20211111_A24_R2_IgM
F83:MRM of 1 channel,ES+
1167.3 > 204.1
1.512e+005

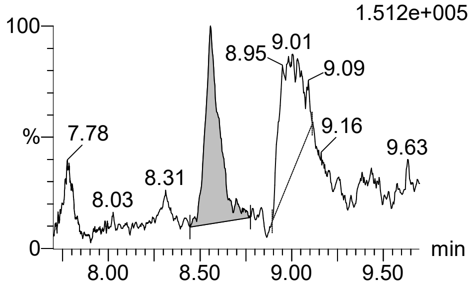

Sample Name: 20211111_L23_R2_IgM
F83:MRM of 1 channel,ES+
1167.3 > 204.1
1.577e+005

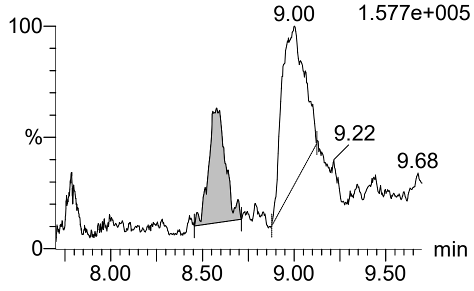

Sample Name: 20211111_L24_R2_IgM
F83:MRM of 1 channel,ES+
1167.3 > 204.1
1.286e+005

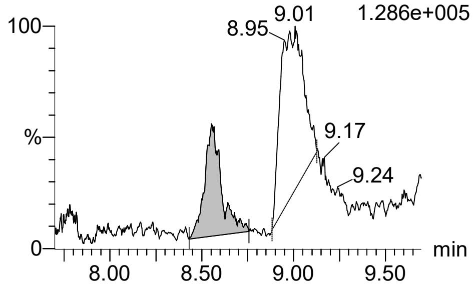

Sample Name: 20211111_H12_R2_IgM
F83:MRM of 1 channel,ES+
1167.3 > 204.1
1.071e+005

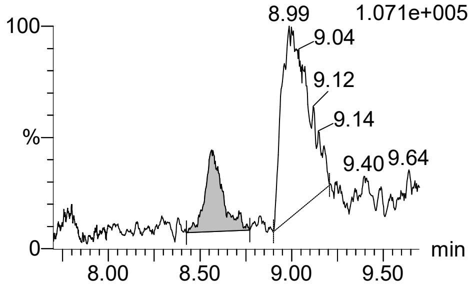

Sample Name: 20211111_QC_R14_IgM
F83:MRM of 1 channel,ES+
1167.3 > 204.1
1.454e+005

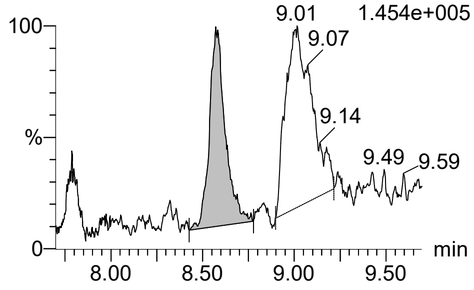

Sample Name: 20211111_A25_R2_IgM
F83:MRM of 1 channel,ES+
1167.3 > 204.1
1.984e+005

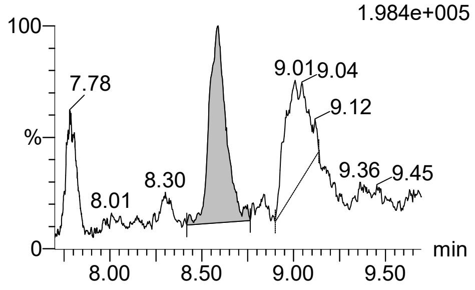

Sample Name: 20211111_A26_R2_IgM
F83:MRM of 1 channel,ES+
1167.3 > 204.1
8.180e+004

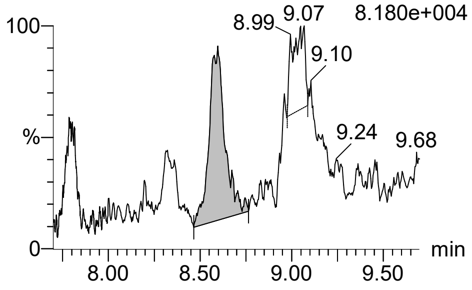

Dataset: C:\MassLynx\10.pro\20211111_clinicalsamples_IgM_IgA.qld

Last Altered: Thursday, March 09, 2023 19:58:25 台北標準時間
Printed: Friday, August 01, 2025 15:17:02 台北標準時間

Sample Name: 20211111_L25_R2_IgM

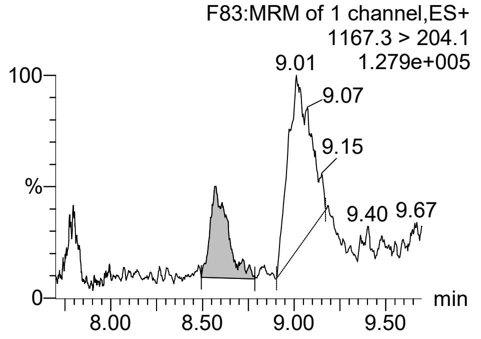

Sample Name: 20211111_L26_R2_IgM

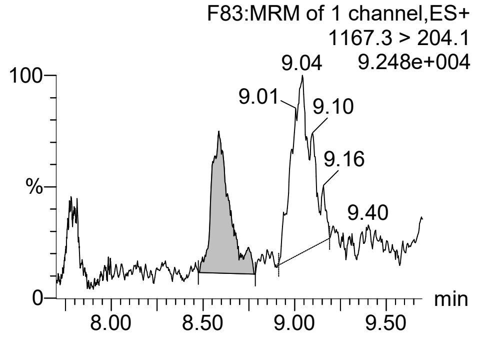

Sample Name: 20211111_H13_R2_IgM

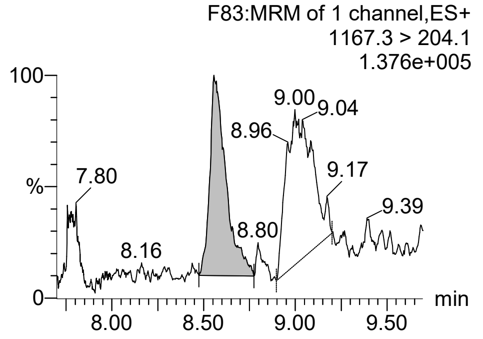

Sample Name: 20211111_A27_R2_IgM

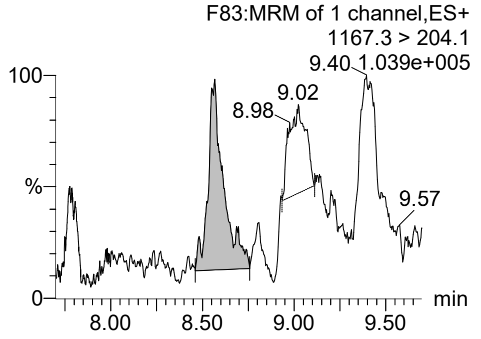

Sample Name: 20211111_A28_R2_IgM

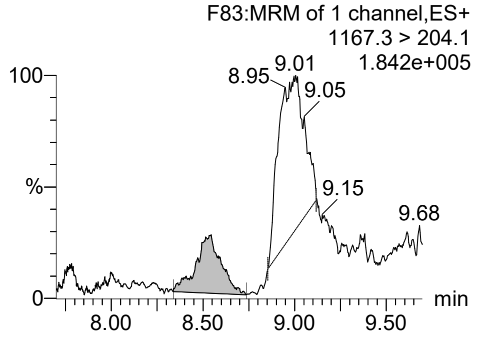

Sample Name: 20211111_L27_R2_IgM

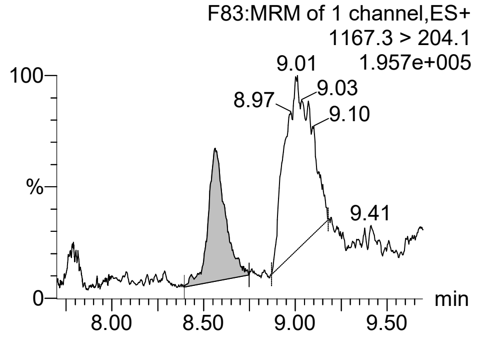

Sample Name: 20211111_L28_R2_IgM

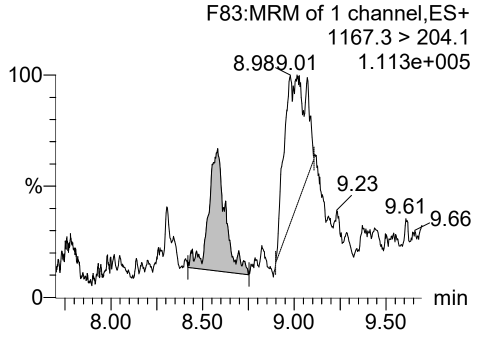

Sample Name: 20211111_H14_R2_IgM

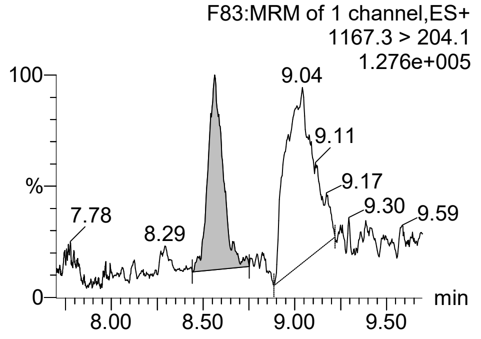

Sample Name: 20211111_A29_R2_IgM

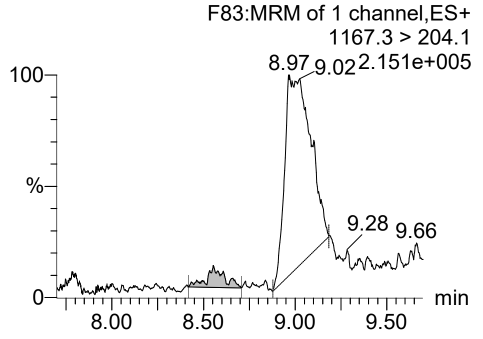

Sample Name: 20211111_A30_R2_IgM

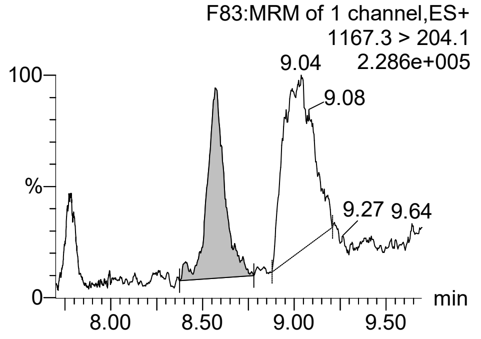

Sample Name: 20211111_QC_R15_IgM

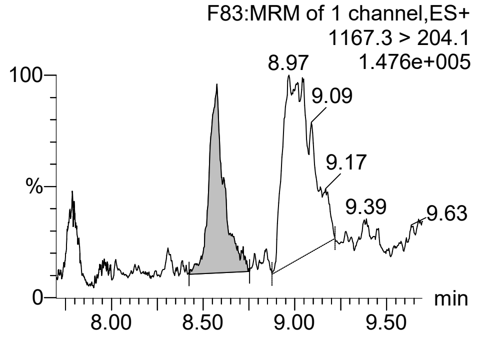

Sample Name: 20211111_L29_R2_IgM

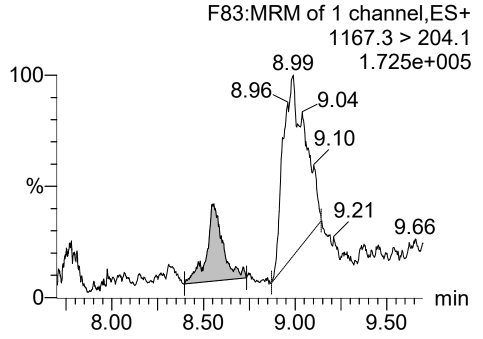

Sample Name: 20211111_L30_R2_IgM

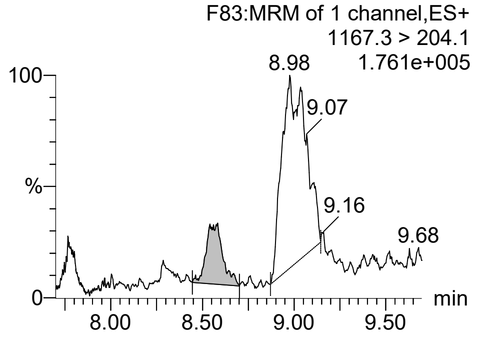

Sample Name: 20211111_H15_R2_IgM

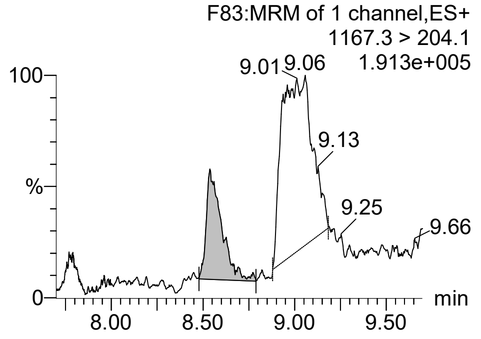

Sample Name: 20211111_A31_R2_IgM

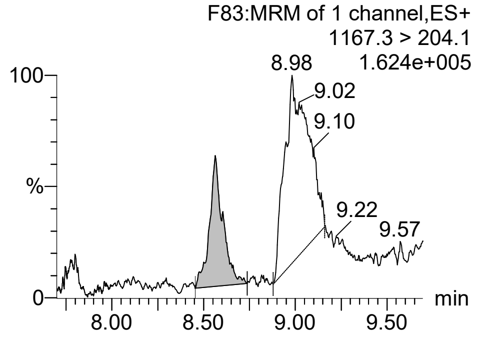

Dataset: C:\MassLynx\10.pro\20211111_clinicalsamples_IgM_IgA.qld

Last Altered: Thursday, March 09, 2023 19:58:25 台北標準時間
Printed: Friday, August 01, 2025 15:17:02 台北標準時間

Sample Name: 20211111_A32_R2_IgM
F83:MRM of 1 channel,ES+
1167.3 > 204.1
1.603e+005

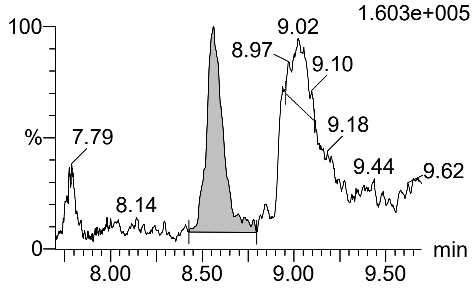

Sample Name: 20211111_L31_R2_IgM
F83:MRM of 1 channel,ES+
1167.3 > 204.1
3.052e+005

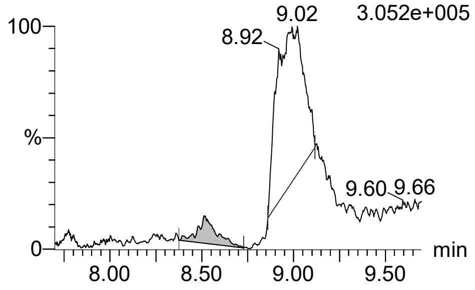

Sample Name: 20211111_L32_R2_IgM
F83:MRM of 1 channel,ES+
1167.3 > 204.1
2.605e+005

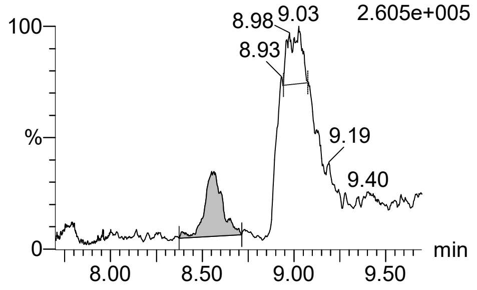

Sample Name: 20211111_H16_R2_IgM
F83:MRM of 1 channel,ES+
1167.3 > 204.1
2.298e+005

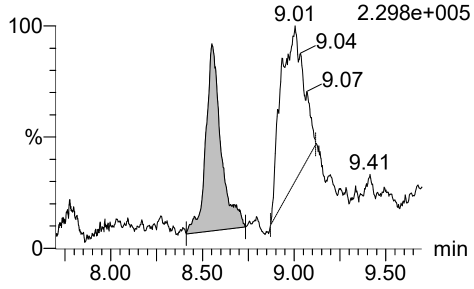

Sample Name: 20211111_A33_R2_IgM
F83:MRM of 1 channel,ES+
1167.3 > 204.1
1.670e+005

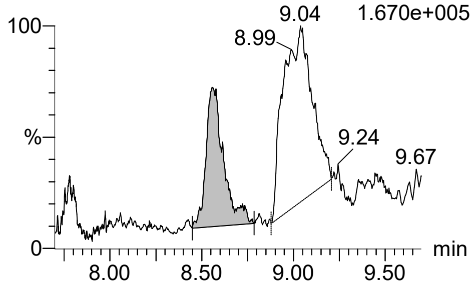

Sample Name: 20211111_A34_R2_IgM
F83:MRM of 1 channel,ES+
1167.3 > 204.1
9.492e+004

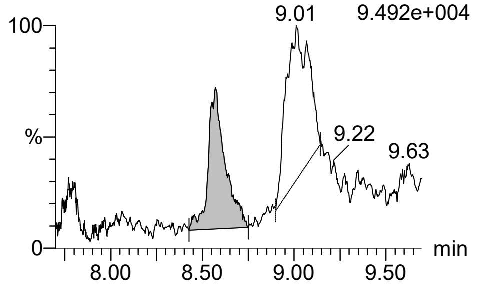

Sample Name: 20211111_H17_R2_IgM
F83:MRM of 1 channel,ES+
1167.3 > 204.1
5.673e+005

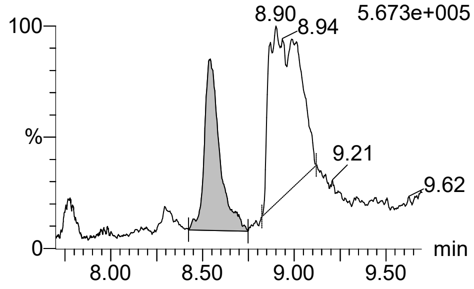

Sample Name: 20211111_A35_R2_IgM
F83:MRM of 1 channel,ES+
1167.3 > 204.1
3.103e+005

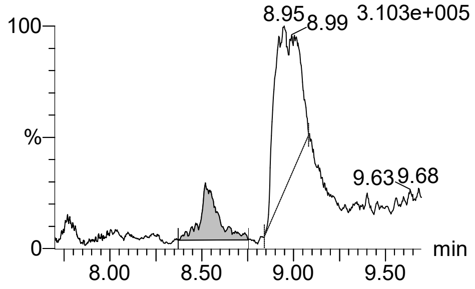

Sample Name: 20211111_QC_R16_IgM
F83:MRM of 1 channel,ES+
1167.3 > 204.1
1.989e+005

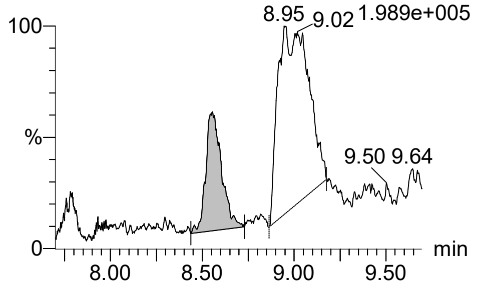

Sample Name: 20211111_A36_R2_IgM
F83:MRM of 1 channel,ES+
1167.3 > 204.1
2.289e+005

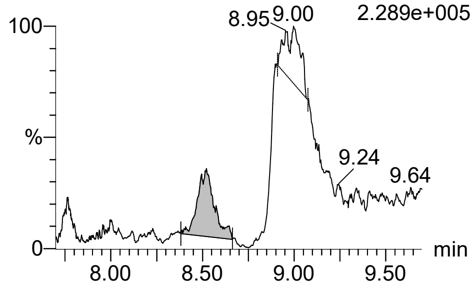

Sample Name: 20211111_H18_R2_IgM
F83:MRM of 1 channel,ES+
1167.3 > 204.1
2.486e+005

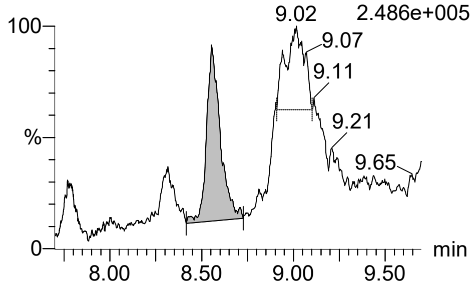

Sample Name: 20211111_A37_R2_IgM
F83:MRM of 1 channel,ES+
1167.3 > 204.1
2.358e+005

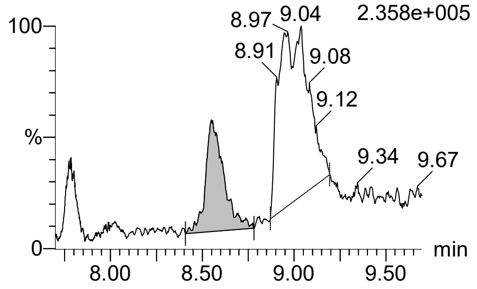

Sample Name: 20211111_A38_R2_IgM
F83:MRM of 1 channel,ES+
1167.3 > 204.1
1.644e+005

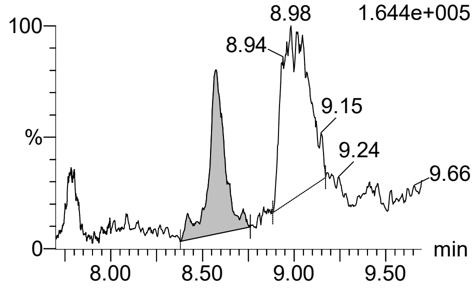

Sample Name: 20211111_H19_R2_IgM
F83:MRM of 1 channel,ES+
1167.3 > 204.1
3.635e+005

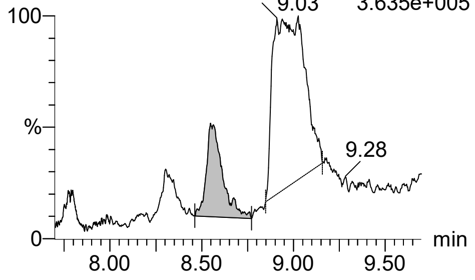

Sample Name: 20211111_A39_R2_IgM
F83:MRM of 1 channel,ES+
1167.3 > 204.1
1.726e+005

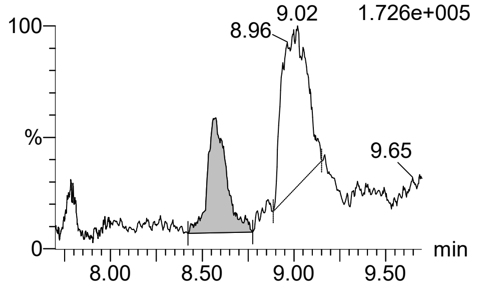

Dataset: C:\MassLynx\10.pro\20211111_clinicalsamples_IgM_IgA.qld

Last Altered: Thursday, March 09, 2023 19:58:25 台北標準時間
Printed: Friday, August 01, 2025 15:17:02 台北標準時間

Sample Name: 20211111_A40_R2_IgM

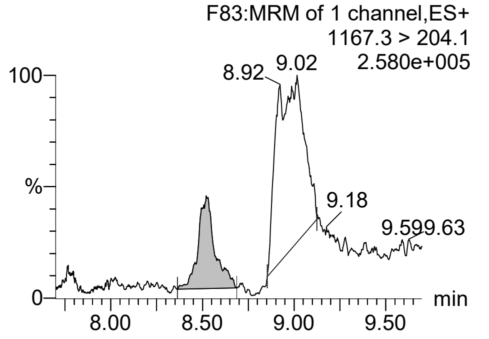

Sample Name: 20211111_H20_R2_IgM

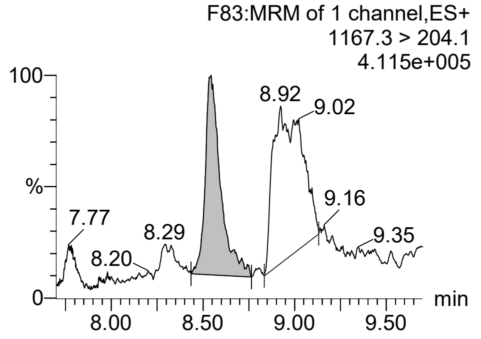

Sample Name: 20211111_A41_R2_IgM

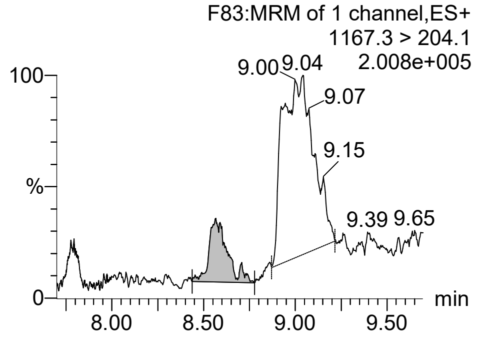

Sample Name: 20211111_A42_R2_IgM

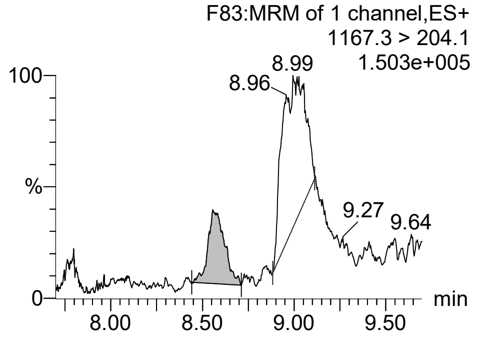

Sample Name: 20211111_A43_R2_IgM

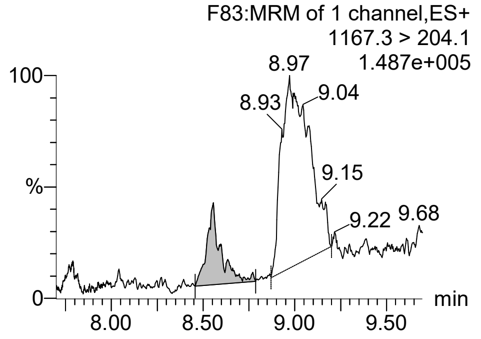

Sample Name: 20211111_A44_R2_IgM

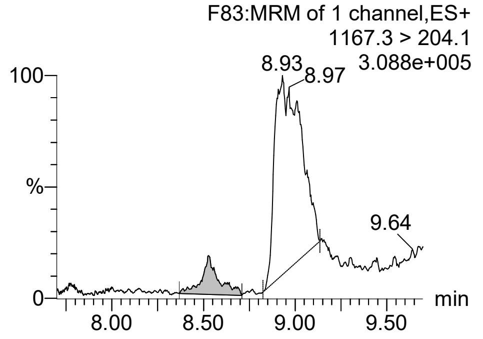

Sample Name: 20211111_QC_R17_IgM

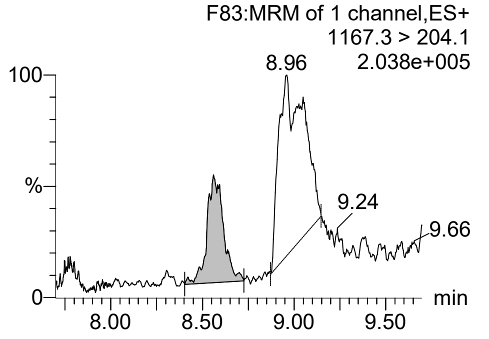

Sample Name: 20211111_L2_R3_IgM

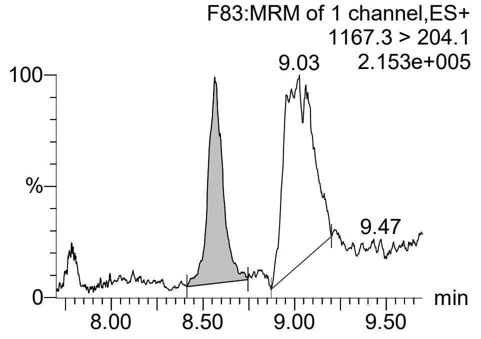

Sample Name: 20211111_H1_R3_IgM

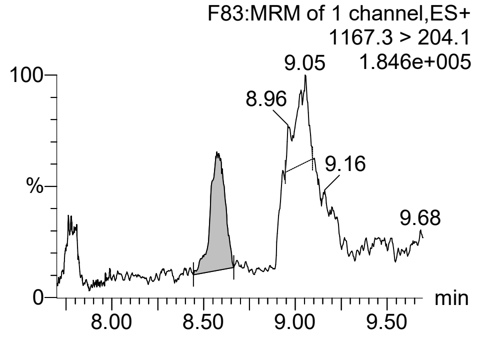

Sample Name: 20211111_A3_R3_IgM

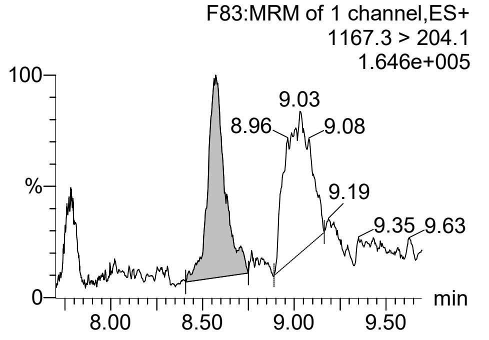

Sample Name: 20211111_A4_R3_IgM

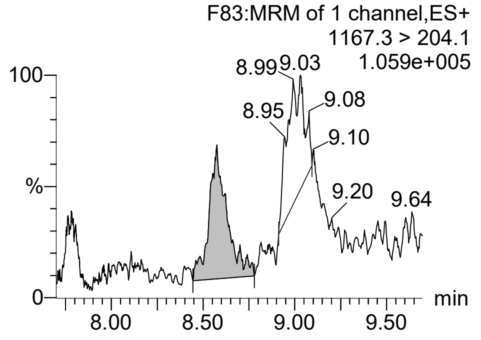

Sample Name: 20211111_L3_R3_IgM

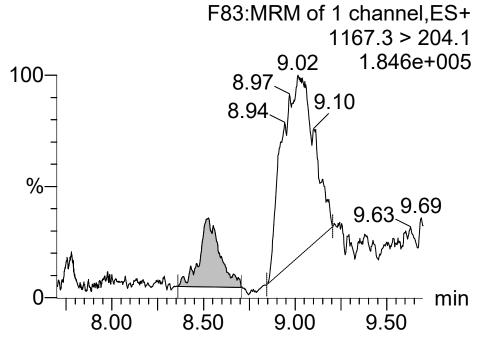

Sample Name: 20211111_L4_R3_IgM

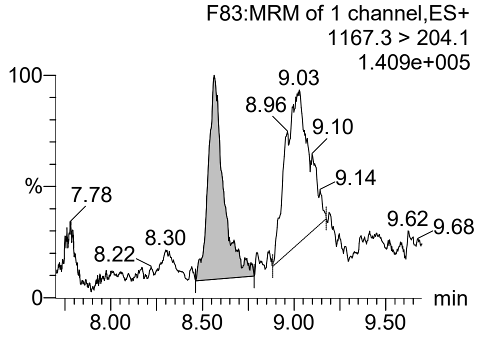

Sample Name: 20211111_H2_R3_IgM

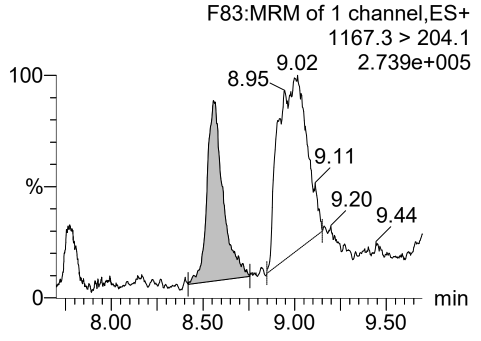

Sample Name: 20211111_A5_R3_IgM

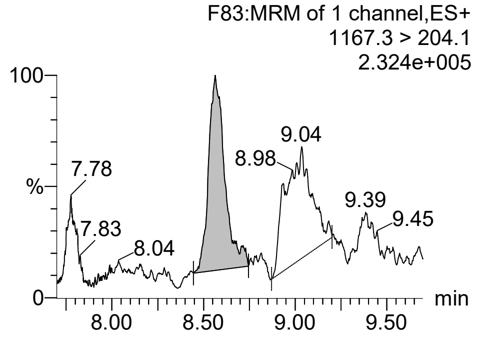

Dataset: C:\MassLynx\10.pro\20211111_clinicalsamples_IgM_IgA.qld

Last Altered: Thursday, March 09, 2023 19:58:25 台北標準時間
Printed: Friday, August 01, 2025 15:17:02 台北標準時間

Sample Name: 20211111_A6_R3_IgM
F83:MRM of 1 channel,ES+
1167.3 > 204.1
1.339e+005

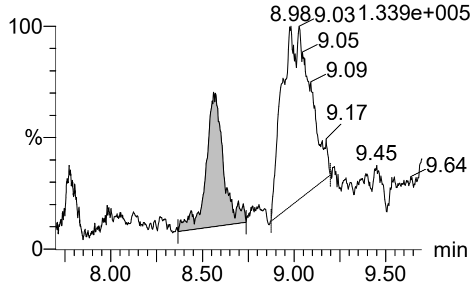

Sample Name: 20211111_QC_R18_IgM
F83:MRM of 1 channel,ES+
1167.3 > 204.1
1.419e+005

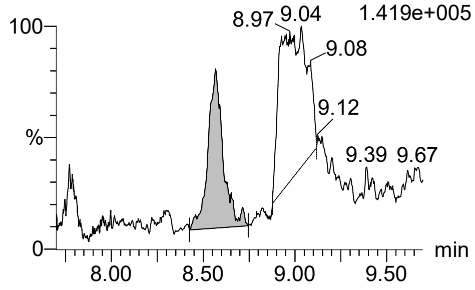

Sample Name: 20211111_L5_R3_IgM
F83:MRM of 1 channel,ES+
1167.3 > 204.1
1.701e+005

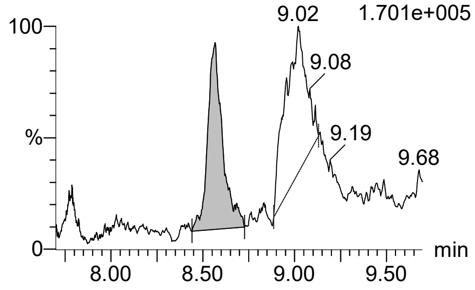

Sample Name: 20211111_L6_R3_IgM
F83:MRM of 1 channel,ES+
1167.3 > 204.1
1.483e+005

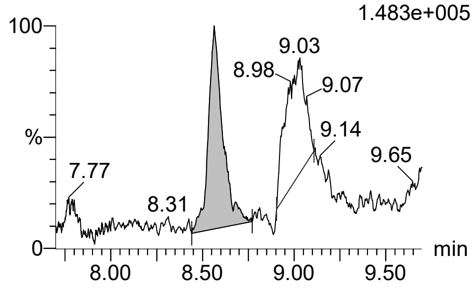

Sample Name: 20211111_H3_R3_IgM
F83:MRM of 1 channel,ES+
1167.3 > 204.1
1.002e+005

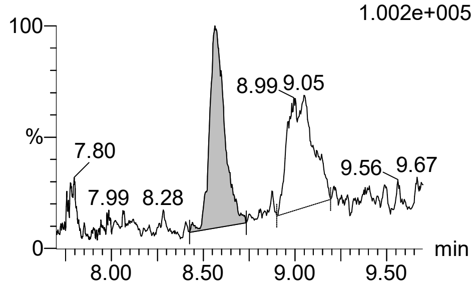

Sample Name: 20211111_A7_R3_IgM
F83:MRM of 1 channel,ES+
1167.3 > 204.1
3.637e+005

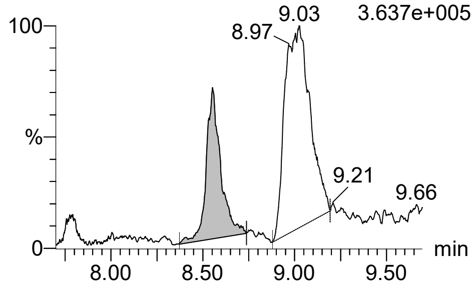

Sample Name: 20211111_A8_R3_IgM
F83:MRM of 1 channel,ES+
1167.3 > 204.1
1.937e+005

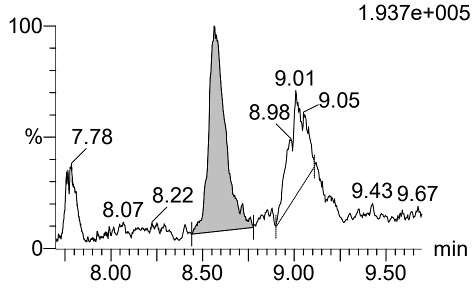

Sample Name: 20211111_L7_R3_IgM
F83:MRM of 1 channel,ES+
1167.3 > 204.1
1.284e+005

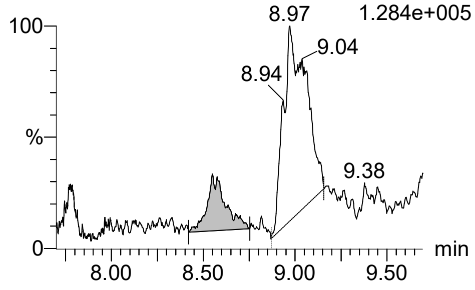

Sample Name: 20211111_L8_R3_IgM
F83:MRM of 1 channel,ES+
1167.3 > 204.1
8.146e+004

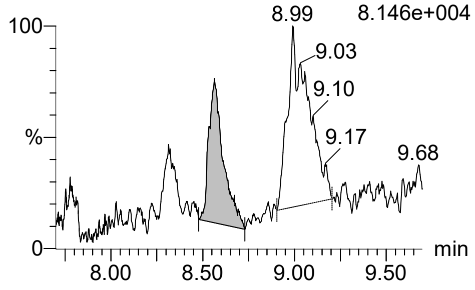

Sample Name: 20211111_H4_R3_IgM
F83:MRM of 1 channel,ES+
1167.3 > 204.1
9.744e+004

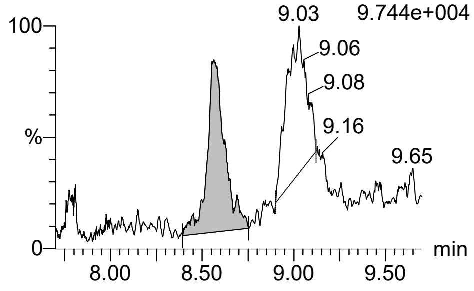

Sample Name: 20211111_A9_R3_IgM
F83:MRM of 1 channel,ES+
1167.3 > 204.1
1.114e+005

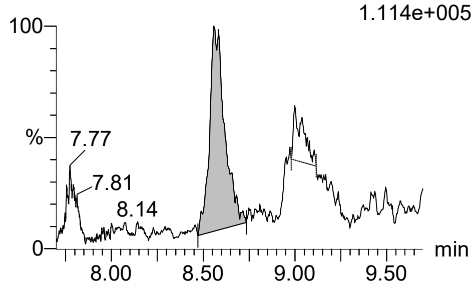

Sample Name: 20211111_A10_R3_IgM
F83:MRM of 1 channel,ES+
1167.3 > 204.1
2.997e+005

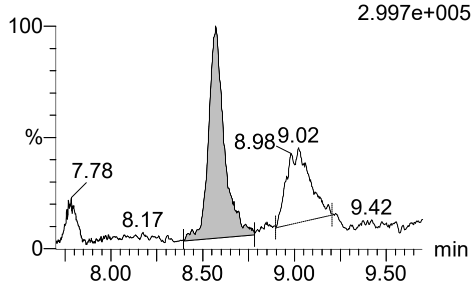

Sample Name: 20211111_L9_R3_IgM
F83:MRM of 1 channel,ES+
1167.3 > 204.1
1.405e+005

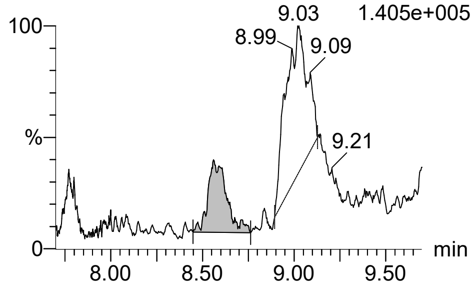

Sample Name: 20211111_L10_R3_IgM
F83:MRM of 1 channel,ES+
1167.3 > 204.1
2.176e+005

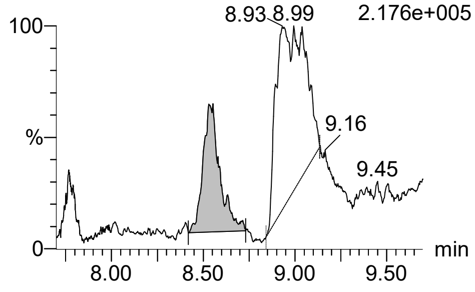

Sample Name: 20211111_QC_R19_IgM
F83:MRM of 1 channel,ES+
1167.3 > 204.1
1.854e+005

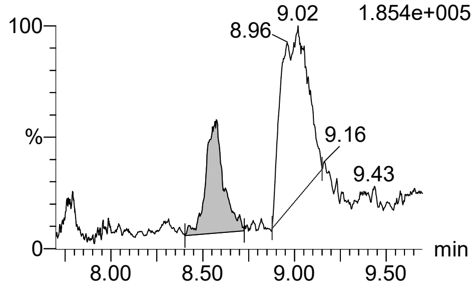

Sample Name: 20211111_H5_R3_IgM

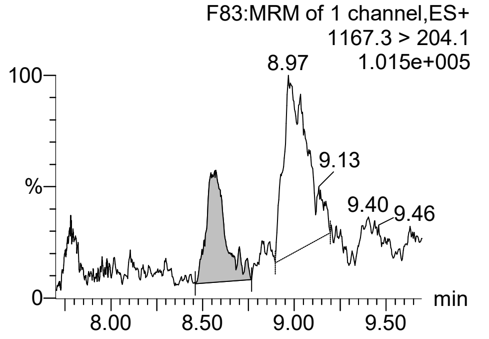

Sample Name: 20211111_A11_R3_IgM

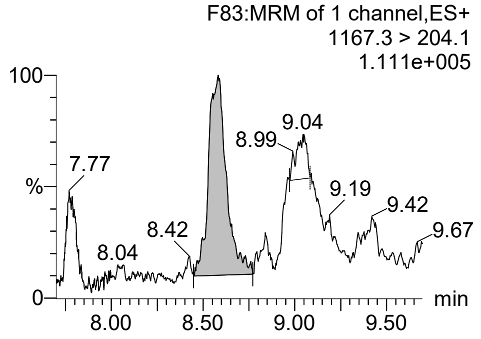

Sample Name: 20211111_A12_R3_IgM

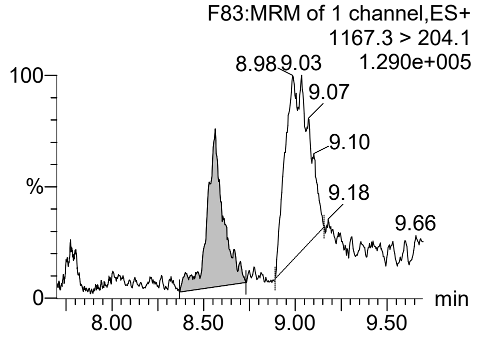

Sample Name: 20211111_L11_R3_IgM

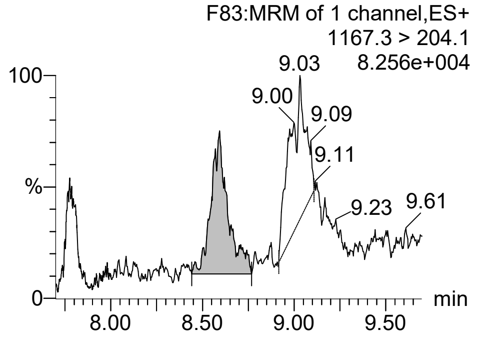

Sample Name: 20211111_L12_R3_IgM

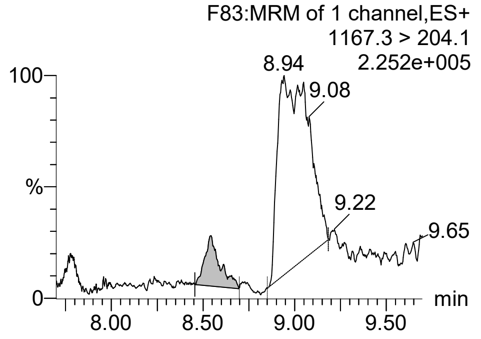

Sample Name: 20211111_H6_R3_IgM

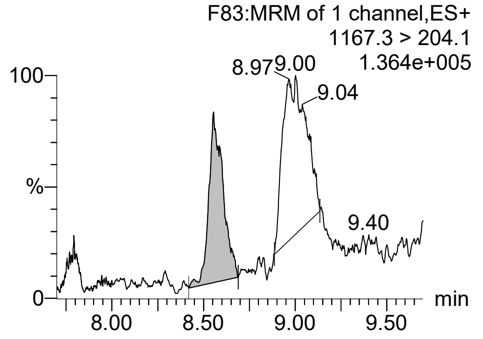

Sample Name: 20211111_A13_R3_IgM

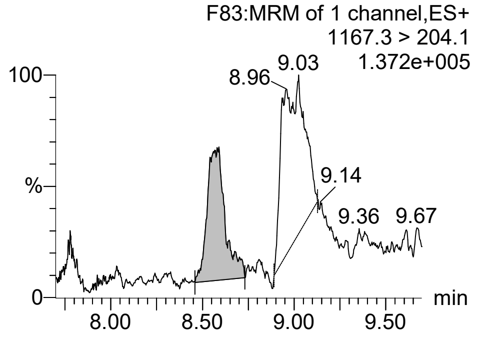

Sample Name: 20211111_A14_R3_IgM

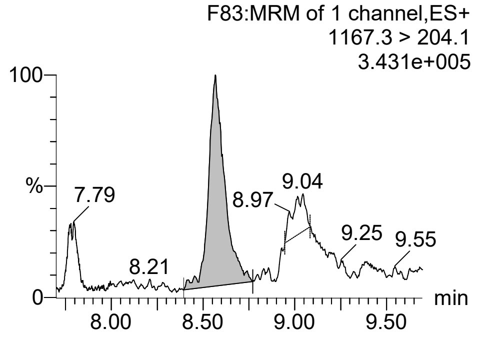

Sample Name: 20211111_L13_R3_IgM

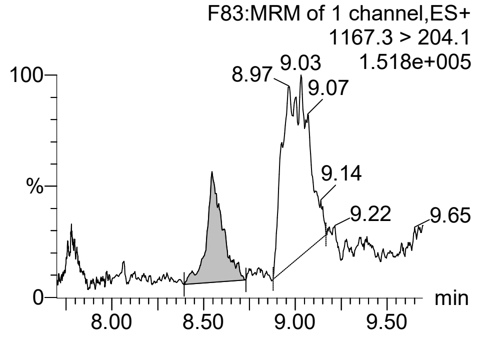

Sample Name: 20211111_L14_R3_IgM

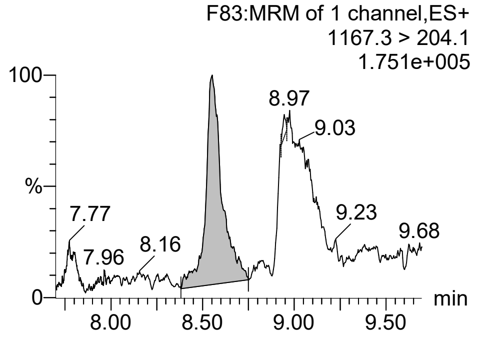

Sample Name: 20211111_H7_R3_IgM

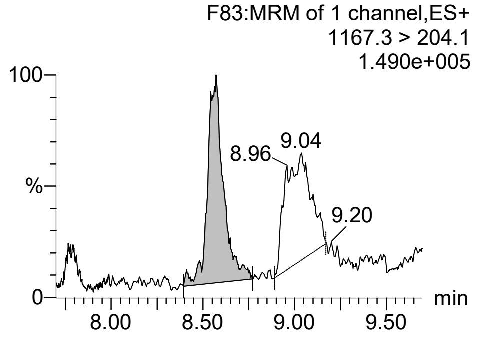

Sample Name: 20211111_A15_R3_IgM

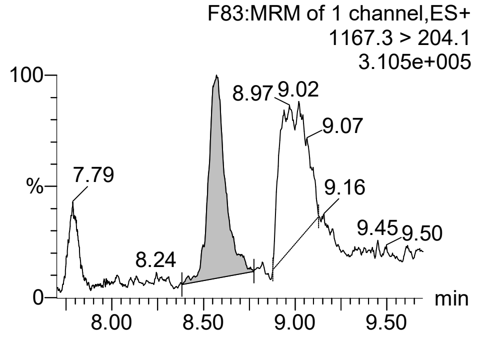

Sample Name: 20211111_QC_R20_IgM

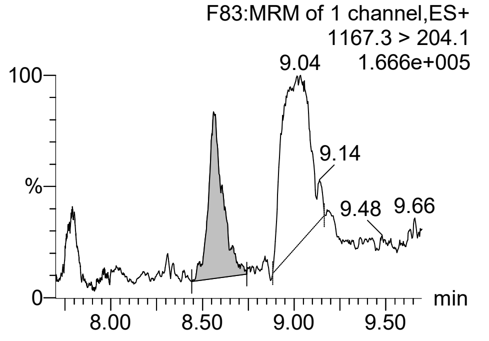

Sample Name: 20211111_A16_R3_IgM

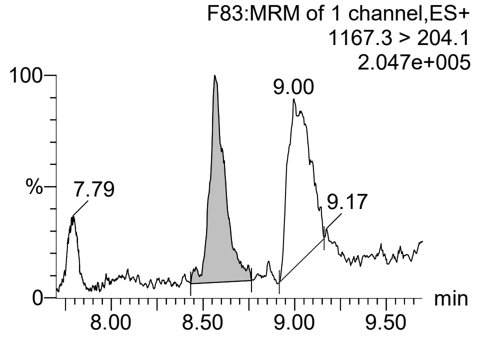

Sample Name: 20211111_L15_R3_IgM

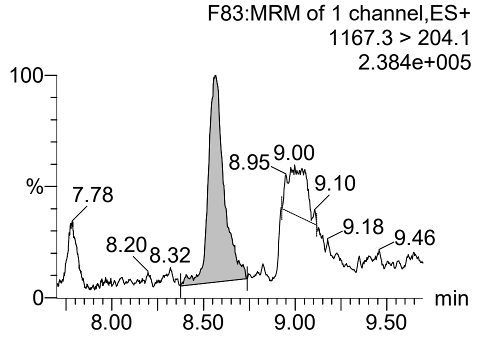

Dataset: C:\MassLynx\10.pro\20211111_clinicalsamples_IgM_IgA.qld

Last Altered: Thursday, March 09, 2023 19:58:25 台北標準時間
Printed: Friday, August 01, 2025 15:17:02 台北標準時間

Sample Name: 20211111_L16_R3_IgM

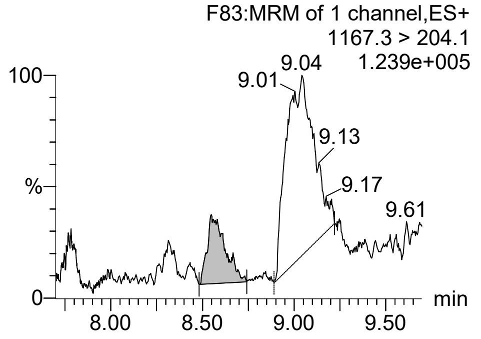

Sample Name: 20211111_H8_R3_IgM

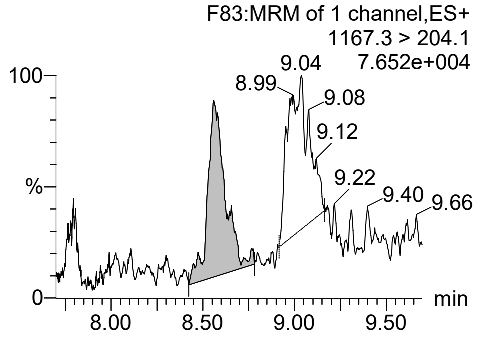

Sample Name: 20211111_A17_R3_IgM

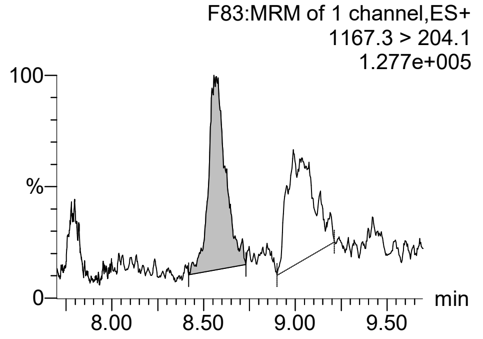

Sample Name: 20211111_A18_R3_IgM

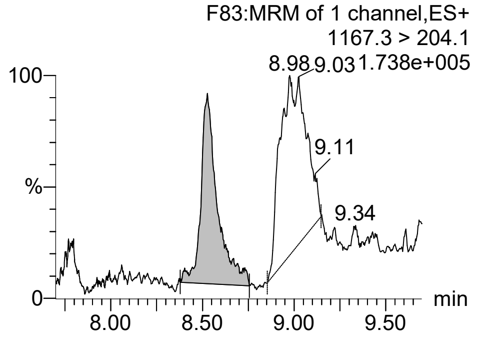

Sample Name: 20211111_L17_R3_IgM

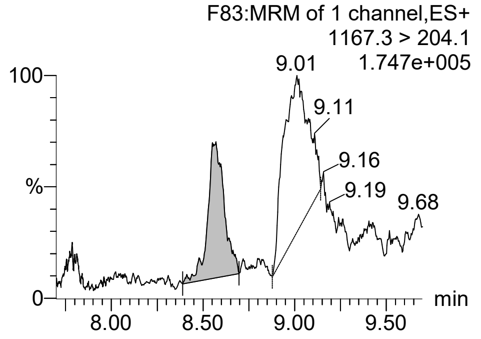

Sample Name: 20211111_L18_R3_IgM

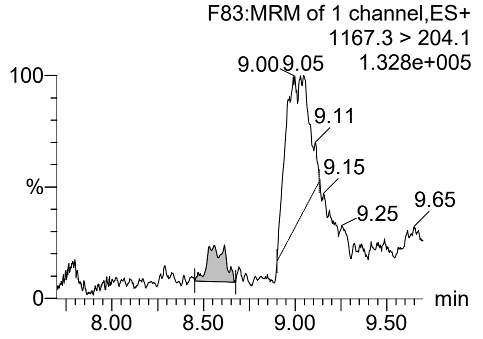

Sample Name: 20211111_H9_R3_IgM

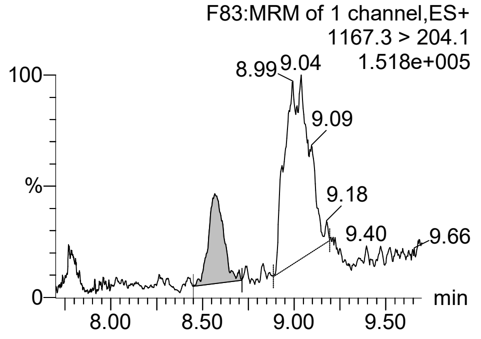

Sample Name: 20211111_A19_R3_IgM

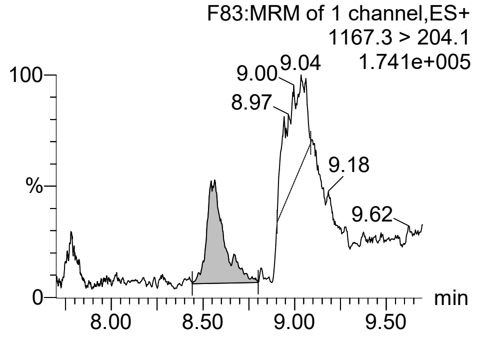

Sample Name: 20211111_A20_R3_IgM

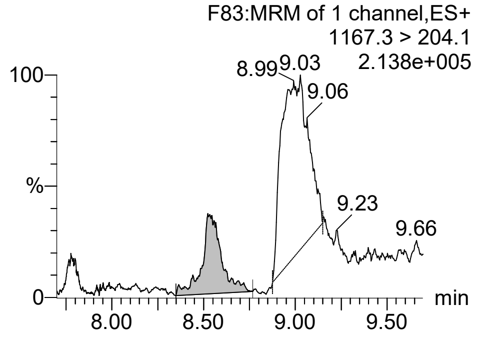

Sample Name: 20211111_L19_R3_IgM

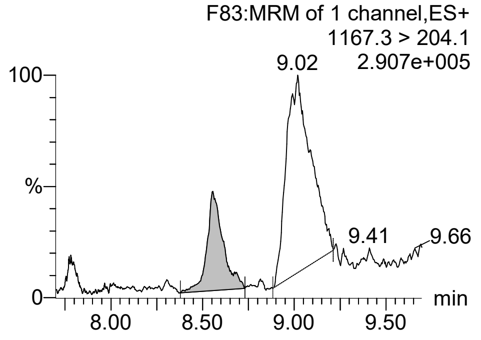

Sample Name: 20211111_QC_R21_IgM

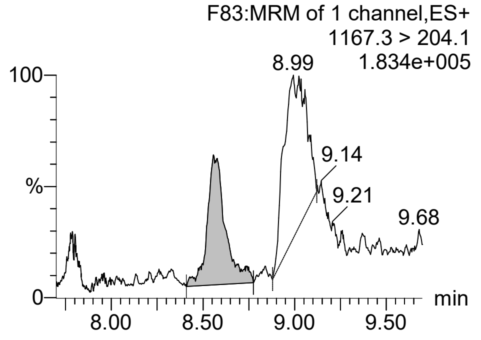

Sample Name: 20211111_L20_R3_IgM

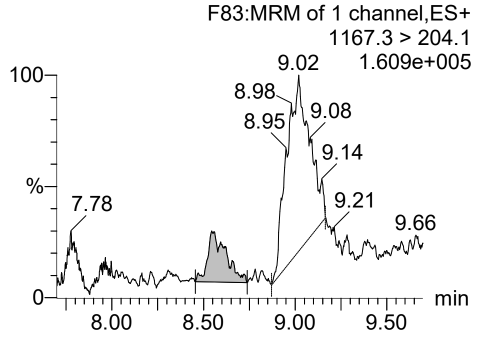

Sample Name: 20211111_H10_R3_IgM

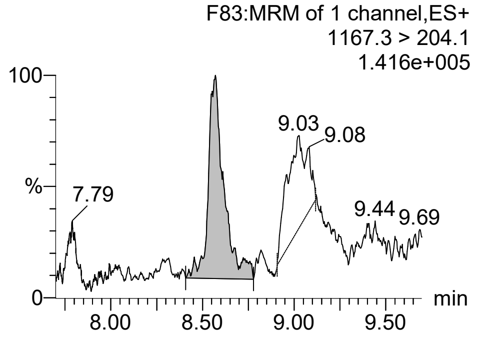

Sample Name: 20211111_A21_R3_IgM

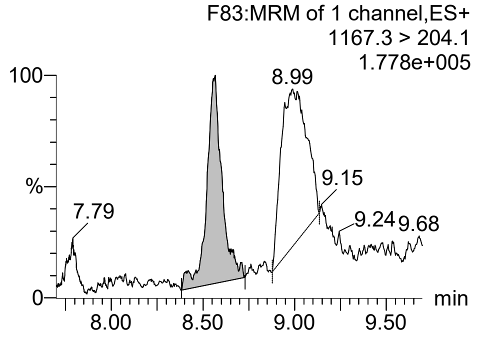

Sample Name: 20211111_A22_R3_IgM

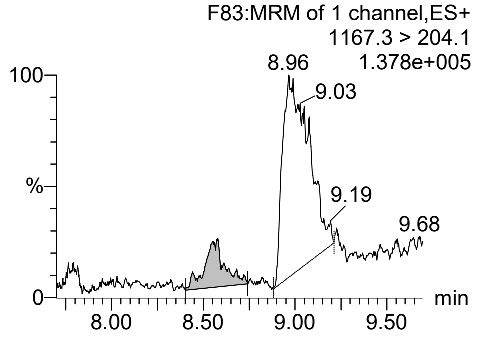

Sample Name: 20211111_L21_R3_IgM

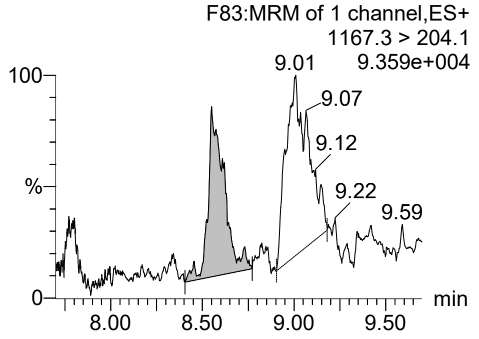

Sample Name: 20211111_L22_R3_IgM

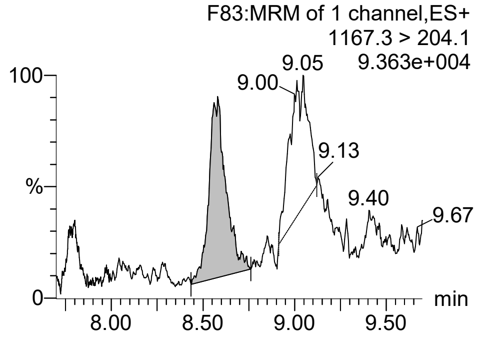

Sample Name: 20211111_H11_R3_IgM

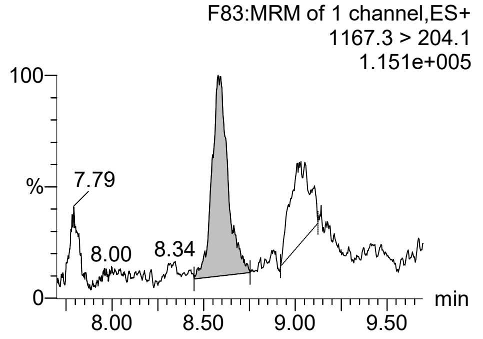

Sample Name: 20211111_A23_R3_IgM

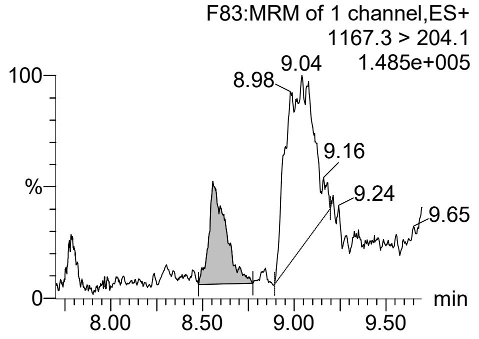

Sample Name: 20211111_A24_R3_IgM

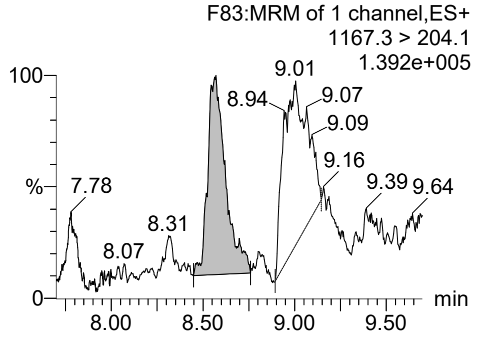

Sample Name: 20211111_L23_R3_IgM

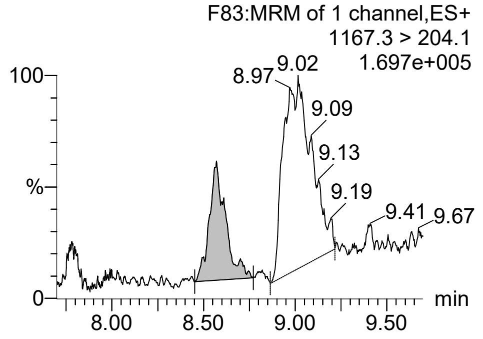

Sample Name: 20211111_L24_R3_IgM

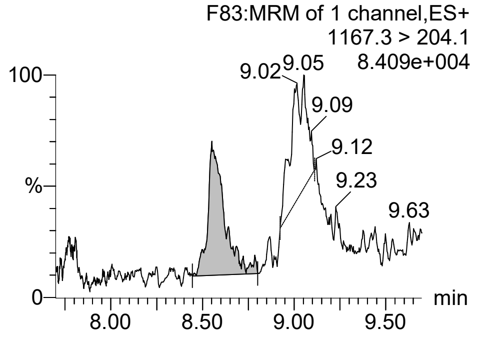

Sample Name: 20211111_H12_R3_IgM

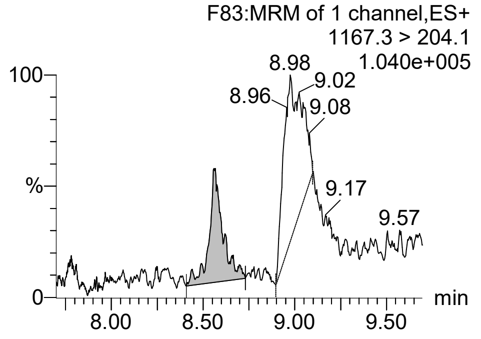

Sample Name: 20211111_QC_R22_IgM

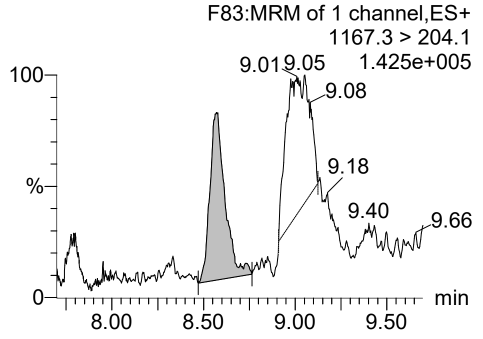

Sample Name: 20211111_A25_R3_IgM

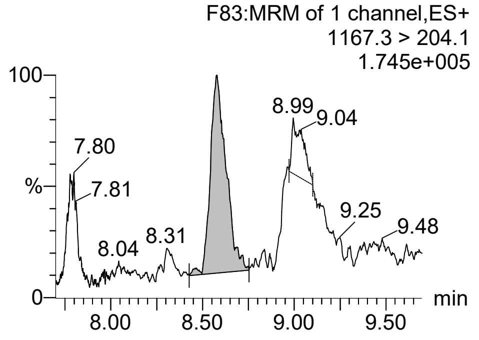

Sample Name: 20211111_A26_R3_IgM

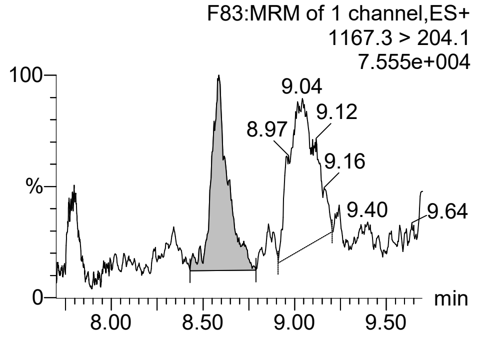

Sample Name: 20211111_L25_R3_IgM

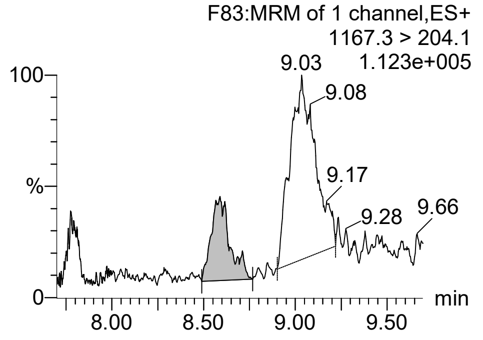

Sample Name: 20211111_L26_R3_IgM

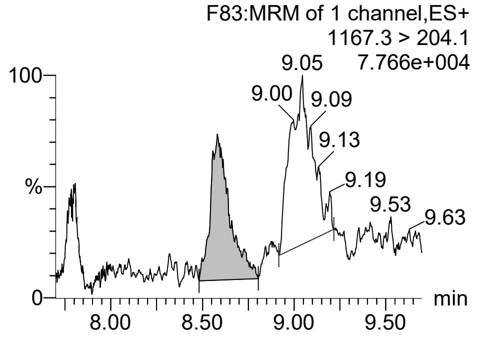

Sample Name: 20211111_H13_R3_IgM

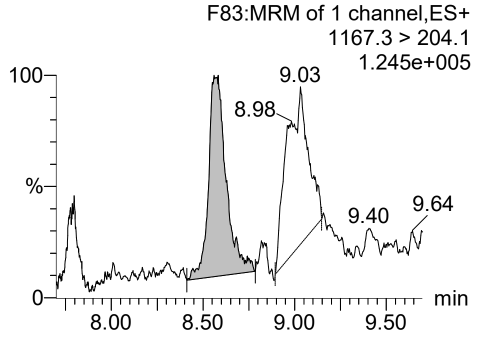

Sample Name: 20211111_A27_R3_IgM

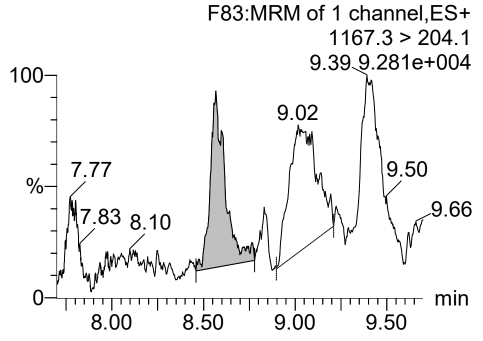

Dataset: C:\MassLynx\10.pro\20211111_clinicalsamples_IgM_IgA.qld

Last Altered: Thursday, March 09, 2023 19:58:25 台北標準時間
Printed: Friday, August 01, 2025 15:17:02 台北標準時間

Sample Name: 20211111_A28_R3_IgM
F83:MRM of 1 channel,ES+
1167.3 > 204.1
9.371e+004

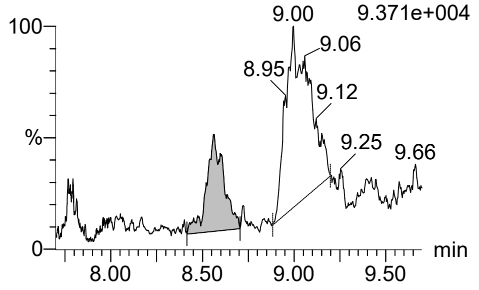

Sample Name: 20211111_L27_R3_IgM
F83:MRM of 1 channel,ES+
1167.3 > 204.1
1.334e+005

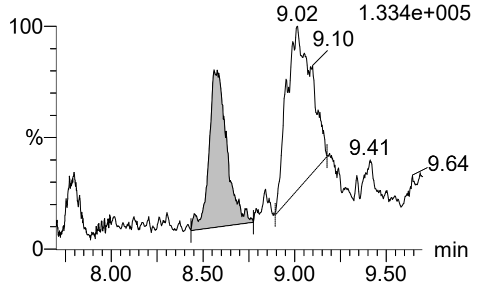

Sample Name: 20211111_L28_R3_IgM
F83:MRM of 1 channel,ES+
1167.3 > 204.1
8.967e+004

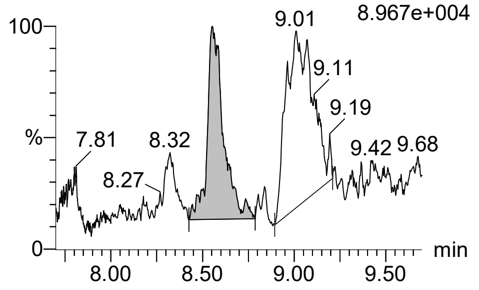

Sample Name: 20211111_H14_R3_IgM
F83:MRM of 1 channel,ES+
1167.3 > 204.1
1.257e+005

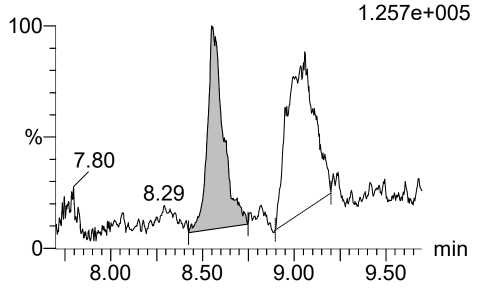

Sample Name: 20211111_A29_R3_IgM
F83:MRM of 1 channel,ES+
1167.3 > 204.1
1.757e+005

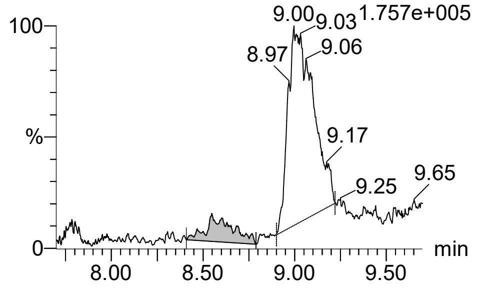

Sample Name: 20211111_A30_R3_IgM
F83:MRM of 1 channel,ES+
1167.3 > 204.1
2.055e+005

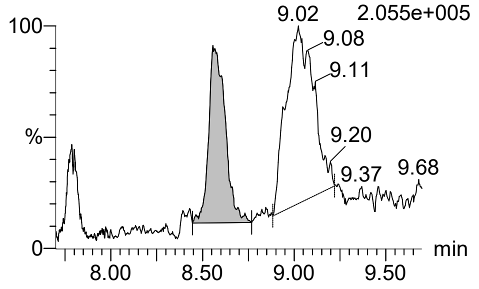

Sample Name: 20211111_QC_R23_IgM
F83:MRM of 1 channel,ES+
1167.3 > 204.1
1.425e+005

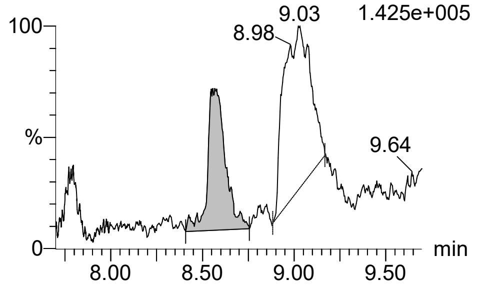

Sample Name: 20211111_L29_R3_IgM
F83:MRM of 1 channel,ES+
1167.3 > 204.1
1.513e+005

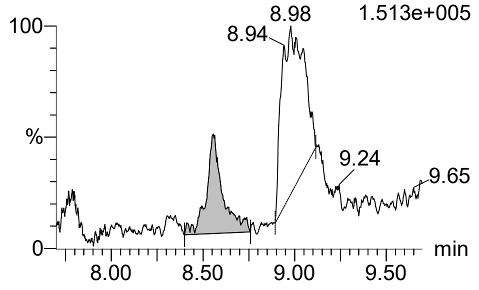

Sample Name: 20211111_L30_R3_IgM
F83:MRM of 1 channel,ES+
1167.3 > 204.1
1.273e+005

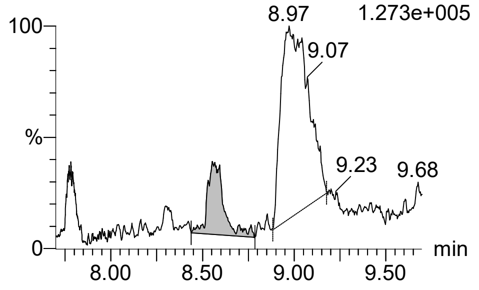

Sample Name: 20211111_H15_R3_IgM
F83:MRM of 1 channel,ES+
1167.3 > 204.1
1.769e+005

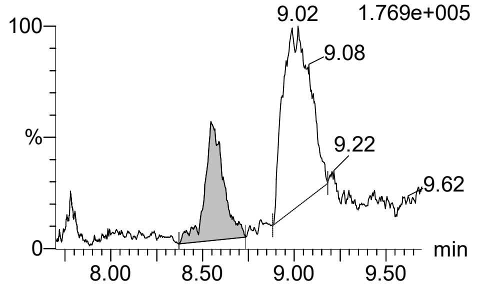

Sample Name: 20211111_A31_R3_IgM
F83:MRM of 1 channel,ES+
1167.3 > 204.1
1.512e+005

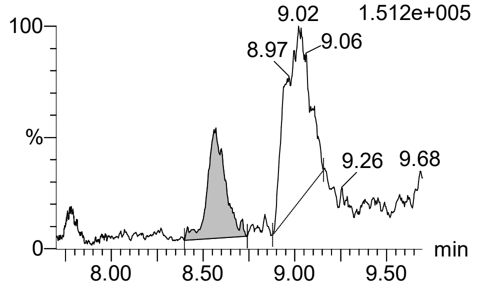

Sample Name: 20211111_A32_R3_IgM
F83:MRM of 1 channel,ES+
1167.3 > 204.1
1.566e+005

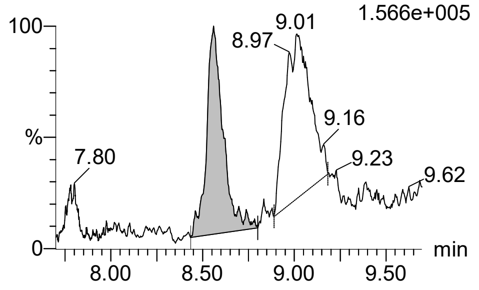

Sample Name: 20211111_L31_R3_IgM
F83:MRM of 1 channel,ES+
1167.3 > 204.1
1.157e+005

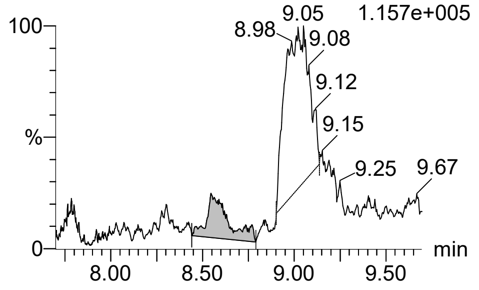

Sample Name: 20211111_L32_R3_IgM
F83:MRM of 1 channel,ES+
1167.3 > 204.1
1.256e+005

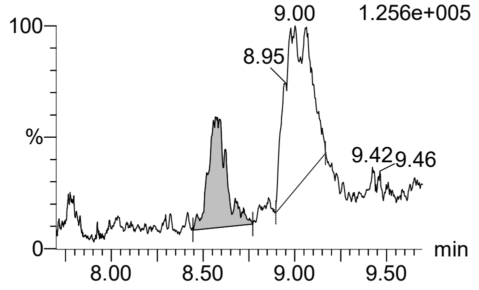

Sample Name: 20211111_H16_R3_IgM
F83:MRM of 1 channel,ES+
1167.3 > 204.1
1.787e+005

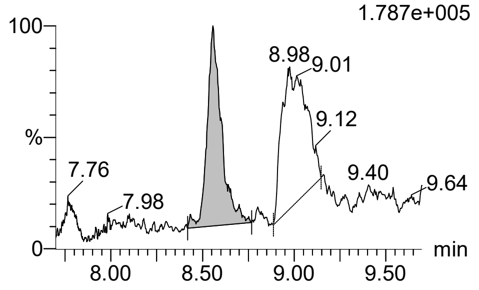

Dataset: C:\MassLynx\10.pro\20211111_clinicalsamples_IgM_IgA.qld

Last Altered: Thursday, March 09, 2023 19:58:25 台北標準時間
Printed: Friday, August 01, 2025 15:17:02 台北標準時間

Sample Name: 20211111_A33_R3_IgM

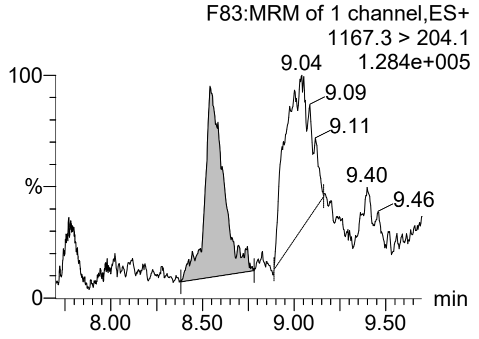

Sample Name: 20211111_A34_R3_IgM

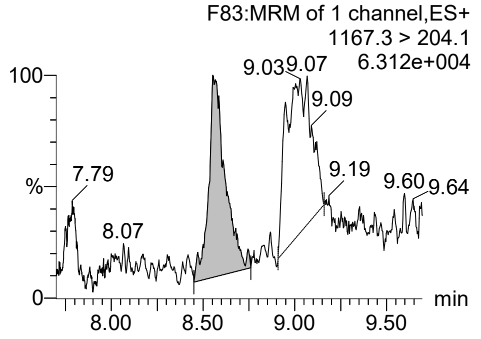

Sample Name: 20211111_H17_R3_IgM

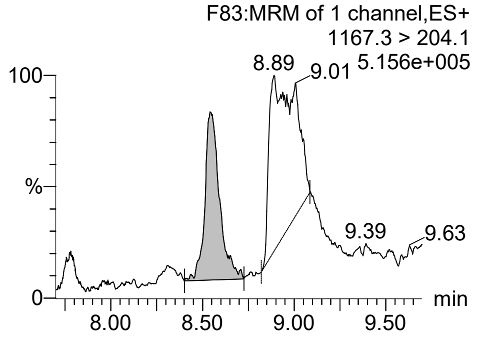

Sample Name: 20211111_A35_R3_IgM

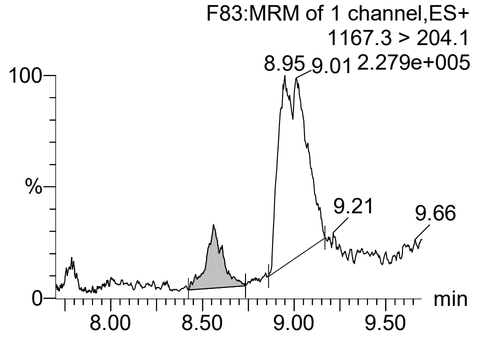

Sample Name: 20211111_QC_R24_IgM

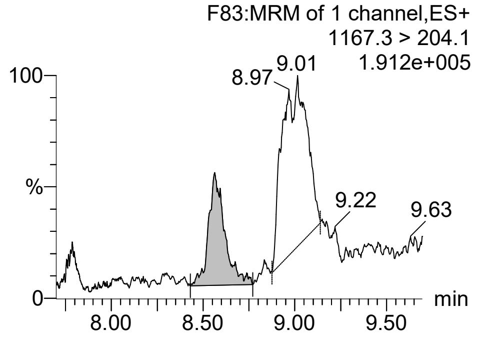

Sample Name: 20211111_A36_R3_IgM

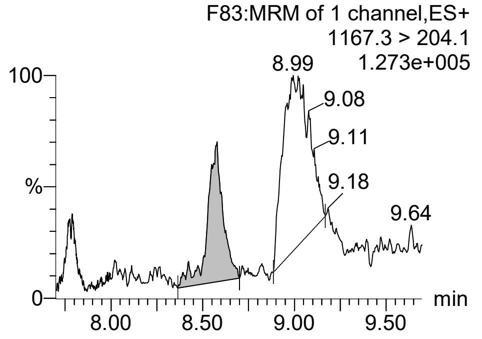

Sample Name: 20211111_H18_R3_IgM

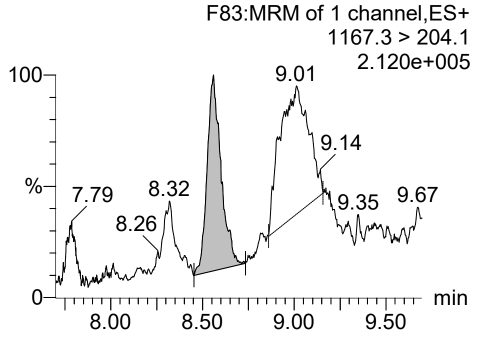

Sample Name: 20211111_A37_R3_IgM

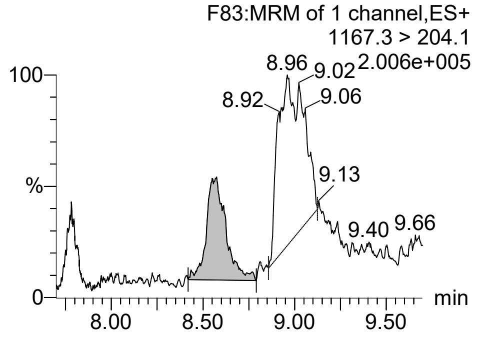

Sample Name: 20211111_A38_R3_IgM

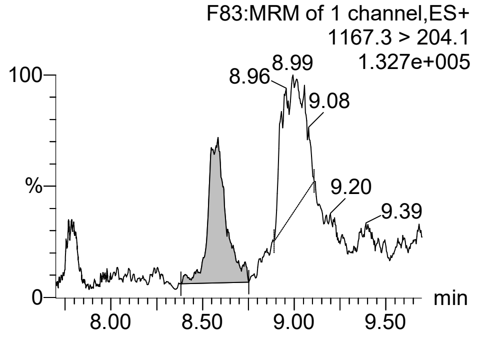

Sample Name: 20211111_H19_R3_IgM

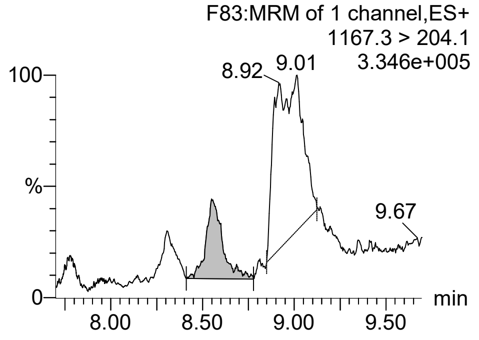

Sample Name: 20211111_A39_R3_IgM

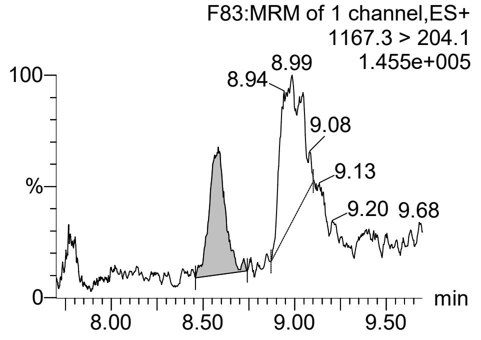

Sample Name: 20211111_A40_R3_IgM

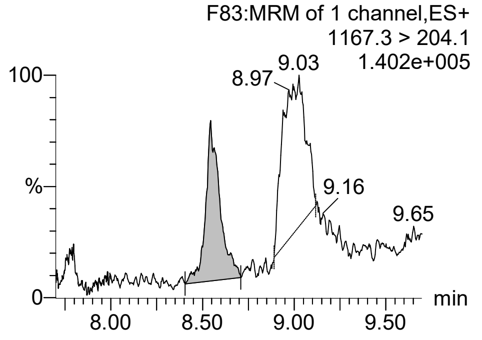

Sample Name: 20211111_H20_R3_IgM

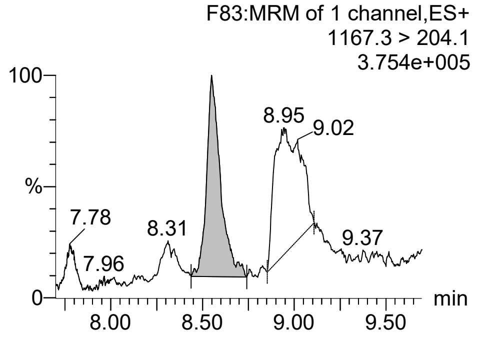

Sample Name: 20211111_A41_R3_IgM

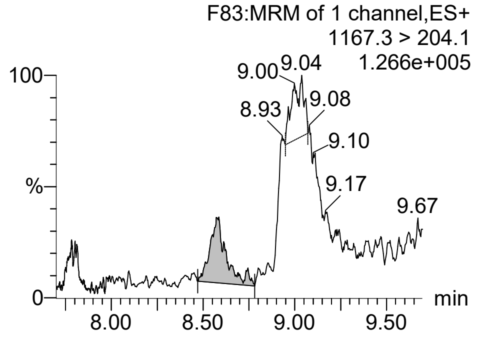

Sample Name: 20211111_A42_R3_IgM

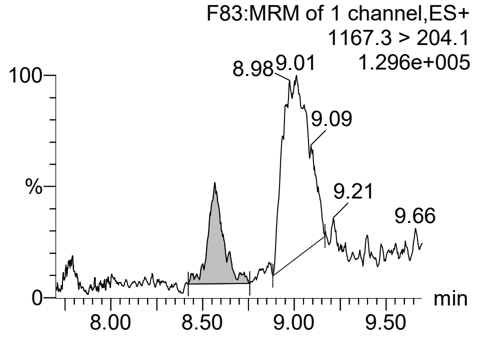

Dataset: C:\MassLynx\10.pro\20211111_clinicalsamples_IgM_IgA.qld

Last Altered: Thursday, March 09, 2023 19:58:25 台北標準時間
Printed: Friday, August 01, 2025 15:17:02 台北標準時間

Sample Name: 20211111_A43_R3_IgM

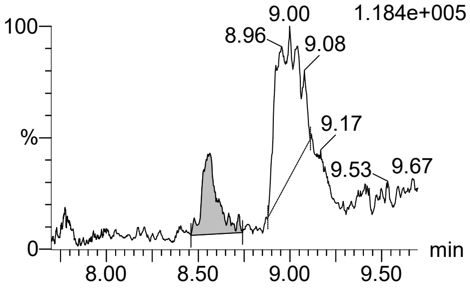

Sample Name: 20211111_A44_R3_IgM

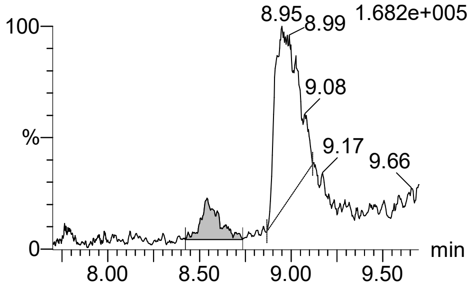

Sample Name: 20211111_QC_R25_IgM

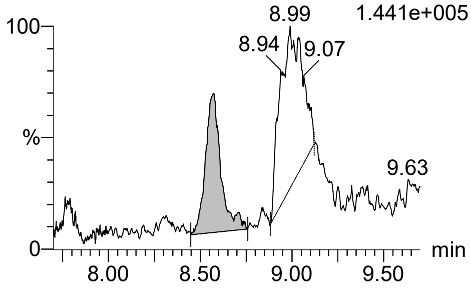

Dataset: C:\MassLynx\10.pro\20211111_clinicalsamples_IgM_IgA.qld

Last Altered: Thursday, March 09, 2023 19:58:25 台北標準時間
Printed: Friday, August 01, 2025 15:17:02 台北標準時間

	#	Name	RT	Area	IS Area	Response	Prima...
1	1	20211112_QC_R001_IgM	8.55	7820.8	15421.4	0.507	MM
2	2	20211112_QC_R002_IgM	8.57	7662.6	13188.8	0.581	MM
3	3	20211111_A1_R2_IgM	8.57	5631.4	11871.0	0.474	MM
4	4	20211111_A1_R3_IgM	8.56	5847.5	12030.5	0.486	MM
5	5	20211111_A2_R2_IgM	8.58	10738.5	11446.8	0.938	MM
6	6	20211111_A2_R3_IgM	8.58	12012.1	11098.4	1.082	MM
7	7	20211111_L1_R2_IgM	8.55	3220.6	11779.4	0.273	MM
8	8	20211111_L1_R3_IgM	8.58	3685.1	11176.2	0.330	MM
9	9	20211111_L2_R2_IgM	8.54	15082.2	11375.0	1.326	MM
10	10	20211111_H1_R2_IgM	8.57	9928.6	13595.2	0.730	MM
11	11	20211111_A3_R2_IgM	8.55	14371.8	14112.3	1.018	MM
12	12	20211111_A4_R2_IgM	8.56	5711.7	11977.5	0.477	MM
13	13	20211111_L3_R2_IgM	8.50	7265.2	12242.1	0.593	MM
14	14	20211111_L4_R2_IgM	8.56	10258.4	12797.0	0.802	MM
15	15	20211111_H2_R2_IgM	8.55	21434.3	10006.0	2.142	MM
16	16	20211111_A5_R2_IgM	8.55	26498.9	11434.6	2.317	MM
17	17	20211111_A6_R2_IgM	8.56	8317.1	13193.7	0.630	MM
18	18	20211111_QC_R10_IgM	8.56	9414.5	12566.1	0.749	MM
19	19	20211111_L5_R2_IgM	8.53	15969.2	13616.4	1.173	MM
20	20	20211111_L6_R2_IgM	8.56	11173.1	13698.5	0.816	MM
21	21	20211111_H3_R2_IgM	8.57	8225.9	15793.1	0.521	MM
22	22	20211111_A7_R2_IgM	8.55	18103.6	12936.9	1.399	MM
23	23	20211111_A8_R2_IgM	8.57	20534.3	13108.0	1.567	MM
24	24	20211111_L7_R2_IgM	8.56	4357.1	13278.5	0.328	MM
25	25	20211111_L8_R2_IgM	8.56	5761.8	15233.6	0.378	MM
26	26	20211111_H4_R2_IgM	8.55	9392.5	16521.9	0.568	MM
27	27	20211111_A9_R2_IgM	8.56	12107.6	16451.6	0.736	MM
28	28	20211111_A10_R2_IgM	8.56	26695.4	13036.0	2.048	MM
29	29	20211111_L9_R2_IgM	8.57	6094.6	14554.1	0.419	MM
30	30	20211111_L10_R2_IgM	8.53	15462.7	12250.4	1.262	MM
31	31	20211111_QC_R11_IgM	8.57	11614.5	11459.2	1.014	MM
32	32	20211111_H5_R2_IgM	8.56	7685.8	14358.2	0.535	MM
33	33	20211111_A11_R2_IgM	8.50	16070.3	16184.1	0.993	MM
34	34	20211111_A12_R2_IgM	8.56	7167.3	13465.4	0.532	MM
35	35	20211111_L11_R2_IgM	8.59	6488.0	13277.9	0.489	MM
36	36	20211111_L12_R2_IgM	8.54	6059.5	15958.8	0.380	MM
37	37	20211111_H6_R2_IgM	8.57	10610.6	14003.5	0.758	MM
38	38	20211111_A13_R2_IgM	8.56	8927.0	13656.1	0.654	MM
39	39	20211111_A14_R2_IgM	8.55	37824.4	14071.3	2.688	MM
40	40	20211111_L13_R2_IgM	8.56	8300.4	14785.6	0.561	MM
41	41	20211111_L14_R2_IgM	8.55	18118.7	14772.2	1.227	MM
42	42	20211111_H7_R2_IgM	8.53	16546.1	15164.4	1.091	MM
43	43	20211111_A15_R2_IgM	8.57	35425.9	15721.5	2.253	MM
44	44	20211111_QC_R12_IgM	8.55	12482.1	13711.5	0.910	MM
45	45	20211111_A16_R2_IgM	8.57	19809.8	14275.6	1.388	MM
46	46	20211111_L15_R2_IgM	8.56	23882.4	12935.8	1.846	MM
47	47	20211111_L16_R2_IgM	8.57	4332.5	13342.8	0.325	MM
48	48	20211111_H8_R2_IgM	8.57	7486.7	15835.1	0.473	MM
49	49	20211111_A17_R2_IgM	8.56	14541.8	15786.7	0.921	MM
50	50	20211111_A18_R2_IgM	8.54	15735.1	16986.8	0.926	MM
51	51	20211111_L17_R2_IgM	8.57	13430.5	13342.7	1.007	MM
52	52	20211111_L18_R2_IgM	8.56	2629.9	14692.0	0.179	MM
53	53	20211111_H9_R2_IgM	8.56	6008.4	15639.7	0.384	MM
54	54	20211111_A19_R2_IgM	8.57	7789.8	14198.7	0.549	MM
55	55	20211111_A20_R2_IgM	8.53	8228.0	14162.7	0.581	MM
56	56	20211111_L19_R2_IgM	8.56	10416.5	9293.4	1.121	MM

	# Name	RT	Area	IS Area	Response	Prima...
57	57 20211111_QC_R13_IgM	8.55	11027.8	13903.0	0.793	MM
58	58 20211111_L20_R2_IgM	8.52	6543.3	12781.6	0.512	MM
59	59 20211111_H10_R2_IgM	8.53	13985.4	14796.4	0.945	MM
60	60 20211111_A21_R2_IgM	8.56	15814.3	13669.0	1.157	MM
61	61 20211111_A22_R2_IgM	8.54	3851.0	14098.3	0.273	MM
62	62 20211111_L21_R2_IgM	8.57	7539.9	15678.2	0.481	MM
63	63 20211111_L22_R2_IgM	8.56	8831.7	15724.5	0.562	MM
64	64 20211111_H11_R2_IgM	8.56	11246.0	15890.2	0.708	MM
65	65 20211111_A23_R2_IgM	8.57	7493.8	13423.2	0.558	MM
66	66 20211111_A24_R2_IgM	8.56	11922.8	12438.0	0.959	MM
67	67 20211111_L23_R2_IgM	8.58	8589.3	14075.0	0.610	MM
68	68 20211111_L24_R2_IgM	8.55	6860.9	15972.1	0.430	MM
69	69 20211111_H12_R2_IgM	8.56	4367.8	16270.9	0.268	MM
70	70 20211111_QC_R14_IgM	8.57	12837.5	15806.8	0.812	MM
71	71 20211111_A25_R2_IgM	8.59	18518.4	14552.1	1.273	MM
72	72 20211111_A26_R2_IgM	8.60	6420.6	11492.7	0.559	MM
73	73 20211111_L25_R2_IgM	8.57	5908.6	14403.3	0.410	MM
74	74 20211111_L26_R2_IgM	8.59	6669.8	16533.9	0.403	MM
75	75 20211111_H13_R2_IgM	8.56	12572.4	15520.1	0.810	MM
76	76 20211111_A27_R2_IgM	8.57	8663.5	13337.4	0.650	MM
77	77 20211111_A28_R2_IgM	8.54	7611.2	11895.6	0.640	MM
78	78 20211111_L27_R2_IgM	8.56	12567.2	14125.7	0.890	MM
79	79 20211111_L28_R2_IgM	8.58	6403.4	11807.5	0.542	MM
80	80 20211111_H14_R2_IgM	8.56	10483.2	15035.7	0.697	MM
81	81 20211111_A29_R2_IgM	8.55	2231.2	13554.4	0.165	MM
82	82 20211111_A30_R2_IgM	8.57	21758.4	12984.1	1.676	MM
83	83 20211111_QC_R15_IgM	8.58	11784.4	12299.5	0.958	MM
84	84 20211111_L29_R2_IgM	8.56	6113.9	13590.7	0.450	MM
85	85 20211111_L30_R2_IgM	8.58	5022.3	12511.4	0.401	MM
86	86 20211111_H15_R2_IgM	8.54	9215.1	11885.0	0.775	MM
87	87 20211111_A31_R2_IgM	8.56	8023.9	13422.1	0.598	MM
88	88 20211111_A32_R2_IgM	8.56	15416.5	11461.3	1.345	MM
89	89 20211111_L31_R2_IgM	8.52	4332.6	13753.4	0.315	MM
90	90 20211111_L32_R2_IgM	8.56	8595.2	12949.0	0.664	MM
91	91 20211111_H16_R2_IgM	8.55	18425.9	11465.4	1.607	MM
92	92 20211111_A33_R2_IgM	8.56	11674.6	10551.5	1.106	MM
93	93 20211111_A34_R2_IgM	8.57	6462.0	12409.9	0.521	MM
94	94 20211111_H17_R2_IgM	8.54	42334.5	10160.9	4.166	MM
95	95 20211111_A35_R2_IgM	8.52	8893.4	10460.6	0.850	MM
96	96 20211111_QC_R16_IgM	8.56	10554.5	10433.5	1.012	MM
97	97 20211111_A36_R2_IgM	8.52	7354.6	12504.8	0.588	MM
98	98 20211111_H18_R2_IgM	8.55	16884.6	9140.4	1.847	MM
99	99 20211111_A37_R2_IgM	8.55	13457.0	9796.9	1.374	MM
100	1... 20211111_A38_R2_IgM	8.57	12350.9	11224.3	1.100	MM
101	1... 20211111_H19_R2_IgM	8.55	15531.9	11739.2	1.323	MM
102	1... 20211111_A39_R2_IgM	8.57	10729.8	12718.9	0.844	MM
103	1... 20211111_A40_R2_IgM	8.52	11381.8	10006.1	1.137	MM
104	1... 20211111_H20_R2_IgM	8.54	35831.9	7988.2	4.486	MM
105	1... 20211111_A41_R2_IgM	8.57	6574.1	11513.3	0.571	MM
106	1... 20211111_A42_R2_IgM	8.56	5209.9	10238.5	0.509	MM
107	1... 20211111_A43_R2_IgM	8.56	4738.5	11098.9	0.427	MM
108	1... 20211111_A44_R2_IgM	8.53	5766.8	10969.9	0.526	MM
109	1... 20211111_QC_R17_IgM	8.56	10149.1	9700.8	1.046	MM
110	1... 20211111_L2_R3_IgM	8.56	18422.6	8528.0	2.160	MM
111	1... 20211111_H1_R3_IgM	8.57	9013.8	9438.2	0.955	MM
112	1... 20211111_A3_R3_IgM	8.57	16066.7	11598.6	1.385	MM

Dataset: C:\MassLynx\10.pro\20211111_clinicalsamples_IgM_IgA.qld

Last Altered: Thursday, March 09, 2023 19:58:25 台北標準時間
Printed: Friday, August 01, 2025 15:17:02 台北標準時間

	# Name	RT	Area	IS Area	Response	Prima...
113	1... 20211111_A4_R3_IgM	8.58	7128.6	11364.1	0.627	MM
114	1... 20211111_L3_R3_IgM	8.53	7409.6	10753.4	0.689	MM
115	1... 20211111_L4_R3_IgM	8.56	12453.8	10291.6	1.210	MM
116	1... 20211111_H2_R3_IgM	8.56	22391.0	9315.8	2.404	MM
117	1... 20211111_A5_R3_IgM	8.56	20736.8	9102.2	2.278	MM
118	1... 20211111_A6_R3_IgM	8.56	8916.1	10356.4	0.861	MM
119	1... 20211111_QC_R18_IgM	8.57	9578.9	8398.3	1.141	MM
120	1... 20211111_L5_R3_IgM	8.57	13321.5	9786.7	1.361	MM
121	1... 20211111_L6_R3_IgM	8.56	12145.2	9911.3	1.225	MM
122	1... 20211111_H3_R3_IgM	8.56	8523.3	12155.5	0.701	MM
123	1... 20211111_A7_R3_IgM	8.55	22289.3	11804.8	1.888	MM
124	1... 20211111_A8_R3_IgM	8.56	18559.6	10510.6	1.766	MM
125	1... 20211111_L7_R3_IgM	8.55	3705.8	12177.9	0.304	MM
126	1... 20211111_L8_R3_IgM	8.56	4784.9	11668.6	0.410	MM
127	1... 20211111_H4_R3_IgM	8.56	8486.2	11192.0	0.758	MM
128	1... 20211111_A9_R3_IgM	8.56	9814.4	14077.4	0.697	MM
129	1... 20211111_A10_R3_IgM	8.57	28108.6	11190.4	2.512	MM
130	1... 20211111_L9_R3_IgM	8.56	5360.9	14649.3	0.366	MM
131	1... 20211111_L10_R3_IgM	8.55	13263.8	9361.3	1.417	MM
132	1... 20211111_QC_R19_IgM	8.57	10488.9	10125.5	1.036	MM
133	1... 20211111_H5_R3_IgM	8.57	6169.7	10010.9	0.616	MM
134	1... 20211111_A11_R3_IgM	8.58	11172.6	12697.2	0.880	MM
135	1... 20211111_A12_R3_IgM	8.56	9267.7	13148.5	0.705	MM
136	1... 20211111_L11_R3_IgM	8.59	5648.9	10786.6	0.524	MM
137	1... 20211111_L12_R3_IgM	8.54	4844.8	11681.8	0.415	MM
138	1... 20211111_H6_R3_IgM	8.55	9309.0	12095.5	0.770	MM
139	1... 20211111_A13_R3_IgM	8.59	9274.0	12908.3	0.718	MM
140	1... 20211111_A14_R3_IgM	8.57	32985.9	11322.6	2.913	MM
141	1... 20211111_L13_R3_IgM	8.55	7602.3	11516.1	0.660	MM
142	1... 20211111_L14_R3_IgM	8.55	16898.6	10790.7	1.566	MM
143	1... 20211111_H7_R3_IgM	8.57	13868.7	11572.1	1.198	MM
144	1... 20211111_A15_R3_IgM	8.57	30059.6	12434.4	2.417	MM
145	1... 20211111_QC_R20_IgM	8.56	11389.5	10701.0	1.064	MM
146	1... 20211111_A16_R3_IgM	8.56	18468.0	11452.1	1.613	MM
147	1... 20211111_L15_R3_IgM	8.56	22054.1	10531.1	2.094	MM
148	1... 20211111_L16_R3_IgM	8.55	4296.5	12049.4	0.357	MM
149	1... 20211111_H8_R3_IgM	8.56	6700.1	13434.9	0.499	MM
150	1... 20211111_A17_R3_IgM	8.56	11965.2	12452.0	0.961	MM
151	1... 20211111_A18_R3_IgM	8.53	15435.4	12506.5	1.234	MM
152	1... 20211111_L17_R3_IgM	8.57	11108.4	9948.9	1.117	MM
153	1... 20211111_L18_R3_IgM	8.61	2470.8	13462.1	0.184	MM
154	1... 20211111_H9_R3_IgM	8.57	5880.9	13178.7	0.446	MM
155	1... 20211111_A19_R3_IgM	8.56	8818.9	11298.6	0.781	MM
156	1... 20211111_A20_R3_IgM	8.52	9410.4	11175.6	0.842	MM
157	1... 20211111_L19_R3_IgM	8.56	12995.2	11558.2	1.124	MM
158	1... 20211111_QC_R21_IgM	8.56	12289.1	9977.0	1.232	MM
159	1... 20211111_L20_R3_IgM	8.54	4273.5	13508.1	0.316	MM
160	1... 20211111_H10_R3_IgM	8.57	12684.3	12374.8	1.025	MM
161	1... 20211111_A21_R3_IgM	8.57	16007.7	12443.6	1.286	MM
162	1... 20211111_A22_R3_IgM	8.58	3692.2	11589.3	0.319	MM
163	1... 20211111_L21_R3_IgM	8.55	7201.4	13107.4	0.549	MM
164	1... 20211111_L22_R3_IgM	8.58	8189.2	12021.5	0.681	MM
165	1... 20211111_H11_R3_IgM	8.58	10395.3	12687.8	0.819	MM
166	1... 20211111_A23_R3_IgM	8.56	7244.8	11611.8	0.624	MM
167	1... 20211111_A24_R3_IgM	8.57	14018.3	10476.8	1.338	MM
168	1... 20211111_L23_R3_IgM	8.57	9308.8	11873.1	0.784	MM

Dataset: C:\MassLynx\10.pro\20211111_clinicalsamples_IgM_IgA.qld

Last Altered: Thursday, March 09, 2023 19:58:25 台北標準時間
Printed: Friday, August 01, 2025 15:17:02 台北標準時間

	# Name	RT	Area	IS Area	Response	Prima...
169	1... 20211111_L24_R3_IgM	8.55	5261.3	13468.0	0.391	MM
170	1... 20211111_H12_R3_IgM	8.57	4574.0	14229.1	0.321	MM
171	1... 20211111_QC_R22_IgM	8.57	10298.2	11264.4	0.914	MM
172	1... 20211111_A25_R3_IgM	8.58	15357.7	11775.7	1.304	MM
173	1... 20211111_A26_R3_IgM	8.59	6863.6	10473.9	0.655	MM
174	1... 20211111_L25_R3_IgM	8.59	4916.5	13256.8	0.371	MM
175	1... 20211111_L26_R3_IgM	8.58	6152.5	13565.0	0.454	MM
176	1... 20211111_H13_R3_IgM	8.58	12524.4	13146.0	0.953	MM
177	1... 20211111_A27_R3_IgM	8.57	6937.9	12025.6	0.577	MM
178	1... 20211111_A28_R3_IgM	8.56	4403.8	11229.1	0.392	MM
179	1... 20211111_L27_R3_IgM	8.56	10648.2	14960.0	0.712	MM
180	1... 20211111_L28_R3_IgM	8.55	7979.4	11359.0	0.702	MM
181	1... 20211111_H14_R3_IgM	8.55	11446.5	12830.8	0.892	MM
182	1... 20211111_A29_R3_IgM	8.55	3489.0	13434.3	0.260	MM
183	1... 20211111_A30_R3_IgM	8.56	16986.5	11272.2	1.507	MM
184	1... 20211111_QC_R23_IgM	8.57	10221.4	11872.2	0.861	MM
185	1... 20211111_L29_R3_IgM	8.56	6955.9	12948.7	0.537	MM
186	1... 20211111_L30_R3_IgM	8.55	4281.1	11026.6	0.388	MM
187	1... 20211111_H15_R3_IgM	8.55	11125.9	10786.3	1.031	MM
188	1... 20211111_A31_R3_IgM	8.57	8542.0	12010.5	0.711	MM
189	1... 20211111_A32_R3_IgM	8.56	15254.1	11107.7	1.373	MM
190	1... 20211111_L31_R3_IgM	8.55	3127.1	11021.8	0.284	MM
191	1... 20211111_L32_R3_IgM	8.57	7228.2	11683.8	0.619	MM
192	1... 20211111_H16_R3_IgM	8.56	14364.5	9831.7	1.461	MM
193	1... 20211111_A33_R3_IgM	8.54	13028.2	11213.1	1.162	MM
194	1... 20211111_A34_R3_IgM	8.55	6013.2	11550.1	0.521	MM
195	1... 20211111_H17_R3_IgM	8.54	35977.2	8874.4	4.054	MM
196	1... 20211111_A35_R3_IgM	8.56	6676.7	9856.3	0.677	MM
197	1... 20211111_QC_R24_IgM	8.56	9904.8	7561.8	1.310	MM
198	1... 20211111_A36_R3_IgM	8.58	7885.9	9562.8	0.825	MM
199	1... 20211111_H18_R3_IgM	8.56	16290.5	8165.3	1.995	MM
200	2... 20211111_A37_R3_IgM	8.57	11379.9	9278.8	1.226	MM
201	2... 20211111_A38_R3_IgM	8.58	9906.1	10920.3	0.907	MM
202	2... 20211111_H19_R3_IgM	8.55	12563.9	9285.3	1.353	MM
203	2... 20211111_A39_R3_IgM	8.58	8366.5	9083.4	0.921	MM
204	2... 20211111_A40_R3_IgM	8.54	9031.7	8382.4	1.077	MM
205	2... 20211111_H20_R3_IgM	8.55	29878.5	7807.9	3.827	MM
206	2... 20211111_A41_R3_IgM	8.59	3869.7	5925.2	0.653	MM
207	2... 20211111_A42_R3_IgM	8.57	5632.9	9352.6	0.602	MM
208	2... 20211111_A43_R3_IgM	8.56	4295.6	10216.3	0.420	MM
209	2... 20211111_A44_R3_IgM	8.54	3511.7	10262.7	0.342	MM
210	2... 20211111_QC_R25_IgM	8.57	8757.0	8575.3	1.021	MM

Method: C:\MassLynx\10_Pro_IgA.PRO\MethDB\20211111_IgM_IgA_DMRM_new.mdb 24 Feb 2022 13:44:14
Calibration: 15 Nov 2021 14:42:11

Sample Name: 20211112_QC_R001_IgM
F70:MRM of 1 channel,ES+
1102.8 > 204.1
4.301e+005

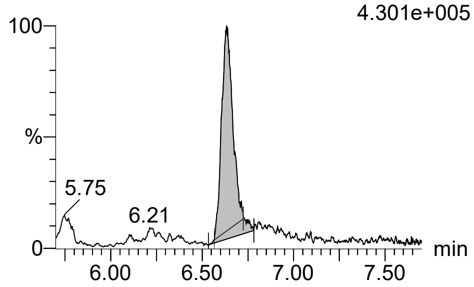

Sample Name: 20211112_QC_R002_IgM
F70:MRM of 1 channel,ES+
1102.8 > 204.1
3.751e+005

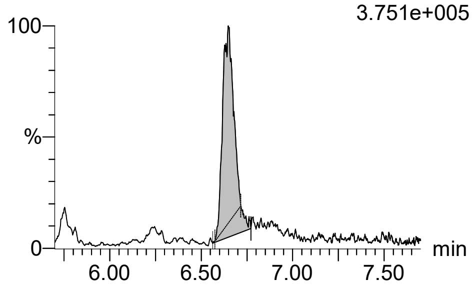

Sample Name: 20211111_A1_R2_IgM
F70:MRM of 1 channel,ES+
1102.8 > 204.1
1.995e+005

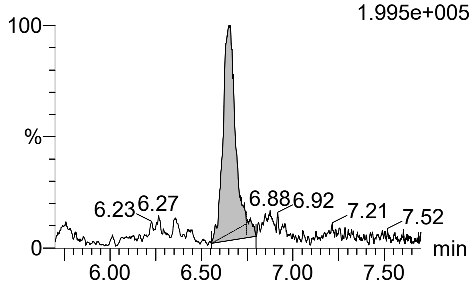

Sample Name: 20211111_A1_R3_IgM
F70:MRM of 1 channel,ES+
1102.8 > 204.1
1.719e+005

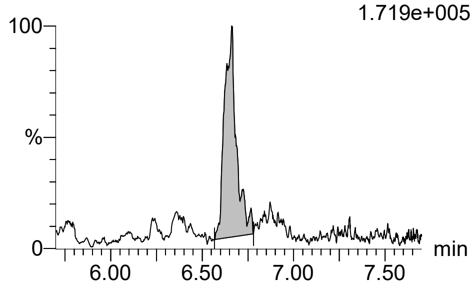

Sample Name: 20211111_A2_R2_IgM
F70:MRM of 1 channel,ES+
1102.8 > 204.1
5.382e+005

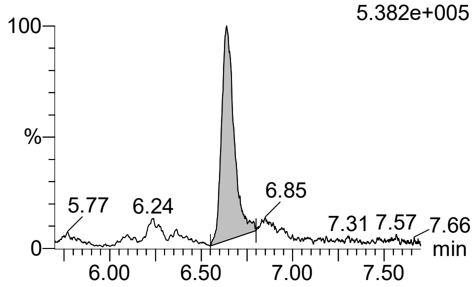

Sample Name: 20211111_A2_R3_IgM
F70:MRM of 1 channel,ES+
1102.8 > 204.1
6.141e+005

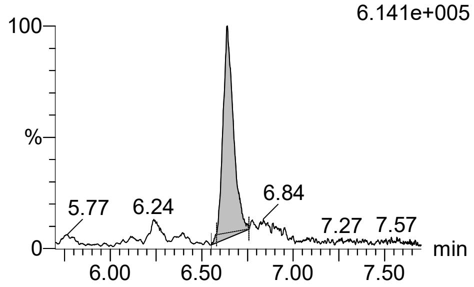

Sample Name: 20211111_L1_R2_IgM
F70:MRM of 1 channel,ES+
1102.8 > 204.1
1.594e+005

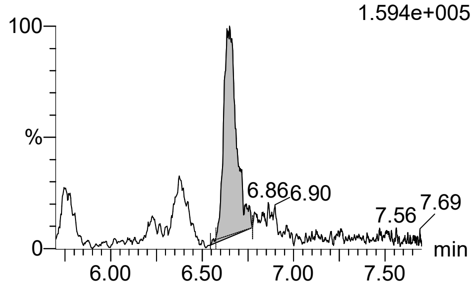

Sample Name: 20211111_L1_R3_IgM
F70:MRM of 1 channel,ES+
1102.8 > 204.1
1.003e+005

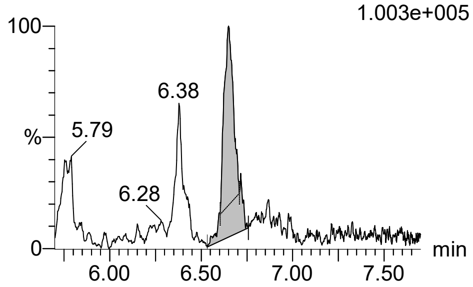

Sample Name: 20211111_L2_R2_IgM
F70:MRM of 1 channel,ES+
1102.8 > 204.1
3.718e+005

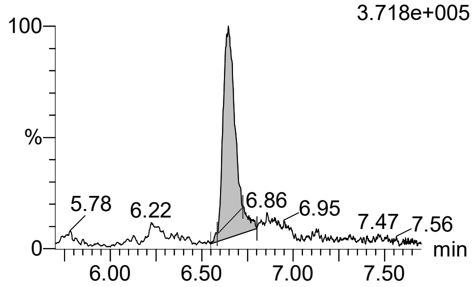

Sample Name: 20211111_H1_R2_IgM
F70:MRM of 1 channel,ES+
1102.8 > 204.1
7.921e+005

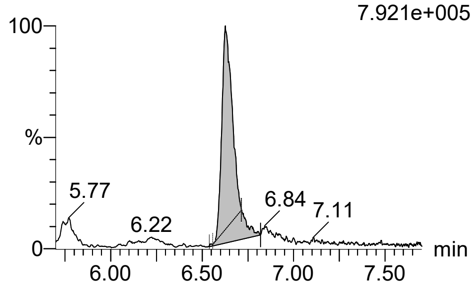

Sample Name: 20211111_A3_R2_IgM
F70:MRM of 1 channel,ES+
1102.8 > 204.1
6.638e+005

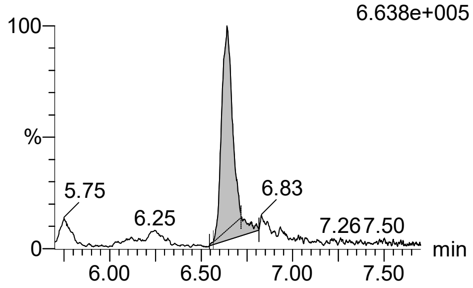

Sample Name: 20211111_A4_R2_IgM
F70:MRM of 1 channel,ES+
1102.8 > 204.1
3.690e+005

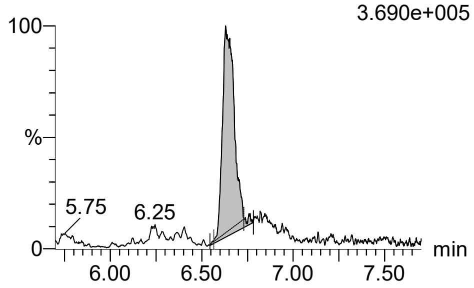

Dataset: C:\MassLynx\10.pro\20211111_clinicalsamples_IgM_IgA.qld

Last Altered: Thursday, March 09, 2023 19:58:25 台北標準時間
Printed: Friday, August 01, 2025 15:17:43 台北標準時間

Sample Name: 20211111_L3_R2_IgM

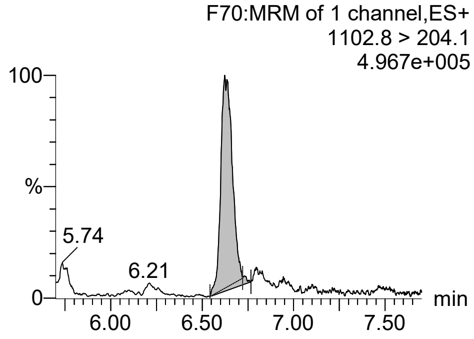

Sample Name: 20211111_L4_R2_IgM

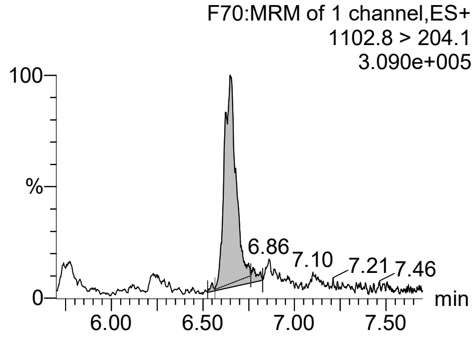

Sample Name: 20211111_H2_R2_IgM

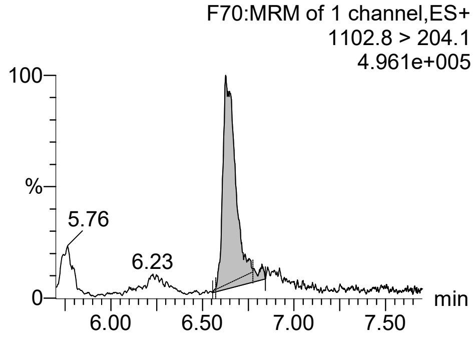

Sample Name: 20211111_A5_R2_IgM

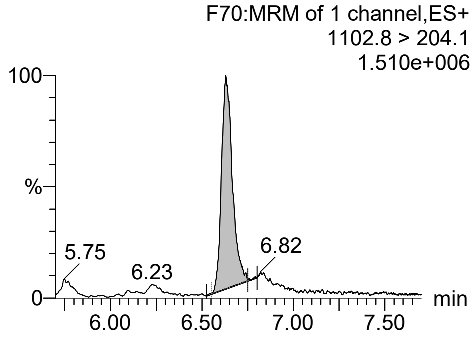

Sample Name: 20211111_A6_R2_IgM

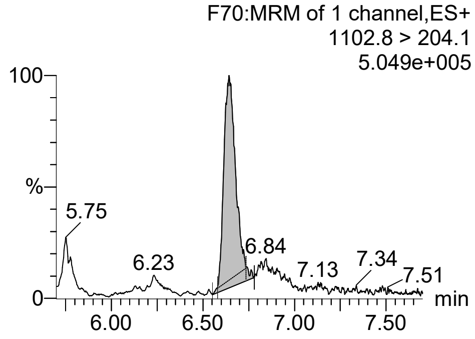

Sample Name: 20211111_QC_R10_IgM

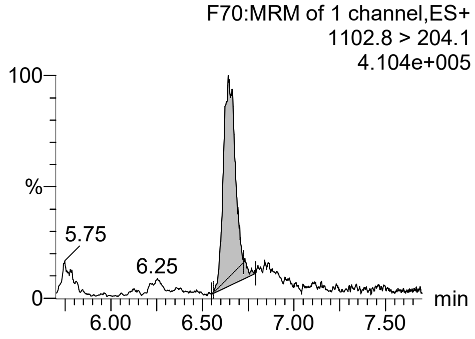

Sample Name: 20211111_L5_R2_IgM

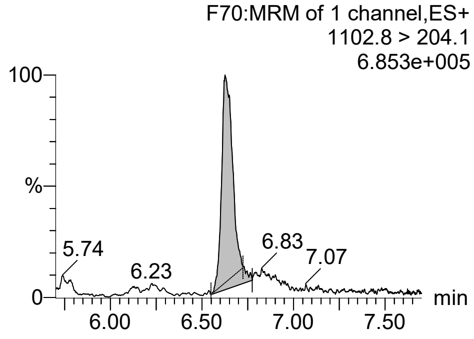

Sample Name: 20211111_L6_R2_IgM

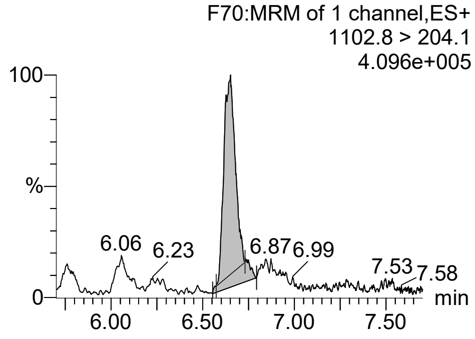

Sample Name: 20211111_H3_R2_IgM

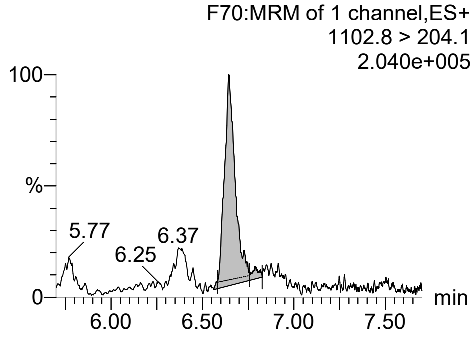

Sample Name: 20211111_A7_R2_IgM

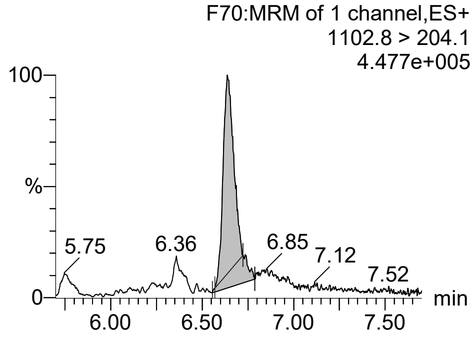

Sample Name: 20211111_A8_R2_IgM

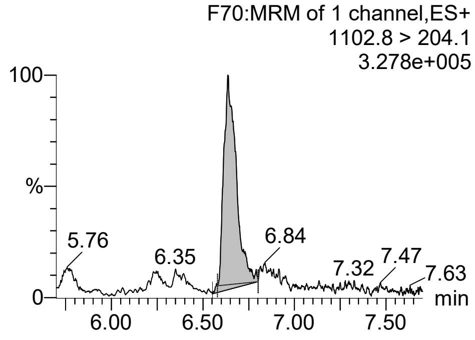

Sample Name: 20211111_L7_R2_IgM

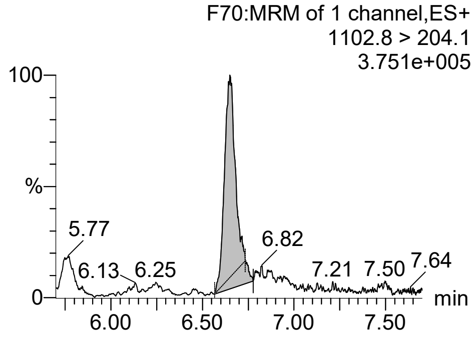

Sample Name: 20211111_L8_R2_IgM

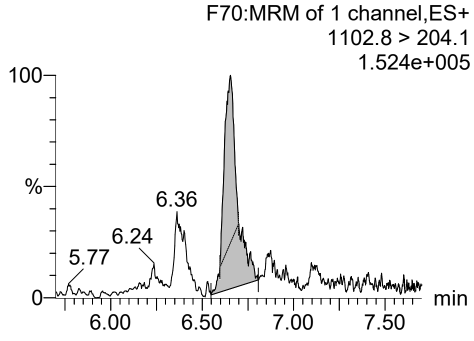

Sample Name: 20211111_H4_R2_IgM

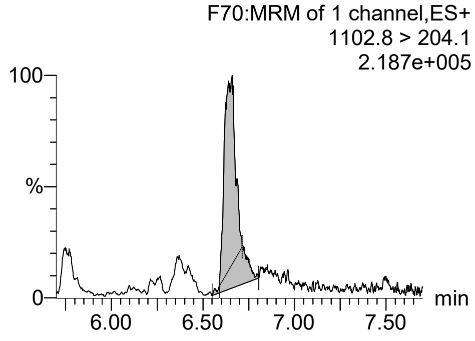

Sample Name: 20211111_A9_R2_IgM

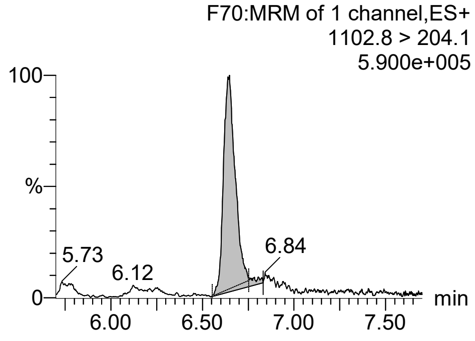

Dataset: C:\MassLynx\10.pro\20211111_clinicalsamples_IgM_IgA.qld

Last Altered: Thursday, March 09, 2023 19:58:25 台北標準時間
Printed: Friday, August 01, 2025 15:17:43 台北標準時間

Sample Name: 20211111_A10_R2_IgM
F70:MRM of 1 channel,ES+
1102.8 > 204.1
6.445e+005

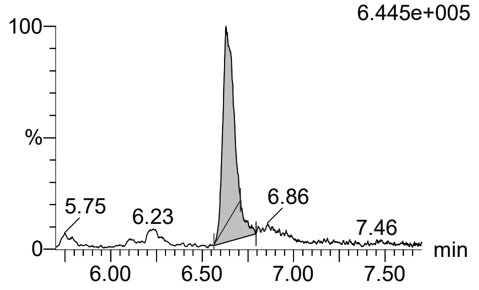

Sample Name: 20211111_L9_R2_IgM
F70:MRM of 1 channel,ES+
1102.8 > 204.1
4.773e+005

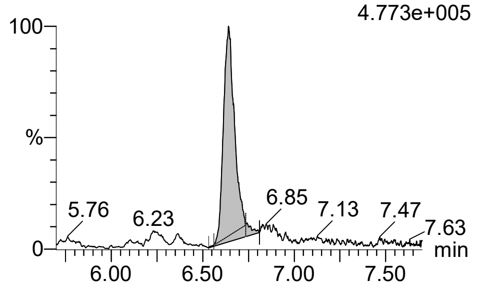

Sample Name: 20211111_L10_R2_IgM
F70:MRM of 1 channel,ES+
1102.8 > 204.1
5.693e+005

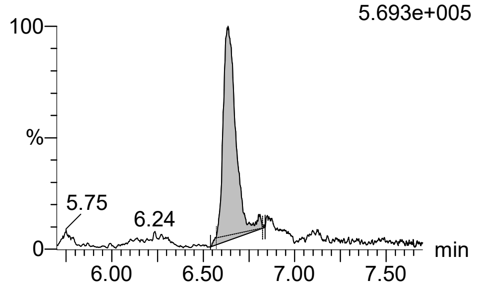

Sample Name: 20211111_QC_R11_IgM
F70:MRM of 1 channel,ES+
1102.8 > 204.1
4.347e+005

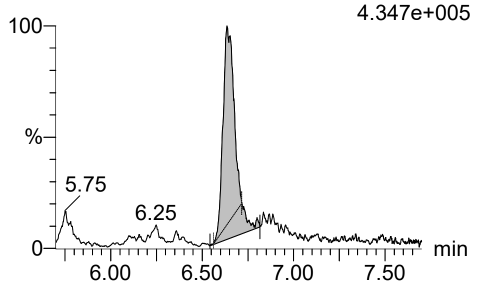

Sample Name: 20211111_H5_R2_IgM
F70:MRM of 1 channel,ES+
1102.8 > 204.1
4.496e+005

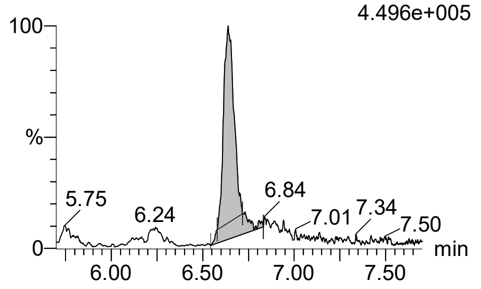

Sample Name: 20211111_A11_R2_IgM
F70:MRM of 1 channel,ES+
1102.8 > 204.1
5.213e+005

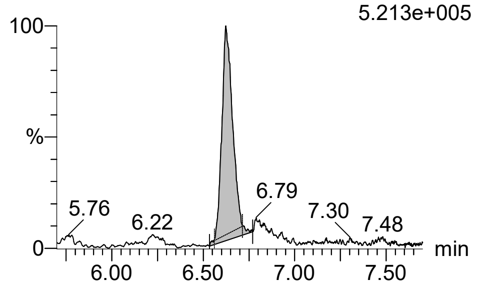

Sample Name: 20211111_A12_R2_IgM
F70:MRM of 1 channel,ES+
1102.8 > 204.1
1.828e+005

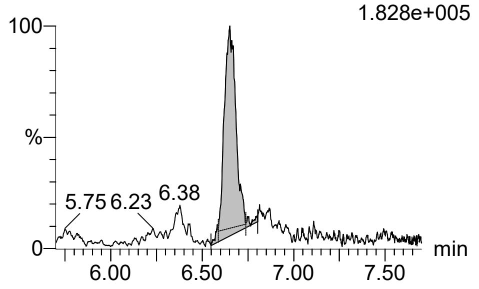

Sample Name: 20211111_L11_R2_IgM
F70:MRM of 1 channel,ES+
1102.8 > 204.1
2.217e+005

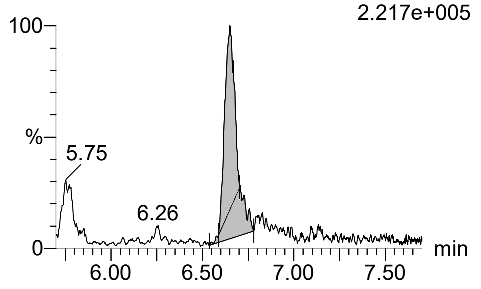

Sample Name: 20211111_L12_R2_IgM
F70:MRM of 1 channel,ES+
1102.8 > 204.1
3.464e+005

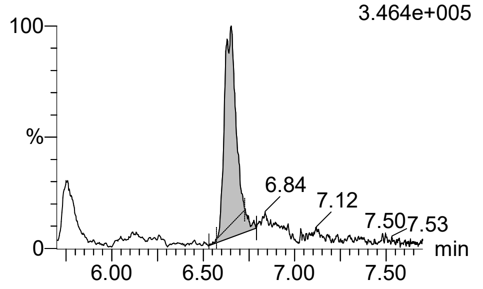

Sample Name: 20211111_H6_R2_IgM
F70:MRM of 1 channel,ES+
1102.8 > 204.1
1.727e+005

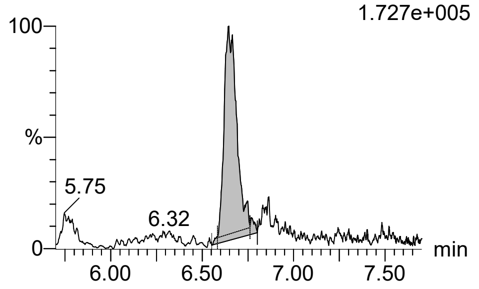

Sample Name: 20211111_A13_R2_IgM
F70:MRM of 1 channel,ES+
1102.8 > 204.1
1.528e+005

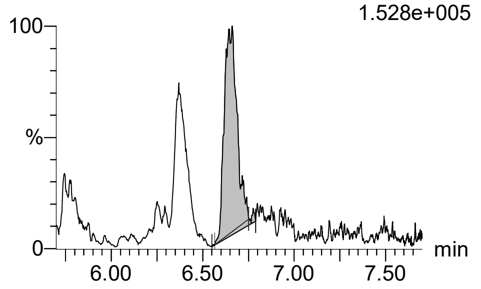

Sample Name: 20211111_A14_R2_IgM
F70:MRM of 1 channel,ES+
1102.8 > 204.1
6.309e+005

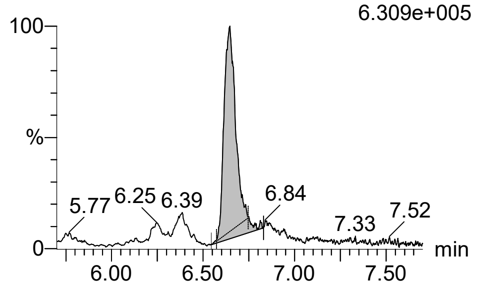

Sample Name: 20211111_L13_R2_IgM
F70:MRM of 1 channel,ES+
1102.8 > 204.1
3.907e+005

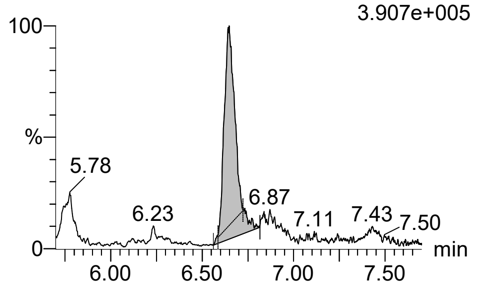

Sample Name: 20211111_L14_R2_IgM
F70:MRM of 1 channel,ES+
1102.8 > 204.1
3.843e+005

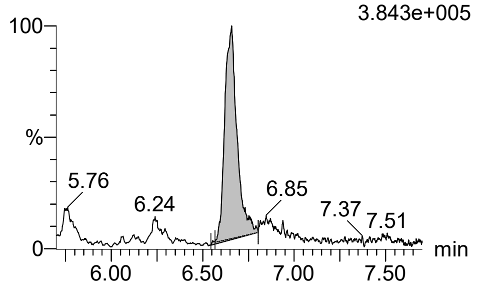

Sample Name: 20211111_H7_R2_IgM
F70:MRM of 1 channel,ES+
1102.8 > 204.1
3.954e+005

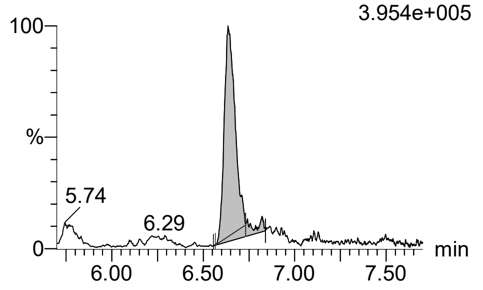

Dataset: C:\MassLynx\10.pro\20211111_clinicalsamples_IgM_IgA.qld

Last Altered: Thursday, March 09, 2023 19:58:25 台北標準時間
Printed: Friday, August 01, 2025 15:17:43 台北標準時間

Sample Name: 20211111_A15_R2_IgM
F70:MRM of 1 channel,ES+
1102.8 > 204.1
6.793e+005

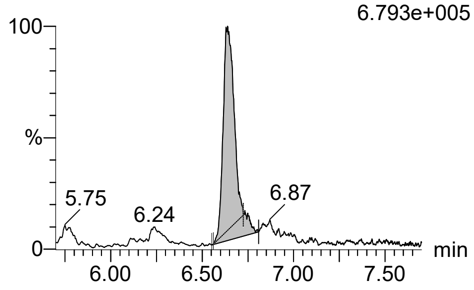

Sample Name: 20211111_QC_R12_IgM
F70:MRM of 1 channel,ES+
1102.8 > 204.1
4.888e+005

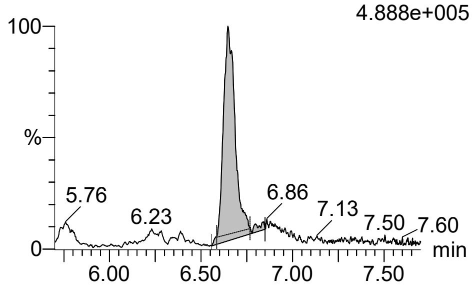

Sample Name: 20211111_A16_R2_IgM
F70:MRM of 1 channel,ES+
1102.8 > 204.1
6.006e+005

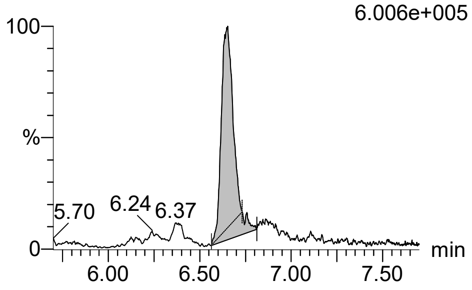

Sample Name: 20211111_L15_R2_IgM
F70:MRM of 1 channel,ES+
1102.8 > 204.1
4.701e+005

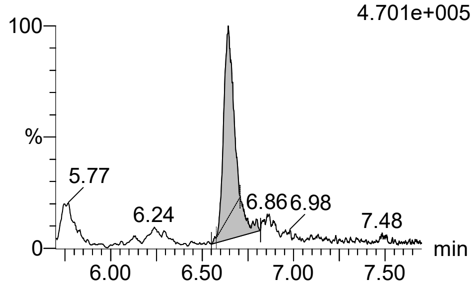

Sample Name: 20211111_L16_R2_IgM
F70:MRM of 1 channel,ES+
1102.8 > 204.1
2.979e+005

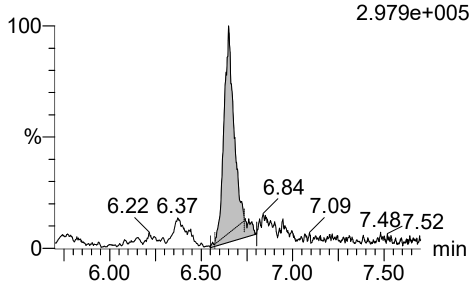

Sample Name: 20211111_H8_R2_IgM
F70:MRM of 1 channel,ES+
1102.8 > 204.1
2.716e+005

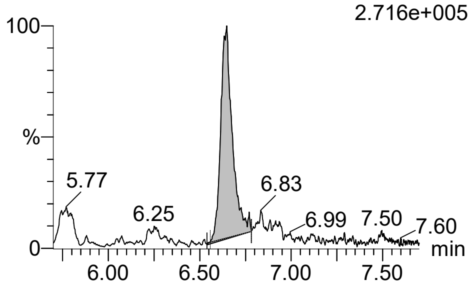

Sample Name: 20211111_A17_R2_IgM
F70:MRM of 1 channel,ES+
1102.8 > 204.1
6.111e+005

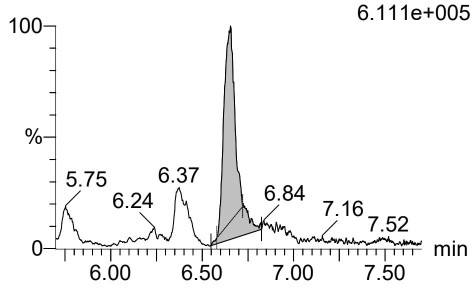

Sample Name: 20211111_A18_R2_IgM
F70:MRM of 1 channel,ES+
1102.8 > 204.1
2.866e+005

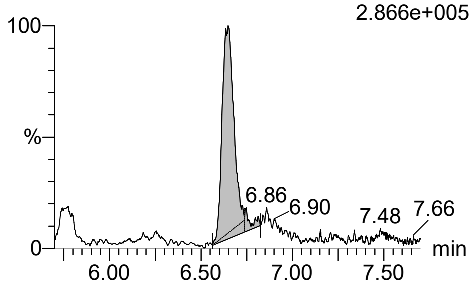

Sample Name: 20211111_L17_R2_IgM
F70:MRM of 1 channel,ES+
1102.8 > 204.1
7.244e+005

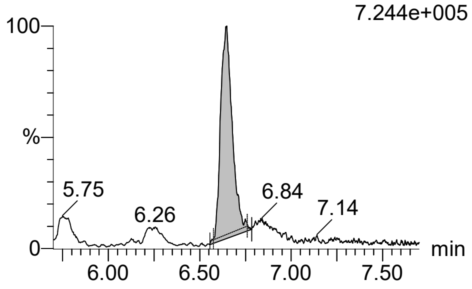

Sample Name: 20211111_L18_R2_IgM
F70:MRM of 1 channel,ES+
1102.8 > 204.1
1.594e+005

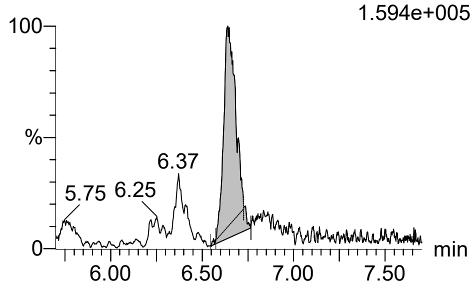

Sample Name: 20211111_H9_R2_IgM
F70:MRM of 1 channel,ES+
1102.8 > 204.1
2.322e+005

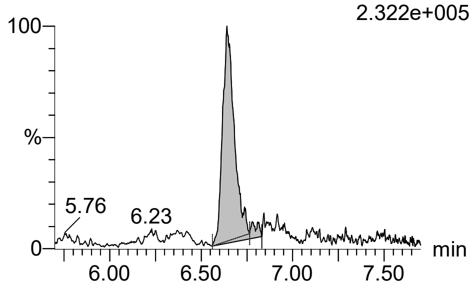

Sample Name: 20211111_A19_R2_IgM
F70:MRM of 1 channel,ES+
1102.8 > 204.1
3.272e+005

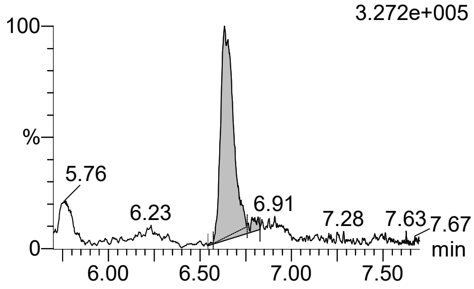

Sample Name: 20211111_A20_R2_IgM
F70:MRM of 1 channel,ES+
1102.8 > 204.1
1.497e+005

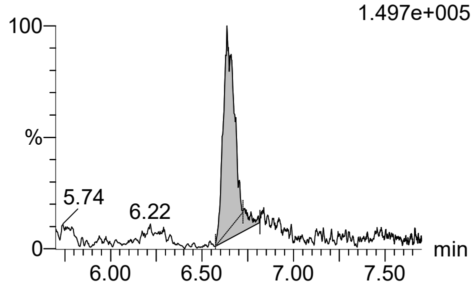

Sample Name: 20211111_L19_R2_IgM
F70:MRM of 1 channel,ES+
1102.8 > 204.1
1.833e+005

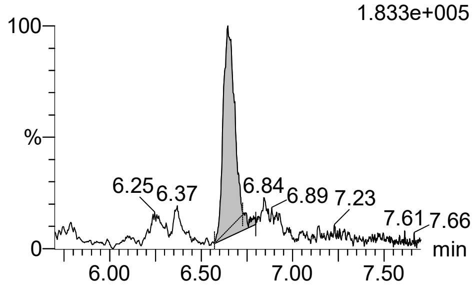

Sample Name: 20211111_QC_R13_IgM
F70:MRM of 1 channel,ES+
1102.8 > 204.1
3.647e+005

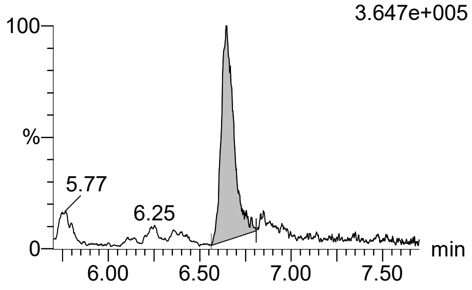

Dataset: C:\MassLynx\10.pro\20211111_clinicalsamples_IgM_IgA.qld

Last Altered: Thursday, March 09, 2023 19:58:25 台北標準時間
Printed: Friday, August 01, 2025 15:17:43 台北標準時間

Sample Name: 20211111_L20_R2_IgM
F70:MRM of 1 channel,ES+
1102.8 > 204.1
5.800e+005

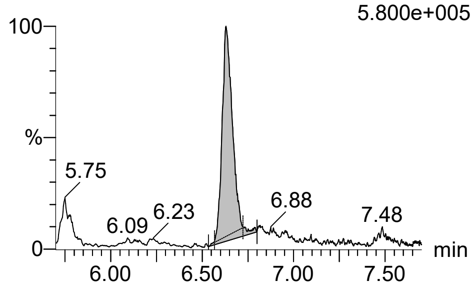

Sample Name: 20211111_H10_R2_IgM
F70:MRM of 1 channel,ES+
1102.8 > 204.1
4.336e+005

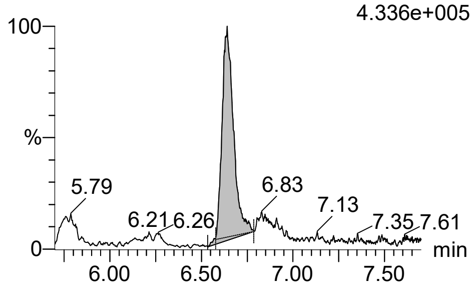

Sample Name: 20211111_A21_R2_IgM
F70:MRM of 1 channel,ES+
1102.8 > 204.1
4.471e+005

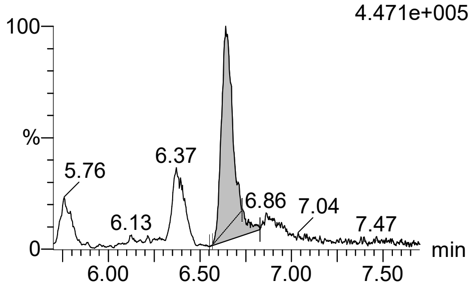

Sample Name: 20211111_A22_R2_IgM
F70:MRM of 1 channel,ES+
1102.8 > 204.1
1.606e+005

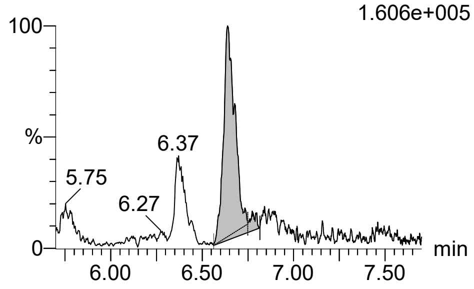

Sample Name: 20211111_L21_R2_IgM
F70:MRM of 1 channel,ES+
1102.8 > 204.1
2.188e+005

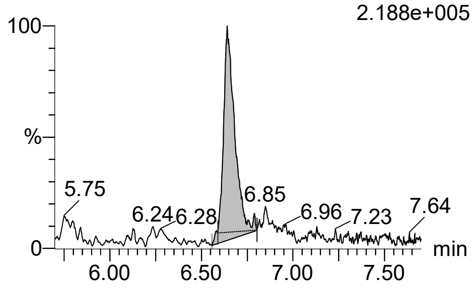

Sample Name: 20211111_L22_R2_IgM
F70:MRM of 1 channel,ES+
1102.8 > 204.1
2.771e+005

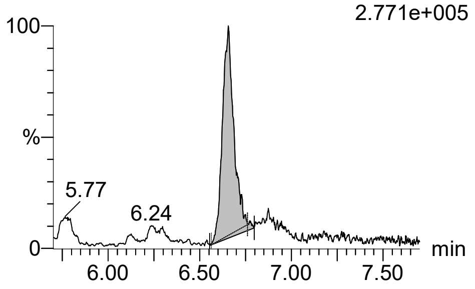

Sample Name: 20211111_H11_R2_IgM
F70:MRM of 1 channel,ES+
1102.8 > 204.1
3.977e+005

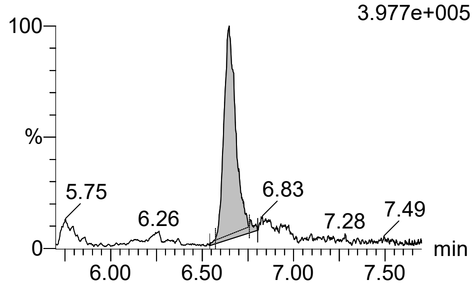

Sample Name: 20211111_A23_R2_IgM
F70:MRM of 1 channel,ES+
1102.8 > 204.1
1.647e+005

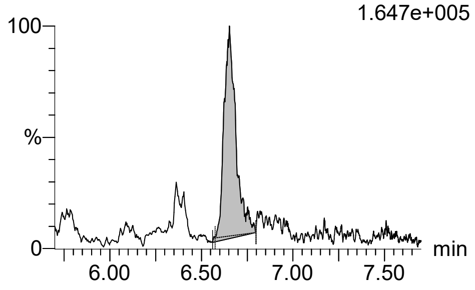

Sample Name: 20211111_A24_R2_IgM
F70:MRM of 1 channel,ES+
1102.8 > 204.1
4.139e+005

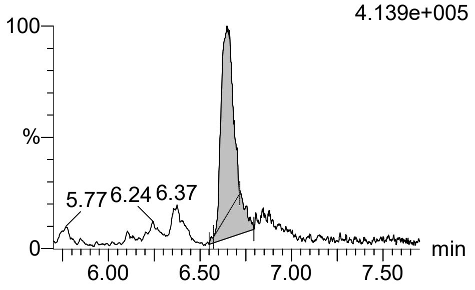

Sample Name: 20211111_L23_R2_IgM
F70:MRM of 1 channel,ES+
1102.8 > 204.1
7.223e+005

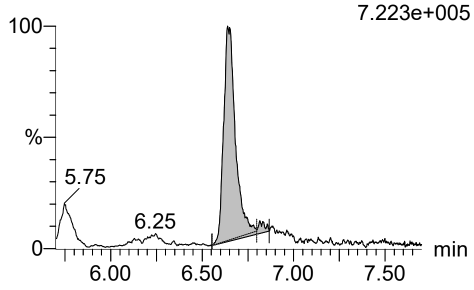

Sample Name: 20211111_L24_R2_IgM
F70:MRM of 1 channel,ES+
1102.8 > 204.1
2.621e+005

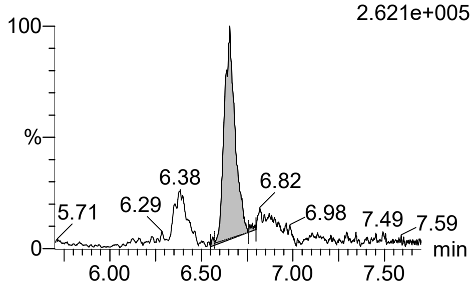

Sample Name: 20211111_H12_R2_IgM
F70:MRM of 1 channel,ES+
1102.8 > 204.1
1.834e+005

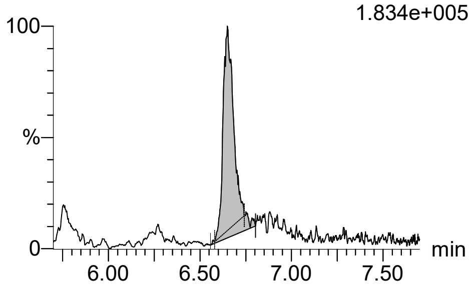

Sample Name: 20211111_QC_R14_IgM
F70:MRM of 1 channel,ES+
1102.8 > 204.1
3.816e+005

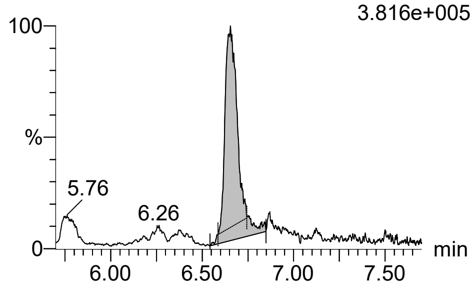

Sample Name: 20211111_A25_R2_IgM
F70:MRM of 1 channel,ES+
1102.8 > 204.1
5.672e+005

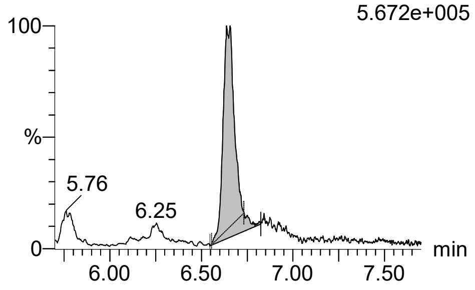

Sample Name: 20211111_A26_R2_IgM
F70:MRM of 1 channel,ES+
1102.8 > 204.1
3.086e+005

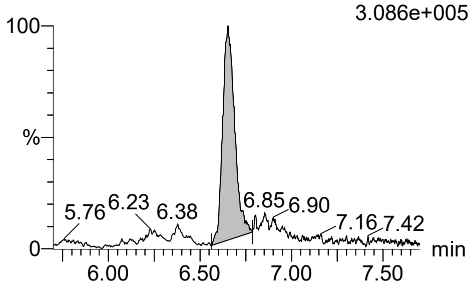

Sample Name: 20211111_L25_R2_IgM
F70:MRM of 1 channel,ES+
1102.8 > 204.1
3.484e+005

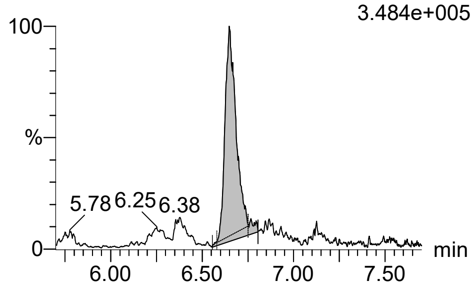

Sample Name: 20211111_L26_R2_IgM
F70:MRM of 1 channel,ES+
1102.8 > 204.1
2.983e+005

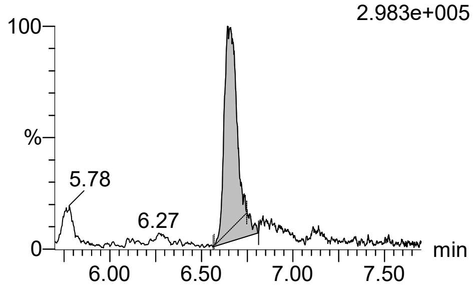

Sample Name: 20211111_H13_R2_IgM
F70:MRM of 1 channel,ES+
1102.8 > 204.1
4.807e+005

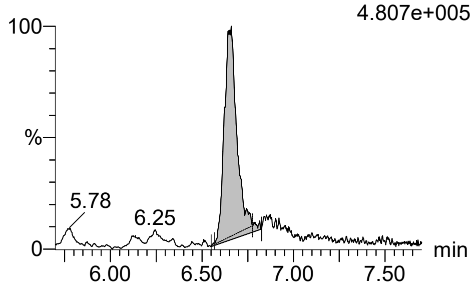

Sample Name: 20211111_A27_R2_IgM
F70:MRM of 1 channel,ES+
1102.8 > 204.1
6.162e+005

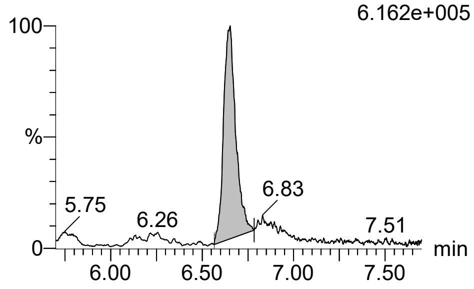

Sample Name: 20211111_A28_R2_IgM
F70:MRM of 1 channel,ES+
1102.8 > 204.1
3.599e+005

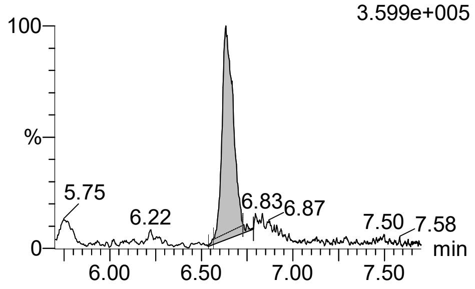

Sample Name: 20211111_L27_R2_IgM
F70:MRM of 1 channel,ES+
1102.8 > 204.1
7.895e+005

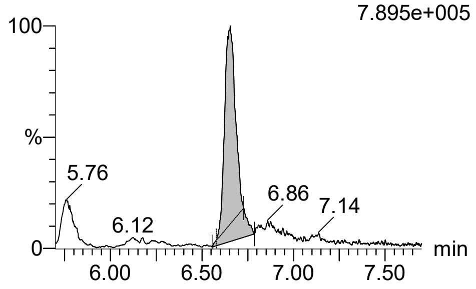

Sample Name: 20211111_L28_R2_IgM
F70:MRM of 1 channel,ES+
1102.8 > 204.1
4.005e+005

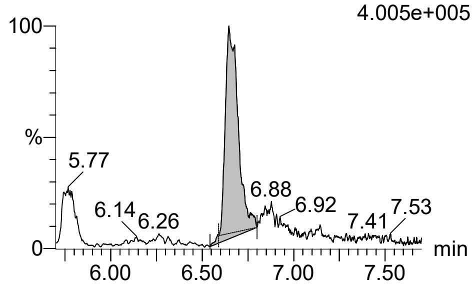

Sample Name: 20211111_H14_R2_IgM
F70:MRM of 1 channel,ES+
1102.8 > 204.1
5.513e+005

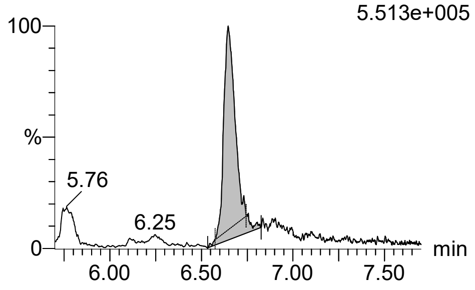

Sample Name: 20211111_A29_R2_IgM
F70:MRM of 1 channel,ES+
1102.8 > 204.1
1.240e+005

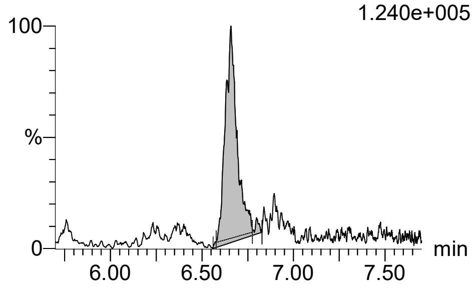

Sample Name: 20211111_A30_R2_IgM
F70:MRM of 1 channel,ES+
1102.8 > 204.1
3.827e+005

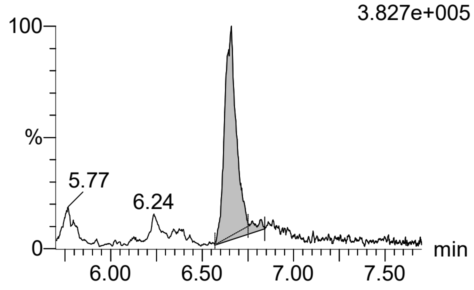

Sample Name: 20211111_QC_R15_IgM
F70:MRM of 1 channel,ES+
1102.8 > 204.1
3.823e+005

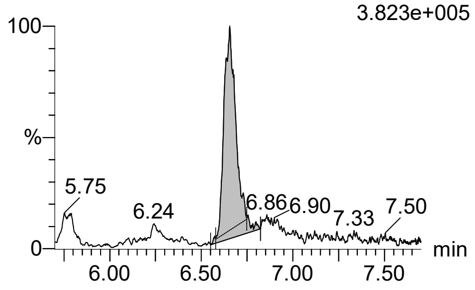

Sample Name: 20211111_L29_R2_IgM
F70:MRM of 1 channel,ES+
1102.8 > 204.1
1.583e+005

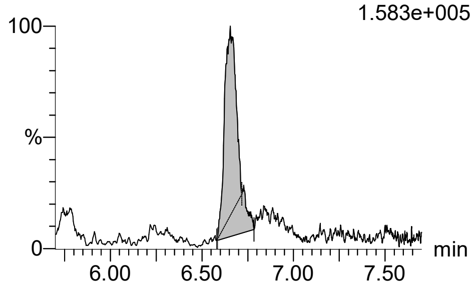

Sample Name: 20211111_L30_R2_IgM
F70:MRM of 1 channel,ES+
1102.8 > 204.1
1.120e+005

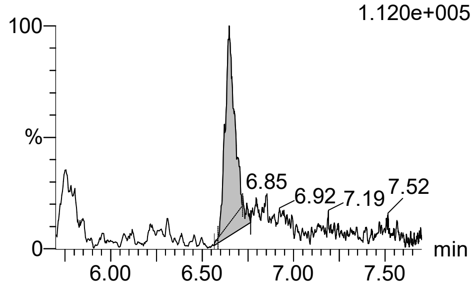

Sample Name: 20211111_H15_R2_IgM
F70:MRM of 1 channel,ES+
1102.8 > 204.1
4.296e+005

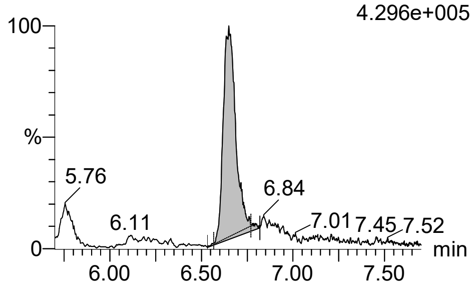

Sample Name: 20211111_A31_R2_IgM
F70:MRM of 1 channel,ES+
1102.8 > 204.1
2.171e+005

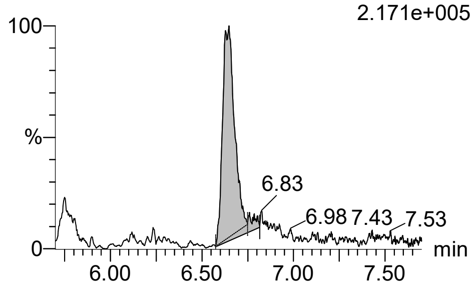

Dataset: C:\MassLynx\10.pro\20211111_clinicalsamples_IgM_IgA.qld

Last Altered: Thursday, March 09, 2023 19:58:25 台北標準時間
Printed: Friday, August 01, 2025 15:17:43 台北標準時間

Sample Name: 20211111_A32_R2_IgM
F70:MRM of 1 channel,ES+
1102.8 > 204.1
3.635e+005

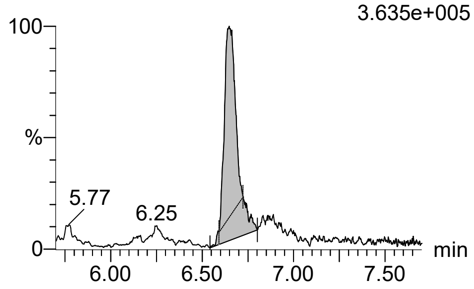

Sample Name: 20211111_L31_R2_IgM
F70:MRM of 1 channel,ES+
1102.8 > 204.1
3.108e+005

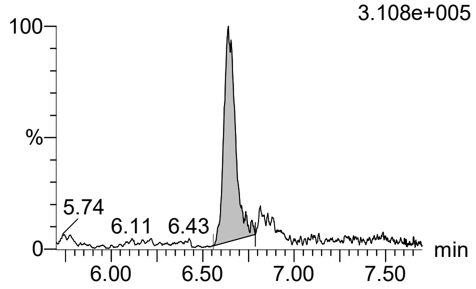

Sample Name: 20211111_L32_R2_IgM
F70:MRM of 1 channel,ES+
1102.8 > 204.1
4.761e+005

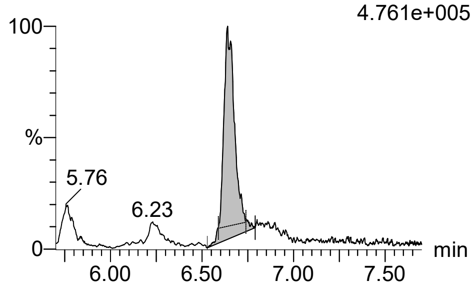

Sample Name: 20211111_H16_R2_IgM
F70:MRM of 1 channel,ES+
1102.8 > 204.1
8.072e+005

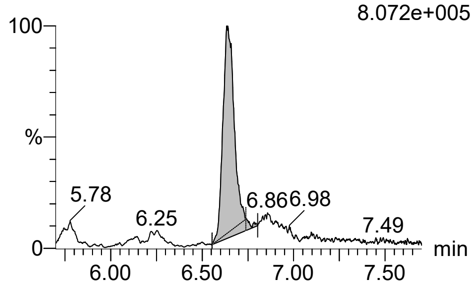

Sample Name: 20211111_A33_R2_IgM
F70:MRM of 1 channel,ES+
1102.8 > 204.1
4.776e+005

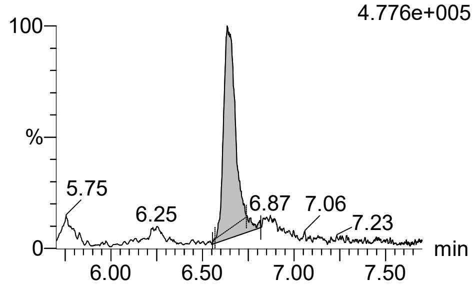

Sample Name: 20211111_A34_R2_IgM
F70:MRM of 1 channel,ES+
1102.8 > 204.1
1.362e+005

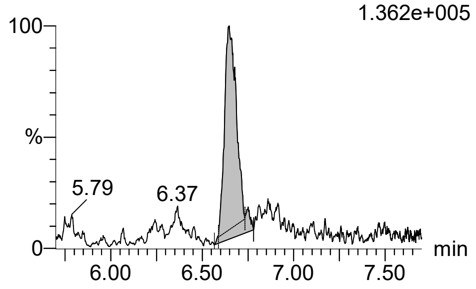

Sample Name: 20211111_H17_R2_IgM
F70:MRM of 1 channel,ES+
1102.8 > 204.1
2.365e+006

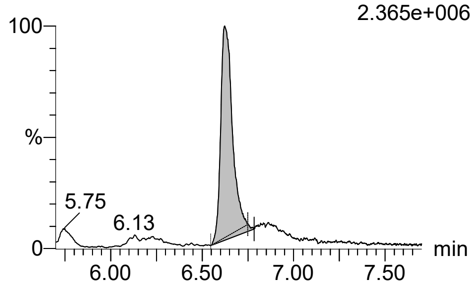

Sample Name: 20211111_A35_R2_IgM
F70:MRM of 1 channel,ES+
1102.8 > 204.1
4.892e+005

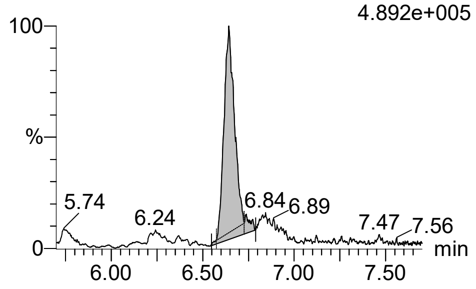

Sample Name: 20211111_QC_R16_IgM
F70:MRM of 1 channel,ES+
1102.8 > 204.1
4.199e+005

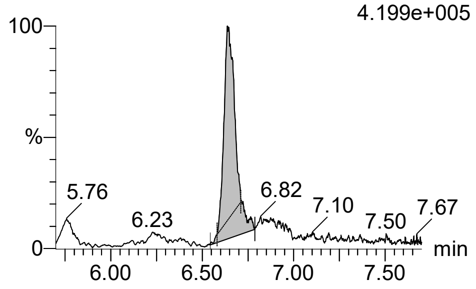

Sample Name: 20211111_A36_R2_IgM
F70:MRM of 1 channel,ES+
1102.8 > 204.1
5.497e+005

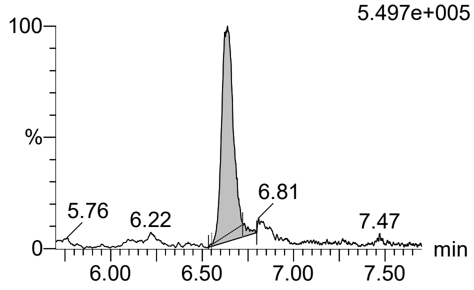

Sample Name: 20211111_H18_R2_IgM
F70:MRM of 1 channel,ES+
1102.8 > 204.1
9.896e+005

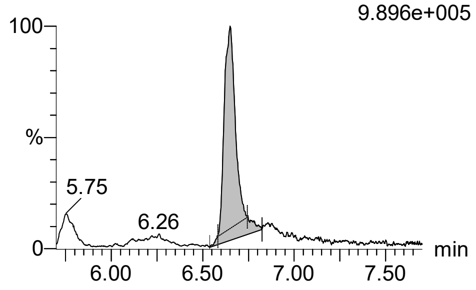

Sample Name: 20211111_A37_R2_IgM
F70:MRM of 1 channel,ES+
1102.8 > 204.1
4.240e+005

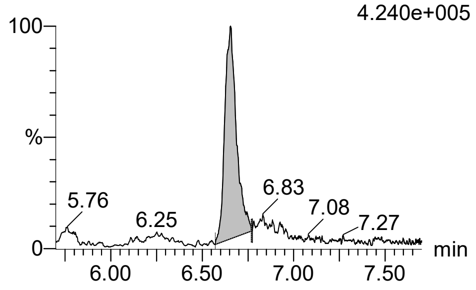

Sample Name: 20211111_A38_R2_IgM
F70:MRM of 1 channel,ES+
1102.8 > 204.1
4.338e+005

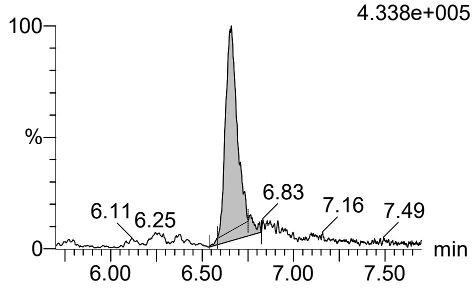

Sample Name: 20211111_H19_R2_IgM
F70:MRM of 1 channel,ES+
1102.8 > 204.1
4.891e+005

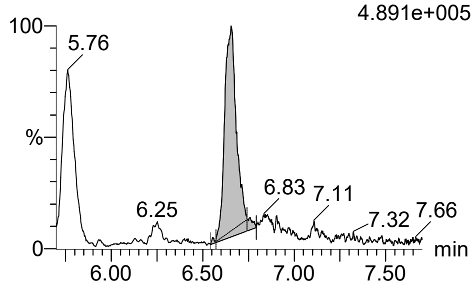

Sample Name: 20211111_A39_R2_IgM
F70:MRM of 1 channel,ES+
1102.8 > 204.1
5.588e+005

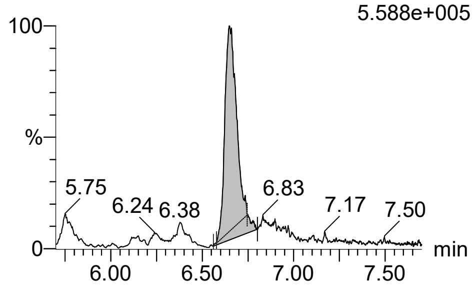

Dataset: C:\MassLynx\10.pro\20211111_clinicalsamples_IgM_IgA.qld

Last Altered: Thursday, March 09, 2023 19:58:25 台北標準時間
Printed: Friday, August 01, 2025 15:17:43 台北標準時間

Sample Name: 20211111_A40_R2_IgM
F70:MRM of 1 channel,ES+
1102.8 > 204.1
2.592e+005

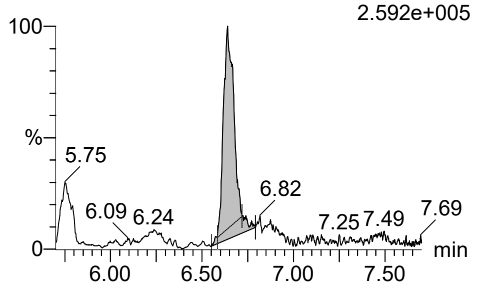

Sample Name: 20211111_H20_R2_IgM
F70:MRM of 1 channel,ES+
1102.8 > 204.1
1.139e+006

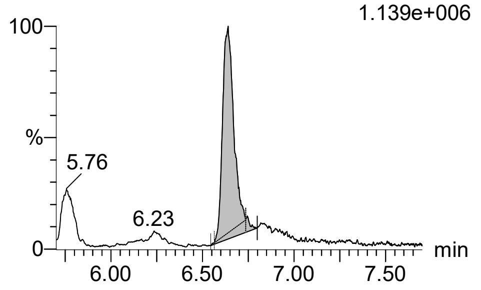

Sample Name: 20211111_A41_R2_IgM
F70:MRM of 1 channel,ES+
1102.8 > 204.1
3.388e+005

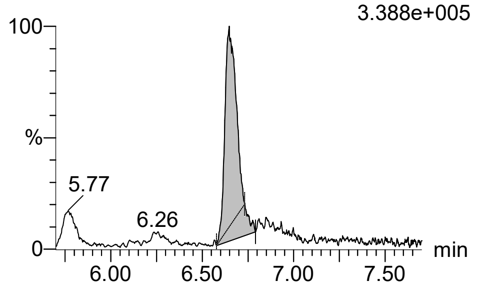

Sample Name: 20211111_A42_R2_IgM
F70:MRM of 1 channel,ES+
1102.8 > 204.1
1.595e+005

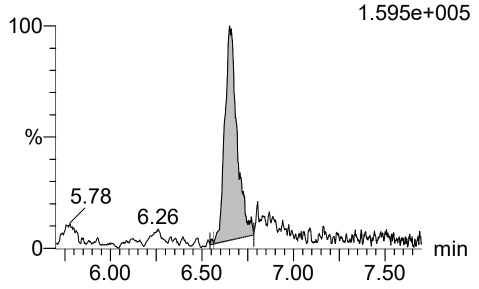

Sample Name: 20211111_A43_R2_IgM
F70:MRM of 1 channel,ES+
1102.8 > 204.1
1.767e+005

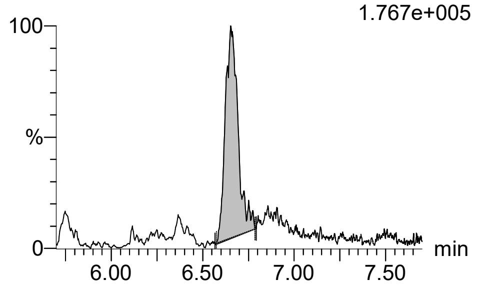

Sample Name: 20211111_A44_R2_IgM
F70:MRM of 1 channel,ES+
1102.8 > 204.1
1.296e+005

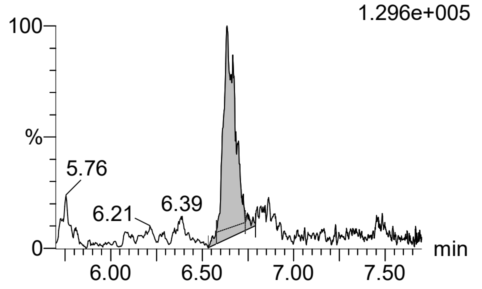

Sample Name: 20211111_QC_R17_IgM
F70:MRM of 1 channel,ES+
1102.8 > 204.1
3.171e+005

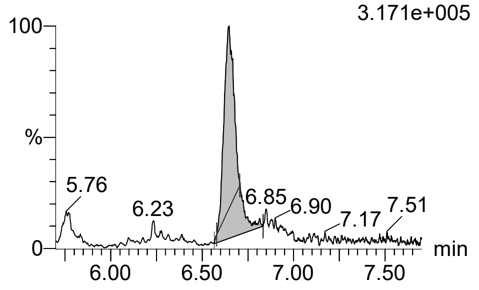

Sample Name: 20211111_L2_R3_IgM
F70:MRM of 1 channel,ES+
1102.8 > 204.1
3.658e+005

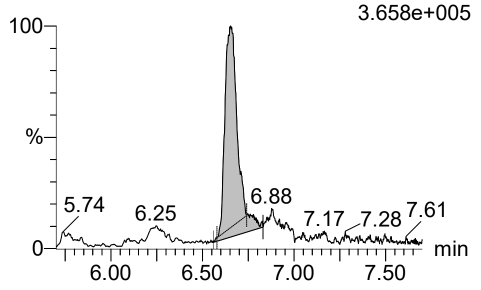

Sample Name: 20211111_H1_R3_IgM
F70:MRM of 1 channel,ES+
1102.8 > 204.1
6.006e+005

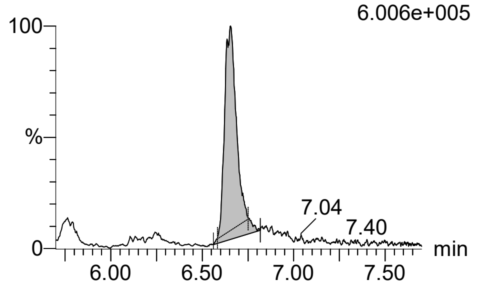

Sample Name: 20211111_A3_R3_IgM
F70:MRM of 1 channel,ES+
1102.8 > 204.1
4.810e+005

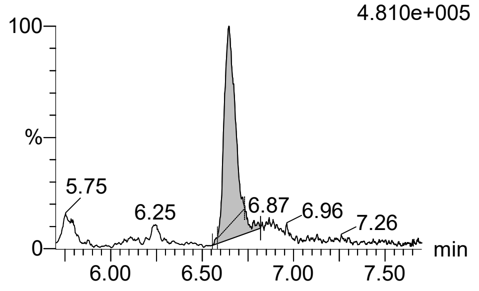

Sample Name: 20211111_A4_R3_IgM
F70:MRM of 1 channel,ES+
1102.8 > 204.1
2.991e+005

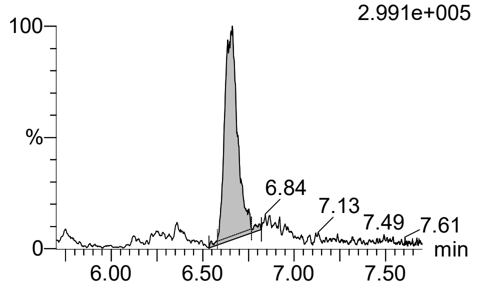

Sample Name: 20211111_L3_R3_IgM
F70:MRM of 1 channel,ES+
1102.8 > 204.1
3.819e+005

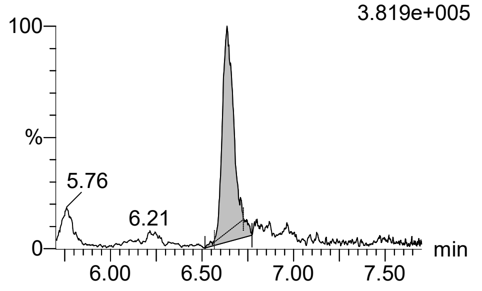

Sample Name: 20211111_L4_R3_IgM
F70:MRM of 1 channel,ES+
1102.8 > 204.1
2.393e+005

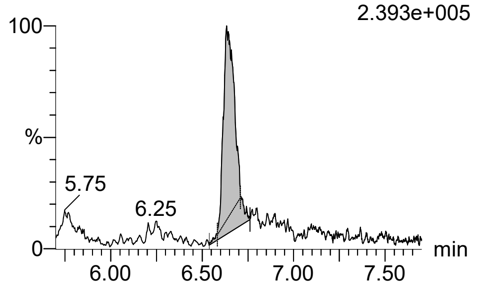

Sample Name: 20211111_H2_R3_IgM
F70:MRM of 1 channel,ES+
1102.8 > 204.1
5.529e+005

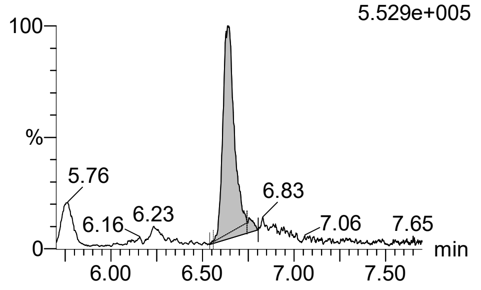

Sample Name: 20211111_A5_R3_IgM
F70:MRM of 1 channel,ES+
1102.8 > 204.1
1.020e+006

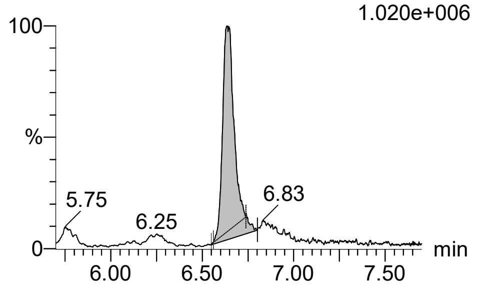

Sample Name: 20211111_A6_R3_IgM

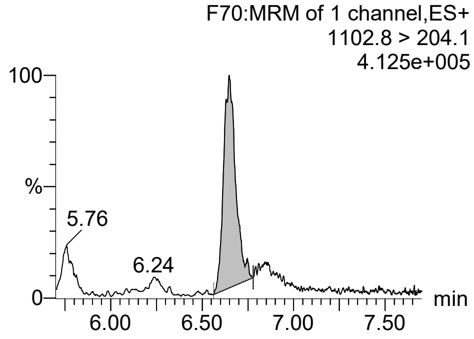

Sample Name: 20211111_QC_R18_IgM

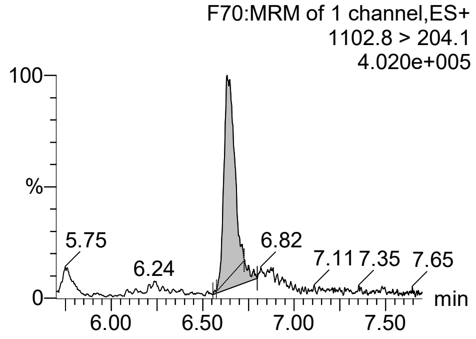

Sample Name: 20211111_L5_R3_IgM

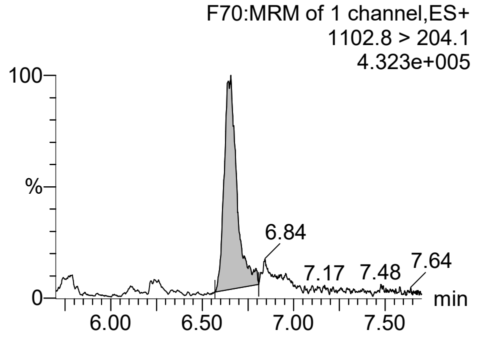

Sample Name: 20211111_L6_R3_IgM

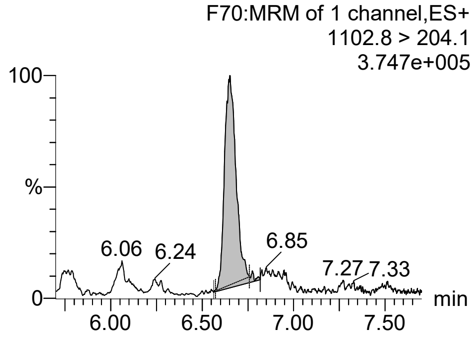

Sample Name: 20211111_H3_R3_IgM

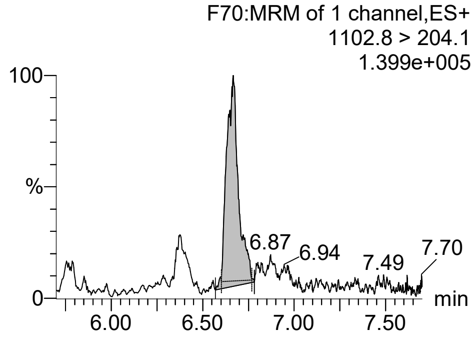

Sample Name: 20211111_A7_R3_IgM

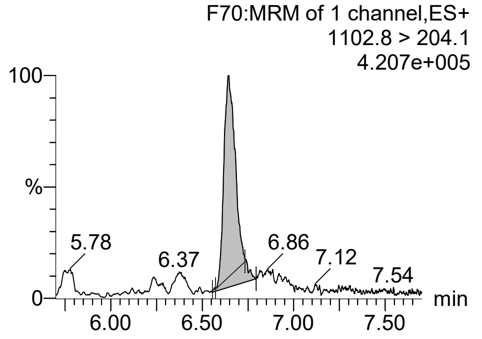

Sample Name: 20211111_A8_R3_IgM

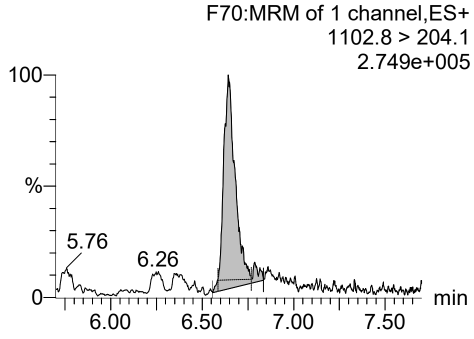

Sample Name: 20211111_L7_R3_IgM

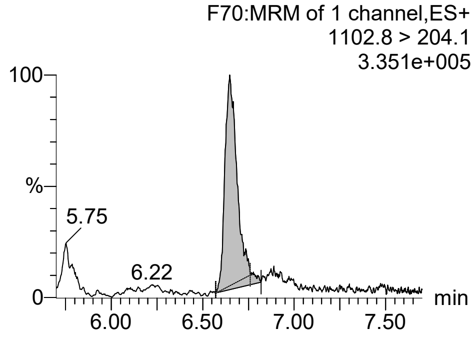

Sample Name: 20211111_L8_R3_IgM

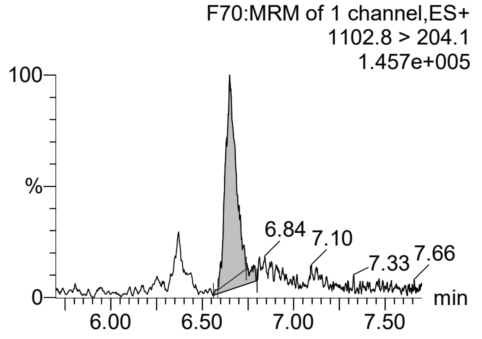

Sample Name: 20211111_H4_R3_IgM

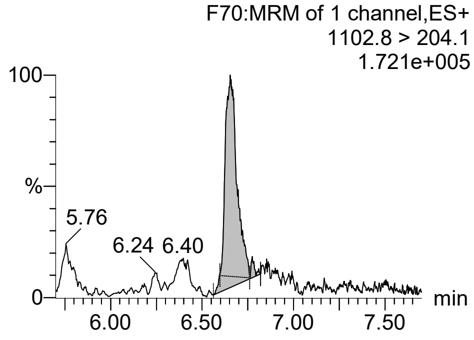

Sample Name: 20211111_A9_R3_IgM

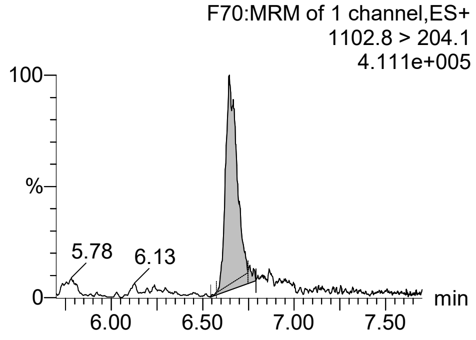

Sample Name: 20211111_A10_R3_IgM

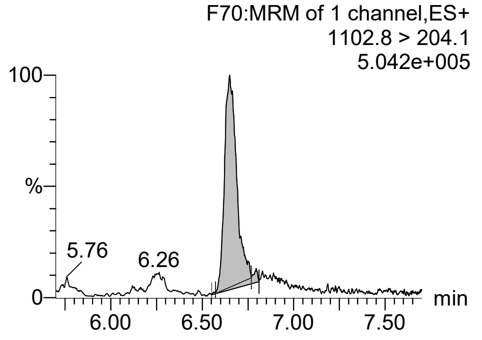

Sample Name: 20211111_L9_R3_IgM

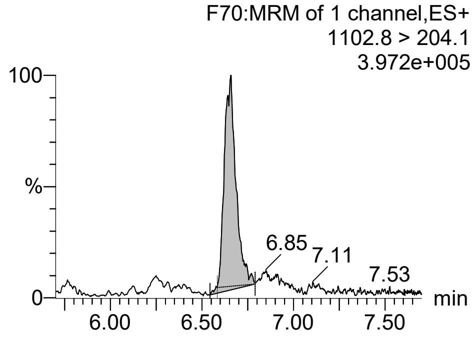

Sample Name: 20211111_L10_R3_IgM

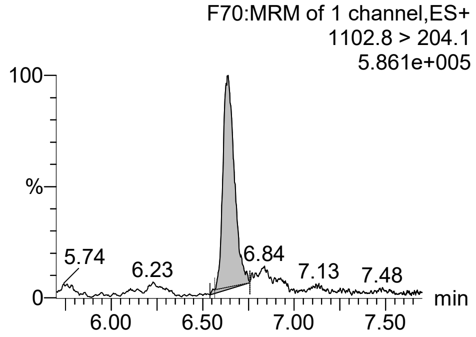

Sample Name: 20211111_QC_R19_IgM

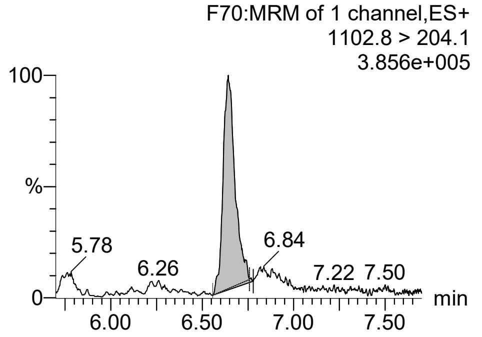

Dataset: C:\MassLynx\10.pro\20211111_clinicalsamples_IgM_IgA.qld

Last Altered: Thursday, March 09, 2023 19:58:25 台北標準時間
Printed: Friday, August 01, 2025 15:17:43 台北標準時間

Sample Name: 20211111_H5_R3_IgM
F70:MRM of 1 channel,ES+
1102.8 > 204.1
3.198e+005

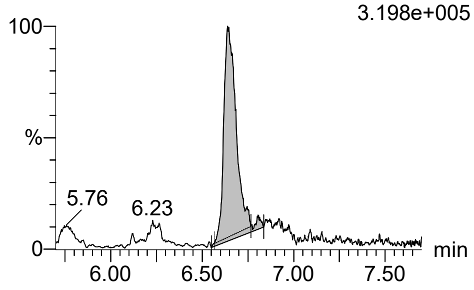

Sample Name: 20211111_A11_R3_IgM
F70:MRM of 1 channel,ES+
1102.8 > 204.1
2.667e+005

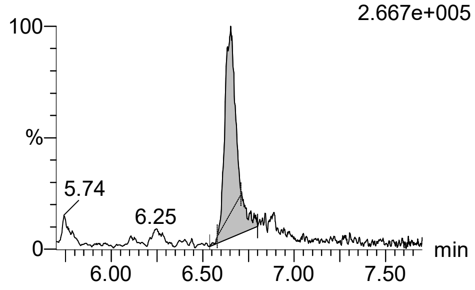

Sample Name: 20211111_A12_R3_IgM
F70:MRM of 1 channel,ES+
1102.8 > 204.1
1.463e+005

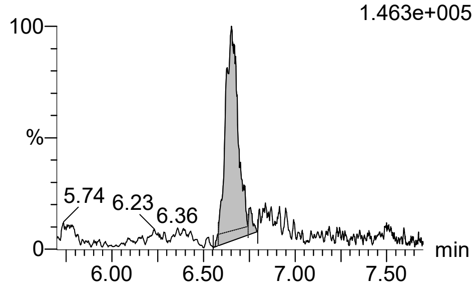

Sample Name: 20211111_L11_R3_IgM
F70:MRM of 1 channel,ES+
1102.8 > 204.1
1.525e+005

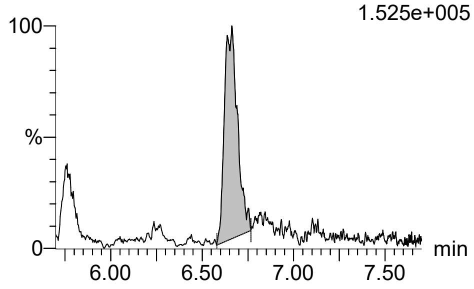

Sample Name: 20211111_L12_R3_IgM
F70:MRM of 1 channel,ES+
1102.8 > 204.1
3.559e+005

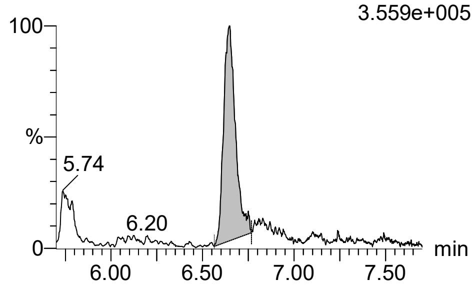

Sample Name: 20211111_H6_R3_IgM
F70:MRM of 1 channel,ES+
1102.8 > 204.1
1.759e+005

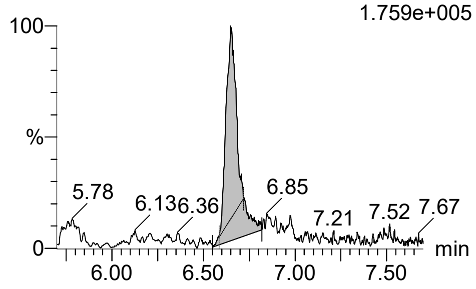

Sample Name: 20211111_A13_R3_IgM
F70:MRM of 1 channel,ES+
1102.8 > 204.1
1.631e+005

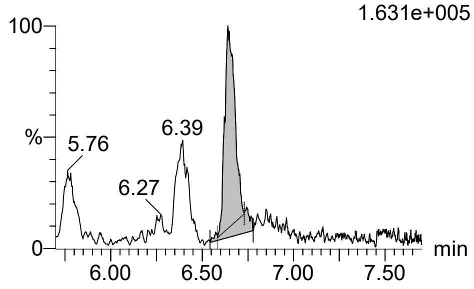

Sample Name: 20211111_A14_R3_IgM
F70:MRM of 1 channel,ES+
1102.8 > 204.1
4.870e+005

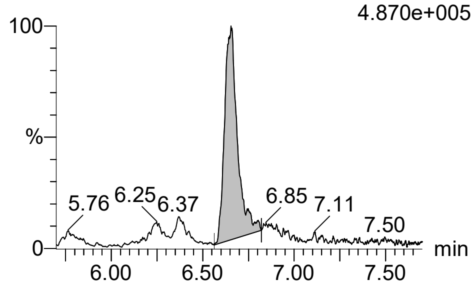

Sample Name: 20211111_L13_R3_IgM
F70:MRM of 1 channel,ES+
1102.8 > 204.1
3.242e+005

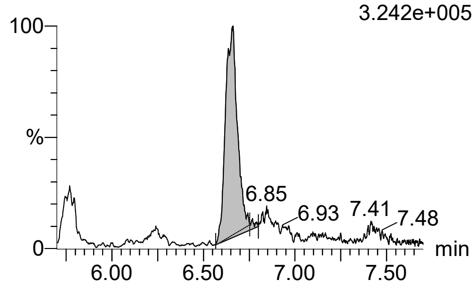

Sample Name: 20211111_L14_R3_IgM
F70:MRM of 1 channel,ES+
1102.8 > 204.1
3.258e+005

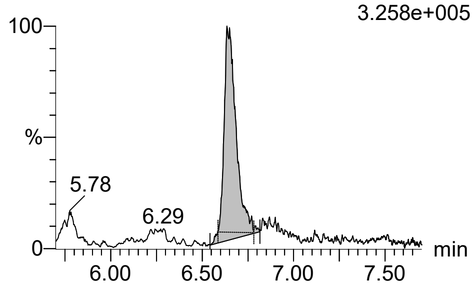

Sample Name: 20211111_H7_R3_IgM
F70:MRM of 1 channel,ES+
1102.8 > 204.1
2.607e+005

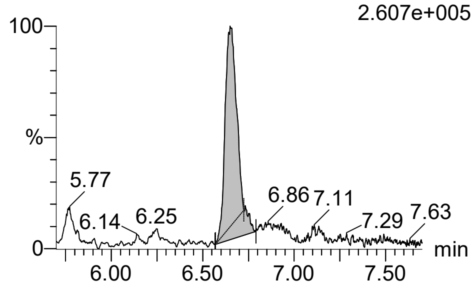

Sample Name: 20211111_A15_R3_IgM
F70:MRM of 1 channel,ES+
1102.8 > 204.1
5.755e+005

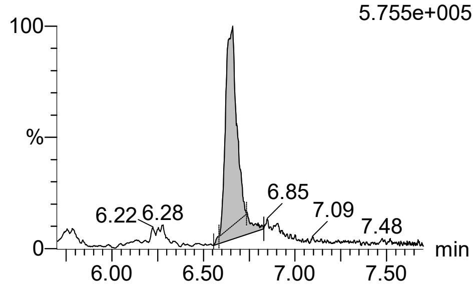

Sample Name: 20211111_QC_R20_IgM
F70:MRM of 1 channel,ES+
1102.8 > 204.1
4.288e+005

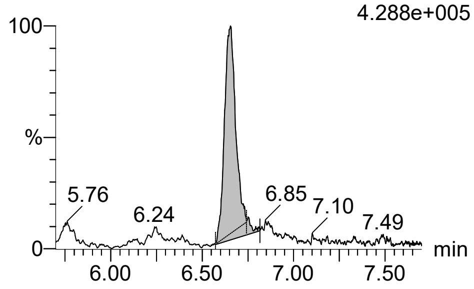

Sample Name: 20211111_A16_R3_IgM
F70:MRM of 1 channel,ES+
1102.8 > 204.1
5.297e+005

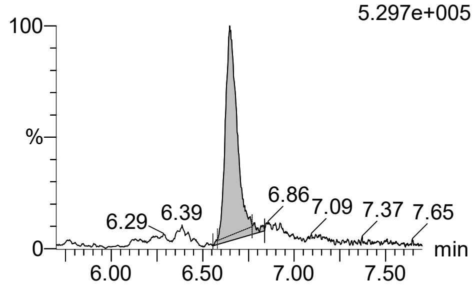

Sample Name: 20211111_L15_R3_IgM
F70:MRM of 1 channel,ES+
1102.8 > 204.1
3.740e+005

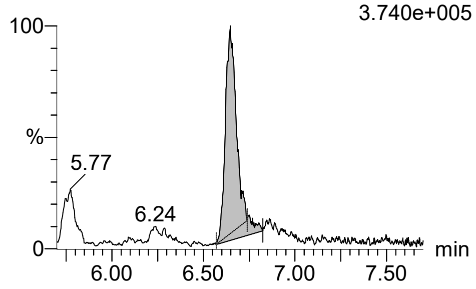

Dataset: C:\MassLynx\10.pro\20211111_clinicalsamples_IgM_IgA.qld

Last Altered: Thursday, March 09, 2023 19:58:25 台北標準時間
Printed: Friday, August 01, 2025 15:17:43 台北標準時間

Sample Name: 20211111_L16_R3_IgM
F70:MRM of 1 channel,ES+
1102.8 > 204.1
2.370e+005

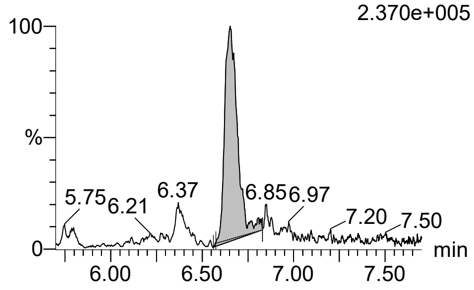

Sample Name: 20211111_H8_R3_IgM
F70:MRM of 1 channel,ES+
1102.8 > 204.1
2.117e+005

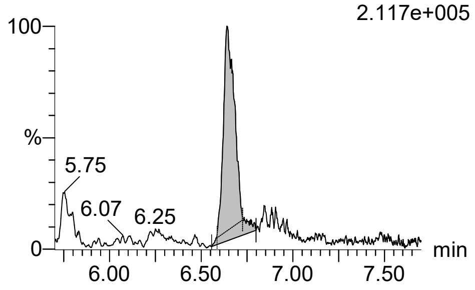

Sample Name: 20211111_A17_R3_IgM
F70:MRM of 1 channel,ES+
1102.8 > 204.1
4.921e+005

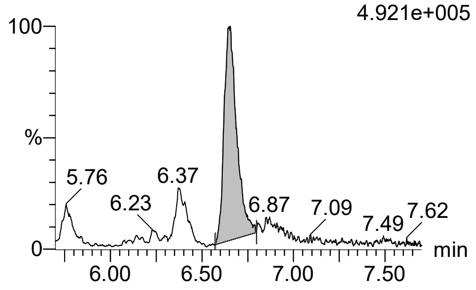

Sample Name: 20211111_A18_R3_IgM
F70:MRM of 1 channel,ES+
1102.8 > 204.1
2.948e+005

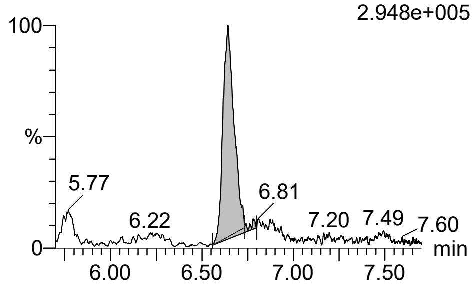

Sample Name: 20211111_L17_R3_IgM
F70:MRM of 1 channel,ES+
1102.8 > 204.1
5.980e+005

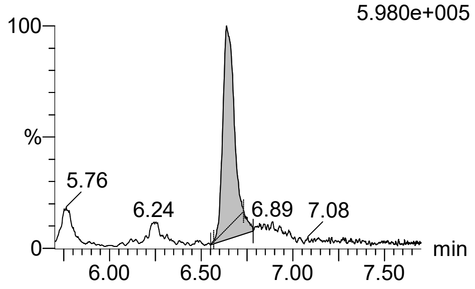

Sample Name: 20211111_L18_R3_IgM
F70:MRM of 1 channel,ES+
1102.8 > 204.1
1.476e+005

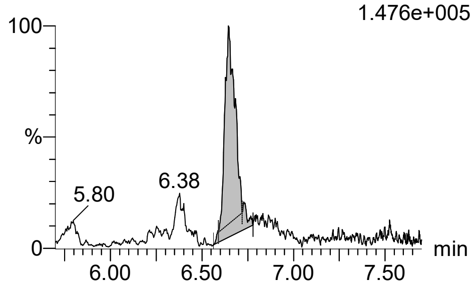

Sample Name: 20211111_H9_R3_IgM
F70:MRM of 1 channel,ES+
1102.8 > 204.1
1.910e+005

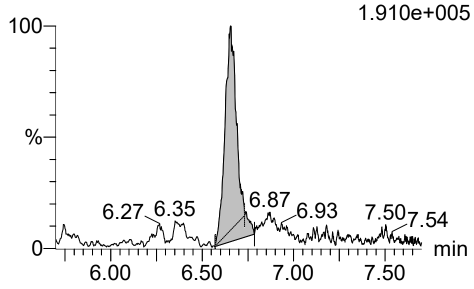

Sample Name: 20211111_A19_R3_IgM
F70:MRM of 1 channel,ES+
1102.8 > 204.1
3.398e+005

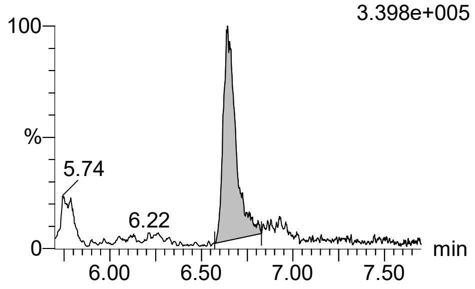

Sample Name: 20211111_A20_R3_IgM
F70:MRM of 1 channel,ES+
1102.8 > 204.1
1.356e+005

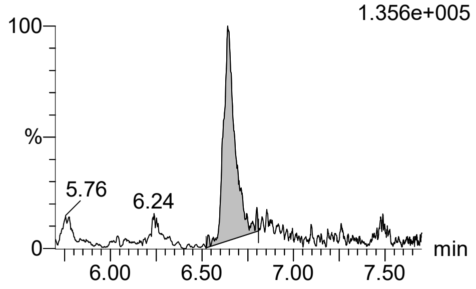

Sample Name: 20211111_L19_R3_IgM
F70:MRM of 1 channel,ES+
1102.8 > 204.1
2.069e+005

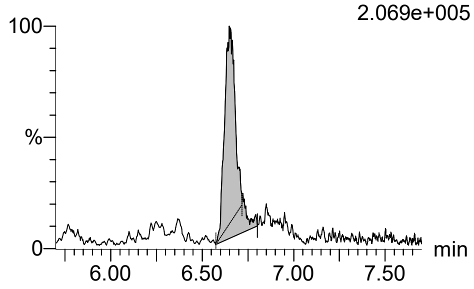

Sample Name: 20211111_QC_R21_IgM
F70:MRM of 1 channel,ES+
1102.8 > 204.1
3.404e+005

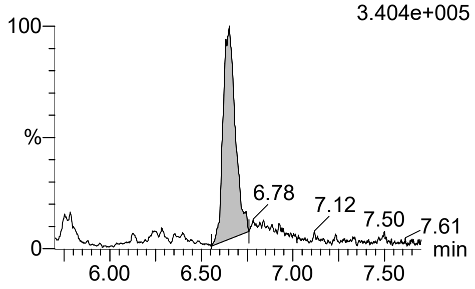

Sample Name: 20211111_L20_R3_IgM
F70:MRM of 1 channel,ES+
1102.8 > 204.1
3.136e+005

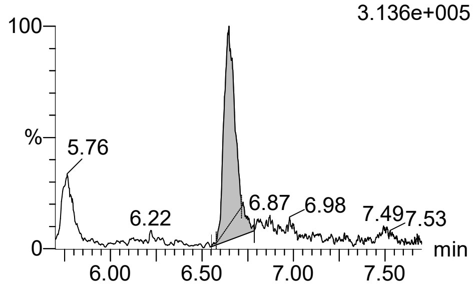

Sample Name: 20211111_H10_R3_IgM
F70:MRM of 1 channel,ES+
1102.8 > 204.1
2.960e+005

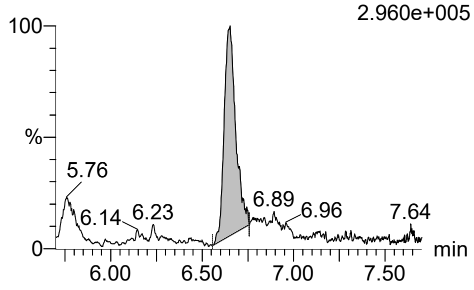

Sample Name: 20211111_A21_R3_IgM
F70:MRM of 1 channel,ES+
1102.8 > 204.1
3.719e+005

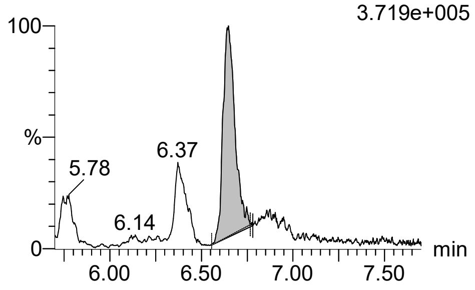

Sample Name: 20211111_A22_R3_IgM
F70:MRM of 1 channel,ES+
1102.8 > 204.1
1.458e+005

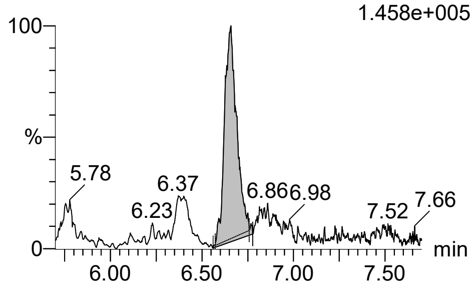

Dataset: C:\MassLynx\10.pro\20211111_clinicalsamples_IgM_IgA.qld

Last Altered: Thursday, March 09, 2023 19:58:25 台北標準時間
Printed: Friday, August 01, 2025 15:17:43 台北標準時間

Sample Name: 20211111_L21_R3_IgM
F70:MRM of 1 channel,ES+
1102.8 > 204.1
1.962e+005

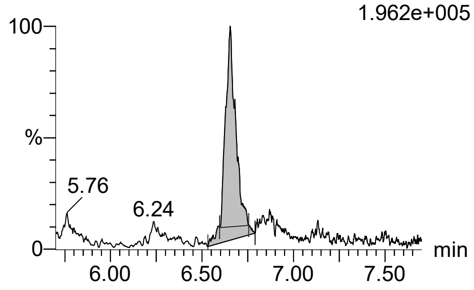

Sample Name: 20211111_L22_R3_IgM
F70:MRM of 1 channel,ES+
1102.8 > 204.1
2.413e+005

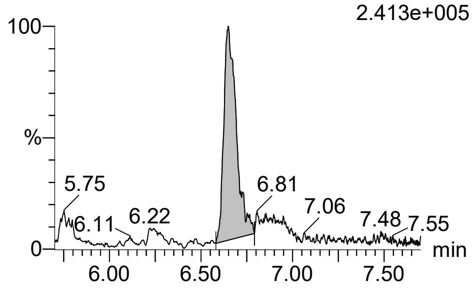

Sample Name: 20211111_H11_R3_IgM
F70:MRM of 1 channel,ES+
1102.8 > 204.1
3.046e+005

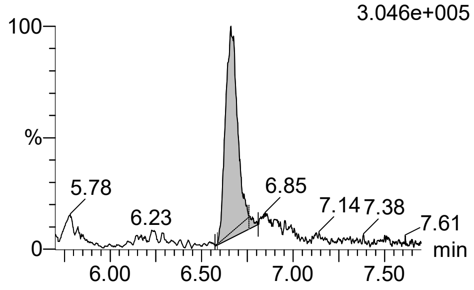

Sample Name: 20211111_A23_R3_IgM
F70:MRM of 1 channel,ES+
1102.8 > 204.1
1.228e+005

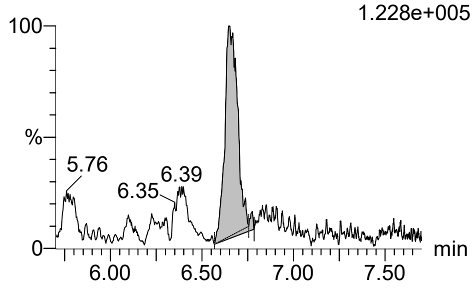

Sample Name: 20211111_A24_R3_IgM
F70:MRM of 1 channel,ES+
1102.8 > 204.1
4.650e+005

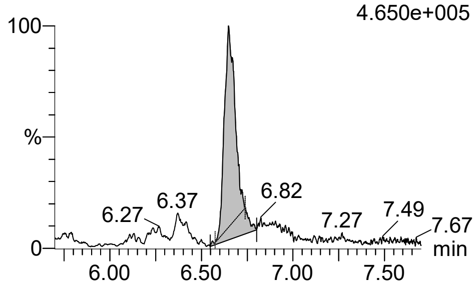

Sample Name: 20211111_L23_R3_IgM
F70:MRM of 1 channel,ES+
1102.8 > 204.1
6.334e+005

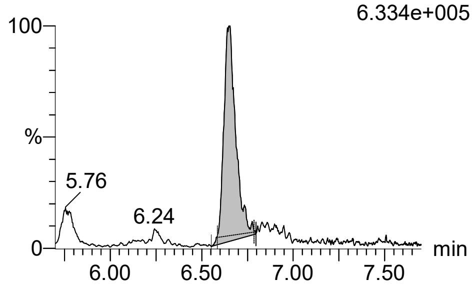

Sample Name: 20211111_L24_R3_IgM
F70:MRM of 1 channel,ES+
1102.8 > 204.1
1.806e+005

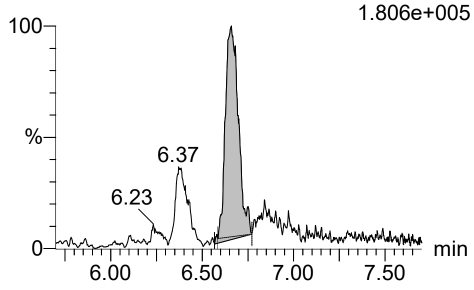

Sample Name: 20211111_H12_R3_IgM
F70:MRM of 1 channel,ES+
1102.8 > 204.1
1.697e+005

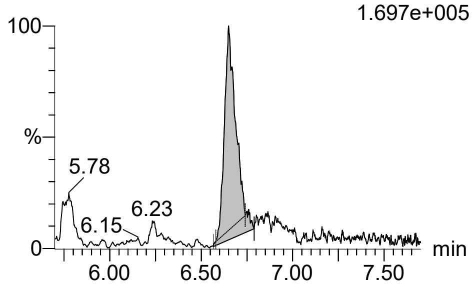

Sample Name: 20211111_QC_R22_IgM
F70:MRM of 1 channel,ES+
1102.8 > 204.1
3.353e+005

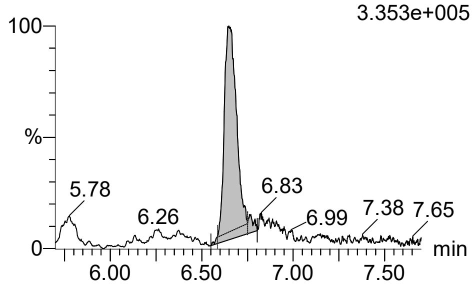

Sample Name: 20211111_A25_R3_IgM
F70:MRM of 1 channel,ES+
1102.8 > 204.1
4.696e+005

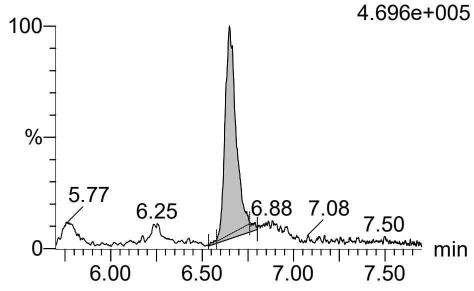

Sample Name: 20211111_A26_R3_IgM
F70:MRM of 1 channel,ES+
1102.8 > 204.1
2.548e+005

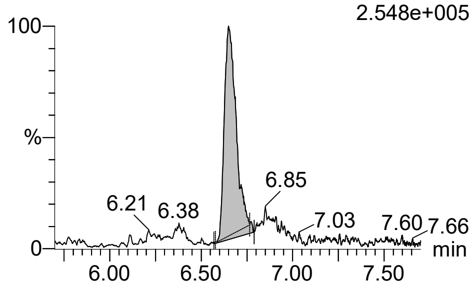

Sample Name: 20211111_L25_R3_IgM
F70:MRM of 1 channel,ES+
1102.8 > 204.1
2.899e+005

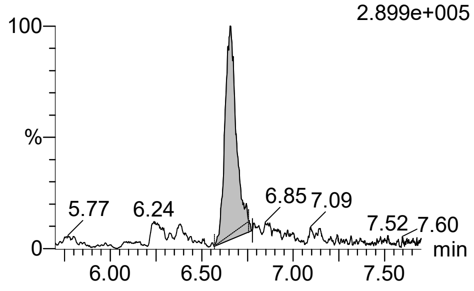

Sample Name: 20211111_L26_R3_IgM
F70:MRM of 1 channel,ES+
1102.8 > 204.1
2.898e+005

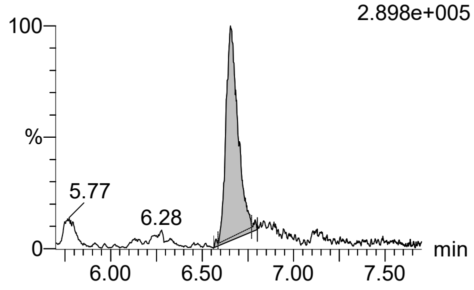

Sample Name: 20211111_H13_R3_IgM
F70:MRM of 1 channel,ES+
1102.8 > 204.1
4.578e+005

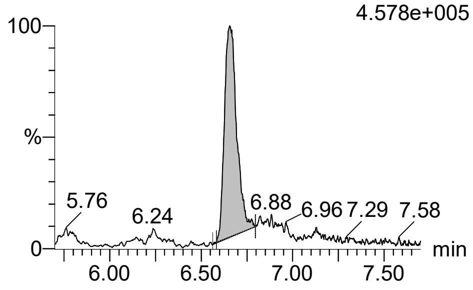

Sample Name: 20211111_A27_R3_IgM
F70:MRM of 1 channel,ES+
1102.8 > 204.1
5.124e+005

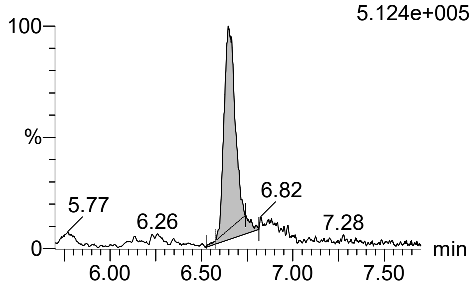

Sample Name: 20211111_A28_R3_IgM
F70:MRM of 1 channel,ES+
1102.8 > 204.1
2.326e+005

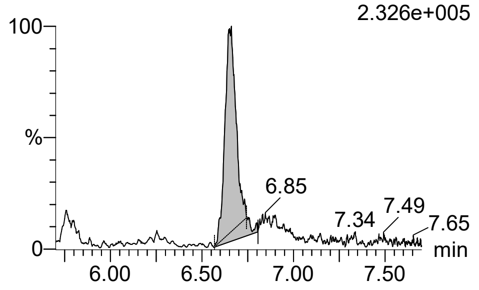

Sample Name: 20211111_L27_R3_IgM
F70:MRM of 1 channel,ES+
1102.8 > 204.1
6.411e+005

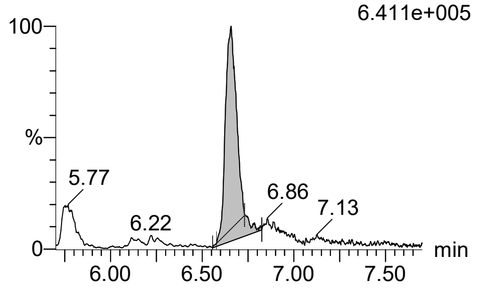

Sample Name: 20211111_L28_R3_IgM
F70:MRM of 1 channel,ES+
1102.8 > 204.1
4.005e+005

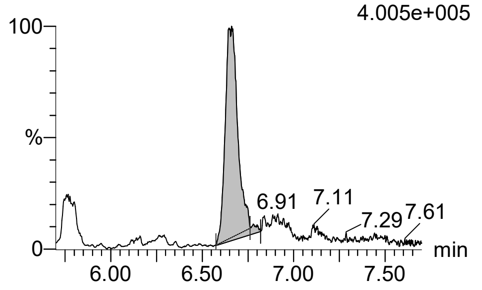

Sample Name: 20211111_H14_R3_IgM
F70:MRM of 1 channel,ES+
1102.8 > 204.1
5.626e+005

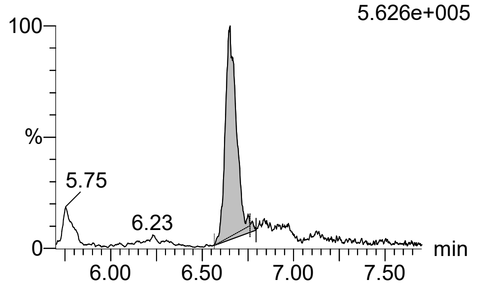

Sample Name: 20211111_A29_R3_IgM
F70:MRM of 1 channel,ES+
1102.8 > 204.1
1.134e+005

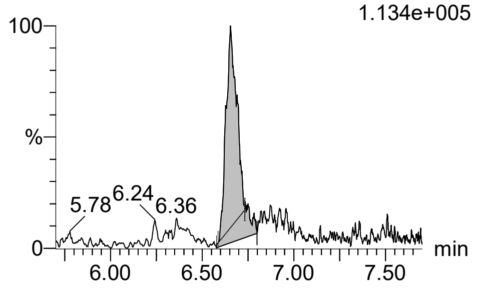

Sample Name: 20211111_A30_R3_IgM
F70:MRM of 1 channel,ES+
1102.8 > 204.1
3.074e+005

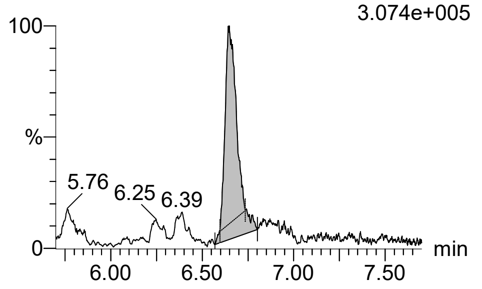

Sample Name: 20211111_QC_R23_IgM
F70:MRM of 1 channel,ES+
1102.8 > 204.1
3.487e+005

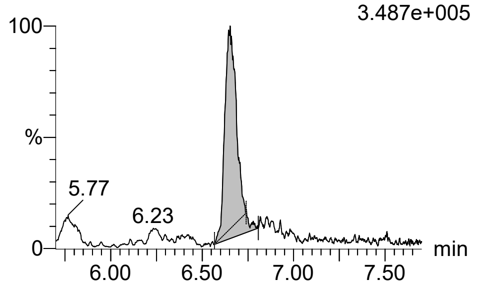

Sample Name: 20211111_L29_R3_IgM
F70:MRM of 1 channel,ES+
1102.8 > 204.1
1.504e+005

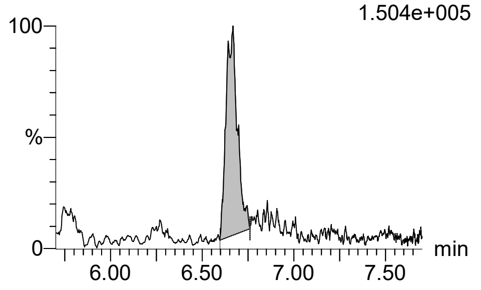

Sample Name: 20211111_L30_R3_IgM
F70:MRM of 1 channel,ES+
1102.8 > 204.1
9.017e+004

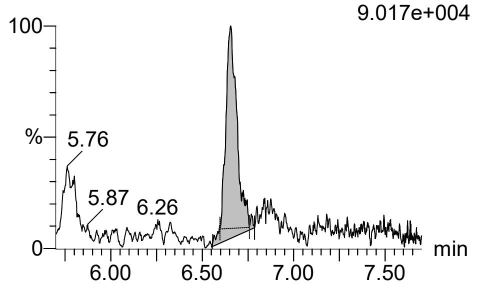

Sample Name: 20211111_H15_R3_IgM
F70:MRM of 1 channel,ES+
1102.8 > 204.1
3.787e+005

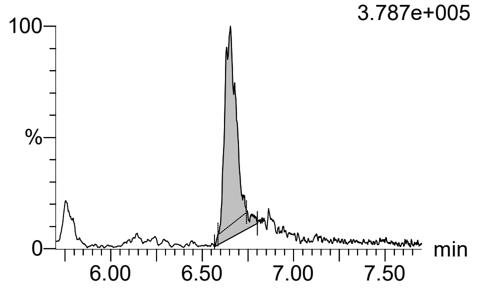

Sample Name: 20211111_A31_R3_IgM
F70:MRM of 1 channel,ES+
1102.8 > 204.1
2.258e+005

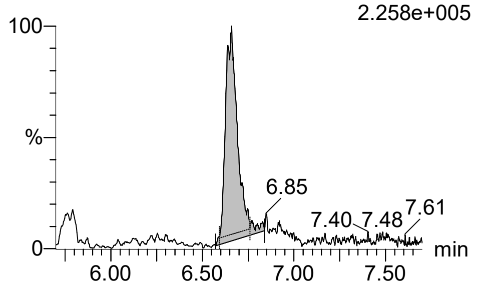

Sample Name: 20211111_A32_R3_IgM
F70:MRM of 1 channel,ES+
1102.8 > 204.1
3.608e+005

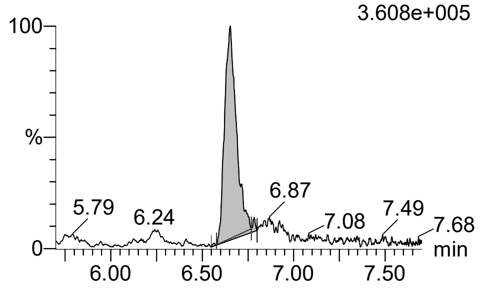

Sample Name: 20211111_L31_R3_IgM
F70:MRM of 1 channel,ES+
1102.8 > 204.1
1.810e+005

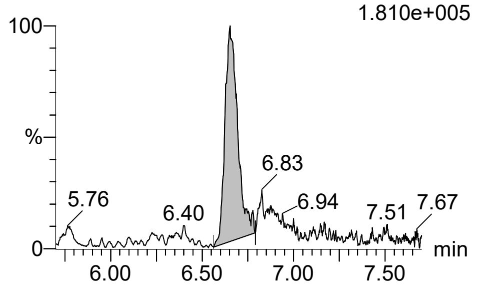

Sample Name: 20211111_L32_R3_IgM
F70:MRM of 1 channel,ES+
1102.8 > 204.1
3.468e+005

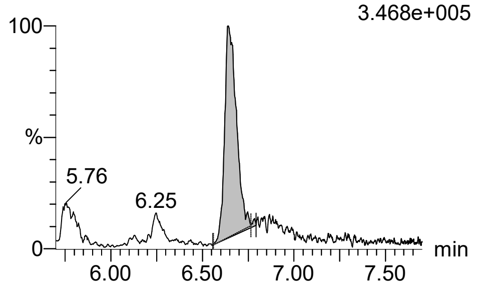

Sample Name: 20211111_H16_R3_IgM
F70:MRM of 1 channel,ES+
1102.8 > 204.1
6.691e+005

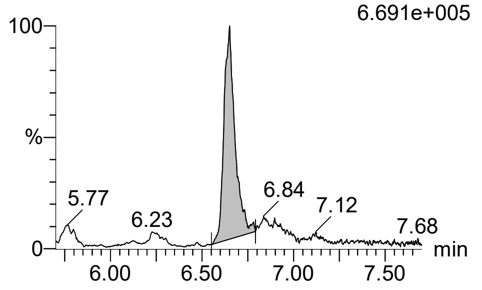

Dataset: C:\MassLynx\10.pro\20211111_clinicalsamples_IgM_IgA.qld

Last Altered: Thursday, March 09, 2023 19:58:25 台北標準時間
Printed: Friday, August 01, 2025 15:17:43 台北標準時間

Sample Name: 20211111_A33_R3_IgM
F70:MRM of 1 channel,ES+
1102.8 > 204.1
4.447e+005

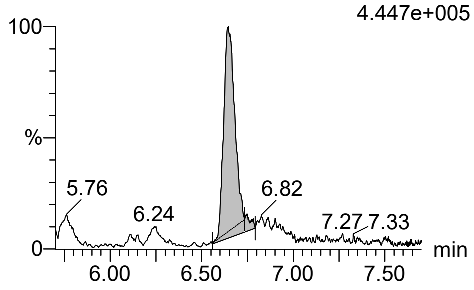

Sample Name: 20211111_A34_R3_IgM
F70:MRM of 1 channel,ES+
1102.8 > 204.1
1.135e+005

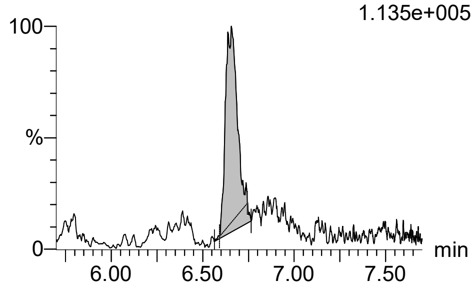

Sample Name: 20211111_H17_R3_IgM
F70:MRM of 1 channel,ES+
1102.8 > 204.1
2.120e+006

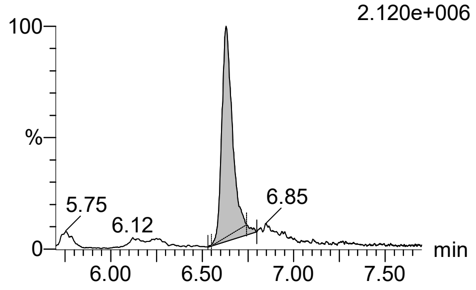

Sample Name: 20211111_A35_R3_IgM
F70:MRM of 1 channel,ES+
1102.8 > 204.1
3.603e+005

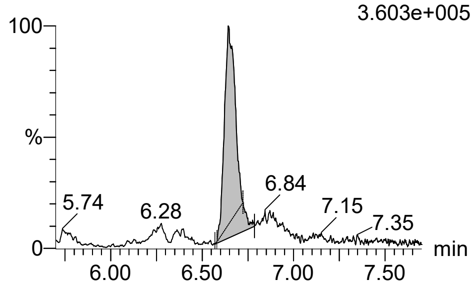

Sample Name: 20211111_QC_R24_IgM
F70:MRM of 1 channel,ES+
1102.8 > 204.1
3.109e+005

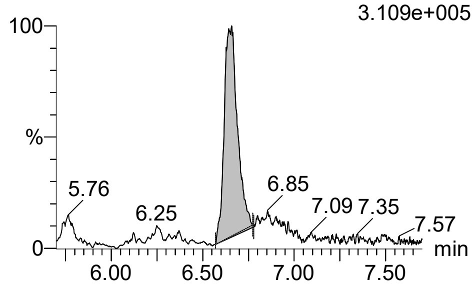

Sample Name: 20211111_A36_R3_IgM
F70:MRM of 1 channel,ES+
1102.8 > 204.1
3.084e+005

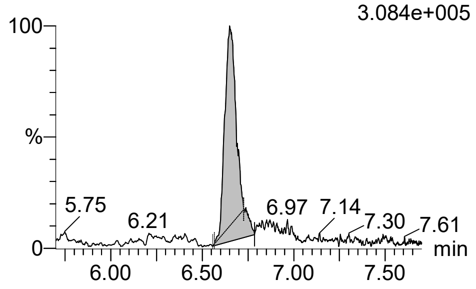

Sample Name: 20211111_H18_R3_IgM
F70:MRM of 1 channel,ES+
1102.8 > 204.1
8.295e+005

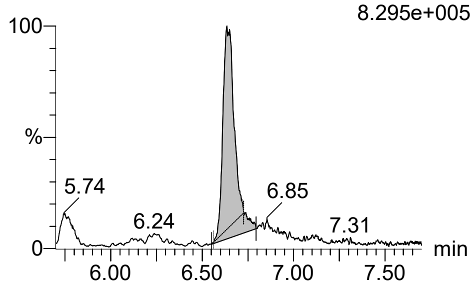

Sample Name: 20211111_A37_R3_IgM
F70:MRM of 1 channel,ES+
1102.8 > 204.1
3.381e+005

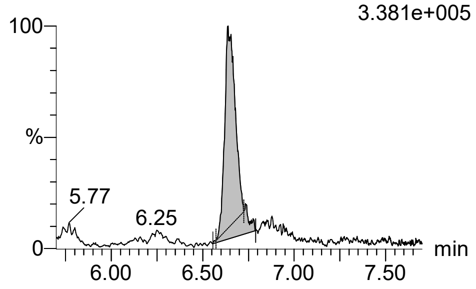

Sample Name: 20211111_A38_R3_IgM
F70:MRM of 1 channel,ES+
1102.8 > 204.1
3.738e+005

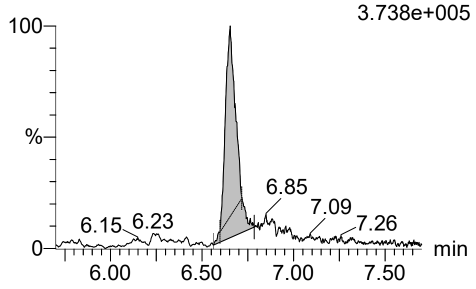

Sample Name: 20211111_H19_R3_IgM
F70:MRM of 1 channel,ES+
1102.8 > 204.1
4.114e+005

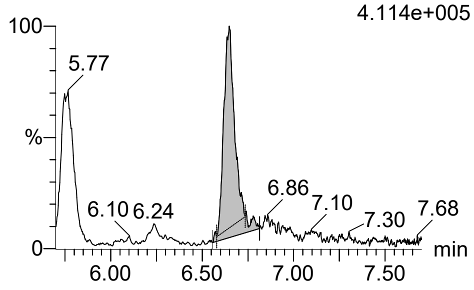

Sample Name: 20211111_A39_R3_IgM
F70:MRM of 1 channel,ES+
1102.8 > 204.1
4.744e+005

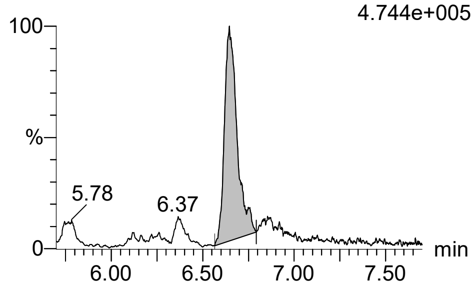

Sample Name: 20211111_A40_R3_IgM
F70:MRM of 1 channel,ES+
1102.8 > 204.1
1.667e+005

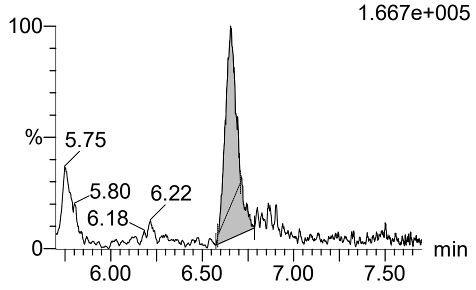

Sample Name: 20211111_H20_R3_IgM
F70:MRM of 1 channel,ES+
1102.8 > 204.1
9.222e+005

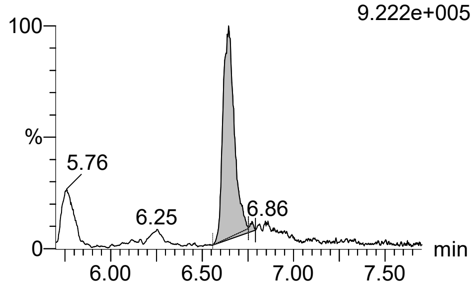

Sample Name: 20211111_A41_R3_IgM
F70:MRM of 1 channel,ES+
1102.8 > 204.1
1.961e+005

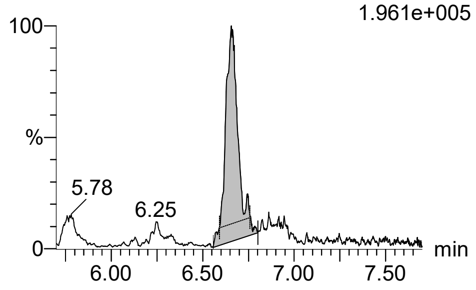

Sample Name: 20211111_A42_R3_IgM
F70:MRM of 1 channel,ES+
1102.8 > 204.1
1.245e+005

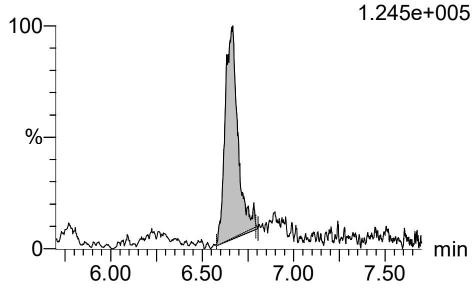

Dataset: C:\MassLynx\10.pro\20211111_clinicalsamples_IgM_IgA.qld

Last Altered: Thursday, March 09, 2023 19:58:25 台北標準時間
Printed: Friday, August 01, 2025 15:17:43 台北標準時間

Sample Name: 20211111_A43_R3_IgM

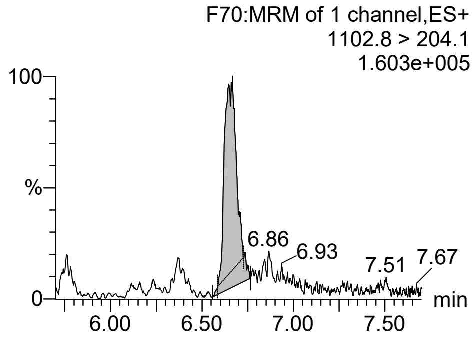

Sample Name: 20211111_A44_R3_IgM

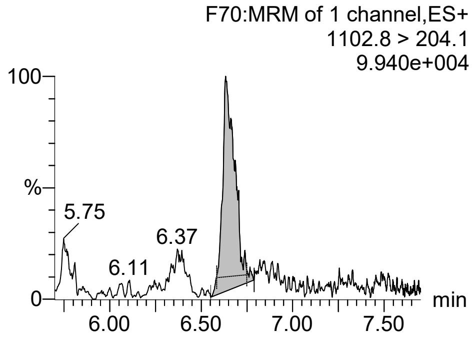

Sample Name: 20211111_QC_R25_IgM

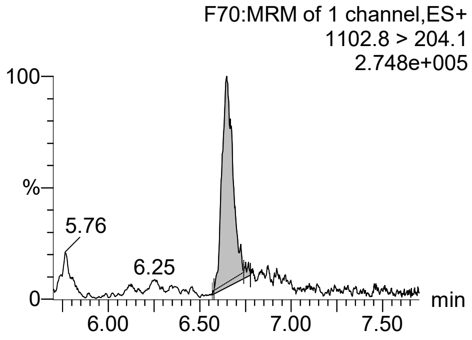

Dataset: C:\MassLynx\10.pro\20211111_clinicalsamples_IgM_IgA.qld

Last Altered: Thursday, March 09, 2023 19:58:25 台北標準時間
Printed: Friday, August 01, 2025 15:17:43 台北標準時間

	#	Name	RT	Area	IS Area	Response	Prima...
1	1	20211112_QC_R001_IgM	6.63	31170.7	15421.4	2.021	MM
2	2	20211112_QC_R002_IgM	6.65	26952.6	13188.8	2.044	MM
3	3	20211111_A1_R2_IgM	6.66	15703.0	11871.0	1.323	MM
4	4	20211111_A1_R3_IgM	6.66	12211.6	12030.5	1.015	bb
5	5	20211111_A2_R2_IgM	6.64	40870.2	11446.8	3.570	bb
6	6	20211111_A2_R3_IgM	6.64	38717.4	11098.4	3.489	MM
7	7	20211111_L1_R2_IgM	6.65	12201.9	11779.4	1.036	MM
8	8	20211111_L1_R3_IgM	6.65	6919.8	11176.2	0.619	MM
9	9	20211111_L2_R2_IgM	6.65	27748.9	11375.0	2.439	MM
10	10	20211111_H1_R2_IgM	6.63	58351.8	13595.2	4.292	MM
11	11	20211111_A3_R2_IgM	6.64	47691.2	14112.3	3.379	MM
12	12	20211111_A4_R2_IgM	6.63	26402.7	11977.5	2.204	MM
13	13	20211111_L3_R2_IgM	6.62	35673.9	12242.1	2.914	MM
14	14	20211111_L4_R2_IgM	6.65	23008.3	12797.0	1.798	MM
15	15	20211111_H2_R2_IgM	6.63	39964.7	10006.0	3.994	MM
16	16	20211111_A5_R2_IgM	6.63	99914.4	11434.6	8.738	MM
17	17	20211111_A6_R2_IgM	6.64	36945.1	13193.7	2.800	MM
18	18	20211111_QC_R10_IgM	6.64	29910.8	12566.1	2.380	MM
19	19	20211111_L5_R2_IgM	6.63	48576.0	13616.4	3.567	MM
20	20	20211111_L6_R2_IgM	6.65	31287.5	13698.5	2.284	MM
21	21	20211111_H3_R2_IgM	6.64	13694.7	15793.1	0.867	MM
22	22	20211111_A7_R2_IgM	6.64	33067.3	12936.9	2.556	MM
23	23	20211111_A8_R2_IgM	6.64	25316.8	13108.0	1.931	MM
24	24	20211111_L7_R2_IgM	6.65	26747.2	13278.5	2.014	MM
25	25	20211111_L8_R2_IgM	6.65	12211.0	15233.6	0.802	MM
26	26	20211111_H4_R2_IgM	6.66	17071.3	16521.9	1.033	MM
27	27	20211111_A9_R2_IgM	6.65	44089.9	16451.6	2.680	MM
28	28	20211111_A10_R2_IgM	6.63	47395.7	13036.0	3.636	MM
29	29	20211111_L9_R2_IgM	6.64	33769.2	14554.1	2.320	MM
30	30	20211111_L10_R2_IgM	6.64	44349.3	12250.4	3.620	MM
31	31	20211111_QC_R11_IgM	6.64	32706.1	11459.2	2.854	MM
32	32	20211111_H5_R2_IgM	6.64	32664.6	14358.2	2.275	MM
33	33	20211111_A11_R2_IgM	6.62	34973.6	16184.1	2.161	MM
34	34	20211111_A12_R2_IgM	6.65	13136.9	13465.4	0.976	MM
35	35	20211111_L11_R2_IgM	6.65	16065.7	13277.9	1.210	MM
36	36	20211111_L12_R2_IgM	6.65	25522.8	15958.8	1.599	MM
37	37	20211111_H6_R2_IgM	6.65	13938.8	14003.5	0.995	MM
38	38	20211111_A13_R2_IgM	6.66	11929.6	13656.1	0.874	MM
39	39	20211111_A14_R2_IgM	6.65	47248.3	14071.3	3.358	MM
40	40	20211111_L13_R2_IgM	6.65	27421.2	14785.6	1.855	MM
41	41	20211111_L14_R2_IgM	6.66	28615.7	14772.2	1.937	MM
42	42	20211111_H7_R2_IgM	6.64	28397.9	15164.4	1.873	MM
43	43	20211111_A15_R2_IgM	6.64	50052.8	15721.5	3.184	MM
44	44	20211111_QC_R12_IgM	6.65	36087.9	13711.5	2.632	MM
45	45	20211111_A16_R2_IgM	6.65	46168.9	14275.6	3.234	MM
46	46	20211111_L15_R2_IgM	6.64	33084.1	12935.8	2.558	MM
47	47	20211111_L16_R2_IgM	6.65	19913.6	13342.8	1.492	MM
48	48	20211111_H8_R2_IgM	6.65	19003.1	15835.1	1.200	MM
49	49	20211111_A17_R2_IgM	6.65	46197.0	15786.7	2.926	MM
50	50	20211111_A18_R2_IgM	6.65	21859.9	16986.8	1.287	MM
51	51	20211111_L17_R2_IgM	6.65	51078.0	13342.7	3.828	MM
52	52	20211111_L18_R2_IgM	6.64	12248.7	14692.0	0.834	MM
53	53	20211111_H9_R2_IgM	6.64	17634.5	15639.7	1.128	MM
54	54	20211111_A19_R2_IgM	6.63	26349.4	14198.7	1.856	MM
55	55	20211111_A20_R2_IgM	6.64	10715.4	14162.7	0.757	MM
56	56	20211111_L19_R2_IgM	6.65	13092.0	9293.4	1.409	MM

Dataset: C:\MassLynx\10.pro\20211111_clinicalsamples_IgM_IgA.qld

Last Altered: Thursday, March 09, 2023 19:58:25 台北標準時間
Printed: Friday, August 01, 2025 15:17:43 台北標準時間

	# Name	RT	Area	IS Area	Response	Prima...
57	57 20211111_QC_R13_IgM	6.65	27917.1	13903.0	2.008	MM
58	58 20211111_L20_R2_IgM	6.63	37526.5	12781.6	2.936	MM
59	59 20211111_H10_R2_IgM	6.64	30249.9	14796.4	2.044	MM
60	60 20211111_A21_R2_IgM	6.64	32828.8	13669.0	2.402	MM
61	61 20211111_A22_R2_IgM	6.64	11800.9	14098.3	0.837	MM
62	62 20211111_L21_R2_IgM	6.64	15209.9	15678.2	0.970	MM
63	63 20211111_L22_R2_IgM	6.66	19269.4	15724.5	1.225	MM
64	64 20211111_H11_R2_IgM	6.65	29228.3	15890.2	1.839	MM
65	65 20211111_A23_R2_IgM	6.65	11782.4	13423.2	0.878	MM
66	66 20211111_A24_R2_IgM	6.65	34729.4	12438.0	2.792	MM
67	67 20211111_L23_R2_IgM	6.64	54140.8	14075.0	3.847	MM
68	68 20211111_L24_R2_IgM	6.65	17037.9	15972.1	1.067	MM
69	69 20211111_H12_R2_IgM	6.65	12322.4	16270.9	0.757	MM
70	70 20211111_QC_R14_IgM	6.65	30648.5	15806.8	1.939	MM
71	71 20211111_A25_R2_IgM	6.64	40816.9	14552.1	2.805	MM
72	72 20211111_A26_R2_IgM	6.65	22389.7	11492.7	1.948	bb
73	73 20211111_L25_R2_IgM	6.65	24755.9	14403.3	1.719	MM
74	74 20211111_L26_R2_IgM	6.64	24571.7	16533.9	1.486	MM
75	75 20211111_H13_R2_IgM	6.66	37392.3	15520.1	2.409	MM
76	76 20211111_A27_R2_IgM	6.65	45338.3	13337.4	3.399	bb
77	77 20211111_A28_R2_IgM	6.63	24781.6	11895.6	2.083	MM
78	78 20211111_L27_R2_IgM	6.65	58638.8	14125.7	4.151	MM
79	79 20211111_L28_R2_IgM	6.65	31426.9	11807.5	2.662	MM
80	80 20211111_H14_R2_IgM	6.64	40991.0	15035.7	2.726	MM
81	81 20211111_A29_R2_IgM	6.66	9215.6	13554.4	0.680	MM
82	82 20211111_A30_R2_IgM	6.66	28332.3	12984.1	2.182	MM
83	83 20211111_QC_R15_IgM	6.66	28500.5	12299.5	2.317	MM
84	84 20211111_L29_R2_IgM	6.65	12382.0	13590.7	0.911	MM
85	85 20211111_L30_R2_IgM	6.65	6789.2	12511.4	0.543	MM
86	86 20211111_H15_R2_IgM	6.65	32918.6	11885.0	2.770	MM
87	87 20211111_A31_R2_IgM	6.65	16773.2	13422.1	1.250	MM
88	88 20211111_A32_R2_IgM	6.65	28674.3	11461.3	2.502	MM
89	89 20211111_L31_R2_IgM	6.64	21850.3	13753.4	1.589	bb
90	90 20211111_L32_R2_IgM	6.64	32249.7	12949.0	2.491	MM
91	91 20211111_H16_R2_IgM	6.64	57024.0	11465.4	4.974	MM
92	92 20211111_A33_R2_IgM	6.63	35876.0	10551.5	3.400	MM
93	93 20211111_A34_R2_IgM	6.65	10468.6	12409.9	0.844	MM
94	94 20211111_H17_R2_IgM	6.62	171489.6	10160.9	16.877	MM
95	95 20211111_A35_R2_IgM	6.64	33404.5	10460.6	3.193	MM
96	96 20211111_QC_R16_IgM	6.64	30174.5	10433.5	2.892	MM
97	97 20211111_A36_R2_IgM	6.64	40664.8	12504.8	3.252	MM
98	98 20211111_H18_R2_IgM	6.65	73851.7	9140.4	8.080	MM
99	99 20211111_A37_R2_IgM	6.65	29703.8	9796.9	3.032	MM
100	1... 20211111_A38_R2_IgM	6.66	33532.9	11224.3	2.988	MM
101	1... 20211111_H19_R2_IgM	6.65	35632.9	11739.2	3.035	MM
102	1... 20211111_A39_R2_IgM	6.65	42469.6	12718.9	3.339	MM
103	1... 20211111_A40_R2_IgM	6.64	17511.2	10006.1	1.750	MM
104	1... 20211111_H20_R2_IgM	6.64	81125.1	7988.2	10.156	MM
105	1... 20211111_A41_R2_IgM	6.65	26010.7	11513.3	2.259	MM
106	1... 20211111_A42_R2_IgM	6.65	11812.8	10238.5	1.154	MM
107	1... 20211111_A43_R2_IgM	6.65	13569.5	11098.9	1.223	MM
108	1... 20211111_A44_R2_IgM	6.64	9579.9	10969.9	0.873	MM
109	1... 20211111_QC_R17_IgM	6.65	23128.7	9700.8	2.384	MM
110	1... 20211111_L2_R3_IgM	6.65	28481.9	8528.0	3.340	MM
111	1... 20211111_H1_R3_IgM	6.65	45630.0	9438.2	4.835	MM
112	1... 20211111_A3_R3_IgM	6.65	34821.5	11598.6	3.002	MM

Dataset: C:\MassLynx\10.pro\20211111_clinicalsamples_IgM_IgA.qld

Last Altered: Thursday, March 09, 2023 19:58:25 台北標準時間
Printed: Friday, August 01, 2025 15:17:43 台北標準時間

	#	Name	RT	Area	IS Area	Response	Prima...
113	1...	20211111_A4_R3_IgM	6.66	23757.7	11364.1	2.091	MM
114	1...	20211111_L3_R3_IgM	6.64	27724.8	10753.4	2.578	MM
115	1...	20211111_L4_R3_IgM	6.63	16758.3	10291.6	1.628	MM
116	1...	20211111_H2_R3_IgM	6.64	40211.7	9315.8	4.317	MM
117	1...	20211111_A5_R3_IgM	6.64	74995.0	9102.2	8.239	MM
118	1...	20211111_A6_R3_IgM	6.65	30186.0	10356.4	2.915	MM
119	1...	20211111_QC_R18_IgM	6.63	28437.8	8398.3	3.386	MM
120	1...	20211111_L5_R3_IgM	6.66	33060.5	9786.7	3.378	bb
121	1...	20211111_L6_R3_IgM	6.65	26524.3	9911.3	2.676	MM
122	1...	20211111_H3_R3_IgM	6.67	9968.5	12155.5	0.820	MM
123	1...	20211111_A7_R3_IgM	6.64	31048.2	11804.8	2.630	MM
124	1...	20211111_A8_R3_IgM	6.64	20303.8	10510.6	1.932	MM
125	1...	20211111_L7_R3_IgM	6.65	25514.4	12177.9	2.095	MM
126	1...	20211111_L8_R3_IgM	6.65	10144.1	11668.6	0.869	MM
127	1...	20211111_H4_R3_IgM	6.65	12903.4	11192.0	1.153	MM
128	1...	20211111_A9_R3_IgM	6.65	29889.3	14077.4	2.123	MM
129	1...	20211111_A10_R3_IgM	6.65	38848.6	11190.4	3.472	MM
130	1...	20211111_L9_R3_IgM	6.66	28677.3	14649.3	1.958	MM
131	1...	20211111_L10_R3_IgM	6.64	41320.9	9361.3	4.414	MM
132	1...	20211111_QC_R19_IgM	6.64	28180.1	10125.5	2.783	MM
133	1...	20211111_H5_R3_IgM	6.64	24915.2	10010.9	2.489	MM
134	1...	20211111_A11_R3_IgM	6.65	19069.3	12697.2	1.502	MM
135	1...	20211111_A12_R3_IgM	6.65	11707.3	13148.5	0.890	MM
136	1...	20211111_L11_R3_IgM	6.66	12017.9	10786.6	1.114	MM
137	1...	20211111_L12_R3_IgM	6.65	25741.1	11681.8	2.204	MM
138	1...	20211111_H6_R3_IgM	6.65	12985.7	12095.5	1.074	MM
139	1...	20211111_A13_R3_IgM	6.64	11207.8	12908.3	0.868	MM
140	1...	20211111_A14_R3_IgM	6.65	37907.2	11322.6	3.348	bb
141	1...	20211111_L13_R3_IgM	6.66	23647.9	11516.1	2.053	MM
142	1...	20211111_L14_R3_IgM	6.64	24330.7	10790.7	2.255	MM
143	1...	20211111_H7_R3_IgM	6.65	19765.9	11572.1	1.708	MM
144	1...	20211111_A15_R3_IgM	6.66	42561.2	12434.4	3.423	MM
145	1...	20211111_QC_R20_IgM	6.65	30960.1	10701.0	2.893	MM
146	1...	20211111_A16_R3_IgM	6.65	38872.0	11452.1	3.394	MM
147	1...	20211111_L15_R3_IgM	6.65	26991.6	10531.1	2.563	MM
148	1...	20211111_L16_R3_IgM	6.65	18392.7	12049.4	1.526	MM
149	1...	20211111_H8_R3_IgM	6.64	15326.3	13434.9	1.141	MM
150	1...	20211111_A17_R3_IgM	6.65	37823.1	12452.0	3.037	MM
151	1...	20211111_A18_R3_IgM	6.64	20420.0	12506.5	1.633	MM
152	1...	20211111_L17_R3_IgM	6.64	43674.2	9948.9	4.390	MM
153	1...	20211111_L18_R3_IgM	6.64	9836.4	13462.1	0.731	MM
154	1...	20211111_H9_R3_IgM	6.65	14202.6	13178.7	1.078	MM
155	1...	20211111_A19_R3_IgM	6.64	25848.5	11298.6	2.288	bb
156	1...	20211111_A20_R3_IgM	6.64	9503.6	11175.6	0.850	bb
157	1...	20211111_L19_R3_IgM	6.65	14876.8	11558.2	1.287	MM
158	1...	20211111_QC_R21_IgM	6.65	24998.6	9977.0	2.506	bb
159	1...	20211111_L20_R3_IgM	6.65	22365.7	13508.1	1.656	MM
160	1...	20211111_H10_R3_IgM	6.65	20306.1	12374.8	1.641	MM
161	1...	20211111_A21_R3_IgM	6.65	26757.5	12443.6	2.150	MM
162	1...	20211111_A22_R3_IgM	6.66	11066.9	11589.3	0.955	MM
163	1...	20211111_L21_R3_IgM	6.65	13085.1	13107.4	0.998	MM
164	1...	20211111_L22_R3_IgM	6.65	18289.8	12021.5	1.521	bb
165	1...	20211111_H11_R3_IgM	6.66	22729.2	12687.8	1.791	MM
166	1...	20211111_A23_R3_IgM	6.65	9697.2	11611.8	0.835	MM
167	1...	20211111_A24_R3_IgM	6.65	33721.9	10476.8	3.219	MM
168	1...	20211111_L23_R3_IgM	6.65	46734.9	11873.1	3.936	MM

Dataset: C:\MassLynx\10.pro\20211111_clinicalsamples_IgM_IgA.qld

Last Altered: Thursday, March 09, 2023 19:58:25 台北標準時間
Printed: Friday, August 01, 2025 15:17:43 台北標準時間

	# Name	RT	Area	IS Area	Response	Prima...
169	1... 20211111_L24_R3_IgM	6.66	14353.2	13468.0	1.066	MM
170	1... 20211111_H12_R3_IgM	6.65	12172.0	14229.1	0.855	MM
171	1... 20211111_QC_R22_IgM	6.65	26351.0	11264.4	2.339	MM
172	1... 20211111_A25_R3_IgM	6.65	32184.5	11775.7	2.733	MM
173	1... 20211111_A26_R3_IgM	6.65	18587.7	10473.9	1.775	MM
174	1... 20211111_L25_R3_IgM	6.66	19979.5	13256.8	1.507	MM
175	1... 20211111_L26_R3_IgM	6.65	21462.9	13565.0	1.582	MM
176	1... 20211111_H13_R3_IgM	6.66	33752.9	13146.0	2.568	MM
177	1... 20211111_A27_R3_IgM	6.65	37636.5	12025.6	3.130	MM
178	1... 20211111_A28_R3_IgM	6.66	17065.6	11229.1	1.520	MM
179	1... 20211111_L27_R3_IgM	6.66	47025.0	14960.0	3.143	MM
180	1... 20211111_L28_R3_IgM	6.66	31492.3	11359.0	2.772	MM
181	1... 20211111_H14_R3_IgM	6.65	38363.9	12830.8	2.990	MM
182	1... 20211111_A29_R3_IgM	6.65	8657.4	13434.3	0.644	MM
183	1... 20211111_A30_R3_IgM	6.65	23032.6	11272.2	2.043	MM
184	1... 20211111_QC_R23_IgM	6.65	25635.7	11872.2	2.159	MM
185	1... 20211111_L29_R3_IgM	6.67	10057.4	12948.7	0.777	MM
186	1... 20211111_L30_R3_IgM	6.65	6358.9	11026.6	0.577	MM
187	1... 20211111_H15_R3_IgM	6.65	27742.8	10786.3	2.572	MM
188	1... 20211111_A31_R3_IgM	6.66	16838.7	12010.5	1.402	MM
189	1... 20211111_A32_R3_IgM	6.65	26401.6	11107.7	2.377	MM
190	1... 20211111_L31_R3_IgM	6.65	13999.4	11021.8	1.270	bb
191	1... 20211111_L32_R3_IgM	6.64	24752.4	11683.8	2.119	MM
192	1... 20211111_H16_R3_IgM	6.65	44448.6	9831.7	4.521	bb
193	1... 20211111_A33_R3_IgM	6.64	31526.1	11213.1	2.812	MM
194	1... 20211111_A34_R3_IgM	6.66	8152.9	11550.1	0.706	MM
195	1... 20211111_H17_R3_IgM	6.63	142380.2	8874.4	16.044	MM
196	1... 20211111_A35_R3_IgM	6.64	25588.0	9856.3	2.596	MM
197	1... 20211111_QC_R24_IgM	6.66	24930.7	7561.8	3.297	MM
198	1... 20211111_A36_R3_IgM	6.65	23396.7	9562.8	2.447	MM
199	1... 20211111_H18_R3_IgM	6.64	60562.3	8165.3	7.417	MM
200	2... 20211111_A37_R3_IgM	6.64	24244.7	9278.8	2.613	MM
201	2... 20211111_A38_R3_IgM	6.65	25040.1	10920.3	2.293	MM
202	2... 20211111_H19_R3_IgM	6.65	28997.0	9285.3	3.123	MM
203	2... 20211111_A39_R3_IgM	6.65	36343.2	9083.4	4.001	bb
204	2... 20211111_A40_R3_IgM	6.65	12116.3	8382.4	1.445	MM
205	2... 20211111_H20_R3_IgM	6.64	64338.3	7807.9	8.240	MM
206	2... 20211111_A41_R3_IgM	6.66	14979.0	5925.2	2.528	MM
207	2... 20211111_A42_R3_IgM	6.67	9645.5	9352.6	1.031	MM
208	2... 20211111_A43_R3_IgM	6.67	12201.9	10216.3	1.194	MM
209	2... 20211111_A44_R3_IgM	6.63	7448.5	10262.7	0.726	MM
210	2... 20211111_QC_R25_IgM	6.65	18500.6	8575.3	2.157	MM

Dataset: C:\MassLynx\10.pro\20211111_clinicalsamples_IgM_IgA.qld

Last Altered: Thursday, March 09, 2023 19:58:25 台北標準時間
Printed: Friday, August 01, 2025 15:18:29 台北標準時間

Method: C:\MassLynx\10_Pro_IgA.PRO\MethDB\20211111_IgM_IgA_DMRM_new.mdb 24 Feb 2022 13:44:14
Calibration: 15 Nov 2021 14:42:11

Sample Name: 20211112_QC_R001_IgM

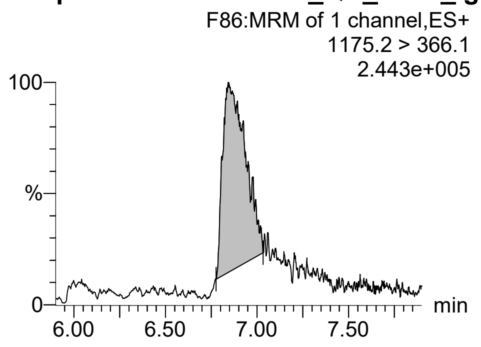

Sample Name: 20211112_QC_R002_IgM

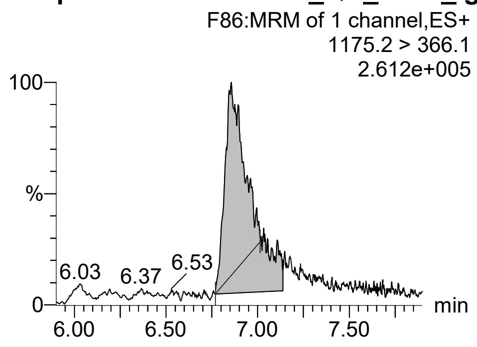

Sample Name: 20211111_A1_R2_IgM

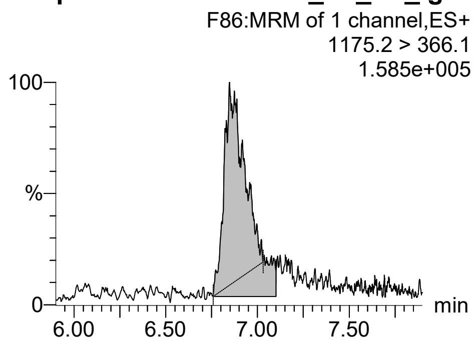

Sample Name: 20211111_A1_R3_IgM

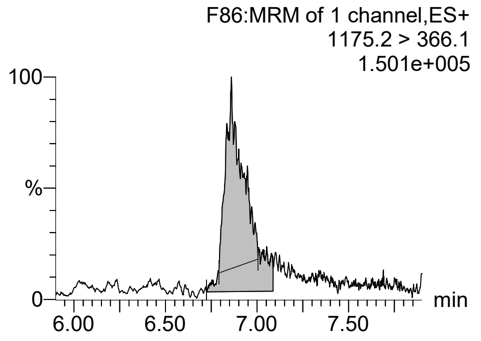

Sample Name: 20211111_A2_R2_IgM

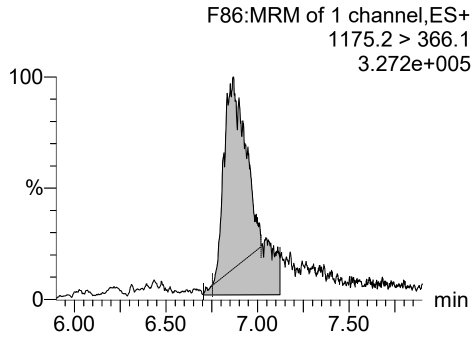

Sample Name: 20211111_A2_R3_IgM

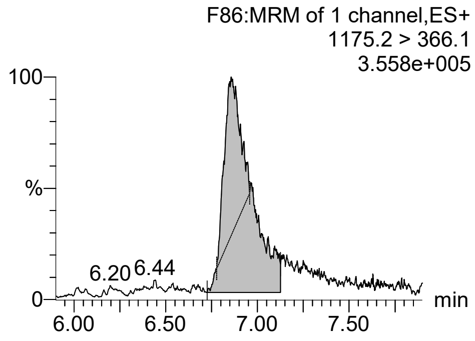

Sample Name: 20211111_L1_R2_IgM

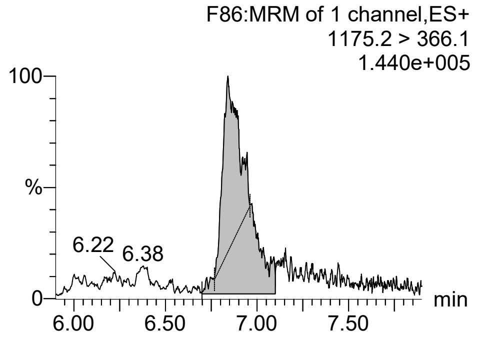

Sample Name: 20211111_L1_R3_IgM

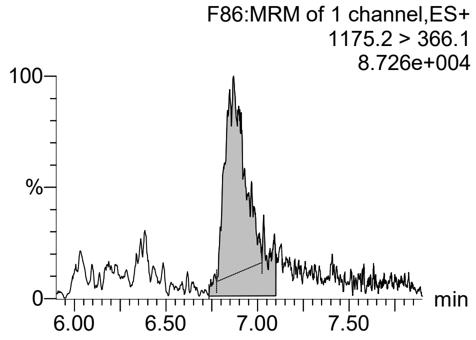

Sample Name: 20211111_L2_R2_IgM

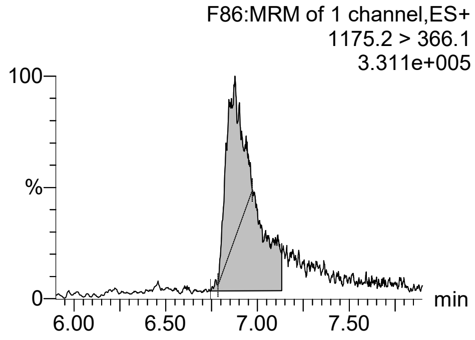

Sample Name: 20211111_H1_R2_IgM

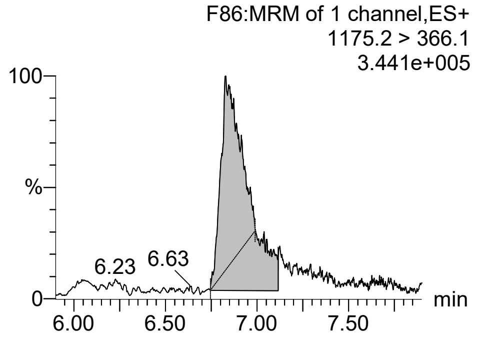

Sample Name: 20211111_A3_R2_IgM

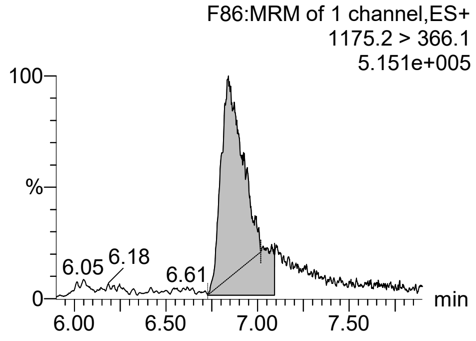

Sample Name: 20211111_A4_R2_IgM

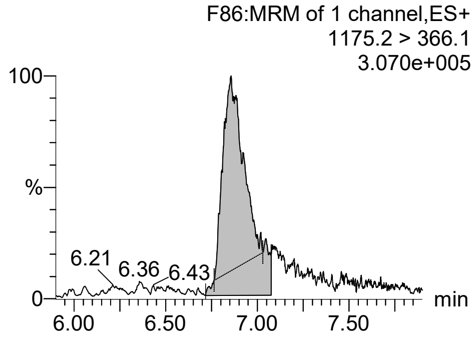

Dataset: C:\MassLynx\10.pro\20211111_clinicalsamples_IgM_IgA.qld

Last Altered: Thursday, March 09, 2023 19:58:25 台北標準時間
Printed: Friday, August 01, 2025 15:18:29 台北標準時間

Sample Name: 20211111_L3_R2_IgM
F86:MRM of 1 channel,ES+
1175.2 > 366.1
3.641e+005

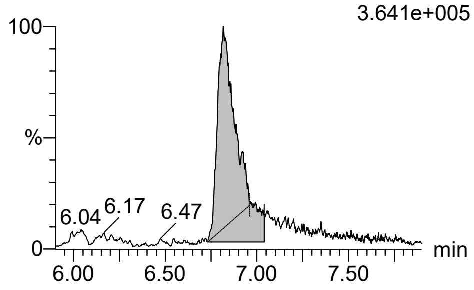

Sample Name: 20211111_L4_R2_IgM
F86:MRM of 1 channel,ES+
1175.2 > 366.1
2.452e+005

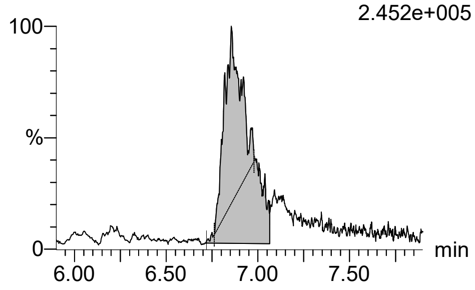

Sample Name: 20211111_H2_R2_IgM
F86:MRM of 1 channel,ES+
1175.2 > 366.1
3.442e+005

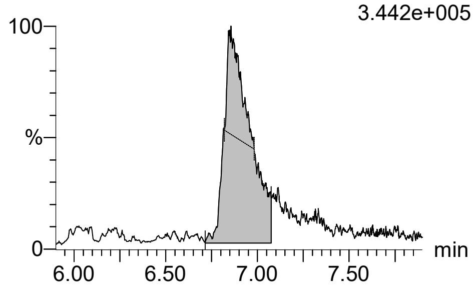

Sample Name: 20211111_A5_R2_IgM
F86:MRM of 1 channel,ES+
1175.2 > 366.1
9.473e+005

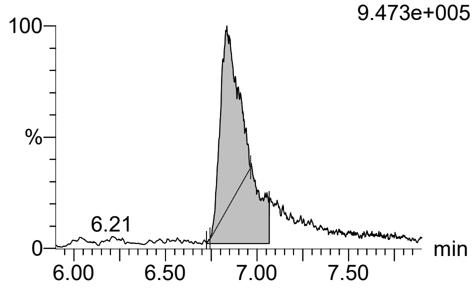

Sample Name: 20211111_A6_R2_IgM
F86:MRM of 1 channel,ES+
1175.2 > 366.1
5.179e+005

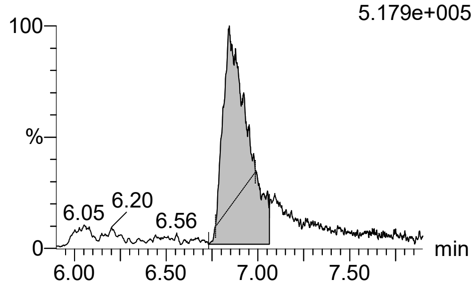

Sample Name: 20211111_QC_R10_IgM
F86:MRM of 1 channel,ES+
1175.2 > 366.1
3.825e+005

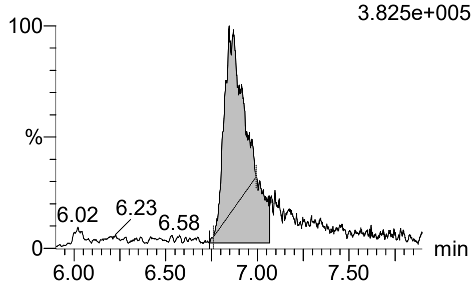

Sample Name: 20211111_L5_R2_IgM
F86:MRM of 1 channel,ES+
1175.2 > 366.1
6.649e+005

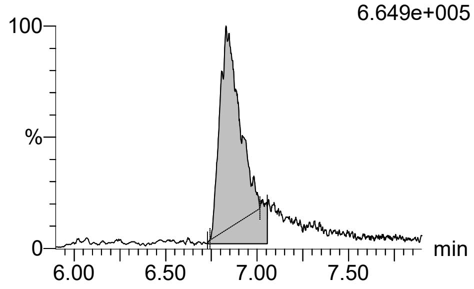

Sample Name: 20211111_L6_R2_IgM
F86:MRM of 1 channel,ES+
1175.2 > 366.1
3.708e+005

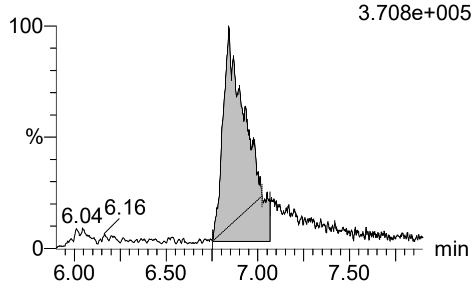

Sample Name: 20211111_H3_R2_IgM
F86:MRM of 1 channel,ES+
1175.2 > 366.1
1.941e+005

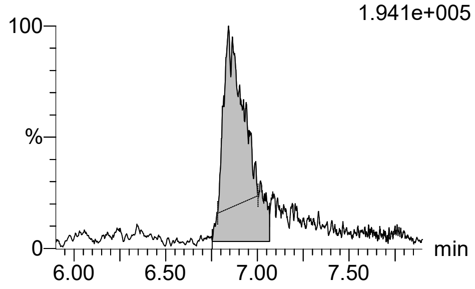

Sample Name: 20211111_A7_R2_IgM
F86:MRM of 1 channel,ES+
1175.2 > 366.1
3.008e+005

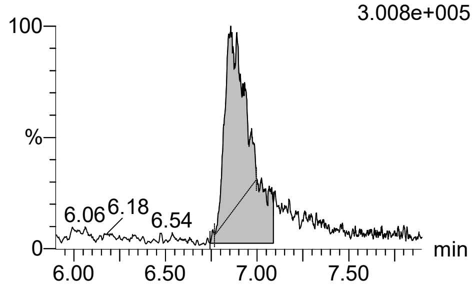

Sample Name: 20211111_A8_R2_IgM
F86:MRM of 1 channel,ES+
1175.2 > 366.1
2.286e+005

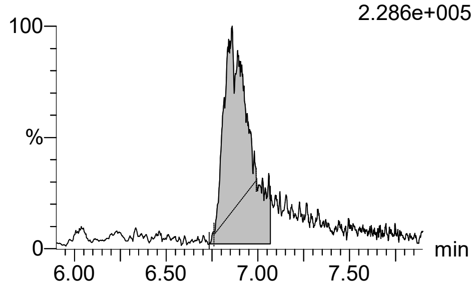

Sample Name: 20211111_L7_R2_IgM
F86:MRM of 1 channel,ES+
1175.2 > 366.1
2.409e+005

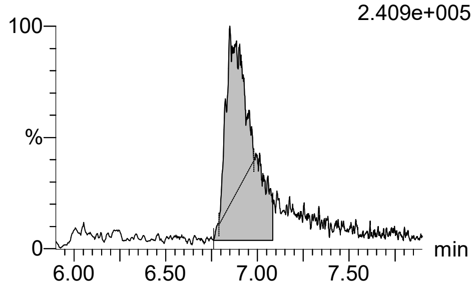

Sample Name: 20211111_L8_R2_IgM
F86:MRM of 1 channel,ES+
1175.2 > 366.1
1.688e+005

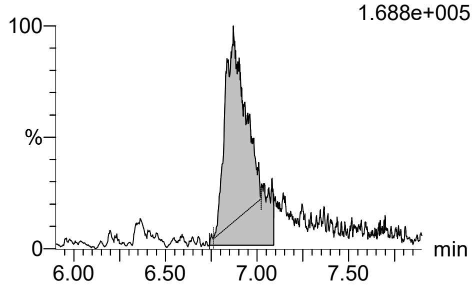

Sample Name: 20211111_H4_R2_IgM
F86:MRM of 1 channel,ES+
1175.2 > 366.1
1.803e+005

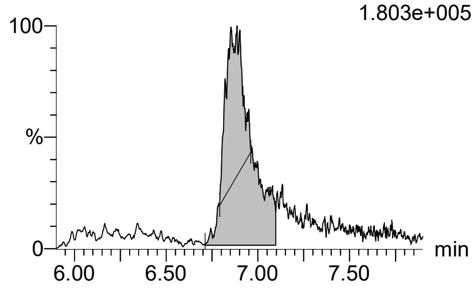

Sample Name: 20211111_A9_R2_IgM
F86:MRM of 1 channel,ES+
1175.2 > 366.1
2.865e+005

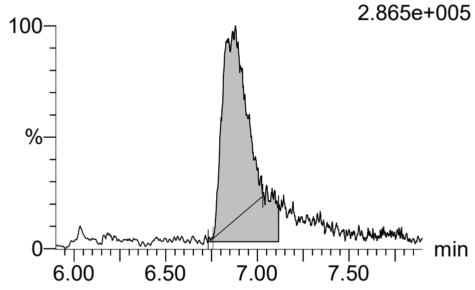

Dataset: C:\MassLynx\10.pro\20211111_clinicalsamples_IgM_IgA.qld

Last Altered: Thursday, March 09, 2023 19:58:25 台北標準時間
Printed: Friday, August 01, 2025 15:18:29 台北標準時間

Sample Name: 20211111_A10_R2_IgM
F86:MRM of 1 channel,ES+
1175.2 > 366.1
3.862e+005

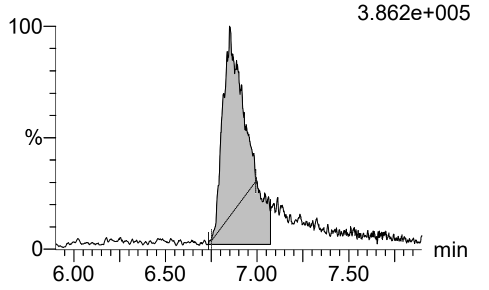

Sample Name: 20211111_L9_R2_IgM
F86:MRM of 1 channel,ES+
1175.2 > 366.1
2.831e+005

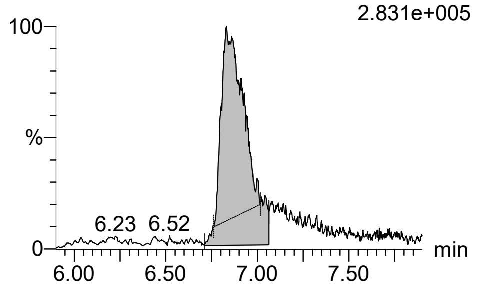

Sample Name: 20211111_L10_R2_IgM
F86:MRM of 1 channel,ES+
1175.2 > 366.1
5.600e+005

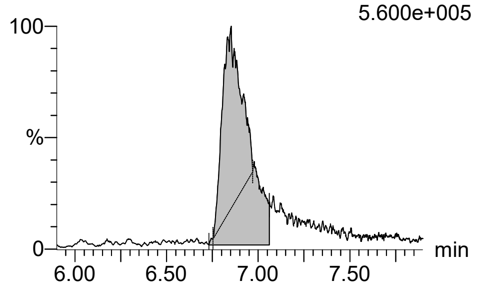

Sample Name: 20211111_QC_R11_IgM
F86:MRM of 1 channel,ES+
1175.2 > 366.1
3.792e+005

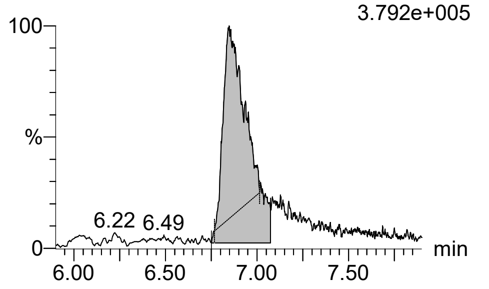

Sample Name: 20211111_H5_R2_IgM
F86:MRM of 1 channel,ES+
1175.2 > 366.1
4.773e+005

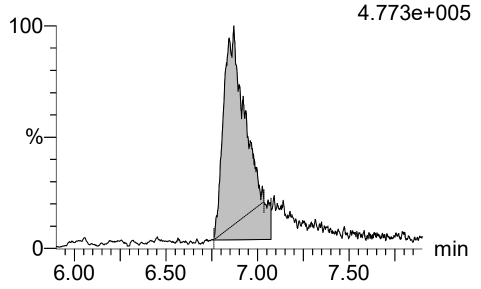

Sample Name: 20211111_A11_R2_IgM
F86:MRM of 1 channel,ES+
1175.2 > 366.1
4.212e+005

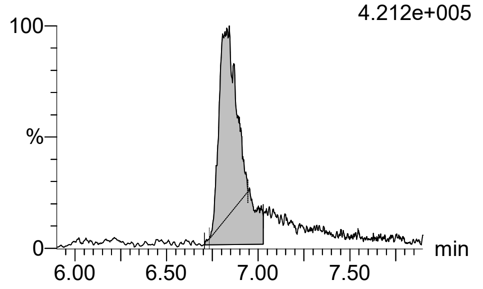

Sample Name: 20211111_A12_R2_IgM
F86:MRM of 1 channel,ES+
1175.2 > 366.1
1.921e+005

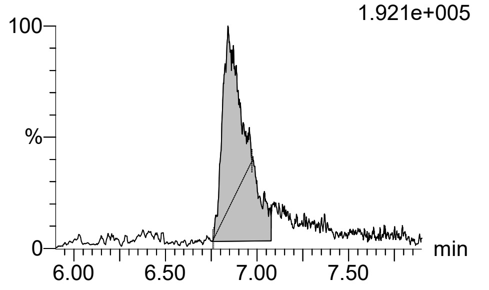

Sample Name: 20211111_L11_R2_IgM
F86:MRM of 1 channel,ES+
1175.2 > 366.1
1.514e+005

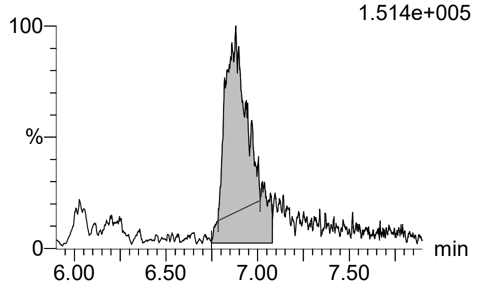

Sample Name: 20211111_L12_R2_IgM
F86:MRM of 1 channel,ES+
1175.2 > 366.1
3.054e+005

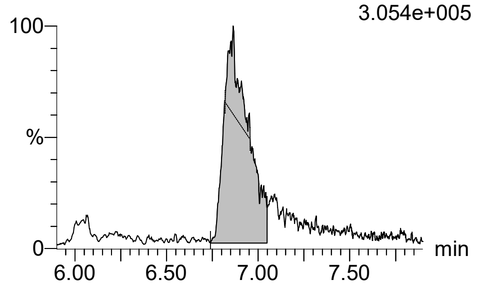

Sample Name: 20211111_H6_R2_IgM
F86:MRM of 1 channel,ES+
1175.2 > 366.1
1.623e+005

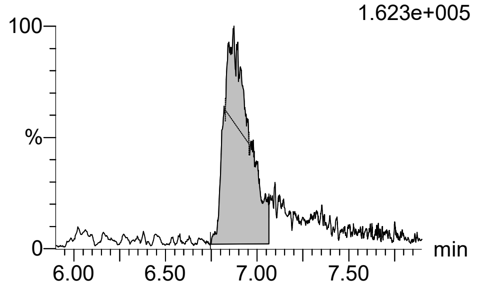

Sample Name: 20211111_A13_R2_IgM
F86:MRM of 1 channel,ES+
1175.2 > 366.1
1.276e+005

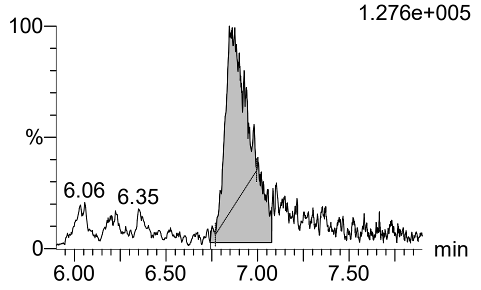

Sample Name: 20211111_A14_R2_IgM
F86:MRM of 1 channel,ES+
1175.2 > 366.1
3.838e+005

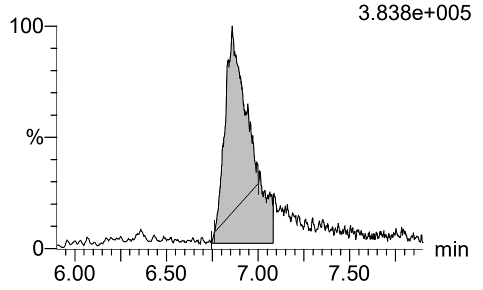

Sample Name: 20211111_L13_R2_IgM
F86:MRM of 1 channel,ES+
1175.2 > 366.1
4.230e+005

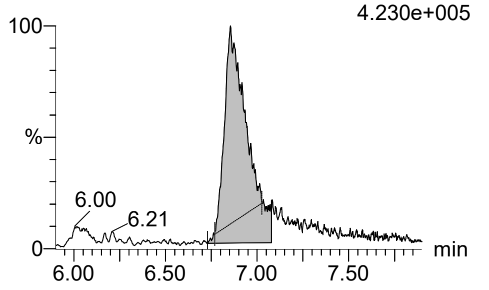

Sample Name: 20211111_L14_R2_IgM
F86:MRM of 1 channel,ES+
1175.2 > 366.1
3.075e+005

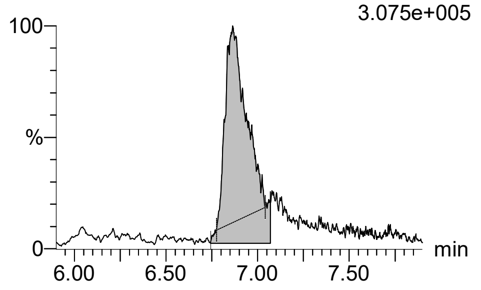

Sample Name: 20211111_H7_R2_IgM
F86:MRM of 1 channel,ES+
1175.2 > 366.1
2.536e+005

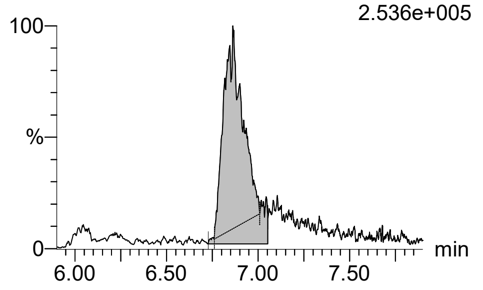

Dataset: C:\MassLynx\10.pro\20211111_clinicalsamples_IgM_IgA.qld

Last Altered: Thursday, March 09, 2023 19:58:25 台北標準時間
Printed: Friday, August 01, 2025 15:18:29 台北標準時間

Sample Name: 20211111_A15_R2_IgM
F86:MRM of 1 channel,ES+
1175.2 > 366.1
3.962e+005

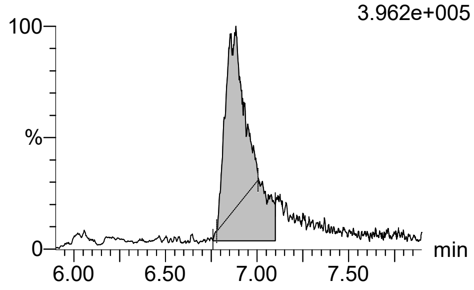

Sample Name: 20211111_QC_R12_IgM
F86:MRM of 1 channel,ES+
1175.2 > 366.1
3.478e+005

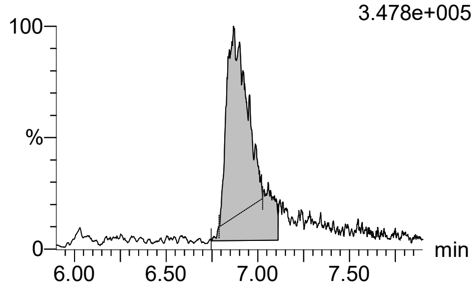

Sample Name: 20211111_A16_R2_IgM
F86:MRM of 1 channel,ES+
1175.2 > 366.1
4.223e+005

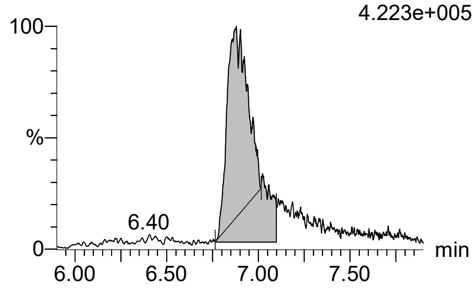

Sample Name: 20211111_L15_R2_IgM
F86:MRM of 1 channel,ES+
1175.2 > 366.1
3.789e+005

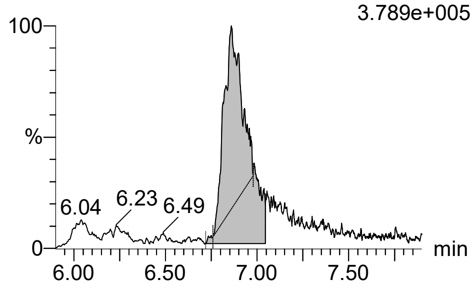

Sample Name: 20211111_L16_R2_IgM
F86:MRM of 1 channel,ES+
1175.2 > 366.1
2.416e+005

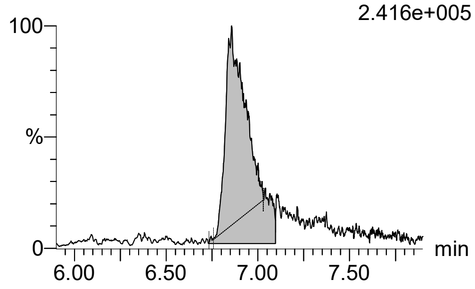

Sample Name: 20211111_H8_R2_IgM
F86:MRM of 1 channel,ES+
1175.2 > 366.1
2.515e+005

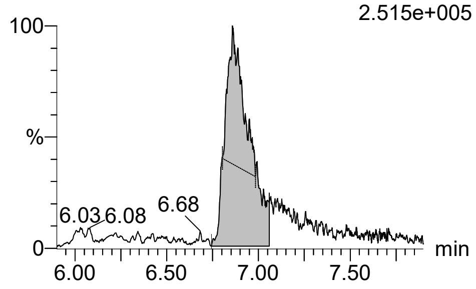

Sample Name: 20211111_A17_R2_IgM
F86:MRM of 1 channel,ES+
1175.2 > 366.1
4.101e+005

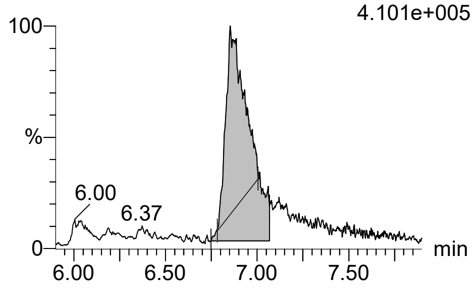

Sample Name: 20211111_A18_R2_IgM
F86:MRM of 1 channel,ES+
1175.2 > 366.1
2.026e+005

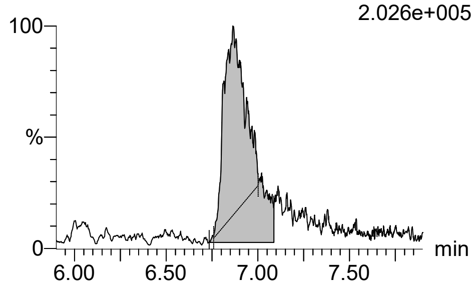

Sample Name: 20211111_L17_R2_IgM
F86:MRM of 1 channel,ES+
1175.2 > 366.1
5.230e+005

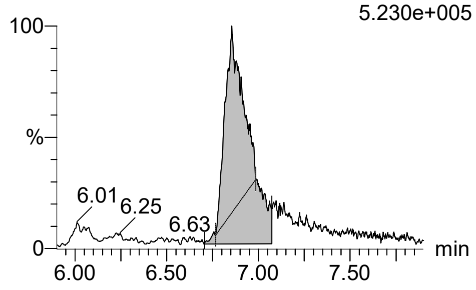

Sample Name: 20211111_L18_R2_IgM
F86:MRM of 1 channel,ES+
1175.2 > 366.1
1.578e+005

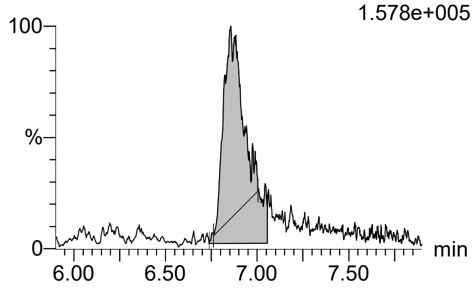

Sample Name: 20211111_H9_R2_IgM
F86:MRM of 1 channel,ES+
1175.2 > 366.1
1.782e+005

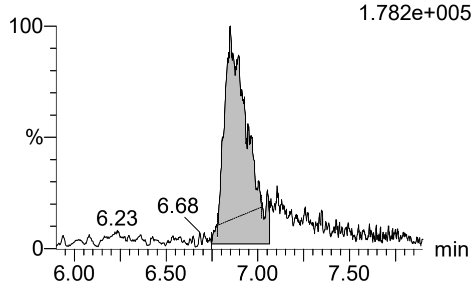

Sample Name: 20211111_A19_R2_IgM
F86:MRM of 1 channel,ES+
1175.2 > 366.1
1.806e+005

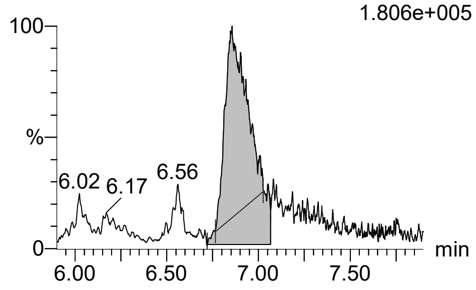

Sample Name: 20211111_A20_R2_IgM
F86:MRM of 1 channel,ES+
1175.2 > 366.1
1.062e+005

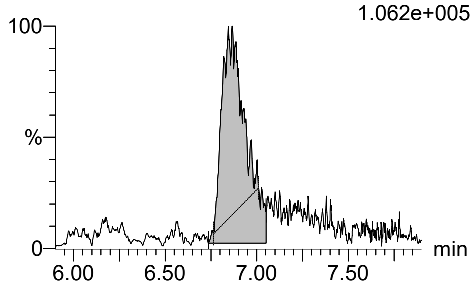

Sample Name: 20211111_L19_R2_IgM
F86:MRM of 1 channel,ES+
1175.2 > 366.1
1.617e+005

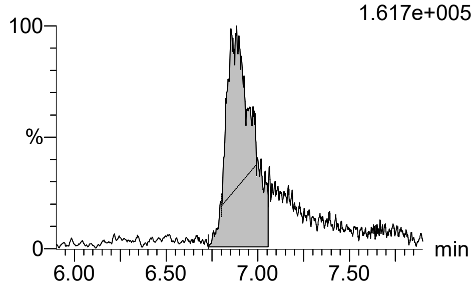

Sample Name: 20211111_QC_R13_IgM
F86:MRM of 1 channel,ES+
1175.2 > 366.1
2.933e+005

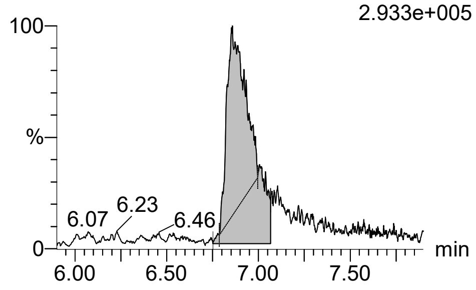

Dataset: C:\MassLynx\10.pro\20211111_clinicalsamples_IgM_IgA.qld

Last Altered: Thursday, March 09, 2023 19:58:25 台北標準時間
Printed: Friday, August 01, 2025 15:18:29 台北標準時間

Sample Name: 20211111_L20_R2_IgM
F86:MRM of 1 channel,ES+
1175.2 > 366.1
3.480e+005

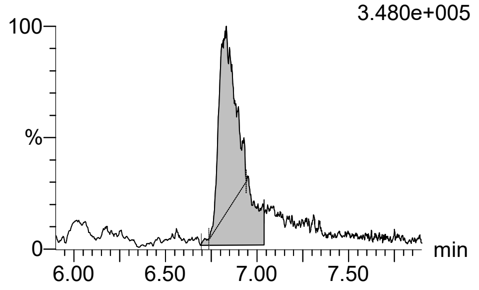

Sample Name: 20211111_H10_R2_IgM
F86:MRM of 1 channel,ES+
1175.2 > 366.1
3.761e+005

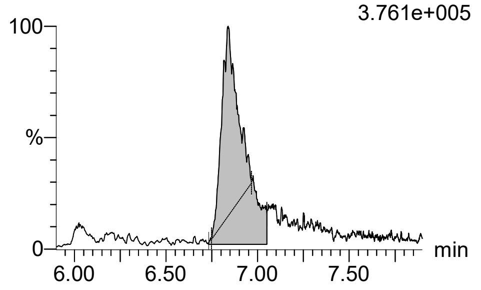

Sample Name: 20211111_A21_R2_IgM
F86:MRM of 1 channel,ES+
1175.2 > 366.1
4.726e+005

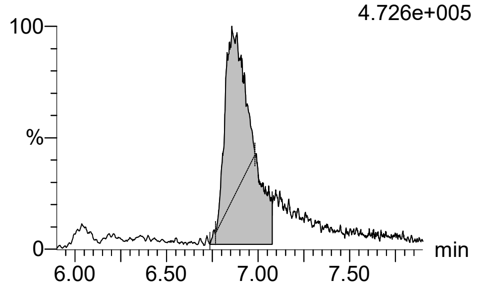

Sample Name: 20211111_A22_R2_IgM
F86:MRM of 1 channel,ES+
1175.2 > 366.1
2.034e+005

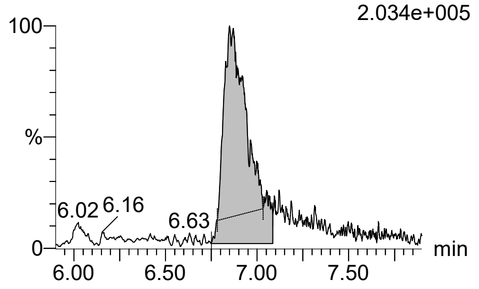

Sample Name: 20211111_L21_R2_IgM
F86:MRM of 1 channel,ES+
1175.2 > 366.1
2.164e+005

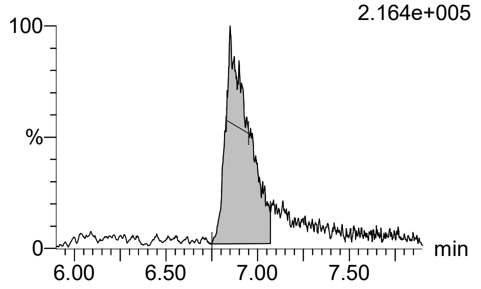

Sample Name: 20211111_L22_R2_IgM
F86:MRM of 1 channel,ES+
1175.2 > 366.1
3.024e+005

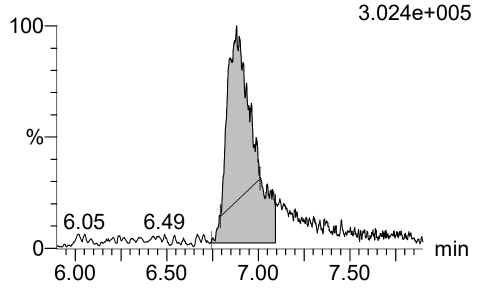

Sample Name: 20211111_H11_R2_IgM
F86:MRM of 1 channel,ES+
1175.2 > 366.1
2.830e+005

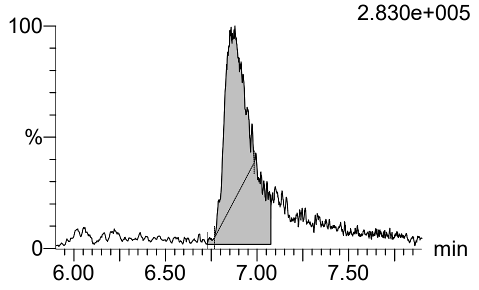

Sample Name: 20211111_A23_R2_IgM
F86:MRM of 1 channel,ES+
1175.2 > 366.1
1.370e+005

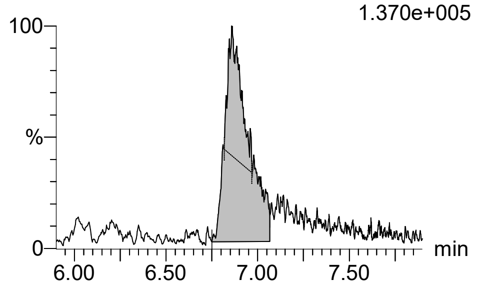

Sample Name: 20211111_A24_R2_IgM
F86:MRM of 1 channel,ES+
1175.2 > 366.1
3.146e+005

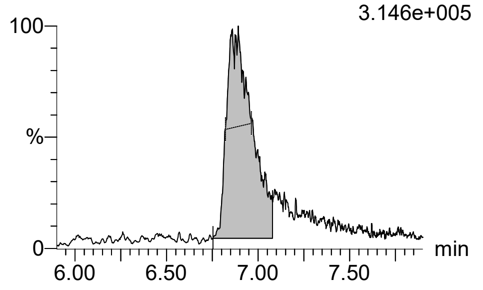

Sample Name: 20211111_L23_R2_IgM
F86:MRM of 1 channel,ES+
1175.2 > 366.1
4.588e+005

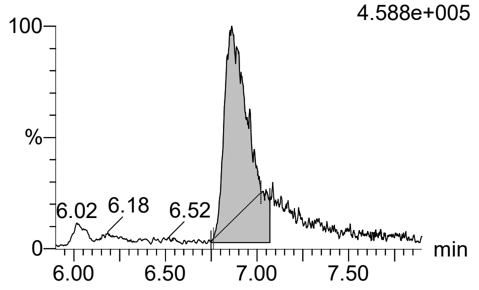

Sample Name: 20211111_L24_R2_IgM
F86:MRM of 1 channel,ES+
1175.2 > 366.1
2.497e+005

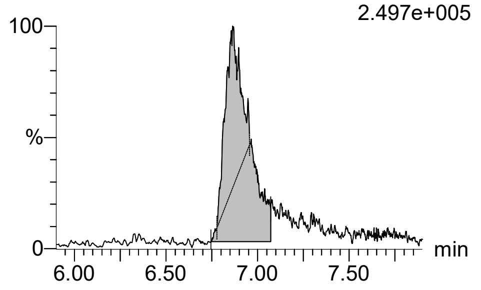

Sample Name: 20211111_H12_R2_IgM
F86:MRM of 1 channel,ES+
1175.2 > 366.1
1.794e+005

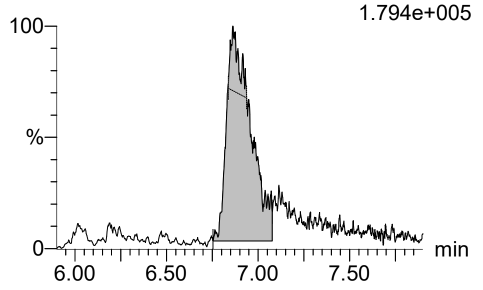

Sample Name: 20211111_QC_R14_IgM
F86:MRM of 1 channel,ES+
1175.2 > 366.1
3.129e+005

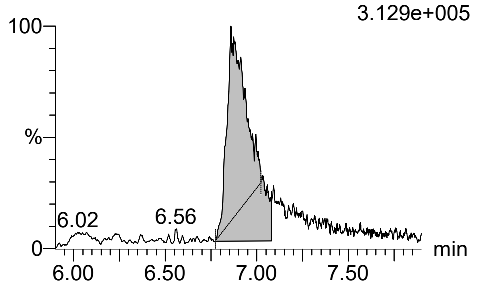

Sample Name: 20211111_A25_R2_IgM
F86:MRM of 1 channel,ES+
1175.2 > 366.1
5.045e+005

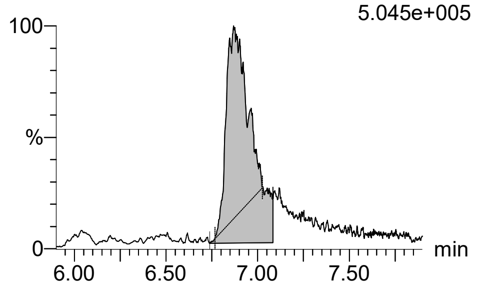

Sample Name: 20211111_A26_R2_IgM
F86:MRM of 1 channel,ES+
1175.2 > 366.1
2.793e+005

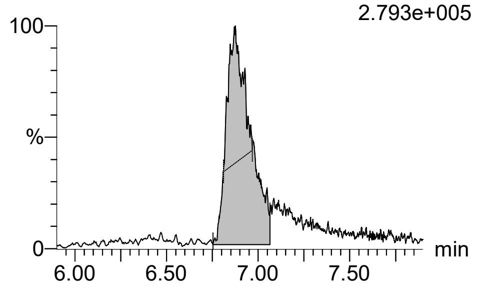

Dataset: C:\MassLynx\10.pro\20211111_clinicalsamples_IgM_IgA.qld

Last Altered: Thursday, March 09, 2023 19:58:25 台北標準時間
Printed: Friday, August 01, 2025 15:18:29 台北標準時間

Sample Name: 20211111_L25_R2_IgM

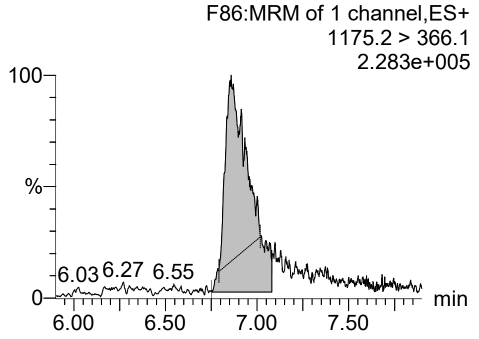

Sample Name: 20211111_L26_R2_IgM

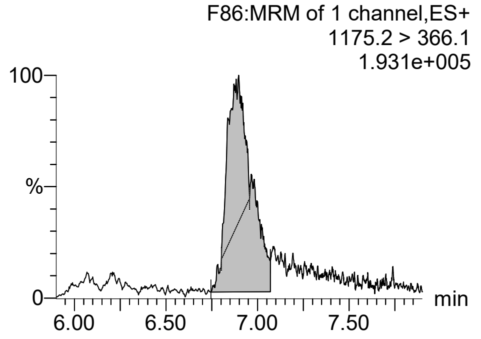

Sample Name: 20211111_H13_R2_IgM

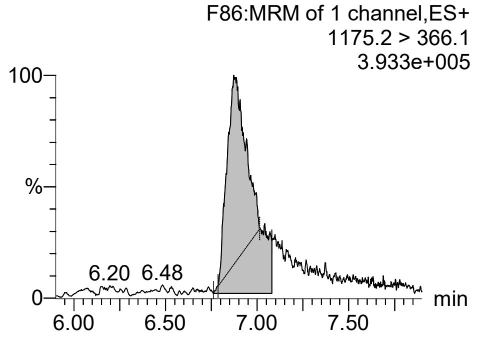

Sample Name: 20211111_A27_R2_IgM

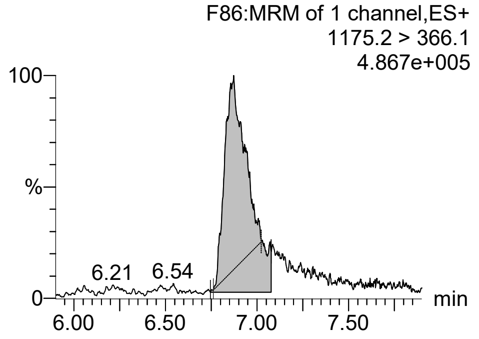

Sample Name: 20211111_A28_R2_IgM

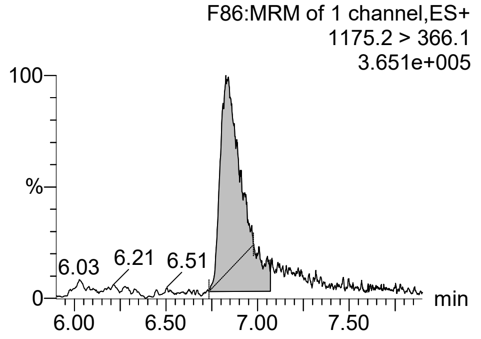

Sample Name: 20211111_L27_R2_IgM

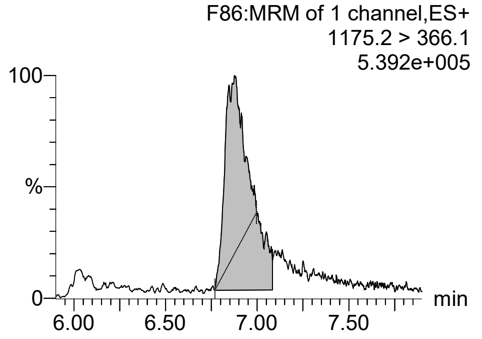

Sample Name: 20211111_L28_R2_IgM

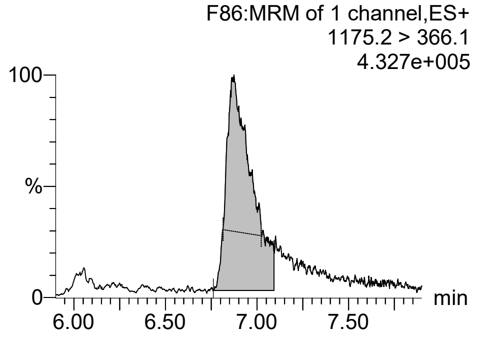

Sample Name: 20211111_H14_R2_IgM

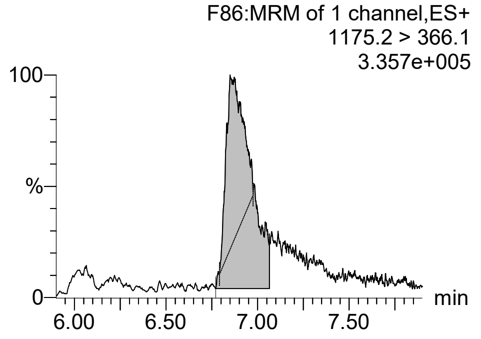

Sample Name: 20211111_A29_R2_IgM

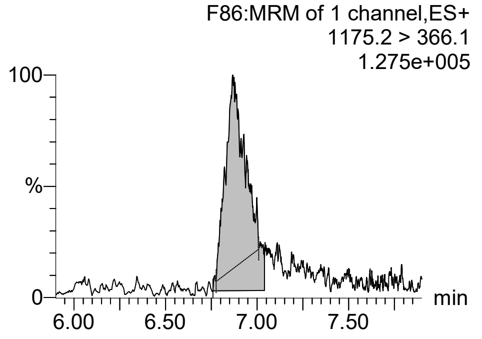

Sample Name: 20211111_A30_R2_IgM

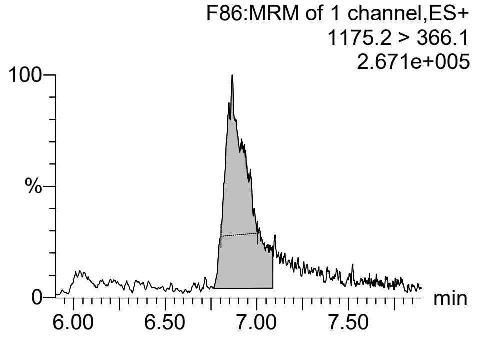

Sample Name: 20211111_QC_R15_IgM

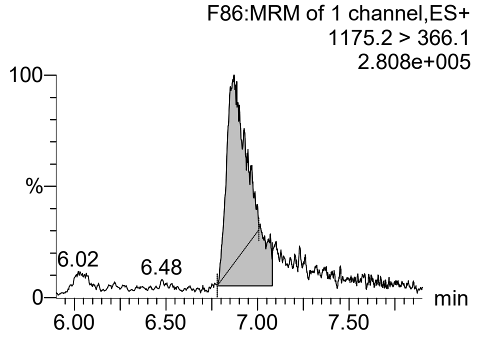

Sample Name: 20211111_L29_R2_IgM

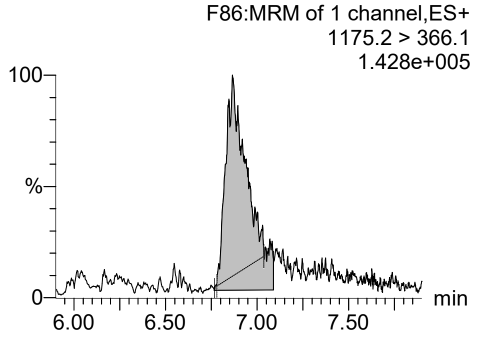

Sample Name: 20211111_L30_R2_IgM

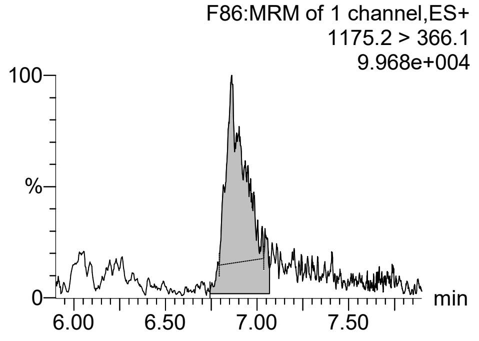

Sample Name: 20211111_H15_R2_IgM

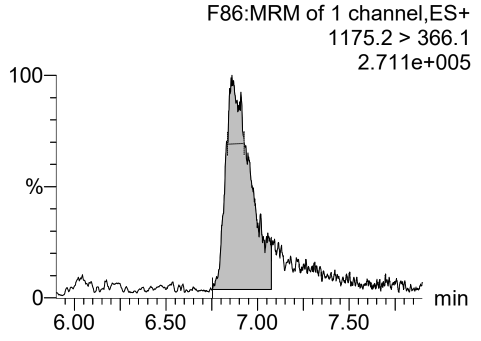

Sample Name: 20211111_A31_R2_IgM

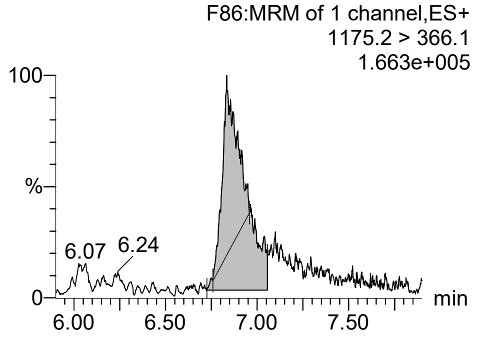

Sample Name: 20211111_A32_R2_IgM

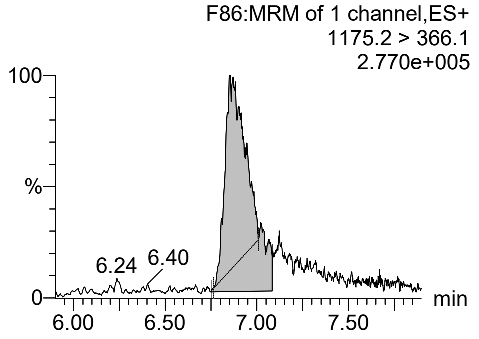

Sample Name: 20211111_L31_R2_IgM

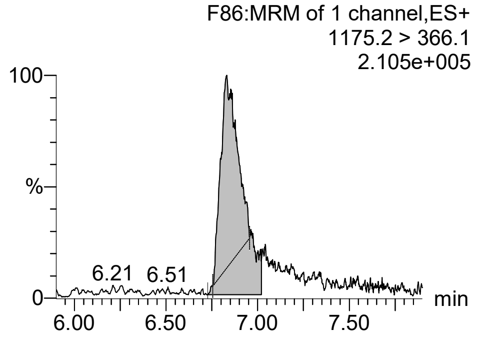

Sample Name: 20211111_L32_R2_IgM

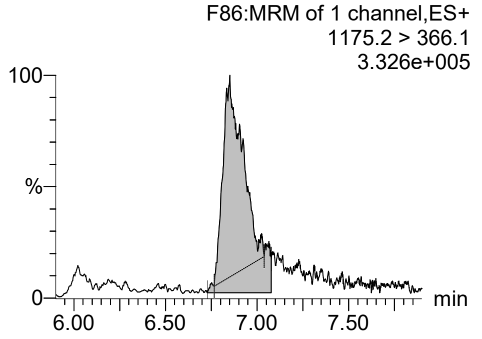

Sample Name: 20211111_H16_R2_IgM

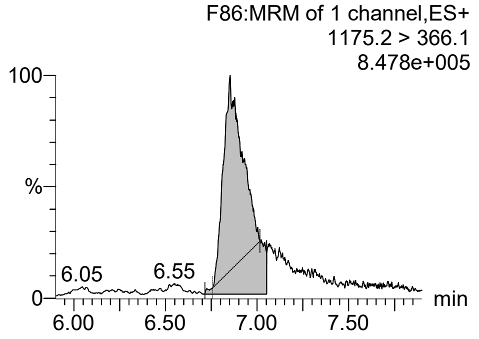

Sample Name: 20211111_A33_R2_IgM

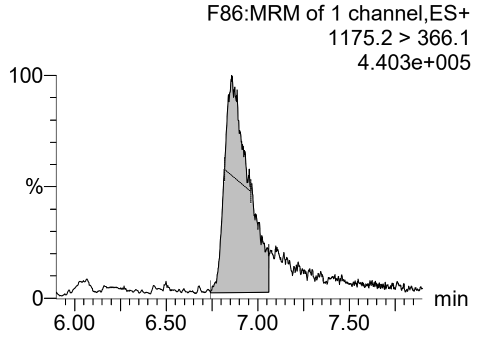

Sample Name: 20211111_A34_R2_IgM

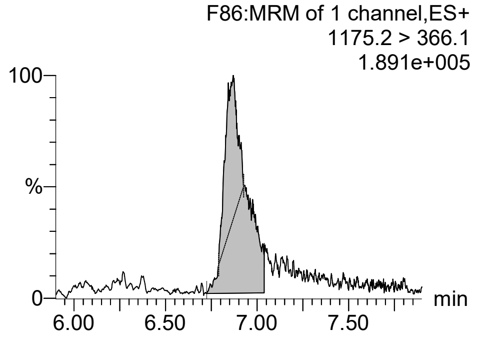

Sample Name: 20211111_H17_R2_IgM

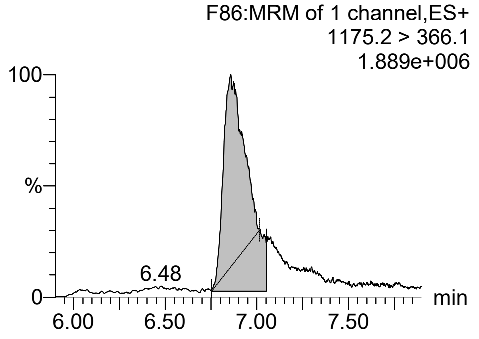

Sample Name: 20211111_A35_R2_IgM

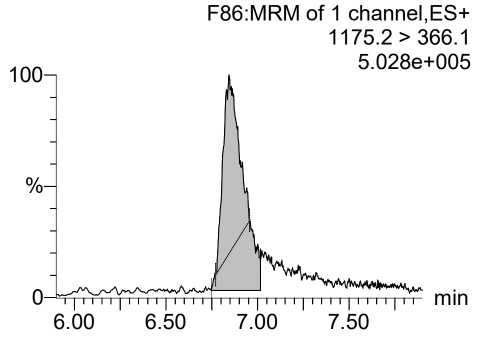

Sample Name: 20211111_QC_R16_IgM

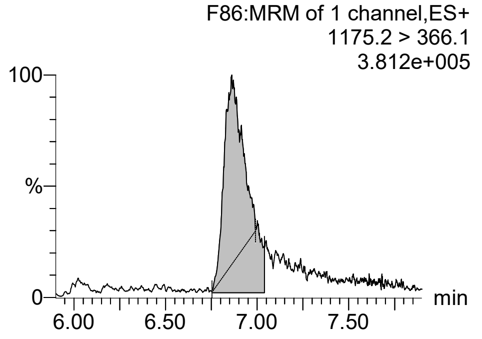

Sample Name: 20211111_A36_R2_IgM

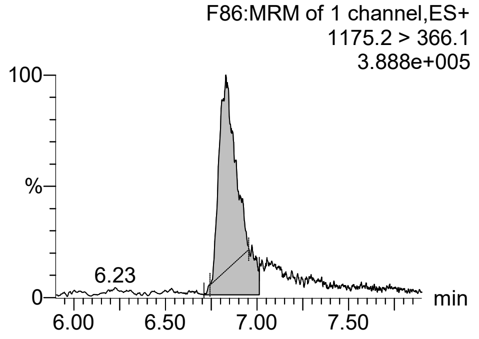

Sample Name: 20211111_H18_R2_IgM

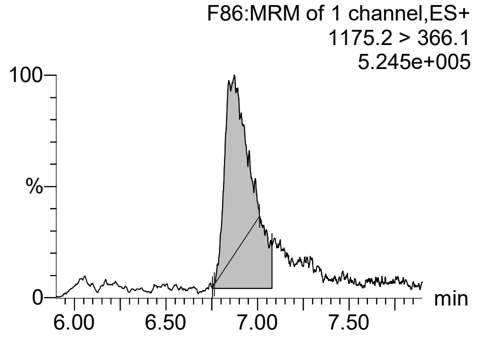

Sample Name: 20211111_A37_R2_IgM

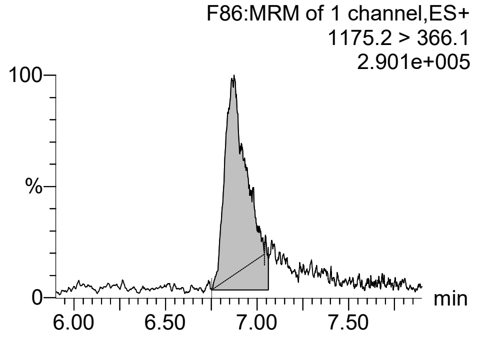

Sample Name: 20211111_A38_R2_IgM

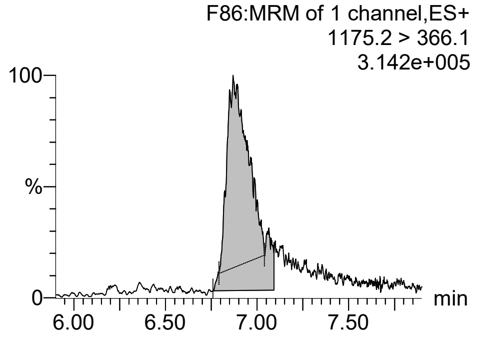

Sample Name: 20211111_H19_R2_IgM

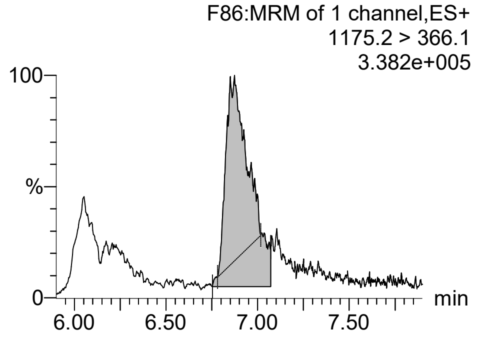

Sample Name: 20211111_A39_R2_IgM

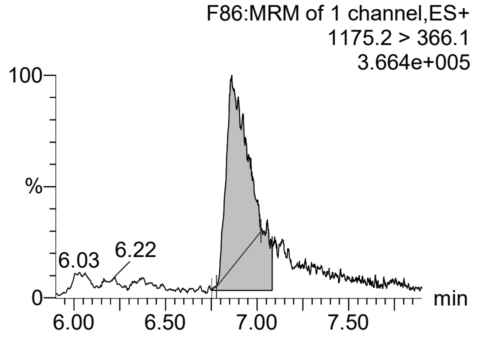

Dataset: C:\MassLynx\10.pro\20211111_clinicalsamples_IgM_IgA.qld

Last Altered: Thursday, March 09, 2023 19:58:25 台北標準時間
Printed: Friday, August 01, 2025 15:18:29 台北標準時間

Sample Name: 20211111_A40_R2_IgM
F86:MRM of 1 channel,ES+
1175.2 > 366.1
1.908e+005

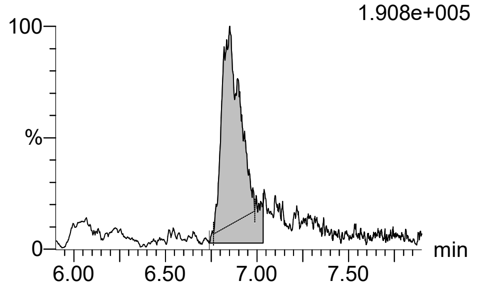

Sample Name: 20211111_H20_R2_IgM
F86:MRM of 1 channel,ES+
1175.2 > 366.1
5.725e+005

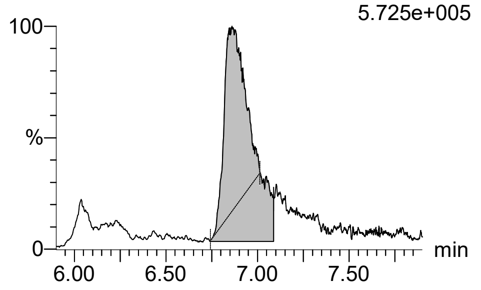

Sample Name: 20211111_A41_R2_IgM
F86:MRM of 1 channel,ES+
1175.2 > 366.1
2.482e+005

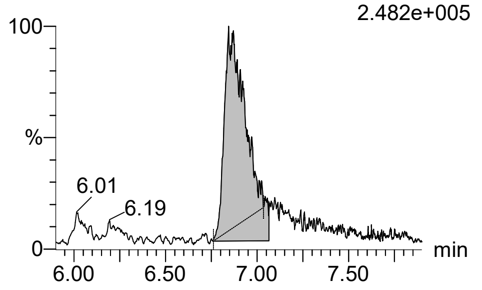

Sample Name: 20211111_A42_R2_IgM
F86:MRM of 1 channel,ES+
1175.2 > 366.1
1.438e+005

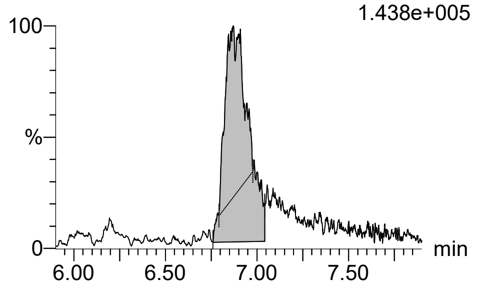

Sample Name: 20211111_A43_R2_IgM
F86:MRM of 1 channel,ES+
1175.2 > 366.1
2.086e+005

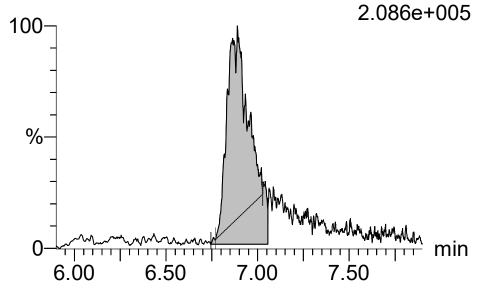

Sample Name: 20211111_A44_R2_IgM
F86:MRM of 1 channel,ES+
1175.2 > 366.1
1.471e+005

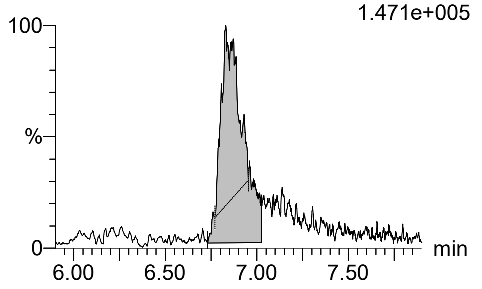

Sample Name: 20211111_QC_R17_IgM
F86:MRM of 1 channel,ES+
1175.2 > 366.1
2.423e+005

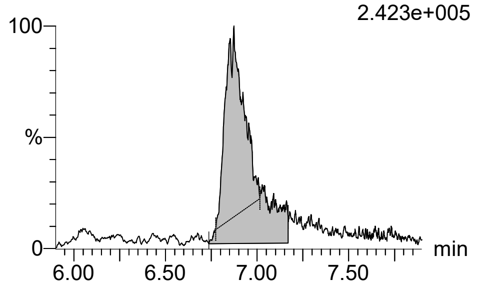

Sample Name: 20211111_L2_R3_IgM
F86:MRM of 1 channel,ES+
1175.2 > 366.1
3.440e+005

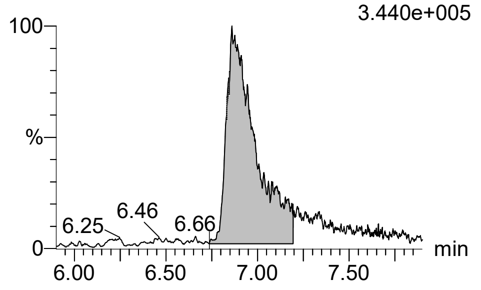

Sample Name: 20211111_H1_R3_IgM
F86:MRM of 1 channel,ES+
1175.2 > 366.1
3.171e+005

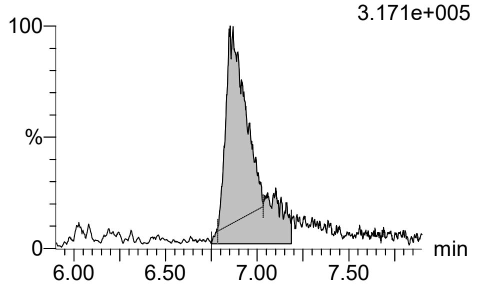

Sample Name: 20211111_A3_R3_IgM
F86:MRM of 1 channel,ES+
1175.2 > 366.1
3.866e+005

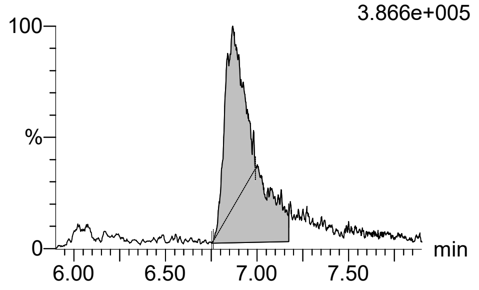

Sample Name: 20211111_A4_R3_IgM
F86:MRM of 1 channel,ES+
1175.2 > 366.1
2.640e+005

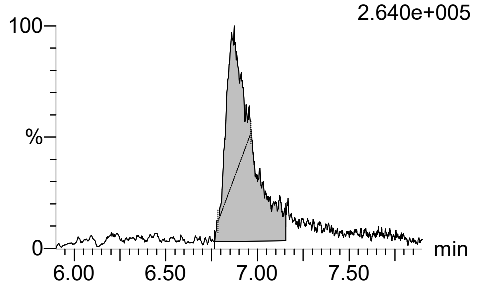

Sample Name: 20211111_L3_R3_IgM
F86:MRM of 1 channel,ES+
1175.2 > 366.1
2.473e+005

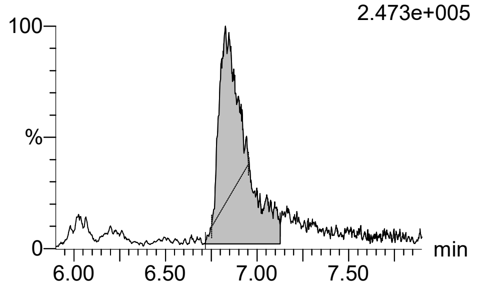

Sample Name: 20211111_L4_R3_IgM
F86:MRM of 1 channel,ES+
1175.2 > 366.1
1.959e+005

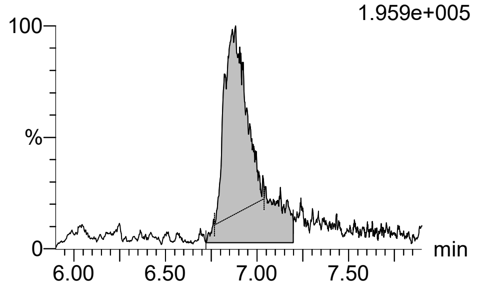

Sample Name: 20211111_H2_R3_IgM
F86:MRM of 1 channel,ES+
1175.2 > 366.1
2.911e+005

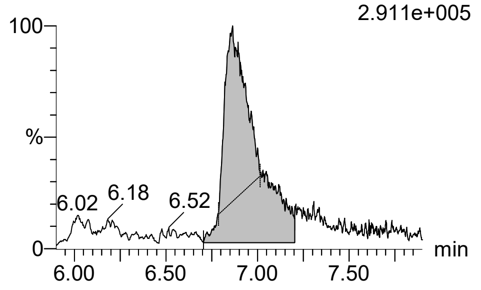

Sample Name: 20211111_A5_R3_IgM
F86:MRM of 1 channel,ES+
1175.2 > 366.1
6.199e+005

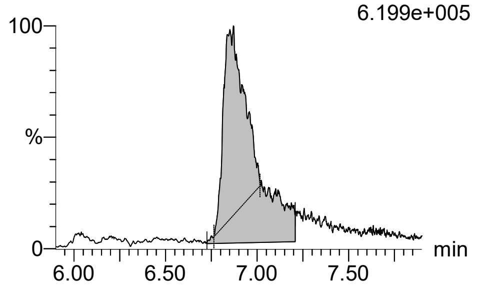

Dataset: C:\MassLynx\10.pro\20211111_clinicalsamples_IgM_IgA.qld

Last Altered: Thursday, March 09, 2023 19:58:25 台北標準時間
Printed: Friday, August 01, 2025 15:18:29 台北標準時間

Sample Name: 20211111_A6_R3_IgM
F86:MRM of 1 channel,ES+
1175.2 > 366.1
4.107e+005

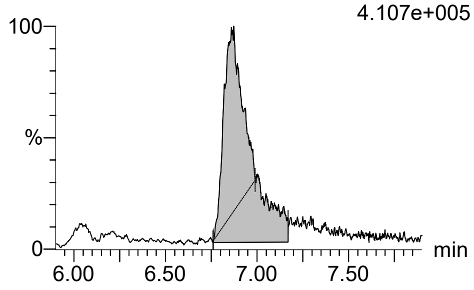

Sample Name: 20211111_QC_R18_IgM
F86:MRM of 1 channel,ES+
1175.2 > 366.1
3.051e+005

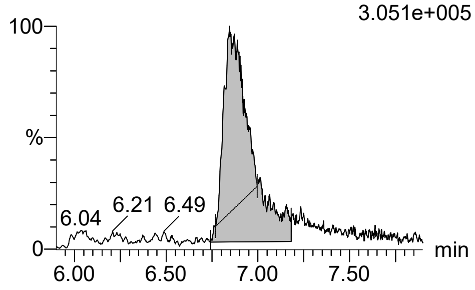

Sample Name: 20211111_L5_R3_IgM
F86:MRM of 1 channel,ES+
1175.2 > 366.1
4.084e+005

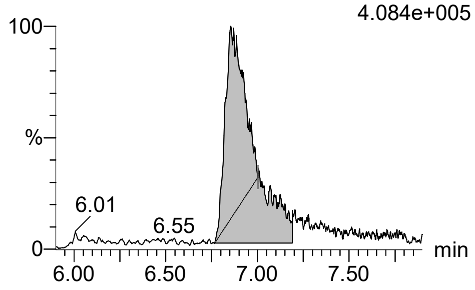

Sample Name: 20211111_L6_R3_IgM
F86:MRM of 1 channel,ES+
1175.2 > 366.1
2.808e+005

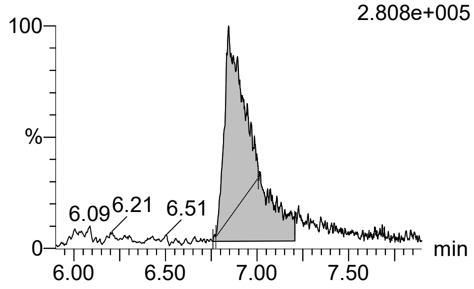

Sample Name: 20211111_H3_R3_IgM
F86:MRM of 1 channel,ES+
1175.2 > 366.1
1.447e+005

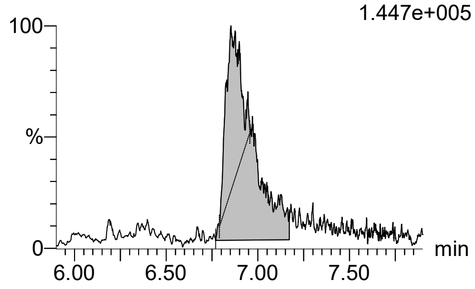

Sample Name: 20211111_A7_R3_IgM
F86:MRM of 1 channel,ES+
1175.2 > 366.1
2.505e+005

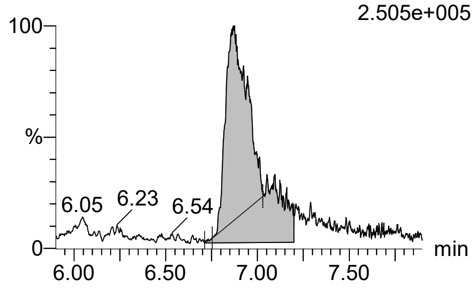

Sample Name: 20211111_A8_R3_IgM
F86:MRM of 1 channel,ES+
1175.2 > 366.1
1.831e+005

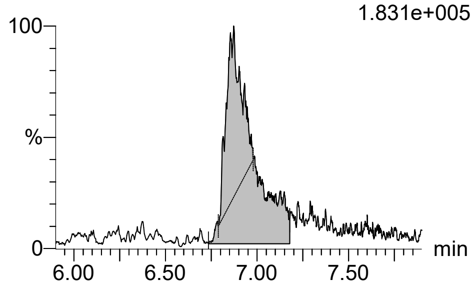

Sample Name: 20211111_L7_R3_IgM
F86:MRM of 1 channel,ES+
1175.2 > 366.1
2.233e+005

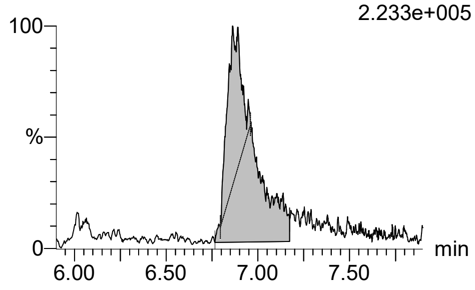

Sample Name: 20211111_L8_R3_IgM
F86:MRM of 1 channel,ES+
1175.2 > 366.1
1.357e+005

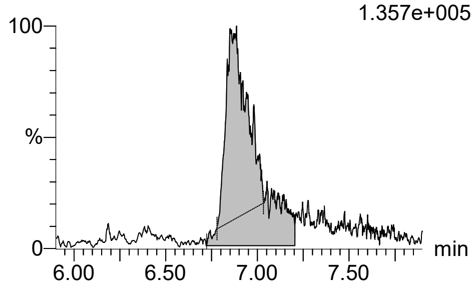

Sample Name: 20211111_H4_R3_IgM
F86:MRM of 1 channel,ES+
1175.2 > 366.1
1.517e+005

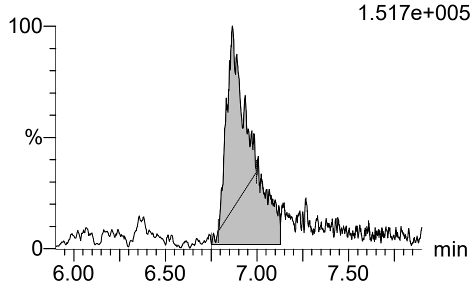

Sample Name: 20211111_A9_R3_IgM
F86:MRM of 1 channel,ES+
1175.2 > 366.1
2.294e+005

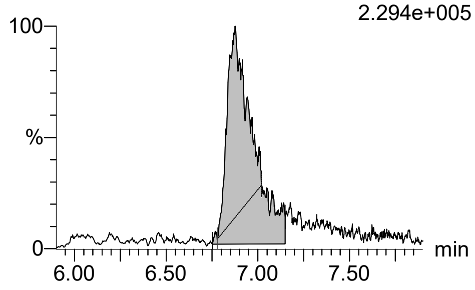

Sample Name: 20211111_A10_R3_IgM
F86:MRM of 1 channel,ES+
1175.2 > 366.1
2.705e+005

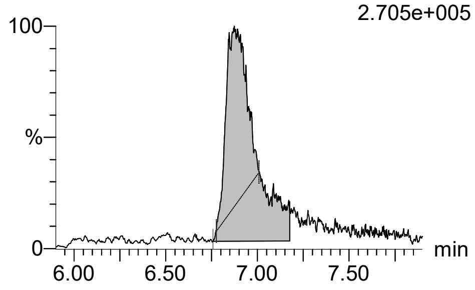

Sample Name: 20211111_L9_R3_IgM
F86:MRM of 1 channel,ES+
1175.2 > 366.1
2.298e+005

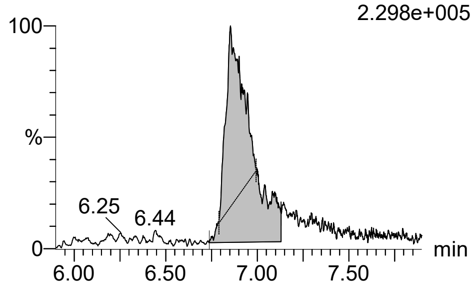

Sample Name: 20211111_L10_R3_IgM
F86:MRM of 1 channel,ES+
1175.2 > 366.1
5.078e+005

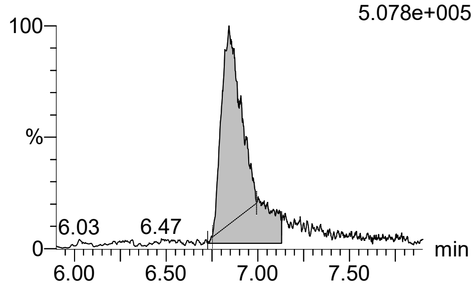

Sample Name: 20211111_QC_R19_IgM
F86:MRM of 1 channel,ES+
1175.2 > 366.1
3.005e+005

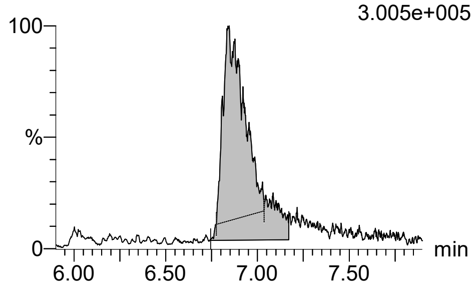

Dataset: C:\MassLynx\10.pro\20211111_clinicalsamples_IgM_IgA.qld

Last Altered: Thursday, March 09, 2023 19:58:25 台北標準時間
Printed: Friday, August 01, 2025 15:18:29 台北標準時間

Sample Name: 20211111_H5_R3_IgM

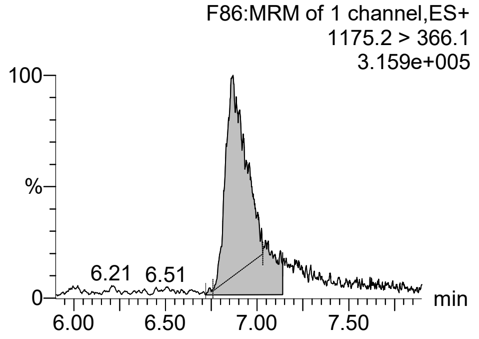

Sample Name: 20211111_A11_R3_IgM

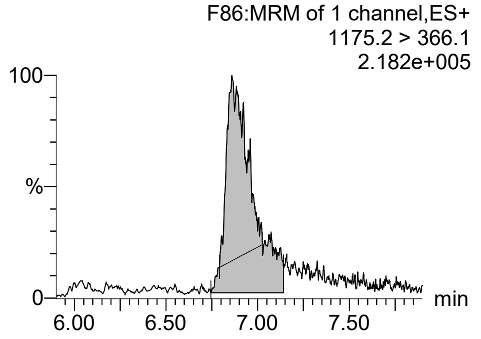

Sample Name: 20211111_A12_R3_IgM

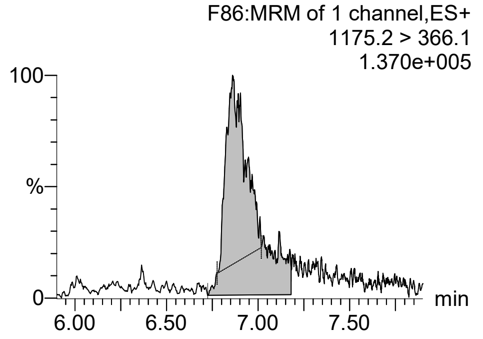

Sample Name: 20211111_L11_R3_IgM

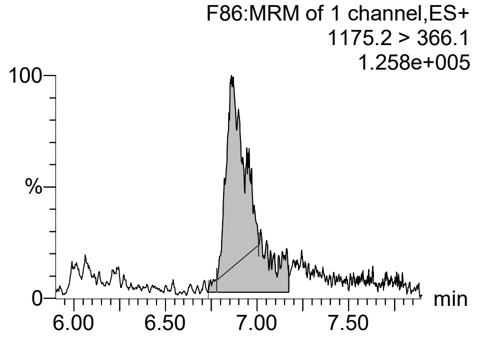

Sample Name: 20211111_L12_R3_IgM

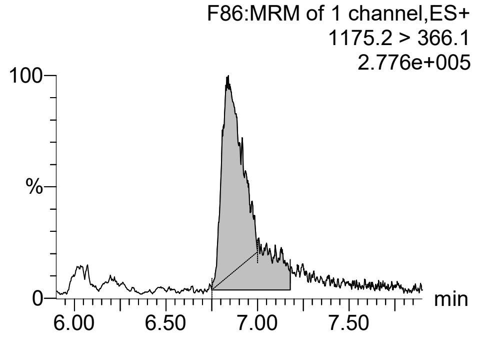

Sample Name: 20211111_H6_R3_IgM

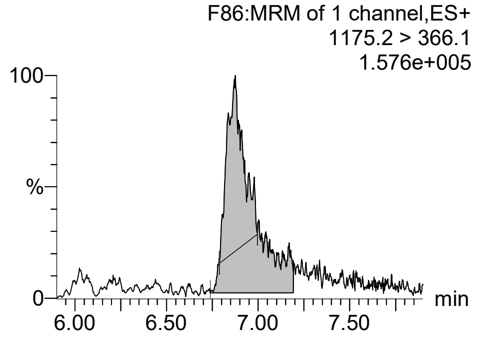

Sample Name: 20211111_A13_R3_IgM

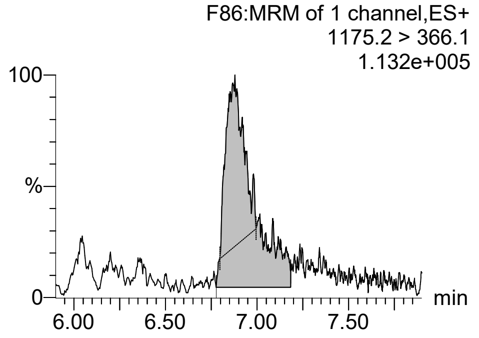

Sample Name: 20211111_A14_R3_IgM

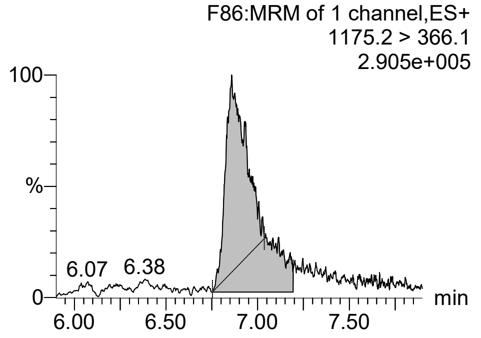

Sample Name: 20211111_L13_R3_IgM

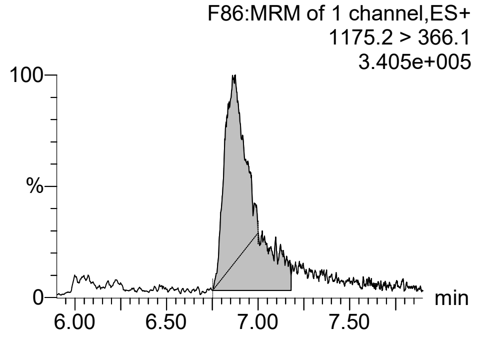

Sample Name: 20211111_L14_R3_IgM

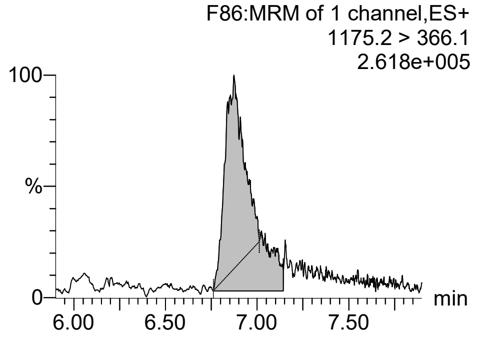

Sample Name: 20211111_H7_R3_IgM

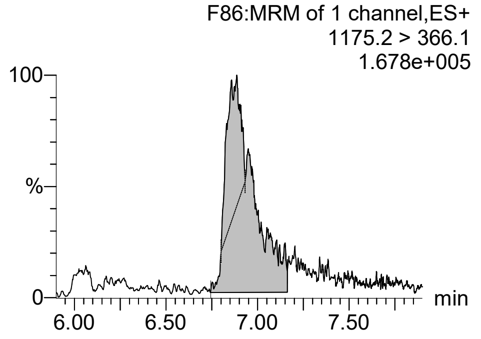

Sample Name: 20211111_A15_R3_IgM

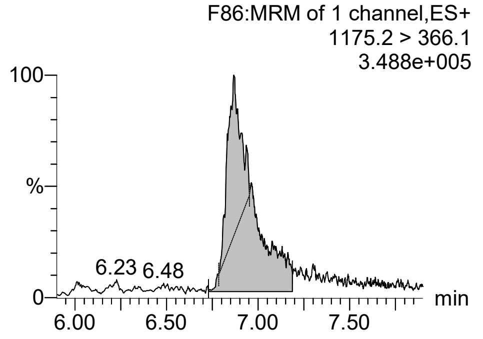

Sample Name: 20211111_QC_R20_IgM

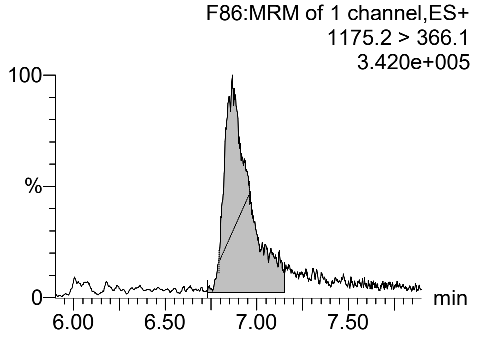

Sample Name: 20211111_A16_R3_IgM

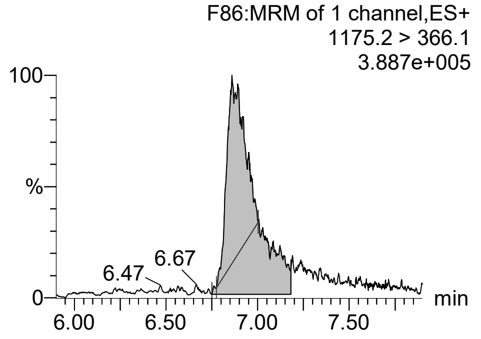

Sample Name: 20211111_L15_R3_IgM

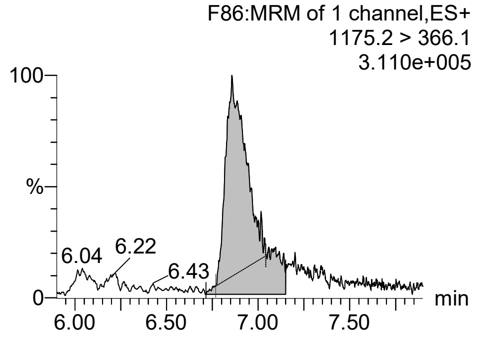

Dataset: C:\MassLynx\10.pro\20211111_clinicalsamples_IgM_IgA.qld

Last Altered: Thursday, March 09, 2023 19:58:25 台北標準時間
Printed: Friday, August 01, 2025 15:18:29 台北標準時間

Sample Name: 20211111_L16_R3_IgM
F86:MRM of 1 channel,ES+
1175.2 > 366.1
2.128e+005

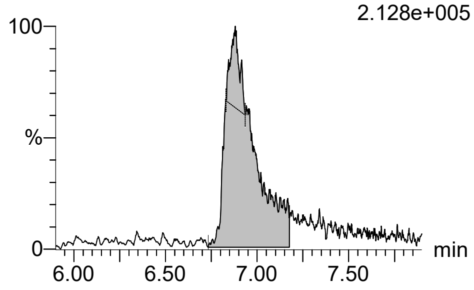

Sample Name: 20211111_H8_R3_IgM
F86:MRM of 1 channel,ES+
1175.2 > 366.1
1.839e+005

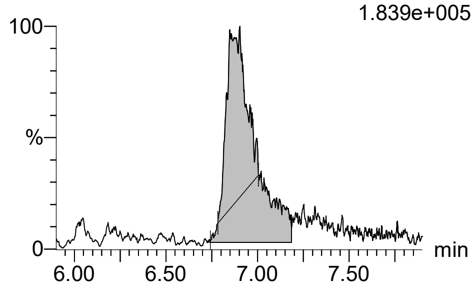

Sample Name: 20211111_A17_R3_IgM
F86:MRM of 1 channel,ES+
1175.2 > 366.1
3.694e+005

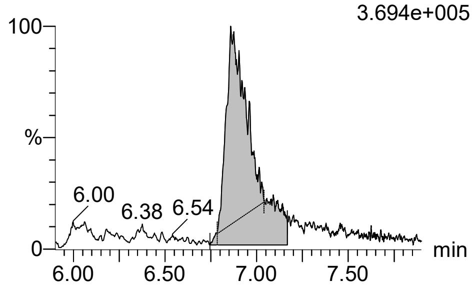

Sample Name: 20211111_A18_R3_IgM
F86:MRM of 1 channel,ES+
1175.2 > 366.1
1.970e+005

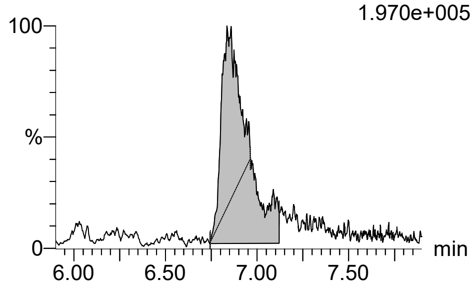

Sample Name: 20211111_L17_R3_IgM
F86:MRM of 1 channel,ES+
1175.2 > 366.1
4.103e+005

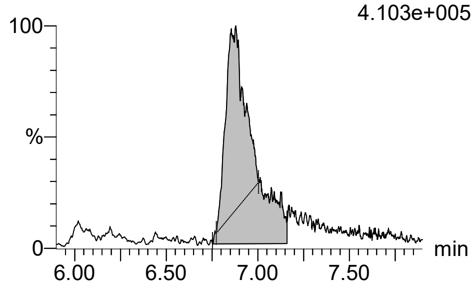

Sample Name: 20211111_L18_R3_IgM
F86:MRM of 1 channel,ES+
1175.2 > 366.1
1.349e+005

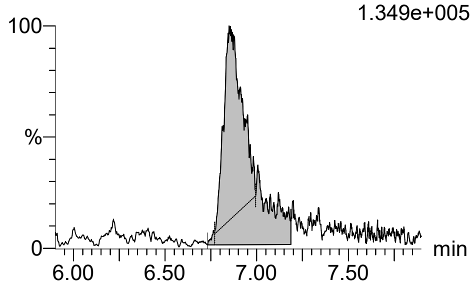

Sample Name: 20211111_H9_R3_IgM
F86:MRM of 1 channel,ES+
1175.2 > 366.1
1.547e+005

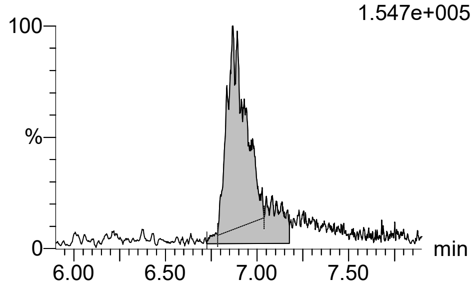

Sample Name: 20211111_A19_R3_IgM
F86:MRM of 1 channel,ES+
1175.2 > 366.1
1.831e+005

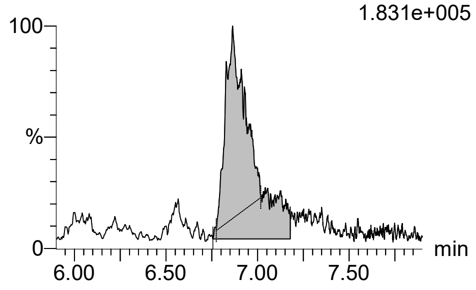

Sample Name: 20211111_A20_R3_IgM
F86:MRM of 1 channel,ES+
1175.2 > 366.1
1.017e+005

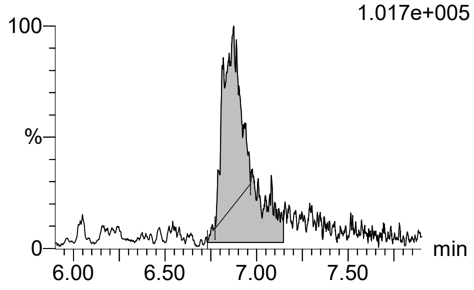

Sample Name: 20211111_L19_R3_IgM
F86:MRM of 1 channel,ES+
1175.2 > 366.1
1.982e+005

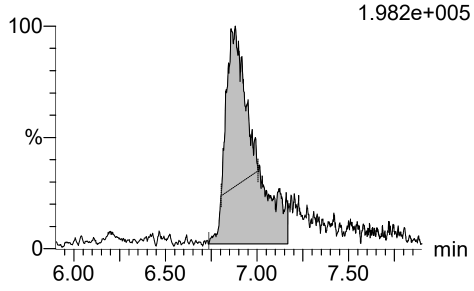

Sample Name: 20211111_QC_R21_IgM
F86:MRM of 1 channel,ES+
1175.2 > 366.1
2.933e+005

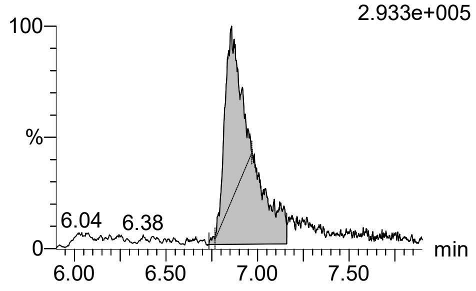

Sample Name: 20211111_L20_R3_IgM
F86:MRM of 1 channel,ES+
1175.2 > 366.1
2.280e+005

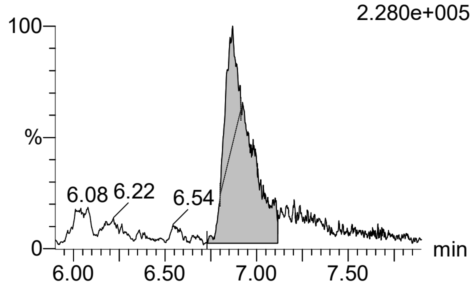

Sample Name: 20211111_H10_R3_IgM
F86:MRM of 1 channel,ES+
1175.2 > 366.1
2.486e+005

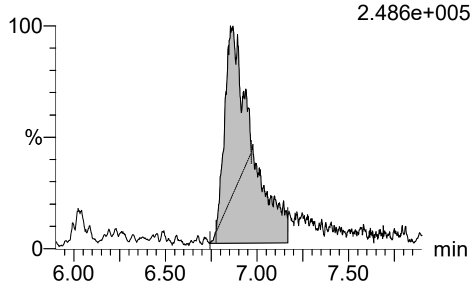

Sample Name: 20211111_A21_R3_IgM
F86:MRM of 1 channel,ES+
1175.2 > 366.1
4.685e+005

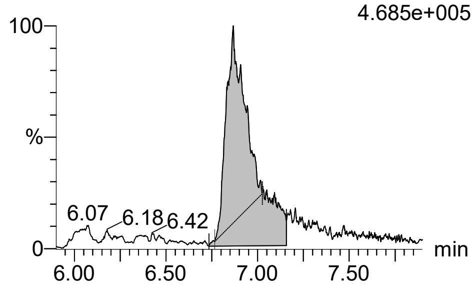

Sample Name: 20211111_A22_R3_IgM
F86:MRM of 1 channel,ES+
1175.2 > 366.1
1.970e+005

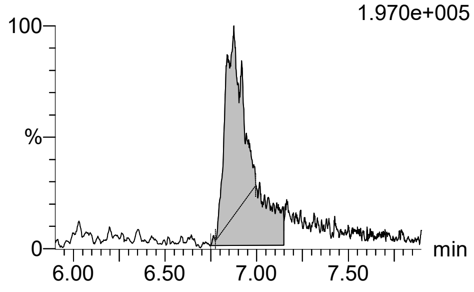

Sample Name: 20211111_L21_R3_IgM

F86:MRM of 1 channel,ES+
1175.2 > 366.1
1.693e+005

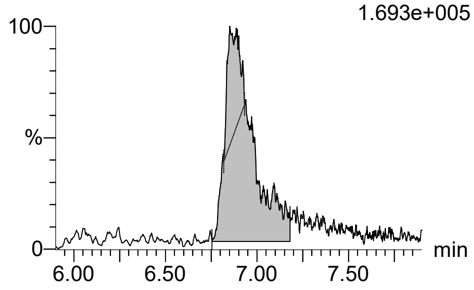

Sample Name: 20211111_L22_R3_IgM

F86:MRM of 1 channel,ES+
1175.2 > 366.1
2.721e+005

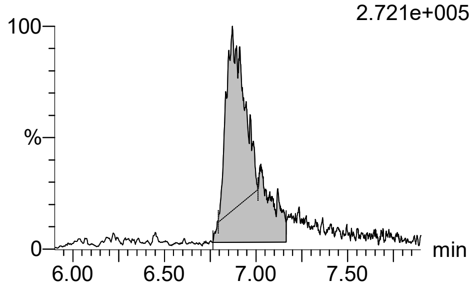

Sample Name: 20211111_H11_R3_IgM

F86:MRM of 1 channel,ES+
1175.2 > 366.1
2.415e+005

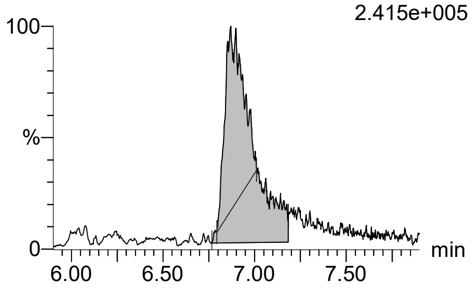

Sample Name: 20211111_A23_R3_IgM

F86:MRM of 1 channel,ES+
1175.2 > 366.1
1.272e+005

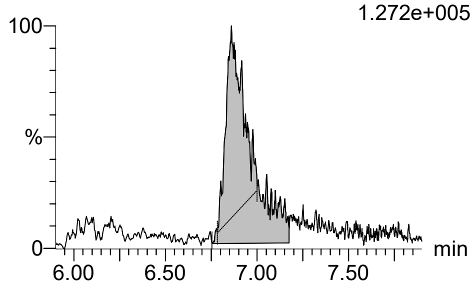

Sample Name: 20211111_A24_R3_IgM

F86:MRM of 1 channel,ES+
1175.2 > 366.1
3.188e+005

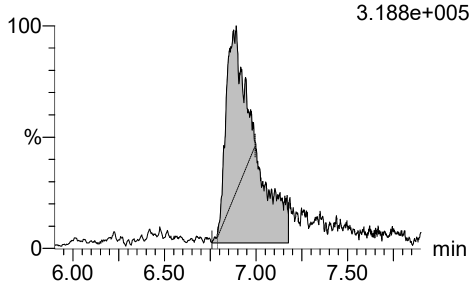

Sample Name: 20211111_L23_R3_IgM

F86:MRM of 1 channel,ES+
1175.2 > 366.1
4.414e+005

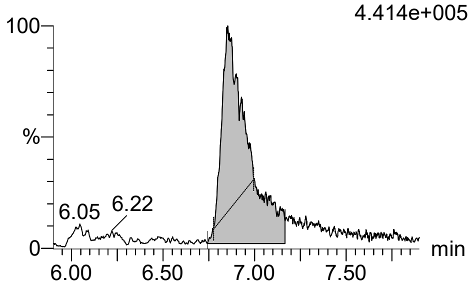

Sample Name: 20211111_L24_R3_IgM

F86:MRM of 1 channel,ES+
1175.2 > 366.1
2.092e+005

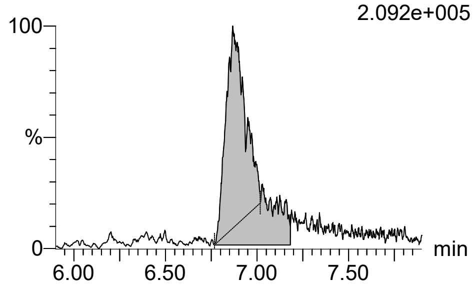

Sample Name: 20211111_H12_R3_IgM

F86:MRM of 1 channel,ES+
1175.2 > 366.1
1.569e+005

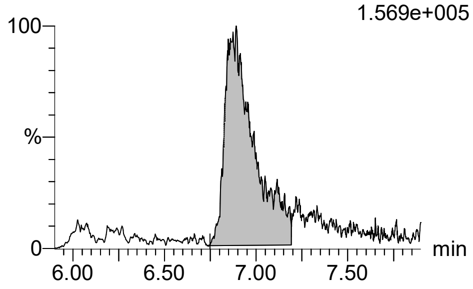

Sample Name: 20211111_QC_R22_IgM

F86:MRM of 1 channel,ES+
1175.2 > 366.1
2.963e+005

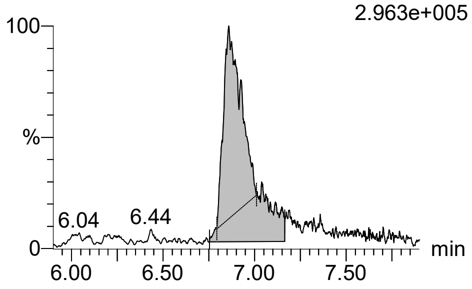

Sample Name: 20211111_A25_R3_IgM

F86:MRM of 1 channel,ES+
1175.2 > 366.1
4.005e+005

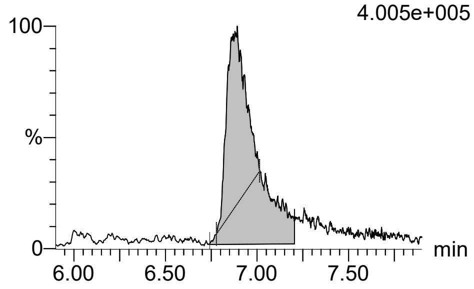

Sample Name: 20211111_A26_R3_IgM

F86:MRM of 1 channel,ES+
1175.2 > 366.1
2.306e+005

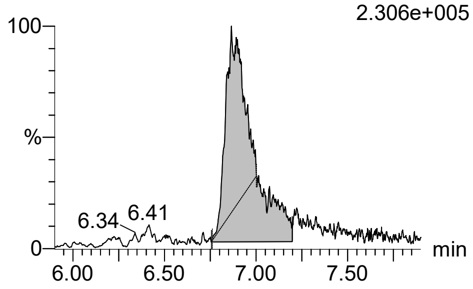

Sample Name: 20211111_L25_R3_IgM

F86:MRM of 1 channel,ES+
1175.2 > 366.1
2.071e+005

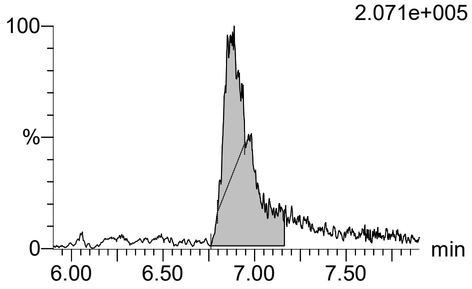

Sample Name: 20211111_L26_R3_IgM

F86:MRM of 1 channel,ES+
1175.2 > 366.1
1.771e+005

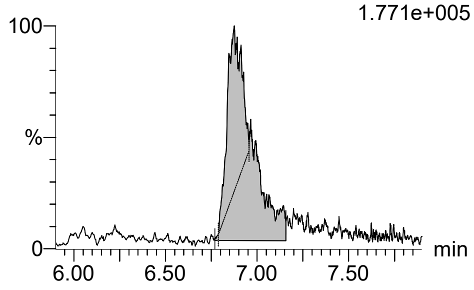

Sample Name: 20211111_H13_R3_IgM

F86:MRM of 1 channel,ES+
1175.2 > 366.1
3.783e+005

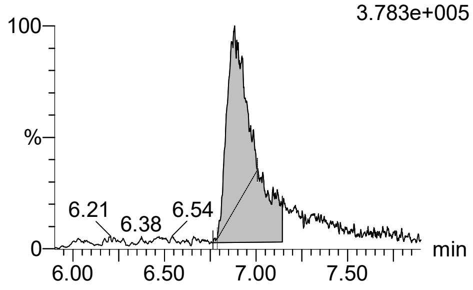

Sample Name: 20211111_A27_R3_IgM

F86:MRM of 1 channel,ES+
1175.2 > 366.1
4.321e+005

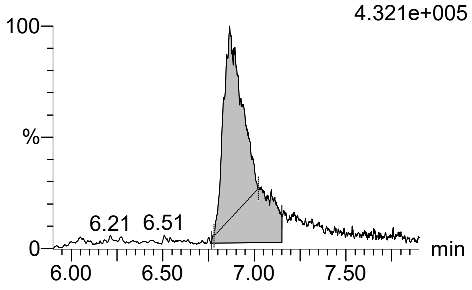

Dataset: C:\MassLynx\10.pro\20211111_clinicalsamples_IgM_IgA.qld

Last Altered: Thursday, March 09, 2023 19:58:25 台北標準時間
Printed: Friday, August 01, 2025 15:18:29 台北標準時間

Sample Name: 20211111_A28_R3_IgM

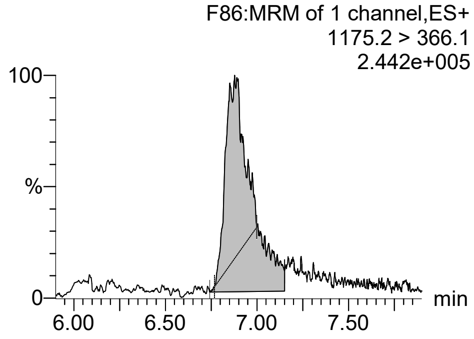

Sample Name: 20211111_L27_R3_IgM

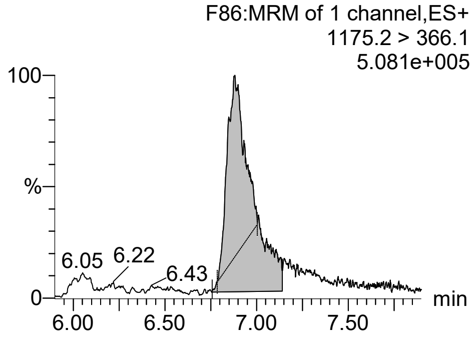

Sample Name: 20211111_L28_R3_IgM

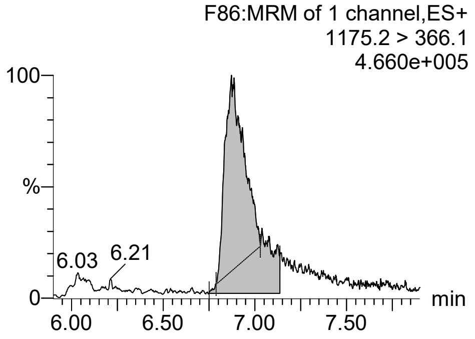

Sample Name: 20211111_H14_R3_IgM

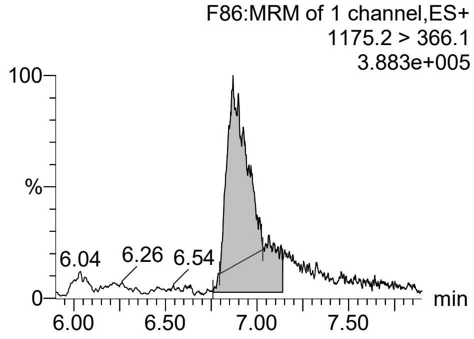

Sample Name: 20211111_A29_R3_IgM

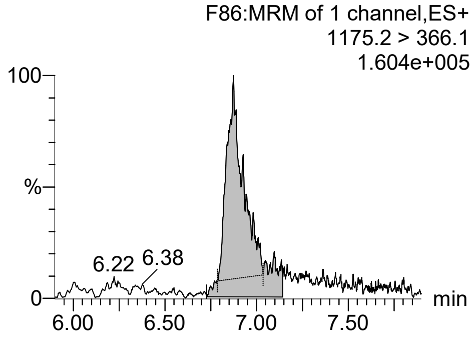

Sample Name: 20211111_A30_R3_IgM

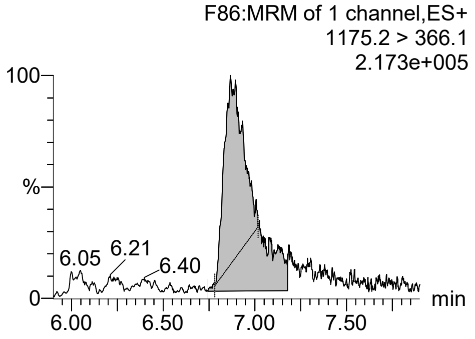

Sample Name: 20211111_QC_R23_IgM

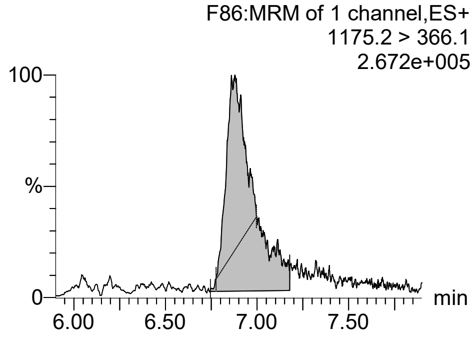

Sample Name: 20211111_L29_R3_IgM

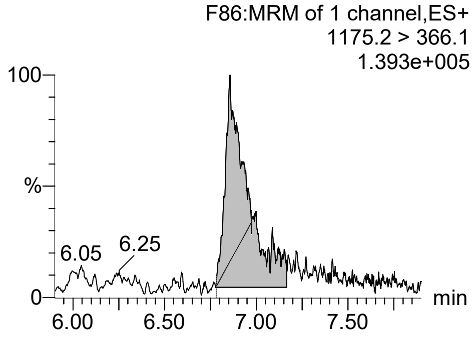

Sample Name: 20211111_L30_R3_IgM

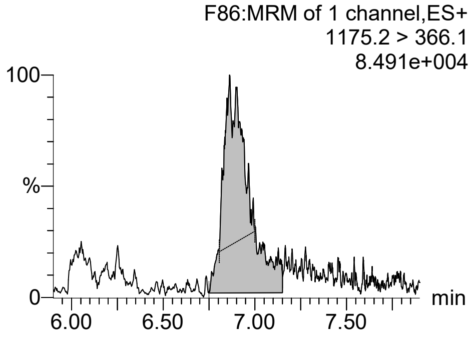

Sample Name: 20211111_H15_R3_IgM

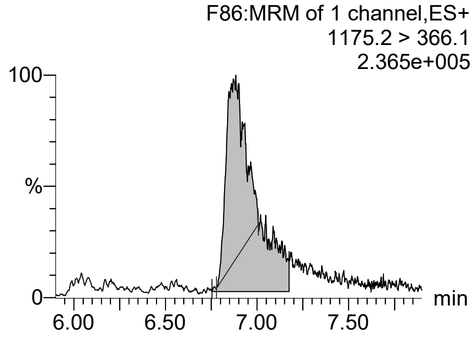

Sample Name: 20211111_A31_R3_IgM

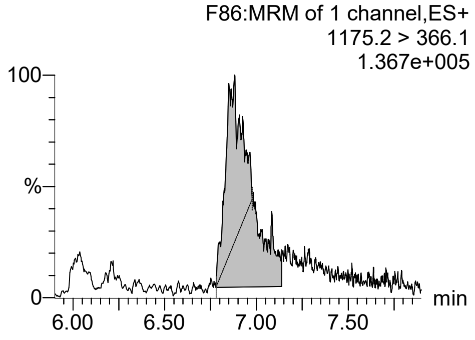

Sample Name: 20211111_A32_R3_IgM

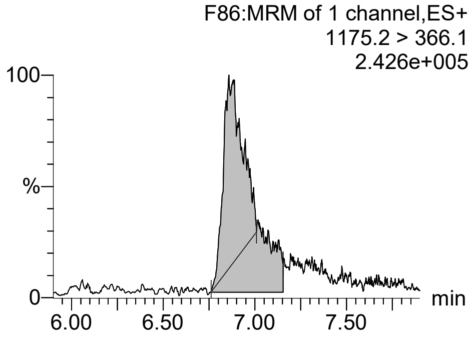

Sample Name: 20211111_L31_R3_IgM

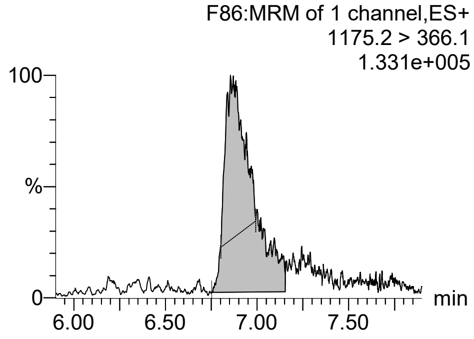

Sample Name: 20211111_L32_R3_IgM

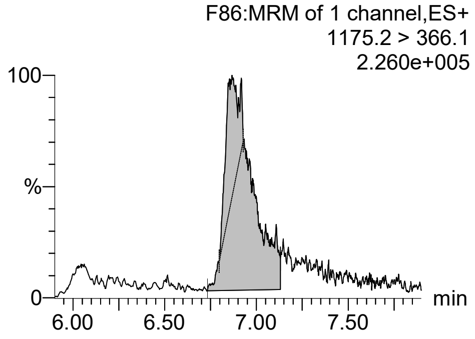

Sample Name: 20211111_H16_R3_IgM

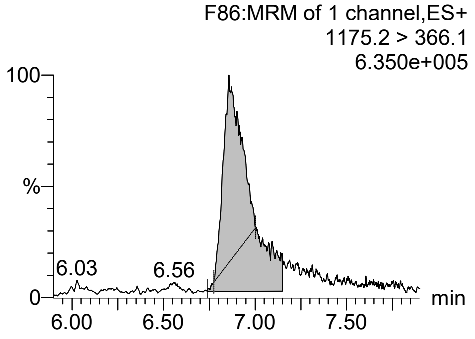

Sample Name: 20211111_A33_R3_IgM

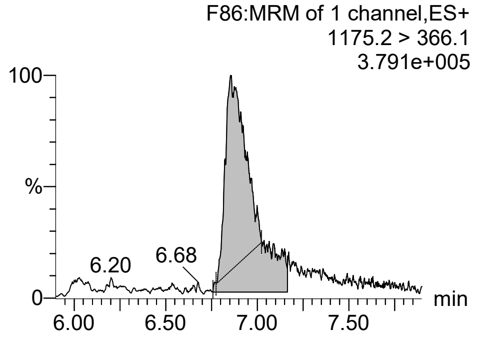

Sample Name: 20211111_A34_R3_IgM

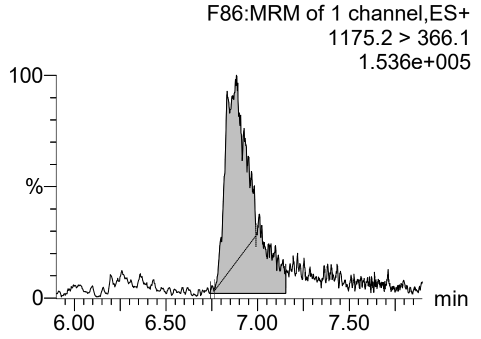

Sample Name: 20211111_H17_R3_IgM

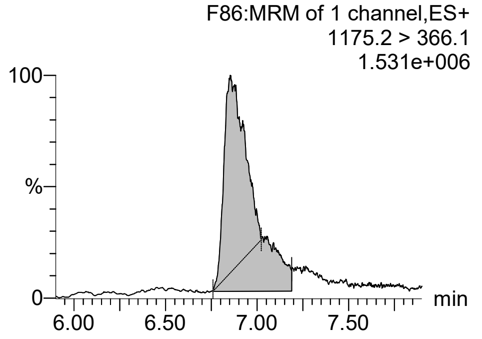

Sample Name: 20211111_A35_R3_IgM

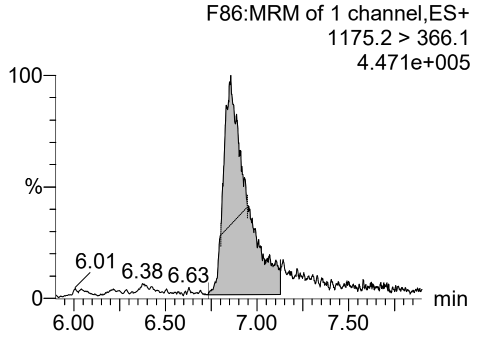

Sample Name: 20211111_QC_R24_IgM

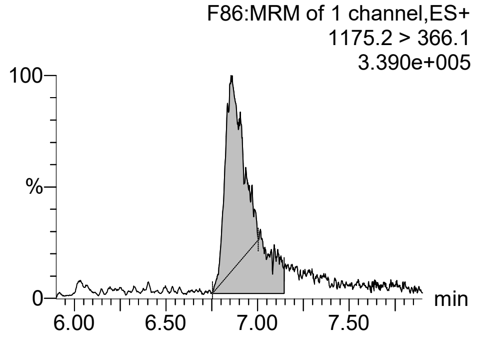

Sample Name: 20211111_A36_R3_IgM

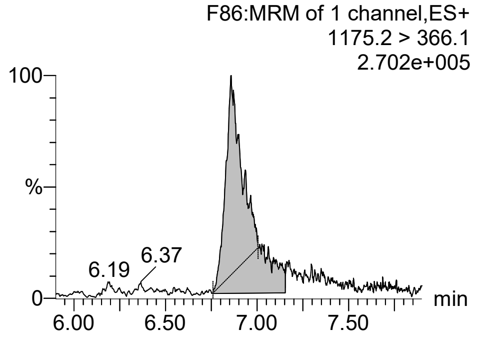

Sample Name: 20211111_H18_R3_IgM

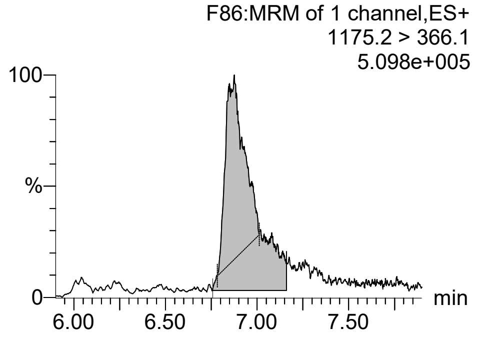

Sample Name: 20211111_A37_R3_IgM

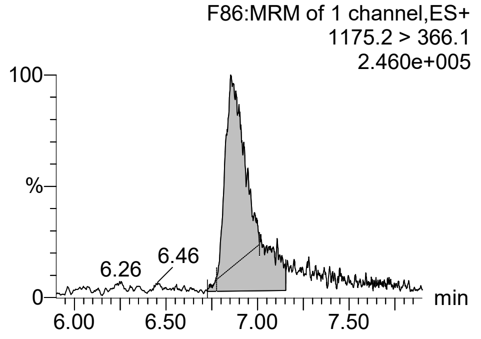

Sample Name: 20211111_A38_R3_IgM

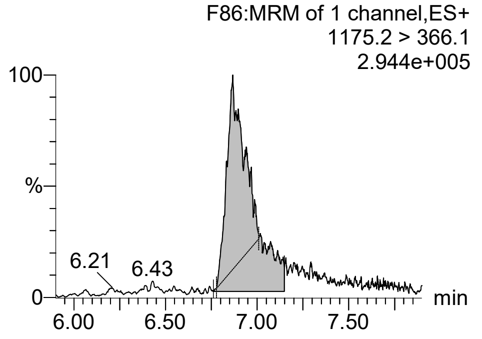

Sample Name: 20211111_H19_R3_IgM

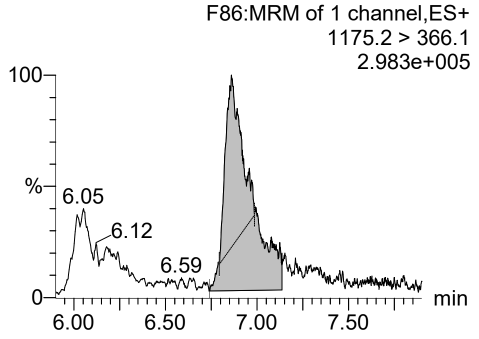

Sample Name: 20211111_A39_R3_IgM

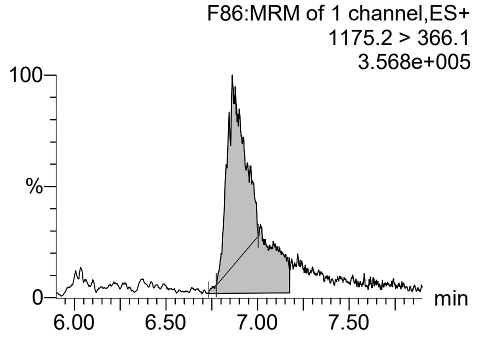

Sample Name: 20211111_A40_R3_IgM

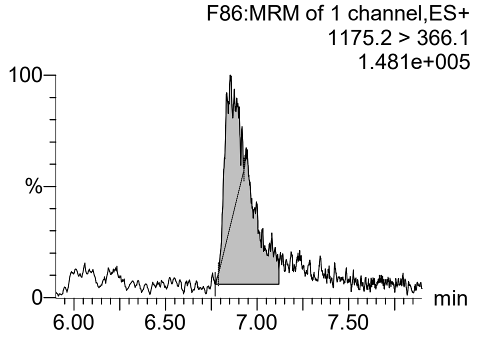

Sample Name: 20211111_H20_R3_IgM

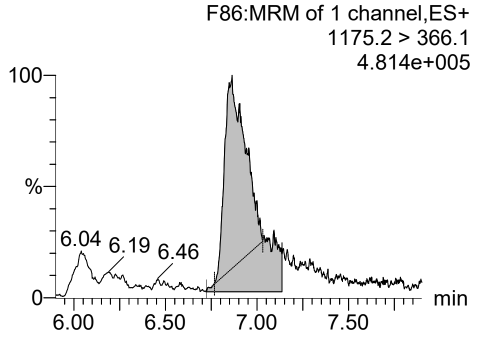

Sample Name: 20211111_A41_R3_IgM

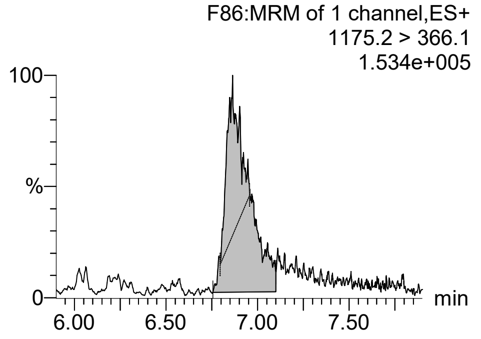

Sample Name: 20211111_A42_R3_IgM

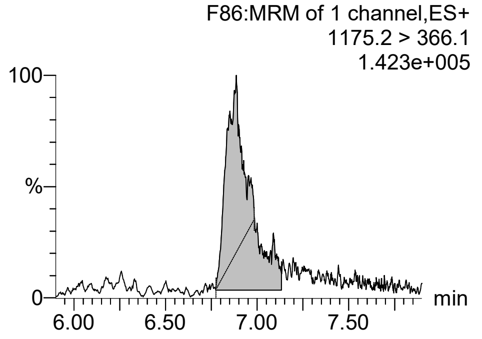

Dataset: C:\MassLynx\10.pro\20211111_clinicalsamples_IgM_IgA.qld

Last Altered: Thursday, March 09, 2023 19:58:25 台北標準時間
Printed: Friday, August 01, 2025 15:18:29 台北標準時間

Sample Name: 20211111_A43_R3_IgM
F86:MRM of 1 channel,ES+
1175.2 > 366.1
1.953e+005

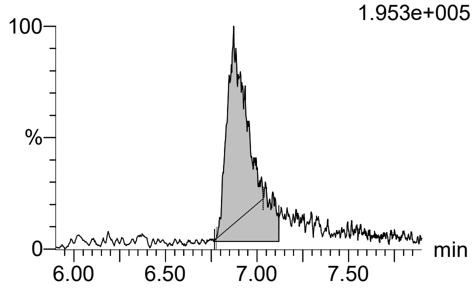

Sample Name: 20211111_A44_R3_IgM
F86:MRM of 1 channel,ES+
1175.2 > 366.1
1.484e+005

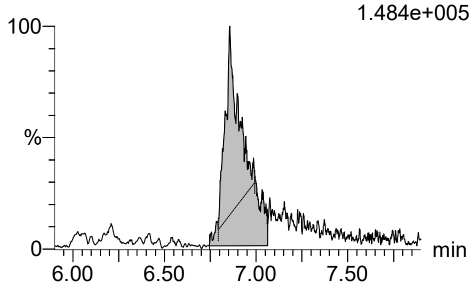

Sample Name: 20211111_QC_R25_IgM
F86:MRM of 1 channel,ES+
1175.2 > 366.1
2.134e+005

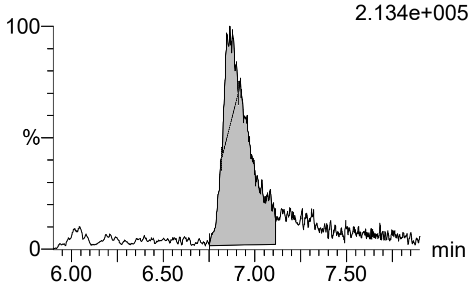

Dataset: C:\MassLynx\10.pro\20211111_clinicalsamples_IgM_IgA.qld

Last Altered: Thursday, March 09, 2023 19:58:25 台北標準時間
Printed: Friday, August 01, 2025 15:18:29 台北標準時間

	#	Name	RT	Area	IS Area	Response	Prima...
1	1	20211112_QC_R001_IgM	6.84	29673.8	15421.4	1.924	bb
2	2	20211112_QC_R002_IgM	6.86	39374.4	13188.8	2.985	MM
3	3	20211111_A1_R2_IgM	6.85	22802.3	11871.0	1.921	MM
4	4	20211111_A1_R3_IgM	6.86	18519.0	12030.5	1.539	MM
5	5	20211111_A2_R2_IgM	6.87	56424.0	11446.8	4.929	MM
6	6	20211111_A2_R3_IgM	6.86	58872.1	11098.4	5.305	MM
7	7	20211111_L1_R2_IgM	6.84	21856.9	11779.4	1.856	MM
8	8	20211111_L1_R3_IgM	6.87	13343.7	11176.2	1.194	MM
9	9	20211111_L2_R2_IgM	6.88	53177.0	11375.0	4.675	MM
10	10	20211111_H1_R2_IgM	6.83	55259.0	13595.2	4.065	MM
11	11	20211111_A3_R2_IgM	6.84	84440.8	14112.3	5.983	MM
12	12	20211111_A4_R2_IgM	6.86	47911.2	11977.5	4.000	MM
13	13	20211111_L3_R2_IgM	6.82	45102.5	12242.1	3.684	MM
14	14	20211111_L4_R2_IgM	6.85	36714.7	12797.0	2.869	MM
15	15	20211111_H2_R2_IgM	6.86	53308.0	10006.0	5.328	MM
16	16	20211111_A5_R2_IgM	6.84	144432.8	11434.6	12.631	MM
17	17	20211111_A6_R2_IgM	6.84	78425.3	13193.7	5.944	MM
18	18	20211111_QC_R10_IgM	6.85	55198.7	12566.1	4.393	MM
19	19	20211111_L5_R2_IgM	6.83	95528.1	13616.4	7.016	MM
20	20	20211111_L6_R2_IgM	6.84	53424.3	13698.5	3.900	MM
21	21	20211111_H3_R2_IgM	6.84	27809.9	15793.1	1.761	MM
22	22	20211111_A7_R2_IgM	6.86	46837.2	12936.9	3.620	MM
23	23	20211111_A8_R2_IgM	6.86	35532.1	13108.0	2.711	MM
24	24	20211111_L7_R2_IgM	6.85	36631.1	13278.5	2.759	MM
25	25	20211111_L8_R2_IgM	6.87	26320.6	15233.6	1.728	MM
26	26	20211111_H4_R2_IgM	6.89	29734.1	16521.9	1.800	MM
27	27	20211111_A9_R2_IgM	6.88	50026.7	16451.6	3.041	MM
28	28	20211111_A10_R2_IgM	6.85	58230.9	13036.0	4.467	MM
29	29	20211111_L9_R2_IgM	6.83	45157.0	14554.1	3.103	MM
30	30	20211111_L10_R2_IgM	6.85	88284.0	12250.4	7.207	MM
31	31	20211111_QC_R11_IgM	6.85	58209.0	11459.2	5.080	MM
32	32	20211111_H5_R2_IgM	6.87	68370.7	14358.2	4.762	MM
33	33	20211111_A11_R2_IgM	6.84	56455.8	16184.1	3.488	MM
34	34	20211111_A12_R2_IgM	6.84	26907.9	13465.4	1.998	MM
35	35	20211111_L11_R2_IgM	6.88	23051.0	13277.9	1.736	MM
36	36	20211111_L12_R2_IgM	6.86	43663.0	15958.8	2.736	MM
37	37	20211111_H6_R2_IgM	6.87	24322.8	14003.5	1.737	MM
38	38	20211111_A13_R2_IgM	6.85	19372.4	13656.1	1.419	MM
39	39	20211111_A14_R2_IgM	6.86	58351.2	14071.3	4.147	MM
40	40	20211111_L13_R2_IgM	6.85	59669.9	14785.6	4.036	MM
41	41	20211111_L14_R2_IgM	6.86	46032.0	14772.2	3.116	MM
42	42	20211111_H7_R2_IgM	6.86	34197.3	15164.4	2.255	MM
43	43	20211111_A15_R2_IgM	6.88	59120.1	15721.5	3.760	MM
44	44	20211111_QC_R12_IgM	6.87	53731.5	13711.5	3.919	MM
45	45	20211111_A16_R2_IgM	6.88	67144.8	14275.6	4.703	MM
46	46	20211111_L15_R2_IgM	6.86	54595.4	12935.8	4.220	MM
47	47	20211111_L16_R2_IgM	6.86	36195.4	13342.8	2.713	MM
48	48	20211111_H8_R2_IgM	6.86	36372.9	15835.1	2.297	MM
49	49	20211111_A17_R2_IgM	6.85	58860.1	15786.7	3.728	MM
50	50	20211111_A18_R2_IgM	6.86	33084.6	16986.8	1.948	MM
51	51	20211111_L17_R2_IgM	6.85	76600.0	13342.7	5.741	MM
52	52	20211111_L18_R2_IgM	6.86	22459.1	14692.0	1.529	MM
53	53	20211111_H9_R2_IgM	6.85	25273.0	15639.7	1.616	MM
54	54	20211111_A19_R2_IgM	6.86	29371.1	14198.7	2.069	MM
55	55	20211111_A20_R2_IgM	6.87	15655.3	14162.7	1.105	MM
56	56	20211111_L19_R2_IgM	6.88	25530.9	9293.4	2.747	MM

Dataset: C:\MassLynx\10.pro\20211111_clinicalsamples_IgM_IgA.qld

Last Altered: Thursday, March 09, 2023 19:58:25 台北標準時間
Printed: Friday, August 01, 2025 15:18:29 台北標準時間

	#	Name	RT	Area	IS Area	Response	Prima...
57	57	20211111_QC_R13_IgM	6.86	44055.3	13903.0	3.169	MM
58	58	20211111_L20_R2_IgM	6.83	47114.5	12781.6	3.686	MM
59	59	20211111_H10_R2_IgM	6.84	49906.5	14796.4	3.373	MM
60	60	20211111_A21_R2_IgM	6.86	77007.3	13669.0	5.634	MM
61	61	20211111_A22_R2_IgM	6.85	31369.9	14098.3	2.225	MM
62	62	20211111_L21_R2_IgM	6.85	30041.8	15678.2	1.916	MM
63	63	20211111_L22_R2_IgM	6.88	47706.1	15724.5	3.034	MM
64	64	20211111_H11_R2_IgM	6.88	45135.9	15890.2	2.840	MM
65	65	20211111_A23_R2_IgM	6.86	18833.6	13423.2	1.403	MM
66	66	20211111_A24_R2_IgM	6.89	47428.1	12438.0	3.813	MM
67	67	20211111_L23_R2_IgM	6.86	69666.1	14075.0	4.950	MM
68	68	20211111_L24_R2_IgM	6.86	36047.2	15972.1	2.257	MM
69	69	20211111_H12_R2_IgM	6.86	26053.6	16270.9	1.601	MM
70	70	20211111_QC_R14_IgM	6.86	44449.7	15806.8	2.812	MM
71	71	20211111_A25_R2_IgM	6.87	79283.1	14552.1	5.448	MM
72	72	20211111_A26_R2_IgM	6.88	40483.7	11492.7	3.523	MM
73	73	20211111_L25_R2_IgM	6.86	34066.0	14403.3	2.365	MM
74	74	20211111_L26_R2_IgM	6.90	28840.4	16533.9	1.744	MM
75	75	20211111_H13_R2_IgM	6.87	59924.4	15520.1	3.861	MM
76	76	20211111_A27_R2_IgM	6.87	72893.8	13337.4	5.465	MM
77	77	20211111_A28_R2_IgM	6.83	49311.5	11895.6	4.145	MM
78	78	20211111_L27_R2_IgM	6.88	83047.2	14125.7	5.879	MM
79	79	20211111_L28_R2_IgM	6.87	66122.1	11807.5	5.600	MM
80	80	20211111_H14_R2_IgM	6.85	50991.1	15035.7	3.391	MM
81	81	20211111_A29_R2_IgM	6.87	17096.6	13554.4	1.261	MM
82	82	20211111_A30_R2_IgM	6.87	36713.3	12984.1	2.828	MM
83	83	20211111_QC_R15_IgM	6.87	39213.9	12299.5	3.188	MM
84	84	20211111_L29_R2_IgM	6.87	19744.7	13590.7	1.453	MM
85	85	20211111_L30_R2_IgM	6.86	13488.9	12511.4	1.078	MM
86	86	20211111_H15_R2_IgM	6.86	40418.7	11885.0	3.401	MM
87	87	20211111_A31_R2_IgM	6.83	22322.7	13422.1	1.663	MM
88	88	20211111_A32_R2_IgM	6.85	42386.1	11461.3	3.698	MM
89	89	20211111_L31_R2_IgM	6.83	28105.2	13753.4	2.044	MM
90	90	20211111_L32_R2_IgM	6.85	48791.9	12949.0	3.768	MM
91	91	20211111_H16_R2_IgM	6.85	123415.2	11465.4	10.764	MM
92	92	20211111_A33_R2_IgM	6.86	64214.9	10551.5	6.086	MM
93	93	20211111_A34_R2_IgM	6.87	25273.0	12409.9	2.037	MM
94	94	20211111_H17_R2_IgM	6.86	286381.6	10160.9	28.185	MM
95	95	20211111_A35_R2_IgM	6.84	63238.7	10460.6	6.045	MM
96	96	20211111_QC_R16_IgM	6.86	54088.6	10433.5	5.184	MM
97	97	20211111_A36_R2_IgM	6.83	47967.9	12504.8	3.836	MM
98	98	20211111_H18_R2_IgM	6.87	84748.1	9140.4	9.272	MM
99	99	20211111_A37_R2_IgM	6.87	40767.1	9796.9	4.161	MM
100	1...	20211111_A38_R2_IgM	6.87	48349.6	11224.3	4.308	MM
101	1...	20211111_H19_R2_IgM	6.87	49527.9	11739.2	4.219	MM
102	1...	20211111_A39_R2_IgM	6.86	56036.8	12718.9	4.406	MM
103	1...	20211111_A40_R2_IgM	6.85	24625.8	10006.1	2.461	MM
104	1...	20211111_H20_R2_IgM	6.86	95930.8	7988.2	12.009	MM
105	1...	20211111_A41_R2_IgM	6.84	33866.8	11513.3	2.942	MM
106	1...	20211111_A42_R2_IgM	6.87	21074.3	10238.5	2.058	MM
107	1...	20211111_A43_R2_IgM	6.89	30178.8	11098.9	2.719	MM
108	1...	20211111_A44_R2_IgM	6.83	20004.0	10969.9	1.824	MM
109	1...	20211111_QC_R17_IgM	6.87	38362.7	9700.8	3.955	MM
110	1...	20211111_L2_R3_IgM	6.86	60792.0	8528.0	7.129	MM
111	1...	20211111_H1_R3_IgM	6.85	51744.3	9438.2	5.482	MM
112	1...	20211111_A3_R3_IgM	6.87	66276.8	11598.6	5.714	MM

Dataset: C:\MassLynx\10.pro\20211111_clinicalsamples_IgM_IgA.qld

Last Altered: Thursday, March 09, 2023 19:58:25 台北標準時間
Printed: Friday, August 01, 2025 15:18:29 台北標準時間

	#	Name	RT	Area	IS Area	Response	Prima...
113	1...	20211111_A4_R3_IgM	6.87	40774.8	11364.1	3.588	MM
114	1...	20211111_L3_R3_IgM	6.83	39583.7	10753.4	3.681	MM
115	1...	20211111_L4_R3_IgM	6.88	35736.2	10291.6	3.472	MM
116	1...	20211111_H2_R3_IgM	6.86	56146.7	9315.8	6.027	MM
117	1...	20211111_A5_R3_IgM	6.87	114665.2	9102.2	12.598	MM
118	1...	20211111_A6_R3_IgM	6.87	65532.2	10356.4	6.328	MM
119	1...	20211111_QC_R18_IgM	6.85	50711.5	8398.3	6.038	MM
120	1...	20211111_L5_R3_IgM	6.85	69397.4	9786.7	7.091	MM
121	1...	20211111_L6_R3_IgM	6.84	46077.7	9911.3	4.649	MM
122	1...	20211111_H3_R3_IgM	6.85	23349.0	12155.5	1.921	MM
123	1...	20211111_A7_R3_IgM	6.88	46481.3	11804.8	3.937	MM
124	1...	20211111_A8_R3_IgM	6.87	29945.9	10510.6	2.849	MM
125	1...	20211111_L7_R3_IgM	6.86	36049.4	12177.9	2.960	MM
126	1...	20211111_L8_R3_IgM	6.89	24734.4	11668.6	2.120	MM
127	1...	20211111_H4_R3_IgM	6.87	22519.2	11192.0	2.012	MM
128	1...	20211111_A9_R3_IgM	6.88	36666.0	14077.4	2.605	MM
129	1...	20211111_A10_R3_IgM	6.87	47630.1	11190.4	4.256	MM
130	1...	20211111_L9_R3_IgM	6.85	35376.3	14649.3	2.415	MM
131	1...	20211111_L10_R3_IgM	6.84	77892.2	9361.3	8.321	MM
132	1...	20211111_QC_R19_IgM	6.85	48615.1	10125.5	4.801	MM
133	1...	20211111_H5_R3_IgM	6.87	50843.1	10010.9	5.079	MM
134	1...	20211111_A11_R3_IgM	6.86	35532.4	12697.2	2.798	MM
135	1...	20211111_A12_R3_IgM	6.86	23621.4	13148.5	1.797	MM
136	1...	20211111_L11_R3_IgM	6.86	19150.6	10786.6	1.775	MM
137	1...	20211111_L12_R3_IgM	6.84	44870.8	11681.8	3.841	MM
138	1...	20211111_H6_R3_IgM	6.88	25312.1	12095.5	2.093	MM
139	1...	20211111_A13_R3_IgM	6.88	18346.9	12908.3	1.421	MM
140	1...	20211111_A14_R3_IgM	6.86	50400.7	11322.6	4.451	MM
141	1...	20211111_L13_R3_IgM	6.88	57909.1	11516.1	5.029	MM
142	1...	20211111_L14_R3_IgM	6.87	40816.2	10790.7	3.783	MM
143	1...	20211111_H7_R3_IgM	6.89	28643.9	11572.1	2.475	MM
144	1...	20211111_A15_R3_IgM	6.87	54505.4	12434.4	4.383	MM
145	1...	20211111_QC_R20_IgM	6.87	52147.7	10701.0	4.873	MM
146	1...	20211111_A16_R3_IgM	6.86	65279.0	11452.1	5.700	MM
147	1...	20211111_L15_R3_IgM	6.86	50700.2	10531.1	4.814	MM
148	1...	20211111_L16_R3_IgM	6.88	37313.3	12049.4	3.097	MM
149	1...	20211111_H8_R3_IgM	6.90	32027.8	13434.9	2.384	MM
150	1...	20211111_A17_R3_IgM	6.86	59066.1	12452.0	4.743	MM
151	1...	20211111_A18_R3_IgM	6.84	31186.9	12506.5	2.494	MM
152	1...	20211111_L17_R3_IgM	6.88	68277.8	9948.9	6.863	MM
153	1...	20211111_L18_R3_IgM	6.85	22228.3	13462.1	1.651	MM
154	1...	20211111_H9_R3_IgM	6.87	22998.3	13178.7	1.745	MM
155	1...	20211111_A19_R3_IgM	6.86	28794.3	11298.6	2.548	MM
156	1...	20211111_A20_R3_IgM	6.87	15513.6	11175.6	1.388	MM
157	1...	20211111_L19_R3_IgM	6.88	34226.4	11558.2	2.961	MM
158	1...	20211111_QC_R21_IgM	6.86	46680.5	9977.0	4.679	MM
159	1...	20211111_L20_R3_IgM	6.87	34307.5	13508.1	2.540	MM
160	1...	20211111_H10_R3_IgM	6.86	41473.7	12374.8	3.351	MM
161	1...	20211111_A21_R3_IgM	6.87	74421.0	12443.6	5.981	MM
162	1...	20211111_A22_R3_IgM	6.88	29760.8	11589.3	2.568	MM
163	1...	20211111_L21_R3_IgM	6.85	28570.6	13107.4	2.180	MM
164	1...	20211111_L22_R3_IgM	6.87	43471.3	12021.5	3.616	MM
165	1...	20211111_H11_R3_IgM	6.87	40916.2	12687.8	3.225	MM
166	1...	20211111_A23_R3_IgM	6.86	19173.1	11611.8	1.651	MM
167	1...	20211111_A24_R3_IgM	6.89	54934.3	10476.8	5.243	MM
168	1...	20211111_L23_R3_IgM	6.85	71153.8	11873.1	5.993	MM

Dataset: C:\MassLynx\10.pro\20211111_clinicalsamples_IgM_IgA.qld

Last Altered: Thursday, March 09, 2023 19:58:25 台北標準時間
Printed: Friday, August 01, 2025 15:18:29 台北標準時間

	#	Name	RT	Area	IS Area	Response	Prima...
169	1...	20211111_L24_R3_IgM	6.87	33978.6	13468.0	2.523	MM
170	1...	20211111_H12_R3_IgM	6.89	28173.0	14229.1	1.980	MM
171	1...	20211111_QC_R22_IgM	6.86	44593.5	11264.4	3.959	MM
172	1...	20211111_A25_R3_IgM	6.89	71122.7	11775.7	6.040	MM
173	1...	20211111_A26_R3_IgM	6.87	37424.2	10473.9	3.573	MM
174	1...	20211111_L25_R3_IgM	6.89	32873.6	13256.8	2.480	MM
175	1...	20211111_L26_R3_IgM	6.88	26510.8	13565.0	1.954	MM
176	1...	20211111_H13_R3_IgM	6.88	59753.0	13146.0	4.545	MM
177	1...	20211111_A27_R3_IgM	6.87	67794.8	12025.6	5.638	MM
178	1...	20211111_A28_R3_IgM	6.88	38717.3	11229.1	3.448	MM
179	1...	20211111_L27_R3_IgM	6.88	77973.7	14960.0	5.212	MM
180	1...	20211111_L28_R3_IgM	6.87	73599.4	11359.0	6.479	MM
181	1...	20211111_H14_R3_IgM	6.87	59952.6	12830.8	4.673	MM
182	1...	20211111_A29_R3_IgM	6.88	20878.0	13434.3	1.554	MM
183	1...	20211111_A30_R3_IgM	6.87	36601.4	11272.2	3.247	MM
184	1...	20211111_QC_R23_IgM	6.88	44216.6	11872.2	3.724	MM
185	1...	20211111_L29_R3_IgM	6.86	18557.4	12948.7	1.433	MM
186	1...	20211111_L30_R3_IgM	6.86	13062.8	11026.6	1.185	MM
187	1...	20211111_H15_R3_IgM	6.88	40739.9	10786.3	3.777	MM
188	1...	20211111_A31_R3_IgM	6.88	20561.1	12010.5	1.712	MM
189	1...	20211111_A32_R3_IgM	6.86	41118.8	11107.7	3.702	MM
190	1...	20211111_L31_R3_IgM	6.85	22458.1	11021.8	2.038	MM
191	1...	20211111_L32_R3_IgM	6.87	38326.6	11683.8	3.280	MM
192	1...	20211111_H16_R3_IgM	6.86	101645.8	9831.7	10.339	MM
193	1...	20211111_A33_R3_IgM	6.86	62888.2	11213.1	5.608	MM
194	1...	20211111_A34_R3_IgM	6.88	25232.4	11550.1	2.185	MM
195	1...	20211111_H17_R3_IgM	6.85	266930.0	8874.4	30.079	MM
196	1...	20211111_A35_R3_IgM	6.86	63485.5	9856.3	6.441	MM
197	1...	20211111_QC_R24_IgM	6.85	52251.4	7561.8	6.910	MM
198	1...	20211111_A36_R3_IgM	6.86	36517.6	9562.8	3.819	MM
199	1...	20211111_H18_R3_IgM	6.88	83812.7	8165.3	10.265	MM
200	2...	20211111_A37_R3_IgM	6.85	38259.2	9278.8	4.123	MM
201	2...	20211111_A38_R3_IgM	6.87	43286.5	10920.3	3.964	MM
202	2...	20211111_H19_R3_IgM	6.86	46460.7	9285.3	5.004	MM
203	2...	20211111_A39_R3_IgM	6.86	57526.8	9083.4	6.333	MM
204	2...	20211111_A40_R3_IgM	6.85	20966.0	8382.4	2.501	MM
205	2...	20211111_H20_R3_IgM	6.86	81925.0	7807.9	10.493	MM
206	2...	20211111_A41_R3_IgM	6.86	20732.1	5925.2	3.499	MM
207	2...	20211111_A42_R3_IgM	6.89	19931.7	9352.6	2.131	MM
208	2...	20211111_A43_R3_IgM	6.87	27212.8	10216.3	2.664	MM
209	2...	20211111_A44_R3_IgM	6.86	17565.1	10262.7	1.712	MM
210	2...	20211111_QC_R25_IgM	6.86	33319.0	8575.3	3.885	MM

Dataset: C:\MassLynx\10.pro\20211111_clinicalsamples_IgM_IgA.qld

Last Altered: Thursday, March 09, 2023 19:58:25 台北標準時間
Printed: Friday, August 01, 2025 15:19:24 台北標準時間

Method: C:\MassLynx\10_Pro_IgA.PRO\MethDB\20211111_IgM_IgA_DMRM_new.mdb 24 Feb 2022 13:44:14
Calibration: 15 Nov 2021 14:42:11

Sample Name: 20211112_QC_R001_IgM

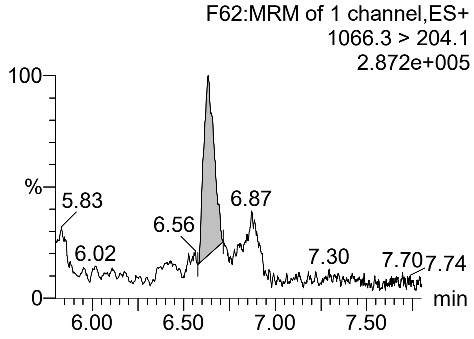

Sample Name: 20211112_QC_R002_IgM

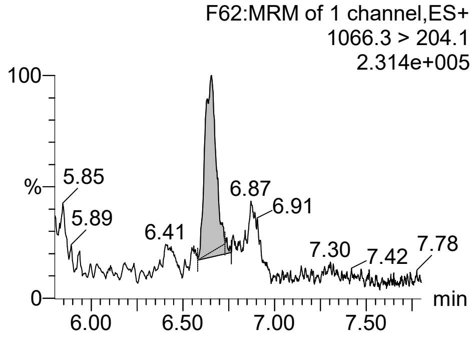

Sample Name: 20211111_A1_R2_IgM

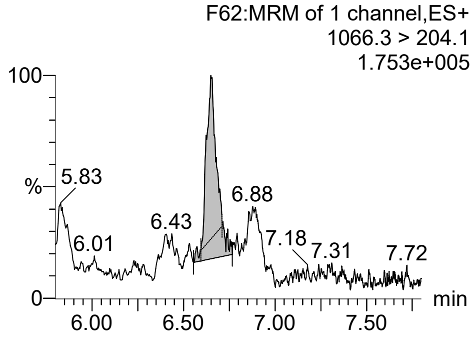

Sample Name: 20211111_A1_R3_IgM

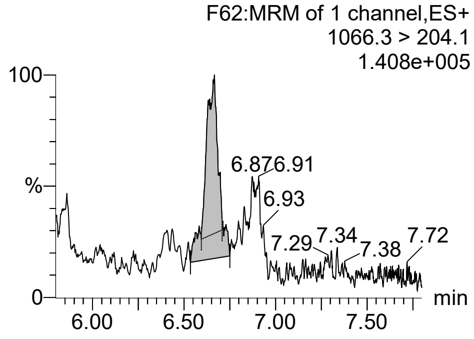

Sample Name: 20211111_A2_R2_IgM

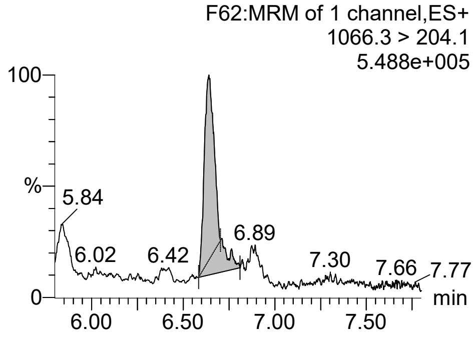

Sample Name: 20211111_A2_R3_IgM

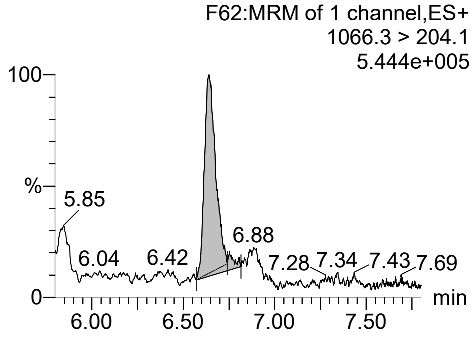

Sample Name: 20211111_L1_R2_IgM

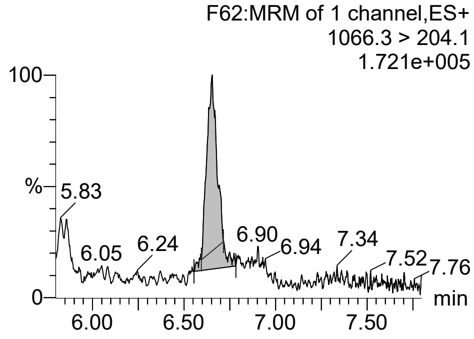

Sample Name: 20211111_L1_R3_IgM

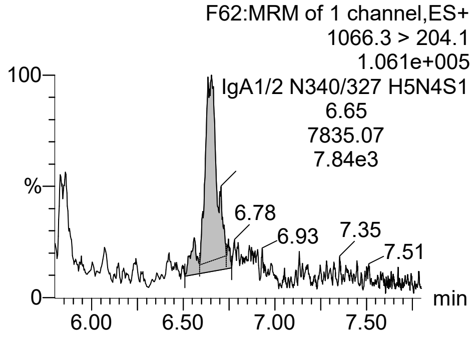

Sample Name: 20211111_L2_R2_IgM

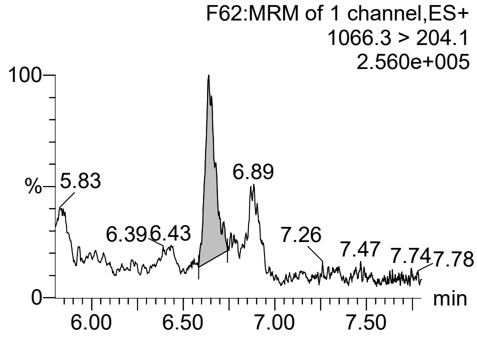

Sample Name: 20211111_H1_R2_IgM

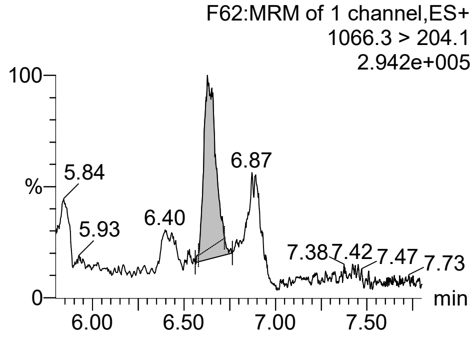

Sample Name: 20211111_A3_R2_IgM

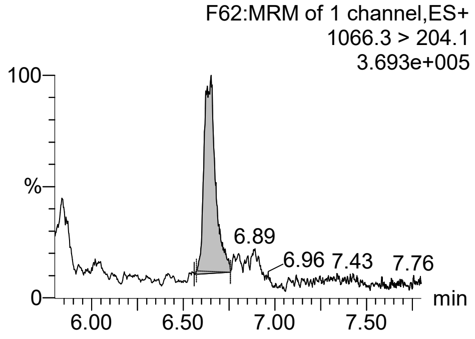

Sample Name: 20211111_A4_R2_IgM

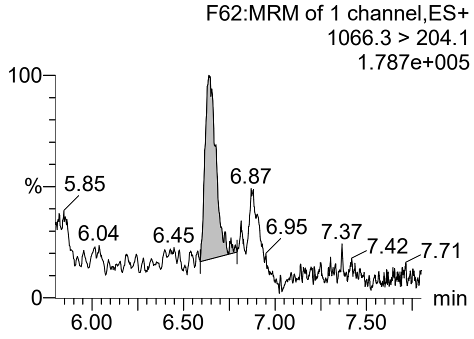

Sample Name: 20211111_L3_R2_IgM
F62:MRM of 1 channel,ES+
1066.3 > 204.1
2.917e+005

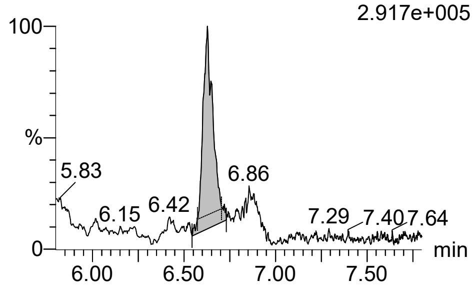

Sample Name: 20211111_L4_R2_IgM
F62:MRM of 1 channel,ES+
1066.3 > 204.1
2.332e+005

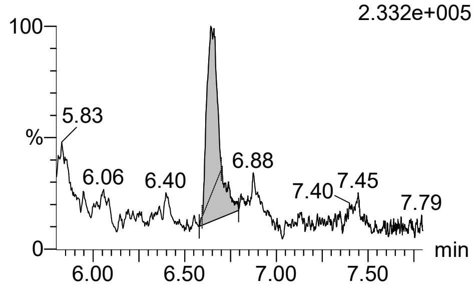

Sample Name: 20211111_H2_R2_IgM
F62:MRM of 1 channel,ES+
1066.3 > 204.1
3.292e+005

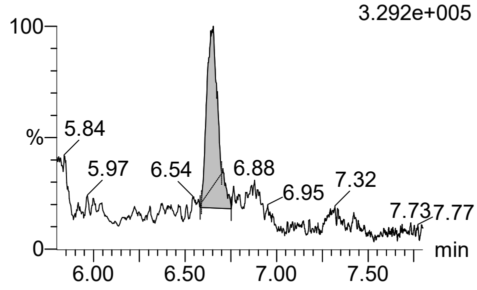

Sample Name: 20211111_A5_R2_IgM
F62:MRM of 1 channel,ES+
1066.3 > 204.1
6.117e+005

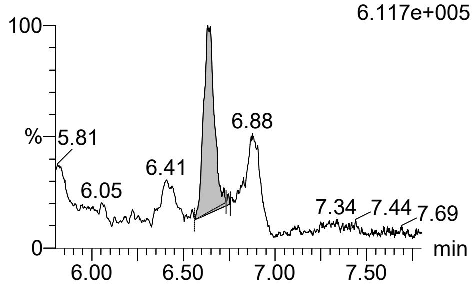

Sample Name: 20211111_A6_R2_IgM
F62:MRM of 1 channel,ES+
1066.3 > 204.1
2.972e+005

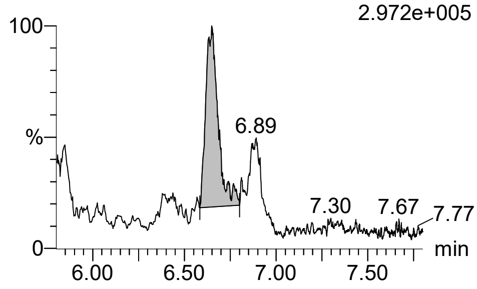

Sample Name: 20211111_QC_R10_IgM
F62:MRM of 1 channel,ES+
1066.3 > 204.1
2.779e+005

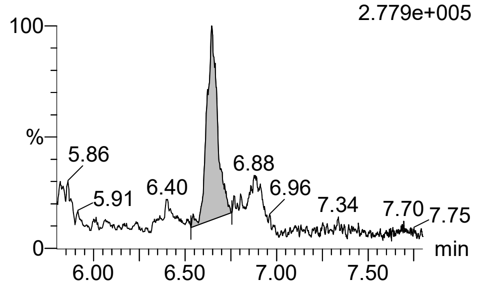

Sample Name: 20211111_L5_R2_IgM
F62:MRM of 1 channel,ES+
1066.3 > 204.1
3.615e+005

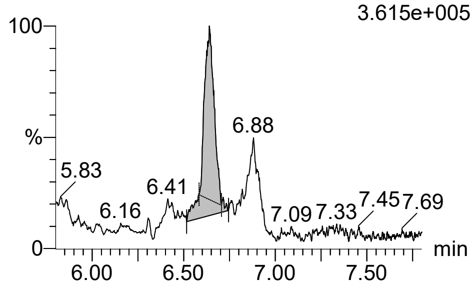

Sample Name: 20211111_L6_R2_IgM
F62:MRM of 1 channel,ES+
1066.3 > 204.1
2.670e+005

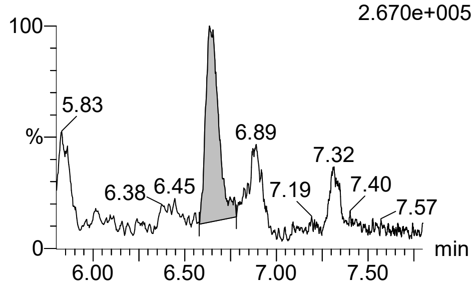

Sample Name: 20211111_H3_R2_IgM
F62:MRM of 1 channel,ES+
1066.3 > 204.1
1.270e+005

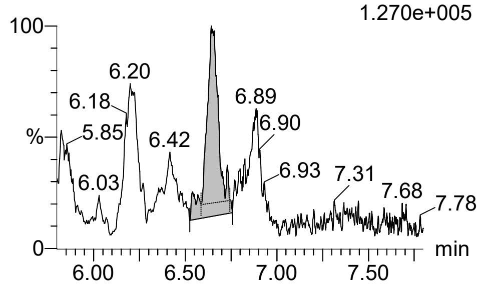

Sample Name: 20211111_A7_R2_IgM
F62:MRM of 1 channel,ES+
1066.3 > 204.1
4.227e+005

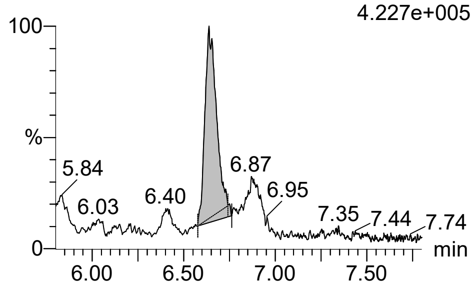

Sample Name: 20211111_A8_R2_IgM
F62:MRM of 1 channel,ES+
1066.3 > 204.1
2.806e+005

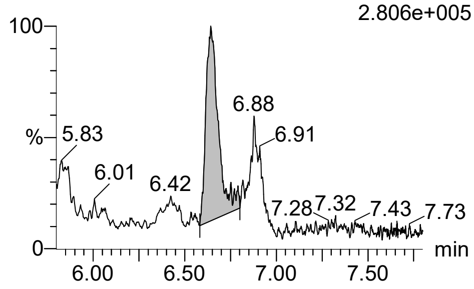

Sample Name: 20211111_L7_R2_IgM
F62:MRM of 1 channel,ES+
1066.3 > 204.1
1.481e+005

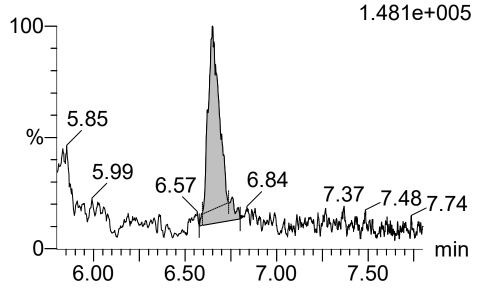

Sample Name: 20211111_L8_R2_IgM
F62:MRM of 1 channel,ES+
1066.3 > 204.1
9.084e+004

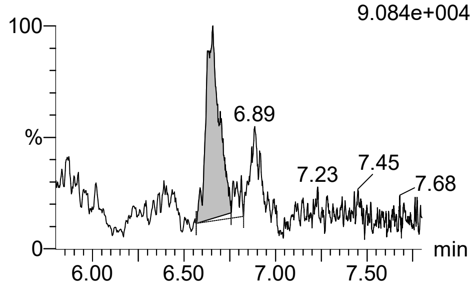

Sample Name: 20211111_H4_R2_IgM
F62:MRM of 1 channel,ES+
1066.3 > 204.1
1.702e+005

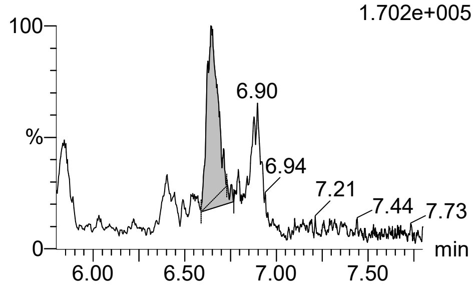

Sample Name: 20211111_A9_R2_IgM
F62:MRM of 1 channel,ES+
1066.3 > 204.1
2.312e+005

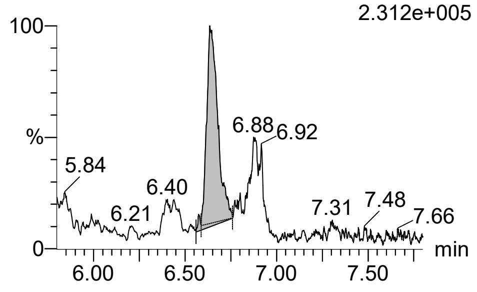

Dataset: C:\MassLynx\10.pro\20211111_clinicalsamples_IgM_IgA.qld

Last Altered: Thursday, March 09, 2023 19:58:25 台北標準時間
Printed: Friday, August 01, 2025 15:19:24 台北標準時間

Sample Name: 20211111_A10_R2_IgM
F62:MRM of 1 channel,ES+
1066.3 > 204.1
3.462e+005

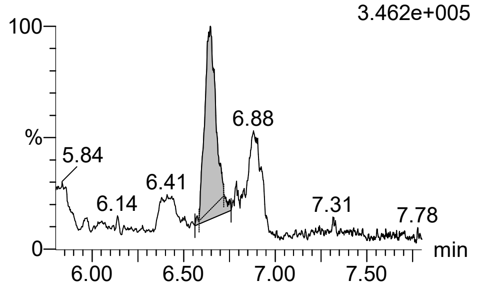

Sample Name: 20211111_L9_R2_IgM
F62:MRM of 1 channel,ES+
1066.3 > 204.1
3.254e+005

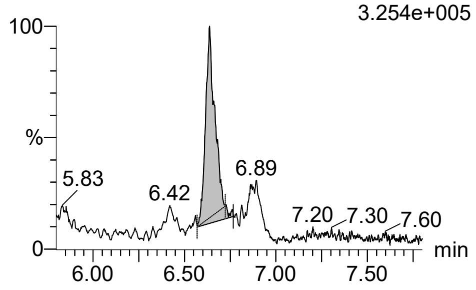

Sample Name: 20211111_L10_R2_IgM
F62:MRM of 1 channel,ES+
1066.3 > 204.1
4.227e+005

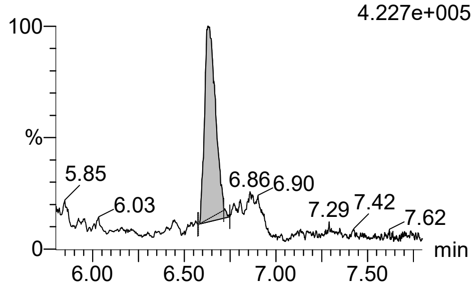

Sample Name: 20211111_QC_R11_IgM
F62:MRM of 1 channel,ES+
1066.3 > 204.1
2.612e+005

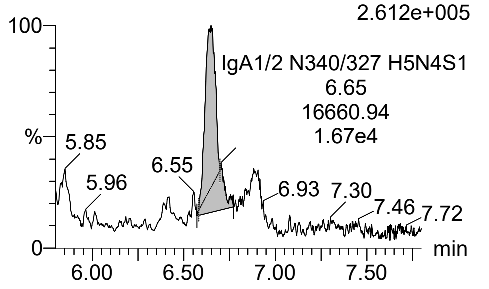

Sample Name: 20211111_H5_R2_IgM
F62:MRM of 1 channel,ES+
1066.3 > 204.1
2.429e+005

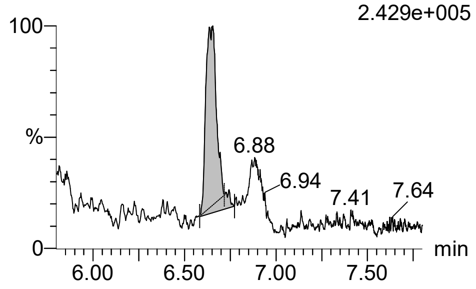

Sample Name: 20211111_A11_R2_IgM
F62:MRM of 1 channel,ES+
1066.3 > 204.1
3.274e+005

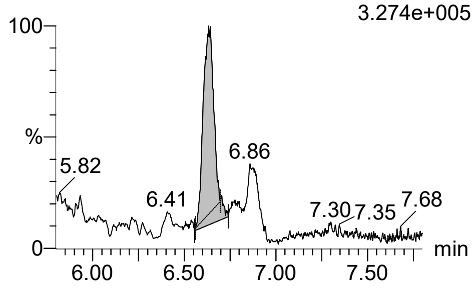

Sample Name: 20211111_A12_R2_IgM
F62:MRM of 1 channel,ES+
1066.3 > 204.1
1.222e+005

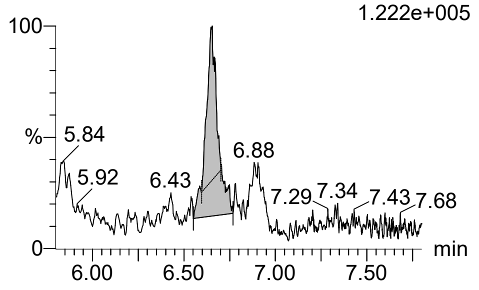

Sample Name: 20211111_L11_R2_IgM
F62:MRM of 1 channel,ES+
1066.3 > 204.1
1.549e+005

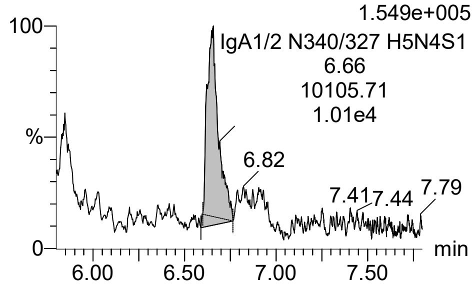

Sample Name: 20211111_L12_R2_IgM
F62:MRM of 1 channel,ES+
1066.3 > 204.1
2.346e+005

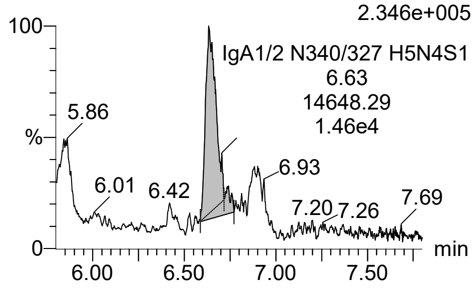

Sample Name: 20211111_H6_R2_IgM
F62:MRM of 1 channel,ES+
1066.3 > 204.1
1.900e+005

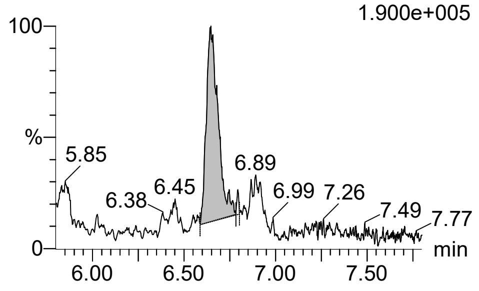

Sample Name: 20211111_A13_R2_IgM
F62:MRM of 1 channel,ES+
1066.3 > 204.1
1.901e+005

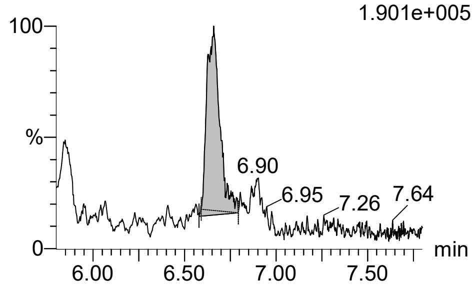

Sample Name: 20211111_A14_R2_IgM
F62:MRM of 1 channel,ES+
1066.3 > 204.1
3.040e+005

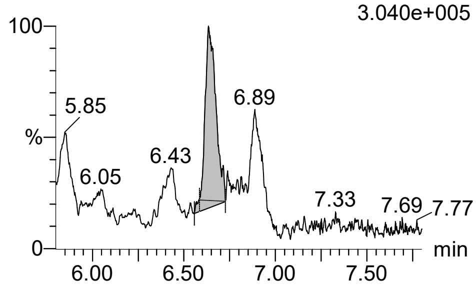

Sample Name: 20211111_L13_R2_IgM
F62:MRM of 1 channel,ES+
1066.3 > 204.1
1.781e+005

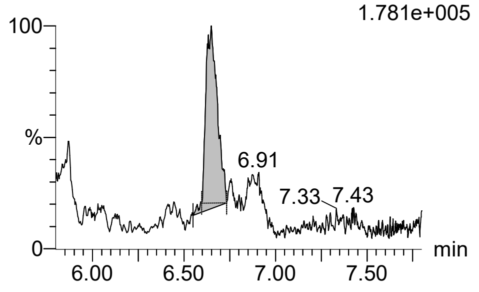

Sample Name: 20211111_L14_R2_IgM
F62:MRM of 1 channel,ES+
1066.3 > 204.1
2.478e+005

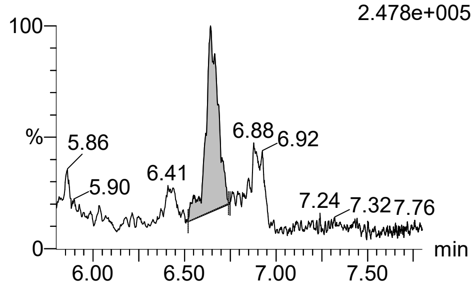

Sample Name: 20211111_H7_R2_IgM
F62:MRM of 1 channel,ES+
1066.3 > 204.1
3.253e+005

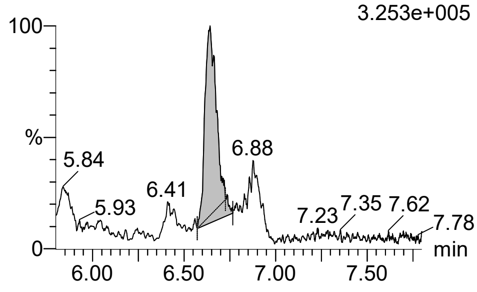

Dataset: C:\MassLynx\10.pro\20211111_clinicalsamples_IgM_IgA.qld

Last Altered: Thursday, March 09, 2023 19:58:25 台北標準時間
Printed: Friday, August 01, 2025 15:19:24 台北標準時間

Sample Name: 20211111_A15_R2_IgM
F62:MRM of 1 channel,ES+
1066.3 > 204.1
6.514e+005

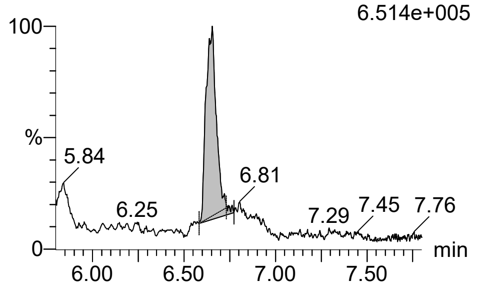

Sample Name: 20211111_QC_R12_IgM
F62:MRM of 1 channel,ES+
1066.3 > 204.1
3.731e+005

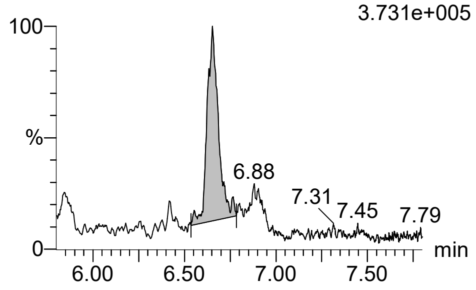

Sample Name: 20211111_A16_R2_IgM
F62:MRM of 1 channel,ES+
1066.3 > 204.1
3.769e+005

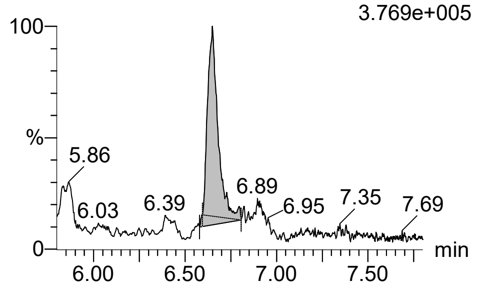

Sample Name: 20211111_L15_R2_IgM
F62:MRM of 1 channel,ES+
1066.3 > 204.1
3.624e+005

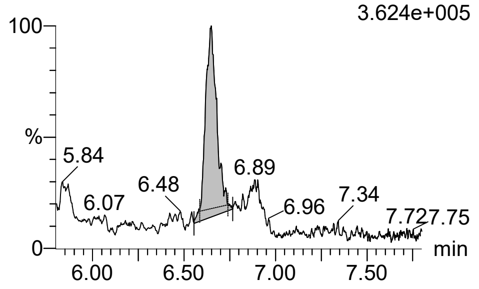

Sample Name: 20211111_L16_R2_IgM
F62:MRM of 1 channel,ES+
1066.3 > 204.1
1.219e+005

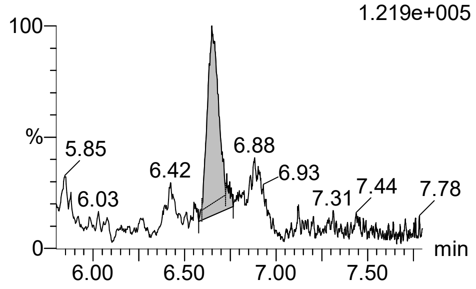

Sample Name: 20211111_H8_R2_IgM
F62:MRM of 1 channel,ES+
1066.3 > 204.1
1.598e+005

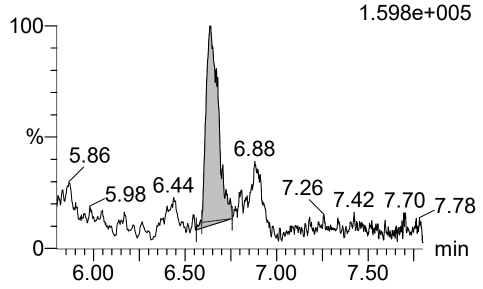

Sample Name: 20211111_A17_R2_IgM
F62:MRM of 1 channel,ES+
1066.3 > 204.1
3.631e+005

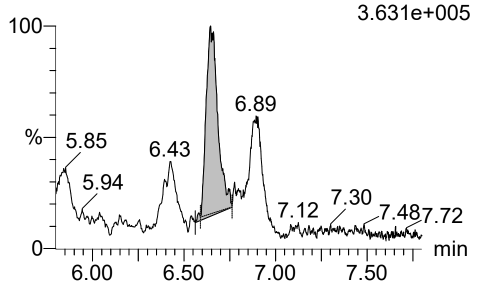

Sample Name: 20211111_A18_R2_IgM
F62:MRM of 1 channel,ES+
1066.3 > 204.1
2.293e+005

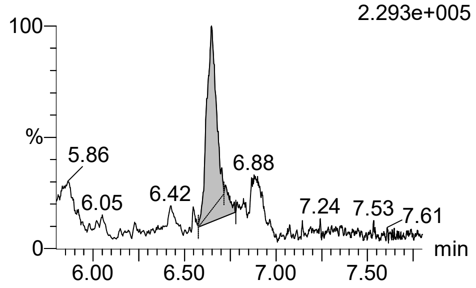

Sample Name: 20211111_L17_R2_IgM
F62:MRM of 1 channel,ES+
1066.3 > 204.1
4.398e+005

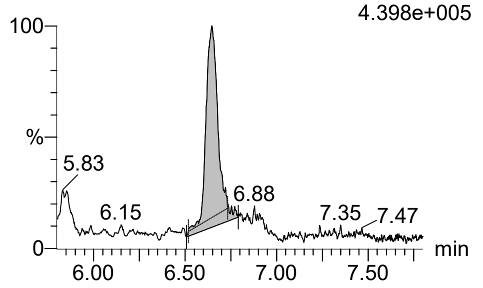

Sample Name: 20211111_L18_R2_IgM
F62:MRM of 1 channel,ES+
1066.3 > 204.1
1.124e+005

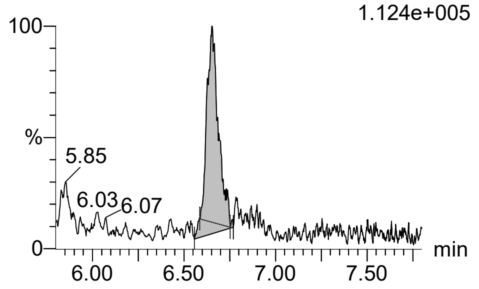

Sample Name: 20211111_H9_R2_IgM
F62:MRM of 1 channel,ES+
1066.3 > 204.1
2.467e+005

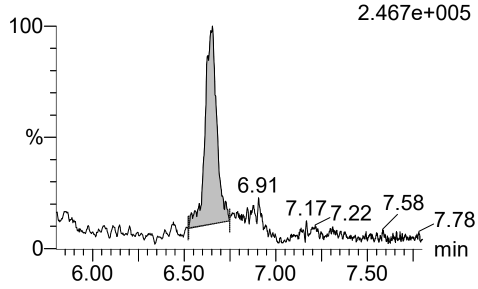

Sample Name: 20211111_A19_R2_IgM
F62:MRM of 1 channel,ES+
1066.3 > 204.1
3.730e+005

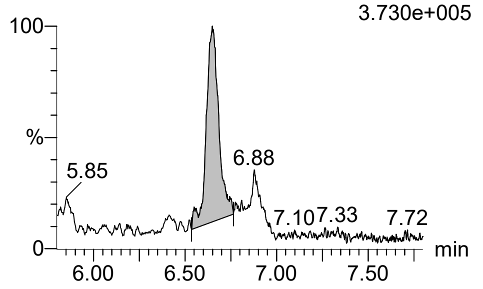

Sample Name: 20211111_A20_R2_IgM
F62:MRM of 1 channel,ES+
1066.3 > 204.1
1.762e+005

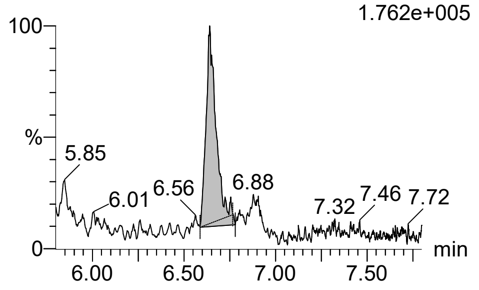

Sample Name: 20211111_L19_R2_IgM
F62:MRM of 1 channel,ES+
1066.3 > 204.1
1.545e+005

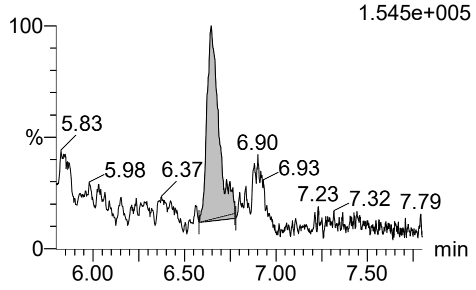

Sample Name: 20211111_QC_R13_IgM
F62:MRM of 1 channel,ES+
1066.3 > 204.1
2.524e+005

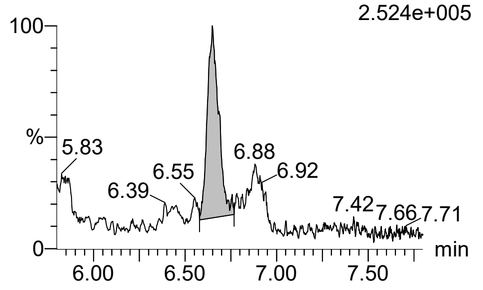

Dataset: C:\MassLynx\10.pro\20211111_clinicalsamples_IgM_IgA.qld

Last Altered: Thursday, March 09, 2023 19:58:25 台北標準時間
Printed: Friday, August 01, 2025 15:19:24 台北標準時間

Sample Name: 20211111_L20_R2_IgM
F62:MRM of 1 channel,ES+
1066.3 > 204.1
2.847e+005

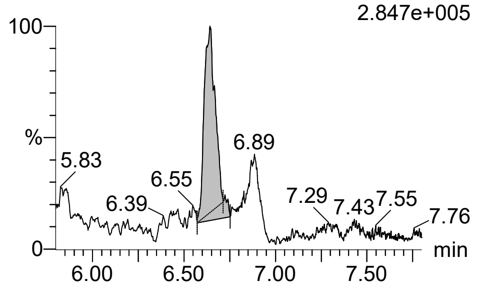

Sample Name: 20211111_H10_R2_IgM
F62:MRM of 1 channel,ES+
1066.3 > 204.1
2.722e+005

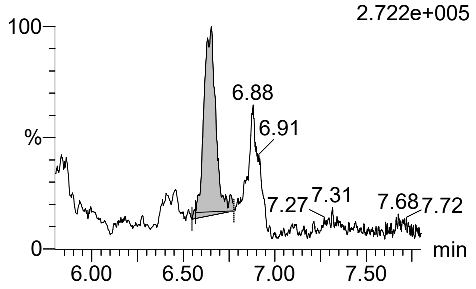

Sample Name: 20211111_A21_R2_IgM
F62:MRM of 1 channel,ES+
1066.3 > 204.1
2.279e+005

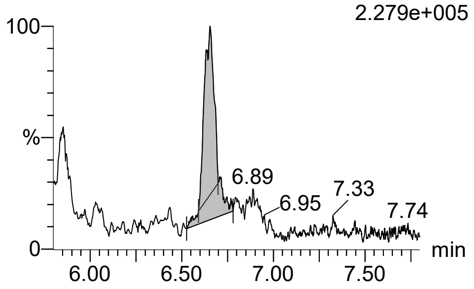

Sample Name: 20211111_A22_R2_IgM
F62:MRM of 1 channel,ES+
1066.3 > 204.1
9.656e+004

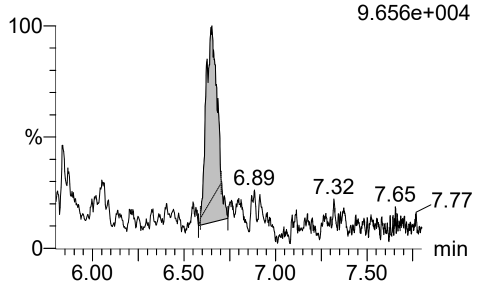

Sample Name: 20211111_L21_R2_IgM
F62:MRM of 1 channel,ES+
1066.3 > 204.1
1.776e+005

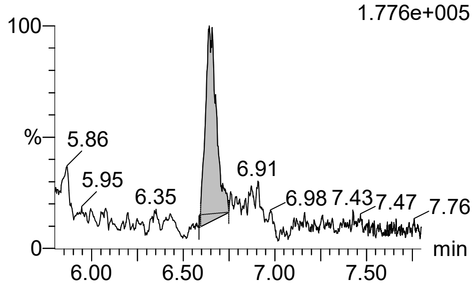

Sample Name: 20211111_L22_R2_IgM
F62:MRM of 1 channel,ES+
1066.3 > 204.1
1.876e+005

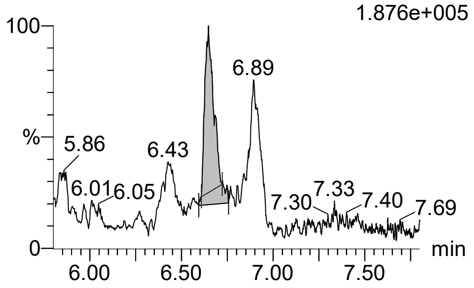

Sample Name: 20211111_H11_R2_IgM
F62:MRM of 1 channel,ES+
1066.3 > 204.1
1.582e+005

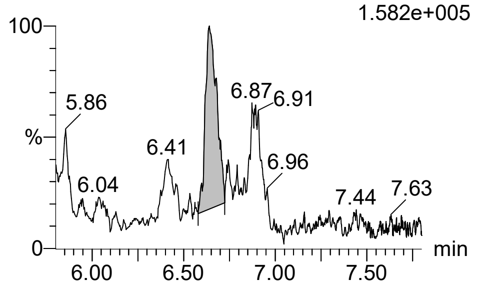

Sample Name: 20211111_A23_R2_IgM
F62:MRM of 1 channel,ES+
1066.3 > 204.1
1.404e+005

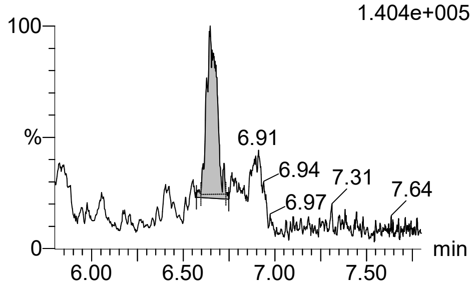

Sample Name: 20211111_A24_R2_IgM
F62:MRM of 1 channel,ES+
1066.3 > 204.1
4.214e+005

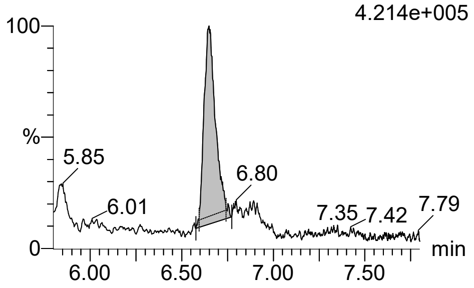

Sample Name: 20211111_L23_R2_IgM
F62:MRM of 1 channel,ES+
1066.3 > 204.1
2.759e+005

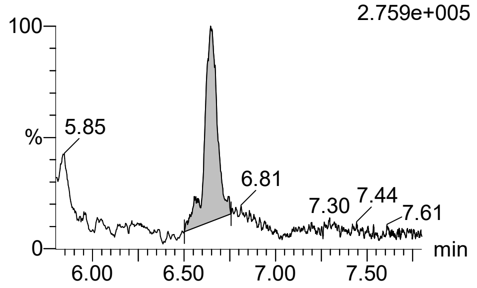

Sample Name: 20211111_L24_R2_IgM
F62:MRM of 1 channel,ES+
1066.3 > 204.1
1.383e+005

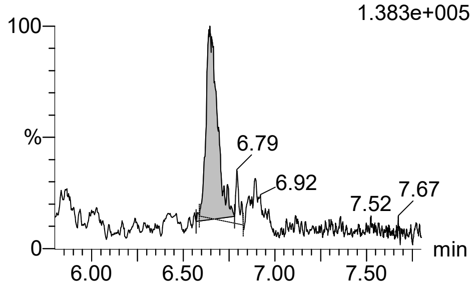

Sample Name: 20211111_H12_R2_IgM
F62:MRM of 1 channel,ES+
1066.3 > 204.1
1.134e+005

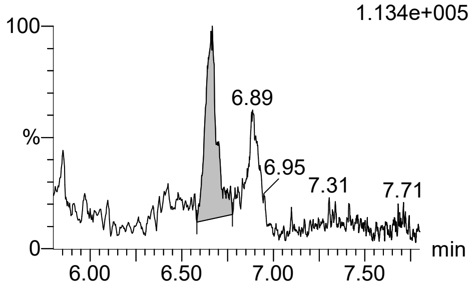

Sample Name: 20211111_QC_R14_IgM
F62:MRM of 1 channel,ES+
1066.3 > 204.1
2.423e+005

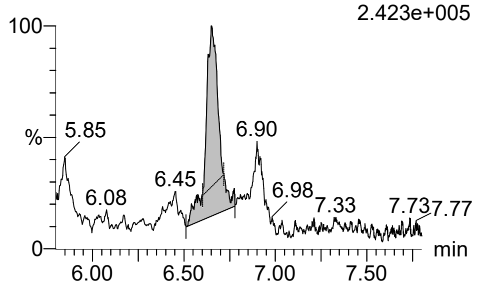

Sample Name: 20211111_A25_R2_IgM
F62:MRM of 1 channel,ES+
1066.3 > 204.1
3.419e+005

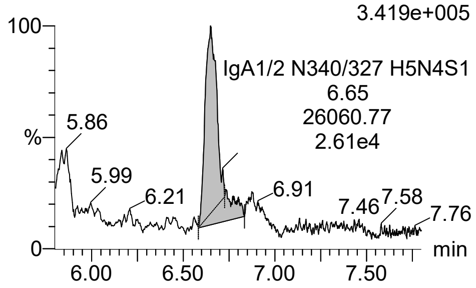

Sample Name: 20211111_A26_R2_IgM
F62:MRM of 1 channel,ES+
1066.3 > 204.1
1.338e+005

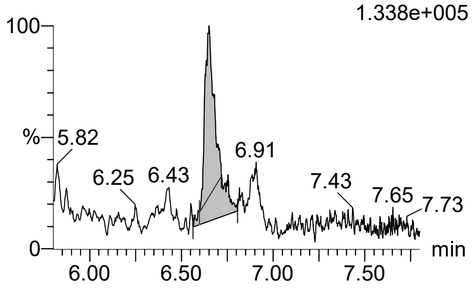

Sample Name: 20211111_L25_R2_IgM
F62:MRM of 1 channel,ES+
1066.3 > 204.1
2.151e+005

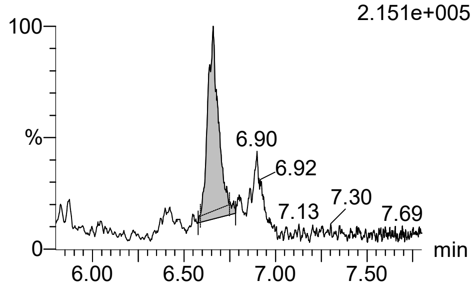

Sample Name: 20211111_L26_R2_IgM
F62:MRM of 1 channel,ES+
1066.3 > 204.1
2.081e+005

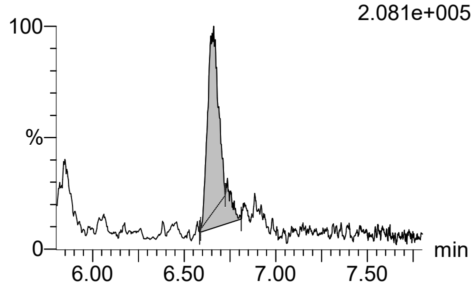

Sample Name: 20211111_H13_R2_IgM
F62:MRM of 1 channel,ES+
1066.3 > 204.1
2.124e+005

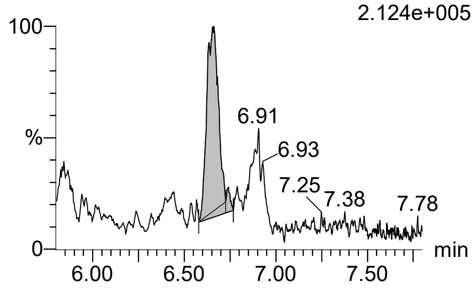

Sample Name: 20211111_A27_R2_IgM
F62:MRM of 1 channel,ES+
1066.3 > 204.1
2.672e+005

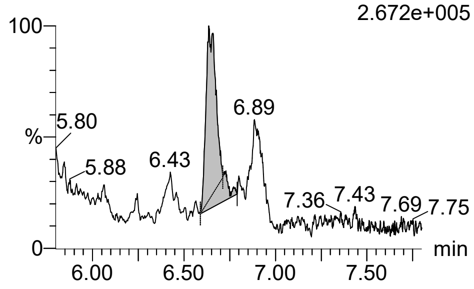

Sample Name: 20211111_A28_R2_IgM
F62:MRM of 1 channel,ES+
1066.3 > 204.1
2.376e+005

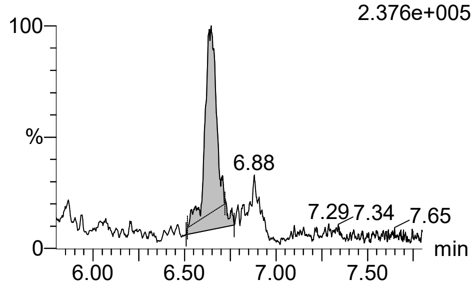

Sample Name: 20211111_L27_R2_IgM
F62:MRM of 1 channel,ES+
1066.3 > 204.1
4.643e+005

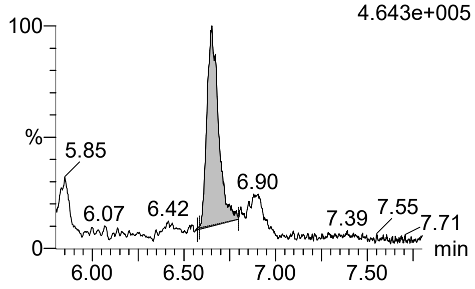

Sample Name: 20211111_L28_R2_IgM
F62:MRM of 1 channel,ES+
1066.3 > 204.1
2.089e+005

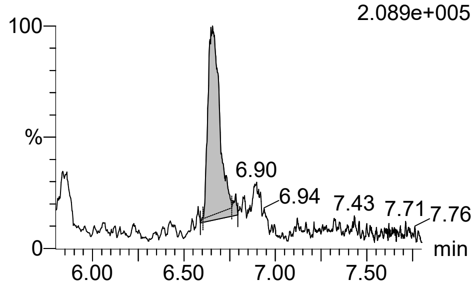

Sample Name: 20211111_H14_R2_IgM
F62:MRM of 1 channel,ES+
1066.3 > 204.1
3.257e+005

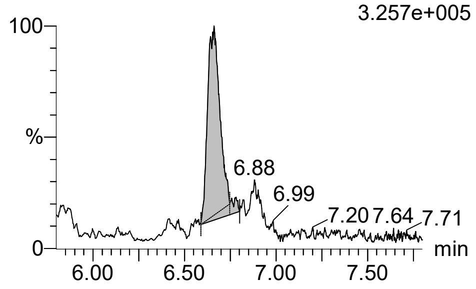

Sample Name: 20211111_A29_R2_IgM
F62:MRM of 1 channel,ES+
1066.3 > 204.1
7.982e+004

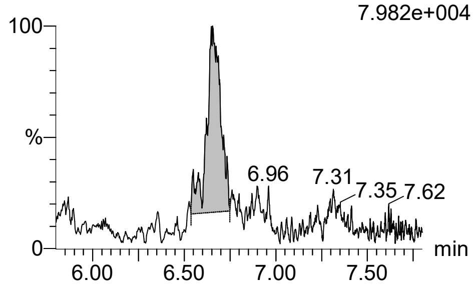

Sample Name: 20211111_A30_R2_IgM
F62:MRM of 1 channel,ES+
1066.3 > 204.1
2.040e+005

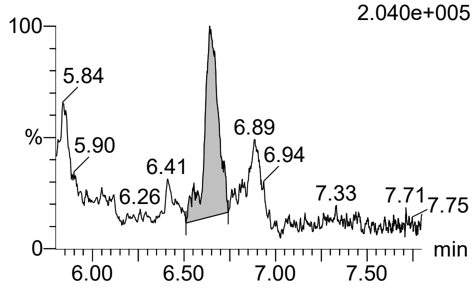

Sample Name: 20211111_QC_R15_IgM
F62:MRM of 1 channel,ES+
1066.3 > 204.1
2.452e+005

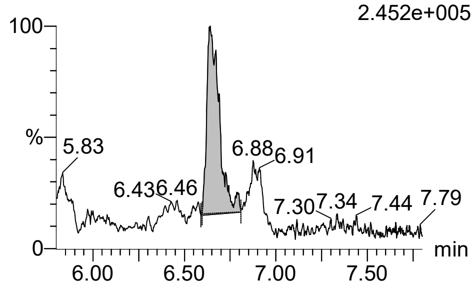

Sample Name: 20211111_L29_R2_IgM
F62:MRM of 1 channel,ES+
1066.3 > 204.1
1.263e+005

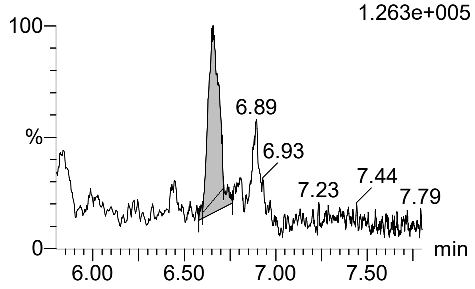

Sample Name: 20211111_L30_R2_IgM
F62:MRM of 1 channel,ES+
1066.3 > 204.1
8.992e+004

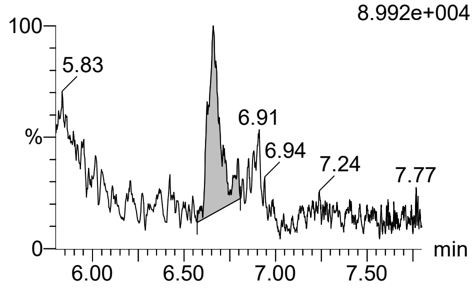

Sample Name: 20211111_H15_R2_IgM
F62:MRM of 1 channel,ES+
1066.3 > 204.1
1.975e+005

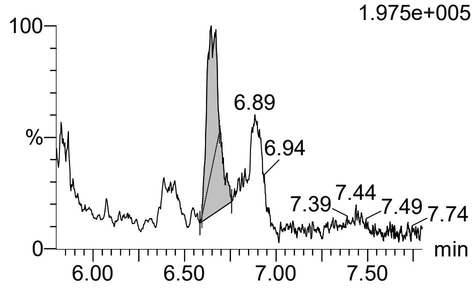

Sample Name: 20211111_A31_R2_IgM
F62:MRM of 1 channel,ES+
1066.3 > 204.1
8.550e+004

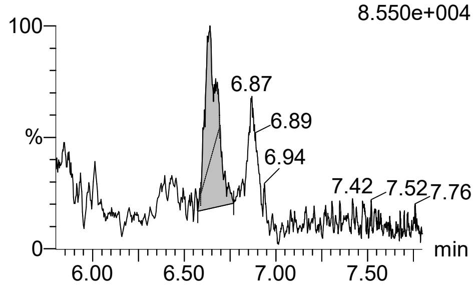

Dataset: C:\MassLynx\10.pro\20211111_clinicalsamples_IgM_IgA.qld

Last Altered: Thursday, March 09, 2023 19:58:25 台北標準時間
Printed: Friday, August 01, 2025 15:19:24 台北標準時間

Sample Name: 20211111_A32_R2_IgM
F62:MRM of 1 channel,ES+
1066.3 > 204.1
2.224e+005

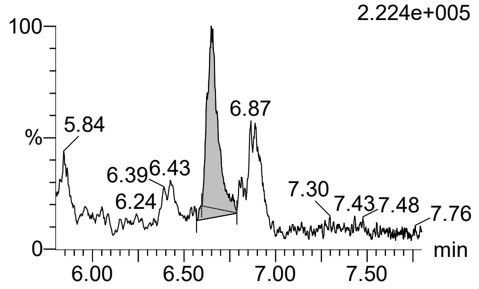

Sample Name: 20211111_L31_R2_IgM
F62:MRM of 1 channel,ES+
1066.3 > 204.1
1.130e+005

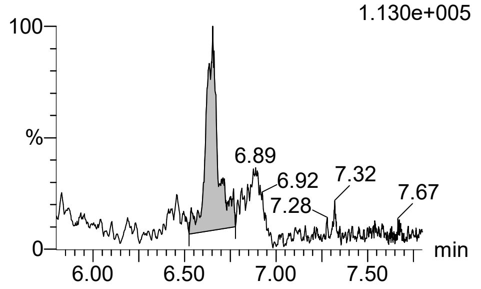

Sample Name: 20211111_L32_R2_IgM
F62:MRM of 1 channel,ES+
1066.3 > 204.1
2.901e+005

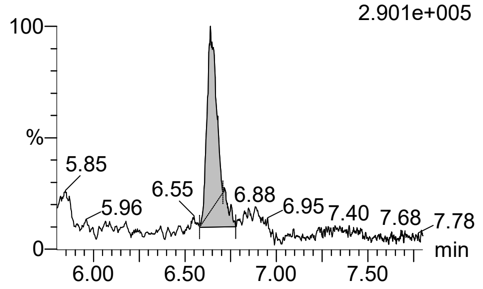

Sample Name: 20211111_H16_R2_IgM
F62:MRM of 1 channel,ES+
1066.3 > 204.1
4.087e+005

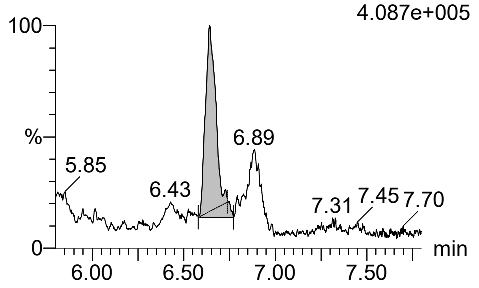

Sample Name: 20211111_A33_R2_IgM
F62:MRM of 1 channel,ES+
1066.3 > 204.1
2.873e+005

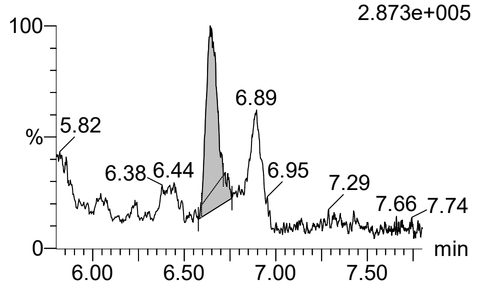

Sample Name: 20211111_A34_R2_IgM
F62:MRM of 1 channel,ES+
1066.3 > 204.1
8.332e+004

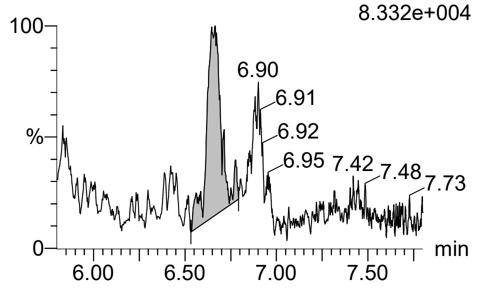

Sample Name: 20211111_H17_R2_IgM
F62:MRM of 1 channel,ES+
1066.3 > 204.1
1.530e+006

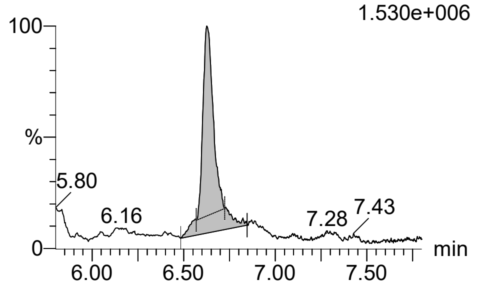

Sample Name: 20211111_A35_R2_IgM
F62:MRM of 1 channel,ES+
1066.3 > 204.1
3.330e+005

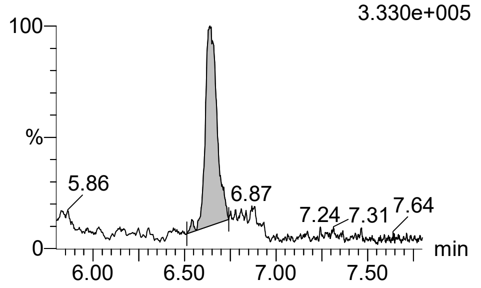

Sample Name: 20211111_QC_R16_IgM
F62:MRM of 1 channel,ES+
1066.3 > 204.1
2.665e+005

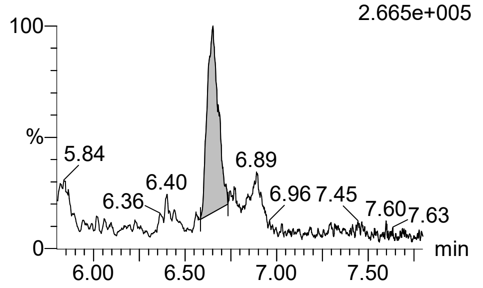

Sample Name: 20211111_A36_R2_IgM
F62:MRM of 1 channel,ES+
1066.3 > 204.1
2.736e+005

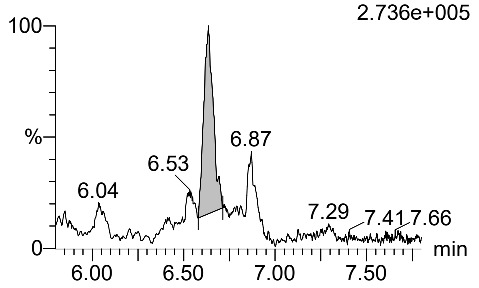

Sample Name: 20211111_H18_R2_IgM
F62:MRM of 1 channel,ES+
1066.3 > 204.1
6.109e+005

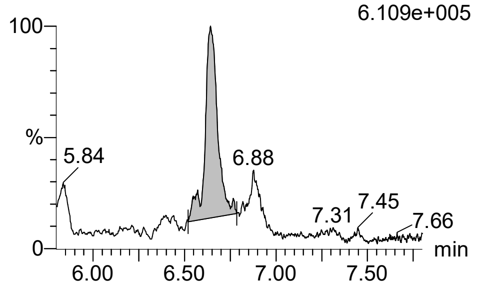

Sample Name: 20211111_A37_R2_IgM
F62:MRM of 1 channel,ES+
1066.3 > 204.1
3.175e+005

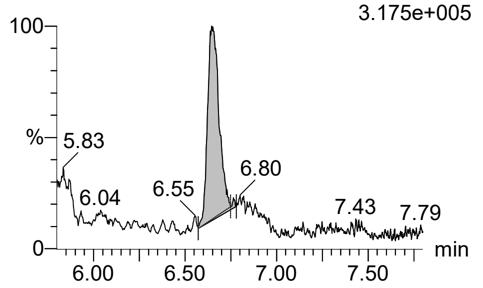

Sample Name: 20211111_A38_R2_IgM
F62:MRM of 1 channel,ES+
1066.3 > 204.1
2.230e+005

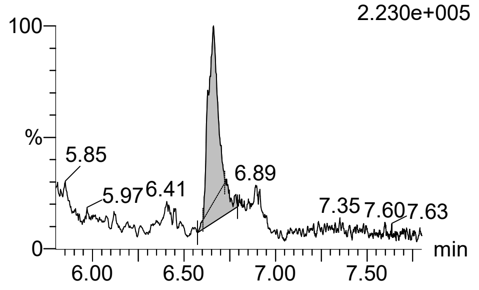

Sample Name: 20211111_H19_R2_IgM
F62:MRM of 1 channel,ES+
1066.3 > 204.1
5.701e+005

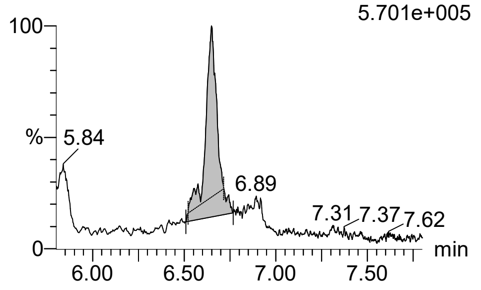

Sample Name: 20211111_A39_R2_IgM
F62:MRM of 1 channel,ES+
1066.3 > 204.1
3.117e+005

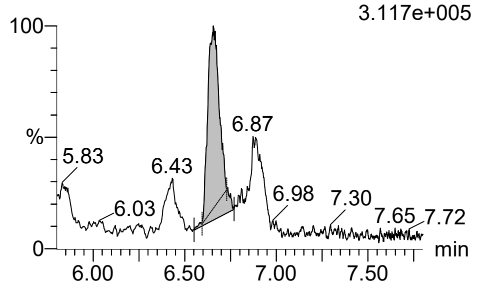

Sample Name: 20211111_A40_R2_IgM
F62:MRM of 1 channel,ES+
1066.3 > 204.1
1.653e+005

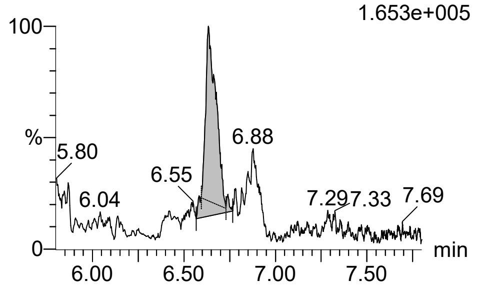

Sample Name: 20211111_H20_R2_IgM
F62:MRM of 1 channel,ES+
1066.3 > 204.1
9.427e+005

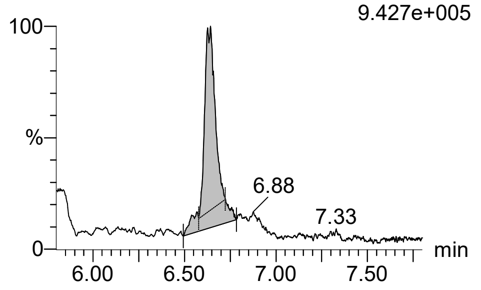

Sample Name: 20211111_A41_R2_IgM
F62:MRM of 1 channel,ES+
1066.3 > 204.1
2.832e+005

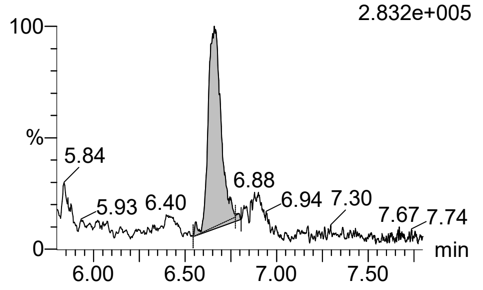

Sample Name: 20211111_A42_R2_IgM
F62:MRM of 1 channel,ES+
1066.3 > 204.1
1.334e+005

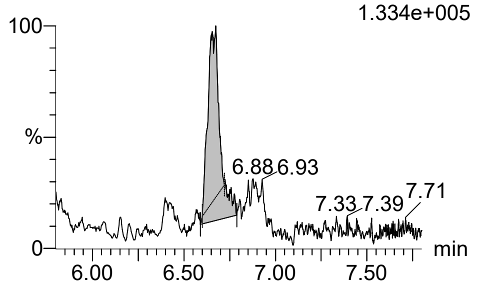

Sample Name: 20211111_A43_R2_IgM
F62:MRM of 1 channel,ES+
1066.3 > 204.1
1.079e+005

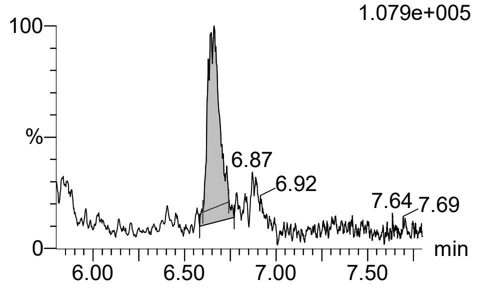

Sample Name: 20211111_A44_R2_IgM
F62:MRM of 1 channel,ES+
1066.3 > 204.1
1.085e+005

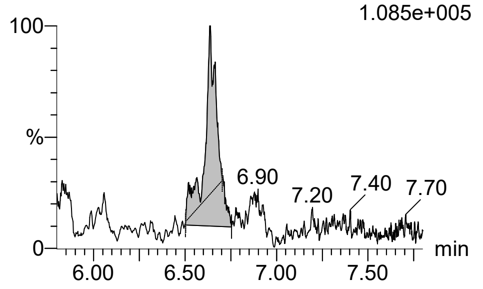

Sample Name: 20211111_QC_R17_IgM
F62:MRM of 1 channel,ES+
1066.3 > 204.1
2.217e+005

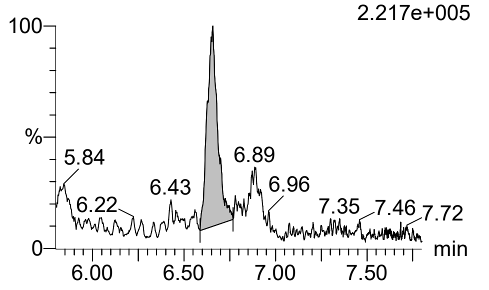

Sample Name: 20211111_L2_R3_IgM
F62:MRM of 1 channel,ES+
1066.3 > 204.1
1.962e+005

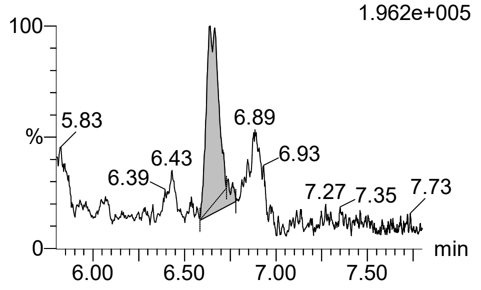

Sample Name: 20211111_H1_R3_IgM
F62:MRM of 1 channel,ES+
1066.3 > 204.1
2.166e+005

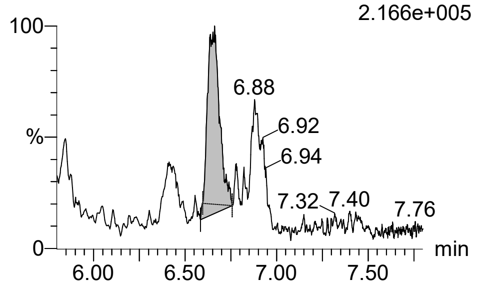

Sample Name: 20211111_A3_R3_IgM
F62:MRM of 1 channel,ES+
1066.3 > 204.1
2.257e+005

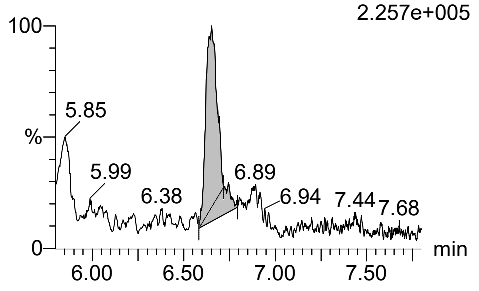

Sample Name: 20211111_A4_R3_IgM
F62:MRM of 1 channel,ES+
1066.3 > 204.1
1.450e+005

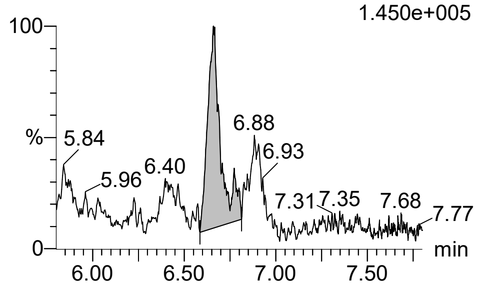

Sample Name: 20211111_L3_R3_IgM
F62:MRM of 1 channel,ES+
1066.3 > 204.1
2.165e+005

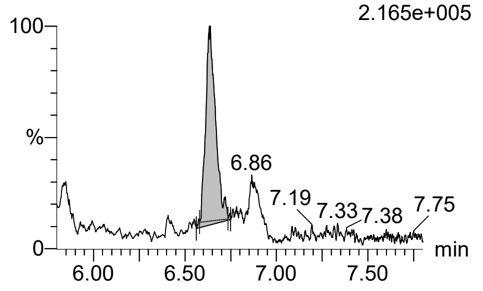

Sample Name: 20211111_L4_R3_IgM
F62:MRM of 1 channel,ES+
1066.3 > 204.1
1.992e+005

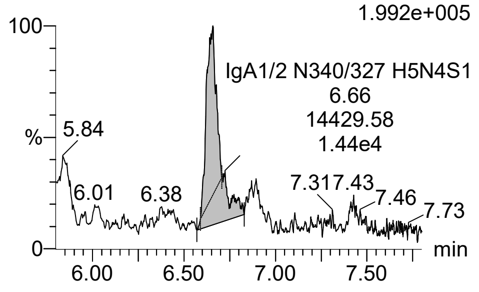

Sample Name: 20211111_H2_R3_IgM
F62:MRM of 1 channel,ES+
1066.3 > 204.1
3.081e+005

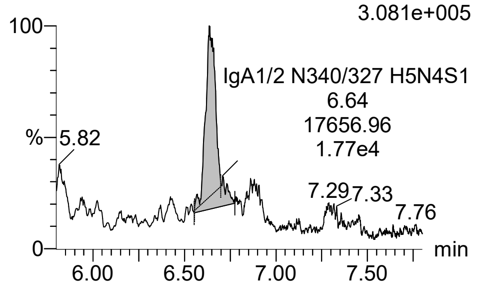

Sample Name: 20211111_A5_R3_IgM
F62:MRM of 1 channel,ES+
1066.3 > 204.1
4.104e+005

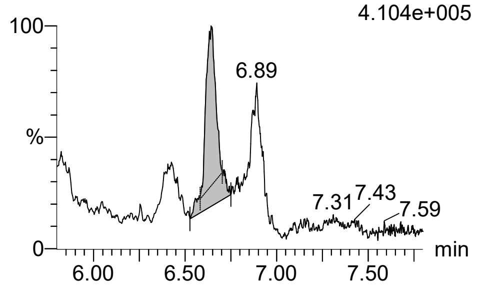

Dataset: C:\MassLynx\10.pro\20211111_clinicalsamples_IgM_IgA.qld

Last Altered: Thursday, March 09, 2023 19:58:25 台北標準時間
Printed: Friday, August 01, 2025 15:19:24 台北標準時間

Sample Name: 20211111_A6_R3_IgM
F62:MRM of 1 channel,ES+
1066.3 > 204.1
2.443e+005

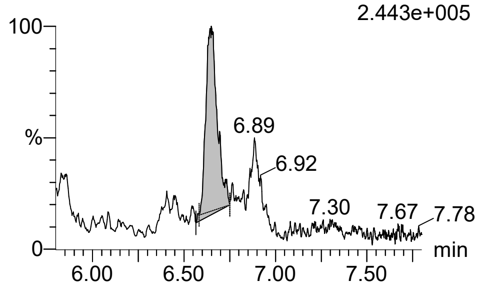

Sample Name: 20211111_QC_R18_IgM
F62:MRM of 1 channel,ES+
1066.3 > 204.1
2.536e+005

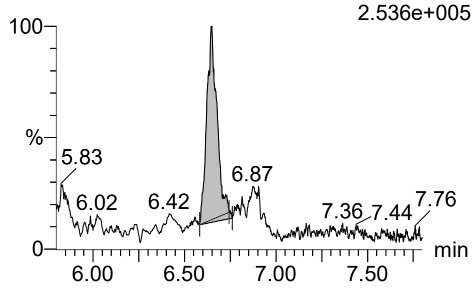

Sample Name: 20211111_L5_R3_IgM
F62:MRM of 1 channel,ES+
1066.3 > 204.1
2.240e+005

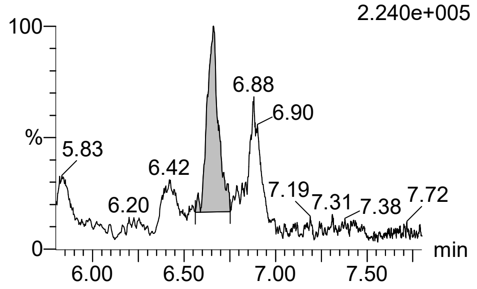

Sample Name: 20211111_L6_R3_IgM
F62:MRM of 1 channel,ES+
1066.3 > 204.1
2.495e+005

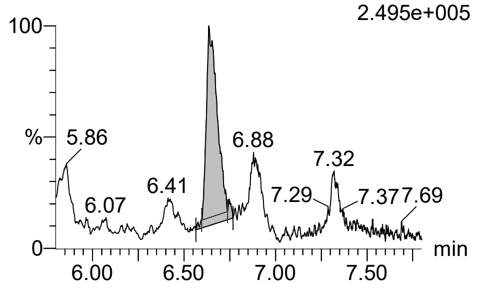

Sample Name: 20211111_H3_R3_IgM
F62:MRM of 1 channel,ES+
1066.3 > 204.1
1.044e+005

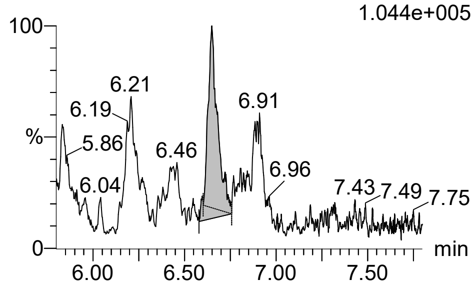

Sample Name: 20211111_A7_R3_IgM
F62:MRM of 1 channel,ES+
1066.3 > 204.1
4.301e+005

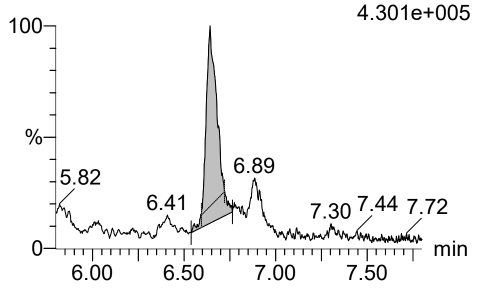

Sample Name: 20211111_A8_R3_IgM
F62:MRM of 1 channel,ES+
1066.3 > 204.1
1.994e+005

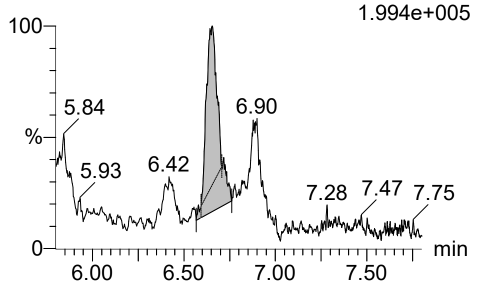

Sample Name: 20211111_L7_R3_IgM
F62:MRM of 1 channel,ES+
1066.3 > 204.1
1.447e+005

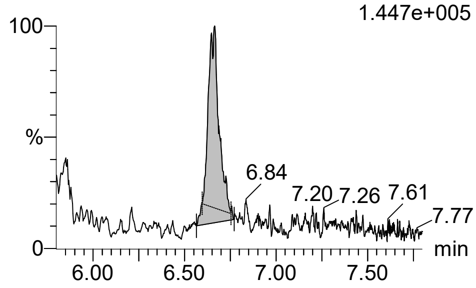

Sample Name: 20211111_L8_R3_IgM
F62:MRM of 1 channel,ES+
1066.3 > 204.1
7.437e+004

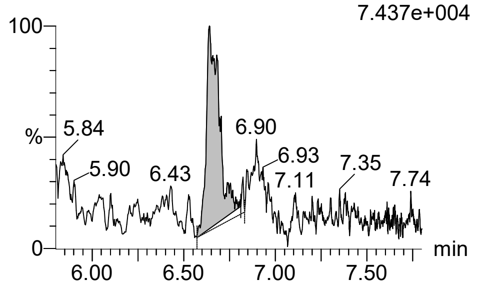

Sample Name: 20211111_H4_R3_IgM
F62:MRM of 1 channel,ES+
1066.3 > 204.1
1.440e+005

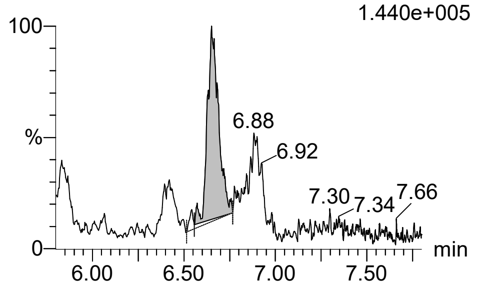

Sample Name: 20211111_A9_R3_IgM
F62:MRM of 1 channel,ES+
1066.3 > 204.1
1.384e+005

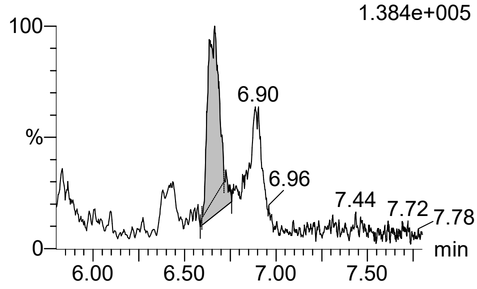

Sample Name: 20211111_A10_R3_IgM
F62:MRM of 1 channel,ES+
1066.3 > 204.1
2.755e+005

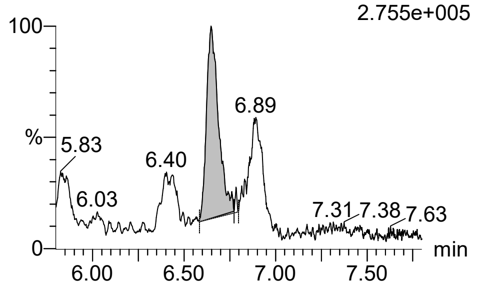

Sample Name: 20211111_L9_R3_IgM
F62:MRM of 1 channel,ES+
1066.3 > 204.1
2.222e+005

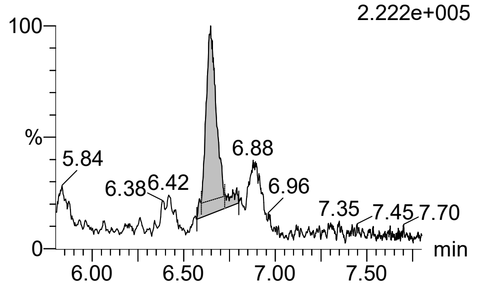

Sample Name: 20211111_L10_R3_IgM
F62:MRM of 1 channel,ES+
1066.3 > 204.1
4.436e+005

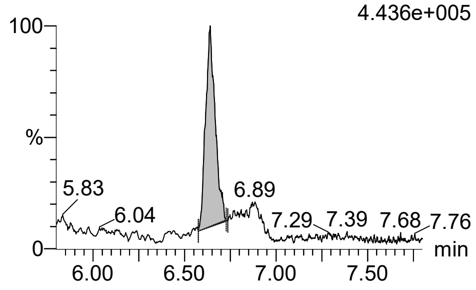

Sample Name: 20211111_QC_R19_IgM
F62:MRM of 1 channel,ES+
1066.3 > 204.1
2.478e+005

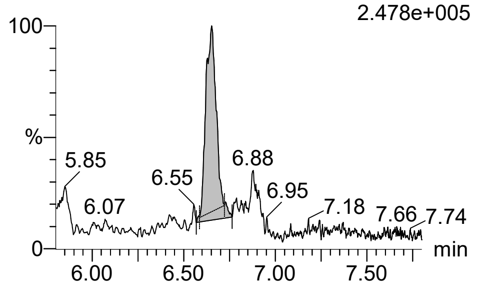

Sample Name: 20211111_H5_R3_IgM
F62:MRM of 1 channel,ES+
1066.3 > 204.1
1.901e+005

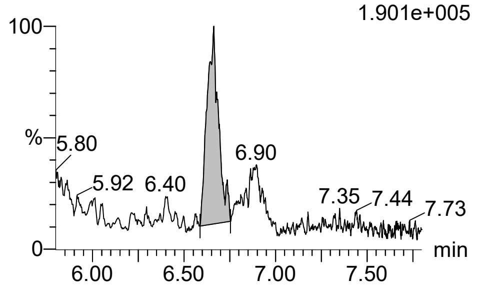

Sample Name: 20211111_A11_R3_IgM
F62:MRM of 1 channel,ES+
1066.3 > 204.1
1.571e+005

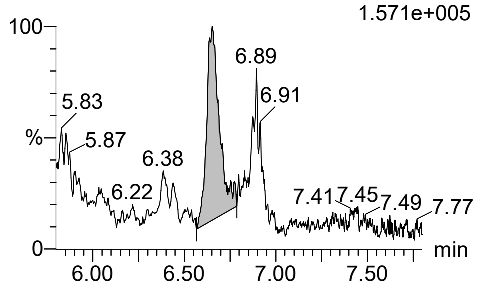

Sample Name: 20211111_A12_R3_IgM
F62:MRM of 1 channel,ES+
1066.3 > 204.1
1.064e+005

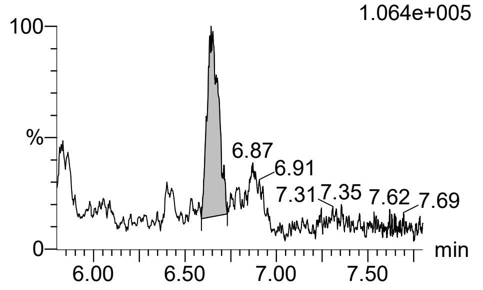

Sample Name: 20211111_L11_R3_IgM
F62:MRM of 1 channel,ES+
1066.3 > 204.1
1.203e+005

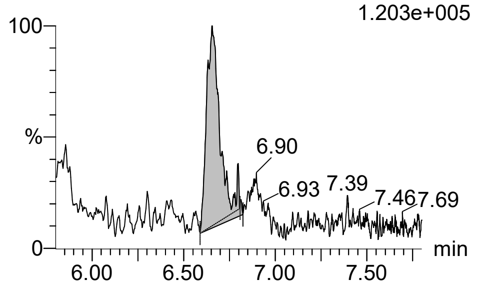

Sample Name: 20211111_L12_R3_IgM
F62:MRM of 1 channel,ES+
1066.3 > 204.1
2.264e+005

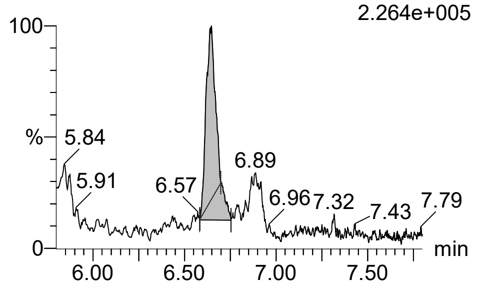

Sample Name: 20211111_H6_R3_IgM
F62:MRM of 1 channel,ES+
1066.3 > 204.1
1.589e+005

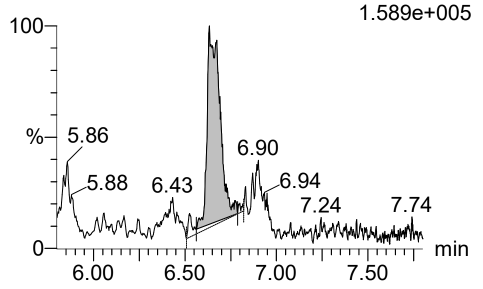

Sample Name: 20211111_A13_R3_IgM
F62:MRM of 1 channel,ES+
1066.3 > 204.1
1.912e+005

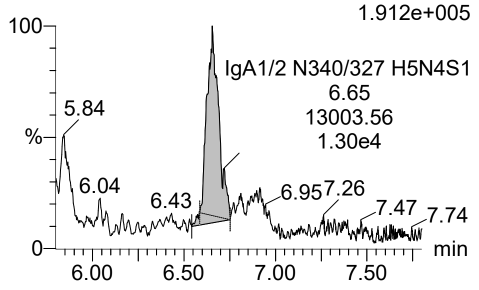

Sample Name: 20211111_A14_R3_IgM
F62:MRM of 1 channel,ES+
1066.3 > 204.1
2.580e+005

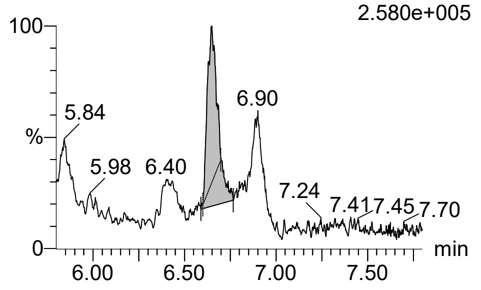

Sample Name: 20211111_L13_R3_IgM
F62:MRM of 1 channel,ES+
1066.3 > 204.1
1.873e+005

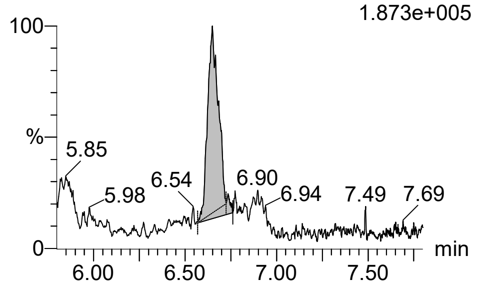

Sample Name: 20211111_L14_R3_IgM
F62:MRM of 1 channel,ES+
1066.3 > 204.1
1.974e+005

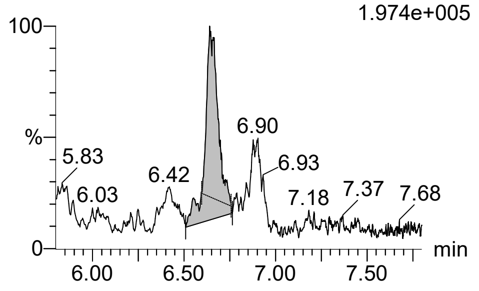

Sample Name: 20211111_H7_R3_IgM
F62:MRM of 1 channel,ES+
1066.3 > 204.1
2.244e+005

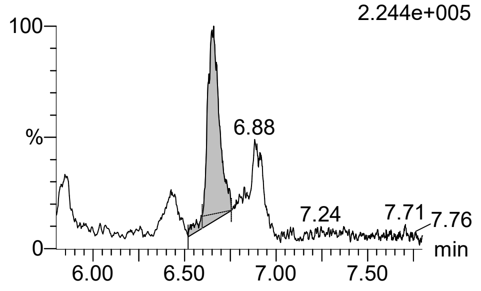

Sample Name: 20211111_A15_R3_IgM
F62:MRM of 1 channel,ES+
1066.3 > 204.1
5.449e+005

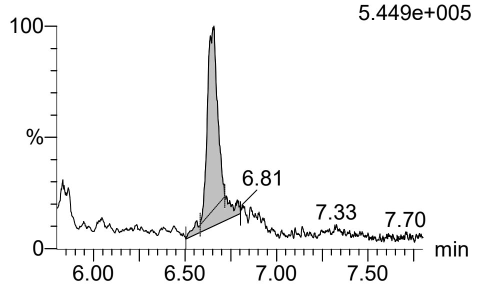

Sample Name: 20211111_QC_R20_IgM
F62:MRM of 1 channel,ES+
1066.3 > 204.1
3.082e+005

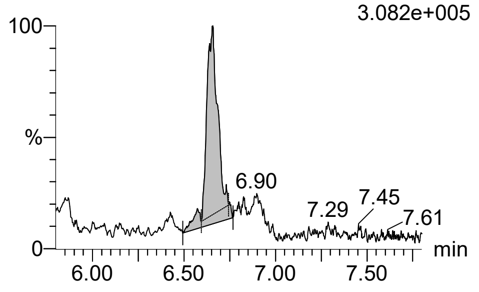

Sample Name: 20211111_A16_R3_IgM
F62:MRM of 1 channel,ES+
1066.3 > 204.1
3.087e+005

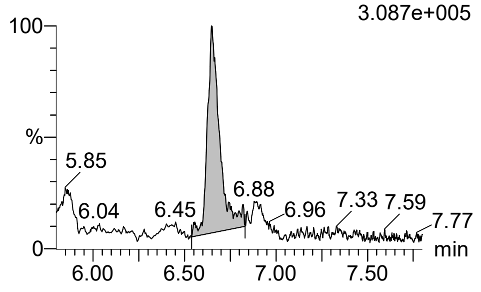

Sample Name: 20211111_L15_R3_IgM
F62:MRM of 1 channel,ES+
1066.3 > 204.1
2.869e+005

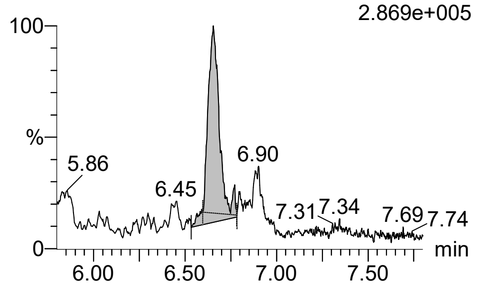

Dataset: C:\MassLynx\10.pro\20211111_clinicalsamples_IgM_IgA.qld

Last Altered: Thursday, March 09, 2023 19:58:25 台北標準時間
Printed: Friday, August 01, 2025 15:19:24 台北標準時間

Sample Name: 20211111_L16_R3_IgM
F62:MRM of 1 channel,ES+
1066.3 > 204.1
1.168e+005

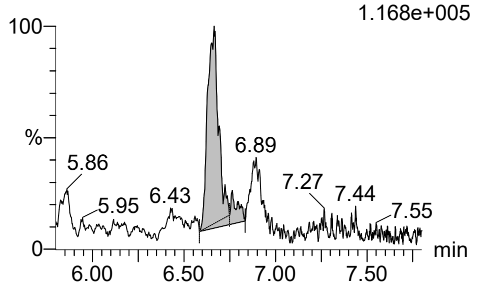

Sample Name: 20211111_H8_R3_IgM
F62:MRM of 1 channel,ES+
1066.3 > 204.1
1.218e+005

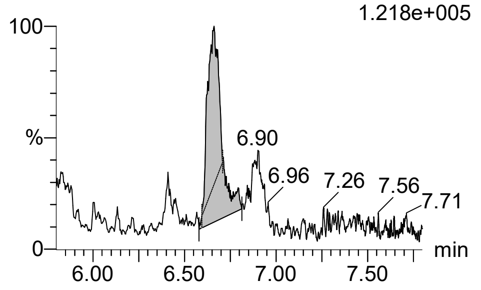

Sample Name: 20211111_A17_R3_IgM
F62:MRM of 1 channel,ES+
1066.3 > 204.1
3.055e+005

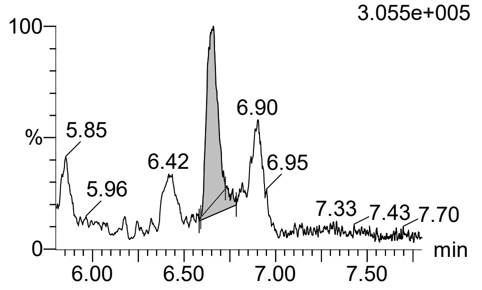

Sample Name: 20211111_A18_R3_IgM
F62:MRM of 1 channel,ES+
1066.3 > 204.1
2.203e+005

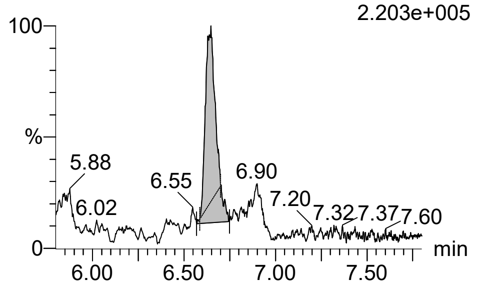

Sample Name: 20211111_L17_R3_IgM
F62:MRM of 1 channel,ES+
1066.3 > 204.1
3.255e+005

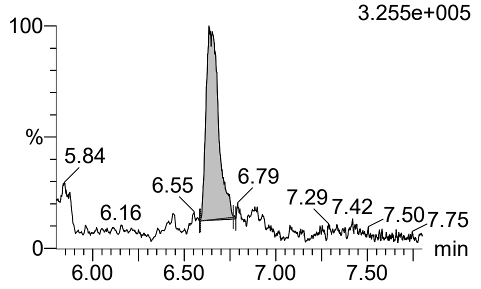

Sample Name: 20211111_L18_R3_IgM
F62:MRM of 1 channel,ES+
1066.3 > 204.1
9.452e+004

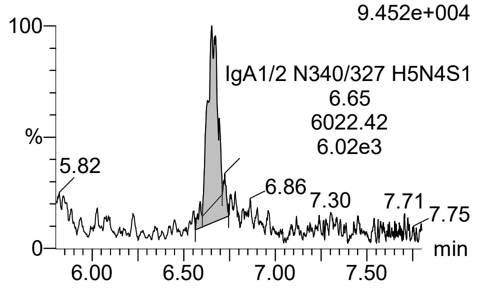

Sample Name: 20211111_H9_R3_IgM
F62:MRM of 1 channel,ES+
1066.3 > 204.1
1.711e+005

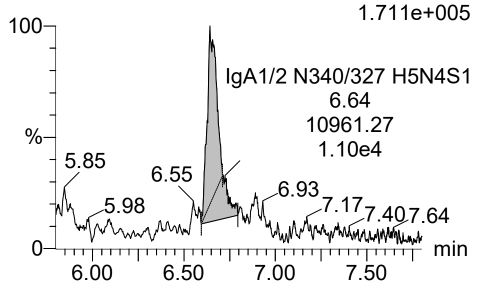

Sample Name: 20211111_A19_R3_IgM
F62:MRM of 1 channel,ES+
1066.3 > 204.1
3.128e+005

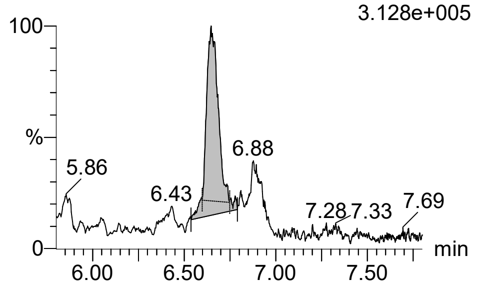

Sample Name: 20211111_A20_R3_IgM
F62:MRM of 1 channel,ES+
1066.3 > 204.1
1.527e+005

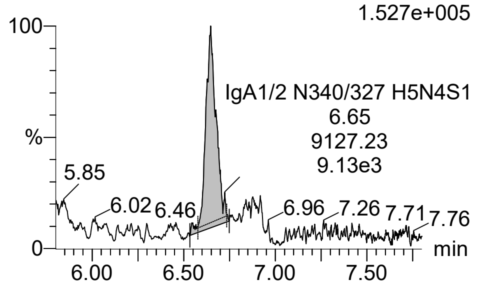

Sample Name: 20211111_L19_R3_IgM
F62:MRM of 1 channel,ES+
1066.3 > 204.1
1.668e+005

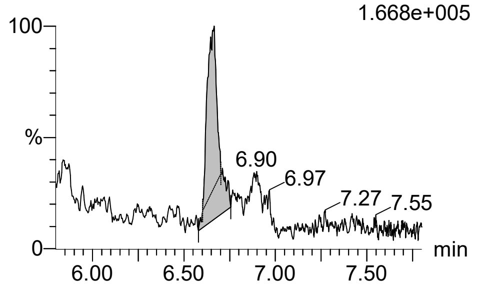

Sample Name: 20211111_QC_R21_IgM
F62:MRM of 1 channel,ES+
1066.3 > 204.1
1.957e+005

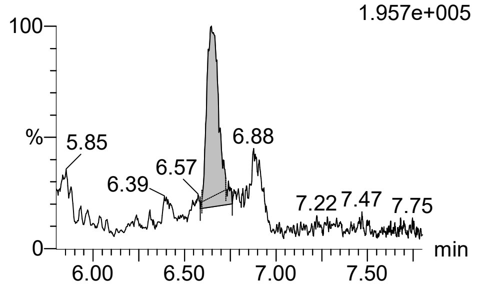

Sample Name: 20211111_L20_R3_IgM
F62:MRM of 1 channel,ES+
1066.3 > 204.1
1.707e+005

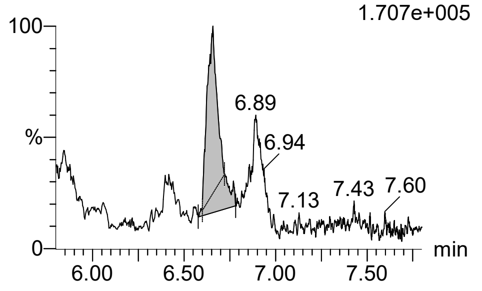

Sample Name: 20211111_H10_R3_IgM
F62:MRM of 1 channel,ES+
1066.3 > 204.1
1.934e+005

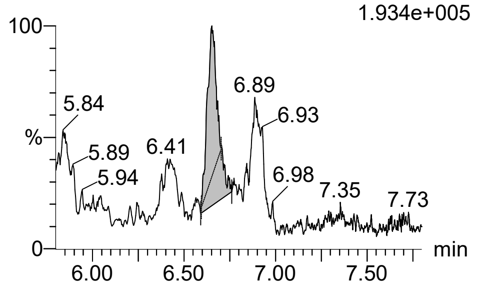

Sample Name: 20211111_A21_R3_IgM
F62:MRM of 1 channel,ES+
1066.3 > 204.1
1.776e+005

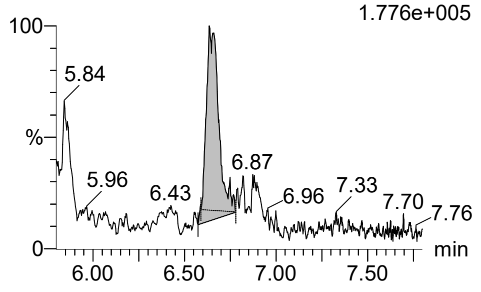

Sample Name: 20211111_A22_R3_IgM
F62:MRM of 1 channel,ES+
1066.3 > 204.1
8.747e+004

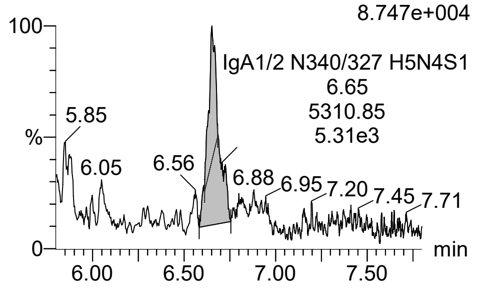

Sample Name: 20211111_L21_R3_IgM
F62:MRM of 1 channel,ES+
1066.3 > 204.1
1.588e+005

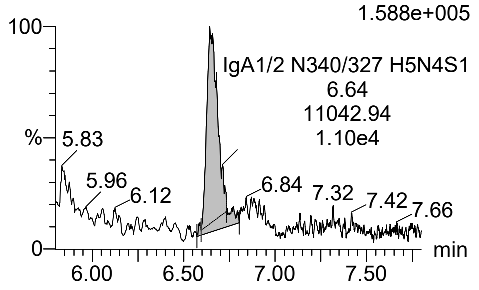

Sample Name: 20211111_L22_R3_IgM
F62:MRM of 1 channel,ES+
1066.3 > 204.1
1.448e+005

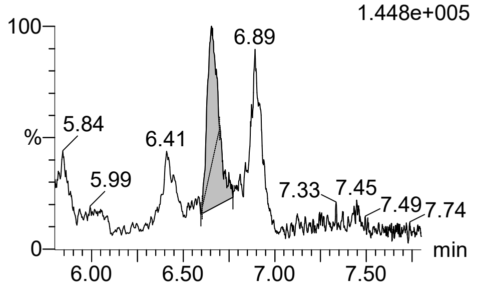

Sample Name: 20211111_H11_R3_IgM
F62:MRM of 1 channel,ES+
1066.3 > 204.1
1.508e+005

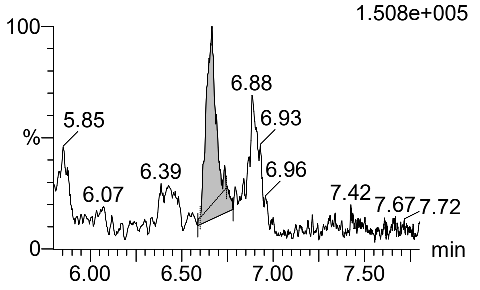

Sample Name: 20211111_A23_R3_IgM
F62:MRM of 1 channel,ES+
1066.3 > 204.1
1.278e+005

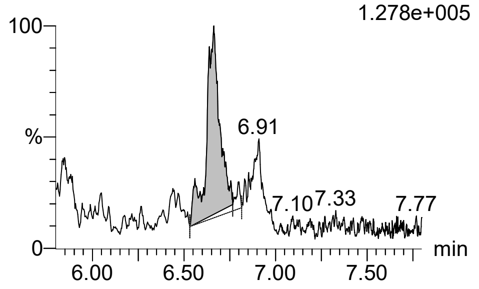

Sample Name: 20211111_A24_R3_IgM
F62:MRM of 1 channel,ES+
1066.3 > 204.1
4.115e+005

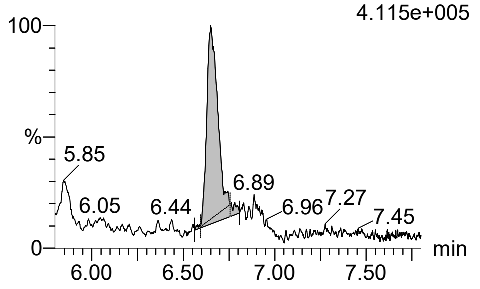

Sample Name: 20211111_L23_R3_IgM
F62:MRM of 1 channel,ES+
1066.3 > 204.1
3.031e+005

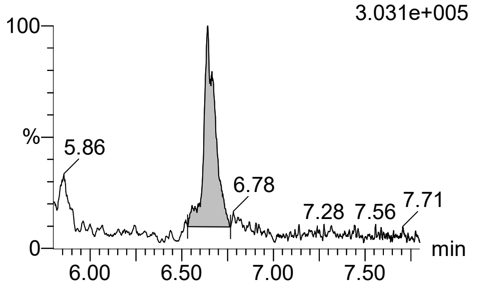

Sample Name: 20211111_L24_R3_IgM
F62:MRM of 1 channel,ES+
1066.3 > 204.1
9.604e+004

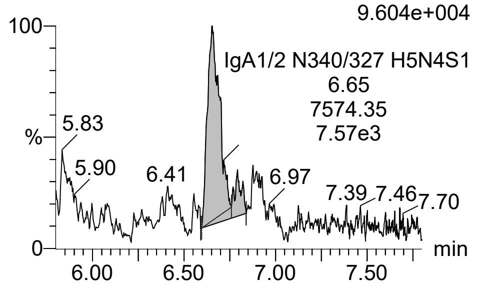

Sample Name: 20211111_H12_R3_IgM
F62:MRM of 1 channel,ES+
1066.3 > 204.1
9.543e+004

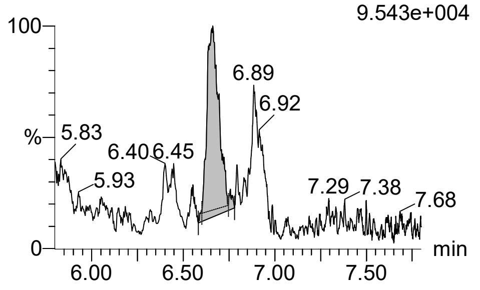

Sample Name: 20211111_QC_R22_IgM
F62:MRM of 1 channel,ES+
1066.3 > 204.1
2.339e+005

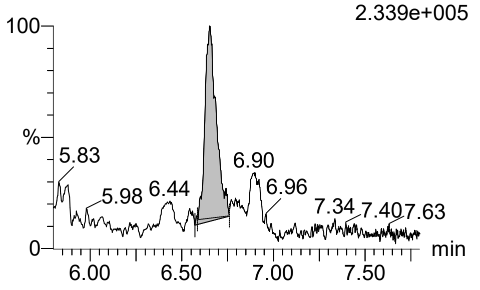

Sample Name: 20211111_A25_R3_IgM
F62:MRM of 1 channel,ES+
1066.3 > 204.1
2.626e+005

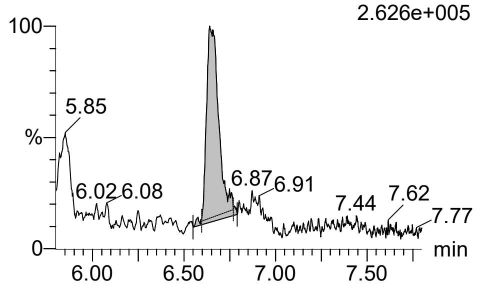

Sample Name: 20211111_A26_R3_IgM
F62:MRM of 1 channel,ES+
1066.3 > 204.1
1.092e+005

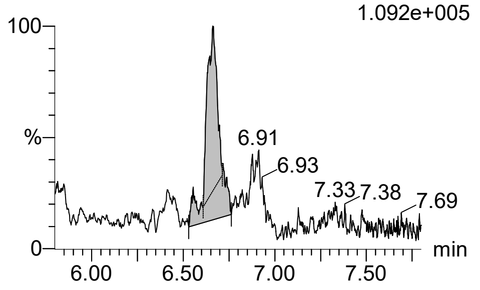

Sample Name: 20211111_L25_R3_IgM
F62:MRM of 1 channel,ES+
1066.3 > 204.1
1.671e+005

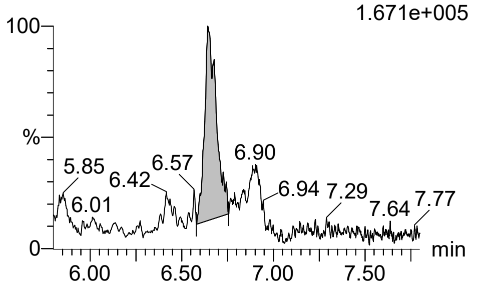

Sample Name: 20211111_L26_R3_IgM
F62:MRM of 1 channel,ES+
1066.3 > 204.1
1.741e+005

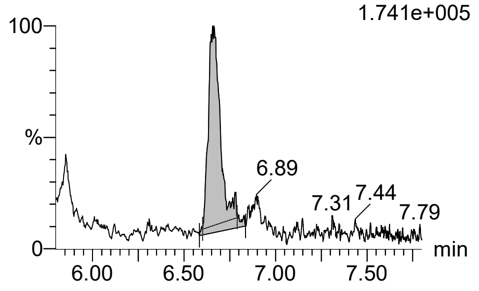

Sample Name: 20211111_H13_R3_IgM
F62:MRM of 1 channel,ES+
1066.3 > 204.1
2.159e+005

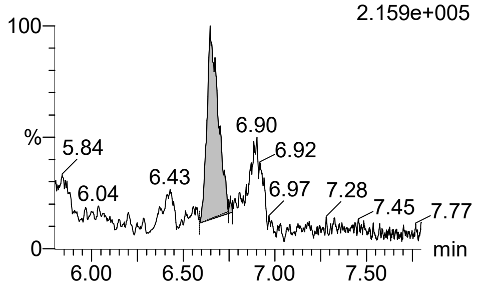

Sample Name: 20211111_A27_R3_IgM
F62:MRM of 1 channel,ES+
1066.3 > 204.1
2.180e+005

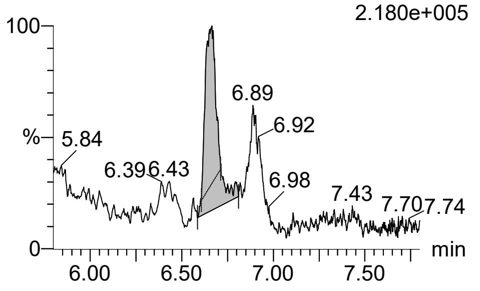

Dataset: C:\MassLynx\10.pro\20211111_clinicalsamples_IgM_IgA.qld

Last Altered: Thursday, March 09, 2023 19:58:25 台北標準時間
Printed: Friday, August 01, 2025 15:19:24 台北標準時間

Sample Name: 20211111_A28_R3_IgM
F62:MRM of 1 channel,ES+
1066.3 > 204.1
1.424e+005

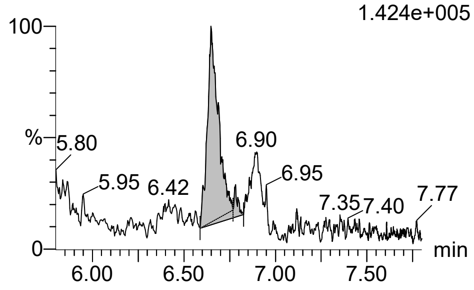

Sample Name: 20211111_L27_R3_IgM
F62:MRM of 1 channel,ES+
1066.3 > 204.1
3.593e+005

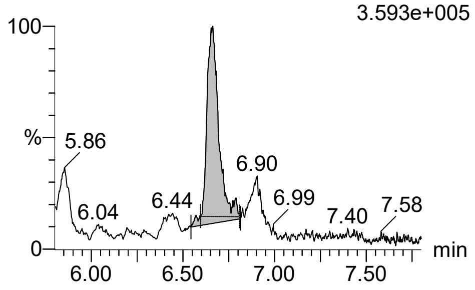

Sample Name: 20211111_L28_R3_IgM
F62:MRM of 1 channel,ES+
1066.3 > 204.1
2.175e+005

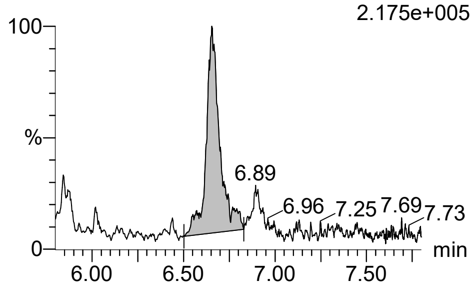

Sample Name: 20211111_H14_R3_IgM
F62:MRM of 1 channel,ES+
1066.3 > 204.1
3.086e+005

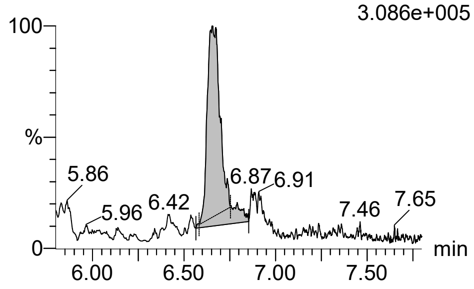

Sample Name: 20211111_A29_R3_IgM
F62:MRM of 1 channel,ES+
1066.3 > 204.1
5.986e+004

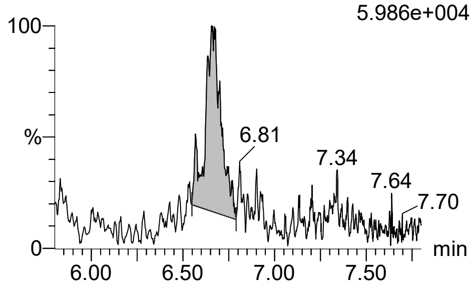

Sample Name: 20211111_A30_R3_IgM
F62:MRM of 1 channel,ES+
1066.3 > 204.1
1.860e+005

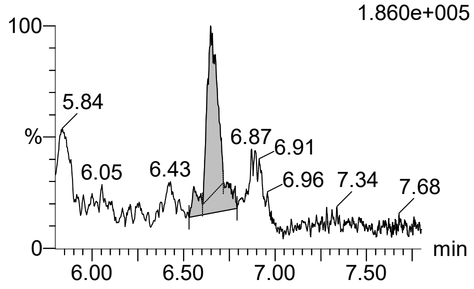

Sample Name: 20211111_QC_R23_IgM
F62:MRM of 1 channel,ES+
1066.3 > 204.1
2.110e+005

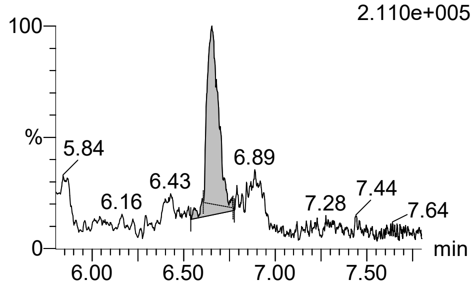

Sample Name: 20211111_L29_R3_IgM
F62:MRM of 1 channel,ES+
1066.3 > 204.1
1.110e+005

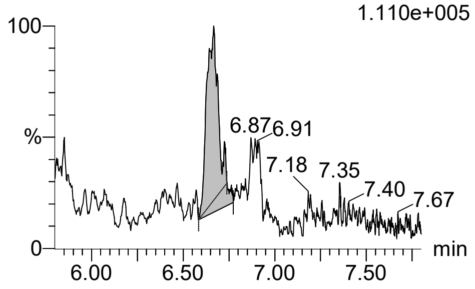

Sample Name: 20211111_L30_R3_IgM
F62:MRM of 1 channel,ES+
1066.3 > 204.1
7.221e+004

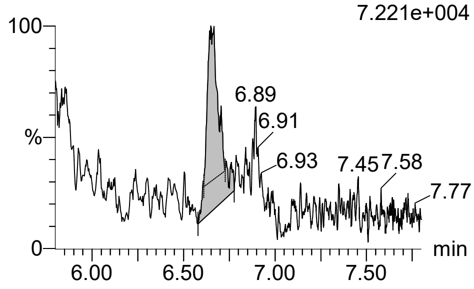

Sample Name: 20211111_H15_R3_IgM
F62:MRM of 1 channel,ES+
1066.3 > 204.1
2.108e+005

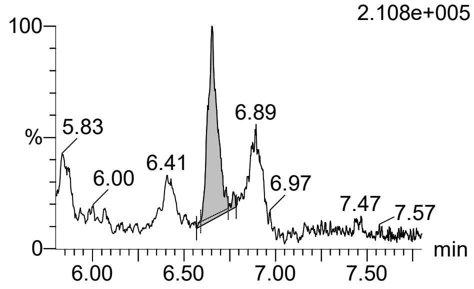

Sample Name: 20211111_A31_R3_IgM
F62:MRM of 1 channel,ES+
1066.3 > 204.1
7.756e+004

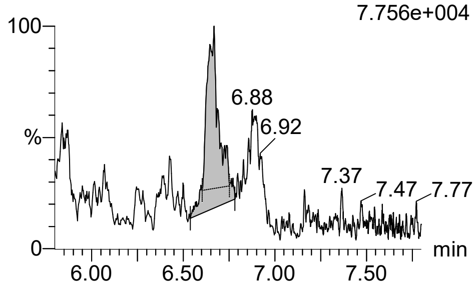

Sample Name: 20211111_A32_R3_IgM
F62:MRM of 1 channel,ES+
1066.3 > 204.1
1.879e+005

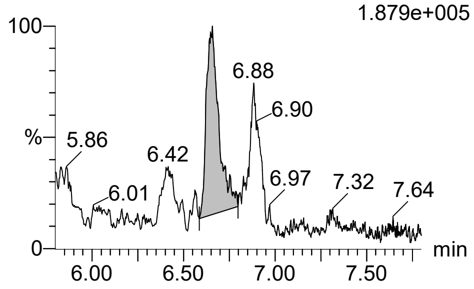

Sample Name: 20211111_L31_R3_IgM
F62:MRM of 1 channel,ES+
1066.3 > 204.1
7.044e+004

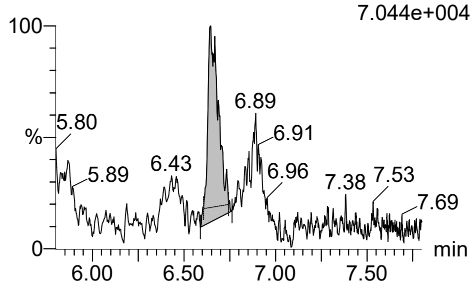

Sample Name: 20211111_L32_R3_IgM
F62:MRM of 1 channel,ES+
1066.3 > 204.1
2.196e+005

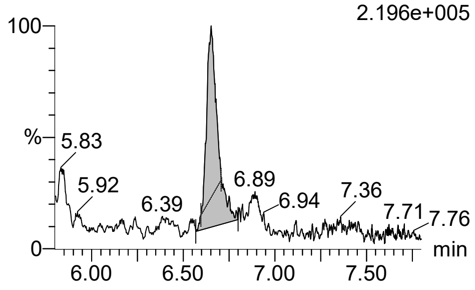

Sample Name: 20211111_H16_R3_IgM
F62:MRM of 1 channel,ES+
1066.3 > 204.1
2.835e+005

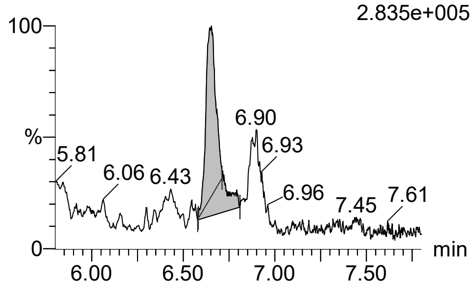

Dataset: C:\MassLynx\10.pro\20211111_clinicalsamples_IgM_IgA.qld

Last Altered: Thursday, March 09, 2023 19:58:25 台北標準時間
Printed: Friday, August 01, 2025 15:19:24 台北標準時間

Sample Name: 20211111_A33_R3_IgM
F62:MRM of 1 channel,ES+
1066.3 > 204.1
2.588e+005

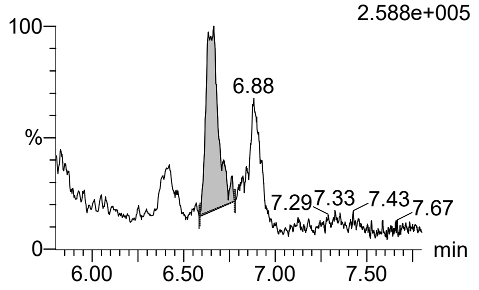

Sample Name: 20211111_A34_R3_IgM
F62:MRM of 1 channel,ES+
1066.3 > 204.1
8.333e+004

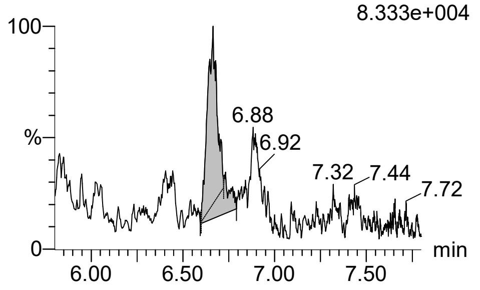

Sample Name: 20211111_H17_R3_IgM
F62:MRM of 1 channel,ES+
1066.3 > 204.1
1.162e+006

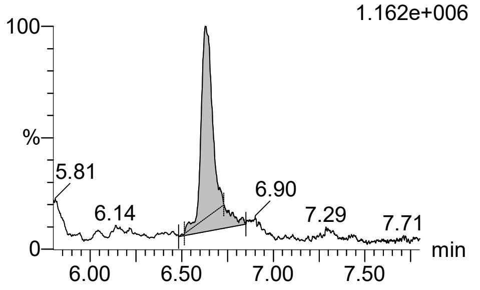

Sample Name: 20211111_A35_R3_IgM
F62:MRM of 1 channel,ES+
1066.3 > 204.1
2.316e+005

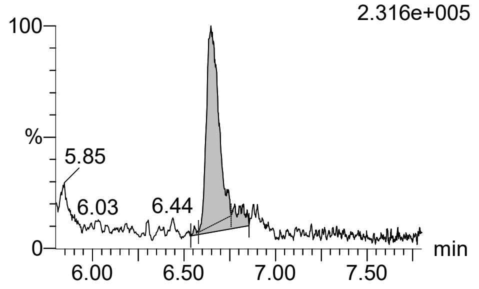

Sample Name: 20211111_QC_R24_IgM
F62:MRM of 1 channel,ES+
1066.3 > 204.1
2.389e+005

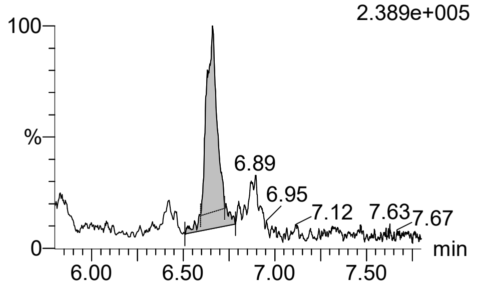

Sample Name: 20211111_A36_R3_IgM
F62:MRM of 1 channel,ES+
1066.3 > 204.1
1.359e+005

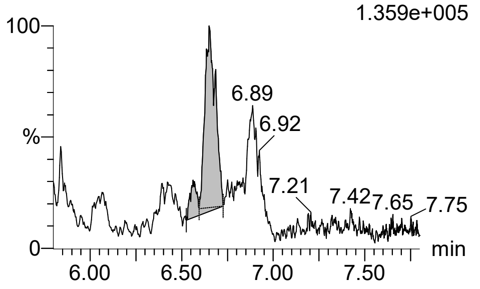

Sample Name: 20211111_H18_R3_IgM
F62:MRM of 1 channel,ES+
1066.3 > 204.1
5.675e+005

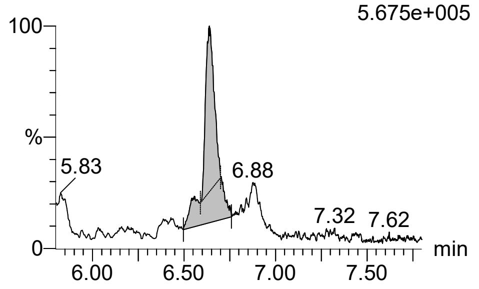

Sample Name: 20211111_A37_R3_IgM
F62:MRM of 1 channel,ES+
1066.3 > 204.1
2.910e+005

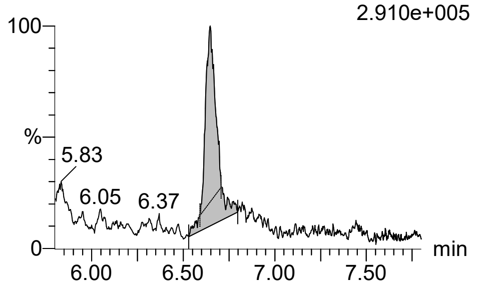

Sample Name: 20211111_A38_R3_IgM
F62:MRM of 1 channel,ES+
1066.3 > 204.1
1.970e+005

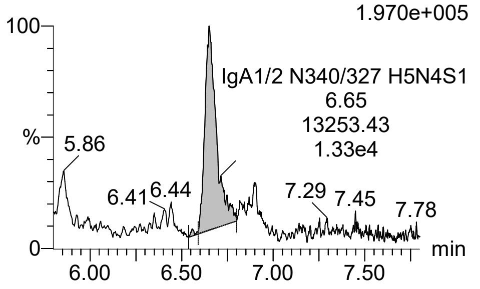

Sample Name: 20211111_H19_R3_IgM
F62:MRM of 1 channel,ES+
1066.3 > 204.1
4.580e+005

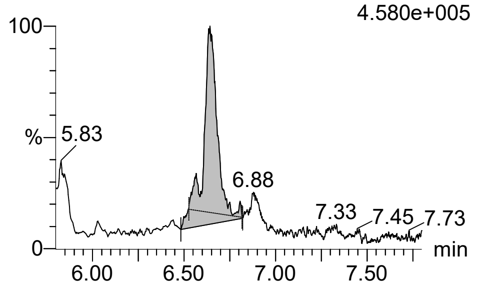

Sample Name: 20211111_A39_R3_IgM
F62:MRM of 1 channel,ES+
1066.3 > 204.1
2.910e+005

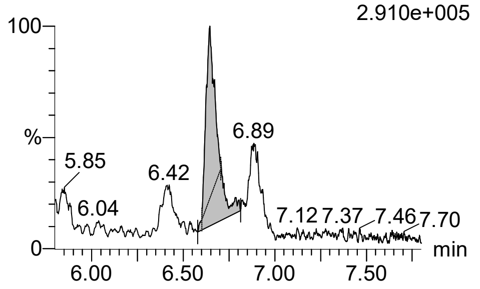

Sample Name: 20211111_A40_R3_IgM
F62:MRM of 1 channel,ES+
1066.3 > 204.1
1.132e+005

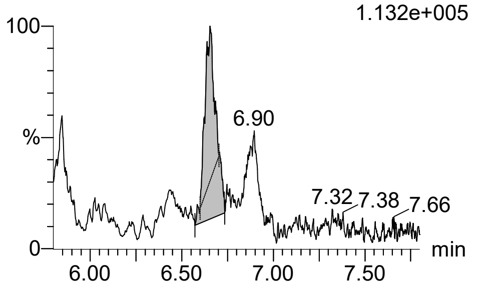

Sample Name: 20211111_H20_R3_IgM
F62:MRM of 1 channel,ES+
1066.3 > 204.1
6.932e+005

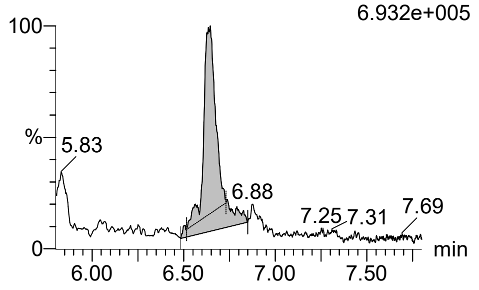

Sample Name: 20211111_A41_R3_IgM
F62:MRM of 1 channel,ES+
1066.3 > 204.1
1.857e+005

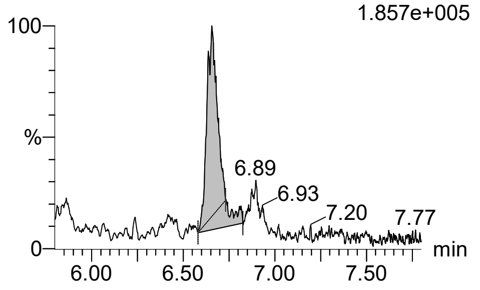

Sample Name: 20211111_A42_R3_IgM
F62:MRM of 1 channel,ES+
1066.3 > 204.1
8.621e+004

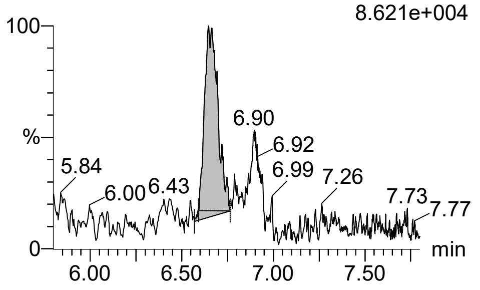

Dataset: C:\MassLynx\10.pro\20211111_clinicalsamples_IgM_IgA.qld

Last Altered: Thursday, March 09, 2023 19:58:25 台北標準時間
Printed: Friday, August 01, 2025 15:19:24 台北標準時間

Sample Name: 20211111_A43_R3_IgM

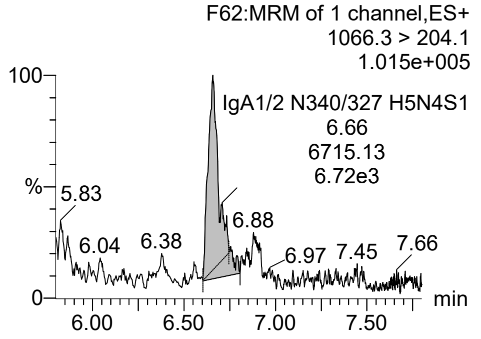

Sample Name: 20211111_A44_R3_IgM

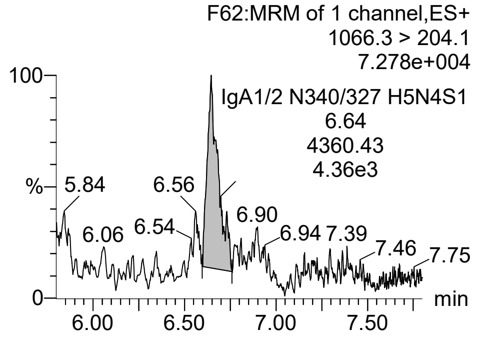

Sample Name: 20211111_QC_R25_IgM

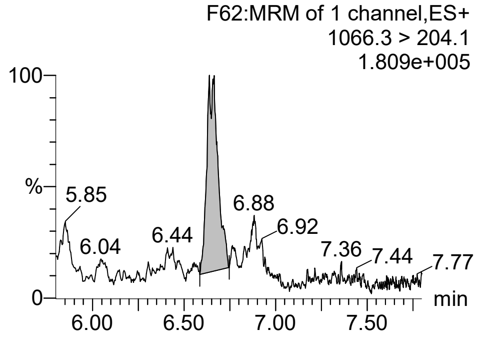

Dataset: C:\MassLynx\10.pro\20211111_clinicalsamples_IgM_IgA.qld

Last Altered: Thursday, March 09, 2023 19:58:25 台北標準時間
Printed: Friday, August 01, 2025 15:19:24 台北標準時間

	# Name	RT	Area	IS Area	Response	Prima...
1	1 20211112_QC_R001_IgM	6.63	15087.4	15421.4	0.978	bb
2	2 20211112_QC_R002_IgM	6.66	13673.4	13188.8	1.037	MM
3	3 20211111_A1_R2_IgM	6.65	9988.4	11871.0	0.841	MM
4	4 20211111_A1_R3_IgM	6.67	9699.1	12030.5	0.806	MM
5	5 20211111_A2_R2_IgM	6.64	36093.3	11446.8	3.153	MM
6	6 20211111_A2_R3_IgM	6.64	37053.6	11098.4	3.339	MM
7	7 20211111_L1_R2_IgM	6.65	10619.4	11779.4	0.902	MM
8	8 20211111_L1_R3_IgM	6.65	7835.1	11176.2	0.701	MM
9	9 20211111_L2_R2_IgM	6.64	13304.3	11375.0	1.170	bb
10	10 20211111_H1_R2_IgM	6.63	18515.8	13595.2	1.362	MM
11	11 20211111_A3_R2_IgM	6.65	23583.7	14112.3	1.671	MM
12	12 20211111_A4_R2_IgM	6.64	10267.6	11977.5	0.857	bb
13	13 20211111_L3_R2_IgM	6.63	17071.5	12242.1	1.394	MM
14	14 20211111_L4_R2_IgM	6.64	15806.2	12797.0	1.235	MM
15	15 20211111_H2_R2_IgM	6.65	19132.3	10006.0	1.912	MM
16	16 20211111_A5_R2_IgM	6.63	35289.1	11434.6	3.086	MM
17	17 20211111_A6_R2_IgM	6.65	18781.4	13193.7	1.424	bb
18	18 20211111_QC_R10_IgM	6.64	16373.3	12566.1	1.303	bb
19	19 20211111_L5_R2_IgM	6.64	21586.3	13616.4	1.585	MM
20	20 20211111_L6_R2_IgM	6.64	19122.4	13698.5	1.396	bb
21	21 20211111_H3_R2_IgM	6.64	8444.3	15793.1	0.535	MM
22	22 20211111_A7_R2_IgM	6.64	27734.0	12936.9	2.144	MM
23	23 20211111_A8_R2_IgM	6.64	19068.3	13108.0	1.455	bb
24	24 20211111_L7_R2_IgM	6.65	10154.2	13278.5	0.765	MM
25	25 20211111_L8_R2_IgM	6.66	6483.5	15233.6	0.426	MM
26	26 20211111_H4_R2_IgM	6.64	10299.2	16521.9	0.623	MM
27	27 20211111_A9_R2_IgM	6.64	14863.2	16451.6	0.903	MM
28	28 20211111_A10_R2_IgM	6.64	22015.4	13036.0	1.689	MM
29	29 20211111_L9_R2_IgM	6.64	17631.6	14554.1	1.211	MM
30	30 20211111_L10_R2_IgM	6.63	25933.5	12250.4	2.117	MM
31	31 20211111_QC_R11_IgM	6.65	16660.9	11459.2	1.454	MM
32	32 20211111_H5_R2_IgM	6.65	14615.5	14358.2	1.018	MM
33	33 20211111_A11_R2_IgM	6.63	20449.3	16184.1	1.264	MM
34	34 20211111_A12_R2_IgM	6.65	7807.5	13465.4	0.580	MM
35	35 20211111_L11_R2_IgM	6.66	10105.7	13277.9	0.761	MM
36	36 20211111_L12_R2_IgM	6.63	14648.3	15958.8	0.918	MM
37	37 20211111_H6_R2_IgM	6.65	12263.3	14003.5	0.876	MM
38	38 20211111_A13_R2_IgM	6.66	13217.3	13656.1	0.968	MM
39	39 20211111_A14_R2_IgM	6.63	17236.5	14071.3	1.225	MM
40	40 20211111_L13_R2_IgM	6.65	10678.8	14785.6	0.722	MM
41	41 20211111_L14_R2_IgM	6.64	15978.4	14772.2	1.082	MM
42	42 20211111_H7_R2_IgM	6.64	21791.3	15164.4	1.437	MM
43	43 20211111_A15_R2_IgM	6.65	37567.3	15721.5	2.390	MM
44	44 20211111_QC_R12_IgM	6.65	23091.6	13711.5	1.684	bb
45	45 20211111_A16_R2_IgM	6.65	23144.2	14275.6	1.621	MM
46	46 20211111_L15_R2_IgM	6.65	22228.4	12935.8	1.718	MM
47	47 20211111_L16_R2_IgM	6.65	7773.6	13342.8	0.583	MM
48	48 20211111_H8_R2_IgM	6.64	11098.9	15835.1	0.701	MM
49	49 20211111_A17_R2_IgM	6.64	22994.2	15786.7	1.457	MM
50	50 20211111_A18_R2_IgM	6.65	13919.2	16986.8	0.819	MM
51	51 20211111_L17_R2_IgM	6.65	31041.9	13342.7	2.327	MM
52	52 20211111_L18_R2_IgM	6.65	8105.3	14692.0	0.552	MM
53	53 20211111_H9_R2_IgM	6.65	16074.1	15639.7	1.028	MM
54	54 20211111_A19_R2_IgM	6.65	25775.2	14198.7	1.815	bb
55	55 20211111_A20_R2_IgM	6.64	10211.6	14162.7	0.721	MM
56	56 20211111_L19_R2_IgM	6.64	10222.5	9293.4	1.100	MM

Dataset: C:\MassLynx\10.pro\20211111_clinicalsamples_IgM_IgA.qld

Last Altered: Thursday, March 09, 2023 19:58:25 台北標準時間
Printed: Friday, August 01, 2025 15:19:24 台北標準時間

	# Name	RT	Area	IS Area	Response	Prima...
57	57 20211111_QC_R13_IgM	6.65	15523.4	13903.0	1.117	bb
58	58 20211111_L20_R2_IgM	6.64	18483.9	12781.6	1.446	MM
59	59 20211111_H10_R2_IgM	6.65	17540.3	14796.4	1.185	MM
60	60 20211111_A21_R2_IgM	6.65	14794.1	13669.0	1.082	MM
61	61 20211111_A22_R2_IgM	6.65	6134.0	14098.3	0.435	MM
62	62 20211111_L21_R2_IgM	6.64	10903.3	15678.2	0.695	MM
63	63 20211111_L22_R2_IgM	6.65	9313.4	15724.5	0.592	MM
64	64 20211111_H11_R2_IgM	6.64	9321.8	15890.2	0.587	bb
65	65 20211111_A23_R2_IgM	6.65	7128.5	13423.2	0.531	MM
66	66 20211111_A24_R2_IgM	6.65	27571.4	12438.0	2.217	MM
67	67 20211111_L23_R2_IgM	6.64	19389.1	14075.0	1.378	bb
68	68 20211111_L24_R2_IgM	6.64	8586.3	15972.1	0.538	MM
69	69 20211111_H12_R2_IgM	6.67	7022.5	16270.9	0.432	bb
70	70 20211111_QC_R14_IgM	6.65	17018.2	15806.8	1.077	MM
71	71 20211111_A25_R2_IgM	6.65	26060.8	14552.1	1.791	MM
72	72 20211111_A26_R2_IgM	6.65	8581.2	11492.7	0.747	MM
73	73 20211111_L25_R2_IgM	6.66	12731.0	14403.3	0.884	MM
74	74 20211111_L26_R2_IgM	6.66	14857.9	16533.9	0.899	MM
75	75 20211111_H13_R2_IgM	6.66	14087.6	15520.1	0.908	MM
76	76 20211111_A27_R2_IgM	6.64	15549.1	13337.4	1.166	MM
77	77 20211111_A28_R2_IgM	6.64	17779.0	11895.6	1.495	MM
78	78 20211111_L27_R2_IgM	6.65	30277.2	14125.7	2.143	MM
79	79 20211111_L28_R2_IgM	6.66	14047.1	11807.5	1.190	MM
80	80 20211111_H14_R2_IgM	6.66	23155.1	15035.7	1.540	MM
81	81 20211111_A29_R2_IgM	6.65	5886.3	13554.4	0.434	MM
82	82 20211111_A30_R2_IgM	6.64	15079.9	12984.1	1.161	bb
83	83 20211111_QC_R15_IgM	6.64	16426.5	12299.5	1.336	MM
84	84 20211111_L29_R2_IgM	6.66	7482.3	13590.7	0.551	MM
85	85 20211111_L30_R2_IgM	6.66	5732.5	12511.4	0.458	bb
86	86 20211111_H15_R2_IgM	6.64	13056.6	11885.0	1.099	MM
87	87 20211111_A31_R2_IgM	6.64	5526.9	13422.1	0.412	MM
88	88 20211111_A32_R2_IgM	6.65	13567.2	11461.3	1.184	MM
89	89 20211111_L31_R2_IgM	6.65	7698.3	13753.4	0.560	bb
90	90 20211111_L32_R2_IgM	6.64	18007.3	12949.0	1.391	MM
91	91 20211111_H16_R2_IgM	6.64	24609.5	11465.4	2.146	MM
92	92 20211111_A33_R2_IgM	6.64	17815.2	10551.5	1.688	MM
93	93 20211111_A34_R2_IgM	6.66	6484.6	12409.9	0.523	bb
94	94 20211111_H17_R2_IgM	6.63	115026.0	10160.9	11.320	MM
95	95 20211111_A35_R2_IgM	6.64	22091.7	10460.6	2.112	bb
96	96 20211111_QC_R16_IgM	6.65	15527.5	10433.5	1.488	bb
97	97 20211111_A36_R2_IgM	6.64	13772.8	12504.8	1.101	bb
98	98 20211111_H18_R2_IgM	6.64	41487.7	9140.4	4.539	bb
99	99 20211111_A37_R2_IgM	6.64	20381.4	9796.9	2.080	MM
100	1... 20211111_A38_R2_IgM	6.66	14072.4	11224.3	1.254	MM
101	1... 20211111_H19_R2_IgM	6.65	37816.7	11739.2	3.221	MM
102	1... 20211111_A39_R2_IgM	6.66	20964.4	12718.9	1.648	MM
103	1... 20211111_A40_R2_IgM	6.63	10033.2	10006.1	1.003	MM
104	1... 20211111_H20_R2_IgM	6.64	68509.3	7988.2	8.576	MM
105	1... 20211111_A41_R2_IgM	6.66	21245.8	11513.3	1.845	MM
106	1... 20211111_A42_R2_IgM	6.67	8948.4	10238.5	0.874	MM
107	1... 20211111_A43_R2_IgM	6.66	7470.6	11098.9	0.673	MM
108	1... 20211111_A44_R2_IgM	6.64	8053.3	10969.9	0.734	MM
109	1... 20211111_QC_R17_IgM	6.66	13154.1	9700.8	1.356	bb
110	1... 20211111_L2_R3_IgM	6.64	13891.9	8528.0	1.629	MM
111	1... 20211111_H1_R3_IgM	6.66	13997.7	9438.2	1.483	MM
112	1... 20211111_A3_R3_IgM	6.65	15792.8	11598.6	1.362	MM

Dataset: C:\MassLynx\10.pro\20211111_clinicalsamples_IgM_IgA.qld

Last Altered: Thursday, March 09, 2023 19:58:25 台北標準時間
Printed: Friday, August 01, 2025 15:19:24 台北標準時間

	#	Name	RT	Area	IS Area	Response	Prima...
113	1...	20211111_A4_R3_IgM	6.66	10607.5	11364.1	0.933	bb
114	1...	20211111_L3_R3_IgM	6.64	12297.3	10753.4	1.144	MM
115	1...	20211111_L4_R3_IgM	6.66	14429.6	10291.6	1.402	MM
116	1...	20211111_H2_R3_IgM	6.64	17657.0	9315.8	1.895	MM
117	1...	20211111_A5_R3_IgM	6.64	24339.8	9102.2	2.674	MM
118	1...	20211111_A6_R3_IgM	6.65	15061.5	10356.4	1.454	MM
119	1...	20211111_QC_R18_IgM	6.65	14638.4	8398.3	1.743	MM
120	1...	20211111_L5_R3_IgM	6.66	13230.9	9786.7	1.352	bb
121	1...	20211111_L6_R3_IgM	6.64	16057.7	9911.3	1.620	MM
122	1...	20211111_H3_R3_IgM	6.65	5702.9	12155.5	0.469	MM
123	1...	20211111_A7_R3_IgM	6.64	26636.7	11804.8	2.256	MM
124	1...	20211111_A8_R3_IgM	6.65	13072.6	10510.6	1.244	MM
125	1...	20211111_L7_R3_IgM	6.66	9339.0	12177.9	0.767	MM
126	1...	20211111_L8_R3_IgM	6.64	5387.1	11668.6	0.462	MM
127	1...	20211111_H4_R3_IgM	6.65	8752.3	11192.0	0.782	MM
128	1...	20211111_A9_R3_IgM	6.66	9075.9	14077.4	0.645	MM
129	1...	20211111_A10_R3_IgM	6.65	17804.9	11190.4	1.591	MM
130	1...	20211111_L9_R3_IgM	6.65	13041.4	14649.3	0.890	MM
131	1...	20211111_L10_R3_IgM	6.64	24102.9	9361.3	2.575	MM
132	1...	20211111_QC_R19_IgM	6.65	14818.9	10125.5	1.464	MM
133	1...	20211111_H5_R3_IgM	6.66	12293.6	10010.9	1.228	bb
134	1...	20211111_A11_R3_IgM	6.65	10952.0	12697.2	0.863	bb
135	1...	20211111_A12_R3_IgM	6.64	6455.6	13148.5	0.491	bb
136	1...	20211111_L11_R3_IgM	6.65	9454.9	10786.6	0.877	MM
137	1...	20211111_L12_R3_IgM	6.65	13323.8	11681.8	1.141	MM
138	1...	20211111_H6_R3_IgM	6.63	11126.7	12095.5	0.920	MM
139	1...	20211111_A13_R3_IgM	6.65	13003.6	12908.3	1.007	MM
140	1...	20211111_A14_R3_IgM	6.65	14058.8	11322.6	1.242	MM
141	1...	20211111_L13_R3_IgM	6.65	11408.9	11516.1	0.991	MM
142	1...	20211111_L14_R3_IgM	6.64	14177.4	10790.7	1.314	MM
143	1...	20211111_H7_R3_IgM	6.66	14948.7	11572.1	1.292	MM
144	1...	20211111_A15_R3_IgM	6.66	37363.9	12434.4	3.005	MM
145	1...	20211111_QC_R20_IgM	6.66	20882.2	10701.0	1.951	MM
146	1...	20211111_A16_R3_IgM	6.65	22065.2	11452.1	1.927	bb
147	1...	20211111_L15_R3_IgM	6.65	18848.2	10531.1	1.790	MM
148	1...	20211111_L16_R3_IgM	6.66	8109.4	12049.4	0.673	MM
149	1...	20211111_H8_R3_IgM	6.66	9293.1	13434.9	0.692	MM
150	1...	20211111_A17_R3_IgM	6.66	19465.1	12452.0	1.563	MM
151	1...	20211111_A18_R3_IgM	6.65	13022.3	12506.5	1.041	MM
152	1...	20211111_L17_R3_IgM	6.63	22327.6	9948.9	2.244	MM
153	1...	20211111_L18_R3_IgM	6.65	6022.4	13462.1	0.447	MM
154	1...	20211111_H9_R3_IgM	6.64	10961.3	13178.7	0.832	MM
155	1...	20211111_A19_R3_IgM	6.65	21050.6	11298.6	1.863	MM
156	1...	20211111_A20_R3_IgM	6.65	9127.2	11175.6	0.817	MM
157	1...	20211111_L19_R3_IgM	6.66	10458.6	11558.2	0.905	MM
158	1...	20211111_QC_R21_IgM	6.65	12323.2	9977.0	1.235	MM
159	1...	20211111_L20_R3_IgM	6.66	10747.8	13508.1	0.796	MM
160	1...	20211111_H10_R3_IgM	6.65	10631.4	12374.8	0.859	MM
161	1...	20211111_A21_R3_IgM	6.64	12408.7	12443.6	0.997	MM
162	1...	20211111_A22_R3_IgM	6.65	5310.8	11589.3	0.458	MM
163	1...	20211111_L21_R3_IgM	6.64	11042.9	13107.4	0.842	MM
164	1...	20211111_L22_R3_IgM	6.66	8495.3	12021.5	0.707	MM
165	1...	20211111_H11_R3_IgM	6.66	8896.0	12687.8	0.701	MM
166	1...	20211111_A23_R3_IgM	6.66	8872.2	11611.8	0.764	MM
167	1...	20211111_A24_R3_IgM	6.65	26966.5	10476.8	2.574	MM
168	1...	20211111_L23_R3_IgM	6.64	19575.3	11873.1	1.649	bb

Dataset: C:\MassLynx\10.pro\20211111_clinicalsamples_IgM_IgA.qld

Last Altered: Thursday, March 09, 2023 19:58:25 台北標準時間
Printed: Friday, August 01, 2025 15:19:24 台北標準時間

	#	Name	RT	Area	IS Area	Response	Prima...
169	1...	20211111_L24_R3_IgM	6.65	7574.4	13468.0	0.562	MM
170	1...	20211111_H12_R3_IgM	6.66	6336.0	14229.1	0.445	MM
171	1...	20211111_QC_R22_IgM	6.65	14798.7	11264.4	1.314	MM
172	1...	20211111_A25_R3_IgM	6.64	18209.3	11775.7	1.546	MM
173	1...	20211111_A26_R3_IgM	6.66	7804.1	10473.9	0.745	MM
174	1...	20211111_L25_R3_IgM	6.64	10778.2	13256.8	0.813	bb
175	1...	20211111_L26_R3_IgM	6.66	12867.5	13565.0	0.949	MM
176	1...	20211111_H13_R3_IgM	6.65	13123.4	13146.0	0.998	MM
177	1...	20211111_A27_R3_IgM	6.66	14084.0	12025.6	1.171	MM
178	1...	20211111_A28_R3_IgM	6.65	9443.7	11229.1	0.841	MM
179	1...	20211111_L27_R3_IgM	6.66	24451.2	14960.0	1.634	MM
180	1...	20211111_L28_R3_IgM	6.65	17016.6	11359.0	1.498	bb
181	1...	20211111_H14_R3_IgM	6.65	24991.1	12830.8	1.948	MM
182	1...	20211111_A29_R3_IgM	6.66	4980.4	13434.3	0.371	MM
183	1...	20211111_A30_R3_IgM	6.65	12847.3	11272.2	1.140	MM
184	1...	20211111_QC_R23_IgM	6.65	13570.3	11872.2	1.143	MM
185	1...	20211111_L29_R3_IgM	6.67	7299.8	12948.7	0.564	MM
186	1...	20211111_L30_R3_IgM	6.65	4724.0	11026.6	0.428	MM
187	1...	20211111_H15_R3_IgM	6.65	11360.0	10786.3	1.053	MM
188	1...	20211111_A31_R3_IgM	6.67	5223.8	12010.5	0.435	MM
189	1...	20211111_A32_R3_IgM	6.66	12498.2	11107.7	1.125	bb
190	1...	20211111_L31_R3_IgM	6.65	4229.4	11021.8	0.384	MM
191	1...	20211111_L32_R3_IgM	6.65	14031.7	11683.8	1.201	MM
192	1...	20211111_H16_R3_IgM	6.65	18563.3	9831.7	1.888	MM
193	1...	20211111_A33_R3_IgM	6.66	16890.3	11213.1	1.506	MM
194	1...	20211111_A34_R3_IgM	6.66	5105.1	11550.1	0.442	MM
195	1...	20211111_H17_R3_IgM	6.63	89985.1	8874.4	10.140	MM
196	1...	20211111_A35_R3_IgM	6.65	18920.4	9856.3	1.920	MM
197	1...	20211111_QC_R24_IgM	6.66	16187.1	7561.8	2.141	MM
198	1...	20211111_A36_R3_IgM	6.65	8472.3	9562.8	0.886	MM
199	1...	20211111_H18_R3_IgM	6.64	39080.1	8165.3	4.786	MM
200	2...	20211111_A37_R3_IgM	6.65	19258.5	9278.8	2.076	MM
201	2...	20211111_A38_R3_IgM	6.65	13253.4	10920.3	1.214	MM
202	2...	20211111_H19_R3_IgM	6.64	36932.5	9285.3	3.978	MM
203	2...	20211111_A39_R3_IgM	6.64	18354.5	9083.4	2.021	MM
204	2...	20211111_A40_R3_IgM	6.65	7002.6	8382.4	0.835	MM
205	2...	20211111_H20_R3_IgM	6.64	58999.8	7807.9	7.556	MM
206	2...	20211111_A41_R3_IgM	6.66	12928.4	5925.2	2.182	MM
207	2...	20211111_A42_R3_IgM	6.65	6330.0	9352.6	0.677	MM
208	2...	20211111_A43_R3_IgM	6.66	6715.1	10216.3	0.657	MM
209	2...	20211111_A44_R3_IgM	6.64	4360.4	10262.7	0.425	bb
210	2...	20211111_QC_R25_IgM	6.64	11122.0	8575.3	1.297	bb

Dataset: C:\MassLynx\10.pro\20211111_clinicalsamples_IgM_IgA.qld

Last Altered: Thursday, March 09, 2023 19:58:25 台北標準時間
Printed: Friday, August 01, 2025 15:20:28 台北標準時間

Method: C:\MassLynx\10_Pro_IgA.PRO\MethDB\20211111_IgM_IgA_DMRM_new.mdb 24 Feb 2022 13:44:14
Calibration: 15 Nov 2021 14:42:11

Sample Name: 20211112_QC_R001_IgM

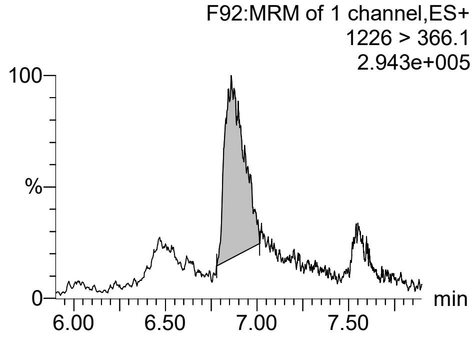

Sample Name: 20211112_QC_R002_IgM

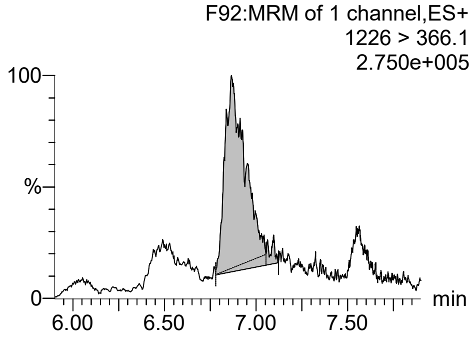

Sample Name: 20211111_A1_R2_IgM

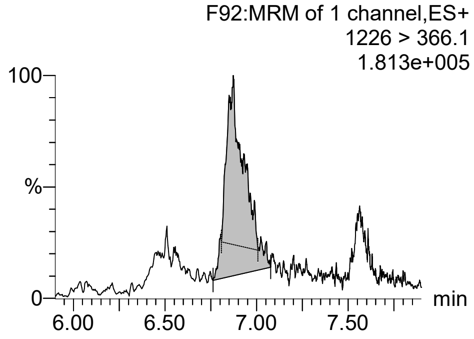

Sample Name: 20211111_A1_R3_IgM

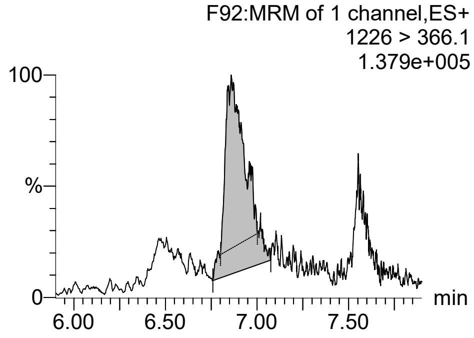

Sample Name: 20211111_A2_R2_IgM

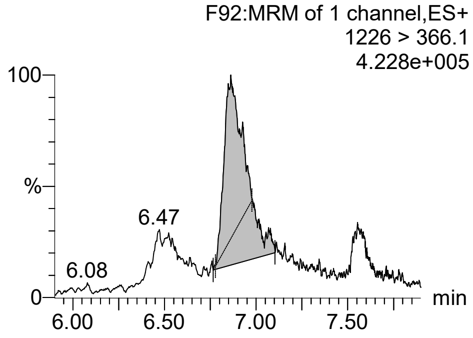

Sample Name: 20211111_A2_R3_IgM

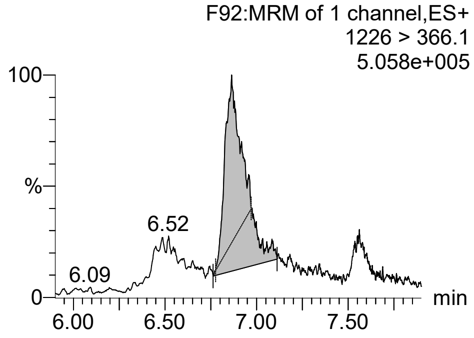

Sample Name: 20211111_L1_R2_IgM

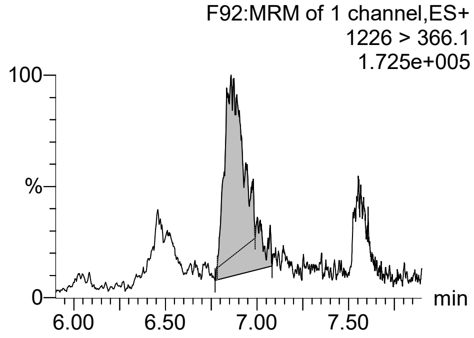

Sample Name: 20211111_L1_R3_IgM

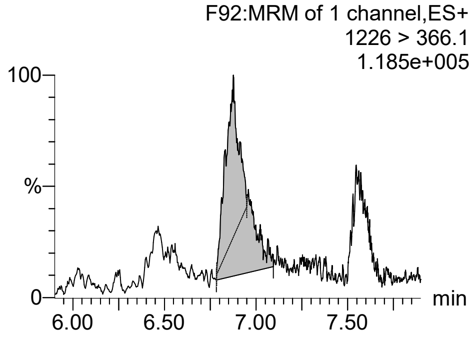

Sample Name: 20211111_L2_R2_IgM

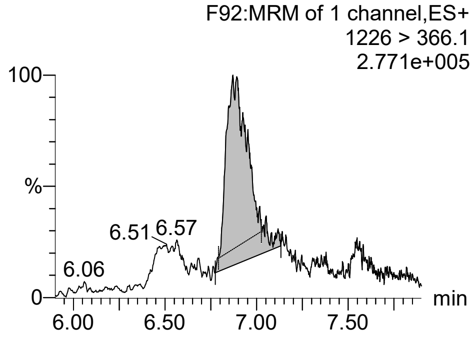

Sample Name: 20211111_H1_R2_IgM

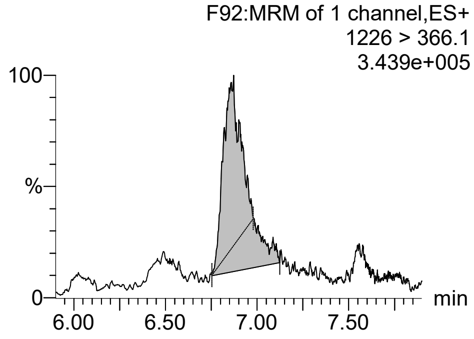

Sample Name: 20211111_A3_R2_IgM

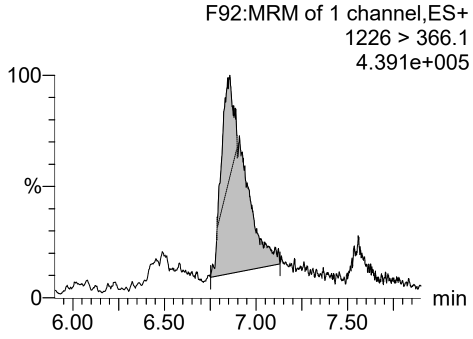

Sample Name: 20211111_A4_R2_IgM

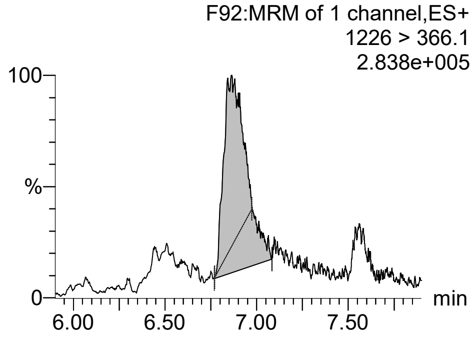

Dataset: C:\MassLynx\10.pro\20211111_clinicalsamples_IgM_IgA.qld

Last Altered: Thursday, March 09, 2023 19:58:25 台北標準時間
Printed: Friday, August 01, 2025 15:20:28 台北標準時間

Sample Name: 20211111_L3_R2_IgM
F92:MRM of 1 channel,ES+
1226 > 366.1
3.525e+005

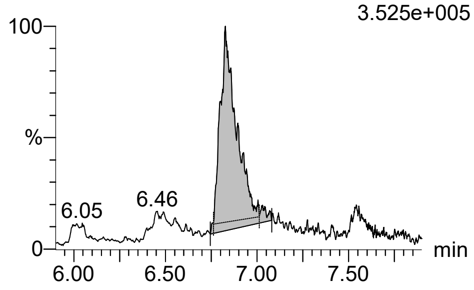

Sample Name: 20211111_L4_R2_IgM
F92:MRM of 1 channel,ES+
1226 > 366.1
3.017e+005

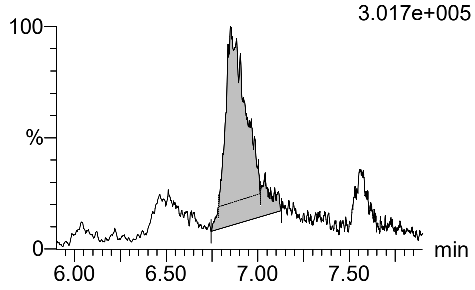

Sample Name: 20211111_H2_R2_IgM
F92:MRM of 1 channel,ES+
1226 > 366.1
4.480e+005

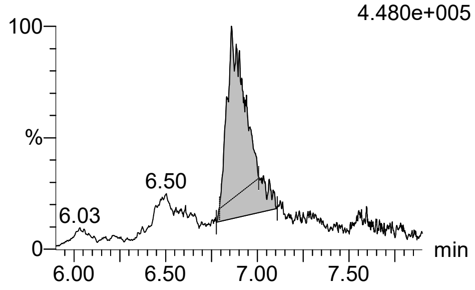

Sample Name: 20211111_A5_R2_IgM
F92:MRM of 1 channel,ES+
1226 > 366.1
1.229e+006

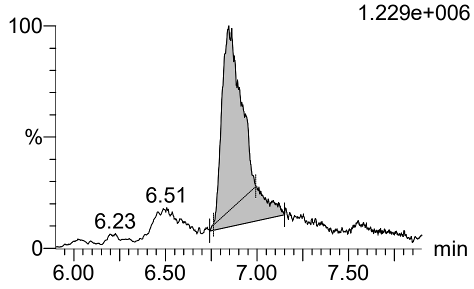

Sample Name: 20211111_A6_R2_IgM
F92:MRM of 1 channel,ES+
1226 > 366.1
5.638e+005

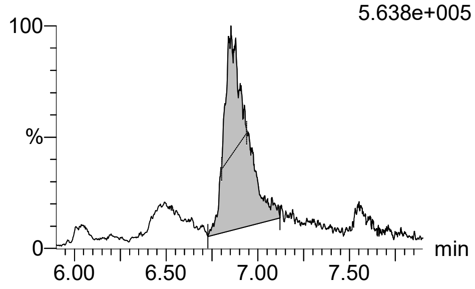

Sample Name: 20211111_QC_R10_IgM
F92:MRM of 1 channel,ES+
1226 > 366.1
3.719e+005

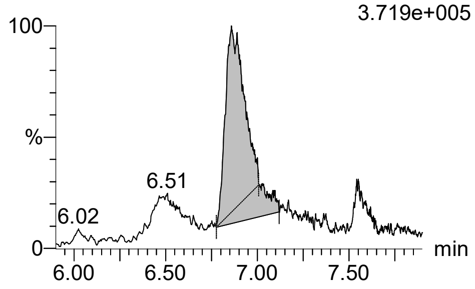

Sample Name: 20211111_L5_R2_IgM
F92:MRM of 1 channel,ES+
1226 > 366.1
4.898e+005

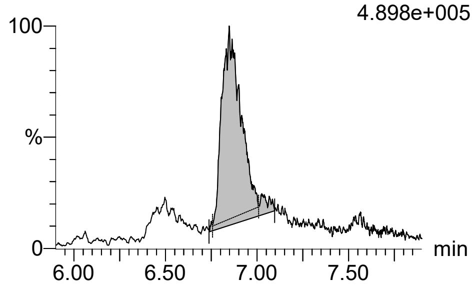

Sample Name: 20211111_L6_R2_IgM
F92:MRM of 1 channel,ES+
1226 > 366.1
3.257e+005

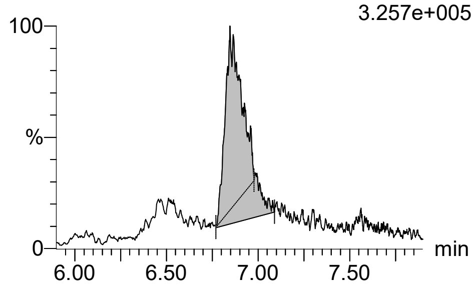

Sample Name: 20211111_H3_R2_IgM
F92:MRM of 1 channel,ES+
1226 > 366.1
2.213e+005

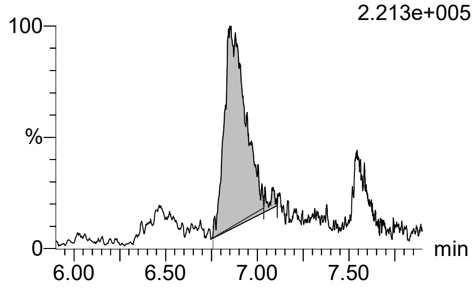

Sample Name: 20211111_A7_R2_IgM
F92:MRM of 1 channel,ES+
1226 > 366.1
2.459e+005

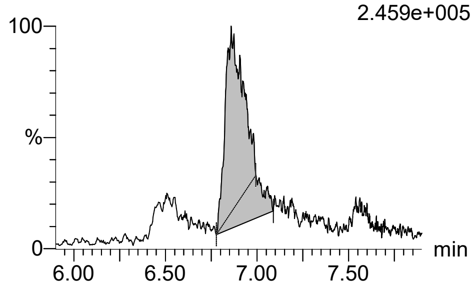

Sample Name: 20211111_A8_R2_IgM
F92:MRM of 1 channel,ES+
1226 > 366.1
3.067e+005

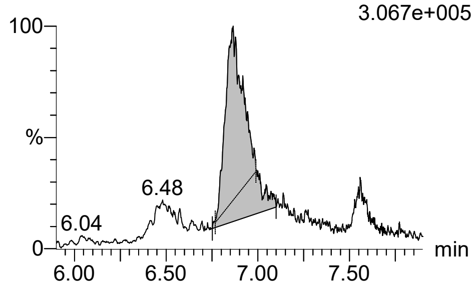

Sample Name: 20211111_L7_R2_IgM
F92:MRM of 1 channel,ES+
1226 > 366.1
2.215e+005

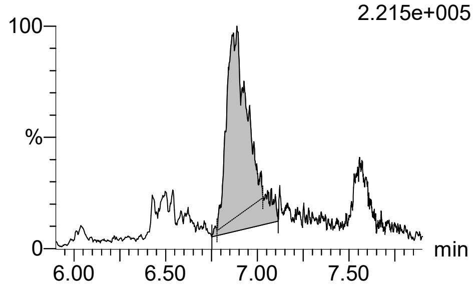

Sample Name: 20211111_L8_R2_IgM
F92:MRM of 1 channel,ES+
1226 > 366.1
1.679e+005

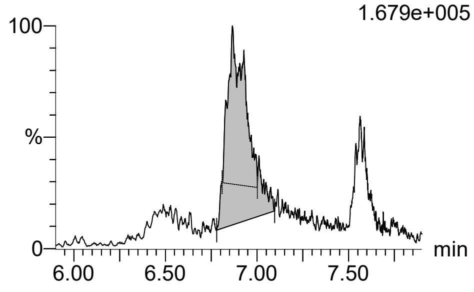

Sample Name: 20211111_H4_R2_IgM
F92:MRM of 1 channel,ES+
1226 > 366.1
1.735e+005

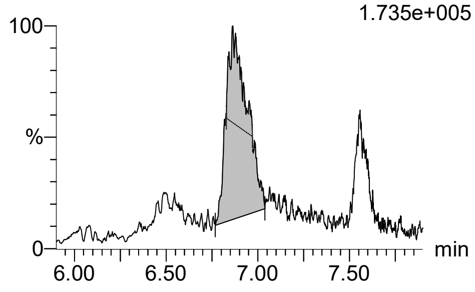

Sample Name: 20211111_A9_R2_IgM
F92:MRM of 1 channel,ES+
1226 > 366.1
3.858e+005

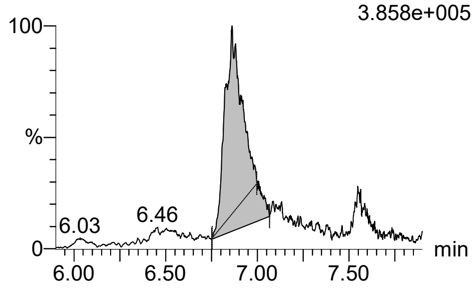

Dataset: C:\MassLynx\10.pro\20211111_clinicalsamples_IgM_IgA.qld

Last Altered: Thursday, March 09, 2023 19:58:25 台北標準時間
Printed: Friday, August 01, 2025 15:20:28 台北標準時間

Sample Name: 20211111_A10_R2_IgM
F92:MRM of 1 channel,ES+
1226 > 366.1
3.530e+005

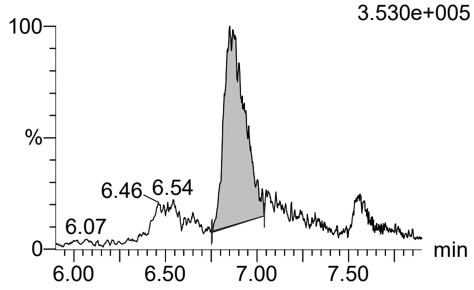

Sample Name: 20211111_L9_R2_IgM
F92:MRM of 1 channel,ES+
1226 > 366.1
1.994e+005

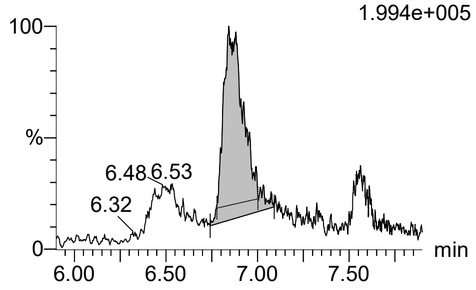

Sample Name: 20211111_L10_R2_IgM
F92:MRM of 1 channel,ES+
1226 > 366.1
4.862e+005

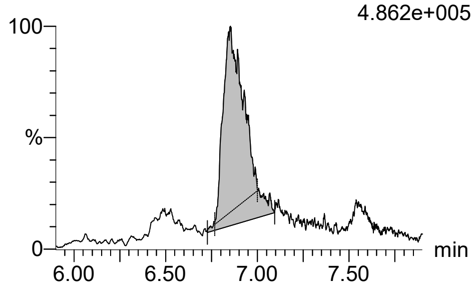

Sample Name: 20211111_QC_R11_IgM
F92:MRM of 1 channel,ES+
1226 > 366.1
3.712e+005

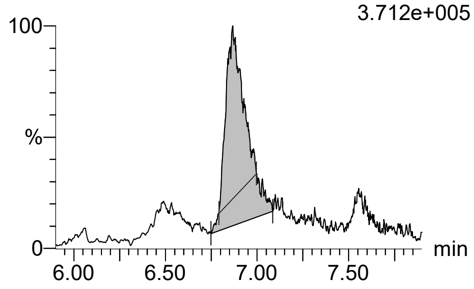

Sample Name: 20211111_H5_R2_IgM
F92:MRM of 1 channel,ES+
1226 > 366.1
4.399e+005

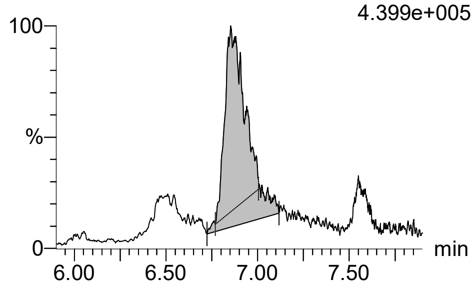

Sample Name: 20211111_A11_R2_IgM
F92:MRM of 1 channel,ES+
1226 > 366.1
7.822e+005

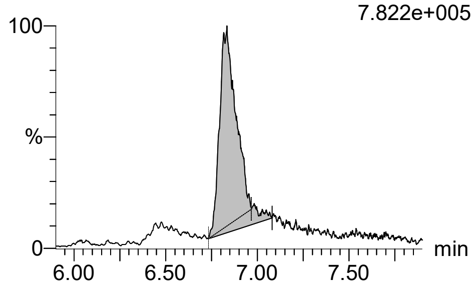

Sample Name: 20211111_A12_R2_IgM
F92:MRM of 1 channel,ES+
1226 > 366.1
2.871e+005

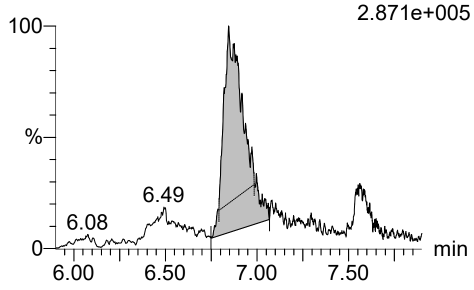

Sample Name: 20211111_L11_R2_IgM
F92:MRM of 1 channel,ES+
1226 > 366.1
2.025e+005

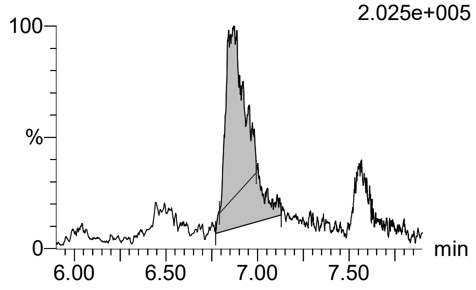

Sample Name: 20211111_L12_R2_IgM
F92:MRM of 1 channel,ES+
1226 > 366.1
2.897e+005

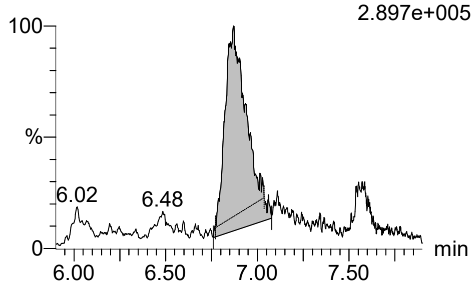

Sample Name: 20211111_H6_R2_IgM
F92:MRM of 1 channel,ES+
1226 > 366.1
1.758e+005

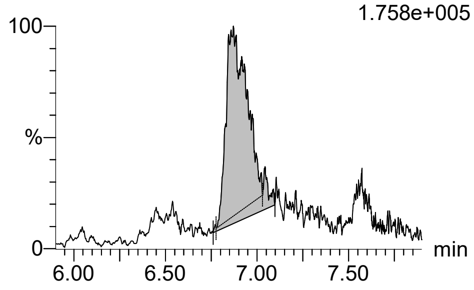

Sample Name: 20211111_A13_R2_IgM
F92:MRM of 1 channel,ES+
1226 > 366.1
1.804e+005

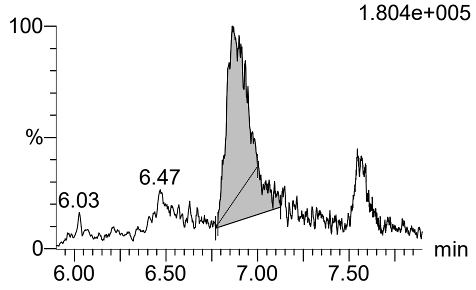

Sample Name: 20211111_A14_R2_IgM
F92:MRM of 1 channel,ES+
1226 > 366.1
5.317e+005

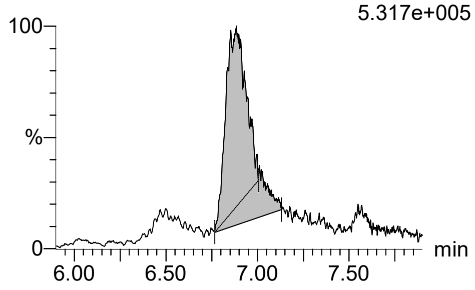

Sample Name: 20211111_L13_R2_IgM
F92:MRM of 1 channel,ES+
1226 > 366.1
3.196e+005

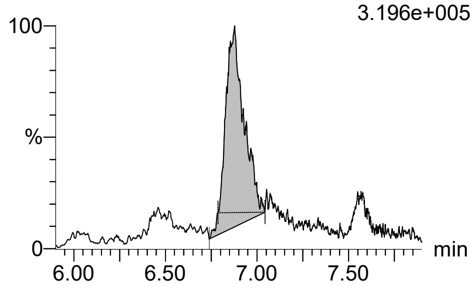

Sample Name: 20211111_L14_R2_IgM
F92:MRM of 1 channel,ES+
1226 > 366.1
2.678e+005

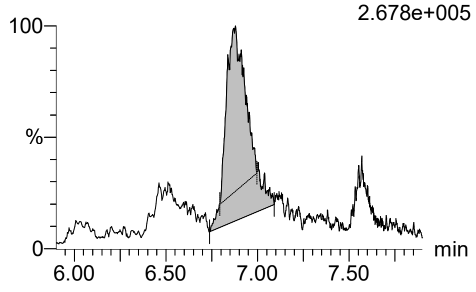

Sample Name: 20211111_H7_R2_IgM
F92:MRM of 1 channel,ES+
1226 > 366.1
2.781e+005

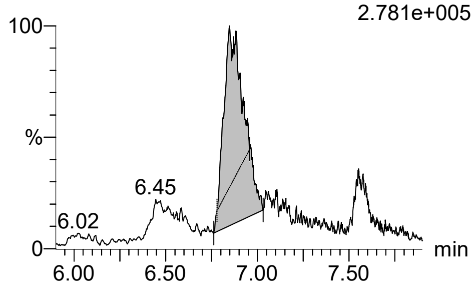

Dataset: C:\MassLynx\10.pro\20211111_clinicalsamples_IgM_IgA.qld

Last Altered: Thursday, March 09, 2023 19:58:25 台北標準時間
Printed: Friday, August 01, 2025 15:20:28 台北標準時間

Sample Name: 20211111_A15_R2_IgM
F92:MRM of 1 channel,ES+
1226 > 366.1
4.874e+005

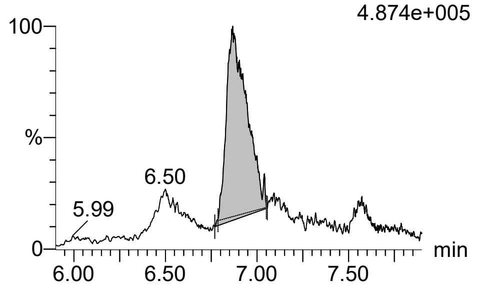

Sample Name: 20211111_QC_R12_IgM
F92:MRM of 1 channel,ES+
1226 > 366.1
3.860e+005

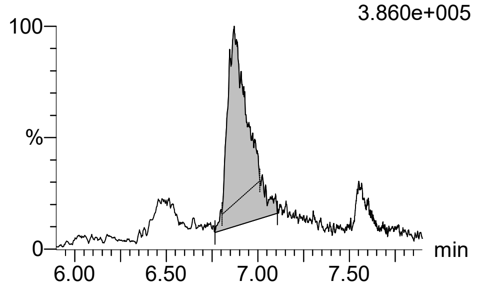

Sample Name: 20211111_A16_R2_IgM
F92:MRM of 1 channel,ES+
1226 > 366.1
3.741e+005

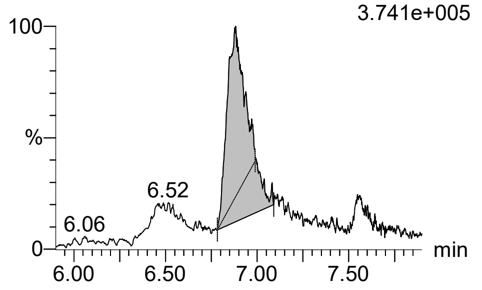

Sample Name: 20211111_L15_R2_IgM
F92:MRM of 1 channel,ES+
1226 > 366.1
5.032e+005

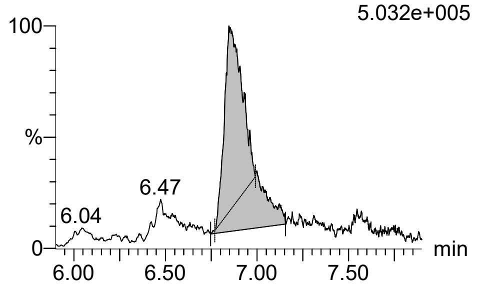

Sample Name: 20211111_L16_R2_IgM
F92:MRM of 1 channel,ES+
1226 > 366.1
2.170e+005

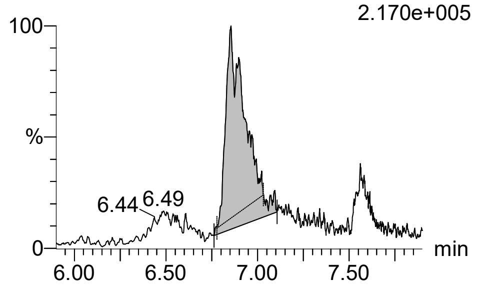

Sample Name: 20211111_H8_R2_IgM
F92:MRM of 1 channel,ES+
1226 > 366.1
2.067e+005

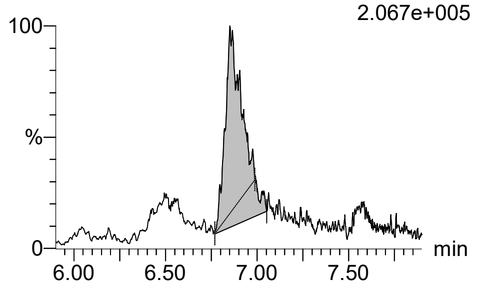

Sample Name: 20211111_A17_R2_IgM
F92:MRM of 1 channel,ES+
1226 > 366.1
3.930e+005

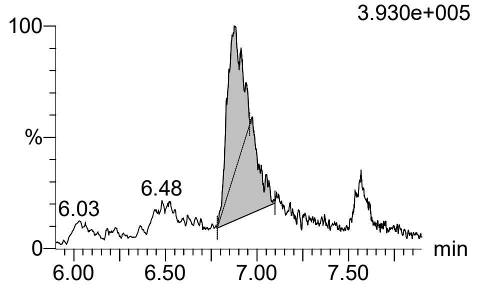

Sample Name: 20211111_A18_R2_IgM
F92:MRM of 1 channel,ES+
1226 > 366.1
2.482e+005

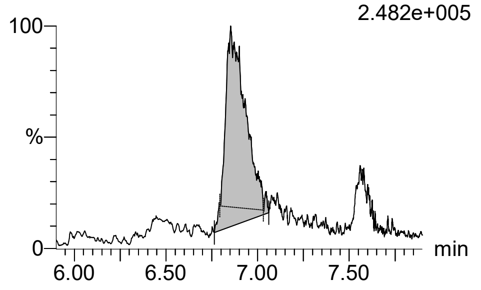

Sample Name: 20211111_L17_R2_IgM
F92:MRM of 1 channel,ES+
1226 > 366.1
3.246e+005

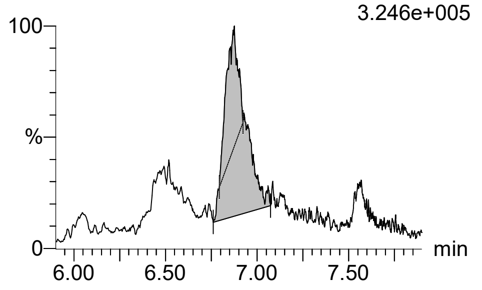

Sample Name: 20211111_L18_R2_IgM
F92:MRM of 1 channel,ES+
1226 > 366.1
1.306e+005

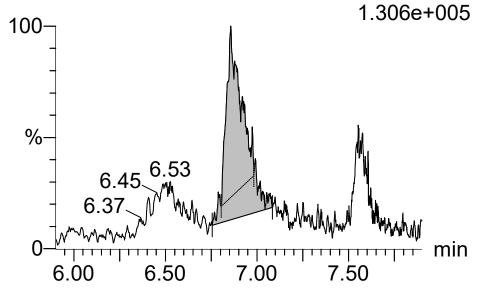

Sample Name: 20211111_H9_R2_IgM
F92:MRM of 1 channel,ES+
1226 > 366.1
1.529e+005

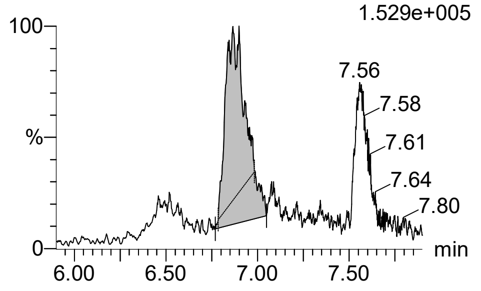

Sample Name: 20211111_A19_R2_IgM
F92:MRM of 1 channel,ES+
1226 > 366.1
2.217e+005

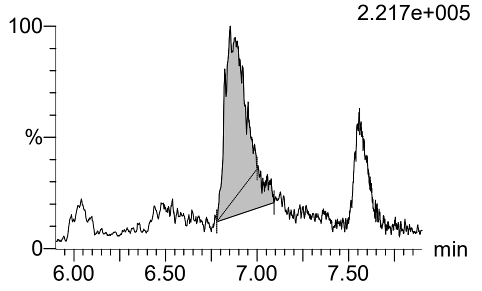

Sample Name: 20211111_A20_R2_IgM
F92:MRM of 1 channel,ES+
1226 > 366.1
1.495e+005

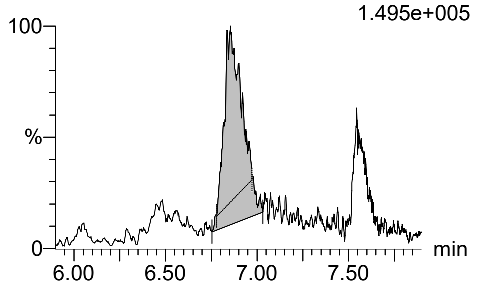

Sample Name: 20211111_L19_R2_IgM
F92:MRM of 1 channel,ES+
1226 > 366.1
1.850e+005

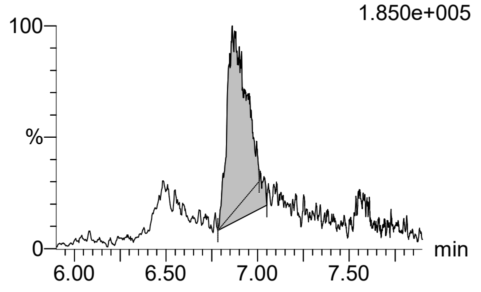

Sample Name: 20211111_QC_R13_IgM
F92:MRM of 1 channel,ES+
1226 > 366.1
2.793e+005

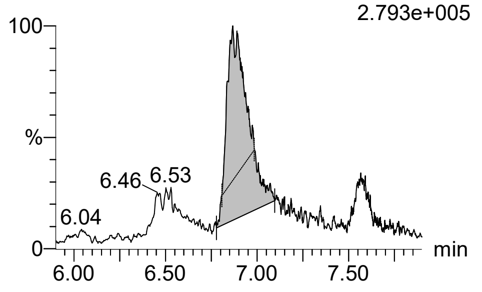

Dataset: C:\MassLynx\10.pro\20211111_clinicalsamples_IgM_IgA.qld

Last Altered: Thursday, March 09, 2023 19:58:25 台北標準時間
Printed: Friday, August 01, 2025 15:20:28 台北標準時間

Sample Name: 20211111_L20_R2_IgM
F92:MRM of 1 channel,ES+
1226 > 366.1
3.354e+005

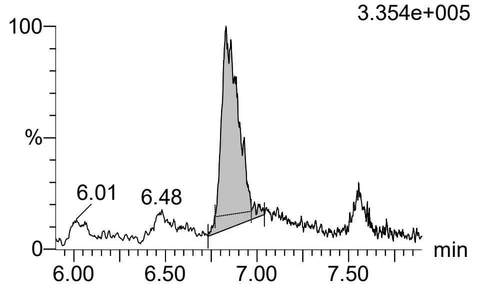

Sample Name: 20211111_H10_R2_IgM
F92:MRM of 1 channel,ES+
1226 > 366.1
5.858e+005

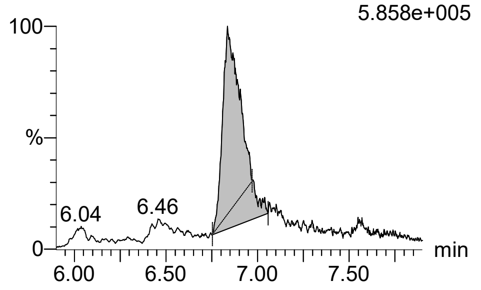

Sample Name: 20211111_A21_R2_IgM
F92:MRM of 1 channel,ES+
1226 > 366.1
4.589e+005

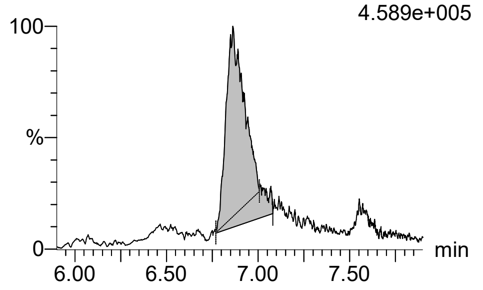

Sample Name: 20211111_A22_R2_IgM
F92:MRM of 1 channel,ES+
1226 > 366.1
2.129e+005

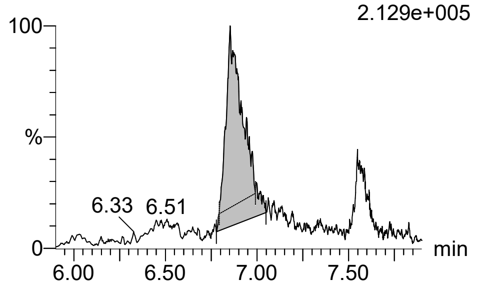

Sample Name: 20211111_L21_R2_IgM
F92:MRM of 1 channel,ES+
1226 > 366.1
2.516e+005

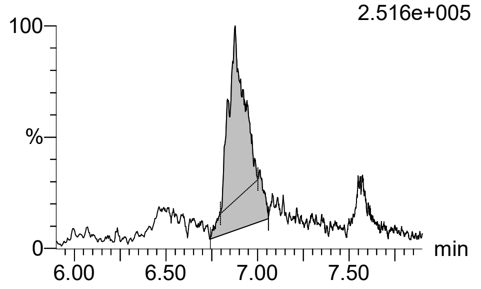

Sample Name: 20211111_L22_R2_IgM
F92:MRM of 1 channel,ES+
1226 > 366.1
2.169e+005

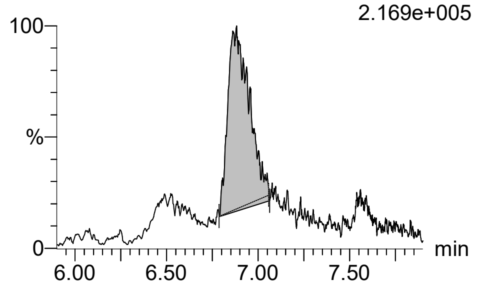

Sample Name: 20211111_H11_R2_IgM
F92:MRM of 1 channel,ES+
1226 > 366.1
2.877e+005

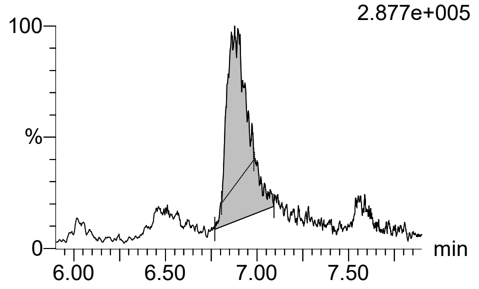

Sample Name: 20211111_A23_R2_IgM
F92:MRM of 1 channel,ES+
1226 > 366.1
1.560e+005

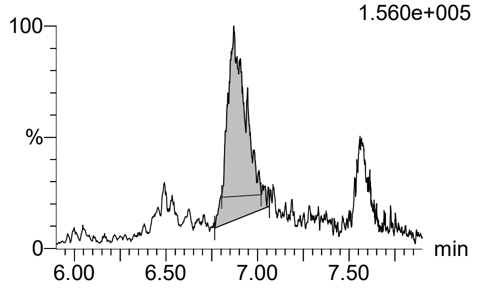

Sample Name: 20211111_A24_R2_IgM
F92:MRM of 1 channel,ES+
1226 > 366.1
3.766e+005

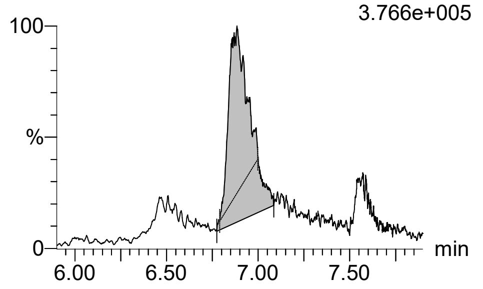

Sample Name: 20211111_L23_R2_IgM
F92:MRM of 1 channel,ES+
1226 > 366.1
4.485e+005

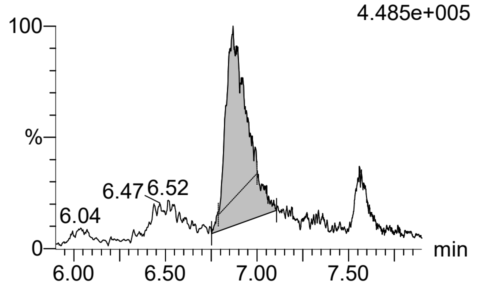

Sample Name: 20211111_L24_R2_IgM
F92:MRM of 1 channel,ES+
1226 > 366.1
2.432e+005

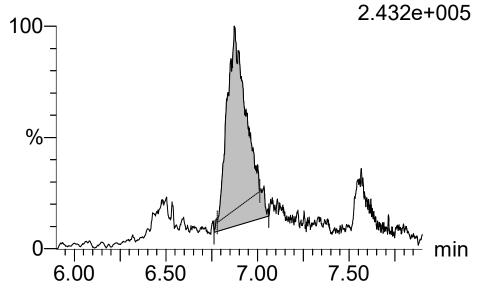

Sample Name: 20211111_H12_R2_IgM
F92:MRM of 1 channel,ES+
1226 > 366.1
1.829e+005

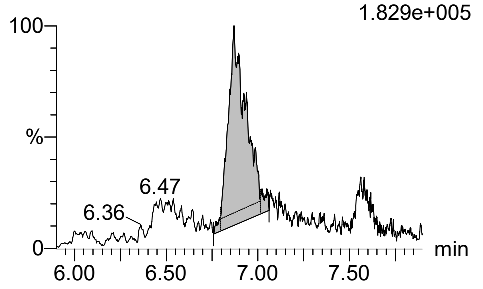

Sample Name: 20211111_QC_R14_IgM
F92:MRM of 1 channel,ES+
1226 > 366.1
2.863e+005

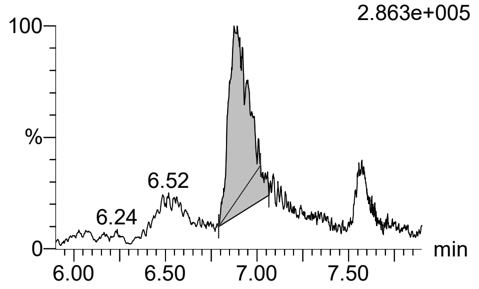

Sample Name: 20211111_A25_R2_IgM
F92:MRM of 1 channel,ES+
1226 > 366.1
4.795e+005

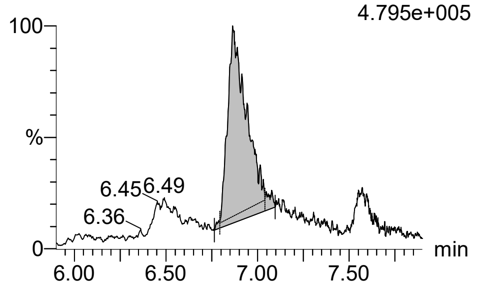

Sample Name: 20211111_A26_R2_IgM
F92:MRM of 1 channel,ES+
1226 > 366.1
2.514e+005

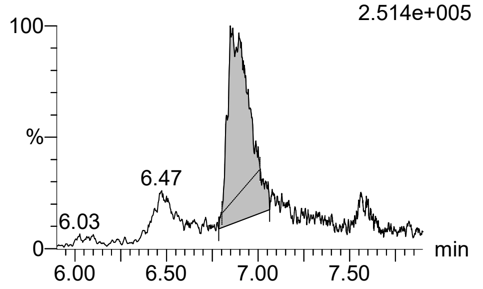

Sample Name: 20211111_L25_R2_IgM

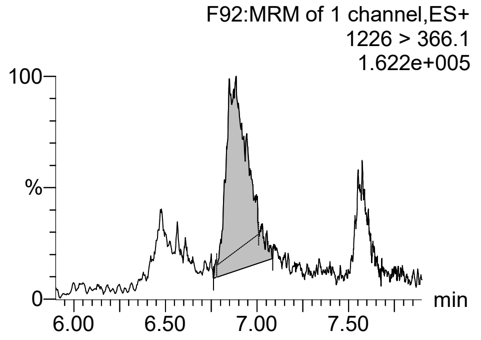

Sample Name: 20211111_L26_R2_IgM

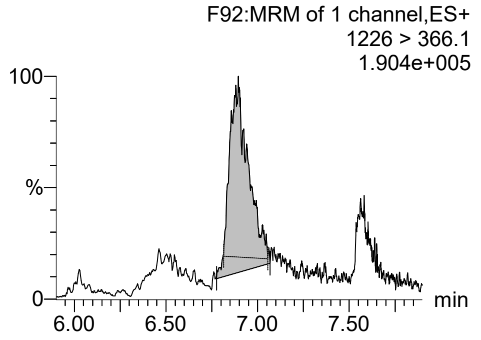

Sample Name: 20211111_H13_R2_IgM

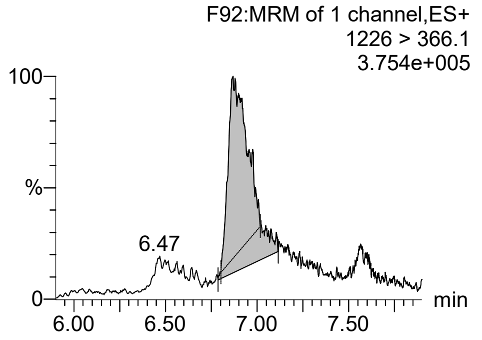

Sample Name: 20211111_A27_R2_IgM

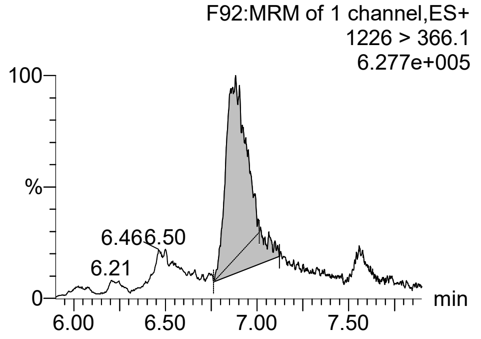

Sample Name: 20211111_A28_R2_IgM

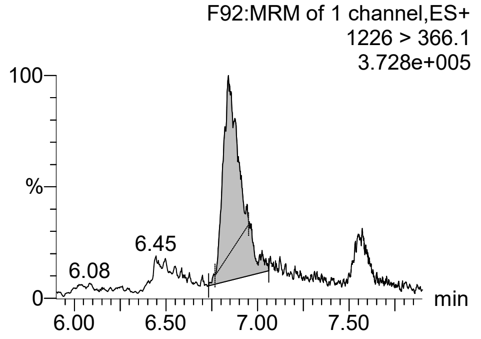

Sample Name: 20211111_L27_R2_IgM

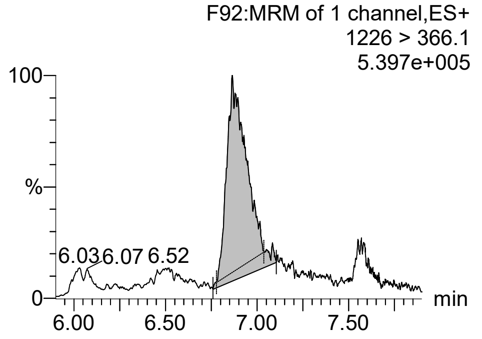

Sample Name: 20211111_L28_R2_IgM

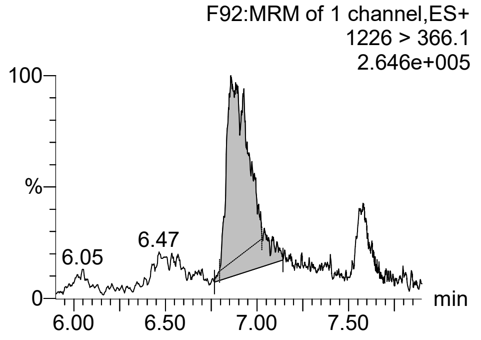

Sample Name: 20211111_H14_R2_IgM

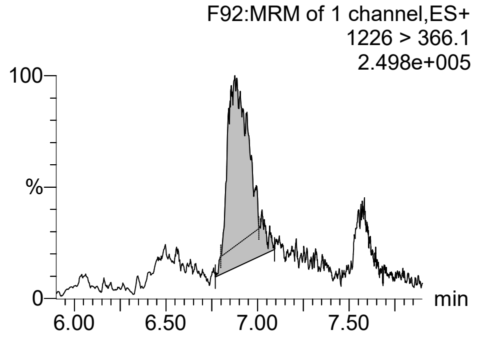

Sample Name: 20211111_A29_R2_IgM

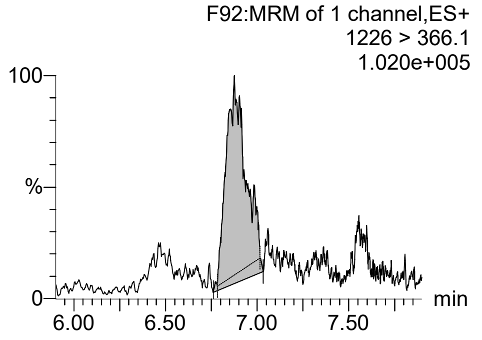

Sample Name: 20211111_A30_R2_IgM

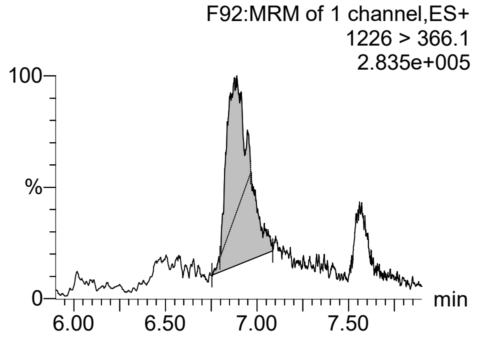

Sample Name: 20211111_QC_R15_IgM

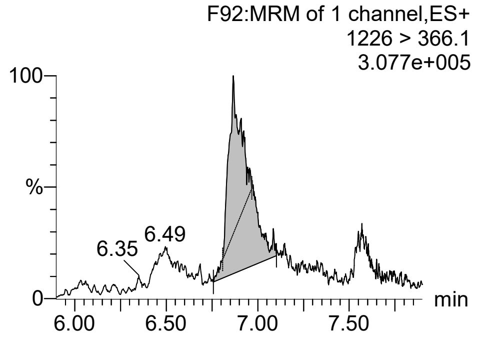

Sample Name: 20211111_L29_R2_IgM

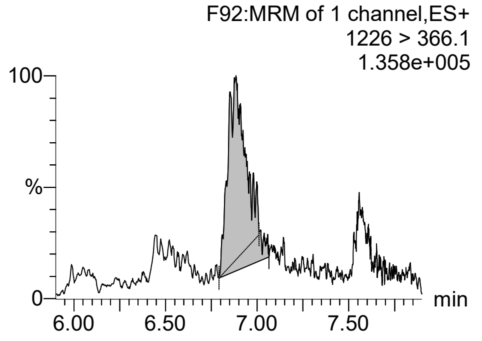

Sample Name: 20211111_L30_R2_IgM

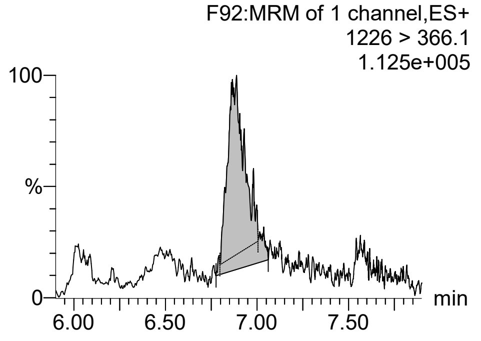

Sample Name: 20211111_H15_R2_IgM

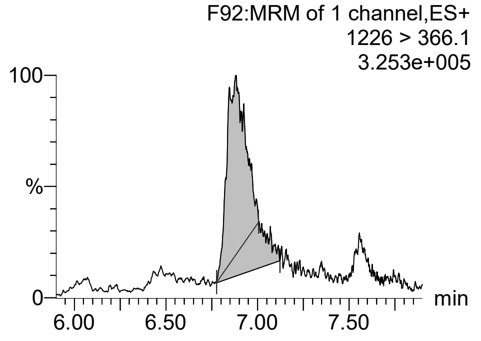

Sample Name: 20211111_A31_R2_IgM

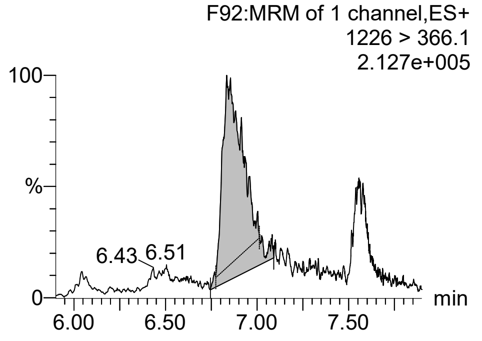

Dataset: C:\MassLynx\10.pro\20211111_clinicalsamples_IgM_IgA.qld

Last Altered: Thursday, March 09, 2023 19:58:25 台北標準時間
Printed: Friday, August 01, 2025 15:20:28 台北標準時間

Sample Name: 20211111_A32_R2_IgM
F92:MRM of 1 channel,ES+
1226 > 366.1
3.056e+005

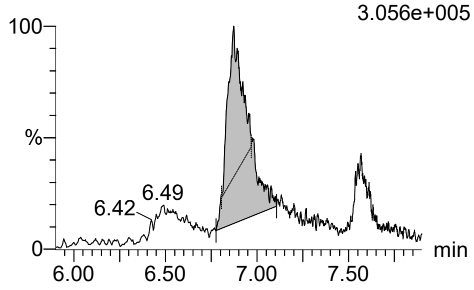

Sample Name: 20211111_L31_R2_IgM
F92:MRM of 1 channel,ES+
1226 > 366.1
1.655e+005

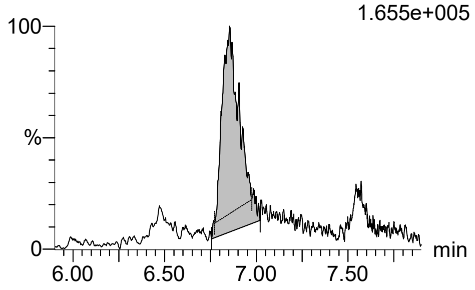

Sample Name: 20211111_L32_R2_IgM
F92:MRM of 1 channel,ES+
1226 > 366.1
2.341e+005

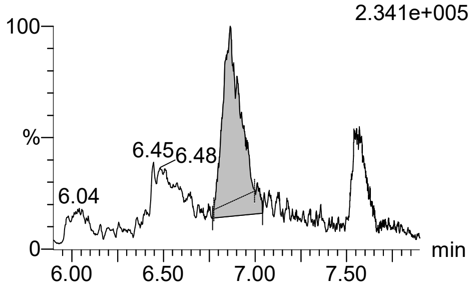

Sample Name: 20211111_H16_R2_IgM
F92:MRM of 1 channel,ES+
1226 > 366.1
6.019e+005

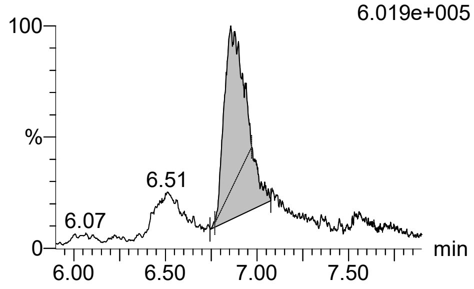

Sample Name: 20211111_A33_R2_IgM
F92:MRM of 1 channel,ES+
1226 > 366.1
6.091e+005

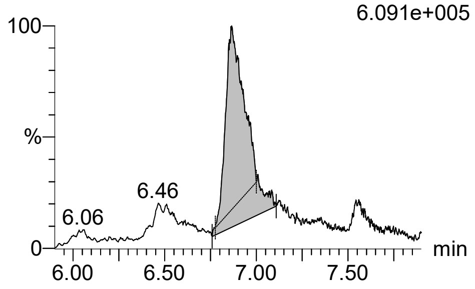

Sample Name: 20211111_A34_R2_IgM
F92:MRM of 1 channel,ES+
1226 > 366.1
2.217e+005

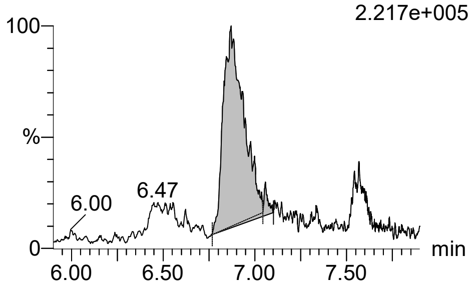

Sample Name: 20211111_H17_R2_IgM
F92:MRM of 1 channel,ES+
1226 > 366.1
1.616e+006

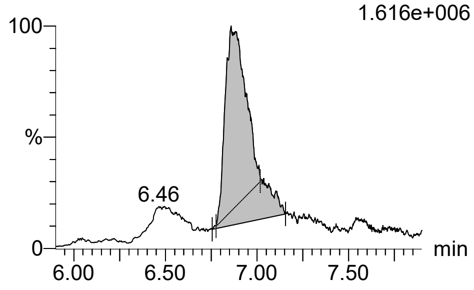

Sample Name: 20211111_A35_R2_IgM
F92:MRM of 1 channel,ES+
1226 > 366.1
5.148e+005

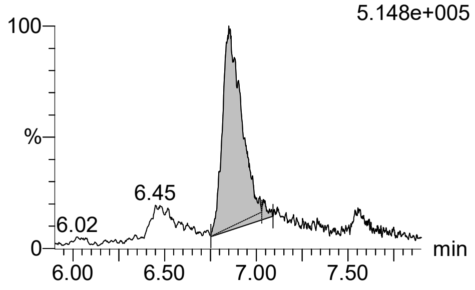

Sample Name: 20211111_QC_R16_IgM
F92:MRM of 1 channel,ES+
1226 > 366.1
3.800e+005

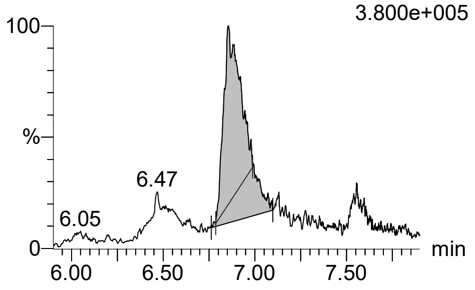

Sample Name: 20211111_A36_R2_IgM
F92:MRM of 1 channel,ES+
1226 > 366.1
3.828e+005

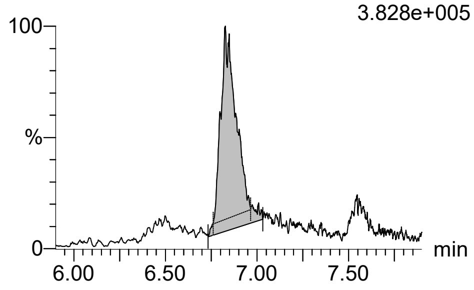

Sample Name: 20211111_H18_R2_IgM
F92:MRM of 1 channel,ES+
1226 > 366.1
4.340e+005

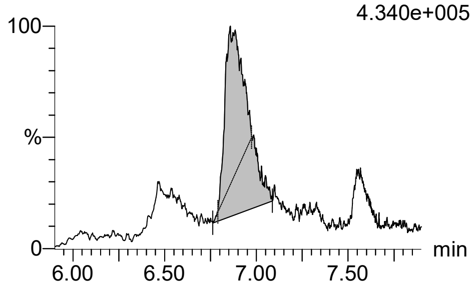

Sample Name: 20211111_A37_R2_IgM
F92:MRM of 1 channel,ES+
1226 > 366.1
3.206e+005

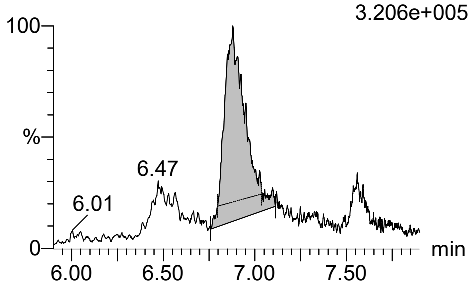

Sample Name: 20211111_A38_R2_IgM
F92:MRM of 1 channel,ES+
1226 > 366.1
2.605e+005

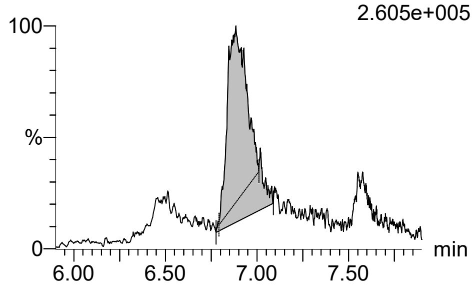

Sample Name: 20211111_H19_R2_IgM
F92:MRM of 1 channel,ES+
1226 > 366.1
3.664e+005

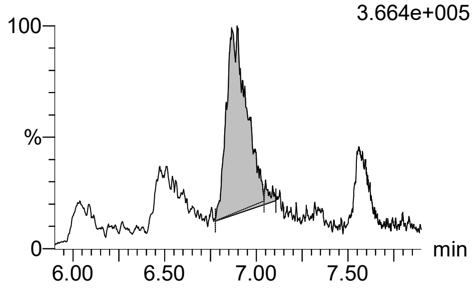

Sample Name: 20211111_A39_R2_IgM
F92:MRM of 1 channel,ES+
1226 > 366.1
4.092e+005

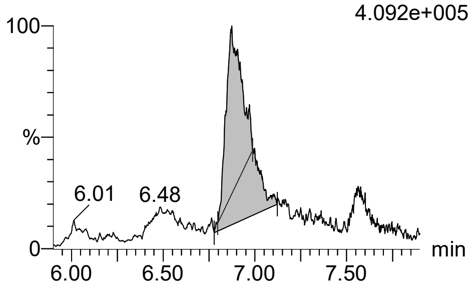

Dataset: C:\MassLynx\10.pro\20211111_clinicalsamples_IgM_IgA.qld

Last Altered: Thursday, March 09, 2023 19:58:25 台北標準時間
Printed: Friday, August 01, 2025 15:20:28 台北標準時間

Sample Name: 20211111_A40_R2_IgM
F92:MRM of 1 channel,ES+
1226 > 366.1
2.374e+005

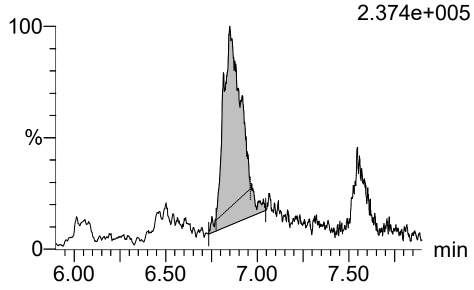

Sample Name: 20211111_H20_R2_IgM
F92:MRM of 1 channel,ES+
1226 > 366.1
6.231e+005

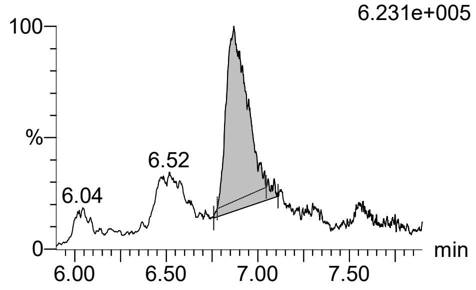

Sample Name: 20211111_A41_R2_IgM
F92:MRM of 1 channel,ES+
1226 > 366.1
2.706e+005

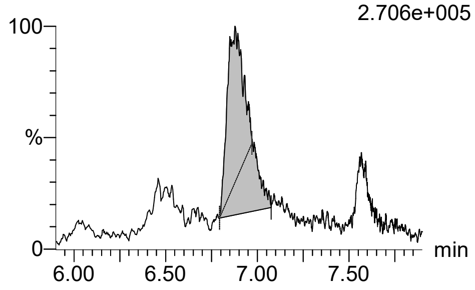

Sample Name: 20211111_A42_R2_IgM
F92:MRM of 1 channel,ES+
1226 > 366.1
1.757e+005

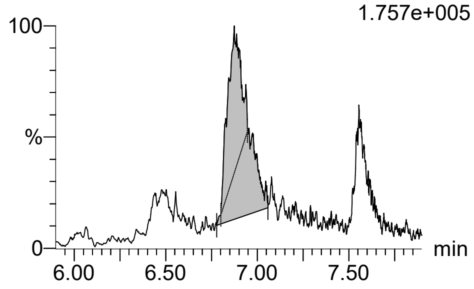

Sample Name: 20211111_A43_R2_IgM
F92:MRM of 1 channel,ES+
1226 > 366.1
1.821e+005

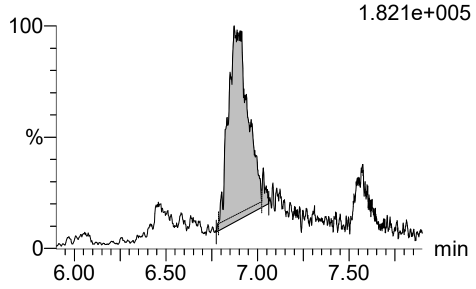

Sample Name: 20211111_A44_R2_IgM
F92:MRM of 1 channel,ES+
1226 > 366.1
1.445e+005

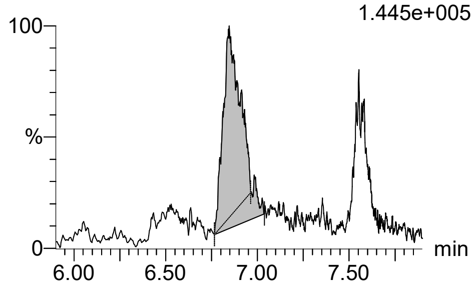

Sample Name: 20211111_QC_R17_IgM
F92:MRM of 1 channel,ES+
1226 > 366.1
2.400e+005

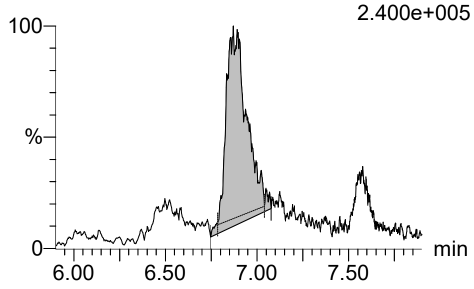

Sample Name: 20211111_L2_R3_IgM
F92:MRM of 1 channel,ES+
1226 > 366.1
2.982e+005

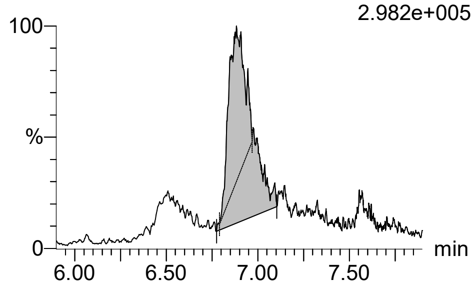

Sample Name: 20211111_H1_R3_IgM
F92:MRM of 1 channel,ES+
1226 > 366.1
2.980e+005

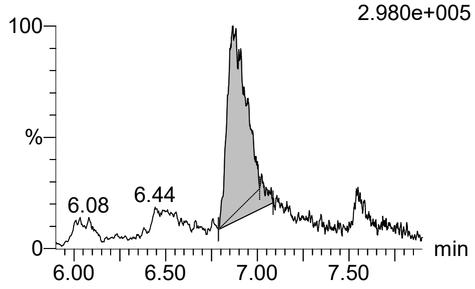

Sample Name: 20211111_A3_R3_IgM
F92:MRM of 1 channel,ES+
1226 > 366.1
3.377e+005

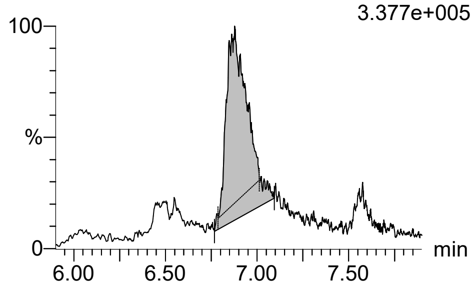

Sample Name: 20211111_A4_R3_IgM
F92:MRM of 1 channel,ES+
1226 > 366.1
2.538e+005

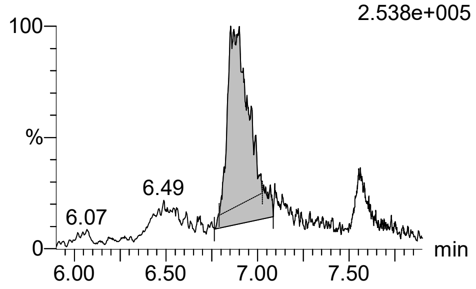

Sample Name: 20211111_L3_R3_IgM
F92:MRM of 1 channel,ES+
1226 > 366.1
2.483e+005

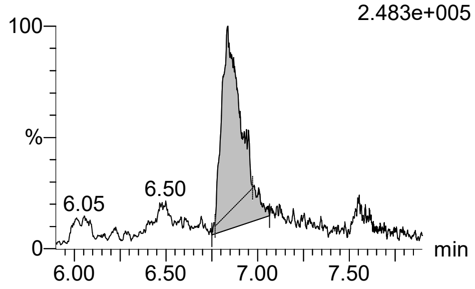

Sample Name: 20211111_L4_R3_IgM
F92:MRM of 1 channel,ES+
1226 > 366.1
2.462e+005

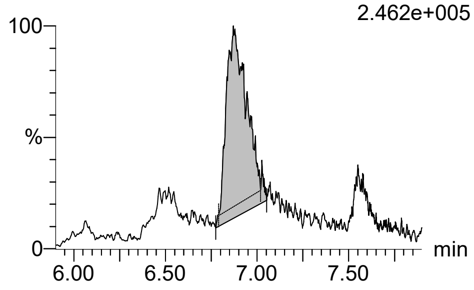

Sample Name: 20211111_H2_R3_IgM
F92:MRM of 1 channel,ES+
1226 > 366.1
3.952e+005

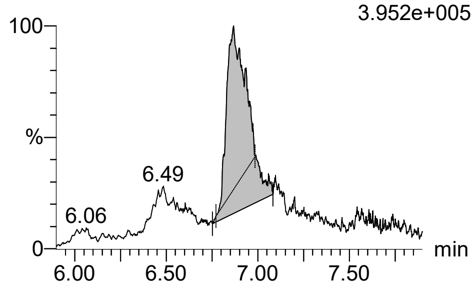

Sample Name: 20211111_A5_R3_IgM
F92:MRM of 1 channel,ES+
1226 > 366.1
9.274e+005

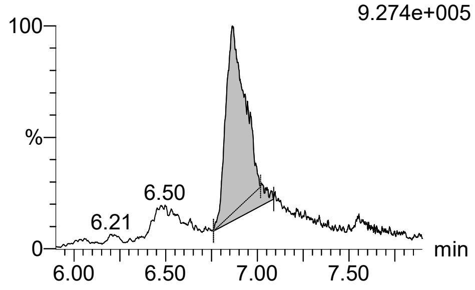

Dataset: C:\MassLynx\10.pro\20211111_clinicalsamples_IgM_IgA.qld

Last Altered: Thursday, March 09, 2023 19:58:25 台北標準時間
Printed: Friday, August 01, 2025 15:20:28 台北標準時間

Sample Name: 20211111_A6_R3_IgM
F92:MRM of 1 channel,ES+
1226 > 366.1
4.387e+005

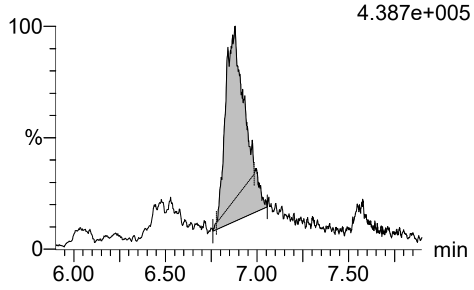

Sample Name: 20211111_QC_R18_IgM
F92:MRM of 1 channel,ES+
1226 > 366.1
3.418e+005

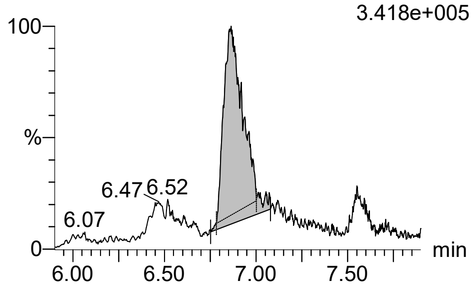

Sample Name: 20211111_L5_R3_IgM
F92:MRM of 1 channel,ES+
1226 > 366.1
3.449e+005

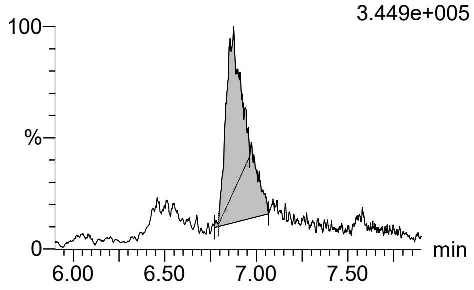

Sample Name: 20211111_L6_R3_IgM
F92:MRM of 1 channel,ES+
1226 > 366.1
2.520e+005

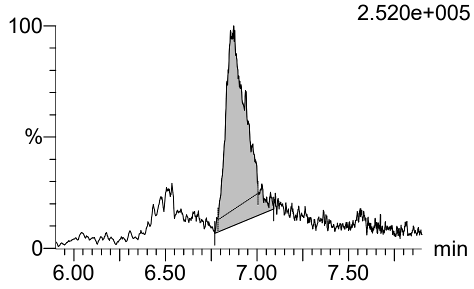

Sample Name: 20211111_H3_R3_IgM
F92:MRM of 1 channel,ES+
1226 > 366.1
1.835e+005

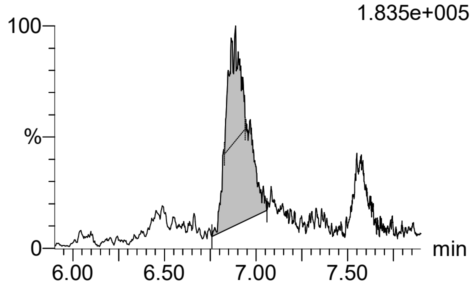

Sample Name: 20211111_A7_R3_IgM
F92:MRM of 1 channel,ES+
1226 > 366.1
2.008e+005

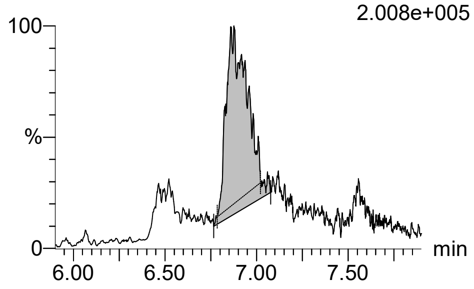

Sample Name: 20211111_A8_R3_IgM
F92:MRM of 1 channel,ES+
1226 > 366.1
2.451e+005

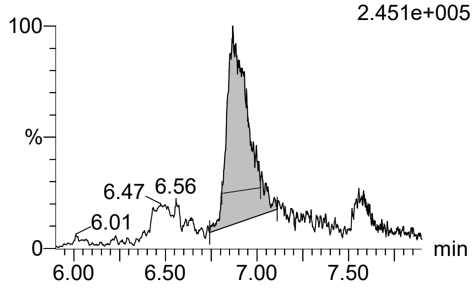

Sample Name: 20211111_L7_R3_IgM
F92:MRM of 1 channel,ES+
1226 > 366.1
2.088e+005

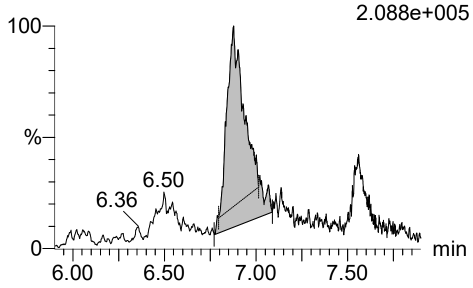

Sample Name: 20211111_L8_R3_IgM
F92:MRM of 1 channel,ES+
1226 > 366.1
1.440e+005

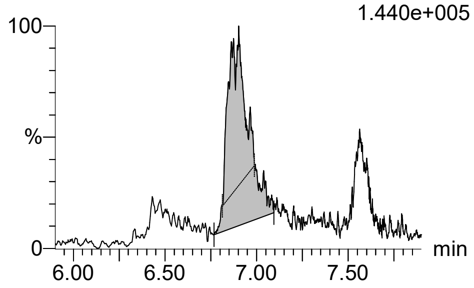

Sample Name: 20211111_H4_R3_IgM
F92:MRM of 1 channel,ES+
1226 > 366.1
1.397e+005

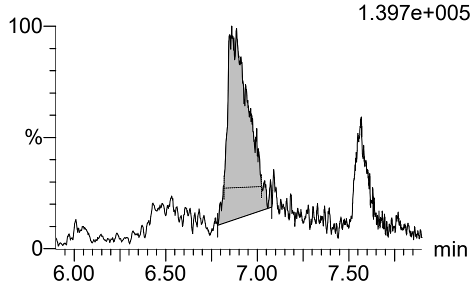

Sample Name: 20211111_A9_R3_IgM
F92:MRM of 1 channel,ES+
1226 > 366.1
2.551e+005

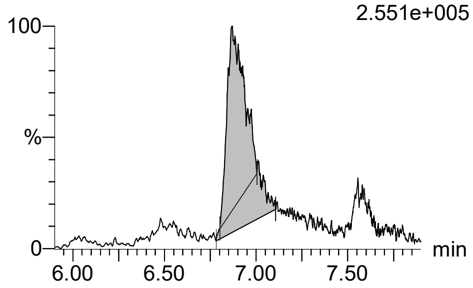

Sample Name: 20211111_A10_R3_IgM
F92:MRM of 1 channel,ES+
1226 > 366.1
2.709e+005

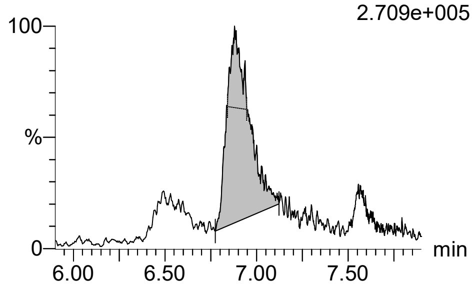

Sample Name: 20211111_L9_R3_IgM
F92:MRM of 1 channel,ES+
1226 > 366.1
1.629e+005

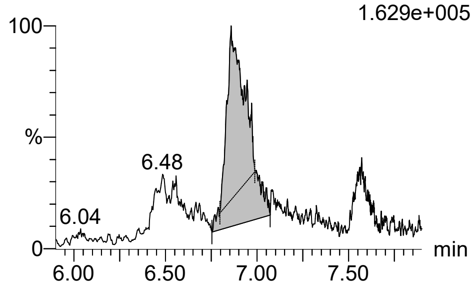

Sample Name: 20211111_L10_R3_IgM
F92:MRM of 1 channel,ES+
1226 > 366.1
4.819e+005

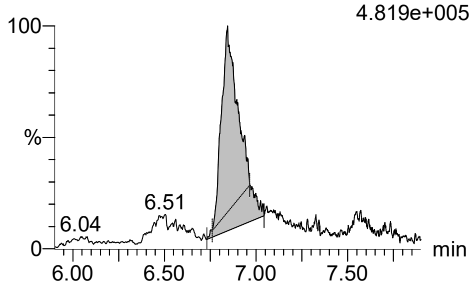

Sample Name: 20211111_QC_R19_IgM
F92:MRM of 1 channel,ES+
1226 > 366.1
3.289e+005

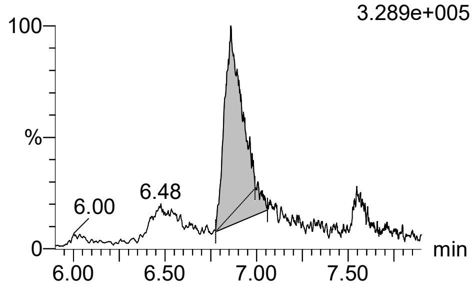

Dataset: C:\MassLynx\10.pro\20211111_clinicalsamples_IgM_IgA.qld

Last Altered: Thursday, March 09, 2023 19:58:25 台北標準時間
Printed: Friday, August 01, 2025 15:20:28 台北標準時間

Sample Name: 20211111_H5_R3_IgM

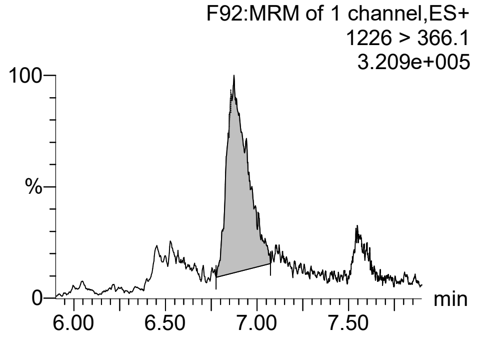

Sample Name: 20211111_A11_R3_IgM

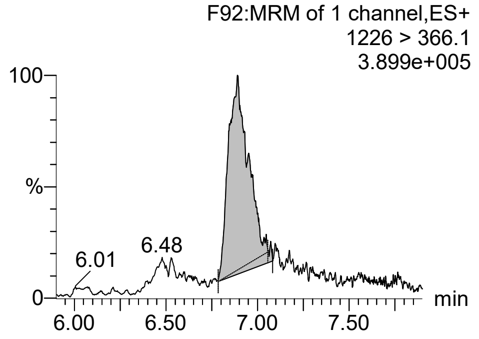

Sample Name: 20211111_A12_R3_IgM

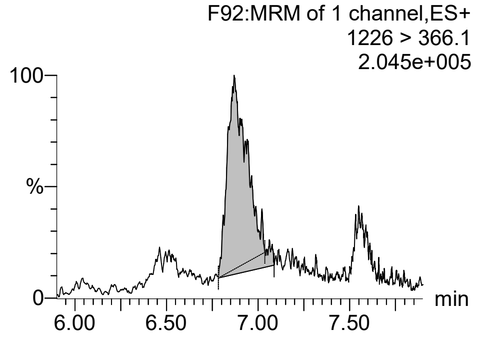

Sample Name: 20211111_L11_R3_IgM

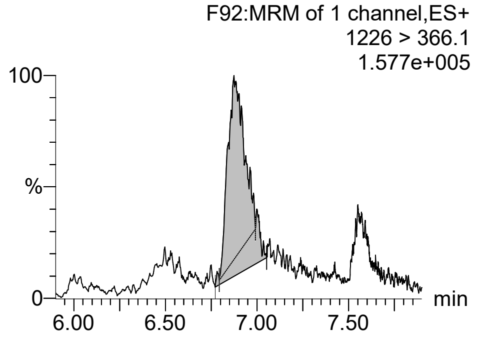

Sample Name: 20211111_L12_R3_IgM

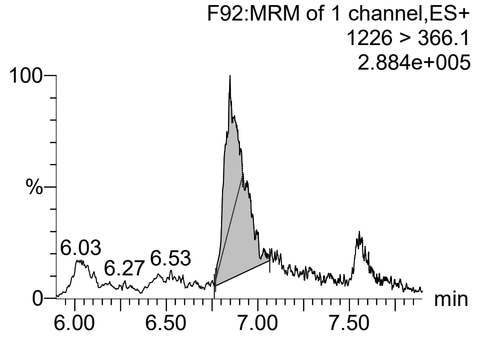

Sample Name: 20211111_H6_R3_IgM

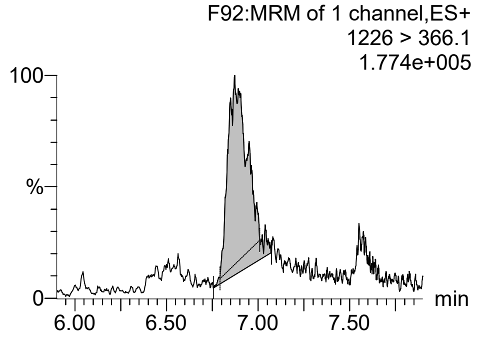

Sample Name: 20211111_A13_R3_IgM

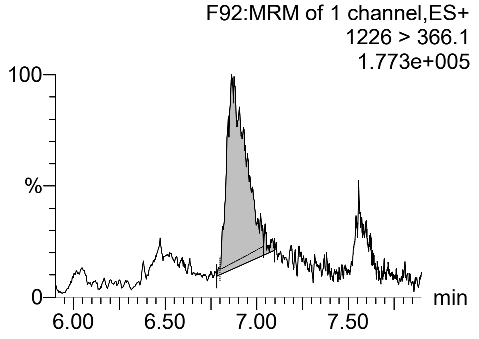

Sample Name: 20211111_A14_R3_IgM

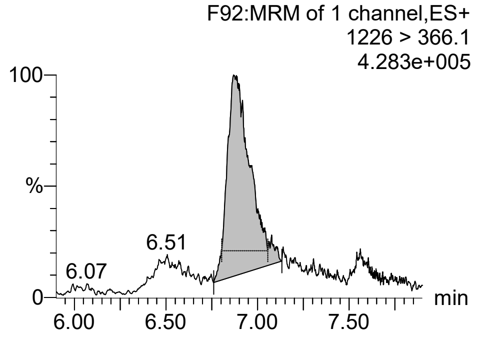

Sample Name: 20211111_L13_R3_IgM

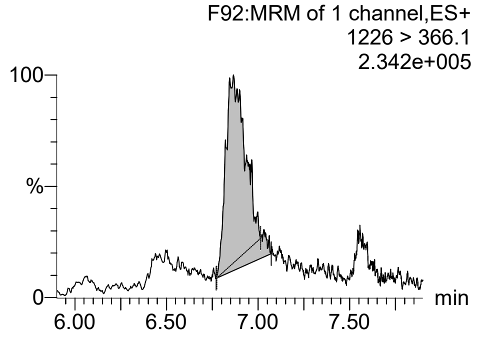

Sample Name: 20211111_L14_R3_IgM

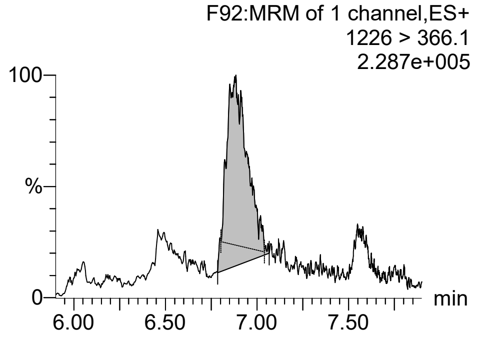

Sample Name: 20211111_H7_R3_IgM

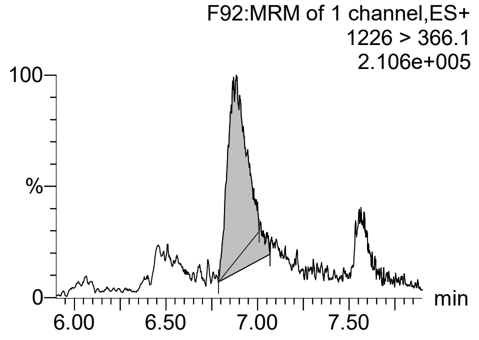

Sample Name: 20211111_A15_R3_IgM

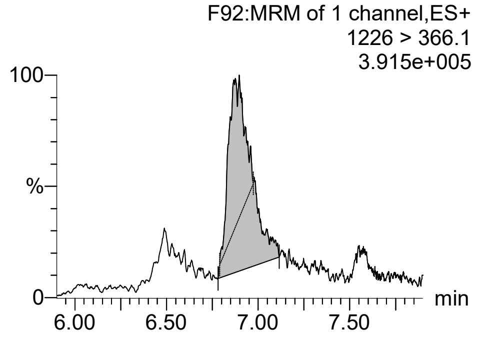

Sample Name: 20211111_QC_R20_IgM

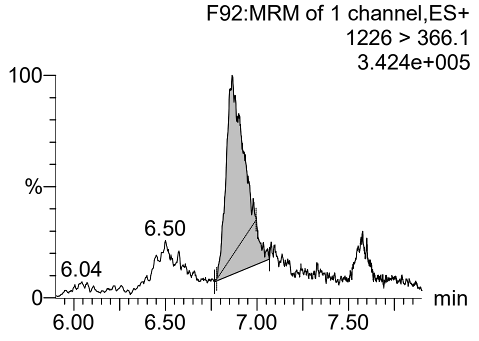

Sample Name: 20211111_A16_R3_IgM

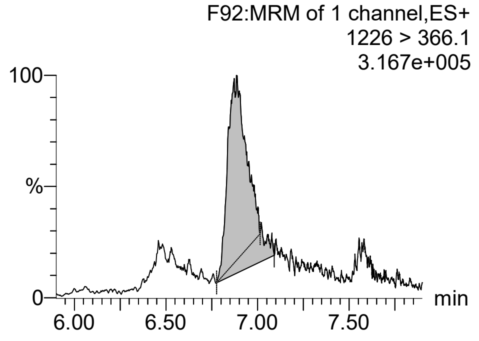

Sample Name: 20211111_L15_R3_IgM

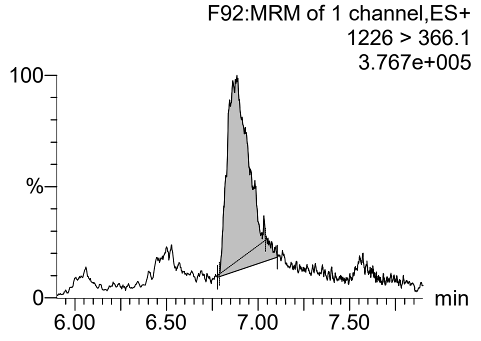

Dataset: C:\MassLynx\10.pro\20211111_clinicalsamples_IgM_IgA.qld

Last Altered: Thursday, March 09, 2023 19:58:25 台北標準時間
Printed: Friday, August 01, 2025 15:20:28 台北標準時間

Sample Name: 20211111_L16_R3_IgM

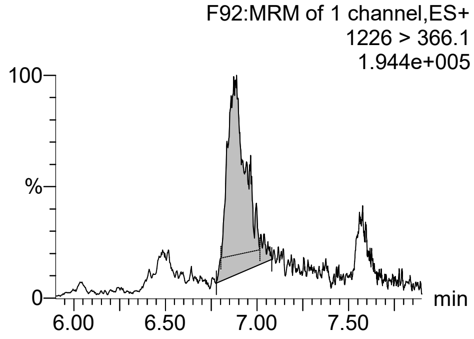

Sample Name: 20211111_H8_R3_IgM

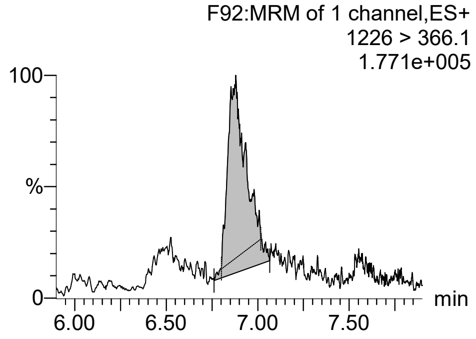

Sample Name: 20211111_A17_R3_IgM

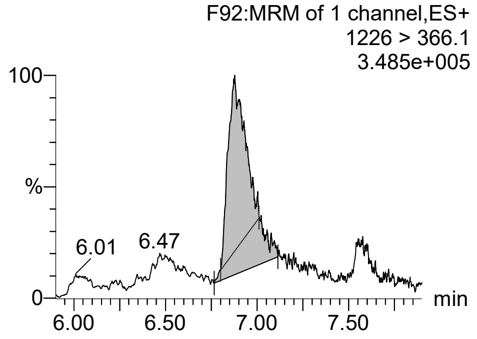

Sample Name: 20211111_A18_R3_IgM

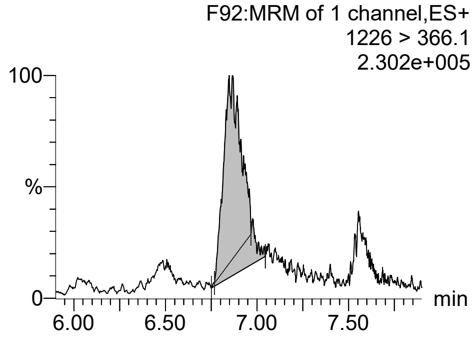

Sample Name: 20211111_L17_R3_IgM

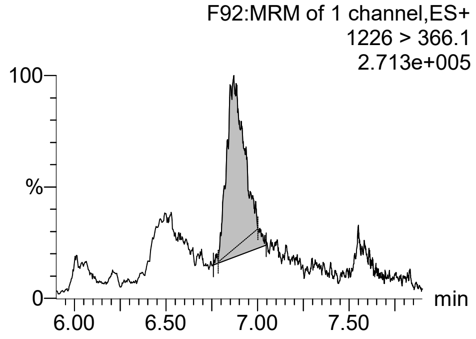

Sample Name: 20211111_L18_R3_IgM

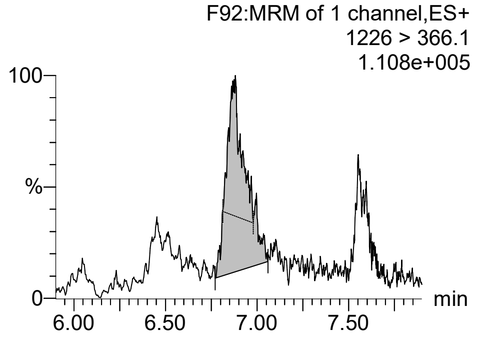

Sample Name: 20211111_H9_R3_IgM

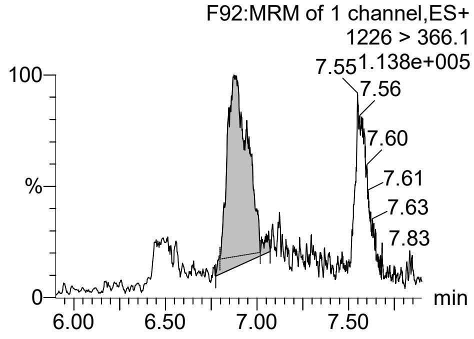

Sample Name: 20211111_A19_R3_IgM

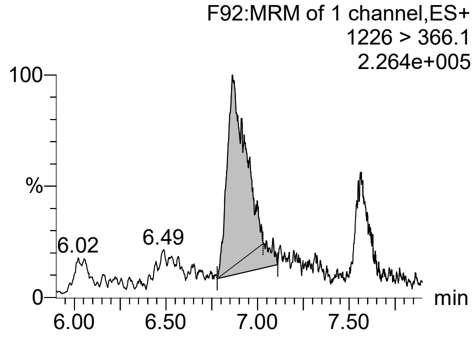

Sample Name: 20211111_A20_R3_IgM

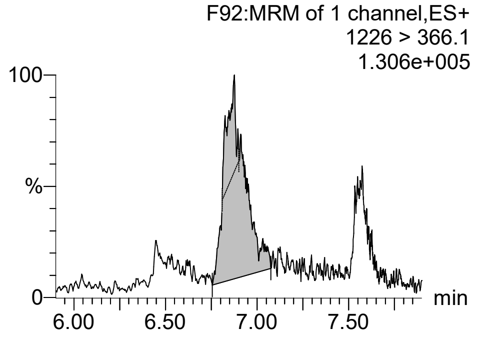

Sample Name: 20211111_L19_R3_IgM

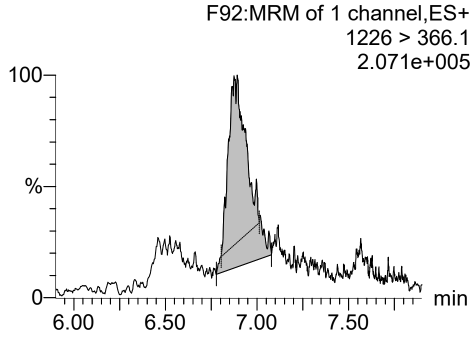

Sample Name: 20211111_QC_R21_IgM

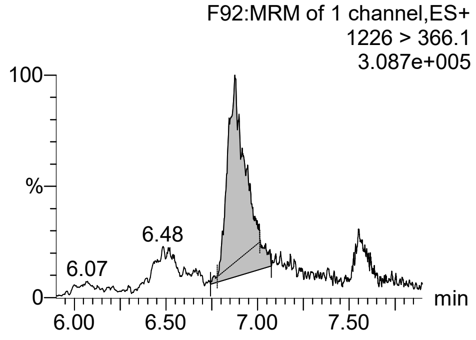

Sample Name: 20211111_L20_R3_IgM

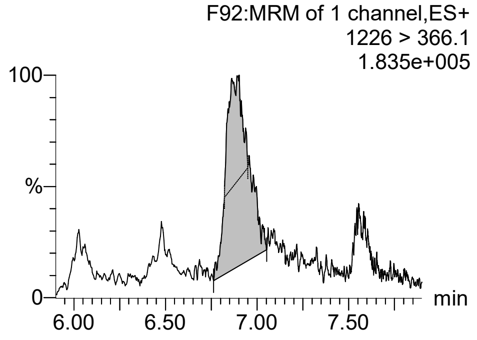

Sample Name: 20211111_H10_R3_IgM

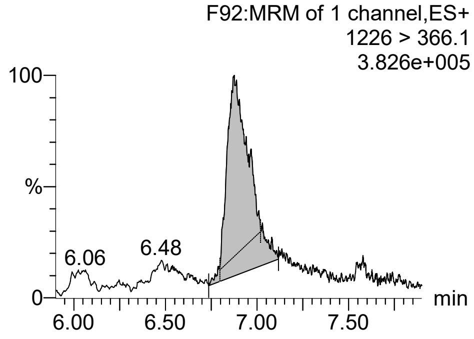

Sample Name: 20211111_A21_R3_IgM

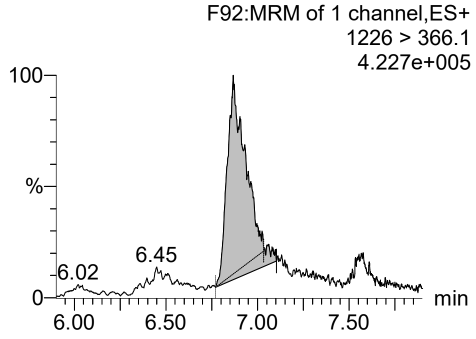

Sample Name: 20211111_A22_R3_IgM

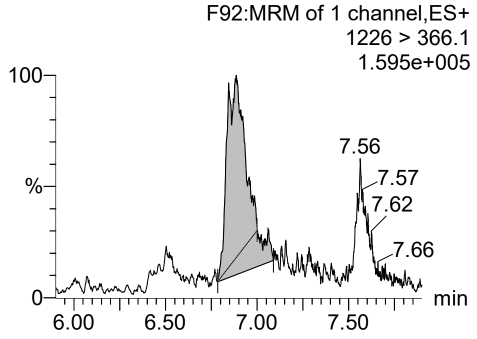

Dataset: C:\MassLynx\10.pro\20211111_clinicalsamples_IgM_IgA.qld

Last Altered: Thursday, March 09, 2023 19:58:25 台北標準時間
Printed: Friday, August 01, 2025 15:20:28 台北標準時間

Sample Name: 20211111_L21_R3_IgM
F92:MRM of 1 channel,ES+
1226 > 366.1
2.023e+005

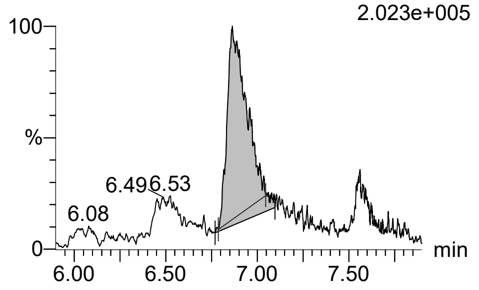

Sample Name: 20211111_L22_R3_IgM
F92:MRM of 1 channel,ES+
1226 > 366.1
2.058e+005

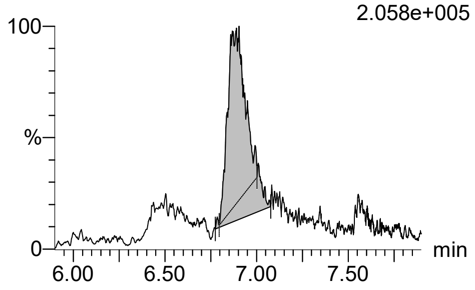

Sample Name: 20211111_H11_R3_IgM
F92:MRM of 1 channel,ES+
1226 > 366.1
2.388e+005

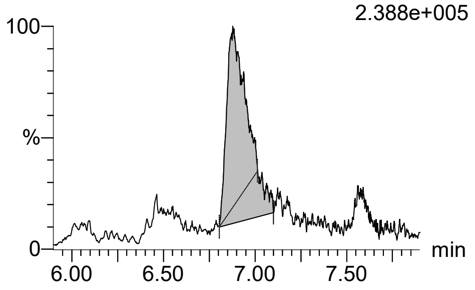

Sample Name: 20211111_A23_R3_IgM
F92:MRM of 1 channel,ES+
1226 > 366.1
1.403e+005

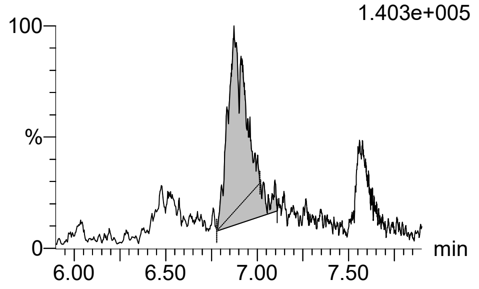

Sample Name: 20211111_A24_R3_IgM
F92:MRM of 1 channel,ES+
1226 > 366.1
3.663e+005

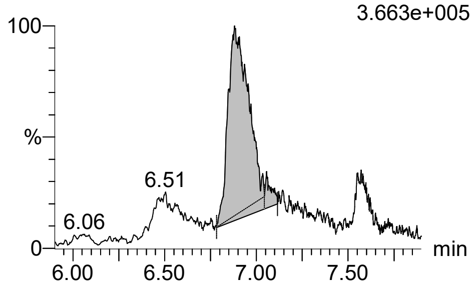

Sample Name: 20211111_L23_R3_IgM
F92:MRM of 1 channel,ES+
1226 > 366.1
3.939e+005

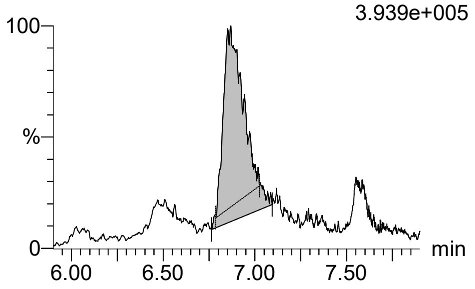

Sample Name: 20211111_L24_R3_IgM
F92:MRM of 1 channel,ES+
1226 > 366.1
2.028e+005

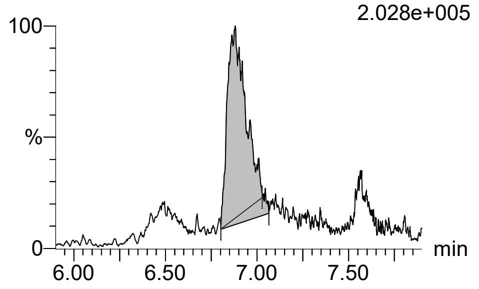

Sample Name: 20211111_H12_R3_IgM
F92:MRM of 1 channel,ES+
1226 > 366.1
1.579e+005

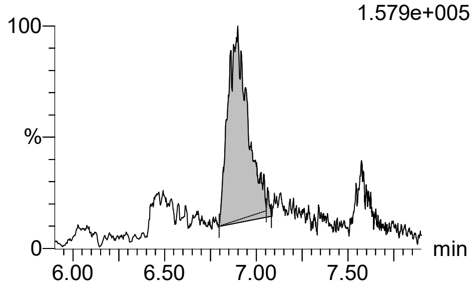

Sample Name: 20211111_QC_R22_IgM
F92:MRM of 1 channel,ES+
1226 > 366.1
2.790e+005

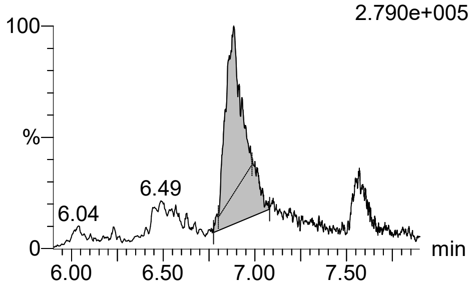

Sample Name: 20211111_A25_R3_IgM
F92:MRM of 1 channel,ES+
1226 > 366.1
3.605e+005

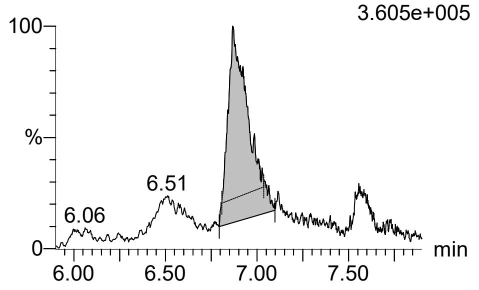

Sample Name: 20211111_A26_R3_IgM
F92:MRM of 1 channel,ES+
1226 > 366.1
2.224e+005

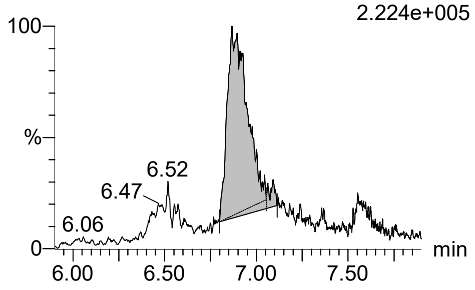

Sample Name: 20211111_L25_R3_IgM
F92:MRM of 1 channel,ES+
1226 > 366.1
1.476e+005

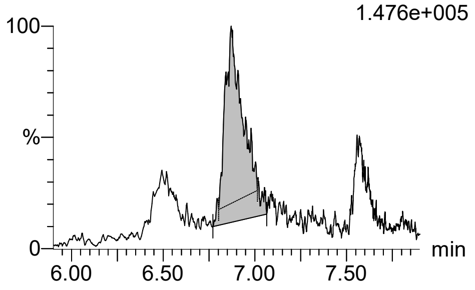

Sample Name: 20211111_L26_R3_IgM
F92:MRM of 1 channel,ES+
1226 > 366.1
1.665e+005

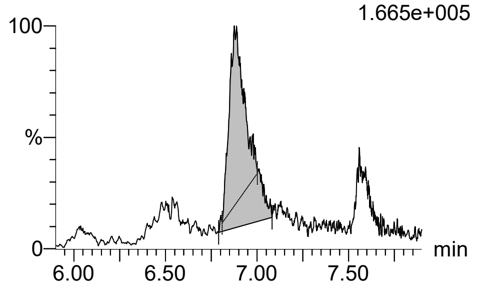

Sample Name: 20211111_H13_R3_IgM
F92:MRM of 1 channel,ES+
1226 > 366.1
3.670e+005

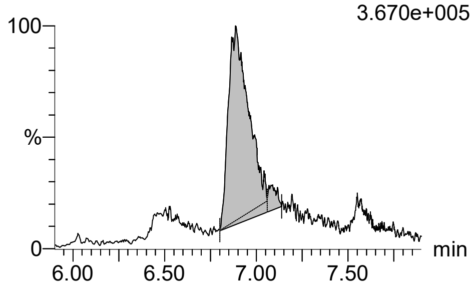

Sample Name: 20211111_A27_R3_IgM
F92:MRM of 1 channel,ES+
1226 > 366.1
5.263e+005

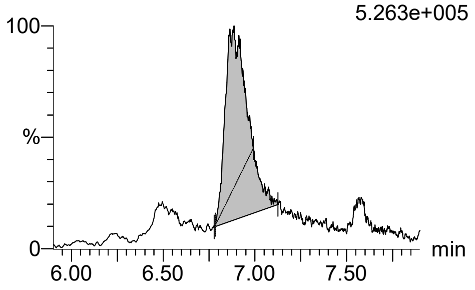

Dataset: C:\MassLynx\10.pro\20211111_clinicalsamples_IgM_IgA.qld

Last Altered: Thursday, March 09, 2023 19:58:25 台北標準時間
Printed: Friday, August 01, 2025 15:20:28 台北標準時間

Sample Name: 20211111_A28_R3_IgM
F92:MRM of 1 channel,ES+
1226 > 366.1
2.373e+005

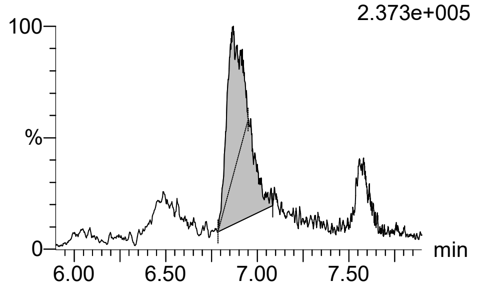

Sample Name: 20211111_L27_R3_IgM
F92:MRM of 1 channel,ES+
1226 > 366.1
4.722e+005

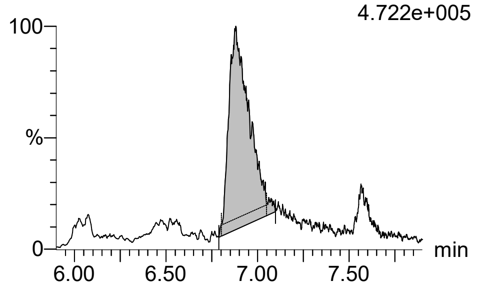

Sample Name: 20211111_L28_R3_IgM
F92:MRM of 1 channel,ES+
1226 > 366.1
2.682e+005

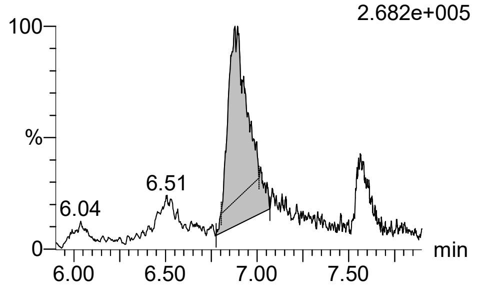

Sample Name: 20211111_H14_R3_IgM
F92:MRM of 1 channel,ES+
1226 > 366.1
2.557e+005

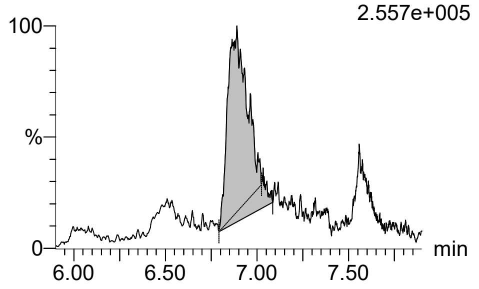

Sample Name: 20211111_A29_R3_IgM
F92:MRM of 1 channel,ES+
1226 > 366.1
1.128e+005

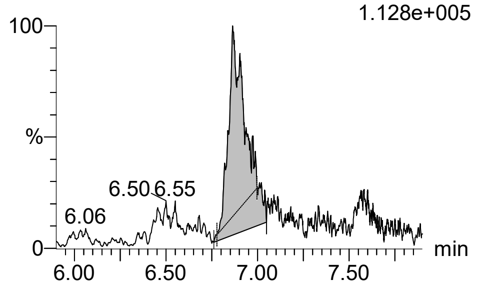

Sample Name: 20211111_A30_R3_IgM
F92:MRM of 1 channel,ES+
1226 > 366.1
2.732e+005

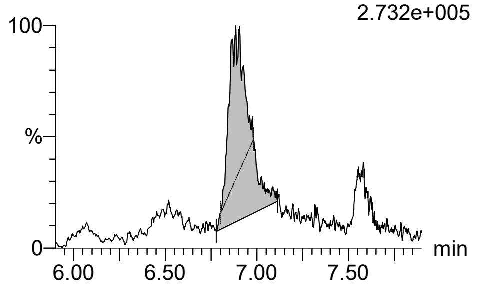

Sample Name: 20211111_QC_R23_IgM
F92:MRM of 1 channel,ES+
1226 > 366.1
2.732e+005

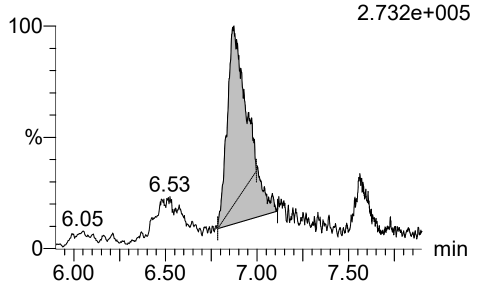

Sample Name: 20211111_L29_R3_IgM
F92:MRM of 1 channel,ES+
1226 > 366.1
1.170e+005

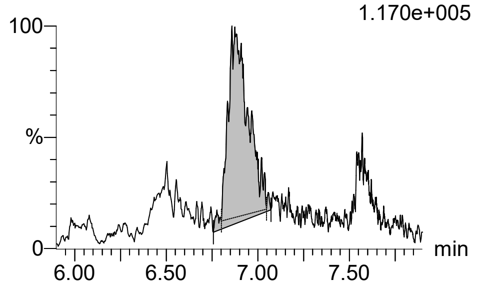

Sample Name: 20211111_L30_R3_IgM
F92:MRM of 1 channel,ES+
1226 > 366.1
8.595e+004

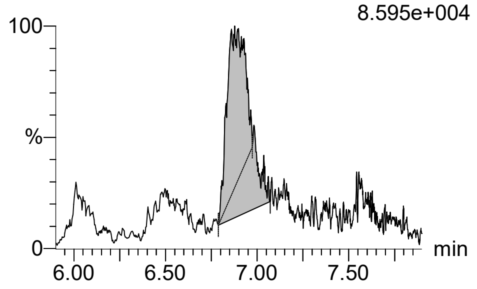

Sample Name: 20211111_H15_R3_IgM
F92:MRM of 1 channel,ES+
1226 > 366.1
2.911e+005

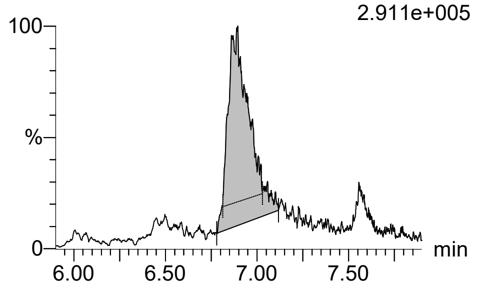

Sample Name: 20211111_A31_R3_IgM
F92:MRM of 1 channel,ES+
1226 > 366.1
2.085e+005

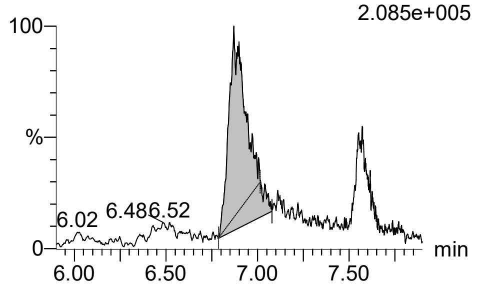

Sample Name: 20211111_A32_R3_IgM
F92:MRM of 1 channel,ES+
1226 > 366.1
2.722e+005

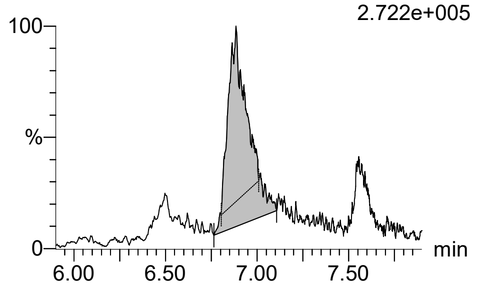

Sample Name: 20211111_L31_R3_IgM
F92:MRM of 1 channel,ES+
1226 > 366.1
1.260e+005

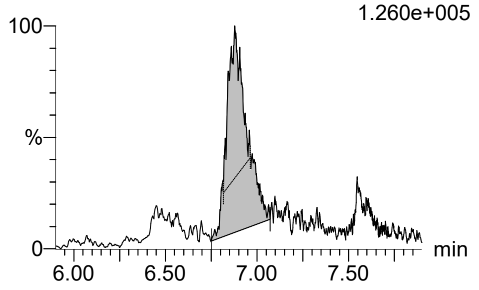

Sample Name: 20211111_L32_R3_IgM
F92:MRM of 1 channel,ES+
1226 > 366.1
1.650e+005

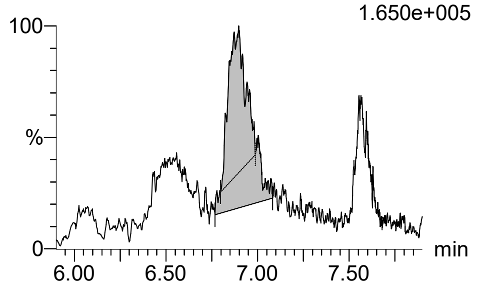

Sample Name: 20211111_H16_R3_IgM
F92:MRM of 1 channel,ES+
1226 > 366.1
4.780e+005

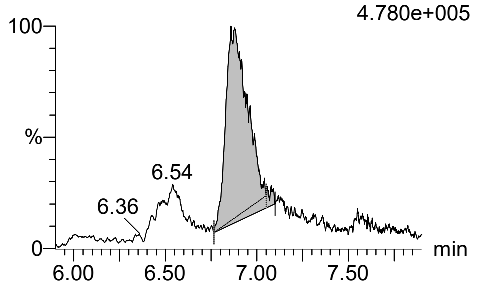

Sample Name: 20211111_A33_R3_IgM
F92:MRM of 1 channel,ES+
1226 > 366.1
5.775e+005

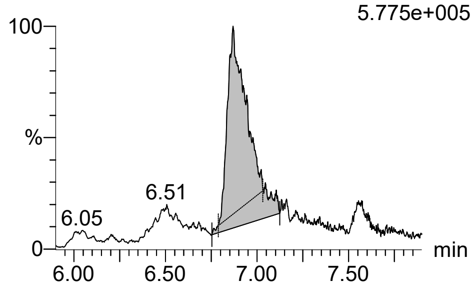

Sample Name: 20211111_A34_R3_IgM
F92:MRM of 1 channel,ES+
1226 > 366.1
1.921e+005

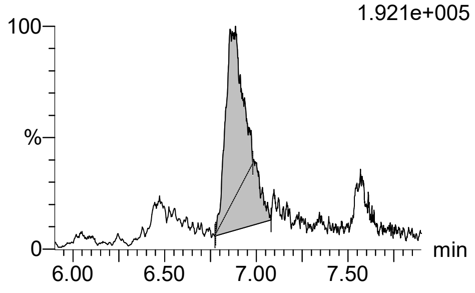

Sample Name: 20211111_H17_R3_IgM
F92:MRM of 1 channel,ES+
1226 > 366.1
1.402e+006

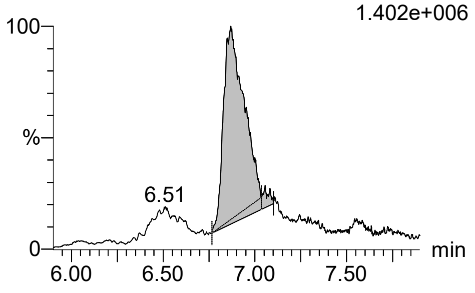

Sample Name: 20211111_A35_R3_IgM
F92:MRM of 1 channel,ES+
1226 > 366.1
4.366e+005

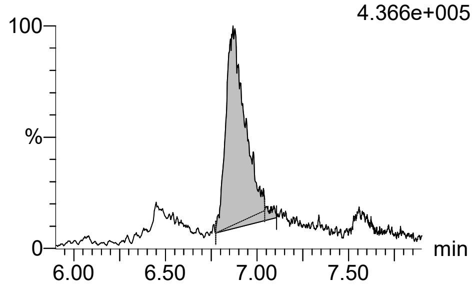

Sample Name: 20211111_QC_R24_IgM
F92:MRM of 1 channel,ES+
1226 > 366.1
3.240e+005

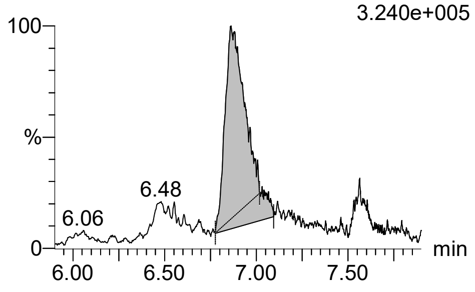

Sample Name: 20211111_A36_R3_IgM
F92:MRM of 1 channel,ES+
1226 > 366.1
2.035e+005

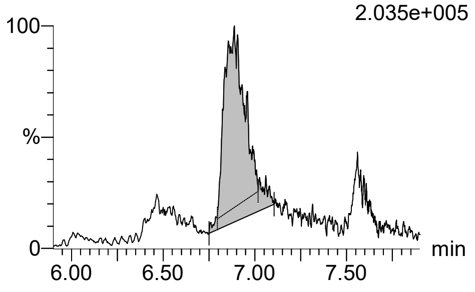

Sample Name: 20211111_H18_R3_IgM
F92:MRM of 1 channel,ES+
1226 > 366.1
3.913e+005

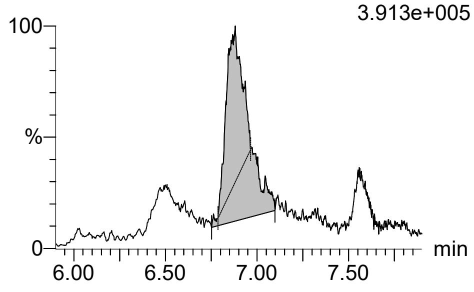

Sample Name: 20211111_A37_R3_IgM
F92:MRM of 1 channel,ES+
1226 > 366.1
2.472e+005

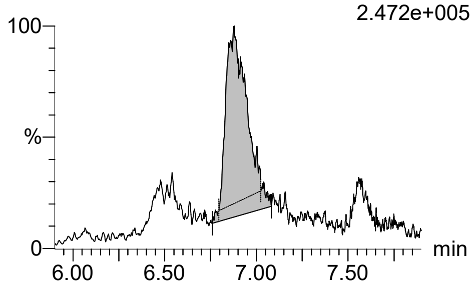

Sample Name: 20211111_A38_R3_IgM
F92:MRM of 1 channel,ES+
1226 > 366.1
2.535e+005

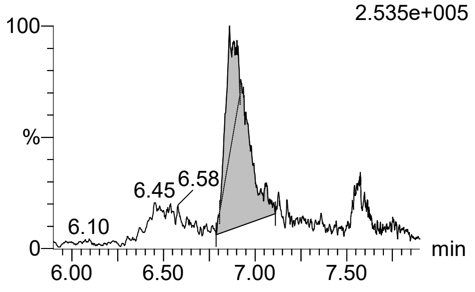

Sample Name: 20211111_H19_R3_IgM
F92:MRM of 1 channel,ES+
1226 > 366.1
3.203e+005

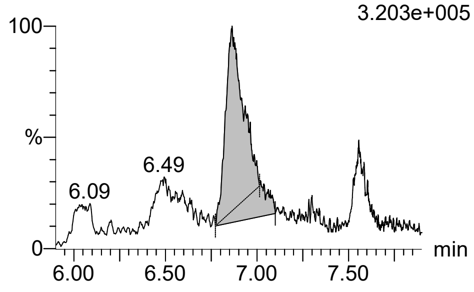

Sample Name: 20211111_A39_R3_IgM
F92:MRM of 1 channel,ES+
1226 > 366.1
3.955e+005

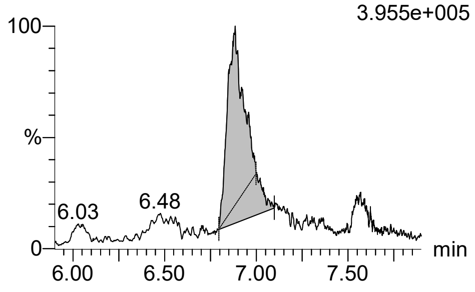

Sample Name: 20211111_A40_R3_IgM
F92:MRM of 1 channel,ES+
1226 > 366.1
1.805e+005

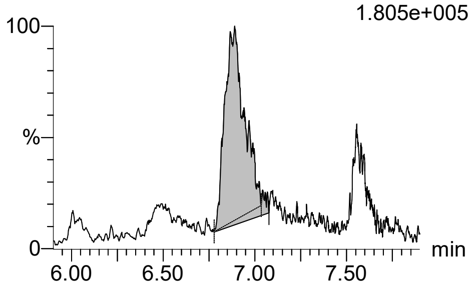

Sample Name: 20211111_H20_R3_IgM
F92:MRM of 1 channel,ES+
1226 > 366.1
5.012e+005

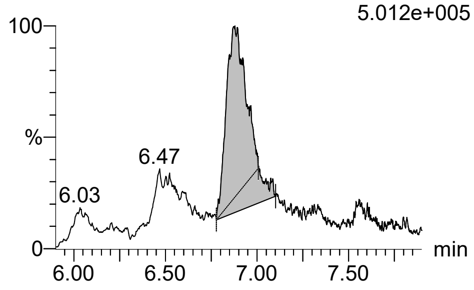

Sample Name: 20211111_A41_R3_IgM
F92:MRM of 1 channel,ES+
1226 > 366.1
1.746e+005

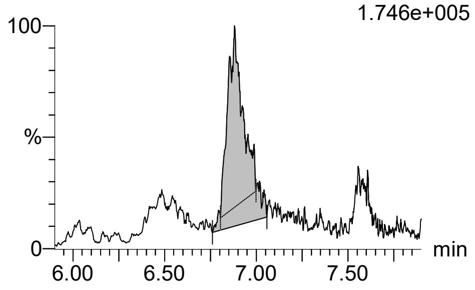

Sample Name: 20211111_A42_R3_IgM
F92:MRM of 1 channel,ES+
1226 > 366.1
1.593e+005

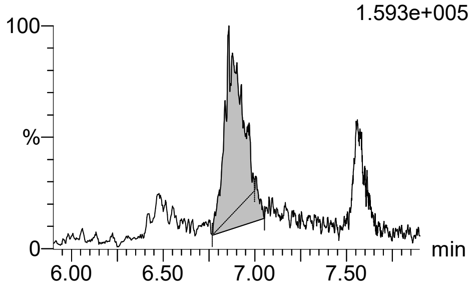

Dataset: C:\MassLynx\10.pro\20211111_clinicalsamples_IgM_IgA.qld

Last Altered: Thursday, March 09, 2023 19:58:25 台北標準時間
Printed: Friday, August 01, 2025 15:20:28 台北標準時間

Sample Name: 20211111_A43_R3_IgM

F92:MRM of 1 channel,ES+
1226 > 366.1
1.579e+005

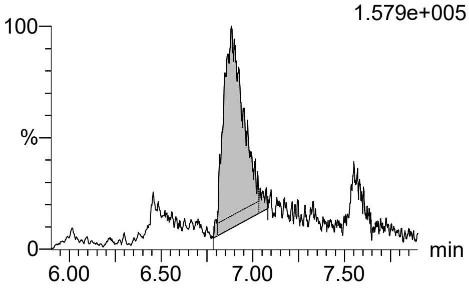

Sample Name: 20211111_A44_R3_IgM

F92:MRM of 1 channel,ES+
1226 > 366.1
1.170e+005

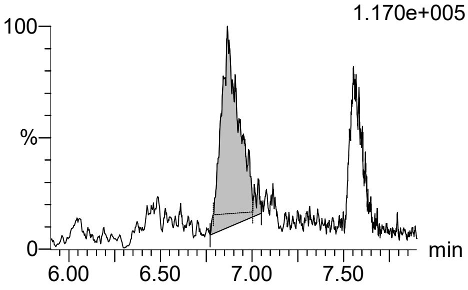

Sample Name: 20211111_QC_R25_IgM

F92:MRM of 1 channel,ES+
1226 > 366.1
2.093e+005

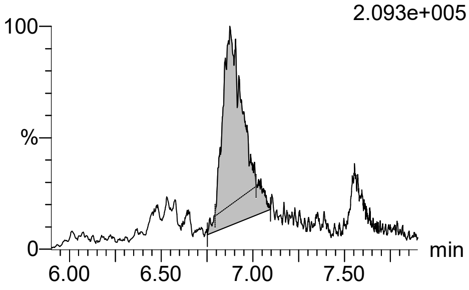

Dataset: C:\MassLynx\10.pro\20211111_clinicalsamples_IgM_IgA.qld

Last Altered: Thursday, March 09, 2023 19:58:25 台北標準時間
Printed: Friday, August 01, 2025 15:20:28 台北標準時間

	#	Name	RT	Area	IS Area	Response	Prima...
1	1	20211112_QC_R001_IgM	6.86	28839.7	15421.4	1.870	bb
2	2	20211112_QC_R002_IgM	6.87	32291.8	13188.8	2.448	MM
3	3	20211111_A1_R2_IgM	6.87	19407.6	11871.0	1.635	MM
4	4	20211111_A1_R3_IgM	6.86	16438.1	12030.5	1.366	MM
5	5	20211111_A2_R2_IgM	6.86	49140.3	11446.8	4.293	MM
6	6	20211111_A2_R3_IgM	6.86	55370.2	11098.4	4.989	MM
7	7	20211111_L1_R2_IgM	6.86	21375.3	11779.4	1.815	MM
8	8	20211111_L1_R3_IgM	6.88	12775.5	11176.2	1.143	MM
9	9	20211111_L2_R2_IgM	6.87	33811.0	11375.0	2.972	MM
10	10	20211111_H1_R2_IgM	6.87	42373.3	13595.2	3.117	MM
11	11	20211111_A3_R2_IgM	6.86	56320.0	14112.3	3.991	MM
12	12	20211111_A4_R2_IgM	6.86	36089.1	11977.5	3.013	MM
13	13	20211111_L3_R2_IgM	6.83	34917.4	12242.1	2.852	MM
14	14	20211111_L4_R2_IgM	6.85	38898.6	12797.0	3.040	MM
15	15	20211111_H2_R2_IgM	6.86	50329.3	10006.0	5.030	MM
16	16	20211111_A5_R2_IgM	6.85	159157.0	11434.6	13.919	MM
17	17	20211111_A6_R2_IgM	6.85	70819.9	13193.7	5.368	MM
18	18	20211111_QC_R10_IgM	6.86	46247.1	12566.1	3.680	MM
19	19	20211111_L5_R2_IgM	6.85	55471.3	13616.4	4.074	MM
20	20	20211111_L6_R2_IgM	6.85	35546.5	13698.5	2.595	MM
21	21	20211111_H3_R2_IgM	6.85	27610.0	15793.1	1.748	MM
22	22	20211111_A7_R2_IgM	6.86	29966.3	12936.9	2.316	MM
23	23	20211111_A8_R2_IgM	6.87	36050.0	13108.0	2.750	MM
24	24	20211111_L7_R2_IgM	6.89	29042.4	13278.5	2.187	MM
25	25	20211111_L8_R2_IgM	6.87	19728.6	15233.6	1.295	MM
26	26	20211111_H4_R2_IgM	6.86	19359.8	16521.9	1.172	MM
27	27	20211111_A9_R2_IgM	6.86	44527.2	16451.6	2.707	MM
28	28	20211111_A10_R2_IgM	6.85	41163.9	13036.0	3.158	MM
29	29	20211111_L9_R2_IgM	6.84	22472.5	14554.1	1.544	MM
30	30	20211111_L10_R2_IgM	6.85	60725.5	12250.4	4.957	MM
31	31	20211111_QC_R11_IgM	6.87	43347.8	11459.2	3.783	MM
32	32	20211111_H5_R2_IgM	6.85	56731.6	14358.2	3.951	MM
33	33	20211111_A11_R2_IgM	6.83	81556.2	16184.1	5.039	MM
34	34	20211111_A12_R2_IgM	6.84	34290.8	13465.4	2.547	MM
35	35	20211111_L11_R2_IgM	6.87	26375.5	13277.9	1.986	MM
36	36	20211111_L12_R2_IgM	6.87	36817.2	15958.8	2.307	MM
37	37	20211111_H6_R2_IgM	6.87	22055.3	14003.5	1.575	MM
38	38	20211111_A13_R2_IgM	6.86	22967.7	13656.1	1.682	MM
39	39	20211111_A14_R2_IgM	6.89	72703.9	14071.3	5.167	MM
40	40	20211111_L13_R2_IgM	6.88	33712.9	14785.6	2.280	MM
41	41	20211111_L14_R2_IgM	6.88	32874.1	14772.2	2.225	MM
42	42	20211111_H7_R2_IgM	6.85	31438.0	15164.4	2.073	MM
43	43	20211111_A15_R2_IgM	6.87	54450.9	15721.5	3.463	MM
44	44	20211111_QC_R12_IgM	6.87	45976.3	13711.5	3.353	MM
45	45	20211111_A16_R2_IgM	6.88	43079.6	14275.6	3.018	MM
46	46	20211111_L15_R2_IgM	6.85	69653.1	12935.8	5.385	MM
47	47	20211111_L16_R2_IgM	6.85	24971.7	13342.8	1.872	MM
48	48	20211111_H8_R2_IgM	6.85	21240.5	15835.1	1.341	MM
49	49	20211111_A17_R2_IgM	6.88	47428.4	15786.7	3.004	MM
50	50	20211111_A18_R2_IgM	6.85	28497.4	16986.8	1.678	MM
51	51	20211111_L17_R2_IgM	6.88	35100.2	13342.7	2.631	MM
52	52	20211111_L18_R2_IgM	6.86	12965.5	14692.0	0.882	MM
53	53	20211111_H9_R2_IgM	6.90	17785.0	15639.7	1.137	MM
54	54	20211111_A19_R2_IgM	6.85	26079.8	14198.7	1.837	MM
55	55	20211111_A20_R2_IgM	6.85	15645.1	14162.7	1.105	MM
56	56	20211111_L19_R2_IgM	6.86	21103.7	9293.4	2.271	MM

Dataset: C:\MassLynx\10.pro\20211111_clinicalsamples_IgM_IgA.qld

Last Altered: Thursday, March 09, 2023 19:58:25 台北標準時間
Printed: Friday, August 01, 2025 15:20:28 台北標準時間

	# Name	RT	Area	IS Area	Response	Prima...
57	57 20211111_QC_R13_IgM	6.87	32121.0	13903.0	2.310	MM
58	58 20211111_L20_R2_IgM	6.83	32799.8	12781.6	2.566	MM
59	59 20211111_H10_R2_IgM	6.84	64302.6	14796.4	4.346	MM
60	60 20211111_A21_R2_IgM	6.86	54016.6	13669.0	3.952	MM
61	61 20211111_A22_R2_IgM	6.85	20898.8	14098.3	1.482	MM
62	62 20211111_L21_R2_IgM	6.88	28885.3	15678.2	1.842	MM
63	63 20211111_L22_R2_IgM	6.88	24517.6	15724.5	1.559	MM
64	64 20211111_H11_R2_IgM	6.88	33663.9	15890.2	2.119	MM
65	65 20211111_A23_R2_IgM	6.87	16348.1	13423.2	1.218	MM
66	66 20211111_A24_R2_IgM	6.88	44783.5	12438.0	3.601	MM
67	67 20211111_L23_R2_IgM	6.87	54072.7	14075.0	3.842	MM
68	68 20211111_L24_R2_IgM	6.87	27690.1	15972.1	1.734	MM
69	69 20211111_H12_R2_IgM	6.87	18805.9	16270.9	1.156	MM
70	70 20211111_QC_R14_IgM	6.87	31756.1	15806.8	2.009	MM
71	71 20211111_A25_R2_IgM	6.86	51864.4	14552.1	3.564	MM
72	72 20211111_A26_R2_IgM	6.85	30617.3	11492.7	2.664	MM
73	73 20211111_L25_R2_IgM	6.89	19650.4	14403.3	1.364	MM
74	74 20211111_L26_R2_IgM	6.89	20051.9	16533.9	1.213	MM
75	75 20211111_H13_R2_IgM	6.87	45638.2	15520.1	2.941	MM
76	76 20211111_A27_R2_IgM	6.88	79932.3	13337.4	5.993	MM
77	77 20211111_A28_R2_IgM	6.84	38246.3	11895.6	3.215	MM
78	78 20211111_L27_R2_IgM	6.86	64755.9	14125.7	4.584	MM
79	79 20211111_L28_R2_IgM	6.86	35607.9	11807.5	3.016	MM
80	80 20211111_H14_R2_IgM	6.88	30439.3	15035.7	2.024	MM
81	81 20211111_A29_R2_IgM	6.88	11944.1	13554.4	0.881	MM
82	82 20211111_A30_R2_IgM	6.89	34287.3	12984.1	2.641	MM
83	83 20211111_QC_R15_IgM	6.87	32809.7	12299.5	2.668	MM
84	84 20211111_L29_R2_IgM	6.88	14534.5	13590.7	1.069	MM
85	85 20211111_L30_R2_IgM	6.89	11695.9	12511.4	0.935	MM
86	86 20211111_H15_R2_IgM	6.88	42616.6	11885.0	3.586	MM
87	87 20211111_A31_R2_IgM	6.83	26863.3	13422.1	2.001	MM
88	88 20211111_A32_R2_IgM	6.87	34229.7	11461.3	2.987	MM
89	89 20211111_L31_R2_IgM	6.85	17226.0	13753.4	1.252	MM
90	90 20211111_L32_R2_IgM	6.86	23536.1	12949.0	1.818	MM
91	91 20211111_H16_R2_IgM	6.86	73130.2	11465.4	6.378	MM
92	92 20211111_A33_R2_IgM	6.87	72923.3	10551.5	6.911	MM
93	93 20211111_A34_R2_IgM	6.87	26529.2	12409.9	2.138	MM
94	94 20211111_H17_R2_IgM	6.86	227544.7	10160.9	22.394	MM
95	95 20211111_A35_R2_IgM	6.85	58815.2	10460.6	5.623	MM
96	96 20211111_QC_R16_IgM	6.85	44185.1	10433.5	4.235	MM
97	97 20211111_A36_R2_IgM	6.83	36342.7	12504.8	2.906	MM
98	98 20211111_H18_R2_IgM	6.86	53075.7	9140.4	5.807	MM
99	99 20211111_A37_R2_IgM	6.88	37055.3	9796.9	3.782	MM
100	1... 20211111_A38_R2_IgM	6.88	32077.4	11224.3	2.858	MM
101	1... 20211111_H19_R2_IgM	6.90	40441.9	11739.2	3.445	MM
102	1... 20211111_A39_R2_IgM	6.87	48463.8	12718.9	3.810	MM
103	1... 20211111_A40_R2_IgM	6.85	24611.5	10006.1	2.460	MM
104	1... 20211111_H20_R2_IgM	6.87	72391.3	7988.2	9.062	MM
105	1... 20211111_A41_R2_IgM	6.88	30141.3	11513.3	2.618	MM
106	1... 20211111_A42_R2_IgM	6.87	18899.2	10238.5	1.846	MM
107	1... 20211111_A43_R2_IgM	6.87	20233.0	11098.9	1.823	MM
108	1... 20211111_A44_R2_IgM	6.85	15400.7	10969.9	1.404	MM
109	1... 20211111_QC_R17_IgM	6.87	28255.1	9700.8	2.913	MM
110	1... 20211111_L2_R3_IgM	6.88	37736.3	8528.0	4.425	MM
111	1... 20211111_H1_R3_IgM	6.87	33620.6	9438.2	3.562	MM
112	1... 20211111_A3_R3_IgM	6.88	39660.8	11598.6	3.419	MM

Dataset: C:\MassLynx\10.pro\20211111_clinicalsamples_IgM_IgA.qld

Last Altered: Thursday, March 09, 2023 19:58:25 台北標準時間
Printed: Friday, August 01, 2025 15:20:28 台北標準時間

	# Name	RT	Area	IS Area	Response	Prima...
113	1... 20211111_A4_R3_IgM	6.85	32017.9	11364.1	2.817	MM
114	1... 20211111_L3_R3_IgM	6.84	28368.9	10753.4	2.638	MM
115	1... 20211111_L4_R3_IgM	6.87	27742.2	10291.6	2.696	MM
116	1... 20211111_H2_R3_IgM	6.87	45218.2	9315.8	4.854	MM
117	1... 20211111_A5_R3_IgM	6.86	105077.0	9102.2	11.544	MM
118	1... 20211111_A6_R3_IgM	6.88	47678.3	10356.4	4.604	MM
119	1... 20211111_QC_R18_IgM	6.86	38389.1	8398.3	4.571	MM
120	1... 20211111_L5_R3_IgM	6.88	37300.8	9786.7	3.811	MM
121	1... 20211111_L6_R3_IgM	6.87	28937.3	9911.3	2.920	MM
122	1... 20211111_H3_R3_IgM	6.89	20421.1	12155.5	1.680	MM
123	1... 20211111_A7_R3_IgM	6.88	23136.9	11804.8	1.960	MM
124	1... 20211111_A8_R3_IgM	6.87	28801.8	10510.6	2.740	MM
125	1... 20211111_L7_R3_IgM	6.88	23467.0	12177.9	1.927	MM
126	1... 20211111_L8_R3_IgM	6.90	16519.1	11668.6	1.416	MM
127	1... 20211111_H4_R3_IgM	6.86	16229.0	11192.0	1.450	MM
128	1... 20211111_A9_R3_IgM	6.87	31958.5	14077.4	2.270	MM
129	1... 20211111_A10_R3_IgM	6.88	32820.7	11190.4	2.933	MM
130	1... 20211111_L9_R3_IgM	6.86	19222.8	14649.3	1.312	MM
131	1... 20211111_L10_R3_IgM	6.84	51624.6	9361.3	5.515	MM
132	1... 20211111_QC_R19_IgM	6.86	34641.6	10125.5	3.421	MM
133	1... 20211111_H5_R3_IgM	6.87	36402.6	10010.9	3.636	MM
134	1... 20211111_A11_R3_IgM	6.89	45287.8	12697.2	3.567	MM
135	1... 20211111_A12_R3_IgM	6.87	23734.2	13148.5	1.805	MM
136	1... 20211111_L11_R3_IgM	6.87	17185.8	10786.6	1.593	MM
137	1... 20211111_L12_R3_IgM	6.85	29891.7	11681.8	2.559	MM
138	1... 20211111_H6_R3_IgM	6.87	20118.9	12095.5	1.663	MM
139	1... 20211111_A13_R3_IgM	6.86	19016.8	12908.3	1.473	MM
140	1... 20211111_A14_R3_IgM	6.87	56700.7	11322.6	5.008	MM
141	1... 20211111_L13_R3_IgM	6.87	27444.8	11516.1	2.383	MM
142	1... 20211111_L14_R3_IgM	6.88	26034.4	10790.7	2.413	MM
143	1... 20211111_H7_R3_IgM	6.88	24092.8	11572.1	2.082	MM
144	1... 20211111_A15_R3_IgM	6.90	49398.2	12434.4	3.973	MM
145	1... 20211111_QC_R20_IgM	6.86	36508.9	10701.0	3.412	MM
146	1... 20211111_A16_R3_IgM	6.88	36996.1	11452.1	3.231	MM
147	1... 20211111_L15_R3_IgM	6.88	46163.8	10531.1	4.384	MM
148	1... 20211111_L16_R3_IgM	6.89	20921.1	12049.4	1.736	MM
149	1... 20211111_H8_R3_IgM	6.88	18591.0	13434.9	1.384	MM
150	1... 20211111_A17_R3_IgM	6.88	42407.2	12452.0	3.406	MM
151	1... 20211111_A18_R3_IgM	6.85	25235.2	12506.5	2.018	MM
152	1... 20211111_L17_R3_IgM	6.87	25803.4	9948.9	2.594	MM
153	1... 20211111_L18_R3_IgM	6.88	11535.1	13462.1	0.857	MM
154	1... 20211111_H9_R3_IgM	6.87	13056.8	13178.7	0.991	MM
155	1... 20211111_A19_R3_IgM	6.86	26713.9	11298.6	2.364	MM
156	1... 20211111_A20_R3_IgM	6.88	15047.8	11175.6	1.346	MM
157	1... 20211111_L19_R3_IgM	6.89	23101.4	11558.2	1.999	MM
158	1... 20211111_QC_R21_IgM	6.88	33183.8	9977.0	3.326	MM
159	1... 20211111_L20_R3_IgM	6.90	22099.4	13508.1	1.636	MM
160	1... 20211111_H10_R3_IgM	6.88	49516.8	12374.8	4.001	MM
161	1... 20211111_A21_R3_IgM	6.87	47582.4	12443.6	3.824	MM
162	1... 20211111_A22_R3_IgM	6.89	18665.3	11589.3	1.611	MM
163	1... 20211111_L21_R3_IgM	6.86	23527.5	13107.4	1.795	MM
164	1... 20211111_L22_R3_IgM	6.90	22504.2	12021.5	1.872	MM
165	1... 20211111_H11_R3_IgM	6.88	28431.8	12687.8	2.241	MM
166	1... 20211111_A23_R3_IgM	6.87	15723.5	11611.8	1.354	MM
167	1... 20211111_A24_R3_IgM	6.88	44294.4	10476.8	4.228	MM
168	1... 20211111_L23_R3_IgM	6.87	46891.5	11873.1	3.949	MM

Dataset: C:\MassLynx\10.pro\20211111_clinicalsamples_IgM_IgA.qld

Last Altered: Thursday, March 09, 2023 19:58:25 台北標準時間
Printed: Friday, August 01, 2025 15:20:28 台北標準時間

	# Name	RT	Area	IS Area	Response	Prima...
169	1... 20211111_L24_R3_IgM	6.88	22462.7	13468.0	1.668	MM
170	1... 20211111_H12_R3_IgM	6.90	17644.2	14229.1	1.240	MM
171	1... 20211111_QC_R22_IgM	6.88	29857.3	11264.4	2.651	MM
172	1... 20211111_A25_R3_IgM	6.87	40546.7	11775.7	3.443	MM
173	1... 20211111_A26_R3_IgM	6.87	25277.2	10473.9	2.413	MM
174	1... 20211111_L25_R3_IgM	6.87	15551.0	13256.8	1.173	MM
175	1... 20211111_L26_R3_IgM	6.88	17948.3	13565.0	1.323	MM
176	1... 20211111_H13_R3_IgM	6.89	44825.5	13146.0	3.410	MM
177	1... 20211111_A27_R3_IgM	6.89	64590.3	12025.6	5.371	MM
178	1... 20211111_A28_R3_IgM	6.87	27498.7	11229.1	2.449	MM
179	1... 20211111_L27_R3_IgM	6.88	55644.9	14960.0	3.720	MM
180	1... 20211111_L28_R3_IgM	6.88	31140.9	11359.0	2.742	MM
181	1... 20211111_H14_R3_IgM	6.89	29805.6	12830.8	2.323	MM
182	1... 20211111_A29_R3_IgM	6.86	11837.1	13434.3	0.881	MM
183	1... 20211111_A30_R3_IgM	6.88	30574.7	11272.2	2.712	MM
184	1... 20211111_QC_R23_IgM	6.87	31775.4	11872.2	2.676	MM
185	1... 20211111_L29_R3_IgM	6.86	13485.2	12948.7	1.041	MM
186	1... 20211111_L30_R3_IgM	6.88	10504.7	11026.6	0.953	MM
187	1... 20211111_H15_R3_IgM	6.89	35413.2	10786.3	3.283	MM
188	1... 20211111_A31_R3_IgM	6.87	22243.8	12010.5	1.852	MM
189	1... 20211111_A32_R3_IgM	6.88	31308.0	11107.7	2.819	MM
190	1... 20211111_L31_R3_IgM	6.88	14144.9	11021.8	1.283	MM
191	1... 20211111_L32_R3_IgM	6.90	18899.7	11683.8	1.618	MM
192	1... 20211111_H16_R3_IgM	6.86	59937.2	9831.7	6.096	MM
193	1... 20211111_A33_R3_IgM	6.87	69869.1	11213.1	6.231	MM
194	1... 20211111_A34_R3_IgM	6.89	23575.1	11550.1	2.041	MM
195	1... 20211111_H17_R3_IgM	6.87	169636.7	8874.4	19.115	MM
196	1... 20211111_A35_R3_IgM	6.87	49630.5	9856.3	5.035	MM
197	1... 20211111_QC_R24_IgM	6.86	40529.1	7561.8	5.360	MM
198	1... 20211111_A36_R3_IgM	6.89	24869.3	9562.8	2.601	MM
199	1... 20211111_H18_R3_IgM	6.88	47471.6	8165.3	5.814	MM
200	2... 20211111_A37_R3_IgM	6.88	28783.0	9278.8	3.102	MM
201	2... 20211111_A38_R3_IgM	6.86	29899.8	10920.3	2.738	MM
202	2... 20211111_H19_R3_IgM	6.86	36913.4	9285.3	3.975	MM
203	2... 20211111_A39_R3_IgM	6.88	41686.4	9083.4	4.589	MM
204	2... 20211111_A40_R3_IgM	6.89	20840.4	8382.4	2.486	MM
205	2... 20211111_H20_R3_IgM	6.88	59206.7	7807.9	7.583	MM
206	2... 20211111_A41_R3_IgM	6.88	17726.2	5925.2	2.992	MM
207	2... 20211111_A42_R3_IgM	6.86	16817.1	9352.6	1.798	MM
208	2... 20211111_A43_R3_IgM	6.88	18208.9	10216.3	1.782	MM
209	2... 20211111_A44_R3_IgM	6.87	11892.4	10262.7	1.159	MM
210	2... 20211111_QC_R25_IgM	6.87	25043.1	8575.3	2.920	MM

Dataset: C:\MassLynx\10.pro\20211111_clinicalsamples_IgM_IgA.qld

Last Altered: Thursday, March 09, 2023 19:58:25 台北標準時間
Printed: Friday, August 01, 2025 15:21:31 台北標準時間

Method: C:\MassLynx\10_Pro_IgA.PRO\MethDB\20211111_IgM_IgA_DMRM_new.mdb 24 Feb 2022 13:44:14
Calibration: 15 Nov 2021 14:42:11

Sample Name: 20211112_QC_R001_IgM

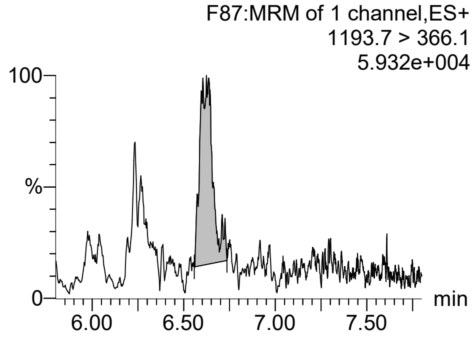

Sample Name: 20211112_QC_R002_IgM

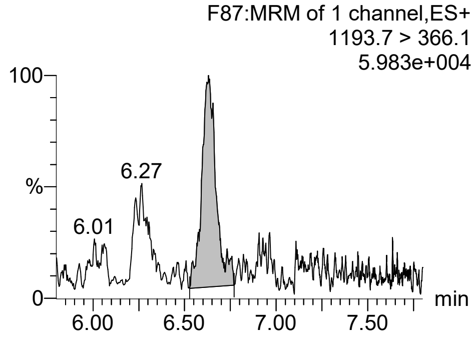

Sample Name: 20211111_A1_R2_IgM

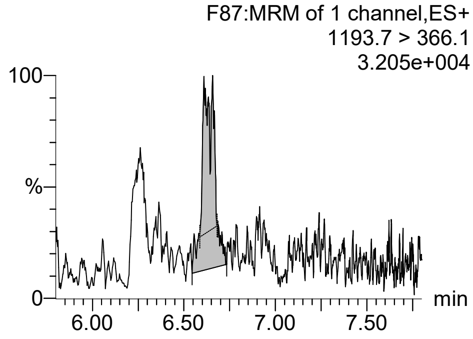

Sample Name: 20211111_A1_R3_IgM

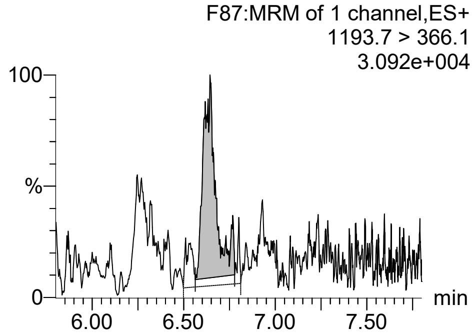

Sample Name: 20211111_A2_R2_IgM

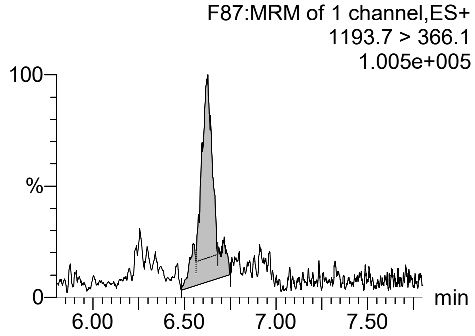

Sample Name: 20211111_A2_R3_IgM

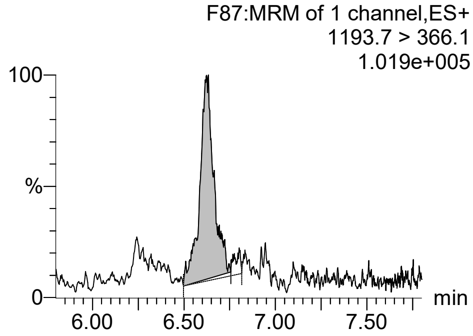

Sample Name: 20211111_L1_R2_IgM

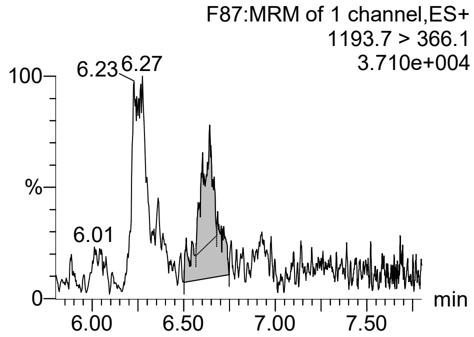

Sample Name: 20211111_L1_R3_IgM

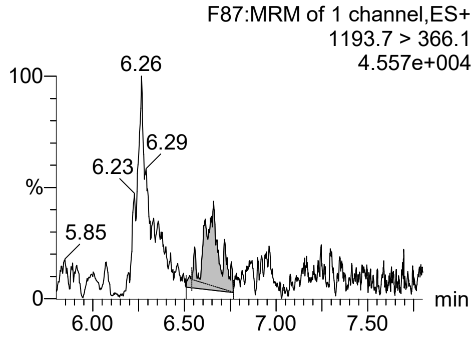

Sample Name: 20211111_L2_R2_IgM

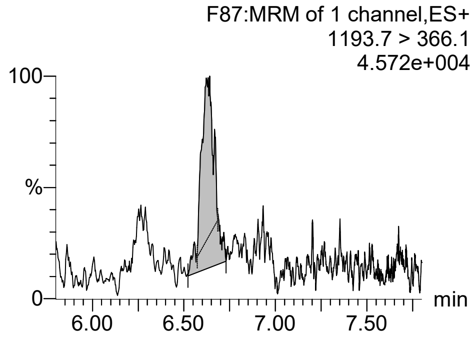

Sample Name: 20211111_H1_R2_IgM

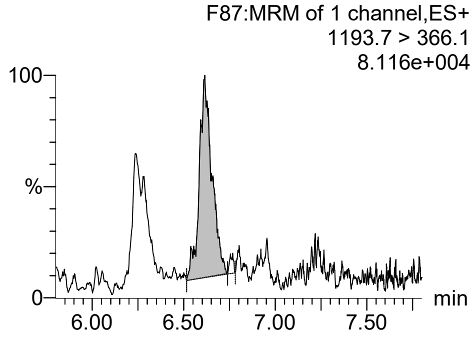

Sample Name: 20211111_A3_R2_IgM

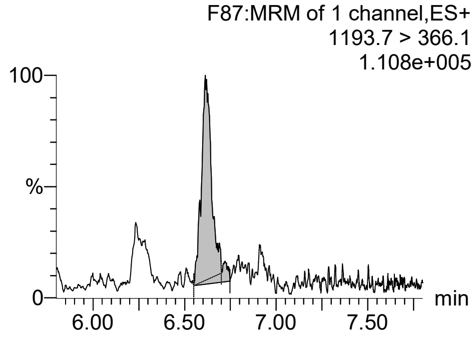

Sample Name: 20211111_A4_R2_IgM

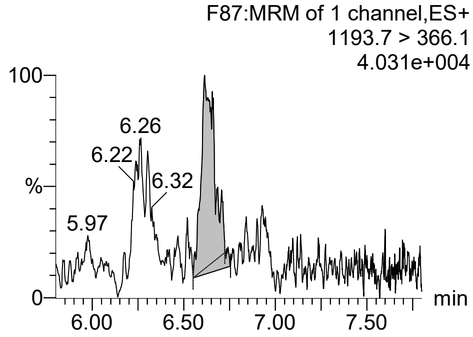

Sample Name: 20211111_L3_R2_IgM
F87:MRM of 1 channel,ES+
1193.7 > 366.1
5.780e+004

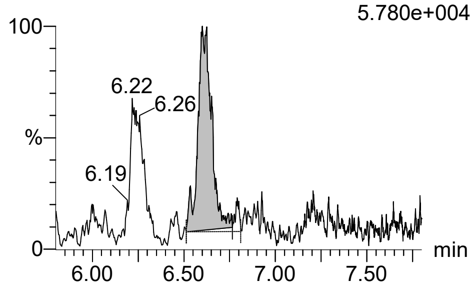

Sample Name: 20211111_L4_R2_IgM
F87:MRM of 1 channel,ES+
1193.7 > 366.1
5.983e+004

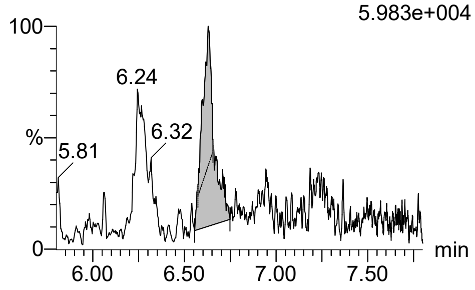

Sample Name: 20211111_H2_R2_IgM
F87:MRM of 1 channel,ES+
1193.7 > 366.1
7.698e+004

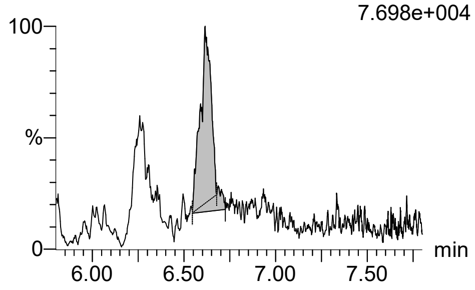

Sample Name: 20211111_A5_R2_IgM
F87:MRM of 1 channel,ES+
1193.7 > 366.1
2.170e+005

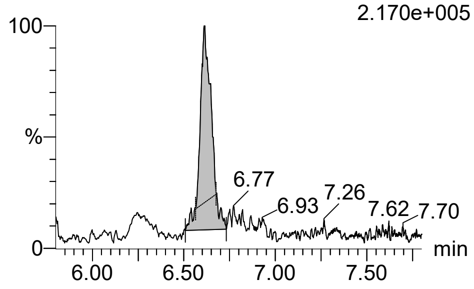

Sample Name: 20211111_A6_R2_IgM
F87:MRM of 1 channel,ES+
1193.7 > 366.1
8.656e+004

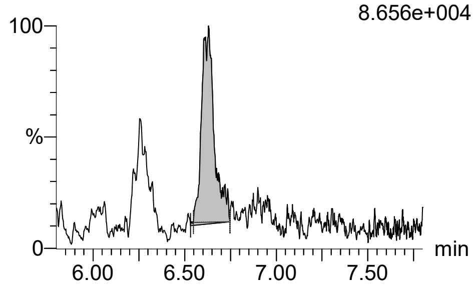

Sample Name: 20211111_QC_R10_IgM
F87:MRM of 1 channel,ES+
1193.7 > 366.1
7.815e+004

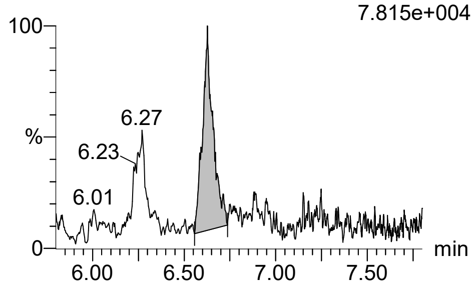

Sample Name: 20211111_L5_R2_IgM
F87:MRM of 1 channel,ES+
1193.7 > 366.1
1.200e+005

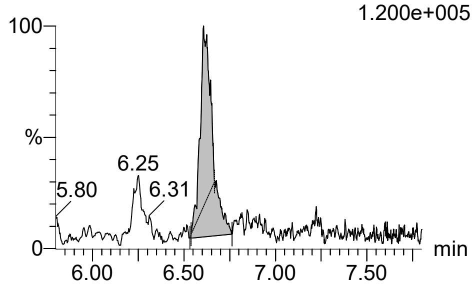

Sample Name: 20211111_L6_R2_IgM
F87:MRM of 1 channel,ES+
1193.7 > 366.1
1.076e+005

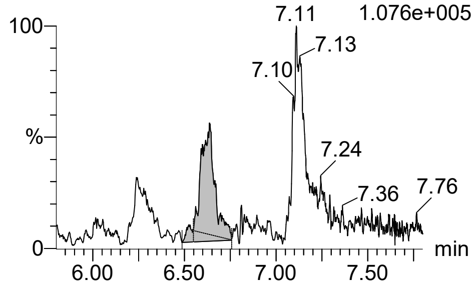

Sample Name: 20211111_H3_R2_IgM
F87:MRM of 1 channel,ES+
1193.7 > 366.1
3.622e+004

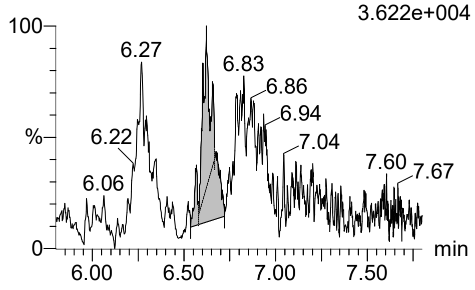

Sample Name: 20211111_A7_R2_IgM
F87:MRM of 1 channel,ES+
1193.7 > 366.1
8.510e+004

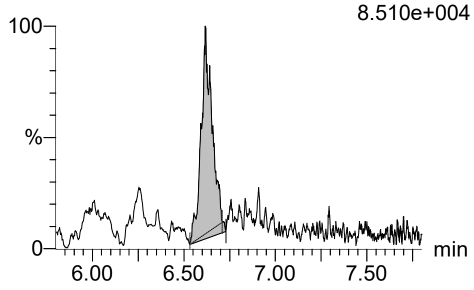

Sample Name: 20211111_A8_R2_IgM
F87:MRM of 1 channel,ES+
1193.7 > 366.1
5.806e+004

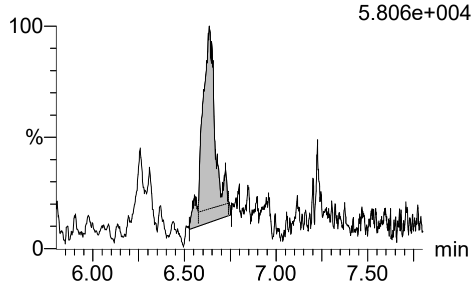

Sample Name: 20211111_L7_R2_IgM
F87:MRM of 1 channel,ES+
1193.7 > 366.1
5.341e+004

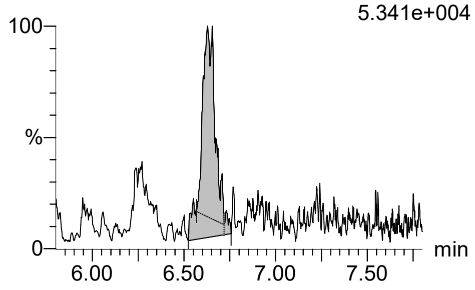

Sample Name: 20211111_L8_R2_IgM
F87:MRM of 1 channel,ES+
1193.7 > 366.1
3.364e+004

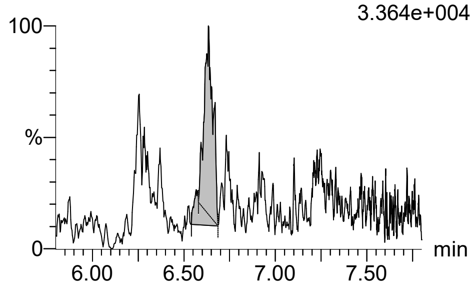

Sample Name: 20211111_H4_R2_IgM
F87:MRM of 1 channel,ES+
1193.7 > 366.1
5.238e+004

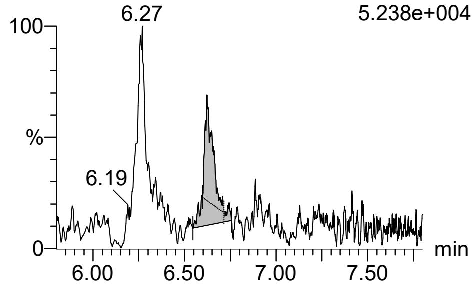

Sample Name: 20211111_A9_R2_IgM
F87:MRM of 1 channel,ES+
1193.7 > 366.1
5.923e+004

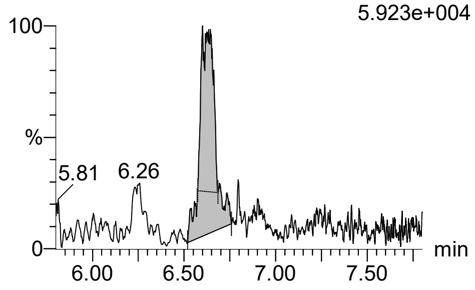

Dataset: C:\MassLynx\10.pro\20211111_clinicalsamples_IgM_IgA.qld

Last Altered: Thursday, March 09, 2023 19:58:25 台北標準時間
Printed: Friday, August 01, 2025 15:21:31 台北標準時間

Sample Name: 20211111_A10_R2_IgM
F87:MRM of 1 channel,ES+
1193.7 > 366.1
7.555e+004

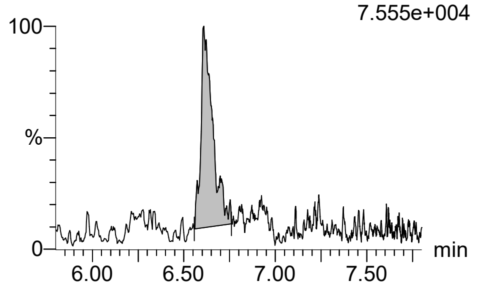

Sample Name: 20211111_L9_R2_IgM
F87:MRM of 1 channel,ES+
1193.7 > 366.1
6.018e+004

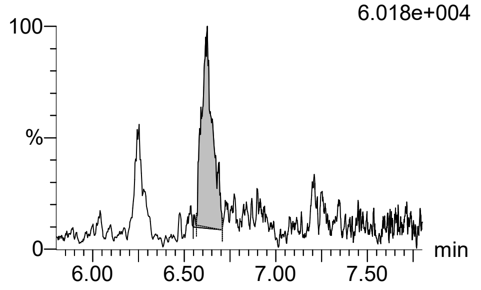

Sample Name: 20211111_L10_R2_IgM
F87:MRM of 1 channel,ES+
1193.7 > 366.1
1.716e+005

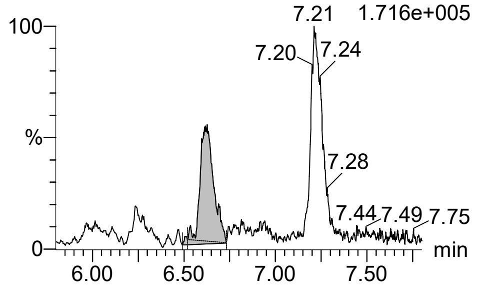

Sample Name: 20211111_QC_R11_IgM
F87:MRM of 1 channel,ES+
1193.7 > 366.1
9.194e+004

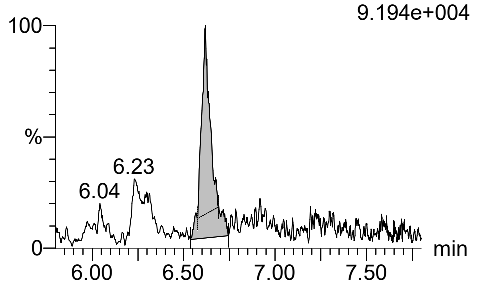

Sample Name: 20211111_H5_R2_IgM
F87:MRM of 1 channel,ES+
1193.7 > 366.1
8.331e+004

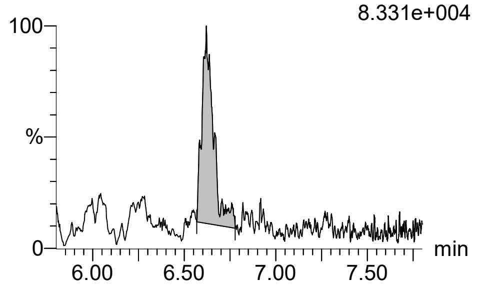

Sample Name: 20211111_A11_R2_IgM
F87:MRM of 1 channel,ES+
1193.7 > 366.1
9.166e+004

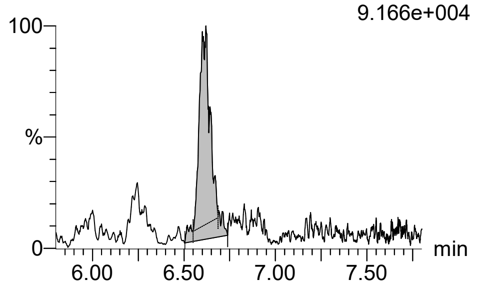

Sample Name: 20211111_A12_R2_IgM
F87:MRM of 1 channel,ES+
1193.7 > 366.1
3.132e+004

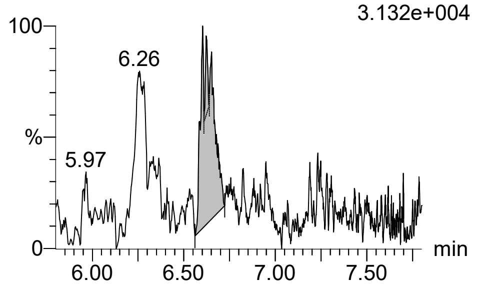

Sample Name: 20211111_L11_R2_IgM
F87:MRM of 1 channel,ES+
1193.7 > 366.1
2.620e+004

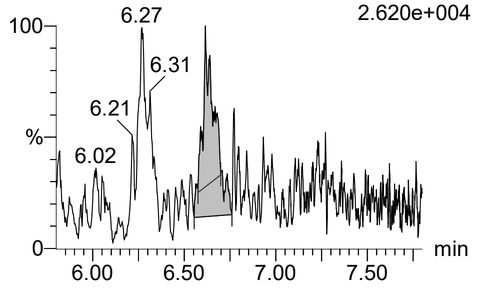

Sample Name: 20211111_L12_R2_IgM
F87:MRM of 1 channel,ES+
1193.7 > 366.1
5.415e+004

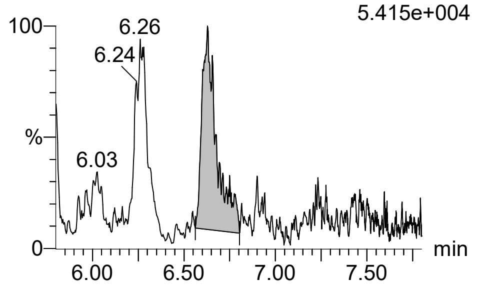

Sample Name: 20211111_H6_R2_IgM
F87:MRM of 1 channel,ES+
1193.7 > 366.1
3.128e+004

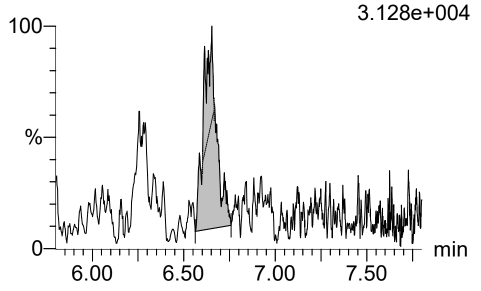

Sample Name: 20211111_A13_R2_IgM
F87:MRM of 1 channel,ES+
1193.7 > 366.1
4.451e+004

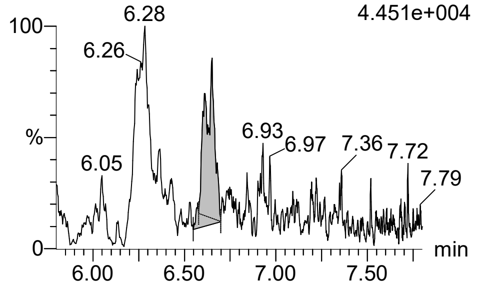

Sample Name: 20211111_A14_R2_IgM
F87:MRM of 1 channel,ES+
1193.7 > 366.1
1.064e+005

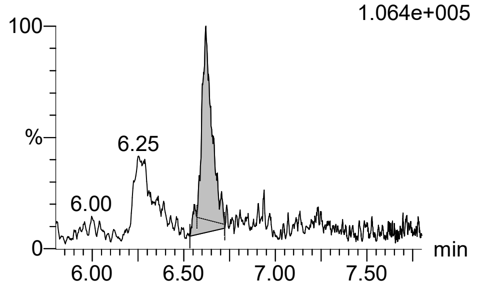

Sample Name: 20211111_L13_R2_IgM
F87:MRM of 1 channel,ES+
1193.7 > 366.1
6.006e+004

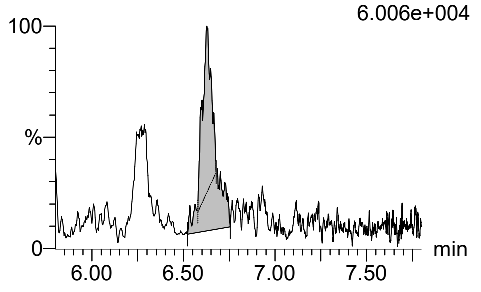

Sample Name: 20211111_L14_R2_IgM
F87:MRM of 1 channel,ES+
1193.7 > 366.1
6.968e+004

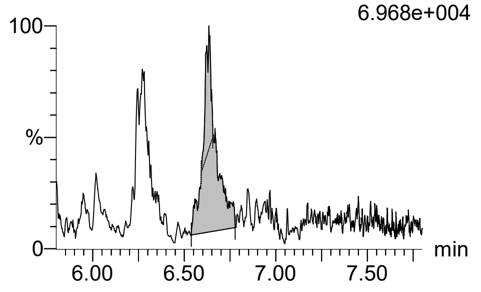

Sample Name: 20211111_H7_R2_IgM
F87:MRM of 1 channel,ES+
1193.7 > 366.1
6.742e+004

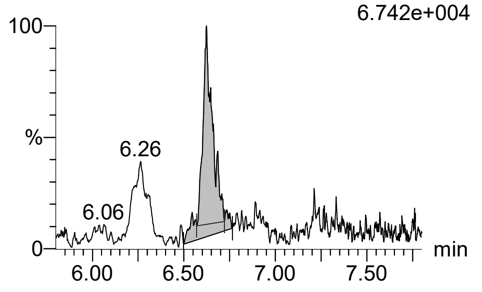

Dataset: C:\MassLynx\10.pro\20211111_clinicalsamples_IgM_IgA.qld

Last Altered: Thursday, March 09, 2023 19:58:25 台北標準時間
Printed: Friday, August 01, 2025 15:21:31 台北標準時間

Sample Name: 20211111_A15_R2_IgM
F87:MRM of 1 channel,ES+
1193.7 > 366.1
9.717e+004

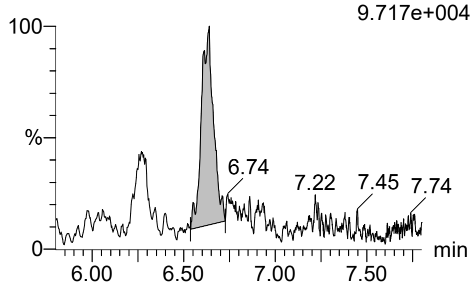

Sample Name: 20211111_QC_R12_IgM
F87:MRM of 1 channel,ES+
1193.7 > 366.1
7.422e+004

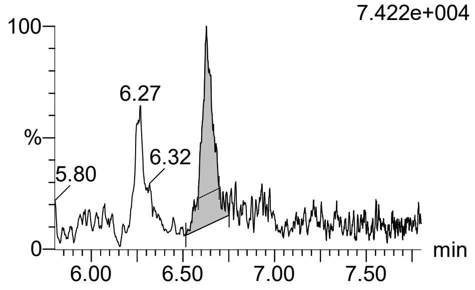

Sample Name: 20211111_A16_R2_IgM
F87:MRM of 1 channel,ES+
1193.7 > 366.1
1.007e+005

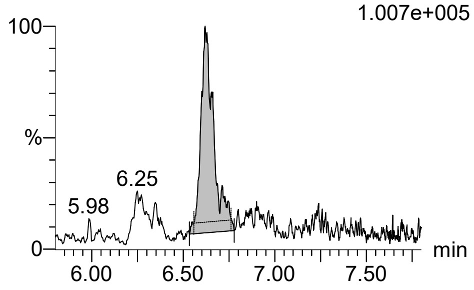

Sample Name: 20211111_L15_R2_IgM
F87:MRM of 1 channel,ES+
1193.7 > 366.1
8.565e+004

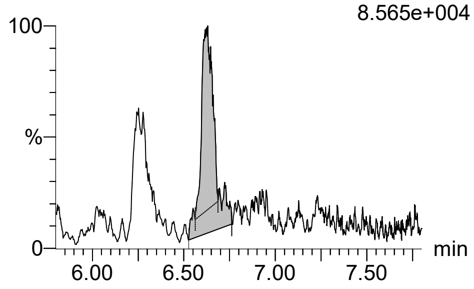

Sample Name: 20211111_L16_R2_IgM
F87:MRM of 1 channel,ES+
1193.7 > 366.1
5.081e+004

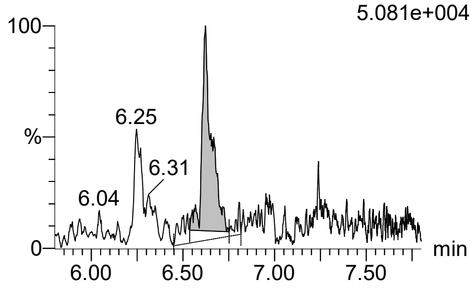

Sample Name: 20211111_H8_R2_IgM
F87:MRM of 1 channel,ES+
1193.7 > 366.1
4.075e+004

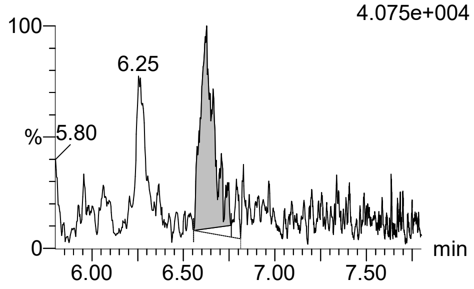

Sample Name: 20211111_A17_R2_IgM
F87:MRM of 1 channel,ES+
1193.7 > 366.1
9.982e+004

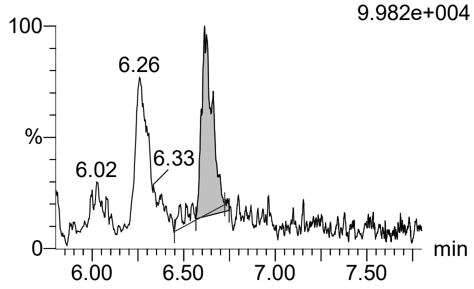

Sample Name: 20211111_A18_R2_IgM
F87:MRM of 1 channel,ES+
1193.7 > 366.1
4.199e+004

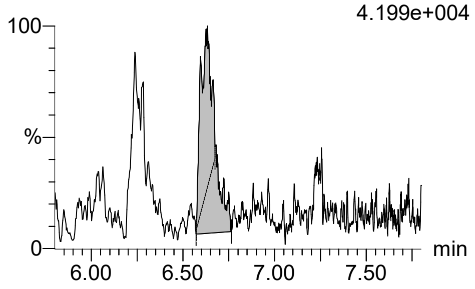

Sample Name: 20211111_L17_R2_IgM
F87:MRM of 1 channel,ES+
1193.7 > 366.1
9.209e+004

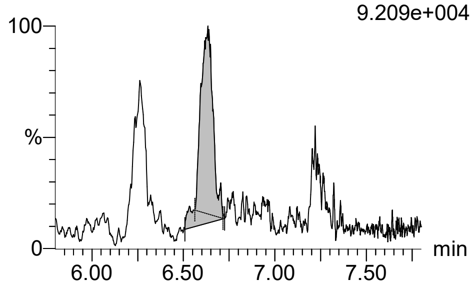

Sample Name: 20211111_L18_R2_IgM
F87:MRM of 1 channel,ES+
1193.7 > 366.1
2.843e+004

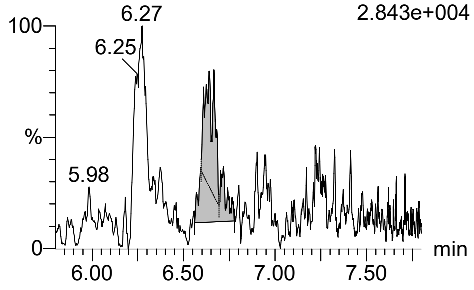

Sample Name: 20211111_H9_R2_IgM
F87:MRM of 1 channel,ES+
1193.7 > 366.1
4.069e+004

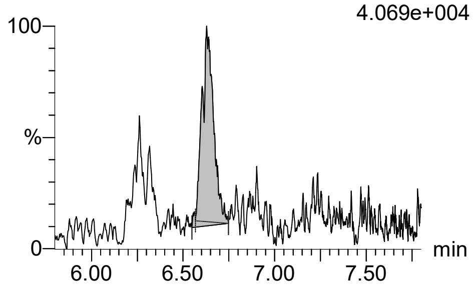

Sample Name: 20211111_A19_R2_IgM
F87:MRM of 1 channel,ES+
1193.7 > 366.1
5.632e+004

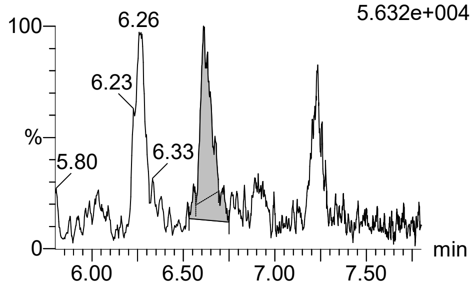

Sample Name: 20211111_A20_R2_IgM
F87:MRM of 1 channel,ES+
1193.7 > 366.1
2.578e+004

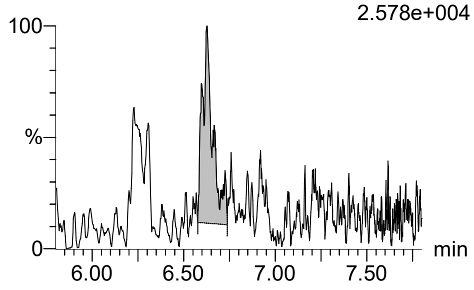

Sample Name: 20211111_L19_R2_IgM
F87:MRM of 1 channel,ES+
1193.7 > 366.1
2.491e+004

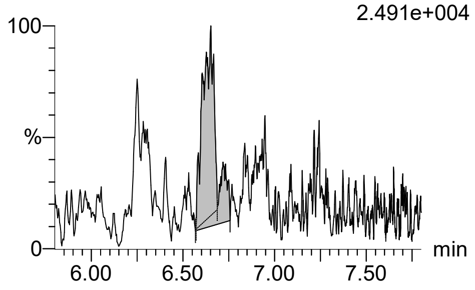

Sample Name: 20211111_QC_R13_IgM
F87:MRM of 1 channel,ES+
1193.7 > 366.1
5.899e+004

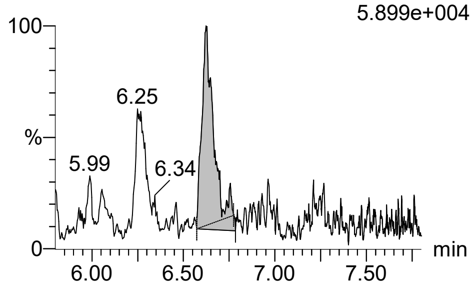

Dataset: C:\MassLynx\10.pro\20211111_clinicalsamples_IgM_IgA.qld

Last Altered: Thursday, March 09, 2023 19:58:25 台北標準時間
Printed: Friday, August 01, 2025 15:21:31 台北標準時間

Sample Name: 20211111_L20_R2_IgM

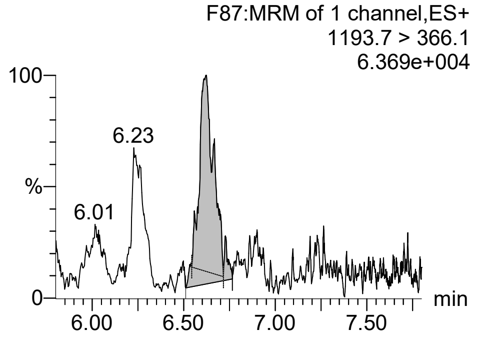

Sample Name: 20211111_H10_R2_IgM

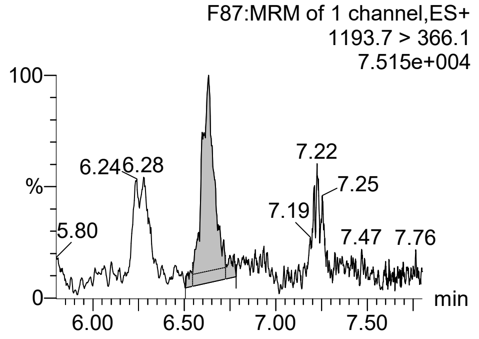

Sample Name: 20211111_A21_R2_IgM

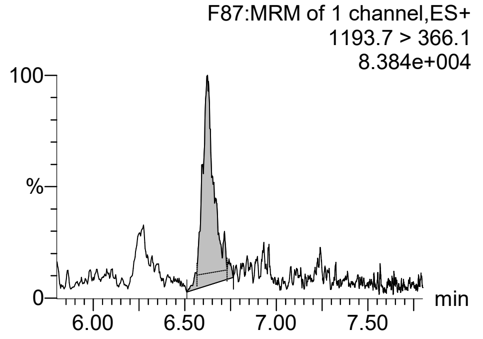

Sample Name: 20211111_A22_R2_IgM

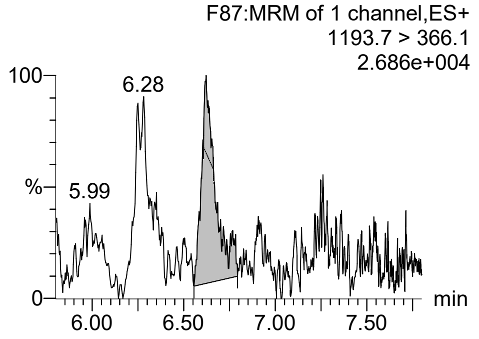

Sample Name: 20211111_L21_R2_IgM

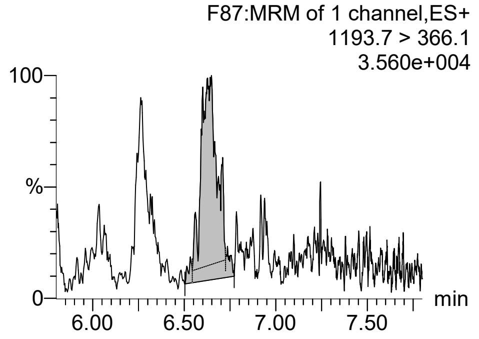

Sample Name: 20211111_L22_R2_IgM

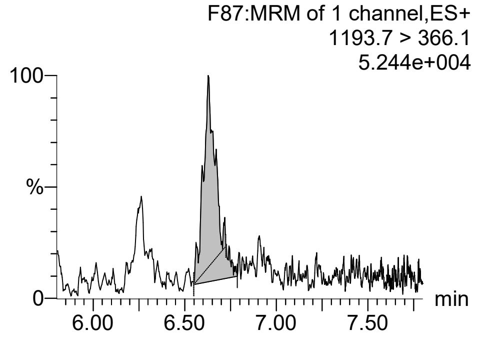

Sample Name: 20211111_H11_R2_IgM

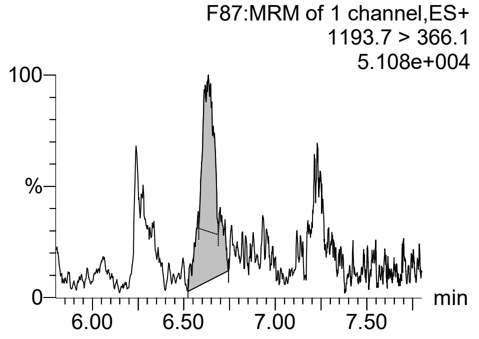

Sample Name: 20211111_A23_R2_IgM

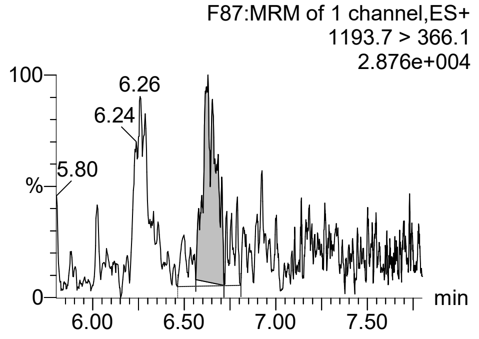

Sample Name: 20211111_A24_R2_IgM

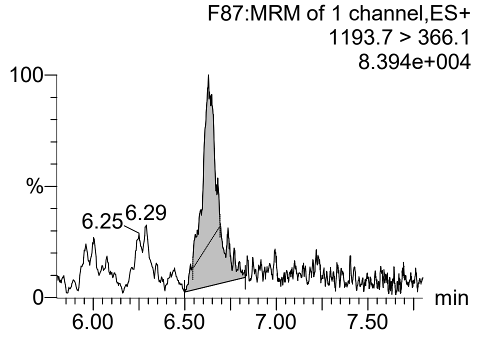

Sample Name: 20211111_L23_R2_IgM

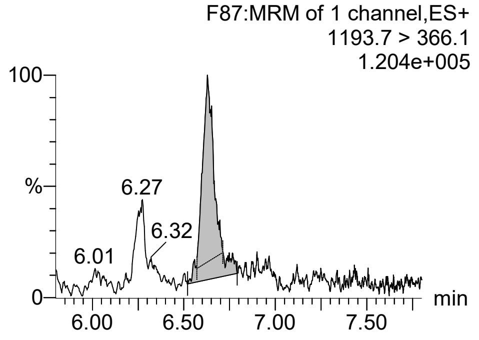

Sample Name: 20211111_L24_R2_IgM

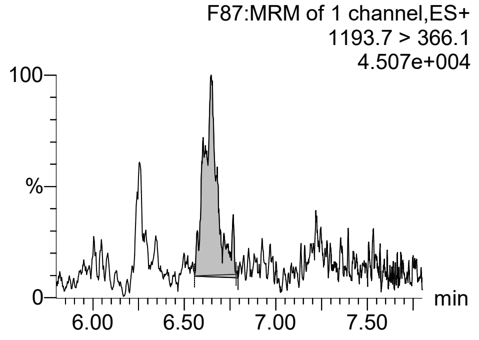

Sample Name: 20211111_H12_R2_IgM

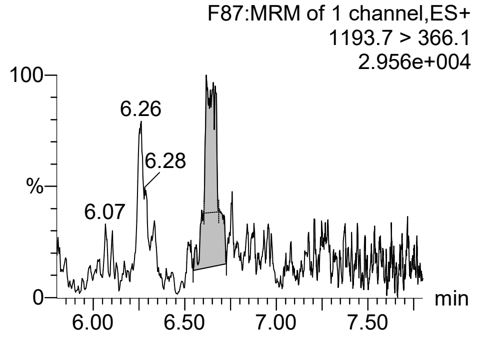

Sample Name: 20211111_QC_R14_IgM

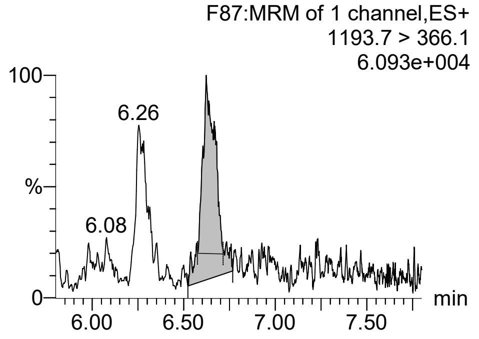

Sample Name: 20211111_A25_R2_IgM

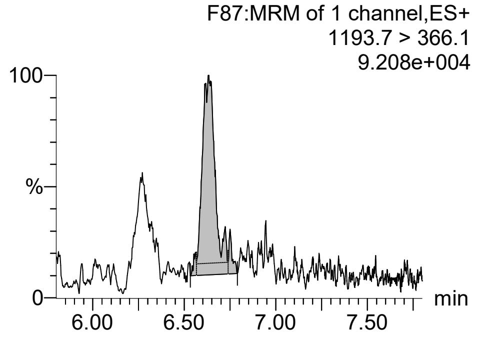

Sample Name: 20211111_A26_R2_IgM

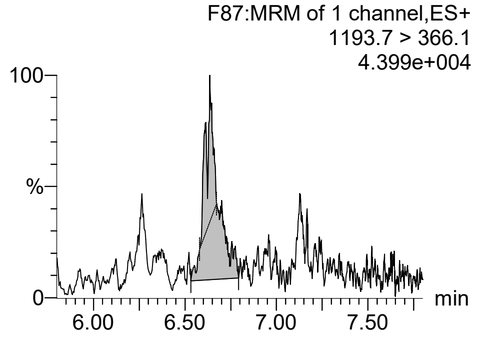

Sample Name: 20211111_L25_R2_IgM
F87:MRM of 1 channel,ES+
1193.7 > 366.1
4.865e+004

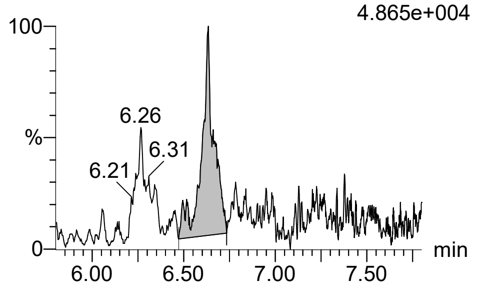

Sample Name: 20211111_L26_R2_IgM
F87:MRM of 1 channel,ES+
1193.7 > 366.1
3.954e+004

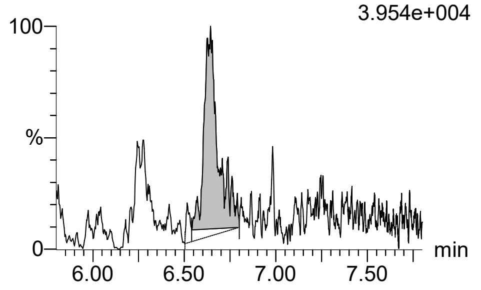

Sample Name: 20211111_H13_R2_IgM
F87:MRM of 1 channel,ES+
1193.7 > 366.1
8.123e+004

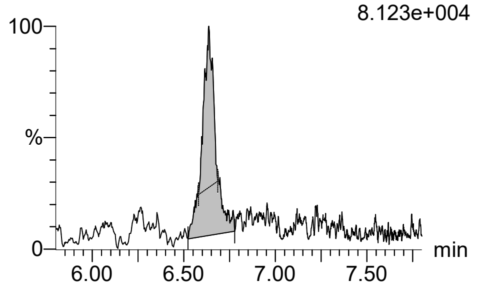

Sample Name: 20211111_A27_R2_IgM
F87:MRM of 1 channel,ES+
1193.7 > 366.1
8.823e+004

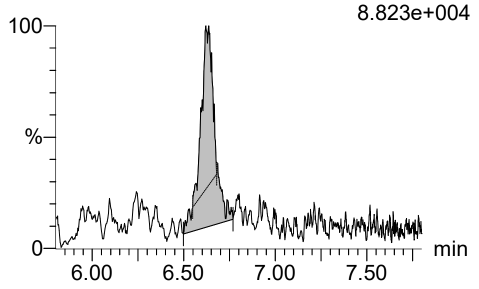

Sample Name: 20211111_A28_R2_IgM
F87:MRM of 1 channel,ES+
1193.7 > 366.1
6.138e+004

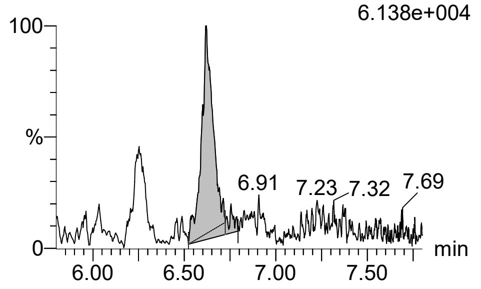

Sample Name: 20211111_L27_R2_IgM
F87:MRM of 1 channel,ES+
1193.7 > 366.1
1.114e+005

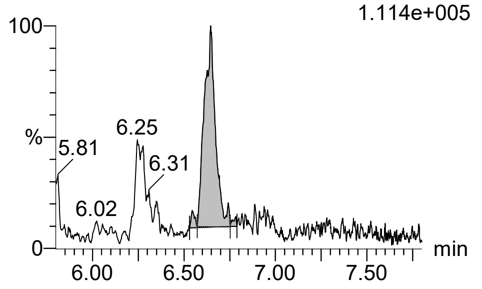

Sample Name: 20211111_L28_R2_IgM
F87:MRM of 1 channel,ES+
1193.7 > 366.1
6.827e+004

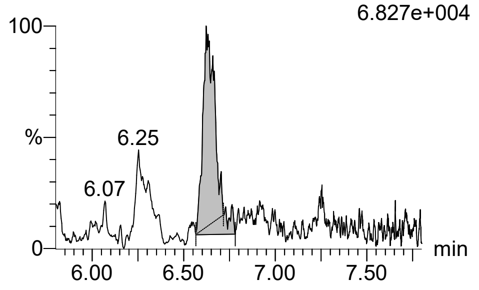

Sample Name: 20211111_H14_R2_IgM
F87:MRM of 1 channel,ES+
1193.7 > 366.1
7.291e+004

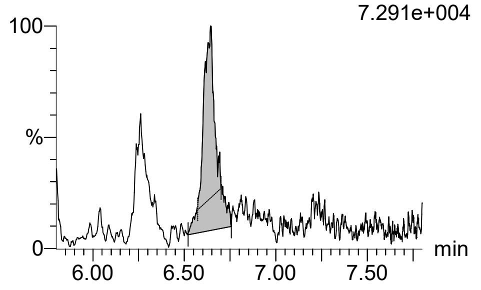

Sample Name: 20211111_A29_R2_IgM
F87:MRM of 1 channel,ES+
1193.7 > 366.1
2.037e+004

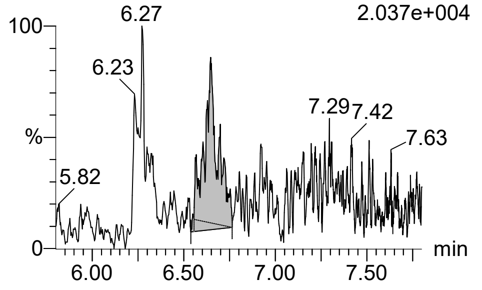

Sample Name: 20211111_A30_R2_IgM
F87:MRM of 1 channel,ES+
1193.7 > 366.1
4.689e+004

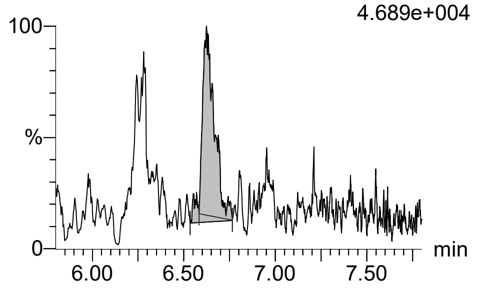

Sample Name: 20211111_QC_R15_IgM
F87:MRM of 1 channel,ES+
1193.7 > 366.1
5.501e+004

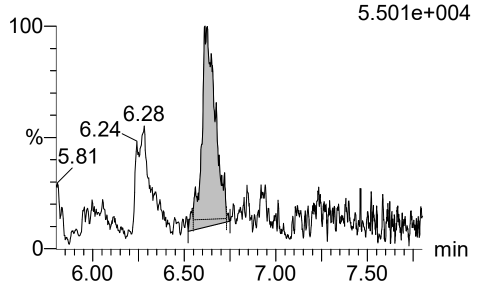

Sample Name: 20211111_L29_R2_IgM
F87:MRM of 1 channel,ES+
1193.7 > 366.1
2.773e+004

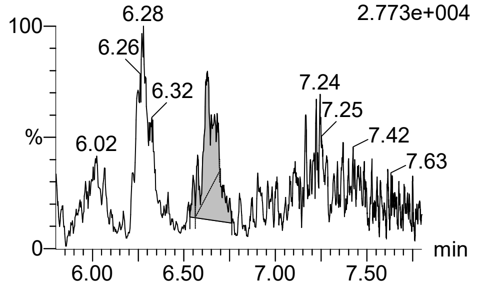

Sample Name: 20211111_L30_R2_IgM
F87:MRM of 1 channel,ES+
1193.7 > 366.1
1.827e+004

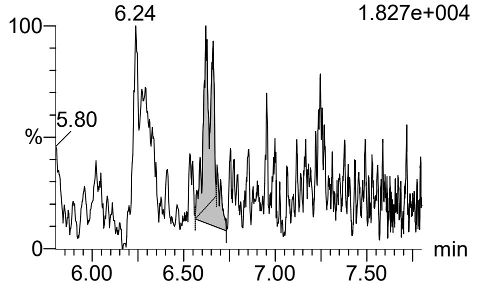

Sample Name: 20211111_H15_R2_IgM
F87:MRM of 1 channel,ES+
1193.7 > 366.1
5.646e+004

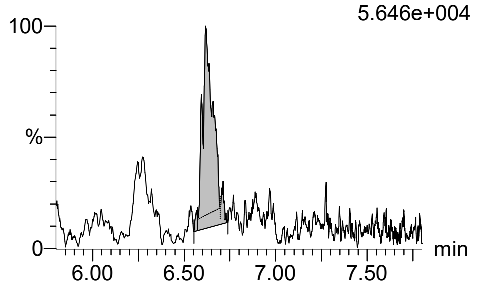

Sample Name: 20211111_A31_R2_IgM
F87:MRM of 1 channel,ES+
1193.7 > 366.1
2.877e+004

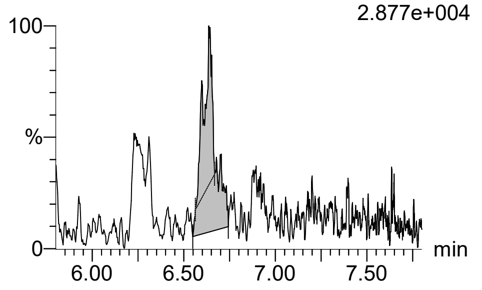

Sample Name: 20211111_A32_R2_IgM
F87:MRM of 1 channel,ES+
1193.7 > 366.1
5.709e+004

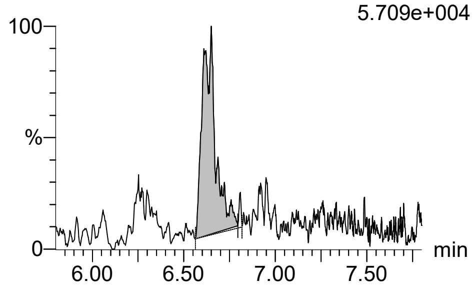

Sample Name: 20211111_L31_R2_IgM
F87:MRM of 1 channel,ES+
1193.7 > 366.1
3.909e+004

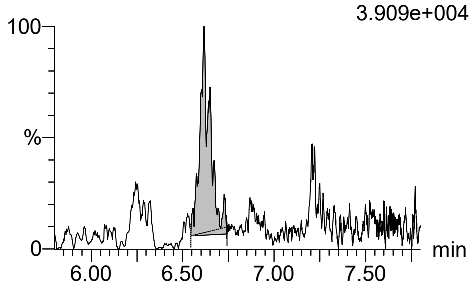

Sample Name: 20211111_L32_R2_IgM
F87:MRM of 1 channel,ES+
1193.7 > 366.1
6.529e+004

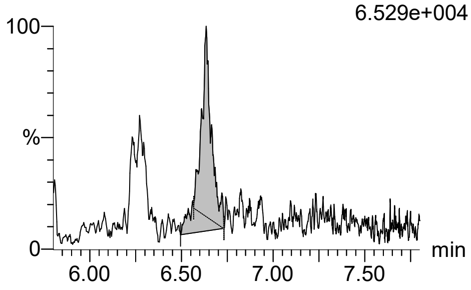

Sample Name: 20211111_H16_R2_IgM
F87:MRM of 1 channel,ES+
1193.7 > 366.1
1.397e+005

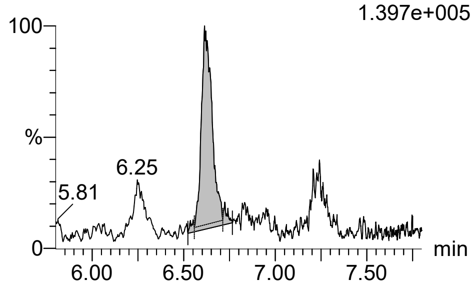

Sample Name: 20211111_A33_R2_IgM
F87:MRM of 1 channel,ES+
1193.7 > 366.1
8.575e+004

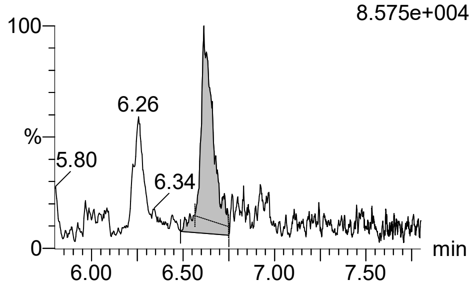

Sample Name: 20211111_A34_R2_IgM
F87:MRM of 1 channel,ES+
1193.7 > 366.1
3.949e+004

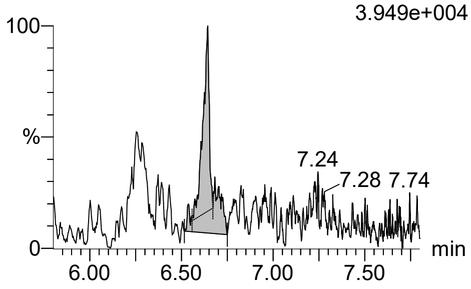

Sample Name: 20211111_H17_R2_IgM
F87:MRM of 1 channel,ES+
1193.7 > 366.1
4.296e+005

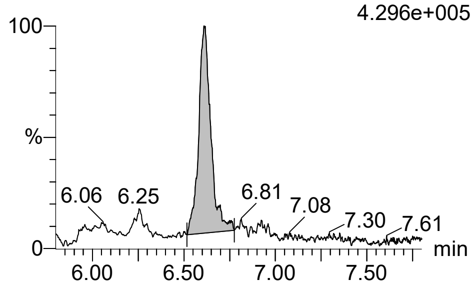

Sample Name: 20211111_A35_R2_IgM
F87:MRM of 1 channel,ES+
1193.7 > 366.1
8.069e+004

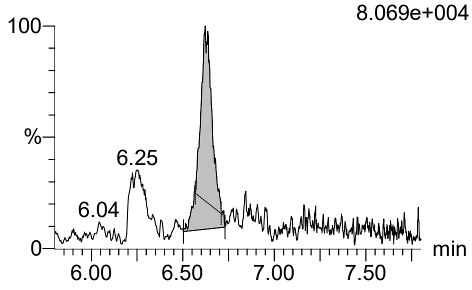

Sample Name: 20211111_QC_R16_IgM
F87:MRM of 1 channel,ES+
1193.7 > 366.1
7.649e+004

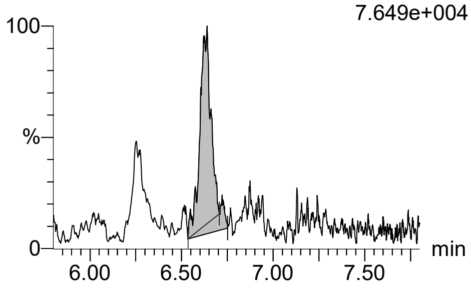

Sample Name: 20211111_A36_R2_IgM
F87:MRM of 1 channel,ES+
1193.7 > 366.1
7.691e+004

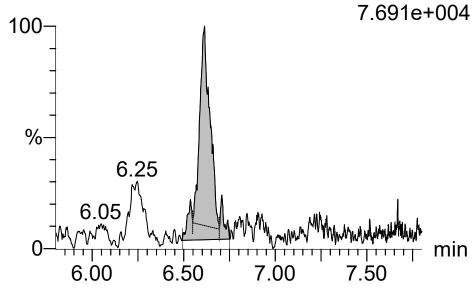

Sample Name: 20211111_H18_R2_IgM
F87:MRM of 1 channel,ES+
1193.7 > 366.1
1.349e+005

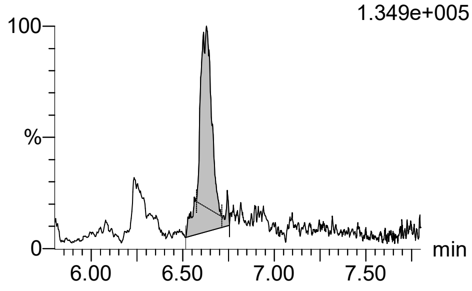

Sample Name: 20211111_A37_R2_IgM
F87:MRM of 1 channel,ES+
1193.7 > 366.1
7.066e+004

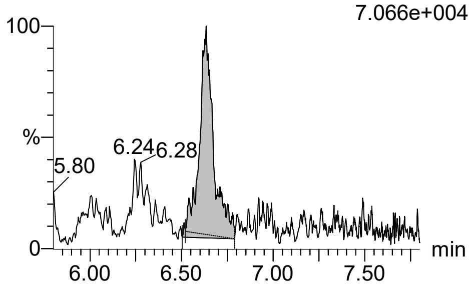

Sample Name: 20211111_A38_R2_IgM
F87:MRM of 1 channel,ES+
1193.7 > 366.1
6.191e+004

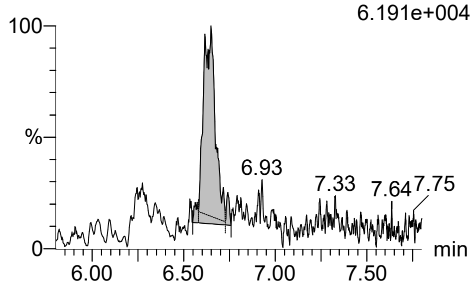

Sample Name: 20211111_H19_R2_IgM
F87:MRM of 1 channel,ES+
1193.7 > 366.1
1.586e+005

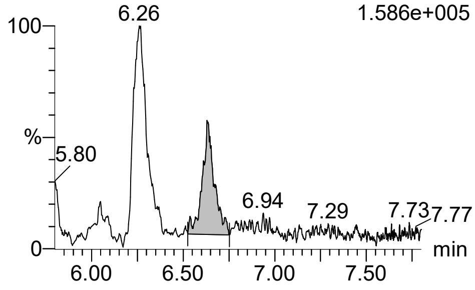

Sample Name: 20211111_A39_R2_IgM
F87:MRM of 1 channel,ES+
1193.7 > 366.1
8.775e+004

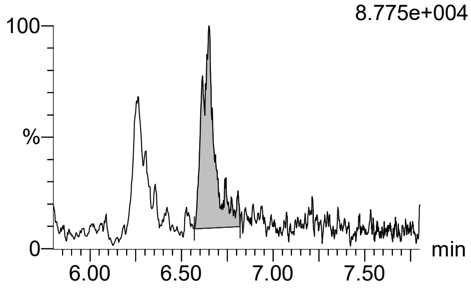

Dataset: C:\MassLynx\10.pro\20211111_clinicalsamples_IgM_IgA.qld

Last Altered: Thursday, March 09, 2023 19:58:25 台北標準時間
Printed: Friday, August 01, 2025 15:21:31 台北標準時間

Sample Name: 20211111_A40_R2_IgM

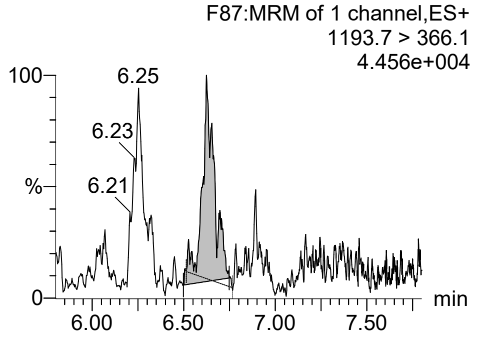

Sample Name: 20211111_H20_R2_IgM

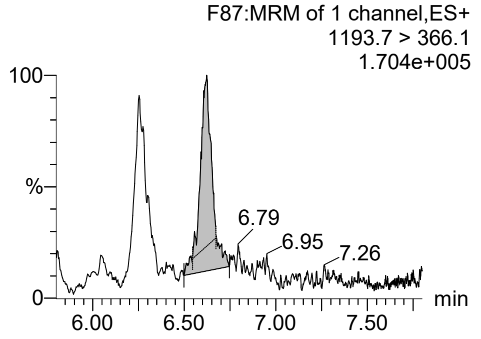

Sample Name: 20211111_A41_R2_IgM

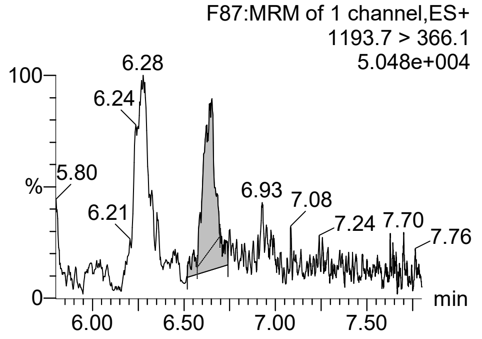

Sample Name: 20211111_A42_R2_IgM

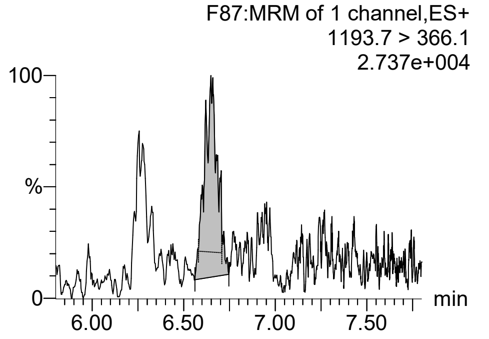

Sample Name: 20211111_A43_R2_IgM

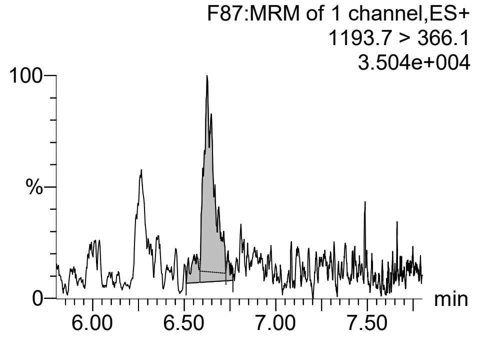

Sample Name: 20211111_A44_R2_IgM

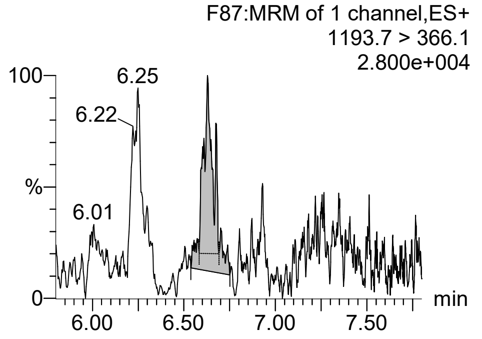

Sample Name: 20211111_QC_R17_IgM

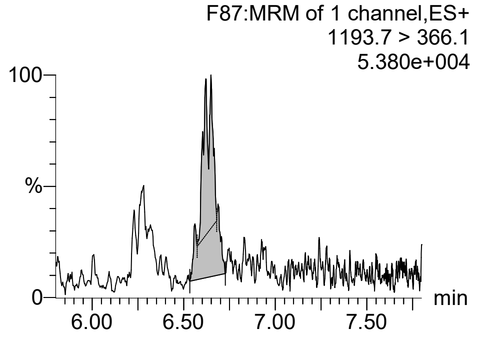

Sample Name: 20211111_L2_R3_IgM

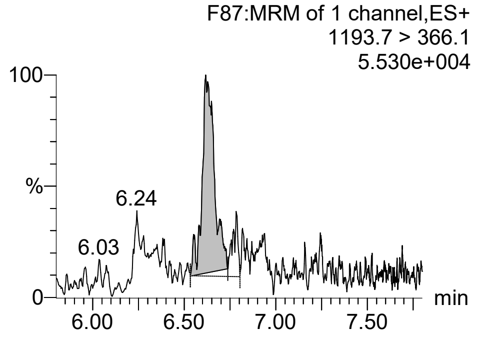

Sample Name: 20211111_H1_R3_IgM

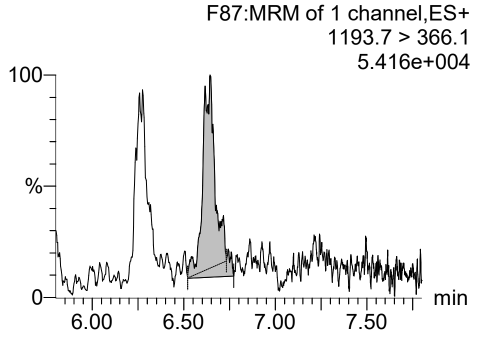

Sample Name: 20211111_A3_R3_IgM

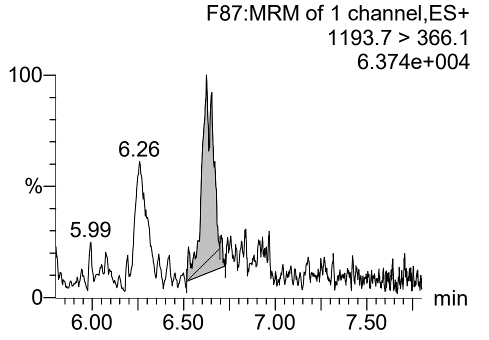

Sample Name: 20211111_A4_R3_IgM

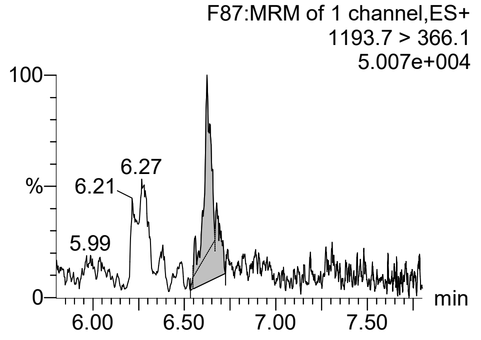

Sample Name: 20211111_L3_R3_IgM

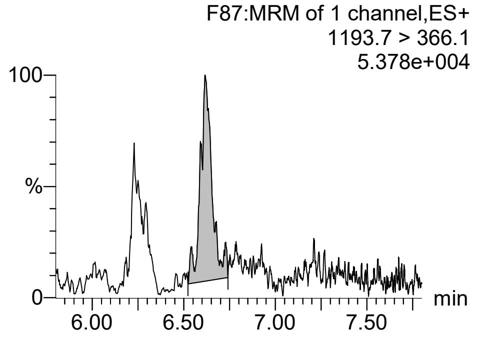

Sample Name: 20211111_L4_R3_IgM

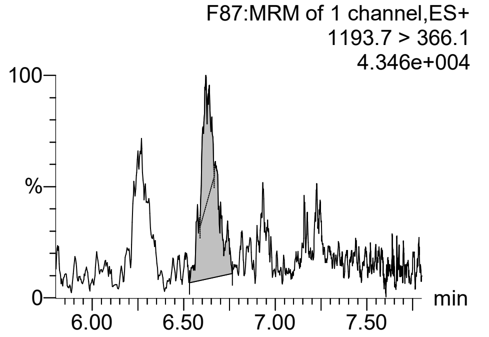

Sample Name: 20211111_H2_R3_IgM

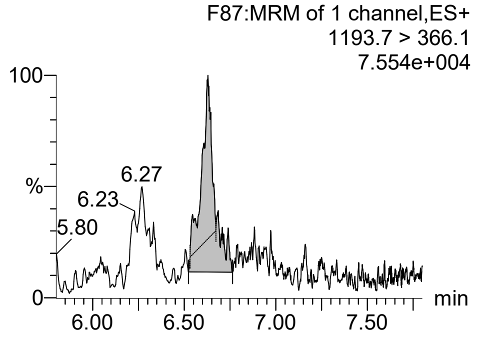

Sample Name: 20211111_A5_R3_IgM

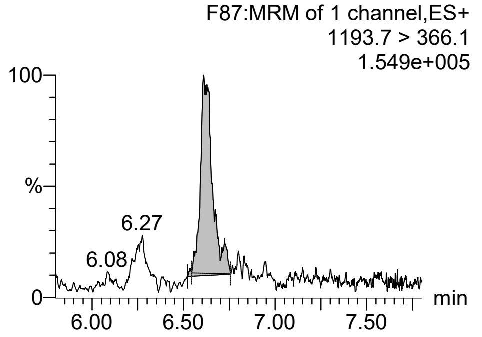

Sample Name: 20211111_A6_R3_IgM
F87:MRM of 1 channel,ES+
1193.7 > 366.1
6.661e+004

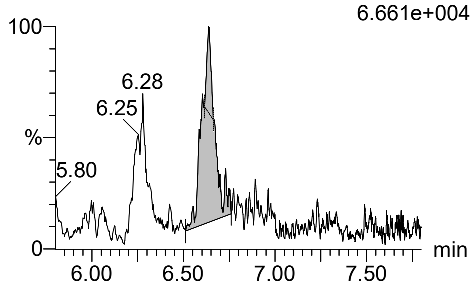

Sample Name: 20211111_QC_R18_IgM
F87:MRM of 1 channel,ES+
1193.7 > 366.1
5.429e+004

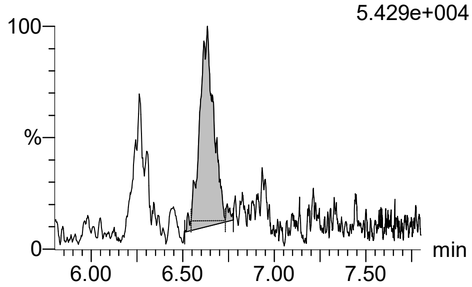

Sample Name: 20211111_L5_R3_IgM
F87:MRM of 1 channel,ES+
1193.7 > 366.1
7.123e+004

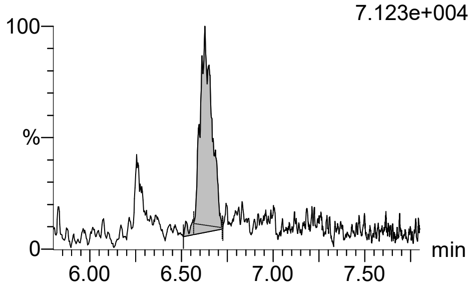

Sample Name: 20211111_L6_R3_IgM
F87:MRM of 1 channel,ES+
1193.7 > 366.1
8.976e+004

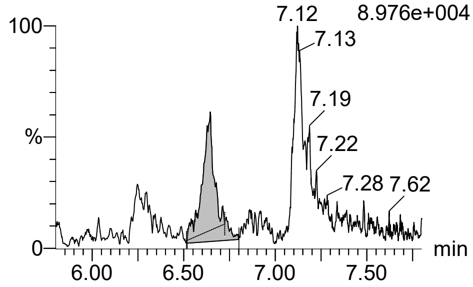

Sample Name: 20211111_H3_R3_IgM
F87:MRM of 1 channel,ES+
1193.7 > 366.1
2.666e+004

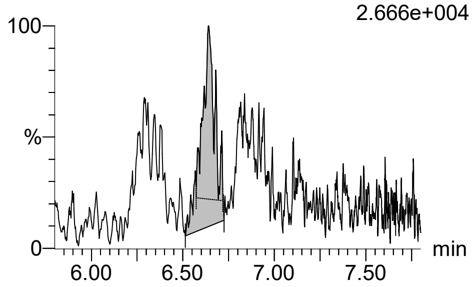

Sample Name: 20211111_A7_R3_IgM
F87:MRM of 1 channel,ES+
1193.7 > 366.1
6.882e+004

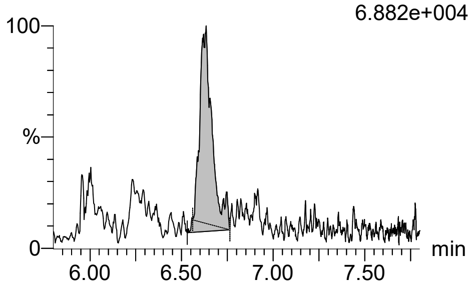

Sample Name: 20211111_A8_R3_IgM
F87:MRM of 1 channel,ES+
1193.7 > 366.1
5.264e+004

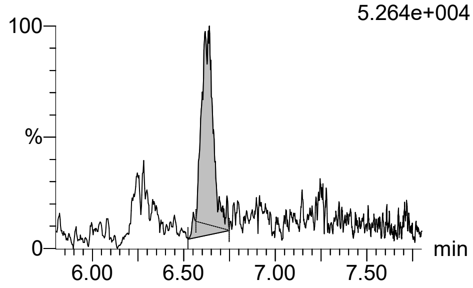

Sample Name: 20211111_L7_R3_IgM
F87:MRM of 1 channel,ES+
1193.7 > 366.1
4.551e+004

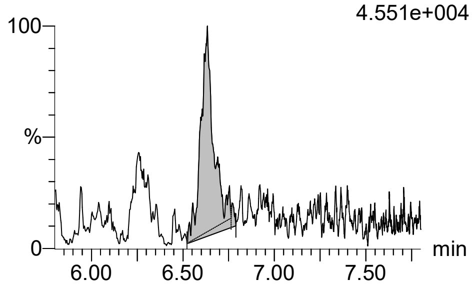

Sample Name: 20211111_L8_R3_IgM
F87:MRM of 1 channel,ES+
1193.7 > 366.1
2.314e+004

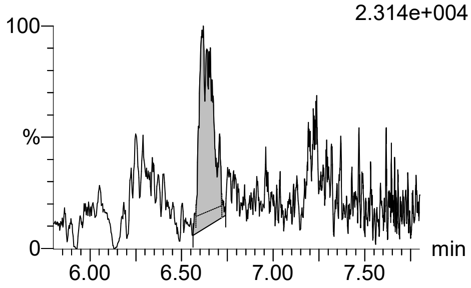

Sample Name: 20211111_H4_R3_IgM
F87:MRM of 1 channel,ES+
1193.7 > 366.1
2.531e+004

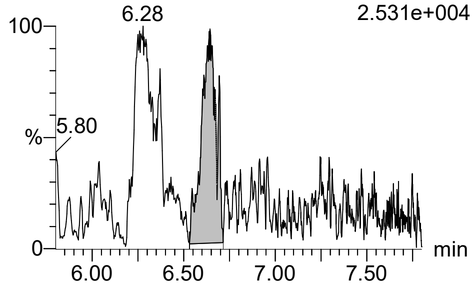

Sample Name: 20211111_A9_R3_IgM
F87:MRM of 1 channel,ES+
1193.7 > 366.1
4.770e+004

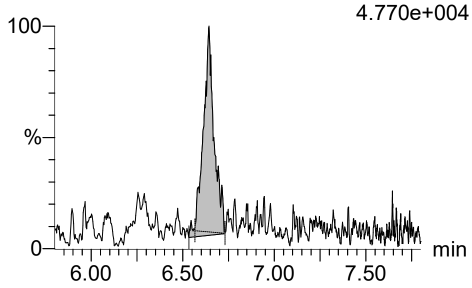

Sample Name: 20211111_A10_R3_IgM
F87:MRM of 1 channel,ES+
1193.7 > 366.1
5.473e+004

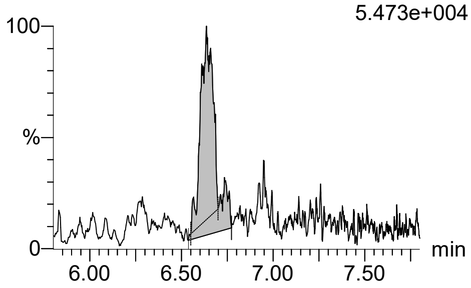

Sample Name: 20211111_L9_R3_IgM
F87:MRM of 1 channel,ES+
1193.7 > 366.1
4.166e+004

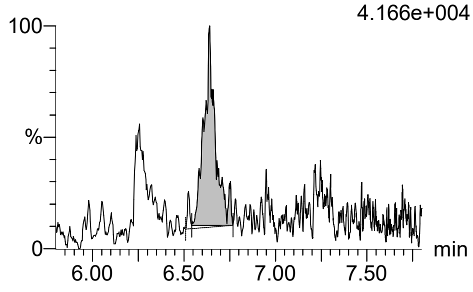

Sample Name: 20211111_L10_R3_IgM
F87:MRM of 1 channel,ES+
1193.7 > 366.1
1.441e+005

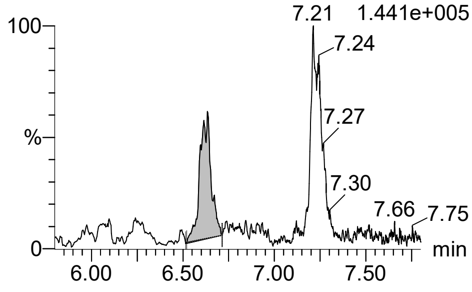

Sample Name: 20211111_QC_R19_IgM
F87:MRM of 1 channel,ES+
1193.7 > 366.1
5.552e+004

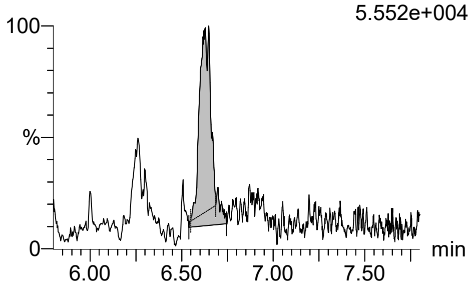

Dataset: C:\MassLynx\10.pro\20211111_clinicalsamples_IgM_IgA.qld

Last Altered: Thursday, March 09, 2023 19:58:25 台北標準時間
Printed: Friday, August 01, 2025 15:21:31 台北標準時間

Sample Name: 20211111_H5_R3_IgM
F87:MRM of 1 channel,ES+
1193.7 > 366.1
6.223e+004

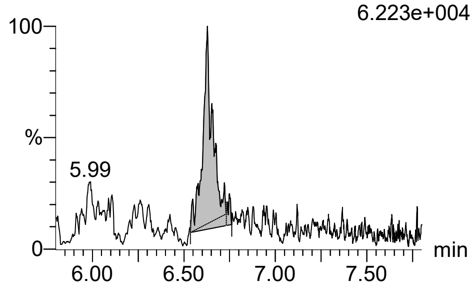

Sample Name: 20211111_A11_R3_IgM
F87:MRM of 1 channel,ES+
1193.7 > 366.1
4.220e+004

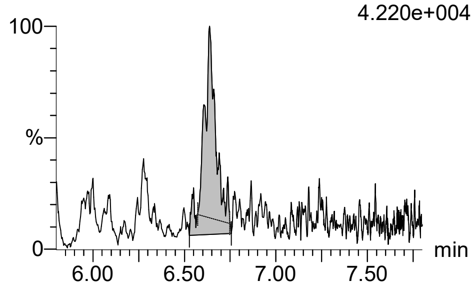

Sample Name: 20211111_A12_R3_IgM
F87:MRM of 1 channel,ES+
1193.7 > 366.1
3.563e+004

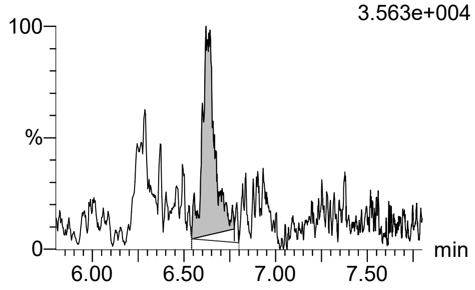

Sample Name: 20211111_L11_R3_IgM
F87:MRM of 1 channel,ES+
1193.7 > 366.1
2.242e+004

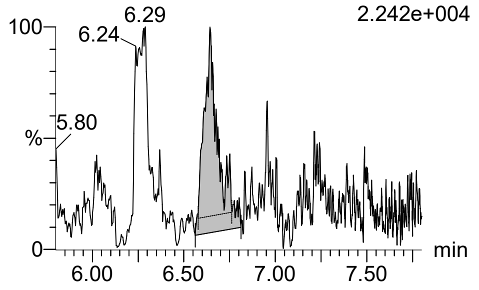

Sample Name: 20211111_L12_R3_IgM
F87:MRM of 1 channel,ES+
1193.7 > 366.1
5.479e+004

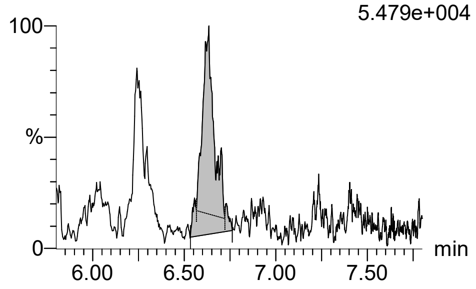

Sample Name: 20211111_H6_R3_IgM
F87:MRM of 1 channel,ES+
1193.7 > 366.1
3.248e+004

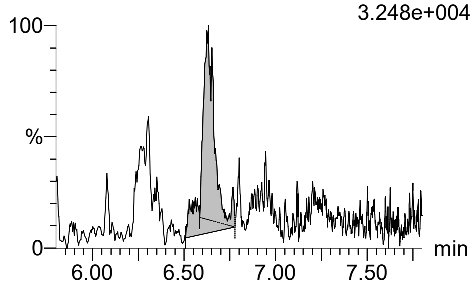

Sample Name: 20211111_A13_R3_IgM
F87:MRM of 1 channel,ES+
1193.7 > 366.1
4.612e+004

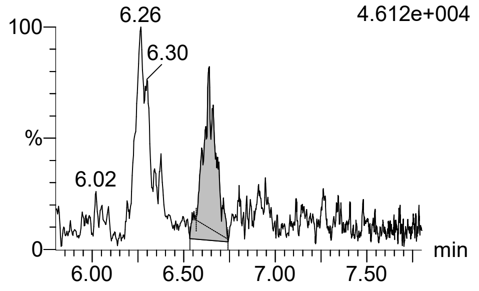

Sample Name: 20211111_A14_R3_IgM
F87:MRM of 1 channel,ES+
1193.7 > 366.1
6.684e+004

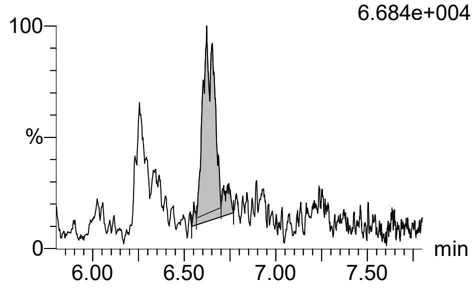

Sample Name: 20211111_L13_R3_IgM
F87:MRM of 1 channel,ES+
1193.7 > 366.1
6.679e+004

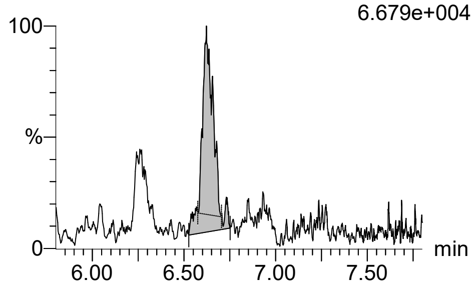

Sample Name: 20211111_L14_R3_IgM
F87:MRM of 1 channel,ES+
1193.7 > 366.1
5.794e+004

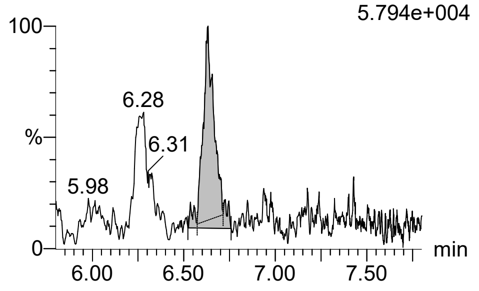

Sample Name: 20211111_H7_R3_IgM
F87:MRM of 1 channel,ES+
1193.7 > 366.1
4.221e+004

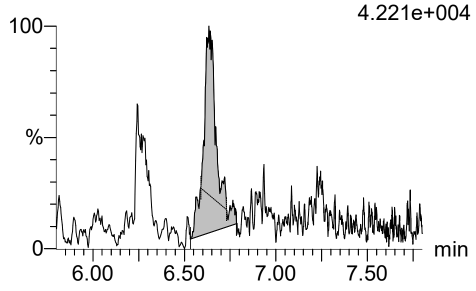

Sample Name: 20211111_A15_R3_IgM
F87:MRM of 1 channel,ES+
1193.7 > 366.1
9.915e+004

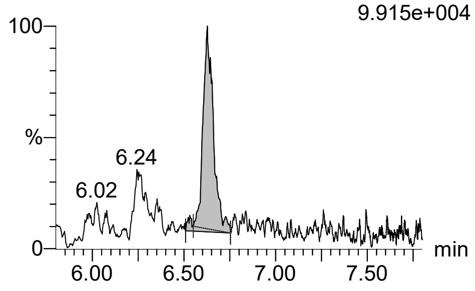

Sample Name: 20211111_QC_R20_IgM
F87:MRM of 1 channel,ES+
1193.7 > 366.1
7.300e+004

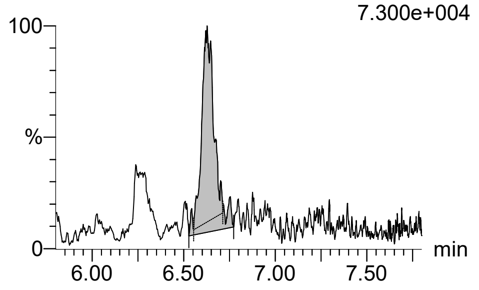

Sample Name: 20211111_A16_R3_IgM
F87:MRM of 1 channel,ES+
1193.7 > 366.1
8.019e+004

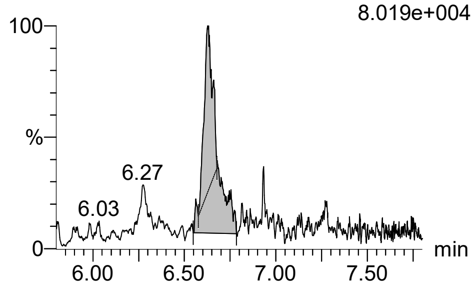

Sample Name: 20211111_L15_R3_IgM
F87:MRM of 1 channel,ES+
1193.7 > 366.1
8.018e+004

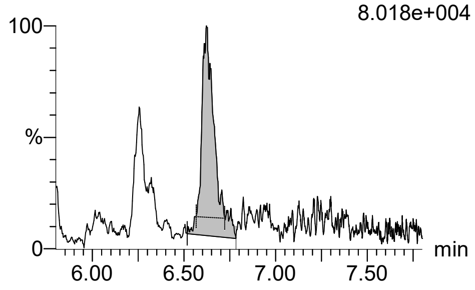

Dataset: C:\MassLynx\10.pro\20211111_clinicalsamples_IgM_IgA.qld

Last Altered: Thursday, March 09, 2023 19:58:25 台北標準時間
Printed: Friday, August 01, 2025 15:21:31 台北標準時間

Sample Name: 20211111_L16_R3_IgM
F87:MRM of 1 channel,ES+
1193.7 > 366.1
3.500e+004

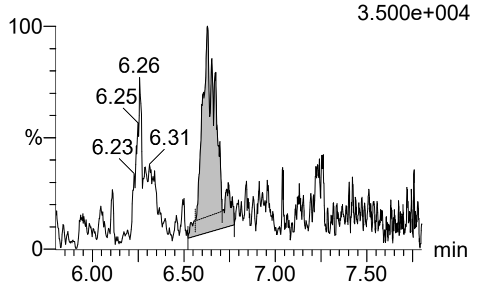

Sample Name: 20211111_H8_R3_IgM
F87:MRM of 1 channel,ES+
1193.7 > 366.1
4.292e+004

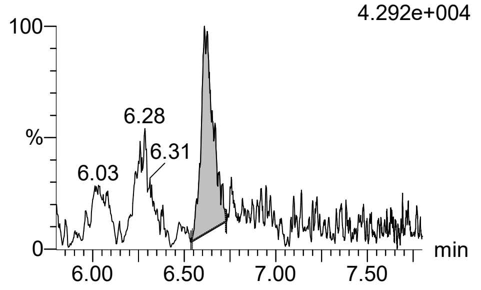

Sample Name: 20211111_A17_R3_IgM
F87:MRM of 1 channel,ES+
1193.7 > 366.1
7.937e+004

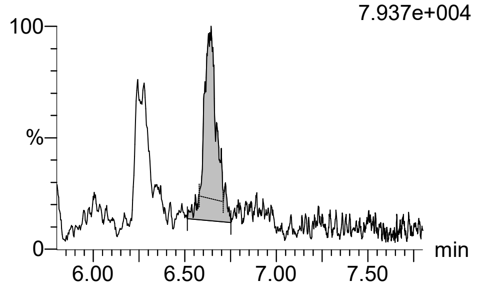

Sample Name: 20211111_A18_R3_IgM
F87:MRM of 1 channel,ES+
1193.7 > 366.1
4.325e+004

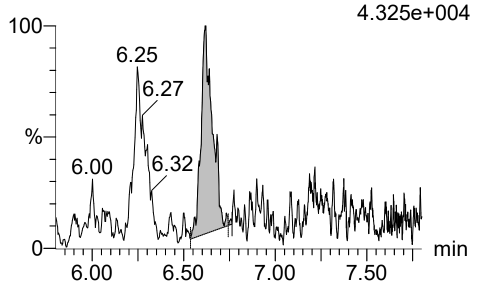

Sample Name: 20211111_L17_R3_IgM
F87:MRM of 1 channel,ES+
1193.7 > 366.1
8.629e+004

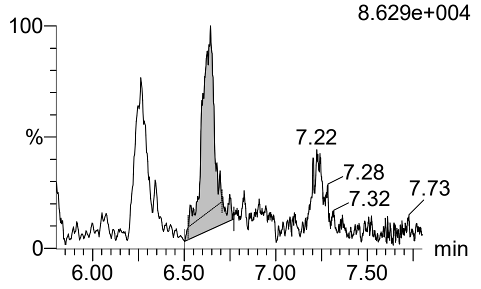

Sample Name: 20211111_L18_R3_IgM
F87:MRM of 1 channel,ES+
1193.7 > 366.1
2.633e+004

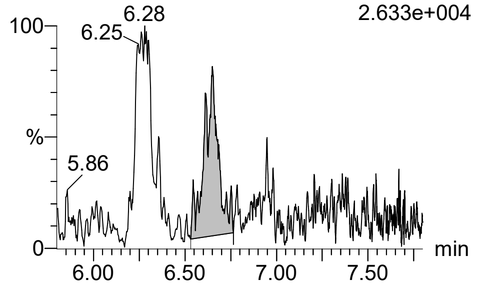

Sample Name: 20211111_H9_R3_IgM
F87:MRM of 1 channel,ES+
1193.7 > 366.1
3.836e+004

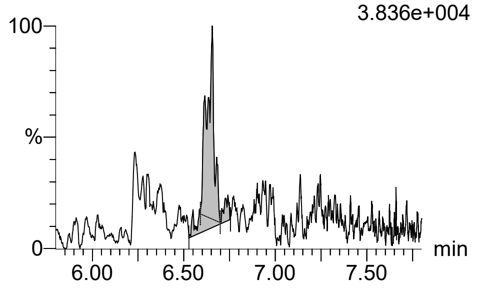

Sample Name: 20211111_A19_R3_IgM
F87:MRM of 1 channel,ES+
1193.7 > 366.1
6.642e+004

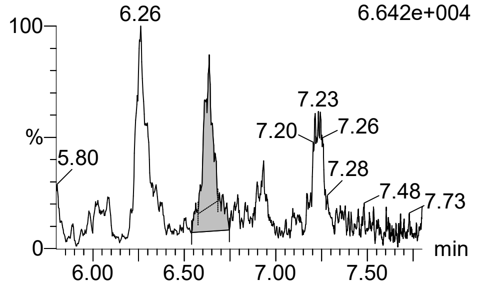

Sample Name: 20211111_A20_R3_IgM
F87:MRM of 1 channel,ES+
1193.7 > 366.1
2.417e+004

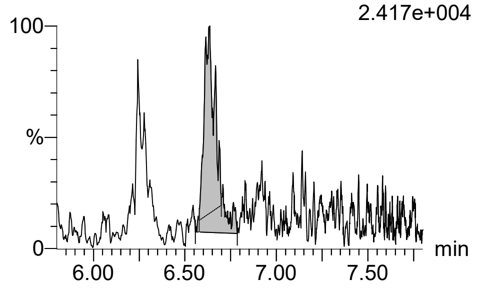

Sample Name: 20211111_L19_R3_IgM
F87:MRM of 1 channel,ES+
1193.7 > 366.1
3.172e+004

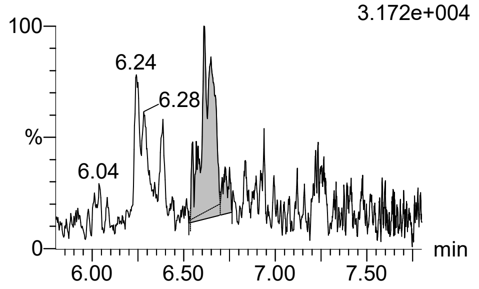

Sample Name: 20211111_QC_R21_IgM
F87:MRM of 1 channel,ES+
1193.7 > 366.1
6.270e+004

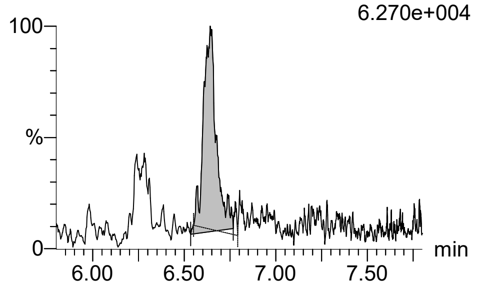

Sample Name: 20211111_L20_R3_IgM
F87:MRM of 1 channel,ES+
1193.7 > 366.1
4.293e+004

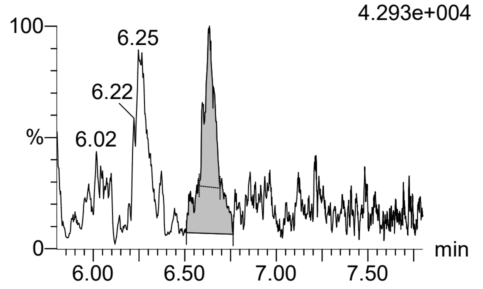

Sample Name: 20211111_H10_R3_IgM
F87:MRM of 1 channel,ES+
1193.7 > 366.1
6.372e+004

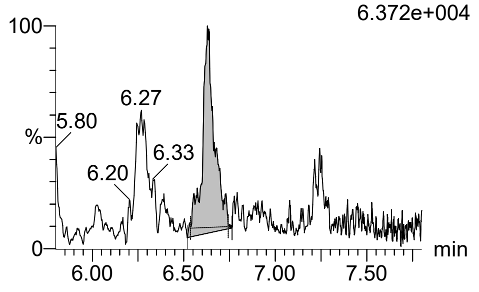

Sample Name: 20211111_A21_R3_IgM
F87:MRM of 1 channel,ES+
1193.7 > 366.1
6.005e+004

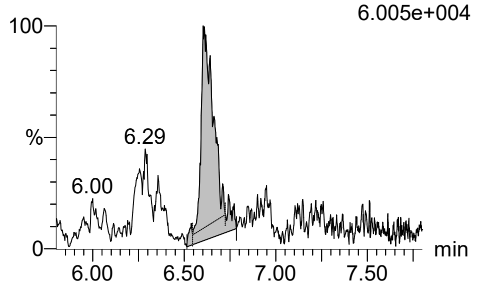

Sample Name: 20211111_A22_R3_IgM
F87:MRM of 1 channel,ES+
1193.7 > 366.1
2.983e+004

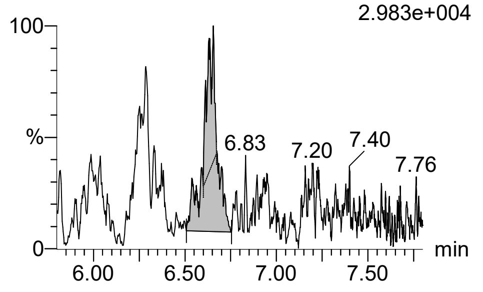

Dataset: C:\MassLynx\10.pro\20211111_clinicalsamples_IgM_IgA.qld

Last Altered: Thursday, March 09, 2023 19:58:25 台北標準時間
Printed: Friday, August 01, 2025 15:21:31 台北標準時間

Sample Name: 20211111_L21_R3_IgM
F87:MRM of 1 channel,ES+
1193.7 > 366.1
4.124e+004

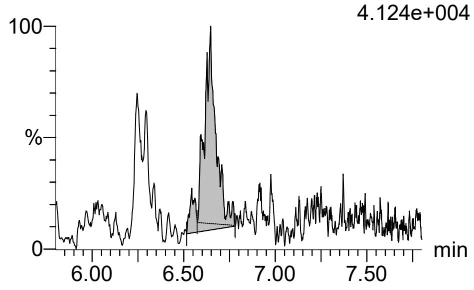

Sample Name: 20211111_L22_R3_IgM
F87:MRM of 1 channel,ES+
1193.7 > 366.1
5.298e+004

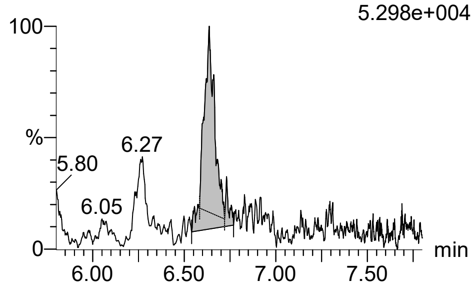

Sample Name: 20211111_H11_R3_IgM
F87:MRM of 1 channel,ES+
1193.7 > 366.1
5.718e+004

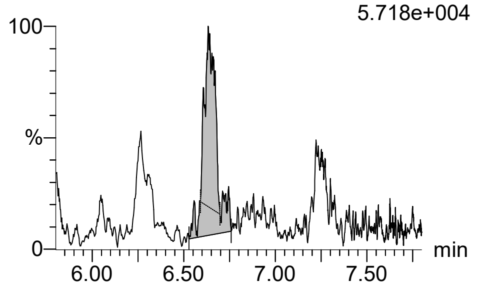

Sample Name: 20211111_A23_R3_IgM
F87:MRM of 1 channel,ES+
1193.7 > 366.1
2.883e+004

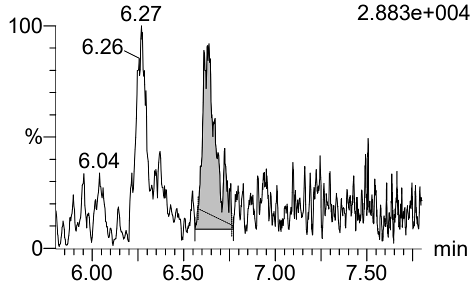

Sample Name: 20211111_A24_R3_IgM
F87:MRM of 1 channel,ES+
1193.7 > 366.1
9.629e+004

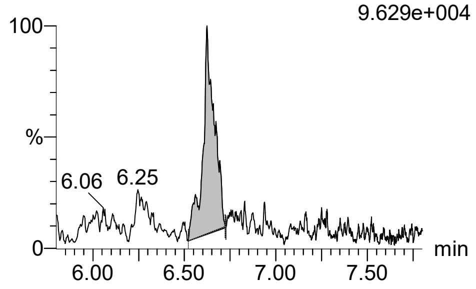

Sample Name: 20211111_L23_R3_IgM
F87:MRM of 1 channel,ES+
1193.7 > 366.1
1.137e+005

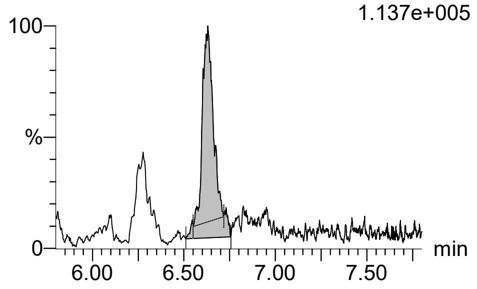

Sample Name: 20211111_L24_R3_IgM
F87:MRM of 1 channel,ES+
1193.7 > 366.1
4.000e+004

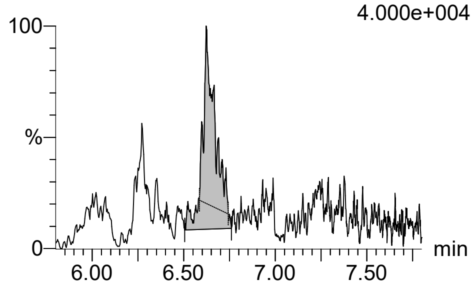

Sample Name: 20211111_H12_R3_IgM
F87:MRM of 1 channel,ES+
1193.7 > 366.1
3.553e+004

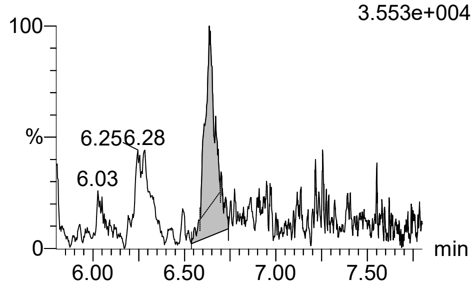

Sample Name: 20211111_QC_R22_IgM
F87:MRM of 1 channel,ES+
1193.7 > 366.1
5.588e+004

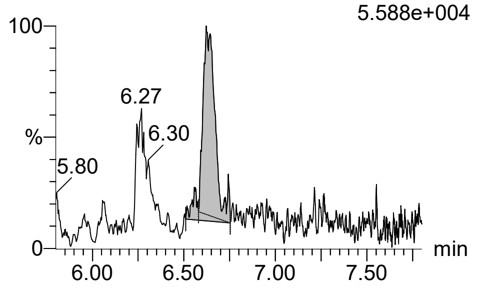

Sample Name: 20211111_A25_R3_IgM
F87:MRM of 1 channel,ES+
1193.7 > 366.1
9.235e+004

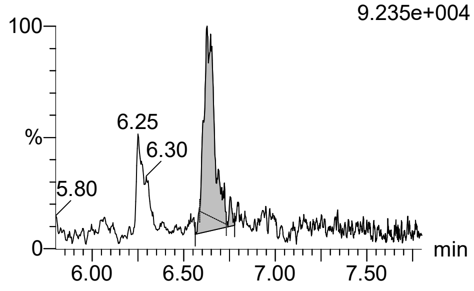

Sample Name: 20211111_A26_R3_IgM
F87:MRM of 1 channel,ES+
1193.7 > 366.1
3.741e+004

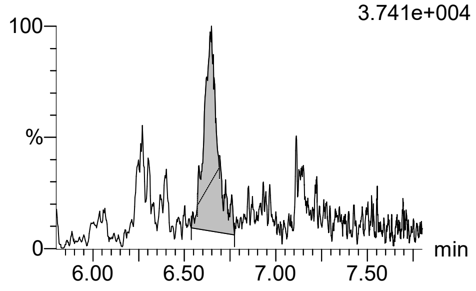

Sample Name: 20211111_L25_R3_IgM
F87:MRM of 1 channel,ES+
1193.7 > 366.1
3.466e+004

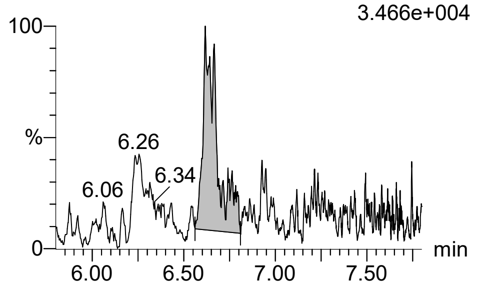

Sample Name: 20211111_L26_R3_IgM
F87:MRM of 1 channel,ES+
1193.7 > 366.1
3.115e+004

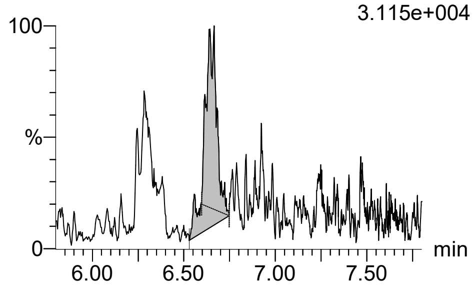

Sample Name: 20211111_H13_R3_IgM
F87:MRM of 1 channel,ES+
1193.7 > 366.1
7.362e+004

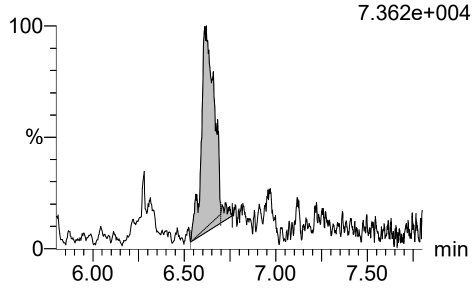

Sample Name: 20211111_A27_R3_IgM
F87:MRM of 1 channel,ES+
1193.7 > 366.1
7.663e+004

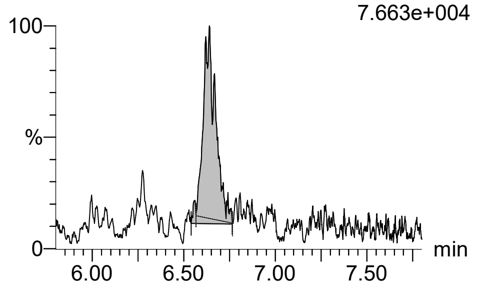

Sample Name: 20211111_A28_R3_IgM
F87:MRM of 1 channel,ES+
1193.7 > 366.1
3.855e+004

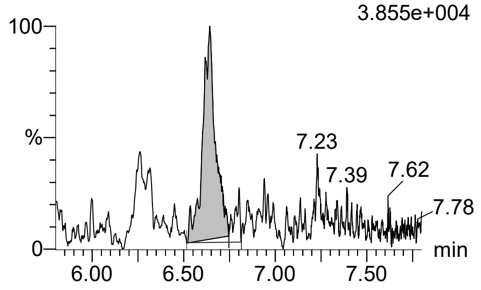

Sample Name: 20211111_L27_R3_IgM
F87:MRM of 1 channel,ES+
1193.7 > 366.1
8.422e+004

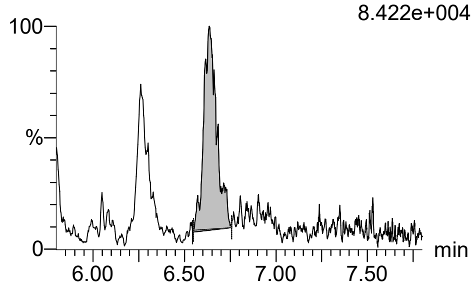

Sample Name: 20211111_L28_R3_IgM
F87:MRM of 1 channel,ES+
1193.7 > 366.1
5.738e+004

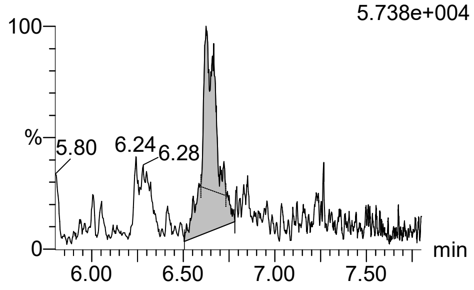

Sample Name: 20211111_H14_R3_IgM
F87:MRM of 1 channel,ES+
1193.7 > 366.1
6.848e+004

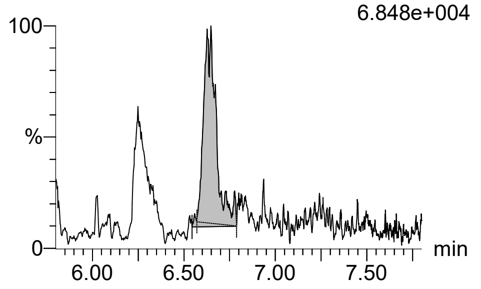

Sample Name: 20211111_A29_R3_IgM
F87:MRM of 1 channel,ES+
1193.7 > 366.1
1.922e+004

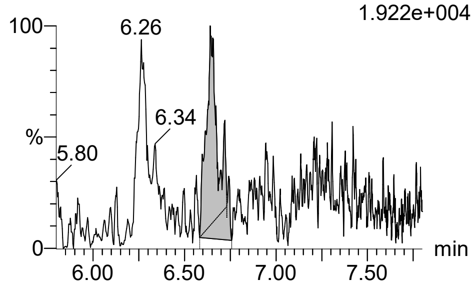

Sample Name: 20211111_A30_R3_IgM
F87:MRM of 1 channel,ES+
1193.7 > 366.1
4.661e+004

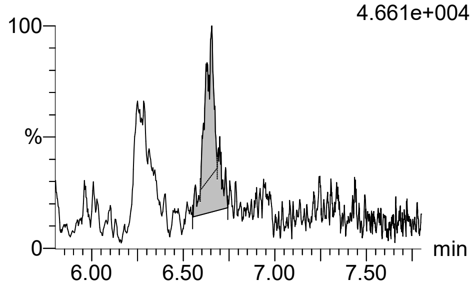

Sample Name: 20211111_QC_R23_IgM
F87:MRM of 1 channel,ES+
1193.7 > 366.1
6.407e+004

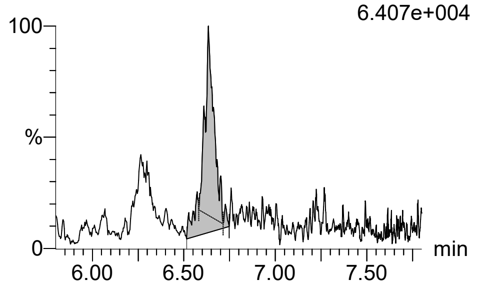

Sample Name: 20211111_L29_R3_IgM
F87:MRM of 1 channel,ES+
1193.7 > 366.1
3.150e+004

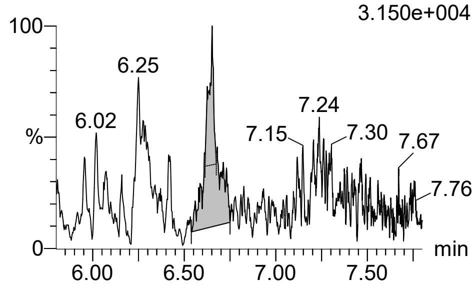

Sample Name: 20211111_L30_R3_IgM
F87:MRM of 1 channel,ES+
1193.7 > 366.1
2.142e+004

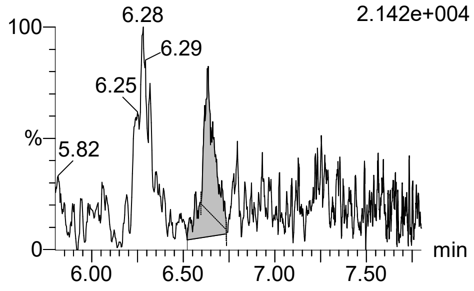

Sample Name: 20211111_H15_R3_IgM
F87:MRM of 1 channel,ES+
1193.7 > 366.1
4.821e+004

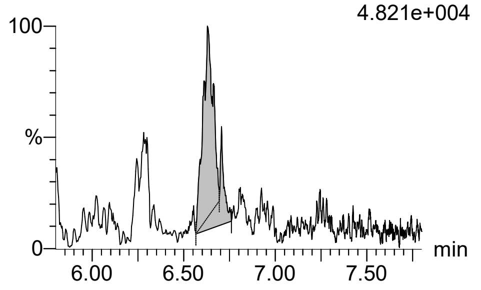

Sample Name: 20211111_A31_R3_IgM
F87:MRM of 1 channel,ES+
1193.7 > 366.1
2.421e+004

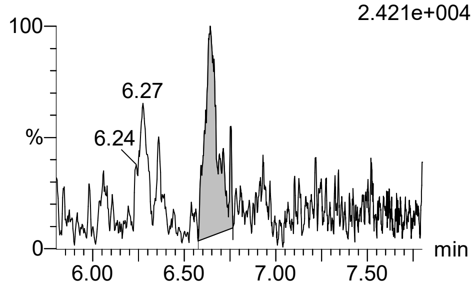

Sample Name: 20211111_A32_R3_IgM
F87:MRM of 1 channel,ES+
1193.7 > 366.1
5.122e+004

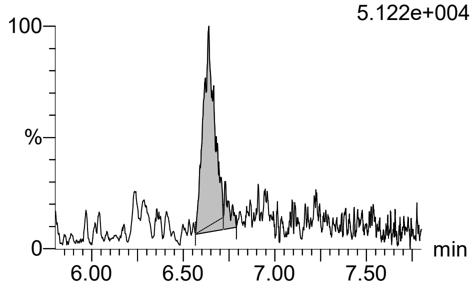

Sample Name: 20211111_L31_R3_IgM
F87:MRM of 1 channel,ES+
1193.7 > 366.1
2.733e+004

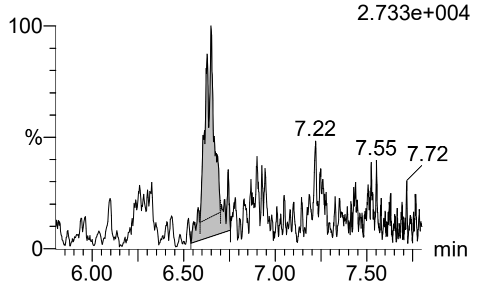

Sample Name: 20211111_L32_R3_IgM
F87:MRM of 1 channel,ES+
1193.7 > 366.1
4.495e+004

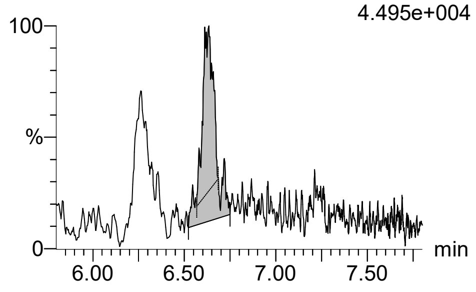

Sample Name: 20211111_H16_R3_IgM
F87:MRM of 1 channel,ES+
1193.7 > 366.1
9.683e+004

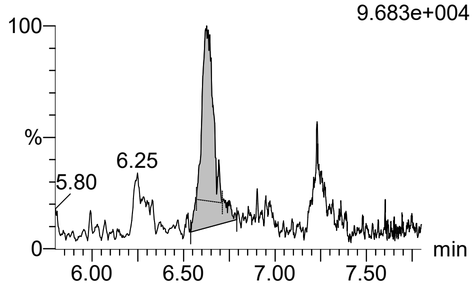

Sample Name: 20211111_A33_R3_IgM

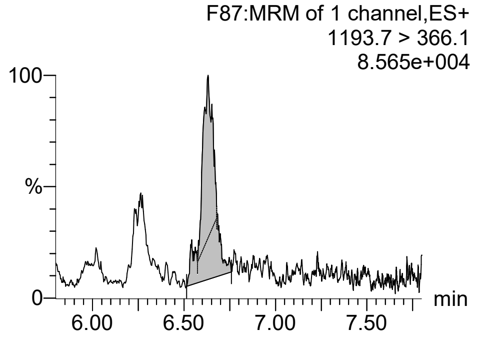

Sample Name: 20211111_A34_R3_IgM

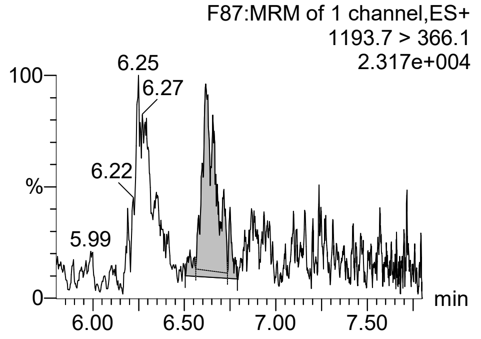

Sample Name: 20211111_H17_R3_IgM

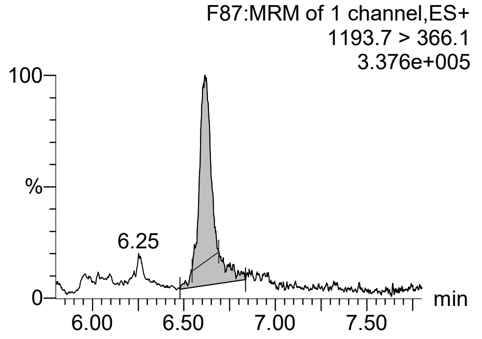

Sample Name: 20211111_A35_R3_IgM

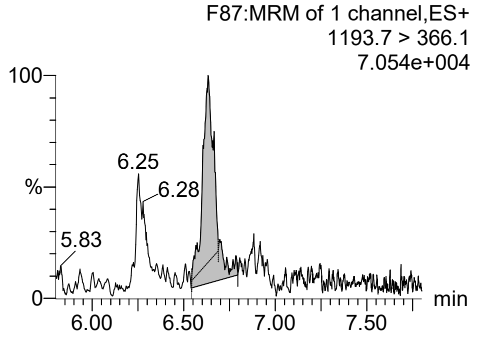

Sample Name: 20211111_QC_R24_IgM

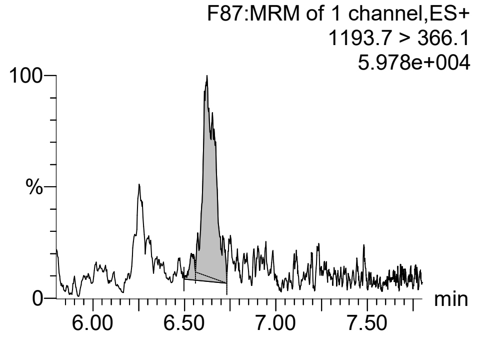

Sample Name: 20211111_A36_R3_IgM

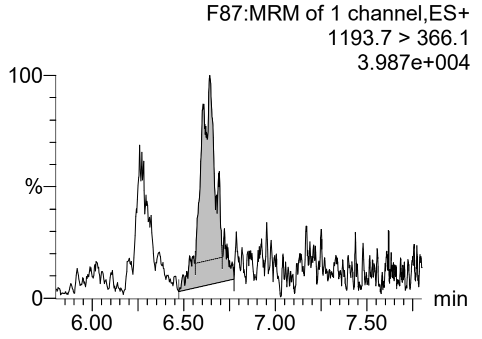

Sample Name: 20211111_H18_R3_IgM

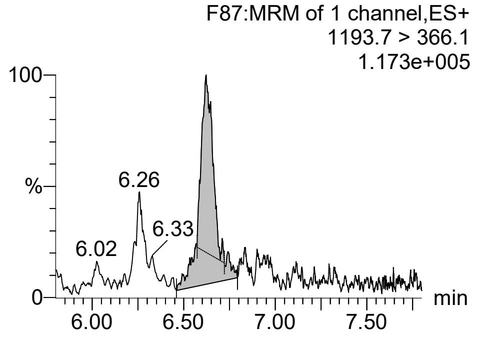

Sample Name: 20211111_A37_R3_IgM

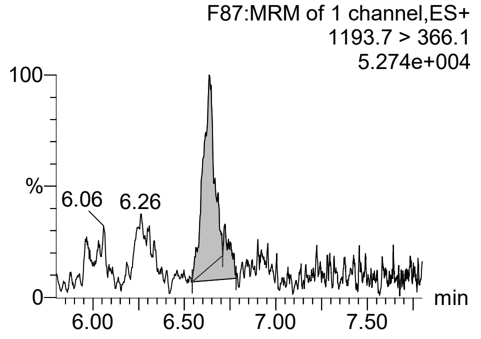

Sample Name: 20211111_A38_R3_IgM

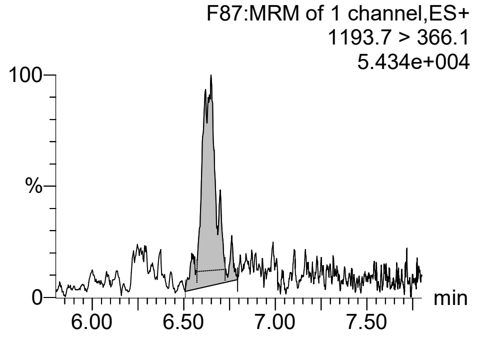

Sample Name: 20211111_H19_R3_IgM

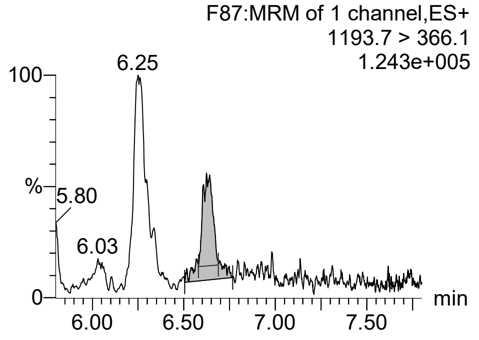

Sample Name: 20211111_A39_R3_IgM

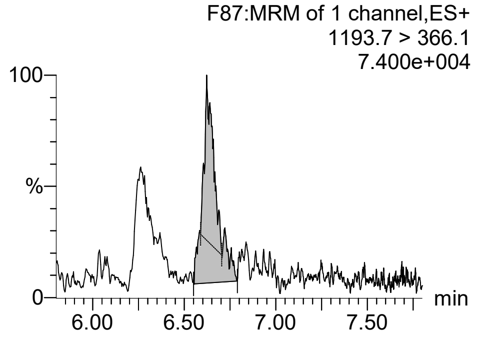

Sample Name: 20211111_A40_R3_IgM

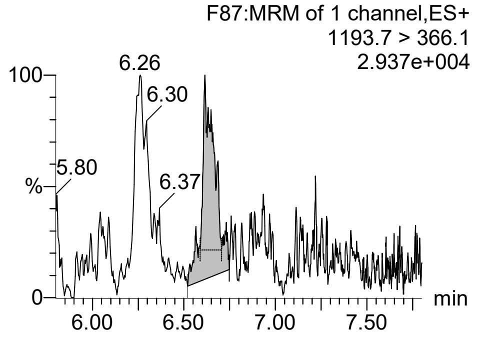

Sample Name: 20211111_H20_R3_IgM

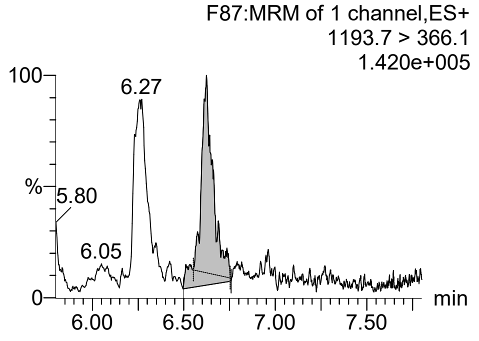

Sample Name: 20211111_A41_R3_IgM

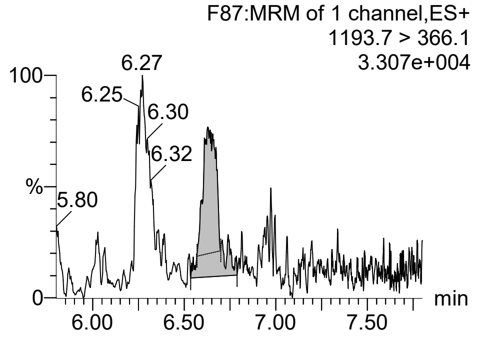

Sample Name: 20211111_A42_R3_IgM

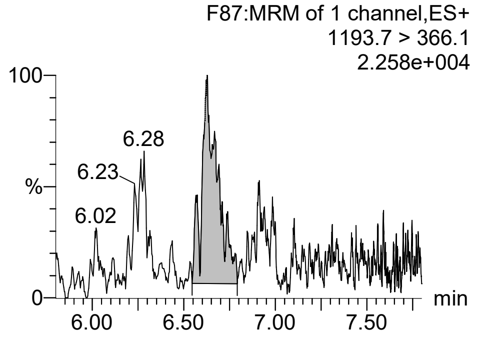

Dataset: C:\MassLynx\10.pro\20211111_clinicalsamples_IgM_IgA.qld

Last Altered: Thursday, March 09, 2023 19:58:25 台北標準時間
Printed: Friday, August 01, 2025 15:21:31 台北標準時間

Sample Name: 20211111_A43_R3_IgM

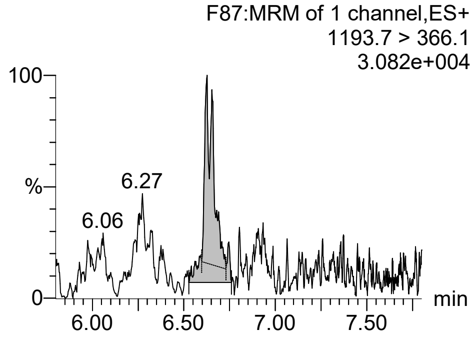

Sample Name: 20211111_A44_R3_IgM

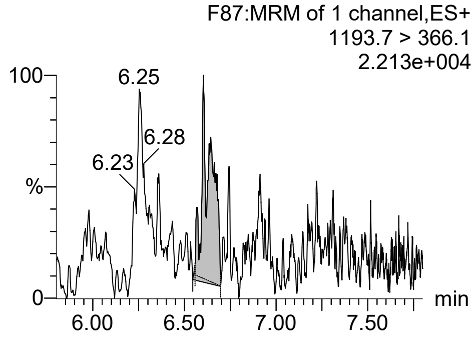

Sample Name: 20211111_QC_R25_IgM

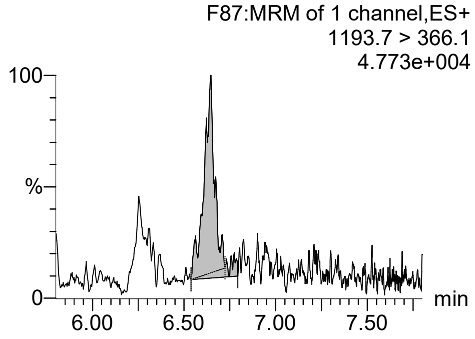

Dataset: C:\MassLynx\10.pro\20211111_clinicalsamples_IgM_IgA.qld

Last Altered: Thursday, March 09, 2023 19:58:25 台北標準時間
Printed: Friday, August 01, 2025 15:21:31 台北標準時間

	# Name	RT	Area	IS Area	Response	Prima...
1	1 20211112_QC_R001_IgM	6.62	4070.7	15421.4	0.264	bb
2	2 20211112_QC_R002_IgM	6.63	5091.9	13188.8	0.386	bb
3	3 20211111_A1_R2_IgM	6.66	2127.5	11871.0	0.179	MM
4	4 20211111_A1_R3_IgM	6.64	2169.4	12030.5	0.180	MM
5	5 20211111_A2_R2_IgM	6.63	7776.4	11446.8	0.679	MM
6	6 20211111_A2_R3_IgM	6.63	8218.9	11098.4	0.741	MM
7	7 20211111_L1_R2_IgM	6.64	2479.3	11779.4	0.210	MM
8	8 20211111_L1_R3_IgM	6.66	1784.1	11176.2	0.160	MM
9	9 20211111_L2_R2_IgM	6.64	3350.9	11375.0	0.295	MM
10	10 20211111_H1_R2_IgM	6.61	5828.3	13595.2	0.429	MM
11	11 20211111_A3_R2_IgM	6.61	7025.2	14112.3	0.498	MM
12	12 20211111_A4_R2_IgM	6.61	3217.9	11977.5	0.269	MM
13	13 20211111_L3_R2_IgM	6.60	4529.4	12242.1	0.370	MM
14	14 20211111_L4_R2_IgM	6.63	3981.2	12797.0	0.311	MM
15	15 20211111_H2_R2_IgM	6.61	4671.8	10006.0	0.467	MM
16	16 20211111_A5_R2_IgM	6.61	14440.0	11434.6	1.263	MM
17	17 20211111_A6_R2_IgM	6.63	6178.4	13193.7	0.468	MM
18	18 20211111_QC_R10_IgM	6.63	4691.1	12566.1	0.373	bb
19	19 20211111_L5_R2_IgM	6.61	8862.5	13616.4	0.651	MM
20	20 20211111_L6_R2_IgM	6.64	5370.9	13698.5	0.392	MM
21	21 20211111_H3_R2_IgM	6.62	2139.4	15793.1	0.135	MM
22	22 20211111_A7_R2_IgM	6.62	5657.6	12936.9	0.437	MM
23	23 20211111_A8_R2_IgM	6.63	4191.7	13108.0	0.320	MM
24	24 20211111_L7_R2_IgM	6.65	4531.8	13278.5	0.341	MM
25	25 20211111_L8_R2_IgM	6.63	1866.9	15233.6	0.123	MM
26	26 20211111_H4_R2_IgM	6.62	1942.4	16521.9	0.118	MM
27	27 20211111_A9_R2_IgM	6.60	5327.2	16451.6	0.324	MM
28	28 20211111_A10_R2_IgM	6.61	5063.7	13036.0	0.388	bb
29	29 20211111_L9_R2_IgM	6.62	3703.5	14554.1	0.254	MM
30	30 20211111_L10_R2_IgM	6.63	8112.3	12250.4	0.662	MM
31	31 20211111_QC_R11_IgM	6.62	5548.4	11459.2	0.484	MM
32	32 20211111_H5_R2_IgM	6.62	5276.3	14358.2	0.367	bb
33	33 20211111_A11_R2_IgM	6.62	6635.2	16184.1	0.410	MM
34	34 20211111_A12_R2_IgM	6.60	2083.7	13465.4	0.155	MM
35	35 20211111_L11_R2_IgM	6.61	1768.9	13277.9	0.133	MM
36	36 20211111_L12_R2_IgM	6.63	4528.3	15958.8	0.284	bb
37	37 20211111_H6_R2_IgM	6.65	2250.4	14003.5	0.161	MM
38	38 20211111_A13_R2_IgM	6.65	2122.7	13656.1	0.155	MM
39	39 20211111_A14_R2_IgM	6.62	6671.7	14071.3	0.474	MM
40	40 20211111_L13_R2_IgM	6.63	4570.9	14785.6	0.309	MM
41	41 20211111_L14_R2_IgM	6.63	4992.9	14772.2	0.338	MM
42	42 20211111_H7_R2_IgM	6.62	4516.6	15164.4	0.298	MM
43	43 20211111_A15_R2_IgM	6.64	6590.7	15721.5	0.419	bb
44	44 20211111_QC_R12_IgM	6.63	4759.6	13711.5	0.347	MM
45	45 20211111_A16_R2_IgM	6.62	7489.5	14275.6	0.525	MM
46	46 20211111_L15_R2_IgM	6.63	6864.0	12935.8	0.531	MM
47	47 20211111_L16_R2_IgM	6.62	2794.2	13342.8	0.209	MM
48	48 20211111_H8_R2_IgM	6.63	3136.6	15835.1	0.198	MM
49	49 20211111_A17_R2_IgM	6.61	5840.2	15786.7	0.370	MM
50	50 20211111_A18_R2_IgM	6.63	3446.0	16986.8	0.203	MM
51	51 20211111_L17_R2_IgM	6.63	6682.1	13342.7	0.501	MM
52	52 20211111_L18_R2_IgM	6.66	1725.0	14692.0	0.117	MM
53	53 20211111_H9_R2_IgM	6.63	2697.4	15639.7	0.172	MM
54	54 20211111_A19_R2_IgM	6.61	3772.4	14198.7	0.266	MM
55	55 20211111_A20_R2_IgM	6.63	1487.5	14162.7	0.105	MM
56	56 20211111_L19_R2_IgM	6.65	1878.5	9293.4	0.202	MM

	# Name	RT	Area	IS Area	Response	Prima...
57	57 20211111_QC_R13_IgM	6.62	4181.0	13903.0	0.301	MM
58	58 20211111_L20_R2_IgM	6.62	5964.3	12781.6	0.467	MM
59	59 20211111_H10_R2_IgM	6.63	5951.9	14796.4	0.402	MM
60	60 20211111_A21_R2_IgM	6.62	5928.3	13669.0	0.434	MM
61	61 20211111_A22_R2_IgM	6.62	2183.6	14098.3	0.155	MM
62	62 20211111_L21_R2_IgM	6.65	3570.9	15678.2	0.228	MM
63	63 20211111_L22_R2_IgM	6.63	4033.2	15724.5	0.256	MM
64	64 20211111_H11_R2_IgM	6.63	4567.4	15890.2	0.287	MM
65	65 20211111_A23_R2_IgM	6.63	2155.5	13423.2	0.161	MM
66	66 20211111_A24_R2_IgM	6.63	7843.8	12438.0	0.631	MM
67	67 20211111_L23_R2_IgM	6.63	8906.0	14075.0	0.633	MM
68	68 20211111_L24_R2_IgM	6.65	3447.8	15972.1	0.216	MM
69	69 20211111_H12_R2_IgM	6.62	2035.0	16270.9	0.125	MM
70	70 20211111_QC_R14_IgM	6.62	5019.1	15806.8	0.318	MM
71	71 20211111_A25_R2_IgM	6.63	7270.9	14552.1	0.500	MM
72	72 20211111_A26_R2_IgM	6.64	3266.4	11492.7	0.284	MM
73	73 20211111_L25_R2_IgM	6.63	3469.5	14403.3	0.241	bb
74	74 20211111_L26_R2_IgM	6.64	3124.0	16533.9	0.189	MM
75	75 20211111_H13_R2_IgM	6.64	6302.7	15520.1	0.406	MM
76	76 20211111_A27_R2_IgM	6.64	7203.8	13337.4	0.540	MM
77	77 20211111_A28_R2_IgM	6.62	4708.8	11895.6	0.396	MM
78	78 20211111_L27_R2_IgM	6.65	7328.4	14125.7	0.519	MM
79	79 20211111_L28_R2_IgM	6.62	5145.3	11807.5	0.436	MM
80	80 20211111_H14_R2_IgM	6.64	5459.9	15035.7	0.363	MM
81	81 20211111_A29_R2_IgM	6.65	1373.2	13554.4	0.101	MM
82	82 20211111_A30_R2_IgM	6.62	3219.1	12984.1	0.248	MM
83	83 20211111_QC_R15_IgM	6.61	4310.1	12299.5	0.350	MM
84	84 20211111_L29_R2_IgM	6.63	1622.1	13590.7	0.119	MM
85	85 20211111_L30_R2_IgM	6.62	1021.0	12511.4	0.082	MM
86	86 20211111_H15_R2_IgM	6.62	3596.4	11885.0	0.303	MM
87	87 20211111_A31_R2_IgM	6.64	2055.2	13422.1	0.153	MM
88	88 20211111_A32_R2_IgM	6.65	4383.8	11461.3	0.382	MM
89	89 20211111_L31_R2_IgM	6.62	2348.0	13753.4	0.171	MM
90	90 20211111_L32_R2_IgM	6.63	4265.0	12949.0	0.329	MM
91	91 20211111_H16_R2_IgM	6.61	9037.2	11465.4	0.788	MM
92	92 20211111_A33_R2_IgM	6.61	6356.6	10551.5	0.602	MM
93	93 20211111_A34_R2_IgM	6.64	2181.0	12409.9	0.176	MM
94	94 20211111_H17_R2_IgM	6.61	30831.2	10160.9	3.034	bb
95	95 20211111_A35_R2_IgM	6.62	5891.6	10460.6	0.563	MM
96	96 20211111_QC_R16_IgM	6.64	5421.7	10433.5	0.520	MM
97	97 20211111_A36_R2_IgM	6.61	5809.4	12504.8	0.465	MM
98	98 20211111_H18_R2_IgM	6.63	10196.4	9140.4	1.116	MM
99	99 20211111_A37_R2_IgM	6.63	6006.4	9796.9	0.613	MM
100	1... 20211111_A38_R2_IgM	6.65	4418.3	11224.3	0.394	MM
101	1... 20211111_H19_R2_IgM	6.63	6098.8	11739.2	0.520	bb
102	1... 20211111_A39_R2_IgM	6.65	6268.9	12718.9	0.493	MM
103	1... 20211111_A40_R2_IgM	6.62	3173.7	10006.1	0.317	MM
104	1... 20211111_H20_R2_IgM	6.62	11349.7	7988.2	1.421	MM
105	1... 20211111_A41_R2_IgM	6.65	3191.7	11513.3	0.277	MM
106	1... 20211111_A42_R2_IgM	6.65	1984.2	10238.5	0.194	MM
107	1... 20211111_A43_R2_IgM	6.62	2409.1	11098.9	0.217	MM
108	1... 20211111_A44_R2_IgM	6.63	1742.7	10969.9	0.159	MM
109	1... 20211111_QC_R17_IgM	6.65	3814.9	9700.8	0.393	MM
110	1... 20211111_L2_R3_IgM	6.62	3807.0	8528.0	0.446	MM
111	1... 20211111_H1_R3_IgM	6.64	4132.7	9438.2	0.438	MM
112	1... 20211111_A3_R3_IgM	6.62	4248.0	11598.6	0.366	MM

Dataset: C:\MassLynx\10.pro\20211111_clinicalsamples_IgM_IgA.qld

Last Altered: Thursday, March 09, 2023 19:58:25 台北標準時間
Printed: Friday, August 01, 2025 15:21:31 台北標準時間

	# Name	RT	Area	IS Area	Response	Prima...
113	1... 20211111_A4_R3_IgM	6.62	2834.5	11364.1	0.249	MM
114	1... 20211111_L3_R3_IgM	6.62	3666.5	10753.4	0.341	bb
115	1... 20211111_L4_R3_IgM	6.62	3686.6	10291.6	0.358	MM
116	1... 20211111_H2_R3_IgM	6.63	5510.3	9315.8	0.592	MM
117	1... 20211111_A5_R3_IgM	6.61	10778.0	9102.2	1.184	MM
118	1... 20211111_A6_R3_IgM	6.64	4677.0	10356.4	0.452	MM
119	1... 20211111_QC_R18_IgM	6.63	4395.6	8398.3	0.523	MM
120	1... 20211111_L5_R3_IgM	6.63	4986.7	9786.7	0.510	MM
121	1... 20211111_L6_R3_IgM	6.64	4423.7	9911.3	0.446	MM
122	1... 20211111_H3_R3_IgM	6.64	2075.0	12155.5	0.171	MM
123	1... 20211111_A7_R3_IgM	6.63	5072.7	11804.8	0.430	MM
124	1... 20211111_A8_R3_IgM	6.64	3862.9	10510.6	0.368	MM
125	1... 20211111_L7_R3_IgM	6.63	3627.0	12177.9	0.298	MM
126	1... 20211111_L8_R3_IgM	6.62	1772.1	11668.6	0.152	MM
127	1... 20211111_H4_R3_IgM	6.64	2246.9	11192.0	0.201	MM
128	1... 20211111_A9_R3_IgM	6.64	3067.0	14077.4	0.218	MM
129	1... 20211111_A10_R3_IgM	6.64	4696.2	11190.4	0.420	MM
130	1... 20211111_L9_R3_IgM	6.64	2633.3	14649.3	0.180	MM
131	1... 20211111_L10_R3_IgM	6.63	5799.6	9361.3	0.620	MM
132	1... 20211111_QC_R19_IgM	6.65	3853.7	10125.5	0.381	MM
133	1... 20211111_H5_R3_IgM	6.63	3772.5	10010.9	0.377	MM
134	1... 20211111_A11_R3_IgM	6.64	3113.4	12697.2	0.245	MM
135	1... 20211111_A12_R3_IgM	6.62	2547.0	13148.5	0.194	MM
136	1... 20211111_L11_R3_IgM	6.64	1830.8	10786.6	0.170	MM
137	1... 20211111_L12_R3_IgM	6.63	4286.0	11681.8	0.367	MM
138	1... 20211111_H6_R3_IgM	6.63	2423.2	12095.5	0.200	MM
139	1... 20211111_A13_R3_IgM	6.64	2854.5	12908.3	0.221	MM
140	1... 20211111_A14_R3_IgM	6.62	4735.7	11322.6	0.418	MM
141	1... 20211111_L13_R3_IgM	6.62	4511.2	11516.1	0.392	MM
142	1... 20211111_L14_R3_IgM	6.63	3968.8	10790.7	0.368	MM
143	1... 20211111_H7_R3_IgM	6.63	3192.1	11572.1	0.276	MM
144	1... 20211111_A15_R3_IgM	6.63	6179.3	12434.4	0.497	MM
145	1... 20211111_QC_R20_IgM	6.63	5583.3	10701.0	0.522	MM
146	1... 20211111_A16_R3_IgM	6.63	6221.4	11452.1	0.543	MM
147	1... 20211111_L15_R3_IgM	6.62	6131.5	10531.1	0.582	MM
148	1... 20211111_L16_R3_IgM	6.63	2886.7	12049.4	0.240	MM
149	1... 20211111_H8_R3_IgM	6.61	3023.9	13434.9	0.225	MM
150	1... 20211111_A17_R3_IgM	6.64	5585.4	12452.0	0.449	MM
151	1... 20211111_A18_R3_IgM	6.62	2916.8	12506.5	0.233	MM
152	1... 20211111_L17_R3_IgM	6.64	6527.9	9948.9	0.656	MM
153	1... 20211111_L18_R3_IgM	6.65	1764.2	13462.1	0.131	bb
154	1... 20211111_H9_R3_IgM	6.66	1943.2	13178.7	0.147	MM
155	1... 20211111_A19_R3_IgM	6.64	3606.2	11298.6	0.319	MM
156	1... 20211111_A20_R3_IgM	6.64	1748.4	11175.6	0.156	MM
157	1... 20211111_L19_R3_IgM	6.61	2434.6	11558.2	0.211	MM
158	1... 20211111_QC_R21_IgM	6.64	4751.6	9977.0	0.476	MM
159	1... 20211111_L20_R3_IgM	6.63	3654.6	13508.1	0.271	MM
160	1... 20211111_H10_R3_IgM	6.63	4492.2	12374.8	0.363	MM
161	1... 20211111_A21_R3_IgM	6.60	5071.7	12443.6	0.408	MM
162	1... 20211111_A22_R3_IgM	6.66	2194.2	11589.3	0.189	MM
163	1... 20211111_L21_R3_IgM	6.65	3013.2	13107.4	0.230	MM
164	1... 20211111_L22_R3_IgM	6.64	3657.4	12021.5	0.304	MM
165	1... 20211111_H11_R3_IgM	6.63	4298.8	12687.8	0.339	MM
166	1... 20211111_A23_R3_IgM	6.64	2047.1	11611.8	0.176	MM
167	1... 20211111_A24_R3_IgM	6.62	6495.4	10476.8	0.620	MM
168	1... 20211111_L23_R3_IgM	6.63	8486.1	11873.1	0.715	MM

Dataset: C:\MassLynx\10.pro\20211111_clinicalsamples_IgM_IgA.qld

Last Altered: Thursday, March 09, 2023 19:58:25 台北標準時間
Printed: Friday, August 01, 2025 15:21:31 台北標準時間

	# Name	RT	Area	IS Area	Response	Prima...
169	1... 20211111_L24_R3_IgM	6.62	2952.5	13468.0	0.219	MM
170	1... 20211111_H12_R3_IgM	6.64	2307.1	14229.1	0.162	MM
171	1... 20211111_QC_R22_IgM	6.62	4024.3	11264.4	0.357	MM
172	1... 20211111_A25_R3_IgM	6.63	6120.7	11775.7	0.520	MM
173	1... 20211111_A26_R3_IgM	6.65	2896.1	10473.9	0.277	MM
174	1... 20211111_L25_R3_IgM	6.62	2781.6	13256.8	0.210	bb
175	1... 20211111_L26_R3_IgM	6.66	2138.4	13565.0	0.158	MM
176	1... 20211111_H13_R3_IgM	6.62	5666.1	13146.0	0.431	MM
177	1... 20211111_A27_R3_IgM	6.64	5487.6	12025.6	0.456	MM
178	1... 20211111_A28_R3_IgM	6.64	2970.1	11229.1	0.265	MM
179	1... 20211111_L27_R3_IgM	6.64	6286.8	14960.0	0.420	MM
180	1... 20211111_L28_R3_IgM	6.62	4900.8	11359.0	0.431	MM
181	1... 20211111_H14_R3_IgM	6.65	5017.0	12830.8	0.391	MM
182	1... 20211111_A29_R3_IgM	6.64	1402.2	13434.3	0.104	MM
183	1... 20211111_A30_R3_IgM	6.66	2736.1	11272.2	0.243	MM
184	1... 20211111_QC_R23_IgM	6.63	4120.7	11872.2	0.347	MM
185	1... 20211111_L29_R3_IgM	6.65	1864.7	12948.7	0.144	MM
186	1... 20211111_L30_R3_IgM	6.64	1246.6	11026.6	0.113	MM
187	1... 20211111_H15_R3_IgM	6.63	3458.7	10786.3	0.321	MM
188	1... 20211111_A31_R3_IgM	6.64	1920.9	12010.5	0.160	bb
189	1... 20211111_A32_R3_IgM	6.64	3727.2	11107.7	0.336	MM
190	1... 20211111_L31_R3_IgM	6.65	1712.0	11021.8	0.155	MM
191	1... 20211111_L32_R3_IgM	6.63	3350.5	11683.8	0.287	MM
192	1... 20211111_H16_R3_IgM	6.63	7367.1	9831.7	0.749	MM
193	1... 20211111_A33_R3_IgM	6.63	6533.9	11213.1	0.583	MM
194	1... 20211111_A34_R3_IgM	6.62	1919.0	11550.1	0.166	MM
195	1... 20211111_H17_R3_IgM	6.61	27366.5	8874.4	3.084	MM
196	1... 20211111_A35_R3_IgM	6.63	5207.6	9856.3	0.528	MM
197	1... 20211111_QC_R24_IgM	6.62	4613.5	7561.8	0.610	MM
198	1... 20211111_A36_R3_IgM	6.64	3728.7	9562.8	0.390	MM
199	1... 20211111_H18_R3_IgM	6.62	10331.5	8165.3	1.265	MM
200	2... 20211111_A37_R3_IgM	6.64	4186.4	9278.8	0.451	MM
201	2... 20211111_A38_R3_IgM	6.65	4780.0	10920.3	0.438	MM
202	2... 20211111_H19_R3_IgM	6.62	4933.3	9285.3	0.531	MM
203	2... 20211111_A39_R3_IgM	6.62	5613.3	9083.4	0.618	MM
204	2... 20211111_A40_R3_IgM	6.61	2191.1	8382.4	0.261	MM
205	2... 20211111_H20_R3_IgM	6.62	10789.0	7807.9	1.382	MM
206	2... 20211111_A41_R3_IgM	6.63	2261.4	5925.2	0.382	MM
207	2... 20211111_A42_R3_IgM	6.63	2085.6	9352.6	0.223	MM
208	2... 20211111_A43_R3_IgM	6.63	1930.3	10216.3	0.189	MM
209	2... 20211111_A44_R3_IgM	6.60	1281.7	10262.7	0.125	MM
210	2... 20211111_QC_R25_IgM	6.65	3117.5	8575.3	0.364	MM

Dataset: C:\MassLynx\10.pro\20211111_clinicalsamples_IgM_IgA.qld

Last Altered: Thursday, March 09, 2023 19:58:25 台北標準時間
Printed: Friday, August 01, 2025 15:22:23 台北標準時間

Method: C:\MassLynx\10_Pro_IgA.PRO\MethDB\20211111_IgM_IgA_DMRM_new.mdb 24 Feb 2022 13:44:14
Calibration: 15 Nov 2021 14:42:11

Sample Name: 20211112_QC_R001_IgM

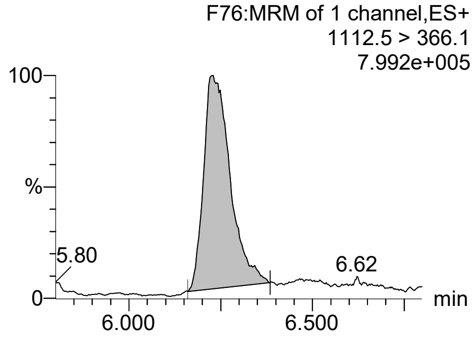

Sample Name: 20211112_QC_R002_IgM

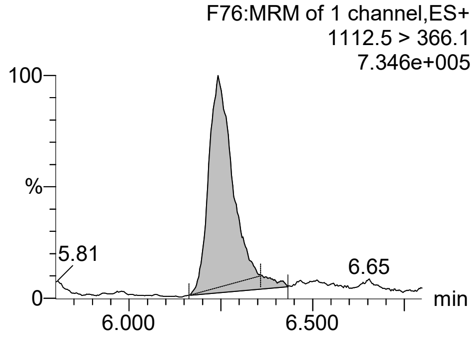

Sample Name: 20211111_A1_R2_IgM

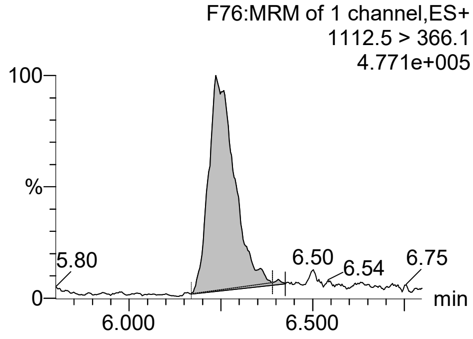

Sample Name: 20211111_A1_R3_IgM

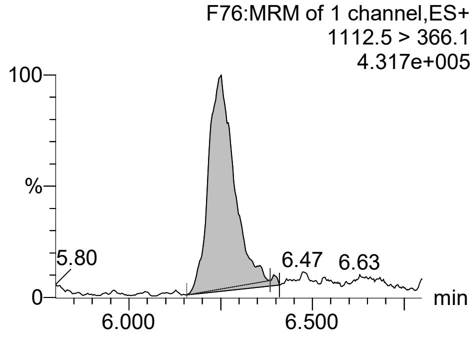

Sample Name: 20211111_A2_R2_IgM

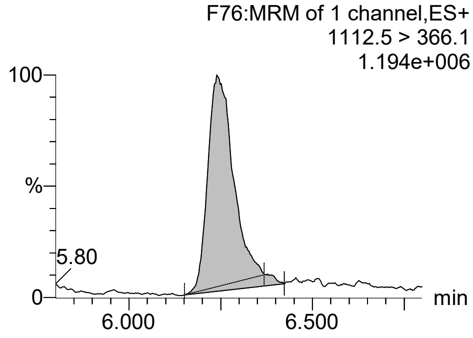

Sample Name: 20211111_A2_R3_IgM

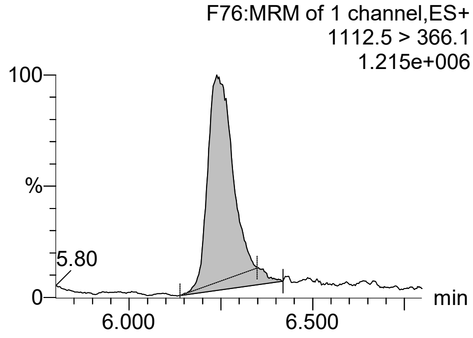

Sample Name: 20211111_L1_R2_IgM

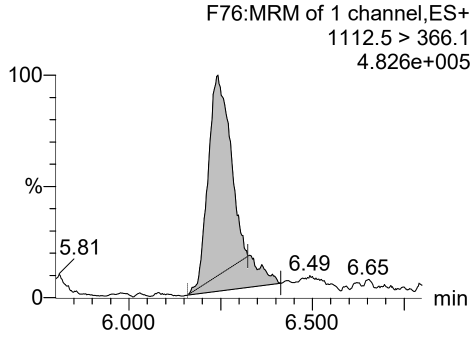

Sample Name: 20211111_L1_R3_IgM

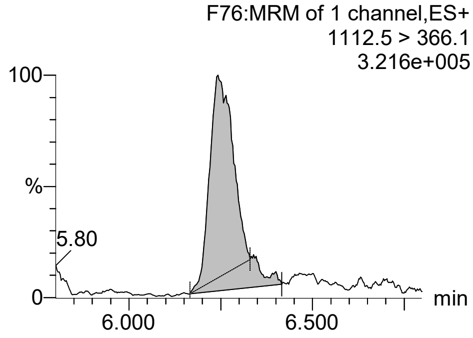

Sample Name: 20211111_L2_R2_IgM

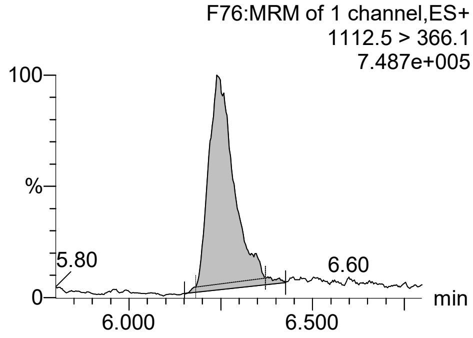

Sample Name: 20211111_H1_R2_IgM

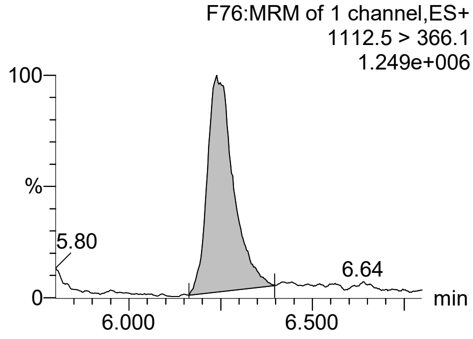

Sample Name: 20211111_A3_R2_IgM

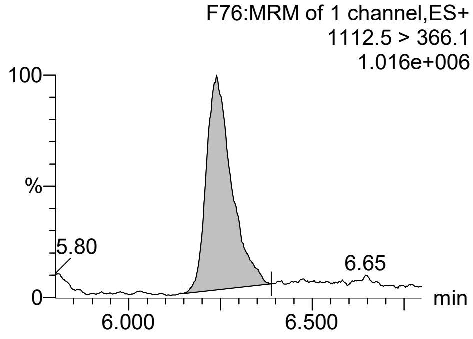

Sample Name: 20211111_A4_R2_IgM

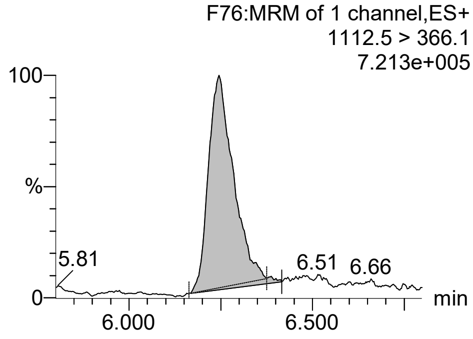

Dataset: C:\MassLynx\10.pro\20211111_clinicalsamples_IgM_IgA.qld

Last Altered: Thursday, March 09, 2023 19:58:25 台北標準時間
Printed: Friday, August 01, 2025 15:22:23 台北標準時間

Sample Name: 20211111_L3_R2_IgM
F76:MRM of 1 channel,ES+
1112.5 > 366.1
6.746e+005

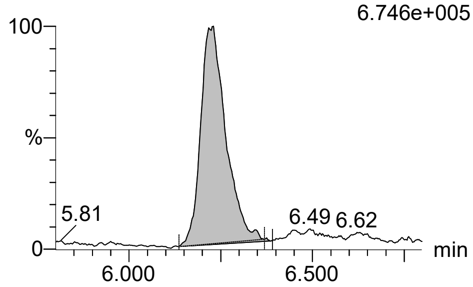

Sample Name: 20211111_L4_R2_IgM
F76:MRM of 1 channel,ES+
1112.5 > 366.1
8.185e+005

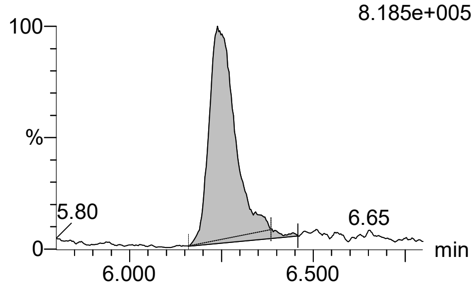

Sample Name: 20211111_H2_R2_IgM
F76:MRM of 1 channel,ES+
1112.5 > 366.1
1.702e+006

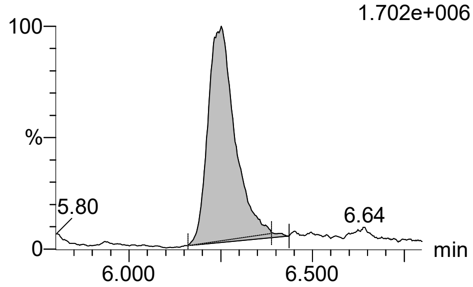

Sample Name: 20211111_A5_R2_IgM
F76:MRM of 1 channel,ES+
1112.5 > 366.1
2.040e+006

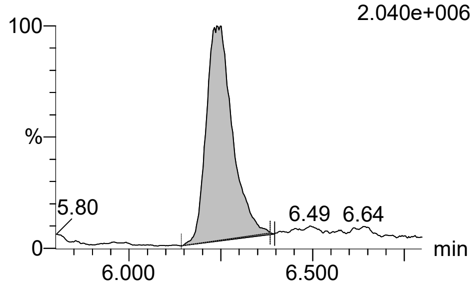

Sample Name: 20211111_A6_R2_IgM
F76:MRM of 1 channel,ES+
1112.5 > 366.1
8.432e+005

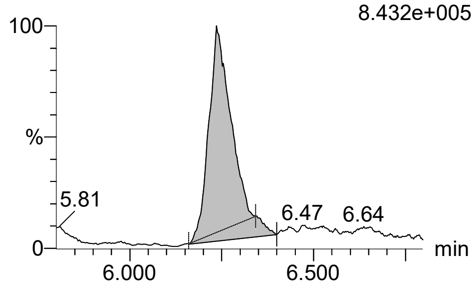

Sample Name: 20211111_QC_R10_IgM
F76:MRM of 1 channel,ES+
1112.5 > 366.1
7.241e+005

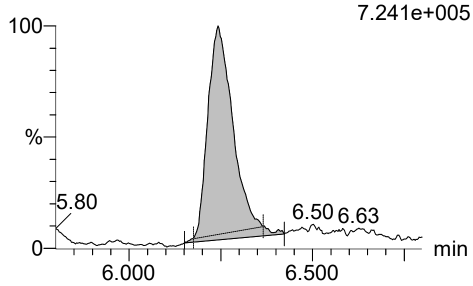

Sample Name: 20211111_L5_R2_IgM
F76:MRM of 1 channel,ES+
1112.5 > 366.1
6.425e+005

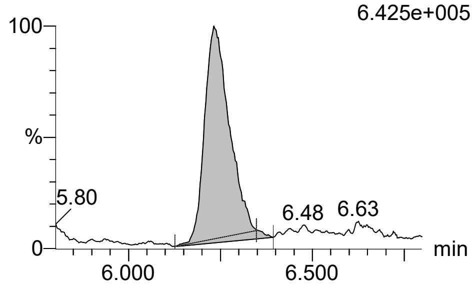

Sample Name: 20211111_L6_R2_IgM
F76:MRM of 1 channel,ES+
1112.5 > 366.1
7.879e+005

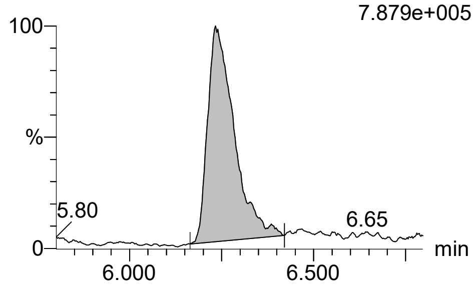

Sample Name: 20211111_H3_R2_IgM
F76:MRM of 1 channel,ES+
1112.5 > 366.1
2.803e+005

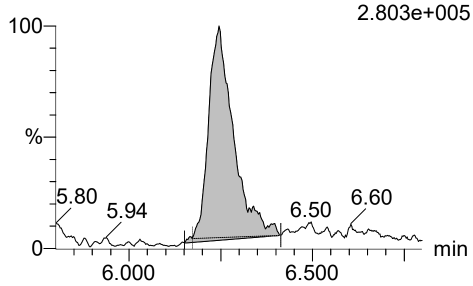

Sample Name: 20211111_A7_R2_IgM
F76:MRM of 1 channel,ES+
1112.5 > 366.1
5.766e+005

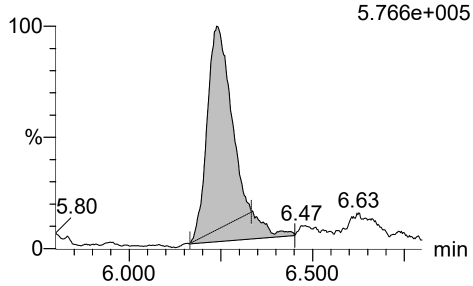

Sample Name: 20211111_A8_R2_IgM
F76:MRM of 1 channel,ES+
1112.5 > 366.1
8.957e+005

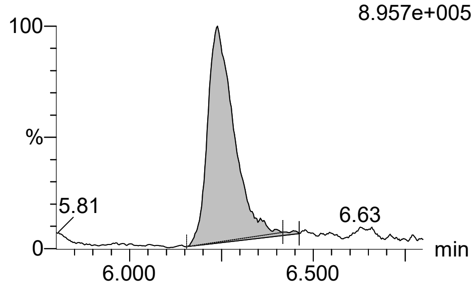

Sample Name: 20211111_L7_R2_IgM
F76:MRM of 1 channel,ES+
1112.5 > 366.1
5.327e+005

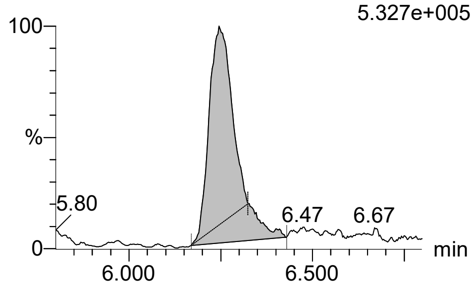

Sample Name: 20211111_L8_R2_IgM
F76:MRM of 1 channel,ES+
1112.5 > 366.1
2.328e+005

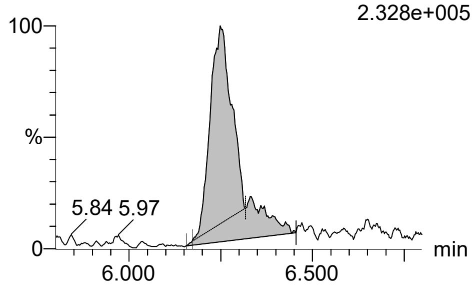

Sample Name: 20211111_H4_R2_IgM
F76:MRM of 1 channel,ES+
1112.5 > 366.1
4.013e+005

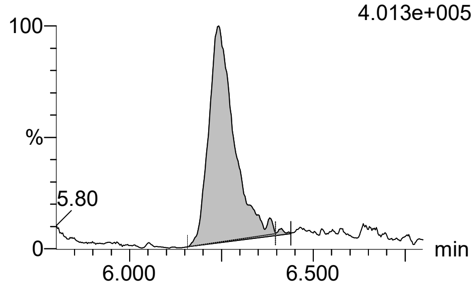

Sample Name: 20211111_A9_R2_IgM
F76:MRM of 1 channel,ES+
1112.5 > 366.1
4.363e+005

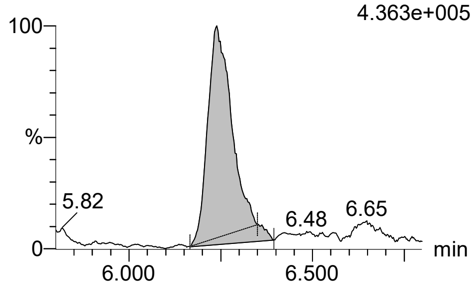

Sample Name: 20211111_A10_R2_IgM
F76:MRM of 1 channel,ES+
1112.5 > 366.1
1.075e+006

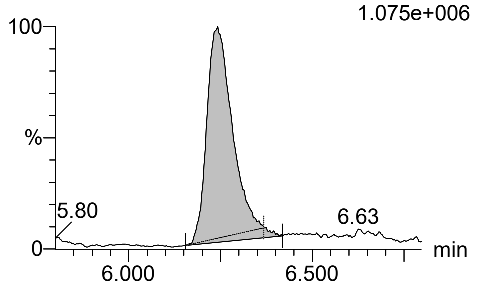

Sample Name: 20211111_L9_R2_IgM
F76:MRM of 1 channel,ES+
1112.5 > 366.1
6.040e+005

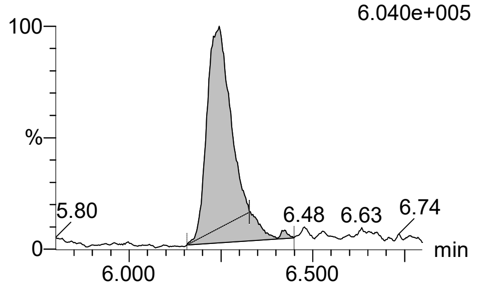

Sample Name: 20211111_L10_R2_IgM
F76:MRM of 1 channel,ES+
1112.5 > 366.1
6.565e+005

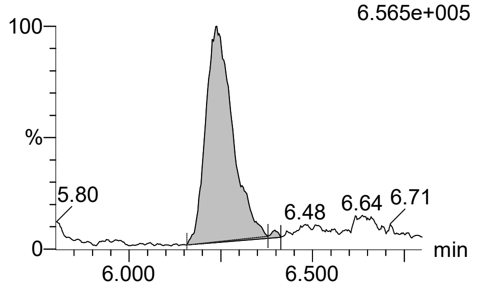

Sample Name: 20211111_QC_R11_IgM
F76:MRM of 1 channel,ES+
1112.5 > 366.1
7.541e+005

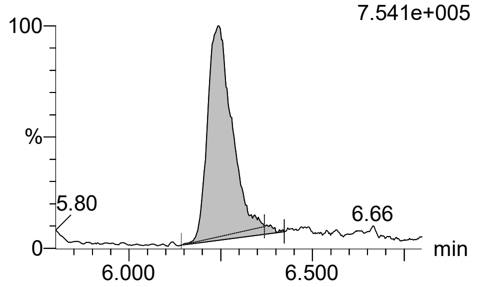

Sample Name: 20211111_H5_R2_IgM
F76:MRM of 1 channel,ES+
1112.5 > 366.1
9.131e+005

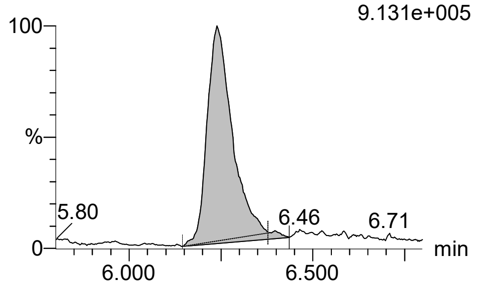

Sample Name: 20211111_A11_R2_IgM
F76:MRM of 1 channel,ES+
1112.5 > 366.1
1.009e+006

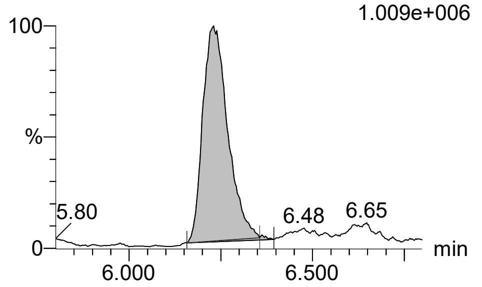

Sample Name: 20211111_A12_R2_IgM
F76:MRM of 1 channel,ES+
1112.5 > 366.1
4.337e+005

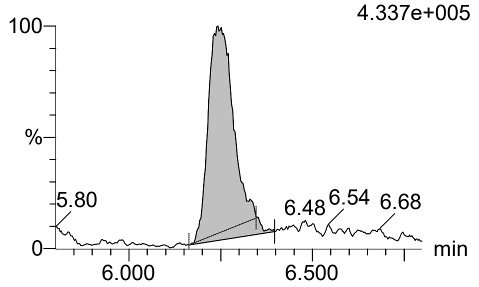

Sample Name: 20211111_L11_R2_IgM
F76:MRM of 1 channel,ES+
1112.5 > 366.1
5.262e+005

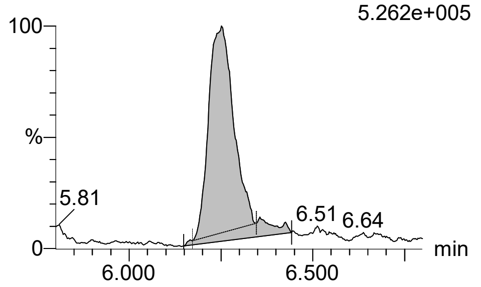

Sample Name: 20211111_L12_R2_IgM
F76:MRM of 1 channel,ES+
1112.5 > 366.1
3.642e+005

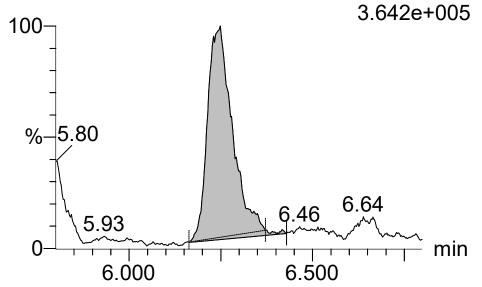

Sample Name: 20211111_H6_R2_IgM
F76:MRM of 1 channel,ES+
1112.5 > 366.1
3.043e+005

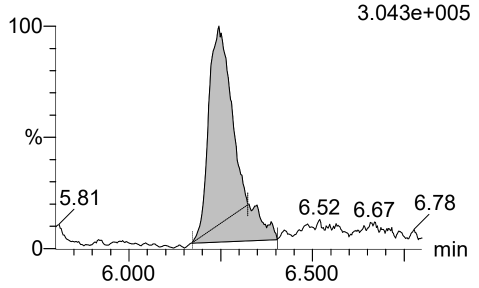

Sample Name: 20211111_A13_R2_IgM
F76:MRM of 1 channel,ES+
1112.5 > 366.1
4.798e+005

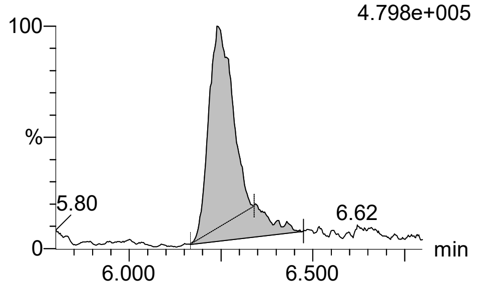

Sample Name: 20211111_A14_R2_IgM
F76:MRM of 1 channel,ES+
1112.5 > 366.1
1.945e+006

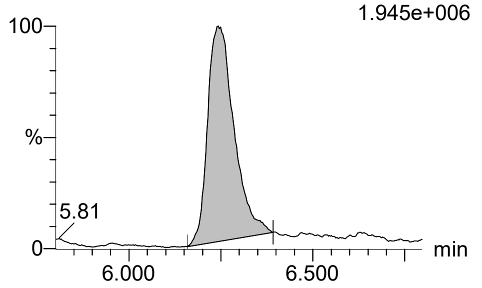

Sample Name: 20211111_L13_R2_IgM
F76:MRM of 1 channel,ES+
1112.5 > 366.1
5.478e+005

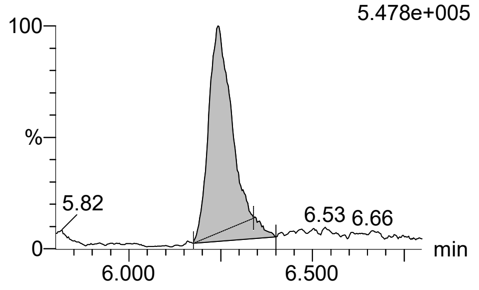

Sample Name: 20211111_L14_R2_IgM
F76:MRM of 1 channel,ES+
1112.5 > 366.1
9.675e+005

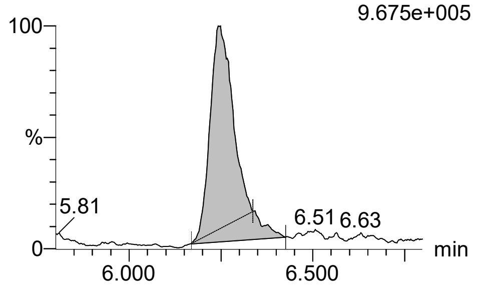

Sample Name: 20211111_H7_R2_IgM
F76:MRM of 1 channel,ES+
1112.5 > 366.1
7.589e+005

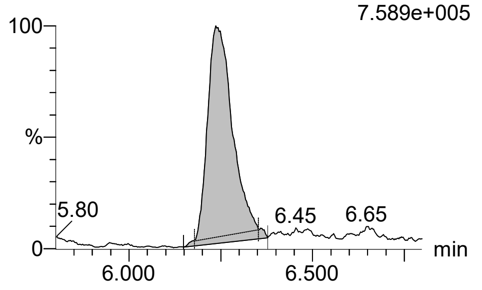

Sample Name: 20211111_A15_R2_IgM
F76:MRM of 1 channel,ES+
1112.5 > 366.1
2.013e+006

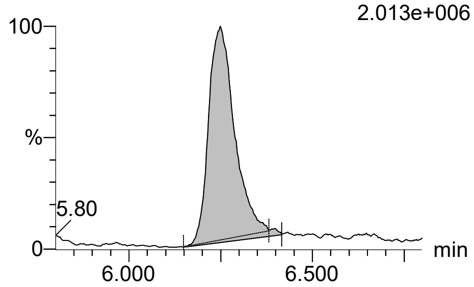

Sample Name: 20211111_QC_R12_IgM
F76:MRM of 1 channel,ES+
1112.5 > 366.1
9.054e+005

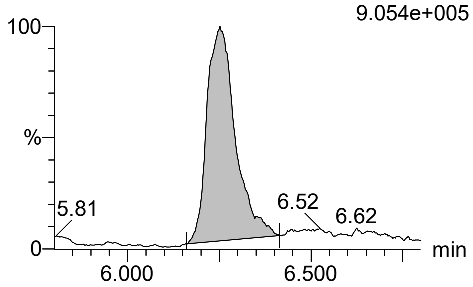

Sample Name: 20211111_A16_R2_IgM
F76:MRM of 1 channel,ES+
1112.5 > 366.1
1.020e+006

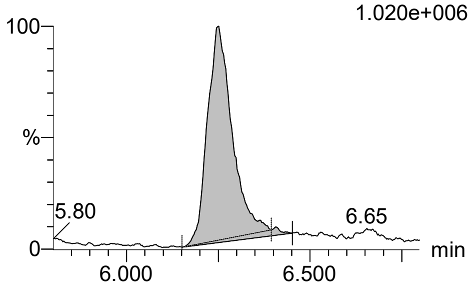

Sample Name: 20211111_L15_R2_IgM
F76:MRM of 1 channel,ES+
1112.5 > 366.1
1.025e+006

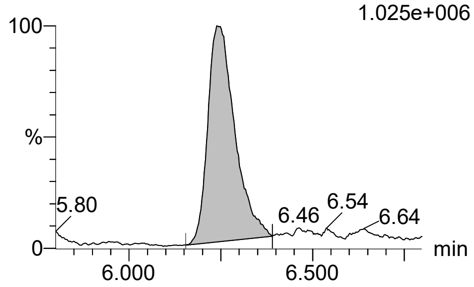

Sample Name: 20211111_L16_R2_IgM
F76:MRM of 1 channel,ES+
1112.5 > 366.1
3.296e+005

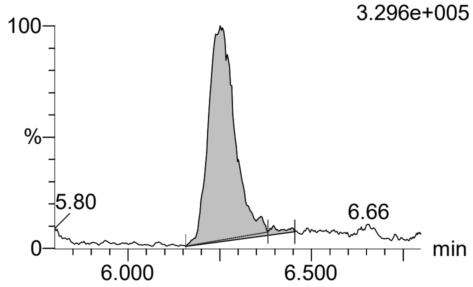

Sample Name: 20211111_H8_R2_IgM
F76:MRM of 1 channel,ES+
1112.5 > 366.1
4.256e+005

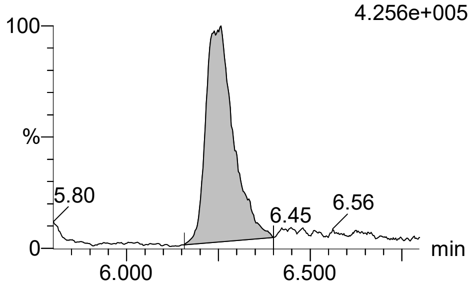

Sample Name: 20211111_A17_R2_IgM
F76:MRM of 1 channel,ES+
1112.5 > 366.1
9.813e+005

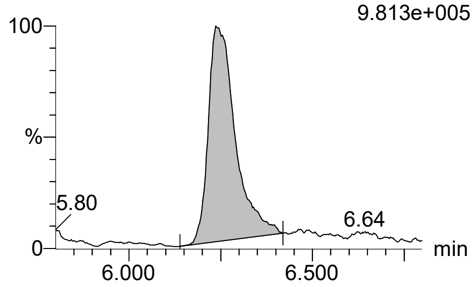

Sample Name: 20211111_A18_R2_IgM
F76:MRM of 1 channel,ES+
1112.5 > 366.1
4.326e+005

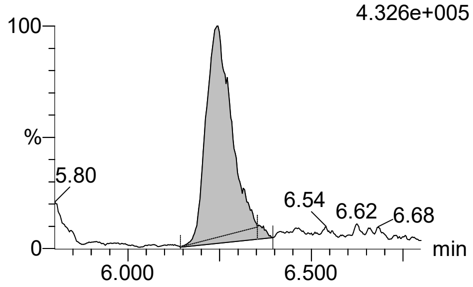

Sample Name: 20211111_L17_R2_IgM
F76:MRM of 1 channel,ES+
1112.5 > 366.1
1.377e+006

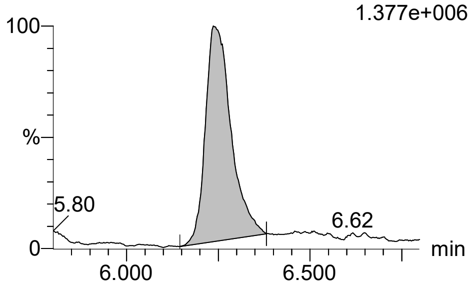

Sample Name: 20211111_L18_R2_IgM
F76:MRM of 1 channel,ES+
1112.5 > 366.1
3.927e+005

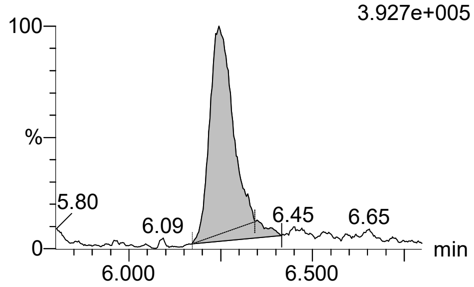

Sample Name: 20211111_H9_R2_IgM
F76:MRM of 1 channel,ES+
1112.5 > 366.1
3.821e+005

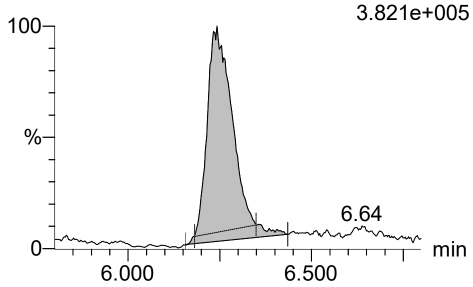

Sample Name: 20211111_A19_R2_IgM
F76:MRM of 1 channel,ES+
1112.5 > 366.1
7.505e+005

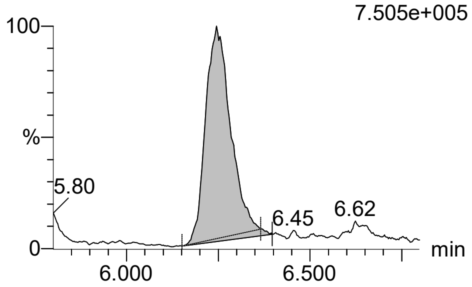

Sample Name: 20211111_A20_R2_IgM
F76:MRM of 1 channel,ES+
1112.5 > 366.1
3.438e+005

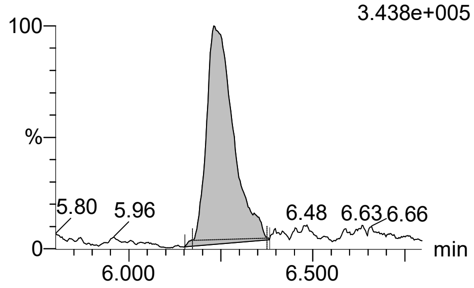

Sample Name: 20211111_L19_R2_IgM
F76:MRM of 1 channel,ES+
1112.5 > 366.1
6.084e+005

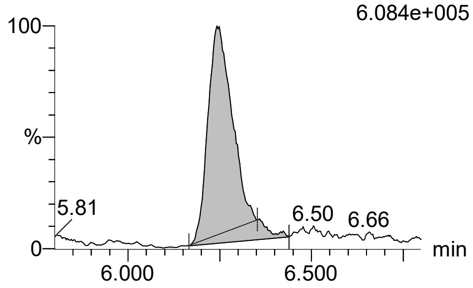

Sample Name: 20211111_QC_R13_IgM
F76:MRM of 1 channel,ES+
1112.5 > 366.1
7.759e+005

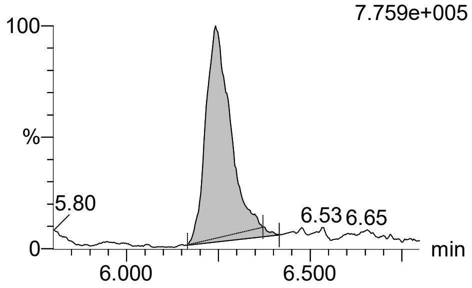

Sample Name: 20211111_L20_R2_IgM
F76:MRM of 1 channel,ES+
1112.5 > 366.1
5.197e+005

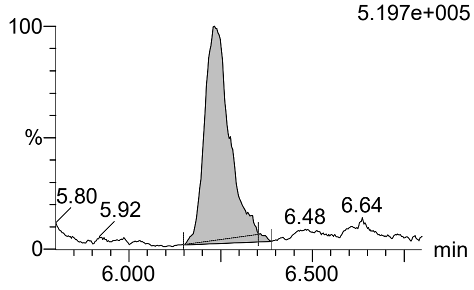

Sample Name: 20211111_H10_R2_IgM
F76:MRM of 1 channel,ES+
1112.5 > 366.1
6.834e+005

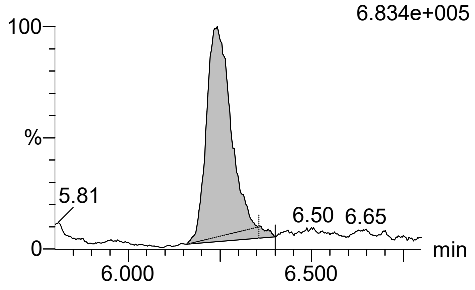

Sample Name: 20211111_A21_R2_IgM
F76:MRM of 1 channel,ES+
1112.5 > 366.1
3.931e+005

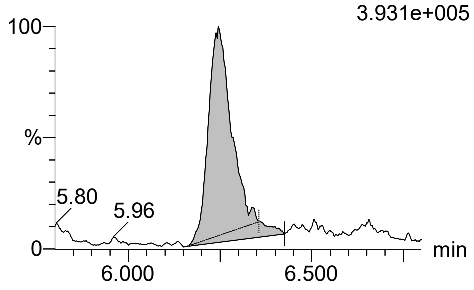

Sample Name: 20211111_A22_R2_IgM
F76:MRM of 1 channel,ES+
1112.5 > 366.1
1.666e+005

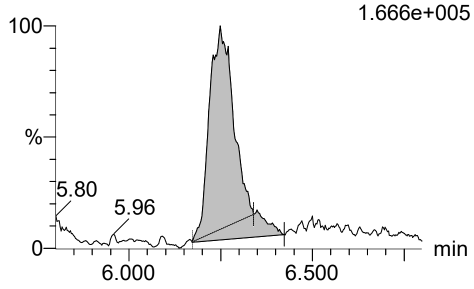

Sample Name: 20211111_L21_R2_IgM
F76:MRM of 1 channel,ES+
1112.5 > 366.1
5.785e+005

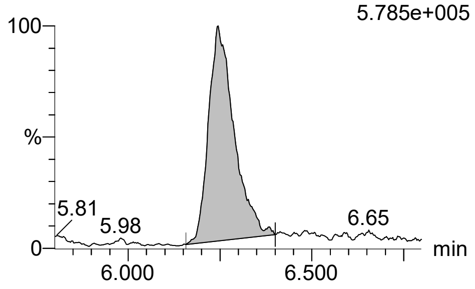

Sample Name: 20211111_L22_R2_IgM
F76:MRM of 1 channel,ES+
1112.5 > 366.1
3.610e+005

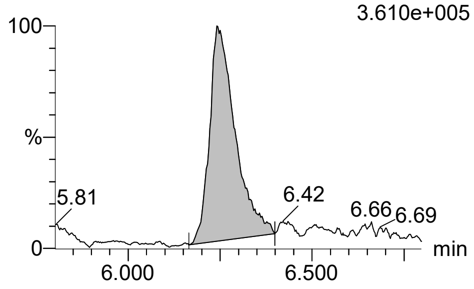

Sample Name: 20211111_H11_R2_IgM
F76:MRM of 1 channel,ES+
1112.5 > 366.1
6.151e+005

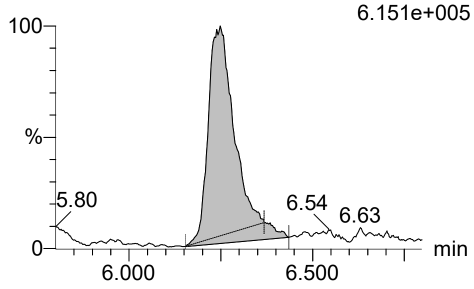

Sample Name: 20211111_A23_R2_IgM
F76:MRM of 1 channel,ES+
1112.5 > 366.1
4.190e+005

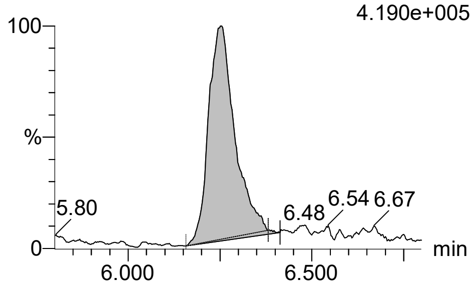

Sample Name: 20211111_A24_R2_IgM
F76:MRM of 1 channel,ES+
1112.5 > 366.1
8.628e+005

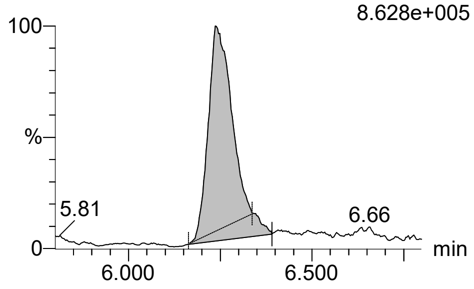

Sample Name: 20211111_L23_R2_IgM
F76:MRM of 1 channel,ES+
1112.5 > 366.1
1.136e+006

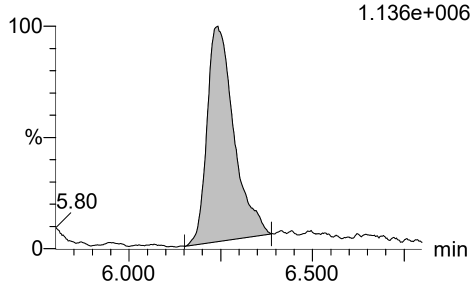

Sample Name: 20211111_L24_R2_IgM
F76:MRM of 1 channel,ES+
1112.5 > 366.1
2.950e+005

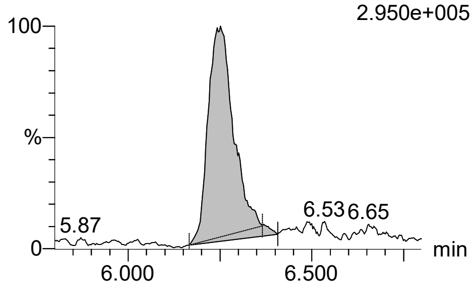

Sample Name: 20211111_H12_R2_IgM
F76:MRM of 1 channel,ES+
1112.5 > 366.1
3.043e+005

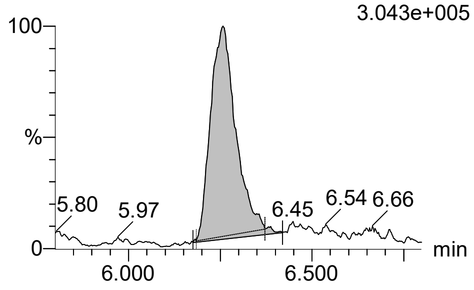

Sample Name: 20211111_QC_R14_IgM
F76:MRM of 1 channel,ES+
1112.5 > 366.1
8.144e+005

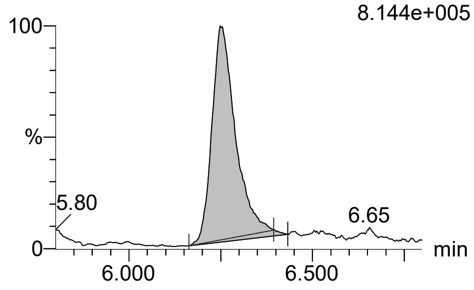

Sample Name: 20211111_A25_R2_IgM
F76:MRM of 1 channel,ES+
1112.5 > 366.1
1.078e+006

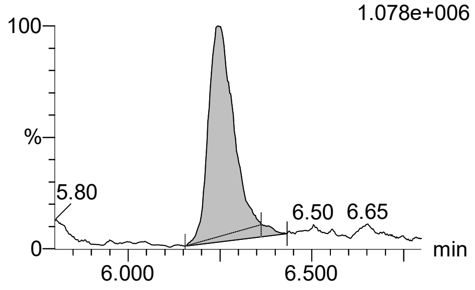

Sample Name: 20211111_A26_R2_IgM
F76:MRM of 1 channel,ES+
1112.5 > 366.1
5.766e+005

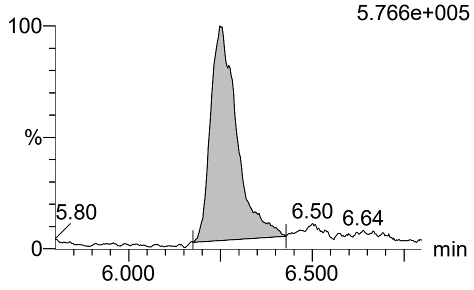

Sample Name: 20211111_L25_R2_IgM
F76:MRM of 1 channel,ES+
1112.5 > 366.1
6.386e+005

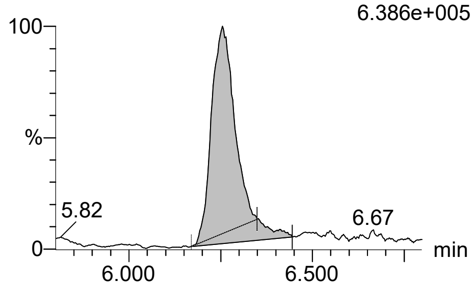

Sample Name: 20211111_L26_R2_IgM
F76:MRM of 1 channel,ES+
1112.5 > 366.1
6.778e+005

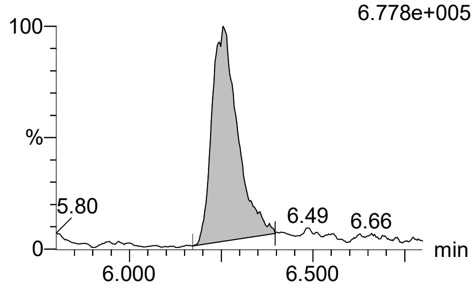

Sample Name: 20211111_H13_R2_IgM
F76:MRM of 1 channel,ES+
1112.5 > 366.1
8.062e+005

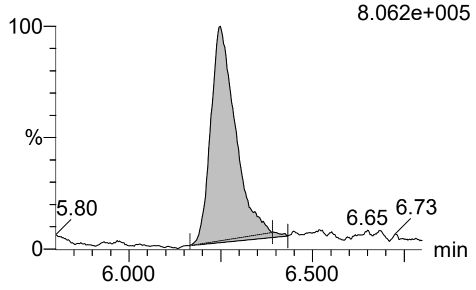

Sample Name: 20211111_A27_R2_IgM
F76:MRM of 1 channel,ES+
1112.5 > 366.1
1.001e+006

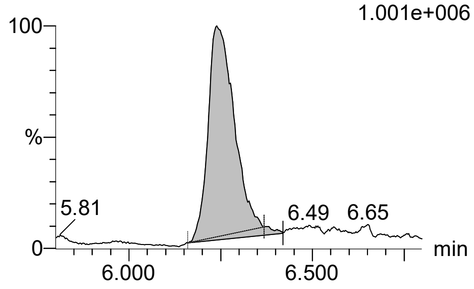

Sample Name: 20211111_A28_R2_IgM
F76:MRM of 1 channel,ES+
1112.5 > 366.1
4.179e+005

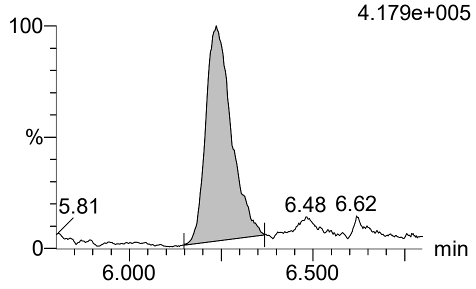

Sample Name: 20211111_L27_R2_IgM
F76:MRM of 1 channel,ES+
1112.5 > 366.1
5.167e+005

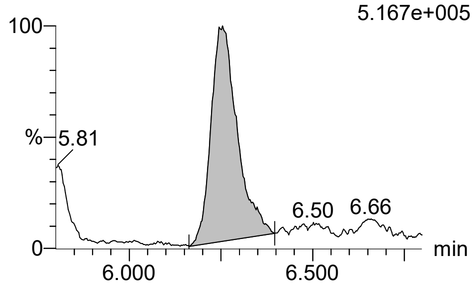

Sample Name: 20211111_L28_R2_IgM
F76:MRM of 1 channel,ES+
1112.5 > 366.1
3.066e+005

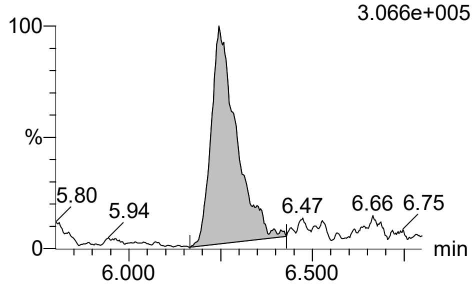

Sample Name: 20211111_H14_R2_IgM
F76:MRM of 1 channel,ES+
1112.5 > 366.1
5.935e+005

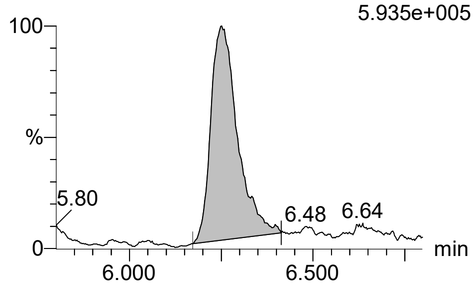

Sample Name: 20211111_A29_R2_IgM
F76:MRM of 1 channel,ES+
1112.5 > 366.1
1.914e+005

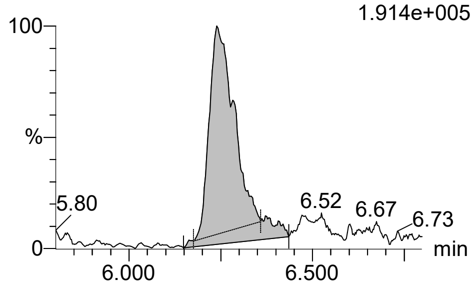

Sample Name: 20211111_A30_R2_IgM
F76:MRM of 1 channel,ES+
1112.5 > 366.1
1.047e+006

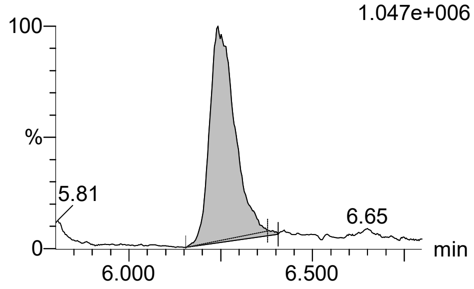

Sample Name: 20211111_QC_R15_IgM
F76:MRM of 1 channel,ES+
1112.5 > 366.1
7.533e+005

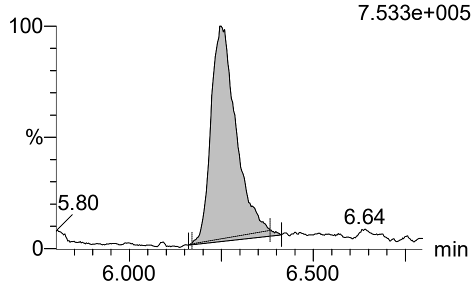

Sample Name: 20211111_L29_R2_IgM
F76:MRM of 1 channel,ES+
1112.5 > 366.1
3.938e+005

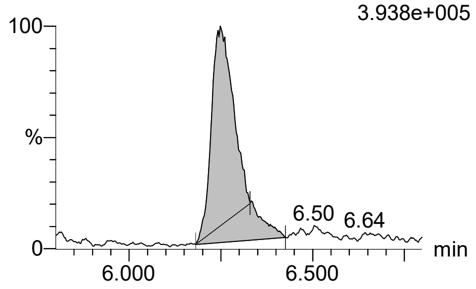

Sample Name: 20211111_L30_R2_IgM
F76:MRM of 1 channel,ES+
1112.5 > 366.1
2.077e+005

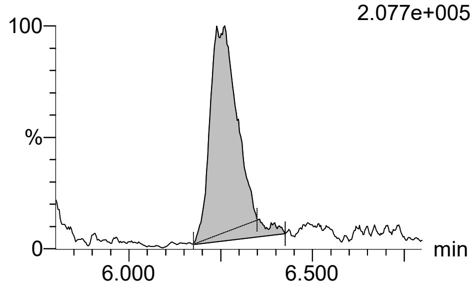

Sample Name: 20211111_H15_R2_IgM
F76:MRM of 1 channel,ES+
1112.5 > 366.1
4.601e+005

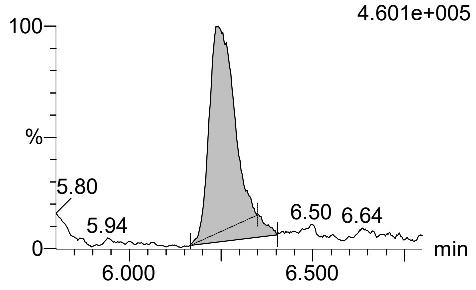

Sample Name: 20211111_A31_R2_IgM
F76:MRM of 1 channel,ES+
1112.5 > 366.1
3.512e+005

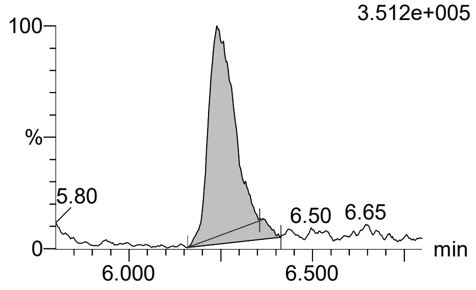

Sample Name: 20211111_A32_R2_IgM

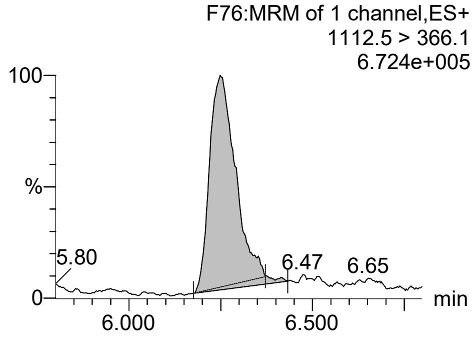

Sample Name: 20211111_L31_R2_IgM

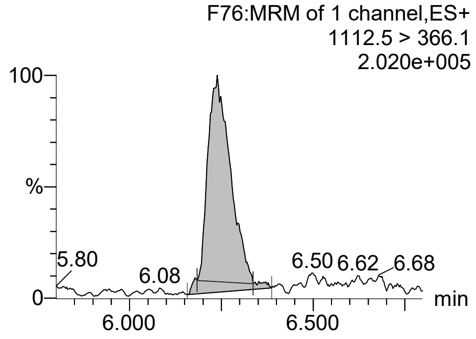

Sample Name: 20211111_L32_R2_IgM

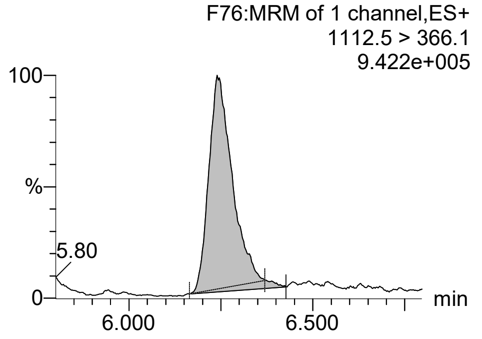

Sample Name: 20211111_H16_R2_IgM

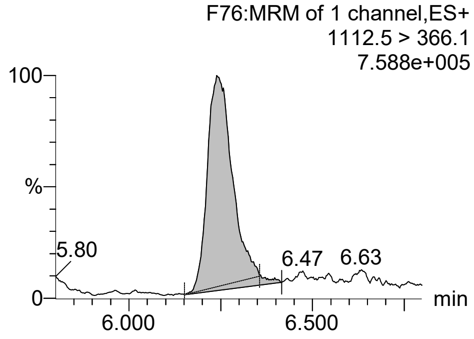

Sample Name: 20211111_A33_R2_IgM

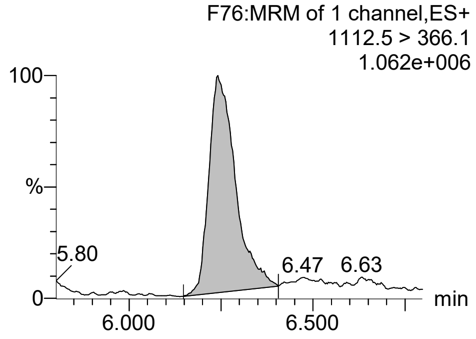

Sample Name: 20211111_A34_R2_IgM

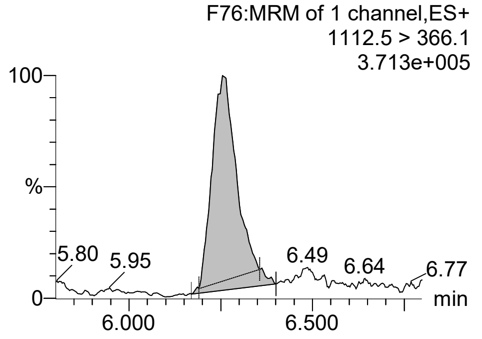

Sample Name: 20211111_H17_R2_IgM

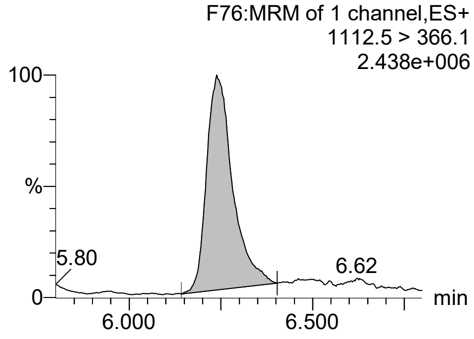

Sample Name: 20211111_A35_R2_IgM

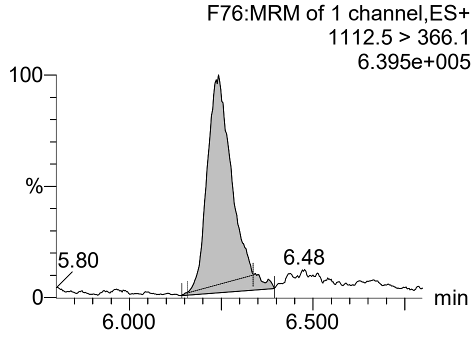

Sample Name: 20211111_QC_R16_IgM

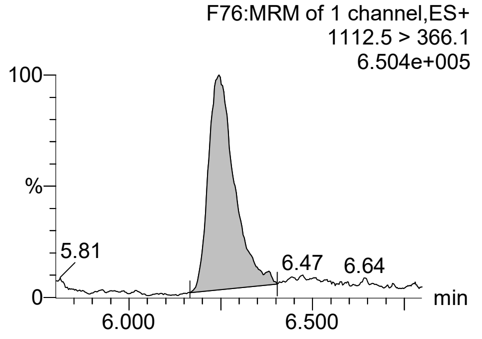

Sample Name: 20211111_A36_R2_IgM

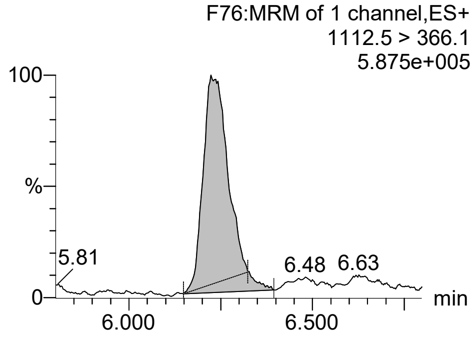

Sample Name: 20211111_H18_R2_IgM

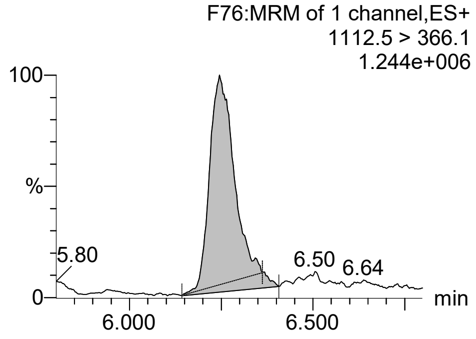

Sample Name: 20211111_A37_R2_IgM

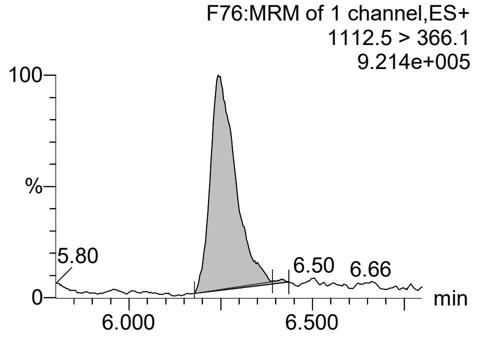

Sample Name: 20211111_A38_R2_IgM

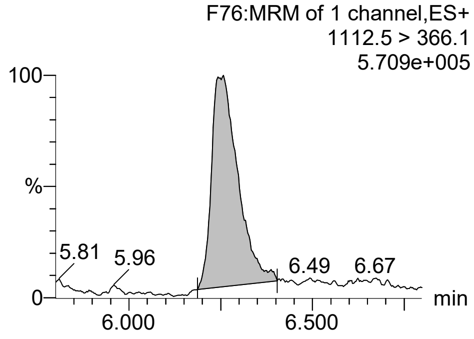

Sample Name: 20211111_H19_R2_IgM

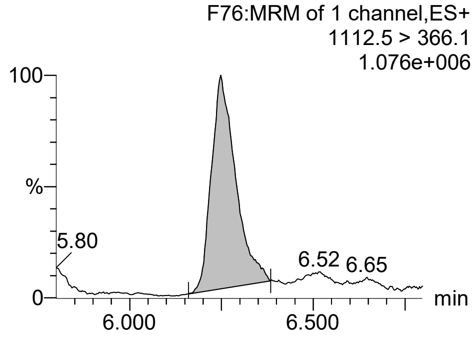

Sample Name: 20211111_A39_R2_IgM

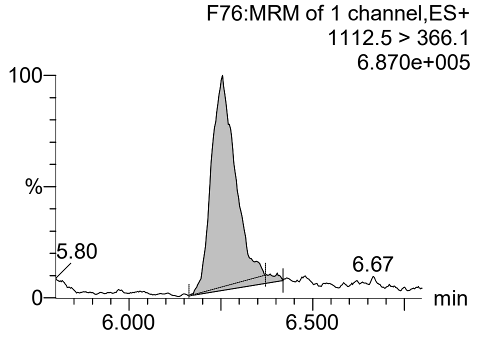

Dataset: C:\MassLynx\10.pro\20211111_clinicalsamples_IgM_IgA.qld

Last Altered: Thursday, March 09, 2023 19:58:25 台北標準時間
Printed: Friday, August 01, 2025 15:22:23 台北標準時間

Sample Name: 20211111_A40_R2_IgM

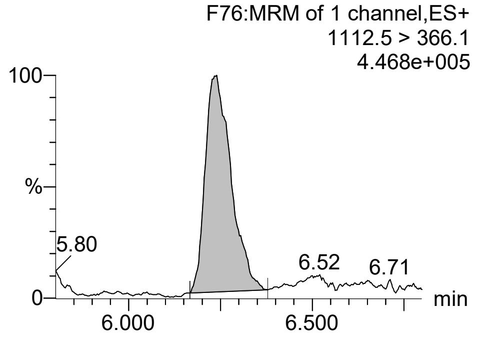

Sample Name: 20211111_H20_R2_IgM

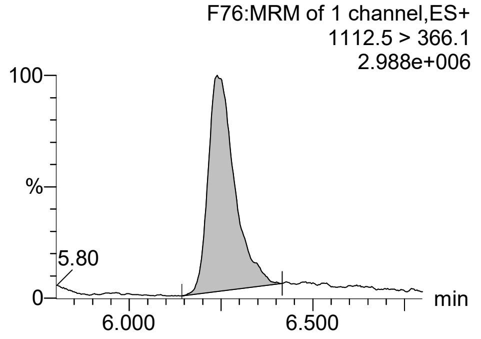

Sample Name: 20211111_A41_R2_IgM

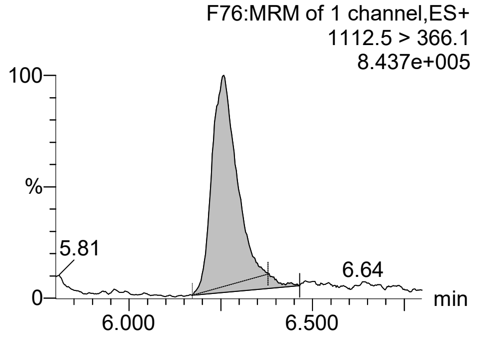

Sample Name: 20211111_A42_R2_IgM

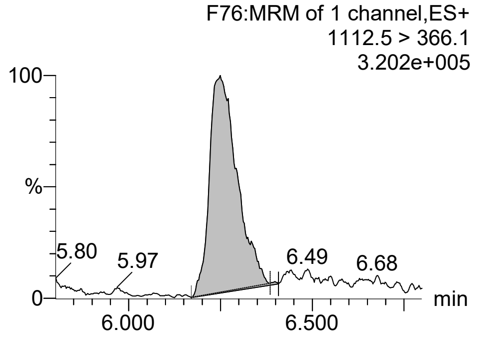

Sample Name: 20211111_A43_R2_IgM

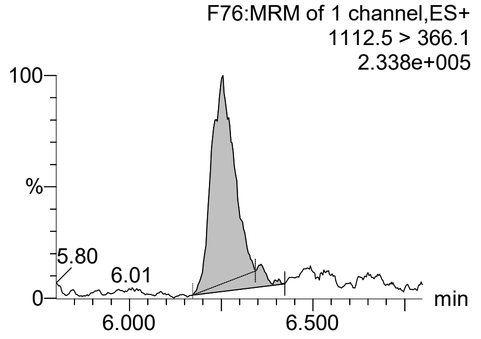

Sample Name: 20211111_A44_R2_IgM

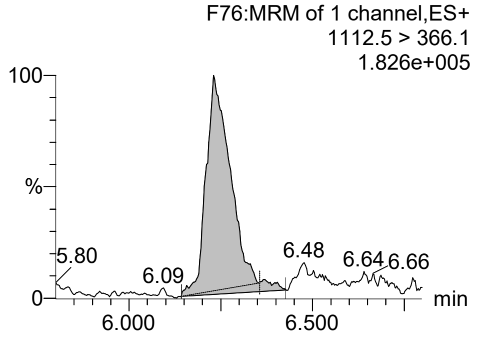

Sample Name: 20211111_QC_R17_IgM

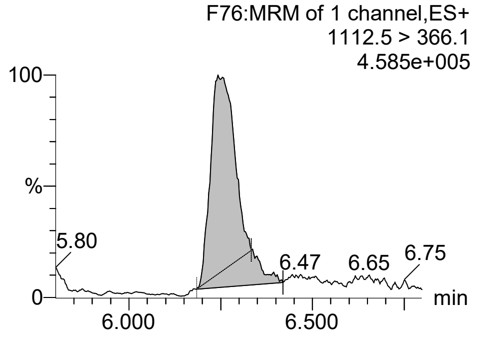

Sample Name: 20211111_L2_R3_IgM

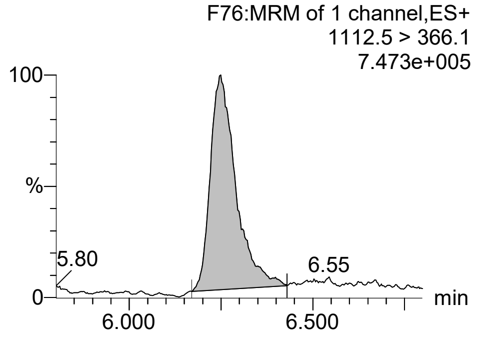

Sample Name: 20211111_H1_R3_IgM

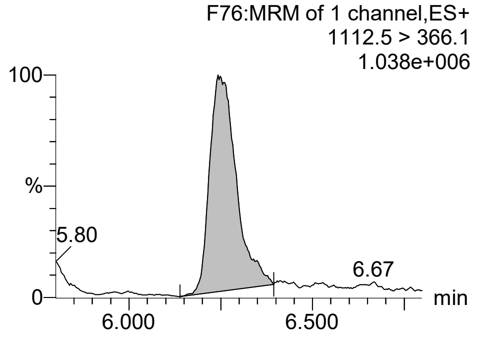

Sample Name: 20211111_A3_R3_IgM

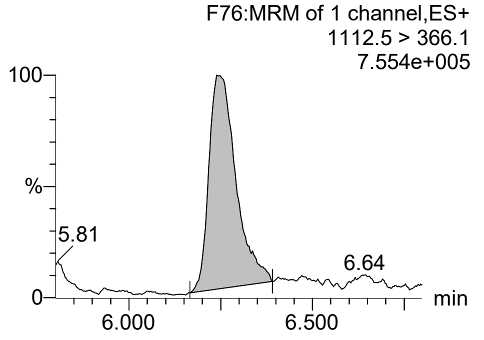

Sample Name: 20211111_A4_R3_IgM

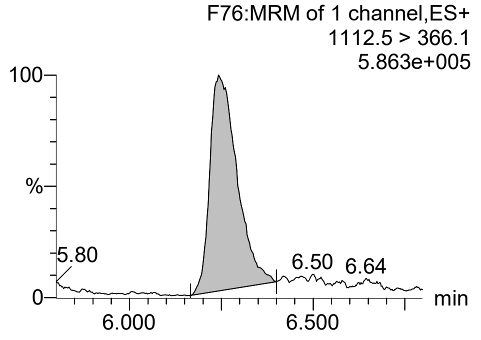

Sample Name: 20211111_L3_R3_IgM

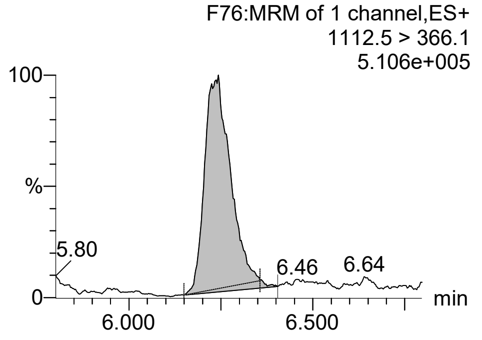

Sample Name: 20211111_L4_R3_IgM

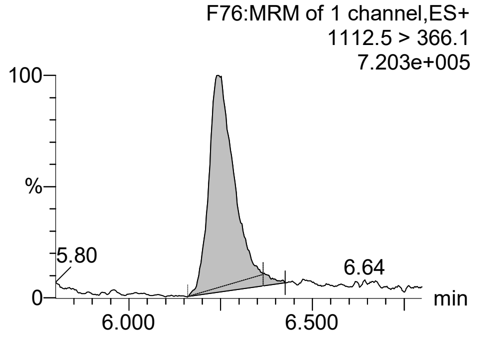

Sample Name: 20211111_H2_R3_IgM

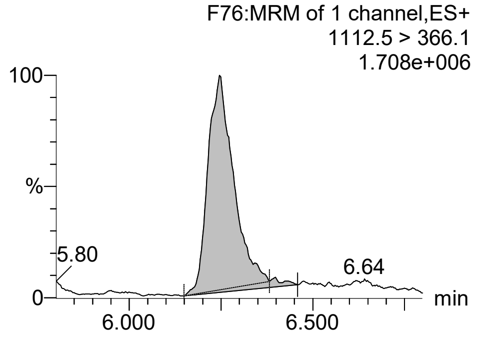

Sample Name: 20211111_A5_R3_IgM

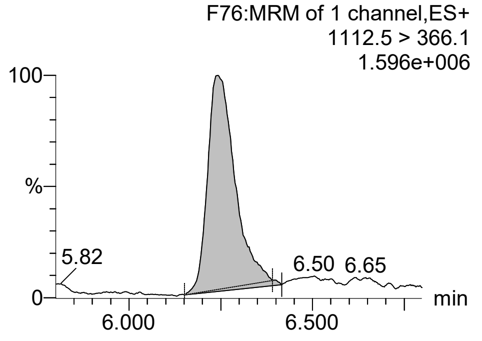

Sample Name: 20211111_A6_R3_IgM
F76:MRM of 1 channel,ES+
1112.5 > 366.1
6.710e+005

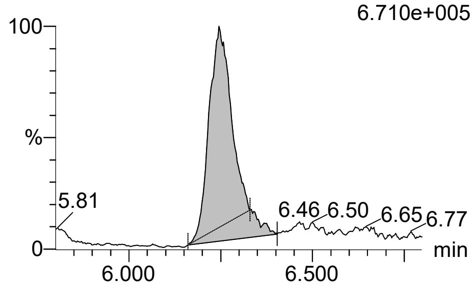

Sample Name: 20211111_QC_R18_IgM
F76:MRM of 1 channel,ES+
1112.5 > 366.1
6.351e+005

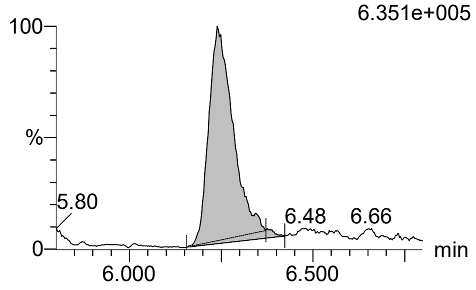

Sample Name: 20211111_L5_R3_IgM
F76:MRM of 1 channel,ES+
1112.5 > 366.1
4.728e+005

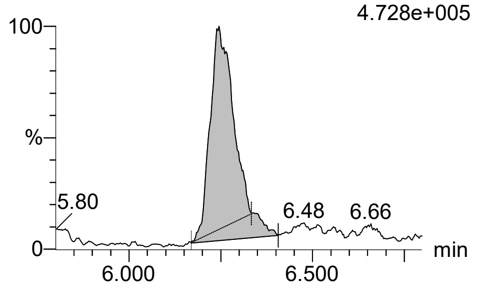

Sample Name: 20211111_L6_R3_IgM
F76:MRM of 1 channel,ES+
1112.5 > 366.1
6.111e+005

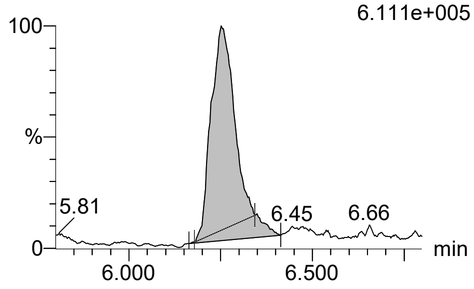

Sample Name: 20211111_H3_R3_IgM
F76:MRM of 1 channel,ES+
1112.5 > 366.1
2.217e+005

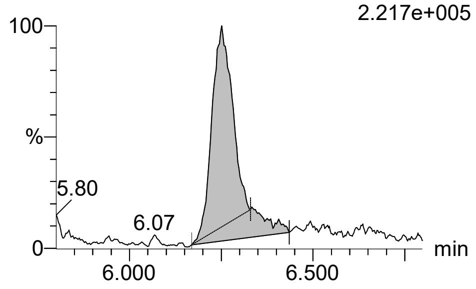

Sample Name: 20211111_A7_R3_IgM
F76:MRM of 1 channel,ES+
1112.5 > 366.1
5.711e+005

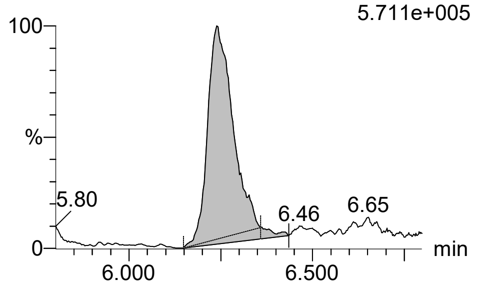

Sample Name: 20211111_A8_R3_IgM
F76:MRM of 1 channel,ES+
1112.5 > 366.1
6.863e+005

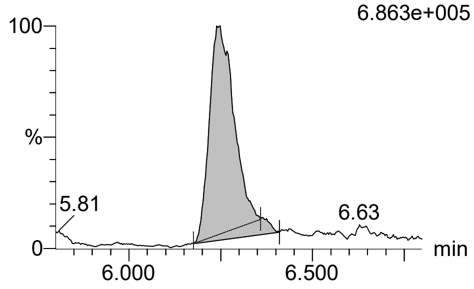

Sample Name: 20211111_L7_R3_IgM
F76:MRM of 1 channel,ES+
1112.5 > 366.1
4.735e+005

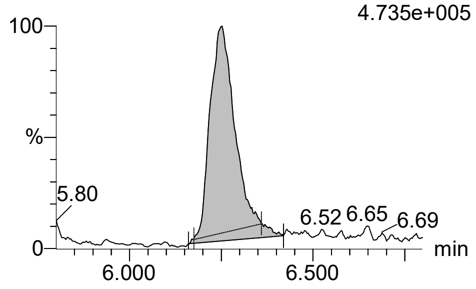

Sample Name: 20211111_L8_R3_IgM
F76:MRM of 1 channel,ES+
1112.5 > 366.1
1.951e+005

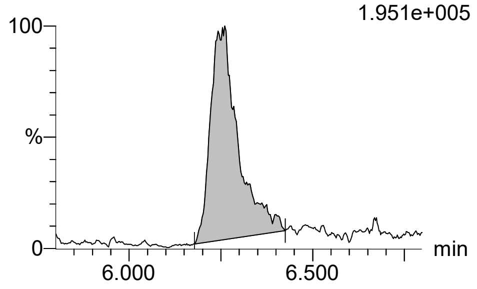

Sample Name: 20211111_H4_R3_IgM
F76:MRM of 1 channel,ES+
1112.5 > 366.1
2.906e+005

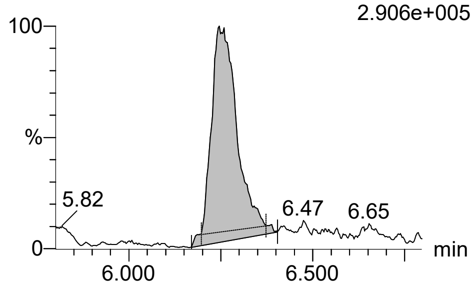

Sample Name: 20211111_A9_R3_IgM
F76:MRM of 1 channel,ES+
1112.5 > 366.1
3.251e+005

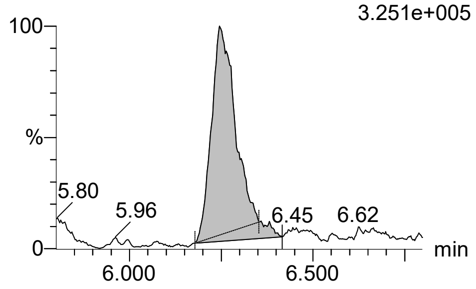

Sample Name: 20211111_A10_R3_IgM
F76:MRM of 1 channel,ES+
1112.5 > 366.1
9.415e+005

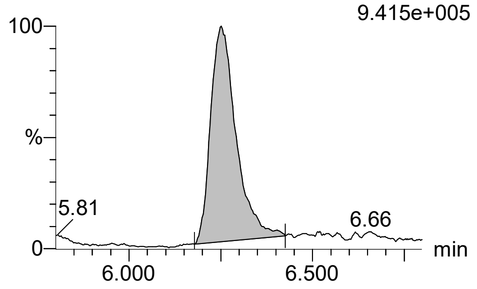

Sample Name: 20211111_L9_R3_IgM
F76:MRM of 1 channel,ES+
1112.5 > 366.1
5.615e+005

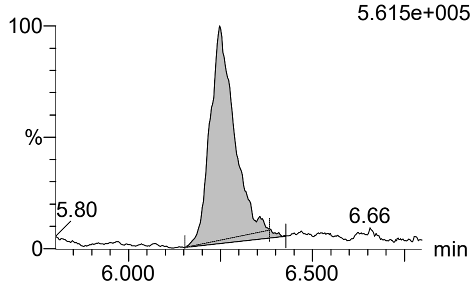

Sample Name: 20211111_L10_R3_IgM
F76:MRM of 1 channel,ES+
1112.5 > 366.1
5.431e+005

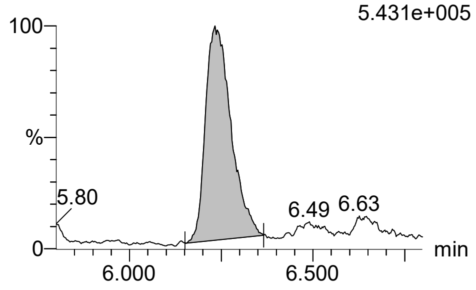

Sample Name: 20211111_QC_R19_IgM
F76:MRM of 1 channel,ES+
1112.5 > 366.1
5.717e+005

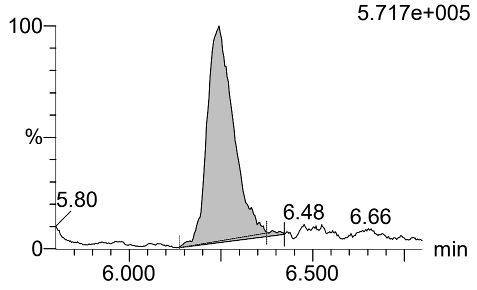

Sample Name: 20211111_H5_R3_IgM
F76:MRM of 1 channel,ES+
1112.5 > 366.1
6.392e+005

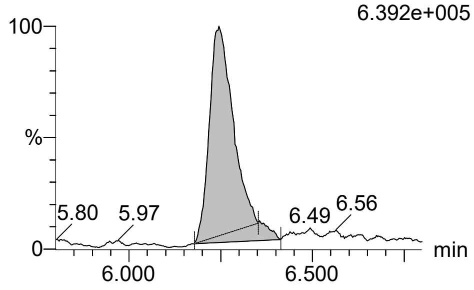

Sample Name: 20211111_A11_R3_IgM
F76:MRM of 1 channel,ES+
1112.5 > 366.1
6.550e+005

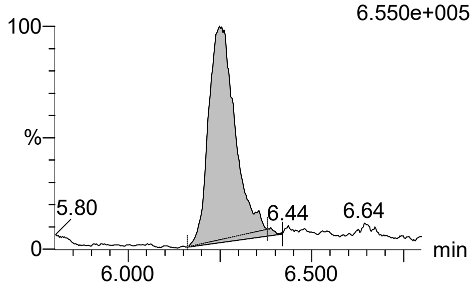

Sample Name: 20211111_A12_R3_IgM
F76:MRM of 1 channel,ES+
1112.5 > 366.1
4.253e+005

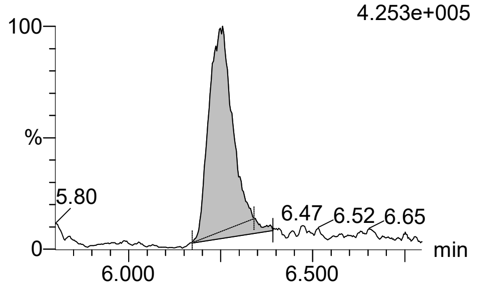

Sample Name: 20211111_L11_R3_IgM
F76:MRM of 1 channel,ES+
1112.5 > 366.1
4.493e+005

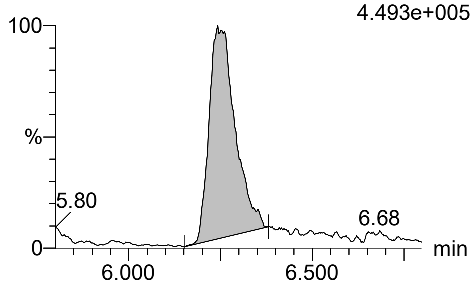

Sample Name: 20211111_L12_R3_IgM
F76:MRM of 1 channel,ES+
1112.5 > 366.1
2.809e+005

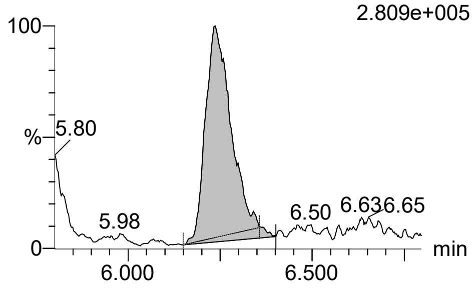

Sample Name: 20211111_H6_R3_IgM
F76:MRM of 1 channel,ES+
1112.5 > 366.1
2.620e+005

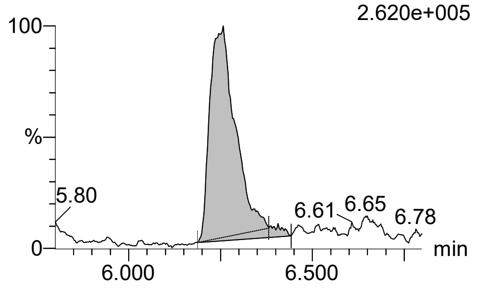

Sample Name: 20211111_A13_R3_IgM
F76:MRM of 1 channel,ES+
1112.5 > 366.1
4.523e+005

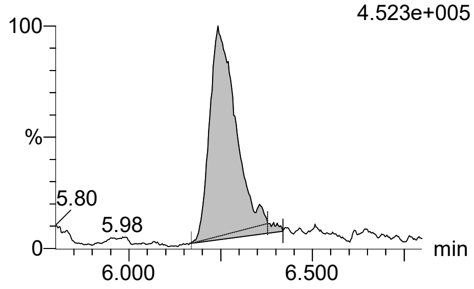

Sample Name: 20211111_A14_R3_IgM
F76:MRM of 1 channel,ES+
1112.5 > 366.1
1.620e+006

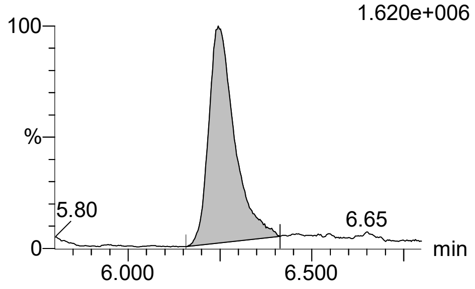

Sample Name: 20211111_L13_R3_IgM
F76:MRM of 1 channel,ES+
1112.5 > 366.1
4.161e+005

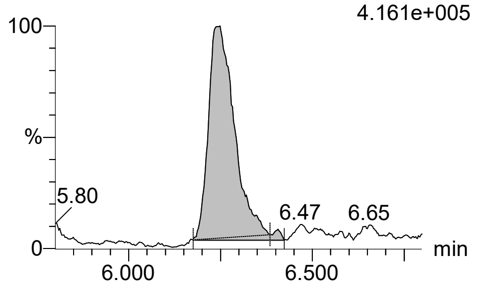

Sample Name: 20211111_L14_R3_IgM
F76:MRM of 1 channel,ES+
1112.5 > 366.1
7.586e+005

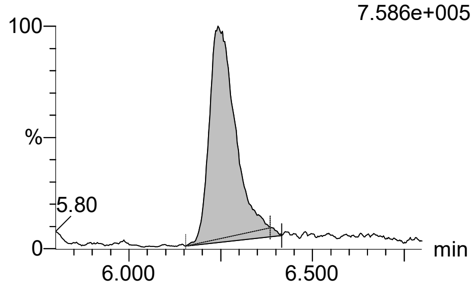

Sample Name: 20211111_H7_R3_IgM
F76:MRM of 1 channel,ES+
1112.5 > 366.1
6.071e+005

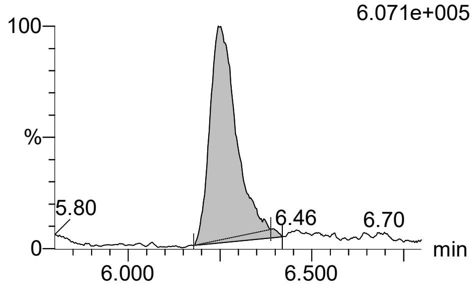

Sample Name: 20211111_A15_R3_IgM
F76:MRM of 1 channel,ES+
1112.5 > 366.1
1.644e+006

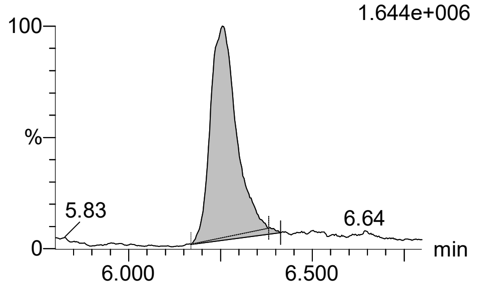

Sample Name: 20211111_QC_R20_IgM
F76:MRM of 1 channel,ES+
1112.5 > 366.1
8.691e+005

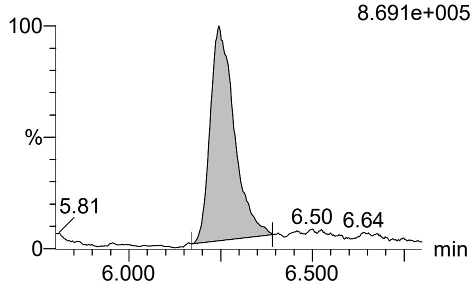

Sample Name: 20211111_A16_R3_IgM
F76:MRM of 1 channel,ES+
1112.5 > 366.1
8.403e+005

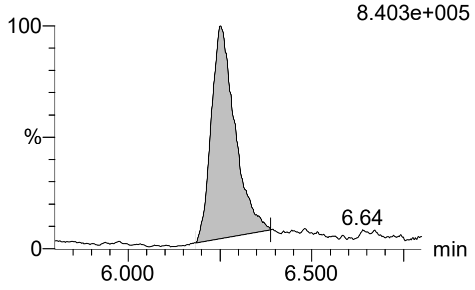

Sample Name: 20211111_L15_R3_IgM
F76:MRM of 1 channel,ES+
1112.5 > 366.1
9.303e+005

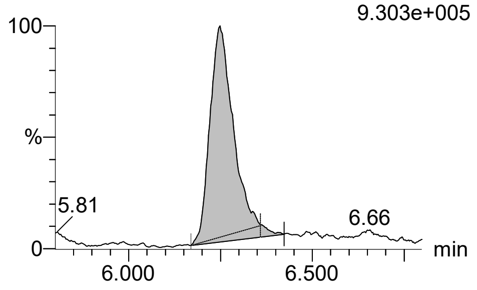

Sample Name: 20211111_L16_R3_IgM
F76:MRM of 1 channel,ES+
1112.5 > 366.1
2.681e+005

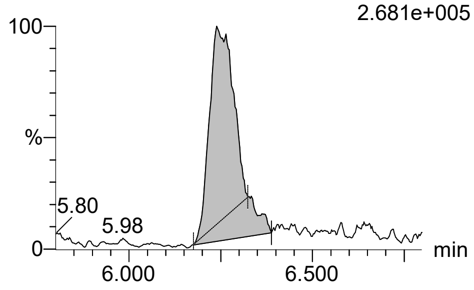

Sample Name: 20211111_H8_R3_IgM
F76:MRM of 1 channel,ES+
1112.5 > 366.1
3.699e+005

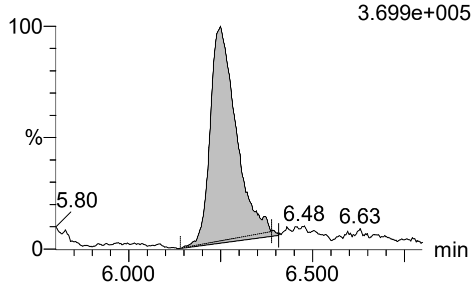

Sample Name: 20211111_A17_R3_IgM
F76:MRM of 1 channel,ES+
1112.5 > 366.1
8.815e+005

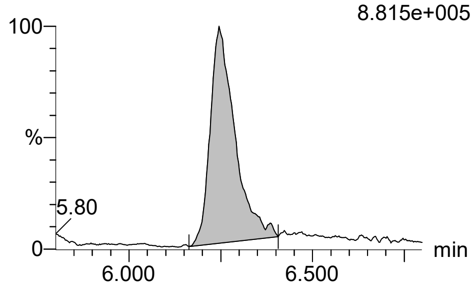

Sample Name: 20211111_A18_R3_IgM
F76:MRM of 1 channel,ES+
1112.5 > 366.1
3.707e+005

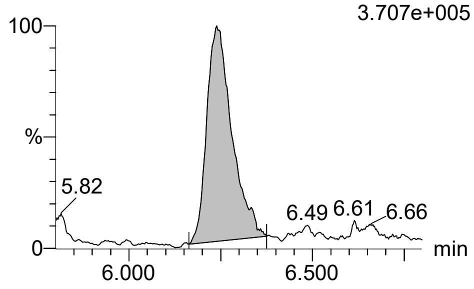

Sample Name: 20211111_L17_R3_IgM
F76:MRM of 1 channel,ES+
1112.5 > 366.1
1.176e+006

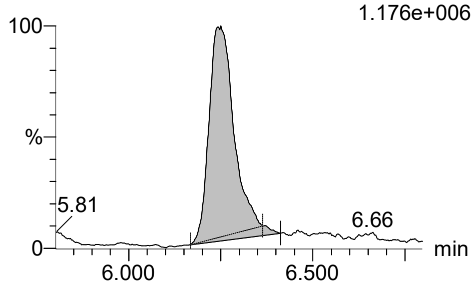

Sample Name: 20211111_L18_R3_IgM
F76:MRM of 1 channel,ES+
1112.5 > 366.1
3.169e+005

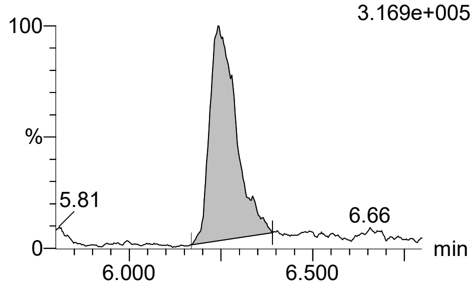

Sample Name: 20211111_H9_R3_IgM
F76:MRM of 1 channel,ES+
1112.5 > 366.1
2.980e+005

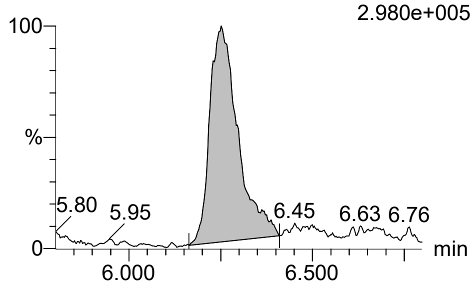

Sample Name: 20211111_A19_R3_IgM
F76:MRM of 1 channel,ES+
1112.5 > 366.1
6.832e+005

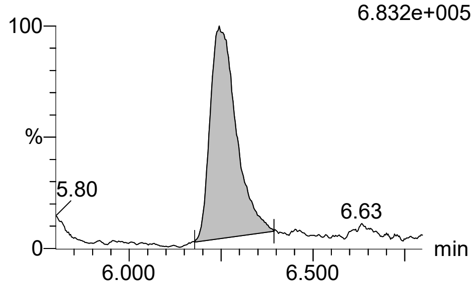

Sample Name: 20211111_A20_R3_IgM
F76:MRM of 1 channel,ES+
1112.5 > 366.1
3.349e+005

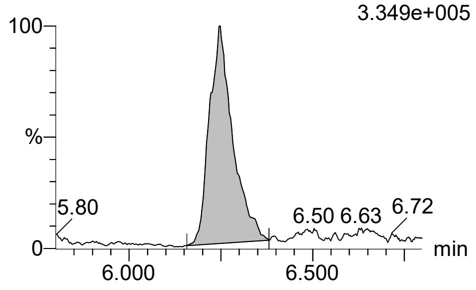

Sample Name: 20211111_L19_R3_IgM
F76:MRM of 1 channel,ES+
1112.5 > 366.1
6.741e+005

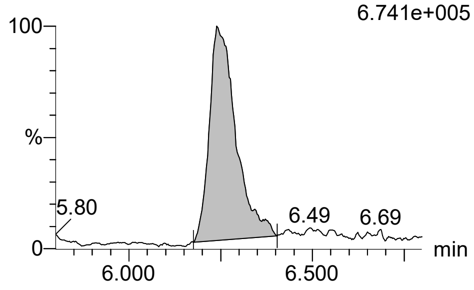

Sample Name: 20211111_QC_R21_IgM
F76:MRM of 1 channel,ES+
1112.5 > 366.1
6.159e+005

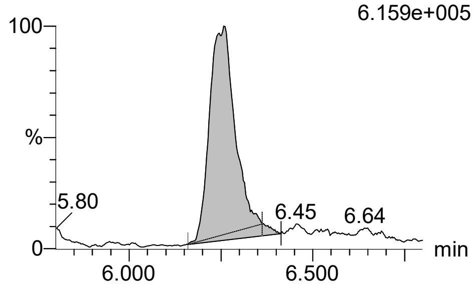

Sample Name: 20211111_L20_R3_IgM
F76:MRM of 1 channel,ES+
1112.5 > 366.1
4.188e+005

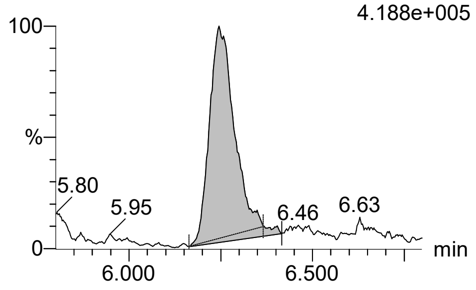

Sample Name: 20211111_H10_R3_IgM
F76:MRM of 1 channel,ES+
1112.5 > 366.1
5.530e+005

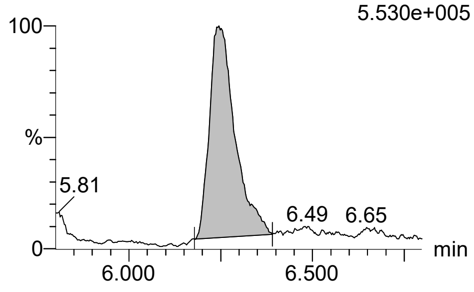

Sample Name: 20211111_A21_R3_IgM
F76:MRM of 1 channel,ES+
1112.5 > 366.1
3.072e+005

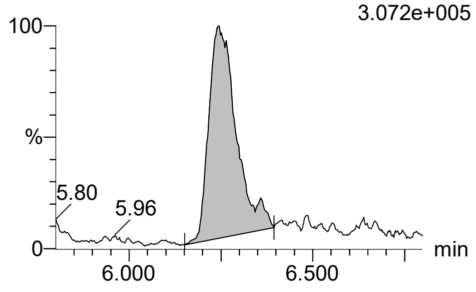

Sample Name: 20211111_A22_R3_IgM
F76:MRM of 1 channel,ES+
1112.5 > 366.1
1.473e+005

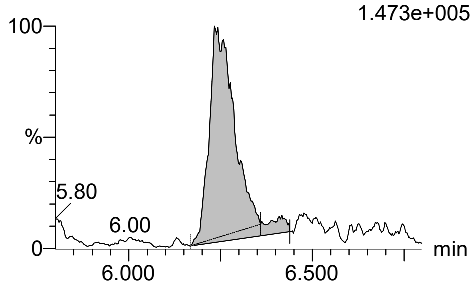

Sample Name: 20211111_L21_R3_IgM

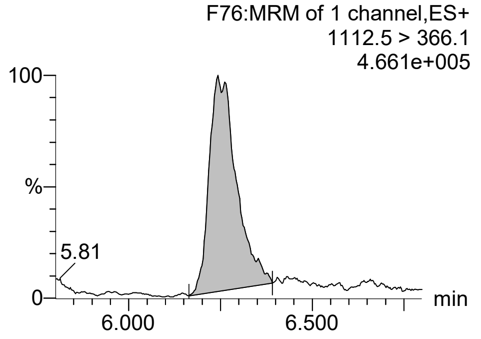

Sample Name: 20211111_L22_R3_IgM

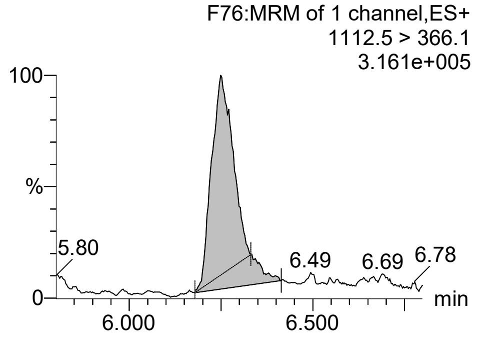

Sample Name: 20211111_H11_R3_IgM

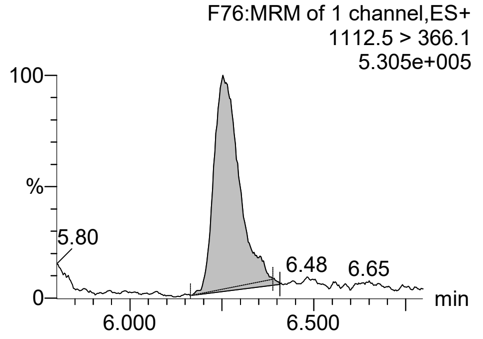

Sample Name: 20211111_A23_R3_IgM

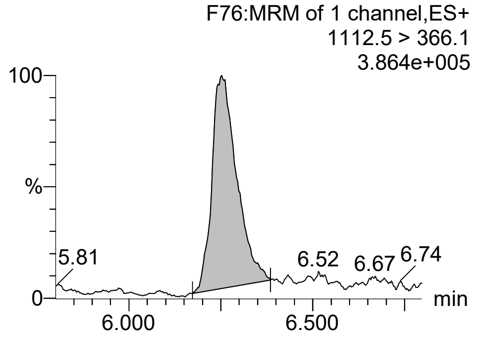

Sample Name: 20211111_A24_R3_IgM

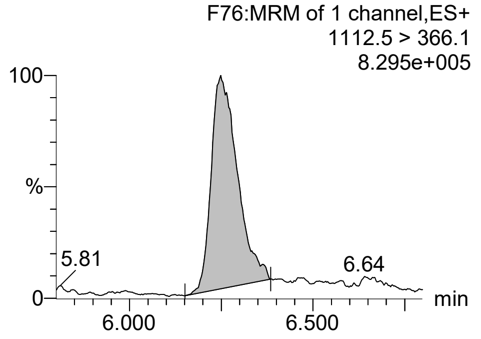

Sample Name: 20211111_L23_R3_IgM

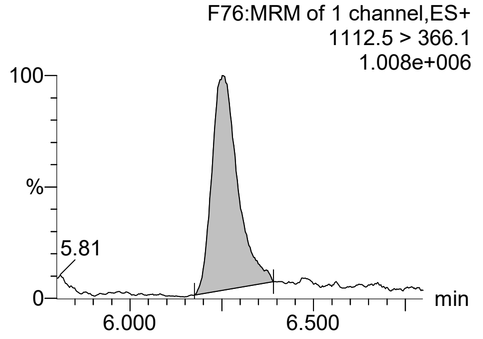

Sample Name: 20211111_L24_R3_IgM

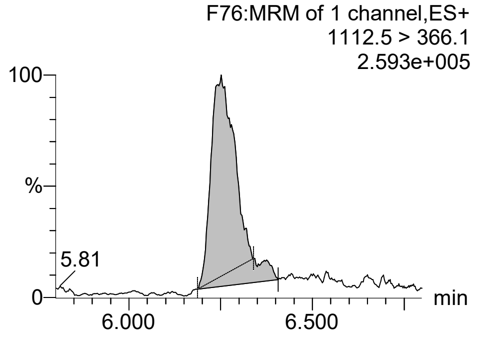

Sample Name: 20211111_H12_R3_IgM

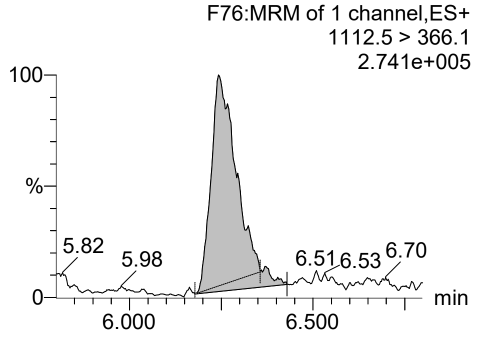

Sample Name: 20211111_QC_R22_IgM

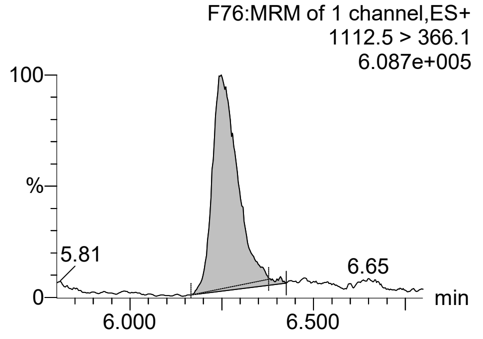

Sample Name: 20211111_A25_R3_IgM

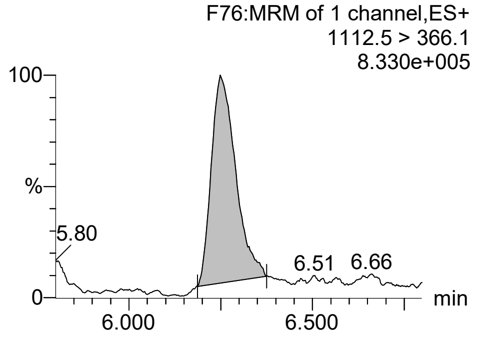

Sample Name: 20211111_A26_R3_IgM

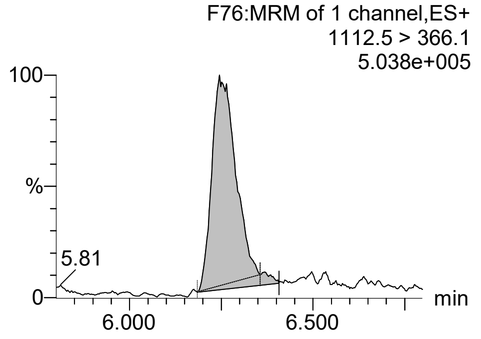

Sample Name: 20211111_L25_R3_IgM

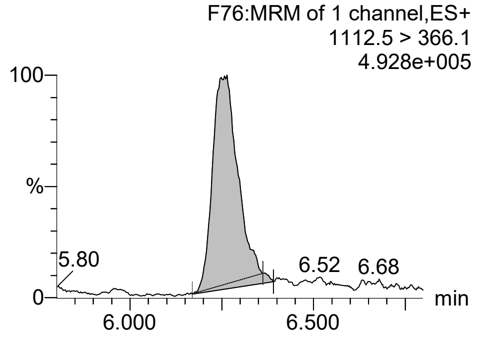

Sample Name: 20211111_L26_R3_IgM

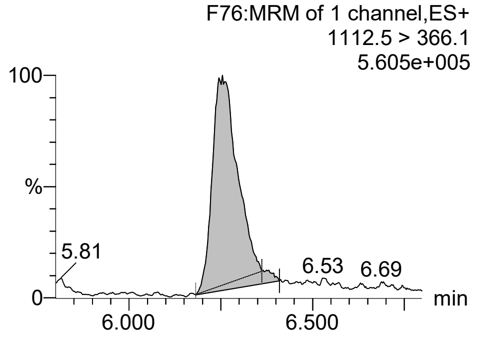

Sample Name: 20211111_H13_R3_IgM

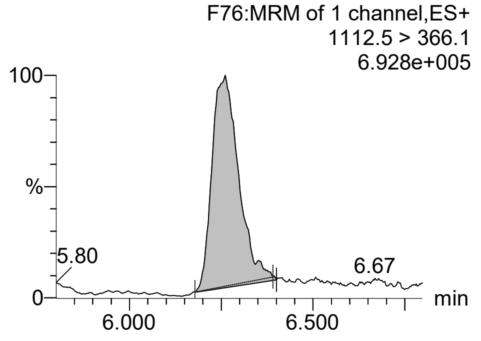

Sample Name: 20211111_A27_R3_IgM

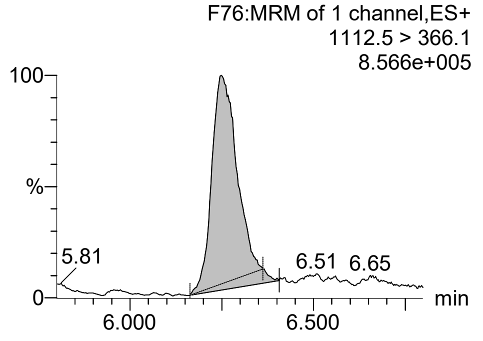

Dataset: C:\MassLynx\10.pro\20211111_clinicalsamples_IgM_IgA.qld

Last Altered: Thursday, March 09, 2023 19:58:25 台北標準時間
Printed: Friday, August 01, 2025 15:22:23 台北標準時間

Sample Name: 20211111_A28_R3_IgM

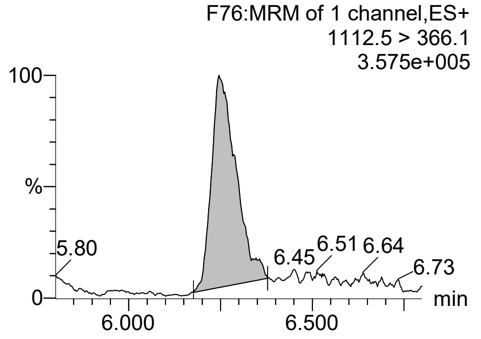

Sample Name: 20211111_L27_R3_IgM

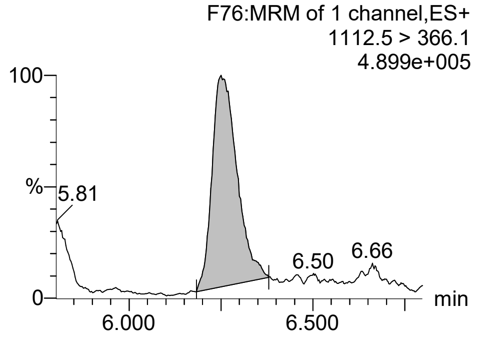

Sample Name: 20211111_L28_R3_IgM

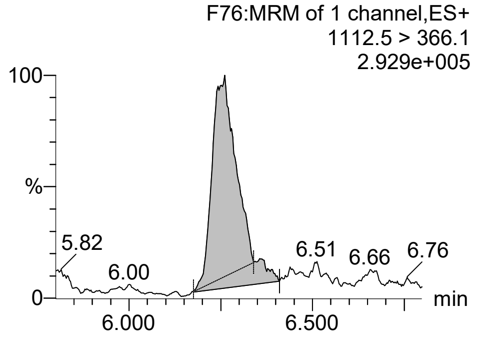

Sample Name: 20211111_H14_R3_IgM

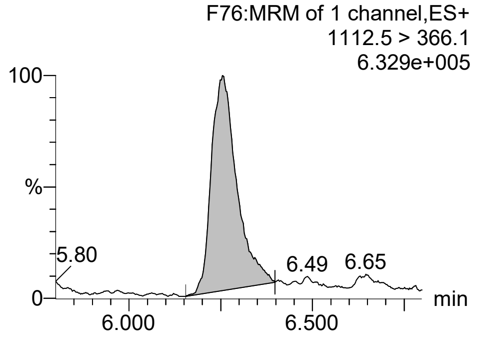

Sample Name: 20211111_A29_R3_IgM

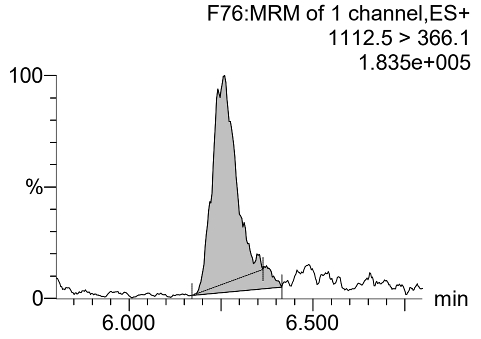

Sample Name: 20211111_A30_R3_IgM

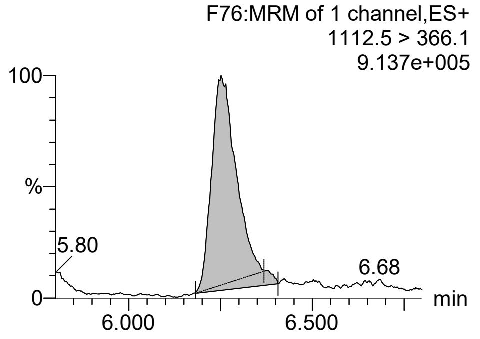

Sample Name: 20211111_QC_R23_IgM

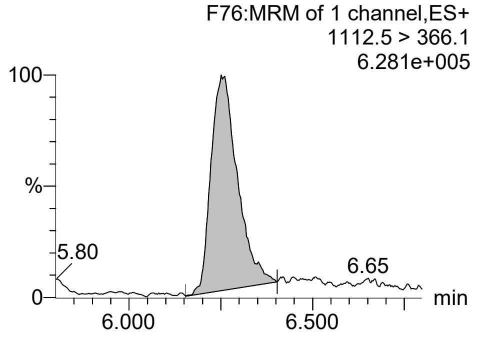

Sample Name: 20211111_L29_R3_IgM

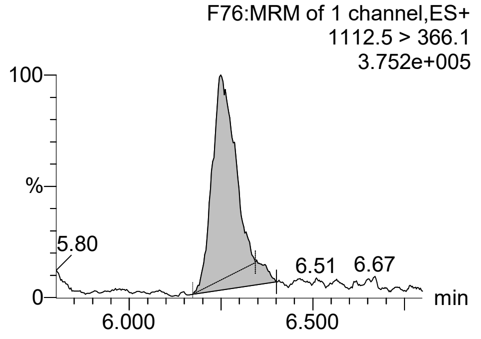

Sample Name: 20211111_L30_R3_IgM

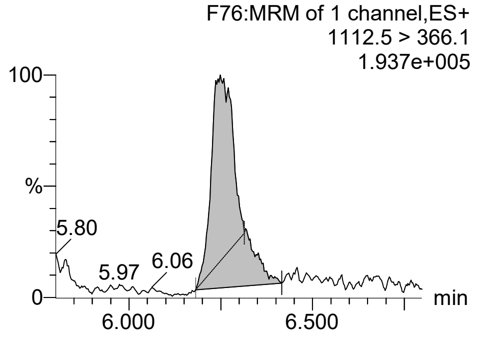

Sample Name: 20211111_H15_R3_IgM

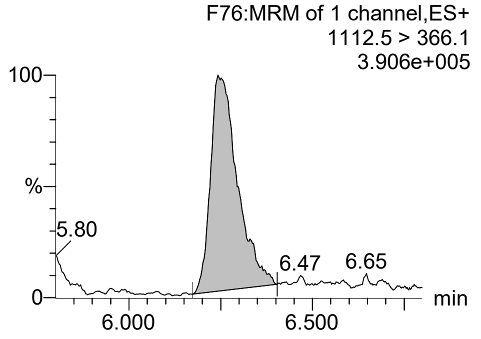

Sample Name: 20211111_A31_R3_IgM

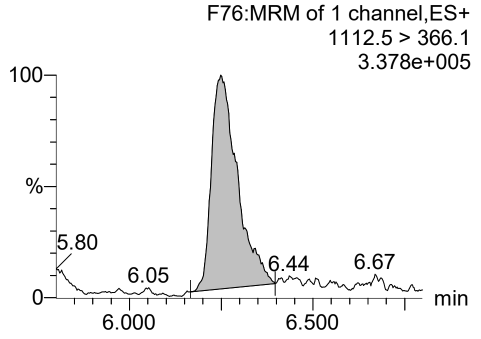

Sample Name: 20211111_A32_R3_IgM

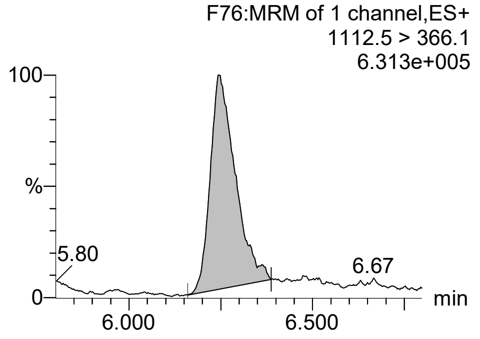

Sample Name: 20211111_L31_R3_IgM

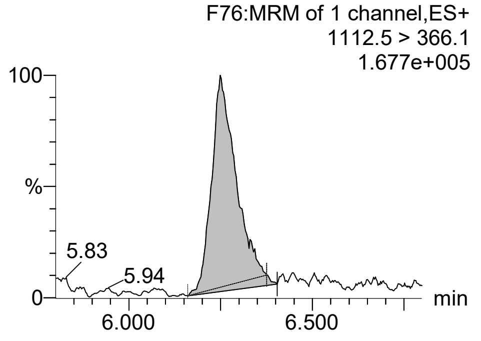

Sample Name: 20211111_L32_R3_IgM

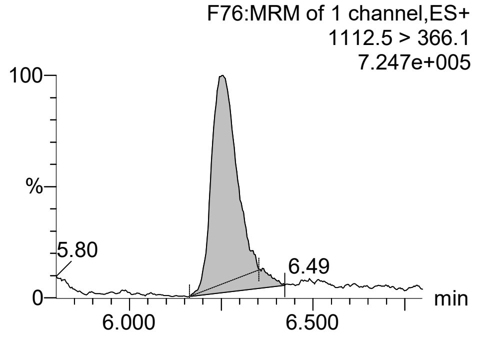

Sample Name: 20211111_H16_R3_IgM

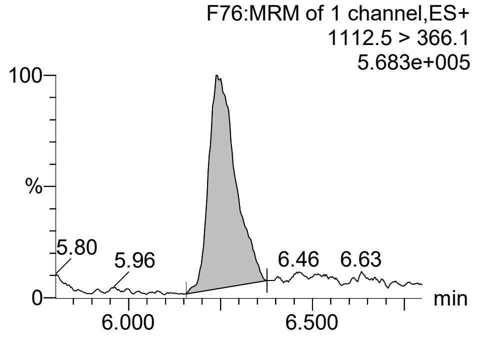

Dataset: C:\MassLynx\10.pro\20211111_clinicalsamples_IgM_IgA.qld

Last Altered: Thursday, March 09, 2023 19:58:25 台北標準時間
Printed: Friday, August 01, 2025 15:22:23 台北標準時間

Sample Name: 20211111_A33_R3_IgM

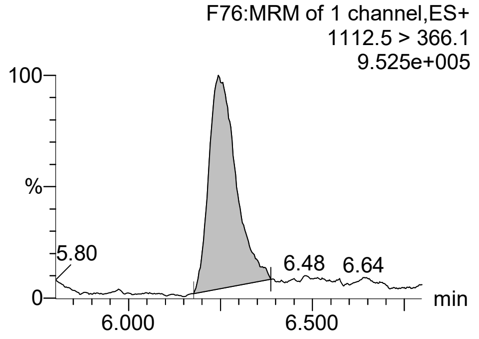

Sample Name: 20211111_A34_R3_IgM

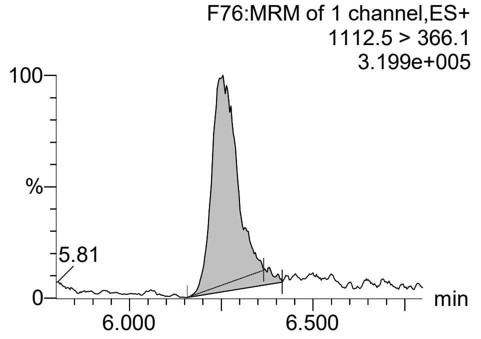

Sample Name: 20211111_H17_R3_IgM

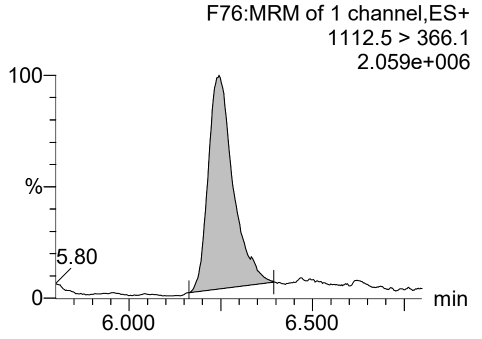

Sample Name: 20211111_A35_R3_IgM

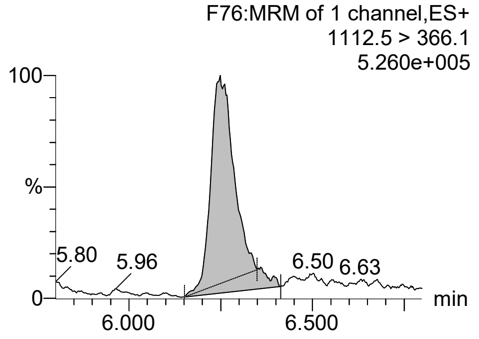

Sample Name: 20211111_QC_R24_IgM

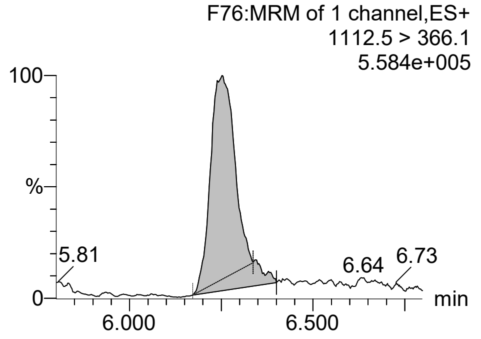

Sample Name: 20211111_A36_R3_IgM

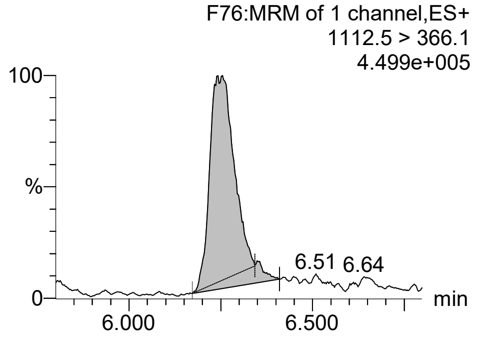

Sample Name: 20211111_H18_R3_IgM

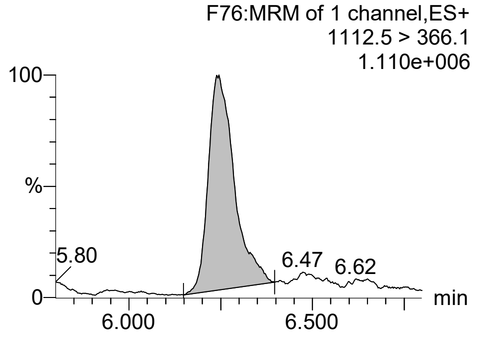

Sample Name: 20211111_A37_R3_IgM

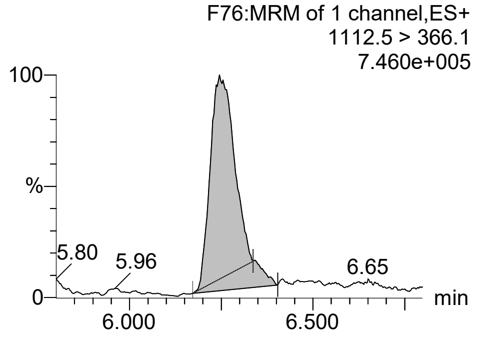

Sample Name: 20211111_A38_R3_IgM

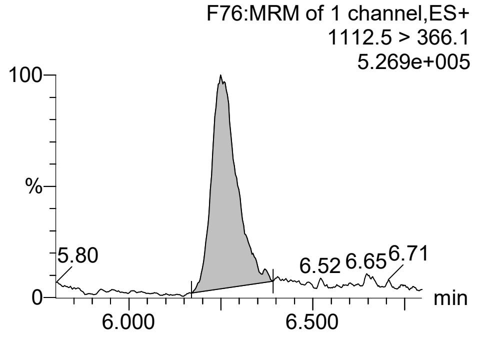

Sample Name: 20211111_H19_R3_IgM

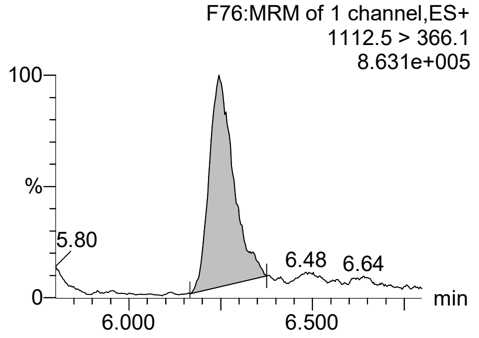

Sample Name: 20211111_A39_R3_IgM

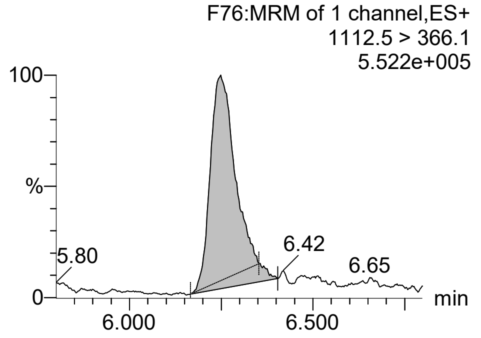

Sample Name: 20211111_A40_R3_IgM

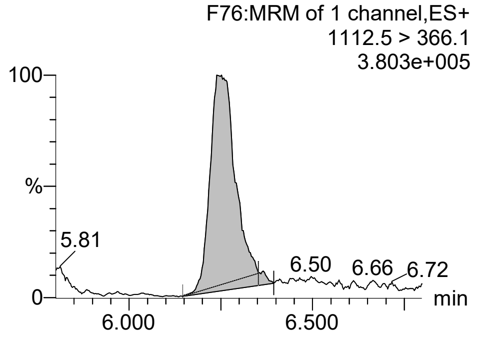

Sample Name: 20211111_H20_R3_IgM

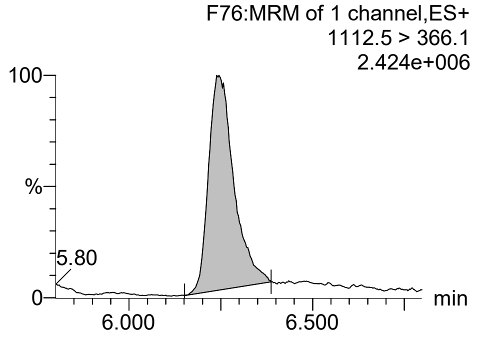

Sample Name: 20211111_A41_R3_IgM

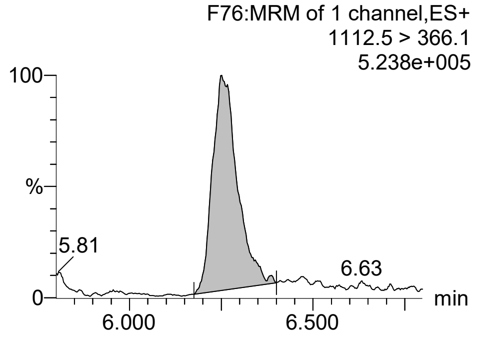

Sample Name: 20211111_A42_R3_IgM

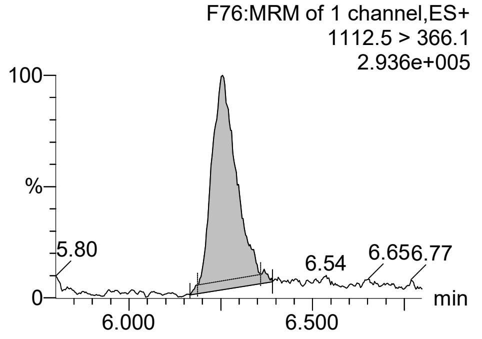

Dataset: C:\MassLynx\10.pro\20211111_clinicalsamples_IgM_IgA.qld

Last Altered: Thursday, March 09, 2023 19:58:25 台北標準時間
Printed: Friday, August 01, 2025 15:22:23 台北標準時間

Sample Name: 20211111_A43_R3_IgM

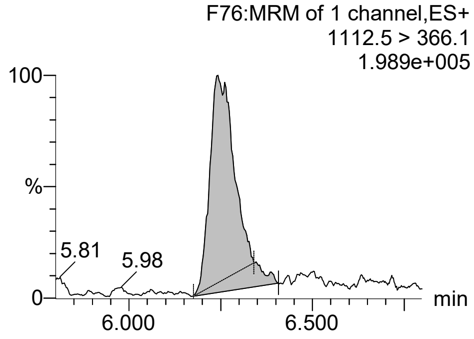

Sample Name: 20211111_A44_R3_IgM

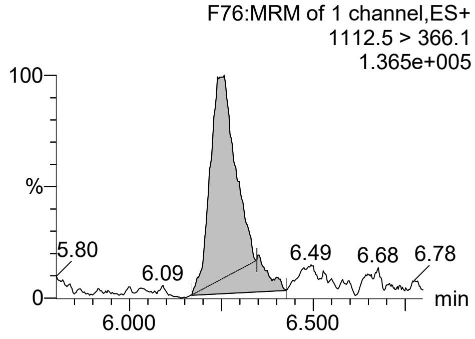

Sample Name: 20211111_QC_R25_IgM

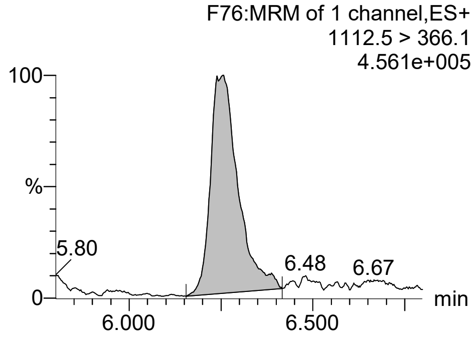

Dataset: C:\MassLynx\10.pro\20211111_clinicalsamples_IgM_IgA.qld

Last Altered: Thursday, March 09, 2023 19:58:25 台北標準時間
Printed: Friday, August 01, 2025 15:22:23 台北標準時間

	#	Name	RT	Area	IS Area	Response	Prima...
1	1	20211112_QC_R001_IgM	6.23	64027.4	15421.4	4.152	bb
2	2	20211112_QC_R002_IgM	6.24	56950.6	13188.8	4.318	MM
3	3	20211111_A1_R2_IgM	6.24	38047.3	11871.0	3.205	MM
4	4	20211111_A1_R3_IgM	6.25	34803.4	12030.5	2.893	MM
5	5	20211111_A2_R2_IgM	6.24	98462.7	11446.8	8.602	MM
6	6	20211111_A2_R3_IgM	6.24	98931.3	11098.4	8.914	MM
7	7	20211111_L1_R2_IgM	6.24	38525.9	11779.4	3.271	MM
8	8	20211111_L1_R3_IgM	6.24	26234.7	11176.2	2.347	MM
9	9	20211111_L2_R2_IgM	6.24	59158.5	11375.0	5.201	MM
10	10	20211111_H1_R2_IgM	6.24	100089.8	13595.2	7.362	bb
11	11	20211111_A3_R2_IgM	6.24	77694.1	14112.3	5.505	bb
12	12	20211111_A4_R2_IgM	6.25	56330.9	11977.5	4.703	MM
13	13	20211111_L3_R2_IgM	6.23	52797.1	12242.1	4.313	MM
14	14	20211111_L4_R2_IgM	6.24	68226.2	12797.0	5.331	MM
15	15	20211111_H2_R2_IgM	6.25	142553.0	10006.0	14.247	MM
16	16	20211111_A5_R2_IgM	6.24	163733.5	11434.6	14.319	MM
17	17	20211111_A6_R2_IgM	6.24	61918.5	13193.7	4.693	MM
18	18	20211111_QC_R10_IgM	6.24	56797.4	12566.1	4.520	MM
19	19	20211111_L5_R2_IgM	6.23	50437.6	13616.4	3.704	MM
20	20	20211111_L6_R2_IgM	6.23	63389.5	13698.5	4.627	bb
21	21	20211111_H3_R2_IgM	6.25	21543.1	15793.1	1.364	MM
22	22	20211111_A7_R2_IgM	6.24	47801.6	12936.9	3.695	MM
23	23	20211111_A8_R2_IgM	6.24	73656.7	13108.0	5.619	MM
24	24	20211111_L7_R2_IgM	6.25	43892.6	13278.5	3.306	MM
25	25	20211111_L8_R2_IgM	6.25	19293.2	15233.6	1.266	MM
26	26	20211111_H4_R2_IgM	6.24	31585.3	16521.9	1.912	MM
27	27	20211111_A9_R2_IgM	6.24	34630.6	16451.6	2.105	MM
28	28	20211111_A10_R2_IgM	6.24	86771.1	13036.0	6.656	MM
29	29	20211111_L9_R2_IgM	6.25	49301.6	14554.1	3.387	MM
30	30	20211111_L10_R2_IgM	6.24	54854.4	12250.4	4.478	MM
31	31	20211111_QC_R11_IgM	6.25	57741.4	11459.2	5.039	MM
32	32	20211111_H5_R2_IgM	6.24	72966.3	14358.2	5.082	MM
33	33	20211111_A11_R2_IgM	6.23	77712.8	16184.1	4.802	MM
34	34	20211111_A12_R2_IgM	6.24	34999.7	13465.4	2.599	MM
35	35	20211111_L11_R2_IgM	6.25	44454.4	13277.9	3.348	MM
36	36	20211111_L12_R2_IgM	6.25	27865.5	15958.8	1.746	MM
37	37	20211111_H6_R2_IgM	6.25	24933.2	14003.5	1.781	MM
38	38	20211111_A13_R2_IgM	6.24	41812.4	13656.1	3.062	MM
39	39	20211111_A14_R2_IgM	6.24	156715.0	14071.3	11.137	bb
40	40	20211111_L13_R2_IgM	6.24	41093.3	14785.6	2.779	MM
41	41	20211111_L14_R2_IgM	6.25	76610.6	14772.2	5.186	MM
42	42	20211111_H7_R2_IgM	6.24	60092.5	15164.4	3.963	MM
43	43	20211111_A15_R2_IgM	6.25	164435.8	15721.5	10.459	MM
44	44	20211111_QC_R12_IgM	6.25	78101.3	13711.5	5.696	bb
45	45	20211111_A16_R2_IgM	6.25	80084.7	14275.6	5.610	MM
46	46	20211111_L15_R2_IgM	6.24	84573.0	12935.8	6.538	bb
47	47	20211111_L16_R2_IgM	6.25	27288.4	13342.8	2.045	MM
48	48	20211111_H8_R2_IgM	6.26	35964.9	15835.1	2.271	bb
49	49	20211111_A17_R2_IgM	6.24	80947.3	15786.7	5.128	bb
50	50	20211111_A18_R2_IgM	6.24	35455.9	16986.8	2.087	MM
51	51	20211111_L17_R2_IgM	6.24	107685.0	13342.7	8.071	bb
52	52	20211111_L18_R2_IgM	6.25	30028.9	14692.0	2.044	MM
53	53	20211111_H9_R2_IgM	6.24	30386.0	15639.7	1.943	MM
54	54	20211111_A19_R2_IgM	6.25	58469.6	14198.7	4.118	MM
55	55	20211111_A20_R2_IgM	6.23	28529.8	14162.7	2.014	MM
56	56	20211111_L19_R2_IgM	6.24	50254.8	9293.4	5.408	MM

Dataset: C:\MassLynx\10.pro\20211111_clinicalsamples_IgM_IgA.qld

Last Altered: Thursday, March 09, 2023 19:58:25 台北標準時間
Printed: Friday, August 01, 2025 15:22:23 台北標準時間

	# Name	RT	Area	IS Area	Response	Prima...
57	57 20211111_QC_R13_IgM	6.24	60390.1	13903.0	4.344	MM
58	58 20211111_L20_R2_IgM	6.23	40740.8	12781.6	3.187	MM
59	59 20211111_H10_R2_IgM	6.24	53336.3	14796.4	3.605	MM
60	60 20211111_A21_R2_IgM	6.25	30148.4	13669.0	2.206	MM
61	61 20211111_A22_R2_IgM	6.25	13645.3	14098.3	0.968	MM
62	62 20211111_L21_R2_IgM	6.25	44935.1	15678.2	2.866	bb
63	63 20211111_L22_R2_IgM	6.24	28032.8	15724.5	1.783	bb
64	64 20211111_H11_R2_IgM	6.25	52422.9	15890.2	3.299	MM
65	65 20211111_A23_R2_IgM	6.25	33989.5	13423.2	2.532	MM
66	66 20211111_A24_R2_IgM	6.24	67530.3	12438.0	5.429	MM
67	67 20211111_L23_R2_IgM	6.24	92912.3	14075.0	6.601	bb
68	68 20211111_L24_R2_IgM	6.25	23837.4	15972.1	1.492	MM
69	69 20211111_H12_R2_IgM	6.26	23670.5	16270.9	1.455	MM
70	70 20211111_QC_R14_IgM	6.25	60092.0	15806.8	3.802	MM
71	71 20211111_A25_R2_IgM	6.25	86543.5	14552.1	5.947	MM
72	72 20211111_A26_R2_IgM	6.25	45904.0	11492.7	3.994	bb
73	73 20211111_L25_R2_IgM	6.25	50343.3	14403.3	3.495	MM
74	74 20211111_L26_R2_IgM	6.25	52896.5	16533.9	3.199	bb
75	75 20211111_H13_R2_IgM	6.25	62268.7	15520.1	4.012	MM
76	76 20211111_A27_R2_IgM	6.24	82152.8	13337.4	6.160	MM
77	77 20211111_A28_R2_IgM	6.24	32529.4	11895.6	2.735	bb
78	78 20211111_L27_R2_IgM	6.25	41407.1	14125.7	2.931	bb
79	79 20211111_L28_R2_IgM	6.25	23898.3	11807.5	2.024	bb
80	80 20211111_H14_R2_IgM	6.25	48083.4	15035.7	3.198	bb
81	81 20211111_A29_R2_IgM	6.24	16781.8	13554.4	1.238	MM
82	82 20211111_A30_R2_IgM	6.24	82015.2	12984.1	6.317	MM
83	83 20211111_QC_R15_IgM	6.25	58788.1	12299.5	4.780	MM
84	84 20211111_L29_R2_IgM	6.25	31564.2	13590.7	2.322	MM
85	85 20211111_L30_R2_IgM	6.26	18111.1	12511.4	1.448	MM
86	86 20211111_H15_R2_IgM	6.24	38435.9	11885.0	3.234	MM
87	87 20211111_A31_R2_IgM	6.24	30149.8	13422.1	2.246	MM
88	88 20211111_A32_R2_IgM	6.25	54777.3	11461.3	4.779	MM
89	89 20211111_L31_R2_IgM	6.24	14851.6	13753.4	1.080	MM
90	90 20211111_L32_R2_IgM	6.24	70674.7	12949.0	5.458	MM
91	91 20211111_H16_R2_IgM	6.24	59958.8	11465.4	5.230	MM
92	92 20211111_A33_R2_IgM	6.24	86056.5	10551.5	8.156	bb
93	93 20211111_A34_R2_IgM	6.25	29104.3	12409.9	2.345	MM
94	94 20211111_H17_R2_IgM	6.24	192760.8	10160.9	18.971	bb
95	95 20211111_A35_R2_IgM	6.24	49911.3	10460.6	4.771	MM
96	96 20211111_QC_R16_IgM	6.25	51751.5	10433.5	4.960	bb
97	97 20211111_A36_R2_IgM	6.22	47573.0	12504.8	3.804	MM
98	98 20211111_H18_R2_IgM	6.25	100645.0	9140.4	11.011	MM
99	99 20211111_A37_R2_IgM	6.24	71692.4	9796.9	7.318	MM
100	1... 20211111_A38_R2_IgM	6.26	45158.1	11224.3	4.023	bb
101	1... 20211111_H19_R2_IgM	6.25	78840.1	11739.2	6.716	bb
102	1... 20211111_A39_R2_IgM	6.25	52258.7	12718.9	4.109	MM
103	1... 20211111_A40_R2_IgM	6.24	35483.1	10006.1	3.546	bb
104	1... 20211111_H20_R2_IgM	6.24	242330.2	7988.2	30.336	bb
105	1... 20211111_A41_R2_IgM	6.26	69237.7	11513.3	6.014	MM
106	1... 20211111_A42_R2_IgM	6.25	27423.9	10238.5	2.678	MM
107	1... 20211111_A43_R2_IgM	6.25	17712.7	11098.9	1.596	MM
108	1... 20211111_A44_R2_IgM	6.23	14403.9	10969.9	1.313	MM
109	1... 20211111_QC_R17_IgM	6.24	38004.3	9700.8	3.918	MM
110	1... 20211111_L2_R3_IgM	6.25	57999.9	8528.0	6.801	bb
111	1... 20211111_H1_R3_IgM	6.24	84554.6	9438.2	8.959	bb
112	1... 20211111_A3_R3_IgM	6.24	61796.6	11598.6	5.328	bb

Dataset: C:\MassLynx\10.pro\20211111_clinicalsamples_IgM_IgA.qld

Last Altered: Thursday, March 09, 2023 19:58:25 台北標準時間
Printed: Friday, August 01, 2025 15:22:23 台北標準時間

	#	Name	RT	Area	IS Area	Response	Prima...
113	1...	20211111_A4_R3_IgM	6.24	48781.5	11364.1	4.293	bb
114	1...	20211111_L3_R3_IgM	6.24	41381.2	10753.4	3.848	MM
115	1...	20211111_L4_R3_IgM	6.24	57356.9	10291.6	5.573	MM
116	1...	20211111_H2_R3_IgM	6.25	140131.1	9315.8	15.042	MM
117	1...	20211111_A5_R3_IgM	6.24	137193.5	9102.2	15.073	MM
118	1...	20211111_A6_R3_IgM	6.25	51134.4	10356.4	4.937	MM
119	1...	20211111_QC_R18_IgM	6.24	49132.8	8398.3	5.850	MM
120	1...	20211111_L5_R3_IgM	6.25	36636.4	9786.7	3.743	MM
121	1...	20211111_L6_R3_IgM	6.25	47651.1	9911.3	4.808	MM
122	1...	20211111_H3_R3_IgM	6.25	16696.9	12155.5	1.374	MM
123	1...	20211111_A7_R3_IgM	6.24	47111.9	11804.8	3.991	MM
124	1...	20211111_A8_R3_IgM	6.25	55273.7	10510.6	5.259	MM
125	1...	20211111_L7_R3_IgM	6.25	37485.0	12177.9	3.078	MM
126	1...	20211111_L8_R3_IgM	6.26	16288.6	11668.6	1.396	bb
127	1...	20211111_H4_R3_IgM	6.25	23330.4	11192.0	2.085	MM
128	1...	20211111_A9_R3_IgM	6.25	25501.1	14077.4	1.811	MM
129	1...	20211111_A10_R3_IgM	6.25	73327.4	11190.4	6.553	bb
130	1...	20211111_L9_R3_IgM	6.25	43294.4	14649.3	2.955	MM
131	1...	20211111_L10_R3_IgM	6.23	42007.3	9361.3	4.487	bb
132	1...	20211111_QC_R19_IgM	6.24	46650.2	10125.5	4.607	MM
133	1...	20211111_H5_R3_IgM	6.25	50569.4	10010.9	5.051	MM
134	1...	20211111_A11_R3_IgM	6.25	54421.9	12697.2	4.286	MM
135	1...	20211111_A12_R3_IgM	6.26	31383.8	13148.5	2.387	MM
136	1...	20211111_L11_R3_IgM	6.24	35084.1	10786.6	3.253	bb
137	1...	20211111_L12_R3_IgM	6.24	22820.0	11681.8	1.953	MM
138	1...	20211111_H6_R3_IgM	6.26	21390.4	12095.5	1.768	MM
139	1...	20211111_A13_R3_IgM	6.24	36892.4	12908.3	2.858	MM
140	1...	20211111_A14_R3_IgM	6.25	128500.8	11322.6	11.349	bb
141	1...	20211111_L13_R3_IgM	6.25	33842.0	11516.1	2.939	MM
142	1...	20211111_L14_R3_IgM	6.24	62222.6	10790.7	5.766	MM
143	1...	20211111_H7_R3_IgM	6.25	50609.7	11572.1	4.373	MM
144	1...	20211111_A15_R3_IgM	6.25	133312.1	12434.4	10.721	MM
145	1...	20211111_QC_R20_IgM	6.25	63311.3	10701.0	5.916	bb
146	1...	20211111_A16_R3_IgM	6.25	59759.3	11452.1	5.218	bb
147	1...	20211111_L15_R3_IgM	6.25	69030.9	10531.1	6.555	MM
148	1...	20211111_L16_R3_IgM	6.24	22592.5	12049.4	1.875	MM
149	1...	20211111_H8_R3_IgM	6.25	29748.4	13434.9	2.214	MM
150	1...	20211111_A17_R3_IgM	6.25	66886.7	12452.0	5.372	bb
151	1...	20211111_A18_R3_IgM	6.24	28335.5	12506.5	2.266	bb
152	1...	20211111_L17_R3_IgM	6.25	92153.6	9948.9	9.263	MM
153	1...	20211111_L18_R3_IgM	6.24	25593.0	13462.1	1.901	bb
154	1...	20211111_H9_R3_IgM	6.25	25621.9	13178.7	1.944	bb
155	1...	20211111_A19_R3_IgM	6.25	53529.0	11298.6	4.738	bb
156	1...	20211111_A20_R3_IgM	6.25	24057.4	11175.6	2.153	bb
157	1...	20211111_L19_R3_IgM	6.24	54255.1	11558.2	4.694	bb
158	1...	20211111_QC_R21_IgM	6.26	49799.7	9977.0	4.991	MM
159	1...	20211111_L20_R3_IgM	6.25	33239.8	13508.1	2.461	MM
160	1...	20211111_H10_R3_IgM	6.25	42374.5	12374.8	3.424	bb
161	1...	20211111_A21_R3_IgM	6.25	25266.6	12443.6	2.030	bb
162	1...	20211111_A22_R3_IgM	6.23	12171.2	11589.3	1.050	MM
163	1...	20211111_L21_R3_IgM	6.24	38781.6	13107.4	2.959	bb
164	1...	20211111_L22_R3_IgM	6.25	23553.0	12021.5	1.959	MM
165	1...	20211111_H11_R3_IgM	6.25	41762.5	12687.8	3.292	MM
166	1...	20211111_A23_R3_IgM	6.25	28898.0	11611.8	2.489	bb
167	1...	20211111_A24_R3_IgM	6.25	65510.7	10476.8	6.253	bb
168	1...	20211111_L23_R3_IgM	6.25	79137.5	11873.1	6.665	bb

Dataset: C:\MassLynx\10.pro\20211111_clinicalsamples_IgM_IgA.qld

Last Altered: Thursday, March 09, 2023 19:58:25 台北標準時間
Printed: Friday, August 01, 2025 15:22:23 台北標準時間

	# Name	RT	Area	IS Area	Response	Prima...
169	1... 20211111_L24_R3_IgM	6.25	20473.0	13468.0	1.520	MM
170	1... 20211111_H12_R3_IgM	6.24	22506.5	14229.1	1.582	MM
171	1... 20211111_QC_R22_IgM	6.25	47777.3	11264.4	4.241	MM
172	1... 20211111_A25_R3_IgM	6.25	59385.5	11775.7	5.043	bb
173	1... 20211111_A26_R3_IgM	6.25	38278.9	10473.9	3.655	MM
174	1... 20211111_L25_R3_IgM	6.26	38894.8	13256.8	2.934	MM
175	1... 20211111_L26_R3_IgM	6.25	45018.6	13565.0	3.319	MM
176	1... 20211111_H13_R3_IgM	6.26	55991.8	13146.0	4.259	MM
177	1... 20211111_A27_R3_IgM	6.25	69185.3	12025.6	5.753	MM
178	1... 20211111_A28_R3_IgM	6.25	26359.6	11229.1	2.347	bb
179	1... 20211111_L27_R3_IgM	6.25	36451.6	14960.0	2.437	bb
180	1... 20211111_L28_R3_IgM	6.26	23238.5	11359.0	2.046	MM
181	1... 20211111_H14_R3_IgM	6.25	48834.9	12830.8	3.806	bb
182	1... 20211111_A29_R3_IgM	6.26	14638.9	13434.3	1.090	MM
183	1... 20211111_A30_R3_IgM	6.25	72684.6	11272.2	6.448	MM
184	1... 20211111_QC_R23_IgM	6.25	50664.6	11872.2	4.268	bb
185	1... 20211111_L29_R3_IgM	6.25	29026.6	12948.7	2.242	MM
186	1... 20211111_L30_R3_IgM	6.25	15671.4	11026.6	1.421	MM
187	1... 20211111_H15_R3_IgM	6.24	32195.5	10786.3	2.985	bb
188	1... 20211111_A31_R3_IgM	6.25	26602.9	12010.5	2.215	bb
189	1... 20211111_A32_R3_IgM	6.24	48201.9	11107.7	4.340	bb
190	1... 20211111_L31_R3_IgM	6.25	12916.0	11021.8	1.172	MM
191	1... 20211111_L32_R3_IgM	6.25	60818.1	11683.8	5.205	MM
192	1... 20211111_H16_R3_IgM	6.24	46119.4	9831.7	4.691	bb
193	1... 20211111_A33_R3_IgM	6.24	77837.3	11213.1	6.942	bb
194	1... 20211111_A34_R3_IgM	6.25	26505.6	11550.1	2.295	MM
195	1... 20211111_H17_R3_IgM	6.25	154630.5	8874.4	17.424	bb
196	1... 20211111_A35_R3_IgM	6.25	41478.7	9856.3	4.208	MM
197	1... 20211111_QC_R24_IgM	6.25	45497.3	7561.8	6.017	MM
198	1... 20211111_A36_R3_IgM	6.25	35939.6	9562.8	3.758	MM
199	1... 20211111_H18_R3_IgM	6.25	87823.4	8165.3	10.756	bb
200	2... 20211111_A37_R3_IgM	6.25	61468.6	9278.8	6.625	MM
201	2... 20211111_A38_R3_IgM	6.25	40055.9	10920.3	3.668	bb
202	2... 20211111_H19_R3_IgM	6.25	62796.9	9285.3	6.763	bb
203	2... 20211111_A39_R3_IgM	6.25	44140.6	9083.4	4.859	MM
204	2... 20211111_A40_R3_IgM	6.24	30467.7	8382.4	3.635	MM
205	2... 20211111_H20_R3_IgM	6.25	186704.3	7807.9	23.912	bb
206	2... 20211111_A41_R3_IgM	6.25	39795.4	5925.2	6.716	bb
207	2... 20211111_A42_R3_IgM	6.25	22598.4	9352.6	2.416	MM
208	2... 20211111_A43_R3_IgM	6.24	16236.2	10216.3	1.589	MM
209	2... 20211111_A44_R3_IgM	6.26	12337.3	10262.7	1.202	MM
210	2... 20211111_QC_R25_IgM	6.26	38822.3	8575.3	4.527	bb

Dataset: C:\MassLynx\10.pro\20211111_clinicalsamples_IgM_IgA.qld

Last Altered: Thursday, March 09, 2023 19:58:25 台北標準時間
Printed: Friday, August 01, 2025 15:23:22 台北標準時間

Method: C:\MassLynx\10_Pro_IgA.PRO\MethDB\20211111_IgM_IgA_DMRM_new.mdb 24 Feb 2022 13:44:14
Calibration: 15 Nov 2021 14:42:11

Sample Name: 20211112_QC_R001_IgM
F38:MRM of 2 channels,ES+
948.4 > 366.1
1.838e+005

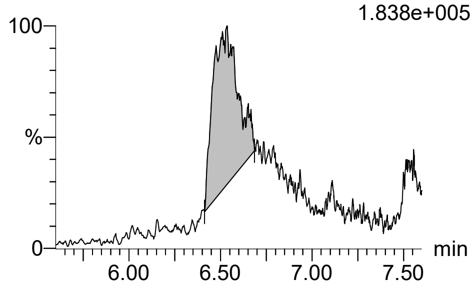

Sample Name: 20211112_QC_R001_IgM
F38:MRM of 2 channels,ES+
1580 > 366.1
1.604e+005

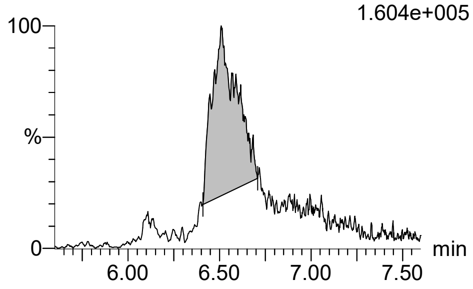

Sample Name: 20211112_QC_R002_IgM
F38:MRM of 2 channels,ES+
948.4 > 366.1
1.733e+005

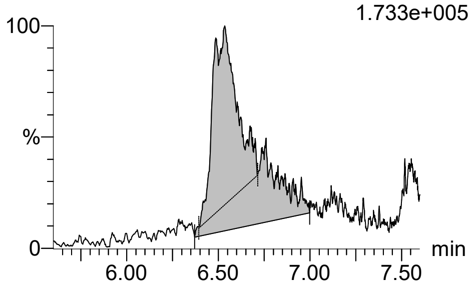

Sample Name: 20211112_QC_R002_IgM
F38:MRM of 2 channels,ES+
1580 > 366.1
1.581e+005

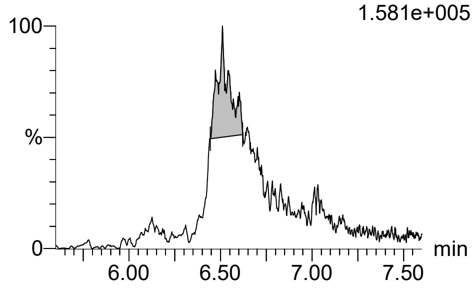

Sample Name: 20211111_A1_R2_IgM
F38:MRM of 2 channels,ES+
948.4 > 366.1
1.579e+005

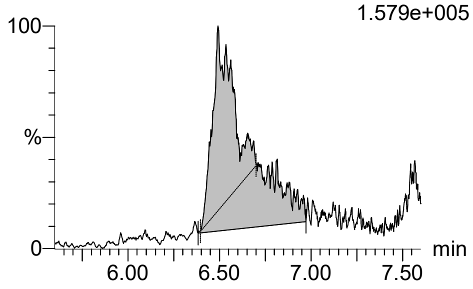

Sample Name: 20211111_A1_R2_IgM
F38:MRM of 2 channels,ES+
1580 > 366.1
1.148e+005

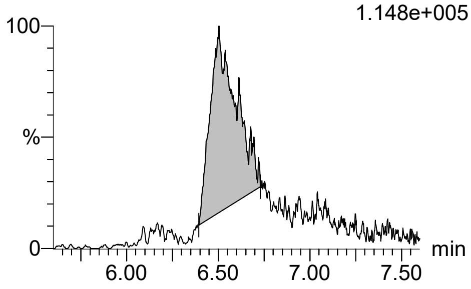

Sample Name: 20211111_A1_R3_IgM
F38:MRM of 2 channels,ES+
948.4 > 366.1
1.513e+005

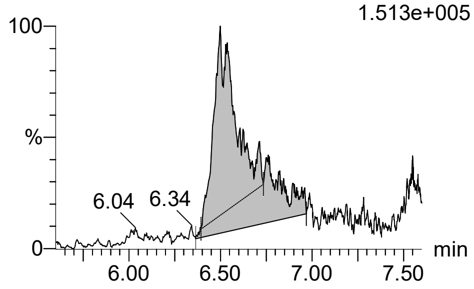

Sample Name: 20211111_A1_R3_IgM
F38:MRM of 2 channels,ES+
1580 > 366.1
1.136e+005

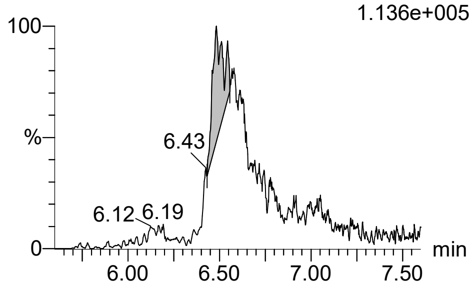

Sample Name: 20211111_A2_R2_IgM
F38:MRM of 2 channels,ES+
948.4 > 366.1
2.437e+005

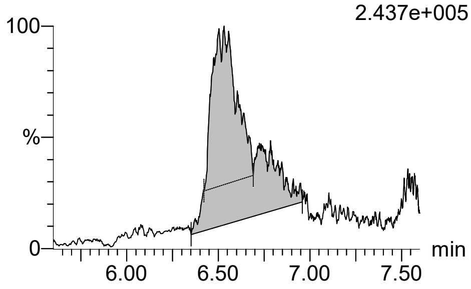

Sample Name: 20211111_A2_R2_IgM
F38:MRM of 2 channels,ES+
1580 > 366.1
2.860e+005

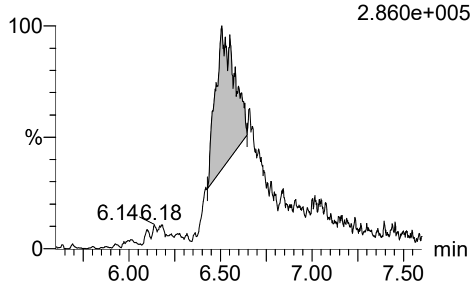

Sample Name: 20211111_A2_R3_IgM
F38:MRM of 2 channels,ES+
948.4 > 366.1
2.553e+005

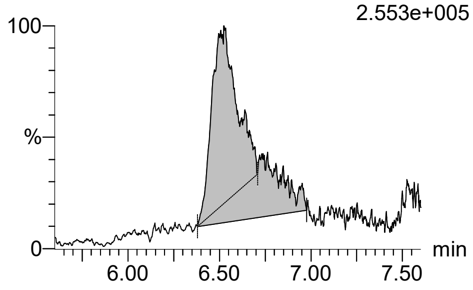

Sample Name: 20211111_A2_R3_IgM
F38:MRM of 2 channels,ES+
1580 > 366.1
2.861e+005

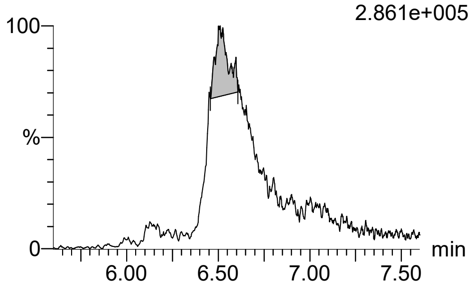

Dataset: C:\MassLynx\10.pro\20211111_clinicalsamples_IgM_IgA.qld

Last Altered: Thursday, March 09, 2023 19:58:25 台北標準時間
Printed: Friday, August 01, 2025 15:23:22 台北標準時間

Sample Name: 20211111_L1_R2_IgM
F38:MRM of 2 channels,ES+
948.4 > 366.1
1.430e+005

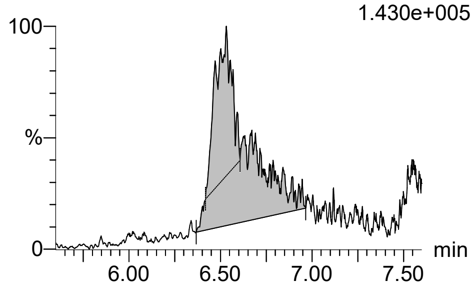

Sample Name: 20211111_L1_R2_IgM
F38:MRM of 2 channels,ES+
1580 > 366.1
1.167e+005

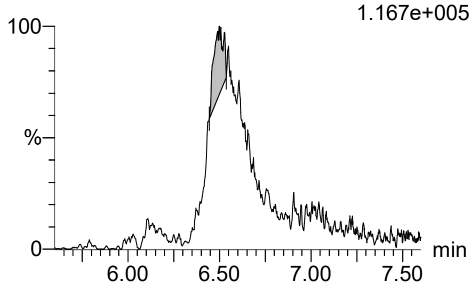

Sample Name: 20211111_L1_R3_IgM
F38:MRM of 2 channels,ES+
948.4 > 366.1
1.010e+005

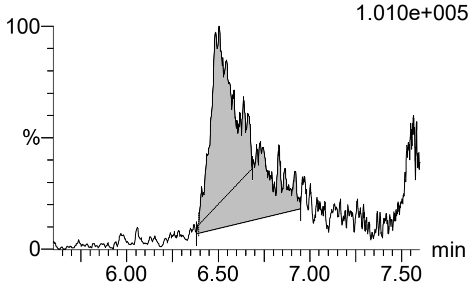

Sample Name: 20211111_L1_R3_IgM
F38:MRM of 2 channels,ES+
1580 > 366.1
6.672e+004

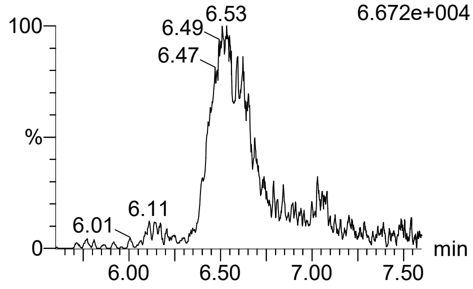

Sample Name: 20211111_L2_R2_IgM
F38:MRM of 2 channels,ES+
948.4 > 366.1
1.869e+005

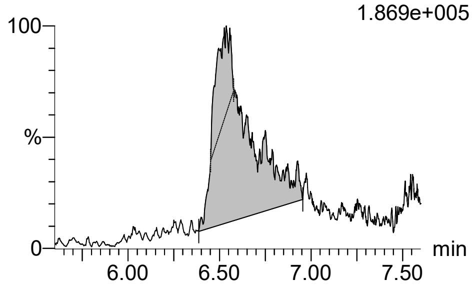

Sample Name: 20211111_L2_R2_IgM
F38:MRM of 2 channels,ES+
1580 > 366.1
2.093e+005

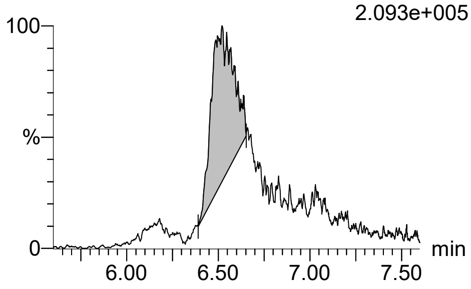

Sample Name: 20211111_H1_R2_IgM
F38:MRM of 2 channels,ES+
948.4 > 366.1
1.930e+005

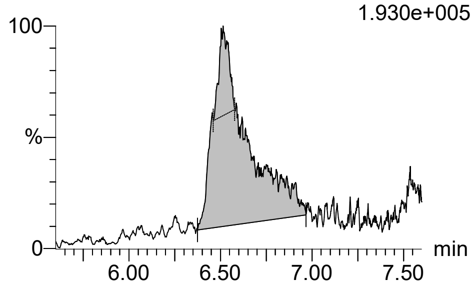

Sample Name: 20211111_H1_R2_IgM
F38:MRM of 2 channels,ES+
1580 > 366.1
1.961e+005

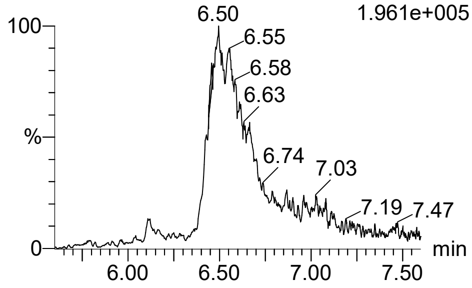

Sample Name: 20211111_A3_R2_IgM
F38:MRM of 2 channels,ES+
948.4 > 366.1
2.179e+005

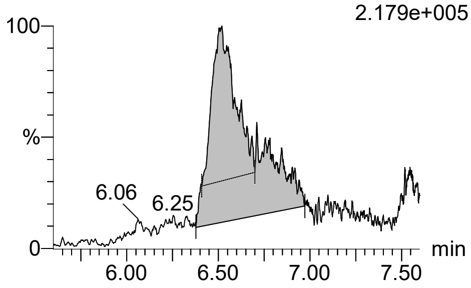

Sample Name: 20211111_A3_R2_IgM
F38:MRM of 2 channels,ES+
1580 > 366.1
2.523e+005

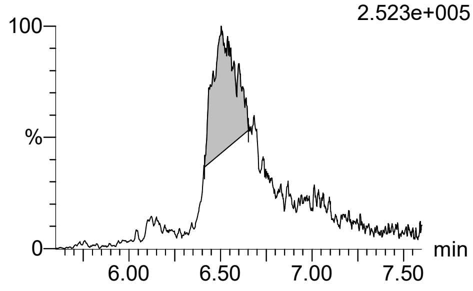

Sample Name: 20211111_A4_R2_IgM
F38:MRM of 2 channels,ES+
948.4 > 366.1
2.029e+005

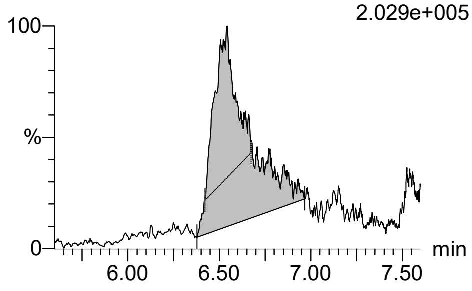

Sample Name: 20211111_A4_R2_IgM
F38:MRM of 2 channels,ES+
1580 > 366.1
2.030e+005

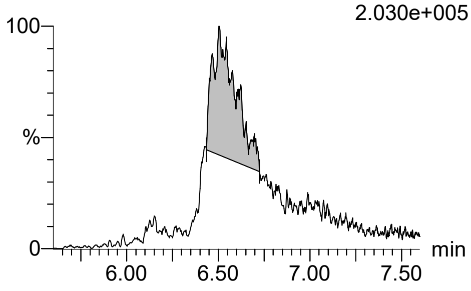

Sample Name: 20211111_L3_R2_IgM
F38:MRM of 2 channels,ES+
948.4 > 366.1
1.304e+005

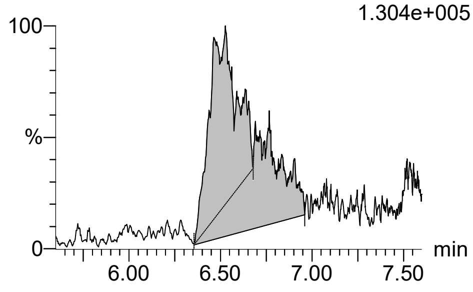

Sample Name: 20211111_L3_R2_IgM
F38:MRM of 2 channels,ES+
1580 > 366.1
1.413e+005

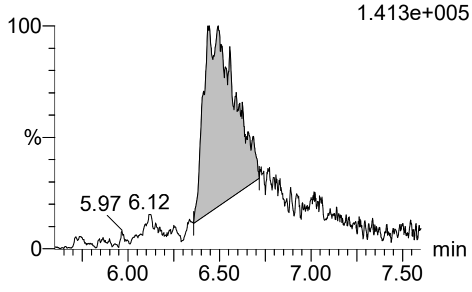

Sample Name: 20211111_L4_R2_IgM
F38:MRM of 2 channels,ES+
948.4 > 366.1
1.899e+005

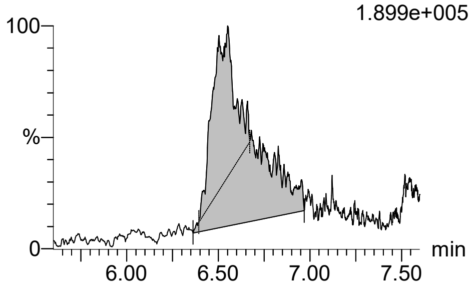

Dataset: C:\MassLynx\10.pro\20211111_clinicalsamples_IgM_IgA.qld

Last Altered: Thursday, March 09, 2023 19:58:25 台北標準時間
Printed: Friday, August 01, 2025 15:23:22 台北標準時間

Sample Name: 20211111_L4_R2_IgM
F38:MRM of 2 channels,ES+
1580 > 366.1
2.264e+005

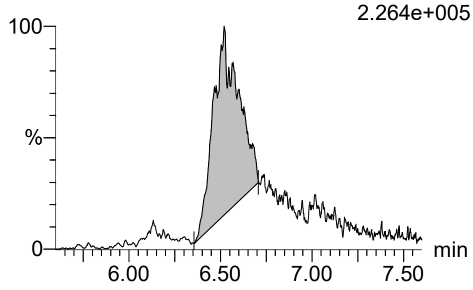

Sample Name: 20211111_H2_R2_IgM
F38:MRM of 2 channels,ES+
948.4 > 366.1
2.927e+005

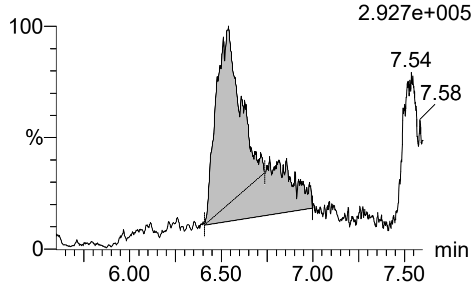

Sample Name: 20211111_H2_R2_IgM
F38:MRM of 2 channels,ES+
1580 > 366.1
3.359e+005

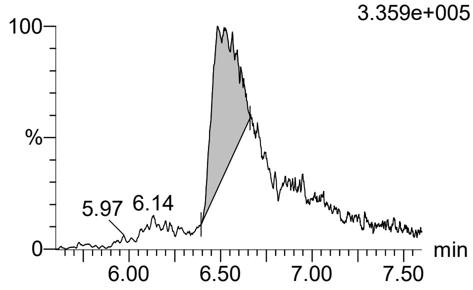

Sample Name: 20211111_A5_R2_IgM
F38:MRM of 2 channels,ES+
948.4 > 366.1
5.398e+005

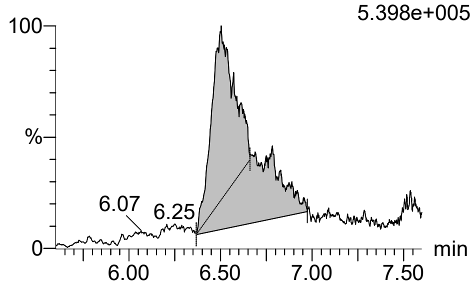

Sample Name: 20211111_A5_R2_IgM
F38:MRM of 2 channels,ES+
1580 > 366.1
7.698e+005

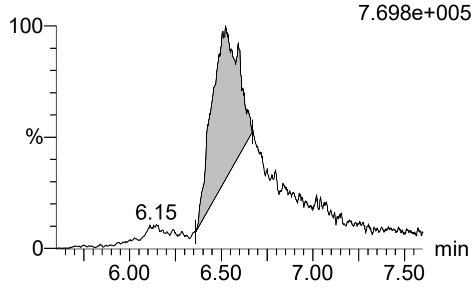

Sample Name: 20211111_A6_R2_IgM
F38:MRM of 2 channels,ES+
948.4 > 366.1
2.595e+005

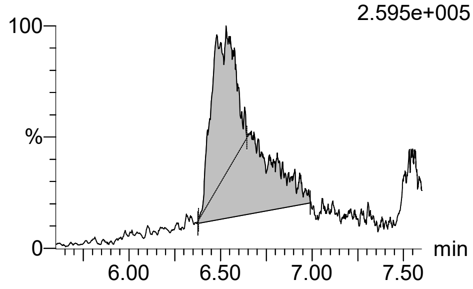

Sample Name: 20211111_A6_R2_IgM
F38:MRM of 2 channels,ES+
1580 > 366.1
3.818e+005

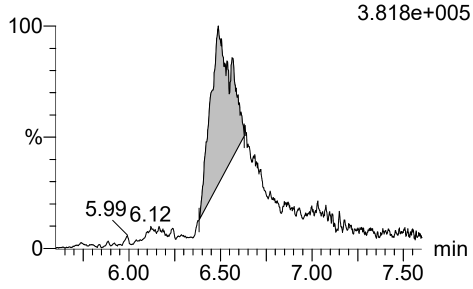

Sample Name: 20211111_QC_R10_IgM
F38:MRM of 2 channels,ES+
948.4 > 366.1
2.267e+005

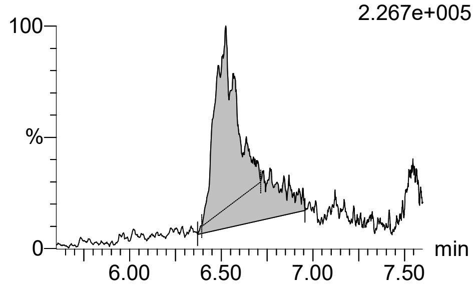

Sample Name: 20211111_QC_R10_IgM
F38:MRM of 2 channels,ES+
1580 > 366.1
2.527e+005

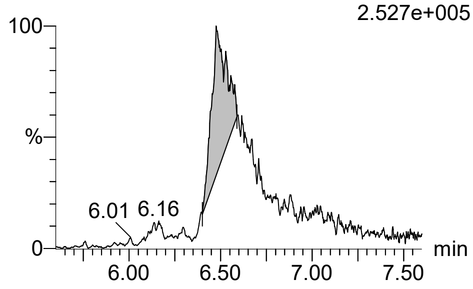

Sample Name: 20211111_L5_R2_IgM
F38:MRM of 2 channels,ES+
948.4 > 366.1
1.869e+005

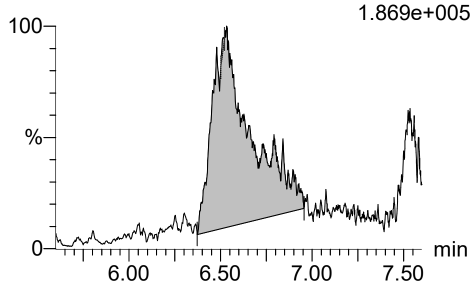

Sample Name: 20211111_L5_R2_IgM
F38:MRM of 2 channels,ES+
1580 > 366.1
2.116e+005

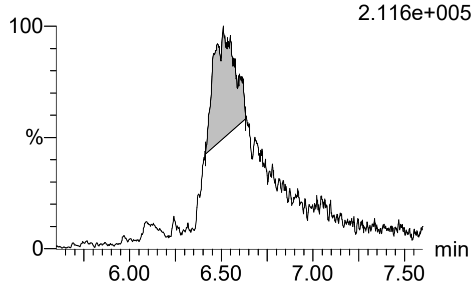

Sample Name: 20211111_L6_R2_IgM
F38:MRM of 2 channels,ES+
948.4 > 366.1
1.518e+005

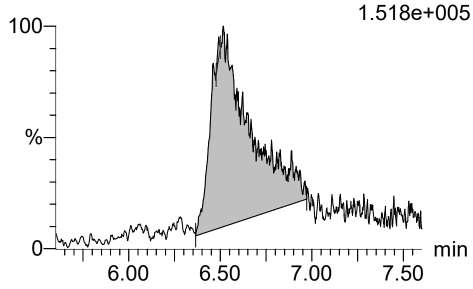

Sample Name: 20211111_L6_R2_IgM
F38:MRM of 2 channels,ES+
1580 > 366.1
1.855e+005

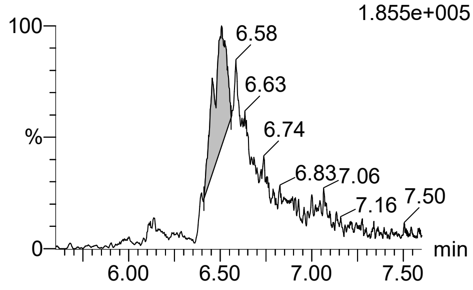

Sample Name: 20211111_H3_R2_IgM
F38:MRM of 2 channels,ES+
948.4 > 366.1
1.133e+005

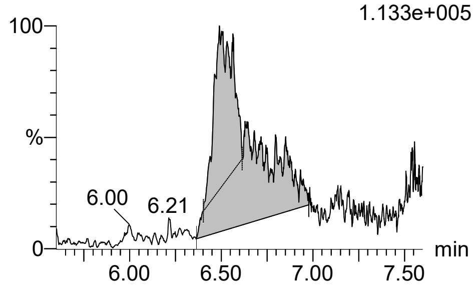

Sample Name: 20211111_H3_R2_IgM
F38:MRM of 2 channels,ES+
1580 > 366.1
9.540e+004

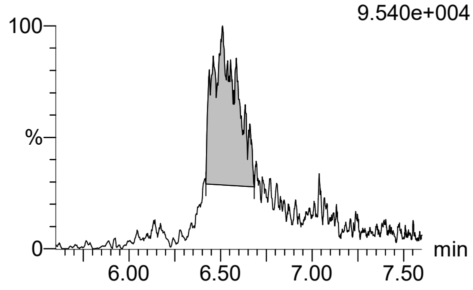

Dataset: C:\MassLynx\10.pro\20211111_clinicalsamples_IgM_IgA.qld

Last Altered: Thursday, March 09, 2023 19:58:25 台北標準時間
Printed: Friday, August 01, 2025 15:23:22 台北標準時間

Sample Name: 20211111_A7_R2_IgM
F38:MRM of 2 channels,ES+
948.4 > 366.1
1.784e+005

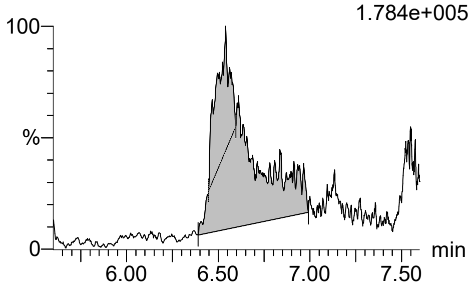

Sample Name: 20211111_A7_R2_IgM
F38:MRM of 2 channels,ES+
1580 > 366.1
1.818e+005

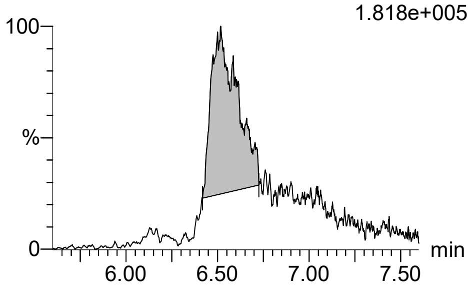

Sample Name: 20211111_A8_R2_IgM
F38:MRM of 2 channels,ES+
948.4 > 366.1
1.937e+005

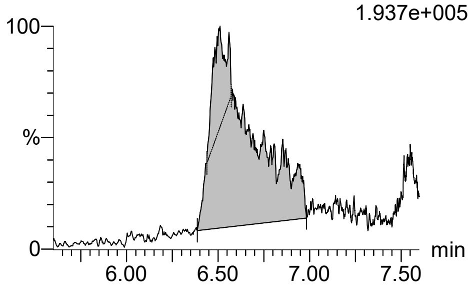

Sample Name: 20211111_A8_R2_IgM
F38:MRM of 2 channels,ES+
1580 > 366.1
2.231e+005

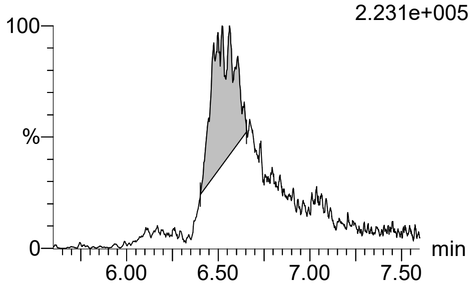

Sample Name: 20211111_L7_R2_IgM
F38:MRM of 2 channels,ES+
948.4 > 366.1
1.240e+005

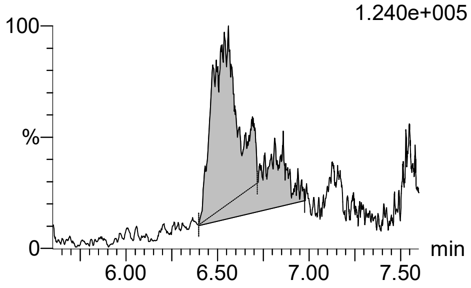

Sample Name: 20211111_L7_R2_IgM
F38:MRM of 2 channels,ES+
1580 > 366.1
1.450e+005

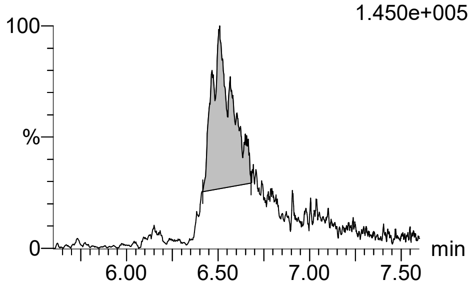

Sample Name: 20211111_L8_R2_IgM
F38:MRM of 2 channels,ES+
948.4 > 366.1
1.041e+005

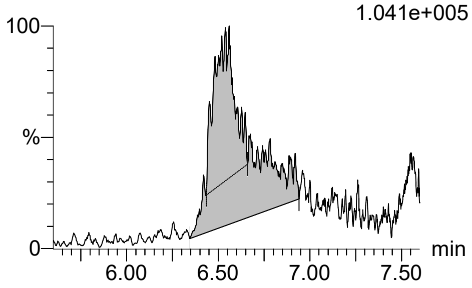

Sample Name: 20211111_L8_R2_IgM
F38:MRM of 2 channels,ES+
1580 > 366.1
7.528e+004

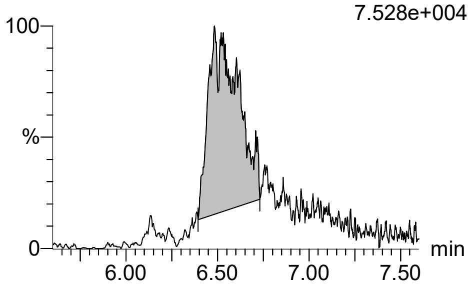

Sample Name: 20211111_H4_R2_IgM
F38:MRM of 2 channels,ES+
948.4 > 366.1
1.094e+005

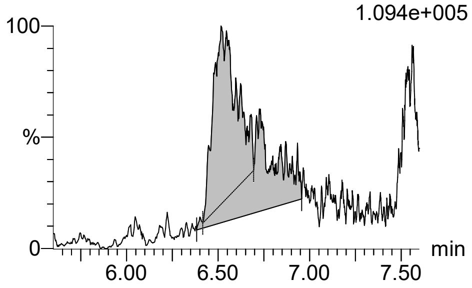

Sample Name: 20211111_H4_R2_IgM
F38:MRM of 2 channels,ES+
1580 > 366.1
1.039e+005

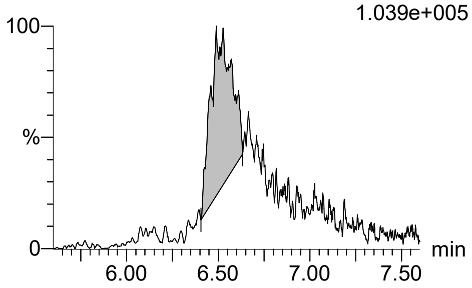

Sample Name: 20211111_A9_R2_IgM
F38:MRM of 2 channels,ES+
948.4 > 366.1
9.897e+004

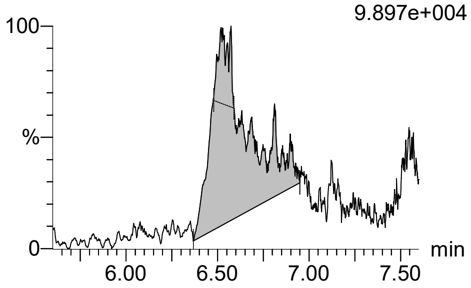

Sample Name: 20211111_A9_R2_IgM
F38:MRM of 2 channels,ES+
1580 > 366.1
9.540e+004

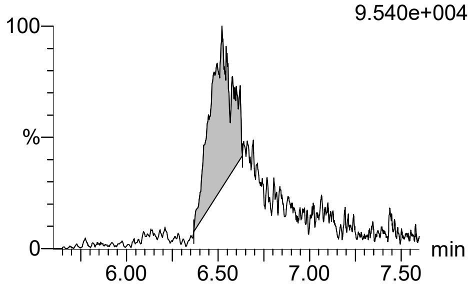

Sample Name: 20211111_A10_R2_IgM
F38:MRM of 2 channels,ES+
948.4 > 366.1
2.051e+005

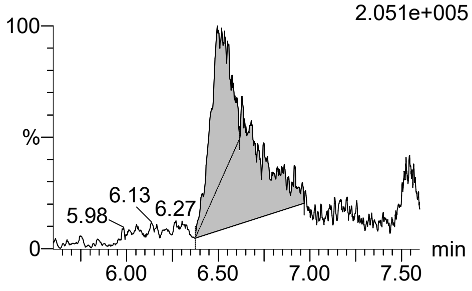

Sample Name: 20211111_A10_R2_IgM
F38:MRM of 2 channels,ES+
1580 > 366.1
2.167e+005

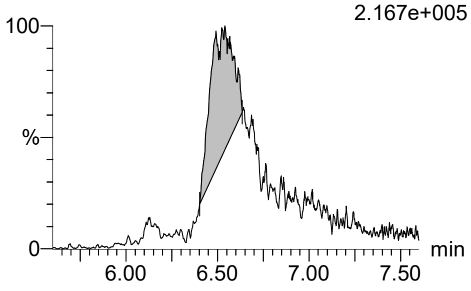

Sample Name: 20211111_L9_R2_IgM
F38:MRM of 2 channels,ES+
948.4 > 366.1
1.298e+005

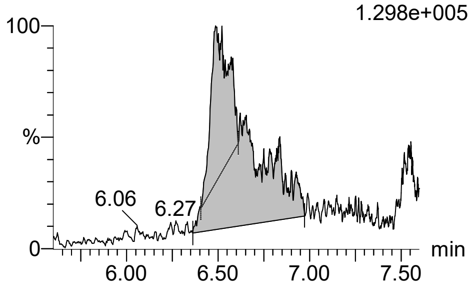

Dataset: C:\MassLynx\10.pro\20211111_clinicalsamples_IgM_IgA.qld

Last Altered: Thursday, March 09, 2023 19:58:25 台北標準時間
Printed: Friday, August 01, 2025 15:23:22 台北標準時間

Sample Name: 20211111_L9_R2_IgM
F38:MRM of 2 channels,ES+
1580 > 366.1
1.362e+005

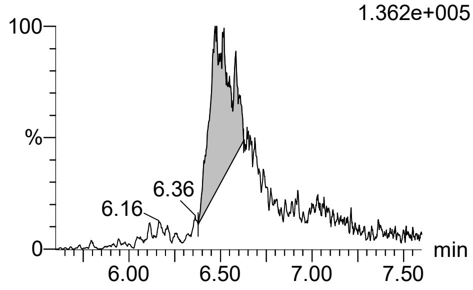

Sample Name: 20211111_L10_R2_IgM
F38:MRM of 2 channels,ES+
948.4 > 366.1
1.718e+005

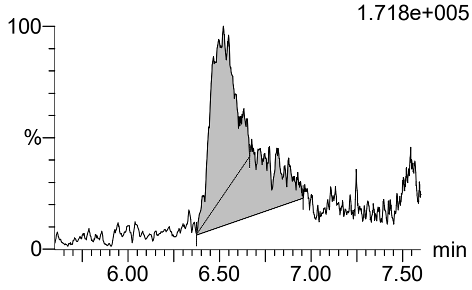

Sample Name: 20211111_L10_R2_IgM
F38:MRM of 2 channels,ES+
1580 > 366.1
2.240e+005

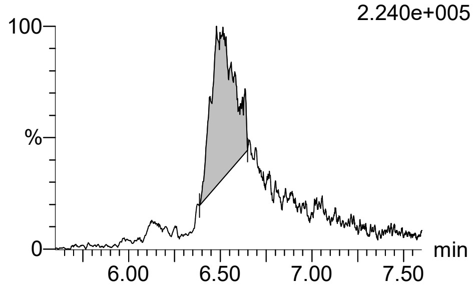

Sample Name: 20211111_QC_R11_IgM
F38:MRM of 2 channels,ES+
948.4 > 366.1
1.915e+005

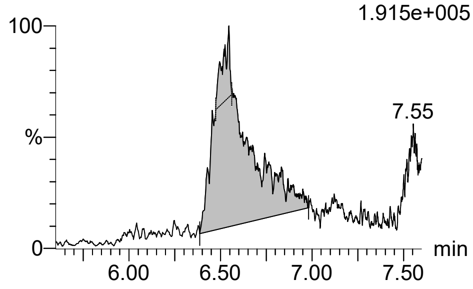

Sample Name: 20211111_QC_R11_IgM
F38:MRM of 2 channels,ES+
1580 > 366.1
1.932e+005

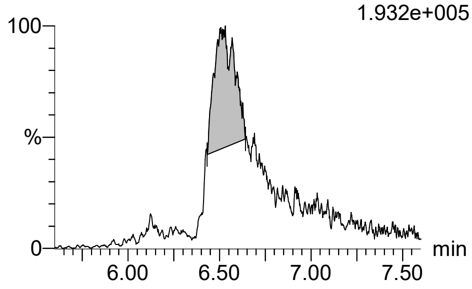

Sample Name: 20211111_H5_R2_IgM
F38:MRM of 2 channels,ES+
948.4 > 366.1
2.240e+005

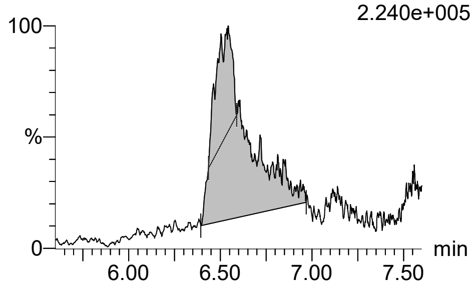

Sample Name: 20211111_H5_R2_IgM
F38:MRM of 2 channels,ES+
1580 > 366.1
2.851e+005

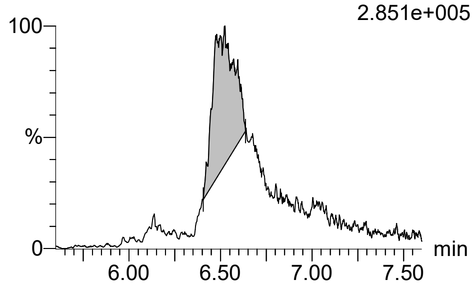

Sample Name: 20211111_A11_R2_IgM
F38:MRM of 2 channels,ES+
948.4 > 366.1
2.394e+005

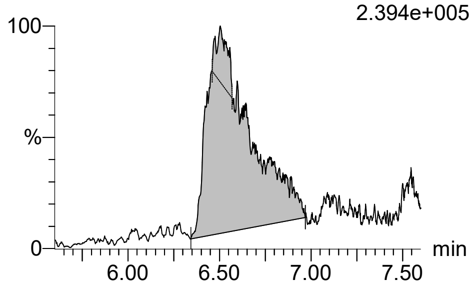

Sample Name: 20211111_A11_R2_IgM
F38:MRM of 2 channels,ES+
1580 > 366.1
3.453e+005

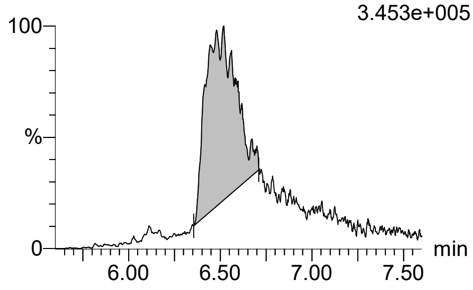

Sample Name: 20211111_A12_R2_IgM
F38:MRM of 2 channels,ES+
948.4 > 366.1
1.431e+005

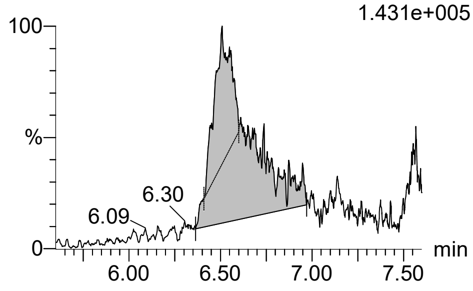

Sample Name: 20211111_A12_R2_IgM
F38:MRM of 2 channels,ES+
1580 > 366.1
1.633e+005

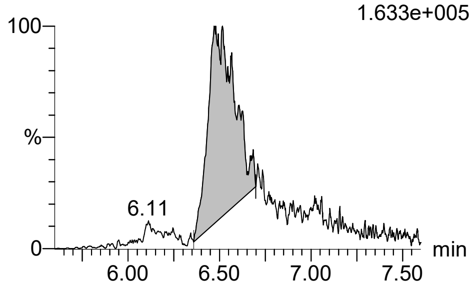

Sample Name: 20211111_L11_R2_IgM
F38:MRM of 2 channels,ES+
948.4 > 366.1
1.279e+005

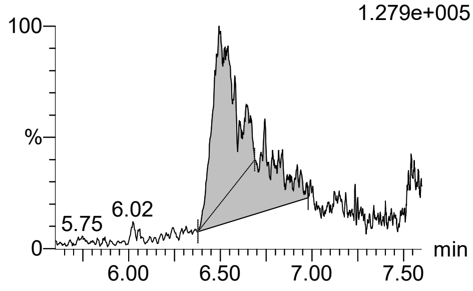

Sample Name: 20211111_L11_R2_IgM
F38:MRM of 2 channels,ES+
1580 > 366.1
1.274e+005

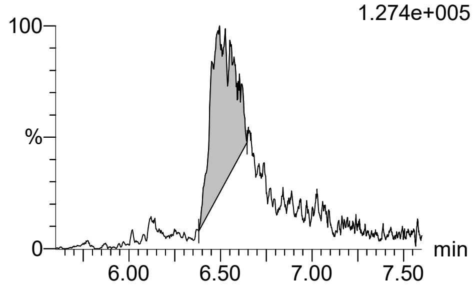

Sample Name: 20211111_L12_R2_IgM
F38:MRM of 2 channels,ES+
948.4 > 366.1
9.882e+004

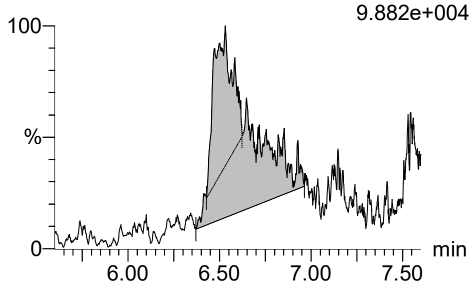

Sample Name: 20211111_L12_R2_IgM
F38:MRM of 2 channels,ES+
1580 > 366.1
1.029e+005

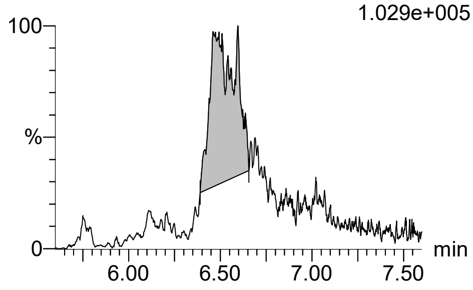

Sample Name: 20211111_H6_R2_IgM
F38:MRM of 2 channels,ES+
948.4 > 366.1
1.089e+005

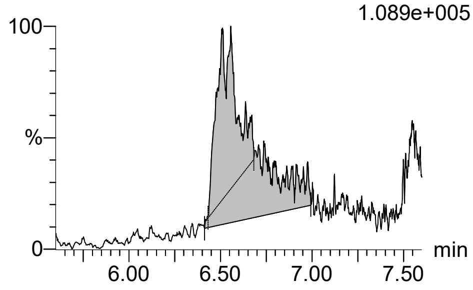

Sample Name: 20211111_H6_R2_IgM
F38:MRM of 2 channels,ES+
1580 > 366.1
9.936e+004

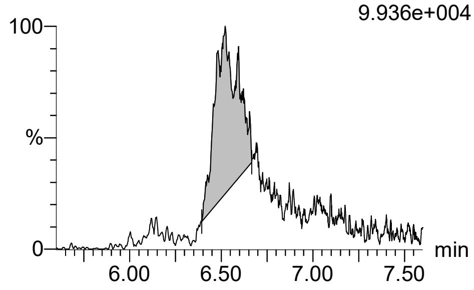

Sample Name: 20211111_A13_R2_IgM
F38:MRM of 2 channels,ES+
948.4 > 366.1
1.211e+005

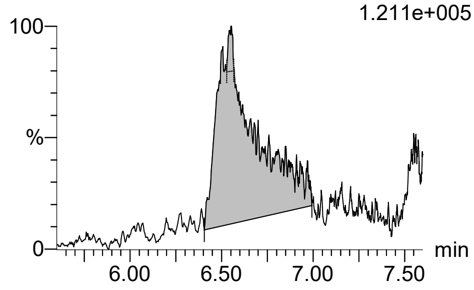

Sample Name: 20211111_A13_R2_IgM
F38:MRM of 2 channels,ES+
1580 > 366.1
1.328e+005

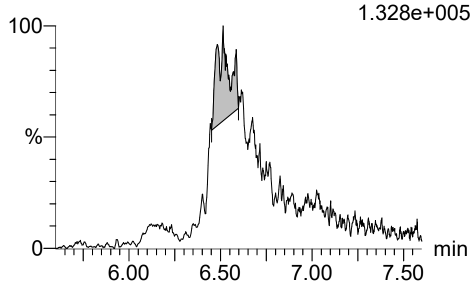

Sample Name: 20211111_A14_R2_IgM
F38:MRM of 2 channels,ES+
948.4 > 366.1
2.961e+005

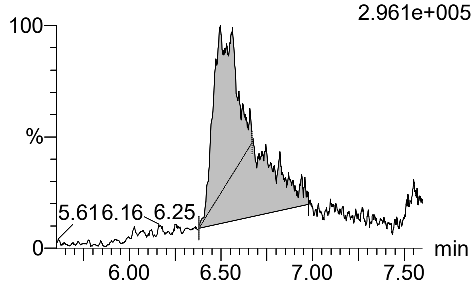

Sample Name: 20211111_A14_R2_IgM
F38:MRM of 2 channels,ES+
1580 > 366.1
4.192e+005

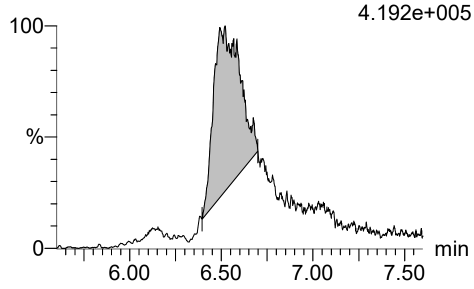

Sample Name: 20211111_L13_R2_IgM
F38:MRM of 2 channels,ES+
948.4 > 366.1
1.361e+005

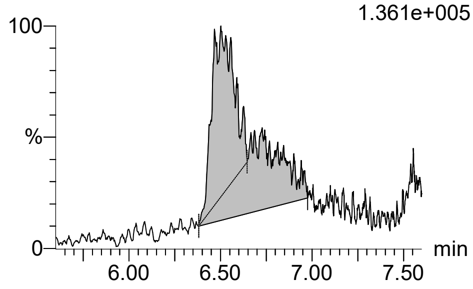

Sample Name: 20211111_L13_R2_IgM
F38:MRM of 2 channels,ES+
1580 > 366.1
1.640e+005

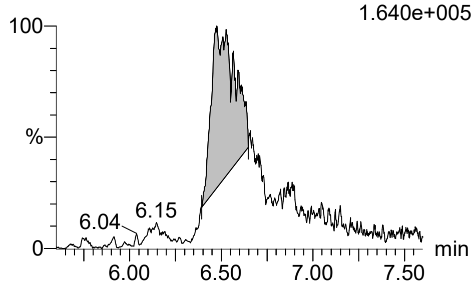

Sample Name: 20211111_L14_R2_IgM
F38:MRM of 2 channels,ES+
948.4 > 366.1
1.770e+005

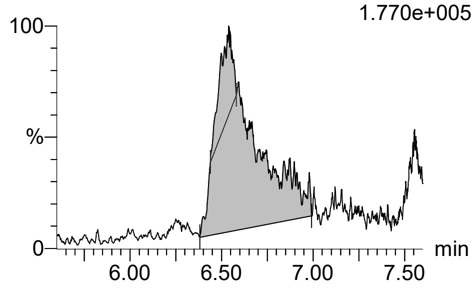

Sample Name: 20211111_L14_R2_IgM
F38:MRM of 2 channels,ES+
1580 > 366.1
2.445e+005

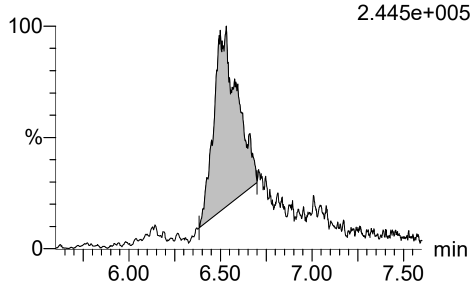

Sample Name: 20211111_H7_R2_IgM
F38:MRM of 2 channels,ES+
948.4 > 366.1
1.574e+005

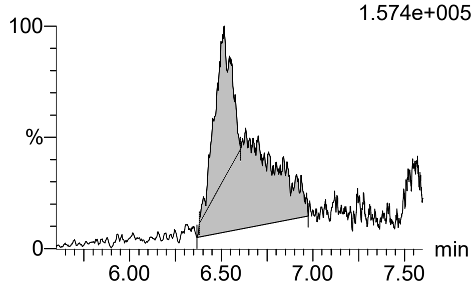

Sample Name: 20211111_H7_R2_IgM
F38:MRM of 2 channels,ES+
1580 > 366.1
1.835e+005

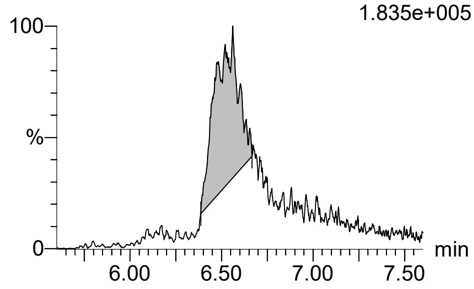

Sample Name: 20211111_A15_R2_IgM
F38:MRM of 2 channels,ES+
948.4 > 366.1
3.448e+005

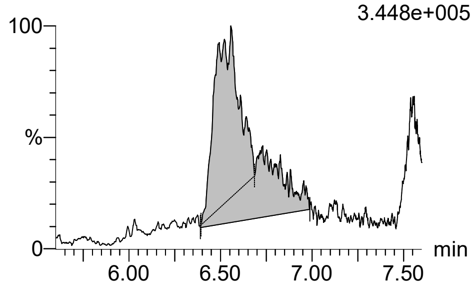

Sample Name: 20211111_A15_R2_IgM
F38:MRM of 2 channels,ES+
1580 > 366.1
5.222e+005

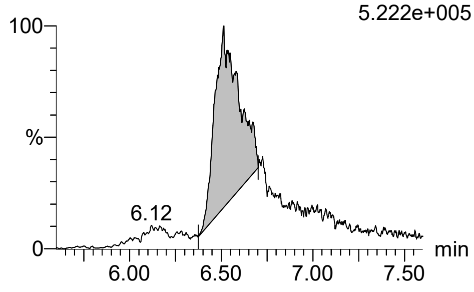

Sample Name: 20211111_QC_R12_IgM
F38:MRM of 2 channels,ES+
948.4 > 366.1
2.225e+005

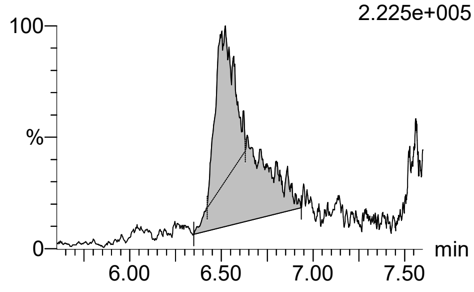

Sample Name: 20211111_QC_R12_IgM
F38:MRM of 2 channels,ES+
1580 > 366.1
2.647e+005

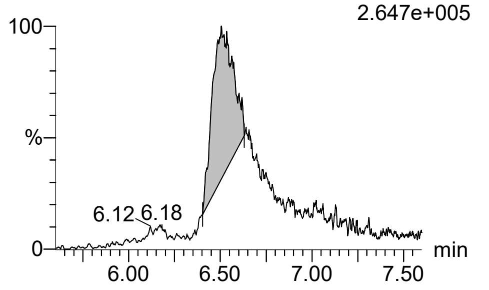

Sample Name: 20211111_A16_R2_IgM
F38:MRM of 2 channels,ES+
948.4 > 366.1
1.732e+005

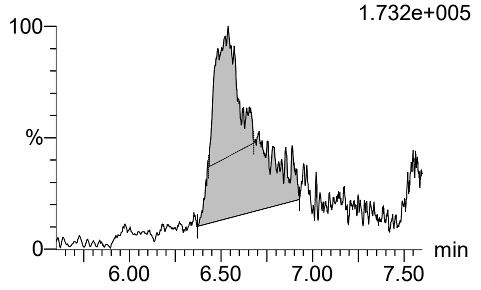

Sample Name: 20211111_A16_R2_IgM
F38:MRM of 2 channels,ES+
1580 > 366.1
2.232e+005

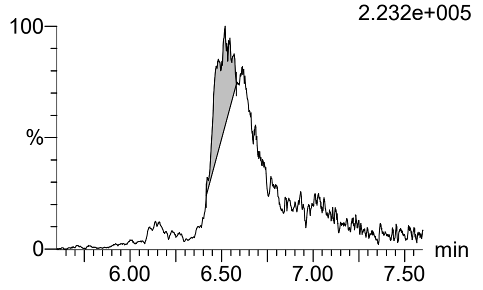

Sample Name: 20211111_L15_R2_IgM
F38:MRM of 2 channels,ES+
948.4 > 366.1
2.143e+005

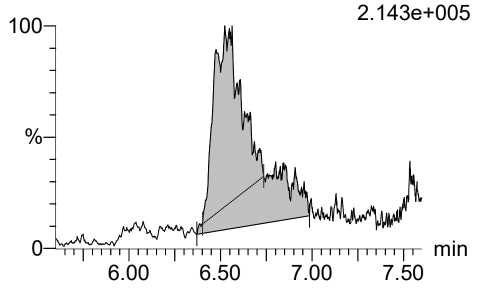

Sample Name: 20211111_L15_R2_IgM
F38:MRM of 2 channels,ES+
1580 > 366.1
2.674e+005

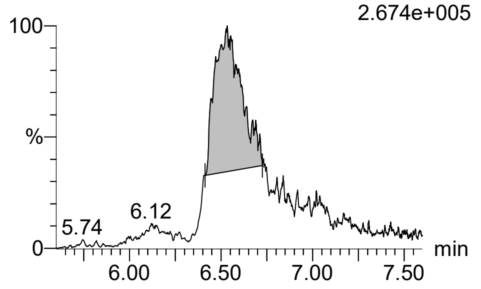

Sample Name: 20211111_L16_R2_IgM
F38:MRM of 2 channels,ES+
948.4 > 366.1
1.030e+005

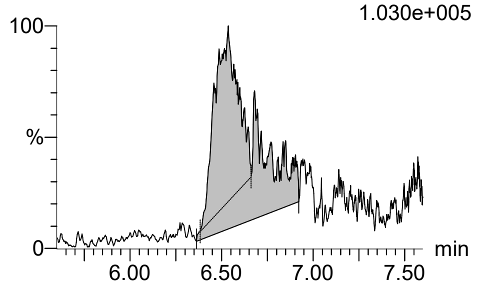

Sample Name: 20211111_L16_R2_IgM
F38:MRM of 2 channels,ES+
1580 > 366.1
1.252e+005

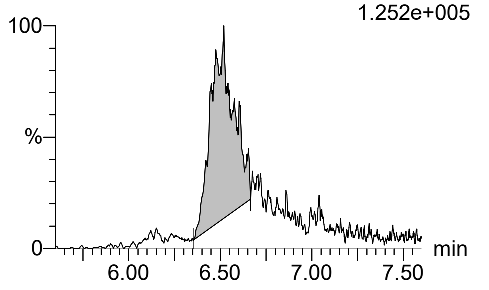

Sample Name: 20211111_H8_R2_IgM
F38:MRM of 2 channels,ES+
948.4 > 366.1
1.277e+005

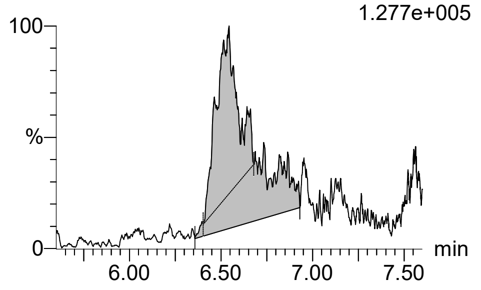

Sample Name: 20211111_H8_R2_IgM
F38:MRM of 2 channels,ES+
1580 > 366.1
1.291e+005

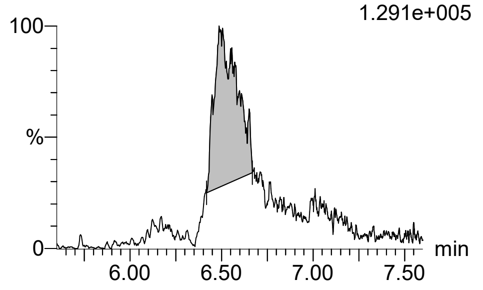

Sample Name: 20211111_A17_R2_IgM
F38:MRM of 2 channels,ES+
948.4 > 366.1
1.836e+005

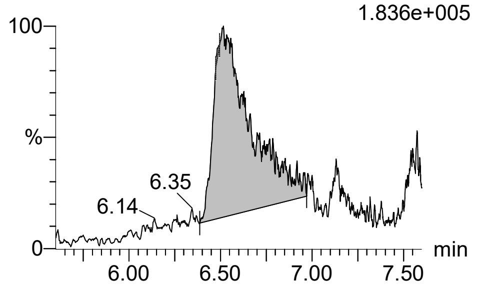

Sample Name: 20211111_A17_R2_IgM
F38:MRM of 2 channels,ES+
1580 > 366.1
2.214e+005

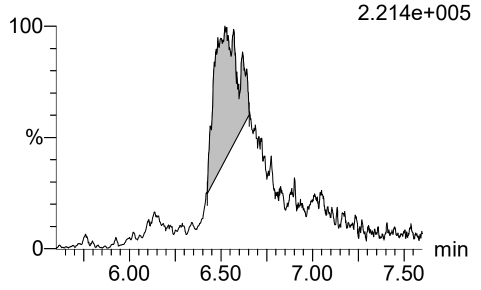

Sample Name: 20211111_A18_R2_IgM
F38:MRM of 2 channels,ES+
948.4 > 366.1
1.109e+005

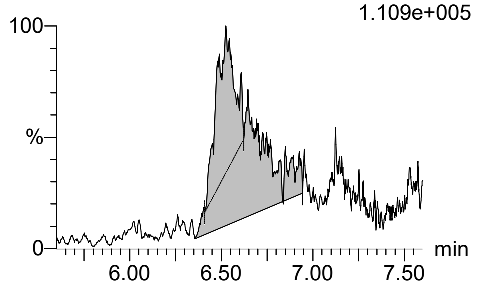

Sample Name: 20211111_A18_R2_IgM
F38:MRM of 2 channels,ES+
1580 > 366.1
1.370e+005

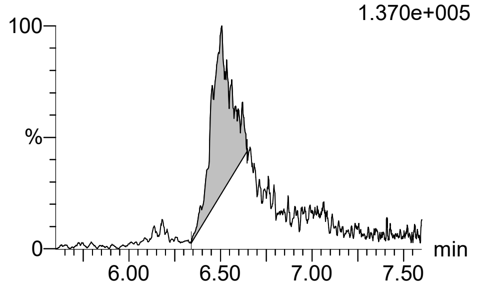

Sample Name: 20211111_L17_R2_IgM
F38:MRM of 2 channels,ES+
948.4 > 366.1
2.074e+005

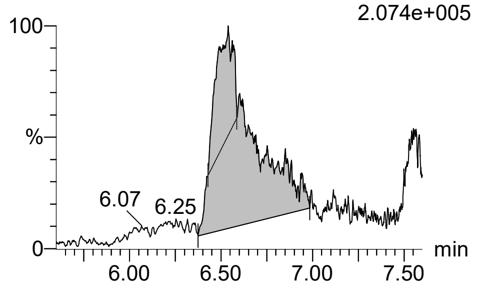

Sample Name: 20211111_L17_R2_IgM
F38:MRM of 2 channels,ES+
1580 > 366.1
2.929e+005

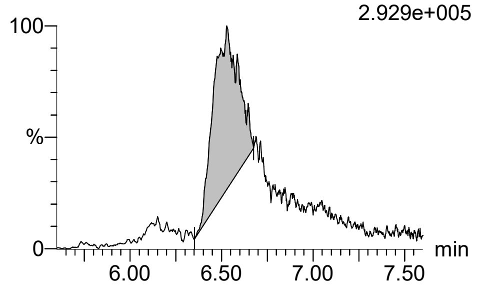

Dataset: C:\MassLynx\10.pro\20211111_clinicalsamples_IgM_IgA.qld

Last Altered: Thursday, March 09, 2023 19:58:25 台北標準時間
Printed: Friday, August 01, 2025 15:23:22 台北標準時間

Sample Name: 20211111_L18_R2_IgM
F38:MRM of 2 channels,ES+
948.4 > 366.1
1.139e+005

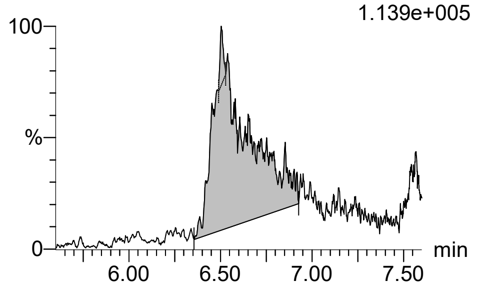

Sample Name: 20211111_L18_R2_IgM
F38:MRM of 2 channels,ES+
1580 > 366.1
9.985e+004

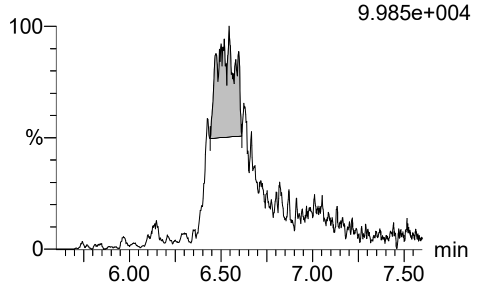

Sample Name: 20211111_H9_R2_IgM
F38:MRM of 2 channels,ES+
948.4 > 366.1
1.074e+005

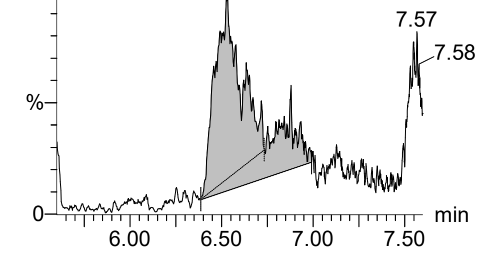

Sample Name: 20211111_H9_R2_IgM
F38:MRM of 2 channels,ES+
1580 > 366.1
1.067e+005

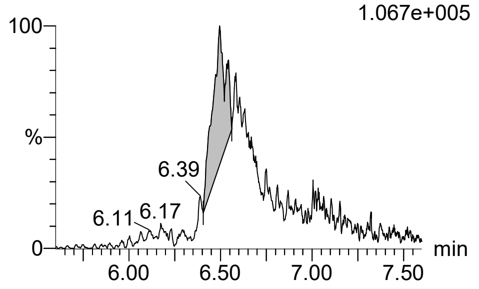

Sample Name: 20211111_A19_R2_IgM
F38:MRM of 2 channels,ES+
948.4 > 366.1
1.444e+005

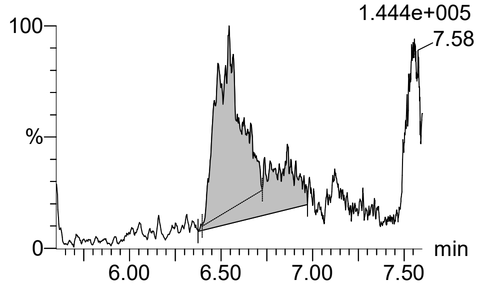

Sample Name: 20211111_A19_R2_IgM
F38:MRM of 2 channels,ES+
1580 > 366.1
1.670e+005

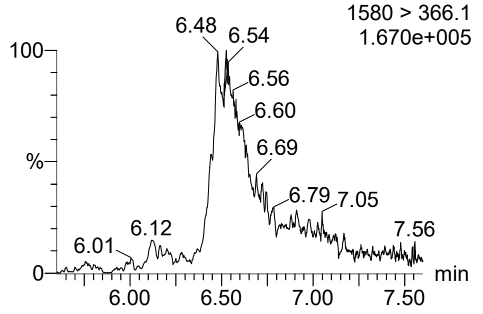

Sample Name: 20211111_A20_R2_IgM
F38:MRM of 2 channels,ES+
948.4 > 366.1
1.149e+005

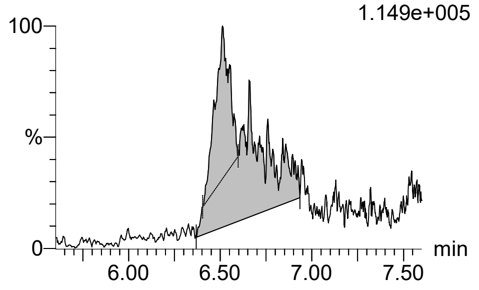

Sample Name: 20211111_A20_R2_IgM
F38:MRM of 2 channels,ES+
1580 > 366.1
9.403e+004

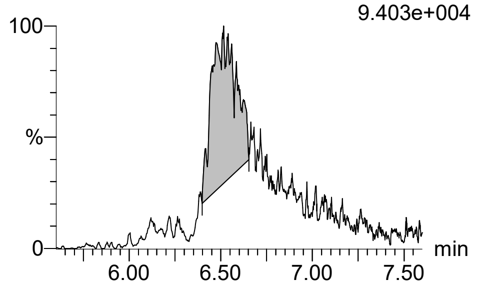

Sample Name: 20211111_L19_R2_IgM
F38:MRM of 2 channels,ES+
948.4 > 366.1
1.492e+005

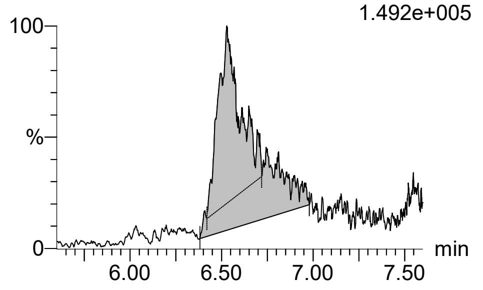

Sample Name: 20211111_L19_R2_IgM
F38:MRM of 2 channels,ES+
1580 > 366.1
1.573e+005

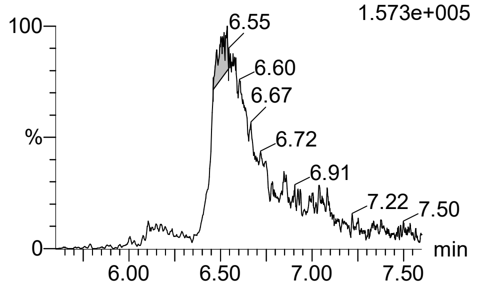

Sample Name: 20211111_QC_R13_IgM
F38:MRM of 2 channels,ES+
948.4 > 366.1
1.560e+005

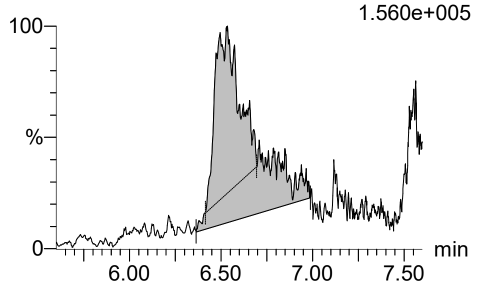

Sample Name: 20211111_QC_R13_IgM
F38:MRM of 2 channels,ES+
1580 > 366.1
2.137e+005

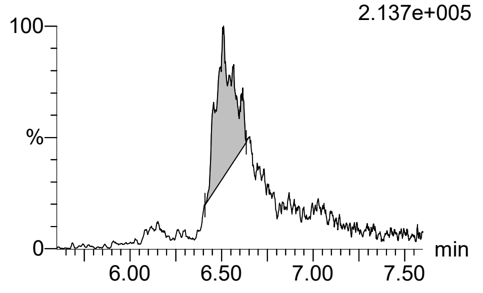

Sample Name: 20211111_L20_R2_IgM
F38:MRM of 2 channels,ES+
948.4 > 366.1
1.112e+005

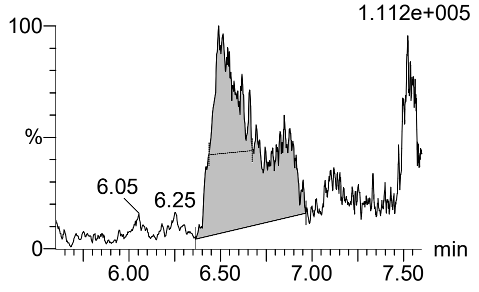

Sample Name: 20211111_L20_R2_IgM
F38:MRM of 2 channels,ES+
1580 > 366.1
1.278e+005

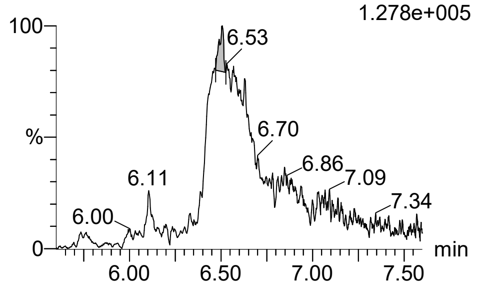

Sample Name: 20211111_H10_R2_IgM
F38:MRM of 2 channels,ES+
948.4 > 366.1
1.579e+005

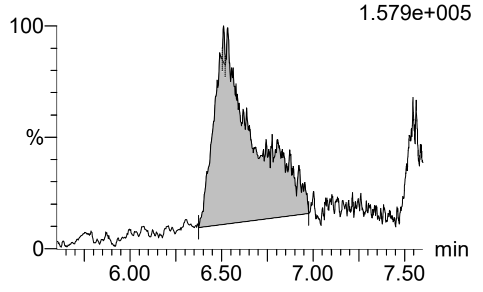

Sample Name: 20211111_H10_R2_IgM
F38:MRM of 2 channels,ES+
1580 > 366.1
1.835e+005

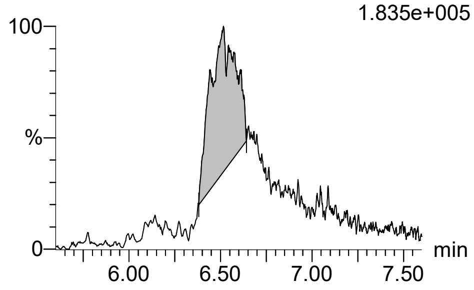

Sample Name: 20211111_A21_R2_IgM
F38:MRM of 2 channels,ES+
948.4 > 366.1
1.275e+005

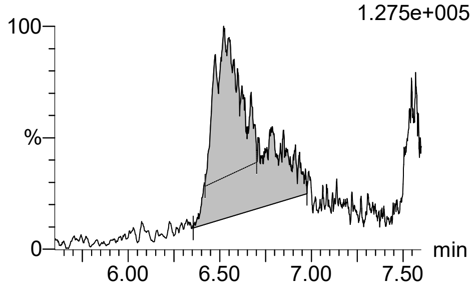

Sample Name: 20211111_A21_R2_IgM
F38:MRM of 2 channels,ES+
1580 > 366.1
1.482e+005

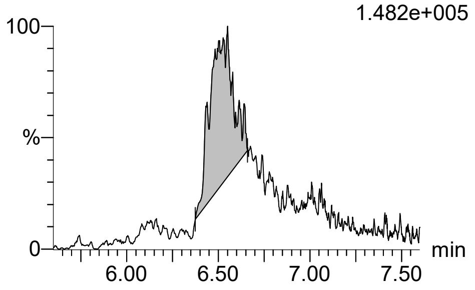

Sample Name: 20211111_A22_R2_IgM
F38:MRM of 2 channels,ES+
948.4 > 366.1
8.629e+004

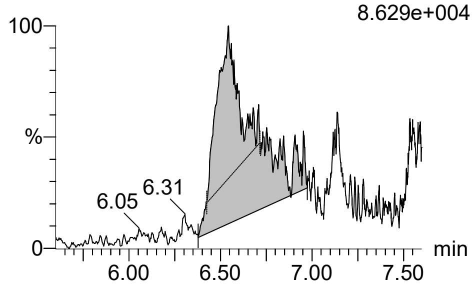

Sample Name: 20211111_A22_R2_IgM
F38:MRM of 2 channels,ES+
1580 > 366.1
7.573e+004

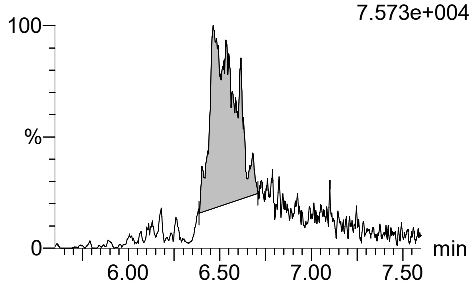

Sample Name: 20211111_L21_R2_IgM
F38:MRM of 2 channels,ES+
948.4 > 366.1
1.398e+005

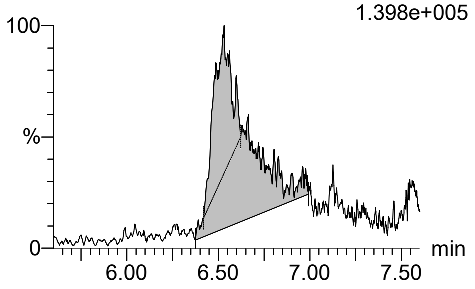

Sample Name: 20211111_L21_R2_IgM
F38:MRM of 2 channels,ES+
1580 > 366.1
1.507e+005

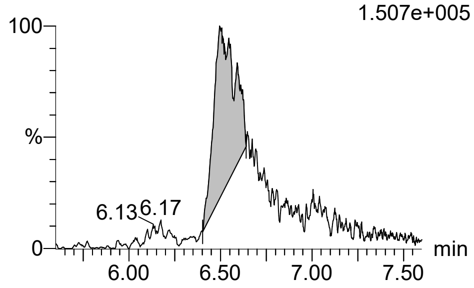

Sample Name: 20211111_L22_R2_IgM
F38:MRM of 2 channels,ES+
948.4 > 366.1
1.138e+005

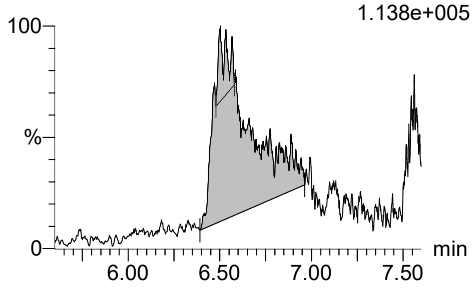

Sample Name: 20211111_L22_R2_IgM
F38:MRM of 2 channels,ES+
1580 > 366.1
1.255e+005

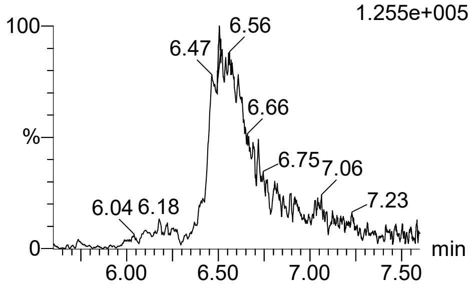

Sample Name: 20211111_H11_R2_IgM
F38:MRM of 2 channels,ES+
948.4 > 366.1
1.428e+005

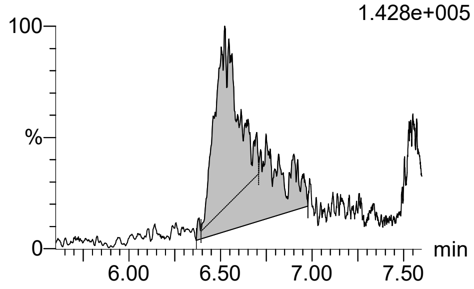

Sample Name: 20211111_H11_R2_IgM
F38:MRM of 2 channels,ES+
1580 > 366.1
1.402e+005

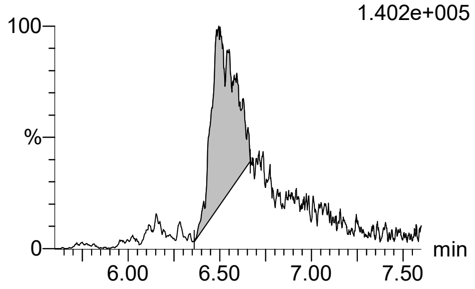

Sample Name: 20211111_A23_R2_IgM
F38:MRM of 2 channels,ES+
948.4 > 366.1
1.168e+005

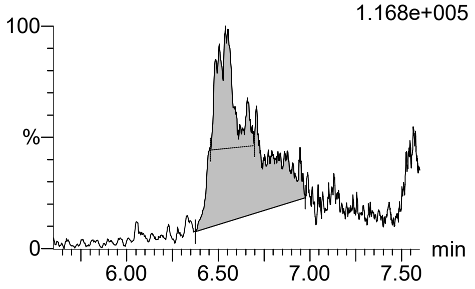

Sample Name: 20211111_A23_R2_IgM
F38:MRM of 2 channels,ES+
1580 > 366.1
1.152e+005

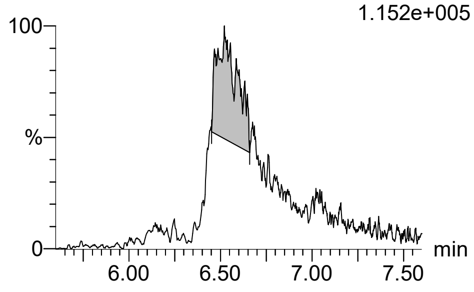

Sample Name: 20211111_A24_R2_IgM
F38:MRM of 2 channels,ES+
948.4 > 366.1
1.763e+005

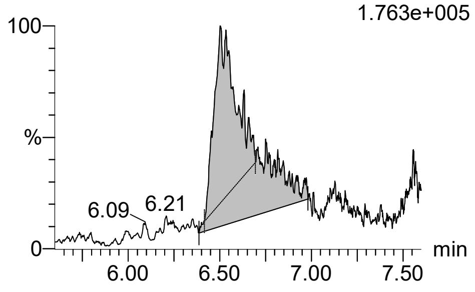

Sample Name: 20211111_A24_R2_IgM
F38:MRM of 2 channels,ES+
1580 > 366.1
2.068e+005

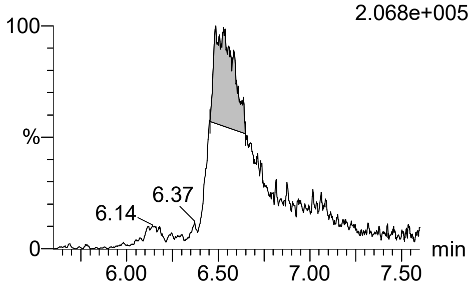

Dataset: C:\MassLynx\10.pro\20211111_clinicalsamples_IgM_IgA.qld

Last Altered: Thursday, March 09, 2023 19:58:25 台北標準時間
Printed: Friday, August 01, 2025 15:23:22 台北標準時間

Sample Name: 20211111_L23_R2_IgM
F38:MRM of 2 channels,ES+
948.4 > 366.1
1.849e+005

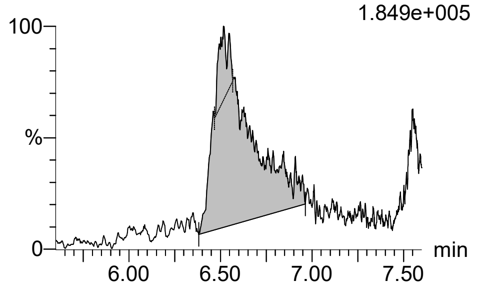

Sample Name: 20211111_L23_R2_IgM
F38:MRM of 2 channels,ES+
1580 > 366.1
2.355e+005

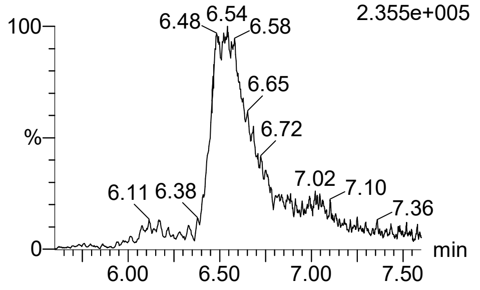

Sample Name: 20211111_L24_R2_IgM
F38:MRM of 2 channels,ES+
948.4 > 366.1
1.004e+005

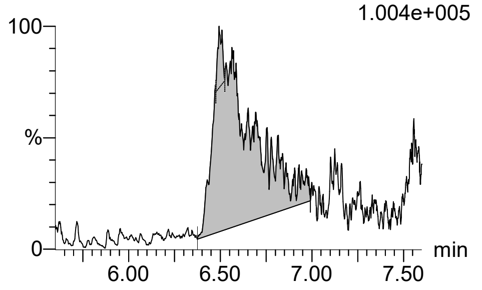

Sample Name: 20211111_L24_R2_IgM
F38:MRM of 2 channels,ES+
1580 > 366.1
1.078e+005

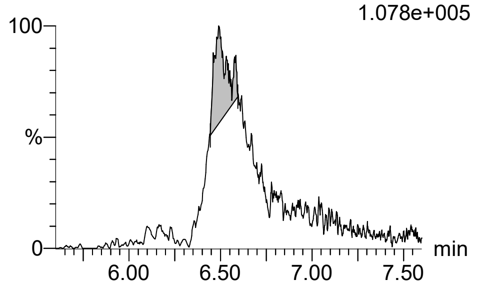

Sample Name: 20211111_H12_R2_IgM
F38:MRM of 2 channels,ES+
948.4 > 366.1
1.049e+005

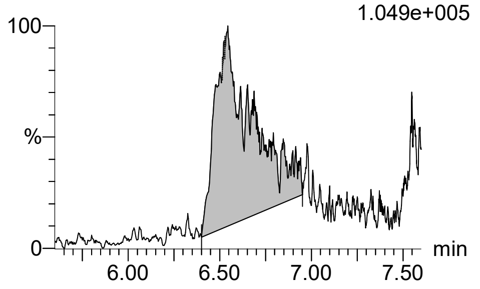

Sample Name: 20211111_H12_R2_IgM
F38:MRM of 2 channels,ES+
1580 > 366.1
1.138e+005

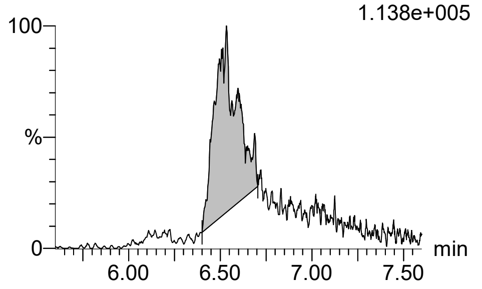

Sample Name: 20211111_QC_R14_IgM
F38:MRM of 2 channels,ES+
948.4 > 366.1
1.518e+005

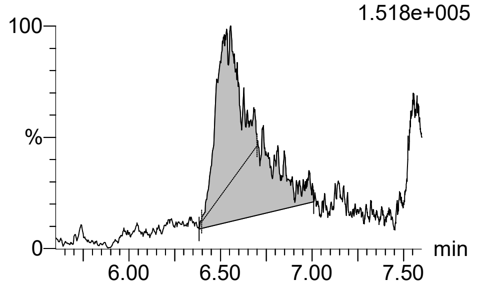

Sample Name: 20211111_QC_R14_IgM
F38:MRM of 2 channels,ES+
1580 > 366.1
1.998e+005

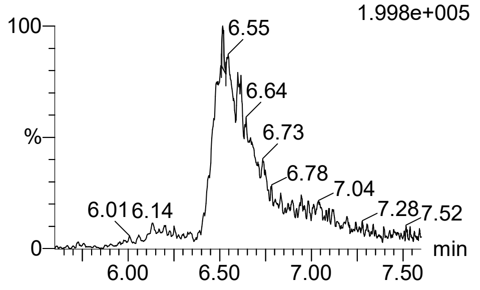

Sample Name: 20211111_A25_R2_IgM
F38:MRM of 2 channels,ES+
948.4 > 366.1
2.221e+005

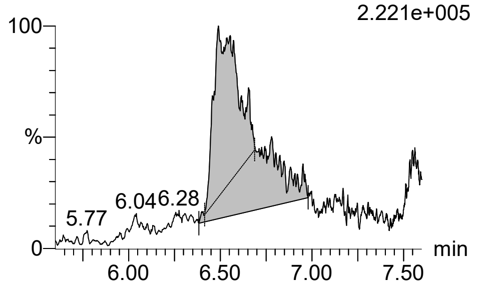

Sample Name: 20211111_A25_R2_IgM
F38:MRM of 2 channels,ES+
1580 > 366.1
3.163e+005

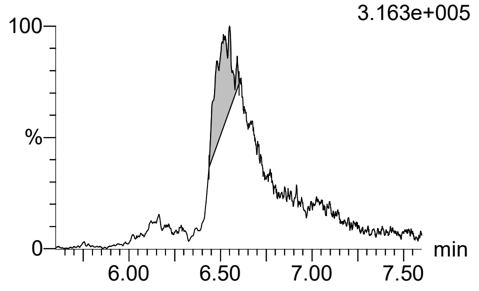

Sample Name: 20211111_A26_R2_IgM
F38:MRM of 2 channels,ES+
948.4 > 366.1
1.553e+005

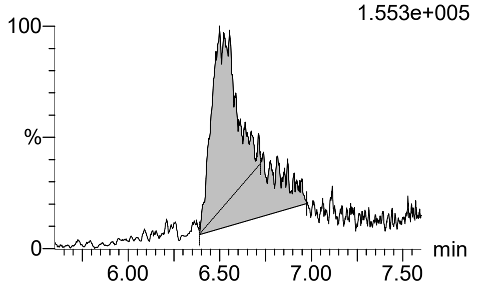

Sample Name: 20211111_A26_R2_IgM
F38:MRM of 2 channels,ES+
1580 > 366.1
1.780e+005

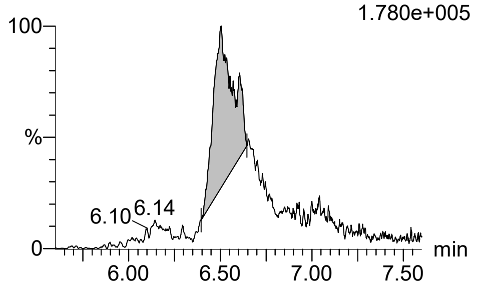

Sample Name: 20211111_L25_R2_IgM
F38:MRM of 2 channels,ES+
948.4 > 366.1
1.235e+005

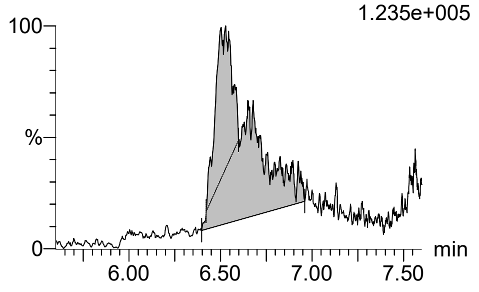

Sample Name: 20211111_L25_R2_IgM
F38:MRM of 2 channels,ES+
1580 > 366.1
1.332e+005

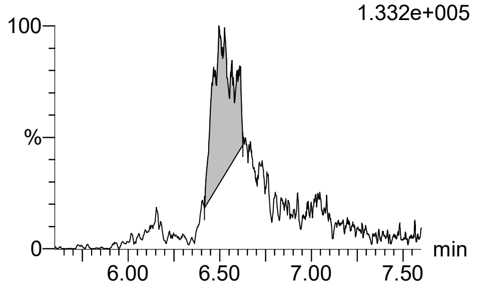

Sample Name: 20211111_L26_R2_IgM
F38:MRM of 2 channels,ES+
948.4 > 366.1
1.140e+005

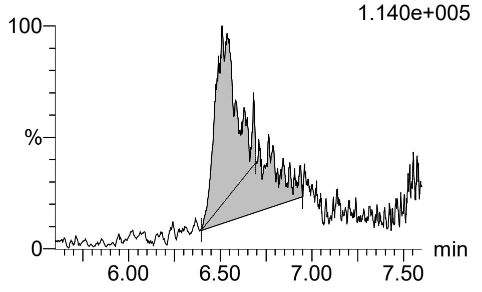

Dataset: C:\MassLynx\10.pro\20211111_clinicalsamples_IgM_IgA.qld

Last Altered: Thursday, March 09, 2023 19:58:25 台北標準時間
Printed: Friday, August 01, 2025 15:23:22 台北標準時間

Sample Name: 20211111_L26_R2_IgM
F38:MRM of 2 channels,ES+
1580 > 366.1
1.049e+005

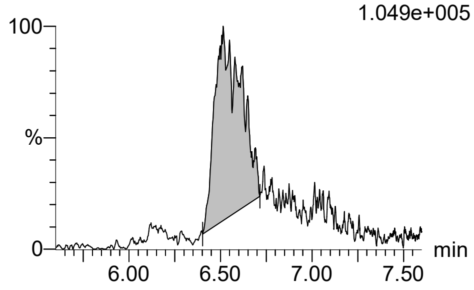

Sample Name: 20211111_H13_R2_IgM
F38:MRM of 2 channels,ES+
948.4 > 366.1
1.503e+005

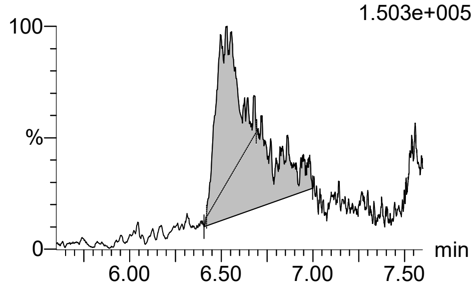

Sample Name: 20211111_H13_R2_IgM
F38:MRM of 2 channels,ES+
1580 > 366.1
1.887e+005

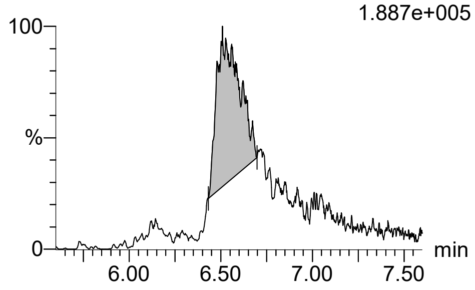

Sample Name: 20211111_A27_R2_IgM
F38:MRM of 2 channels,ES+
948.4 > 366.1
2.787e+005

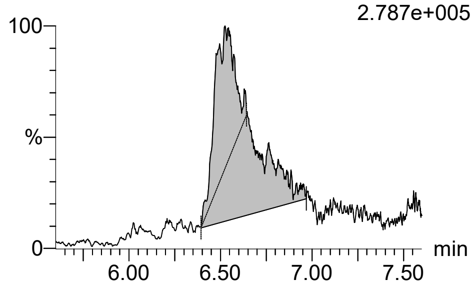

Sample Name: 20211111_A27_R2_IgM
F38:MRM of 2 channels,ES+
1580 > 366.1
3.779e+005

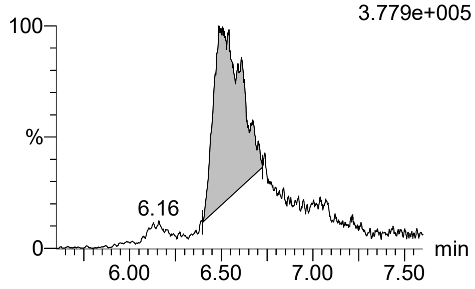

Sample Name: 20211111_A28_R2_IgM
F38:MRM of 2 channels,ES+
948.4 > 366.1
1.526e+005

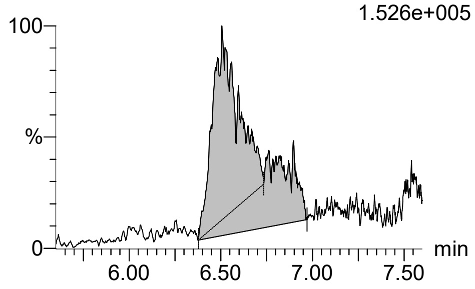

Sample Name: 20211111_A28_R2_IgM
F38:MRM of 2 channels,ES+
1580 > 366.1
1.713e+005

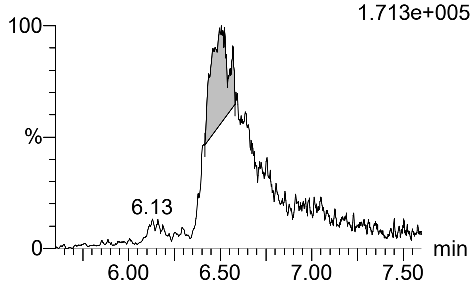

Sample Name: 20211111_L27_R2_IgM
F38:MRM of 2 channels,ES+
948.4 > 366.1
1.453e+005

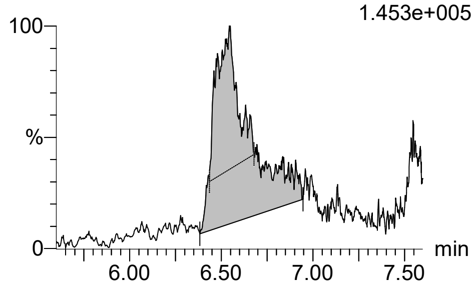

Sample Name: 20211111_L27_R2_IgM
F38:MRM of 2 channels,ES+
1580 > 366.1
1.493e+005

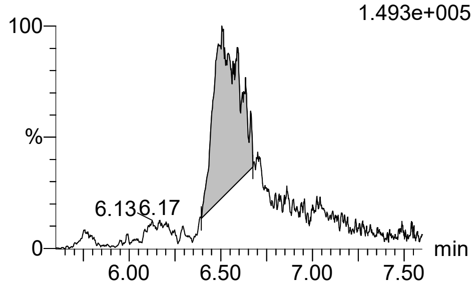

Sample Name: 20211111_L28_R2_IgM
F38:MRM of 2 channels,ES+
948.4 > 366.1
1.160e+005

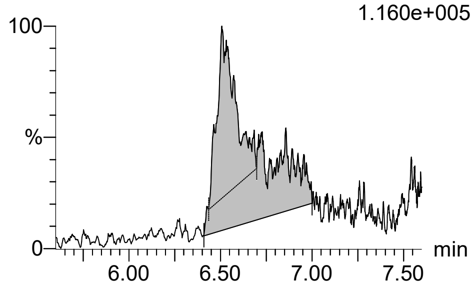

Sample Name: 20211111_L28_R2_IgM
F38:MRM of 2 channels,ES+
1580 > 366.1
1.032e+005

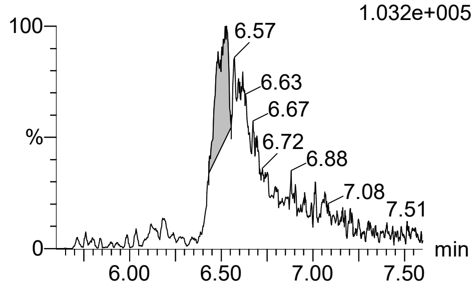

Sample Name: 20211111_H14_R2_IgM
F38:MRM of 2 channels,ES+
948.4 > 366.1
1.294e+005

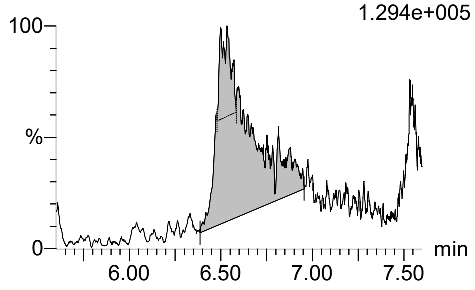

Sample Name: 20211111_H14_R2_IgM
F38:MRM of 2 channels,ES+
1580 > 366.1
1.231e+005

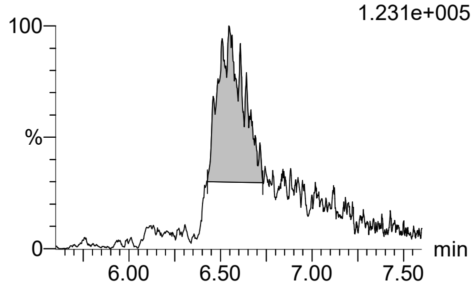

Sample Name: 20211111_A29_R2_IgM
F38:MRM of 2 channels,ES+
948.4 > 366.1
8.244e+004

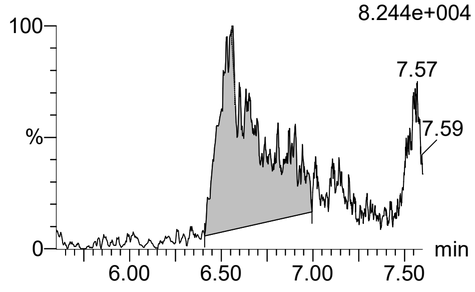

Sample Name: 20211111_A29_R2_IgM
F38:MRM of 2 channels,ES+
1580 > 366.1
5.902e+004

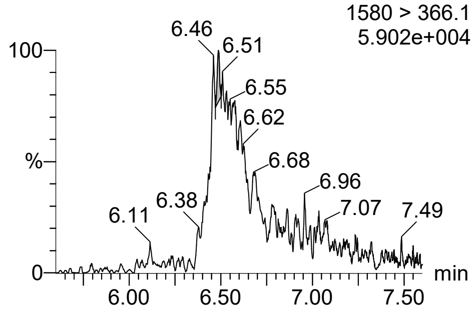

Sample Name: 20211111_A30_R2_IgM
F38:MRM of 2 channels,ES+
948.4 > 366.1
1.782e+005

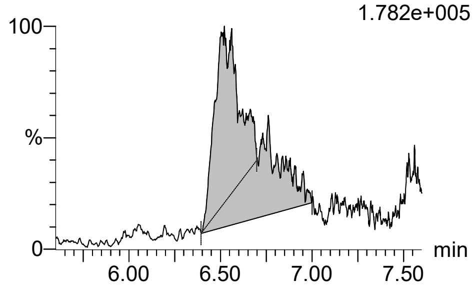

Sample Name: 20211111_A30_R2_IgM
F38:MRM of 2 channels,ES+
1580 > 366.1
2.112e+005

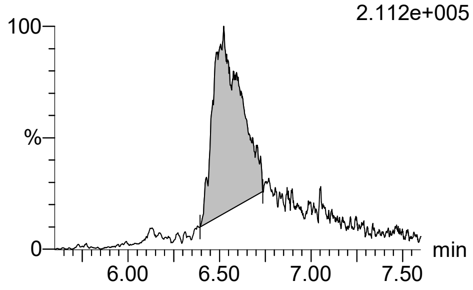

Sample Name: 20211111_QC_R15_IgM
F38:MRM of 2 channels,ES+
948.4 > 366.1
1.706e+005

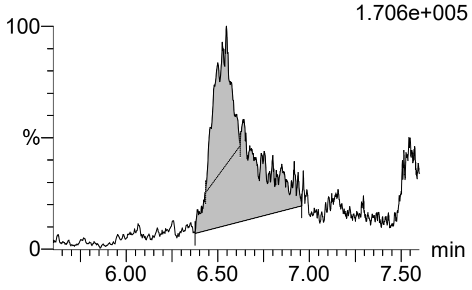

Sample Name: 20211111_QC_R15_IgM
F38:MRM of 2 channels,ES+
1580 > 366.1
1.619e+005

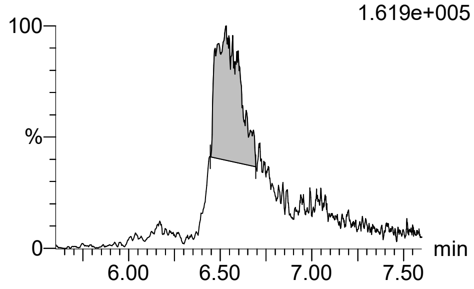

Sample Name: 20211111_L29_R2_IgM
F38:MRM of 2 channels,ES+
948.4 > 366.1
9.920e+004

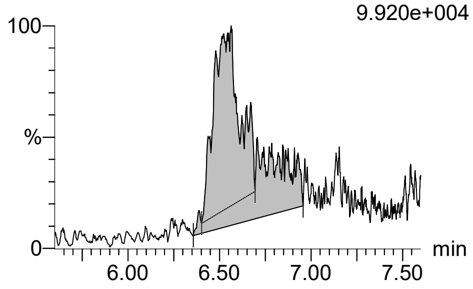

Sample Name: 20211111_L29_R2_IgM
F38:MRM of 2 channels,ES+
1580 > 366.1
9.255e+004

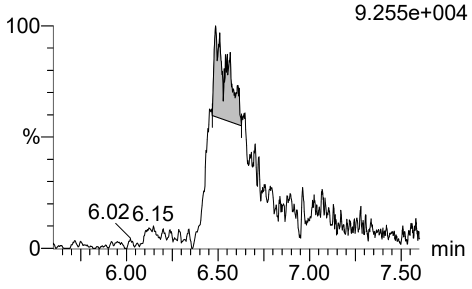

Sample Name: 20211111_L30_R2_IgM
F38:MRM of 2 channels,ES+
948.4 > 366.1
8.300e+004

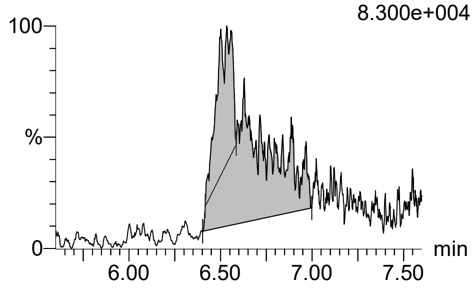

Sample Name: 20211111_L30_R2_IgM
F38:MRM of 2 channels,ES+
1580 > 366.1
6.208e+004

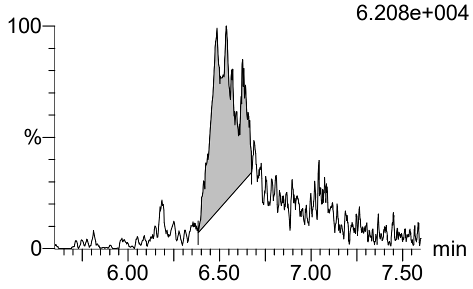

Sample Name: 20211111_H15_R2_IgM
F38:MRM of 2 channels,ES+
948.4 > 366.1
1.158e+005

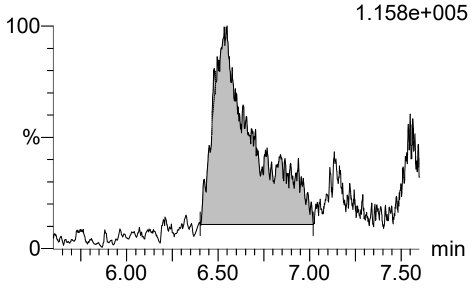

Sample Name: 20211111_H15_R2_IgM
F38:MRM of 2 channels,ES+
1580 > 366.1
1.079e+005

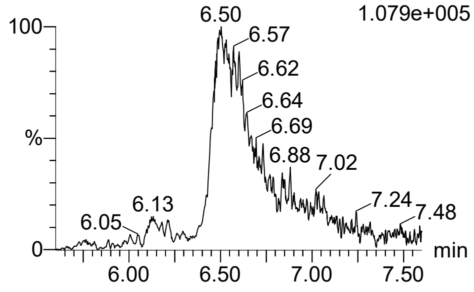

Sample Name: 20211111_A31_R2_IgM
F38:MRM of 2 channels,ES+
948.4 > 366.1
1.099e+005

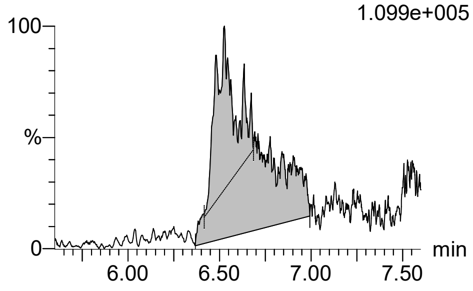

Sample Name: 20211111_A31_R2_IgM
F38:MRM of 2 channels,ES+
1580 > 366.1
8.699e+004

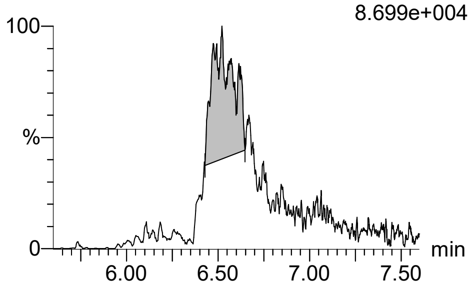

Sample Name: 20211111_A32_R2_IgM
F38:MRM of 2 channels,ES+
948.4 > 366.1
1.456e+005

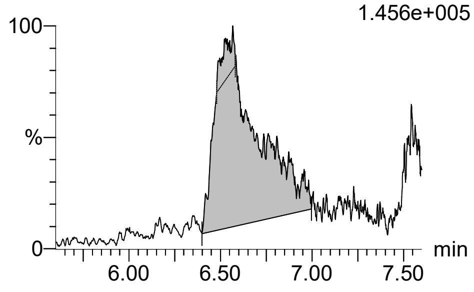

Sample Name: 20211111_A32_R2_IgM
F38:MRM of 2 channels,ES+
1580 > 366.1
1.702e+005

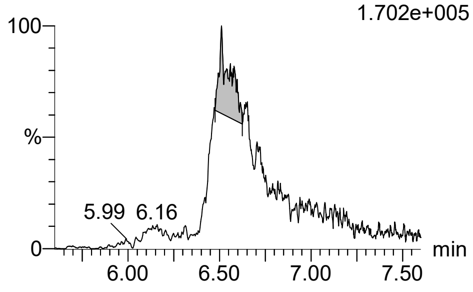

Sample Name: 20211111_L31_R2_IgM
F38:MRM of 2 channels,ES+
948.4 > 366.1
7.126e+004

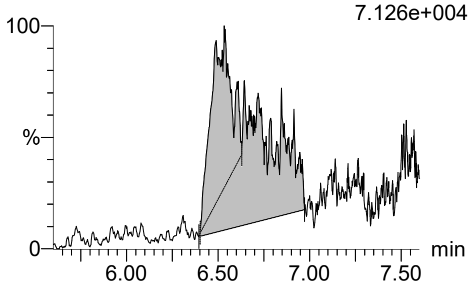

Dataset: C:\MassLynx\10.pro\20211111_clinicalsamples_IgM_IgA.qld

Last Altered: Thursday, March 09, 2023 19:58:25 台北標準時間
Printed: Friday, August 01, 2025 15:23:22 台北標準時間

Sample Name: 20211111_L31_R2_IgM
F38:MRM of 2 channels,ES+
1580 > 366.1
5.520e+004

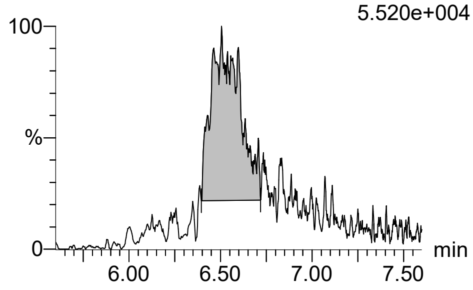

Sample Name: 20211111_L32_R2_IgM
F38:MRM of 2 channels,ES+
948.4 > 366.1
1.720e+005

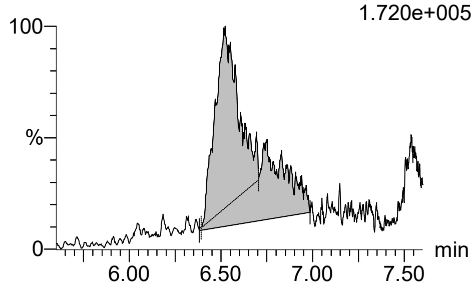

Sample Name: 20211111_L32_R2_IgM
F38:MRM of 2 channels,ES+
1580 > 366.1
1.949e+005

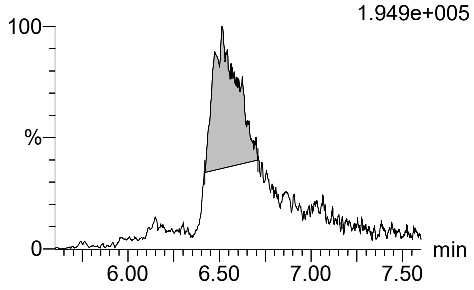

Sample Name: 20211111_H16_R2_IgM
F38:MRM of 2 channels,ES+
948.4 > 366.1
2.313e+005

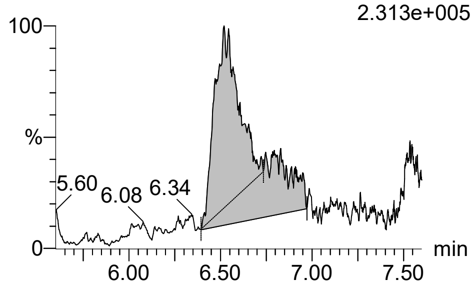

Sample Name: 20211111_H16_R2_IgM
F38:MRM of 2 channels,ES+
1580 > 366.1
3.177e+005

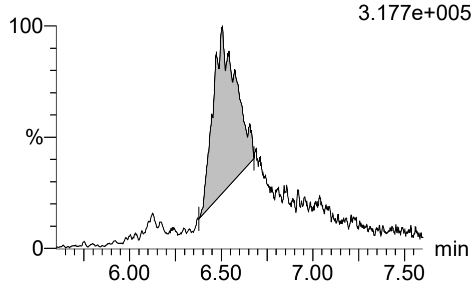

Sample Name: 20211111_A33_R2_IgM
F38:MRM of 2 channels,ES+
948.4 > 366.1
2.717e+005

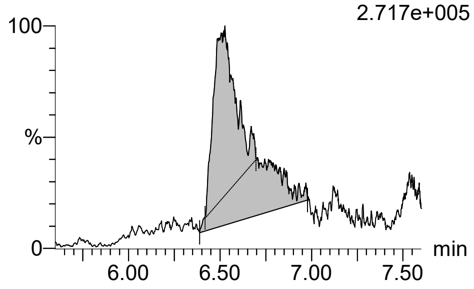

Sample Name: 20211111_A33_R2_IgM
F38:MRM of 2 channels,ES+
1580 > 366.1
3.214e+005

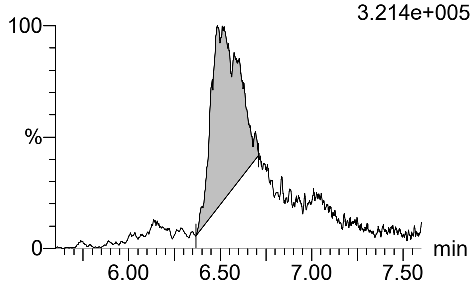

Sample Name: 20211111_A34_R2_IgM
F38:MRM of 2 channels,ES+
948.4 > 366.1
1.338e+005

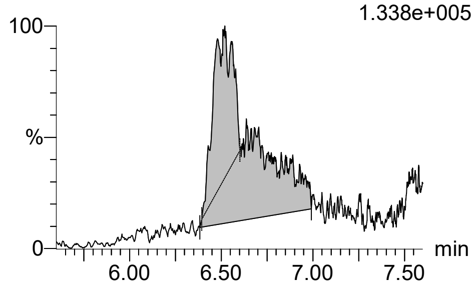

Sample Name: 20211111_A34_R2_IgM
F38:MRM of 2 channels,ES+
1580 > 366.1
1.392e+005

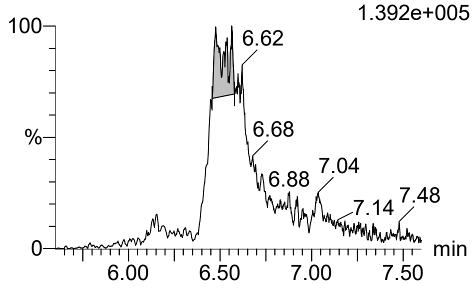

Sample Name: 20211111_H17_R2_IgM
F38:MRM of 2 channels,ES+
948.4 > 366.1
4.696e+005

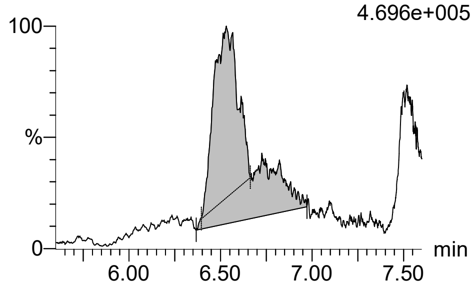

Sample Name: 20211111_H17_R2_IgM
F38:MRM of 2 channels,ES+
1580 > 366.1
6.970e+005

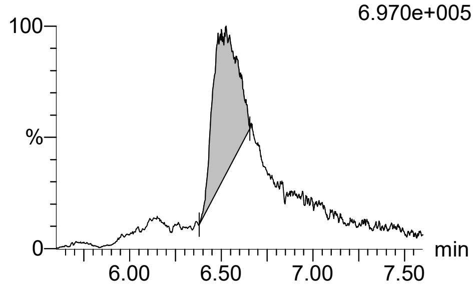

Sample Name: 20211111_A35_R2_IgM
F38:MRM of 2 channels,ES+
948.4 > 366.1
1.710e+005

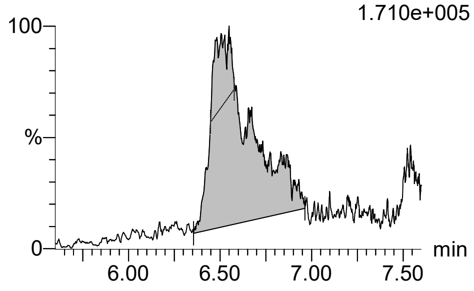

Sample Name: 20211111_A35_R2_IgM
F38:MRM of 2 channels,ES+
1580 > 366.1
2.126e+005

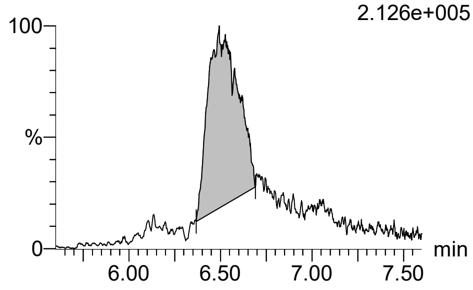

Sample Name: 20211111_QC_R16_IgM
F38:MRM of 2 channels,ES+
948.4 > 366.1
1.725e+005

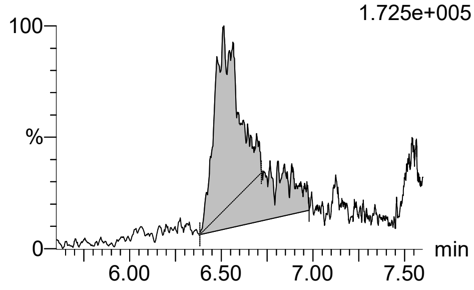

Sample Name: 20211111_QC_R16_IgM
F38:MRM of 2 channels,ES+
1580 > 366.1
2.004e+005

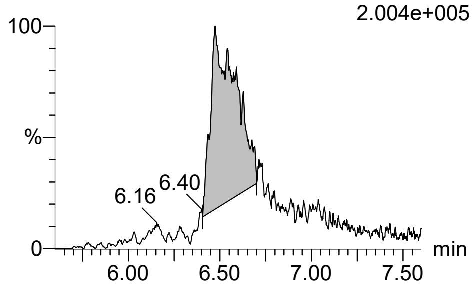

Dataset: C:\MassLynx\10.pro\20211111_clinicalsamples_IgM_IgA.qld

Last Altered: Thursday, March 09, 2023 19:58:25 台北標準時間
Printed: Friday, August 01, 2025 15:23:22 台北標準時間

Sample Name: 20211111_A36_R2_IgM
F38:MRM of 2 channels,ES+
948.4 > 366.1
1.139e+005

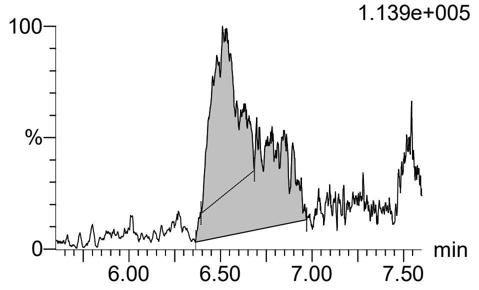

Sample Name: 20211111_A36_R2_IgM
F38:MRM of 2 channels,ES+
1580 > 366.1
1.359e+005

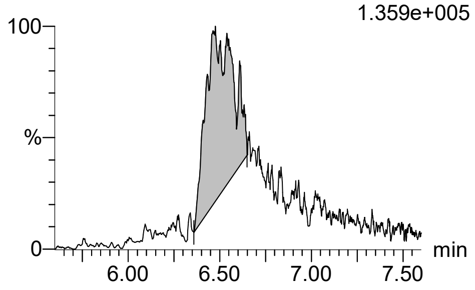

Sample Name: 20211111_H18_R2_IgM
F38:MRM of 2 channels,ES+
948.4 > 366.1
2.467e+005

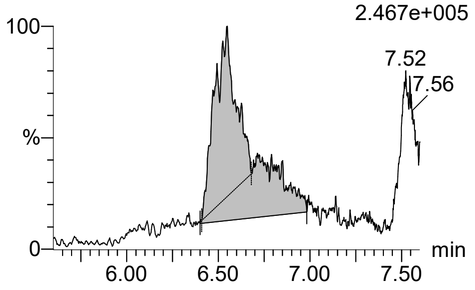

Sample Name: 20211111_H18_R2_IgM
F38:MRM of 2 channels,ES+
1580 > 366.1
3.326e+005

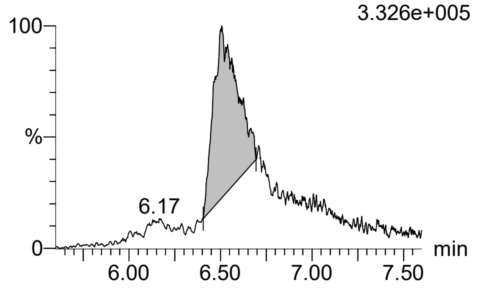

Sample Name: 20211111_A37_R2_IgM
F38:MRM of 2 channels,ES+
948.4 > 366.1
1.662e+005

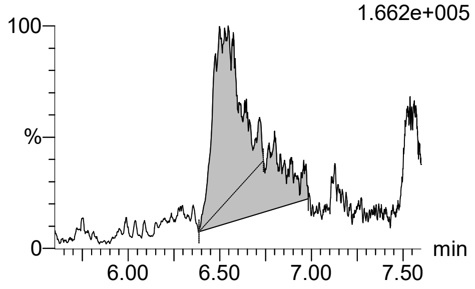

Sample Name: 20211111_A37_R2_IgM
F38:MRM of 2 channels,ES+
1580 > 366.1
2.226e+005

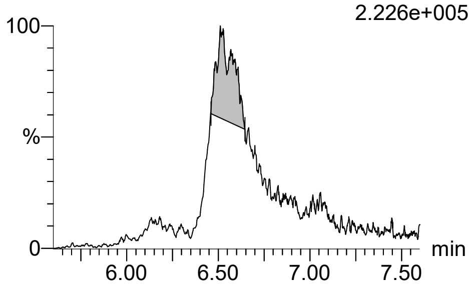

Sample Name: 20211111_A38_R2_IgM
F38:MRM of 2 channels,ES+
948.4 > 366.1
1.421e+005

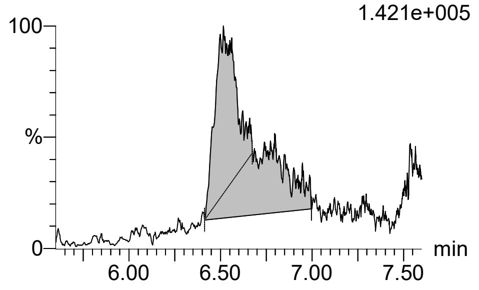

Sample Name: 20211111_A38_R2_IgM
F38:MRM of 2 channels,ES+
1580 > 366.1
1.346e+005

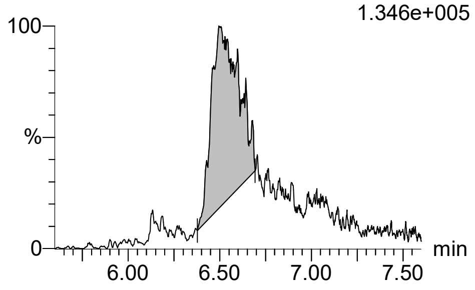

Sample Name: 20211111_H19_R2_IgM
F38:MRM of 2 channels,ES+
948.4 > 366.1
2.478e+005

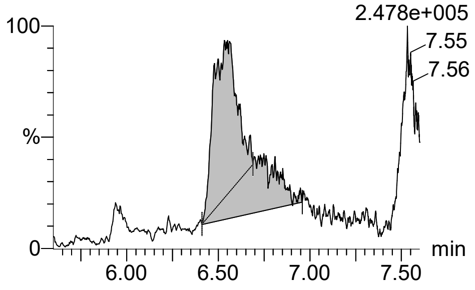

Sample Name: 20211111_H19_R2_IgM
F38:MRM of 2 channels,ES+
1580 > 366.1
3.020e+005

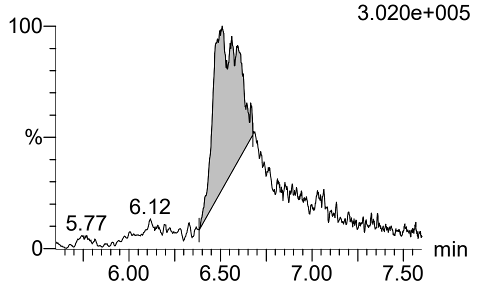

Sample Name: 20211111_A39_R2_IgM
F38:MRM of 2 channels,ES+
948.4 > 366.1
1.718e+005

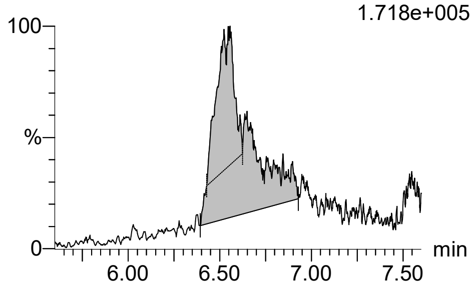

Sample Name: 20211111_A39_R2_IgM
F38:MRM of 2 channels,ES+
1580 > 366.1
1.753e+005

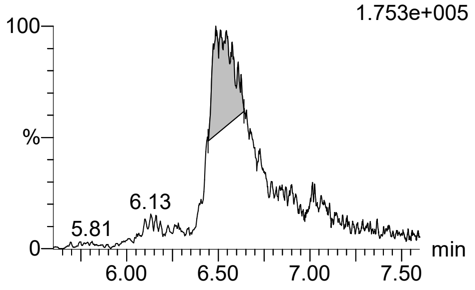

Sample Name: 20211111_A40_R2_IgM
F38:MRM of 2 channels,ES+
948.4 > 366.1
1.223e+005

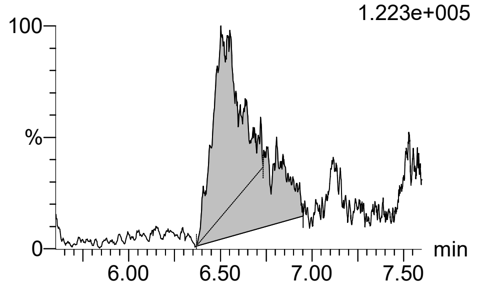

Sample Name: 20211111_A40_R2_IgM
F38:MRM of 2 channels,ES+
1580 > 366.1
1.370e+005

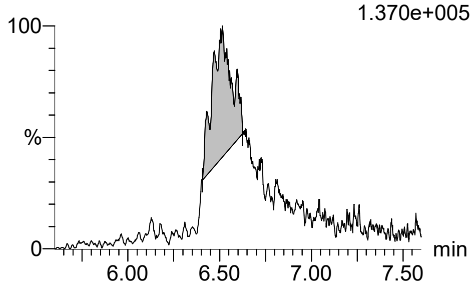

Sample Name: 20211111_H20_R2_IgM
F38:MRM of 2 channels,ES+
948.4 > 366.1
4.701e+005

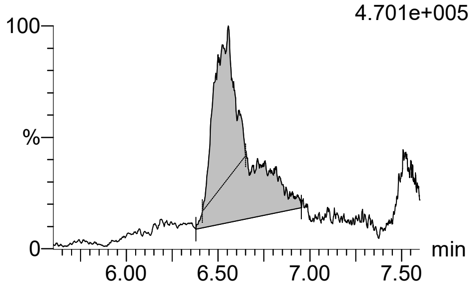

Sample Name: 20211111_H20_R2_IgM
F38:MRM of 2 channels,ES+
1580 > 366.1
6.902e+005

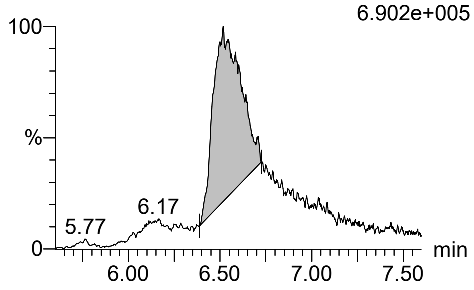

Sample Name: 20211111_A41_R2_IgM
F38:MRM of 2 channels,ES+
948.4 > 366.1
1.759e+005

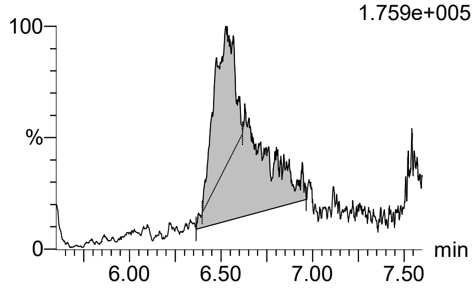

Sample Name: 20211111_A41_R2_IgM
F38:MRM of 2 channels,ES+
1580 > 366.1
2.115e+005

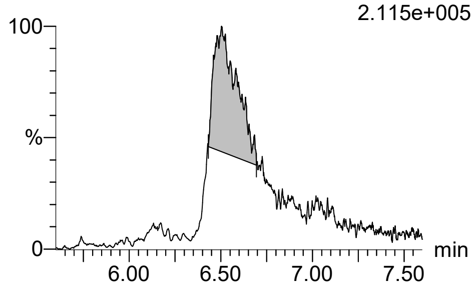

Sample Name: 20211111_A42_R2_IgM
F38:MRM of 2 channels,ES+
948.4 > 366.1
1.119e+005

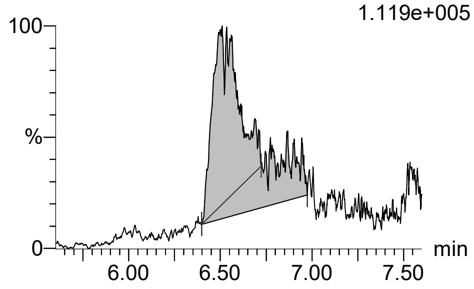

Sample Name: 20211111_A42_R2_IgM
F38:MRM of 2 channels,ES+
1580 > 366.1
1.110e+005

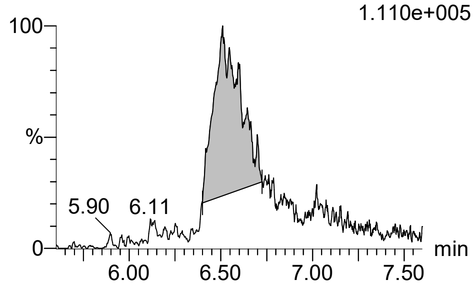

Sample Name: 20211111_A43_R2_IgM
F38:MRM of 2 channels,ES+
948.4 > 366.1
1.032e+005

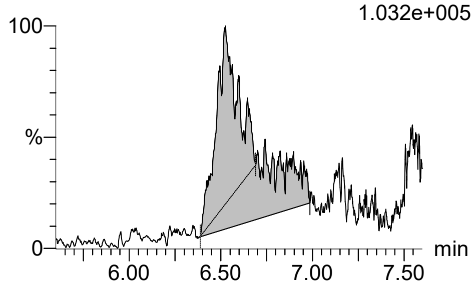

Sample Name: 20211111_A43_R2_IgM
F38:MRM of 2 channels,ES+
1580 > 366.1
1.003e+005

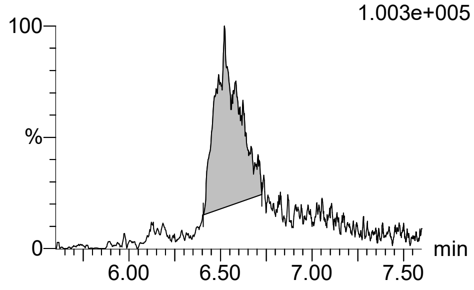

Sample Name: 20211111_A44_R2_IgM
F38:MRM of 2 channels,ES+
948.4 > 366.1
8.770e+004

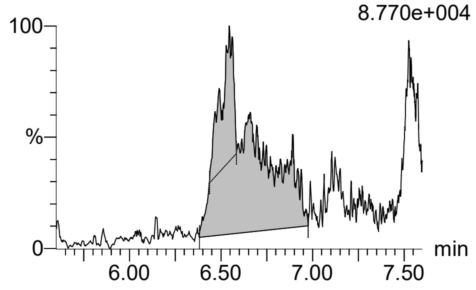

Sample Name: 20211111_A44_R2_IgM
F38:MRM of 2 channels,ES+
1580 > 366.1
6.489e+004

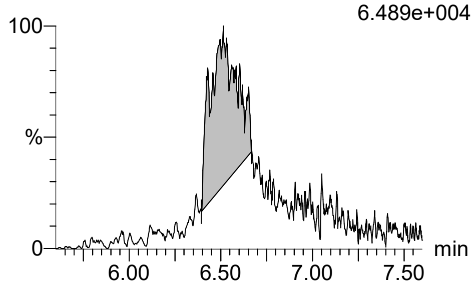

Sample Name: 20211111_QC_R17_IgM
F38:MRM of 2 channels,ES+
948.4 > 366.1
1.331e+005

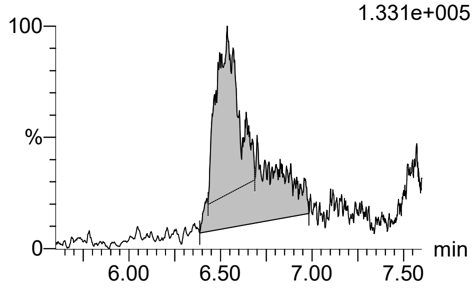

Sample Name: 20211111_QC_R17_IgM
F38:MRM of 2 channels,ES+
1580 > 366.1
1.253e+005

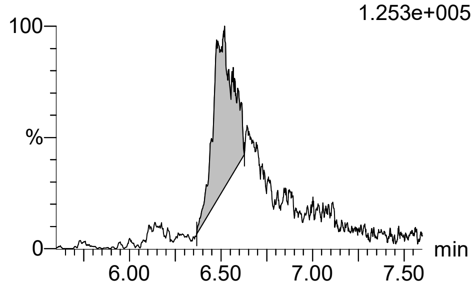

Sample Name: 20211111_L2_R3_IgM
F38:MRM of 2 channels,ES+
948.4 > 366.1
1.769e+005

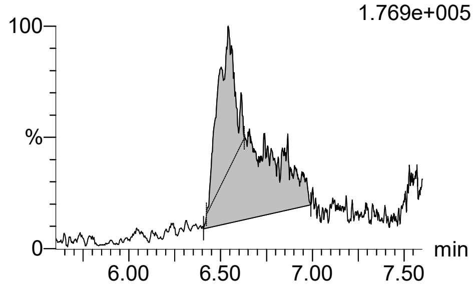

Sample Name: 20211111_L2_R3_IgM
F38:MRM of 2 channels,ES+
1580 > 366.1
1.972e+005

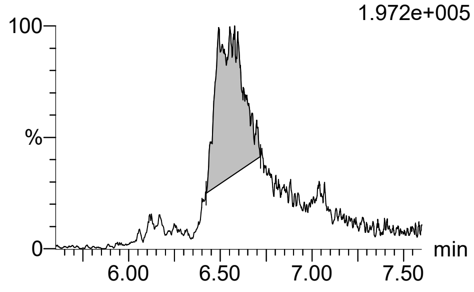

Sample Name: 20211111_H1_R3_IgM
F38:MRM of 2 channels,ES+
948.4 > 366.1
1.648e+005

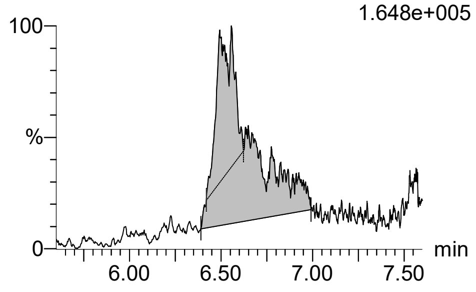

Sample Name: 20211111_H1_R3_IgM
F38:MRM of 2 channels,ES+
1580 > 366.1
1.878e+005

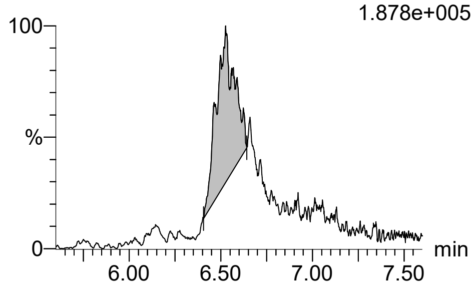

Dataset: C:\MassLynx\10.pro\20211111_clinicalsamples_IgM_IgA.qld

Last Altered: Thursday, March 09, 2023 19:58:25 台北標準時間
Printed: Friday, August 01, 2025 15:23:22 台北標準時間

Sample Name: 20211111_A3_R3_IgM
F38:MRM of 2 channels,ES+
948.4 > 366.1
1.955e+005

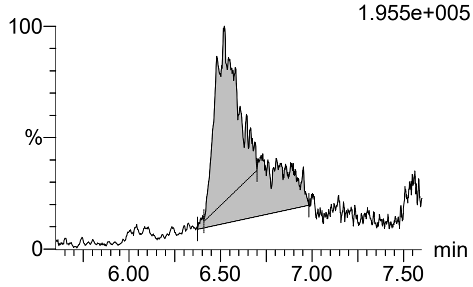

Sample Name: 20211111_A3_R3_IgM
F38:MRM of 2 channels,ES+
1580 > 366.1
2.004e+005

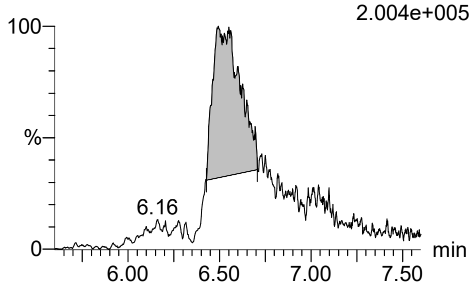

Sample Name: 20211111_A4_R3_IgM
F38:MRM of 2 channels,ES+
948.4 > 366.1
1.528e+005

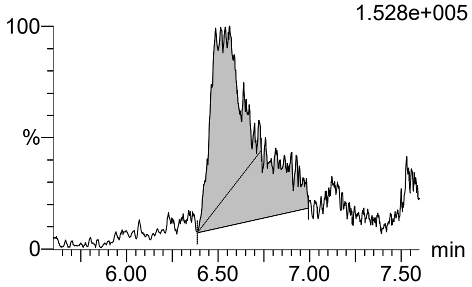

Sample Name: 20211111_A4_R3_IgM
F38:MRM of 2 channels,ES+
1580 > 366.1
1.855e+005

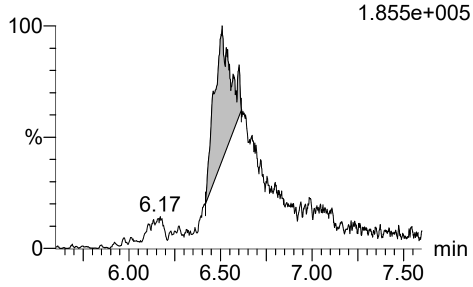

Sample Name: 20211111_L3_R3_IgM
F38:MRM of 2 channels,ES+
948.4 > 366.1
1.244e+005

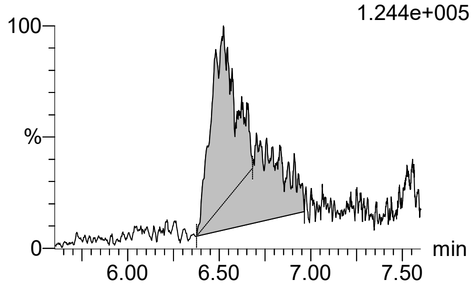

Sample Name: 20211111_L3_R3_IgM
F38:MRM of 2 channels,ES+
1580 > 366.1
1.247e+005

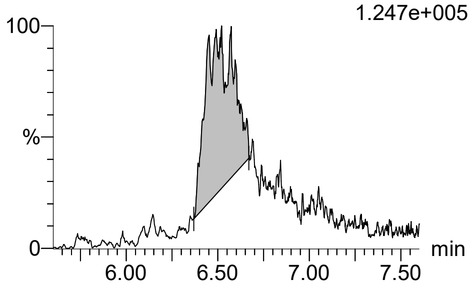

Sample Name: 20211111_L4_R3_IgM
F38:MRM of 2 channels,ES+
948.4 > 366.1
1.671e+005

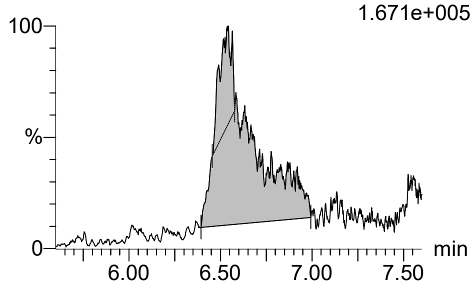

Sample Name: 20211111_L4_R3_IgM
F38:MRM of 2 channels,ES+
1580 > 366.1
1.688e+005

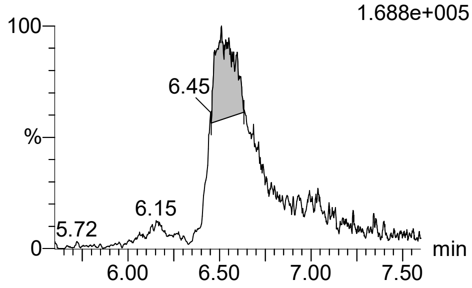

Sample Name: 20211111_H2_R3_IgM
F38:MRM of 2 channels,ES+
948.4 > 366.1
2.832e+005

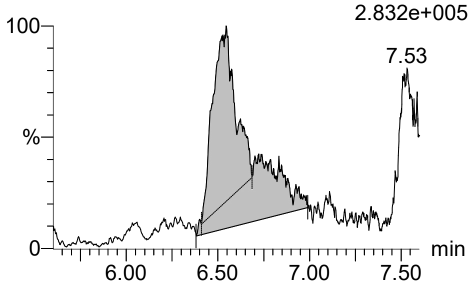

Sample Name: 20211111_H2_R3_IgM
F38:MRM of 2 channels,ES+
1580 > 366.1
3.656e+005

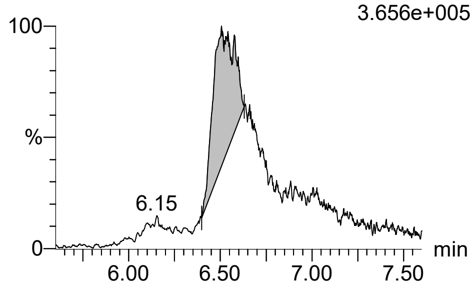

Sample Name: 20211111_A5_R3_IgM
F38:MRM of 2 channels,ES+
948.4 > 366.1
4.139e+005

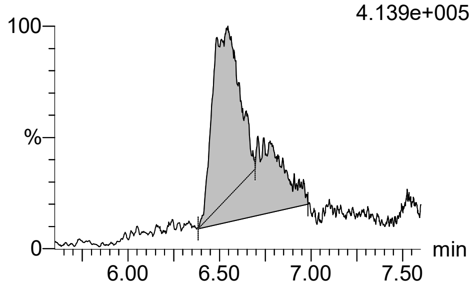

Sample Name: 20211111_A5_R3_IgM
F38:MRM of 2 channels,ES+
1580 > 366.1
6.230e+005

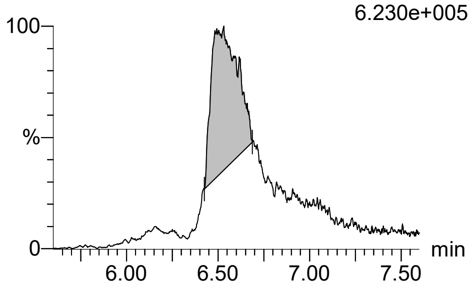

Sample Name: 20211111_A6_R3_IgM
F38:MRM of 2 channels,ES+
948.4 > 366.1
2.190e+005

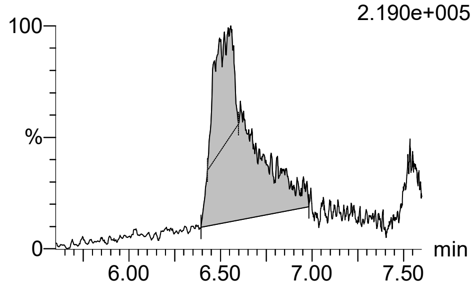

Sample Name: 20211111_A6_R3_IgM
F38:MRM of 2 channels,ES+
1580 > 366.1
2.954e+005

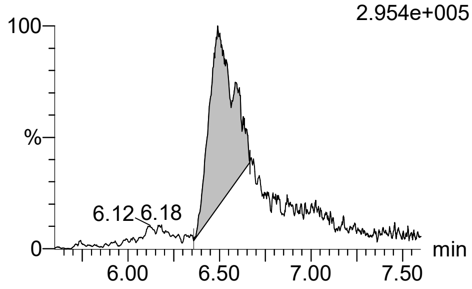

Sample Name: 20211111_QC_R18_IgM
F38:MRM of 2 channels,ES+
948.4 > 366.1
1.661e+005

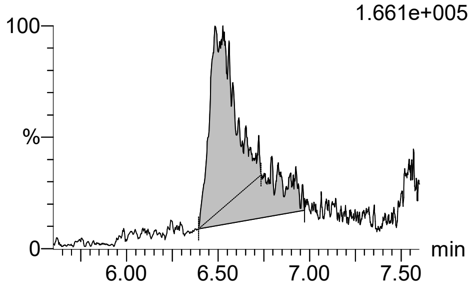

Sample Name: 20211111_QC_R18_IgM
F38:MRM of 2 channels,ES+
1580 > 366.1
1.923e+005

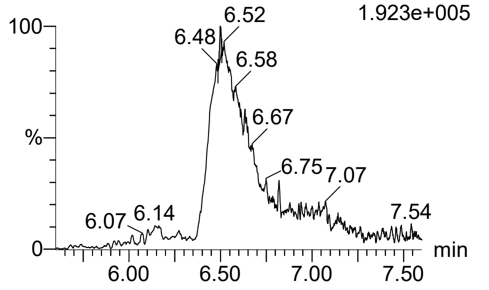

Sample Name: 20211111_L5_R3_IgM
F38:MRM of 2 channels,ES+
948.4 > 366.1
1.559e+005

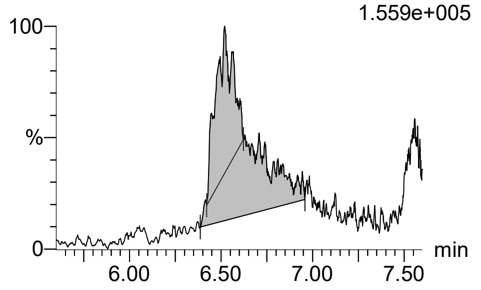

Sample Name: 20211111_L5_R3_IgM
F38:MRM of 2 channels,ES+
1580 > 366.1
1.758e+005

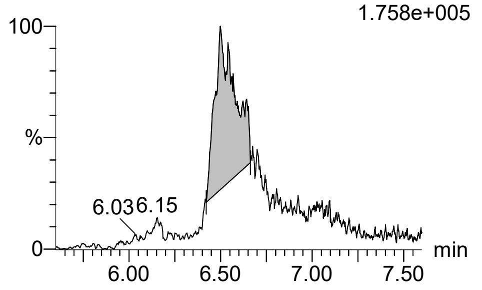

Sample Name: 20211111_L6_R3_IgM
F38:MRM of 2 channels,ES+
948.4 > 366.1
1.333e+005

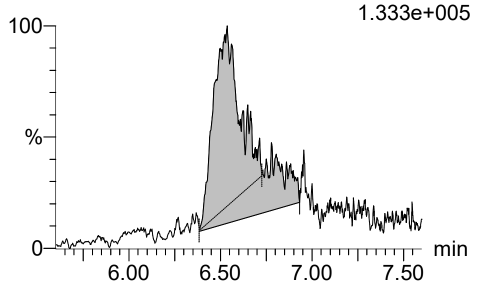

Sample Name: 20211111_L6_R3_IgM
F38:MRM of 2 channels,ES+
1580 > 366.1
1.358e+005

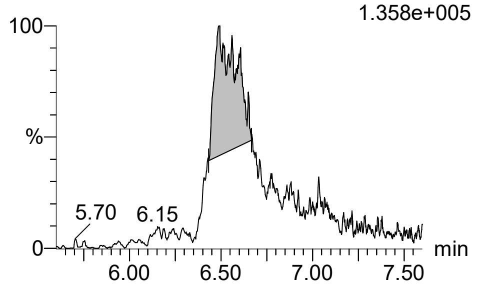

Sample Name: 20211111_H3_R3_IgM
F38:MRM of 2 channels,ES+
948.4 > 366.1
9.204e+004

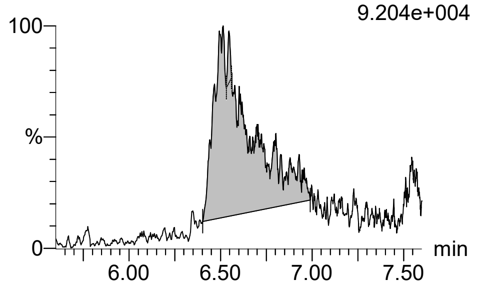

Sample Name: 20211111_H3_R3_IgM
F38:MRM of 2 channels,ES+
1580 > 366.1
7.367e+004

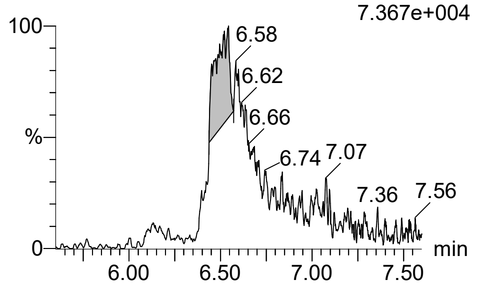

Sample Name: 20211111_A7_R3_IgM
F38:MRM of 2 channels,ES+
948.4 > 366.1
1.443e+005

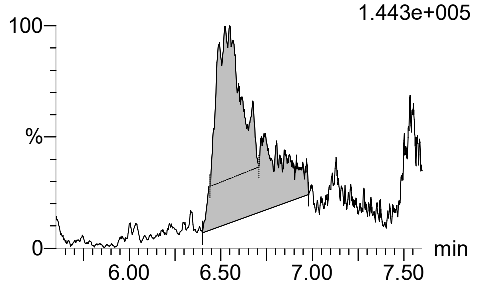

Sample Name: 20211111_A7_R3_IgM
F38:MRM of 2 channels,ES+
1580 > 366.1
1.747e+005

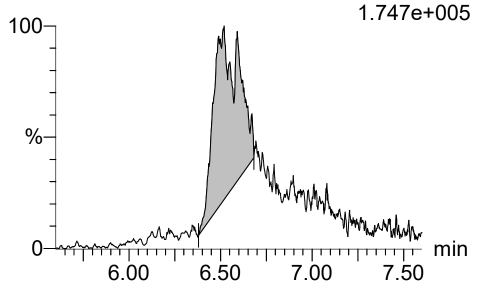

Sample Name: 20211111_A8_R3_IgM
F38:MRM of 2 channels,ES+
948.4 > 366.1
1.531e+005

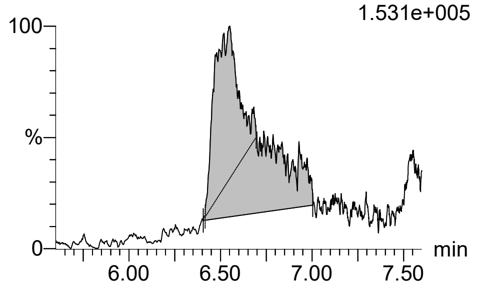

Sample Name: 20211111_A8_R3_IgM
F38:MRM of 2 channels,ES+
1580 > 366.1
1.936e+005

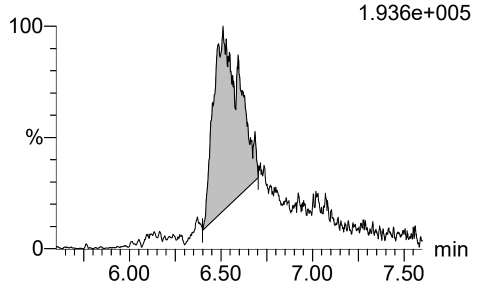

Sample Name: 20211111_L7_R3_IgM
F38:MRM of 2 channels,ES+
948.4 > 366.1
1.089e+005

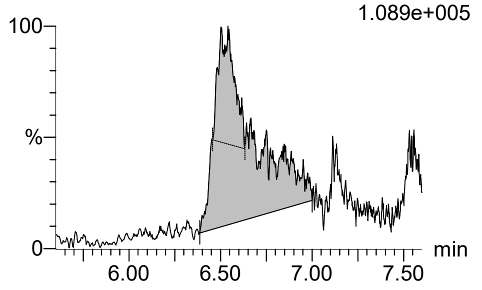

Sample Name: 20211111_L7_R3_IgM
F38:MRM of 2 channels,ES+
1580 > 366.1
1.080e+005

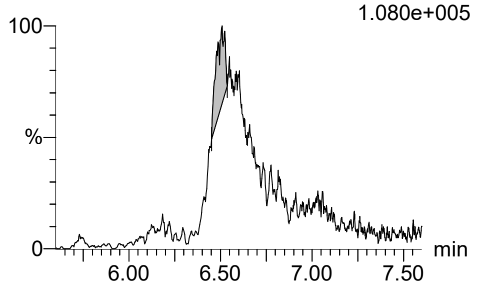

Sample Name: 20211111_L8_R3_IgM
F38:MRM of 2 channels,ES+
948.4 > 366.1
1.003e+005

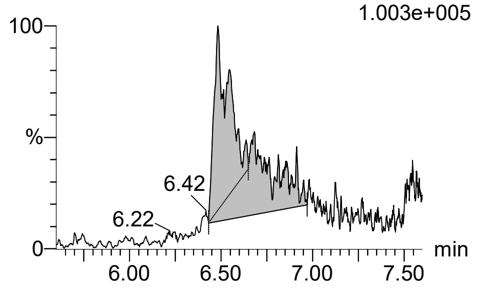

Sample Name: 20211111_L8_R3_IgM
F38:MRM of 2 channels,ES+
1580 > 366.1
6.358e+004

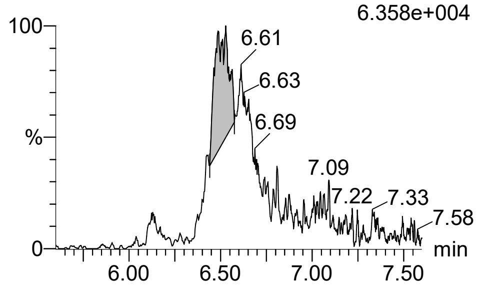

Dataset: C:\MassLynx\10.pro\20211111_clinicalsamples_IgM_IgA.qld

Last Altered: Thursday, March 09, 2023 19:58:25 台北標準時間
Printed: Friday, August 01, 2025 15:23:22 台北標準時間

Sample Name: 20211111_H4_R3_IgM
F38:MRM of 2 channels,ES+
948.4 > 366.1
1.011e+005

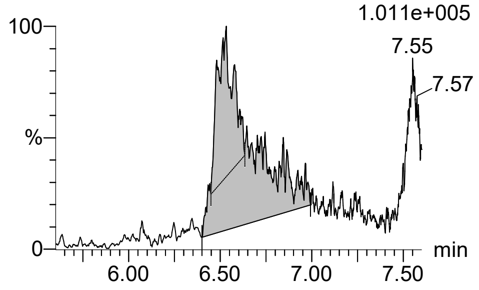

Sample Name: 20211111_H4_R3_IgM
F38:MRM of 2 channels,ES+
1580 > 366.1
8.186e+004

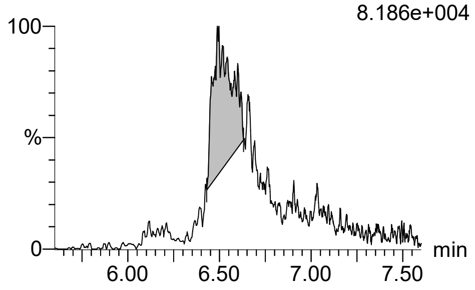

Sample Name: 20211111_A9_R3_IgM
F38:MRM of 2 channels,ES+
948.4 > 366.1
9.794e+004

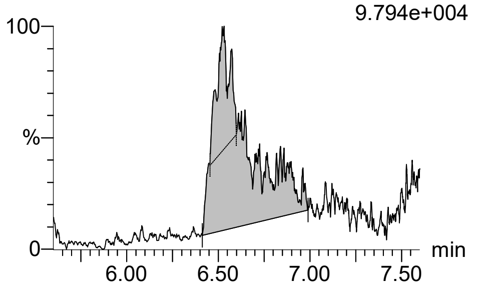

Sample Name: 20211111_A9_R3_IgM
F38:MRM of 2 channels,ES+
1580 > 366.1
7.089e+004

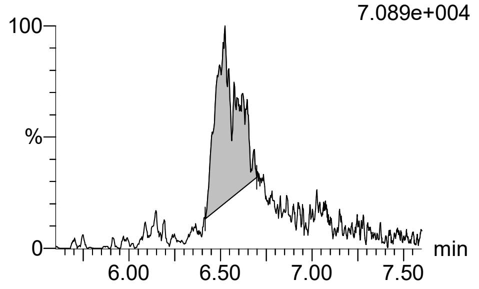

Sample Name: 20211111_A10_R3_IgM
F38:MRM of 2 channels,ES+
948.4 > 366.1
1.734e+005

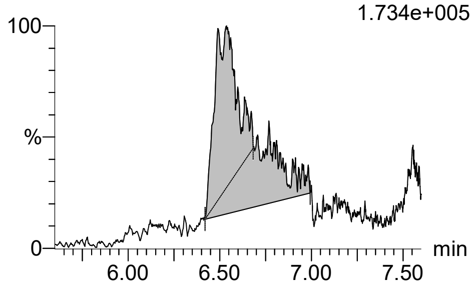

Sample Name: 20211111_A10_R3_IgM
F38:MRM of 2 channels,ES+
1580 > 366.1
2.182e+005

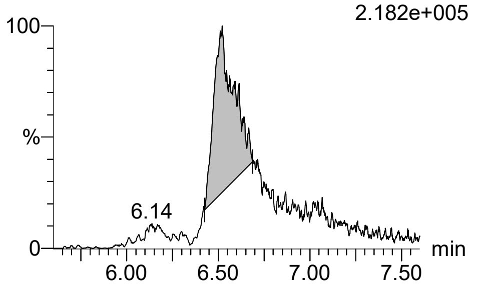

Sample Name: 20211111_L9_R3_IgM
F38:MRM of 2 channels,ES+
948.4 > 366.1
1.213e+005

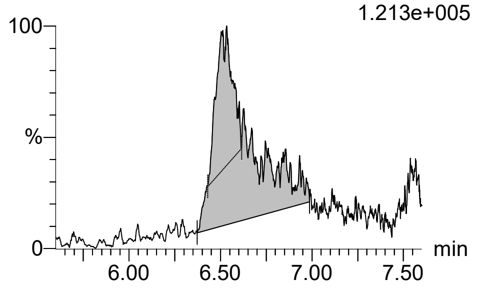

Sample Name: 20211111_L9_R3_IgM
F38:MRM of 2 channels,ES+
1580 > 366.1
1.062e+005

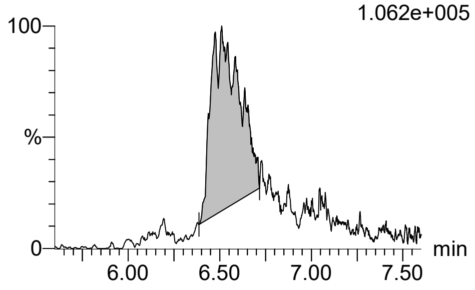

Sample Name: 20211111_L10_R3_IgM
F38:MRM of 2 channels,ES+
948.4 > 366.1
1.542e+005

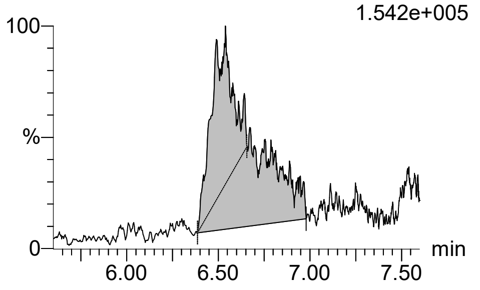

Sample Name: 20211111_L10_R3_IgM
F38:MRM of 2 channels,ES+
1580 > 366.1
1.586e+005

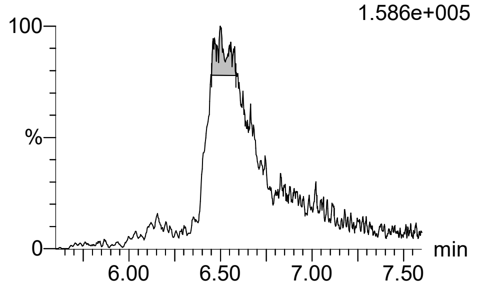

Sample Name: 20211111_QC_R19_IgM
F38:MRM of 2 channels,ES+
948.4 > 366.1
1.374e+005

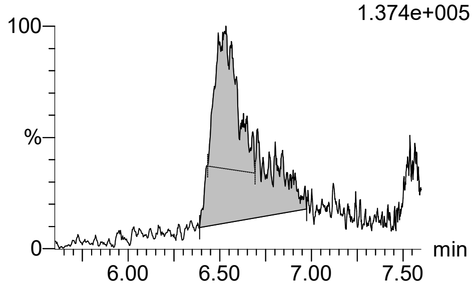

Sample Name: 20211111_QC_R19_IgM
F38:MRM of 2 channels,ES+
1580 > 366.1
1.601e+005

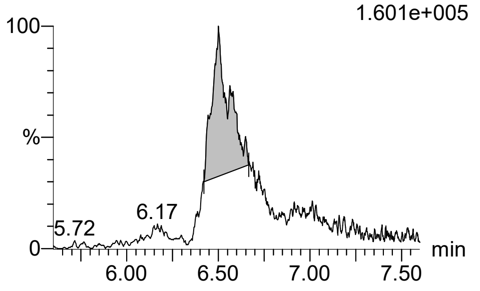

Sample Name: 20211111_H5_R3_IgM
F38:MRM of 2 channels,ES+
948.4 > 366.1
1.704e+005

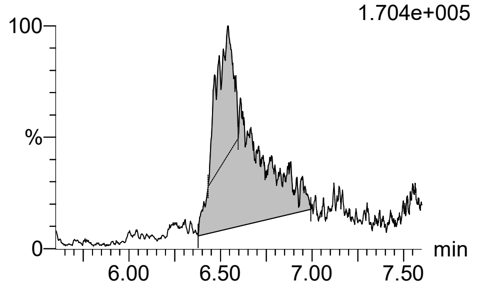

Sample Name: 20211111_H5_R3_IgM
F38:MRM of 2 channels,ES+
1580 > 366.1
1.885e+005

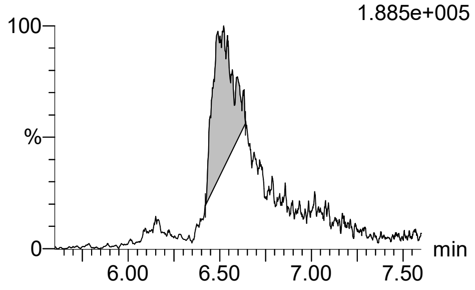

Sample Name: 20211111_A11_R3_IgM
F38:MRM of 2 channels,ES+
948.4 > 366.1
2.116e+005

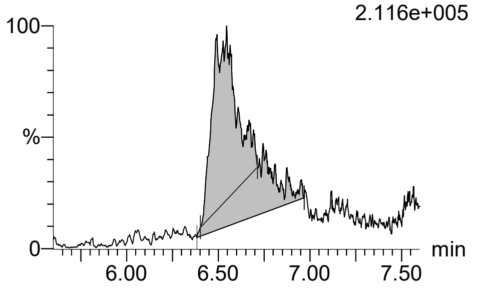

Dataset: C:\MassLynx\10.pro\20211111_clinicalsamples_IgM_IgA.qld

Last Altered: Thursday, March 09, 2023 19:58:25 台北標準時間
Printed: Friday, August 01, 2025 15:23:22 台北標準時間

Sample Name: 20211111_A11_R3_IgM
F38:MRM of 2 channels,ES+
1580 > 366.1
2.508e+005

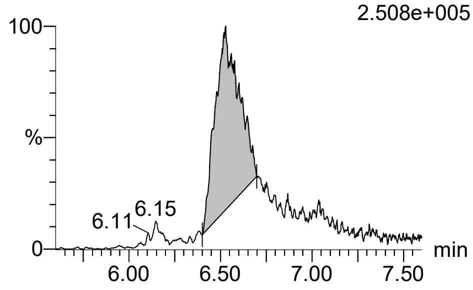

Sample Name: 20211111_A12_R3_IgM
F38:MRM of 2 channels,ES+
948.4 > 366.1
1.291e+005

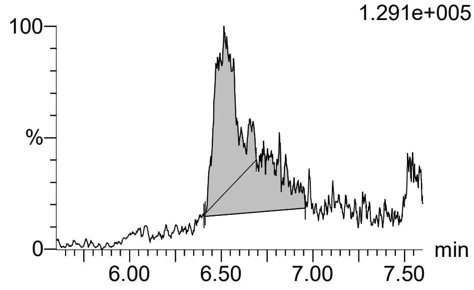

Sample Name: 20211111_A12_R3_IgM
F38:MRM of 2 channels,ES+
1580 > 366.1
1.338e+005

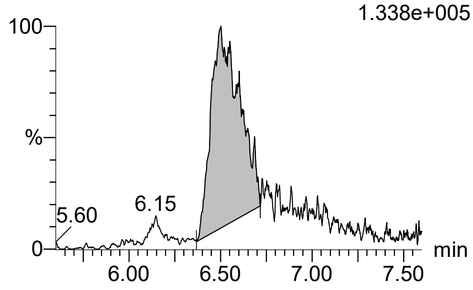

Sample Name: 20211111_L11_R3_IgM
F38:MRM of 2 channels,ES+
948.4 > 366.1
1.094e+005

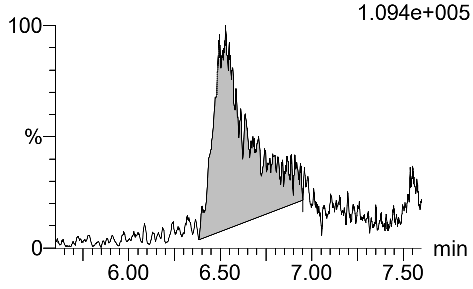

Sample Name: 20211111_L11_R3_IgM
F38:MRM of 2 channels,ES+
1580 > 366.1
1.170e+005

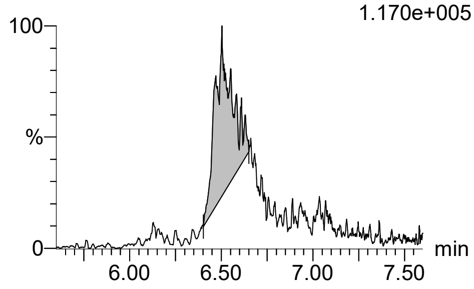

Sample Name: 20211111_L12_R3_IgM
F38:MRM of 2 channels,ES+
948.4 > 366.1
9.578e+004

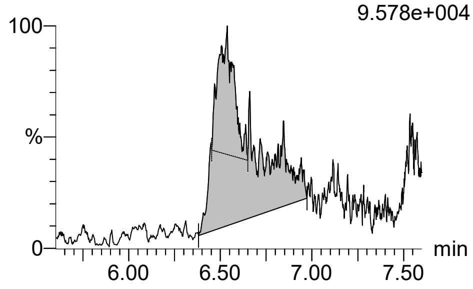

Sample Name: 20211111_L12_R3_IgM
F38:MRM of 2 channels,ES+
1580 > 366.1
1.005e+005

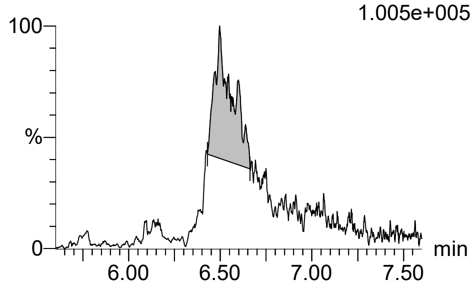

Sample Name: 20211111_H6_R3_IgM
F38:MRM of 2 channels,ES+
948.4 > 366.1
8.813e+004

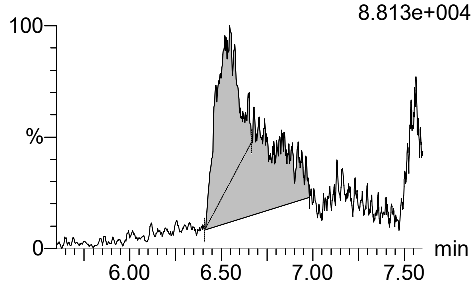

Sample Name: 20211111_H6_R3_IgM
F38:MRM of 2 channels,ES+
1580 > 366.1
9.447e+004

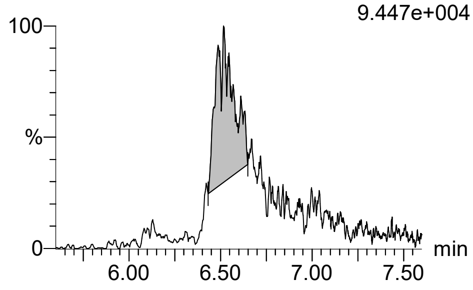

Sample Name: 20211111_A13_R3_IgM
F38:MRM of 2 channels,ES+
948.4 > 366.1
1.171e+005

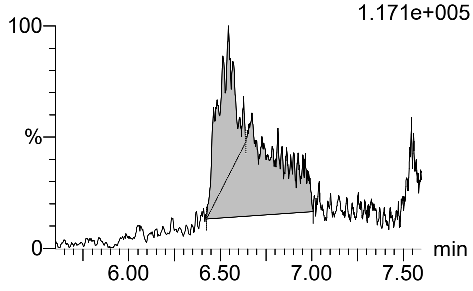

Sample Name: 20211111_A13_R3_IgM
F38:MRM of 2 channels,ES+
1580 > 366.1
1.173e+005

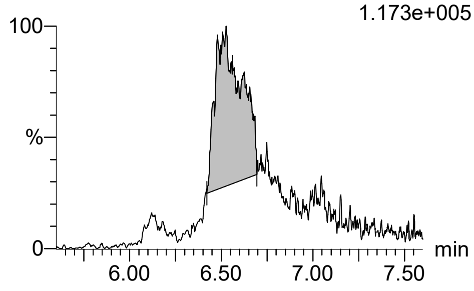

Sample Name: 20211111_A14_R3_IgM
F38:MRM of 2 channels,ES+
948.4 > 366.1
2.425e+005

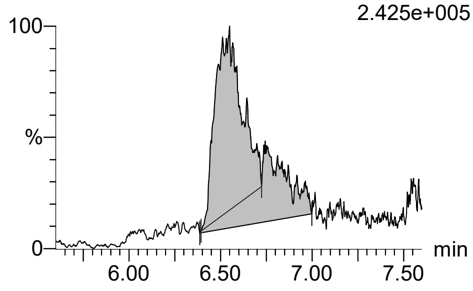

Sample Name: 20211111_A14_R3_IgM
F38:MRM of 2 channels,ES+
1580 > 366.1
3.318e+005

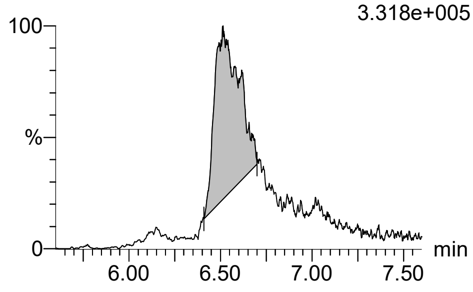

Sample Name: 20211111_L13_R3_IgM
F38:MRM of 2 channels,ES+
948.4 > 366.1
1.287e+005

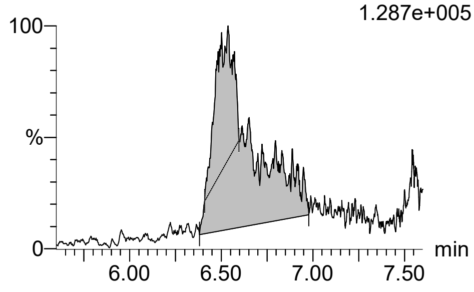

Sample Name: 20211111_L13_R3_IgM
F38:MRM of 2 channels,ES+
1580 > 366.1
1.461e+005

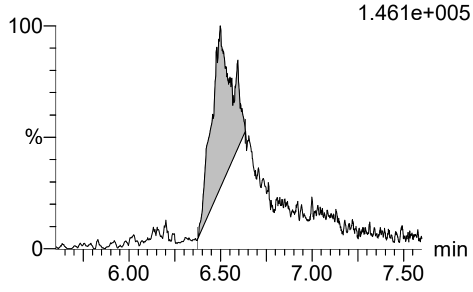

Dataset: C:\MassLynx\10.pro\20211111_clinicalsamples_IgM_IgA.qld

Last Altered: Thursday, March 09, 2023 19:58:25 台北標準時間
Printed: Friday, August 01, 2025 15:23:22 台北標準時間

Sample Name: 20211111_L14_R3_IgM
F38:MRM of 2 channels,ES+
948.4 > 366.1
1.534e+005

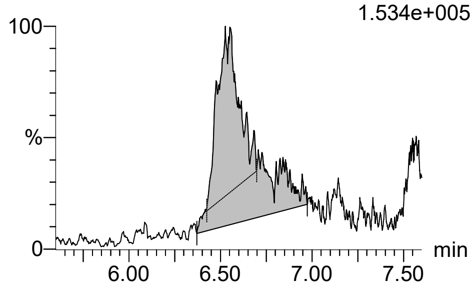

Sample Name: 20211111_L14_R3_IgM
F38:MRM of 2 channels,ES+
1580 > 366.1
1.780e+005

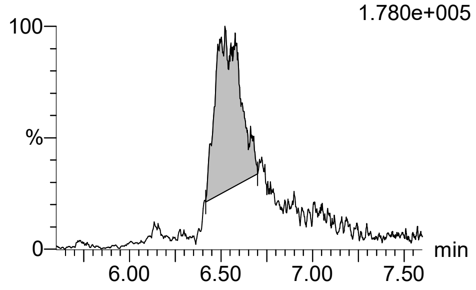

Sample Name: 20211111_H7_R3_IgM
F38:MRM of 2 channels,ES+
948.4 > 366.1
1.282e+005

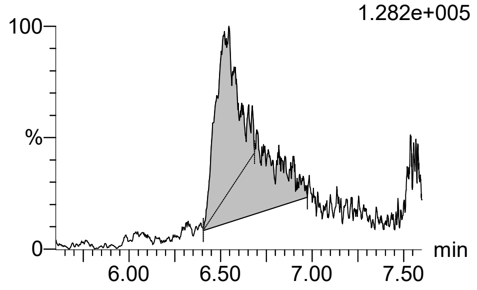

Sample Name: 20211111_H7_R3_IgM
F38:MRM of 2 channels,ES+
1580 > 366.1
1.417e+005

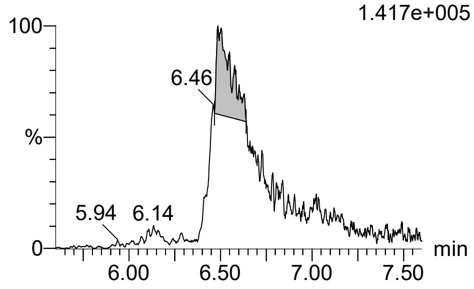

Sample Name: 20211111_A15_R3_IgM
F38:MRM of 2 channels,ES+
948.4 > 366.1
2.814e+005

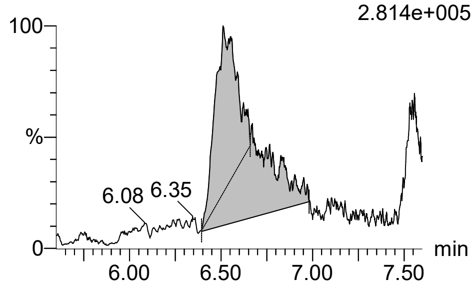

Sample Name: 20211111_A15_R3_IgM
F38:MRM of 2 channels,ES+
1580 > 366.1
4.172e+005

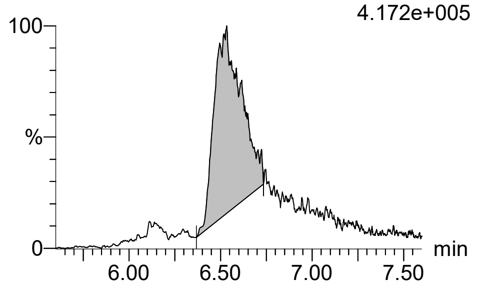

Sample Name: 20211111_QC_R20_IgM
F38:MRM of 2 channels,ES+
948.4 > 366.1
1.740e+005

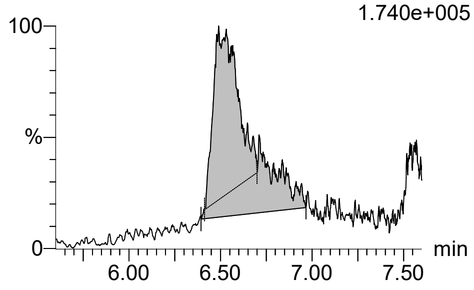

Sample Name: 20211111_QC_R20_IgM
F38:MRM of 2 channels,ES+
1580 > 366.1
2.218e+005

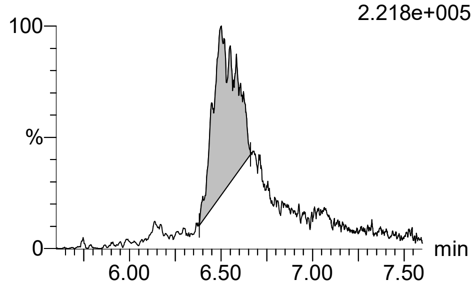

Sample Name: 20211111_A16_R3_IgM
F38:MRM of 2 channels,ES+
948.4 > 366.1
1.566e+005

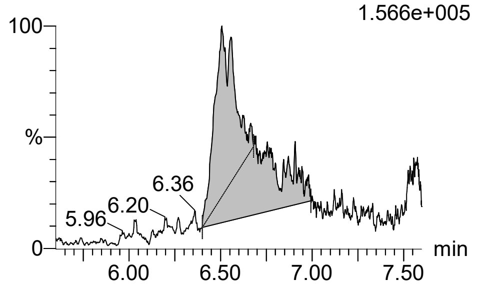

Sample Name: 20211111_A16_R3_IgM
F38:MRM of 2 channels,ES+
1580 > 366.1
1.870e+005

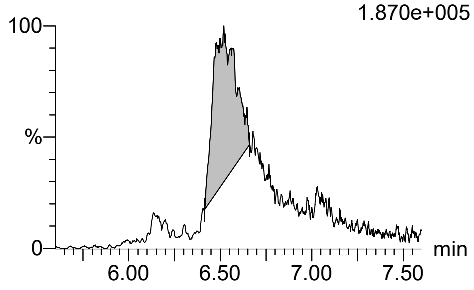

Sample Name: 20211111_L15_R3_IgM
F38:MRM of 2 channels,ES+
948.4 > 366.1
1.839e+005

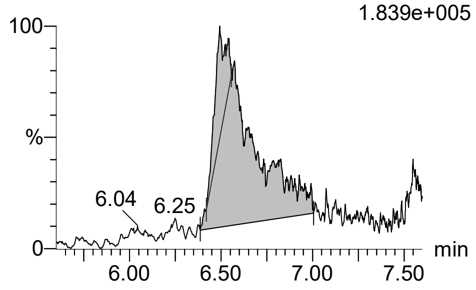

Sample Name: 20211111_L15_R3_IgM
F38:MRM of 2 channels,ES+
1580 > 366.1
2.390e+005

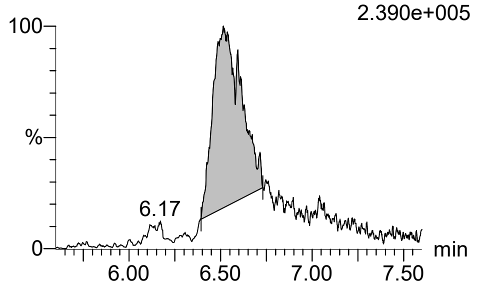

Sample Name: 20211111_L16_R3_IgM
F38:MRM of 2 channels,ES+
948.4 > 366.1
9.707e+004

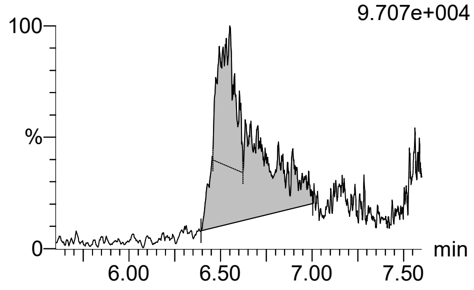

Sample Name: 20211111_L16_R3_IgM
F38:MRM of 2 channels,ES+
1580 > 366.1
1.019e+005

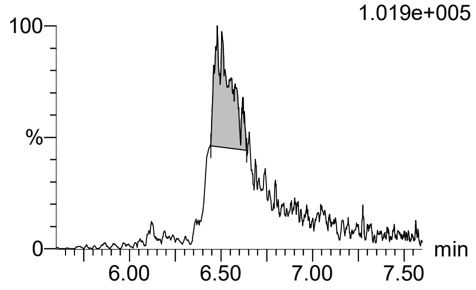

Sample Name: 20211111_H8_R3_IgM
F38:MRM of 2 channels,ES+
948.4 > 366.1
1.207e+005

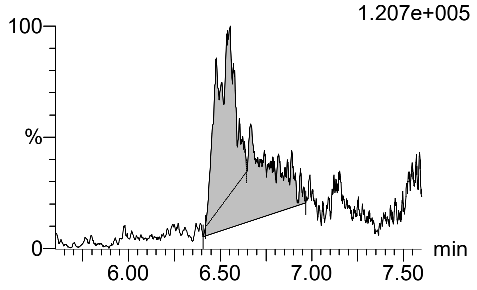

Dataset: C:\MassLynx\10.pro\20211111_clinicalsamples_IgM_IgA.qld

Last Altered: Thursday, March 09, 2023 19:58:25 台北標準時間
Printed: Friday, August 01, 2025 15:23:22 台北標準時間

Sample Name: 20211111_H8_R3_IgM
F38:MRM of 2 channels,ES+
1580 > 366.1
1.171e+005

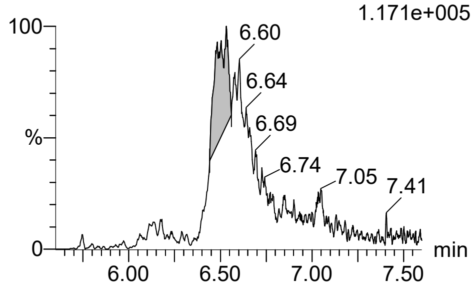

Sample Name: 20211111_A17_R3_IgM
F38:MRM of 2 channels,ES+
948.4 > 366.1
1.664e+005

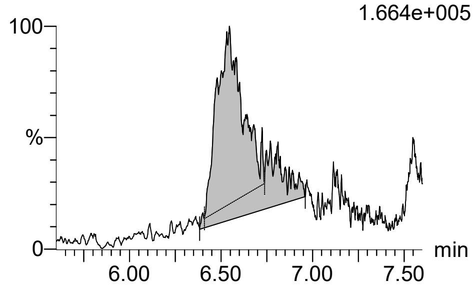

Sample Name: 20211111_A17_R3_IgM
F38:MRM of 2 channels,ES+
1580 > 366.1
1.956e+005

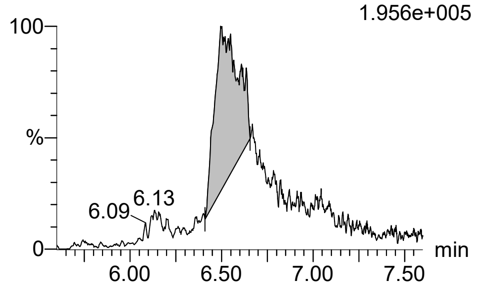

Sample Name: 20211111_A18_R3_IgM
F38:MRM of 2 channels,ES+
948.4 > 366.1
1.045e+005

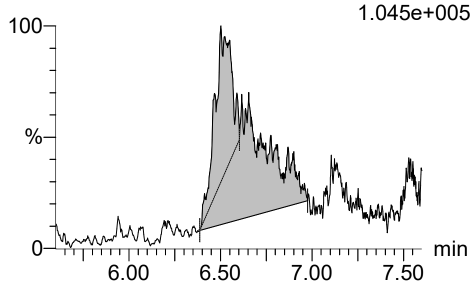

Sample Name: 20211111_A18_R3_IgM
F38:MRM of 2 channels,ES+
1580 > 366.1
1.020e+005

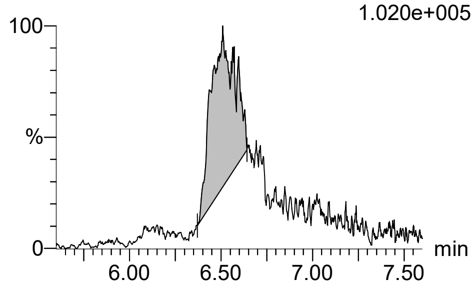

Sample Name: 20211111_L17_R3_IgM
F38:MRM of 2 channels,ES+
948.4 > 366.1
1.951e+005

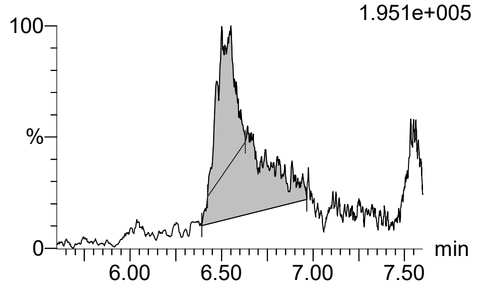

Sample Name: 20211111_L17_R3_IgM
F38:MRM of 2 channels,ES+
1580 > 366.1
2.575e+005

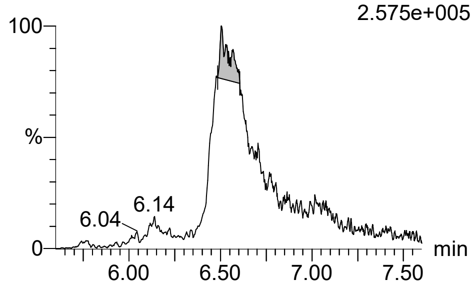

Sample Name: 20211111_L18_R3_IgM
F38:MRM of 2 channels,ES+
948.4 > 366.1
8.999e+004

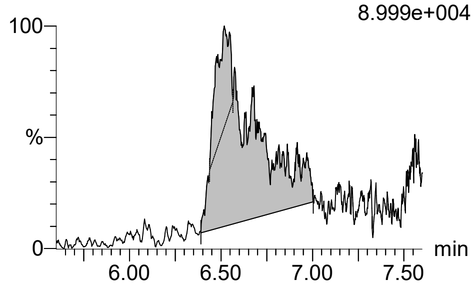

Sample Name: 20211111_L18_R3_IgM
F38:MRM of 2 channels,ES+
1580 > 366.1
9.426e+004

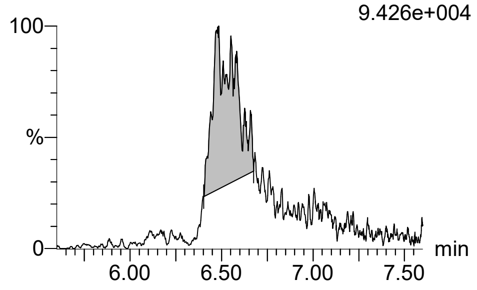

Sample Name: 20211111_H9_R3_IgM
F38:MRM of 2 channels,ES+
948.4 > 366.1
8.648e+004

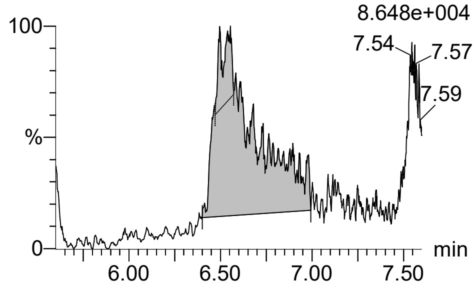

Sample Name: 20211111_H9_R3_IgM
F38:MRM of 2 channels,ES+
1580 > 366.1
7.293e+004

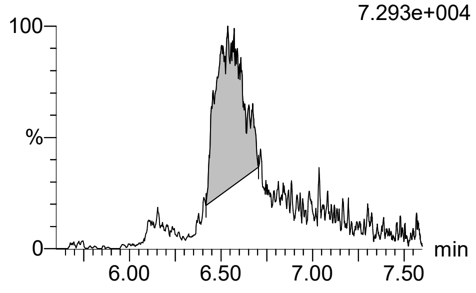

Sample Name: 20211111_A19_R3_IgM
F38:MRM of 2 channels,ES+
948.4 > 366.1
1.556e+005

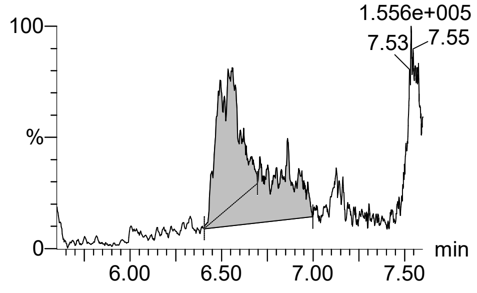

Sample Name: 20211111_A19_R3_IgM
F38:MRM of 2 channels,ES+
1580 > 366.1
1.579e+005

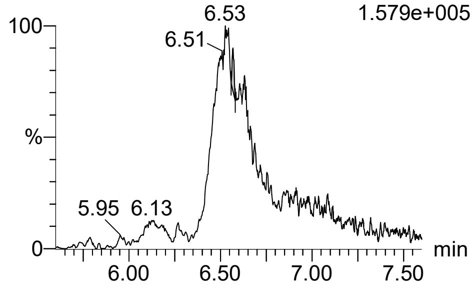

Sample Name: 20211111_A20_R3_IgM
F38:MRM of 2 channels,ES+
948.4 > 366.1
9.831e+004

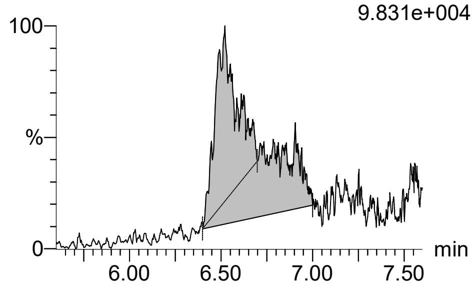

Sample Name: 20211111_A20_R3_IgM
F38:MRM of 2 channels,ES+
1580 > 366.1
8.525e+004

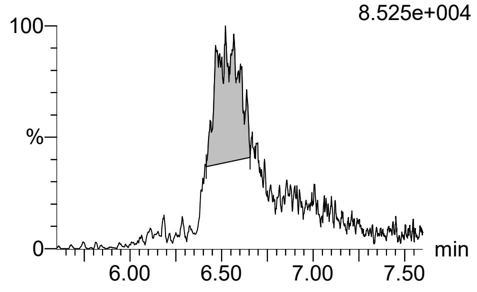

Sample Name: 20211111_L19_R3_IgM
F38:MRM of 2 channels,ES+
948.4 > 366.1
1.446e+005

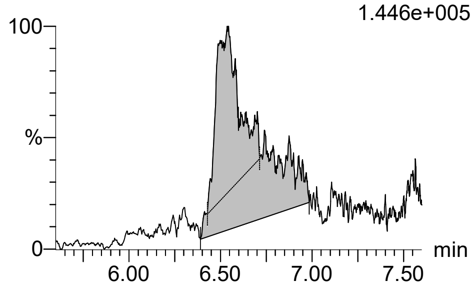

Sample Name: 20211111_L19_R3_IgM
F38:MRM of 2 channels,ES+
1580 > 366.1
1.871e+005

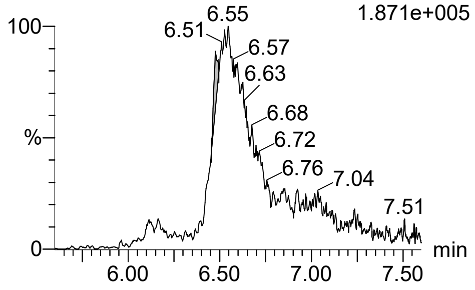

Sample Name: 20211111_QC_R21_IgM
F38:MRM of 2 channels,ES+
948.4 > 366.1
1.574e+005

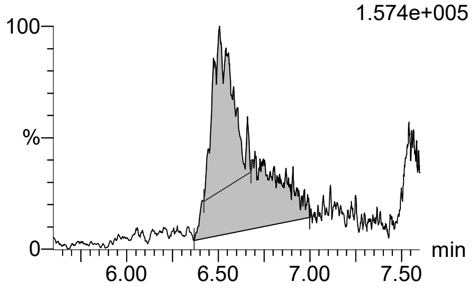

Sample Name: 20211111_QC_R21_IgM
F38:MRM of 2 channels,ES+
1580 > 366.1
1.789e+005

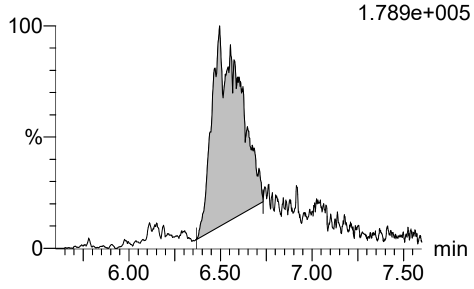

Sample Name: 20211111_L20_R3_IgM
F38:MRM of 2 channels,ES+
948.4 > 366.1
1.065e+005

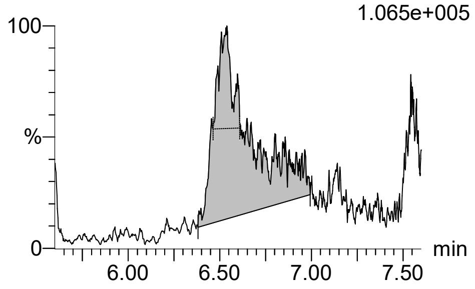

Sample Name: 20211111_L20_R3_IgM
F38:MRM of 2 channels,ES+
1580 > 366.1
1.231e+005

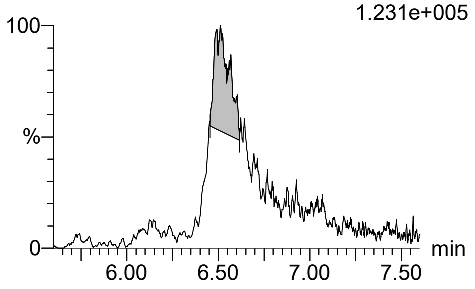

Sample Name: 20211111_H10_R3_IgM
F38:MRM of 2 channels,ES+
948.4 > 366.1
1.390e+005

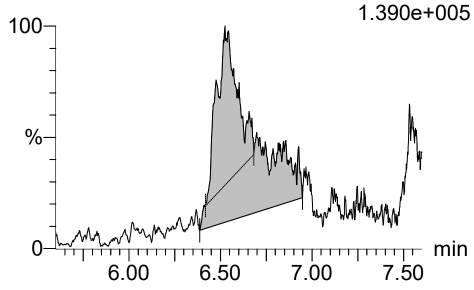

Sample Name: 20211111_H10_R3_IgM
F38:MRM of 2 channels,ES+
1580 > 366.1
1.656e+005

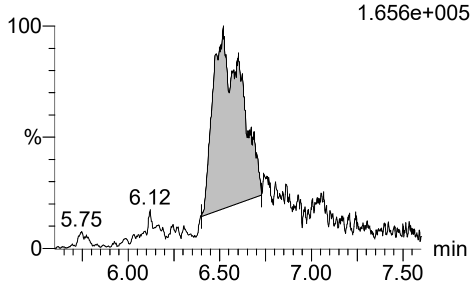

Sample Name: 20211111_A21_R3_IgM
F38:MRM of 2 channels,ES+
948.4 > 366.1
1.266e+005

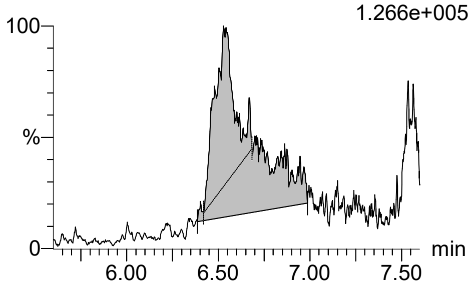

Sample Name: 20211111_A21_R3_IgM
F38:MRM of 2 channels,ES+
1580 > 366.1
1.334e+005

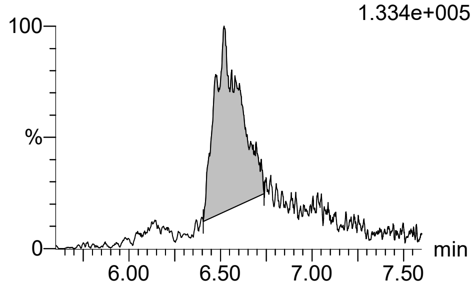

Sample Name: 20211111_A22_R3_IgM
F38:MRM of 2 channels,ES+
948.4 > 366.1
7.940e+004

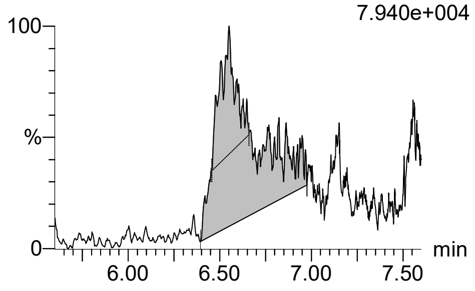

Sample Name: 20211111_A22_R3_IgM
F38:MRM of 2 channels,ES+
1580 > 366.1
6.721e+004

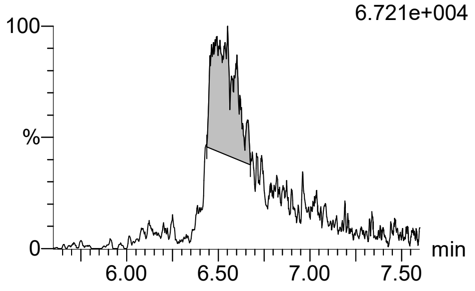

Sample Name: 20211111_L21_R3_IgM
F38:MRM of 2 channels,ES+
948.4 > 366.1
1.246e+005

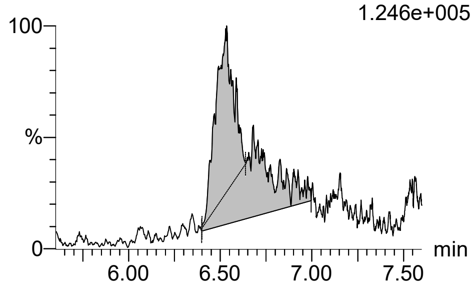

Sample Name: 20211111_L21_R3_IgM
F38:MRM of 2 channels,ES+
1580 > 366.1
1.207e+005

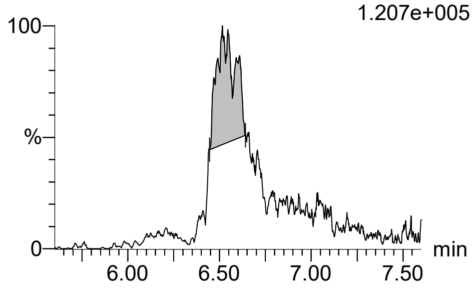

Sample Name: 20211111_L22_R3_IgM
F38:MRM of 2 channels,ES+
948.4 > 366.1
1.059e+005

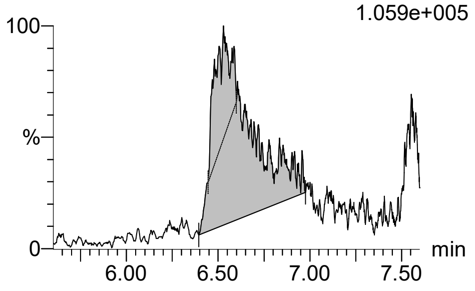

Dataset: C:\MassLynx\10.pro\20211111_clinicalsamples_IgM_IgA.qld

Last Altered: Thursday, March 09, 2023 19:58:25 台北標準時間
Printed: Friday, August 01, 2025 15:23:22 台北標準時間

Sample Name: 20211111_L22_R3_IgM
F38:MRM of 2 channels,ES+
1580 > 366.1
1.046e+005

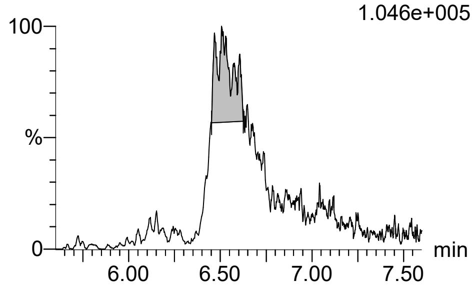

Sample Name: 20211111_H11_R3_IgM
F38:MRM of 2 channels,ES+
948.4 > 366.1
1.133e+005

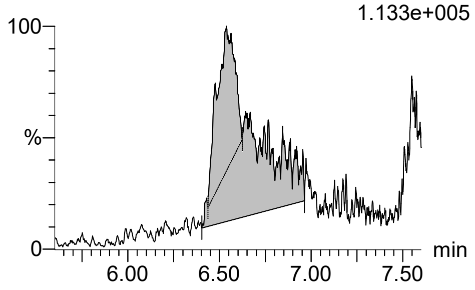

Sample Name: 20211111_H11_R3_IgM
F38:MRM of 2 channels,ES+
1580 > 366.1
1.170e+005

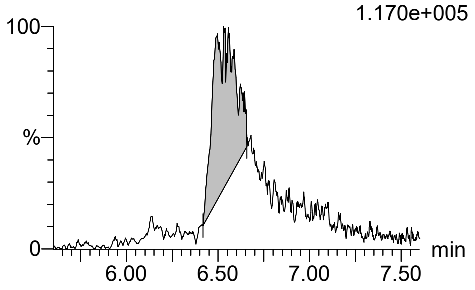

Sample Name: 20211111_A23_R3_IgM
F38:MRM of 2 channels,ES+
948.4 > 366.1
1.181e+005

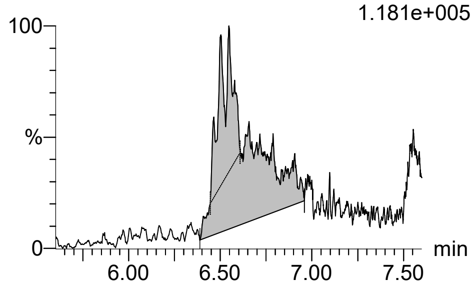

Sample Name: 20211111_A23_R3_IgM
F38:MRM of 2 channels,ES+
1580 > 366.1
1.063e+005

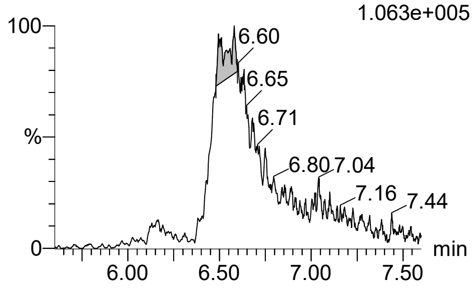

Sample Name: 20211111_A24_R3_IgM
F38:MRM of 2 channels,ES+
948.4 > 366.1
1.819e+005

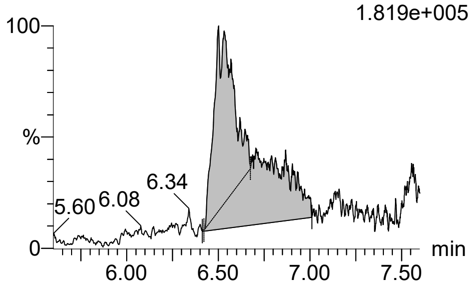

Sample Name: 20211111_A24_R3_IgM
F38:MRM of 2 channels,ES+
1580 > 366.1
2.326e+005

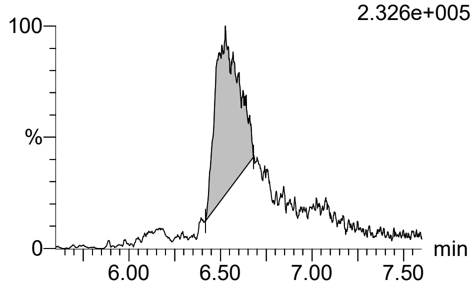

Sample Name: 20211111_L23_R3_IgM
F38:MRM of 2 channels,ES+
948.4 > 366.1
1.639e+005

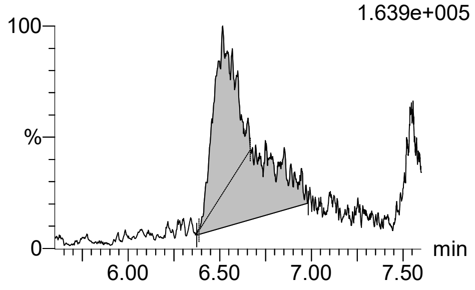

Sample Name: 20211111_L23_R3_IgM
F38:MRM of 2 channels,ES+
1580 > 366.1
2.222e+005

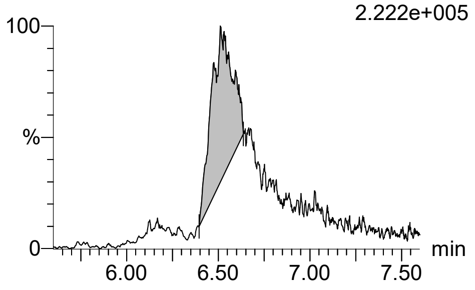

Sample Name: 20211111_L24_R3_IgM
F38:MRM of 2 channels,ES+
948.4 > 366.1
9.153e+004

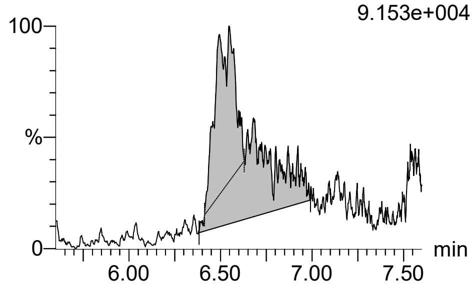

Sample Name: 20211111_L24_R3_IgM
F38:MRM of 2 channels,ES+
1580 > 366.1
1.000e+005

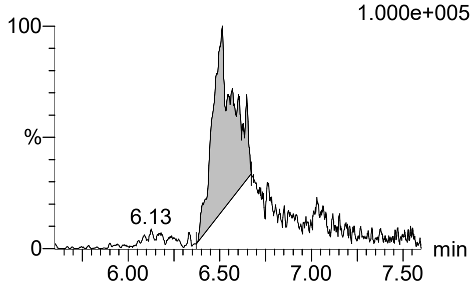

Sample Name: 20211111_H12_R3_IgM
F38:MRM of 2 channels,ES+
948.4 > 366.1
1.026e+005

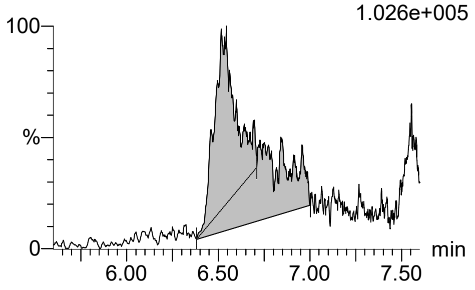

Sample Name: 20211111_H12_R3_IgM
F38:MRM of 2 channels,ES+
1580 > 366.1
9.075e+004

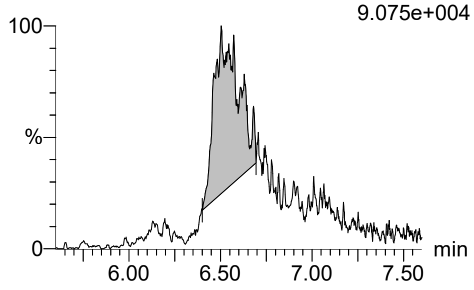

Sample Name: 20211111_QC_R22_IgM
F38:MRM of 2 channels,ES+
948.4 > 366.1
1.369e+005

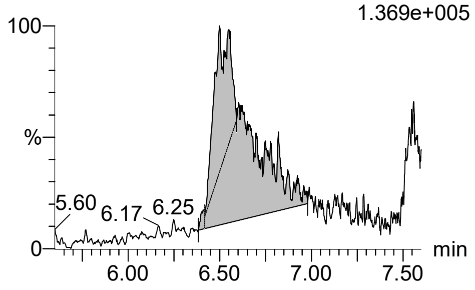

Sample Name: 20211111_QC_R22_IgM
F38:MRM of 2 channels,ES+
1580 > 366.1
1.537e+005

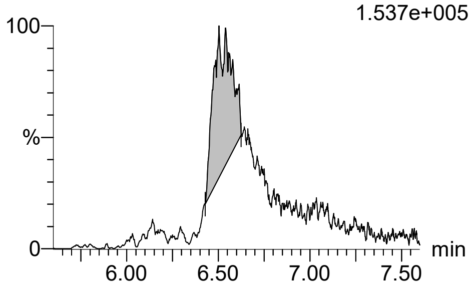

Sample Name: 20211111_A25_R3_IgM
F38:MRM of 2 channels,ES+
948.4 > 366.1
2.002e+005

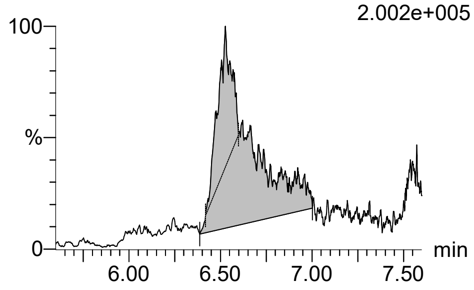

Sample Name: 20211111_A25_R3_IgM
F38:MRM of 2 channels,ES+
1580 > 366.1
2.284e+005

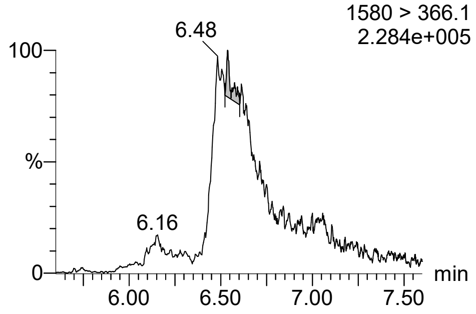

Sample Name: 20211111_A26_R3_IgM
F38:MRM of 2 channels,ES+
948.4 > 366.1
1.304e+005

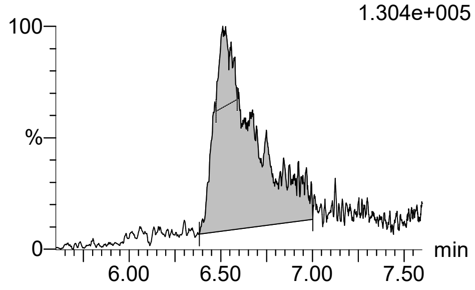

Sample Name: 20211111_A26_R3_IgM
F38:MRM of 2 channels,ES+
1580 > 366.1
1.340e+005

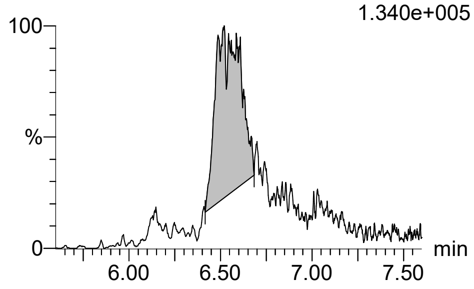

Sample Name: 20211111_L25_R3_IgM
F38:MRM of 2 channels,ES+
948.4 > 366.1
1.062e+005

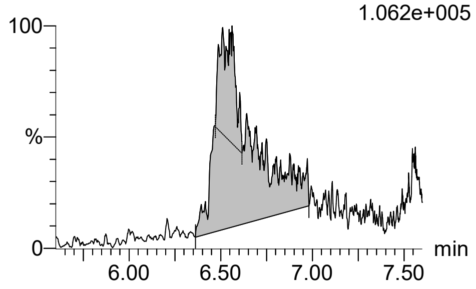

Sample Name: 20211111_L25_R3_IgM
F38:MRM of 2 channels,ES+
1580 > 366.1
1.145e+005

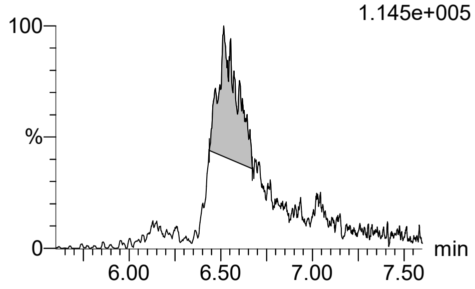

Sample Name: 20211111_L26_R3_IgM
F38:MRM of 2 channels,ES+
948.4 > 366.1
1.074e+005

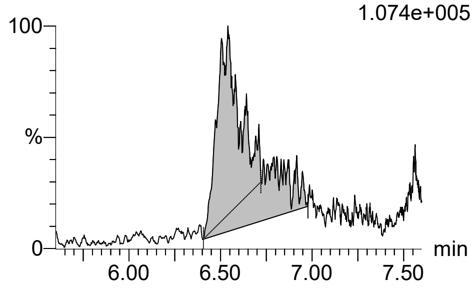

Sample Name: 20211111_L26_R3_IgM
F38:MRM of 2 channels,ES+
1580 > 366.1
1.037e+005

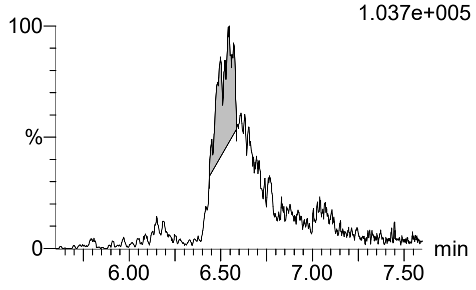

Sample Name: 20211111_H13_R3_IgM
F38:MRM of 2 channels,ES+
948.4 > 366.1
1.567e+005

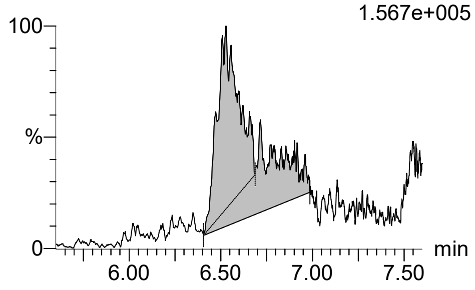

Sample Name: 20211111_H13_R3_IgM
F38:MRM of 2 channels,ES+
1580 > 366.1
1.932e+005

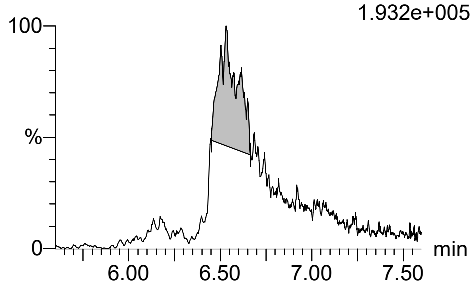

Sample Name: 20211111_A27_R3_IgM
F38:MRM of 2 channels,ES+
948.4 > 366.1
2.591e+005

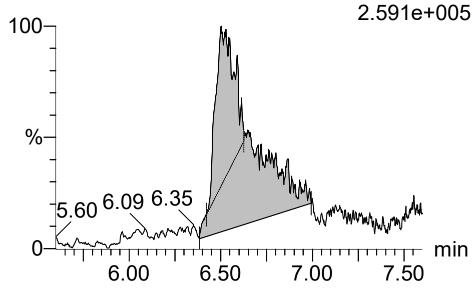

Sample Name: 20211111_A27_R3_IgM
F38:MRM of 2 channels,ES+
1580 > 366.1
3.456e+005

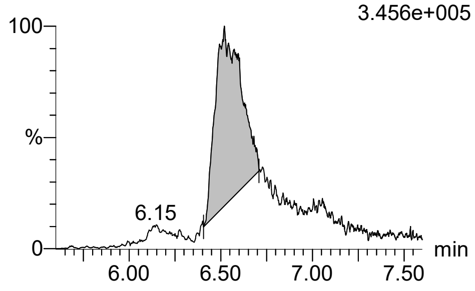

Sample Name: 20211111_A28_R3_IgM
F38:MRM of 2 channels,ES+
948.4 > 366.1
1.283e+005

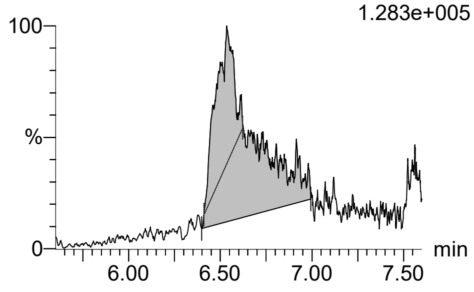

Sample Name: 20211111_A28_R3_IgM
F38:MRM of 2 channels,ES+
1580 > 366.1
1.635e+005

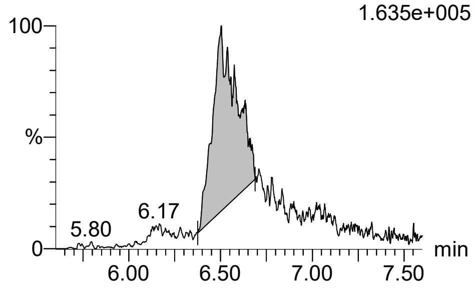

Sample Name: 20211111_L27_R3_IgM
F38:MRM of 2 channels,ES+
948.4 > 366.1
1.397e+005

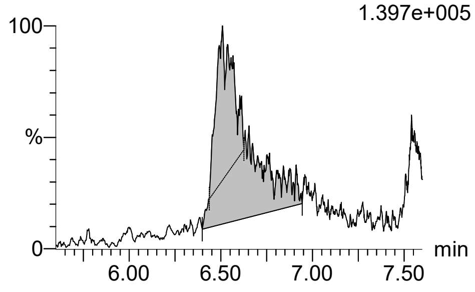

Sample Name: 20211111_L27_R3_IgM
F38:MRM of 2 channels,ES+
1580 > 366.1
1.549e+005

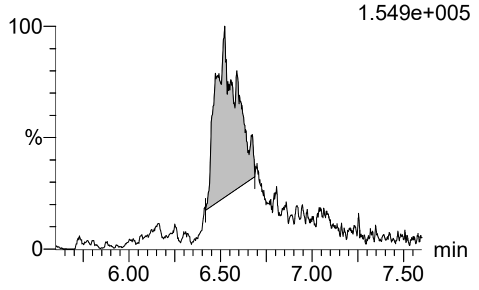

Sample Name: 20211111_L28_R3_IgM
F38:MRM of 2 channels,ES+
948.4 > 366.1
1.099e+005

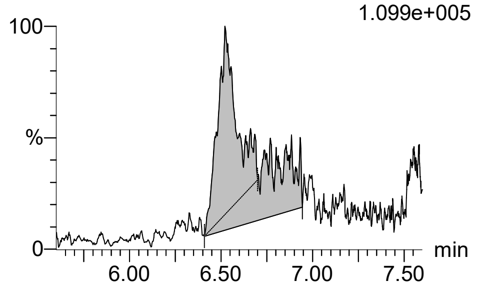

Sample Name: 20211111_L28_R3_IgM
F38:MRM of 2 channels,ES+
1580 > 366.1
1.066e+005

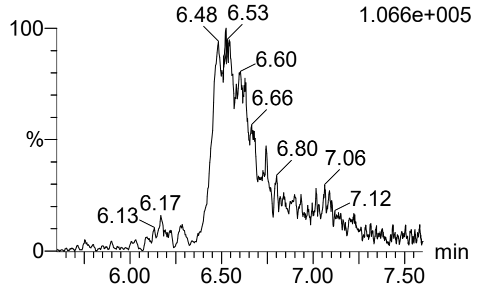

Sample Name: 20211111_H14_R3_IgM
F38:MRM of 2 channels,ES+
948.4 > 366.1
1.153e+005

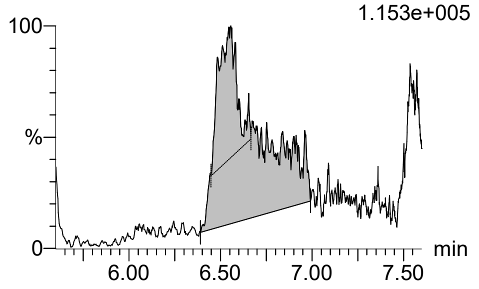

Sample Name: 20211111_H14_R3_IgM
F38:MRM of 2 channels,ES+
1580 > 366.1
1.299e+005

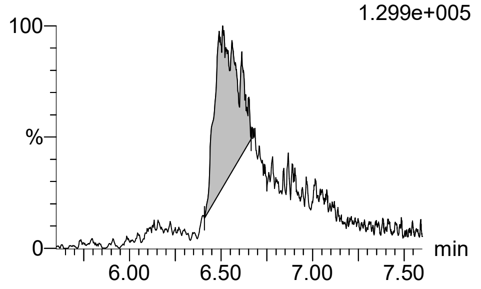

Sample Name: 20211111_A29_R3_IgM
F38:MRM of 2 channels,ES+
948.4 > 366.1
7.680e+004

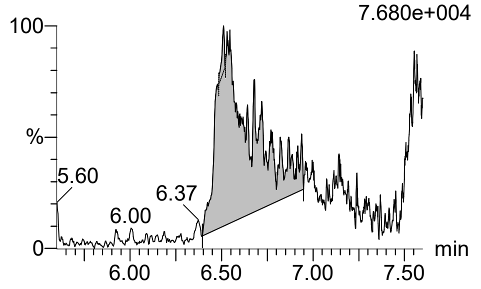

Sample Name: 20211111_A29_R3_IgM
F38:MRM of 2 channels,ES+
1580 > 366.1
6.403e+004

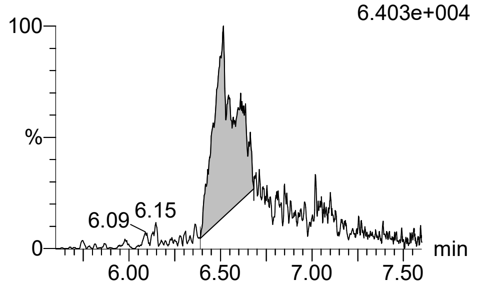

Sample Name: 20211111_A30_R3_IgM
F38:MRM of 2 channels,ES+
948.4 > 366.1
1.615e+005

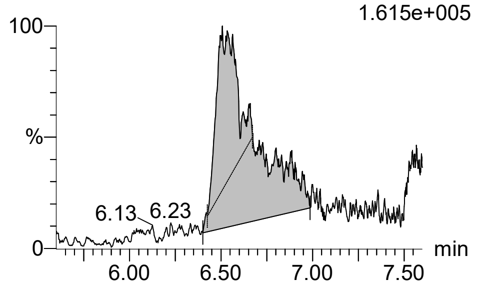

Sample Name: 20211111_A30_R3_IgM
F38:MRM of 2 channels,ES+
1580 > 366.1
1.803e+005

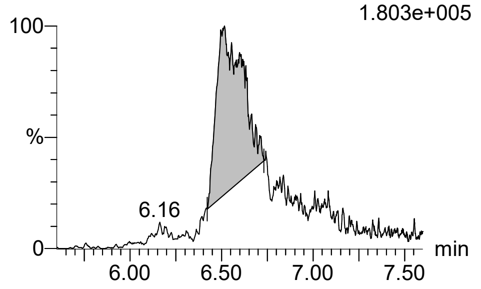

Sample Name: 20211111_QC_R23_IgM
F38:MRM of 2 channels,ES+
948.4 > 366.1
1.343e+005

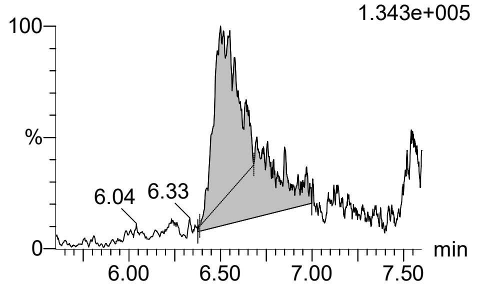

Sample Name: 20211111_QC_R23_IgM
F38:MRM of 2 channels,ES+
1580 > 366.1
1.548e+005

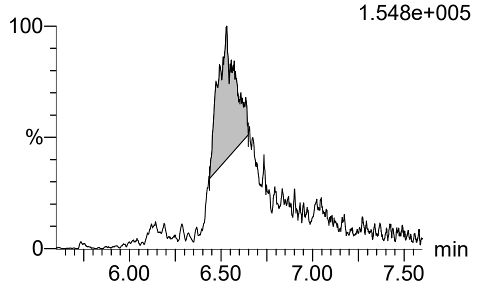

Sample Name: 20211111_L29_R3_IgM
F38:MRM of 2 channels,ES+
948.4 > 366.1
9.875e+004

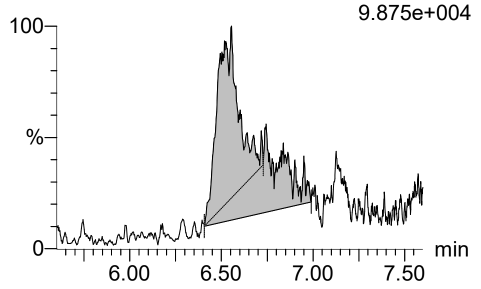

Sample Name: 20211111_L29_R3_IgM
F38:MRM of 2 channels,ES+
1580 > 366.1
9.498e+004

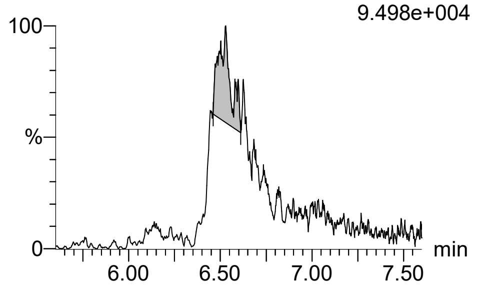

Sample Name: 20211111_L30_R3_IgM
F38:MRM of 2 channels,ES+
948.4 > 366.1
6.762e+004

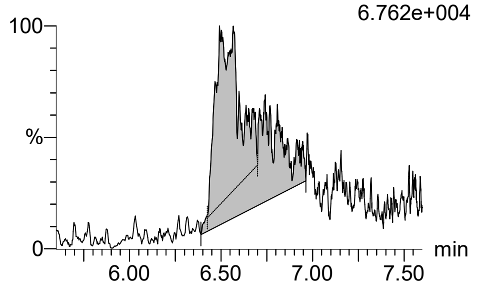

Sample Name: 20211111_L30_R3_IgM
F38:MRM of 2 channels,ES+
1580 > 366.1
6.036e+004

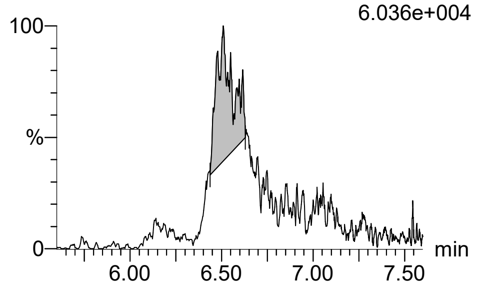

Sample Name: 20211111_H15_R3_IgM
F38:MRM of 2 channels,ES+
948.4 > 366.1
1.272e+005

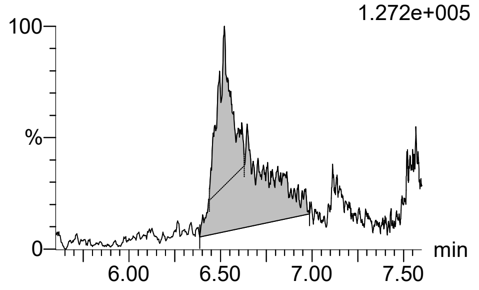

Sample Name: 20211111_H15_R3_IgM
F38:MRM of 2 channels,ES+
1580 > 366.1
9.484e+004

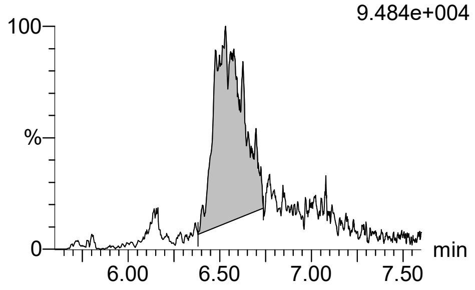

Sample Name: 20211111_A31_R3_IgM
F38:MRM of 2 channels,ES+
948.4 > 366.1
1.023e+005

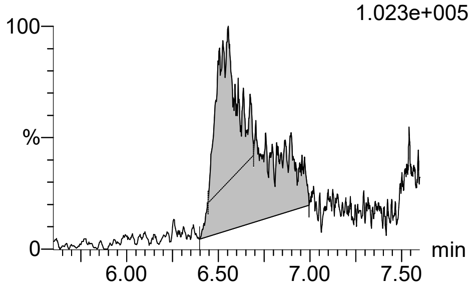

Sample Name: 20211111_A31_R3_IgM
F38:MRM of 2 channels,ES+
1580 > 366.1
7.956e+004

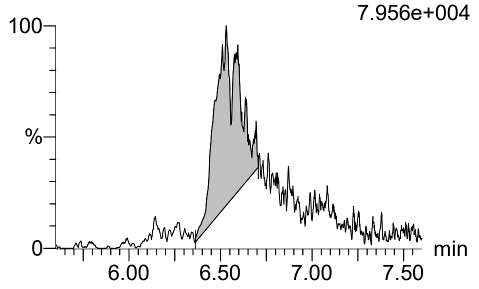

Sample Name: 20211111_A32_R3_IgM
F38:MRM of 2 channels,ES+
948.4 > 366.1
1.378e+005

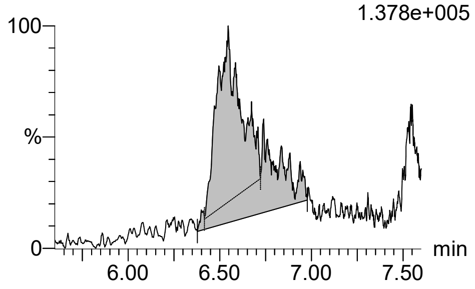

Sample Name: 20211111_A32_R3_IgM
F38:MRM of 2 channels,ES+
1580 > 366.1
1.321e+005

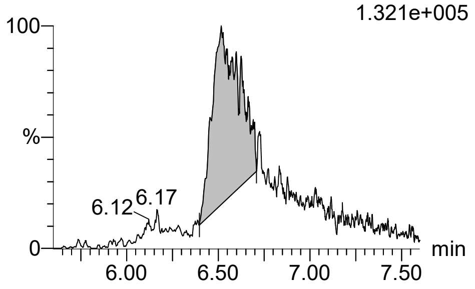

Sample Name: 20211111_L31_R3_IgM
F38:MRM of 2 channels,ES+
948.4 > 366.1
6.730e+004

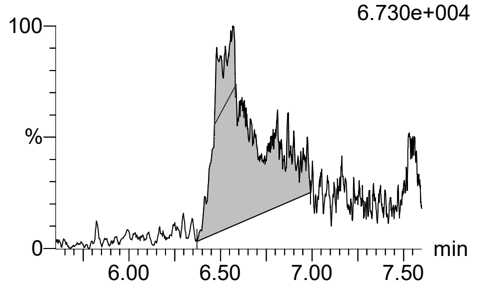

Sample Name: 20211111_L31_R3_IgM
F38:MRM of 2 channels,ES+
1580 > 366.1
4.890e+004

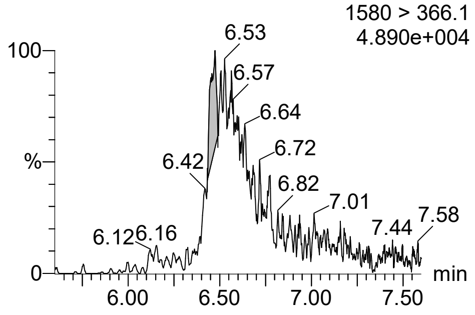

Sample Name: 20211111_L32_R3_IgM
F38:MRM of 2 channels,ES+
948.4 > 366.1
1.413e+005

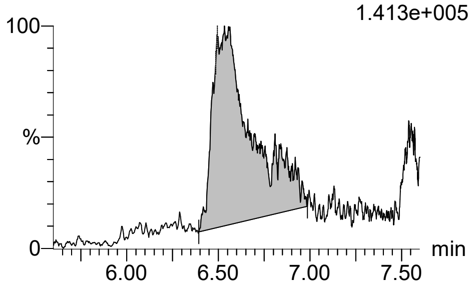

Sample Name: 20211111_L32_R3_IgM
F38:MRM of 2 channels,ES+
1580 > 366.1
1.673e+005

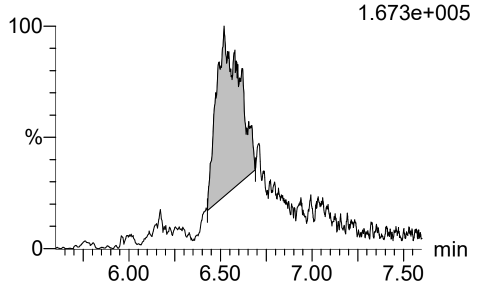

Sample Name: 20211111_H16_R3_IgM
F38:MRM of 2 channels,ES+
948.4 > 366.1
1.872e+005

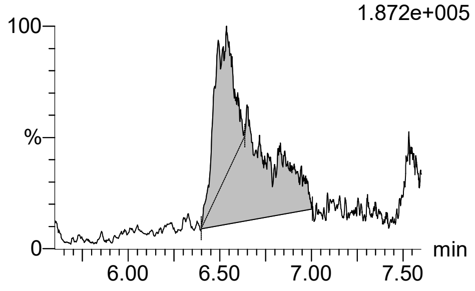

Sample Name: 20211111_H16_R3_IgM
F38:MRM of 2 channels,ES+
1580 > 366.1
2.271e+005

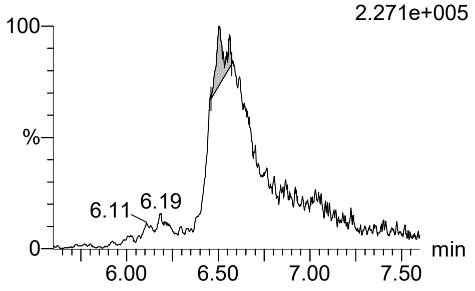

Sample Name: 20211111_A33_R3_IgM
F38:MRM of 2 channels,ES+
948.4 > 366.1
2.488e+005

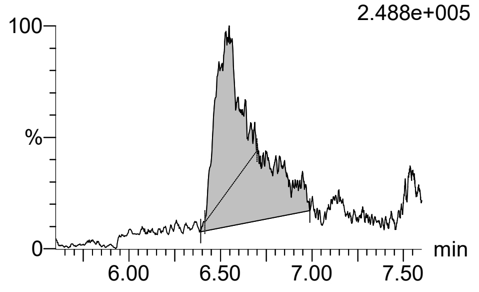

Sample Name: 20211111_A33_R3_IgM
F38:MRM of 2 channels,ES+
1580 > 366.1
3.084e+005

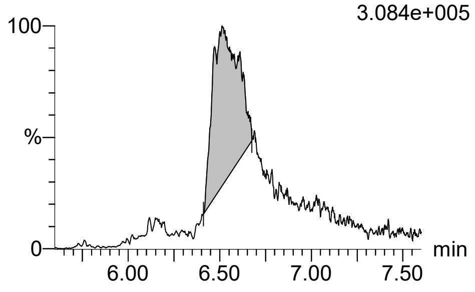

Sample Name: 20211111_A34_R3_IgM
F38:MRM of 2 channels,ES+
948.4 > 366.1
1.218e+005

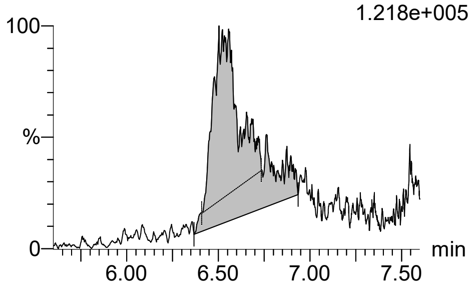

Dataset: C:\MassLynx\10.pro\20211111_clinicalsamples_IgM_IgA.qld

Last Altered: Thursday, March 09, 2023 19:58:25 台北標準時間
Printed: Friday, August 01, 2025 15:23:22 台北標準時間

Sample Name: 20211111_A34_R3_IgM
F38:MRM of 2 channels,ES+
1580 > 366.1
1.254e+005

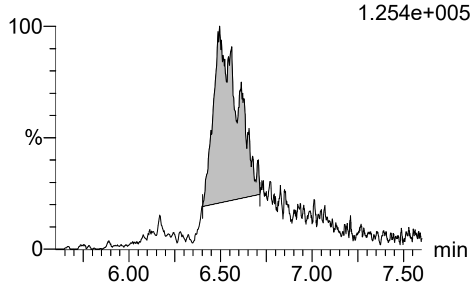

Sample Name: 20211111_H17_R3_IgM
F38:MRM of 2 channels,ES+
948.4 > 366.1
4.078e+005

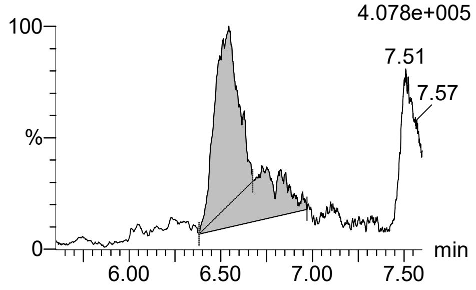

Sample Name: 20211111_H17_R3_IgM
F38:MRM of 2 channels,ES+
1580 > 366.1
5.595e+005

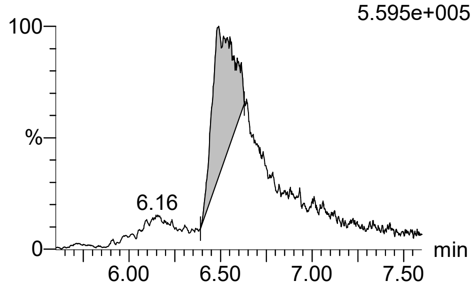

Sample Name: 20211111_A35_R3_IgM
F38:MRM of 2 channels,ES+
948.4 > 366.1
1.428e+005

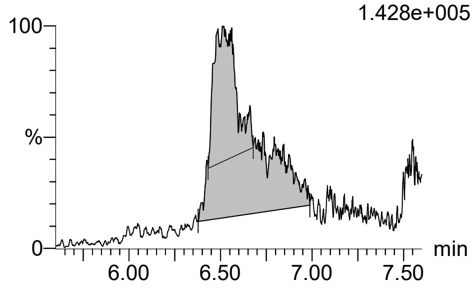

Sample Name: 20211111_A35_R3_IgM
F38:MRM of 2 channels,ES+
1580 > 366.1
1.964e+005

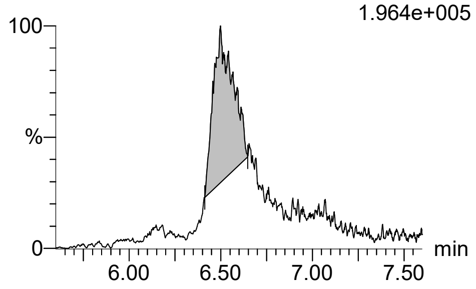

Sample Name: 20211111_QC_R24_IgM
F38:MRM of 2 channels,ES+
948.4 > 366.1
1.527e+005

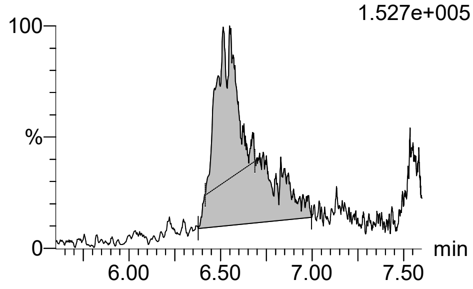

Sample Name: 20211111_QC_R24_IgM
F38:MRM of 2 channels,ES+
1580 > 366.1
1.518e+005

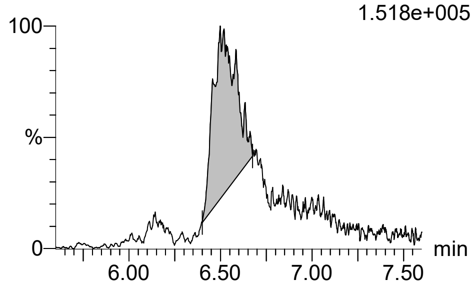

Sample Name: 20211111_A36_R3_IgM
F38:MRM of 2 channels,ES+
948.4 > 366.1
1.145e+005

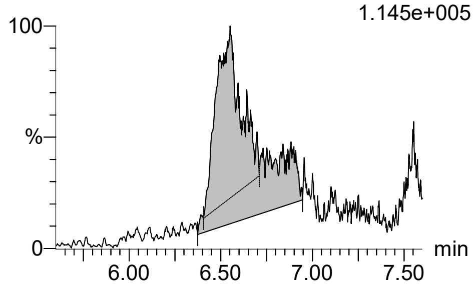

Sample Name: 20211111_A36_R3_IgM
F38:MRM of 2 channels,ES+
1580 > 366.1
1.167e+005

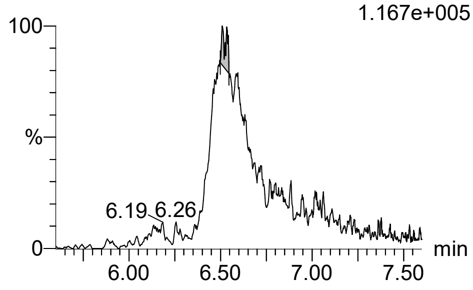

Sample Name: 20211111_H18_R3_IgM
F38:MRM of 2 channels,ES+
948.4 > 366.1
2.116e+005

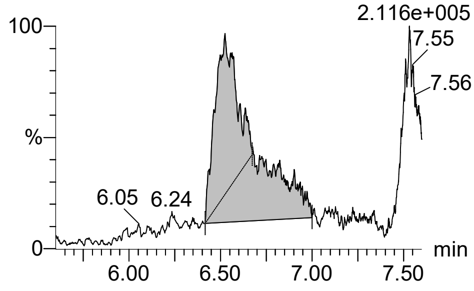

Sample Name: 20211111_H18_R3_IgM
F38:MRM of 2 channels,ES+
1580 > 366.1
2.628e+005

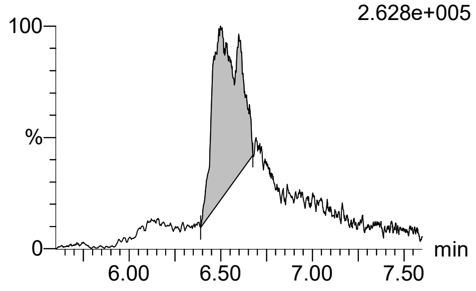

Sample Name: 20211111_A37_R3_IgM
F38:MRM of 2 channels,ES+
948.4 > 366.1
1.481e+005

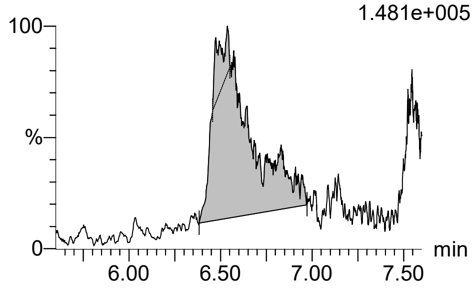

Sample Name: 20211111_A37_R3_IgM
F38:MRM of 2 channels,ES+
1580 > 366.1
1.896e+005

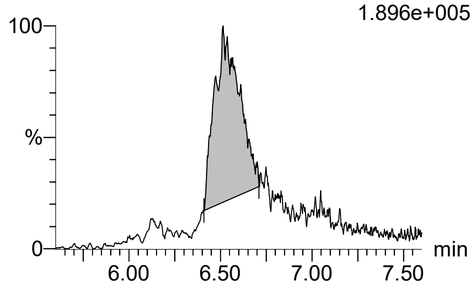

Sample Name: 20211111_A38_R3_IgM
F38:MRM of 2 channels,ES+
948.4 > 366.1
1.196e+005

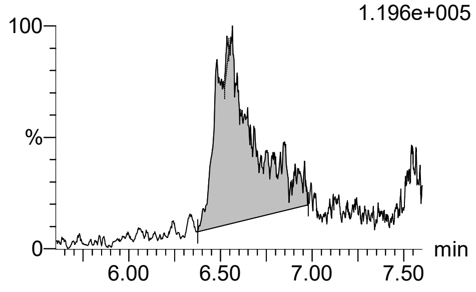

Sample Name: 20211111_A38_R3_IgM
F38:MRM of 2 channels,ES+
1580 > 366.1
1.202e+005

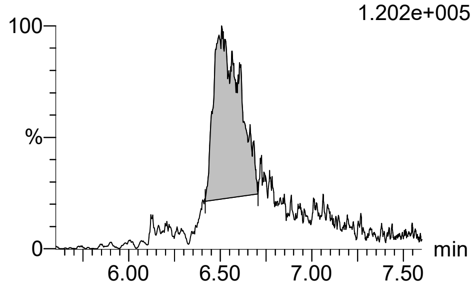

Sample Name: 20211111_H19_R3_IgM
F38:MRM of 2 channels,ES+
948.4 > 366.1
2.269e+005

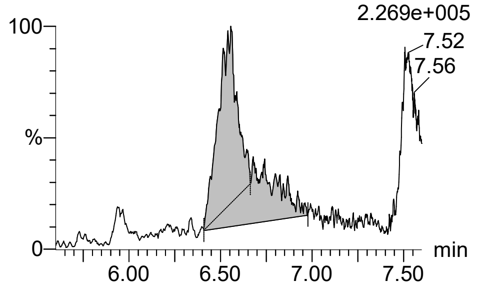

Sample Name: 20211111_H19_R3_IgM
F38:MRM of 2 channels,ES+
1580 > 366.1
2.444e+005

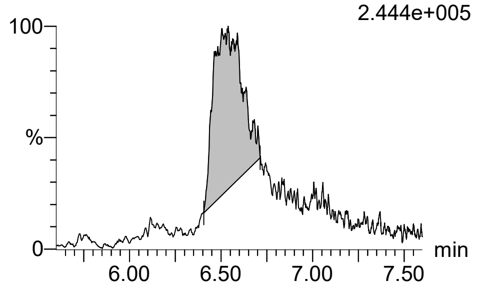

Sample Name: 20211111_A39_R3_IgM
F38:MRM of 2 channels,ES+
948.4 > 366.1
1.431e+005

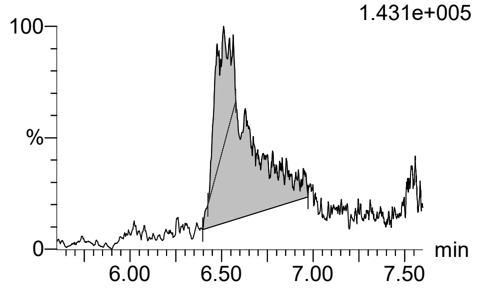

Sample Name: 20211111_A39_R3_IgM
F38:MRM of 2 channels,ES+
1580 > 366.1
1.634e+005

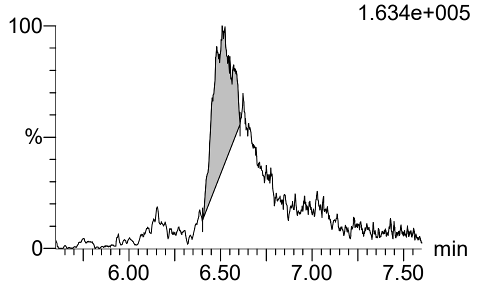

Sample Name: 20211111_A40_R3_IgM
F38:MRM of 2 channels,ES+
948.4 > 366.1
1.300e+005

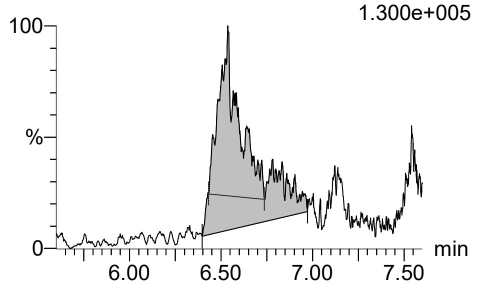

Sample Name: 20211111_A40_R3_IgM
F38:MRM of 2 channels,ES+
1580 > 366.1
9.195e+004

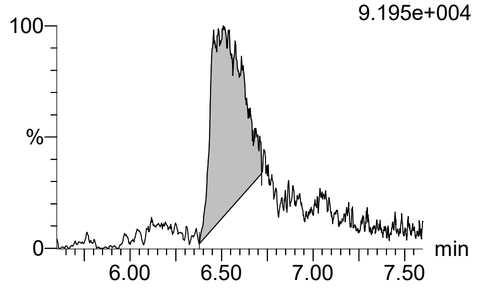

Sample Name: 20211111_H20_R3_IgM
F38:MRM of 2 channels,ES+
948.4 > 366.1
3.577e+005

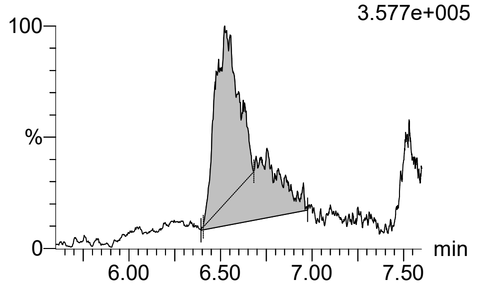

Sample Name: 20211111_H20_R3_IgM
F38:MRM of 2 channels,ES+
1580 > 366.1
5.279e+005

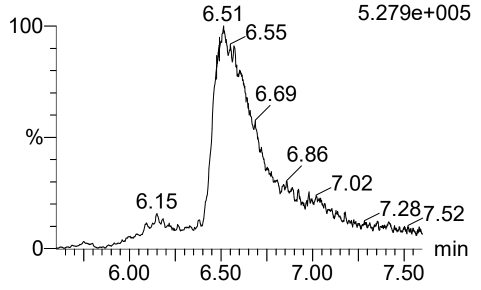

Sample Name: 20211111_A41_R3_IgM
F38:MRM of 2 channels,ES+
948.4 > 366.1
1.022e+005

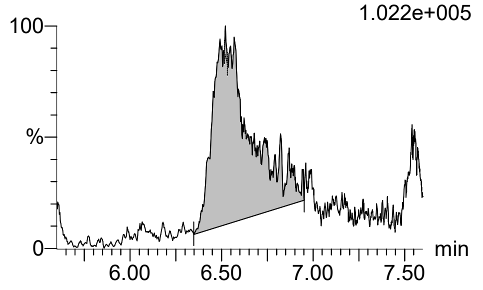

Sample Name: 20211111_A41_R3_IgM
F38:MRM of 2 channels,ES+
1580 > 366.1
1.193e+005

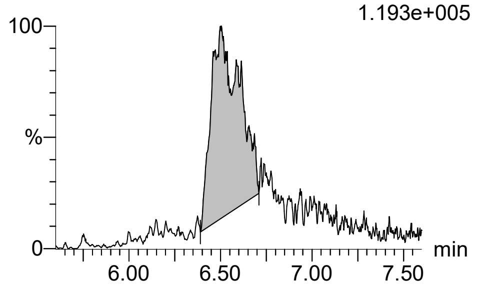

Sample Name: 20211111_A42_R3_IgM
F38:MRM of 2 channels,ES+
948.4 > 366.1
1.066e+005

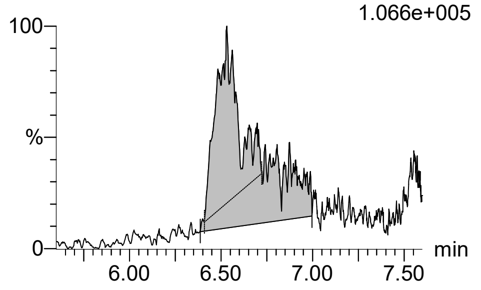

Sample Name: 20211111_A42_R3_IgM
F38:MRM of 2 channels,ES+
1580 > 366.1
9.793e+004

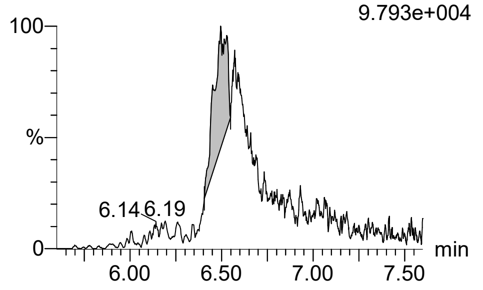

Sample Name: 20211111_A43_R3_IgM
F38:MRM of 2 channels,ES+
948.4 > 366.1
9.466e+004

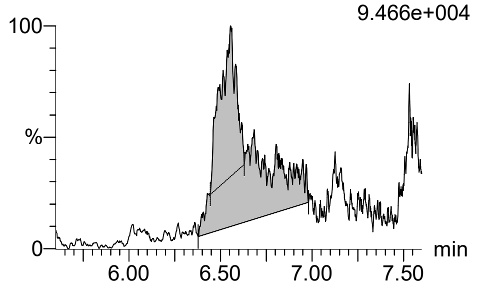

Sample Name: 20211111_A43_R3_IgM
F38:MRM of 2 channels,ES+
1580 > 366.1
8.161e+004

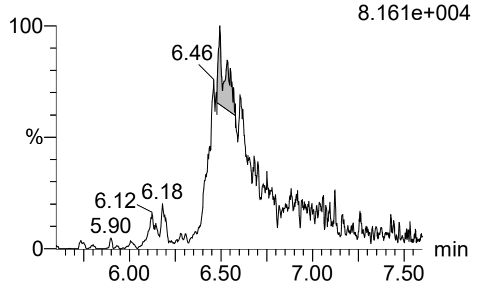

Sample Name: 20211111_A44_R3_IgM
F38:MRM of 2 channels,ES+
948.4 > 366.1
7.643e+004

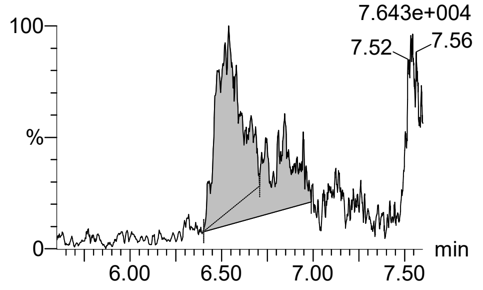

Dataset: C:\MassLynx\10.pro\20211111_clinicalsamples_IgM_IgA.qld

Last Altered: Thursday, March 09, 2023 19:58:25 台北標準時間
Printed: Friday, August 01, 2025 15:23:22 台北標準時間

Sample Name: 20211111_A44_R3_IgM

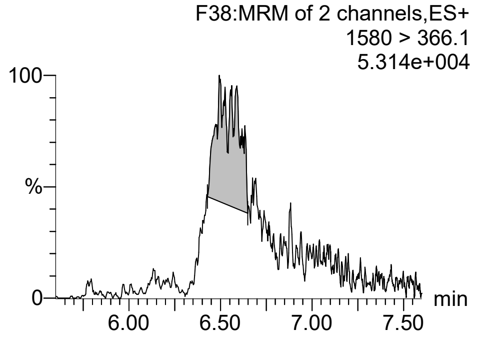

Sample Name: 20211111_QC_R25_IgM

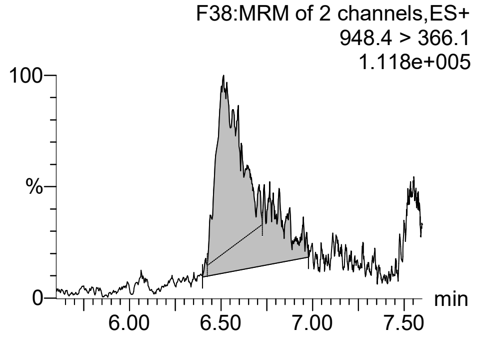

Sample Name: 20211111_QC_R25_IgM

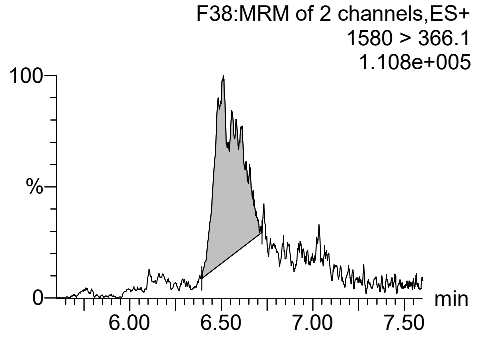

Dataset: C:\MassLynx\10.pro\20211111_clinicalsamples_IgM_IgA.qld

Last Altered: Thursday, March 09, 2023 19:58:25 台北標準時間
Printed: Friday, August 01, 2025 15:23:22 台北標準時間

	#	Name	RT	Area	IS Area	Response	Prima...
1	1	20211112_QC_R001_IgM	6.54	20562.7	15421.4	1.333	bb
2	2	20211112_QC_R002_IgM	6.54	36708.6	13188.8	2.783	MM
3	3	20211111_A1_R2_IgM	6.49	30525.5	11871.0	2.571	MM
4	4	20211111_A1_R3_IgM	6.50	27974.1	12030.5	2.325	MM
5	5	20211111_A2_R2_IgM	6.53	50792.5	11446.8	4.437	MM
6	6	20211111_A2_R3_IgM	6.53	51341.2	11098.4	4.626	MM
7	7	20211111_L1_R2_IgM	6.53	25824.0	11779.4	2.192	MM
8	8	20211111_L1_R3_IgM	6.50	20025.8	11176.2	1.792	MM
9	9	20211111_L2_R2_IgM	6.54	36211.3	11375.0	3.183	MM
10	10	20211111_H1_R2_IgM	6.51	36534.5	13595.2	2.687	MM
11	11	20211111_A3_R2_IgM	6.52	46423.9	14112.3	3.290	MM
12	12	20211111_A4_R2_IgM	6.54	39162.4	11977.5	3.270	MM
13	13	20211111_L3_R2_IgM	6.53	33543.9	12242.1	2.740	MM
14	14	20211111_L4_R2_IgM	6.55	41133.9	12797.0	3.214	MM
15	15	20211111_H2_R2_IgM	6.54	56817.9	10006.0	5.678	MM
16	16	20211111_A5_R2_IgM	6.50	113142.5	11434.6	9.895	MM
17	17	20211111_A6_R2_IgM	6.53	54810.0	13193.7	4.154	MM
18	18	20211111_QC_R10_IgM	6.52	39304.4	12566.1	3.128	MM
19	19	20211111_L5_R2_IgM	6.53	40119.0	13616.4	2.946	MM
20	20	20211111_L6_R2_IgM	6.51	33056.5	13698.5	2.413	MM
21	21	20211111_H3_R2_IgM	6.49	24507.9	15793.1	1.552	MM
22	22	20211111_A7_R2_IgM	6.54	34557.1	12936.9	2.671	MM
23	23	20211111_A8_R2_IgM	6.51	45885.8	13108.0	3.501	MM
24	24	20211111_L7_R2_IgM	6.56	23263.3	13278.5	1.752	MM
25	25	20211111_L8_R2_IgM	6.56	20749.3	15233.6	1.362	MM
26	26	20211111_H4_R2_IgM	6.52	22286.7	16521.9	1.349	MM
27	27	20211111_A9_R2_IgM	6.57	19485.3	16451.6	1.184	MM
28	28	20211111_A10_R2_IgM	6.50	43542.6	13036.0	3.340	MM
29	29	20211111_L9_R2_IgM	6.49	28508.7	14554.1	1.959	MM
30	30	20211111_L10_R2_IgM	6.52	35366.8	12250.4	2.887	MM
31	31	20211111_QC_R11_IgM	6.55	34561.2	11459.2	3.016	MM
32	32	20211111_H5_R2_IgM	6.54	41949.6	14358.2	2.922	MM
33	33	20211111_A11_R2_IgM	6.50	59103.6	16184.1	3.652	MM
34	34	20211111_A12_R2_IgM	6.51	28193.3	13465.4	2.094	MM
35	35	20211111_L11_R2_IgM	6.49	24843.3	13277.9	1.871	MM
36	36	20211111_L12_R2_IgM	6.53	19382.6	15958.8	1.215	MM
37	37	20211111_H6_R2_IgM	6.56	20009.8	14003.5	1.429	MM
38	38	20211111_A13_R2_IgM	6.55	25238.3	13656.1	1.848	MM
39	39	20211111_A14_R2_IgM	6.50	60641.2	14071.3	4.310	MM
40	40	20211111_L13_R2_IgM	6.50	28002.4	14785.6	1.894	MM
41	41	20211111_L14_R2_IgM	6.54	39097.7	14772.2	2.647	MM
42	42	20211111_H7_R2_IgM	6.52	33512.2	15164.4	2.210	MM
43	43	20211111_A15_R2_IgM	6.56	69093.1	15721.5	4.395	MM
44	44	20211111_QC_R12_IgM	6.52	41482.8	13711.5	3.025	MM
45	45	20211111_A16_R2_IgM	6.54	35632.6	14275.6	2.496	MM
46	46	20211111_L15_R2_IgM	6.52	47792.9	12935.8	3.695	MM
47	47	20211111_L16_R2_IgM	6.54	21825.2	13342.8	1.636	MM
48	48	20211111_H8_R2_IgM	6.54	24581.5	15835.1	1.552	MM
49	49	20211111_A17_R2_IgM	6.52	36313.1	15786.7	2.300	MM
50	50	20211111_A18_R2_IgM	6.52	22427.9	16986.8	1.320	MM
51	51	20211111_L17_R2_IgM	6.54	46331.1	13342.7	3.472	MM
52	52	20211111_L18_R2_IgM	6.50	21636.3	14692.0	1.473	MM
53	53	20211111_H9_R2_IgM	6.53	20793.9	15639.7	1.330	MM
54	54	20211111_A19_R2_IgM	6.54	26863.6	14198.7	1.892	MM
55	55	20211111_A20_R2_IgM	6.51	21717.6	14162.7	1.533	MM
56	56	20211111_L19_R2_IgM	6.53	28972.6	9293.4	3.118	MM

Dataset: C:\MassLynx\10.pro\20211111_clinicalsamples_IgM_IgA.qld

Last Altered: Thursday, March 09, 2023 19:58:25 台北標準時間
Printed: Friday, August 01, 2025 15:23:22 台北標準時間

	# Name	RT	Area	IS Area	Response	Prima...
57	57 20211111_QC_R13_IgM	6.54	31490.1	13903.0	2.265	MM
58	58 20211111_L20_R2_IgM	6.49	26894.6	12781.6	2.104	MM
59	59 20211111_H10_R2_IgM	6.51	34153.0	14796.4	2.308	MM
60	60 20211111_A21_R2_IgM	6.52	27808.2	13669.0	2.034	MM
61	61 20211111_A22_R2_IgM	6.54	17430.1	14098.3	1.236	MM
62	62 20211111_L21_R2_IgM	6.53	26394.5	15678.2	1.684	MM
63	63 20211111_L22_R2_IgM	6.50	21673.5	15724.5	1.378	MM
64	64 20211111_H11_R2_IgM	6.52	29423.8	15890.2	1.852	MM
65	65 20211111_A23_R2_IgM	6.54	23597.2	13423.2	1.758	MM
66	66 20211111_A24_R2_IgM	6.50	33450.3	12438.0	2.689	MM
67	67 20211111_L23_R2_IgM	6.52	37724.6	14075.0	2.680	MM
68	68 20211111_L24_R2_IgM	6.49	21029.3	15972.1	1.317	MM
69	69 20211111_H12_R2_IgM	6.54	21780.4	16270.9	1.339	MM
70	70 20211111_QC_R14_IgM	6.56	31208.9	15806.8	1.974	MM
71	71 20211111_A25_R2_IgM	6.49	44672.2	14552.1	3.070	MM
72	72 20211111_A26_R2_IgM	6.50	31567.9	11492.7	2.747	MM
73	73 20211111_L25_R2_IgM	6.53	23757.2	14403.3	1.649	MM
74	74 20211111_L26_R2_IgM	6.51	20200.8	16533.9	1.222	MM
75	75 20211111_H13_R2_IgM	6.53	30414.9	15520.1	1.960	MM
76	76 20211111_A27_R2_IgM	6.52	54807.0	13337.4	4.109	MM
77	77 20211111_A28_R2_IgM	6.51	34833.6	11895.6	2.928	MM
78	78 20211111_L27_R2_IgM	6.55	28175.6	14125.7	1.995	MM
79	79 20211111_L28_R2_IgM	6.51	23226.8	11807.5	1.967	MM
80	80 20211111_H14_R2_IgM	6.53	23475.3	15035.7	1.561	MM
81	81 20211111_A29_R2_IgM	6.56	18383.4	13554.4	1.356	MM
82	82 20211111_A30_R2_IgM	6.52	38390.4	12984.1	2.957	MM
83	83 20211111_QC_R15_IgM	6.55	31335.7	12299.5	2.548	MM
84	84 20211111_L29_R2_IgM	6.56	21486.6	13590.7	1.581	MM
85	85 20211111_L30_R2_IgM	6.53	18197.0	12511.4	1.454	MM
86	86 20211111_H15_R2_IgM	6.55	26024.0	11885.0	2.190	MM
87	87 20211111_A31_R2_IgM	6.53	25803.3	13422.1	1.922	MM
88	88 20211111_A32_R2_IgM	6.57	33591.9	11461.3	2.931	MM
89	89 20211111_L31_R2_IgM	6.53	17894.4	13753.4	1.301	MM
90	90 20211111_L32_R2_IgM	6.52	35205.9	12949.0	2.719	MM
91	91 20211111_H16_R2_IgM	6.52	47370.4	11465.4	4.132	MM
92	92 20211111_A33_R2_IgM	6.53	51466.0	10551.5	4.878	MM
93	93 20211111_A34_R2_IgM	6.52	28940.8	12409.9	2.332	MM
94	94 20211111_H17_R2_IgM	6.53	91255.2	10160.9	8.981	MM
95	95 20211111_A35_R2_IgM	6.55	38079.1	10460.6	3.640	MM
96	96 20211111_QC_R16_IgM	6.51	34749.0	10433.5	3.331	MM
97	97 20211111_A36_R2_IgM	6.51	29827.4	12504.8	2.385	MM
98	98 20211111_H18_R2_IgM	6.55	45397.5	9140.4	4.967	MM
99	99 20211111_A37_R2_IgM	6.55	36483.3	9796.9	3.724	MM
100	1... 20211111_A38_R2_IgM	6.52	28107.9	11224.3	2.504	MM
101	1... 20211111_H19_R2_IgM	6.55	42331.6	11739.2	3.606	MM
102	1... 20211111_A39_R2_IgM	6.56	30545.2	12718.9	2.402	MM
103	1... 20211111_A40_R2_IgM	6.50	28942.8	10006.1	2.893	MM
104	1... 20211111_H20_R2_IgM	6.55	83354.9	7988.2	10.435	MM
105	1... 20211111_A41_R2_IgM	6.53	36316.8	11513.3	3.154	MM
106	1... 20211111_A42_R2_IgM	6.51	22683.8	10238.5	2.216	MM
107	1... 20211111_A43_R2_IgM	6.53	20703.2	11098.9	1.865	MM
108	1... 20211111_A44_R2_IgM	6.54	18923.4	10969.9	1.725	MM
109	1... 20211111_QC_R17_IgM	6.54	26963.1	9700.8	2.779	MM
110	1... 20211111_L2_R3_IgM	6.54	34442.2	8528.0	4.039	MM
111	1... 20211111_H1_R3_IgM	6.56	31837.6	9438.2	3.373	MM
112	1... 20211111_A3_R3_IgM	6.52	37706.6	11598.6	3.251	MM

	#	Name	RT	Area	IS Area	Response	Prima...
113	1...	20211111_A4_R3_IgM	6.56	36842.4	11364.1	3.242	MM
114	1...	20211111_L3_R3_IgM	6.52	27862.6	10753.4	2.591	MM
115	1...	20211111_L4_R3_IgM	6.54	33913.4	10291.6	3.295	MM
116	1...	20211111_H2_R3_IgM	6.54	56284.0	9315.8	6.042	MM
117	1...	20211111_A5_R3_IgM	6.55	91191.4	9102.2	10.019	MM
118	1...	20211111_A6_R3_IgM	6.56	45563.9	10356.4	4.400	MM
119	1...	20211111_QC_R18_IgM	6.53	32784.5	8398.3	3.904	MM
120	1...	20211111_L5_R3_IgM	6.52	27938.4	9786.7	2.855	MM
121	1...	20211111_L6_R3_IgM	6.54	25945.6	9911.3	2.618	MM
122	1...	20211111_H3_R3_IgM	6.51	17619.2	12155.5	1.449	MM
123	1...	20211111_A7_R3_IgM	6.55	30857.8	11804.8	2.614	MM
124	1...	20211111_A8_R3_IgM	6.55	34740.4	10510.6	3.305	MM
125	1...	20211111_L7_R3_IgM	6.54	22900.0	12177.9	1.880	MM
126	1...	20211111_L8_R3_IgM	6.48	15613.0	11668.6	1.338	MM
127	1...	20211111_H4_R3_IgM	6.53	19744.2	11192.0	1.764	MM
128	1...	20211111_A9_R3_IgM	6.53	18816.1	14077.4	1.337	MM
129	1...	20211111_A10_R3_IgM	6.54	32889.4	11190.4	2.939	MM
130	1...	20211111_L9_R3_IgM	6.53	23604.9	14649.3	1.611	MM
131	1...	20211111_L10_R3_IgM	6.54	34408.2	9361.3	3.676	MM
132	1...	20211111_QC_R19_IgM	6.53	28102.4	10125.5	2.775	MM
133	1...	20211111_H5_R3_IgM	6.54	35618.2	10010.9	3.558	MM
134	1...	20211111_A11_R3_IgM	6.55	39838.8	12697.2	3.138	MM
135	1...	20211111_A12_R3_IgM	6.51	22849.6	13148.5	1.738	MM
136	1...	20211111_L11_R3_IgM	6.53	21825.6	10786.6	2.023	MM
137	1...	20211111_L12_R3_IgM	6.54	18740.8	11681.8	1.604	MM
138	1...	20211111_H6_R3_IgM	6.55	19261.7	12095.5	1.592	MM
139	1...	20211111_A13_R3_IgM	6.54	22939.0	12908.3	1.777	MM
140	1...	20211111_A14_R3_IgM	6.55	52450.7	11322.6	4.632	MM
141	1...	20211111_L13_R3_IgM	6.54	27731.8	11516.1	2.408	MM
142	1...	20211111_L14_R3_IgM	6.53	28701.2	10790.7	2.660	MM
143	1...	20211111_H7_R3_IgM	6.55	24956.5	11572.1	2.157	MM
144	1...	20211111_A15_R3_IgM	6.51	57487.7	12434.4	4.623	MM
145	1...	20211111_QC_R20_IgM	6.49	32860.1	10701.0	3.071	MM
146	1...	20211111_A16_R3_IgM	6.51	30282.0	11452.1	2.644	MM
147	1...	20211111_L15_R3_IgM	6.49	38201.2	10531.1	3.627	MM
148	1...	20211111_L16_R3_IgM	6.55	19130.4	12049.4	1.588	MM
149	1...	20211111_H8_R3_IgM	6.55	22215.1	13434.9	1.654	MM
150	1...	20211111_A17_R3_IgM	6.55	30970.2	12452.0	2.487	MM
151	1...	20211111_A18_R3_IgM	6.50	21166.5	12506.5	1.692	MM
152	1...	20211111_L17_R3_IgM	6.55	34741.2	9948.9	3.492	MM
153	1...	20211111_L18_R3_IgM	6.52	20485.3	13462.1	1.522	MM
154	1...	20211111_H9_R3_IgM	6.55	18248.7	13178.7	1.385	MM
155	1...	20211111_A19_R3_IgM	6.56	26403.9	11298.6	2.337	MM
156	1...	20211111_A20_R3_IgM	6.52	20732.4	11175.6	1.855	MM
157	1...	20211111_L19_R3_IgM	6.54	32280.3	11558.2	2.793	MM
158	1...	20211111_QC_R21_IgM	6.51	33178.7	9977.0	3.326	MM
159	1...	20211111_L20_R3_IgM	6.54	20478.1	13508.1	1.516	MM
160	1...	20211111_H10_R3_IgM	6.52	27580.8	12374.8	2.229	MM
161	1...	20211111_A21_R3_IgM	6.53	24236.6	12443.6	1.948	MM
162	1...	20211111_A22_R3_IgM	6.55	15861.1	11589.3	1.369	MM
163	1...	20211111_L21_R3_IgM	6.53	21388.6	13107.4	1.632	MM
164	1...	20211111_L22_R3_IgM	6.53	21836.7	12021.5	1.816	MM
165	1...	20211111_H11_R3_IgM	6.54	23304.6	12687.8	1.837	MM
166	1...	20211111_A23_R3_IgM	6.55	21222.2	11611.8	1.828	MM
167	1...	20211111_A24_R3_IgM	6.50	37114.3	10476.8	3.543	MM
168	1...	20211111_L23_R3_IgM	6.52	33686.4	11873.1	2.837	MM

Dataset: C:\MassLynx\10.pro\20211111_clinicalsamples_IgM_IgA.qld

Last Altered: Thursday, March 09, 2023 19:58:25 台北標準時間
Printed: Friday, August 01, 2025 15:23:22 台北標準時間

	# Name	RT	Area	IS Area	Response	Prima...
169	1... 20211111_L24_R3_IgM	6.55	18301.9	13468.0	1.359	MM
170	1... 20211111_H12_R3_IgM	6.54	21399.6	14229.1	1.504	MM
171	1... 20211111_QC_R22_IgM	6.50	26970.6	11264.4	2.394	MM
172	1... 20211111_A25_R3_IgM	6.53	37066.3	11775.7	3.148	MM
173	1... 20211111_A26_R3_IgM	6.51	30286.7	10473.9	2.892	MM
174	1... 20211111_L25_R3_IgM	6.56	22217.1	13256.8	1.676	MM
175	1... 20211111_L26_R3_IgM	6.54	20043.3	13565.0	1.478	MM
176	1... 20211111_H13_R3_IgM	6.53	29854.6	13146.0	2.271	MM
177	1... 20211111_A27_R3_IgM	6.50	53246.1	12025.6	4.428	MM
178	1... 20211111_A28_R3_IgM	6.53	25934.6	11229.1	2.310	MM
179	1... 20211111_L27_R3_IgM	6.51	23870.3	14960.0	1.596	MM
180	1... 20211111_L28_R3_IgM	6.52	19617.3	11359.0	1.727	MM
181	1... 20211111_H14_R3_IgM	6.56	26128.8	12830.8	2.036	MM
182	1... 20211111_A29_R3_IgM	6.51	14515.9	13434.3	1.081	MM
183	1... 20211111_A30_R3_IgM	6.51	34451.3	11272.2	3.056	MM
184	1... 20211111_QC_R23_IgM	6.50	27931.5	11872.2	2.353	MM
185	1... 20211111_L29_R3_IgM	6.55	18230.2	12948.7	1.408	MM
186	1... 20211111_L30_R3_IgM	6.49	14141.4	11026.6	1.282	MM
187	1... 20211111_H15_R3_IgM	6.52	21883.4	10786.3	2.029	MM
188	1... 20211111_A31_R3_IgM	6.55	22535.0	12010.5	1.876	MM
189	1... 20211111_A32_R3_IgM	6.55	26716.3	11107.7	2.405	MM
190	1... 20211111_L31_R3_IgM	6.57	15154.4	11021.8	1.375	MM
191	1... 20211111_L32_R3_IgM	6.53	31167.9	11683.8	2.668	MM
192	1... 20211111_H16_R3_IgM	6.54	40016.9	9831.7	4.070	MM
193	1... 20211111_A33_R3_IgM	6.55	52054.2	11213.1	4.642	MM
194	1... 20211111_A34_R3_IgM	6.50	22926.2	11550.1	1.985	MM
195	1... 20211111_H17_R3_IgM	6.54	76082.5	8874.4	8.573	MM
196	1... 20211111_A35_R3_IgM	6.51	31112.7	9856.3	3.157	MM
197	1... 20211111_QC_R24_IgM	6.55	29940.3	7561.8	3.959	MM
198	1... 20211111_A36_R3_IgM	6.55	23173.4	9562.8	2.423	MM
199	1... 20211111_H18_R3_IgM	6.52	41631.7	8165.3	5.099	MM
200	2... 20211111_A37_R3_IgM	6.54	29607.4	9278.8	3.191	MM
201	2... 20211111_A38_R3_IgM	6.56	23968.1	10920.3	2.195	MM
202	2... 20211111_H19_R3_IgM	6.56	36955.8	9285.3	3.980	MM
203	2... 20211111_A39_R3_IgM	6.51	26635.4	9083.4	2.932	MM
204	2... 20211111_A40_R3_IgM	6.54	22585.0	8382.4	2.694	MM
205	2... 20211111_H20_R3_IgM	6.52	69271.4	7807.9	8.872	MM
206	2... 20211111_A41_R3_IgM	6.52	21122.8	5925.2	3.565	MM
207	2... 20211111_A42_R3_IgM	6.53	22284.7	9352.6	2.383	MM
208	2... 20211111_A43_R3_IgM	6.55	17567.9	10216.3	1.720	MM
209	2... 20211111_A44_R3_IgM	6.54	15577.3	10262.7	1.518	MM
210	2... 20211111_QC_R25_IgM	6.51	22263.2	8575.3	2.596	MM

Dataset: C:\MassLynx\10.pro\20211111_clinicalsamples_IgM_IgA.qld

Last Altered: Thursday, March 09, 2023 19:58:25 台北標準時間
Printed: Friday, August 01, 2025 15:24:29 台北標準時間

Method: C:\MassLynx\10_Pro_IgA.PRO\MethDB\20211111_IgM_IgA_DMRM_new.mdb 24 Feb 2022 13:44:14
Calibration: 15 Nov 2021 14:42:11

Sample Name: 20211112_QC_R001_IgM
F61:MRM of 1 channel,ES+
1061.7 > 366.1
7.513e+005

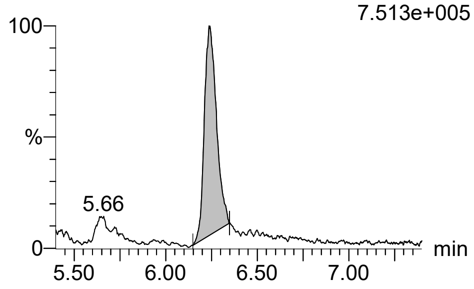

Sample Name: 20211112_QC_R002_IgM
F61:MRM of 1 channel,ES+
1061.7 > 366.1
6.184e+005

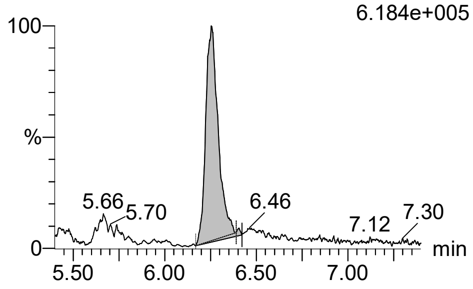

Sample Name: 20211111_A1_R2_IgM
F61:MRM of 1 channel,ES+
1061.7 > 366.1
3.627e+005

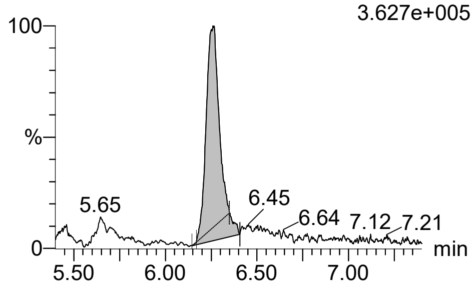

Sample Name: 20211111_A1_R3_IgM
F61:MRM of 1 channel,ES+
1061.7 > 366.1
3.308e+005

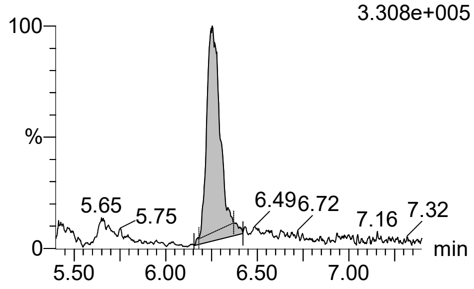

Sample Name: 20211111_A2_R2_IgM
F61:MRM of 1 channel,ES+
1061.7 > 366.1
8.903e+005

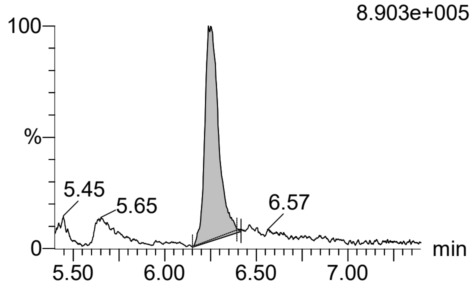

Sample Name: 20211111_A2_R3_IgM
F61:MRM of 1 channel,ES+
1061.7 > 366.1
8.946e+005

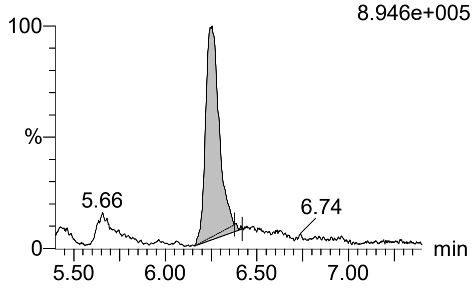

Sample Name: 20211111_L1_R2_IgM
F61:MRM of 1 channel,ES+
1061.7 > 366.1
3.072e+005

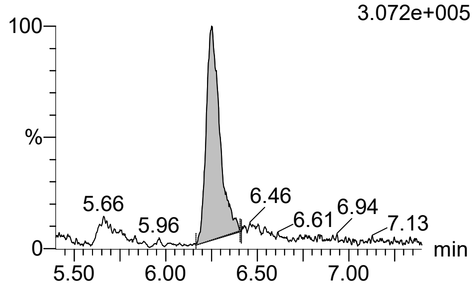

Sample Name: 20211111_L1_R3_IgM
F61:MRM of 1 channel,ES+
1061.7 > 366.1
2.169e+005

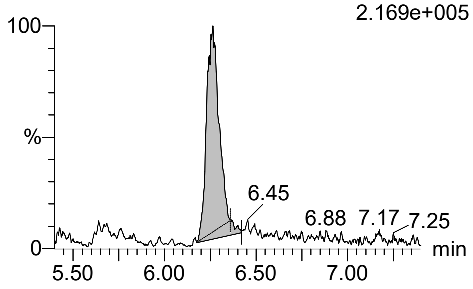

Sample Name: 20211111_L2_R2_IgM
F61:MRM of 1 channel,ES+
1061.7 > 366.1
6.584e+005

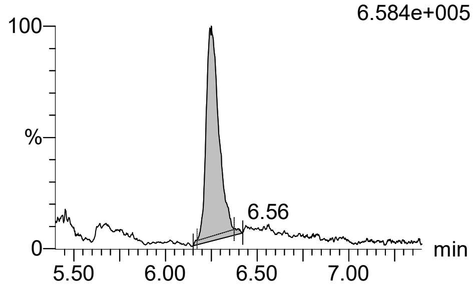

Sample Name: 20211111_H1_R2_IgM
F61:MRM of 1 channel,ES+
1061.7 > 366.1
1.198e+006

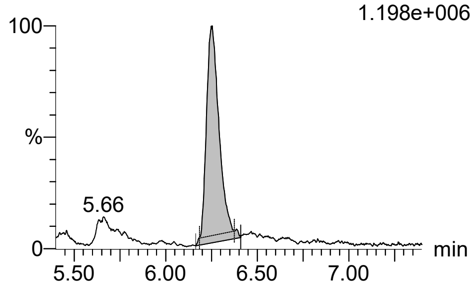

Sample Name: 20211111_A3_R2_IgM
F61:MRM of 1 channel,ES+
1061.7 > 366.1
9.098e+005

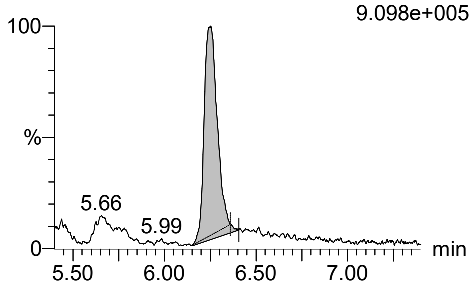

Sample Name: 20211111_A4_R2_IgM
F61:MRM of 1 channel,ES+
1061.7 > 366.1
6.244e+005

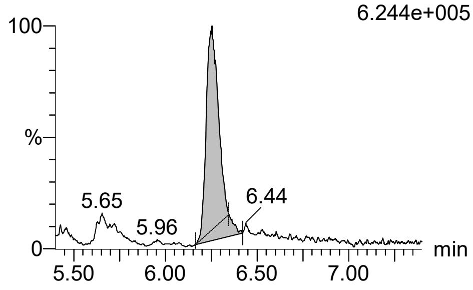

Dataset: C:\MassLynx\10.pro\20211111_clinicalsamples_IgM_IgA.qld

Last Altered: Thursday, March 09, 2023 19:58:25 台北標準時間
Printed: Friday, August 01, 2025 15:24:29 台北標準時間

Sample Name: 20211111_L3_R2_IgM
F61:MRM of 1 channel,ES+
1061.7 > 366.1
6.311e+005

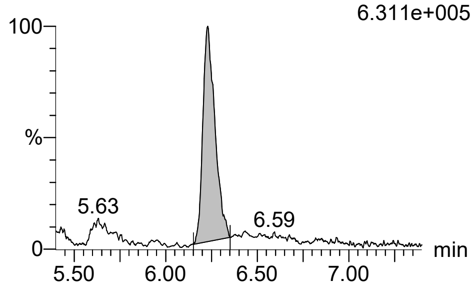

Sample Name: 20211111_L4_R2_IgM
F61:MRM of 1 channel,ES+
1061.7 > 366.1
5.429e+005

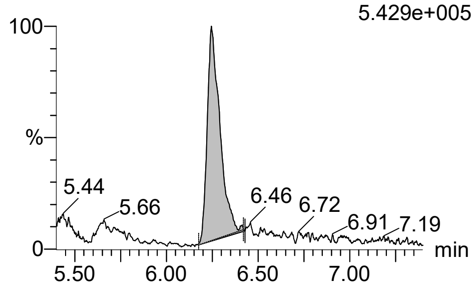

Sample Name: 20211111_H2_R2_IgM
F61:MRM of 1 channel,ES+
1061.7 > 366.1
1.183e+006

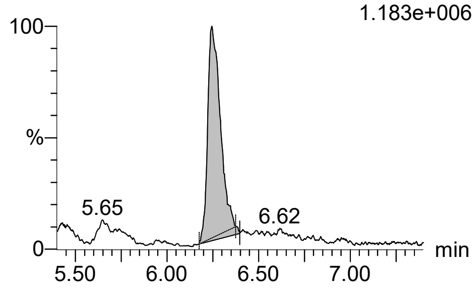

Sample Name: 20211111_A5_R2_IgM
F61:MRM of 1 channel,ES+
1061.7 > 366.1
2.003e+006

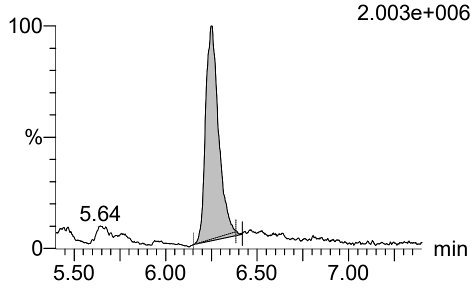

Sample Name: 20211111_A6_R2_IgM
F61:MRM of 1 channel,ES+
1061.7 > 366.1
8.241e+005

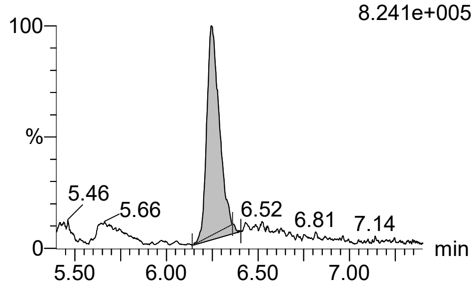

Sample Name: 20211111_QC_R10_IgM
F61:MRM of 1 channel,ES+
1061.7 > 366.1
6.209e+005

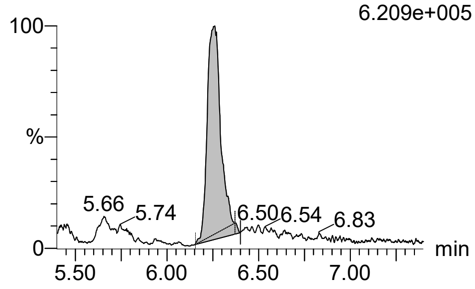

Sample Name: 20211111_L5_R2_IgM
F61:MRM of 1 channel,ES+
1061.7 > 366.1
7.295e+005

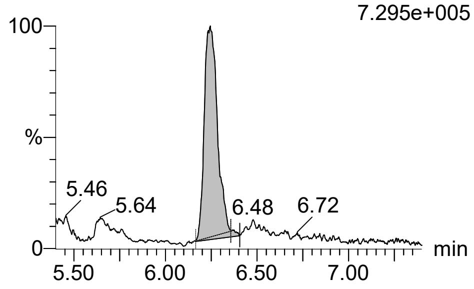

Sample Name: 20211111_L6_R2_IgM
F61:MRM of 1 channel,ES+
1061.7 > 366.1
7.218e+005

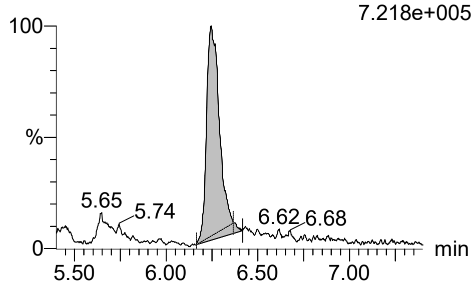

Sample Name: 20211111_H3_R2_IgM
F61:MRM of 1 channel,ES+
1061.7 > 366.1
1.993e+005

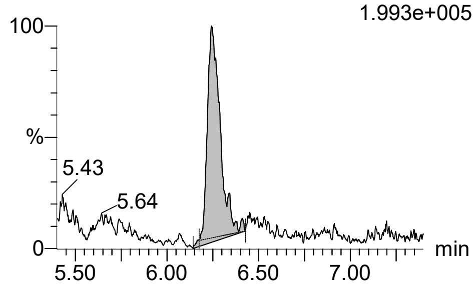

Sample Name: 20211111_A7_R2_IgM
F61:MRM of 1 channel,ES+
1061.7 > 366.1
5.575e+005

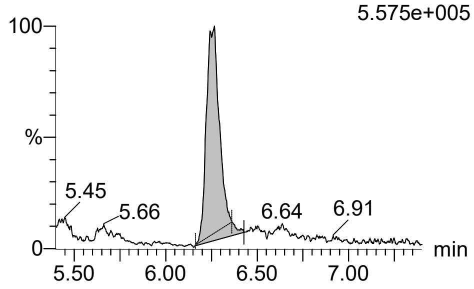

Sample Name: 20211111_A8_R2_IgM
F61:MRM of 1 channel,ES+
1061.7 > 366.1
6.409e+005

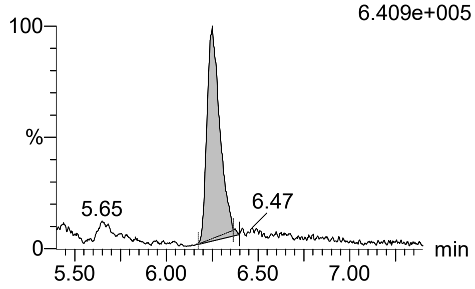

Sample Name: 20211111_L7_R2_IgM
F61:MRM of 1 channel,ES+
1061.7 > 366.1
4.739e+005

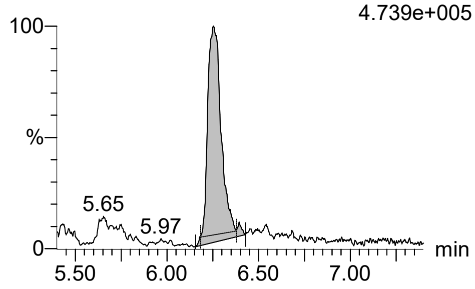

Sample Name: 20211111_L8_R2_IgM
F61:MRM of 1 channel,ES+
1061.7 > 366.1
1.768e+005

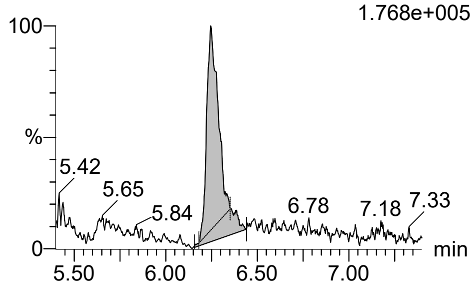

Sample Name: 20211111_H4_R2_IgM
F61:MRM of 1 channel,ES+
1061.7 > 366.1
3.108e+005

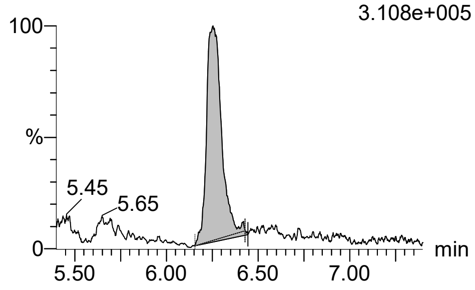

Sample Name: 20211111_A9_R2_IgM
F61:MRM of 1 channel,ES+
1061.7 > 366.1
3.806e+005

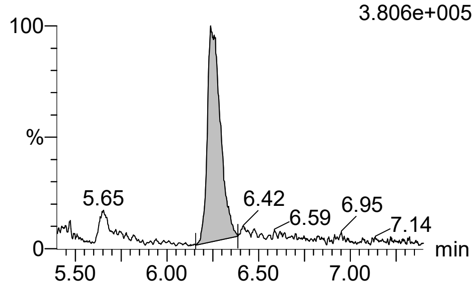

Sample Name: 20211111_A10_R2_IgM
F61:MRM of 1 channel,ES+
1061.7 > 366.1
1.114e+006

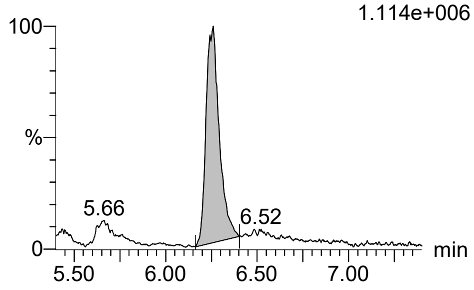

Sample Name: 20211111_L9_R2_IgM
F61:MRM of 1 channel,ES+
1061.7 > 366.1
9.503e+005

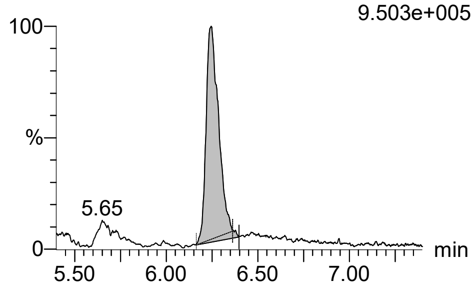

Sample Name: 20211111_L10_R2_IgM
F61:MRM of 1 channel,ES+
1061.7 > 366.1
7.237e+005

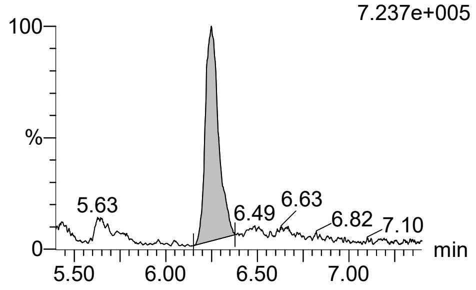

Sample Name: 20211111_QC_R11_IgM
F61:MRM of 1 channel,ES+
1061.7 > 366.1
7.446e+005

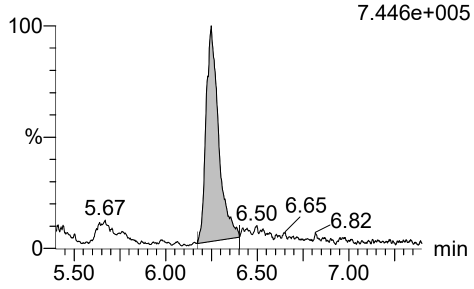

Sample Name: 20211111_H5_R2_IgM
F61:MRM of 1 channel,ES+
1061.7 > 366.1
8.454e+005

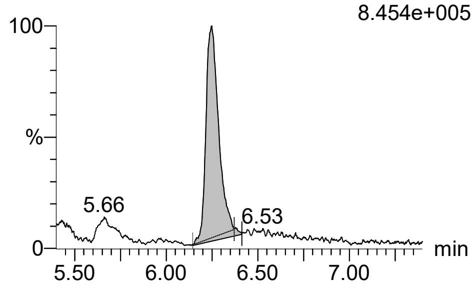

Sample Name: 20211111_A11_R2_IgM
F61:MRM of 1 channel,ES+
1061.7 > 366.1
6.341e+005

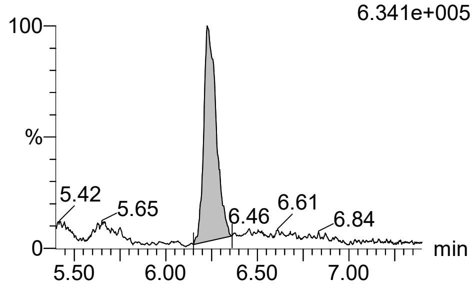

Sample Name: 20211111_A12_R2_IgM
F61:MRM of 1 channel,ES+
1061.7 > 366.1
2.975e+005

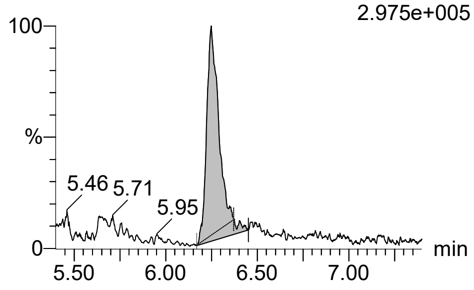

Sample Name: 20211111_L11_R2_IgM
F61:MRM of 1 channel,ES+
1061.7 > 366.1
3.965e+005

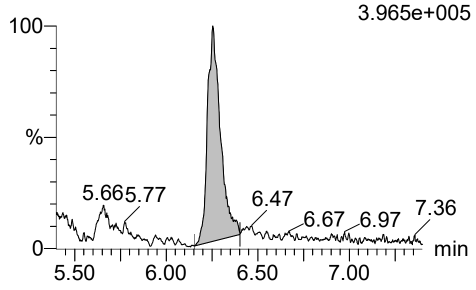

Sample Name: 20211111_L12_R2_IgM
F61:MRM of 1 channel,ES+
1061.7 > 366.1
3.312e+005

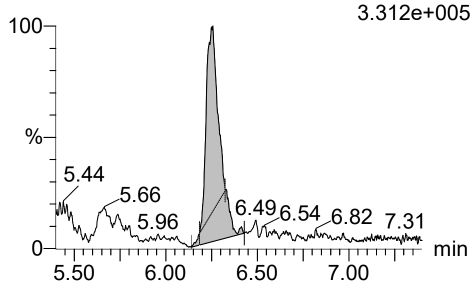

Sample Name: 20211111_H6_R2_IgM
F61:MRM of 1 channel,ES+
1061.7 > 366.1
1.925e+005

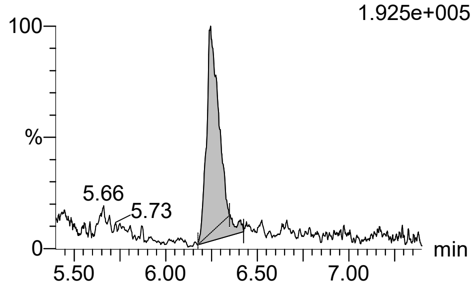

Sample Name: 20211111_A13_R2_IgM
F61:MRM of 1 channel,ES+
1061.7 > 366.1
2.731e+005

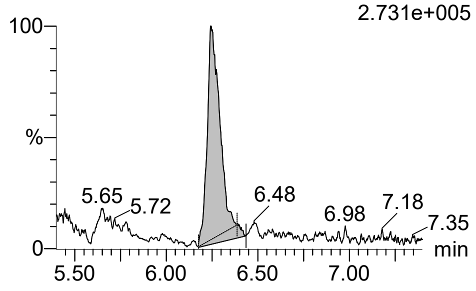

Sample Name: 20211111_A14_R2_IgM
F61:MRM of 1 channel,ES+
1061.7 > 366.1
1.225e+006

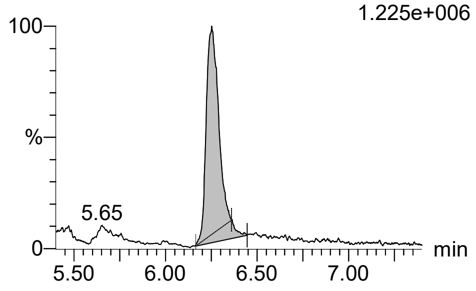

Sample Name: 20211111_L13_R2_IgM
F61:MRM of 1 channel,ES+
1061.7 > 366.1
5.125e+005

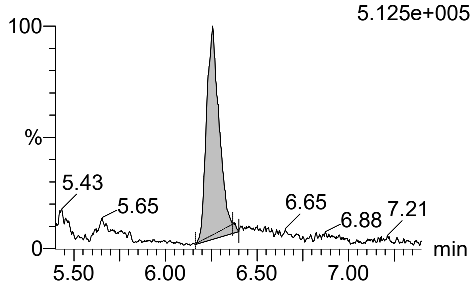

Sample Name: 20211111_L14_R2_IgM
F61:MRM of 1 channel,ES+
1061.7 > 366.1
9.866e+005

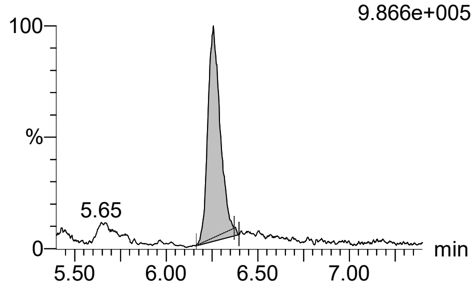

Sample Name: 20211111_H7_R2_IgM
F61:MRM of 1 channel,ES+
1061.7 > 366.1
5.967e+005

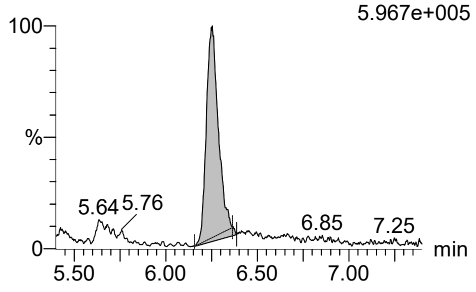

Dataset: C:\MassLynx\10.pro\20211111_clinicalsamples_IgM_IgA.qld

Last Altered: Thursday, March 09, 2023 19:58:25 台北標準時間
Printed: Friday, August 01, 2025 15:24:29 台北標準時間

Sample Name: 20211111_A15_R2_IgM
F61:MRM of 1 channel,ES+
1061.7 > 366.1
1.259e+006

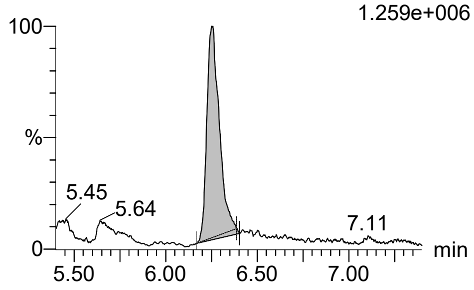

Sample Name: 20211111_QC_R12_IgM
F61:MRM of 1 channel,ES+
1061.7 > 366.1
7.717e+005

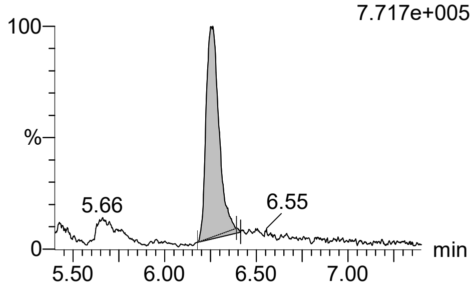

Sample Name: 20211111_A16_R2_IgM
F61:MRM of 1 channel,ES+
1061.7 > 366.1
1.007e+006

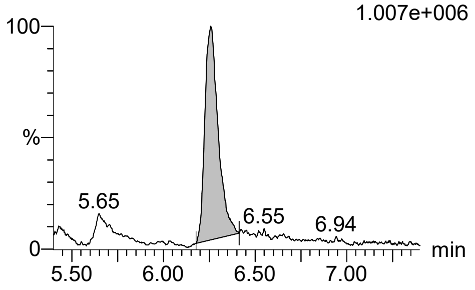

Sample Name: 20211111_L15_R2_IgM
F61:MRM of 1 channel,ES+
1061.7 > 366.1
6.214e+005

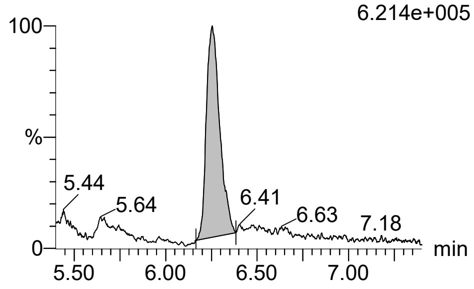

Sample Name: 20211111_L16_R2_IgM
F61:MRM of 1 channel,ES+
1061.7 > 366.1
3.324e+005

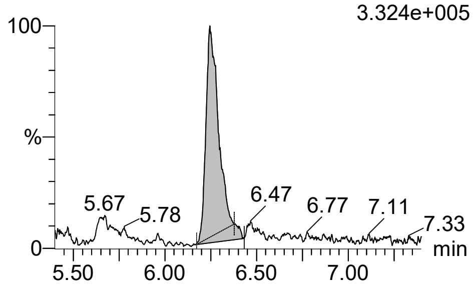

Sample Name: 20211111_H8_R2_IgM
F61:MRM of 1 channel,ES+
1061.7 > 366.1
4.770e+005

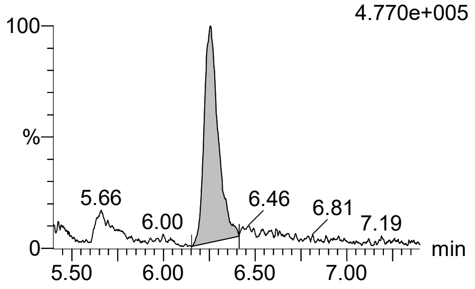

Sample Name: 20211111_A17_R2_IgM
F61:MRM of 1 channel,ES+
1061.7 > 366.1
8.940e+005

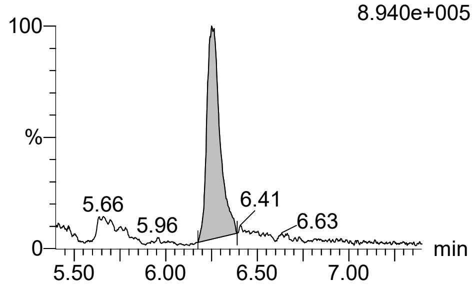

Sample Name: 20211111_A18_R2_IgM
F61:MRM of 1 channel,ES+
1061.7 > 366.1
3.263e+005

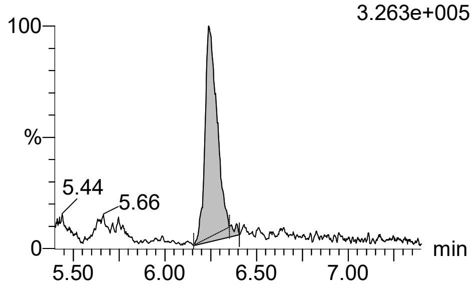

Sample Name: 20211111_L17_R2_IgM
F61:MRM of 1 channel,ES+
1061.7 > 366.1
2.090e+006

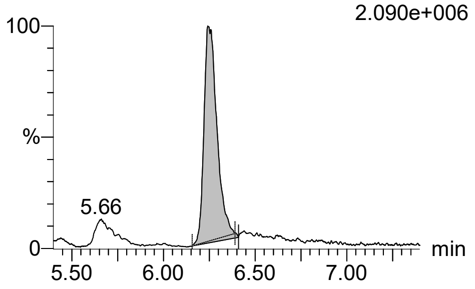

Sample Name: 20211111_L18_R2_IgM
F61:MRM of 1 channel,ES+
1061.7 > 366.1
4.177e+005

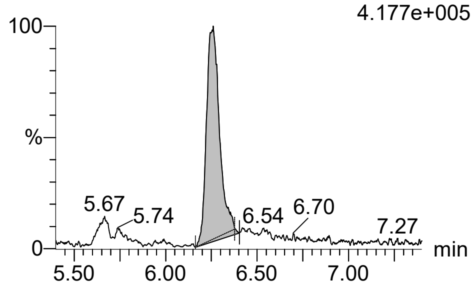

Sample Name: 20211111_H9_R2_IgM
F61:MRM of 1 channel,ES+
1061.7 > 366.1
3.041e+005

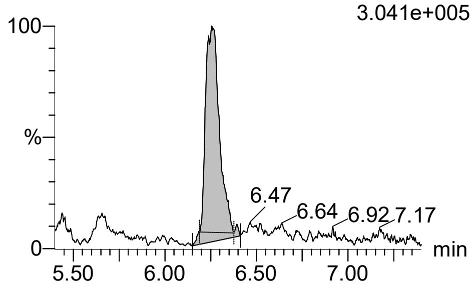

Sample Name: 20211111_A19_R2_IgM
F61:MRM of 1 channel,ES+
1061.7 > 366.1
5.464e+005

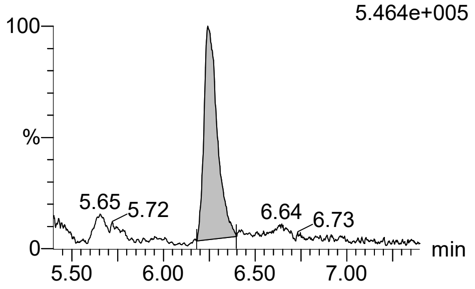

Sample Name: 20211111_A20_R2_IgM
F61:MRM of 1 channel,ES+
1061.7 > 366.1
2.164e+005

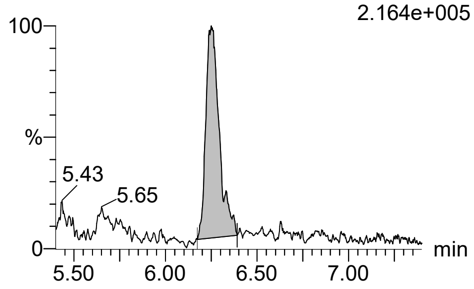

Sample Name: 20211111_L19_R2_IgM
F61:MRM of 1 channel,ES+
1061.7 > 366.1
4.205e+005

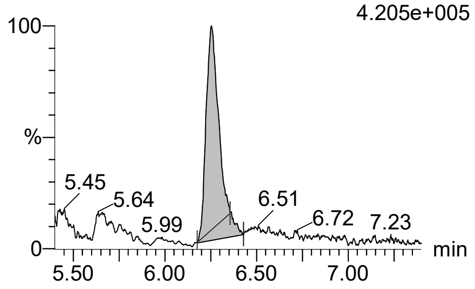

Sample Name: 20211111_QC_R13_IgM
F61:MRM of 1 channel,ES+
1061.7 > 366.1
6.699e+005

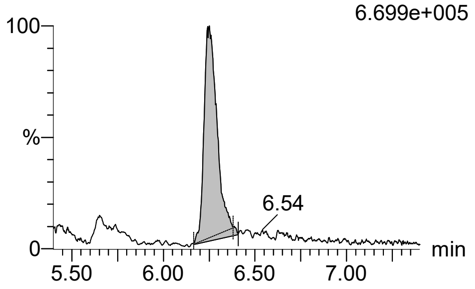

Dataset: C:\MassLynx\10.pro\20211111_clinicalsamples_IgM_IgA.qld

Last Altered: Thursday, March 09, 2023 19:58:25 台北標準時間
Printed: Friday, August 01, 2025 15:24:29 台北標準時間

Sample Name: 20211111_L20_R2_IgM
F61:MRM of 1 channel,ES+
1061.7 > 366.1
4.993e+005

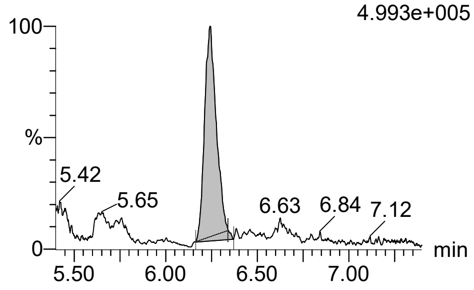

Sample Name: 20211111_H10_R2_IgM
F61:MRM of 1 channel,ES+
1061.7 > 366.1
3.804e+005

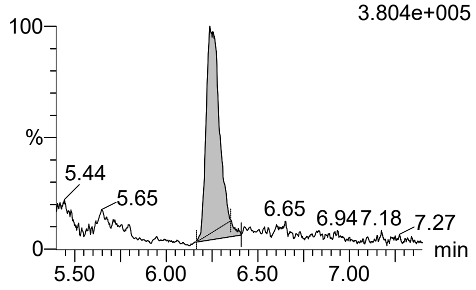

Sample Name: 20211111_A21_R2_IgM
F61:MRM of 1 channel,ES+
1061.7 > 366.1
3.354e+005

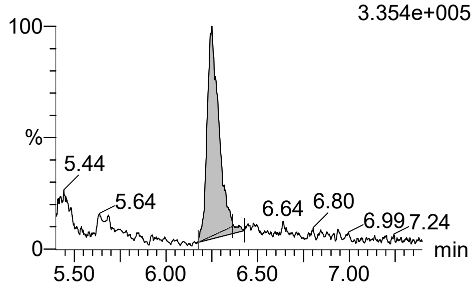

Sample Name: 20211111_A22_R2_IgM
F61:MRM of 1 channel,ES+
1061.7 > 366.1
1.571e+005

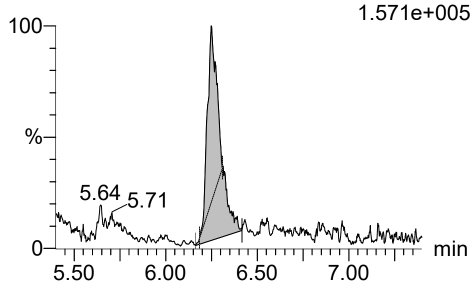

Sample Name: 20211111_L21_R2_IgM
F61:MRM of 1 channel,ES+
1061.7 > 366.1
3.686e+005

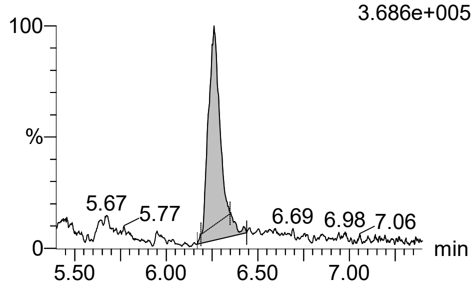

Sample Name: 20211111_L22_R2_IgM
F61:MRM of 1 channel,ES+
1061.7 > 366.1
4.382e+005

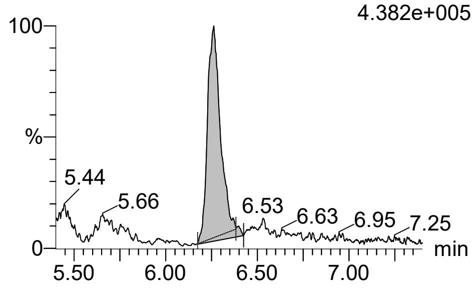

Sample Name: 20211111_H11_R2_IgM
F61:MRM of 1 channel,ES+
1061.7 > 366.1
6.150e+005

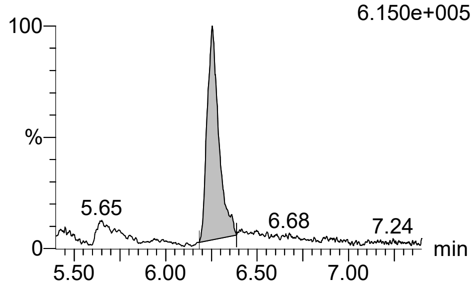

Sample Name: 20211111_A23_R2_IgM
F61:MRM of 1 channel,ES+
1061.7 > 366.1
2.894e+005

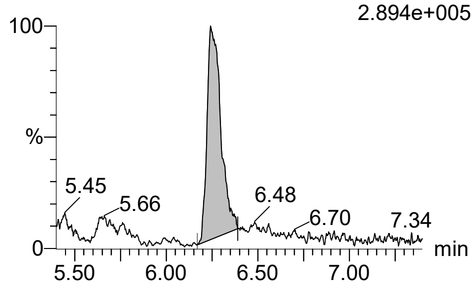

Sample Name: 20211111_A24_R2_IgM
F61:MRM of 1 channel,ES+
1061.7 > 366.1
6.788e+005

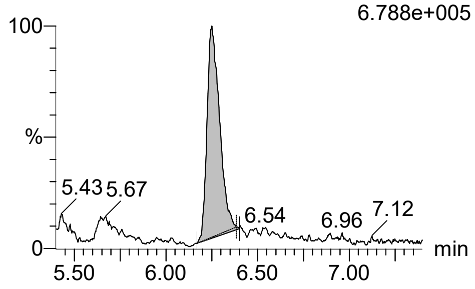

Sample Name: 20211111_L23_R2_IgM
F61:MRM of 1 channel,ES+
1061.7 > 366.1
1.125e+006

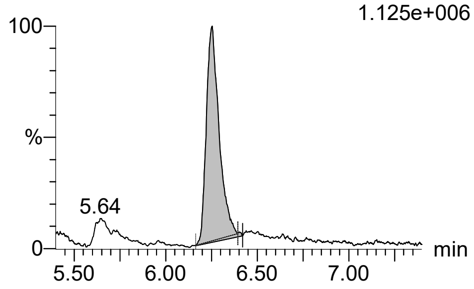

Sample Name: 20211111_L24_R2_IgM
F61:MRM of 1 channel,ES+
1061.7 > 366.1
2.726e+005

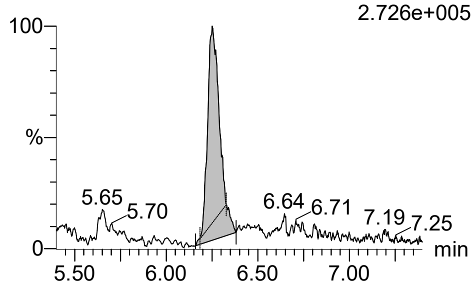

Sample Name: 20211111_H12_R2_IgM
F61:MRM of 1 channel,ES+
1061.7 > 366.1
3.459e+005

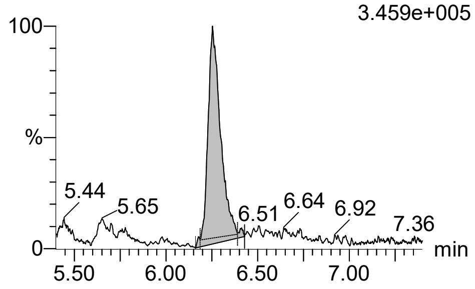

Sample Name: 20211111_QC_R14_IgM
F61:MRM of 1 channel,ES+
1061.7 > 366.1
6.893e+005

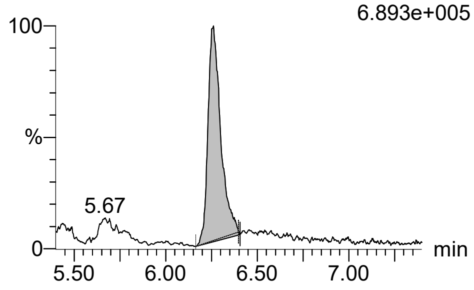

Sample Name: 20211111_A25_R2_IgM
F61:MRM of 1 channel,ES+
1061.7 > 366.1
1.004e+006

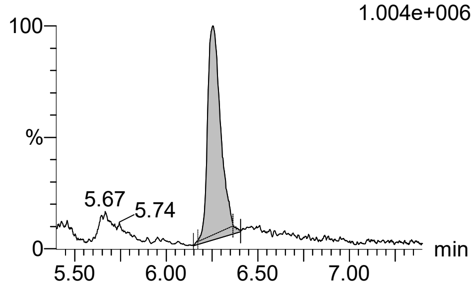

Sample Name: 20211111_A26_R2_IgM
F61:MRM of 1 channel,ES+
1061.7 > 366.1
5.487e+005

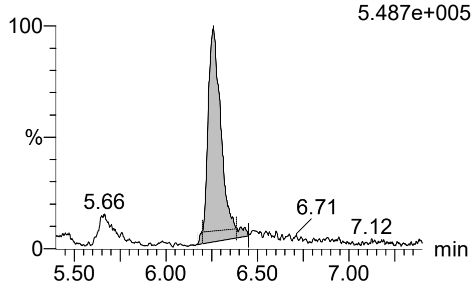

Sample Name: 20211111_L25_R2_IgM
F61:MRM of 1 channel,ES+
1061.7 > 366.1
9.678e+005

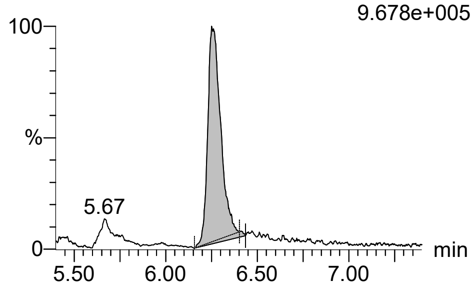

Sample Name: 20211111_L26_R2_IgM
F61:MRM of 1 channel,ES+
1061.7 > 366.1
6.099e+005

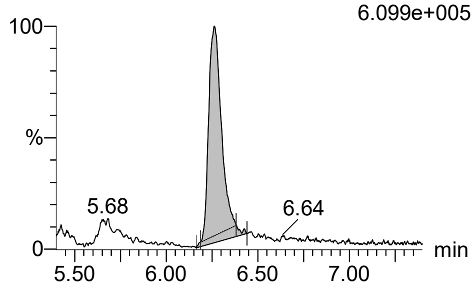

Sample Name: 20211111_H13_R2_IgM
F61:MRM of 1 channel,ES+
1061.7 > 366.1
6.904e+005

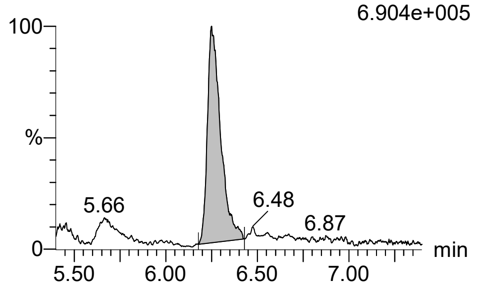

Sample Name: 20211111_A27_R2_IgM
F61:MRM of 1 channel,ES+
1061.7 > 366.1
9.425e+005

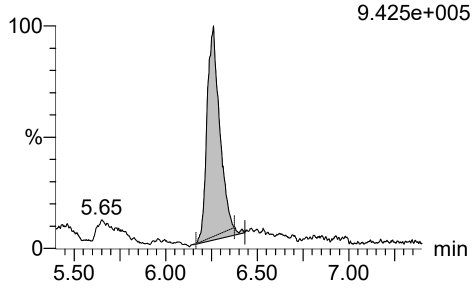

Sample Name: 20211111_A28_R2_IgM
F61:MRM of 1 channel,ES+
1061.7 > 366.1
4.510e+005

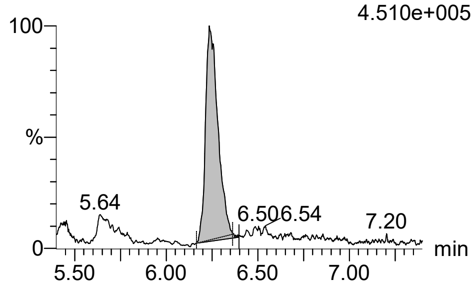

Sample Name: 20211111_L27_R2_IgM
F61:MRM of 1 channel,ES+
1061.7 > 366.1
5.257e+005

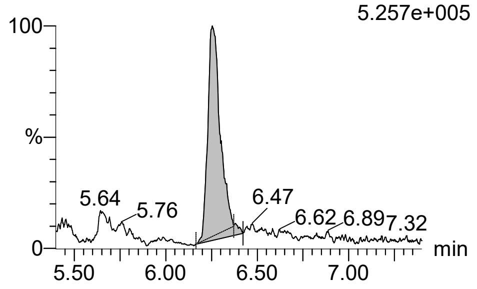

Sample Name: 20211111_L28_R2_IgM
F61:MRM of 1 channel,ES+
1061.7 > 366.1
3.966e+005

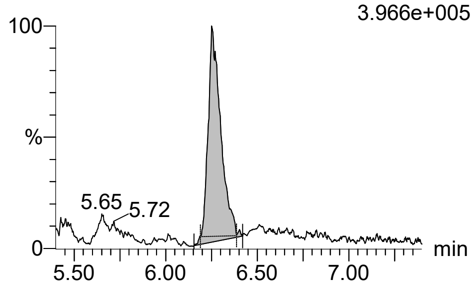

Sample Name: 20211111_H14_R2_IgM
F61:MRM of 1 channel,ES+
1061.7 > 366.1
6.674e+005

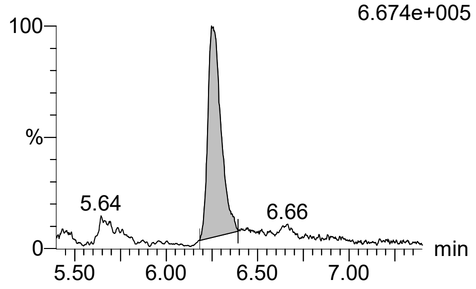

Sample Name: 20211111_A29_R2_IgM
F61:MRM of 1 channel,ES+
1061.7 > 366.1
1.716e+005

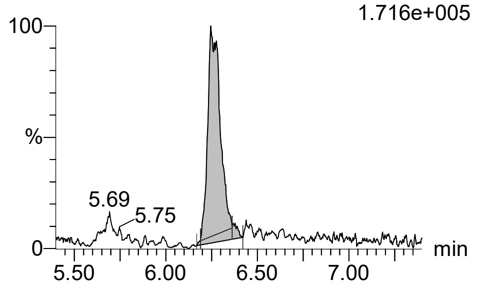

Sample Name: 20211111_A30_R2_IgM
F61:MRM of 1 channel,ES+
1061.7 > 366.1
6.862e+005

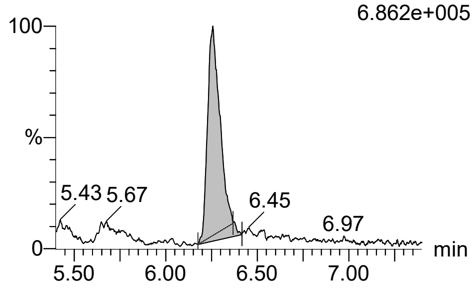

Sample Name: 20211111_QC_R15_IgM
F61:MRM of 1 channel,ES+
1061.7 > 366.1
5.987e+005

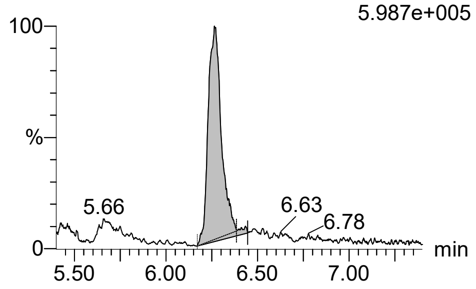

Sample Name: 20211111_L29_R2_IgM
F61:MRM of 1 channel,ES+
1061.7 > 366.1
3.502e+005

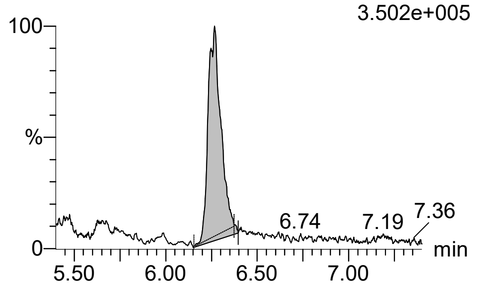

Sample Name: 20211111_L30_R2_IgM
F61:MRM of 1 channel,ES+
1061.7 > 366.1
1.572e+005

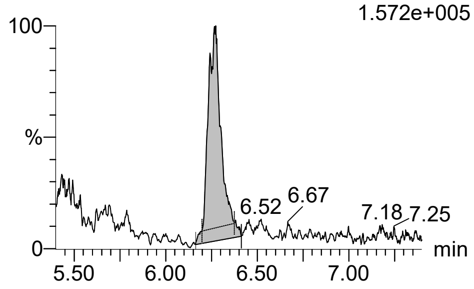

Sample Name: 20211111_H15_R2_IgM
F61:MRM of 1 channel,ES+
1061.7 > 366.1
3.529e+005

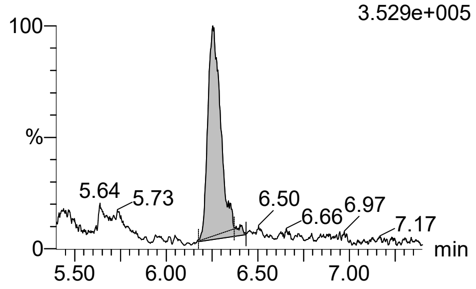

Sample Name: 20211111_A31_R2_IgM
F61:MRM of 1 channel,ES+
1061.7 > 366.1
2.255e+005

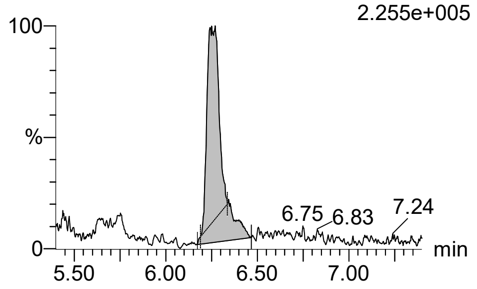

Sample Name: 20211111_A32_R2_IgM
F61:MRM of 1 channel,ES+
1061.7 > 366.1
6.141e+005

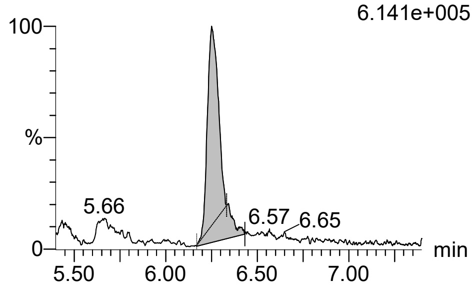

Sample Name: 20211111_L31_R2_IgM
F61:MRM of 1 channel,ES+
1061.7 > 366.1
2.213e+005

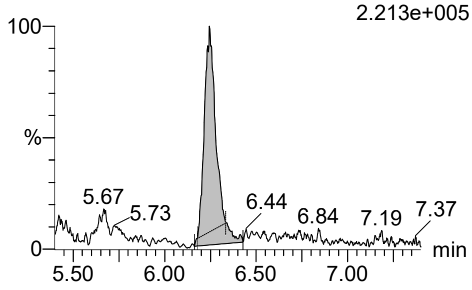

Sample Name: 20211111_L32_R2_IgM
F61:MRM of 1 channel,ES+
1061.7 > 366.1
1.246e+006

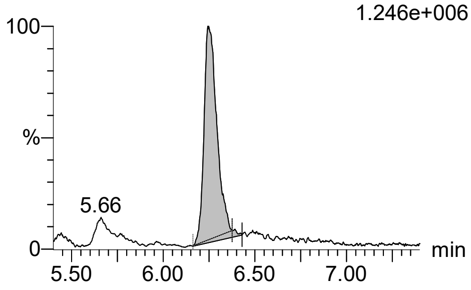

Sample Name: 20211111_H16_R2_IgM
F61:MRM of 1 channel,ES+
1061.7 > 366.1
8.420e+005

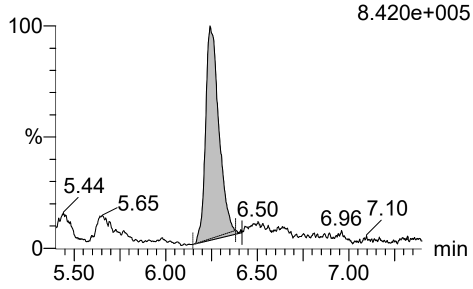

Sample Name: 20211111_A33_R2_IgM
F61:MRM of 1 channel,ES+
1061.7 > 366.1
7.254e+005

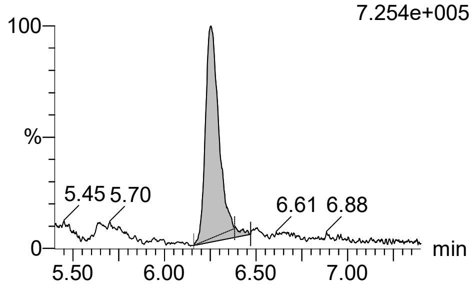

Sample Name: 20211111_A34_R2_IgM
F61:MRM of 1 channel,ES+
1061.7 > 366.1
2.554e+005

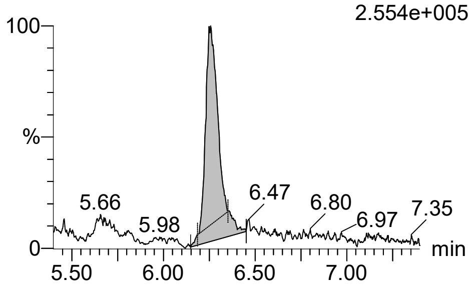

Sample Name: 20211111_H17_R2_IgM
F61:MRM of 1 channel,ES+
1061.7 > 366.1
2.032e+006

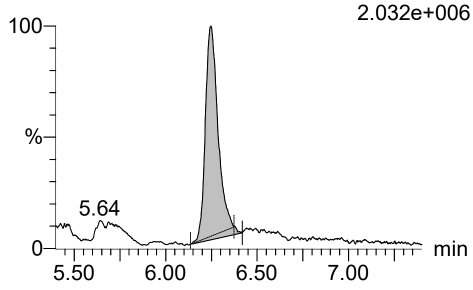

Sample Name: 20211111_A35_R2_IgM
F61:MRM of 1 channel,ES+
1061.7 > 366.1
3.964e+005

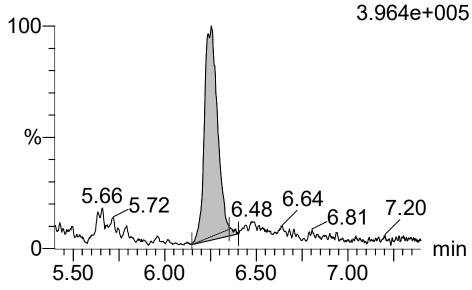

Sample Name: 20211111_QC_R16_IgM
F61:MRM of 1 channel,ES+
1061.7 > 366.1
5.484e+005

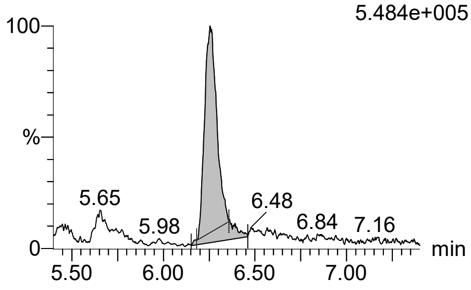

Sample Name: 20211111_A36_R2_IgM
F61:MRM of 1 channel,ES+
1061.7 > 366.1
6.007e+005

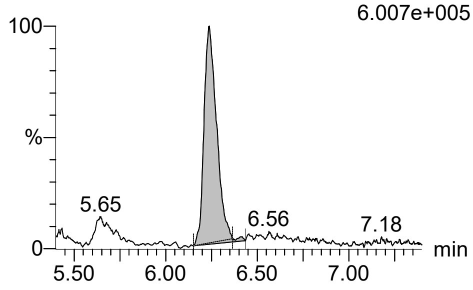

Sample Name: 20211111_H18_R2_IgM
F61:MRM of 1 channel,ES+
1061.7 > 366.1
1.396e+006

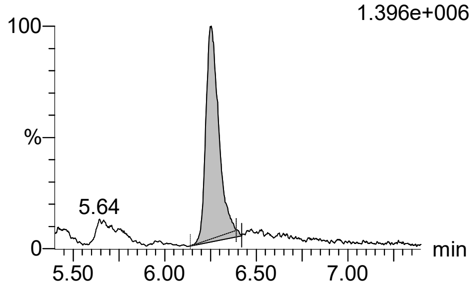

Sample Name: 20211111_A37_R2_IgM
F61:MRM of 1 channel,ES+
1061.7 > 366.1
6.015e+005

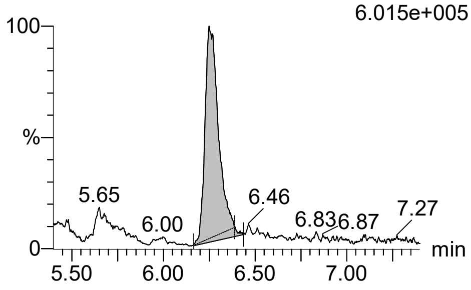

Sample Name: 20211111_A38_R2_IgM
F61:MRM of 1 channel,ES+
1061.7 > 366.1
5.230e+005

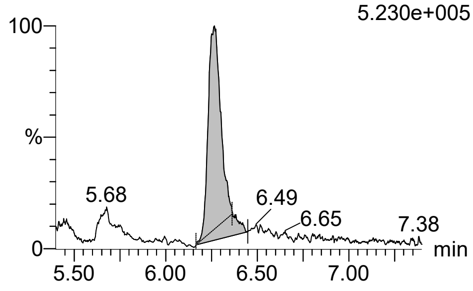

Sample Name: 20211111_H19_R2_IgM
F61:MRM of 1 channel,ES+
1061.7 > 366.1
7.196e+005

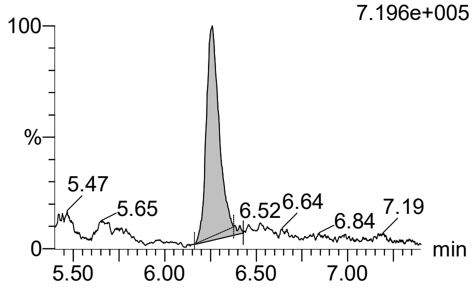

Sample Name: 20211111_A39_R2_IgM
F61:MRM of 1 channel,ES+
1061.7 > 366.1
5.675e+005

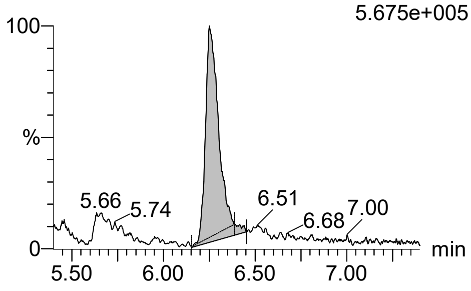

Dataset: C:\MassLynx\10.pro\20211111_clinicalsamples_IgM_IgA.qld

Last Altered: Thursday, March 09, 2023 19:58:25 台北標準時間
Printed: Friday, August 01, 2025 15:24:29 台北標準時間

Sample Name: 20211111_A40_R2_IgM

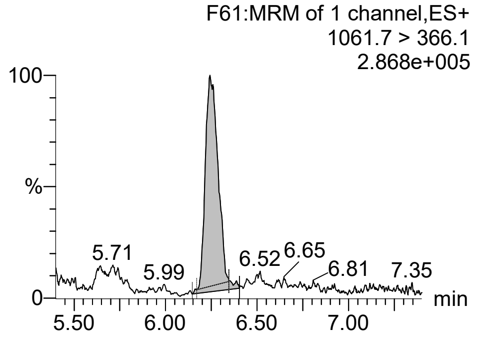

Sample Name: 20211111_H20_R2_IgM

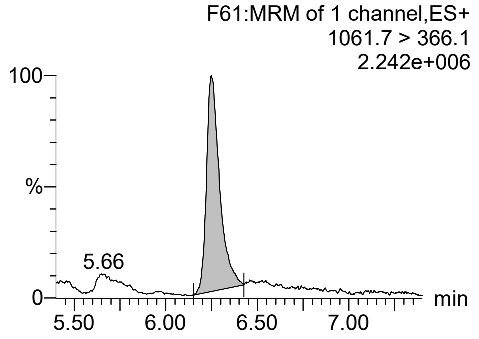

Sample Name: 20211111_A41_R2_IgM

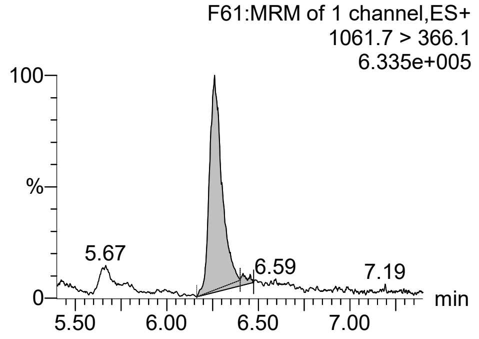

Sample Name: 20211111_A42_R2_IgM

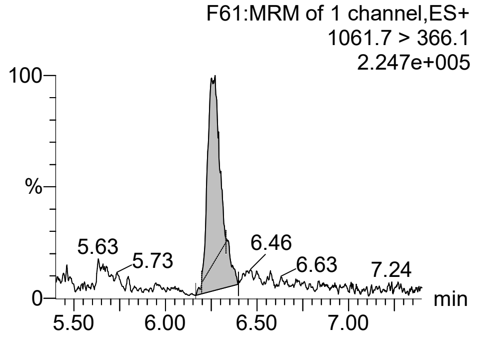

Sample Name: 20211111_A43_R2_IgM

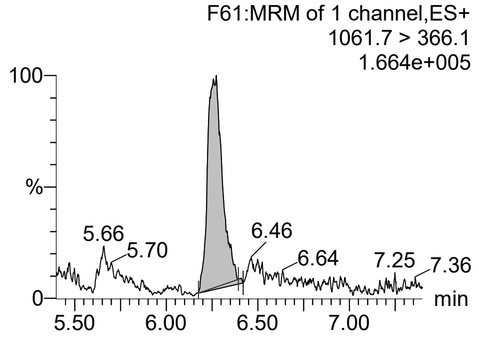

Sample Name: 20211111_A44_R2_IgM

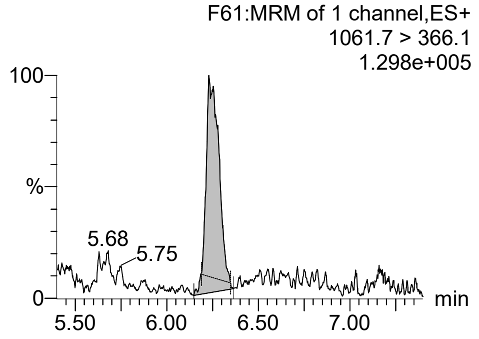

Sample Name: 20211111_QC_R17_IgM

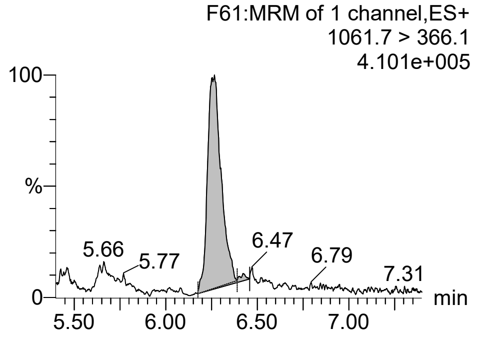

Sample Name: 20211111_L2_R3_IgM

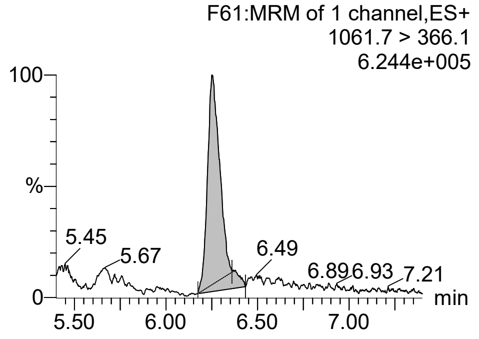

Sample Name: 20211111_H1_R3_IgM

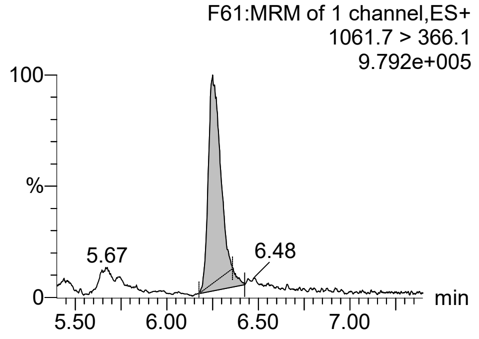

Sample Name: 20211111_A3_R3_IgM

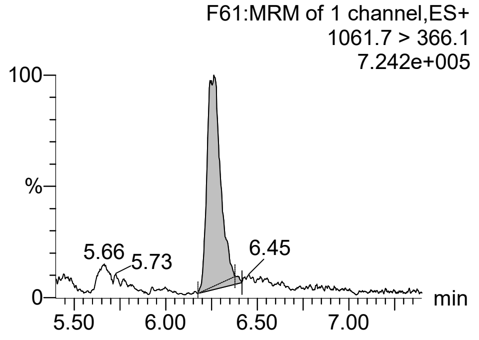

Sample Name: 20211111_A4_R3_IgM

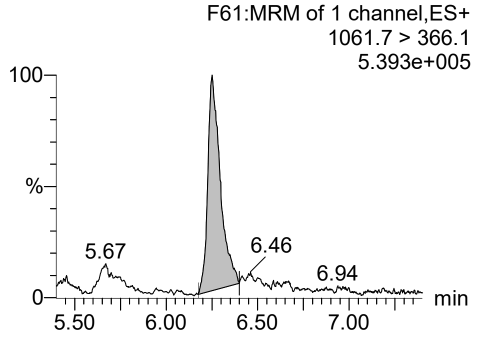

Sample Name: 20211111_L3_R3_IgM

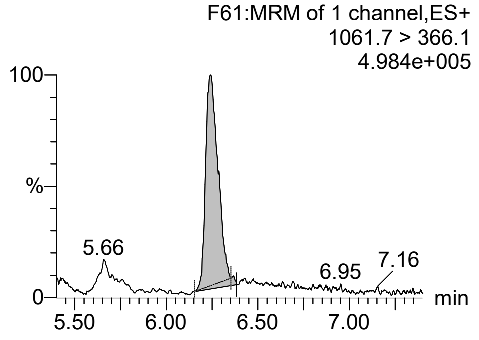

Sample Name: 20211111_L4_R3_IgM

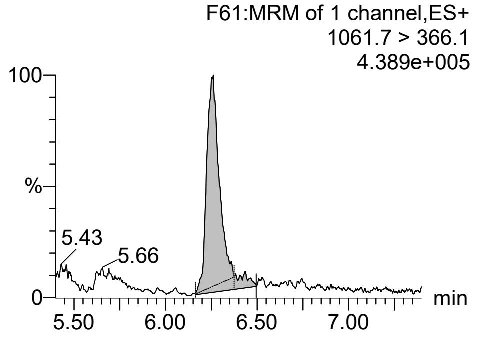

Sample Name: 20211111_H2_R3_IgM

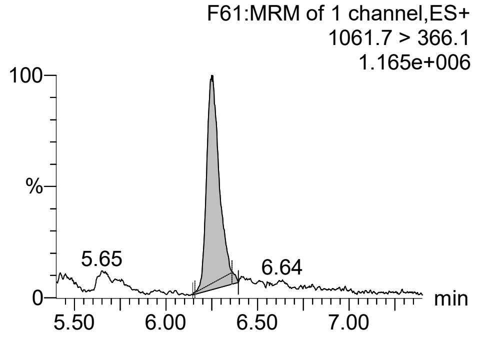

Sample Name: 20211111_A5_R3_IgM

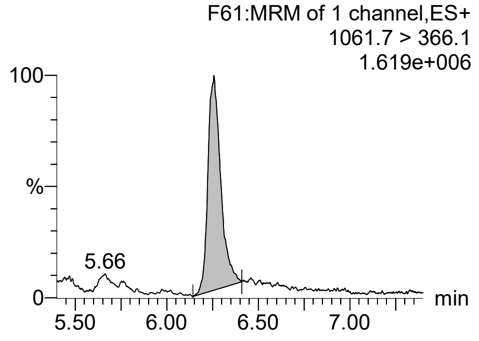

Dataset: C:\MassLynx\10.pro\20211111_clinicalsamples_IgM_IgA.qld

Last Altered: Thursday, March 09, 2023 19:58:25 台北標準時間
Printed: Friday, August 01, 2025 15:24:29 台北標準時間

Sample Name: 20211111_A6_R3_IgM

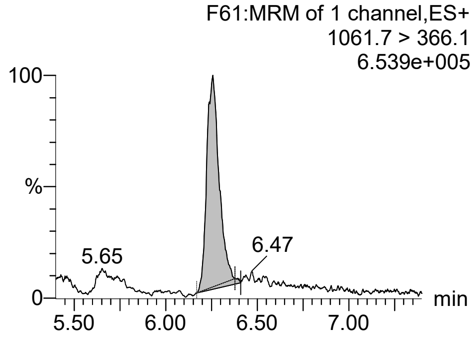

Sample Name: 20211111_QC_R18_IgM

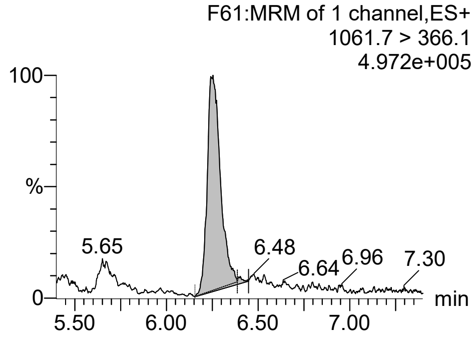

Sample Name: 20211111_L5_R3_IgM

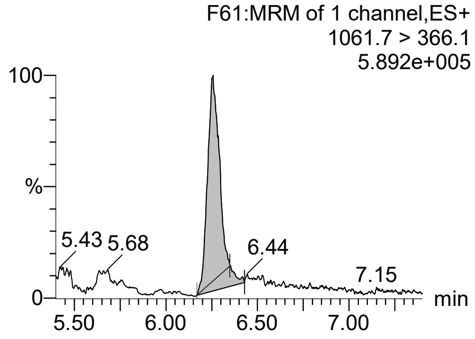

Sample Name: 20211111_L6_R3_IgM

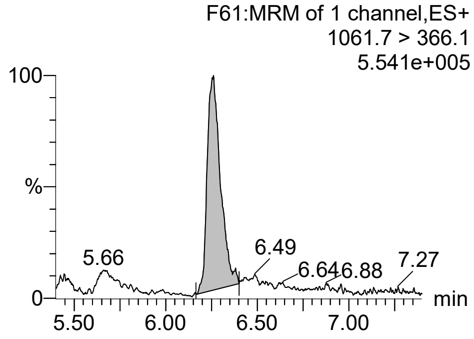

Sample Name: 20211111_H3_R3_IgM

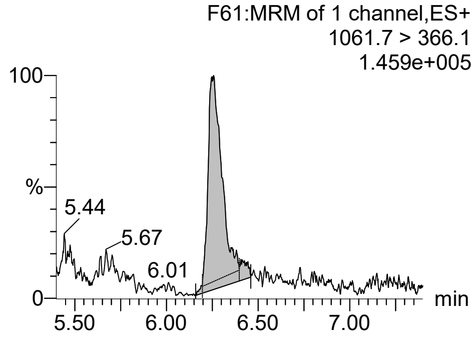

Sample Name: 20211111_A7_R3_IgM

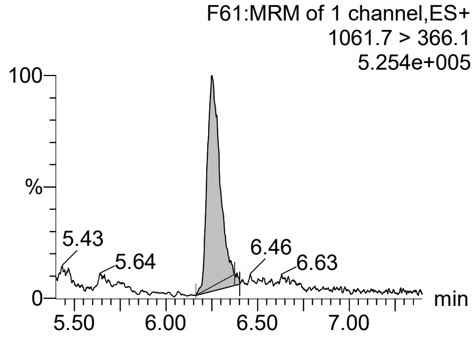

Sample Name: 20211111_A8_R3_IgM

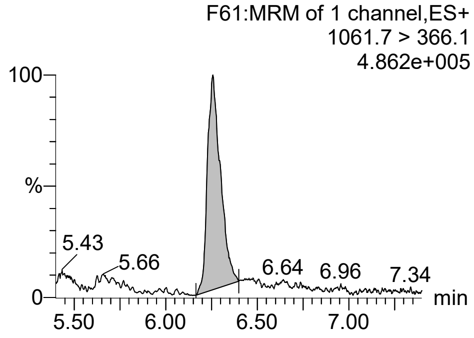

Sample Name: 20211111_L7_R3_IgM

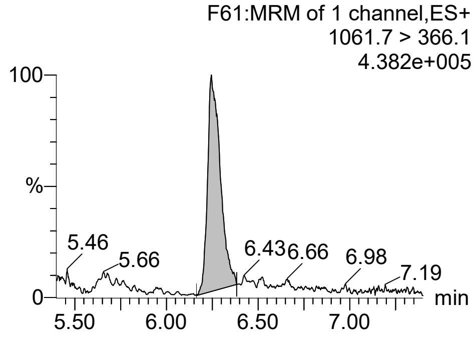

Sample Name: 20211111_L8_R3_IgM

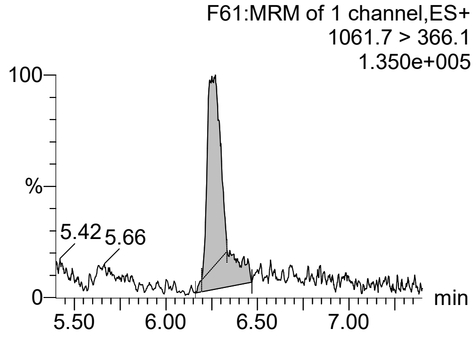

Sample Name: 20211111_H4_R3_IgM

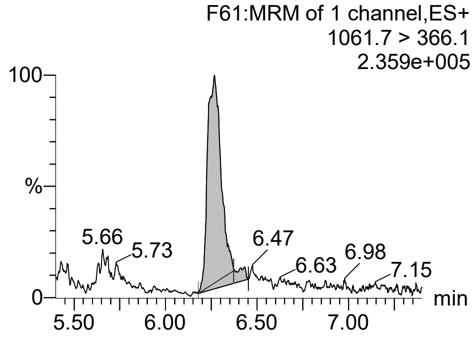

Sample Name: 20211111_A9_R3_IgM

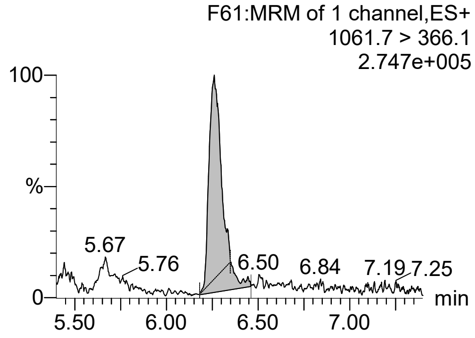

Sample Name: 20211111_A10_R3_IgM

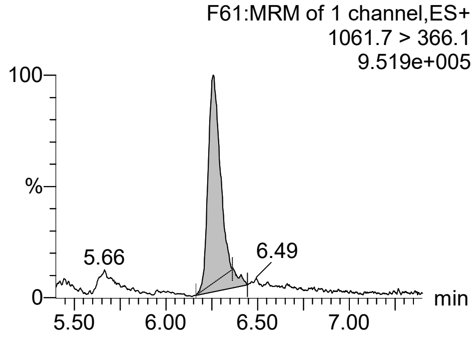

Sample Name: 20211111_L9_R3_IgM

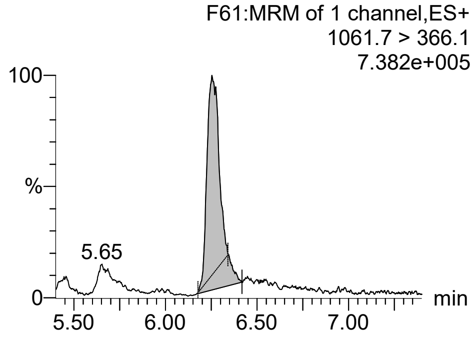

Sample Name: 20211111_L10_R3_IgM

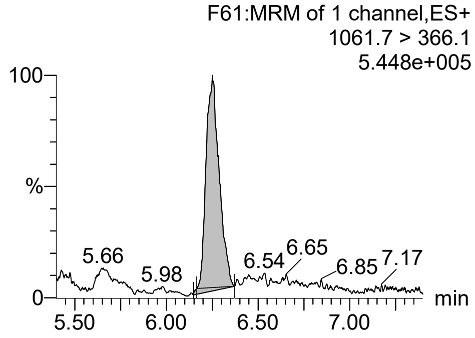

Sample Name: 20211111_QC_R19_IgM

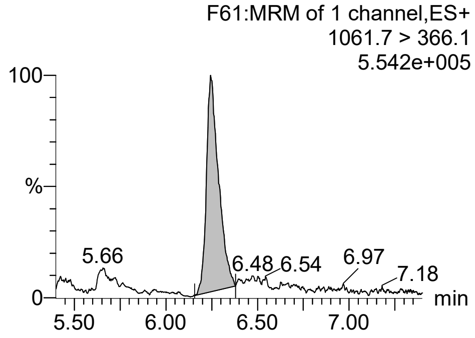

Sample Name: 20211111_H5_R3_IgM
F61:MRM of 1 channel,ES+
1061.7 > 366.1
5.664e+005

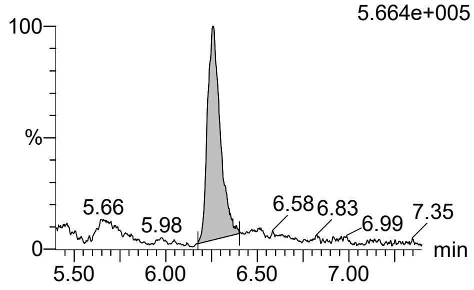

Sample Name: 20211111_A11_R3_IgM
F61:MRM of 1 channel,ES+
1061.7 > 366.1
4.077e+005

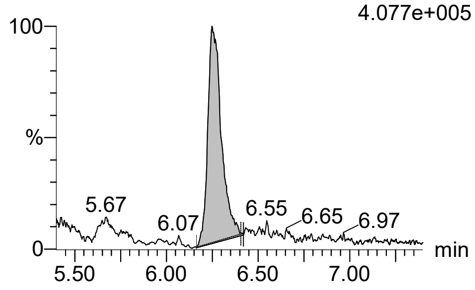

Sample Name: 20211111_A12_R3_IgM
F61:MRM of 1 channel,ES+
1061.7 > 366.1
2.609e+005

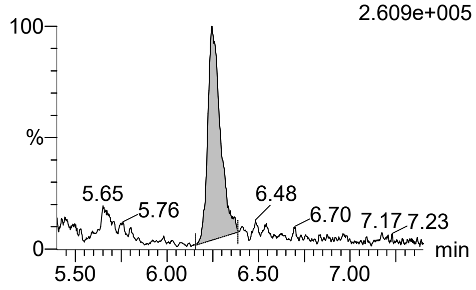

Sample Name: 20211111_L11_R3_IgM
F61:MRM of 1 channel,ES+
1061.7 > 366.1
2.747e+005

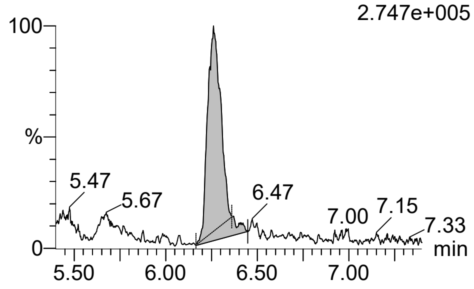

Sample Name: 20211111_L12_R3_IgM
F61:MRM of 1 channel,ES+
1061.7 > 366.1
2.774e+005

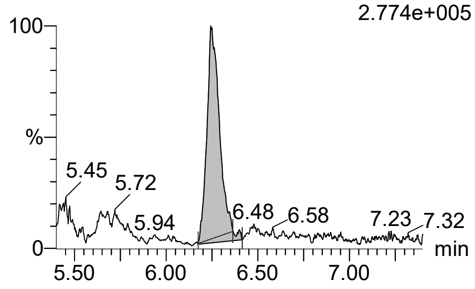

Sample Name: 20211111_H6_R3_IgM
F61:MRM of 1 channel,ES+
1061.7 > 366.1
1.711e+005

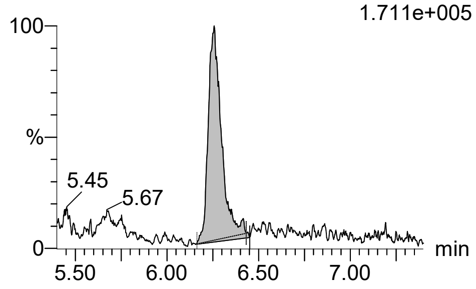

Sample Name: 20211111_A13_R3_IgM
F61:MRM of 1 channel,ES+
1061.7 > 366.1
2.345e+005

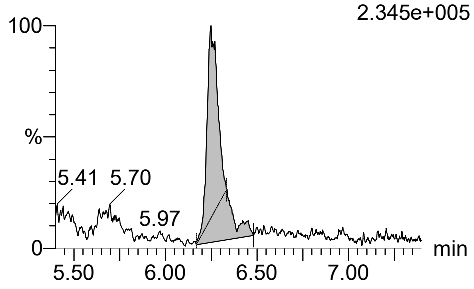

Sample Name: 20211111_A14_R3_IgM
F61:MRM of 1 channel,ES+
1061.7 > 366.1
9.238e+005

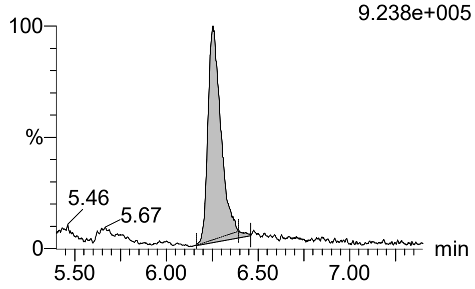

Sample Name: 20211111_L13_R3_IgM
F61:MRM of 1 channel,ES+
1061.7 > 366.1
4.486e+005

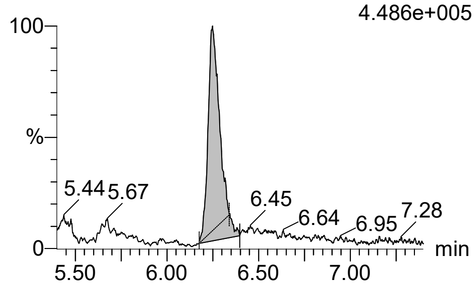

Sample Name: 20211111_L14_R3_IgM
F61:MRM of 1 channel,ES+
1061.7 > 366.1
7.406e+005

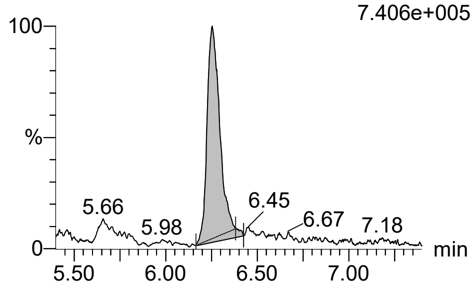

Sample Name: 20211111_H7_R3_IgM
F61:MRM of 1 channel,ES+
1061.7 > 366.1
4.523e+005

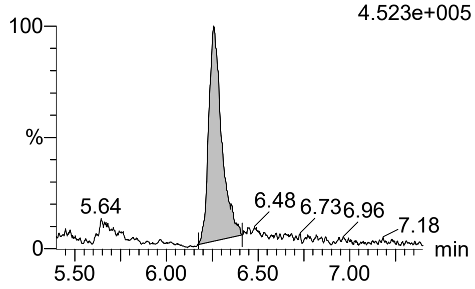

Sample Name: 20211111_A15_R3_IgM
F61:MRM of 1 channel,ES+
1061.7 > 366.1
9.968e+005

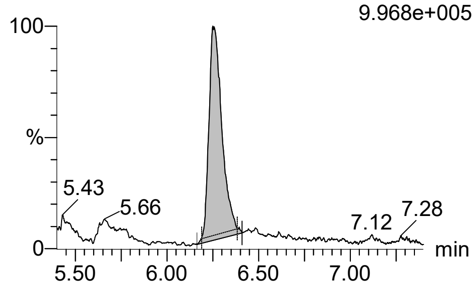

Sample Name: 20211111_QC_R20_IgM
F61:MRM of 1 channel,ES+
1061.7 > 366.1
6.169e+005

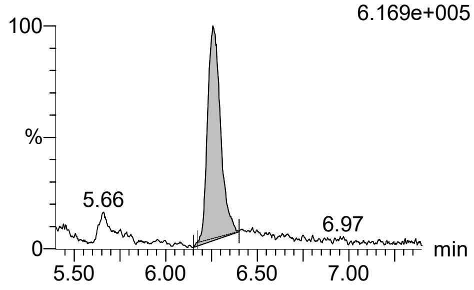

Sample Name: 20211111_A16_R3_IgM
F61:MRM of 1 channel,ES+
1061.7 > 366.1
7.963e+005

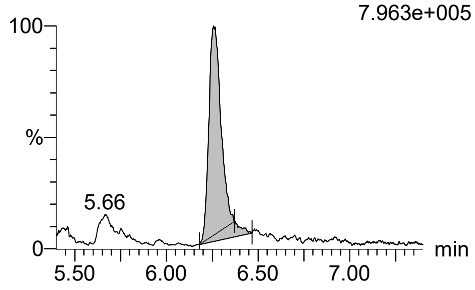

Sample Name: 20211111_L15_R3_IgM
F61:MRM of 1 channel,ES+
1061.7 > 366.1
5.531e+005

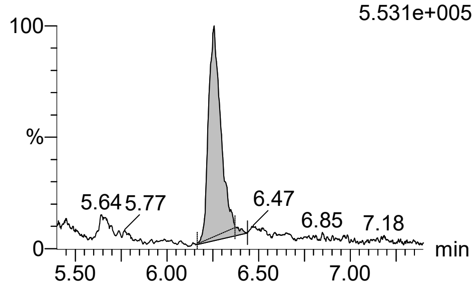

Sample Name: 20211111_L16_R3_IgM
F61:MRM of 1 channel,ES+
1061.7 > 366.1
2.919e+005

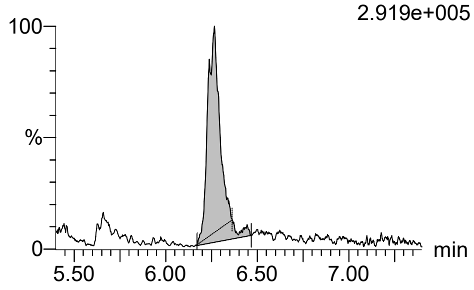

Sample Name: 20211111_H8_R3_IgM
F61:MRM of 1 channel,ES+
1061.7 > 366.1
4.006e+005

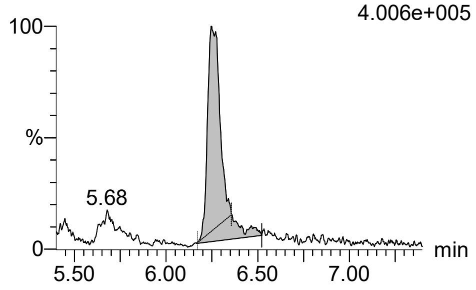

Sample Name: 20211111_A17_R3_IgM
F61:MRM of 1 channel,ES+
1061.7 > 366.1
7.378e+005

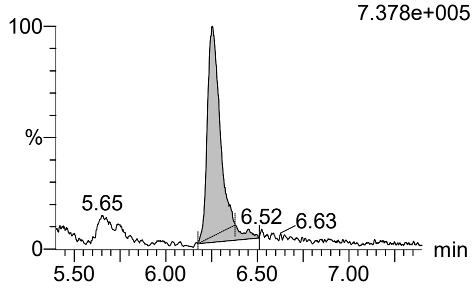

Sample Name: 20211111_A18_R3_IgM
F61:MRM of 1 channel,ES+
1061.7 > 366.1
2.644e+005

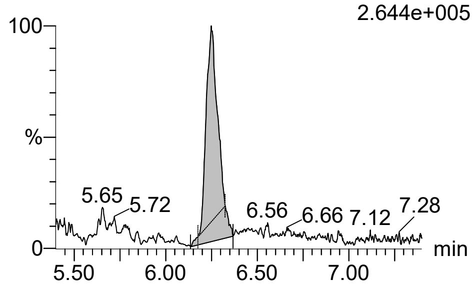

Sample Name: 20211111_L17_R3_IgM
F61:MRM of 1 channel,ES+
1061.7 > 366.1
1.700e+006

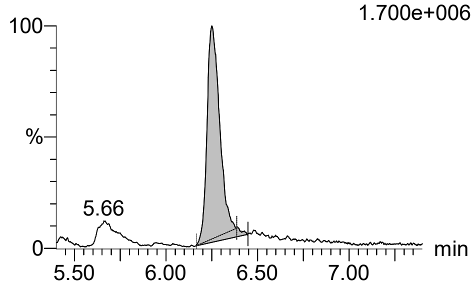

Sample Name: 20211111_L18_R3_IgM
F61:MRM of 1 channel,ES+
1061.7 > 366.1
3.626e+005

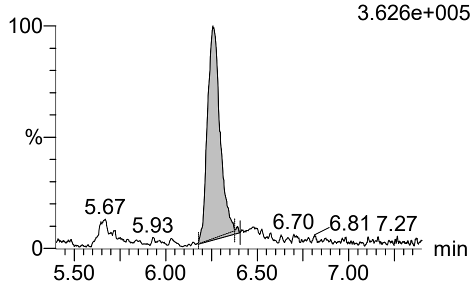

Sample Name: 20211111_H9_R3_IgM
F61:MRM of 1 channel,ES+
1061.7 > 366.1
2.471e+005

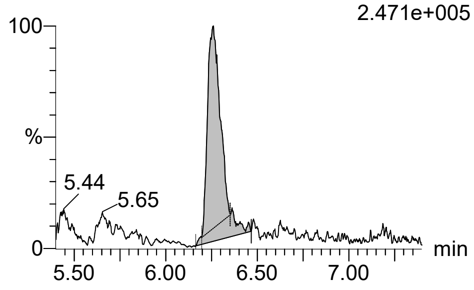

Sample Name: 20211111_A19_R3_IgM
F61:MRM of 1 channel,ES+
1061.7 > 366.1
5.165e+005

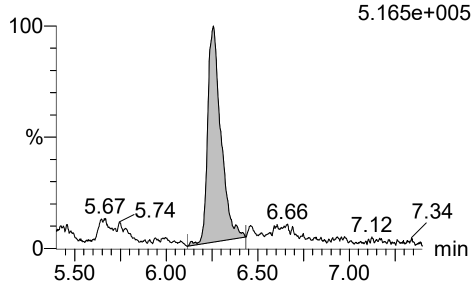

Sample Name: 20211111_A20_R3_IgM
F61:MRM of 1 channel,ES+
1061.7 > 366.1
1.949e+005

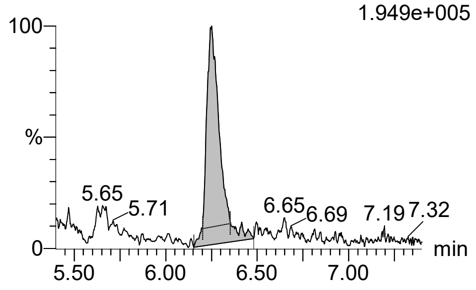

Sample Name: 20211111_L19_R3_IgM
F61:MRM of 1 channel,ES+
1061.7 > 366.1
4.889e+005

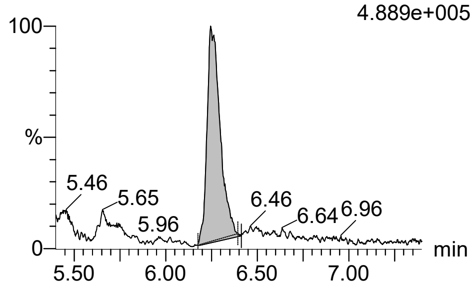

Sample Name: 20211111_QC_R21_IgM
F61:MRM of 1 channel,ES+
1061.7 > 366.1
5.648e+005

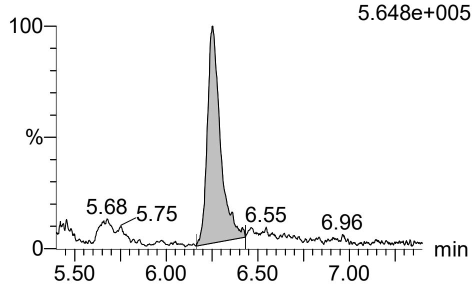

Sample Name: 20211111_L20_R3_IgM
F61:MRM of 1 channel,ES+
1061.7 > 366.1
4.126e+005

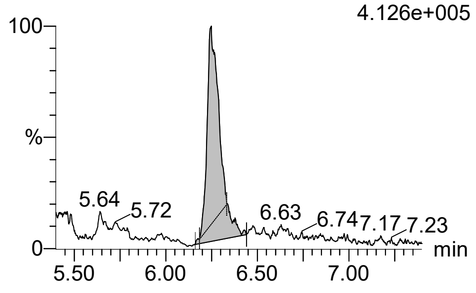

Sample Name: 20211111_H10_R3_IgM
F61:MRM of 1 channel,ES+
1061.7 > 366.1
3.106e+005

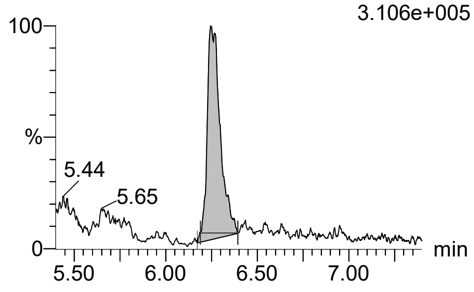

Sample Name: 20211111_A21_R3_IgM
F61:MRM of 1 channel,ES+
1061.7 > 366.1
3.162e+005

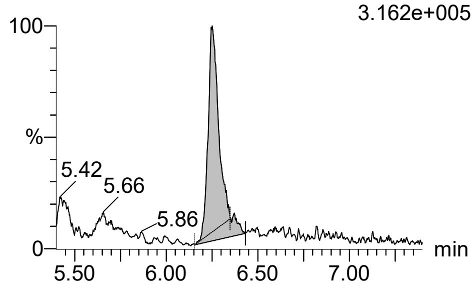

Sample Name: 20211111_A22_R3_IgM
F61:MRM of 1 channel,ES+
1061.7 > 366.1
1.343e+005

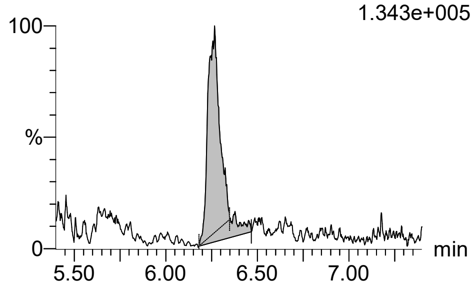

Dataset: C:\MassLynx\10.pro\20211111_clinicalsamples_IgM_IgA.qld

Last Altered: Thursday, March 09, 2023 19:58:25 台北標準時間
Printed: Friday, August 01, 2025 15:24:29 台北標準時間

Sample Name: 20211111_L21_R3_IgM
F61:MRM of 1 channel,ES+
1061.7 > 366.1
3.161e+005

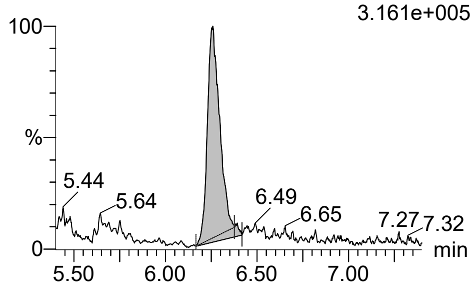

Sample Name: 20211111_L22_R3_IgM
F61:MRM of 1 channel,ES+
1061.7 > 366.1
3.295e+005

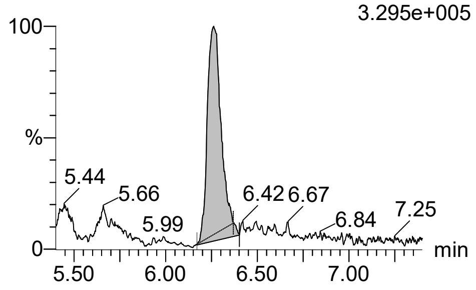

Sample Name: 20211111_H11_R3_IgM
F61:MRM of 1 channel,ES+
1061.7 > 366.1
4.643e+005

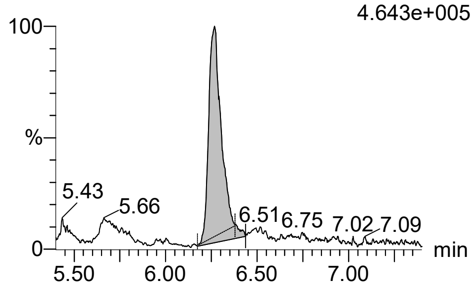

Sample Name: 20211111_A23_R3_IgM
F61:MRM of 1 channel,ES+
1061.7 > 366.1
2.777e+005

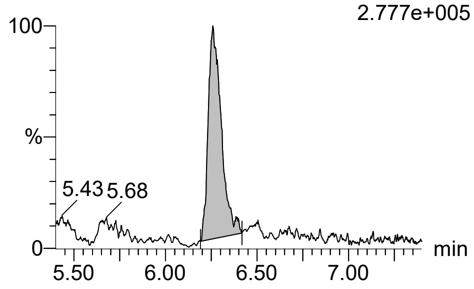

Sample Name: 20211111_A24_R3_IgM
F61:MRM of 1 channel,ES+
1061.7 > 366.1
6.726e+005

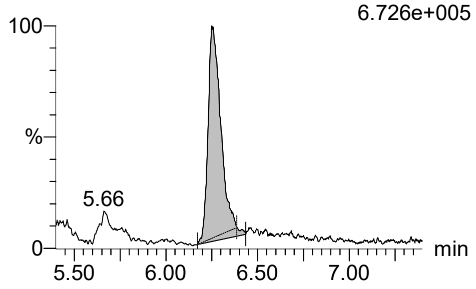

Sample Name: 20211111_L23_R3_IgM
F61:MRM of 1 channel,ES+
1061.7 > 366.1
1.013e+006

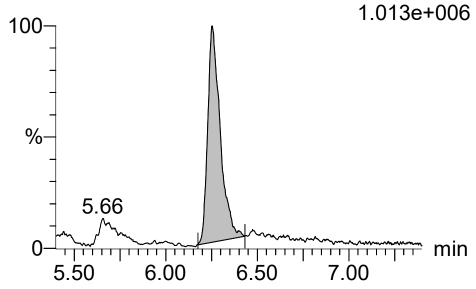

Sample Name: 20211111_L24_R3_IgM
F61:MRM of 1 channel,ES+
1061.7 > 366.1
2.331e+005

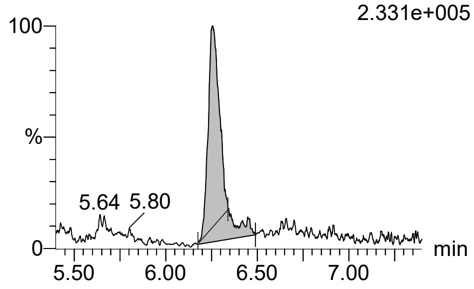

Sample Name: 20211111_H12_R3_IgM
F61:MRM of 1 channel,ES+
1061.7 > 366.1
3.273e+005

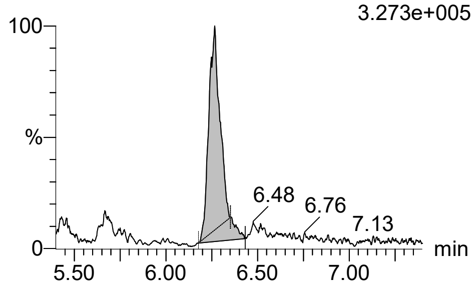

Sample Name: 20211111_QC_R22_IgM
F61:MRM of 1 channel,ES+
1061.7 > 366.1
5.454e+005

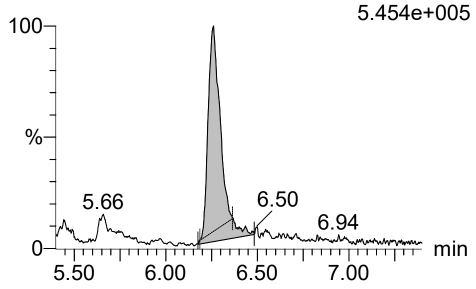

Sample Name: 20211111_A25_R3_IgM
F61:MRM of 1 channel,ES+
1061.7 > 366.1
7.411e+005

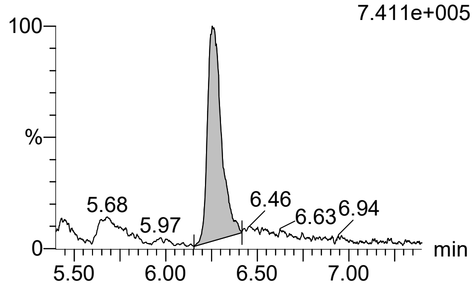

Sample Name: 20211111_A26_R3_IgM
F61:MRM of 1 channel,ES+
1061.7 > 366.1
4.566e+005

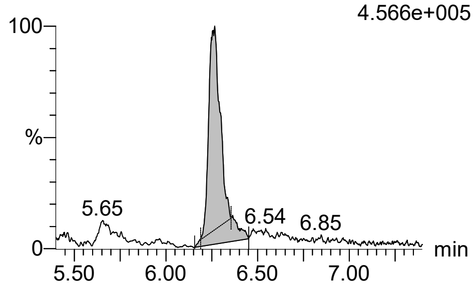

Sample Name: 20211111_L25_R3_IgM
F61:MRM of 1 channel,ES+
1061.7 > 366.1
7.519e+005

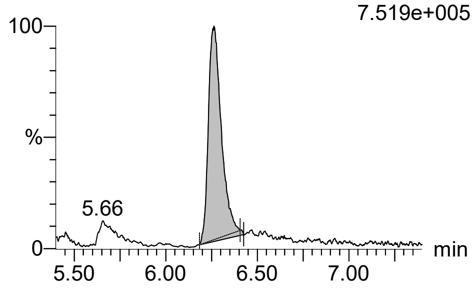

Sample Name: 20211111_L26_R3_IgM
F61:MRM of 1 channel,ES+
1061.7 > 366.1
5.021e+005

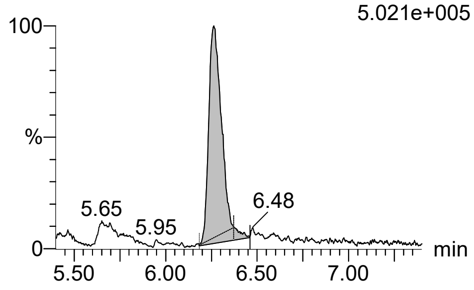

Sample Name: 20211111_H13_R3_IgM
F61:MRM of 1 channel,ES+
1061.7 > 366.1
6.440e+005

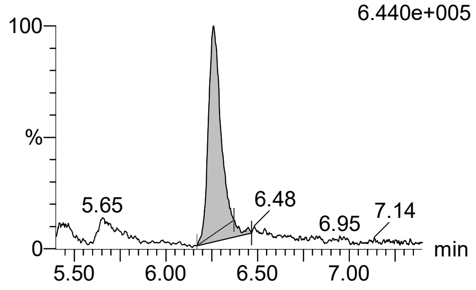

Sample Name: 20211111_A27_R3_IgM
F61:MRM of 1 channel,ES+
1061.7 > 366.1
7.689e+005

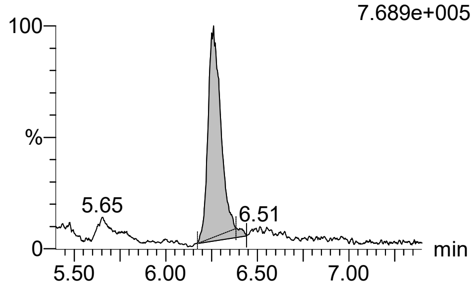

Sample Name: 20211111_A28_R3_IgM
F61:MRM of 1 channel,ES+
1061.7 > 366.1
3.554e+005

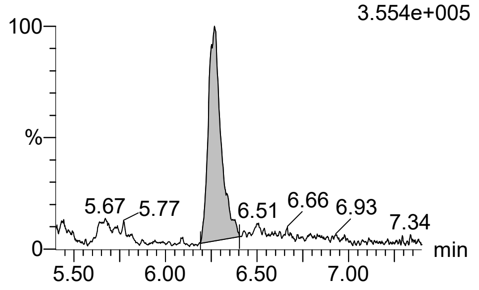

Sample Name: 20211111_L27_R3_IgM
F61:MRM of 1 channel,ES+
1061.7 > 366.1
5.135e+005

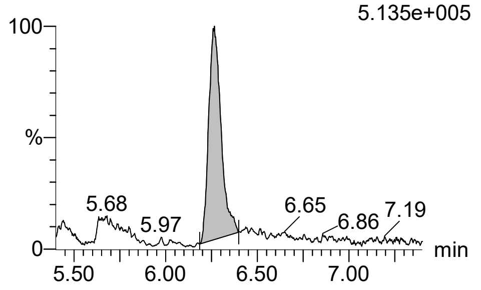

Sample Name: 20211111_L28_R3_IgM
F61:MRM of 1 channel,ES+
1061.7 > 366.1
4.080e+005

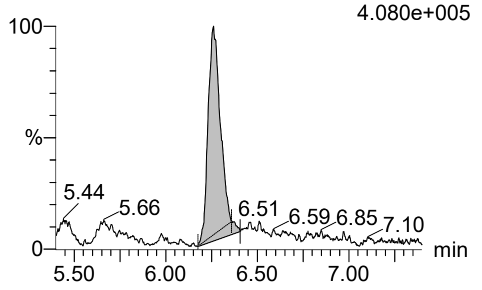

Sample Name: 20211111_H14_R3_IgM
F61:MRM of 1 channel,ES+
1061.7 > 366.1
6.732e+005

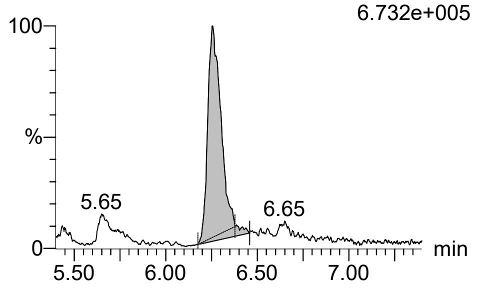

Sample Name: 20211111_A29_R3_IgM
F61:MRM of 1 channel,ES+
1061.7 > 366.1
1.563e+005

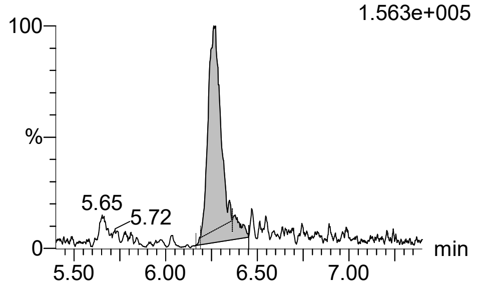

Sample Name: 20211111_A30_R3_IgM
F61:MRM of 1 channel,ES+
1061.7 > 366.1
6.119e+005

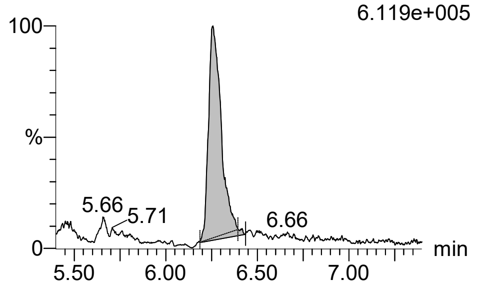

Sample Name: 20211111_QC_R23_IgM
F61:MRM of 1 channel,ES+
1061.7 > 366.1
5.707e+005

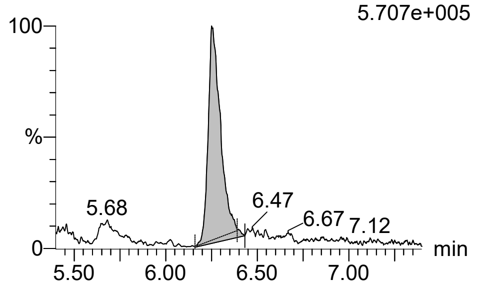

Sample Name: 20211111_L29_R3_IgM
F61:MRM of 1 channel,ES+
1061.7 > 366.1
3.209e+005

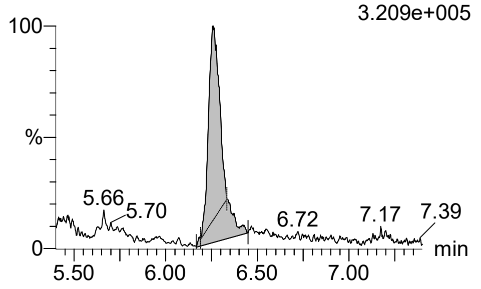

Sample Name: 20211111_L30_R3_IgM
F61:MRM of 1 channel,ES+
1061.7 > 366.1
1.387e+005

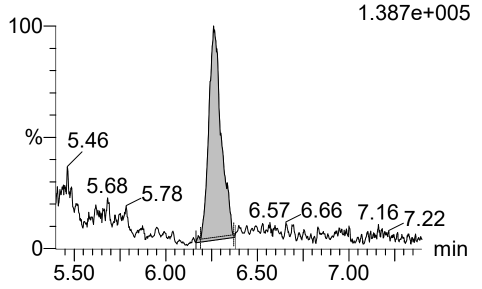

Sample Name: 20211111_H15_R3_IgM
F61:MRM of 1 channel,ES+
1061.7 > 366.1
2.963e+005

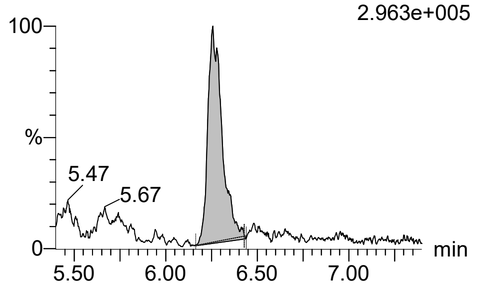

Sample Name: 20211111_A31_R3_IgM
F61:MRM of 1 channel,ES+
1061.7 > 366.1
2.084e+005

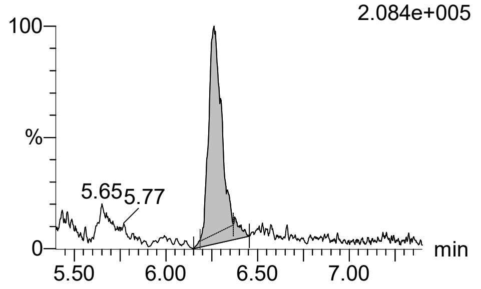

Sample Name: 20211111_A32_R3_IgM
F61:MRM of 1 channel,ES+
1061.7 > 366.1
5.333e+005

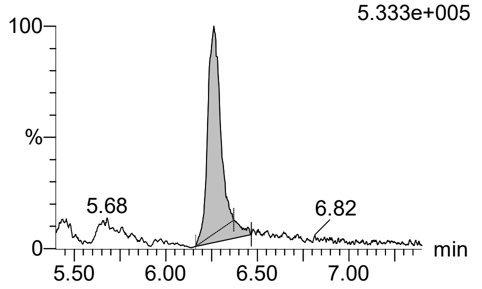

Sample Name: 20211111_L31_R3_IgM
F61:MRM of 1 channel,ES+
1061.7 > 366.1
1.662e+005

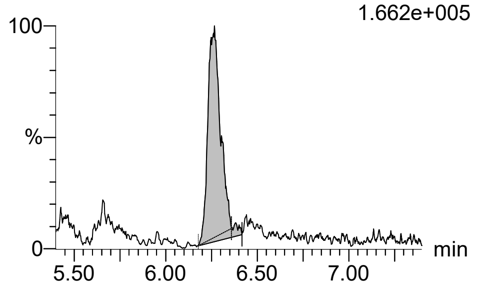

Sample Name: 20211111_L32_R3_IgM
F61:MRM of 1 channel,ES+
1061.7 > 366.1
1.028e+006

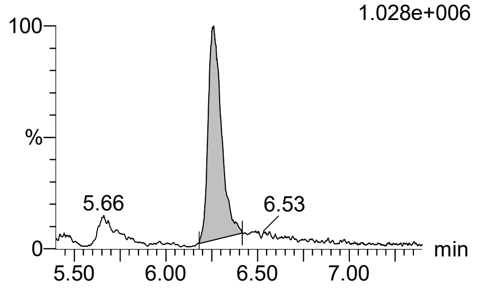

Sample Name: 20211111_H16_R3_IgM
F61:MRM of 1 channel,ES+
1061.7 > 366.1
7.186e+005

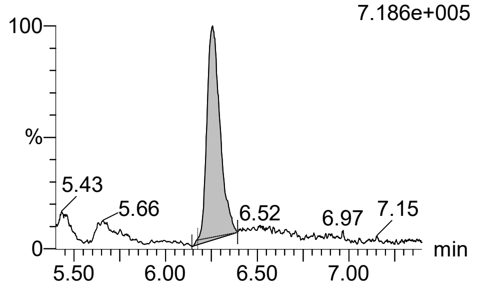

Sample Name: 20211111_A33_R3_IgM

F61:MRM of 1 channel,ES+
1061.7 > 366.1
7.202e+005

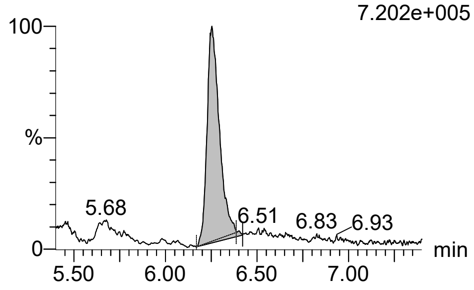

Sample Name: 20211111_A34_R3_IgM

F61:MRM of 1 channel,ES+
1061.7 > 366.1
2.213e+005

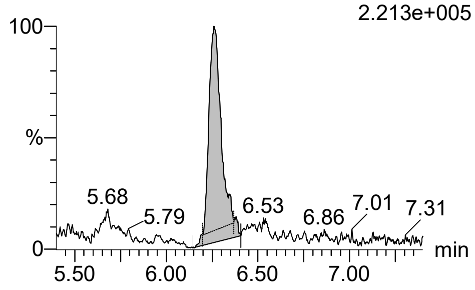

Sample Name: 20211111_H17_R3_IgM

F61:MRM of 1 channel,ES+
1061.7 > 366.1
1.714e+006

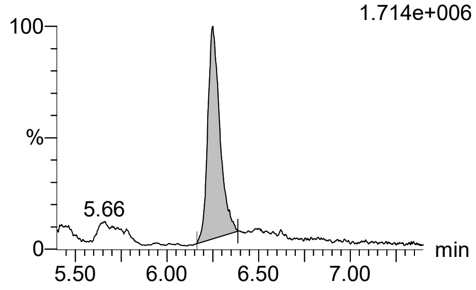

Sample Name: 20211111_A35_R3_IgM

F61:MRM of 1 channel,ES+
1061.7 > 366.1
3.341e+005

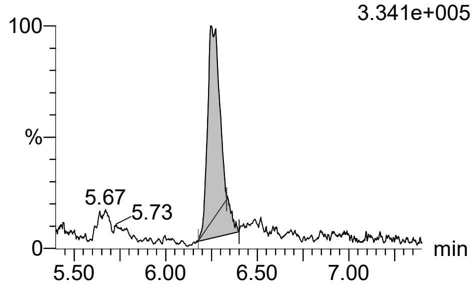

Sample Name: 20211111_QC_R24_IgM

F61:MRM of 1 channel,ES+
1061.7 > 366.1
4.628e+005

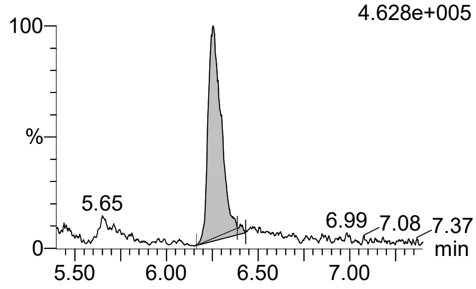

Sample Name: 20211111_A36_R3_IgM

F61:MRM of 1 channel,ES+
1061.7 > 366.1
4.373e+005

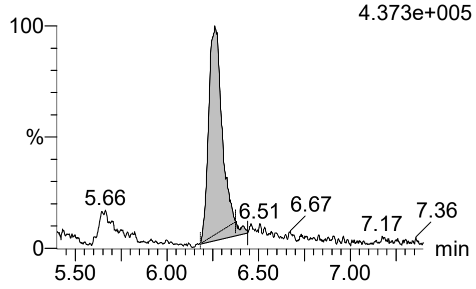

Sample Name: 20211111_H18_R3_IgM

F61:MRM of 1 channel,ES+
1061.7 > 366.1
1.207e+006

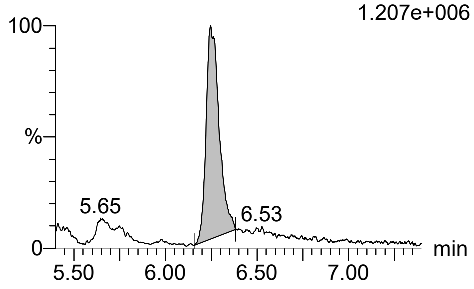

Sample Name: 20211111_A37_R3_IgM

F61:MRM of 1 channel,ES+
1061.7 > 366.1
5.655e+005

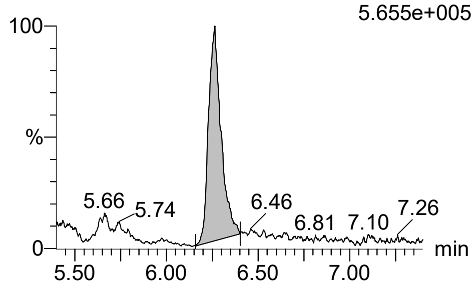

Sample Name: 20211111_A38_R3_IgM

F61:MRM of 1 channel,ES+
1061.7 > 366.1
4.972e+005

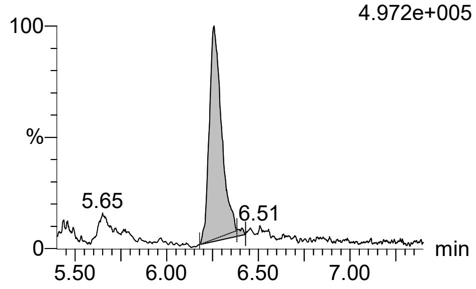

Sample Name: 20211111_H19_R3_IgM

F61:MRM of 1 channel,ES+
1061.7 > 366.1
6.353e+005

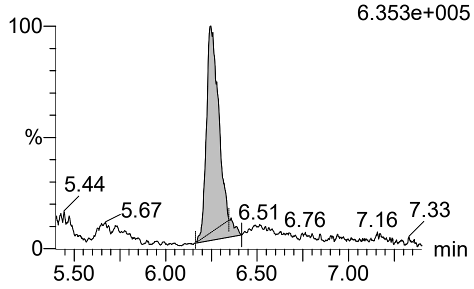

Sample Name: 20211111_A39_R3_IgM

F61:MRM of 1 channel,ES+
1061.7 > 366.1
5.302e+005

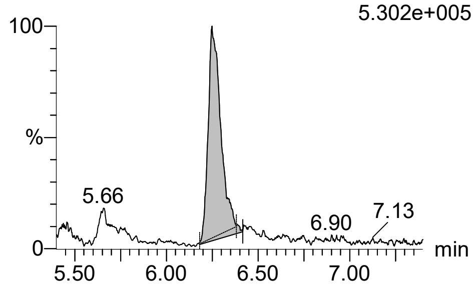

Sample Name: 20211111_A40_R3_IgM

F61:MRM of 1 channel,ES+
1061.7 > 366.1
2.472e+005

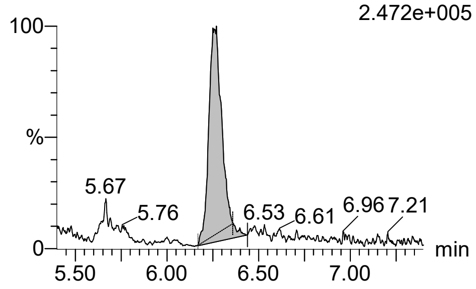

Sample Name: 20211111_H20_R3_IgM

F61:MRM of 1 channel,ES+
1061.7 > 366.1
1.930e+006

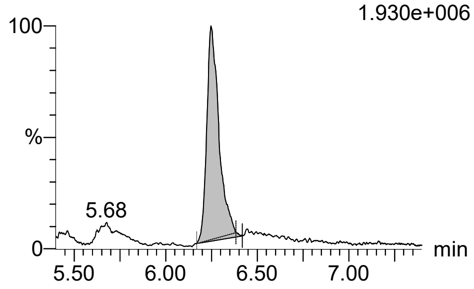

Sample Name: 20211111_A41_R3_IgM

F61:MRM of 1 channel,ES+
1061.7 > 366.1
3.690e+005

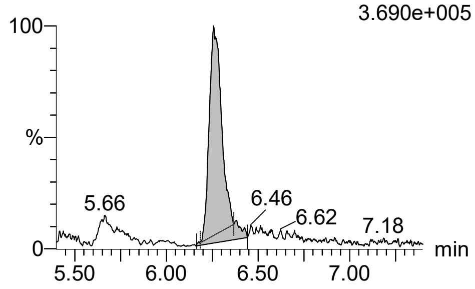

Sample Name: 20211111_A42_R3_IgM

F61:MRM of 1 channel,ES+
1061.7 > 366.1
1.974e+005

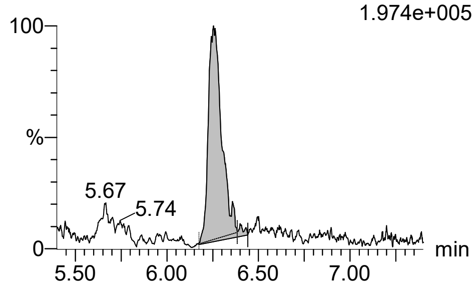

Dataset: C:\MassLynx\10.pro\20211111_clinicalsamples_IgM_IgA.qld

Last Altered: Thursday, March 09, 2023 19:58:25 台北標準時間
Printed: Friday, August 01, 2025 15:24:29 台北標準時間

Sample Name: 20211111_A43_R3_IgM

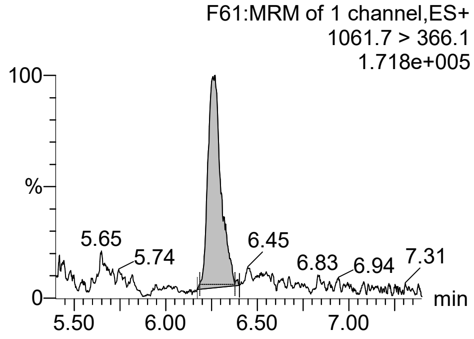

Sample Name: 20211111_A44_R3_IgM

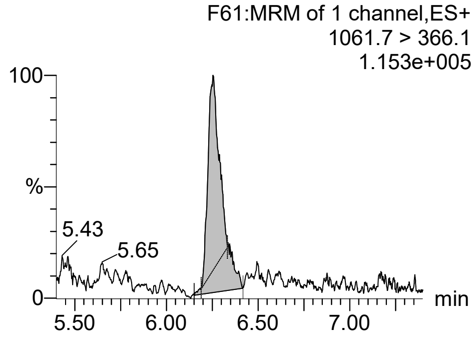

Sample Name: 20211111_QC_R25_IgM

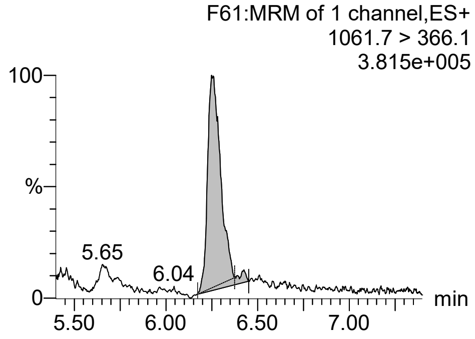

Dataset: C:\MassLynx\10.pro\20211111_clinicalsamples_IgM_IgA.qld

Last Altered: Thursday, March 09, 2023 19:58:25 台北標準時間
Printed: Friday, August 01, 2025 15:24:29 台北標準時間

	#	Name	RT	Area	IS Area	Response	Prima...
1	1	20211112_QC_R001_IgM	6.24	54053.0	15421.4	3.505	bb
2	2	20211112_QC_R002_IgM	6.25	48212.6	13188.8	3.656	MM
3	3	20211111_A1_R2_IgM	6.26	31020.6	11871.0	2.613	MM
4	4	20211111_A1_R3_IgM	6.25	27699.1	12030.5	2.302	MM
5	5	20211111_A2_R2_IgM	6.24	75043.3	11446.8	6.556	MM
6	6	20211111_A2_R3_IgM	6.25	75467.8	11098.4	6.800	MM
7	7	20211111_L1_R2_IgM	6.25	25385.9	11779.4	2.155	MM
8	8	20211111_L1_R3_IgM	6.26	17203.1	11176.2	1.539	MM
9	9	20211111_L2_R2_IgM	6.25	52164.9	11375.0	4.586	MM
10	10	20211111_H1_R2_IgM	6.25	95301.9	13595.2	7.010	MM
11	11	20211111_A3_R2_IgM	6.25	74234.0	14112.3	5.260	MM
12	12	20211111_A4_R2_IgM	6.25	50329.2	11977.5	4.202	MM
13	13	20211111_L3_R2_IgM	6.23	46588.0	12242.1	3.806	bb
14	14	20211111_L4_R2_IgM	6.24	42077.9	12797.0	3.288	MM
15	15	20211111_H2_R2_IgM	6.24	93116.2	10006.0	9.306	MM
16	16	20211111_A5_R2_IgM	6.25	158914.6	11434.6	13.898	MM
17	17	20211111_A6_R2_IgM	6.24	64657.0	13193.7	4.901	MM
18	18	20211111_QC_R10_IgM	6.26	52088.8	12566.1	4.145	MM
19	19	20211111_L5_R2_IgM	6.24	59259.3	13616.4	4.352	MM
20	20	20211111_L6_R2_IgM	6.25	57637.3	13698.5	4.208	MM
21	21	20211111_H3_R2_IgM	6.24	15932.8	15793.1	1.009	MM
22	22	20211111_A7_R2_IgM	6.27	46070.6	12936.9	3.561	MM
23	23	20211111_A8_R2_IgM	6.25	50466.5	13108.0	3.850	MM
24	24	20211111_L7_R2_IgM	6.25	40446.9	13278.5	3.046	MM
25	25	20211111_L8_R2_IgM	6.24	14286.8	15233.6	0.938	MM
26	26	20211111_H4_R2_IgM	6.25	28911.1	16521.9	1.750	MM
27	27	20211111_A9_R2_IgM	6.24	30064.1	16451.6	1.827	bb
28	28	20211111_A10_R2_IgM	6.26	90442.7	13036.0	6.938	bb
29	29	20211111_L9_R2_IgM	6.24	74112.4	14554.1	5.092	MM
30	30	20211111_L10_R2_IgM	6.25	57870.7	12250.4	4.724	bb
31	31	20211111_QC_R11_IgM	6.25	56815.5	11459.2	4.958	bb
32	32	20211111_H5_R2_IgM	6.25	67498.4	14358.2	4.701	MM
33	33	20211111_A11_R2_IgM	6.23	48043.3	16184.1	2.969	bb
34	34	20211111_A12_R2_IgM	6.25	23409.2	13465.4	1.738	MM
35	35	20211111_L11_R2_IgM	6.25	31356.1	13277.9	2.362	bb
36	36	20211111_L12_R2_IgM	6.26	27926.4	15958.8	1.750	MM
37	37	20211111_H6_R2_IgM	6.24	15289.6	14003.5	1.092	MM
38	38	20211111_A13_R2_IgM	6.24	22599.4	13656.1	1.655	MM
39	39	20211111_A14_R2_IgM	6.25	100091.4	14071.3	7.113	MM
40	40	20211111_L13_R2_IgM	6.26	39625.9	14785.6	2.680	MM
41	41	20211111_L14_R2_IgM	6.26	75021.7	14772.2	5.079	MM
42	42	20211111_H7_R2_IgM	6.25	44484.4	15164.4	2.933	MM
43	43	20211111_A15_R2_IgM	6.26	97178.1	15721.5	6.181	MM
44	44	20211111_QC_R12_IgM	6.26	60716.2	13711.5	4.428	MM
45	45	20211111_A16_R2_IgM	6.26	80967.3	14275.6	5.672	bb
46	46	20211111_L15_R2_IgM	6.25	49568.5	12935.8	3.832	bb
47	47	20211111_L16_R2_IgM	6.24	27295.8	13342.8	2.046	MM
48	48	20211111_H8_R2_IgM	6.26	40831.5	15835.1	2.579	bb
49	49	20211111_A17_R2_IgM	6.25	70354.6	15786.7	4.457	bb
50	50	20211111_A18_R2_IgM	6.24	25095.7	16986.8	1.477	MM
51	51	20211111_L17_R2_IgM	6.24	169273.9	13342.7	12.687	MM
52	52	20211111_L18_R2_IgM	6.26	33728.7	14692.0	2.296	MM
53	53	20211111_H9_R2_IgM	6.25	27097.6	15639.7	1.733	MM
54	54	20211111_A19_R2_IgM	6.24	43940.1	14198.7	3.095	bb
55	55	20211111_A20_R2_IgM	6.25	17422.5	14162.7	1.230	bb
56	56	20211111_L19_R2_IgM	6.25	34475.4	9293.4	3.710	MM

Dataset: C:\MassLynx\10.pro\20211111_clinicalsamples_IgM_IgA.qld

Last Altered: Thursday, March 09, 2023 19:58:25 台北標準時間
Printed: Friday, August 01, 2025 15:24:29 台北標準時間

	#	Name	RT	Area	IS Area	Response	Prima...
57	57	20211111_QC_R13_IgM	6.25	54046.5	13903.0	3.887	MM
58	58	20211111_L20_R2_IgM	6.24	36655.9	12781.6	2.868	MM
59	59	20211111_H10_R2_IgM	6.24	31478.6	14796.4	2.127	MM
60	60	20211111_A21_R2_IgM	6.25	24229.8	13669.0	1.773	MM
61	61	20211111_A22_R2_IgM	6.25	12139.0	14098.3	0.861	MM
62	62	20211111_L21_R2_IgM	6.26	27667.3	15678.2	1.765	MM
63	63	20211111_L22_R2_IgM	6.26	35581.0	15724.5	2.263	MM
64	64	20211111_H11_R2_IgM	6.25	43419.3	15890.2	2.732	bb
65	65	20211111_A23_R2_IgM	6.24	23968.6	13423.2	1.786	bb
66	66	20211111_A24_R2_IgM	6.25	51248.5	12438.0	4.120	MM
67	67	20211111_L23_R2_IgM	6.25	89484.1	14075.0	6.358	MM
68	68	20211111_L24_R2_IgM	6.25	20493.0	15972.1	1.283	MM
69	69	20211111_H12_R2_IgM	6.25	27110.3	16270.9	1.666	MM
70	70	20211111_QC_R14_IgM	6.26	53869.7	15806.8	3.408	MM
71	71	20211111_A25_R2_IgM	6.25	80880.5	14552.1	5.558	MM
72	72	20211111_A26_R2_IgM	6.26	44149.9	11492.7	3.842	MM
73	73	20211111_L25_R2_IgM	6.25	78256.2	14403.3	5.433	MM
74	74	20211111_L26_R2_IgM	6.26	49921.6	16533.9	3.019	MM
75	75	20211111_H13_R2_IgM	6.25	55915.2	15520.1	3.603	bb
76	76	20211111_A27_R2_IgM	6.26	74064.2	13337.4	5.553	MM
77	77	20211111_A28_R2_IgM	6.24	34195.2	11895.6	2.875	MM
78	78	20211111_L27_R2_IgM	6.25	41179.4	14125.7	2.915	MM
79	79	20211111_L28_R2_IgM	6.25	31093.9	11807.5	2.633	MM
80	80	20211111_H14_R2_IgM	6.25	52570.4	15035.7	3.496	bb
81	81	20211111_A29_R2_IgM	6.24	13889.9	13554.4	1.025	MM
82	82	20211111_A30_R2_IgM	6.26	54474.3	12984.1	4.195	MM
83	83	20211111_QC_R15_IgM	6.26	49380.5	12299.5	4.015	MM
84	84	20211111_L29_R2_IgM	6.27	27779.6	13590.7	2.044	MM
85	85	20211111_L30_R2_IgM	6.27	12714.3	12511.4	1.016	MM
86	86	20211111_H15_R2_IgM	6.25	28209.3	11885.0	2.374	MM
87	87	20211111_A31_R2_IgM	6.27	20380.6	13422.1	1.518	MM
88	88	20211111_A32_R2_IgM	6.25	48414.3	11461.3	4.224	MM
89	89	20211111_L31_R2_IgM	6.24	16772.4	13753.4	1.220	MM
90	90	20211111_L32_R2_IgM	6.24	100993.0	12949.0	7.799	MM
91	91	20211111_H16_R2_IgM	6.24	68364.5	11465.4	5.963	MM
92	92	20211111_A33_R2_IgM	6.25	61514.7	10551.5	5.830	MM
93	93	20211111_A34_R2_IgM	6.26	21119.3	12409.9	1.702	MM
94	94	20211111_H17_R2_IgM	6.25	160321.6	10160.9	15.778	MM
95	95	20211111_A35_R2_IgM	6.25	32076.2	10460.6	3.066	MM
96	96	20211111_QC_R16_IgM	6.25	46055.4	10433.5	4.414	MM
97	97	20211111_A36_R2_IgM	6.24	47430.9	12504.8	3.793	MM
98	98	20211111_H18_R2_IgM	6.25	112798.0	9140.4	12.341	MM
99	99	20211111_A37_R2_IgM	6.25	50919.1	9796.9	5.197	MM
100	1...	20211111_A38_R2_IgM	6.27	44639.9	11224.3	3.977	MM
101	1...	20211111_H19_R2_IgM	6.26	57720.7	11739.2	4.917	MM
102	1...	20211111_A39_R2_IgM	6.25	47218.7	12718.9	3.712	MM
103	1...	20211111_A40_R2_IgM	6.24	22993.3	10006.1	2.298	MM
104	1...	20211111_H20_R2_IgM	6.25	180734.9	7988.2	22.625	bb
105	1...	20211111_A41_R2_IgM	6.26	50226.9	11513.3	4.362	MM
106	1...	20211111_A42_R2_IgM	6.27	18860.3	10238.5	1.842	MM
107	1...	20211111_A43_R2_IgM	6.27	14963.7	11098.9	1.348	MM
108	1...	20211111_A44_R2_IgM	6.23	10427.3	10969.9	0.951	MM
109	1...	20211111_QC_R17_IgM	6.27	34610.1	9700.8	3.568	MM
110	1...	20211111_L2_R3_IgM	6.25	50824.7	8528.0	5.960	MM
111	1...	20211111_H1_R3_IgM	6.25	77826.3	9438.2	8.246	MM
112	1...	20211111_A3_R3_IgM	6.26	60885.9	11598.6	5.249	MM

Dataset: C:\MassLynx\10.pro\20211111_clinicalsamples_IgM_IgA.qld

Last Altered: Thursday, March 09, 2023 19:58:25 台北標準時間
Printed: Friday, August 01, 2025 15:24:29 台北標準時間

	#	Name	RT	Area	IS Area	Response	Prima...
113	1...	20211111_A4_R3_IgM	6.25	41186.7	11364.1	3.624	bb
114	1...	20211111_L3_R3_IgM	6.24	38577.0	10753.4	3.587	MM
115	1...	20211111_L4_R3_IgM	6.26	37544.1	10291.6	3.648	MM
116	1...	20211111_H2_R3_IgM	6.25	91351.5	9315.8	9.806	MM
117	1...	20211111_A5_R3_IgM	6.26	125682.2	9102.2	13.808	bb
118	1...	20211111_A6_R3_IgM	6.26	49690.4	10356.4	4.798	MM
119	1...	20211111_QC_R18_IgM	6.25	40877.4	8398.3	4.867	MM
120	1...	20211111_L5_R3_IgM	6.26	46037.9	9786.7	4.704	MM
121	1...	20211111_L6_R3_IgM	6.26	44824.2	9911.3	4.523	bb
122	1...	20211111_H3_R3_IgM	6.26	13233.8	12155.5	1.089	MM
123	1...	20211111_A7_R3_IgM	6.25	42206.3	11804.8	3.575	MM
124	1...	20211111_A8_R3_IgM	6.26	38592.2	10510.6	3.672	bb
125	1...	20211111_L7_R3_IgM	6.24	34034.0	12177.9	2.795	bb
126	1...	20211111_L8_R3_IgM	6.27	13388.6	11668.6	1.147	MM
127	1...	20211111_H4_R3_IgM	6.27	20753.3	11192.0	1.854	MM
128	1...	20211111_A9_R3_IgM	6.26	22577.3	14077.4	1.604	MM
129	1...	20211111_A10_R3_IgM	6.26	77094.5	11190.4	6.889	MM
130	1...	20211111_L9_R3_IgM	6.25	61004.2	14649.3	4.164	MM
131	1...	20211111_L10_R3_IgM	6.25	44104.7	9361.3	4.711	MM
132	1...	20211111_QC_R19_IgM	6.24	41214.2	10125.5	4.070	bb
133	1...	20211111_H5_R3_IgM	6.26	43481.2	10010.9	4.343	bb
134	1...	20211111_A11_R3_IgM	6.25	33974.7	12697.2	2.676	MM
135	1...	20211111_A12_R3_IgM	6.25	19816.1	13148.5	1.507	MM
136	1...	20211111_L11_R3_IgM	6.26	23872.4	10786.6	2.213	MM
137	1...	20211111_L12_R3_IgM	6.24	22065.3	11681.8	1.889	MM
138	1...	20211111_H6_R3_IgM	6.26	14025.2	12095.5	1.160	MM
139	1...	20211111_A13_R3_IgM	6.25	20168.0	12908.3	1.562	MM
140	1...	20211111_A14_R3_IgM	6.25	75288.0	11322.6	6.649	MM
141	1...	20211111_L13_R3_IgM	6.25	33041.2	11516.1	2.869	MM
142	1...	20211111_L14_R3_IgM	6.25	59560.0	10790.7	5.520	MM
143	1...	20211111_H7_R3_IgM	6.26	35347.6	11572.1	3.055	bb
144	1...	20211111_A15_R3_IgM	6.25	79477.6	12434.4	6.392	MM
145	1...	20211111_QC_R20_IgM	6.26	48792.4	10701.0	4.560	MM
146	1...	20211111_A16_R3_IgM	6.26	63987.9	11452.1	5.587	MM
147	1...	20211111_L15_R3_IgM	6.26	42641.3	10531.1	4.049	MM
148	1...	20211111_L16_R3_IgM	6.27	23449.9	12049.4	1.946	MM
149	1...	20211111_H8_R3_IgM	6.25	35157.8	13434.9	2.617	MM
150	1...	20211111_A17_R3_IgM	6.25	61875.1	12452.0	4.969	MM
151	1...	20211111_A18_R3_IgM	6.25	20180.4	12506.5	1.614	MM
152	1...	20211111_L17_R3_IgM	6.25	137182.4	9948.9	13.789	MM
153	1...	20211111_L18_R3_IgM	6.26	27929.3	13462.1	2.075	MM
154	1...	20211111_H9_R3_IgM	6.26	21502.4	13178.7	1.632	MM
155	1...	20211111_A19_R3_IgM	6.26	41803.3	11298.6	3.700	bb
156	1...	20211111_A20_R3_IgM	6.25	16402.4	11175.6	1.468	MM
157	1...	20211111_L19_R3_IgM	6.24	39504.9	11558.2	3.418	MM
158	1...	20211111_QC_R21_IgM	6.25	45307.2	9977.0	4.541	bb
159	1...	20211111_L20_R3_IgM	6.25	32884.4	13508.1	2.434	MM
160	1...	20211111_H10_R3_IgM	6.24	24627.8	12374.8	1.990	MM
161	1...	20211111_A21_R3_IgM	6.25	22710.4	12443.6	1.825	MM
162	1...	20211111_A22_R3_IgM	6.27	10877.4	11589.3	0.939	MM
163	1...	20211111_L21_R3_IgM	6.26	24918.3	13107.4	1.901	MM
164	1...	20211111_L22_R3_IgM	6.26	28113.8	12021.5	2.339	MM
165	1...	20211111_H11_R3_IgM	6.27	37249.9	12687.8	2.936	MM
166	1...	20211111_A23_R3_IgM	6.26	21848.5	11611.8	1.882	bb
167	1...	20211111_A24_R3_IgM	6.25	53781.4	10476.8	5.133	MM
168	1...	20211111_L23_R3_IgM	6.25	77673.2	11873.1	6.542	bb

Dataset: C:\MassLynx\10.pro\20211111_clinicalsamples_IgM_IgA.qld

Last Altered: Thursday, March 09, 2023 19:58:25 台北標準時間
Printed: Friday, August 01, 2025 15:24:29 台北標準時間

	# Name	RT	Area	IS Area	Response	Prima...
169	1... 20211111_L24_R3_IgM	6.25	19127.9	13468.0	1.420	MM
170	1... 20211111_H12_R3_IgM	6.27	24727.2	14229.1	1.738	MM
171	1... 20211111_QC_R22_IgM	6.26	43395.1	11264.4	3.852	MM
172	1... 20211111_A25_R3_IgM	6.25	62675.4	11775.7	5.322	bb
173	1... 20211111_A26_R3_IgM	6.27	37425.4	10473.9	3.573	MM
174	1... 20211111_L25_R3_IgM	6.26	60208.4	13256.8	4.542	MM
175	1... 20211111_L26_R3_IgM	6.26	40894.3	13565.0	3.015	MM
176	1... 20211111_H13_R3_IgM	6.26	52120.7	13146.0	3.965	MM
177	1... 20211111_A27_R3_IgM	6.26	61810.7	12025.6	5.140	MM
178	1... 20211111_A28_R3_IgM	6.27	27298.6	11229.1	2.431	bb
179	1... 20211111_L27_R3_IgM	6.27	38082.6	14960.0	2.546	bb
180	1... 20211111_L28_R3_IgM	6.26	31678.7	11359.0	2.789	MM
181	1... 20211111_H14_R3_IgM	6.25	56345.1	12830.8	4.391	MM
182	1... 20211111_A29_R3_IgM	6.26	13490.1	13434.3	1.004	MM
183	1... 20211111_A30_R3_IgM	6.26	48222.6	11272.2	4.278	MM
184	1... 20211111_QC_R23_IgM	6.25	44556.0	11872.2	3.753	MM
185	1... 20211111_L29_R3_IgM	6.26	26438.2	12948.7	2.042	MM
186	1... 20211111_L30_R3_IgM	6.26	10440.8	11026.6	0.947	MM
187	1... 20211111_H15_R3_IgM	6.26	26170.9	10786.3	2.426	MM
188	1... 20211111_A31_R3_IgM	6.26	17936.7	12010.5	1.493	MM
189	1... 20211111_A32_R3_IgM	6.26	45332.1	11107.7	4.081	MM
190	1... 20211111_L31_R3_IgM	6.27	13289.4	11021.8	1.206	MM
191	1... 20211111_L32_R3_IgM	6.26	82184.9	11683.8	7.034	bb
192	1... 20211111_H16_R3_IgM	6.26	57146.0	9831.7	5.812	MM
193	1... 20211111_A33_R3_IgM	6.25	55307.7	11213.1	4.932	MM
194	1... 20211111_A34_R3_IgM	6.26	17611.4	11550.1	1.525	MM
195	1... 20211111_H17_R3_IgM	6.25	124184.0	8874.4	13.993	bb
196	1... 20211111_A35_R3_IgM	6.24	27020.2	9856.3	2.741	MM
197	1... 20211111_QC_R24_IgM	6.25	37770.4	7561.8	4.995	MM
198	1... 20211111_A36_R3_IgM	6.26	37281.3	9562.8	3.899	MM
199	1... 20211111_H18_R3_IgM	6.24	95147.1	8165.3	11.653	bb
200	2... 20211111_A37_R3_IgM	6.26	42842.3	9278.8	4.617	bb
201	2... 20211111_A38_R3_IgM	6.26	38147.0	10920.3	3.493	MM
202	2... 20211111_H19_R3_IgM	6.25	50087.5	9285.3	5.394	MM
203	2... 20211111_A39_R3_IgM	6.25	41443.6	9083.4	4.563	MM
204	2... 20211111_A40_R3_IgM	6.27	20084.2	8382.4	2.396	MM
205	2... 20211111_H20_R3_IgM	6.25	144558.1	7807.9	18.514	MM
206	2... 20211111_A41_R3_IgM	6.26	30634.8	5925.2	5.170	MM
207	2... 20211111_A42_R3_IgM	6.25	16514.3	9352.6	1.766	MM
208	2... 20211111_A43_R3_IgM	6.27	13608.4	10216.3	1.332	MM
209	2... 20211111_A44_R3_IgM	6.25	9756.1	10262.7	0.951	MM
210	2... 20211111_QC_R25_IgM	6.25	31654.9	8575.3	3.691	MM

Dataset: C:\MassLynx\10.pro\20211111_clinicalsamples_IgM_IgA.qld

Last Altered: Thursday, March 09, 2023 19:58:25 台北標準時間
Printed: Friday, August 01, 2025 15:25:12 台北標準時間

Method: C:\MassLynx\10_Pro_IgA.PRO\MethDB\20211111_IgM_IgA_DMRM_new.mdb 24 Feb 2022 13:44:14
Calibration: 15 Nov 2021 14:42:11

Sample Name: 20211112_QC_R001_IgM

F80:MRM of 1 channel,ES+
1134.5 > 204.1
6.218e+005

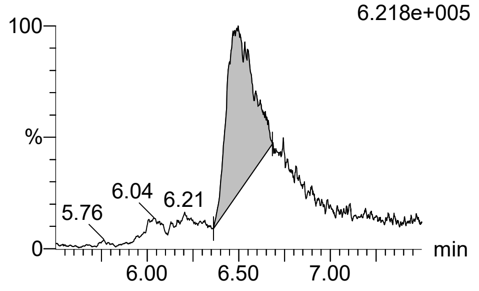

Sample Name: 20211112_QC_R002_IgM

F80:MRM of 1 channel,ES+
1134.5 > 204.1
5.839e+005

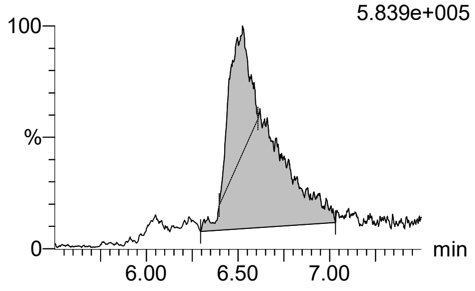

Sample Name: 20211111_A1_R2_IgM

F80:MRM of 1 channel,ES+
1134.5 > 204.1
4.198e+005

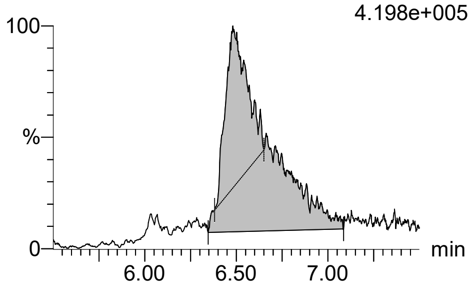

Sample Name: 20211111_A1_R3_IgM

F80:MRM of 1 channel,ES+
1134.5 > 204.1
3.870e+005

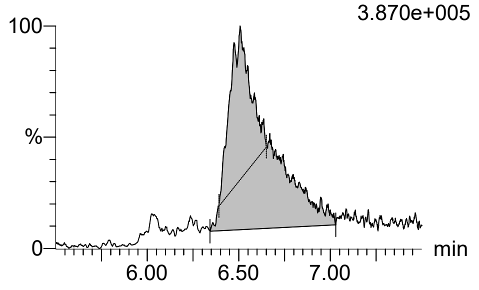

Sample Name: 20211111_A2_R2_IgM

F80:MRM of 1 channel,ES+
1134.5 > 204.1
8.298e+005

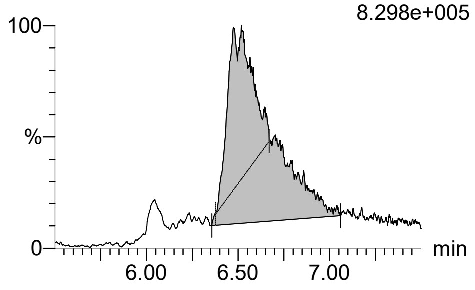

Sample Name: 20211111_A2_R3_IgM

F80:MRM of 1 channel,ES+
1134.5 > 204.1
8.759e+005

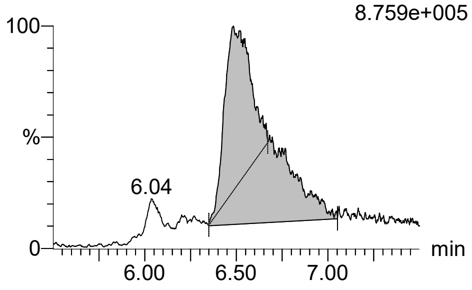

Sample Name: 20211111_L1_R2_IgM

F80:MRM of 1 channel,ES+
1134.5 > 204.1
3.542e+005

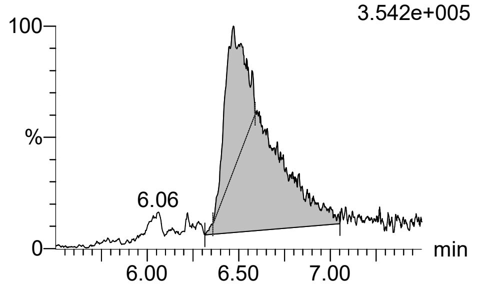

Sample Name: 20211111_L1_R3_IgM

F80:MRM of 1 channel,ES+
1134.5 > 204.1
2.505e+005

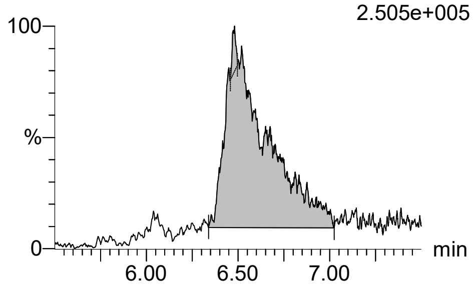

Sample Name: 20211111_L2_R2_IgM

F80:MRM of 1 channel,ES+
1134.5 > 204.1
8.052e+005

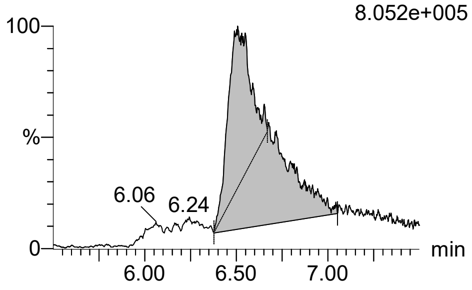

Sample Name: 20211111_H1_R2_IgM

F80:MRM of 1 channel,ES+
1134.5 > 204.1
7.092e+005

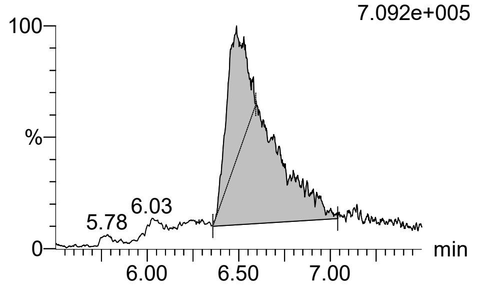

Sample Name: 20211111_A3_R2_IgM

F80:MRM of 1 channel,ES+
1134.5 > 204.1
9.669e+005

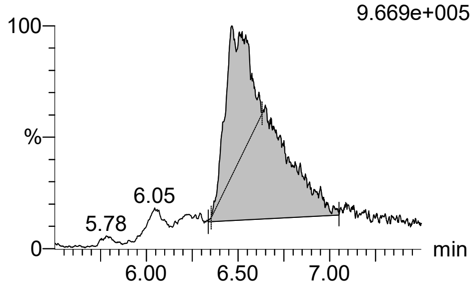

Sample Name: 20211111_A4_R2_IgM

F80:MRM of 1 channel,ES+
1134.5 > 204.1
6.562e+005

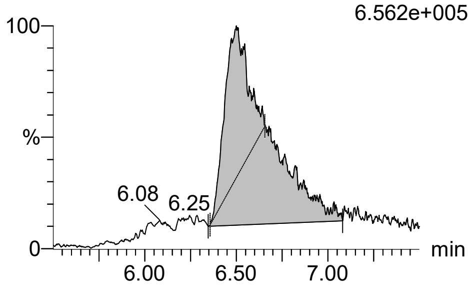

Sample Name: 20211111_L3_R2_IgM

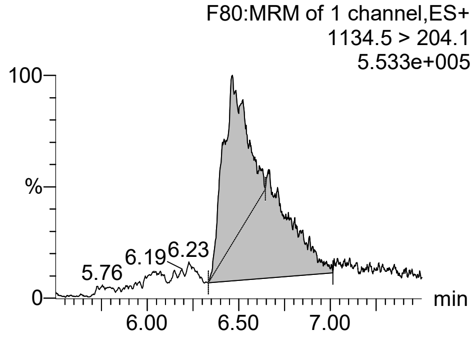

Sample Name: 20211111_L4_R2_IgM

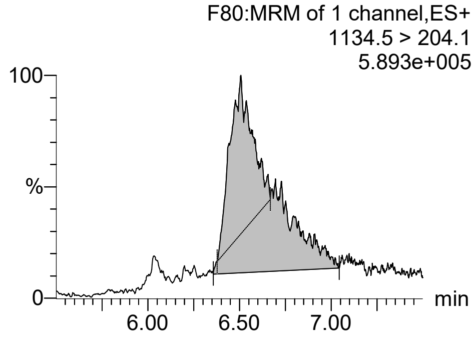

Sample Name: 20211111_H2_R2_IgM

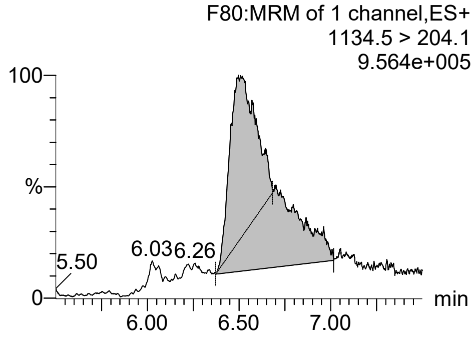

Sample Name: 20211111_A5_R2_IgM

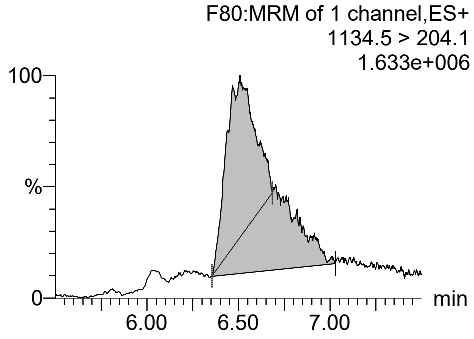

Sample Name: 20211111_A6_R2_IgM

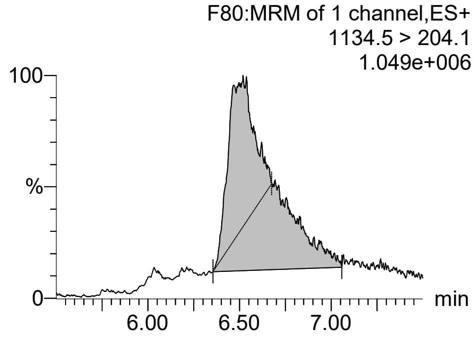

Sample Name: 20211111_QC_R10_IgM

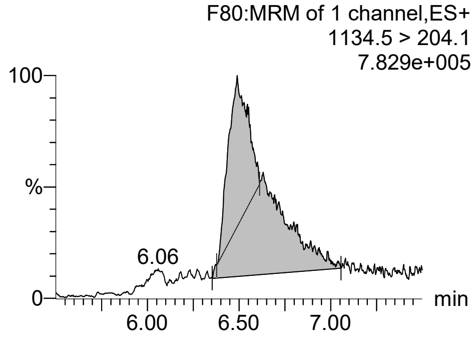

Sample Name: 20211111_L5_R2_IgM

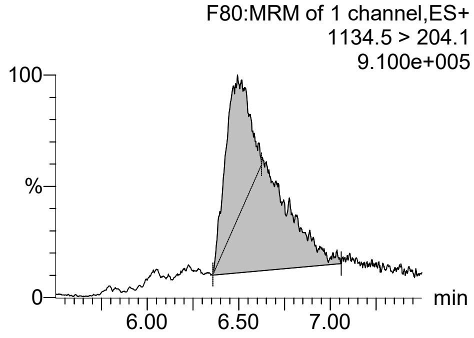

Sample Name: 20211111_L6_R2_IgM

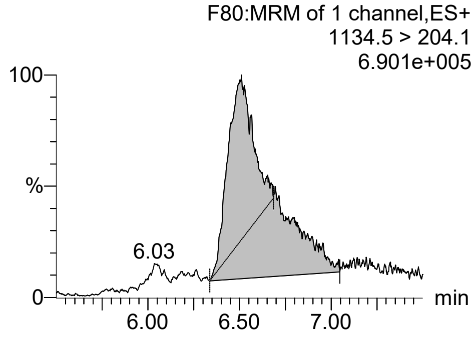

Sample Name: 20211111_H3_R2_IgM

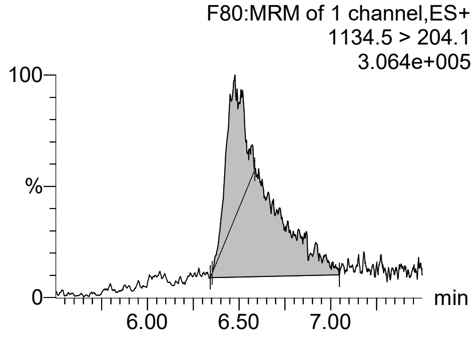

Sample Name: 20211111_A7_R2_IgM

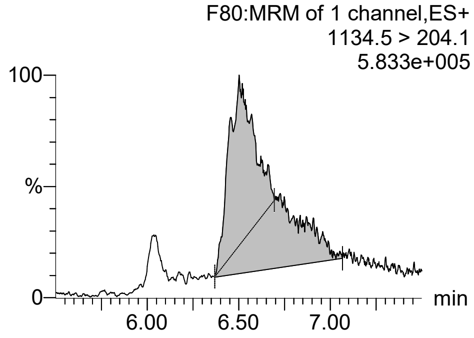

Sample Name: 20211111_A8_R2_IgM

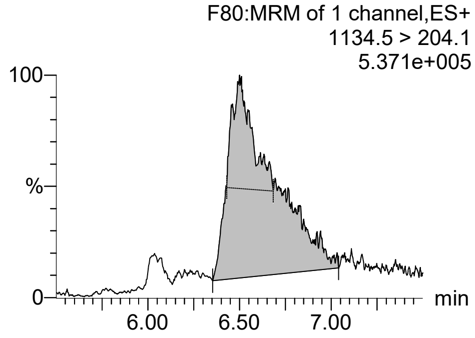

Sample Name: 20211111_L7_R2_IgM

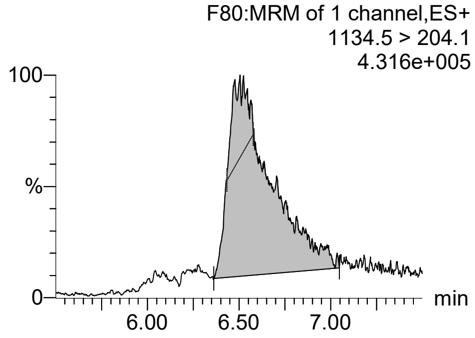

Sample Name: 20211111_L8_R2_IgM

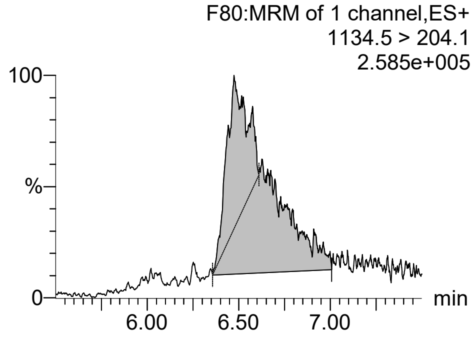

Sample Name: 20211111_H4_R2_IgM

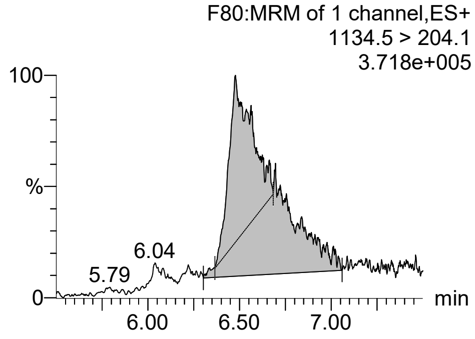

Sample Name: 20211111_A9_R2_IgM

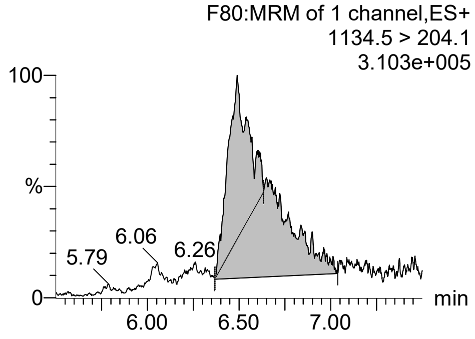

Dataset: C:\MassLynx\10.pro\20211111_clinicalsamples_IgM_IgA.qld

Last Altered: Thursday, March 09, 2023 19:58:25 台北標準時間
Printed: Friday, August 01, 2025 15:25:12 台北標準時間

Sample Name: 20211111_A10_R2_IgM
F80:MRM of 1 channel,ES+
1134.5 > 204.1
9.304e+005

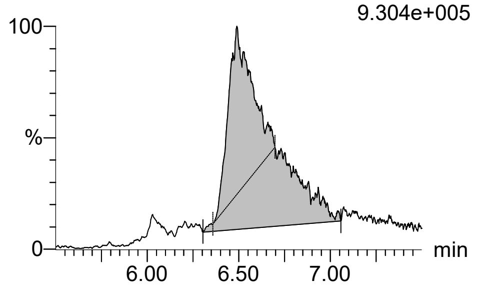

Sample Name: 20211111_L9_R2_IgM
F80:MRM of 1 channel,ES+
1134.5 > 204.1
7.222e+005

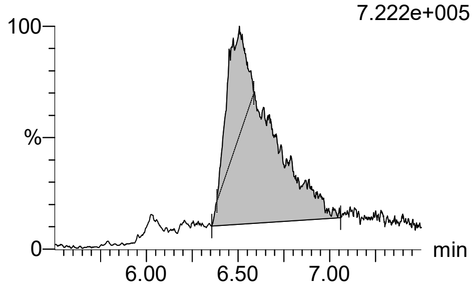

Sample Name: 20211111_L10_R2_IgM
F80:MRM of 1 channel,ES+
1134.5 > 204.1
8.712e+005

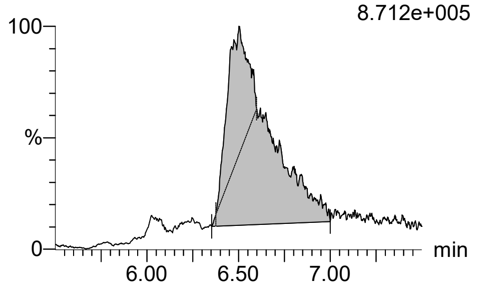

Sample Name: 20211111_QC_R11_IgM
F80:MRM of 1 channel,ES+
1134.5 > 204.1
7.052e+005

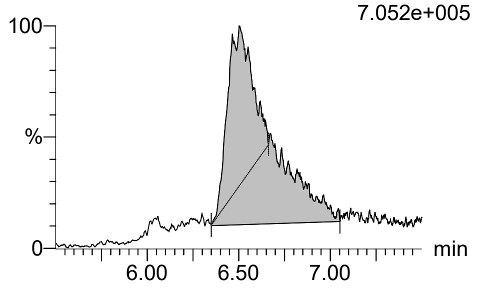

Sample Name: 20211111_H5_R2_IgM
F80:MRM of 1 channel,ES+
1134.5 > 204.1
9.042e+005

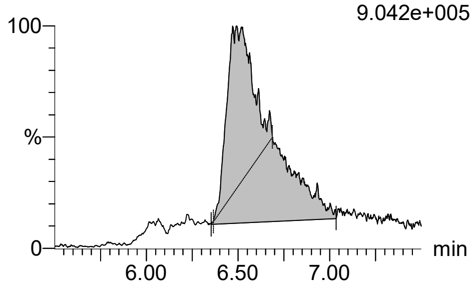

Sample Name: 20211111_A11_R2_IgM
F80:MRM of 1 channel,ES+
1134.5 > 204.1
6.864e+005

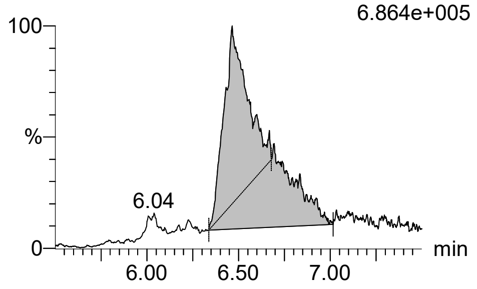

Sample Name: 20211111_A12_R2_IgM
F80:MRM of 1 channel,ES+
1134.5 > 204.1
3.706e+005

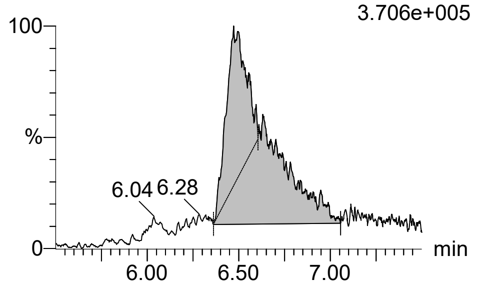

Sample Name: 20211111_L11_R2_IgM
F80:MRM of 1 channel,ES+
1134.5 > 204.1
3.012e+005

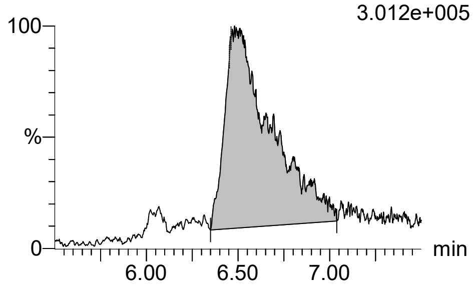

Sample Name: 20211111_L12_R2_IgM
F80:MRM of 1 channel,ES+
1134.5 > 204.1
3.277e+005

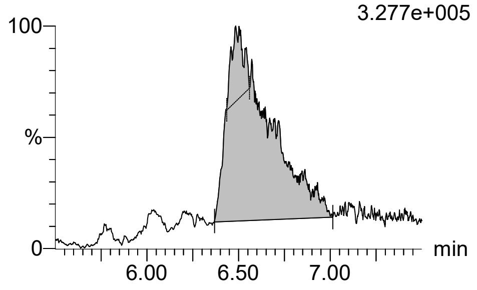

Sample Name: 20211111_H6_R2_IgM
F80:MRM of 1 channel,ES+
1134.5 > 204.1
2.726e+005

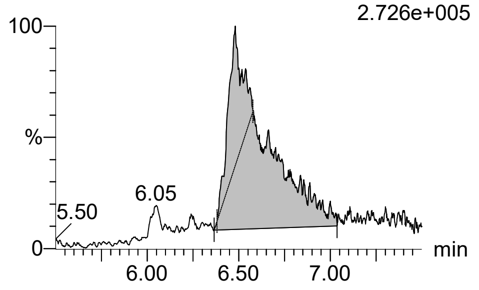

Sample Name: 20211111_A13_R2_IgM
F80:MRM of 1 channel,ES+
1134.5 > 204.1
2.534e+005

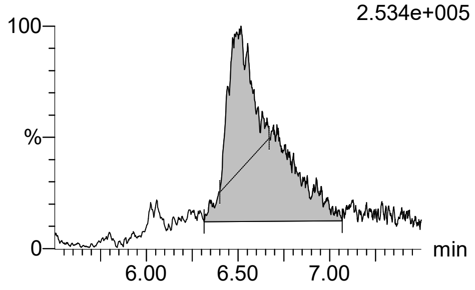

Sample Name: 20211111_A14_R2_IgM
F80:MRM of 1 channel,ES+
1134.5 > 204.1
8.245e+005

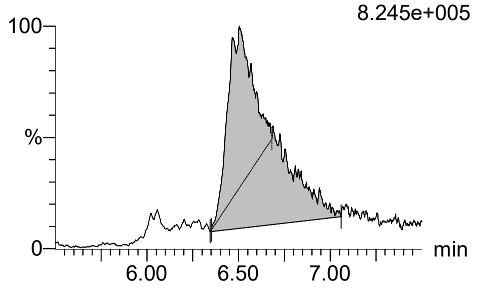

Sample Name: 20211111_L13_R2_IgM
F80:MRM of 1 channel,ES+
1134.5 > 204.1
6.676e+005

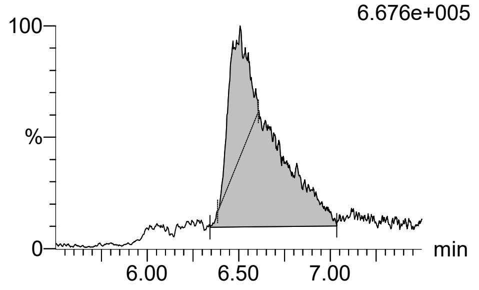

Sample Name: 20211111_L14_R2_IgM
F80:MRM of 1 channel,ES+
1134.5 > 204.1
8.250e+005

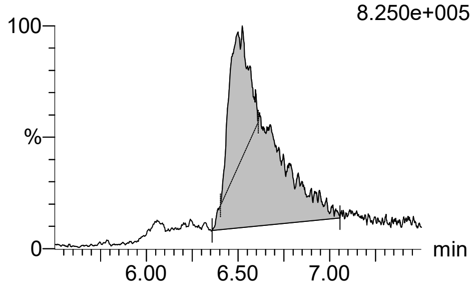

Sample Name: 20211111_H7_R2_IgM
F80:MRM of 1 channel,ES+
1134.5 > 204.1
4.568e+005

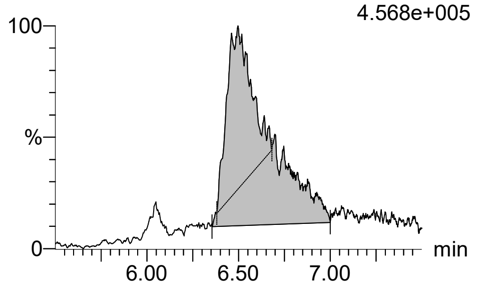

Sample Name: 20211111_A15_R2_IgM
F80:MRM of 1 channel,ES+
1134.5 > 204.1
9.899e+005

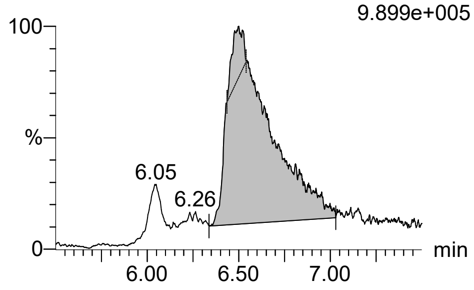

Sample Name: 20211111_QC_R12_IgM
F80:MRM of 1 channel,ES+
1134.5 > 204.1
7.467e+005

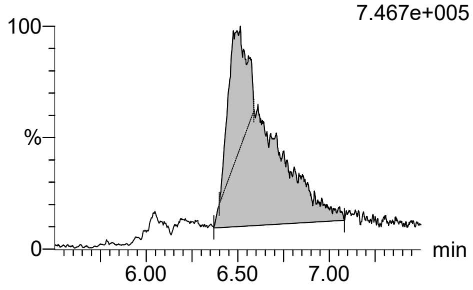

Sample Name: 20211111_A16_R2_IgM
F80:MRM of 1 channel,ES+
1134.5 > 204.1
8.218e+005

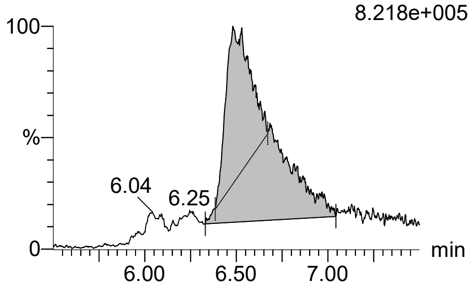

Sample Name: 20211111_L15_R2_IgM
F80:MRM of 1 channel,ES+
1134.5 > 204.1
7.494e+005

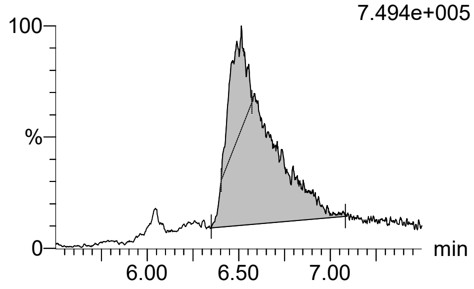

Sample Name: 20211111_L16_R2_IgM
F80:MRM of 1 channel,ES+
1134.5 > 204.1
3.782e+005

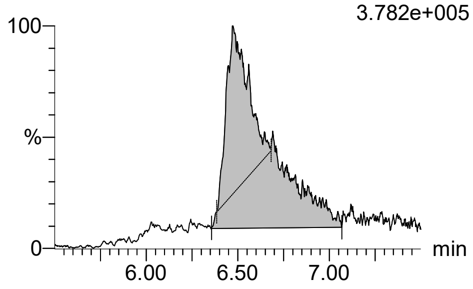

Sample Name: 20211111_H8_R2_IgM
F80:MRM of 1 channel,ES+
1134.5 > 204.1
5.083e+005

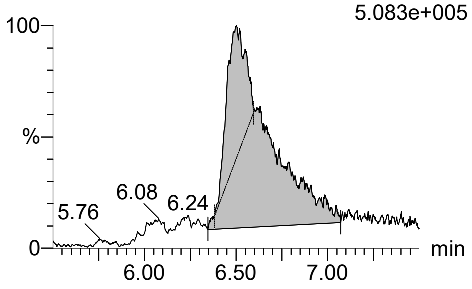

Sample Name: 20211111_A17_R2_IgM
F80:MRM of 1 channel,ES+
1134.5 > 204.1
7.202e+005

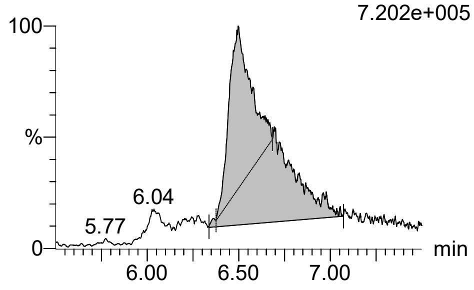

Sample Name: 20211111_A18_R2_IgM
F80:MRM of 1 channel,ES+
1134.5 > 204.1
3.330e+005

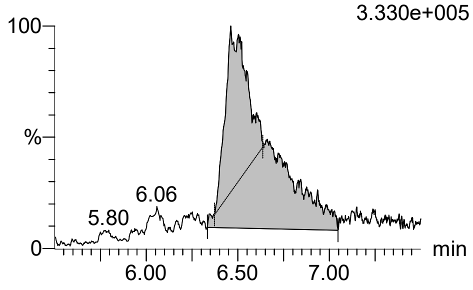

Sample Name: 20211111_L17_R2_IgM
F80:MRM of 1 channel,ES+
1134.5 > 204.1
1.600e+006

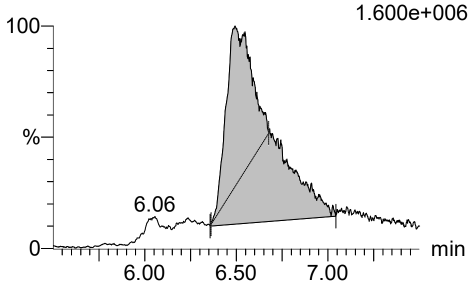

Sample Name: 20211111_L18_R2_IgM
F80:MRM of 1 channel,ES+
1134.5 > 204.1
5.131e+005

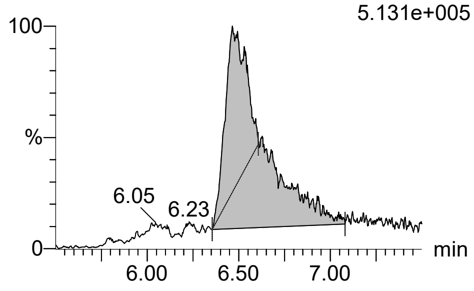

Sample Name: 20211111_H9_R2_IgM
F80:MRM of 1 channel,ES+
1134.5 > 204.1
3.623e+005

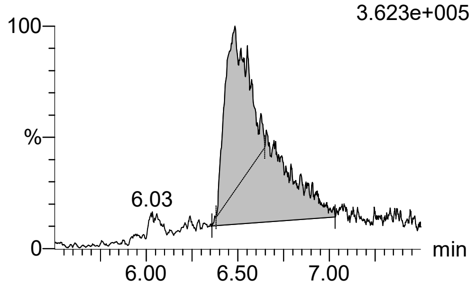

Sample Name: 20211111_A19_R2_IgM
F80:MRM of 1 channel,ES+
1134.5 > 204.1
4.237e+005

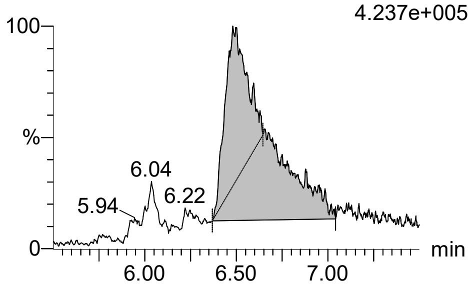

Sample Name: 20211111_A20_R2_IgM
F80:MRM of 1 channel,ES+
1134.5 > 204.1
2.431e+005

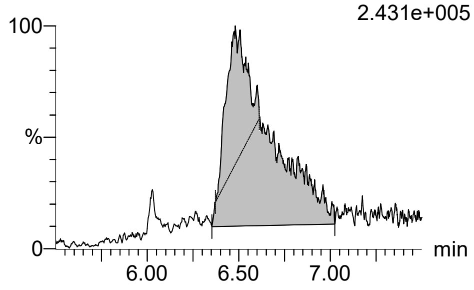

Sample Name: 20211111_L19_R2_IgM
F80:MRM of 1 channel,ES+
1134.5 > 204.1
5.393e+005

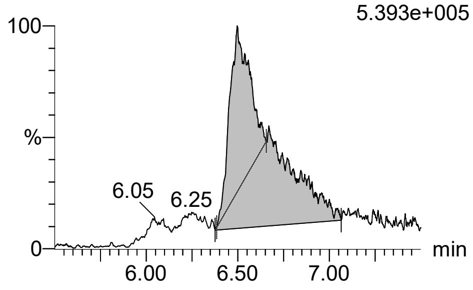

Sample Name: 20211111_QC_R13_IgM
F80:MRM of 1 channel,ES+
1134.5 > 204.1
6.372e+005

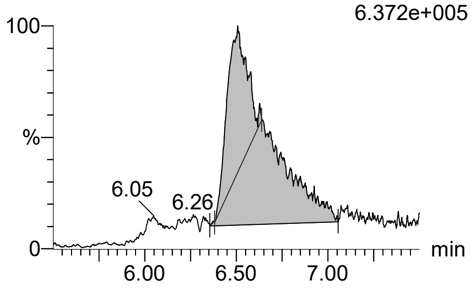

Dataset: C:\MassLynx\10.pro\20211111_clinicalsamples_IgM_IgA.qld

Last Altered: Thursday, March 09, 2023 19:58:25 台北標準時間
Printed: Friday, August 01, 2025 15:25:12 台北標準時間

Sample Name: 20211111_L20_R2_IgM
F80:MRM of 1 channel,ES+
1134.5 > 204.1
4.124e+005

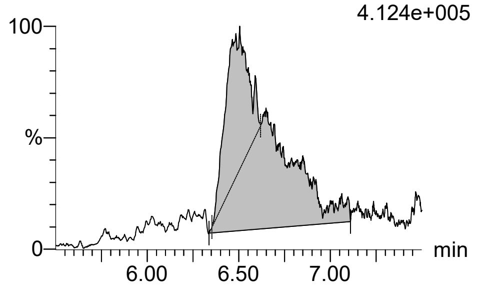

Sample Name: 20211111_H10_R2_IgM
F80:MRM of 1 channel,ES+
1134.5 > 204.1
3.902e+005

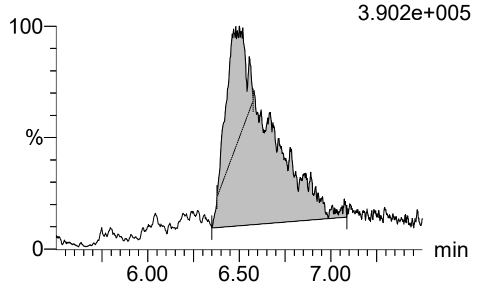

Sample Name: 20211111_A21_R2_IgM
F80:MRM of 1 channel,ES+
1134.5 > 204.1
4.573e+005

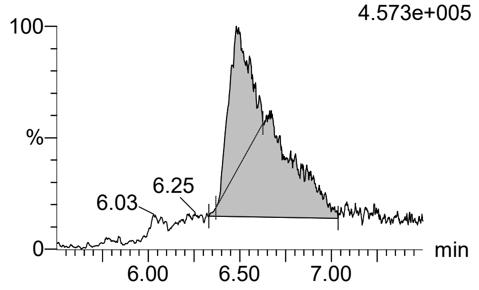

Sample Name: 20211111_A22_R2_IgM
F80:MRM of 1 channel,ES+
1134.5 > 204.1
2.447e+005

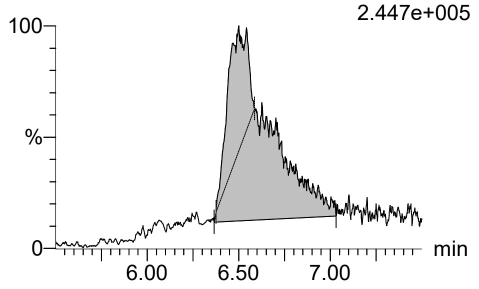

Sample Name: 20211111_L21_R2_IgM
F80:MRM of 1 channel,ES+
1134.5 > 204.1
4.093e+005

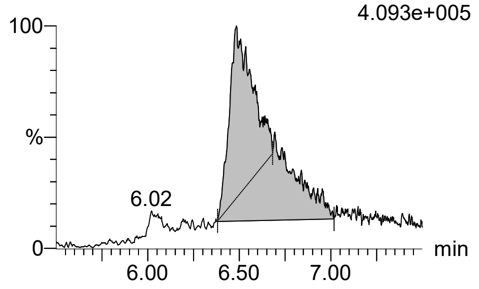

Sample Name: 20211111_L22_R2_IgM
F80:MRM of 1 channel,ES+
1134.5 > 204.1
5.311e+005

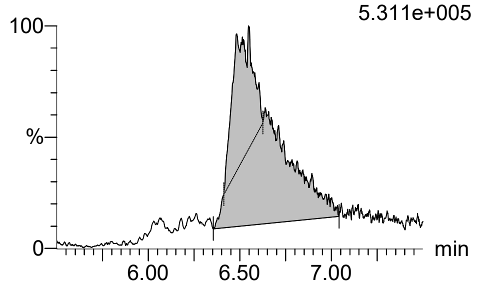

Sample Name: 20211111_H11_R2_IgM
F80:MRM of 1 channel,ES+
1134.5 > 204.1
4.641e+005

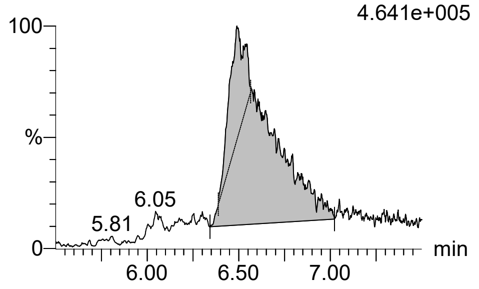

Sample Name: 20211111_A23_R2_IgM
F80:MRM of 1 channel,ES+
1134.5 > 204.1
3.321e+005

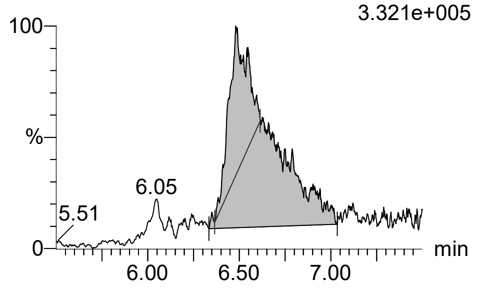

Sample Name: 20211111_A24_R2_IgM
F80:MRM of 1 channel,ES+
1134.5 > 204.1
5.949e+005

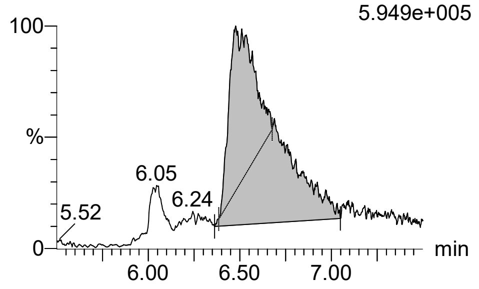

Sample Name: 20211111_L23_R2_IgM
F80:MRM of 1 channel,ES+
1134.5 > 204.1
8.544e+005

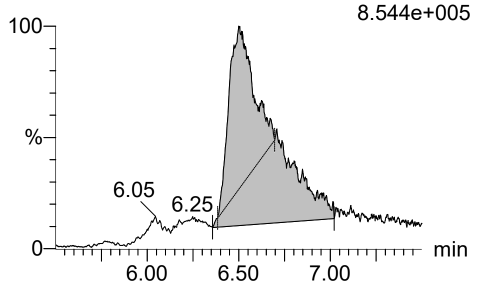

Sample Name: 20211111_L24_R2_IgM
F80:MRM of 1 channel,ES+
1134.5 > 204.1
3.431e+005

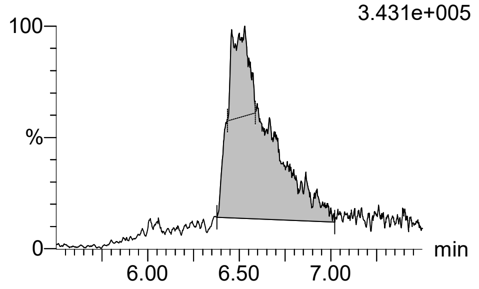

Sample Name: 20211111_H12_R2_IgM
F80:MRM of 1 channel,ES+
1134.5 > 204.1
3.898e+005

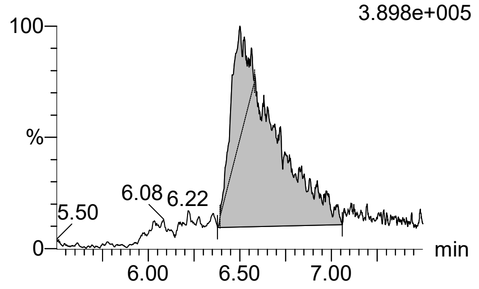

Sample Name: 20211111_QC_R14_IgM
F80:MRM of 1 channel,ES+
1134.5 > 204.1
6.270e+005

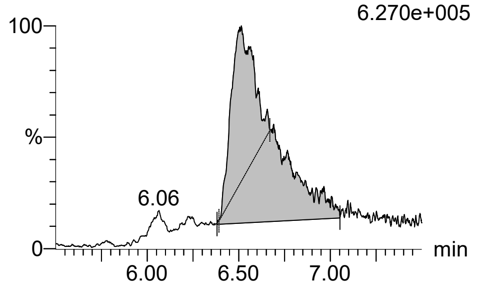

Sample Name: 20211111_A25_R2_IgM
F80:MRM of 1 channel,ES+
1134.5 > 204.1
1.164e+006

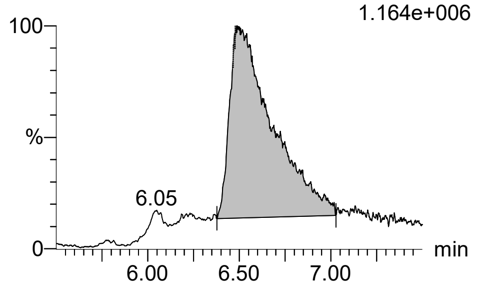

Sample Name: 20211111_A26_R2_IgM
F80:MRM of 1 channel,ES+
1134.5 > 204.1
5.728e+005

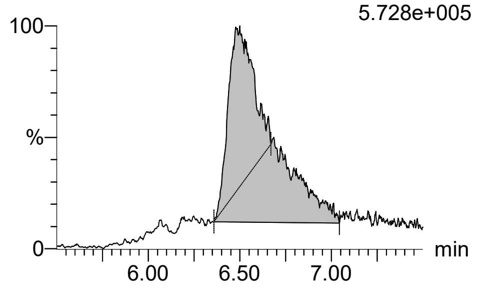

Dataset: C:\MassLynx\10.pro\20211111_clinicalsamples_IgM_IgA.qld

Last Altered: Thursday, March 09, 2023 19:58:25 台北標準時間
Printed: Friday, August 01, 2025 15:25:12 台北標準時間

Sample Name: 20211111_L25_R2_IgM
F80:MRM of 1 channel,ES+
1134.5 > 204.1
7.747e+005

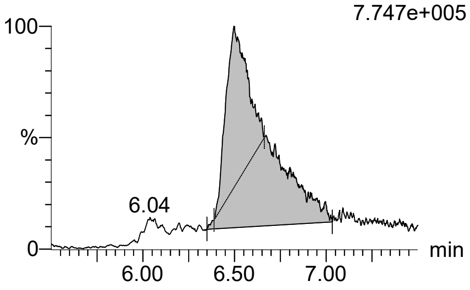

Sample Name: 20211111_L26_R2_IgM
F80:MRM of 1 channel,ES+
1134.5 > 204.1
4.738e+005

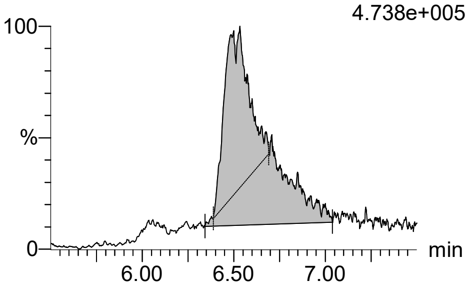

Sample Name: 20211111_H13_R2_IgM
F80:MRM of 1 channel,ES+
1134.5 > 204.1
6.204e+005

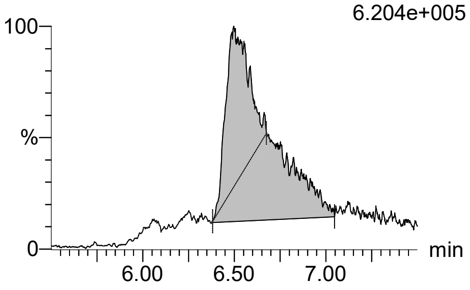

Sample Name: 20211111_A27_R2_IgM
F80:MRM of 1 channel,ES+
1134.5 > 204.1
8.995e+005

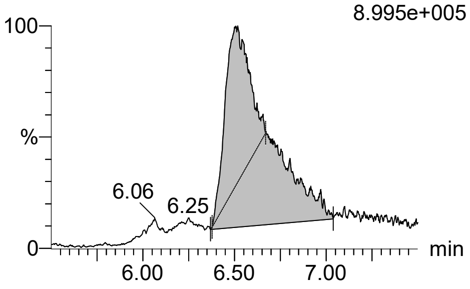

Sample Name: 20211111_A28_R2_IgM
F80:MRM of 1 channel,ES+
1134.5 > 204.1
5.144e+005

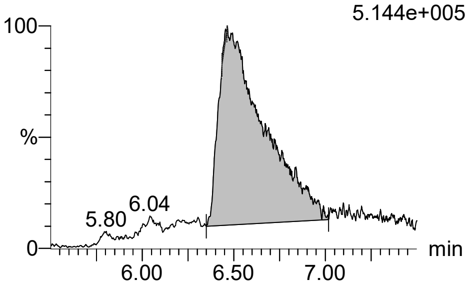

Sample Name: 20211111_L27_R2_IgM
F80:MRM of 1 channel,ES+
1134.5 > 204.1
5.805e+005

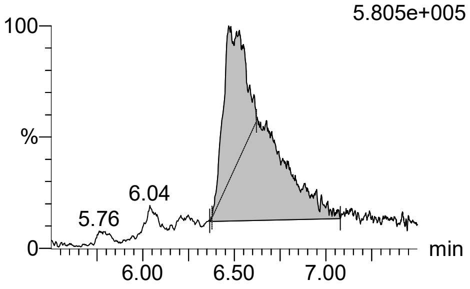

Sample Name: 20211111_L28_R2_IgM
F80:MRM of 1 channel,ES+
1134.5 > 204.1
5.864e+005

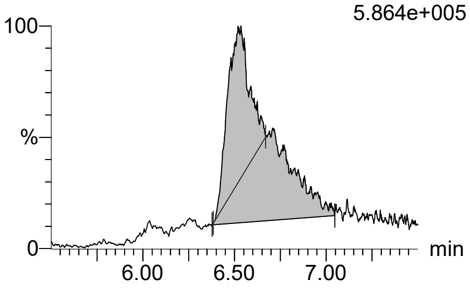

Sample Name: 20211111_H14_R2_IgM
F80:MRM of 1 channel,ES+
1134.5 > 204.1
5.888e+005

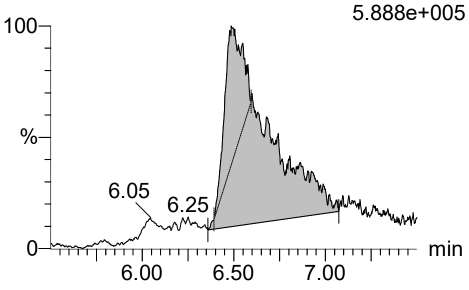

Sample Name: 20211111_A29_R2_IgM
F80:MRM of 1 channel,ES+
1134.5 > 204.1
2.170e+005

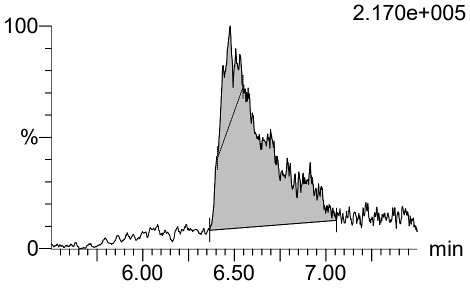

Sample Name: 20211111_A30_R2_IgM
F80:MRM of 1 channel,ES+
1134.5 > 204.1
5.699e+005

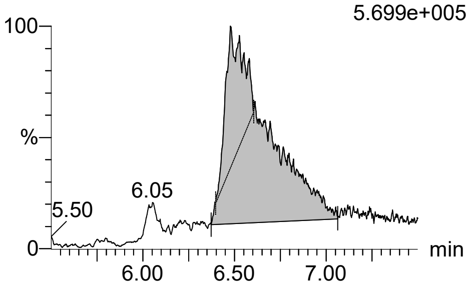

Sample Name: 20211111_QC_R15_IgM
F80:MRM of 1 channel,ES+
1134.5 > 204.1
5.525e+005

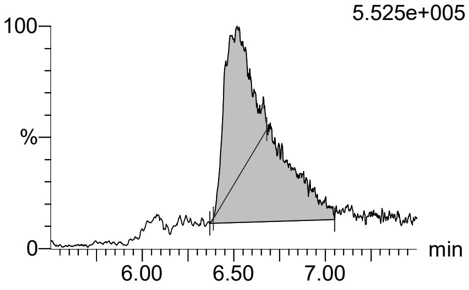

Sample Name: 20211111_L29_R2_IgM
F80:MRM of 1 channel,ES+
1134.5 > 204.1
3.507e+005

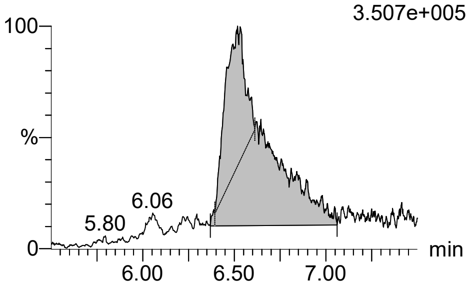

Sample Name: 20211111_L30_R2_IgM
F80:MRM of 1 channel,ES+
1134.5 > 204.1
1.922e+005

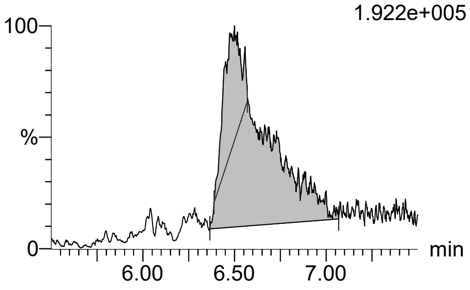

Sample Name: 20211111_H15_R2_IgM
F80:MRM of 1 channel,ES+
1134.5 > 204.1
2.991e+005

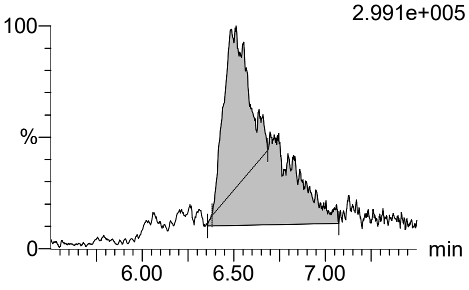

Sample Name: 20211111_A31_R2_IgM
F80:MRM of 1 channel,ES+
1134.5 > 204.1
1.901e+005

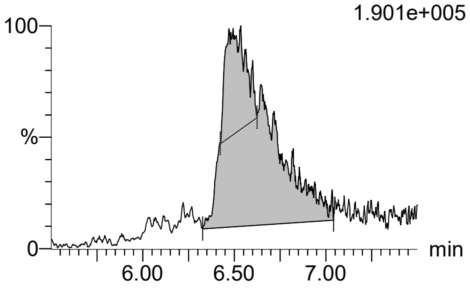

Sample Name: 20211111_A32_R2_IgM
F80:MRM of 1 channel,ES+
1134.5 > 204.1
5.200e+005

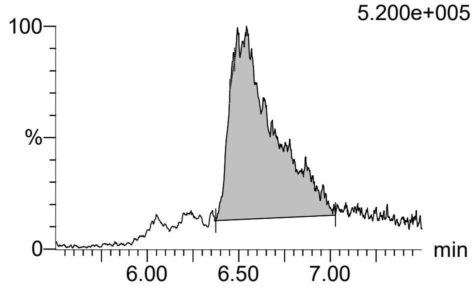

Sample Name: 20211111_L31_R2_IgM
F80:MRM of 1 channel,ES+
1134.5 > 204.1
2.293e+005

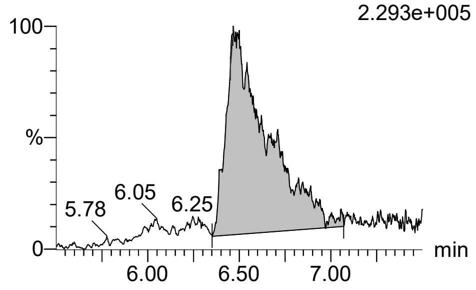

Sample Name: 20211111_L32_R2_IgM
F80:MRM of 1 channel,ES+
1134.5 > 204.1
1.109e+006

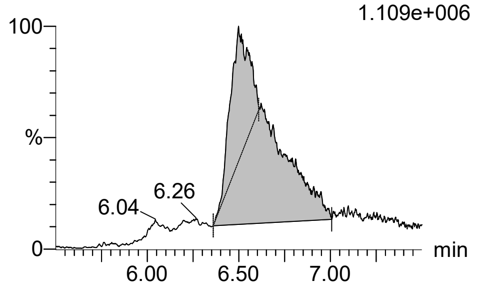

Sample Name: 20211111_H16_R2_IgM
F80:MRM of 1 channel,ES+
1134.5 > 204.1
1.256e+006

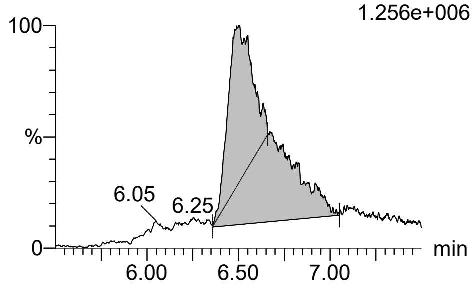

Sample Name: 20211111_A33_R2_IgM
F80:MRM of 1 channel,ES+
1134.5 > 204.1
8.113e+005

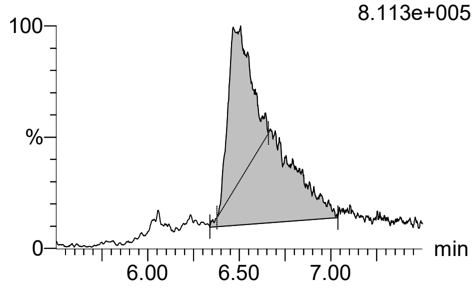

Sample Name: 20211111_A34_R2_IgM
F80:MRM of 1 channel,ES+
1134.5 > 204.1
3.536e+005

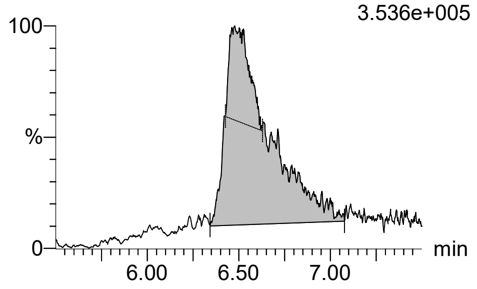

Sample Name: 20211111_H17_R2_IgM
F80:MRM of 1 channel,ES+
1134.5 > 204.1
2.499e+006

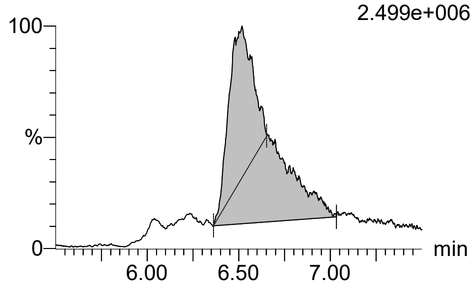

Sample Name: 20211111_A35_R2_IgM
F80:MRM of 1 channel,ES+
1134.5 > 204.1
6.684e+005

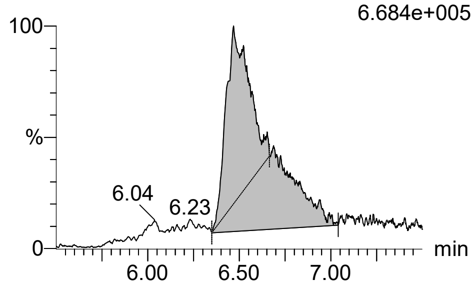

Sample Name: 20211111_QC_R16_IgM
F80:MRM of 1 channel,ES+
1134.5 > 204.1
5.854e+005

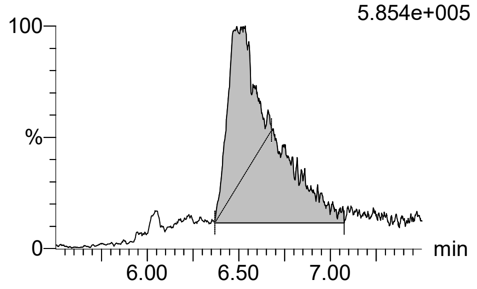

Sample Name: 20211111_A36_R2_IgM
F80:MRM of 1 channel,ES+
1134.5 > 204.1
4.172e+005

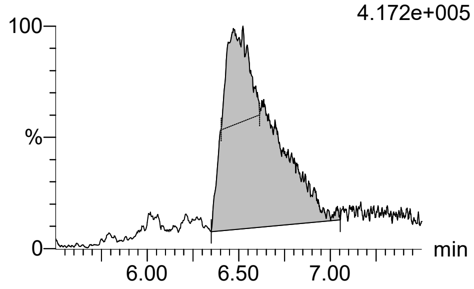

Sample Name: 20211111_H18_R2_IgM
F80:MRM of 1 channel,ES+
1134.5 > 204.1
1.226e+006

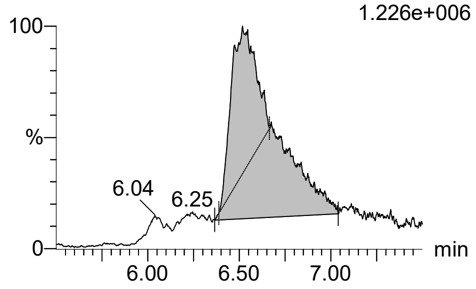

Sample Name: 20211111_A37_R2_IgM
F80:MRM of 1 channel,ES+
1134.5 > 204.1
6.046e+005

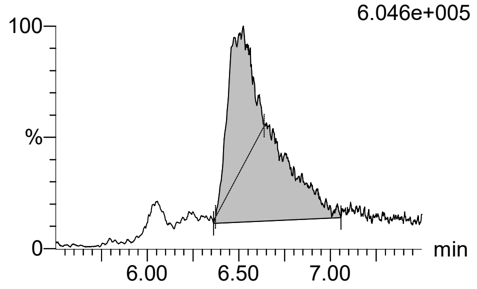

Sample Name: 20211111_A38_R2_IgM
F80:MRM of 1 channel,ES+
1134.5 > 204.1
5.233e+005

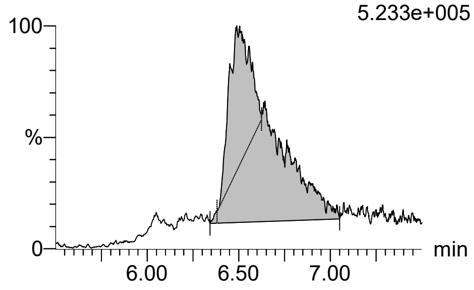

Sample Name: 20211111_H19_R2_IgM
F80:MRM of 1 channel,ES+
1134.5 > 204.1
9.515e+005

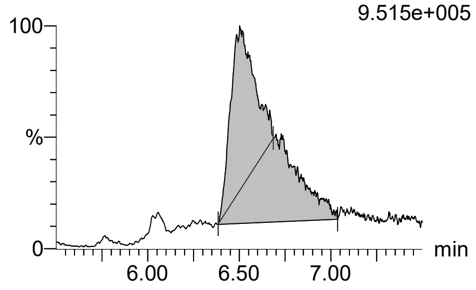

Sample Name: 20211111_A39_R2_IgM
F80:MRM of 1 channel,ES+
1134.5 > 204.1
5.539e+005

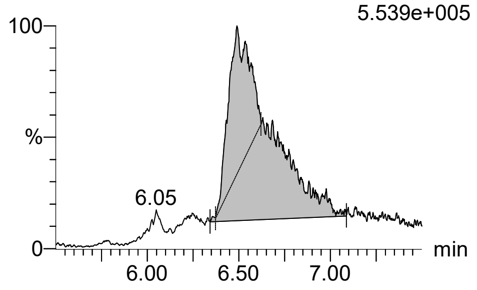

Dataset: C:\MassLynx\10.pro\20211111_clinicalsamples_IgM_IgA.qld

Last Altered: Thursday, March 09, 2023 19:58:25 台北標準時間
Printed: Friday, August 01, 2025 15:25:12 台北標準時間

Sample Name: 20211111_A40_R2_IgM
F80:MRM of 1 channel,ES+
1134.5 > 204.1
3.361e+005

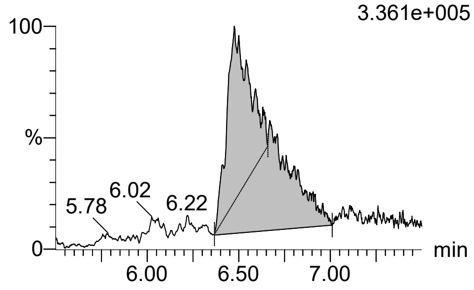

Sample Name: 20211111_H20_R2_IgM
F80:MRM of 1 channel,ES+
1134.5 > 204.1
1.886e+006

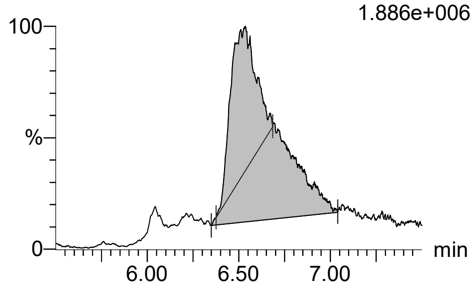

Sample Name: 20211111_A41_R2_IgM
F80:MRM of 1 channel,ES+
1134.5 > 204.1
6.239e+005

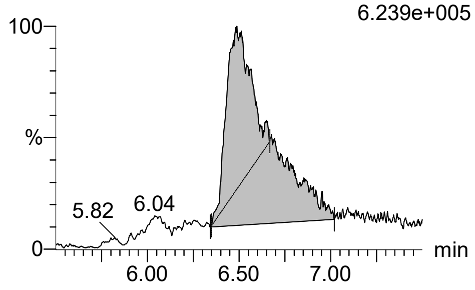

Sample Name: 20211111_A42_R2_IgM
F80:MRM of 1 channel,ES+
1134.5 > 204.1
3.233e+005

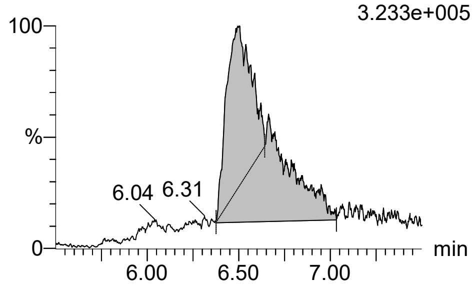

Sample Name: 20211111_A43_R2_IgM
F80:MRM of 1 channel,ES+
1134.5 > 204.1
3.058e+005

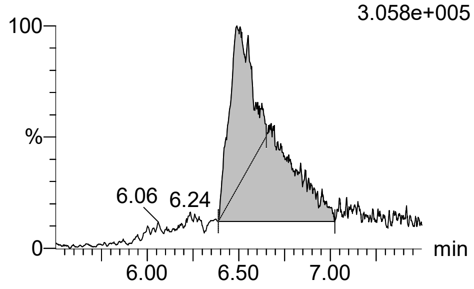

Sample Name: 20211111_A44_R2_IgM
F80:MRM of 1 channel,ES+
1134.5 > 204.1
2.030e+005

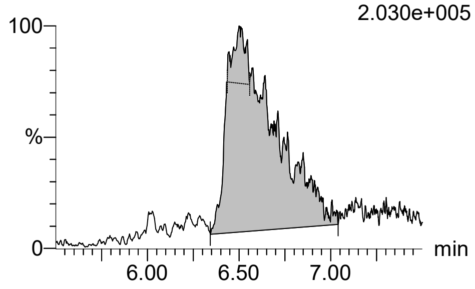

Sample Name: 20211111_QC_R17_IgM
F80:MRM of 1 channel,ES+
1134.5 > 204.1
4.618e+005

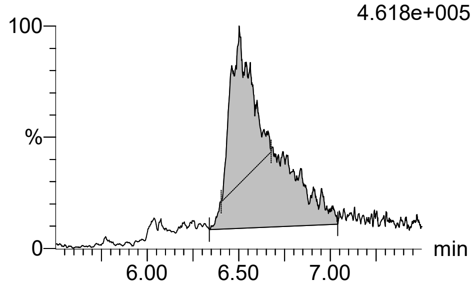

Sample Name: 20211111_L2_R3_IgM
F80:MRM of 1 channel,ES+
1134.5 > 204.1
7.493e+005

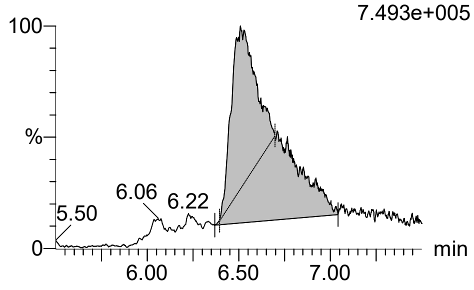

Sample Name: 20211111_H1_R3_IgM
F80:MRM of 1 channel,ES+
1134.5 > 204.1
6.168e+005

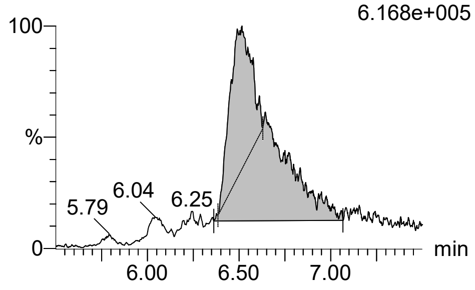

Sample Name: 20211111_A3_R3_IgM
F80:MRM of 1 channel,ES+
1134.5 > 204.1
7.892e+005

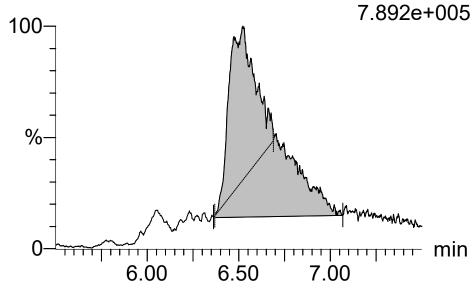

Sample Name: 20211111_A4_R3_IgM
F80:MRM of 1 channel,ES+
1134.5 > 204.1
5.747e+005

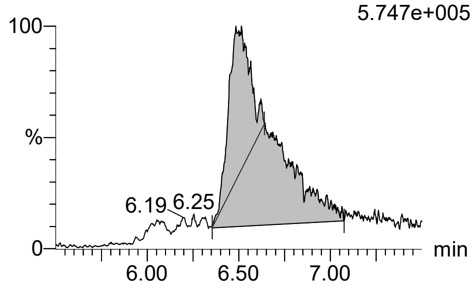

Sample Name: 20211111_L3_R3_IgM
F80:MRM of 1 channel,ES+
1134.5 > 204.1
4.753e+005

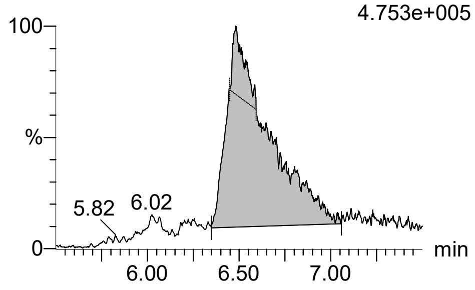

Sample Name: 20211111_L4_R3_IgM
F80:MRM of 1 channel,ES+
1134.5 > 204.1
4.334e+005

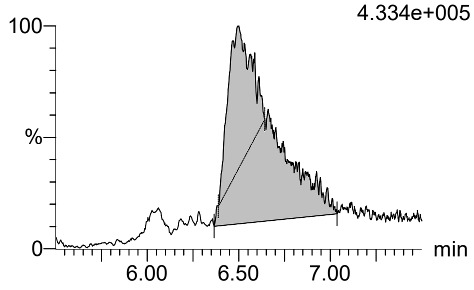

Sample Name: 20211111_H2_R3_IgM
F80:MRM of 1 channel,ES+
1134.5 > 204.1
9.257e+005

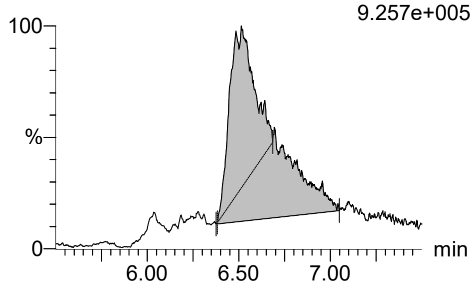

Sample Name: 20211111_A5_R3_IgM
F80:MRM of 1 channel,ES+
1134.5 > 204.1
1.322e+006

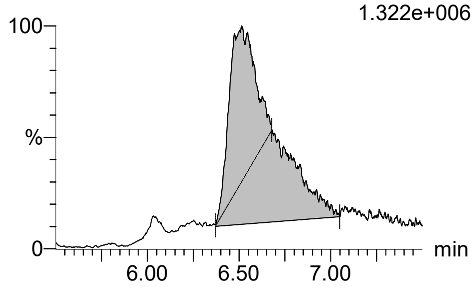

Sample Name: 20211111_A6_R3_IgM
F80:MRM of 1 channel,ES+
1134.5 > 204.1
8.322e+005

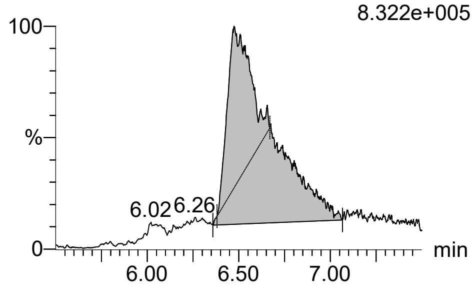

Sample Name: 20211111_QC_R18_IgM
F80:MRM of 1 channel,ES+
1134.5 > 204.1
6.091e+005

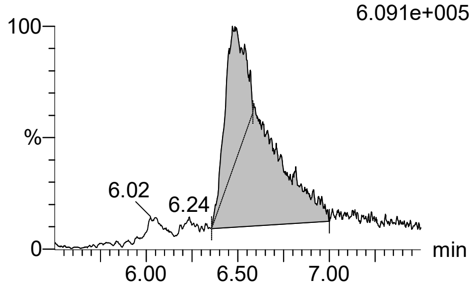

Sample Name: 20211111_L5_R3_IgM
F80:MRM of 1 channel,ES+
1134.5 > 204.1
6.975e+005

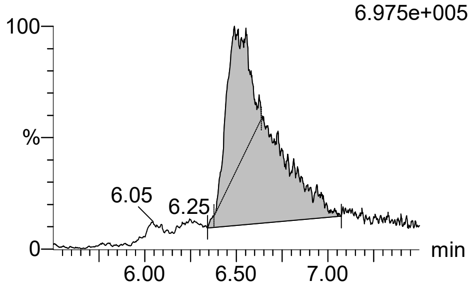

Sample Name: 20211111_L6_R3_IgM
F80:MRM of 1 channel,ES+
1134.5 > 204.1
5.461e+005

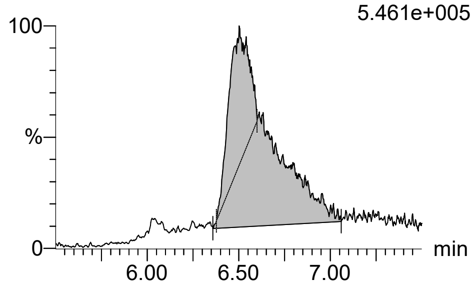

Sample Name: 20211111_H3_R3_IgM
F80:MRM of 1 channel,ES+
1134.5 > 204.1
2.460e+005

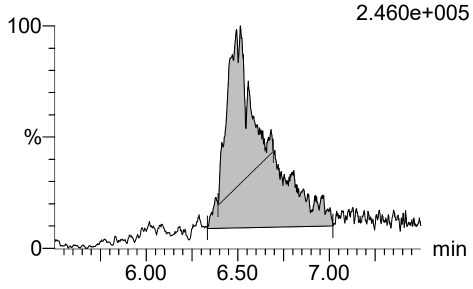

Sample Name: 20211111_A7_R3_IgM
F80:MRM of 1 channel,ES+
1134.5 > 204.1
5.269e+005

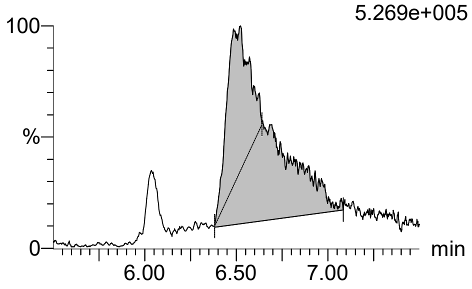

Sample Name: 20211111_A8_R3_IgM
F80:MRM of 1 channel,ES+
1134.5 > 204.1
4.260e+005

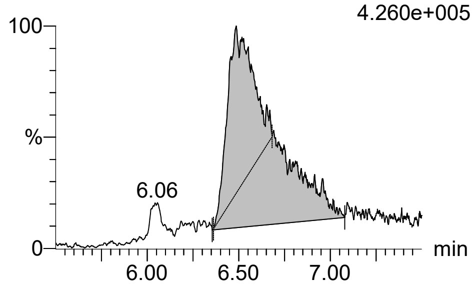

Sample Name: 20211111_L7_R3_IgM
F80:MRM of 1 channel,ES+
1134.5 > 204.1
3.780e+005

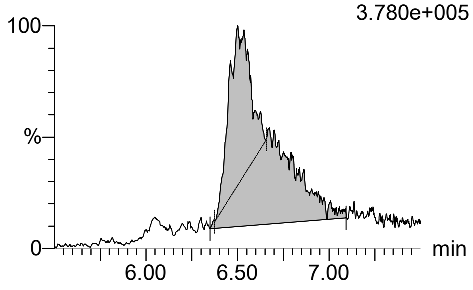

Sample Name: 20211111_L8_R3_IgM
F80:MRM of 1 channel,ES+
1134.5 > 204.1
2.326e+005

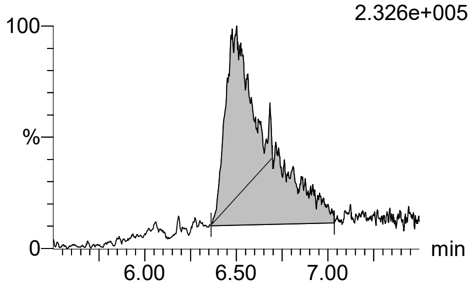

Sample Name: 20211111_H4_R3_IgM
F80:MRM of 1 channel,ES+
1134.5 > 204.1
2.739e+005

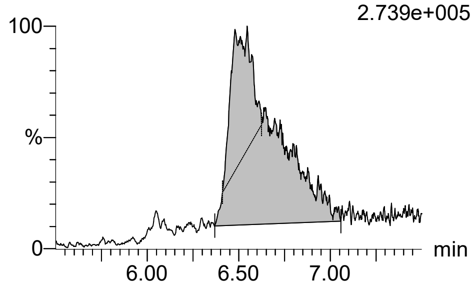

Sample Name: 20211111_A9_R3_IgM
F80:MRM of 1 channel,ES+
1134.5 > 204.1
2.312e+005

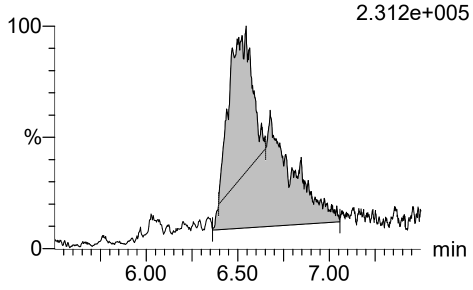

Sample Name: 20211111_A10_R3_IgM
F80:MRM of 1 channel,ES+
1134.5 > 204.1
7.660e+005

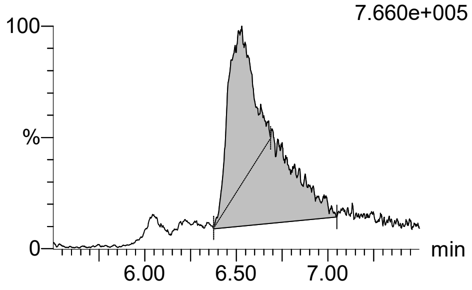

Sample Name: 20211111_L9_R3_IgM
F80:MRM of 1 channel,ES+
1134.5 > 204.1
6.569e+005

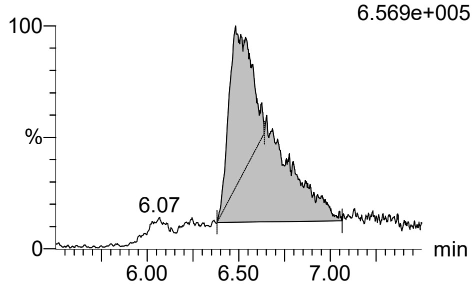

Sample Name: 20211111_L10_R3_IgM
F80:MRM of 1 channel,ES+
1134.5 > 204.1
6.868e+005

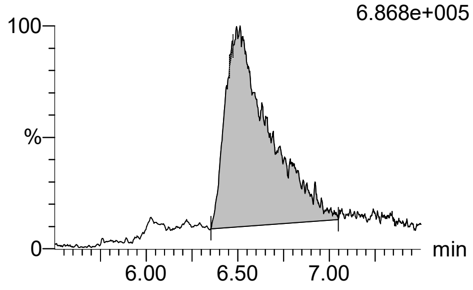

Sample Name: 20211111_QC_R19_IgM
F80:MRM of 1 channel,ES+
1134.5 > 204.1
5.906e+005

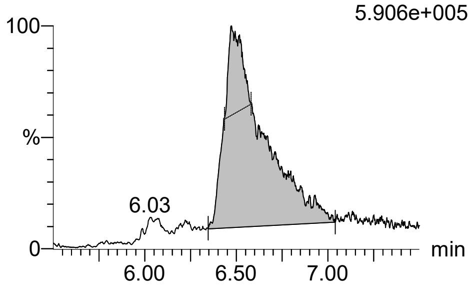

Sample Name: 20211111_H5_R3_IgM
F80:MRM of 1 channel,ES+
1134.5 > 204.1
6.583e+005

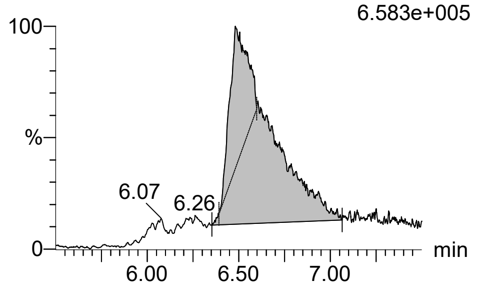

Sample Name: 20211111_A11_R3_IgM
F80:MRM of 1 channel,ES+
1134.5 > 204.1
4.983e+005

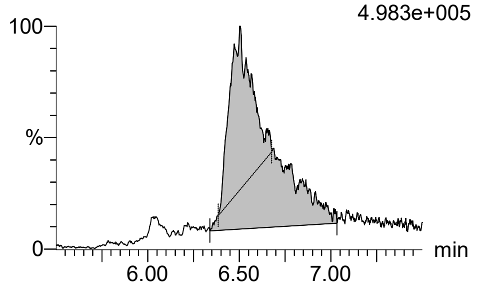

Sample Name: 20211111_A12_R3_IgM
F80:MRM of 1 channel,ES+
1134.5 > 204.1
2.920e+005

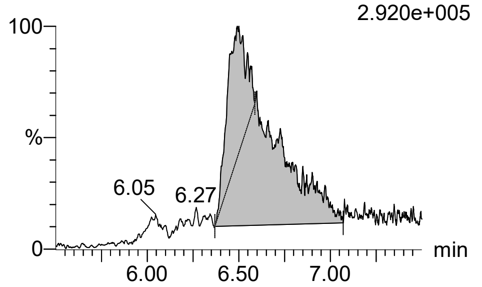

Sample Name: 20211111_L11_R3_IgM
F80:MRM of 1 channel,ES+
1134.5 > 204.1
2.809e+005

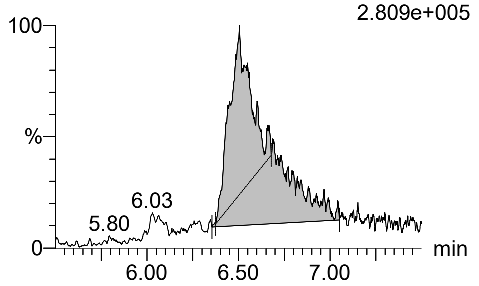

Sample Name: 20211111_L12_R3_IgM
F80:MRM of 1 channel,ES+
1134.5 > 204.1
2.877e+005

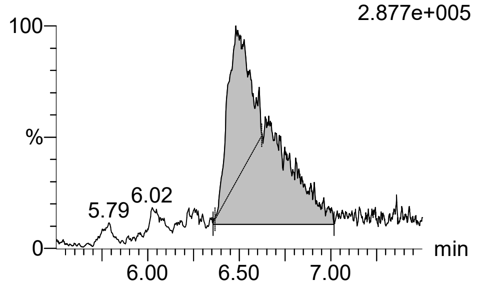

Sample Name: 20211111_H6_R3_IgM
F80:MRM of 1 channel,ES+
1134.5 > 204.1
2.210e+005

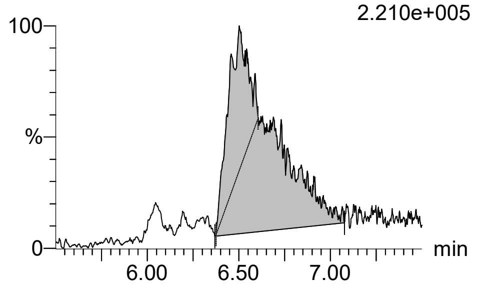

Sample Name: 20211111_A13_R3_IgM
F80:MRM of 1 channel,ES+
1134.5 > 204.1
2.217e+005

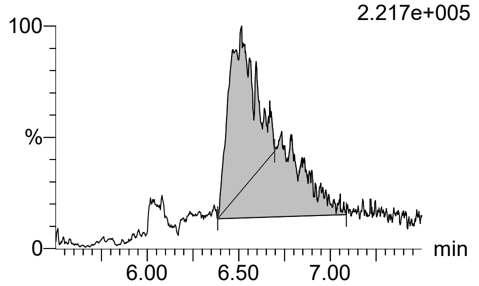

Sample Name: 20211111_A14_R3_IgM
F80:MRM of 1 channel,ES+
1134.5 > 204.1
6.673e+005

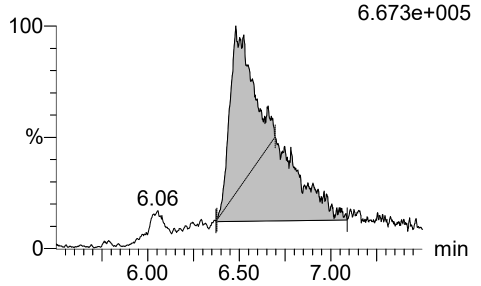

Sample Name: 20211111_L13_R3_IgM
F80:MRM of 1 channel,ES+
1134.5 > 204.1
5.471e+005

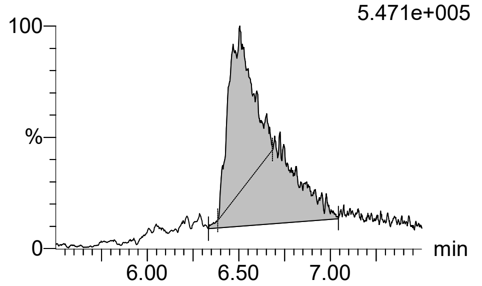

Sample Name: 20211111_L14_R3_IgM
F80:MRM of 1 channel,ES+
1134.5 > 204.1
6.369e+005

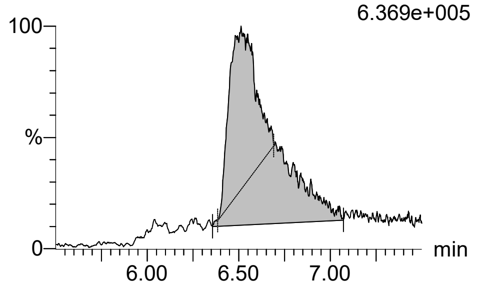

Sample Name: 20211111_H7_R3_IgM
F80:MRM of 1 channel,ES+
1134.5 > 204.1
3.693e+005

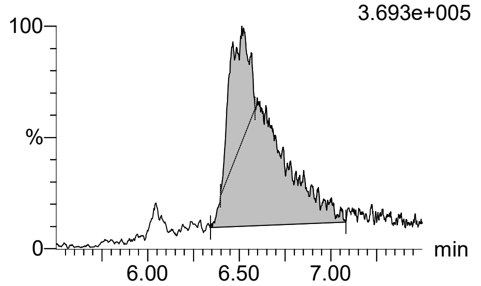

Sample Name: 20211111_A15_R3_IgM
F80:MRM of 1 channel,ES+
1134.5 > 204.1
8.866e+005

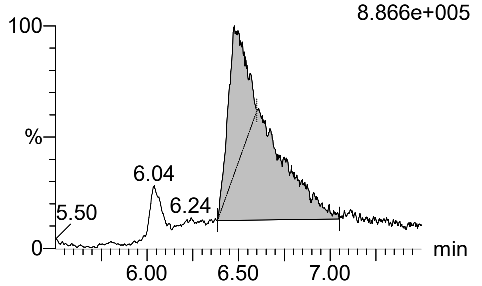

Sample Name: 20211111_QC_R20_IgM
F80:MRM of 1 channel,ES+
1134.5 > 204.1
6.311e+005

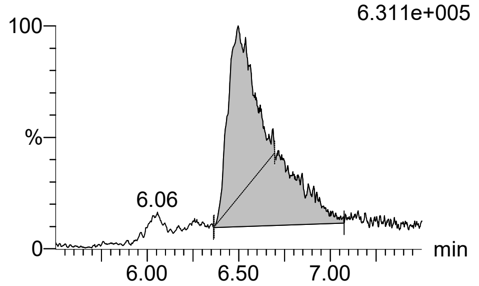

Sample Name: 20211111_A16_R3_IgM
F80:MRM of 1 channel,ES+
1134.5 > 204.1
7.275e+005

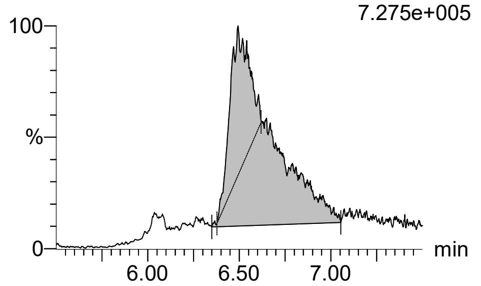

Sample Name: 20211111_L15_R3_IgM
F80:MRM of 1 channel,ES+
1134.5 > 204.1
5.679e+005

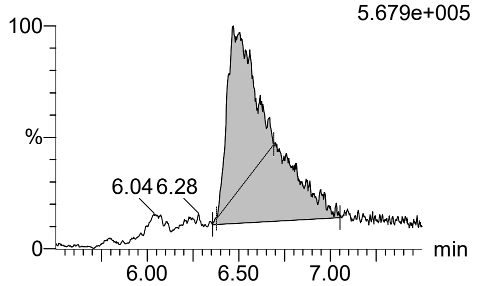

Dataset: C:\MassLynx\10.pro\20211111_clinicalsamples_IgM_IgA.qld

Last Altered: Thursday, March 09, 2023 19:58:25 台北標準時間
Printed: Friday, August 01, 2025 15:25:12 台北標準時間

Sample Name: 20211111_L16_R3_IgM
F80:MRM of 1 channel,ES+
1134.5 > 204.1
2.900e+005

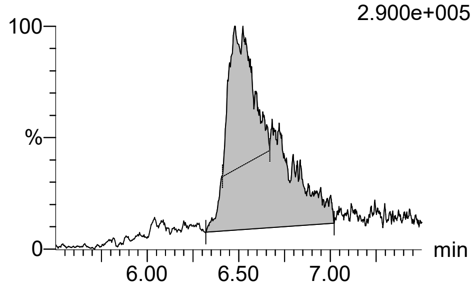

Sample Name: 20211111_H8_R3_IgM
F80:MRM of 1 channel,ES+
1134.5 > 204.1
4.163e+005

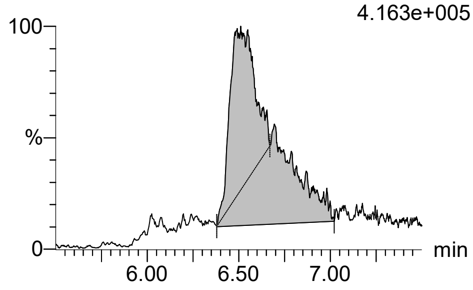

Sample Name: 20211111_A17_R3_IgM
F80:MRM of 1 channel,ES+
1134.5 > 204.1
5.631e+005

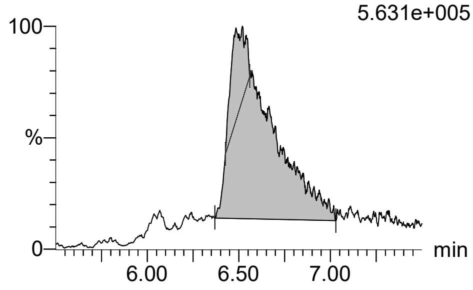

Sample Name: 20211111_A18_R3_IgM
F80:MRM of 1 channel,ES+
1134.5 > 204.1
2.761e+005

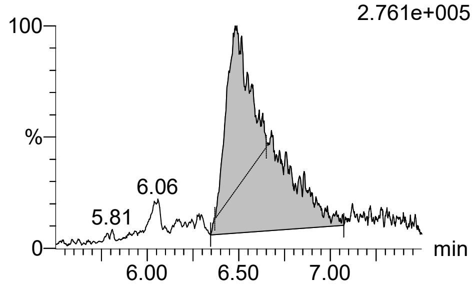

Sample Name: 20211111_L17_R3_IgM
F80:MRM of 1 channel,ES+
1134.5 > 204.1
1.382e+006

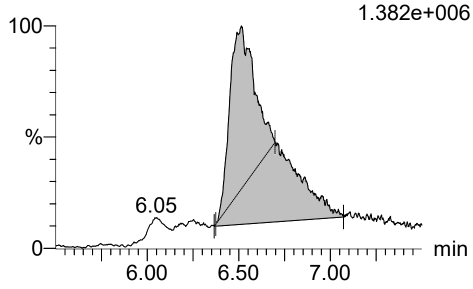

Sample Name: 20211111_L18_R3_IgM
F80:MRM of 1 channel,ES+
1134.5 > 204.1
4.010e+005

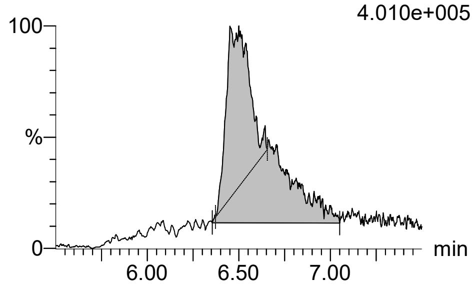

Sample Name: 20211111_H9_R3_IgM
F80:MRM of 1 channel,ES+
1134.5 > 204.1
2.929e+005

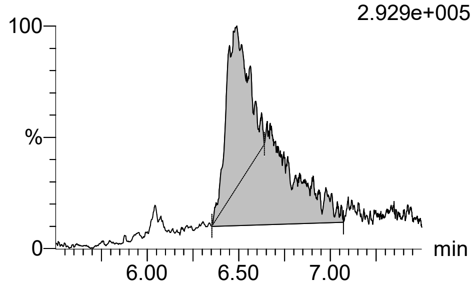

Sample Name: 20211111_A19_R3_IgM
F80:MRM of 1 channel,ES+
1134.5 > 204.1
3.619e+005

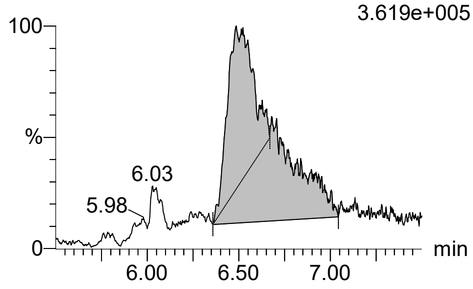

Sample Name: 20211111_A20_R3_IgM
F80:MRM of 1 channel,ES+
1134.5 > 204.1
2.190e+005

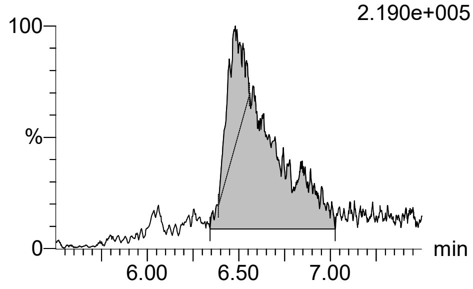

Sample Name: 20211111_L19_R3_IgM
F80:MRM of 1 channel,ES+
1134.5 > 204.1
5.122e+005

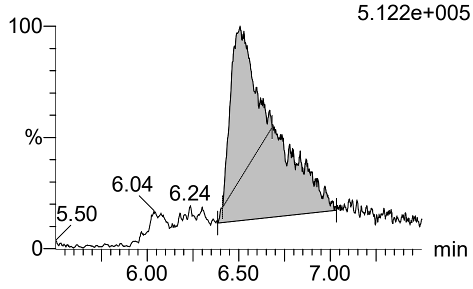

Sample Name: 20211111_QC_R21_IgM
F80:MRM of 1 channel,ES+
1134.5 > 204.1
5.925e+005

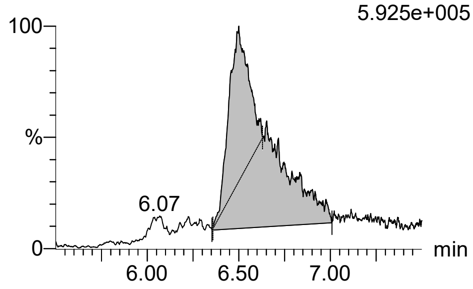

Sample Name: 20211111_L20_R3_IgM
F80:MRM of 1 channel,ES+
1134.5 > 204.1
3.327e+005

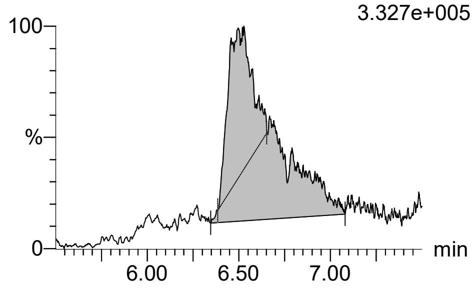

Sample Name: 20211111_H10_R3_IgM
F80:MRM of 1 channel,ES+
1134.5 > 204.1
3.312e+005

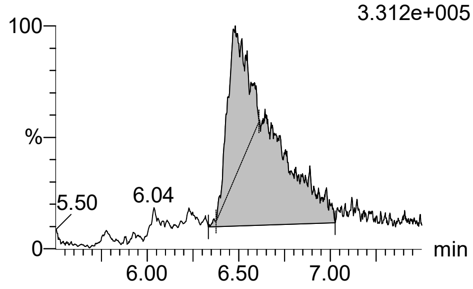

Sample Name: 20211111_A21_R3_IgM
F80:MRM of 1 channel,ES+
1134.5 > 204.1
3.993e+005

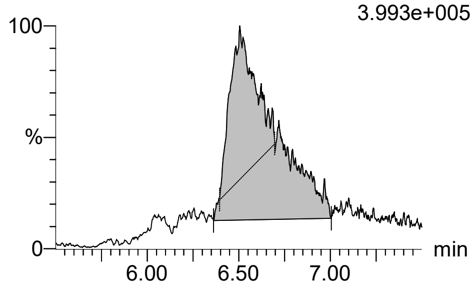

Sample Name: 20211111_A22_R3_IgM
F80:MRM of 1 channel,ES+
1134.5 > 204.1
2.252e+005

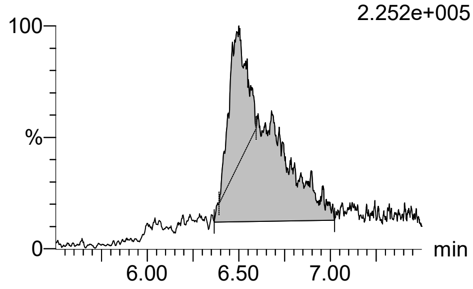

Sample Name: 20211111_L21_R3_IgM
F80:MRM of 1 channel,ES+
1134.5 > 204.1
3.305e+005

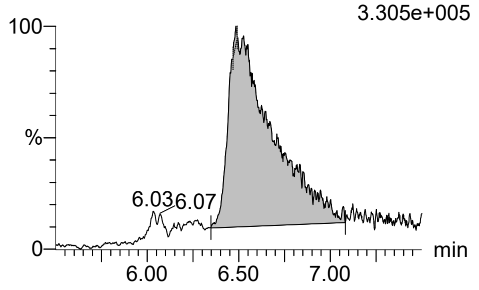

Sample Name: 20211111_L22_R3_IgM
F80:MRM of 1 channel,ES+
1134.5 > 204.1
4.506e+005

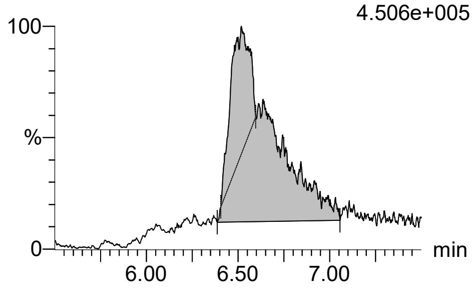

Sample Name: 20211111_H11_R3_IgM
F80:MRM of 1 channel,ES+
1134.5 > 204.1
3.880e+005

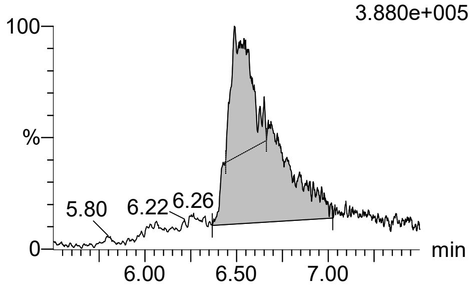

Sample Name: 20211111_A23_R3_IgM
F80:MRM of 1 channel,ES+
1134.5 > 204.1
2.907e+005

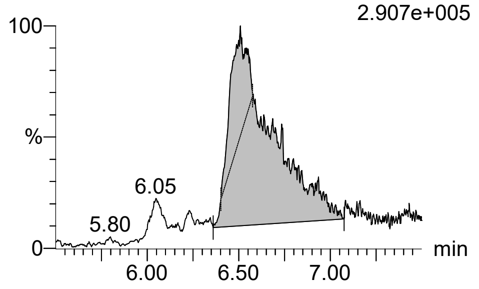

Sample Name: 20211111_A24_R3_IgM
F80:MRM of 1 channel,ES+
1134.5 > 204.1
6.047e+005

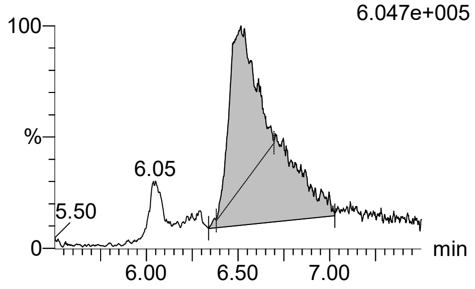

Sample Name: 20211111_L23_R3_IgM
F80:MRM of 1 channel,ES+
1134.5 > 204.1
7.130e+005

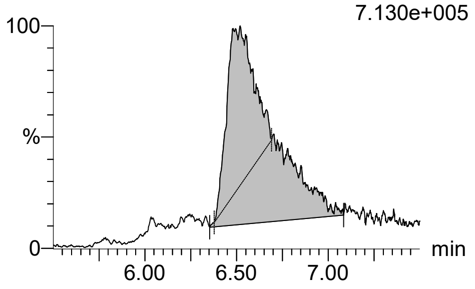

Sample Name: 20211111_L24_R3_IgM
F80:MRM of 1 channel,ES+
1134.5 > 204.1
3.287e+005

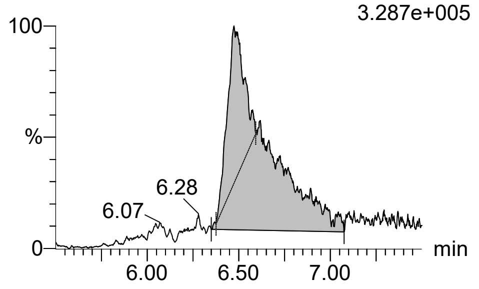

Sample Name: 20211111_H12_R3_IgM
F80:MRM of 1 channel,ES+
1134.5 > 204.1
3.517e+005

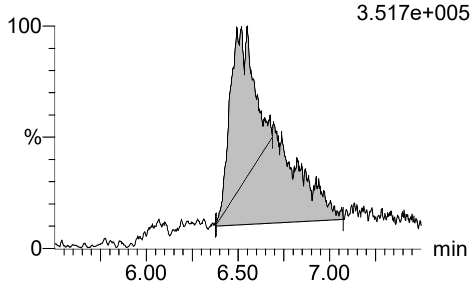

Sample Name: 20211111_QC_R22_IgM
F80:MRM of 1 channel,ES+
1134.5 > 204.1
5.513e+005

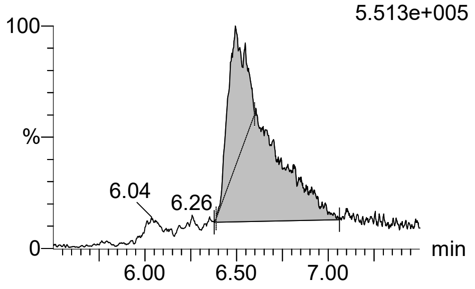

Sample Name: 20211111_A25_R3_IgM
F80:MRM of 1 channel,ES+
1134.5 > 204.1
8.876e+005

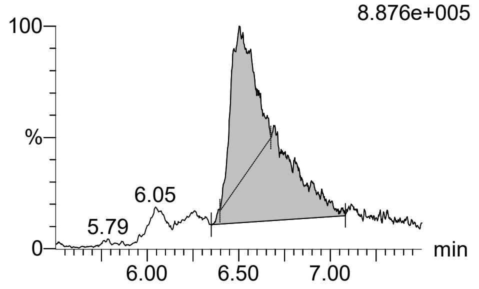

Sample Name: 20211111_A26_R3_IgM
F80:MRM of 1 channel,ES+
1134.5 > 204.1
5.149e+005

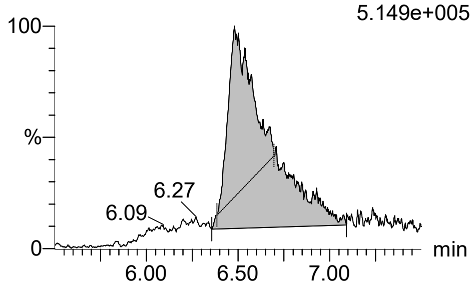

Sample Name: 20211111_L25_R3_IgM
F80:MRM of 1 channel,ES+
1134.5 > 204.1
6.439e+005

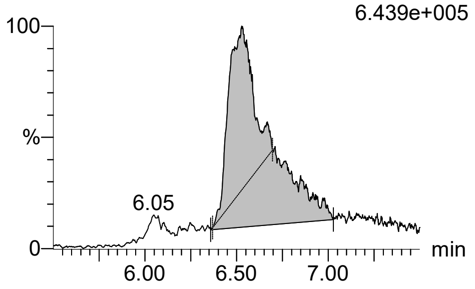

Sample Name: 20211111_L26_R3_IgM
F80:MRM of 1 channel,ES+
1134.5 > 204.1
3.964e+005

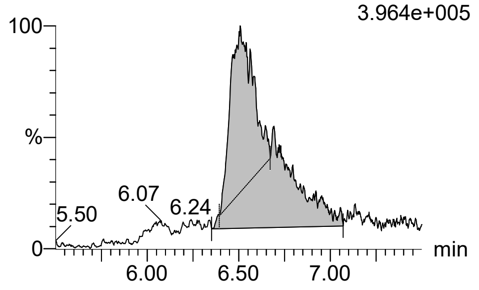

Sample Name: 20211111_H13_R3_IgM
F80:MRM of 1 channel,ES+
1134.5 > 204.1
5.955e+005

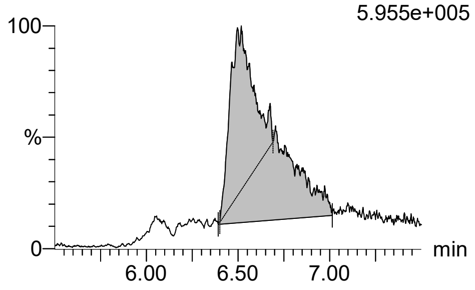

Sample Name: 20211111_A27_R3_IgM
F80:MRM of 1 channel,ES+
1134.5 > 204.1
7.565e+005

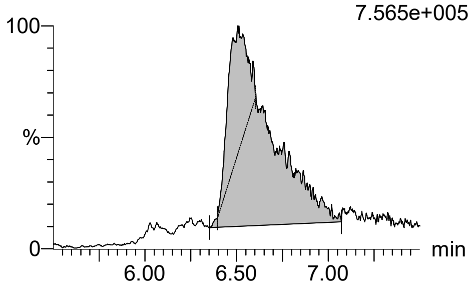

Sample Name: 20211111_A28_R3_IgM
F80:MRM of 1 channel,ES+
1134.5 > 204.1
4.548e+005

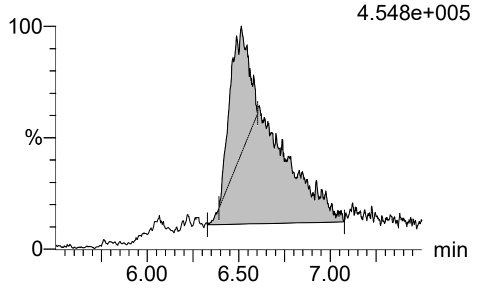

Sample Name: 20211111_L27_R3_IgM
F80:MRM of 1 channel,ES+
1134.5 > 204.1
5.513e+005

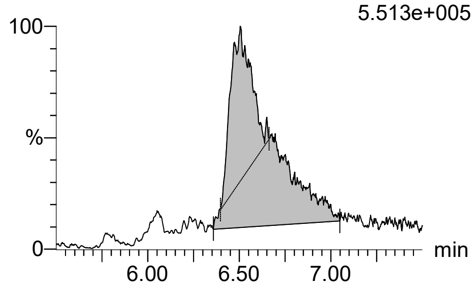

Sample Name: 20211111_L28_R3_IgM
F80:MRM of 1 channel,ES+
1134.5 > 204.1
5.665e+005

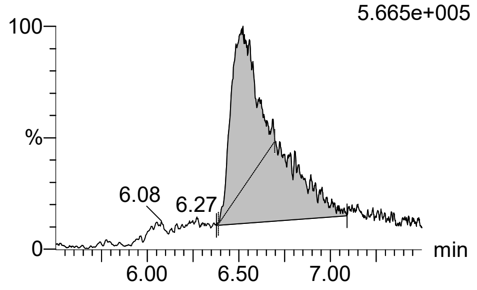

Sample Name: 20211111_H14_R3_IgM
F80:MRM of 1 channel,ES+
1134.5 > 204.1
5.702e+005

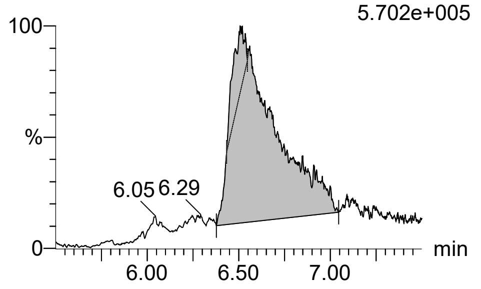

Sample Name: 20211111_A29_R3_IgM
F80:MRM of 1 channel,ES+
1134.5 > 204.1
2.051e+005

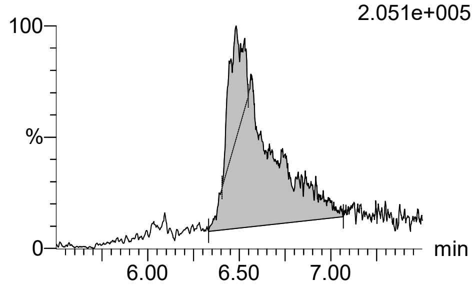

Sample Name: 20211111_A30_R3_IgM
F80:MRM of 1 channel,ES+
1134.5 > 204.1
4.773e+005

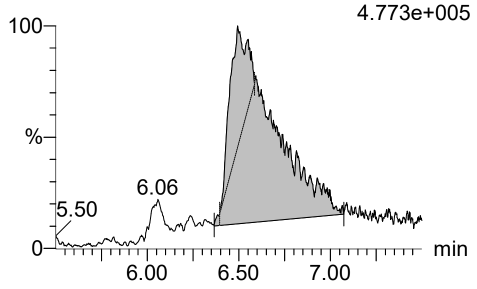

Sample Name: 20211111_QC_R23_IgM
F80:MRM of 1 channel,ES+
1134.5 > 204.1
5.055e+005

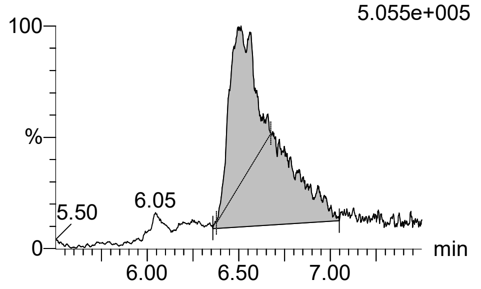

Sample Name: 20211111_L29_R3_IgM
F80:MRM of 1 channel,ES+
1134.5 > 204.1
3.170e+005

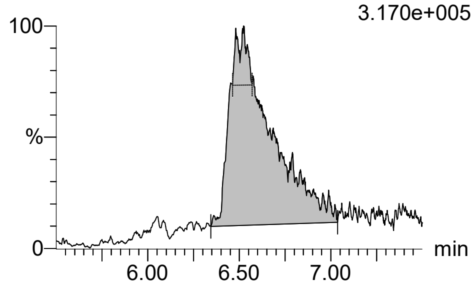

Sample Name: 20211111_L30_R3_IgM
F80:MRM of 1 channel,ES+
1134.5 > 204.1
1.711e+005

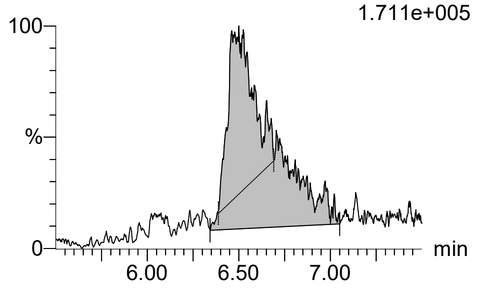

Sample Name: 20211111_H15_R3_IgM
F80:MRM of 1 channel,ES+
1134.5 > 204.1
2.639e+005

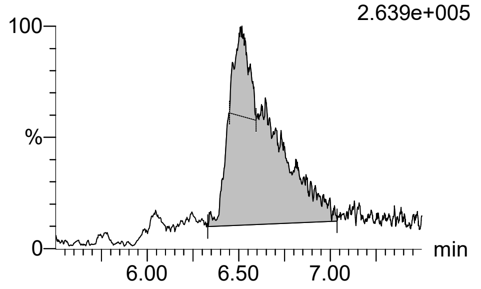

Sample Name: 20211111_A31_R3_IgM
F80:MRM of 1 channel,ES+
1134.5 > 204.1
1.822e+005

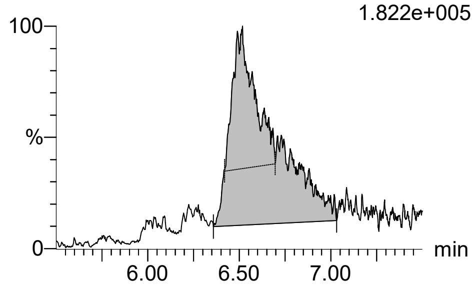

Sample Name: 20211111_A32_R3_IgM
F80:MRM of 1 channel,ES+
1134.5 > 204.1
4.458e+005

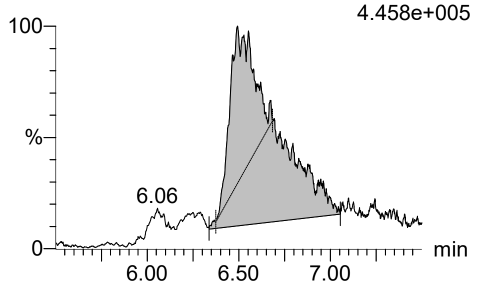

Sample Name: 20211111_L31_R3_IgM
F80:MRM of 1 channel,ES+
1134.5 > 204.1
1.929e+005

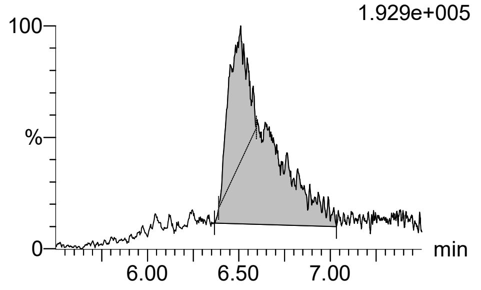

Sample Name: 20211111_L32_R3_IgM
F80:MRM of 1 channel,ES+
1134.5 > 204.1
9.782e+005

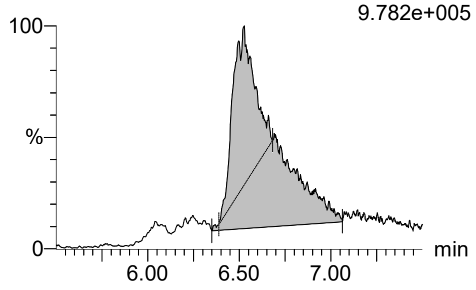

Sample Name: 20211111_H16_R3_IgM
F80:MRM of 1 channel,ES+
1134.5 > 204.1
1.017e+006

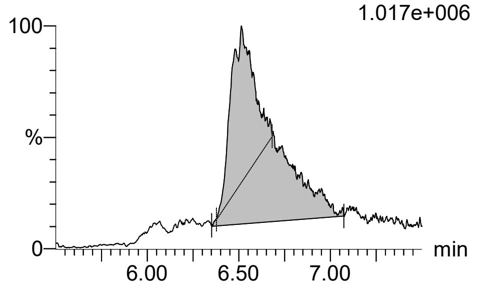

Sample Name: 20211111_A33_R3_IgM
F80:MRM of 1 channel,ES+
1134.5 > 204.1
7.129e+005

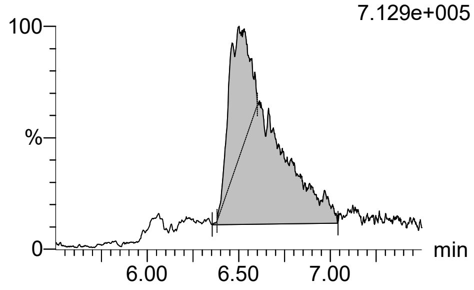

Sample Name: 20211111_A34_R3_IgM
F80:MRM of 1 channel,ES+
1134.5 > 204.1
3.298e+005

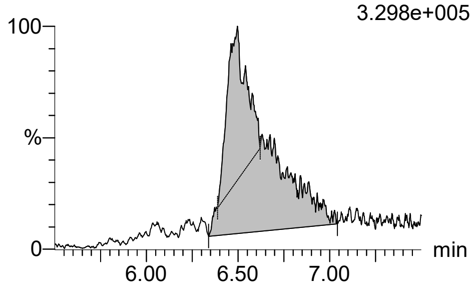

Sample Name: 20211111_H17_R3_IgM
F80:MRM of 1 channel,ES+
1134.5 > 204.1
2.015e+006

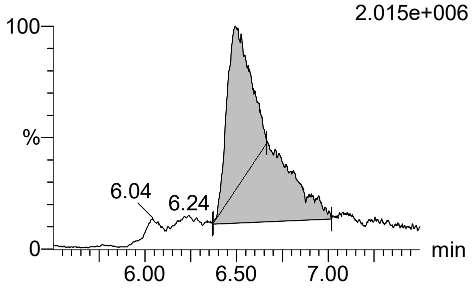

Sample Name: 20211111_A35_R3_IgM
F80:MRM of 1 channel,ES+
1134.5 > 204.1
5.417e+005

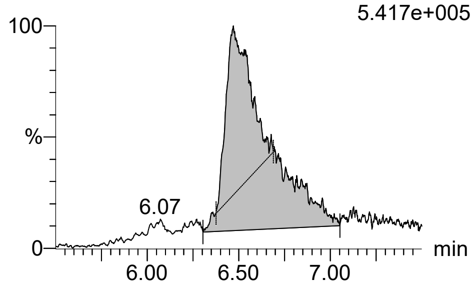

Sample Name: 20211111_QC_R24_IgM
F80:MRM of 1 channel,ES+
1134.5 > 204.1
5.279e+005

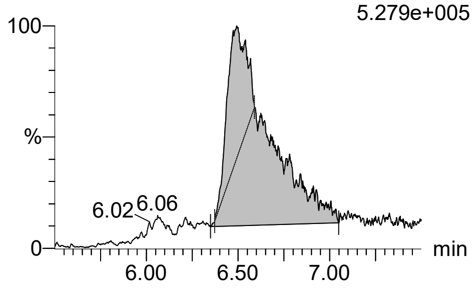

Sample Name: 20211111_A36_R3_IgM
F80:MRM of 1 channel,ES+
1134.5 > 204.1
3.816e+005

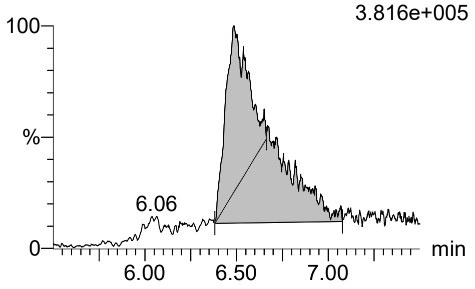

Sample Name: 20211111_H18_R3_IgM
F80:MRM of 1 channel,ES+
1134.5 > 204.1
1.115e+006

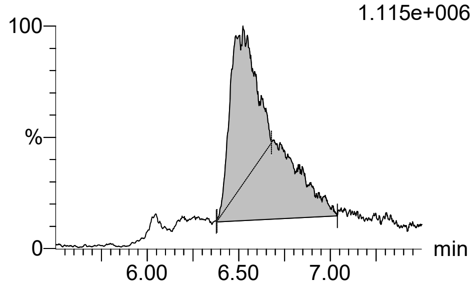

Sample Name: 20211111_A37_R3_IgM
F80:MRM of 1 channel,ES+
1134.5 > 204.1
5.123e+005

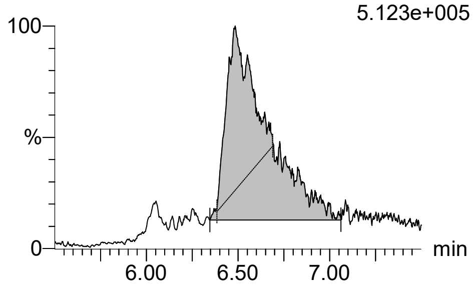

Sample Name: 20211111_A38_R3_IgM
F80:MRM of 1 channel,ES+
1134.5 > 204.1
4.407e+005

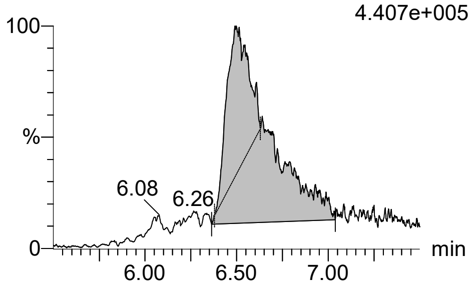

Sample Name: 20211111_H19_R3_IgM
F80:MRM of 1 channel,ES+
1134.5 > 204.1
8.038e+005

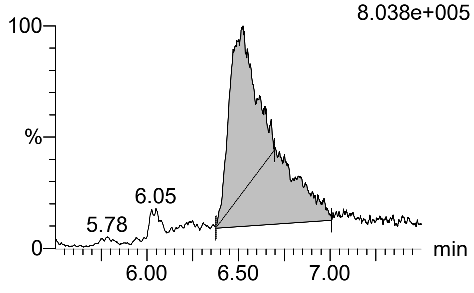

Sample Name: 20211111_A39_R3_IgM
F80:MRM of 1 channel,ES+
1134.5 > 204.1
4.697e+005

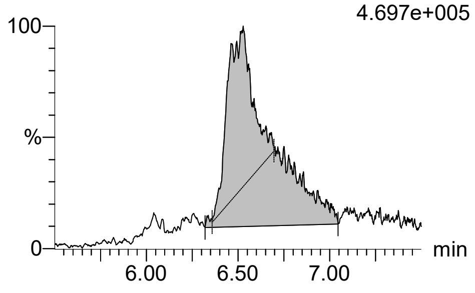

Sample Name: 20211111_A40_R3_IgM
F80:MRM of 1 channel,ES+
1134.5 > 204.1
2.755e+005

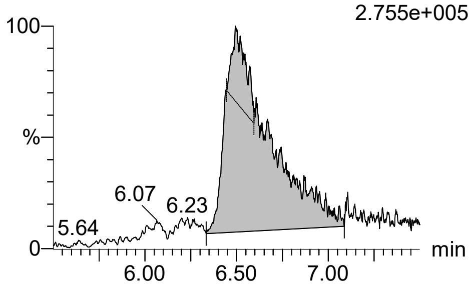

Sample Name: 20211111_H20_R3_IgM
F80:MRM of 1 channel,ES+
1134.5 > 204.1
1.574e+006

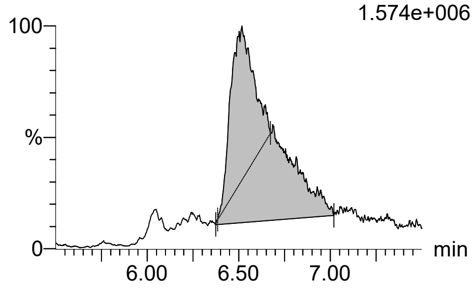

Sample Name: 20211111_A41_R3_IgM
F80:MRM of 1 channel,ES+
1134.5 > 204.1
3.895e+005

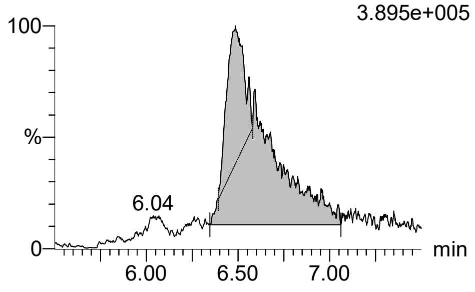

Sample Name: 20211111_A42_R3_IgM
F80:MRM of 1 channel,ES+
1134.5 > 204.1
2.885e+005

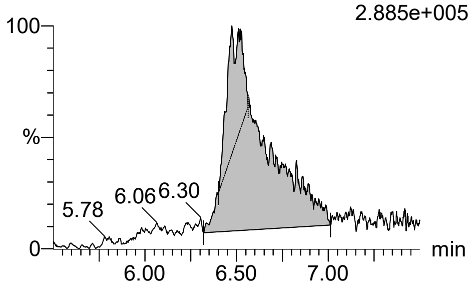

Dataset: C:\MassLynx\10.pro\20211111_clinicalsamples_IgM_IgA.qld

Last Altered: Thursday, March 09, 2023 19:58:25 台北標準時間
Printed: Friday, August 01, 2025 15:25:12 台北標準時間

Sample Name: 20211111_A43_R3_IgM

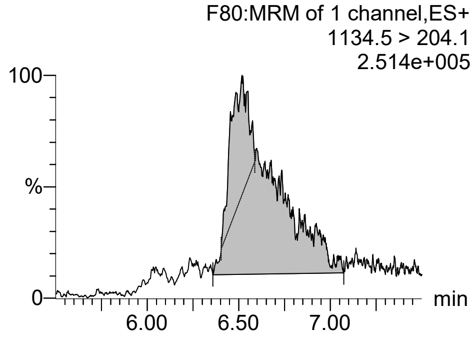

Sample Name: 20211111_A44_R3_IgM

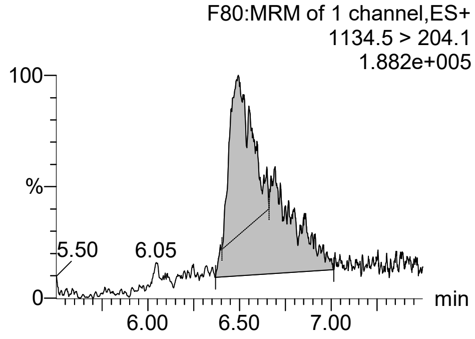

Sample Name: 20211111_QC_R25_IgM

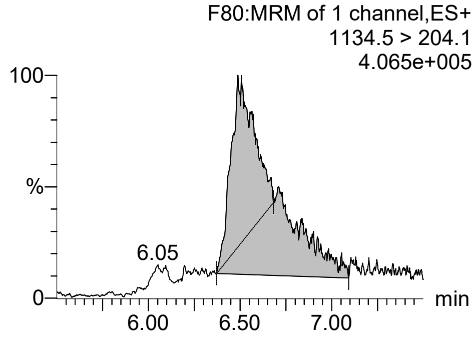

Dataset: C:\MassLynx\10.pro\20211111_clinicalsamples_IgM_IgA.qld

Last Altered: Thursday, March 09, 2023 19:58:25 台北標準時間
Printed: Friday, August 01, 2025 15:25:12 台北標準時間

	#	Name	RT	Area	IS Area	Response	Prima...
1	1	20211112_QC_R001_IgM	6.50	76876.0	15421.4	4.985	bb
2	2	20211112_QC_R002_IgM	6.52	139153.8	13188.8	10.551	MM
3	3	20211111_A1_R2_IgM	6.48	102031.4	11871.0	8.595	MM
4	4	20211111_A1_R3_IgM	6.51	86186.7	12030.5	7.164	MM
5	5	20211111_A2_R2_IgM	6.52	195451.0	11446.8	17.075	MM
6	6	20211111_A2_R3_IgM	6.48	212489.3	11098.4	19.146	MM
7	7	20211111_L1_R2_IgM	6.47	88239.3	11779.4	7.491	MM
8	8	20211111_L1_R3_IgM	6.48	56023.9	11176.2	5.013	MM
9	9	20211111_L2_R2_IgM	6.51	188223.6	11375.0	16.547	MM
10	10	20211111_H1_R2_IgM	6.49	161747.3	13595.2	11.897	MM
11	11	20211111_A3_R2_IgM	6.46	241769.2	14112.3	17.132	MM
12	12	20211111_A4_R2_IgM	6.50	159149.5	11977.5	13.287	MM
13	13	20211111_L3_R2_IgM	6.46	137117.8	12242.1	11.200	MM
14	14	20211111_L4_R2_IgM	6.51	131136.2	12797.0	10.247	MM
15	15	20211111_H2_R2_IgM	6.50	227520.6	10006.0	22.738	MM
16	16	20211111_A5_R2_IgM	6.51	407181.3	11434.6	35.610	MM
17	17	20211111_A6_R2_IgM	6.52	251656.6	13193.7	19.074	MM
18	18	20211111_QC_R10_IgM	6.49	170109.3	12566.1	13.537	MM
19	19	20211111_L5_R2_IgM	6.49	225332.5	13616.4	16.549	MM
20	20	20211111_L6_R2_IgM	6.51	166810.3	13698.5	12.177	MM
21	21	20211111_H3_R2_IgM	6.48	67508.0	15793.1	4.275	MM
22	22	20211111_A7_R2_IgM	6.50	132701.0	12936.9	10.258	MM
23	23	20211111_A8_R2_IgM	6.50	135035.5	13108.0	10.302	MM
24	24	20211111_L7_R2_IgM	6.50	102107.4	13278.5	7.690	MM
25	25	20211111_L8_R2_IgM	6.47	60553.8	15233.6	3.975	MM
26	26	20211111_H4_R2_IgM	6.48	90480.2	16521.9	5.476	MM
27	27	20211111_A9_R2_IgM	6.49	69505.9	16451.6	4.225	MM
28	28	20211111_A10_R2_IgM	6.49	222369.5	13036.0	17.058	MM
29	29	20211111_L9_R2_IgM	6.51	175181.7	14554.1	12.037	MM
30	30	20211111_L10_R2_IgM	6.50	206297.8	12250.4	16.840	MM
31	31	20211111_QC_R11_IgM	6.50	166687.8	11459.2	14.546	MM
32	32	20211111_H5_R2_IgM	6.49	215485.8	14358.2	15.008	MM
33	33	20211111_A11_R2_IgM	6.46	154088.4	16184.1	9.521	MM
34	34	20211111_A12_R2_IgM	6.47	83244.8	13465.4	6.182	MM
35	35	20211111_L11_R2_IgM	6.48	78849.7	13277.9	5.938	MM
36	36	20211111_L12_R2_IgM	6.48	76629.1	15958.8	4.802	MM
37	37	20211111_H6_R2_IgM	6.48	57825.8	14003.5	4.129	MM
38	38	20211111_A13_R2_IgM	6.51	61520.8	13656.1	4.505	MM
39	39	20211111_A14_R2_IgM	6.50	199930.8	14071.3	14.208	MM
40	40	20211111_L13_R2_IgM	6.51	159670.3	14785.6	10.799	MM
41	41	20211111_L14_R2_IgM	6.52	188176.7	14772.2	12.739	MM
42	42	20211111_H7_R2_IgM	6.50	106142.2	15164.4	6.999	MM
43	43	20211111_A15_R2_IgM	6.50	237853.3	15721.5	15.129	MM
44	44	20211111_QC_R12_IgM	6.51	176284.3	13711.5	12.857	MM
45	45	20211111_A16_R2_IgM	6.48	198713.2	14275.6	13.920	MM
46	46	20211111_L15_R2_IgM	6.51	165027.5	12935.8	12.757	MM
47	47	20211111_L16_R2_IgM	6.47	87360.2	13342.8	6.547	MM
48	48	20211111_H8_R2_IgM	6.50	124331.4	15835.1	7.852	MM
49	49	20211111_A17_R2_IgM	6.50	161796.8	15786.7	10.249	MM
50	50	20211111_A18_R2_IgM	6.46	79508.0	16986.8	4.681	MM
51	51	20211111_L17_R2_IgM	6.49	389622.1	13342.7	29.201	MM
52	52	20211111_L18_R2_IgM	6.46	110420.7	14692.0	7.516	MM
53	53	20211111_H9_R2_IgM	6.48	81452.7	15639.7	5.208	MM
54	54	20211111_A19_R2_IgM	6.48	96398.2	14198.7	6.789	MM
55	55	20211111_A20_R2_IgM	6.48	60790.1	14162.7	4.292	MM
56	56	20211111_L19_R2_IgM	6.50	121279.8	9293.4	13.050	MM

	# Name	RT	Area	IS Area	Response	Prima...
57	57 20211111_QC_R13_IgM	6.51	145287.7	13903.0	10.450	MM
58	58 20211111_L20_R2_IgM	6.50	112364.3	12781.6	8.791	MM
59	59 20211111_H10_R2_IgM	6.50	97048.2	14796.4	6.559	MM
60	60 20211111_A21_R2_IgM	6.48	106948.8	13669.0	7.824	MM
61	61 20211111_A22_R2_IgM	6.50	56828.0	14098.3	4.031	MM
62	62 20211111_L21_R2_IgM	6.48	87276.6	15678.2	5.567	MM
63	63 20211111_L22_R2_IgM	6.55	126351.0	15724.5	8.035	MM
64	64 20211111_H11_R2_IgM	6.49	108198.5	15890.2	6.809	MM
65	65 20211111_A23_R2_IgM	6.48	78608.6	13423.2	5.856	MM
66	66 20211111_A24_R2_IgM	6.48	150698.4	12438.0	12.116	MM
67	67 20211111_L23_R2_IgM	6.50	204503.8	14075.0	14.530	MM
68	68 20211111_L24_R2_IgM	6.53	75769.3	15972.1	4.744	MM
69	69 20211111_H12_R2_IgM	6.50	94763.8	16270.9	5.824	MM
70	70 20211111_QC_R14_IgM	6.51	143106.5	15806.8	9.053	MM
71	71 20211111_A25_R2_IgM	6.48	269599.4	14552.1	18.527	MM
72	72 20211111_A26_R2_IgM	6.50	134304.5	11492.7	11.686	MM
73	73 20211111_L25_R2_IgM	6.50	176111.4	14403.3	12.227	MM
74	74 20211111_L26_R2_IgM	6.53	105937.5	16533.9	6.407	MM
75	75 20211111_H13_R2_IgM	6.50	143718.1	15520.1	9.260	MM
76	76 20211111_A27_R2_IgM	6.52	217556.4	13337.4	16.312	MM
77	77 20211111_A28_R2_IgM	6.46	129545.5	11895.6	10.890	MM
78	78 20211111_L27_R2_IgM	6.47	136307.6	14125.7	9.650	MM
79	79 20211111_L28_R2_IgM	6.53	130818.0	11807.5	11.079	MM
80	80 20211111_H14_R2_IgM	6.49	143802.5	15035.7	9.564	MM
81	81 20211111_A29_R2_IgM	6.48	51124.0	13554.4	3.772	MM
82	82 20211111_A30_R2_IgM	6.48	135433.9	12984.1	10.431	MM
83	83 20211111_QC_R15_IgM	6.52	137433.7	12299.5	11.174	MM
84	84 20211111_L29_R2_IgM	6.52	80344.0	13590.7	5.912	MM
85	85 20211111_L30_R2_IgM	6.50	45983.1	12511.4	3.675	MM
86	86 20211111_H15_R2_IgM	6.51	74086.6	11885.0	6.234	MM
87	87 20211111_A31_R2_IgM	6.53	50952.5	13422.1	3.796	MM
88	88 20211111_A32_R2_IgM	6.54	122494.6	11461.3	10.688	MM
89	89 20211111_L31_R2_IgM	6.47	54956.5	13753.4	3.996	MM
90	90 20211111_L32_R2_IgM	6.50	257426.9	12949.0	19.880	MM
91	91 20211111_H16_R2_IgM	6.50	304250.0	11465.4	26.536	MM
92	92 20211111_A33_R2_IgM	6.51	192259.5	10551.5	18.221	MM
93	93 20211111_A34_R2_IgM	6.48	85983.6	12409.9	6.929	MM
94	94 20211111_H17_R2_IgM	6.52	564952.8	10160.9	55.601	MM
95	95 20211111_A35_R2_IgM	6.47	153274.0	10460.6	14.653	MM
96	96 20211111_QC_R16_IgM	6.53	147434.7	10433.5	14.131	MM
97	97 20211111_A36_R2_IgM	6.52	115287.7	12504.8	9.219	MM
98	98 20211111_H18_R2_IgM	6.52	289570.7	9140.4	31.680	MM
99	99 20211111_A37_R2_IgM	6.53	143258.9	9796.9	14.623	MM
100	1... 20211111_A38_R2_IgM	6.49	126456.8	11224.3	11.266	MM
101	1... 20211111_H19_R2_IgM	6.50	220150.0	11739.2	18.753	MM
102	1... 20211111_A39_R2_IgM	6.49	126941.3	12718.9	9.980	MM
103	1... 20211111_A40_R2_IgM	6.47	80133.4	10006.1	8.008	MM
104	1... 20211111_H20_R2_IgM	6.54	459932.4	7988.2	57.577	MM
105	1... 20211111_A41_R2_IgM	6.49	146397.1	11513.3	12.715	MM
106	1... 20211111_A42_R2_IgM	6.50	77035.3	10238.5	7.524	MM
107	1... 20211111_A43_R2_IgM	6.49	70117.0	11098.9	6.317	MM
108	1... 20211111_A44_R2_IgM	6.50	55752.9	10969.9	5.082	MM
109	1... 20211111_QC_R17_IgM	6.50	103975.3	9700.8	10.718	MM
110	1... 20211111_L2_R3_IgM	6.51	176659.0	8528.0	20.715	MM
111	1... 20211111_H1_R3_IgM	6.51	141039.0	9438.2	14.943	MM
112	1... 20211111_A3_R3_IgM	6.52	184731.7	11598.6	15.927	MM

Dataset: C:\MassLynx\10.pro\20211111_clinicalsamples_IgM_IgA.qld

Last Altered: Thursday, March 09, 2023 19:58:25 台北標準時間
Printed: Friday, August 01, 2025 15:25:12 台北標準時間

	# Name	RT	Area	IS Area	Response	Prima...
113	1... 20211111_A4_R3_IgM	6.48	138431.9	11364.1	12.182	MM
114	1... 20211111_L3_R3_IgM	6.48	111063.9	10753.4	10.328	MM
115	1... 20211111_L4_R3_IgM	6.50	108002.6	10291.6	10.494	MM
116	1... 20211111_H2_R3_IgM	6.51	210736.0	9315.8	22.621	MM
117	1... 20211111_A5_R3_IgM	6.51	324705.1	9102.2	35.673	MM
118	1... 20211111_A6_R3_IgM	6.47	200314.4	10356.4	19.342	MM
119	1... 20211111_QC_R18_IgM	6.47	142940.2	8398.3	17.020	MM
120	1... 20211111_L5_R3_IgM	6.49	169043.9	9786.7	17.273	MM
121	1... 20211111_L6_R3_IgM	6.50	128316.4	9911.3	12.947	MM
122	1... 20211111_H3_R3_IgM	6.51	53850.3	12155.5	4.430	MM
123	1... 20211111_A7_R3_IgM	6.52	128614.1	11804.8	10.895	MM
124	1... 20211111_A8_R3_IgM	6.49	108283.0	10510.6	10.302	MM
125	1... 20211111_L7_R3_IgM	6.50	88398.8	12177.9	7.259	MM
126	1... 20211111_L8_R3_IgM	6.50	52550.5	11668.6	4.504	MM
127	1... 20211111_H4_R3_IgM	6.55	68789.0	11192.0	6.146	MM
128	1... 20211111_A9_R3_IgM	6.54	55478.7	14077.4	3.941	MM
129	1... 20211111_A10_R3_IgM	6.53	181522.1	11190.4	16.221	MM
130	1... 20211111_L9_R3_IgM	6.48	150747.3	14649.3	10.290	MM
131	1... 20211111_L10_R3_IgM	6.51	169319.4	9361.3	18.087	MM
132	1... 20211111_QC_R19_IgM	6.47	130901.4	10125.5	12.928	MM
133	1... 20211111_H5_R3_IgM	6.48	150353.2	10010.9	15.019	MM
134	1... 20211111_A11_R3_IgM	6.50	111928.1	12697.2	8.815	MM
135	1... 20211111_A12_R3_IgM	6.49	72163.2	13148.5	5.488	MM
136	1... 20211111_L11_R3_IgM	6.50	57597.2	10786.6	5.340	MM
137	1... 20211111_L12_R3_IgM	6.48	68372.5	11681.8	5.853	MM
138	1... 20211111_H6_R3_IgM	6.50	56738.0	12095.5	4.691	MM
139	1... 20211111_A13_R3_IgM	6.51	51717.4	12908.3	4.007	MM
140	1... 20211111_A14_R3_IgM	6.48	152026.0	11322.6	13.427	MM
141	1... 20211111_L13_R3_IgM	6.50	125805.2	11516.1	10.924	MM
142	1... 20211111_L14_R3_IgM	6.51	153296.4	10790.7	14.206	MM
143	1... 20211111_H7_R3_IgM	6.51	90369.4	11572.1	7.809	MM
144	1... 20211111_A15_R3_IgM	6.48	190148.3	12434.4	15.292	MM
145	1... 20211111_QC_R20_IgM	6.50	147112.9	10701.0	13.748	MM
146	1... 20211111_A16_R3_IgM	6.49	168314.7	11452.1	14.697	MM
147	1... 20211111_L15_R3_IgM	6.47	134221.8	10531.1	12.745	MM
148	1... 20211111_L16_R3_IgM	6.52	74219.6	12049.4	6.160	MM
149	1... 20211111_H8_R3_IgM	6.51	99248.7	13434.9	7.387	MM
150	1... 20211111_A17_R3_IgM	6.52	130134.8	12452.0	10.451	MM
151	1... 20211111_A18_R3_IgM	6.48	67624.4	12506.5	5.407	MM
152	1... 20211111_L17_R3_IgM	6.51	320114.2	9948.9	32.176	MM
153	1... 20211111_L18_R3_IgM	6.50	87967.3	13462.1	6.534	MM
154	1... 20211111_H9_R3_IgM	6.49	70593.4	13178.7	5.357	MM
155	1... 20211111_A19_R3_IgM	6.49	92389.4	11298.6	8.177	MM
156	1... 20211111_A20_R3_IgM	6.48	54369.0	11175.6	4.865	MM
157	1... 20211111_L19_R3_IgM	6.51	118417.3	11558.2	10.245	MM
158	1... 20211111_QC_R21_IgM	6.50	131592.3	9977.0	13.190	MM
159	1... 20211111_L20_R3_IgM	6.53	82096.9	13508.1	6.078	MM
160	1... 20211111_H10_R3_IgM	6.48	80678.0	12374.8	6.520	MM
161	1... 20211111_A21_R3_IgM	6.51	96128.6	12443.6	7.725	MM
162	1... 20211111_A22_R3_IgM	6.50	50350.0	11589.3	4.345	MM
163	1... 20211111_L21_R3_IgM	6.48	80432.5	13107.4	6.136	MM
164	1... 20211111_L22_R3_IgM	6.52	105963.7	12021.5	8.815	MM
165	1... 20211111_H11_R3_IgM	6.49	88177.5	12687.8	6.950	MM
166	1... 20211111_A23_R3_IgM	6.51	68021.0	11611.8	5.858	MM
167	1... 20211111_A24_R3_IgM	6.51	147132.5	10476.8	14.044	MM
168	1... 20211111_L23_R3_IgM	6.52	177935.2	11873.1	14.986	MM

Dataset: C:\MassLynx\10.pro\20211111_clinicalsamples_IgM_IgA.qld

Last Altered: Thursday, March 09, 2023 19:58:25 台北標準時間
Printed: Friday, August 01, 2025 15:25:12 台北標準時間

	#	Name	RT	Area	IS Area	Response	Prima...
169	1...	20211111_L24_R3_IgM	6.47	73922.3	13468.0	5.489	MM
170	1...	20211111_H12_R3_IgM	6.55	83888.9	14229.1	5.896	MM
171	1...	20211111_QC_R22_IgM	6.49	117267.3	11264.4	10.410	MM
172	1...	20211111_A25_R3_IgM	6.50	207020.2	11775.7	17.580	MM
173	1...	20211111_A26_R3_IgM	6.48	117327.7	10473.9	11.202	MM
174	1...	20211111_L25_R3_IgM	6.53	145022.9	13256.8	10.940	MM
175	1...	20211111_L26_R3_IgM	6.51	91186.0	13565.0	6.722	MM
176	1...	20211111_H13_R3_IgM	6.52	133610.1	13146.0	10.164	MM
177	1...	20211111_A27_R3_IgM	6.51	184781.6	12025.6	15.366	MM
178	1...	20211111_A28_R3_IgM	6.51	108189.9	11229.1	9.635	MM
179	1...	20211111_L27_R3_IgM	6.50	124905.6	14960.0	8.349	MM
180	1...	20211111_L28_R3_IgM	6.52	129526.5	11359.0	11.403	MM
181	1...	20211111_H14_R3_IgM	6.51	139490.5	12830.8	10.872	MM
182	1...	20211111_A29_R3_IgM	6.48	46018.8	13434.3	3.425	MM
183	1...	20211111_A30_R3_IgM	6.49	119161.6	11272.2	10.571	MM
184	1...	20211111_QC_R23_IgM	6.51	124128.9	11872.2	10.455	MM
185	1...	20211111_L29_R3_IgM	6.52	73892.8	12948.7	5.707	MM
186	1...	20211111_L30_R3_IgM	6.50	41866.1	11026.6	3.797	MM
187	1...	20211111_H15_R3_IgM	6.52	62611.5	10786.3	5.805	MM
188	1...	20211111_A31_R3_IgM	6.52	41967.8	12010.5	3.494	MM
189	1...	20211111_A32_R3_IgM	6.49	113727.9	11107.7	10.239	MM
190	1...	20211111_L31_R3_IgM	6.51	42575.7	11021.8	3.863	MM
191	1...	20211111_L32_R3_IgM	6.53	224234.7	11683.8	19.192	MM
192	1...	20211111_H16_R3_IgM	6.51	233271.8	9831.7	23.727	MM
193	1...	20211111_A33_R3_IgM	6.50	175561.3	11213.1	15.657	MM
194	1...	20211111_A34_R3_IgM	6.50	75930.8	11550.1	6.574	MM
195	1...	20211111_H17_R3_IgM	6.49	444249.0	8874.4	50.059	MM
196	1...	20211111_A35_R3_IgM	6.47	130126.6	9856.3	13.202	MM
197	1...	20211111_QC_R24_IgM	6.49	124707.6	7561.8	16.492	MM
198	1...	20211111_A36_R3_IgM	6.48	87701.5	9562.8	9.171	MM
199	1...	20211111_H18_R3_IgM	6.52	255921.5	8165.3	31.343	MM
200	2...	20211111_A37_R3_IgM	6.48	114942.2	9278.8	12.388	MM
201	2...	20211111_A38_R3_IgM	6.50	103071.6	10920.3	9.439	MM
202	2...	20211111_H19_R3_IgM	6.52	184101.0	9285.3	19.827	MM
203	2...	20211111_A39_R3_IgM	6.53	113180.0	9083.4	12.460	MM
204	2...	20211111_A40_R3_IgM	6.49	68927.1	8382.4	8.223	MM
205	2...	20211111_H20_R3_IgM	6.52	359755.8	7807.9	46.076	MM
206	2...	20211111_A41_R3_IgM	6.49	88172.2	5925.2	14.881	MM
207	2...	20211111_A42_R3_IgM	6.47	66162.7	9352.6	7.074	MM
208	2...	20211111_A43_R3_IgM	6.52	61685.3	10216.3	6.038	MM
209	2...	20211111_A44_R3_IgM	6.49	42788.5	10262.7	4.169	MM
210	2...	20211111_QC_R25_IgM	6.49	95237.5	8575.3	11.106	MM

Dataset: C:\MassLynx\10.pro\20211111_clinicalsamples_IgM_IgA.qld

Last Altered: Thursday, March 09, 2023 19:58:25 台北標準時間
Printed: Friday, August 01, 2025 15:26:09 台北標準時間

Method: C:\MassLynx\10_Pro_IgA.PRO\MethDB\20211111_IgM_IgA_DMRM_new.mdb 24 Feb 2022 13:44:14
Calibration: 15 Nov 2021 14:42:11

Sample Name: 20211112_QC_R001_IgM

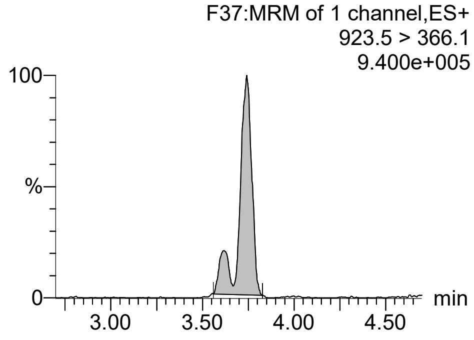

Sample Name: 20211112_QC_R002_IgM

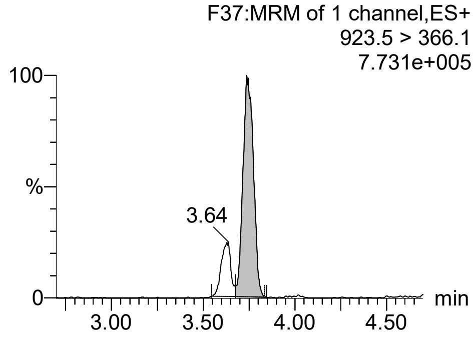

Sample Name: 20211111_A1_R2_IgM

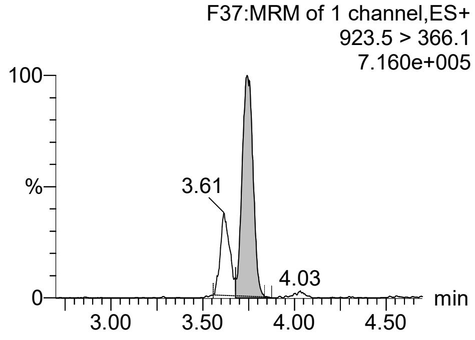

Sample Name: 20211111_A1_R3_IgM

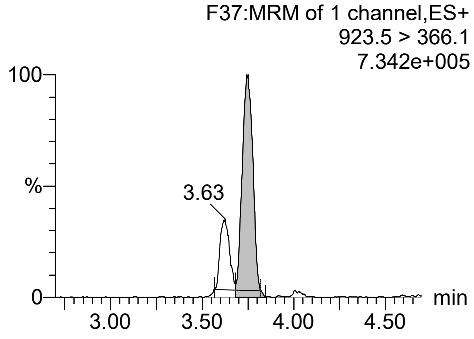

Sample Name: 20211111_A2_R2_IgM

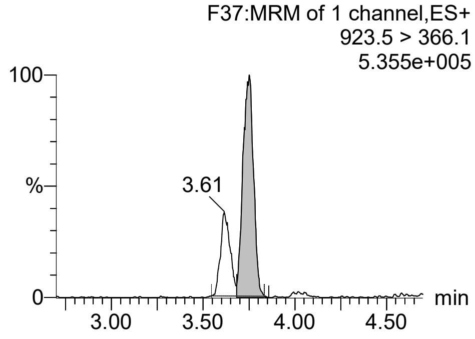

Sample Name: 20211111_A2_R3_IgM

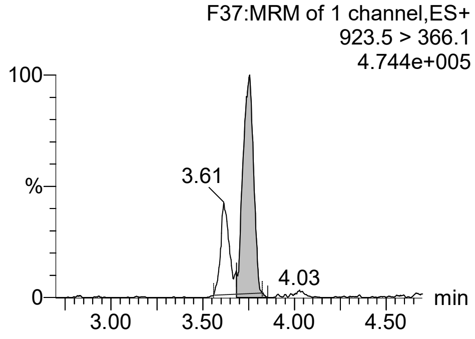

Sample Name: 20211111_L1_R2_IgM

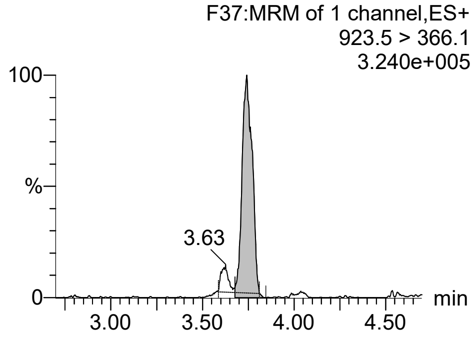

Sample Name: 20211111_L1_R3_IgM

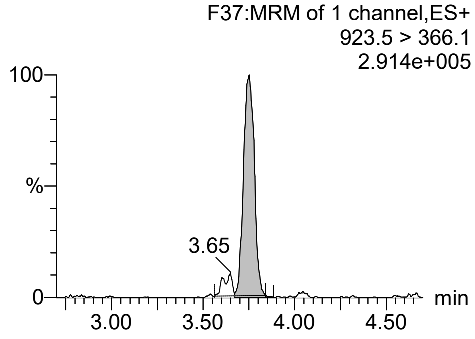

Sample Name: 20211111_L2_R2_IgM

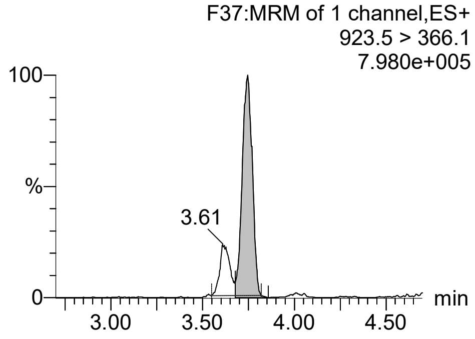

Sample Name: 20211111_H1_R2_IgM

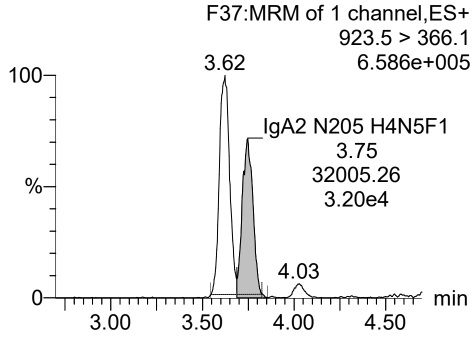

Sample Name: 20211111_A3_R2_IgM

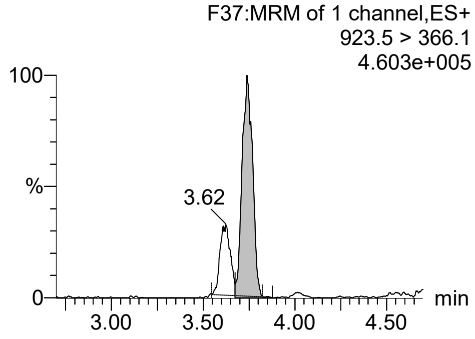

Sample Name: 20211111_A4_R2_IgM

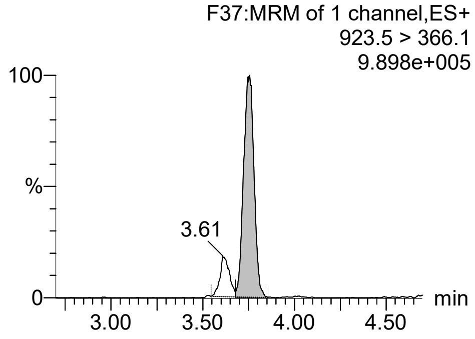

Sample Name: 20211111_L3_R2_IgM

F37:MRM of 1 channel,ES+
923.5 > 366.1
3.157e+005

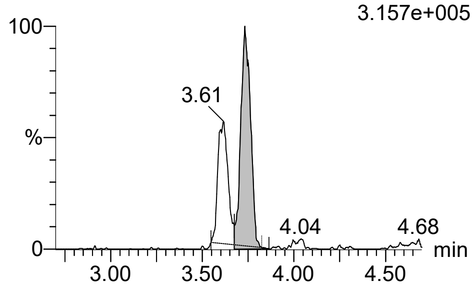

Sample Name: 20211111_L4_R2_IgM

F37:MRM of 1 channel,ES+
923.5 > 366.1
4.362e+005

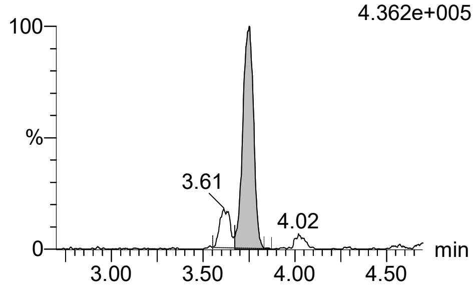

Sample Name: 20211111_H2_R2_IgM

F37:MRM of 1 channel,ES+
923.5 > 366.1
1.068e+006

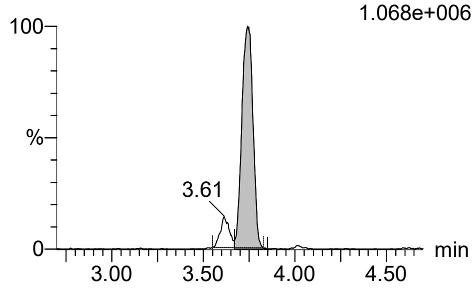

Sample Name: 20211111_A5_R2_IgM

F37:MRM of 1 channel,ES+
923.5 > 366.1
1.022e+006

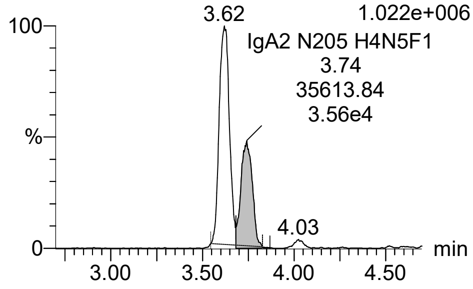

Sample Name: 20211111_A6_R2_IgM

F37:MRM of 1 channel,ES+
923.5 > 366.1
1.091e+006

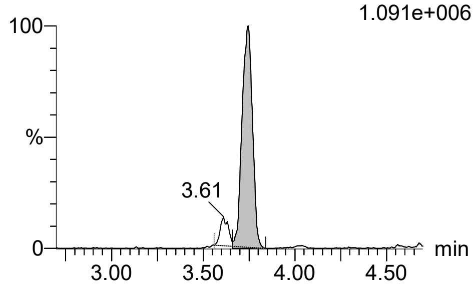

Sample Name: 20211111_QC_R10_IgM

F37:MRM of 1 channel,ES+
923.5 > 366.1
6.884e+005

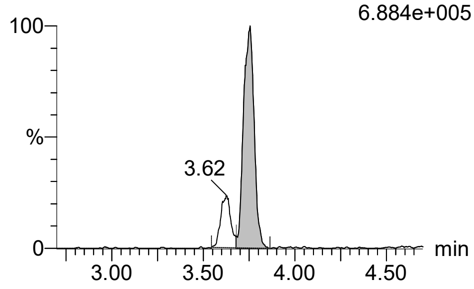

Sample Name: 20211111_L5_R2_IgM

F37:MRM of 1 channel,ES+
923.5 > 366.1
6.172e+005

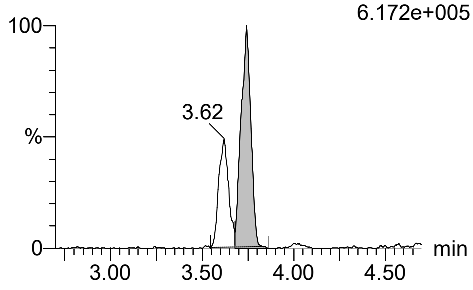

Sample Name: 20211111_L6_R2_IgM

F37:MRM of 1 channel,ES+
923.5 > 366.1
2.264e+005

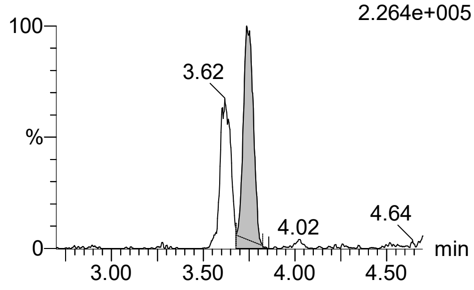

Sample Name: 20211111_H3_R2_IgM

F37:MRM of 1 channel,ES+
923.5 > 366.1
9.123e+005

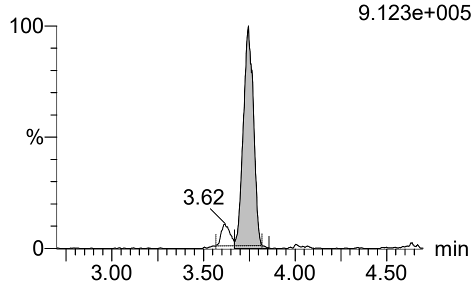

Sample Name: 20211111_A7_R2_IgM

F37:MRM of 1 channel,ES+
923.5 > 366.1
1.002e+006

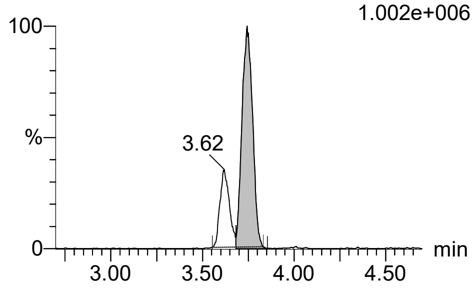

Sample Name: 20211111_A8_R2_IgM

F37:MRM of 1 channel,ES+
923.5 > 366.1
5.447e+005

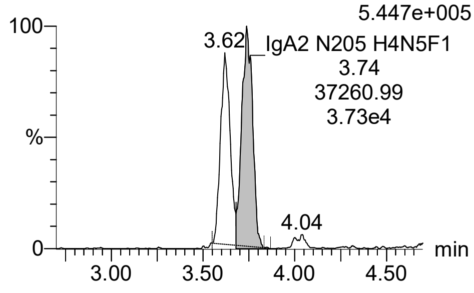

Sample Name: 20211111_L7_R2_IgM

F37:MRM of 1 channel,ES+
923.5 > 366.1
7.750e+005

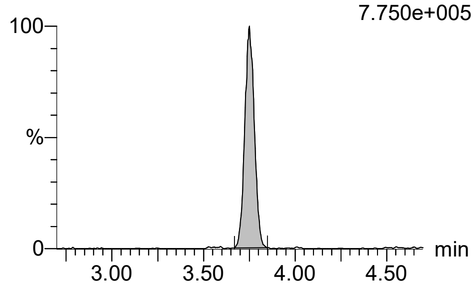

Sample Name: 20211111_L8_R2_IgM

F37:MRM of 1 channel,ES+
923.5 > 366.1
5.400e+005

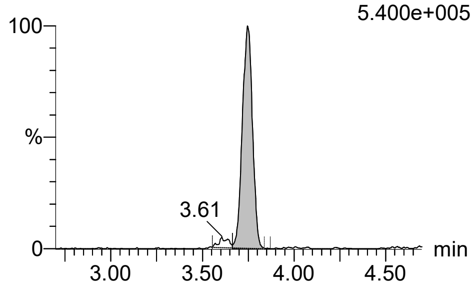

Sample Name: 20211111_H4_R2_IgM

F37:MRM of 1 channel,ES+
923.5 > 366.1
5.019e+005

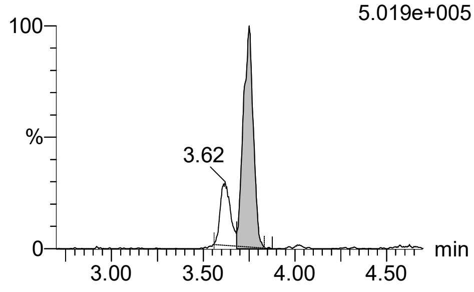

Sample Name: 20211111_A9_R2_IgM

F37:MRM of 1 channel,ES+
923.5 > 366.1
5.785e+005

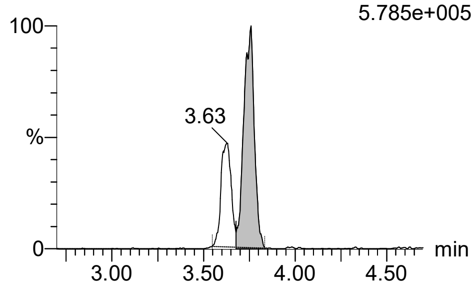

Dataset: C:\MassLynx\10.pro\20211111_clinicalsamples_IgM_IgA.qld

Last Altered: Thursday, March 09, 2023 19:58:25 台北標準時間
Printed: Friday, August 01, 2025 15:26:09 台北標準時間

Sample Name: 20211111_A10_R2_IgM
F37:MRM of 1 channel,ES+
923.5 > 366.1
7.053e+005

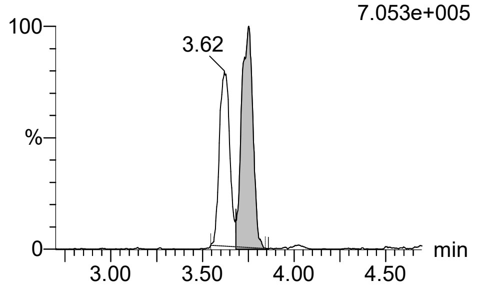

Sample Name: 20211111_L9_R2_IgM
F37:MRM of 1 channel,ES+
923.5 > 366.1
8.396e+005

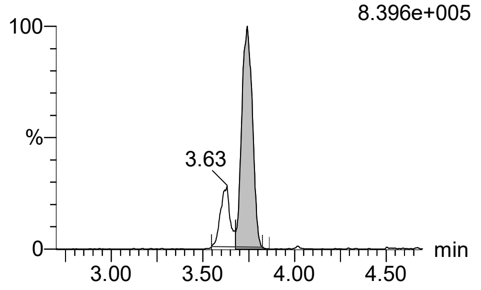

Sample Name: 20211111_L10_R2_IgM
F37:MRM of 1 channel,ES+
923.5 > 366.1
3.677e+005

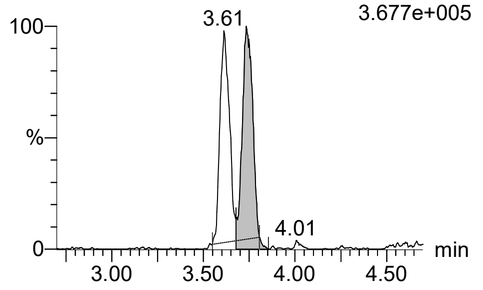

Sample Name: 20211111_QC_R11_IgM
F37:MRM of 1 channel,ES+
923.5 > 366.1
7.269e+005

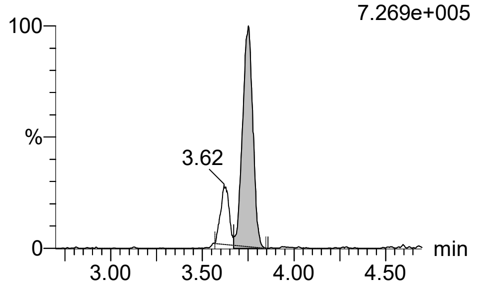

Sample Name: 20211111_H5_R2_IgM
F37:MRM of 1 channel,ES+
923.5 > 366.1
5.837e+005

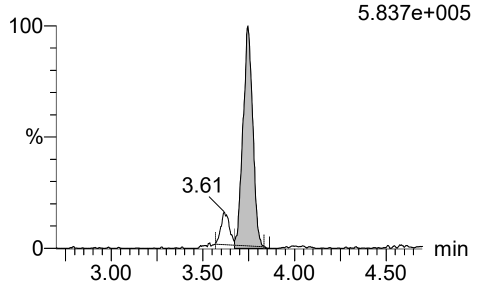

Sample Name: 20211111_A11_R2_IgM
F37:MRM of 1 channel,ES+
923.5 > 366.1
4.307e+005

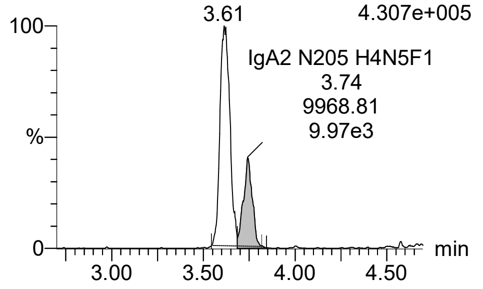

Sample Name: 20211111_A12_R2_IgM
F37:MRM of 1 channel,ES+
923.5 > 366.1
4.985e+005

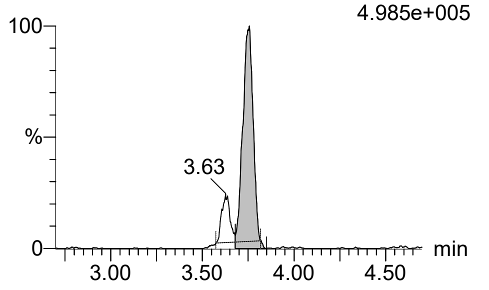

Sample Name: 20211111_L11_R2_IgM
F37:MRM of 1 channel,ES+
923.5 > 366.1
3.113e+005

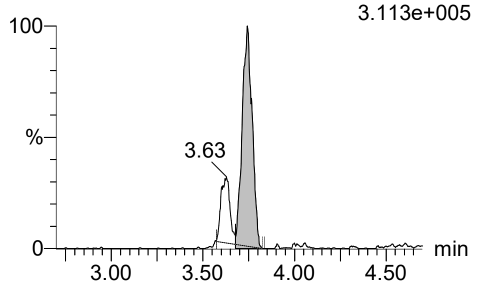

Sample Name: 20211111_L12_R2_IgM
F37:MRM of 1 channel,ES+
923.5 > 366.1
2.750e+005

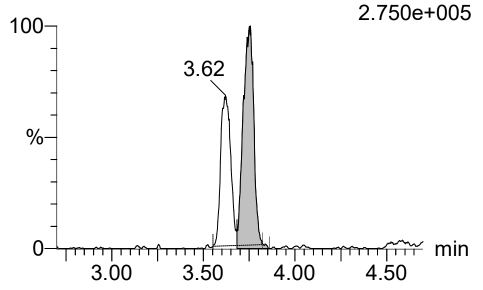

Sample Name: 20211111_H6_R2_IgM
F37:MRM of 1 channel,ES+
923.5 > 366.1
9.414e+005

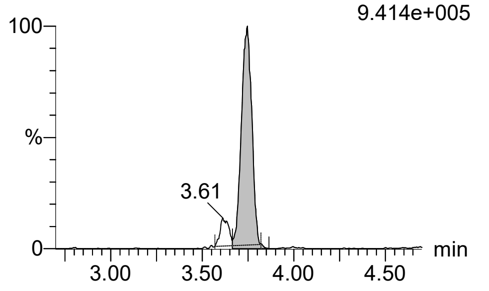

Sample Name: 20211111_A13_R2_IgM
F37:MRM of 1 channel,ES+
923.5 > 366.1
6.968e+005

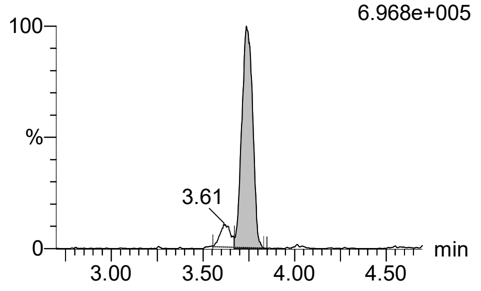

Sample Name: 20211111_A14_R2_IgM
F37:MRM of 1 channel,ES+
923.5 > 366.1
1.061e+006

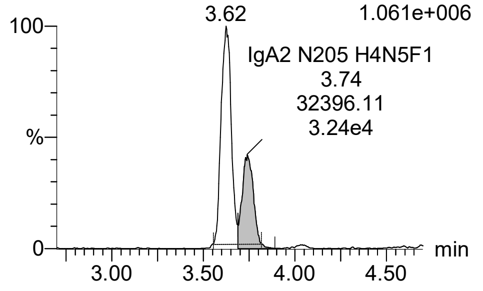

Sample Name: 20211111_L13_R2_IgM
F37:MRM of 1 channel,ES+
923.5 > 366.1
7.684e+005

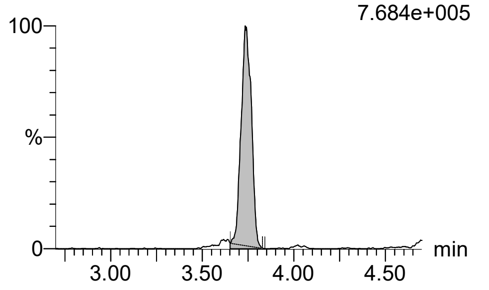

Sample Name: 20211111_L14_R2_IgM
F37:MRM of 1 channel,ES+
923.5 > 366.1
5.201e+005

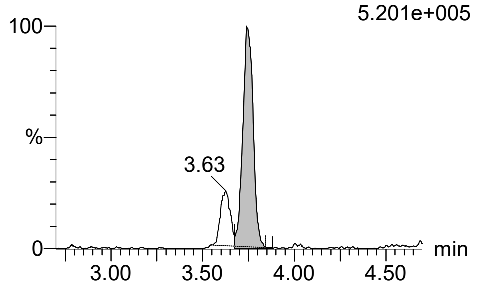

Sample Name: 20211111_H7_R2_IgM
F37:MRM of 1 channel,ES+
923.5 > 366.1
6.589e+005

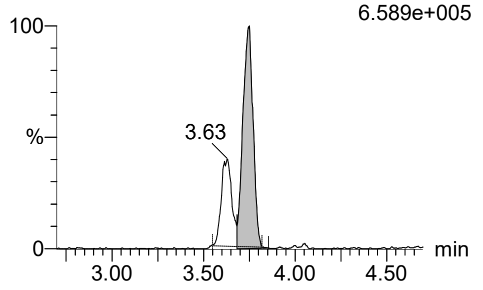

Dataset: C:\MassLynx\10.pro\20211111_clinicalsamples_IgM_IgA.qld

Last Altered: Thursday, March 09, 2023 19:58:25 台北標準時間
Printed: Friday, August 01, 2025 15:26:09 台北標準時間

Sample Name: 20211111_A15_R2_IgM
F37:MRM of 1 channel,ES+
923.5 > 366.1
6.678e+005

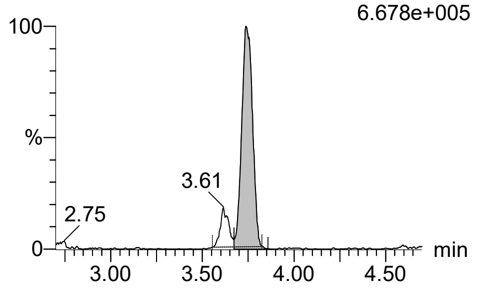

Sample Name: 20211111_QC_R12_IgM
F37:MRM of 1 channel,ES+
923.5 > 366.1
7.327e+005

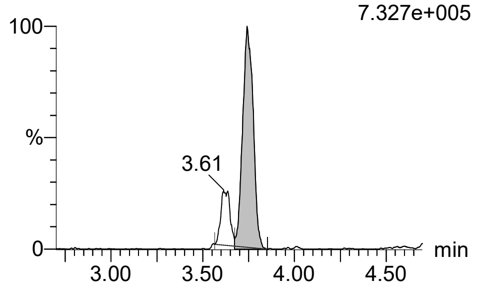

Sample Name: 20211111_A16_R2_IgM
F37:MRM of 1 channel,ES+
923.5 > 366.1
4.362e+005

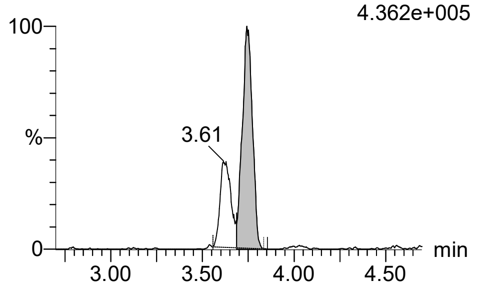

Sample Name: 20211111_L15_R2_IgM
F37:MRM of 1 channel,ES+
923.5 > 366.1
7.596e+005

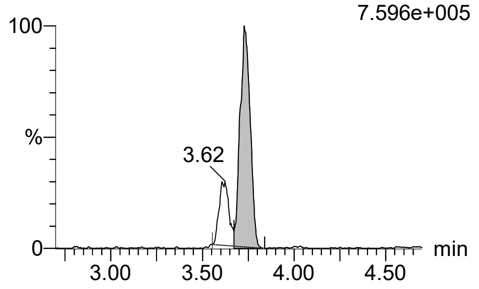

Sample Name: 20211111_L16_R2_IgM
F37:MRM of 1 channel,ES+
923.5 > 366.1
5.698e+005

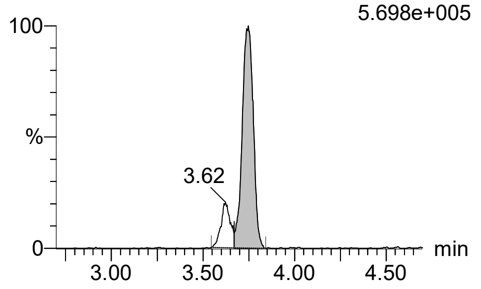

Sample Name: 20211111_H8_R2_IgM
F37:MRM of 1 channel,ES+
923.5 > 366.1
5.904e+005

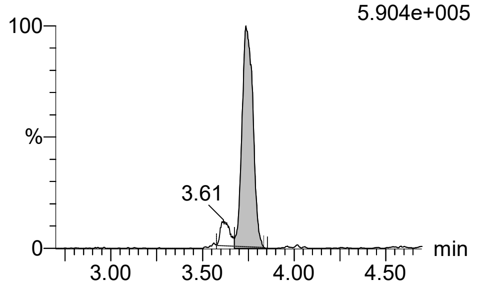

Sample Name: 20211111_A17_R2_IgM
F37:MRM of 1 channel,ES+
923.5 > 366.1
7.736e+005

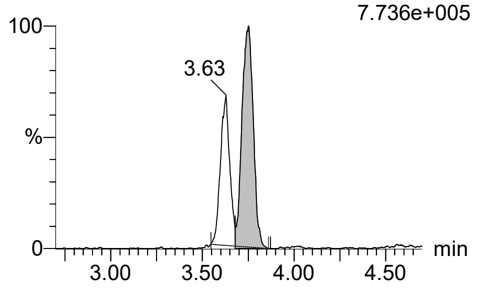

Sample Name: 20211111_A18_R2_IgM
F37:MRM of 1 channel,ES+
923.5 > 366.1
6.309e+005

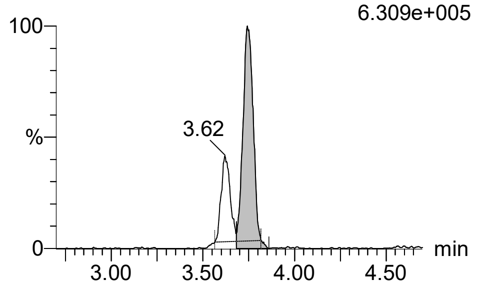

Sample Name: 20211111_L17_R2_IgM
F37:MRM of 1 channel,ES+
923.5 > 366.1
7.411e+005

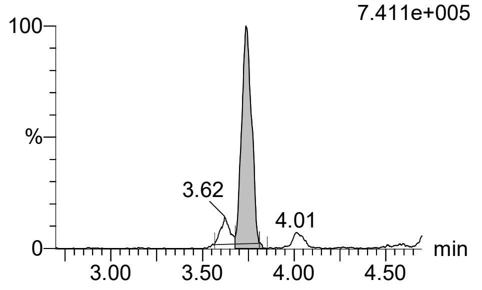

Sample Name: 20211111_L18_R2_IgM
F37:MRM of 1 channel,ES+
923.5 > 366.1
1.505e+005

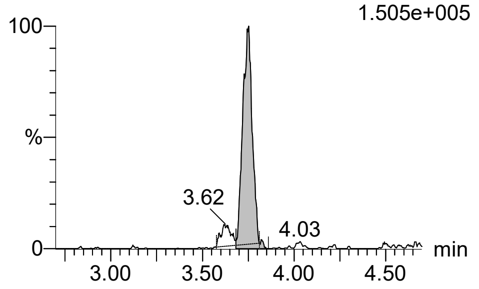

Sample Name: 20211111_H9_R2_IgM
F37:MRM of 1 channel,ES+
923.5 > 366.1
6.704e+005

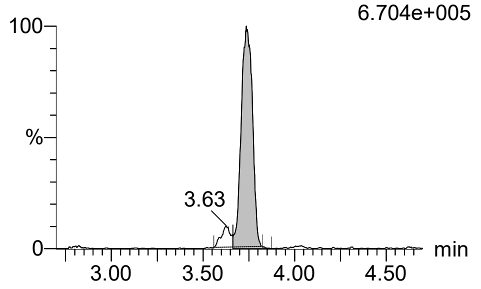

Sample Name: 20211111_A19_R2_IgM
F37:MRM of 1 channel,ES+
923.5 > 366.1
8.000e+005

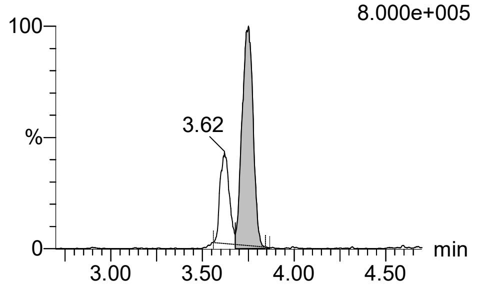

Sample Name: 20211111_A20_R2_IgM
F37:MRM of 1 channel,ES+
923.5 > 366.1
2.315e+005

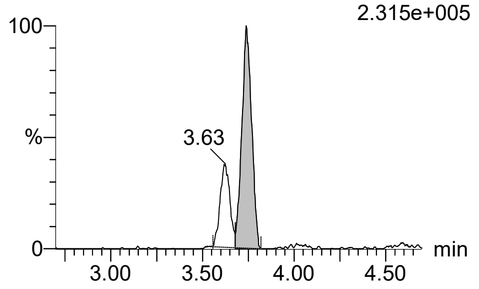

Sample Name: 20211111_L19_R2_IgM
F37:MRM of 1 channel,ES+
923.5 > 366.1
3.144e+005

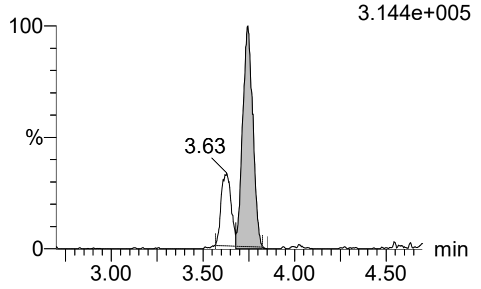

Sample Name: 20211111_QC_R13_IgM
F37:MRM of 1 channel,ES+
923.5 > 366.1
7.163e+005

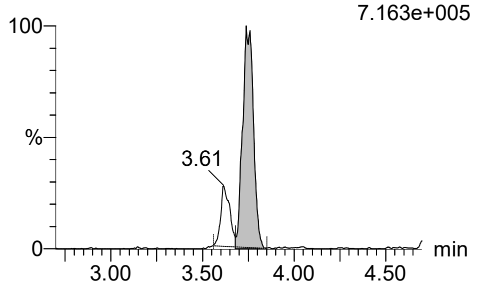

Sample Name: 20211111_L20_R2_IgM
F37:MRM of 1 channel,ES+
923.5 > 366.1
3.895e+005

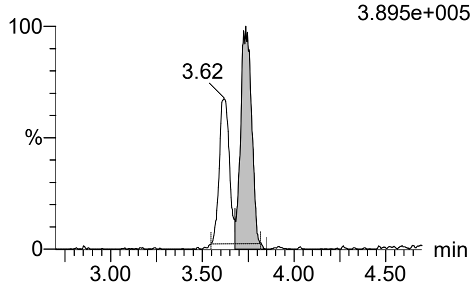

Sample Name: 20211111_H10_R2_IgM
F37:MRM of 1 channel,ES+
923.5 > 366.1
3.827e+005

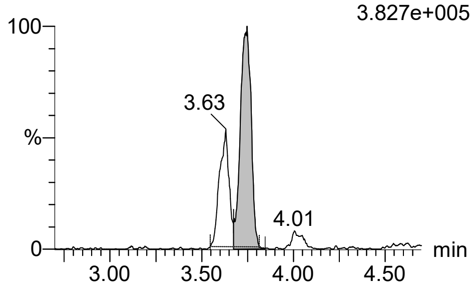

Sample Name: 20211111_A21_R2_IgM
F37:MRM of 1 channel,ES+
923.5 > 366.1
1.221e+006

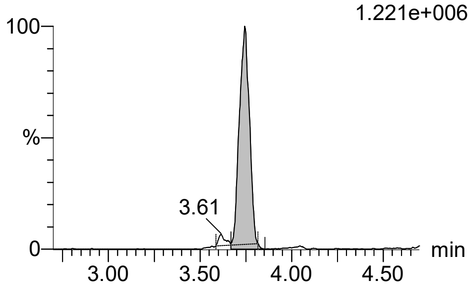

Sample Name: 20211111_A22_R2_IgM
F37:MRM of 1 channel,ES+
923.5 > 366.1
3.984e+005

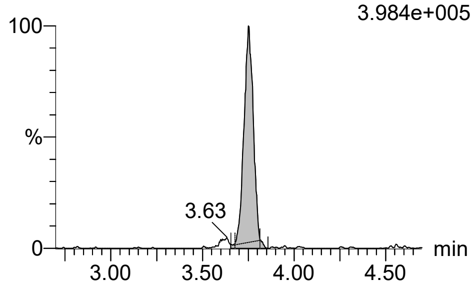

Sample Name: 20211111_L21_R2_IgM
F37:MRM of 1 channel,ES+
923.5 > 366.1
2.681e+005

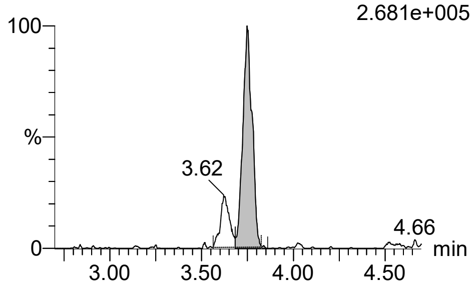

Sample Name: 20211111_L22_R2_IgM
F37:MRM of 1 channel,ES+
923.5 > 366.1
4.114e+005

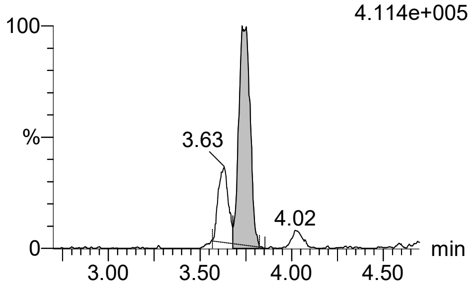

Sample Name: 20211111_H11_R2_IgM
F37:MRM of 1 channel,ES+
923.5 > 366.1
1.117e+006

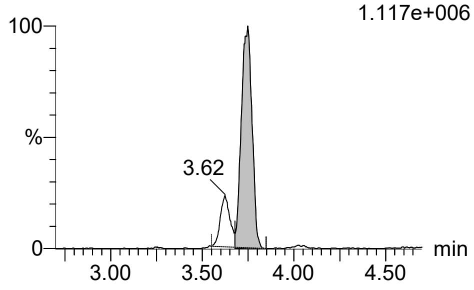

Sample Name: 20211111_A23_R2_IgM
F37:MRM of 1 channel,ES+
923.5 > 366.1
5.499e+005

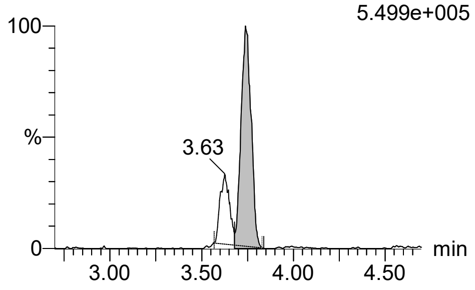

Sample Name: 20211111_A24_R2_IgM
F37:MRM of 1 channel,ES+
923.5 > 366.1
2.354e+005

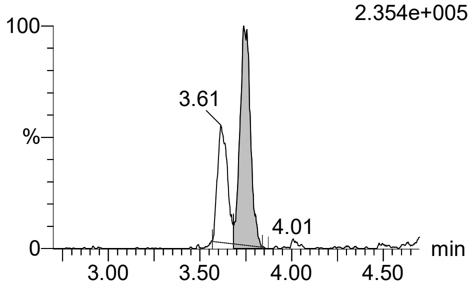

Sample Name: 20211111_L23_R2_IgM
F37:MRM of 1 channel,ES+
923.5 > 366.1
1.002e+006

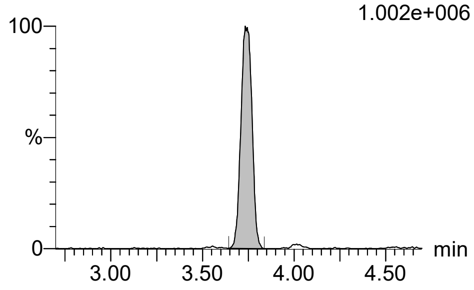

Sample Name: 20211111_L24_R2_IgM
F37:MRM of 1 channel,ES+
923.5 > 366.1
4.380e+005

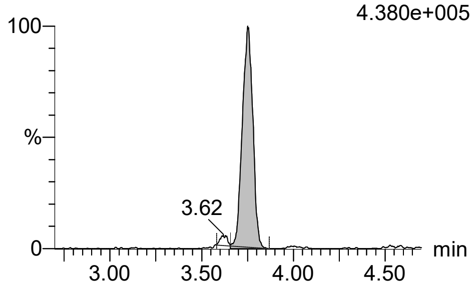

Sample Name: 20211111_H12_R2_IgM
F37:MRM of 1 channel,ES+
923.5 > 366.1
4.351e+005

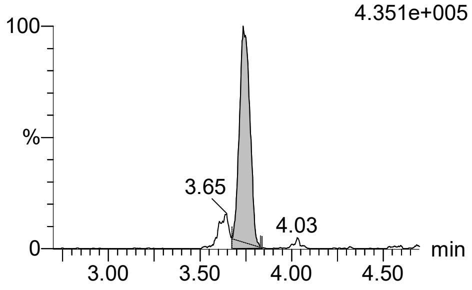

Sample Name: 20211111_QC_R14_IgM
F37:MRM of 1 channel,ES+
923.5 > 366.1
7.839e+005

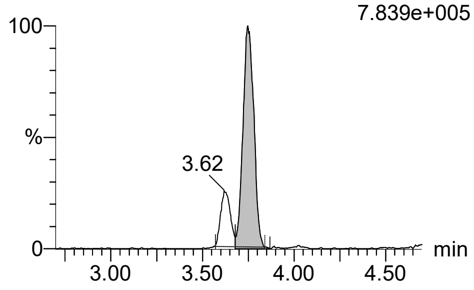

Sample Name: 20211111_A25_R2_IgM
F37:MRM of 1 channel,ES+
923.5 > 366.1
3.710e+005

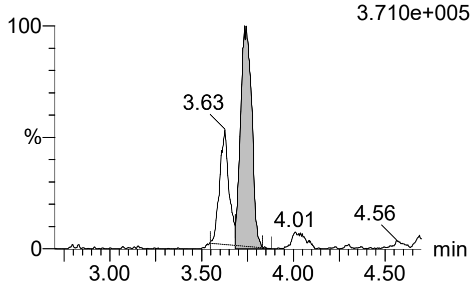

Sample Name: 20211111_A26_R2_IgM
F37:MRM of 1 channel,ES+
923.5 > 366.1
2.968e+005

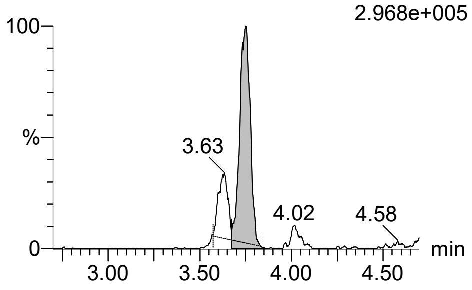

Sample Name: 20211111_L25_R2_IgM

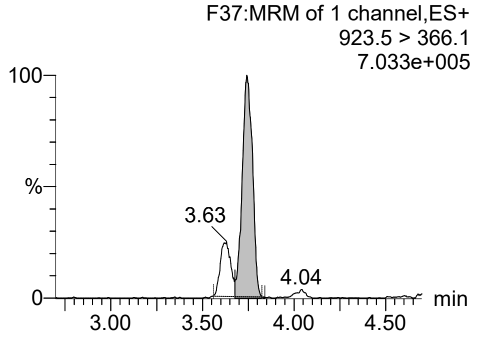

Sample Name: 20211111_L26_R2_IgM

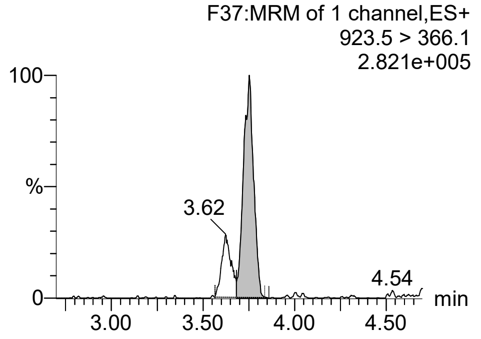

Sample Name: 20211111_H13_R2_IgM

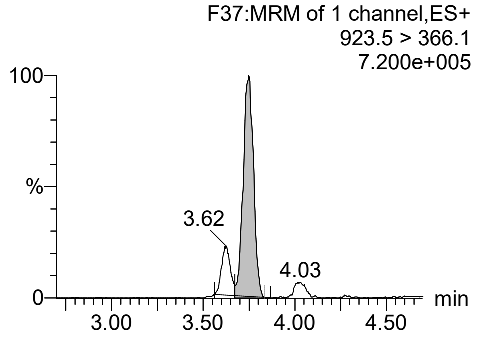

Sample Name: 20211111_A27_R2_IgM

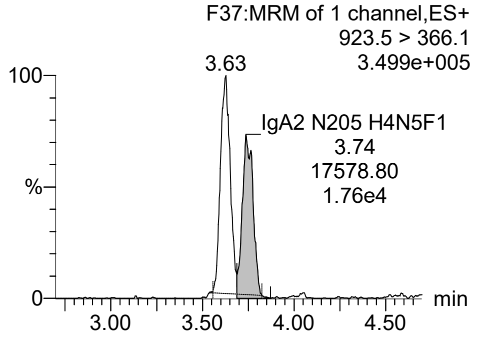

Sample Name: 20211111_A28_R2_IgM

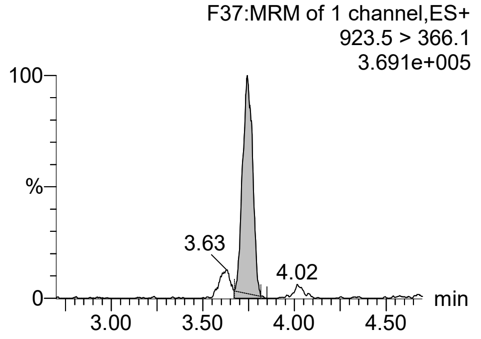

Sample Name: 20211111_L27_R2_IgM

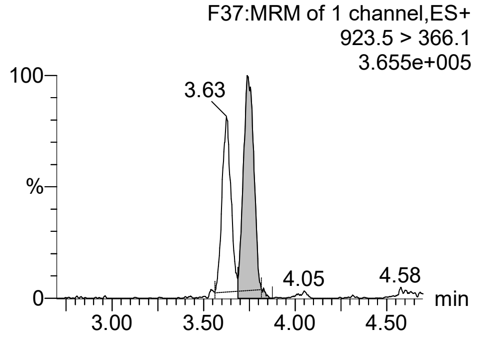

Sample Name: 20211111_L28_R2_IgM

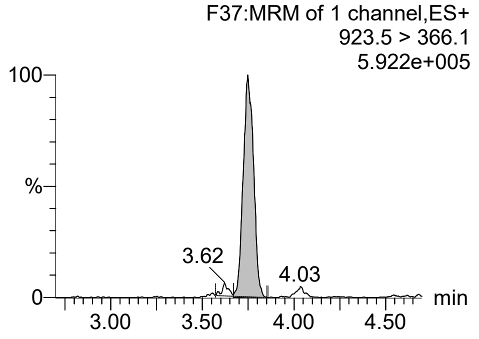

Sample Name: 20211111_H14_R2_IgM

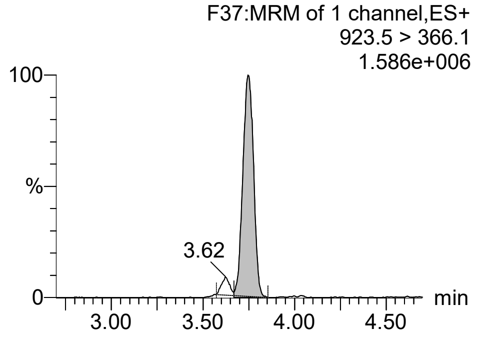

Sample Name: 20211111_A29_R2_IgM

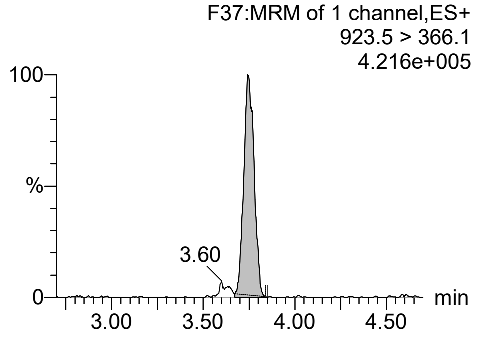

Sample Name: 20211111_A30_R2_IgM

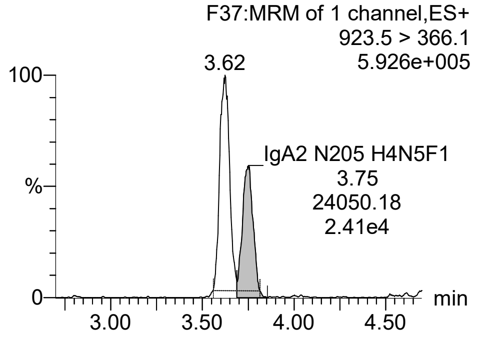

Sample Name: 20211111_QC_R15_IgM

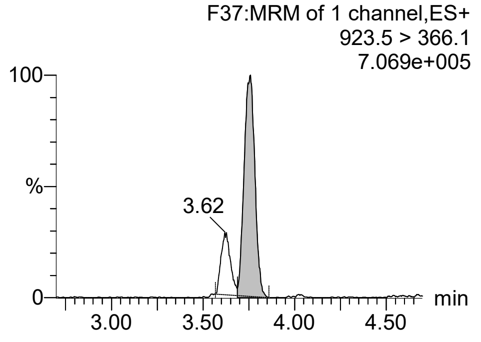

Sample Name: 20211111_L29_R2_IgM

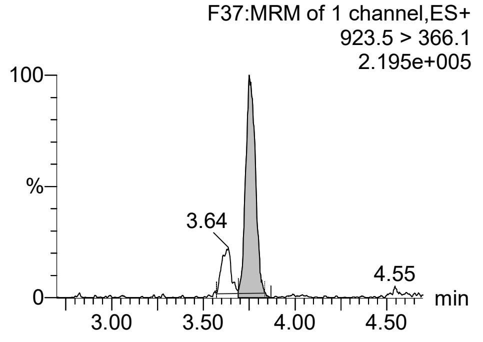

Sample Name: 20211111_L30_R2_IgM

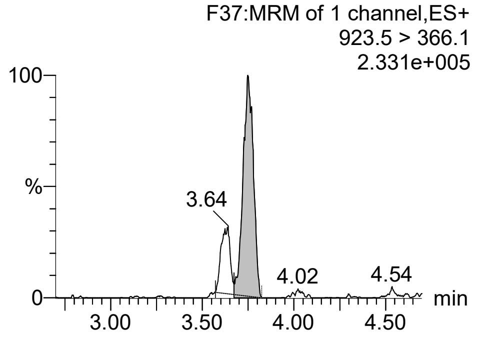

Sample Name: 20211111_H15_R2_IgM

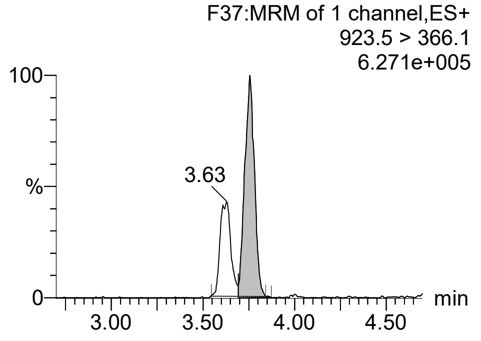

Sample Name: 20211111_A31_R2_IgM

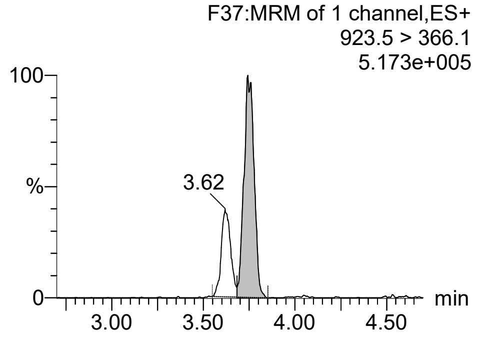

Sample Name: 20211111_A32_R2_IgM

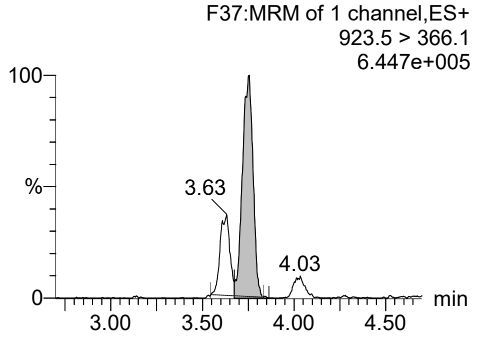

Sample Name: 20211111_L31_R2_IgM

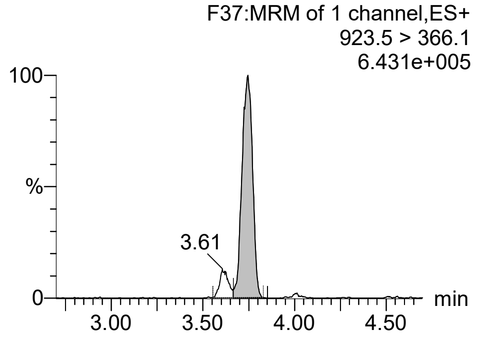

Sample Name: 20211111_L32_R2_IgM

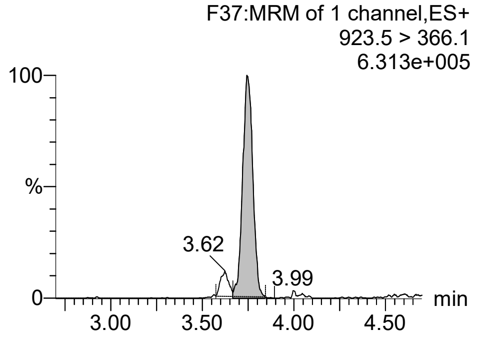

Sample Name: 20211111_H16_R2_IgM

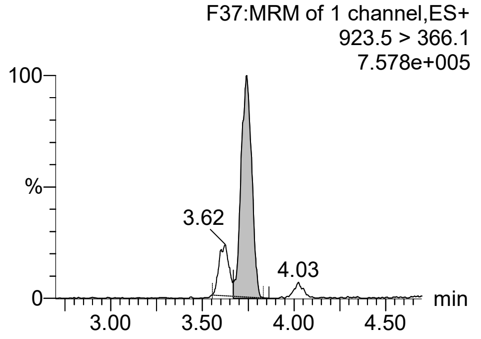

Sample Name: 20211111_A33_R2_IgM

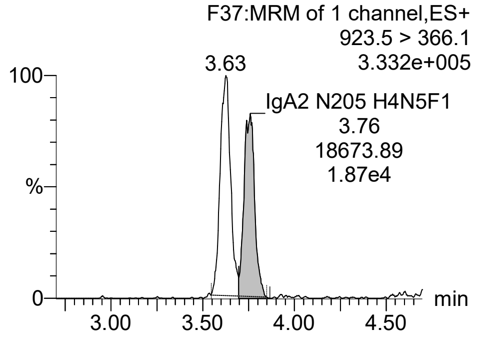

Sample Name: 20211111_A34_R2_IgM

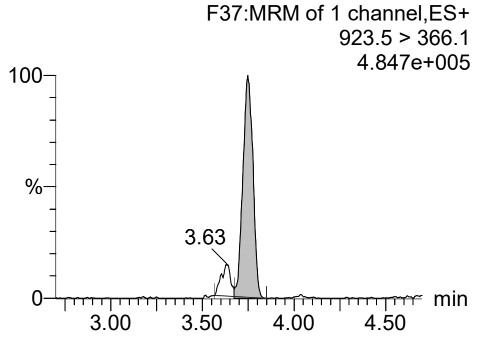

Sample Name: 20211111_H17_R2_IgM

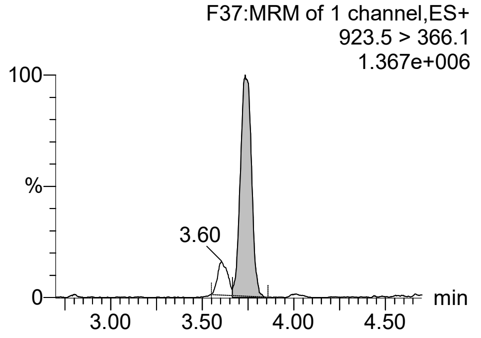

Sample Name: 20211111_A35_R2_IgM

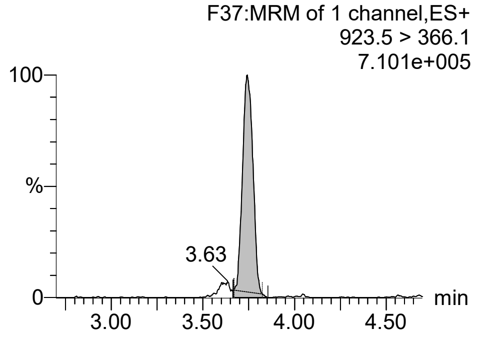

Sample Name: 20211111_QC_R16_IgM

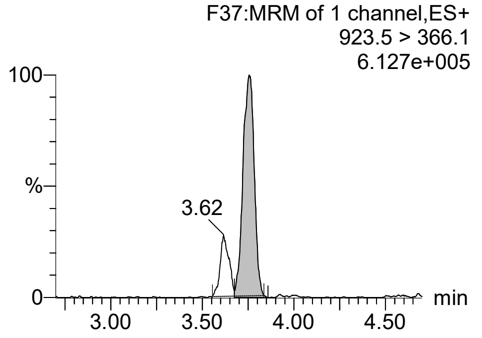

Sample Name: 20211111_A36_R2_IgM

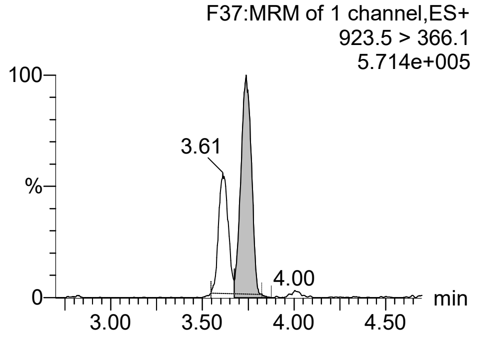

Sample Name: 20211111_H18_R2_IgM

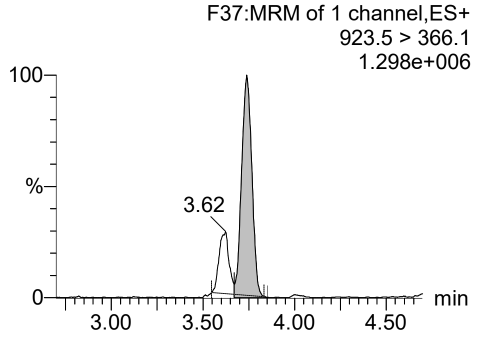

Sample Name: 20211111_A37_R2_IgM

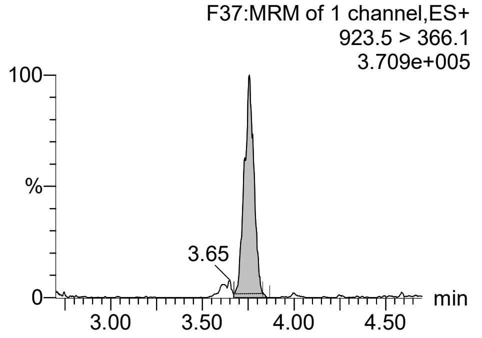

Sample Name: 20211111_A38_R2_IgM

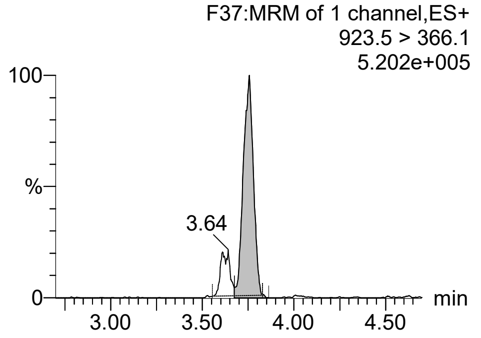

Sample Name: 20211111_H19_R2_IgM

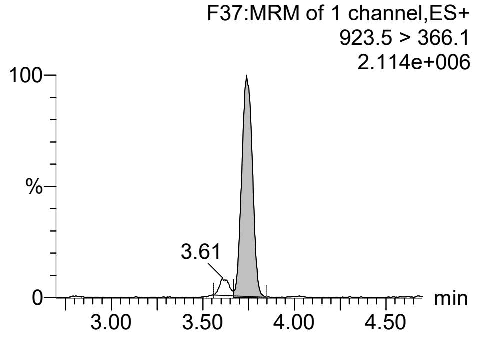

Sample Name: 20211111_A39_R2_IgM

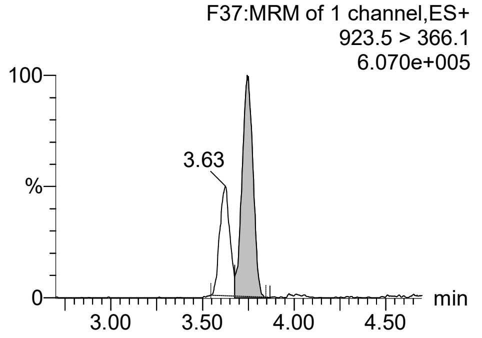

Dataset: C:\MassLynx\10.pro\20211111_clinicalsamples_IgM_IgA.qld

Last Altered: Thursday, March 09, 2023 19:58:25 台北標準時間
Printed: Friday, August 01, 2025 15:26:09 台北標準時間

Sample Name: 20211111_A40_R2_IgM

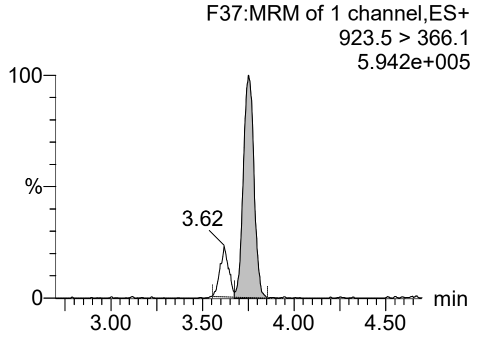

Sample Name: 20211111_H20_R2_IgM

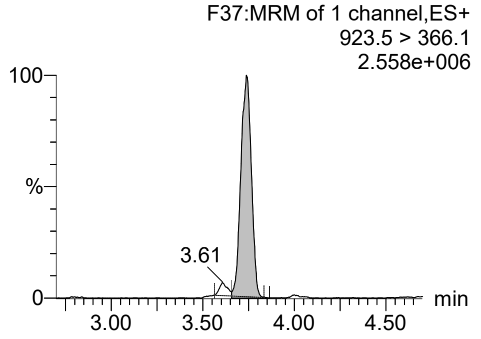

Sample Name: 20211111_A41_R2_IgM

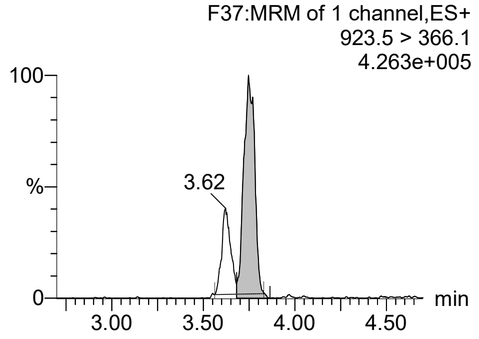

Sample Name: 20211111_A42_R2_IgM

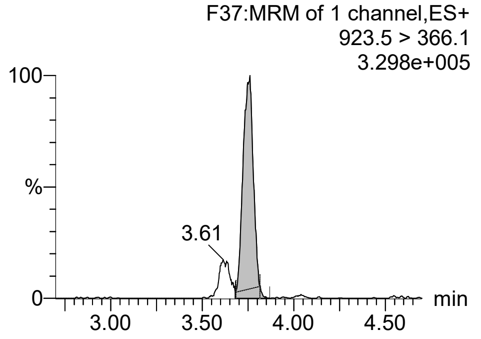

Sample Name: 20211111_A43_R2_IgM

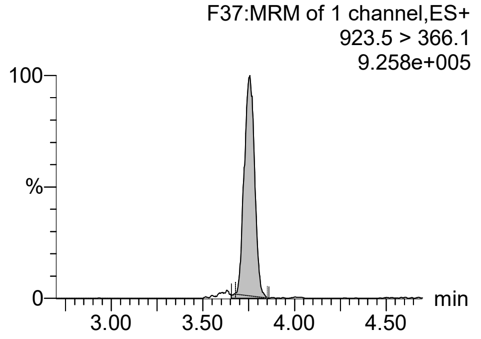

Sample Name: 20211111_A44_R2_IgM

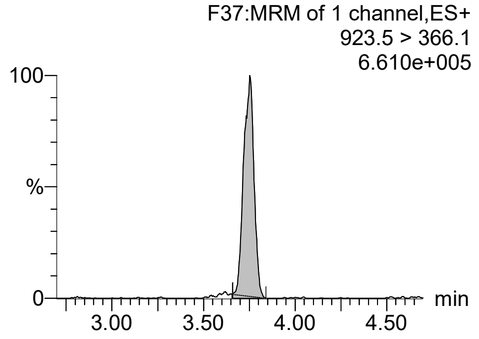

Sample Name: 20211111_QC_R17_IgM

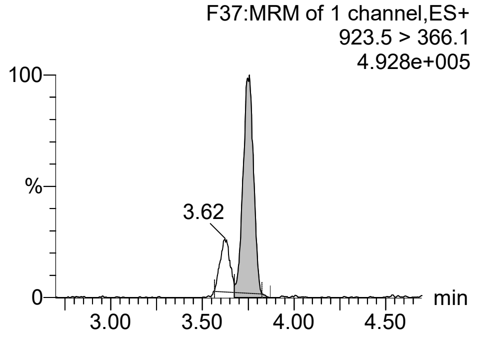

Sample Name: 20211111_L2_R3_IgM

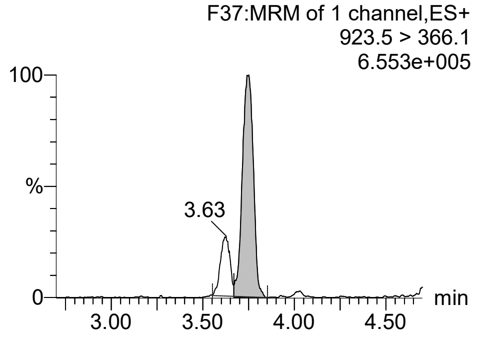

Sample Name: 20211111_H1_R3_IgM

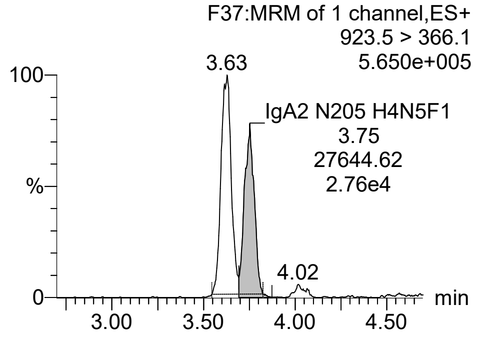

Sample Name: 20211111_A3_R3_IgM

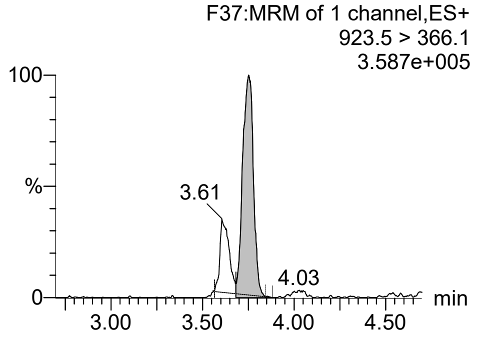

Sample Name: 20211111_A4_R3_IgM

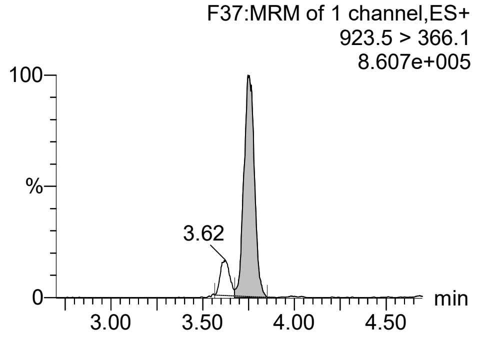

Sample Name: 20211111_L3_R3_IgM

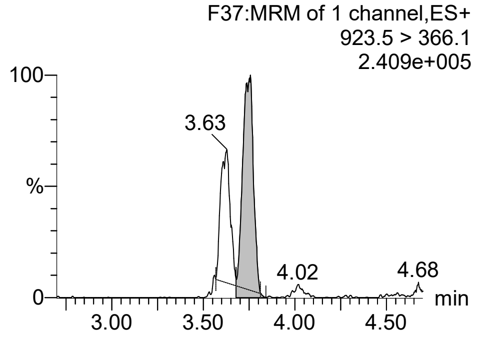

Sample Name: 20211111_L4_R3_IgM

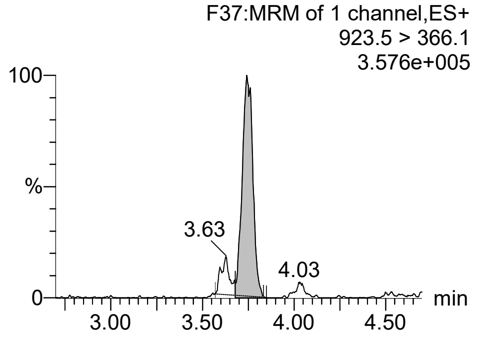

Sample Name: 20211111_H2_R3_IgM

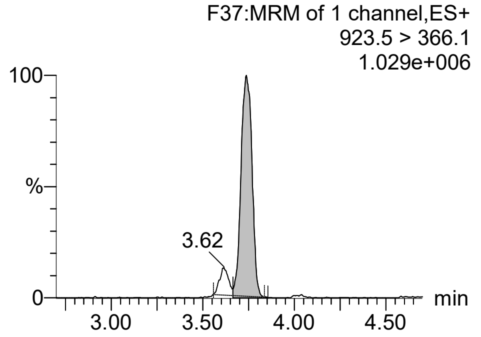

Sample Name: 20211111_A5_R3_IgM

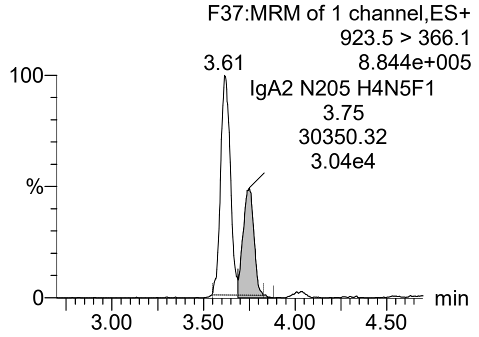

Sample Name: 20211111_A6_R3_IgM
F37:MRM of 1 channel,ES+
923.5 > 366.1
8.835e+005

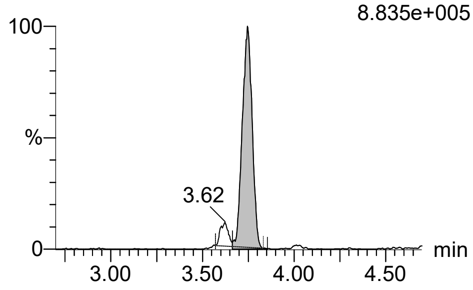

Sample Name: 20211111_QC_R18_IgM
F37:MRM of 1 channel,ES+
923.5 > 366.1
5.095e+005

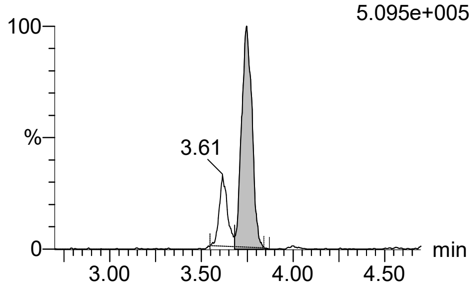

Sample Name: 20211111_L5_R3_IgM
F37:MRM of 1 channel,ES+
923.5 > 366.1
5.013e+005

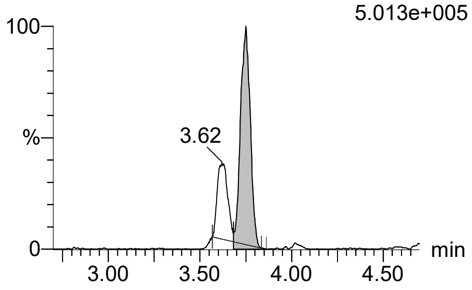

Sample Name: 20211111_L6_R3_IgM
F37:MRM of 1 channel,ES+
923.5 > 366.1
2.092e+005

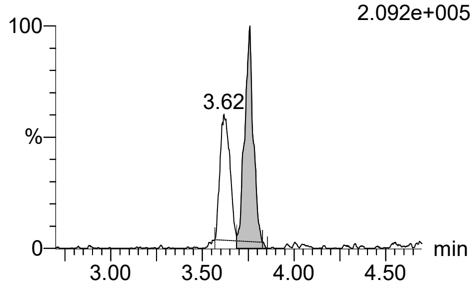

Sample Name: 20211111_H3_R3_IgM
F37:MRM of 1 channel,ES+
923.5 > 366.1
7.053e+005

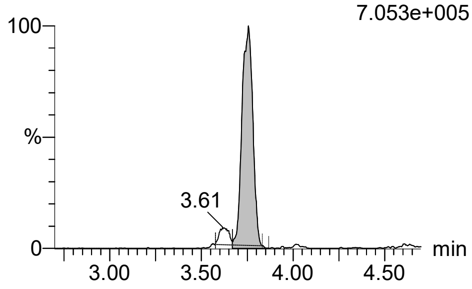

Sample Name: 20211111_A7_R3_IgM
F37:MRM of 1 channel,ES+
923.5 > 366.1
9.531e+005

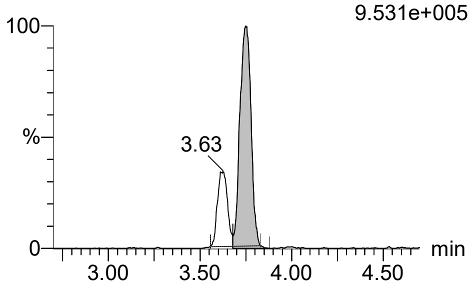

Sample Name: 20211111_A8_R3_IgM
F37:MRM of 1 channel,ES+
923.5 > 366.1
4.652e+005

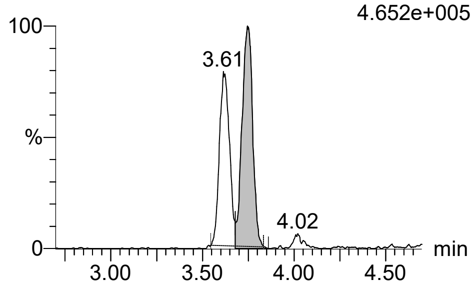

Sample Name: 20211111_L7_R3_IgM
F37:MRM of 1 channel,ES+
923.5 > 366.1
6.488e+005

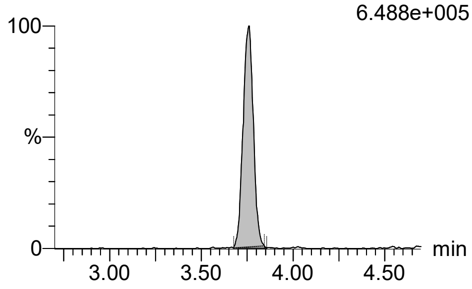

Sample Name: 20211111_L8_R3_IgM
F37:MRM of 1 channel,ES+
923.5 > 366.1
4.381e+005

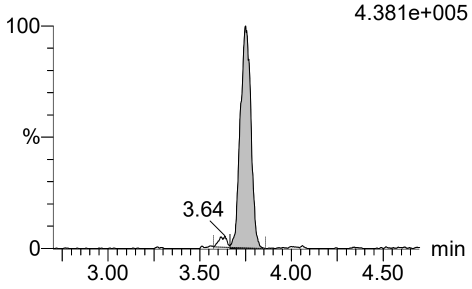

Sample Name: 20211111_H4_R3_IgM
F37:MRM of 1 channel,ES+
923.5 > 366.1
3.815e+005

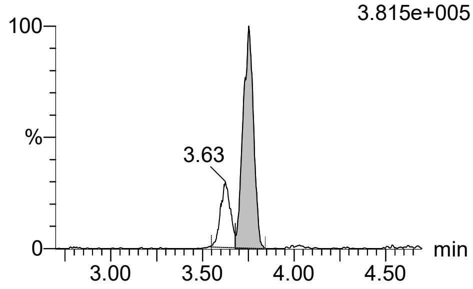

Sample Name: 20211111_A9_R3_IgM
F37:MRM of 1 channel,ES+
923.5 > 366.1
4.785e+005

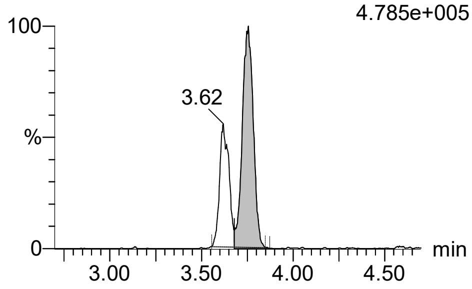

Sample Name: 20211111_A10_R3_IgM
F37:MRM of 1 channel,ES+
923.5 > 366.1
6.585e+005

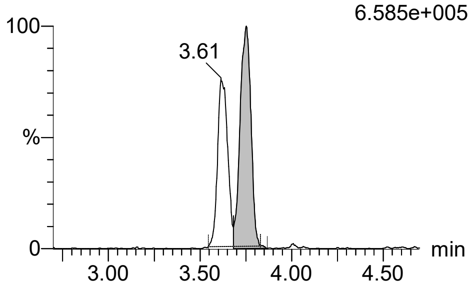

Sample Name: 20211111_L9_R3_IgM
F37:MRM of 1 channel,ES+
923.5 > 366.1
7.523e+005

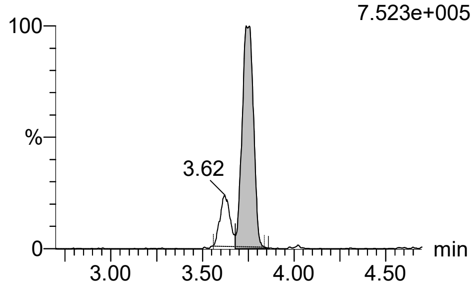

Sample Name: 20211111_L10_R3_IgM
F37:MRM of 1 channel,ES+
923.5 > 366.1
3.194e+005

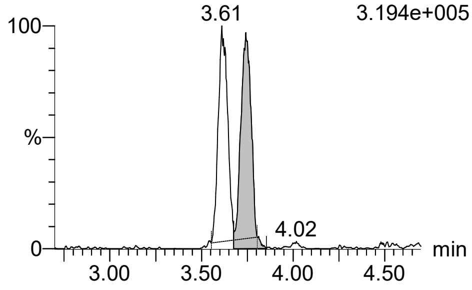

Sample Name: 20211111_QC_R19_IgM
F37:MRM of 1 channel,ES+
923.5 > 366.1
5.566e+005

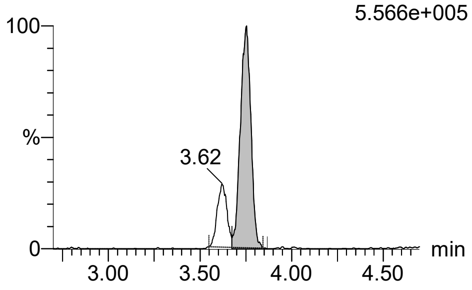

Sample Name: 20211111_H5_R3_IgM

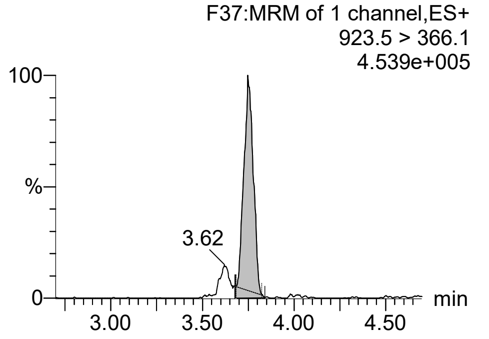

Sample Name: 20211111_A11_R3_IgM

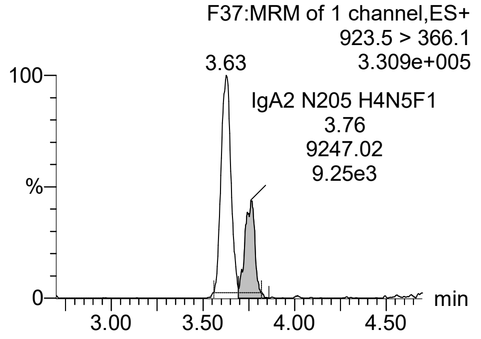

Sample Name: 20211111_A12_R3_IgM

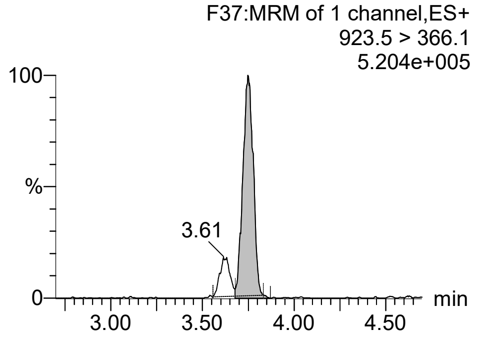

Sample Name: 20211111_L11_R3_IgM

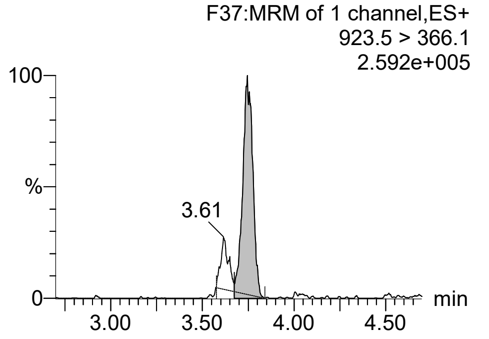

Sample Name: 20211111_L12_R3_IgM

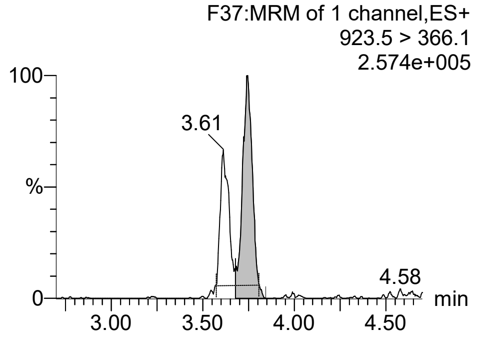

Sample Name: 20211111_H6_R3_IgM

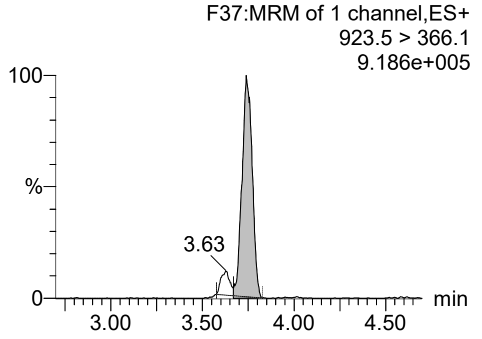

Sample Name: 20211111_A13_R3_IgM

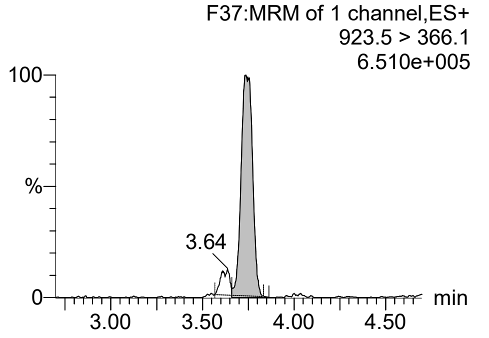

Sample Name: 20211111_A14_R3_IgM

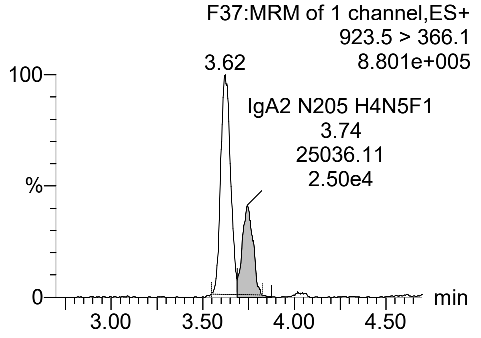

Sample Name: 20211111_L13_R3_IgM

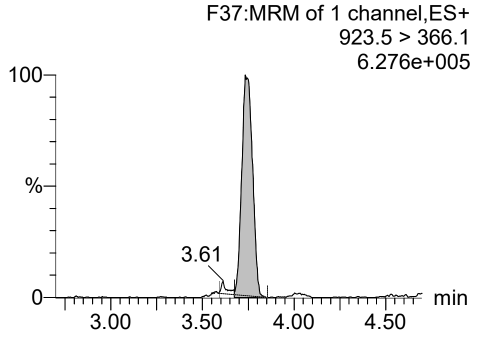

Sample Name: 20211111_L14_R3_IgM

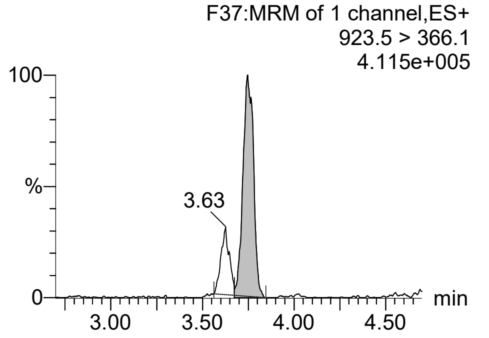

Sample Name: 20211111_H7_R3_IgM

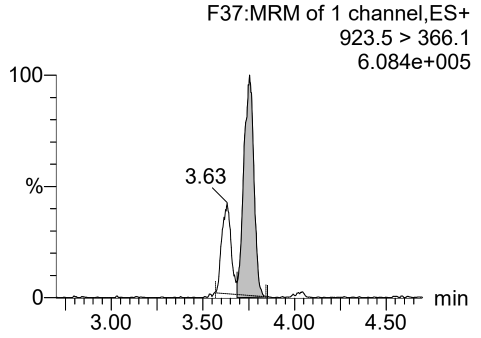

Sample Name: 20211111_A15_R3_IgM

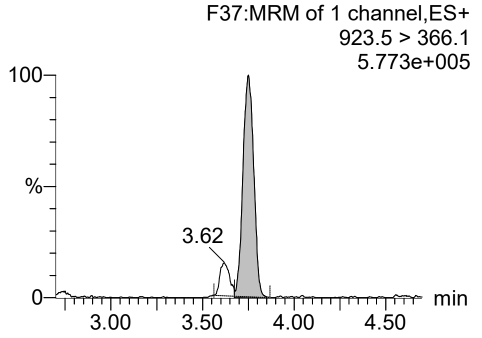

Sample Name: 20211111_QC_R20_IgM

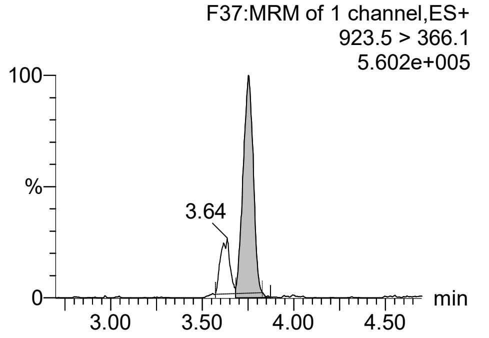

Sample Name: 20211111_A16_R3_IgM

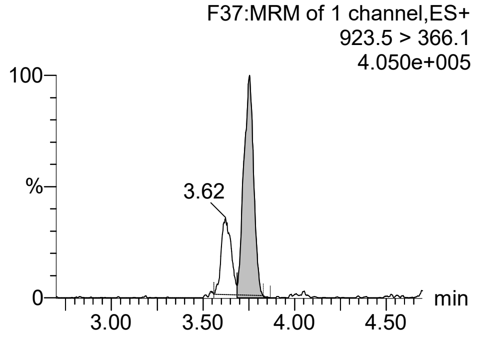

Sample Name: 20211111_L15_R3_IgM

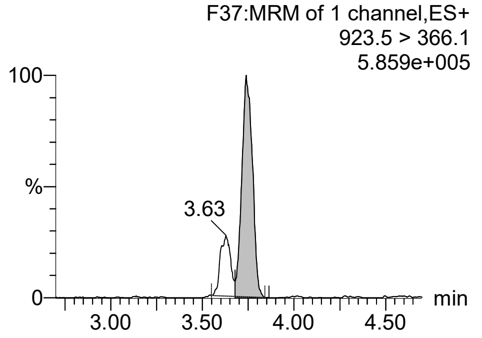

Dataset: C:\MassLynx\10.pro\20211111_clinicalsamples_IgM_IgA.qld

Last Altered: Thursday, March 09, 2023 19:58:25 台北標準時間
Printed: Friday, August 01, 2025 15:26:09 台北標準時間

Sample Name: 20211111_L16_R3_IgM
F37:MRM of 1 channel,ES+
923.5 > 366.1
5.375e+005

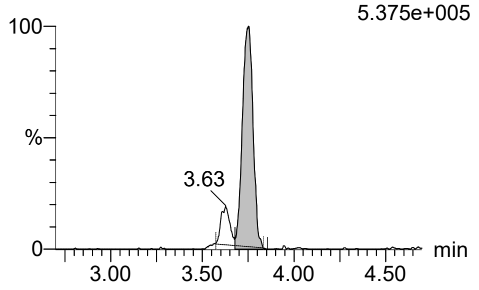

Sample Name: 20211111_H8_R3_IgM
F37:MRM of 1 channel,ES+
923.5 > 366.1
5.553e+005

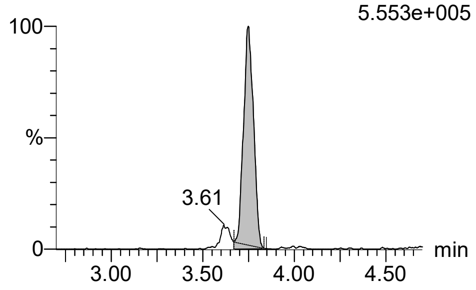

Sample Name: 20211111_A17_R3_IgM
F37:MRM of 1 channel,ES+
923.5 > 366.1
7.800e+005

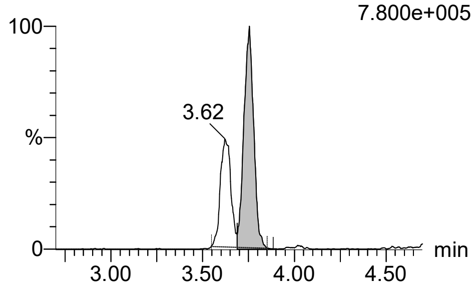

Sample Name: 20211111_A18_R3_IgM
F37:MRM of 1 channel,ES+
923.5 > 366.1
5.835e+005

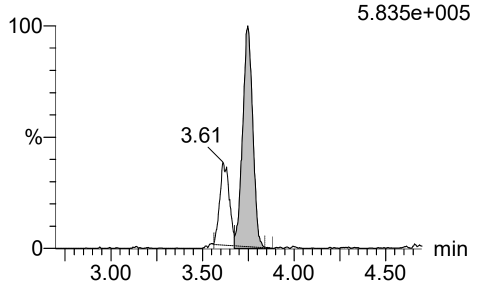

Sample Name: 20211111_L17_R3_IgM
F37:MRM of 1 channel,ES+
923.5 > 366.1
6.446e+005

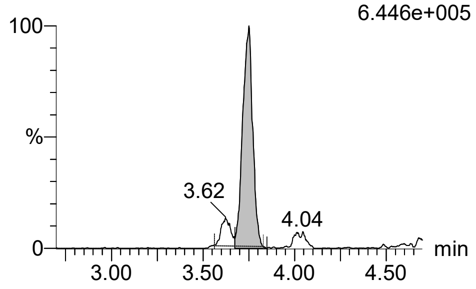

Sample Name: 20211111_L18_R3_IgM
F37:MRM of 1 channel,ES+
923.5 > 366.1
1.476e+005

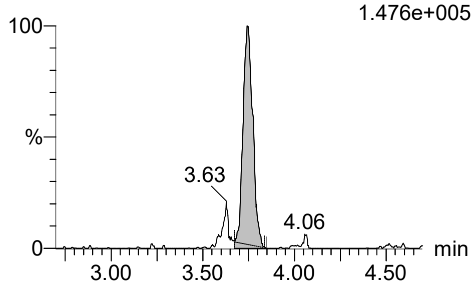

Sample Name: 20211111_H9_R3_IgM
F37:MRM of 1 channel,ES+
923.5 > 366.1
6.273e+005

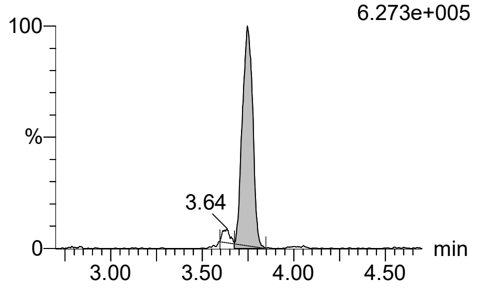

Sample Name: 20211111_A19_R3_IgM
F37:MRM of 1 channel,ES+
923.5 > 366.1
8.518e+005

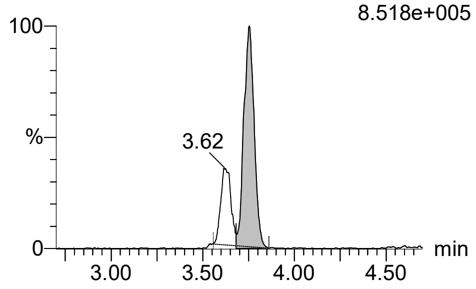

Sample Name: 20211111_A20_R3_IgM
F37:MRM of 1 channel,ES+
923.5 > 366.1
2.042e+005

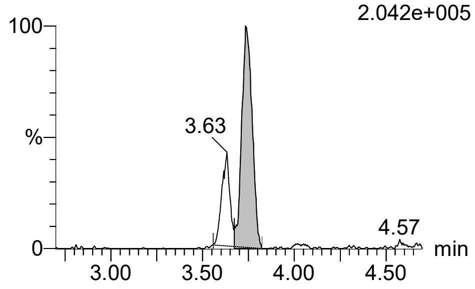

Sample Name: 20211111_L19_R3_IgM
F37:MRM of 1 channel,ES+
923.5 > 366.1
3.593e+005

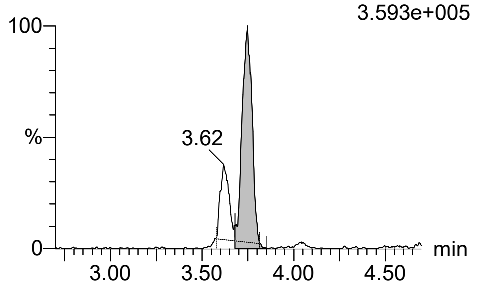

Sample Name: 20211111_QC_R21_IgM
F37:MRM of 1 channel,ES+
923.5 > 366.1
5.239e+005

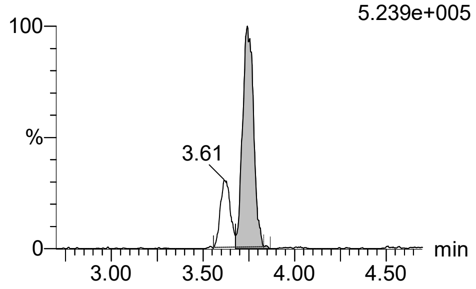

Sample Name: 20211111_L20_R3_IgM
F37:MRM of 1 channel,ES+
923.5 > 366.1
3.638e+005

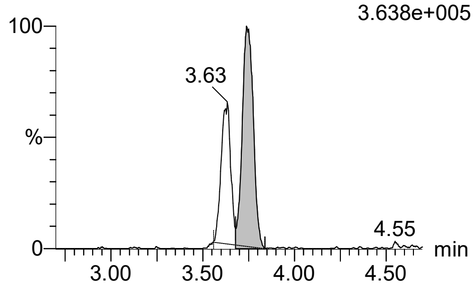

Sample Name: 20211111_H10_R3_IgM
F37:MRM of 1 channel,ES+
923.5 > 366.1
3.701e+005

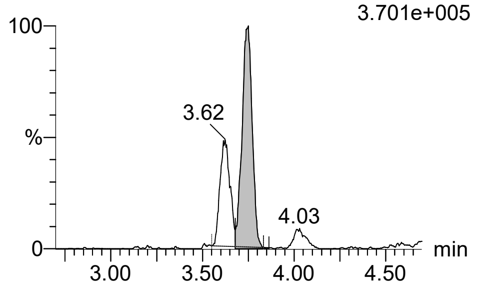

Sample Name: 20211111_A21_R3_IgM
F37:MRM of 1 channel,ES+
923.5 > 366.1
1.156e+006

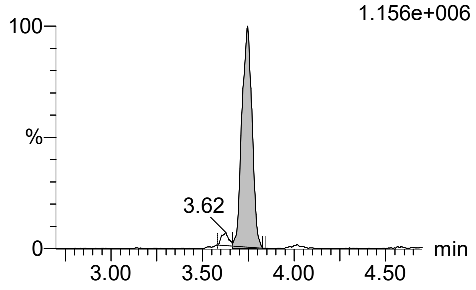

Sample Name: 20211111_A22_R3_IgM
F37:MRM of 1 channel,ES+
923.5 > 366.1
3.708e+005

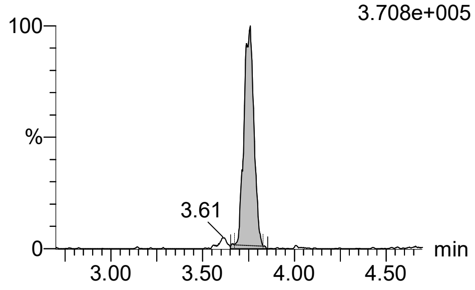

Dataset: C:\MassLynx\10.pro\20211111_clinicalsamples_IgM_IgA.qld

Last Altered: Thursday, March 09, 2023 19:58:25 台北標準時間
Printed: Friday, August 01, 2025 15:26:09 台北標準時間

Sample Name: 20211111_L21_R3_IgM
F37:MRM of 1 channel,ES+
923.5 > 366.1
2.236e+005

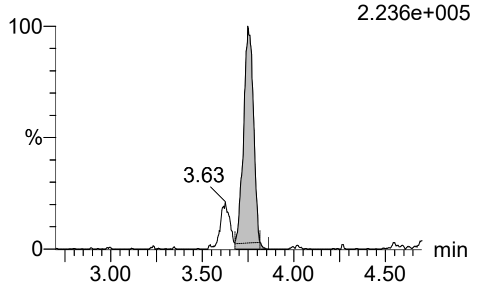

Sample Name: 20211111_L22_R3_IgM
F37:MRM of 1 channel,ES+
923.5 > 366.1
3.769e+005

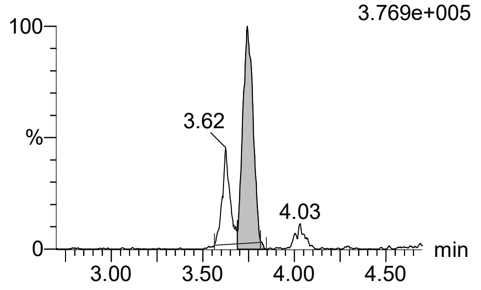

Sample Name: 20211111_H11_R3_IgM
F37:MRM of 1 channel,ES+
923.5 > 366.1
1.010e+006

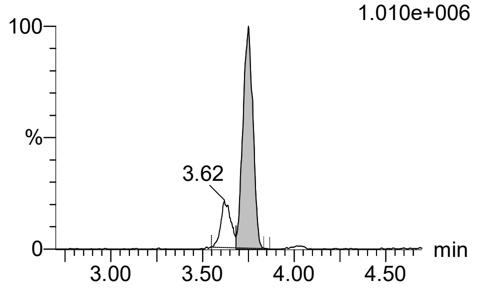

Sample Name: 20211111_A23_R3_IgM
F37:MRM of 1 channel,ES+
923.5 > 366.1
4.772e+005

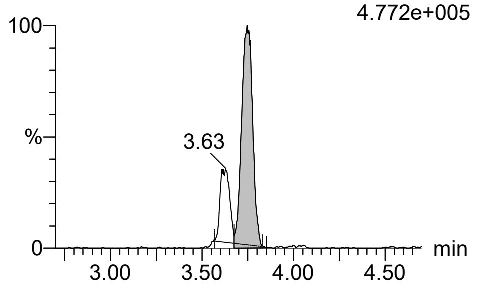

Sample Name: 20211111_A24_R3_IgM
F37:MRM of 1 channel,ES+
923.5 > 366.1
2.543e+005

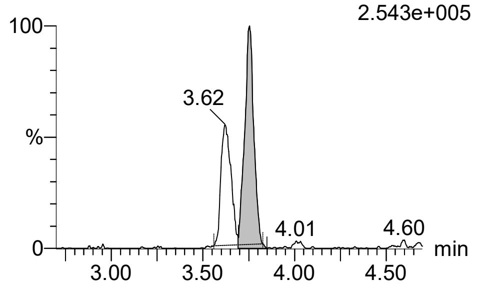

Sample Name: 20211111_L23_R3_IgM
F37:MRM of 1 channel,ES+
923.5 > 366.1
9.540e+005

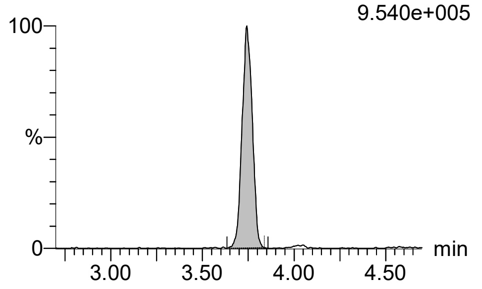

Sample Name: 20211111_L24_R3_IgM
F37:MRM of 1 channel,ES+
923.5 > 366.1
3.843e+005

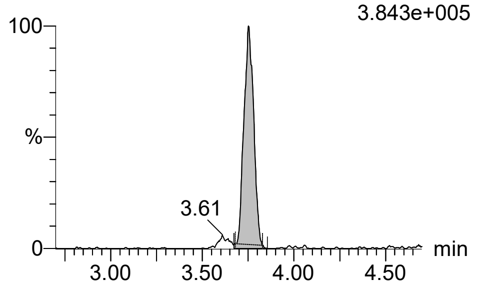

Sample Name: 20211111_H12_R3_IgM
F37:MRM of 1 channel,ES+
923.5 > 366.1
4.238e+005

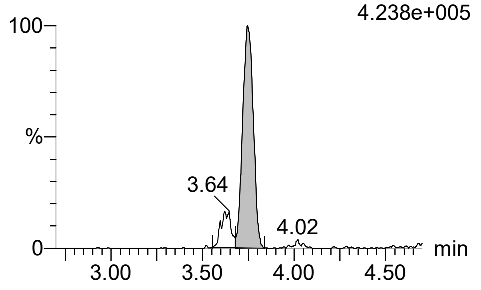

Sample Name: 20211111_QC_R22_IgM
F37:MRM of 1 channel,ES+
923.5 > 366.1
5.961e+005

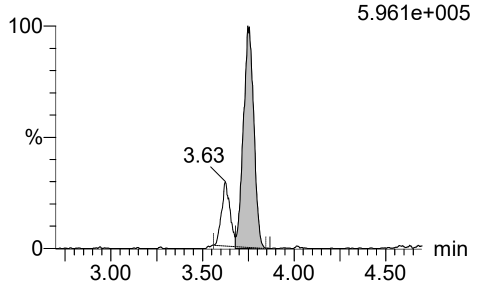

Sample Name: 20211111_A25_R3_IgM
F37:MRM of 1 channel,ES+
923.5 > 366.1
3.165e+005

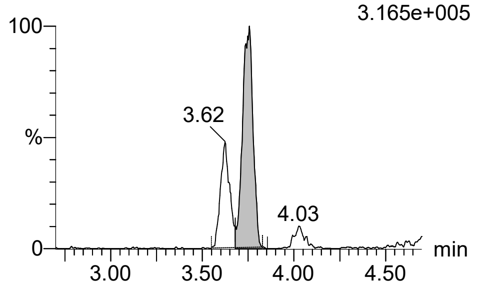

Sample Name: 20211111_A26_R3_IgM
F37:MRM of 1 channel,ES+
923.5 > 366.1
2.580e+005

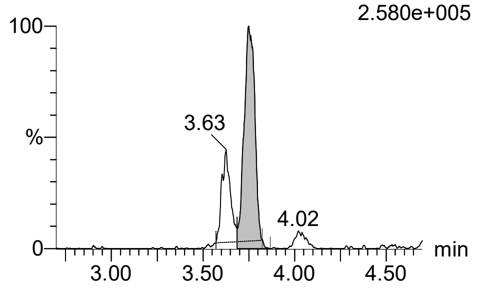

Sample Name: 20211111_L25_R3_IgM
F37:MRM of 1 channel,ES+
923.5 > 366.1
5.541e+005

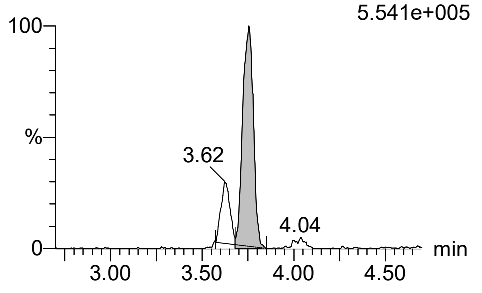

Sample Name: 20211111_L26_R3_IgM
F37:MRM of 1 channel,ES+
923.5 > 366.1
2.290e+005

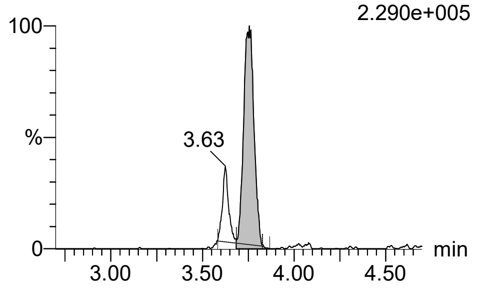

Sample Name: 20211111_H13_R3_IgM
F37:MRM of 1 channel,ES+
923.5 > 366.1
6.698e+005

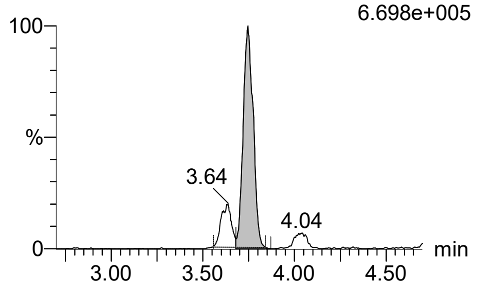

Sample Name: 20211111_A27_R3_IgM
F37:MRM of 1 channel,ES+
923.5 > 366.1
3.070e+005

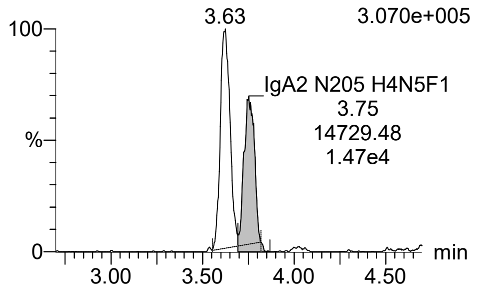

Sample Name: 20211111_A28_R3_IgM

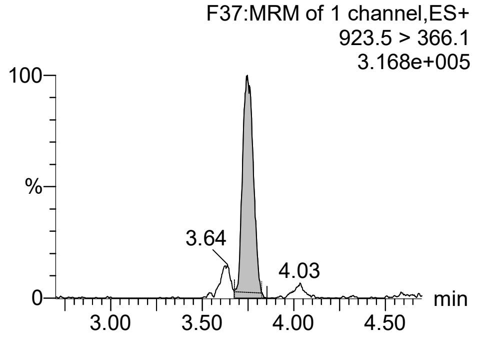

Sample Name: 20211111_L27_R3_IgM

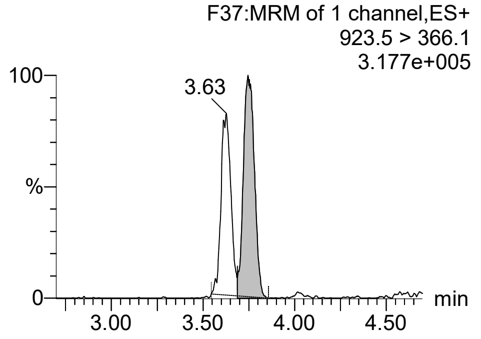

Sample Name: 20211111_L28_R3_IgM

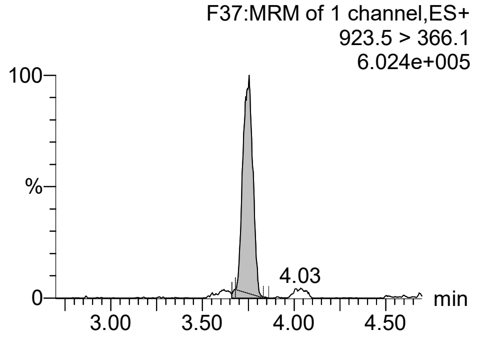

Sample Name: 20211111_H14_R3_IgM

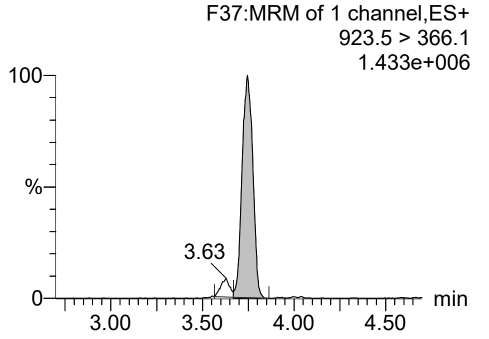

Sample Name: 20211111_A29_R3_IgM

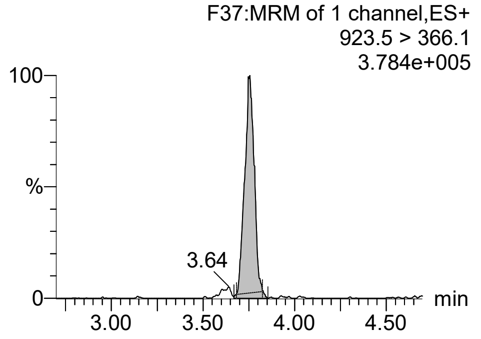

Sample Name: 20211111_A30_R3_IgM

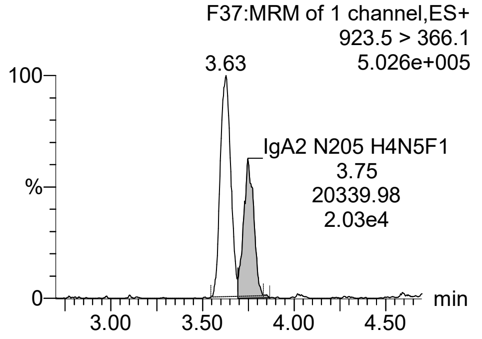

Sample Name: 20211111_QC_R23_IgM

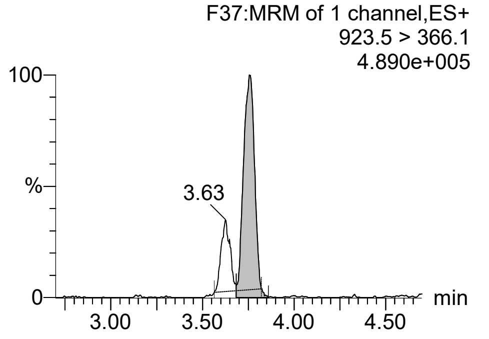

Sample Name: 20211111_L29_R3_IgM

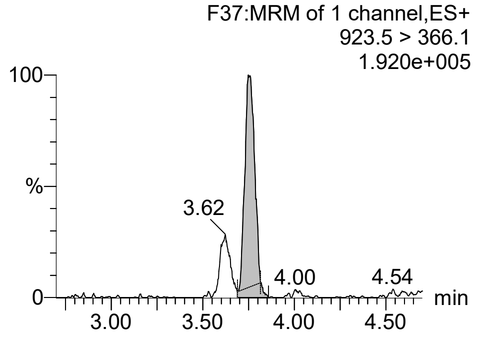

Sample Name: 20211111_L30_R3_IgM

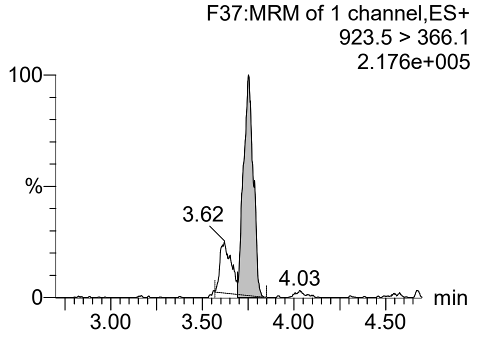

Sample Name: 20211111_H15_R3_IgM

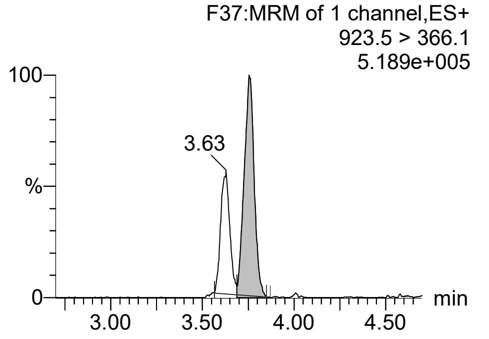

Sample Name: 20211111_A31_R3_IgM

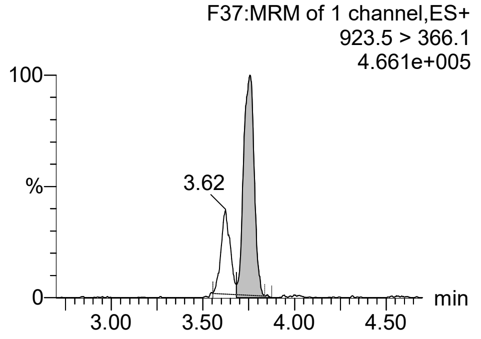

Sample Name: 20211111_A32_R3_IgM

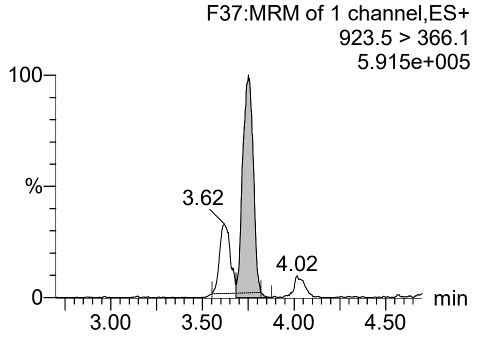

Sample Name: 20211111_L31_R3_IgM

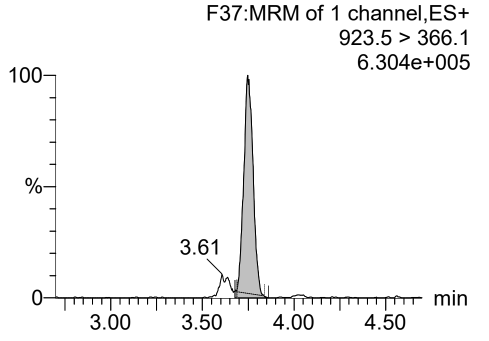

Sample Name: 20211111_L32_R3_IgM

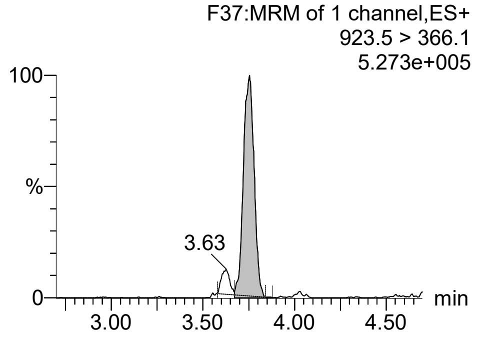

Sample Name: 20211111_H16_R3_IgM

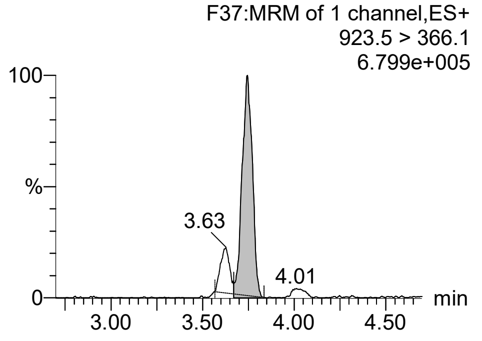

Sample Name: 20211111_A33_R3_IgM

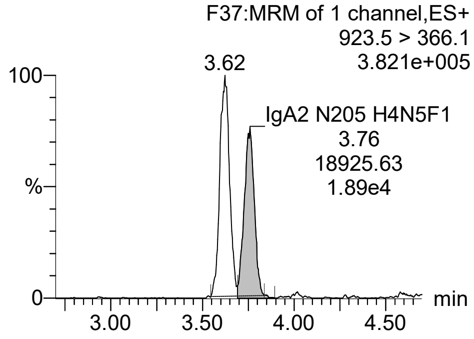

Sample Name: 20211111_A34_R3_IgM

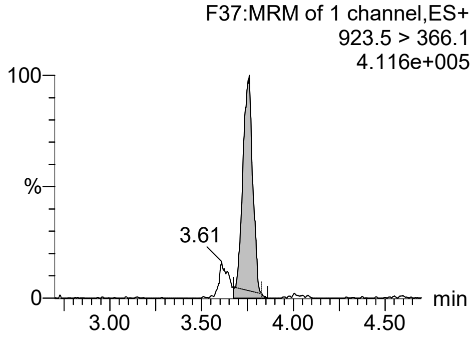

Sample Name: 20211111_H17_R3_IgM

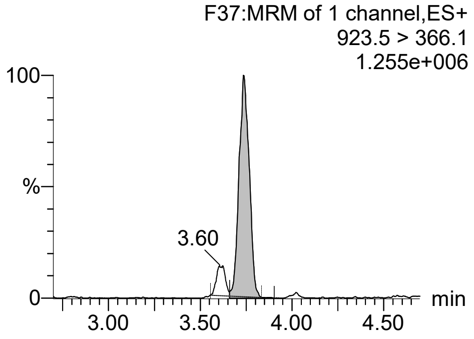

Sample Name: 20211111_A35_R3_IgM

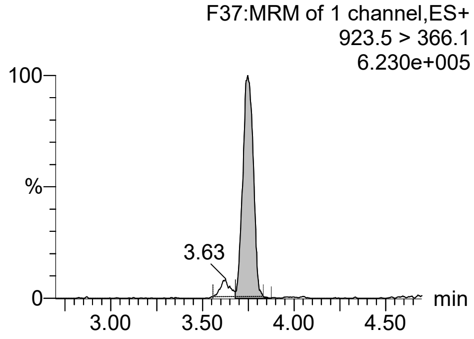

Sample Name: 20211111_QC_R24_IgM

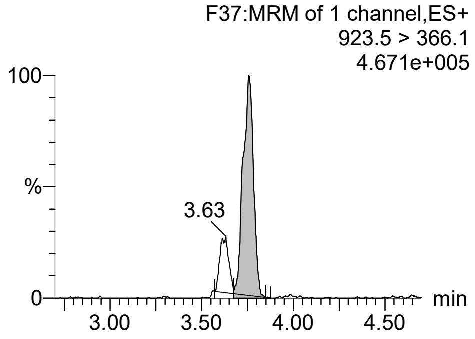

Sample Name: 20211111_A36_R3_IgM

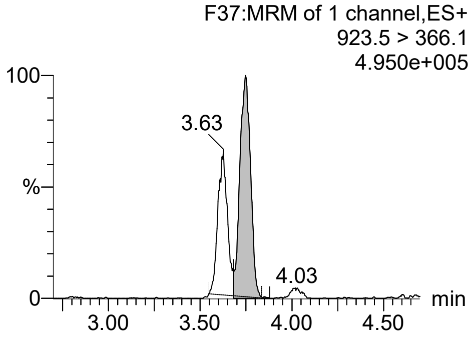

Sample Name: 20211111_H18_R3_IgM

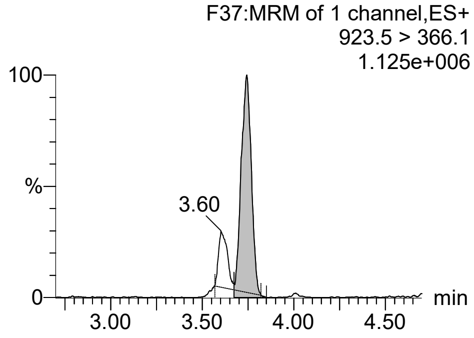

Sample Name: 20211111_A37_R3_IgM

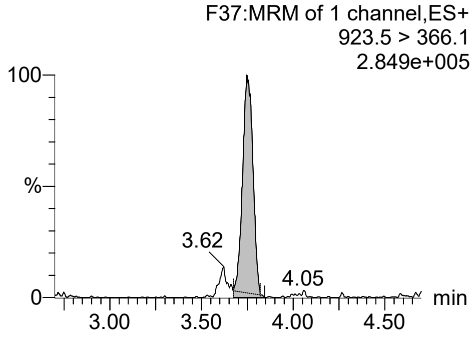

Sample Name: 20211111_A38_R3_IgM

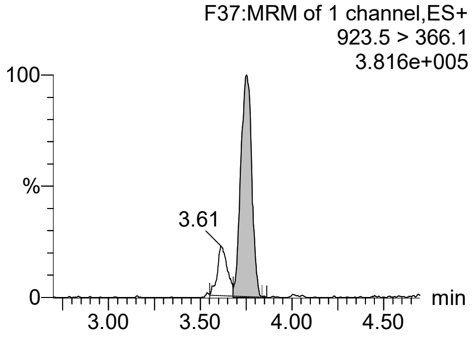

Sample Name: 20211111_H19_R3_IgM

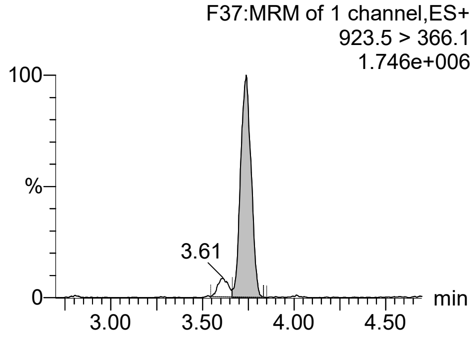

Sample Name: 20211111_A39_R3_IgM

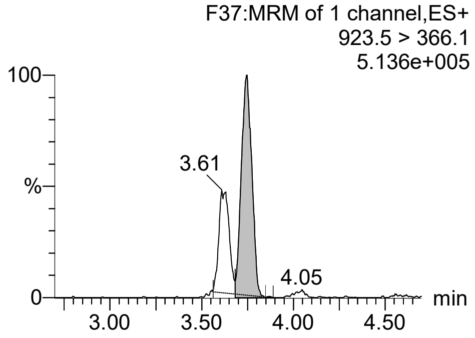

Sample Name: 20211111_A40_R3_IgM

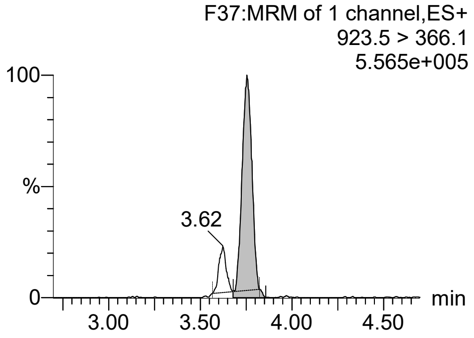

Sample Name: 20211111_H20_R3_IgM

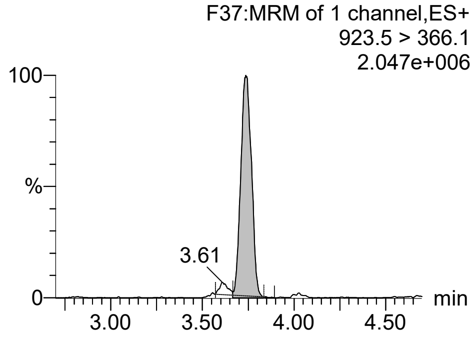

Sample Name: 20211111_A41_R3_IgM

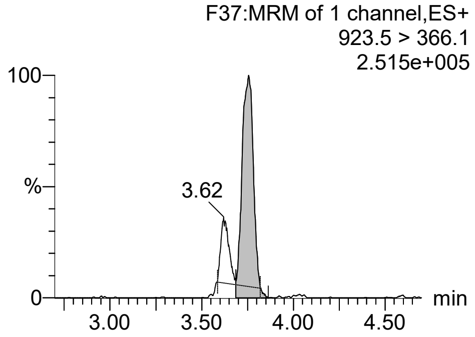

Sample Name: 20211111_A42_R3_IgM

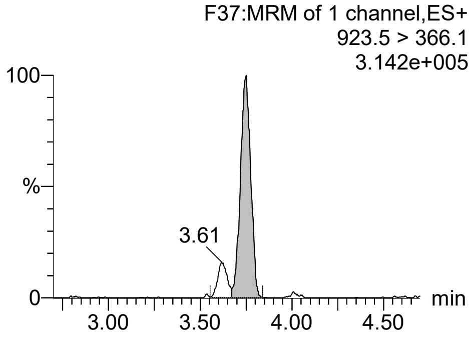

Dataset: C:\MassLynx\10.pro\20211111_clinicalsamples_IgM_IgA.qld

Last Altered: Thursday, March 09, 2023 19:58:25 台北標準時間
Printed: Friday, August 01, 2025 15:26:09 台北標準時間

Sample Name: 20211111_A43_R3_IgM

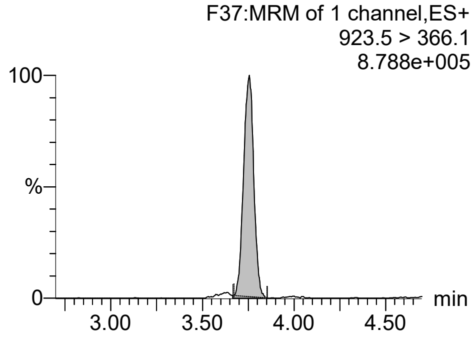

Sample Name: 20211111_A44_R3_IgM

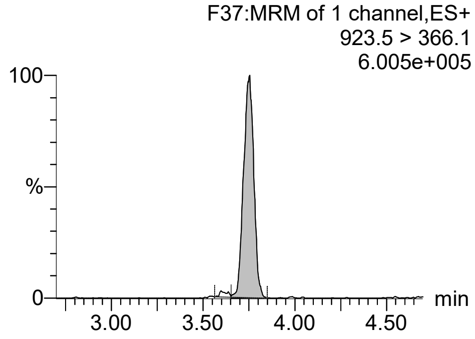

Sample Name: 20211111_QC_R25_IgM

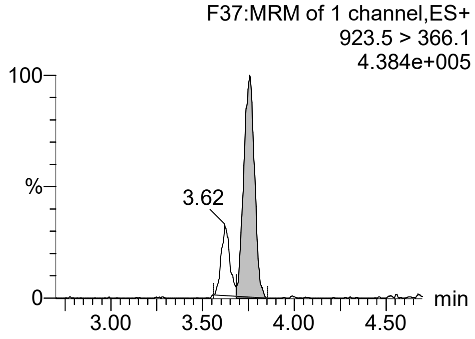

Dataset: C:\MassLynx\10.pro\20211111_clinicalsamples_IgM_IgA.qld

Last Altered: Thursday, March 09, 2023 19:58:25 台北標準時間
Printed: Friday, August 01, 2025 15:26:09 台北標準時間

	#	Name	RT	Area	IS Area	Response	Prima...
1	1	20211112_QC_R001_IgM	3.74	73274.7	15421.4	4.751	bb
2	2	20211112_QC_R002_IgM	3.74	50959.5	13188.8	3.864	MM
3	3	20211111_A1_R2_IgM	3.74	49659.3	11871.0	4.183	MM
4	4	20211111_A1_R3_IgM	3.74	50119.7	12030.5	4.166	MM
5	5	20211111_A2_R2_IgM	3.75	35809.4	11446.8	3.128	MM
6	6	20211111_A2_R3_IgM	3.76	31960.9	11098.4	2.880	MM
7	7	20211111_L1_R2_IgM	3.74	20890.7	11779.4	1.774	MM
8	8	20211111_L1_R3_IgM	3.75	19982.3	11176.2	1.788	MM
9	9	20211111_L2_R2_IgM	3.75	51157.4	11375.0	4.497	MM
10	10	20211111_H1_R2_IgM	3.75	32005.3	13595.2	2.354	MM
11	11	20211111_A3_R2_IgM	3.74	30407.4	14112.3	2.155	MM
12	12	20211111_A4_R2_IgM	3.76	64998.4	11977.5	5.427	MM
13	13	20211111_L3_R2_IgM	3.73	19440.9	12242.1	1.588	MM
14	14	20211111_L4_R2_IgM	3.75	29653.8	12797.0	2.317	MM
15	15	20211111_H2_R2_IgM	3.74	74984.5	10006.0	7.494	MM
16	16	20211111_A5_R2_IgM	3.74	35613.8	11434.6	3.115	MM
17	17	20211111_A6_R2_IgM	3.74	71719.8	13193.7	5.436	MM
18	18	20211111_QC_R10_IgM	3.75	45997.5	12566.1	3.660	MM
19	19	20211111_L5_R2_IgM	3.74	38143.0	13616.4	2.801	MM
20	20	20211111_L6_R2_IgM	3.74	14751.0	13698.5	1.077	MM
21	21	20211111_H3_R2_IgM	3.74	59007.5	15793.1	3.736	MM
22	22	20211111_A7_R2_IgM	3.74	67159.1	12936.9	5.191	MM
23	23	20211111_A8_R2_IgM	3.74	37261.0	13108.0	2.843	MM
24	24	20211111_L7_R2_IgM	3.75	46336.8	13278.5	3.490	bb
25	25	20211111_L8_R2_IgM	3.74	34599.0	15233.6	2.271	MM
26	26	20211111_H4_R2_IgM	3.75	31606.1	16521.9	1.913	MM
27	27	20211111_A9_R2_IgM	3.76	38458.9	16451.6	2.338	MM
28	28	20211111_A10_R2_IgM	3.75	50419.1	13036.0	3.868	MM
29	29	20211111_L9_R2_IgM	3.74	56952.8	14554.1	3.913	MM
30	30	20211111_L10_R2_IgM	3.73	25000.3	12250.4	2.041	MM
31	31	20211111_QC_R11_IgM	3.75	47555.8	11459.2	4.150	MM
32	32	20211111_H5_R2_IgM	3.75	36052.5	14358.2	2.511	MM
33	33	20211111_A11_R2_IgM	3.74	9968.8	16184.1	0.616	MM
34	34	20211111_A12_R2_IgM	3.76	33070.8	13465.4	2.456	MM
35	35	20211111_L11_R2_IgM	3.74	19675.7	13277.9	1.482	MM
36	36	20211111_L12_R2_IgM	3.76	18512.0	15958.8	1.160	MM
37	37	20211111_H6_R2_IgM	3.75	63066.6	14003.5	4.504	MM
38	38	20211111_A13_R2_IgM	3.74	47774.2	13656.1	3.498	MM
39	39	20211111_A14_R2_IgM	3.74	32396.1	14071.3	2.302	MM
40	40	20211111_L13_R2_IgM	3.73	50922.2	14785.6	3.444	MM
41	41	20211111_L14_R2_IgM	3.74	34236.6	14772.2	2.318	MM
42	42	20211111_H7_R2_IgM	3.75	42499.1	15164.4	2.803	MM
43	43	20211111_A15_R2_IgM	3.74	45931.9	15721.5	2.922	MM
44	44	20211111_QC_R12_IgM	3.74	48736.5	13711.5	3.554	MM
45	45	20211111_A16_R2_IgM	3.74	27952.7	14275.6	1.958	MM
46	46	20211111_L15_R2_IgM	3.73	48460.4	12935.8	3.746	MM
47	47	20211111_L16_R2_IgM	3.75	39629.6	13342.8	2.970	MM
48	48	20211111_H8_R2_IgM	3.74	40781.9	15835.1	2.575	MM
49	49	20211111_A17_R2_IgM	3.75	55208.2	15786.7	3.497	MM
50	50	20211111_A18_R2_IgM	3.74	41558.1	16986.8	2.446	MM
51	51	20211111_L17_R2_IgM	3.74	47243.1	13342.7	3.541	MM
52	52	20211111_L18_R2_IgM	3.75	9191.0	14692.0	0.626	MM
53	53	20211111_H9_R2_IgM	3.74	47847.3	15639.7	3.059	MM
54	54	20211111_A19_R2_IgM	3.75	56055.4	14198.7	3.948	MM
55	55	20211111_A20_R2_IgM	3.74	14228.1	14162.7	1.005	MM
56	56	20211111_L19_R2_IgM	3.74	20313.2	9293.4	2.186	MM

Dataset: C:\MassLynx\10.pro\20211111_clinicalsamples_IgM_IgA.qld

Last Altered: Thursday, March 09, 2023 19:58:25 台北標準時間
Printed: Friday, August 01, 2025 15:26:09 台北標準時間

	# Name	RT	Area	IS Area	Response	Prima...
57	57 20211111_QC_R13_IgM	3.74	48559.4	13903.0	3.493	MM
58	58 20211111_L20_R2_IgM	3.74	27116.2	12781.6	2.121	MM
59	59 20211111_H10_R2_IgM	3.75	25908.0	14796.4	1.751	MM
60	60 20211111_A21_R2_IgM	3.74	79274.5	13669.0	5.800	MM
61	61 20211111_A22_R2_IgM	3.75	24188.9	14098.3	1.716	MM
62	62 20211111_L21_R2_IgM	3.75	15681.0	15678.2	1.000	MM
63	63 20211111_L22_R2_IgM	3.73	29586.0	15724.5	1.882	MM
64	64 20211111_H11_R2_IgM	3.75	75457.5	15890.2	4.749	MM
65	65 20211111_A23_R2_IgM	3.74	34902.2	13423.2	2.600	MM
66	66 20211111_A24_R2_IgM	3.74	15733.8	12438.0	1.265	MM
67	67 20211111_L23_R2_IgM	3.73	69920.9	14075.0	4.968	bb
68	68 20211111_L24_R2_IgM	3.75	29099.2	15972.1	1.822	MM
69	69 20211111_H12_R2_IgM	3.74	29231.0	16270.9	1.797	MM
70	70 20211111_QC_R14_IgM	3.75	53898.8	15806.8	3.410	MM
71	71 20211111_A25_R2_IgM	3.73	26170.0	14552.1	1.798	MM
72	72 20211111_A26_R2_IgM	3.75	20374.0	11492.7	1.773	MM
73	73 20211111_L25_R2_IgM	3.74	45854.5	14403.3	3.184	MM
74	74 20211111_L26_R2_IgM	3.75	17993.6	16533.9	1.088	MM
75	75 20211111_H13_R2_IgM	3.75	47211.7	15520.1	3.042	MM
76	76 20211111_A27_R2_IgM	3.74	17578.8	13337.4	1.318	MM
77	77 20211111_A28_R2_IgM	3.74	23970.9	11895.6	2.015	MM
78	78 20211111_L27_R2_IgM	3.74	24977.6	14125.7	1.768	MM
79	79 20211111_L28_R2_IgM	3.75	38598.5	11807.5	3.269	MM
80	80 20211111_H14_R2_IgM	3.74	107663.1	15035.7	7.161	MM
81	81 20211111_A29_R2_IgM	3.74	27627.1	13554.4	2.038	MM
82	82 20211111_A30_R2_IgM	3.75	24050.2	12984.1	1.852	MM
83	83 20211111_QC_R15_IgM	3.76	48145.4	12299.5	3.914	MM
84	84 20211111_L29_R2_IgM	3.75	13591.5	13590.7	1.000	MM
85	85 20211111_L30_R2_IgM	3.75	15272.4	12511.4	1.221	MM
86	86 20211111_H15_R2_IgM	3.76	38366.0	11885.0	3.228	MM
87	87 20211111_A31_R2_IgM	3.74	33284.2	13422.1	2.480	MM
88	88 20211111_A32_R2_IgM	3.76	43335.9	11461.3	3.781	MM
89	89 20211111_L31_R2_IgM	3.74	42921.8	13753.4	3.121	MM
90	90 20211111_L32_R2_IgM	3.74	40856.2	12949.0	3.155	MM
91	91 20211111_H16_R2_IgM	3.74	50965.8	11465.4	4.445	MM
92	92 20211111_A33_R2_IgM	3.76	18673.9	10551.5	1.770	MM
93	93 20211111_A34_R2_IgM	3.75	30992.9	12409.9	2.497	MM
94	94 20211111_H17_R2_IgM	3.73	95956.1	10160.9	9.444	MM
95	95 20211111_A35_R2_IgM	3.74	48064.0	10460.6	4.595	MM
96	96 20211111_QC_R16_IgM	3.75	43683.2	10433.5	4.187	MM
97	97 20211111_A36_R2_IgM	3.74	38375.7	12504.8	3.069	MM
98	98 20211111_H18_R2_IgM	3.74	84518.8	9140.4	9.247	MM
99	99 20211111_A37_R2_IgM	3.76	22750.3	9796.9	2.322	MM
100	1... 20211111_A38_R2_IgM	3.76	33253.1	11224.3	2.963	MM
101	1... 20211111_H19_R2_IgM	3.74	142228.3	11739.2	12.116	MM
102	1... 20211111_A39_R2_IgM	3.74	41909.7	12718.9	3.295	MM
103	1... 20211111_A40_R2_IgM	3.75	39932.9	10006.1	3.991	MM
104	1... 20211111_H20_R2_IgM	3.74	170299.0	7988.2	21.319	MM
105	1... 20211111_A41_R2_IgM	3.75	29640.3	11513.3	2.574	MM
106	1... 20211111_A42_R2_IgM	3.76	21896.0	10238.5	2.139	MM
107	1... 20211111_A43_R2_IgM	3.76	61902.2	11098.9	5.577	MM
108	1... 20211111_A44_R2_IgM	3.75	43717.5	10969.9	3.985	MM
109	1... 20211111_QC_R17_IgM	3.76	33068.2	9700.8	3.409	MM
110	1... 20211111_L2_R3_IgM	3.75	45724.9	8528.0	5.362	MM
111	1... 20211111_H1_R3_IgM	3.75	27644.6	9438.2	2.929	MM
112	1... 20211111_A3_R3_IgM	3.75	24505.9	11598.6	2.113	MM

Dataset: C:\MassLynx\10.pro\20211111_clinicalsamples_IgM_IgA.qld

Last Altered: Thursday, March 09, 2023 19:58:25 台北標準時間
Printed: Friday, August 01, 2025 15:26:09 台北標準時間

	#	Name	RT	Area	IS Area	Response	Prima...
113	1...	20211111_A4_R3_IgM	3.75	57222.8	11364.1	5.035	MM
114	1...	20211111_L3_R3_IgM	3.76	16667.6	10753.4	1.550	MM
115	1...	20211111_L4_R3_IgM	3.74	24011.8	10291.6	2.333	MM
116	1...	20211111_H2_R3_IgM	3.74	71688.7	9315.8	7.695	MM
117	1...	20211111_A5_R3_IgM	3.75	30350.3	9102.2	3.334	MM
118	1...	20211111_A6_R3_IgM	3.74	55155.6	10356.4	5.326	MM
119	1...	20211111_QC_R18_IgM	3.75	33646.8	8398.3	4.006	MM
120	1...	20211111_L5_R3_IgM	3.75	31248.3	9786.7	3.193	MM
121	1...	20211111_L6_R3_IgM	3.76	11672.3	9911.3	1.178	MM
122	1...	20211111_H3_R3_IgM	3.76	48634.4	12155.5	4.001	MM
123	1...	20211111_A7_R3_IgM	3.75	67049.9	11804.8	5.680	MM
124	1...	20211111_A8_R3_IgM	3.74	31448.8	10510.6	2.992	MM
125	1...	20211111_L7_R3_IgM	3.76	41986.4	12177.9	3.448	MM
126	1...	20211111_L8_R3_IgM	3.75	29243.2	11668.6	2.506	MM
127	1...	20211111_H4_R3_IgM	3.75	25156.5	11192.0	2.248	MM
128	1...	20211111_A9_R3_IgM	3.76	33069.7	14077.4	2.349	MM
129	1...	20211111_A10_R3_IgM	3.75	45369.9	11190.4	4.054	MM
130	1...	20211111_L9_R3_IgM	3.74	54042.9	14649.3	3.689	MM
131	1...	20211111_L10_R3_IgM	3.74	20659.5	9361.3	2.207	MM
132	1...	20211111_QC_R19_IgM	3.75	36845.8	10125.5	3.639	MM
133	1...	20211111_H5_R3_IgM	3.75	27941.2	10010.9	2.791	MM
134	1...	20211111_A11_R3_IgM	3.76	9247.0	12697.2	0.728	MM
135	1...	20211111_A12_R3_IgM	3.75	34398.1	13148.5	2.616	MM
136	1...	20211111_L11_R3_IgM	3.74	16471.6	10786.6	1.527	MM
137	1...	20211111_L12_R3_IgM	3.75	16177.6	11681.8	1.385	MM
138	1...	20211111_H6_R3_IgM	3.74	59225.8	12095.5	4.897	MM
139	1...	20211111_A13_R3_IgM	3.74	47470.4	12908.3	3.678	MM
140	1...	20211111_A14_R3_IgM	3.74	25036.1	11322.6	2.211	MM
141	1...	20211111_L13_R3_IgM	3.73	42764.0	11516.1	3.713	MM
142	1...	20211111_L14_R3_IgM	3.75	27521.6	10790.7	2.550	MM
143	1...	20211111_H7_R3_IgM	3.75	38545.3	11572.1	3.331	MM
144	1...	20211111_A15_R3_IgM	3.75	39415.1	12434.4	3.170	MM
145	1...	20211111_QC_R20_IgM	3.75	36497.1	10701.0	3.411	MM
146	1...	20211111_A16_R3_IgM	3.75	25451.9	11452.1	2.222	MM
147	1...	20211111_L15_R3_IgM	3.74	39343.8	10531.1	3.736	MM
148	1...	20211111_L16_R3_IgM	3.75	36373.1	12049.4	3.019	MM
149	1...	20211111_H8_R3_IgM	3.75	35633.9	13434.9	2.652	MM
150	1...	20211111_A17_R3_IgM	3.75	48079.3	12452.0	3.861	MM
151	1...	20211111_A18_R3_IgM	3.75	37330.4	12506.5	2.985	MM
152	1...	20211111_L17_R3_IgM	3.75	40778.7	9948.9	4.099	MM
153	1...	20211111_L18_R3_IgM	3.74	9208.4	13462.1	0.684	MM
154	1...	20211111_H9_R3_IgM	3.75	41503.5	13178.7	3.149	MM
155	1...	20211111_A19_R3_IgM	3.75	55599.6	11298.6	4.921	MM
156	1...	20211111_A20_R3_IgM	3.73	13468.4	11175.6	1.205	MM
157	1...	20211111_L19_R3_IgM	3.75	22744.2	11558.2	1.968	MM
158	1...	20211111_QC_R21_IgM	3.74	36552.9	9977.0	3.664	MM
159	1...	20211111_L20_R3_IgM	3.74	25462.8	13508.1	1.885	MM
160	1...	20211111_H10_R3_IgM	3.75	23741.2	12374.8	1.919	MM
161	1...	20211111_A21_R3_IgM	3.75	74216.1	12443.6	5.964	MM
162	1...	20211111_A22_R3_IgM	3.76	23772.3	11589.3	2.051	MM
163	1...	20211111_L21_R3_IgM	3.75	14291.5	13107.4	1.090	MM
164	1...	20211111_L22_R3_IgM	3.74	24775.8	12021.5	2.061	MM
165	1...	20211111_H11_R3_IgM	3.75	63939.6	12687.8	5.039	MM
166	1...	20211111_A23_R3_IgM	3.75	33331.2	11611.8	2.870	MM
167	1...	20211111_A24_R3_IgM	3.75	14895.6	10476.8	1.422	MM
168	1...	20211111_L23_R3_IgM	3.74	61613.2	11873.1	5.189	MM

Dataset: C:\MassLynx\10.pro\20211111_clinicalsamples_IgM_IgA.qld

Last Altered: Thursday, March 09, 2023 19:58:25 台北標準時間
Printed: Friday, August 01, 2025 15:26:09 台北標準時間

	#	Name	RT	Area	IS Area	Response	Prima...
169	1...	20211111_L24_R3_IgM	3.75	24057.2	13468.0	1.786	MM
170	1...	20211111_H12_R3_IgM	3.75	28544.2	14229.1	2.006	MM
171	1...	20211111_QC_R22_IgM	3.75	39237.9	11264.4	3.483	MM
172	1...	20211111_A25_R3_IgM	3.76	20932.8	11775.7	1.778	MM
173	1...	20211111_A26_R3_IgM	3.75	17887.1	10473.9	1.708	MM
174	1...	20211111_L25_R3_IgM	3.75	37421.2	13256.8	2.823	MM
175	1...	20211111_L26_R3_IgM	3.76	15110.4	13565.0	1.114	MM
176	1...	20211111_H13_R3_IgM	3.75	42378.0	13146.0	3.224	MM
177	1...	20211111_A27_R3_IgM	3.75	14729.5	12025.6	1.225	MM
178	1...	20211111_A28_R3_IgM	3.75	21912.5	11229.1	1.951	MM
179	1...	20211111_L27_R3_IgM	3.74	22203.0	14960.0	1.484	MM
180	1...	20211111_L28_R3_IgM	3.75	38711.3	11359.0	3.408	MM
181	1...	20211111_H14_R3_IgM	3.74	97373.0	12830.8	7.589	MM
182	1...	20211111_A29_R3_IgM	3.76	23461.5	13434.3	1.746	MM
183	1...	20211111_A30_R3_IgM	3.75	20340.0	11272.2	1.804	MM
184	1...	20211111_QC_R23_IgM	3.76	34639.9	11872.2	2.918	MM
185	1...	20211111_L29_R3_IgM	3.75	12495.0	12948.7	0.965	MM
186	1...	20211111_L30_R3_IgM	3.75	13401.0	11026.6	1.215	MM
187	1...	20211111_H15_R3_IgM	3.75	33030.5	10786.3	3.062	MM
188	1...	20211111_A31_R3_IgM	3.76	31619.8	12010.5	2.633	MM
189	1...	20211111_A32_R3_IgM	3.75	39894.0	11107.7	3.592	MM
190	1...	20211111_L31_R3_IgM	3.75	38033.1	11021.8	3.451	MM
191	1...	20211111_L32_R3_IgM	3.75	35833.5	11683.8	3.067	MM
192	1...	20211111_H16_R3_IgM	3.74	44245.0	9831.7	4.500	MM
193	1...	20211111_A33_R3_IgM	3.76	18925.6	11213.1	1.688	MM
194	1...	20211111_A34_R3_IgM	3.76	26202.0	11550.1	2.269	MM
195	1...	20211111_H17_R3_IgM	3.74	81481.6	8874.4	9.182	MM
196	1...	20211111_A35_R3_IgM	3.75	41630.4	9856.3	4.224	MM
197	1...	20211111_QC_R24_IgM	3.76	31115.4	7561.8	4.115	MM
198	1...	20211111_A36_R3_IgM	3.75	33432.3	9562.8	3.496	MM
199	1...	20211111_H18_R3_IgM	3.74	73282.3	8165.3	8.975	MM
200	2...	20211111_A37_R3_IgM	3.75	18989.1	9278.8	2.047	MM
201	2...	20211111_A38_R3_IgM	3.75	26450.9	10920.3	2.422	MM
202	2...	20211111_H19_R3_IgM	3.74	115949.8	9285.3	12.487	MM
203	2...	20211111_A39_R3_IgM	3.75	34197.4	9083.4	3.765	MM
204	2...	20211111_A40_R3_IgM	3.75	35508.2	8382.4	4.236	MM
205	2...	20211111_H20_R3_IgM	3.74	139102.5	7807.9	17.816	MM
206	2...	20211111_A41_R3_IgM	3.76	18273.6	5925.2	3.084	MM
207	2...	20211111_A42_R3_IgM	3.75	20282.1	9352.6	2.169	MM
208	2...	20211111_A43_R3_IgM	3.76	56813.0	10216.3	5.561	MM
209	2...	20211111_A44_R3_IgM	3.75	38835.8	10262.7	3.784	MM
210	2...	20211111_QC_R25_IgM	3.76	29692.9	8575.3	3.463	MM

Dataset: C:\MassLynx\10.pro\20211111_clinicalsamples_IgM_IgA.qld

Last Altered: Thursday, March 09, 2023 19:58:25 台北標準時間
Printed: Friday, August 01, 2025 15:26:50 台北標準時間

Method: C:\MassLynx\10_Pro_IgA.PRO\MethDB\20211111_IgM_IgA_DMRM_new.mdb 24 Feb 2022 13:44:14
Calibration: 15 Nov 2021 14:42:11

Sample Name: 20211112_QC_R001_IgM

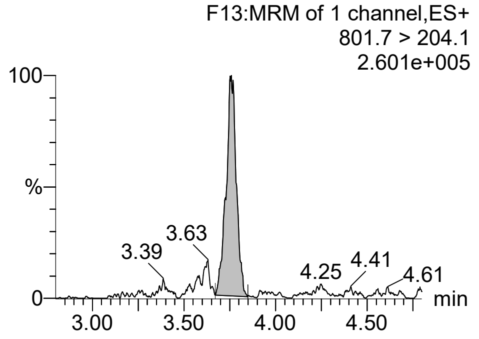

Sample Name: 20211112_QC_R002_IgM

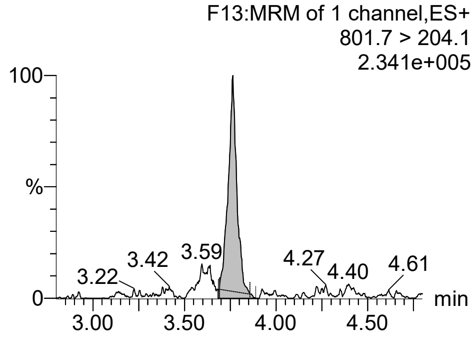

Sample Name: 20211111_A1_R2_IgM

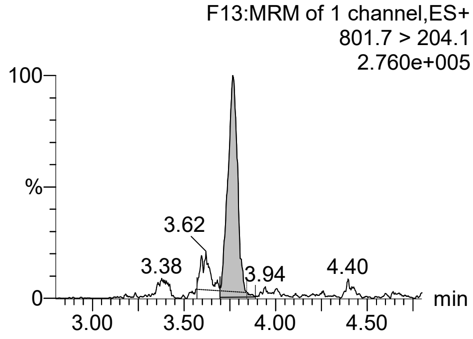

Sample Name: 20211111_A1_R3_IgM

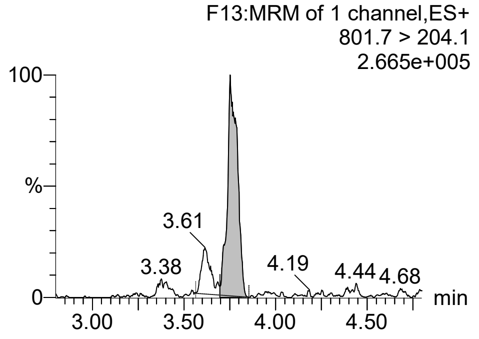

Sample Name: 20211111_A2_R2_IgM

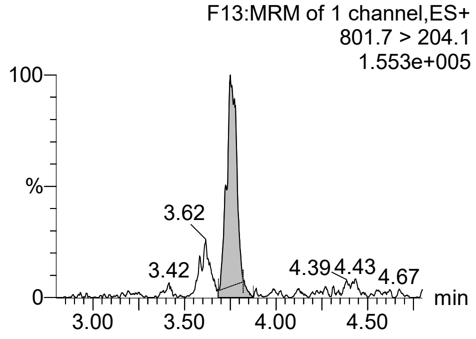

Sample Name: 20211111_A2_R3_IgM

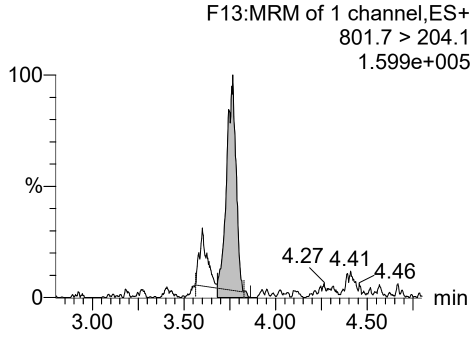

Sample Name: 20211111_L1_R2_IgM

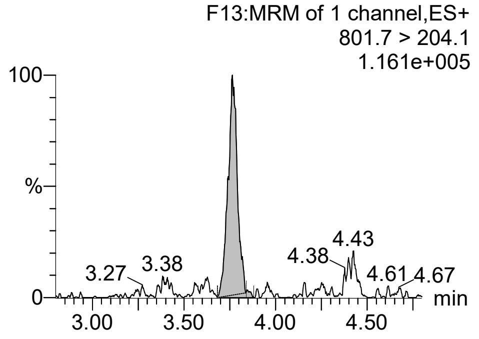

Sample Name: 20211111_L1_R3_IgM

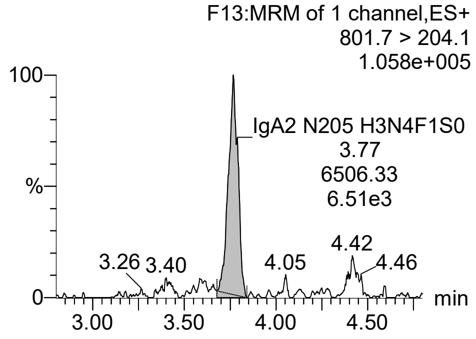

Sample Name: 20211111_L2_R2_IgM

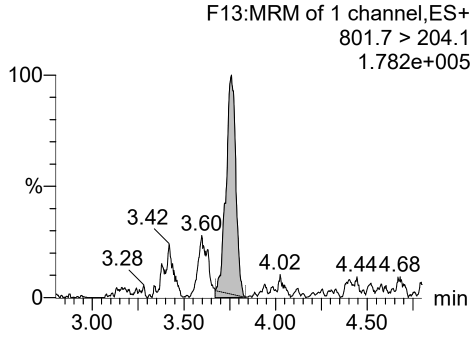

Sample Name: 20211111_H1_R2_IgM

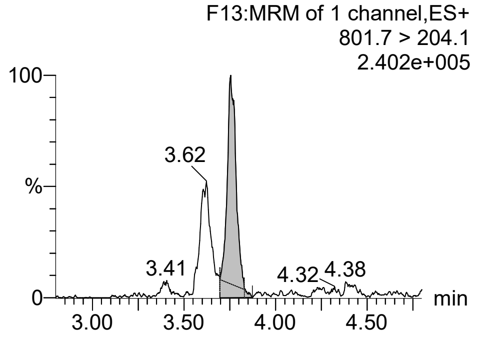

Sample Name: 20211111_A3_R2_IgM

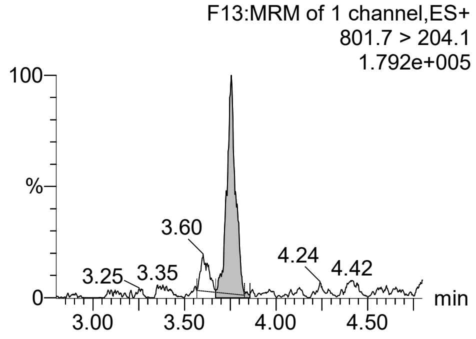

Sample Name: 20211111_A4_R2_IgM

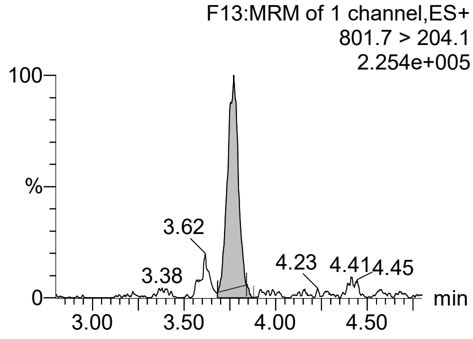

Sample Name: 20211111_L3_R2_IgM

F13:MRM of 1 channel,ES+
801.7 > 204.1
1.218e+005

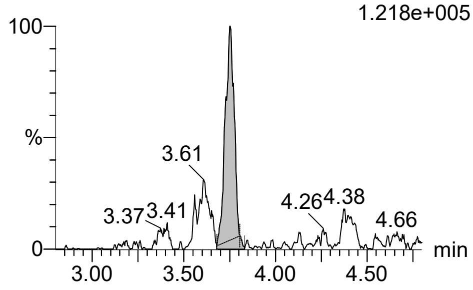

Sample Name: 20211111_L4_R2_IgM

F13:MRM of 1 channel,ES+
801.7 > 204.1
1.325e+005

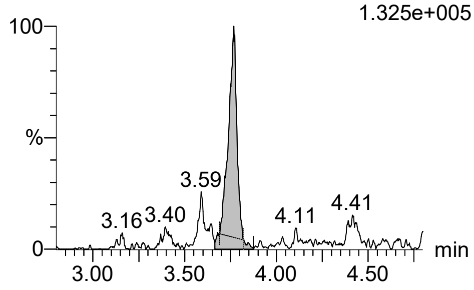

Sample Name: 20211111_H2_R2_IgM

F13:MRM of 1 channel,ES+
801.7 > 204.1
2.711e+005

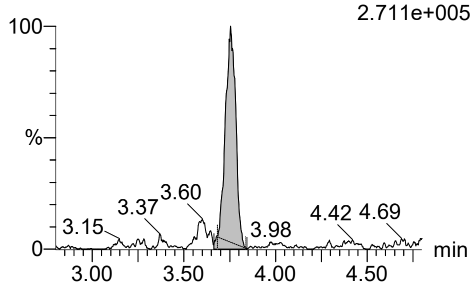

Sample Name: 20211111_A5_R2_IgM

F13:MRM of 1 channel,ES+
801.7 > 204.1
2.891e+005

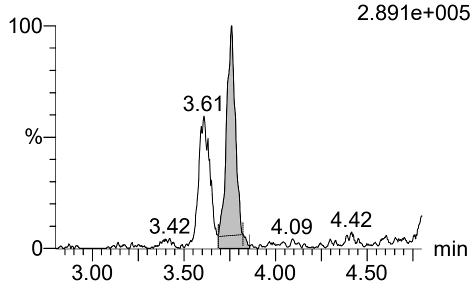

Sample Name: 20211111_A6_R2_IgM

F13:MRM of 1 channel,ES+
801.7 > 204.1
2.417e+005

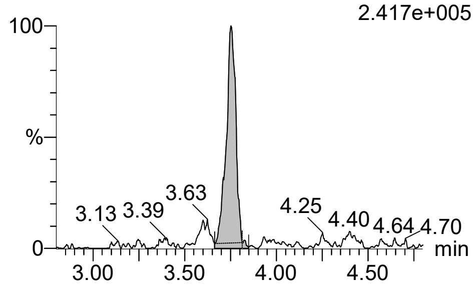

Sample Name: 20211111_QC_R10_IgM

F13:MRM of 1 channel,ES+
801.7 > 204.1
1.938e+005

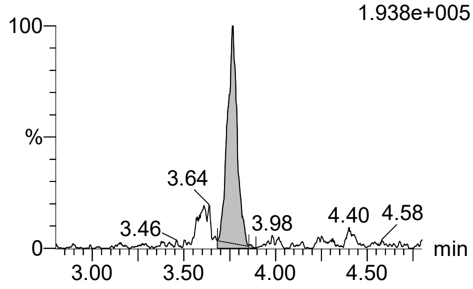

Sample Name: 20211111_L5_R2_IgM

F13:MRM of 1 channel,ES+
801.7 > 204.1
1.813e+005

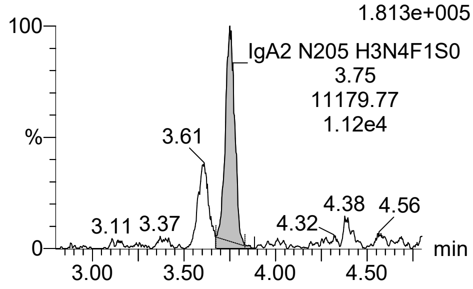

Sample Name: 20211111_L6_R2_IgM

F13:MRM of 1 channel,ES+
801.7 > 204.1
1.044e+005

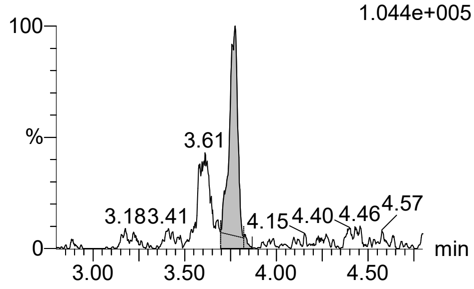

Sample Name: 20211111_H3_R2_IgM

F13:MRM of 1 channel,ES+
801.7 > 204.1
2.107e+005

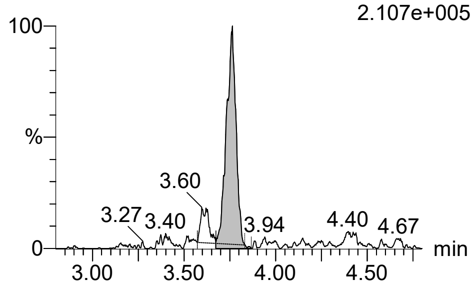

Sample Name: 20211111_A7_R2_IgM

F13:MRM of 1 channel,ES+
801.7 > 204.1
3.286e+005

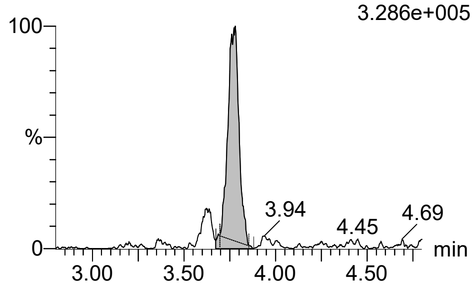

Sample Name: 20211111_A8_R2_IgM

F13:MRM of 1 channel,ES+
801.7 > 204.1
2.079e+005

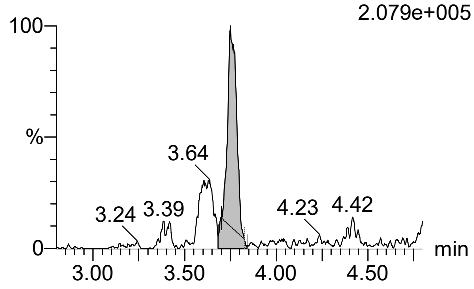

Sample Name: 20211111_L7_R2_IgM

F13:MRM of 1 channel,ES+
801.7 > 204.1
1.866e+005

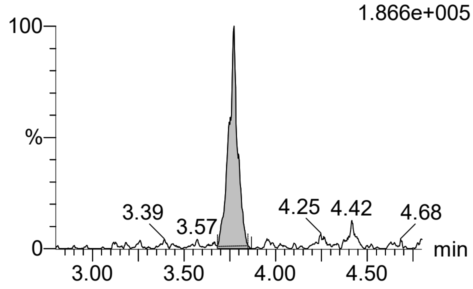

Sample Name: 20211111_L8_R2_IgM

F13:MRM of 1 channel,ES+
801.7 > 204.1
1.195e+005

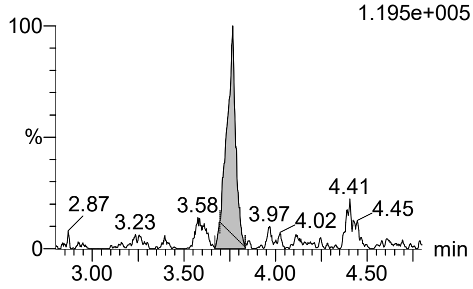

Sample Name: 20211111_H4_R2_IgM

F13:MRM of 1 channel,ES+
801.7 > 204.1
1.486e+005

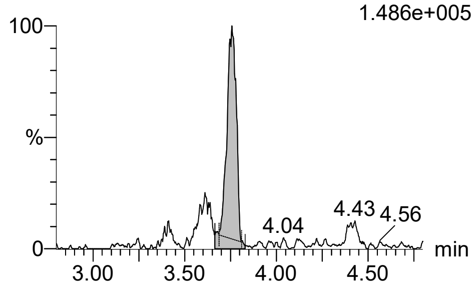

Sample Name: 20211111_A9_R2_IgM

F13:MRM of 1 channel,ES+
801.7 > 204.1
2.264e+005

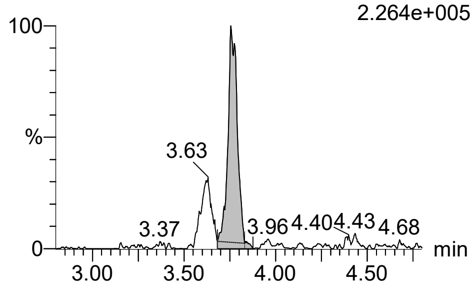

Dataset: C:\MassLynx\10.pro\20211111_clinicalsamples_IgM_IgA.qld

Last Altered: Thursday, March 09, 2023 19:58:25 台北標準時間
Printed: Friday, August 01, 2025 15:26:50 台北標準時間

Sample Name: 20211111_A10_R2_IgM
F13:MRM of 1 channel,ES+
801.7 > 204.1
3.094e+005

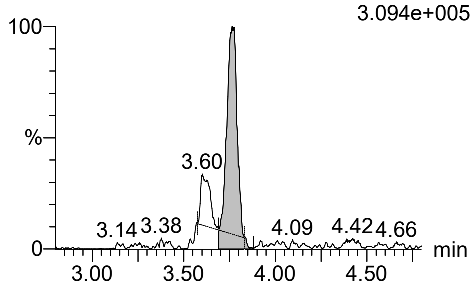

Sample Name: 20211111_L9_R2_IgM
F13:MRM of 1 channel,ES+
801.7 > 204.1
2.431e+005

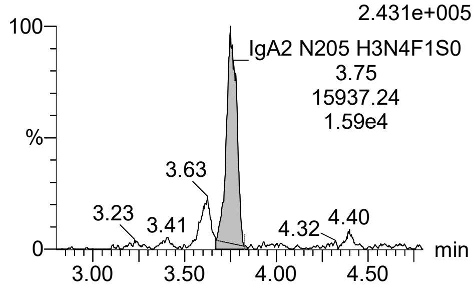

Sample Name: 20211111_L10_R2_IgM
F13:MRM of 1 channel,ES+
801.7 > 204.1
1.167e+005

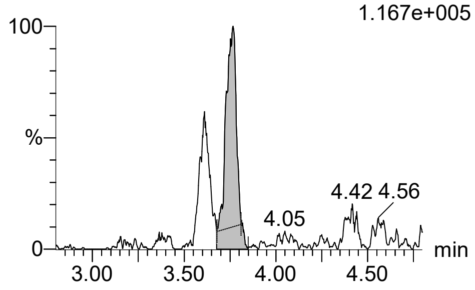

Sample Name: 20211111_QC_R11_IgM
F13:MRM of 1 channel,ES+
801.7 > 204.1
1.769e+005

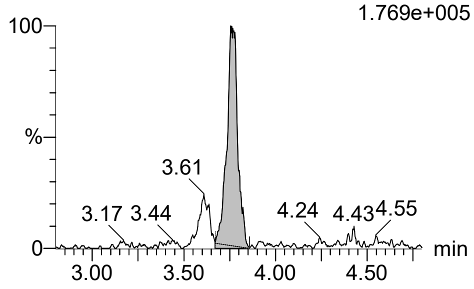

Sample Name: 20211111_H5_R2_IgM
F13:MRM of 1 channel,ES+
801.7 > 204.1
1.326e+005

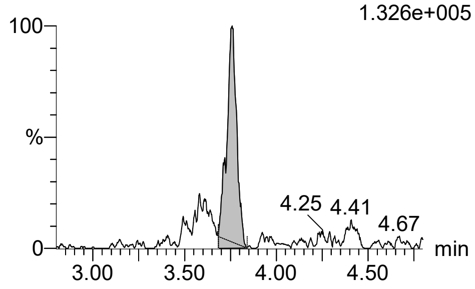

Sample Name: 20211111_A11_R2_IgM
F13:MRM of 1 channel,ES+
801.7 > 204.1
1.167e+005

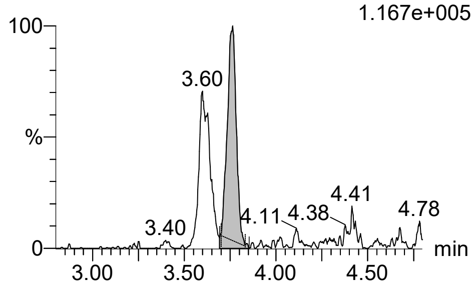

Sample Name: 20211111_A12_R2_IgM
F13:MRM of 1 channel,ES+
801.7 > 204.1
1.533e+005

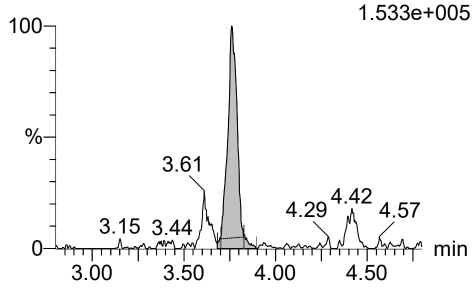

Sample Name: 20211111_L11_R2_IgM
F13:MRM of 1 channel,ES+
801.7 > 204.1
9.855e+004

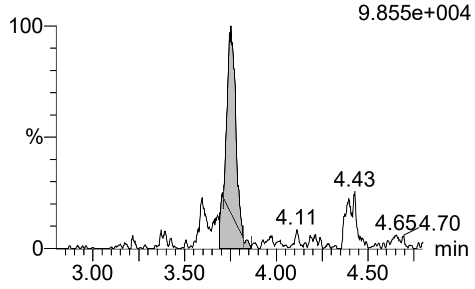

Sample Name: 20211111_L12_R2_IgM
F13:MRM of 1 channel,ES+
801.7 > 204.1
1.228e+005

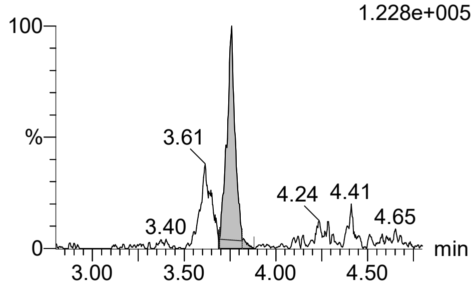

Sample Name: 20211111_H6_R2_IgM
F13:MRM of 1 channel,ES+
801.7 > 204.1
2.263e+005

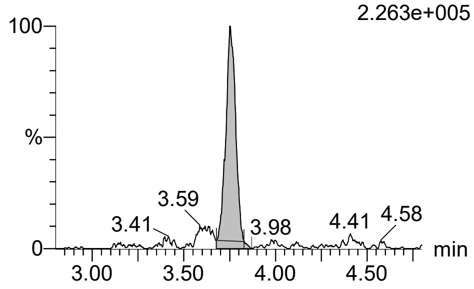

Sample Name: 20211111_A13_R2_IgM
F13:MRM of 1 channel,ES+
801.7 > 204.1
1.687e+005

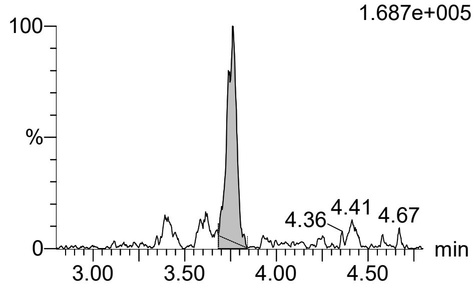

Sample Name: 20211111_A14_R2_IgM
F13:MRM of 1 channel,ES+
801.7 > 204.1
3.068e+005

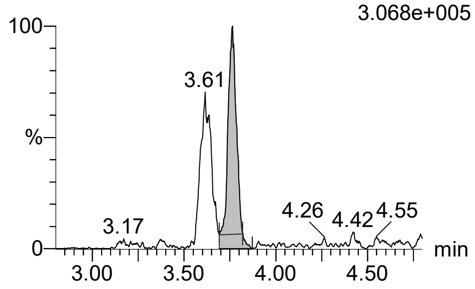

Sample Name: 20211111_L13_R2_IgM
F13:MRM of 1 channel,ES+
801.7 > 204.1
1.334e+005

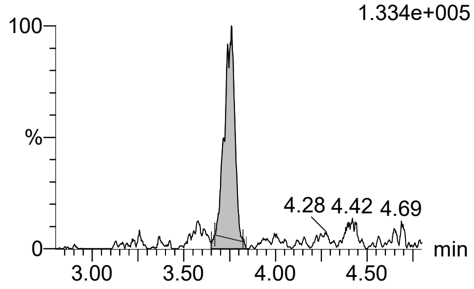

Sample Name: 20211111_L14_R2_IgM
F13:MRM of 1 channel,ES+
801.7 > 204.1
1.212e+005

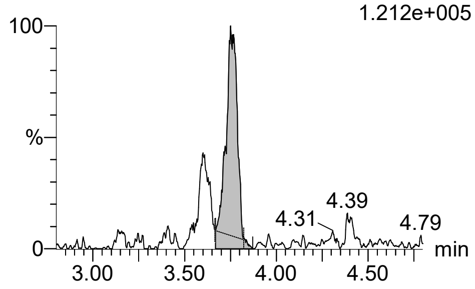

Sample Name: 20211111_H7_R2_IgM
F13:MRM of 1 channel,ES+
801.7 > 204.1
1.810e+005

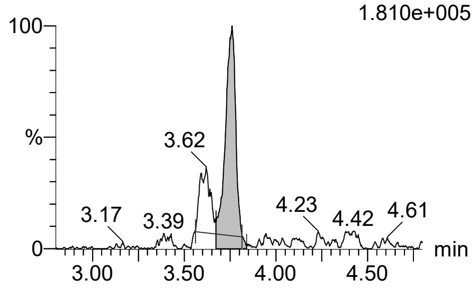

Dataset: C:\MassLynx\10.pro\20211111_clinicalsamples_IgM_IgA.qld

Last Altered: Thursday, March 09, 2023 19:58:25 台北標準時間
Printed: Friday, August 01, 2025 15:26:50 台北標準時間

Sample Name: 20211111_A15_R2_IgM
F13:MRM of 1 channel,ES+
801.7 > 204.1
2.134e+005

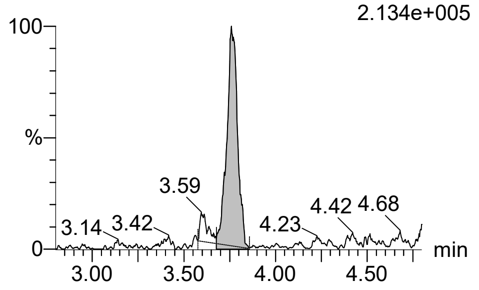

Sample Name: 20211111_QC_R12_IgM
F13:MRM of 1 channel,ES+
801.7 > 204.1
2.136e+005

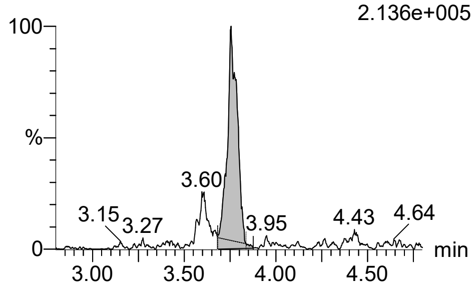

Sample Name: 20211111_A16_R2_IgM
F13:MRM of 1 channel,ES+
801.7 > 204.1
1.882e+005

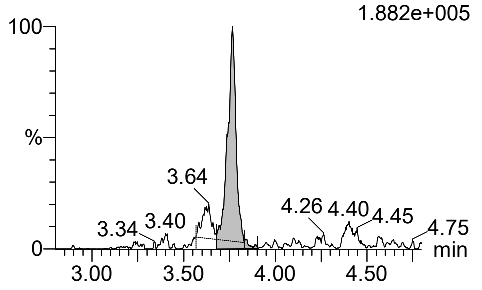

Sample Name: 20211111_L15_R2_IgM
F13:MRM of 1 channel,ES+
801.7 > 204.1
1.939e+005

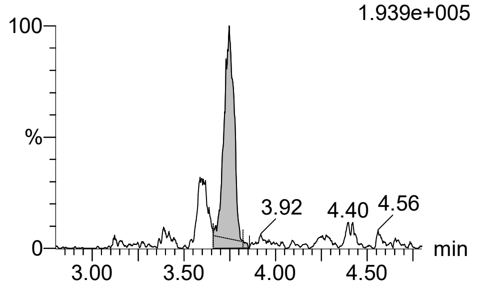

Sample Name: 20211111_L16_R2_IgM
F13:MRM of 1 channel,ES+
801.7 > 204.1
1.344e+005

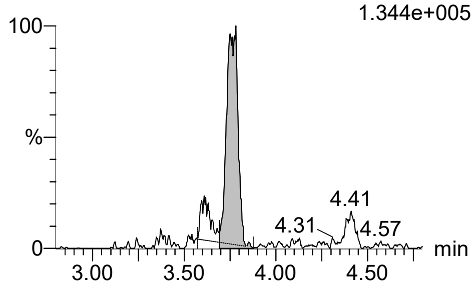

Sample Name: 20211111_H8_R2_IgM
F13:MRM of 1 channel,ES+
801.7 > 204.1
1.392e+005

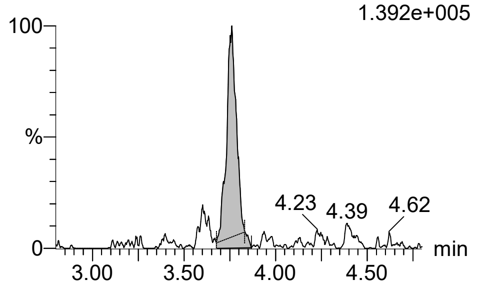

Sample Name: 20211111_A17_R2_IgM
F13:MRM of 1 channel,ES+
801.7 > 204.1
2.565e+005

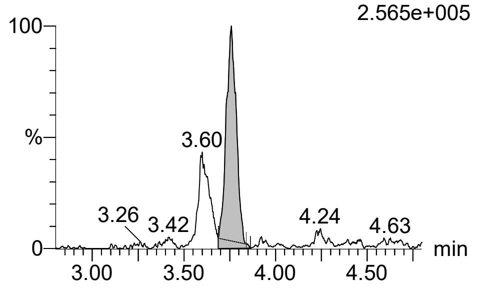

Sample Name: 20211111_A18_R2_IgM
F13:MRM of 1 channel,ES+
801.7 > 204.1
2.250e+005

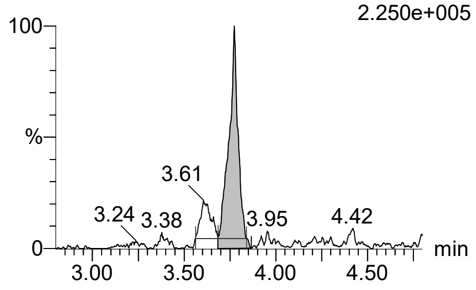

Sample Name: 20211111_L17_R2_IgM
F13:MRM of 1 channel,ES+
801.7 > 204.1
1.913e+005

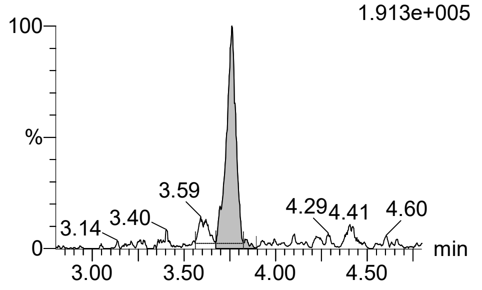

Sample Name: 20211111_L18_R2_IgM
F13:MRM of 1 channel,ES+
801.7 > 204.1
5.248e+004

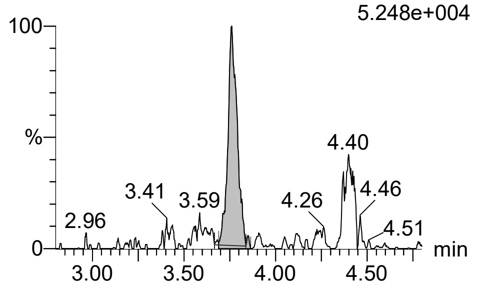

Sample Name: 20211111_H9_R2_IgM
F13:MRM of 1 channel,ES+
801.7 > 204.1
2.214e+005

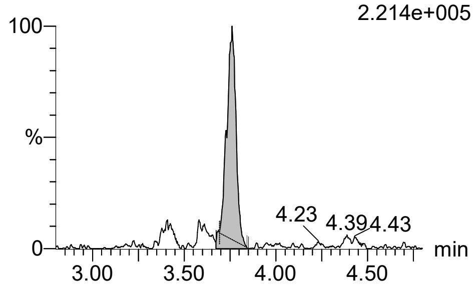

Sample Name: 20211111_A19_R2_IgM
F13:MRM of 1 channel,ES+
801.7 > 204.1
2.154e+005

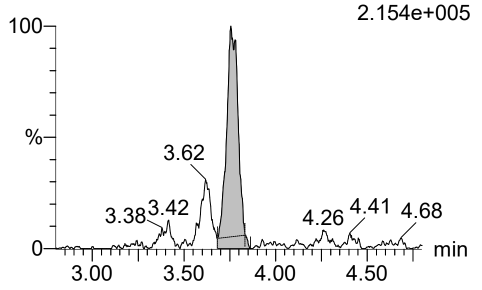

Sample Name: 20211111_A20_R2_IgM
F13:MRM of 1 channel,ES+
801.7 > 204.1
6.872e+004

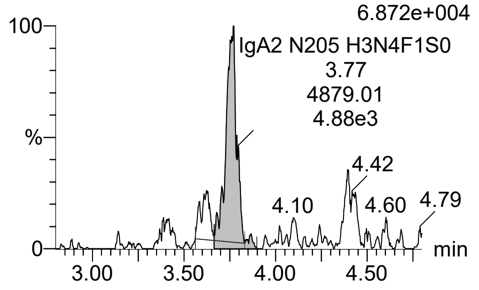

Sample Name: 20211111_L19_R2_IgM
F13:MRM of 1 channel,ES+
801.7 > 204.1
8.657e+004

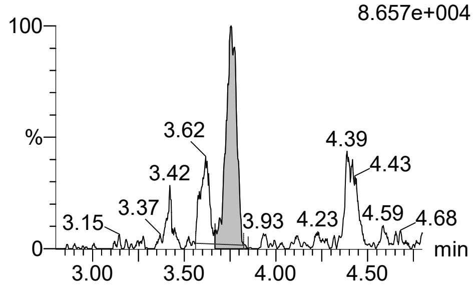

Sample Name: 20211111_QC_R13_IgM
F13:MRM of 1 channel,ES+
801.7 > 204.1
1.710e+005

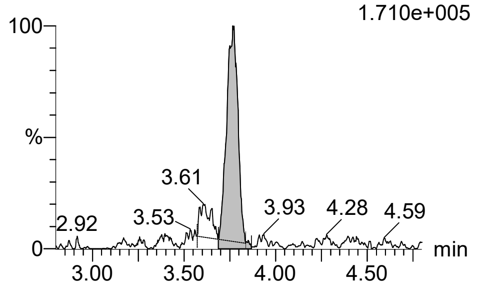

Sample Name: 20211111_L20_R2_IgM
F13:MRM of 1 channel,ES+
801.7 > 204.1
1.416e+005

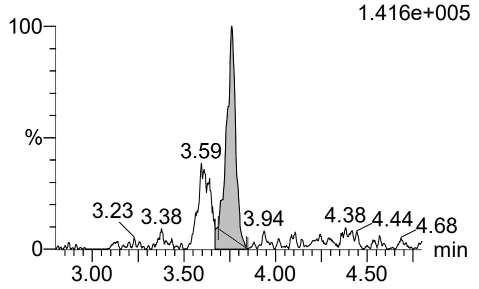

Sample Name: 20211111_H10_R2_IgM
F13:MRM of 1 channel,ES+
801.7 > 204.1
1.110e+005

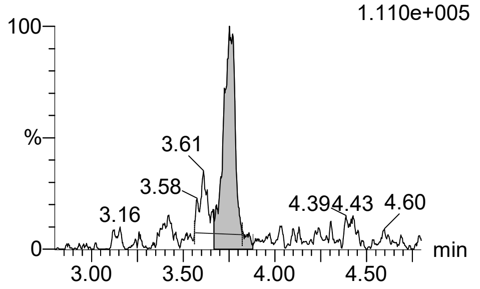

Sample Name: 20211111_A21_R2_IgM
F13:MRM of 1 channel,ES+
801.7 > 204.1
2.773e+005

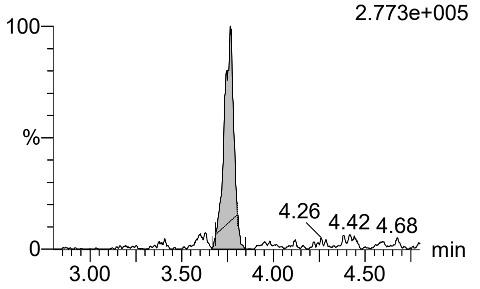

Sample Name: 20211111_A22_R2_IgM
F13:MRM of 1 channel,ES+
801.7 > 204.1
5.379e+004

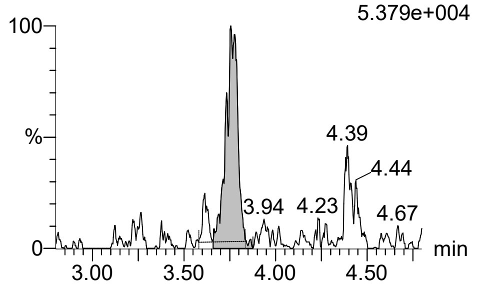

Sample Name: 20211111_L21_R2_IgM
F13:MRM of 1 channel,ES+
801.7 > 204.1
7.207e+004

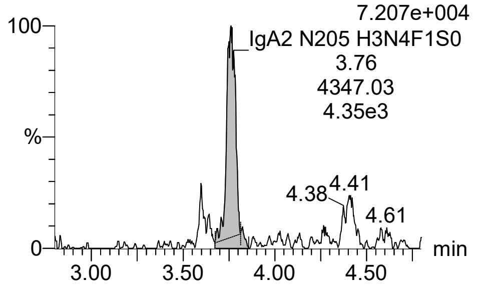

Sample Name: 20211111_L22_R2_IgM
F13:MRM of 1 channel,ES+
801.7 > 204.1
1.551e+005

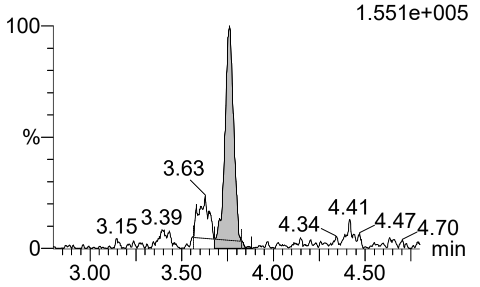

Sample Name: 20211111_H11_R2_IgM
F13:MRM of 1 channel,ES+
801.7 > 204.1
3.346e+005

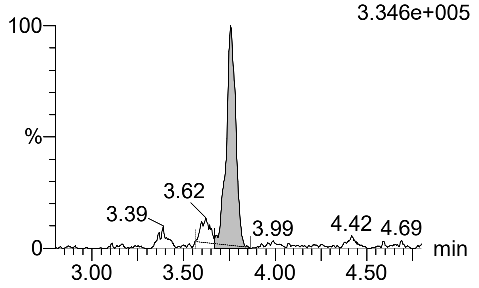

Sample Name: 20211111_A23_R2_IgM
F13:MRM of 1 channel,ES+
801.7 > 204.1
2.269e+005

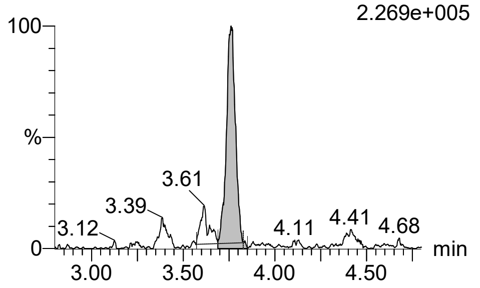

Sample Name: 20211111_A24_R2_IgM
F13:MRM of 1 channel,ES+
801.7 > 204.1
1.124e+005

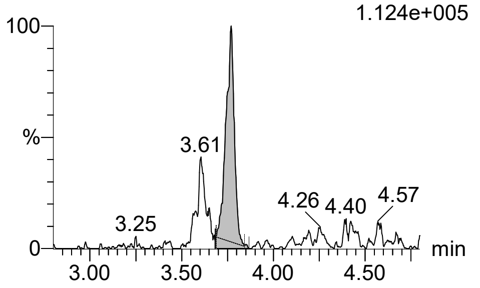

Sample Name: 20211111_L23_R2_IgM
F13:MRM of 1 channel,ES+
801.7 > 204.1
2.259e+005

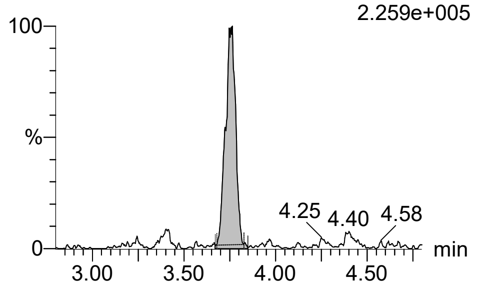

Sample Name: 20211111_L24_R2_IgM
F13:MRM of 1 channel,ES+
801.7 > 204.1
7.115e+004

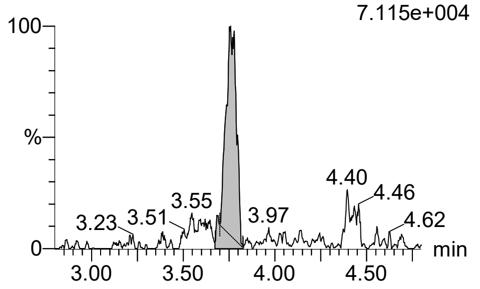

Sample Name: 20211111_H12_R2_IgM
F13:MRM of 1 channel,ES+
801.7 > 204.1
1.178e+005

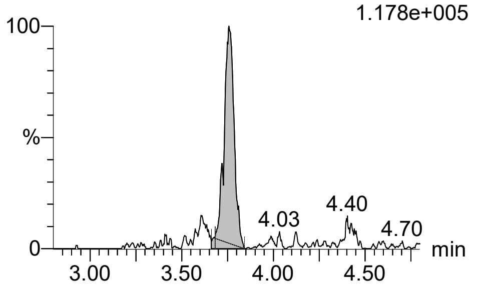

Sample Name: 20211111_QC_R14_IgM
F13:MRM of 1 channel,ES+
801.7 > 204.1
2.008e+005

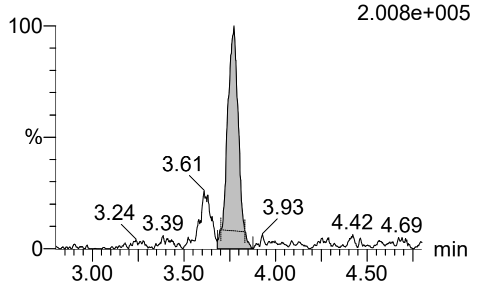

Sample Name: 20211111_A25_R2_IgM
F13:MRM of 1 channel,ES+
801.7 > 204.1
1.565e+005

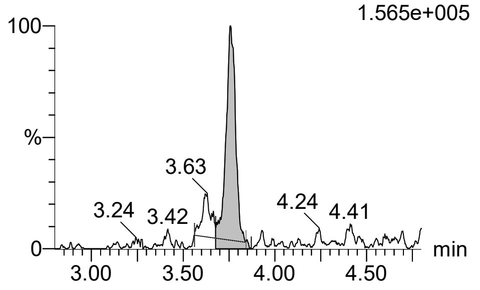

Sample Name: 20211111_A26_R2_IgM
F13:MRM of 1 channel,ES+
801.7 > 204.1
8.423e+004

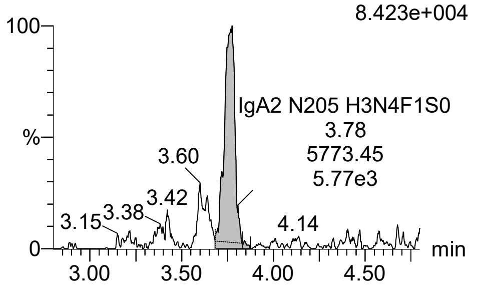

Dataset: C:\MassLynx\10.pro\20211111_clinicalsamples_IgM_IgA.qld

Last Altered: Thursday, March 09, 2023 19:58:25 台北標準時間
Printed: Friday, August 01, 2025 15:26:50 台北標準時間

Sample Name: 20211111_L25_R2_IgM

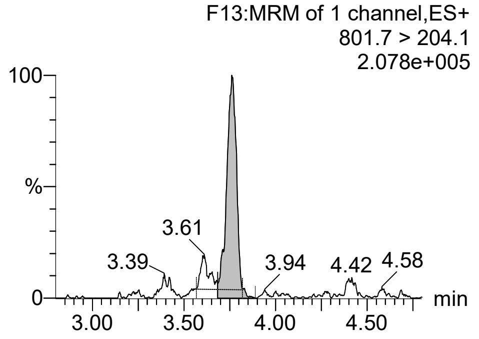

Sample Name: 20211111_L26_R2_IgM

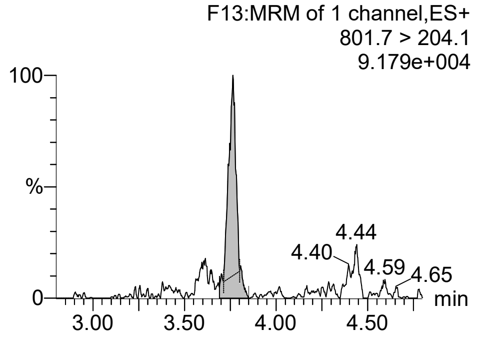

Sample Name: 20211111_H13_R2_IgM

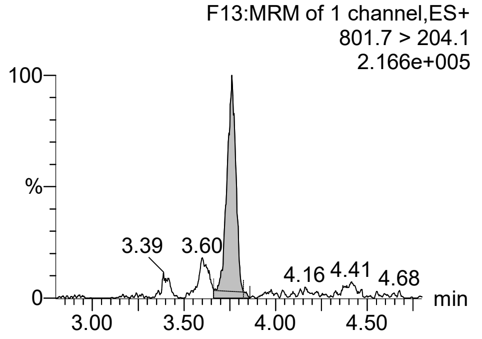

Sample Name: 20211111_A27_R2_IgM

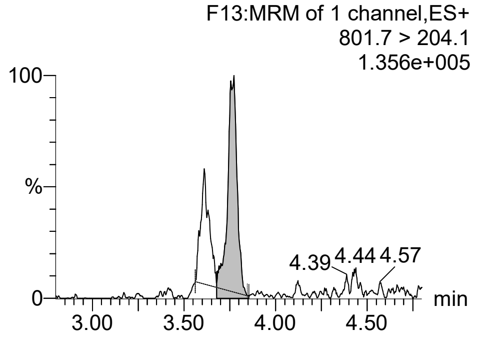

Sample Name: 20211111_A28_R2_IgM

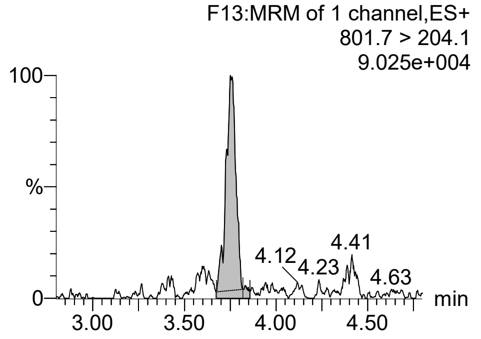

Sample Name: 20211111_L27_R2_IgM

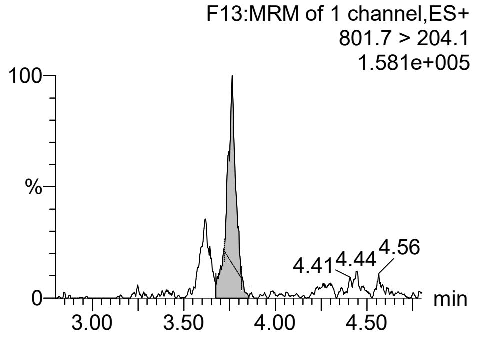

Sample Name: 20211111_L28_R2_IgM

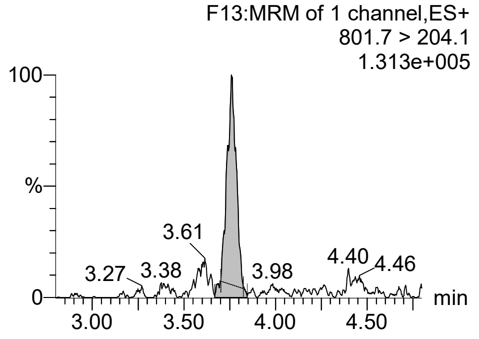

Sample Name: 20211111_H14_R2_IgM

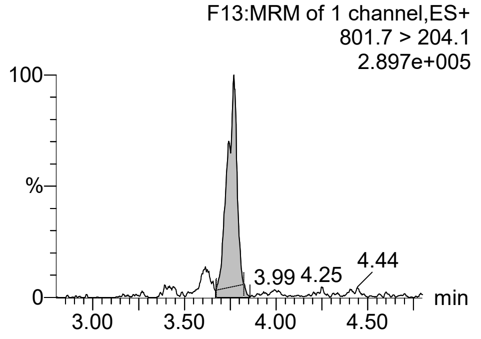

Sample Name: 20211111_A29_R2_IgM

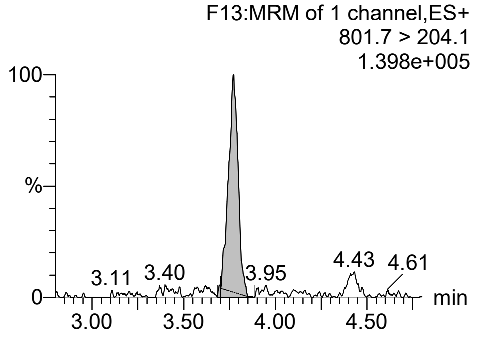

Sample Name: 20211111_A30_R2_IgM

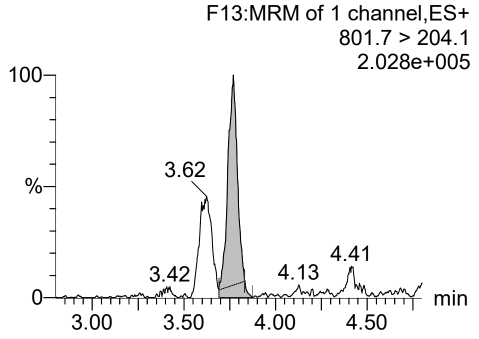

Sample Name: 20211111_QC_R15_IgM

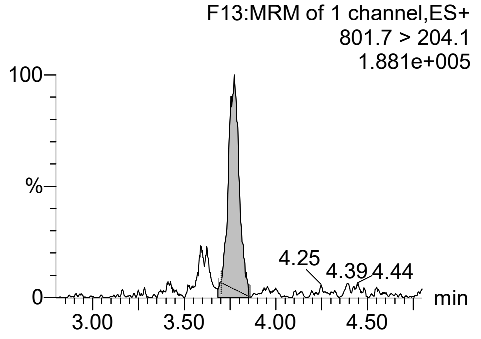

Sample Name: 20211111_L29_R2_IgM

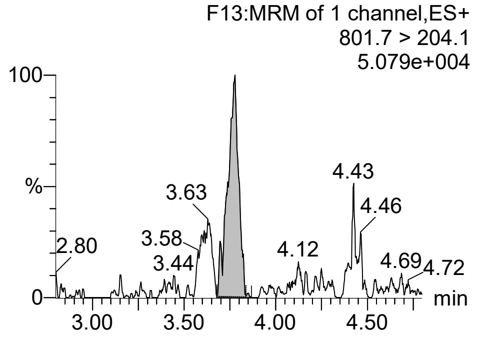

Sample Name: 20211111_L30_R2_IgM

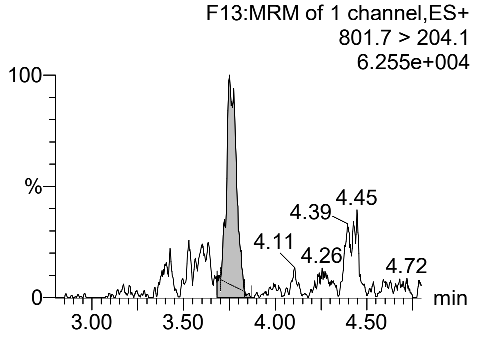

Sample Name: 20211111_H15_R2_IgM

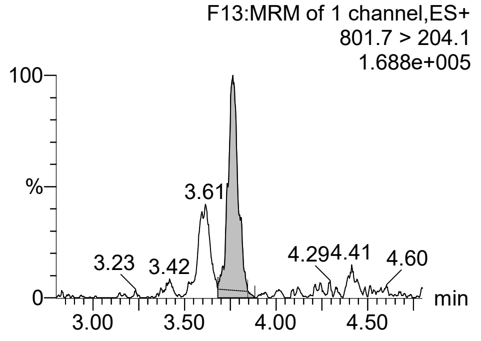

Sample Name: 20211111_A31_R2_IgM

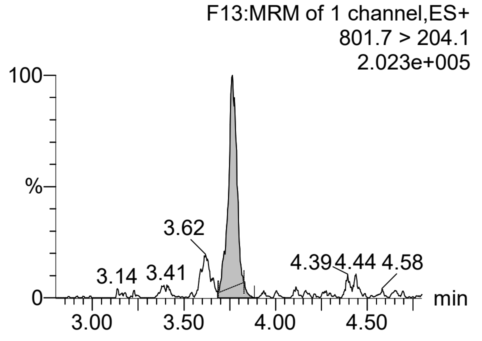

Sample Name: 20211111_A32_R2_IgM

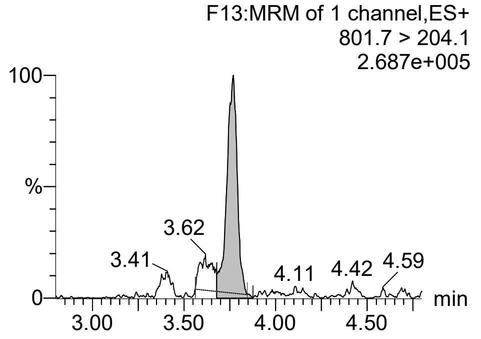

Sample Name: 20211111_L31_R2_IgM

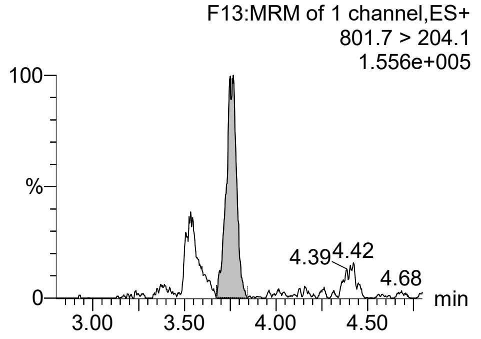

Sample Name: 20211111_L32_R2_IgM

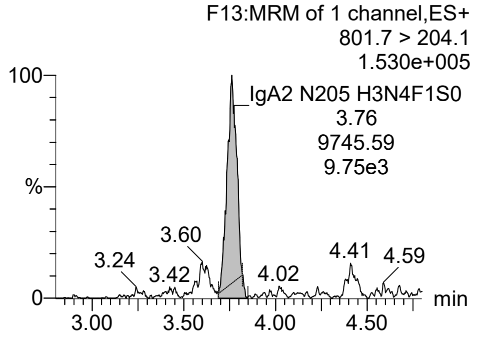

Sample Name: 20211111_H16_R2_IgM

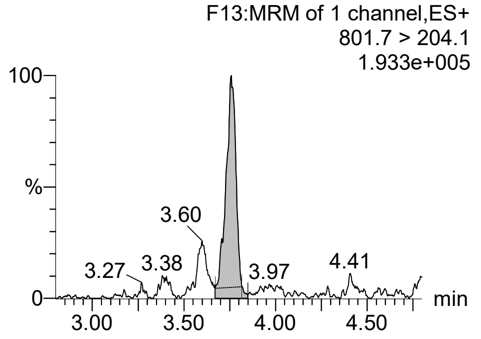

Sample Name: 20211111_A33_R2_IgM

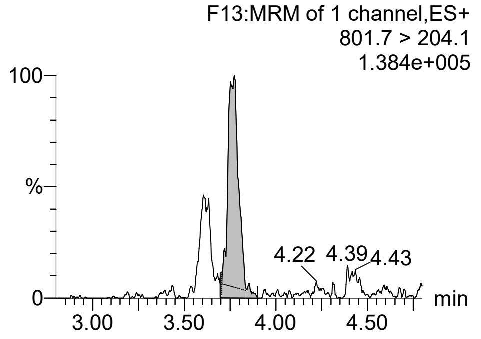

Sample Name: 20211111_A34_R2_IgM

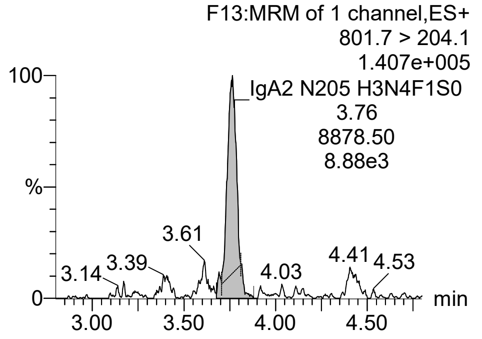

Sample Name: 20211111_H17_R2_IgM

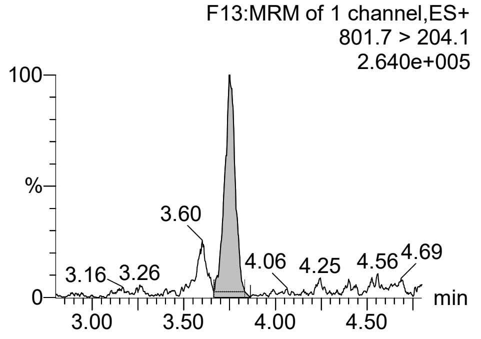

Sample Name: 20211111_A35_R2_IgM

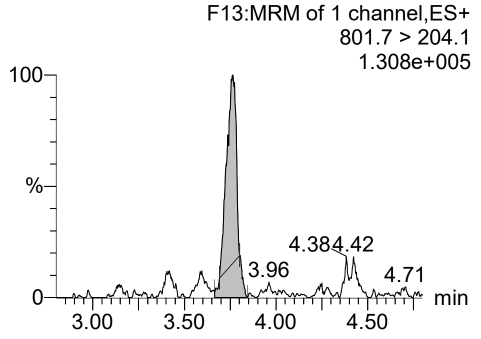

Sample Name: 20211111_QC_R16_IgM

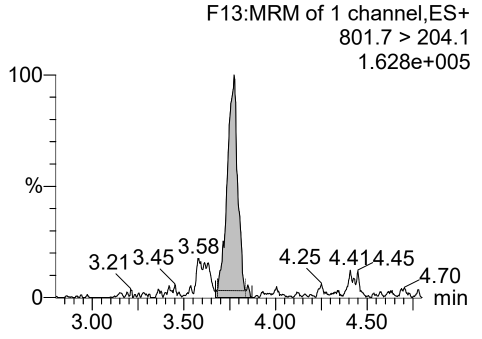

Sample Name: 20211111_A36_R2_IgM

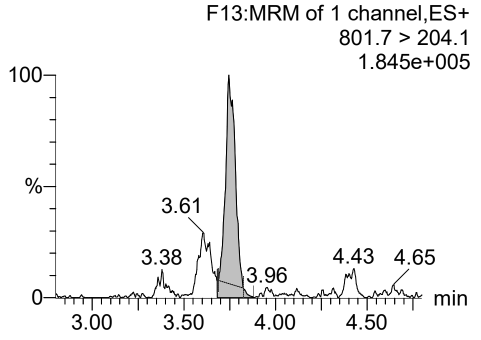

Sample Name: 20211111_H18_R2_IgM

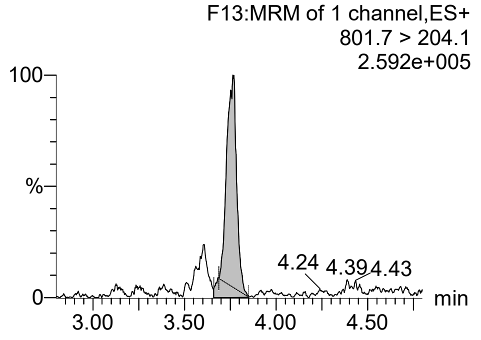

Sample Name: 20211111_A37_R2_IgM

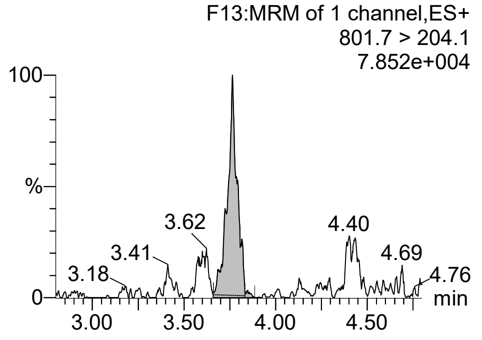

Sample Name: 20211111_A38_R2_IgM

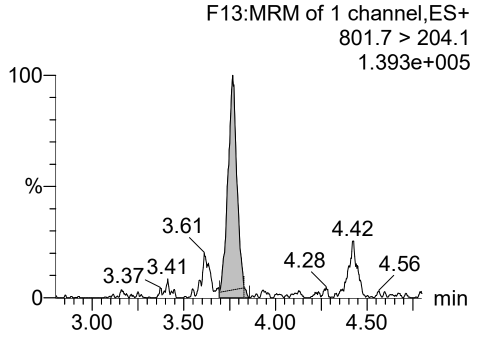

Sample Name: 20211111_H19_R2_IgM

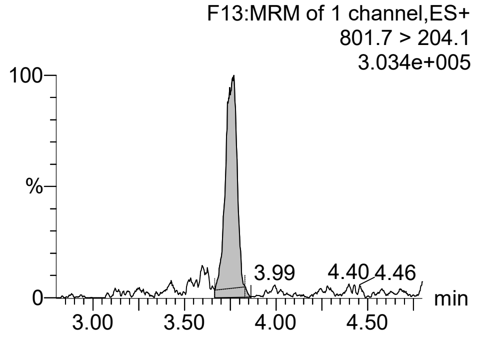

Sample Name: 20211111_A39_R2_IgM

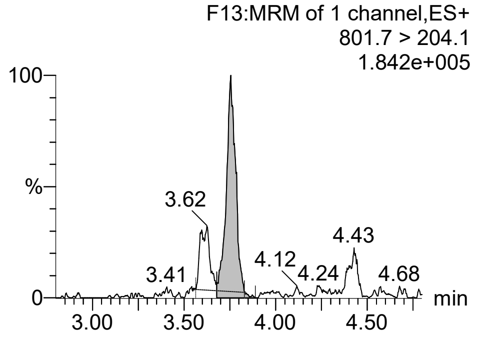

Dataset: C:\MassLynx\10.pro\20211111_clinicalsamples_IgM_IgA.qld

Last Altered: Thursday, March 09, 2023 19:58:25 台北標準時間
Printed: Friday, August 01, 2025 15:26:50 台北標準時間

Sample Name: 20211111_A40_R2_IgM

F13:MRM of 1 channel,ES+
801.7 > 204.1
1.567e+005

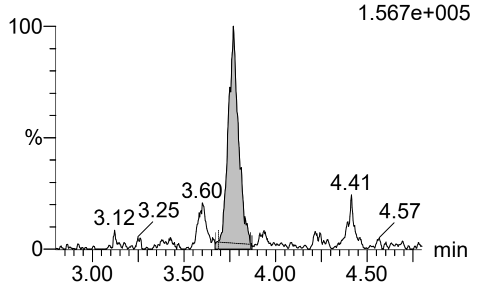

Sample Name: 20211111_H20_R2_IgM

F13:MRM of 1 channel,ES+
801.7 > 204.1
3.131e+005

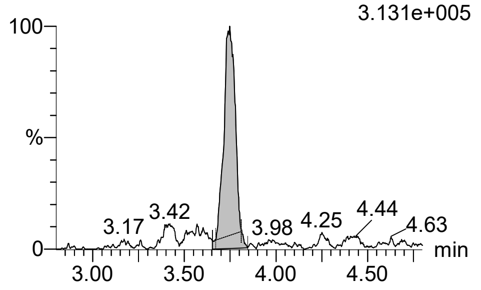

Sample Name: 20211111_A41_R2_IgM

F13:MRM of 1 channel,ES+
801.7 > 204.1
1.268e+005

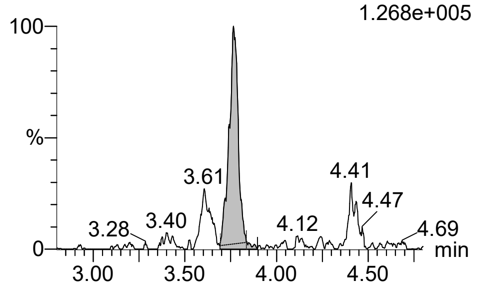

Sample Name: 20211111_A42_R2_IgM

F13:MRM of 1 channel,ES+
801.7 > 204.1
8.121e+004

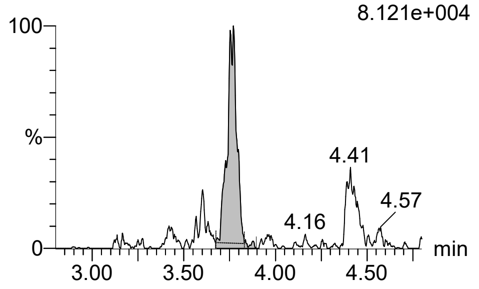

Sample Name: 20211111_A43_R2_IgM

F13:MRM of 1 channel,ES+
801.7 > 204.1
1.729e+005

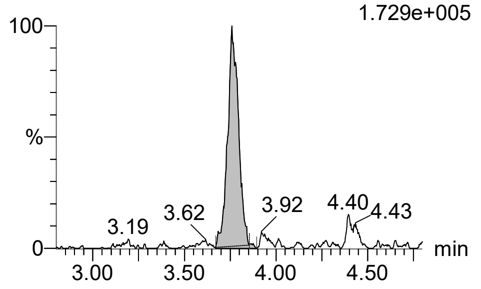

Sample Name: 20211111_A44_R2_IgM

F13:MRM of 1 channel,ES+
801.7 > 204.1
9.564e+004

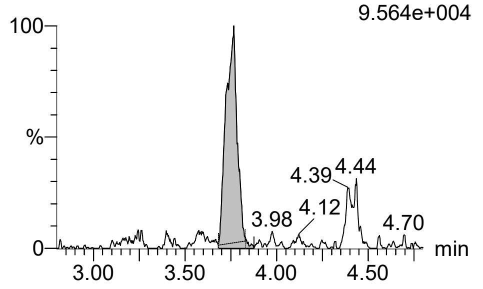

Sample Name: 20211111_QC_R17_IgM

F13:MRM of 1 channel,ES+
801.7 > 204.1
1.307e+005

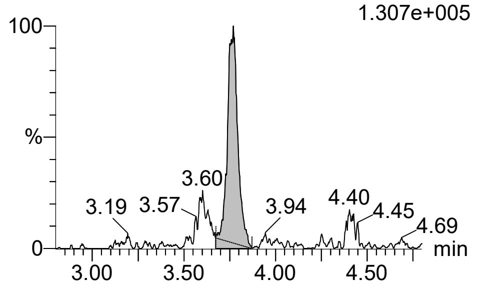

Sample Name: 20211111_L2_R3_IgM

F13:MRM of 1 channel,ES+
801.7 > 204.1
1.504e+005

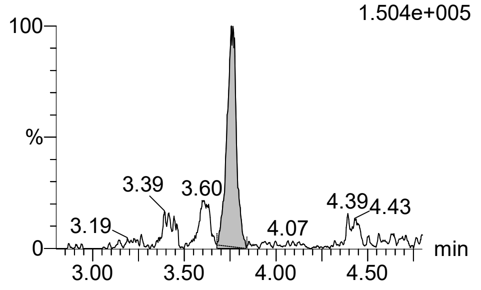

Sample Name: 20211111_H1_R3_IgM

F13:MRM of 1 channel,ES+
801.7 > 204.1
1.639e+005

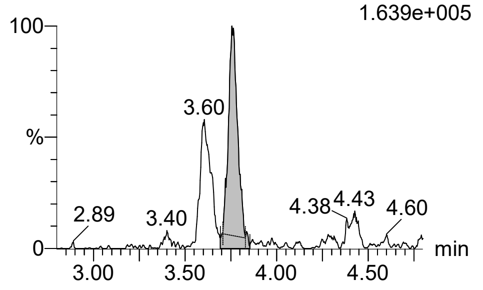

Sample Name: 20211111_A3_R3_IgM

F13:MRM of 1 channel,ES+
801.7 > 204.1
1.332e+005

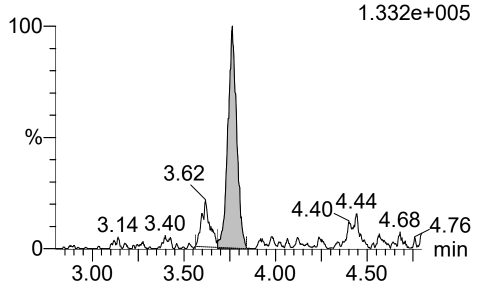

Sample Name: 20211111_A4_R3_IgM

F13:MRM of 1 channel,ES+
801.7 > 204.1
1.868e+005

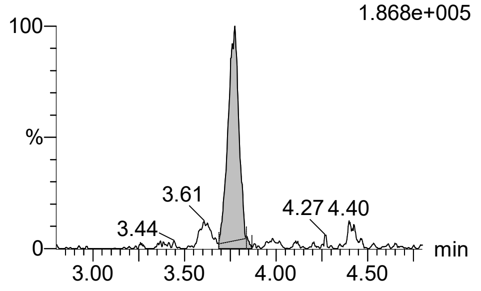

Sample Name: 20211111_L3_R3_IgM

F13:MRM of 1 channel,ES+
801.7 > 204.1
1.025e+005

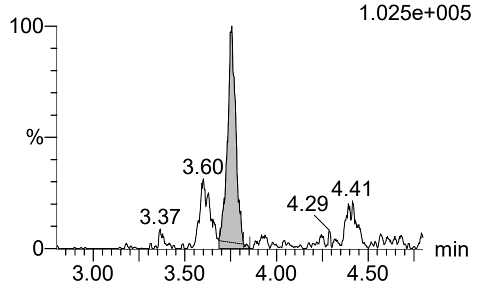

Sample Name: 20211111_L4_R3_IgM

F13:MRM of 1 channel,ES+
801.7 > 204.1
8.237e+004

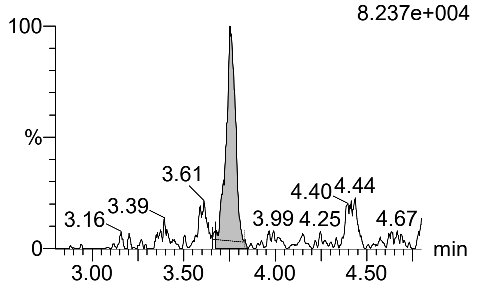

Sample Name: 20211111_H2_R3_IgM

F13:MRM of 1 channel,ES+
801.7 > 204.1
2.334e+005

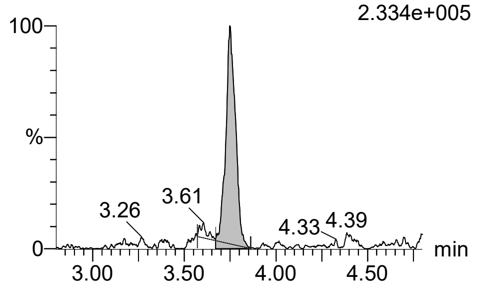

Sample Name: 20211111_A5_R3_IgM

F13:MRM of 1 channel,ES+
801.7 > 204.1
2.100e+005

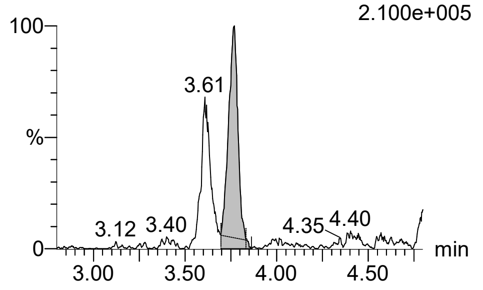

Dataset: C:\MassLynx\10.pro\20211111_clinicalsamples_IgM_IgA.qld

Last Altered: Thursday, March 09, 2023 19:58:25 台北標準時間
Printed: Friday, August 01, 2025 15:26:50 台北標準時間

Sample Name: 20211111_A6_R3_IgM
F13:MRM of 1 channel,ES+
801.7 > 204.1
1.561e+005

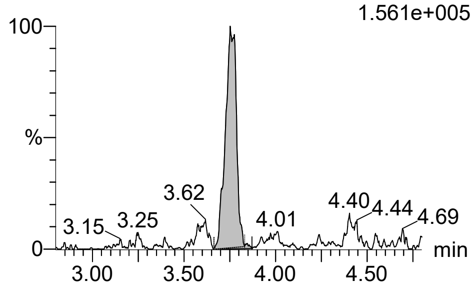

Sample Name: 20211111_QC_R18_IgM
F13:MRM of 1 channel,ES+
801.7 > 204.1
1.350e+005

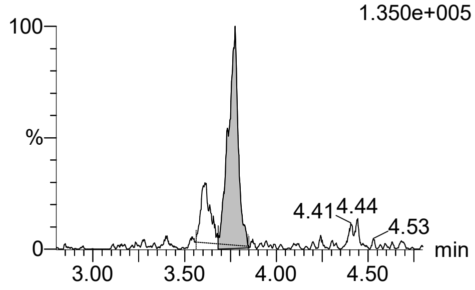

Sample Name: 20211111_L5_R3_IgM
F13:MRM of 1 channel,ES+
801.7 > 204.1
1.442e+005

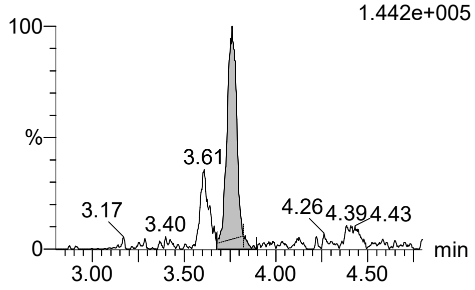

Sample Name: 20211111_L6_R3_IgM
F13:MRM of 1 channel,ES+
801.7 > 204.1
8.236e+004

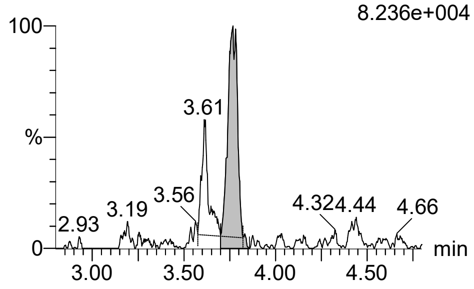

Sample Name: 20211111_H3_R3_IgM
F13:MRM of 1 channel,ES+
801.7 > 204.1
1.404e+005

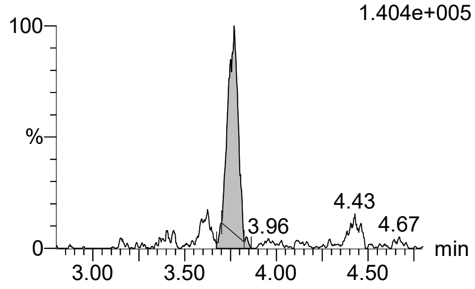

Sample Name: 20211111_A7_R3_IgM
F13:MRM of 1 channel,ES+
801.7 > 204.1
2.997e+005

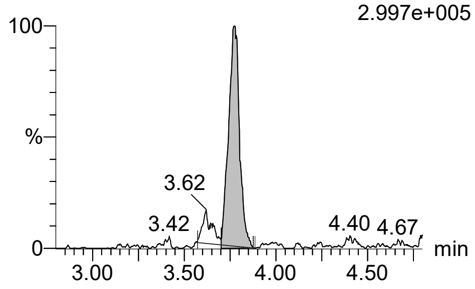

Sample Name: 20211111_A8_R3_IgM
F13:MRM of 1 channel,ES+
801.7 > 204.1
1.381e+005

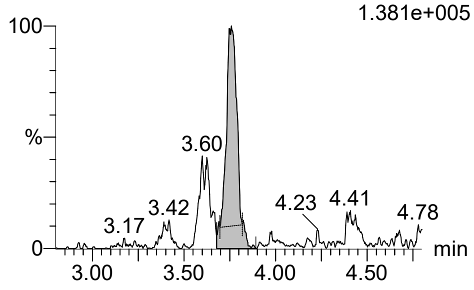

Sample Name: 20211111_L7_R3_IgM
F13:MRM of 1 channel,ES+
801.7 > 204.1
1.274e+005

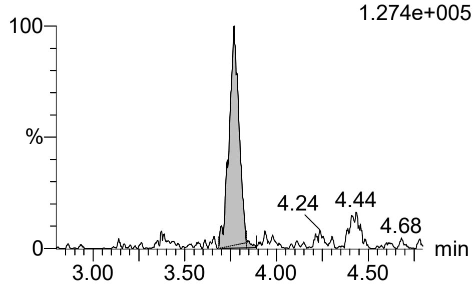

Sample Name: 20211111_L8_R3_IgM
F13:MRM of 1 channel,ES+
801.7 > 204.1
7.250e+004

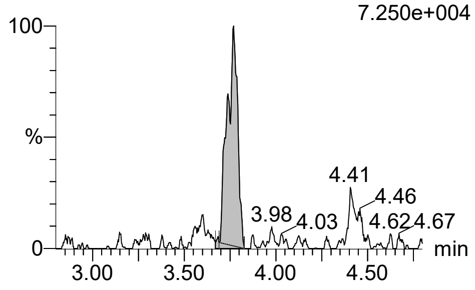

Sample Name: 20211111_H4_R3_IgM
F13:MRM of 1 channel,ES+
801.7 > 204.1
1.088e+005

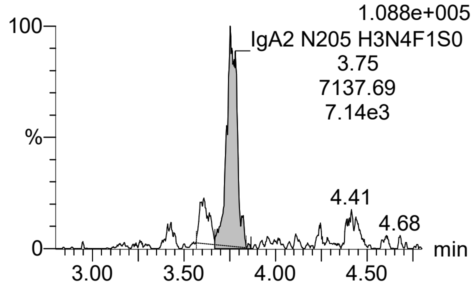

Sample Name: 20211111_A9_R3_IgM
F13:MRM of 1 channel,ES+
801.7 > 204.1
1.562e+005

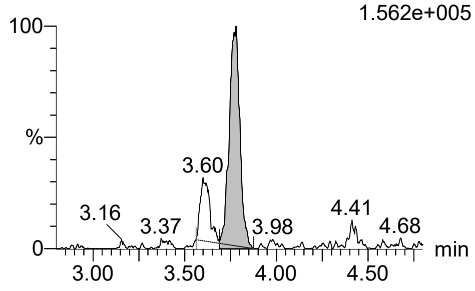

Sample Name: 20211111_A10_R3_IgM
F13:MRM of 1 channel,ES+
801.7 > 204.1
3.071e+005

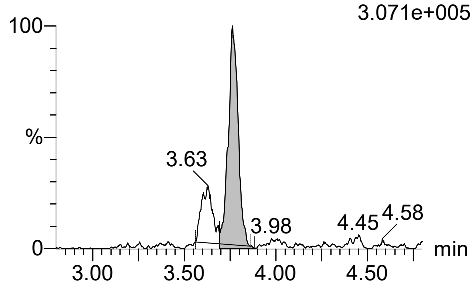

Sample Name: 20211111_L9_R3_IgM
F13:MRM of 1 channel,ES+
801.7 > 204.1
2.076e+005

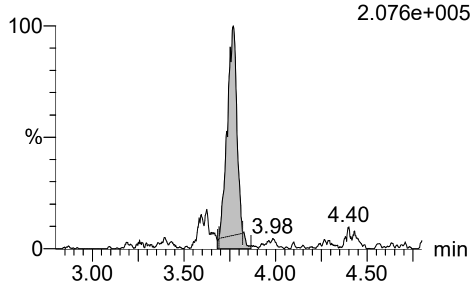

Sample Name: 20211111_L10_R3_IgM
F13:MRM of 1 channel,ES+
801.7 > 204.1
1.176e+005

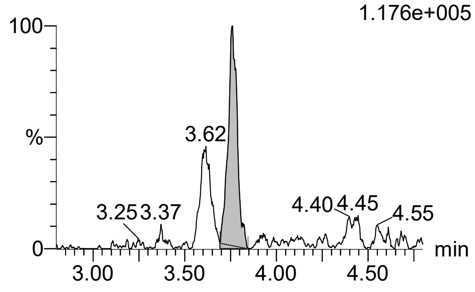

Sample Name: 20211111_QC_R19_IgM
F13:MRM of 1 channel,ES+
801.7 > 204.1
1.260e+005

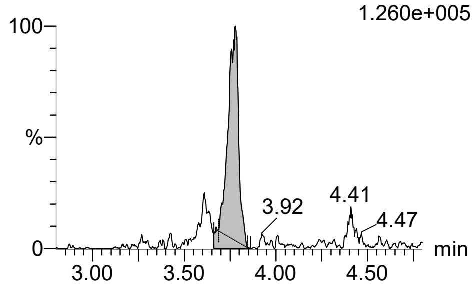

Dataset: C:\MassLynx\10.pro\20211111_clinicalsamples_IgM_IgA.qld

Last Altered: Thursday, March 09, 2023 19:58:25 台北標準時間
Printed: Friday, August 01, 2025 15:26:50 台北標準時間

Sample Name: 20211111_H5_R3_IgM
F13:MRM of 1 channel,ES+
801.7 > 204.1
1.145e+005

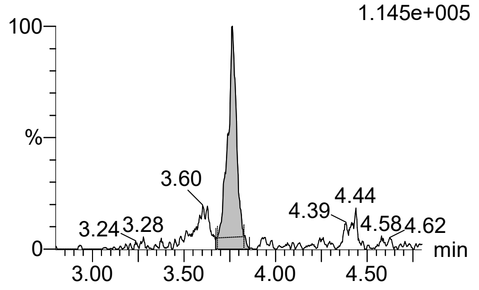

Sample Name: 20211111_A11_R3_IgM
F13:MRM of 1 channel,ES+
801.7 > 204.1
1.175e+005

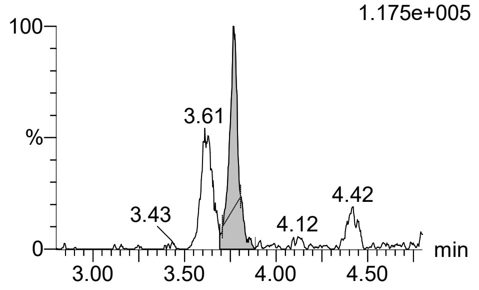

Sample Name: 20211111_A12_R3_IgM
F13:MRM of 1 channel,ES+
801.7 > 204.1
1.513e+005

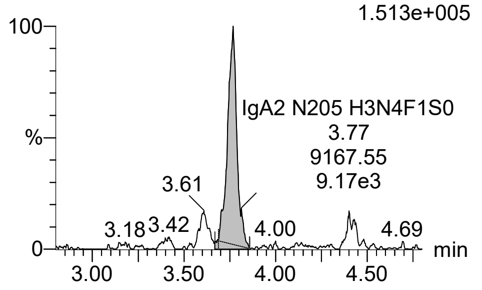

Sample Name: 20211111_L11_R3_IgM
F13:MRM of 1 channel,ES+
801.7 > 204.1
7.762e+004

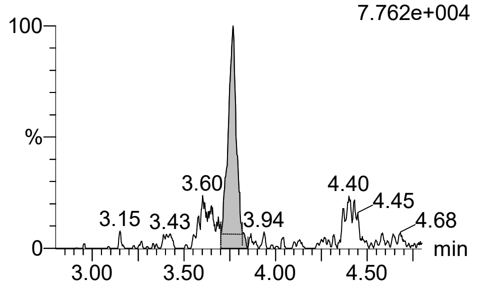

Sample Name: 20211111_L12_R3_IgM
F13:MRM of 1 channel,ES+
801.7 > 204.1
8.579e+004

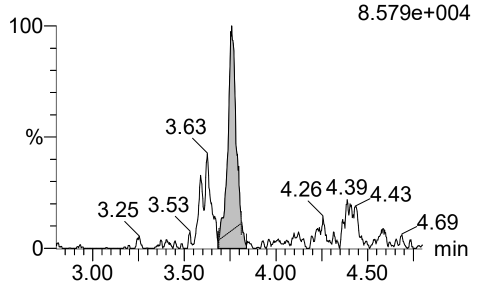

Sample Name: 20211111_H6_R3_IgM
F13:MRM of 1 channel,ES+
801.7 > 204.1
2.075e+005

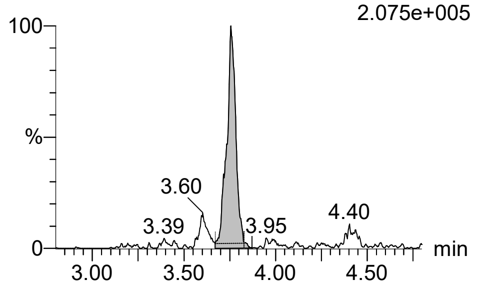

Sample Name: 20211111_A13_R3_IgM
F13:MRM of 1 channel,ES+
801.7 > 204.1
1.355e+005

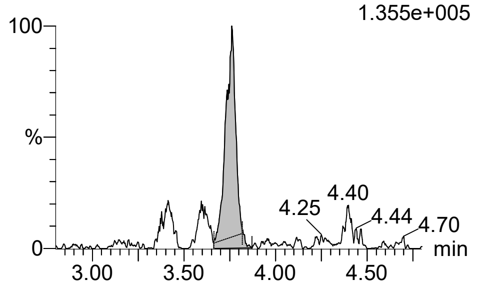

Sample Name: 20211111_A14_R3_IgM
F13:MRM of 1 channel,ES+
801.7 > 204.1
2.162e+005

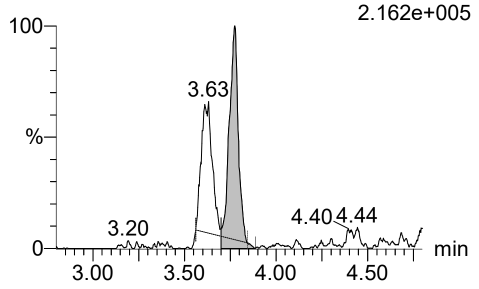

Sample Name: 20211111_L13_R3_IgM
F13:MRM of 1 channel,ES+
801.7 > 204.1
9.732e+004

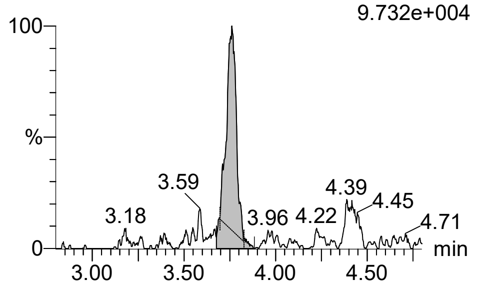

Sample Name: 20211111_L14_R3_IgM
F13:MRM of 1 channel,ES+
801.7 > 204.1
9.670e+004

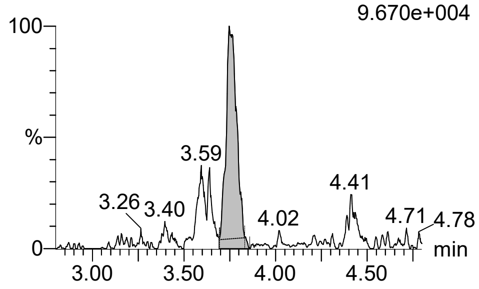

Sample Name: 20211111_H7_R3_IgM
F13:MRM of 1 channel,ES+
801.7 > 204.1
1.583e+005

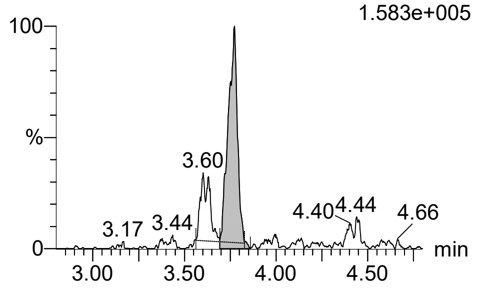

Sample Name: 20211111_A15_R3_IgM
F13:MRM of 1 channel,ES+
801.7 > 204.1
1.520e+005

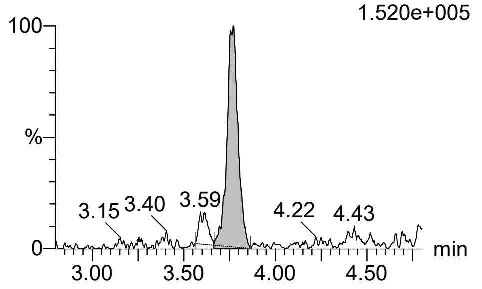

Sample Name: 20211111_QC_R20_IgM
F13:MRM of 1 channel,ES+
801.7 > 204.1
1.517e+005

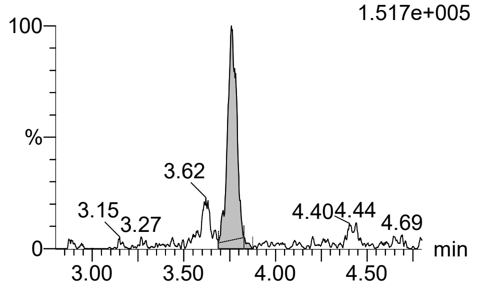

Sample Name: 20211111_A16_R3_IgM
F13:MRM of 1 channel,ES+
801.7 > 204.1
1.230e+005

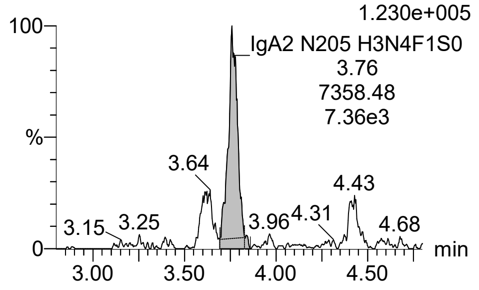

Sample Name: 20211111_L15_R3_IgM
F13:MRM of 1 channel,ES+
801.7 > 204.1
1.184e+005

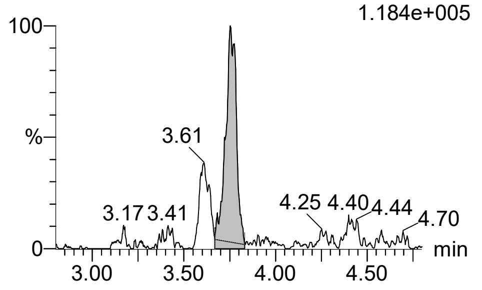

Sample Name: 20211111_L16_R3_IgM

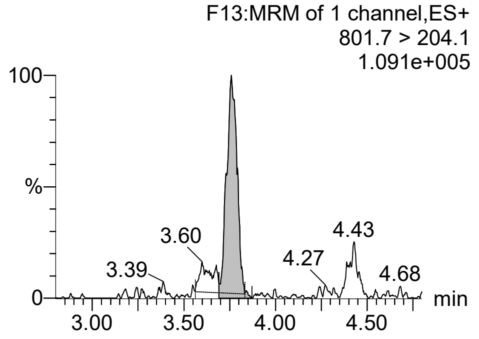

Sample Name: 20211111_H8_R3_IgM

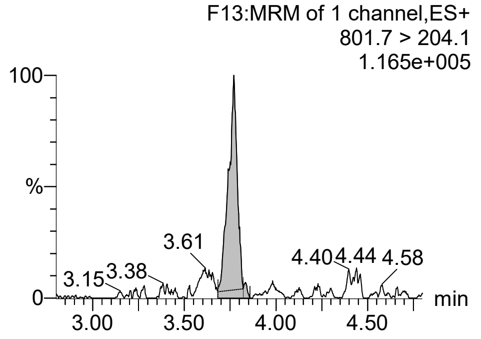

Sample Name: 20211111_A17_R3_IgM

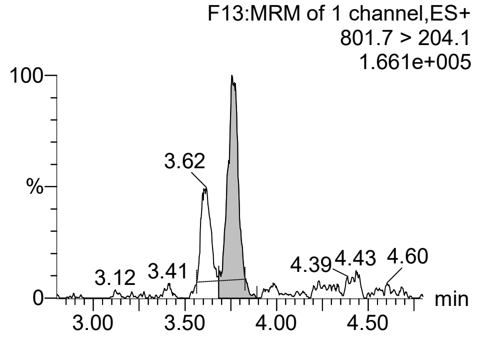

Sample Name: 20211111_A18_R3_IgM

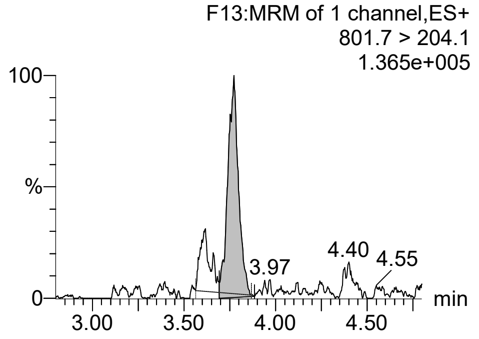

Sample Name: 20211111_L17_R3_IgM

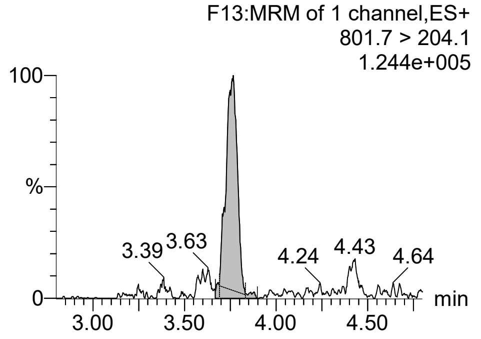

Sample Name: 20211111_L18_R3_IgM

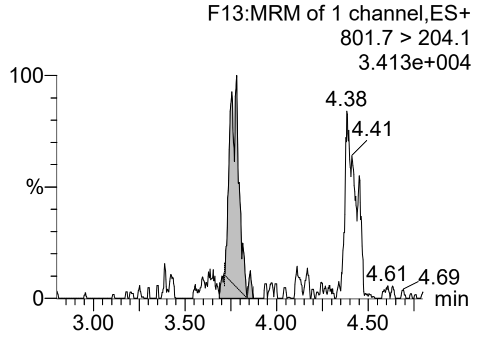

Sample Name: 20211111_H9_R3_IgM

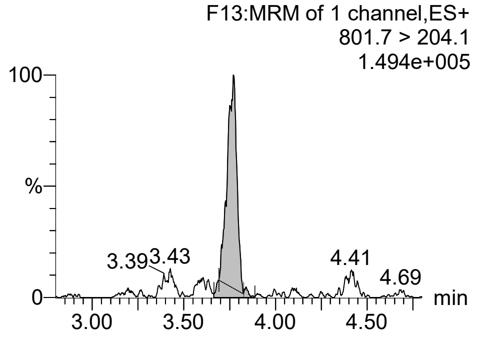

Sample Name: 20211111_A19_R3_IgM

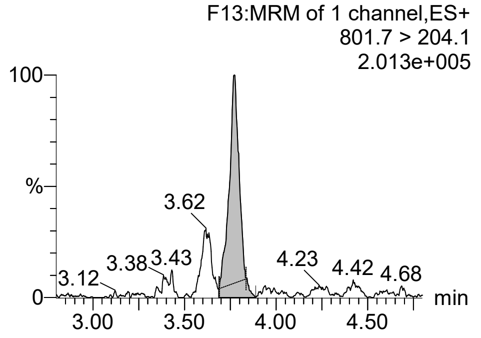

Sample Name: 20211111_A20_R3_IgM

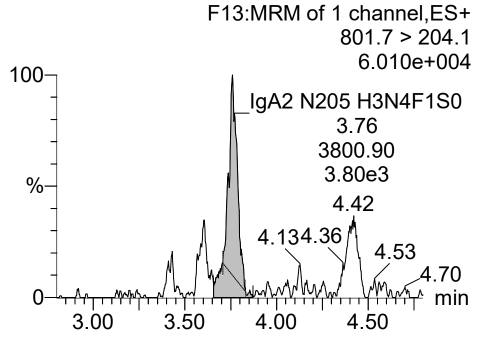

Sample Name: 20211111_L19_R3_IgM

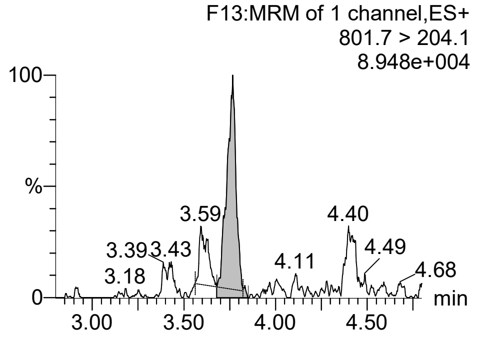

Sample Name: 20211111_QC_R21_IgM

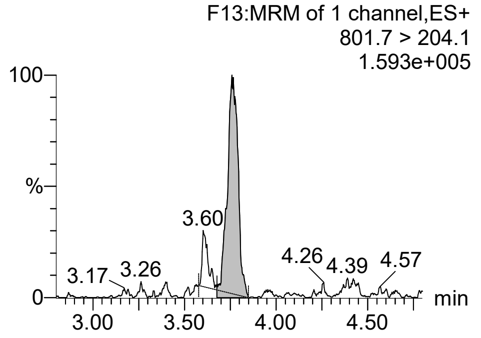

Sample Name: 20211111_L20_R3_IgM

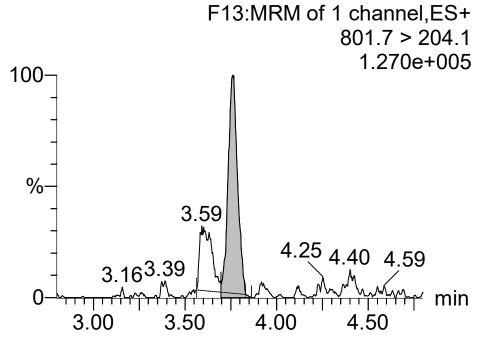

Sample Name: 20211111_H10_R3_IgM

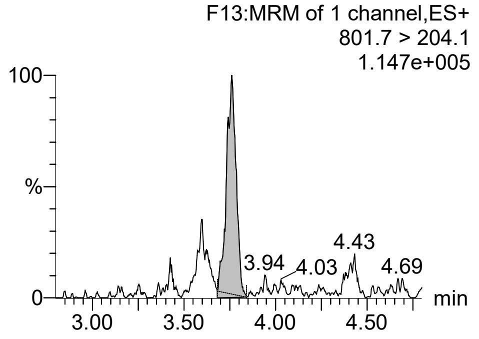

Sample Name: 20211111_A21_R3_IgM

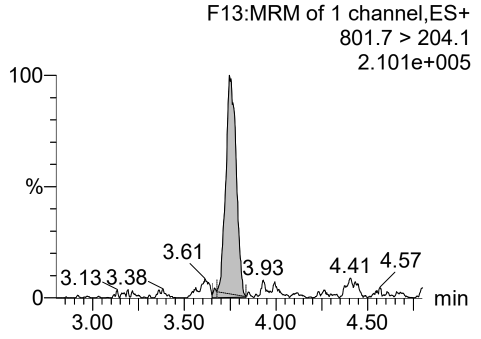

Sample Name: 20211111_A22_R3_IgM

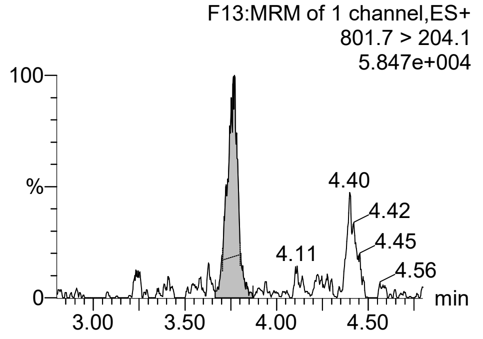

Sample Name: 20211111_L21_R3_IgM
F13:MRM of 1 channel,ES+
801.7 > 204.1
5.411e+004

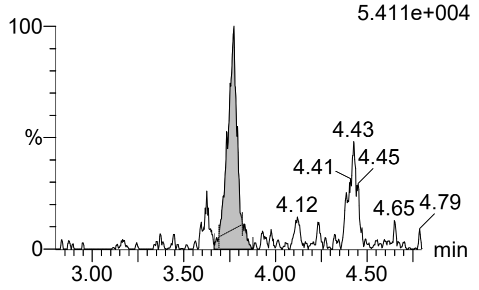

Sample Name: 20211111_L22_R3_IgM
F13:MRM of 1 channel,ES+
801.7 > 204.1
1.360e+005

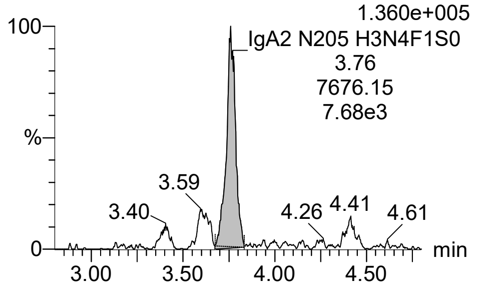

Sample Name: 20211111_H11_R3_IgM
F13:MRM of 1 channel,ES+
801.7 > 204.1
2.797e+005

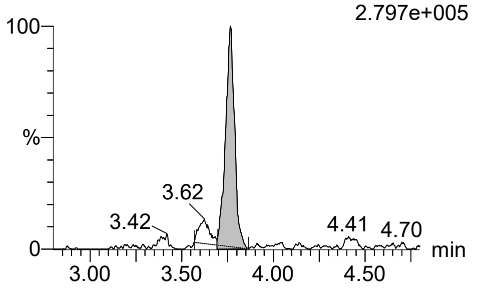

Sample Name: 20211111_A23_R3_IgM
F13:MRM of 1 channel,ES+
801.7 > 204.1
2.011e+005

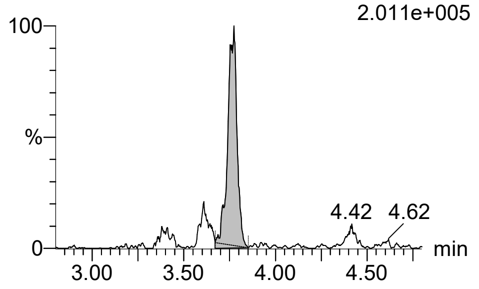

Sample Name: 20211111_A24_R3_IgM
F13:MRM of 1 channel,ES+
801.7 > 204.1
1.078e+005

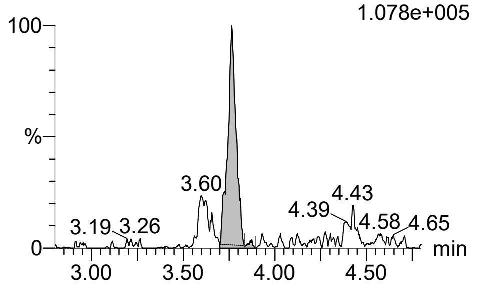

Sample Name: 20211111_L23_R3_IgM
F13:MRM of 1 channel,ES+
801.7 > 204.1
1.599e+005

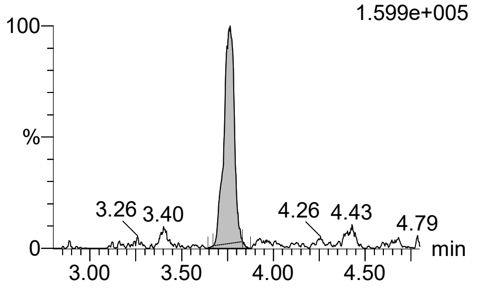

Sample Name: 20211111_L24_R3_IgM
F13:MRM of 1 channel,ES+
801.7 > 204.1
6.394e+004

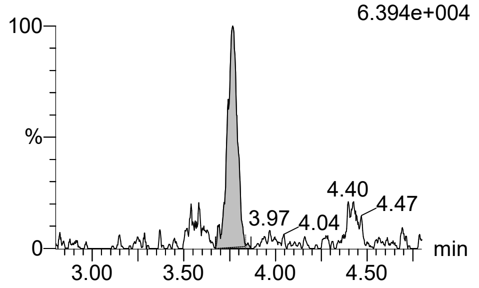

Sample Name: 20211111_H12_R3_IgM
F13:MRM of 1 channel,ES+
801.7 > 204.1
1.071e+005

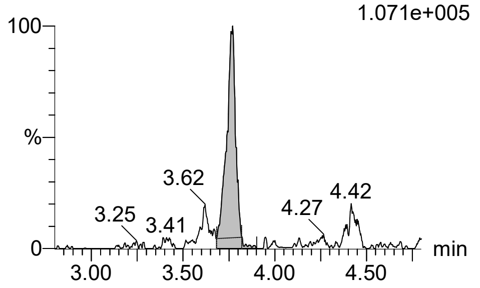

Sample Name: 20211111_QC_R22_IgM
F13:MRM of 1 channel,ES+
801.7 > 204.1
1.356e+005

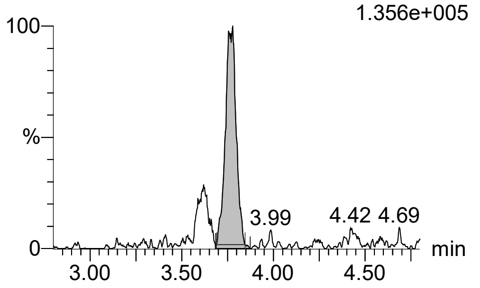

Sample Name: 20211111_A25_R3_IgM
F13:MRM of 1 channel,ES+
801.7 > 204.1
1.022e+005

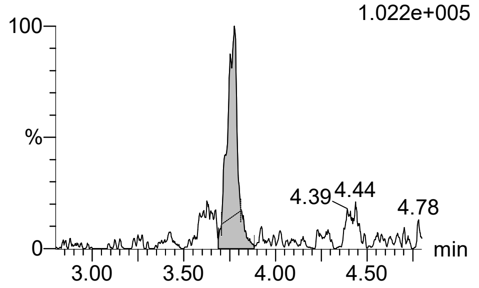

Sample Name: 20211111_A26_R3_IgM
F13:MRM of 1 channel,ES+
801.7 > 204.1
8.464e+004

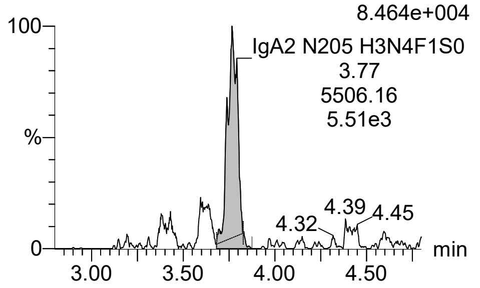

Sample Name: 20211111_L25_R3_IgM
F13:MRM of 1 channel,ES+
801.7 > 204.1
1.697e+005

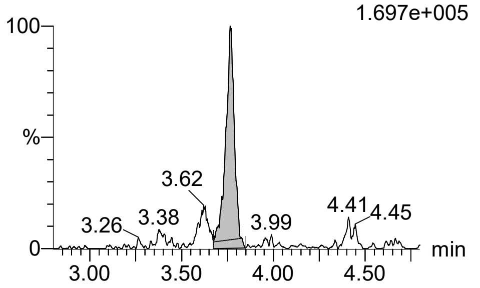

Sample Name: 20211111_L26_R3_IgM
F13:MRM of 1 channel,ES+
801.7 > 204.1
6.316e+004

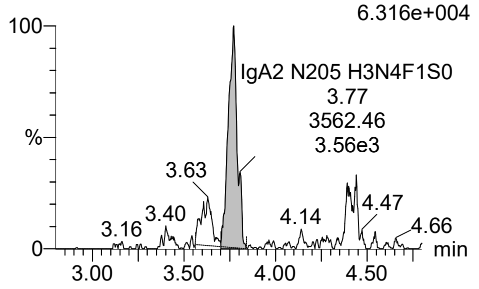

Sample Name: 20211111_H13_R3_IgM
F13:MRM of 1 channel,ES+
801.7 > 204.1
1.531e+005

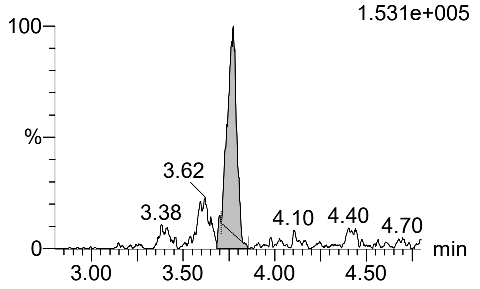

Sample Name: 20211111_A27_R3_IgM
F13:MRM of 1 channel,ES+
801.7 > 204.1
1.251e+005

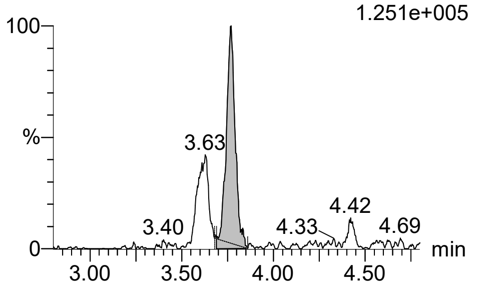

Sample Name: 20211111_A28_R3_IgM
F13:MRM of 1 channel,ES+
801.7 > 204.1
1.042e+005

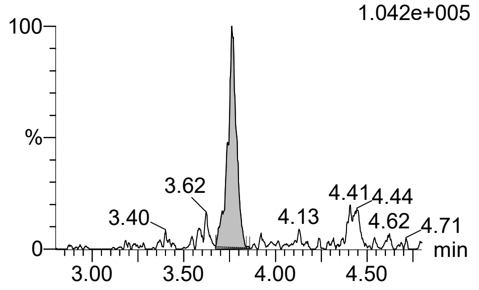

Sample Name: 20211111_L27_R3_IgM
F13:MRM of 1 channel,ES+
801.7 > 204.1
1.216e+005

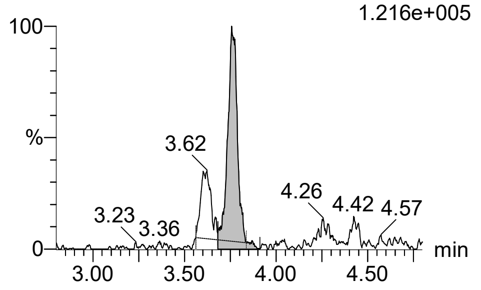

Sample Name: 20211111_L28_R3_IgM
F13:MRM of 1 channel,ES+
801.7 > 204.1
8.718e+004

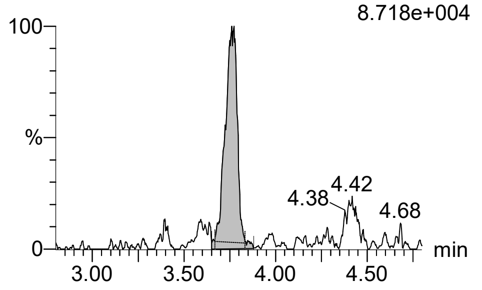

Sample Name: 20211111_H14_R3_IgM
F13:MRM of 1 channel,ES+
801.7 > 204.1
2.375e+005

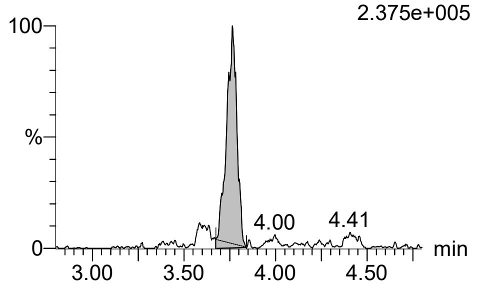

Sample Name: 20211111_A29_R3_IgM
F13:MRM of 1 channel,ES+
801.7 > 204.1
1.107e+005

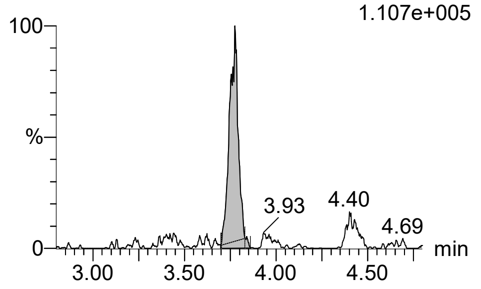

Sample Name: 20211111_A30_R3_IgM
F13:MRM of 1 channel,ES+
801.7 > 204.1
1.676e+005

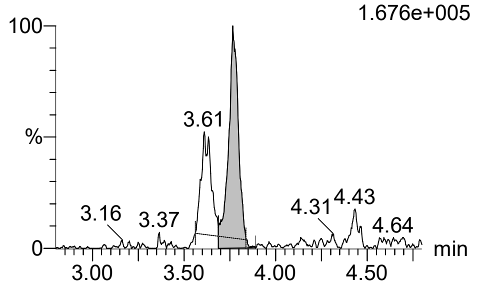

Sample Name: 20211111_QC_R23_IgM
F13:MRM of 1 channel,ES+
801.7 > 204.1
1.365e+005

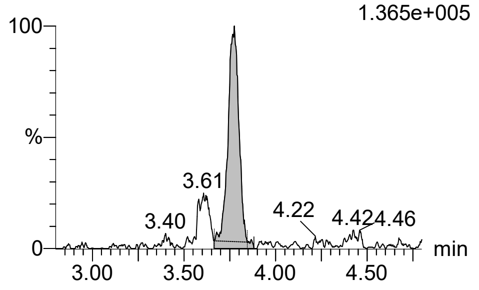

Sample Name: 20211111_L29_R3_IgM
F13:MRM of 1 channel,ES+
801.7 > 204.1
5.903e+004

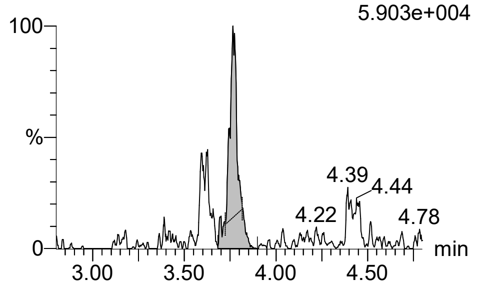

Sample Name: 20211111_L30_R3_IgM
F13:MRM of 1 channel,ES+
801.7 > 204.1
5.137e+004

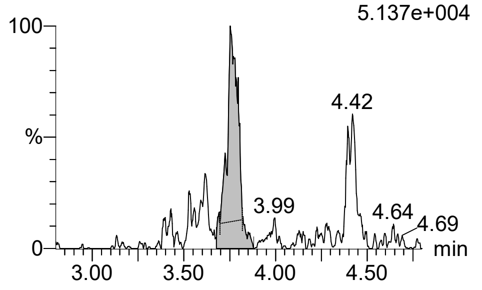

Sample Name: 20211111_H15_R3_IgM
F13:MRM of 1 channel,ES+
801.7 > 204.1
1.380e+005

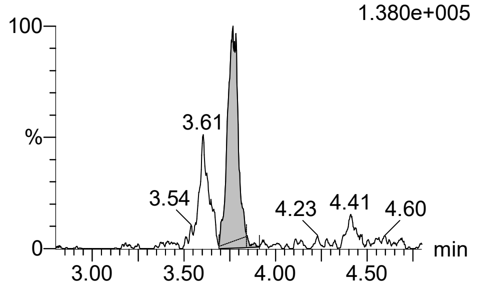

Sample Name: 20211111_A31_R3_IgM
F13:MRM of 1 channel,ES+
801.7 > 204.1
1.665e+005

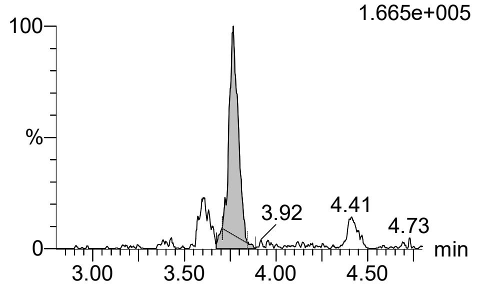

Sample Name: 20211111_A32_R3_IgM
F13:MRM of 1 channel,ES+
801.7 > 204.1
2.184e+005

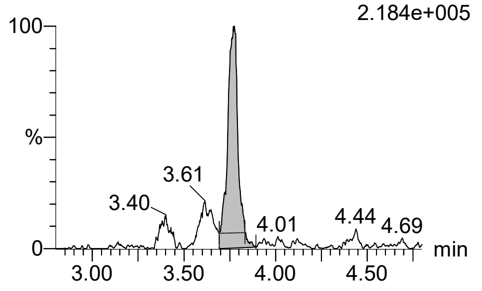

Sample Name: 20211111_L31_R3_IgM
F13:MRM of 1 channel,ES+
801.7 > 204.1
1.289e+005

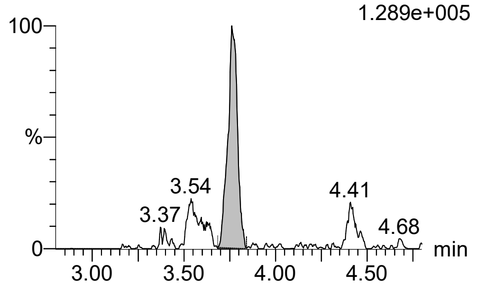

Sample Name: 20211111_L32_R3_IgM
F13:MRM of 1 channel,ES+
801.7 > 204.1
1.238e+005

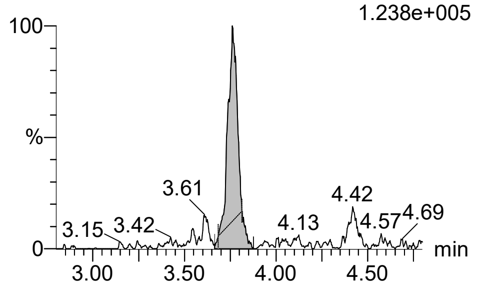

Sample Name: 20211111_H16_R3_IgM
F13:MRM of 1 channel,ES+
801.7 > 204.1
1.777e+005

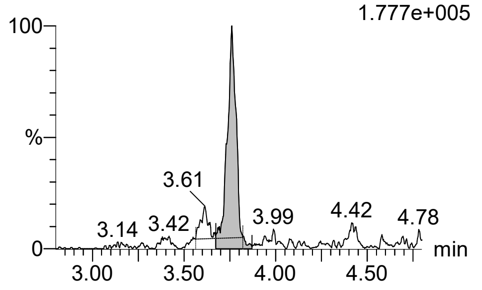

Dataset: C:\MassLynx\10.pro\20211111_clinicalsamples_IgM_IgA.qld

Last Altered: Thursday, March 09, 2023 19:58:25 台北標準時間
Printed: Friday, August 01, 2025 15:26:50 台北標準時間

Sample Name: 20211111_A33_R3_IgM
F13:MRM of 1 channel,ES+
801.7 > 204.1
1.247e+005

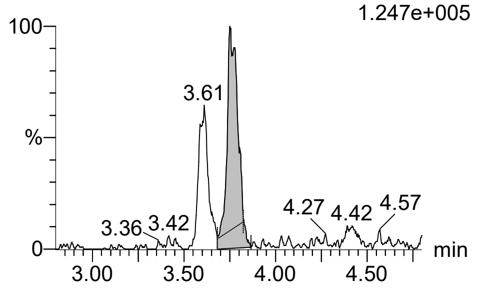

Sample Name: 20211111_A34_R3_IgM
F13:MRM of 1 channel,ES+
801.7 > 204.1
9.854e+004

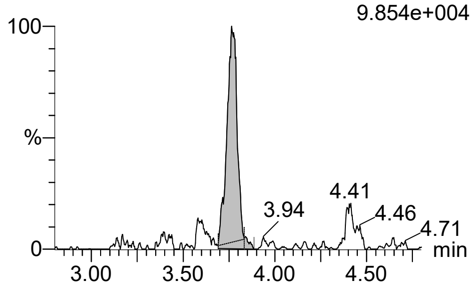

Sample Name: 20211111_H17_R3_IgM
F13:MRM of 1 channel,ES+
801.7 > 204.1
1.945e+005

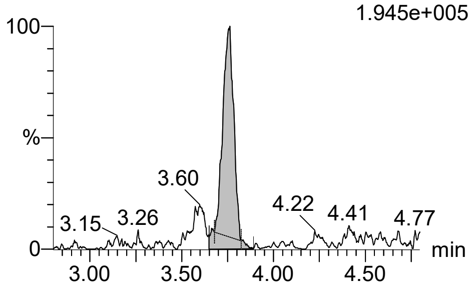

Sample Name: 20211111_A35_R3_IgM
F13:MRM of 1 channel,ES+
801.7 > 204.1
1.342e+005

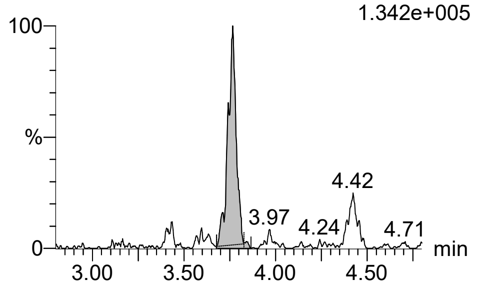

Sample Name: 20211111_QC_R24_IgM
F13:MRM of 1 channel,ES+
801.7 > 204.1
1.078e+005

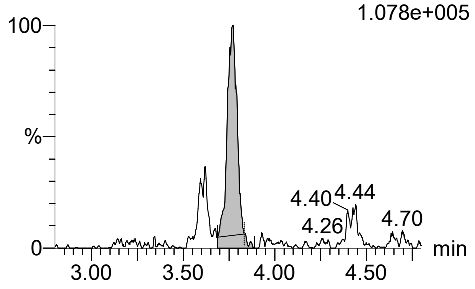

Sample Name: 20211111_A36_R3_IgM
F13:MRM of 1 channel,ES+
801.7 > 204.1
1.661e+005

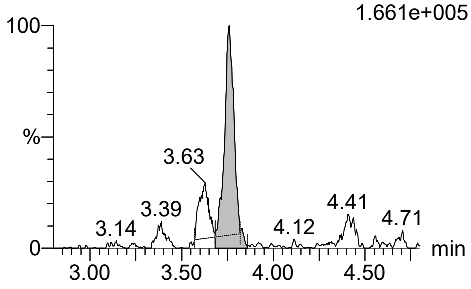

Sample Name: 20211111_H18_R3_IgM
F13:MRM of 1 channel,ES+
801.7 > 204.1
2.121e+005

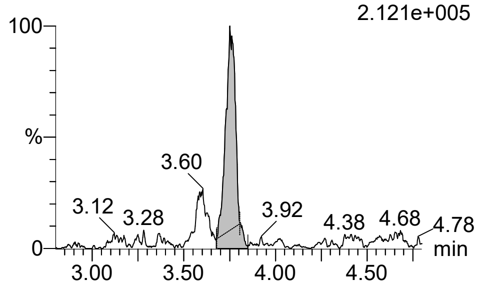

Sample Name: 20211111_A37_R3_IgM
F13:MRM of 1 channel,ES+
801.7 > 204.1
4.859e+004

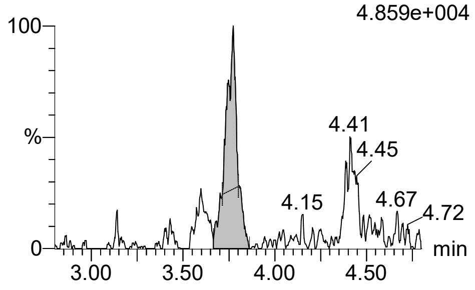

Sample Name: 20211111_A38_R3_IgM
F13:MRM of 1 channel,ES+
801.7 > 204.1
1.025e+005

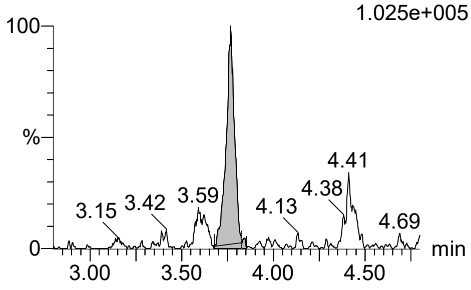

Sample Name: 20211111_H19_R3_IgM
F13:MRM of 1 channel,ES+
801.7 > 204.1
2.601e+005

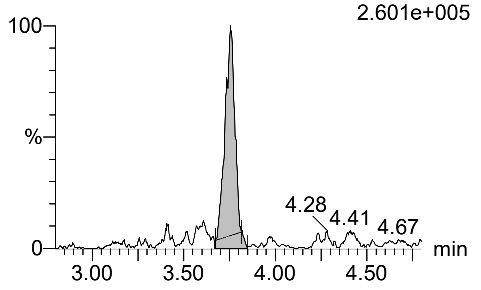

Sample Name: 20211111_A39_R3_IgM
F13:MRM of 1 channel,ES+
801.7 > 204.1
1.377e+005

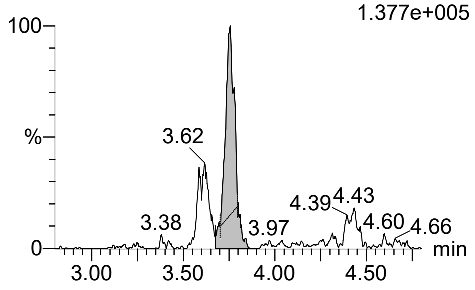

Sample Name: 20211111_A40_R3_IgM
F13:MRM of 1 channel,ES+
801.7 > 204.1
1.186e+005

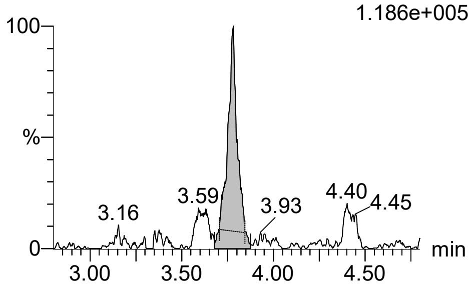

Sample Name: 20211111_H20_R3_IgM
F13:MRM of 1 channel,ES+
801.7 > 204.1
2.449e+005

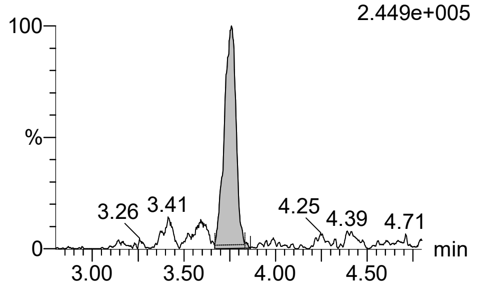

Sample Name: 20211111_A41_R3_IgM
F13:MRM of 1 channel,ES+
801.7 > 204.1
6.108e+004

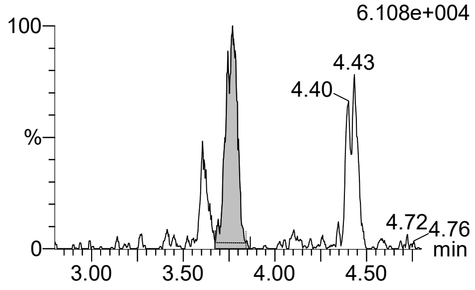

Sample Name: 20211111_A42_R3_IgM
F13:MRM of 1 channel,ES+
801.7 > 204.1
7.580e+004

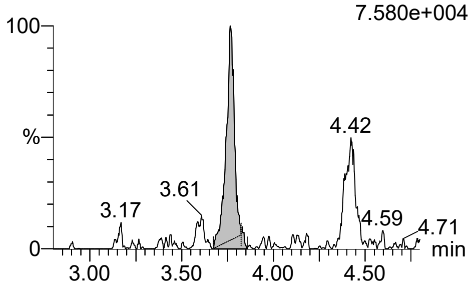

Dataset: C:\MassLynx\10.pro\20211111_clinicalsamples_IgM_IgA.qld

Last Altered: Thursday, March 09, 2023 19:58:25 台北標準時間
Printed: Friday, August 01, 2025 15:26:50 台北標準時間

Sample Name: 20211111_A43_R3_IgM

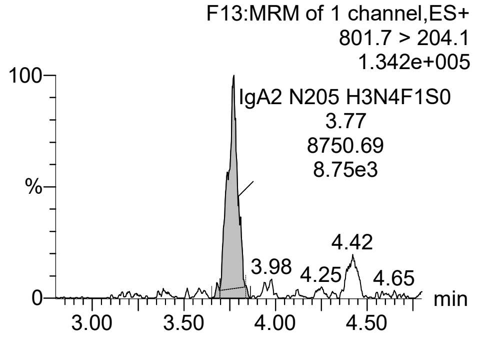

Sample Name: 20211111_A44_R3_IgM

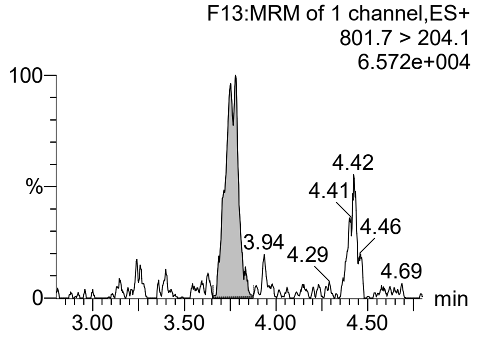

Sample Name: 20211111_QC_R25_IgM

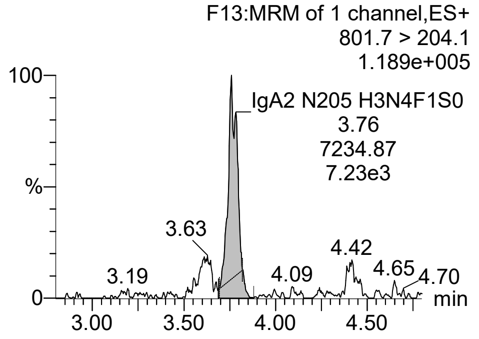

Dataset: C:\MassLynx\10.pro\20211111_clinicalsamples_IgM_IgA.qld

Last Altered: Thursday, March 09, 2023 19:58:25 台北標準時間
Printed: Friday, August 01, 2025 15:26:50 台北標準時間

	#	Name	RT	Area	IS Area	Response	Prima...
1	1	20211112_QC_R001_IgM	3.76	16556.9	15421.4	1.074	bb
2	2	20211112_QC_R002_IgM	3.76	13346.8	13188.8	1.012	MM
3	3	20211111_A1_R2_IgM	3.77	16998.2	11871.0	1.432	MM
4	4	20211111_A1_R3_IgM	3.75	16680.2	12030.5	1.386	MM
5	5	20211111_A2_R2_IgM	3.75	10417.4	11446.8	0.910	MM
6	6	20211111_A2_R3_IgM	3.77	9798.8	11098.4	0.883	MM
7	7	20211111_L1_R2_IgM	3.76	7001.9	11779.4	0.594	MM
8	8	20211111_L1_R3_IgM	3.77	6506.3	11176.2	0.582	MM
9	9	20211111_L2_R2_IgM	3.76	11134.8	11375.0	0.979	MM
10	10	20211111_H1_R2_IgM	3.76	14312.0	13595.2	1.053	MM
11	11	20211111_A3_R2_IgM	3.76	10015.9	14112.3	0.710	MM
12	12	20211111_A4_R2_IgM	3.77	15927.6	11977.5	1.330	MM
13	13	20211111_L3_R2_IgM	3.75	7088.3	12242.1	0.579	MM
14	14	20211111_L4_R2_IgM	3.77	7693.0	12797.0	0.601	MM
15	15	20211111_H2_R2_IgM	3.75	18104.7	10006.0	1.809	MM
16	16	20211111_A5_R2_IgM	3.76	17197.9	11434.6	1.504	MM
17	17	20211111_A6_R2_IgM	3.75	15081.4	13193.7	1.143	MM
18	18	20211111_QC_R10_IgM	3.76	12301.8	12566.1	0.979	MM
19	19	20211111_L5_R2_IgM	3.75	11179.8	13616.4	0.821	MM
20	20	20211111_L6_R2_IgM	3.77	6030.7	13698.5	0.440	MM
21	21	20211111_H3_R2_IgM	3.76	12950.7	15793.1	0.820	MM
22	22	20211111_A7_R2_IgM	3.78	24096.3	12936.9	1.863	MM
23	23	20211111_A8_R2_IgM	3.75	12955.7	13108.0	0.988	MM
24	24	20211111_L7_R2_IgM	3.77	10391.8	13278.5	0.783	MM
25	25	20211111_L8_R2_IgM	3.77	6813.7	15233.6	0.447	MM
26	26	20211111_H4_R2_IgM	3.76	9309.8	16521.9	0.563	MM
27	27	20211111_A9_R2_IgM	3.76	13860.8	16451.6	0.843	MM
28	28	20211111_A10_R2_IgM	3.76	21232.6	13036.0	1.629	MM
29	29	20211111_L9_R2_IgM	3.75	15937.2	14554.1	1.095	MM
30	30	20211111_L10_R2_IgM	3.77	8225.6	12250.4	0.671	MM
31	31	20211111_QC_R11_IgM	3.76	12202.6	11459.2	1.065	MM
32	32	20211111_H5_R2_IgM	3.76	8488.0	14358.2	0.591	MM
33	33	20211111_A11_R2_IgM	3.76	6891.5	16184.1	0.426	MM
34	34	20211111_A12_R2_IgM	3.76	9473.6	13465.4	0.704	MM
35	35	20211111_L11_R2_IgM	3.75	6228.6	13277.9	0.469	MM
36	36	20211111_L12_R2_IgM	3.76	6642.2	15958.8	0.416	MM
37	37	20211111_H6_R2_IgM	3.75	14565.6	14003.5	1.040	MM
38	38	20211111_A13_R2_IgM	3.76	10393.7	13656.1	0.761	MM
39	39	20211111_A14_R2_IgM	3.76	17092.7	14071.3	1.215	MM
40	40	20211111_L13_R2_IgM	3.76	9172.8	14785.6	0.620	MM
41	41	20211111_L14_R2_IgM	3.75	8934.8	14772.2	0.605	MM
42	42	20211111_H7_R2_IgM	3.76	12197.9	15164.4	0.804	MM
43	43	20211111_A15_R2_IgM	3.76	14601.9	15721.5	0.929	MM
44	44	20211111_QC_R12_IgM	3.76	13762.1	13711.5	1.004	MM
45	45	20211111_A16_R2_IgM	3.77	10580.4	14275.6	0.741	MM
46	46	20211111_L15_R2_IgM	3.75	12845.0	12935.8	0.993	MM
47	47	20211111_L16_R2_IgM	3.78	9643.6	13342.8	0.723	MM
48	48	20211111_H8_R2_IgM	3.76	9216.8	15835.1	0.582	MM
49	49	20211111_A17_R2_IgM	3.76	16750.6	15786.7	1.061	MM
50	50	20211111_A18_R2_IgM	3.77	13358.0	16986.8	0.786	MM
51	51	20211111_L17_R2_IgM	3.76	11811.7	13342.7	0.885	MM
52	52	20211111_L18_R2_IgM	3.76	3130.5	14692.0	0.213	MM
53	53	20211111_H9_R2_IgM	3.76	13532.8	15639.7	0.865	MM
54	54	20211111_A19_R2_IgM	3.76	16069.3	14198.7	1.132	MM
55	55	20211111_A20_R2_IgM	3.77	4879.0	14162.7	0.344	MM
56	56	20211111_L19_R2_IgM	3.76	5849.8	9293.4	0.629	MM

Dataset: C:\MassLynx\10.pro\20211111_clinicalsamples_IgM_IgA.qld

Last Altered: Thursday, March 09, 2023 19:58:25 台北標準時間
Printed: Friday, August 01, 2025 15:26:50 台北標準時間

	# Name	RT	Area	IS Area	Response	Prima...
57	57 20211111_QC_R13_IgM	3.77	12596.6	13903.0	0.906	MM
58	58 20211111_L20_R2_IgM	3.76	8506.8	12781.6	0.666	MM
59	59 20211111_H10_R2_IgM	3.75	8626.2	14796.4	0.583	MM
60	60 20211111_A21_R2_IgM	3.76	17022.6	13669.0	1.245	MM
61	61 20211111_A22_R2_IgM	3.76	4112.4	14098.3	0.292	MM
62	62 20211111_L21_R2_IgM	3.76	4347.0	15678.2	0.277	MM
63	63 20211111_L22_R2_IgM	3.76	8818.8	15724.5	0.561	MM
64	64 20211111_H11_R2_IgM	3.76	20316.8	15890.2	1.279	MM
65	65 20211111_A23_R2_IgM	3.76	13260.4	13423.2	0.988	MM
66	66 20211111_A24_R2_IgM	3.77	6261.2	12438.0	0.503	MM
67	67 20211111_L23_R2_IgM	3.76	14693.0	14075.0	1.044	MM
68	68 20211111_L24_R2_IgM	3.76	5033.4	15972.1	0.315	MM
69	69 20211111_H12_R2_IgM	3.76	7691.6	16270.9	0.473	MM
70	70 20211111_QC_R14_IgM	3.77	13810.2	15806.8	0.874	MM
71	71 20211111_A25_R2_IgM	3.76	9945.9	14552.1	0.683	MM
72	72 20211111_A26_R2_IgM	3.78	5773.4	11492.7	0.502	MM
73	73 20211111_L25_R2_IgM	3.76	13072.5	14403.3	0.908	MM
74	74 20211111_L26_R2_IgM	3.76	4827.0	16533.9	0.292	MM
75	75 20211111_H13_R2_IgM	3.76	12663.4	15520.1	0.816	MM
76	76 20211111_A27_R2_IgM	3.77	8397.1	13337.4	0.630	MM
77	77 20211111_A28_R2_IgM	3.75	5978.8	11895.6	0.503	MM
78	78 20211111_L27_R2_IgM	3.76	8812.8	14125.7	0.624	MM
79	79 20211111_L28_R2_IgM	3.76	8174.5	11807.5	0.692	MM
80	80 20211111_H14_R2_IgM	3.77	18672.9	15035.7	1.242	MM
81	81 20211111_A29_R2_IgM	3.77	8788.1	13554.4	0.648	MM
82	82 20211111_A30_R2_IgM	3.77	12897.8	12984.1	0.993	MM
83	83 20211111_QC_R15_IgM	3.77	13021.1	12299.5	1.059	MM
84	84 20211111_L29_R2_IgM	3.78	3486.0	13590.7	0.256	MM
85	85 20211111_L30_R2_IgM	3.75	4100.5	12511.4	0.328	MM
86	86 20211111_H15_R2_IgM	3.76	11177.0	11885.0	0.940	MM
87	87 20211111_A31_R2_IgM	3.76	11495.9	13422.1	0.856	MM
88	88 20211111_A32_R2_IgM	3.77	17863.5	11461.3	1.559	MM
89	89 20211111_L31_R2_IgM	3.77	10145.4	13753.4	0.738	MM
90	90 20211111_L32_R2_IgM	3.76	9745.6	12949.0	0.753	MM
91	91 20211111_H16_R2_IgM	3.76	12557.8	11465.4	1.095	MM
92	92 20211111_A33_R2_IgM	3.77	9744.7	10551.5	0.924	MM
93	93 20211111_A34_R2_IgM	3.76	8878.5	12409.9	0.715	MM
94	94 20211111_H17_R2_IgM	3.75	18023.1	10160.9	1.774	MM
95	95 20211111_A35_R2_IgM	3.76	9096.5	10460.6	0.870	MM
96	96 20211111_QC_R16_IgM	3.77	10989.2	10433.5	1.053	MM
97	97 20211111_A36_R2_IgM	3.74	12049.1	12504.8	0.964	MM
98	98 20211111_H18_R2_IgM	3.76	17555.7	9140.4	1.921	MM
99	99 20211111_A37_R2_IgM	3.76	4801.1	9796.9	0.490	MM
100	1... 20211111_A38_R2_IgM	3.77	8133.0	11224.3	0.725	MM
101	1... 20211111_H19_R2_IgM	3.77	22906.9	11739.2	1.951	MM
102	1... 20211111_A39_R2_IgM	3.76	11106.2	12718.9	0.873	MM
103	1... 20211111_A40_R2_IgM	3.77	10423.4	10006.1	1.042	MM
104	1... 20211111_H20_R2_IgM	3.75	23270.2	7988.2	2.913	MM
105	1... 20211111_A41_R2_IgM	3.77	7547.6	11513.3	0.656	MM
106	1... 20211111_A42_R2_IgM	3.77	5081.4	10238.5	0.496	MM
107	1... 20211111_A43_R2_IgM	3.76	11983.6	11098.9	1.080	MM
108	1... 20211111_A44_R2_IgM	3.77	6834.3	10969.9	0.623	MM
109	1... 20211111_QC_R17_IgM	3.77	8759.5	9700.8	0.903	MM
110	1... 20211111_L2_R3_IgM	3.76	9150.4	8528.0	1.073	MM
111	1... 20211111_H1_R3_IgM	3.76	10119.5	9438.2	1.072	MM
112	1... 20211111_A3_R3_IgM	3.76	7390.6	11598.6	0.637	MM

Dataset: C:\MassLynx\10.pro\20211111_clinicalsamples_IgM_IgA.qld

Last Altered: Thursday, March 09, 2023 19:58:25 台北標準時間
Printed: Friday, August 01, 2025 15:26:50 台北標準時間

	# Name	RT	Area	IS Area	Response	Prima...
113	1... 20211111_A4_R3_IgM	3.77	13152.0	11364.1	1.157	MM
114	1... 20211111_L3_R3_IgM	3.76	5504.4	10753.4	0.512	MM
115	1... 20211111_L4_R3_IgM	3.75	5039.8	10291.6	0.490	MM
116	1... 20211111_H2_R3_IgM	3.75	14616.4	9315.8	1.569	MM
117	1... 20211111_A5_R3_IgM	3.77	13033.0	9102.2	1.432	MM
118	1... 20211111_A6_R3_IgM	3.75	10884.4	10356.4	1.051	MM
119	1... 20211111_QC_R18_IgM	3.78	8388.5	8398.3	0.999	MM
120	1... 20211111_L5_R3_IgM	3.76	9142.9	9786.7	0.934	MM
121	1... 20211111_L6_R3_IgM	3.77	5287.3	9911.3	0.533	MM
122	1... 20211111_H3_R3_IgM	3.77	9754.0	12155.5	0.802	MM
123	1... 20211111_A7_R3_IgM	3.77	20808.3	11804.8	1.763	MM
124	1... 20211111_A8_R3_IgM	3.76	9889.1	10510.6	0.941	MM
125	1... 20211111_L7_R3_IgM	3.77	8105.2	12177.9	0.666	MM
126	1... 20211111_L8_R3_IgM	3.77	4852.8	11668.6	0.416	MM
127	1... 20211111_H4_R3_IgM	3.75	7137.7	11192.0	0.638	MM
128	1... 20211111_A9_R3_IgM	3.78	10577.4	14077.4	0.751	MM
129	1... 20211111_A10_R3_IgM	3.76	19447.5	11190.4	1.738	MM
130	1... 20211111_L9_R3_IgM	3.77	13561.1	14649.3	0.926	MM
131	1... 20211111_L10_R3_IgM	3.76	6882.0	9361.3	0.735	MM
132	1... 20211111_QC_R19_IgM	3.78	9001.9	10125.5	0.889	MM
133	1... 20211111_H5_R3_IgM	3.76	6674.3	10010.9	0.667	MM
134	1... 20211111_A11_R3_IgM	3.77	6565.8	12697.2	0.517	MM
135	1... 20211111_A12_R3_IgM	3.77	9167.6	13148.5	0.697	MM
136	1... 20211111_L11_R3_IgM	3.77	4206.2	10786.6	0.390	MM
137	1... 20211111_L12_R3_IgM	3.76	4710.4	11681.8	0.403	MM
138	1... 20211111_H6_R3_IgM	3.76	12051.4	12095.5	0.996	MM
139	1... 20211111_A13_R3_IgM	3.76	8838.6	12908.3	0.685	MM
140	1... 20211111_A14_R3_IgM	3.78	12769.2	11322.6	1.128	MM
141	1... 20211111_L13_R3_IgM	3.76	6740.4	11516.1	0.585	MM
142	1... 20211111_L14_R3_IgM	3.75	6645.9	10790.7	0.616	MM
143	1... 20211111_H7_R3_IgM	3.77	9499.6	11572.1	0.821	MM
144	1... 20211111_A15_R3_IgM	3.77	10762.3	12434.4	0.866	MM
145	1... 20211111_QC_R20_IgM	3.76	9102.2	10701.0	0.851	MM
146	1... 20211111_A16_R3_IgM	3.76	7358.5	11452.1	0.643	MM
147	1... 20211111_L15_R3_IgM	3.75	8235.4	10531.1	0.782	MM
148	1... 20211111_L16_R3_IgM	3.76	7298.8	12049.4	0.606	MM
149	1... 20211111_H8_R3_IgM	3.77	6873.2	13434.9	0.512	MM
150	1... 20211111_A17_R3_IgM	3.76	11761.6	12452.0	0.945	MM
151	1... 20211111_A18_R3_IgM	3.77	9158.8	12506.5	0.732	MM
152	1... 20211111_L17_R3_IgM	3.77	9360.0	9948.9	0.941	MM
153	1... 20211111_L18_R3_IgM	3.78	2187.1	13462.1	0.162	MM
154	1... 20211111_H9_R3_IgM	3.77	9924.9	13178.7	0.753	MM
155	1... 20211111_A19_R3_IgM	3.77	13462.4	11298.6	1.192	MM
156	1... 20211111_A20_R3_IgM	3.76	3800.9	11175.6	0.340	MM
157	1... 20211111_L19_R3_IgM	3.77	5356.8	11558.2	0.463	MM
158	1... 20211111_QC_R21_IgM	3.76	10828.7	9977.0	1.085	MM
159	1... 20211111_L20_R3_IgM	3.76	7687.2	13508.1	0.569	MM
160	1... 20211111_H10_R3_IgM	3.76	7040.3	12374.8	0.569	MM
161	1... 20211111_A21_R3_IgM	3.75	14078.0	12443.6	1.131	MM
162	1... 20211111_A22_R3_IgM	3.77	3886.4	11589.3	0.335	MM
163	1... 20211111_L21_R3_IgM	3.77	3318.4	13107.4	0.253	MM
164	1... 20211111_L22_R3_IgM	3.76	7676.1	12021.5	0.639	MM
165	1... 20211111_H11_R3_IgM	3.77	16034.3	12687.8	1.264	MM
166	1... 20211111_A23_R3_IgM	3.77	11855.0	11611.8	1.021	MM
167	1... 20211111_A24_R3_IgM	3.76	5840.3	10476.8	0.557	MM
168	1... 20211111_L23_R3_IgM	3.76	10772.3	11873.1	0.907	MM

Dataset: C:\MassLynx\10.pro\20211111_clinicalsamples_IgM_IgA.qld

Last Altered: Thursday, March 09, 2023 19:58:25 台北標準時間
Printed: Friday, August 01, 2025 15:26:50 台北標準時間

	# Name	RT	Area	IS Area	Response	Prima...
169	1... 20211111_L24_R3_IgM	3.77	4051.4	13468.0	0.301	MM
170	1... 20211111_H12_R3_IgM	3.77	6335.1	14229.1	0.445	MM
171	1... 20211111_QC_R22_IgM	3.78	9360.7	11264.4	0.831	MM
172	1... 20211111_A25_R3_IgM	3.78	6784.8	11775.7	0.576	MM
173	1... 20211111_A26_R3_IgM	3.77	5506.2	10473.9	0.526	MM
174	1... 20211111_L25_R3_IgM	3.76	9498.0	13256.8	0.716	MM
175	1... 20211111_L26_R3_IgM	3.77	3562.5	13565.0	0.263	MM
176	1... 20211111_H13_R3_IgM	3.77	9664.8	13146.0	0.735	MM
177	1... 20211111_A27_R3_IgM	3.77	7071.2	12025.6	0.588	MM
178	1... 20211111_A28_R3_IgM	3.76	5769.2	11229.1	0.514	MM
179	1... 20211111_L27_R3_IgM	3.76	7730.9	14960.0	0.517	MM
180	1... 20211111_L28_R3_IgM	3.77	6816.3	11359.0	0.600	MM
181	1... 20211111_H14_R3_IgM	3.76	15559.5	12830.8	1.213	MM
182	1... 20211111_A29_R3_IgM	3.78	6238.1	13434.3	0.464	MM
183	1... 20211111_A30_R3_IgM	3.77	10483.7	11272.2	0.930	MM
184	1... 20211111_QC_R23_IgM	3.77	9775.8	11872.2	0.823	MM
185	1... 20211111_L29_R3_IgM	3.76	3255.9	12948.7	0.251	MM
186	1... 20211111_L30_R3_IgM	3.75	3823.1	11026.6	0.347	MM
187	1... 20211111_H15_R3_IgM	3.77	9433.2	10786.3	0.875	MM
188	1... 20211111_A31_R3_IgM	3.77	10372.7	12010.5	0.864	MM
189	1... 20211111_A32_R3_IgM	3.78	14336.2	11107.7	1.291	MM
190	1... 20211111_L31_R3_IgM	3.76	7806.5	11021.8	0.708	MM
191	1... 20211111_L32_R3_IgM	3.76	8244.1	11683.8	0.706	MM
192	1... 20211111_H16_R3_IgM	3.76	10227.2	9831.7	1.040	MM
193	1... 20211111_A33_R3_IgM	3.75	8751.6	11213.1	0.780	MM
194	1... 20211111_A34_R3_IgM	3.76	6594.0	11550.1	0.571	MM
195	1... 20211111_H17_R3_IgM	3.76	14584.3	8874.4	1.643	MM
196	1... 20211111_A35_R3_IgM	3.77	7169.2	9856.3	0.727	MM
197	1... 20211111_QC_R24_IgM	3.77	7066.9	7561.8	0.935	MM
198	1... 20211111_A36_R3_IgM	3.76	10552.4	9562.8	1.103	MM
199	1... 20211111_H18_R3_IgM	3.75	13822.4	8165.3	1.693	MM
200	2... 20211111_A37_R3_IgM	3.77	3485.8	9278.8	0.376	MM
201	2... 20211111_A38_R3_IgM	3.77	5521.2	10920.3	0.506	MM
202	2... 20211111_H19_R3_IgM	3.76	17051.0	9285.3	1.836	MM
203	2... 20211111_A39_R3_IgM	3.76	8622.6	9083.4	0.949	MM
204	2... 20211111_A40_R3_IgM	3.78	7621.0	8382.4	0.909	MM
205	2... 20211111_H20_R3_IgM	3.76	18244.3	7807.9	2.337	MM
206	2... 20211111_A41_R3_IgM	3.77	4476.5	5925.2	0.755	MM
207	2... 20211111_A42_R3_IgM	3.76	4181.3	9352.6	0.447	MM
208	2... 20211111_A43_R3_IgM	3.77	8750.7	10216.3	0.857	MM
209	2... 20211111_A44_R3_IgM	3.78	5462.0	10262.7	0.532	MM
210	2... 20211111_QC_R25_IgM	3.76	7234.9	8575.3	0.844	MM

Dataset: C:\MassLynx\10.pro\20211111_clinicalsamples_IgM_IgA.qld

Last Altered: Thursday, March 09, 2023 19:58:25 台北標準時間
Printed: Friday, August 01, 2025 15:27:43 台北標準時間

Method: C:\MassLynx\10_Pro_IgA.PRO\MethDB\20211111_IgM_IgA_DMRM_new.mdb 24 Feb 2022 13:44:14
Calibration: 15 Nov 2021 14:42:11

Sample Name: 20211112_QC_R001_IgM
F29:MRM of 2 channels,ES+
869.3 > 204.1
5.721e+006

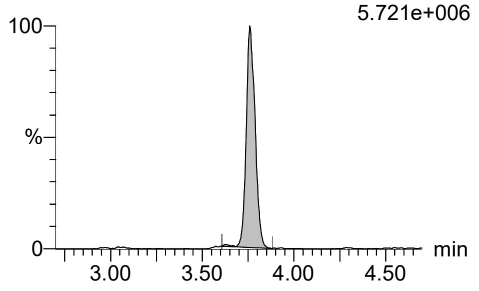

Sample Name: 20211112_QC_R001_IgM
F29:MRM of 2 channels,ES+
1303.5 > 366.1
6.518e+004

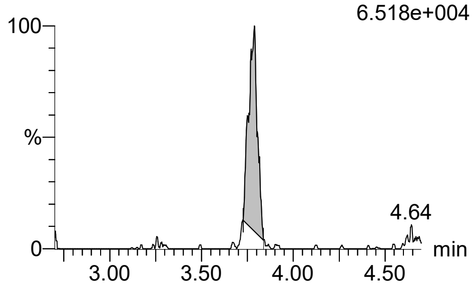

Sample Name: 20211112_QC_R002_IgM
F29:MRM of 2 channels,ES+
869.3 > 204.1
4.526e+006

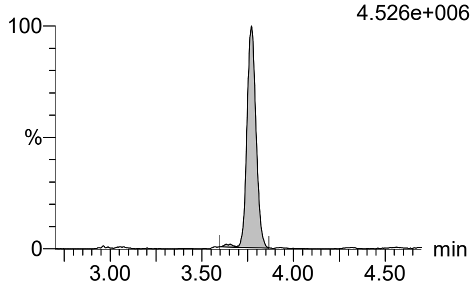

Sample Name: 20211112_QC_R002_IgM
F29:MRM of 2 channels,ES+
1303.5 > 366.1
6.096e+004

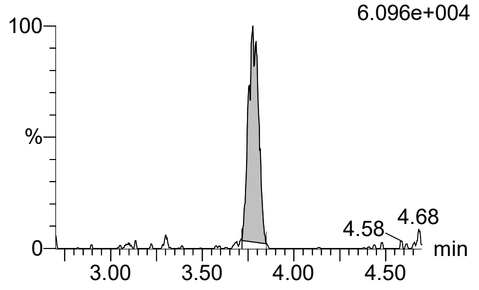

Sample Name: 20211111_A1_R2_IgM
F29:MRM of 2 channels,ES+
869.3 > 204.1
6.812e+006

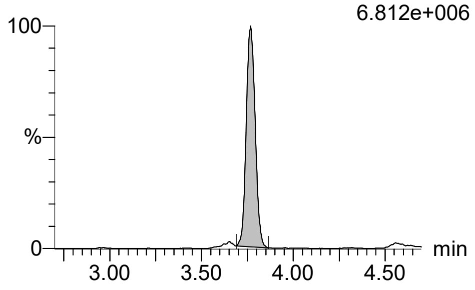

Sample Name: 20211111_A1_R2_IgM
F29:MRM of 2 channels,ES+
1303.5 > 366.1
9.256e+004

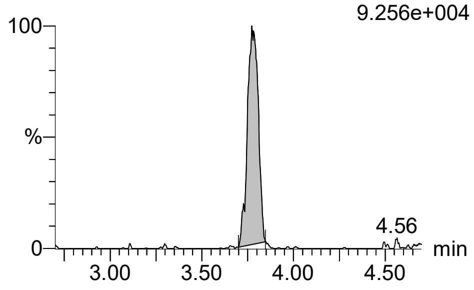

Sample Name: 20211111_A1_R3_IgM
F29:MRM of 2 channels,ES+
869.3 > 204.1
6.648e+006

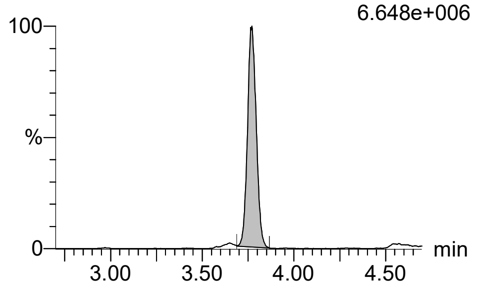

Sample Name: 20211111_A1_R3_IgM
F29:MRM of 2 channels,ES+
1303.5 > 366.1
7.065e+004

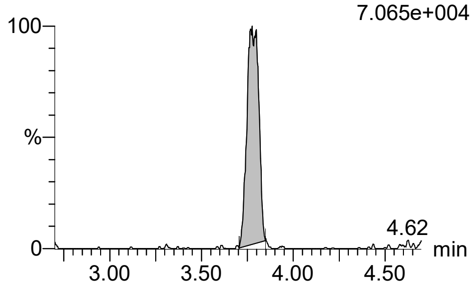

Sample Name: 20211111_A2_R2_IgM
F29:MRM of 2 channels,ES+
869.3 > 204.1
2.310e+006

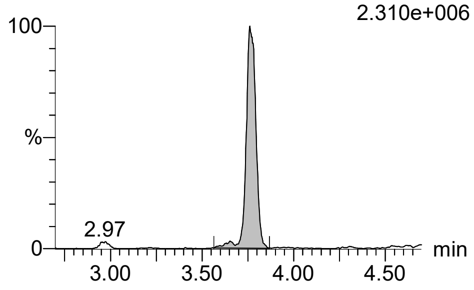

Sample Name: 20211111_A2_R2_IgM
F29:MRM of 2 channels,ES+
1303.5 > 366.1
3.913e+004

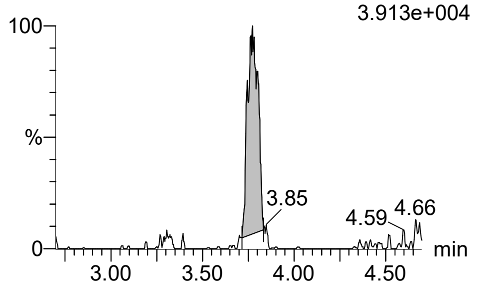

Sample Name: 20211111_A2_R3_IgM
F29:MRM of 2 channels,ES+
869.3 > 204.1
2.350e+006

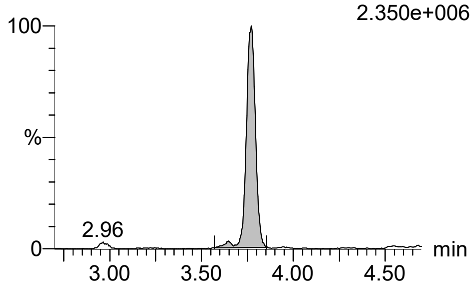

Sample Name: 20211111_A2_R3_IgM
F29:MRM of 2 channels,ES+
1303.5 > 366.1
4.208e+004

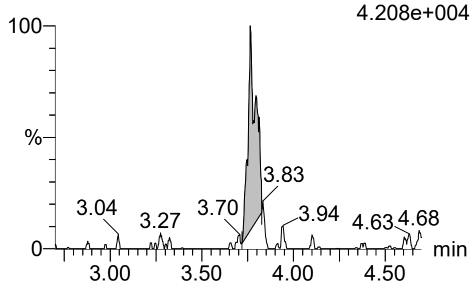

Sample Name: 20211111_L1_R2_IgM

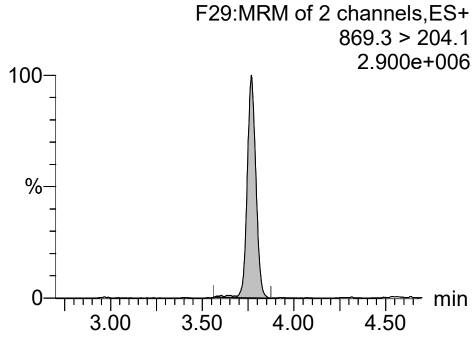

Sample Name: 20211111_L1_R2_IgM

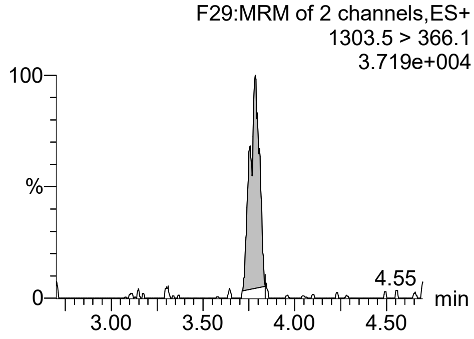

Sample Name: 20211111_L1_R3_IgM

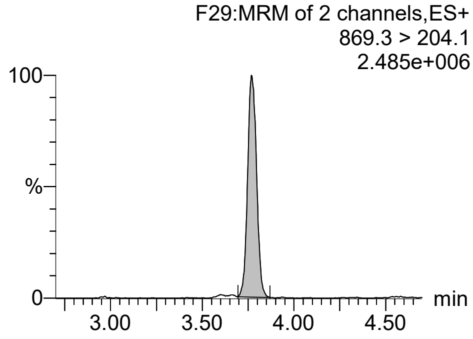

Sample Name: 20211111_L1_R3_IgM

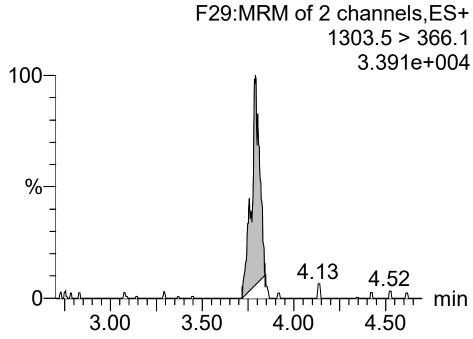

Sample Name: 20211111_L2_R2_IgM

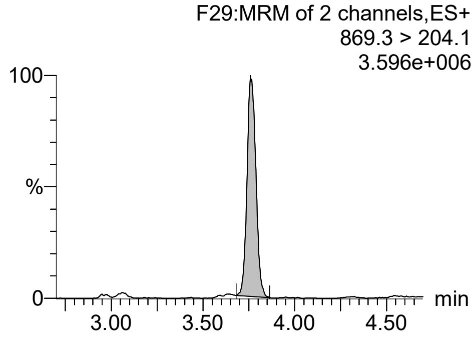

Sample Name: 20211111_L2_R2_IgM

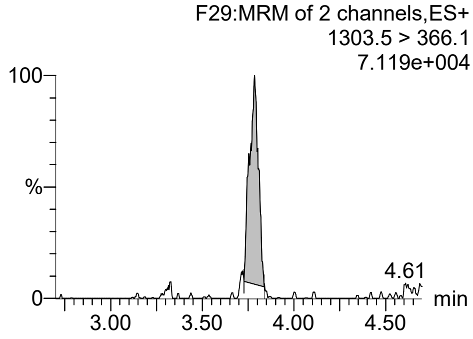

Sample Name: 20211111_H1_R2_IgM

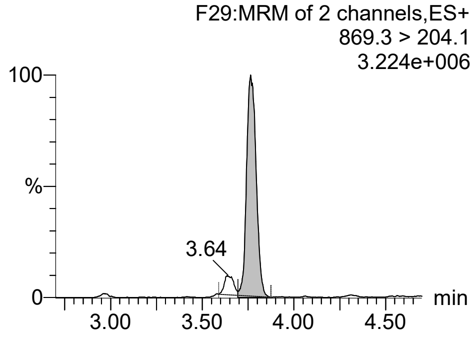

Sample Name: 20211111_H1_R2_IgM

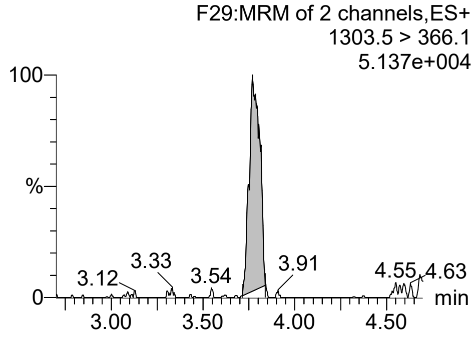

Sample Name: 20211111_A3_R2_IgM

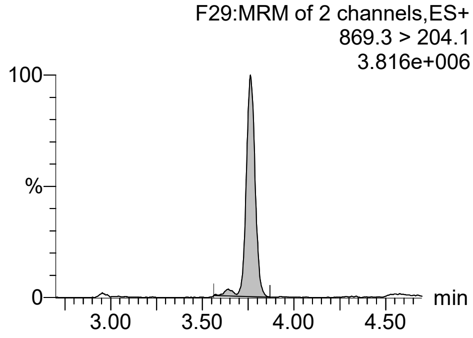

Sample Name: 20211111_A3_R2_IgM

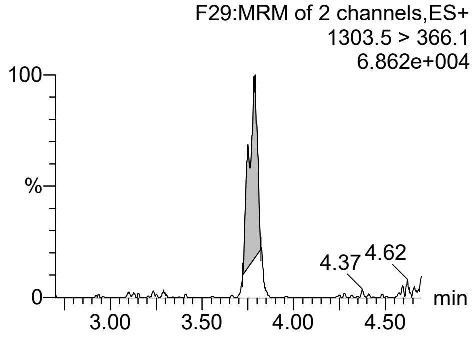

Sample Name: 20211111_A4_R2_IgM

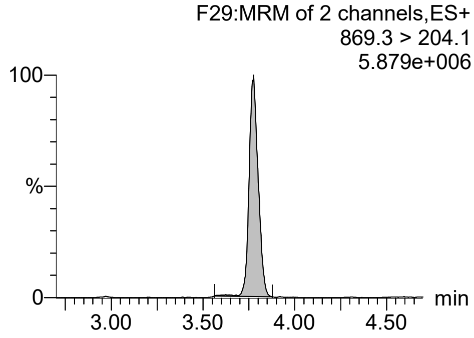

Sample Name: 20211111_A4_R2_IgM

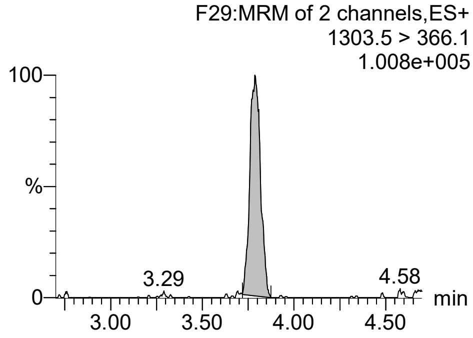

Sample Name: 20211111_L3_R2_IgM

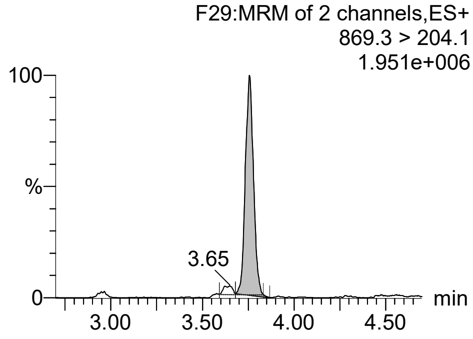

Sample Name: 20211111_L3_R2_IgM

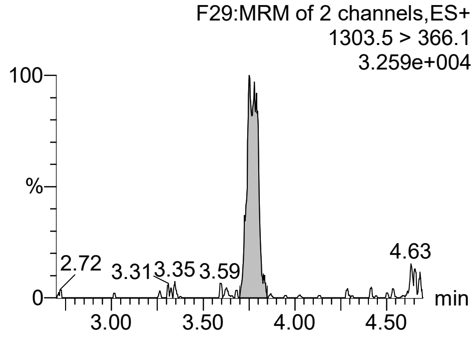

Sample Name: 20211111_L4_R2_IgM

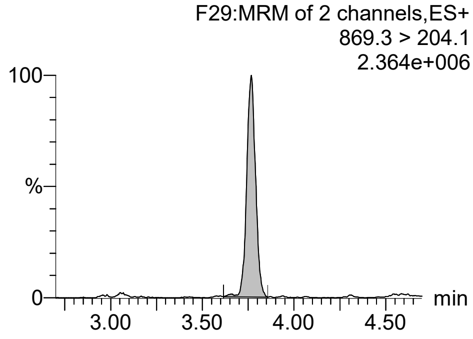

Sample Name: 20211111_L4_R2_IgM
F29:MRM of 2 channels,ES+
1303.5 > 366.1
4.935e+004

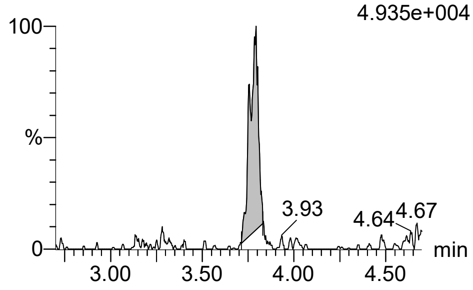

Sample Name: 20211111_H2_R2_IgM
F29:MRM of 2 channels,ES+
869.3 > 204.1
6.293e+006

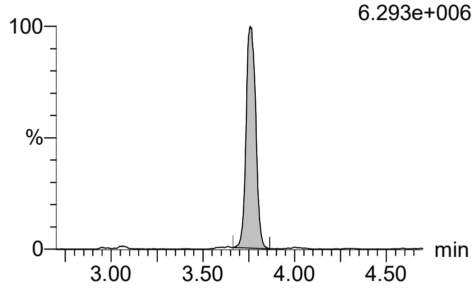

Sample Name: 20211111_H2_R2_IgM
F29:MRM of 2 channels,ES+
1303.5 > 366.1
1.174e+005

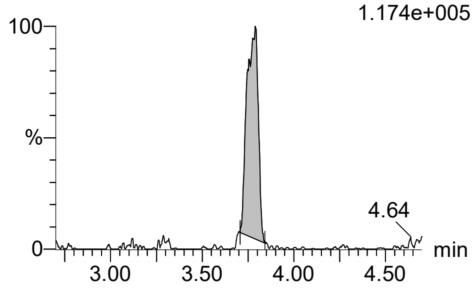

Sample Name: 20211111_A5_R2_IgM
F29:MRM of 2 channels,ES+
869.3 > 204.1
3.721e+006

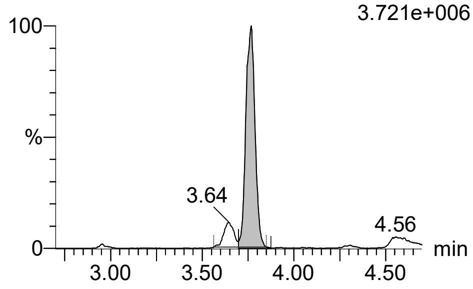

Sample Name: 20211111_A5_R2_IgM
F29:MRM of 2 channels,ES+
1303.5 > 366.1
8.274e+004

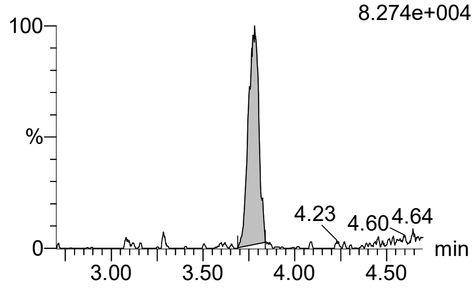

Sample Name: 20211111_A6_R2_IgM
F29:MRM of 2 channels,ES+
869.3 > 204.1
3.708e+006

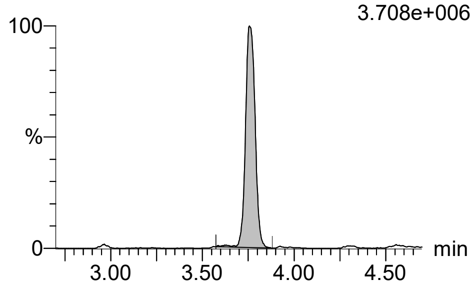

Sample Name: 20211111_A6_R2_IgM
F29:MRM of 2 channels,ES+
1303.5 > 366.1
8.910e+004

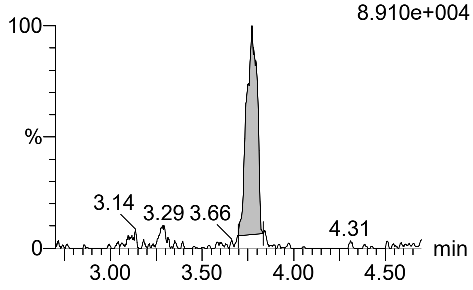

Sample Name: 20211111_QC_R10_IgM
F29:MRM of 2 channels,ES+
869.3 > 204.1
3.603e+006

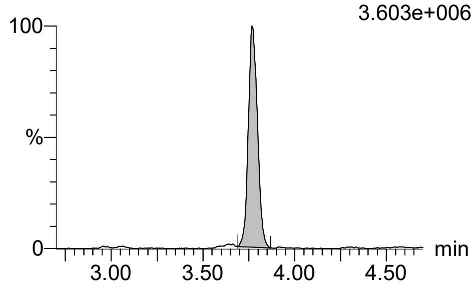

Sample Name: 20211111_QC_R10_IgM
F29:MRM of 2 channels,ES+
1303.5 > 366.1
4.655e+004

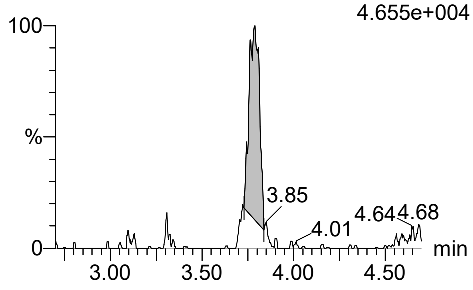

Sample Name: 20211111_L5_R2_IgM
F29:MRM of 2 channels,ES+
869.3 > 204.1
3.281e+006

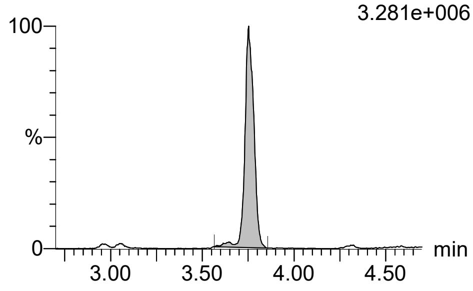

Sample Name: 20211111_L5_R2_IgM
F29:MRM of 2 channels,ES+
1303.5 > 366.1
7.129e+004

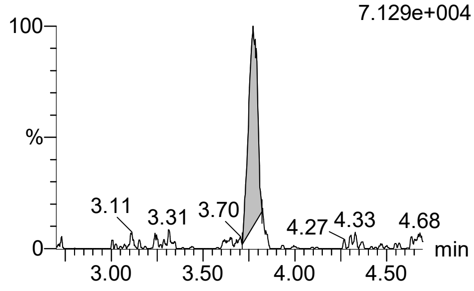

Sample Name: 20211111_L6_R2_IgM
F29:MRM of 2 channels,ES+
869.3 > 204.1
8.157e+005

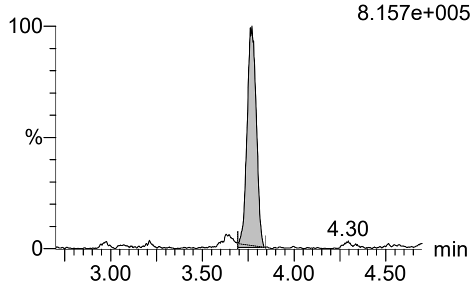

Sample Name: 20211111_L6_R2_IgM
F29:MRM of 2 channels,ES+
1303.5 > 366.1
1.293e+004

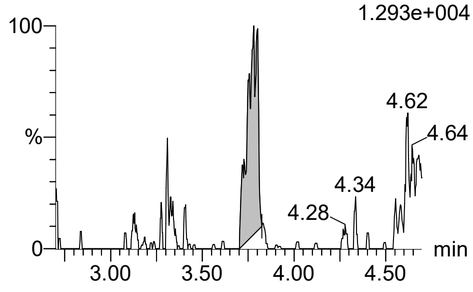

Sample Name: 20211111_H3_R2_IgM
F29:MRM of 2 channels,ES+
869.3 > 204.1
3.805e+006

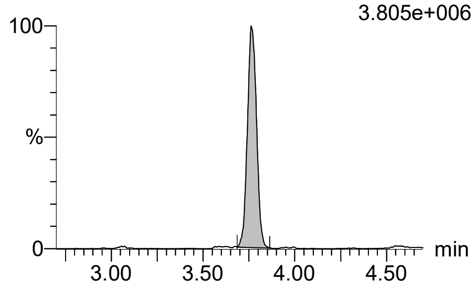

Sample Name: 20211111_H3_R2_IgM
F29:MRM of 2 channels,ES+
1303.5 > 366.1
8.046e+004

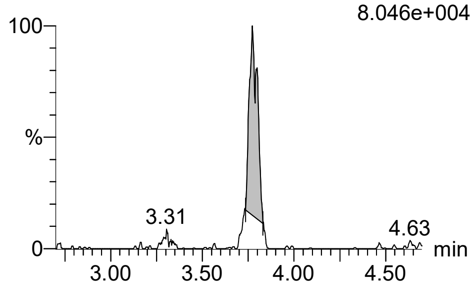

Dataset: C:\MassLynx\10.pro\20211111_clinicalsamples_IgM_IgA.qld

Last Altered: Thursday, March 09, 2023 19:58:25 台北標準時間
Printed: Friday, August 01, 2025 15:27:43 台北標準時間

Sample Name: 20211111_A7_R2_IgM
F29:MRM of 2 channels,ES+
869.3 > 204.1
7.650e+006

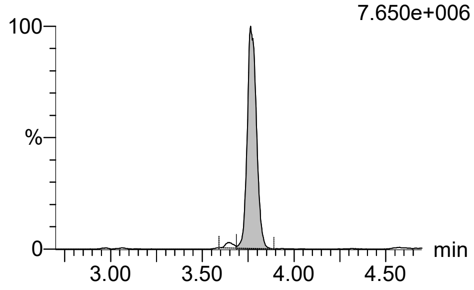

Sample Name: 20211111_A7_R2_IgM
F29:MRM of 2 channels,ES+
1303.5 > 366.1
1.215e+005

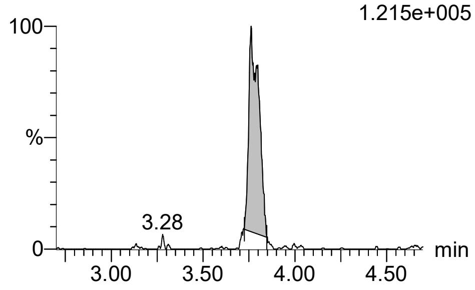

Sample Name: 20211111_A8_R2_IgM
F29:MRM of 2 channels,ES+
869.3 > 204.1
3.910e+006

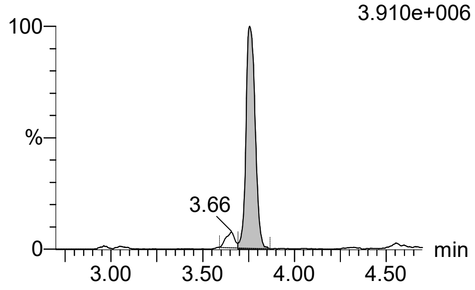

Sample Name: 20211111_A8_R2_IgM
F29:MRM of 2 channels,ES+
1303.5 > 366.1
8.482e+004

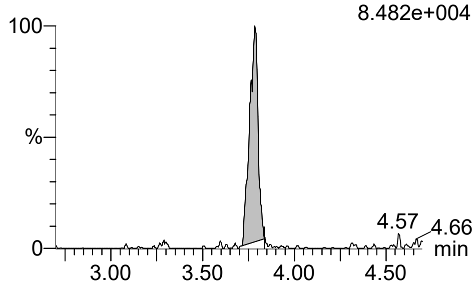

Sample Name: 20211111_L7_R2_IgM
F29:MRM of 2 channels,ES+
869.3 > 204.1
3.838e+006

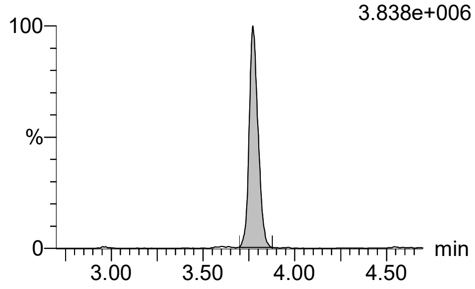

Sample Name: 20211111_L7_R2_IgM
F29:MRM of 2 channels,ES+
1303.5 > 366.1
7.173e+004

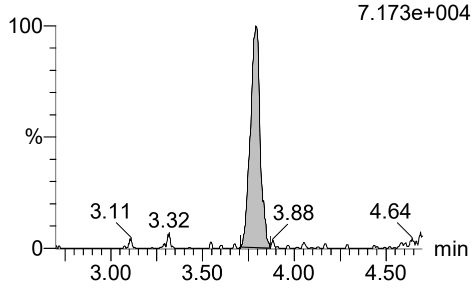

Sample Name: 20211111_L8_R2_IgM
F29:MRM of 2 channels,ES+
869.3 > 204.1
1.628e+006

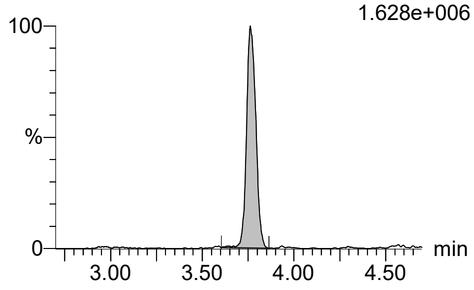

Sample Name: 20211111_L8_R2_IgM
F29:MRM of 2 channels,ES+
1303.5 > 366.1
2.633e+004

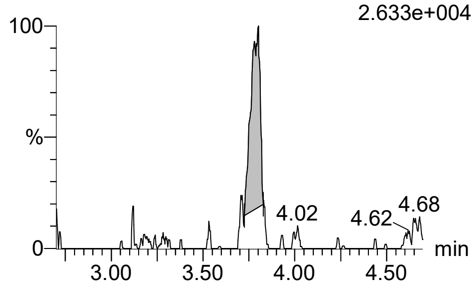

Sample Name: 20211111_H4_R2_IgM
F29:MRM of 2 channels,ES+
869.3 > 204.1
2.368e+006

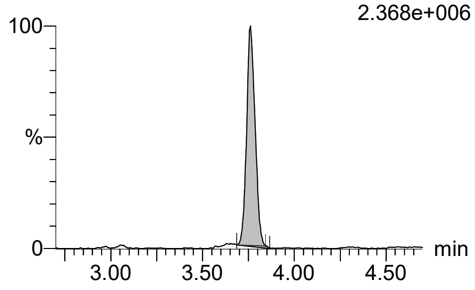

Sample Name: 20211111_H4_R2_IgM
F29:MRM of 2 channels,ES+
1303.5 > 366.1
3.977e+004

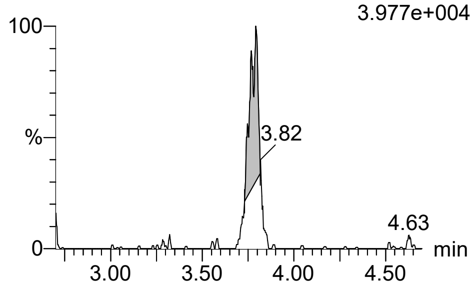

Sample Name: 20211111_A9_R2_IgM
F29:MRM of 2 channels,ES+
869.3 > 204.1
5.058e+006

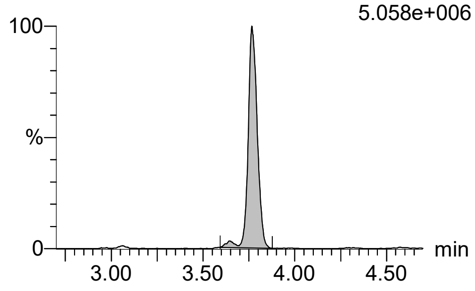

Sample Name: 20211111_A9_R2_IgM
F29:MRM of 2 channels,ES+
1303.5 > 366.1
7.685e+004

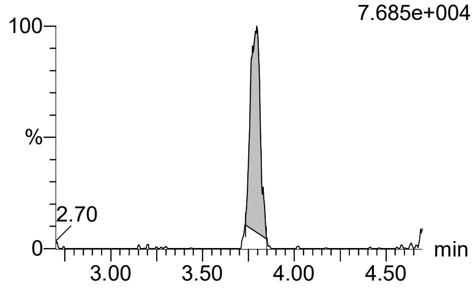

Sample Name: 20211111_A10_R2_IgM
F29:MRM of 2 channels,ES+
869.3 > 204.1
7.405e+006

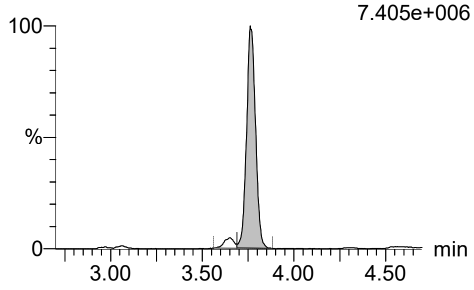

Sample Name: 20211111_A10_R2_IgM
F29:MRM of 2 channels,ES+
1303.5 > 366.1
1.180e+005

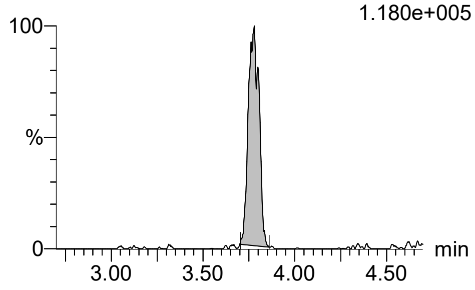

Sample Name: 20211111_L9_R2_IgM
F29:MRM of 2 channels,ES+
869.3 > 204.1
5.750e+006

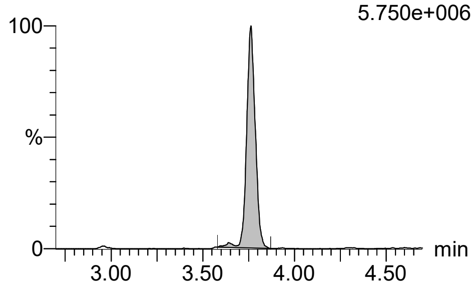

Dataset: C:\MassLynx\10.pro\20211111_clinicalsamples_IgM_IgA.qld

Last Altered: Thursday, March 09, 2023 19:58:25 台北標準時間
Printed: Friday, August 01, 2025 15:27:43 台北標準時間

Sample Name: 20211111_L9_R2_IgM
F29:MRM of 2 channels,ES+
1303.5 > 366.1
7.256e+004

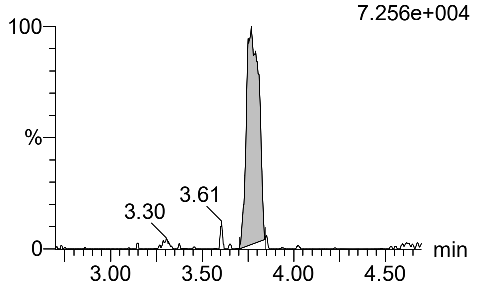

Sample Name: 20211111_L10_R2_IgM
F29:MRM of 2 channels,ES+
869.3 > 204.1
2.706e+006

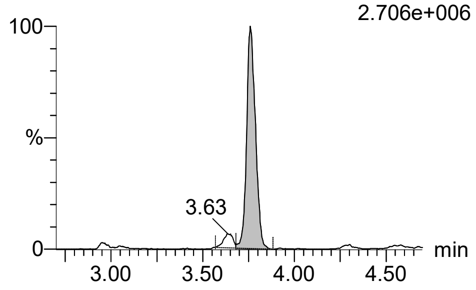

Sample Name: 20211111_L10_R2_IgM
F29:MRM of 2 channels,ES+
1303.5 > 366.1
6.628e+004

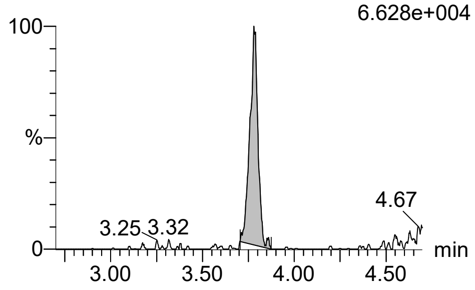

Sample Name: 20211111_QC_R11_IgM
F29:MRM of 2 channels,ES+
869.3 > 204.1
3.791e+006

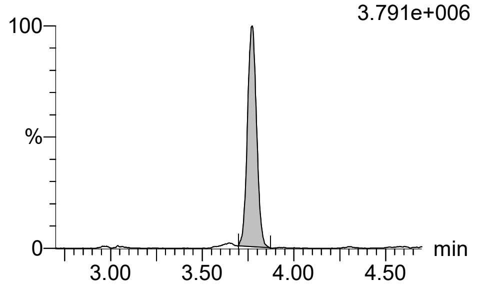

Sample Name: 20211111_QC_R11_IgM
F29:MRM of 2 channels,ES+
1303.5 > 366.1
7.182e+004

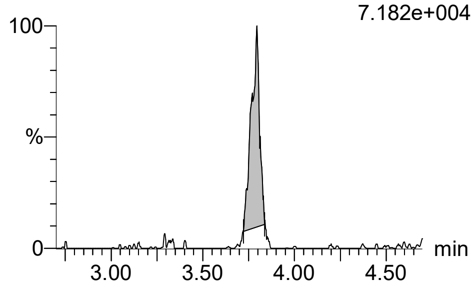

Sample Name: 20211111_H5_R2_IgM
F29:MRM of 2 channels,ES+
869.3 > 204.1
1.925e+006

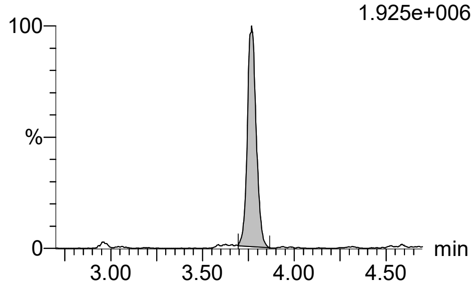

Sample Name: 20211111_H5_R2_IgM
F29:MRM of 2 channels,ES+
1303.5 > 366.1
2.554e+004

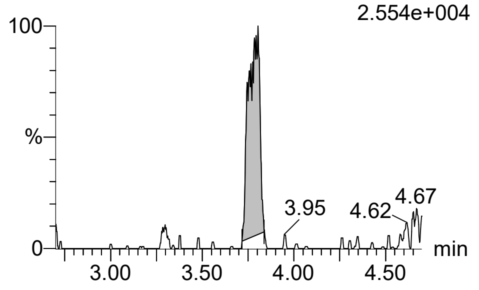

Sample Name: 20211111_A11_R2_IgM
F29:MRM of 2 channels,ES+
869.3 > 204.1
1.170e+006

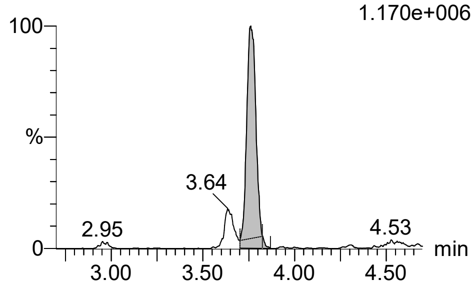

Sample Name: 20211111_A11_R2_IgM
F29:MRM of 2 channels,ES+
1303.5 > 366.1
2.307e+004

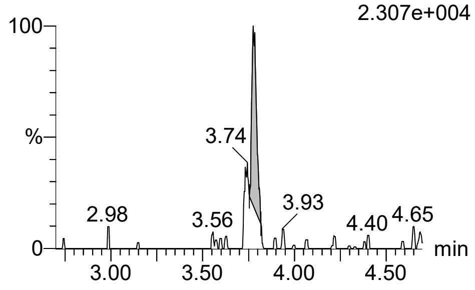

Sample Name: 20211111_A12_R2_IgM
F29:MRM of 2 channels,ES+
869.3 > 204.1
3.972e+006

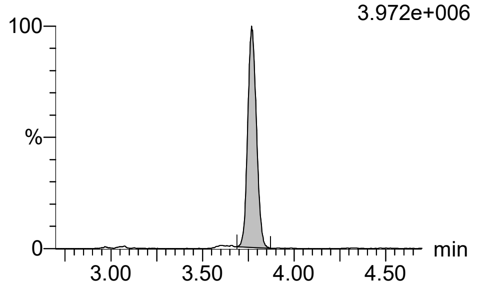

Sample Name: 20211111_A12_R2_IgM
F29:MRM of 2 channels,ES+
1303.5 > 366.1
5.088e+004

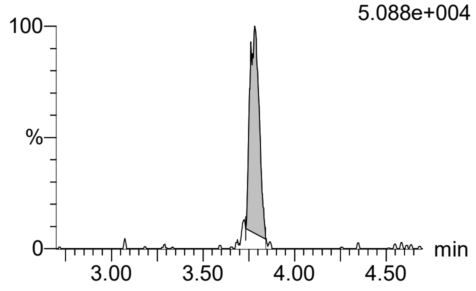

Sample Name: 20211111_L11_R2_IgM
F29:MRM of 2 channels,ES+
869.3 > 204.1
2.258e+006

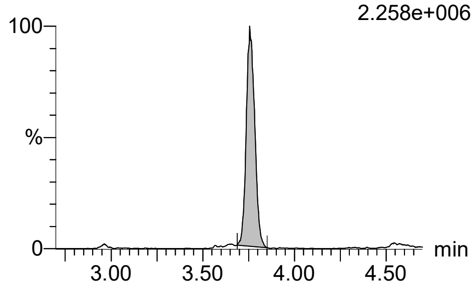

Sample Name: 20211111_L11_R2_IgM
F29:MRM of 2 channels,ES+
1303.5 > 366.1
3.037e+004

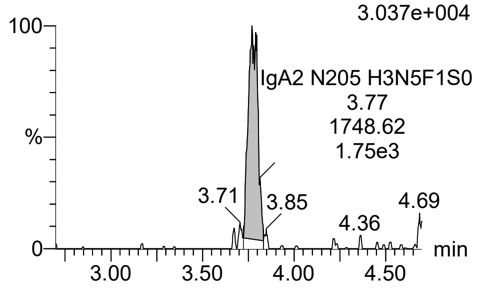

Sample Name: 20211111_L12_R2_IgM
F29:MRM of 2 channels,ES+
869.3 > 204.1
1.347e+006

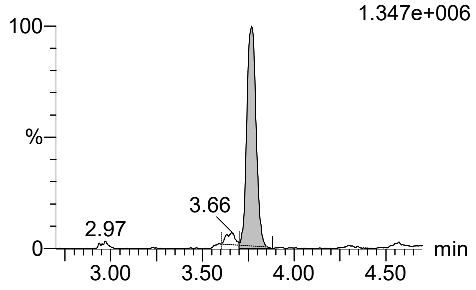

Sample Name: 20211111_L12_R2_IgM
F29:MRM of 2 channels,ES+
1303.5 > 366.1
3.163e+004

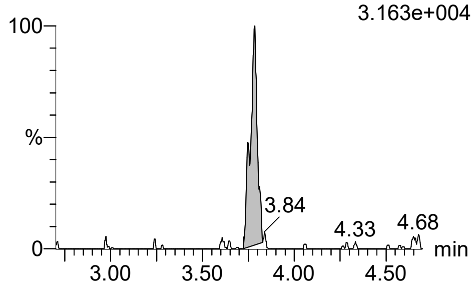

Dataset: C:\MassLynx\10.pro\20211111_clinicalsamples_IgM_IgA.qld

Last Altered: Thursday, March 09, 2023 19:58:25 台北標準時間
Printed: Friday, August 01, 2025 15:27:43 台北標準時間

Sample Name: 20211111_H6_R2_IgM
F29:MRM of 2 channels,ES+
869.3 > 204.1
5.082e+006

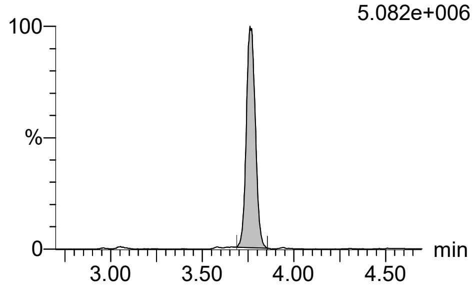

Sample Name: 20211111_H6_R2_IgM
F29:MRM of 2 channels,ES+
1303.5 > 366.1
7.924e+004

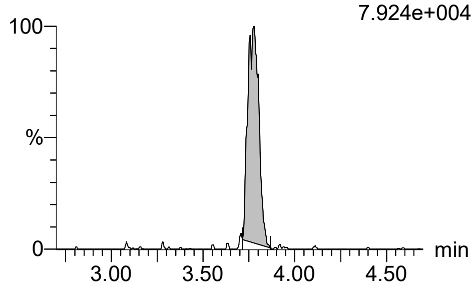

Sample Name: 20211111_A13_R2_IgM
F29:MRM of 2 channels,ES+
869.3 > 204.1
3.370e+006

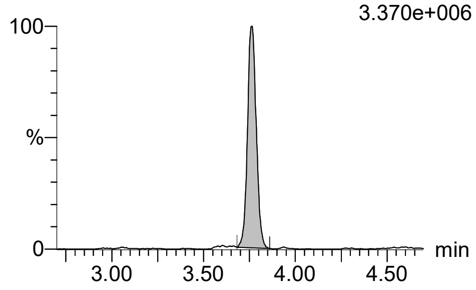

Sample Name: 20211111_A13_R2_IgM
F29:MRM of 2 channels,ES+
1303.5 > 366.1
5.867e+004

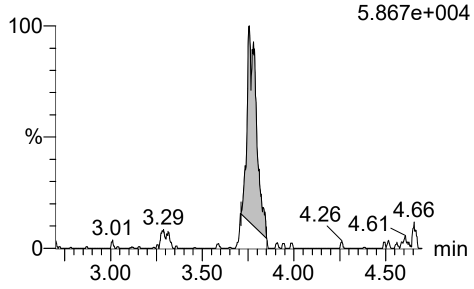

Sample Name: 20211111_A14_R2_IgM
F29:MRM of 2 channels,ES+
869.3 > 204.1
4.876e+006

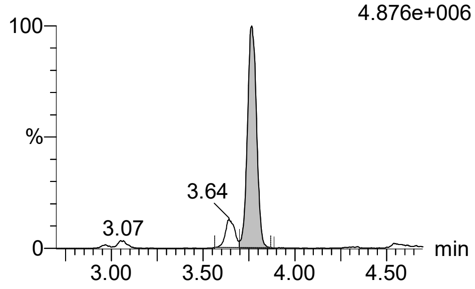

Sample Name: 20211111_A14_R2_IgM
F29:MRM of 2 channels,ES+
1303.5 > 366.1
9.191e+004

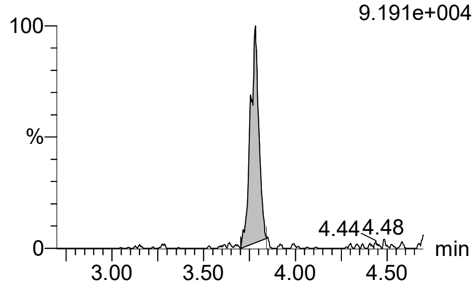

Sample Name: 20211111_L13_R2_IgM
F29:MRM of 2 channels,ES+
869.3 > 204.1
2.470e+006

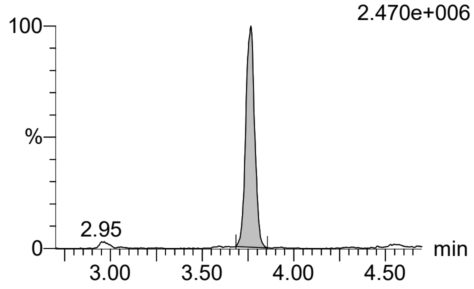

Sample Name: 20211111_L13_R2_IgM
F29:MRM of 2 channels,ES+
1303.5 > 366.1
4.097e+004

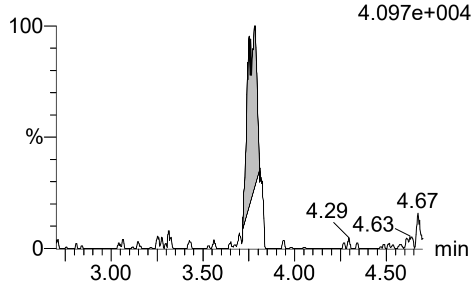

Sample Name: 20211111_L14_R2_IgM
F29:MRM of 2 channels,ES+
869.3 > 204.1
1.991e+006

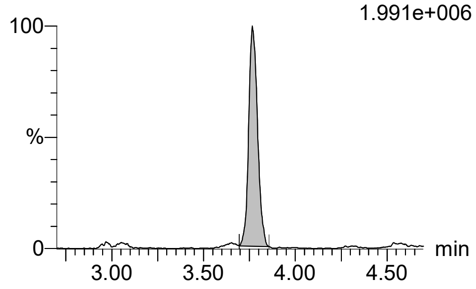

Sample Name: 20211111_L14_R2_IgM
F29:MRM of 2 channels,ES+
1303.5 > 366.1
3.542e+004

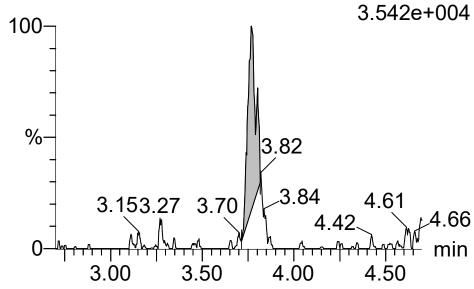

Sample Name: 20211111_H7_R2_IgM
F29:MRM of 2 channels,ES+
869.3 > 204.1
2.836e+006

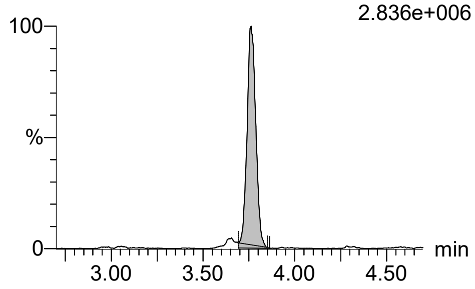

Sample Name: 20211111_H7_R2_IgM
F29:MRM of 2 channels,ES+
1303.5 > 366.1
4.529e+004

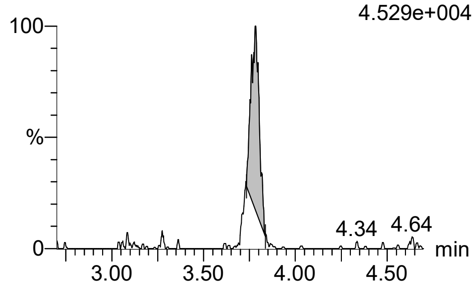

Sample Name: 20211111_A15_R2_IgM
F29:MRM of 2 channels,ES+
869.3 > 204.1
4.675e+006

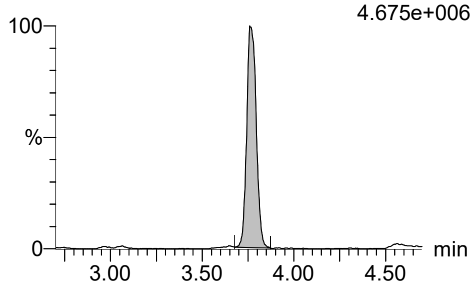

Sample Name: 20211111_A15_R2_IgM
F29:MRM of 2 channels,ES+
1303.5 > 366.1
8.547e+004

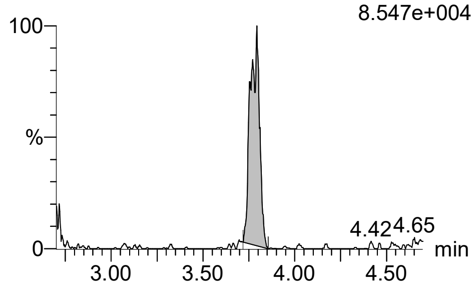

Sample Name: 20211111_QC_R12_IgM
F29:MRM of 2 channels,ES+
869.3 > 204.1
4.317e+006

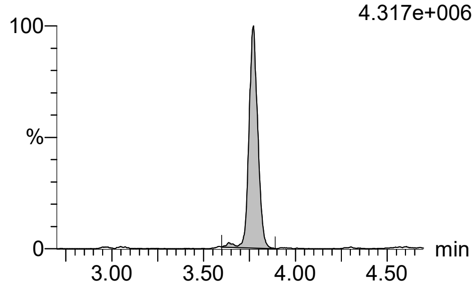

Dataset: C:\MassLynx\10.pro\20211111_clinicalsamples_IgM_IgA.qld

Last Altered: Thursday, March 09, 2023 19:58:25 台北標準時間
Printed: Friday, August 01, 2025 15:27:43 台北標準時間

Sample Name: 20211111_QC_R12_IgM
F29:MRM of 2 channels,ES+
1303.5 > 366.1
6.059e+004

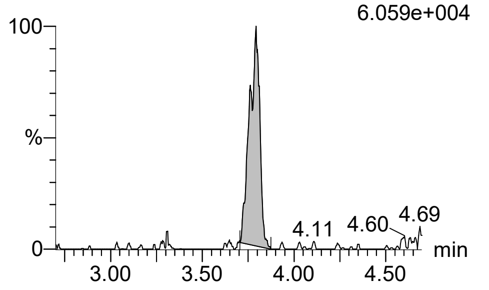

Sample Name: 20211111_A16_R2_IgM
F29:MRM of 2 channels,ES+
869.3 > 204.1
3.617e+006

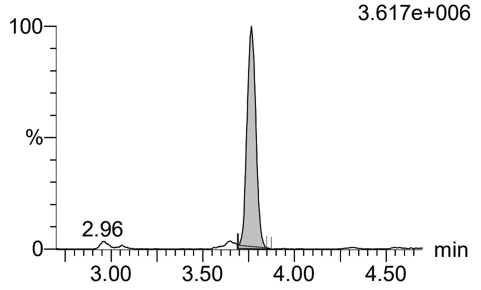

Sample Name: 20211111_A16_R2_IgM
F29:MRM of 2 channels,ES+
1303.5 > 366.1
5.002e+004

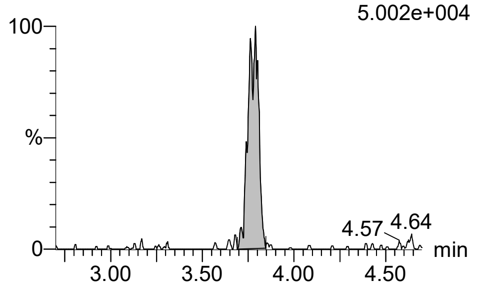

Sample Name: 20211111_L15_R2_IgM
F29:MRM of 2 channels,ES+
869.3 > 204.1
2.999e+006

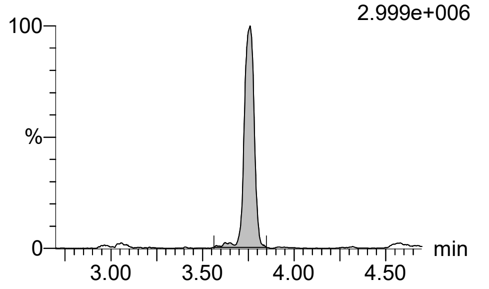

Sample Name: 20211111_L15_R2_IgM
F29:MRM of 2 channels,ES+
1303.5 > 366.1
4.747e+004

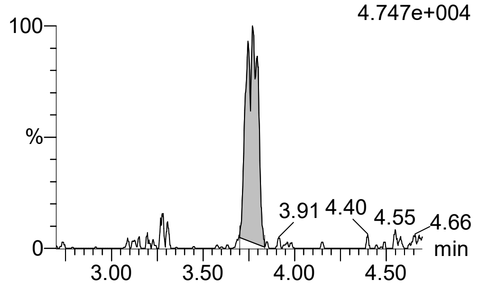

Sample Name: 20211111_L16_R2_IgM
F29:MRM of 2 channels,ES+
869.3 > 204.1
3.609e+006

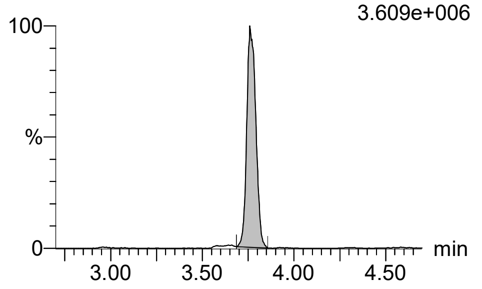

Sample Name: 20211111_L16_R2_IgM
F29:MRM of 2 channels,ES+
1303.5 > 366.1
5.778e+004

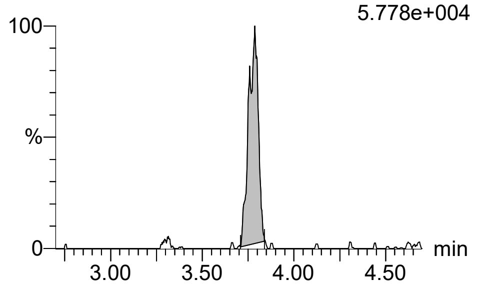

Sample Name: 20211111_H8_R2_IgM
F29:MRM of 2 channels,ES+
869.3 > 204.1
2.652e+006

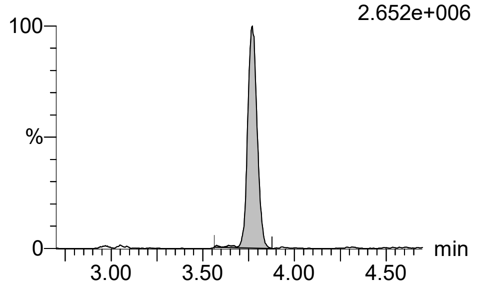

Sample Name: 20211111_H8_R2_IgM
F29:MRM of 2 channels,ES+
1303.5 > 366.1
5.103e+004

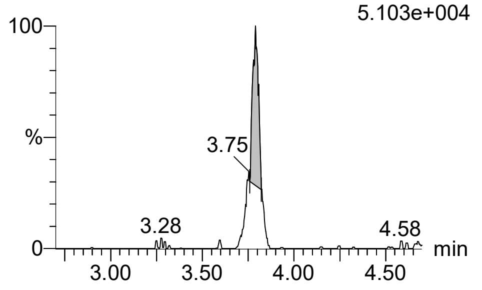

Sample Name: 20211111_A17_R2_IgM
F29:MRM of 2 channels,ES+
869.3 > 204.1
3.539e+006

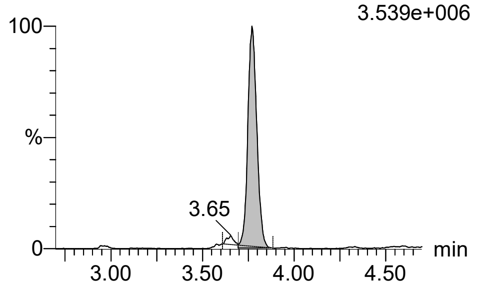

Sample Name: 20211111_A17_R2_IgM
F29:MRM of 2 channels,ES+
1303.5 > 366.1
6.328e+004

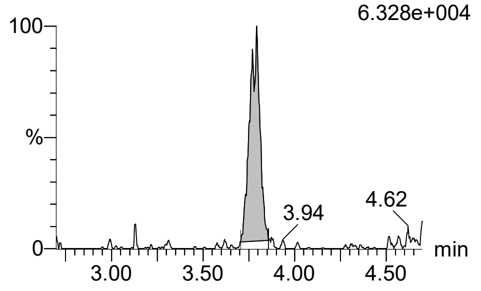

Sample Name: 20211111_A18_R2_IgM
F29:MRM of 2 channels,ES+
869.3 > 204.1
3.777e+006

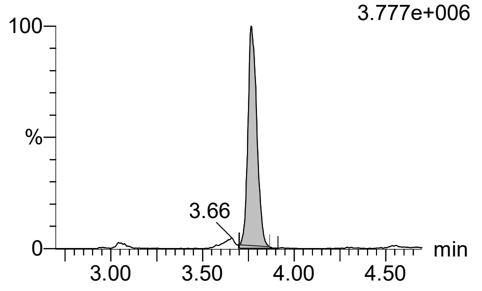

Sample Name: 20211111_A18_R2_IgM
F29:MRM of 2 channels,ES+
1303.5 > 366.1
7.281e+004

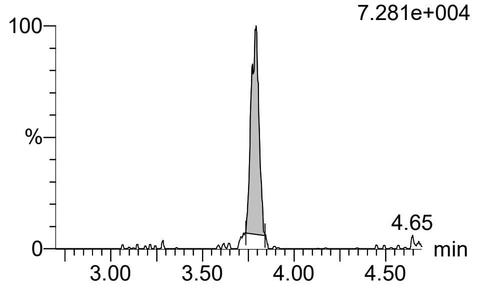

Sample Name: 20211111_L17_R2_IgM
F29:MRM of 2 channels,ES+
869.3 > 204.1
4.208e+006

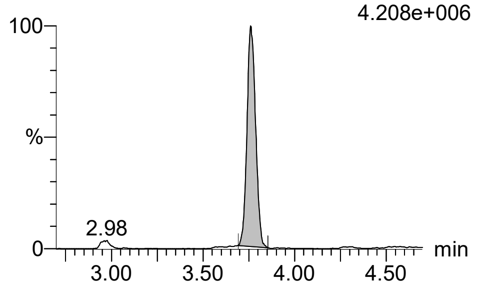

Sample Name: 20211111_L17_R2_IgM
F29:MRM of 2 channels,ES+
1303.5 > 366.1
7.370e+004

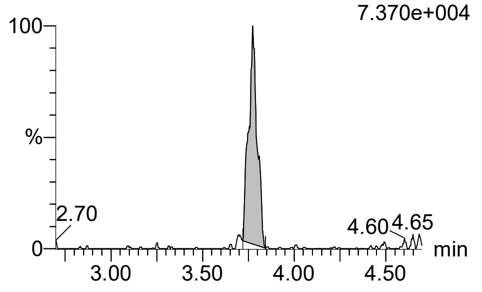

Dataset: C:\MassLynx\10.pro\20211111_clinicalsamples_IgM_IgA.qld

Last Altered: Thursday, March 09, 2023 19:58:25 台北標準時間
Printed: Friday, August 01, 2025 15:27:43 台北標準時間

Sample Name: 20211111_L18_R2_IgM
F29:MRM of 2 channels,ES+
869.3 > 204.1
1.247e+006

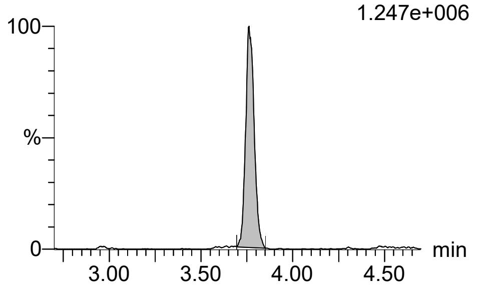

Sample Name: 20211111_L18_R2_IgM
F29:MRM of 2 channels,ES+
1303.5 > 366.1
1.891e+004

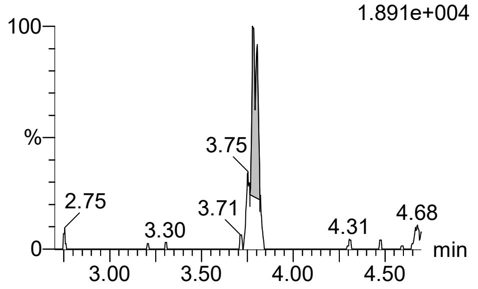

Sample Name: 20211111_H9_R2_IgM
F29:MRM of 2 channels,ES+
869.3 > 204.1
4.996e+006

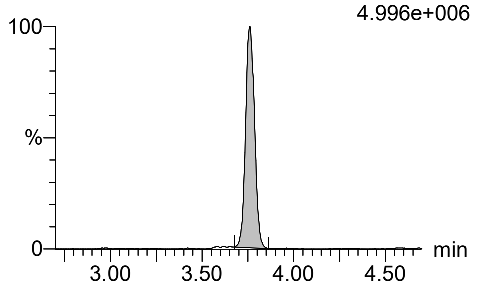

Sample Name: 20211111_H9_R2_IgM
F29:MRM of 2 channels,ES+
1303.5 > 366.1
8.224e+004

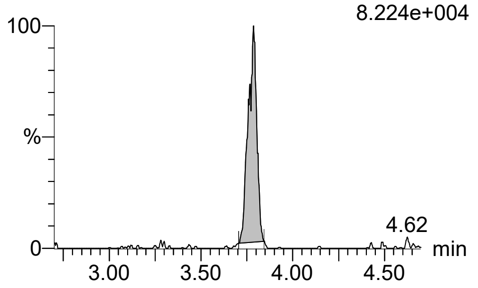

Sample Name: 20211111_A19_R2_IgM
F29:MRM of 2 channels,ES+
869.3 > 204.1
5.395e+006

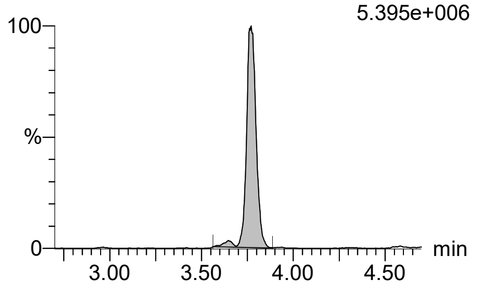

Sample Name: 20211111_A19_R2_IgM
F29:MRM of 2 channels,ES+
1303.5 > 366.1
1.029e+005

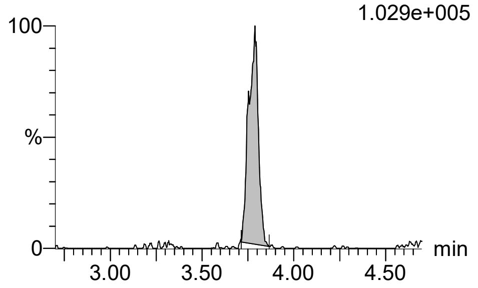

Sample Name: 20211111_A20_R2_IgM
F29:MRM of 2 channels,ES+
869.3 > 204.1
1.152e+006

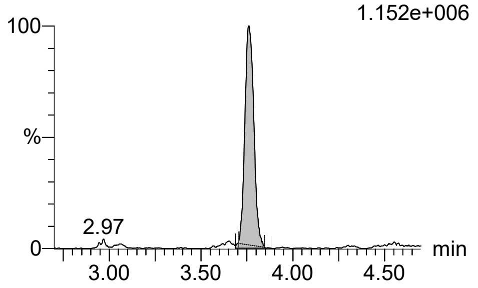

Sample Name: 20211111_A20_R2_IgM
F29:MRM of 2 channels,ES+
1303.5 > 366.1
1.795e+004

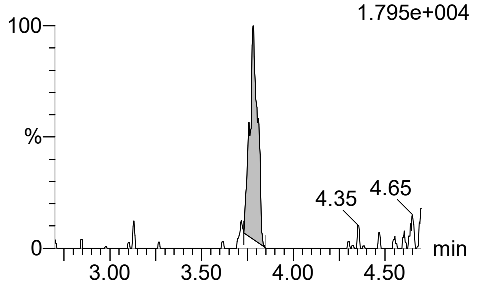

Sample Name: 20211111_L19_R2_IgM
F29:MRM of 2 channels,ES+
869.3 > 204.1
1.478e+006

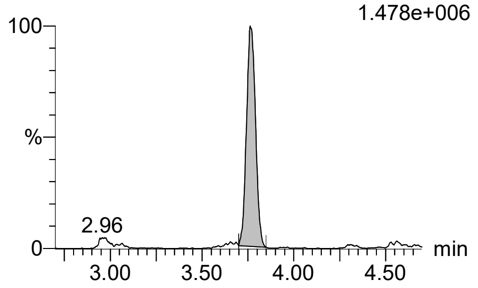

Sample Name: 20211111_L19_R2_IgM
F29:MRM of 2 channels,ES+
1303.5 > 366.1
2.160e+004

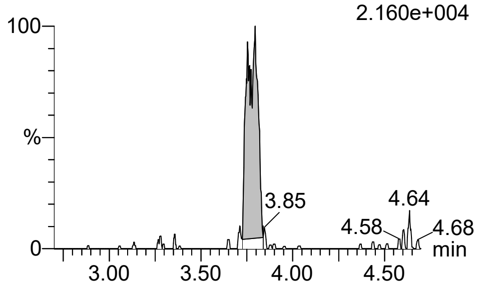

Sample Name: 20211111_QC_R13_IgM
F29:MRM of 2 channels,ES+
869.3 > 204.1
3.959e+006

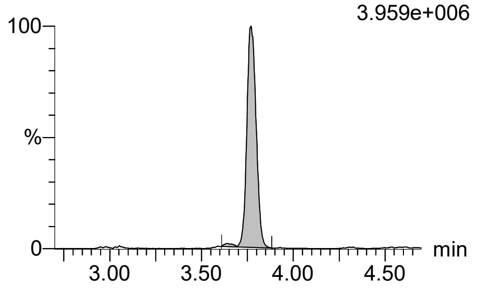

Sample Name: 20211111_QC_R13_IgM
F29:MRM of 2 channels,ES+
1303.5 > 366.1
7.549e+004

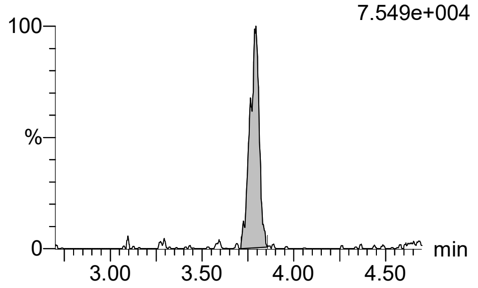

Sample Name: 20211111_L20_R2_IgM
F29:MRM of 2 channels,ES+
869.3 > 204.1
2.450e+006

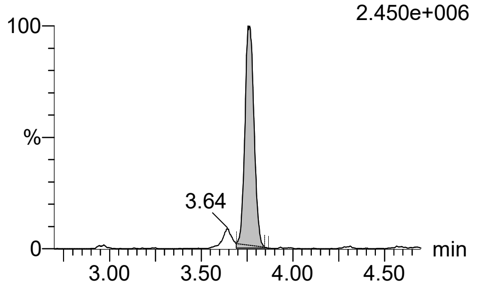

Sample Name: 20211111_L20_R2_IgM
F29:MRM of 2 channels,ES+
1303.5 > 366.1
5.284e+004

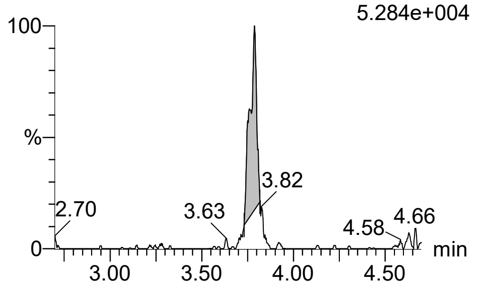

Sample Name: 20211111_H10_R2_IgM
F29:MRM of 2 channels,ES+
869.3 > 204.1
1.658e+006

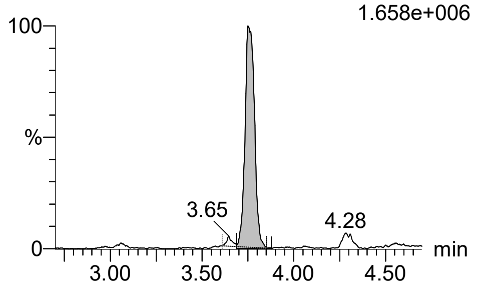

Sample Name: 20211111_H10_R2_IgM
F29:MRM of 2 channels,ES+
1303.5 > 366.1
3.930e+004

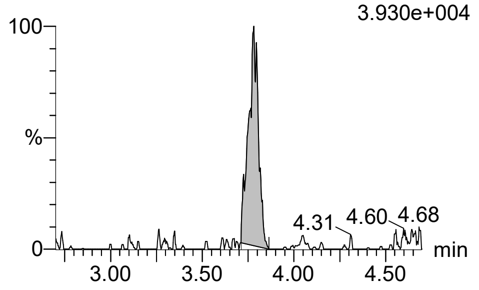

Sample Name: 20211111_A21_R2_IgM
F29:MRM of 2 channels,ES+
869.3 > 204.1
5.851e+006

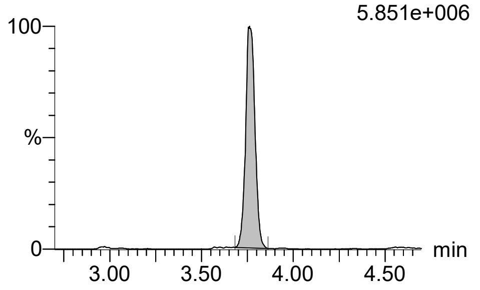

Sample Name: 20211111_A21_R2_IgM
F29:MRM of 2 channels,ES+
1303.5 > 366.1
1.124e+005

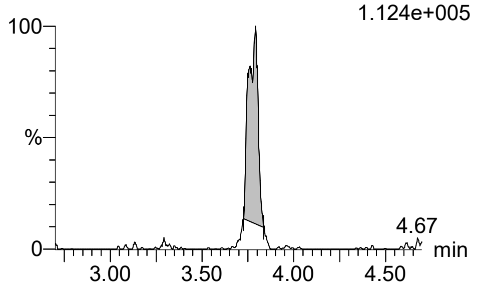

Sample Name: 20211111_A22_R2_IgM
F29:MRM of 2 channels,ES+
869.3 > 204.1
1.293e+006

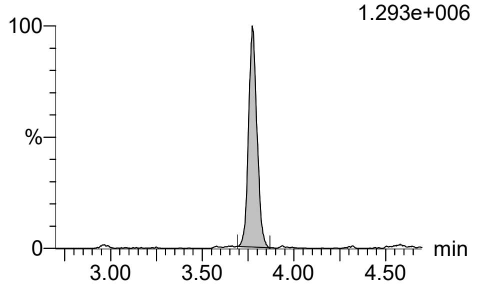

Sample Name: 20211111_A22_R2_IgM
F29:MRM of 2 channels,ES+
1303.5 > 366.1
2.246e+004

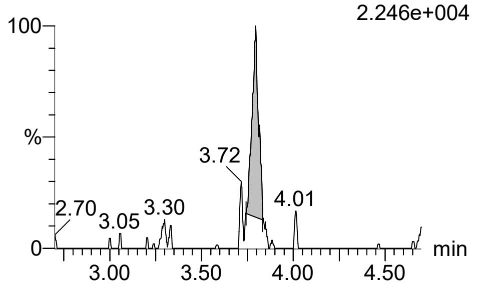

Sample Name: 20211111_L21_R2_IgM
F29:MRM of 2 channels,ES+
869.3 > 204.1
1.259e+006

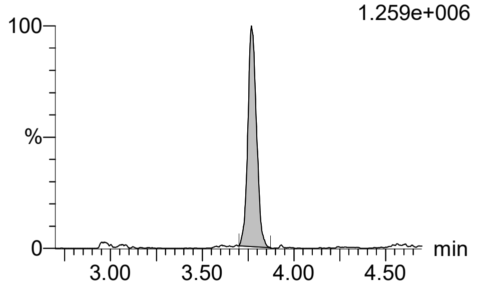

Sample Name: 20211111_L21_R2_IgM
F29:MRM of 2 channels,ES+
1303.5 > 366.1
2.882e+004

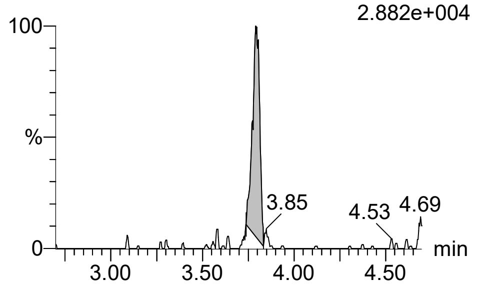

Sample Name: 20211111_L22_R2_IgM
F29:MRM of 2 channels,ES+
869.3 > 204.1
2.429e+006

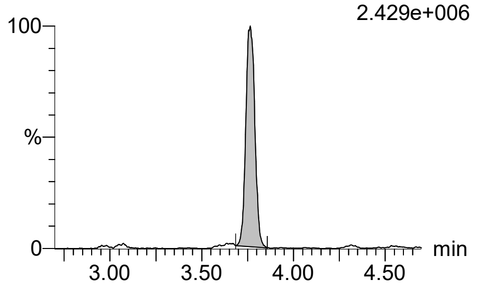

Sample Name: 20211111_L22_R2_IgM
F29:MRM of 2 channels,ES+
1303.5 > 366.1
4.277e+004

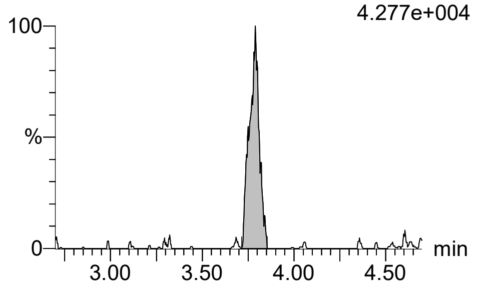

Sample Name: 20211111_H11_R2_IgM
F29:MRM of 2 channels,ES+
869.3 > 204.1
8.848e+006

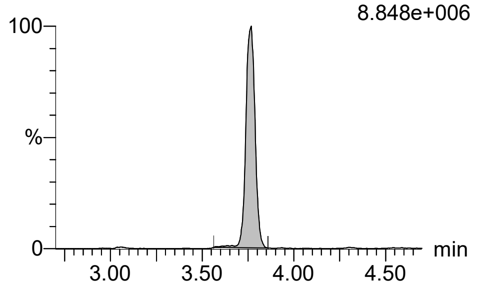

Sample Name: 20211111_H11_R2_IgM
F29:MRM of 2 channels,ES+
1303.5 > 366.1
1.538e+005

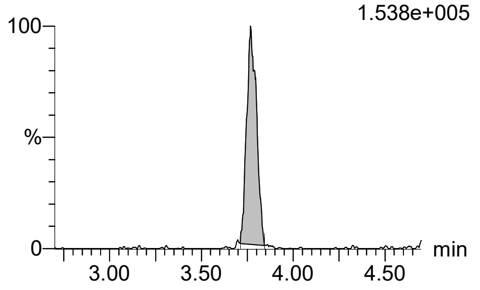

Sample Name: 20211111_A23_R2_IgM
F29:MRM of 2 channels,ES+
869.3 > 204.1
4.810e+006

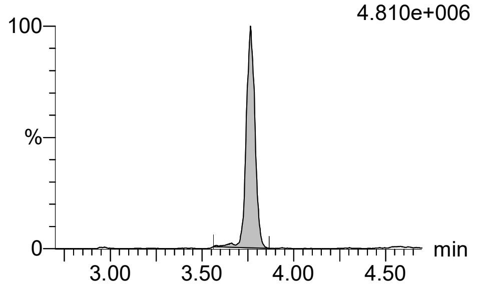

Sample Name: 20211111_A23_R2_IgM
F29:MRM of 2 channels,ES+
1303.5 > 366.1
6.682e+004

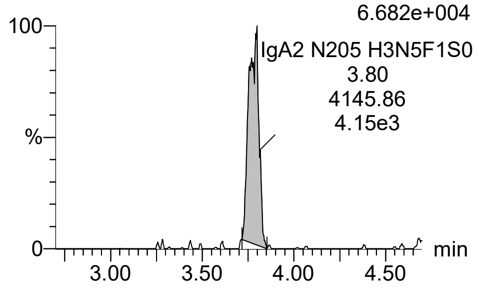

Sample Name: 20211111_A24_R2_IgM
F29:MRM of 2 channels,ES+
869.3 > 204.1
1.049e+006

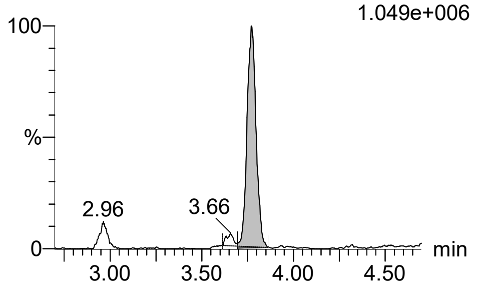

Sample Name: 20211111_A24_R2_IgM
F29:MRM of 2 channels,ES+
1303.5 > 366.1
1.947e+004

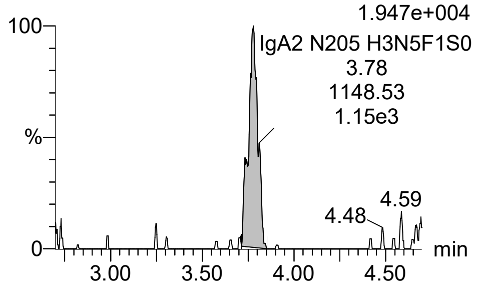

Dataset: C:\MassLynx\10.pro\20211111_clinicalsamples_IgM_IgA.qld

Last Altered: Thursday, March 09, 2023 19:58:25 台北標準時間
Printed: Friday, August 01, 2025 15:27:43 台北標準時間

Sample Name: 20211111_L23_R2_IgM
F29:MRM of 2 channels,ES+
869.3 > 204.1
6.193e+006

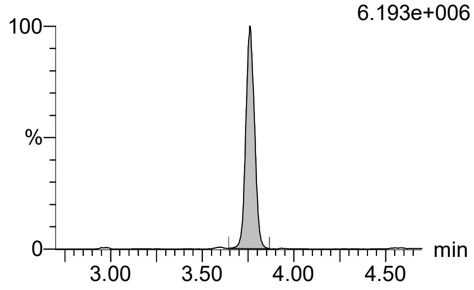

Sample Name: 20211111_L23_R2_IgM
F29:MRM of 2 channels,ES+
1303.5 > 366.1
9.177e+004

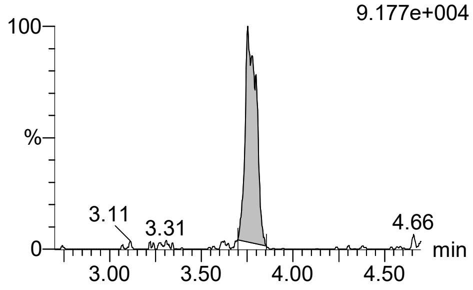

Sample Name: 20211111_L24_R2_IgM
F29:MRM of 2 channels,ES+
869.3 > 204.1
1.470e+006

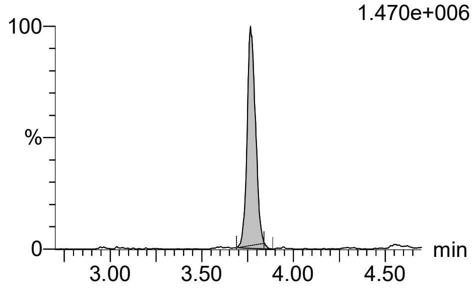

Sample Name: 20211111_L24_R2_IgM
F29:MRM of 2 channels,ES+
1303.5 > 366.1
3.051e+004

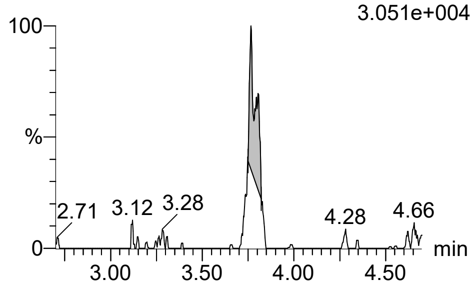

Sample Name: 20211111_H12_R2_IgM
F29:MRM of 2 channels,ES+
869.3 > 204.1
1.880e+006

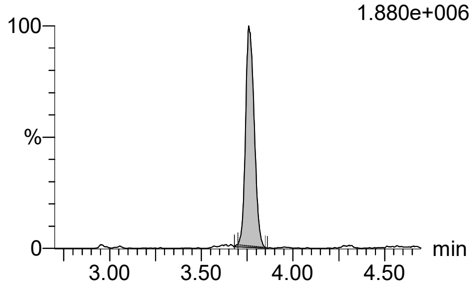

Sample Name: 20211111_H12_R2_IgM
F29:MRM of 2 channels,ES+
1303.5 > 366.1
2.567e+004

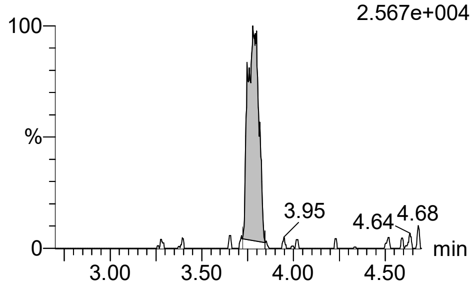

Sample Name: 20211111_QC_R14_IgM
F29:MRM of 2 channels,ES+
869.3 > 204.1
4.538e+006

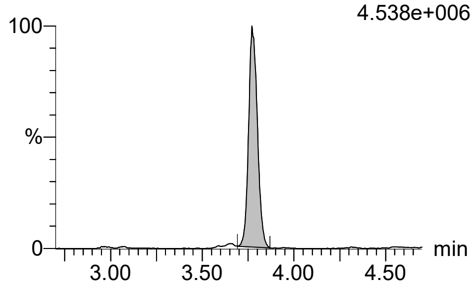

Sample Name: 20211111_QC_R14_IgM
F29:MRM of 2 channels,ES+
1303.5 > 366.1
5.749e+004

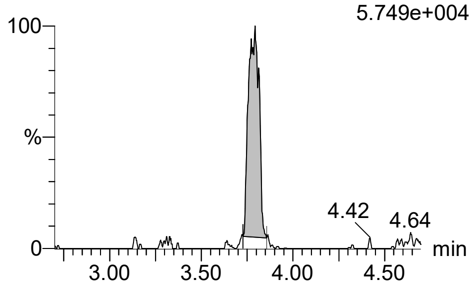

Sample Name: 20211111_A25_R2_IgM
F29:MRM of 2 channels,ES+
869.3 > 204.1
3.546e+006

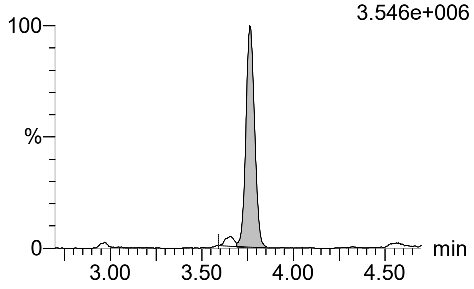

Sample Name: 20211111_A25_R2_IgM
F29:MRM of 2 channels,ES+
1303.5 > 366.1
7.075e+004

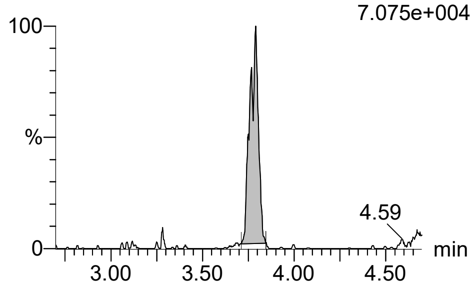

Sample Name: 20211111_A26_R2_IgM
F29:MRM of 2 channels,ES+
869.3 > 204.1
1.816e+006

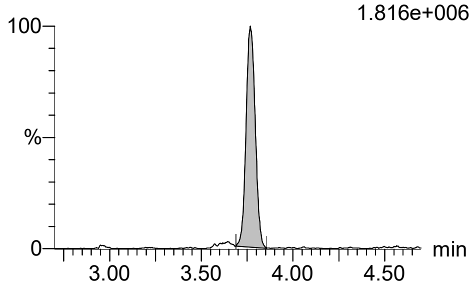

Sample Name: 20211111_A26_R2_IgM
F29:MRM of 2 channels,ES+
1303.5 > 366.1
2.844e+004

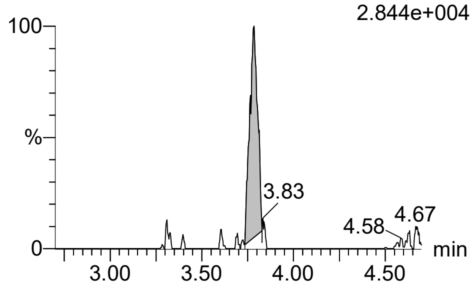

Sample Name: 20211111_L25_R2_IgM
F29:MRM of 2 channels,ES+
869.3 > 204.1
4.489e+006

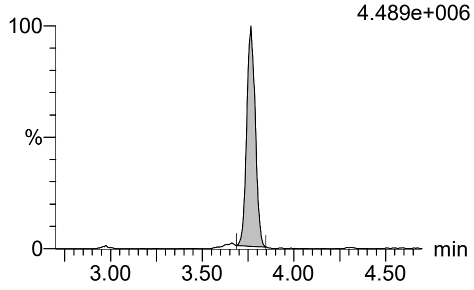

Sample Name: 20211111_L25_R2_IgM
F29:MRM of 2 channels,ES+
1303.5 > 366.1
5.449e+004

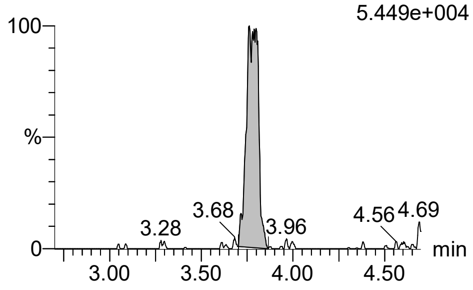

Sample Name: 20211111_L26_R2_IgM
F29:MRM of 2 channels,ES+
869.3 > 204.1
1.800e+006

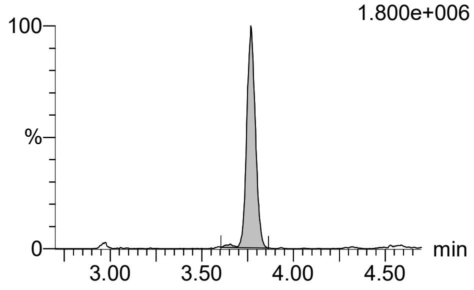

Sample Name: 20211111_L26_R2_IgM
F29:MRM of 2 channels,ES+
1303.5 > 366.1
2.580e+004

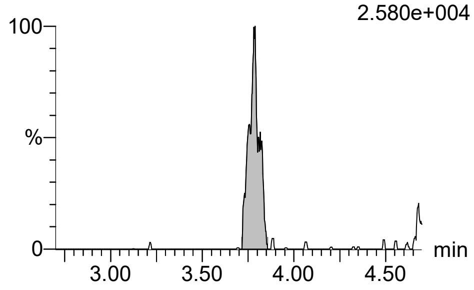

Sample Name: 20211111_H13_R2_IgM
F29:MRM of 2 channels,ES+
869.3 > 204.1
3.375e+006

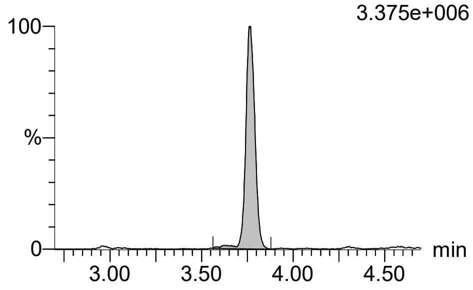

Sample Name: 20211111_H13_R2_IgM
F29:MRM of 2 channels,ES+
1303.5 > 366.1
5.373e+004

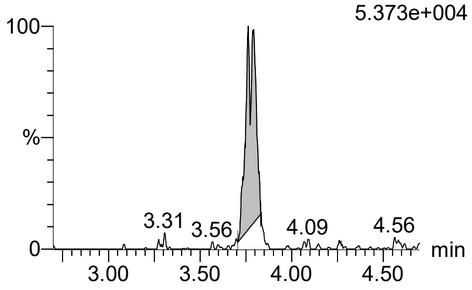

Sample Name: 20211111_A27_R2_IgM
F29:MRM of 2 channels,ES+
869.3 > 204.1
1.597e+006

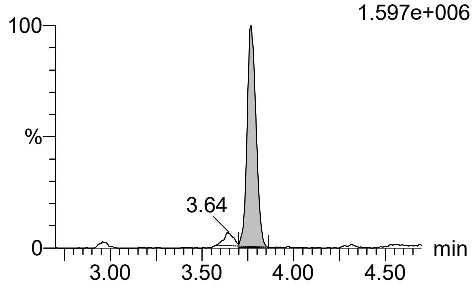

Sample Name: 20211111_A27_R2_IgM
F29:MRM of 2 channels,ES+
1303.5 > 366.1
2.732e+004

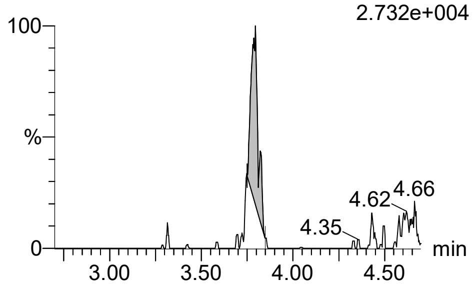

Sample Name: 20211111_A28_R2_IgM
F29:MRM of 2 channels,ES+
869.3 > 204.1
1.497e+006

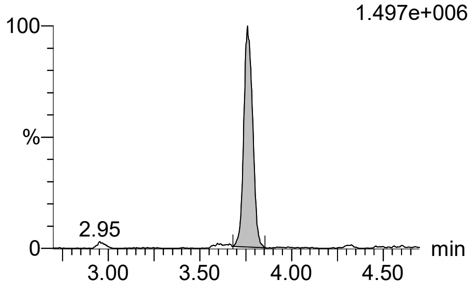

Sample Name: 20211111_A28_R2_IgM
F29:MRM of 2 channels,ES+
1303.5 > 366.1
2.380e+004

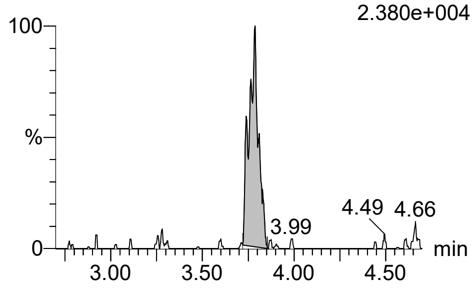

Sample Name: 20211111_L27_R2_IgM
F29:MRM of 2 channels,ES+
869.3 > 204.1
1.925e+006

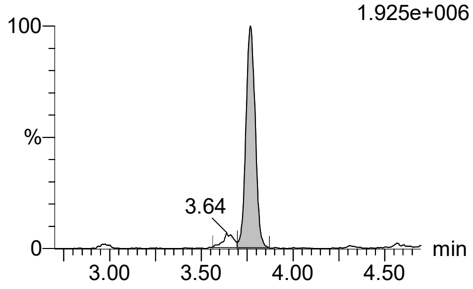

Sample Name: 20211111_L27_R2_IgM
F29:MRM of 2 channels,ES+
1303.5 > 366.1
3.810e+004

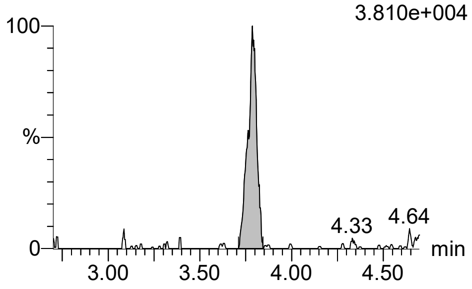

Sample Name: 20211111_L28_R2_IgM
F29:MRM of 2 channels,ES+
869.3 > 204.1
1.917e+006

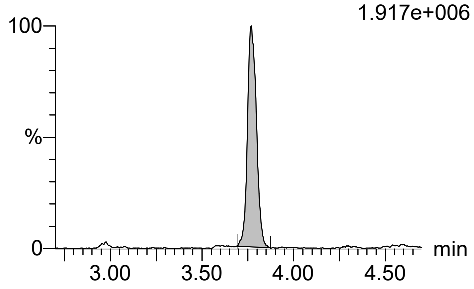

Sample Name: 20211111_L28_R2_IgM
F29:MRM of 2 channels,ES+
1303.5 > 366.1
3.081e+004

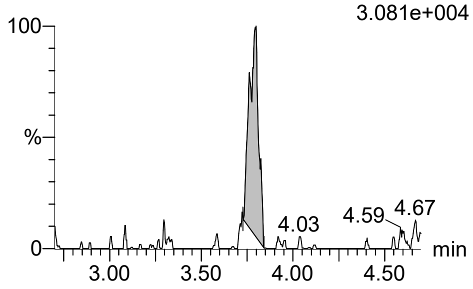

Sample Name: 20211111_H14_R2_IgM
F29:MRM of 2 channels,ES+
869.3 > 204.1
5.461e+006

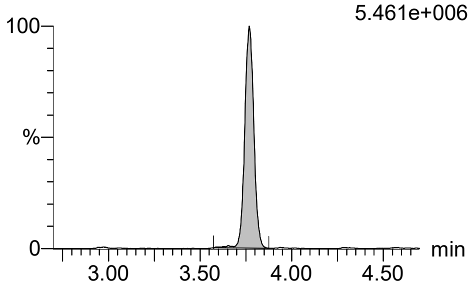

Sample Name: 20211111_H14_R2_IgM
F29:MRM of 2 channels,ES+
1303.5 > 366.1
1.038e+005

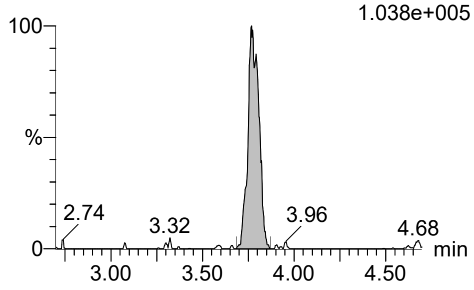

Sample Name: 20211111_A29_R2_IgM
F29:MRM of 2 channels,ES+
869.3 > 204.1
3.806e+006

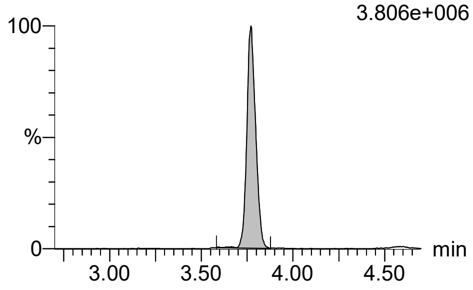

Sample Name: 20211111_A29_R2_IgM
F29:MRM of 2 channels,ES+
1303.5 > 366.1
5.411e+004

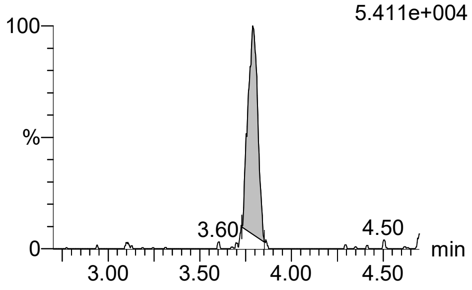

Dataset: C:\MassLynx\10.pro\20211111_clinicalsamples_IgM_IgA.qld

Last Altered: Thursday, March 09, 2023 19:58:25 台北標準時間
Printed: Friday, August 01, 2025 15:27:43 台北標準時間

Sample Name: 20211111_A30_R2_IgM
F29:MRM of 2 channels,ES+
869.3 > 204.1
3.506e+006

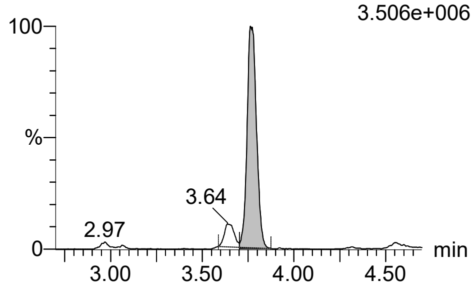

Sample Name: 20211111_A30_R2_IgM
F29:MRM of 2 channels,ES+
1303.5 > 366.1
5.936e+004

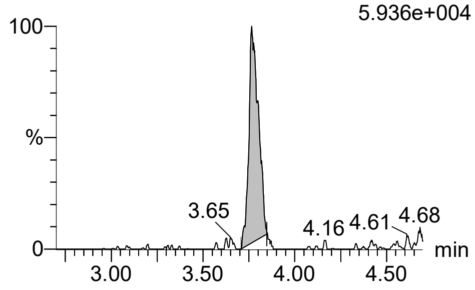

Sample Name: 20211111_QC_R15_IgM
F29:MRM of 2 channels,ES+
869.3 > 204.1
4.122e+006

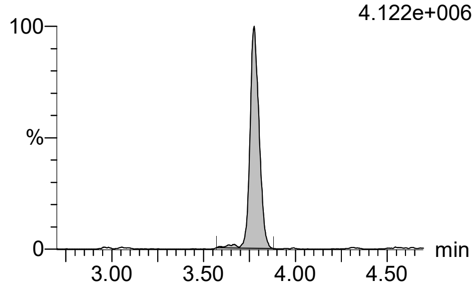

Sample Name: 20211111_QC_R15_IgM
F29:MRM of 2 channels,ES+
1303.5 > 366.1
6.240e+004

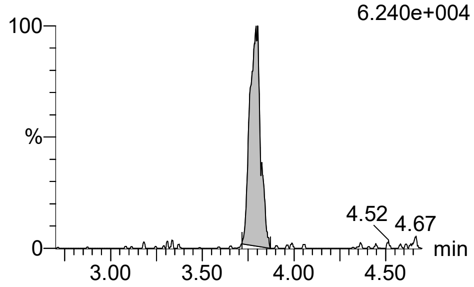

Sample Name: 20211111_L29_R2_IgM
F29:MRM of 2 channels,ES+
869.3 > 204.1
8.386e+005

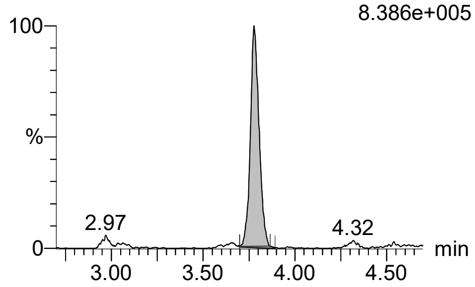

Sample Name: 20211111_L29_R2_IgM
F29:MRM of 2 channels,ES+
1303.5 > 366.1
1.791e+004

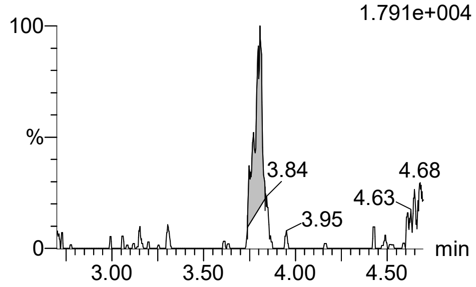

Sample Name: 20211111_L30_R2_IgM
F29:MRM of 2 channels,ES+
869.3 > 204.1
1.075e+006

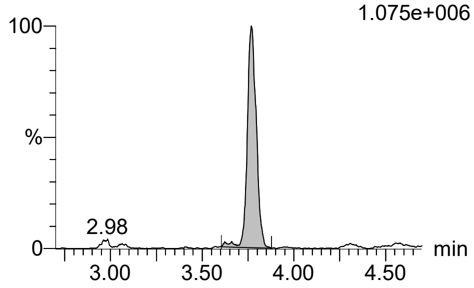

Sample Name: 20211111_L30_R2_IgM
F29:MRM of 2 channels,ES+
1303.5 > 366.1
1.687e+004

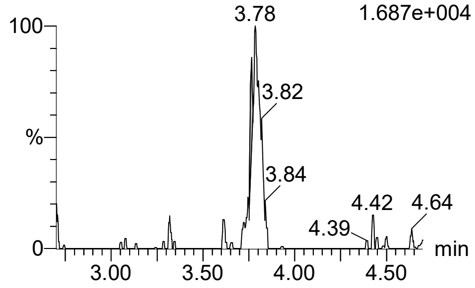

Sample Name: 20211111_H15_R2_IgM
F29:MRM of 2 channels,ES+
869.3 > 204.1
3.084e+006

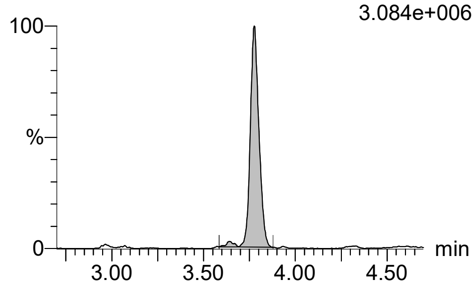

Sample Name: 20211111_H15_R2_IgM
F29:MRM of 2 channels,ES+
1303.5 > 366.1
5.505e+004

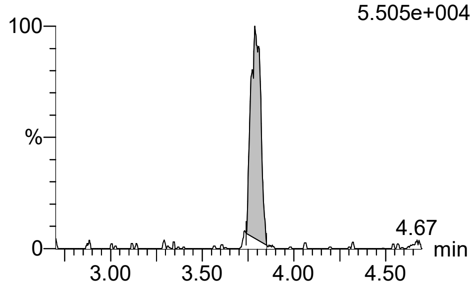

Sample Name: 20211111_A31_R2_IgM
F29:MRM of 2 channels,ES+
869.3 > 204.1
4.680e+006

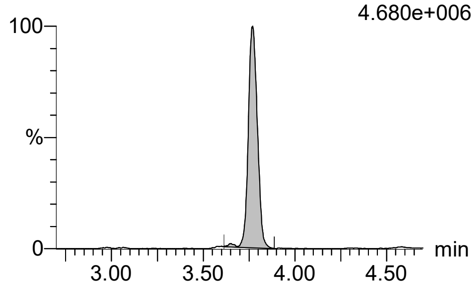

Sample Name: 20211111_A31_R2_IgM
F29:MRM of 2 channels,ES+
1303.5 > 366.1
5.906e+004

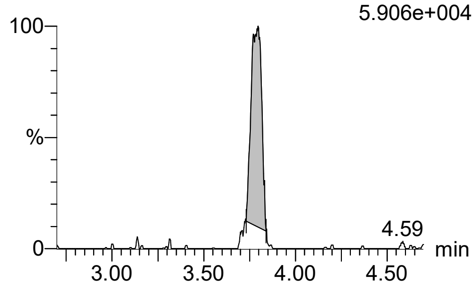

Sample Name: 20211111_A32_R2_IgM
F29:MRM of 2 channels,ES+
869.3 > 204.1
6.974e+006

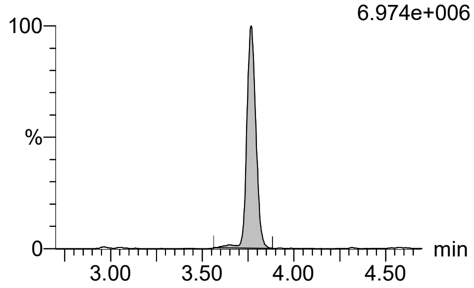

Sample Name: 20211111_A32_R2_IgM
F29:MRM of 2 channels,ES+
1303.5 > 366.1
1.317e+005

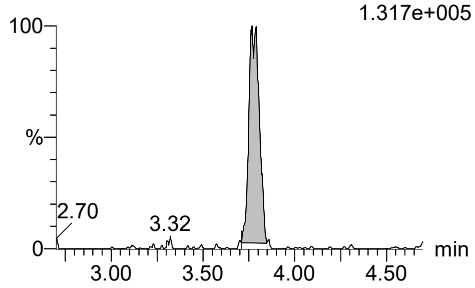

Sample Name: 20211111_L31_R2_IgM
F29:MRM of 2 channels,ES+
869.3 > 204.1
3.879e+006

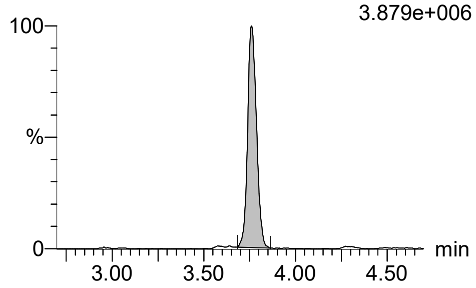

Dataset: C:\MassLynx\10.pro\20211111_clinicalsamples_IgM_IgA.qld

Last Altered: Thursday, March 09, 2023 19:58:25 台北標準時間
Printed: Friday, August 01, 2025 15:27:43 台北標準時間

Sample Name: 20211111_L31_R2_IgM
F29:MRM of 2 channels,ES+
1303.5 > 366.1
5.895e+004

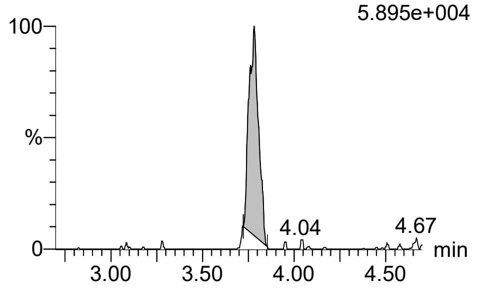

Sample Name: 20211111_L32_R2_IgM
F29:MRM of 2 channels,ES+
869.3 > 204.1
3.292e+006

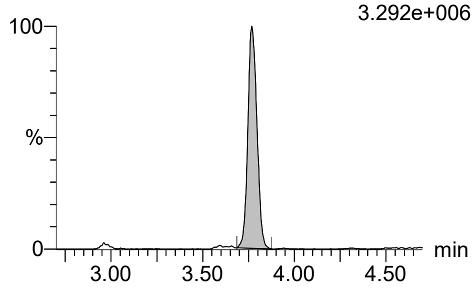

Sample Name: 20211111_L32_R2_IgM
F29:MRM of 2 channels,ES+
1303.5 > 366.1
5.699e+004

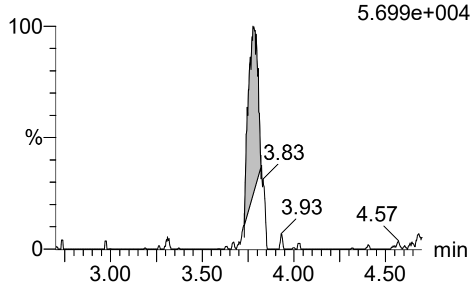

Sample Name: 20211111_H16_R2_IgM
F29:MRM of 2 channels,ES+
869.3 > 204.1
4.010e+006

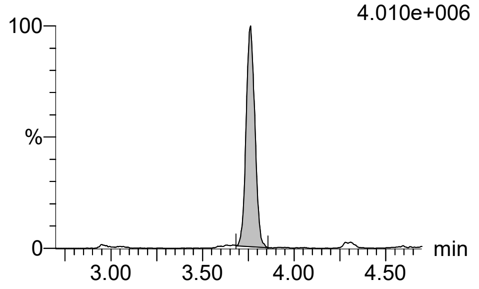

Sample Name: 20211111_H16_R2_IgM
F29:MRM of 2 channels,ES+
1303.5 > 366.1
7.087e+004

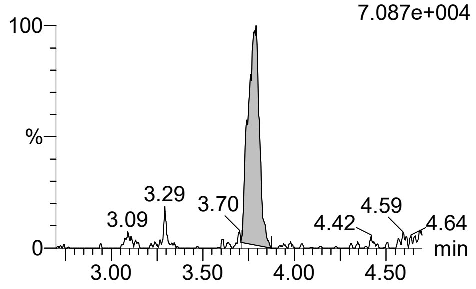

Sample Name: 20211111_A33_R2_IgM
F29:MRM of 2 channels,ES+
869.3 > 204.1
1.977e+006

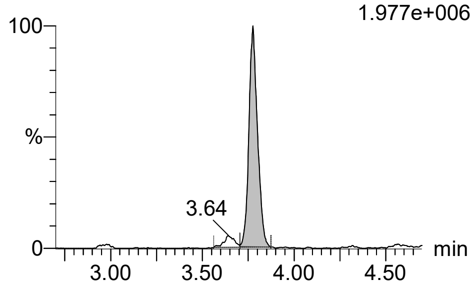

Sample Name: 20211111_A33_R2_IgM
F29:MRM of 2 channels,ES+
1303.5 > 366.1
3.512e+004

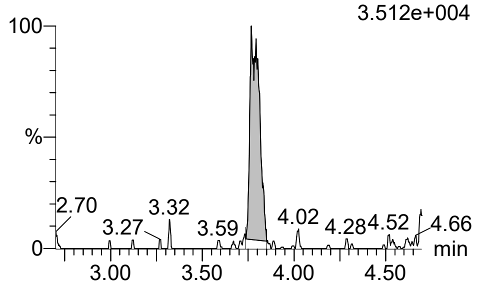

Sample Name: 20211111_A34_R2_IgM
F29:MRM of 2 channels,ES+
869.3 > 204.1
3.875e+006

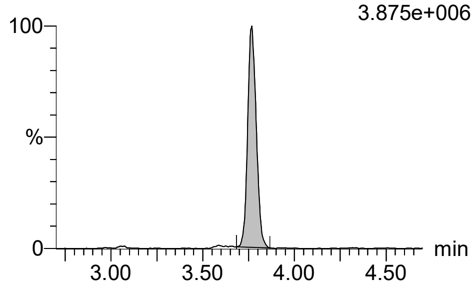

Sample Name: 20211111_A34_R2_IgM
F29:MRM of 2 channels,ES+
1303.5 > 366.1
7.428e+004

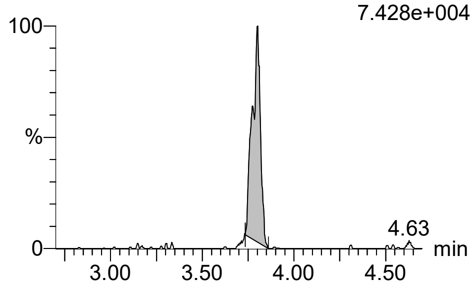

Sample Name: 20211111_H17_R2_IgM
F29:MRM of 2 channels,ES+
869.3 > 204.1
6.096e+006

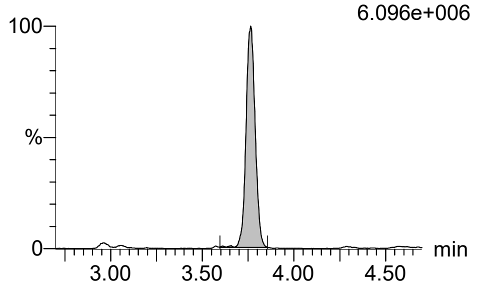

Sample Name: 20211111_H17_R2_IgM
F29:MRM of 2 channels,ES+
1303.5 > 366.1
1.359e+005

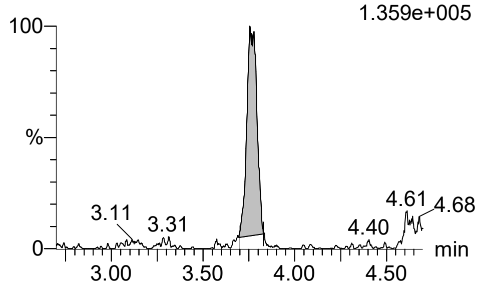

Sample Name: 20211111_A35_R2_IgM
F29:MRM of 2 channels,ES+
869.3 > 204.1
3.225e+006

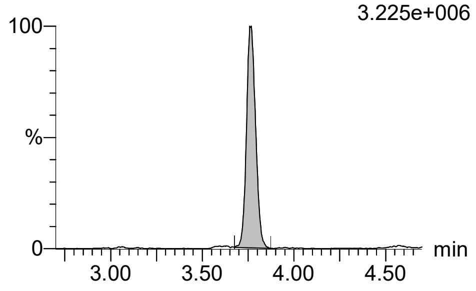

Sample Name: 20211111_A35_R2_IgM
F29:MRM of 2 channels,ES+
1303.5 > 366.1
5.667e+004

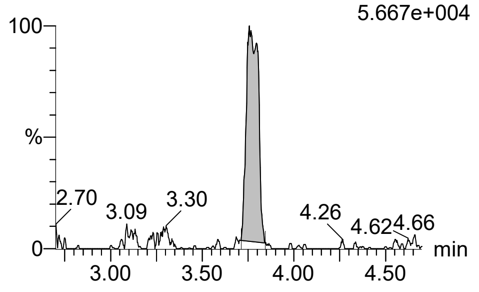

Sample Name: 20211111_QC_R16_IgM
F29:MRM of 2 channels,ES+
869.3 > 204.1
3.441e+006

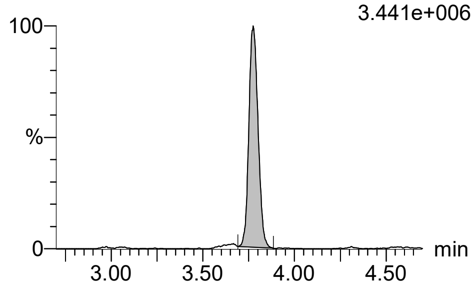

Sample Name: 20211111_QC_R16_IgM
F29:MRM of 2 channels,ES+
1303.5 > 366.1
8.285e+004

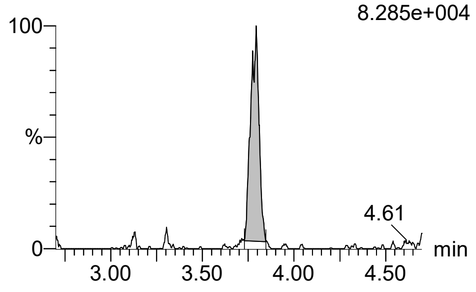

Dataset: C:\MassLynx\10.pro\20211111_clinicalsamples_IgM_IgA.qld

Last Altered: Thursday, March 09, 2023 19:58:25 台北標準時間
Printed: Friday, August 01, 2025 15:27:43 台北標準時間

Sample Name: 20211111_A36_R2_IgM
F29:MRM of 2 channels,ES+
869.3 > 204.1
4.996e+006

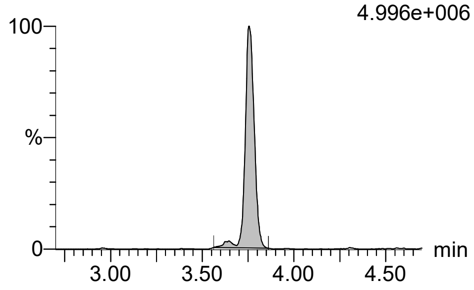

Sample Name: 20211111_A36_R2_IgM
F29:MRM of 2 channels,ES+
1303.5 > 366.1
9.922e+004

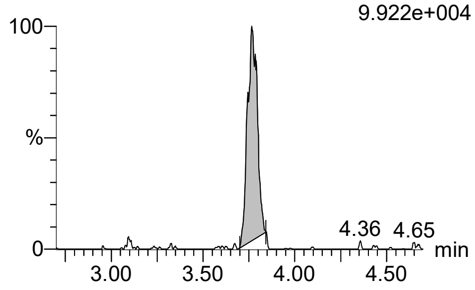

Sample Name: 20211111_H18_R2_IgM
F29:MRM of 2 channels,ES+
869.3 > 204.1
5.929e+006

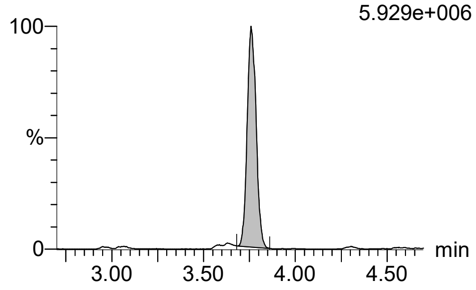

Sample Name: 20211111_H18_R2_IgM
F29:MRM of 2 channels,ES+
1303.5 > 366.1
1.617e+005

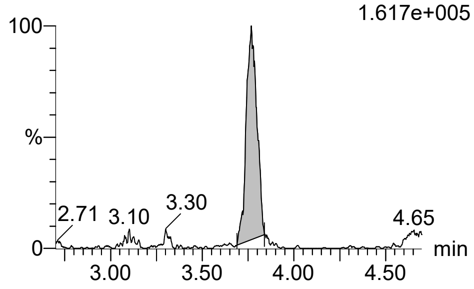

Sample Name: 20211111_A37_R2_IgM
F29:MRM of 2 channels,ES+
869.3 > 204.1
1.443e+006

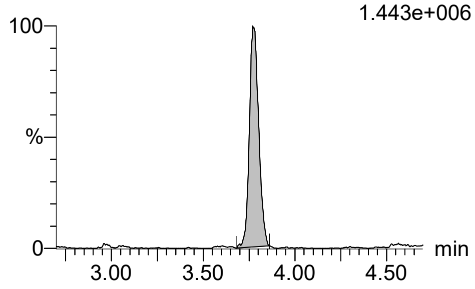

Sample Name: 20211111_A37_R2_IgM
F29:MRM of 2 channels,ES+
1303.5 > 366.1
3.072e+004

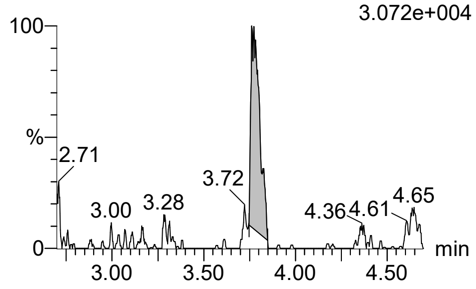

Sample Name: 20211111_A38_R2_IgM
F29:MRM of 2 channels,ES+
869.3 > 204.1
3.501e+006

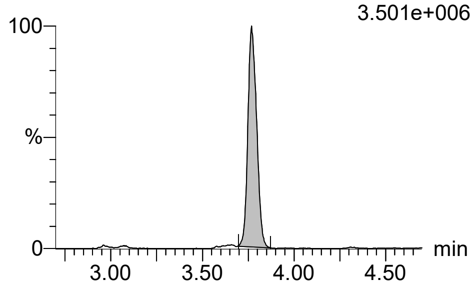

Sample Name: 20211111_A38_R2_IgM
F29:MRM of 2 channels,ES+
1303.5 > 366.1
5.499e+004

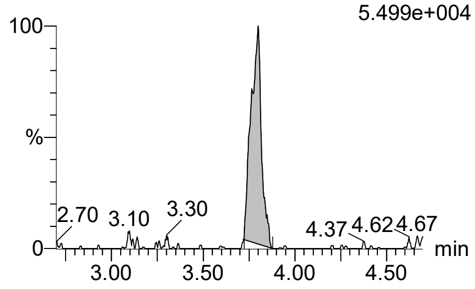

Sample Name: 20211111_H19_R2_IgM
F29:MRM of 2 channels,ES+
869.3 > 204.1
7.046e+006

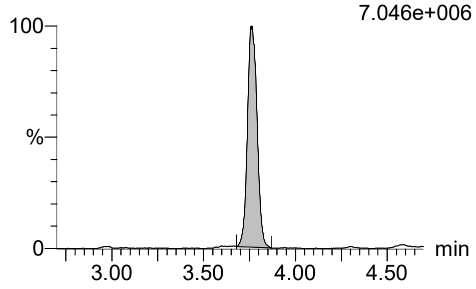

Sample Name: 20211111_H19_R2_IgM
F29:MRM of 2 channels,ES+
1303.5 > 366.1
2.113e+005

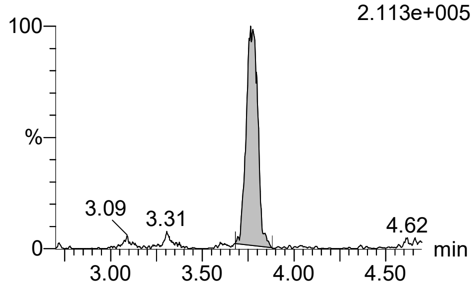

Sample Name: 20211111_A39_R2_IgM
F29:MRM of 2 channels,ES+
869.3 > 204.1
2.374e+006

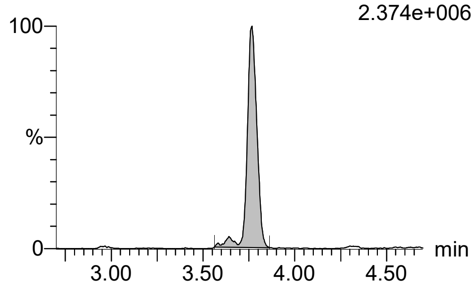

Sample Name: 20211111_A39_R2_IgM
F29:MRM of 2 channels,ES+
1303.5 > 366.1
5.496e+004

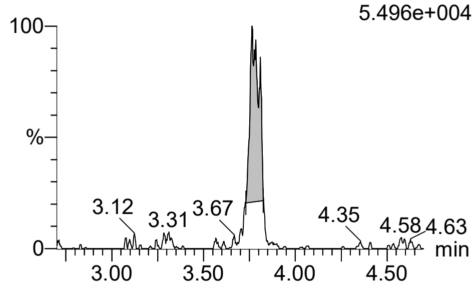

Sample Name: 20211111_A40_R2_IgM
F29:MRM of 2 channels,ES+
869.3 > 204.1
4.184e+006

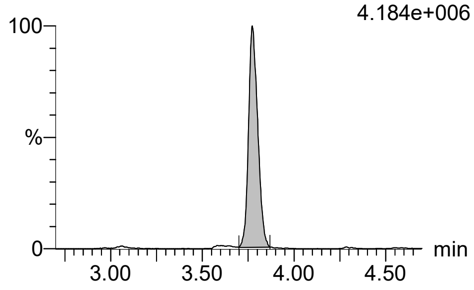

Sample Name: 20211111_A40_R2_IgM
F29:MRM of 2 channels,ES+
1303.5 > 366.1
8.384e+004

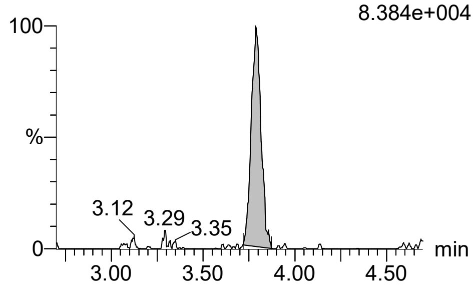

Sample Name: 20211111_H20_R2_IgM
F29:MRM of 2 channels,ES+
869.3 > 204.1
8.297e+006

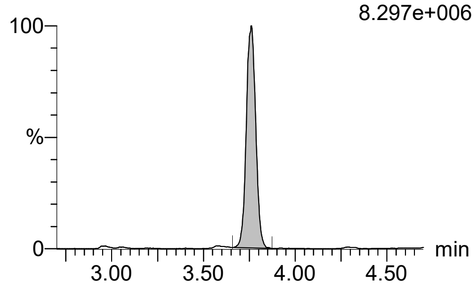

Dataset: C:\MassLynx\10.pro\20211111_clinicalsamples_IgM_IgA.qld

Last Altered: Thursday, March 09, 2023 19:58:25 台北標準時間
Printed: Friday, August 01, 2025 15:27:43 台北標準時間

Sample Name: 20211111_H20_R2_IgM
F29:MRM of 2 channels,ES+
1303.5 > 366.1
3.143e+005

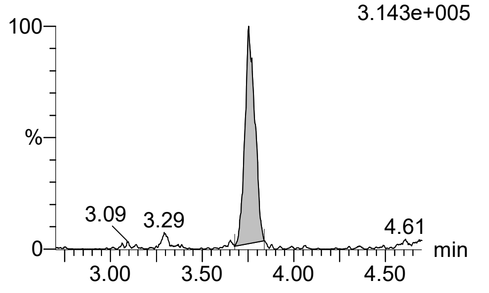

Sample Name: 20211111_A41_R2_IgM
F29:MRM of 2 channels,ES+
869.3 > 204.1
2.805e+006

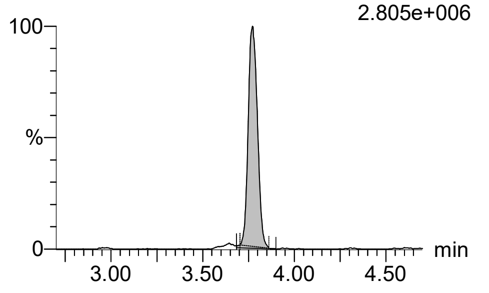

Sample Name: 20211111_A41_R2_IgM
F29:MRM of 2 channels,ES+
1303.5 > 366.1
5.535e+004

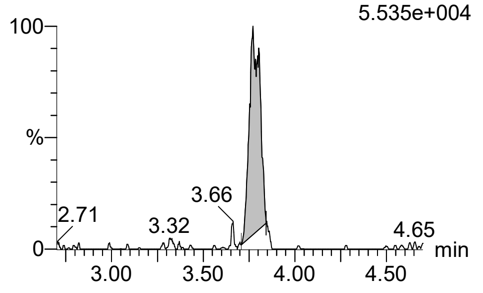

Sample Name: 20211111_A42_R2_IgM
F29:MRM of 2 channels,ES+
869.3 > 204.1
1.653e+006

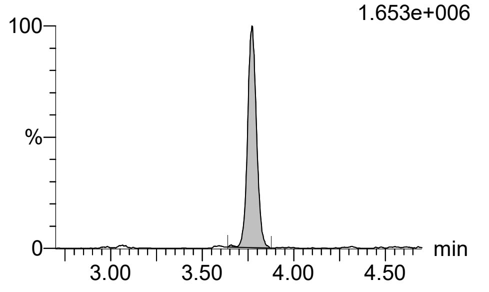

Sample Name: 20211111_A42_R2_IgM
F29:MRM of 2 channels,ES+
1303.5 > 366.1
2.350e+004

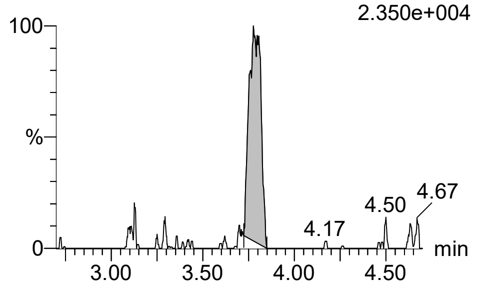

Sample Name: 20211111_A43_R2_IgM
F29:MRM of 2 channels,ES+
869.3 > 204.1
4.231e+006

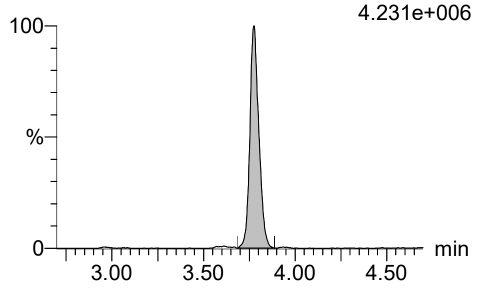

Sample Name: 20211111_A43_R2_IgM
F29:MRM of 2 channels,ES+
1303.5 > 366.1
9.040e+004

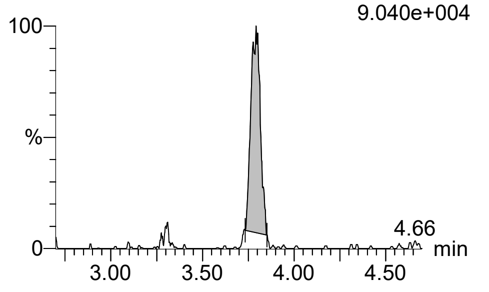

Sample Name: 20211111_A44_R2_IgM
F29:MRM of 2 channels,ES+
869.3 > 204.1
1.947e+006

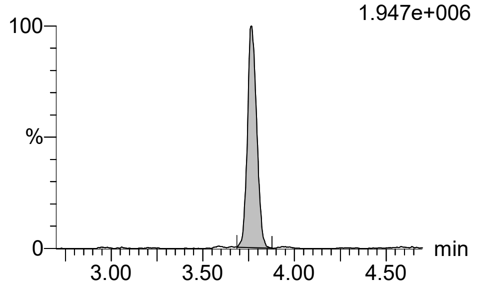

Sample Name: 20211111_A44_R2_IgM
F29:MRM of 2 channels,ES+
1303.5 > 366.1
4.181e+004

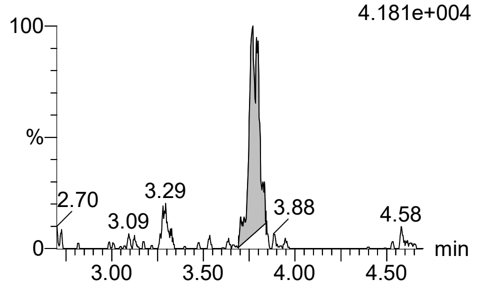

Sample Name: 20211111_QC_R17_IgM
F29:MRM of 2 channels,ES+
869.3 > 204.1
2.707e+006

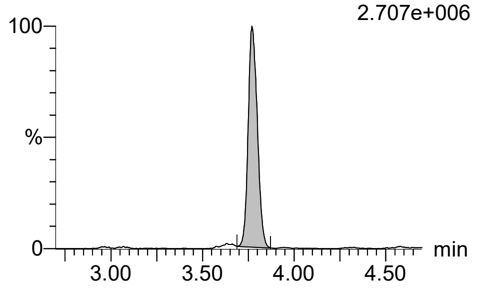

Sample Name: 20211111_QC_R17_IgM
F29:MRM of 2 channels,ES+
1303.5 > 366.1
4.185e+004

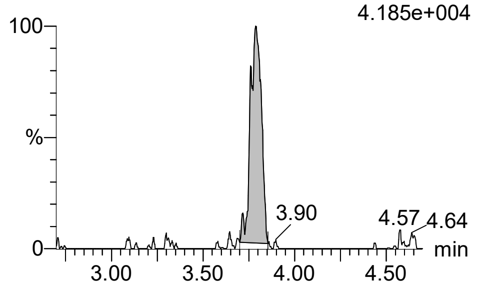

Sample Name: 20211111_L2_R3_IgM
F29:MRM of 2 channels,ES+
869.3 > 204.1
2.978e+006

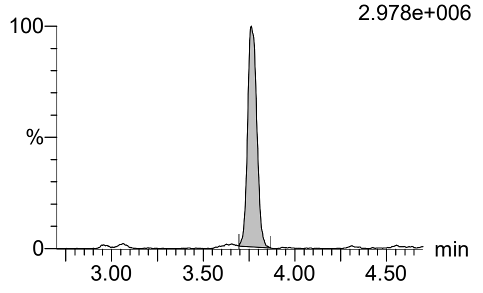

Sample Name: 20211111_L2_R3_IgM
F29:MRM of 2 channels,ES+
1303.5 > 366.1
6.898e+004

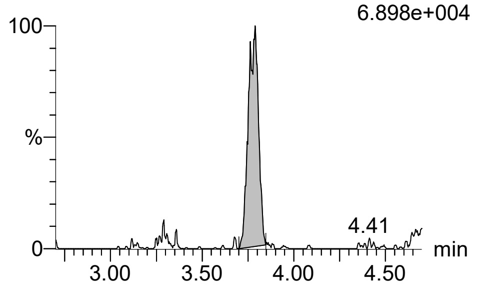

Sample Name: 20211111_H1_R3_IgM
F29:MRM of 2 channels,ES+
869.3 > 204.1
2.798e+006

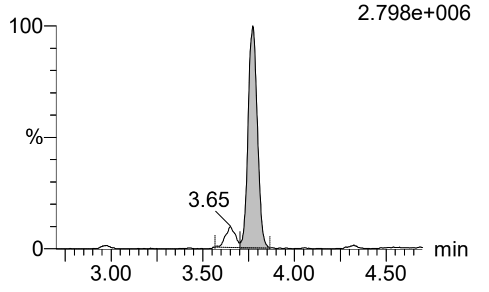

Sample Name: 20211111_H1_R3_IgM
F29:MRM of 2 channels,ES+
1303.5 > 366.1
4.770e+004

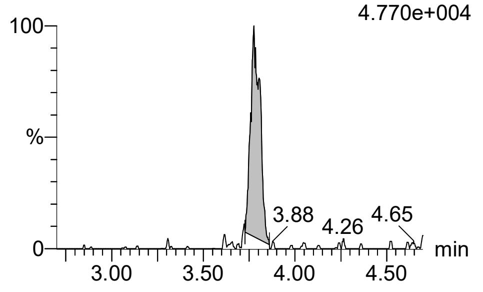

Dataset: C:\MassLynx\10.pro\20211111_clinicalsamples_IgM_IgA.qld

Last Altered: Thursday, March 09, 2023 19:58:25 台北標準時間
Printed: Friday, August 01, 2025 15:27:43 台北標準時間

Sample Name: 20211111_A3_R3_IgM
F29:MRM of 2 channels,ES+
869.3 > 204.1
3.146e+006

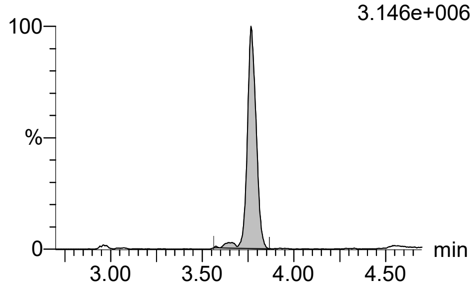

Sample Name: 20211111_A3_R3_IgM
F29:MRM of 2 channels,ES+
1303.5 > 366.1
5.253e+004

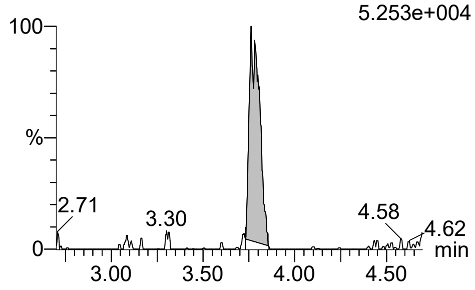

Sample Name: 20211111_A4_R3_IgM
F29:MRM of 2 channels,ES+
869.3 > 204.1
5.072e+006

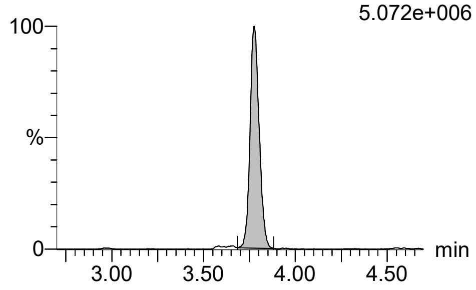

Sample Name: 20211111_A4_R3_IgM
F29:MRM of 2 channels,ES+
1303.5 > 366.1
9.482e+004

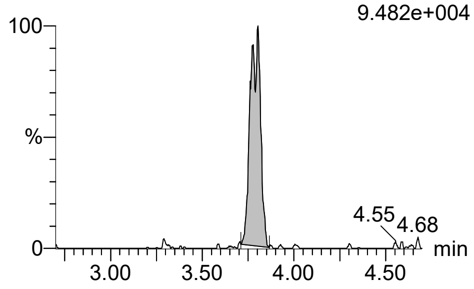

Sample Name: 20211111_L3_R3_IgM
F29:MRM of 2 channels,ES+
869.3 > 204.1
1.752e+006

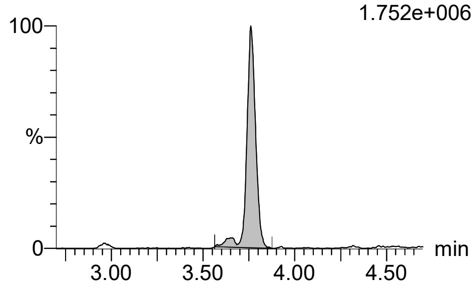

Sample Name: 20211111_L3_R3_IgM
F29:MRM of 2 channels,ES+
1303.5 > 366.1
3.256e+004

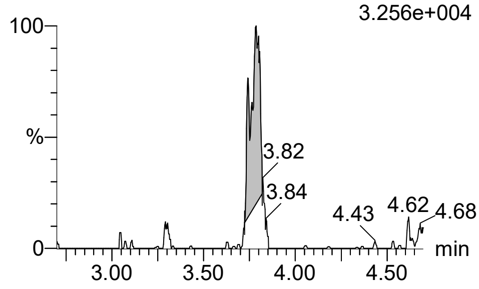

Sample Name: 20211111_L4_R3_IgM
F29:MRM of 2 channels,ES+
869.3 > 204.1
1.899e+006

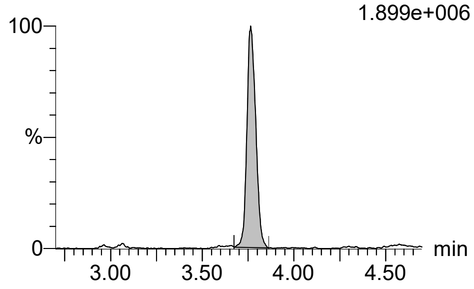

Sample Name: 20211111_L4_R3_IgM
F29:MRM of 2 channels,ES+
1303.5 > 366.1
3.604e+004

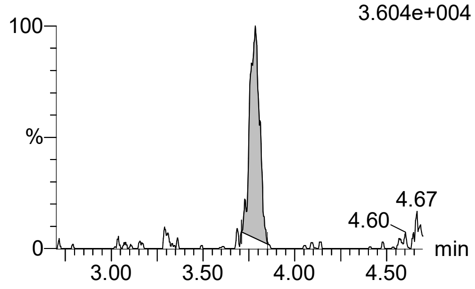

Sample Name: 20211111_H2_R3_IgM
F29:MRM of 2 channels,ES+
869.3 > 204.1
6.455e+006

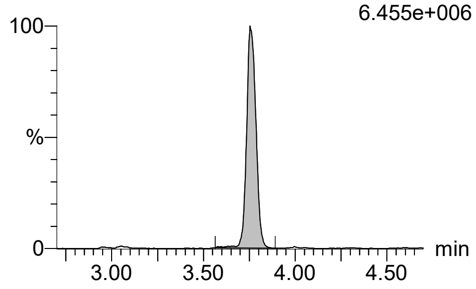

Sample Name: 20211111_H2_R3_IgM
F29:MRM of 2 channels,ES+
1303.5 > 366.1
1.267e+005

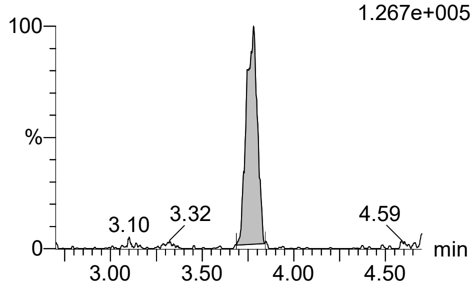

Sample Name: 20211111_A5_R3_IgM
F29:MRM of 2 channels,ES+
869.3 > 204.1
3.191e+006

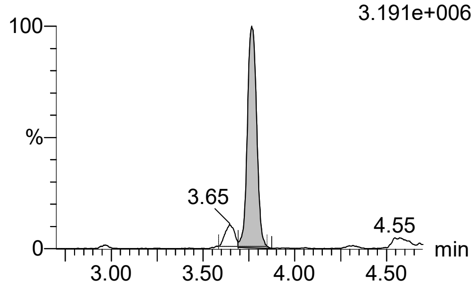

Sample Name: 20211111_A5_R3_IgM
F29:MRM of 2 channels,ES+
1303.5 > 366.1
7.605e+004

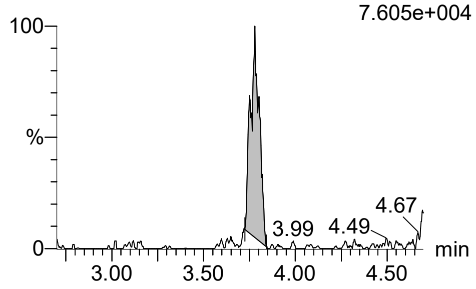

Sample Name: 20211111_A6_R3_IgM
F29:MRM of 2 channels,ES+
869.3 > 204.1
3.122e+006

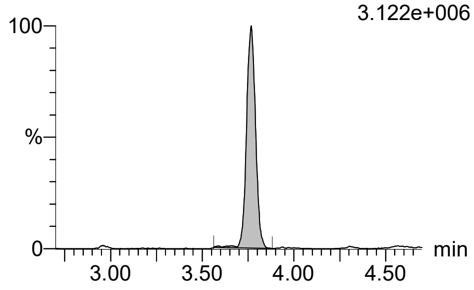

Sample Name: 20211111_A6_R3_IgM
F29:MRM of 2 channels,ES+
1303.5 > 366.1
7.235e+004

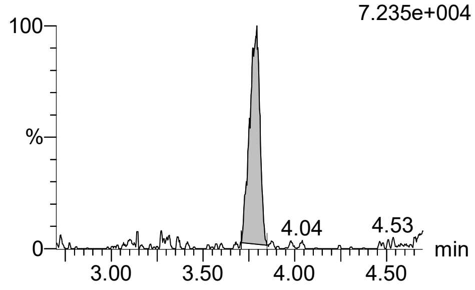

Sample Name: 20211111_QC_R18_IgM
F29:MRM of 2 channels,ES+
869.3 > 204.1
2.742e+006

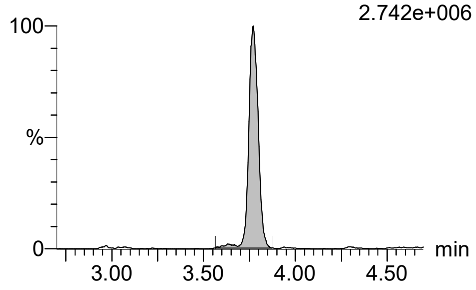

Dataset: C:\MassLynx\10.pro\20211111_clinicalsamples_IgM_IgA.qld

Last Altered: Thursday, March 09, 2023 19:58:25 台北標準時間
Printed: Friday, August 01, 2025 15:27:43 台北標準時間

Sample Name: 20211111_QC_R18_IgM
F29:MRM of 2 channels,ES+
1303.5 > 366.1
4.526e+004

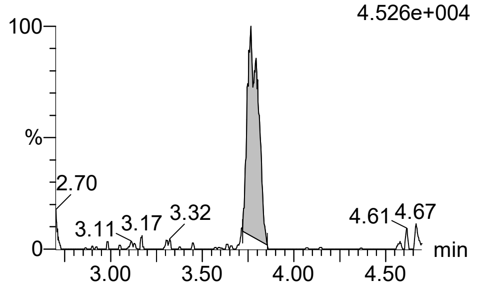

Sample Name: 20211111_L5_R3_IgM
F29:MRM of 2 channels,ES+
869.3 > 204.1
2.739e+006

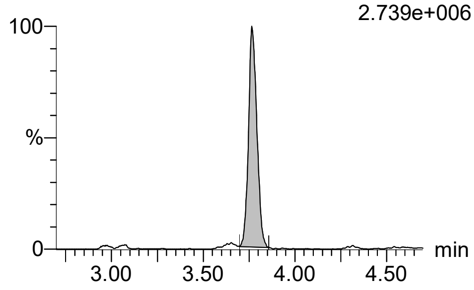

Sample Name: 20211111_L5_R3_IgM
F29:MRM of 2 channels,ES+
1303.5 > 366.1
5.555e+004

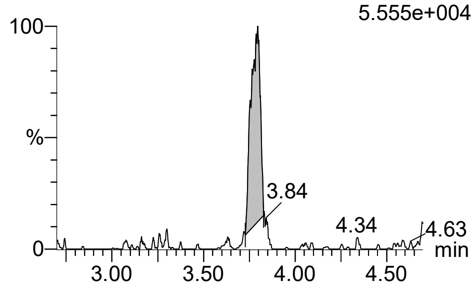

Sample Name: 20211111_L6_R3_IgM
F29:MRM of 2 channels,ES+
869.3 > 204.1
6.264e+005

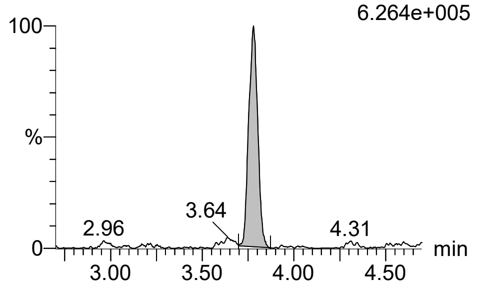

Sample Name: 20211111_L6_R3_IgM
F29:MRM of 2 channels,ES+
1303.5 > 366.1
1.164e+004

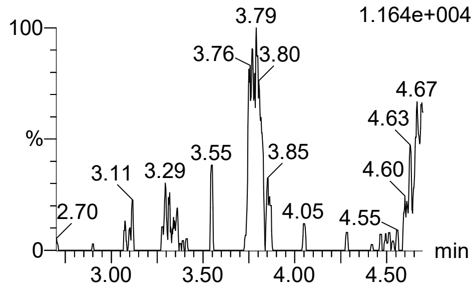

Sample Name: 20211111_H3_R3_IgM
F29:MRM of 2 channels,ES+
869.3 > 204.1
3.221e+006

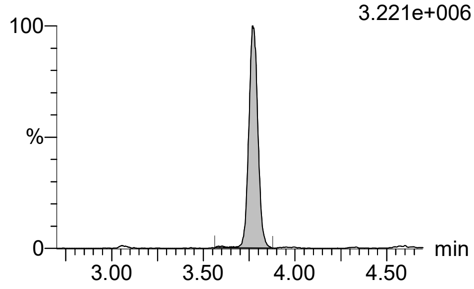

Sample Name: 20211111_H3_R3_IgM
F29:MRM of 2 channels,ES+
1303.5 > 366.1
5.687e+004

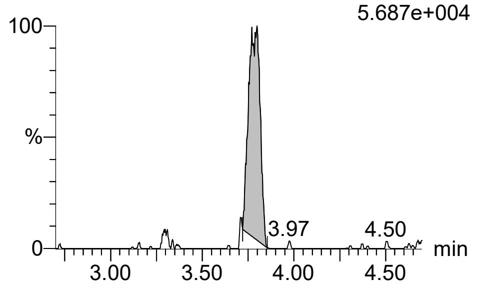

Sample Name: 20211111_A7_R3_IgM
F29:MRM of 2 channels,ES+
869.3 > 204.1
7.816e+006

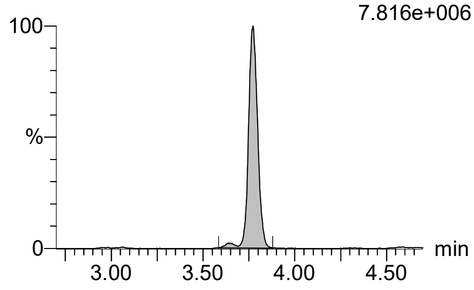

Sample Name: 20211111_A7_R3_IgM
F29:MRM of 2 channels,ES+
1303.5 > 366.1
1.361e+005

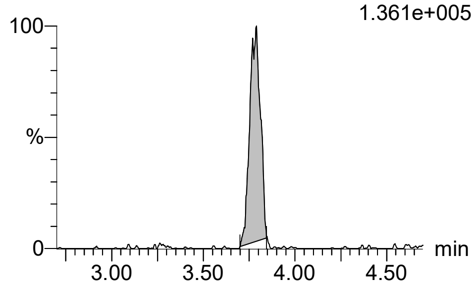

Sample Name: 20211111_A8_R3_IgM
F29:MRM of 2 channels,ES+
869.3 > 204.1
3.188e+006

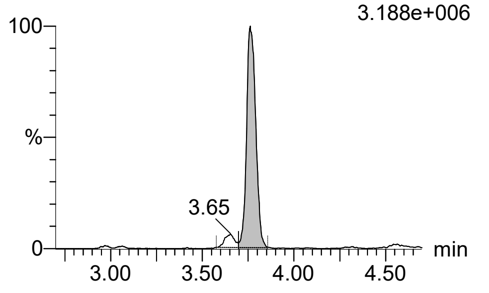

Sample Name: 20211111_A8_R3_IgM
F29:MRM of 2 channels,ES+
1303.5 > 366.1
5.540e+004

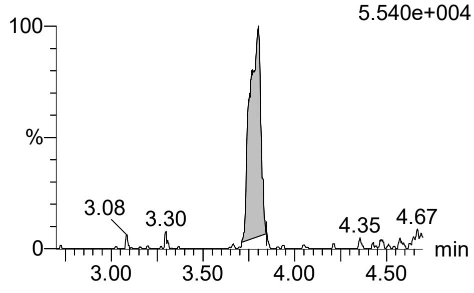

Sample Name: 20211111_L7_R3_IgM
F29:MRM of 2 channels,ES+
869.3 > 204.1
3.558e+006

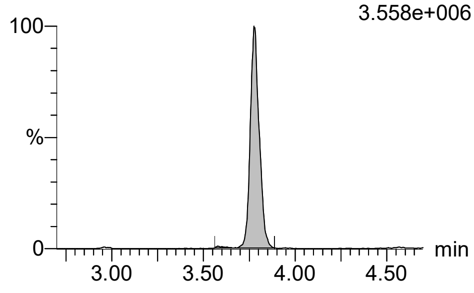

Sample Name: 20211111_L7_R3_IgM
F29:MRM of 2 channels,ES+
1303.5 > 366.1
7.586e+004

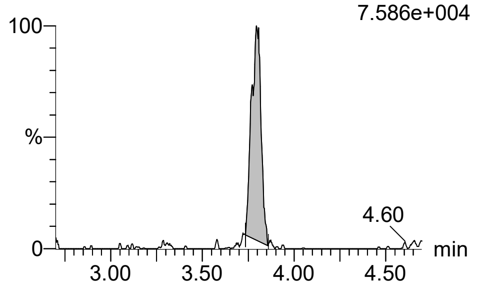

Sample Name: 20211111_L8_R3_IgM
F29:MRM of 2 channels,ES+
869.3 > 204.1
1.473e+006

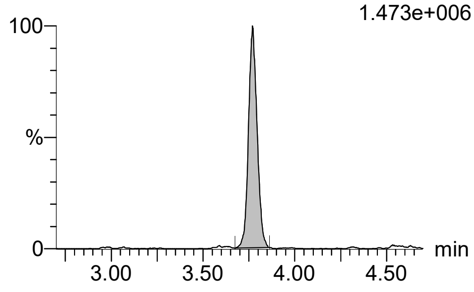

Sample Name: 20211111_L8_R3_IgM
F29:MRM of 2 channels,ES+
1303.5 > 366.1
2.406e+004

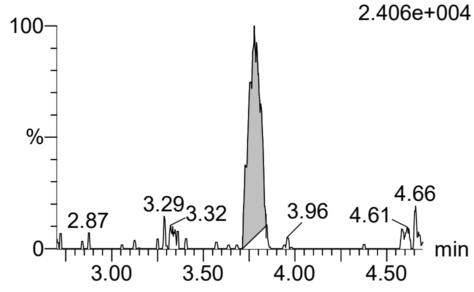

Dataset: C:\MassLynx\10.pro\20211111_clinicalsamples_IgM_IgA.qld

Last Altered: Thursday, March 09, 2023 19:58:25 台北標準時間
Printed: Friday, August 01, 2025 15:27:43 台北標準時間

Sample Name: 20211111_H4_R3_IgM
F29:MRM of 2 channels,ES+
869.3 > 204.1
1.837e+006

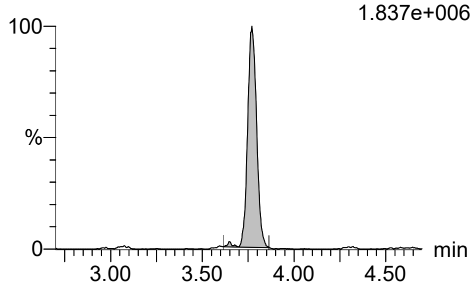

Sample Name: 20211111_H4_R3_IgM
F29:MRM of 2 channels,ES+
1303.5 > 366.1
3.271e+004

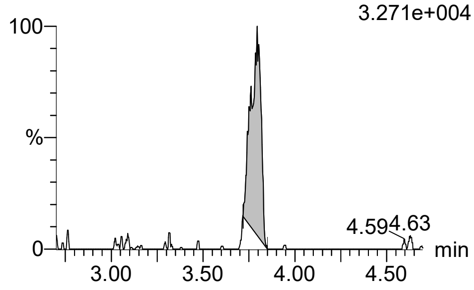

Sample Name: 20211111_A9_R3_IgM
F29:MRM of 2 channels,ES+
869.3 > 204.1
3.950e+006

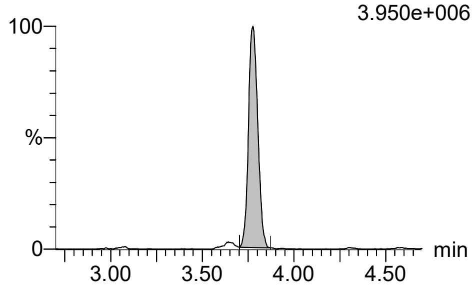

Sample Name: 20211111_A9_R3_IgM
F29:MRM of 2 channels,ES+
1303.5 > 366.1
7.468e+004

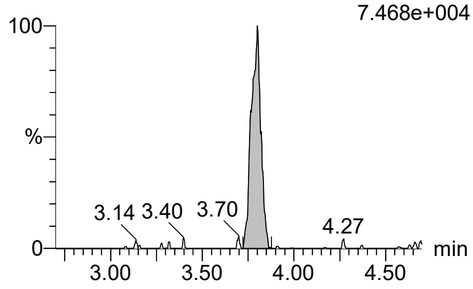

Sample Name: 20211111_A10_R3_IgM
F29:MRM of 2 channels,ES+
869.3 > 204.1
7.085e+006

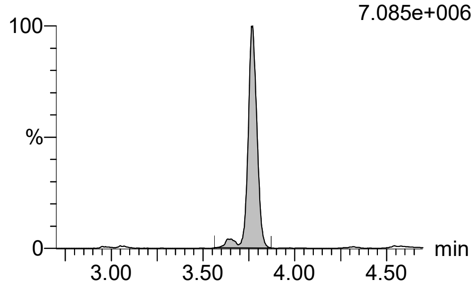

Sample Name: 20211111_A10_R3_IgM
F29:MRM of 2 channels,ES+
1303.5 > 366.1
1.291e+005

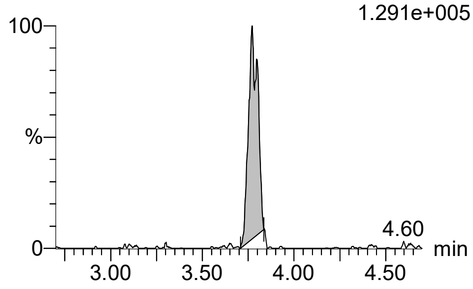

Sample Name: 20211111_L9_R3_IgM
F29:MRM of 2 channels,ES+
869.3 > 204.1
4.866e+006

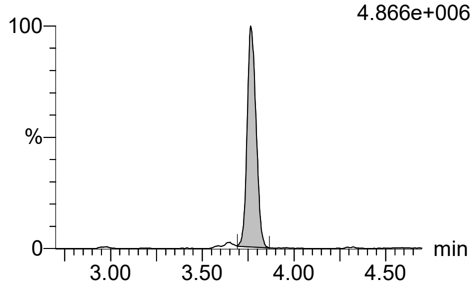

Sample Name: 20211111_L9_R3_IgM
F29:MRM of 2 channels,ES+
1303.5 > 366.1
9.804e+004

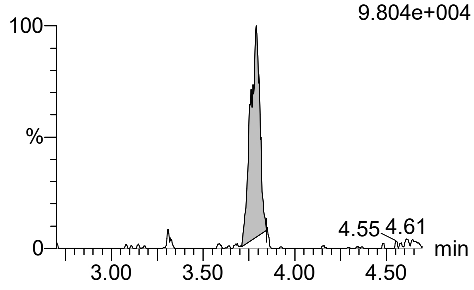

Sample Name: 20211111_L10_R3_IgM
F29:MRM of 2 channels,ES+
869.3 > 204.1
2.403e+006

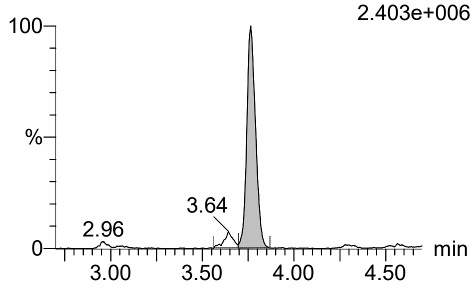

Sample Name: 20211111_L10_R3_IgM
F29:MRM of 2 channels,ES+
1303.5 > 366.1
4.113e+004

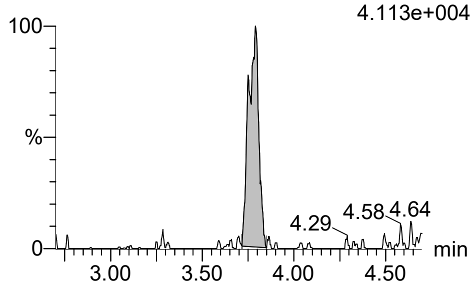

Sample Name: 20211111_QC_R19_IgM
F29:MRM of 2 channels,ES+
869.3 > 204.1
3.128e+006

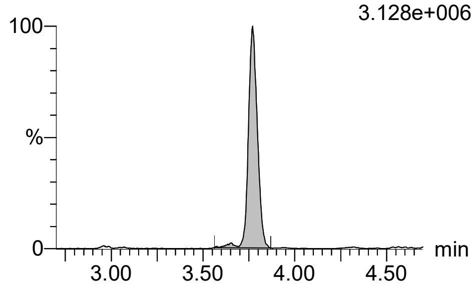

Sample Name: 20211111_QC_R19_IgM
F29:MRM of 2 channels,ES+
1303.5 > 366.1
4.427e+004

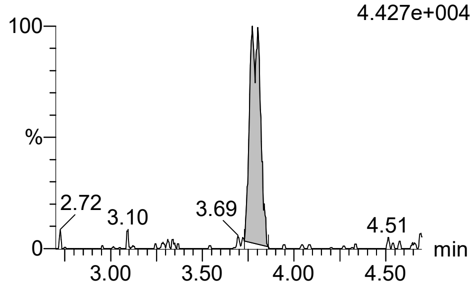

Sample Name: 20211111_H5_R3_IgM
F29:MRM of 2 channels,ES+
869.3 > 204.1
1.459e+006

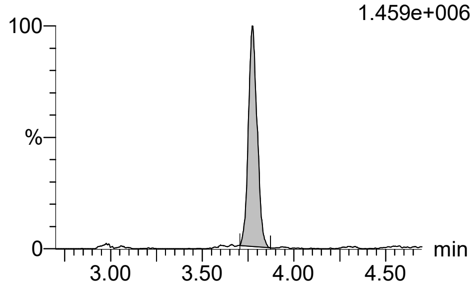

Sample Name: 20211111_H5_R3_IgM
F29:MRM of 2 channels,ES+
1303.5 > 366.1
1.850e+004

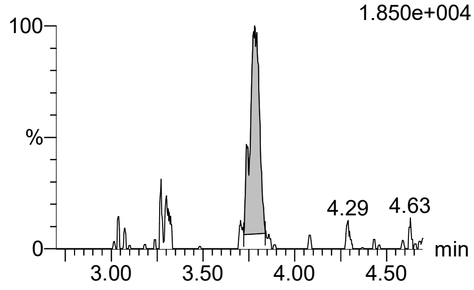

Sample Name: 20211111_A11_R3_IgM
F29:MRM of 2 channels,ES+
869.3 > 204.1
1.041e+006

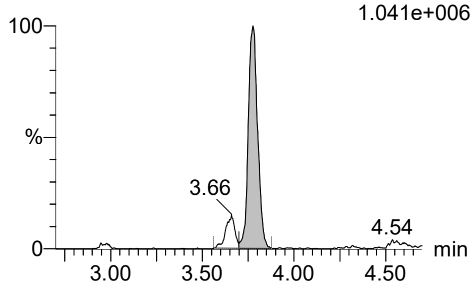

Sample Name: 20211111_A11_R3_IgM
F29:MRM of 2 channels,ES+
1303.5 > 366.1
2.569e+004

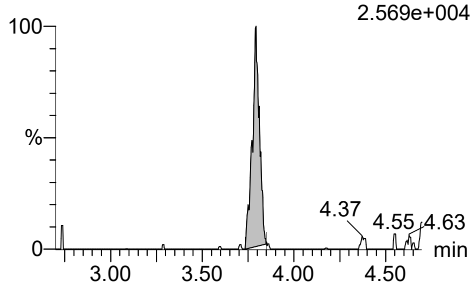

Sample Name: 20211111_A12_R3_IgM
F29:MRM of 2 channels,ES+
869.3 > 204.1
3.826e+006

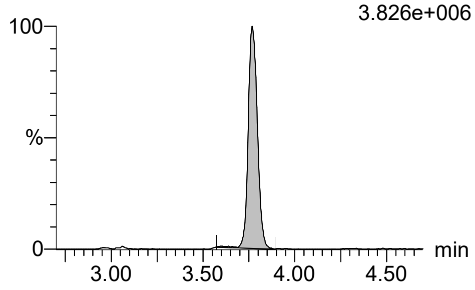

Sample Name: 20211111_A12_R3_IgM
F29:MRM of 2 channels,ES+
1303.5 > 366.1
7.516e+004

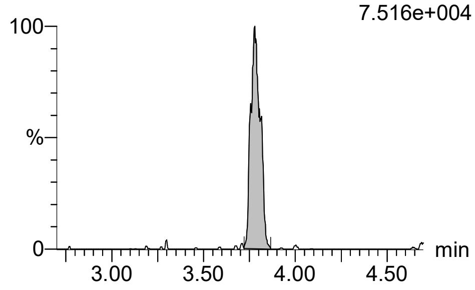

Sample Name: 20211111_L11_R3_IgM
F29:MRM of 2 channels,ES+
869.3 > 204.1
1.803e+006

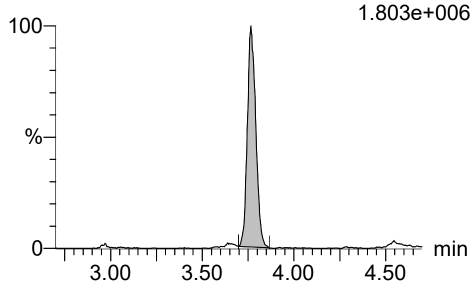

Sample Name: 20211111_L11_R3_IgM
F29:MRM of 2 channels,ES+
1303.5 > 366.1
2.916e+004

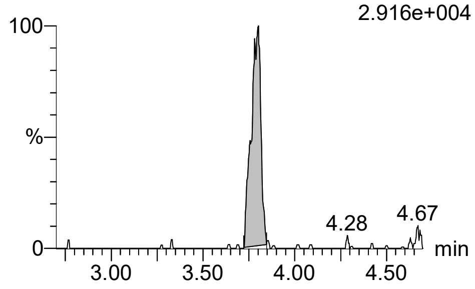

Sample Name: 20211111_L12_R3_IgM
F29:MRM of 2 channels,ES+
869.3 > 204.1
1.187e+006

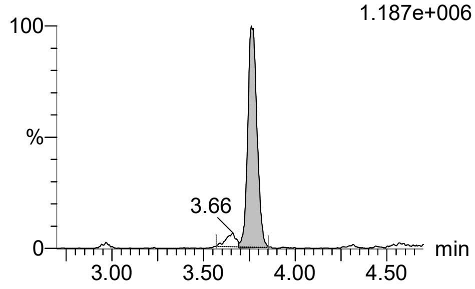

Sample Name: 20211111_L12_R3_IgM
F29:MRM of 2 channels,ES+
1303.5 > 366.1
2.772e+004

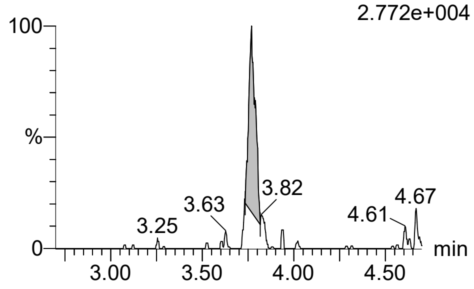

Sample Name: 20211111_H6_R3_IgM
F29:MRM of 2 channels,ES+
869.3 > 204.1
4.893e+006

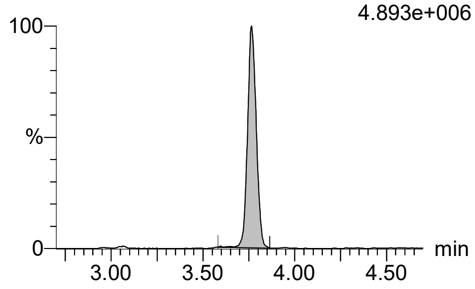

Sample Name: 20211111_H6_R3_IgM
F29:MRM of 2 channels,ES+
1303.5 > 366.1
7.082e+004

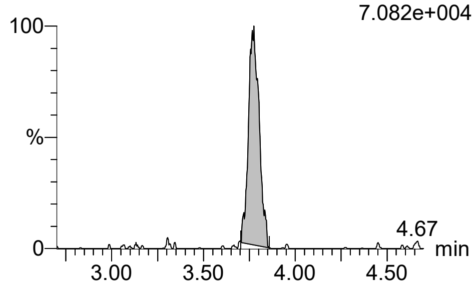

Sample Name: 20211111_A13_R3_IgM
F29:MRM of 2 channels,ES+
869.3 > 204.1
3.120e+006

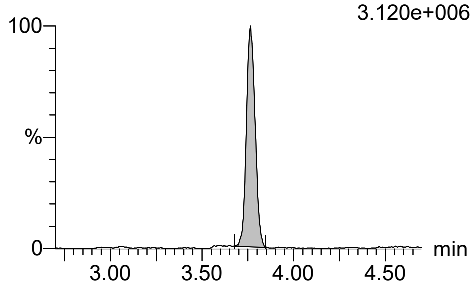

Sample Name: 20211111_A13_R3_IgM
F29:MRM of 2 channels,ES+
1303.5 > 366.1
6.508e+004

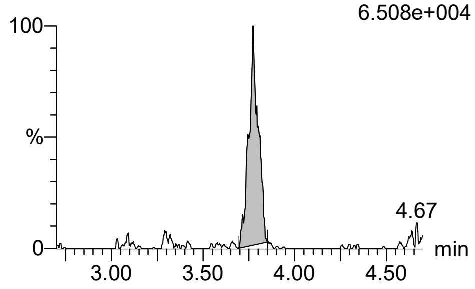

Sample Name: 20211111_A14_R3_IgM
F29:MRM of 2 channels,ES+
869.3 > 204.1
3.986e+006

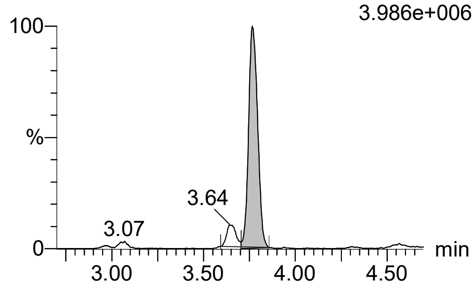

Sample Name: 20211111_A14_R3_IgM
F29:MRM of 2 channels,ES+
1303.5 > 366.1
6.506e+004

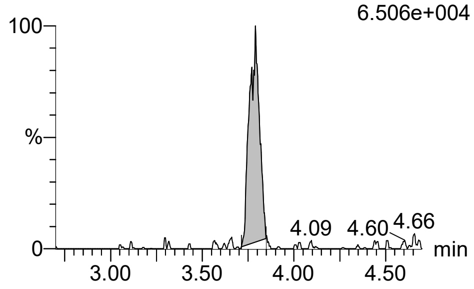

Sample Name: 20211111_L13_R3_IgM
F29:MRM of 2 channels,ES+
869.3 > 204.1
2.118e+006

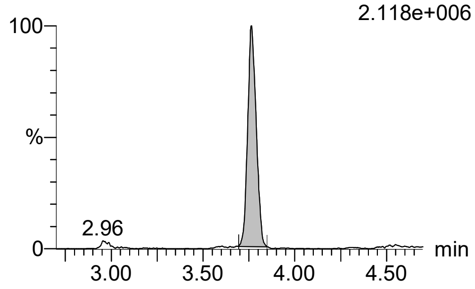

Sample Name: 20211111_L13_R3_IgM
F29:MRM of 2 channels,ES+
1303.5 > 366.1
2.628e+004

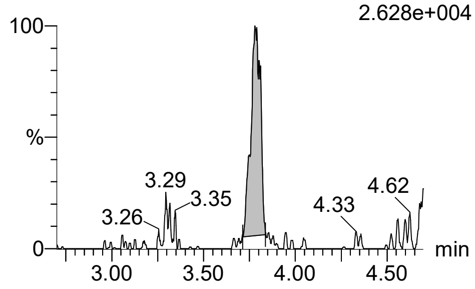

Dataset: C:\MassLynx\10.pro\20211111_clinicalsamples_IgM_IgA.qld

Last Altered: Thursday, March 09, 2023 19:58:25 台北標準時間
Printed: Friday, August 01, 2025 15:27:43 台北標準時間

Sample Name: 20211111_L14_R3_IgM

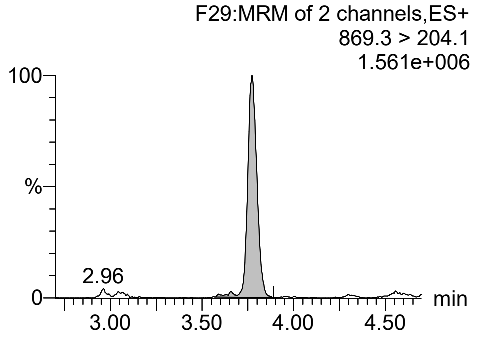

Sample Name: 20211111_L14_R3_IgM

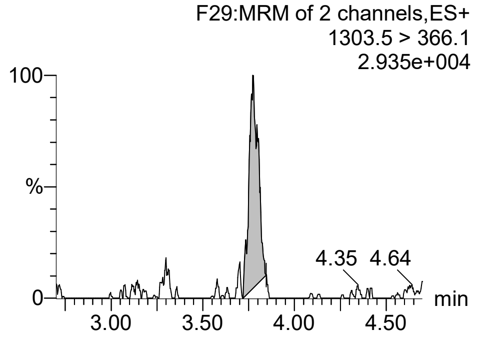

Sample Name: 20211111_H7_R3_IgM

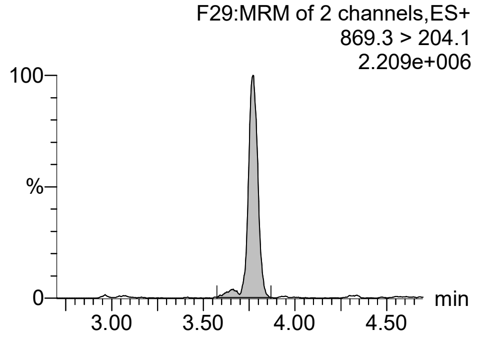

Sample Name: 20211111_H7_R3_IgM

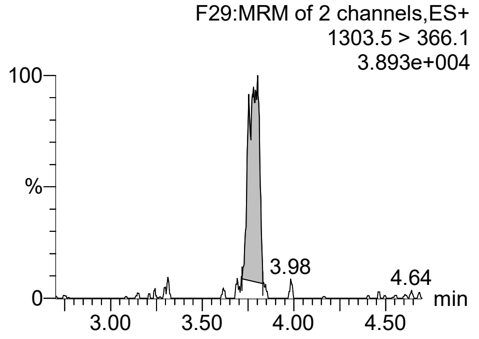

Sample Name: 20211111_A15_R3_IgM

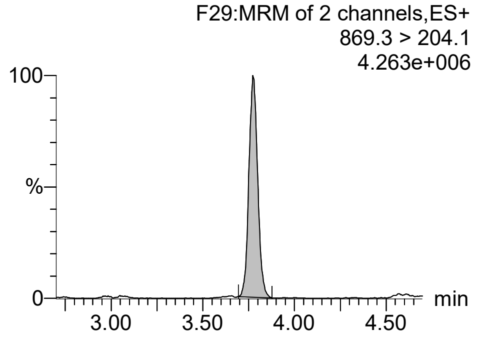

Sample Name: 20211111_A15_R3_IgM

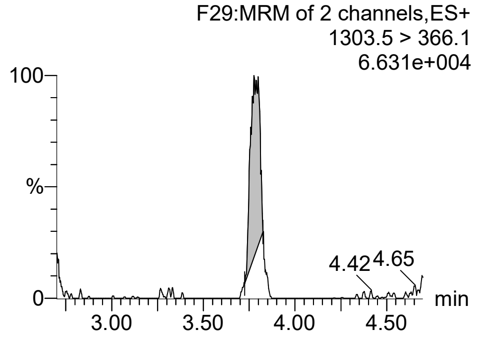

Sample Name: 20211111_QC_R20_IgM

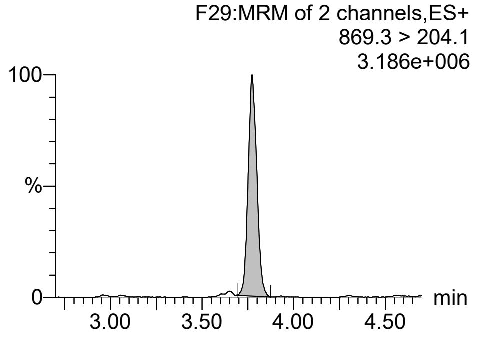

Sample Name: 20211111_QC_R20_IgM

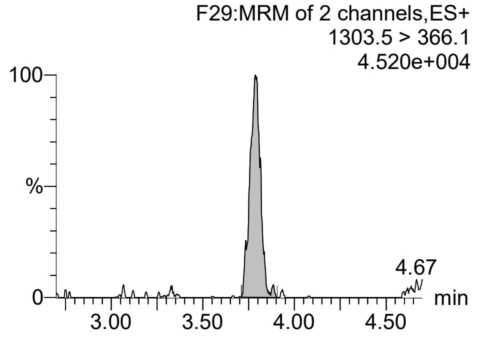

Sample Name: 20211111_A16_R3_IgM

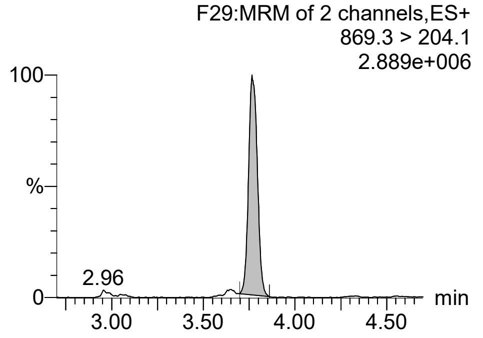

Sample Name: 20211111_A16_R3_IgM

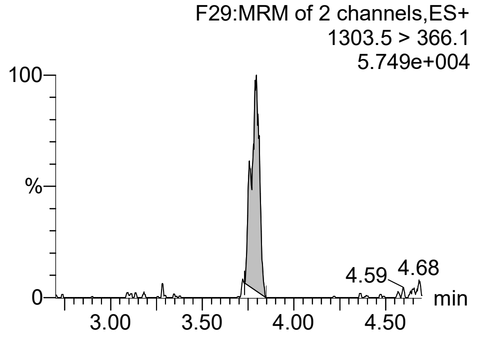

Sample Name: 20211111_L15_R3_IgM

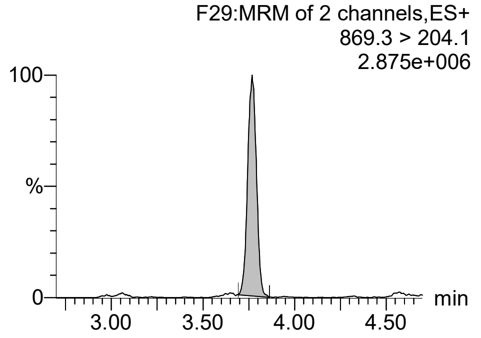

Sample Name: 20211111_L15_R3_IgM

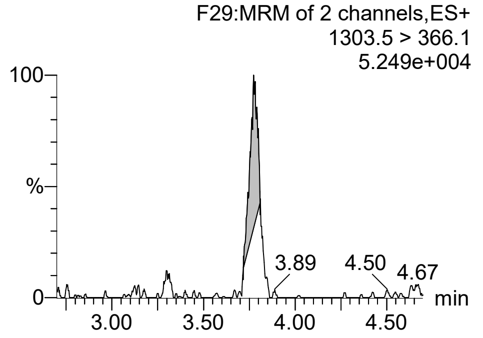

Sample Name: 20211111_L16_R3_IgM

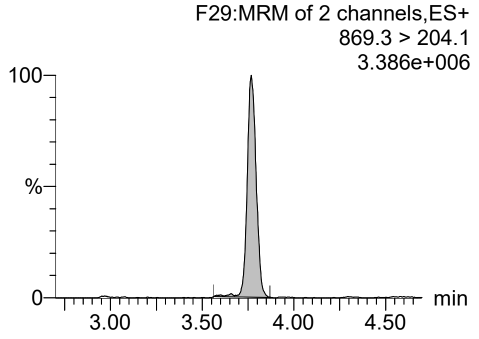

Sample Name: 20211111_L16_R3_IgM

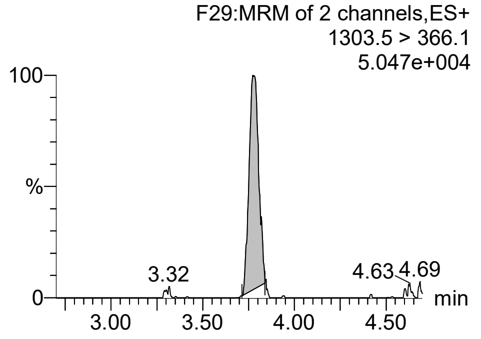

Sample Name: 20211111_H8_R3_IgM

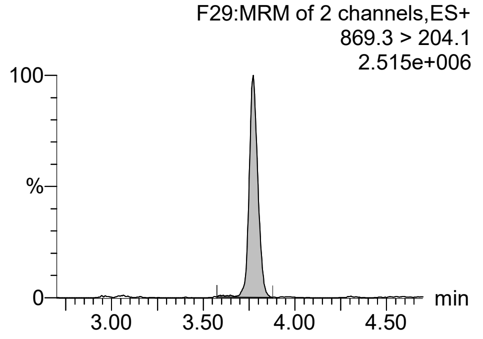

Sample Name: 20211111_H8_R3_IgM
F29:MRM of 2 channels,ES+
1303.5 > 366.1
4.799e+004

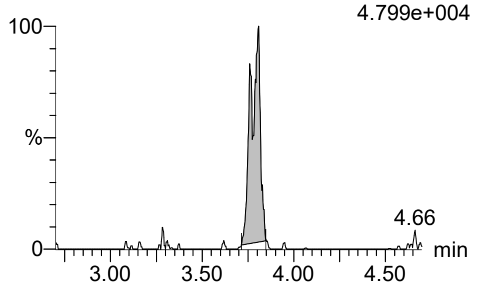

Sample Name: 20211111_A17_R3_IgM
F29:MRM of 2 channels,ES+
869.3 > 204.1
2.797e+006

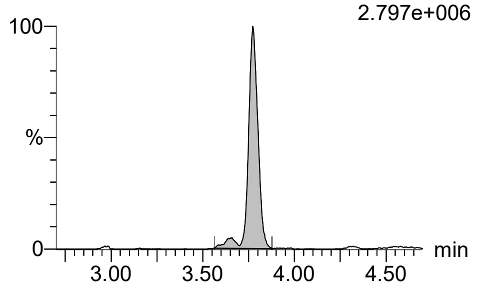

Sample Name: 20211111_A17_R3_IgM
F29:MRM of 2 channels,ES+
1303.5 > 366.1
5.866e+004

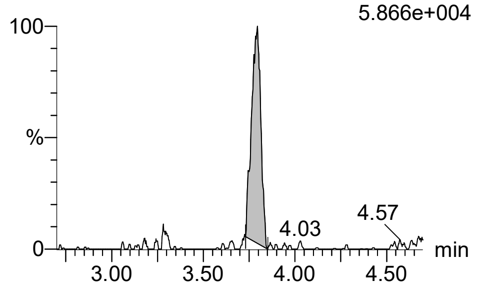

Sample Name: 20211111_A18_R3_IgM
F29:MRM of 2 channels,ES+
869.3 > 204.1
3.244e+006

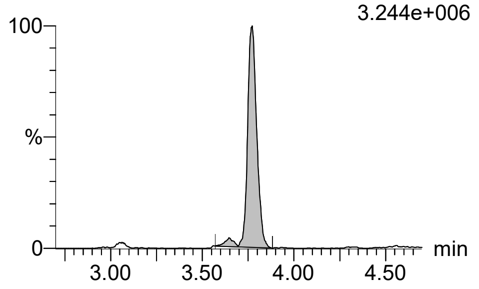

Sample Name: 20211111_A18_R3_IgM
F29:MRM of 2 channels,ES+
1303.5 > 366.1
5.942e+004

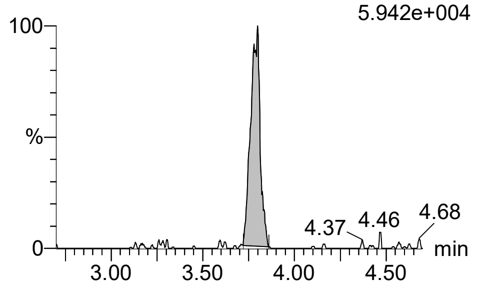

Sample Name: 20211111_L17_R3_IgM
F29:MRM of 2 channels,ES+
869.3 > 204.1
3.102e+006

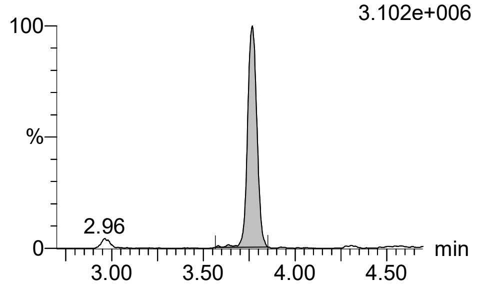

Sample Name: 20211111_L17_R3_IgM
F29:MRM of 2 channels,ES+
1303.5 > 366.1
5.568e+004

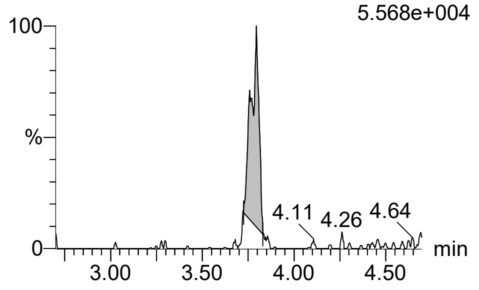

Sample Name: 20211111_L18_R3_IgM
F29:MRM of 2 channels,ES+
869.3 > 204.1
1.120e+006

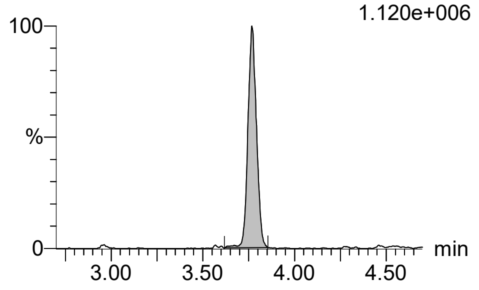

Sample Name: 20211111_L18_R3_IgM
F29:MRM of 2 channels,ES+
1303.5 > 366.1
1.953e+004

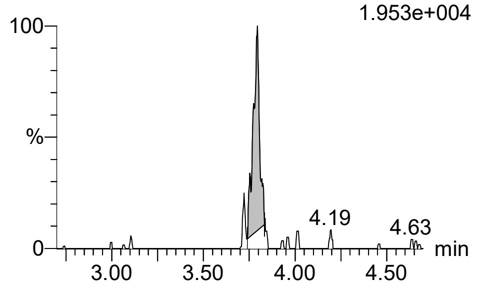

Sample Name: 20211111_H9_R3_IgM
F29:MRM of 2 channels,ES+
869.3 > 204.1
4.371e+006

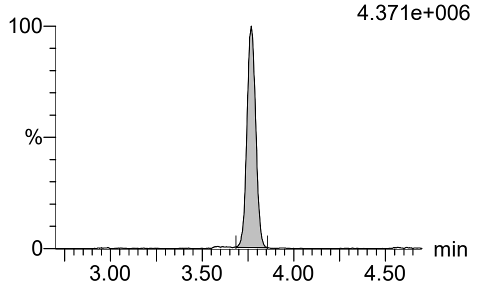

Sample Name: 20211111_H9_R3_IgM
F29:MRM of 2 channels,ES+
1303.5 > 366.1
7.479e+004

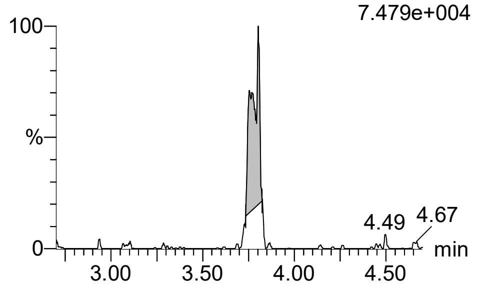

Sample Name: 20211111_A19_R3_IgM
F29:MRM of 2 channels,ES+
869.3 > 204.1
5.200e+006

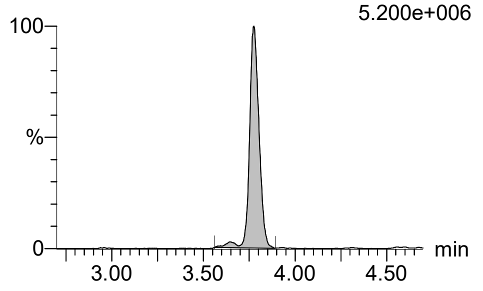

Sample Name: 20211111_A19_R3_IgM
F29:MRM of 2 channels,ES+
1303.5 > 366.1
9.391e+004

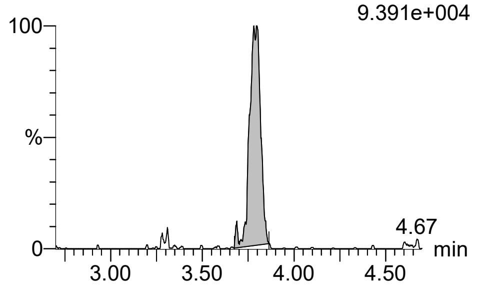

Sample Name: 20211111_A20_R3_IgM
F29:MRM of 2 channels,ES+
869.3 > 204.1
1.091e+006

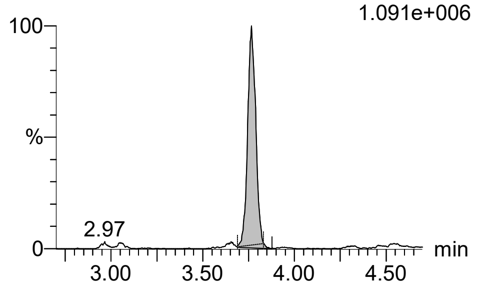

Sample Name: 20211111_A20_R3_IgM
F29:MRM of 2 channels,ES+
1303.5 > 366.1
1.731e+004

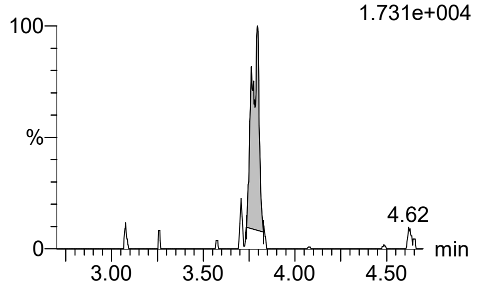

Dataset: C:\MassLynx\10.pro\20211111_clinicalsamples_IgM_IgA.qld

Last Altered: Thursday, March 09, 2023 19:58:25 台北標準時間
Printed: Friday, August 01, 2025 15:27:43 台北標準時間

Sample Name: 20211111_L19_R3_IgM
F29:MRM of 2 channels,ES+
869.3 > 204.1
1.605e+006

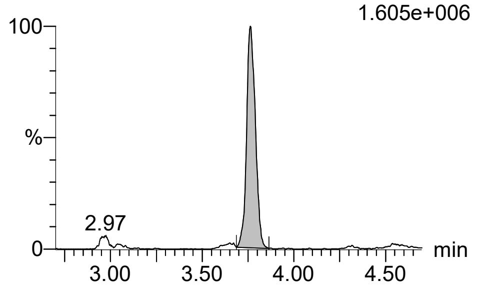

Sample Name: 20211111_L19_R3_IgM
F29:MRM of 2 channels,ES+
1303.5 > 366.1
3.295e+004

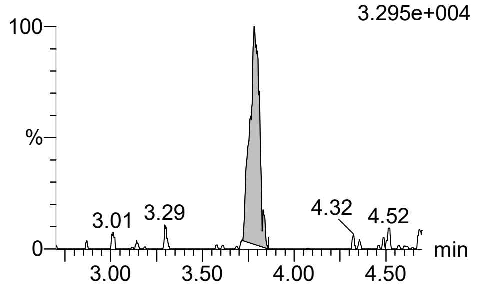

Sample Name: 20211111_QC_R21_IgM
F29:MRM of 2 channels,ES+
869.3 > 204.1
3.087e+006

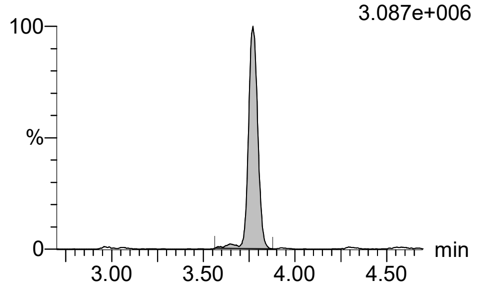

Sample Name: 20211111_QC_R21_IgM
F29:MRM of 2 channels,ES+
1303.5 > 366.1
4.363e+004

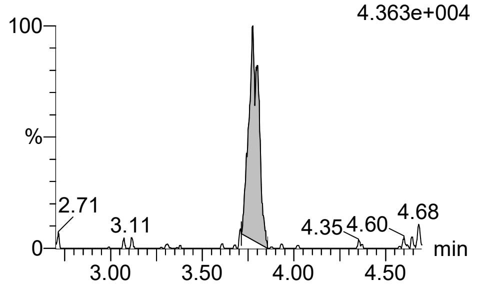

Sample Name: 20211111_L20_R3_IgM
F29:MRM of 2 channels,ES+
869.3 > 204.1
2.249e+006

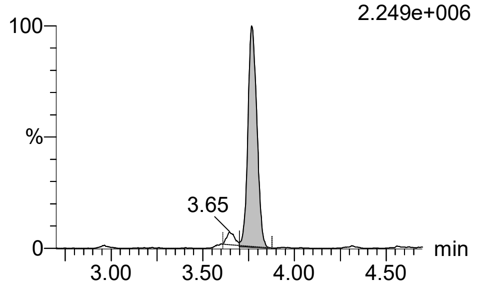

Sample Name: 20211111_L20_R3_IgM
F29:MRM of 2 channels,ES+
1303.5 > 366.1
3.407e+004

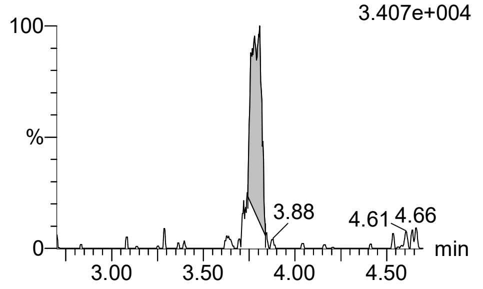

Sample Name: 20211111_H10_R3_IgM
F29:MRM of 2 channels,ES+
869.3 > 204.1
1.673e+006

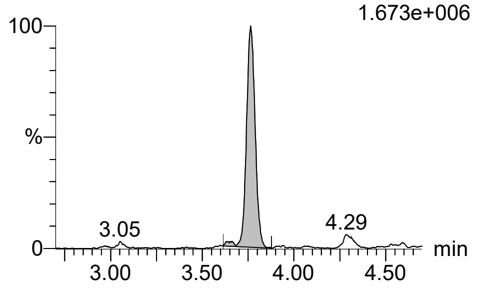

Sample Name: 20211111_H10_R3_IgM
F29:MRM of 2 channels,ES+
1303.5 > 366.1
3.002e+004

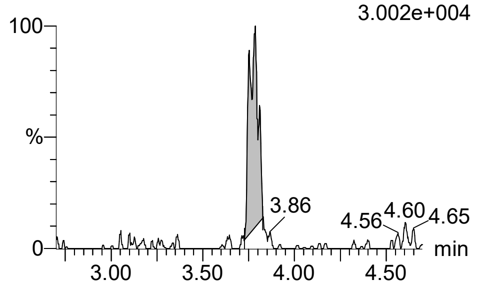

Sample Name: 20211111_A21_R3_IgM
F29:MRM of 2 channels,ES+
869.3 > 204.1
5.648e+006

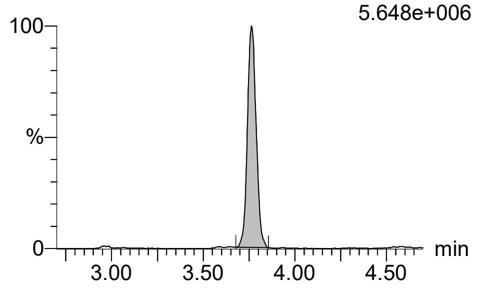

Sample Name: 20211111_A21_R3_IgM
F29:MRM of 2 channels,ES+
1303.5 > 366.1
1.014e+005

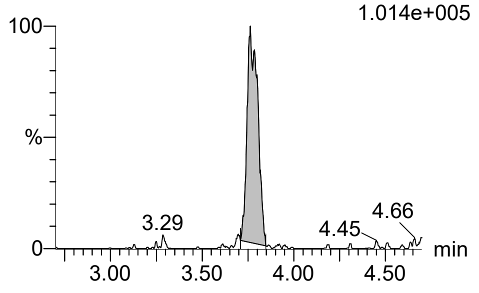

Sample Name: 20211111_A22_R3_IgM
F29:MRM of 2 channels,ES+
869.3 > 204.1
1.171e+006

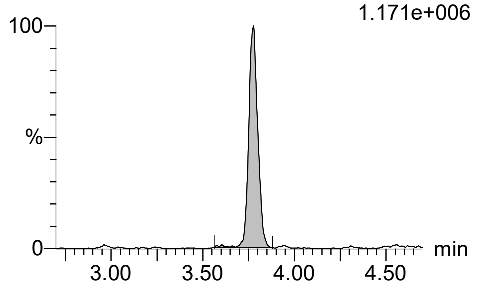

Sample Name: 20211111_A22_R3_IgM
F29:MRM of 2 channels,ES+
1303.5 > 366.1
2.224e+004

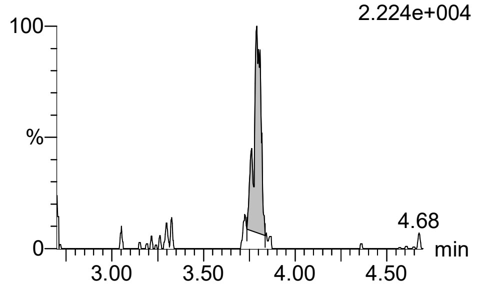

Sample Name: 20211111_L21_R3_IgM
F29:MRM of 2 channels,ES+
869.3 > 204.1
1.083e+006

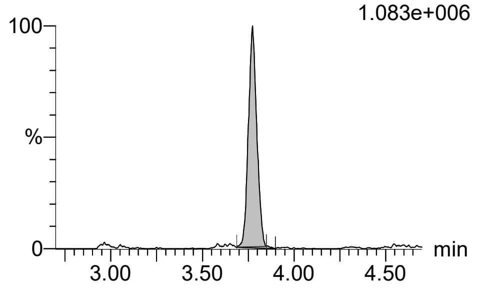

Sample Name: 20211111_L21_R3_IgM
F29:MRM of 2 channels,ES+
1303.5 > 366.1
2.454e+004

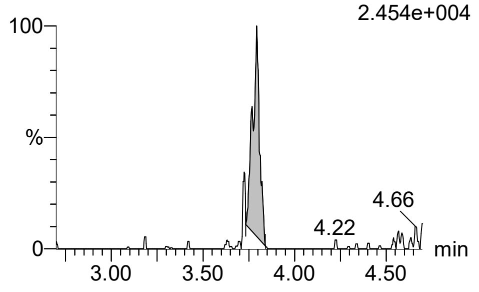

Sample Name: 20211111_L22_R3_IgM
F29:MRM of 2 channels,ES+
869.3 > 204.1
2.095e+006

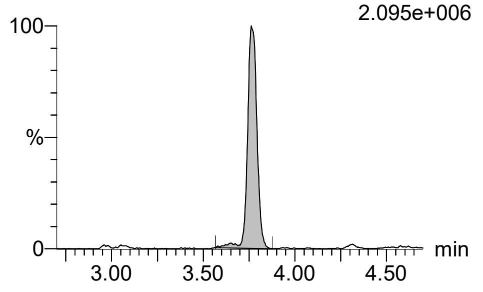

Dataset: C:\MassLynx\10.pro\20211111_clinicalsamples_IgM_IgA.qld

Last Altered: Thursday, March 09, 2023 19:58:25 台北標準時間
Printed: Friday, August 01, 2025 15:27:43 台北標準時間

Sample Name: 20211111_L22_R3_IgM
F29:MRM of 2 channels,ES+
1303.5 > 366.1
3.238e+004

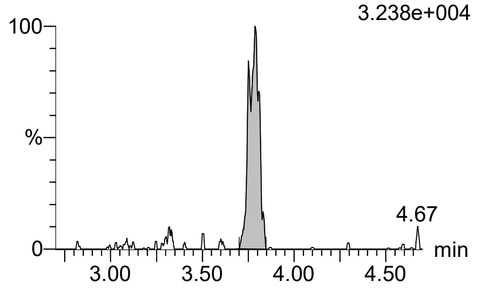

Sample Name: 20211111_H11_R3_IgM
F29:MRM of 2 channels,ES+
869.3 > 204.1
7.641e+006

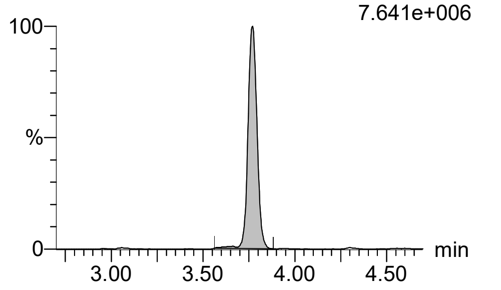

Sample Name: 20211111_H11_R3_IgM
F29:MRM of 2 channels,ES+
1303.5 > 366.1
1.267e+005

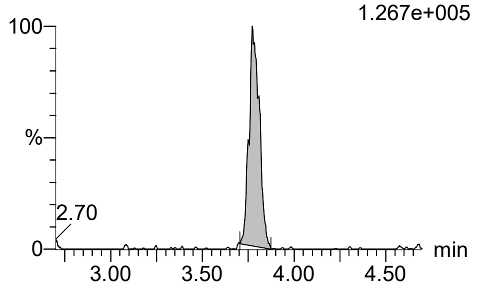

Sample Name: 20211111_A23_R3_IgM
F29:MRM of 2 channels,ES+
869.3 > 204.1
4.539e+006

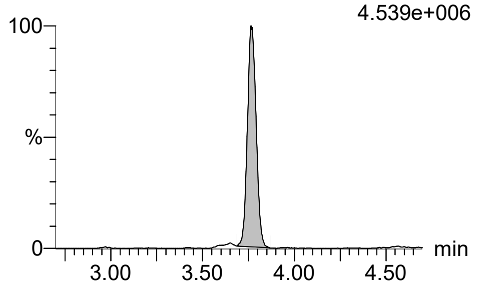

Sample Name: 20211111_A23_R3_IgM
F29:MRM of 2 channels,ES+
1303.5 > 366.1
7.829e+004

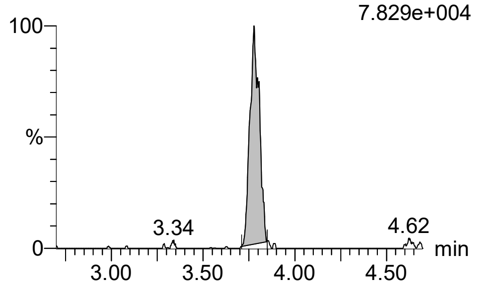

Sample Name: 20211111_A24_R3_IgM
F29:MRM of 2 channels,ES+
869.3 > 204.1
9.275e+005

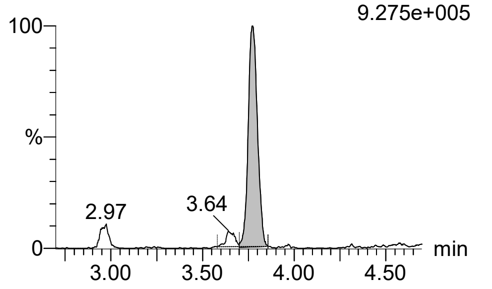

Sample Name: 20211111_A24_R3_IgM
F29:MRM of 2 channels,ES+
1303.5 > 366.1
2.466e+004

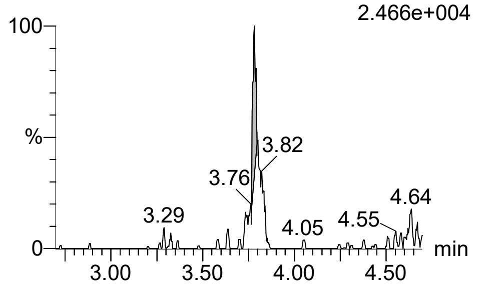

Sample Name: 20211111_L23_R3_IgM
F29:MRM of 2 channels,ES+
869.3 > 204.1
4.880e+006

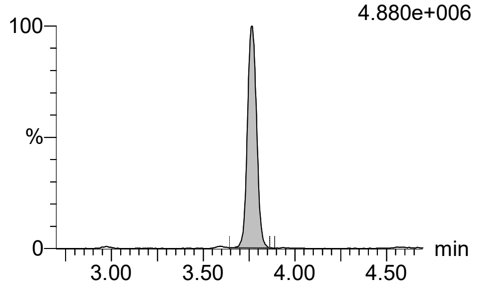

Sample Name: 20211111_L23_R3_IgM
F29:MRM of 2 channels,ES+
1303.5 > 366.1
7.140e+004

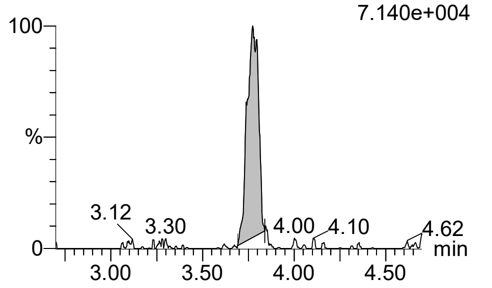

Sample Name: 20211111_L24_R3_IgM
F29:MRM of 2 channels,ES+
869.3 > 204.1
1.173e+006

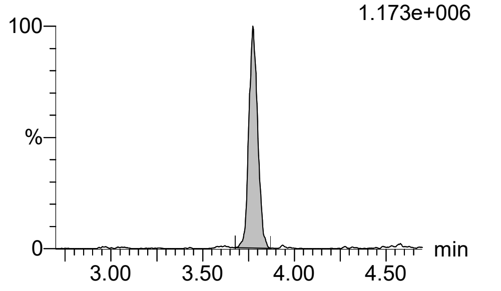

Sample Name: 20211111_L24_R3_IgM
F29:MRM of 2 channels,ES+
1303.5 > 366.1
2.219e+004

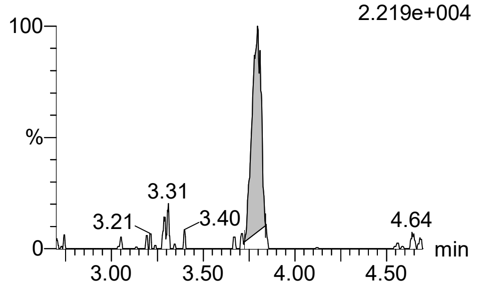

Sample Name: 20211111_H12_R3_IgM
F29:MRM of 2 channels,ES+
869.3 > 204.1
1.741e+006

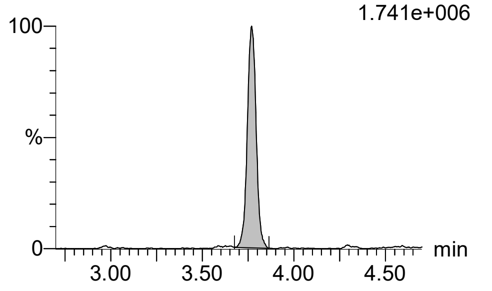

Sample Name: 20211111_H12_R3_IgM
F29:MRM of 2 channels,ES+
1303.5 > 366.1
2.482e+004

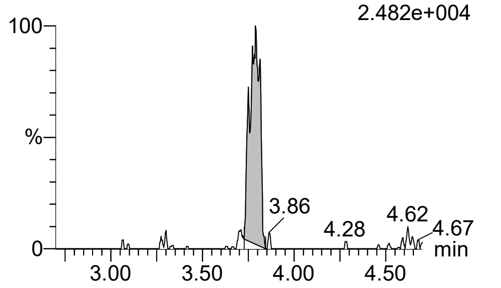

Sample Name: 20211111_QC_R22_IgM
F29:MRM of 2 channels,ES+
869.3 > 204.1
3.191e+006

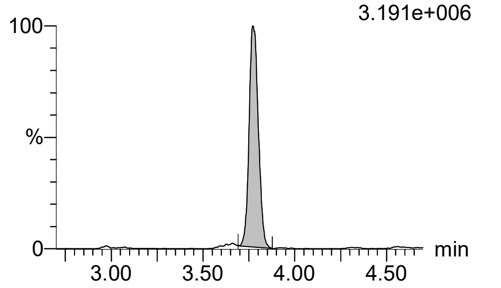

Sample Name: 20211111_QC_R22_IgM
F29:MRM of 2 channels,ES+
1303.5 > 366.1
5.073e+004

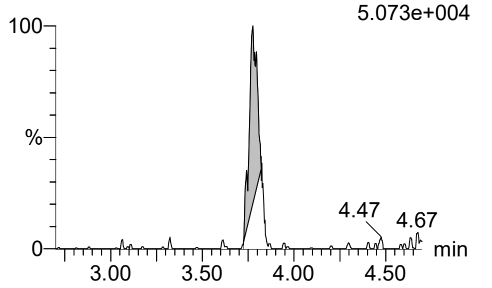

Dataset: C:\MassLynx\10.pro\20211111_clinicalsamples_IgM_IgA.qld

Last Altered: Thursday, March 09, 2023 19:58:25 台北標準時間
Printed: Friday, August 01, 2025 15:27:43 台北標準時間

Sample Name: 20211111_A25_R3_IgM
F29:MRM of 2 channels,ES+
869.3 > 204.1
2.572e+006

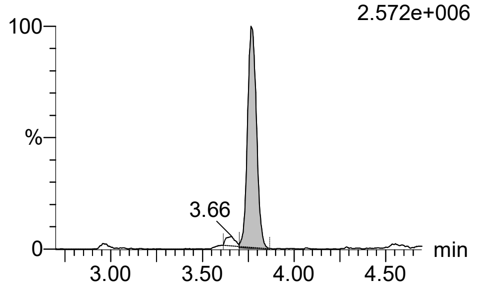

Sample Name: 20211111_A25_R3_IgM
F29:MRM of 2 channels,ES+
1303.5 > 366.1
5.008e+004

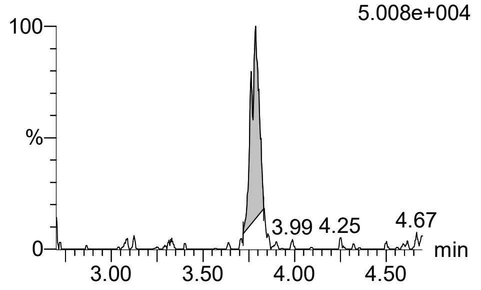

Sample Name: 20211111_A26_R3_IgM
F29:MRM of 2 channels,ES+
869.3 > 204.1
1.683e+006

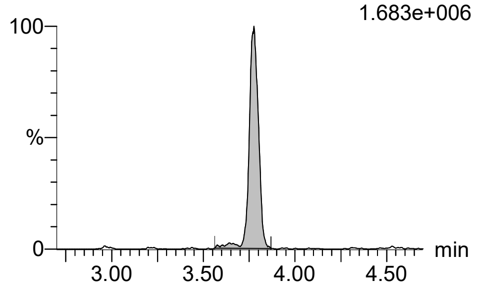

Sample Name: 20211111_A26_R3_IgM
F29:MRM of 2 channels,ES+
1303.5 > 366.1
2.542e+004

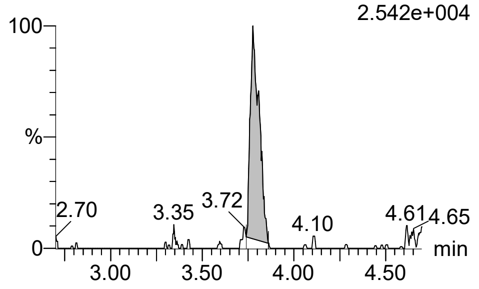

Sample Name: 20211111_L25_R3_IgM
F29:MRM of 2 channels,ES+
869.3 > 204.1
3.614e+006

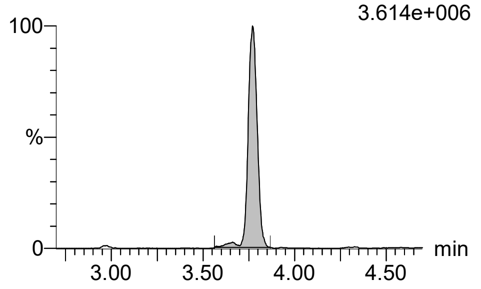

Sample Name: 20211111_L25_R3_IgM
F29:MRM of 2 channels,ES+
1303.5 > 366.1
5.665e+004

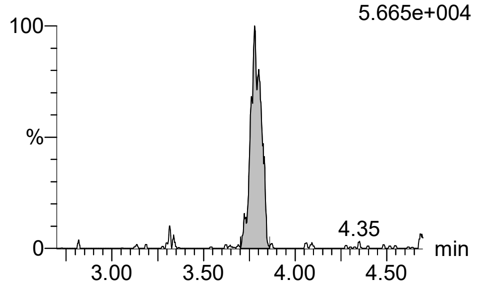

Sample Name: 20211111_L26_R3_IgM
F29:MRM of 2 channels,ES+
869.3 > 204.1
1.500e+006

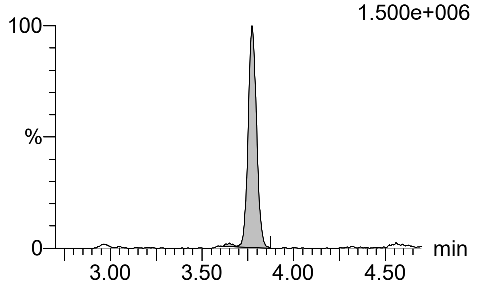

Sample Name: 20211111_L26_R3_IgM
F29:MRM of 2 channels,ES+
1303.5 > 366.1
1.878e+004

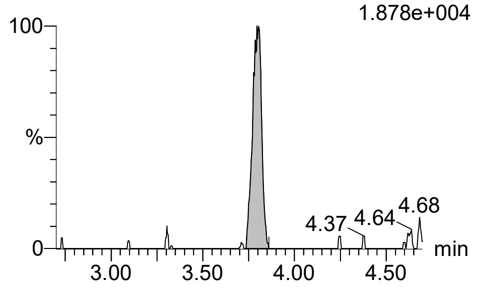

Sample Name: 20211111_H13_R3_IgM
F29:MRM of 2 channels,ES+
869.3 > 204.1
3.126e+006

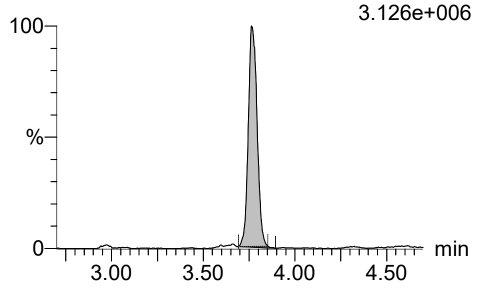

Sample Name: 20211111_H13_R3_IgM
F29:MRM of 2 channels,ES+
1303.5 > 366.1
5.781e+004

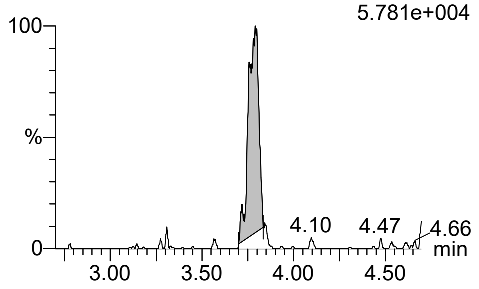

Sample Name: 20211111_A27_R3_IgM
F29:MRM of 2 channels,ES+
869.3 > 204.1
1.241e+006

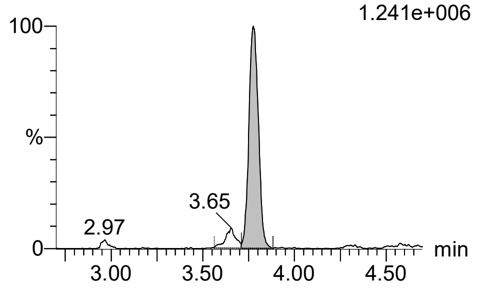

Sample Name: 20211111_A27_R3_IgM
F29:MRM of 2 channels,ES+
1303.5 > 366.1
1.739e+004

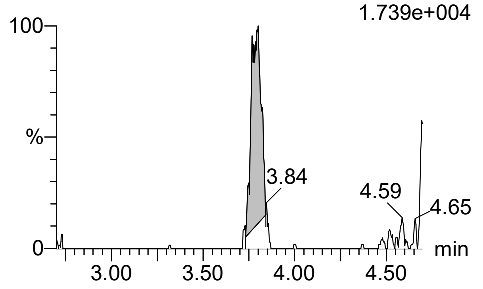

Sample Name: 20211111_A28_R3_IgM
F29:MRM of 2 channels,ES+
869.3 > 204.1
1.264e+006

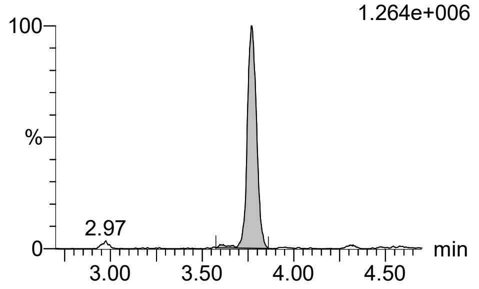

Sample Name: 20211111_A28_R3_IgM
F29:MRM of 2 channels,ES+
1303.5 > 366.1
2.261e+004

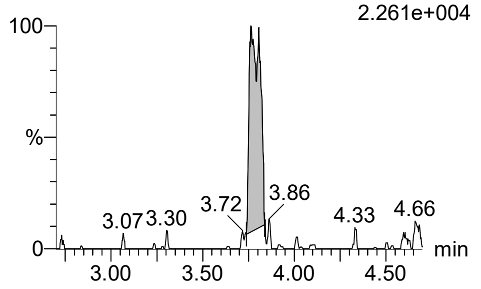

Sample Name: 20211111_L27_R3_IgM
F29:MRM of 2 channels,ES+
869.3 > 204.1
1.814e+006

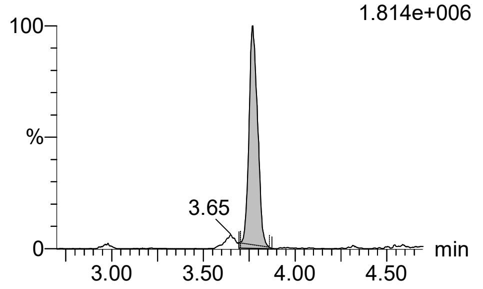

Dataset: C:\MassLynx\10.pro\20211111_clinicalsamples_IgM_IgA.qld

Last Altered: Thursday, March 09, 2023 19:58:25 台北標準時間
Printed: Friday, August 01, 2025 15:27:43 台北標準時間

Sample Name: 20211111_L27_R3_IgM
F29:MRM of 2 channels,ES+
1303.5 > 366.1
3.640e+004

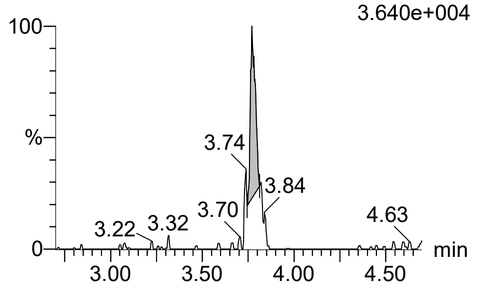

Sample Name: 20211111_L28_R3_IgM
F29:MRM of 2 channels,ES+
869.3 > 204.1
1.939e+006

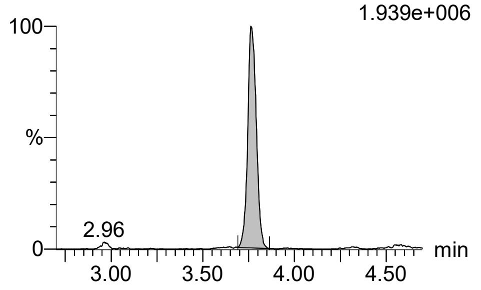

Sample Name: 20211111_L28_R3_IgM
F29:MRM of 2 channels,ES+
1303.5 > 366.1
3.605e+004

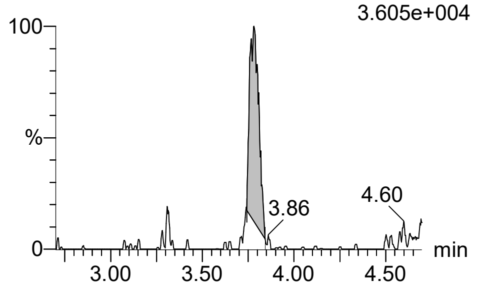

Sample Name: 20211111_H14_R3_IgM
F29:MRM of 2 channels,ES+
869.3 > 204.1
4.517e+006

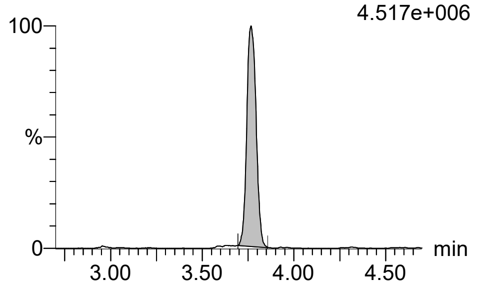

Sample Name: 20211111_H14_R3_IgM
F29:MRM of 2 channels,ES+
1303.5 > 366.1
9.557e+004

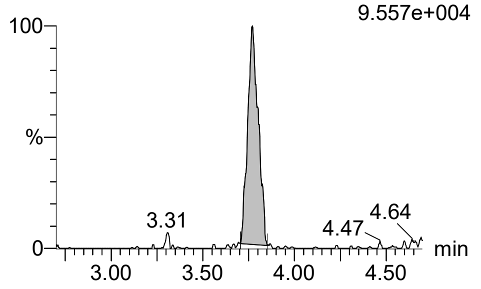

Sample Name: 20211111_A29_R3_IgM
F29:MRM of 2 channels,ES+
869.3 > 204.1
3.127e+006

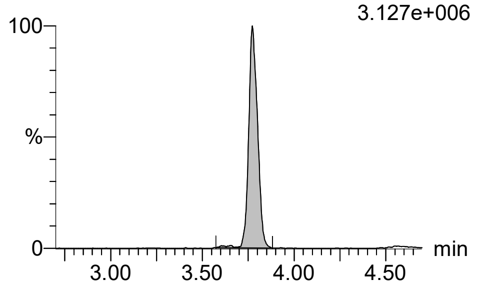

Sample Name: 20211111_A29_R3_IgM
F29:MRM of 2 channels,ES+
1303.5 > 366.1
5.239e+004

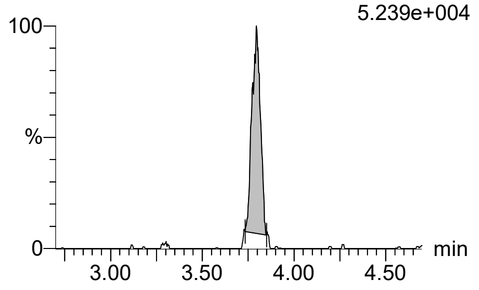

Sample Name: 20211111_A30_R3_IgM
F29:MRM of 2 channels,ES+
869.3 > 204.1
2.927e+006

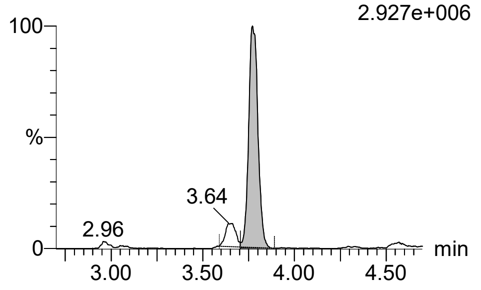

Sample Name: 20211111_A30_R3_IgM
F29:MRM of 2 channels,ES+
1303.5 > 366.1
6.352e+004

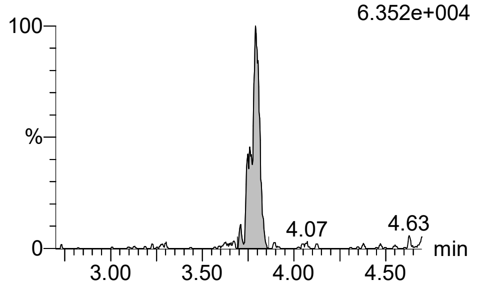

Sample Name: 20211111_QC_R23_IgM
F29:MRM of 2 channels,ES+
869.3 > 204.1
2.993e+006

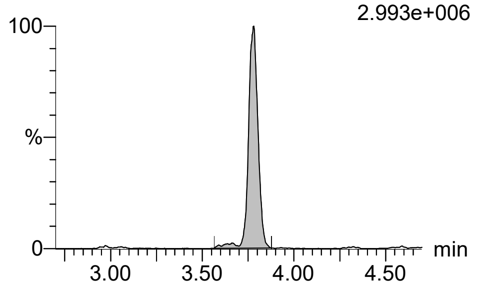

Sample Name: 20211111_QC_R23_IgM
F29:MRM of 2 channels,ES+
1303.5 > 366.1
4.413e+004

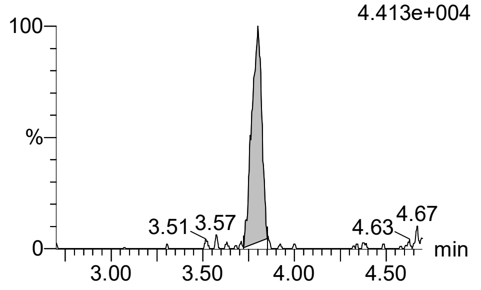

Sample Name: 20211111_L29_R3_IgM
F29:MRM of 2 channels,ES+
869.3 > 204.1
8.269e+005

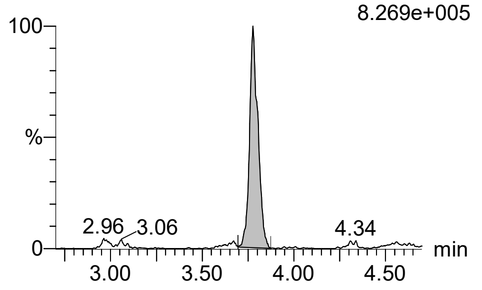

Sample Name: 20211111_L29_R3_IgM
F29:MRM of 2 channels,ES+
1303.5 > 366.1
1.562e+004

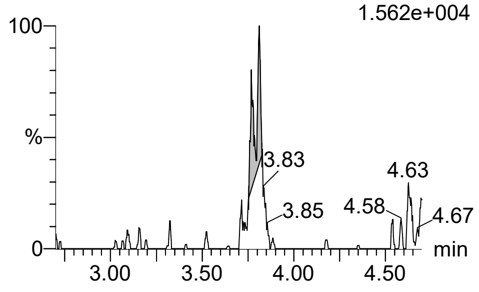

Sample Name: 20211111_L30_R3_IgM
F29:MRM of 2 channels,ES+
869.3 > 204.1
9.196e+005

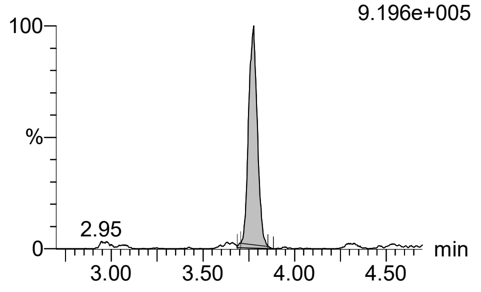

Sample Name: 20211111_L30_R3_IgM
F29:MRM of 2 channels,ES+
1303.5 > 366.1
1.825e+004

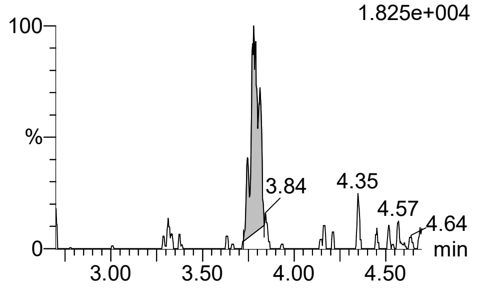

Sample Name: 20211111_H15_R3_IgM
F29:MRM of 2 channels,ES+
869.3 > 204.1
2.424e+006

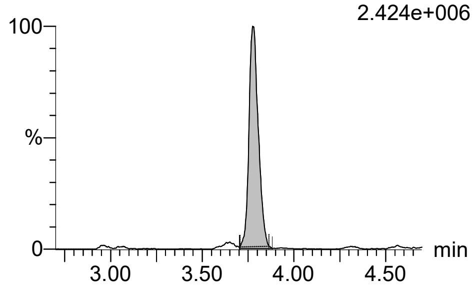

Sample Name: 20211111_H15_R3_IgM
F29:MRM of 2 channels,ES+
1303.5 > 366.1
5.507e+004

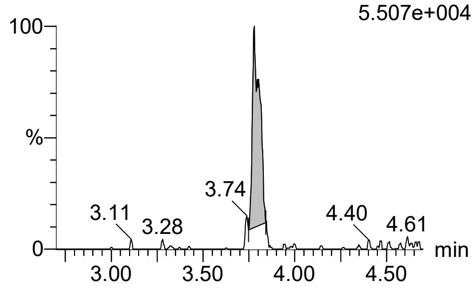

Sample Name: 20211111_A31_R3_IgM
F29:MRM of 2 channels,ES+
869.3 > 204.1
4.296e+006

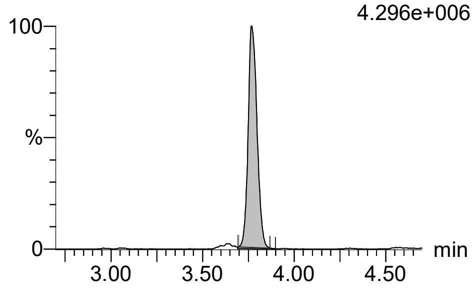

Sample Name: 20211111_A31_R3_IgM
F29:MRM of 2 channels,ES+
1303.5 > 366.1
7.993e+004

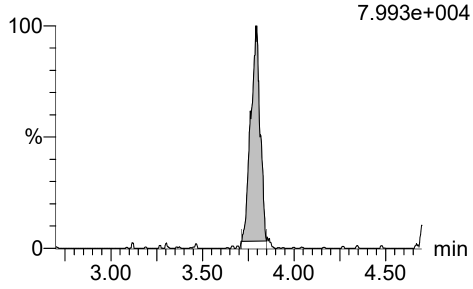

Sample Name: 20211111_A32_R3_IgM
F29:MRM of 2 channels,ES+
869.3 > 204.1
6.205e+006

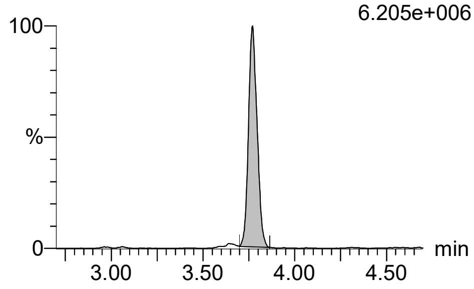

Sample Name: 20211111_A32_R3_IgM
F29:MRM of 2 channels,ES+
1303.5 > 366.1
1.279e+005

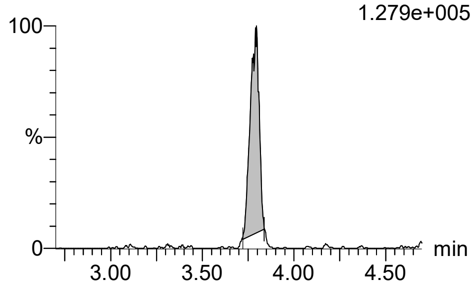

Sample Name: 20211111_L31_R3_IgM
F29:MRM of 2 channels,ES+
869.3 > 204.1
3.417e+006

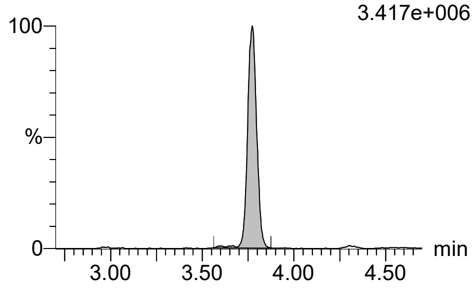

Sample Name: 20211111_L31_R3_IgM
F29:MRM of 2 channels,ES+
1303.5 > 366.1
6.473e+004

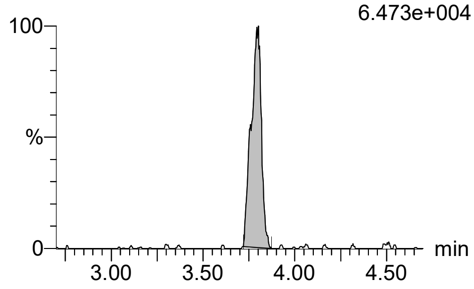

Sample Name: 20211111_L32_R3_IgM
F29:MRM of 2 channels,ES+
869.3 > 204.1
2.888e+006

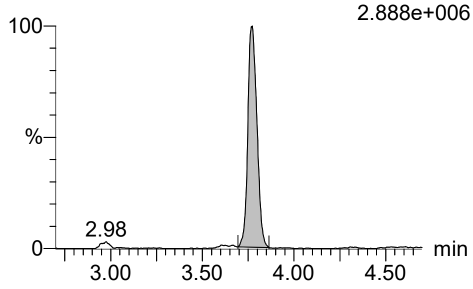

Sample Name: 20211111_L32_R3_IgM
F29:MRM of 2 channels,ES+
1303.5 > 366.1
5.595e+004

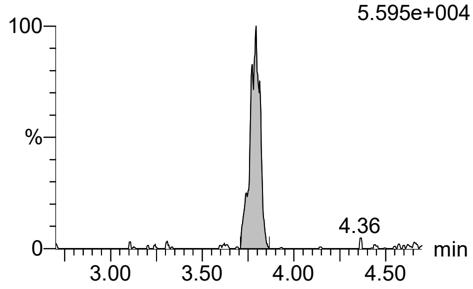

Sample Name: 20211111_H16_R3_IgM
F29:MRM of 2 channels,ES+
869.3 > 204.1
3.062e+006

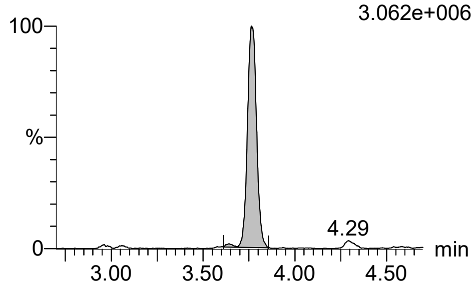

Sample Name: 20211111_H16_R3_IgM
F29:MRM of 2 channels,ES+
1303.5 > 366.1
6.204e+004

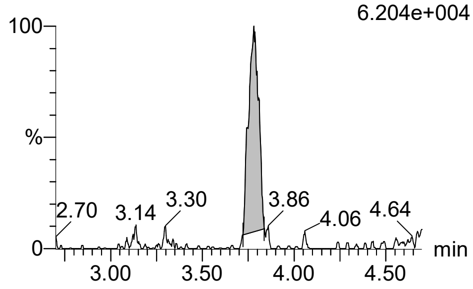

Sample Name: 20211111_A33_R3_IgM
F29:MRM of 2 channels,ES+
869.3 > 204.1
1.762e+006

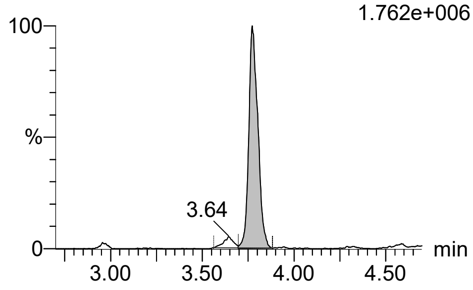

Sample Name: 20211111_A33_R3_IgM
F29:MRM of 2 channels,ES+
1303.5 > 366.1
4.363e+004

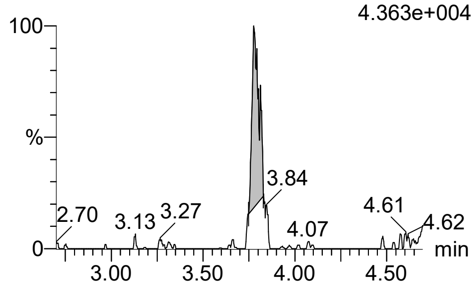

Sample Name: 20211111_A34_R3_IgM
F29:MRM of 2 channels,ES+
869.3 > 204.1
2.938e+006

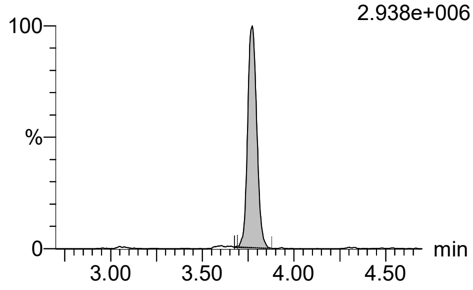

Dataset: C:\MassLynx\10.pro\20211111_clinicalsamples_IgM_IgA.qld

Last Altered: Thursday, March 09, 2023 19:58:25 台北標準時間
Printed: Friday, August 01, 2025 15:27:43 台北標準時間

Sample Name: 20211111_A34_R3_IgM
F29:MRM of 2 channels,ES+
1303.5 > 366.1
4.981e+004

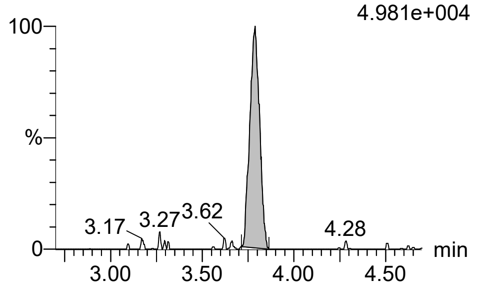

Sample Name: 20211111_H17_R3_IgM
F29:MRM of 2 channels,ES+
869.3 > 204.1
5.051e+006

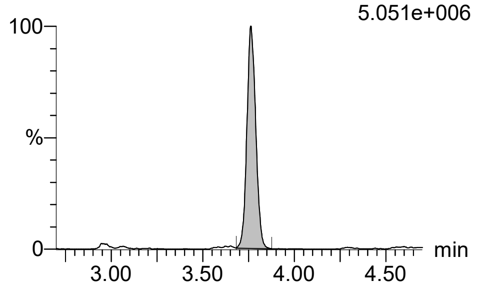

Sample Name: 20211111_H17_R3_IgM
F29:MRM of 2 channels,ES+
1303.5 > 366.1
1.091e+005

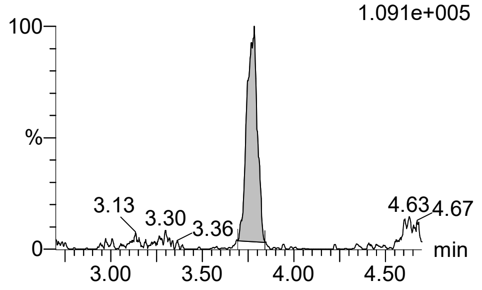

Sample Name: 20211111_A35_R3_IgM
F29:MRM of 2 channels,ES+
869.3 > 204.1
2.919e+006

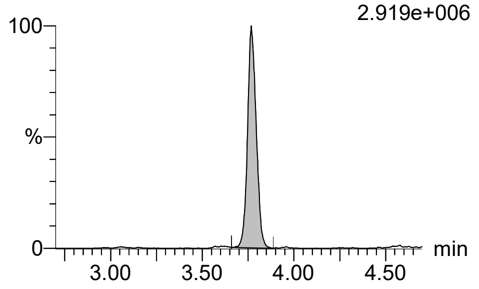

Sample Name: 20211111_A35_R3_IgM
F29:MRM of 2 channels,ES+
1303.5 > 366.1
6.802e+004

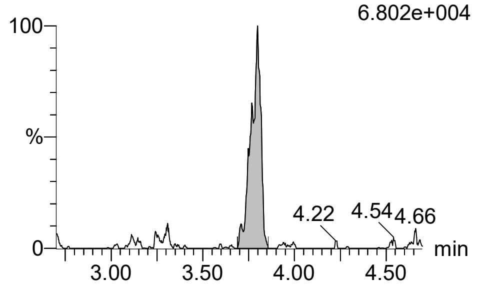

Sample Name: 20211111_QC_R24_IgM
F29:MRM of 2 channels,ES+
869.3 > 204.1
2.387e+006

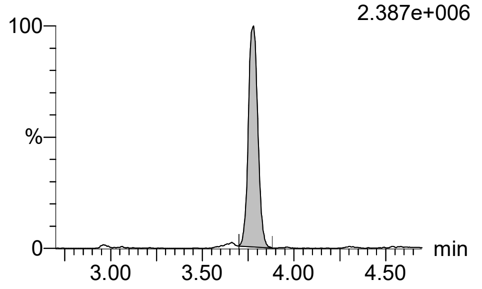

Sample Name: 20211111_QC_R24_IgM
F29:MRM of 2 channels,ES+
1303.5 > 366.1
4.948e+004

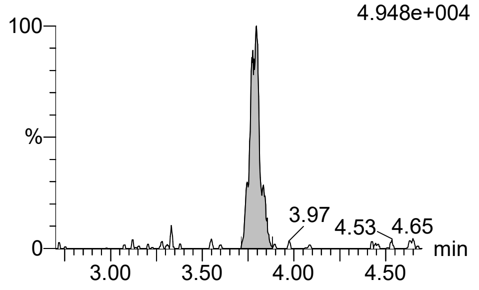

Sample Name: 20211111_A36_R3_IgM
F29:MRM of 2 channels,ES+
869.3 > 204.1
4.342e+006

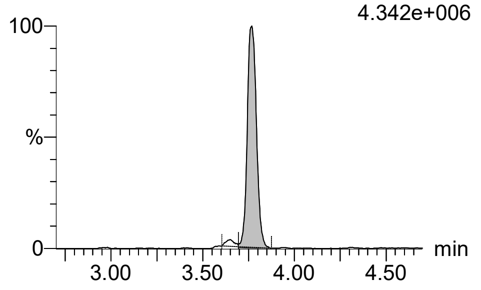

Sample Name: 20211111_A36_R3_IgM
F29:MRM of 2 channels,ES+
1303.5 > 366.1
6.651e+004

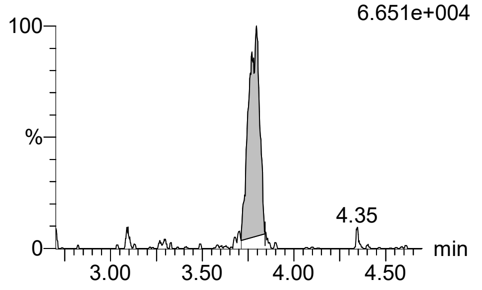

Sample Name: 20211111_H18_R3_IgM
F29:MRM of 2 channels,ES+
869.3 > 204.1
5.370e+006

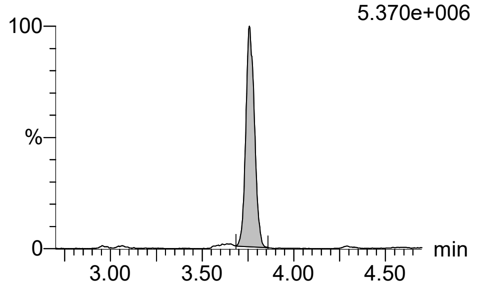

Sample Name: 20211111_H18_R3_IgM
F29:MRM of 2 channels,ES+
1303.5 > 366.1
1.297e+005

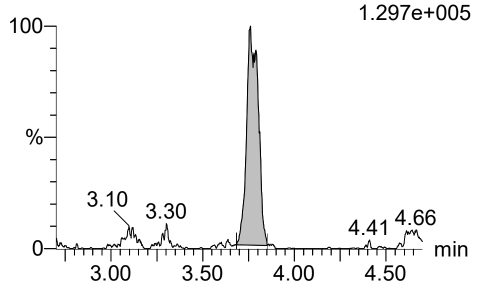

Sample Name: 20211111_A37_R3_IgM
F29:MRM of 2 channels,ES+
869.3 > 204.1
1.129e+006

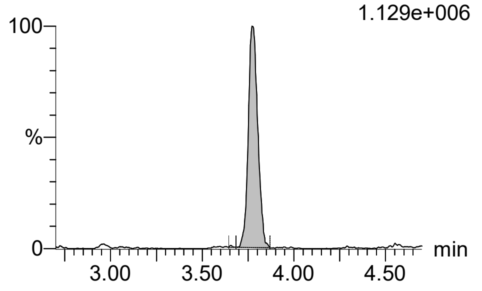

Sample Name: 20211111_A37_R3_IgM
F29:MRM of 2 channels,ES+
1303.5 > 366.1
2.321e+004

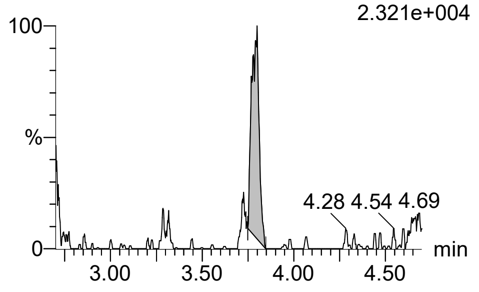

Sample Name: 20211111_A38_R3_IgM
F29:MRM of 2 channels,ES+
869.3 > 204.1
2.870e+006

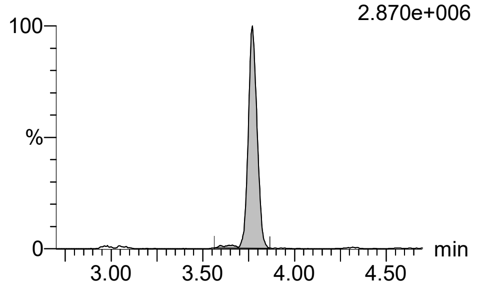

Sample Name: 20211111_A38_R3_IgM
F29:MRM of 2 channels,ES+
1303.5 > 366.1
6.732e+004

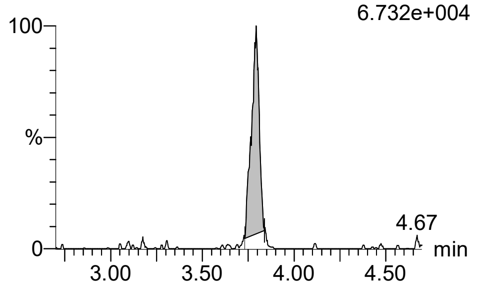

Dataset: C:\MassLynx\10.pro\20211111_clinicalsamples_IgM_IgA.qld

Last Altered: Thursday, March 09, 2023 19:58:25 台北標準時間
Printed: Friday, August 01, 2025 15:27:43 台北標準時間

Sample Name: 20211111_H19_R3_IgM
F29:MRM of 2 channels,ES+
869.3 > 204.1
5.653e+006

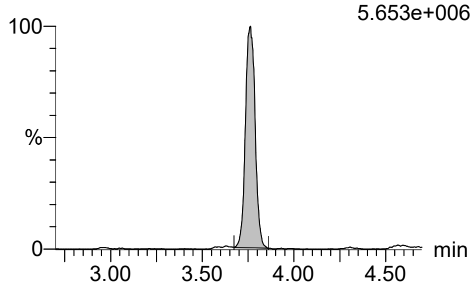

Sample Name: 20211111_H19_R3_IgM
F29:MRM of 2 channels,ES+
1303.5 > 366.1
1.460e+005

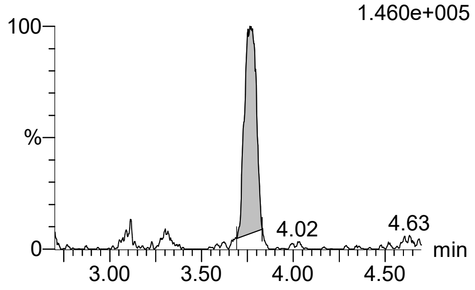

Sample Name: 20211111_A39_R3_IgM
F29:MRM of 2 channels,ES+
869.3 > 204.1
2.087e+006

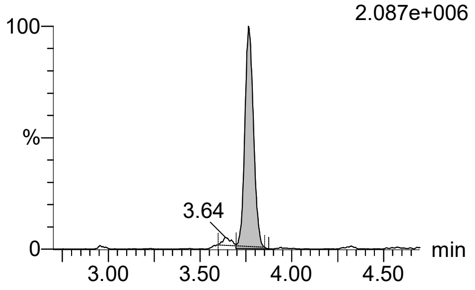

Sample Name: 20211111_A39_R3_IgM
F29:MRM of 2 channels,ES+
1303.5 > 366.1
4.153e+004

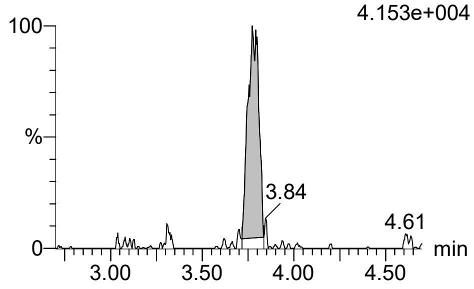

Sample Name: 20211111_A40_R3_IgM
F29:MRM of 2 channels,ES+
869.3 > 204.1
3.890e+006

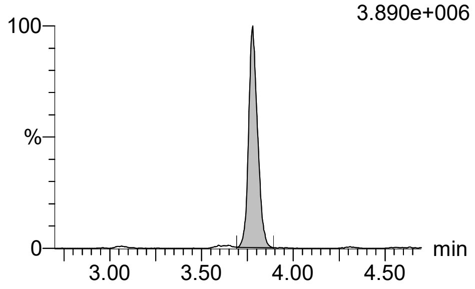

Sample Name: 20211111_A40_R3_IgM
F29:MRM of 2 channels,ES+
1303.5 > 366.1
8.231e+004

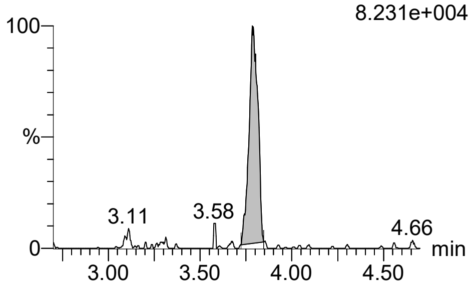

Sample Name: 20211111_H20_R3_IgM
F29:MRM of 2 channels,ES+
869.3 > 204.1
6.496e+006

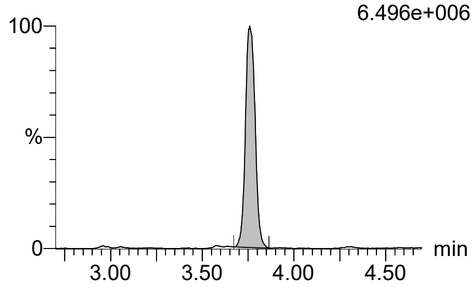

Sample Name: 20211111_H20_R3_IgM
F29:MRM of 2 channels,ES+
1303.5 > 366.1
2.444e+005

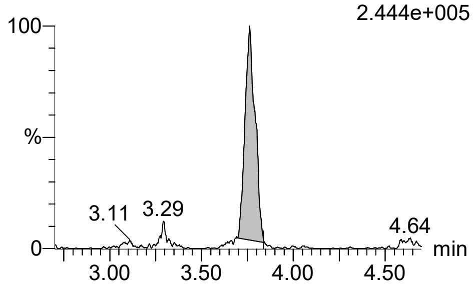

Sample Name: 20211111_A41_R3_IgM
F29:MRM of 2 channels,ES+
869.3 > 204.1
1.678e+006

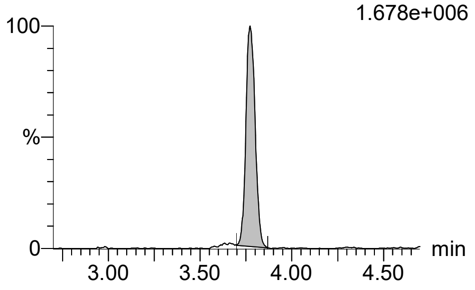

Sample Name: 20211111_A41_R3_IgM
F29:MRM of 2 channels,ES+
1303.5 > 366.1
3.461e+004

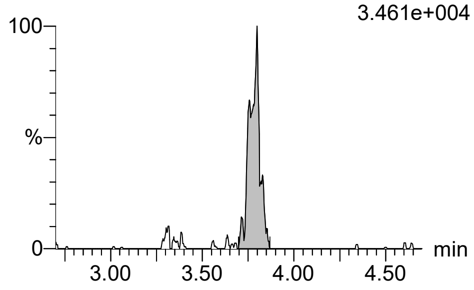

Sample Name: 20211111_A42_R3_IgM
F29:MRM of 2 channels,ES+
869.3 > 204.1
1.498e+006

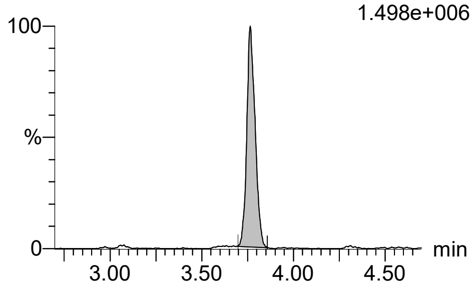

Sample Name: 20211111_A42_R3_IgM
F29:MRM of 2 channels,ES+
1303.5 > 366.1
3.116e+004

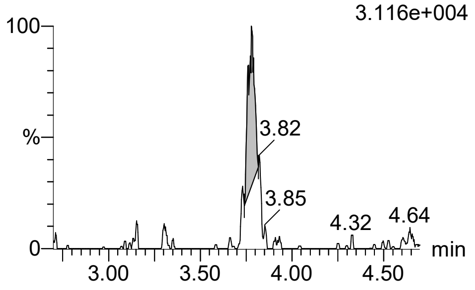

Sample Name: 20211111_A43_R3_IgM
F29:MRM of 2 channels,ES+
869.3 > 204.1
3.537e+006

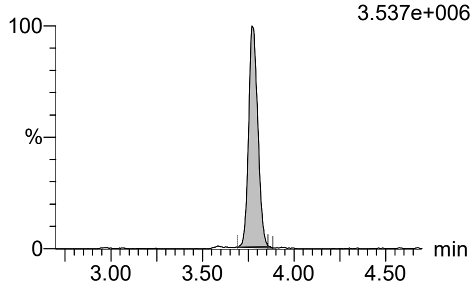

Sample Name: 20211111_A43_R3_IgM
F29:MRM of 2 channels,ES+
1303.5 > 366.1
7.797e+004

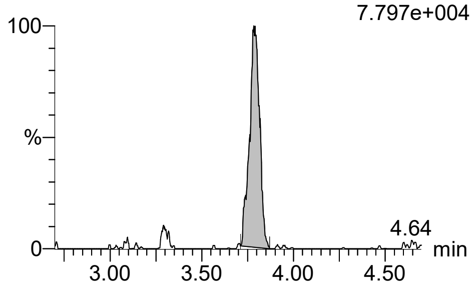

Sample Name: 20211111_A44_R3_IgM
F29:MRM of 2 channels,ES+
869.3 > 204.1
1.707e+006

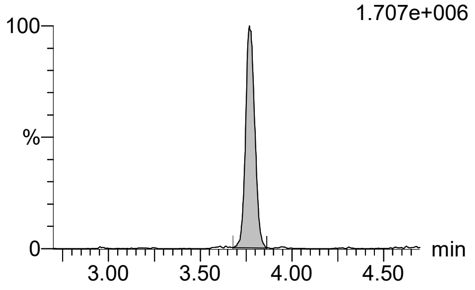

Dataset: C:\MassLynx\10.pro\20211111_clinicalsamples_IgM_IgA.qld

Last Altered: Thursday, March 09, 2023 19:58:25 台北標準時間
Printed: Friday, August 01, 2025 15:27:43 台北標準時間

Sample Name: 20211111_A44_R3_IgM

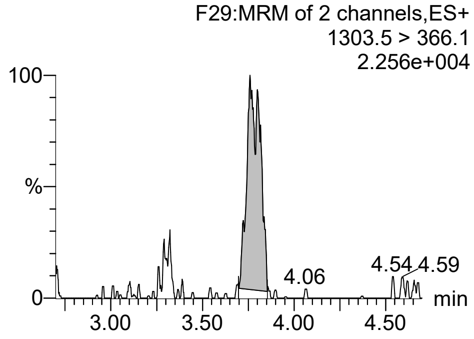

Sample Name: 20211111_QC_R25_IgM

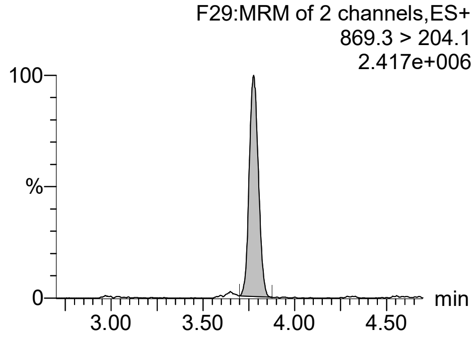

Sample Name: 20211111_QC_R25_IgM

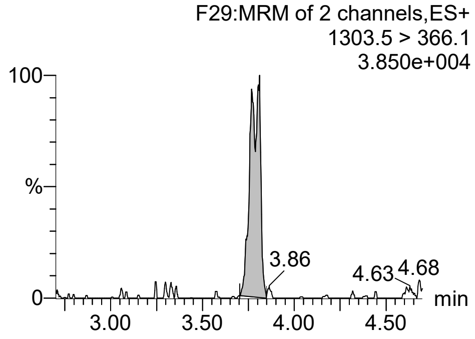

Dataset: C:\MassLynx\10.pro\20211111_clinicalsamples_IgM_IgA.qld

Last Altered: Thursday, March 09, 2023 19:58:25 台北標準時間
Printed: Friday, August 01, 2025 15:27:43 台北標準時間

	#	Name	RT	Area	IS Area	Response	Prima...
1	1	20211112_QC_R001_IgM	3.76	317326.4	15421.4	20.577	bb
2	2	20211112_QC_R002_IgM	3.77	256799.3	13188.8	19.471	bb
3	3	20211111_A1_R2_IgM	3.77	378957.3	11871.0	31.923	bb
4	4	20211111_A1_R3_IgM	3.77	362274.1	12030.5	30.113	bb
5	5	20211111_A2_R2_IgM	3.76	140683.9	11446.8	12.290	bb
6	6	20211111_A2_R3_IgM	3.77	134763.5	11098.4	12.143	bb
7	7	20211111_L1_R2_IgM	3.77	157902.1	11779.4	13.405	bb
8	8	20211111_L1_R3_IgM	3.77	140047.3	11176.2	12.531	bb
9	9	20211111_L2_R2_IgM	3.76	196918.4	11375.0	17.312	bb
10	10	20211111_H1_R2_IgM	3.76	195623.2	13595.2	14.389	MM
11	11	20211111_A3_R2_IgM	3.76	214650.4	14112.3	15.210	bb
12	12	20211111_A4_R2_IgM	3.78	332653.3	11977.5	27.773	bb
13	13	20211111_L3_R2_IgM	3.76	99415.5	12242.1	8.121	MM
14	14	20211111_L4_R2_IgM	3.77	131391.1	12797.0	10.267	bb
15	15	20211111_H2_R2_IgM	3.76	378569.9	10006.0	37.834	bb
16	16	20211111_A5_R2_IgM	3.77	215939.2	11434.6	18.885	MM
17	17	20211111_A6_R2_IgM	3.76	222775.4	13193.7	16.885	bb
18	18	20211111_QC_R10_IgM	3.77	205975.4	12566.1	16.391	bb
19	19	20211111_L5_R2_IgM	3.75	182011.0	13616.4	13.367	bb
20	20	20211111_L6_R2_IgM	3.77	46881.6	13698.5	3.422	MM
21	21	20211111_H3_R2_IgM	3.76	215937.6	15793.1	13.673	bb
22	22	20211111_A7_R2_IgM	3.76	442530.2	12936.9	34.207	MM
23	23	20211111_A8_R2_IgM	3.76	225524.1	13108.0	17.205	MM
24	24	20211111_L7_R2_IgM	3.77	211725.8	13278.5	15.945	bb
25	25	20211111_L8_R2_IgM	3.76	94730.1	15233.6	6.219	bb
26	26	20211111_H4_R2_IgM	3.76	125006.3	16521.9	7.566	MM
27	27	20211111_A9_R2_IgM	3.76	289087.0	16451.6	17.572	bb
28	28	20211111_A10_R2_IgM	3.76	414193.4	13036.0	31.773	MM
29	29	20211111_L9_R2_IgM	3.76	311002.2	14554.1	21.369	bb
30	30	20211111_L10_R2_IgM	3.76	149526.2	12250.4	12.206	MM
31	31	20211111_QC_R11_IgM	3.77	215977.8	11459.2	18.848	bb
32	32	20211111_H5_R2_IgM	3.77	105598.7	14358.2	7.355	bb
33	33	20211111_A11_R2_IgM	3.76	69889.2	16184.1	4.318	MM
34	34	20211111_A12_R2_IgM	3.77	216818.5	13465.4	16.102	bb
35	35	20211111_L11_R2_IgM	3.76	117698.5	13277.9	8.864	bb
36	36	20211111_L12_R2_IgM	3.77	79808.2	15958.8	5.001	MM
37	37	20211111_H6_R2_IgM	3.76	285657.6	14003.5	20.399	bb
38	38	20211111_A13_R2_IgM	3.76	184651.5	13656.1	13.522	bb
39	39	20211111_A14_R2_IgM	3.76	279725.7	14071.3	19.879	MM
40	40	20211111_L13_R2_IgM	3.77	137202.3	14785.6	9.279	bb
41	41	20211111_L14_R2_IgM	3.76	109131.3	14772.2	7.388	bb
42	42	20211111_H7_R2_IgM	3.76	157065.8	15164.4	10.358	MM
43	43	20211111_A15_R2_IgM	3.76	277002.0	15721.5	17.619	bb
44	44	20211111_QC_R12_IgM	3.77	237671.2	13711.5	17.334	bb
45	45	20211111_A16_R2_IgM	3.76	203115.2	14275.6	14.228	MM
46	46	20211111_L15_R2_IgM	3.76	186935.0	12935.8	14.451	bb
47	47	20211111_L16_R2_IgM	3.76	202776.6	13342.8	15.197	bb
48	48	20211111_H8_R2_IgM	3.77	158021.7	15835.1	9.979	bb
49	49	20211111_A17_R2_IgM	3.77	199573.2	15786.7	12.642	MM
50	50	20211111_A18_R2_IgM	3.76	216060.4	16986.8	12.719	MM
51	51	20211111_L17_R2_IgM	3.76	225987.1	13342.7	16.937	bb
52	52	20211111_L18_R2_IgM	3.76	66489.0	14692.0	4.526	bb
53	53	20211111_H9_R2_IgM	3.76	280931.1	15639.7	17.963	bb
54	54	20211111_A19_R2_IgM	3.77	327343.4	14198.7	23.054	bb
55	55	20211111_A20_R2_IgM	3.76	66024.1	14162.7	4.662	MM
56	56	20211111_L19_R2_IgM	3.76	82662.9	9293.4	8.895	bb

Dataset: C:\MassLynx\10.pro\20211111_clinicalsamples_IgM_IgA.qld

Last Altered: Thursday, March 09, 2023 19:58:25 台北標準時間
Printed: Friday, August 01, 2025 15:27:43 台北標準時間

	# Name	RT	Area	IS Area	Response	Prima...
57	57 20211111_QC_R13_IgM	3.77	231664.8	13903.0	16.663	bb
58	58 20211111_L20_R2_IgM	3.76	142239.7	12781.6	11.128	MM
59	59 20211111_H10_R2_IgM	3.75	98808.7	14796.4	6.678	MM
60	60 20211111_A21_R2_IgM	3.76	341862.3	13669.0	25.010	bb
61	61 20211111_A22_R2_IgM	3.77	67625.0	14098.3	4.797	bb
62	62 20211111_L21_R2_IgM	3.77	68052.4	15678.2	4.341	bb
63	63 20211111_L22_R2_IgM	3.76	136692.9	15724.5	8.693	bb
64	64 20211111_H11_R2_IgM	3.77	510261.8	15890.2	32.112	bb
65	65 20211111_A23_R2_IgM	3.76	262180.3	13423.2	19.532	bb
66	66 20211111_A24_R2_IgM	3.77	59182.4	12438.0	4.758	MM
67	67 20211111_L23_R2_IgM	3.76	337759.7	14075.0	23.997	bb
68	68 20211111_L24_R2_IgM	3.76	78678.1	15972.1	4.926	MM
69	69 20211111_H12_R2_IgM	3.76	102319.6	16270.9	6.289	MM
70	70 20211111_QC_R14_IgM	3.77	247741.1	15806.8	15.673	bb
71	71 20211111_A25_R2_IgM	3.76	193561.2	14552.1	13.301	MM
72	72 20211111_A26_R2_IgM	3.77	103581.4	11492.7	9.013	bb
73	73 20211111_L25_R2_IgM	3.76	237632.9	14403.3	16.498	bb
74	74 20211111_L26_R2_IgM	3.77	97335.3	16533.9	5.887	bb
75	75 20211111_H13_R2_IgM	3.76	194488.6	15520.1	12.531	bb
76	76 20211111_A27_R2_IgM	3.77	89764.6	13337.4	6.730	MM
77	77 20211111_A28_R2_IgM	3.76	82618.8	11895.6	6.945	bb
78	78 20211111_L27_R2_IgM	3.77	112113.3	14125.7	7.937	MM
79	79 20211111_L28_R2_IgM	3.77	107469.6	11807.5	9.102	bb
80	80 20211111_H14_R2_IgM	3.77	310946.6	15035.7	20.681	bb
81	81 20211111_A29_R2_IgM	3.77	209826.7	13554.4	15.480	bb
82	82 20211111_A30_R2_IgM	3.76	207558.6	12984.1	15.986	MM
83	83 20211111_QC_R15_IgM	3.78	235613.0	12299.5	19.156	bb
84	84 20211111_L29_R2_IgM	3.78	45934.4	13590.7	3.380	MM
85	85 20211111_L30_R2_IgM	3.77	61101.6	12511.4	4.884	bb
86	86 20211111_H15_R2_IgM	3.78	169558.0	11885.0	14.267	bb
87	87 20211111_A31_R2_IgM	3.77	266671.9	13422.1	19.868	bb
88	88 20211111_A32_R2_IgM	3.77	396547.2	11461.3	34.599	bb
89	89 20211111_L31_R2_IgM	3.76	214904.7	13753.4	15.626	bb
90	90 20211111_L32_R2_IgM	3.77	186833.1	12949.0	14.428	bb
91	91 20211111_H16_R2_IgM	3.76	218569.7	11465.4	19.063	bb
92	92 20211111_A33_R2_IgM	3.78	105155.6	10551.5	9.966	MM
93	93 20211111_A34_R2_IgM	3.77	209045.2	12409.9	16.845	bb
94	94 20211111_H17_R2_IgM	3.76	347804.1	10160.9	34.230	bb
95	95 20211111_A35_R2_IgM	3.76	183709.6	10460.6	17.562	bb
96	96 20211111_QC_R16_IgM	3.77	197385.6	10433.5	18.918	bb
97	97 20211111_A36_R2_IgM	3.75	283813.4	12504.8	22.696	bb
98	98 20211111_H18_R2_IgM	3.76	328686.5	9140.4	35.960	bb
99	99 20211111_A37_R2_IgM	3.77	83876.5	9796.9	8.562	bb
100	1... 20211111_A38_R2_IgM	3.77	191192.8	11224.3	17.034	bb
101	1... 20211111_H19_R2_IgM	3.76	423860.5	11739.2	36.106	bb
102	1... 20211111_A39_R2_IgM	3.77	143935.5	12718.9	11.317	bb
103	1... 20211111_A40_R2_IgM	3.77	239125.2	10006.1	23.898	bb
104	1... 20211111_H20_R2_IgM	3.76	489082.7	7988.2	61.226	bb
105	1... 20211111_A41_R2_IgM	3.77	163892.2	11513.3	14.235	MM
106	1... 20211111_A42_R2_IgM	3.77	92305.3	10238.5	9.015	bb
107	1... 20211111_A43_R2_IgM	3.78	237903.3	11098.9	21.435	bb
108	1... 20211111_A44_R2_IgM	3.76	112806.8	10969.9	10.283	bb
109	1... 20211111_QC_R17_IgM	3.77	154911.3	9700.8	15.969	bb
110	1... 20211111_L2_R3_IgM	3.76	170532.3	8528.0	19.997	bb
111	1... 20211111_H1_R3_IgM	3.77	162204.1	9438.2	17.186	MM
112	1... 20211111_A3_R3_IgM	3.76	176236.3	11598.6	15.195	bb

Dataset: C:\MassLynx\10.pro\20211111_clinicalsamples_IgM_IgA.qld

Last Altered: Thursday, March 09, 2023 19:58:25 台北標準時間
Printed: Friday, August 01, 2025 15:27:43 台北標準時間

	#	Name	RT	Area	IS Area	Response	Prima...
113	1...	20211111_A4_R3_IgM	3.78	288505.5	11364.1	25.388	bb
114	1...	20211111_L3_R3_IgM	3.76	97344.8	10753.4	9.053	bb
115	1...	20211111_L4_R3_IgM	3.76	104566.1	10291.6	10.160	bb
116	1...	20211111_H2_R3_IgM	3.75	377278.2	9315.8	40.499	bb
117	1...	20211111_A5_R3_IgM	3.76	186602.8	9102.2	20.501	MM
118	1...	20211111_A6_R3_IgM	3.77	176390.3	10356.4	17.032	bb
119	1...	20211111_QC_R18_IgM	3.77	161331.7	8398.3	19.210	bb
120	1...	20211111_L5_R3_IgM	3.76	141803.2	9786.7	14.489	bb
121	1...	20211111_L6_R3_IgM	3.78	34989.7	9911.3	3.530	bb
122	1...	20211111_H3_R3_IgM	3.77	184535.3	12155.5	15.181	bb
123	1...	20211111_A7_R3_IgM	3.77	449056.6	11804.8	38.040	bb
124	1...	20211111_A8_R3_IgM	3.76	182335.8	10510.6	17.348	MM
125	1...	20211111_L7_R3_IgM	3.77	200223.9	12177.9	16.442	bb
126	1...	20211111_L8_R3_IgM	3.77	78559.7	11668.6	6.733	bb
127	1...	20211111_H4_R3_IgM	3.77	102919.0	11192.0	9.196	bb
128	1...	20211111_A9_R3_IgM	3.78	228845.1	14077.4	16.256	bb
129	1...	20211111_A10_R3_IgM	3.77	400920.2	11190.4	35.827	bb
130	1...	20211111_L9_R3_IgM	3.76	269853.8	14649.3	18.421	bb
131	1...	20211111_L10_R3_IgM	3.76	131403.7	9361.3	14.037	MM
132	1...	20211111_QC_R19_IgM	3.77	175409.7	10125.5	17.324	bb
133	1...	20211111_H5_R3_IgM	3.77	79698.7	10010.9	7.961	bb
134	1...	20211111_A11_R3_IgM	3.78	61034.3	12697.2	4.807	MM
135	1...	20211111_A12_R3_IgM	3.77	219945.4	13148.5	16.728	bb
136	1...	20211111_L11_R3_IgM	3.76	94922.4	10786.6	8.800	bb
137	1...	20211111_L12_R3_IgM	3.76	68835.4	11681.8	5.893	MM
138	1...	20211111_H6_R3_IgM	3.76	267207.9	12095.5	22.092	bb
139	1...	20211111_A13_R3_IgM	3.76	169284.9	12908.3	13.114	bb
140	1...	20211111_A14_R3_IgM	3.76	226043.7	11322.6	19.964	MM
141	1...	20211111_L13_R3_IgM	3.76	112700.5	11516.1	9.786	bb
142	1...	20211111_L14_R3_IgM	3.77	91125.6	10790.7	8.445	bb
143	1...	20211111_H7_R3_IgM	3.77	134319.9	11572.1	11.607	bb
144	1...	20211111_A15_R3_IgM	3.77	233489.4	12434.4	18.778	bb
145	1...	20211111_QC_R20_IgM	3.77	172774.5	10701.0	16.146	bb
146	1...	20211111_A16_R3_IgM	3.76	157517.4	11452.1	13.754	bb
147	1...	20211111_L15_R3_IgM	3.77	157012.9	10531.1	14.909	bb
148	1...	20211111_L16_R3_IgM	3.77	189933.2	12049.4	15.763	bb
149	1...	20211111_H8_R3_IgM	3.77	136820.6	13434.9	10.184	bb
150	1...	20211111_A17_R3_IgM	3.77	167777.5	12452.0	13.474	bb
151	1...	20211111_A18_R3_IgM	3.77	189990.6	12506.5	15.191	bb
152	1...	20211111_L17_R3_IgM	3.77	180463.8	9948.9	18.139	bb
153	1...	20211111_L18_R3_IgM	3.77	59874.8	13462.1	4.448	bb
154	1...	20211111_H9_R3_IgM	3.77	238719.5	13178.7	18.114	bb
155	1...	20211111_A19_R3_IgM	3.78	309547.5	11298.6	27.397	bb
156	1...	20211111_A20_R3_IgM	3.77	56021.8	11175.6	5.013	MM
157	1...	20211111_L19_R3_IgM	3.76	88166.6	11558.2	7.628	bb
158	1...	20211111_QC_R21_IgM	3.77	180520.3	9977.0	18.094	bb
159	1...	20211111_L20_R3_IgM	3.76	126805.3	13508.1	9.387	MM
160	1...	20211111_H10_R3_IgM	3.76	91287.7	12374.8	7.377	bb
161	1...	20211111_A21_R3_IgM	3.76	308799.5	12443.6	24.816	bb
162	1...	20211111_A22_R3_IgM	3.78	66583.5	11589.3	5.745	bb
163	1...	20211111_L21_R3_IgM	3.77	57672.4	13107.4	4.400	MM
164	1...	20211111_L22_R3_IgM	3.76	122967.6	12021.5	10.229	bb
165	1...	20211111_H11_R3_IgM	3.77	433922.8	12687.8	34.200	bb
166	1...	20211111_A23_R3_IgM	3.77	248476.0	11611.8	21.399	bb
167	1...	20211111_A24_R3_IgM	3.77	53433.7	10476.8	5.100	MM
168	1...	20211111_L23_R3_IgM	3.76	281418.8	11873.1	23.702	MM

Dataset: C:\MassLynx\10.pro\20211111_clinicalsamples_IgM_IgA.qld

Last Altered: Thursday, March 09, 2023 19:58:25 台北標準時間
Printed: Friday, August 01, 2025 15:27:43 台北標準時間

	#	Name	RT	Area	IS Area	Response	Prima...
169	1...	20211111_L24_R3_IgM	3.77	64701.1	13468.0	4.804	bb
170	1...	20211111_H12_R3_IgM	3.77	96078.4	14229.1	6.752	bb
171	1...	20211111_QC_R22_IgM	3.77	183533.7	11264.4	16.293	bb
172	1...	20211111_A25_R3_IgM	3.77	145118.8	11775.7	12.324	MM
173	1...	20211111_A26_R3_IgM	3.78	97452.7	10473.9	9.304	bb
174	1...	20211111_L25_R3_IgM	3.77	205319.4	13256.8	15.488	bb
175	1...	20211111_L26_R3_IgM	3.77	80002.8	13565.0	5.898	bb
176	1...	20211111_H13_R3_IgM	3.76	173448.8	13146.0	13.194	MM
177	1...	20211111_A27_R3_IgM	3.78	74066.0	12025.6	6.159	MM
178	1...	20211111_A28_R3_IgM	3.77	73570.6	11229.1	6.552	bb
179	1...	20211111_L27_R3_IgM	3.77	99931.8	14960.0	6.680	MM
180	1...	20211111_L28_R3_IgM	3.76	103819.8	11359.0	9.140	bb
181	1...	20211111_H14_R3_IgM	3.76	262035.8	12830.8	20.422	bb
182	1...	20211111_A29_R3_IgM	3.77	173272.7	13434.3	12.898	bb
183	1...	20211111_A30_R3_IgM	3.77	170462.0	11272.2	15.122	MM
184	1...	20211111_QC_R23_IgM	3.78	178079.1	11872.2	15.000	bb
185	1...	20211111_L29_R3_IgM	3.78	43078.2	12948.7	3.327	bb
186	1...	20211111_L30_R3_IgM	3.78	51269.6	11026.6	4.650	MM
187	1...	20211111_H15_R3_IgM	3.77	143452.8	10786.3	13.300	MM
188	1...	20211111_A31_R3_IgM	3.77	242756.9	12010.5	20.212	MM
189	1...	20211111_A32_R3_IgM	3.77	331646.5	11107.7	29.857	bb
190	1...	20211111_L31_R3_IgM	3.77	194745.0	11021.8	17.669	bb
191	1...	20211111_L32_R3_IgM	3.77	163864.0	11683.8	14.025	bb
192	1...	20211111_H16_R3_IgM	3.76	175051.8	9831.7	17.805	bb
193	1...	20211111_A33_R3_IgM	3.77	99772.9	11213.1	8.898	MM
194	1...	20211111_A34_R3_IgM	3.77	168082.8	11550.1	14.553	MM
195	1...	20211111_H17_R3_IgM	3.76	280468.2	8874.4	31.604	bb
196	1...	20211111_A35_R3_IgM	3.77	158645.0	9856.3	16.096	bb
197	1...	20211111_QC_R24_IgM	3.78	141288.4	7561.8	18.684	bb
198	1...	20211111_A36_R3_IgM	3.77	249979.1	9562.8	26.141	MM
199	1...	20211111_H18_R3_IgM	3.76	289346.8	8165.3	35.436	bb
200	2...	20211111_A37_R3_IgM	3.77	66906.6	9278.8	7.211	MM
201	2...	20211111_A38_R3_IgM	3.77	160043.5	10920.3	14.656	bb
202	2...	20211111_H19_R3_IgM	3.76	342608.8	9285.3	36.898	bb
203	2...	20211111_A39_R3_IgM	3.76	114345.9	9083.4	12.588	MM
204	2...	20211111_A40_R3_IgM	3.78	215512.8	8382.4	25.710	bb
205	2...	20211111_H20_R3_IgM	3.76	392122.7	7807.9	50.221	bb
206	2...	20211111_A41_R3_IgM	3.77	95971.8	5925.2	16.197	bb
207	2...	20211111_A42_R3_IgM	3.76	77464.7	9352.6	8.283	bb
208	2...	20211111_A43_R3_IgM	3.77	204003.2	10216.3	19.968	MM
209	2...	20211111_A44_R3_IgM	3.77	97862.6	10262.7	9.536	bb
210	2...	20211111_QC_R25_IgM	3.78	138782.7	8575.3	16.184	bb

Dataset: C:\MassLynx\10.pro\20211111_clinicalsamples_IgM_IgA.qld

Last Altered: Thursday, March 09, 2023 19:58:25 台北標準時間
Printed: Friday, August 01, 2025 15:28:32 台北標準時間

Method: C:\MassLynx\10_Pro_IgA.PRO\MethDB\20211111_IgM_IgA_DMRM_new.mdb 24 Feb 2022 13:44:14
Calibration: 15 Nov 2021 14:42:11

Sample Name: 20211112_QC_R001_IgM
F40:MRM of 2 channels,ES+
952.7 > 204.1
2.406e+005

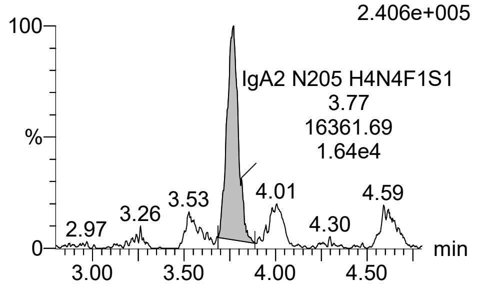

Sample Name: 20211112_QC_R001_IgM
F40:MRM of 2 channels,ES+
952.7 > 274.1
6.079e+004

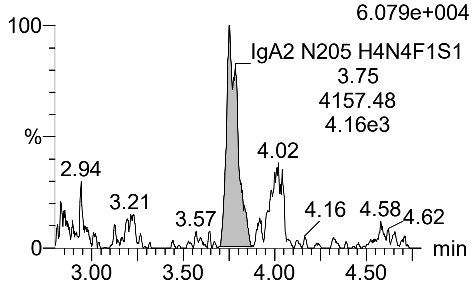

Sample Name: 20211112_QC_R002_IgM
F40:MRM of 2 channels,ES+
952.7 > 204.1
3.157e+005

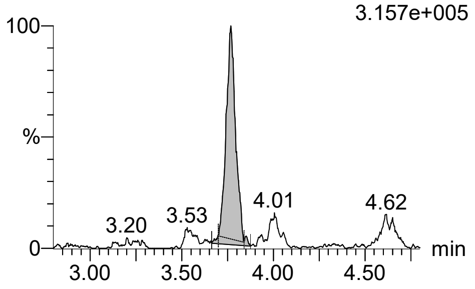

Sample Name: 20211112_QC_R002_IgM
F40:MRM of 2 channels,ES+
952.7 > 274.1
5.844e+004

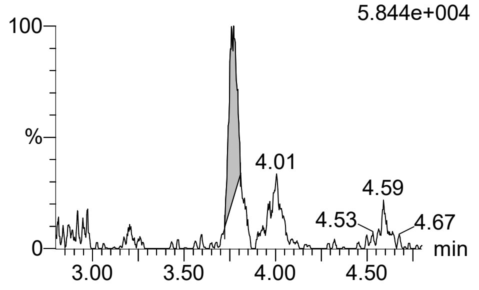

Sample Name: 20211111_A1_R2_IgM
F40:MRM of 2 channels,ES+
952.7 > 204.1
3.443e+005

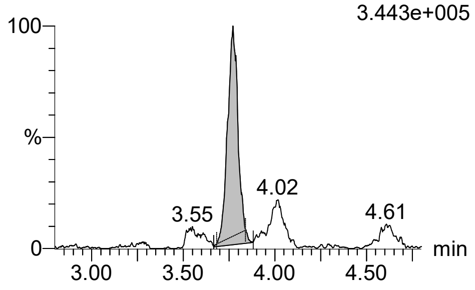

Sample Name: 20211111_A1_R2_IgM
F40:MRM of 2 channels,ES+
952.7 > 274.1
7.245e+004

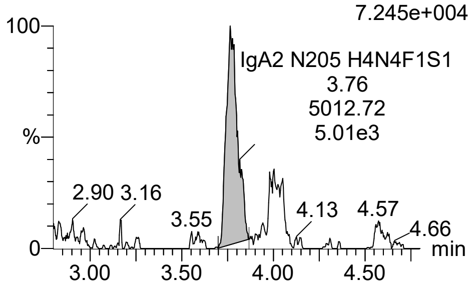

Sample Name: 20211111_A1_R3_IgM
F40:MRM of 2 channels,ES+
952.7 > 204.1
3.483e+005

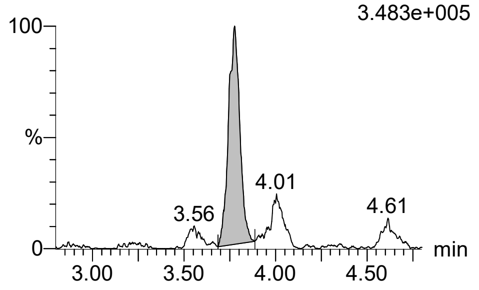

Sample Name: 20211111_A1_R3_IgM
F40:MRM of 2 channels,ES+
952.7 > 274.1
7.429e+004

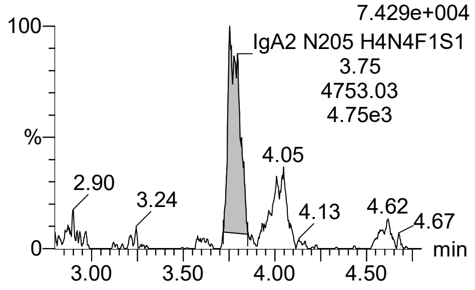

Sample Name: 20211111_A2_R2_IgM
F40:MRM of 2 channels,ES+
952.7 > 204.1
4.911e+005

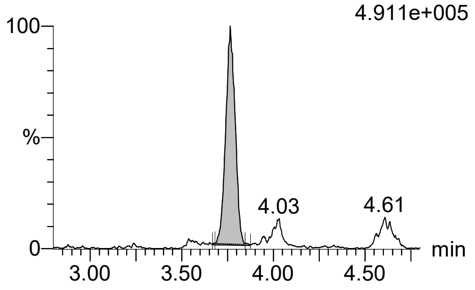

Sample Name: 20211111_A2_R2_IgM
F40:MRM of 2 channels,ES+
952.7 > 274.1
1.201e+005

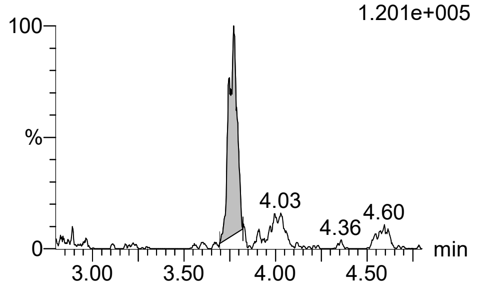

Sample Name: 20211111_A2_R3_IgM
F40:MRM of 2 channels,ES+
952.7 > 204.1
4.449e+005

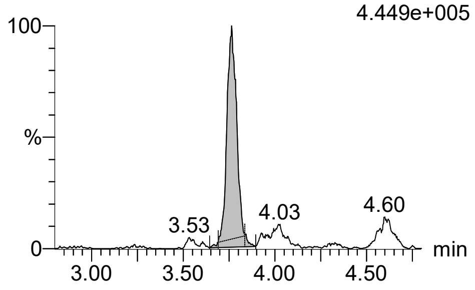

Sample Name: 20211111_A2_R3_IgM
F40:MRM of 2 channels,ES+
952.7 > 274.1
9.551e+004

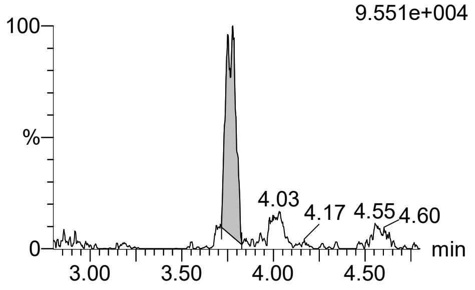

Dataset: C:\MassLynx\10.pro\20211111_clinicalsamples_IgM_IgA.qld

Last Altered: Thursday, March 09, 2023 19:58:25 台北標準時間
Printed: Friday, August 01, 2025 15:28:32 台北標準時間

Sample Name: 20211111_L1_R2_IgM
F40:MRM of 2 channels,ES+
952.7 > 204.1
1.026e+005

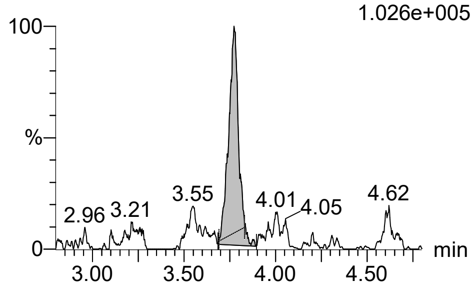

Sample Name: 20211111_L1_R2_IgM
F40:MRM of 2 channels,ES+
952.7 > 274.1
1.346e+004

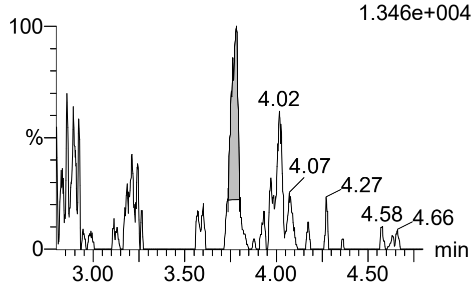

Sample Name: 20211111_L1_R3_IgM
F40:MRM of 2 channels,ES+
952.7 > 204.1
9.307e+004

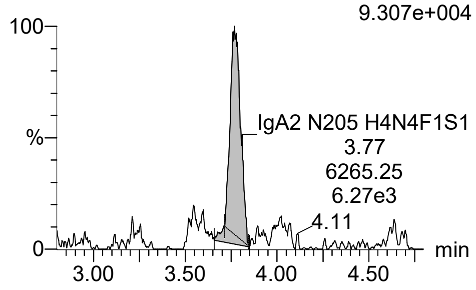

Sample Name: 20211111_L1_R3_IgM
F40:MRM of 2 channels,ES+
952.7 > 274.1
1.100e+004

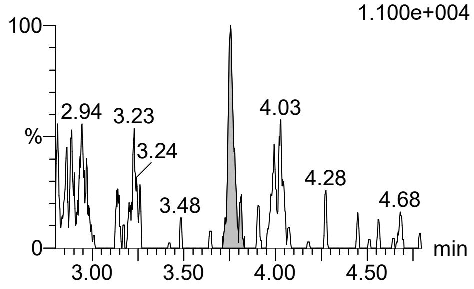

Sample Name: 20211111_L2_R2_IgM
F40:MRM of 2 channels,ES+
952.7 > 204.1
3.230e+005

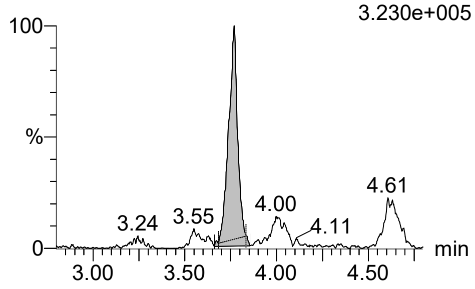

Sample Name: 20211111_L2_R2_IgM
F40:MRM of 2 channels,ES+
952.7 > 274.1
5.030e+004

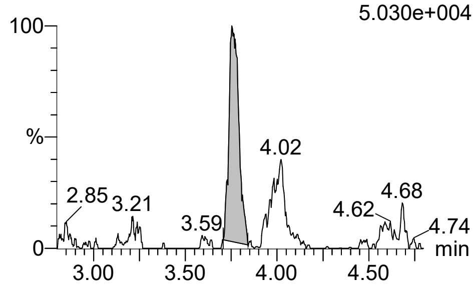

Sample Name: 20211111_H1_R2_IgM
F40:MRM of 2 channels,ES+
952.7 > 204.1
5.908e+005

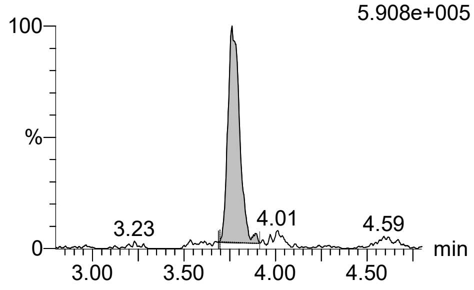

Sample Name: 20211111_H1_R2_IgM
F40:MRM of 2 channels,ES+
952.7 > 274.1
1.578e+005

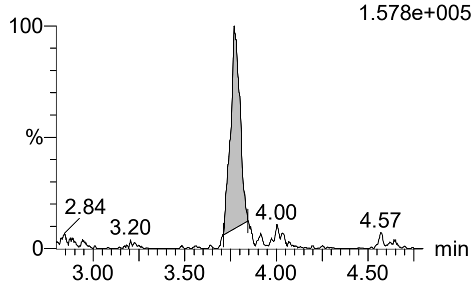

Sample Name: 20211111_A3_R2_IgM
F40:MRM of 2 channels,ES+
952.7 > 204.1
1.823e+005

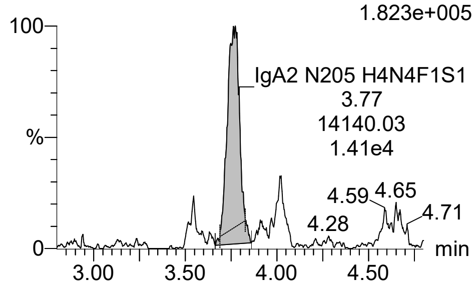

Sample Name: 20211111_A3_R2_IgM
F40:MRM of 2 channels,ES+
952.7 > 274.1
4.917e+004

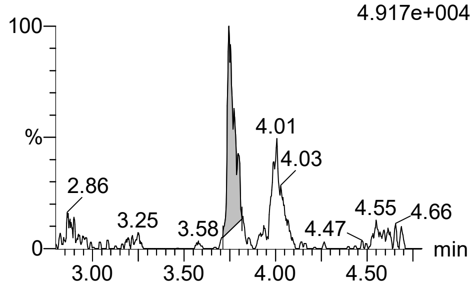

Sample Name: 20211111_A4_R2_IgM
F40:MRM of 2 channels,ES+
952.7 > 204.1
3.483e+005

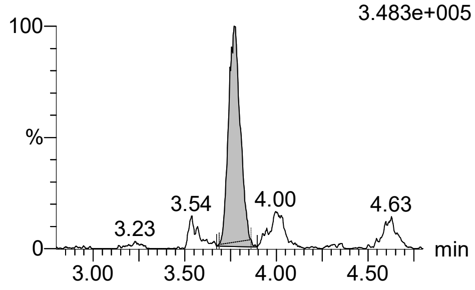

Sample Name: 20211111_A4_R2_IgM
F40:MRM of 2 channels,ES+
952.7 > 274.1
5.348e+004

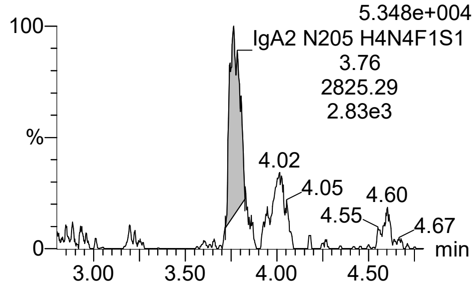

Sample Name: 20211111_L3_R2_IgM
F40:MRM of 2 channels,ES+
952.7 > 204.1
3.144e+005

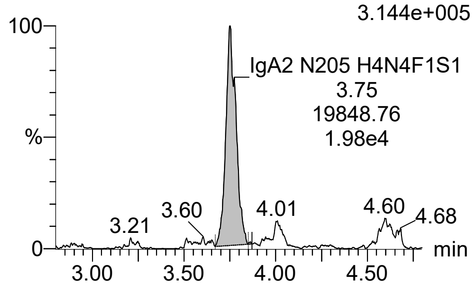

Sample Name: 20211111_L3_R2_IgM
F40:MRM of 2 channels,ES+
952.7 > 274.1
8.549e+004

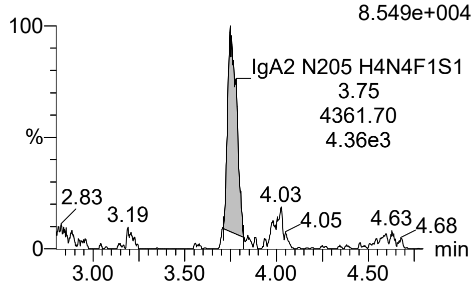

Sample Name: 20211111_L4_R2_IgM
F40:MRM of 2 channels,ES+
952.7 > 204.1
1.735e+005

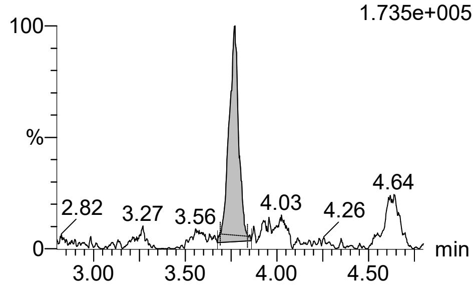

Sample Name: 20211111_L4_R2_IgM
F40:MRM of 2 channels,ES+
952.7 > 274.1
3.773e+004

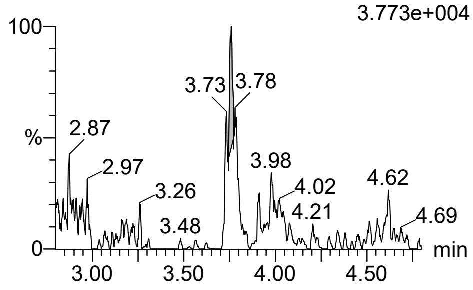

Sample Name: 20211111_H2_R2_IgM
F40:MRM of 2 channels,ES+
952.7 > 204.1
3.080e+005

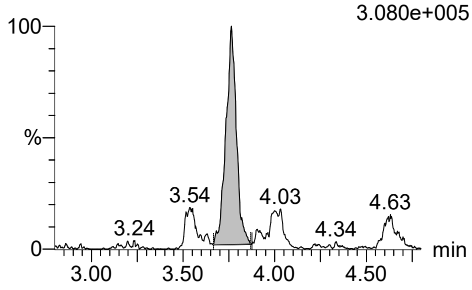

Sample Name: 20211111_H2_R2_IgM
F40:MRM of 2 channels,ES+
952.7 > 274.1
5.385e+004

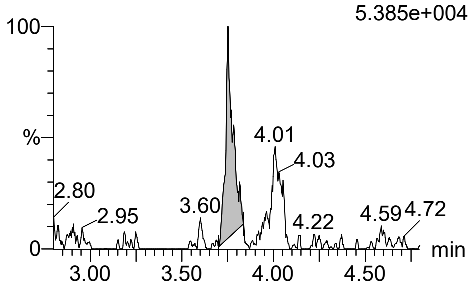

Sample Name: 20211111_A5_R2_IgM
F40:MRM of 2 channels,ES+
952.7 > 204.1
9.972e+005

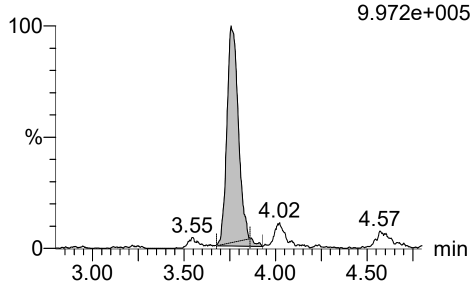

Sample Name: 20211111_A5_R2_IgM
F40:MRM of 2 channels,ES+
952.7 > 274.1
2.886e+005

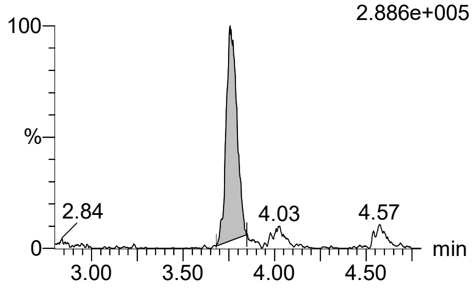

Sample Name: 20211111_A6_R2_IgM
F40:MRM of 2 channels,ES+
952.7 > 204.1
4.400e+005

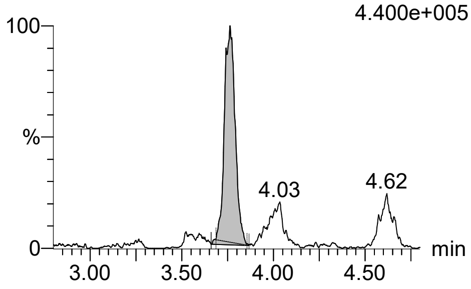

Sample Name: 20211111_A6_R2_IgM
F40:MRM of 2 channels,ES+
952.7 > 274.1
7.693e+004

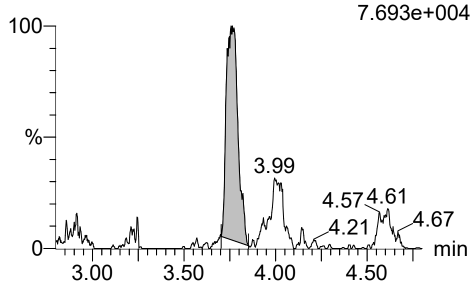

Sample Name: 20211111_QC_R10_IgM
F40:MRM of 2 channels,ES+
952.7 > 204.1
3.149e+005

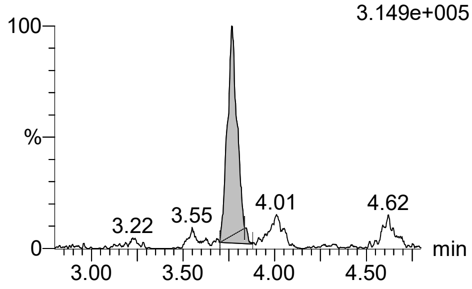

Sample Name: 20211111_QC_R10_IgM
F40:MRM of 2 channels,ES+
952.7 > 274.1
6.801e+004

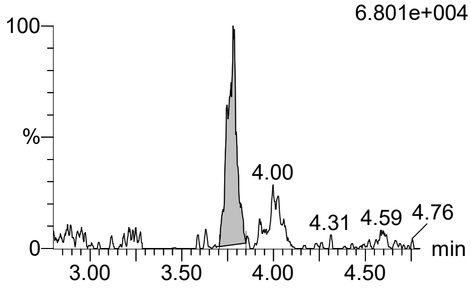

Sample Name: 20211111_L5_R2_IgM
F40:MRM of 2 channels,ES+
952.7 > 204.1
3.733e+005

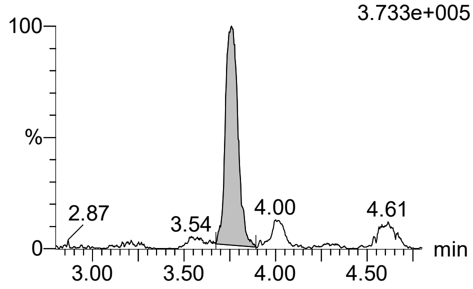

Sample Name: 20211111_L5_R2_IgM
F40:MRM of 2 channels,ES+
952.7 > 274.1
1.054e+005

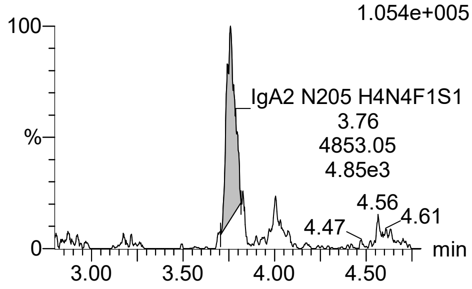

Sample Name: 20211111_L6_R2_IgM
F40:MRM of 2 channels,ES+
952.7 > 204.1
3.141e+005

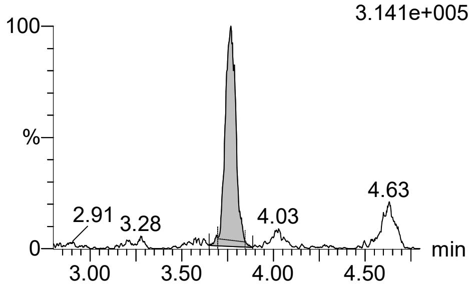

Sample Name: 20211111_L6_R2_IgM
F40:MRM of 2 channels,ES+
952.7 > 274.1
9.279e+004

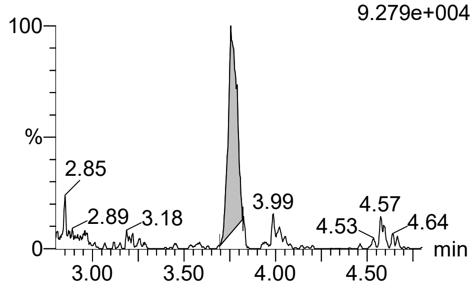

Sample Name: 20211111_H3_R2_IgM
F40:MRM of 2 channels,ES+
952.7 > 204.1
2.424e+005

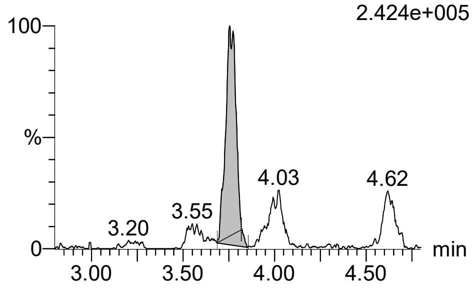

Sample Name: 20211111_H3_R2_IgM
F40:MRM of 2 channels,ES+
952.7 > 274.1
4.214e+004

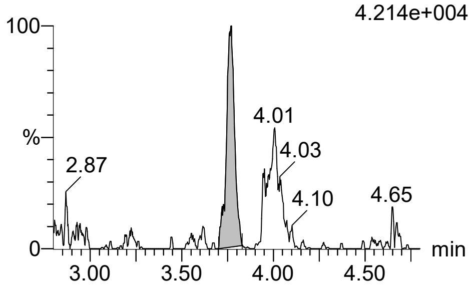

Dataset: C:\MassLynx\10.pro\20211111_clinicalsamples_IgM_IgA.qld

Last Altered: Thursday, March 09, 2023 19:58:25 台北標準時間
Printed: Friday, August 01, 2025 15:28:32 台北標準時間

Sample Name: 20211111_A7_R2_IgM

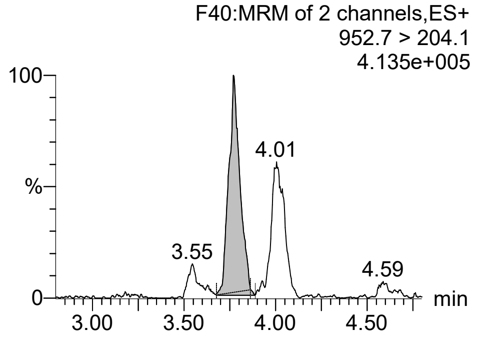

Sample Name: 20211111_A7_R2_IgM

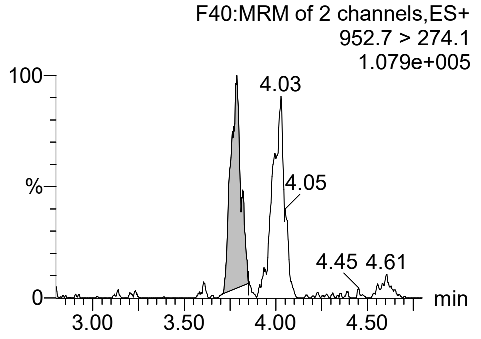

Sample Name: 20211111_A8_R2_IgM

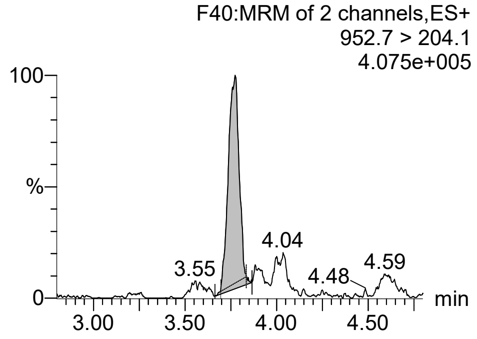

Sample Name: 20211111_A8_R2_IgM

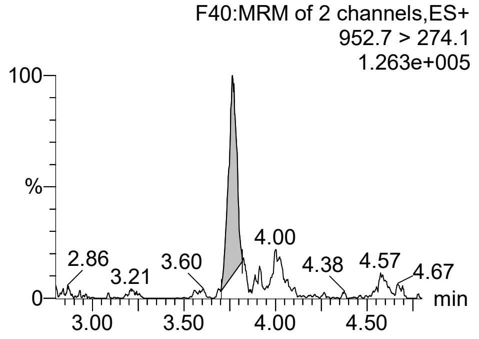

Sample Name: 20211111_L7_R2_IgM

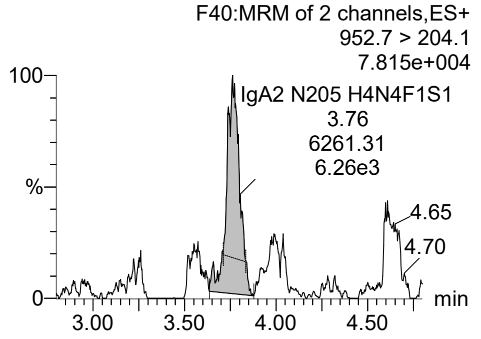

Sample Name: 20211111_L7_R2_IgM

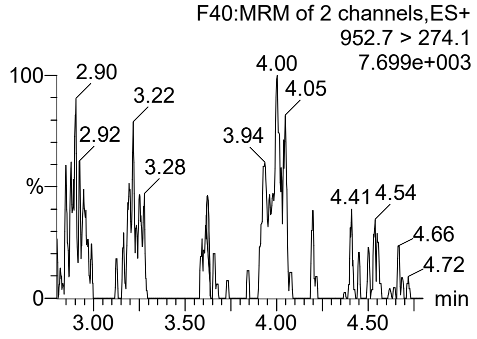

Sample Name: 20211111_L8_R2_IgM

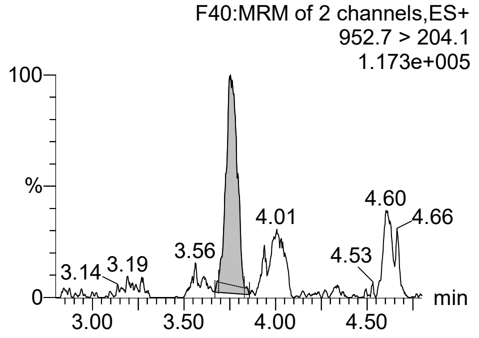

Sample Name: 20211111_L8_R2_IgM

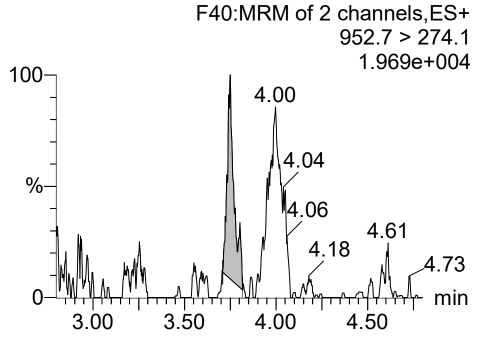

Sample Name: 20211111_H4_R2_IgM

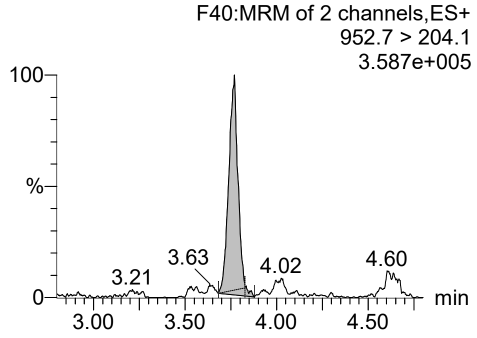

Sample Name: 20211111_H4_R2_IgM

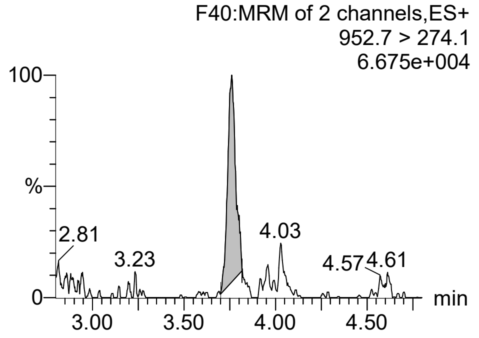

Sample Name: 20211111_A9_R2_IgM

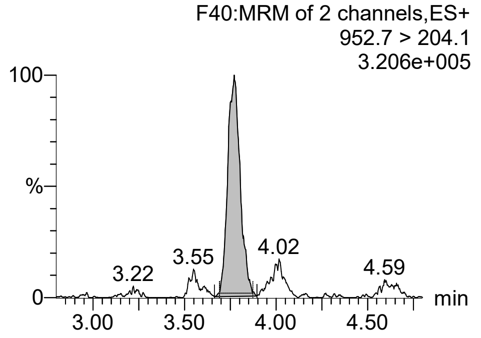

Sample Name: 20211111_A9_R2_IgM

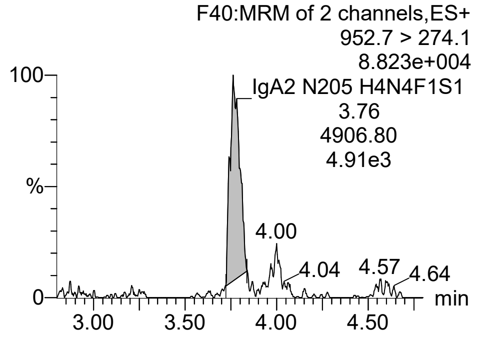

Sample Name: 20211111_A10_R2_IgM

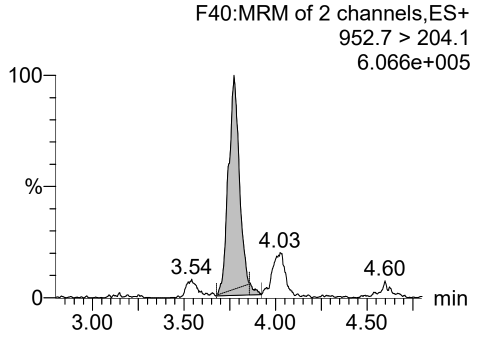

Sample Name: 20211111_A10_R2_IgM

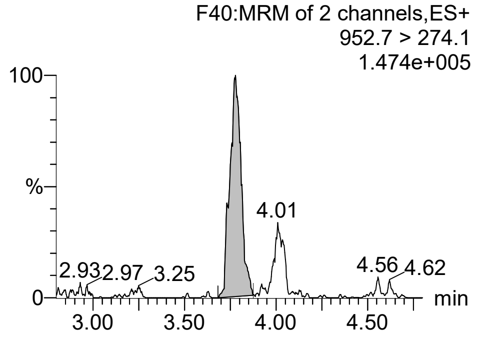

Sample Name: 20211111_L9_R2_IgM

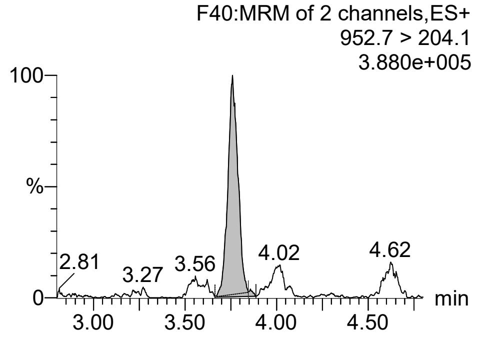

Dataset: C:\MassLynx\10.pro\20211111_clinicalsamples_IgM_IgA.qld

Last Altered: Thursday, March 09, 2023 19:58:25 台北標準時間
Printed: Friday, August 01, 2025 15:28:32 台北標準時間

Sample Name: 20211111_L9_R2_IgM
F40:MRM of 2 channels,ES+
952.7 > 274.1
5.257e+004

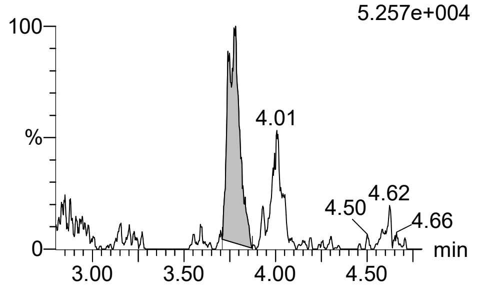

Sample Name: 20211111_L10_R2_IgM
F40:MRM of 2 channels,ES+
952.7 > 204.1
3.768e+005

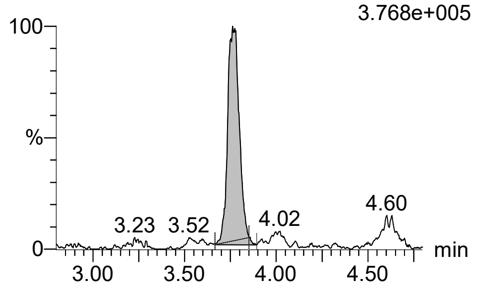

Sample Name: 20211111_L10_R2_IgM
F40:MRM of 2 channels,ES+
952.7 > 274.1
9.533e+004

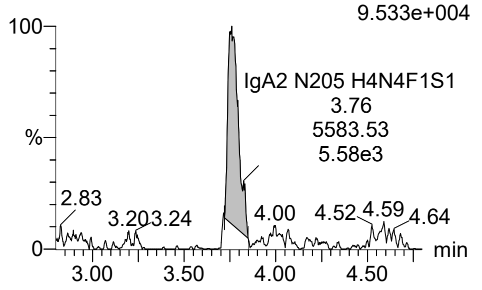

Sample Name: 20211111_QC_R11_IgM
F40:MRM of 2 channels,ES+
952.7 > 204.1
3.138e+005

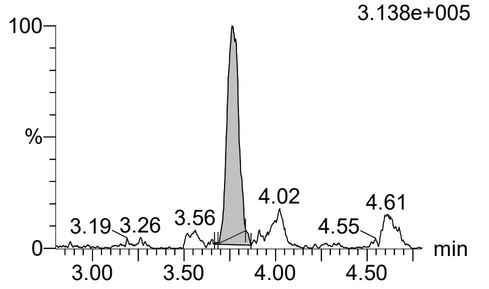

Sample Name: 20211111_QC_R11_IgM
F40:MRM of 2 channels,ES+
952.7 > 274.1
6.969e+004

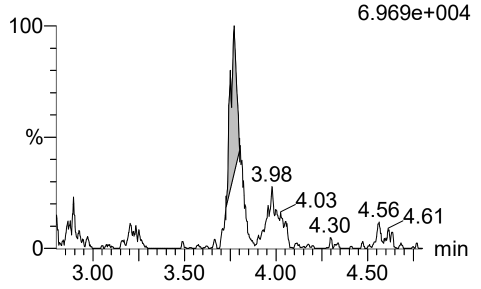

Sample Name: 20211111_H5_R2_IgM
F40:MRM of 2 channels,ES+
952.7 > 204.1
2.574e+005

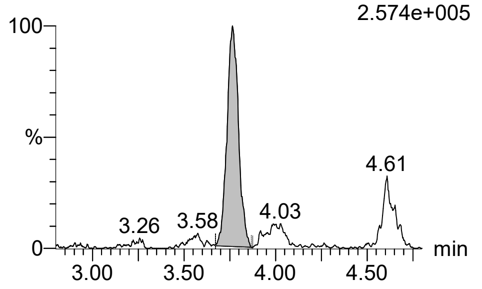

Sample Name: 20211111_H5_R2_IgM
F40:MRM of 2 channels,ES+
952.7 > 274.1
7.191e+004

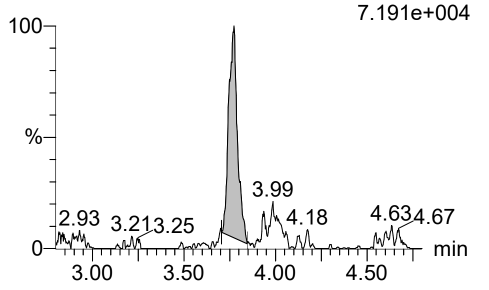

Sample Name: 20211111_A11_R2_IgM
F40:MRM of 2 channels,ES+
952.7 > 204.1
4.789e+005

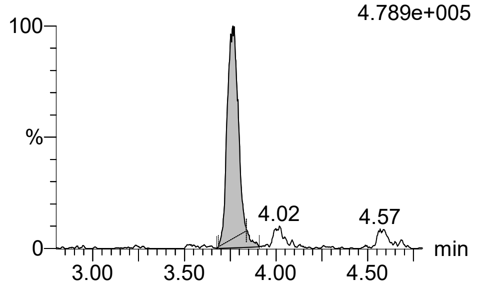

Sample Name: 20211111_A11_R2_IgM
F40:MRM of 2 channels,ES+
952.7 > 274.1
1.359e+005

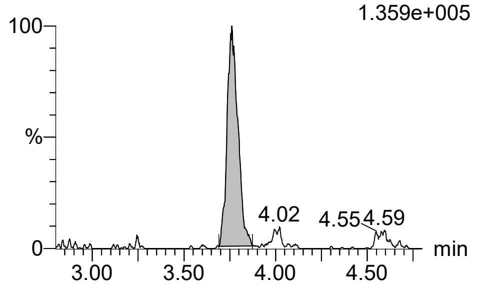

Sample Name: 20211111_A12_R2_IgM
F40:MRM of 2 channels,ES+
952.7 > 204.1
1.846e+005

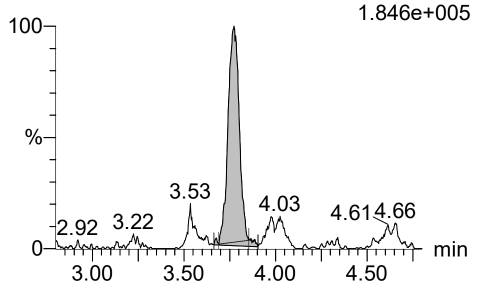

Sample Name: 20211111_A12_R2_IgM
F40:MRM of 2 channels,ES+
952.7 > 274.1
2.892e+004

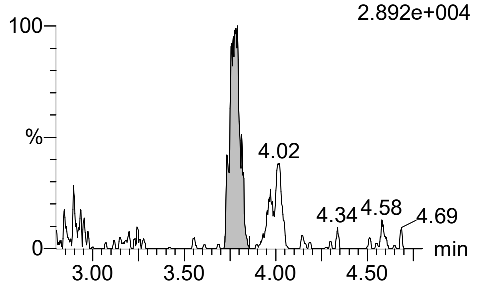

Sample Name: 20211111_L11_R2_IgM
F40:MRM of 2 channels,ES+
952.7 > 204.1
1.436e+005

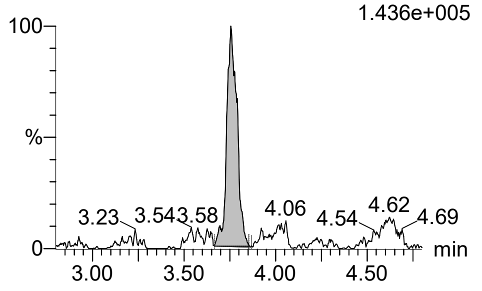

Sample Name: 20211111_L11_R2_IgM
F40:MRM of 2 channels,ES+
952.7 > 274.1
2.648e+004

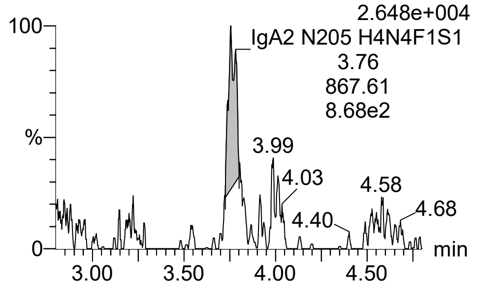

Sample Name: 20211111_L12_R2_IgM
F40:MRM of 2 channels,ES+
952.7 > 204.1
3.445e+005

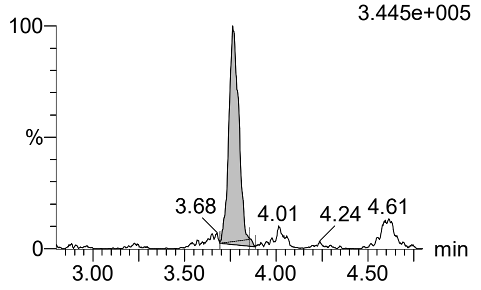

Sample Name: 20211111_L12_R2_IgM
F40:MRM of 2 channels,ES+
952.7 > 274.1
9.205e+004

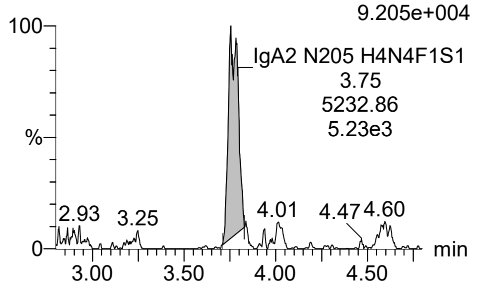

Dataset: C:\MassLynx\10.pro\20211111_clinicalsamples_IgM_IgA.qld

Last Altered: Thursday, March 09, 2023 19:58:25 台北標準時間
Printed: Friday, August 01, 2025 15:28:32 台北標準時間

Sample Name: 20211111_H6_R2_IgM
F40:MRM of 2 channels,ES+
952.7 > 204.1
2.985e+005

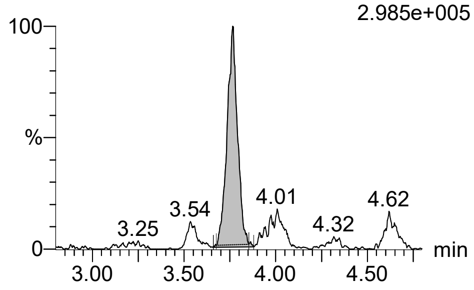

Sample Name: 20211111_H6_R2_IgM
F40:MRM of 2 channels,ES+
952.7 > 274.1
4.838e+004

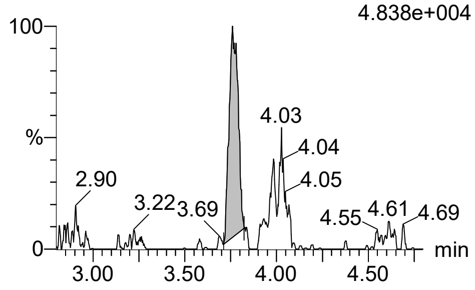

Sample Name: 20211111_A13_R2_IgM
F40:MRM of 2 channels,ES+
952.7 > 204.1
2.288e+005

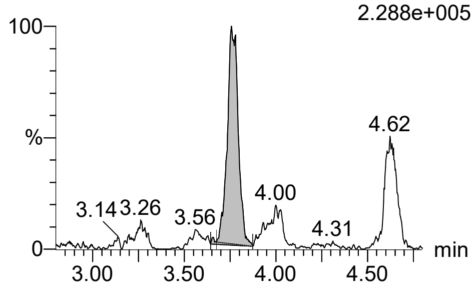

Sample Name: 20211111_A13_R2_IgM
F40:MRM of 2 channels,ES+
952.7 > 274.1
3.933e+004

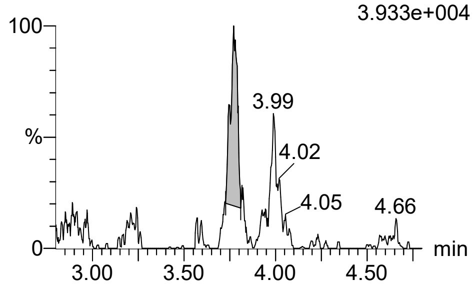

Sample Name: 20211111_A14_R2_IgM
F40:MRM of 2 channels,ES+
952.7 > 204.1
7.651e+005

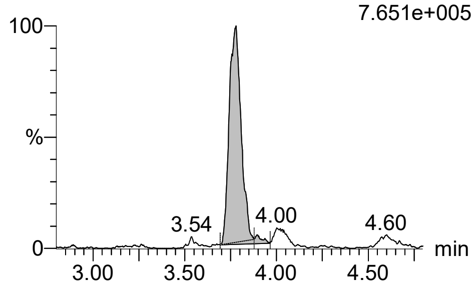

Sample Name: 20211111_A14_R2_IgM
F40:MRM of 2 channels,ES+
952.7 > 274.1
2.085e+005

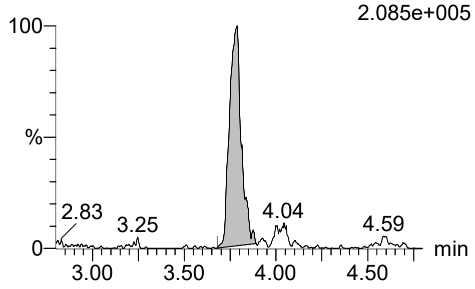

Sample Name: 20211111_L13_R2_IgM
F40:MRM of 2 channels,ES+
952.7 > 204.1
1.565e+005

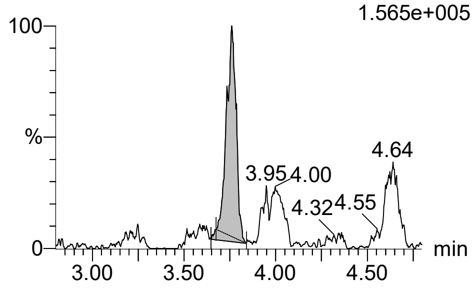

Sample Name: 20211111_L13_R2_IgM
F40:MRM of 2 channels,ES+
952.7 > 274.1
2.729e+004

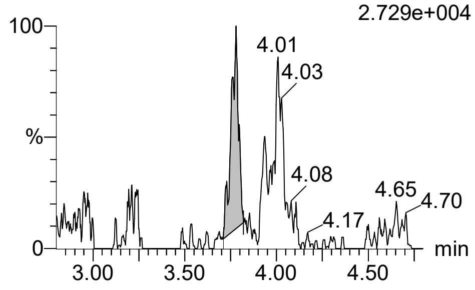

Sample Name: 20211111_L14_R2_IgM
F40:MRM of 2 channels,ES+
952.7 > 204.1
3.239e+005

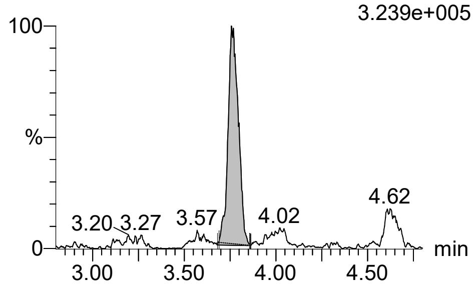

Sample Name: 20211111_L14_R2_IgM
F40:MRM of 2 channels,ES+
952.7 > 274.1
8.921e+004

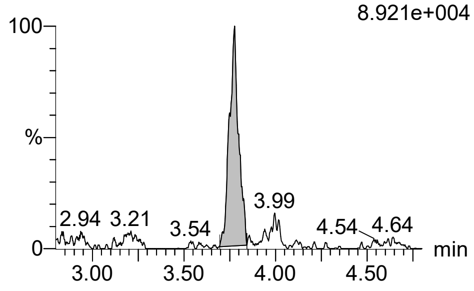

Sample Name: 20211111_H7_R2_IgM
F40:MRM of 2 channels,ES+
952.7 > 204.1
6.230e+005

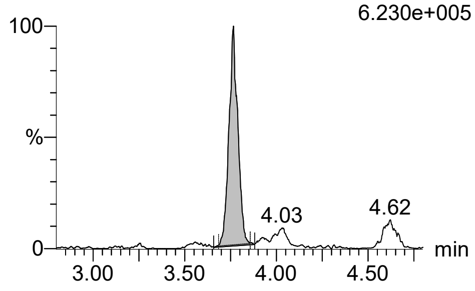

Sample Name: 20211111_H7_R2_IgM
F40:MRM of 2 channels,ES+
952.7 > 274.1
1.482e+005

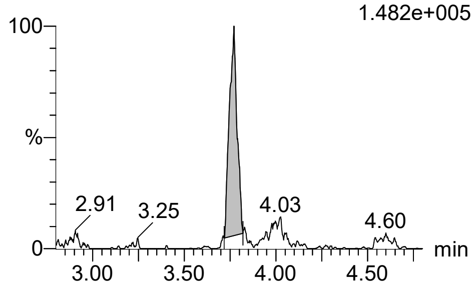

Sample Name: 20211111_A15_R2_IgM
F40:MRM of 2 channels,ES+
952.7 > 204.1
2.319e+005

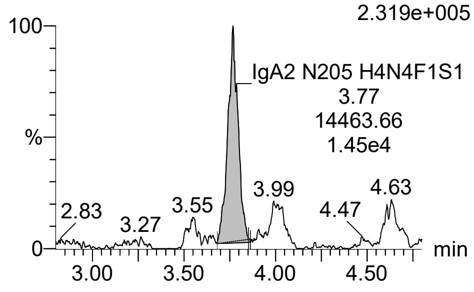

Sample Name: 20211111_A15_R2_IgM
F40:MRM of 2 channels,ES+
952.7 > 274.1
3.433e+004

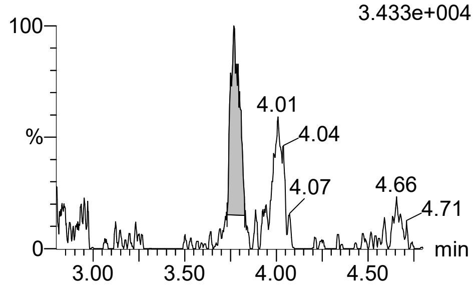

Sample Name: 20211111_QC_R12_IgM
F40:MRM of 2 channels,ES+
952.7 > 204.1
3.573e+005

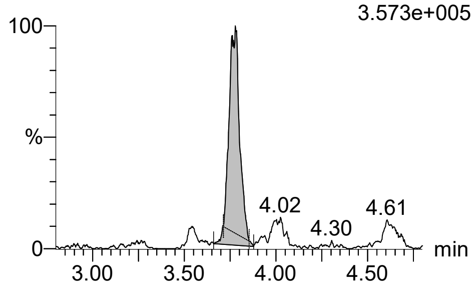

Dataset: C:\MassLynx\10.pro\20211111_clinicalsamples_IgM_IgA.qld

Last Altered: Thursday, March 09, 2023 19:58:25 台北標準時間
Printed: Friday, August 01, 2025 15:28:32 台北標準時間

Sample Name: 20211111_QC_R12_IgM
F40:MRM of 2 channels,ES+
952.7 > 274.1
9.381e+004

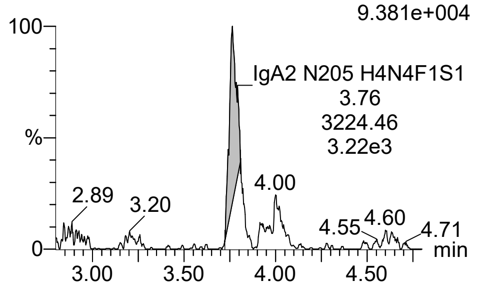

Sample Name: 20211111_A16_R2_IgM
F40:MRM of 2 channels,ES+
952.7 > 204.1
2.130e+005

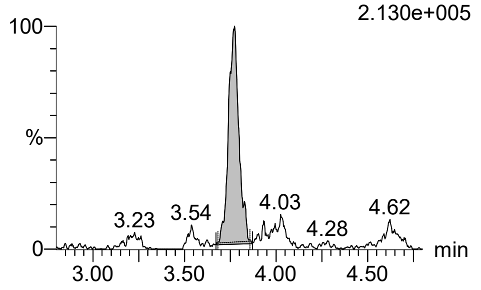

Sample Name: 20211111_A16_R2_IgM
F40:MRM of 2 channels,ES+
952.7 > 274.1
4.166e+004

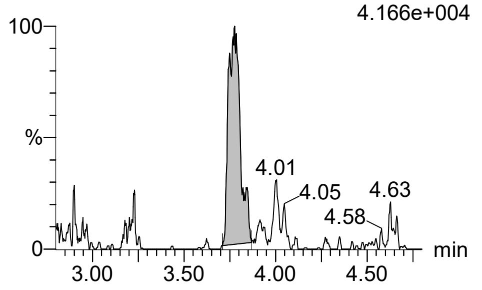

Sample Name: 20211111_L15_R2_IgM
F40:MRM of 2 channels,ES+
952.7 > 204.1
4.116e+005

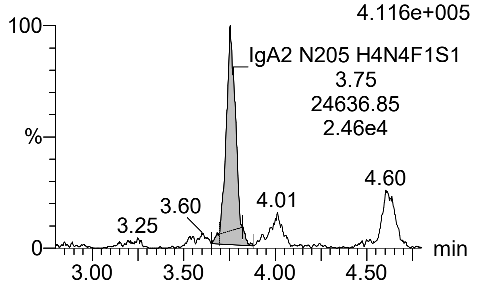

Sample Name: 20211111_L15_R2_IgM
F40:MRM of 2 channels,ES+
952.7 > 274.1
9.258e+004

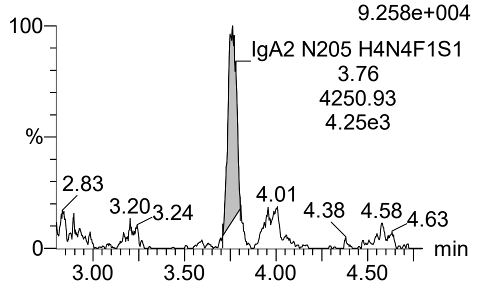

Sample Name: 20211111_L16_R2_IgM
F40:MRM of 2 channels,ES+
952.7 > 204.1
2.110e+005

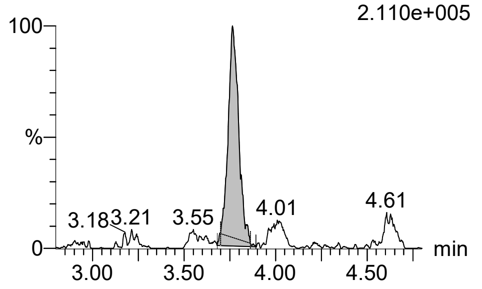

Sample Name: 20211111_L16_R2_IgM
F40:MRM of 2 channels,ES+
952.7 > 274.1
4.205e+004

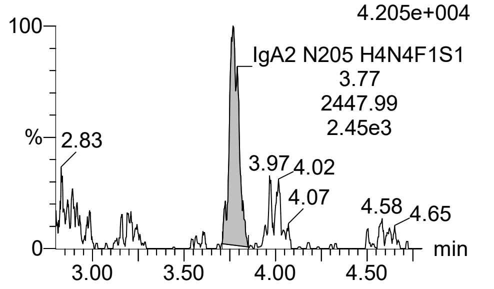

Sample Name: 20211111_H8_R2_IgM
F40:MRM of 2 channels,ES+
952.7 > 204.1
1.812e+005

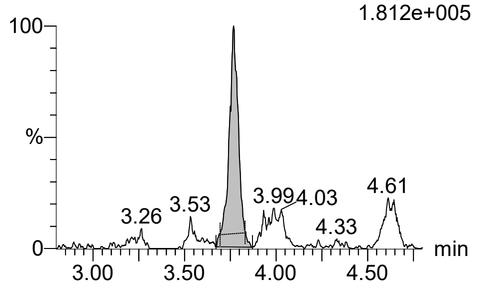

Sample Name: 20211111_H8_R2_IgM
F40:MRM of 2 channels,ES+
952.7 > 274.1
2.144e+004

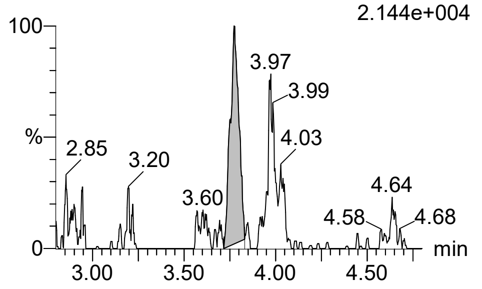

Sample Name: 20211111_A17_R2_IgM
F40:MRM of 2 channels,ES+
952.7 > 204.1
8.932e+005

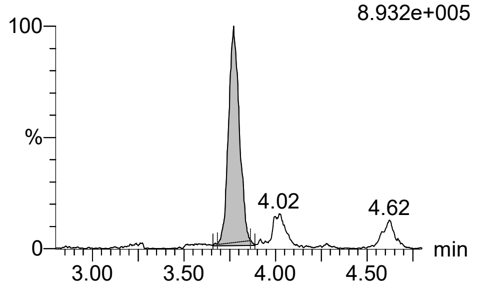

Sample Name: 20211111_A17_R2_IgM
F40:MRM of 2 channels,ES+
952.7 > 274.1
2.368e+005

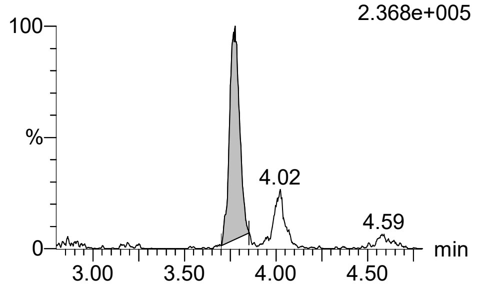

Sample Name: 20211111_A18_R2_IgM
F40:MRM of 2 channels,ES+
952.7 > 204.1
2.348e+005

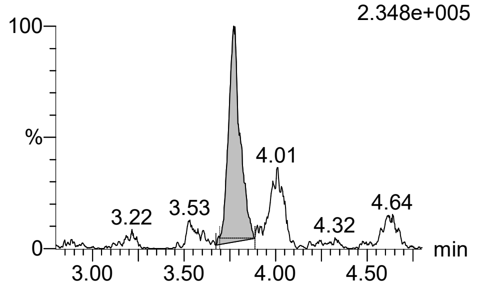

Sample Name: 20211111_A18_R2_IgM
F40:MRM of 2 channels,ES+
952.7 > 274.1
4.684e+004

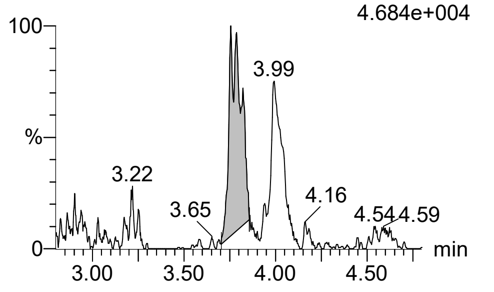

Sample Name: 20211111_L17_R2_IgM
F40:MRM of 2 channels,ES+
952.7 > 204.1
2.747e+005

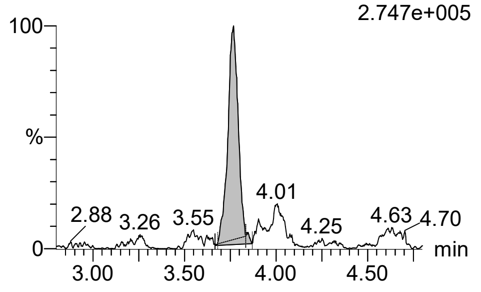

Sample Name: 20211111_L17_R2_IgM
F40:MRM of 2 channels,ES+
952.7 > 274.1
5.916e+004

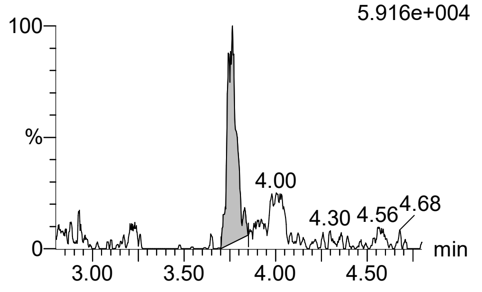

Dataset: C:\MassLynx\10.pro\20211111_clinicalsamples_IgM_IgA.qld

Last Altered: Thursday, March 09, 2023 19:58:25 台北標準時間
Printed: Friday, August 01, 2025 15:28:32 台北標準時間

Sample Name: 20211111_L18_R2_IgM
F40:MRM of 2 channels,ES+
952.7 > 204.1
5.962e+004

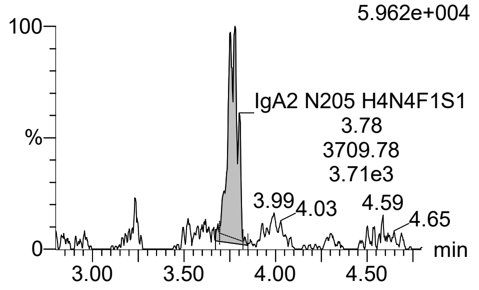

Sample Name: 20211111_L18_R2_IgM
F40:MRM of 2 channels,ES+
952.7 > 274.1
1.486e+004

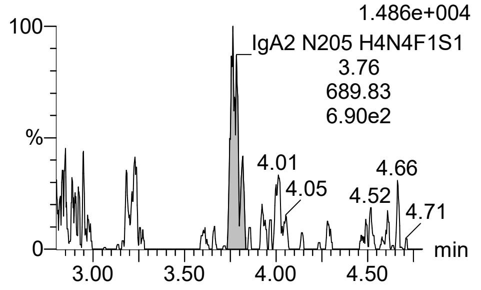

Sample Name: 20211111_H9_R2_IgM
F40:MRM of 2 channels,ES+
952.7 > 204.1
2.489e+005

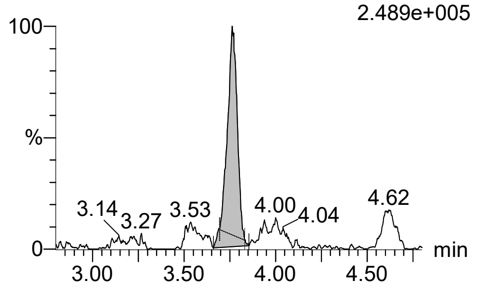

Sample Name: 20211111_H9_R2_IgM
F40:MRM of 2 channels,ES+
952.7 > 274.1
2.590e+004

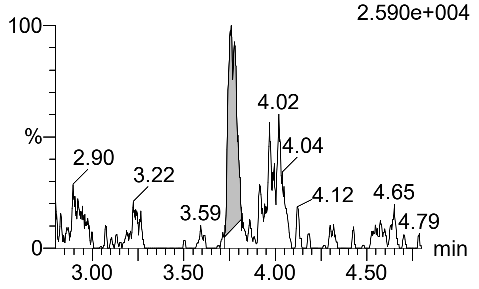

Sample Name: 20211111_A19_R2_IgM
F40:MRM of 2 channels,ES+
952.7 > 204.1
4.992e+005

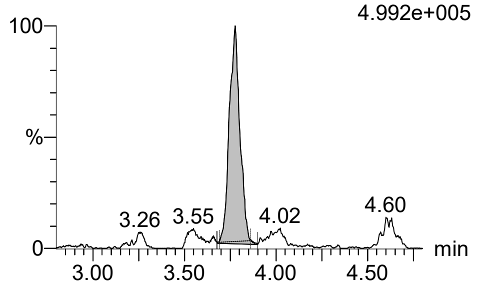

Sample Name: 20211111_A19_R2_IgM
F40:MRM of 2 channels,ES+
952.7 > 274.1
1.131e+005

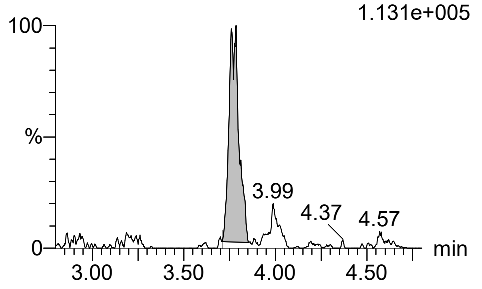

Sample Name: 20211111_A20_R2_IgM
F40:MRM of 2 channels,ES+
952.7 > 204.1
1.507e+005

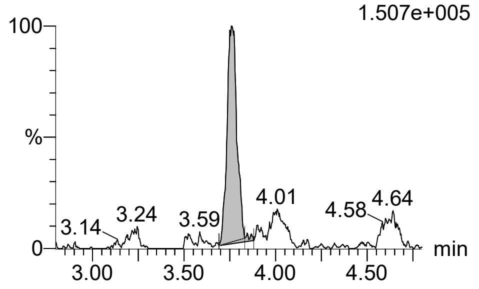

Sample Name: 20211111_A20_R2_IgM
F40:MRM of 2 channels,ES+
952.7 > 274.1
3.630e+004

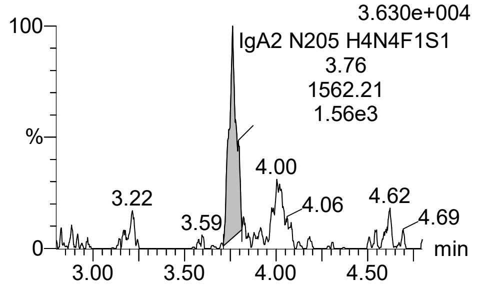

Sample Name: 20211111_L19_R2_IgM
F40:MRM of 2 channels,ES+
952.7 > 204.1
1.785e+005

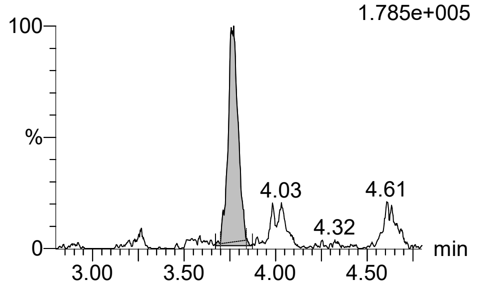

Sample Name: 20211111_L19_R2_IgM
F40:MRM of 2 channels,ES+
952.7 > 274.1
4.095e+004

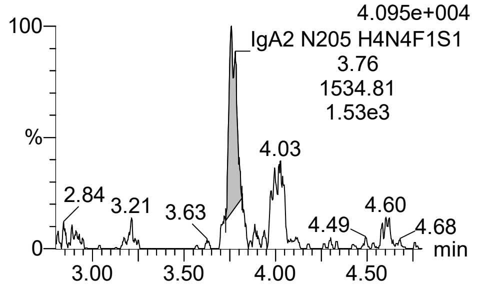

Sample Name: 20211111_QC_R13_IgM
F40:MRM of 2 channels,ES+
952.7 > 204.1
3.591e+005

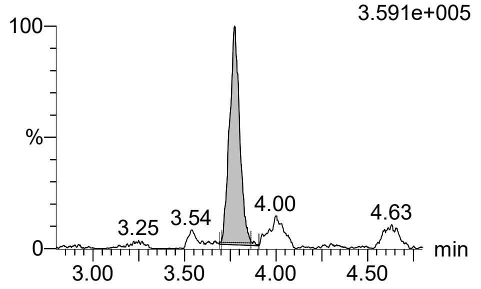

Sample Name: 20211111_QC_R13_IgM
F40:MRM of 2 channels,ES+
952.7 > 274.1
7.458e+004

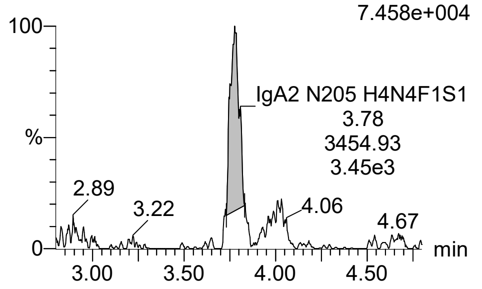

Sample Name: 20211111_L20_R2_IgM
F40:MRM of 2 channels,ES+
952.7 > 204.1
4.247e+005

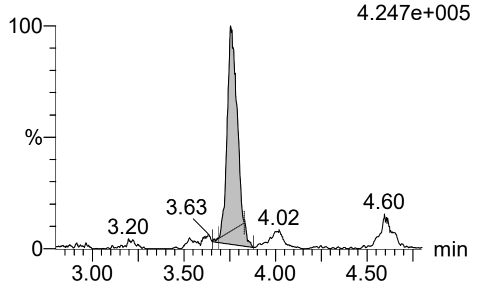

Sample Name: 20211111_L20_R2_IgM
F40:MRM of 2 channels,ES+
952.7 > 274.1
1.077e+005

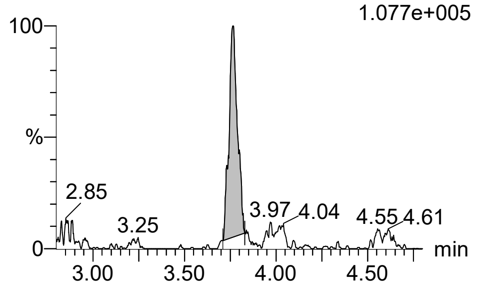

Sample Name: 20211111_H10_R2_IgM
F40:MRM of 2 channels,ES+
952.7 > 204.1
4.614e+005

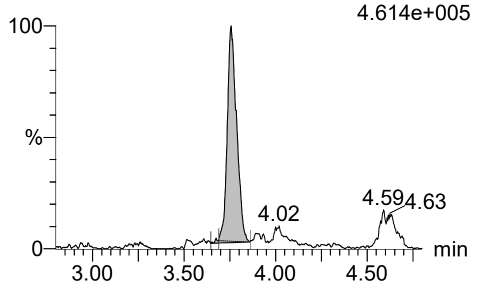

Sample Name: 20211111_H10_R2_IgM
F40:MRM of 2 channels,ES+
952.7 > 274.1
1.027e+005

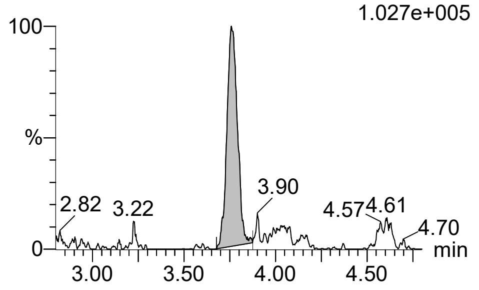

Sample Name: 20211111_A21_R2_IgM
F40:MRM of 2 channels,ES+
952.7 > 204.1
1.899e+005

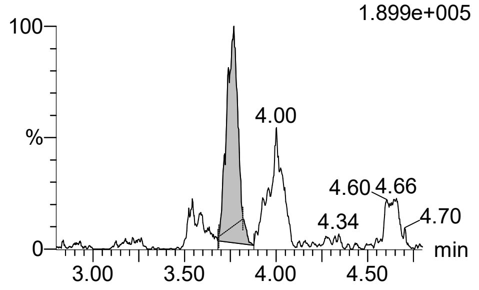

Sample Name: 20211111_A21_R2_IgM
F40:MRM of 2 channels,ES+
952.7 > 274.1
3.742e+004

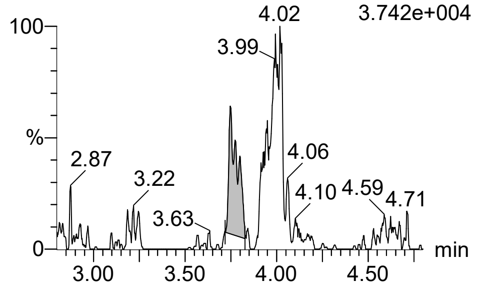

Sample Name: 20211111_A22_R2_IgM
F40:MRM of 2 channels,ES+
952.7 > 204.1
7.359e+004

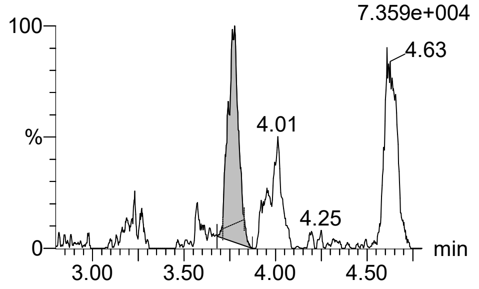

Sample Name: 20211111_A22_R2_IgM
F40:MRM of 2 channels,ES+
952.7 > 274.1
1.202e+004

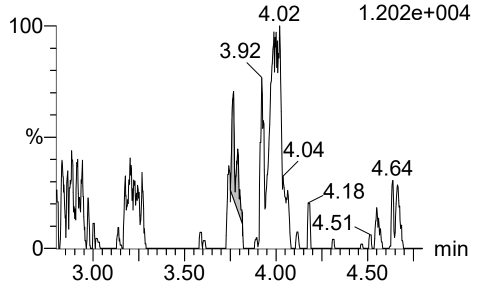

Sample Name: 20211111_L21_R2_IgM
F40:MRM of 2 channels,ES+
952.7 > 204.1
1.200e+005

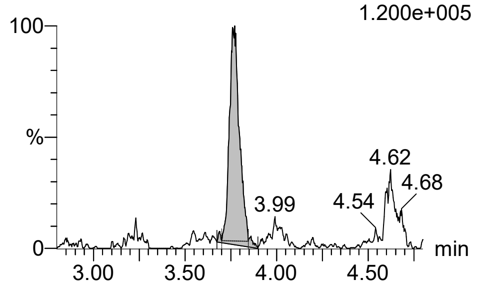

Sample Name: 20211111_L21_R2_IgM
F40:MRM of 2 channels,ES+
952.7 > 274.1
2.091e+004

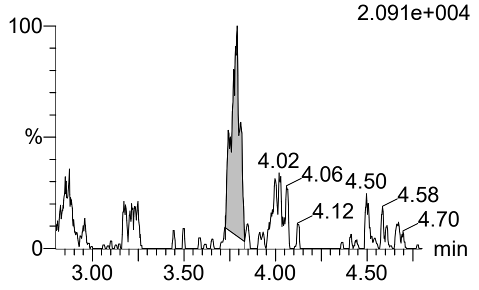

Sample Name: 20211111_L22_R2_IgM
F40:MRM of 2 channels,ES+
952.7 > 204.1
2.890e+005

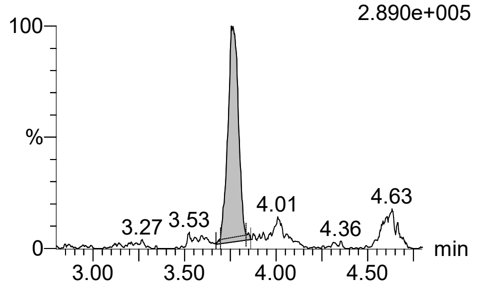

Sample Name: 20211111_L22_R2_IgM
F40:MRM of 2 channels,ES+
952.7 > 274.1
7.346e+004

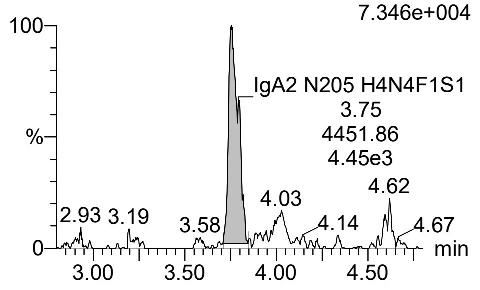

Sample Name: 20211111_H11_R2_IgM
F40:MRM of 2 channels,ES+
952.7 > 204.1
3.681e+005

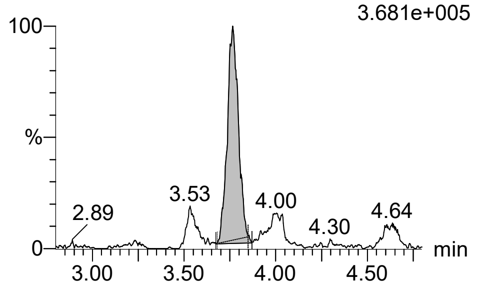

Sample Name: 20211111_H11_R2_IgM
F40:MRM of 2 channels,ES+
952.7 > 274.1
7.338e+004

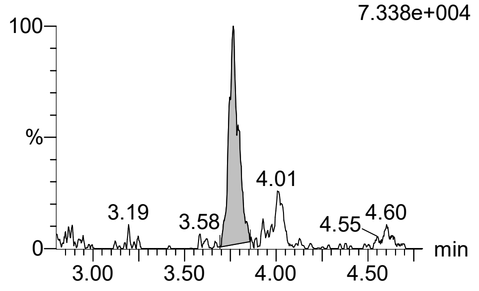

Sample Name: 20211111_A23_R2_IgM
F40:MRM of 2 channels,ES+
952.7 > 204.1
3.575e+005

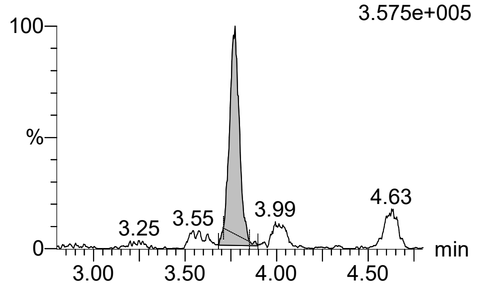

Sample Name: 20211111_A23_R2_IgM
F40:MRM of 2 channels,ES+
952.7 > 274.1
8.733e+004

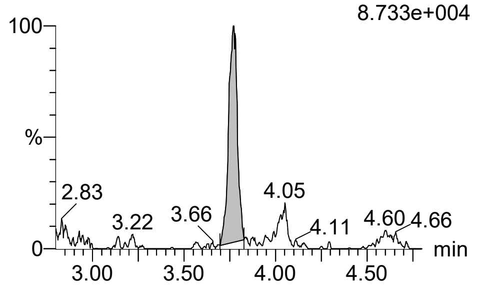

Sample Name: 20211111_A24_R2_IgM
F40:MRM of 2 channels,ES+
952.7 > 204.1
3.276e+005

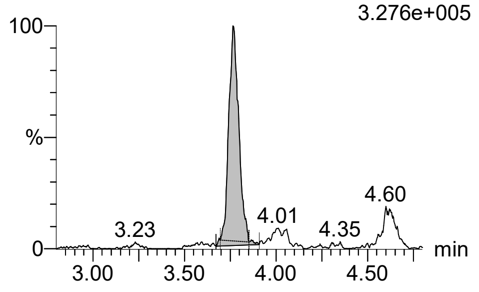

Sample Name: 20211111_A24_R2_IgM
F40:MRM of 2 channels,ES+
952.7 > 274.1
1.192e+005

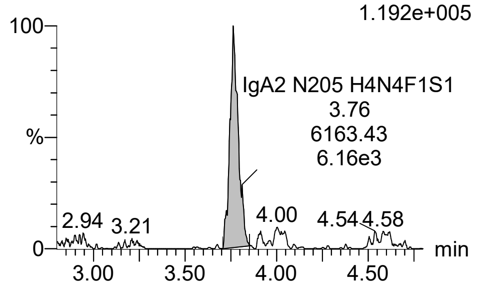

Sample Name: 20211111_L23_R2_IgM

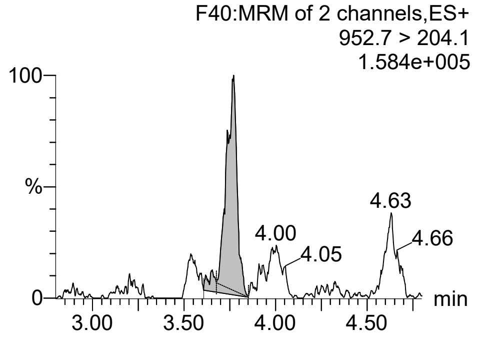

Sample Name: 20211111_L23_R2_IgM

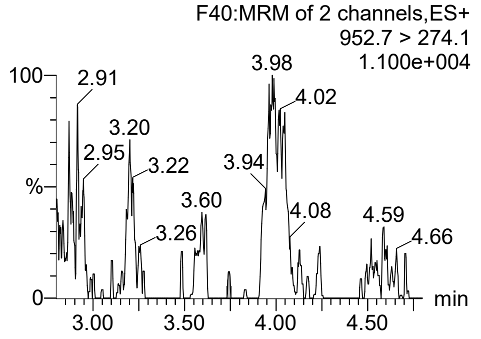

Sample Name: 20211111_L24_R2_IgM

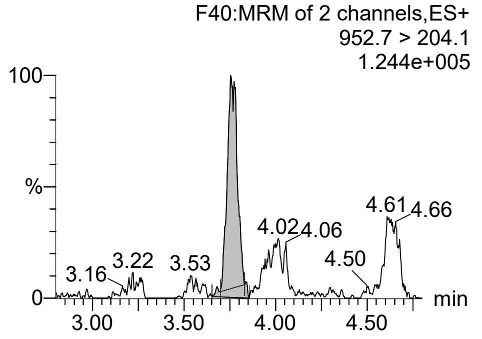

Sample Name: 20211111_L24_R2_IgM

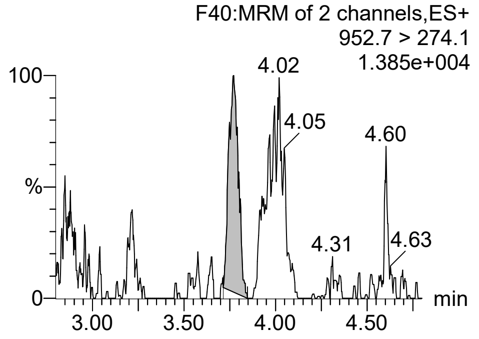

Sample Name: 20211111_H12_R2_IgM

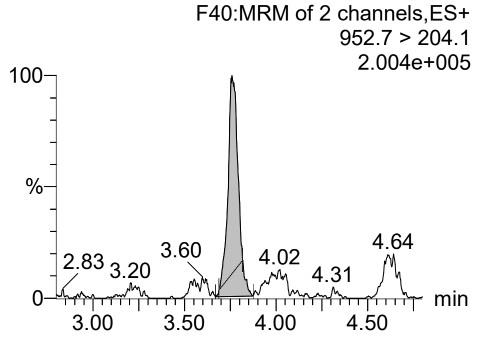

Sample Name: 20211111_H12_R2_IgM

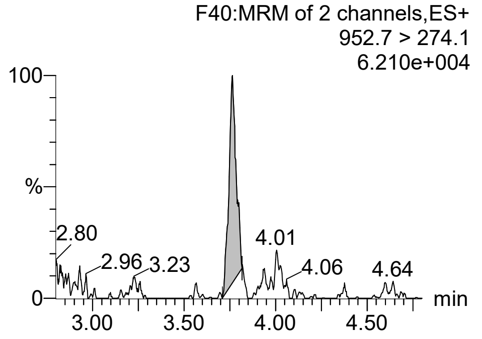

Sample Name: 20211111_QC_R14_IgM

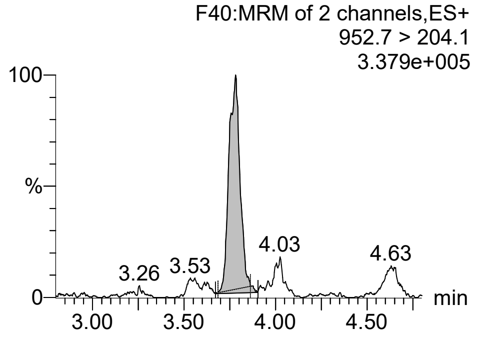

Sample Name: 20211111_QC_R14_IgM

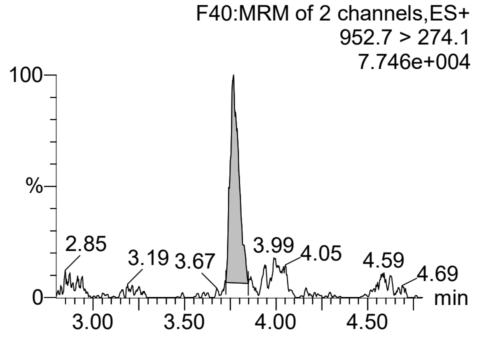

Sample Name: 20211111_A25_R2_IgM

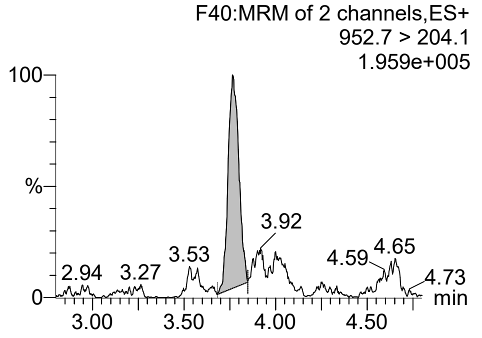

Sample Name: 20211111_A25_R2_IgM

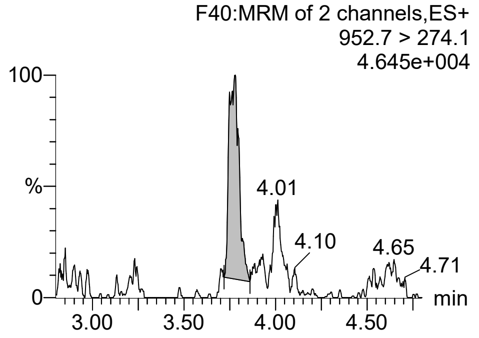

Sample Name: 20211111_A26_R2_IgM

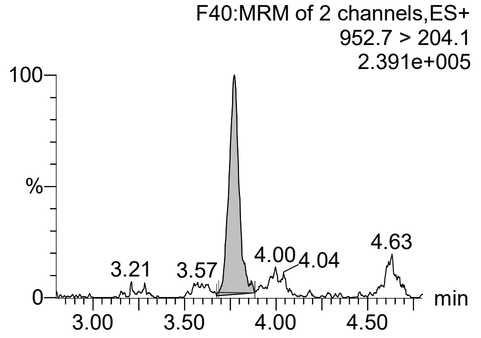

Sample Name: 20211111_A26_R2_IgM

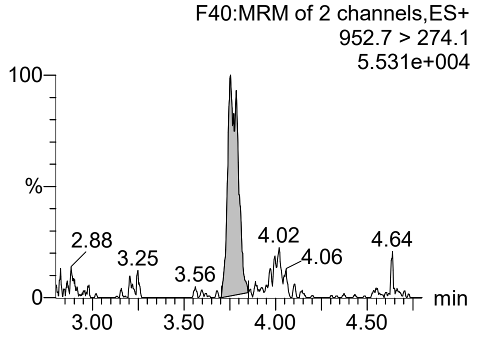

Sample Name: 20211111_L25_R2_IgM

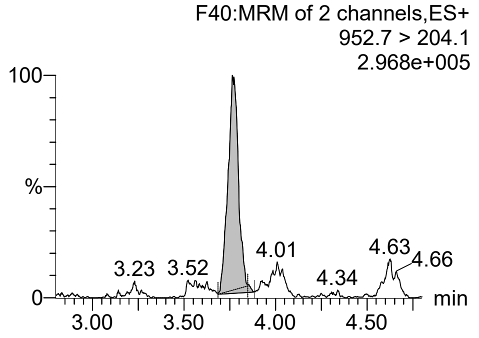

Sample Name: 20211111_L25_R2_IgM

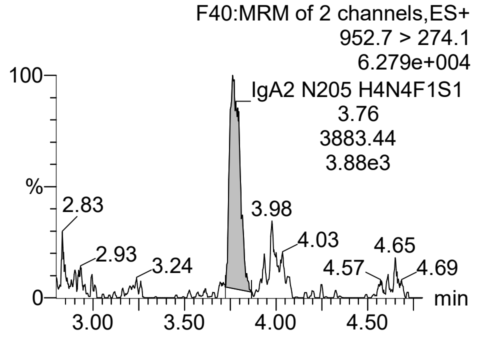

Sample Name: 20211111_L26_R2_IgM

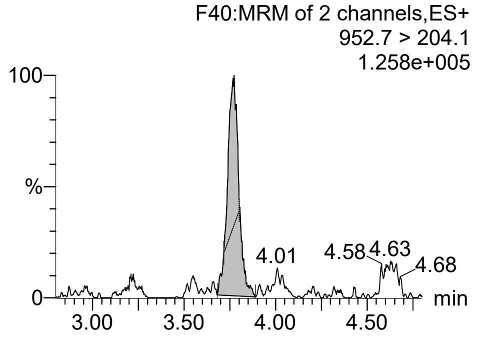

Sample Name: 20211111_L26_R2_IgM
F40:MRM of 2 channels,ES+
952.7 > 274.1
2.568e+004

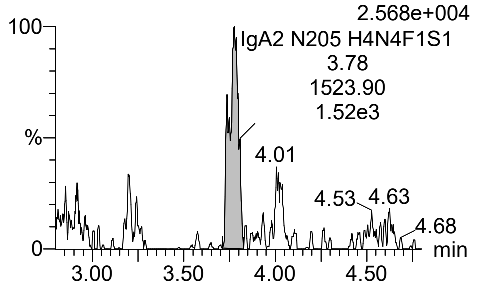

Sample Name: 20211111_H13_R2_IgM
F40:MRM of 2 channels,ES+
952.7 > 204.1
3.299e+005

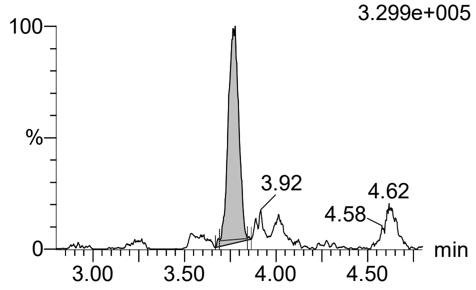

Sample Name: 20211111_H13_R2_IgM
F40:MRM of 2 channels,ES+
952.7 > 274.1
8.897e+004

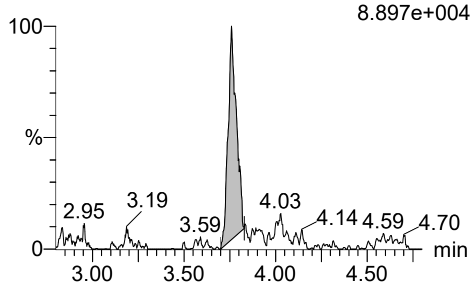

Sample Name: 20211111_A27_R2_IgM
F40:MRM of 2 channels,ES+
952.7 > 204.1
5.394e+005

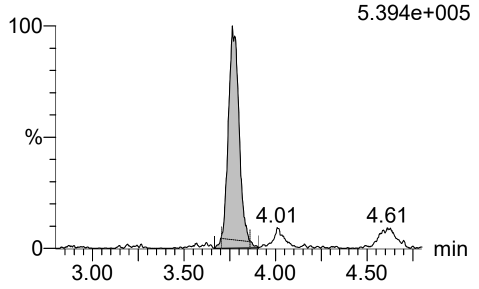

Sample Name: 20211111_A27_R2_IgM
F40:MRM of 2 channels,ES+
952.7 > 274.1
1.653e+005

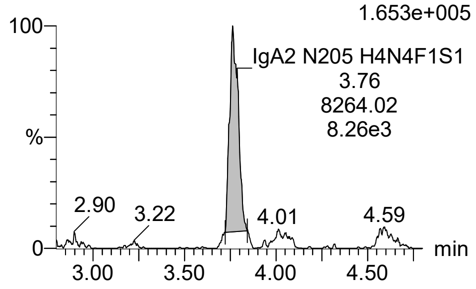

Sample Name: 20211111_A28_R2_IgM
F40:MRM of 2 channels,ES+
952.7 > 204.1
2.191e+005

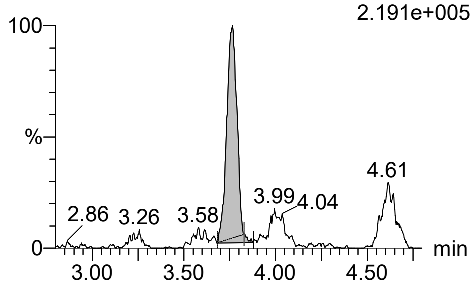

Sample Name: 20211111_A28_R2_IgM
F40:MRM of 2 channels,ES+
952.7 > 274.1
5.954e+004

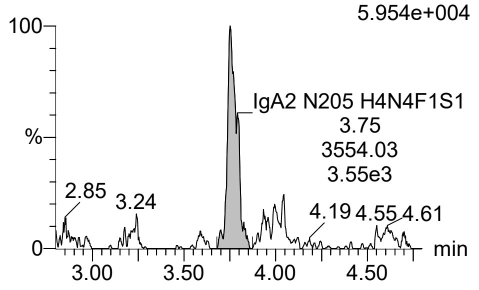

Sample Name: 20211111_L27_R2_IgM
F40:MRM of 2 channels,ES+
952.7 > 204.1
4.267e+005

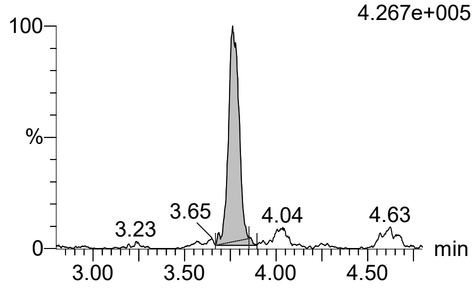

Sample Name: 20211111_L27_R2_IgM
F40:MRM of 2 channels,ES+
952.7 > 274.1
1.122e+005

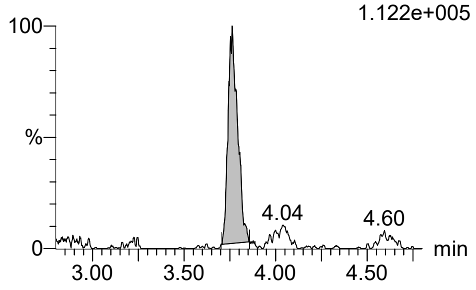

Sample Name: 20211111_L28_R2_IgM
F40:MRM of 2 channels,ES+
952.7 > 204.1
1.092e+005

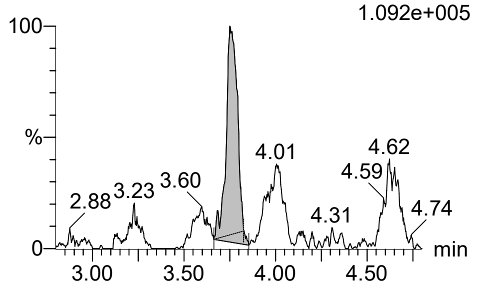

Sample Name: 20211111_L28_R2_IgM
F40:MRM of 2 channels,ES+
952.7 > 274.1
1.967e+004

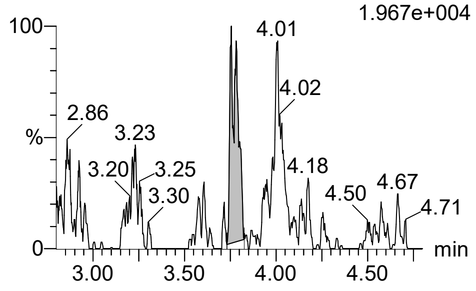

Sample Name: 20211111_H14_R2_IgM
F40:MRM of 2 channels,ES+
952.7 > 204.1
3.049e+005

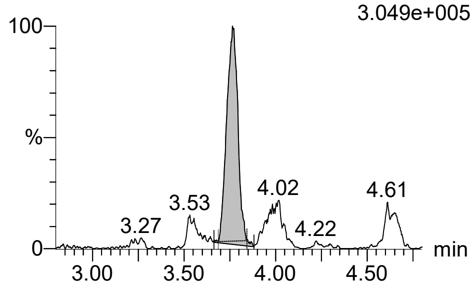

Sample Name: 20211111_H14_R2_IgM
F40:MRM of 2 channels,ES+
952.7 > 274.1
4.901e+004

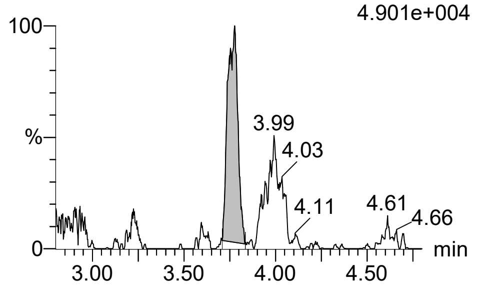

Sample Name: 20211111_A29_R2_IgM
F40:MRM of 2 channels,ES+
952.7 > 204.1
9.613e+004

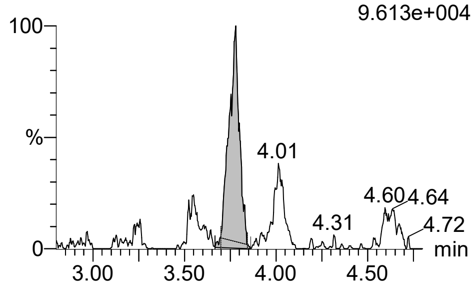

Sample Name: 20211111_A29_R2_IgM
F40:MRM of 2 channels,ES+
952.7 > 274.1
9.100e+003

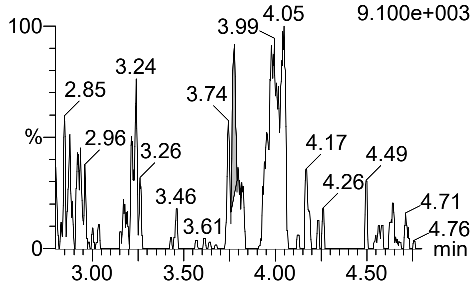

Dataset: C:\MassLynx\10.pro\20211111_clinicalsamples_IgM_IgA.qld

Last Altered: Thursday, March 09, 2023 19:58:25 台北標準時間
Printed: Friday, August 01, 2025 15:28:32 台北標準時間

Sample Name: 20211111_A30_R2_IgM
F40:MRM of 2 channels,ES+
952.7 > 204.1
4.519e+005

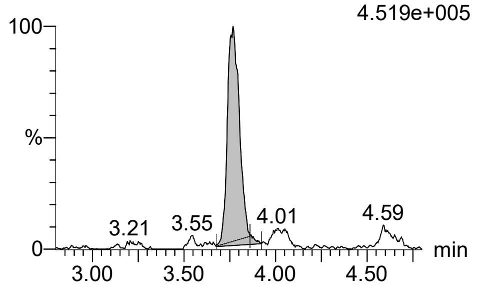

Sample Name: 20211111_A30_R2_IgM
F40:MRM of 2 channels,ES+
952.7 > 274.1
1.369e+005

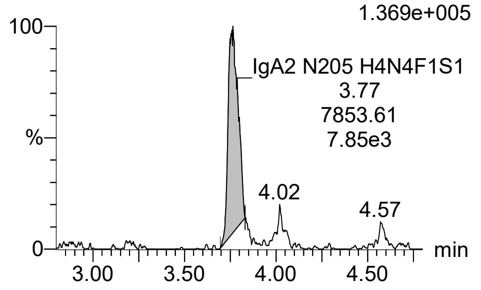

Sample Name: 20211111_QC_R15_IgM
F40:MRM of 2 channels,ES+
952.7 > 204.1
3.314e+005

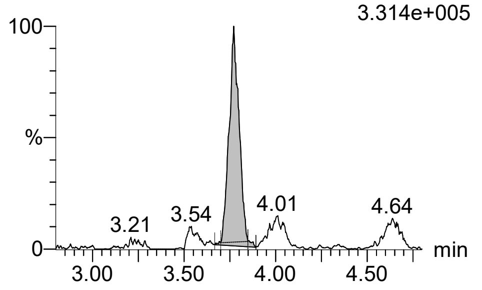

Sample Name: 20211111_QC_R15_IgM
F40:MRM of 2 channels,ES+
952.7 > 274.1
5.952e+004

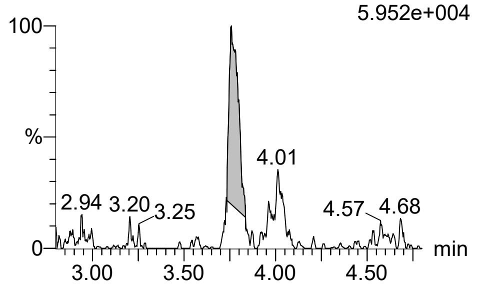

Sample Name: 20211111_L29_R2_IgM
F40:MRM of 2 channels,ES+
952.7 > 204.1
1.095e+005

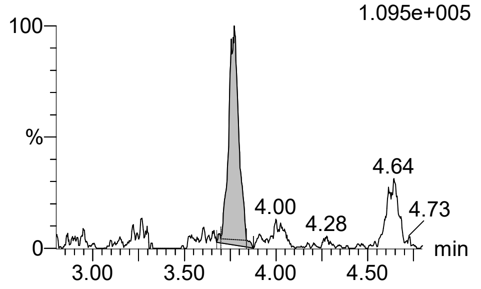

Sample Name: 20211111_L29_R2_IgM
F40:MRM of 2 channels,ES+
952.7 > 274.1
2.767e+004

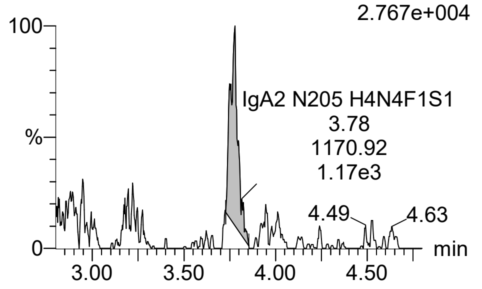

Sample Name: 20211111_L30_R2_IgM
F40:MRM of 2 channels,ES+
952.7 > 204.1
1.279e+005

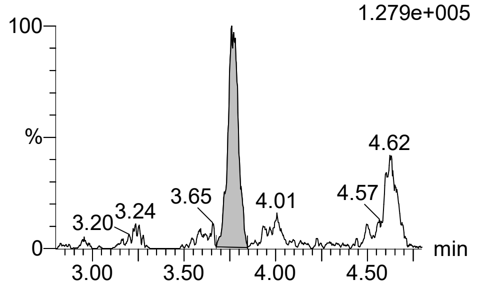

Sample Name: 20211111_L30_R2_IgM
F40:MRM of 2 channels,ES+
952.7 > 274.1
3.266e+004

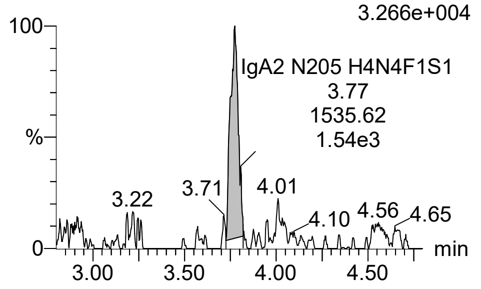

Sample Name: 20211111_H15_R2_IgM
F40:MRM of 2 channels,ES+
952.7 > 204.1
4.475e+005

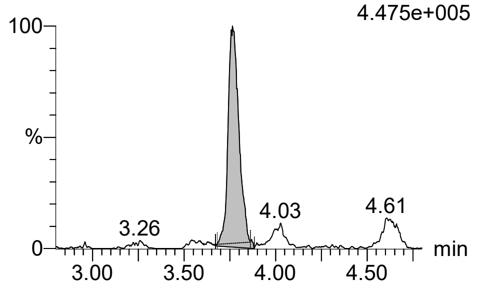

Sample Name: 20211111_H15_R2_IgM
F40:MRM of 2 channels,ES+
952.7 > 274.1
1.282e+005

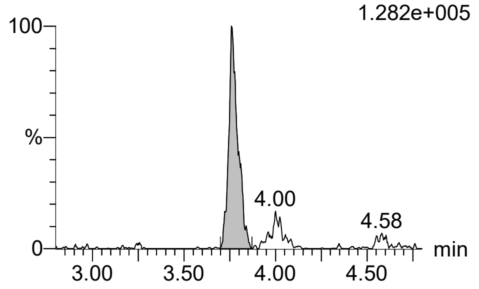

Sample Name: 20211111_A31_R2_IgM
F40:MRM of 2 channels,ES+
952.7 > 204.1
2.645e+005

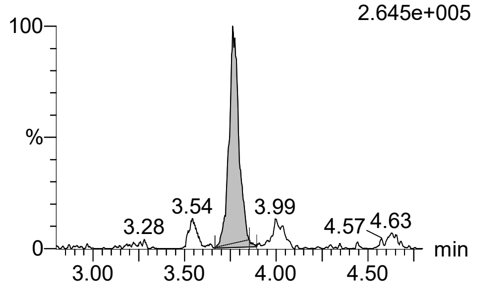

Sample Name: 20211111_A31_R2_IgM
F40:MRM of 2 channels,ES+
952.7 > 274.1
5.159e+004

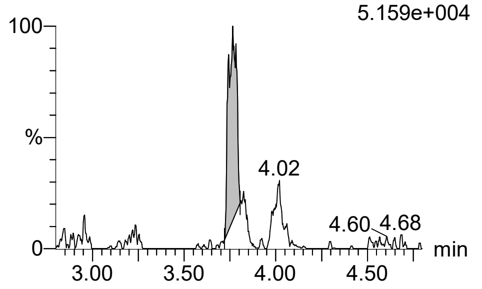

Sample Name: 20211111_A32_R2_IgM
F40:MRM of 2 channels,ES+
952.7 > 204.1
3.620e+005

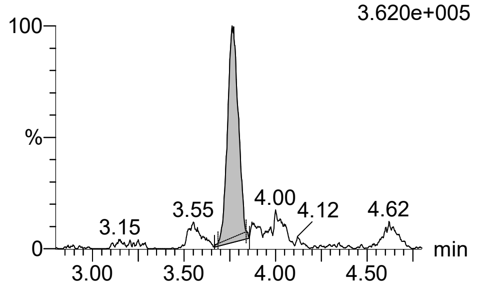

Sample Name: 20211111_A32_R2_IgM
F40:MRM of 2 channels,ES+
952.7 > 274.1
7.703e+004

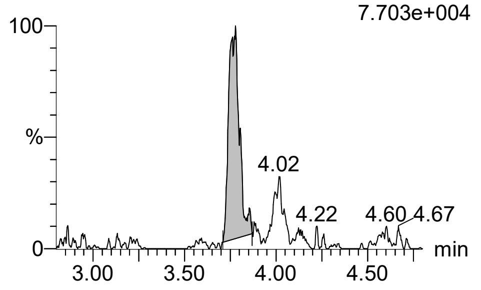

Sample Name: 20211111_L31_R2_IgM
F40:MRM of 2 channels,ES+
952.7 > 204.1
1.421e+005

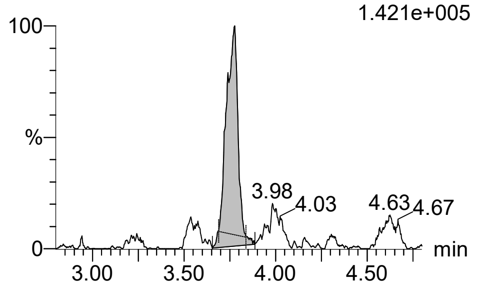

Sample Name: 20211111_L31_R2_IgM
F40:MRM of 2 channels,ES+
952.7 > 274.1
2.764e+004

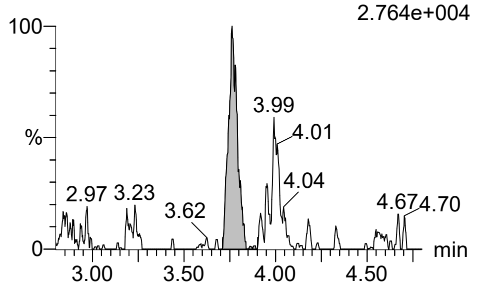

Sample Name: 20211111_L32_R2_IgM
F40:MRM of 2 channels,ES+
952.7 > 204.1
1.922e+005

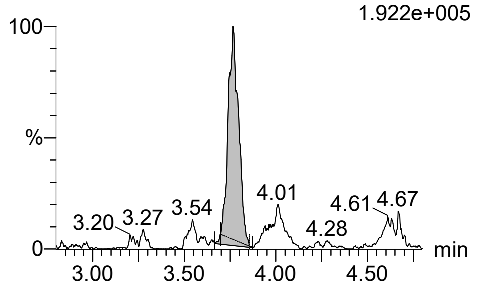

Sample Name: 20211111_L32_R2_IgM
F40:MRM of 2 channels,ES+
952.7 > 274.1
3.188e+004

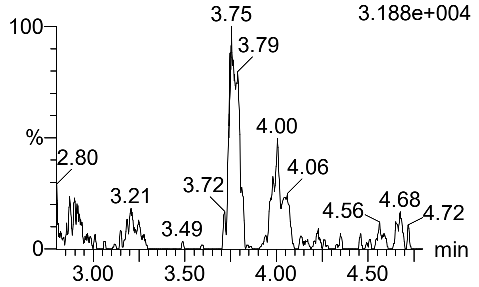

Sample Name: 20211111_H16_R2_IgM
F40:MRM of 2 channels,ES+
952.7 > 204.1
4.528e+005

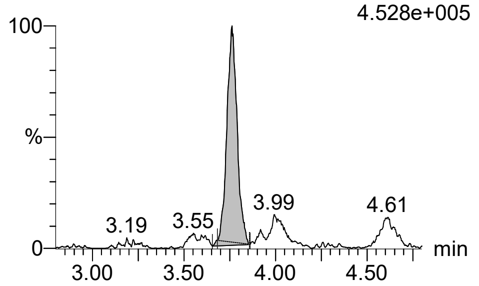

Sample Name: 20211111_H16_R2_IgM
F40:MRM of 2 channels,ES+
952.7 > 274.1
1.039e+005

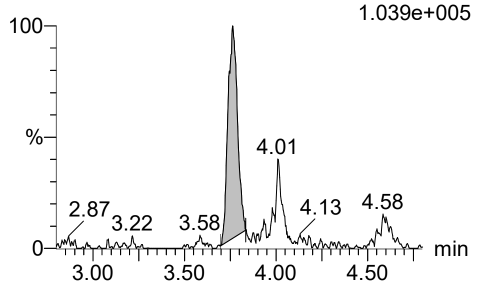

Sample Name: 20211111_A33_R2_IgM
F40:MRM of 2 channels,ES+
952.7 > 204.1
5.871e+005

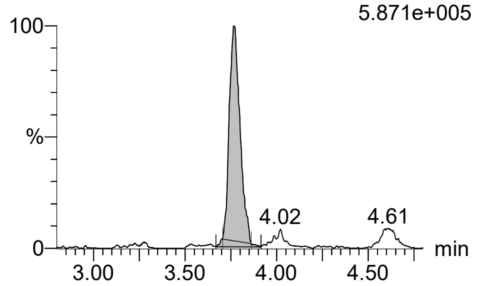

Sample Name: 20211111_A33_R2_IgM
F40:MRM of 2 channels,ES+
952.7 > 274.1
1.677e+005

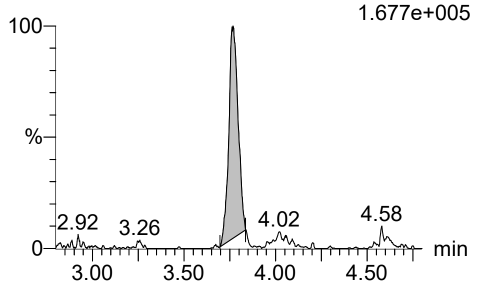

Sample Name: 20211111_A34_R2_IgM
F40:MRM of 2 channels,ES+
952.7 > 204.1
1.529e+005

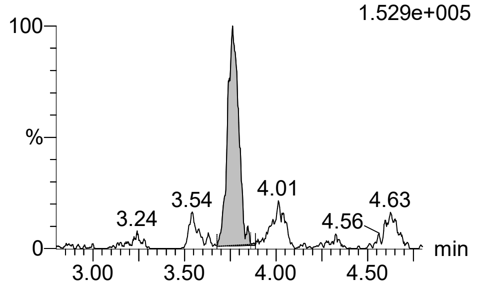

Sample Name: 20211111_A34_R2_IgM
F40:MRM of 2 channels,ES+
952.7 > 274.1
2.452e+004

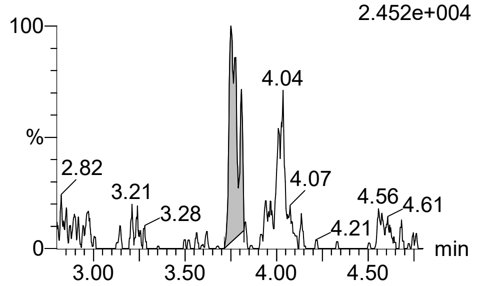

Sample Name: 20211111_H17_R2_IgM
F40:MRM of 2 channels,ES+
952.7 > 204.1
3.762e+005

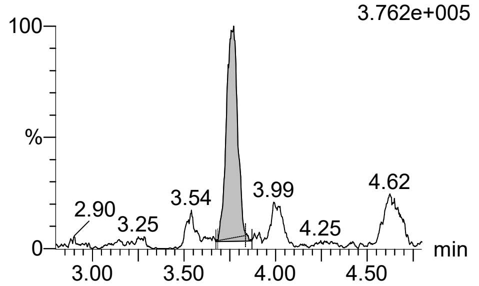

Sample Name: 20211111_H17_R2_IgM
F40:MRM of 2 channels,ES+
952.7 > 274.1
8.686e+004

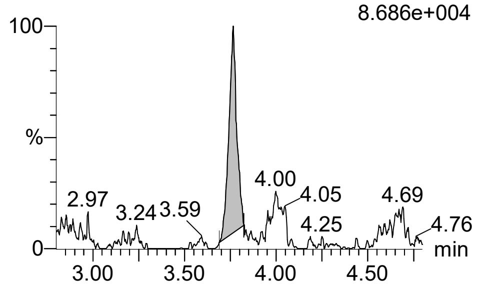

Sample Name: 20211111_A35_R2_IgM
F40:MRM of 2 channels,ES+
952.7 > 204.1
1.268e+005

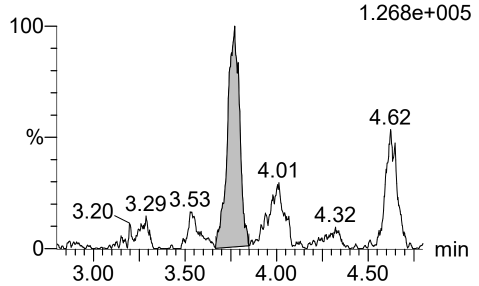

Sample Name: 20211111_A35_R2_IgM
F40:MRM of 2 channels,ES+
952.7 > 274.1
1.384e+004

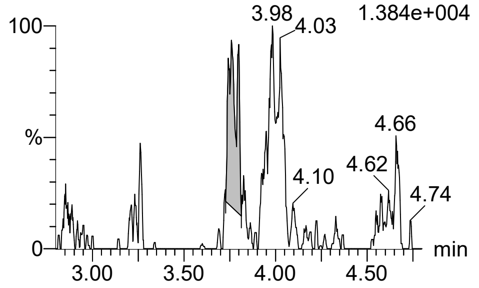

Sample Name: 20211111_QC_R16_IgM
F40:MRM of 2 channels,ES+
952.7 > 204.1
2.774e+005

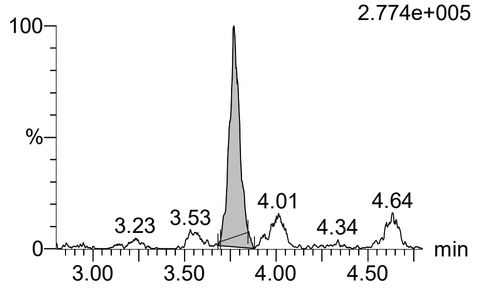

Sample Name: 20211111_QC_R16_IgM
F40:MRM of 2 channels,ES+
952.7 > 274.1
5.070e+004

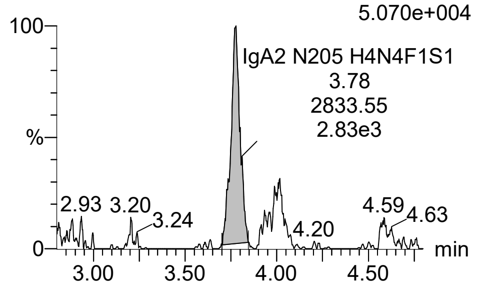

Sample Name: 20211111_A36_R2_IgM
F40:MRM of 2 channels,ES+
952.7 > 204.1
3.479e+005

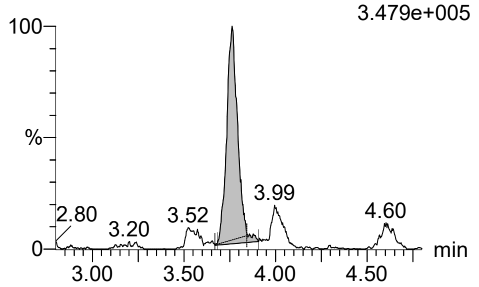

Sample Name: 20211111_A36_R2_IgM
F40:MRM of 2 channels,ES+
952.7 > 274.1
6.940e+004

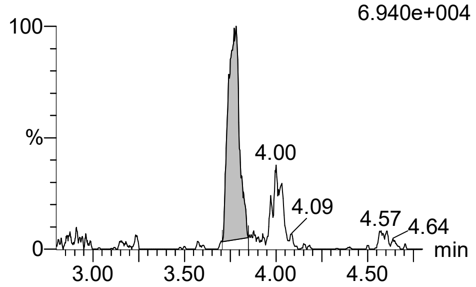

Sample Name: 20211111_H18_R2_IgM
F40:MRM of 2 channels,ES+
952.7 > 204.1
5.057e+005

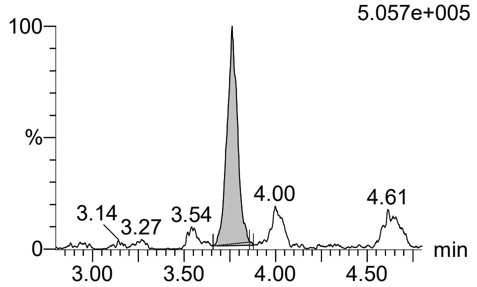

Sample Name: 20211111_H18_R2_IgM
F40:MRM of 2 channels,ES+
952.7 > 274.1
9.653e+004

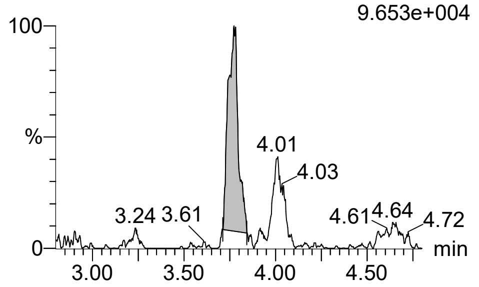

Sample Name: 20211111_A37_R2_IgM
F40:MRM of 2 channels,ES+
952.7 > 204.1
1.063e+005

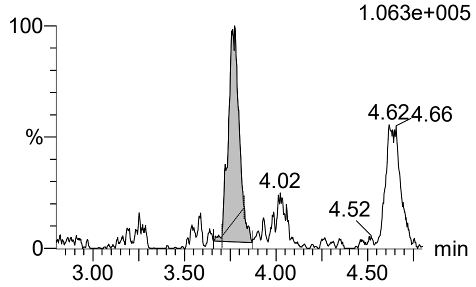

Sample Name: 20211111_A37_R2_IgM
F40:MRM of 2 channels,ES+
952.7 > 274.1
1.457e+004

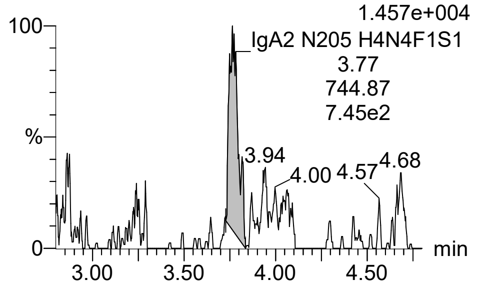

Sample Name: 20211111_A38_R2_IgM
F40:MRM of 2 channels,ES+
952.7 > 204.1
1.478e+005

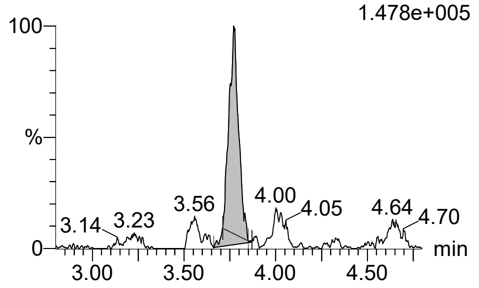

Sample Name: 20211111_A38_R2_IgM
F40:MRM of 2 channels,ES+
952.7 > 274.1
1.494e+004

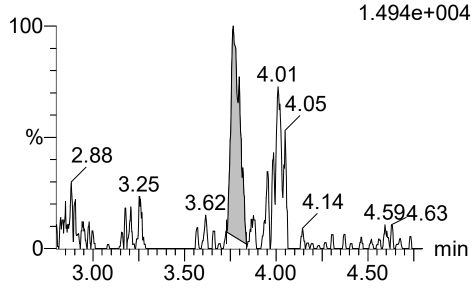

Sample Name: 20211111_H19_R2_IgM
F40:MRM of 2 channels,ES+
952.7 > 204.1
3.811e+005

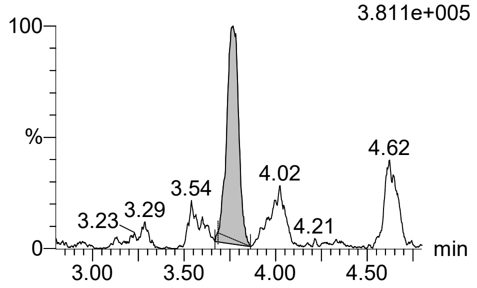

Sample Name: 20211111_H19_R2_IgM
F40:MRM of 2 channels,ES+
952.7 > 274.1
7.918e+004

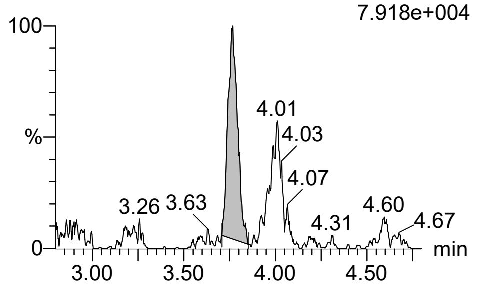

Sample Name: 20211111_A39_R2_IgM
F40:MRM of 2 channels,ES+
952.7 > 204.1
5.477e+005

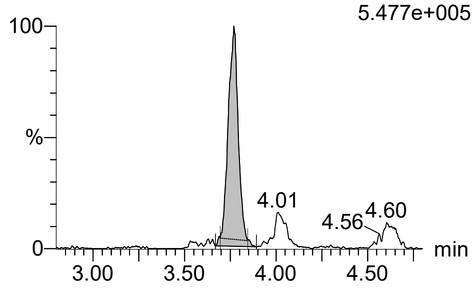

Sample Name: 20211111_A39_R2_IgM
F40:MRM of 2 channels,ES+
952.7 > 274.1
1.448e+005

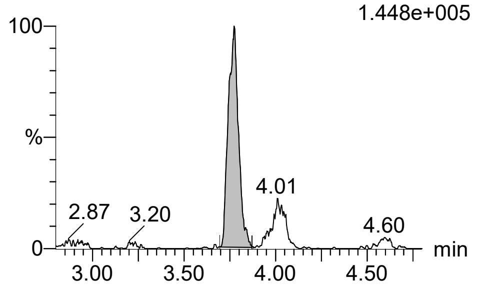

Sample Name: 20211111_A40_R2_IgM
F40:MRM of 2 channels,ES+
952.7 > 204.1
1.576e+005

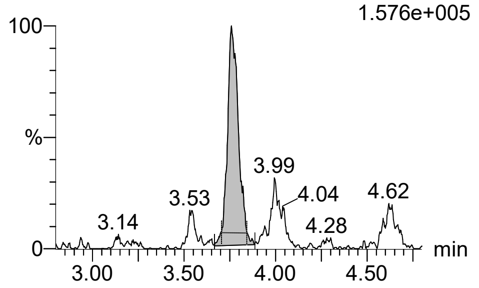

Sample Name: 20211111_A40_R2_IgM
F40:MRM of 2 channels,ES+
952.7 > 274.1
2.600e+004

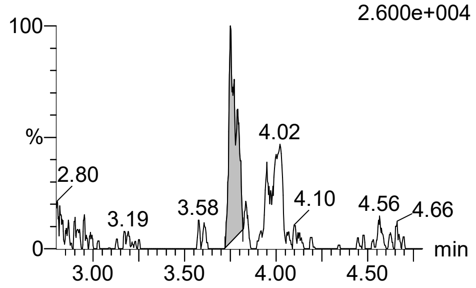

Sample Name: 20211111_H20_R2_IgM
F40:MRM of 2 channels,ES+
952.7 > 204.1
3.900e+005

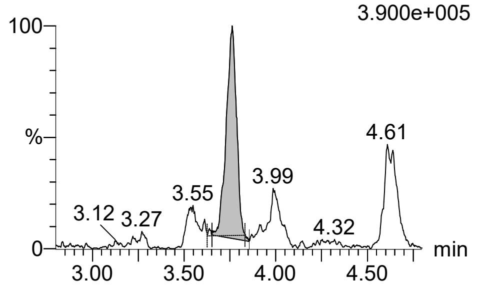

Dataset: C:\MassLynx\10.pro\20211111_clinicalsamples_IgM_IgA.qld

Last Altered: Thursday, March 09, 2023 19:58:25 台北標準時間
Printed: Friday, August 01, 2025 15:28:32 台北標準時間

Sample Name: 20211111_H20_R2_IgM
F40:MRM of 2 channels,ES+
952.7 > 274.1
4.604e+004

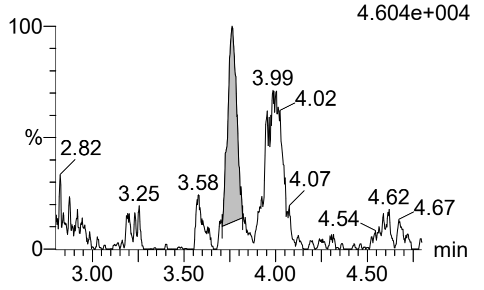

Sample Name: 20211111_A41_R2_IgM
F40:MRM of 2 channels,ES+
952.7 > 204.1
2.275e+005

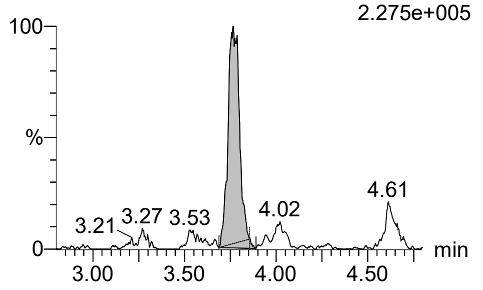

Sample Name: 20211111_A41_R2_IgM
F40:MRM of 2 channels,ES+
952.7 > 274.1
4.574e+004

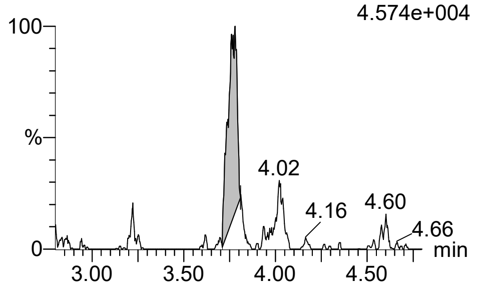

Sample Name: 20211111_A42_R2_IgM
F40:MRM of 2 channels,ES+
952.7 > 204.1
1.224e+005

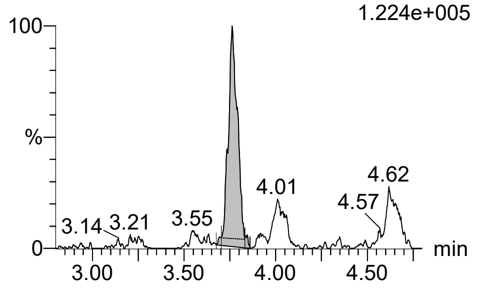

Sample Name: 20211111_A42_R2_IgM
F40:MRM of 2 channels,ES+
952.7 > 274.1
2.418e+004

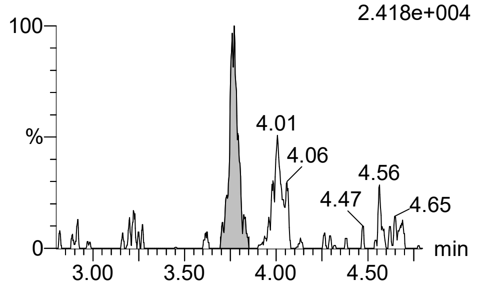

Sample Name: 20211111_A43_R2_IgM
F40:MRM of 2 channels,ES+
952.7 > 204.1
1.335e+005

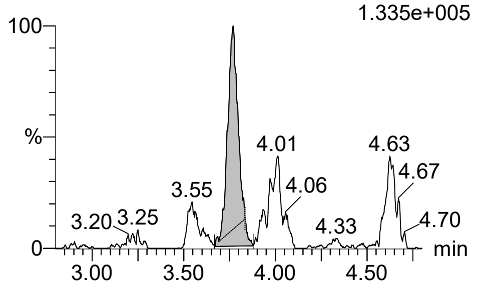

Sample Name: 20211111_A43_R2_IgM
F40:MRM of 2 channels,ES+
952.7 > 274.1
2.210e+004

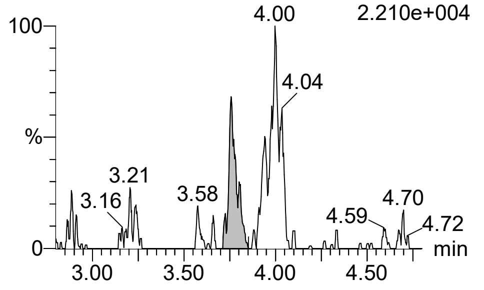

Sample Name: 20211111_A44_R2_IgM
F40:MRM of 2 channels,ES+
952.7 > 204.1
1.039e+005

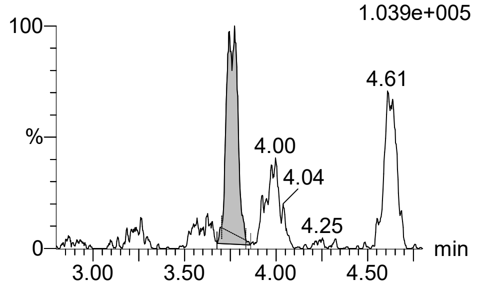

Sample Name: 20211111_A44_R2_IgM
F40:MRM of 2 channels,ES+
952.7 > 274.1
1.469e+004

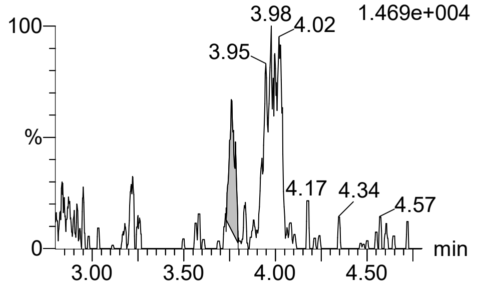

Sample Name: 20211111_QC_R17_IgM
F40:MRM of 2 channels,ES+
952.7 > 204.1
2.360e+005

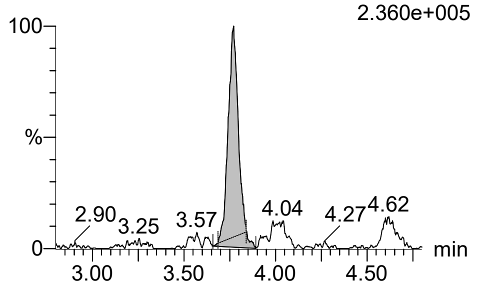

Sample Name: 20211111_QC_R17_IgM
F40:MRM of 2 channels,ES+
952.7 > 274.1
5.093e+004

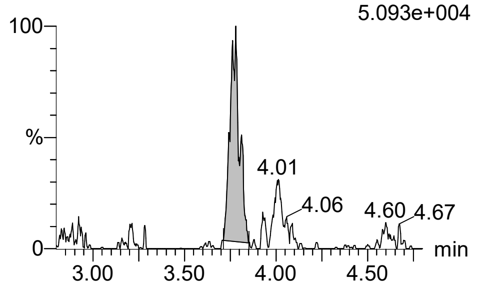

Sample Name: 20211111_L2_R3_IgM
F40:MRM of 2 channels,ES+
952.7 > 204.1
2.038e+005

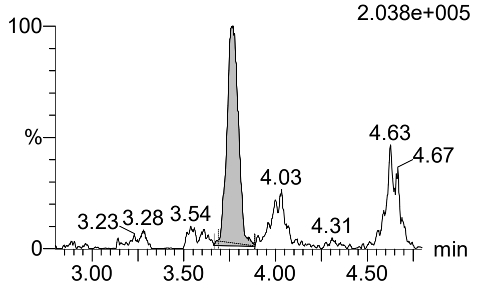

Sample Name: 20211111_L2_R3_IgM
F40:MRM of 2 channels,ES+
952.7 > 274.1
5.007e+004

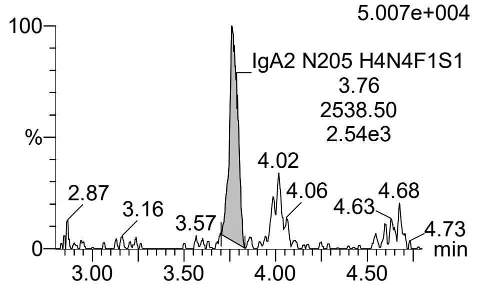

Sample Name: 20211111_H1_R3_IgM
F40:MRM of 2 channels,ES+
952.7 > 204.1
4.498e+005

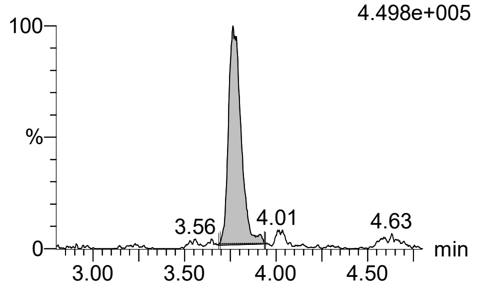

Sample Name: 20211111_H1_R3_IgM
F40:MRM of 2 channels,ES+
952.7 > 274.1
1.104e+005

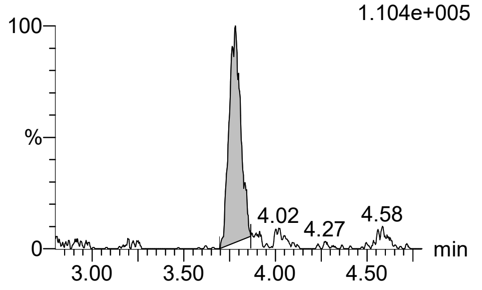

Dataset: C:\MassLynx\10.pro\20211111_clinicalsamples_IgM_IgA.qld

Last Altered: Thursday, March 09, 2023 19:58:25 台北標準時間
Printed: Friday, August 01, 2025 15:28:32 台北標準時間

Sample Name: 20211111_A3_R3_IgM
F40:MRM of 2 channels,ES+
952.7 > 204.1
1.564e+005

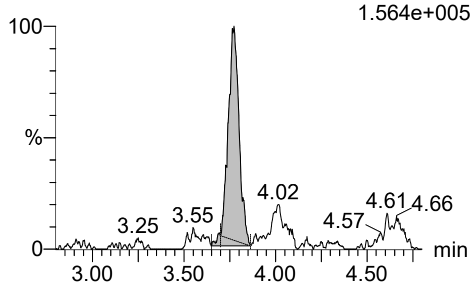

Sample Name: 20211111_A3_R3_IgM
F40:MRM of 2 channels,ES+
952.7 > 274.1
3.520e+004

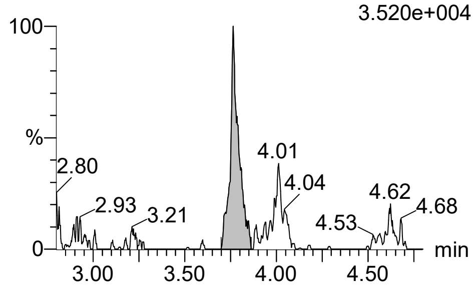

Sample Name: 20211111_A4_R3_IgM
F40:MRM of 2 channels,ES+
952.7 > 204.1
2.903e+005

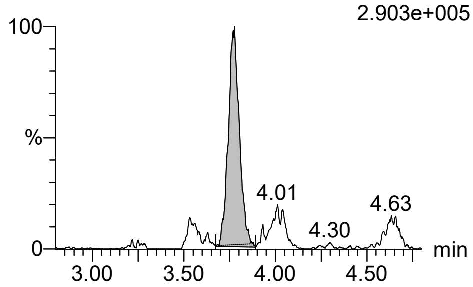

Sample Name: 20211111_A4_R3_IgM
F40:MRM of 2 channels,ES+
952.7 > 274.1
6.314e+004

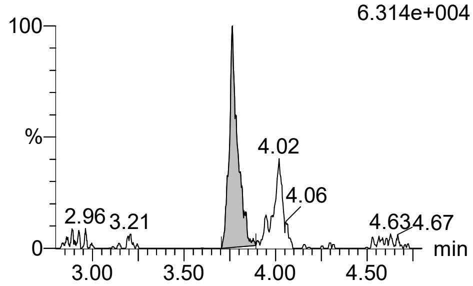

Sample Name: 20211111_L3_R3_IgM
F40:MRM of 2 channels,ES+
952.7 > 204.1
2.912e+005

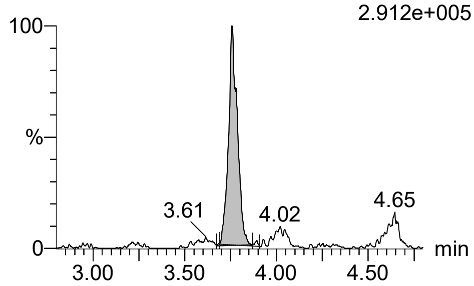

Sample Name: 20211111_L3_R3_IgM
F40:MRM of 2 channels,ES+
952.7 > 274.1
7.660e+004

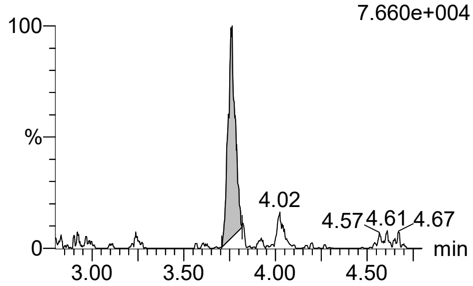

Sample Name: 20211111_L4_R3_IgM
F40:MRM of 2 channels,ES+
952.7 > 204.1
1.247e+005

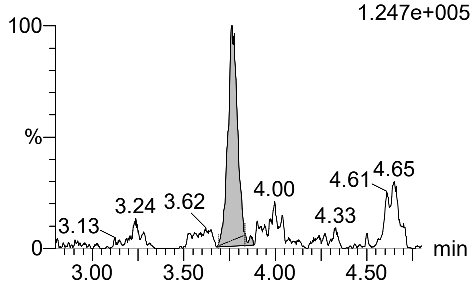

Sample Name: 20211111_L4_R3_IgM
F40:MRM of 2 channels,ES+
952.7 > 274.1
2.416e+004

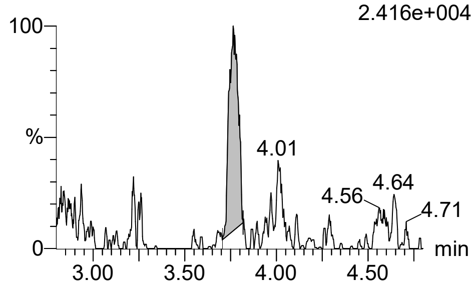

Sample Name: 20211111_H2_R3_IgM
F40:MRM of 2 channels,ES+
952.7 > 204.1
2.204e+005

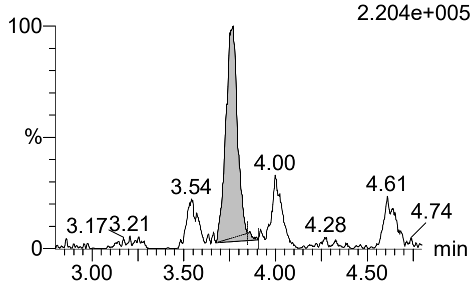

Sample Name: 20211111_H2_R3_IgM
F40:MRM of 2 channels,ES+
952.7 > 274.1
4.883e+004

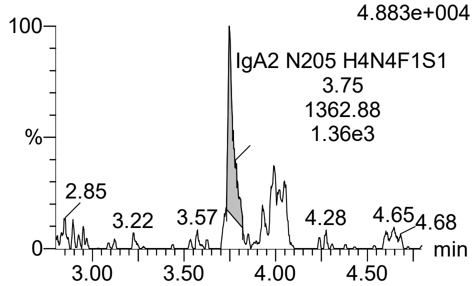

Sample Name: 20211111_A5_R3_IgM
F40:MRM of 2 channels,ES+
952.7 > 204.1
8.175e+005

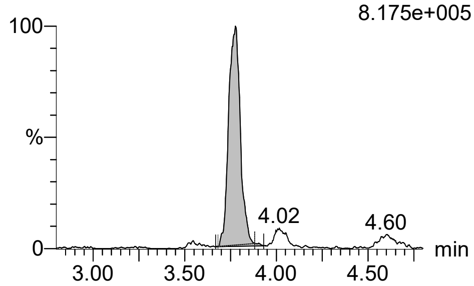

Sample Name: 20211111_A5_R3_IgM
F40:MRM of 2 channels,ES+
952.7 > 274.1
2.167e+005

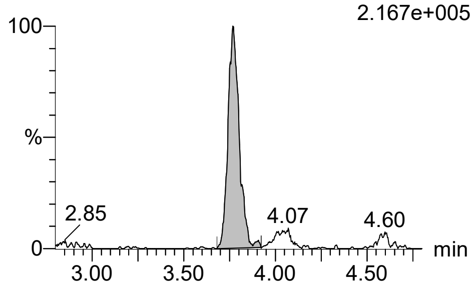

Sample Name: 20211111_A6_R3_IgM
F40:MRM of 2 channels,ES+
952.7 > 204.1
3.389e+005

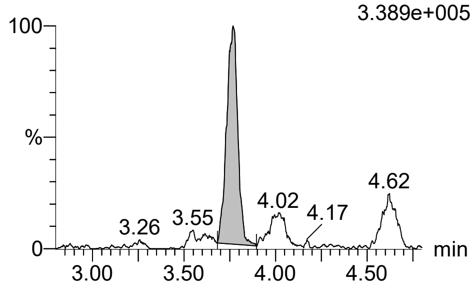

Sample Name: 20211111_A6_R3_IgM
F40:MRM of 2 channels,ES+
952.7 > 274.1
6.845e+004

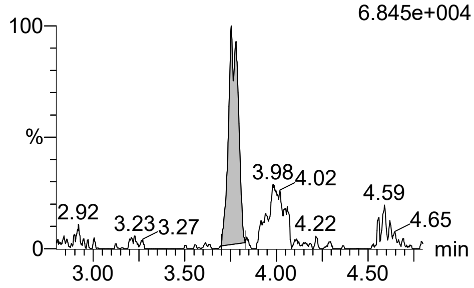

Sample Name: 20211111_QC_R18_IgM
F40:MRM of 2 channels,ES+
952.7 > 204.1
2.030e+005

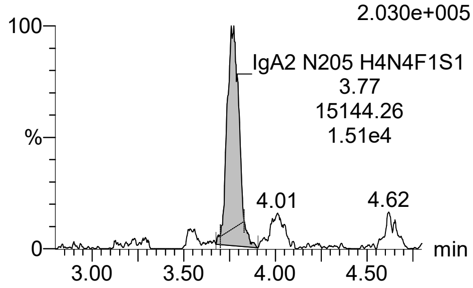

Dataset: C:\MassLynx\10.pro\20211111_clinicalsamples_IgM_IgA.qld

Last Altered: Thursday, March 09, 2023 19:58:25 台北標準時間
Printed: Friday, August 01, 2025 15:28:32 台北標準時間

Sample Name: 20211111_QC_R18_IgM
F40:MRM of 2 channels,ES+
952.7 > 274.1
4.983e+004

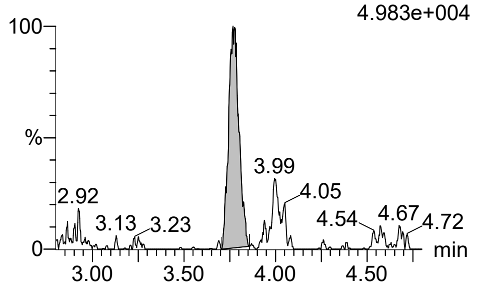

Sample Name: 20211111_L5_R3_IgM
F40:MRM of 2 channels,ES+
952.7 > 204.1
3.398e+005

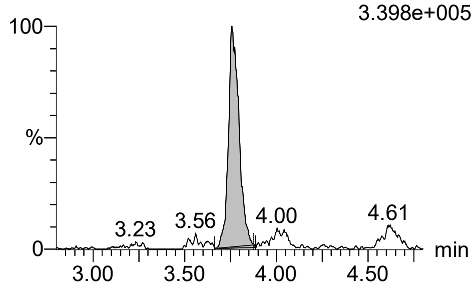

Sample Name: 20211111_L5_R3_IgM
F40:MRM of 2 channels,ES+
952.7 > 274.1
7.188e+004

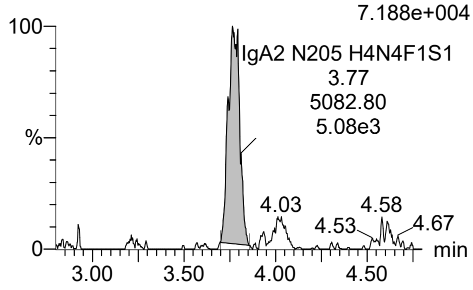

Sample Name: 20211111_L6_R3_IgM
F40:MRM of 2 channels,ES+
952.7 > 204.1
2.538e+005

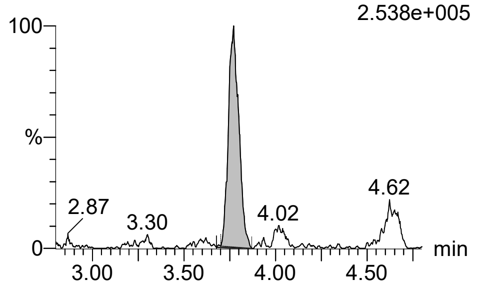

Sample Name: 20211111_L6_R3_IgM
F40:MRM of 2 channels,ES+
952.7 > 274.1
6.618e+004

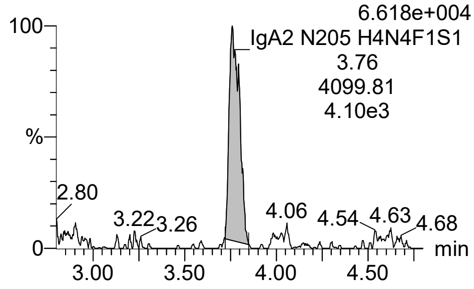

Sample Name: 20211111_H3_R3_IgM
F40:MRM of 2 channels,ES+
952.7 > 204.1
1.781e+005

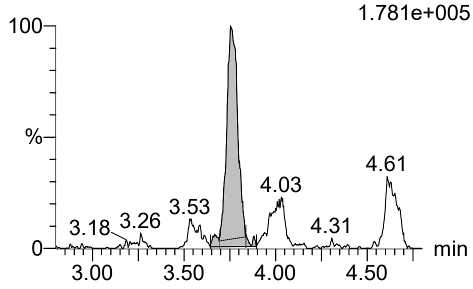

Sample Name: 20211111_H3_R3_IgM
F40:MRM of 2 channels,ES+
952.7 > 274.1
2.309e+004

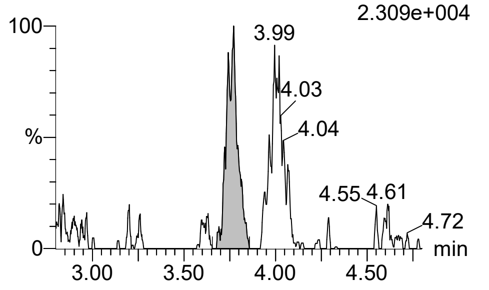

Sample Name: 20211111_A7_R3_IgM
F40:MRM of 2 channels,ES+
952.7 > 204.1
3.278e+005

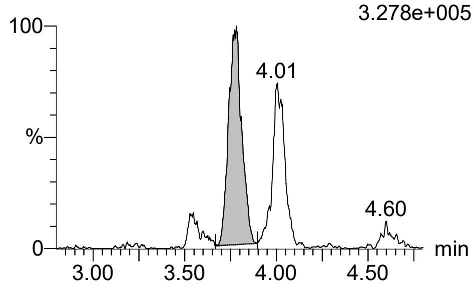

Sample Name: 20211111_A7_R3_IgM
F40:MRM of 2 channels,ES+
952.7 > 274.1
1.065e+005

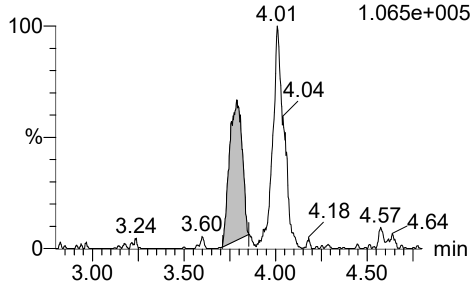

Sample Name: 20211111_A8_R3_IgM
F40:MRM of 2 channels,ES+
952.7 > 204.1
3.465e+005

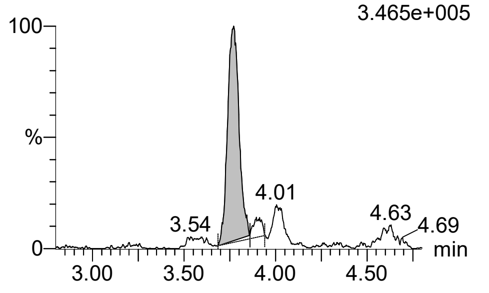

Sample Name: 20211111_A8_R3_IgM
F40:MRM of 2 channels,ES+
952.7 > 274.1
1.044e+005

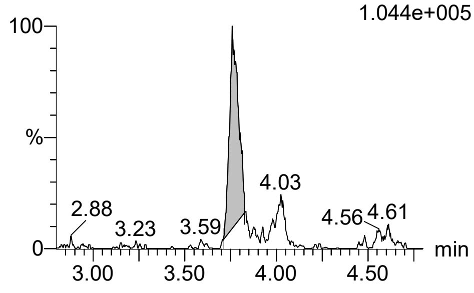

Sample Name: 20211111_L7_R3_IgM
F40:MRM of 2 channels,ES+
952.7 > 204.1
7.582e+004

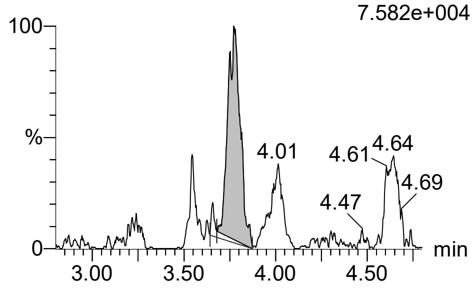

Sample Name: 20211111_L7_R3_IgM
F40:MRM of 2 channels,ES+
952.7 > 274.1
1.169e+004

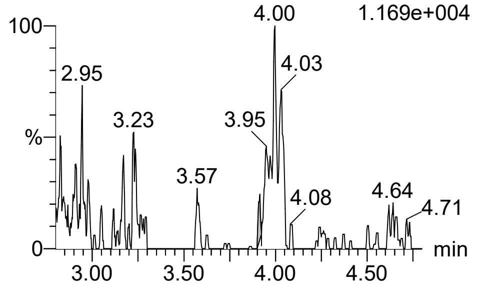

Sample Name: 20211111_L8_R3_IgM
F40:MRM of 2 channels,ES+
952.7 > 204.1
1.080e+005

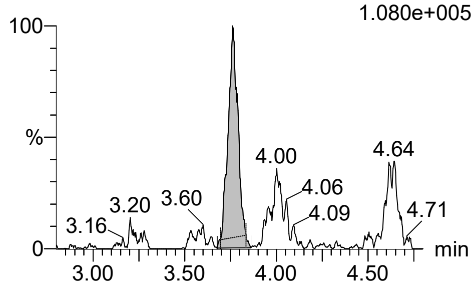

Sample Name: 20211111_L8_R3_IgM
F40:MRM of 2 channels,ES+
952.7 > 274.1
1.836e+004

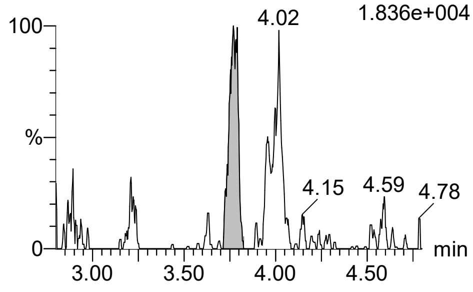

Dataset: C:\MassLynx\10.pro\20211111_clinicalsamples_IgM_IgA.qld

Last Altered: Thursday, March 09, 2023 19:58:25 台北標準時間
Printed: Friday, August 01, 2025 15:28:32 台北標準時間

Sample Name: 20211111_H4_R3_IgM
F40:MRM of 2 channels,ES+
952.7 > 204.1
2.428e+005

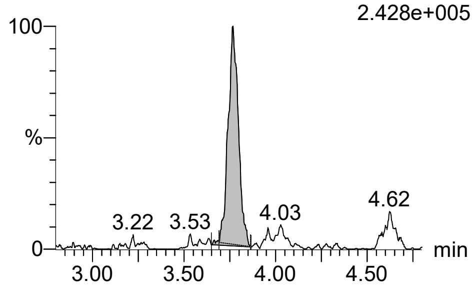

Sample Name: 20211111_H4_R3_IgM
F40:MRM of 2 channels,ES+
952.7 > 274.1
6.561e+004

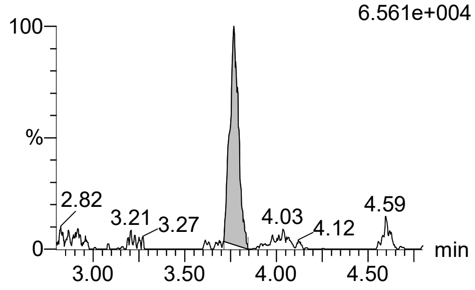

Sample Name: 20211111_A9_R3_IgM
F40:MRM of 2 channels,ES+
952.7 > 204.1
2.589e+005

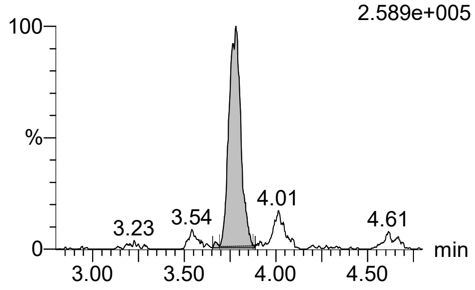

Sample Name: 20211111_A9_R3_IgM
F40:MRM of 2 channels,ES+
952.7 > 274.1
6.974e+004

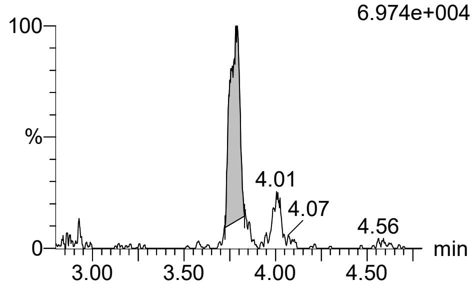

Sample Name: 20211111_A10_R3_IgM
F40:MRM of 2 channels,ES+
952.7 > 204.1
4.880e+005

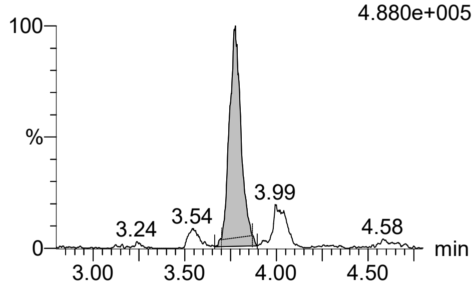

Sample Name: 20211111_A10_R3_IgM
F40:MRM of 2 channels,ES+
952.7 > 274.1
1.220e+005

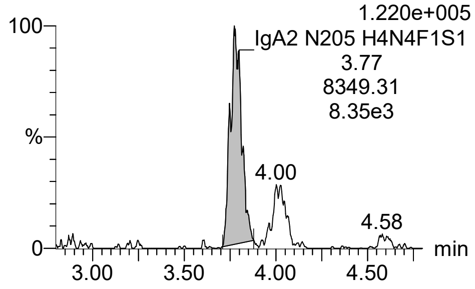

Sample Name: 20211111_L9_R3_IgM
F40:MRM of 2 channels,ES+
952.7 > 204.1
3.086e+005

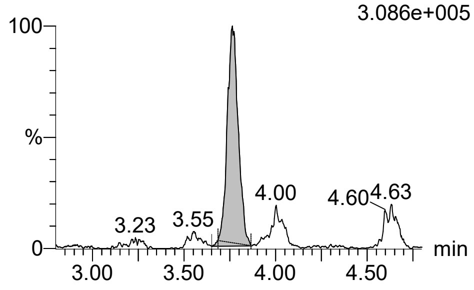

Sample Name: 20211111_L9_R3_IgM
F40:MRM of 2 channels,ES+
952.7 > 274.1
5.818e+004

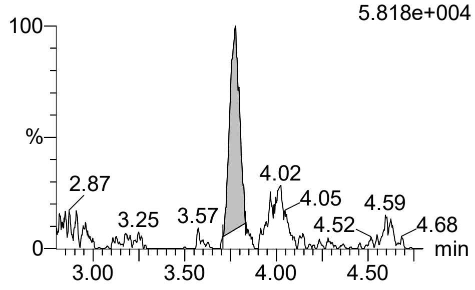

Sample Name: 20211111_L10_R3_IgM
F40:MRM of 2 channels,ES+
952.7 > 204.1
2.860e+005

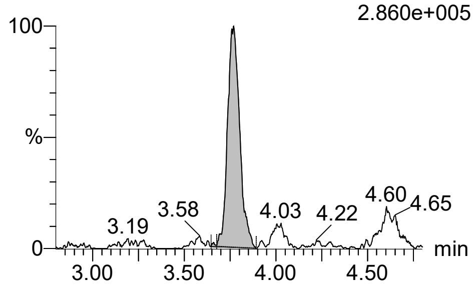

Sample Name: 20211111_L10_R3_IgM
F40:MRM of 2 channels,ES+
952.7 > 274.1
7.523e+004

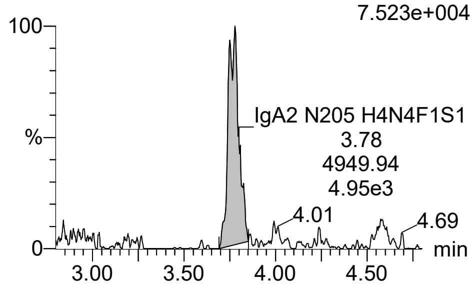

Sample Name: 20211111_QC_R19_IgM
F40:MRM of 2 channels,ES+
952.7 > 204.1
2.236e+005

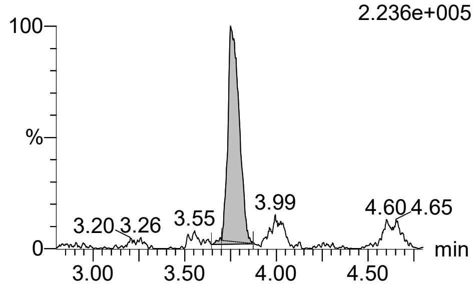

Sample Name: 20211111_QC_R19_IgM
F40:MRM of 2 channels,ES+
952.7 > 274.1
4.645e+004

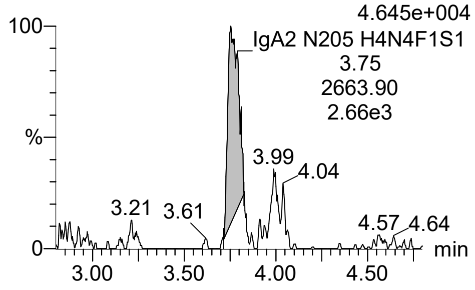

Sample Name: 20211111_H5_R3_IgM
F40:MRM of 2 channels,ES+
952.7 > 204.1
2.192e+005

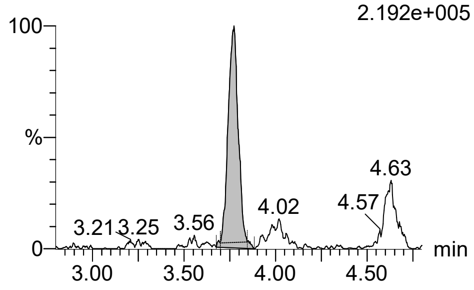

Sample Name: 20211111_H5_R3_IgM
F40:MRM of 2 channels,ES+
952.7 > 274.1
5.186e+004

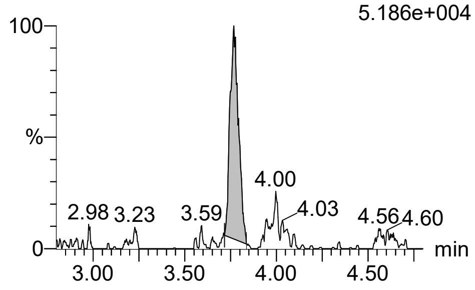

Sample Name: 20211111_A11_R3_IgM
F40:MRM of 2 channels,ES+
952.7 > 204.1
4.085e+005

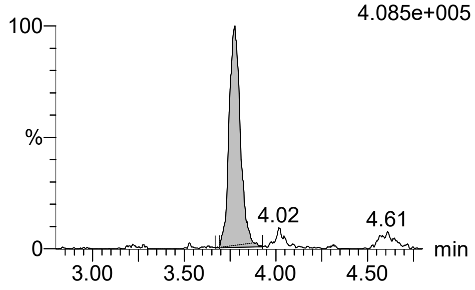

Sample Name: 20211111_A11_R3_IgM
F40:MRM of 2 channels,ES+
952.7 > 274.1
1.061e+005

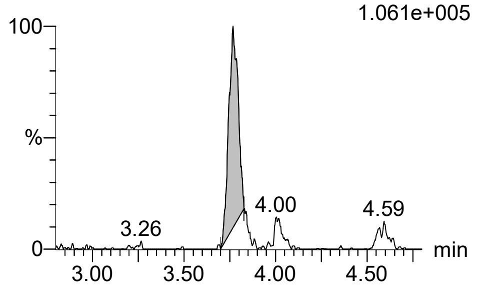

Sample Name: 20211111_A12_R3_IgM
F40:MRM of 2 channels,ES+
952.7 > 204.1
1.642e+005

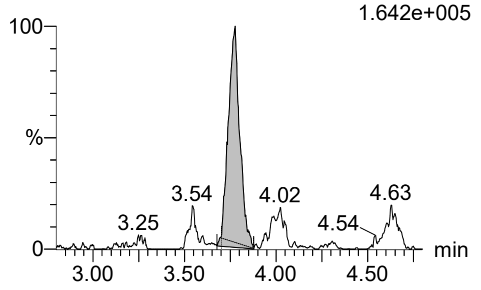

Sample Name: 20211111_A12_R3_IgM
F40:MRM of 2 channels,ES+
952.7 > 274.1
2.664e+004

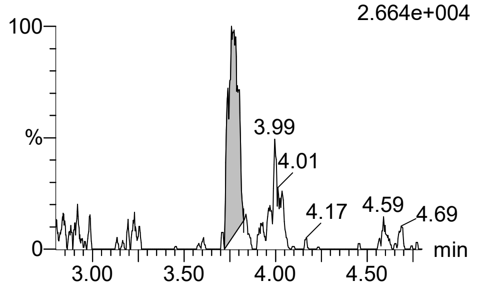

Sample Name: 20211111_L11_R3_IgM
F40:MRM of 2 channels,ES+
952.7 > 204.1
1.092e+005

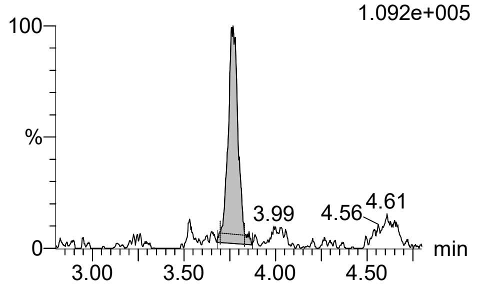

Sample Name: 20211111_L11_R3_IgM
F40:MRM of 2 channels,ES+
952.7 > 274.1
2.060e+004

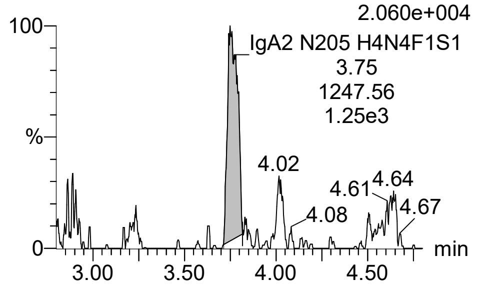

Sample Name: 20211111_L12_R3_IgM
F40:MRM of 2 channels,ES+
952.7 > 204.1
2.404e+005

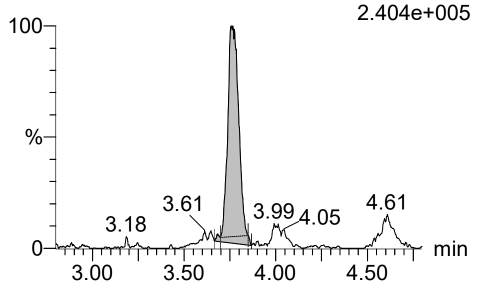

Sample Name: 20211111_L12_R3_IgM
F40:MRM of 2 channels,ES+
952.7 > 274.1
6.940e+004

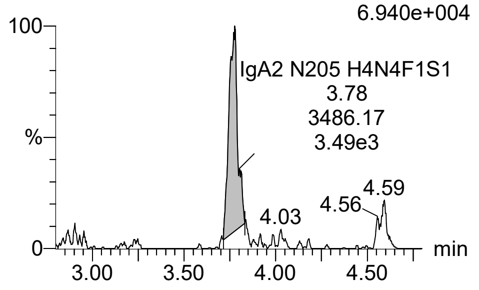

Sample Name: 20211111_H6_R3_IgM
F40:MRM of 2 channels,ES+
952.7 > 204.1
2.503e+005

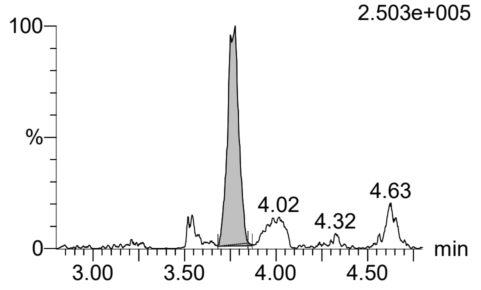

Sample Name: 20211111_H6_R3_IgM
F40:MRM of 2 channels,ES+
952.7 > 274.1
3.332e+004

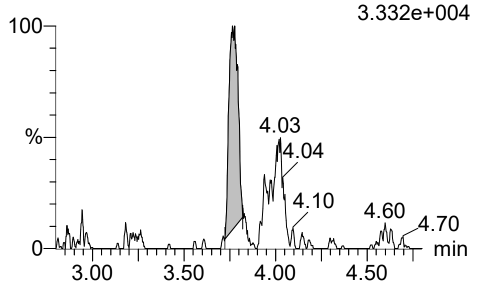

Sample Name: 20211111_A13_R3_IgM
F40:MRM of 2 channels,ES+
952.7 > 204.1
2.052e+005

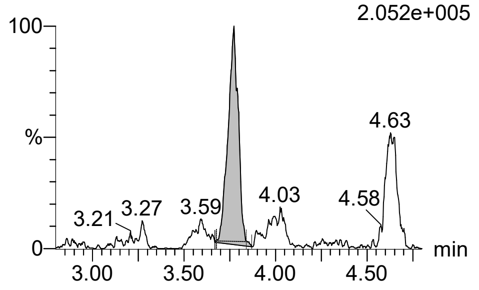

Sample Name: 20211111_A13_R3_IgM
F40:MRM of 2 channels,ES+
952.7 > 274.1
2.594e+004

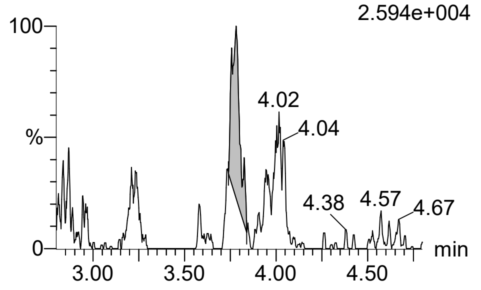

Sample Name: 20211111_A14_R3_IgM
F40:MRM of 2 channels,ES+
952.7 > 204.1
6.114e+005

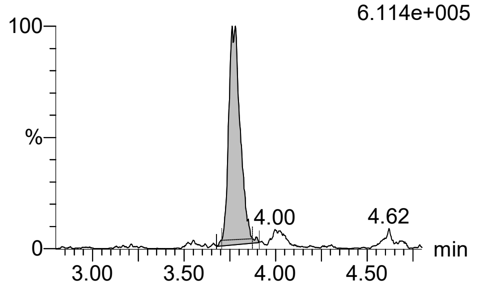

Sample Name: 20211111_A14_R3_IgM
F40:MRM of 2 channels,ES+
952.7 > 274.1
1.727e+005

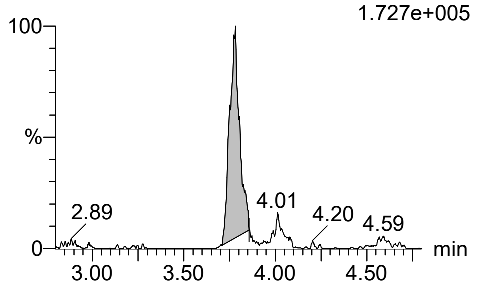

Sample Name: 20211111_L13_R3_IgM
F40:MRM of 2 channels,ES+
952.7 > 204.1
1.264e+005

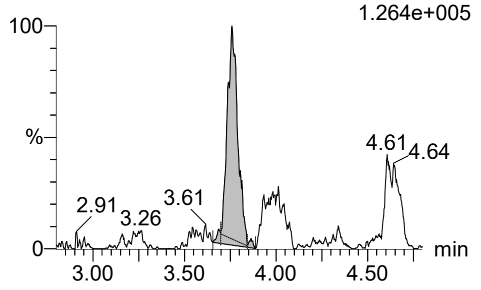

Sample Name: 20211111_L13_R3_IgM
F40:MRM of 2 channels,ES+
952.7 > 274.1
2.588e+004

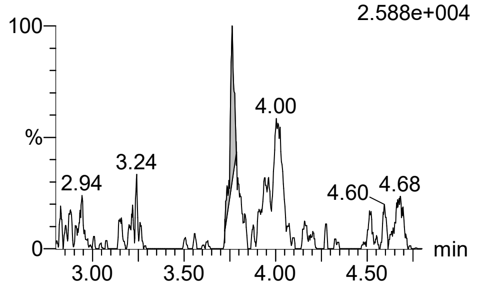

Sample Name: 20211111_L14_R3_IgM
F40:MRM of 2 channels,ES+
952.7 > 204.1
2.237e+005

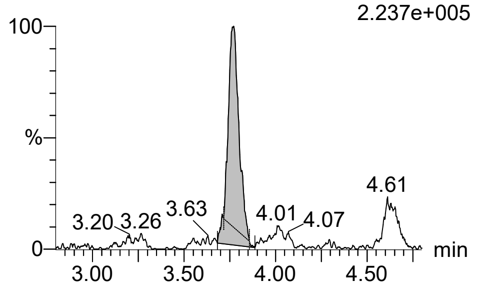

Sample Name: 20211111_L14_R3_IgM
F40:MRM of 2 channels,ES+
952.7 > 274.1
5.866e+004

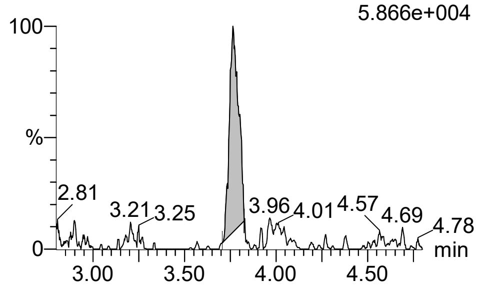

Sample Name: 20211111_H7_R3_IgM
F40:MRM of 2 channels,ES+
952.7 > 204.1
4.172e+005

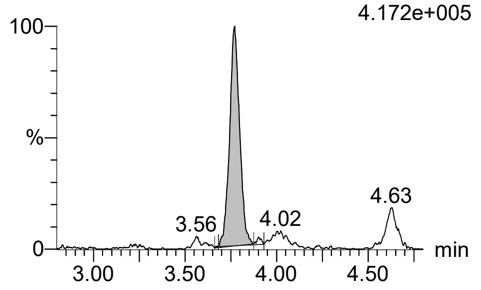

Sample Name: 20211111_H7_R3_IgM
F40:MRM of 2 channels,ES+
952.7 > 274.1
1.262e+005

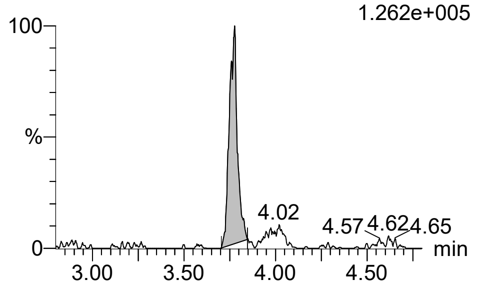

Sample Name: 20211111_A15_R3_IgM
F40:MRM of 2 channels,ES+
952.7 > 204.1
1.500e+005

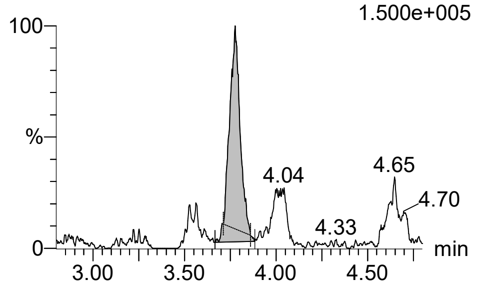

Sample Name: 20211111_A15_R3_IgM
F40:MRM of 2 channels,ES+
952.7 > 274.1
2.236e+004

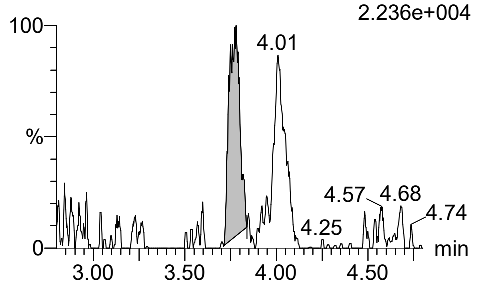

Sample Name: 20211111_QC_R20_IgM
F40:MRM of 2 channels,ES+
952.7 > 204.1
2.903e+005

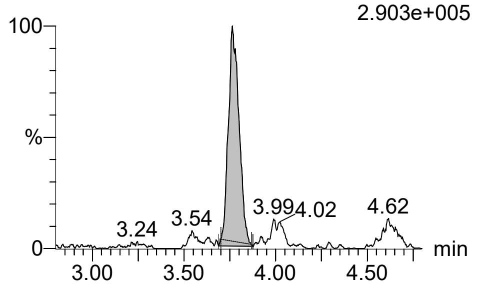

Sample Name: 20211111_QC_R20_IgM
F40:MRM of 2 channels,ES+
952.7 > 274.1
5.840e+004

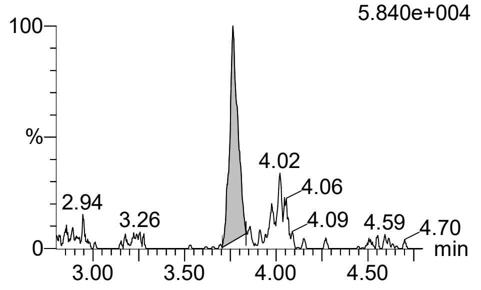

Sample Name: 20211111_A16_R3_IgM
F40:MRM of 2 channels,ES+
952.7 > 204.1
1.479e+005

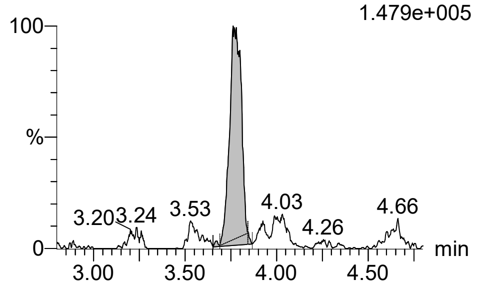

Sample Name: 20211111_A16_R3_IgM
F40:MRM of 2 channels,ES+
952.7 > 274.1
3.093e+004

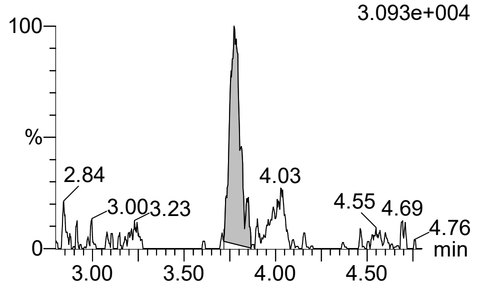

Sample Name: 20211111_L15_R3_IgM
F40:MRM of 2 channels,ES+
952.7 > 204.1
2.690e+005

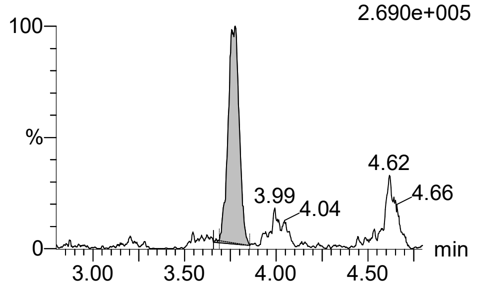

Sample Name: 20211111_L15_R3_IgM
F40:MRM of 2 channels,ES+
952.7 > 274.1
7.335e+004

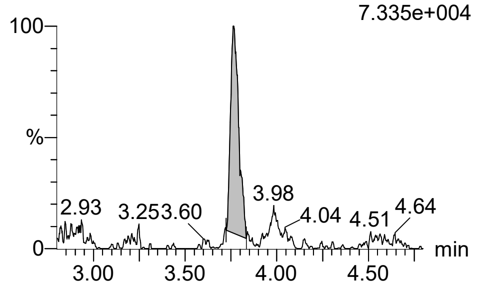

Sample Name: 20211111_L16_R3_IgM
F40:MRM of 2 channels,ES+
952.7 > 204.1
1.833e+005

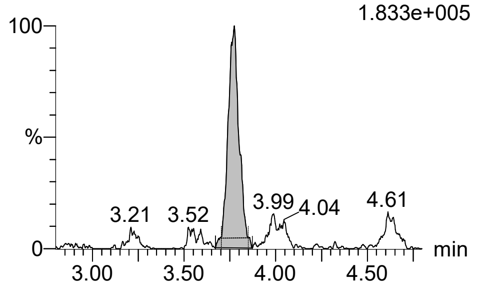

Sample Name: 20211111_L16_R3_IgM
F40:MRM of 2 channels,ES+
952.7 > 274.1
3.557e+004

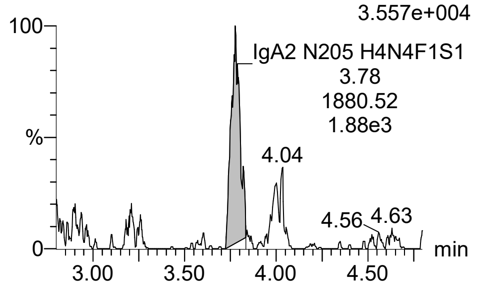

Sample Name: 20211111_H8_R3_IgM
F40:MRM of 2 channels,ES+
952.7 > 204.1
1.423e+005

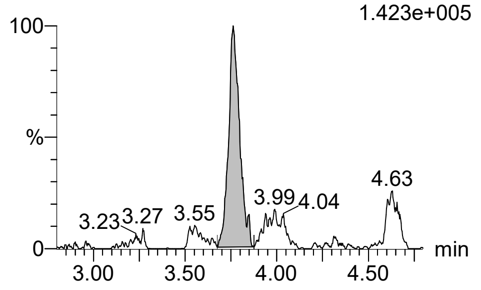

Dataset: C:\MassLynx\10.pro\20211111_clinicalsamples_IgM_IgA.qld

Last Altered: Thursday, March 09, 2023 19:58:25 台北標準時間
Printed: Friday, August 01, 2025 15:28:32 台北標準時間

Sample Name: 20211111_H8_R3_IgM
F40:MRM of 2 channels,ES+
952.7 > 274.1
2.239e+004

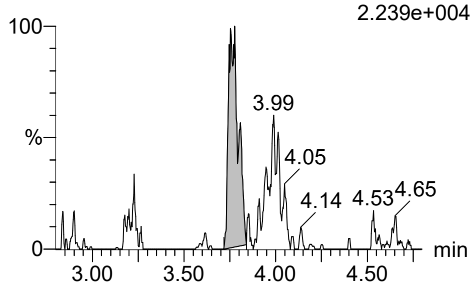

Sample Name: 20211111_A17_R3_IgM
F40:MRM of 2 channels,ES+
952.7 > 204.1
6.910e+005

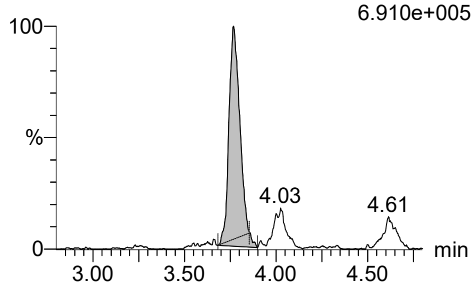

Sample Name: 20211111_A17_R3_IgM
F40:MRM of 2 channels,ES+
952.7 > 274.1
1.989e+005

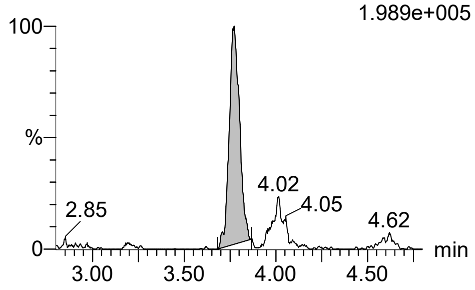

Sample Name: 20211111_A18_R3_IgM
F40:MRM of 2 channels,ES+
952.7 > 204.1
1.696e+005

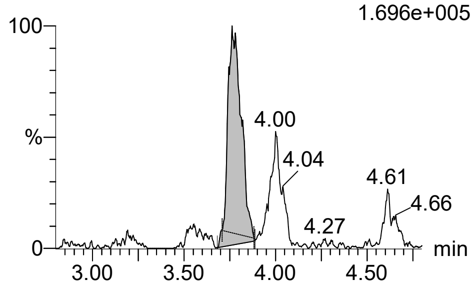

Sample Name: 20211111_A18_R3_IgM
F40:MRM of 2 channels,ES+
952.7 > 274.1
3.972e+004

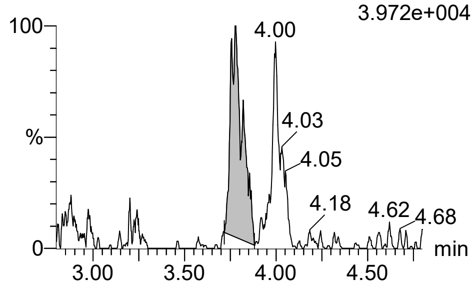

Sample Name: 20211111_L17_R3_IgM
F40:MRM of 2 channels,ES+
952.7 > 204.1
2.216e+005

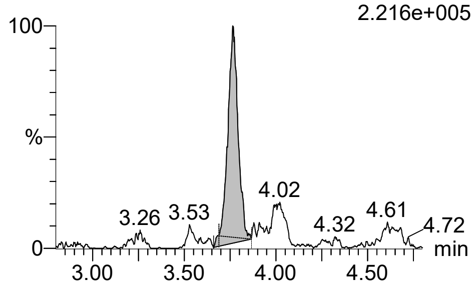

Sample Name: 20211111_L17_R3_IgM
F40:MRM of 2 channels,ES+
952.7 > 274.1
4.598e+004

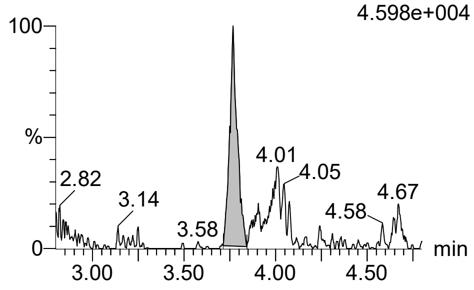

Sample Name: 20211111_L18_R3_IgM
F40:MRM of 2 channels,ES+
952.7 > 204.1
4.444e+004

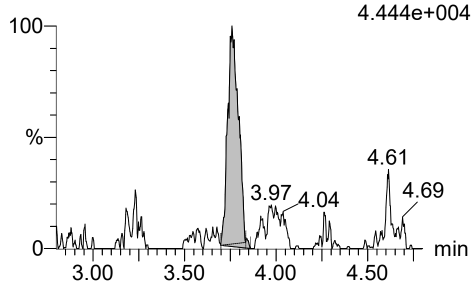

Sample Name: 20211111_L18_R3_IgM
F40:MRM of 2 channels,ES+
952.7 > 274.1
8.640e+003

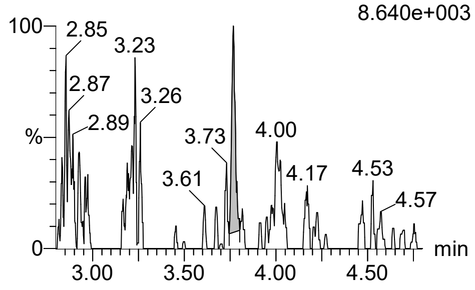

Sample Name: 20211111_H9_R3_IgM
F40:MRM of 2 channels,ES+
952.7 > 204.1
1.966e+005

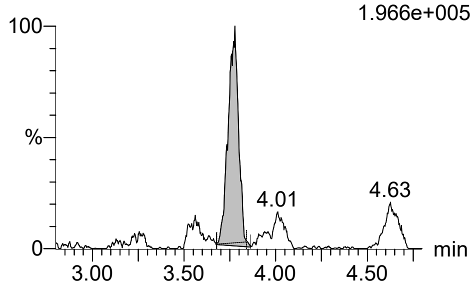

Sample Name: 20211111_H9_R3_IgM
F40:MRM of 2 channels,ES+
952.7 > 274.1
2.839e+004

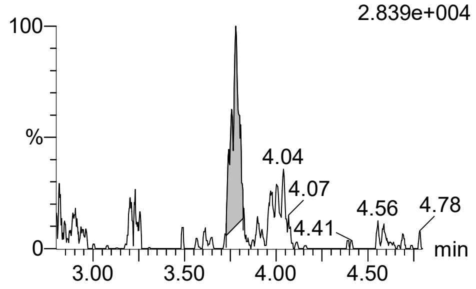

Sample Name: 20211111_A19_R3_IgM
F40:MRM of 2 channels,ES+
952.7 > 204.1
3.892e+005

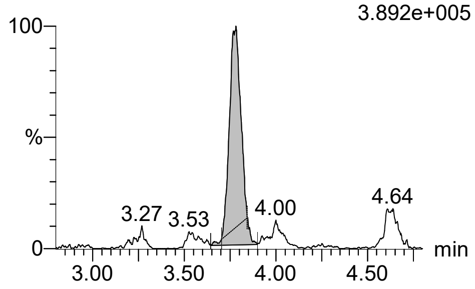

Sample Name: 20211111_A19_R3_IgM
F40:MRM of 2 channels,ES+
952.7 > 274.1
1.072e+005

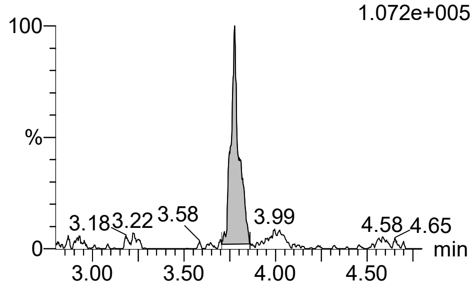

Sample Name: 20211111_A20_R3_IgM
F40:MRM of 2 channels,ES+
952.7 > 204.1
1.338e+005

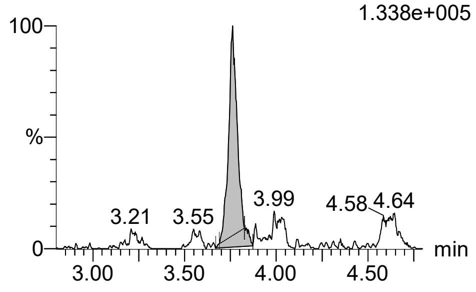

Sample Name: 20211111_A20_R3_IgM
F40:MRM of 2 channels,ES+
952.7 > 274.1
3.523e+004

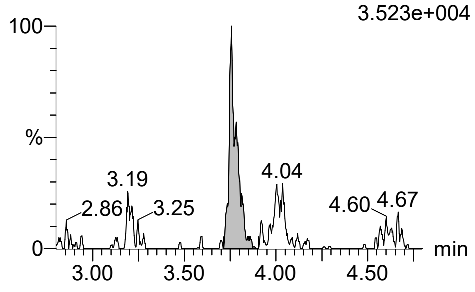

Dataset: C:\MassLynx\10.pro\20211111_clinicalsamples_IgM_IgA.qld

Last Altered: Thursday, March 09, 2023 19:58:25 台北標準時間
Printed: Friday, August 01, 2025 15:28:32 台北標準時間

Sample Name: 20211111_L19_R3_IgM
F40:MRM of 2 channels,ES+
952.7 > 204.1
1.665e+005

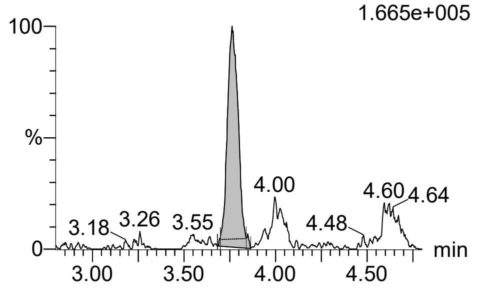

Sample Name: 20211111_L19_R3_IgM
F40:MRM of 2 channels,ES+
952.7 > 274.1
5.051e+004

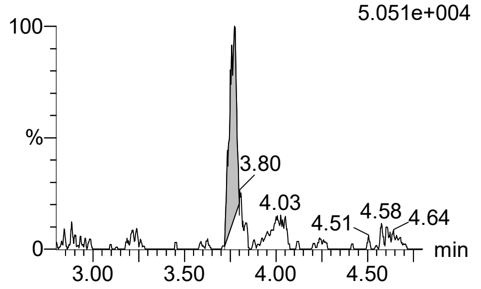

Sample Name: 20211111_QC_R21_IgM
F40:MRM of 2 channels,ES+
952.7 > 204.1
2.654e+005

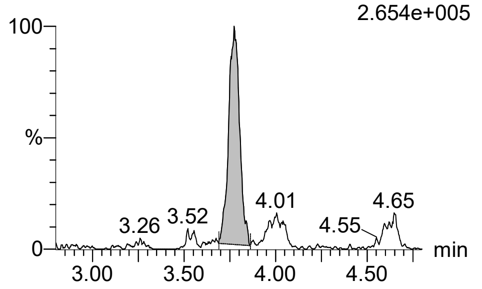

Sample Name: 20211111_QC_R21_IgM
F40:MRM of 2 channels,ES+
952.7 > 274.1
5.651e+004

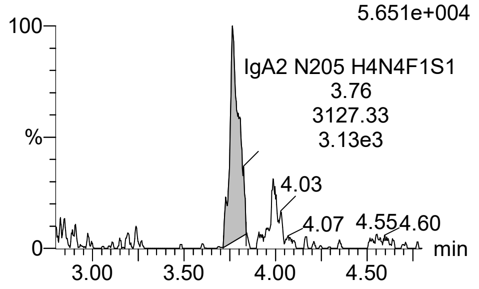

Sample Name: 20211111_L20_R3_IgM
F40:MRM of 2 channels,ES+
952.7 > 204.1
3.274e+005

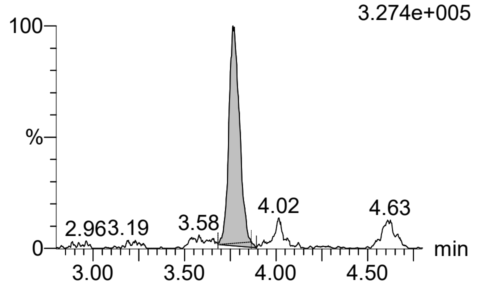

Sample Name: 20211111_L20_R3_IgM
F40:MRM of 2 channels,ES+
952.7 > 274.1
7.317e+004

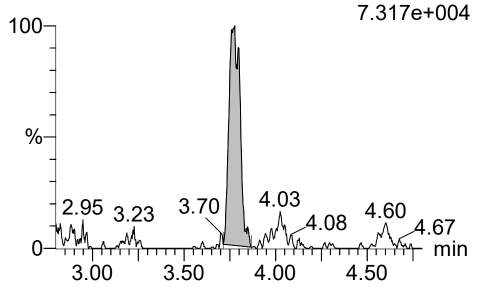

Sample Name: 20211111_H10_R3_IgM
F40:MRM of 2 channels,ES+
952.7 > 204.1
4.251e+005

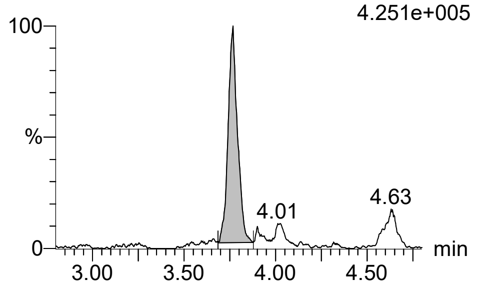

Sample Name: 20211111_H10_R3_IgM
F40:MRM of 2 channels,ES+
952.7 > 274.1
9.669e+004

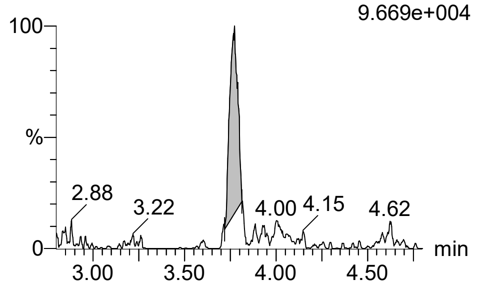

Sample Name: 20211111_A21_R3_IgM
F40:MRM of 2 channels,ES+
952.7 > 204.1
2.119e+005

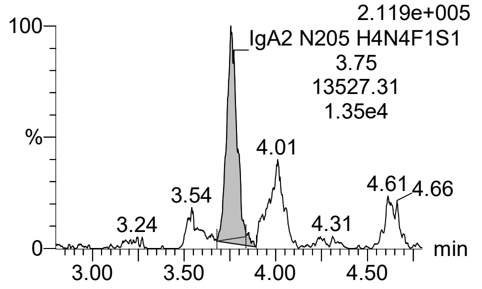

Sample Name: 20211111_A21_R3_IgM
F40:MRM of 2 channels,ES+
952.7 > 274.1
3.232e+004

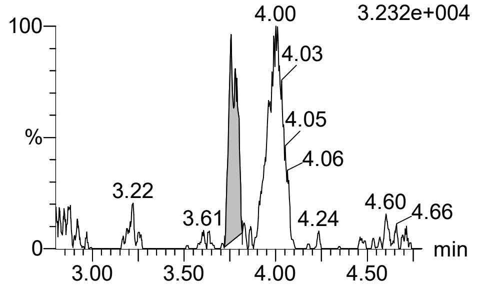

Sample Name: 20211111_A22_R3_IgM
F40:MRM of 2 channels,ES+
952.7 > 204.1
8.519e+004

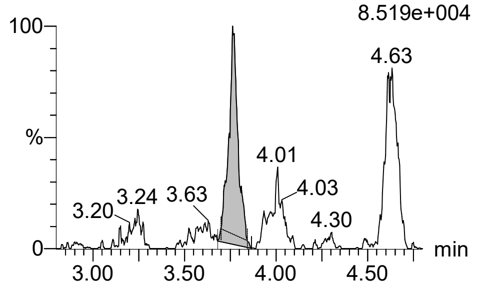

Sample Name: 20211111_A22_R3_IgM
F40:MRM of 2 channels,ES+
952.7 > 274.1
1.303e+004

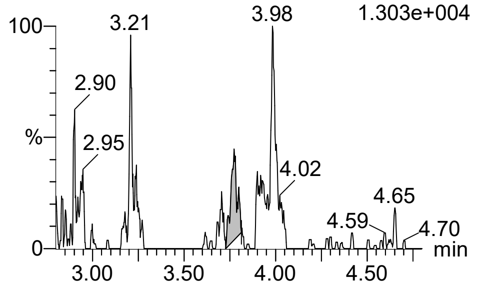

Sample Name: 20211111_L21_R3_IgM
F40:MRM of 2 channels,ES+
952.7 > 204.1
8.441e+004

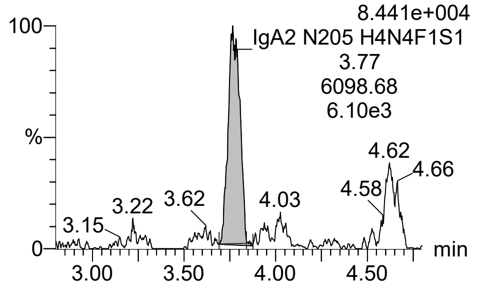

Sample Name: 20211111_L21_R3_IgM
F40:MRM of 2 channels,ES+
952.7 > 274.1
2.083e+004

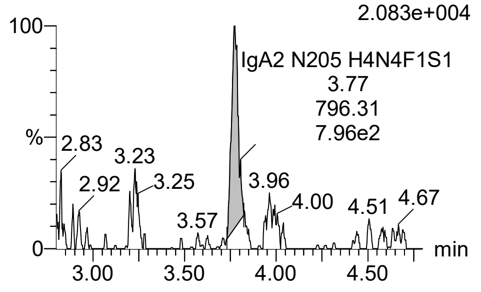

Sample Name: 20211111_L22_R3_IgM
F40:MRM of 2 channels,ES+
952.7 > 204.1
2.481e+005

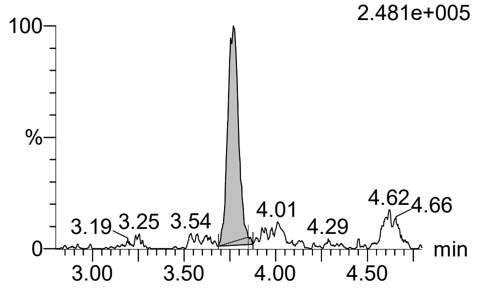

Sample Name: 20211111_L22_R3_IgM

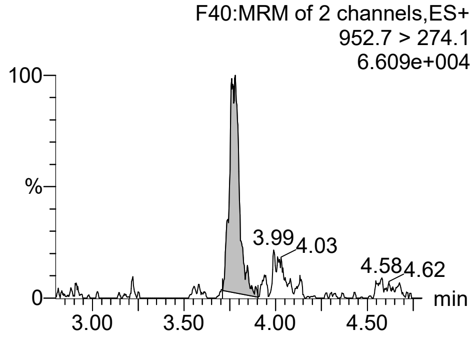

Sample Name: 20211111_H11_R3_IgM

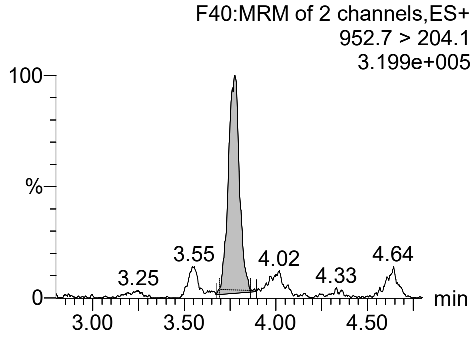

Sample Name: 20211111_H11_R3_IgM

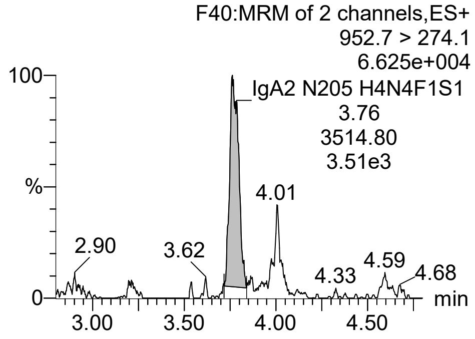

Sample Name: 20211111_A23_R3_IgM

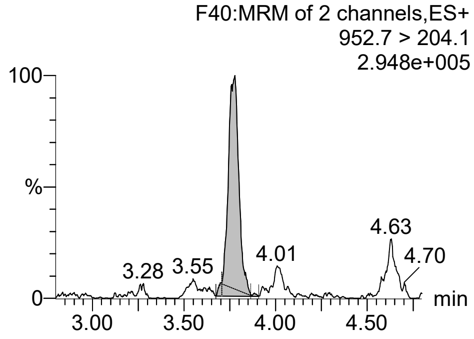

Sample Name: 20211111_A23_R3_IgM

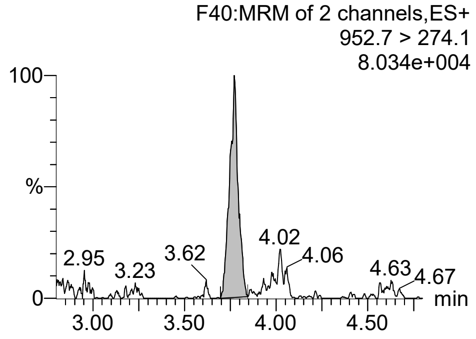

Sample Name: 20211111_A24_R3_IgM

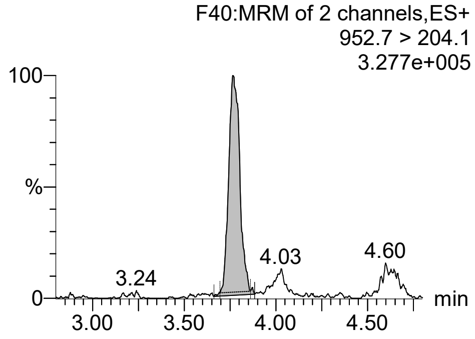

Sample Name: 20211111_A24_R3_IgM

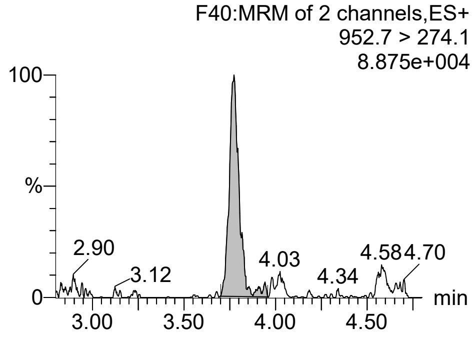

Sample Name: 20211111_L23_R3_IgM

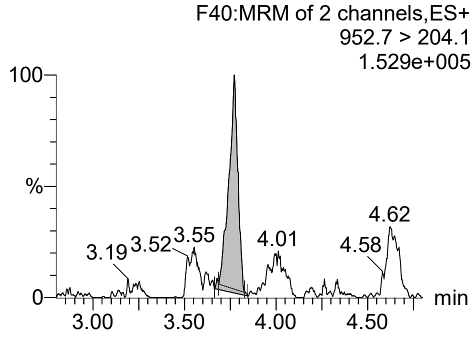

Sample Name: 20211111_L23_R3_IgM

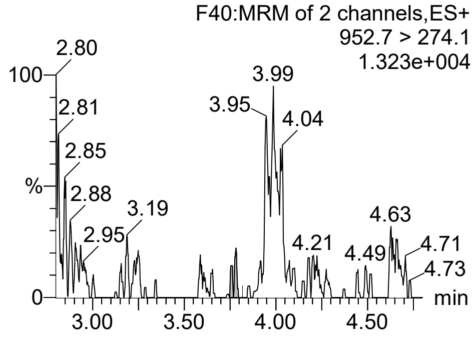

Sample Name: 20211111_L24_R3_IgM

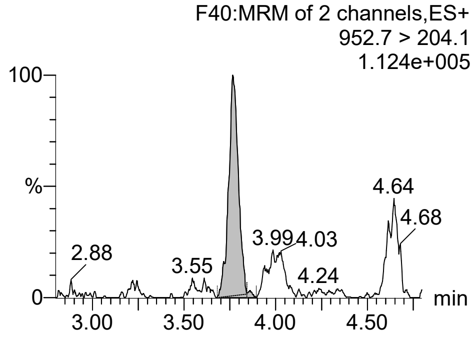

Sample Name: 20211111_L24_R3_IgM

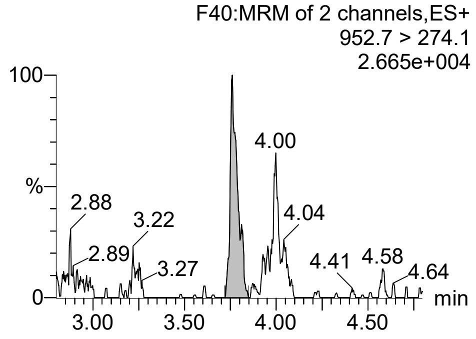

Sample Name: 20211111_H12_R3_IgM

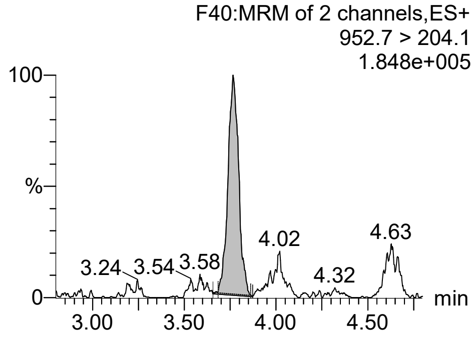

Sample Name: 20211111_H12_R3_IgM

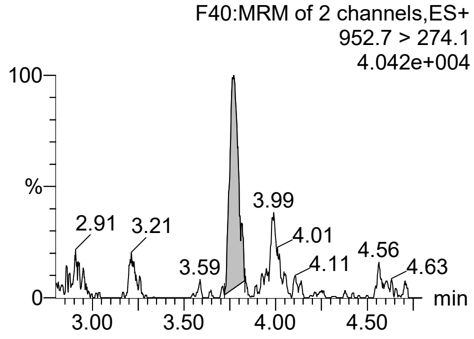

Sample Name: 20211111_QC_R22_IgM

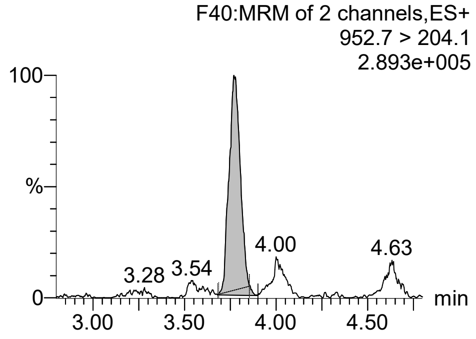

Sample Name: 20211111_QC_R22_IgM

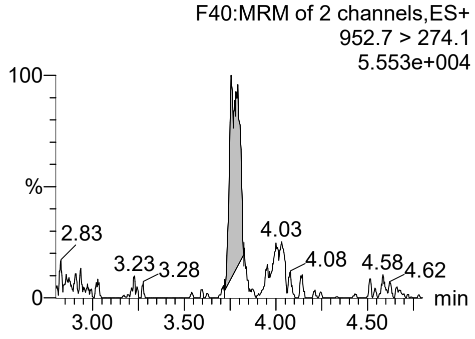

Sample Name: 20211111_A25_R3_IgM
F40:MRM of 2 channels,ES+
952.7 > 204.1
1.614e+005

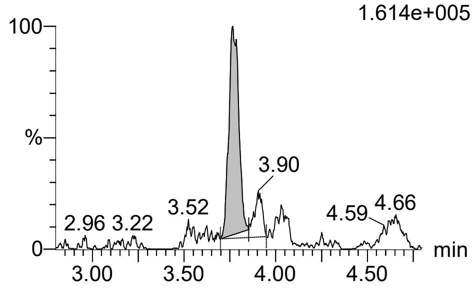

Sample Name: 20211111_A25_R3_IgM
F40:MRM of 2 channels,ES+
952.7 > 274.1
4.108e+004

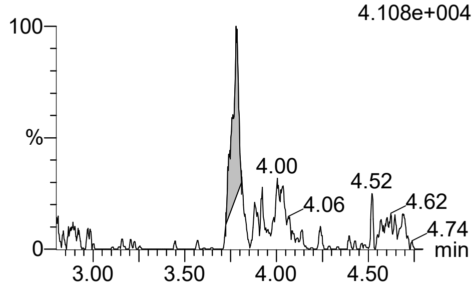

Sample Name: 20211111_A26_R3_IgM
F40:MRM of 2 channels,ES+
952.7 > 204.1
2.016e+005

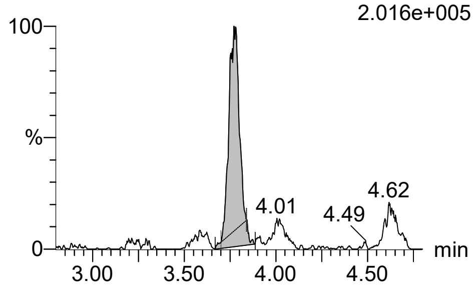

Sample Name: 20211111_A26_R3_IgM
F40:MRM of 2 channels,ES+
952.7 > 274.1
3.981e+004

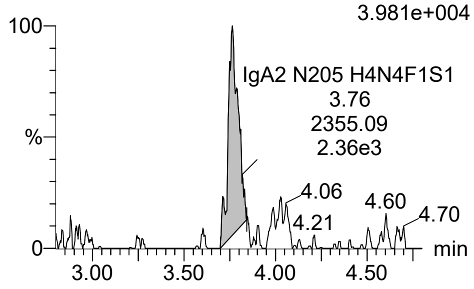

Sample Name: 20211111_L25_R3_IgM
F40:MRM of 2 channels,ES+
952.7 > 204.1
2.564e+005

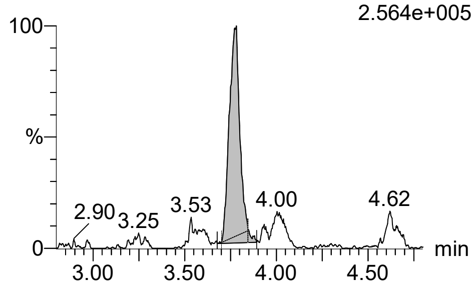

Sample Name: 20211111_L25_R3_IgM
F40:MRM of 2 channels,ES+
952.7 > 274.1
5.955e+004

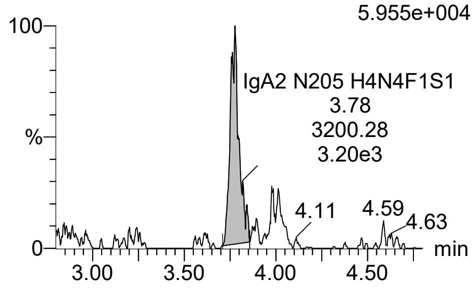

Sample Name: 20211111_L26_R3_IgM
F40:MRM of 2 channels,ES+
952.7 > 204.1
1.112e+005

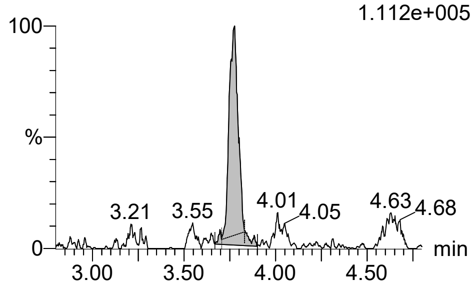

Sample Name: 20211111_L26_R3_IgM
F40:MRM of 2 channels,ES+
952.7 > 274.1
3.058e+004

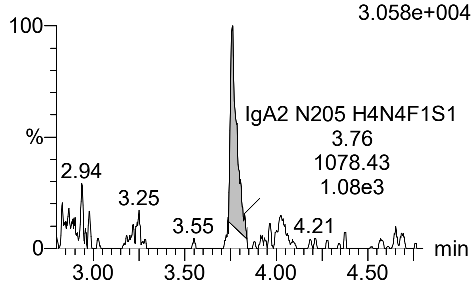

Sample Name: 20211111_H13_R3_IgM
F40:MRM of 2 channels,ES+
952.7 > 204.1
3.176e+005

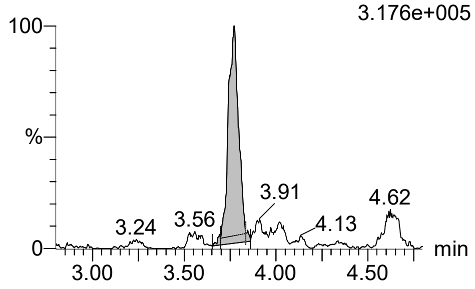

Sample Name: 20211111_H13_R3_IgM
F40:MRM of 2 channels,ES+
952.7 > 274.1
7.140e+004

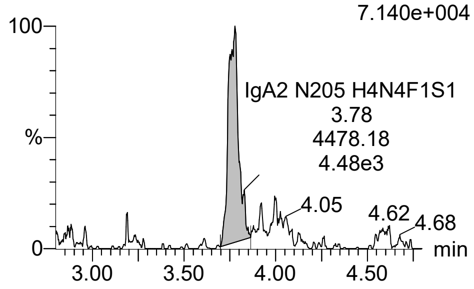

Sample Name: 20211111_A27_R3_IgM
F40:MRM of 2 channels,ES+
952.7 > 204.1
5.026e+005

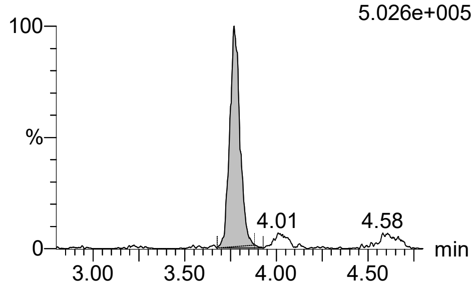

Sample Name: 20211111_A27_R3_IgM
F40:MRM of 2 channels,ES+
952.7 > 274.1
1.228e+005

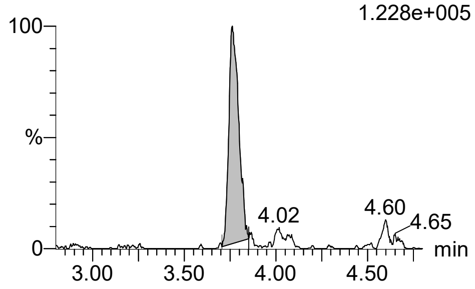

Sample Name: 20211111_A28_R3_IgM
F40:MRM of 2 channels,ES+
952.7 > 204.1
2.277e+005

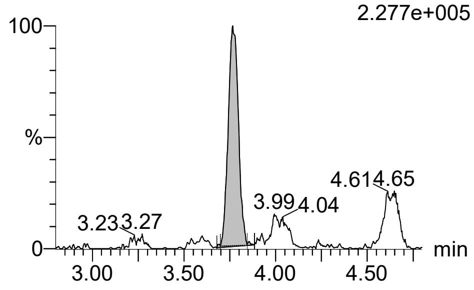

Sample Name: 20211111_A28_R3_IgM
F40:MRM of 2 channels,ES+
952.7 > 274.1
5.485e+004

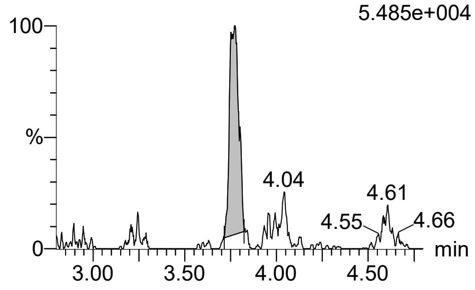

Sample Name: 20211111_L27_R3_IgM
F40:MRM of 2 channels,ES+
952.7 > 204.1
3.889e+005

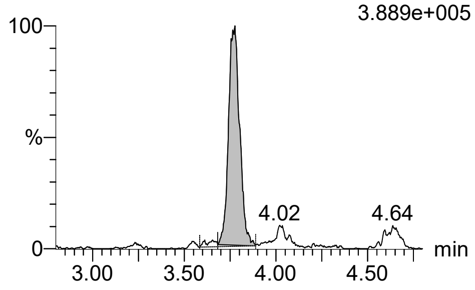

Dataset: C:\MassLynx\10.pro\20211111_clinicalsamples_IgM_IgA.qld

Last Altered: Thursday, March 09, 2023 19:58:25 台北標準時間
Printed: Friday, August 01, 2025 15:28:32 台北標準時間

Sample Name: 20211111_L27_R3_IgM
F40:MRM of 2 channels,ES+
952.7 > 274.1
1.064e+005

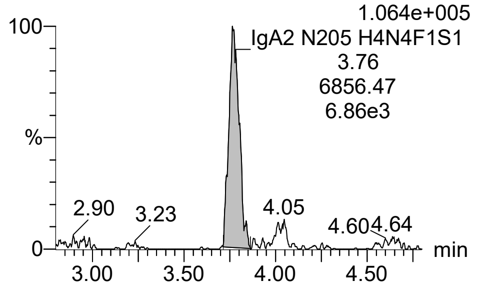

Sample Name: 20211111_L28_R3_IgM
F40:MRM of 2 channels,ES+
952.7 > 204.1
1.315e+005

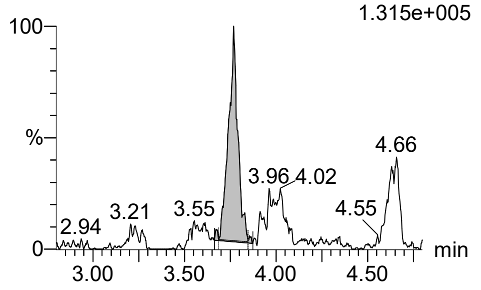

Sample Name: 20211111_L28_R3_IgM
F40:MRM of 2 channels,ES+
952.7 > 274.1
1.973e+004

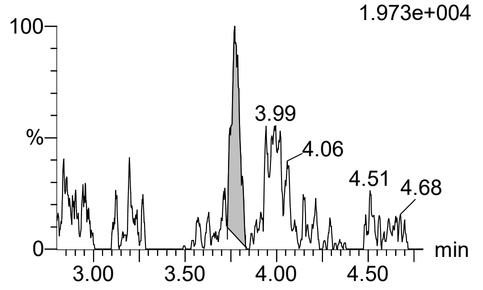

Sample Name: 20211111_H14_R3_IgM
F40:MRM of 2 channels,ES+
952.7 > 204.1
3.183e+005

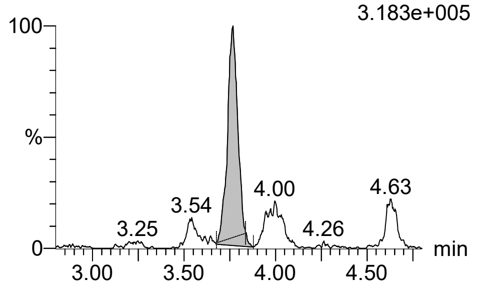

Sample Name: 20211111_H14_R3_IgM
F40:MRM of 2 channels,ES+
952.7 > 274.1
5.990e+004

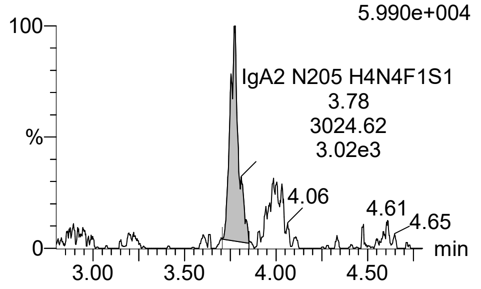

Sample Name: 20211111_A29_R3_IgM
F40:MRM of 2 channels,ES+
952.7 > 204.1
9.248e+004

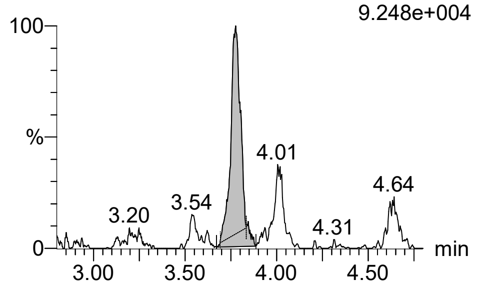

Sample Name: 20211111_A29_R3_IgM
F40:MRM of 2 channels,ES+
952.7 > 274.1
1.019e+004

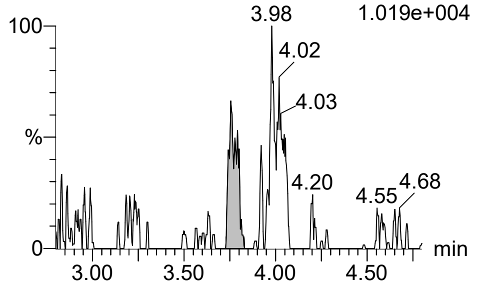

Sample Name: 20211111_A30_R3_IgM
F40:MRM of 2 channels,ES+
952.7 > 204.1
5.133e+005

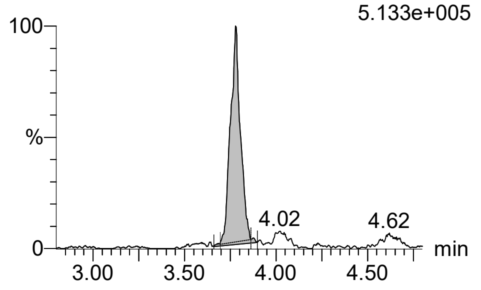

Sample Name: 20211111_A30_R3_IgM
F40:MRM of 2 channels,ES+
952.7 > 274.1
1.399e+005

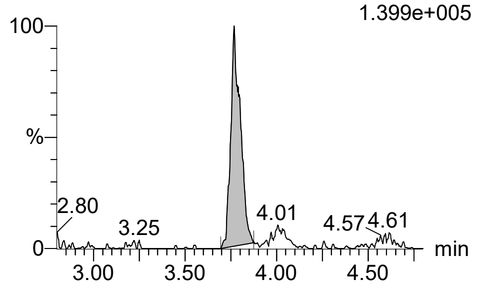

Sample Name: 20211111_QC_R23_IgM
F40:MRM of 2 channels,ES+
952.7 > 204.1
2.265e+005

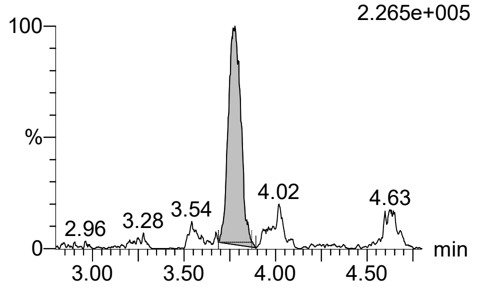

Sample Name: 20211111_QC_R23_IgM
F40:MRM of 2 channels,ES+
952.7 > 274.1
5.363e+004

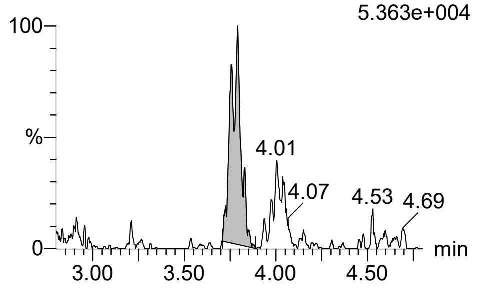

Sample Name: 20211111_L29_R3_IgM
F40:MRM of 2 channels,ES+
952.7 > 204.1
1.232e+005

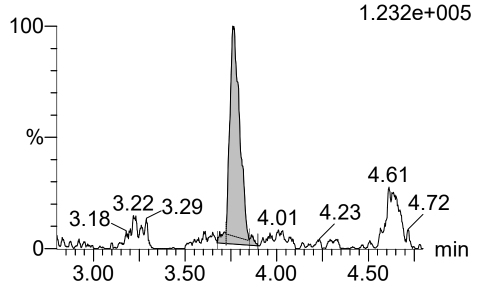

Sample Name: 20211111_L29_R3_IgM
F40:MRM of 2 channels,ES+
952.7 > 274.1
2.737e+004

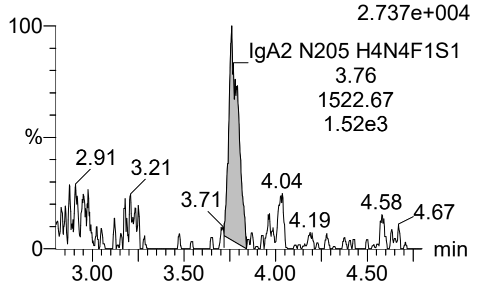

Sample Name: 20211111_L30_R3_IgM
F40:MRM of 2 channels,ES+
952.7 > 204.1
1.231e+005

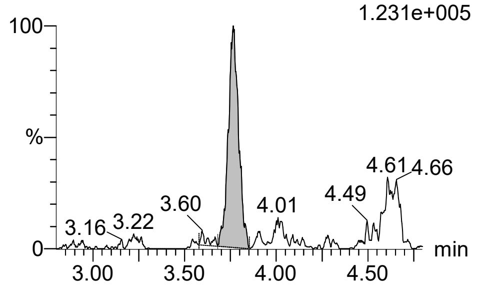

Sample Name: 20211111_L30_R3_IgM
F40:MRM of 2 channels,ES+
952.7 > 274.1
2.700e+004

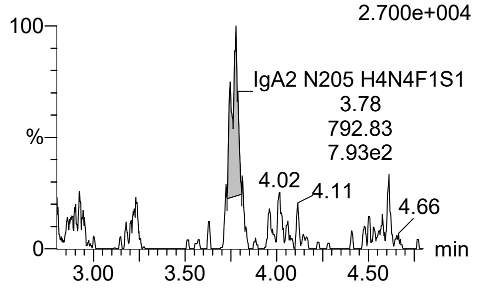

Sample Name: 20211111_H15_R3_IgM
F40:MRM of 2 channels,ES+
952.7 > 204.1
4.045e+005

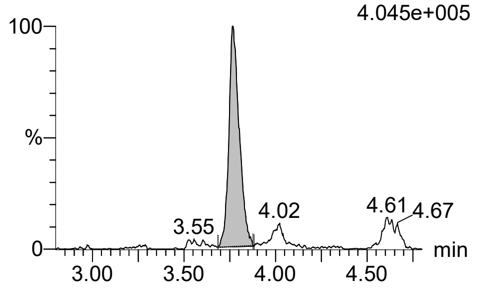

Sample Name: 20211111_H15_R3_IgM
F40:MRM of 2 channels,ES+
952.7 > 274.1
9.463e+004

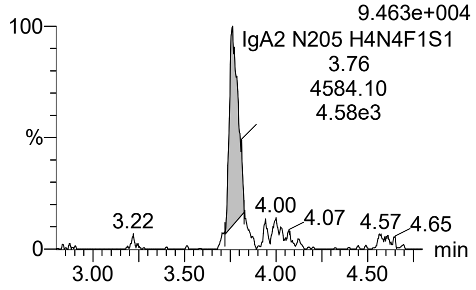

Sample Name: 20211111_A31_R3_IgM
F40:MRM of 2 channels,ES+
952.7 > 204.1
2.508e+005

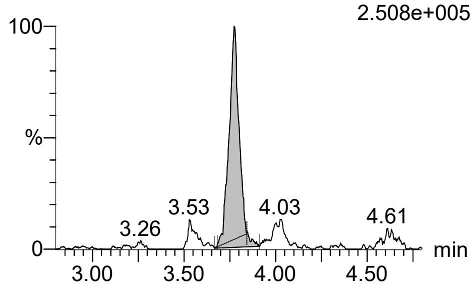

Sample Name: 20211111_A31_R3_IgM
F40:MRM of 2 channels,ES+
952.7 > 274.1
5.520e+004

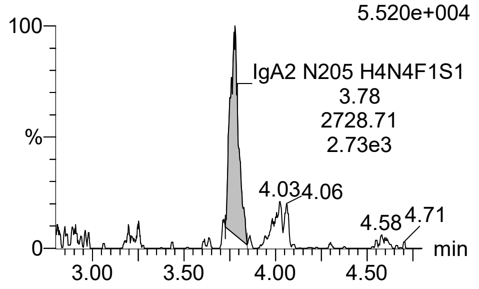

Sample Name: 20211111_A32_R3_IgM
F40:MRM of 2 channels,ES+
952.7 > 204.1
3.406e+005

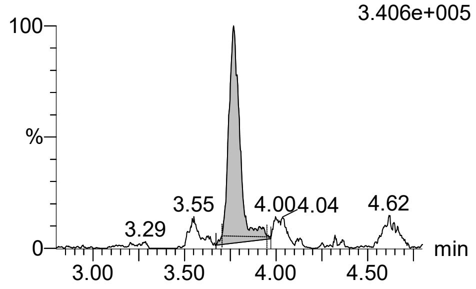

Sample Name: 20211111_A32_R3_IgM
F40:MRM of 2 channels,ES+
952.7 > 274.1
6.932e+004

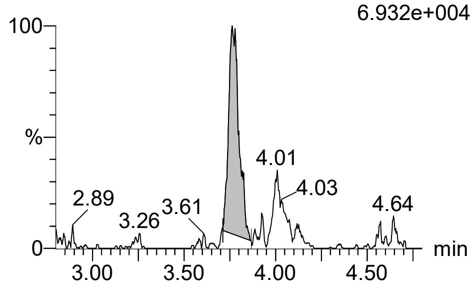

Sample Name: 20211111_L31_R3_IgM
F40:MRM of 2 channels,ES+
952.7 > 204.1
1.575e+005

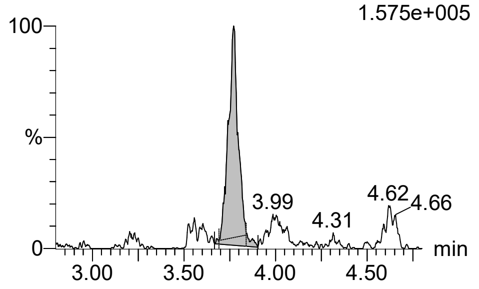

Sample Name: 20211111_L31_R3_IgM
F40:MRM of 2 channels,ES+
952.7 > 274.1
2.898e+004

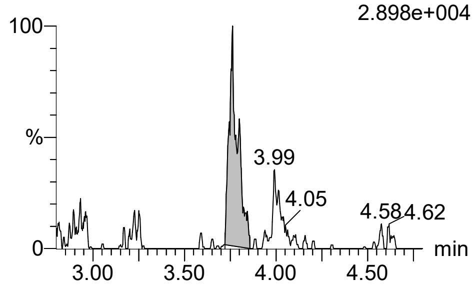

Sample Name: 20211111_L32_R3_IgM
F40:MRM of 2 channels,ES+
952.7 > 204.1
2.081e+005

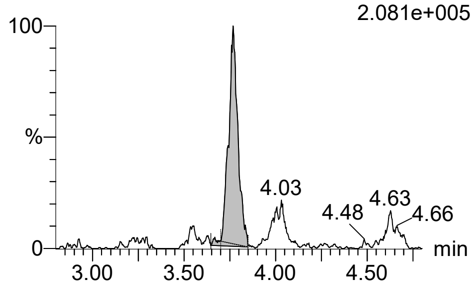

Sample Name: 20211111_L32_R3_IgM
F40:MRM of 2 channels,ES+
952.7 > 274.1
4.313e+004

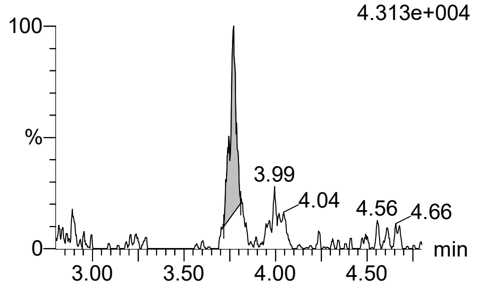

Sample Name: 20211111_H16_R3_IgM
F40:MRM of 2 channels,ES+
952.7 > 204.1
3.298e+005

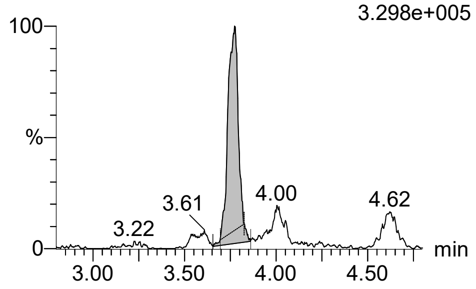

Sample Name: 20211111_H16_R3_IgM
F40:MRM of 2 channels,ES+
952.7 > 274.1
6.884e+004

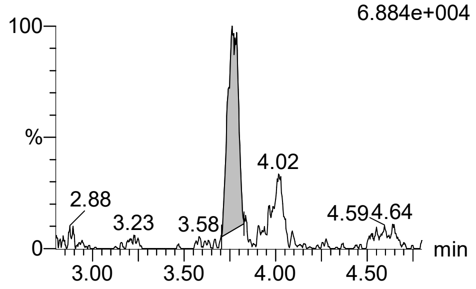

Sample Name: 20211111_A33_R3_IgM
F40:MRM of 2 channels,ES+
952.7 > 204.1
6.023e+005

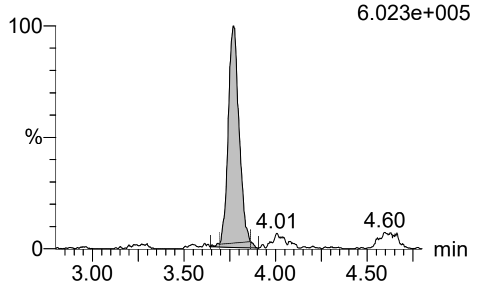

Sample Name: 20211111_A33_R3_IgM
F40:MRM of 2 channels,ES+
952.7 > 274.1
1.754e+005

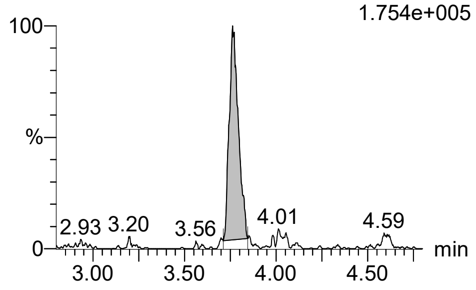

Sample Name: 20211111_A34_R3_IgM
F40:MRM of 2 channels,ES+
952.7 > 204.1
1.355e+005

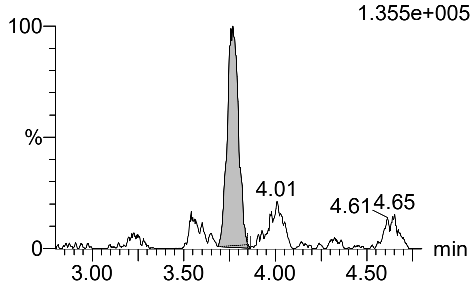

Dataset: C:\MassLynx\10.pro\20211111_clinicalsamples_IgM_IgA.qld

Last Altered: Thursday, March 09, 2023 19:58:25 台北標準時間
Printed: Friday, August 01, 2025 15:28:32 台北標準時間

Sample Name: 20211111_A34_R3_IgM
F40:MRM of 2 channels,ES+
952.7 > 274.1
3.000e+004

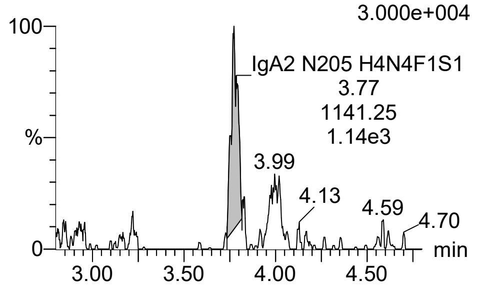

Sample Name: 20211111_H17_R3_IgM
F40:MRM of 2 channels,ES+
952.7 > 204.1
3.311e+005

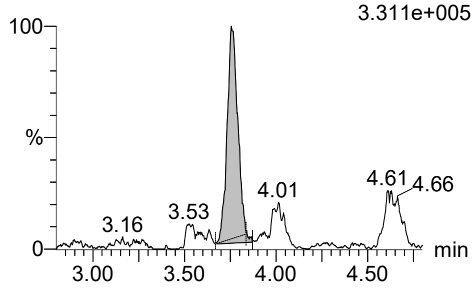

Sample Name: 20211111_H17_R3_IgM
F40:MRM of 2 channels,ES+
952.7 > 274.1
6.422e+004

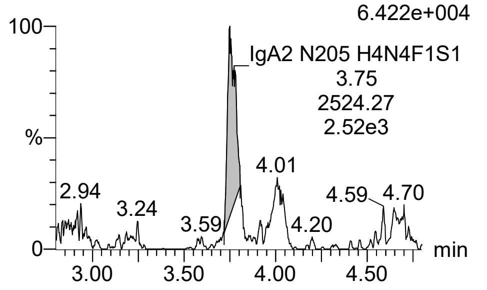

Sample Name: 20211111_A35_R3_IgM
F40:MRM of 2 channels,ES+
952.7 > 204.1
1.206e+005

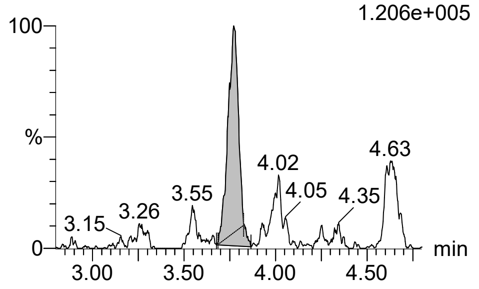

Sample Name: 20211111_A35_R3_IgM
F40:MRM of 2 channels,ES+
952.7 > 274.1
1.768e+004

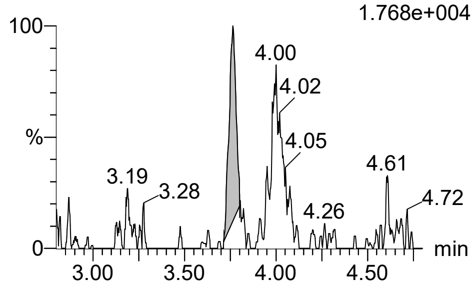

Sample Name: 20211111_QC_R24_IgM
F40:MRM of 2 channels,ES+
952.7 > 204.1
1.988e+005

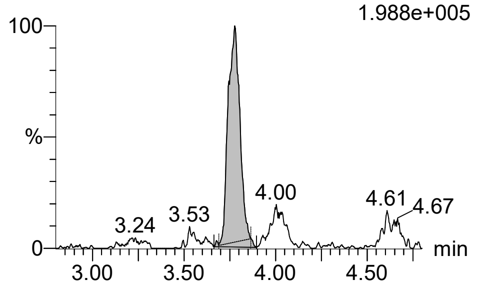

Sample Name: 20211111_QC_R24_IgM
F40:MRM of 2 channels,ES+
952.7 > 274.1
5.797e+004

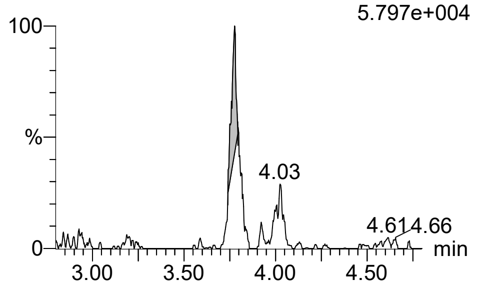

Sample Name: 20211111_A36_R3_IgM
F40:MRM of 2 channels,ES+
952.7 > 204.1
3.028e+005

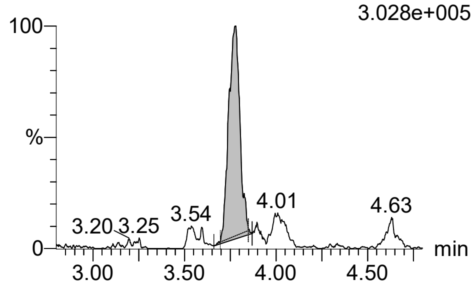

Sample Name: 20211111_A36_R3_IgM
F40:MRM of 2 channels,ES+
952.7 > 274.1
6.741e+004

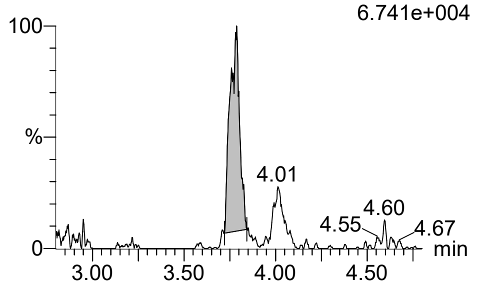

Sample Name: 20211111_H18_R3_IgM
F40:MRM of 2 channels,ES+
952.7 > 204.1
5.251e+005

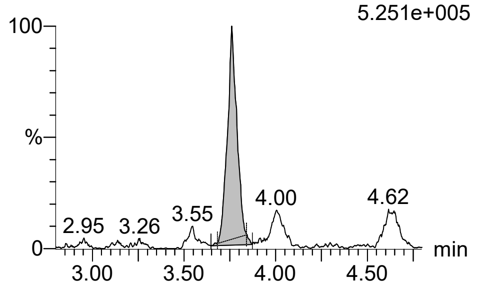

Sample Name: 20211111_H18_R3_IgM
F40:MRM of 2 channels,ES+
952.7 > 274.1
9.345e+004

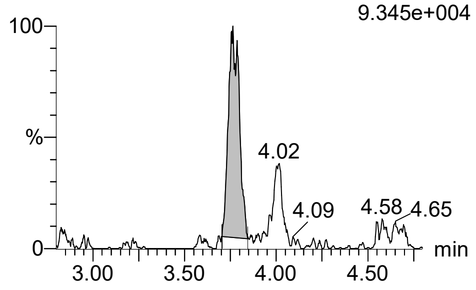

Sample Name: 20211111_A37_R3_IgM
F40:MRM of 2 channels,ES+
952.7 > 204.1
8.225e+004

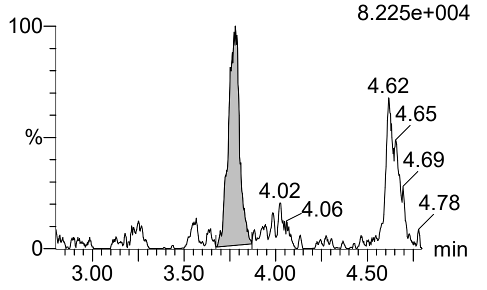

Sample Name: 20211111_A37_R3_IgM
F40:MRM of 2 channels,ES+
952.7 > 274.1
1.420e+004

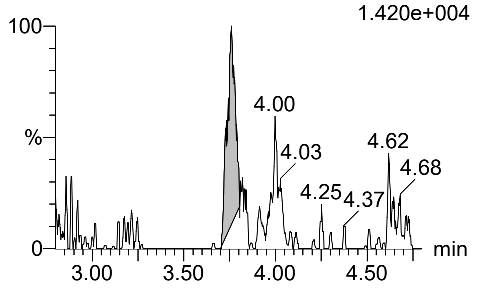

Sample Name: 20211111_A38_R3_IgM
F40:MRM of 2 channels,ES+
952.7 > 204.1
1.234e+005

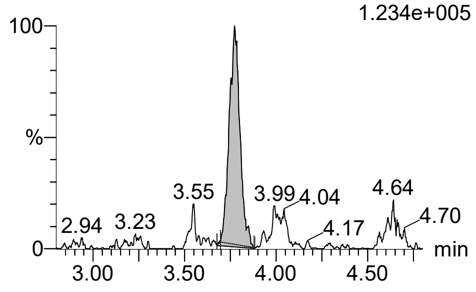

Sample Name: 20211111_A38_R3_IgM
F40:MRM of 2 channels,ES+
952.7 > 274.1
1.849e+004

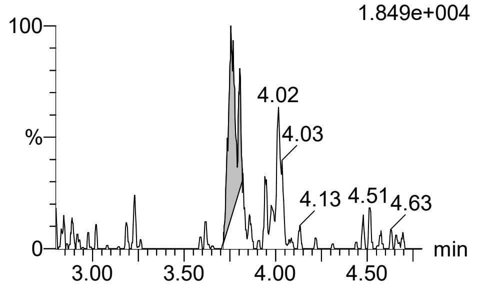

Dataset: C:\MassLynx\10.pro\20211111_clinicalsamples_IgM_IgA.qld

Last Altered: Thursday, March 09, 2023 19:58:25 台北標準時間
Printed: Friday, August 01, 2025 15:28:32 台北標準時間

Sample Name: 20211111_H19_R3_IgM
F40:MRM of 2 channels,ES+
952.7 > 204.1
3.830e+005

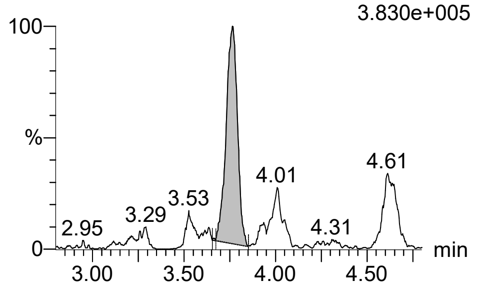

Sample Name: 20211111_H19_R3_IgM
F40:MRM of 2 channels,ES+
952.7 > 274.1
7.415e+004

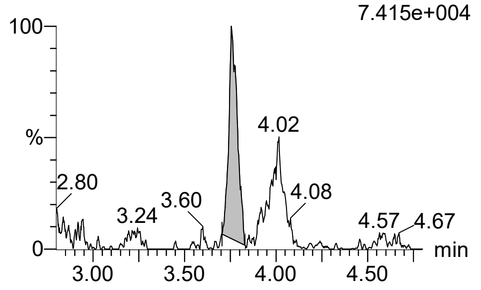

Sample Name: 20211111_A39_R3_IgM
F40:MRM of 2 channels,ES+
952.7 > 204.1
4.863e+005

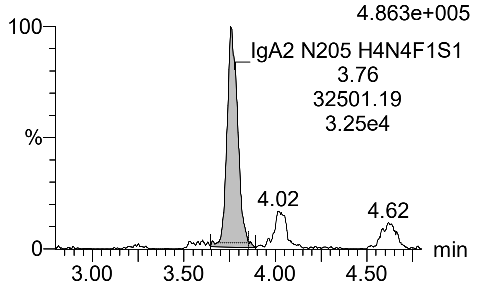

Sample Name: 20211111_A39_R3_IgM
F40:MRM of 2 channels,ES+
952.7 > 274.1
1.164e+005

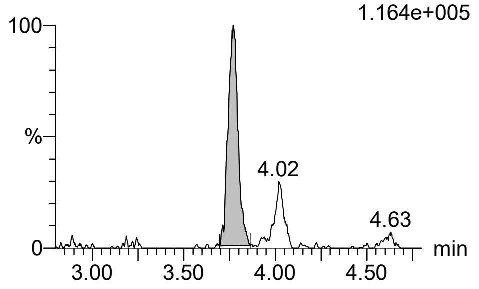

Sample Name: 20211111_A40_R3_IgM
F40:MRM of 2 channels,ES+
952.7 > 204.1
1.562e+005

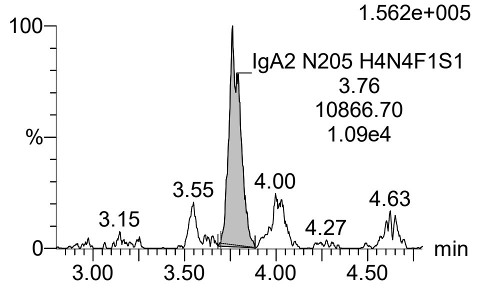

Sample Name: 20211111_A40_R3_IgM
F40:MRM of 2 channels,ES+
952.7 > 274.1
3.386e+004

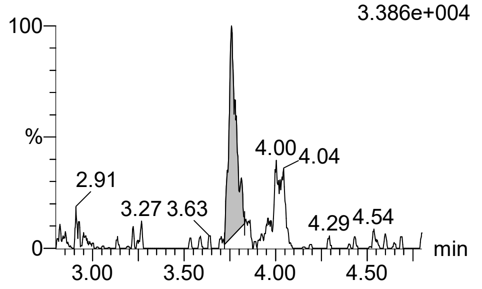

Sample Name: 20211111_H20_R3_IgM
F40:MRM of 2 channels,ES+
952.7 > 204.1
3.501e+005

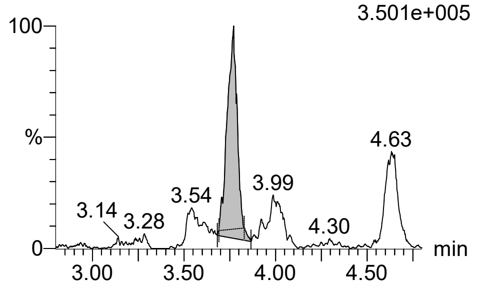

Sample Name: 20211111_H20_R3_IgM
F40:MRM of 2 channels,ES+
952.7 > 274.1
5.192e+004

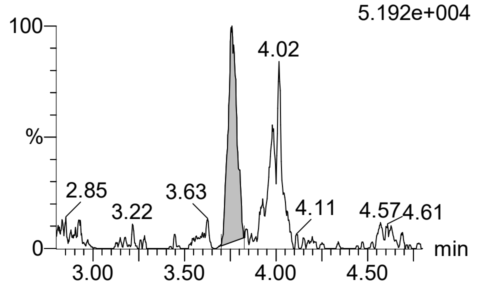

Sample Name: 20211111_A41_R3_IgM
F40:MRM of 2 channels,ES+
952.7 > 204.1
1.936e+005

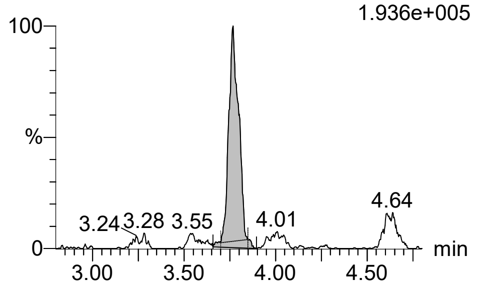

Sample Name: 20211111_A41_R3_IgM
F40:MRM of 2 channels,ES+
952.7 > 274.1
3.657e+004

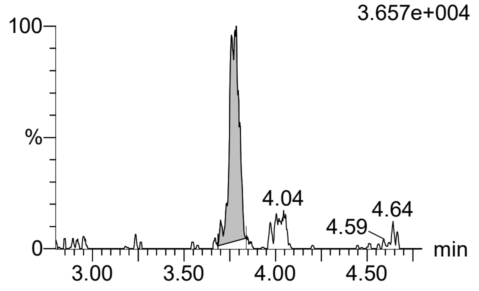

Sample Name: 20211111_A42_R3_IgM
F40:MRM of 2 channels,ES+
952.7 > 204.1
1.244e+005

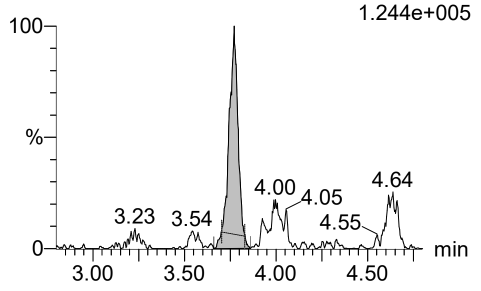

Sample Name: 20211111_A42_R3_IgM
F40:MRM of 2 channels,ES+
952.7 > 274.1
2.340e+004

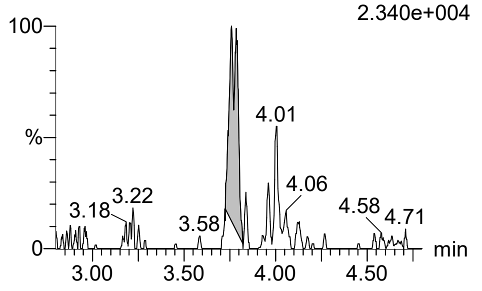

Sample Name: 20211111_A43_R3_IgM
F40:MRM of 2 channels,ES+
952.7 > 204.1
1.175e+005

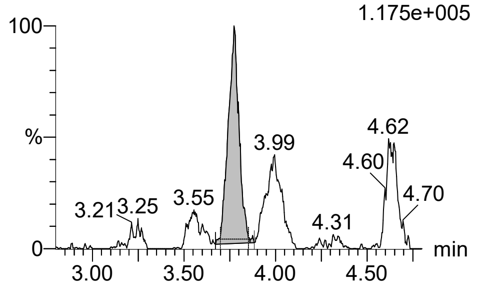

Sample Name: 20211111_A43_R3_IgM
F40:MRM of 2 channels,ES+
952.7 > 274.1
1.561e+004

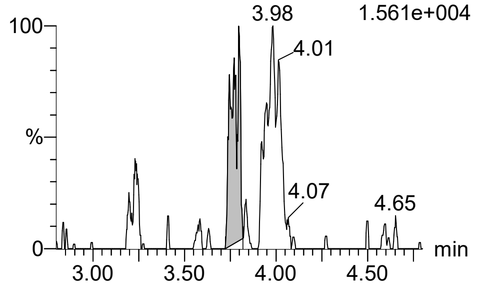

Sample Name: 20211111_A44_R3_IgM
F40:MRM of 2 channels,ES+
952.7 > 204.1
1.054e+005

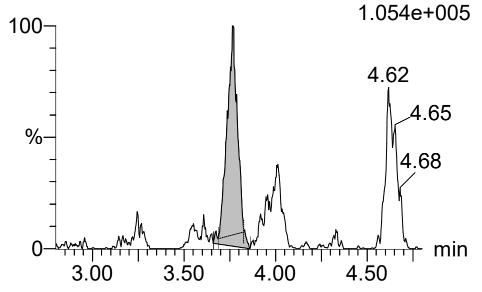

Dataset: C:\MassLynx\10.pro\20211111_clinicalsamples_IgM_IgA.qld

Last Altered: Thursday, March 09, 2023 19:58:25 台北標準時間
Printed: Friday, August 01, 2025 15:28:32 台北標準時間

Sample Name: 20211111_A44_R3_IgM

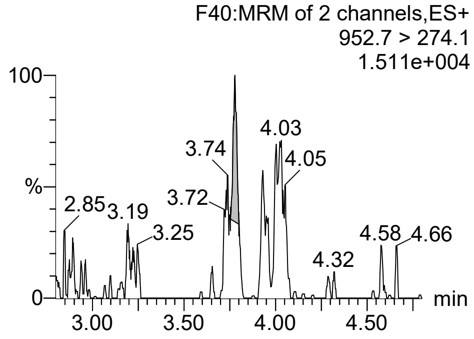

Sample Name: 20211111_QC_R25_IgM

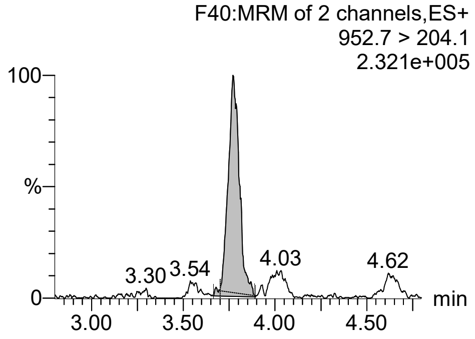

Sample Name: 20211111_QC_R25_IgM

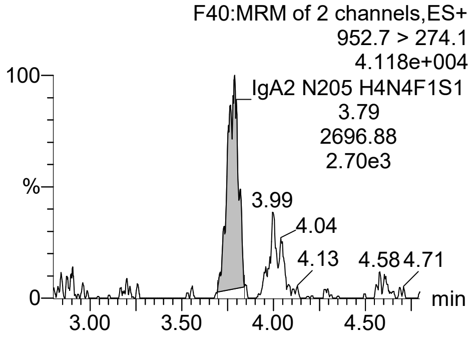

Dataset: C:\MassLynx\10.pro\20211111_clinicalsamples_IgM_IgA.qld

Last Altered: Thursday, March 09, 2023 19:58:25 台北標準時間
Printed: Friday, August 01, 2025 15:28:32 台北標準時間

	# Name	RT	Area	IS Area	Response	Prima...
1	1 20211112_QC_R001_IgM	3.77	16361.7	15421.4	1.061	bb
2	2 20211112_QC_R002_IgM	3.77	19197.9	13188.8	1.456	MM
3	3 20211111_A1_R2_IgM	3.77	22620.5	11871.0	1.906	MM
4	4 20211111_A1_R3_IgM	3.78	24281.7	12030.5	2.018	bb
5	5 20211111_A2_R2_IgM	3.76	28607.3	11446.8	2.499	MM
6	6 20211111_A2_R3_IgM	3.76	28090.5	11098.4	2.531	MM
7	7 20211111_L1_R2_IgM	3.77	6375.3	11779.4	0.541	MM
8	8 20211111_L1_R3_IgM	3.77	6265.3	11176.2	0.561	MM
9	9 20211111_L2_R2_IgM	3.77	18811.1	11375.0	1.654	MM
10	10 20211111_H1_R2_IgM	3.76	42483.3	13595.2	3.125	MM
11	11 20211111_A3_R2_IgM	3.77	14140.0	14112.3	1.002	MM
12	12 20211111_A4_R2_IgM	3.77	25923.2	11977.5	2.164	MM
13	13 20211111_L3_R2_IgM	3.75	19848.8	12242.1	1.621	MM
14	14 20211111_L4_R2_IgM	3.77	10230.9	12797.0	0.799	MM
15	15 20211111_H2_R2_IgM	3.76	19908.8	10006.0	1.990	MM
16	16 20211111_A5_R2_IgM	3.76	73585.4	11434.6	6.435	MM
17	17 20211111_A6_R2_IgM	3.76	29696.8	13193.7	2.251	MM
18	18 20211111_QC_R10_IgM	3.77	19555.4	12566.1	1.556	MM
19	19 20211111_L5_R2_IgM	3.76	29168.0	13616.4	2.142	bb
20	20 20211111_L6_R2_IgM	3.77	20890.0	13698.5	1.525	MM
21	21 20211111_H3_R2_IgM	3.75	16034.8	15793.1	1.015	MM
22	22 20211111_A7_R2_IgM	3.77	28996.3	12936.9	2.241	MM
23	23 20211111_A8_R2_IgM	3.77	27084.3	13108.0	2.066	MM
24	24 20211111_L7_R2_IgM	3.76	6261.3	13278.5	0.472	MM
25	25 20211111_L8_R2_IgM	3.75	8330.7	15233.6	0.547	MM
26	26 20211111_H4_R2_IgM	3.77	20734.3	16521.9	1.255	MM
27	27 20211111_A9_R2_IgM	3.77	24954.1	16451.6	1.517	MM
28	28 20211111_A10_R2_IgM	3.77	43050.4	13036.0	3.302	MM
29	29 20211111_L9_R2_IgM	3.76	24889.8	14554.1	1.710	MM
30	30 20211111_L10_R2_IgM	3.76	27454.6	12250.4	2.241	MM
31	31 20211111_QC_R11_IgM	3.76	23152.3	11459.2	2.020	MM
32	32 20211111_H5_R2_IgM	3.77	18505.5	14358.2	1.289	MM
33	33 20211111_A11_R2_IgM	3.76	35018.8	16184.1	2.164	MM
34	34 20211111_A12_R2_IgM	3.77	13279.1	13465.4	0.986	MM
35	35 20211111_L11_R2_IgM	3.76	9209.2	13277.9	0.694	MM
36	36 20211111_L12_R2_IgM	3.76	21341.5	15958.8	1.337	MM
37	37 20211111_H6_R2_IgM	3.77	19545.5	14003.5	1.396	MM
38	38 20211111_A13_R2_IgM	3.76	15651.0	13656.1	1.146	MM
39	39 20211111_A14_R2_IgM	3.78	57931.1	14071.3	4.117	MM
40	40 20211111_L13_R2_IgM	3.76	9563.0	14785.6	0.647	MM
41	41 20211111_L14_R2_IgM	3.76	20857.4	14772.2	1.412	MM
42	42 20211111_H7_R2_IgM	3.76	34450.6	15164.4	2.272	MM
43	43 20211111_A15_R2_IgM	3.77	14463.7	15721.5	0.920	MM
44	44 20211111_QC_R12_IgM	3.78	24301.0	13711.5	1.772	MM
45	45 20211111_A16_R2_IgM	3.77	13702.0	14275.6	0.960	MM
46	46 20211111_L15_R2_IgM	3.75	24636.9	12935.8	1.905	MM
47	47 20211111_L16_R2_IgM	3.76	14290.3	13342.8	1.071	MM
48	48 20211111_H8_R2_IgM	3.77	10602.3	15835.1	0.670	MM
49	49 20211111_A17_R2_IgM	3.77	61035.0	15786.7	3.866	MM
50	50 20211111_A18_R2_IgM	3.77	16662.1	16986.8	0.981	MM
51	51 20211111_L17_R2_IgM	3.77	17921.2	13342.7	1.343	MM
52	52 20211111_L18_R2_IgM	3.78	3709.8	14692.0	0.253	MM
53	53 20211111_H9_R2_IgM	3.76	16117.5	15639.7	1.031	MM
54	54 20211111_A19_R2_IgM	3.78	32674.6	14198.7	2.301	MM
55	55 20211111_A20_R2_IgM	3.76	9293.3	14162.7	0.656	MM
56	56 20211111_L19_R2_IgM	3.77	11334.0	9293.4	1.220	MM

Dataset: C:\MassLynx\10.pro\20211111_clinicalsamples_IgM_IgA.qld

Last Altered: Thursday, March 09, 2023 19:58:25 台北標準時間
Printed: Friday, August 01, 2025 15:28:32 台北標準時間

	# Name	RT	Area	IS Area	Response	Prima...
57	57 20211111_QC_R13_IgM	3.77	22853.3	13903.0	1.644	MM
58	58 20211111_L20_R2_IgM	3.75	27635.4	12781.6	2.162	MM
59	59 20211111_H10_R2_IgM	3.76	26702.9	14796.4	1.805	MM
60	60 20211111_A21_R2_IgM	3.77	13238.5	13669.0	0.969	MM
61	61 20211111_A22_R2_IgM	3.78	4761.7	14098.3	0.338	MM
62	62 20211111_L21_R2_IgM	3.77	7726.9	15678.2	0.493	MM
63	63 20211111_L22_R2_IgM	3.76	18975.8	15724.5	1.207	MM
64	64 20211111_H11_R2_IgM	3.77	25244.7	15890.2	1.589	MM
65	65 20211111_A23_R2_IgM	3.77	22222.7	13423.2	1.656	MM
66	66 20211111_A24_R2_IgM	3.77	20928.7	12438.0	1.683	MM
67	67 20211111_L23_R2_IgM	3.77	11161.3	14075.0	0.793	MM
68	68 20211111_L24_R2_IgM	3.75	8416.3	15972.1	0.527	MM
69	69 20211111_H12_R2_IgM	3.76	13144.9	16270.9	0.808	MM
70	70 20211111_QC_R14_IgM	3.78	23456.7	15806.8	1.484	MM
71	71 20211111_A25_R2_IgM	3.76	12629.4	14552.1	0.868	MM
72	72 20211111_A26_R2_IgM	3.77	15009.0	11492.7	1.306	MM
73	73 20211111_L25_R2_IgM	3.76	19478.9	14403.3	1.352	MM
74	74 20211111_L26_R2_IgM	3.77	8799.7	16533.9	0.532	MM
75	75 20211111_H13_R2_IgM	3.78	21511.4	15520.1	1.386	MM
76	76 20211111_A27_R2_IgM	3.76	37338.0	13337.4	2.799	MM
77	77 20211111_A28_R2_IgM	3.77	14025.6	11895.6	1.179	MM
78	78 20211111_L27_R2_IgM	3.76	28573.9	14125.7	2.023	MM
79	79 20211111_L28_R2_IgM	3.75	7732.5	11807.5	0.655	MM
80	80 20211111_H14_R2_IgM	3.77	21246.1	15035.7	1.413	MM
81	81 20211111_A29_R2_IgM	3.78	6336.3	13554.4	0.467	MM
82	82 20211111_A30_R2_IgM	3.77	34447.6	12984.1	2.653	MM
83	83 20211111_QC_R15_IgM	3.77	20897.0	12299.5	1.699	MM
84	84 20211111_L29_R2_IgM	3.77	6956.6	13590.7	0.512	MM
85	85 20211111_L30_R2_IgM	3.76	8384.8	12511.4	0.670	bb
86	86 20211111_H15_R2_IgM	3.76	31193.4	11885.0	2.625	MM
87	87 20211111_A31_R2_IgM	3.76	16534.2	13422.1	1.232	MM
88	88 20211111_A32_R2_IgM	3.76	24293.2	11461.3	2.120	MM
89	89 20211111_L31_R2_IgM	3.78	10599.2	13753.4	0.771	MM
90	90 20211111_L32_R2_IgM	3.77	12751.7	12949.0	0.985	MM
91	91 20211111_H16_R2_IgM	3.76	29027.9	11465.4	2.532	MM
92	92 20211111_A33_R2_IgM	3.76	41010.4	10551.5	3.887	MM
93	93 20211111_A34_R2_IgM	3.76	10594.2	12409.9	0.854	MM
94	94 20211111_H17_R2_IgM	3.77	25879.1	10160.9	2.547	MM
95	95 20211111_A35_R2_IgM	3.77	9529.0	10460.6	0.911	bb
96	96 20211111_QC_R16_IgM	3.77	18008.0	10433.5	1.726	MM
97	97 20211111_A36_R2_IgM	3.76	23567.4	12504.8	1.885	MM
98	98 20211111_H18_R2_IgM	3.76	32968.4	9140.4	3.607	MM
99	99 20211111_A37_R2_IgM	3.77	7182.9	9796.9	0.733	MM
100	1... 20211111_A38_R2_IgM	3.77	9281.2	11224.3	0.827	MM
101	1... 20211111_H19_R2_IgM	3.77	28484.2	11739.2	2.426	MM
102	1... 20211111_A39_R2_IgM	3.77	35070.1	12718.9	2.757	MM
103	1... 20211111_A40_R2_IgM	3.76	10739.5	10006.1	1.073	MM
104	1... 20211111_H20_R2_IgM	3.76	24343.8	7988.2	3.047	MM
105	1... 20211111_A41_R2_IgM	3.77	16656.7	11513.3	1.447	MM
106	1... 20211111_A42_R2_IgM	3.76	7215.8	10238.5	0.705	MM
107	1... 20211111_A43_R2_IgM	3.77	9179.9	11098.9	0.827	MM
108	1... 20211111_A44_R2_IgM	3.77	7282.9	10969.9	0.664	MM
109	1... 20211111_QC_R17_IgM	3.77	15743.4	9700.8	1.623	MM
110	1... 20211111_L2_R3_IgM	3.77	14644.0	8528.0	1.717	MM
111	1... 20211111_H1_R3_IgM	3.76	34877.0	9438.2	3.695	MM
112	1... 20211111_A3_R3_IgM	3.77	10893.2	11598.6	0.939	MM

Dataset: C:\MassLynx\10.pro\20211111_clinicalsamples_IgM_IgA.qld

Last Altered: Thursday, March 09, 2023 19:58:25 台北標準時間
Printed: Friday, August 01, 2025 15:28:32 台北標準時間

	#	Name	RT	Area	IS Area	Response	Prima...
113	1...	20211111_A4_R3_IgM	3.78	20018.1	11364.1	1.762	MM
114	1...	20211111_L3_R3_IgM	3.76	15964.4	10753.4	1.485	MM
115	1...	20211111_L4_R3_IgM	3.76	7663.1	10291.6	0.745	MM
116	1...	20211111_H2_R3_IgM	3.77	15992.3	9315.8	1.717	MM
117	1...	20211111_A5_R3_IgM	3.78	60032.5	9102.2	6.595	MM
118	1...	20211111_A6_R3_IgM	3.77	23145.9	10356.4	2.235	bb
119	1...	20211111_QC_R18_IgM	3.77	15144.3	8398.3	1.803	MM
120	1...	20211111_L5_R3_IgM	3.76	22859.9	9786.7	2.336	MM
121	1...	20211111_L6_R3_IgM	3.77	16570.4	9911.3	1.672	MM
122	1...	20211111_H3_R3_IgM	3.75	13027.3	12155.5	1.072	MM
123	1...	20211111_A7_R3_IgM	3.78	26391.7	11804.8	2.236	MM
124	1...	20211111_A8_R3_IgM	3.77	24094.5	10510.6	2.292	MM
125	1...	20211111_L7_R3_IgM	3.77	5671.0	12177.9	0.466	MM
126	1...	20211111_L8_R3_IgM	3.76	6818.1	11668.6	0.584	MM
127	1...	20211111_H4_R3_IgM	3.77	15697.5	11192.0	1.403	MM
128	1...	20211111_A9_R3_IgM	3.78	19438.7	14077.4	1.381	MM
129	1...	20211111_A10_R3_IgM	3.78	35872.1	11190.4	3.206	MM
130	1...	20211111_L9_R3_IgM	3.76	21534.7	14649.3	1.470	MM
131	1...	20211111_L10_R3_IgM	3.77	21733.5	9361.3	2.322	MM
132	1...	20211111_QC_R19_IgM	3.75	15796.8	10125.5	1.560	MM
133	1...	20211111_H5_R3_IgM	3.77	14292.6	10010.9	1.428	MM
134	1...	20211111_A11_R3_IgM	3.78	28970.3	12697.2	2.282	MM
135	1...	20211111_A12_R3_IgM	3.78	11868.4	13148.5	0.903	MM
136	1...	20211111_L11_R3_IgM	3.77	6737.0	10786.6	0.625	MM
137	1...	20211111_L12_R3_IgM	3.76	16839.2	11681.8	1.441	MM
138	1...	20211111_H6_R3_IgM	3.78	17540.9	12095.5	1.450	MM
139	1...	20211111_A13_R3_IgM	3.77	13071.9	12908.3	1.013	MM
140	1...	20211111_A14_R3_IgM	3.78	44344.5	11322.6	3.916	MM
141	1...	20211111_L13_R3_IgM	3.76	8545.8	11516.1	0.742	MM
142	1...	20211111_L14_R3_IgM	3.77	15796.4	10790.7	1.464	MM
143	1...	20211111_H7_R3_IgM	3.77	26013.4	11572.1	2.248	MM
144	1...	20211111_A15_R3_IgM	3.78	10102.3	12434.4	0.812	MM
145	1...	20211111_QC_R20_IgM	3.76	19561.3	10701.0	1.828	MM
146	1...	20211111_A16_R3_IgM	3.76	11080.7	11452.1	0.968	MM
147	1...	20211111_L15_R3_IgM	3.78	18576.4	10531.1	1.764	MM
148	1...	20211111_L16_R3_IgM	3.77	12762.6	12049.4	1.059	MM
149	1...	20211111_H8_R3_IgM	3.76	9851.8	13434.9	0.733	bb
150	1...	20211111_A17_R3_IgM	3.77	48619.8	12452.0	3.905	MM
151	1...	20211111_A18_R3_IgM	3.76	14253.5	12506.5	1.140	MM
152	1...	20211111_L17_R3_IgM	3.76	14221.7	9948.9	1.429	MM
153	1...	20211111_L18_R3_IgM	3.76	3006.8	13462.1	0.223	MM
154	1...	20211111_H9_R3_IgM	3.78	12652.0	13178.7	0.960	MM
155	1...	20211111_A19_R3_IgM	3.78	29284.4	11298.6	2.592	MM
156	1...	20211111_A20_R3_IgM	3.76	7708.6	11175.6	0.690	MM
157	1...	20211111_L19_R3_IgM	3.76	11366.4	11558.2	0.983	MM
158	1...	20211111_QC_R21_IgM	3.77	17955.0	9977.0	1.800	MM
159	1...	20211111_L20_R3_IgM	3.76	21964.9	13508.1	1.626	MM
160	1...	20211111_H10_R3_IgM	3.77	24267.0	12374.8	1.961	bb
161	1...	20211111_A21_R3_IgM	3.75	13527.3	12443.6	1.087	MM
162	1...	20211111_A22_R3_IgM	3.76	4933.1	11589.3	0.426	MM
163	1...	20211111_L21_R3_IgM	3.77	6098.7	13107.4	0.465	MM
164	1...	20211111_L22_R3_IgM	3.77	16831.5	12021.5	1.400	MM
165	1...	20211111_H11_R3_IgM	3.78	22251.2	12687.8	1.754	MM
166	1...	20211111_A23_R3_IgM	3.78	19994.6	11611.8	1.722	MM
167	1...	20211111_A24_R3_IgM	3.77	22933.0	10476.8	2.189	MM
168	1...	20211111_L23_R3_IgM	3.77	8717.5	11873.1	0.734	MM

Dataset: C:\MassLynx\10.pro\20211111_clinicalsamples_IgM_IgA.qld

Last Altered: Thursday, March 09, 2023 19:58:25 台北標準時間
Printed: Friday, August 01, 2025 15:28:32 台北標準時間

	# Name	RT	Area	IS Area	Response	Prima...
169	1... 20211111_L24_R3_IgM	3.77	7053.1	13468.0	0.524	MM
170	1... 20211111_H12_R3_IgM	3.77	12412.8	14229.1	0.872	MM
171	1... 20211111_QC_R22_IgM	3.77	20822.8	11264.4	1.849	MM
172	1... 20211111_A25_R3_IgM	3.77	9340.7	11775.7	0.793	MM
173	1... 20211111_A26_R3_IgM	3.77	14072.1	10473.9	1.344	MM
174	1... 20211111_L25_R3_IgM	3.78	17032.6	13256.8	1.285	MM
175	1... 20211111_L26_R3_IgM	3.78	6728.9	13565.0	0.496	MM
176	1... 20211111_H13_R3_IgM	3.77	20385.1	13146.0	1.551	MM
177	1... 20211111_A27_R3_IgM	3.77	32679.2	12025.6	2.717	MM
178	1... 20211111_A28_R3_IgM	3.77	14523.7	11229.1	1.293	MM
179	1... 20211111_L27_R3_IgM	3.78	26653.8	14960.0	1.782	MM
180	1... 20211111_L28_R3_IgM	3.77	7495.4	11359.0	0.660	MM
181	1... 20211111_H14_R3_IgM	3.77	20938.6	12830.8	1.632	MM
182	1... 20211111_A29_R3_IgM	3.78	6083.0	13434.3	0.453	MM
183	1... 20211111_A30_R3_IgM	3.78	32261.0	11272.2	2.862	MM
184	1... 20211111_QC_R23_IgM	3.78	17614.3	11872.2	1.484	MM
185	1... 20211111_L29_R3_IgM	3.76	7848.0	12948.7	0.606	MM
186	1... 20211111_L30_R3_IgM	3.77	7912.9	11026.6	0.718	MM
187	1... 20211111_H15_R3_IgM	3.77	26315.0	10786.3	2.440	MM
188	1... 20211111_A31_R3_IgM	3.77	16261.1	12010.5	1.354	MM
189	1... 20211111_A32_R3_IgM	3.77	23667.8	11107.7	2.131	MM
190	1... 20211111_L31_R3_IgM	3.77	9975.1	11021.8	0.905	MM
191	1... 20211111_L32_R3_IgM	3.77	12262.2	11683.8	1.050	MM
192	1... 20211111_H16_R3_IgM	3.77	21780.9	9831.7	2.215	MM
193	1... 20211111_A33_R3_IgM	3.77	42065.9	11213.1	3.751	MM
194	1... 20211111_A34_R3_IgM	3.77	9634.5	11550.1	0.834	MM
195	1... 20211111_H17_R3_IgM	3.76	22090.2	8874.4	2.489	MM
196	1... 20211111_A35_R3_IgM	3.77	8234.0	9856.3	0.835	MM
197	1... 20211111_QC_R24_IgM	3.78	15152.8	7561.8	2.004	MM
198	1... 20211111_A36_R3_IgM	3.78	20704.1	9562.8	2.165	MM
199	1... 20211111_H18_R3_IgM	3.76	32059.4	8165.3	3.926	MM
200	2... 20211111_A37_R3_IgM	3.78	5622.3	9278.8	0.606	bb
201	2... 20211111_A38_R3_IgM	3.77	8323.4	10920.3	0.762	MM
202	2... 20211111_H19_R3_IgM	3.76	26670.1	9285.3	2.872	MM
203	2... 20211111_A39_R3_IgM	3.76	32501.2	9083.4	3.578	MM
204	2... 20211111_A40_R3_IgM	3.76	10866.7	8382.4	1.296	MM
205	2... 20211111_H20_R3_IgM	3.77	21377.9	7807.9	2.738	MM
206	2... 20211111_A41_R3_IgM	3.77	12626.8	5925.2	2.131	MM
207	2... 20211111_A42_R3_IgM	3.77	7124.3	9352.6	0.762	MM
208	2... 20211111_A43_R3_IgM	3.77	7799.9	10216.3	0.763	MM
209	2... 20211111_A44_R3_IgM	3.77	7080.5	10262.7	0.690	MM
210	2... 20211111_QC_R25_IgM	3.77	16444.3	8575.3	1.918	MM

Dataset: C:\MassLynx\10.pro\20211111_clinicalsamples_IgM_IgA.qld

Last Altered: Thursday, March 09, 2023 19:58:25 台北標準時間
Printed: Friday, August 01, 2025 15:29:29 台北標準時間

Method: C:\MassLynx\10_Pro_IgA.PRO\MethDB\20211111_IgM_IgA_DMRM_new.mdb 24 Feb 2022 13:44:14
Calibration: 15 Nov 2021 14:42:11

Sample Name: 20211112_QC_R001_IgM
F49:MRM of 1 channel,ES+
1006.8 > 366.1
3.238e+006

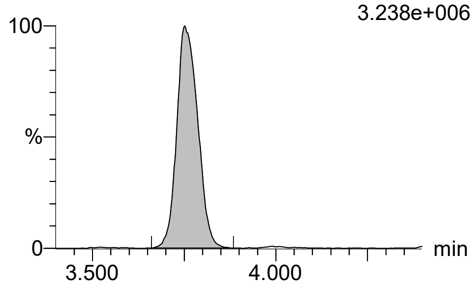

Sample Name: 20211112_QC_R002_IgM
F49:MRM of 1 channel,ES+
1006.8 > 366.1
2.960e+006

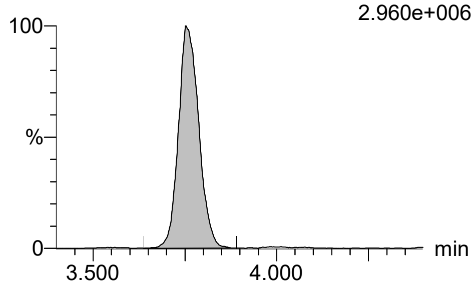

Sample Name: 20211111_A1_R2_IgM
F49:MRM of 1 channel,ES+
1006.8 > 366.1
2.078e+006

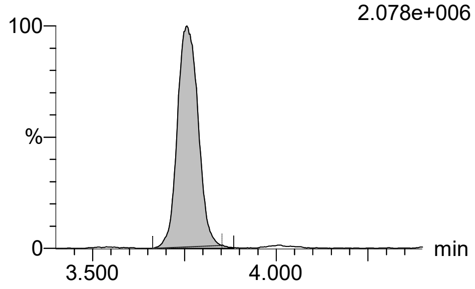

Sample Name: 20211111_A1_R3_IgM
F49:MRM of 1 channel,ES+
1006.8 > 366.1
2.104e+006

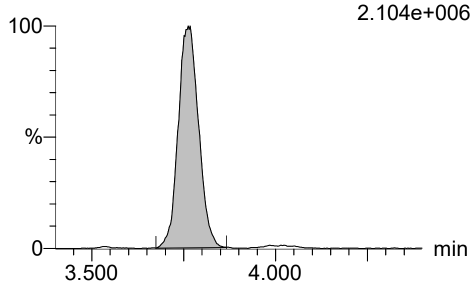

Sample Name: 20211111_A2_R2_IgM
F49:MRM of 1 channel,ES+
1006.8 > 366.1
1.849e+006

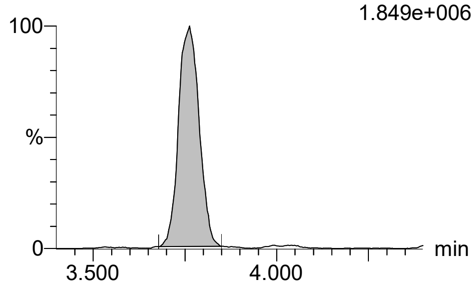

Sample Name: 20211111_A2_R3_IgM
F49:MRM of 1 channel,ES+
1006.8 > 366.1
1.731e+006

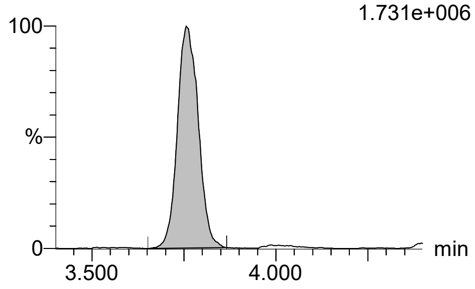

Sample Name: 20211111_L1_R2_IgM
F49:MRM of 1 channel,ES+
1006.8 > 366.1
3.381e+005

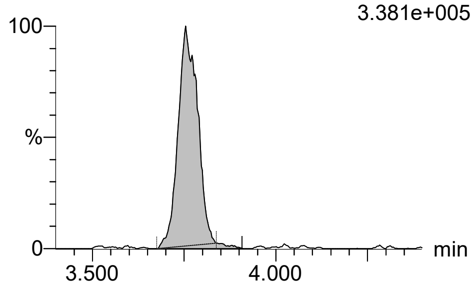

Sample Name: 20211111_L1_R3_IgM
F49:MRM of 1 channel,ES+
1006.8 > 366.1
3.271e+005

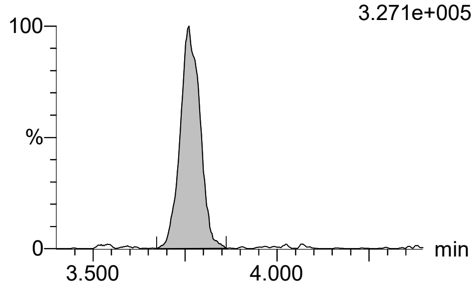

Sample Name: 20211111_L2_R2_IgM
F49:MRM of 1 channel,ES+
1006.8 > 366.1
4.636e+006

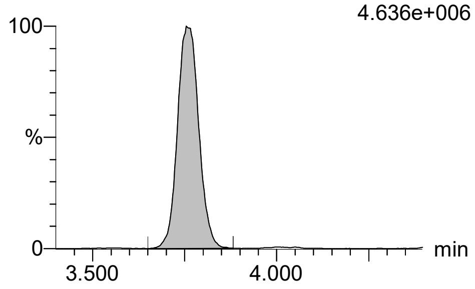

Sample Name: 20211111_H1_R2_IgM
F49:MRM of 1 channel,ES+
1006.8 > 366.1
6.427e+006

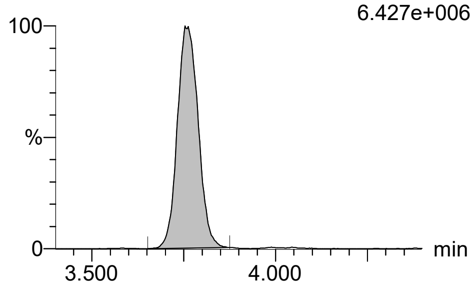

Sample Name: 20211111_A3_R2_IgM
F49:MRM of 1 channel,ES+
1006.8 > 366.1
1.927e+006

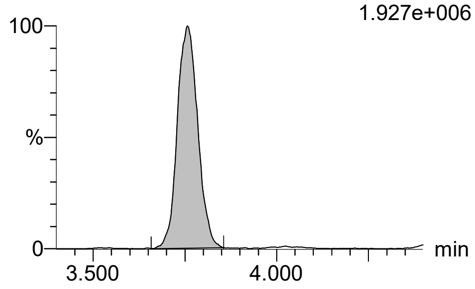

Sample Name: 20211111_A4_R2_IgM
F49:MRM of 1 channel,ES+
1006.8 > 366.1
1.957e+006

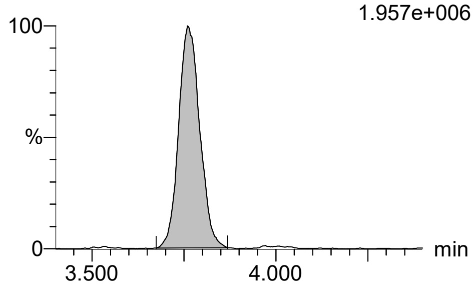

Sample Name: 20211111_L3_R2_IgM
F49:MRM of 1 channel,ES+
1006.8 > 366.1
2.681e+006

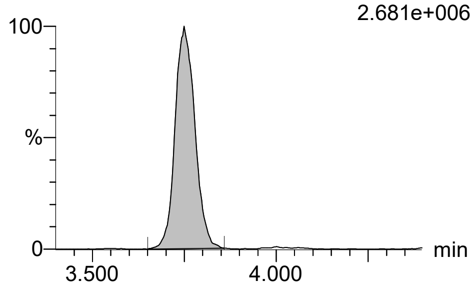

Sample Name: 20211111_L4_R2_IgM
F49:MRM of 1 channel,ES+
1006.8 > 366.1
1.556e+006

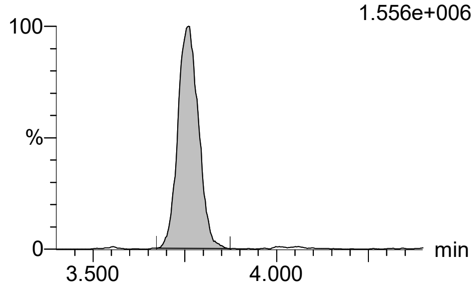

Sample Name: 20211111_H2_R2_IgM
F49:MRM of 1 channel,ES+
1006.8 > 366.1
2.854e+006

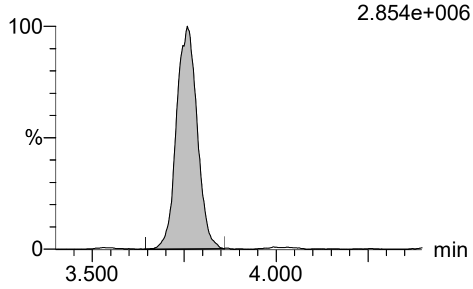

Sample Name: 20211111_A5_R2_IgM
F49:MRM of 1 channel,ES+
1006.8 > 366.1
1.061e+007

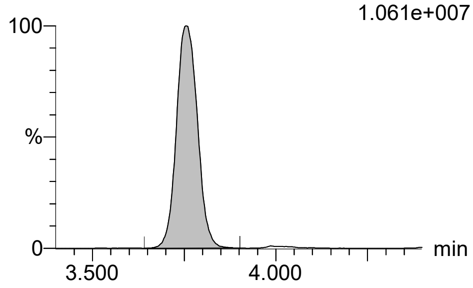

Sample Name: 20211111_A6_R2_IgM
F49:MRM of 1 channel,ES+
1006.8 > 366.1
3.778e+006

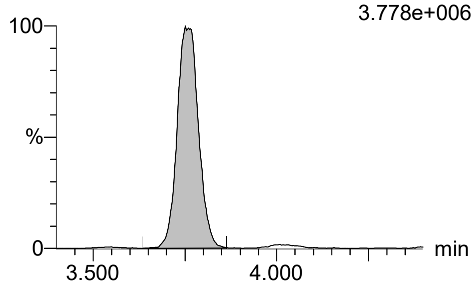

Sample Name: 20211111_QC_R10_IgM
F49:MRM of 1 channel,ES+
1006.8 > 366.1
2.563e+006

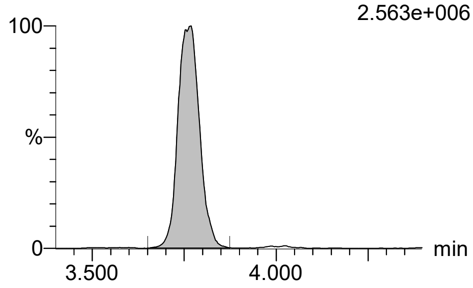

Sample Name: 20211111_L5_R2_IgM
F49:MRM of 1 channel,ES+
1006.8 > 366.1
7.755e+006

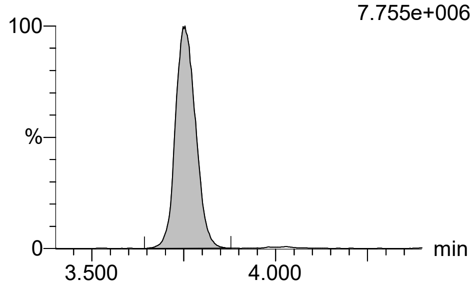

Sample Name: 20211111_L6_R2_IgM
F49:MRM of 1 channel,ES+
1006.8 > 366.1
3.172e+006

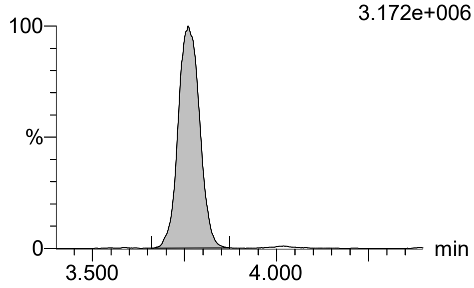

Sample Name: 20211111_H3_R2_IgM
F49:MRM of 1 channel,ES+
1006.8 > 366.1
1.859e+006

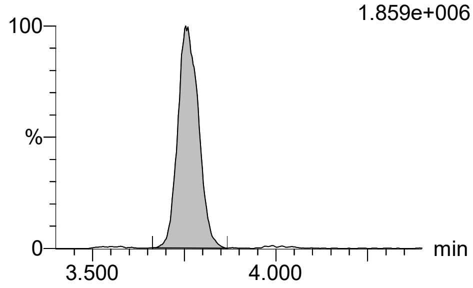

Sample Name: 20211111_A7_R2_IgM
F49:MRM of 1 channel,ES+
1006.8 > 366.1
4.181e+006

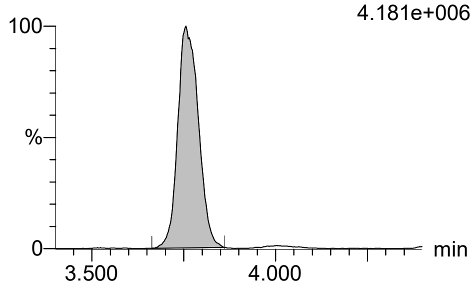

Sample Name: 20211111_A8_R2_IgM
F49:MRM of 1 channel,ES+
1006.8 > 366.1
3.886e+006

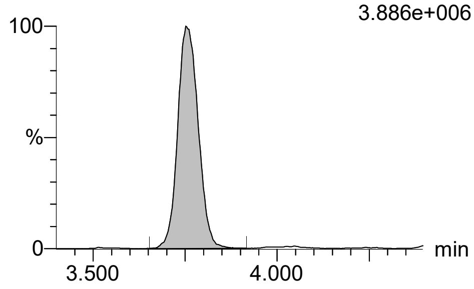

Sample Name: 20211111_L7_R2_IgM
F49:MRM of 1 channel,ES+
1006.8 > 366.1
3.302e+004

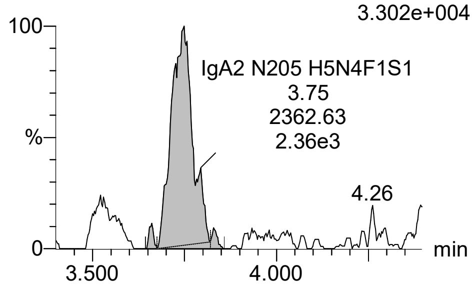

Sample Name: 20211111_L8_R2_IgM
F49:MRM of 1 channel,ES+
1006.8 > 366.1
1.147e+006

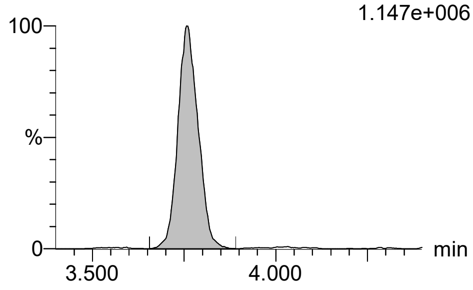

Sample Name: 20211111_H4_R2_IgM
F49:MRM of 1 channel,ES+
1006.8 > 366.1
2.558e+006

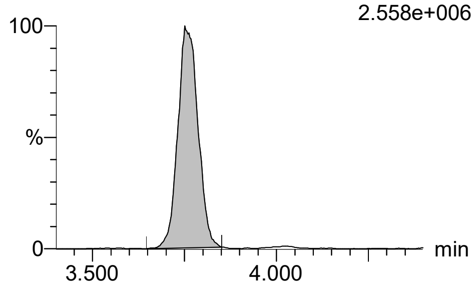

Sample Name: 20211111_A9_R2_IgM
F49:MRM of 1 channel,ES+
1006.8 > 366.1
4.395e+006

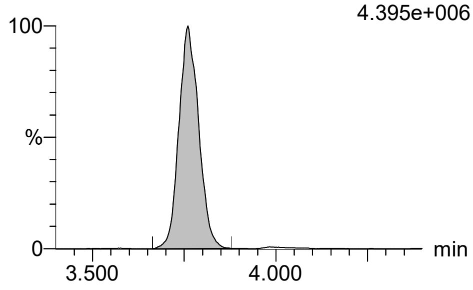

Dataset: C:\MassLynx\10.pro\20211111_clinicalsamples_IgM_IgA.qld

Last Altered: Thursday, March 09, 2023 19:58:25 台北標準時間
Printed: Friday, August 01, 2025 15:29:29 台北標準時間

Sample Name: 20211111_A10_R2_IgM
F49:MRM of 1 channel,ES+
1006.8 > 366.1
8.630e+006

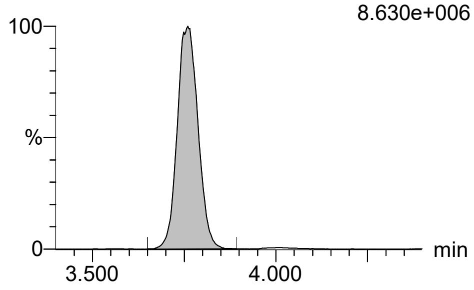

Sample Name: 20211111_L9_R2_IgM
F49:MRM of 1 channel,ES+
1006.8 > 366.1
4.195e+006

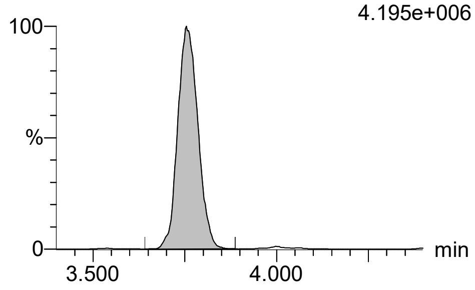

Sample Name: 20211111_L10_R2_IgM
F49:MRM of 1 channel,ES+
1006.8 > 366.1
3.819e+006

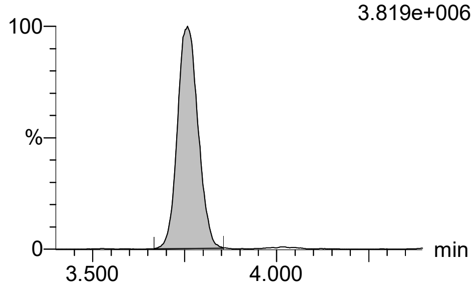

Sample Name: 20211111_QC_R11_IgM
F49:MRM of 1 channel,ES+
1006.8 > 366.1
2.833e+006

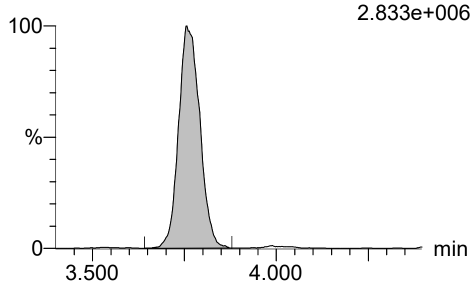

Sample Name: 20211111_H5_R2_IgM
F49:MRM of 1 channel,ES+
1006.8 > 366.1
2.309e+006

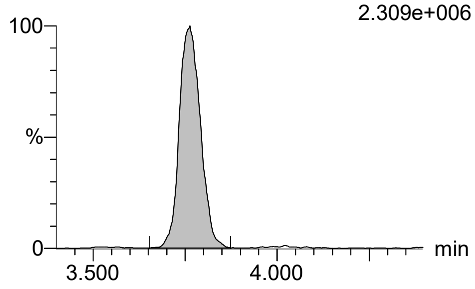

Sample Name: 20211111_A11_R2_IgM
F49:MRM of 1 channel,ES+
1006.8 > 366.1
2.271e+006

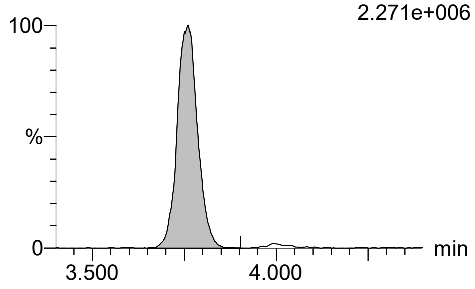

Sample Name: 20211111_A12_R2_IgM
F49:MRM of 1 channel,ES+
1006.8 > 366.1
1.663e+006

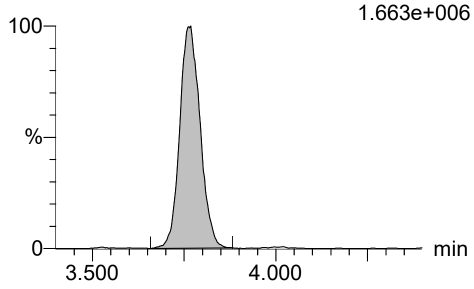

Sample Name: 20211111_L11_R2_IgM
F49:MRM of 1 channel,ES+
1006.8 > 366.1
9.432e+005

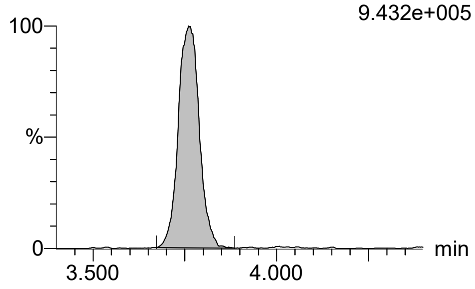

Sample Name: 20211111_L12_R2_IgM
F49:MRM of 1 channel,ES+
1006.8 > 366.1
2.076e+006

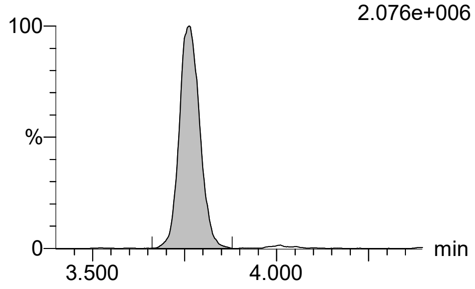

Sample Name: 20211111_H6_R2_IgM
F49:MRM of 1 channel,ES+
1006.8 > 366.1
1.757e+006

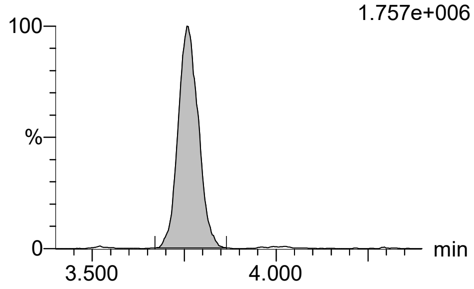

Sample Name: 20211111_A13_R2_IgM
F49:MRM of 1 channel,ES+
1006.8 > 366.1
1.547e+006

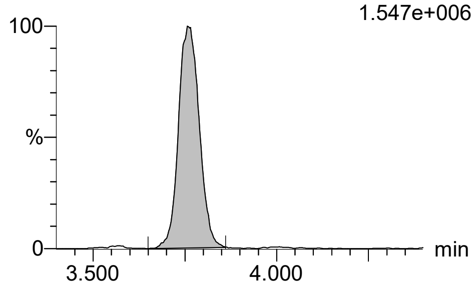

Sample Name: 20211111_A14_R2_IgM
F49:MRM of 1 channel,ES+
1006.8 > 366.1
7.769e+006

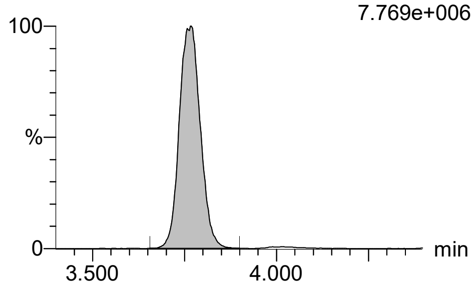

Sample Name: 20211111_L13_R2_IgM
F49:MRM of 1 channel,ES+
1006.8 > 366.1
2.647e+006

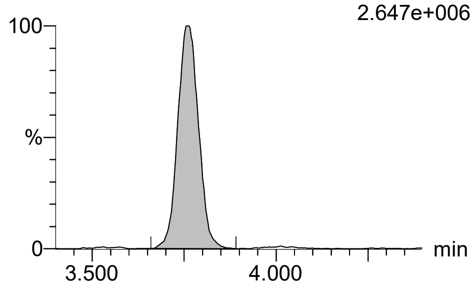

Sample Name: 20211111_L14_R2_IgM
F49:MRM of 1 channel,ES+
1006.8 > 366.1
5.439e+006

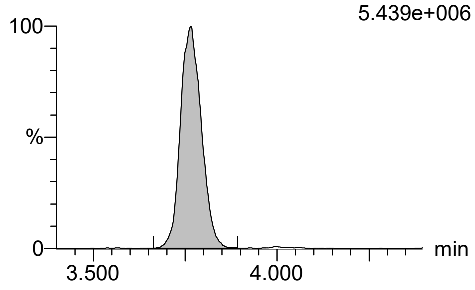

Sample Name: 20211111_H7_R2_IgM
F49:MRM of 1 channel,ES+
1006.8 > 366.1
3.654e+006

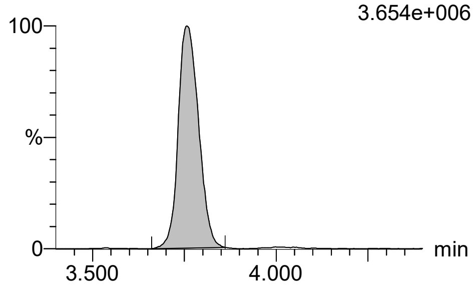

Sample Name: 20211111_A15_R2_IgM
F49:MRM of 1 channel,ES+
1006.8 > 366.1
2.563e+006

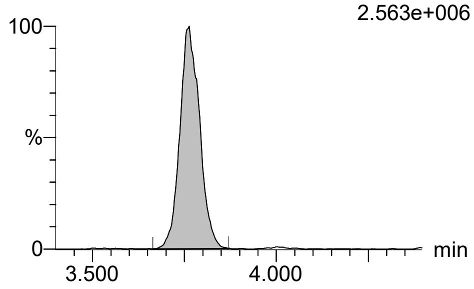

Sample Name: 20211111_QC_R12_IgM
F49:MRM of 1 channel,ES+
1006.8 > 366.1
3.014e+006

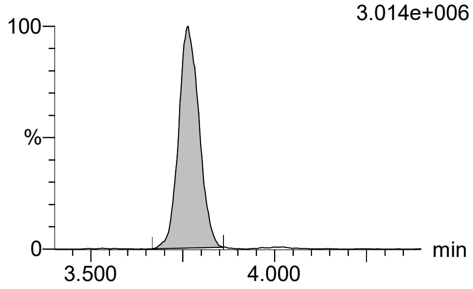

Sample Name: 20211111_A16_R2_IgM
F49:MRM of 1 channel,ES+
1006.8 > 366.1
3.913e+006

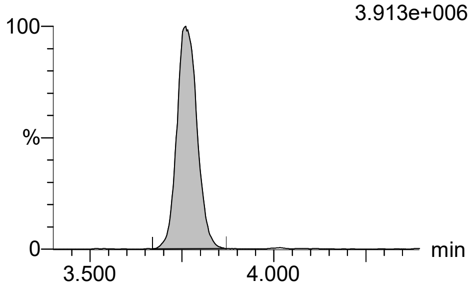

Sample Name: 20211111_L15_R2_IgM
F49:MRM of 1 channel,ES+
1006.8 > 366.1
3.364e+006

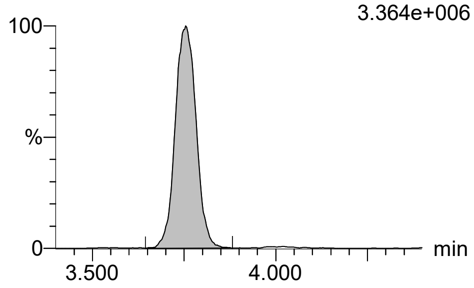

Sample Name: 20211111_L16_R2_IgM
F49:MRM of 1 channel,ES+
1006.8 > 366.1
2.615e+006

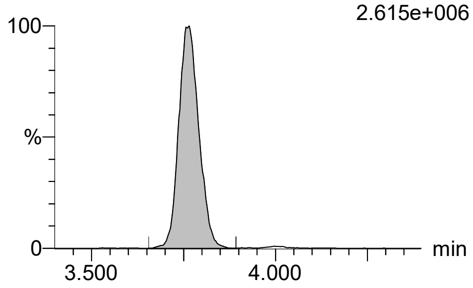

Sample Name: 20211111_H8_R2_IgM
F49:MRM of 1 channel,ES+
1006.8 > 366.1
2.089e+006

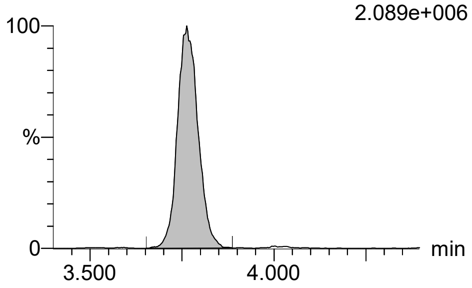

Sample Name: 20211111_A17_R2_IgM
F49:MRM of 1 channel,ES+
1006.8 > 366.1
6.440e+006

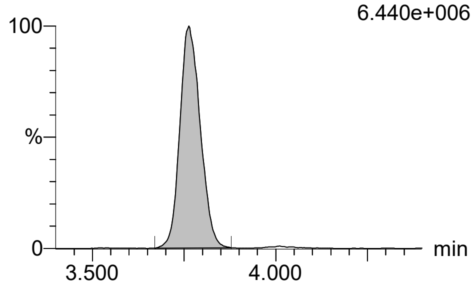

Sample Name: 20211111_A18_R2_IgM
F49:MRM of 1 channel,ES+
1006.8 > 366.1
3.625e+006

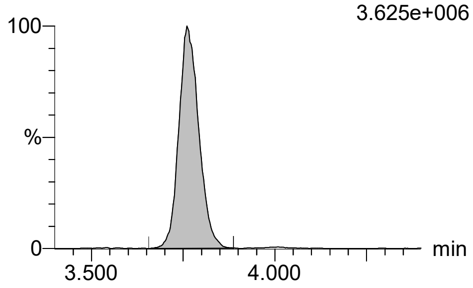

Sample Name: 20211111_L17_R2_IgM
F49:MRM of 1 channel,ES+
1006.8 > 366.1
2.726e+006

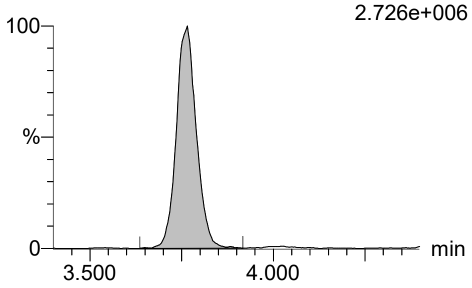

Sample Name: 20211111_L18_R2_IgM
F49:MRM of 1 channel,ES+
1006.8 > 366.1
1.925e+005

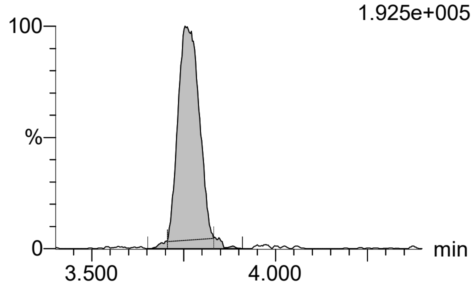

Sample Name: 20211111_H9_R2_IgM
F49:MRM of 1 channel,ES+
1006.8 > 366.1
1.035e+006

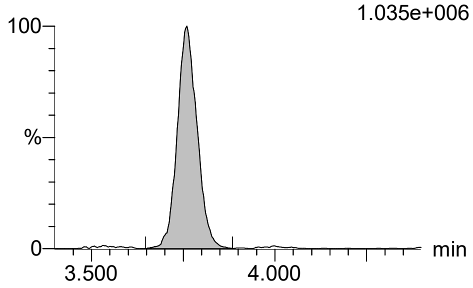

Sample Name: 20211111_A19_R2_IgM
F49:MRM of 1 channel,ES+
1006.8 > 366.1
2.678e+006

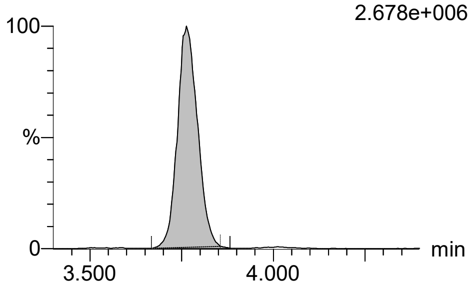

Sample Name: 20211111_A20_R2_IgM
F49:MRM of 1 channel,ES+
1006.8 > 366.1
1.431e+006

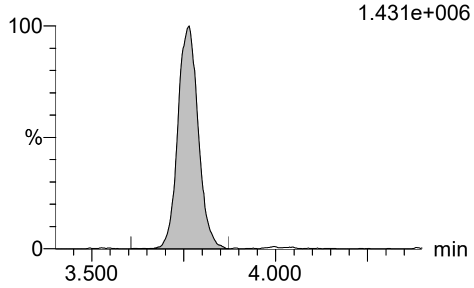

Sample Name: 20211111_L19_R2_IgM
F49:MRM of 1 channel,ES+
1006.8 > 366.1
2.237e+006

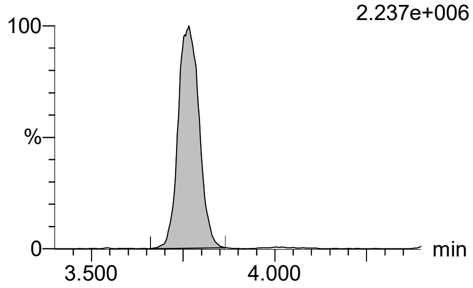

Sample Name: 20211111_QC_R13_IgM
F49:MRM of 1 channel,ES+
1006.8 > 366.1
2.764e+006

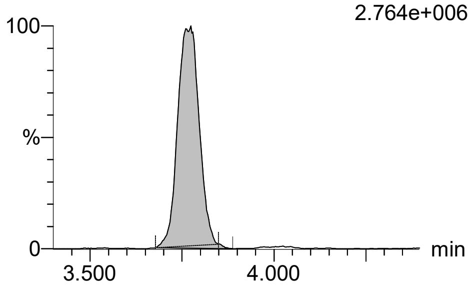

Sample Name: 20211111_L20_R2_IgM
F49:MRM of 1 channel,ES+
1006.8 > 366.1
3.212e+006

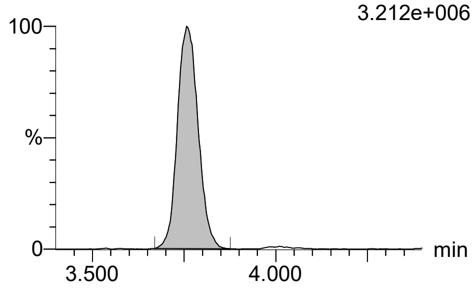

Sample Name: 20211111_H10_R2_IgM
F49:MRM of 1 channel,ES+
1006.8 > 366.1
2.597e+006

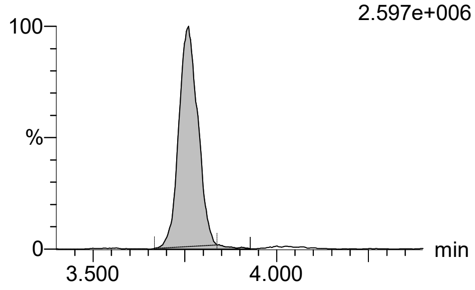

Sample Name: 20211111_A21_R2_IgM
F49:MRM of 1 channel,ES+
1006.8 > 366.1
1.990e+006

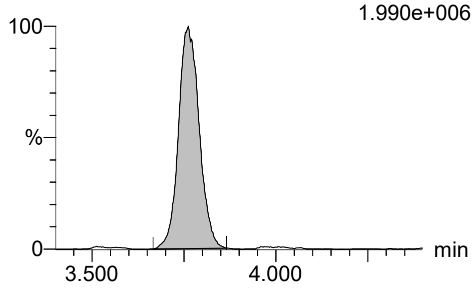

Sample Name: 20211111_A22_R2_IgM
F49:MRM of 1 channel,ES+
1006.8 > 366.1
6.188e+005

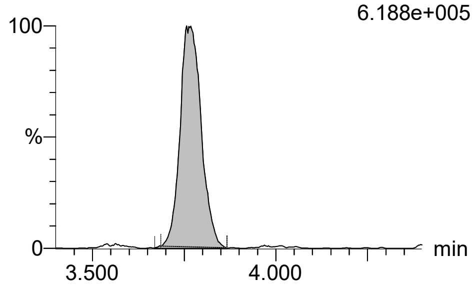

Sample Name: 20211111_L21_R2_IgM
F49:MRM of 1 channel,ES+
1006.8 > 366.1
1.190e+006

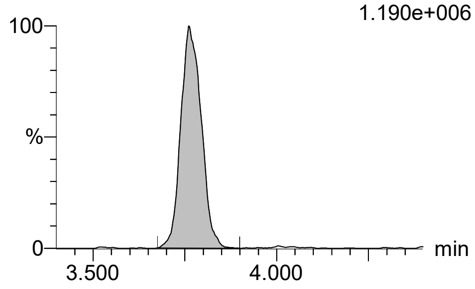

Sample Name: 20211111_L22_R2_IgM
F49:MRM of 1 channel,ES+
1006.8 > 366.1
4.836e+006

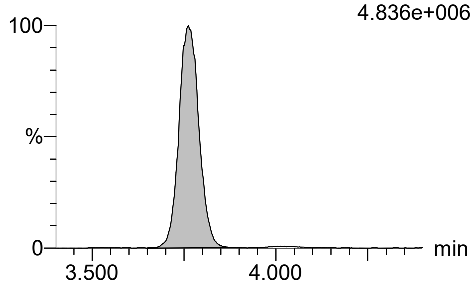

Sample Name: 20211111_H11_R2_IgM
F49:MRM of 1 channel,ES+
1006.8 > 366.1
3.159e+006

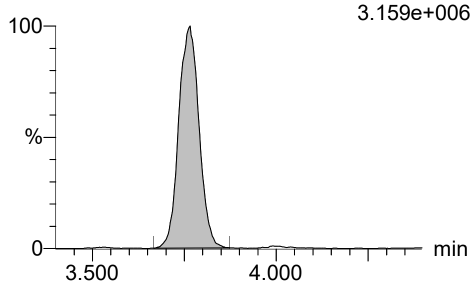

Sample Name: 20211111_A23_R2_IgM
F49:MRM of 1 channel,ES+
1006.8 > 366.1
1.432e+006

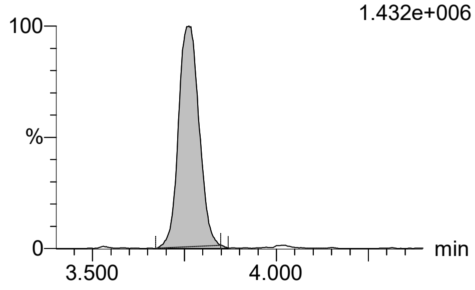

Sample Name: 20211111_A24_R2_IgM
F49:MRM of 1 channel,ES+
1006.8 > 366.1
1.361e+006

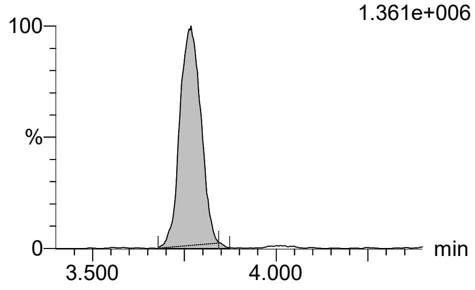

Sample Name: 20211111_L23_R2_IgM
F49:MRM of 1 channel,ES+
1006.8 > 366.1
5.982e+004

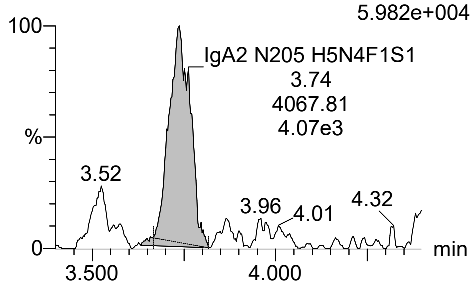

Sample Name: 20211111_L24_R2_IgM
F49:MRM of 1 channel,ES+
1006.8 > 366.1
1.086e+006

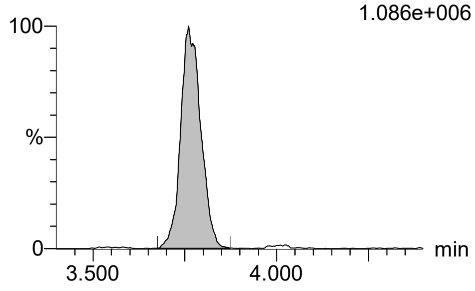

Sample Name: 20211111_H12_R2_IgM
F49:MRM of 1 channel,ES+
1006.8 > 366.1
1.877e+006

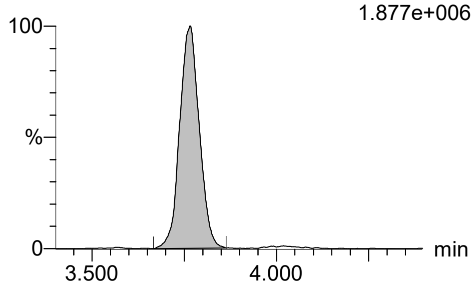

Sample Name: 20211111_QC_R14_IgM
F49:MRM of 1 channel,ES+
1006.8 > 366.1
3.199e+006

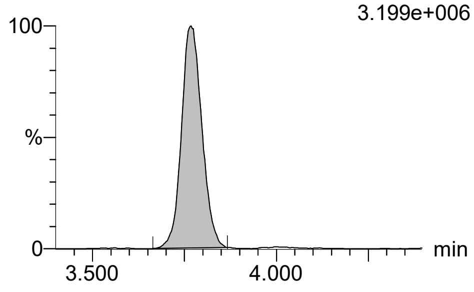

Sample Name: 20211111_A25_R2_IgM
F49:MRM of 1 channel,ES+
1006.8 > 366.1
2.115e+006

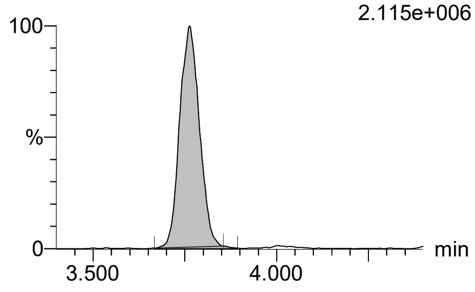

Sample Name: 20211111_A26_R2_IgM
F49:MRM of 1 channel,ES+
1006.8 > 366.1
1.302e+006

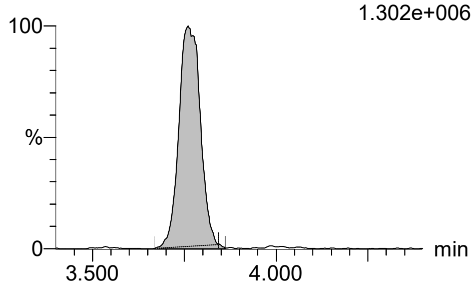

Dataset: C:\MassLynx\10.pro\20211111_clinicalsamples_IgM_IgA.qld

Last Altered: Thursday, March 09, 2023 19:58:25 台北標準時間
Printed: Friday, August 01, 2025 15:29:29 台北標準時間

Sample Name: 20211111_L25_R2_IgM
F49:MRM of 1 channel,ES+
1006.8 > 366.1
3.316e+006

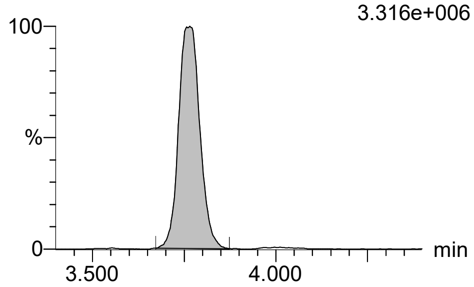

Sample Name: 20211111_L26_R2_IgM
F49:MRM of 1 channel,ES+
1006.8 > 366.1
1.129e+006

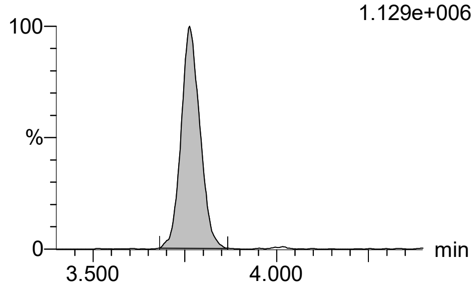

Sample Name: 20211111_H13_R2_IgM
F49:MRM of 1 channel,ES+
1006.8 > 366.1
3.476e+006

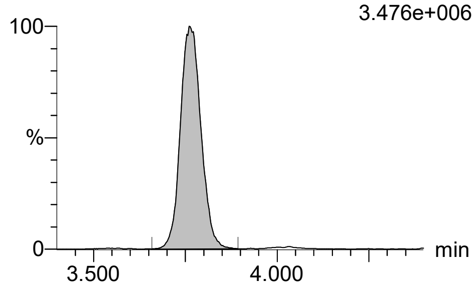

Sample Name: 20211111_A27_R2_IgM
F49:MRM of 1 channel,ES+
1006.8 > 366.1
4.409e+006

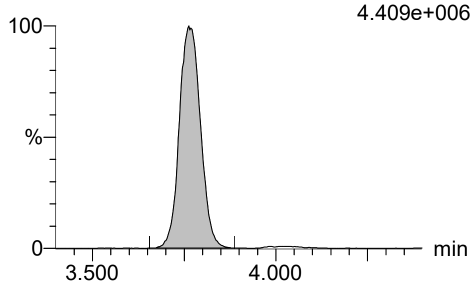

Sample Name: 20211111_A28_R2_IgM
F49:MRM of 1 channel,ES+
1006.8 > 366.1
1.953e+006

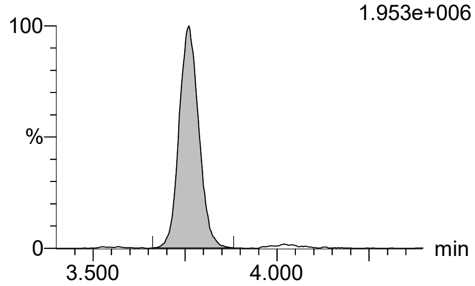

Sample Name: 20211111_L27_R2_IgM
F49:MRM of 1 channel,ES+
1006.8 > 366.1
2.977e+006

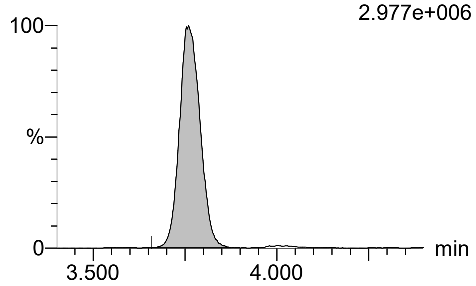

Sample Name: 20211111_L28_R2_IgM
F49:MRM of 1 channel,ES+
1006.8 > 366.1
1.951e+006

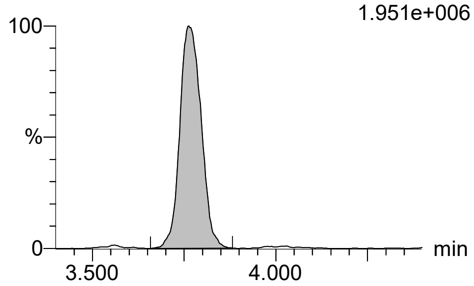

Sample Name: 20211111_H14_R2_IgM
F49:MRM of 1 channel,ES+
1006.8 > 366.1
2.390e+006

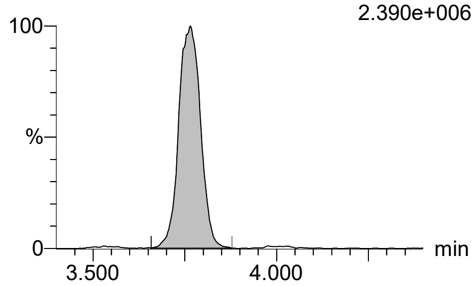

Sample Name: 20211111_A29_R2_IgM
F49:MRM of 1 channel,ES+
1006.8 > 366.1
2.244e+005

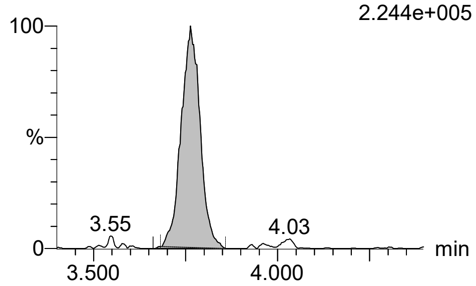

Sample Name: 20211111_A30_R2_IgM
F49:MRM of 1 channel,ES+
1006.8 > 366.1
4.419e+006

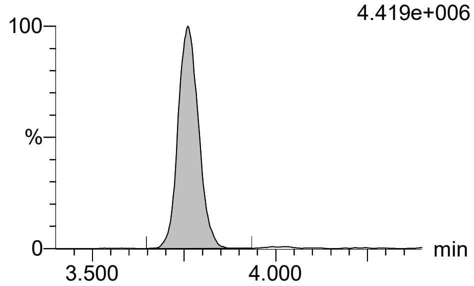

Sample Name: 20211111_QC_R15_IgM
F49:MRM of 1 channel,ES+
1006.8 > 366.1
2.676e+006

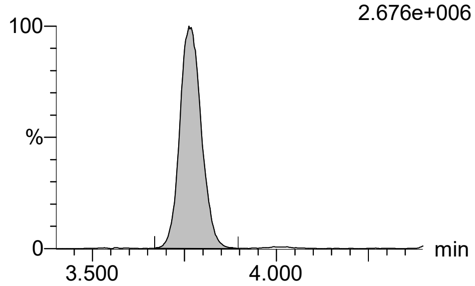

Sample Name: 20211111_L29_R2_IgM
F49:MRM of 1 channel,ES+
1006.8 > 366.1
2.119e+006

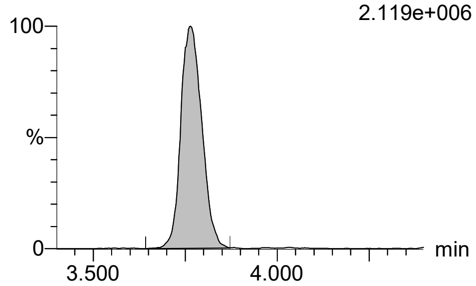

Sample Name: 20211111_L30_R2_IgM
F49:MRM of 1 channel,ES+
1006.8 > 366.1
1.183e+006

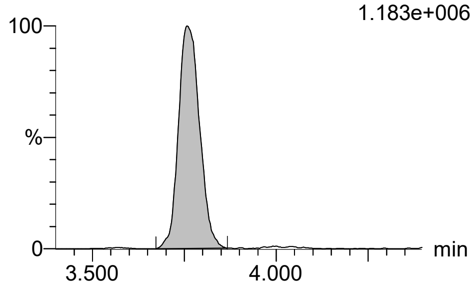

Sample Name: 20211111_H15_R2_IgM
F49:MRM of 1 channel,ES+
1006.8 > 366.1
4.229e+006

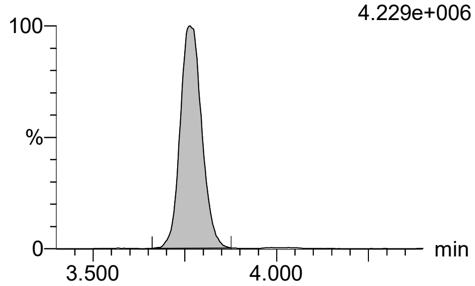

Sample Name: 20211111_A31_R2_IgM
F49:MRM of 1 channel,ES+
1006.8 > 366.1
2.035e+006

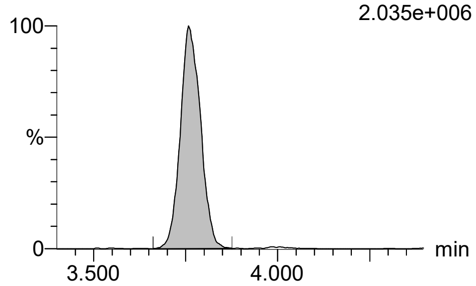

Sample Name: 20211111_A32_R2_IgM

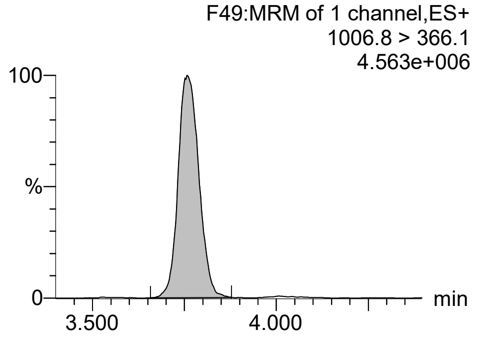

Sample Name: 20211111_L31_R2_IgM

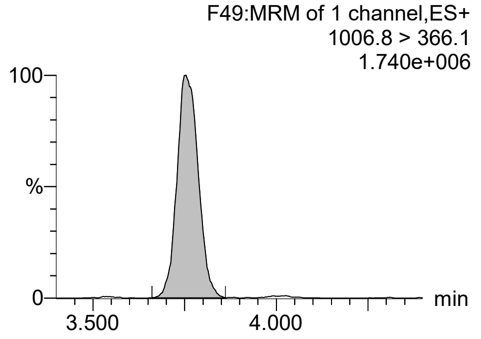

Sample Name: 20211111_L32_R2_IgM

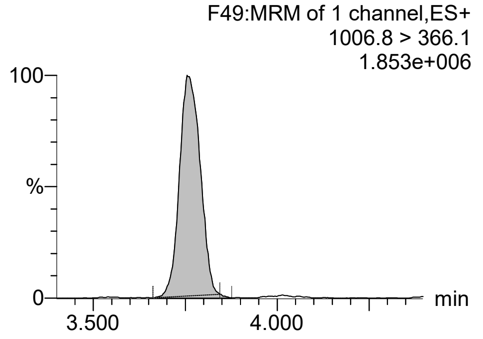

Sample Name: 20211111_H16_R2_IgM

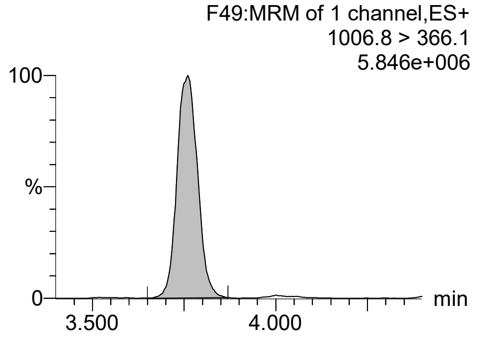

Sample Name: 20211111_A33_R2_IgM

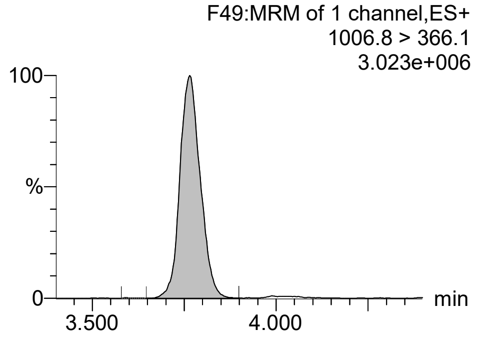

Sample Name: 20211111_A34_R2_IgM

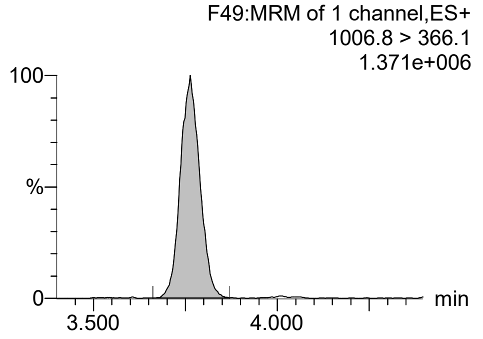

Sample Name: 20211111_H17_R2_IgM

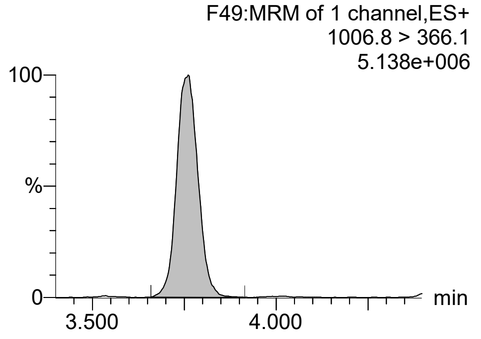

Sample Name: 20211111_A35_R2_IgM

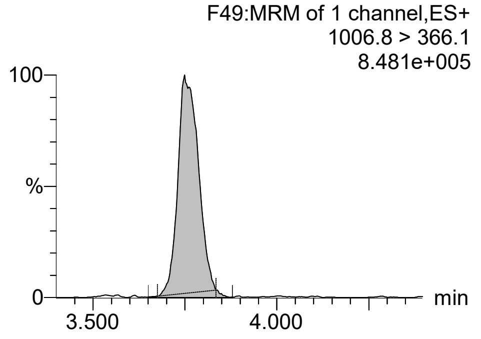

Sample Name: 20211111_QC_R16_IgM

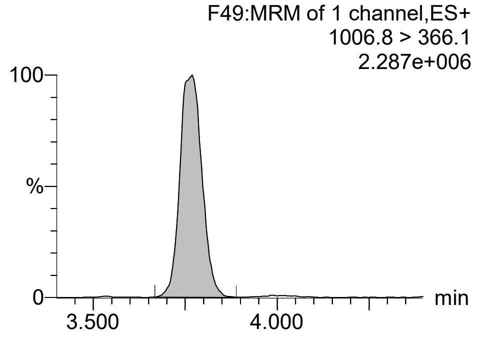

Sample Name: 20211111_A36_R2_IgM

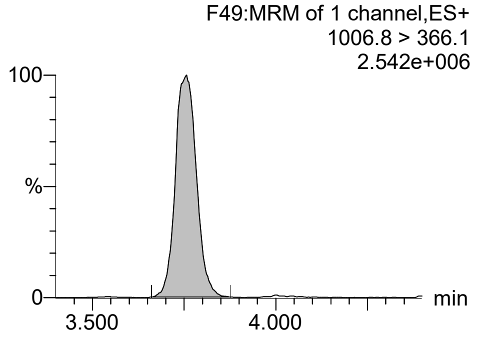

Sample Name: 20211111_H18_R2_IgM

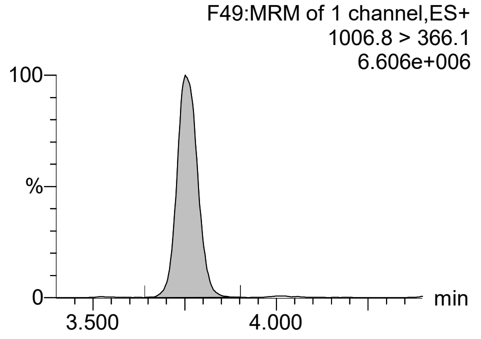

Sample Name: 20211111_A37_R2_IgM

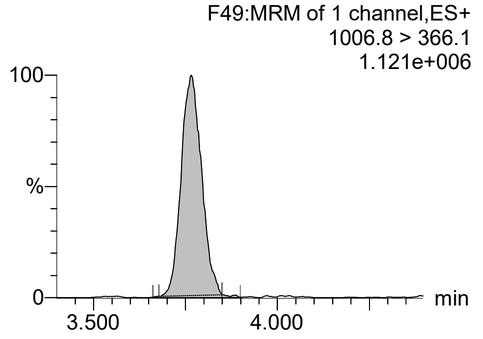

Sample Name: 20211111_A38_R2_IgM

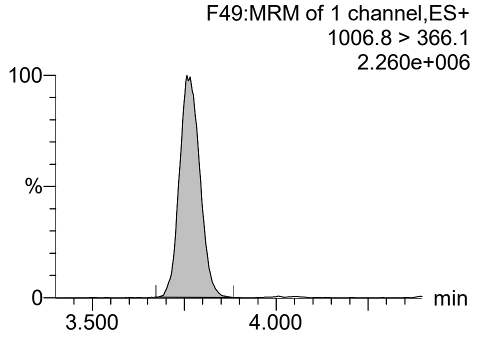

Sample Name: 20211111_H19_R2_IgM

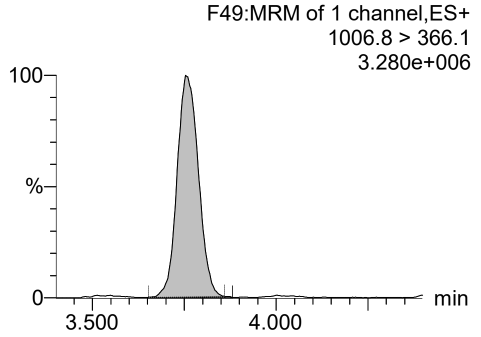

Sample Name: 20211111_A39_R2_IgM

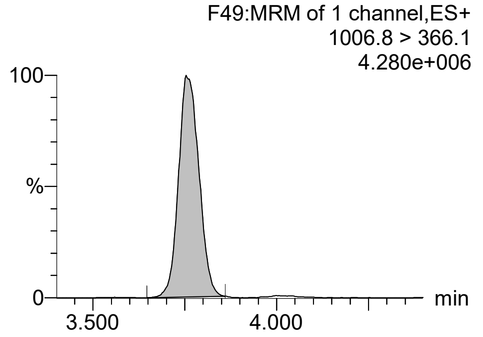

Sample Name: 20211111_A40_R2_IgM

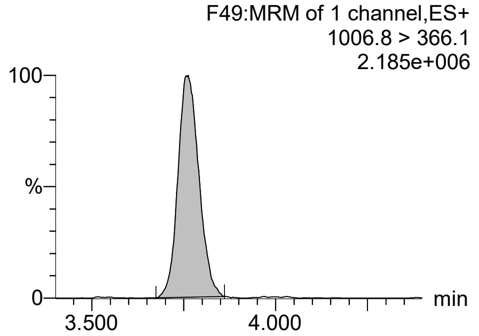

Sample Name: 20211111_H20_R2_IgM

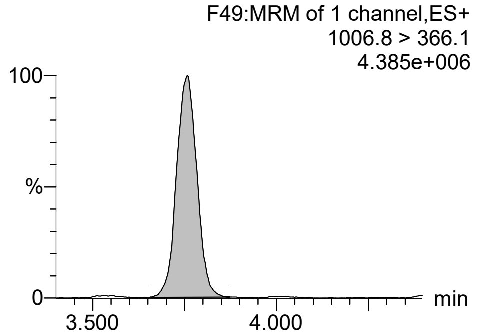

Sample Name: 20211111_A41_R2_IgM

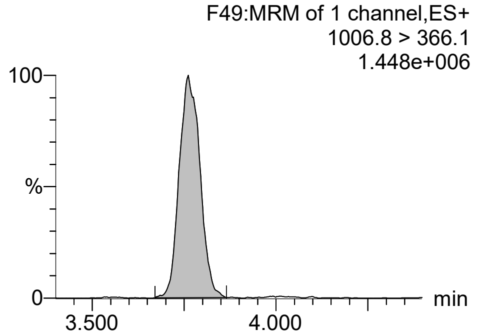

Sample Name: 20211111_A42_R2_IgM

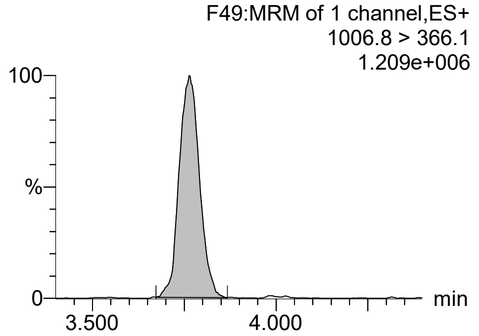

Sample Name: 20211111_A43_R2_IgM

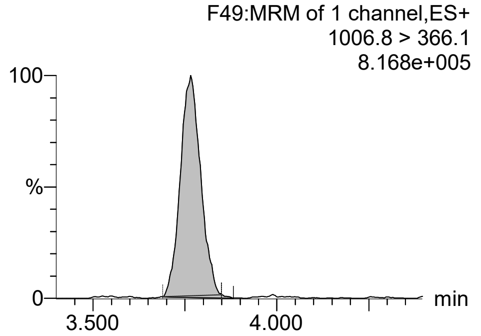

Sample Name: 20211111_A44_R2_IgM

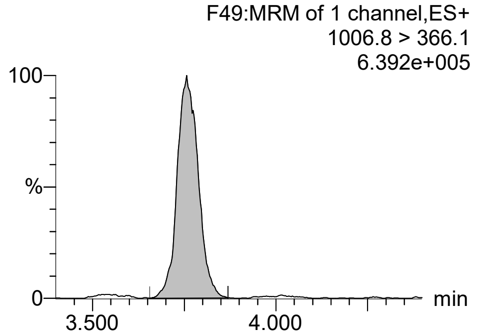

Sample Name: 20211111_QC_R17_IgM

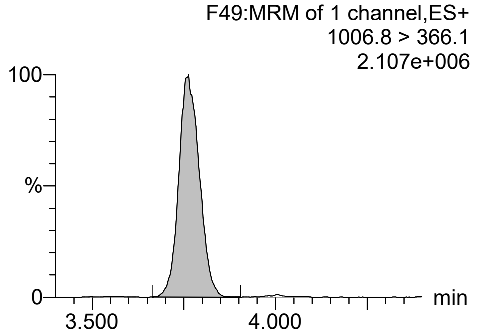

Sample Name: 20211111_L2_R3_IgM

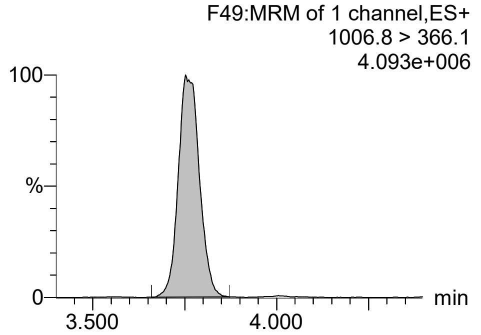

Sample Name: 20211111_H1_R3_IgM

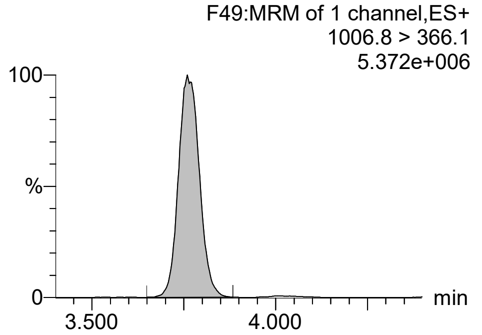

Sample Name: 20211111_A3_R3_IgM

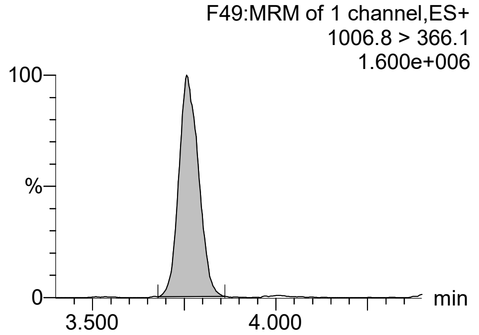

Sample Name: 20211111_A4_R3_IgM

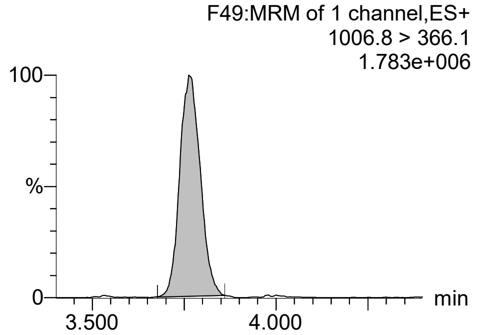

Sample Name: 20211111_L3_R3_IgM

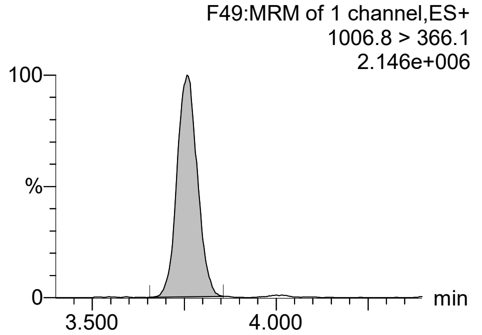

Sample Name: 20211111_L4_R3_IgM

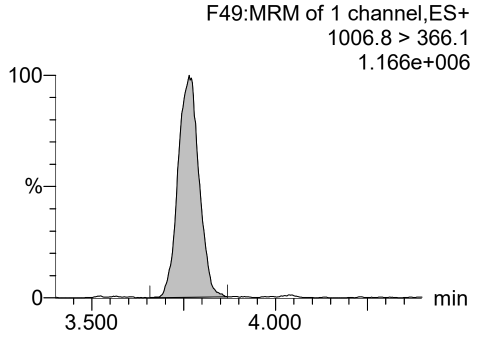

Sample Name: 20211111_H2_R3_IgM

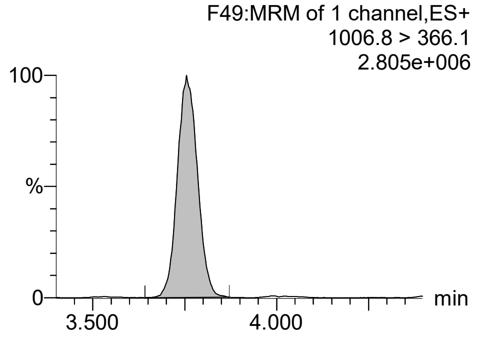

Sample Name: 20211111_A5_R3_IgM

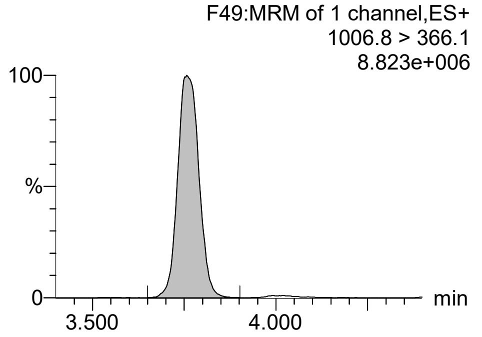

Sample Name: 20211111_A6_R3_IgM
F49:MRM of 1 channel,ES+
1006.8 > 366.1
2.937e+006

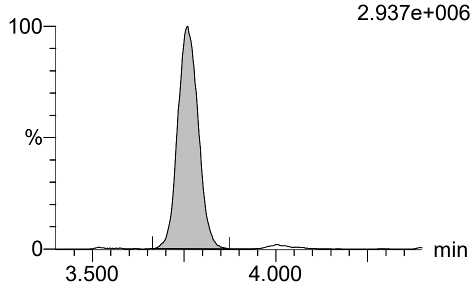

Sample Name: 20211111_QC_R18_IgM
F49:MRM of 1 channel,ES+
1006.8 > 366.1
1.949e+006

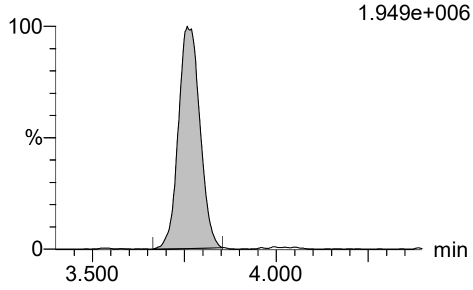

Sample Name: 20211111_L5_R3_IgM
F49:MRM of 1 channel,ES+
1006.8 > 366.1
5.767e+006

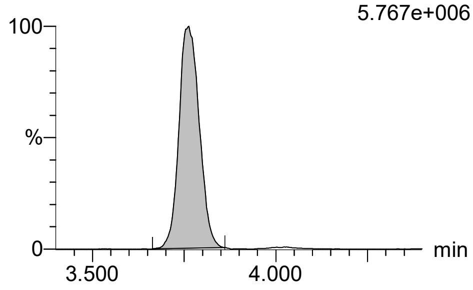

Sample Name: 20211111_L6_R3_IgM
F49:MRM of 1 channel,ES+
1006.8 > 366.1
2.500e+006

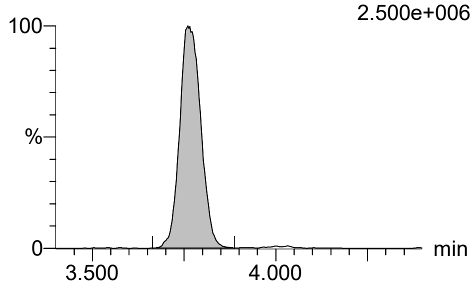

Sample Name: 20211111_H3_R3_IgM
F49:MRM of 1 channel,ES+
1006.8 > 366.1
1.430e+006

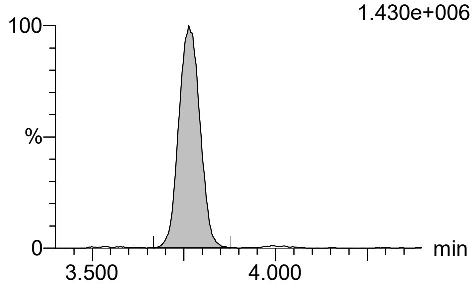

Sample Name: 20211111_A7_R3_IgM
F49:MRM of 1 channel,ES+
1006.8 > 366.1
4.025e+006

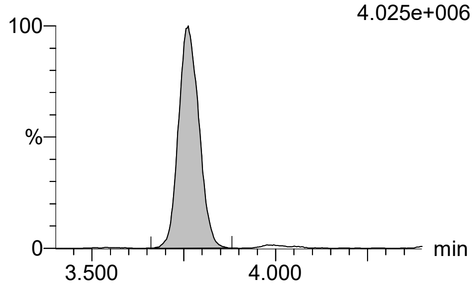

Sample Name: 20211111_A8_R3_IgM
F49:MRM of 1 channel,ES+
1006.8 > 366.1
3.058e+006

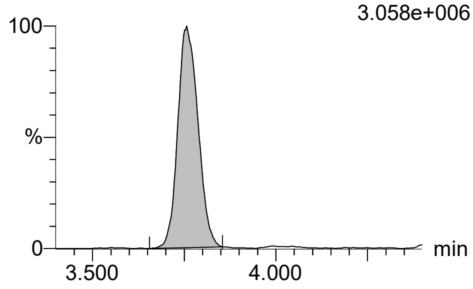

Sample Name: 20211111_L7_R3_IgM
F49:MRM of 1 channel,ES+
1006.8 > 366.1
3.179e+004

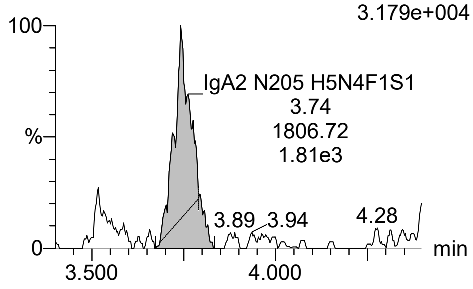

Sample Name: 20211111_L8_R3_IgM
F49:MRM of 1 channel,ES+
1006.8 > 366.1
8.353e+005

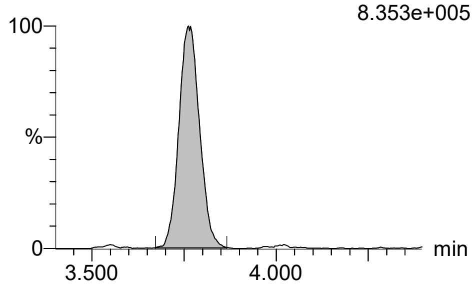

Sample Name: 20211111_H4_R3_IgM
F49:MRM of 1 channel,ES+
1006.8 > 366.1
1.902e+006

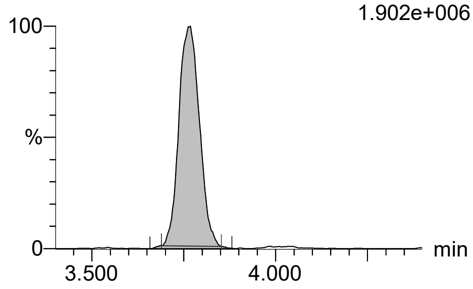

Sample Name: 20211111_A9_R3_IgM
F49:MRM of 1 channel,ES+
1006.8 > 366.1
3.264e+006

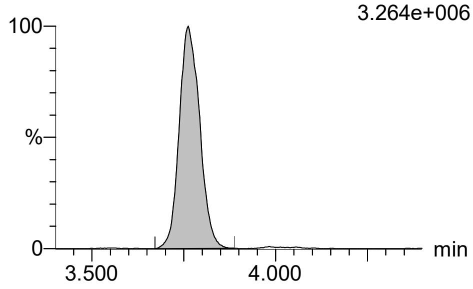

Sample Name: 20211111_A10_R3_IgM
F49:MRM of 1 channel,ES+
1006.8 > 366.1
7.366e+006

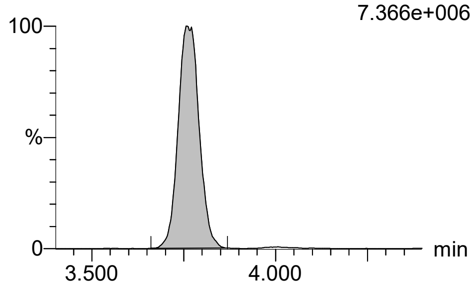

Sample Name: 20211111_L9_R3_IgM
F49:MRM of 1 channel,ES+
1006.8 > 366.1
3.434e+006

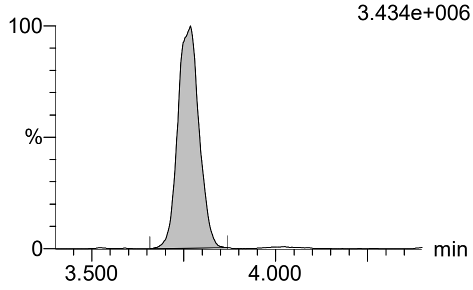

Sample Name: 20211111_L10_R3_IgM
F49:MRM of 1 channel,ES+
1006.8 > 366.1
3.005e+006

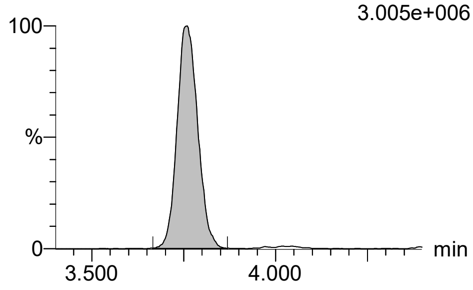

Sample Name: 20211111_QC_R19_IgM
F49:MRM of 1 channel,ES+
1006.8 > 366.1
2.223e+006

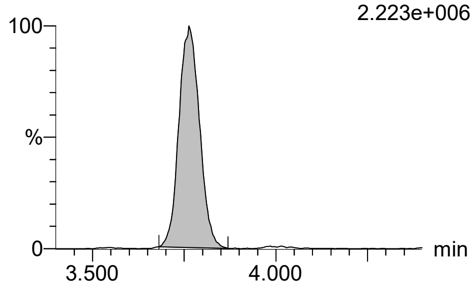

Dataset: C:\MassLynx\10.pro\20211111_clinicalsamples_IgM_IgA.qld

Last Altered: Thursday, March 09, 2023 19:58:25 台北標準時間
Printed: Friday, August 01, 2025 15:29:29 台北標準時間

Sample Name: 20211111_H5_R3_IgM
F49:MRM of 1 channel,ES+
1006.8 > 366.1
1.763e+006

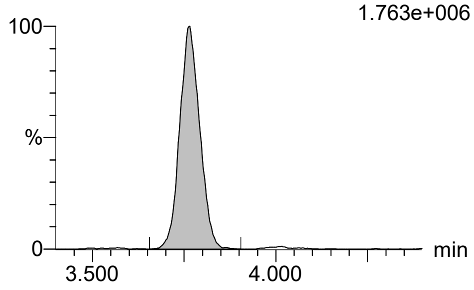

Sample Name: 20211111_A11_R3_IgM
F49:MRM of 1 channel,ES+
1006.8 > 366.1
1.778e+006

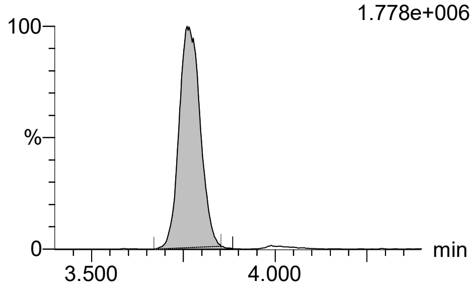

Sample Name: 20211111_A12_R3_IgM
F49:MRM of 1 channel,ES+
1006.8 > 366.1
1.672e+006

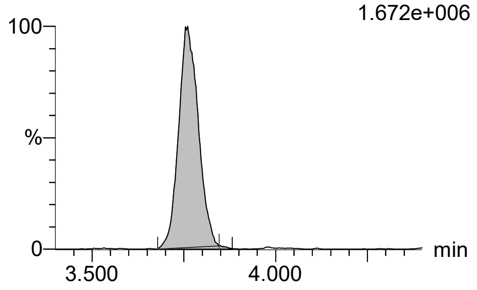

Sample Name: 20211111_L11_R3_IgM
F49:MRM of 1 channel,ES+
1006.8 > 366.1
7.235e+005

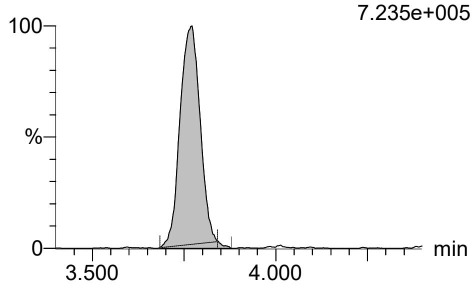

Sample Name: 20211111_L12_R3_IgM
F49:MRM of 1 channel,ES+
1006.8 > 366.1
1.828e+006

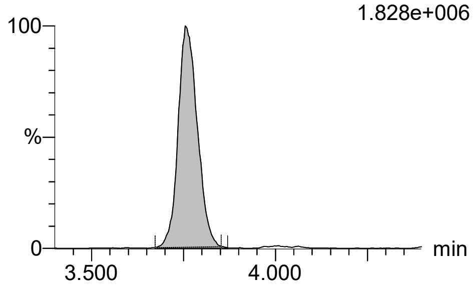

Sample Name: 20211111_H6_R3_IgM
F49:MRM of 1 channel,ES+
1006.8 > 366.1
1.519e+006

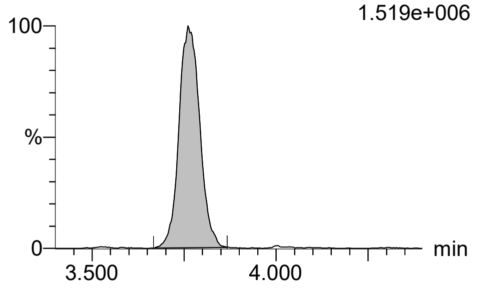

Sample Name: 20211111_A13_R3_IgM
F49:MRM of 1 channel,ES+
1006.8 > 366.1
1.404e+006

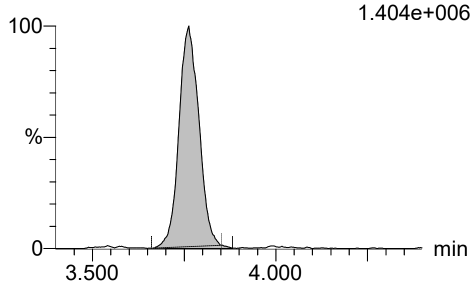

Sample Name: 20211111_A14_R3_IgM
F49:MRM of 1 channel,ES+
1006.8 > 366.1
5.814e+006

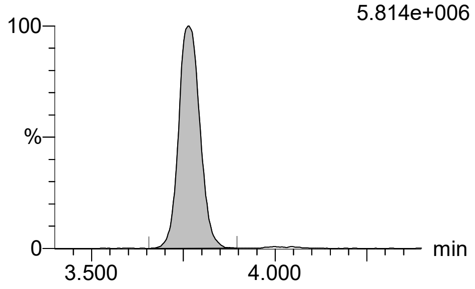

Sample Name: 20211111_L13_R3_IgM
F49:MRM of 1 channel,ES+
1006.8 > 366.1
2.078e+006

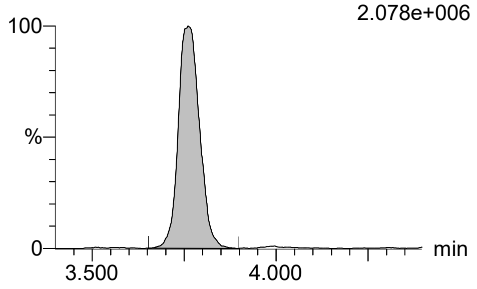

Sample Name: 20211111_L14_R3_IgM
F49:MRM of 1 channel,ES+
1006.8 > 366.1
4.286e+006

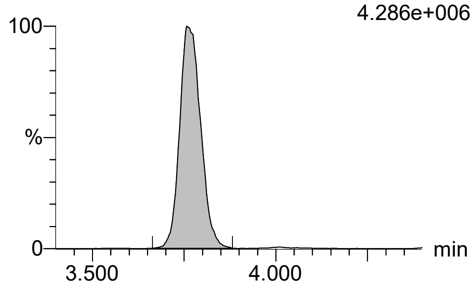

Sample Name: 20211111_H7_R3_IgM
F49:MRM of 1 channel,ES+
1006.8 > 366.1
2.916e+006

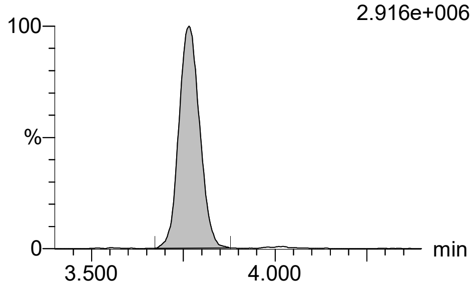

Sample Name: 20211111_A15_R3_IgM
F49:MRM of 1 channel,ES+
1006.8 > 366.1
1.977e+006

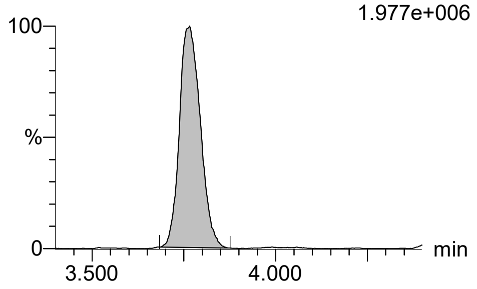

Sample Name: 20211111_QC_R20_IgM
F49:MRM of 1 channel,ES+
1006.8 > 366.1
2.299e+006

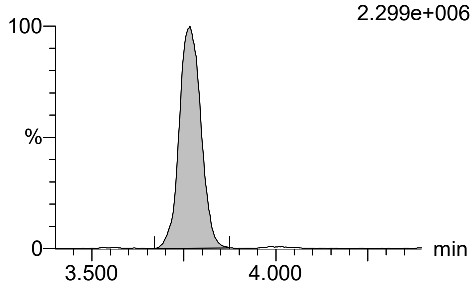

Sample Name: 20211111_A16_R3_IgM
F49:MRM of 1 channel,ES+
1006.8 > 366.1
2.917e+006

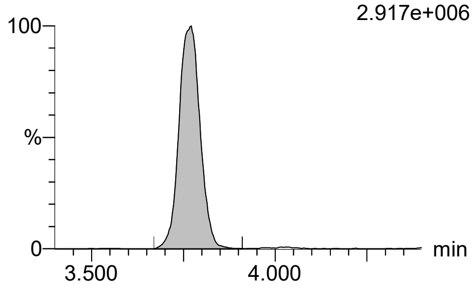

Sample Name: 20211111_L15_R3_IgM
F49:MRM of 1 channel,ES+
1006.8 > 366.1
2.805e+006

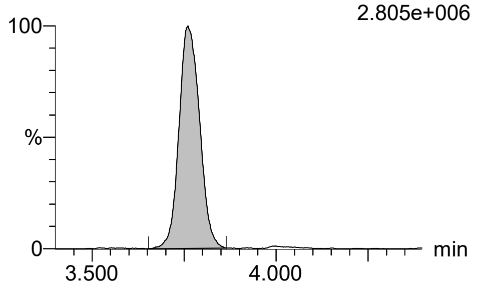

Dataset: C:\MassLynx\10.pro\20211111_clinicalsamples_IgM_IgA.qld

Last Altered: Thursday, March 09, 2023 19:58:25 台北標準時間
Printed: Friday, August 01, 2025 15:29:29 台北標準時間

Sample Name: 20211111_L16_R3_IgM
F49:MRM of 1 channel,ES+
1006.8 > 366.1
2.371e+006

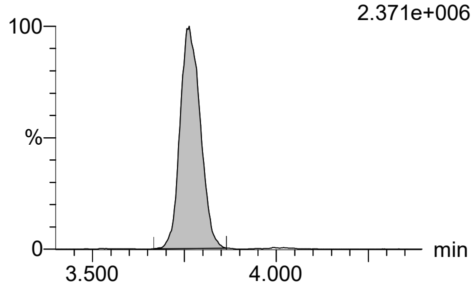

Sample Name: 20211111_H8_R3_IgM
F49:MRM of 1 channel,ES+
1006.8 > 366.1
1.763e+006

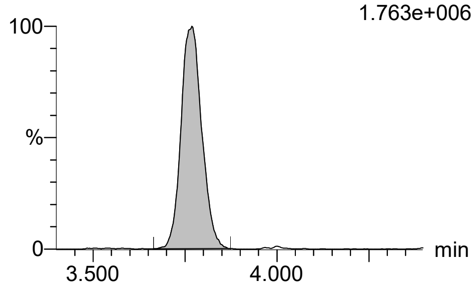

Sample Name: 20211111_A17_R3_IgM
F49:MRM of 1 channel,ES+
1006.8 > 366.1
5.069e+006

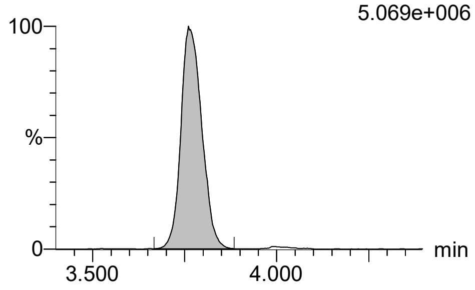

Sample Name: 20211111_A18_R3_IgM
F49:MRM of 1 channel,ES+
1006.8 > 366.1
2.950e+006

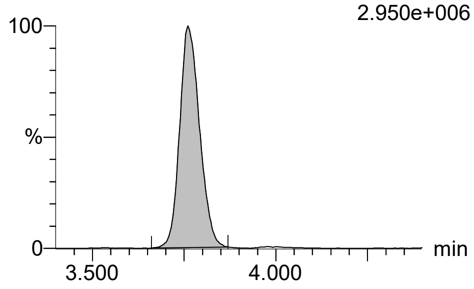

Sample Name: 20211111_L17_R3_IgM
F49:MRM of 1 channel,ES+
1006.8 > 366.1
2.179e+006

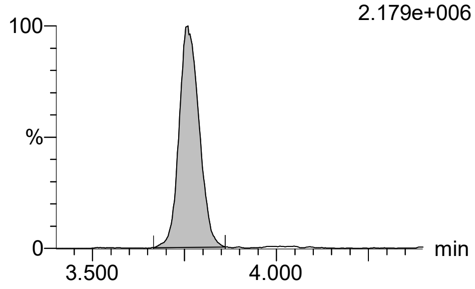

Sample Name: 20211111_L18_R3_IgM
F49:MRM of 1 channel,ES+
1006.8 > 366.1
2.072e+005

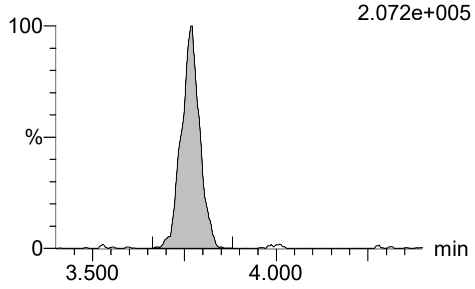

Sample Name: 20211111_H9_R3_IgM
F49:MRM of 1 channel,ES+
1006.8 > 366.1
7.439e+005

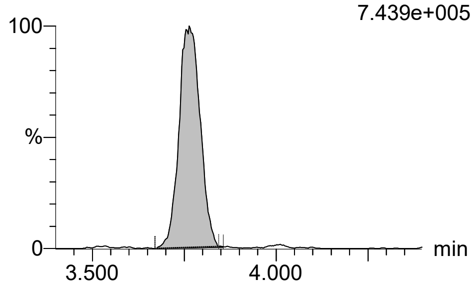

Sample Name: 20211111_A19_R3_IgM
F49:MRM of 1 channel,ES+
1006.8 > 366.1
2.475e+006

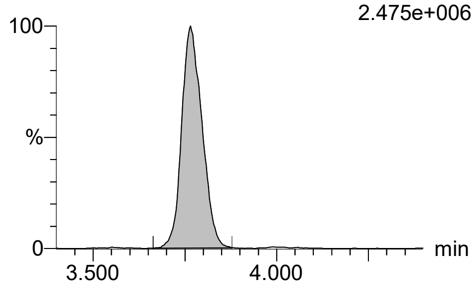

Sample Name: 20211111_A20_R3_IgM
F49:MRM of 1 channel,ES+
1006.8 > 366.1
1.150e+006

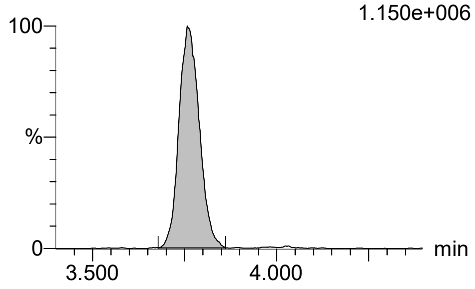

Sample Name: 20211111_L19_R3_IgM
F49:MRM of 1 channel,ES+
1006.8 > 366.1
2.699e+006

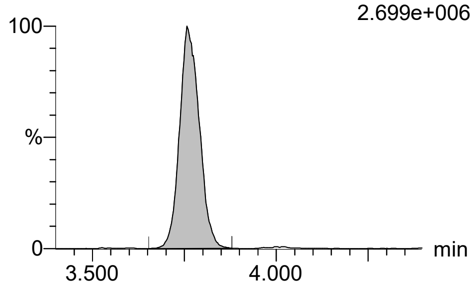

Sample Name: 20211111_QC_R21_IgM
F49:MRM of 1 channel,ES+
1006.8 > 366.1
2.457e+006

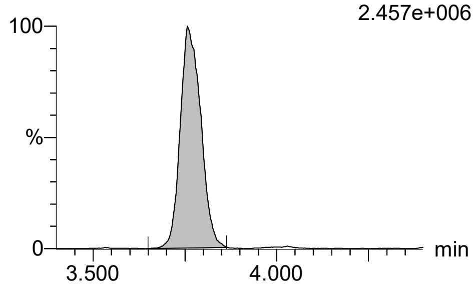

Sample Name: 20211111_L20_R3_IgM
F49:MRM of 1 channel,ES+
1006.8 > 366.1
2.600e+006

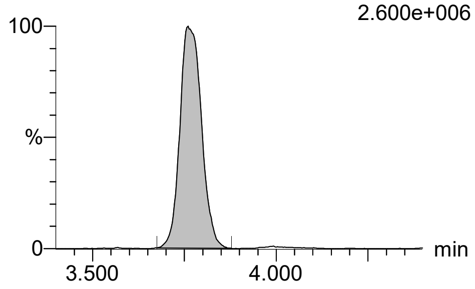

Sample Name: 20211111_H10_R3_IgM
F49:MRM of 1 channel,ES+
1006.8 > 366.1
2.215e+006

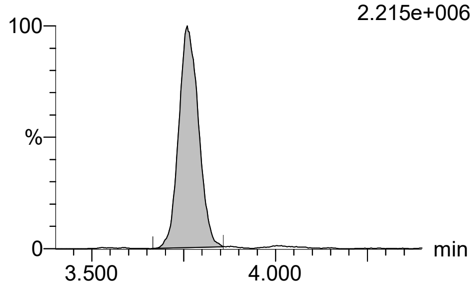

Sample Name: 20211111_A21_R3_IgM
F49:MRM of 1 channel,ES+
1006.8 > 366.1
1.797e+006

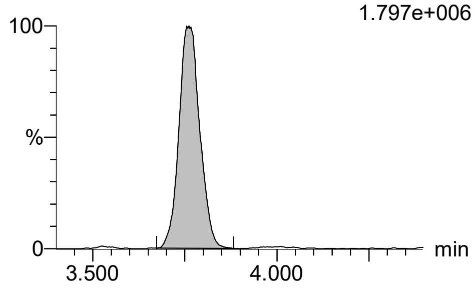

Sample Name: 20211111_A22_R3_IgM
F49:MRM of 1 channel,ES+
1006.8 > 366.1
5.807e+005

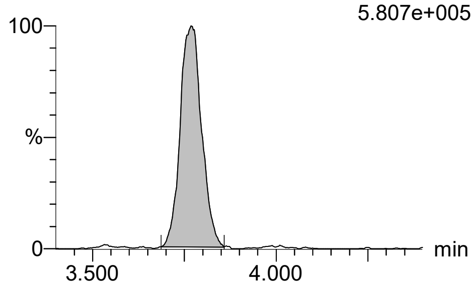

Dataset: C:\MassLynx\10.pro\20211111_clinicalsamples_IgM_IgA.qld

Last Altered: Thursday, March 09, 2023 19:58:25 台北標準時間
Printed: Friday, August 01, 2025 15:29:29 台北標準時間

Sample Name: 20211111_L21_R3_IgM

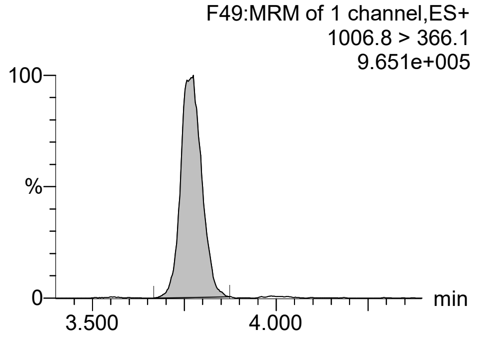

Sample Name: 20211111_L22_R3_IgM

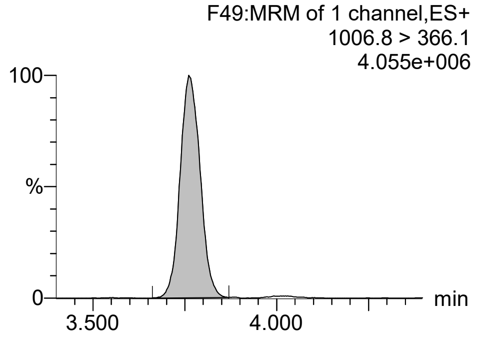

Sample Name: 20211111_H11_R3_IgM

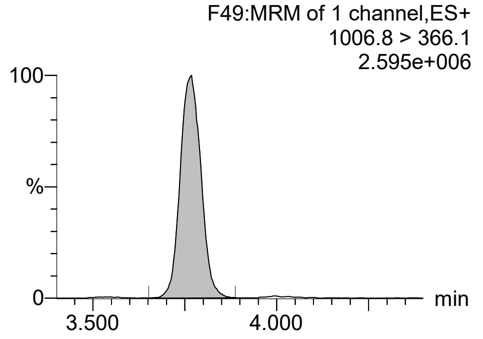

Sample Name: 20211111_A23_R3_IgM

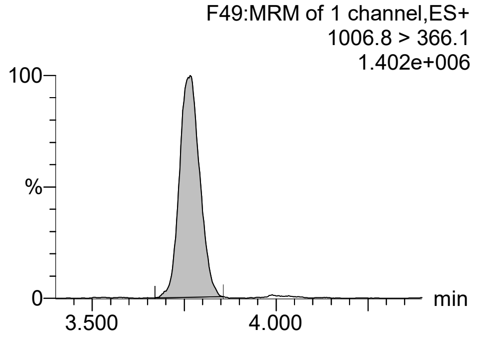

Sample Name: 20211111_A24_R3_IgM

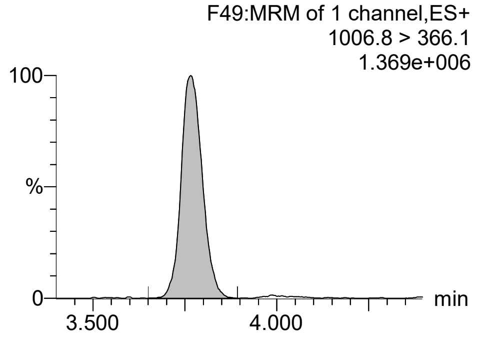

Sample Name: 20211111_L23_R3_IgM

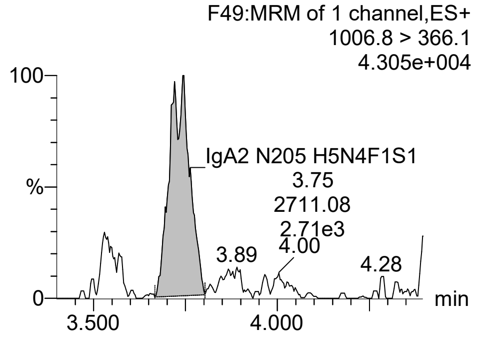

Sample Name: 20211111_L24_R3_IgM

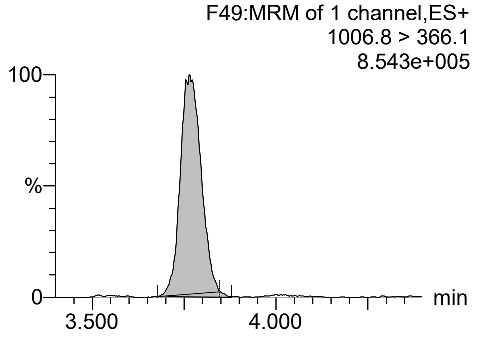

Sample Name: 20211111_H12_R3_IgM

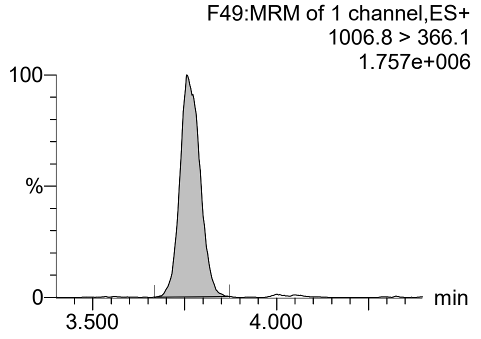

Sample Name: 20211111_QC_R22_IgM

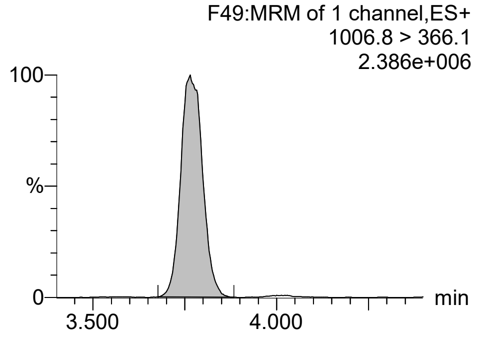

Sample Name: 20211111_A25_R3_IgM

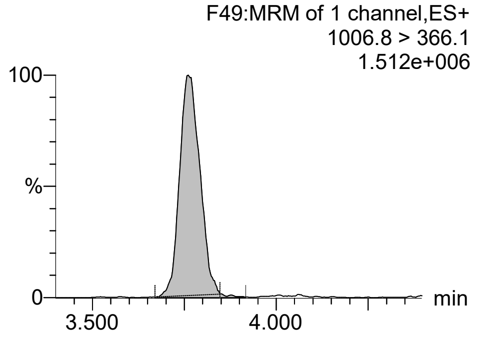

Sample Name: 20211111_A26_R3_IgM

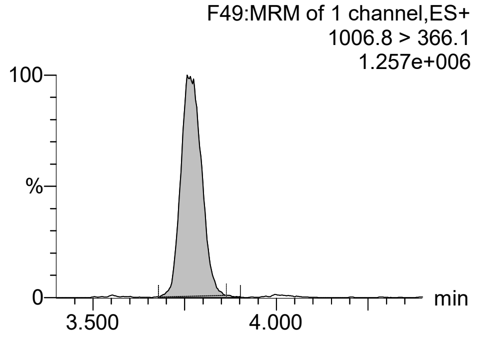

Sample Name: 20211111_L25_R3_IgM

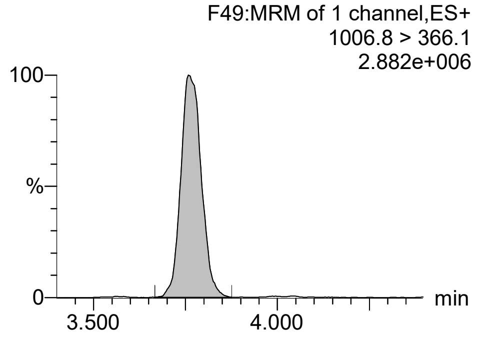

Sample Name: 20211111_L26_R3_IgM

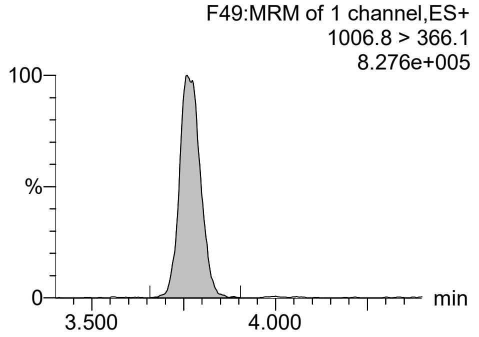

Sample Name: 20211111_H13_R3_IgM

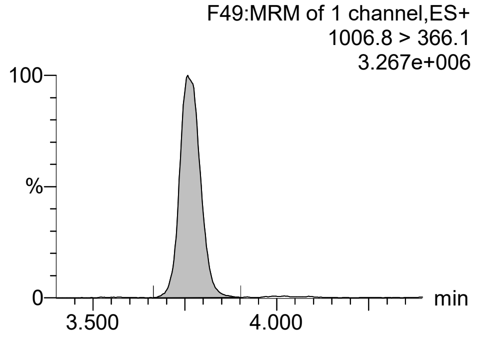

Sample Name: 20211111_A27_R3_IgM

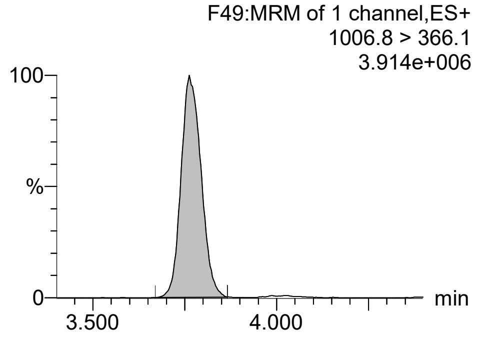

Sample Name: 20211111_A28_R3_IgM
F49:MRM of 1 channel,ES+
1006.8 > 366.1
1.698e+006

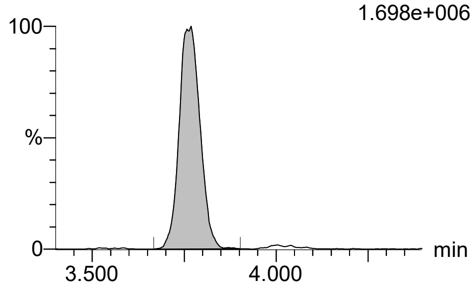

Sample Name: 20211111_L27_R3_IgM
F49:MRM of 1 channel,ES+
1006.8 > 366.1
2.600e+006

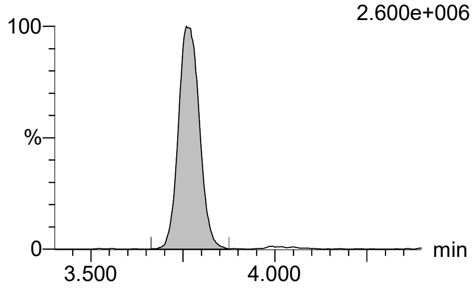

Sample Name: 20211111_L28_R3_IgM
F49:MRM of 1 channel,ES+
1006.8 > 366.1
2.222e+006

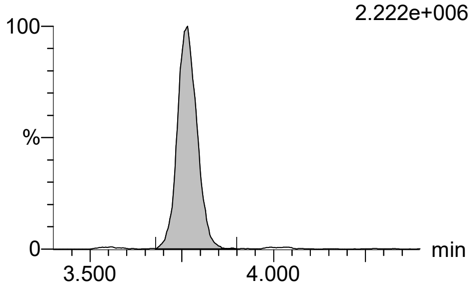

Sample Name: 20211111_H14_R3_IgM
F49:MRM of 1 channel,ES+
1006.8 > 366.1
2.408e+006

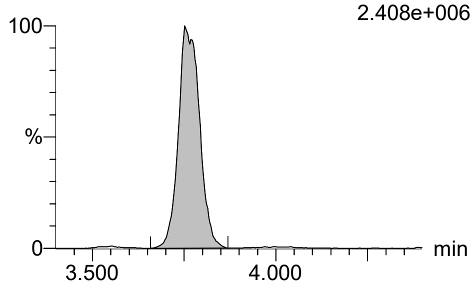

Sample Name: 20211111_A29_R3_IgM
F49:MRM of 1 channel,ES+
1006.8 > 366.1
1.844e+005

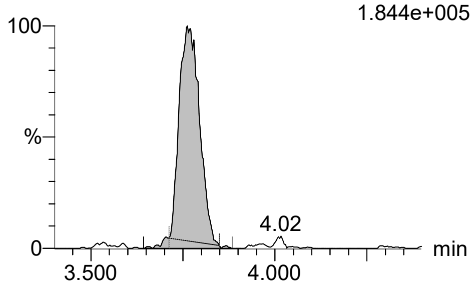

Sample Name: 20211111_A30_R3_IgM
F49:MRM of 1 channel,ES+
1006.8 > 366.1
3.846e+006

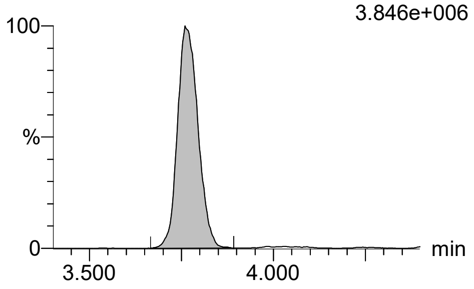

Sample Name: 20211111_QC_R23_IgM
F49:MRM of 1 channel,ES+
1006.8 > 366.1
2.306e+006

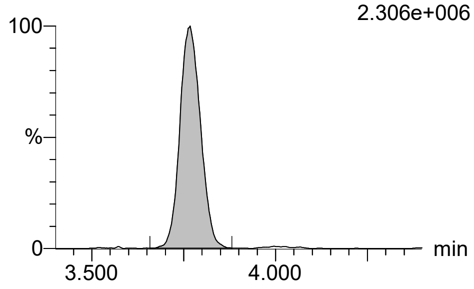

Sample Name: 20211111_L29_R3_IgM
F49:MRM of 1 channel,ES+
1006.8 > 366.1
2.115e+006

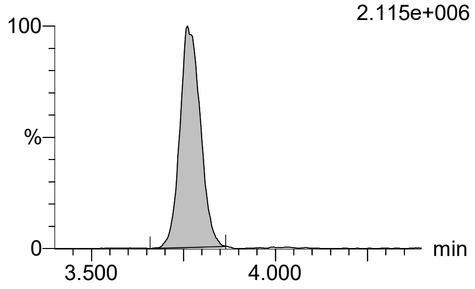

Sample Name: 20211111_L30_R3_IgM
F49:MRM of 1 channel,ES+
1006.8 > 366.1
1.009e+006

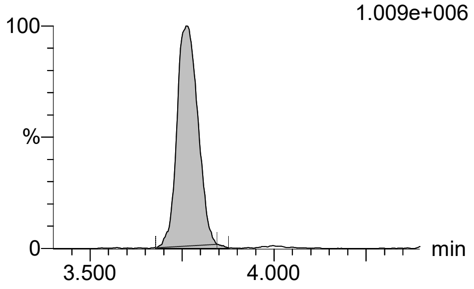

Sample Name: 20211111_H15_R3_IgM
F49:MRM of 1 channel,ES+
1006.8 > 366.1
3.599e+006

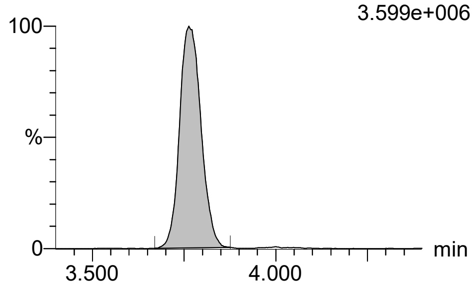

Sample Name: 20211111_A31_R3_IgM
F49:MRM of 1 channel,ES+
1006.8 > 366.1
1.945e+006

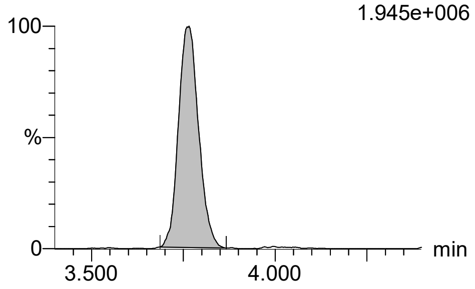

Sample Name: 20211111_A32_R3_IgM
F49:MRM of 1 channel,ES+
1006.8 > 366.1
4.129e+006

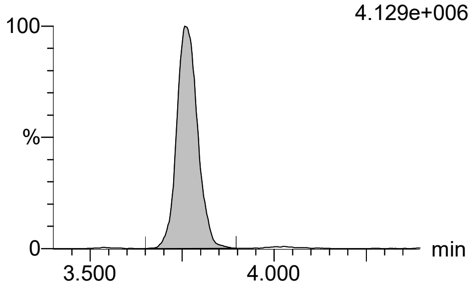

Sample Name: 20211111_L31_R3_IgM
F49:MRM of 1 channel,ES+
1006.8 > 366.1
1.640e+006

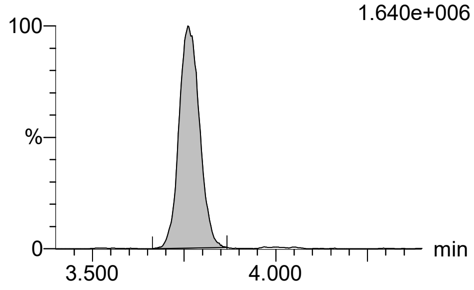

Sample Name: 20211111_L32_R3_IgM
F49:MRM of 1 channel,ES+
1006.8 > 366.1
1.616e+006

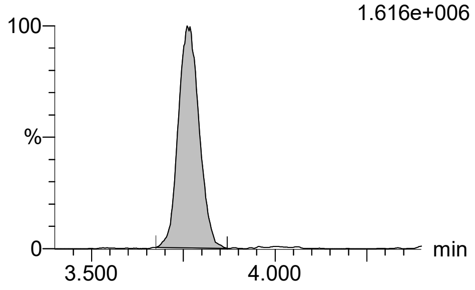

Sample Name: 20211111_H16_R3_IgM
F49:MRM of 1 channel,ES+
1006.8 > 366.1
4.771e+006

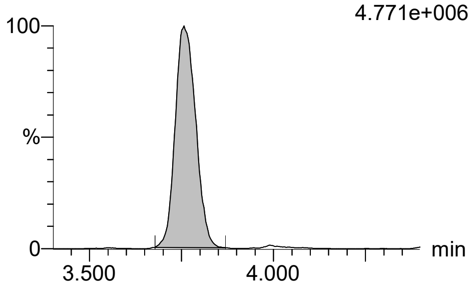

Dataset: C:\MassLynx\10.pro\20211111_clinicalsamples_IgM_IgA.qld

Last Altered: Thursday, March 09, 2023 19:58:25 台北標準時間
Printed: Friday, August 01, 2025 15:29:29 台北標準時間

Sample Name: 20211111_A33_R3_IgM
F49:MRM of 1 channel,ES+
1006.8 > 366.1
2.818e+006

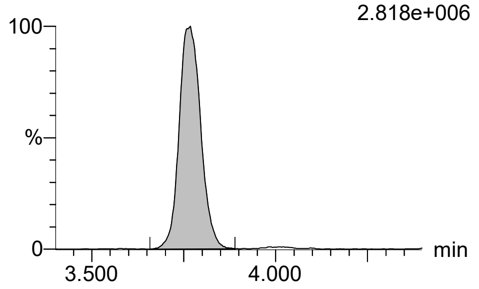

Sample Name: 20211111_A34_R3_IgM
F49:MRM of 1 channel,ES+
1006.8 > 366.1
1.071e+006

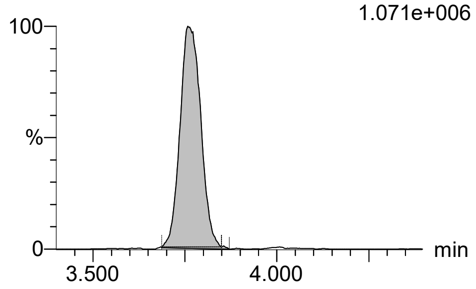

Sample Name: 20211111_H17_R3_IgM
F49:MRM of 1 channel,ES+
1006.8 > 366.1
4.320e+006

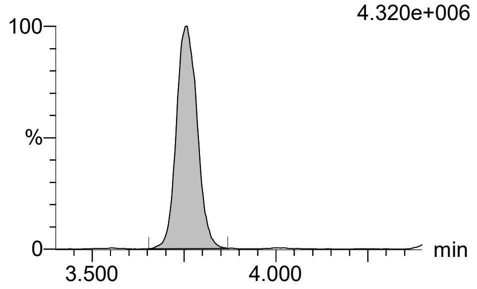

Sample Name: 20211111_A35_R3_IgM
F49:MRM of 1 channel,ES+
1006.8 > 366.1
7.207e+005

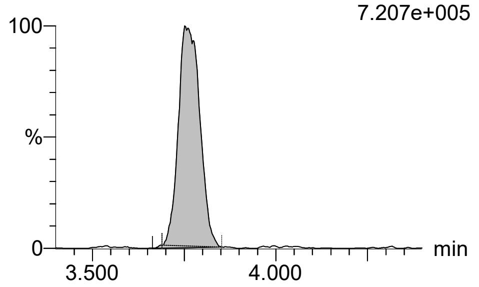

Sample Name: 20211111_QC_R24_IgM
F49:MRM of 1 channel,ES+
1006.8 > 366.1
1.906e+006

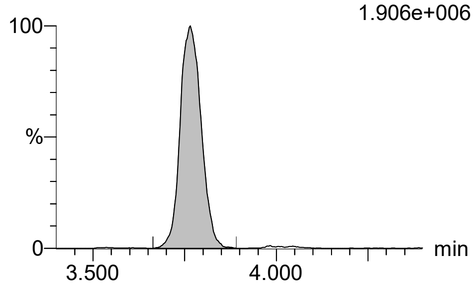

Sample Name: 20211111_A36_R3_IgM
F49:MRM of 1 channel,ES+
1006.8 > 366.1
2.419e+006

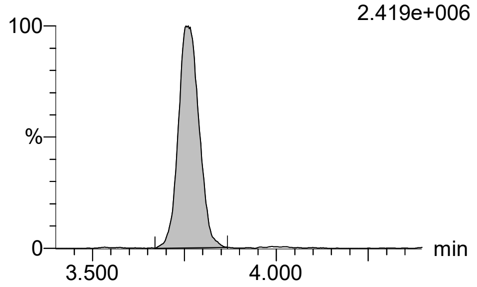

Sample Name: 20211111_H18_R3_IgM
F49:MRM of 1 channel,ES+
1006.8 > 366.1
5.678e+006

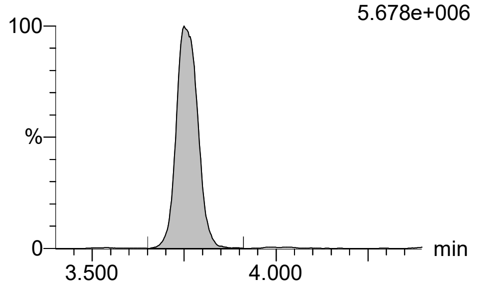

Sample Name: 20211111_A37_R3_IgM
F49:MRM of 1 channel,ES+
1006.8 > 366.1
8.794e+005

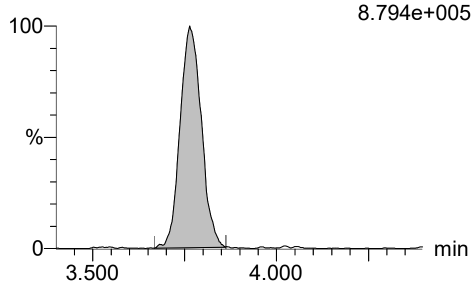

Sample Name: 20211111_A38_R3_IgM
F49:MRM of 1 channel,ES+
1006.8 > 366.1
1.835e+006

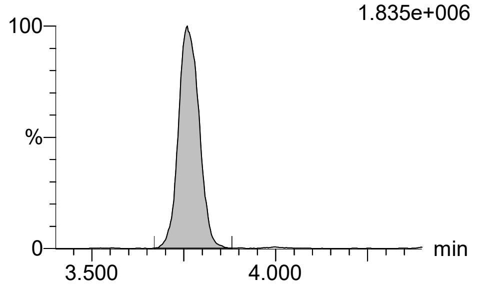

Sample Name: 20211111_H19_R3_IgM
F49:MRM of 1 channel,ES+
1006.8 > 366.1
2.800e+006

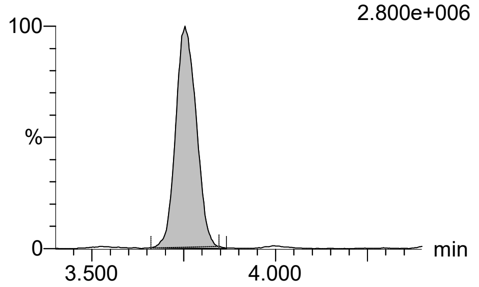

Sample Name: 20211111_A39_R3_IgM
F49:MRM of 1 channel,ES+
1006.8 > 366.1
3.598e+006

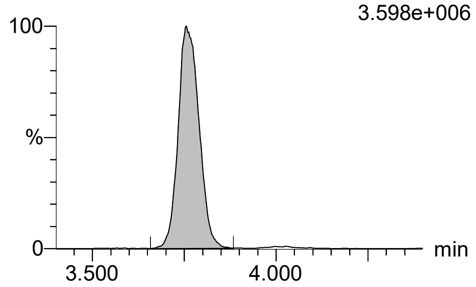

Sample Name: 20211111_A40_R3_IgM
F49:MRM of 1 channel,ES+
1006.8 > 366.1
2.019e+006

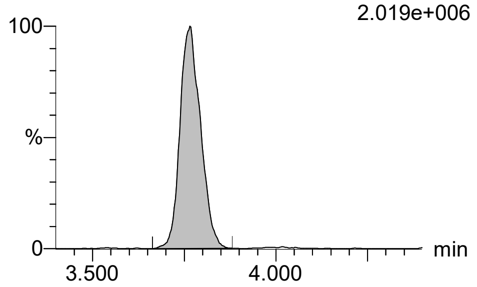

Sample Name: 20211111_H20_R3_IgM
F49:MRM of 1 channel,ES+
1006.8 > 366.1
3.509e+006

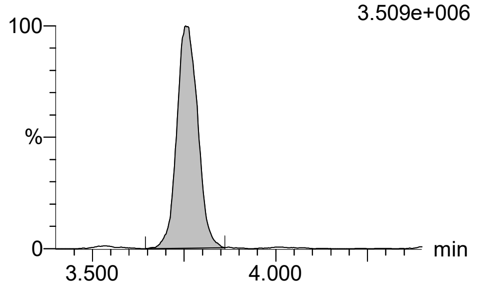

Sample Name: 20211111_A41_R3_IgM
F49:MRM of 1 channel,ES+
1006.8 > 366.1
8.396e+005

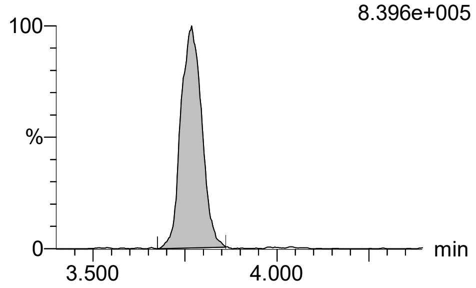

Sample Name: 20211111_A42_R3_IgM
F49:MRM of 1 channel,ES+
1006.8 > 366.1
1.145e+006

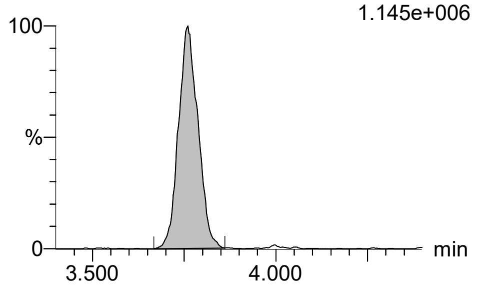

Dataset: C:\MassLynx\10.pro\20211111_clinicalsamples_IgM_IgA.qld

Last Altered: Thursday, March 09, 2023 19:58:25 台北標準時間
Printed: Friday, August 01, 2025 15:29:29 台北標準時間

Sample Name: 20211111_A43_R3_IgM

F49:MRM of 1 channel,ES+
1006.8 > 366.1
7.448e+005

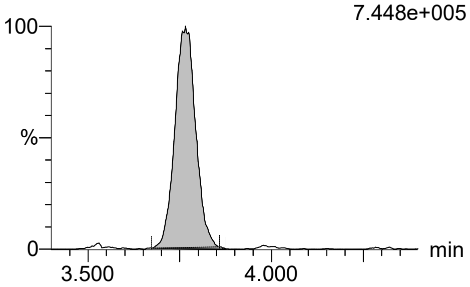

Sample Name: 20211111_A44_R3_IgM

F49:MRM of 1 channel,ES+
1006.8 > 366.1
5.932e+005

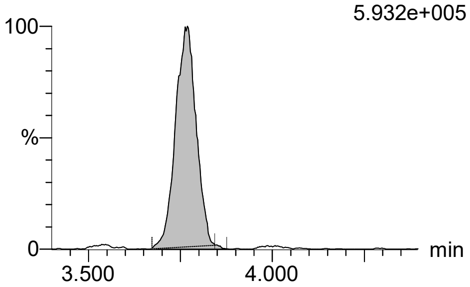

Sample Name: 20211111_QC_R25_IgM

F49:MRM of 1 channel,ES+
1006.8 > 366.1
1.940e+006

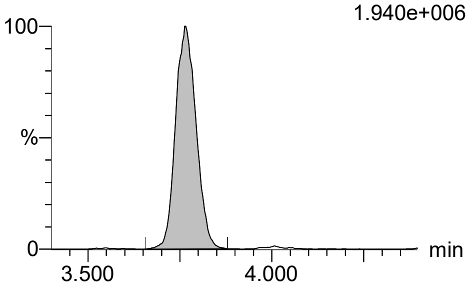

Dataset: C:\MassLynx\10.pro\20211111_clinicalsamples_IgM_IgA.qld

Last Altered: Thursday, March 09, 2023 19:58:25 台北標準時間
Printed: Friday, August 01, 2025 15:29:29 台北標準時間

	#	Name	RT	Area	IS Area	Response	Prima...
1	1	20211112_QC_R001_IgM	3.75	213543.1	15421.4	13.847	bb
2	2	20211112_QC_R002_IgM	3.75	188567.8	13188.8	14.298	bb
3	3	20211111_A1_R2_IgM	3.76	137185.4	11871.0	11.556	MM
4	4	20211111_A1_R3_IgM	3.76	134086.6	12030.5	11.146	bb
5	5	20211111_A2_R2_IgM	3.76	118607.4	11446.8	10.362	bb
6	6	20211111_A2_R3_IgM	3.76	113348.9	11098.4	10.213	bb
7	7	20211111_L1_R2_IgM	3.75	21416.9	11779.4	1.818	MM
8	8	20211111_L1_R3_IgM	3.76	20334.5	11176.2	1.819	bb
9	9	20211111_L2_R2_IgM	3.75	308390.5	11375.0	27.111	bb
10	10	20211111_H1_R2_IgM	3.75	418643.0	13595.2	30.793	bb
11	11	20211111_A3_R2_IgM	3.76	123571.5	14112.3	8.756	bb
12	12	20211111_A4_R2_IgM	3.76	129170.5	11977.5	10.784	bb
13	13	20211111_L3_R2_IgM	3.75	166134.4	12242.1	13.571	bb
14	14	20211111_L4_R2_IgM	3.76	100859.2	12797.0	7.881	bb
15	15	20211111_H2_R2_IgM	3.76	184470.3	10006.0	18.436	bb
16	16	20211111_A5_R2_IgM	3.76	703883.3	11434.6	61.557	bb
17	17	20211111_A6_R2_IgM	3.75	251334.2	13193.7	19.050	bb
18	18	20211111_QC_R10_IgM	3.77	174148.3	12566.1	13.859	bb
19	19	20211111_L5_R2_IgM	3.75	492605.9	13616.4	36.177	bb
20	20	20211111_L6_R2_IgM	3.76	214148.2	13698.5	15.633	bb
21	21	20211111_H3_R2_IgM	3.75	121397.2	15793.1	7.687	bb
22	22	20211111_A7_R2_IgM	3.76	270013.2	12936.9	20.872	bb
23	23	20211111_A8_R2_IgM	3.75	249790.5	13108.0	19.056	bb
24	24	20211111_L7_R2_IgM	3.75	2362.6	13278.5	0.178	MM
25	25	20211111_L8_R2_IgM	3.76	73244.9	15233.6	4.808	bb
26	26	20211111_H4_R2_IgM	3.75	160320.0	16521.9	9.703	bb
27	27	20211111_A9_R2_IgM	3.76	272616.3	16451.6	16.571	bb
28	28	20211111_A10_R2_IgM	3.76	563475.0	13036.0	43.224	bb
29	29	20211111_L9_R2_IgM	3.75	265539.8	14554.1	18.245	bb
30	30	20211111_L10_R2_IgM	3.76	245094.5	12250.4	20.007	bb
31	31	20211111_QC_R11_IgM	3.76	188281.3	11459.2	16.431	bb
32	32	20211111_H5_R2_IgM	3.76	152562.4	14358.2	10.625	bb
33	33	20211111_A11_R2_IgM	3.76	146148.2	16184.1	9.030	bb
34	34	20211111_A12_R2_IgM	3.77	106529.9	13465.4	7.911	bb
35	35	20211111_L11_R2_IgM	3.76	62129.3	13277.9	4.679	bb
36	36	20211111_L12_R2_IgM	3.76	134240.8	15958.8	8.412	bb
37	37	20211111_H6_R2_IgM	3.76	111230.1	14003.5	7.943	bb
38	38	20211111_A13_R2_IgM	3.76	100867.9	13656.1	7.386	bb
39	39	20211111_A14_R2_IgM	3.77	513727.9	14071.3	36.509	bb
40	40	20211111_L13_R2_IgM	3.76	172978.7	14785.6	11.699	bb
41	41	20211111_L14_R2_IgM	3.77	356732.8	14772.2	24.149	bb
42	42	20211111_H7_R2_IgM	3.76	233389.9	15164.4	15.391	bb
43	43	20211111_A15_R2_IgM	3.76	158095.5	15721.5	10.056	bb
44	44	20211111_QC_R12_IgM	3.77	192825.6	13711.5	14.063	bb
45	45	20211111_A16_R2_IgM	3.76	247924.2	14275.6	17.367	bb
46	46	20211111_L15_R2_IgM	3.75	220500.2	12935.8	17.046	bb
47	47	20211111_L16_R2_IgM	3.77	165532.8	13342.8	12.406	bb
48	48	20211111_H8_R2_IgM	3.76	134932.4	15835.1	8.521	bb
49	49	20211111_A17_R2_IgM	3.76	412697.4	15786.7	26.142	bb
50	50	20211111_A18_R2_IgM	3.76	226996.6	16986.8	13.363	bb
51	51	20211111_L17_R2_IgM	3.77	171514.0	13342.7	12.855	bb
52	52	20211111_L18_R2_IgM	3.75	13570.3	14692.0	0.924	MM
53	53	20211111_H9_R2_IgM	3.76	64479.4	15639.7	4.123	bb
54	54	20211111_A19_R2_IgM	3.76	171438.4	14198.7	12.074	MM
55	55	20211111_A20_R2_IgM	3.77	91275.2	14162.7	6.445	bb
56	56	20211111_L19_R2_IgM	3.77	147112.2	9293.4	15.830	bb

Dataset: C:\MassLynx\10.pro\20211111_clinicalsamples_IgM_IgA.qld

Last Altered: Thursday, March 09, 2023 19:58:25 台北標準時間
Printed: Friday, August 01, 2025 15:29:29 台北標準時間

	# Name	RT	Area	IS Area	Response	Prima...
57	57 20211111_QC_R13_IgM	3.77	188388.3	13903.0	13.550	MM
58	58 20211111_L20_R2_IgM	3.76	209176.1	12781.6	16.365	bb
59	59 20211111_H10_R2_IgM	3.76	160237.3	14796.4	10.829	MM
60	60 20211111_A21_R2_IgM	3.76	128412.7	13669.0	9.394	bb
61	61 20211111_A22_R2_IgM	3.76	41670.8	14098.3	2.956	MM
62	62 20211111_L21_R2_IgM	3.76	76687.8	15678.2	4.891	bb
63	63 20211111_L22_R2_IgM	3.76	304472.3	15724.5	19.363	bb
64	64 20211111_H11_R2_IgM	3.77	200170.1	15890.2	12.597	bb
65	65 20211111_A23_R2_IgM	3.76	93602.6	13423.2	6.973	MM
66	66 20211111_A24_R2_IgM	3.77	91738.6	12438.0	7.376	MM
67	67 20211111_L23_R2_IgM	3.74	4067.8	14075.0	0.289	MM
68	68 20211111_L24_R2_IgM	3.76	68443.7	15972.1	4.285	bb
69	69 20211111_H12_R2_IgM	3.77	114766.3	16270.9	7.053	bb
70	70 20211111_QC_R14_IgM	3.77	204621.7	15806.8	12.945	bb
71	71 20211111_A25_R2_IgM	3.76	133126.1	14552.1	9.148	MM
72	72 20211111_A26_R2_IgM	3.76	89297.5	11492.7	7.770	MM
73	73 20211111_L25_R2_IgM	3.77	220839.0	14403.3	15.332	bb
74	74 20211111_L26_R2_IgM	3.76	66489.1	16533.9	4.021	bb
75	75 20211111_H13_R2_IgM	3.76	224648.8	15520.1	14.475	bb
76	76 20211111_A27_R2_IgM	3.76	292640.8	13337.4	21.941	bb
77	77 20211111_A28_R2_IgM	3.76	119923.2	11895.6	10.081	bb
78	78 20211111_L27_R2_IgM	3.76	193203.4	14125.7	13.677	bb
79	79 20211111_L28_R2_IgM	3.76	131233.7	11807.5	11.114	bb
80	80 20211111_H14_R2_IgM	3.77	163938.9	15035.7	10.903	bb
81	81 20211111_A29_R2_IgM	3.76	13419.9	13554.4	0.990	MM
82	82 20211111_A30_R2_IgM	3.76	285628.9	12984.1	21.998	bb
83	83 20211111_QC_R15_IgM	3.76	181012.1	12299.5	14.717	bb
84	84 20211111_L29_R2_IgM	3.76	139829.2	13590.7	10.289	bb
85	85 20211111_L30_R2_IgM	3.76	78449.0	12511.4	6.270	bb
86	86 20211111_H15_R2_IgM	3.76	285914.9	11885.0	24.057	bb
87	87 20211111_A31_R2_IgM	3.76	129138.3	13422.1	9.621	bb
88	88 20211111_A32_R2_IgM	3.76	297732.3	11461.3	25.977	bb
89	89 20211111_L31_R2_IgM	3.75	116762.3	13753.4	8.490	bb
90	90 20211111_L32_R2_IgM	3.76	123144.8	12949.0	9.510	MM
91	91 20211111_H16_R2_IgM	3.76	378293.6	11465.4	32.994	bb
92	92 20211111_A33_R2_IgM	3.77	193456.7	10551.5	18.334	MM
93	93 20211111_A34_R2_IgM	3.76	83134.7	12409.9	6.699	bb
94	94 20211111_H17_R2_IgM	3.76	345139.7	10160.9	33.967	bb
95	95 20211111_A35_R2_IgM	3.75	56279.3	10460.6	5.380	MM
96	96 20211111_QC_R16_IgM	3.77	159208.5	10433.5	15.259	bb
97	97 20211111_A36_R2_IgM	3.76	168554.1	12504.8	13.479	bb
98	98 20211111_H18_R2_IgM	3.75	431499.8	9140.4	47.208	bb
99	99 20211111_A37_R2_IgM	3.77	73440.2	9796.9	7.496	MM
100	1... 20211111_A38_R2_IgM	3.76	144806.7	11224.3	12.901	bb
101	1... 20211111_H19_R2_IgM	3.75	221260.5	11739.2	18.848	MM
102	1... 20211111_A39_R2_IgM	3.75	273625.8	12718.9	21.513	bb
103	1... 20211111_A40_R2_IgM	3.76	143886.9	10006.1	14.380	bb
104	1... 20211111_H20_R2_IgM	3.76	279312.4	7988.2	34.966	bb
105	1... 20211111_A41_R2_IgM	3.76	94491.4	11513.3	8.207	bb
106	1... 20211111_A42_R2_IgM	3.76	76766.1	10238.5	7.498	bb
107	1... 20211111_A43_R2_IgM	3.77	52460.4	11098.9	4.727	MM
108	1... 20211111_A44_R2_IgM	3.76	42051.9	10969.9	3.833	bb
109	1... 20211111_QC_R17_IgM	3.76	136203.1	9700.8	14.040	bb
110	1... 20211111_L2_R3_IgM	3.75	271254.5	8528.0	31.807	bb
111	1... 20211111_H1_R3_IgM	3.76	352189.6	9438.2	37.315	bb
112	1... 20211111_A3_R3_IgM	3.76	98348.1	11598.6	8.479	bb

	#	Name	RT	Area	IS Area	Response	Prima...
113	1...	20211111_A4_R3_IgM	3.76	114392.9	11364.1	10.066	bb
114	1...	20211111_L3_R3_IgM	3.76	136779.9	10753.4	12.720	bb
115	1...	20211111_L4_R3_IgM	3.77	76491.2	10291.6	7.432	bb
116	1...	20211111_H2_R3_IgM	3.76	176898.1	9315.8	18.989	bb
117	1...	20211111_A5_R3_IgM	3.76	590167.4	9102.2	64.838	bb
118	1...	20211111_A6_R3_IgM	3.76	188952.8	10356.4	18.245	bb
119	1...	20211111_QC_R18_IgM	3.76	131219.6	8398.3	15.625	bb
120	1...	20211111_L5_R3_IgM	3.76	367934.2	9786.7	37.595	bb
121	1...	20211111_L6_R3_IgM	3.76	166575.3	9911.3	16.807	bb
122	1...	20211111_H3_R3_IgM	3.76	94816.2	12155.5	7.800	bb
123	1...	20211111_A7_R3_IgM	3.76	258266.0	11804.8	21.878	bb
124	1...	20211111_A8_R3_IgM	3.76	193684.7	10510.6	18.428	bb
125	1...	20211111_L7_R3_IgM	3.74	1806.7	12177.9	0.148	MM
126	1...	20211111_L8_R3_IgM	3.76	54042.6	11668.6	4.631	bb
127	1...	20211111_H4_R3_IgM	3.77	125600.2	11192.0	11.222	MM
128	1...	20211111_A9_R3_IgM	3.76	209903.6	14077.4	14.911	bb
129	1...	20211111_A10_R3_IgM	3.76	488189.9	11190.4	43.626	bb
130	1...	20211111_L9_R3_IgM	3.77	228876.6	14649.3	15.624	bb
131	1...	20211111_L10_R3_IgM	3.76	195126.2	9361.3	20.844	bb
132	1...	20211111_QC_R19_IgM	3.76	143084.8	10125.5	14.131	bb
133	1...	20211111_H5_R3_IgM	3.77	111130.2	10010.9	11.101	bb
134	1...	20211111_A11_R3_IgM	3.76	119785.3	12697.2	9.434	MM
135	1...	20211111_A12_R3_IgM	3.76	105279.8	13148.5	8.007	MM
136	1...	20211111_L11_R3_IgM	3.77	46958.7	10786.6	4.353	MM
137	1...	20211111_L12_R3_IgM	3.75	111308.3	11681.8	9.528	MM
138	1...	20211111_H6_R3_IgM	3.76	98386.7	12095.5	8.134	bb
139	1...	20211111_A13_R3_IgM	3.76	89451.2	12908.3	6.930	MM
140	1...	20211111_A14_R3_IgM	3.76	387638.3	11322.6	34.236	bb
141	1...	20211111_L13_R3_IgM	3.76	138497.4	11516.1	12.026	bb
142	1...	20211111_L14_R3_IgM	3.76	283766.8	10790.7	26.297	bb
143	1...	20211111_H7_R3_IgM	3.77	189847.5	11572.1	16.406	bb
144	1...	20211111_A15_R3_IgM	3.77	129232.2	12434.4	10.393	bb
145	1...	20211111_QC_R20_IgM	3.77	155545.8	10701.0	14.536	bb
146	1...	20211111_A16_R3_IgM	3.77	193467.0	11452.1	16.894	bb
147	1...	20211111_L15_R3_IgM	3.76	180397.0	10531.1	17.130	bb
148	1...	20211111_L16_R3_IgM	3.76	150273.9	12049.4	12.471	bb
149	1...	20211111_H8_R3_IgM	3.77	114862.7	13434.9	8.550	bb
150	1...	20211111_A17_R3_IgM	3.76	329717.9	12452.0	26.479	bb
151	1...	20211111_A18_R3_IgM	3.76	183869.8	12506.5	14.702	bb
152	1...	20211111_L17_R3_IgM	3.76	136171.5	9948.9	13.687	bb
153	1...	20211111_L18_R3_IgM	3.77	11865.5	13462.1	0.881	bb
154	1...	20211111_H9_R3_IgM	3.76	49529.2	13178.7	3.758	MM
155	1...	20211111_A19_R3_IgM	3.77	158133.9	11298.6	13.996	bb
156	1...	20211111_A20_R3_IgM	3.76	72919.0	11175.6	6.525	bb
157	1...	20211111_L19_R3_IgM	3.76	166201.8	11558.2	14.380	bb
158	1...	20211111_QC_R21_IgM	3.76	157842.9	9977.0	15.821	bb
159	1...	20211111_L20_R3_IgM	3.76	176438.1	13508.1	13.062	bb
160	1...	20211111_H10_R3_IgM	3.76	136627.6	12374.8	11.041	bb
161	1...	20211111_A21_R3_IgM	3.76	117192.8	12443.6	9.418	bb
162	1...	20211111_A22_R3_IgM	3.77	38877.0	11589.3	3.355	bb
163	1...	20211111_L21_R3_IgM	3.77	63848.3	13107.4	4.871	bb
164	1...	20211111_L22_R3_IgM	3.76	254546.2	12021.5	21.174	bb
165	1...	20211111_H11_R3_IgM	3.77	168970.4	12687.8	13.318	bb
166	1...	20211111_A23_R3_IgM	3.77	88932.9	11611.8	7.659	bb
167	1...	20211111_A24_R3_IgM	3.77	91168.2	10476.8	8.702	bb
168	1...	20211111_L23_R3_IgM	3.75	2711.1	11873.1	0.228	MM

Dataset: C:\MassLynx\10.pro\20211111_clinicalsamples_IgM_IgA.qld

Last Altered: Thursday, March 09, 2023 19:58:25 台北標準時間
Printed: Friday, August 01, 2025 15:29:29 台北標準時間

	# Name	RT	Area	IS Area	Response	Prima...
169	1... 20211111_L24_R3_IgM	3.77	56620.4	13468.0	4.204	MM
170	1... 20211111_H12_R3_IgM	3.76	110914.7	14229.1	7.795	bb
171	1... 20211111_QC_R22_IgM	3.76	164096.4	11264.4	14.568	bb
172	1... 20211111_A25_R3_IgM	3.76	98806.1	11775.7	8.391	MM
173	1... 20211111_A26_R3_IgM	3.76	83525.7	10473.9	7.975	MM
174	1... 20211111_L25_R3_IgM	3.76	185893.9	13256.8	14.023	bb
175	1... 20211111_L26_R3_IgM	3.76	54774.4	13565.0	4.038	bb
176	1... 20211111_H13_R3_IgM	3.76	212631.5	13146.0	16.175	bb
177	1... 20211111_A27_R3_IgM	3.76	245542.1	12025.6	20.418	bb
178	1... 20211111_A28_R3_IgM	3.77	111550.8	11229.1	9.934	bb
179	1... 20211111_L27_R3_IgM	3.76	171824.8	14960.0	11.486	bb
180	1... 20211111_L28_R3_IgM	3.77	136684.9	11359.0	12.033	bb
181	1... 20211111_H14_R3_IgM	3.75	159707.9	12830.8	12.447	bb
182	1... 20211111_A29_R3_IgM	3.76	12358.9	13434.3	0.920	MM
183	1... 20211111_A30_R3_IgM	3.76	247082.0	11272.2	21.920	bb
184	1... 20211111_QC_R23_IgM	3.77	149764.0	11872.2	12.615	bb
185	1... 20211111_L29_R3_IgM	3.76	135200.9	12948.7	10.441	bb
186	1... 20211111_L30_R3_IgM	3.76	67741.9	11026.6	6.144	MM
187	1... 20211111_H15_R3_IgM	3.76	242126.1	10786.3	22.448	bb
188	1... 20211111_A31_R3_IgM	3.77	122300.0	12010.5	10.183	bb
189	1... 20211111_A32_R3_IgM	3.76	269974.5	11107.7	24.305	bb
190	1... 20211111_L31_R3_IgM	3.76	105692.5	11021.8	9.589	bb
191	1... 20211111_L32_R3_IgM	3.76	105667.8	11683.8	9.044	bb
192	1... 20211111_H16_R3_IgM	3.76	305824.5	9831.7	31.106	bb
193	1... 20211111_A33_R3_IgM	3.77	191181.8	11213.1	17.050	bb
194	1... 20211111_A34_R3_IgM	3.76	70114.0	11550.1	6.070	MM
195	1... 20211111_H17_R3_IgM	3.76	282009.8	8874.4	31.778	bb
196	1... 20211111_A35_R3_IgM	3.75	49203.5	9856.3	4.992	MM
197	1... 20211111_QC_R24_IgM	3.77	126594.1	7561.8	16.741	bb
198	1... 20211111_A36_R3_IgM	3.75	157316.3	9562.8	16.451	bb
199	1... 20211111_H18_R3_IgM	3.75	385934.3	8165.3	47.265	bb
200	2... 20211111_A37_R3_IgM	3.76	58349.7	9278.8	6.289	bb
201	2... 20211111_A38_R3_IgM	3.76	118992.5	10920.3	10.896	bb
202	2... 20211111_H19_R3_IgM	3.75	181077.2	9285.3	19.501	MM
203	2... 20211111_A39_R3_IgM	3.76	235326.1	9083.4	25.907	bb
204	2... 20211111_A40_R3_IgM	3.77	130419.8	8382.4	15.559	bb
205	2... 20211111_H20_R3_IgM	3.75	231280.0	7807.9	29.621	bb
206	2... 20211111_A41_R3_IgM	3.77	54854.9	5925.2	9.258	bb
207	2... 20211111_A42_R3_IgM	3.76	70087.9	9352.6	7.494	bb
208	2... 20211111_A43_R3_IgM	3.77	48821.2	10216.3	4.779	MM
209	2... 20211111_A44_R3_IgM	3.77	39122.3	10262.7	3.812	MM
210	2... 20211111_QC_R25_IgM	3.77	125377.4	8575.3	14.621	bb

Dataset: C:\MassLynx\10.pro\20211111_clinicalsamples_IgM_IgA.qld

Last Altered: Thursday, March 09, 2023 19:58:25 台北標準時間
Printed: Friday, August 01, 2025 15:29:59 台北標準時間

Method: C:\MassLynx\10_Pro_IgA.PRO\MethDB\20211111_IgM_IgA_DMRM_new.mdb 24 Feb 2022 13:44:14
Calibration: 15 Nov 2021 14:42:11

Sample Name: 20211112_QC_R001_IgM

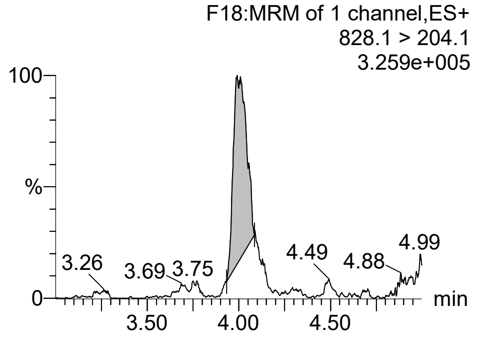

Sample Name: 20211112_QC_R002_IgM

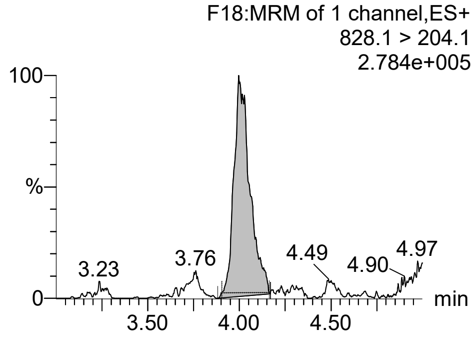

Sample Name: 20211111_A1_R2_IgM

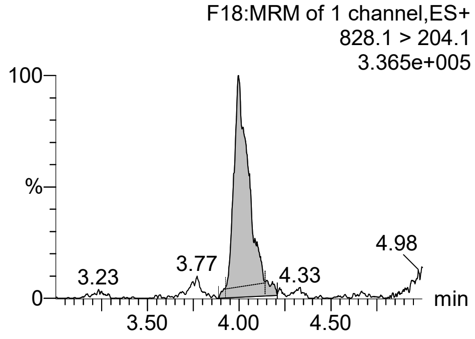

Sample Name: 20211111_A1_R3_IgM

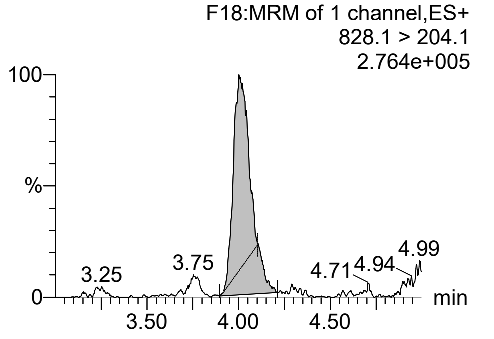

Sample Name: 20211111_A2_R2_IgM

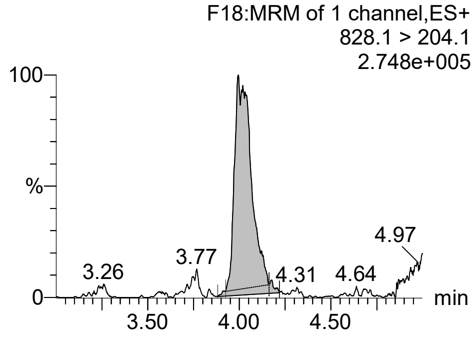

Sample Name: 20211111_A2_R3_IgM

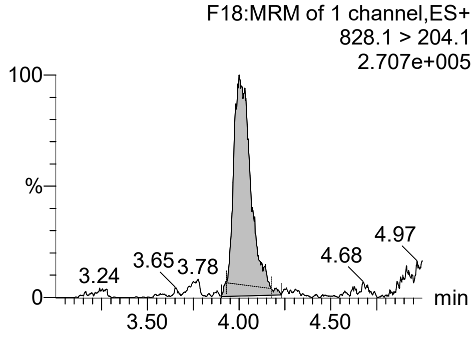

Sample Name: 20211111_L1_R2_IgM

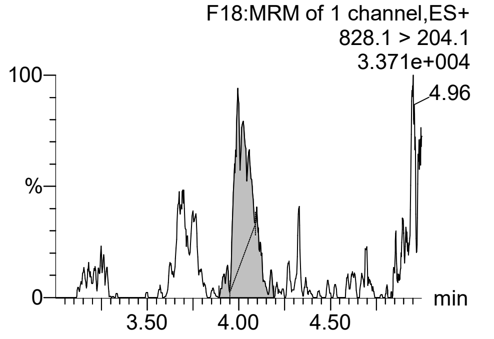

Sample Name: 20211111_L1_R3_IgM

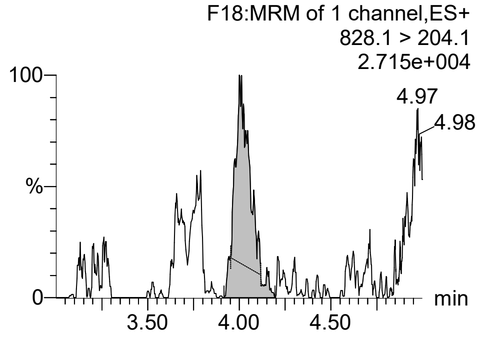

Sample Name: 20211111_L2_R2_IgM

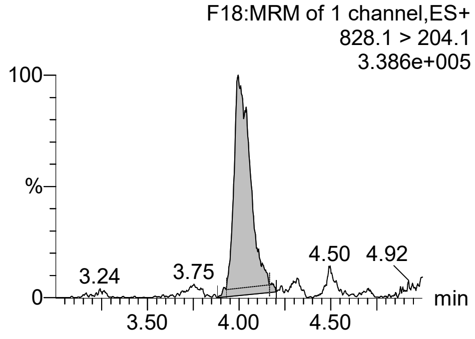

Sample Name: 20211111_H1_R2_IgM

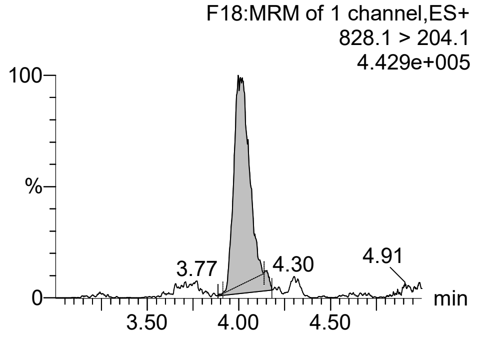

Sample Name: 20211111_A3_R2_IgM

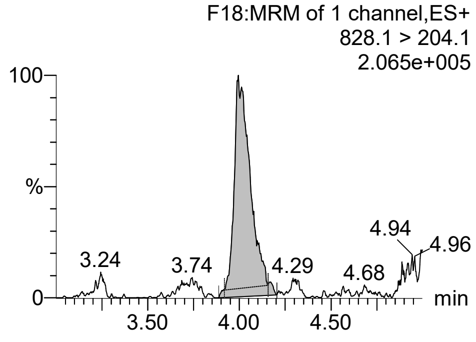

Sample Name: 20211111_A4_R2_IgM

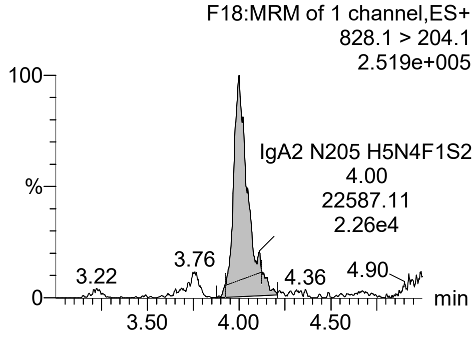

Sample Name: 20211111_L3_R2_IgM
F18:MRM of 1 channel,ES+
828.1 > 204.1
2.885e+005

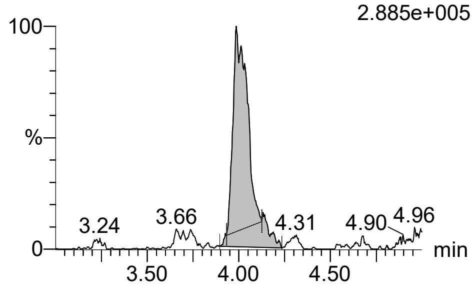

Sample Name: 20211111_L4_R2_IgM
F18:MRM of 1 channel,ES+
828.1 > 204.1
1.329e+005

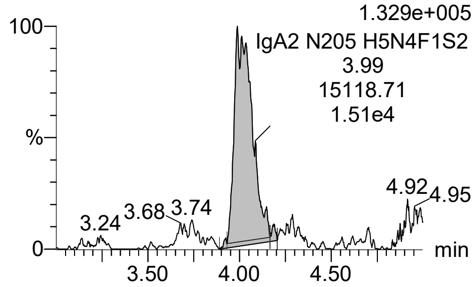

Sample Name: 20211111_H2_R2_IgM
F18:MRM of 1 channel,ES+
828.1 > 204.1
1.881e+005

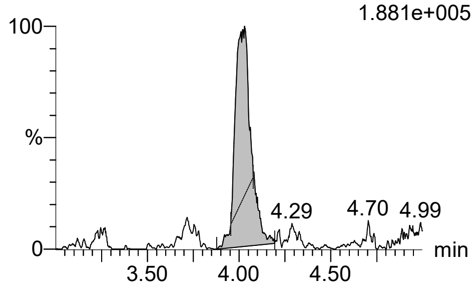

Sample Name: 20211111_A5_R2_IgM
F18:MRM of 1 channel,ES+
828.1 > 204.1
1.151e+006

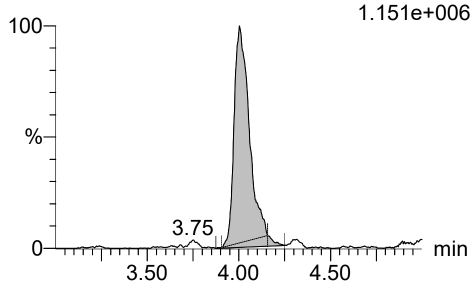

Sample Name: 20211111_A6_R2_IgM
F18:MRM of 1 channel,ES+
828.1 > 204.1
6.098e+005

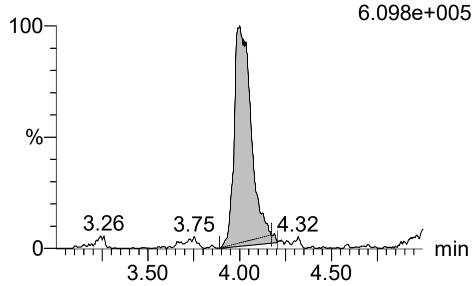

Sample Name: 20211111_QC_R10_IgM
F18:MRM of 1 channel,ES+
828.1 > 204.1
2.420e+005

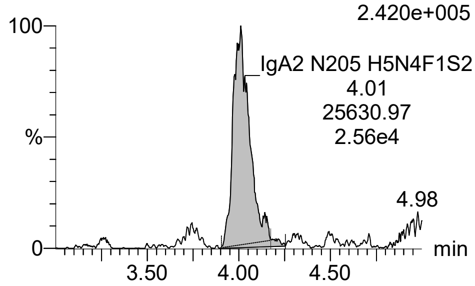

Sample Name: 20211111_L5_R2_IgM
F18:MRM of 1 channel,ES+
828.1 > 204.1
6.427e+005

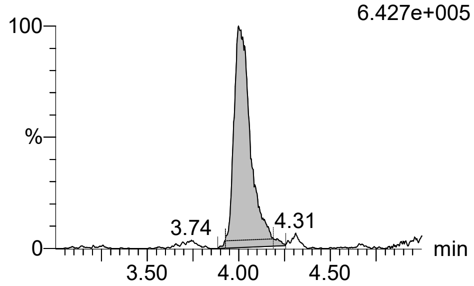

Sample Name: 20211111_L6_R2_IgM
F18:MRM of 1 channel,ES+
828.1 > 204.1
3.130e+005

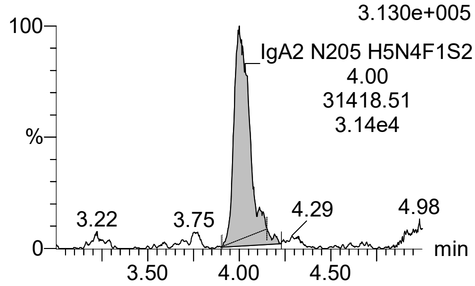

Sample Name: 20211111_H3_R2_IgM
F18:MRM of 1 channel,ES+
828.1 > 204.1
1.696e+005

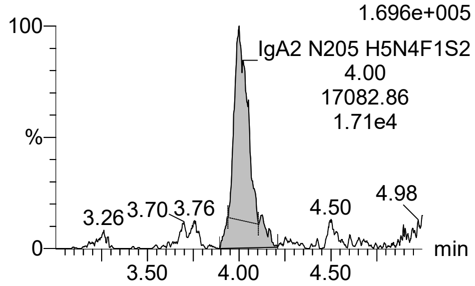

Sample Name: 20211111_A7_R2_IgM
F18:MRM of 1 channel,ES+
828.1 > 204.1
5.387e+005

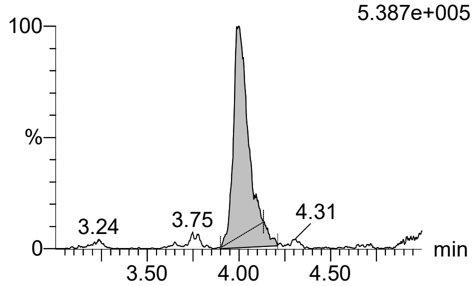

Sample Name: 20211111_A8_R2_IgM
F18:MRM of 1 channel,ES+
828.1 > 204.1
4.095e+005

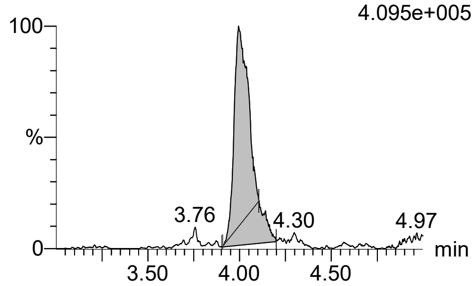

Sample Name: 20211111_L7_R2_IgM
F18:MRM of 1 channel,ES+
828.1 > 204.1
2.626e+004

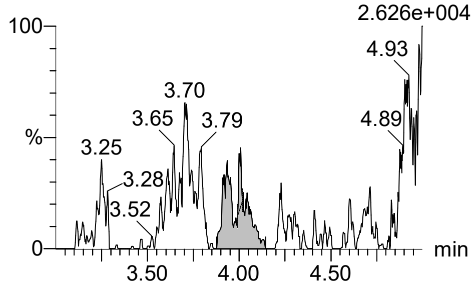

Sample Name: 20211111_L8_R2_IgM
F18:MRM of 1 channel,ES+
828.1 > 204.1
1.254e+005

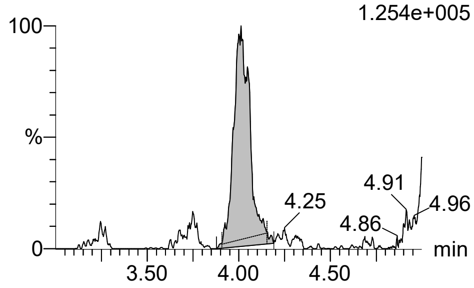

Sample Name: 20211111_H4_R2_IgM
F18:MRM of 1 channel,ES+
828.1 > 204.1
2.825e+005

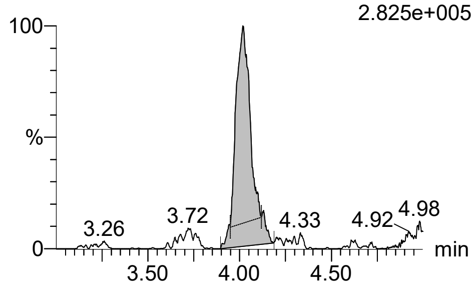

Sample Name: 20211111_A9_R2_IgM
F18:MRM of 1 channel,ES+
828.1 > 204.1
3.226e+005

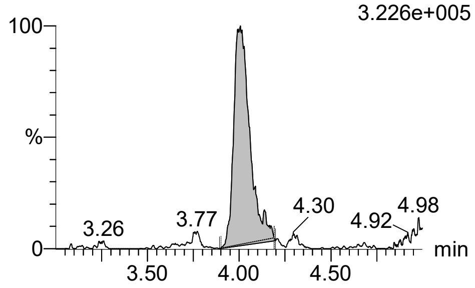

Dataset: C:\MassLynx\10.pro\20211111_clinicalsamples_IgM_IgA.qld

Last Altered: Thursday, March 09, 2023 19:58:25 台北標準時間
Printed: Friday, August 01, 2025 15:29:59 台北標準時間

Sample Name: 20211111_A10_R2_IgM
F18:MRM of 1 channel,ES+
828.1 > 204.1
7.203e+005

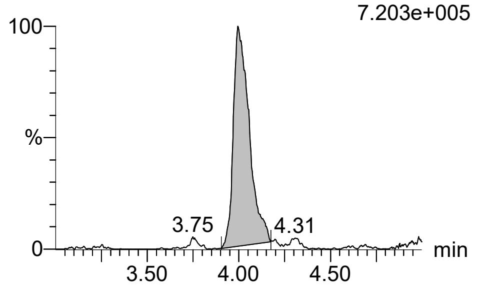

Sample Name: 20211111_L9_R2_IgM
F18:MRM of 1 channel,ES+
828.1 > 204.1
4.014e+005

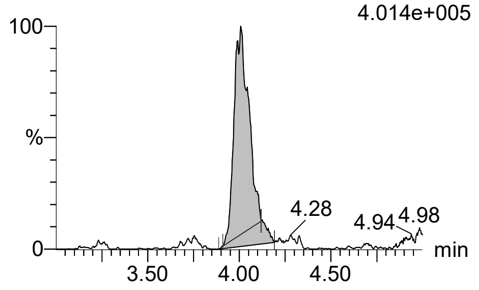

Sample Name: 20211111_L10_R2_IgM
F18:MRM of 1 channel,ES+
828.1 > 204.1
4.538e+005

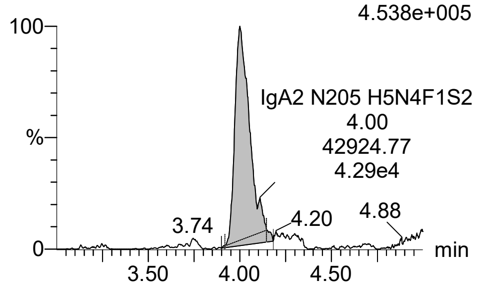

Sample Name: 20211111_QC_R11_IgM
F18:MRM of 1 channel,ES+
828.1 > 204.1
2.595e+005

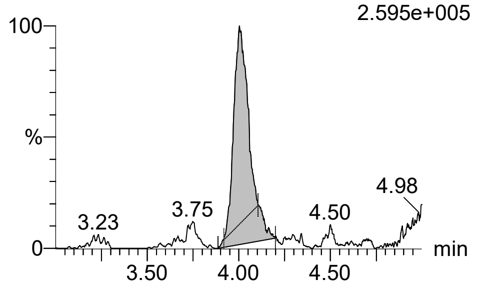

Sample Name: 20211111_H5_R2_IgM
F18:MRM of 1 channel,ES+
828.1 > 204.1
2.544e+005

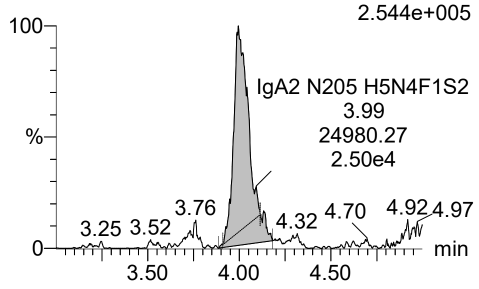

Sample Name: 20211111_A11_R2_IgM
F18:MRM of 1 channel,ES+
828.1 > 204.1
3.552e+005

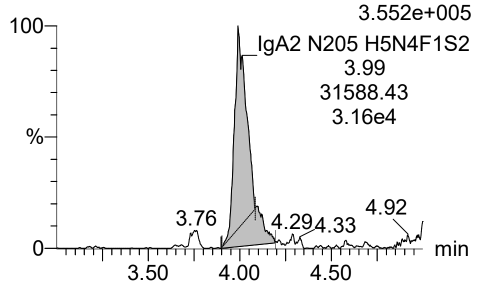

Sample Name: 20211111_A12_R2_IgM
F18:MRM of 1 channel,ES+
828.1 > 204.1
9.934e+004

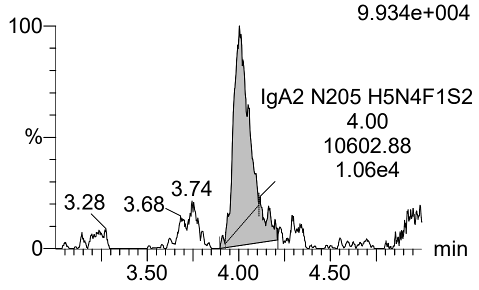

Sample Name: 20211111_L11_R2_IgM
F18:MRM of 1 channel,ES+
828.1 > 204.1
8.157e+004

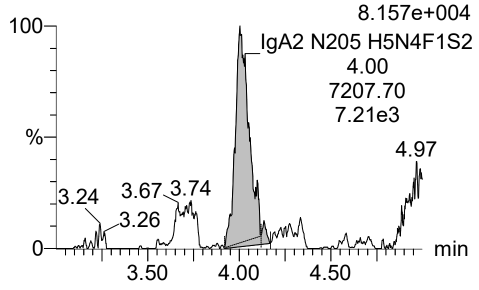

Sample Name: 20211111_L12_R2_IgM
F18:MRM of 1 channel,ES+
828.1 > 204.1
2.500e+005

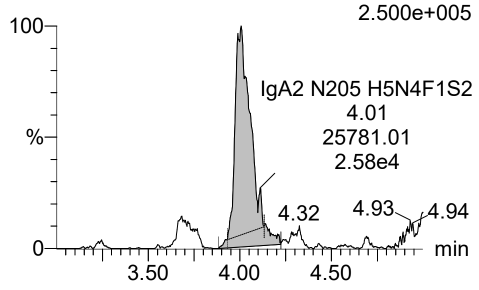

Sample Name: 20211111_H6_R2_IgM
F18:MRM of 1 channel,ES+
828.1 > 204.1
1.495e+005

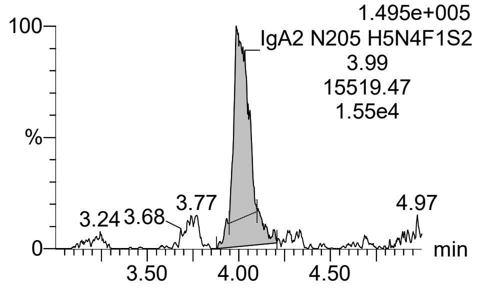

Sample Name: 20211111_A13_R2_IgM
F18:MRM of 1 channel,ES+
828.1 > 204.1
1.001e+005

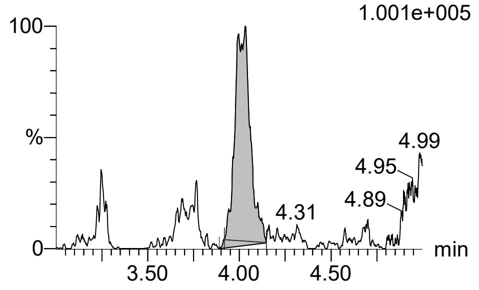

Sample Name: 20211111_A14_R2_IgM
F18:MRM of 1 channel,ES+
828.1 > 204.1
7.441e+005

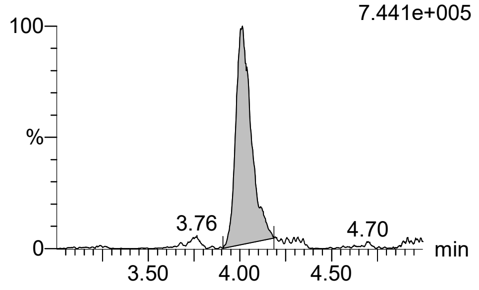

Sample Name: 20211111_L13_R2_IgM
F18:MRM of 1 channel,ES+
828.1 > 204.1
1.964e+005

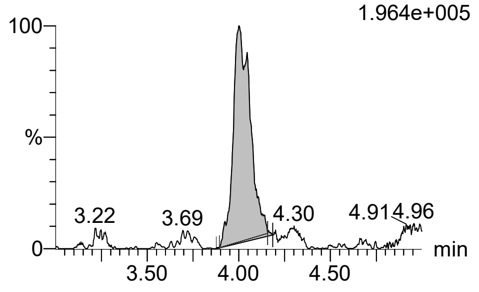

Sample Name: 20211111_L14_R2_IgM
F18:MRM of 1 channel,ES+
828.1 > 204.1
3.136e+005

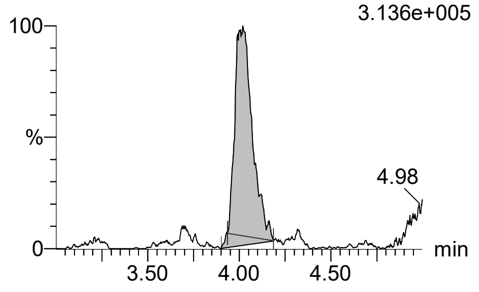

Sample Name: 20211111_H7_R2_IgM
F18:MRM of 1 channel,ES+
828.1 > 204.1
3.566e+005

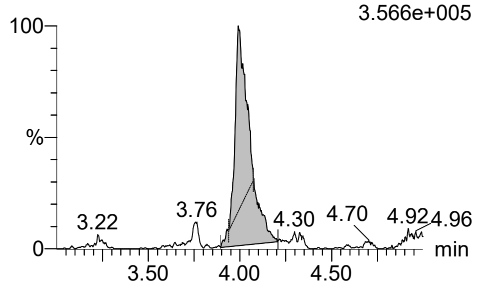

Dataset: C:\MassLynx\10.pro\20211111_clinicalsamples_IgM_IgA.qld

Last Altered: Thursday, March 09, 2023 19:58:25 台北標準時間
Printed: Friday, August 01, 2025 15:29:59 台北標準時間

Sample Name: 20211111_A15_R2_IgM

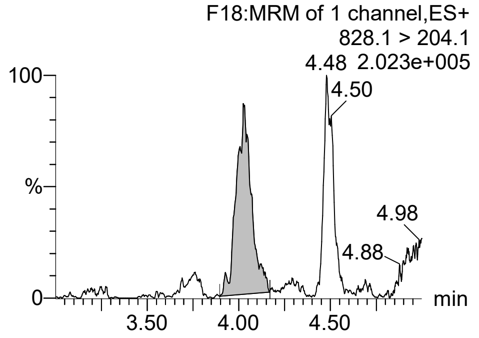

Sample Name: 20211111_QC_R12_IgM

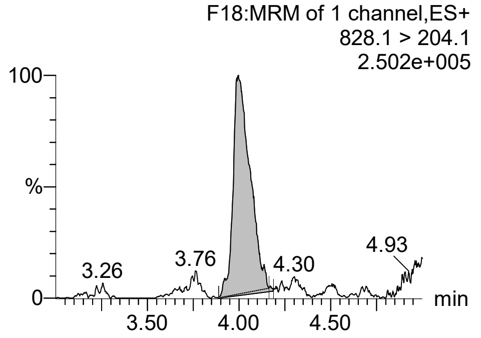

Sample Name: 20211111_A16_R2_IgM

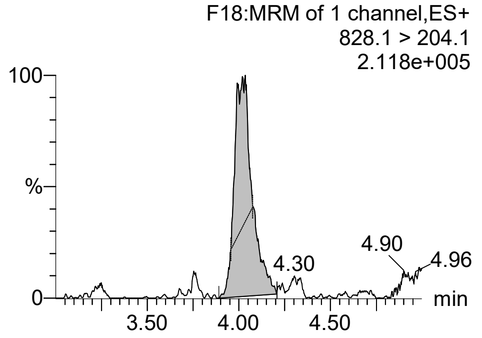

Sample Name: 20211111_L15_R2_IgM

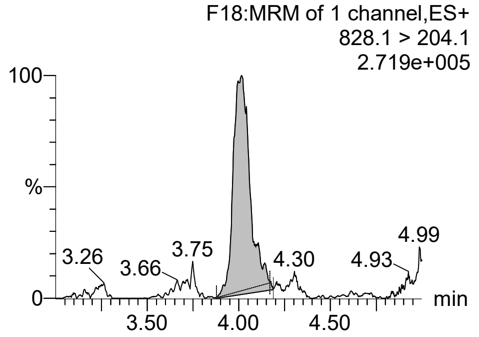

Sample Name: 20211111_L16_R2_IgM

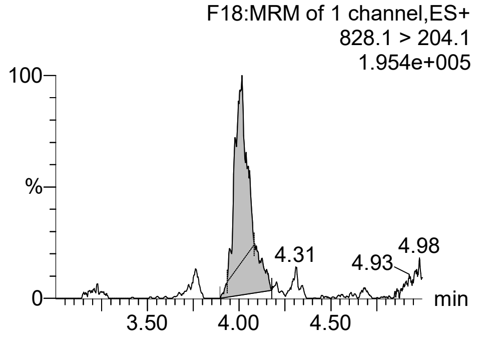

Sample Name: 20211111_H8_R2_IgM

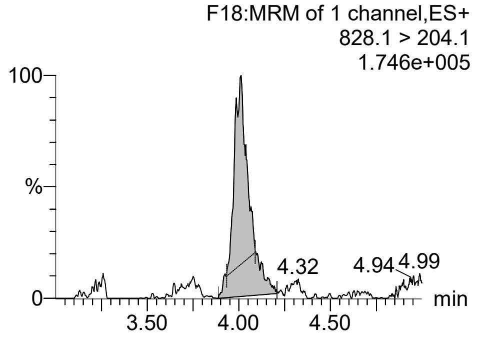

Sample Name: 20211111_A17_R2_IgM

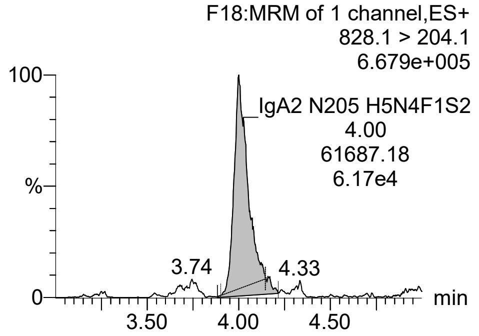

Sample Name: 20211111_A18_R2_IgM

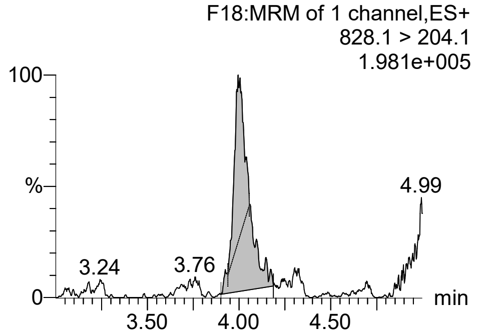

Sample Name: 20211111_L17_R2_IgM

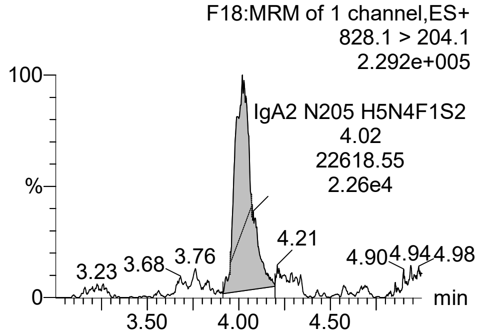

Sample Name: 20211111_L18_R2_IgM

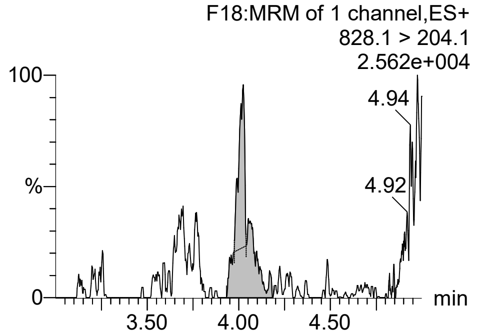

Sample Name: 20211111_H9_R2_IgM

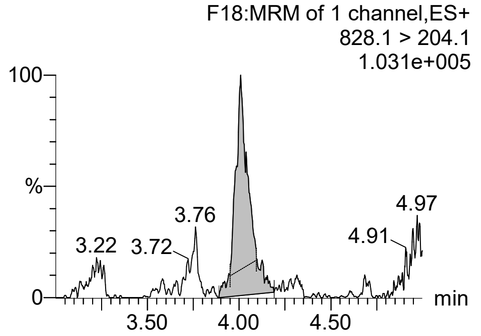

Sample Name: 20211111_A19_R2_IgM

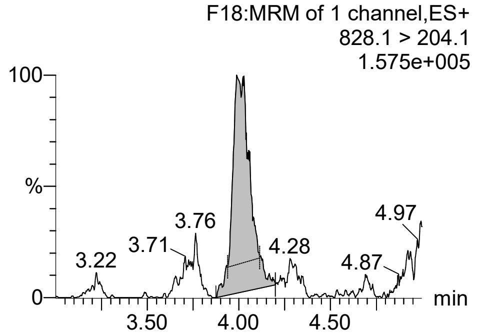

Sample Name: 20211111_A20_R2_IgM

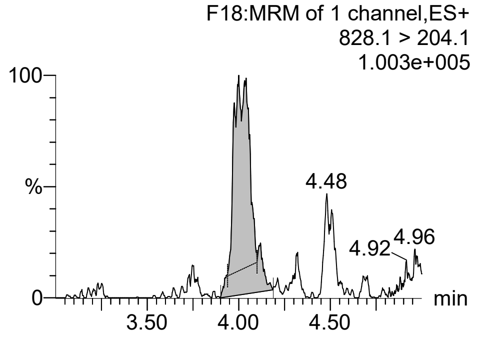

Sample Name: 20211111_L19_R2_IgM

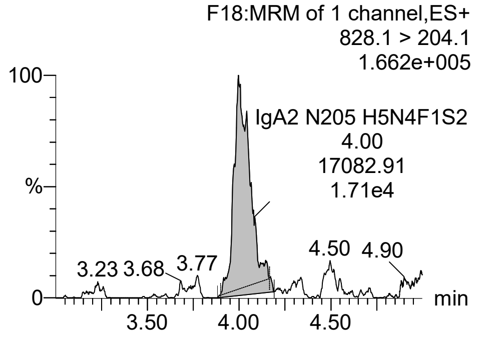

Sample Name: 20211111_QC_R13_IgM

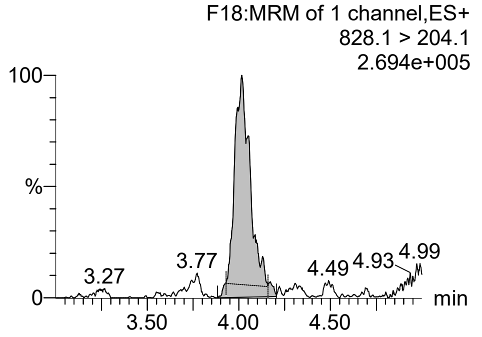

Dataset: C:\MassLynx\10.pro\20211111_clinicalsamples_IgM_IgA.qld

Last Altered: Thursday, March 09, 2023 19:58:25 台北標準時間
Printed: Friday, August 01, 2025 15:29:59 台北標準時間

Sample Name: 20211111_L20_R2_IgM
F18:MRM of 1 channel,ES+
828.1 > 204.1
3.824e+005

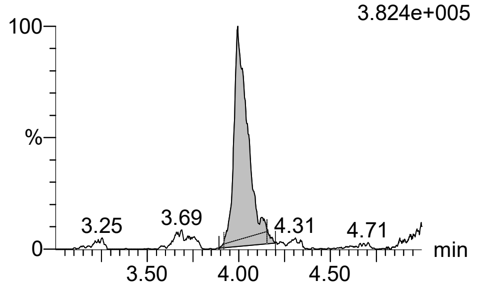

Sample Name: 20211111_H10_R2_IgM
F18:MRM of 1 channel,ES+
828.1 > 204.1
3.282e+005

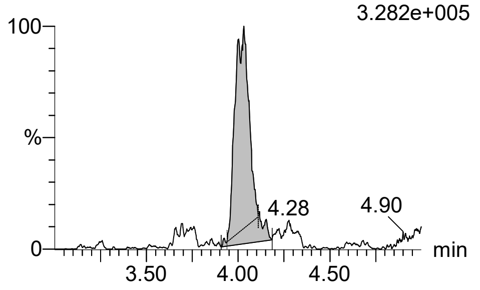

Sample Name: 20211111_A21_R2_IgM
F18:MRM of 1 channel,ES+
828.1 > 204.1
1.674e+005

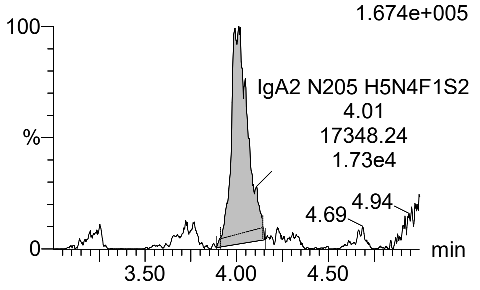

Sample Name: 20211111_A22_R2_IgM
F18:MRM of 1 channel,ES+
828.1 > 204.1
6.871e+004

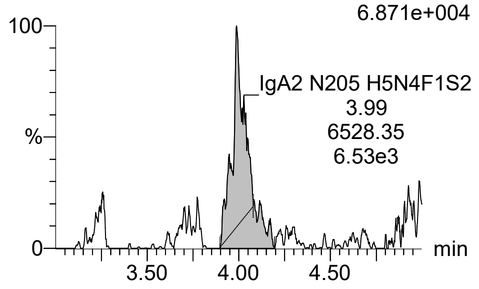

Sample Name: 20211111_L21_R2_IgM
F18:MRM of 1 channel,ES+
828.1 > 204.1
1.220e+005

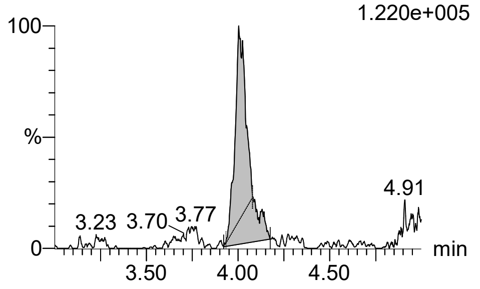

Sample Name: 20211111_L22_R2_IgM
F18:MRM of 1 channel,ES+
828.1 > 204.1
5.134e+005

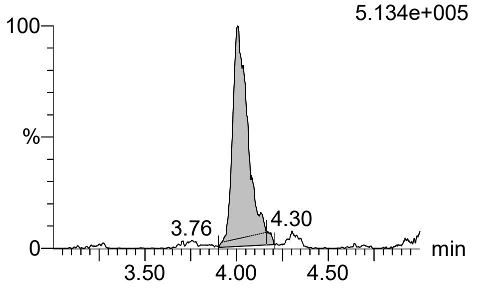

Sample Name: 20211111_H11_R2_IgM
F18:MRM of 1 channel,ES+
828.1 > 204.1
2.675e+005

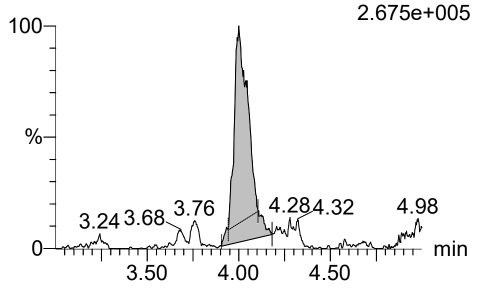

Sample Name: 20211111_A23_R2_IgM
F18:MRM of 1 channel,ES+
828.1 > 204.1
1.835e+005

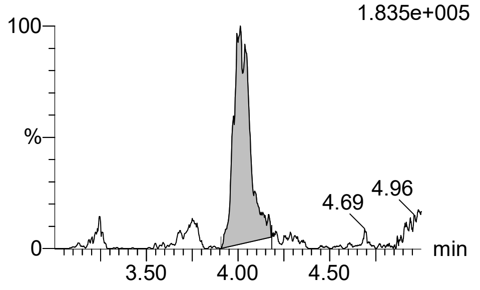

Sample Name: 20211111_A24_R2_IgM
F18:MRM of 1 channel,ES+
828.1 > 204.1
2.200e+005

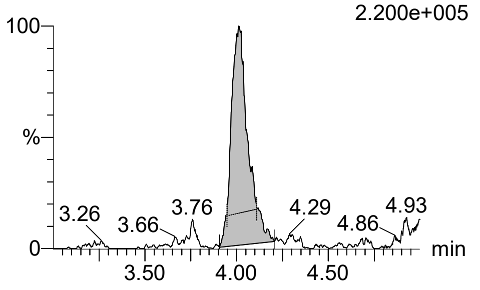

Sample Name: 20211111_L23_R2_IgM
F18:MRM of 1 channel,ES+
828.1 > 204.1
3.296e+004

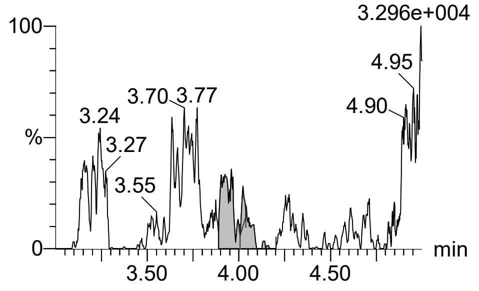

Sample Name: 20211111_L24_R2_IgM
F18:MRM of 1 channel,ES+
828.1 > 204.1
1.340e+005

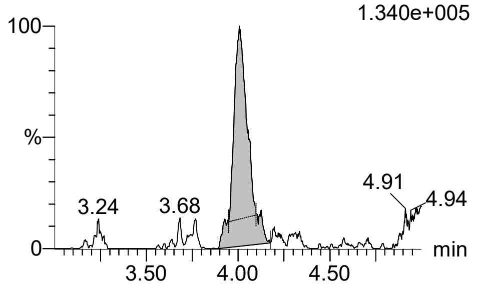

Sample Name: 20211111_H12_R2_IgM
F18:MRM of 1 channel,ES+
828.1 > 204.1
2.473e+005

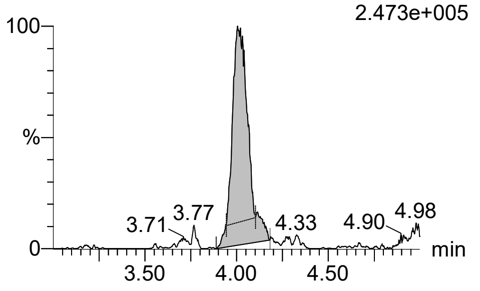

Sample Name: 20211111_QC_R14_IgM
F18:MRM of 1 channel,ES+
828.1 > 204.1
3.099e+005

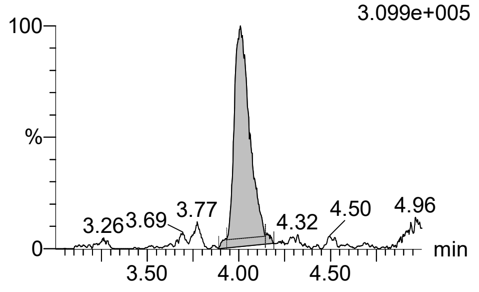

Sample Name: 20211111_A25_R2_IgM
F18:MRM of 1 channel,ES+
828.1 > 204.1
2.319e+005

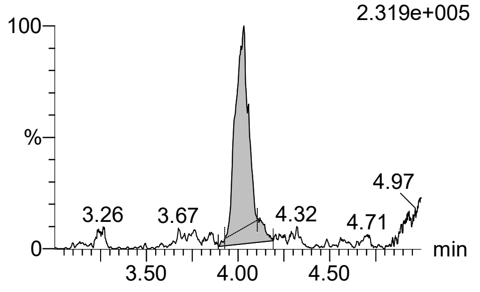

Sample Name: 20211111_A26_R2_IgM
F18:MRM of 1 channel,ES+
828.1 > 204.1
1.776e+005

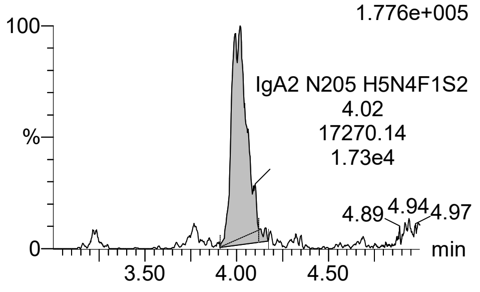

Sample Name: 20211111_L25_R2_IgM
F18:MRM of 1 channel,ES+
828.1 > 204.1
3.354e+005

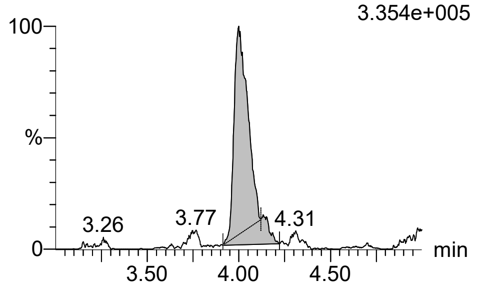

Sample Name: 20211111_L26_R2_IgM
F18:MRM of 1 channel,ES+
828.1 > 204.1
8.108e+004

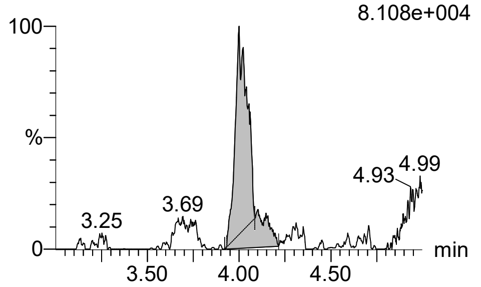

Sample Name: 20211111_H13_R2_IgM
F18:MRM of 1 channel,ES+
828.1 > 204.1
3.707e+005

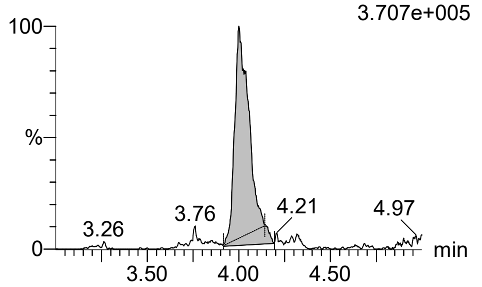

Sample Name: 20211111_A27_R2_IgM
F18:MRM of 1 channel,ES+
828.1 > 204.1
5.354e+005

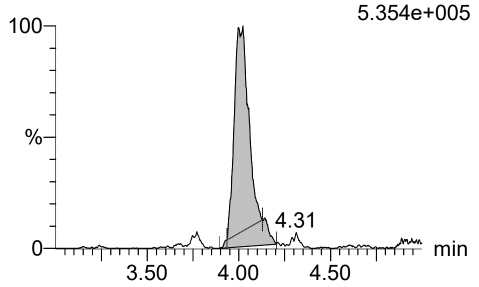

Sample Name: 20211111_A28_R2_IgM
F18:MRM of 1 channel,ES+
828.1 > 204.1
3.933e+005

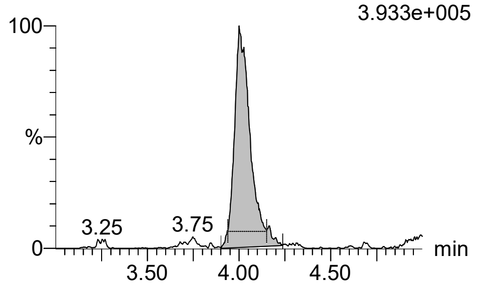

Sample Name: 20211111_L27_R2_IgM
F18:MRM of 1 channel,ES+
828.1 > 204.1
3.900e+005

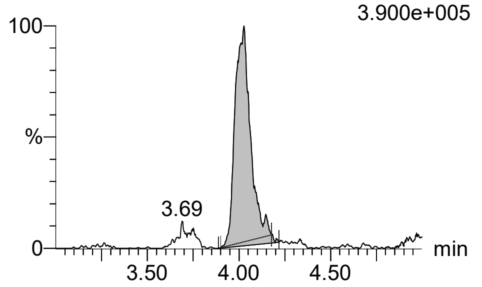

Sample Name: 20211111_L28_R2_IgM
F18:MRM of 1 channel,ES+
828.1 > 204.1
1.730e+005

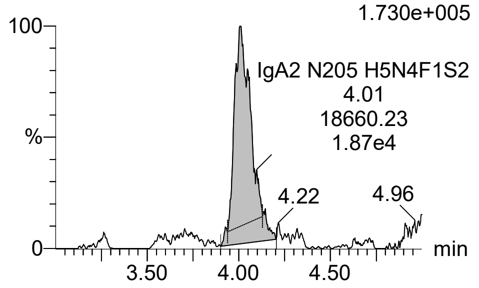

Sample Name: 20211111_H14_R2_IgM
F18:MRM of 1 channel,ES+
828.1 > 204.1
2.364e+005

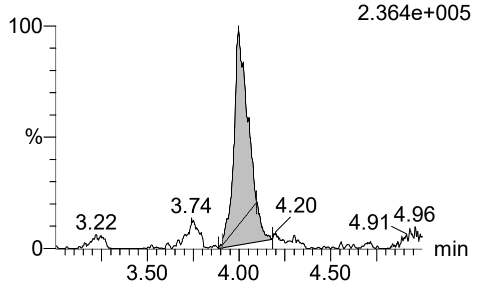

Sample Name: 20211111_A29_R2_IgM
F18:MRM of 1 channel,ES+
828.1 > 204.1
3.059e+004

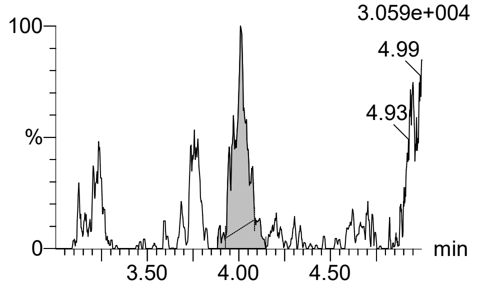

Sample Name: 20211111_A30_R2_IgM
F18:MRM of 1 channel,ES+
828.1 > 204.1
4.220e+005

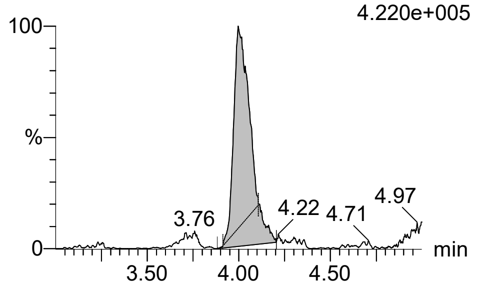

Sample Name: 20211111_QC_R15_IgM
F18:MRM of 1 channel,ES+
828.1 > 204.1
2.576e+005

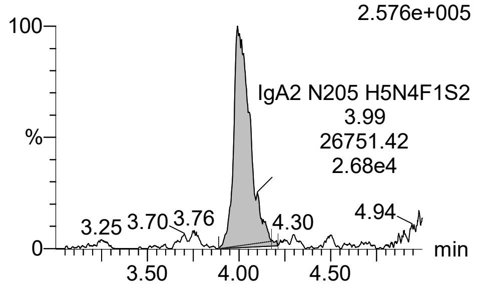

Sample Name: 20211111_L29_R2_IgM
F18:MRM of 1 channel,ES+
828.1 > 204.1
1.576e+005

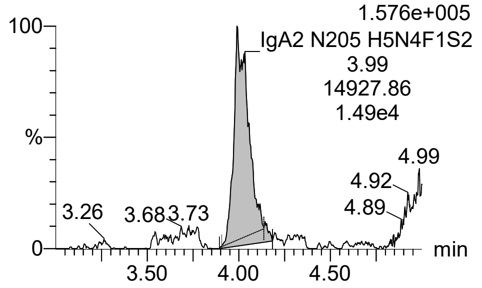

Sample Name: 20211111_L30_R2_IgM
F18:MRM of 1 channel,ES+
828.1 > 204.1
1.163e+005

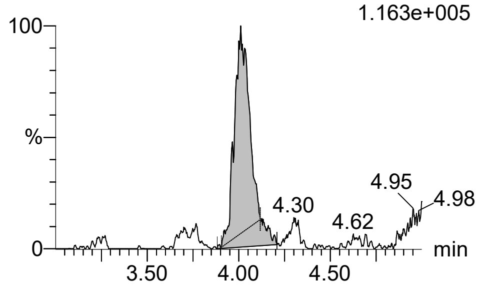

Sample Name: 20211111_H15_R2_IgM
F18:MRM of 1 channel,ES+
828.1 > 204.1
2.991e+005

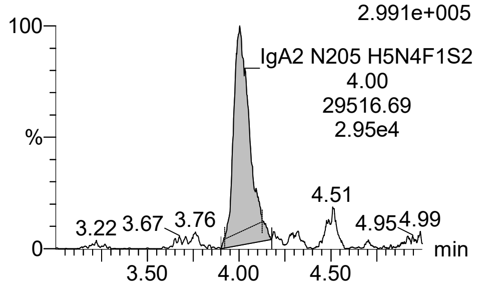

Sample Name: 20211111_A31_R2_IgM
F18:MRM of 1 channel,ES+
828.1 > 204.1
1.606e+005

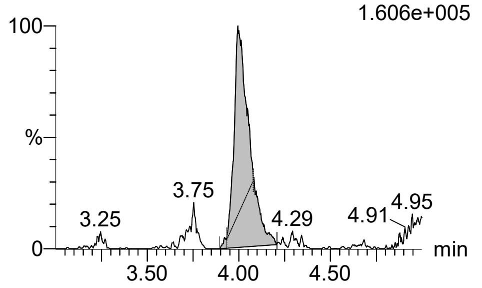

Dataset: C:\MassLynx\10.pro\20211111_clinicalsamples_IgM_IgA.qld

Last Altered: Thursday, March 09, 2023 19:58:25 台北標準時間
Printed: Friday, August 01, 2025 15:29:59 台北標準時間

Sample Name: 20211111_A32_R2_IgM

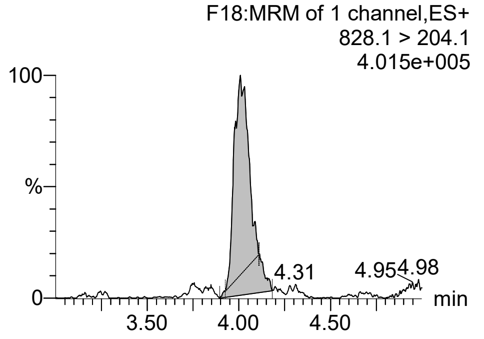

Sample Name: 20211111_L31_R2_IgM

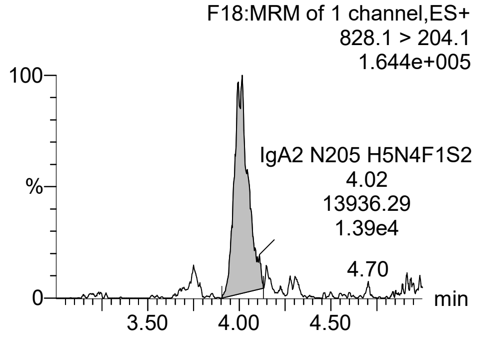

Sample Name: 20211111_L32_R2_IgM

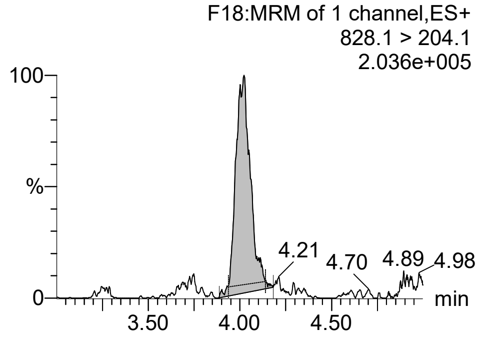

Sample Name: 20211111_H16_R2_IgM

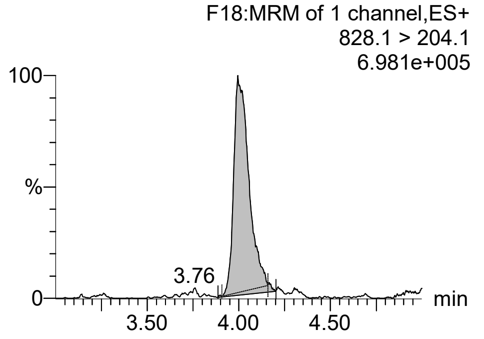

Sample Name: 20211111_A33_R2_IgM

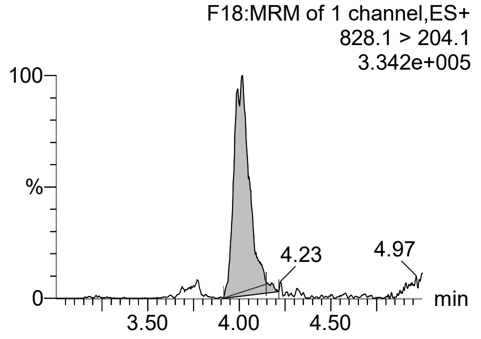

Sample Name: 20211111_A34_R2_IgM

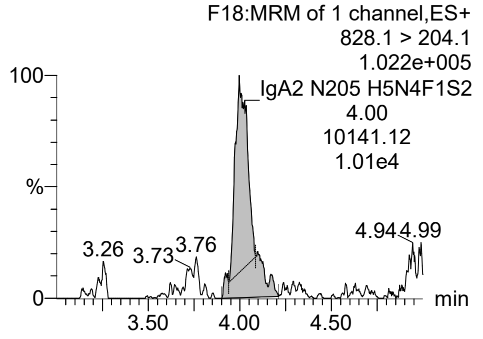

Sample Name: 20211111_H17_R2_IgM

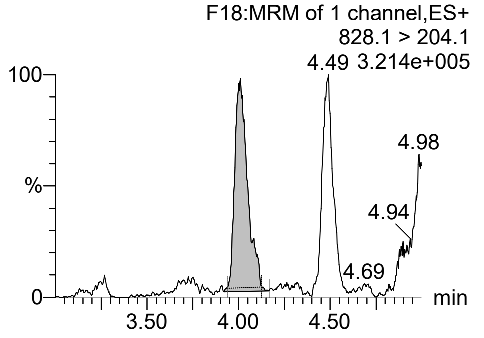

Sample Name: 20211111_A35_R2_IgM

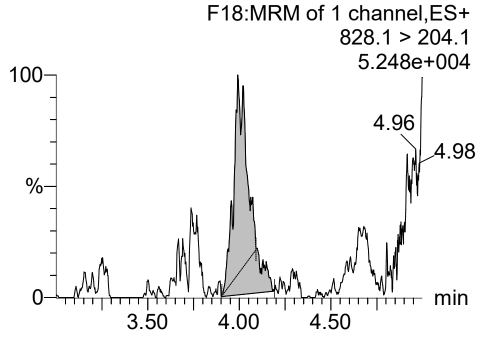

Sample Name: 20211111_QC_R16_IgM

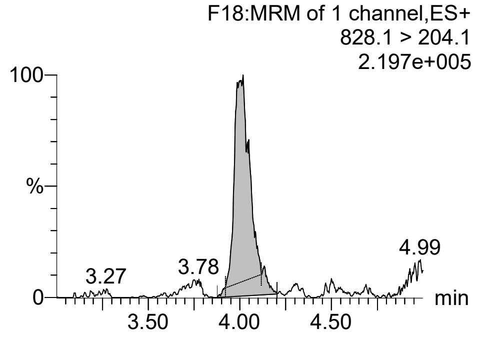

Sample Name: 20211111_A36_R2_IgM

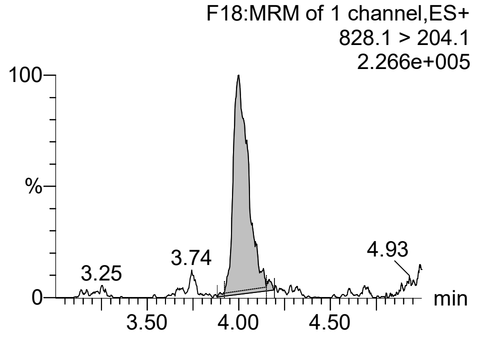

Sample Name: 20211111_H18_R2_IgM

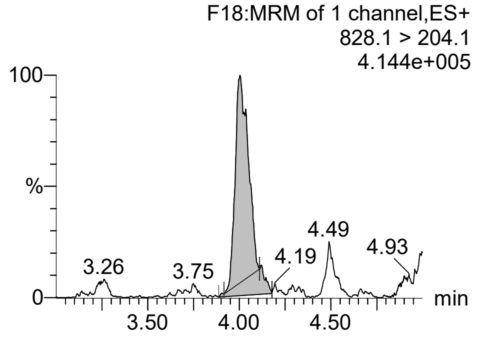

Sample Name: 20211111_A37_R2_IgM

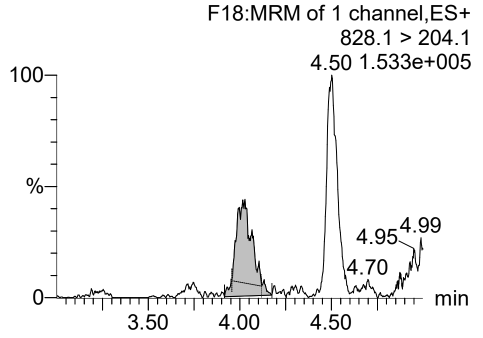

Sample Name: 20211111_A38_R2_IgM

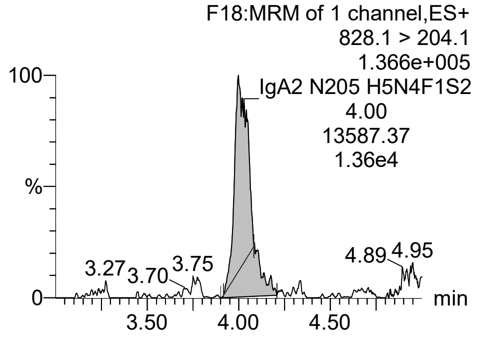

Sample Name: 20211111_H19_R2_IgM

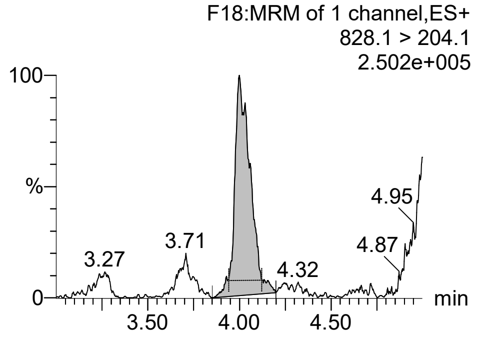

Sample Name: 20211111_A39_R2_IgM

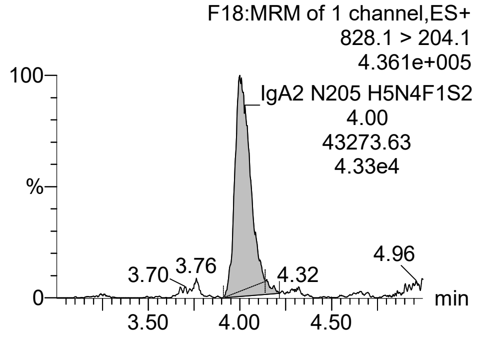

Dataset: C:\MassLynx\10.pro\20211111_clinicalsamples_IgM_IgA.qld

Last Altered: Thursday, March 09, 2023 19:58:25 台北標準時間
Printed: Friday, August 01, 2025 15:29:59 台北標準時間

Sample Name: 20211111_A40_R2_IgM
F18:MRM of 1 channel,ES+
828.1 > 204.1
1.093e+005

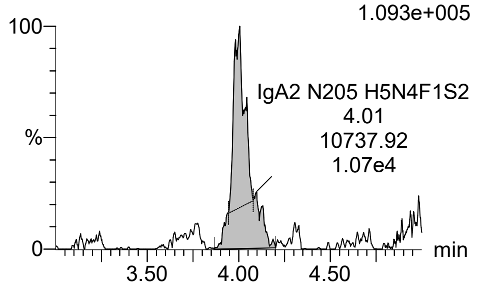

Sample Name: 20211111_H20_R2_IgM
F18:MRM of 1 channel,ES+
828.1 > 204.1
2.177e+005

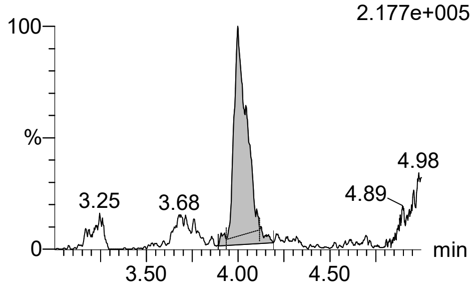

Sample Name: 20211111_A41_R2_IgM
F18:MRM of 1 channel,ES+
828.1 > 204.1
1.211e+005

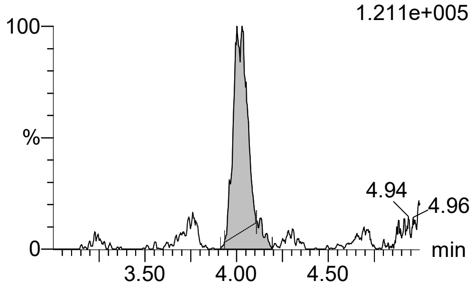

Sample Name: 20211111_A42_R2_IgM
F18:MRM of 1 channel,ES+
828.1 > 204.1
9.261e+004

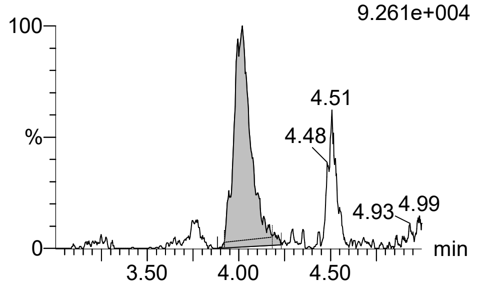

Sample Name: 20211111_A43_R2_IgM
F18:MRM of 1 channel,ES+
828.1 > 204.1
6.823e+004

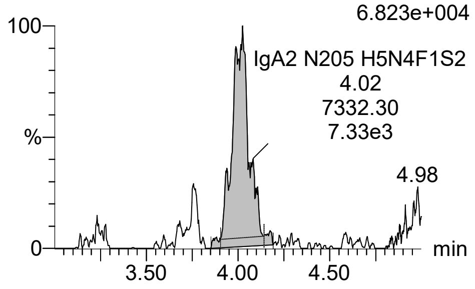

Sample Name: 20211111_A44_R2_IgM
F18:MRM of 1 channel,ES+
828.1 > 204.1
7.143e+004

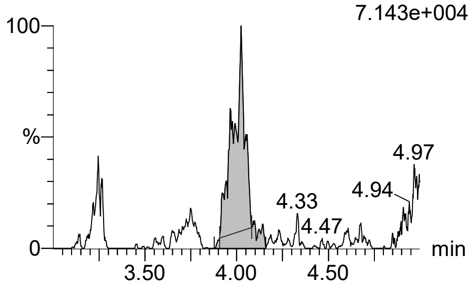

Sample Name: 20211111_QC_R17_IgM
F18:MRM of 1 channel,ES+
828.1 > 204.1
1.800e+005

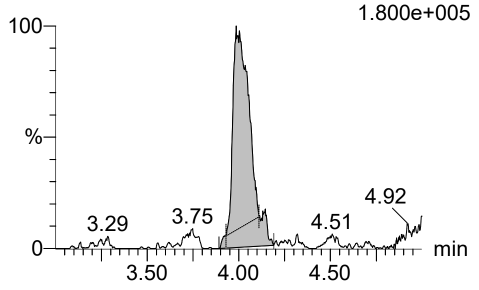

Sample Name: 20211111_L2_R3_IgM
F18:MRM of 1 channel,ES+
828.1 > 204.1
2.716e+005

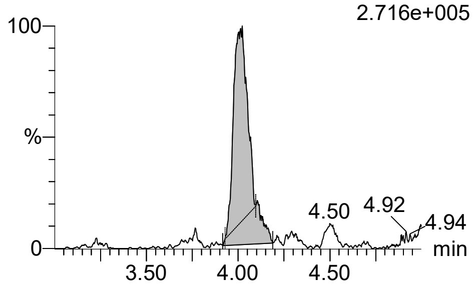

Sample Name: 20211111_H1_R3_IgM
F18:MRM of 1 channel,ES+
828.1 > 204.1
3.245e+005

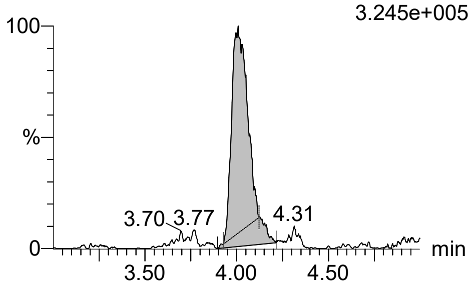

Sample Name: 20211111_A3_R3_IgM
F18:MRM of 1 channel,ES+
828.1 > 204.1
1.661e+005

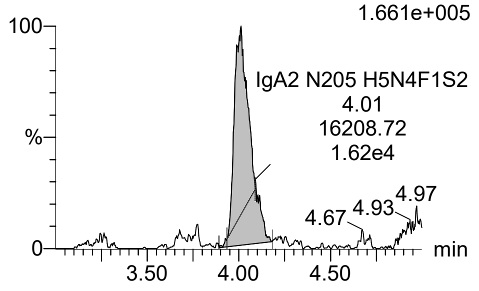

Sample Name: 20211111_A4_R3_IgM
F18:MRM of 1 channel,ES+
828.1 > 204.1
1.747e+005

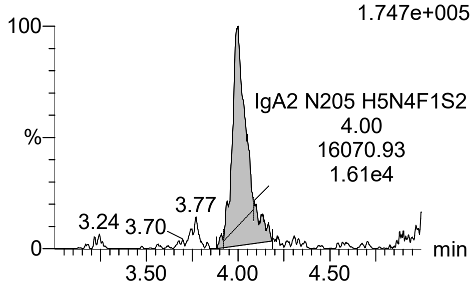

Sample Name: 20211111_L3_R3_IgM
F18:MRM of 1 channel,ES+
828.1 > 204.1
2.449e+005

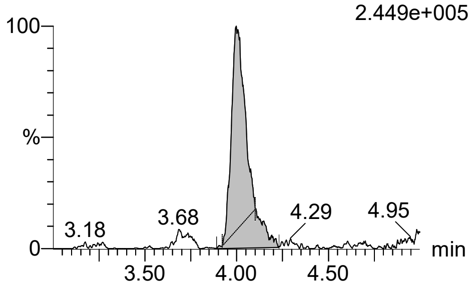

Sample Name: 20211111_L4_R3_IgM
F18:MRM of 1 channel,ES+
828.1 > 204.1
1.133e+005

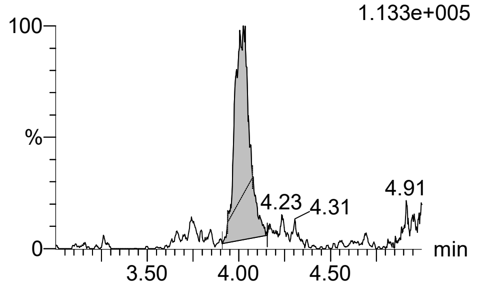
[truncated: 6,039,654 more chars]
